# Integrative multi-omics and single-cell transcriptomics reveal ARHGEF12 driving chemoresistance in bladder cancer

**DOI:** 10.1186/s41065-025-00606-1

**Published:** 2025-11-27

**Authors:** Kunyao Zhu, Zhejiao Zhang, Tinghao Li, Yan Sun, Junlong Zhu, Linfeng Wu, Xiaoyu Zhang, Hang Tong, Zijia Qin, Aimin Wang, Weiyang He

**Affiliations:** 1 Department of Urology, The First Affiliated Hospital of Chongqing Medical University, 1 Youyi Road, Yuzhong District, Chongqing, 400016 P.R. China; 2 Department of Gastroenterology, The First Affiliated Hospital of Chongqing Medical University, Chongqing, 400016 China; 3 Central Laboratory, The First Affiliated Hospital of Chongqing Medical University, Chongqing, 400016 China

**Keywords:** Bladder cancer, Mendelian randomization, ARHGEF12, eQTLs, Chemoresistant

## Abstract

Bladder cancer (BLCA) is the predominant type of urothelial carcinoma in urinary system, and resistance to cisplatin-based chemotherapy substantially worsens clinical outcomes, presenting a major therapeutic obstacle. In this study, we integrated genome-wide association study (GWAS) data with expression quantitative trait loci (eQTL) analyses, and applied Mendelian randomization (MR) to assess the causal effects of eQTLs from 19,942 genes on BLCA. By incorporating scRNA-seq data, our study also identifies differentially expressed genes (DEGs) in cisplatin-resistant BLCA cells and examined their causal associations with BLCA, aiming to elucidate genetic drivers of chemoresistance and tumor progression. Through this integrated approach, we identified the eQTL of the *ARHGEF12* gene as a key mediator of cisplatin resistance. Bioinformatic analysis revealed that elevated *ARHGEF12* expression was strongly associated with activation of the PI3K/Akt signaling pathway. To define *ARHGEF12*’s role in cisplatin resistance, we established a cisplatin-resistant UM-UC-3/DDP model. Silencing *ARHGEF12* markedly reduced chemoresistance, increased apoptotic cell death, and induced pronounced morphological changes. Pharmacological modulation with the ROCK inhibitor Y-27632 and a rescue assay with the Akt activator SC79 supported a model in which *ARHGEF12* drives chemoresistance via RhoA/ROCK-dependent activation of the PI3K/AKT axis. This study is the first to integrate MR with single-cell transcriptomics to explore the genetic contribution to cisplatin resistance in BLCA. Our results uncover a novel mechanistic role of *ARHGEF12* in BLCA progression and chemoresistance and suggest it as a potential therapeutic target for precision treatment strategies.

**Supplementary Information:**

The online version contains supplementary material available at 10.1186/s41065-025-00606-1.

## Introduction

 Bladder cancer (BLCA) is one of the most prevalent malignancies of the urinary tract, with over 430,000 new cases reported globally each year. Both the incidence and mortality rates have shown a persistent upward trend in recent years [1, 2]. Cisplatin-based combination chemotherapy remains the cornerstone of treatment; however, fewer than half of the patients exhibit an initial therapeutic response. In addition, a substantial number of those who initially respond later develop resistance, which severely limits the long-term success of treatment strategies [3]. This highlights the urgent need to elucidate the molecular mechanisms underlying cisplatin resistance and to identify novel therapeutic targets that may enhance clinical outcomes.

Single-cell RNA sequencing has been widely employed to elucidate cellular heterogeneity and to investigate the genetic, transcriptional, and epigenetic characteristics of cancer cell populations and individual tumor cells through multidimensional analyses [4–7]. The limitations of conventional sequencing approaches are overcome by this technology: whereas bulk RNA sequencing averages transcriptional signals across cell populations, scRNA-seq enables complete transcriptome profiling at single-cell resolution [8]. This capability enables detailed analyses of intracellular and intercellular interactions, allows subtle intercellular heterogeneity — difficult to detect in bulk cellular analyses — to be uncovered, supports the identification of novel cell types, and permits monitoring of dynamic changes in cellular states.Enhanced resolution of scRNA-seq facilitates delineation of heterogeneity among drug-resistant tumor cells and within the tumor microenvironment, enables identification of potential therapeutic targets, and provides evidence to support the development of personalized treatment strategies [7]. However, the roles of DEGs linked to heterogeneity in cisplatin-resistant bladder cancer cells remain unclear. Moreover, the causal contribution of DEGs specific to cisplatin-resistant bladder cancer cells to the initiation and progression of bladder urothelial carcinoma has not been fully elucidated.

To address these gaps, this study employs MR to investigate the genetic determinants of disease susceptibility and treatment resistance. MR analysis uses genetic variants associated with a specific exposure as instrumental variables (IVs) to infer the causal effect of that exposure on outcomes, while mitigating bias from confounding and reverse causation.Specifically, using MR analysis, we tested whether expression levels of candidate genes causally influence BLCA risk.Within the DEG analysis framework, we integrated genome-wide association study data with eQTL data to identify genes whose expression variability is associated with complex phenotypes. This study integrated scRNA-seq sequencing with MR analysis to enable high-resolution, cell-type–specific profiling of gene expression effects.Such integration reduces confounding and other sources of bias in causal inference, enabling more precise delineation of disease mechanisms and improved prioritization of candidate therapeutic targets. Building on the analyses above, we aim to clarify how genetic alterations in drug-resistant tumor cells contribute to the development of chemotherapy resistance in BLCA.

## Results

### scRNA-seq integrated with genetically-predicted eQTLs to assess causal mechanisms underlying chemotherapy resistance in bladder cancer

**Fig. 1:**
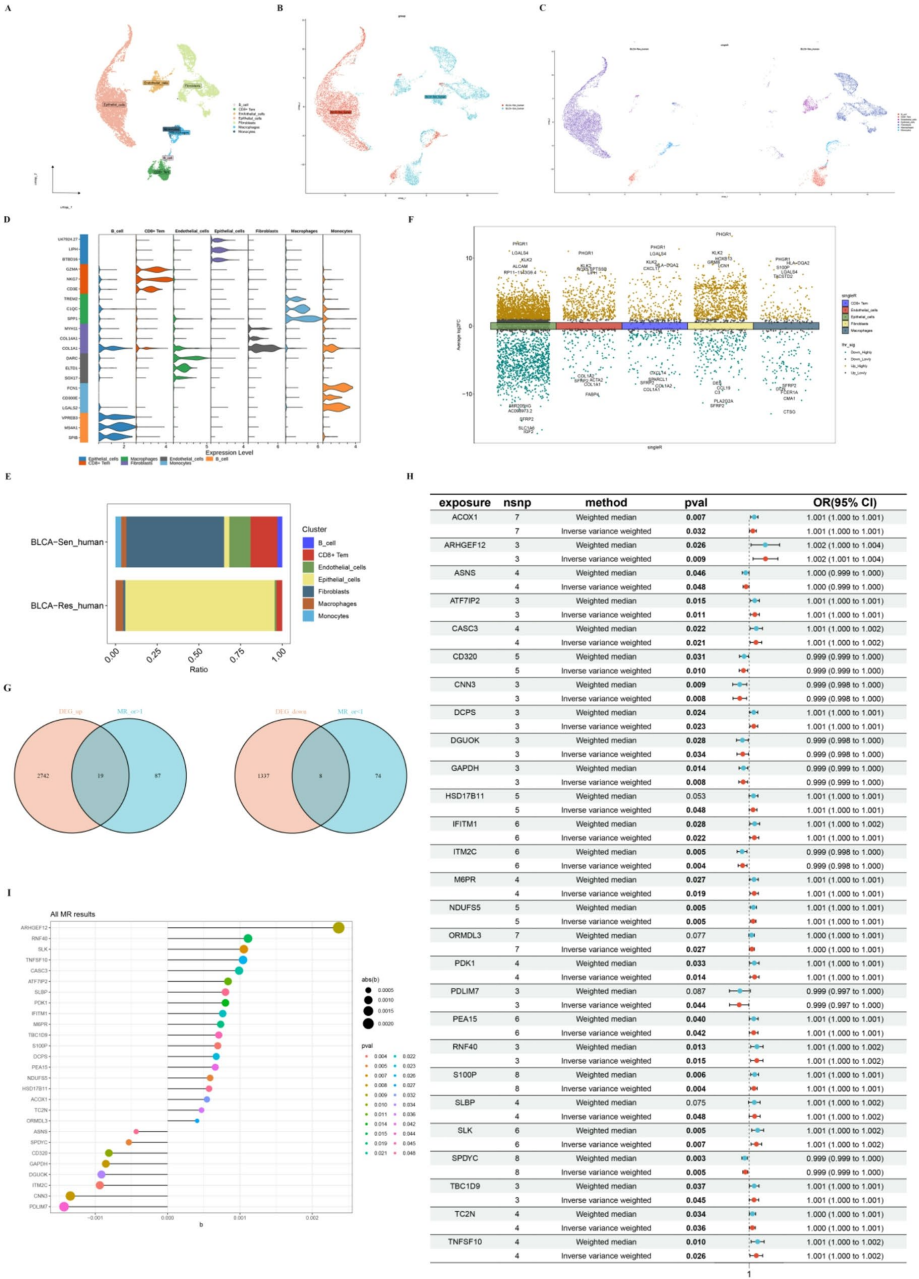
Identification of risk genes through Single-cell RNA sequencing analysis and Mendelian randomization analysis. **A** UMAP plot showing seven distinct cell clusters. **B**,** C** UMAP visualizations of the distribution of cells in cisplatin-resistant and cisplatin-sensitive samples. **D** Violin plot displaying the top three genes for each of the seven cell clusters. **E** Bar graph representing the cell ratio between resistant and sensitive samples. **F** Differentially expressed genes: The top five upregulated and downregulated DEGs across five cell clusters. **G** Intersection of genes identified from DEG analysis of cisplatin-resistant epithelial cell clusters in BLCA and Mendelian randomization results. **H**,** I** Lollipop and forest plots illustrating the MR results for the 27 risk genes

In this study, we interrogated the Gene Expression Omnibus (GEO) database (https://www.ncbi.nlm.nih.gov/geo/) to identify bladder cancer-relevant datasets and retrieved the GSE192575 single-cell RNA sequencing dataset. This dataset comprises transcriptional profiles from two BLCA patients exhibiting contrasting responses to cisplatin-based chemotherapy. Following stringent data normalization and batch effect correction, dimensionality reduction and unsupervised clustering were performed. We subsequently mapped the expression distribution of key marker genes across distinct cellular subpopulations. Cluster analysis was performed using UMAP dimensionality reduction. Through SingleR-based annotation, cells within tumor tissues were classified into seven distinct subtypes (Fig. 1A), encompassing B cells, CD8 + T cells, endothelial cells, epithelial cells, fibroblasts, macrophages, and monocytes. The top three marker genes for each cellular cluster were delineated (Fig. 1D). Notably, each subtype exhibited differential distribution patterns between cisplatin-sensitive and cisplatin-resistant bladder cancer specimens (Fig. 1B-C). Comparative cellular composition analysis revealed significant proportion differences between treatment-responsive and resistant cohorts (Fig. 1E). Transcriptomic profiling identified 4,106 DEGs in epithelial cells from cisplatin-sensitive versus resistant samples (Fig. 1F). To elucidate the functional significance of these DEGs, Mendelian randomization analysis was employed, establishing causal relationships between 19,942 eQTLs and cisplatin resistance. This genetic approach identified 188 eQTL genes with causal associations to bladder cancer pathogenesis. Integration of eQTL and DEG datasets revealed 27 core genes implicated in cisplatin resistance. Among these, 19 candidate genes (e.g., *ARHGEF12*,* RNF40*,* SLK*,* TNFSF11*,* CASC3*) demonstrated both upregulation in resistant epithelium and MR odds ratios (OR) > 1, while 8 candidates (e.g., *PDLIM7*,* CNN3*,* ITM2C*,* DGUOK*,* GAPDH*) showed downregulation with OR < 1 (Fig. 1G).

Next, we visualized the Mendelian randomization results for the 27 core genes using lollipop plots and forest plots (Fig. 1I-H). Specifically, genes with *p* < 0.05 in the IVW analysis were defined as those causally associated with cisplatin chemoresistance. The directionality of the IVW findings was further validated by complementary models, including WM, MR-Egger, Simple Mode, and Weighted Mode analyses. Collectively, these 27 core genes, identified through an integrated approach of single-cell profiling and Mendelian randomization, are not only highly associated with the heritability of bladder cancer pathogenesis but also strongly linked to the incidence of cisplatin chemoresistance, thus warranting further mechanistic investigation.

### Functional enrichment analysis of the core genes

**Fig. 2:**
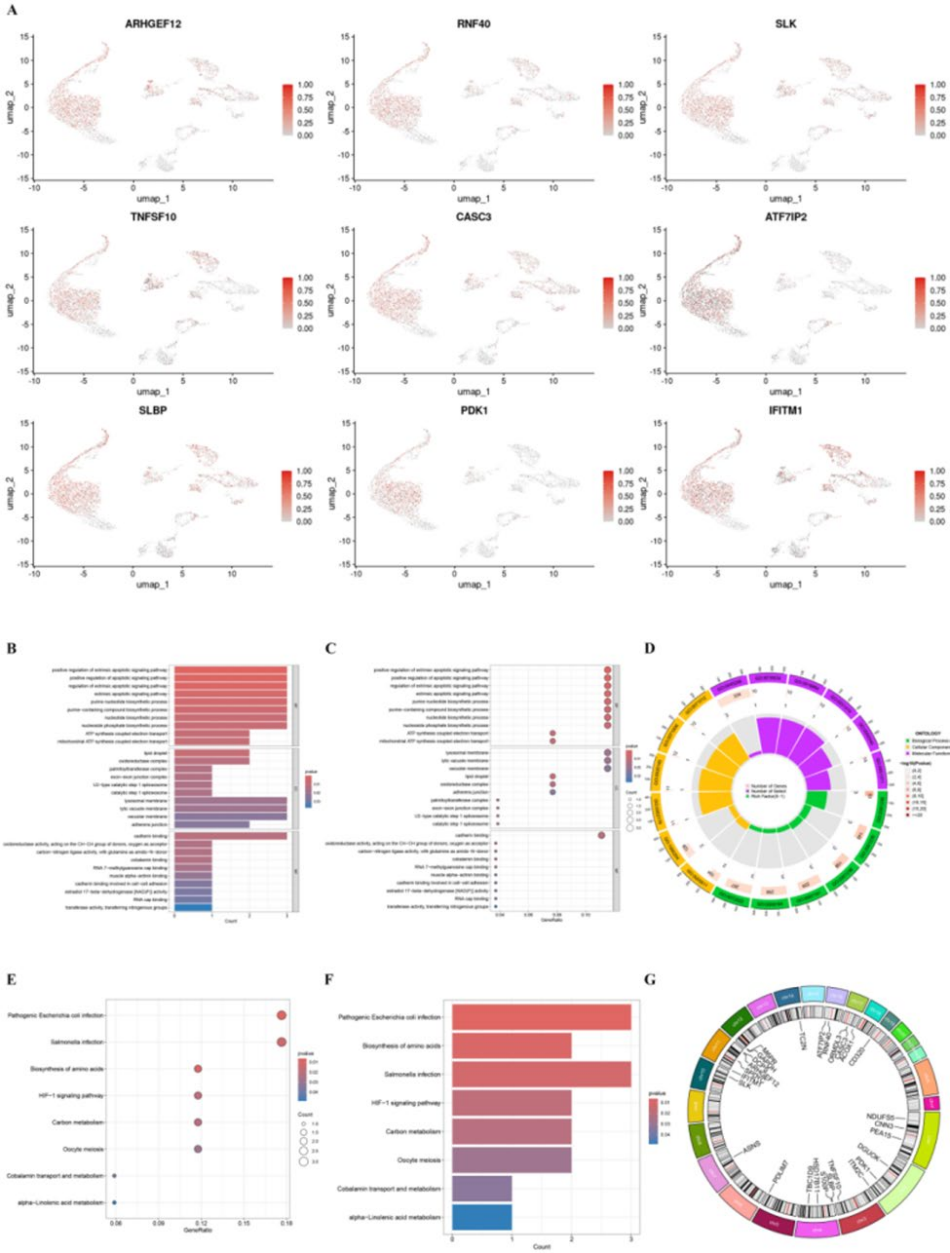
Functional enrichment analysis of 27 risk genes and expression levels in GSE192575. **A** Expression distribution of selected risk genes across various cell types in GSE192575. **B**-**D** GO enrichment analysis of the 27 risk genes. **E**,** F** KEGG enrichment analysis of the 27 risk genes. **G** Chromosomal locations of the 27 risk genes, as illustrated in a circular plot

**Fig. 3:**
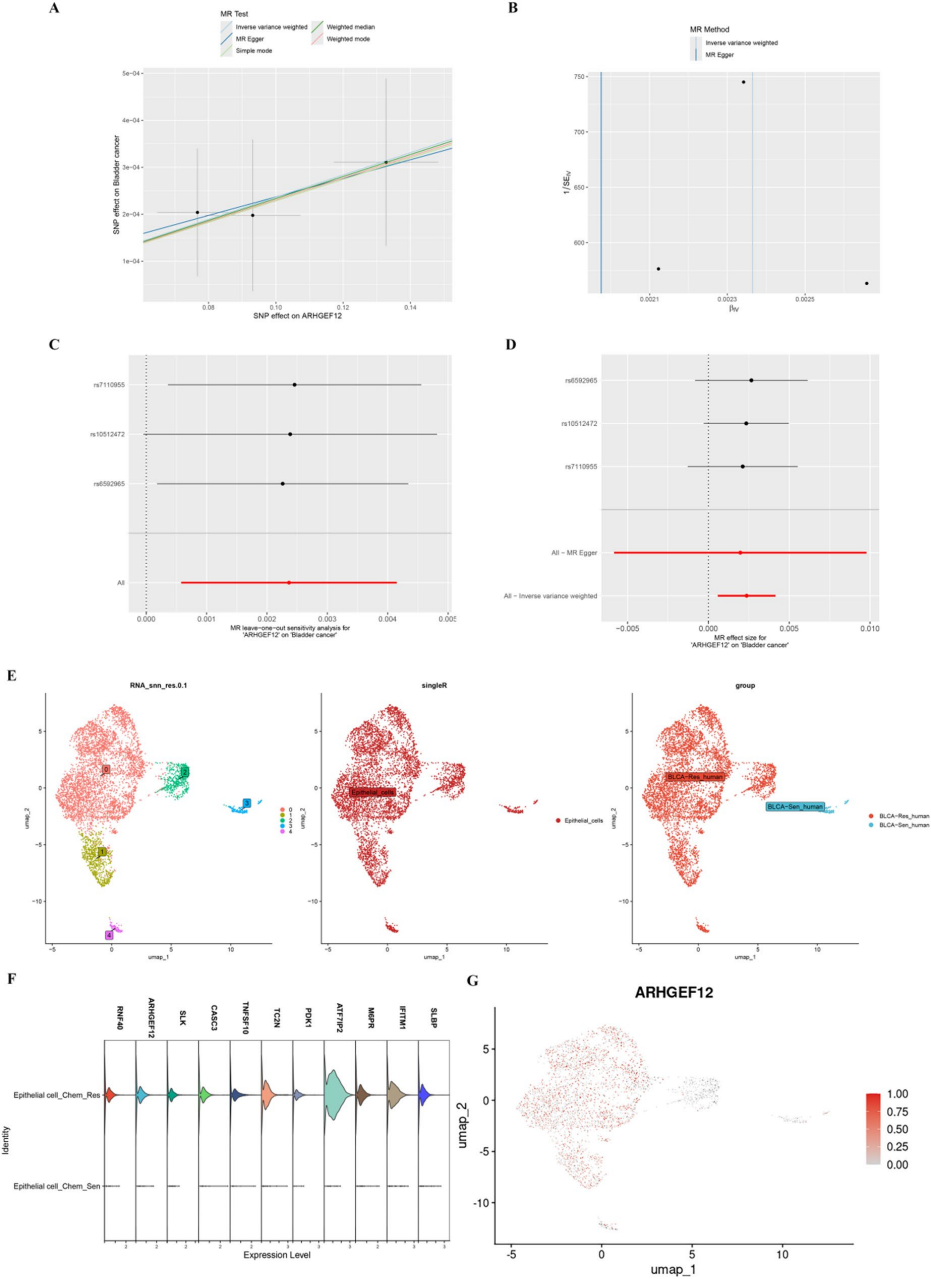
Causal relationship between ARHGEF12 expression and cisplatin chemotherapy resistance in BLCA. **A** Scatter plot illustrating the association between *ARHGEF12* and BLCA using five Mendelian randomization methods. **B** MR funnel plot illustrating the association between *e* and BLCA. **C** Leave-one-out sensitivity analysis of *ARHGEF12* in BLCA. **D** Forest plot illustrating the MR effect size of *ARHGEF12* on BLCA. **E** UMAP visualization of epithelial cell clusters in GSE192575. Epithelial cells were extracted using the Seurat algorithm and re-annotated into five distinct clusters. **F** Violin plot displaying the distribution of selected risk genes in chemotherapy-resistant versus sensitive epithelial clusters. **G** Distribution of *ARHGEF12* expression across epithelial cell clusters

We further analyzed the expression patterns of our core genes within BLCA tissue and found that they are mainly distributed across epithelial cells, endothelial cells, and fibroblasts (Fig. 2A). To better understand the biological roles of these core genes, we performed Gene Ontology (GO) and Kyoto Encyclopedia of Genes and Genomes (KEGG) enrichment analyses. As shown in Fig. 2B–D, GO enrichment analysis revealed the following: (1) In the BP category, the genes were enriched in biological processes such as positive regulation of the apoptotic signaling pathway, purine nucleotide biosynthetic process, respiratory electron transport chain, and deoxyribonucleoside metabolic process. (2) In the CC category, they were linked to the formation or function of lipid droplets, oxidoreductase complexes, and lysosomal membranes. (3) In the MF category, they showed strong associations with cadherin binding, oxidoreductase activity, and cobalamin binding. As shown in Fig. 3E–F, KEGG pathway analysis indicated that these core genes are mainly involved in pathways related to amino acid biosynthesis, the HIF-1 signaling pathway, and alpha-linolenic acid metabolism. To further investigate their chromosomal distribution and structural characteristics, we constructed a circular plot showing the genomic locations of these core genes (Fig. 2G).

### Causal relationship between *ARHGEF12* and cisplatin chemotherapy resistance in BLCA

MR analysis indicated that the eQTL of *ARHGEF12* has a notably stronger causal association with BLCA than the other core genes, implying that *ARHGEF12* expression represents a major risk factor for the disease. The scatter plot shows consistent regression trends across multiple methods (Fig. 3A). The funnel plot illustrates the symmetry of causal effect estimates, supporting the reliability of the results (Fig. 3B). To assess the stability of the MR findings, we conducted a leave-one-out analysis, which showed no substantial influence from any single SNP on the overall causal effect (Fig. 3C). The forest plot details the individual effect sizes of the SNPs within the *ARHGEF12* eQTL (Fig. 3D). MR-Egger intercept analysis of the *ARHGEF12* eQTL yielded a *p* value of 0.936, indicating no significant horizontal pleiotropy, and suggesting that the instrumental variables do not substantially affect the outcome through alternate pathways. Additionally, the Cochran Q test for heterogeneity gave a *p* value of 0.976, suggesting low heterogeneity in the *ARHGEF12* eQTL data.

Concurrently, after identifying *ARHGEF12* as a marker gene, we isolated epithelial cell clusters from the single-cell dataset and performed dimensionality reduction and clustering (Fig. 3E). Visualization of core gene expression across the epithelial cell clusters showed that *ARHGEF12* is markedly overexpressed in cisplatin-resistant epithelial cells (Fig. 3F-G). These results indicate that high *ARHGEF12* expression is not only closely linked to BLCA but may also represent a critical driver of resistance to cisplatin chemotherapy.

### Clinical relevance and functional enrichment analysis of *ARHGEF12* in BLCA

**Fig. 4:**
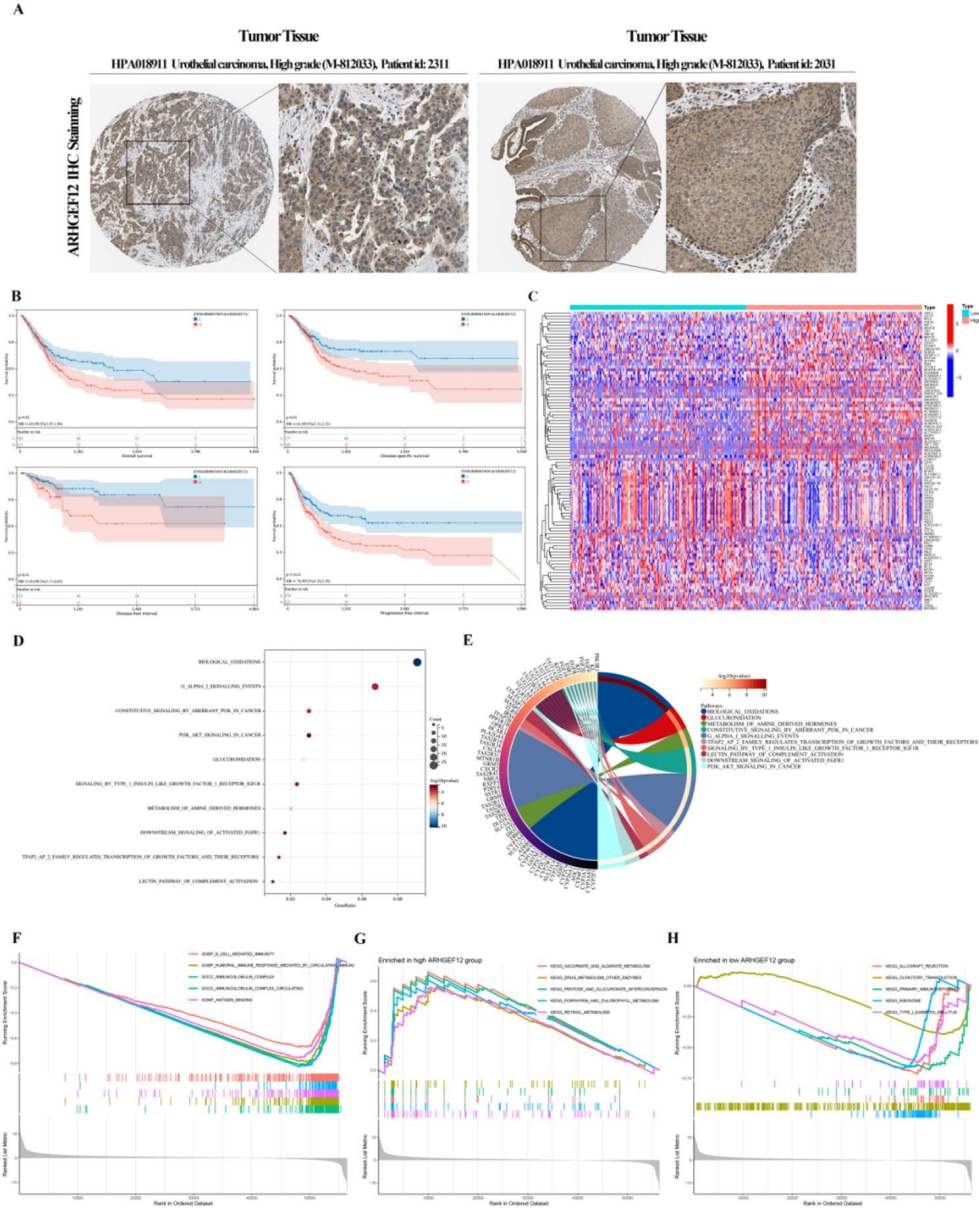
Clinical Relevance and Functional Enrichment Analysis of *ARHGEF12 *in BLCA. **A** Immunohistochemical staining of BLCA tissue (HPA018911) displaying *ARHGEF12* protein expression in urothelial squamous epithelial cells. **B** Associations of *ARHGEF12* with OS, DSS, DFI, and PFI based on TCGA and GTEx data. **C** DEGs identified between *ARHGEF12* low- and high-expression cohorts in the TCGA-BLCA dataset. **D**,** E** Functional enrichment analyses for *ARHGEF12*-associated DEGs. **F** GSEA for *ARHGEF12*. **G** GSEA results for samples with high *ARHGEF12* expression. **H** GSEA results for samples with low *ARHGEF12* expression.

We examined clinical samples from the Human Protein Atlas (HPA) database (Fig. 4 A) and found that e is broadly expressed across tumor tissues. For a more detailed investigation, we conducted bioinformatics analysis using the SangBox 3.0 platform (http://sangerbox.com/home.html), with BLCA datasets obtained from TCGA and GTEx (accessed on October 1, 2024). The association between *ARHGEF12* expression and clinical outcomes in BLCA patients was evaluated through the log-rank test. Results showed that patients with high *ARHGEF12* expression had significantly poorer prognoses than those with low expression levels. The hazard ratios (HR) were as follows: OS: HR = 1.45, *p* = 0.02; DSS: HR = 1.61, *p* = 0.01; DFI: HR = 2.38, *p* = 0.01; PFI: HR = 1.74, *p* = 5.5e-4 (Fig. 4B).

We then used the TCGA-BLCA dataset to compare DEGs between low and high *ARHGEF12* expression groups (Fig. 4 C), aiming to investigate the gene’s functional roles. Enrichment results indicated involvement in pathways such as Biological Oxidation, Glucuronidation, and Metabolism of Amine-Derived Hormones. Importantly, the PI3K-Akt signaling pathway, which plays a central role in tumor initiation and progression, was also enriched (Fig. 4D–E).

To further explore the biological activity of *ARHGEF12*, we conducted GSEA analysis. The results indicated that *ARHGEF12* expression is linked to immune-related processes, including B cell-mediated immunity, immunoglobulin complexes, antigen binding, immunoglobulin receptor binding, and phagocytosis recognition signaling (Fig. 4 F). In the high-expression group, the top five enriched pathways were Ascorbate and Aldarate Metabolism, Drug Metabolism-Other Enzymes, Pentose and Glucuronate Interconversions, and Porphyrin and Chlorophyll Metabolism (Fig. 4G). In contrast, the low-expression group showed enrichment in Allograft Rejection, Olfactory Transduction, Primary Immunodeficiency, Ribosome Activity, and Type I Diabetes Mellitus (Fig. 4H).

### Establishment of cisplatin-resistant UM-UC-3 BLCA cell line

**Fig. 5:**
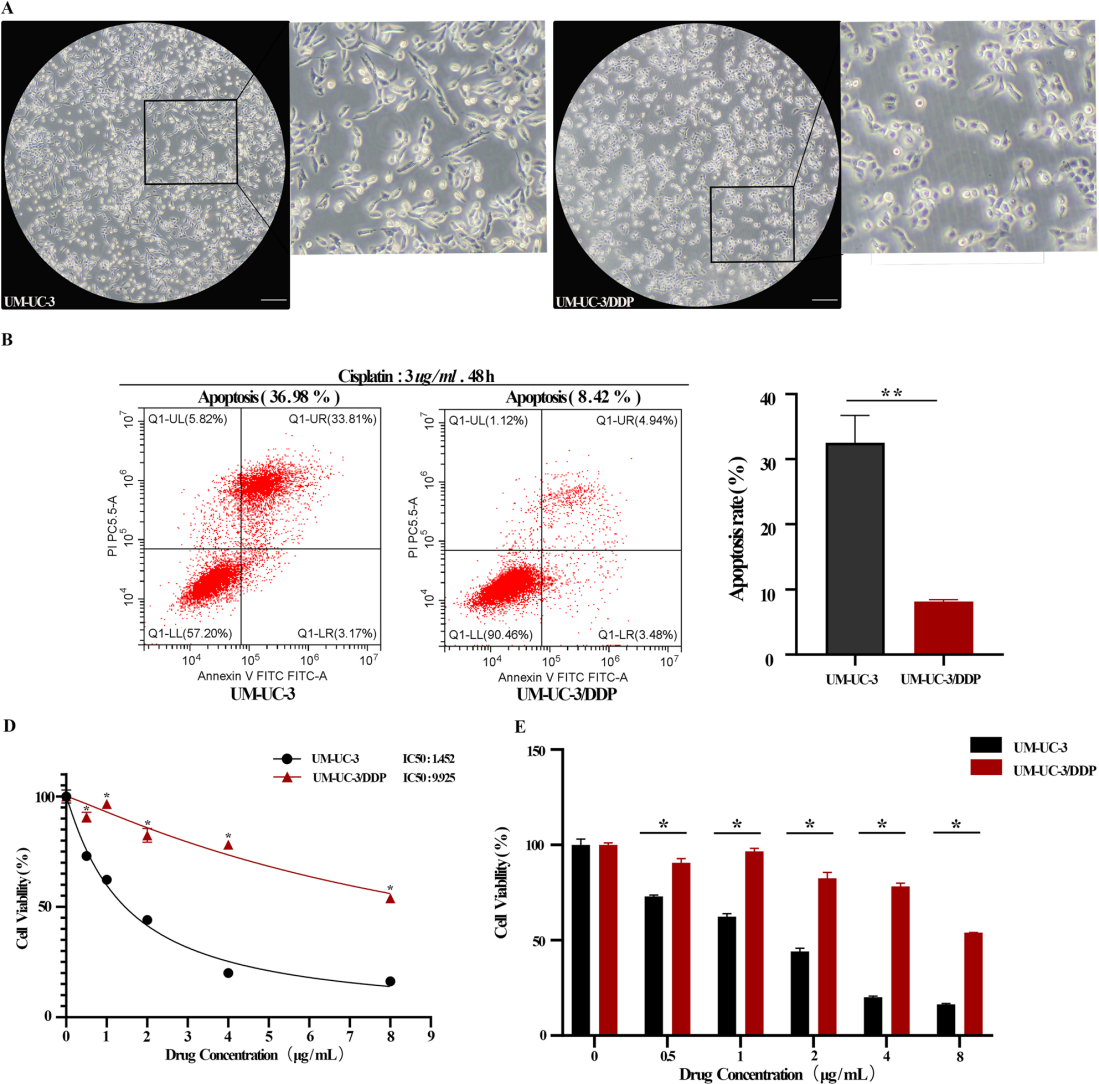
Construction of the UM-UC-3/DDP Cell Line Model. **A** Microscopic morphology of UM-UC-3 and UM-UC-3/DDP (scale bar: 100 μm). **B** Flow cytometry analysis of the apoptosis rate in UM-UC-3 and UM-UC-3/DDP cells. *p < 0.05 indicates statistical significance. **C**,** D** CCK-8 assay determining the IC50 values of UM-UC-3 and UM-UC-3/DDP in response to cisplatin

To explore the role of *ARHGEF12* in BLCA chemotherapy resistance, we established a cisplatin-resistant UM-UC-3/DDP cell line model based on previous work from our group [9]. The resistant cells were able to grow and maintain stable proliferation in medium containing 3 µg/mL cisplatin. Morphologically, UM-UC-3 cells displayed a spindle-shaped appearance with uniform size, well-defined boundaries, and adherent growth. In contrast, UM-UC-3/DDP cells appeared more irregular and shrunken, with visible granules and vacuoles in the cytoplasm (Fig. 5A). After co-treatment with 3 µg/mL cisplatin for 48 h, apoptosis was significantly increased in UM-UC-3 cells compared to UM-UC-3/DDP cells (Fig. 5B). CCK-8 assay results showed that the IC50 of UM-UC-3/DDP cells was substantially higher than that of UM-UC-3 cells, with a resistance index of approximately 9.93 (Fig. 5C–D).

### The role of *ARHGEF12* in the process of drug resistance formation in bladder cancer

**Fig. 6:**
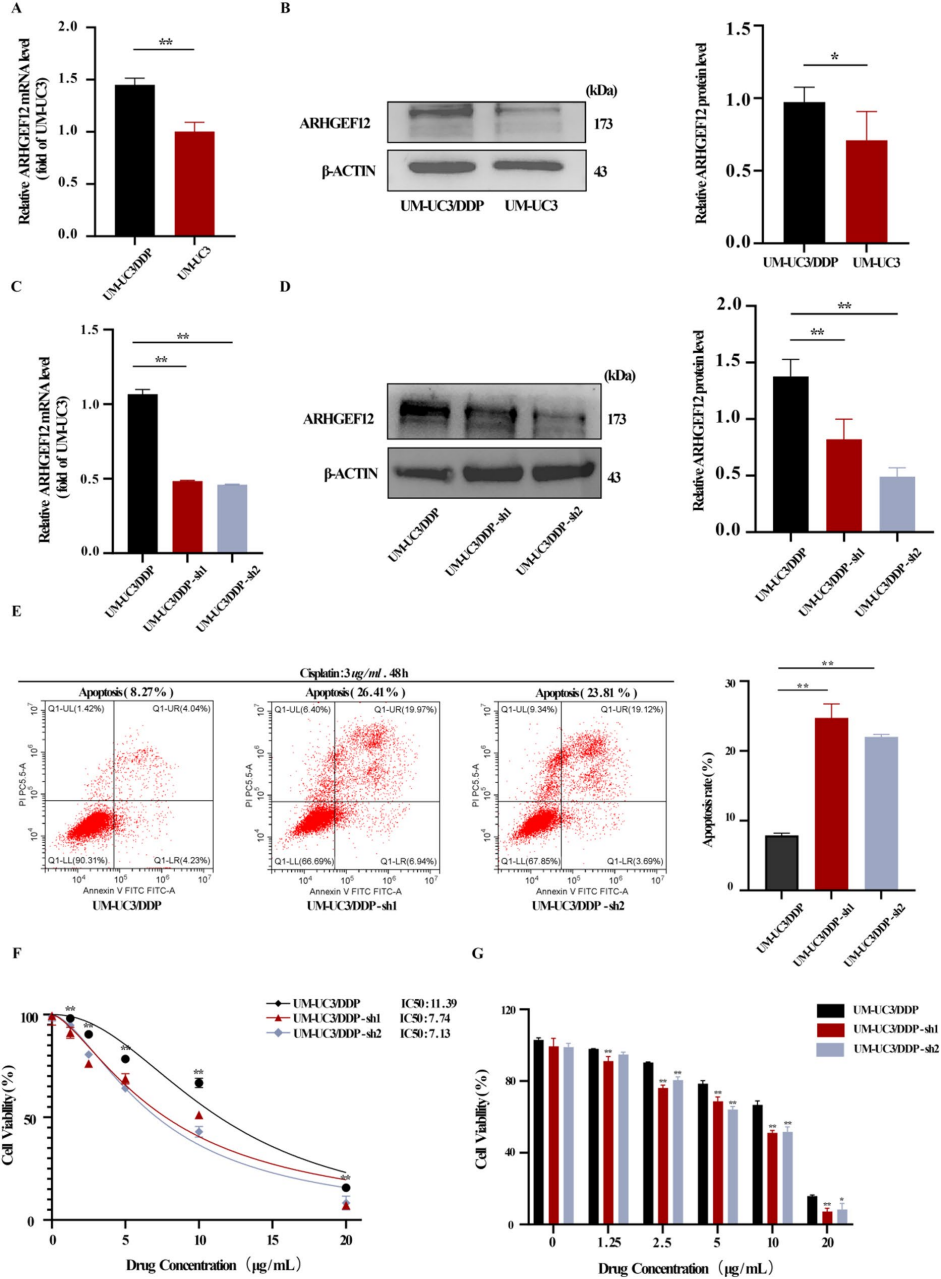
The Role of *ARHGEF12* in BLCA Drug Resistance Development. **A** RT-qPCR analysis of *ARHGEF12* mRNA expression levels in UM-UC-3 and UM-UC-3/DDP. **B** Western blot detection of *e* protein expression in UM-UC-3 and UM-UC-3/DDP. **C** RT-qPCR assessment of *ARHGEF12* silencing efficiency. **D** Western blot detection of *ARHGEF12* silencing efficiency. **E** Flow cytometry measurement of the cell apoptosis rate. **F**,** G** CCK-8 assay measuring the IC50 of the sh*e*-UM-UC-3/DDP for cisplatin

Interestingly, RT-qPCR and Western blotting demonstrated that *ARHGEF12* expression at both the mRNA and protein levels was markedly elevated in UM-UC-3/DDP cells compared with parental UM-UC-3 cells (Fig. 6A-B). These results align with our earlier work and indicate that high *ARHGEF12* expression in the cisplatin-derived UM-UC-3/DDP cell line may contribute to cisplatin resistance in bladder cancer cells. Both shRNAs efficiently reduced *ARHGEF12* expression. Following *ARHGEF12* knockdown, UM-UC-3/DDP cells cultured with 3 µg/mL cisplatin exhibited markedly reduced proliferation, a pronounced increase in apoptosis (Fig. 6E), and a significant reduction in the cisplatin resistance index (Fig. 6F–G). Collectively, these findings indicate that *ARHGEF12* regulates cisplatin resistance in bladder cancer cells and underscore the precision of combining MR analysis with single-cell approaches to pinpoint candidate therapeutic targets.

### *ARHGEF12* promotes cisplatin resistance in BLCA via RhoA/ROCK-mediated activation of the PI3K/Akt pathway

**Fig. 7:**
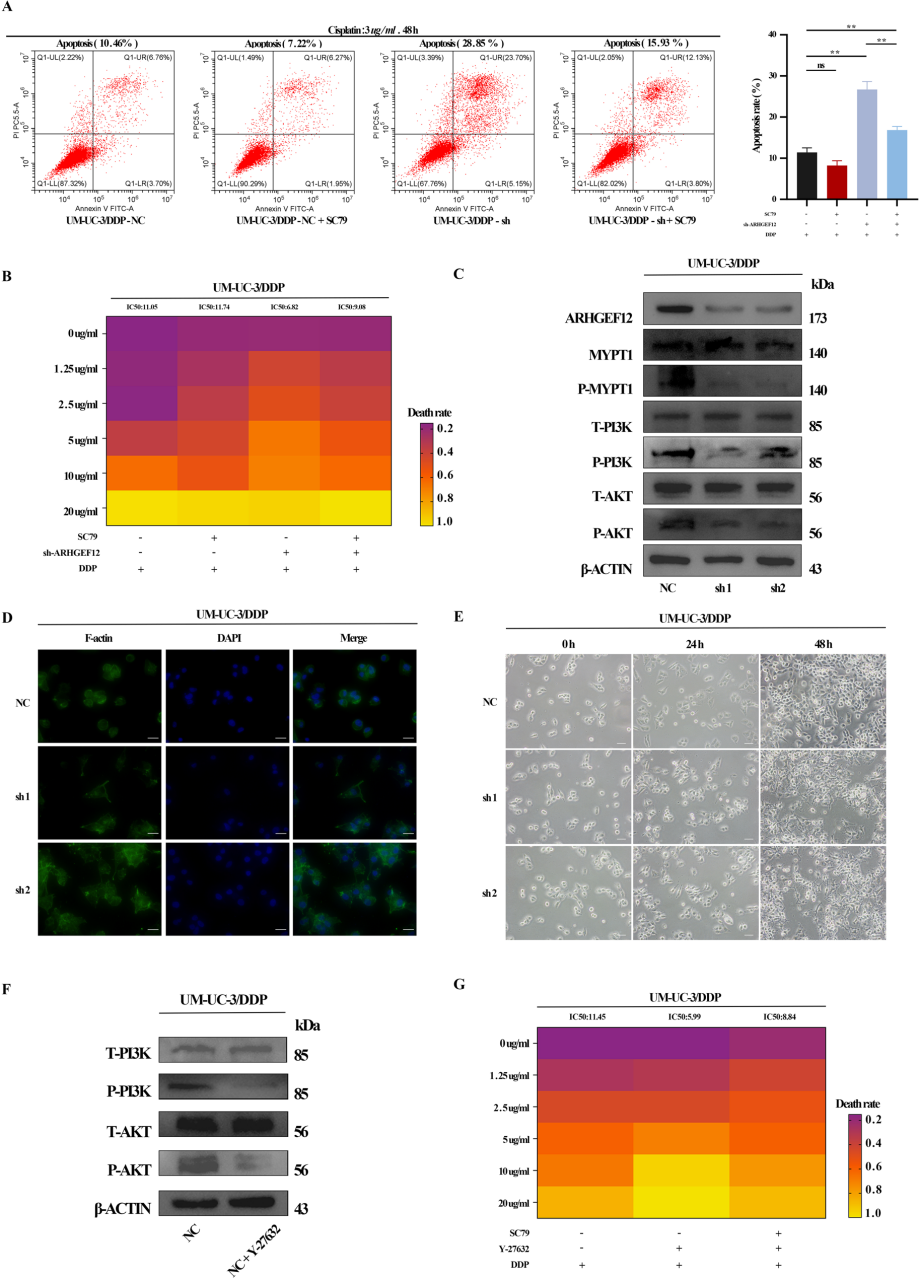
*ARHGEF12* promotes cisplatin resistance in BLCA via RhoA/ROCK-mediated activation of the PI3K/Akt pathway. **A **Apoptosis measured by flow cytometry. **B **CCK-8 assay determining cisplatin IC50 in sh-*ARHGEF12* UM-UC-3/DDP cells. **C **Western blot analysis of RhoA/ROCK and PI3K/Akt signaling following *ARHGEF12* depletion. **D **Fluorescence microscopy of the UM-UC-3/DDP cell cytoskeleton (scale bar: 40 μm). **E **Phase-contrast images showing morphology of *ARHGEF12*-silenced UM-UC-3/DDP cells (scale bar: 100 μm). **F **Western blot analysis of PI3K/Akt pathway after Y-27632 treatment. **G **CCK-8 assay of cisplatin IC50 in UM-UC-3/DDP cells following Y-27632 treatment

Guided by bioinformatic predictions, this study investigated whether *ARHGEF12* mediates cisplatin resistance via the PI3K/Akt pathway. In sh-*ARHGEF12* UM-UC-3/DDP cells, plasmid-mediated *ARHGEF12* knockdown markedly reduced PI3K and Akt phosphorylation (Fig. 7C). Administration of the Akt activator SC79 partially reversed these effects, restoring apoptosis in sh-*ARHGEF12* UM-UC-3/DDP cells (Fig. 7A) and reducing the cisplatin resistance index (Fig. 7B). Multiple reports [10, 11] indicate that RhoA/ROCK signaling can activate PI3K/Akt signaling via downstream effectors, thereby influencing cell survival, proliferation, and apoptosis [12]. Given that ARHGEF12 has been shown to regulate RhoA–ROCK [13], it is therefore proposed that ARHGEF12 may activate PI3K–Akt signaling by modulating this pathway. To test it, phosphorylation of myosin phosphatase target subunit 1 (MYPT1) was measured as a readout of RhoA/ROCK pathway activity [14, 15]. Results demonstrated the decreased MYPT1 phosphorylation in sh-*ARHGEF12* UM-UC-3/DDP cells, indicating suppressed RhoA/ROCK pathway activity (Fig. 7C). Given that ROCK is a key regulator of cytoskeletal signaling and architecture determines cell shape, sh-*ARHGEF12*-treated UM-UC-3/DDP cells exhibited morphological deformation and atrophy accompanied by reorganization of the actin cytoskeleton (Fig. 7D–E). Moreover, treatment with the ROCK inhibitor Y-27632 markedly reduced PI3K and Akt phosphorylation (Fig. 7F) and significantly sensitized UM-UC-3/DDP cells to cisplatin (Fig. 7G). SC79 partially rescued the apoptosis reduced by ROCK inhibition. PI3K/Akt signaling has a key role in drug-resistance phenotypes. Demonstrating that ARHGEF12 activates PI3K/Akt through modulation of the RhoA/ROCK cascade offers novel therapeutic avenues aimed at these signaling components or their downstream effectors. Collectively, our data indicate that *ARHGEF12* activates PI3K/Akt signaling via the RhoA/ROCK axis, contributing to cisplatin resistance in BLCA.

## Discussion

BLCA ranks among the most common malignancies of the urinary tract, driven by a multifaceted interplay of genetic and environmental influences. Both incidence and mortality rates have continued to climb globally, exerting a heavy toll on patient outcomes [5, 6]. In this study, we aimed to uncover novel pathogenic mechanisms and identify therapeutic targets that could contribute to more accurate diagnosis and targeted treatment of BLCA. By integrating GWAS and eQTL datasets with Mendelian randomization (MR), which uses naturally occurring genetic variation as an instrumental variable, we assessed the causal relationship between the eQTLs of 19,942 genes and BLCA. This strategy allowed us to move beyond correlation and added precision to the evaluation of genetic risk factors for BLCA. Additionally, we applied scRNA-seq analysis to explore the molecular mechanisms by which risk genes may drive cisplatin resistance in BLCA, pointing to potential biomarkers and targets with clinical relevance.

Combining GWAS and eQTL data in transcriptome-wide association studies enables the identification of genes whose genetically predicted expression is associated with complex traits, thereby revealing gene-trait links that might otherwise remain hidden [16, 17]. To address the challenge of distinguishing true causal relationships from effects driven by horizontal pleiotropy or confounding, we incorporated MR to estimate the direct impact of gene expression on BLCA risk. MR establishes causality by analyzing how genetic variation at conception influences downstream traits [18, 19]. In recent studies, the combination of MR with GWAS and eQTL datasets [20] has been used to identify disease-associated genes with greater confidence. This approach clarifies the links between genetic variants, transcriptomic changes, and disease phenotypes. MR has already been applied to uncover the genetic architecture of numerous complex conditions, including cardiovascular diseases [21], systemic lupus erythematosus [22], and rheumatoid arthritis [23], highlighting its potential in investigating gene expression effects and pleiotropic roles in diverse traits. Our analysis used 19,942 gene eQTLs as exposures and BLCA as the outcome, identifying 27 risk genes through MR. Among them, genetic susceptibility to the *ARHGEF12* eQTL was strongly associated with increased BLCA risk, suggesting that *ARHGEF12* is a key risk gene. Despite this association, the exact function of *ARHGEF12* in BLCA remains unclear.

To elucidate the role of *ARHGEF12* in bladder cancer initiation and progression, we performed scRNA-seq to map BLCA gene expression profiles at single-cell resolution. scRNA-seq provides transcriptome profiles at single-cell resolution, enabling detection of cell-to-cell variability in gene expression and characterization of intratissue cellular heterogeneity. It therefore overcomes the limitations of bulk RNA sequencing and provides unprecedented opportunities to resolve the functional states of individual cancer cells.This approach has been applied to investigate cisplatin resistance in BLCA. By examining DEGs across cell types, researchers have delineated cell populations associated with sensitivity to chemotherapy and to chemoresistance [24]. Nonetheless, the DEGs specific to cisplatin-resistant BLCA cells, and their causal roles in disease progression, remain poorly understood. Moreover, the precise mechanisms by which these DEGs confer chemoresistance remain undefined. In this study, we integrate MR analyses using GWAS and eQTL datasets with single-cell transcriptomic profiling, enabling associations between single-cell–level gene expression and underlying genetic variants.Such an integrative approach facilitates discovery of regulatory loci that modulate gene expression in specific cell subsets. In parallel, we applied multiple bioinformatics analyses to characterize the biological functions of these DEGs and interrogated TCGA and GTEx cohorts to investigate disease-associated gene functions and underlying mechanisms (Fig. S1).Analysis of the GSE192575 scRNA-seq dataset revealed that *ARHGEF12* is predominantly expressed in cisplatin-resistant malignant epithelial cells. Collectively, these findings indicate that *ARHGEF12* may promote chemoresistance, thereby contributing to BLCA progression and representing a candidate risk gene.

Leukemia-associated RhoGEF (*LARG/ARHGEF12*), a member of the guanine nucleotide exchange factor family, was first identified in acute myeloid leukemia as a fusion partner of mixed-lineage leukemia [25]. To date, the role of *ARHGEF12* in cisplatin chemoresistance remains unexplored in cancer therapy. Previous studies identify LARG as a key regulator of RhoA/ROCK signaling that promotes tumor cell migration and invasion.Further studies indicate that in highly invasive glioblastoma, LARG silencing inactivates RhoA/ROCK signaling, markedly reduces tumor cell viability, and enhances sensitivity to chemotherapeutics such as temozolomide, supporting LARG as a potential therapeutic target [26–28]. LARG has been implicated in immune regulation and cytoskeletal dynamics; for example, lentiviral shRNA-mediated LARG knockdown impairs megakaryocyte maturation and alters actin cytoskeleton organization via RhoA/ROCK signaling, contributing to diverse pathological processes.In our study, high *ARHGEF12* expression correlated with poor prognosis in BLCA patients. In a cisplatin-resistant UM-UC-3/DDP cell line, *ARHGEF12* levels were markedly increased; *ARHGEF12* knockdown reduced the cisplatin resistance index and promoted apoptosis.

To clarify *ARHGEF12*’s role in BLCA, we performed enrichment analysis of DEGs between high- and low-*ARHGEF12* expression groups using the TCGA-BLCA dataset. The results implicate *ARHGEF12* in regulation of the PI3K–Akt signaling pathway in BLCA, whose aberrant activation is closely linked to tumor progression [29]. The PI3K/Akt pathway is central to tumorigenesis and is associated with multiple malignant phenotypes, including chemoresistance [30], cell proliferation [31], and angiogenesis [32]. To test this link, *ARHGEF12* was stably knocked down in UM-UC-3/DDP cells, which markedly reduced PI3K and Akt phosphorylation. Furthermore, treatment with the Akt agonist SC79 partially reversed the apoptosis induced by *ARHGEF12* knockdown. While investigating upstream mechanisms by which *ARHGEF12* regulates PI3K/Akt signaling, *ARHGEF12*-silenced cells exhibited marked morphological changes, suggesting that the RhoA/ROCK pathway may mediate this effect. Treatment with the ROCK inhibitor Y-27632 reduced PI3K and Akt phosphorylation and diminished cisplatin resistance in UM-UC-3/DDP cells, supporting a RhoA/ROCK/PI3K/Akt axis in chemoresistance. Notably, SC79 treatment partially reversed the decrease in apoptosis induced by ROCK inhibition. Together, these findings indicate that *ARHGEF12* activates the RhoA/ROCK cascade to drive PI3K/Akt signaling. This mechanism helps explain how *ARHGEF12* promotes BLCA progression and supports *ARHGEF12* as a potential therapeutic target in bladder cancer.

In conclusion, integrating MR analysis with GWAS and eQTL data identified *ARHGEF12* as a putative causal risk gene for BLCA, thereby expanding understanding of BLCA genetic susceptibility. Furthermore, integrating scRNA sequencing with multi-omics bioinformatics of TCGA and GTEx data, this study identifies *ARHGEF12* as a key regulator of cisplatin resistance in BLCA for the first time, opening new avenues and potential targets for elucidating mechanisms of cisplatin resistance in bladder cancer. Additionally, our study reveals a novel molecular regulatory circuit driving cisplatin resistance in BLCA: *ARHGEF12* activates the PI3K–Akt axis by modulating the RhoA/ROCK pathway, thereby promoting resistance in BLCA cells. Overall, this work delivers the first comprehensive characterization of *ARHGEF12*’s genetic regulatory role in BLCA risk, prognosis, and chemoresistance, highlighting it as a promising target for precision therapy.

This study has some limitations that need to be acknowledged. First, the MR analysis relied on a single GWAS resource, which may limit the generalizability of the results. Second, the GWAS datasets used for MR were derived from individuals of European ancestry, whereas the scRNA-seq data were obtained from Chinese subjects, introducing potential population-specific biases. Considering genetic structural differences between Eurasian populations [33], integrating data across ancestries may introduce multiple layers of population-specific bias. Accordingly, further studies are needed to determine whether the findings of this study generalize across other populations. In addition, most key results were generated using in vitro models, and confirmatory evidence from in vivo systems (e.g. xenograft models) is limited. Therefore, further in vivo studies are required to validate the biological relevance of these findings. To more fully delineate the mechanisms by which *ARHGEF12* promotes chemoresistance in BLCA, future research should integrate multidimensional lines of evidence to corroborate and extend the current experimental results.

## Conclusion

To our knowledge, this is the first time to integrate Mendelian randomization with GWAS and eQTL analyses, along with single-cell sequencing, to clarify the causal relationship between *ARHGEF12* eQTL and chemoresistance in BLCA. Our study indicates that *ARHGEF12* promotes chemoresistance in bladder cancer cells by modulating RhoA/ROCK signaling, which subsequently activates the PI3K/AKT pathway. These results offer new insights into BLCA’s molecular drivers and nominate the *ARHGEF12*/RhoA/ROCK/PI3K/AKT cascade as a promising therapeutic target to overcome treatment resistance in BLCA.

## Method

### Acquisition and processing of scRNA-seq data

We retrieved and downloaded cellular transcriptome data from the GEO database (GSE192575) for analysis. Single-cell RNA sequencing data were processed using the Seurat R package. Data quality control was performed by excluding cells that did not meet specific criteria(gene count per cell ≤ 300 or ≥ 30,000, mitochondrial gene percentage per cell ≥ 20%, and hemoglobin gene percentage per cell ≥ 3%). Batch effect correction was conducted using the “Harmony” R package. Cell clustering was performed using the “FindClusters” and “FindNeighbors” functions in Seurat, followed by dimensionality reduction and visualization with UMAP. Finally, cell clusters were annotated with the “SingleR” R package.

### Identification of DEGs between cisplatin chemotherapy resistant and sensitive BLCA samples

Using the “FindAllMarkers” function in Seurat, we identified seven differentially expressed gene clusters between cisplatin chemotherapy-resistant and chemotherapy-sensitive BLCA samples across seven cell types. DEGs were defined as those with |(logFC)| >1,p val < 0.05. During the DEG analysis, we excluded two cell types, endothelial cells and monocytes, as they were only present in cisplatin-sensitive BLCA samples.

### Exposure data

To strengthen the reliability of the analysis and reduce selection bias, datasets from populations of European ancestry were chosen for all exposure and outcome cohorts. For the exposure data, we extracted SNPs that were significantly associated with the expression of genome-wide DEGs (*p* < 5 × 10⁻⁶) from published GWAS. This dataset encompassed 10,317 SNPs, with cis-eQTL summary statistics sourced from the eQTLGen consortium’s meta-analysis of 19,942 transcripts across 31,684 individuals [34].

### Outcome data

The BLCA-related outcome data were obtained from the summary statistics dataset of GWAS, accessible through the Integrative Epidemiology Unit OpenGWAS project database (accessed on October 1, 2024), with results identified under ID ieu-b-4874. The dataset is available as VCF files or can be accessed using the TwoSampleMR R package. This GWAS subset includes data from 373,295 participants of European ancestry, comprising 1,279 cases and 372,016 controls, and aims to identify risk loci associated with BLCA [35].

### Construction and culture of the UM-UC-3/DDP cell lines

The UM-UC-3 BLCA cell line was obtained from the Cell Bank of the Chinese Academy of Sciences in Shanghai. Cisplatin resistance in UM-UC-3 cells was developed by progressively elevating the cisplatin concentration, which led to the establishment of the UM-UC-3/DDP. The IC50 of cisplatin for both the UM-UC-3 and UM-UC-3/DDP was assessed using the CCK-8 assay. Based on the obtained results, the resistance index (RI) was calculated. The RI was calculated as follows:​

$$\mathrm{RI}=\frac{\mathrm{IC}{50}_{\mathrm{UM}-\mathrm{UC}-3/\mathrm{DDP}}}{\mathrm{IC}{50}_{\mathrm{UM}-\mathrm{UC}-3}}$$

### RT-qPCR assay

ACTB(Human) was used as the internal reference gene. The ARHGEF12 mRNA expression level was evaluated using the relative quantification method (2−∆∆Ct method) with respect to ACTB expression. The primer sequences are shown in Table 1. The primers were designed and synthesized by Tsingke (Beijing, China).

**Table 1:** Primer sequences for RT-qPCR

Gene Name	Forward Sequence	Reverse Sequence
ARHGEF12	5’-GCACACCTCGTACTCTCAATACT-3’	5’- CCCATGTTCAGTCACCCTTTCTA-3’
ACTB	5’-AGAAAATCTGGCACCACACCT-3’	5’-GATAGACAGCCTGGATAGCA-3’

### Transfection of plasmids and shRNA

All target shRNAs were obtained from Tsingke (Beijing, China). Following the manufacturer’s instructions, plasmids and shRNAs were transfected into UM-UC-3 or UM-UC-3/DDP using Lipofectamine 2000 (Invitrogen, Carlsbad, CA, USA). The shRNA sequences are listed in Table 2.

**Table 2:** Sequences of shRNA

Item	Genes	Reverse Sequence
Target Sequences of shRNA	ShARHGEF12-1	5’-GCGTTGCGTAATCATCCAGAA-3’
	ShARHGEF12-2	5’-GCGAGTATCCAGAGAAGGAAT-3’

All primers and shRNAs were designed to target Homo sapiens

### Lentiviral transfection

The plasmids (shNC, shARHGEF12, PAX2, VSVG) were sourced from Tsingke (Beijing, China). In accordance with the manufacturer’s protocol, the plasmids were transfected into 293 T cells using Lipofectamine 8000 (Invitrogen, Carlsbad, CA, USA).Following an 8-hour transfection at 37 °C, the culture medium was changed. Supernatants were collected every 24 h, with the final collection occurring at 72 h. The resulting filtered supernatants were subsequently used for transfecting the UM-UC-3/DDP cells.

### Flow cytometric analysis

Apoptosis was assessed using the FITC Annexin V Apoptosis Detection Kit II (BioScience, San Jose, CA, USA, cat. no. 556570). Following the manufacturer’s protocol, analysis was conducted with a fluorescence-activated cell sorter (FACS; BioScience). The data were then analyzed using Cytexpert V2.3 software (Beckman Coulter, Brea, CA, USA) to determine the apoptosis rate.

### Cell proliferation test

Cell proliferation was evaluated using the CCK-8. A total of 5 × 10³ cells per well were seeded into 96-well plates containing a range of cisplatin concentrations (0, 1.25, 2.5, 5, 10, and 20 µg/mL).The cells were incubated following the manufacturer’s protocol. Absorbance was then measured using a microplate reader (Varioskan LUX; Thermo Fisher Scientific, Waltham, MA, USA).

### Western blot

Cell proteins were extracted using RIPA Lysis Buffer (Meilunbio, Dalian, China). Protein separation was carried out through SDS-PAGE, followed by transfer to a nitrocellulose membrane.After blocking, the membrane was incubated with primary antibodies, including anti-ARHGEF12 (CUSABIO, Cat No. CSB-PA008357), anti-PI3K (Abmart, Cat No. T40064S), anti-p-PI3K (Tyr467/199), anti-AKT (Proteintech, Cat No. 80455-1-RR), anti-p-Akt (Abmart, Cat No. T40067S), anti-MYPT1(HUABIO, Cat No. ER64090), and anti-p-MYPT1(HUABIO, Cat No. HA723049).

### Bioinformatics analysis

The c2.cp.reactome.v7.4.symbols.gmt subset was downloaded from the Molecular Signatures Database to serve as the background dataset. The genes were mapped to this background set, and enrichment analysis was conducted using the clusterProfiler 3.14.3 package to generate the results.

### Statistical analysis

To address potential confounding factors and biases, we utilized MR to evaluate the causal effect of core genes on BLCA incidence. The results were presented as OR with corresponding 95% confidence intervals. To ensure consistent alignment between SNP exposure and SNP outcomes, and to provide a more robust estimate of the causal relationship between genetic variation and BLCA risk, we adhered to the protocol outlined in previous studies [36].

We employed five MR methods: random-effects inverse variance weighting, weighted median, MR-Egger, simple mode, and weighted mode [37, 38]. To investigate potential horizontal pleiotropy, we conducted MR-Egger analysis, despite its lower statistical power compared to IVW. Furthermore, causal heterogeneity across multiple genetic variants was assessed using the Cochran Q test [39]. Forest plots were used to assess the causal effect of individual SNPs. All statistical analyses were performed using R 4.3.2.

We also examined causal estimates between the selected eQTLs and BLCA to validate the relationship between gene profiles and BLCA occurrence. Statistical significance was defined as *p* < 0.05.

All experiments were independently repeated at least three times. A t-test was used for comparisons between two groups. ANOVA was applied for comparisons among multiple groups. Data analysis was conducted using GraphPad Prism 10.0, with results presented as mean ± SD [40].

## Supplementary Information

Supplementary Material 1.

## Data Availability

All data supporting the findings of this study are available within the paper and its Supplementary Information.
