# Supplementary material for: Integrative multi-omics and single-cell transcriptomics reveal ARHGEF12 driving chemoresistance in bladder cancer
Source: Hereditas. 2025 Nov 27;162:234. doi: 10.1186/s41065-025-00606-1 (PMC12661753; doi:10.1186/s41065-025-00606-1)
Supplement: Supplementary file 1 — Supplementary Material 1. [file 41065_2025_606_MOESM1_ESM.zip › Supplementary1/Supplementary - MR/eQTL-MR/MR/pic.forest.pdf]

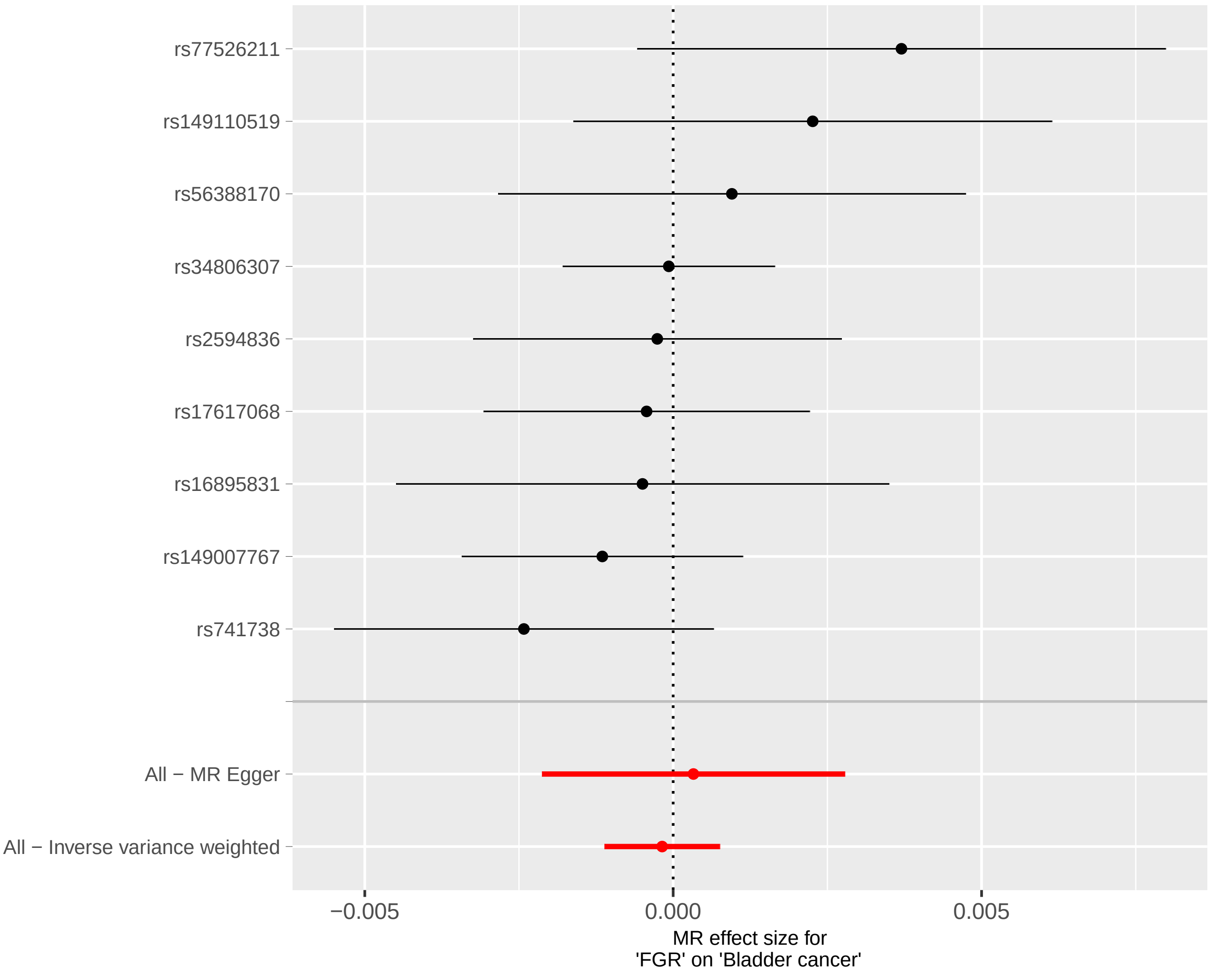
rs77526211
rs149110519
rs56388170
rs34806307
rs2594836
rs17617068
rs16895831
rs149007767
rs741738
All − MR Egger
All − Inverse variance weighted
−0.005
0.000
0.005
MR effect size for
'FGR' on 'Bladder cancer'

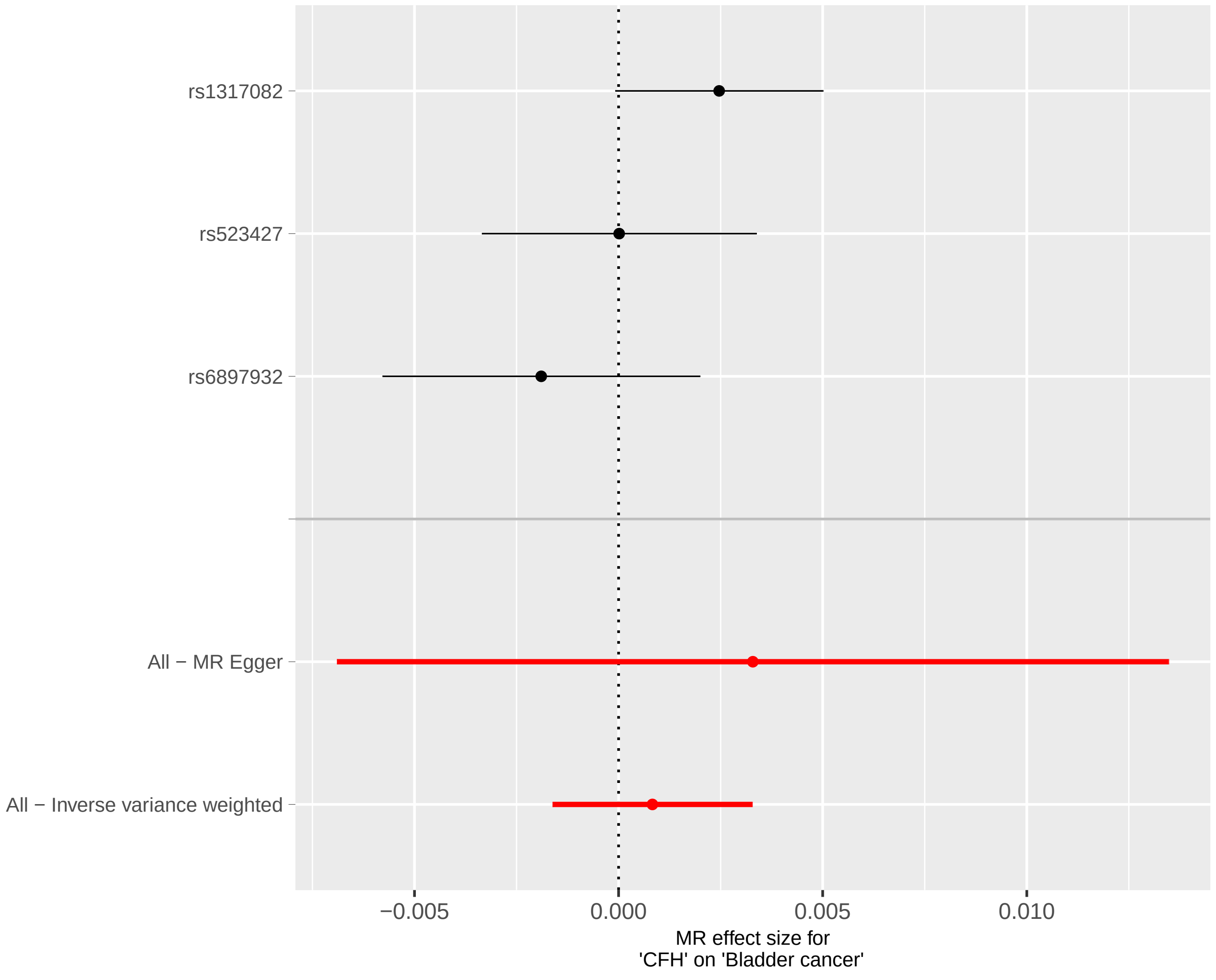

rs1317082
rs523427
rs6897932
All − MR Egger
All − Inverse variance weighted
−0.005
0.000
0.005
0.010
MR effect size for
'CFH' on 'Bladder cancer'

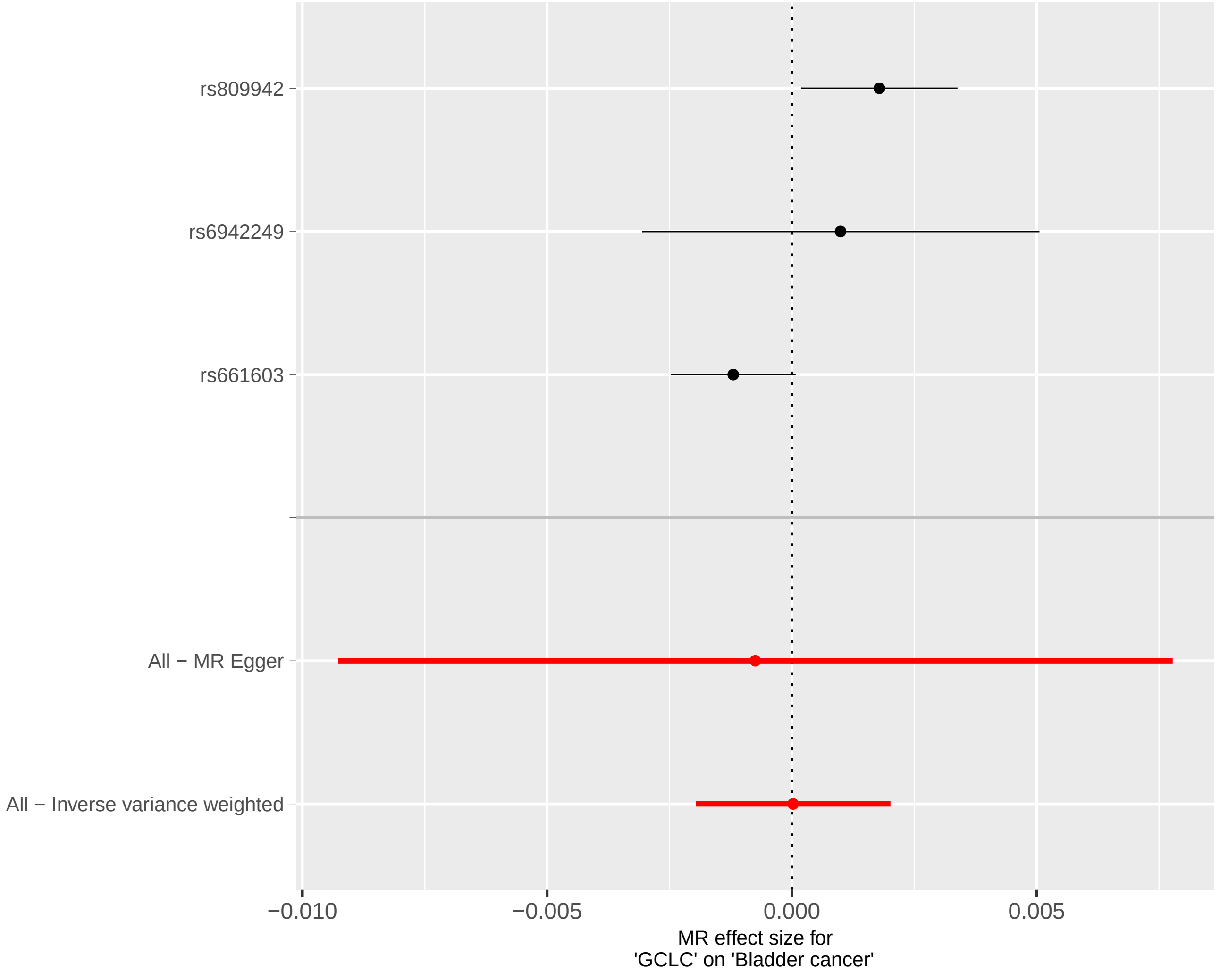
rs809942
rs6942249
rs661603
All − MR Egger
All − Inverse variance weighted
−0.010
−0.005
0.000
0.005
MR effect size for
'GCLC' on 'Bladder cancer'

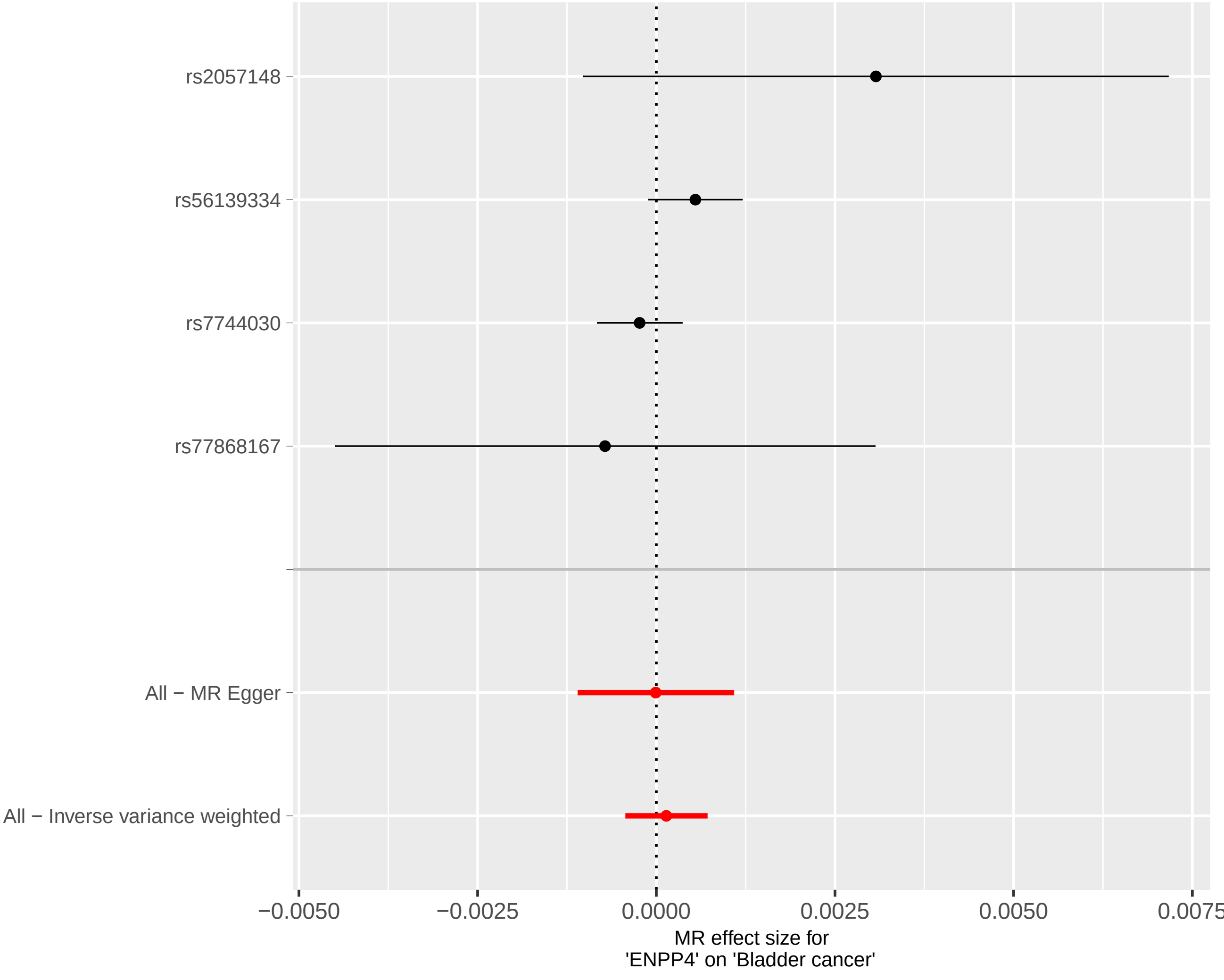
rs2057148
rs56139334
rs7744030
rs77868167
All − MR Egger
All − Inverse variance weighted
−0.0050
−0.0025
0.0000
0.0025
0.0050
0.0075
MR effect size for
'ENPP4' on 'Bladder cancer'

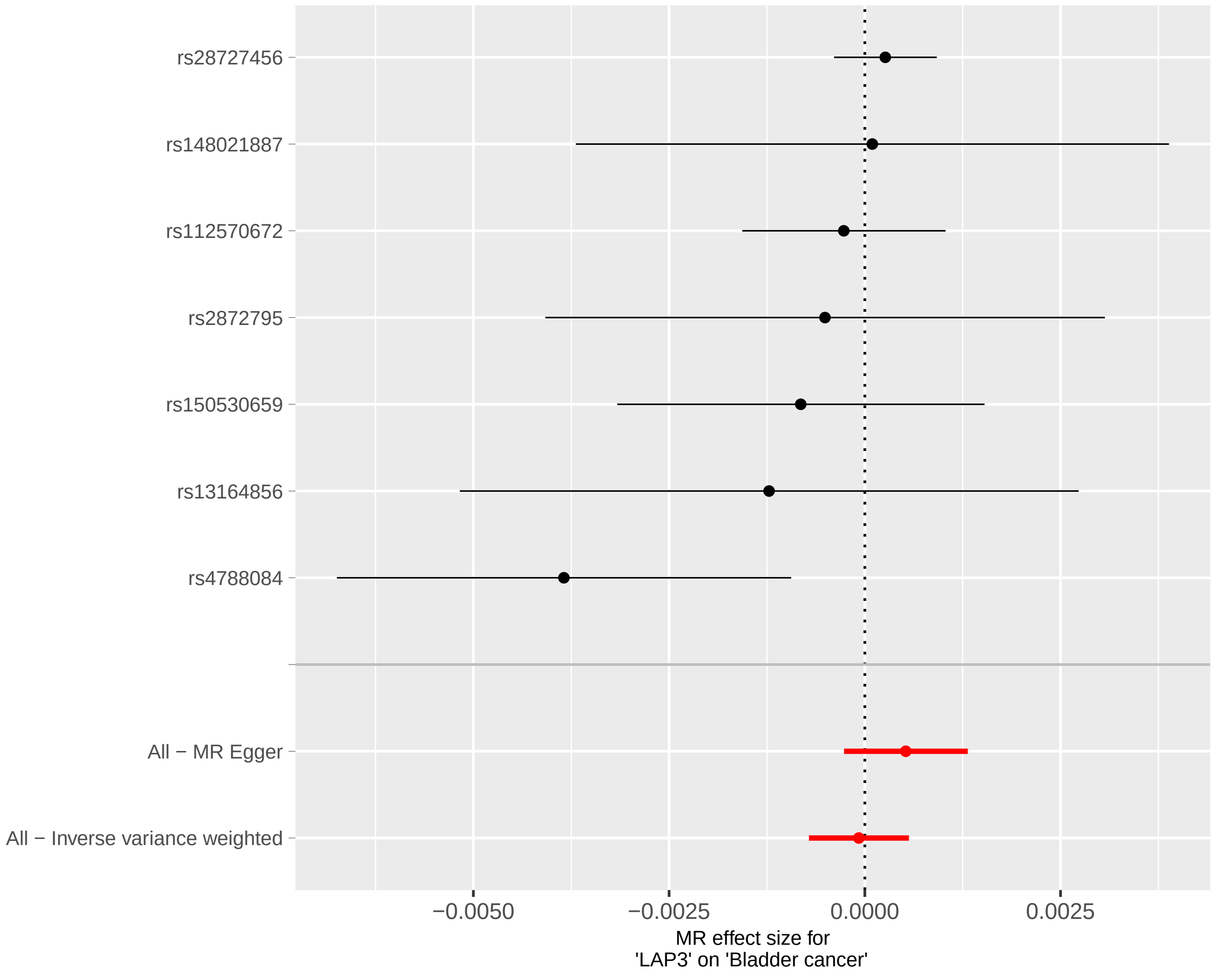
rs28727456
rs148021887
rs112570672
rs2872795
rs150530659
rs13164856
rs4788084
All − MR Egger
All − Inverse variance weighted
−0.0050
−0.0025
0.0000
0.0025
MR effect size for
'LAP3' on 'Bladder cancer'

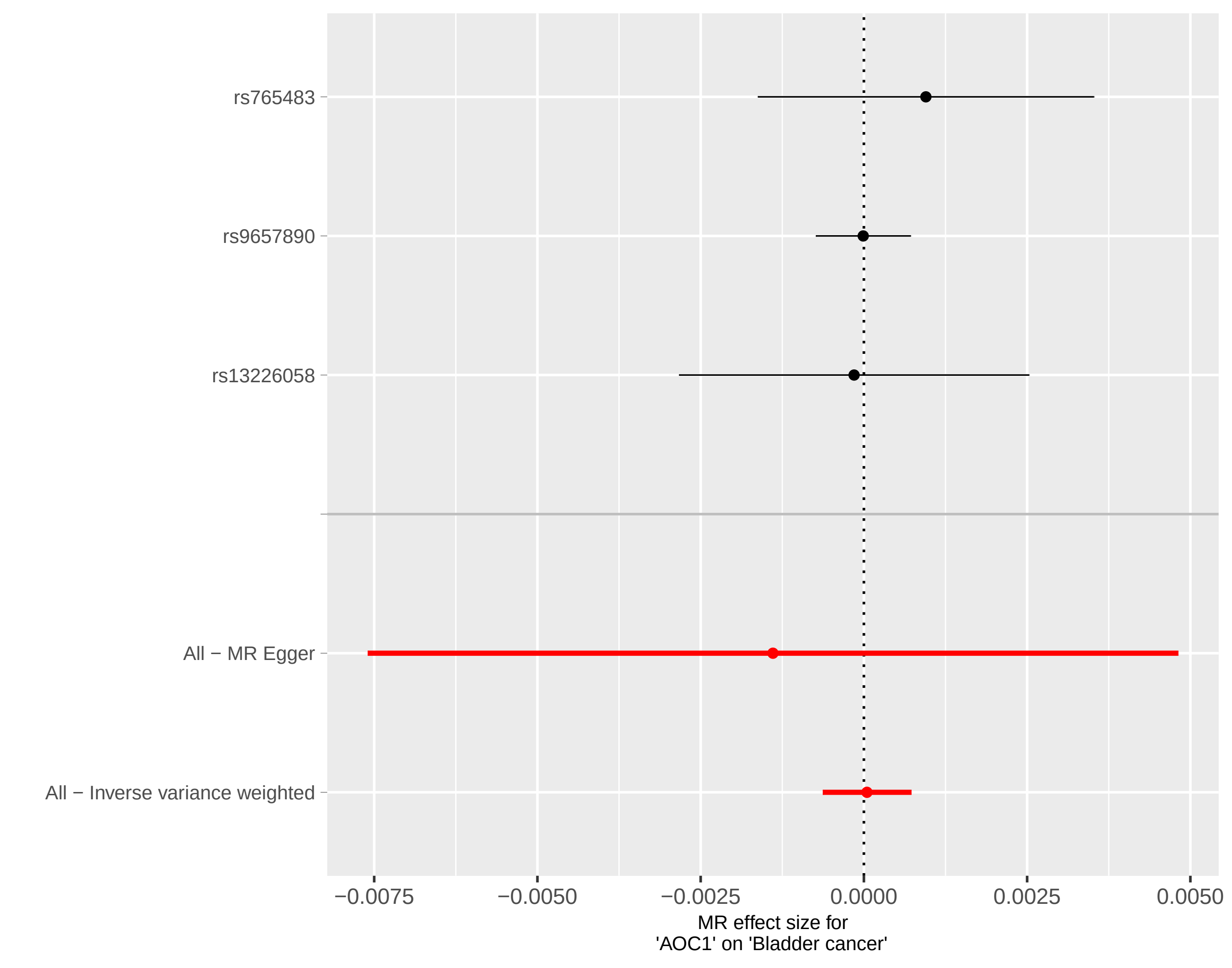

rs765483
rs9657890
rs13226058
All − MR Egger
All − Inverse variance weighted
−0.0075
−0.0050
−0.0025
0.0000
0.0025
0.0050
MR effect size for
'AOC1' on 'Bladder cancer'

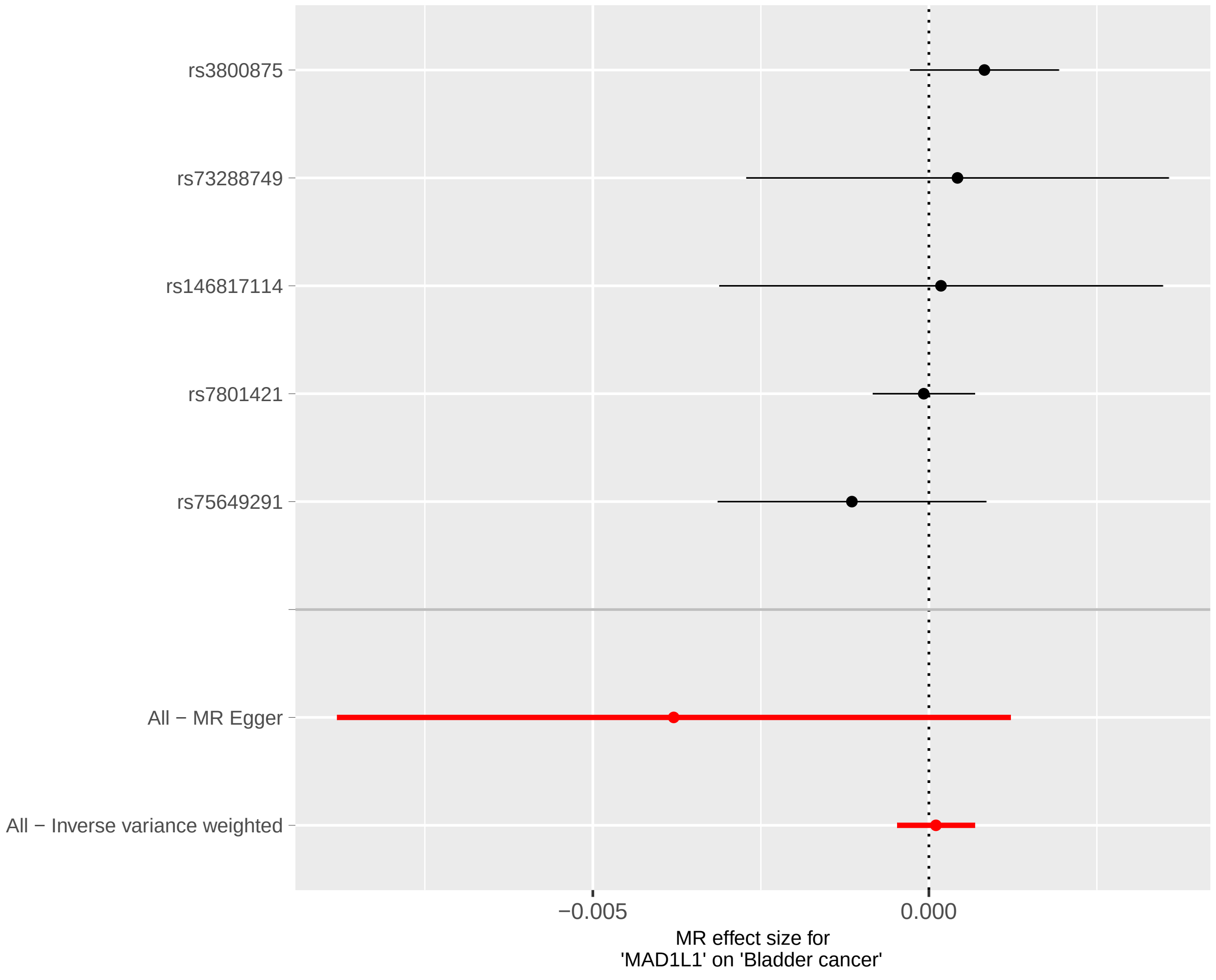
rs3800875
rs73288749
rs146817114
rs7801421
rs75649291
All − MR Egger
All − Inverse variance weighted
−0.005
0.000
MR effect size for
'MAD1L1' on 'Bladder cancer'

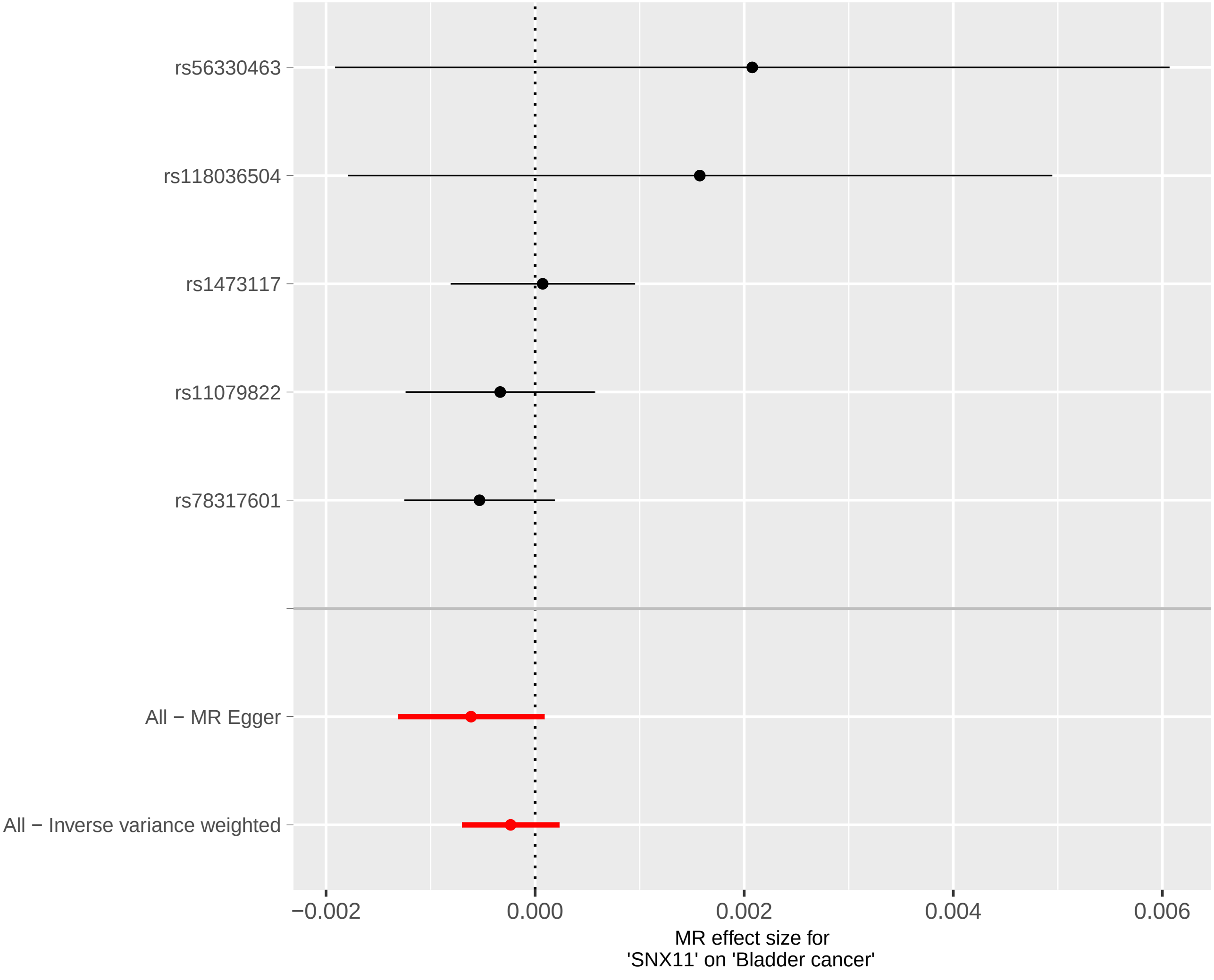
rs56330463
rs118036504
rs1473117
rs11079822
rs78317601
All − MR Egger
All − Inverse variance weighted
−0.002
0.000
0.002
0.004
0.006
MR effect size for
'SNX11' on 'Bladder cancer'

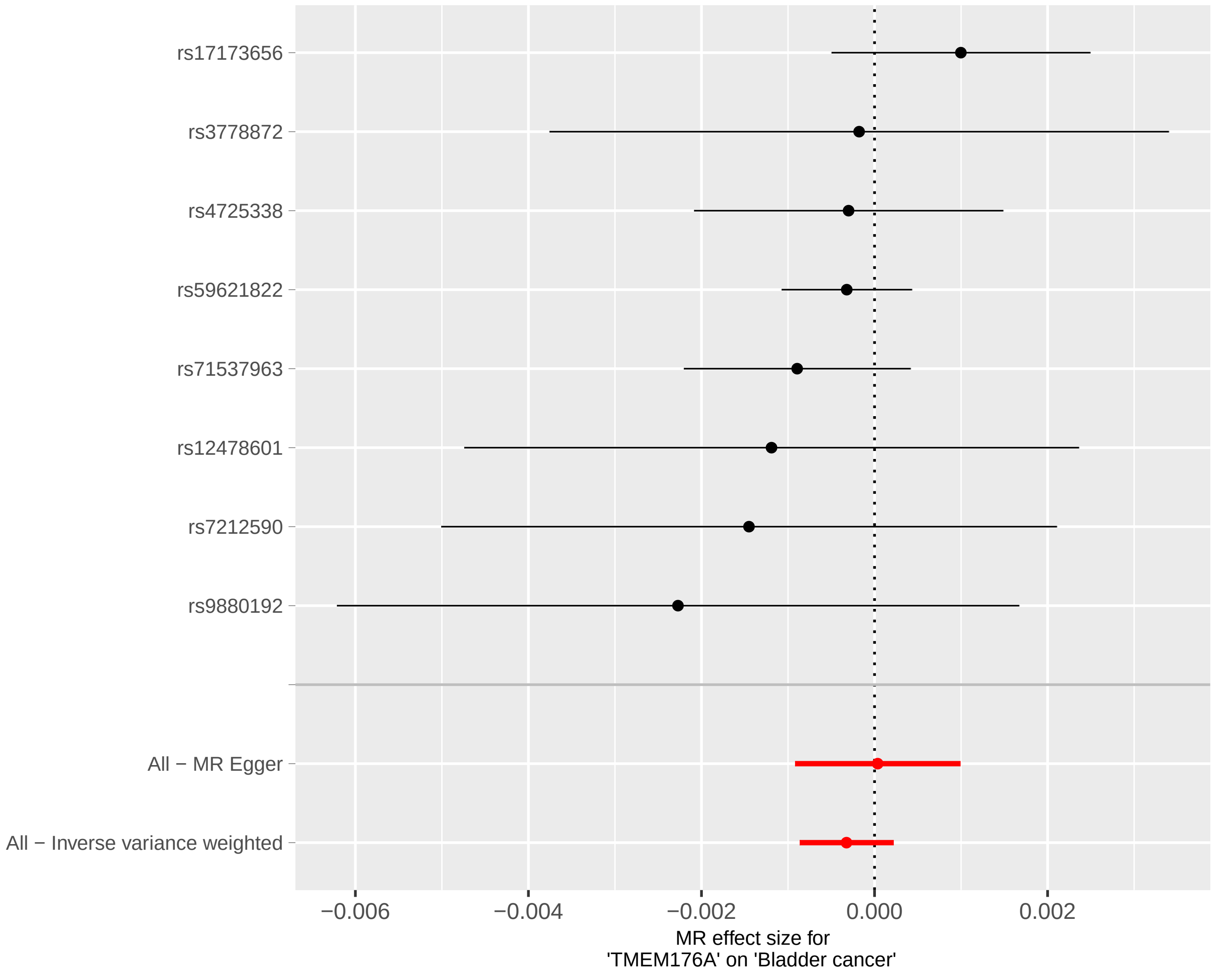
rs17173656
rs3778872
rs4725338
rs59621822
rs71537963
rs12478601
rs7212590
rs9880192
All − MR Egger
All − Inverse variance weighted
−0.006
−0.004
−0.002
0.000
0.002
MR effect size for
'TMEM176A' on 'Bladder cancer'

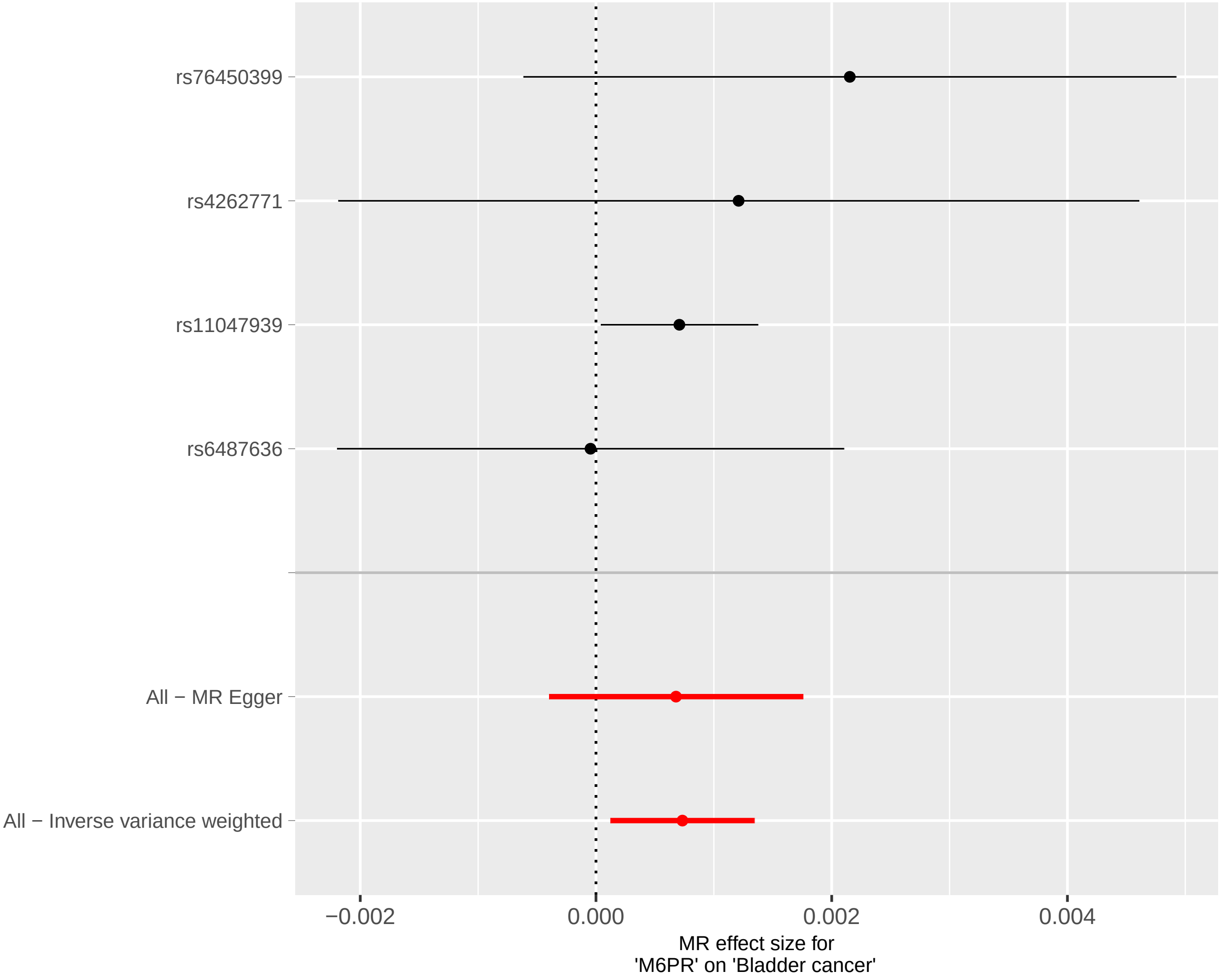
rs76450399
rs4262771
rs11047939
rs6487636
All − MR Egger
All − Inverse variance weighted
−0.002
0.000
0.002
0.004
MR effect size for
'M6PR' on 'Bladder cancer'

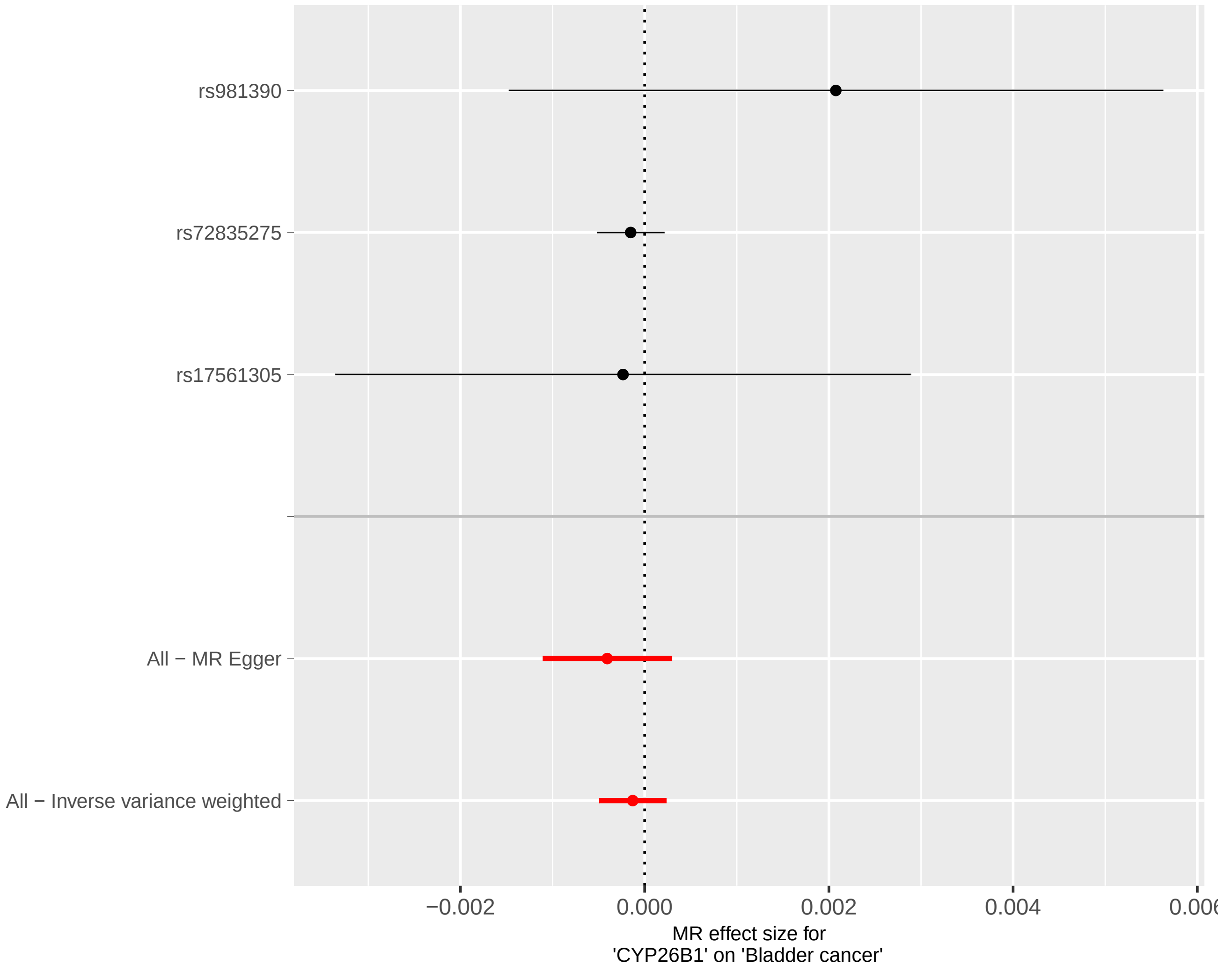
rs981390
rs72835275
rs17561305
All − MR Egger
All − Inverse variance weighted
−0.002
0.000
0.002
0.004
0.006
MR effect size for
'CYP26B1' on 'Bladder cancer'

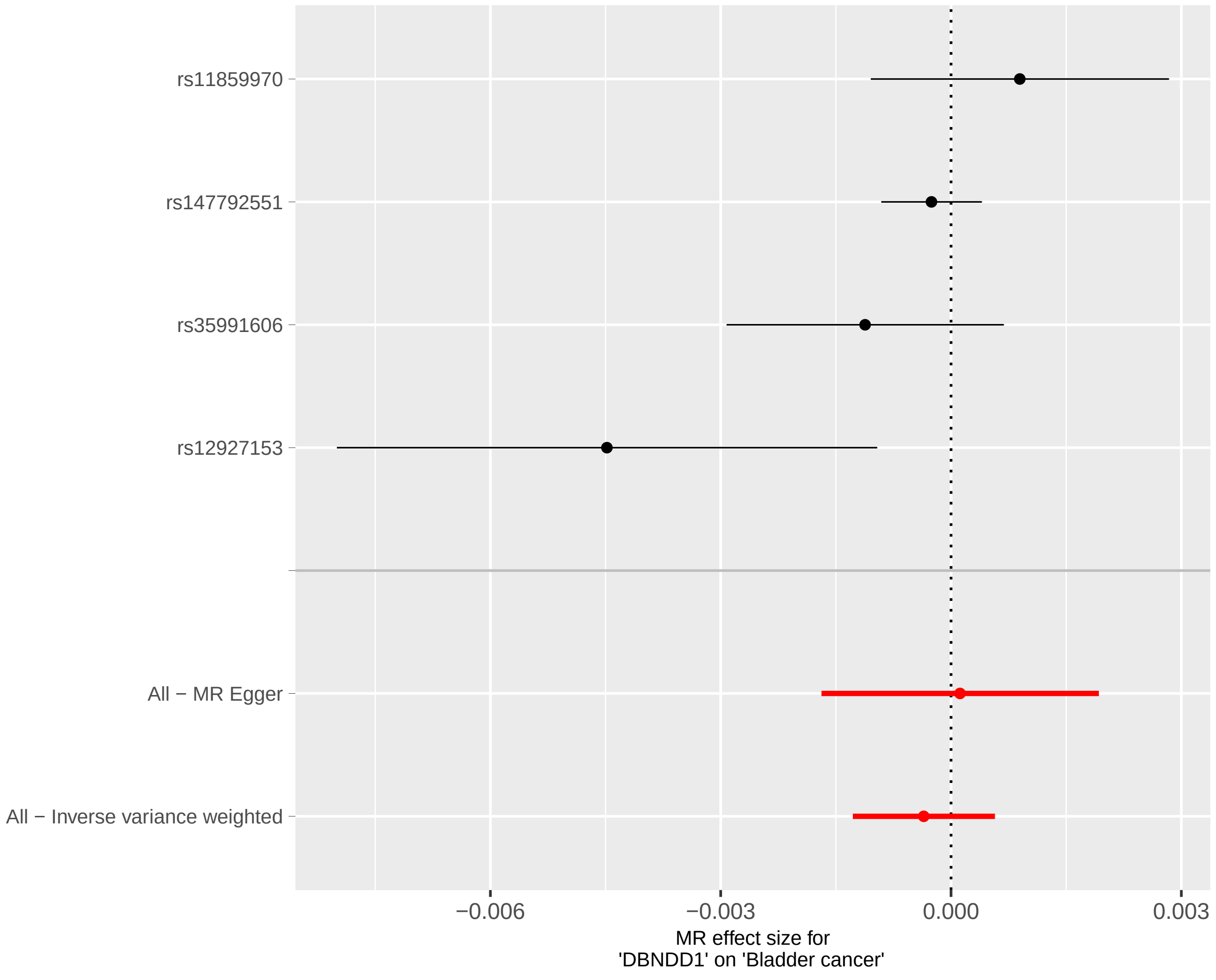
rs11859970
rs147792551
rs35991606
rs12927153
All − MR Egger
All − Inverse variance weighted
−0.006
−0.003
0.000
0.003
MR effect size for
'DBNDD1' on 'Bladder cancer'

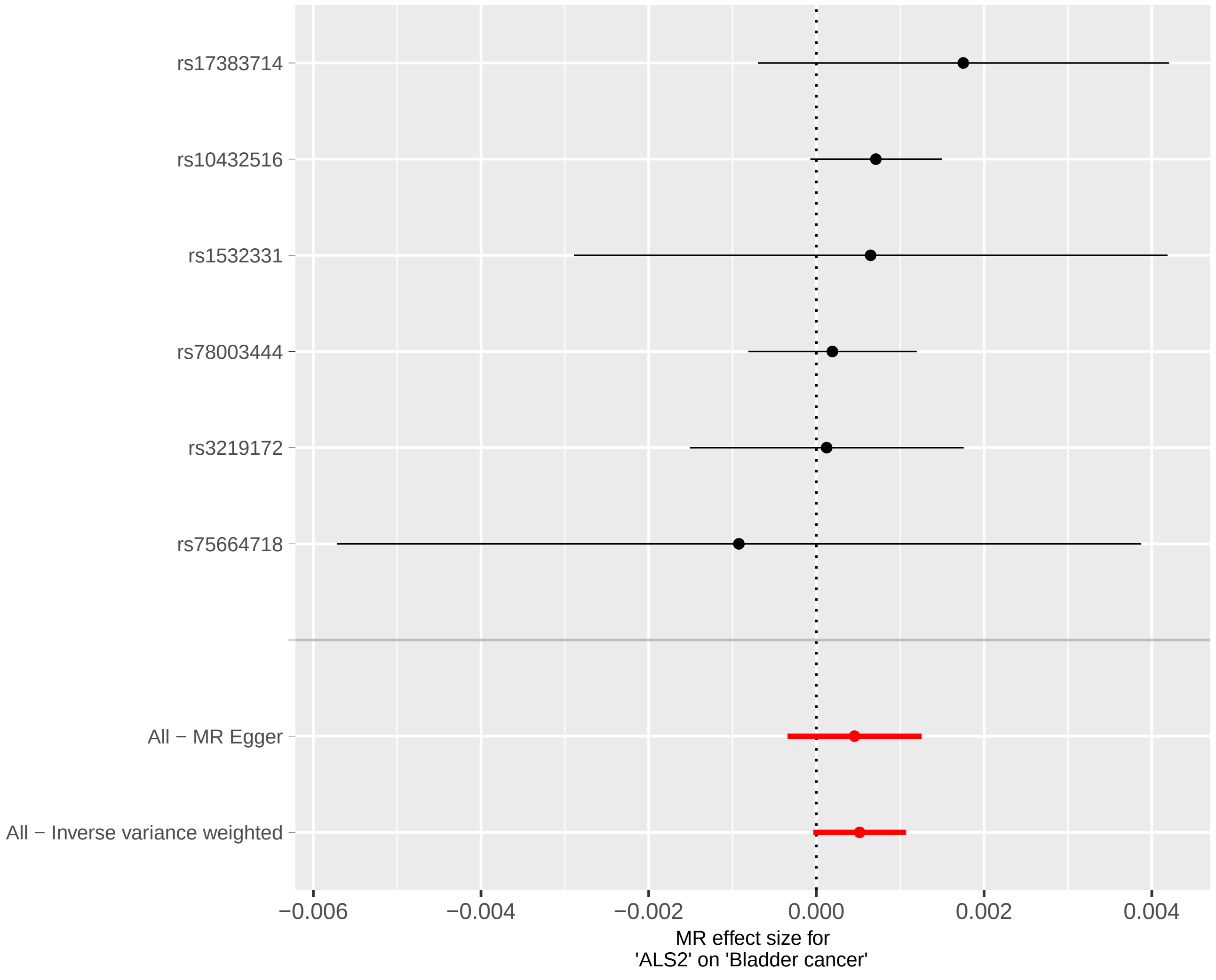
rs17383714
rs10432516
rs1532331
rs78003444
rs3219172
rs75664718
All − MR Egger
All − Inverse variance weighted
−0.006
−0.004
−0.002
0.000
0.002
0.004
MR effect size for
'ALS2' on 'Bladder cancer'

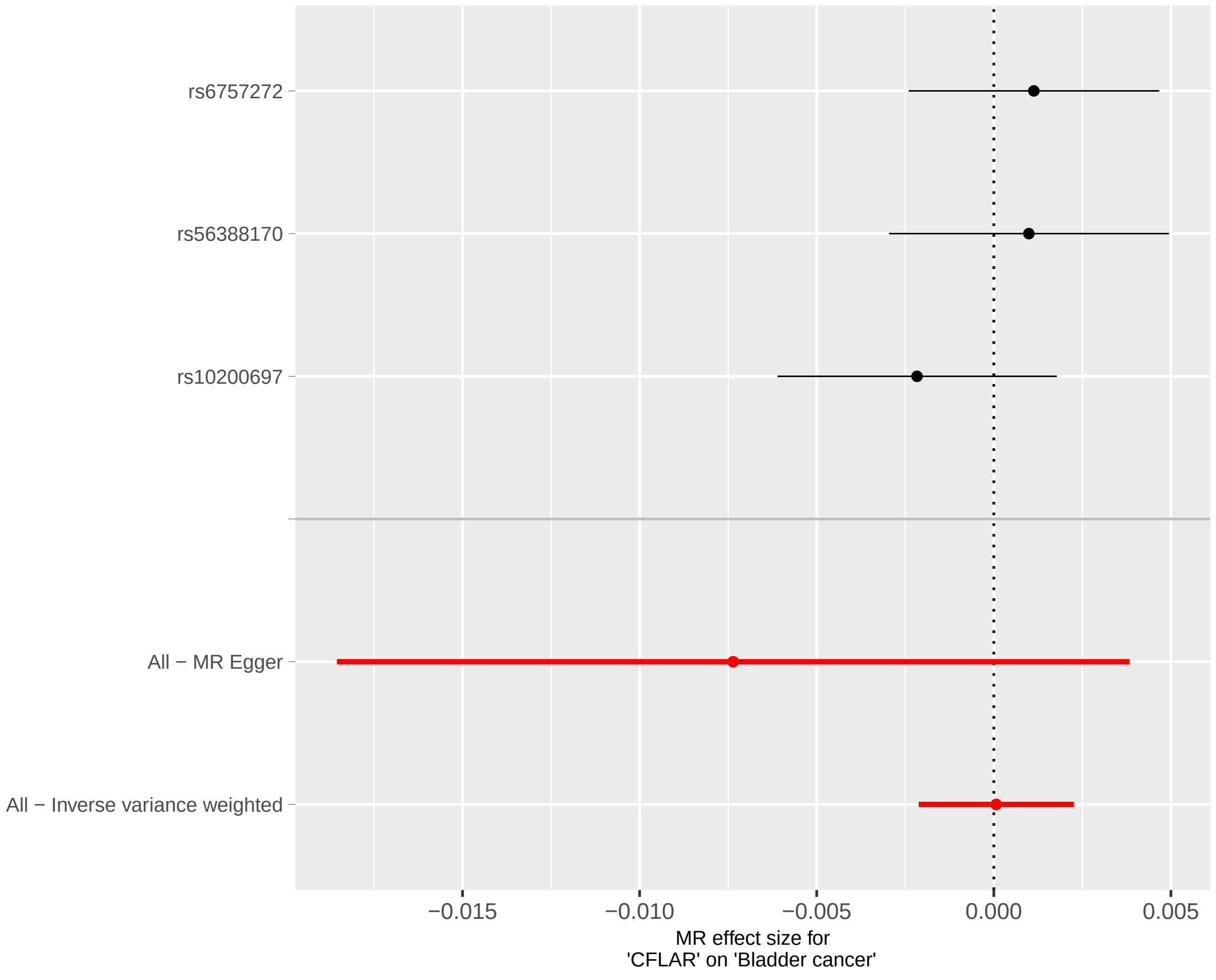
rs6757272
rs56388170
rs10200697
All − MR Egger
All − Inverse variance weighted
−0.015
−0.010
−0.005
0.000
0.005
MR effect size for
'CFLAR' on 'Bladder cancer'

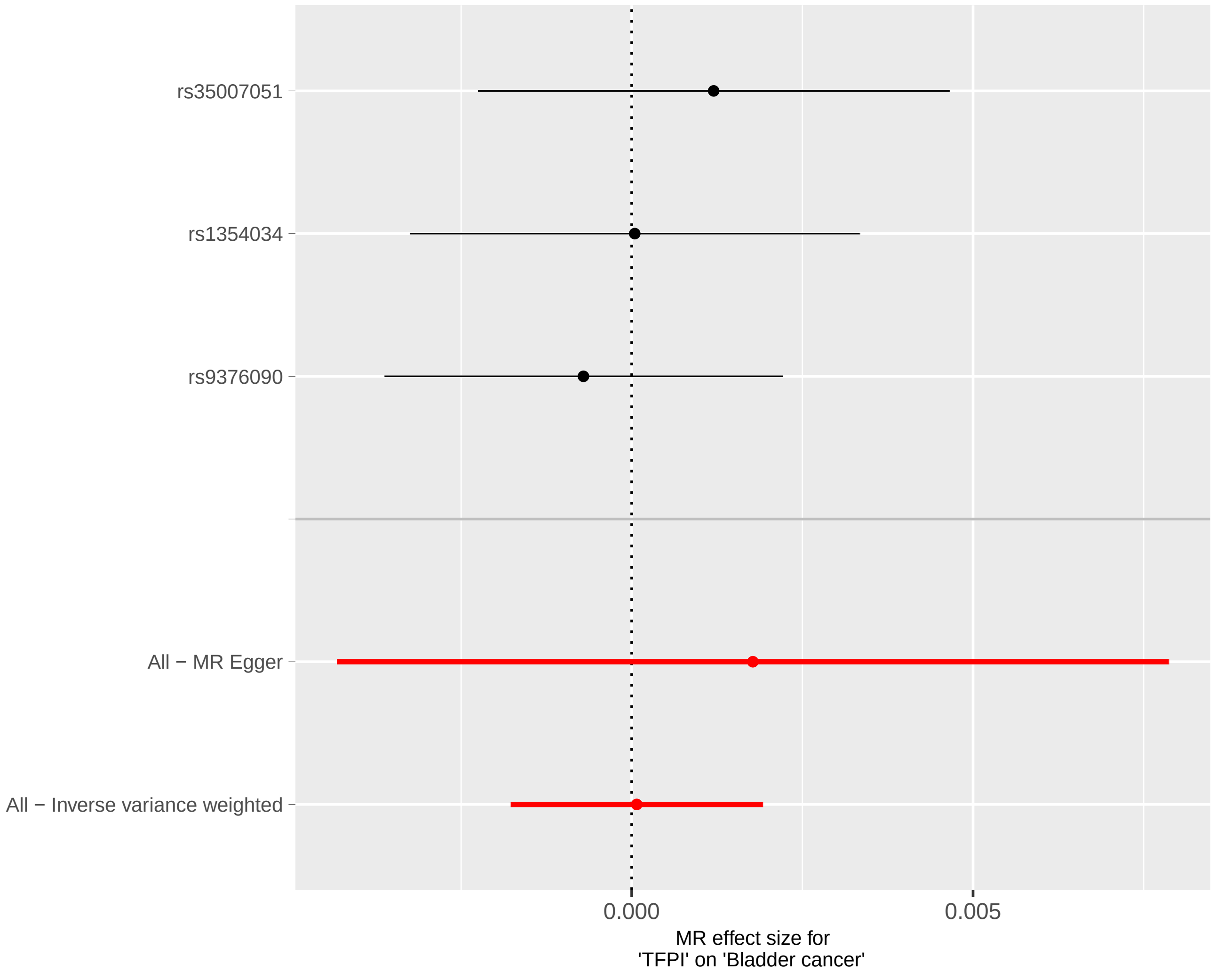
rs35007051
rs1354034
rs9376090
All − MR Egger
All − Inverse variance weighted
0.000
0.005
MR effect size for
'TFPI' on 'Bladder cancer'

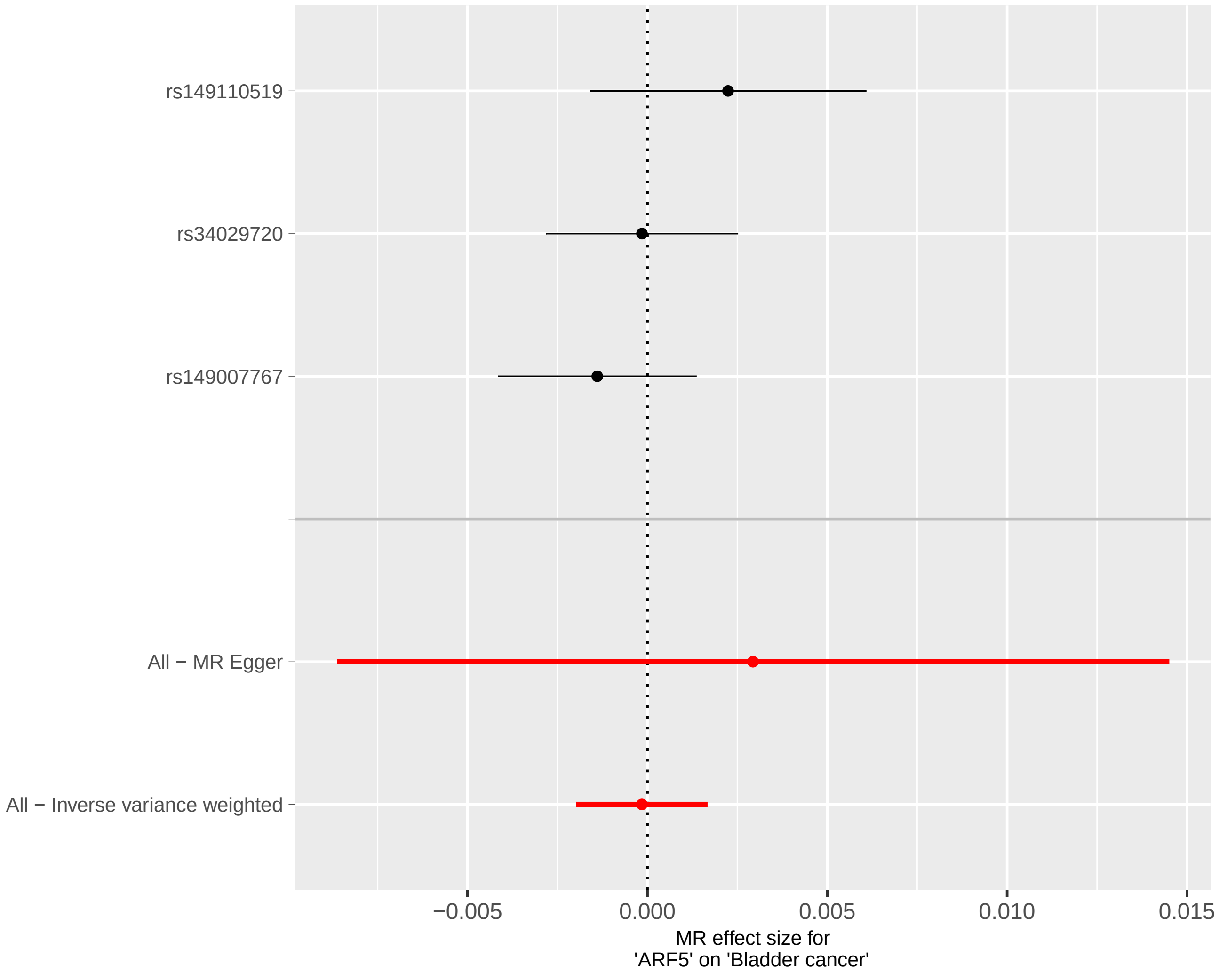
rs149110519
rs34029720
rs149007767
All − MR Egger
All − Inverse variance weighted
−0.005
0.000
0.005
0.010
0.015
MR effect size for
'ARF5' on 'Bladder cancer'

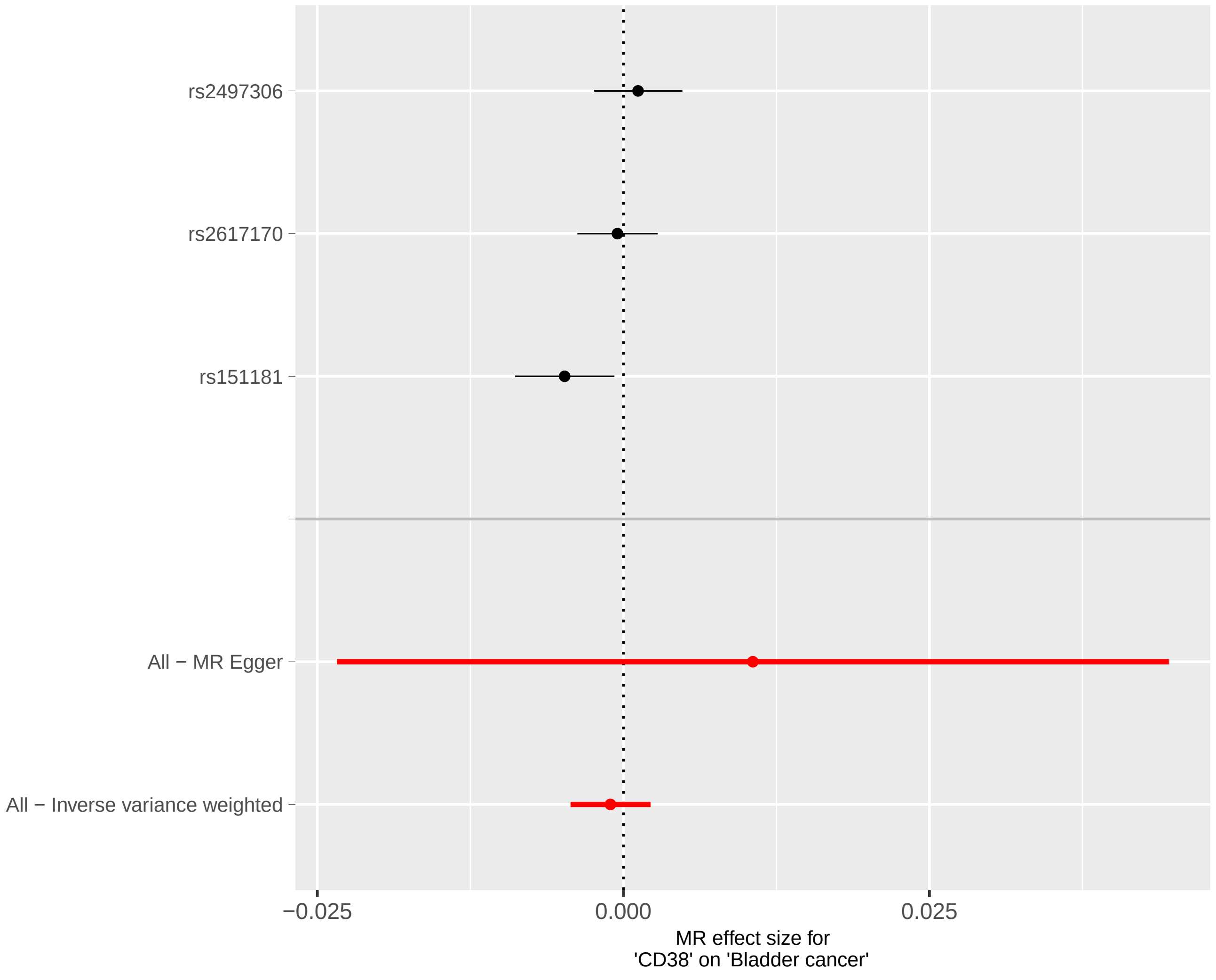
rs2497306
rs2617170
rs151181
All − MR Egger
All − Inverse variance weighted
−0.025
0.000
0.025
MR effect size for
'CD38' on 'Bladder cancer'

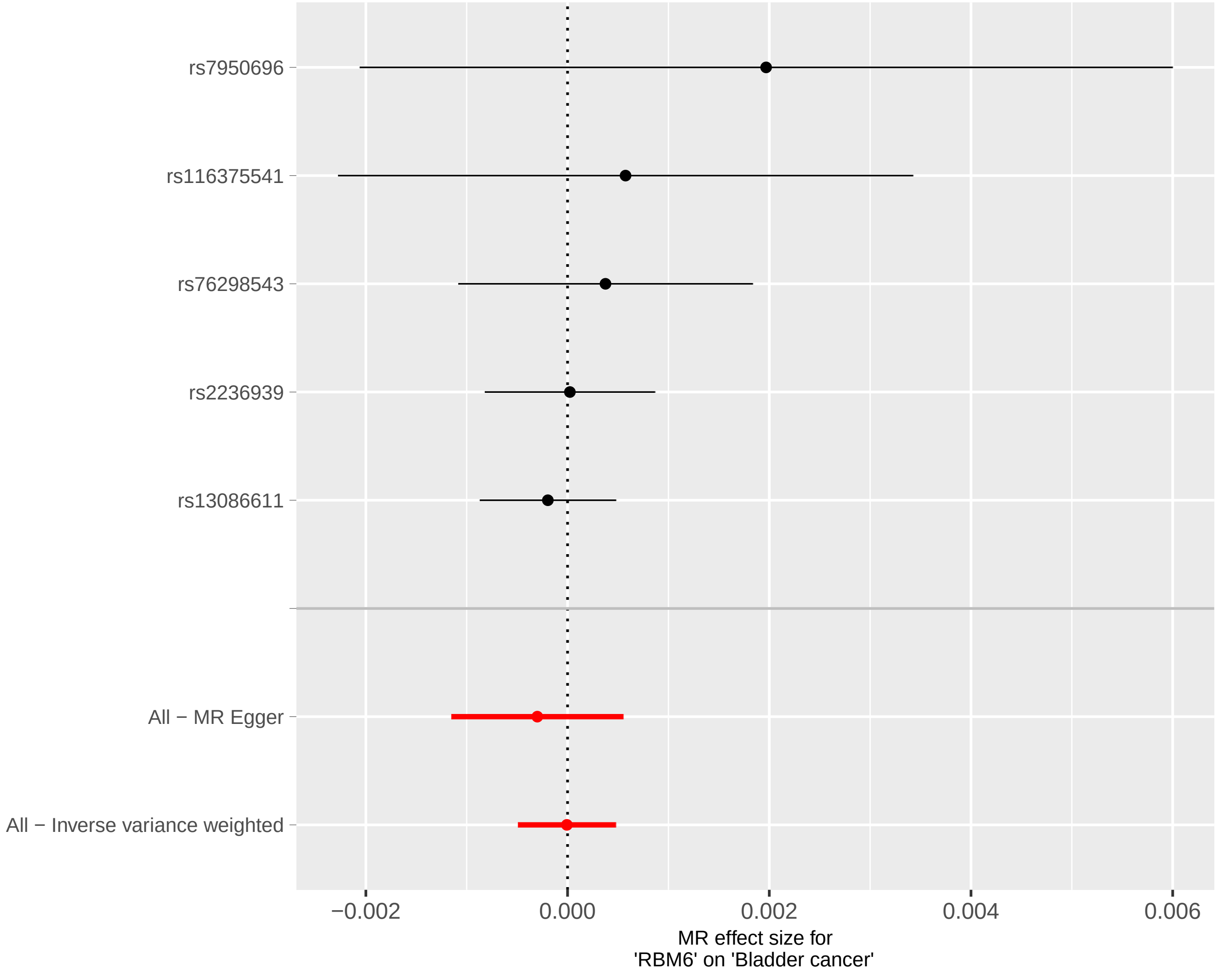

rs7950696
rs116375541
rs76298543
rs2236939
rs13086611
All − MR Egger
All − Inverse variance weighted
−0.002
0.000
0.002
0.004
0.006
MR effect size for
'RBM6' on 'Bladder cancer'

Insufficient number of SNPs

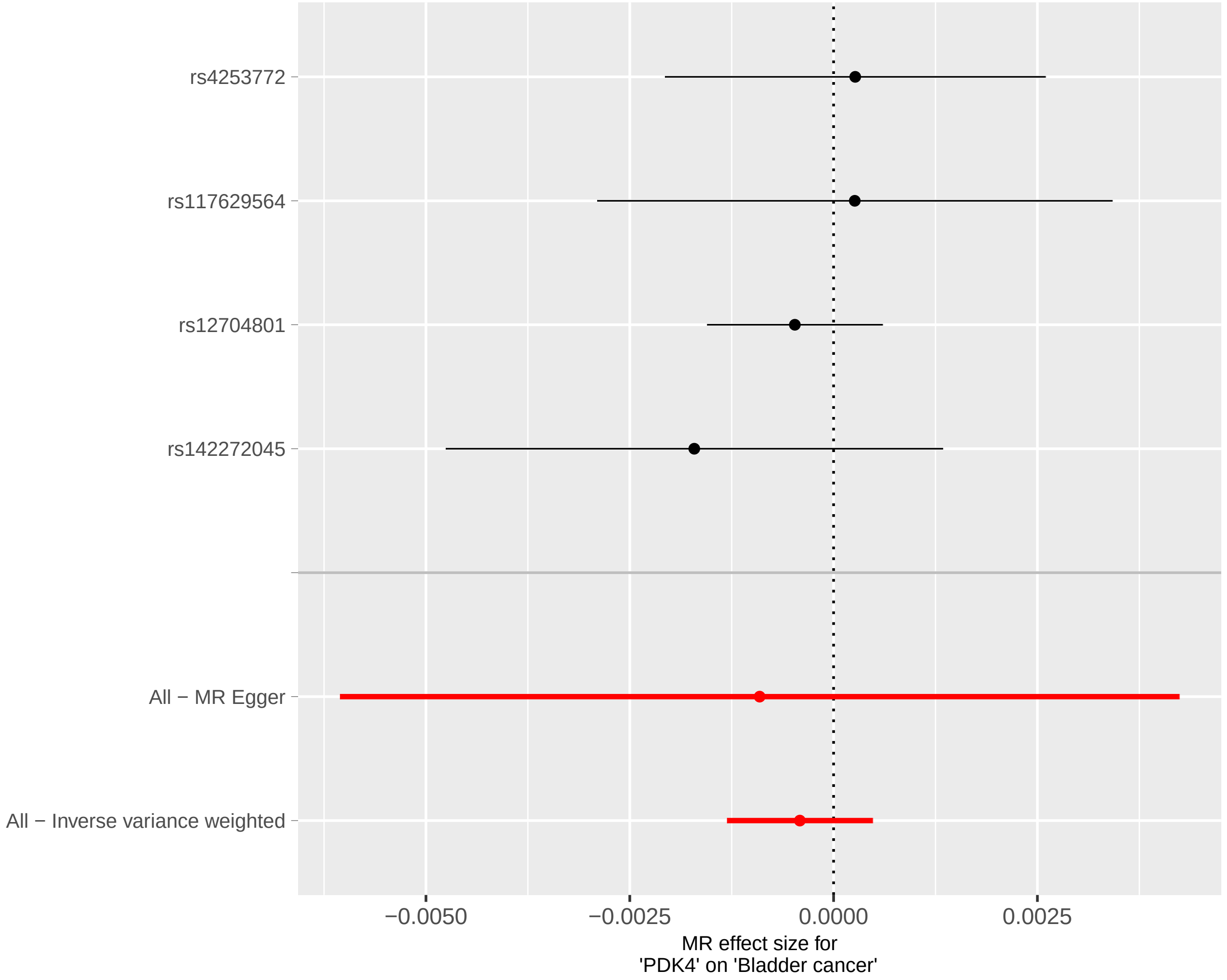
rs4253772
rs117629564
rs12704801
rs142272045
All − MR Egger
All − Inverse variance weighted
−0.0050
−0.0025
0.0000
0.0025
MR effect size for
'PDK4' on 'Bladder cancer'

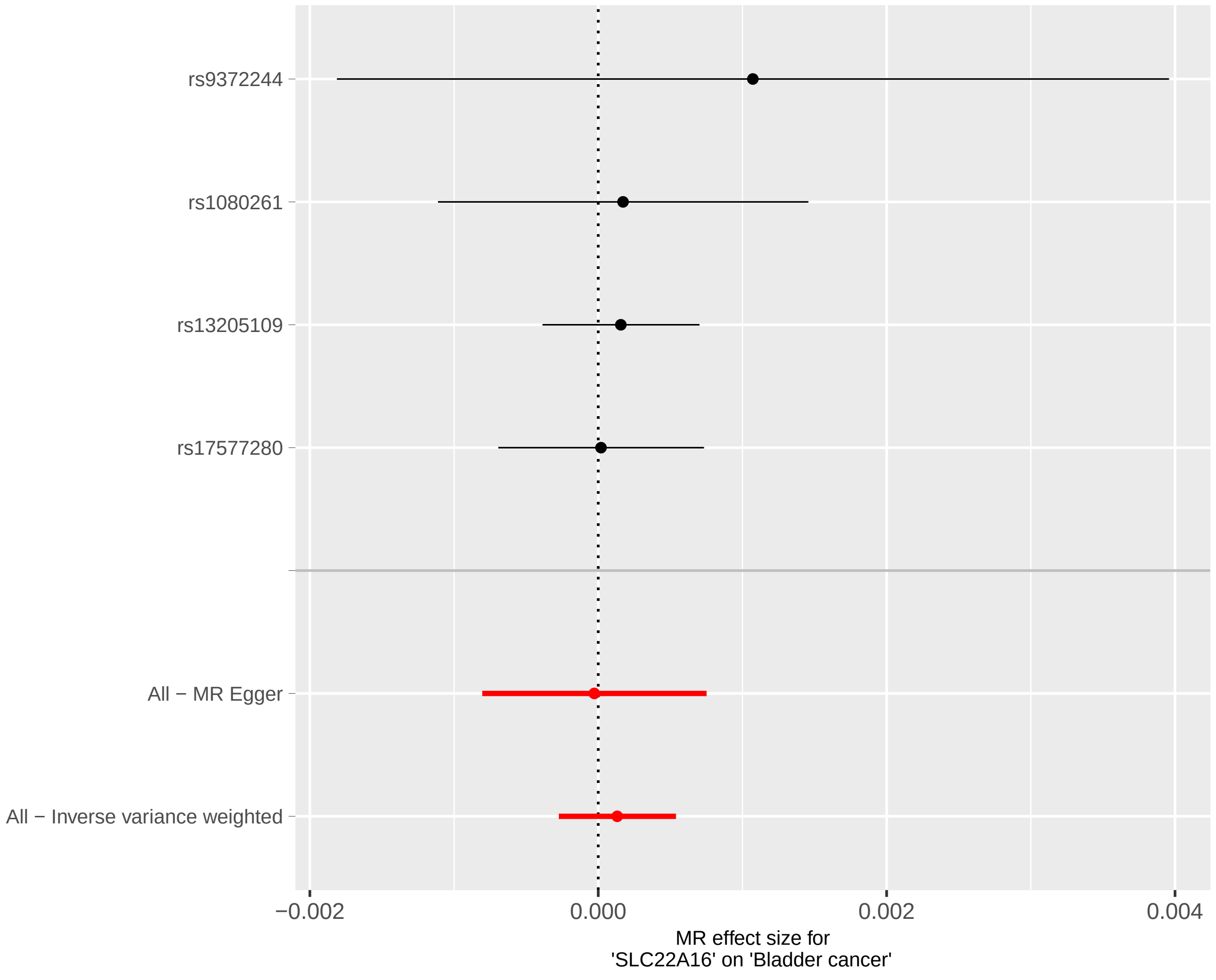
rs9372244
rs1080261
rs13205109
rs17577280
All − MR Egger
All − Inverse variance weighted
−0.002
0.000
0.002
0.004
MR effect size for
'SLC22A16' on 'Bladder cancer'

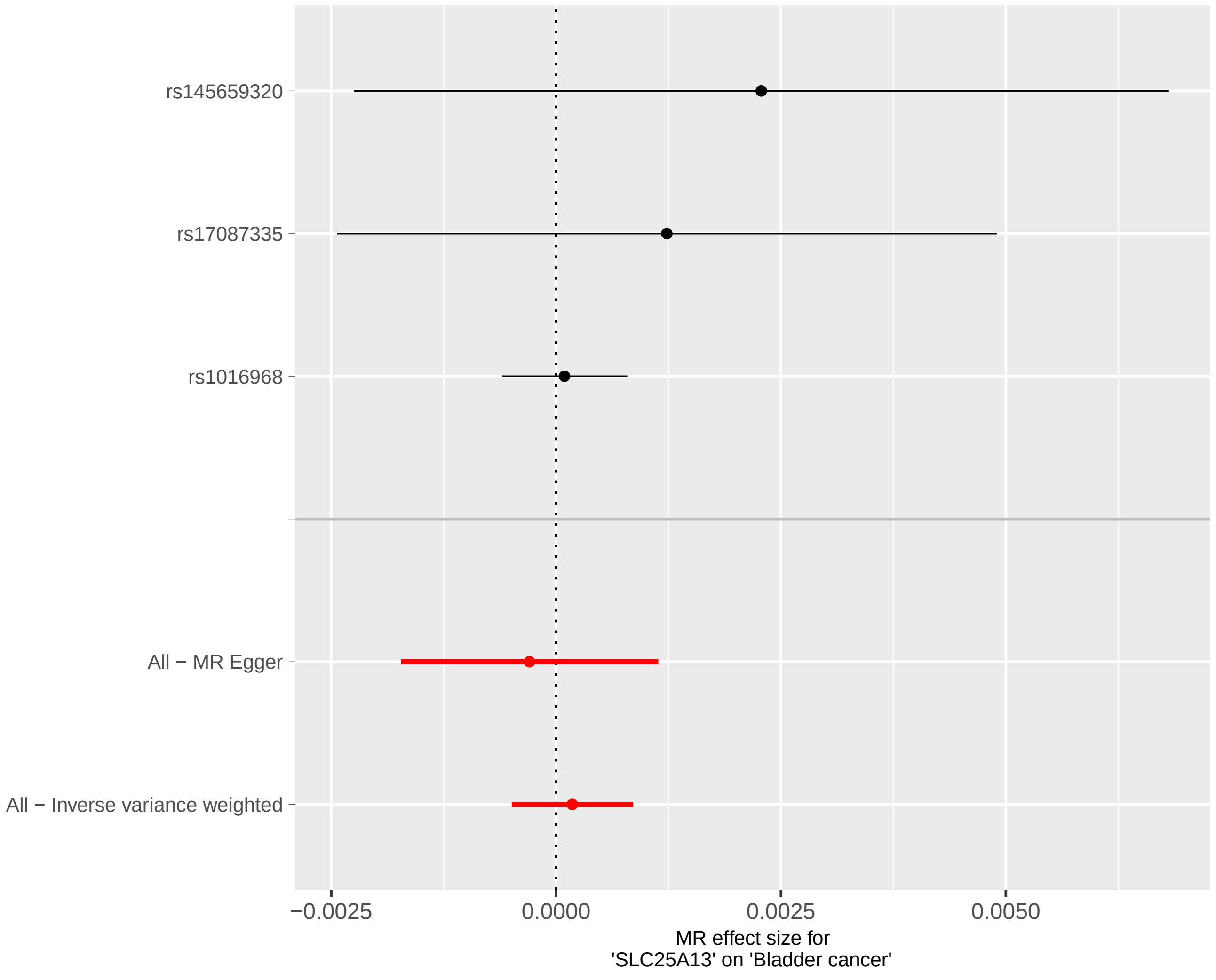

rs145659320
rs17087335
rs1016968
All − MR Egger
All − Inverse variance weighted
−0.0025
0.0000
0.0025
0.0050
MR effect size for
'SLC25A13' on 'Bladder cancer'

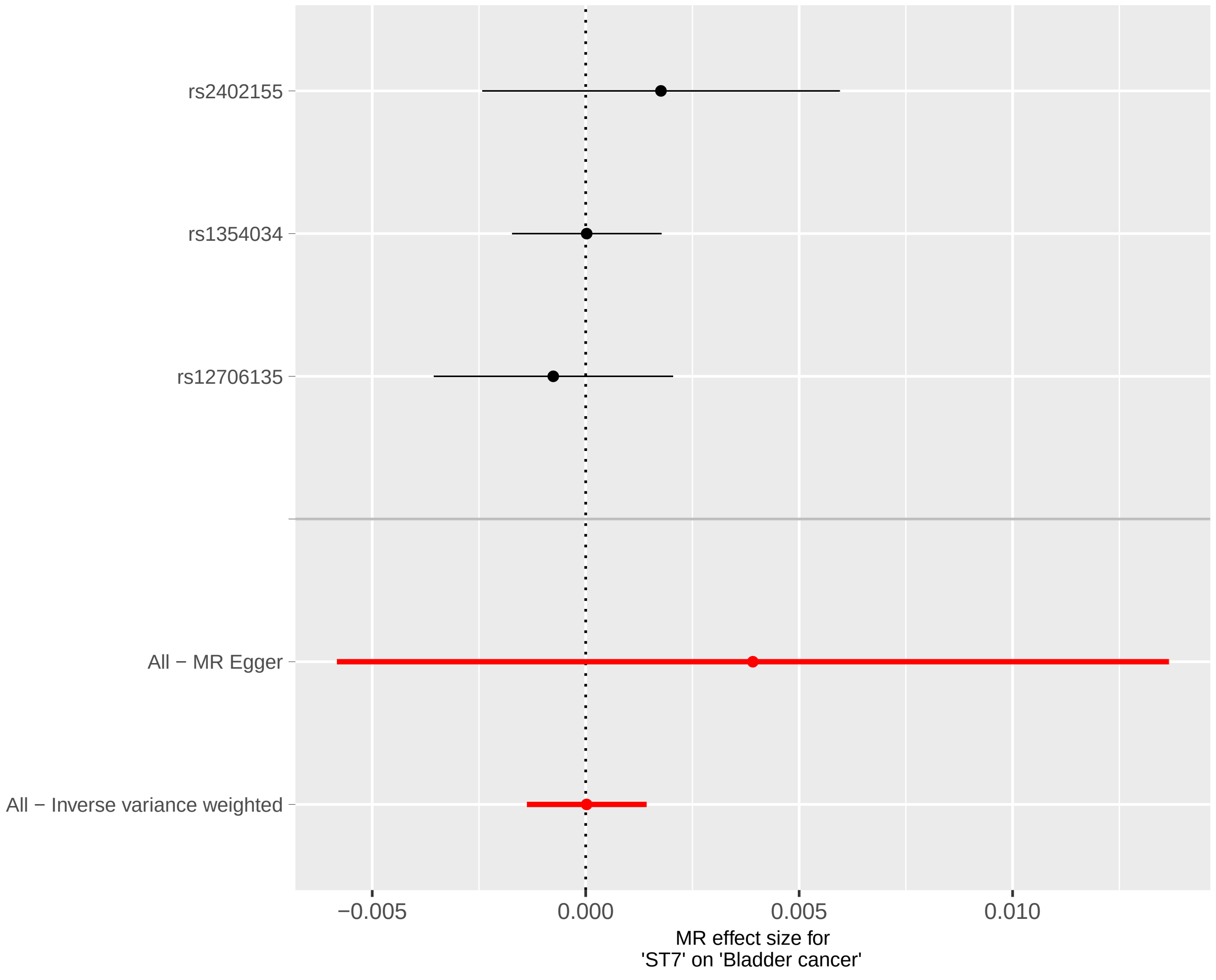

rs2402155
rs1354034
rs12706135
All − MR Egger
All − Inverse variance weighted
−0.005
0.000
0.005
0.010
MR effect size for
'ST7' on 'Bladder cancer'

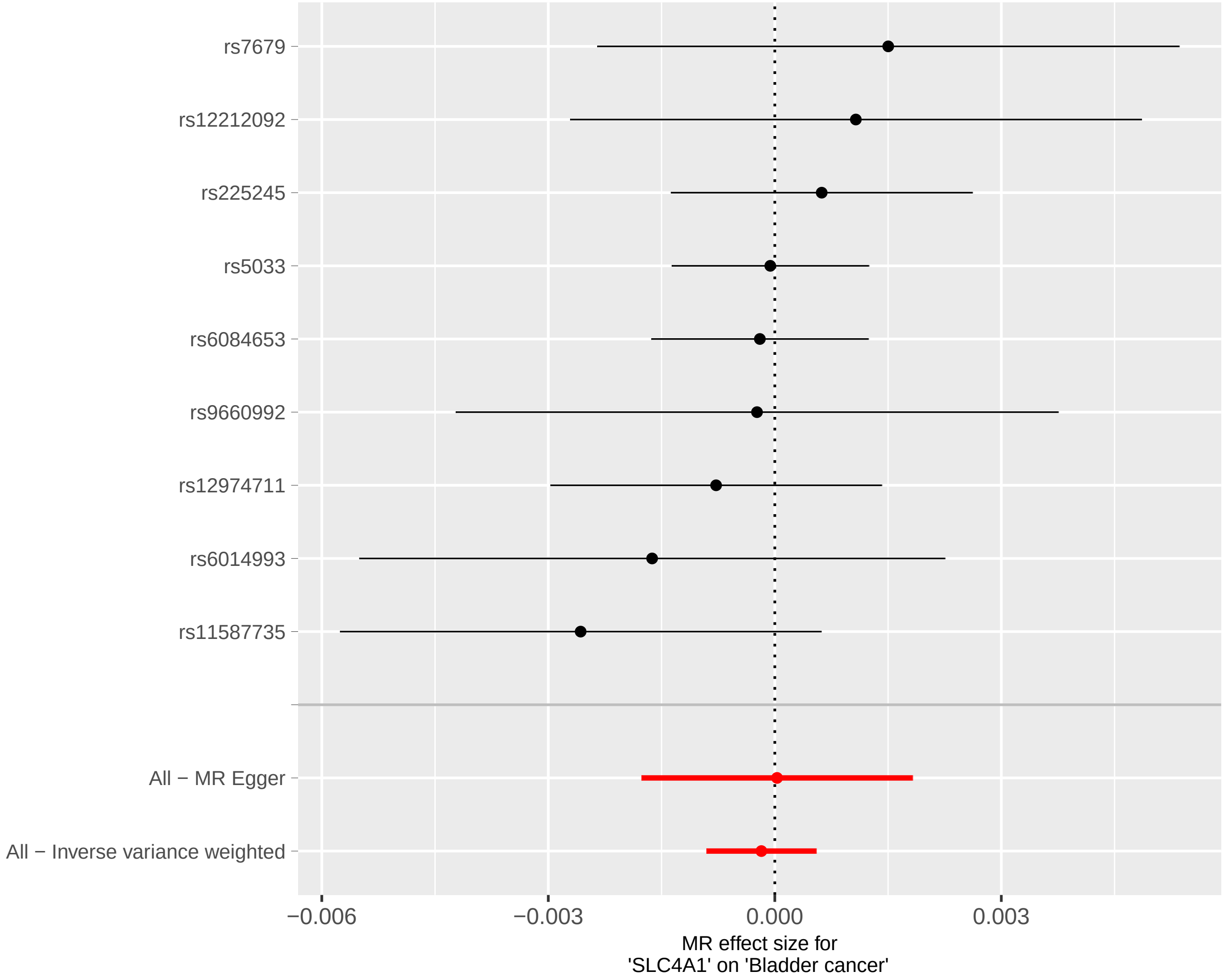
rs7679
rs12212092
rs225245
rs5033
rs6084653
rs9660992
rs12974711
rs6014993
rs11587735
All − MR Egger
All − Inverse variance weighted
−0.006
−0.003
0.000
0.003
MR effect size for
'SLC4A1' on 'Bladder cancer'

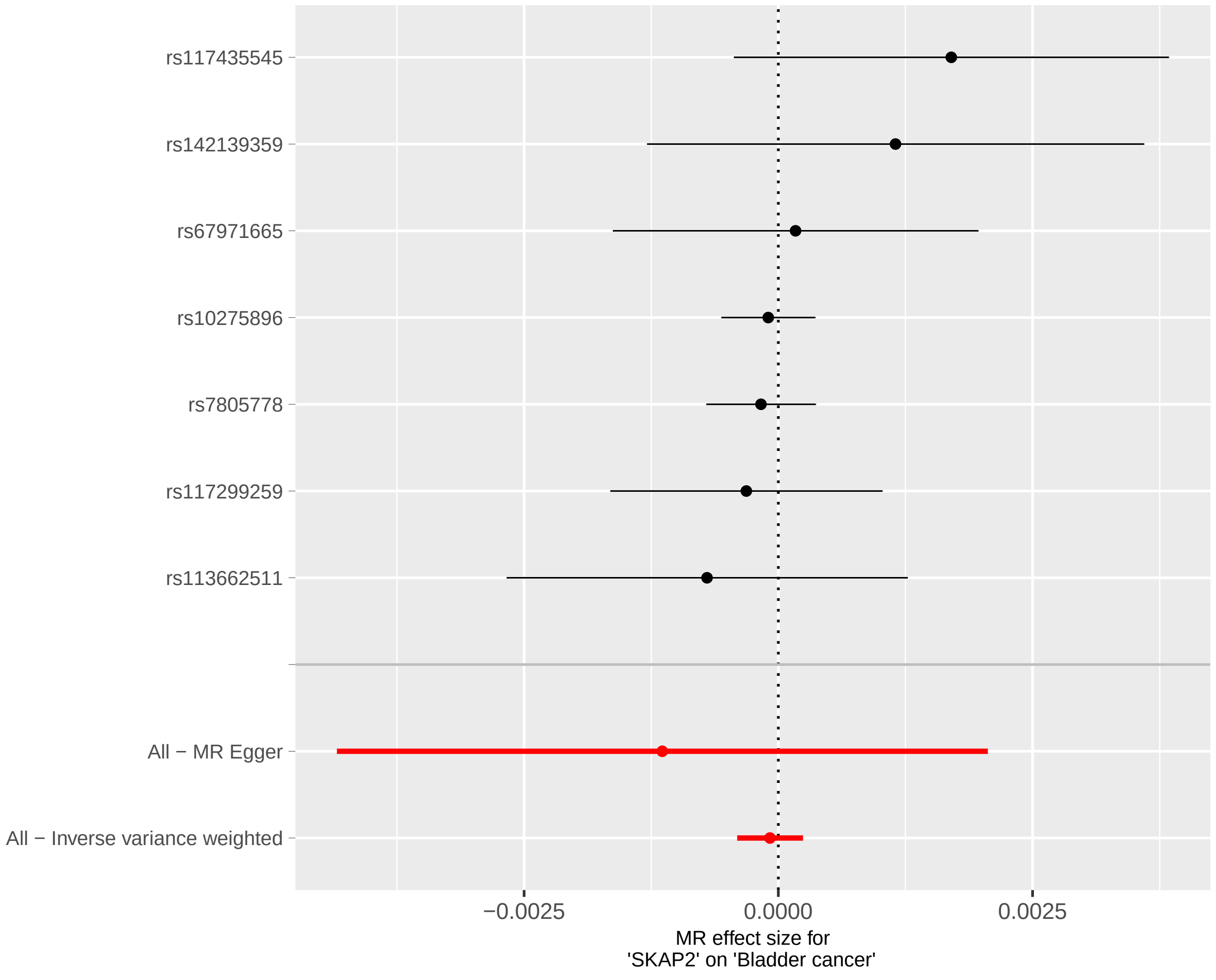

rs117435545
rs142139359
rs67971665
rs10275896
rs7805778
rs117299259
rs113662511
All − MR Egger
All − Inverse variance weighted
−0.0025
0.0000
0.0025
MR effect size for
'SKAP2' on 'Bladder cancer'

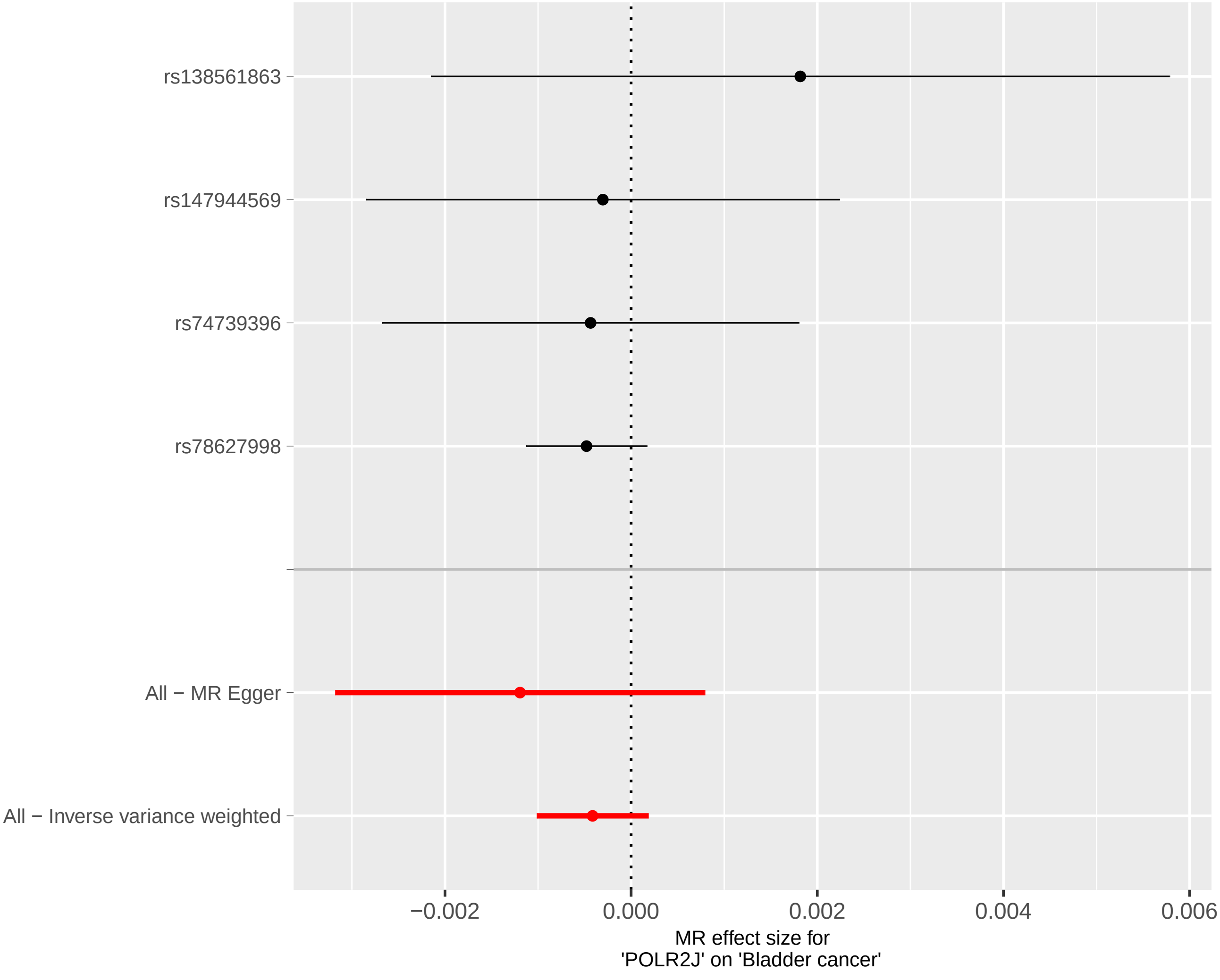

rs138561863
rs147944569
rs74739396
rs78627998
All − MR Egger
All − Inverse variance weighted
−0.002
0.000
0.002
0.004
0.006
MR effect size for
'POLR2J' on 'Bladder cancer'

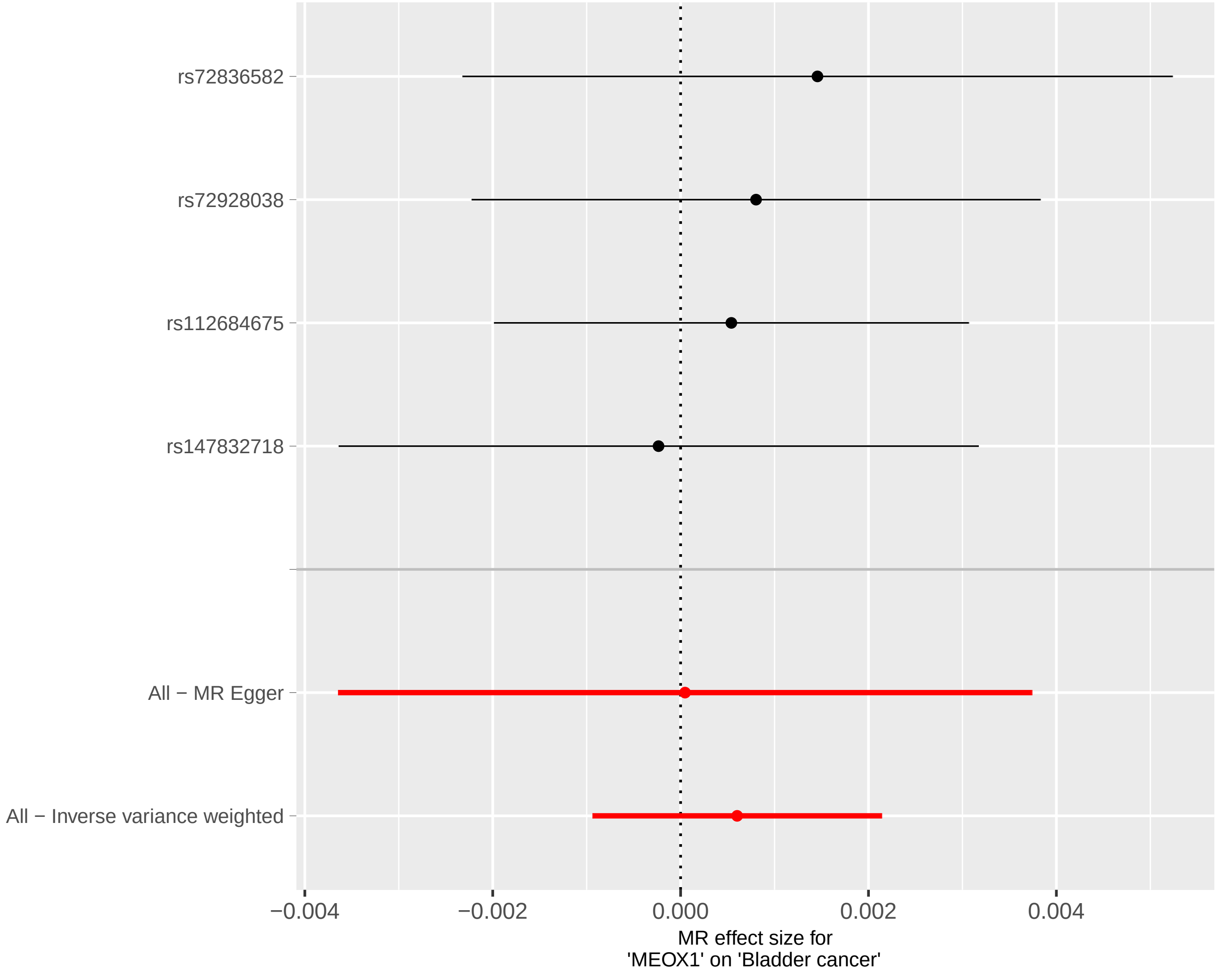

rs72836582
rs72928038
rs112684675
rs147832718
All − MR Egger
All − Inverse variance weighted
−0.004
−0.002
0.000
0.002
0.004
MR effect size for
'MEOX1' on 'Bladder cancer'

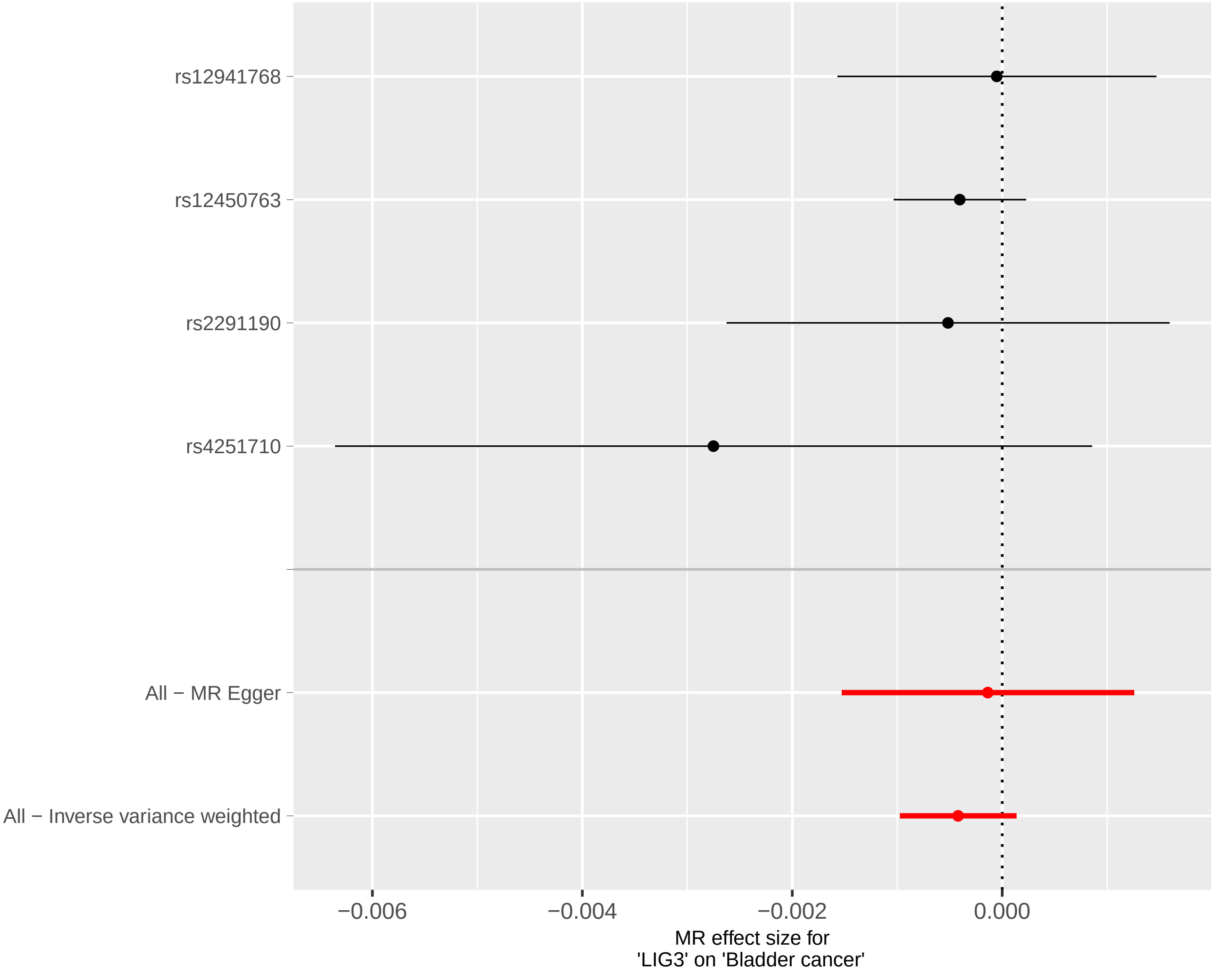
rs12941768
rs12450763
rs2291190
rs4251710
All − MR Egger
All − Inverse variance weighted
−0.006
−0.004
−0.002
0.000
MR effect size for
'LIG3' on 'Bladder cancer'

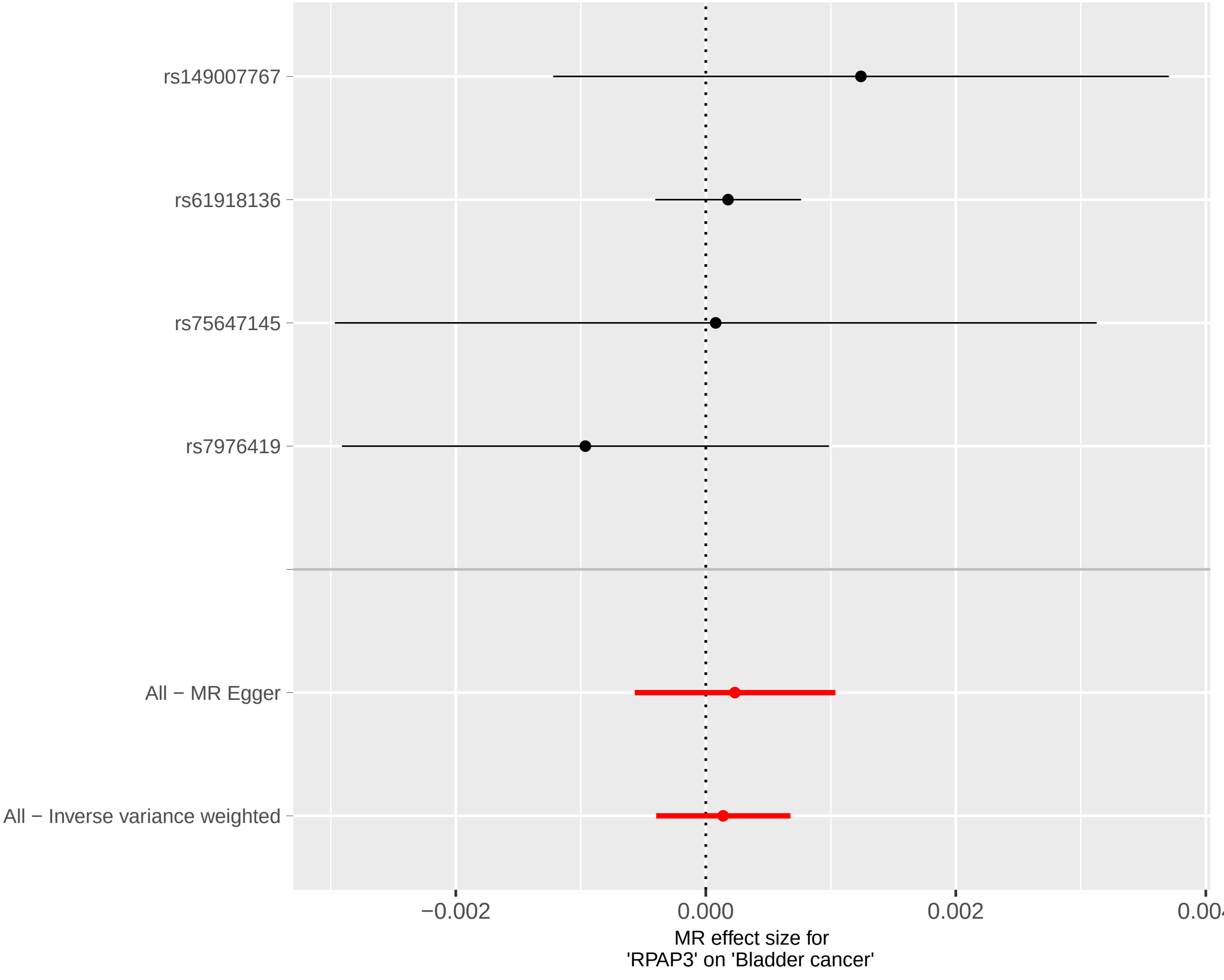
rs149007767
rs61918136
rs75647145
rs7976419
All − MR Egger
All − Inverse variance weighted
−0.002
0.000
0.002
0.004
MR effect size for
'RPAP3' on 'Bladder cancer'

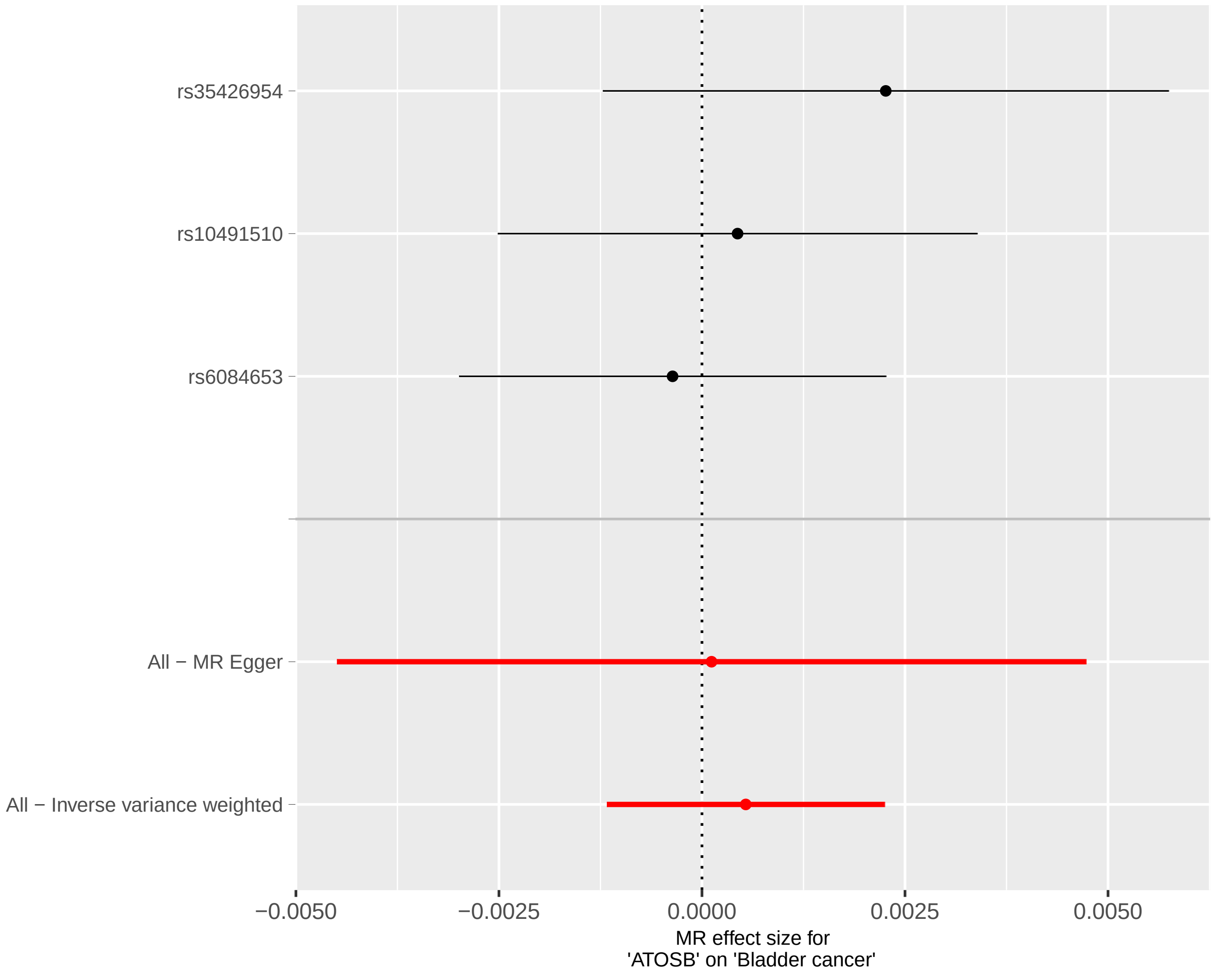

rs35426954
rs10491510
rs6084653
All − MR Egger
All − Inverse variance weighted
−0.0050
−0.0025
0.0000
0.0025
0.0050
MR effect size for
'ATOSB' on 'Bladder cancer'

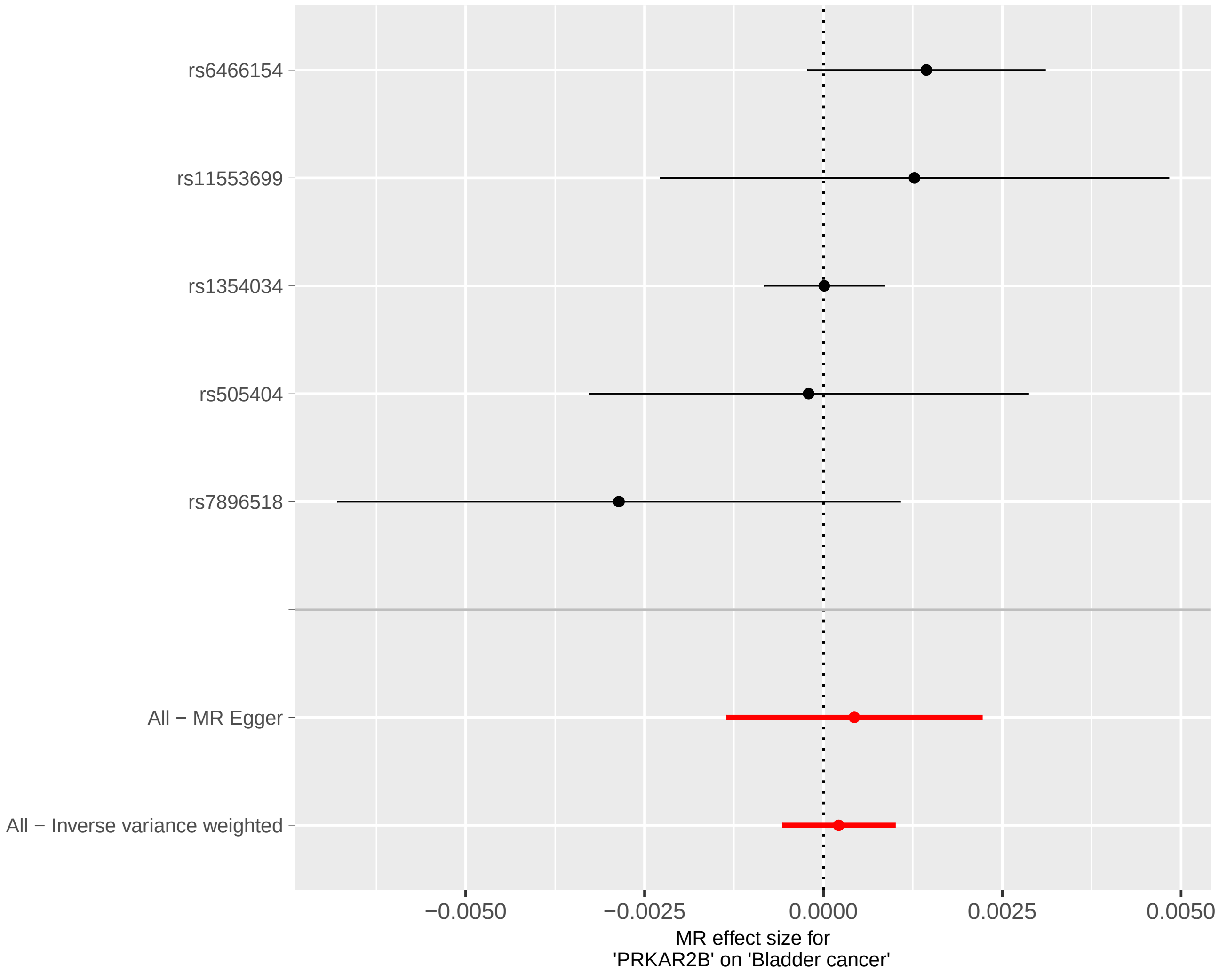
rs6466154
rs11553699
rs1354034
rs505404
rs7896518
All − MR Egger
All − Inverse variance weighted
−0.0050
−0.0025
0.0000
0.0025
0.0050
MR effect size for
'PRKAR2B' on 'Bladder cancer'

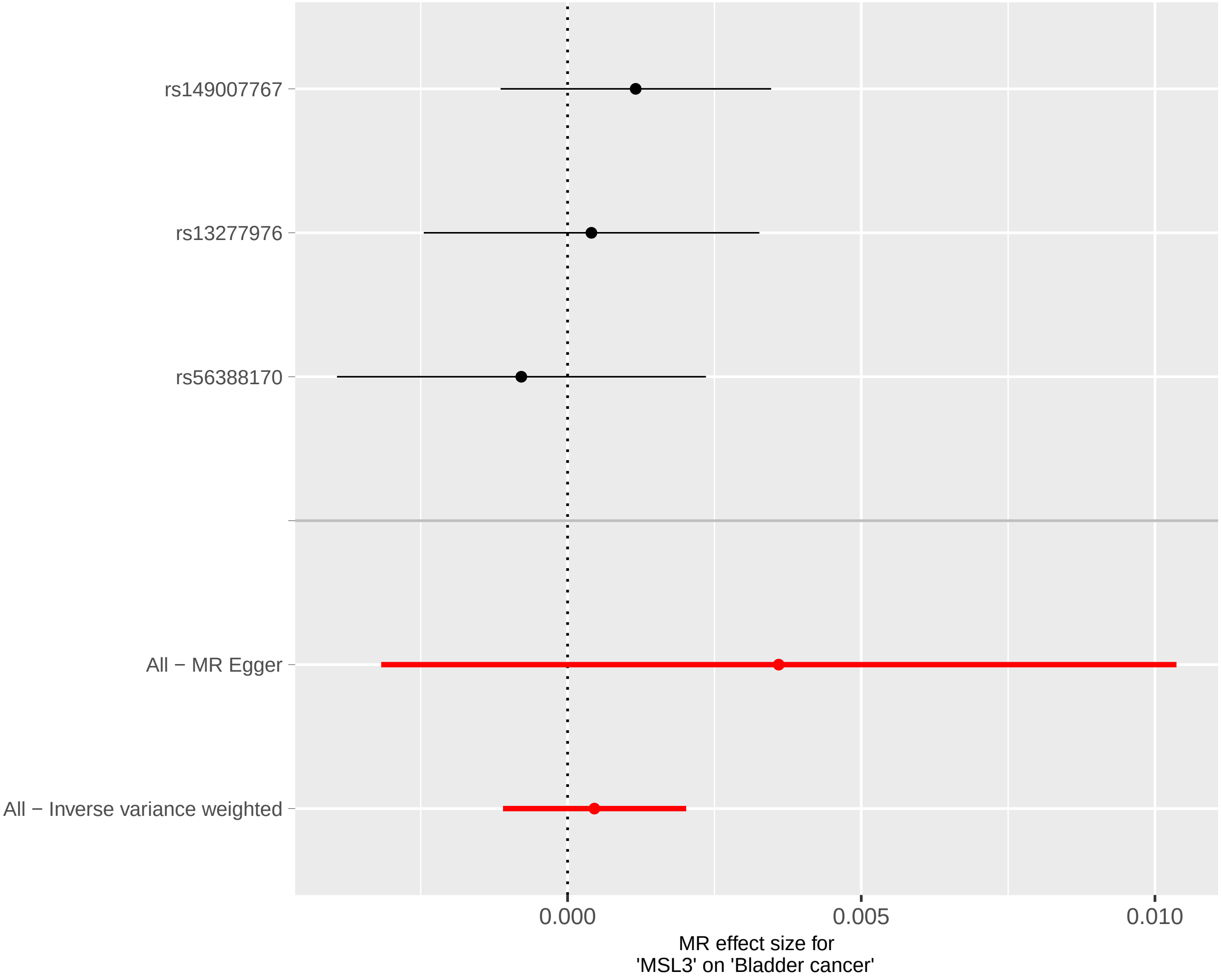
rs149007767
rs13277976
rs56388170
All − MR Egger
All − Inverse variance weighted
0.000
0.005
0.010
MR effect size for
'MSL3' on 'Bladder cancer'

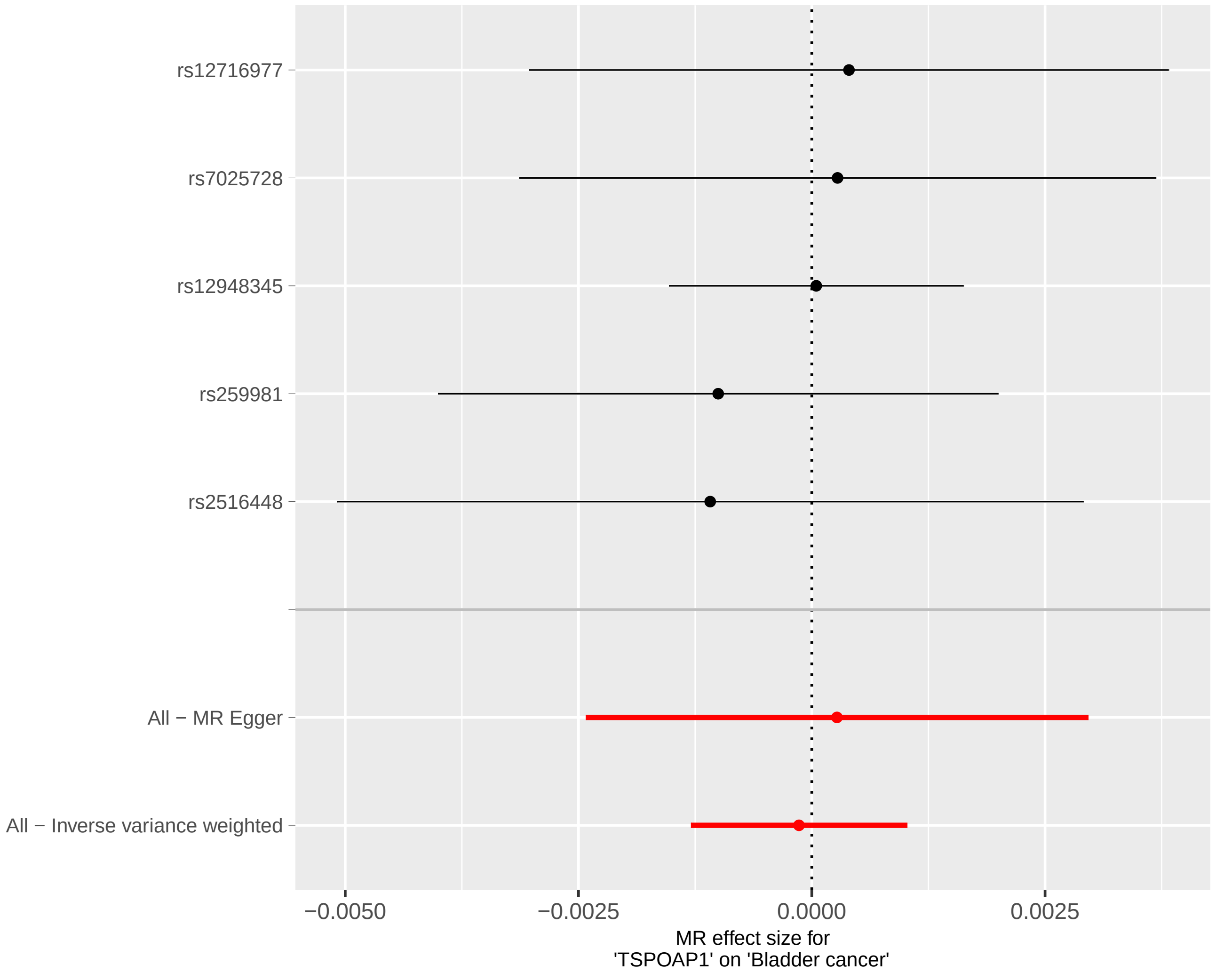

rs12716977
rs7025728
rs12948345
rs259981
rs2516448
All − MR Egger
All − Inverse variance weighted
−0.0050
−0.0025
0.0000
0.0025
MR effect size for
'TSPOAP1' on 'Bladder cancer'

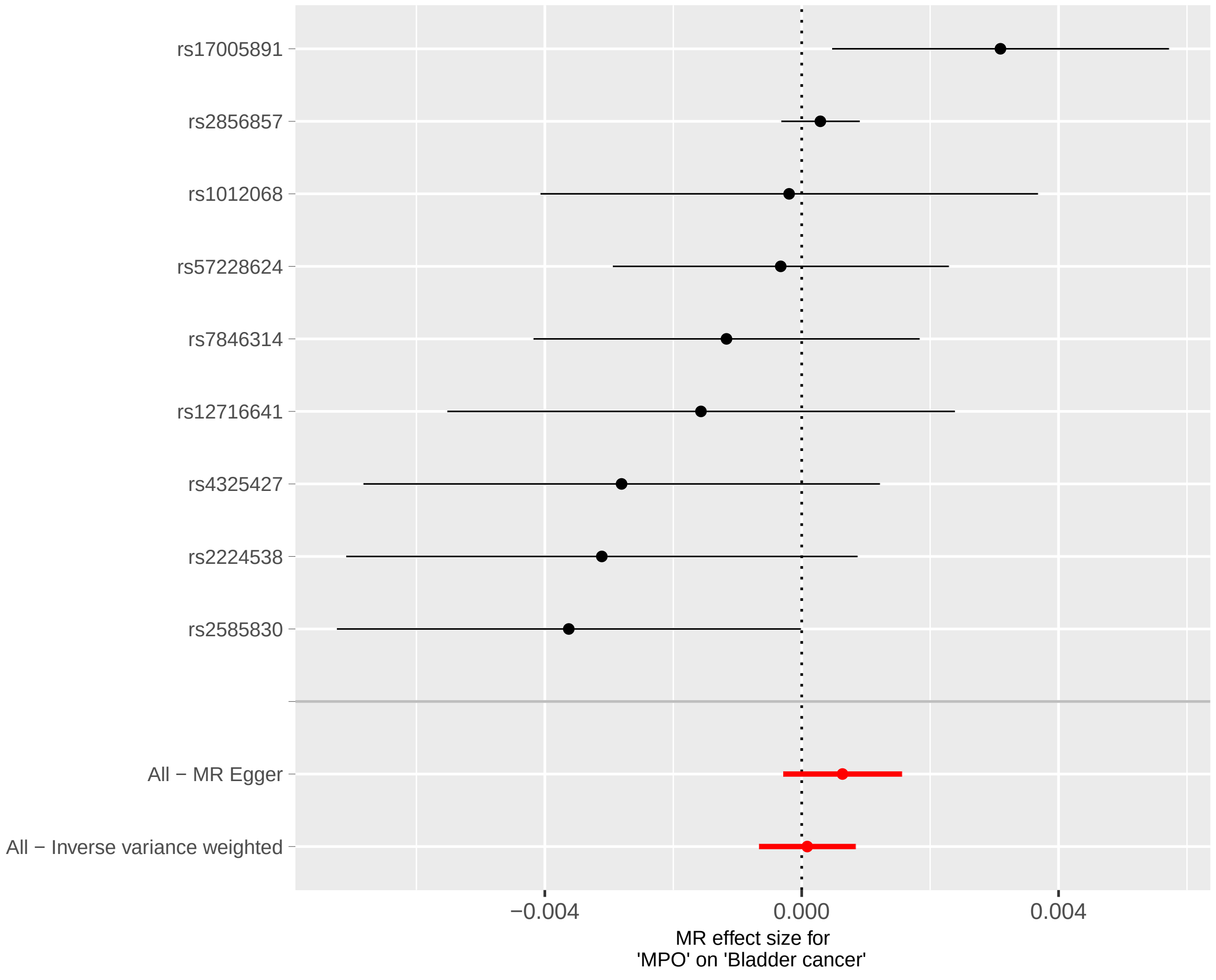
rs17005891
rs2856857
rs1012068
rs57228624
rs7846314
rs12716641
rs4325427
rs2224538
rs2585830
All − MR Egger
All − Inverse variance weighted
−0.004
0.000
0.004
MR effect size for
'MPO' on 'Bladder cancer'

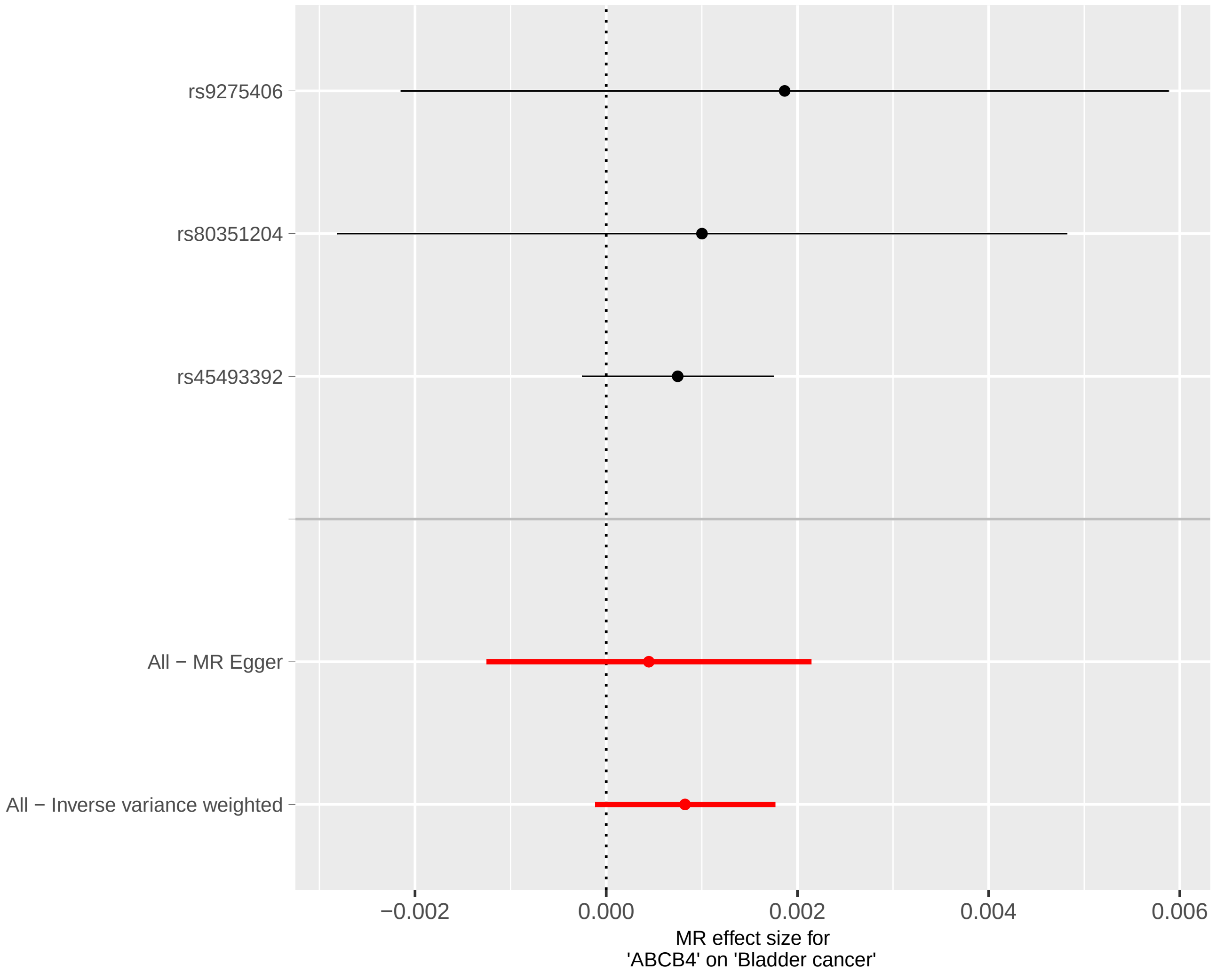

rs9275406
rs80351204
rs45493392
All − MR Egger
All − Inverse variance weighted
−0.002
0.000
0.002
0.004
0.006
MR effect size for
'ABCB4' on 'Bladder cancer'

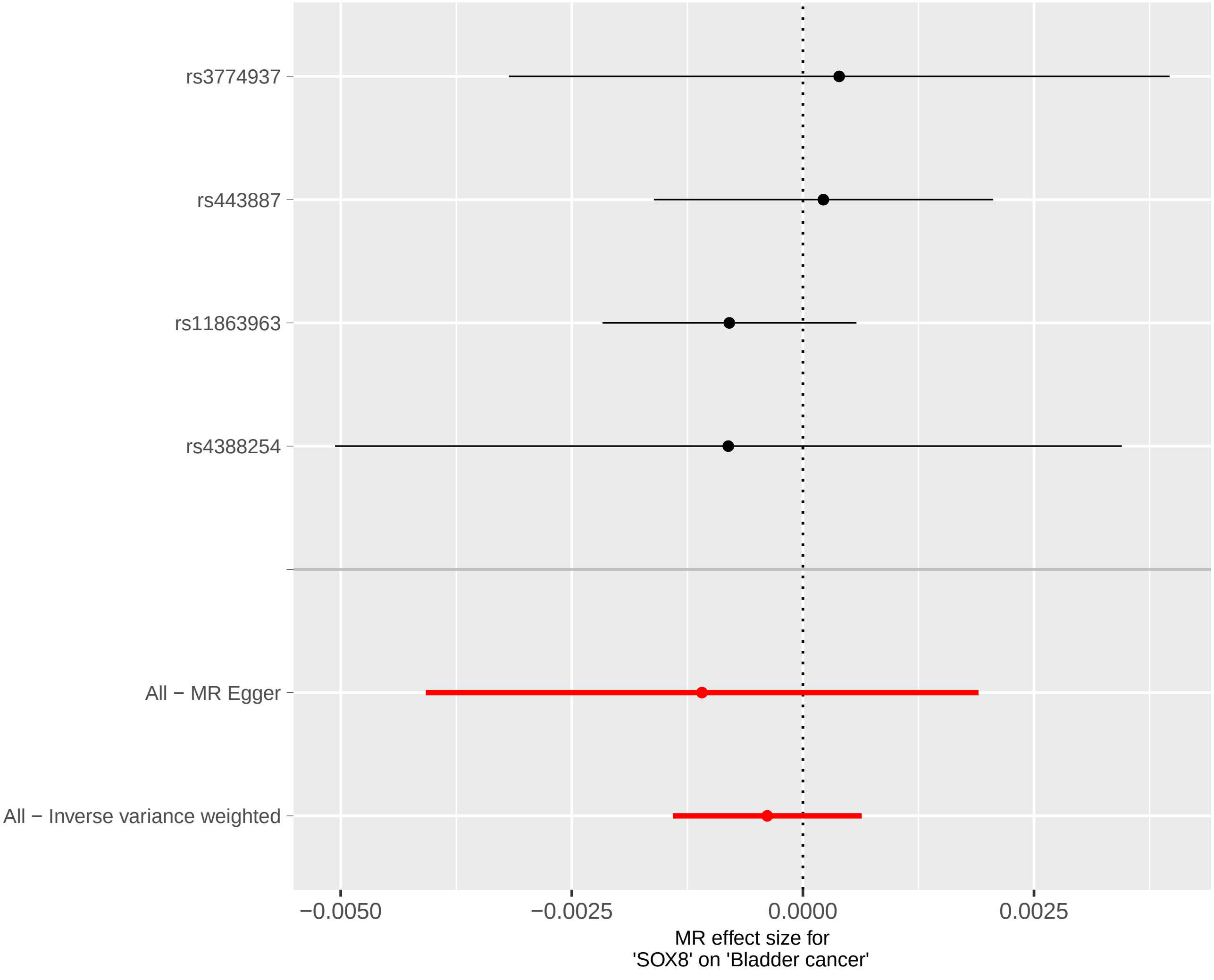

rs3774937
rs443887
rs11863963
rs4388254
All − MR Egger
All − Inverse variance weighted
−0.0050
−0.0025
0.0000
0.0025
MR effect size for
'SOX8' on 'Bladder cancer'

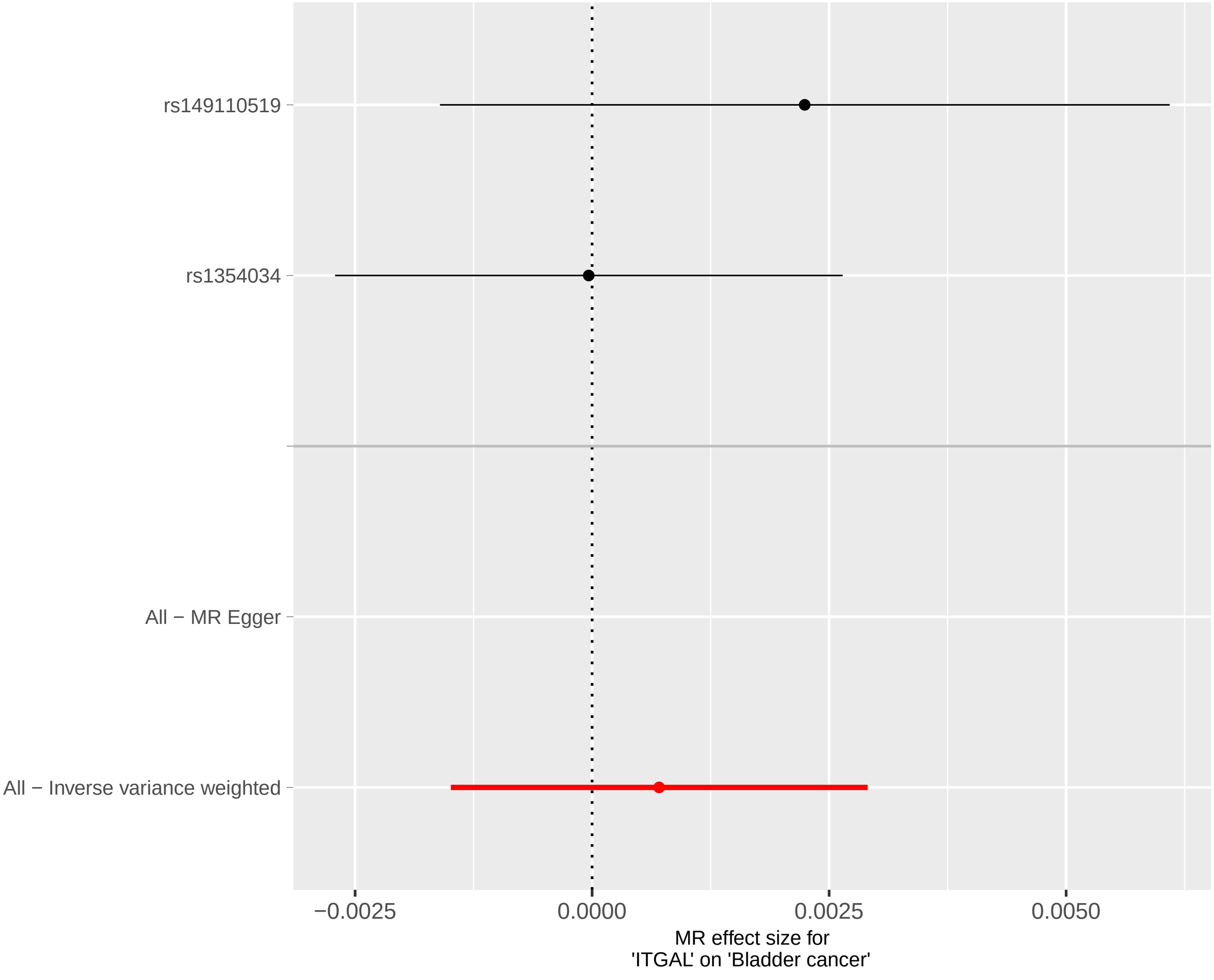
rs149110519
rs1354034
All − MR Egger
All − Inverse variance weighted
−0.0025
0.0000
0.0025
0.0050
MR effect size for
'ITGAL' on 'Bladder cancer'

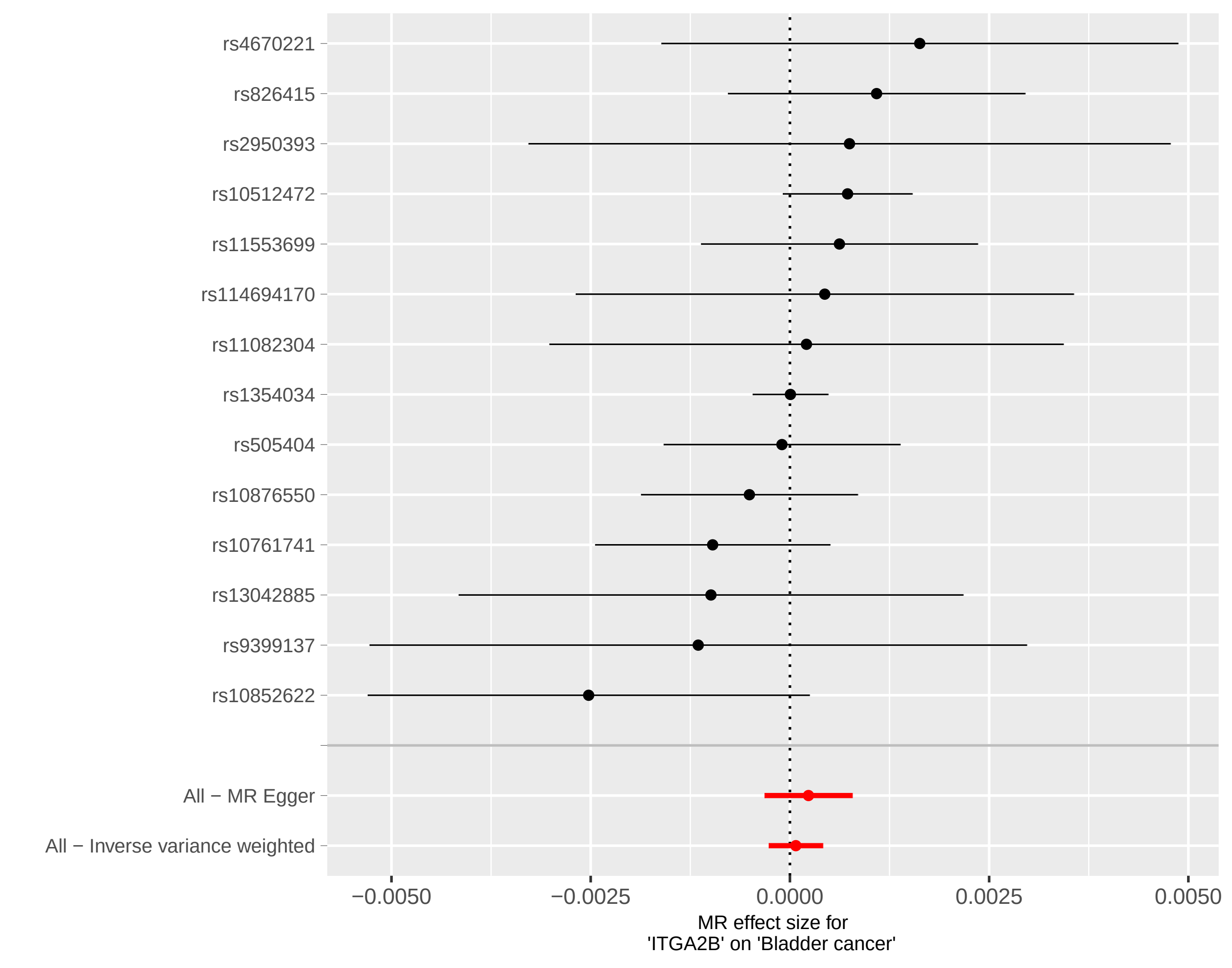

rs4670221
rs826415
rs2950393
rs10512472
rs11553699
rs114694170
rs11082304
rs1354034
rs505404
rs10876550
rs10761741
rs13042885
rs9399137
rs10852622
All − MR Egger
All − Inverse variance weighted
−0.0050
−0.0025
0.0000
0.0025
0.0050
MR effect size for
'ITGA2B' on 'Bladder cancer'

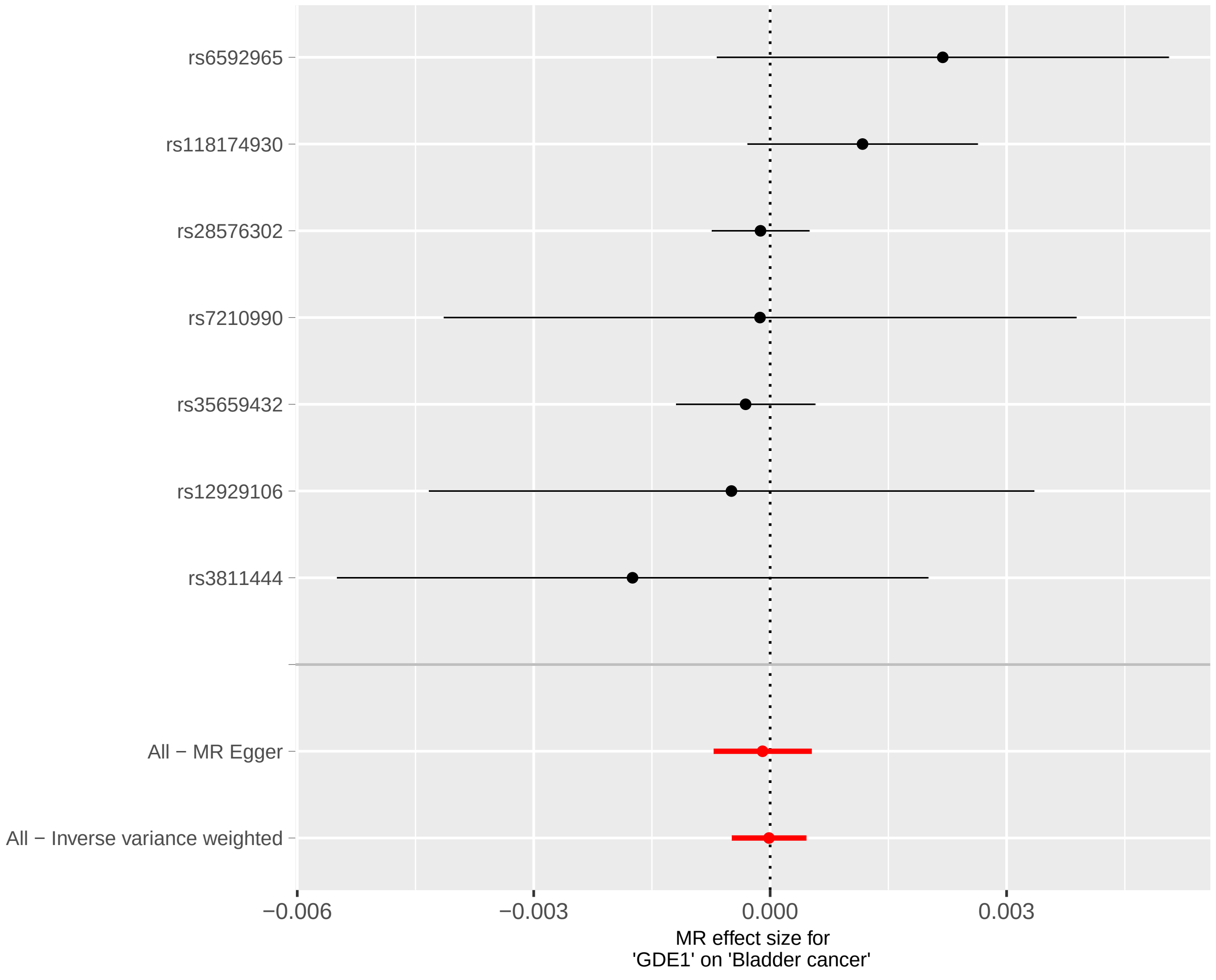

rs6592965
rs118174930
rs28576302
rs7210990
rs35659432
rs12929106
rs3811444
All − MR Egger
All − Inverse variance weighted
−0.006
−0.003
0.000
0.003
MR effect size for
'GDE1' on 'Bladder cancer'

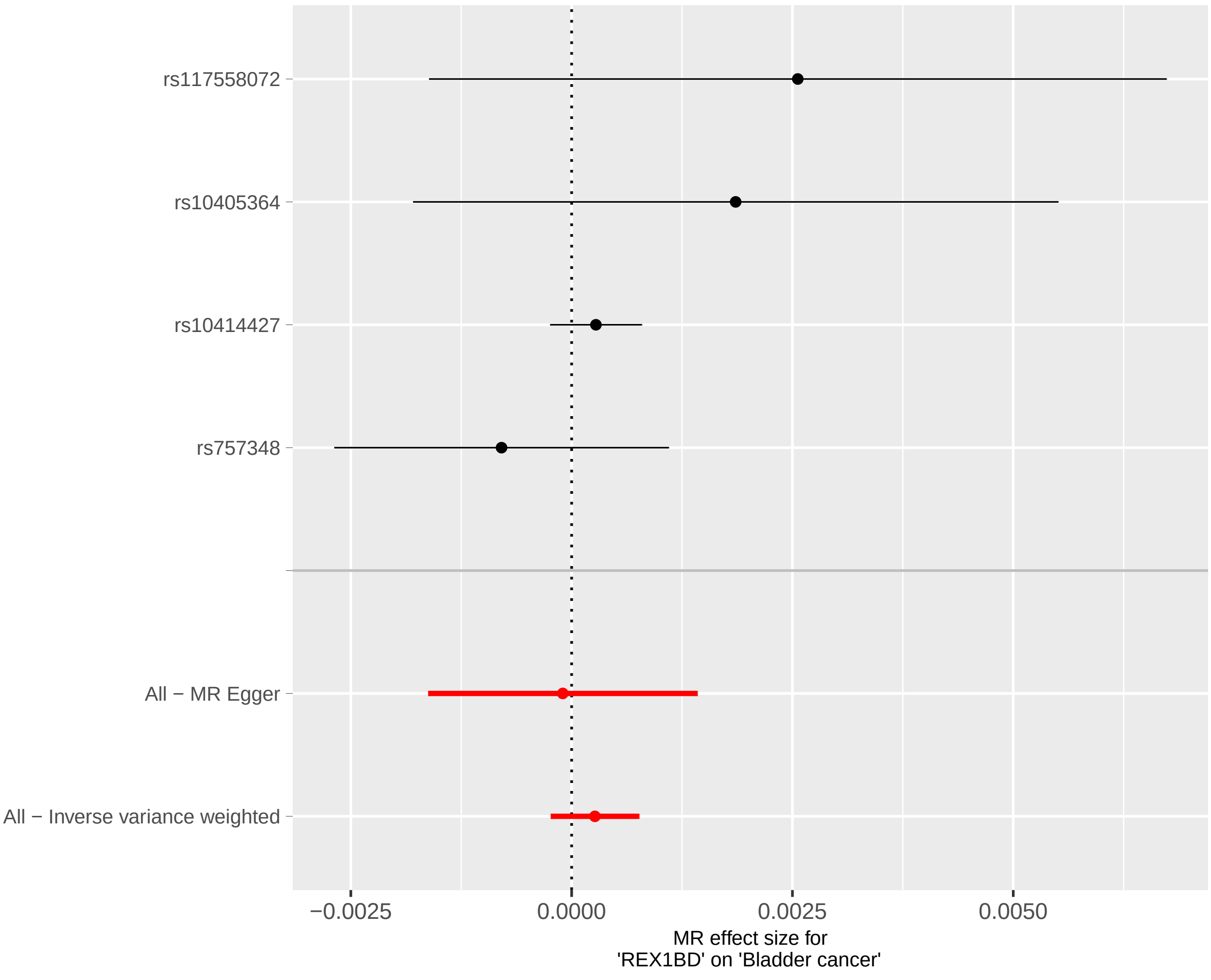

rs117558072
rs10405364
rs10414427
rs757348
All − MR Egger
All − Inverse variance weighted
−0.0025
0.0000
0.0025
0.0050
MR effect size for
'REX1BD' on 'Bladder cancer'

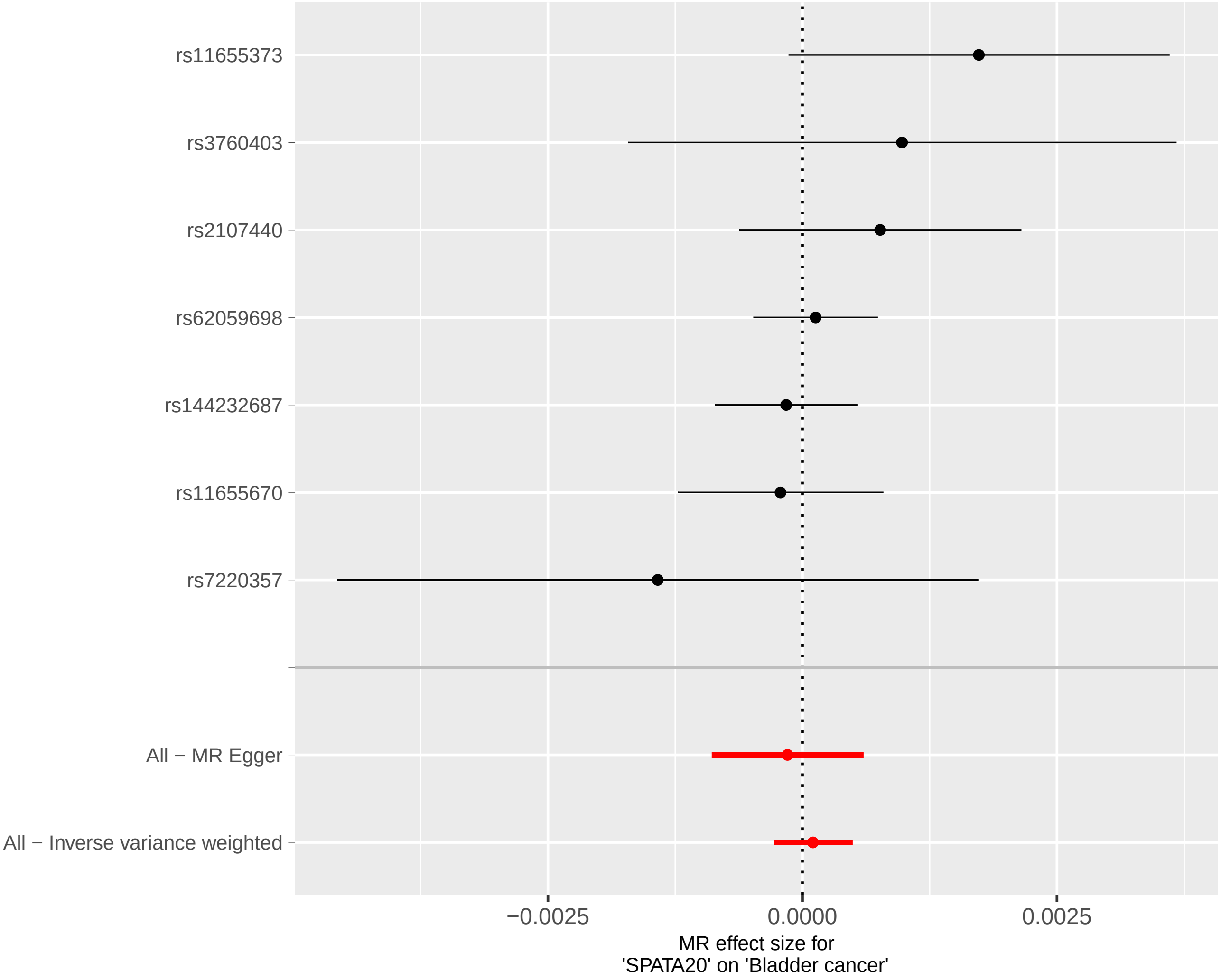
rs11655373
rs3760403
rs2107440
rs62059698
rs144232687
rs11655670
rs7220357
All − MR Egger
All − Inverse variance weighted
−0.0025
0.0000
0.0025
MR effect size for
'SPATA20' on 'Bladder cancer'

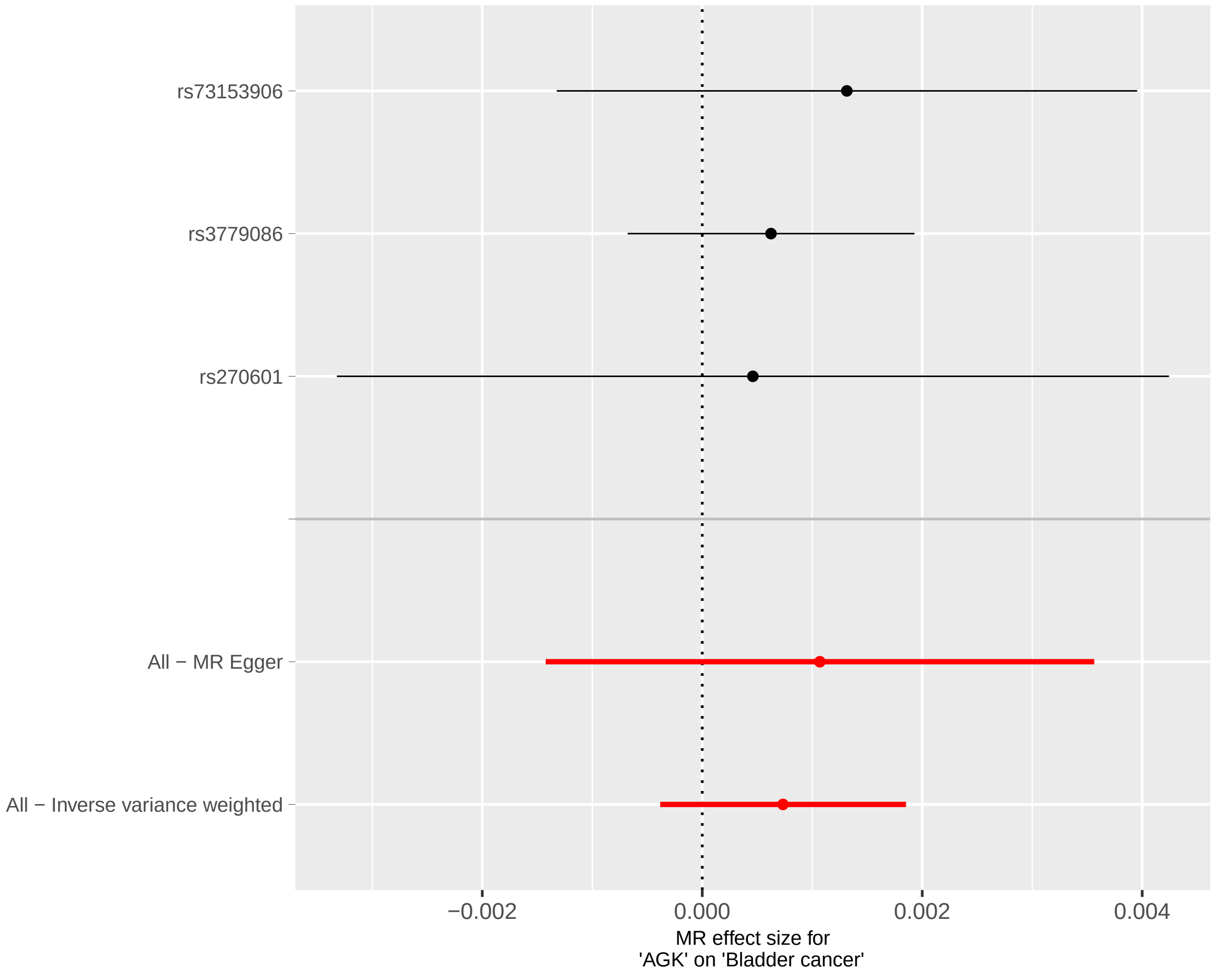

rs73153906
rs3779086
rs270601
All − MR Egger
All − Inverse variance weighted
−0.002
0.000
0.002
0.004
MR effect size for
'AGK' on 'Bladder cancer'

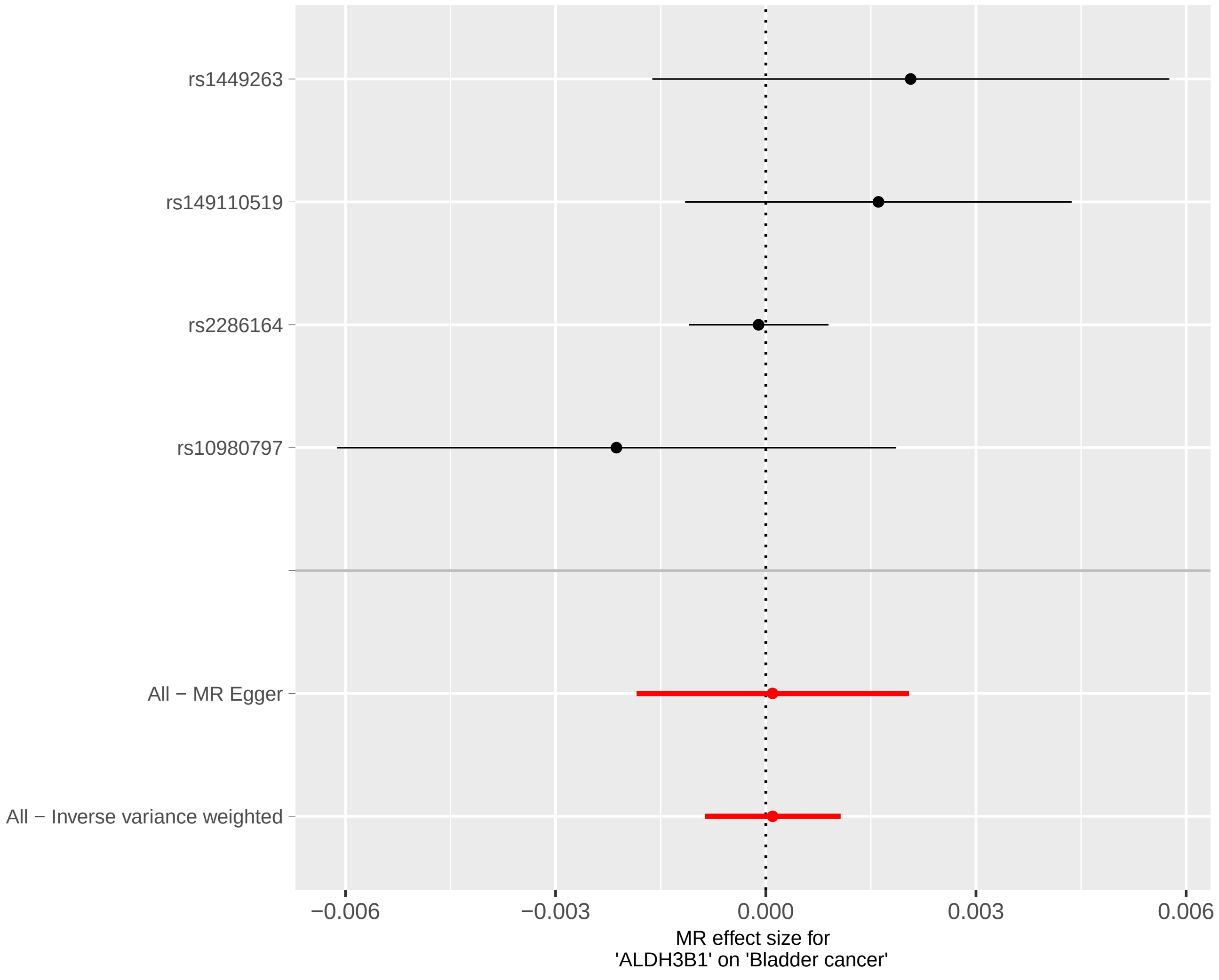
rs1449263
rs149110519
rs2286164
rs10980797
All − MR Egger
All − Inverse variance weighted
−0.006
−0.003
0.000
0.003
0.006
MR effect size for
'ALDH3B1' on 'Bladder cancer'

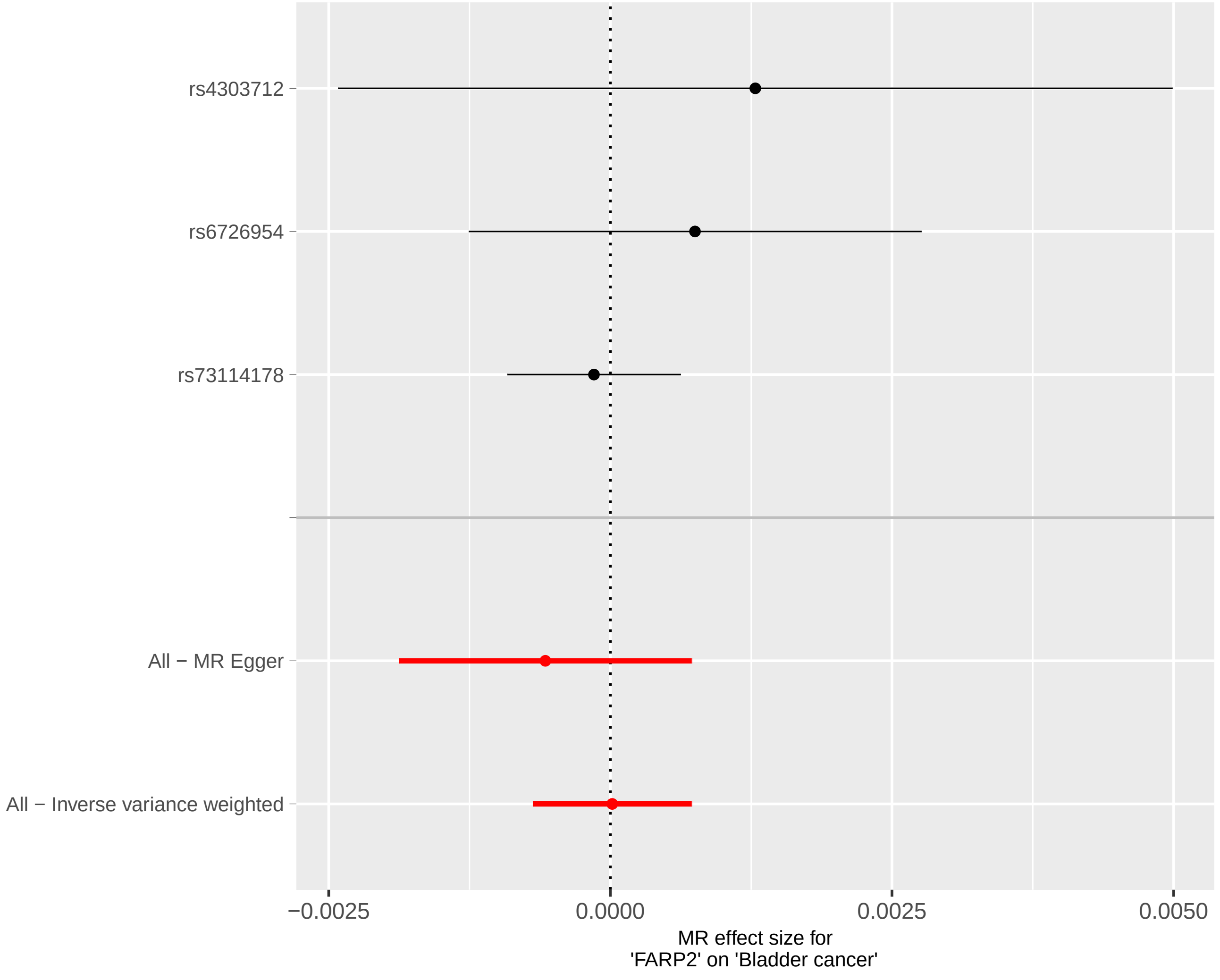
rs4303712
rs6726954
rs73114178
All − MR Egger
All − Inverse variance weighted
−0.0025
0.0000
0.0025
0.0050
MR effect size for
'FARP2' on 'Bladder cancer'

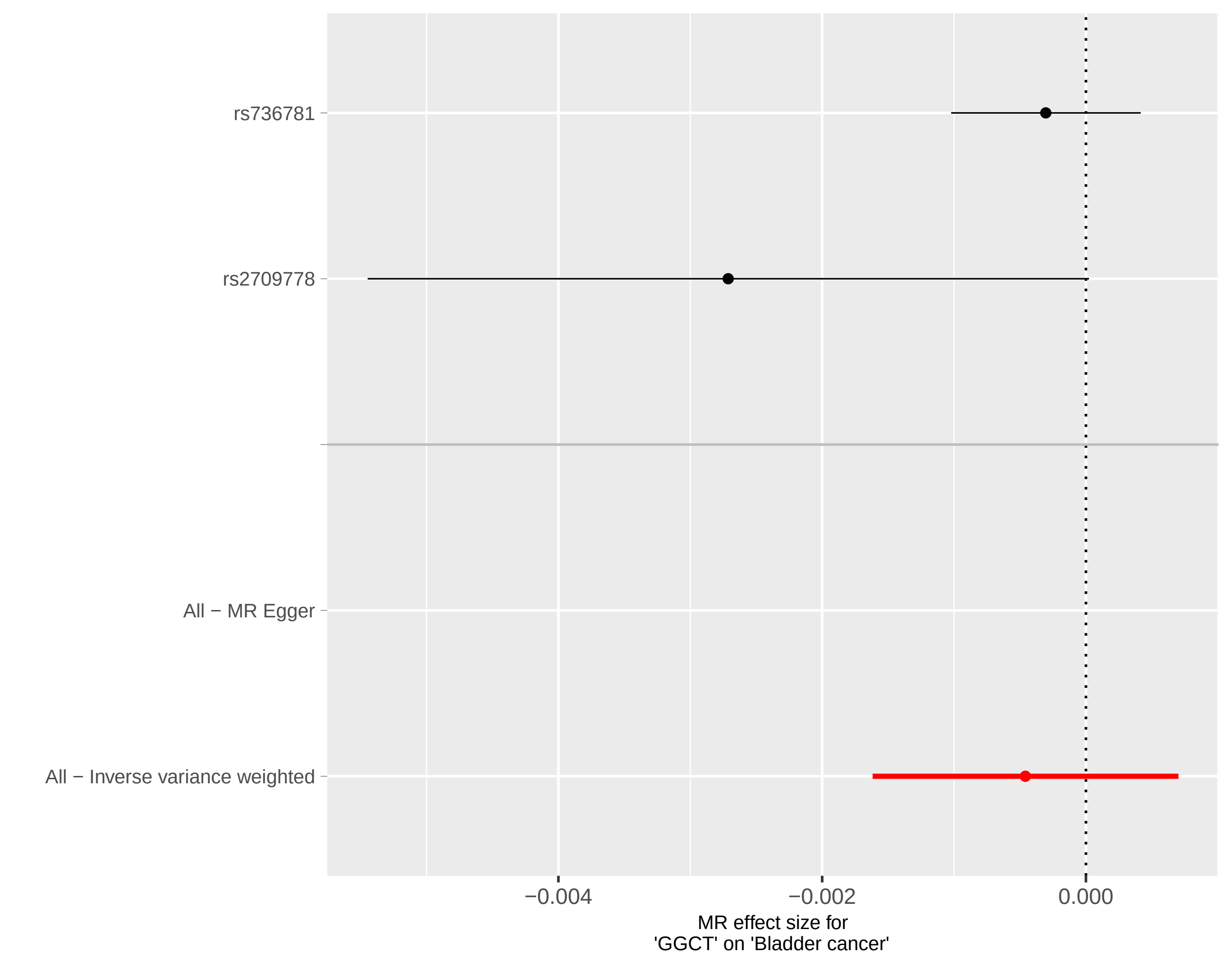

rs736781
rs2709778
All − MR Egger
All − Inverse variance weighted
−0.004
−0.002
0.000
MR effect size for
'GGCT' on 'Bladder cancer'

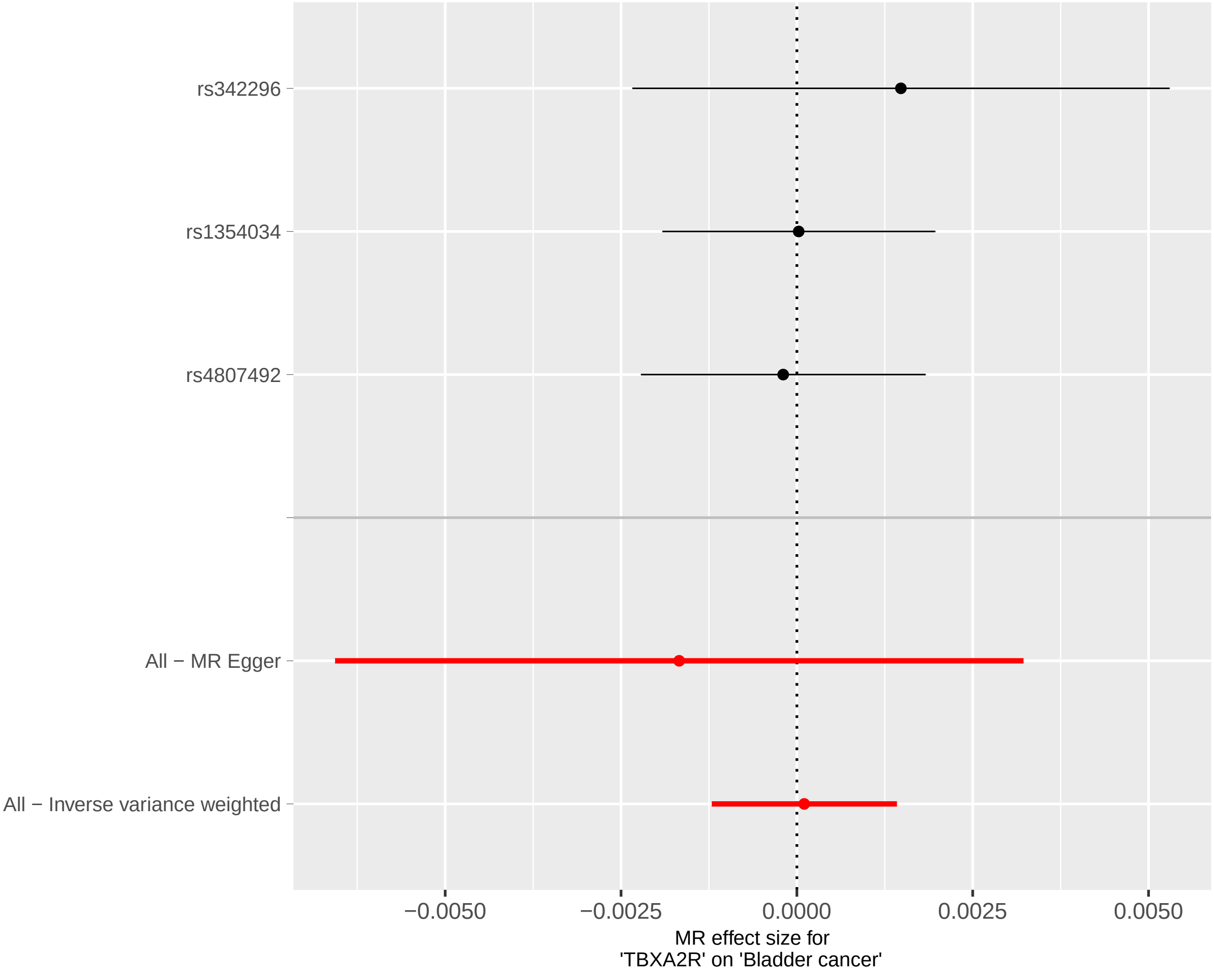
rs342296
rs1354034
rs4807492
All − MR Egger
All − Inverse variance weighted
−0.0050
−0.0025
0.0000
0.0025
0.0050
MR effect size for
'TBXA2R' on 'Bladder cancer'

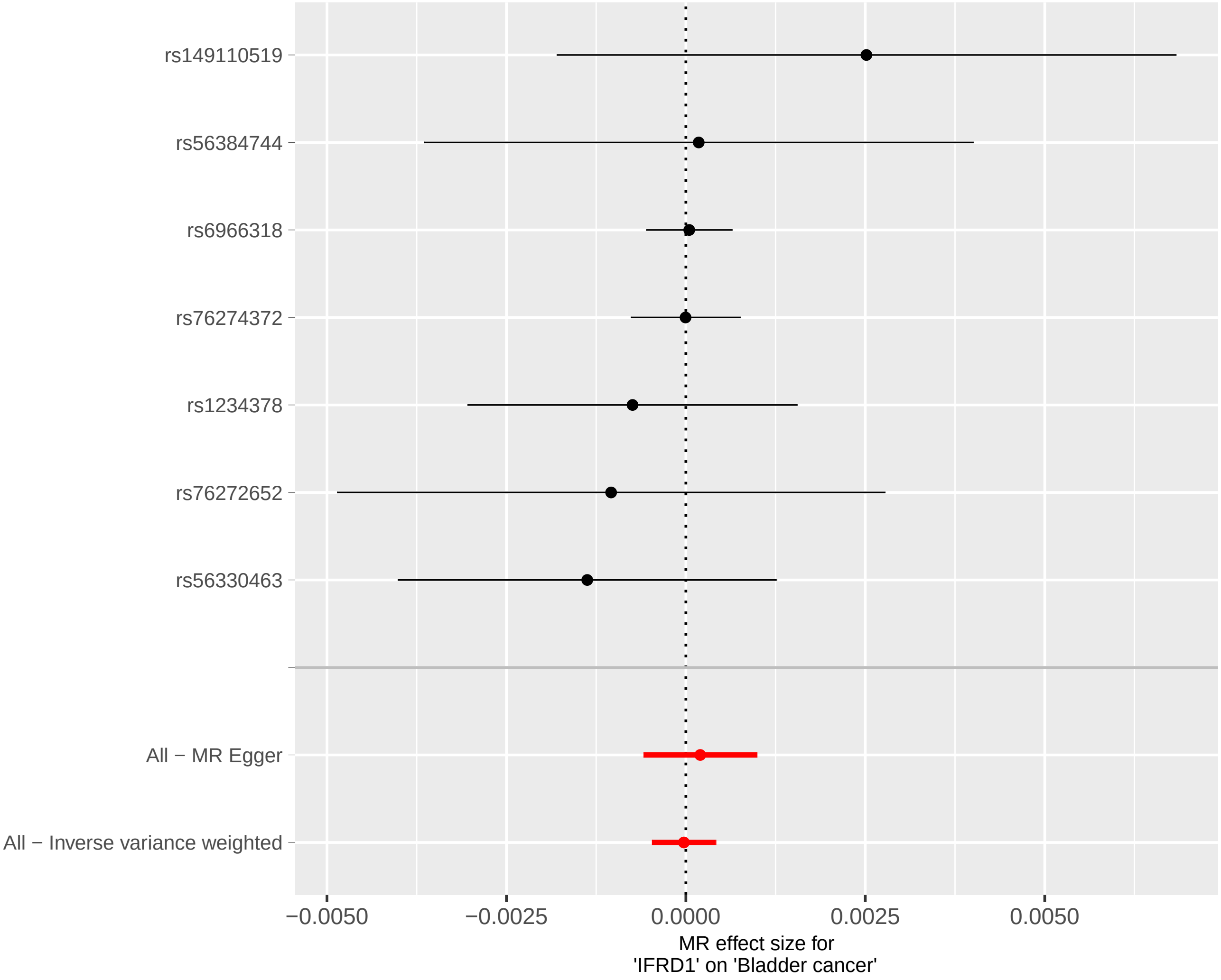
rs149110519
rs56384744
rs6966318
rs76274372
rs1234378
rs76272652
rs56330463
All − MR Egger
All − Inverse variance weighted
−0.0050
−0.0025
0.0000
0.0025
0.0050
MR effect size for
'IFRD1' on 'Bladder cancer'

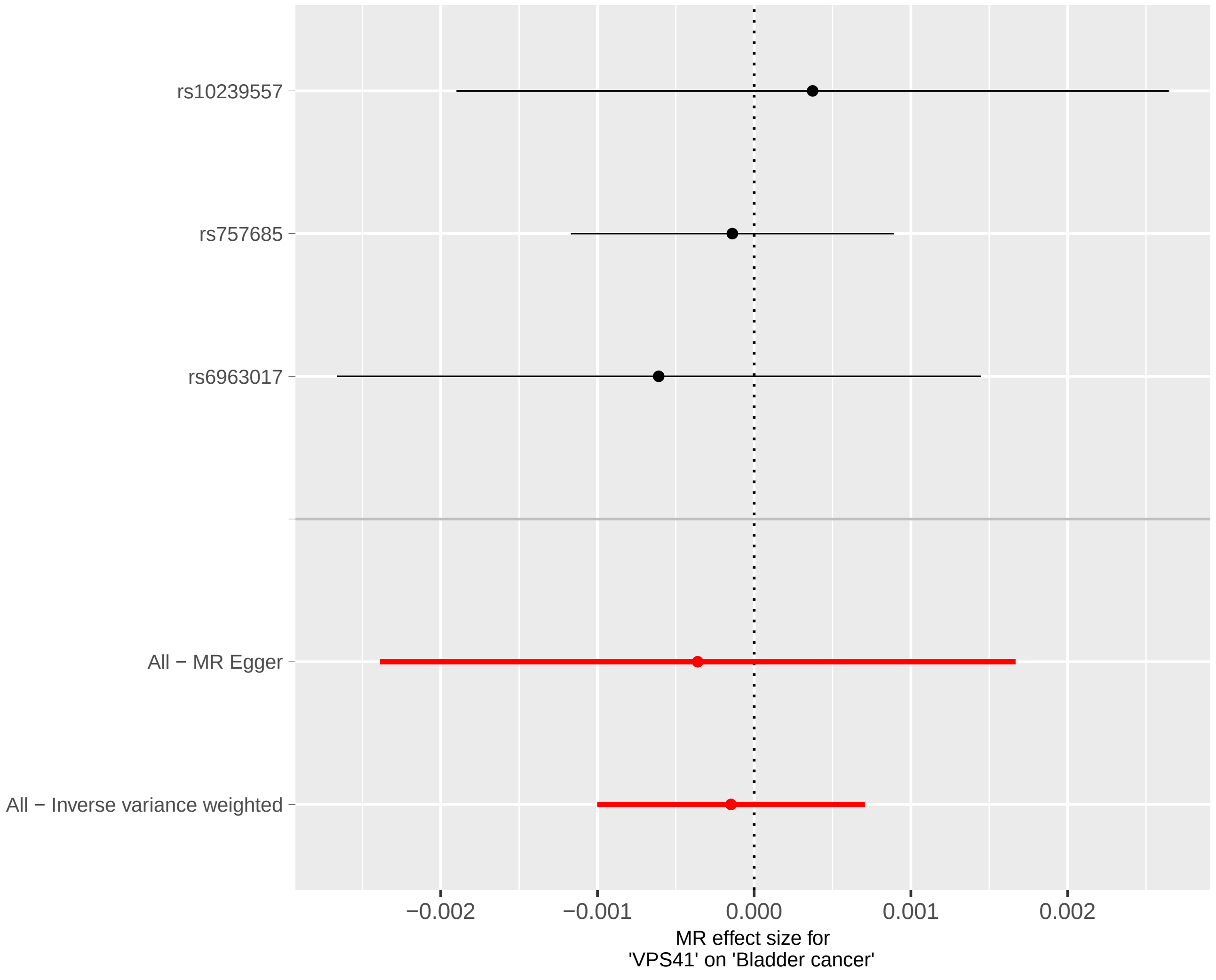

rs10239557
rs757685
rs6963017
All − MR Egger
All − Inverse variance weighted
−0.002
−0.001
0.000
0.001
0.002
MR effect size for
'VPS41' on 'Bladder cancer'

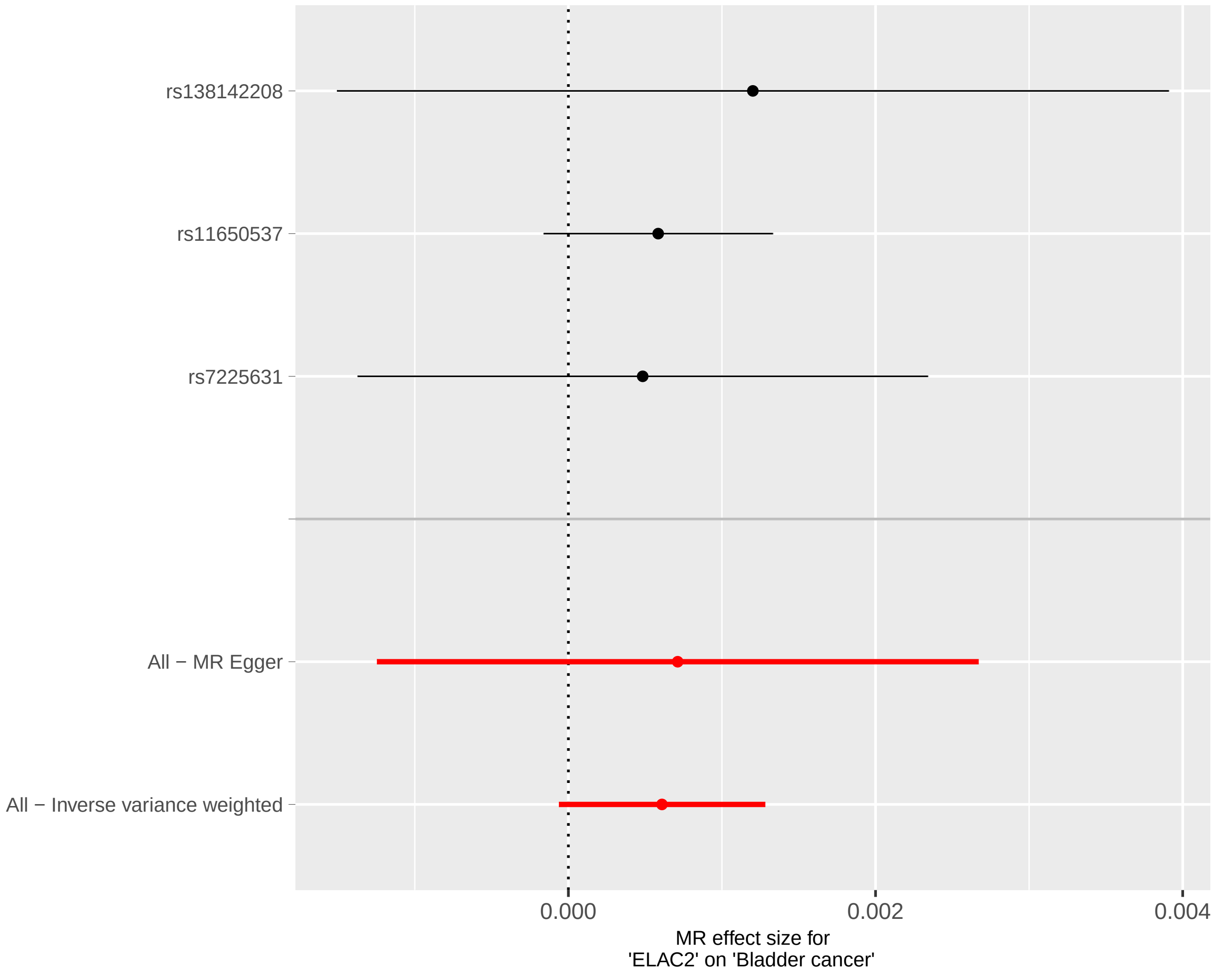

rs138142208
rs11650537
rs7225631
All − MR Egger
All − Inverse variance weighted
0.000
0.002
0.004
MR effect size for
'ELAC2' on 'Bladder cancer'

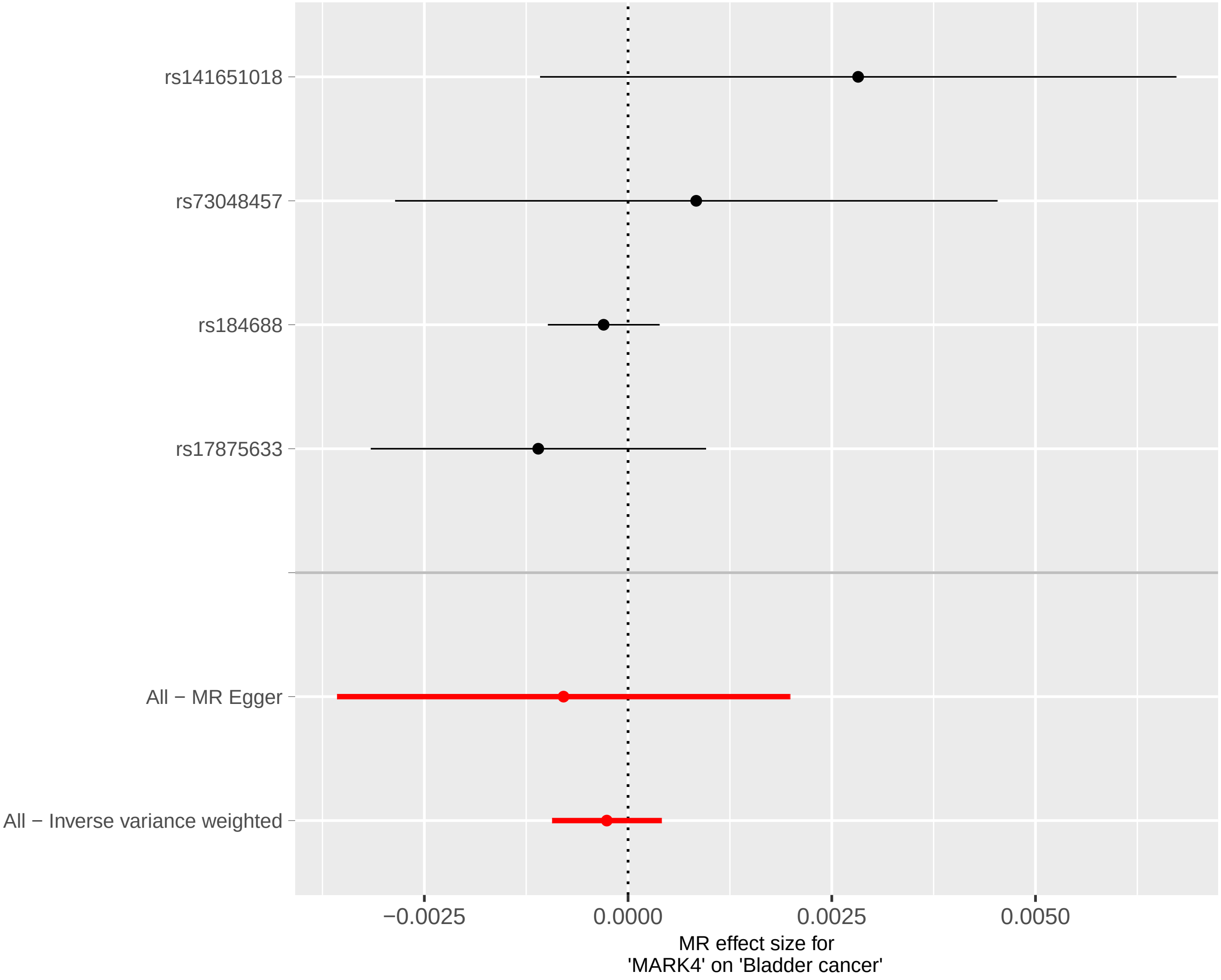
rs141651018
rs73048457
rs184688
rs17875633
All − MR Egger
All − Inverse variance weighted
−0.0025
0.0000
0.0025
0.0050
MR effect size for
'MARK4' on 'Bladder cancer'

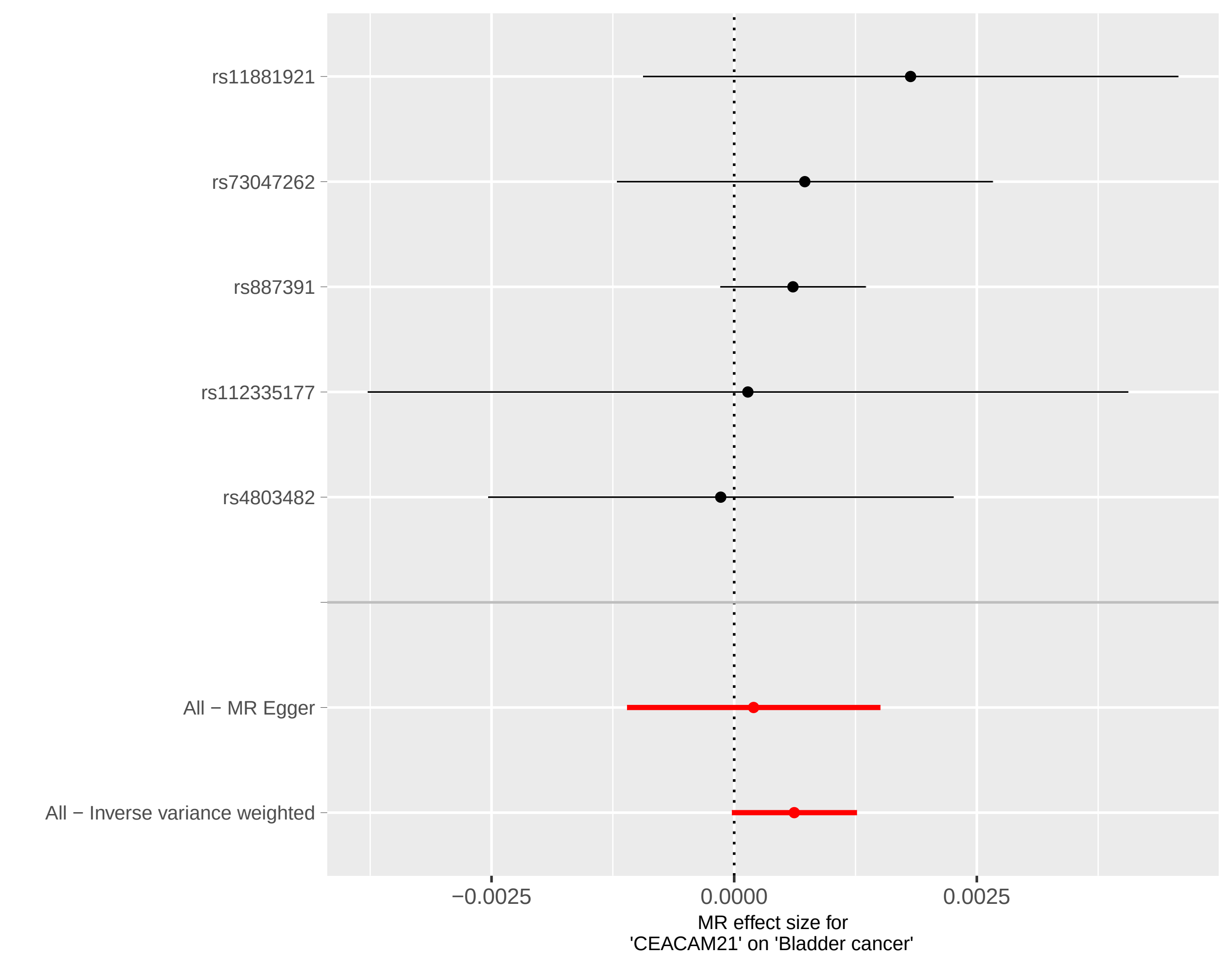
rs11881921
rs73047262
rs887391
rs112335177
rs4803482
All − MR Egger
All − Inverse variance weighted
−0.0025
0.0000
0.0025
MR effect size for
'CEACAM21' on 'Bladder cancer'

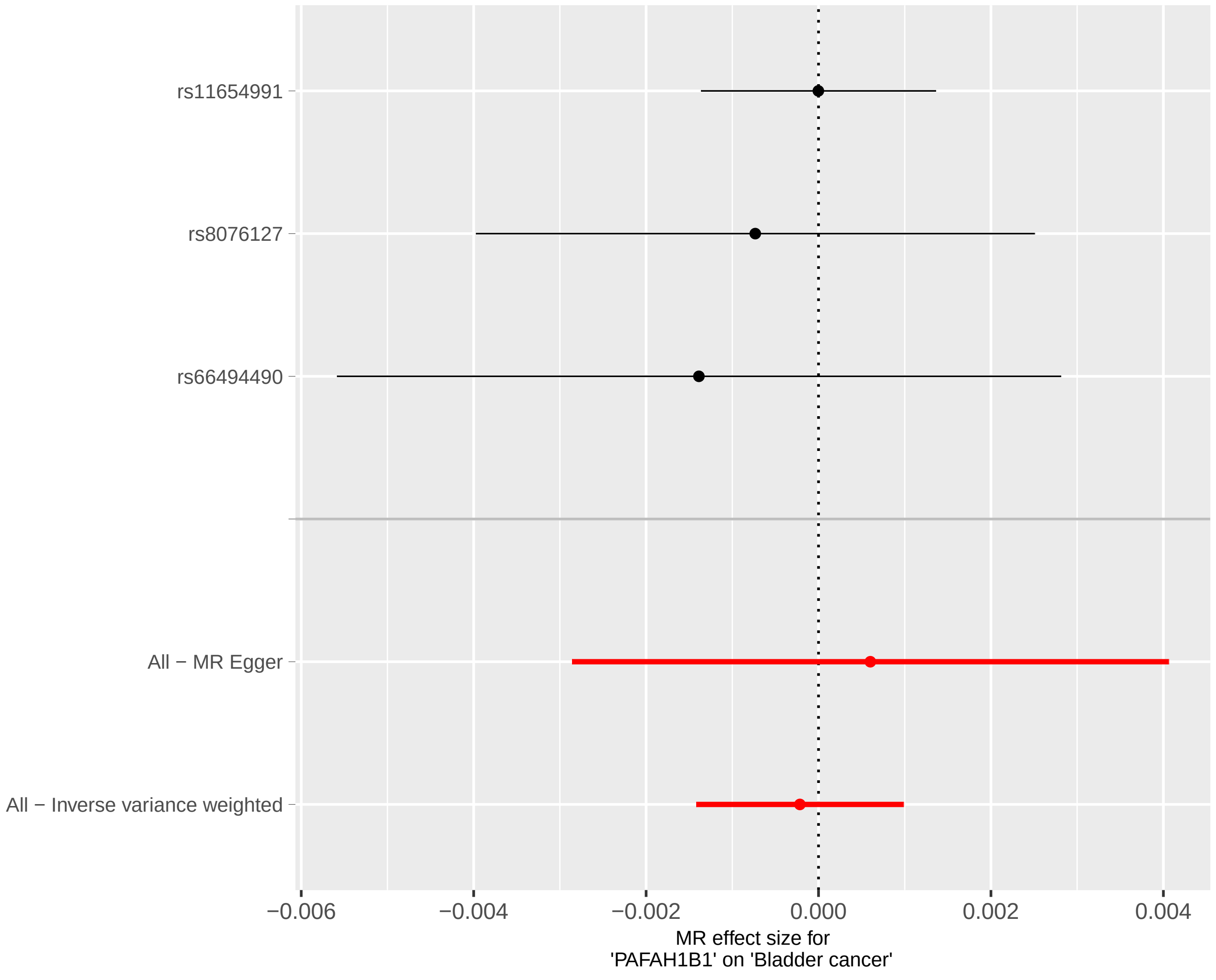

rs11654991
rs8076127
rs66494490
All − MR Egger
All − Inverse variance weighted
−0.006
−0.004
−0.002
0.000
0.002
0.004
MR effect size for
'PAFAH1B1' on 'Bladder cancer'

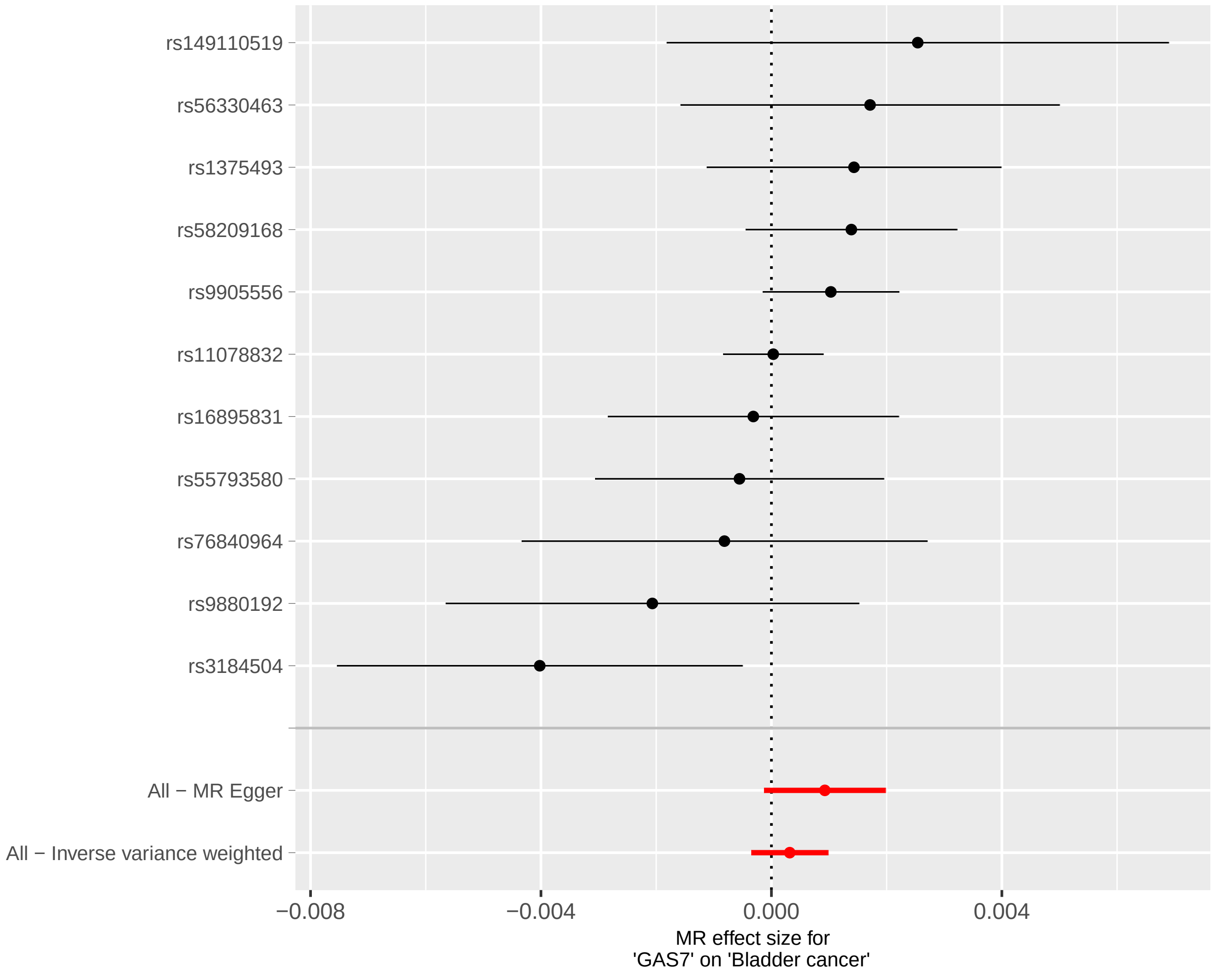

rs149110519
rs56330463
rs1375493
rs58209168
rs9905556
rs11078832
rs16895831
rs55793580
rs76840964
rs9880192
rs3184504
All − MR Egger
All − Inverse variance weighted
−0.008
−0.004
0.000
0.004
MR effect size for
'GAS7' on 'Bladder cancer'

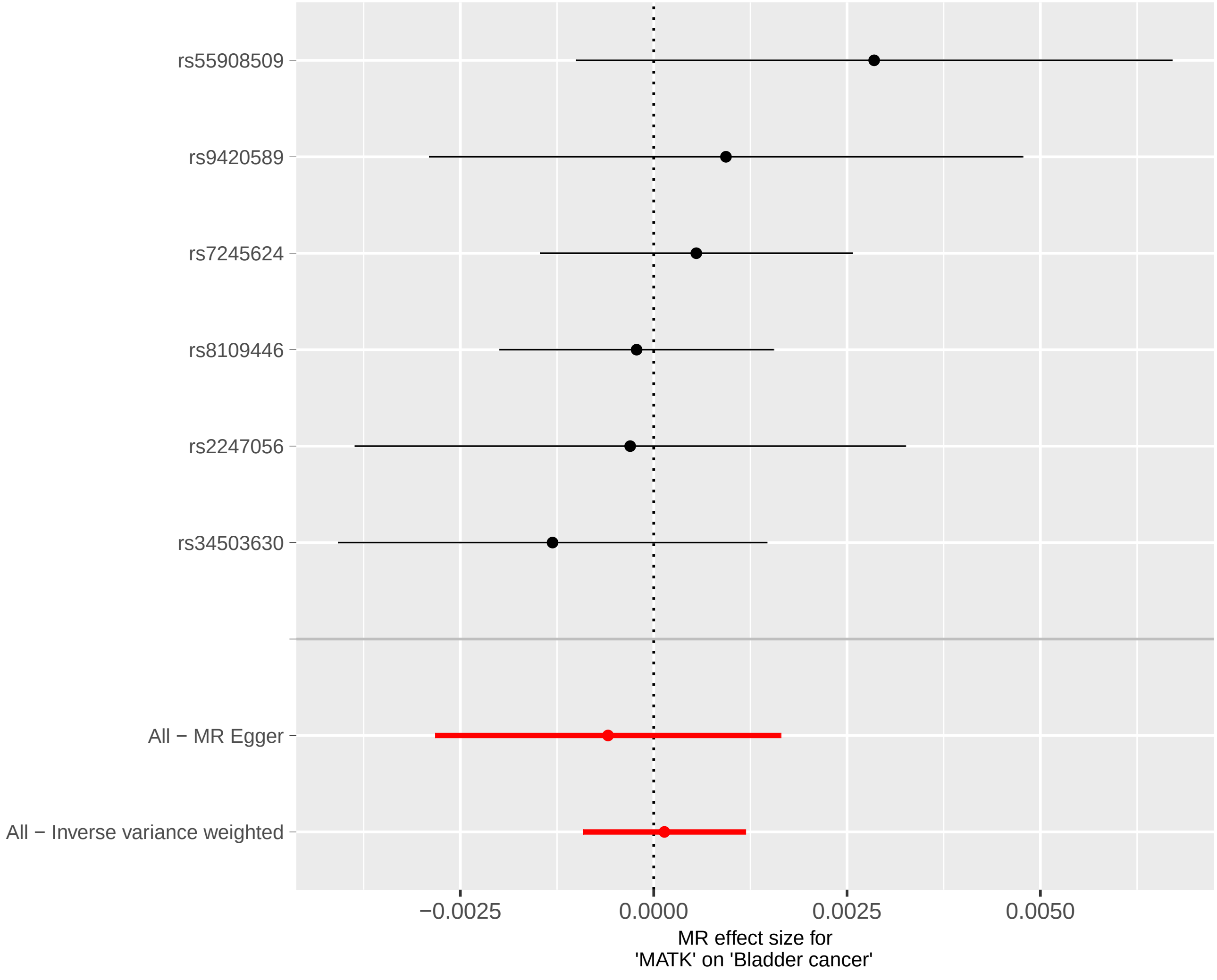
rs55908509
rs9420589
rs7245624
rs8109446
rs2247056
rs34503630
All − MR Egger
All − Inverse variance weighted
−0.0025
0.0000
0.0025
0.0050
MR effect size for
'MATK' on 'Bladder cancer'

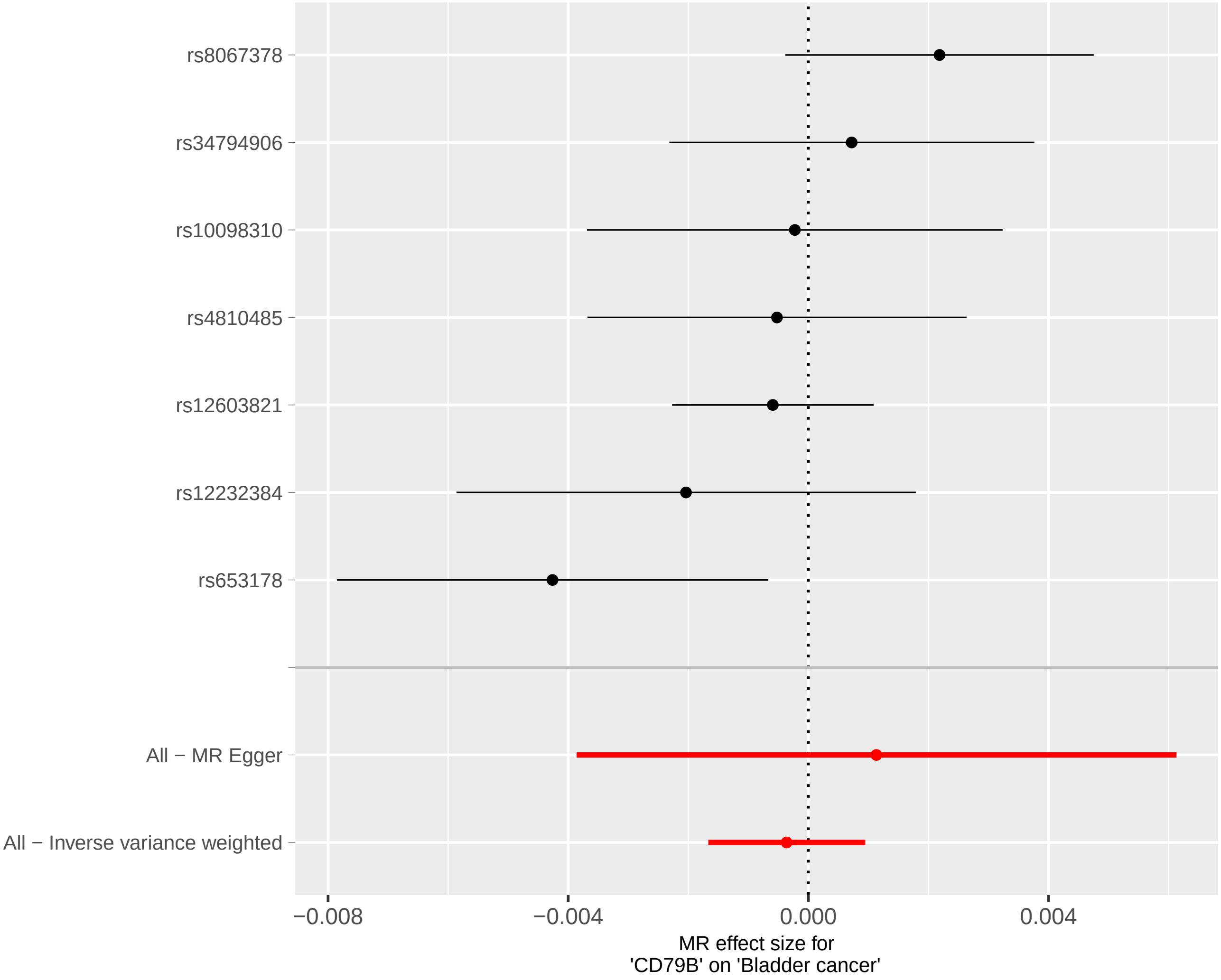

rs8067378
rs34794906
rs10098310
rs4810485
rs12603821
rs12232384
rs653178
All − MR Egger
All − Inverse variance weighted
−0.008
−0.004
0.000
0.004
MR effect size for
'CD79B' on 'Bladder cancer'

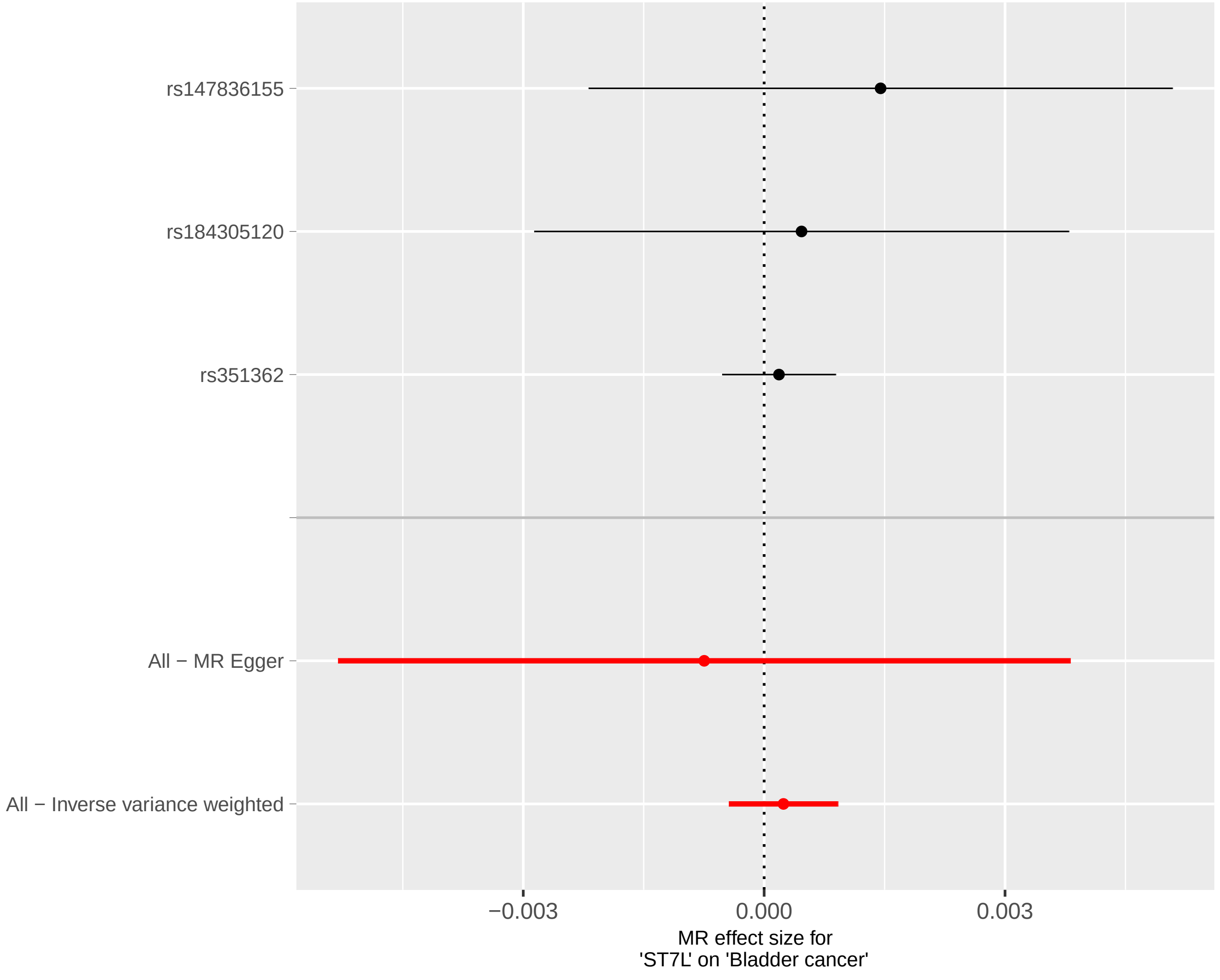

rs147836155
rs184305120
rs351362
All − MR Egger
All − Inverse variance weighted
−0.003
0.000
0.003
MR effect size for
'ST7L' on 'Bladder cancer'

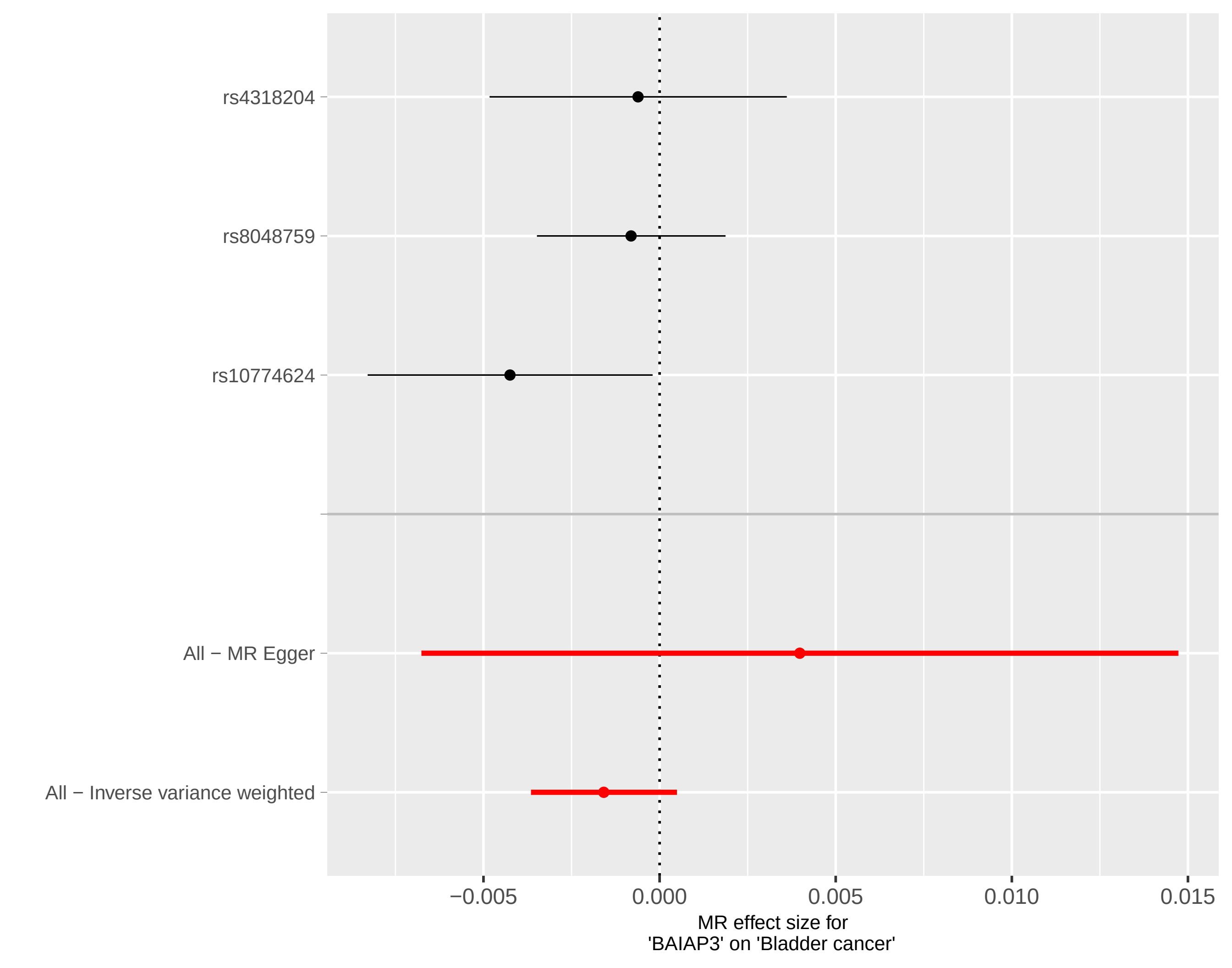
rs4318204
rs8048759
rs10774624
All − MR Egger
All − Inverse variance weighted
−0.005
0.000
0.005
0.010
0.015
MR effect size for
'BAIAP3' on 'Bladder cancer'

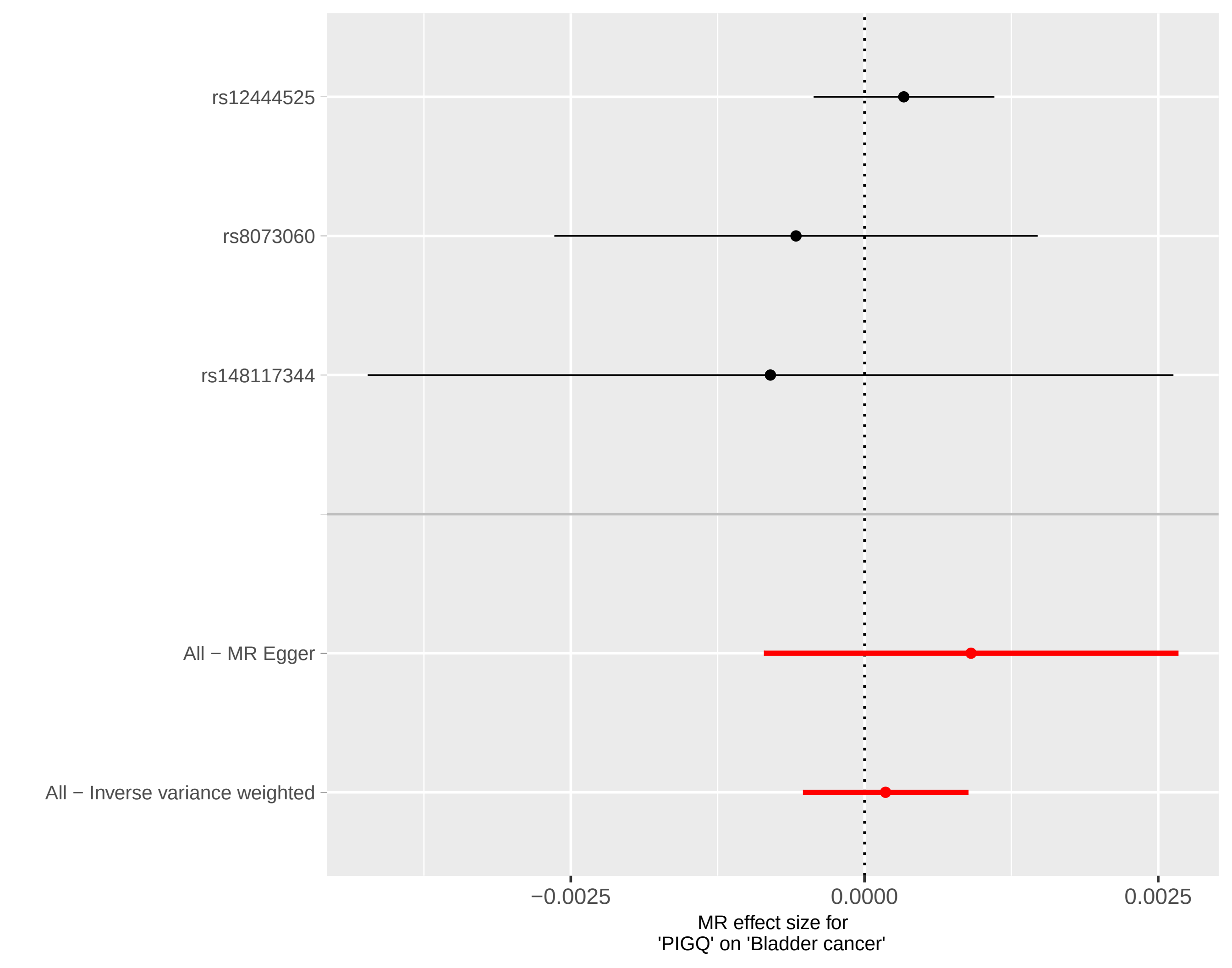

rs12444525
rs8073060
rs148117344
All − MR Egger
All − Inverse variance weighted
−0.0025
0.0000
0.0025
MR effect size for
'PIGQ' on 'Bladder cancer'

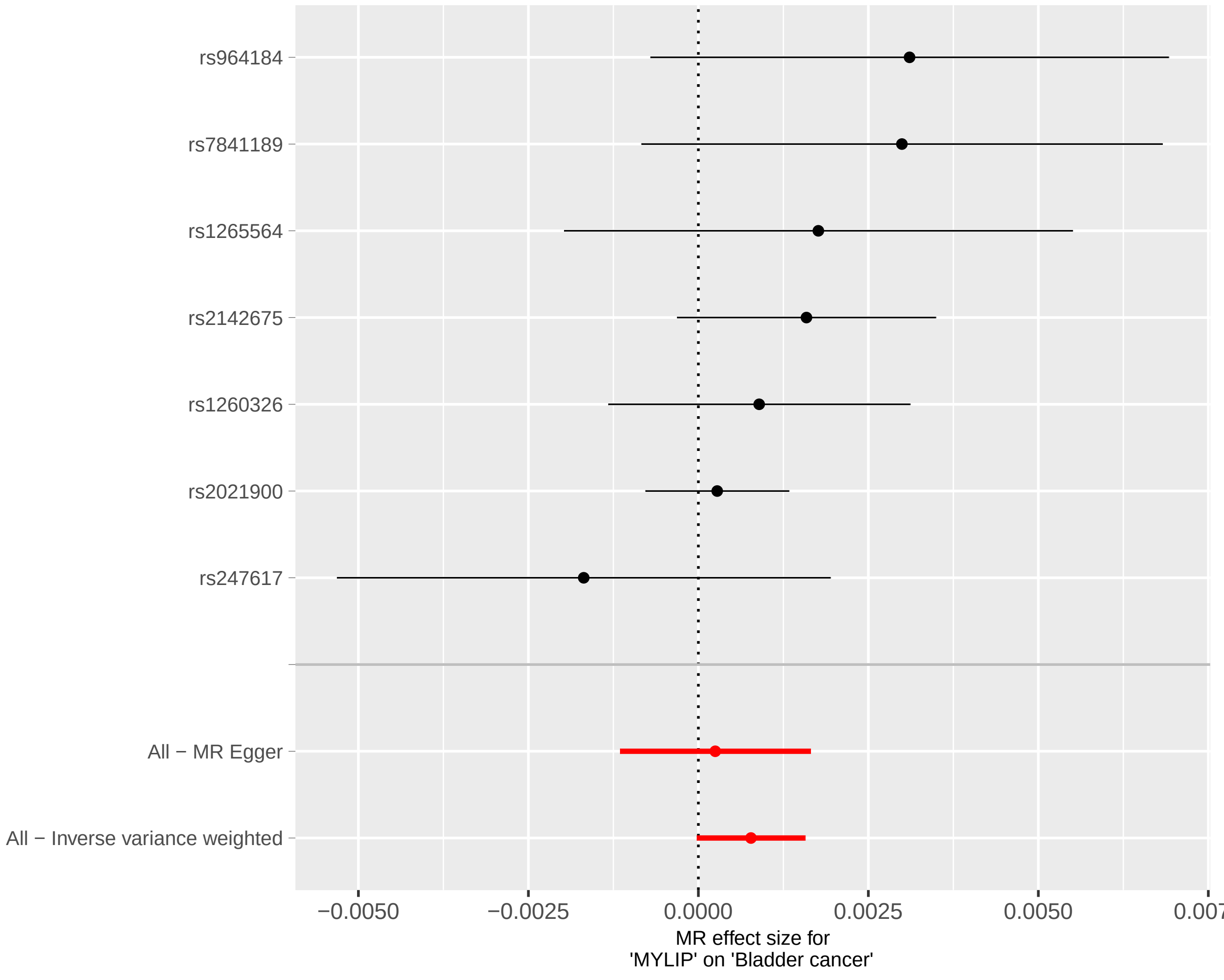

rs964184
rs7841189
rs1265564
rs2142675
rs1260326
rs2021900
rs247617
All − MR Egger
All − Inverse variance weighted
−0.0050
−0.0025
0.0000
0.0025
0.0050
MR effect size for
'MYLIP' on 'Bladder cancer'

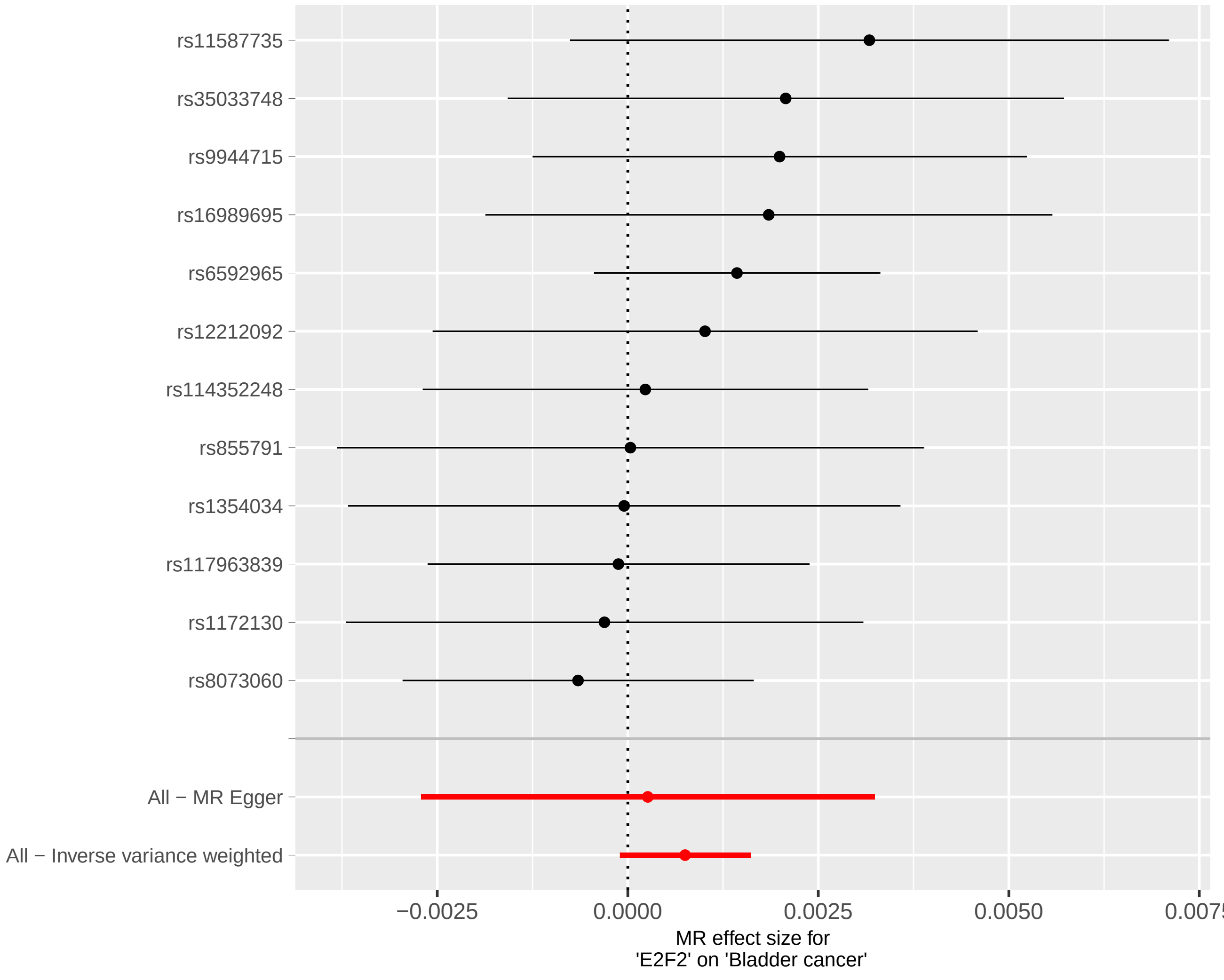

rs11587735
rs35033748
rs9944715
rs16989695
rs6592965
rs12212092
rs114352248
rs855791
rs1354034
rs117963839
rs1172130
rs8073060
All − MR Egger
All − Inverse variance weighted
−0.0025
0.0000
0.0025
0.0050
MR effect size for
'E2F2' on 'Bladder cancer'

Insufficient number of SNPs

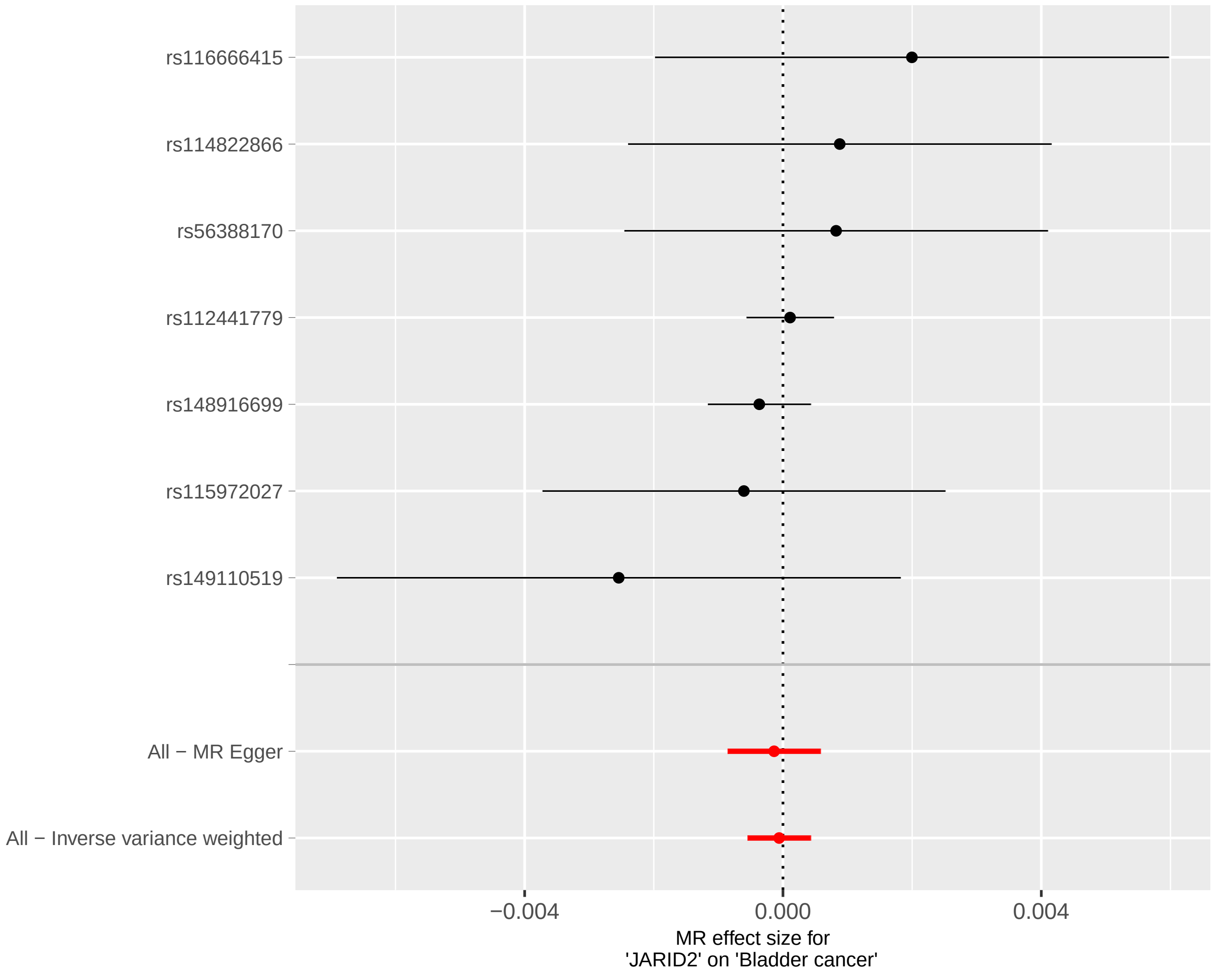
rs116666415
rs114822866
rs56388170
rs112441779
rs148916699
rs115972027
rs149110519
All − MR Egger
All − Inverse variance weighted
−0.004
0.000
0.004
MR effect size for
'JARID2' on 'Bladder cancer'

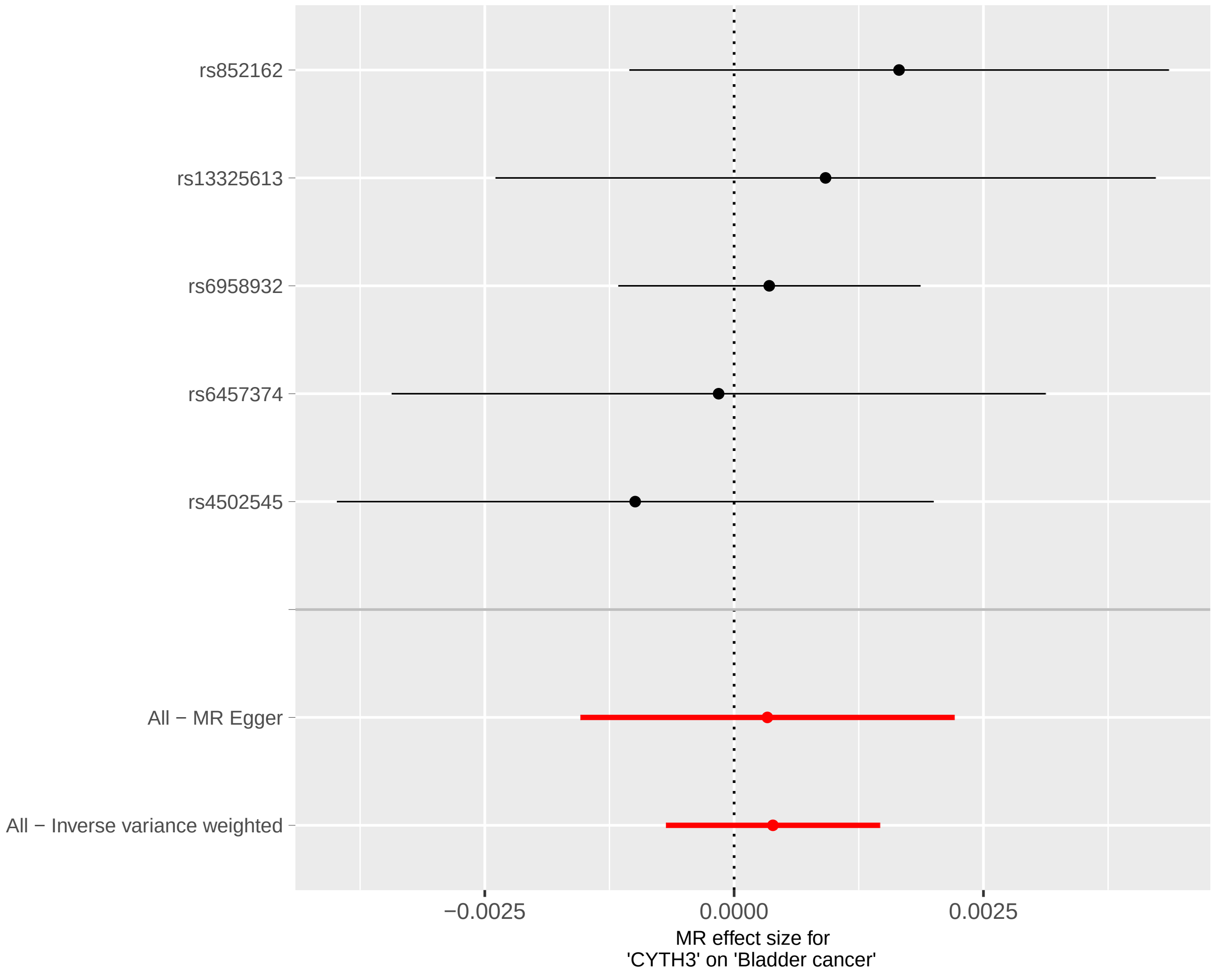
rs852162
rs13325613
rs6958932
rs6457374
rs4502545
All − MR Egger
All − Inverse variance weighted
−0.0025
0.0000
0.0025
MR effect size for
'CYTH3' on 'Bladder cancer'

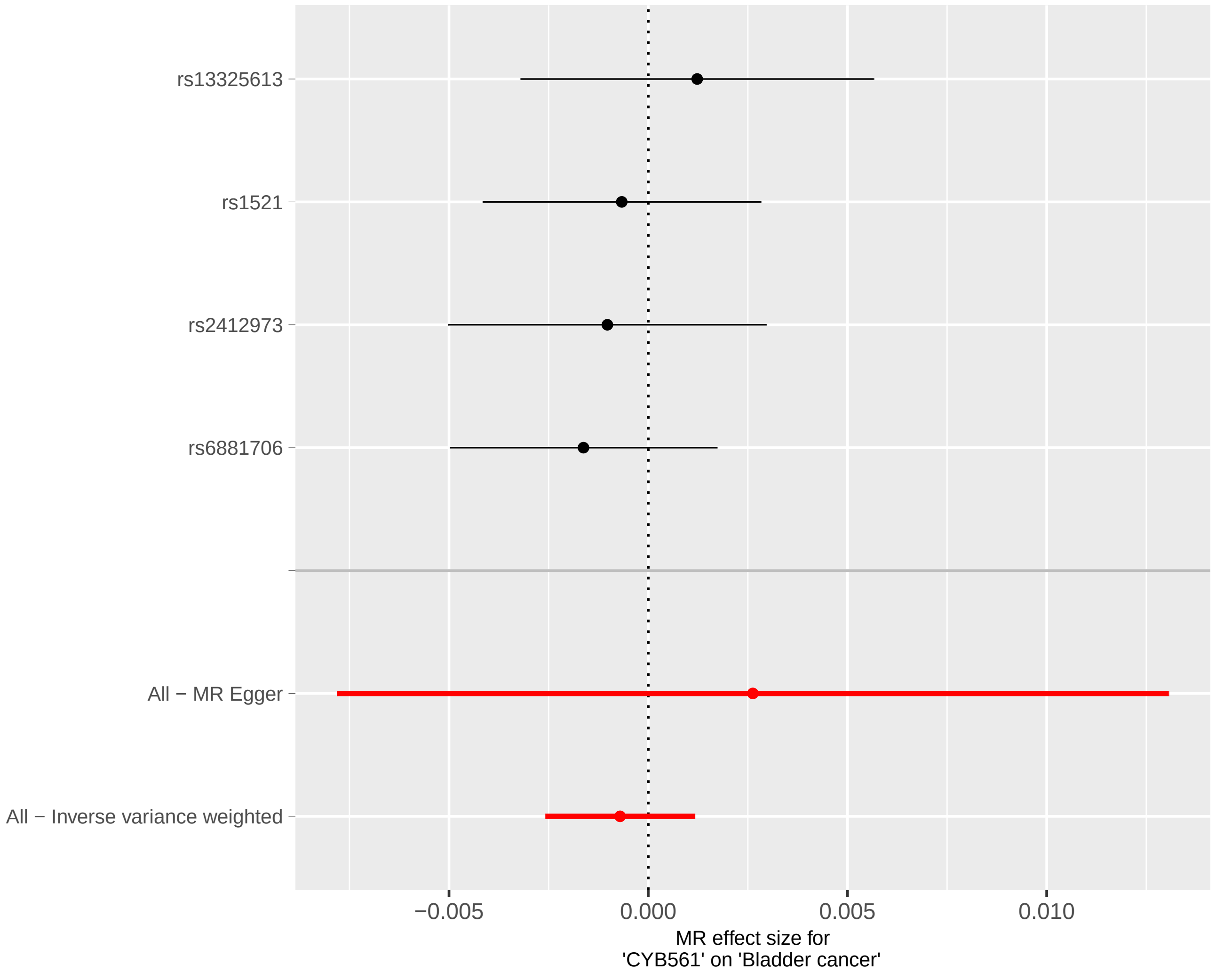

rs13325613
rs1521
rs2412973
rs6881706
All − MR Egger
All − Inverse variance weighted
−0.005
0.000
0.005
0.010
MR effect size for
'CYB561' on 'Bladder cancer'

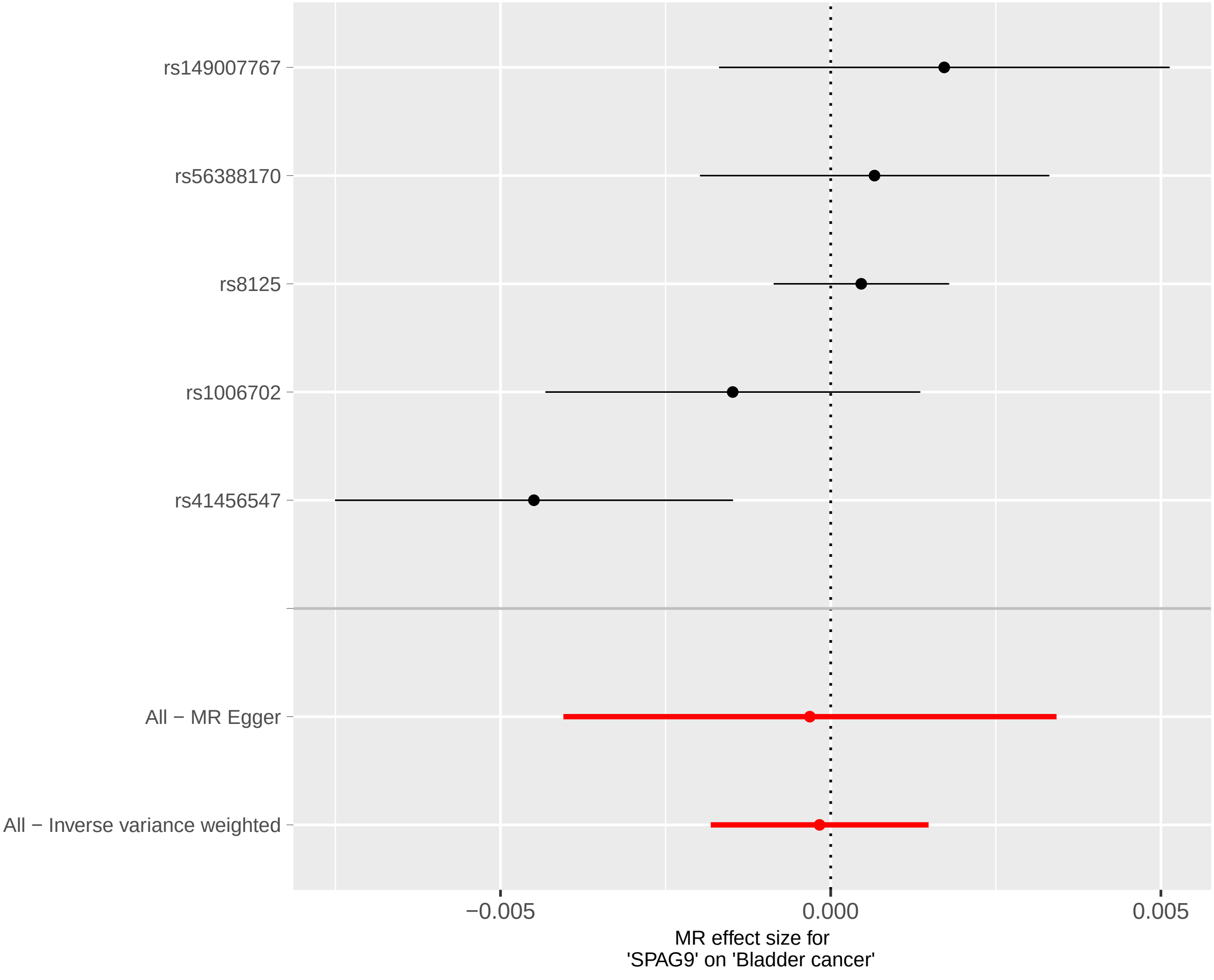
rs149007767
rs56388170
rs8125
rs1006702
rs41456547
All − MR Egger
All − Inverse variance weighted
−0.005
0.000
0.005
MR effect size for
'SPAG9' on 'Bladder cancer'

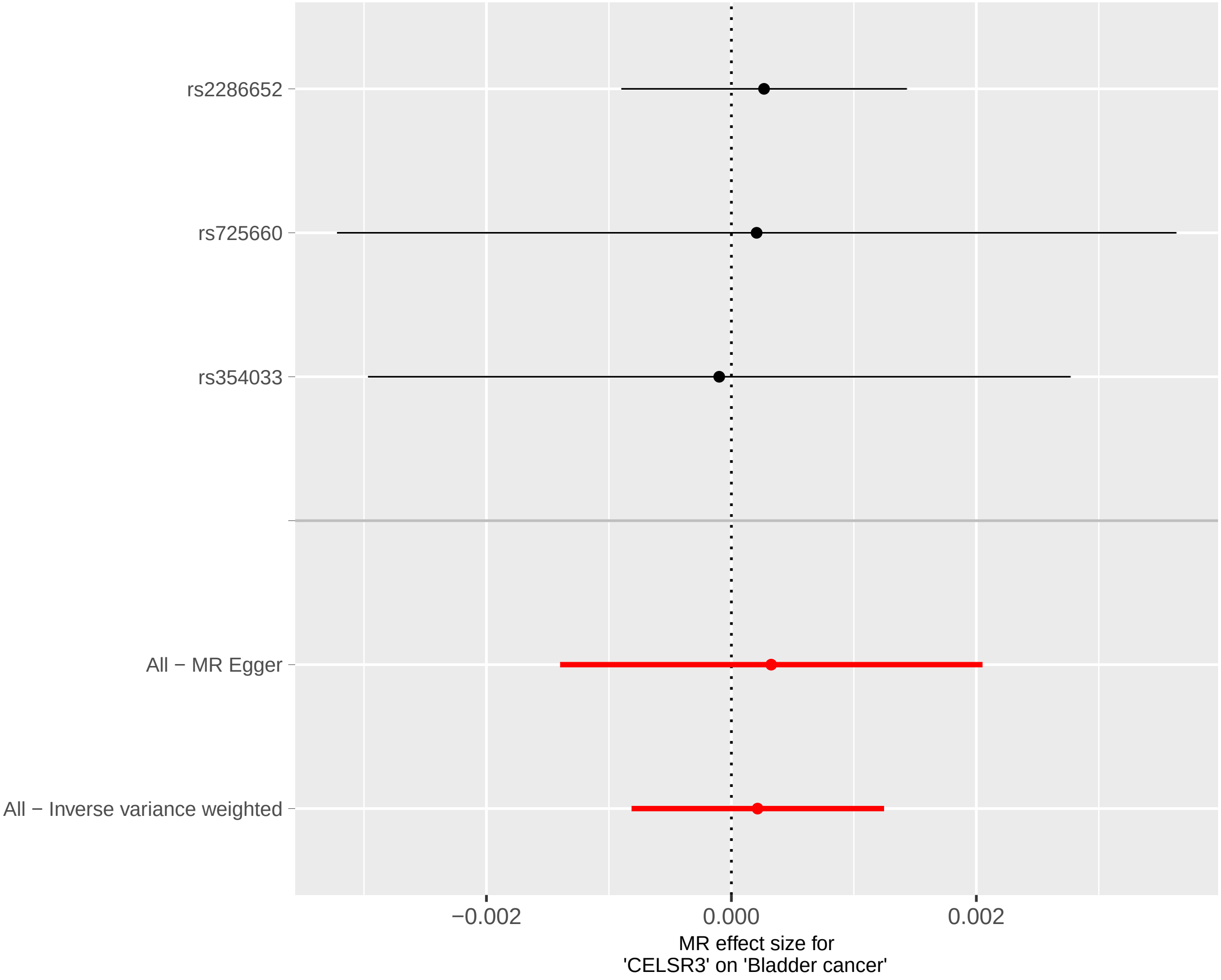
rs2286652
rs725660
rs354033
All − MR Egger
All − Inverse variance weighted
−0.002
0.000
0.002
MR effect size for
'CELSR3' on 'Bladder cancer'

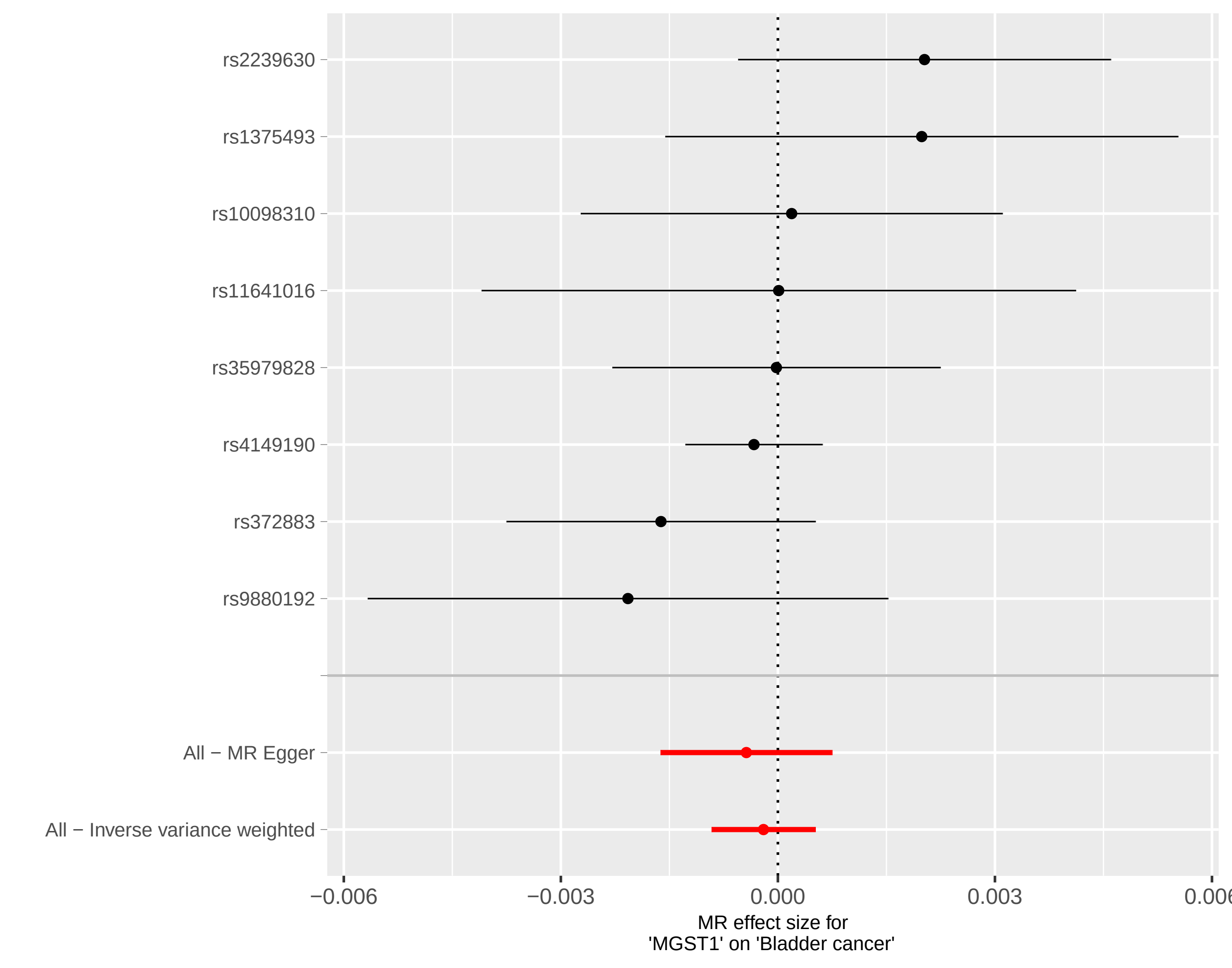

rs2239630
rs1375493
rs10098310
rs11641016
rs35979828
rs4149190
rs372883
rs9880192
All − MR Egger
All − Inverse variance weighted
−0.006
−0.003
0.000
0.003
MR effect size for
'MGST1' on 'Bladder cancer'

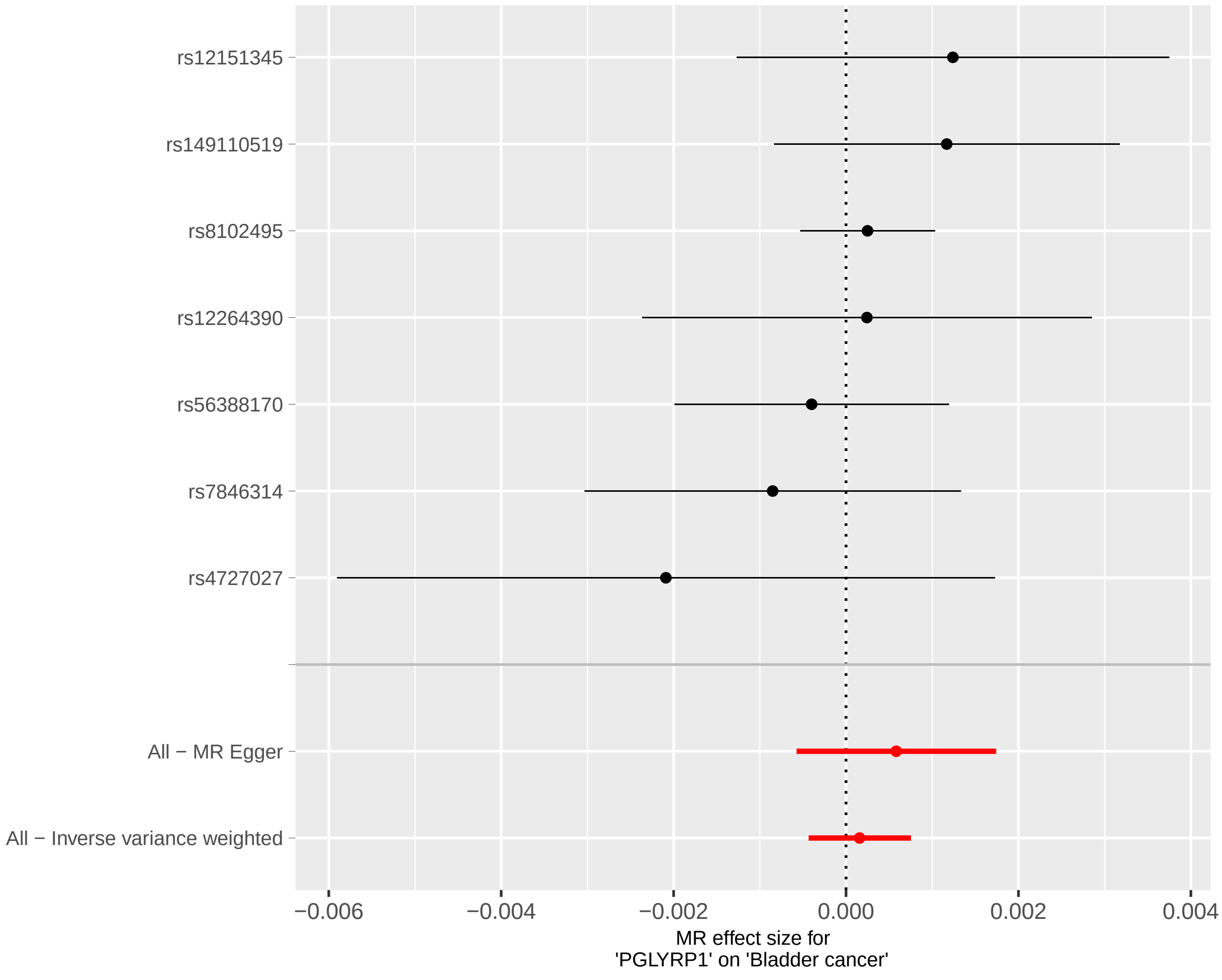
rs12151345
rs149110519
rs8102495
rs12264390
rs56388170
rs7846314
rs4727027
All − MR Egger
All − Inverse variance weighted
−0.006
−0.004
−0.002
0.000
0.002
0.004
MR effect size for
'PGLYRP1' on 'Bladder cancer'

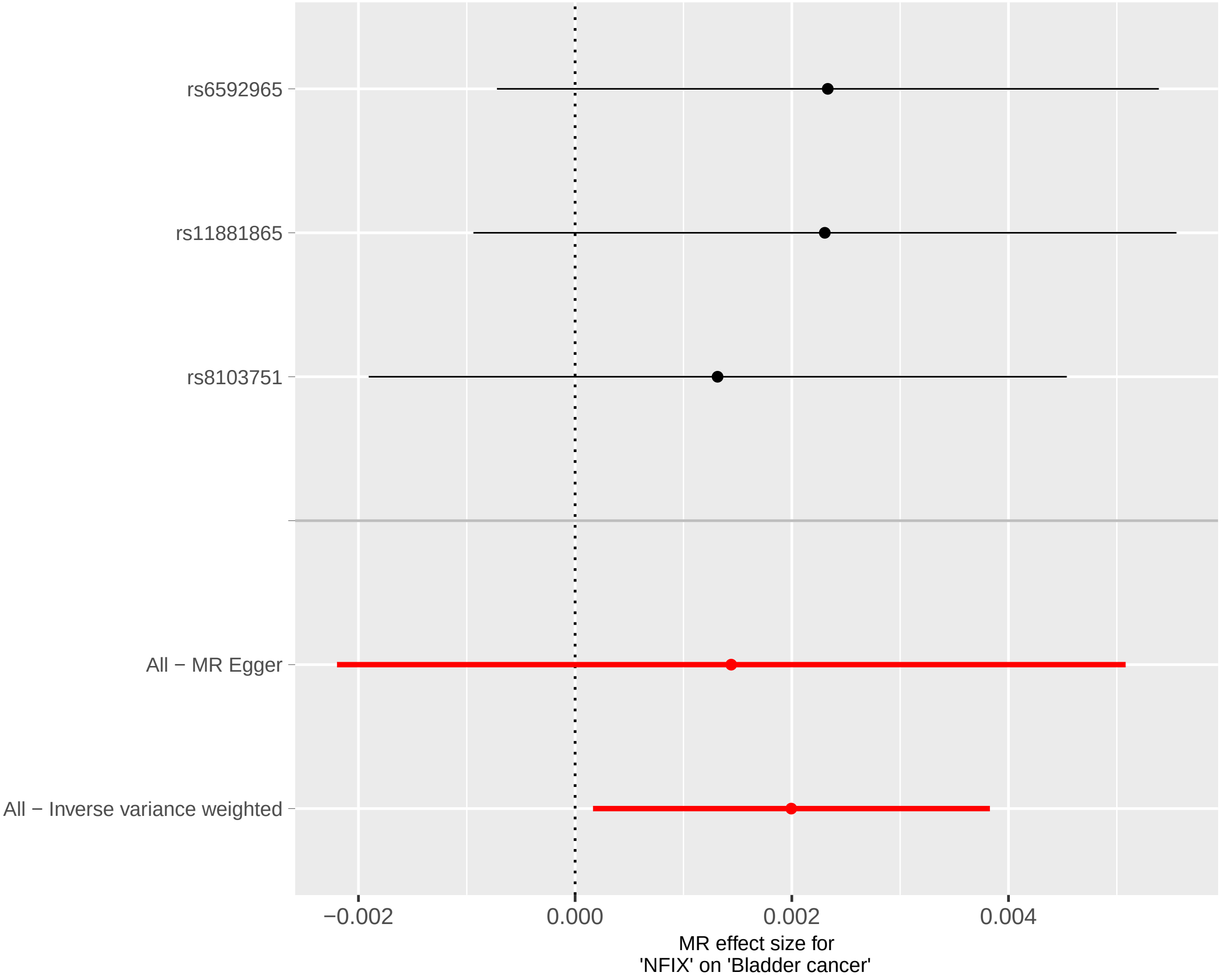
rs6592965
rs11881865
rs8103751
All − MR Egger
All − Inverse variance weighted
−0.002
0.000
0.002
0.004
MR effect size for
'NFIX' on 'Bladder cancer'

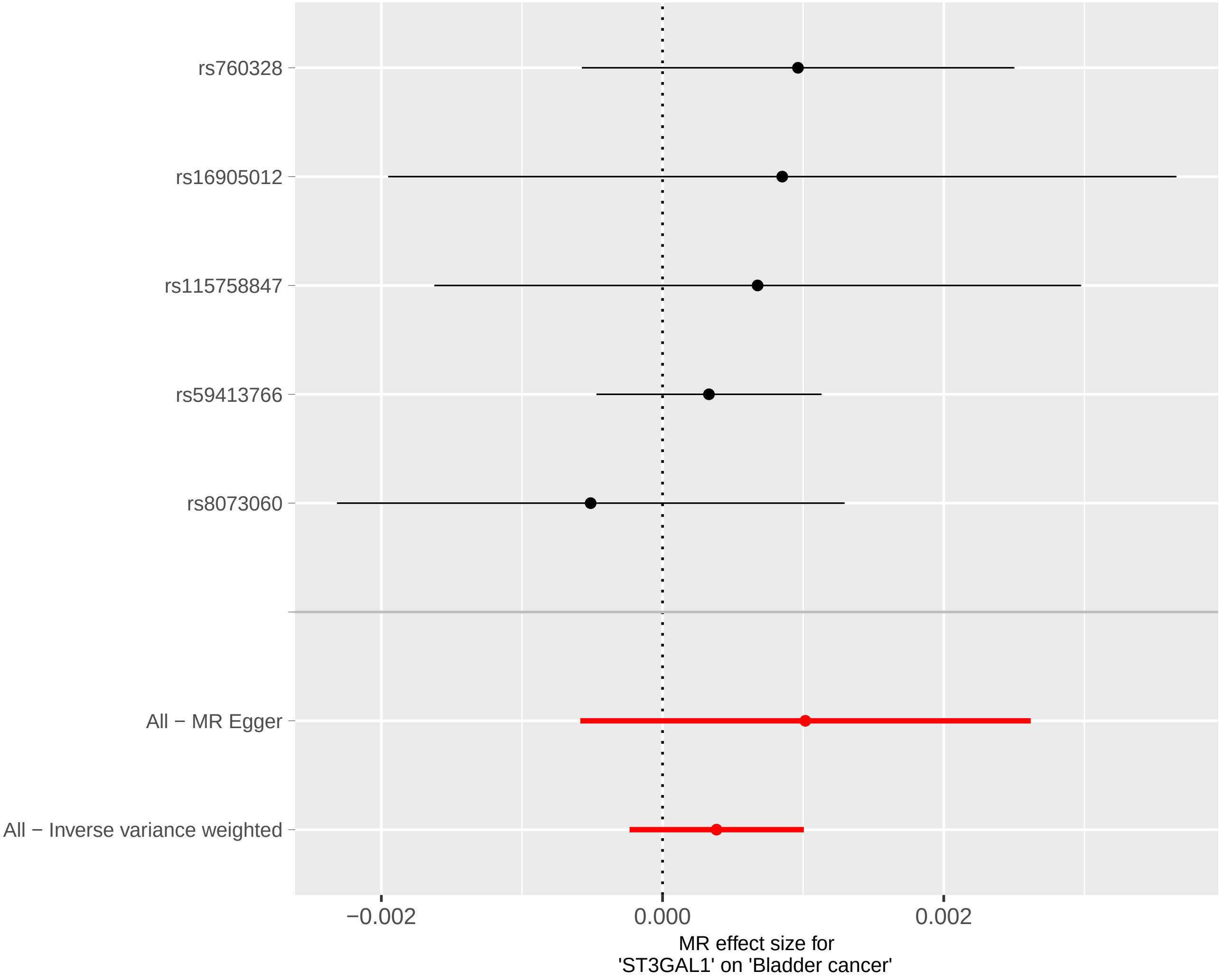
rs760328
rs16905012
rs115758847
rs59413766
rs8073060
All − MR Egger
All − Inverse variance weighted
−0.002
0.000
0.002
MR effect size for
'ST3GAL1' on 'Bladder cancer'

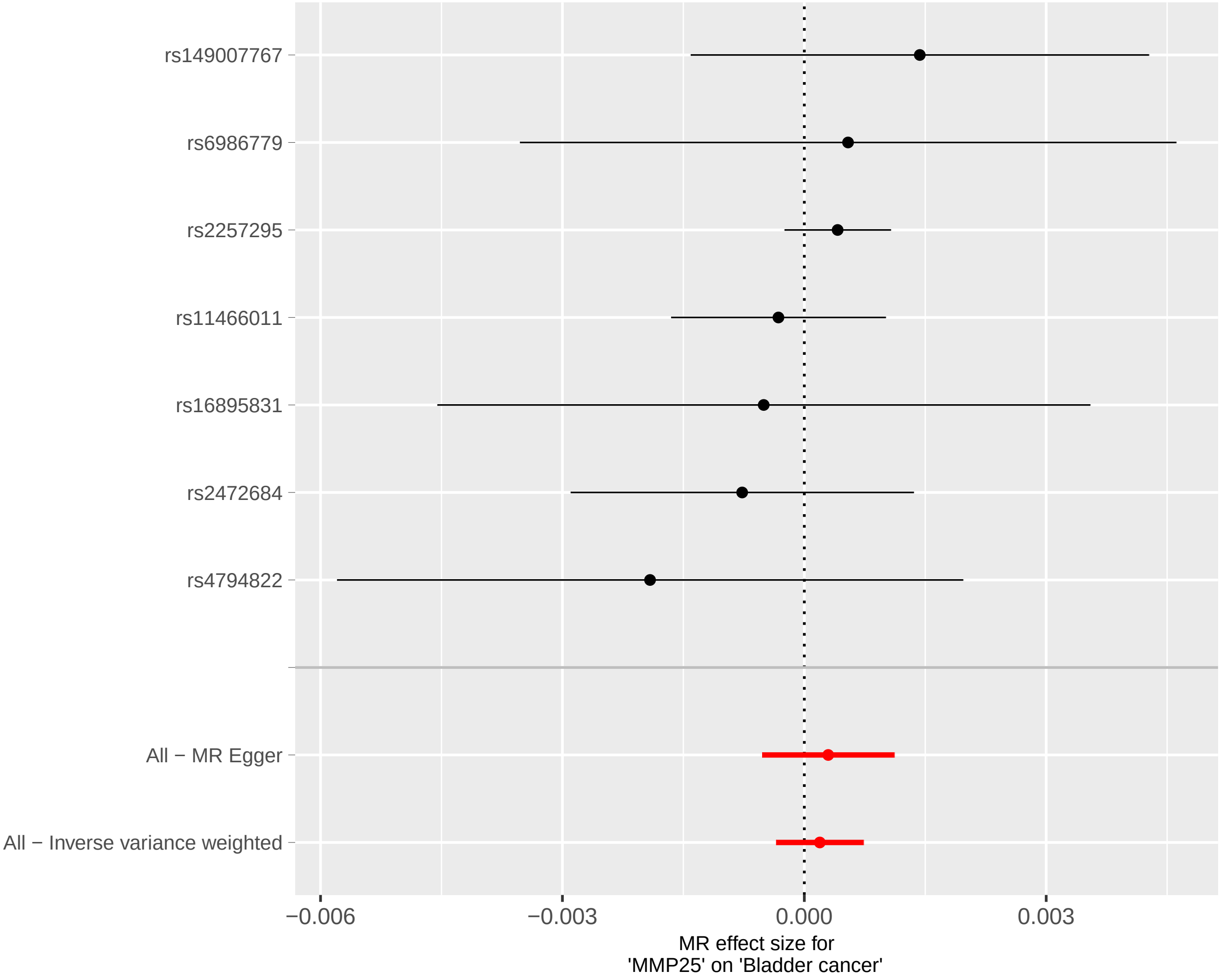
rs149007767
rs6986779
rs2257295
rs11466011
rs16895831
rs2472684
rs4794822
All − MR Egger
All − Inverse variance weighted
−0.006
−0.003
0.000
0.003
MR effect size for
'MMP25' on 'Bladder cancer'

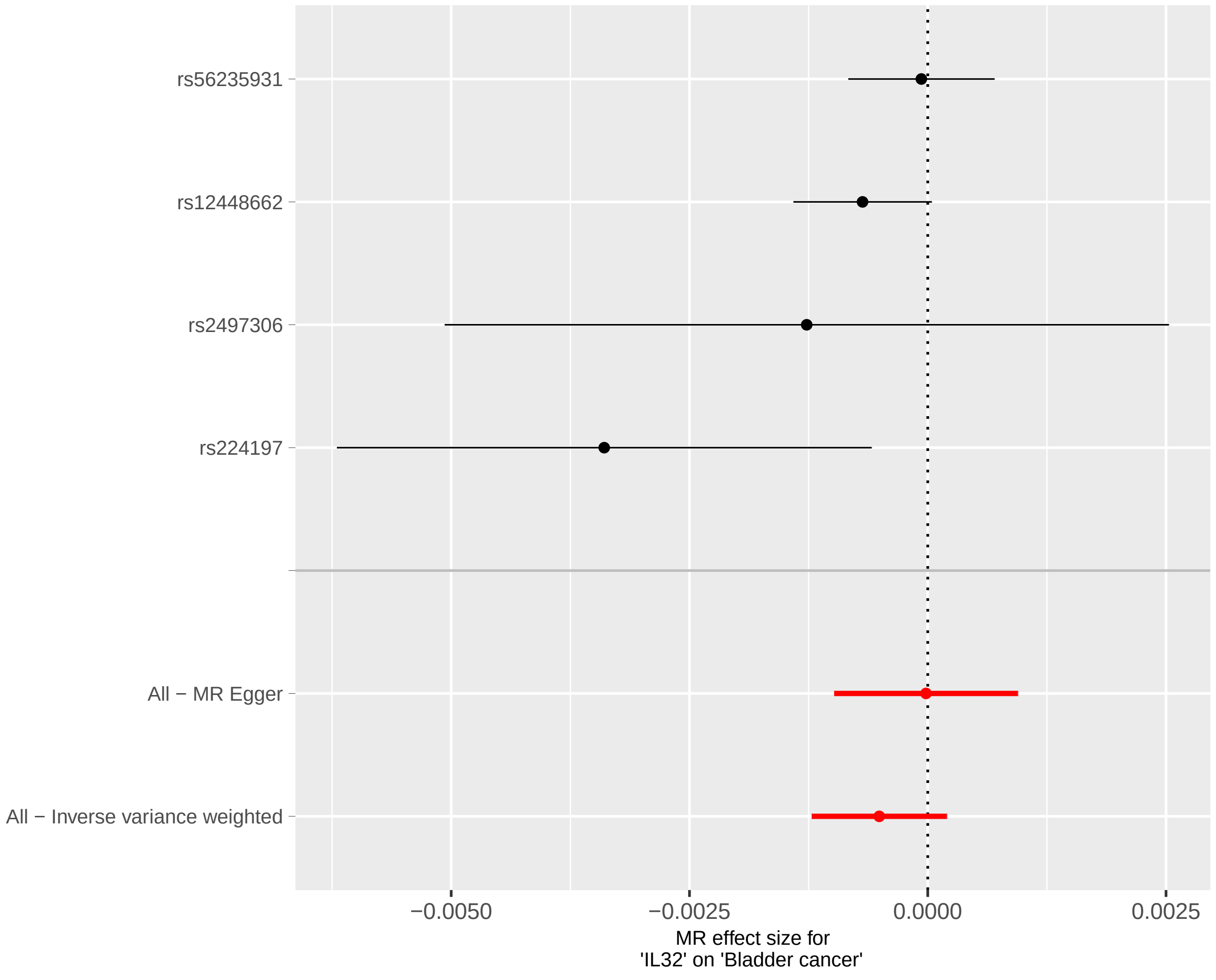
rs56235931
rs12448662
rs2497306
rs224197
All − MR Egger
All − Inverse variance weighted
−0.0050
−0.0025
0.0000
0.0025
MR effect size for
'IL32' on 'Bladder cancer'

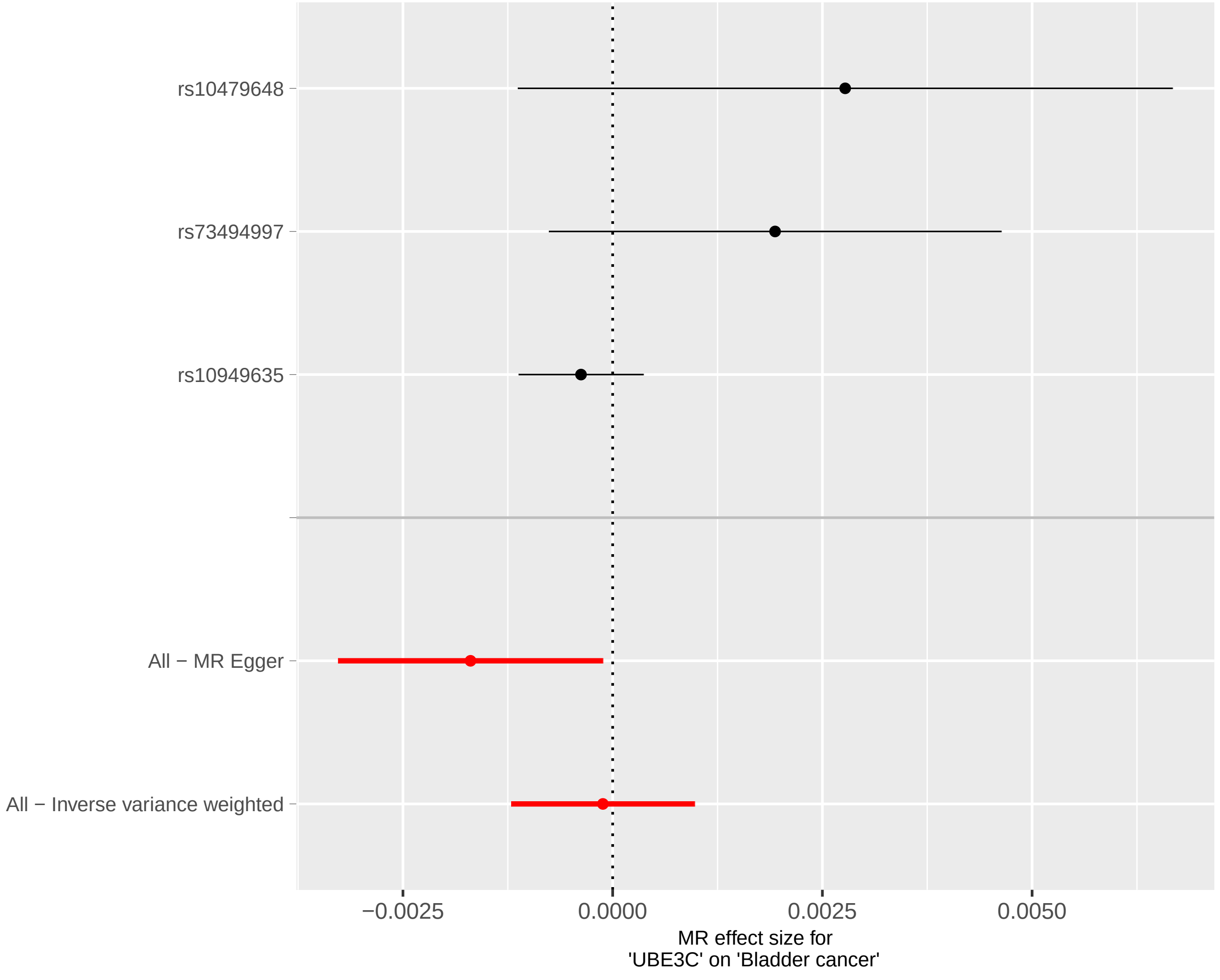

rs10479648
rs73494997
rs10949635
All − MR Egger
All − Inverse variance weighted
−0.0025
0.0000
0.0025
0.0050
MR effect size for
'UBE3C' on 'Bladder cancer'

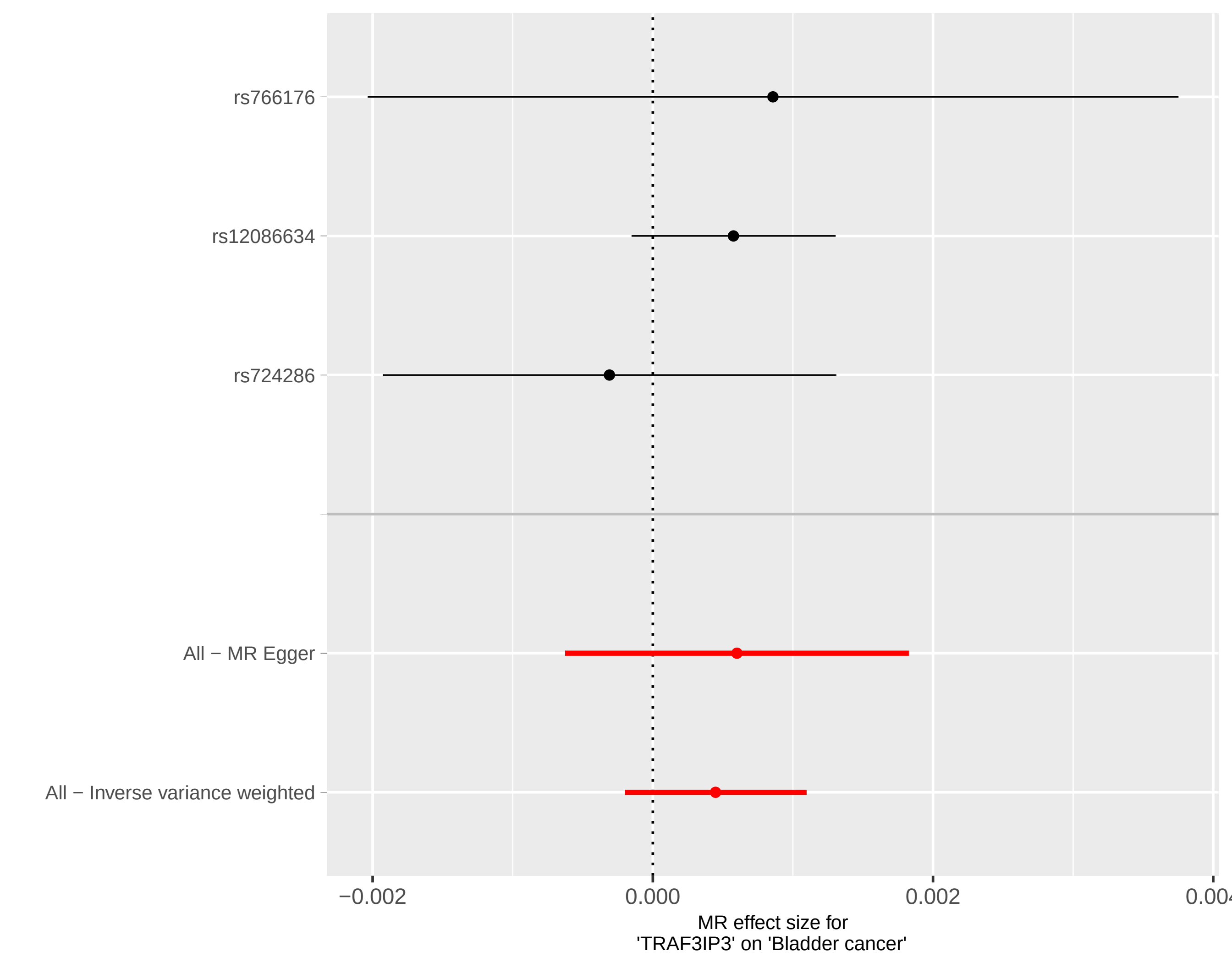
rs766176
rs12086634
rs724286
All − MR Egger
All − Inverse variance weighted
−0.002
0.000
0.002
0.004
MR effect size for
'TRAF3IP3' on 'Bladder cancer'

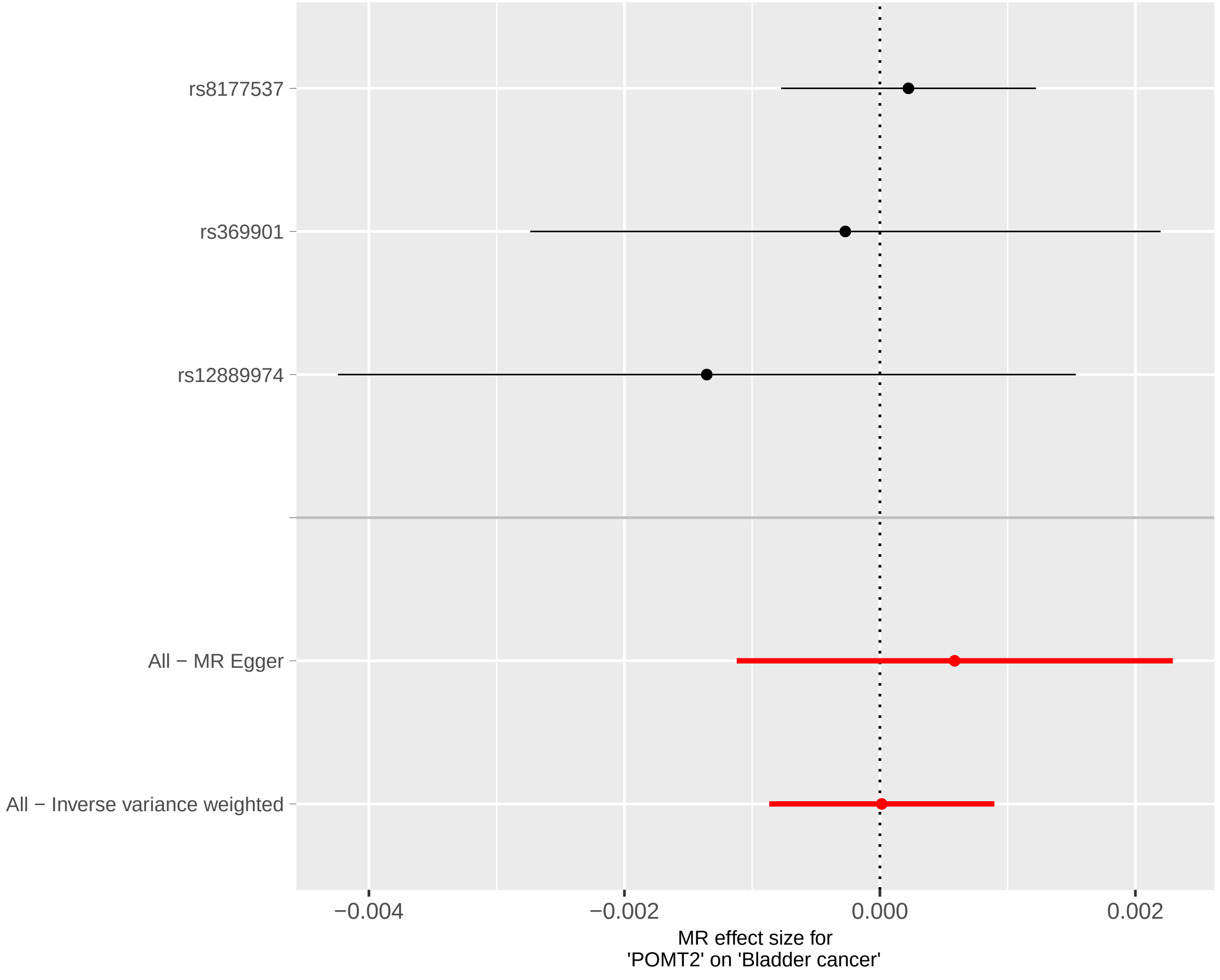
rs8177537
rs369901
rs12889974
All − MR Egger
All − Inverse variance weighted
−0.004
−0.002
0.000
0.002
MR effect size for
'POMT2' on 'Bladder cancer'

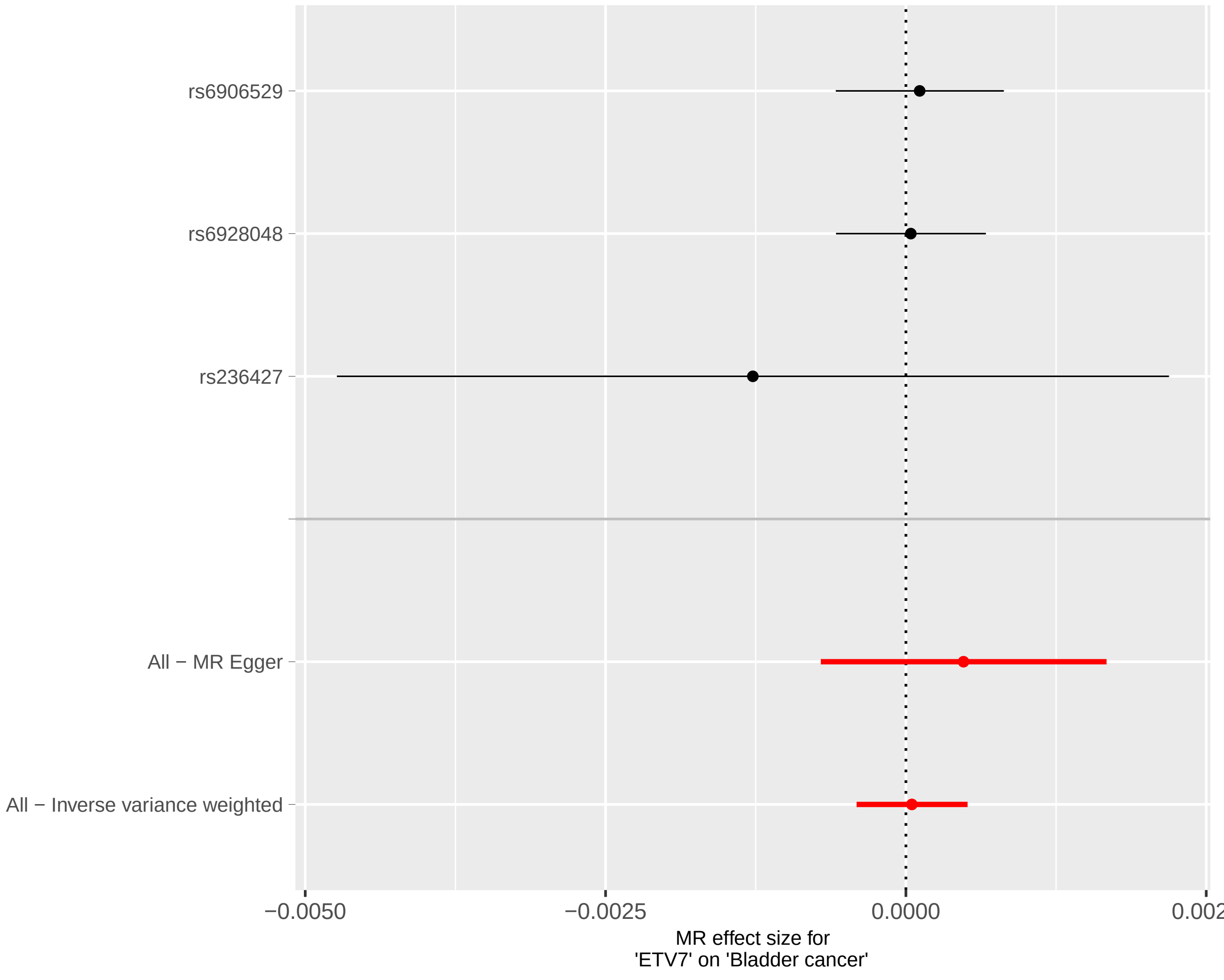
rs6906529
rs6928048
rs236427
All − MR Egger
All − Inverse variance weighted
−0.0050
−0.0025
0.0000
0.002
MR effect size for
'ETV7' on 'Bladder cancer'

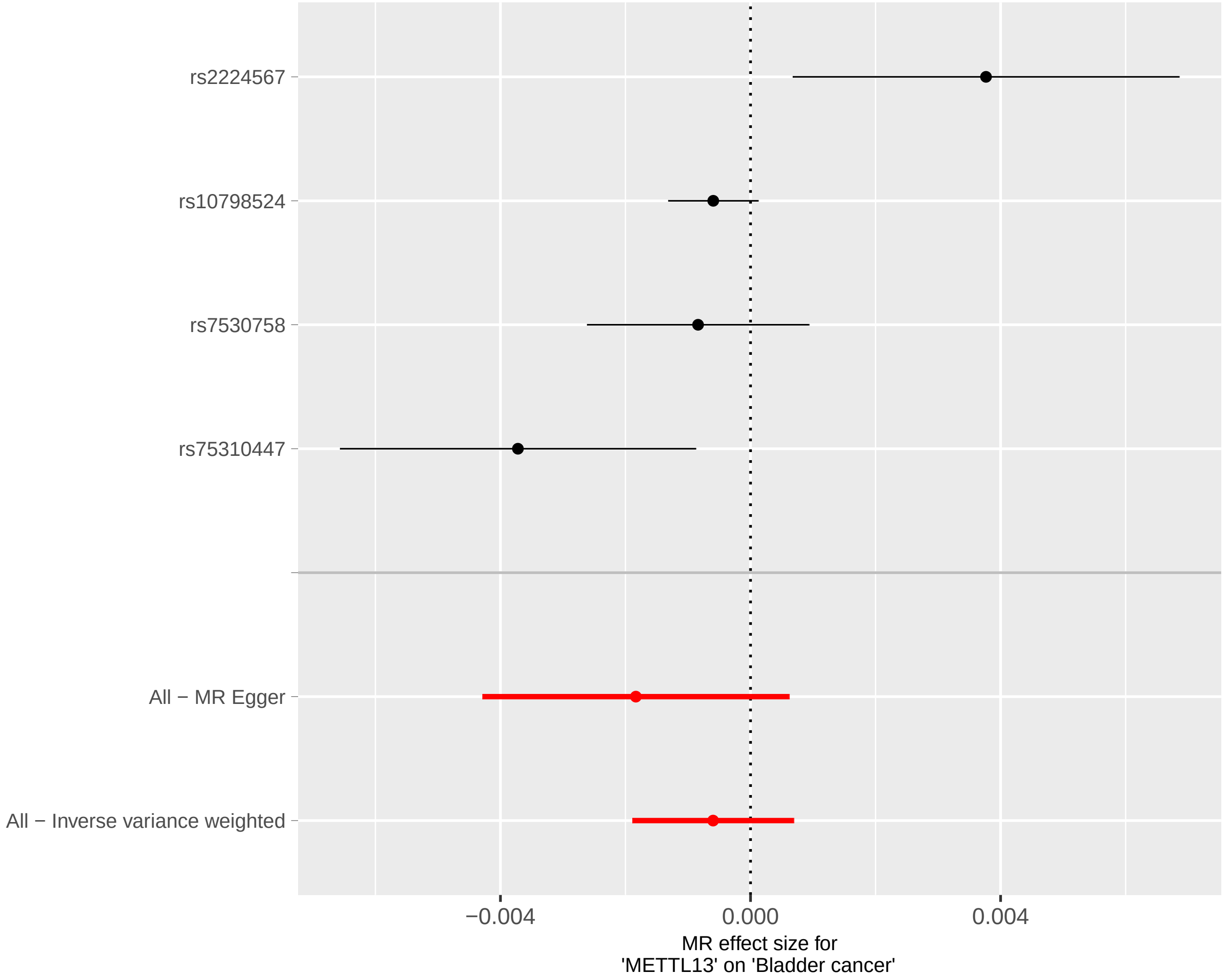

rs2224567
rs10798524
rs7530758
rs75310447
All − MR Egger
All − Inverse variance weighted
−0.004
0.000
0.004
MR effect size for
'METTL13' on 'Bladder cancer'

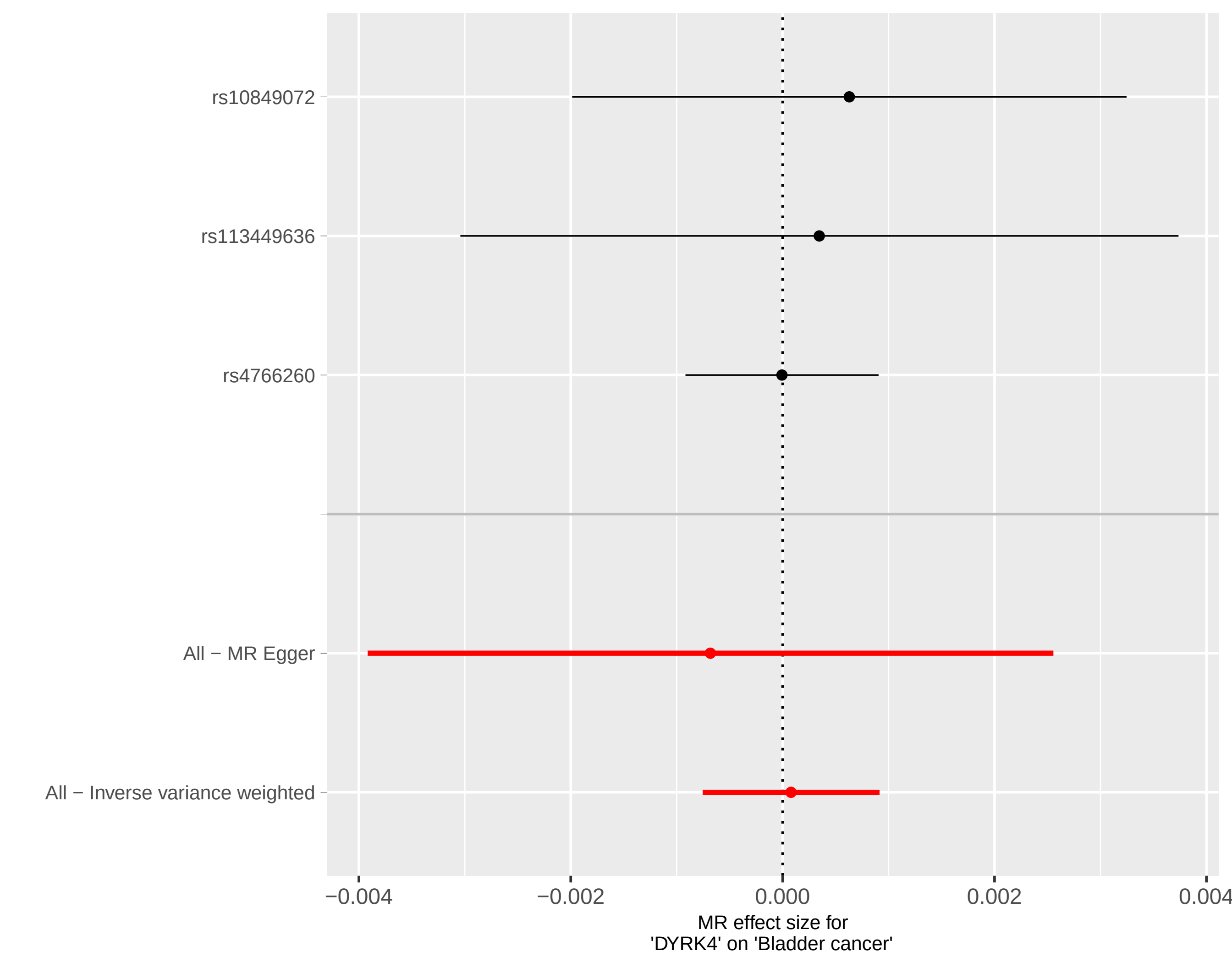

rs10849072
rs113449636
rs4766260
All − MR Egger
All − Inverse variance weighted
−0.004
−0.002
0.000
0.002
0.004
MR effect size for
'DYRK4' on 'Bladder cancer'

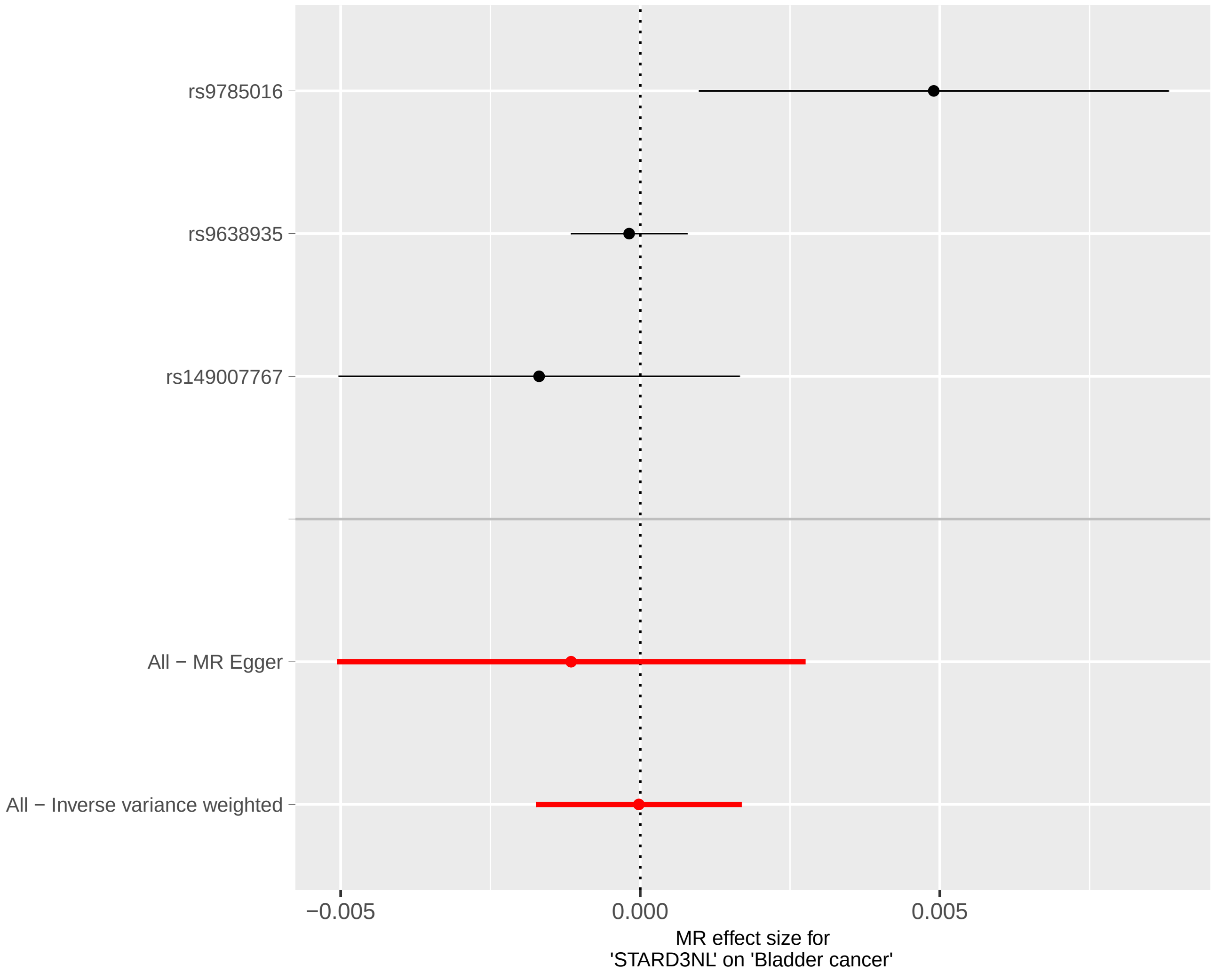
rs9785016
rs9638935
rs149007767
All − MR Egger
All − Inverse variance weighted
−0.005
0.000
0.005
MR effect size for
'STARD3NL' on 'Bladder cancer'

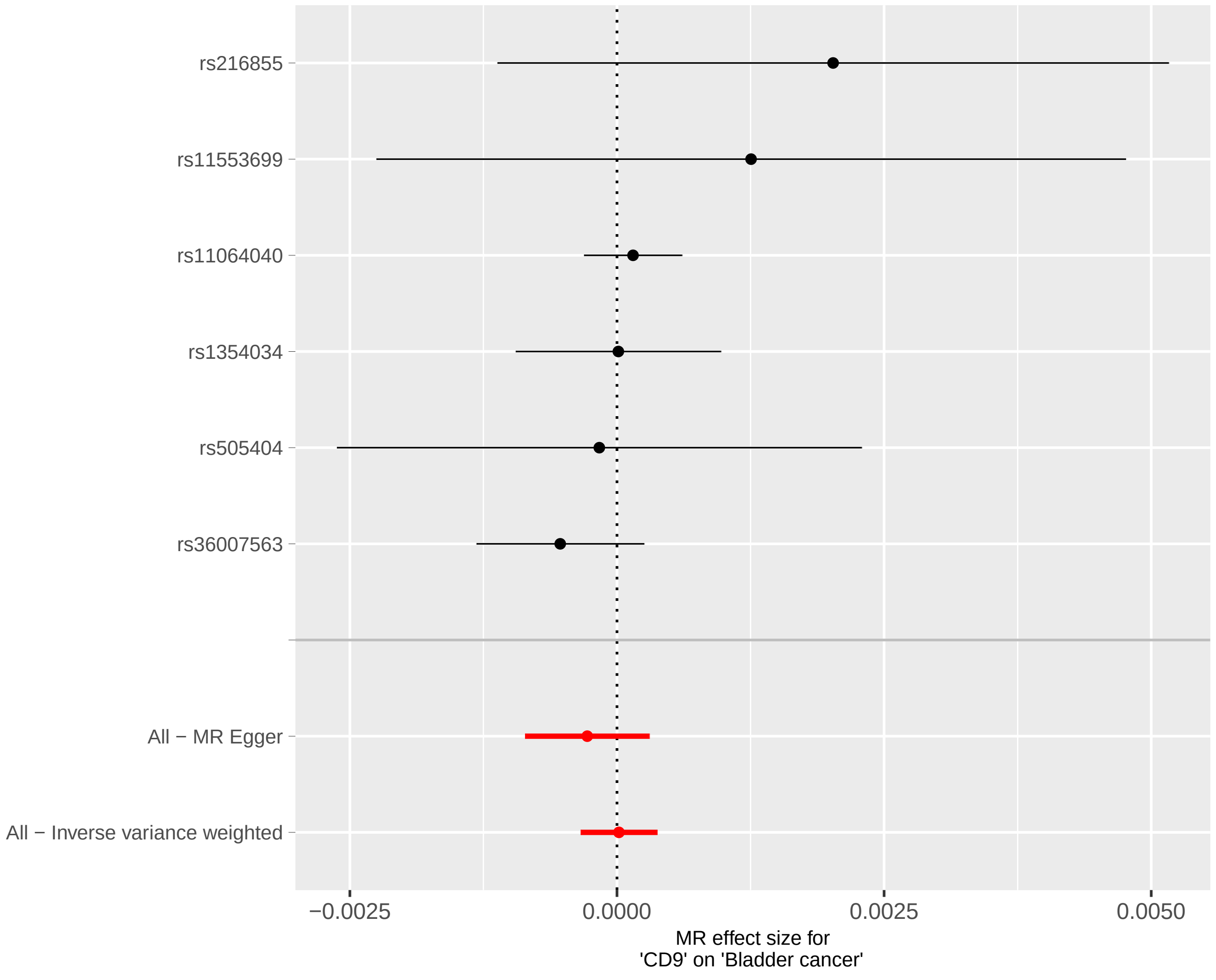
rs216855
rs11553699
rs11064040
rs1354034
rs505404
rs36007563
All − MR Egger
All − Inverse variance weighted
−0.0025
0.0000
0.0025
0.0050
MR effect size for
'CD9' on 'Bladder cancer'

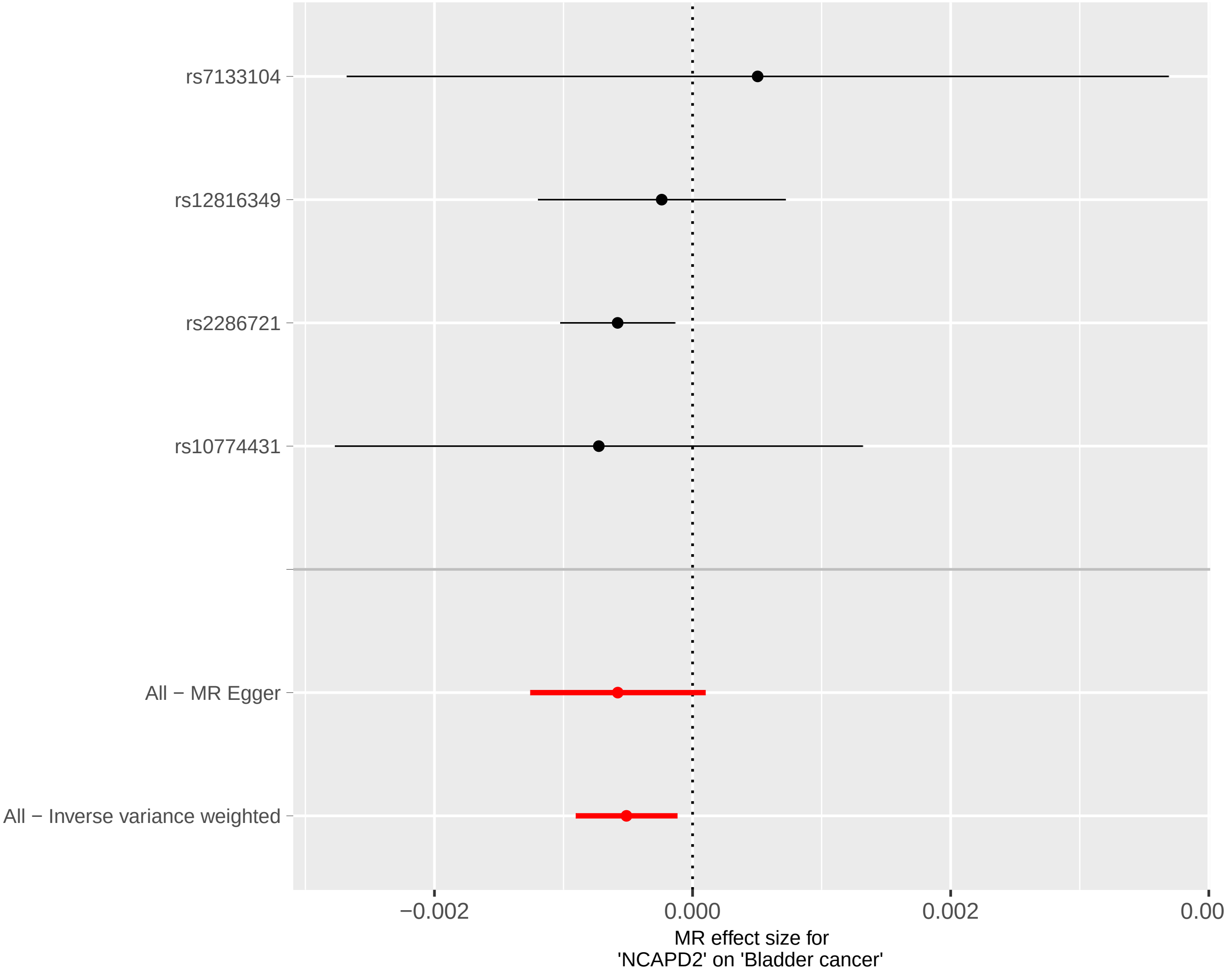
rs7133104
rs12816349
rs2286721
rs10774431
All − MR Egger
All − Inverse variance weighted
−0.002
0.000
0.002
0.00
MR effect size for
'NCAPD2' on 'Bladder cancer'

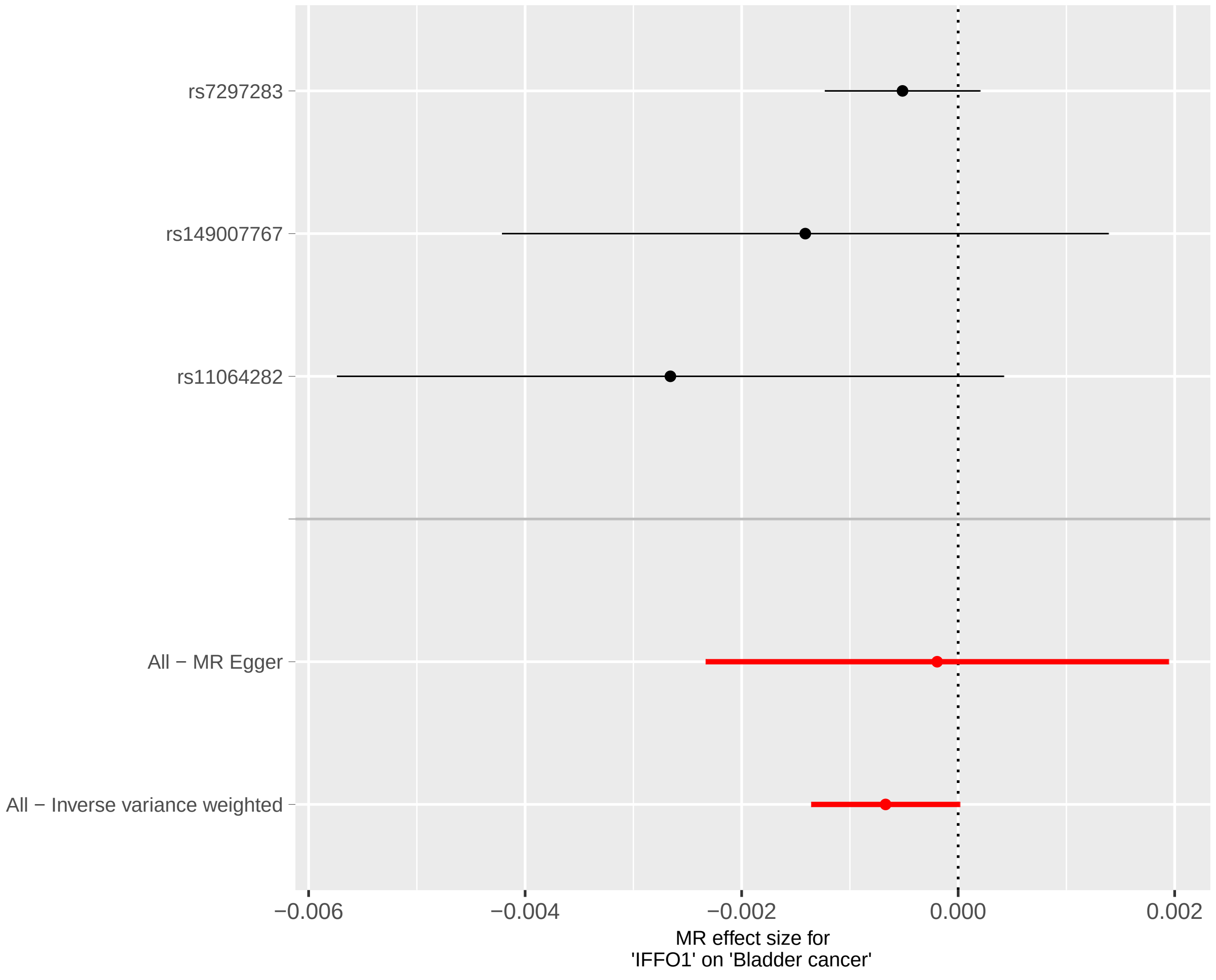

rs7297283
rs149007767
rs11064282
All − MR Egger
All − Inverse variance weighted
−0.006
−0.004
−0.002
0.000
0.002
MR effect size for
'IFFO1' on 'Bladder cancer'

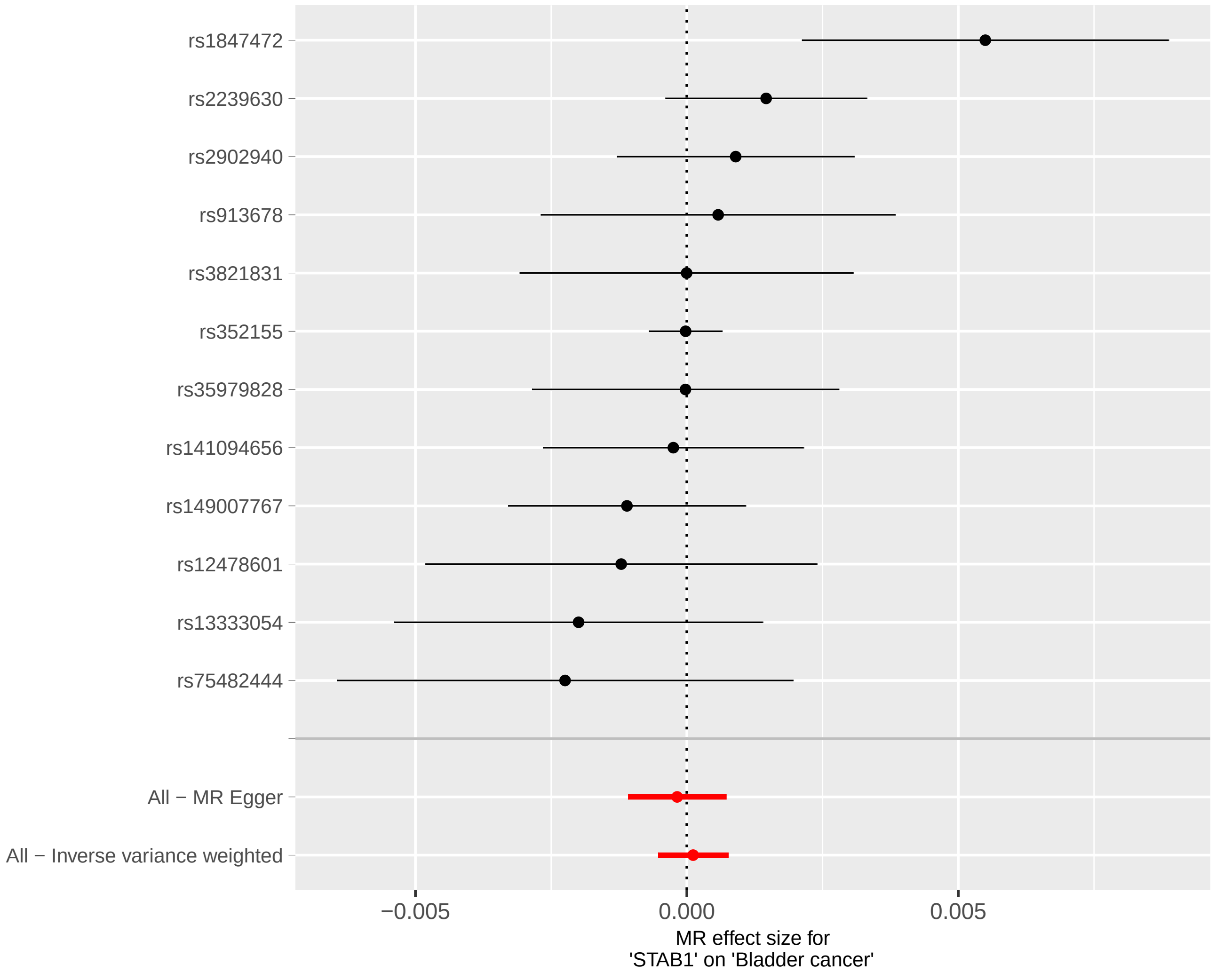
rs1847472
rs2239630
rs2902940
rs913678
rs3821831
rs352155
rs35979828
rs141094656
rs149007767
rs12478601
rs13333054
rs75482444
All − MR Egger
All − Inverse variance weighted
−0.005
0.000
0.005
MR effect size for
'STAB1' on 'Bladder cancer'

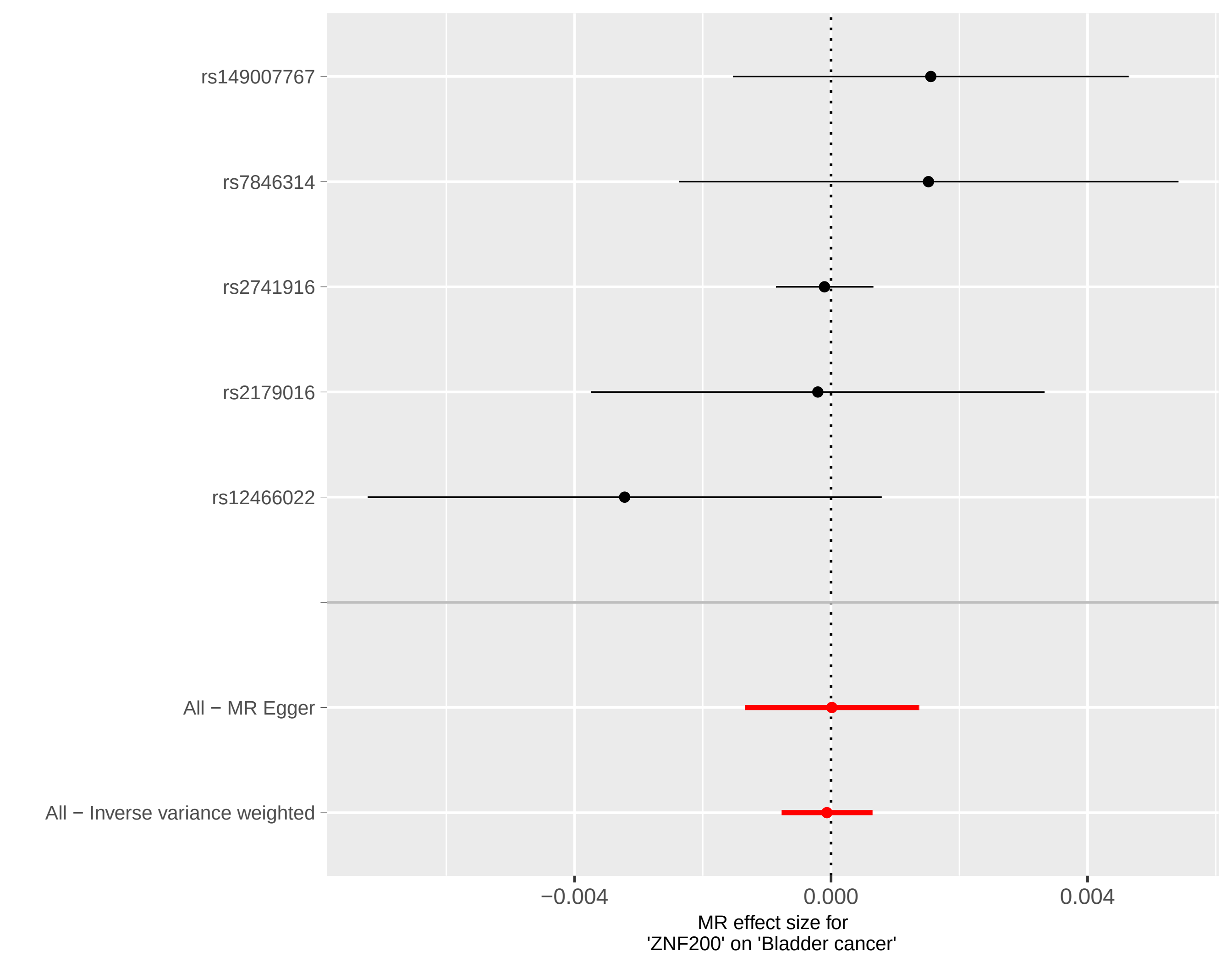
rs149007767
rs7846314
rs2741916
rs2179016
rs12466022
All − MR Egger
All − Inverse variance weighted
−0.004
0.000
0.004
MR effect size for
'ZNF200' on 'Bladder cancer'

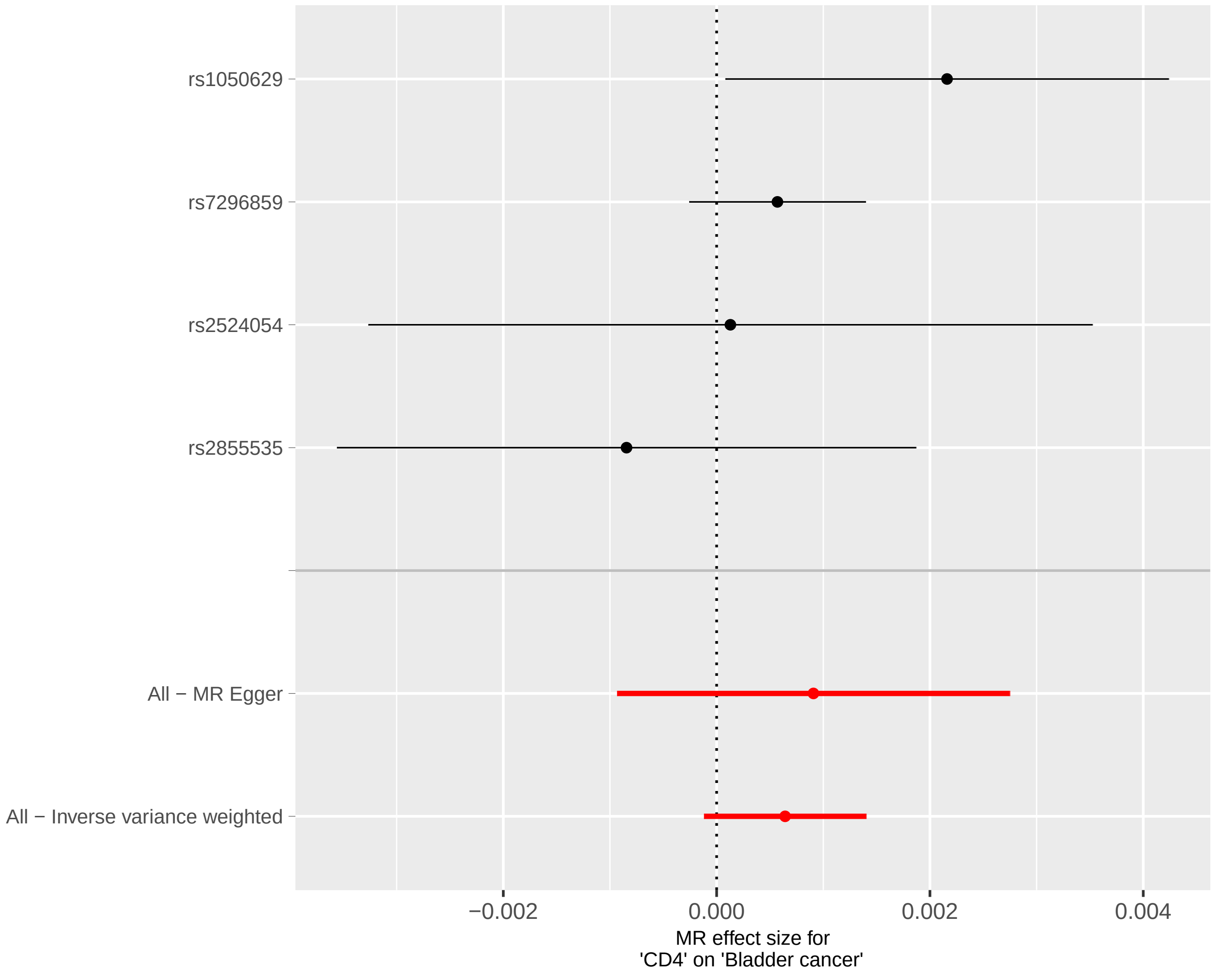
rs1050629
rs7296859
rs2524054
rs2855535
All − MR Egger
All − Inverse variance weighted
−0.002
0.000
0.002
0.004
MR effect size for
'CD4' on 'Bladder cancer'

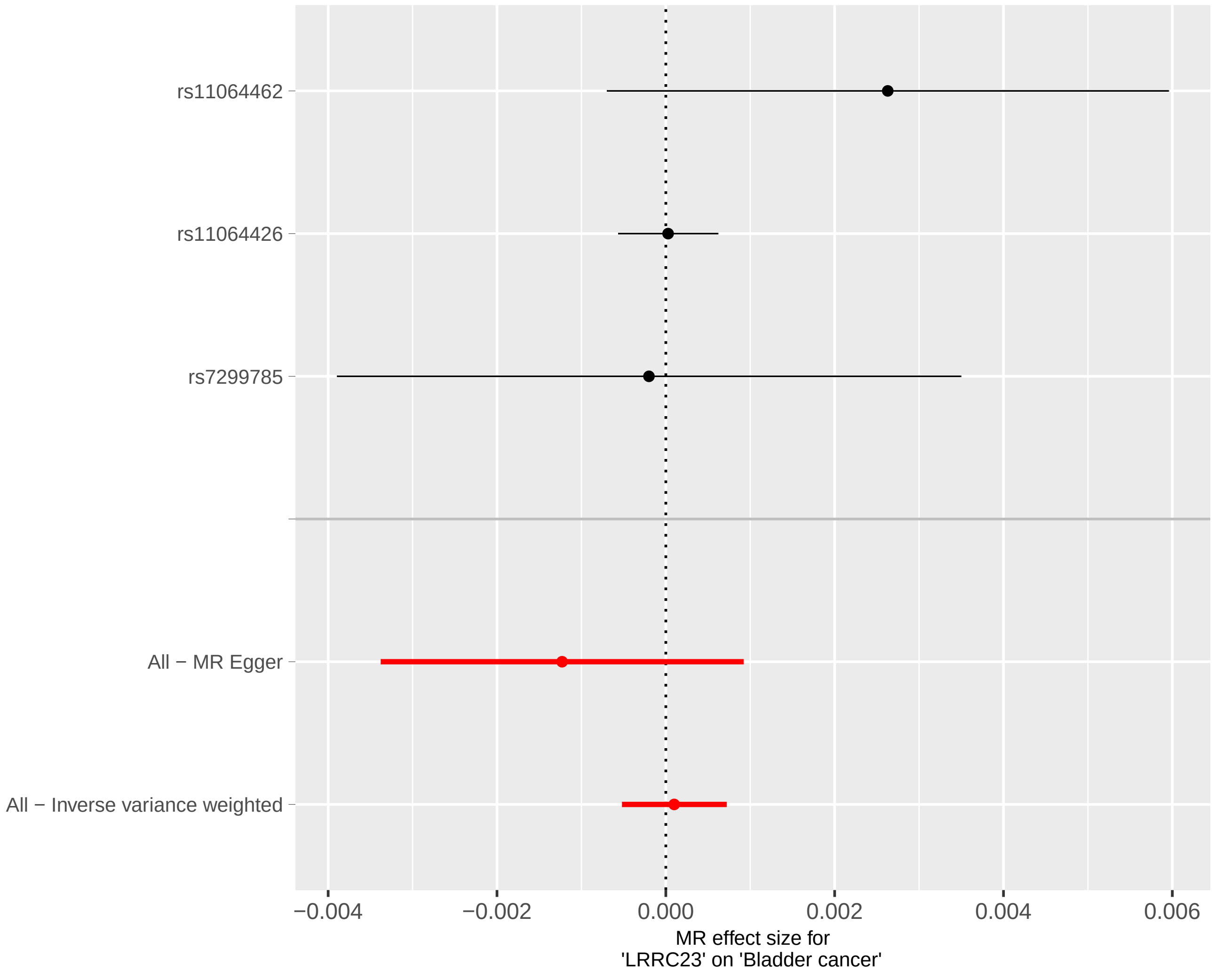

rs11064462
rs11064426
rs7299785
All − MR Egger
All − Inverse variance weighted
−0.004
−0.002
0.000
0.002
0.004
0.006
MR effect size for
'LRRC23' on 'Bladder cancer'

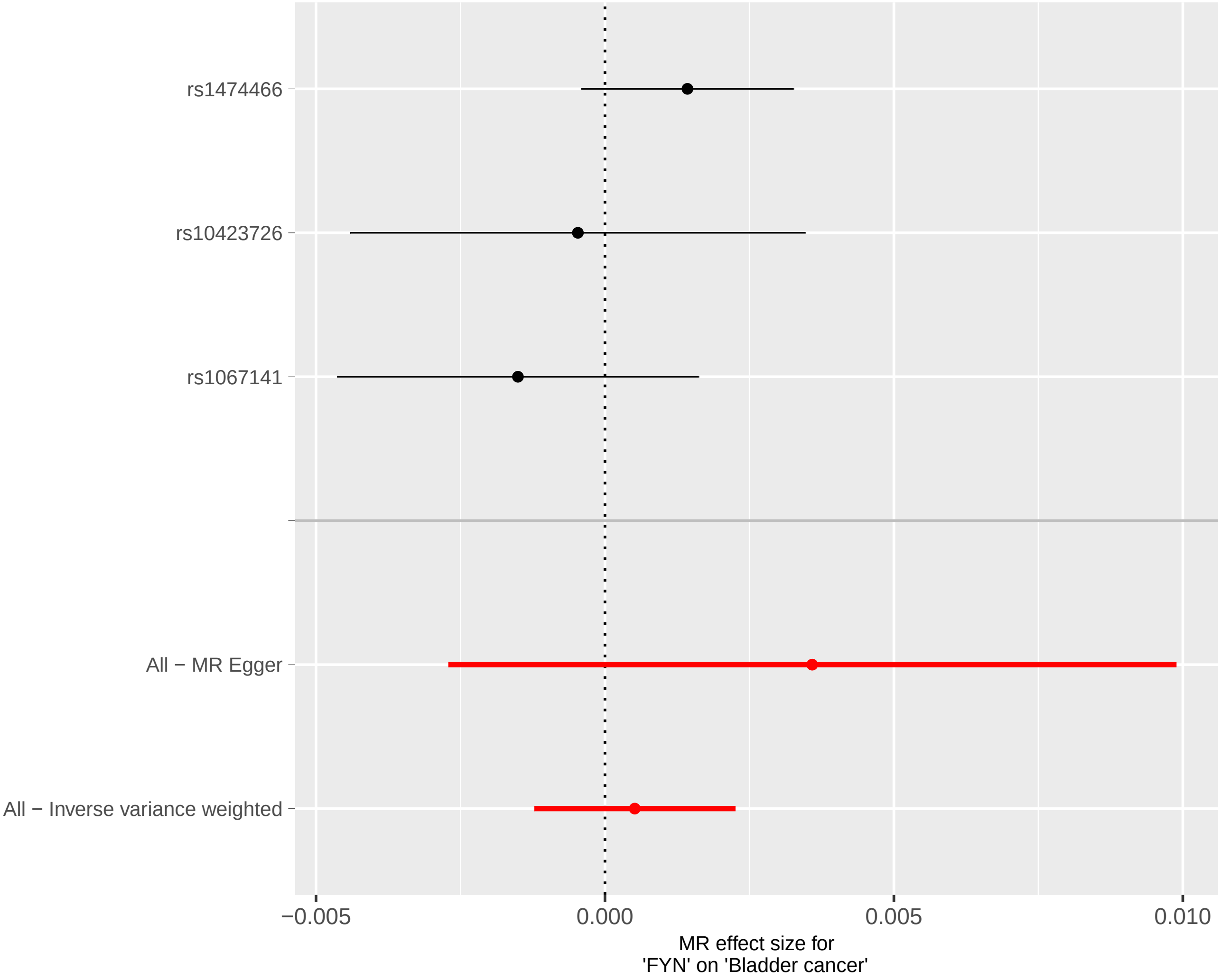
rs1474466
rs10423726
rs1067141
All − MR Egger
All − Inverse variance weighted
−0.005
0.000
0.005
0.010
MR effect size for
'FYN' on 'Bladder cancer'

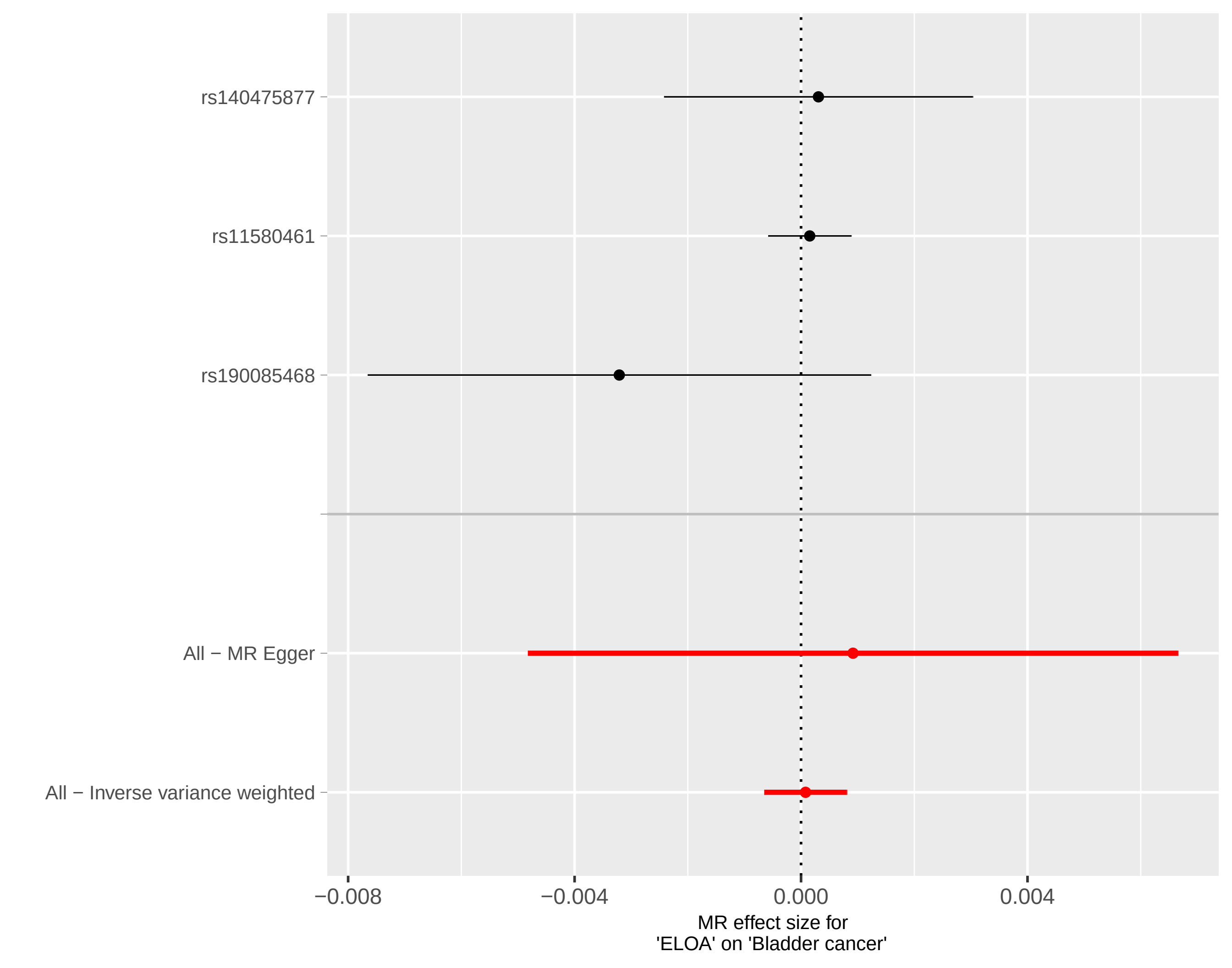

rs140475877
rs11580461
rs190085468
All − MR Egger
All − Inverse variance weighted
−0.008
−0.004
0.000
0.004
MR effect size for
'ELOA' on 'Bladder cancer'

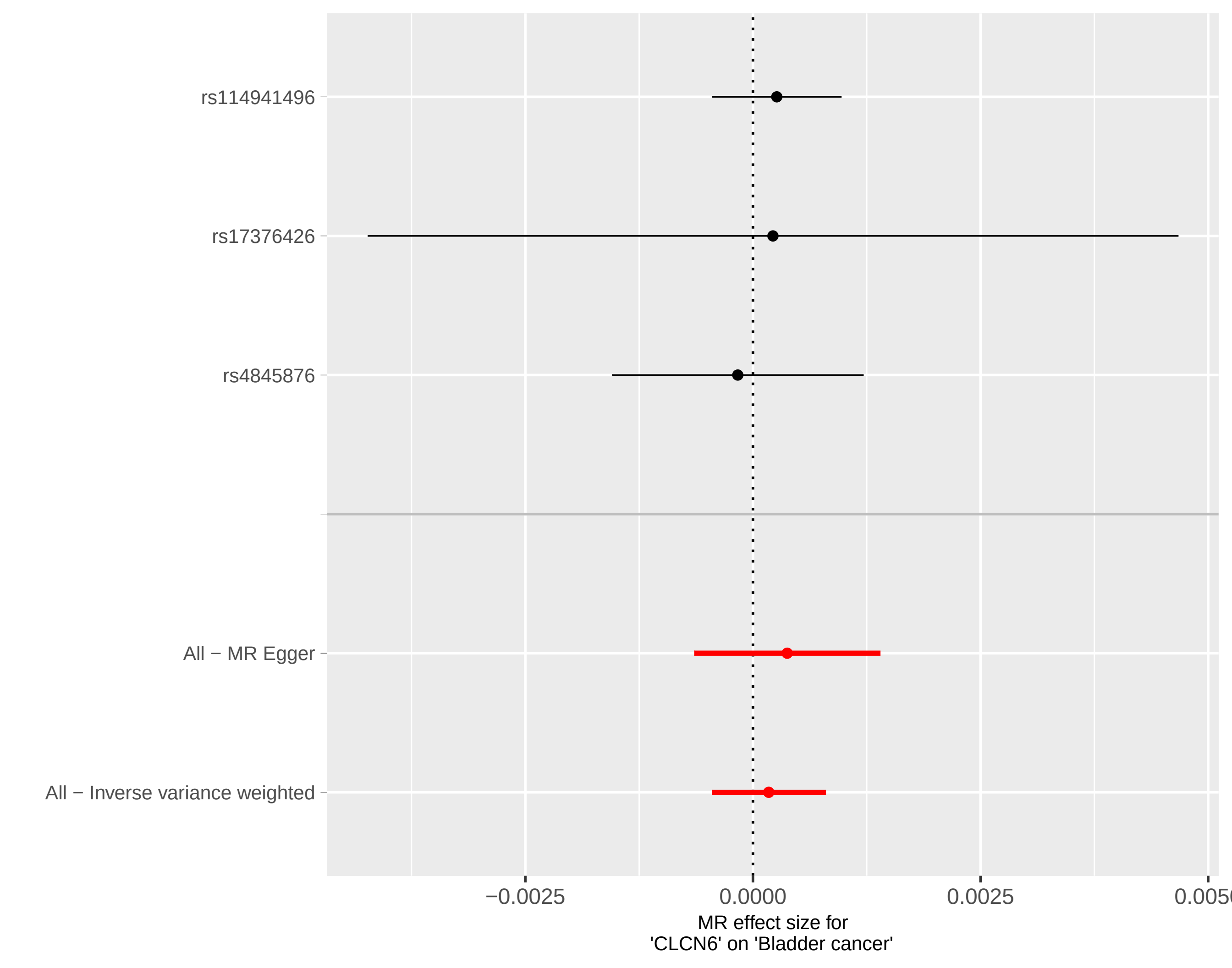
rs114941496
rs17376426
rs4845876
All − MR Egger
All − Inverse variance weighted
−0.0025
0.0000
0.0025
0.005
MR effect size for
'CLCN6' on 'Bladder cancer'

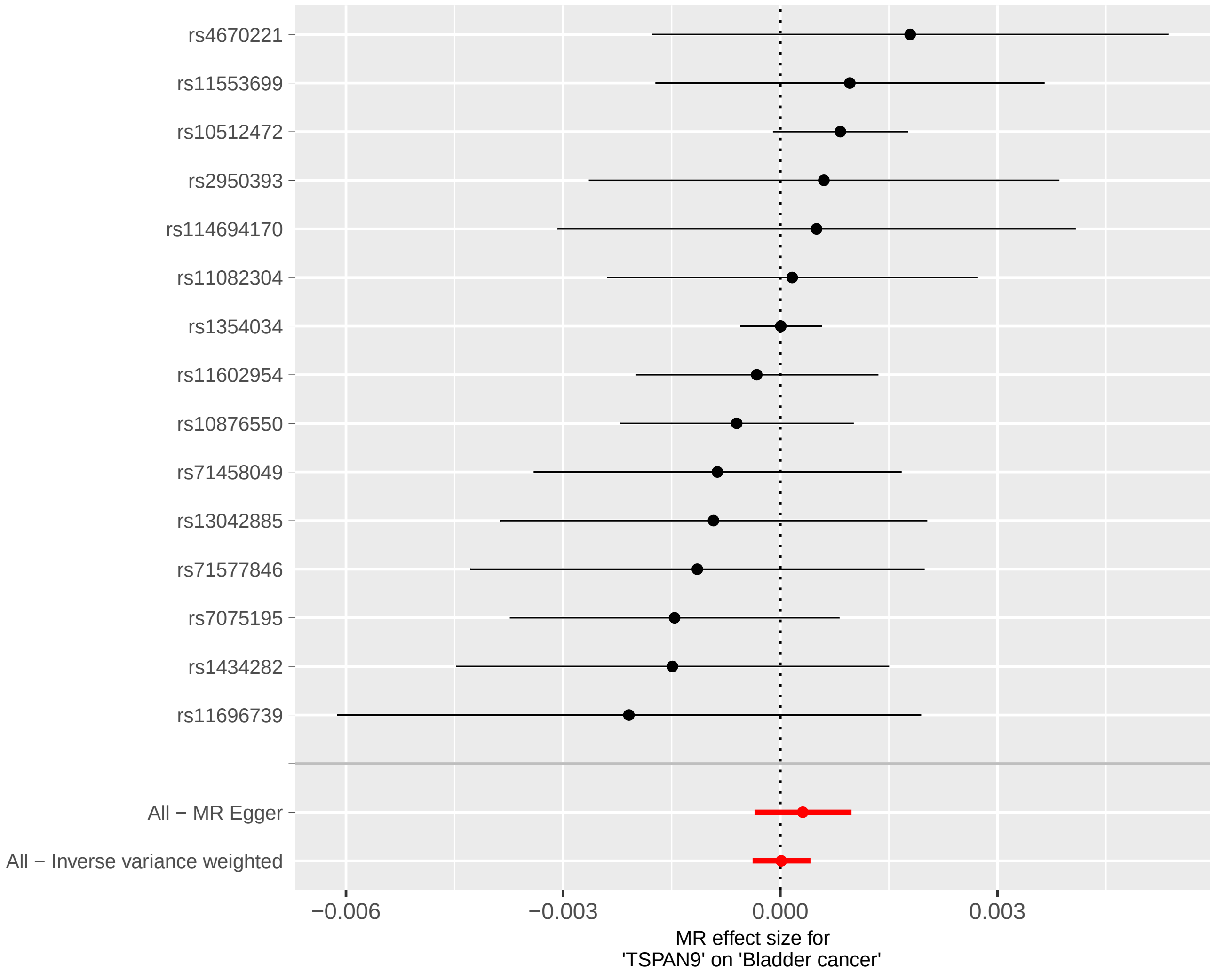

rs4670221
rs11553699
rs10512472
rs2950393
rs114694170
rs11082304
rs1354034
rs11602954
rs10876550
rs71458049
rs13042885
rs71577846
rs7075195
rs1434282
rs11696739
All − MR Egger
All − Inverse variance weighted
−0.006
−0.003
0.000
0.003
MR effect size for
'TSPAN9' on 'Bladder cancer'

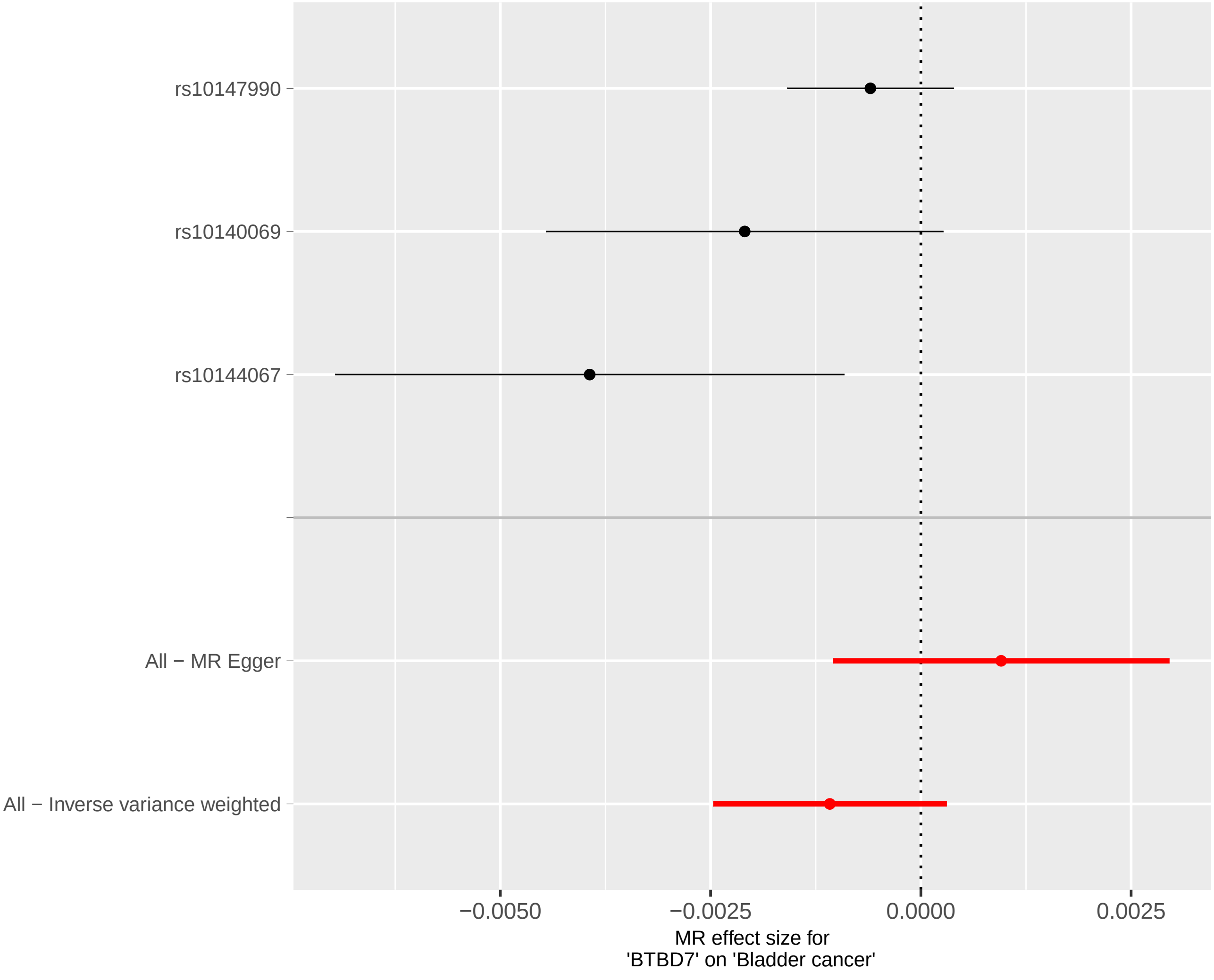

rs10147990
rs10140069
rs10144067
All − MR Egger
All − Inverse variance weighted
−0.0050
−0.0025
0.0000
0.0025
MR effect size for
'BTBD7' on 'Bladder cancer'

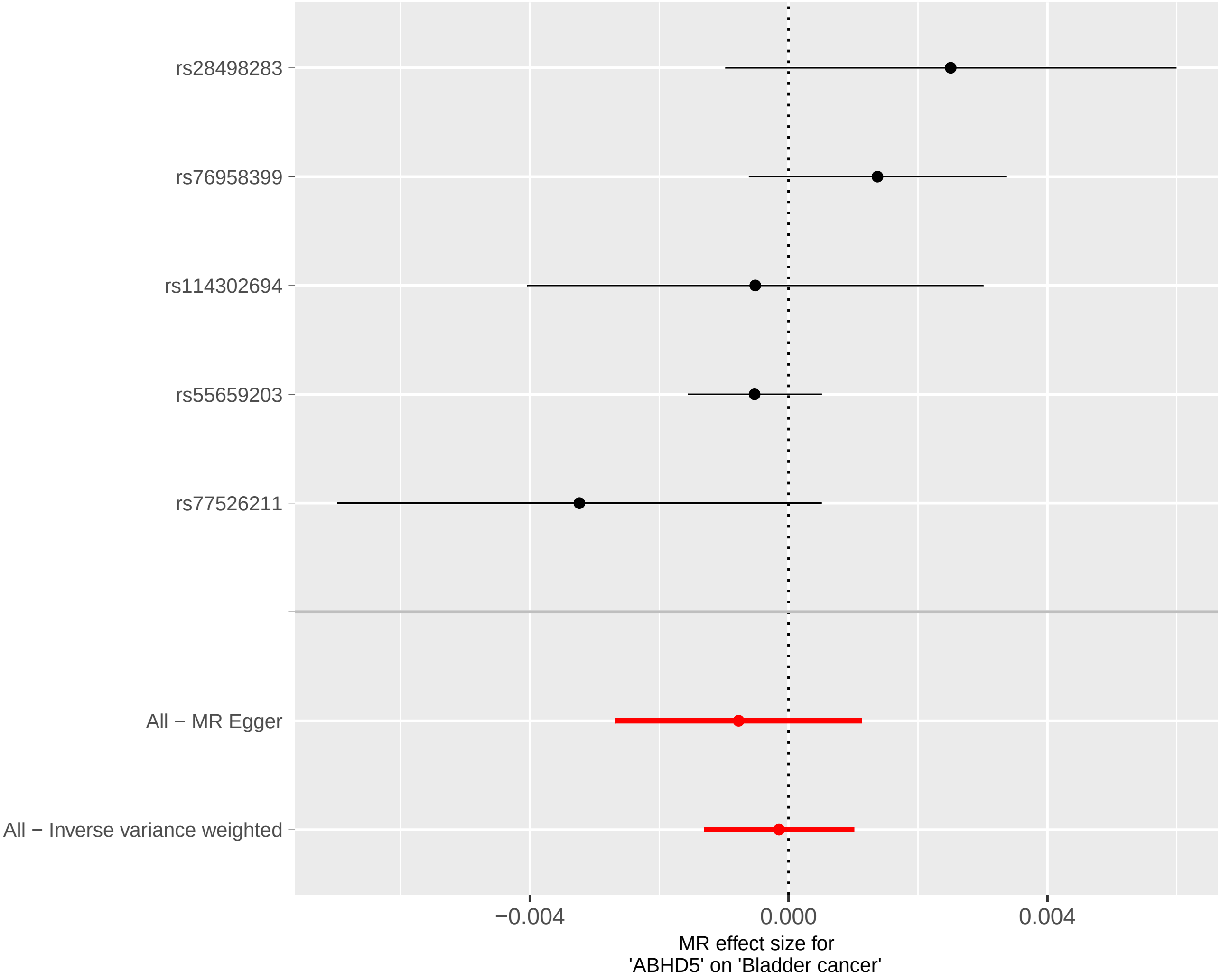

rs28498283
rs76958399
rs114302694
rs55659203
rs77526211
All − MR Egger
All − Inverse variance weighted
−0.004
0.000
0.004
MR effect size for
'ABHD5' on 'Bladder cancer'

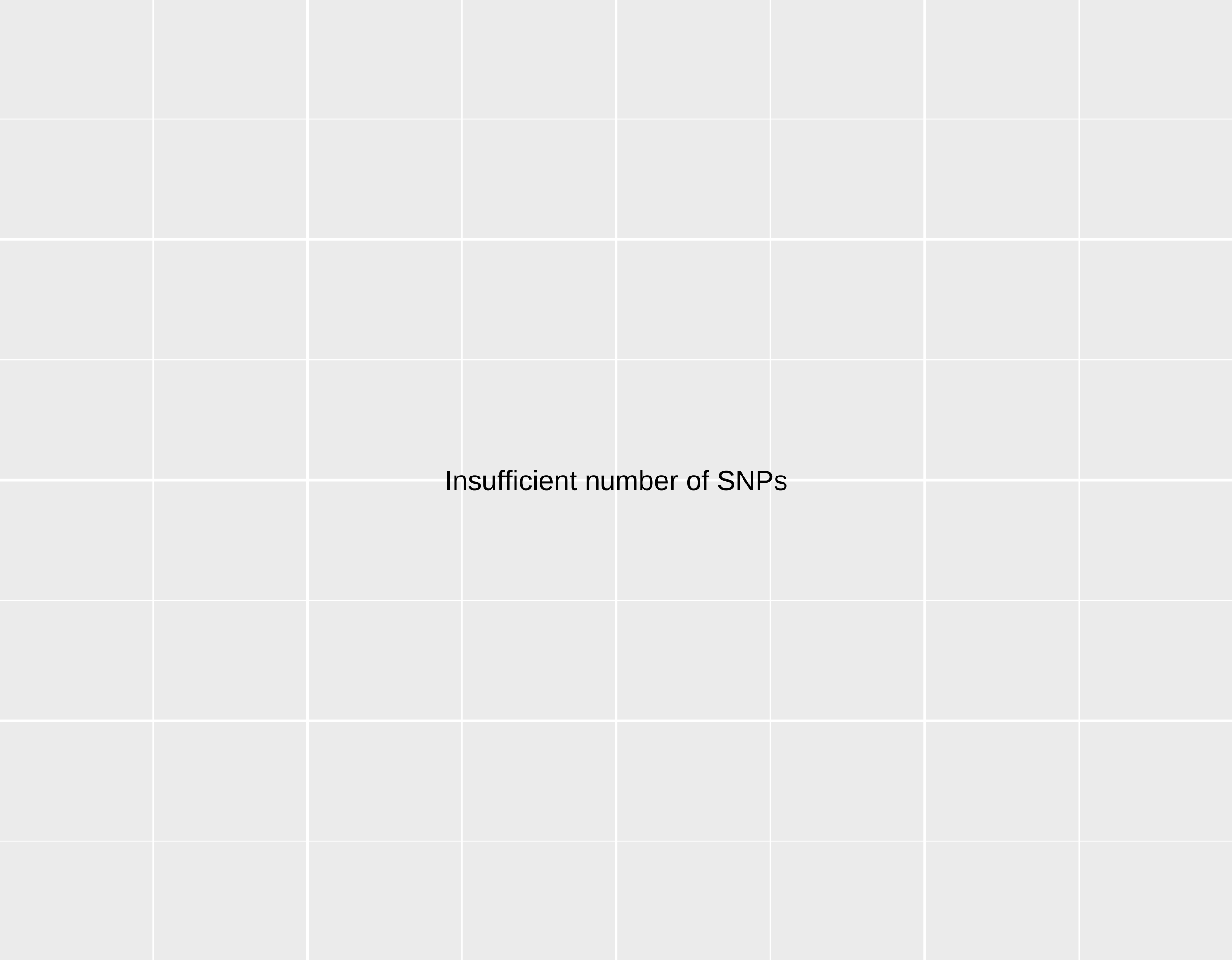
Insufficient number of SNPs

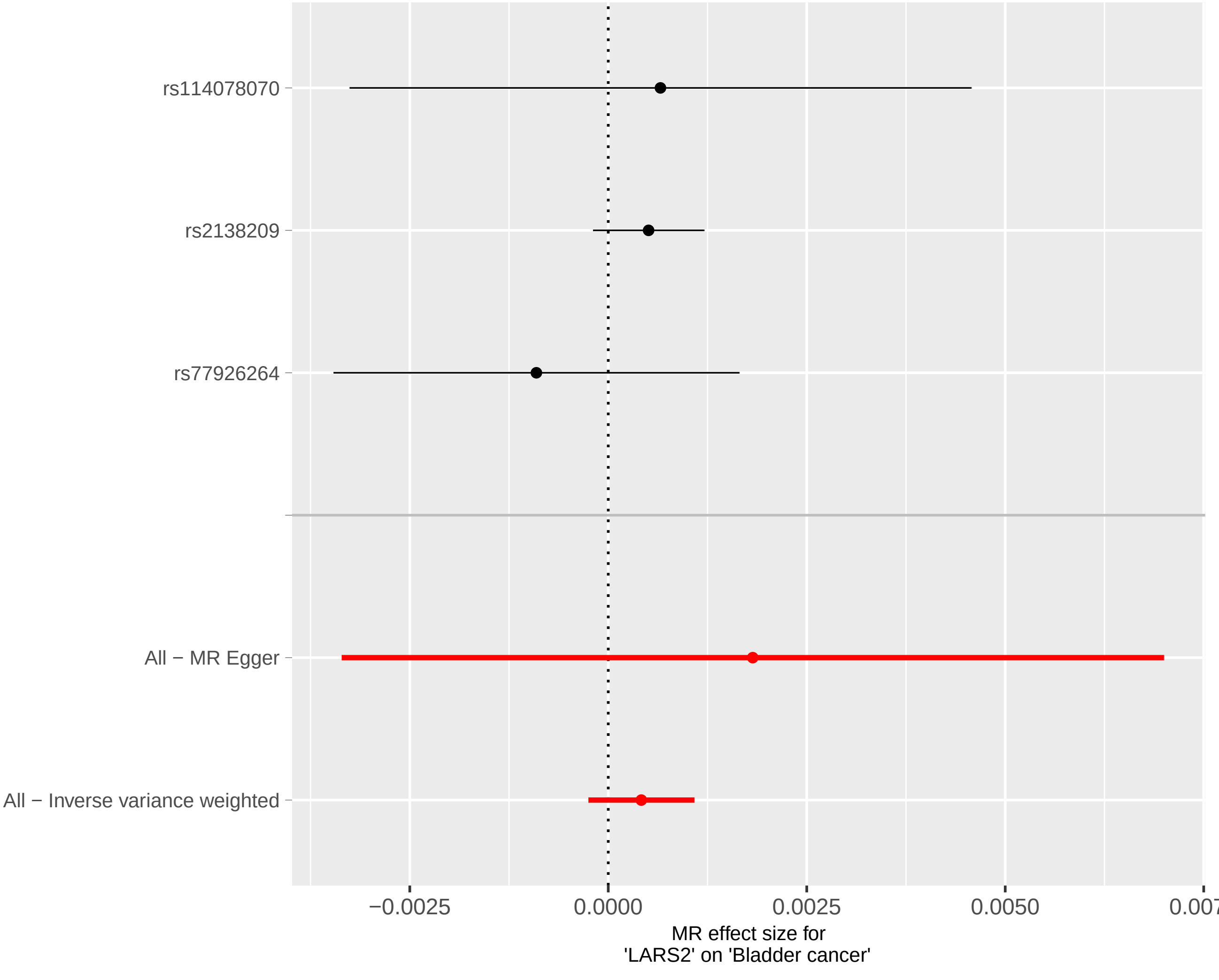
rs114078070
rs2138209
rs77926264
All − MR Egger
All − Inverse variance weighted
−0.0025
0.0000
0.0025
0.0050
MR effect size for
'LARS2' on 'Bladder cancer'

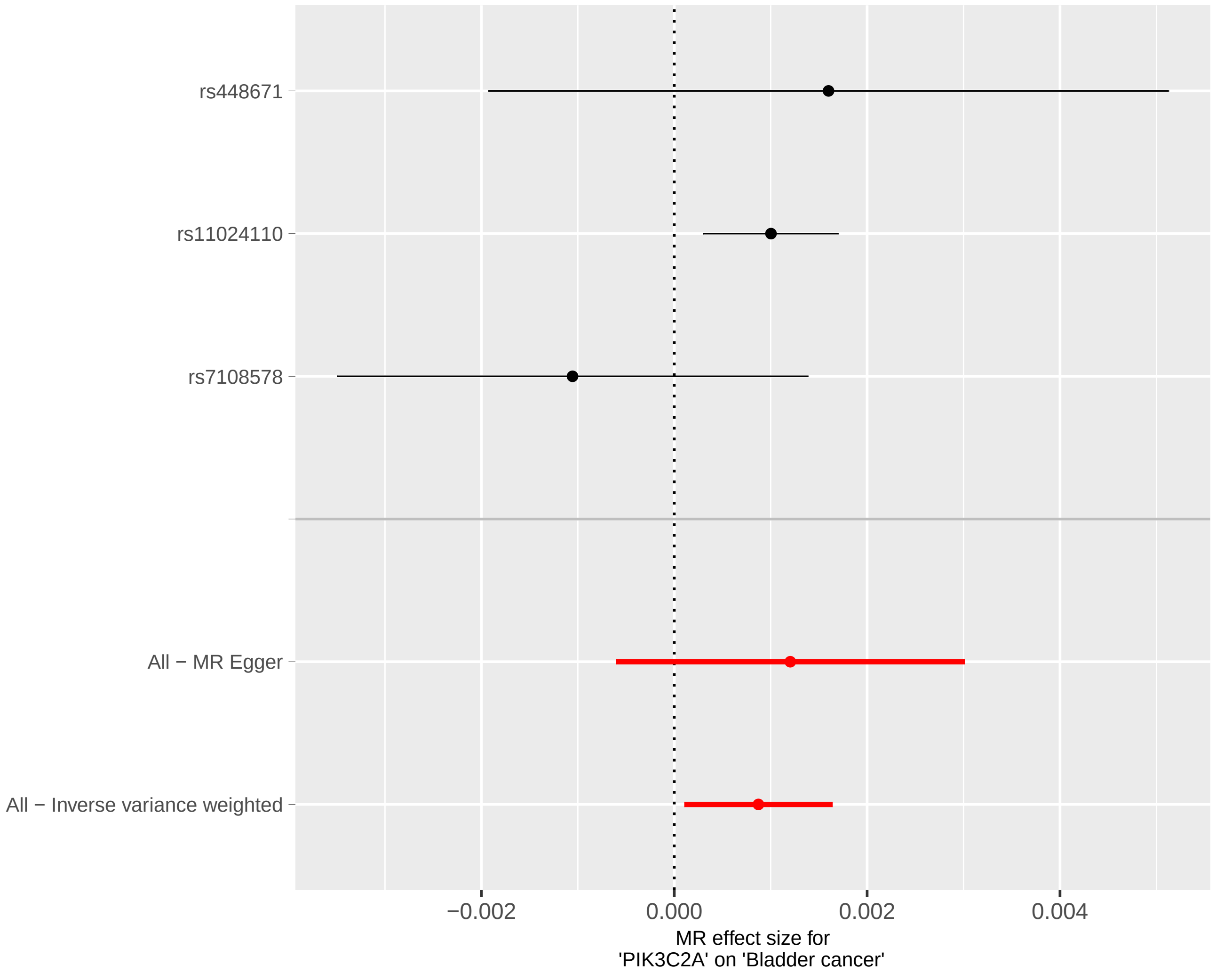

rs448671
rs11024110
rs7108578
All − MR Egger
All − Inverse variance weighted
−0.002
0.000
0.002
0.004
MR effect size for
'PIK3C2A' on 'Bladder cancer'

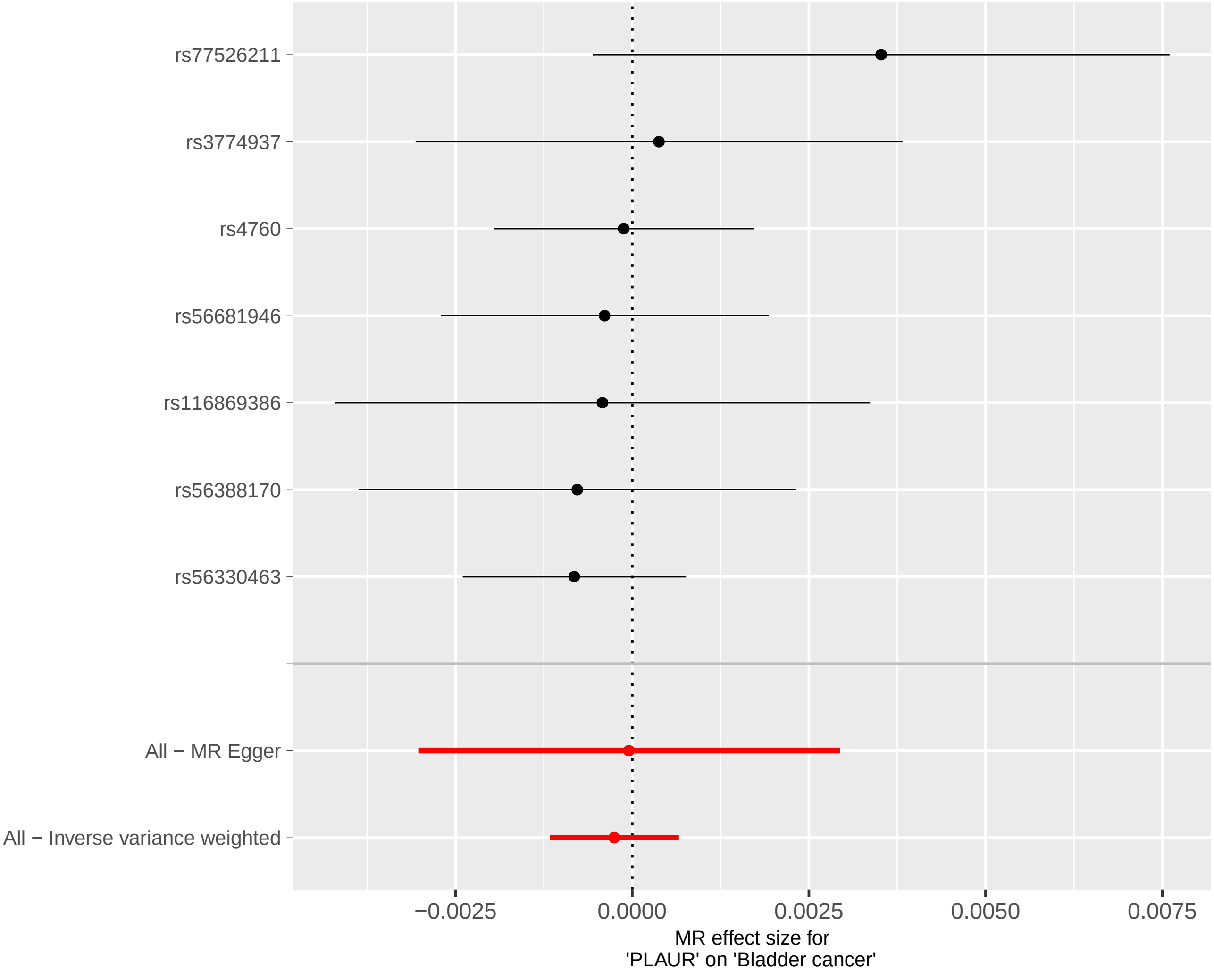
rs77526211
rs3774937
rs4760
rs56681946
rs116869386
rs56388170
rs56330463
All − MR Egger
All − Inverse variance weighted
−0.0025
0.0000
0.0025
0.0050
0.0075
MR effect size for
'PLAUR' on 'Bladder cancer'

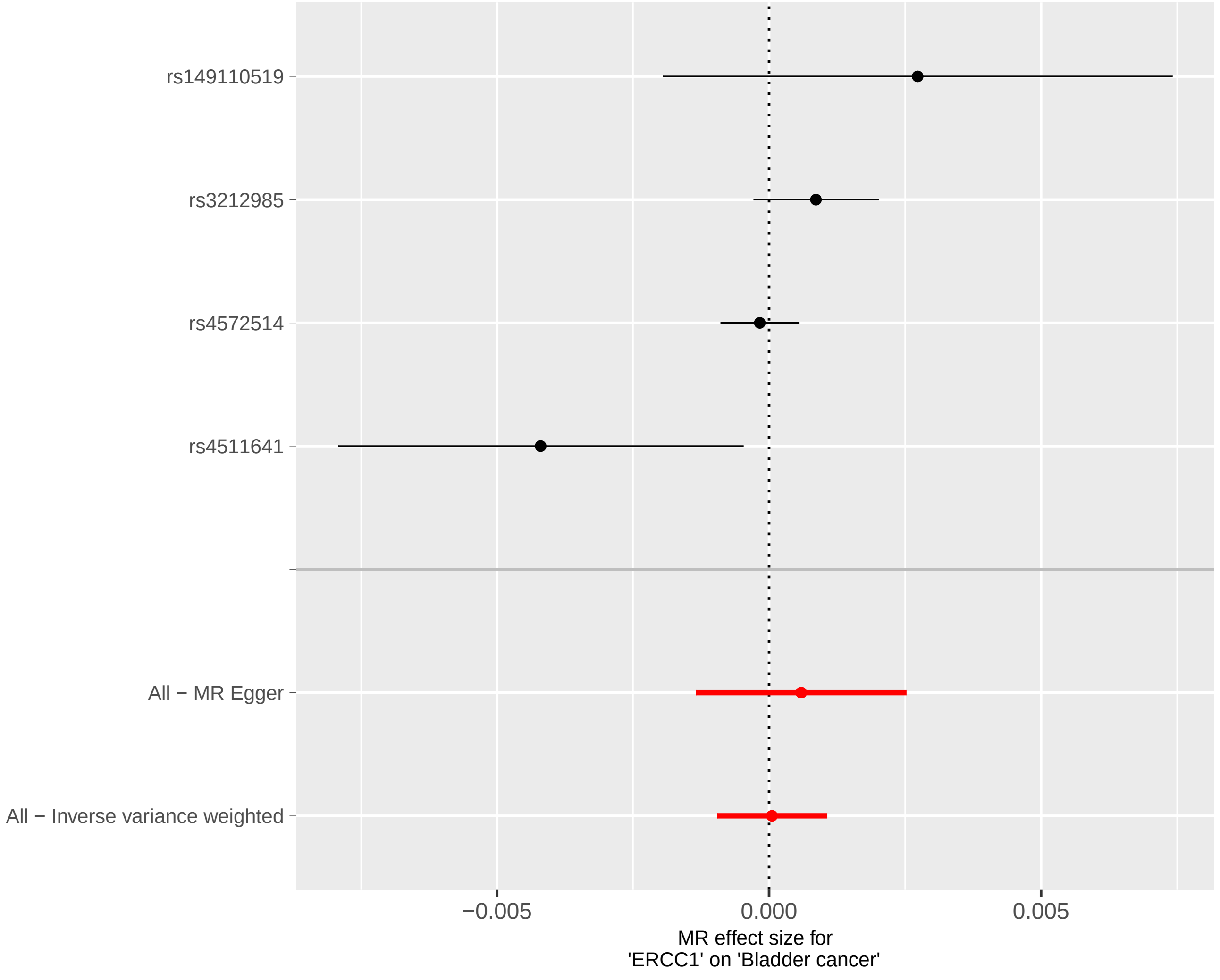
rs149110519
rs3212985
rs4572514
rs4511641
All − MR Egger
All − Inverse variance weighted
−0.005
0.000
0.005
MR effect size for
'ERCC1' on 'Bladder cancer'

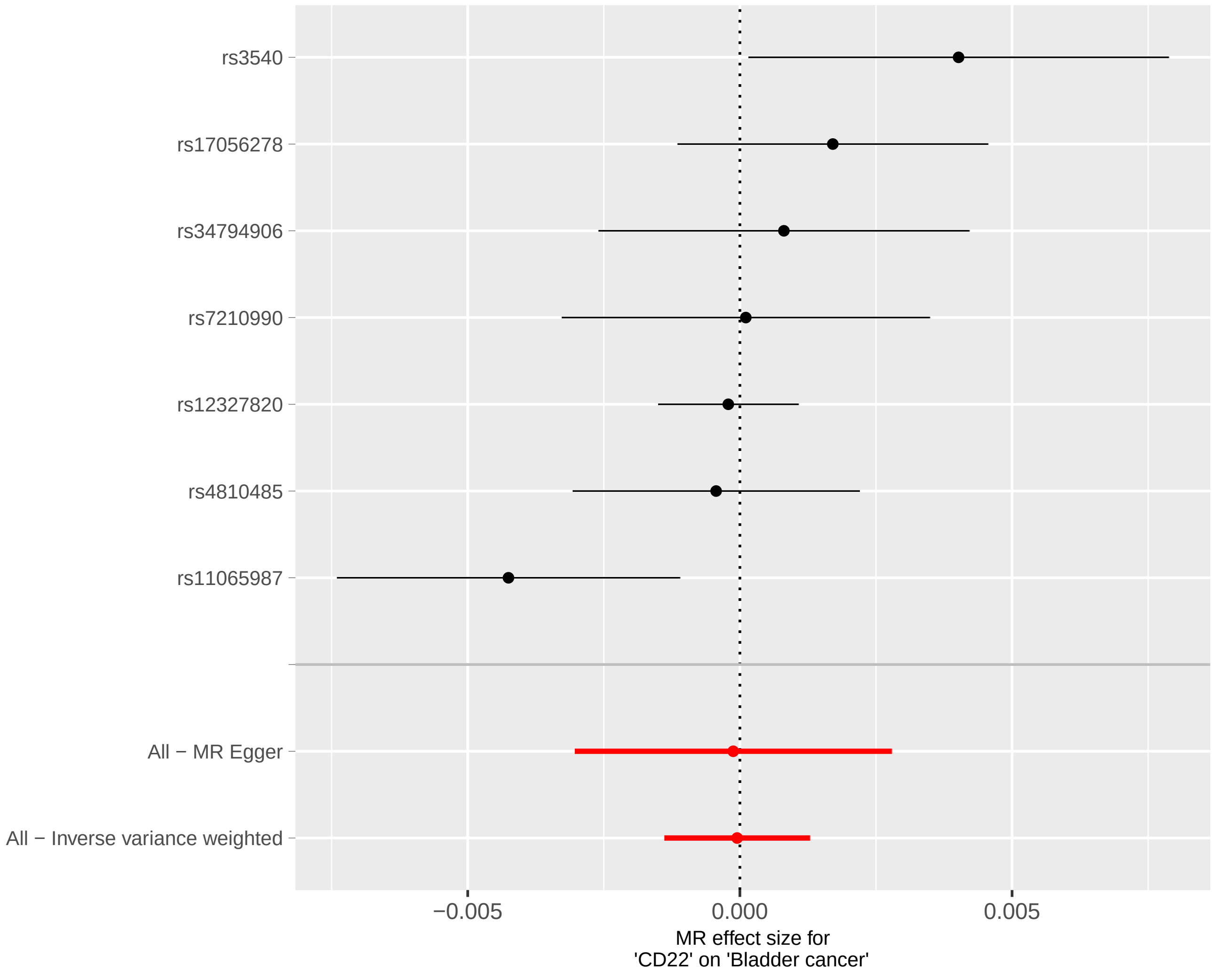

rs3540
rs17056278
rs34794906
rs7210990
rs12327820
rs4810485
rs11065987
All − MR Egger
All − Inverse variance weighted
−0.005
0.000
0.005
MR effect size for
'CD22' on 'Bladder cancer'

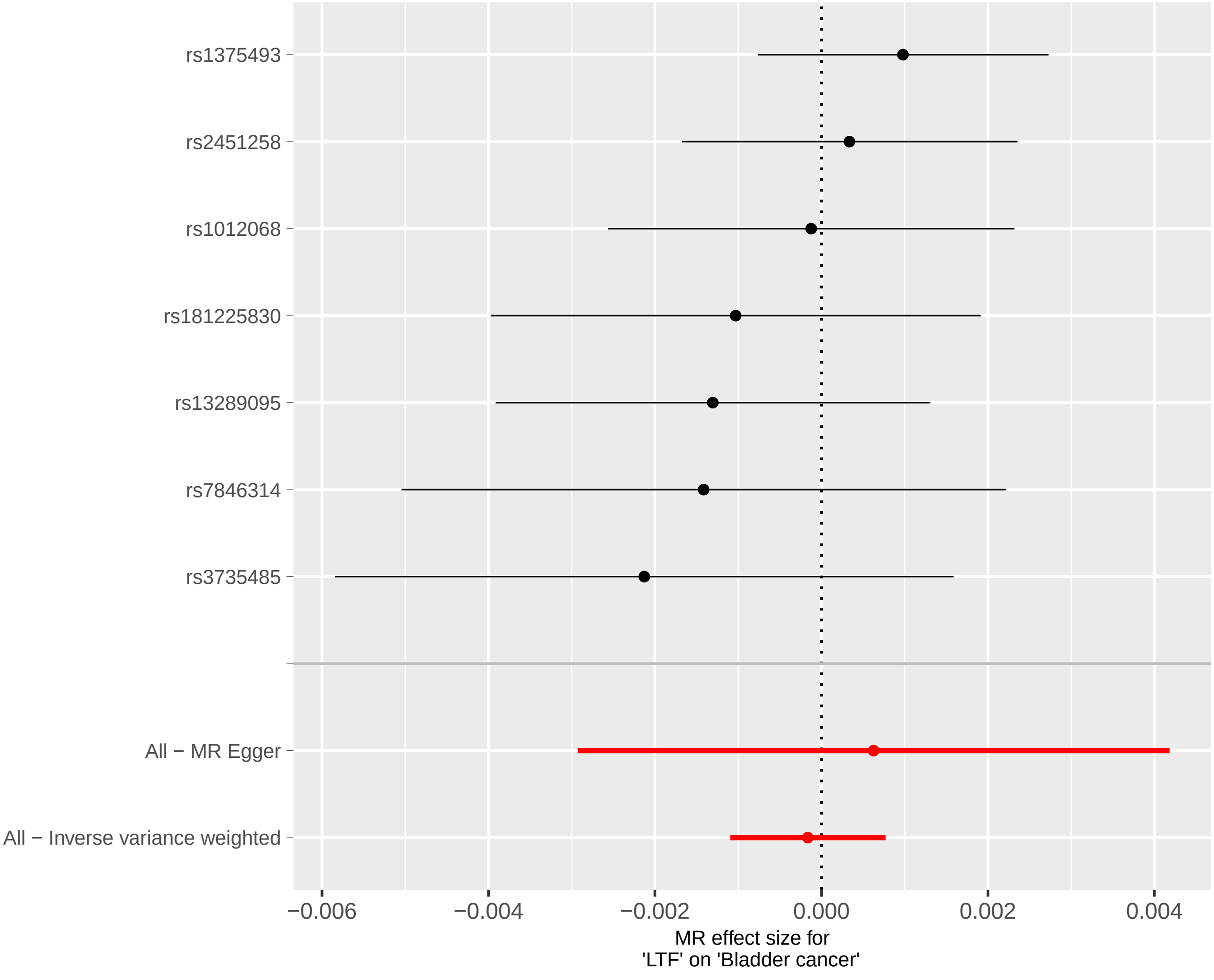

rs1375493
rs2451258
rs1012068
rs181225830
rs13289095
rs7846314
rs3735485
All − MR Egger
All − Inverse variance weighted
−0.006
−0.004
−0.002
0.000
0.002
0.004
MR effect size for
'LTF' on 'Bladder cancer'

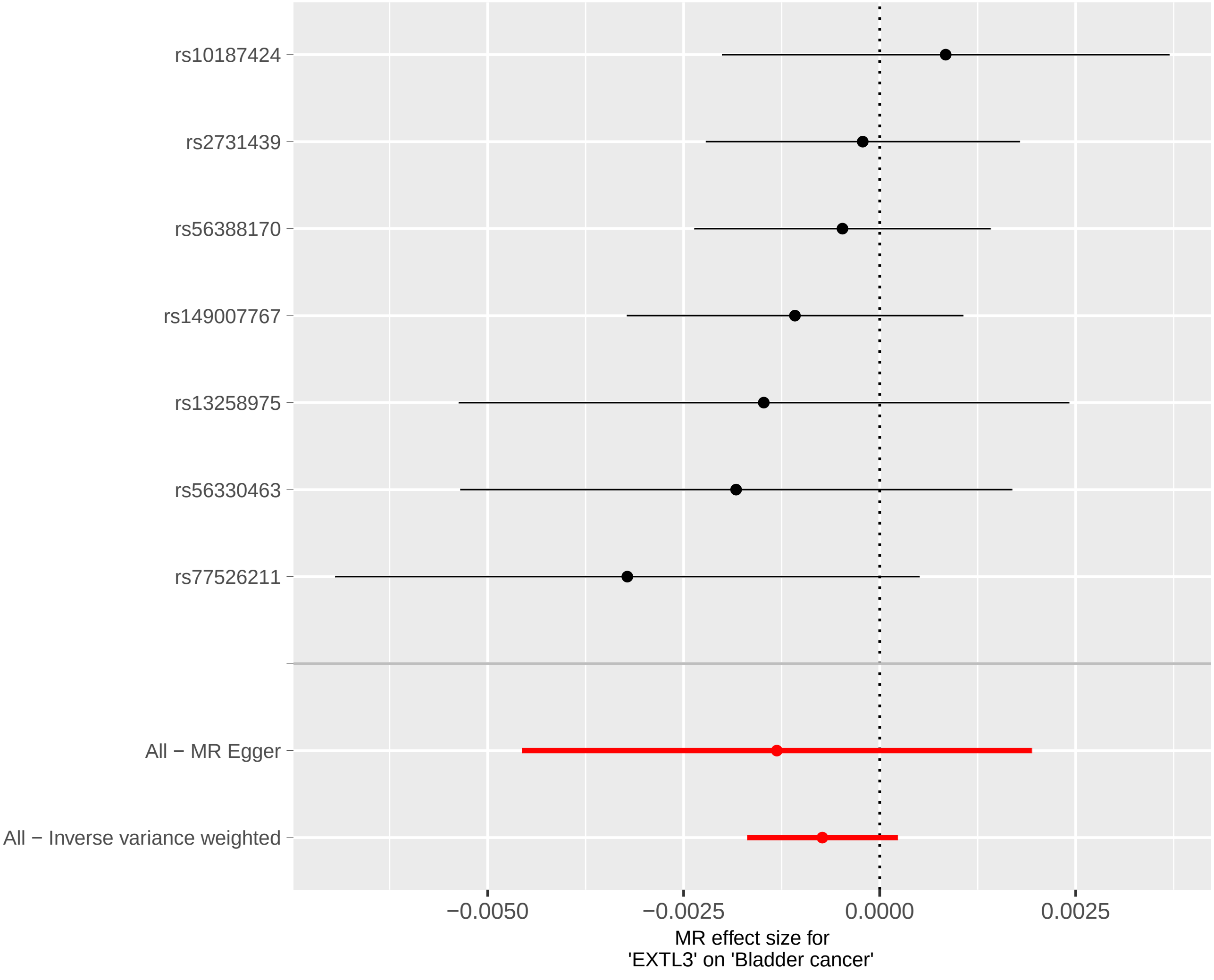

rs10187424
rs2731439
rs56388170
rs149007767
rs13258975
rs56330463
rs77526211
All − MR Egger
All − Inverse variance weighted
−0.0050
−0.0025
0.0000
0.0025
MR effect size for
'EXTL3' on 'Bladder cancer'

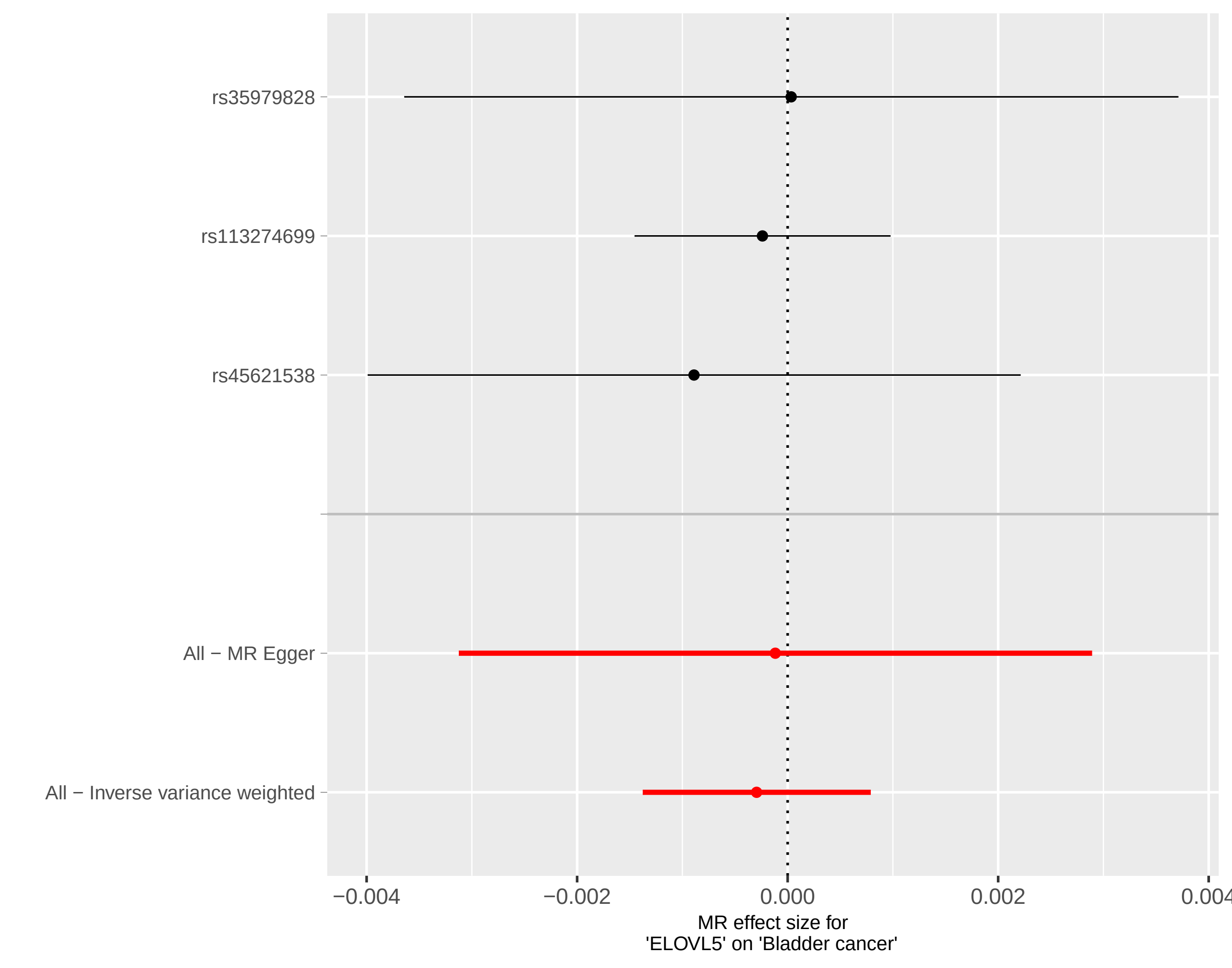
rs35979828
rs113274699
rs45621538
All − MR Egger
All − Inverse variance weighted
−0.004
−0.002
0.000
0.002
0.004
MR effect size for
'ELOVL5' on 'Bladder cancer'

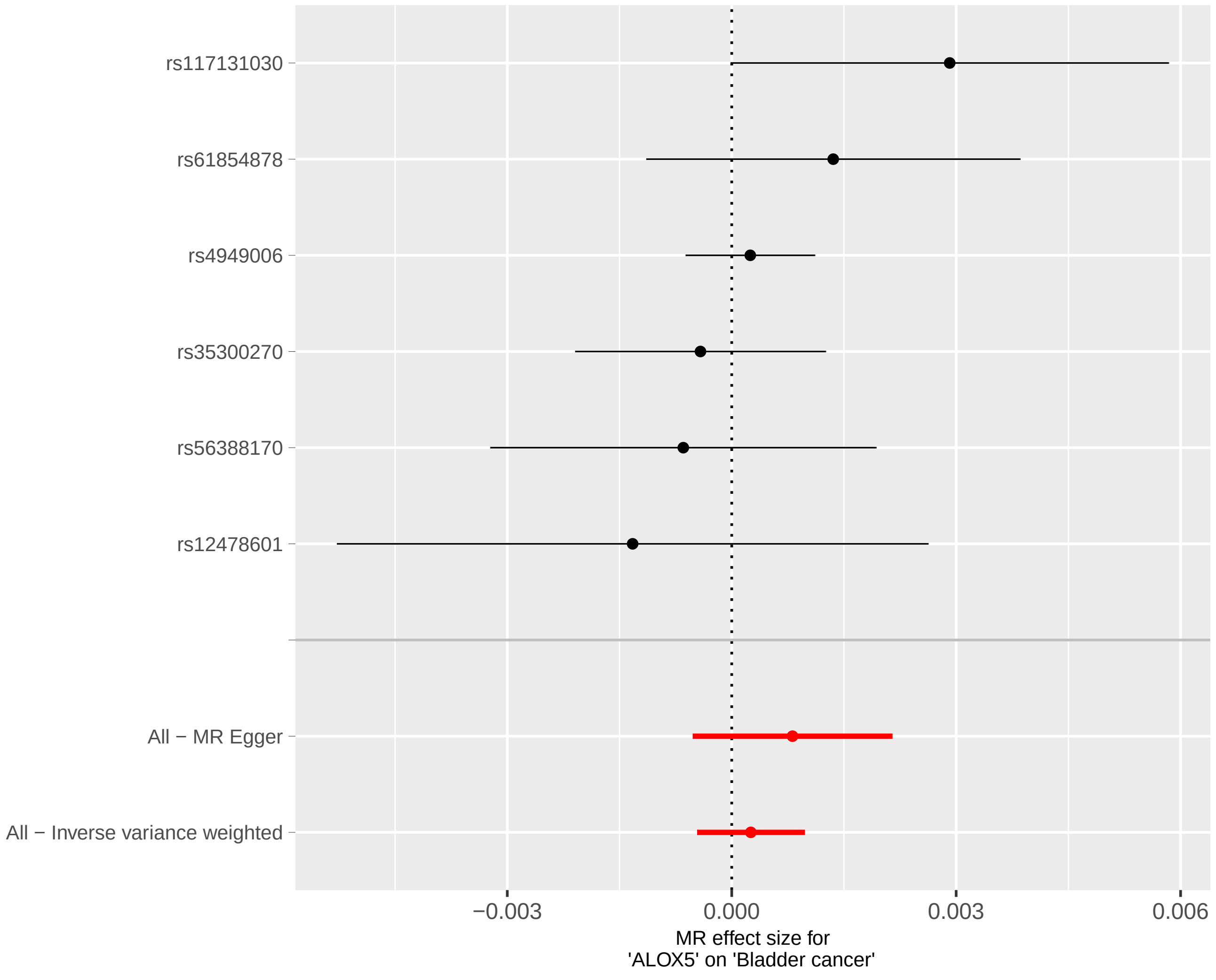
rs117131030
rs61854878
rs4949006
rs35300270
rs56388170
rs12478601
All − MR Egger
All − Inverse variance weighted
−0.003
0.000
0.003
0.006
MR effect size for
'ALOX5' on 'Bladder cancer'

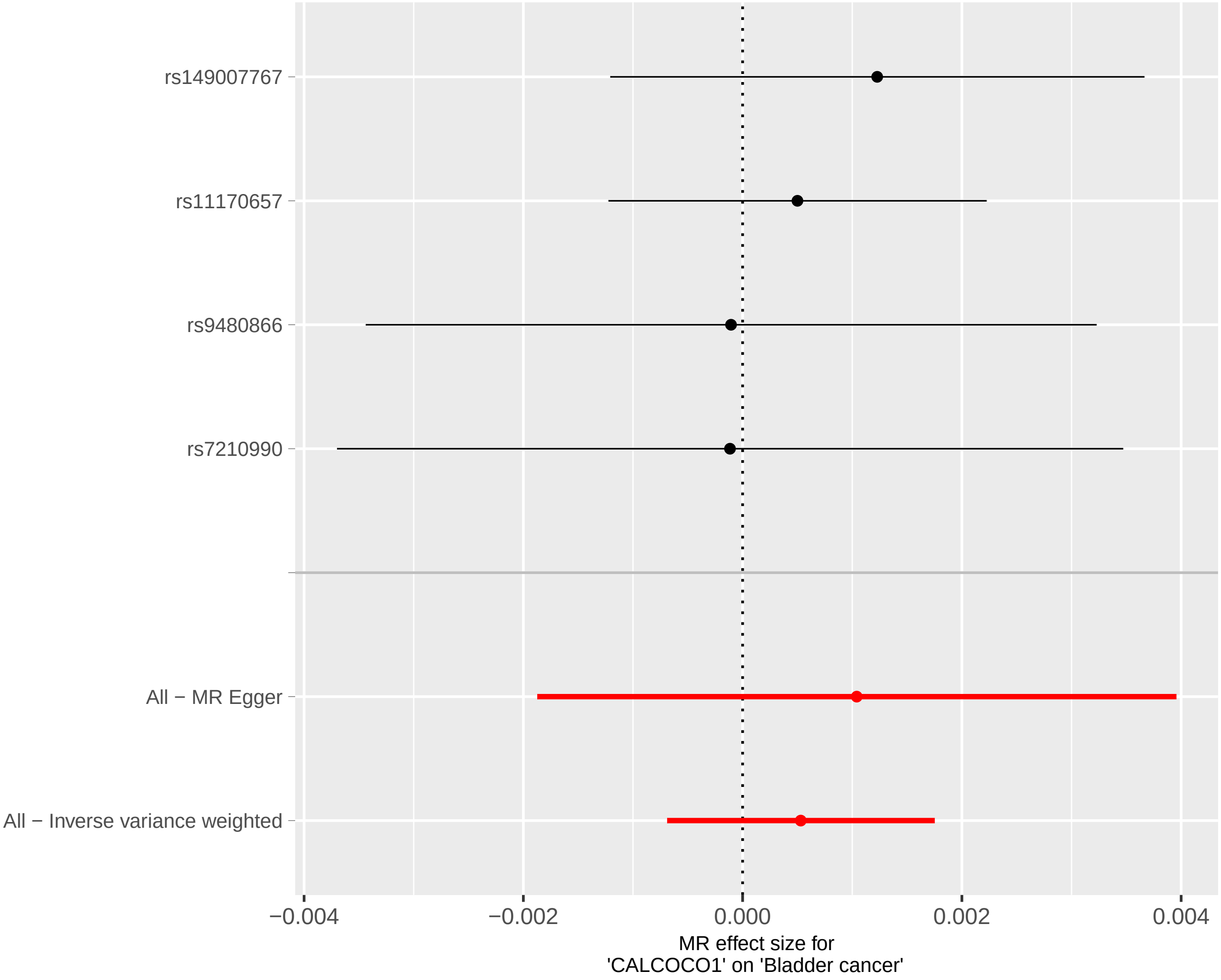
rs149007767
rs11170657
rs9480866
rs7210990
All − MR Egger
All − Inverse variance weighted
−0.004
−0.002
0.000
0.002
0.004
MR effect size for
'CALCOCO1' on 'Bladder cancer'

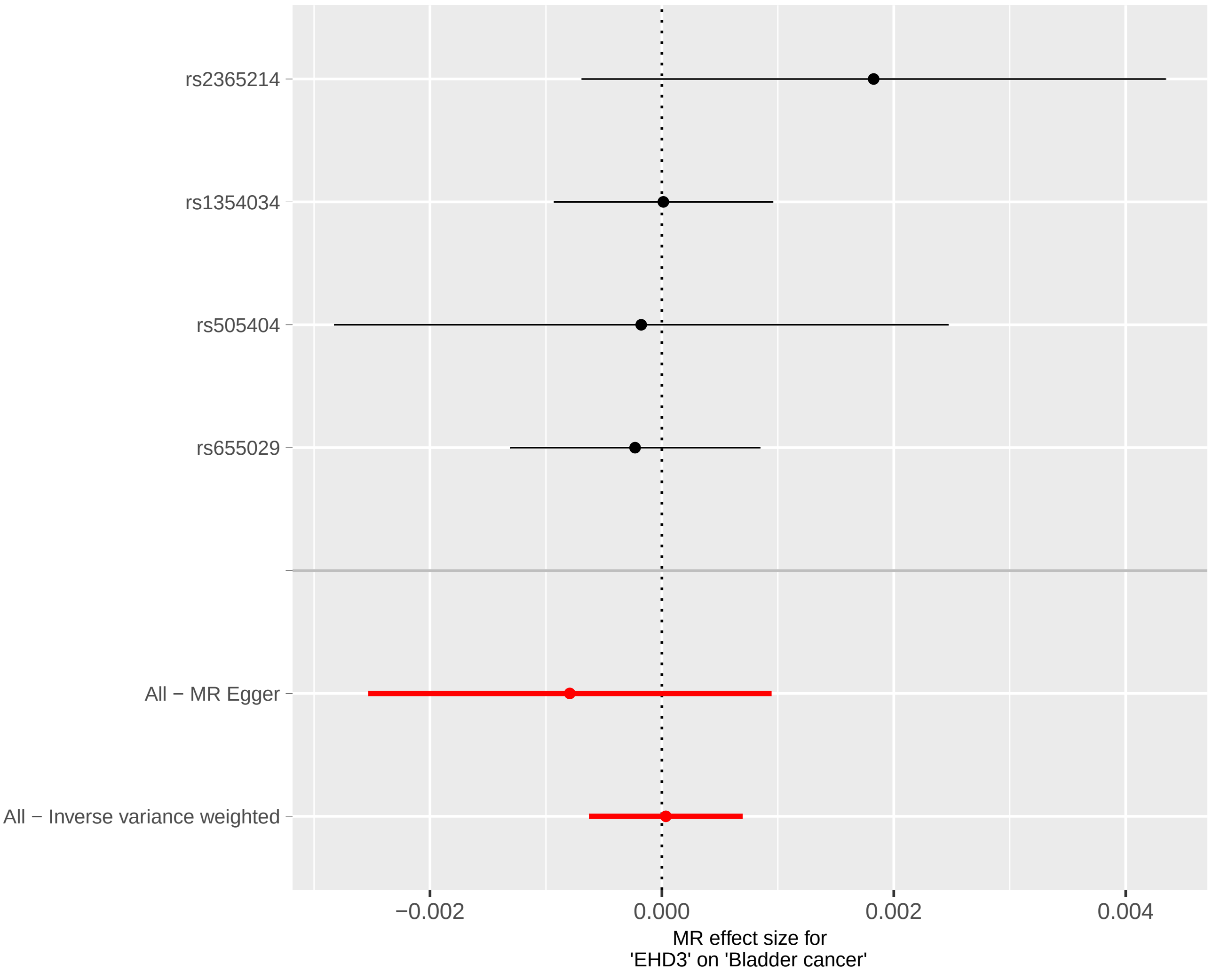
rs2365214
rs1354034
rs505404
rs655029
All − MR Egger
All − Inverse variance weighted
−0.002
0.000
0.002
0.004
MR effect size for
'EHD3' on 'Bladder cancer'

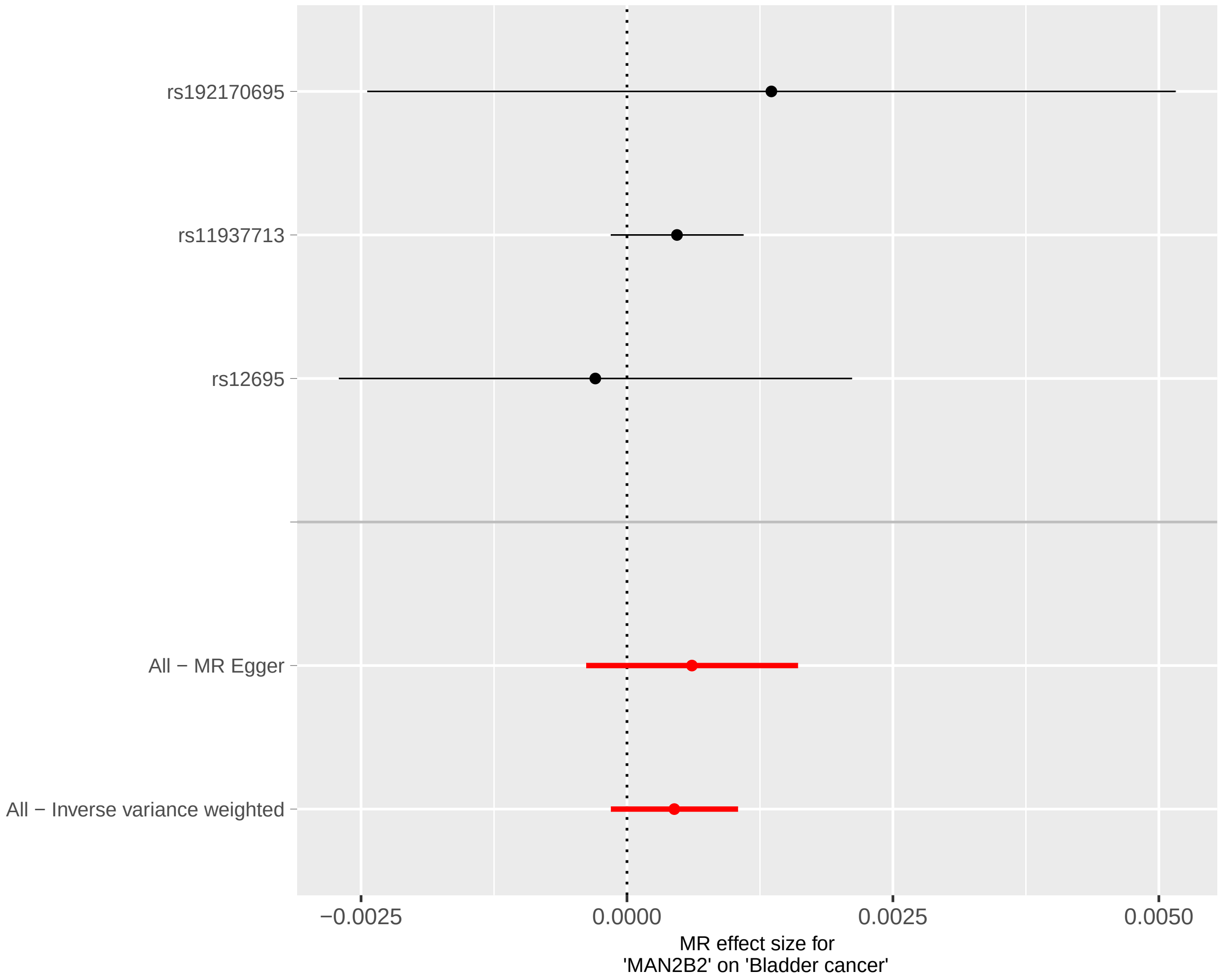

rs192170695
rs11937713
rs12695
All − MR Egger
All − Inverse variance weighted
−0.0025
0.0000
0.0025
0.0050
MR effect size for
'MAN2B2' on 'Bladder cancer'

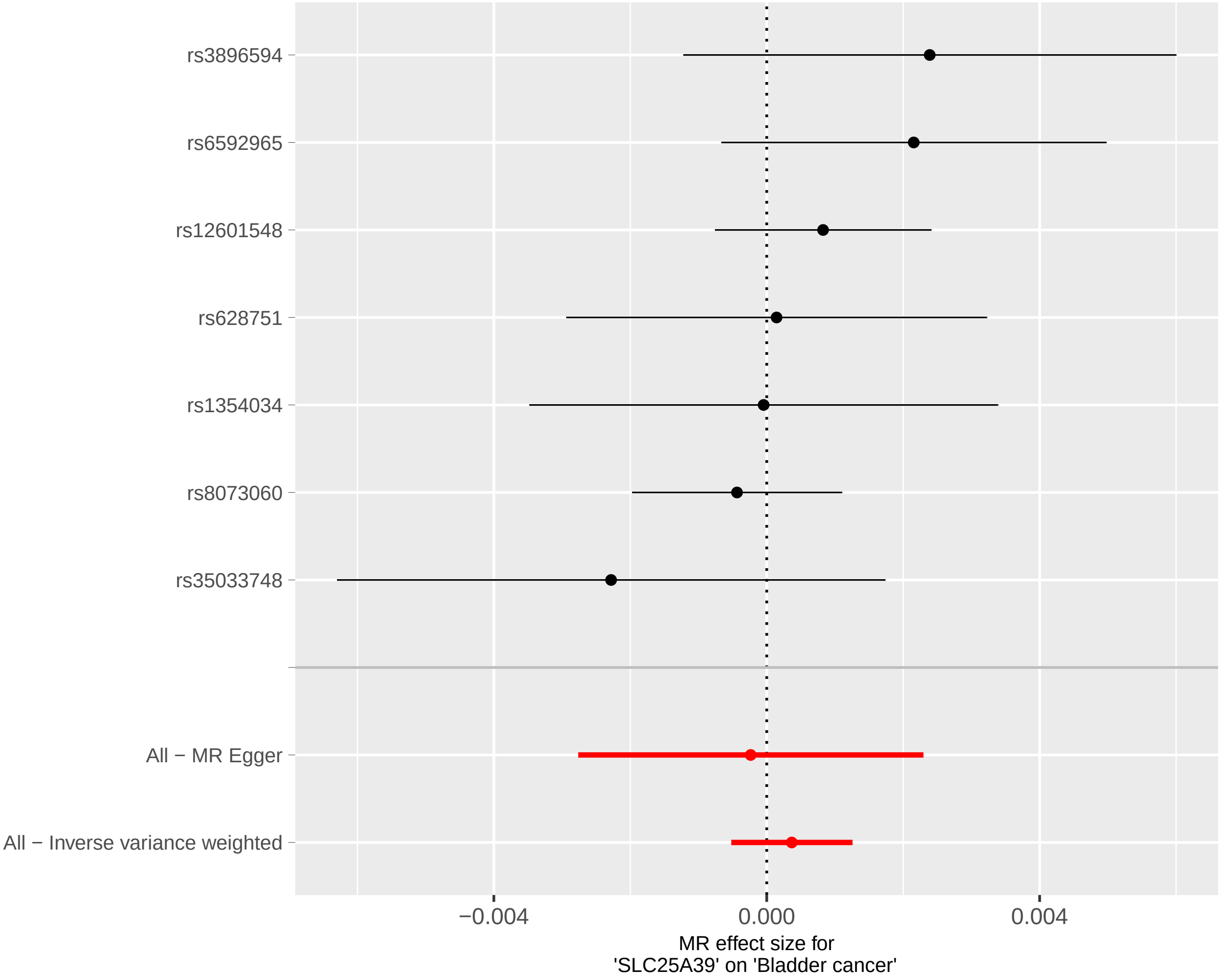

rs3896594
rs6592965
rs12601548
rs628751
rs1354034
rs8073060
rs35033748
All − MR Egger
All − Inverse variance weighted
−0.004
0.000
0.004
MR effect size for
'SLC25A39' on 'Bladder cancer'

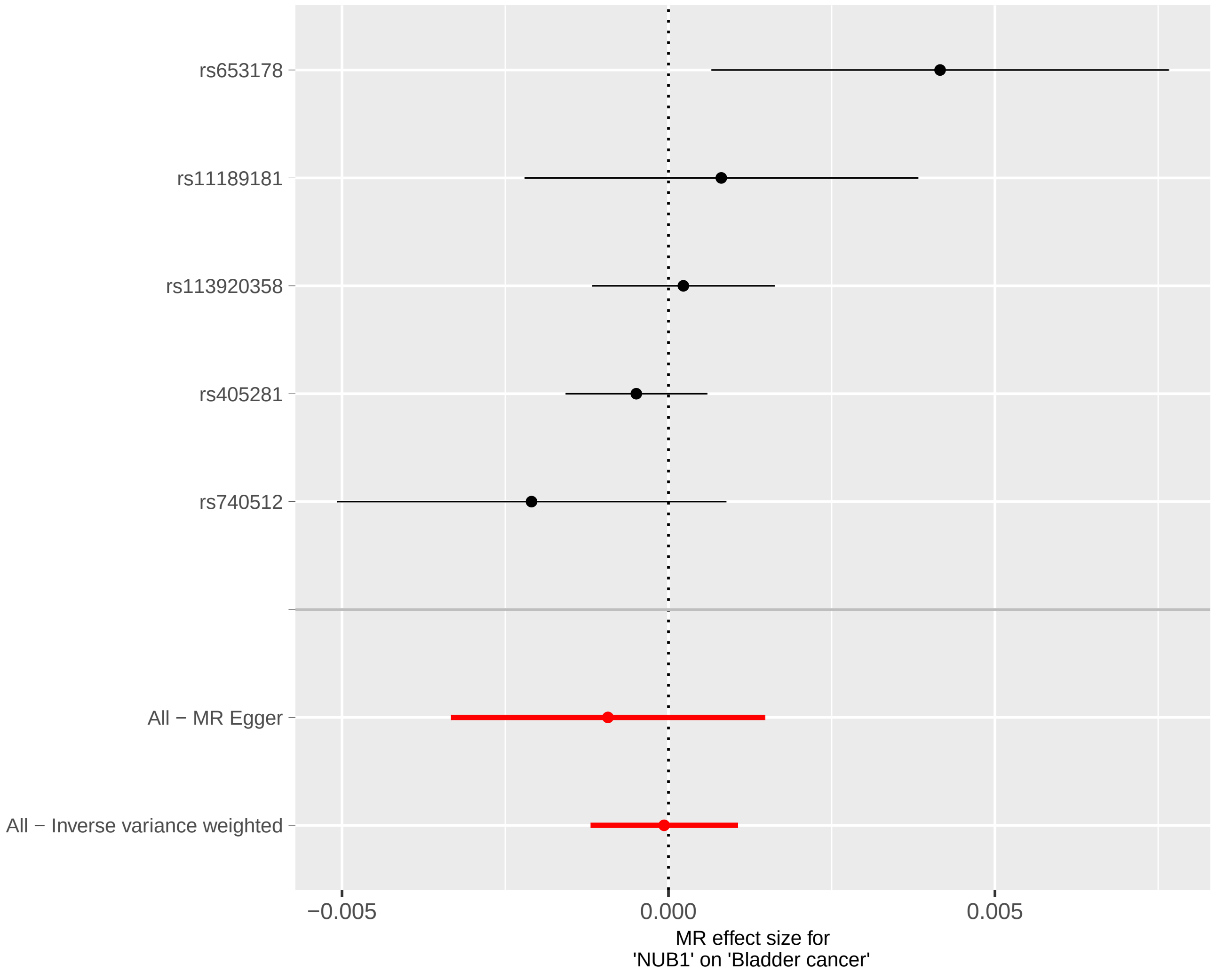
rs653178
rs11189181
rs113920358
rs405281
rs740512
All − MR Egger
All − Inverse variance weighted
−0.005
0.000
0.005
MR effect size for
'NUB1' on 'Bladder cancer'

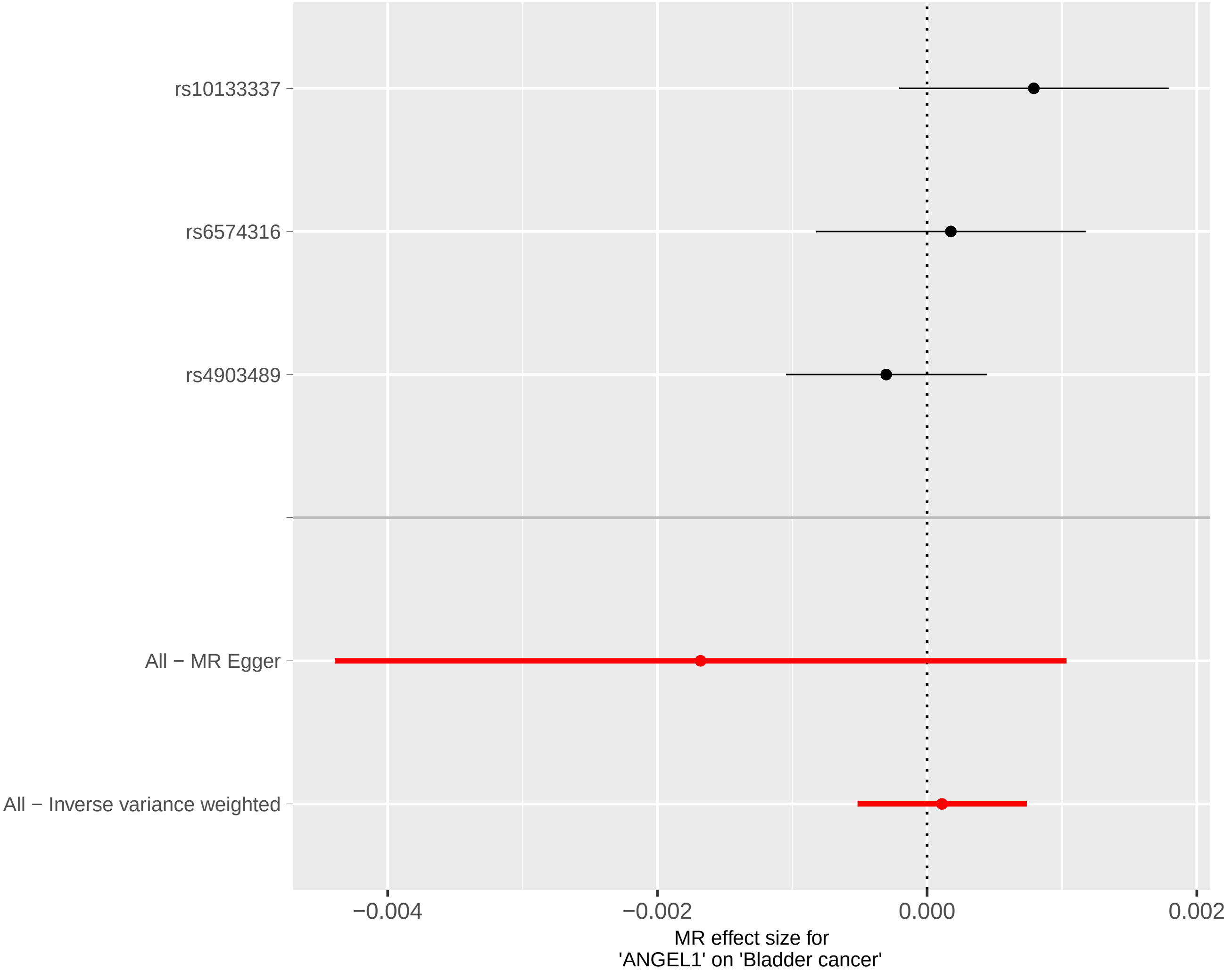

rs10133337
rs6574316
rs4903489
All − MR Egger
All − Inverse variance weighted
−0.004
−0.002
0.000
0.002
MR effect size for
'ANGEL1' on 'Bladder cancer'

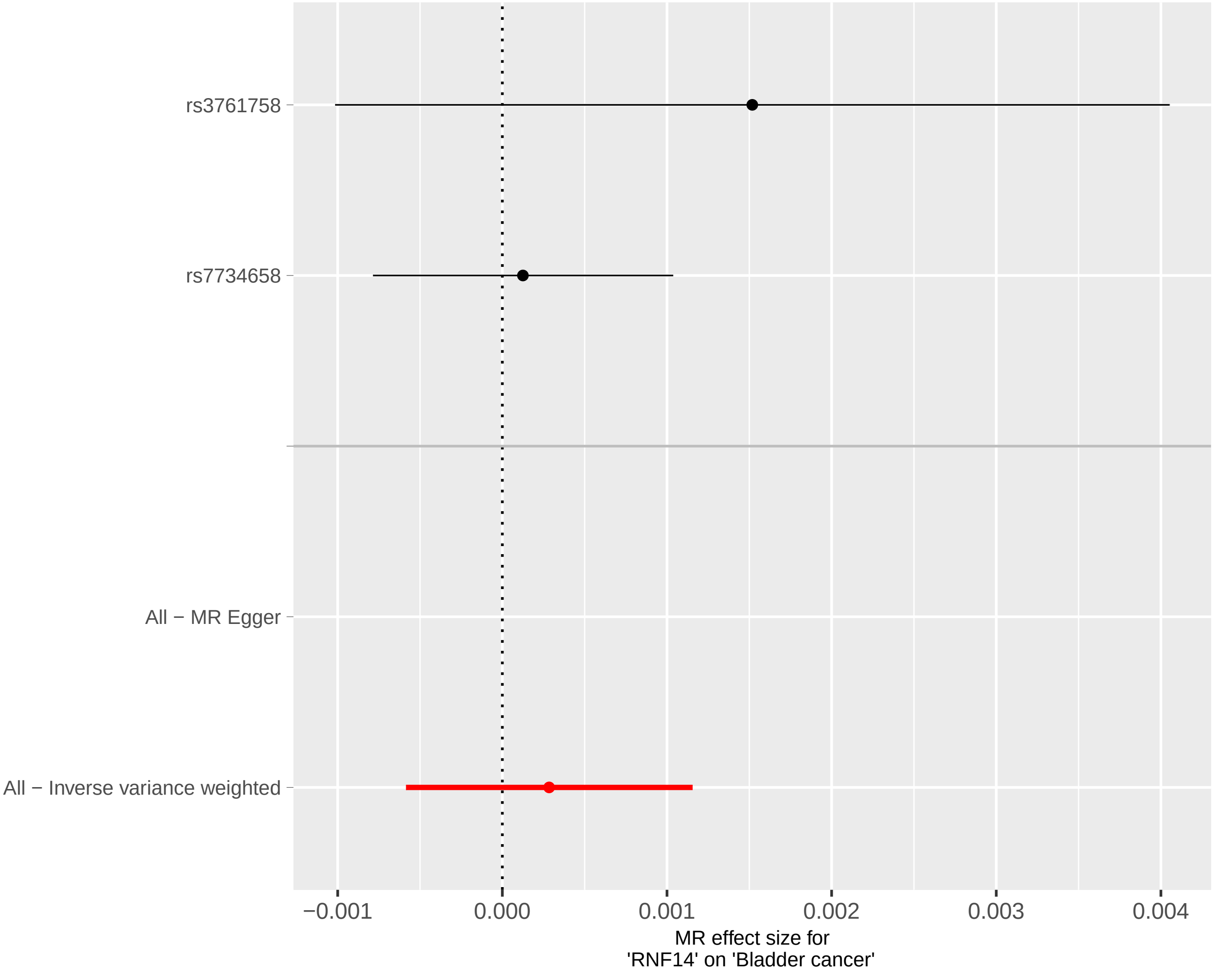

rs3761758
rs7734658
All − MR Egger
All − Inverse variance weighted
−0.001
0.000
0.001
0.002
0.003
0.004
MR effect size for
'RNF14' on 'Bladder cancer'

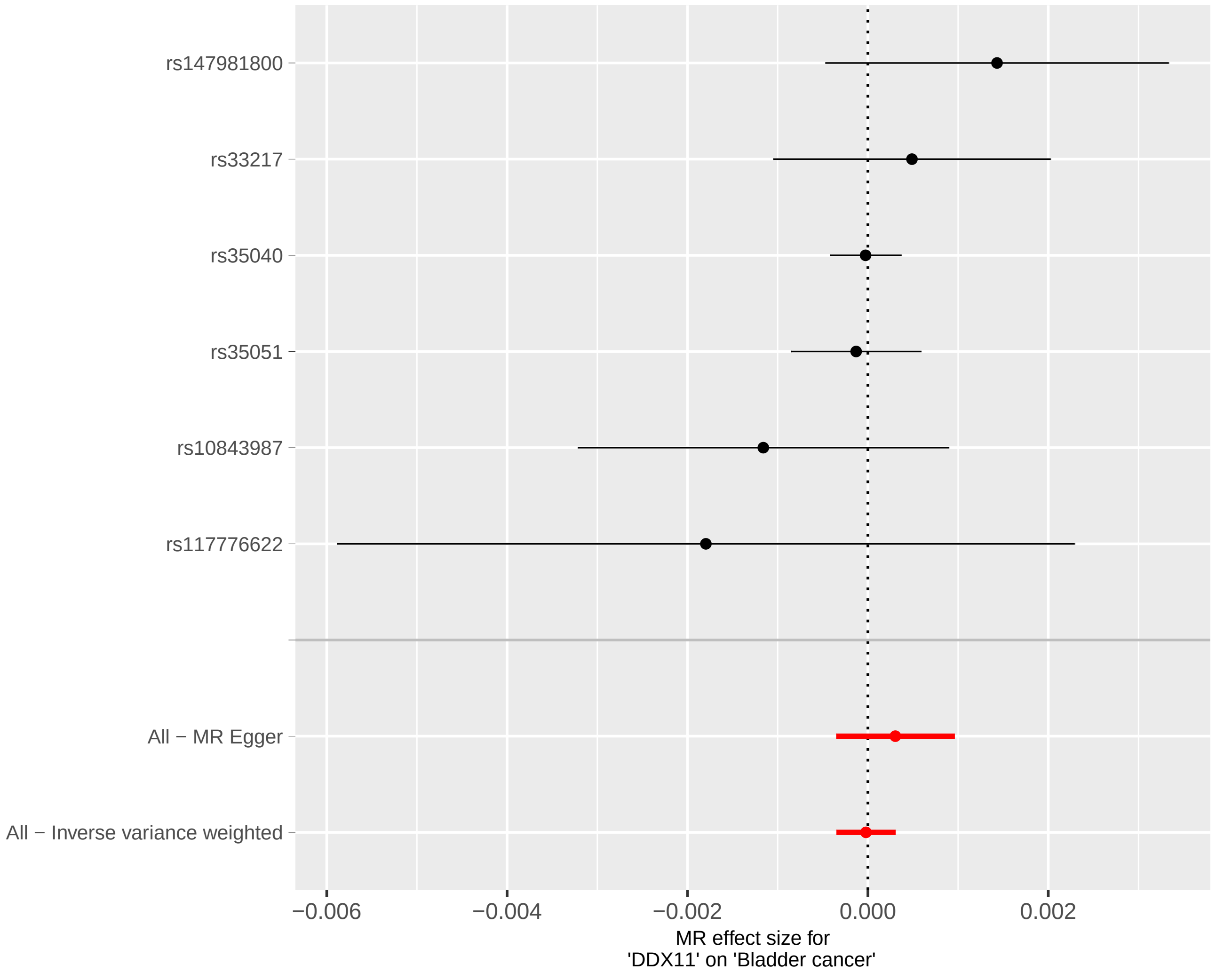

rs147981800
rs33217
rs35040
rs35051
rs10843987
rs117776622
All − MR Egger
All − Inverse variance weighted
−0.006
−0.004
−0.002
0.000
0.002
MR effect size for
'DDX11' on 'Bladder cancer'

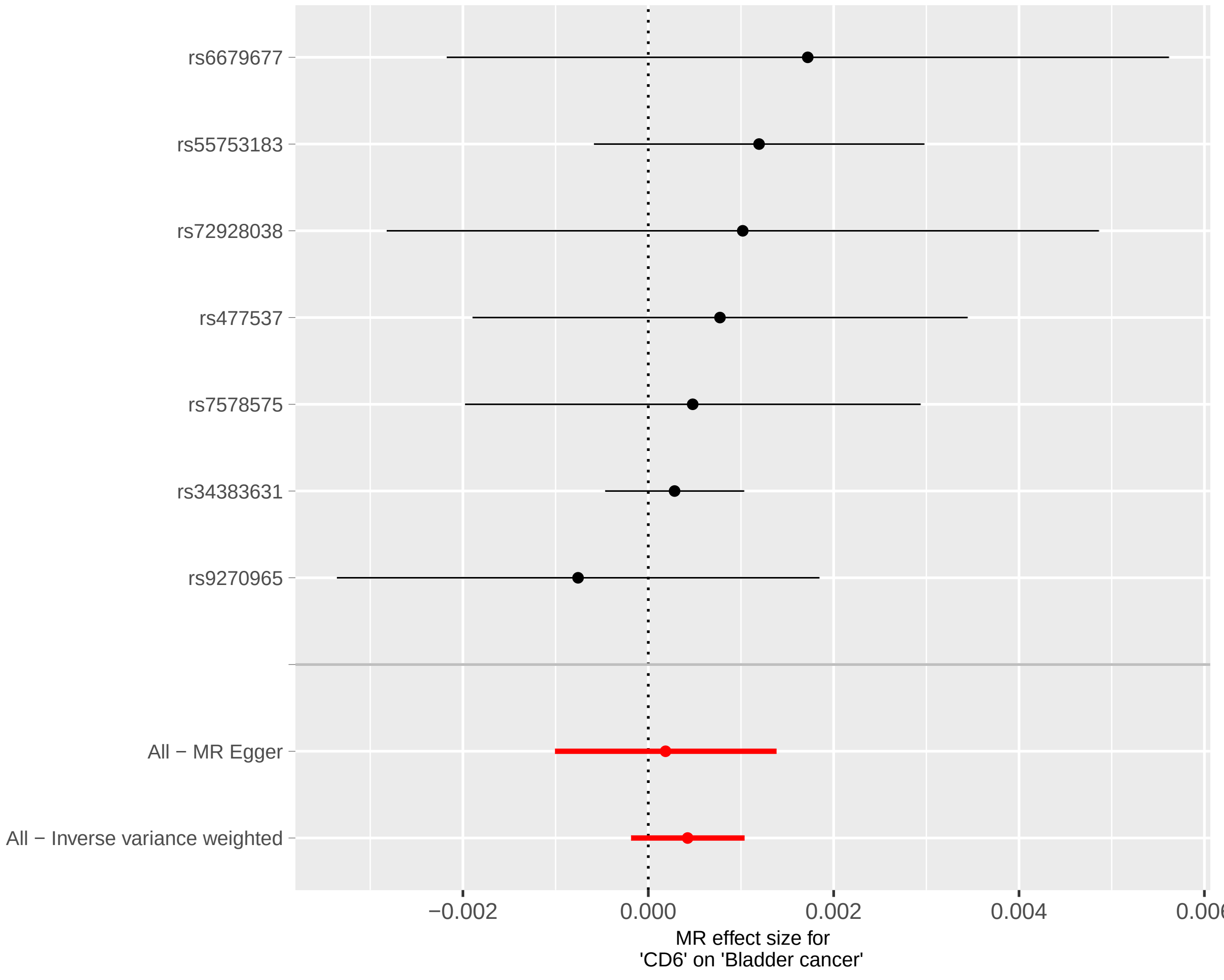

rs6679677
rs55753183
rs72928038
rs477537
rs7578575
rs34383631
rs9270965
All − MR Egger
All − Inverse variance weighted
−0.002
0.000
0.002
0.004
MR effect size for
'CD6' on 'Bladder cancer'

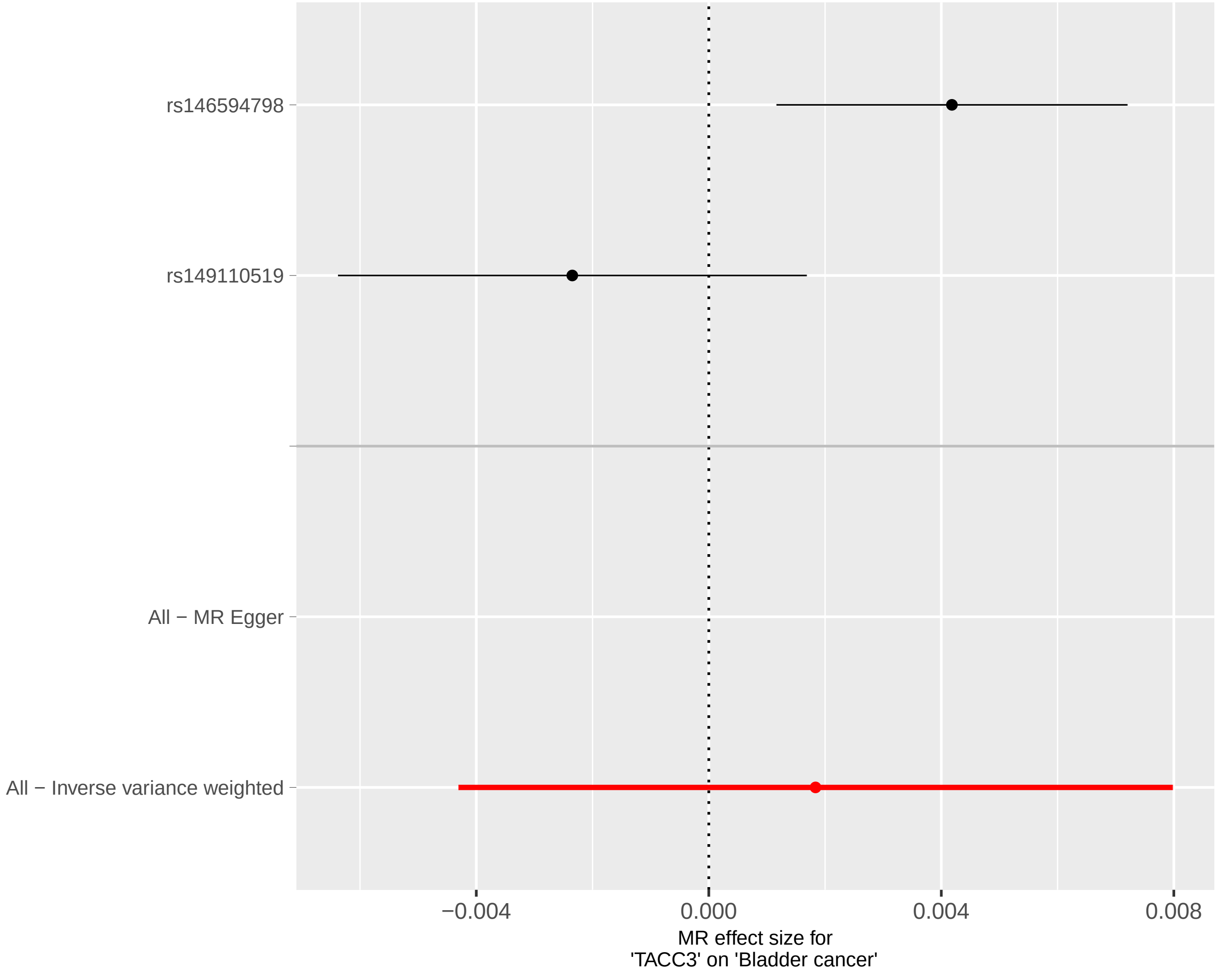
rs146594798
rs149110519
All − MR Egger
All − Inverse variance weighted
−0.004
0.000
0.004
0.008
MR effect size for
'TACC3' on 'Bladder cancer'

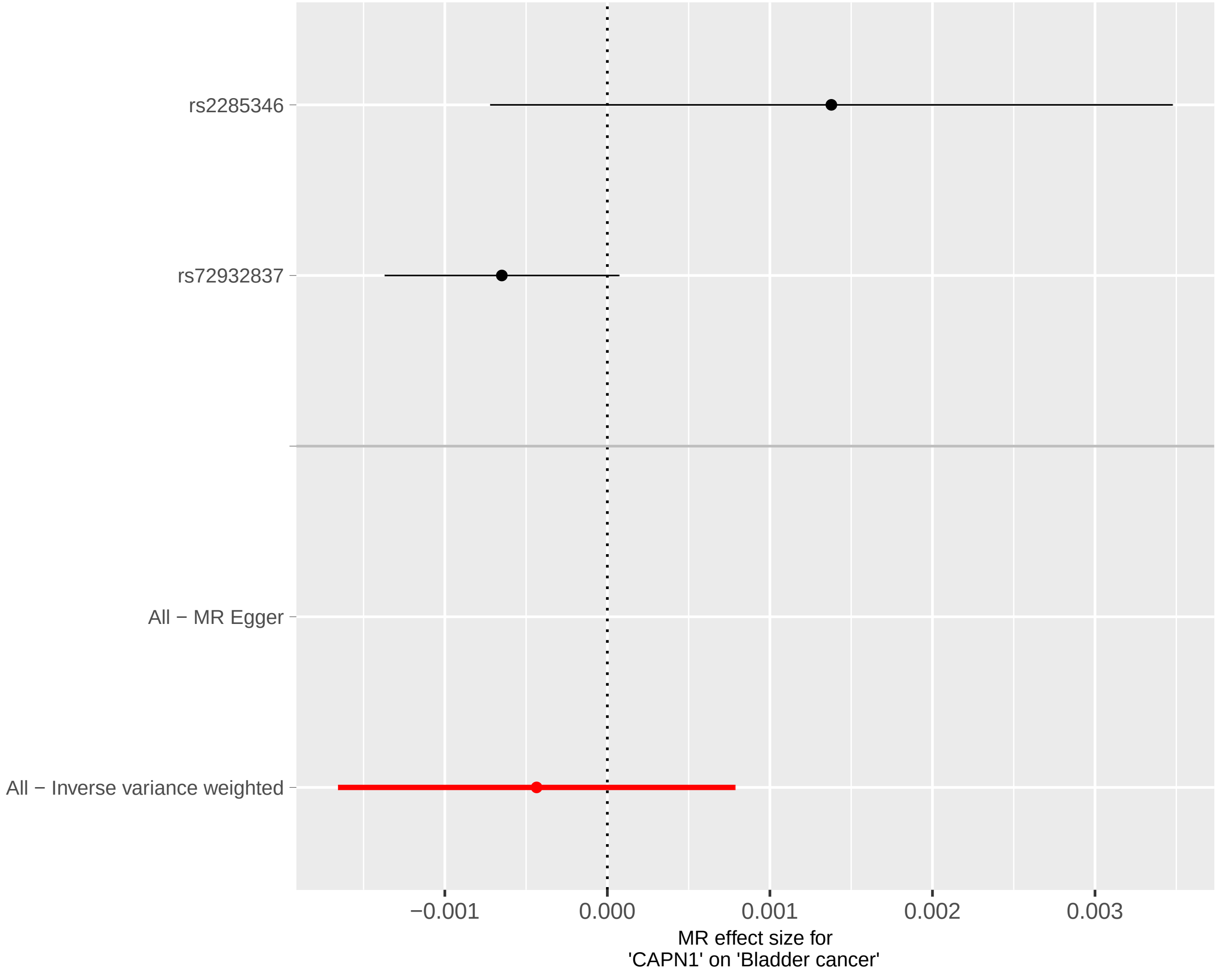
rs2285346
rs72932837
All − MR Egger
All − Inverse variance weighted
−0.001
0.000
0.001
0.002
0.003
MR effect size for
'CAPN1' on 'Bladder cancer'

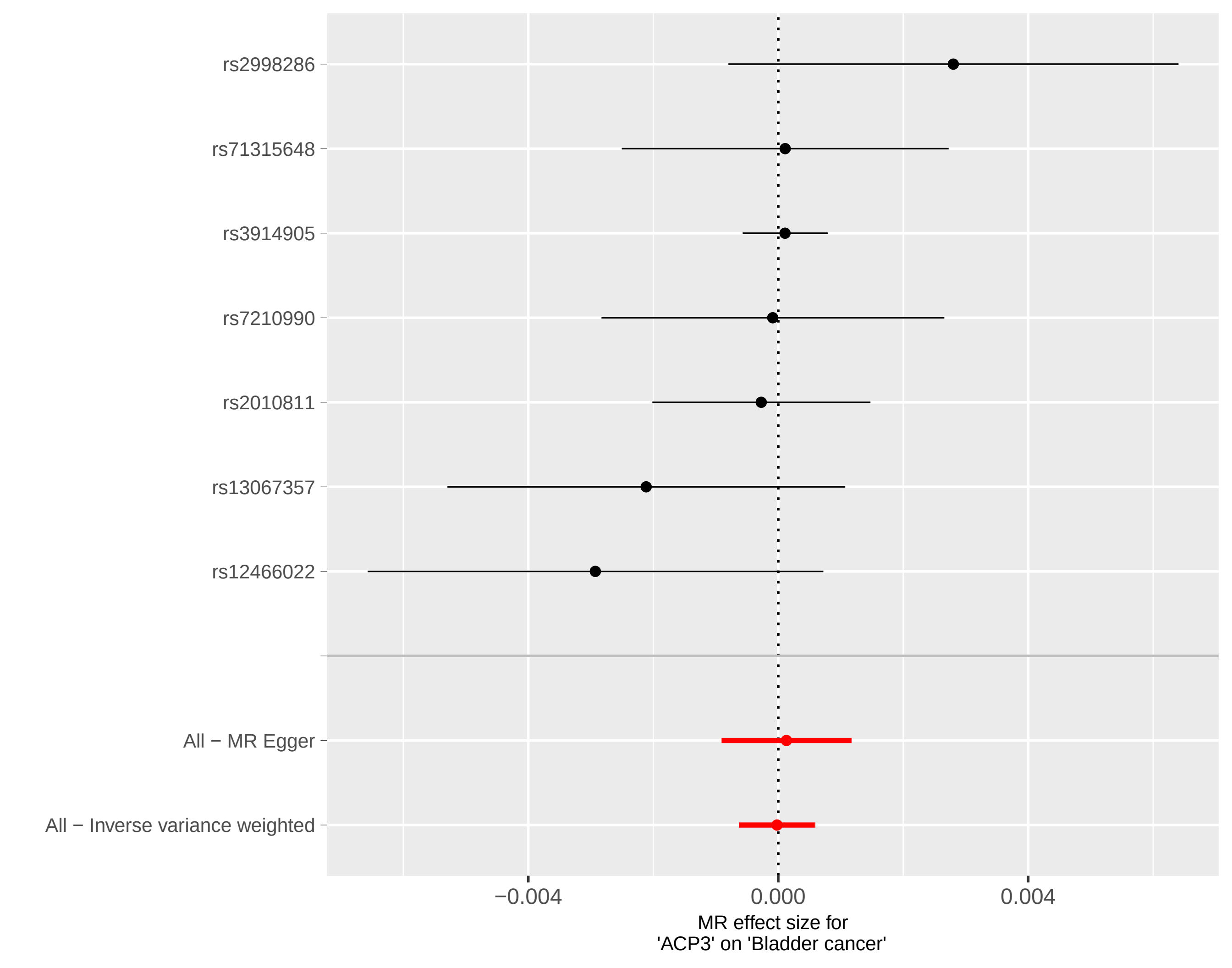
rs2998286
rs71315648
rs3914905
rs7210990
rs2010811
rs13067357
rs12466022
All − MR Egger
All − Inverse variance weighted
−0.004
0.000
0.004
MR effect size for
'ACP3' on 'Bladder cancer'

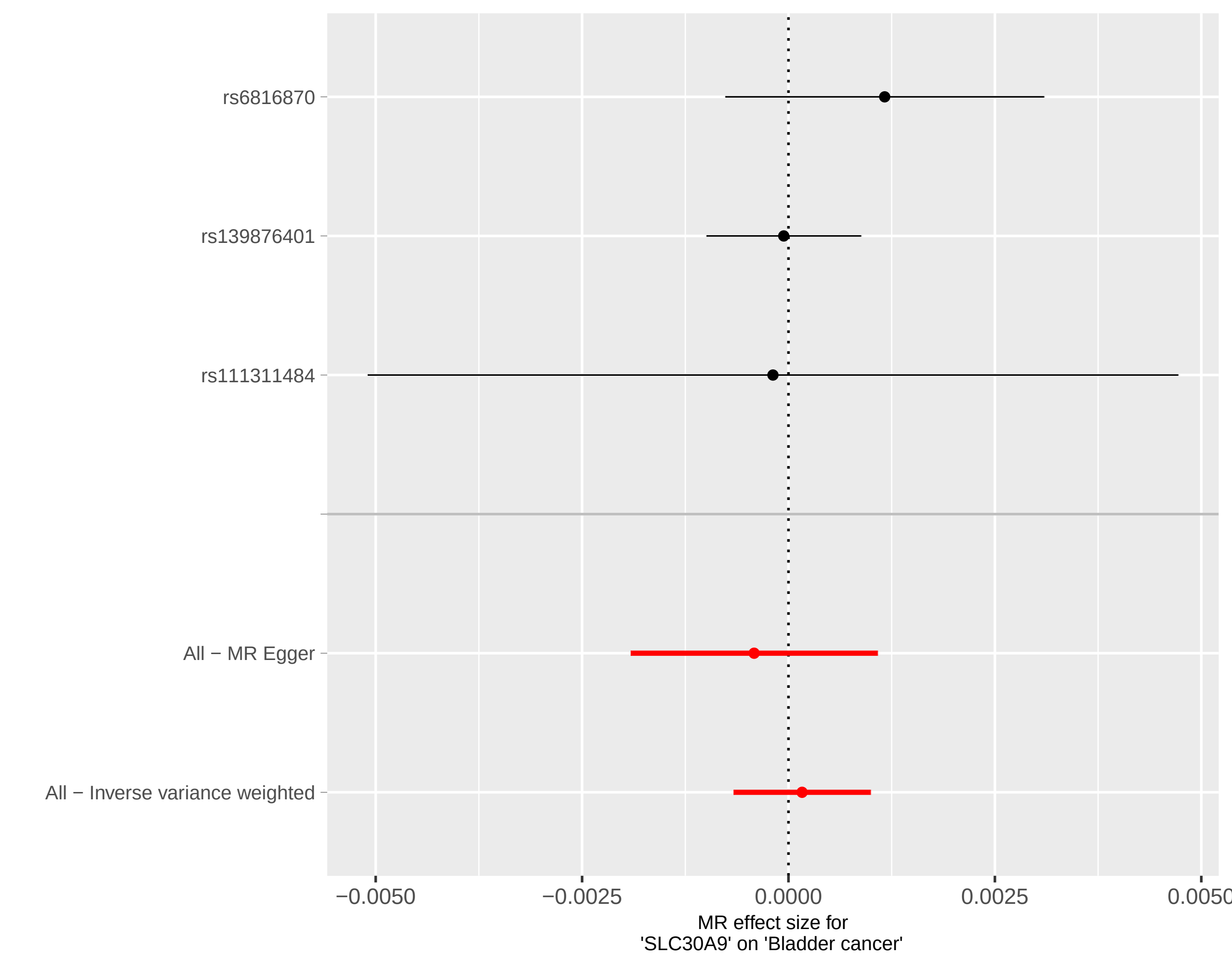
rs6816870
rs139876401
rs111311484
All − MR Egger
All − Inverse variance weighted
−0.0050
−0.0025
0.0000
0.0025
0.0050
MR effect size for
'SLC30A9' on 'Bladder cancer'

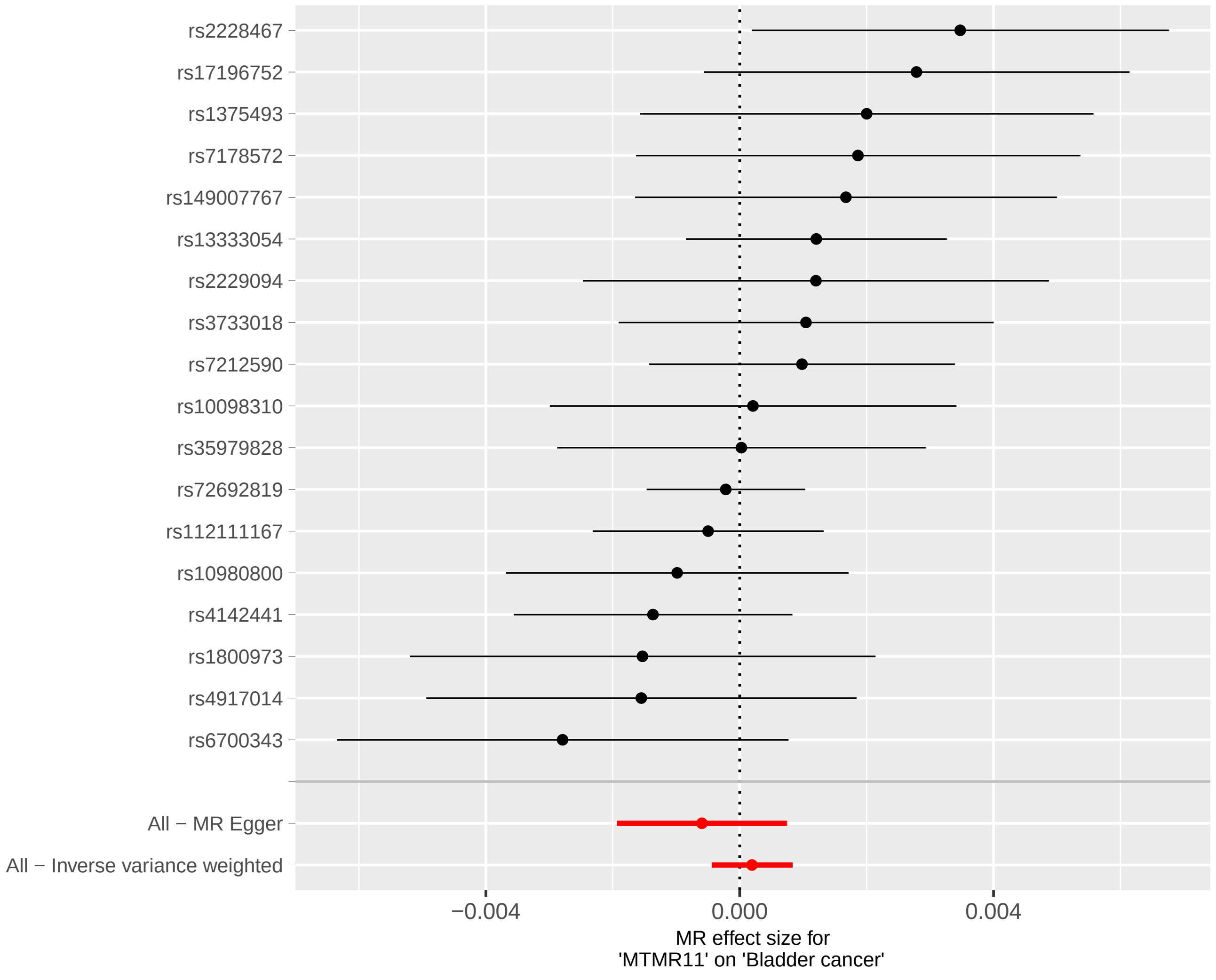

rs2228467
rs17196752
rs1375493
rs7178572
rs149007767
rs13333054
rs2229094
rs3733018
rs7212590
rs10098310
rs35979828
rs72692819
rs112111167
rs10980800
rs4142441
rs1800973
rs4917014
rs6700343
All − MR Egger
All − Inverse variance weighted
−0.004
0.000
0.004
MR effect size for
'MTMR11' on 'Bladder cancer'

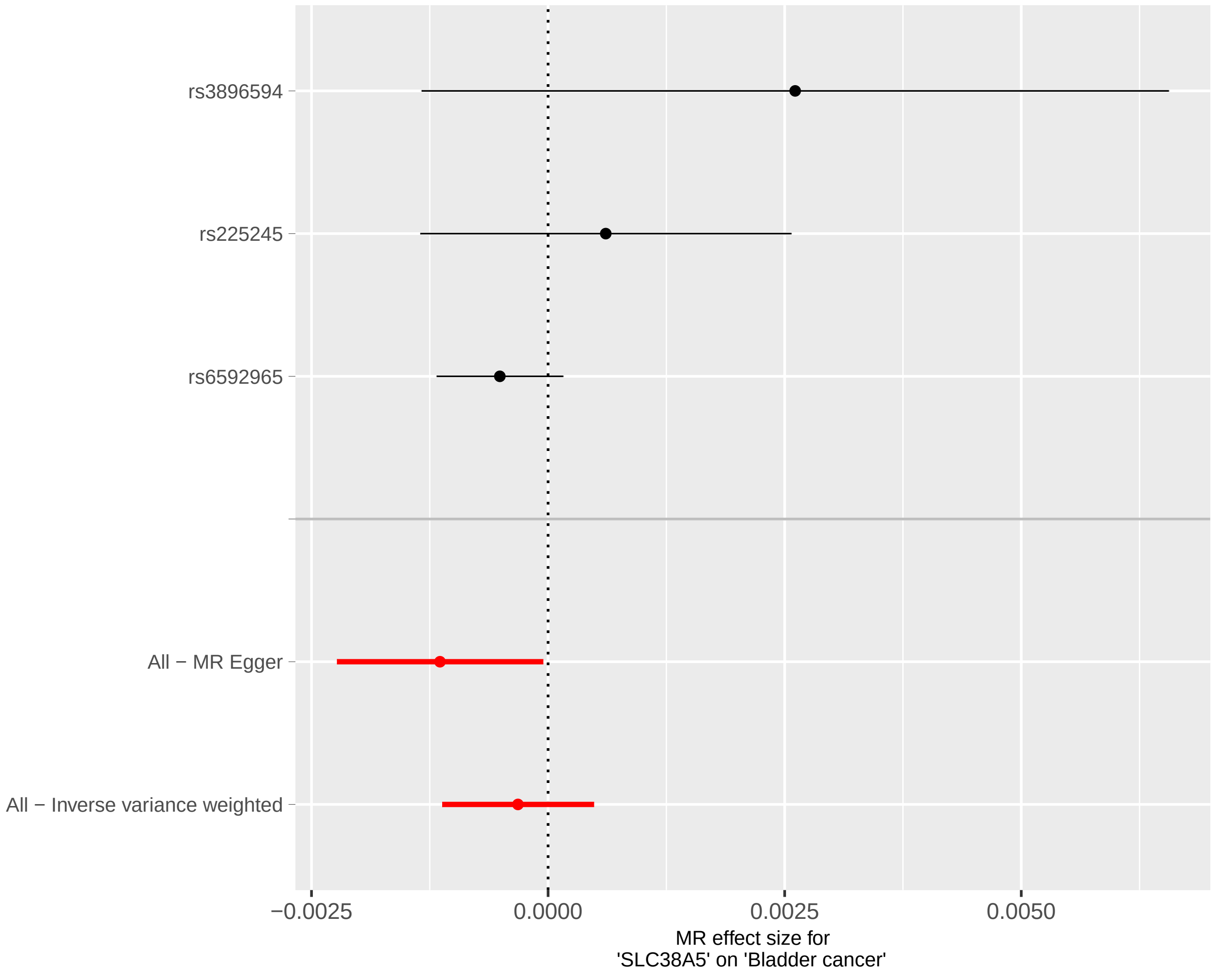
rs3896594
rs225245
rs6592965
All − MR Egger
All − Inverse variance weighted
−0.0025
0.0000
0.0025
0.0050
MR effect size for
'SLC38A5' on 'Bladder cancer'

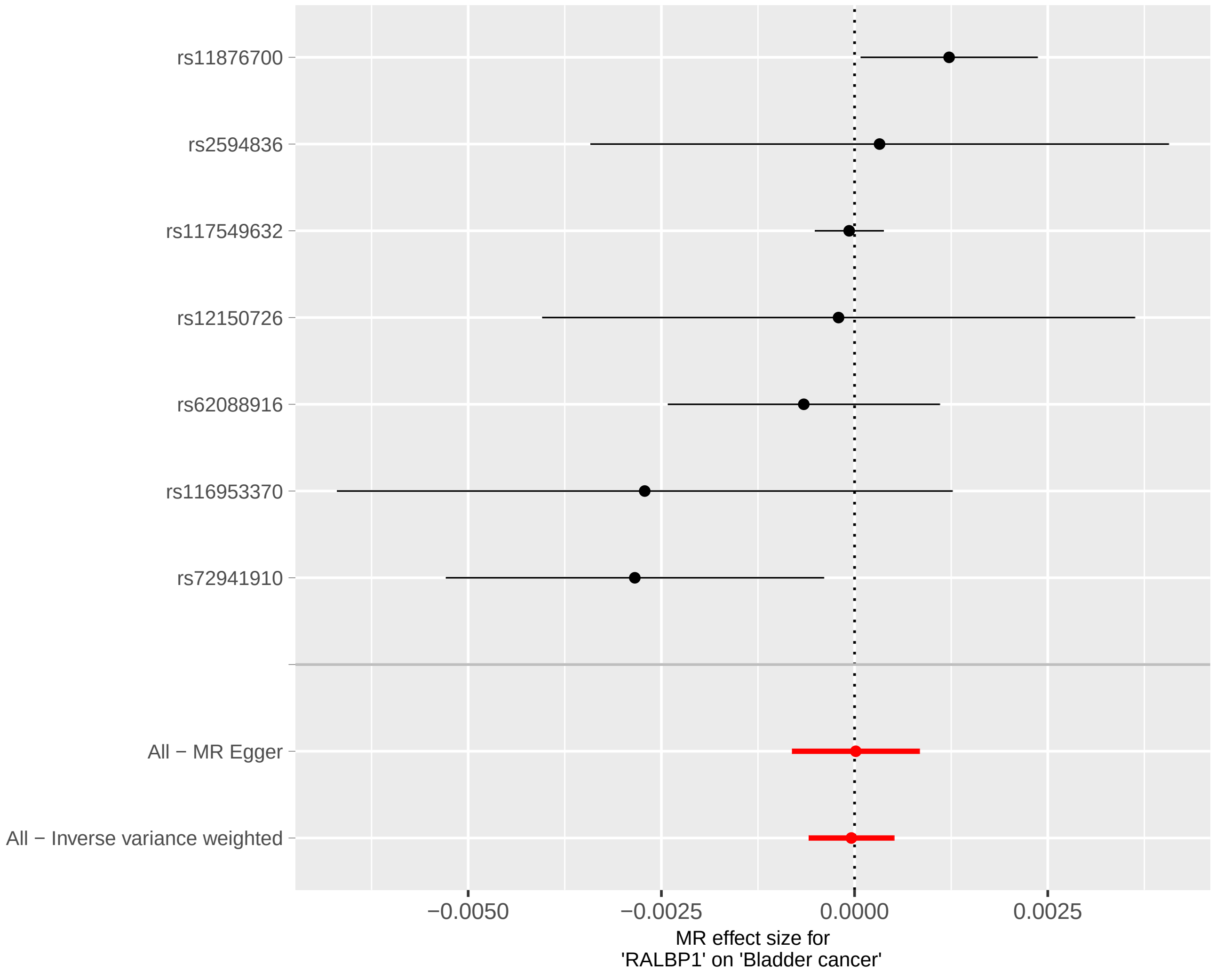
rs11876700
rs2594836
rs117549632
rs12150726
rs62088916
rs116953370
rs72941910
All − MR Egger
All − Inverse variance weighted
−0.0050
−0.0025
0.0000
0.0025
MR effect size for
'RALBP1' on 'Bladder cancer'

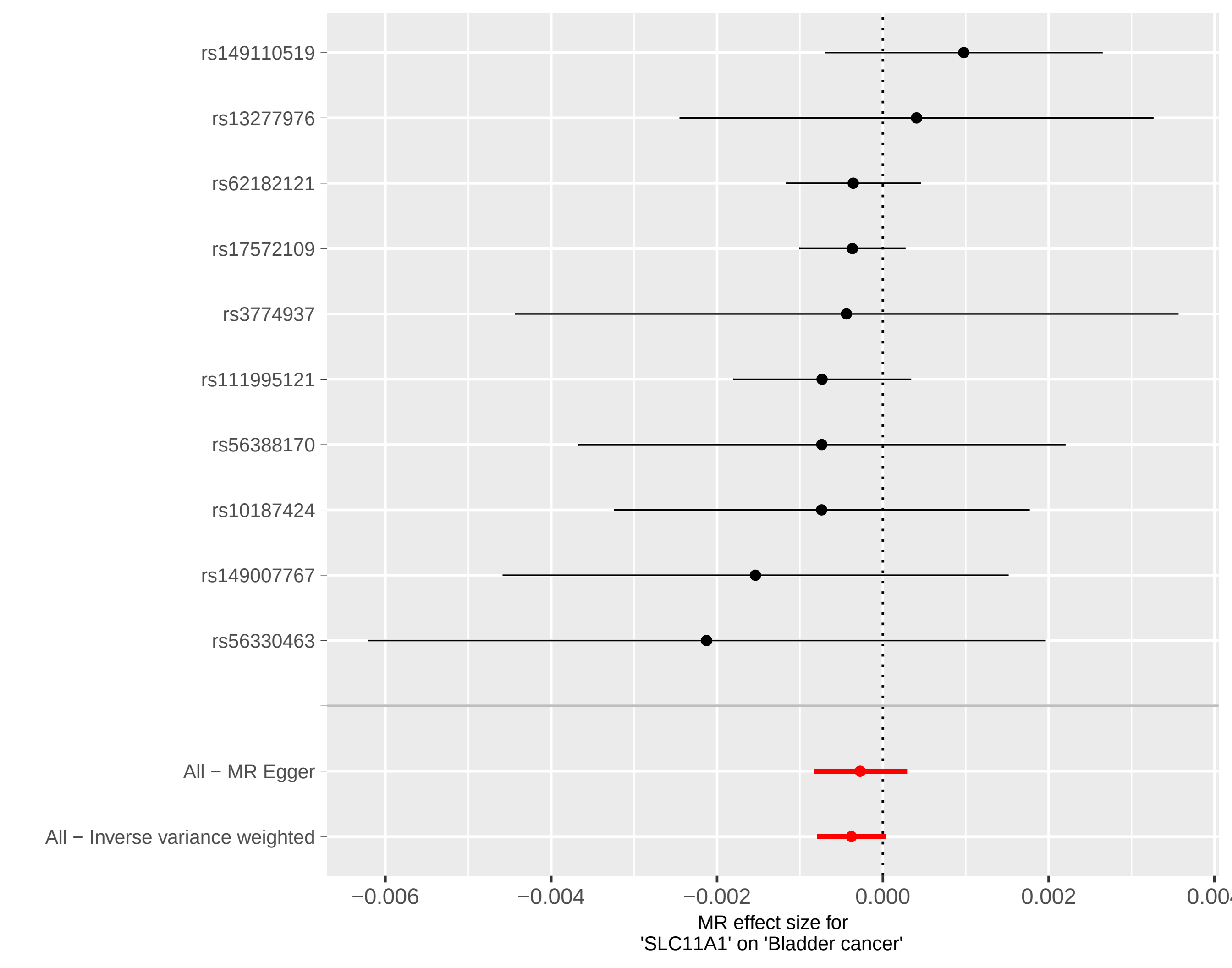

rs149110519
rs13277976
rs62182121
rs17572109
rs3774937
rs111995121
rs56388170
rs10187424
rs149007767
rs56330463
All − MR Egger
All − Inverse variance weighted
−0.006
−0.004
−0.002
0.000
0.002
MR effect size for
'SLC11A1' on 'Bladder cancer'

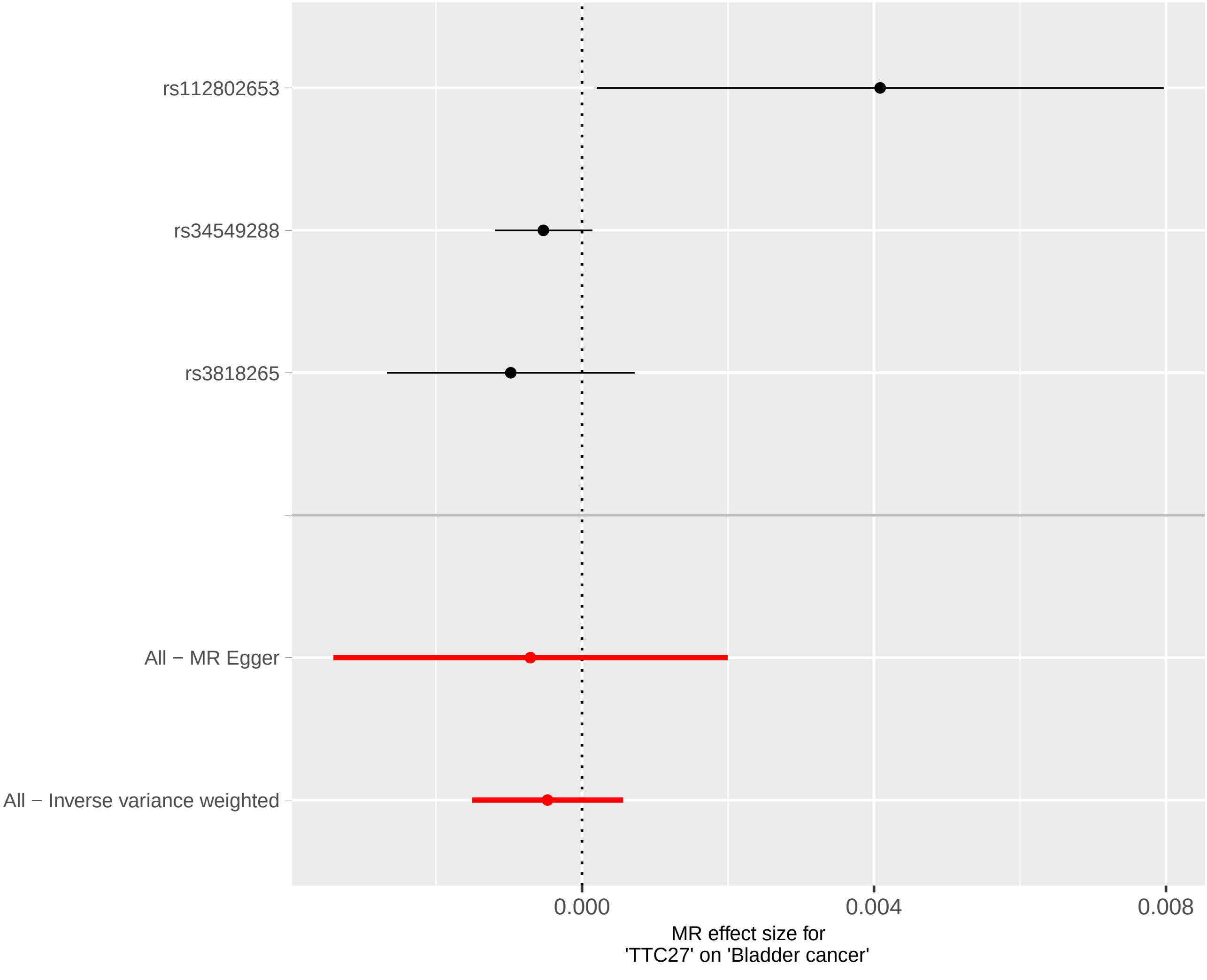
rs112802653
rs34549288
rs3818265
All − MR Egger
All − Inverse variance weighted
0.000
0.004
0.008
MR effect size for
'TTC27' on 'Bladder cancer'

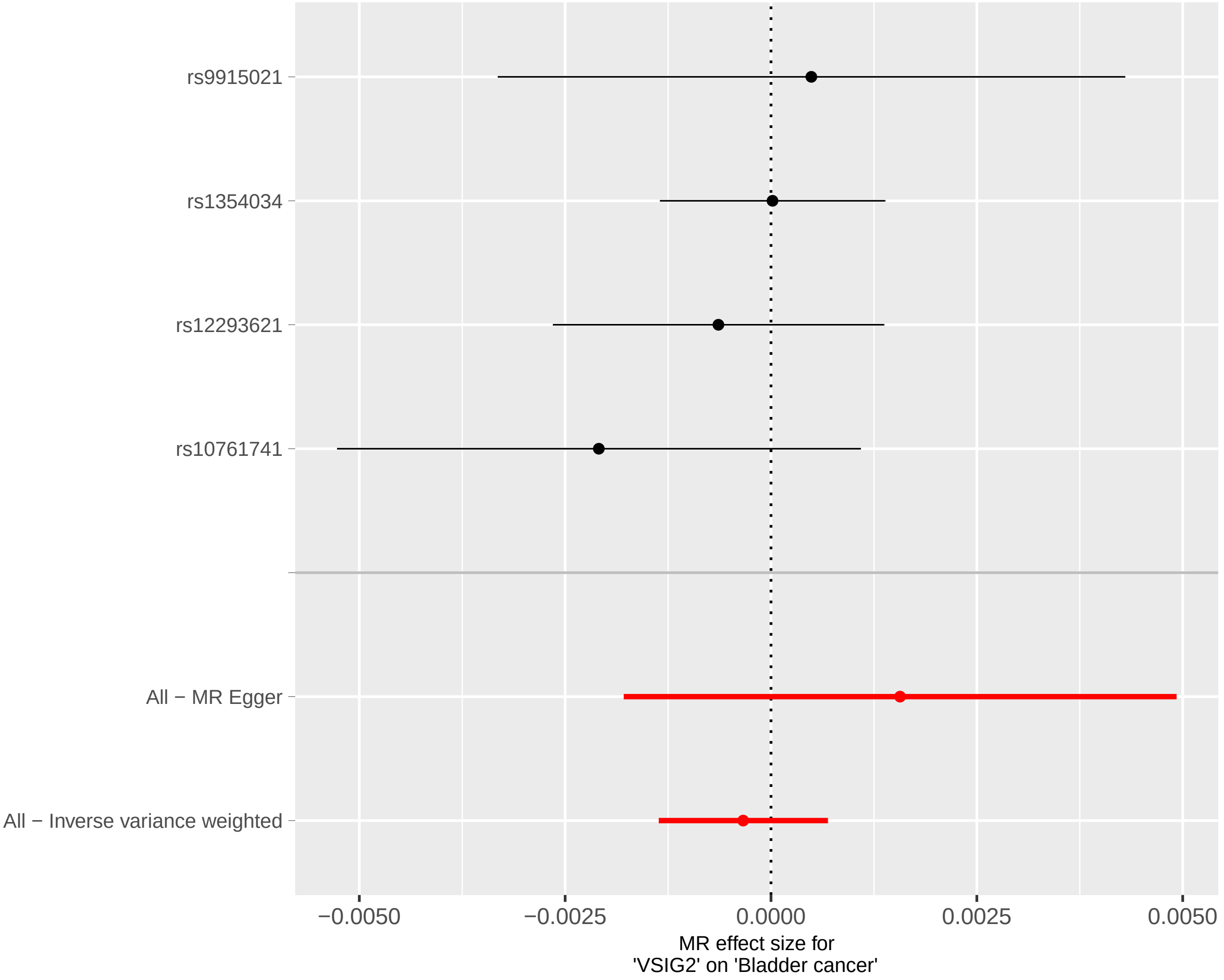

rs9915021
rs1354034
rs12293621
rs10761741
All − MR Egger
All − Inverse variance weighted
−0.0050
−0.0025
0.0000
0.0025
0.0050
MR effect size for
'VSIG2' on 'Bladder cancer'

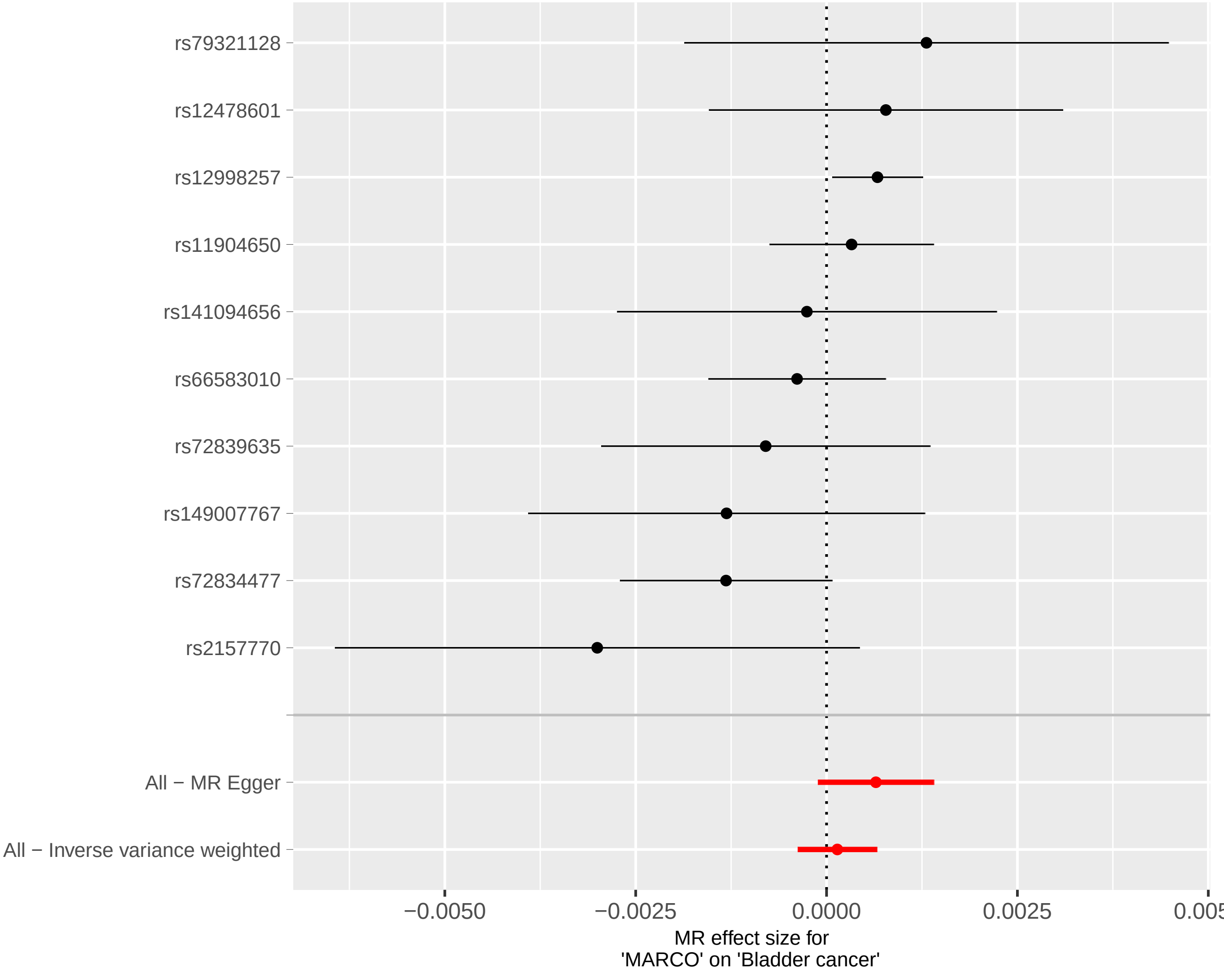

rs79321128
rs12478601
rs12998257
rs11904650
rs141094656
rs66583010
rs72839635
rs149007767
rs72834477
rs2157770
All − MR Egger
All − Inverse variance weighted
−0.0050
−0.0025
0.0000
0.0025
MR effect size for
'MARCO' on 'Bladder cancer'

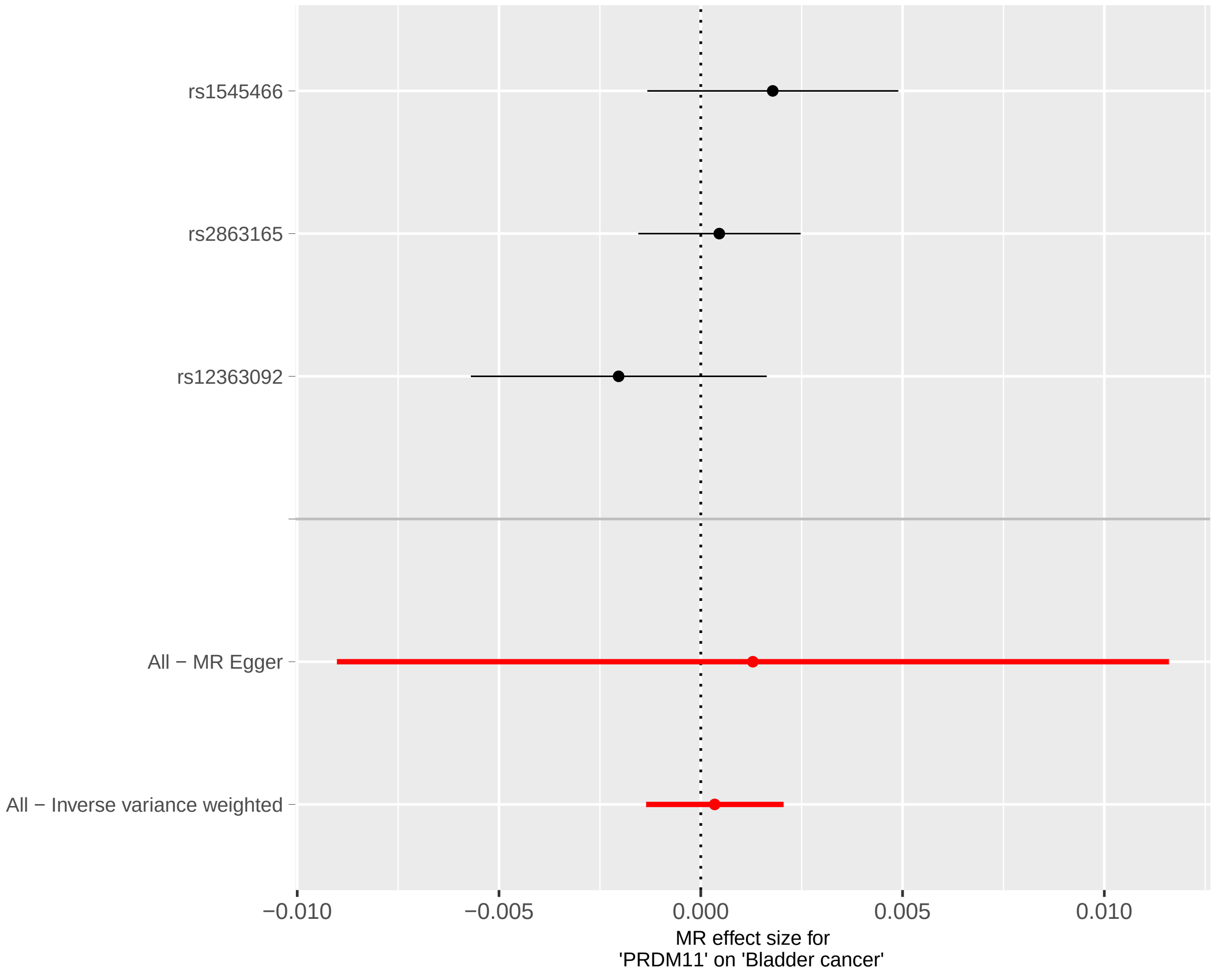
rs1545466
rs2863165
rs12363092
All − MR Egger
All − Inverse variance weighted
−0.010
−0.005
0.000
0.005
0.010
MR effect size for
'PRDM11' on 'Bladder cancer'

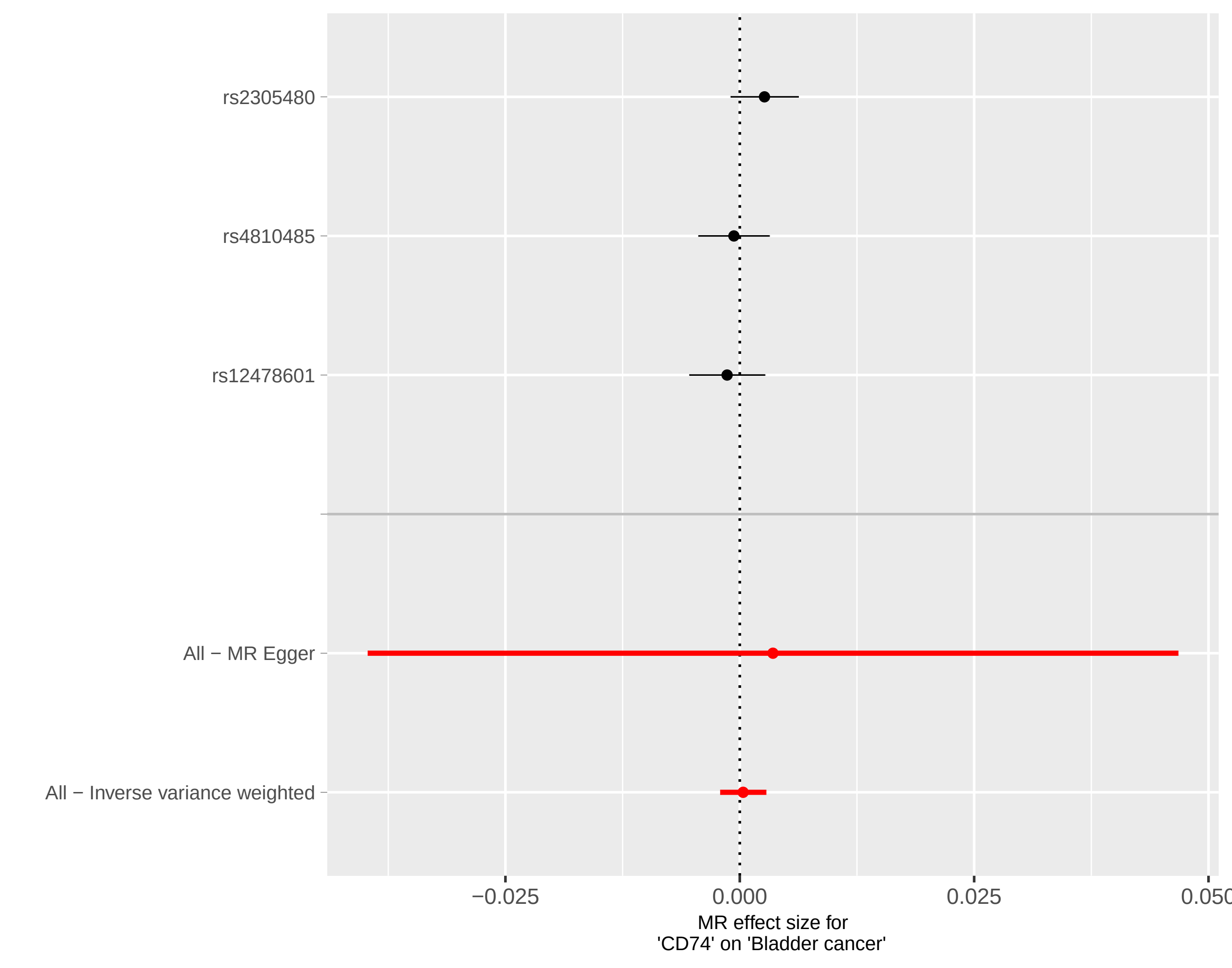
rs2305480
rs4810485
rs12478601
All − MR Egger
All − Inverse variance weighted
−0.025
0.000
0.025
0.050
MR effect size for
'CD74' on 'Bladder cancer'

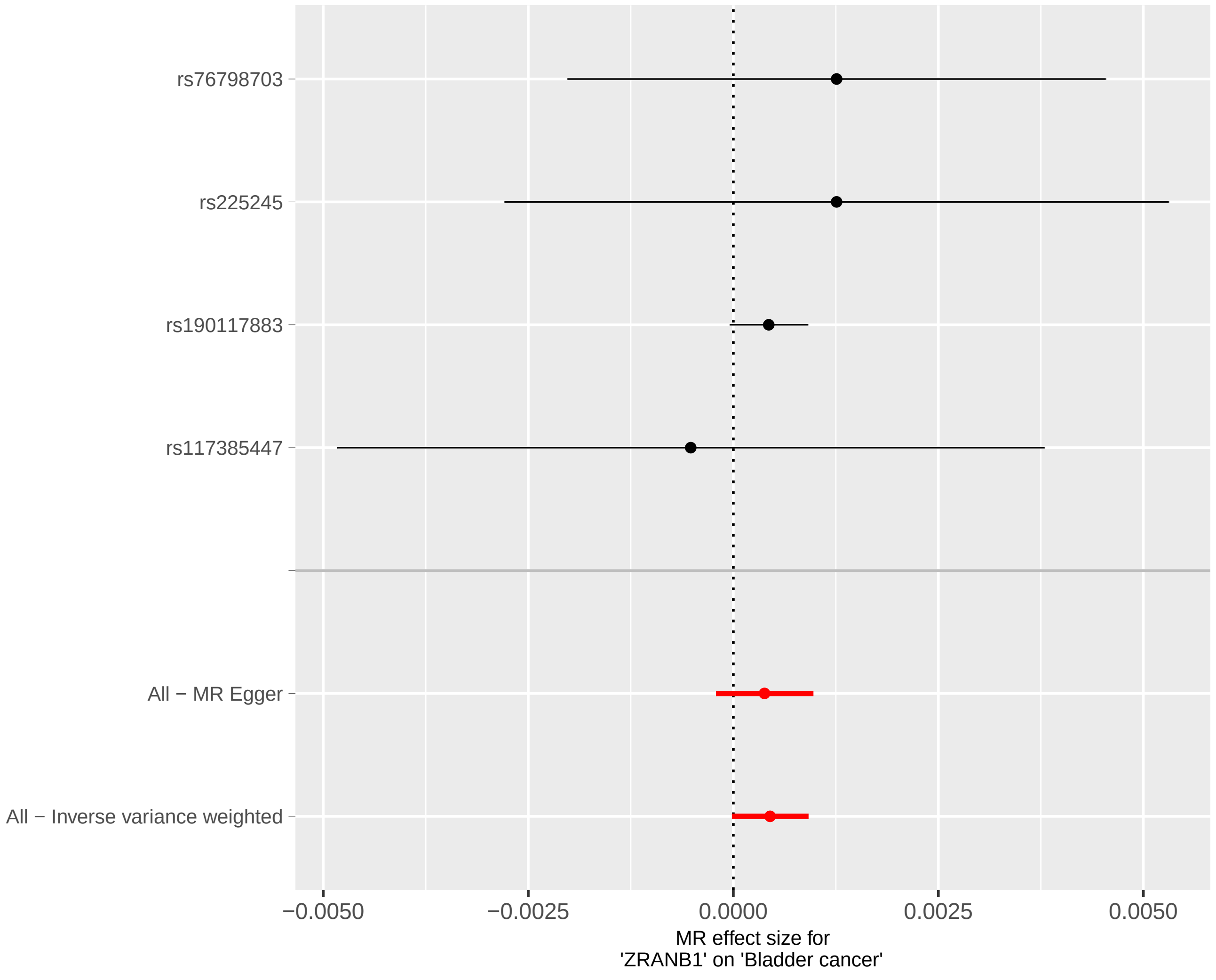
rs76798703
rs225245
rs190117883
rs117385447
All − MR Egger
All − Inverse variance weighted
−0.0050
−0.0025
0.0000
0.0025
0.0050
MR effect size for
'ZRANB1' on 'Bladder cancer'

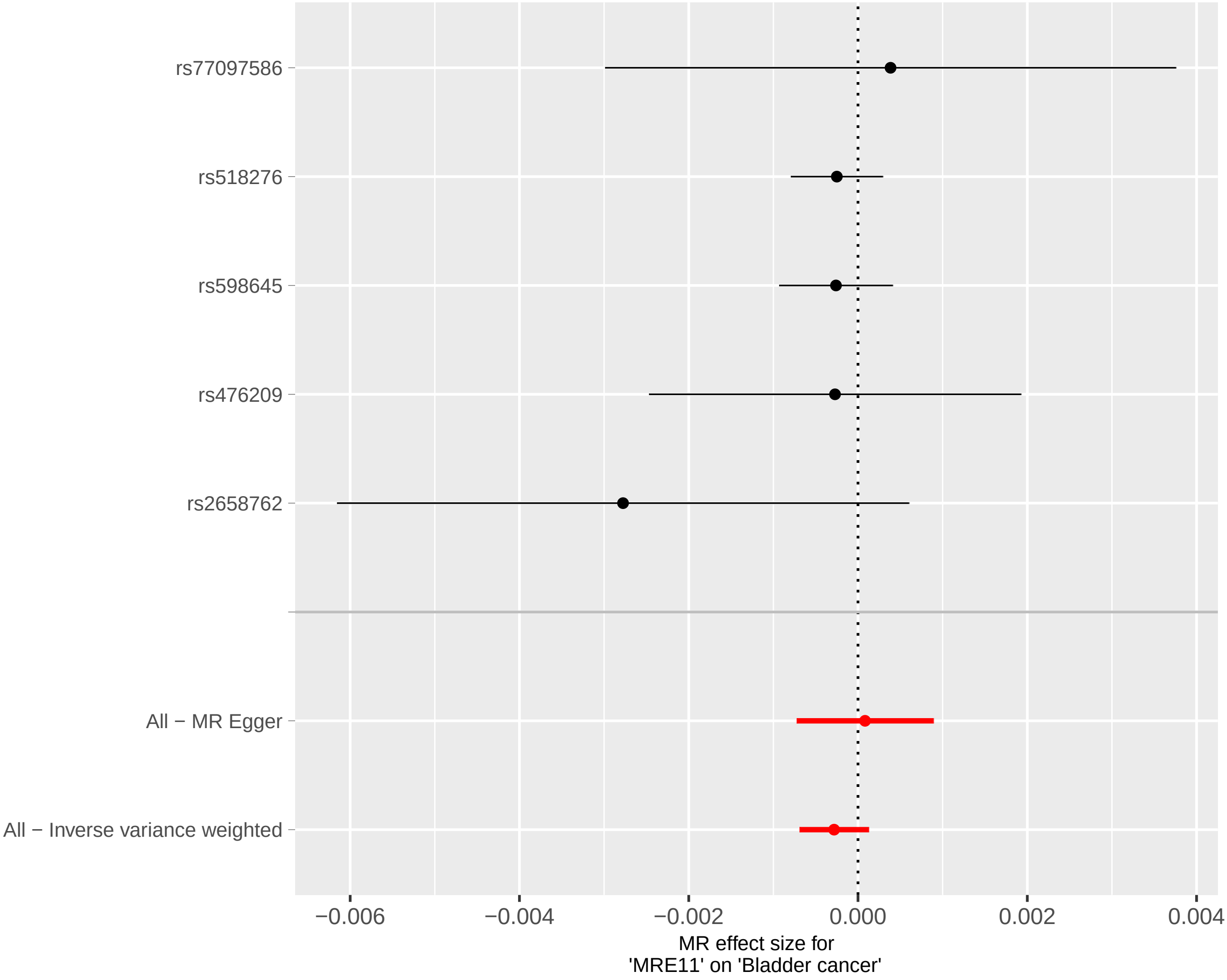

rs77097586
rs518276
rs598645
rs476209
rs2658762
All − MR Egger
All − Inverse variance weighted
−0.006
−0.004
−0.002
0.000
0.002
0.004
MR effect size for
'MRE11' on 'Bladder cancer'

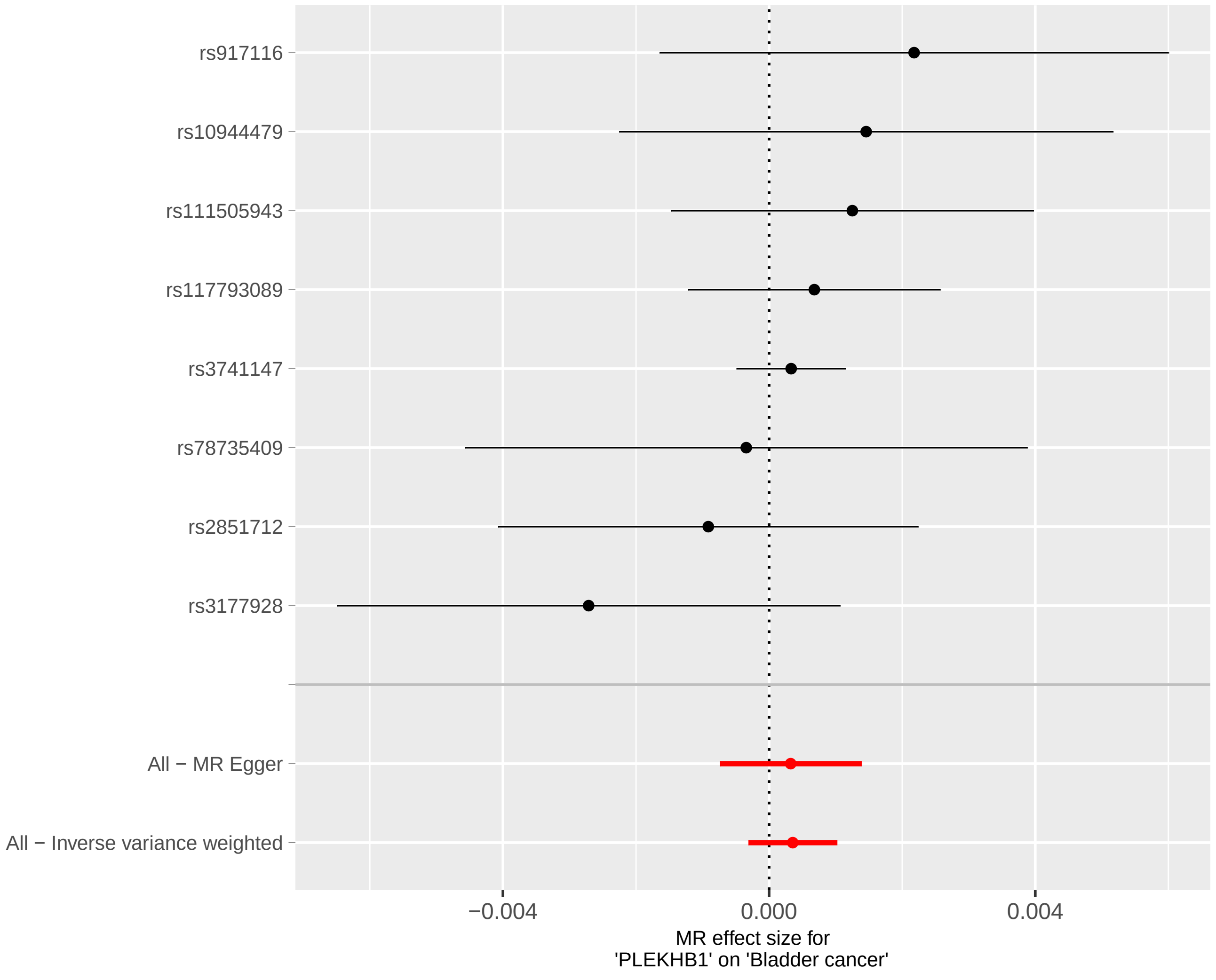
rs917116
rs10944479
rs111505943
rs117793089
rs3741147
rs78735409
rs2851712
rs3177928
All − MR Egger
All − Inverse variance weighted
−0.004
0.000
0.004
MR effect size for
'PLEKHB1' on 'Bladder cancer'

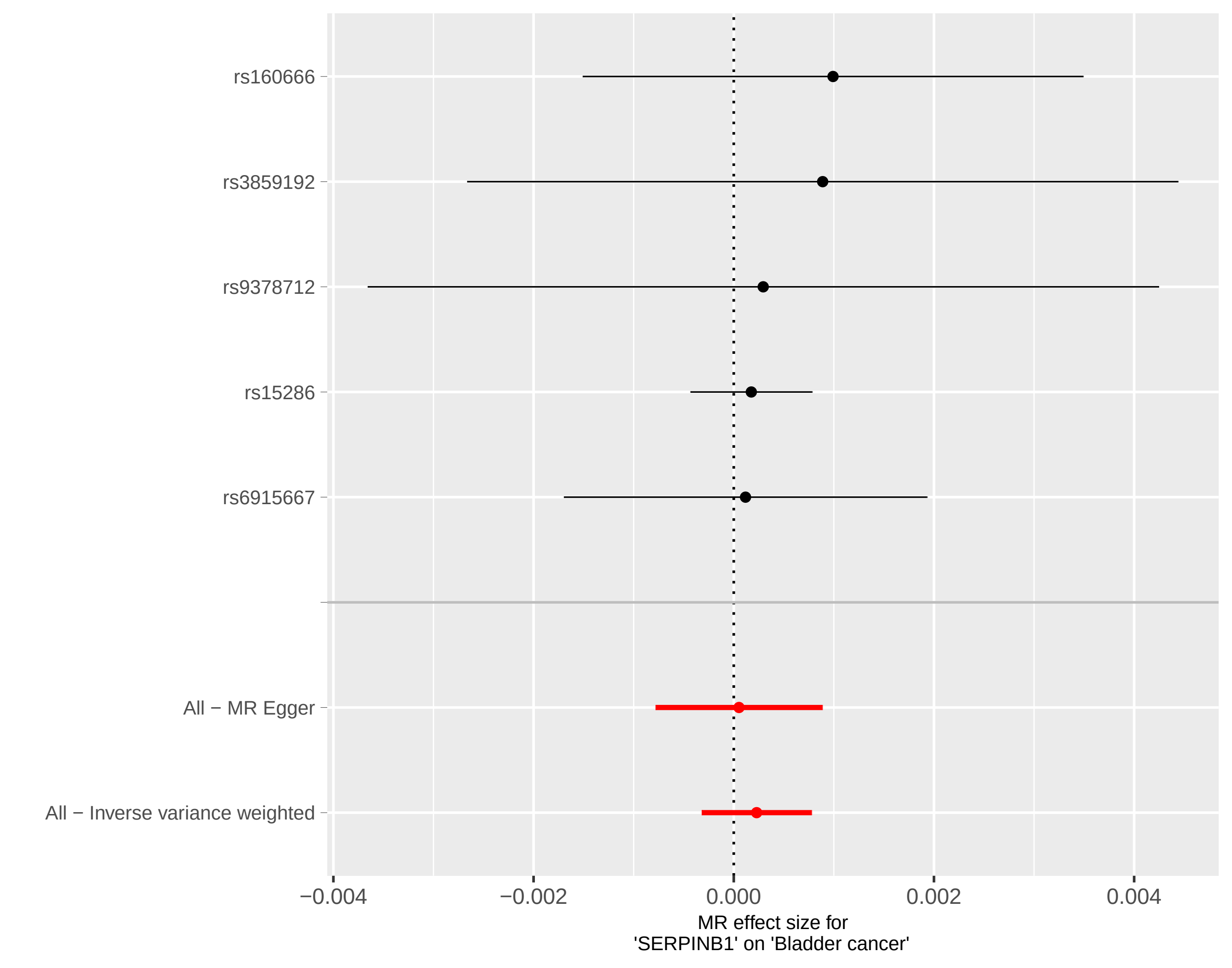
rs160666
rs3859192
rs9378712
rs15286
rs6915667
All − MR Egger
All − Inverse variance weighted
−0.004
−0.002
0.000
0.002
0.004
MR effect size for
'SERPINB1' on 'Bladder cancer'

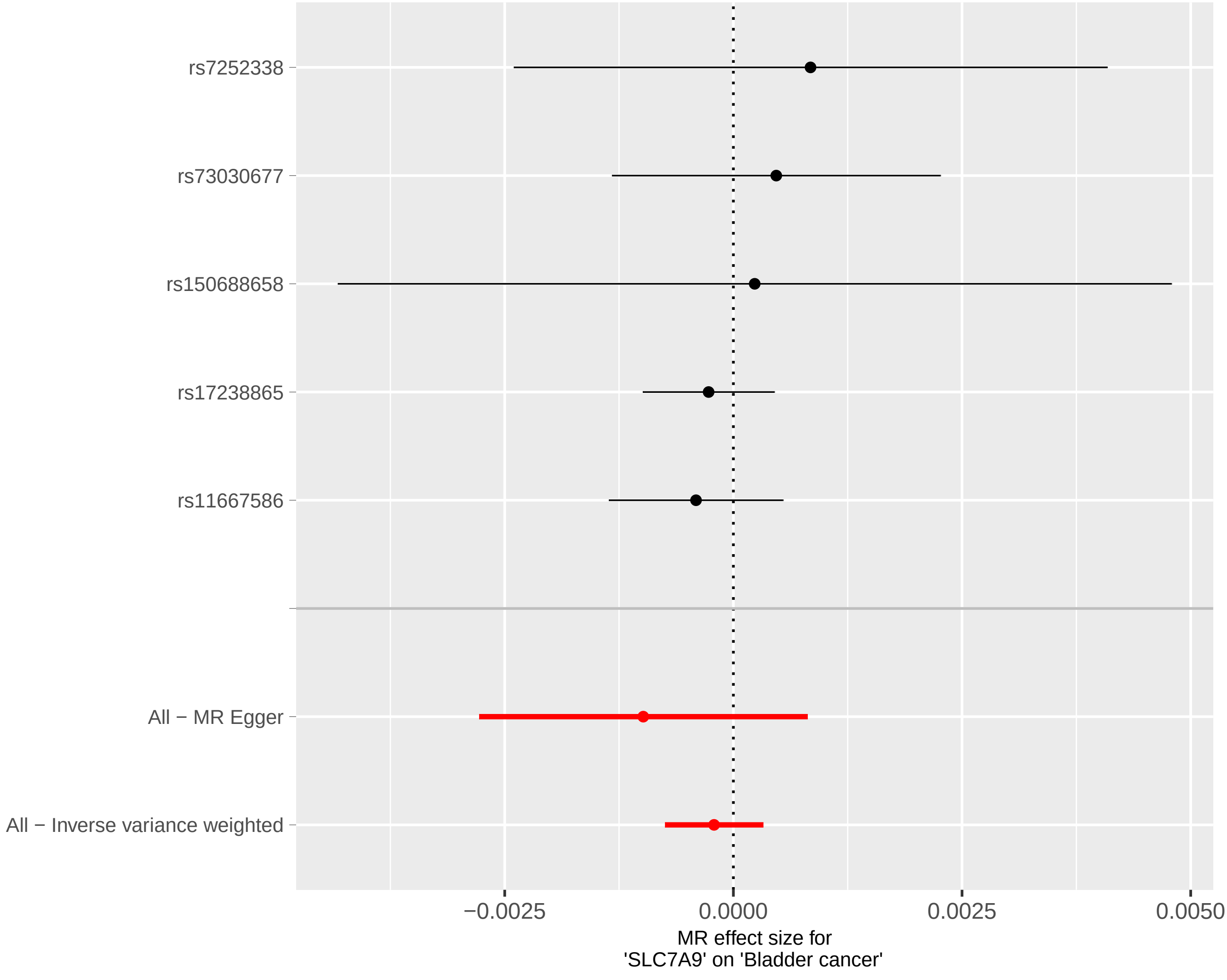

rs7252338
rs73030677
rs150688658
rs17238865
rs11667586
All − MR Egger
All − Inverse variance weighted
−0.0025
0.0000
0.0025
0.0050
MR effect size for
'SLC7A9' on 'Bladder cancer'

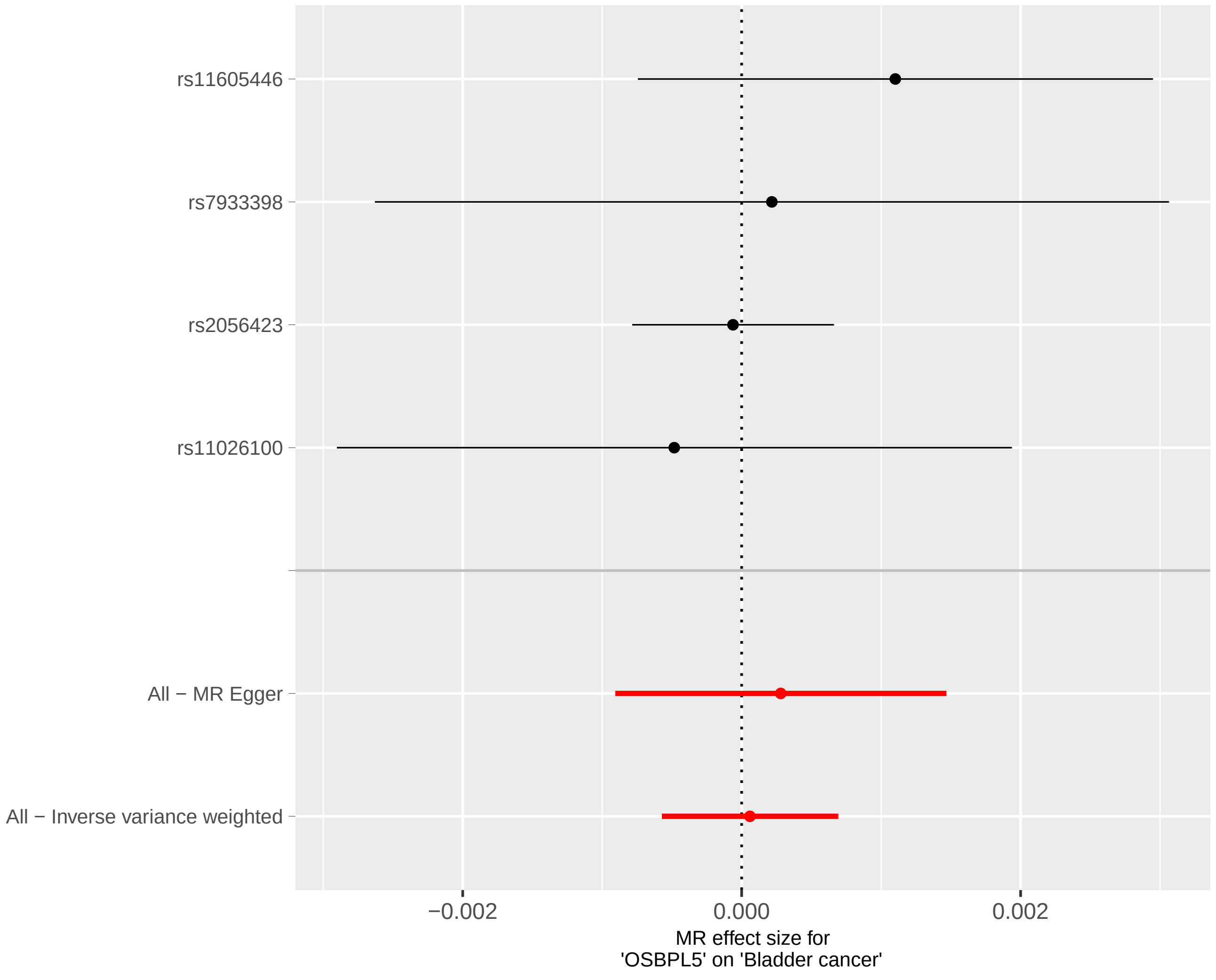
rs11605446
rs7933398
rs2056423
rs11026100
All − MR Egger
All − Inverse variance weighted
−0.002
0.000
0.002
MR effect size for
'OSBPL5' on 'Bladder cancer'

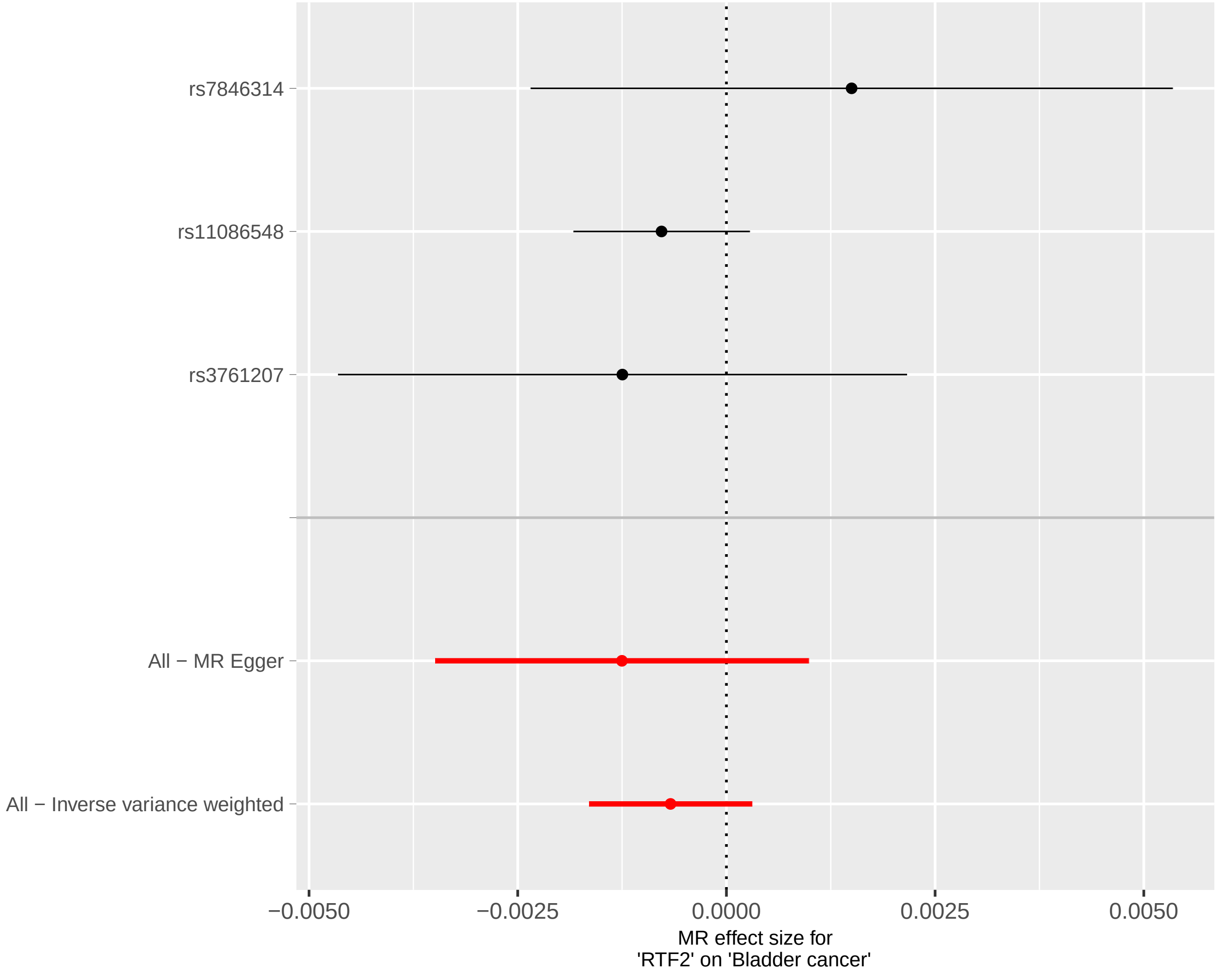
rs7846314
rs11086548
rs3761207
All − MR Egger
All − Inverse variance weighted
−0.0050
−0.0025
0.0000
0.0025
0.0050
MR effect size for
'RTF2' on 'Bladder cancer'

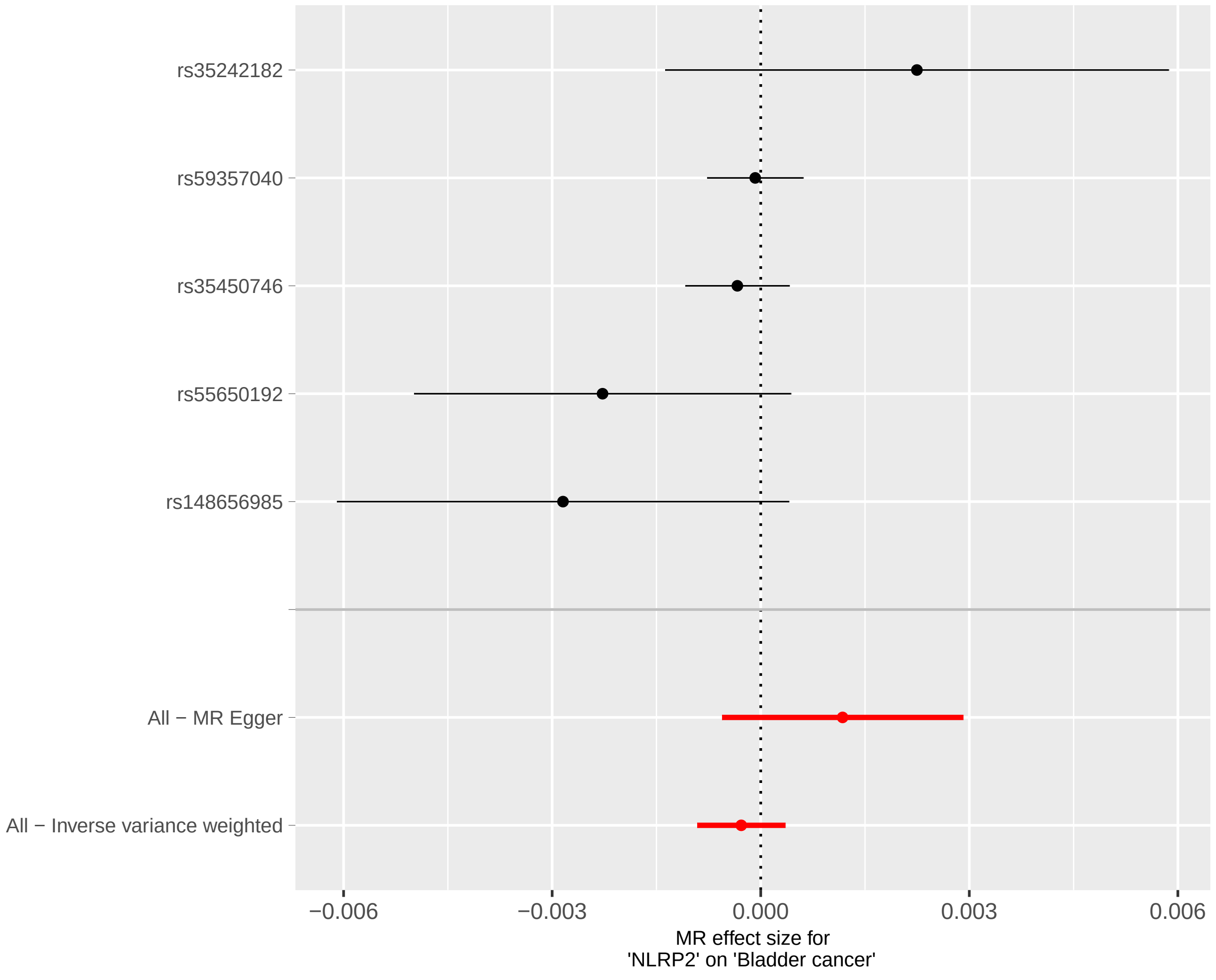
rs35242182
rs59357040
rs35450746
rs55650192
rs148656985
All − MR Egger
All − Inverse variance weighted
−0.006
−0.003
0.000
0.003
0.006
MR effect size for
'NLRP2' on 'Bladder cancer'

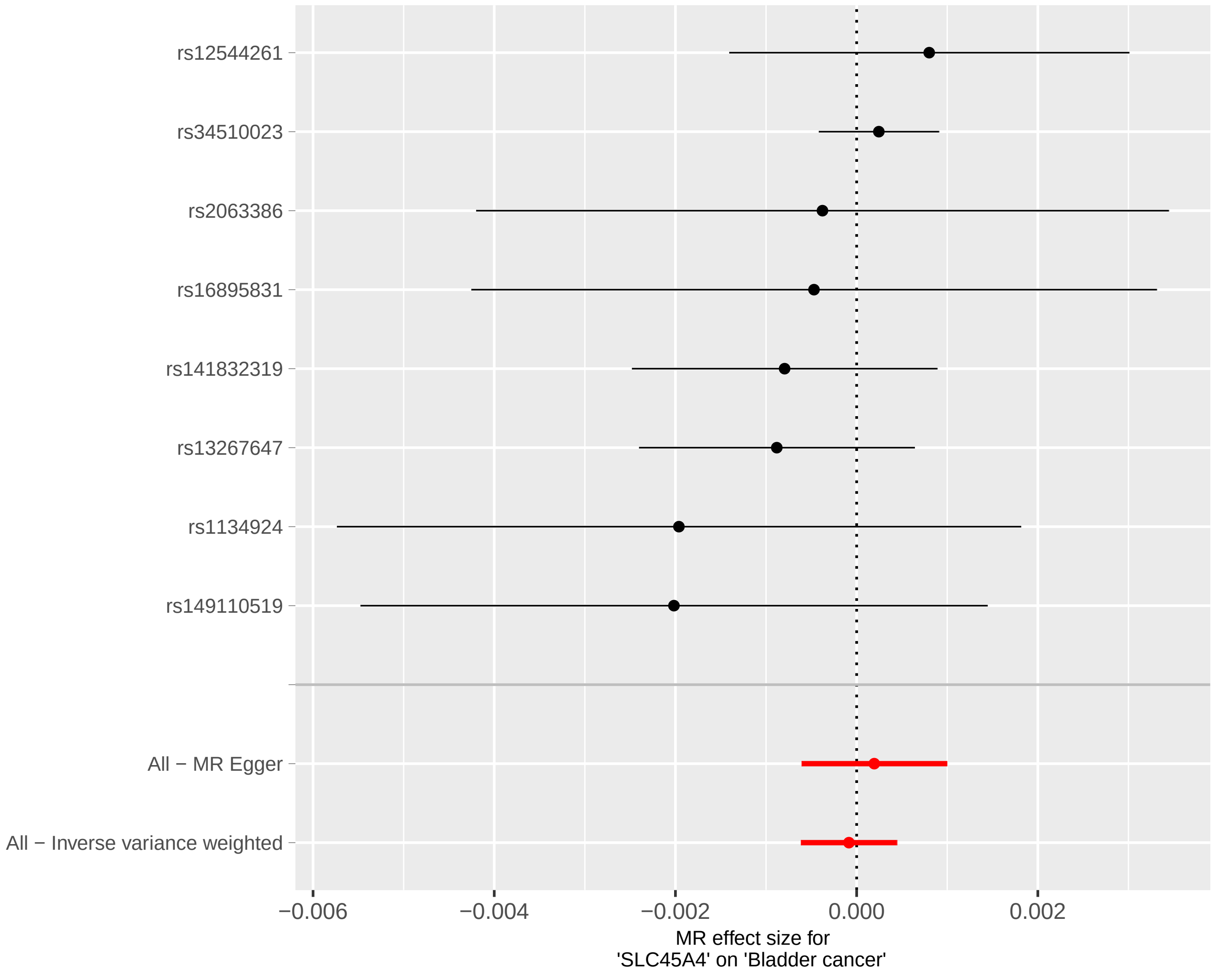

rs12544261
rs34510023
rs2063386
rs16895831
rs141832319
rs13267647
rs1134924
rs149110519
All − MR Egger
All − Inverse variance weighted
−0.006
−0.004
−0.002
0.000
0.002
MR effect size for
'SLC45A4' on 'Bladder cancer'

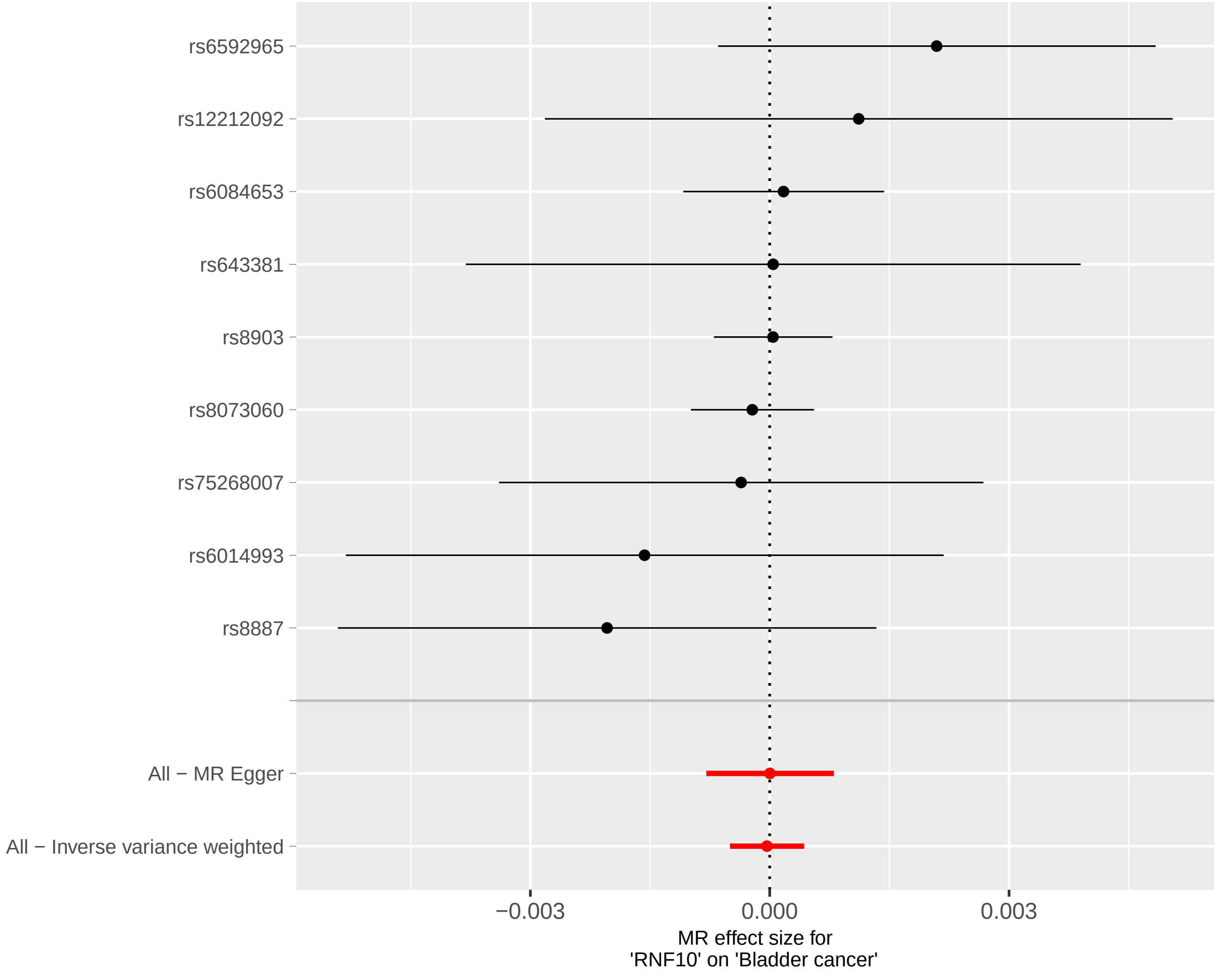

rs6592965
rs12212092
rs6084653
rs643381
rs8903
rs8073060
rs75268007
rs6014993
rs8887
All − MR Egger
All − Inverse variance weighted
−0.003
0.000
0.003
MR effect size for
'RNF10' on 'Bladder cancer'

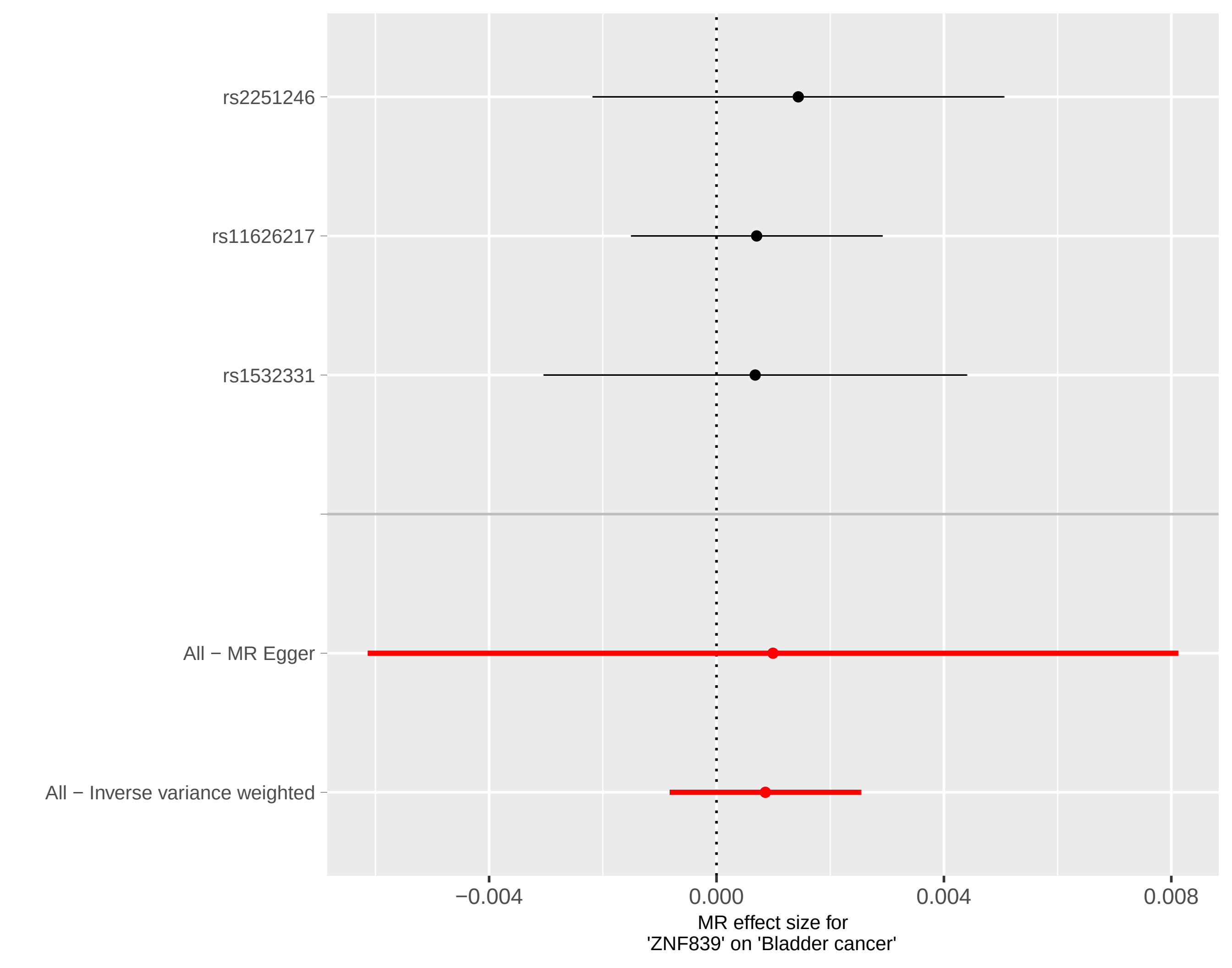

rs2251246
rs11626217
rs1532331
All − MR Egger
All − Inverse variance weighted
−0.004
0.000
0.004
0.008
MR effect size for
'ZNF839' on 'Bladder cancer'

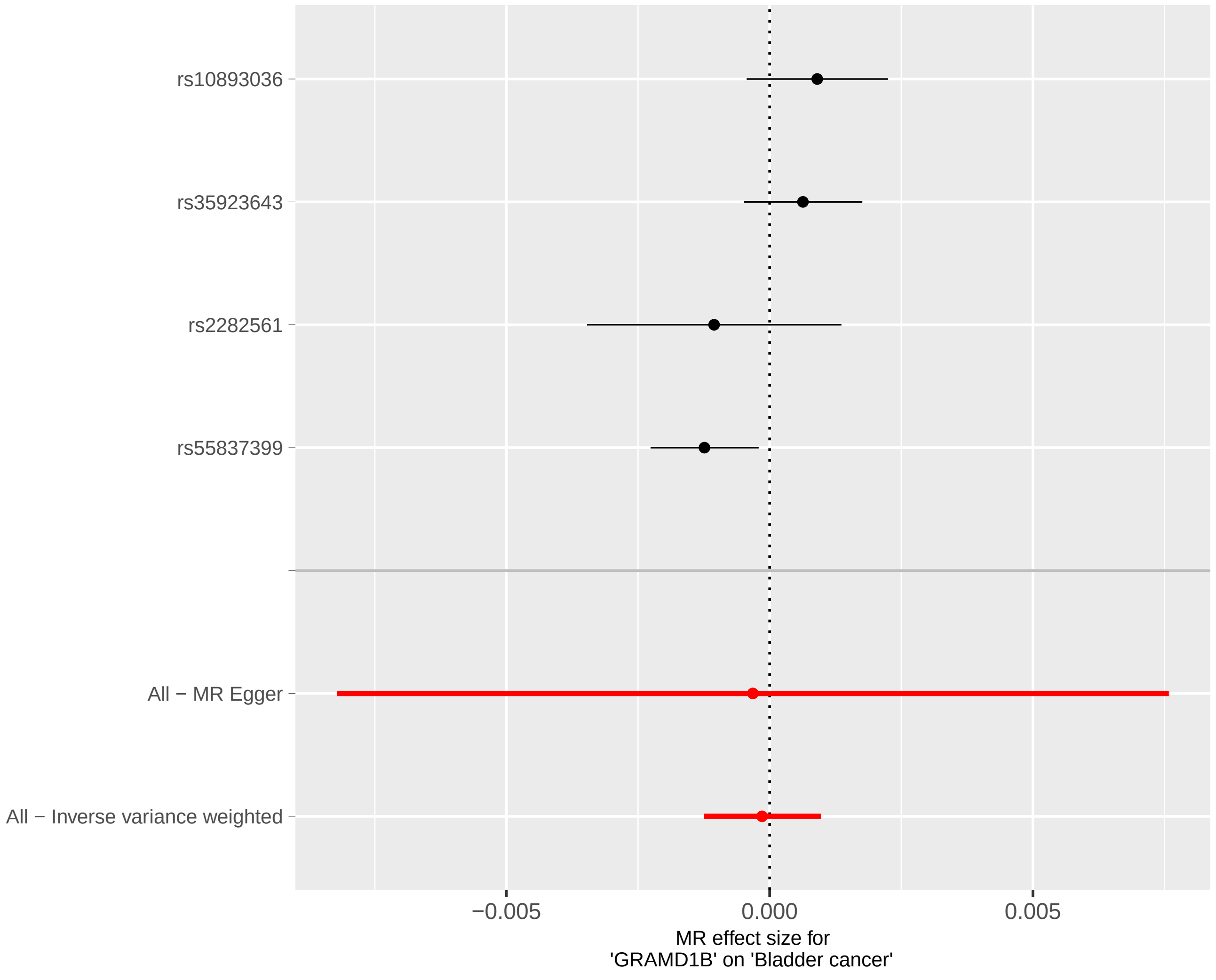
rs10893036
rs35923643
rs2282561
rs55837399
All − MR Egger
All − Inverse variance weighted
−0.005
0.000
0.005
MR effect size for
'GRAMD1B' on 'Bladder cancer'

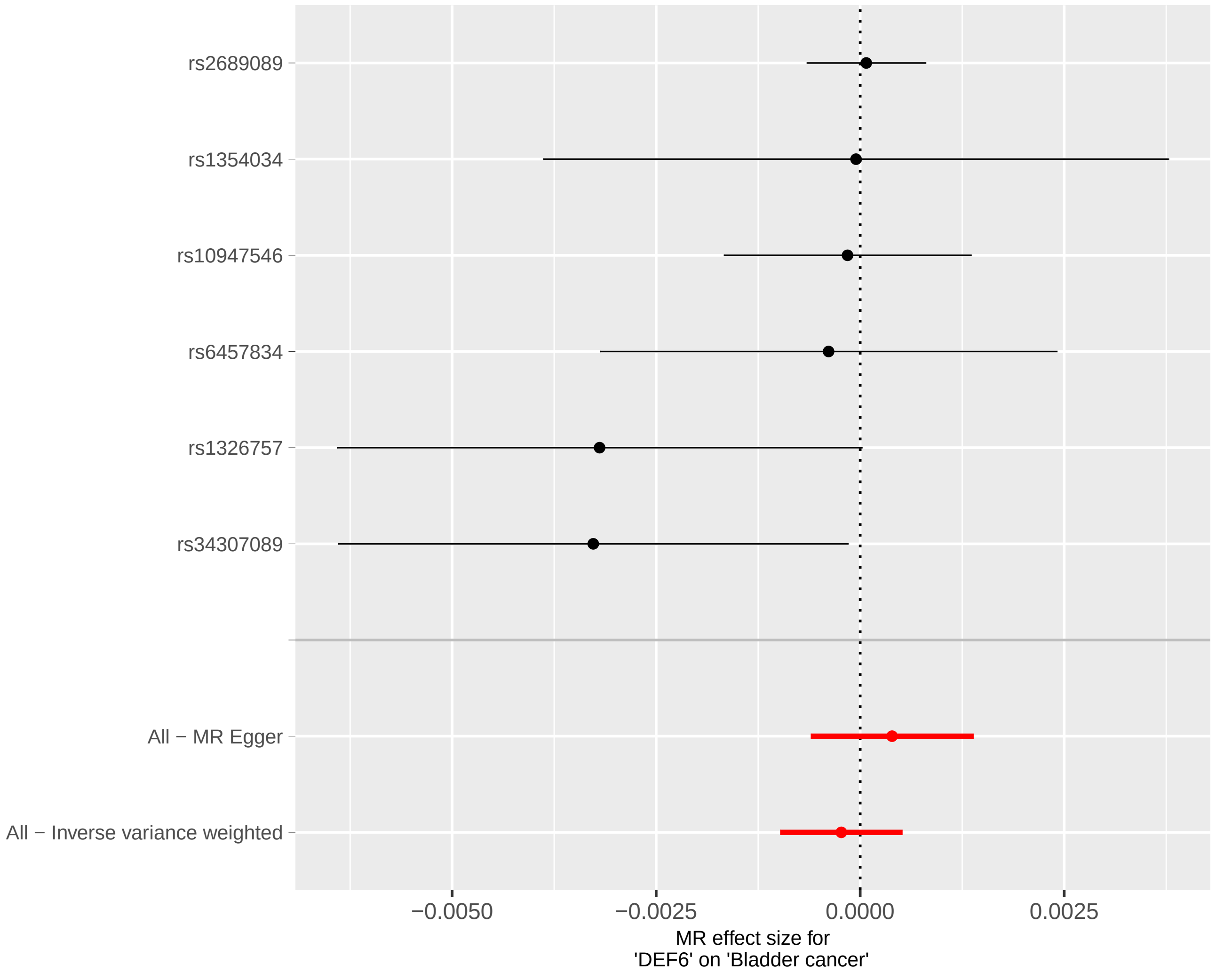
rs2689089
rs1354034
rs10947546
rs6457834
rs1326757
rs34307089
All − MR Egger
All − Inverse variance weighted
−0.0050
−0.0025
0.0000
0.0025
MR effect size for
'DEF6' on 'Bladder cancer'

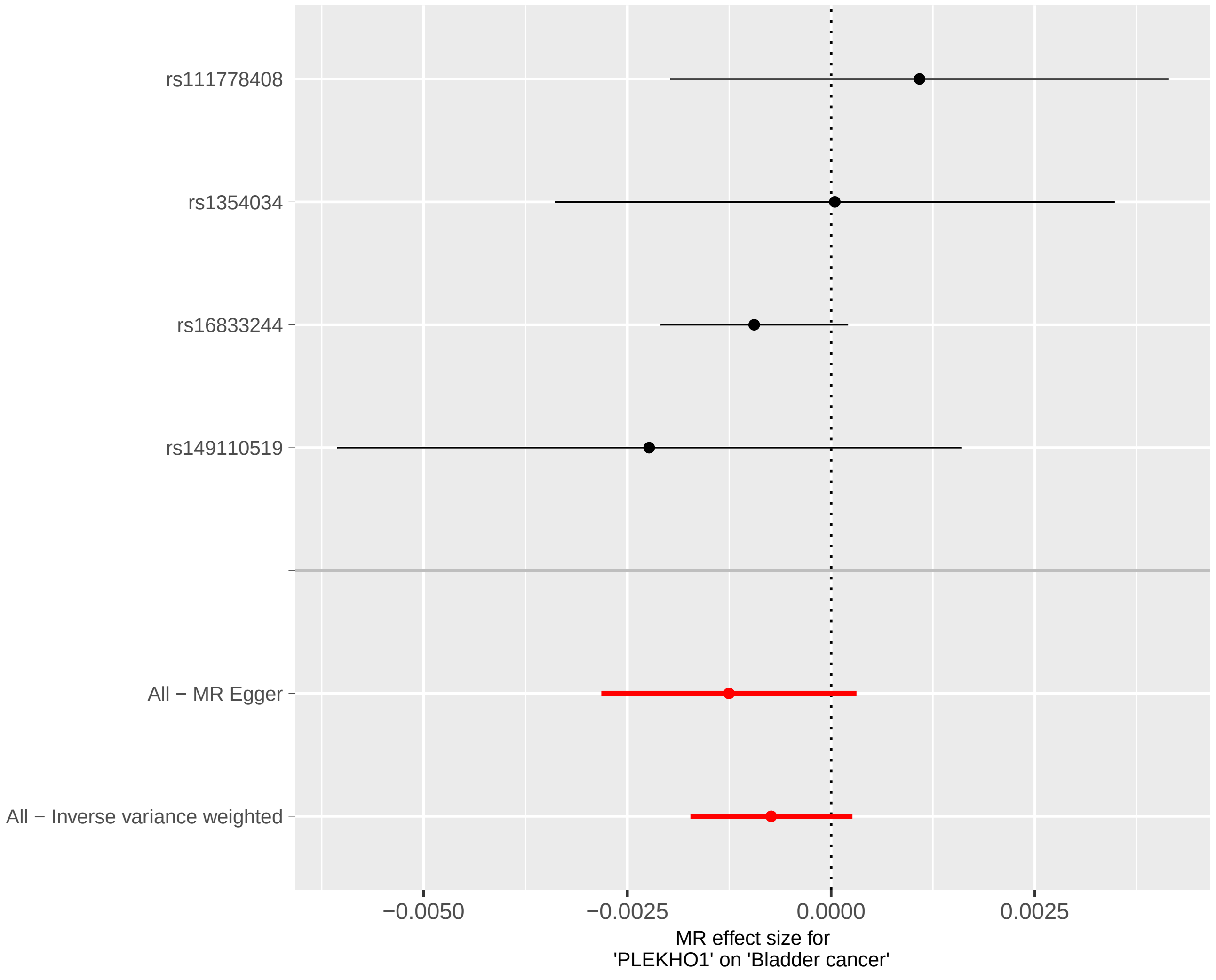

rs111778408
rs1354034
rs16833244
rs149110519
All − MR Egger
All − Inverse variance weighted
−0.0050
−0.0025
0.0000
0.0025
MR effect size for
'PLEKHO1' on 'Bladder cancer'

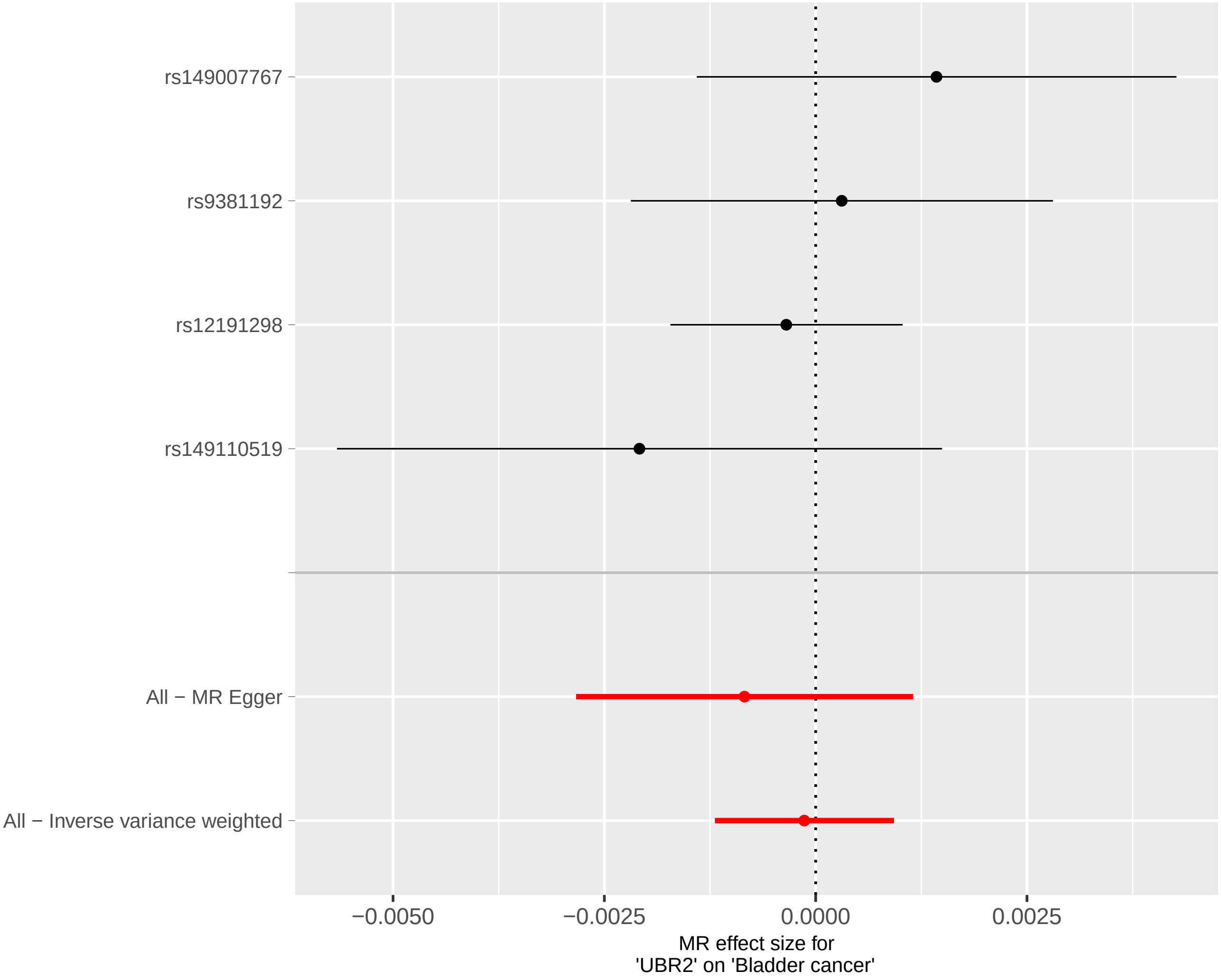
rs149007767
rs9381192
rs12191298
rs149110519
All − MR Egger
All − Inverse variance weighted
−0.0050
−0.0025
0.0000
0.0025
MR effect size for
'UBR2' on 'Bladder cancer'

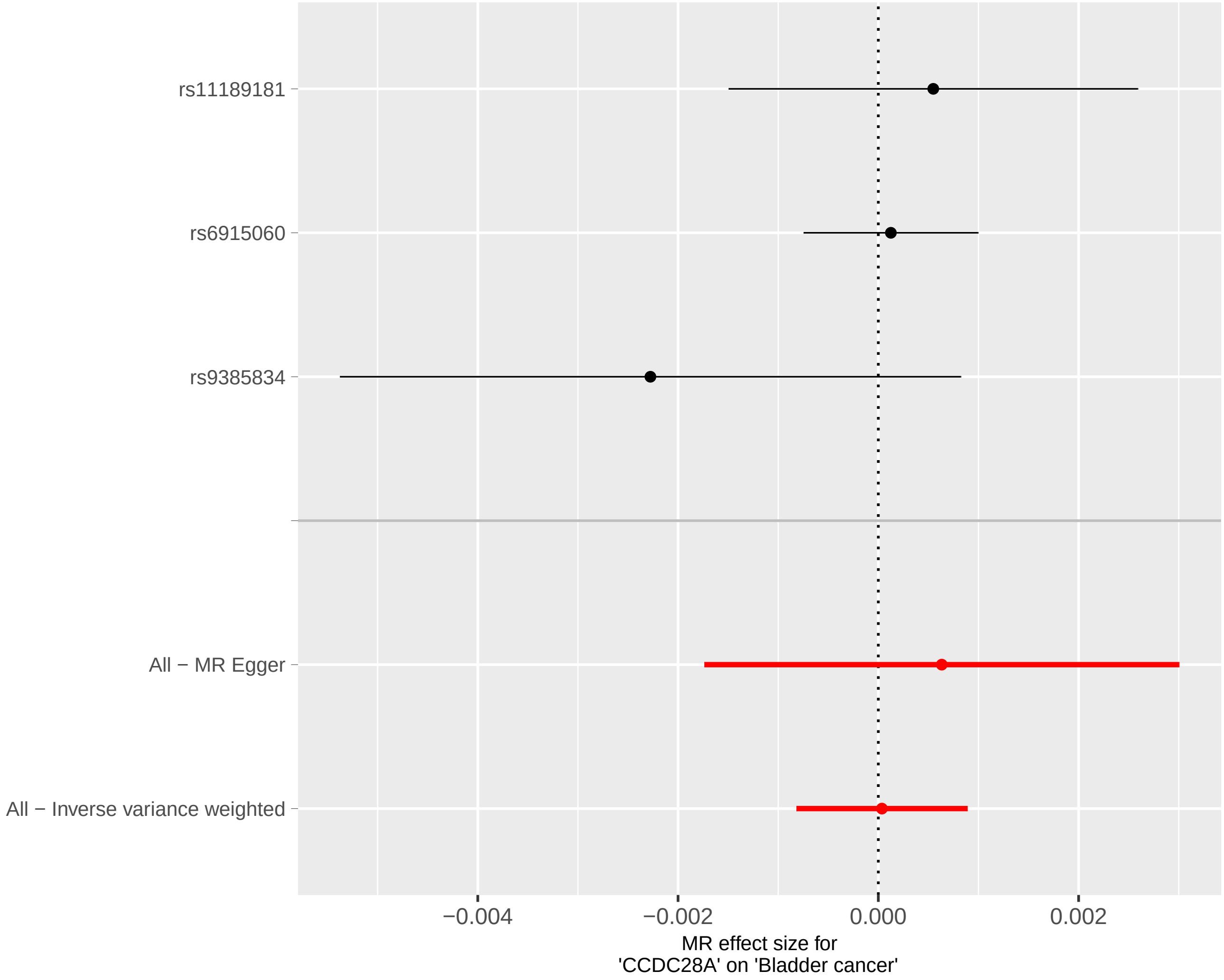
rs11189181
rs6915060
rs9385834
All − MR Egger
All − Inverse variance weighted
−0.004
−0.002
0.000
0.002
MR effect size for
'CCDC28A' on 'Bladder cancer'

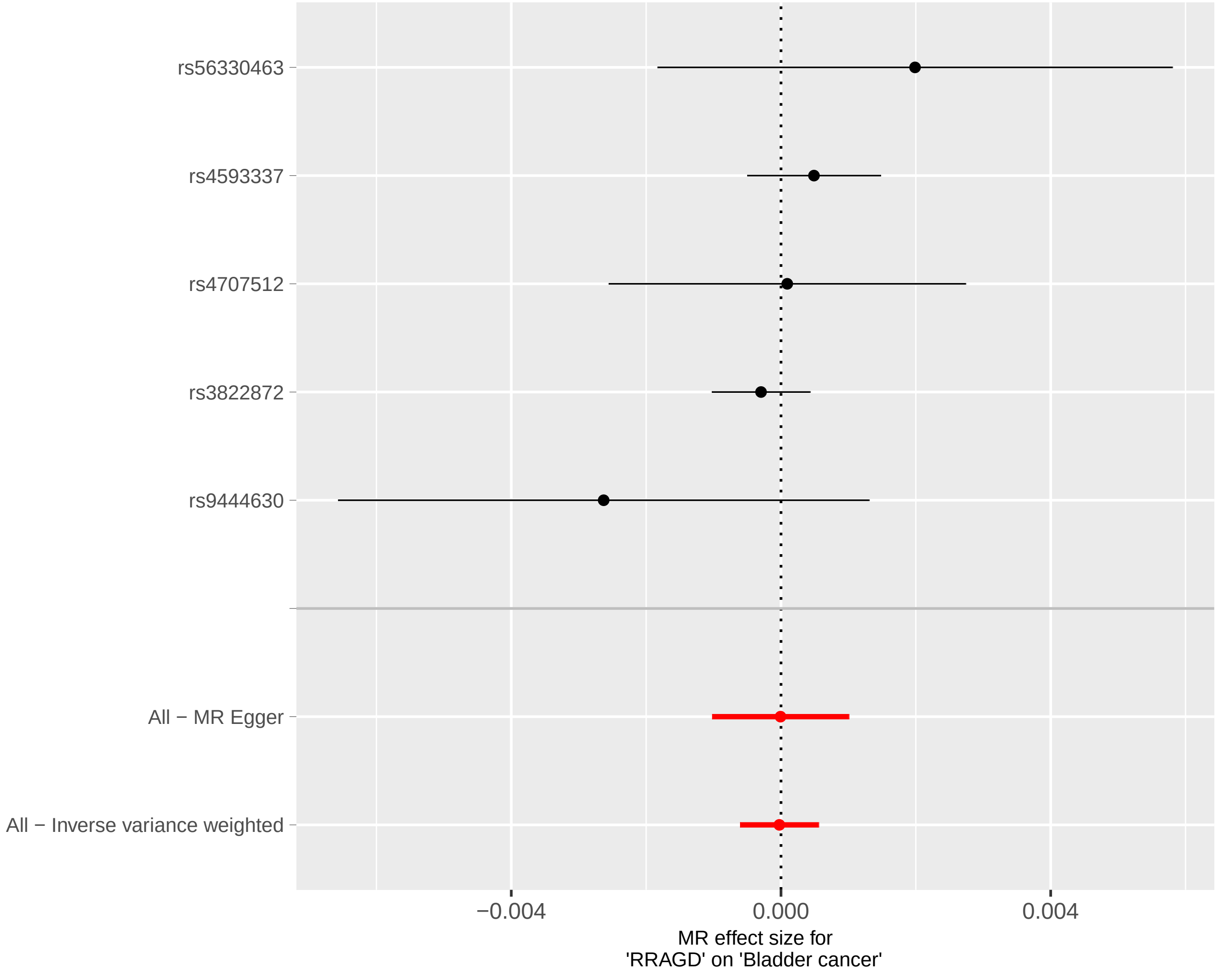
rs56330463
rs4593337
rs4707512
rs3822872
rs9444630
All − MR Egger
All − Inverse variance weighted
−0.004
0.000
0.004
MR effect size for
'RRAGD' on 'Bladder cancer'

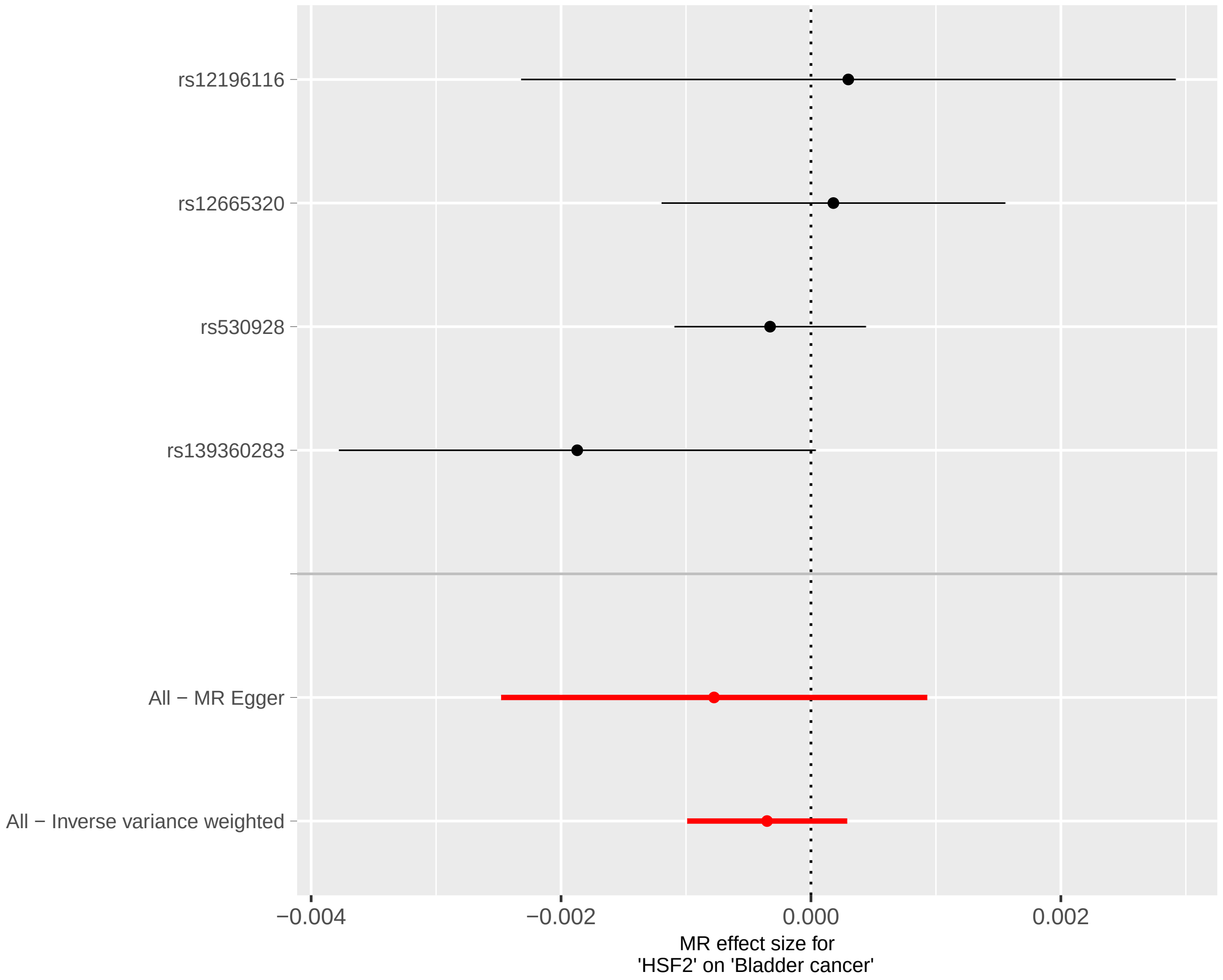
rs12196116
rs12665320
rs530928
rs139360283
All − MR Egger
All − Inverse variance weighted
−0.004
−0.002
0.000
0.002
MR effect size for
'HSF2' on 'Bladder cancer'

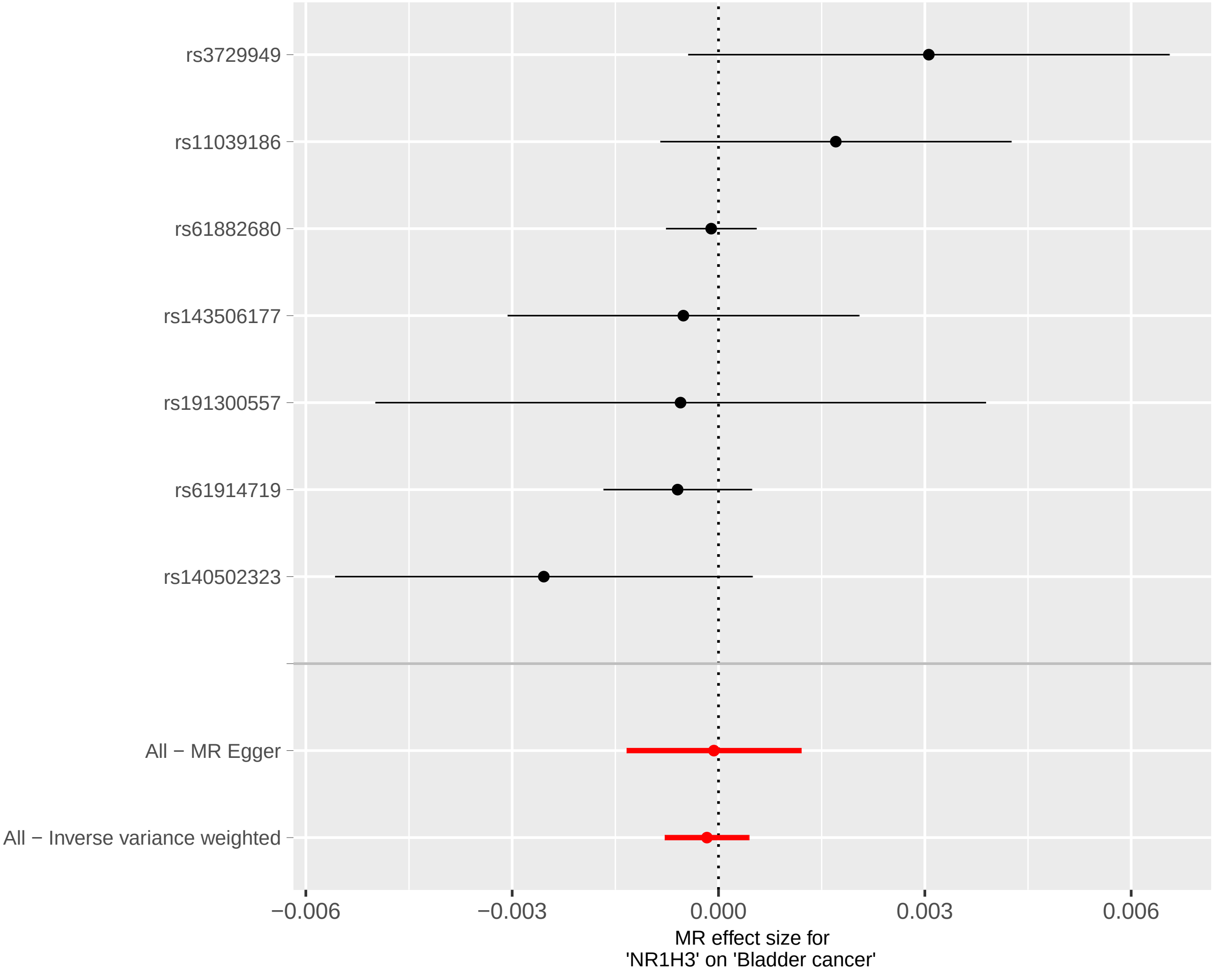
rs3729949
rs11039186
rs61882680
rs143506177
rs191300557
rs61914719
rs140502323
All − MR Egger
All − Inverse variance weighted
−0.006
−0.003
0.000
0.003
0.006
MR effect size for
'NR1H3' on 'Bladder cancer'

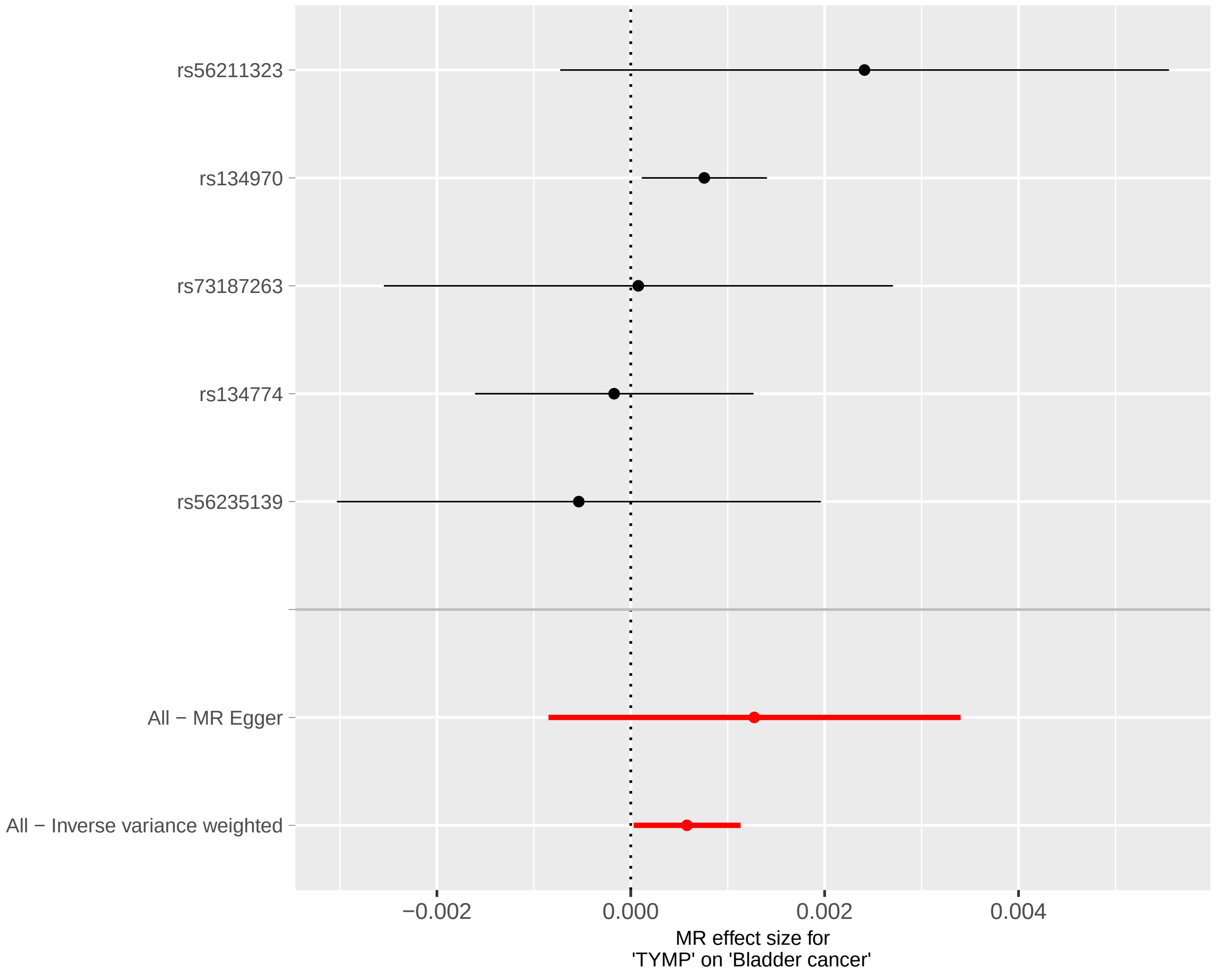
rs56211323
rs134970
rs73187263
rs134774
rs56235139
All − MR Egger
All − Inverse variance weighted
−0.002
0.000
0.002
0.004
MR effect size for
'TYMP' on 'Bladder cancer'

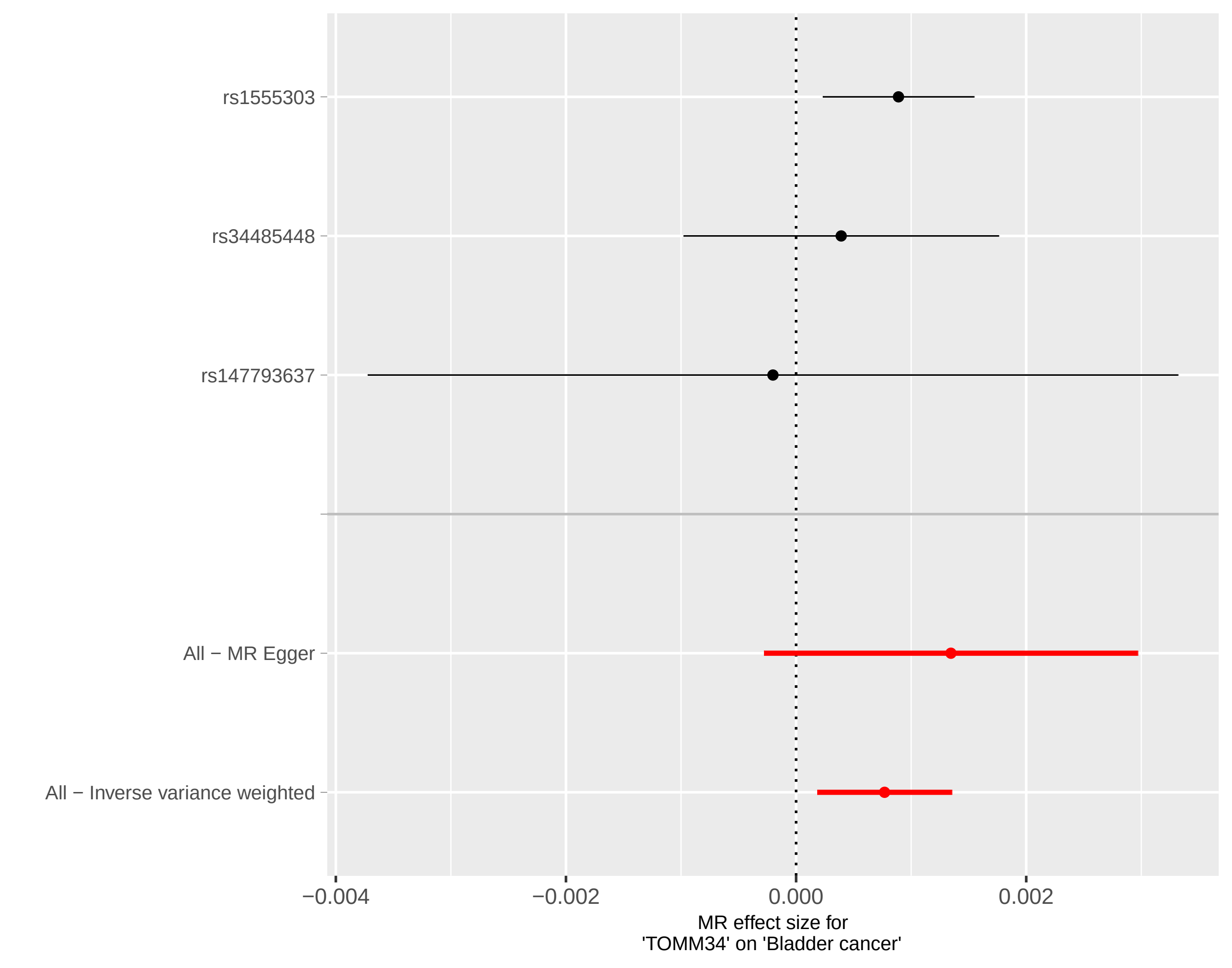

rs1555303
rs34485448
rs147793637
All − MR Egger
All − Inverse variance weighted
−0.004
−0.002
0.000
0.002
MR effect size for
'TOMM34' on 'Bladder cancer'

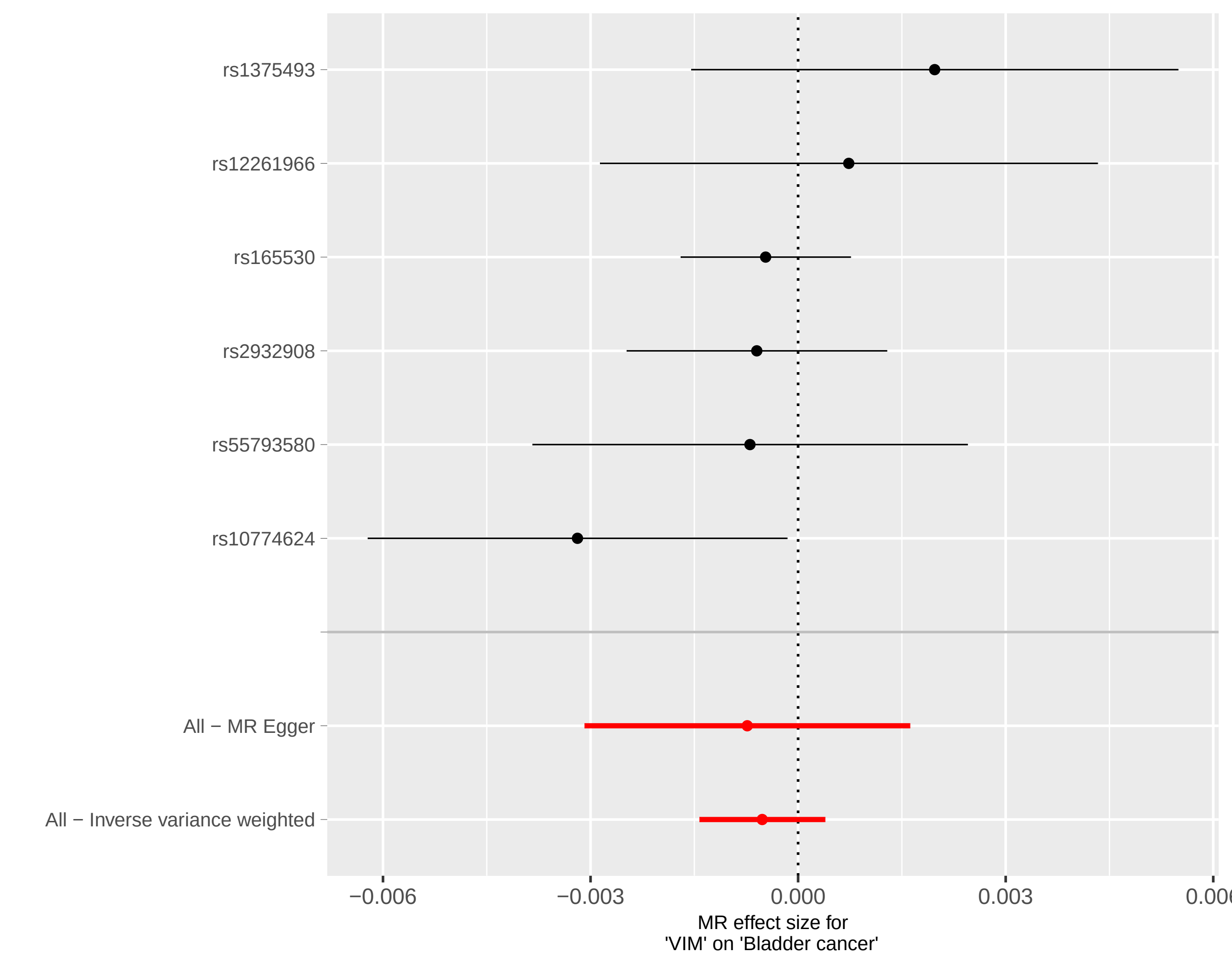
rs1375493
rs12261966
rs165530
rs2932908
rs55793580
rs10774624
All − MR Egger
All − Inverse variance weighted
−0.006
−0.003
0.000
0.003
MR effect size for
'VIM' on 'Bladder cancer'

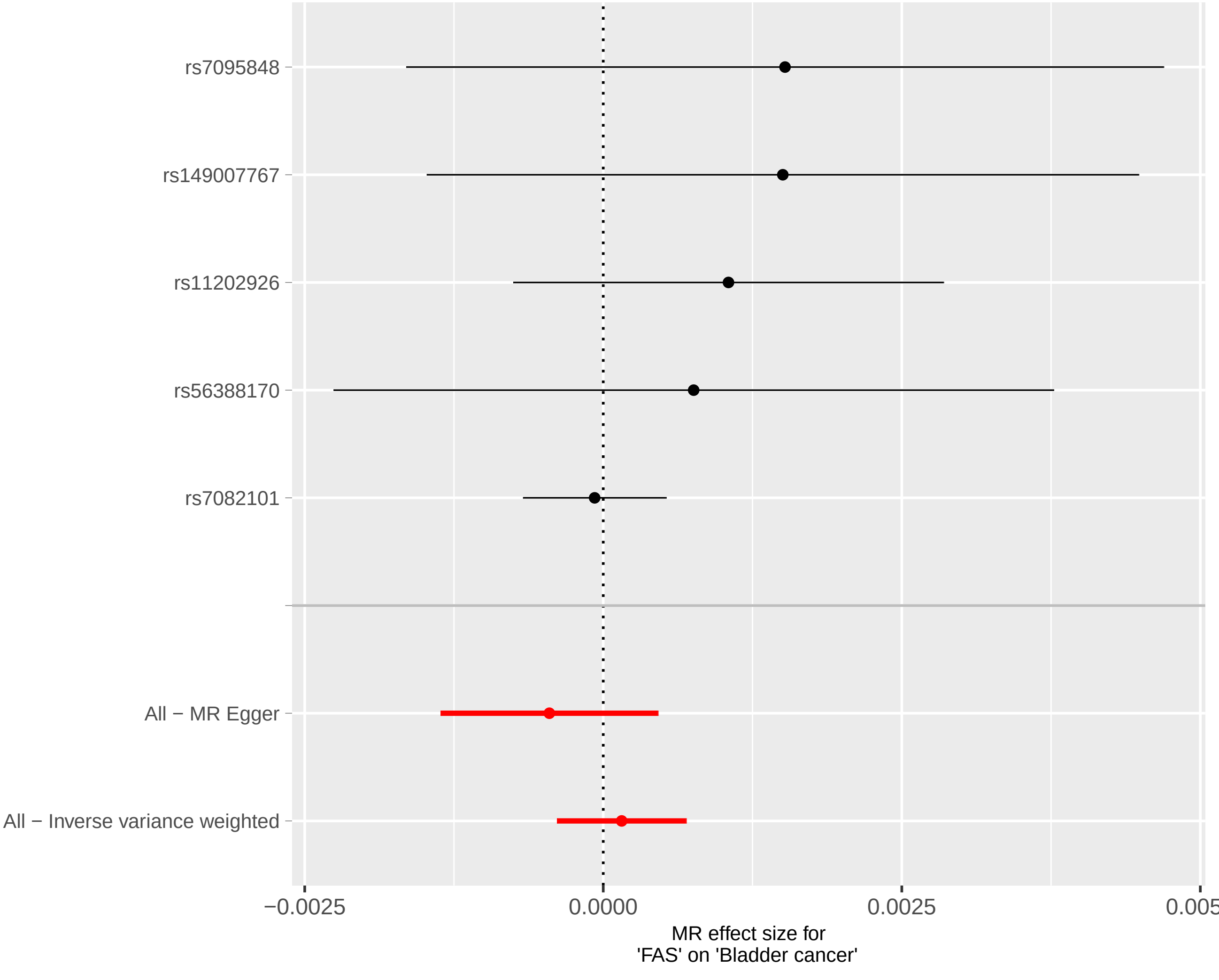

rs7095848
rs149007767
rs11202926
rs56388170
rs7082101
All − MR Egger
All − Inverse variance weighted
−0.0025
0.0000
0.0025
0.005
MR effect size for
'FAS' on 'Bladder cancer'

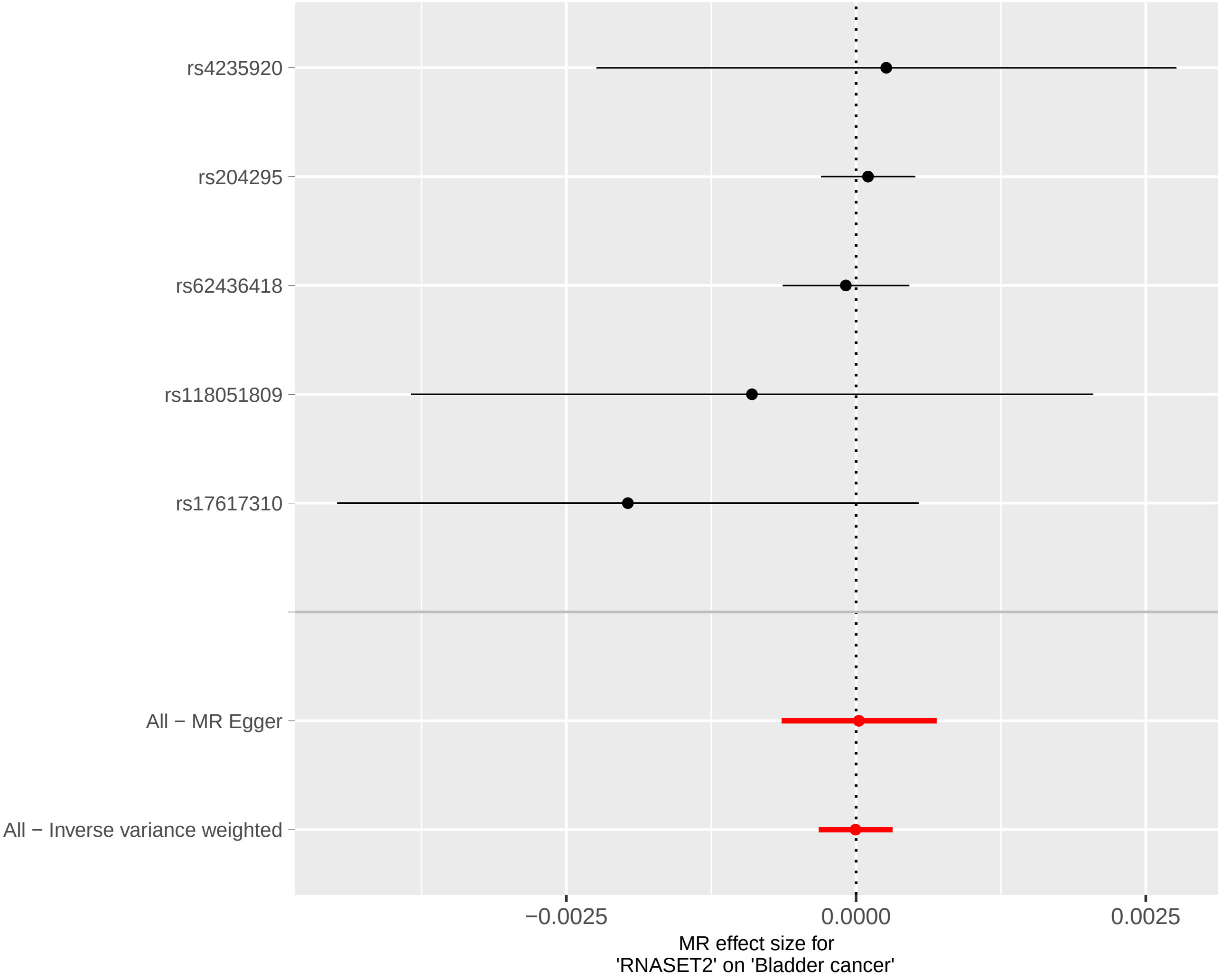

rs4235920
rs204295
rs62436418
rs118051809
rs17617310
All − MR Egger
All − Inverse variance weighted
−0.0025
0.0000
0.0025
MR effect size for
'RNASET2' on 'Bladder cancer'

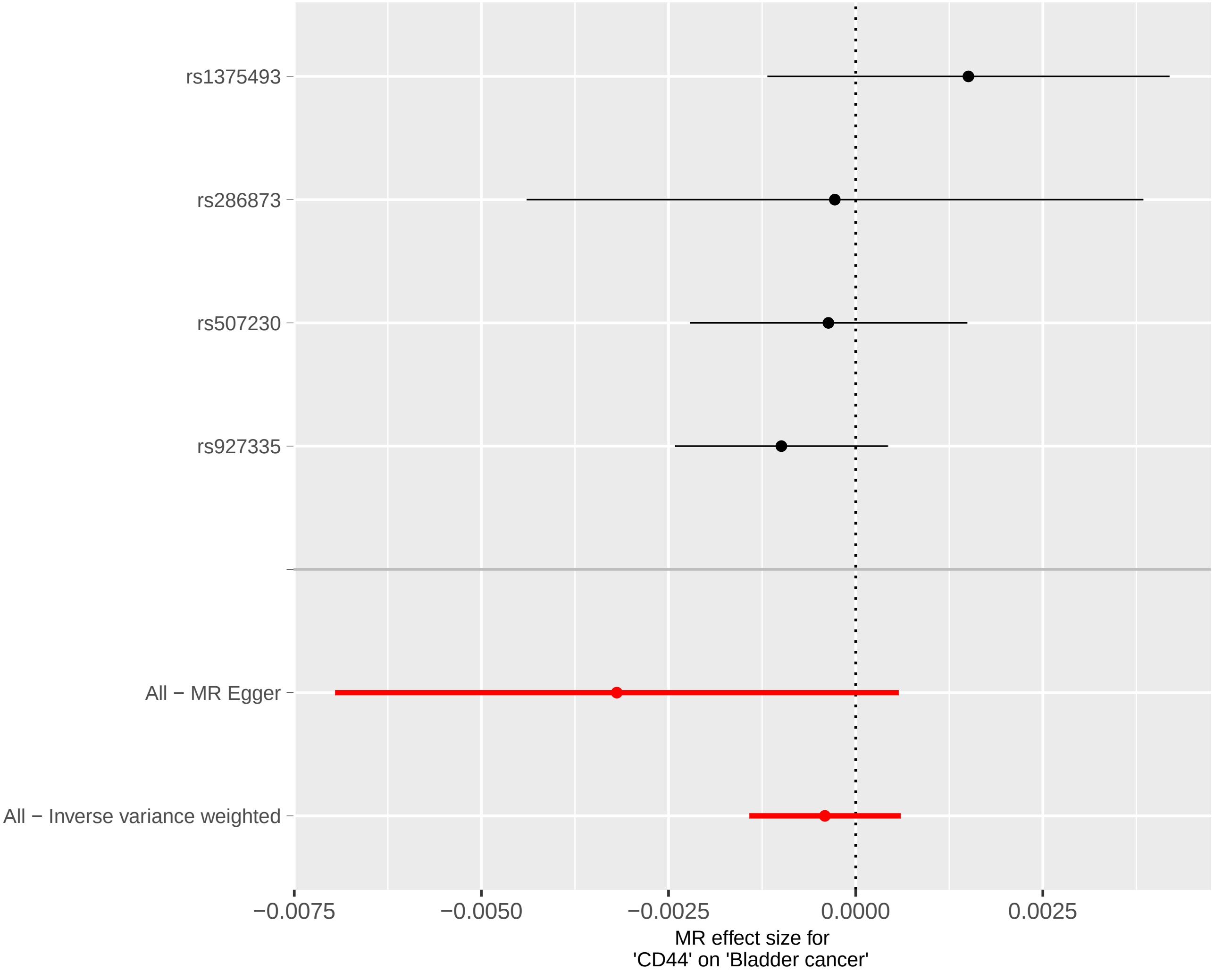
rs1375493
rs286873
rs507230
rs927335
All − MR Egger
All − Inverse variance weighted
−0.0075
−0.0050
−0.0025
0.0000
0.0025
MR effect size for
'CD44' on 'Bladder cancer'

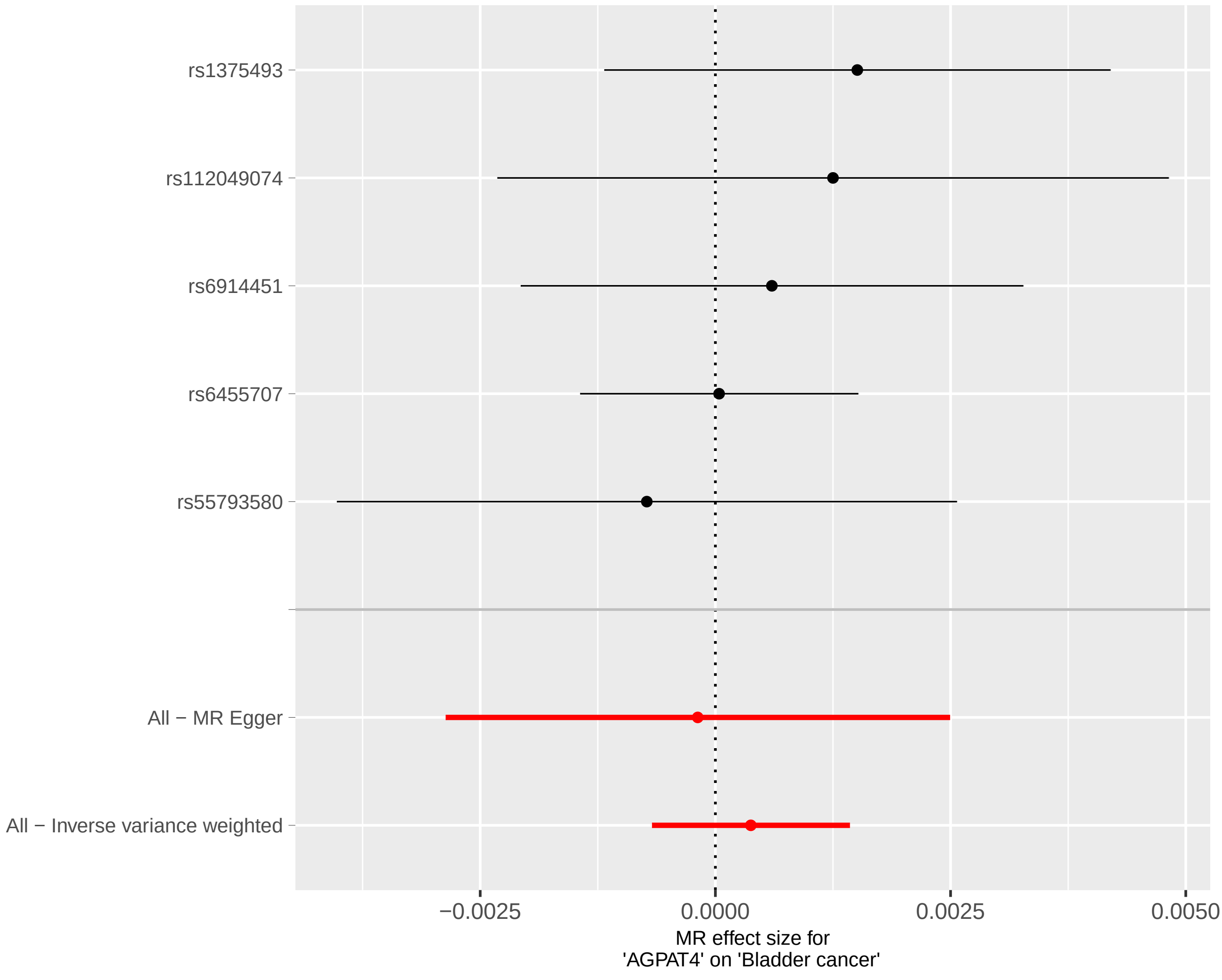

rs1375493
rs112049074
rs6914451
rs6455707
rs55793580
All − MR Egger
All − Inverse variance weighted
−0.0025
0.0000
0.0025
0.0050
MR effect size for
'AGPAT4' on 'Bladder cancer'

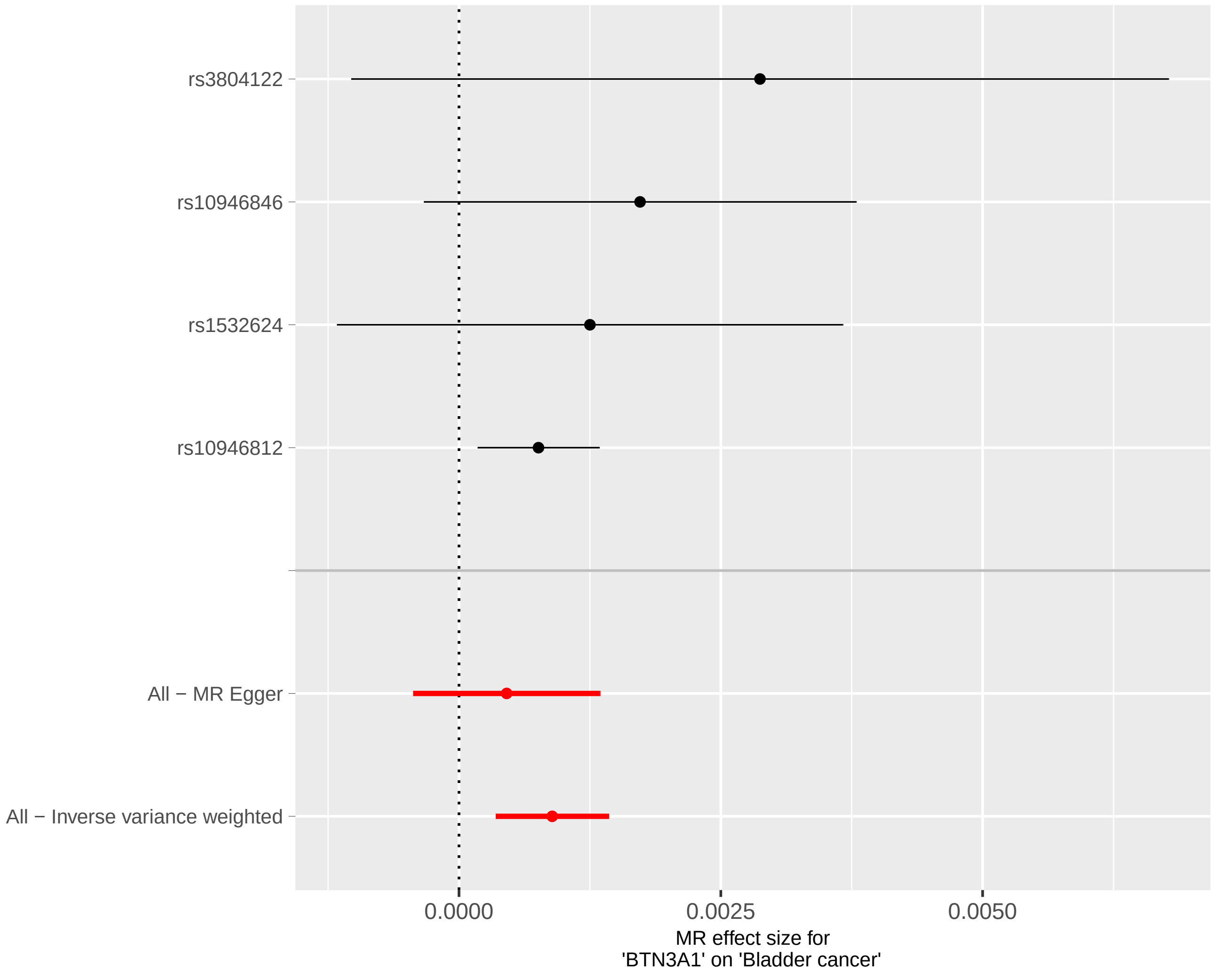

rs3804122
rs10946846
rs1532624
rs10946812
All − MR Egger
All − Inverse variance weighted
0.0000
0.0025
0.0050
MR effect size for
'BTN3A1' on 'Bladder cancer'

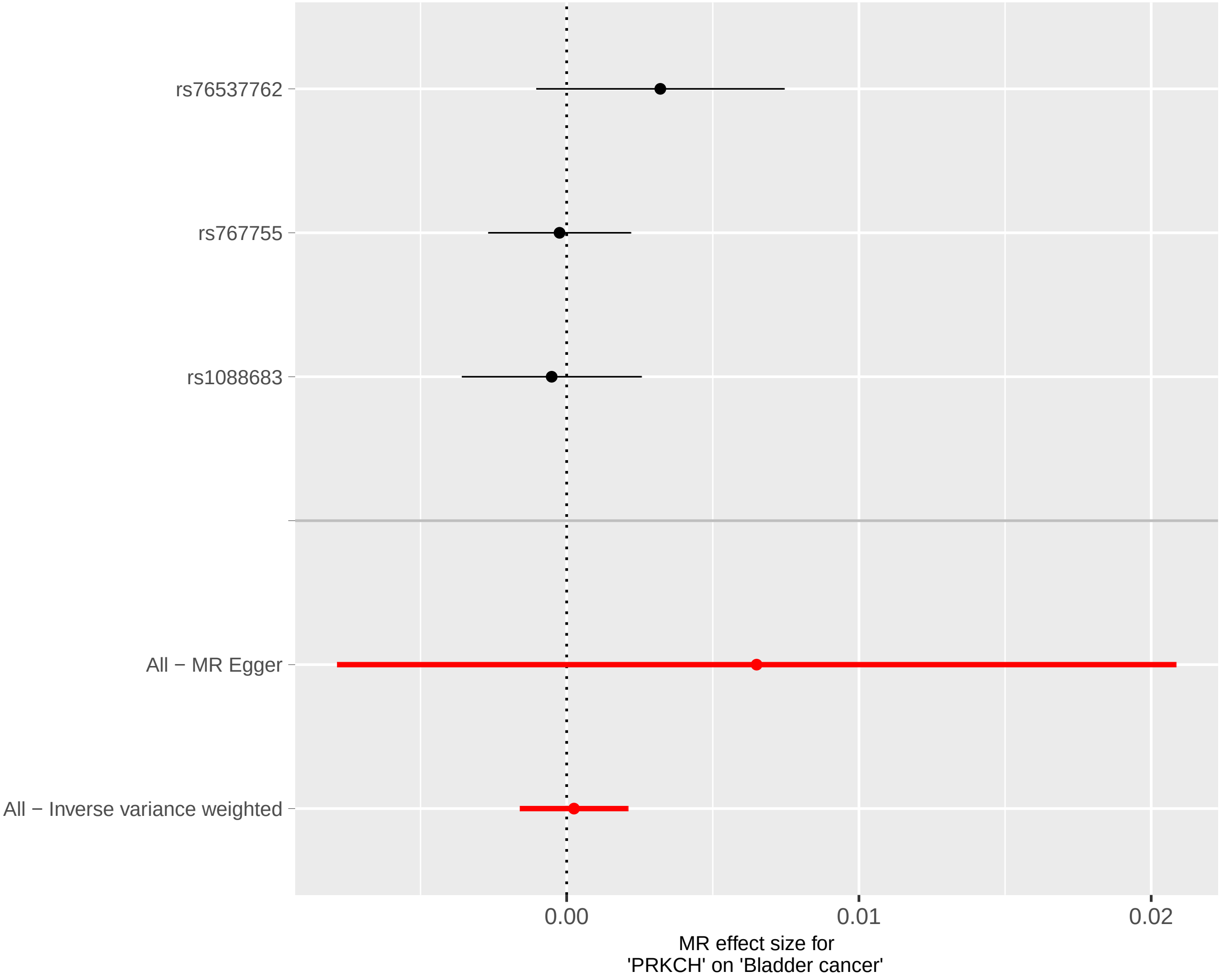

rs76537762
rs767755
rs1088683
All − MR Egger
All − Inverse variance weighted
0.00
0.01
0.02
MR effect size for
'PRKCH' on 'Bladder cancer'

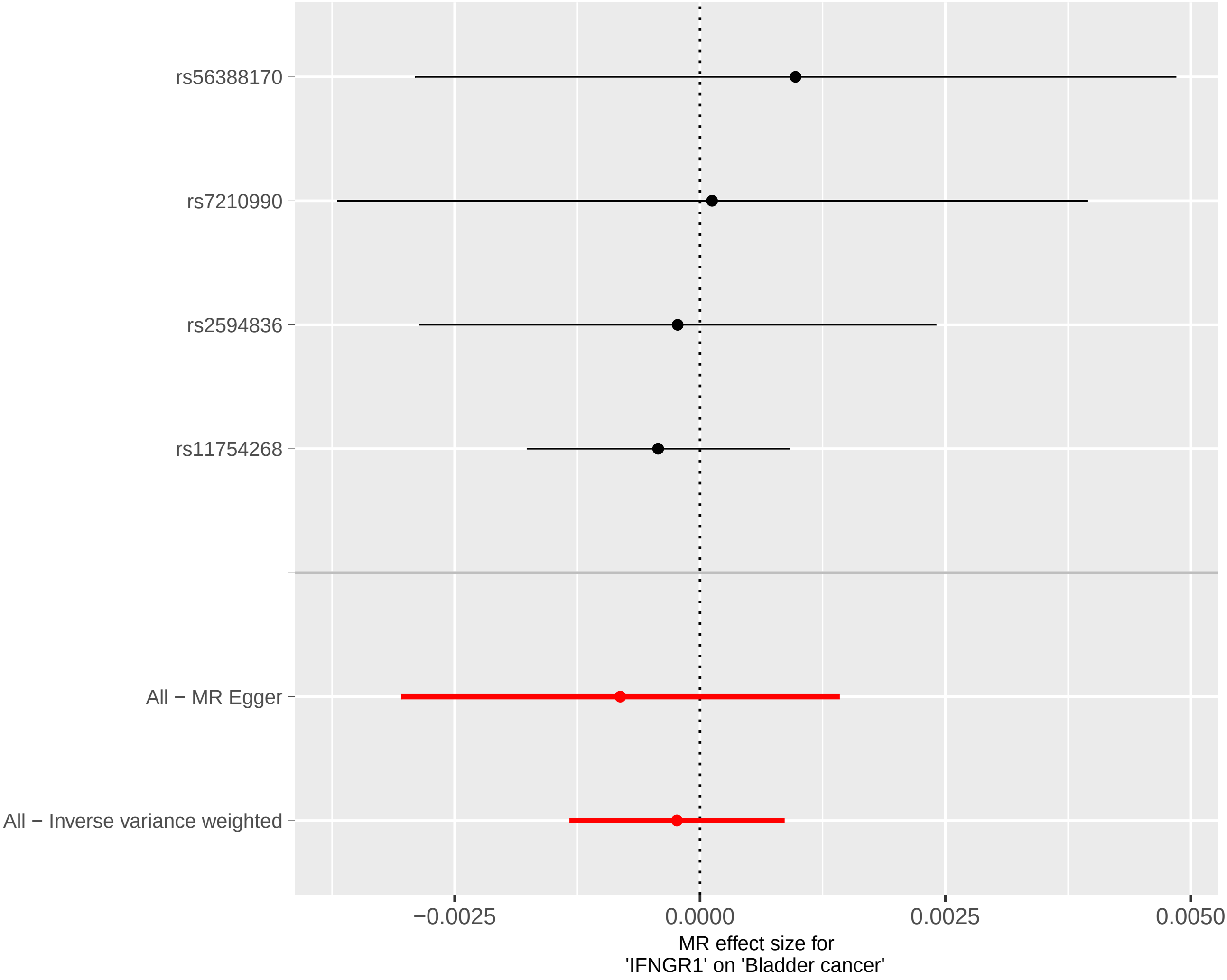
rs56388170
rs7210990
rs2594836
rs11754268
All − MR Egger
All − Inverse variance weighted
−0.0025
0.0000
0.0025
0.0050
MR effect size for
'IFNGR1' on 'Bladder cancer'

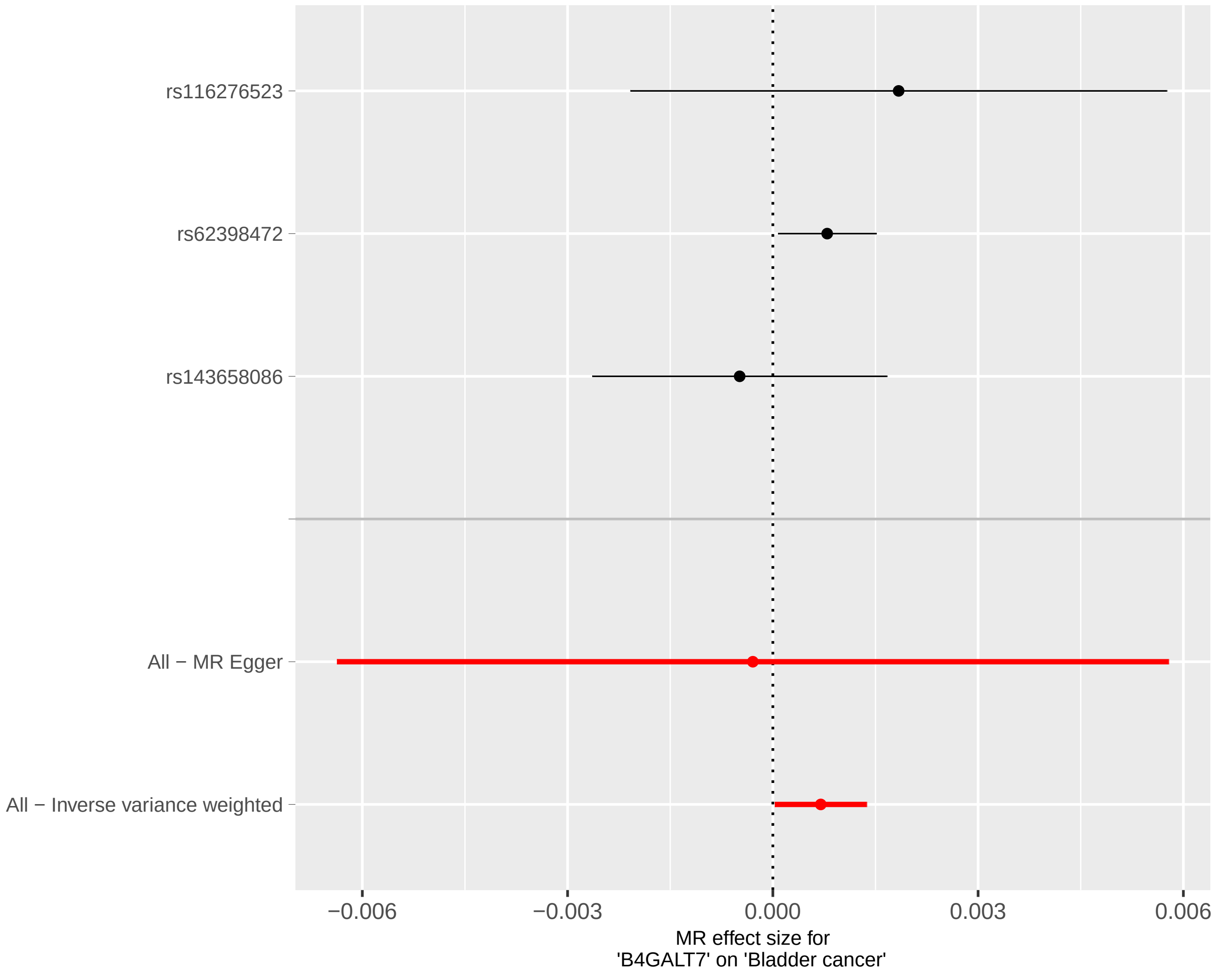

rs116276523
rs62398472
rs143658086
All − MR Egger
All − Inverse variance weighted
−0.006
−0.003
0.000
0.003
0.006
MR effect size for
'B4GALT7' on 'Bladder cancer'

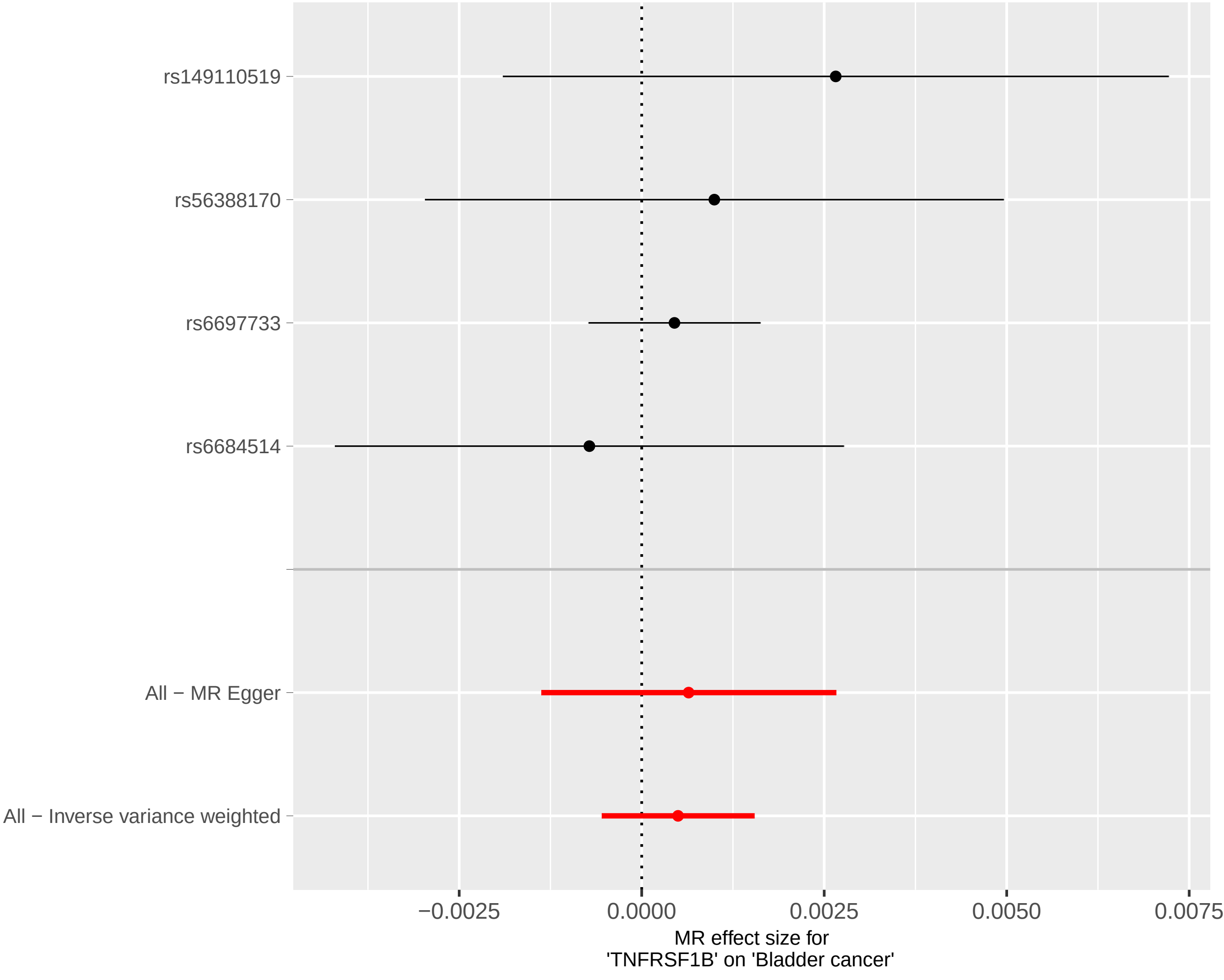
rs149110519
rs56388170
rs6697733
rs6684514
All − MR Egger
All − Inverse variance weighted
−0.0025
0.0000
0.0025
0.0050
0.0075
MR effect size for
'TNFRSF1B' on 'Bladder cancer'

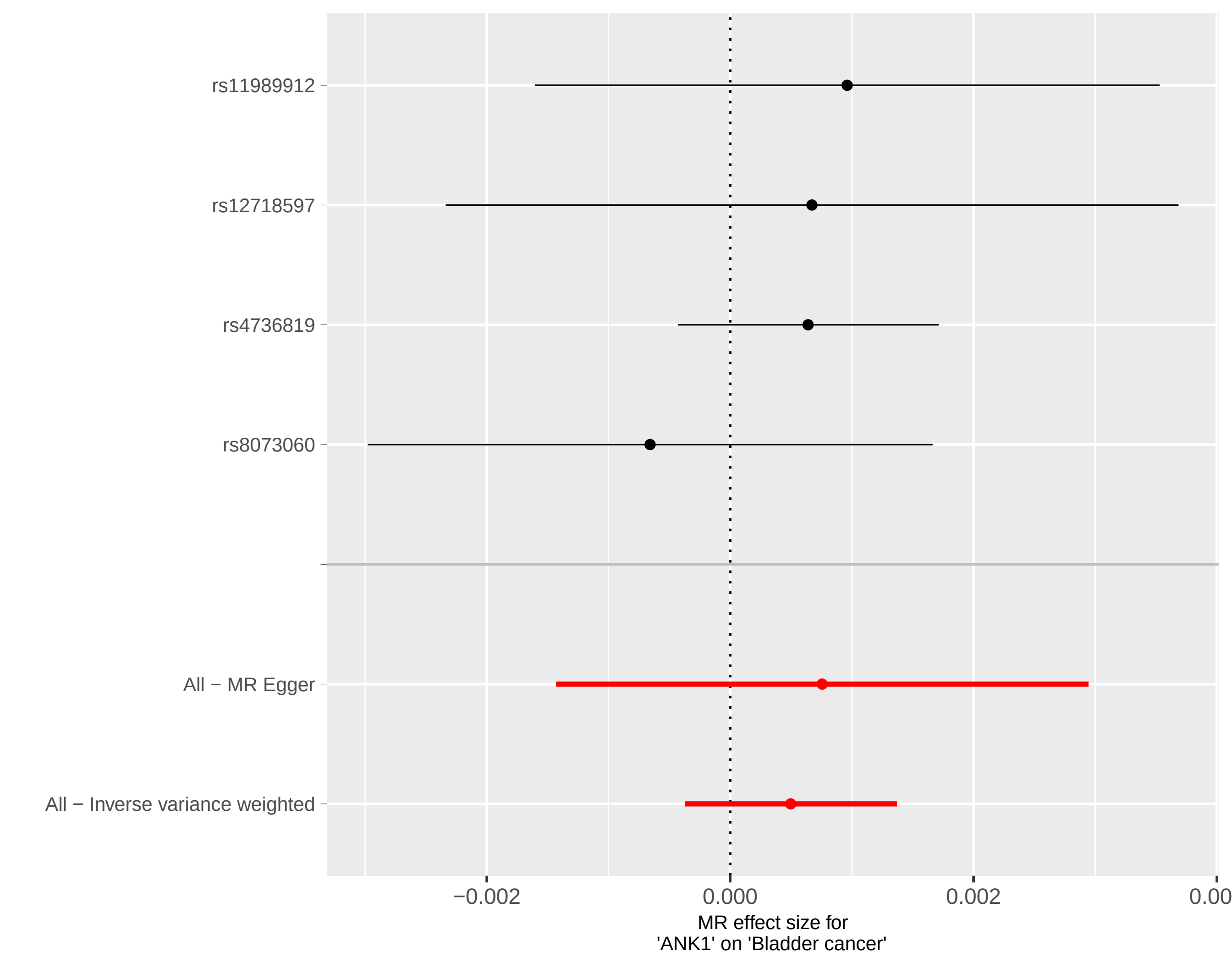
rs11989912
rs12718597
rs4736819
rs8073060
All − MR Egger
All − Inverse variance weighted
−0.002
0.000
0.002
0.00
MR effect size for
'ANK1' on 'Bladder cancer'

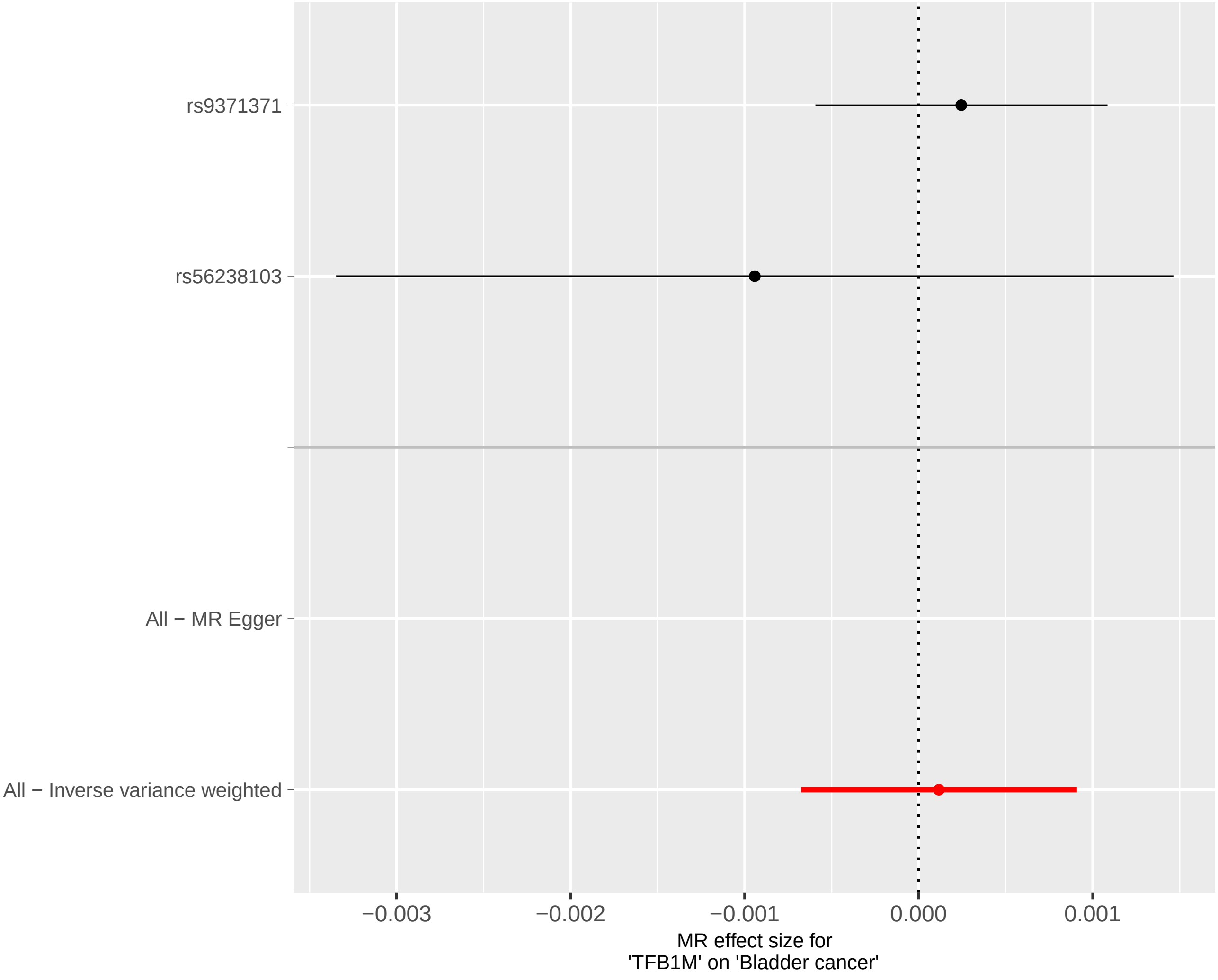

rs9371371
rs56238103
All − MR Egger
All − Inverse variance weighted
−0.003
−0.002
−0.001
0.000
0.001
MR effect size for
'TFB1M' on 'Bladder cancer'

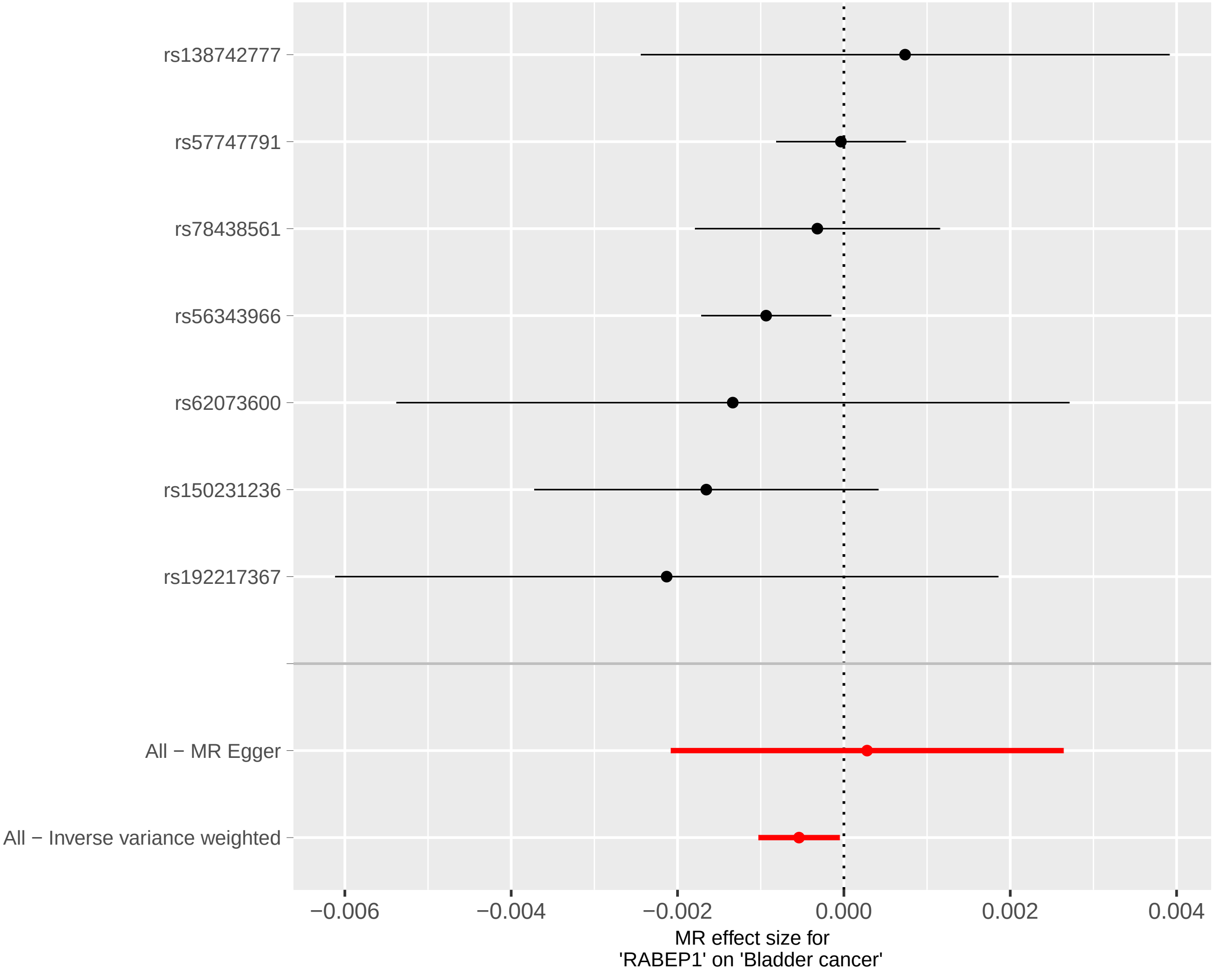

rs138742777
rs57747791
rs78438561
rs56343966
rs62073600
rs150231236
rs192217367
All − MR Egger
All − Inverse variance weighted
−0.006
−0.004
−0.002
0.000
0.002
0.004
MR effect size for
'RABEP1' on 'Bladder cancer'

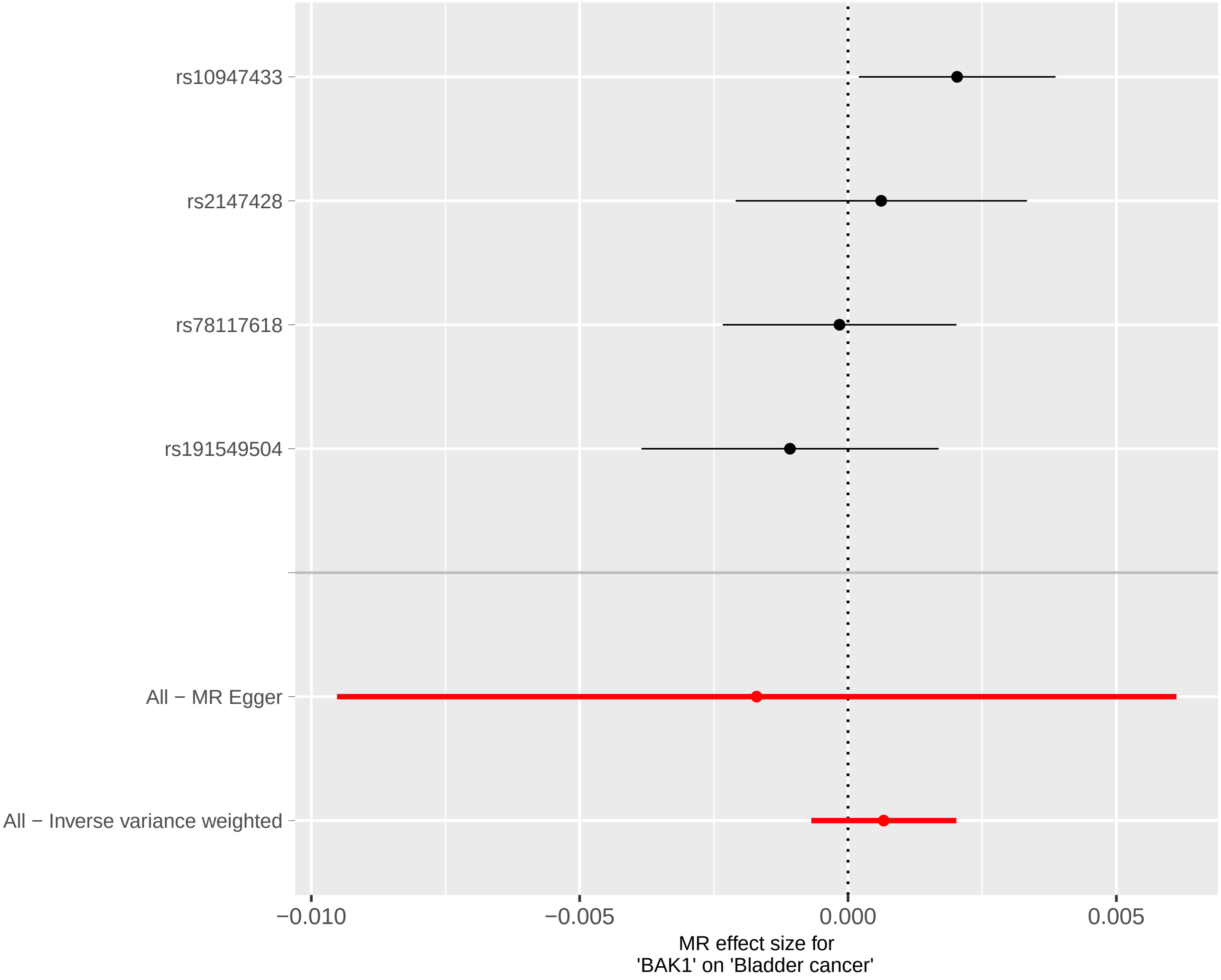

rs10947433
rs2147428
rs78117618
rs191549504
All − MR Egger
All − Inverse variance weighted
−0.010
−0.005
0.000
0.005
MR effect size for
'BAK1' on 'Bladder cancer'

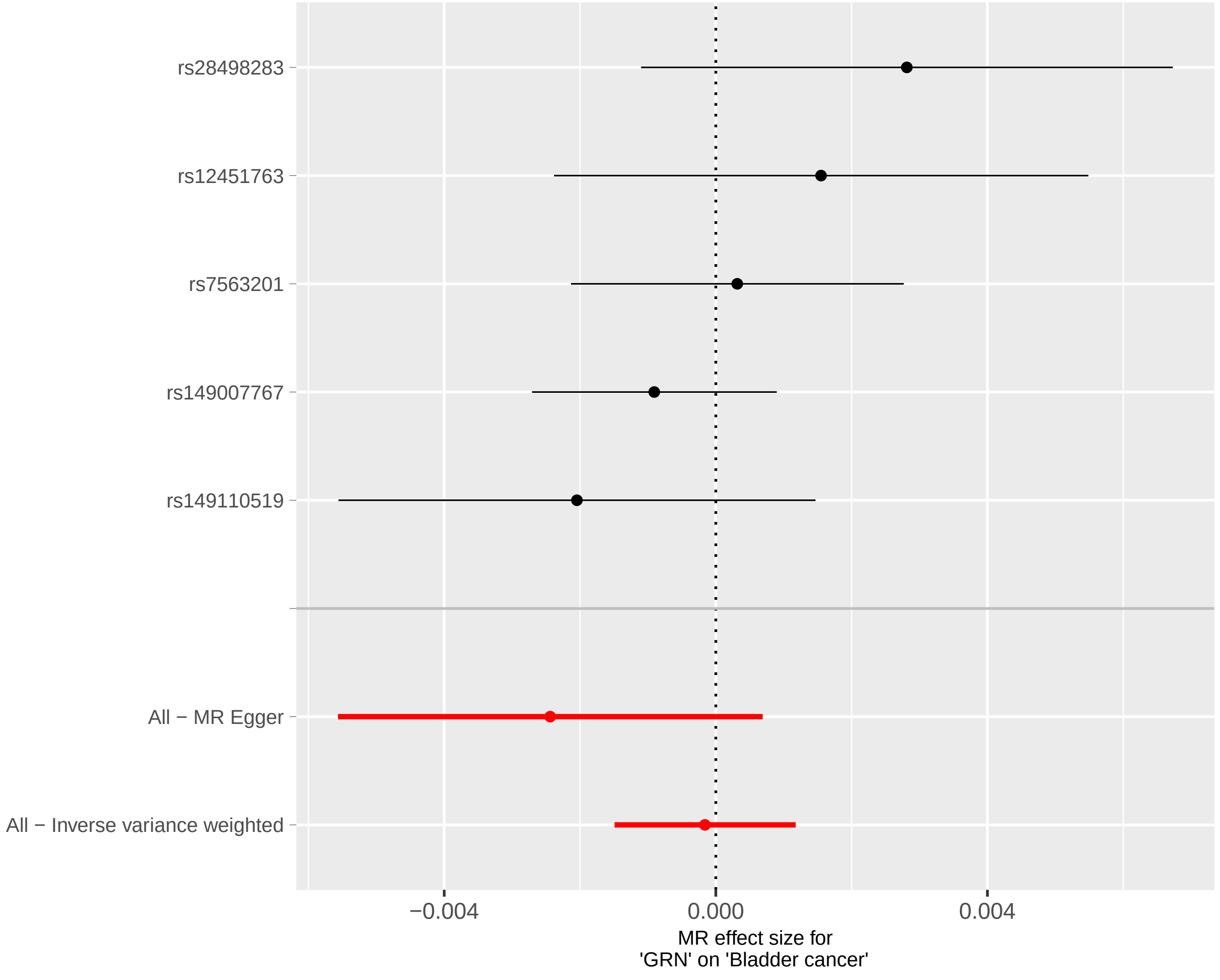
rs28498283
rs12451763
rs7563201
rs149007767
rs149110519
All − MR Egger
All − Inverse variance weighted
−0.004
0.000
0.004
MR effect size for
'GRN' on 'Bladder cancer'

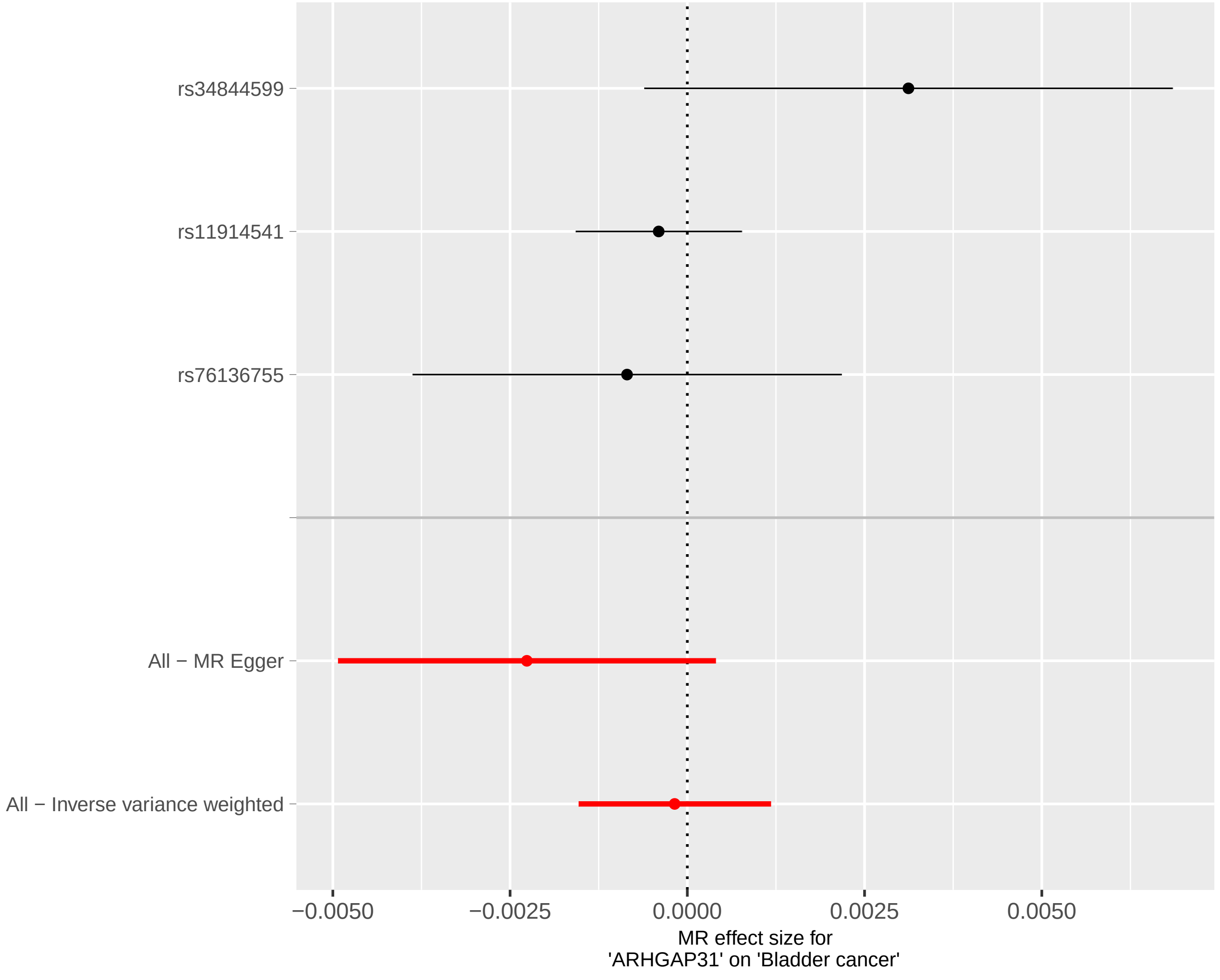
rs34844599
rs11914541
rs76136755
All − MR Egger
All − Inverse variance weighted
−0.0050
−0.0025
0.0000
0.0025
0.0050
MR effect size for
'ARHGAP31' on 'Bladder cancer'

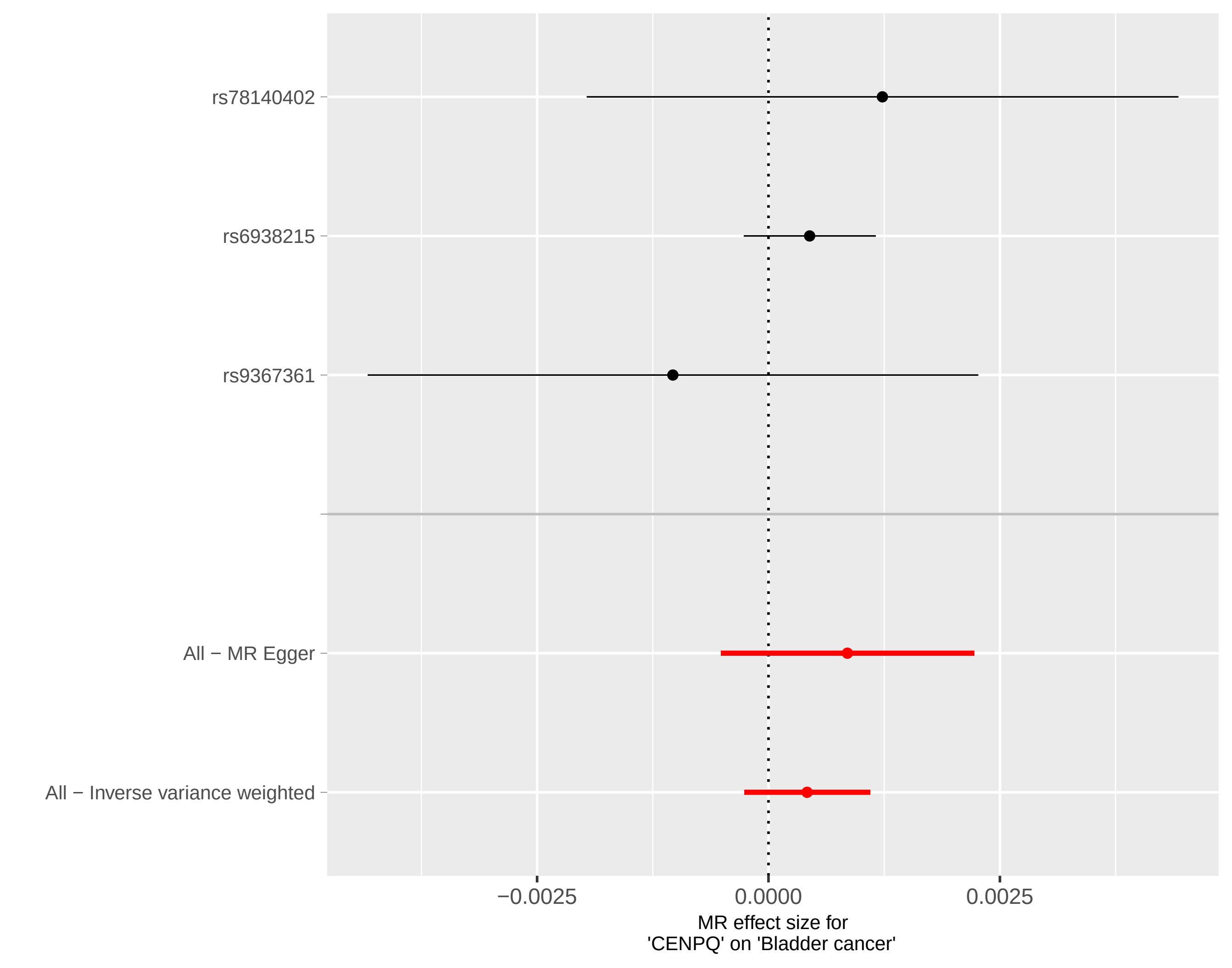
rs78140402
rs6938215
rs9367361
All − MR Egger
All − Inverse variance weighted
−0.0025
0.0000
0.0025
MR effect size for
'CENPQ' on 'Bladder cancer'

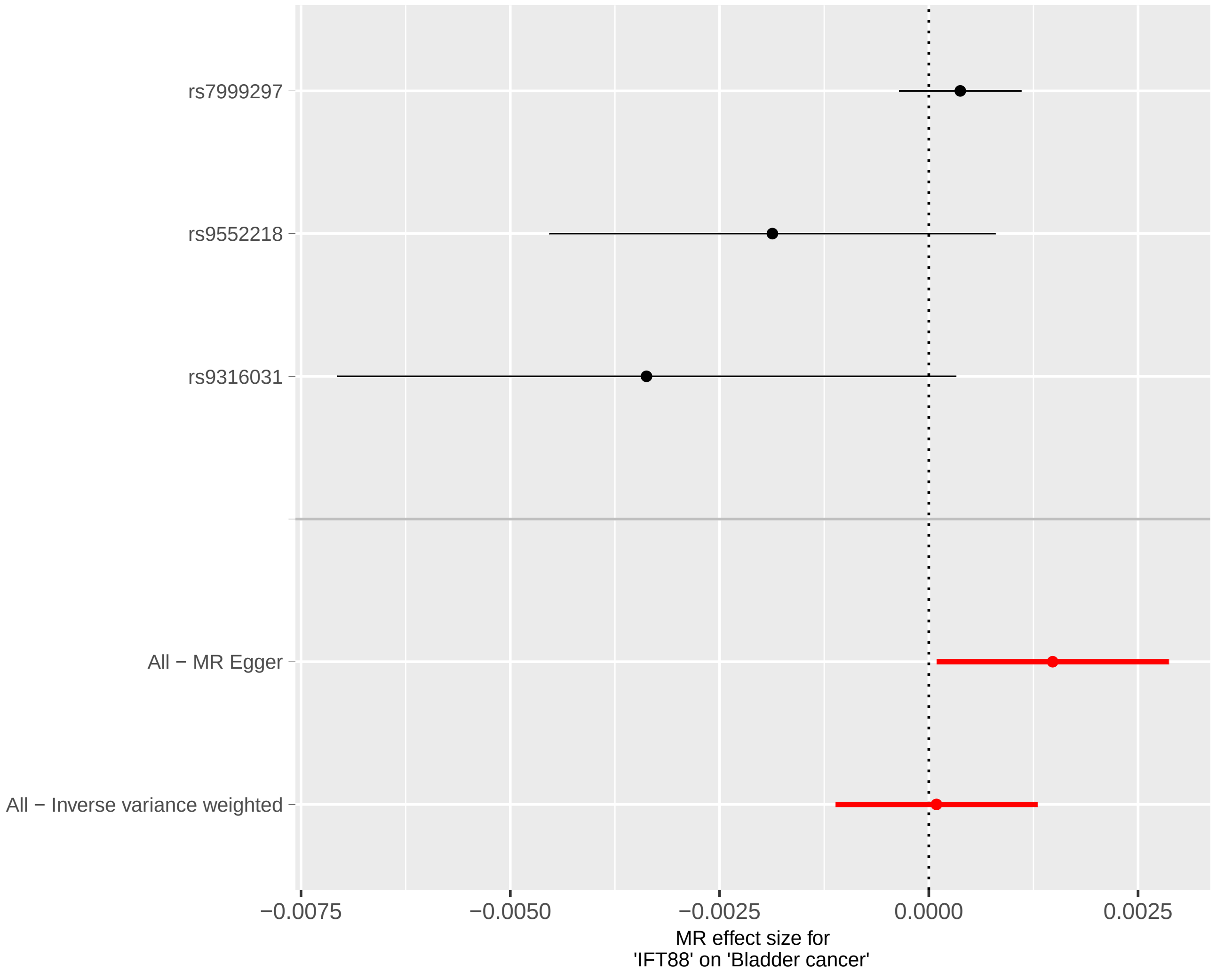

rs7999297
rs9552218
rs9316031
All − MR Egger
All − Inverse variance weighted
−0.0075
−0.0050
−0.0025
0.0000
0.0025
MR effect size for
'IFT88' on 'Bladder cancer'

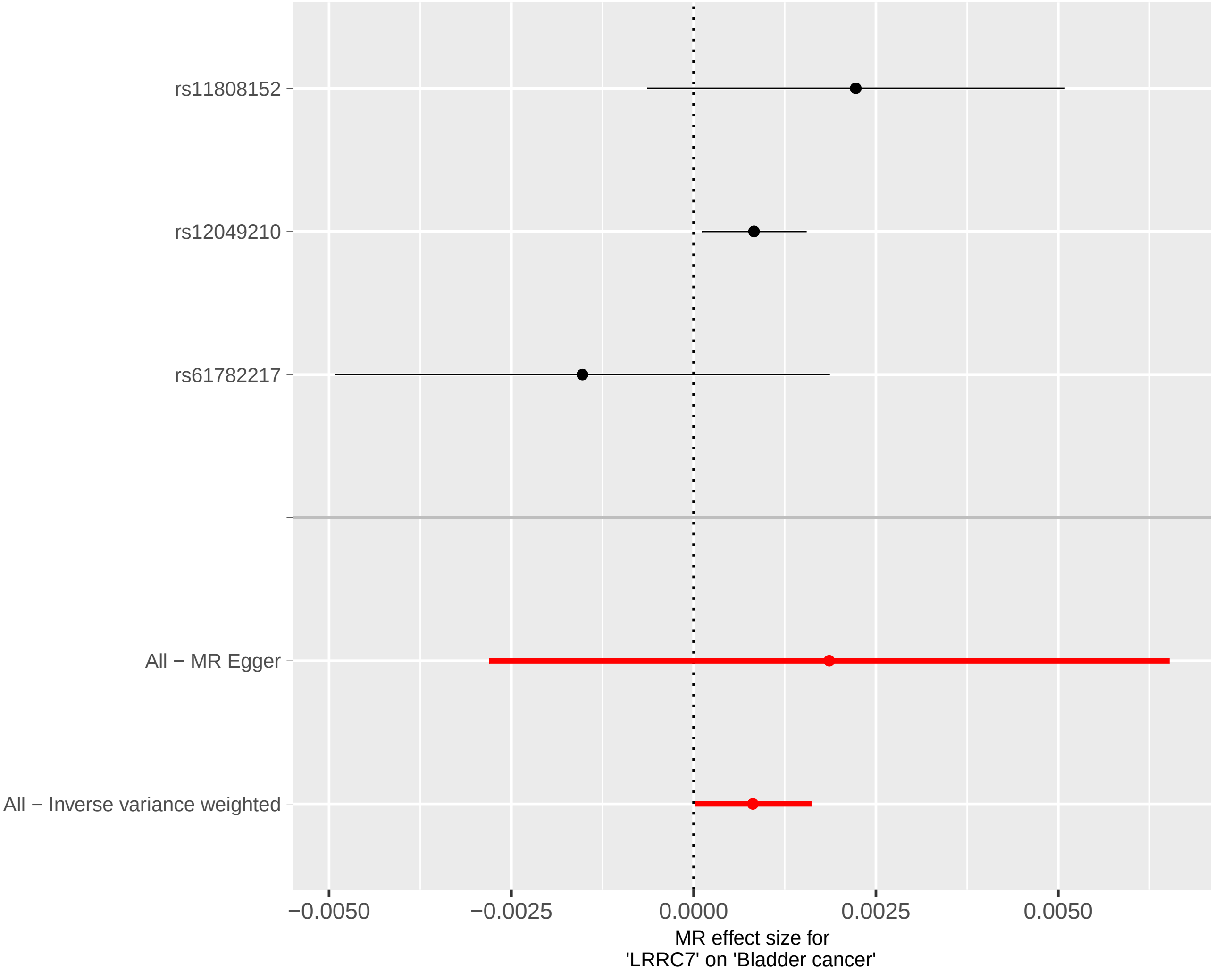
rs11808152
rs12049210
rs61782217
All − MR Egger
All − Inverse variance weighted
−0.0050
−0.0025
0.0000
0.0025
0.0050
MR effect size for
'LRRC7' on 'Bladder cancer'

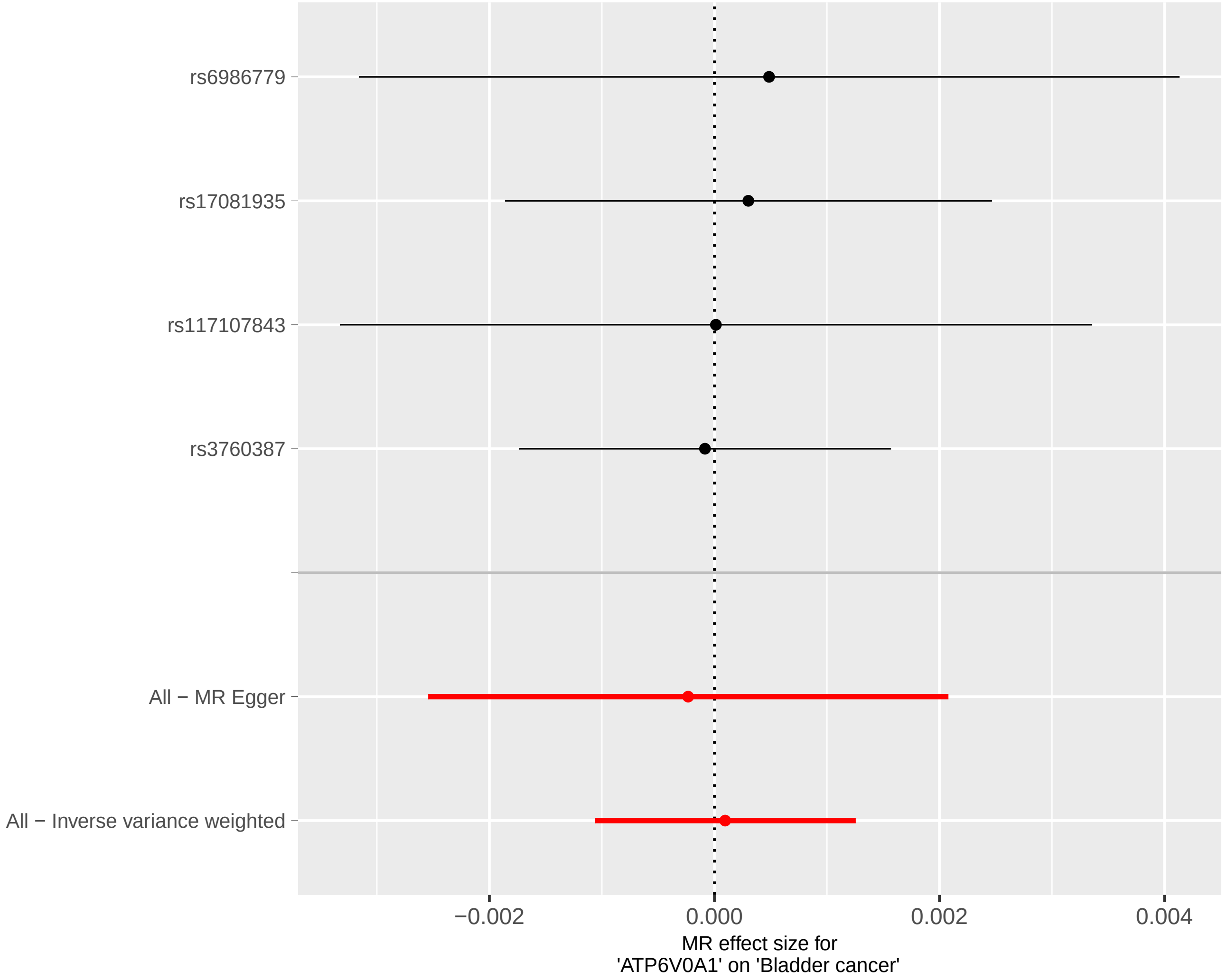

rs6986779
rs17081935
rs117107843
rs3760387
All − MR Egger
All − Inverse variance weighted
−0.002
0.000
0.002
0.004
MR effect size for
'ATP6V0A1' on 'Bladder cancer'

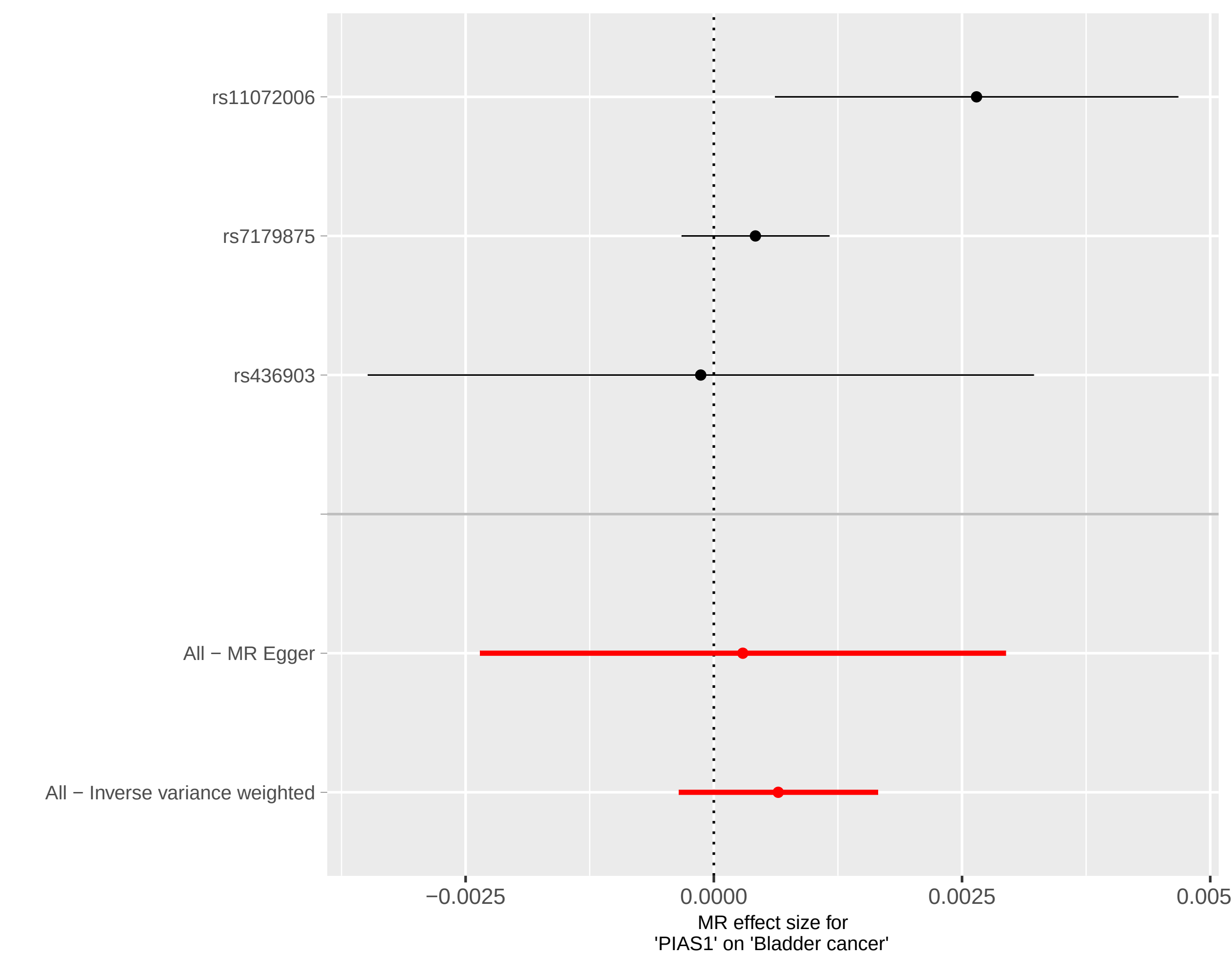
rs11072006
rs7179875
rs436903
All − MR Egger
All − Inverse variance weighted
−0.0025
0.0000
0.0025
0.005
MR effect size for
'PIAS1' on 'Bladder cancer'

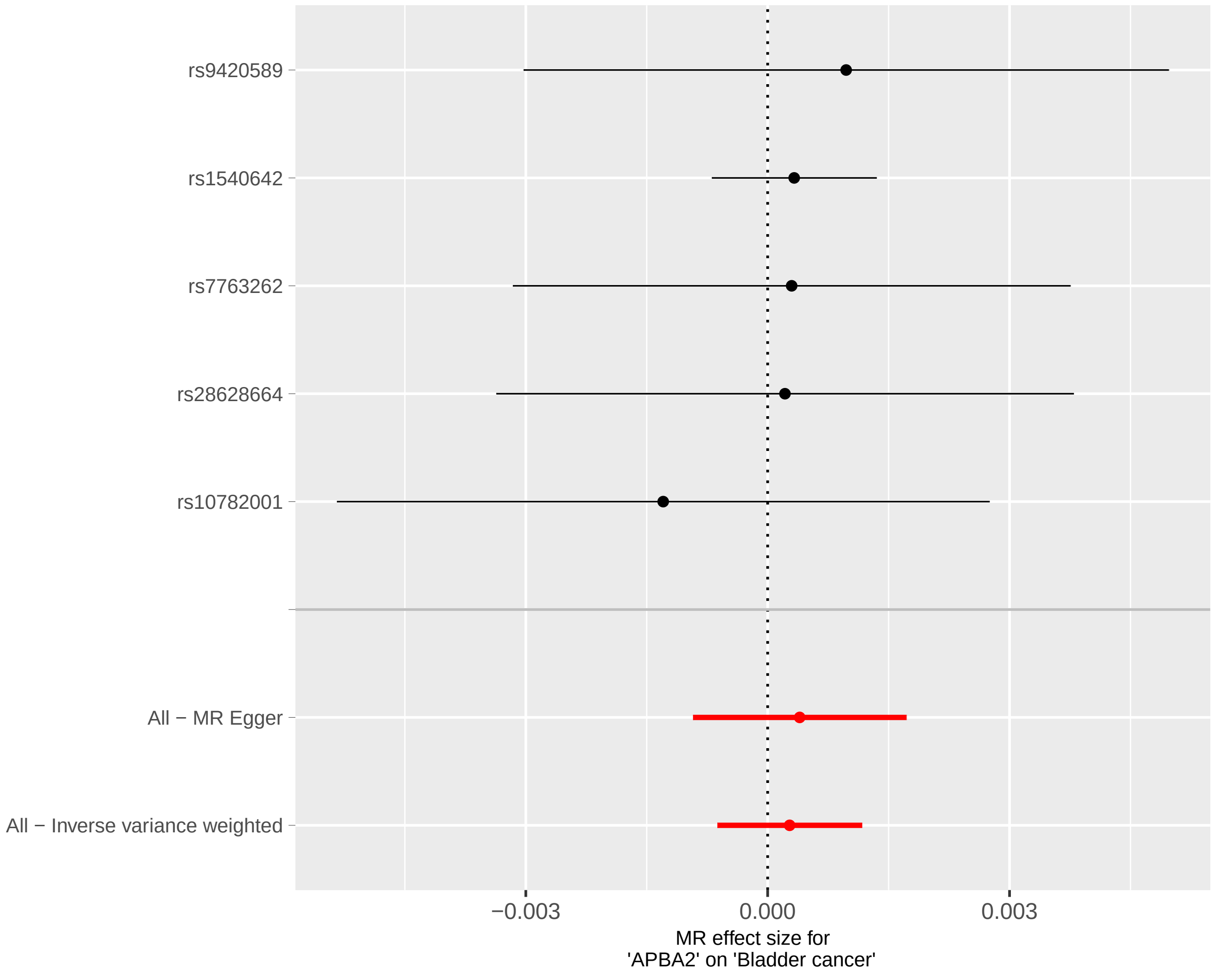
rs9420589
rs1540642
rs7763262
rs28628664
rs10782001
All − MR Egger
All − Inverse variance weighted
−0.003
0.000
0.003
MR effect size for
'APBA2' on 'Bladder cancer'

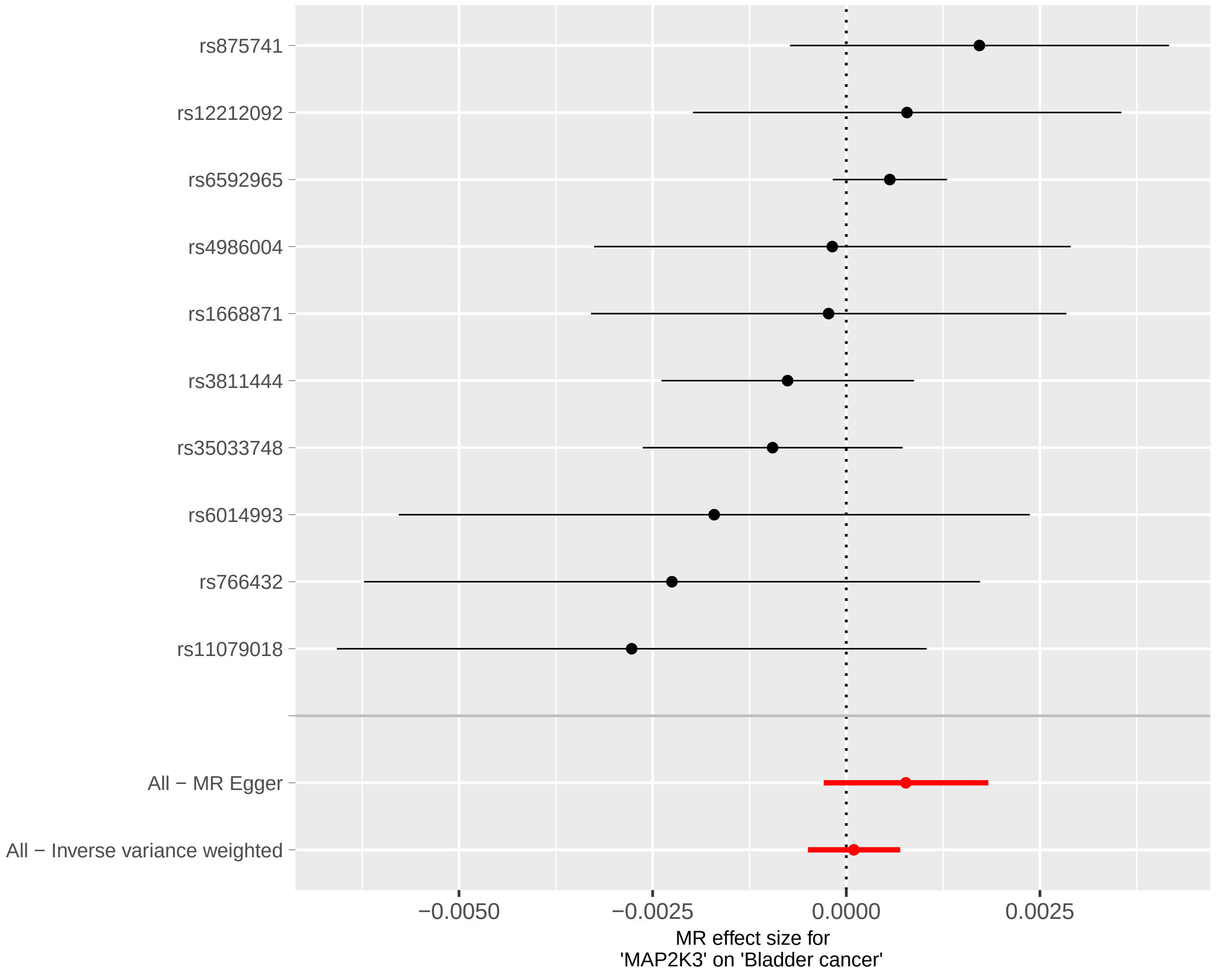
rs875741
rs12212092
rs6592965
rs4986004
rs1668871
rs3811444
rs35033748
rs6014993
rs766432
rs11079018
All − MR Egger
All − Inverse variance weighted
−0.0050
−0.0025
0.0000
0.0025
MR effect size for
'MAP2K3' on 'Bladder cancer'

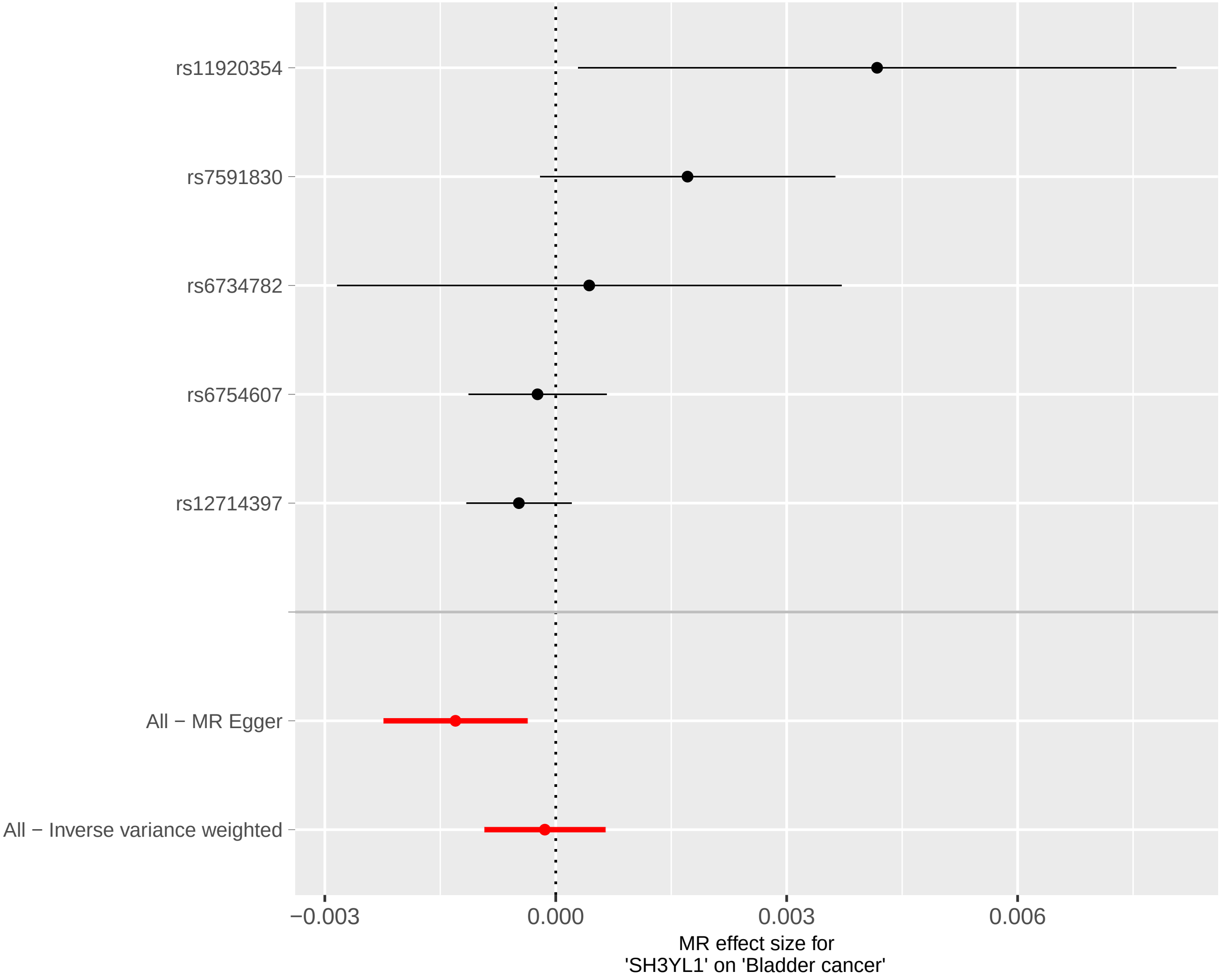
rs11920354
rs7591830
rs6734782
rs6754607
rs12714397
All − MR Egger
All − Inverse variance weighted
−0.003
0.000
0.003
0.006
MR effect size for
'SH3YL1' on 'Bladder cancer'

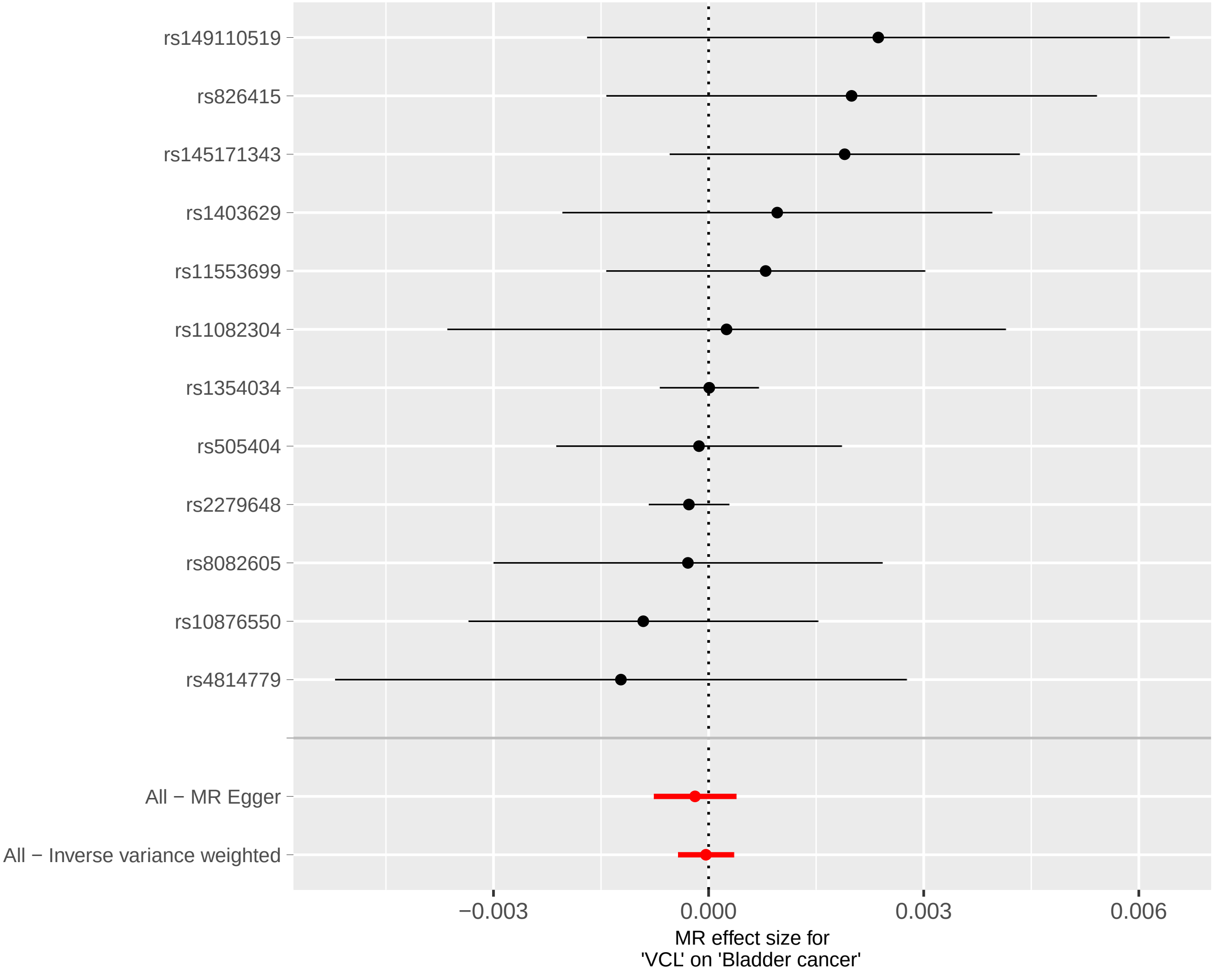

rs149110519
rs826415
rs145171343
rs1403629
rs11553699
rs11082304
rs1354034
rs505404
rs2279648
rs8082605
rs10876550
rs4814779
All − MR Egger
All − Inverse variance weighted
−0.003
0.000
0.003
0.006
MR effect size for
'VCL' on 'Bladder cancer'

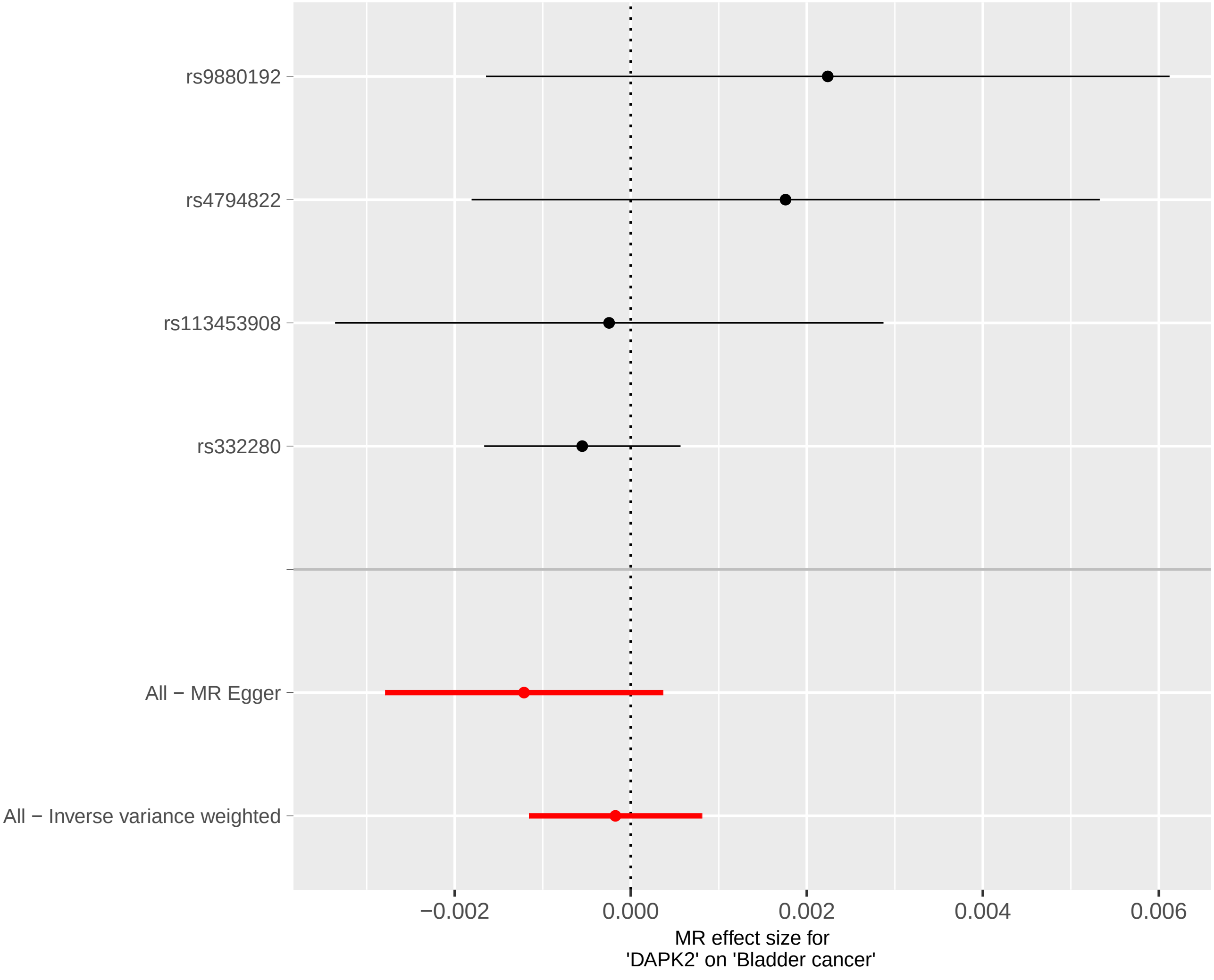

rs9880192
rs4794822
rs113453908
rs332280
All − MR Egger
All − Inverse variance weighted
−0.002
0.000
0.002
0.004
0.006
MR effect size for
'DAPK2' on 'Bladder cancer'

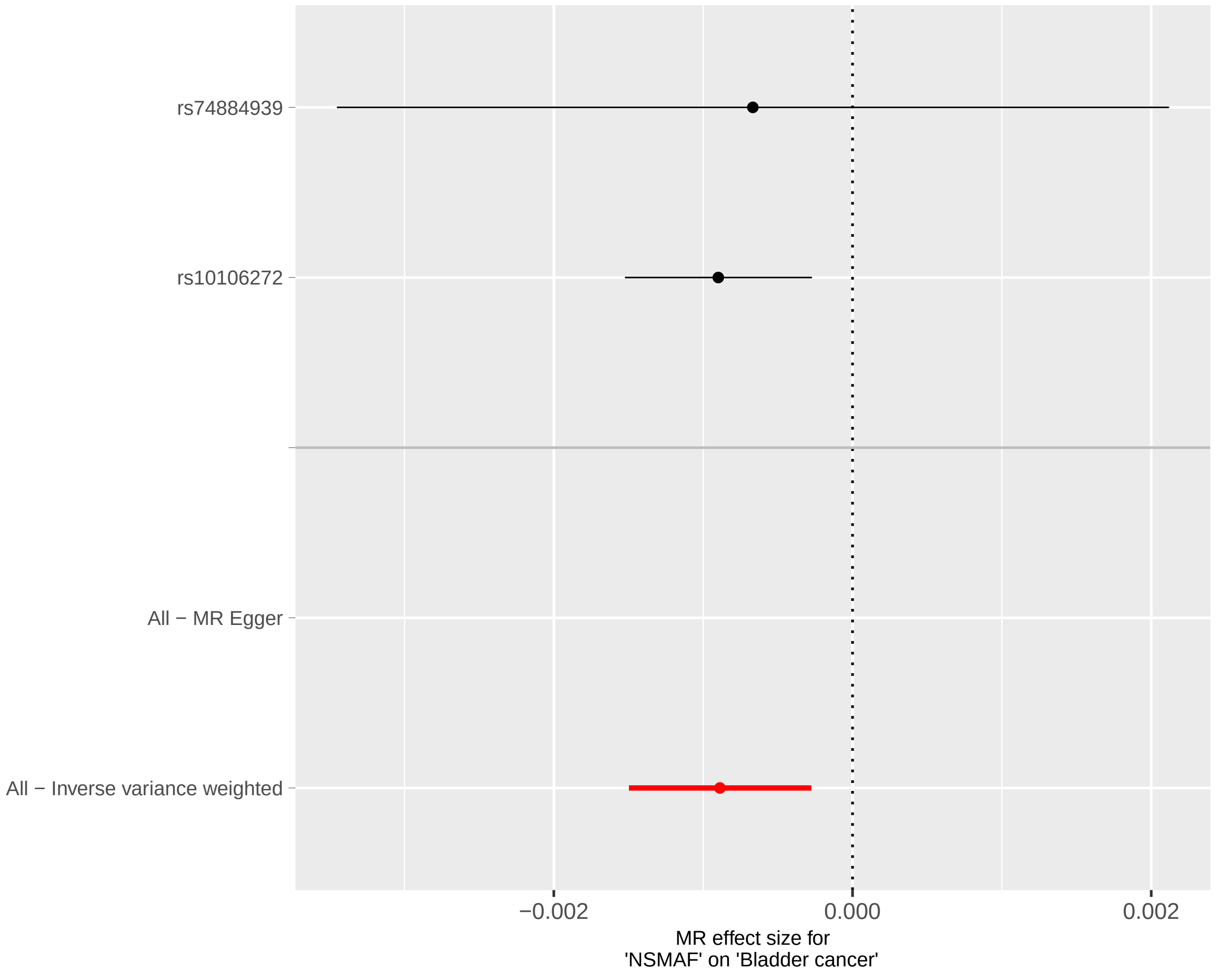
rs74884939
rs10106272
All − MR Egger
All − Inverse variance weighted
−0.002
0.000
0.002
MR effect size for
'NSMAF' on 'Bladder cancer'

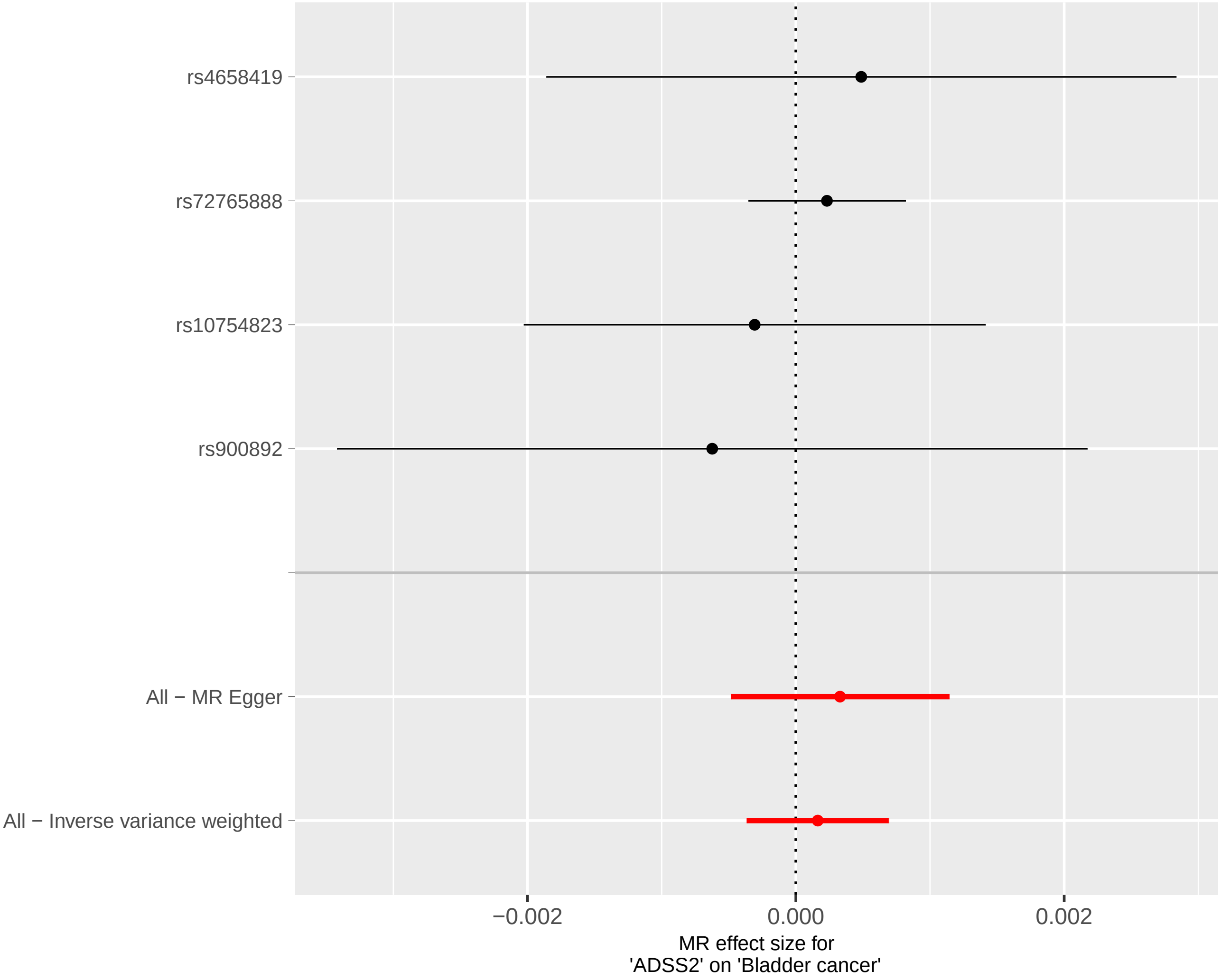
rs4658419
rs72765888
rs10754823
rs900892
All − MR Egger
All − Inverse variance weighted
−0.002
0.000
0.002
MR effect size for
'ADSS2' on 'Bladder cancer'

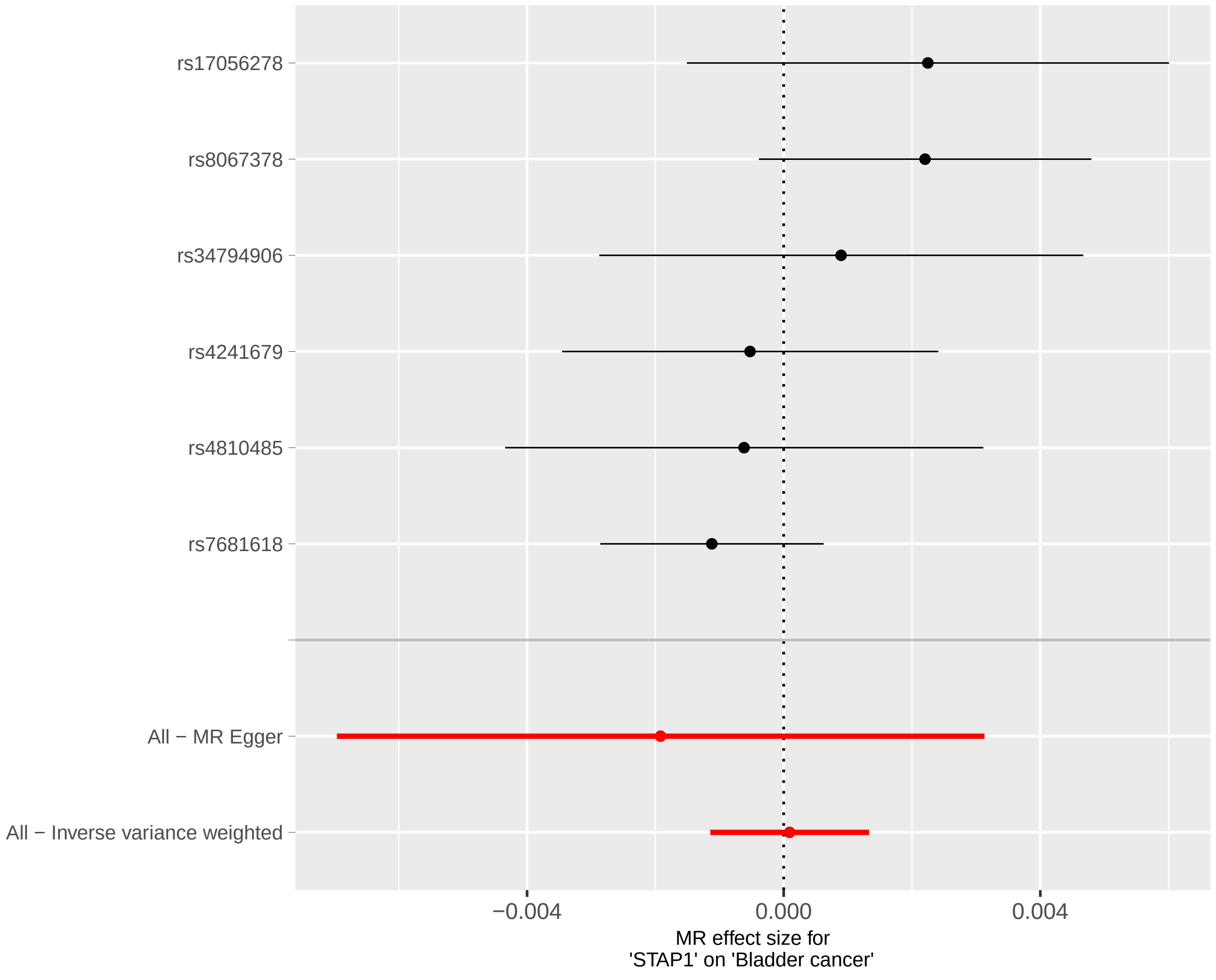
rs17056278
rs8067378
rs34794906
rs4241679
rs4810485
rs7681618
All − MR Egger
All − Inverse variance weighted
−0.004
0.000
0.004
MR effect size for
'STAP1' on 'Bladder cancer'

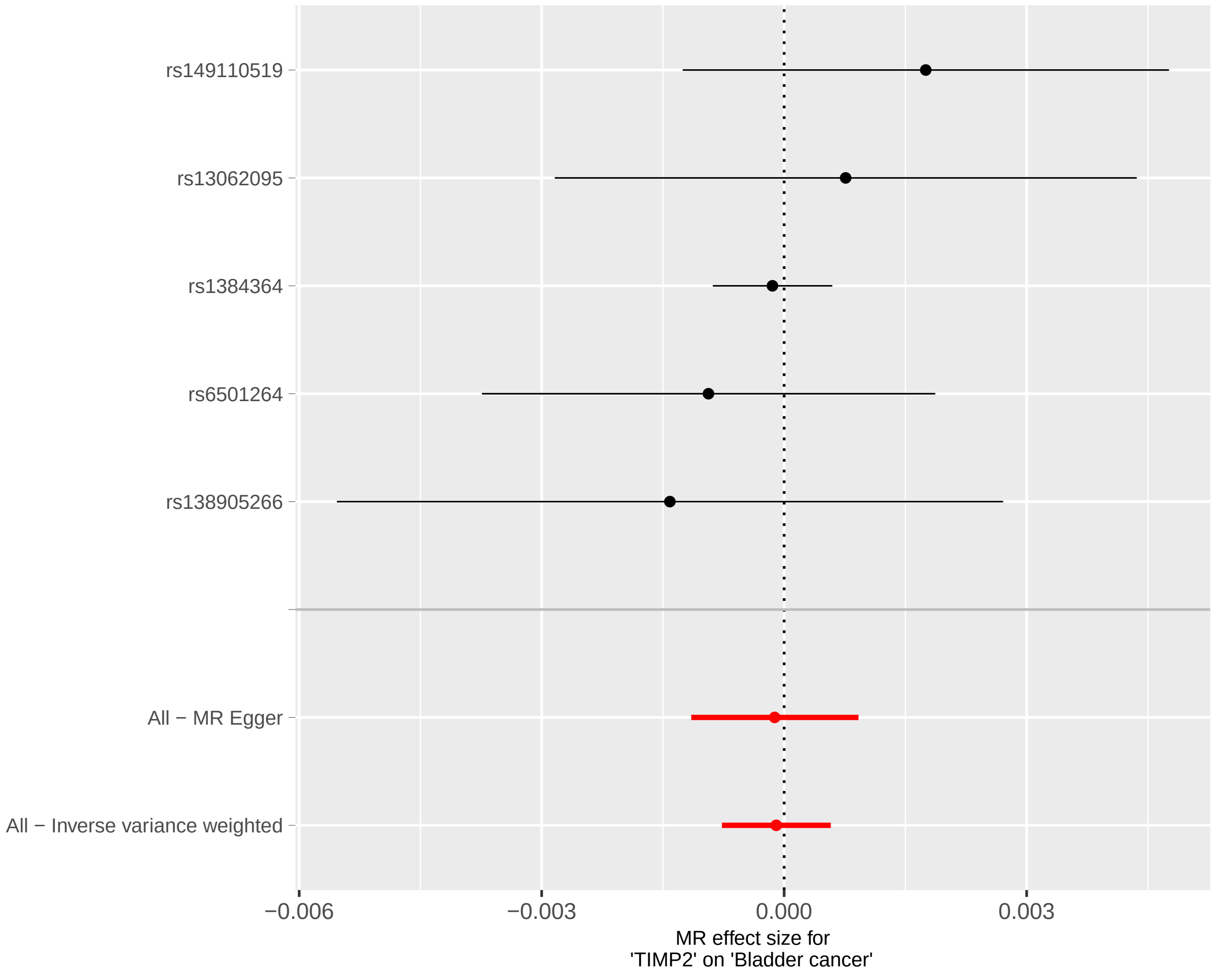

rs149110519
rs13062095
rs1384364
rs6501264
rs138905266
All − MR Egger
All − Inverse variance weighted
−0.006
−0.003
0.000
0.003
MR effect size for
'TIMP2' on 'Bladder cancer'

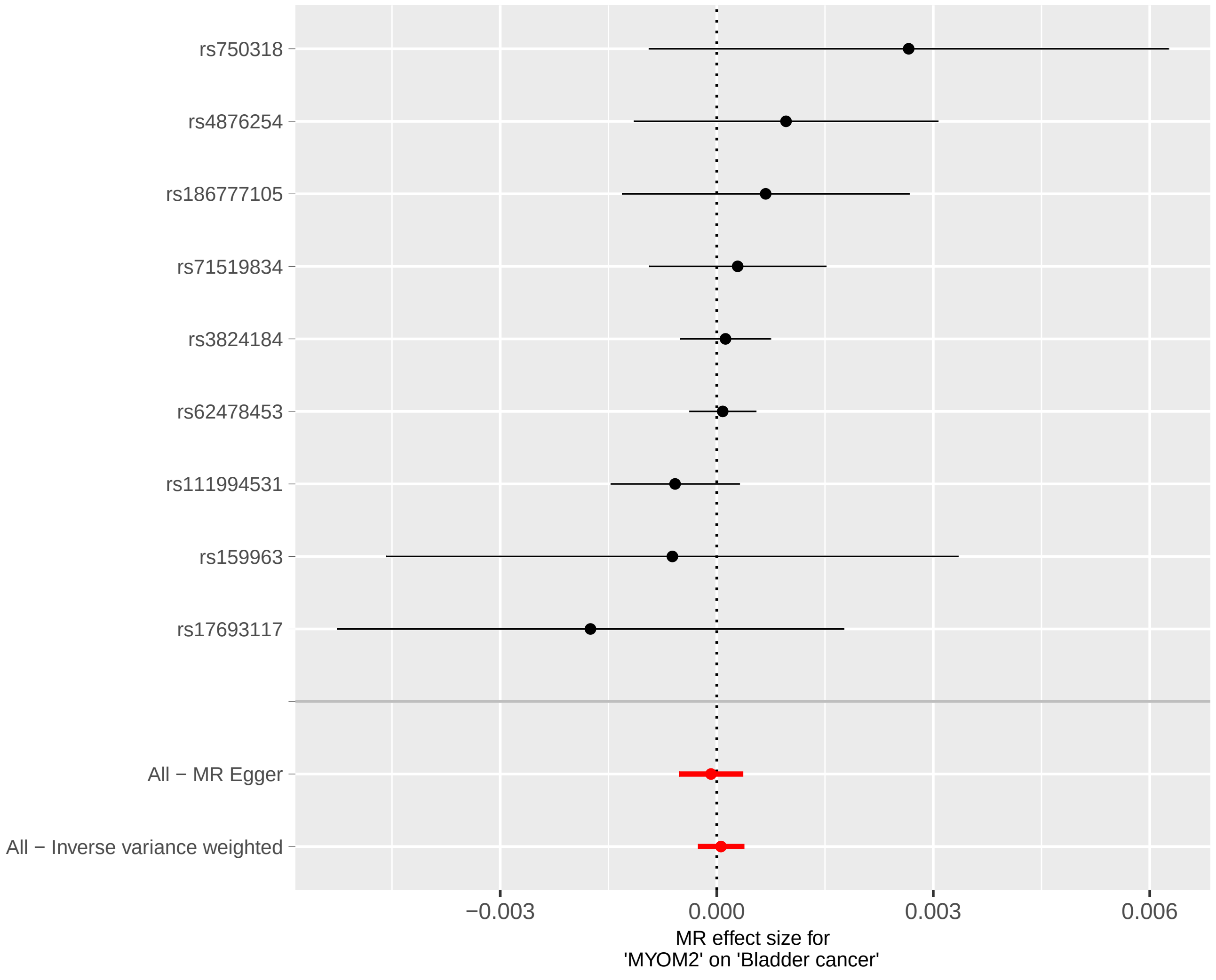

rs750318
rs4876254
rs186777105
rs71519834
rs3824184
rs62478453
rs111994531
rs159963
rs17693117
All − MR Egger
All − Inverse variance weighted
−0.003
0.000
0.003
0.006
MR effect size for
'MYOM2' on 'Bladder cancer'

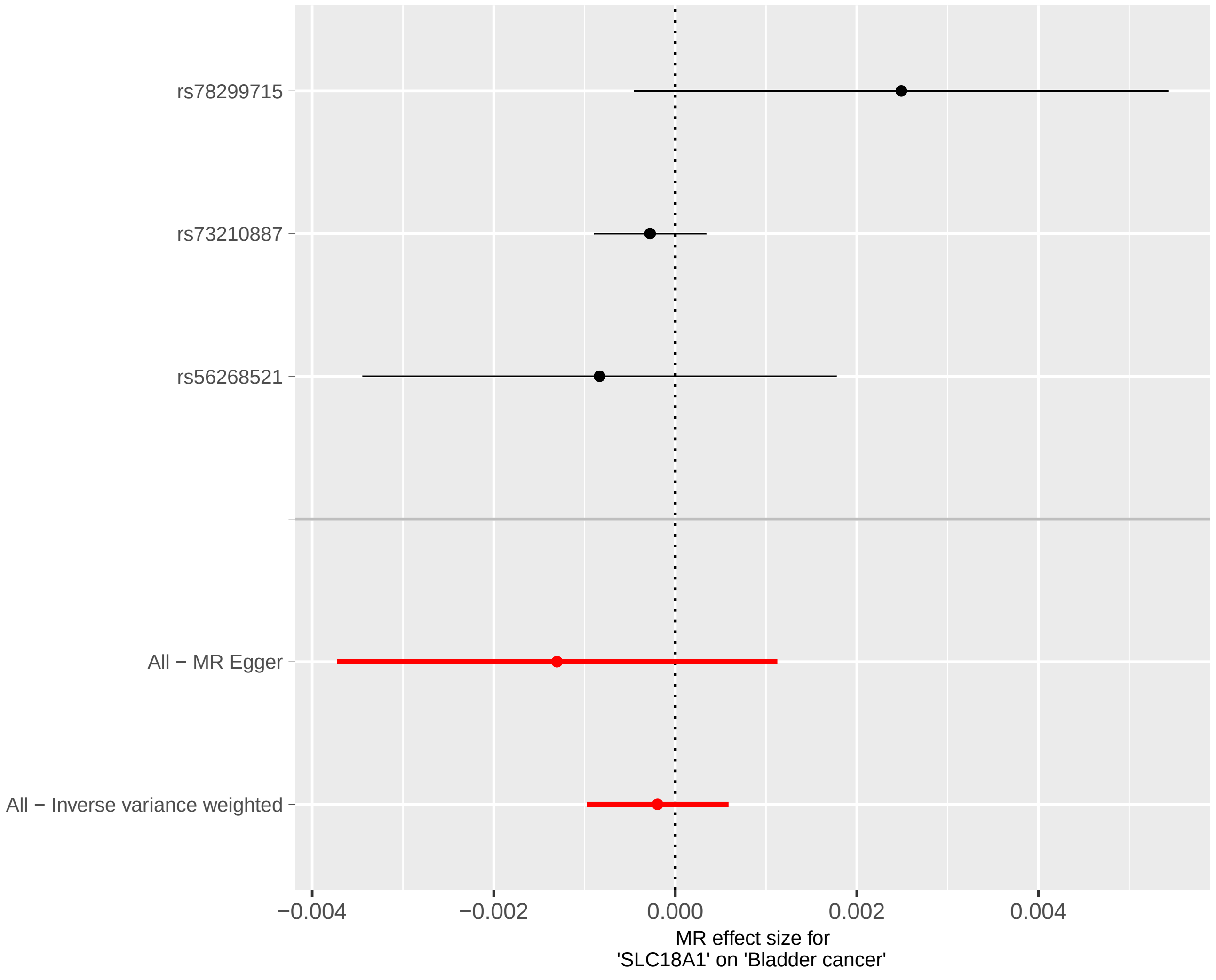

rs78299715
rs73210887
rs56268521
All − MR Egger
All − Inverse variance weighted
−0.004
−0.002
0.000
0.002
0.004
MR effect size for
'SLC18A1' on 'Bladder cancer'

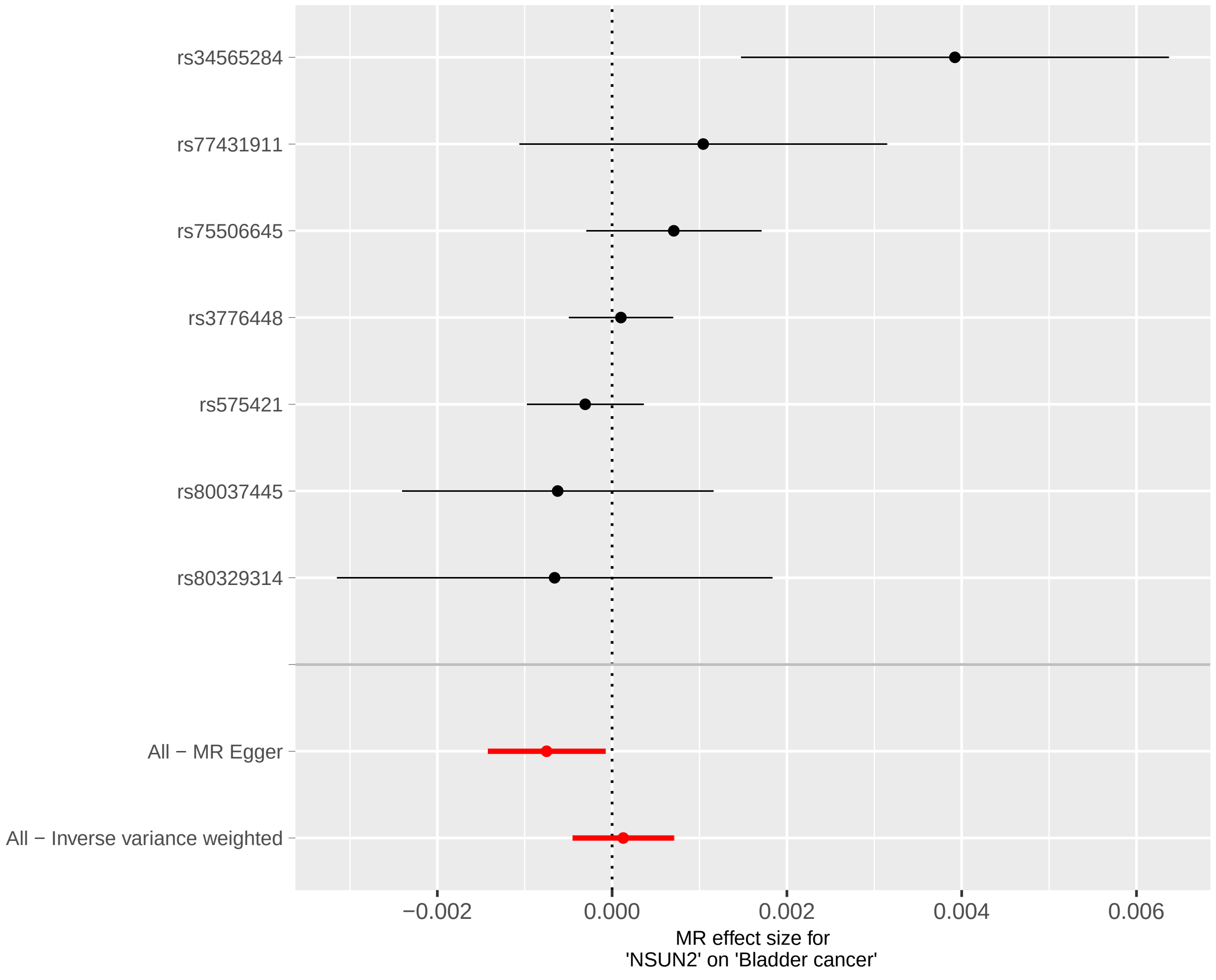
rs34565284
rs77431911
rs75506645
rs3776448
rs575421
rs80037445
rs80329314
All − MR Egger
All − Inverse variance weighted
−0.002
0.000
0.002
0.004
0.006
MR effect size for
'NSUN2' on 'Bladder cancer'

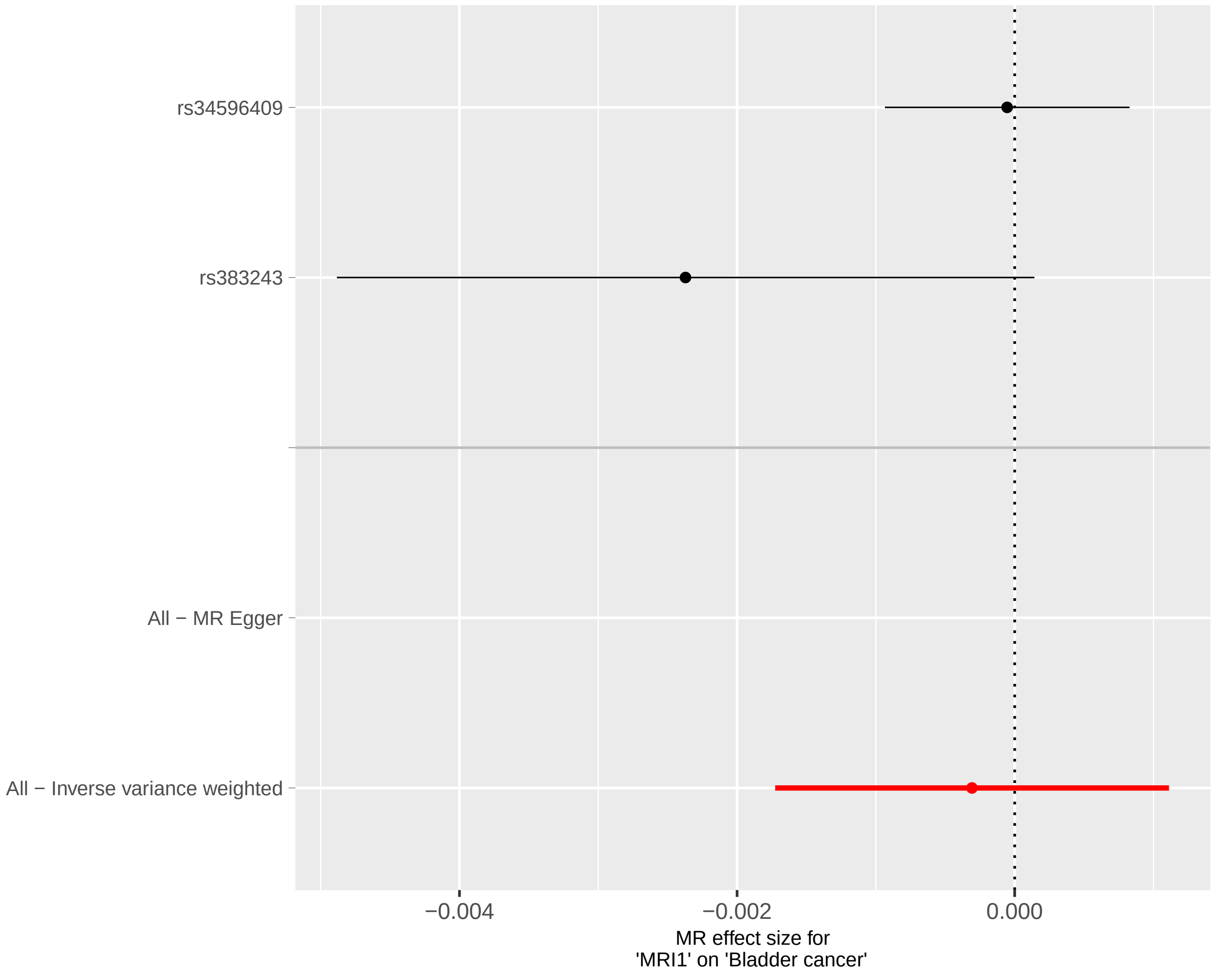

rs34596409
rs383243
All − MR Egger
All − Inverse variance weighted
−0.004
−0.002
0.000
MR effect size for
'MRI1' on 'Bladder cancer'

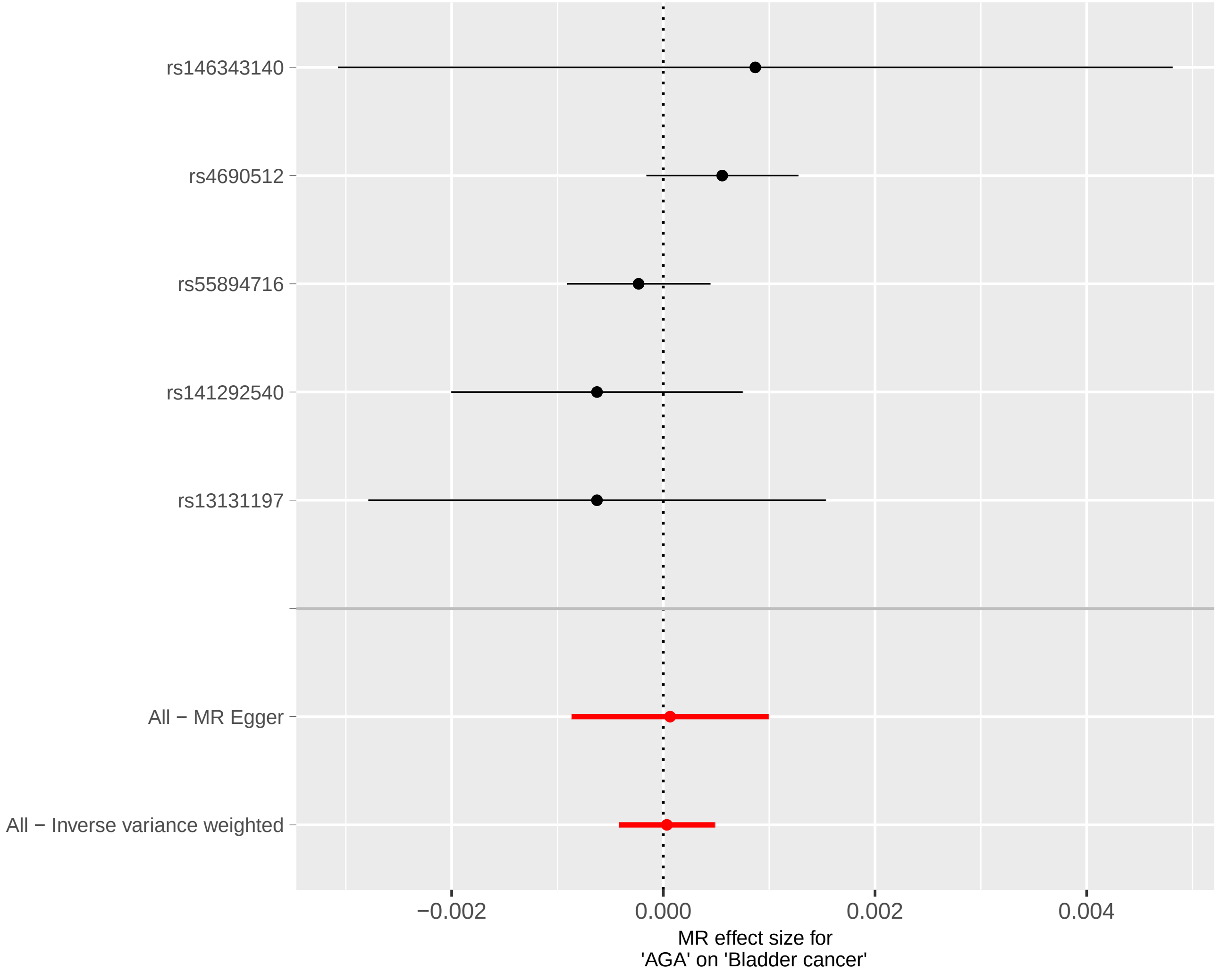

rs146343140
rs4690512
rs55894716
rs141292540
rs13131197
All − MR Egger
All − Inverse variance weighted
−0.002
0.000
0.002
0.004
MR effect size for
'AGA' on 'Bladder cancer'

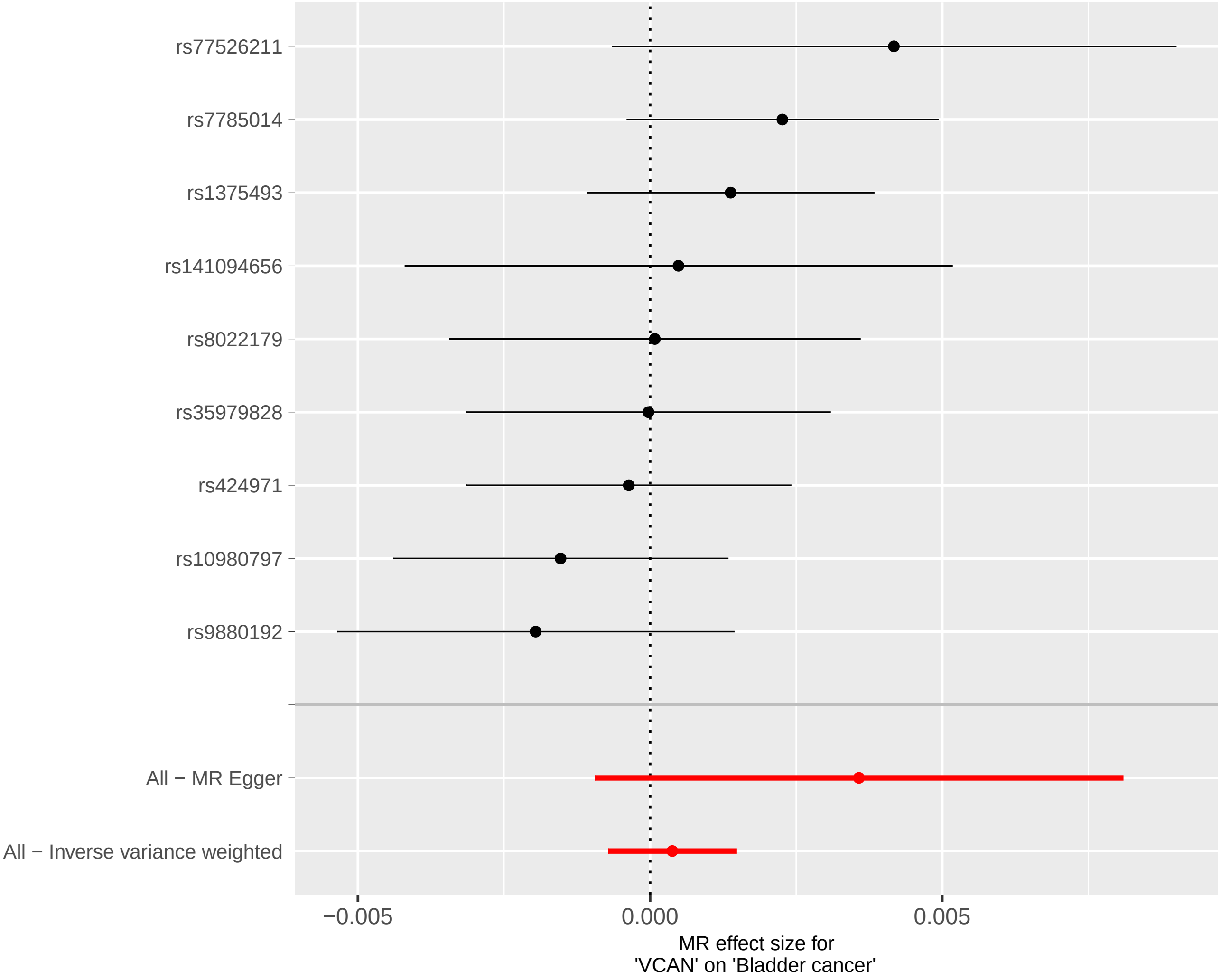
rs77526211
rs7785014
rs1375493
rs141094656
rs8022179
rs35979828
rs424971
rs10980797
rs9880192
All − MR Egger
All − Inverse variance weighted
−0.005
0.000
0.005
MR effect size for
'VCAN' on 'Bladder cancer'

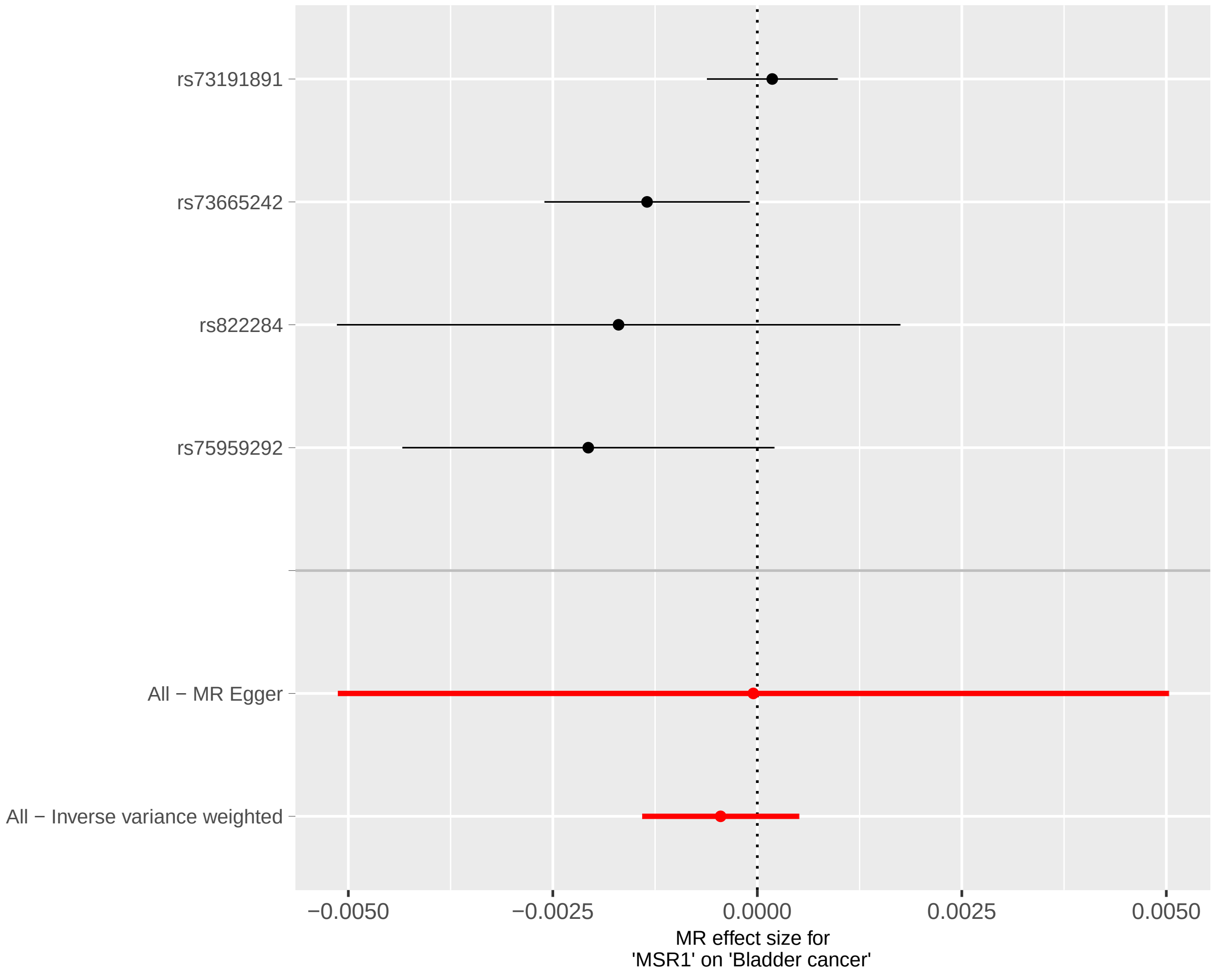

rs73191891
rs73665242
rs822284
rs75959292
All − MR Egger
All − Inverse variance weighted
−0.0050
−0.0025
0.0000
0.0025
0.0050
MR effect size for
'MSR1' on 'Bladder cancer'

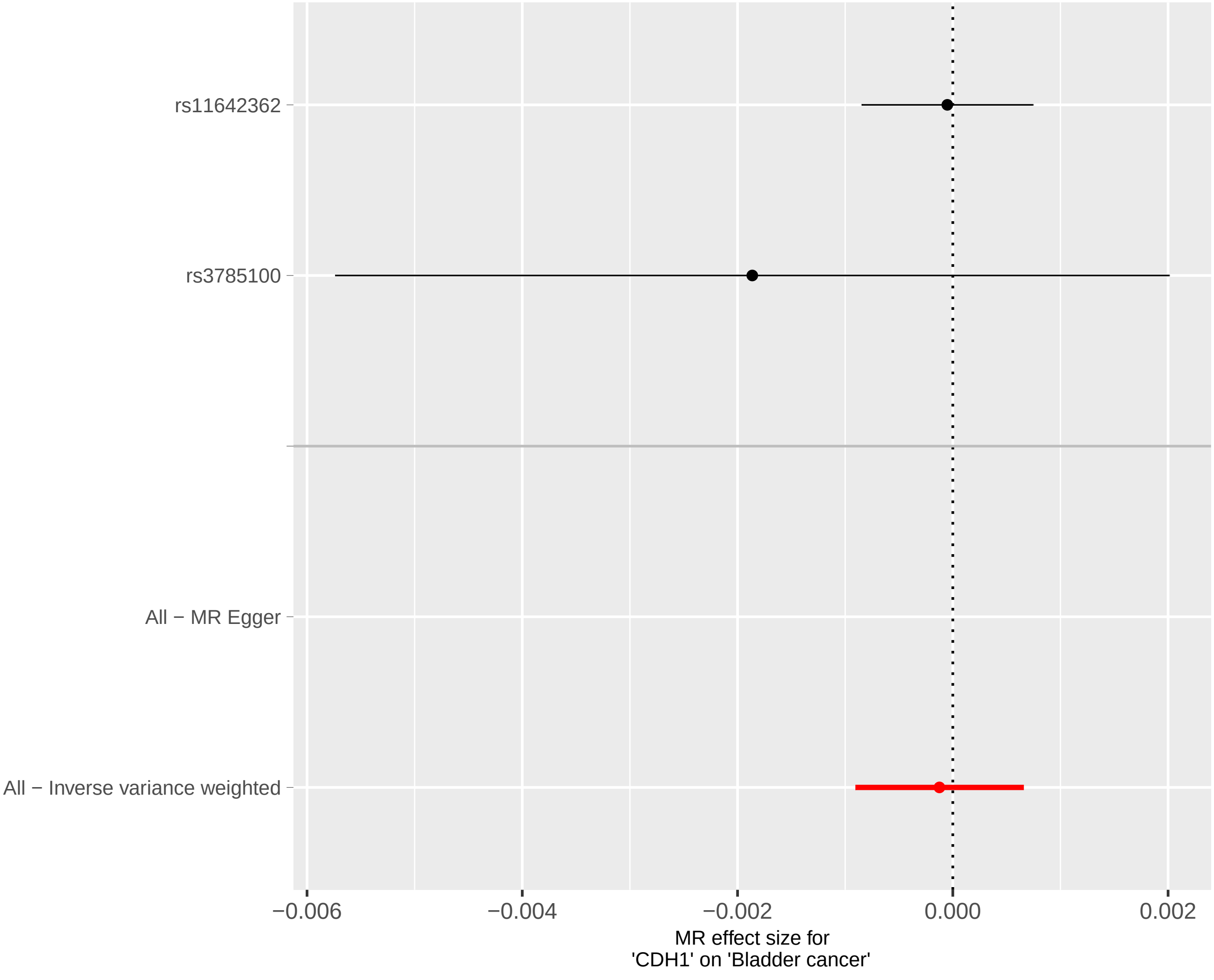

rs11642362
rs3785100
All − MR Egger
All − Inverse variance weighted
−0.006
−0.004
−0.002
0.000
0.002
MR effect size for
'CDH1' on 'Bladder cancer'

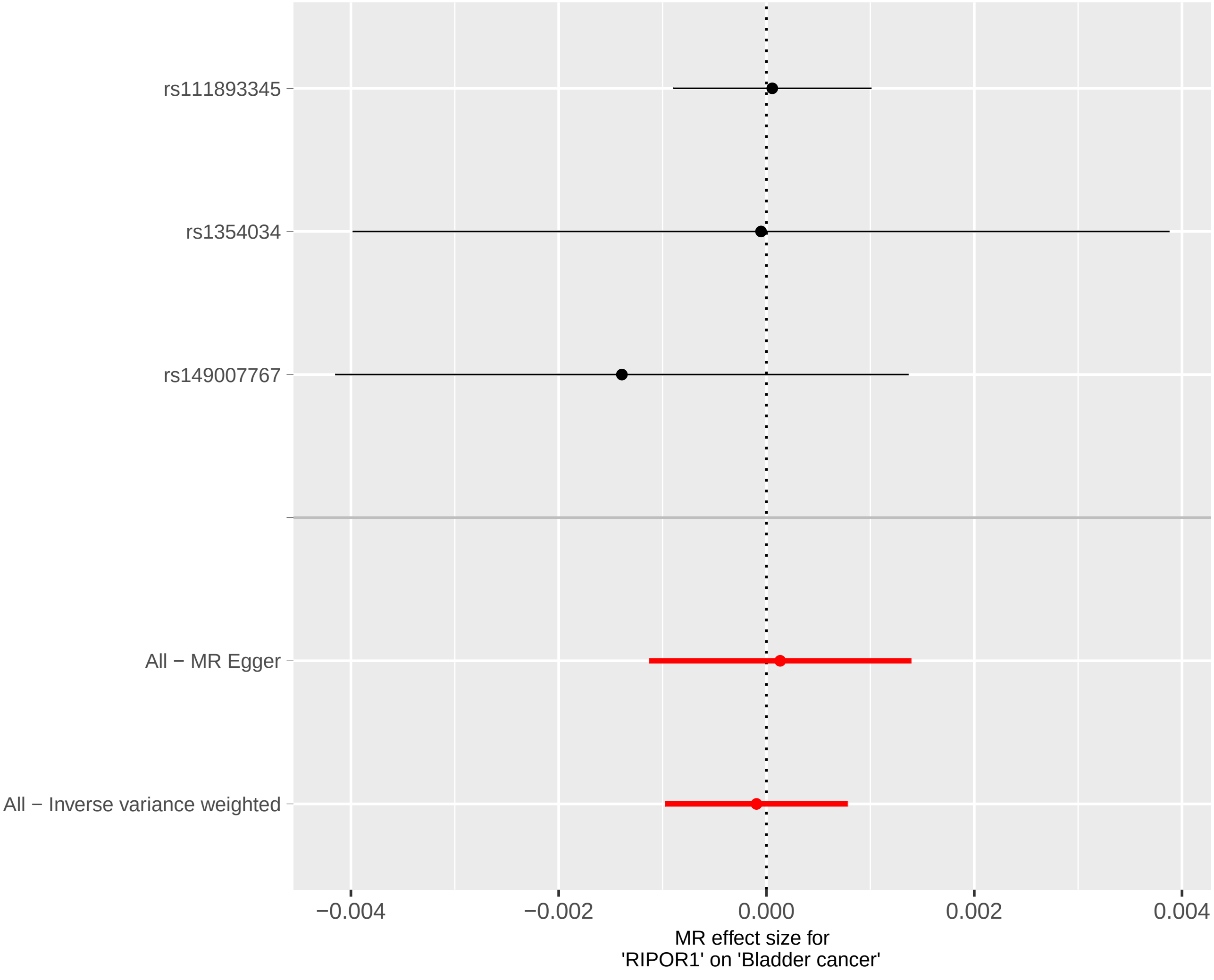
rs111893345
rs1354034
rs149007767
All − MR Egger
All − Inverse variance weighted
−0.004
−0.002
0.000
0.002
0.004
MR effect size for
'RIPOR1' on 'Bladder cancer'

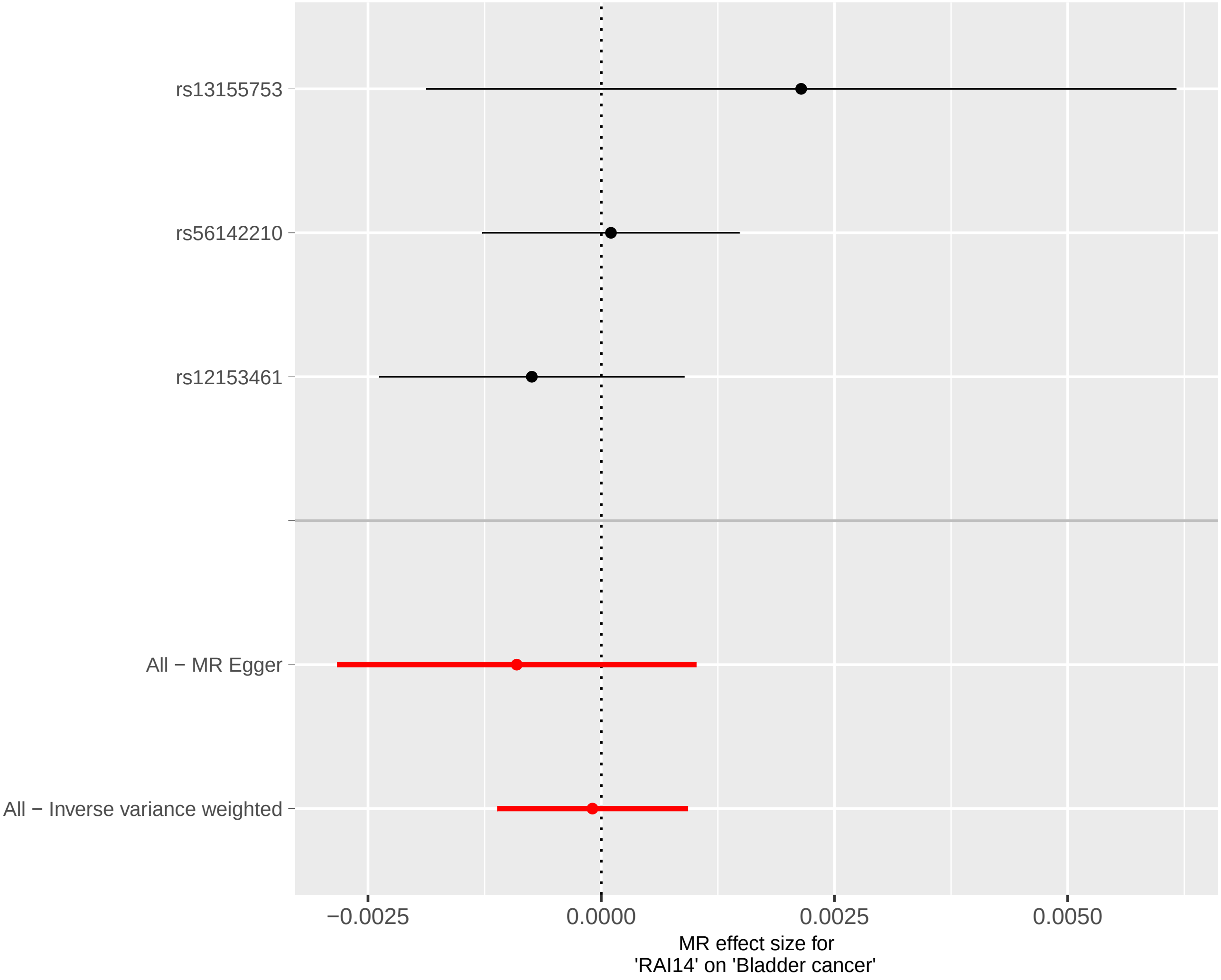
rs13155753
rs56142210
rs12153461
All − MR Egger
All − Inverse variance weighted
−0.0025
0.0000
0.0025
0.0050
MR effect size for
'RAI14' on 'Bladder cancer'

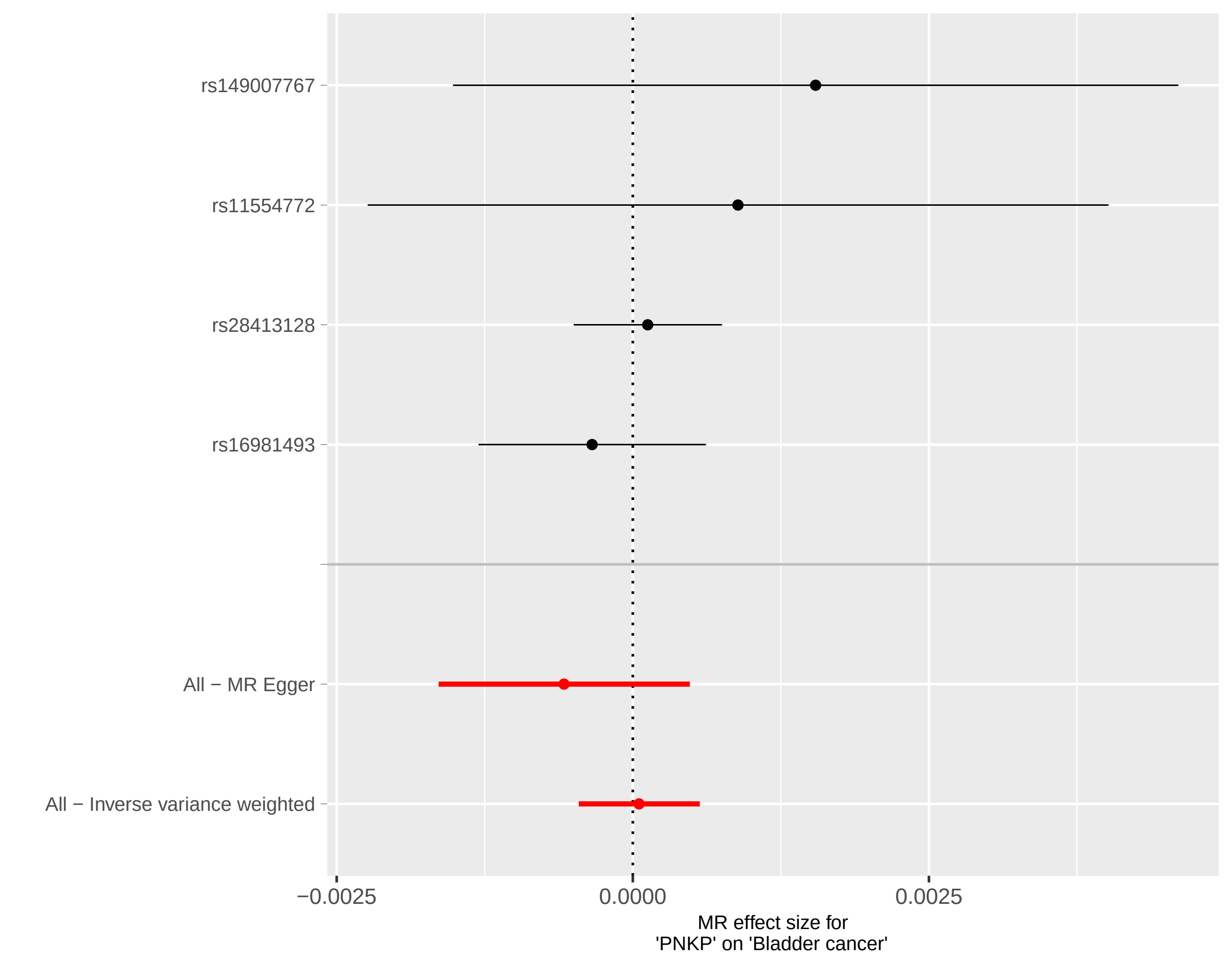

rs149007767
rs11554772
rs28413128
rs16981493
All − MR Egger
All − Inverse variance weighted
−0.0025
0.0000
0.0025
MR effect size for
'PNKP' on 'Bladder cancer'

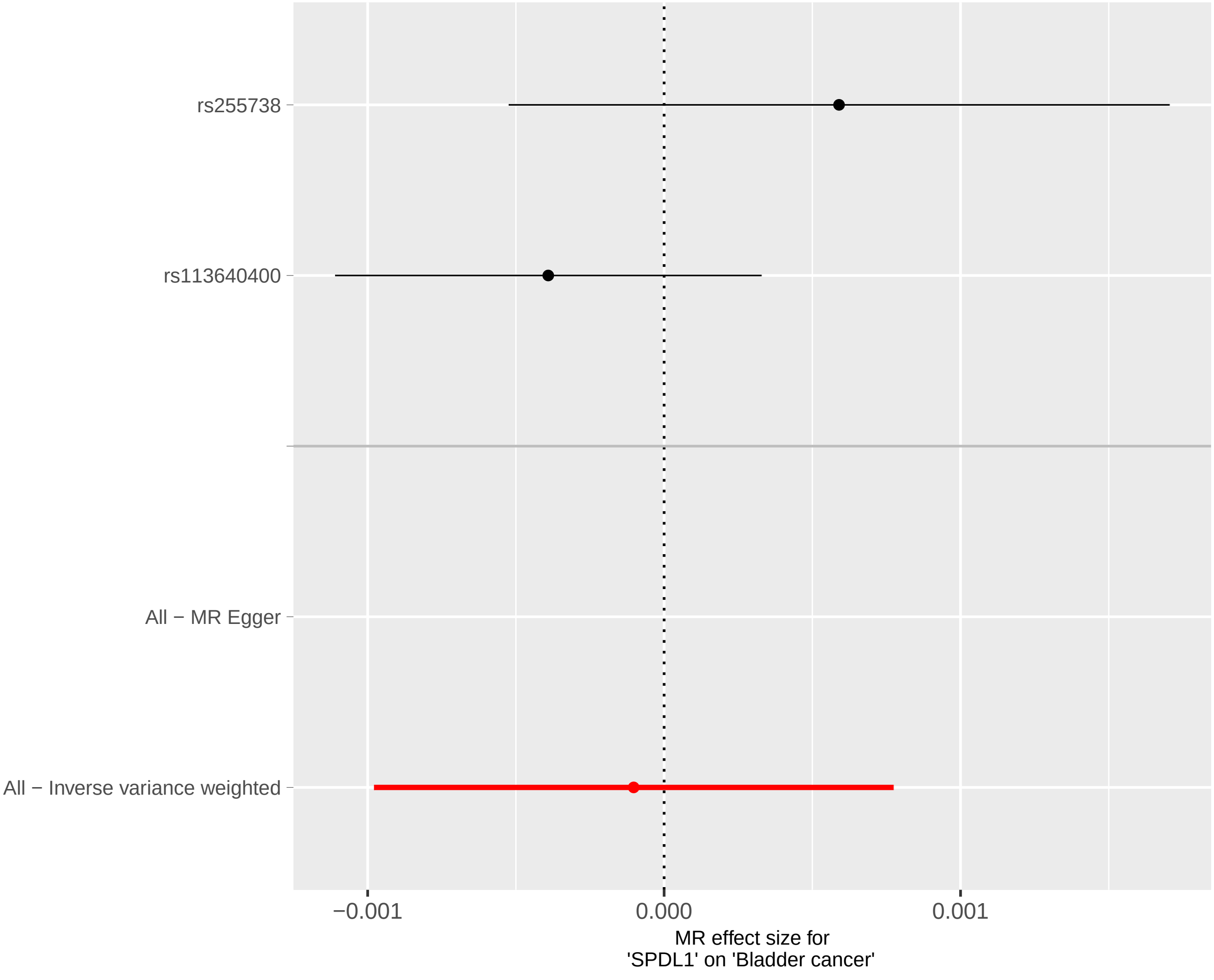
rs255738
rs113640400
All − MR Egger
All − Inverse variance weighted
−0.001
0.000
0.001
MR effect size for
'SPDL1' on 'Bladder cancer'

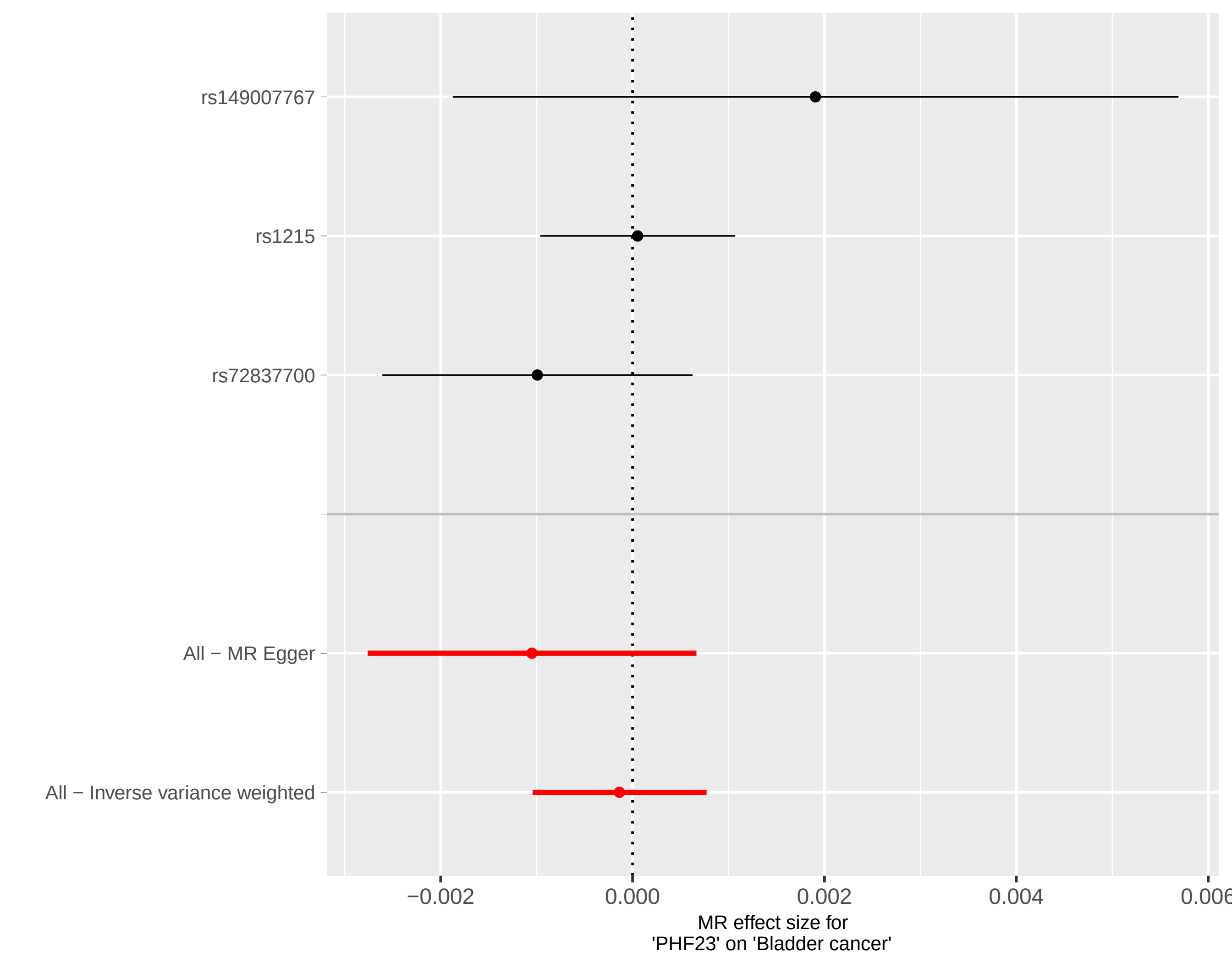
rs149007767
rs1215
rs72837700
All − MR Egger
All − Inverse variance weighted
−0.002
0.000
0.002
0.004
0.006
MR effect size for
'PHF23' on 'Bladder cancer'

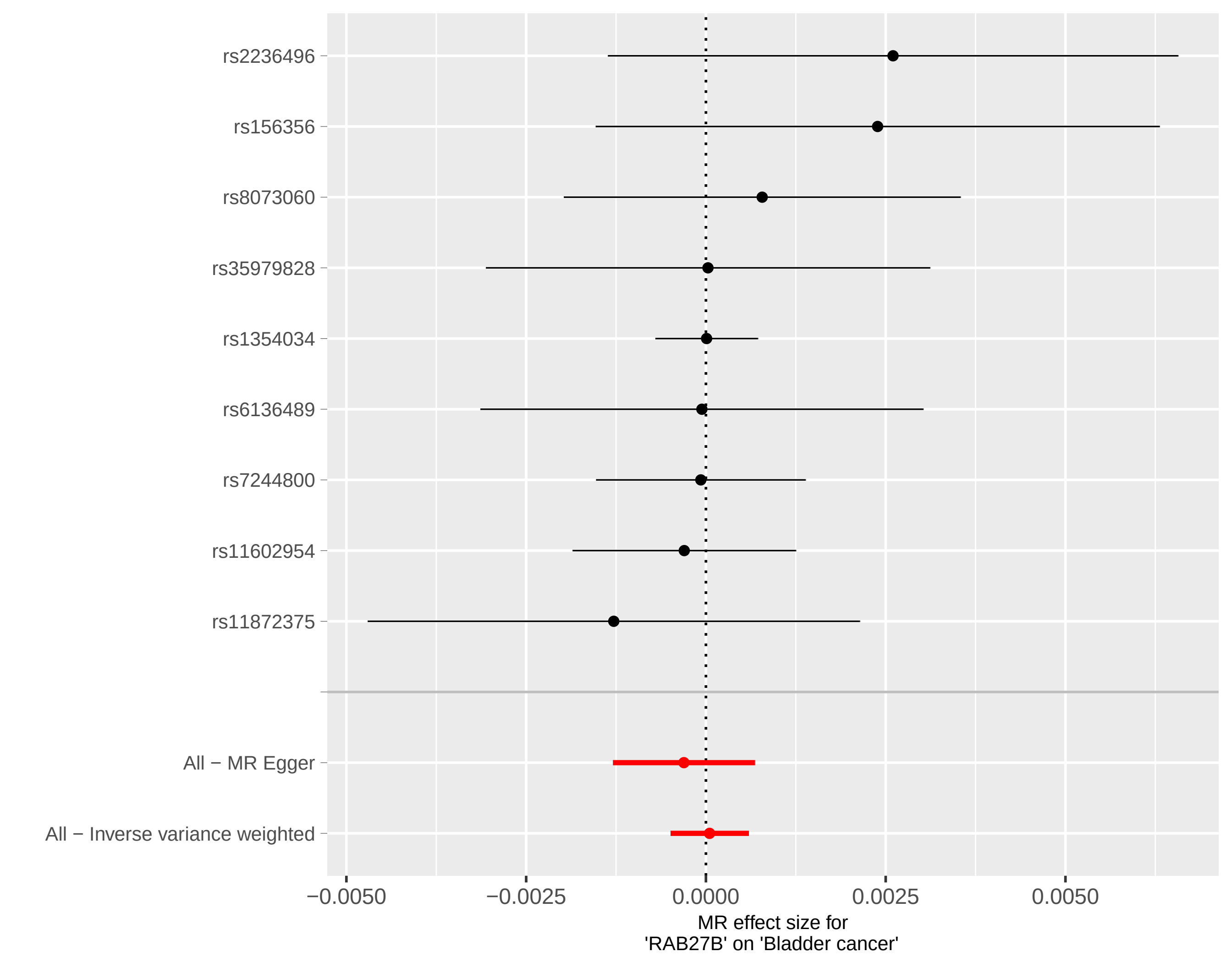
rs2236496
rs156356
rs8073060
rs35979828
rs1354034
rs6136489
rs7244800
rs11602954
rs11872375
All − MR Egger
All − Inverse variance weighted
−0.0050
−0.0025
0.0000
0.0025
0.0050
MR effect size for
'RAB27B' on 'Bladder cancer'

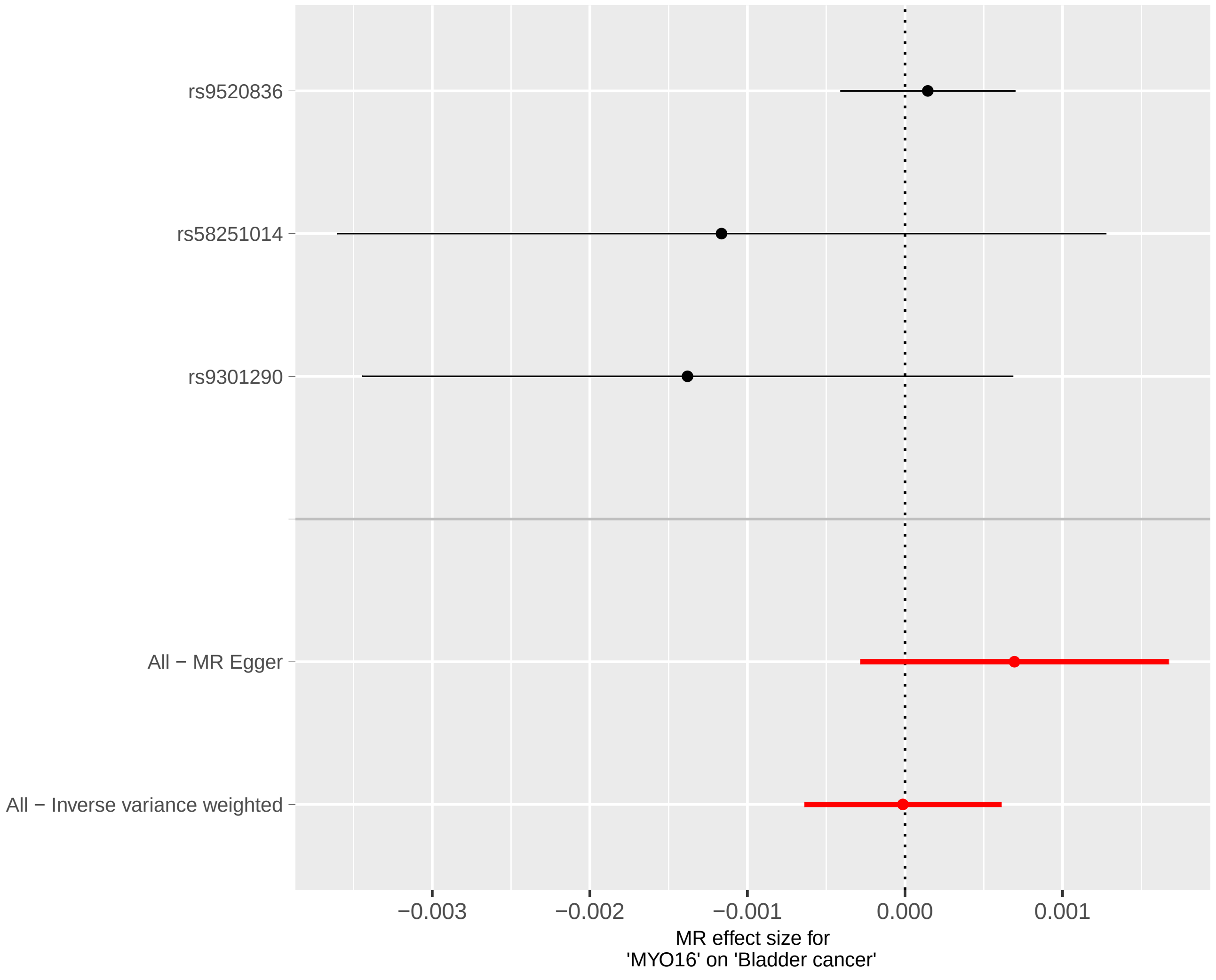

rs9520836
rs58251014
rs9301290
All − MR Egger
All − Inverse variance weighted
−0.003
−0.002
−0.001
0.000
0.001
MR effect size for
'MYO16' on 'Bladder cancer'

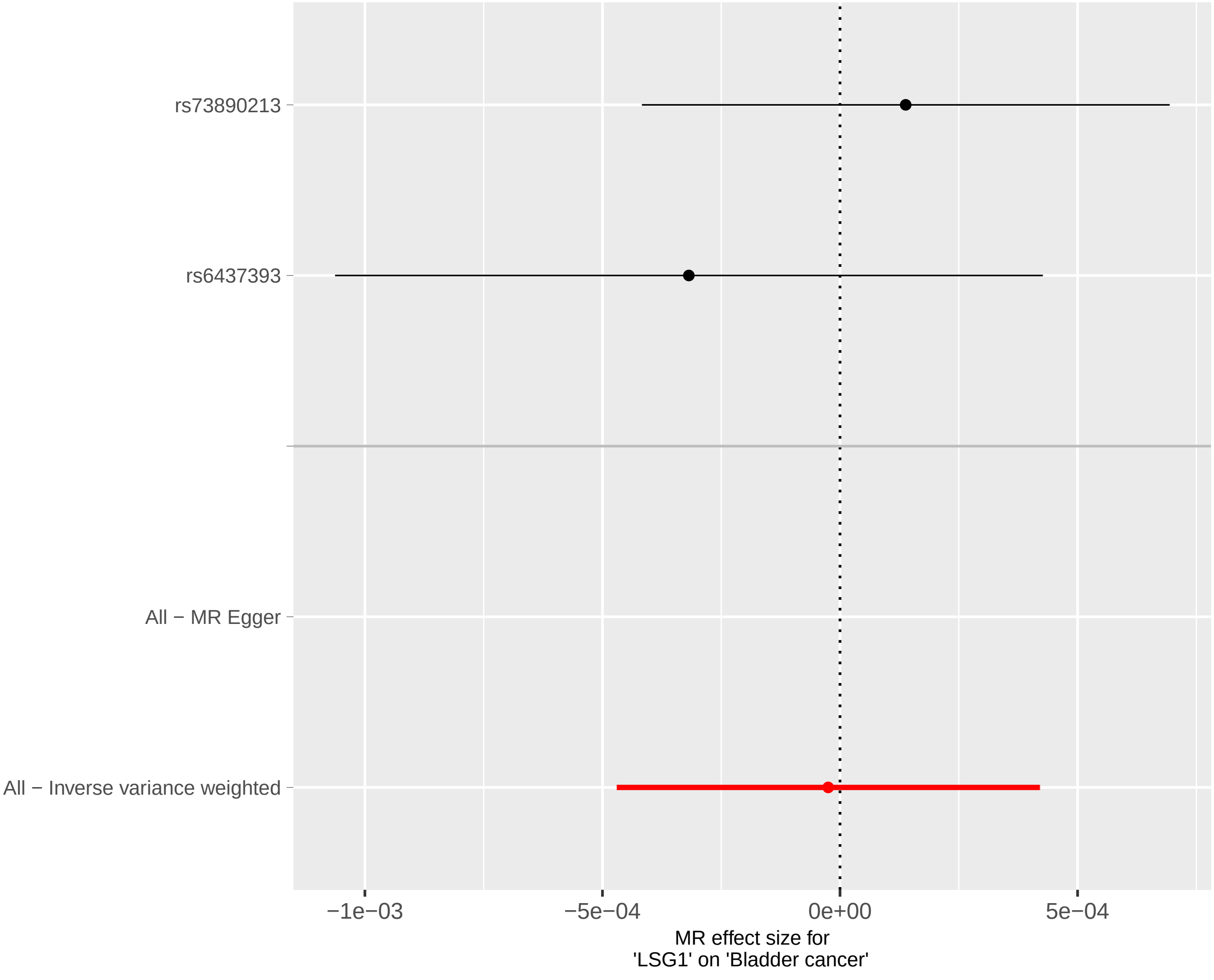
rs73890213
rs6437393
All − MR Egger
All − Inverse variance weighted
−1e−03
−5e−04
0e+00
5e−04
MR effect size for
'LSG1' on 'Bladder cancer'

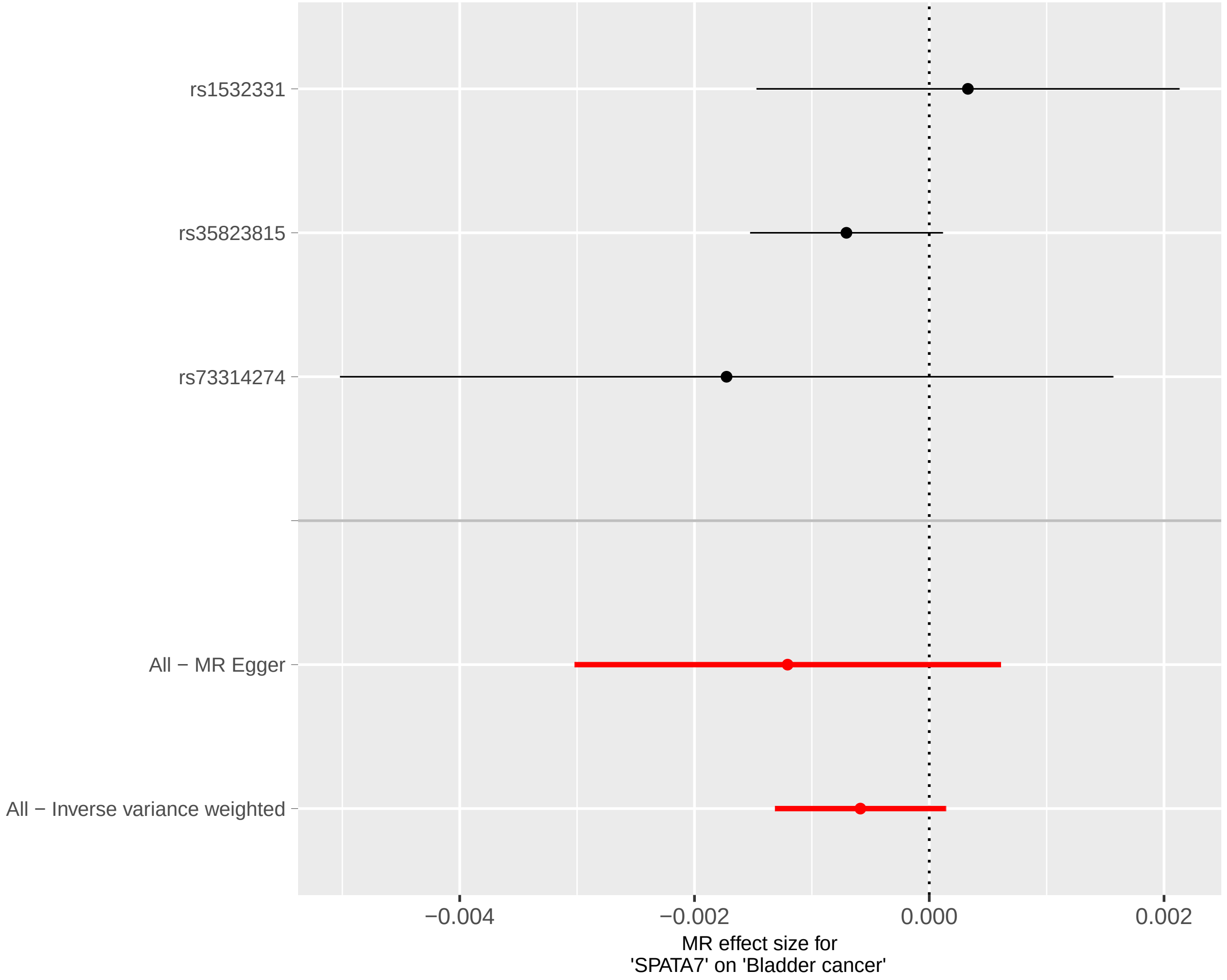
rs1532331
rs35823815
rs73314274
All − MR Egger
All − Inverse variance weighted
−0.004
−0.002
0.000
0.002
MR effect size for
'SPATA7' on 'Bladder cancer'

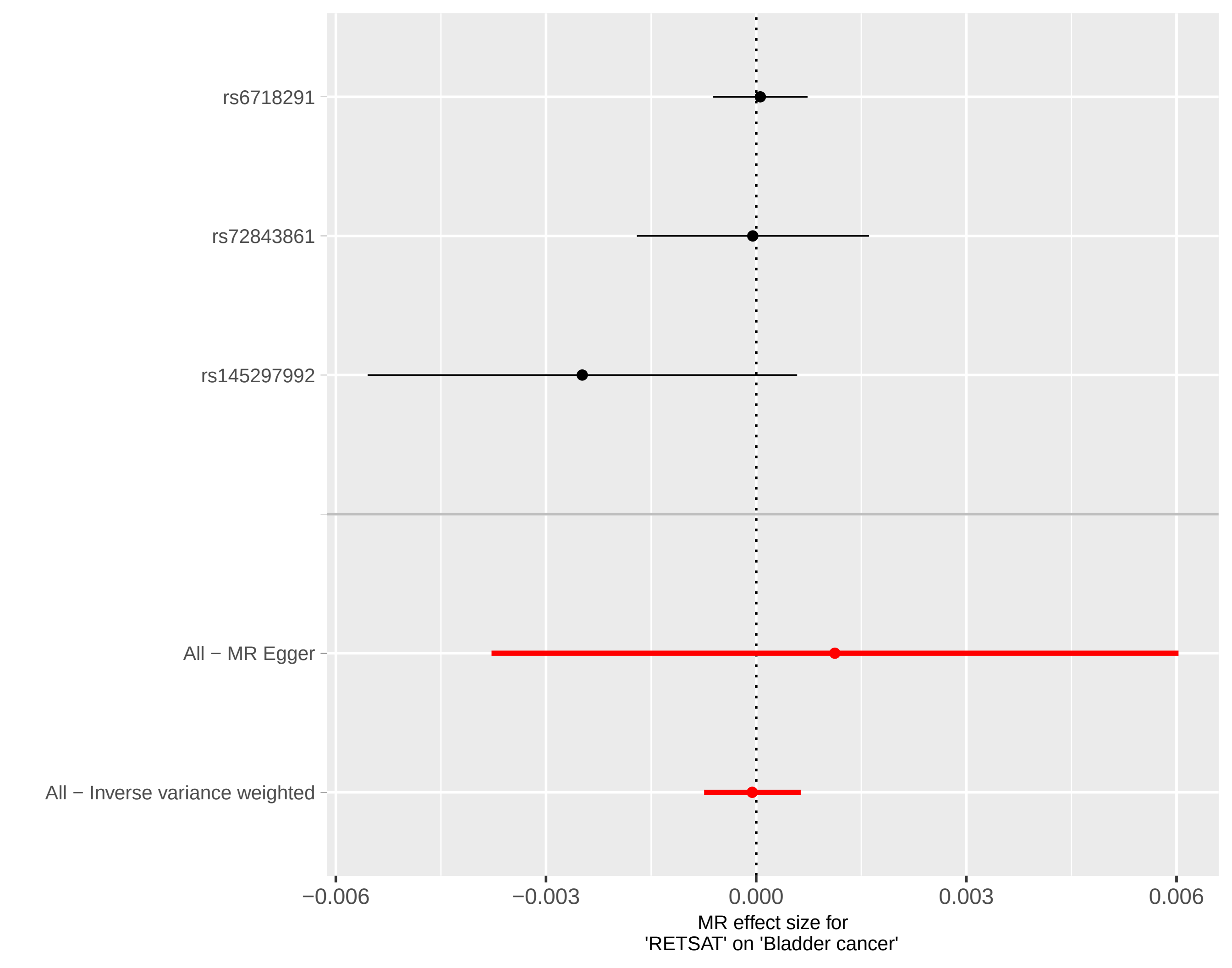
rs6718291
rs72843861
rs145297992
All − MR Egger
All − Inverse variance weighted
−0.006
−0.003
0.000
0.003
0.006
MR effect size for
'RETSAT' on 'Bladder cancer'

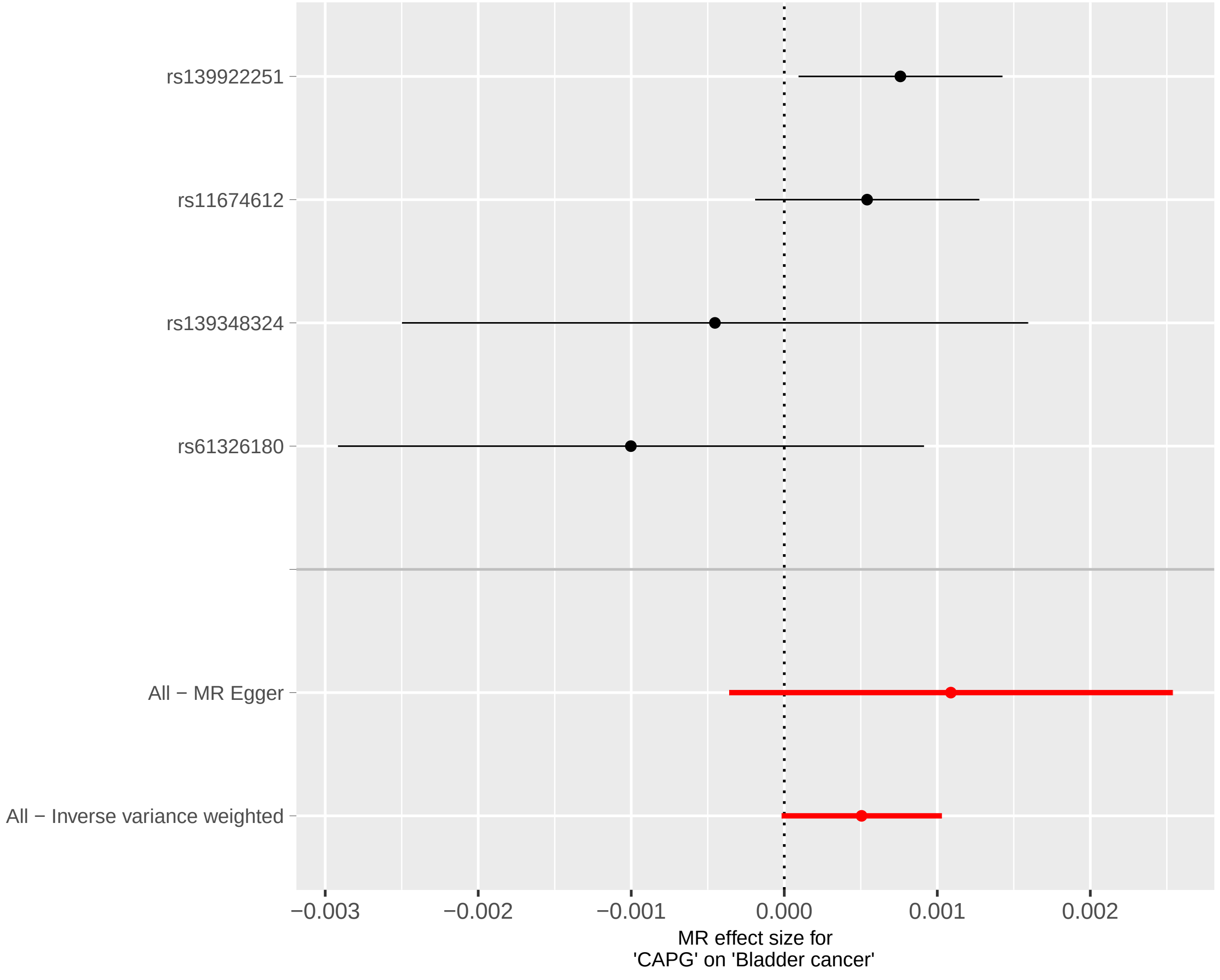
rs139922251
rs11674612
rs139348324
rs61326180
All − MR Egger
All − Inverse variance weighted
−0.003
−0.002
−0.001
0.000
0.001
0.002
MR effect size for
'CAPG' on 'Bladder cancer'

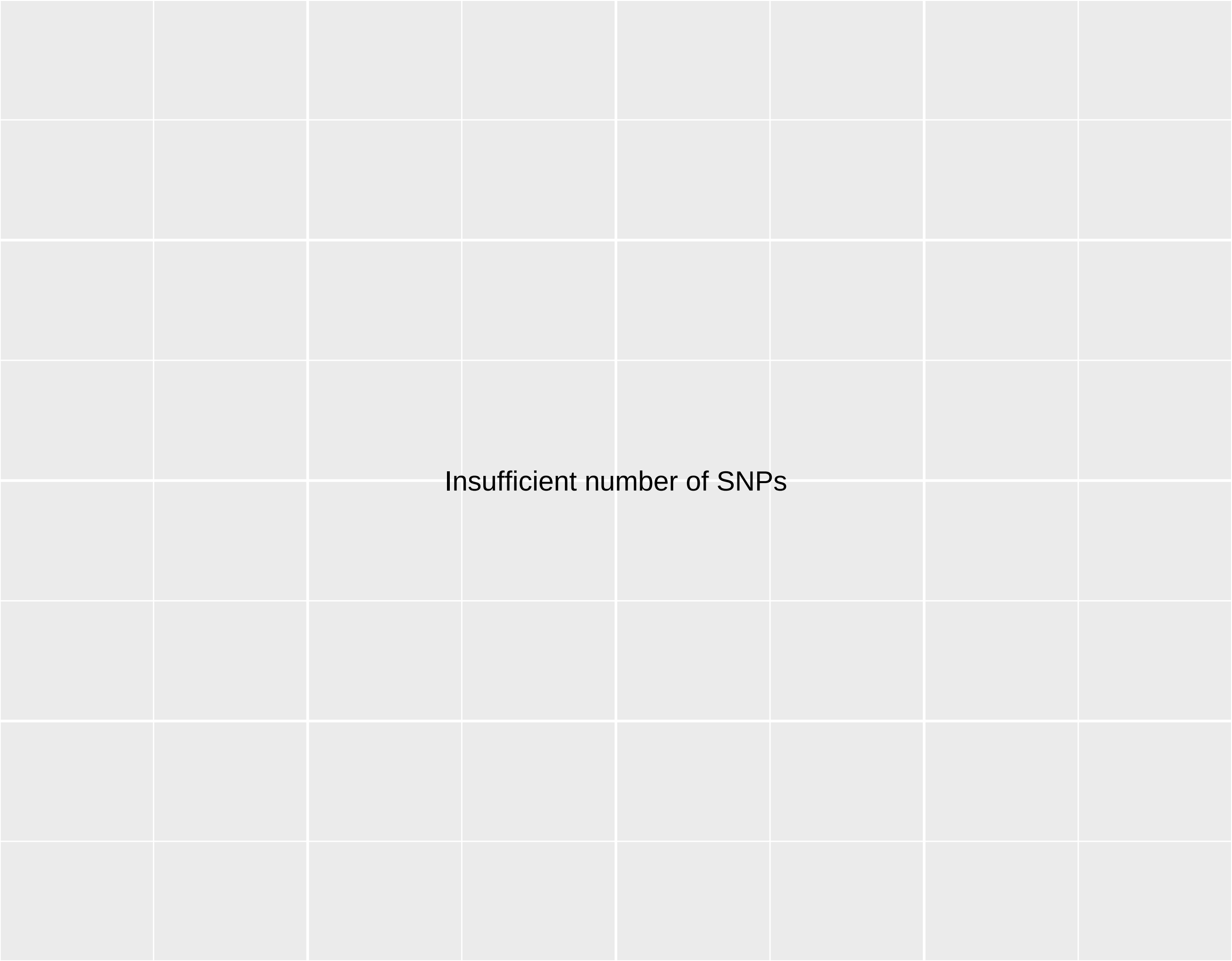
Insufficient number of SNPs

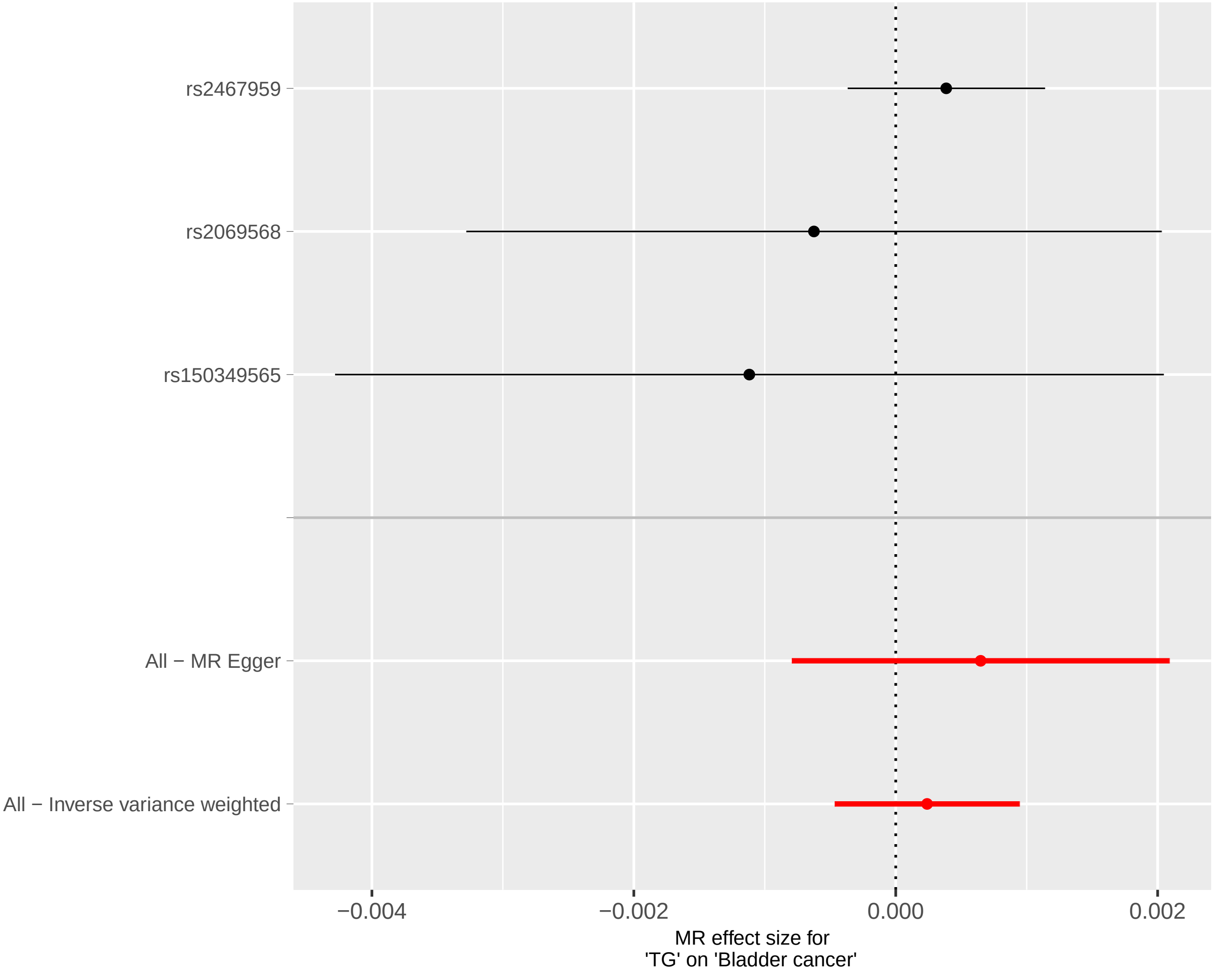
rs2467959
rs2069568
rs150349565
All − MR Egger
All − Inverse variance weighted
−0.004
−0.002
0.000
0.002
MR effect size for
'TG' on 'Bladder cancer'

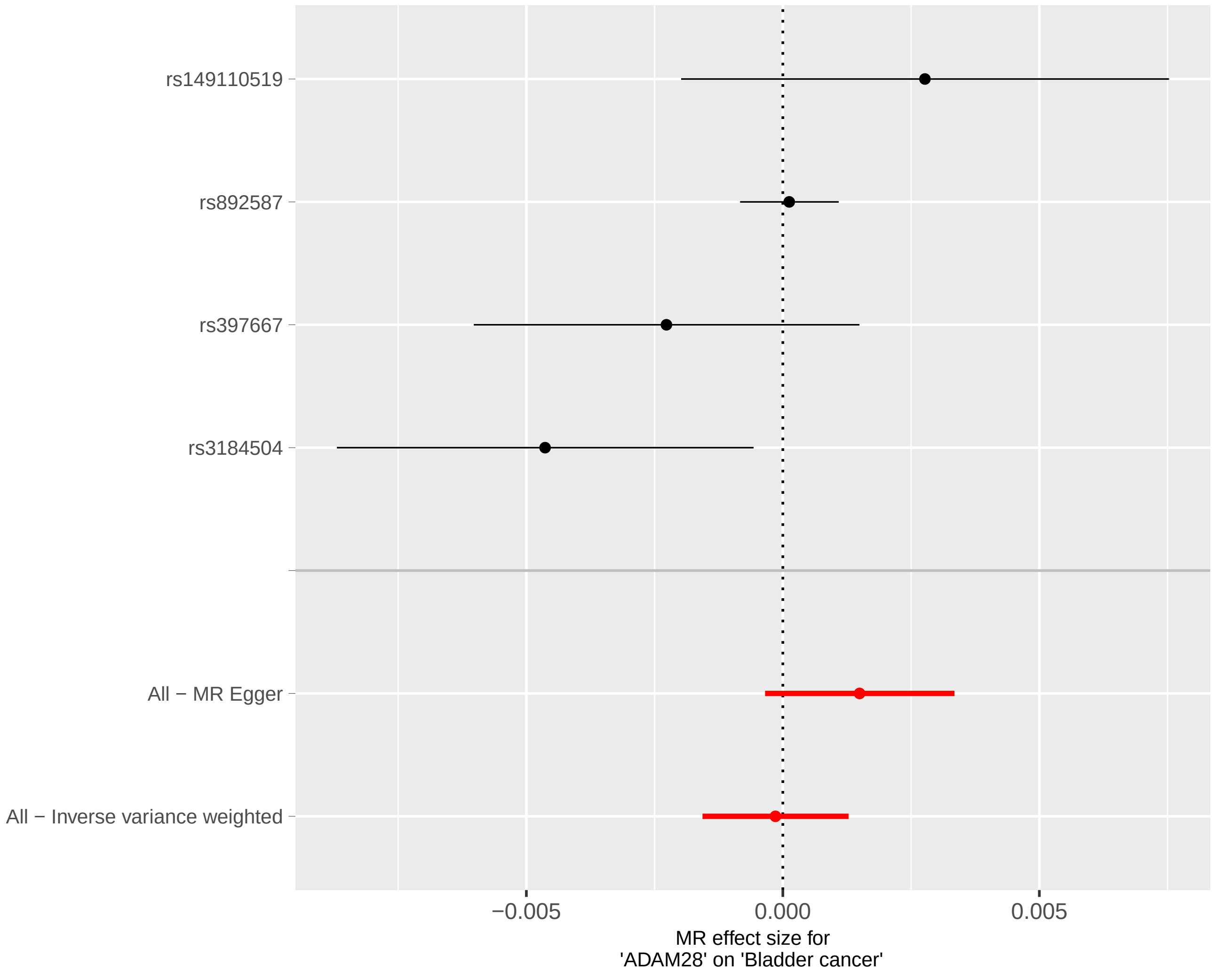
rs149110519
rs892587
rs397667
rs3184504
All − MR Egger
All − Inverse variance weighted
−0.005
0.000
0.005
MR effect size for
'ADAM28' on 'Bladder cancer'

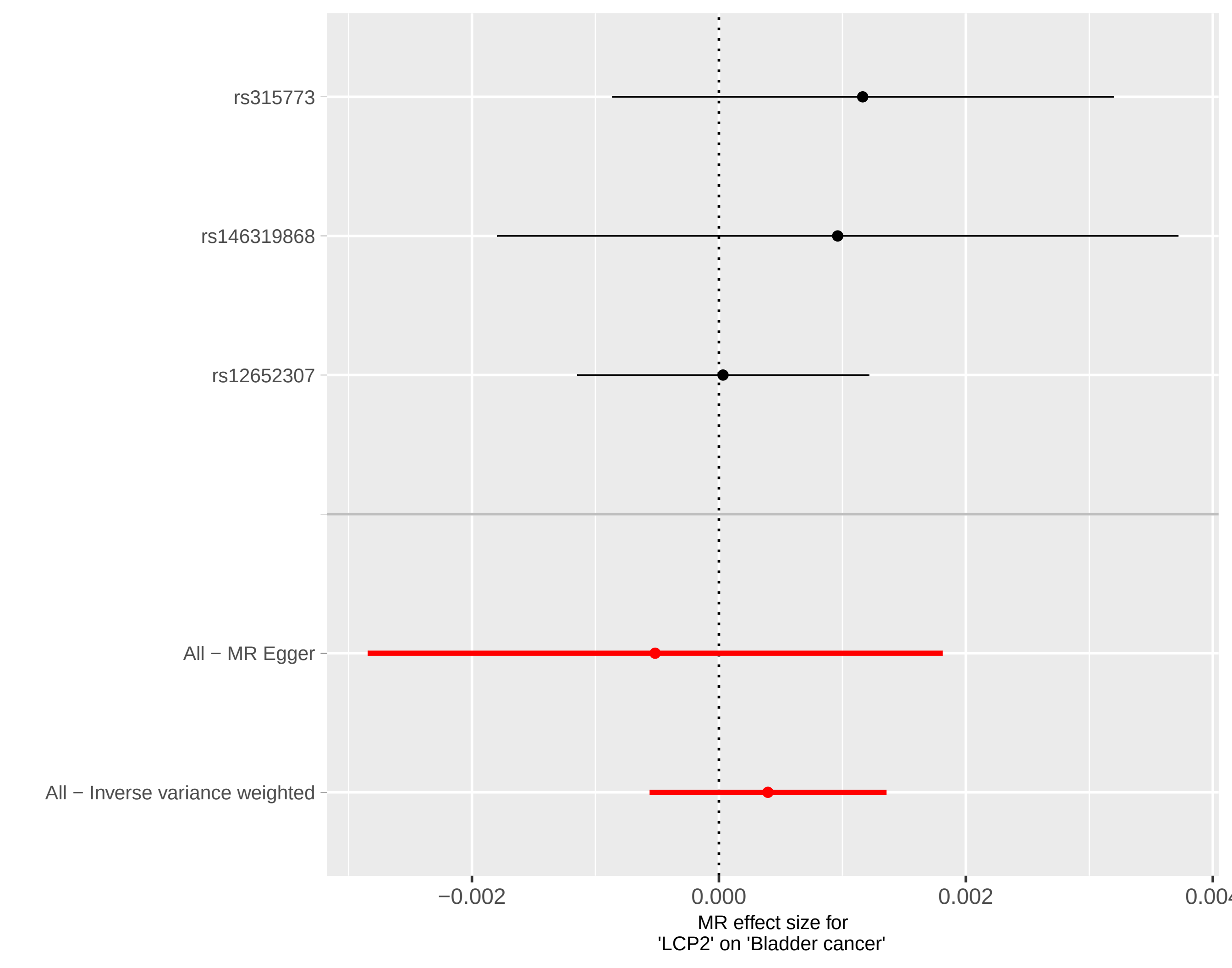

rs315773
rs146319868
rs12652307
All − MR Egger
All − Inverse variance weighted
−0.002
0.000
0.002
MR effect size for
'LCP2' on 'Bladder cancer'

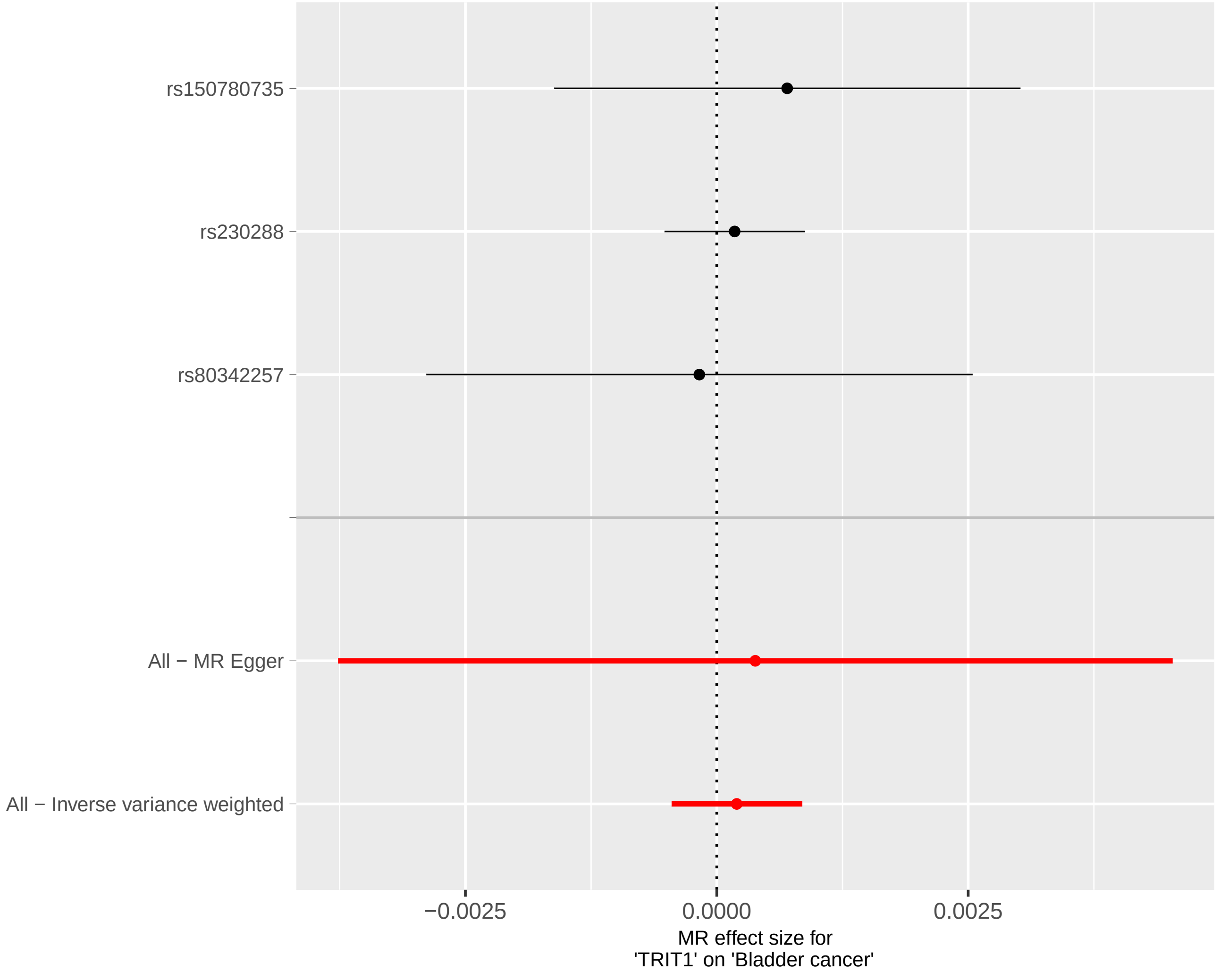
rs150780735
rs230288
rs80342257
All − MR Egger
All − Inverse variance weighted
−0.0025
0.0000
0.0025
MR effect size for
'TRIT1' on 'Bladder cancer'

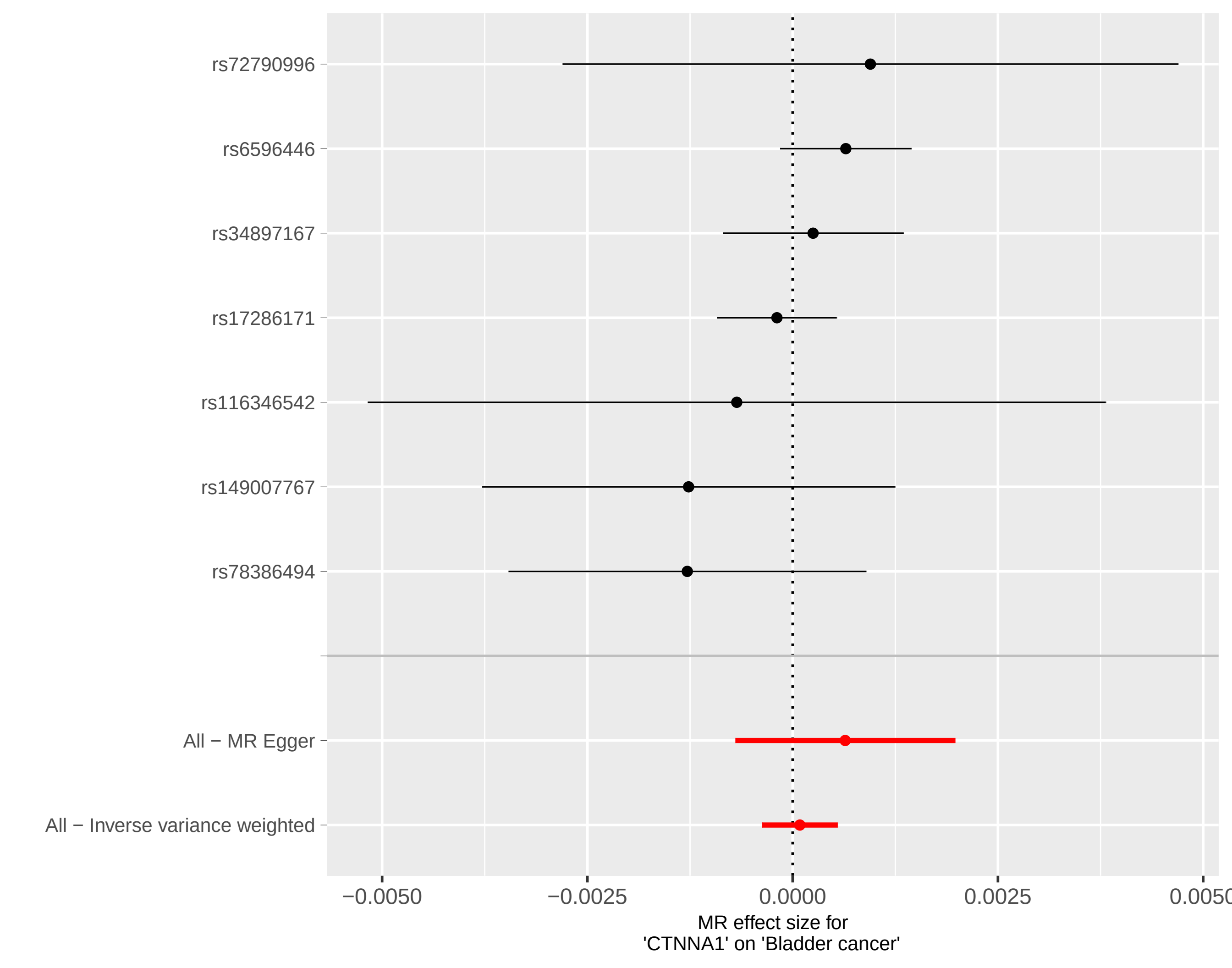

rs72790996
rs6596446
rs34897167
rs17286171
rs116346542
rs149007767
rs78386494
All − MR Egger
All − Inverse variance weighted
−0.0050
−0.0025
0.0000
0.0025
0.0050
MR effect size for
'CTNNA1' on 'Bladder cancer'

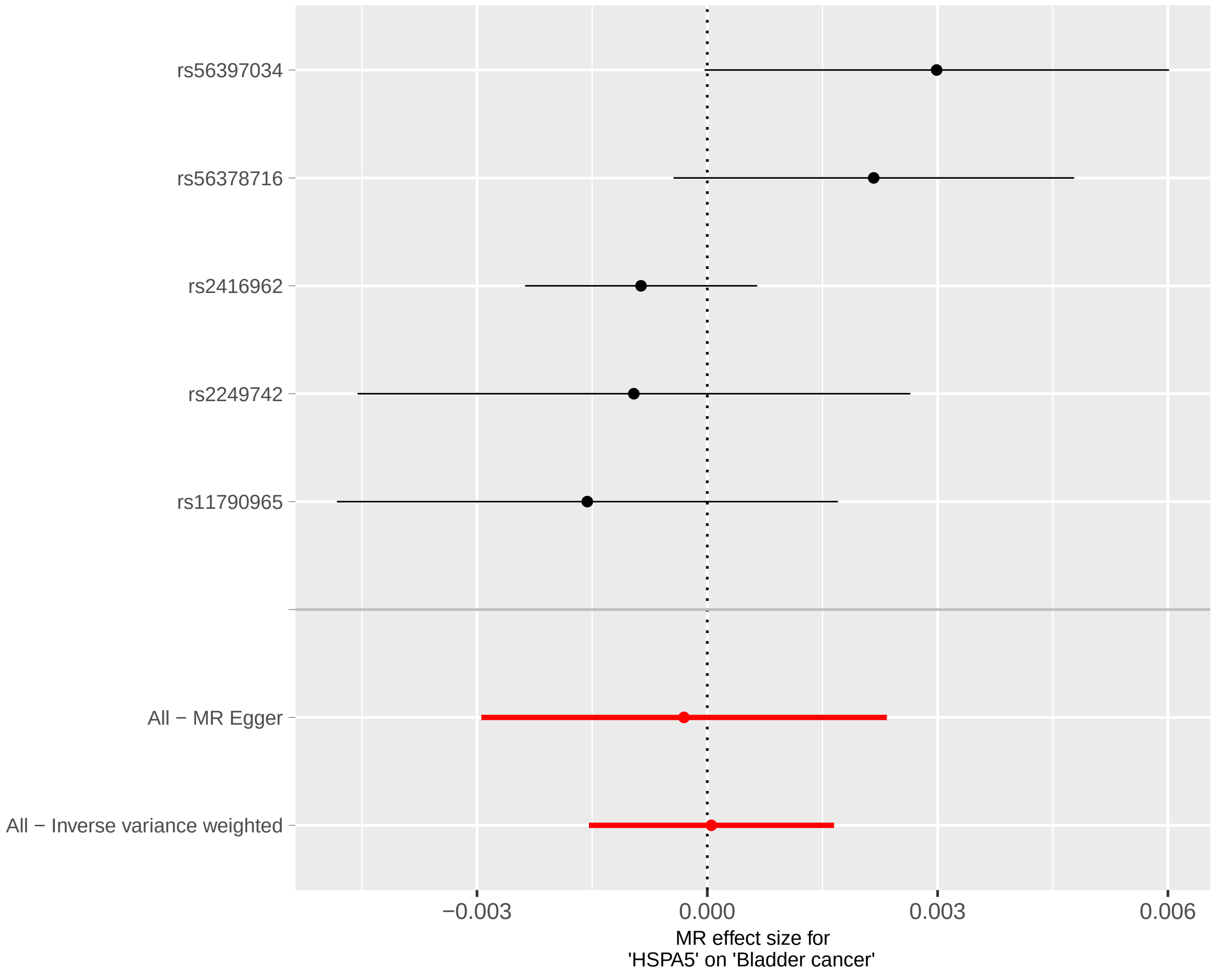

rs56397034
rs56378716
rs2416962
rs2249742
rs11790965
All − MR Egger
All − Inverse variance weighted
−0.003
0.000
0.003
0.006
MR effect size for
'HSPA5' on 'Bladder cancer'

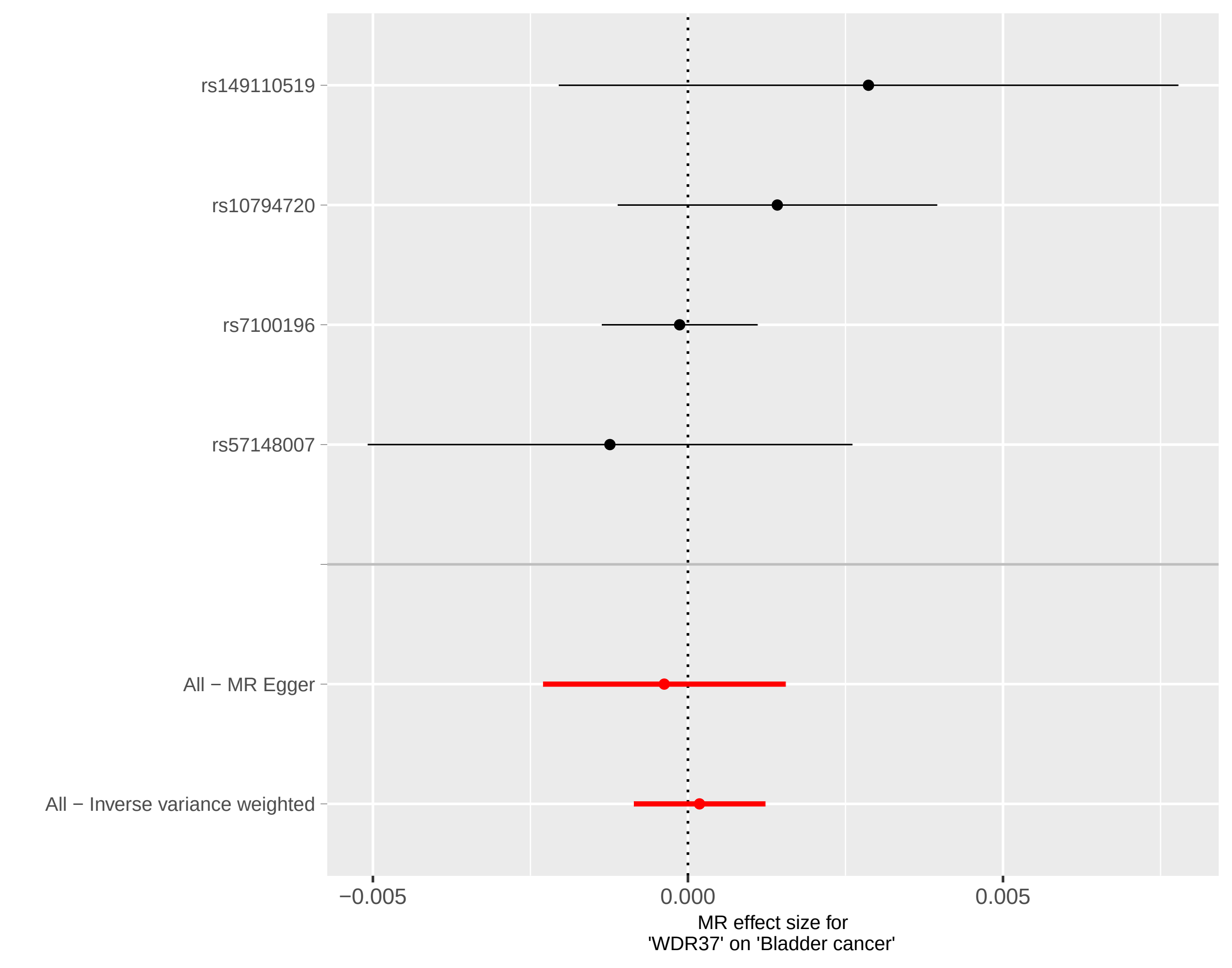
rs149110519
rs10794720
rs7100196
rs57148007
All − MR Egger
All − Inverse variance weighted
−0.005
0.000
0.005
MR effect size for
'WDR37' on 'Bladder cancer'

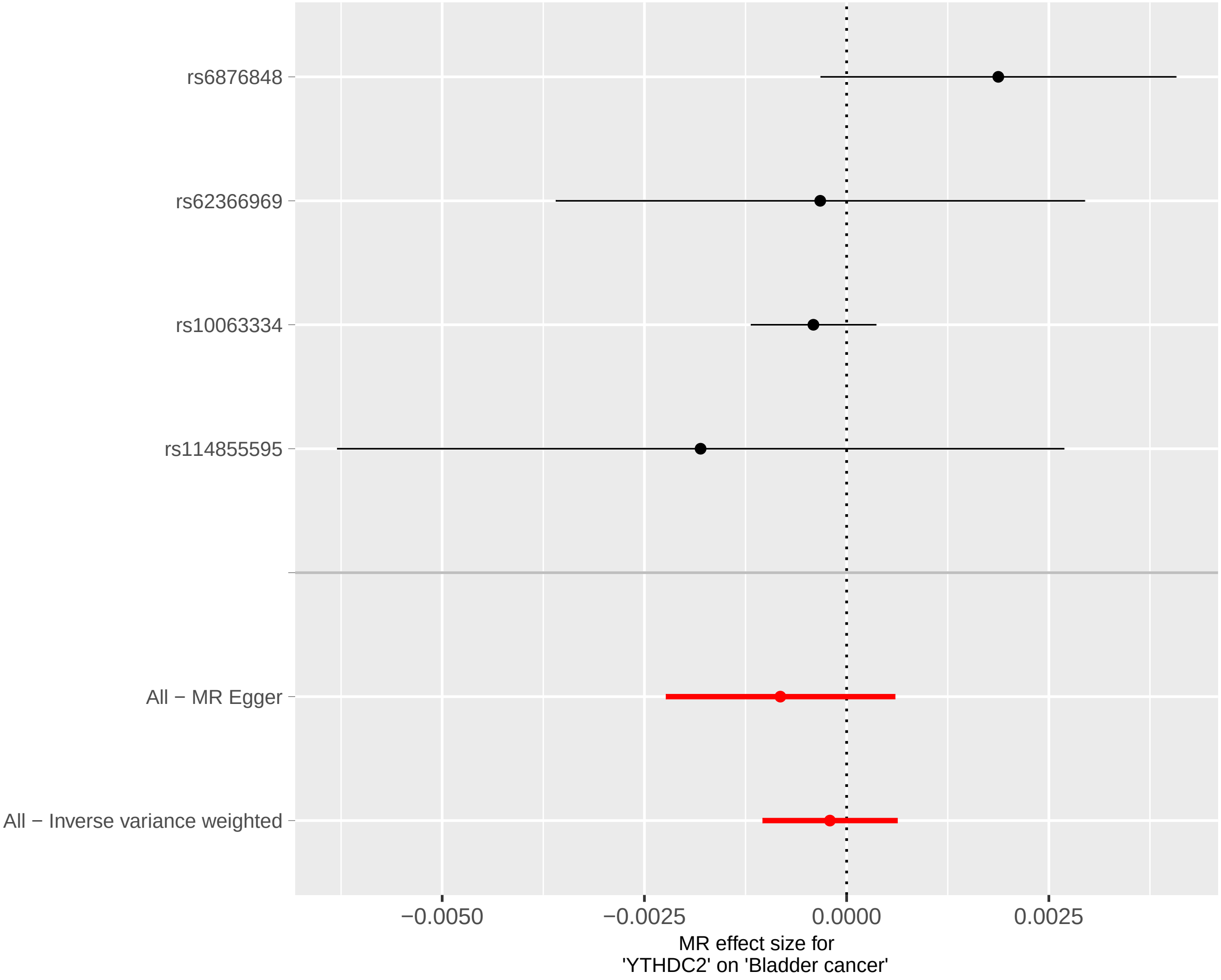
rs6876848
rs62366969
rs10063334
rs114855595
All − MR Egger
All − Inverse variance weighted
−0.0050
−0.0025
0.0000
0.0025
MR effect size for
'YTHDC2' on 'Bladder cancer'

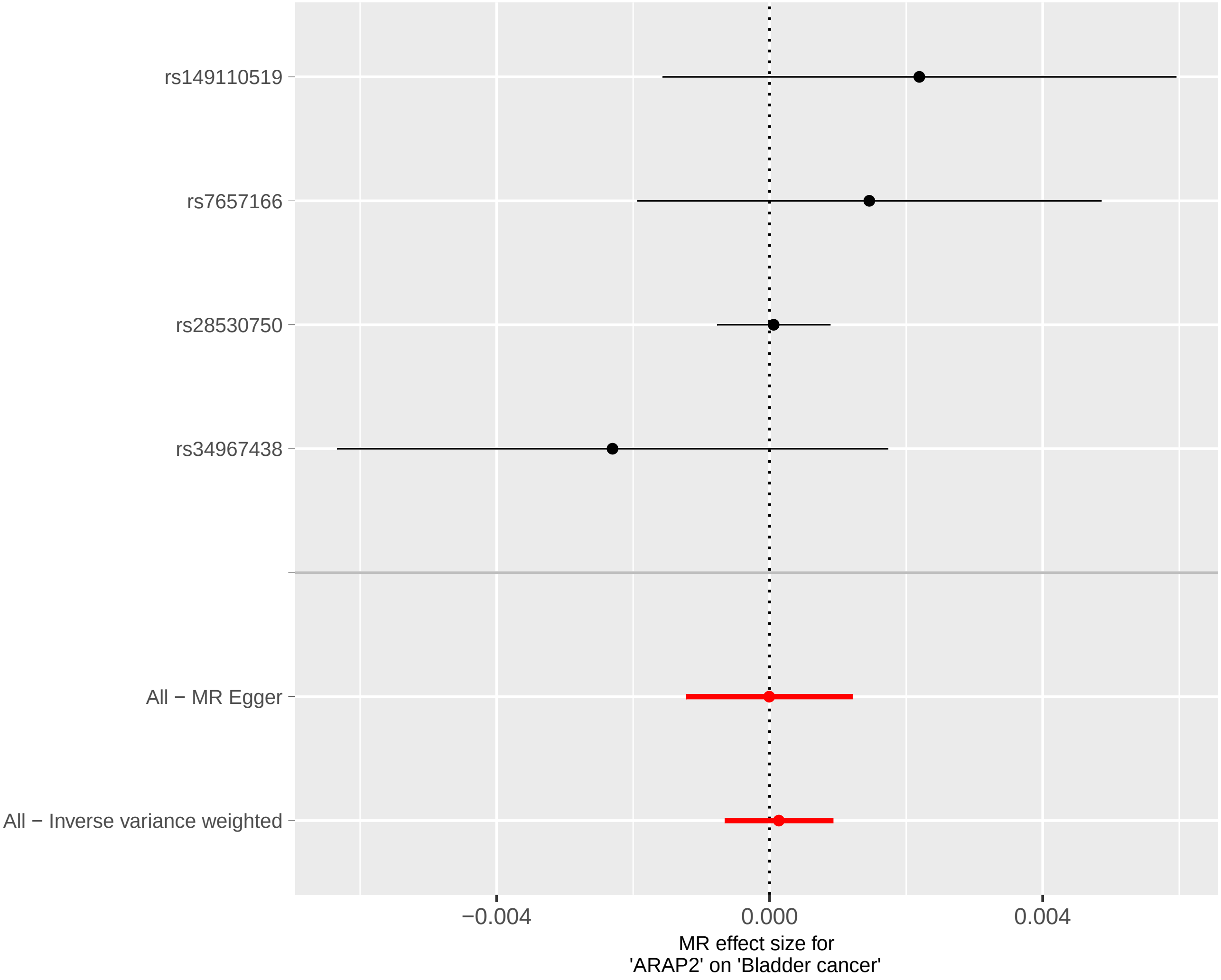
rs149110519
rs7657166
rs28530750
rs34967438
All − MR Egger
All − Inverse variance weighted
−0.004
0.000
0.004
MR effect size for
'ARAP2' on 'Bladder cancer'

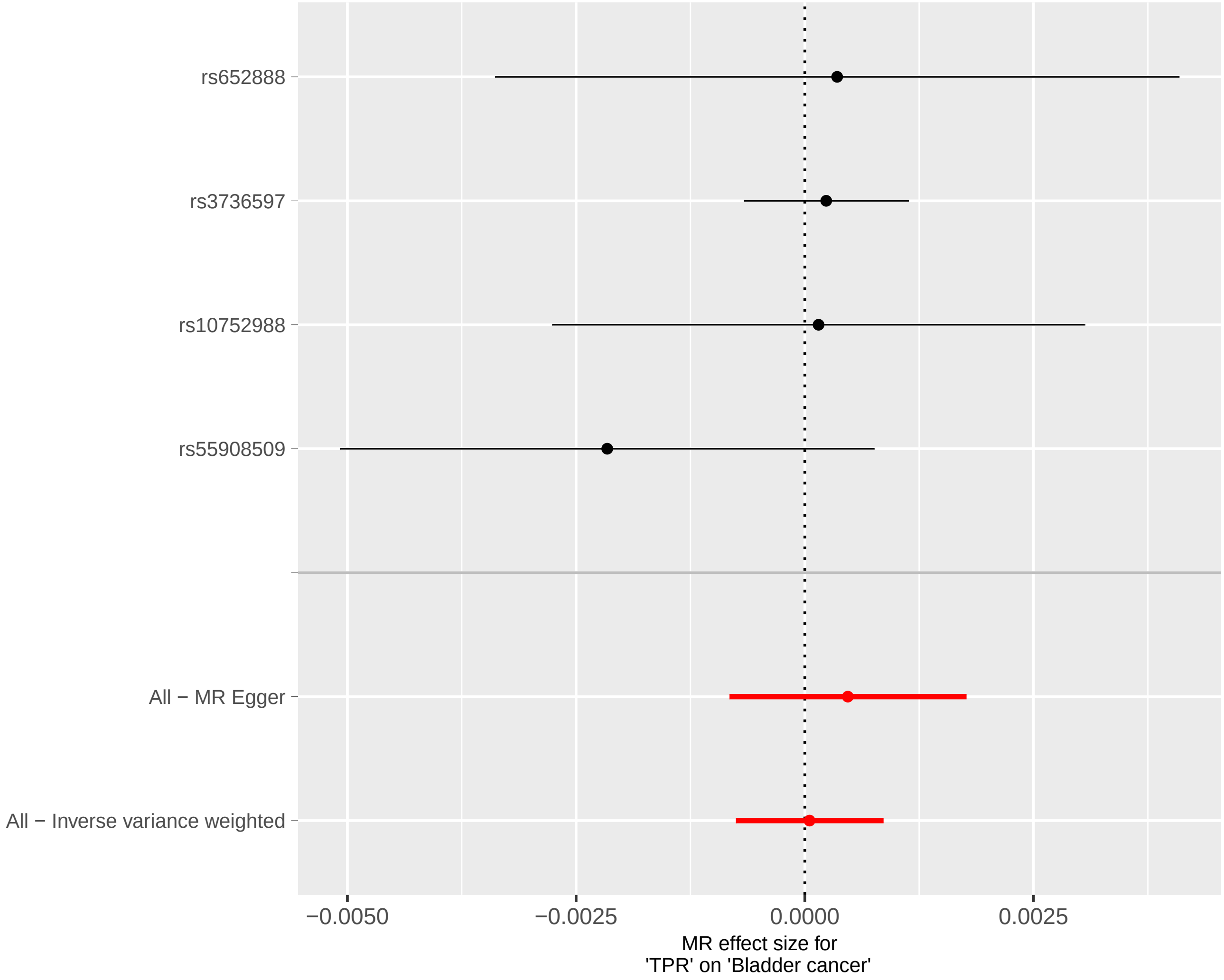

rs652888
rs3736597
rs10752988
rs55908509
All − MR Egger
All − Inverse variance weighted
−0.0050
−0.0025
0.0000
0.0025
MR effect size for
'TPR' on 'Bladder cancer'

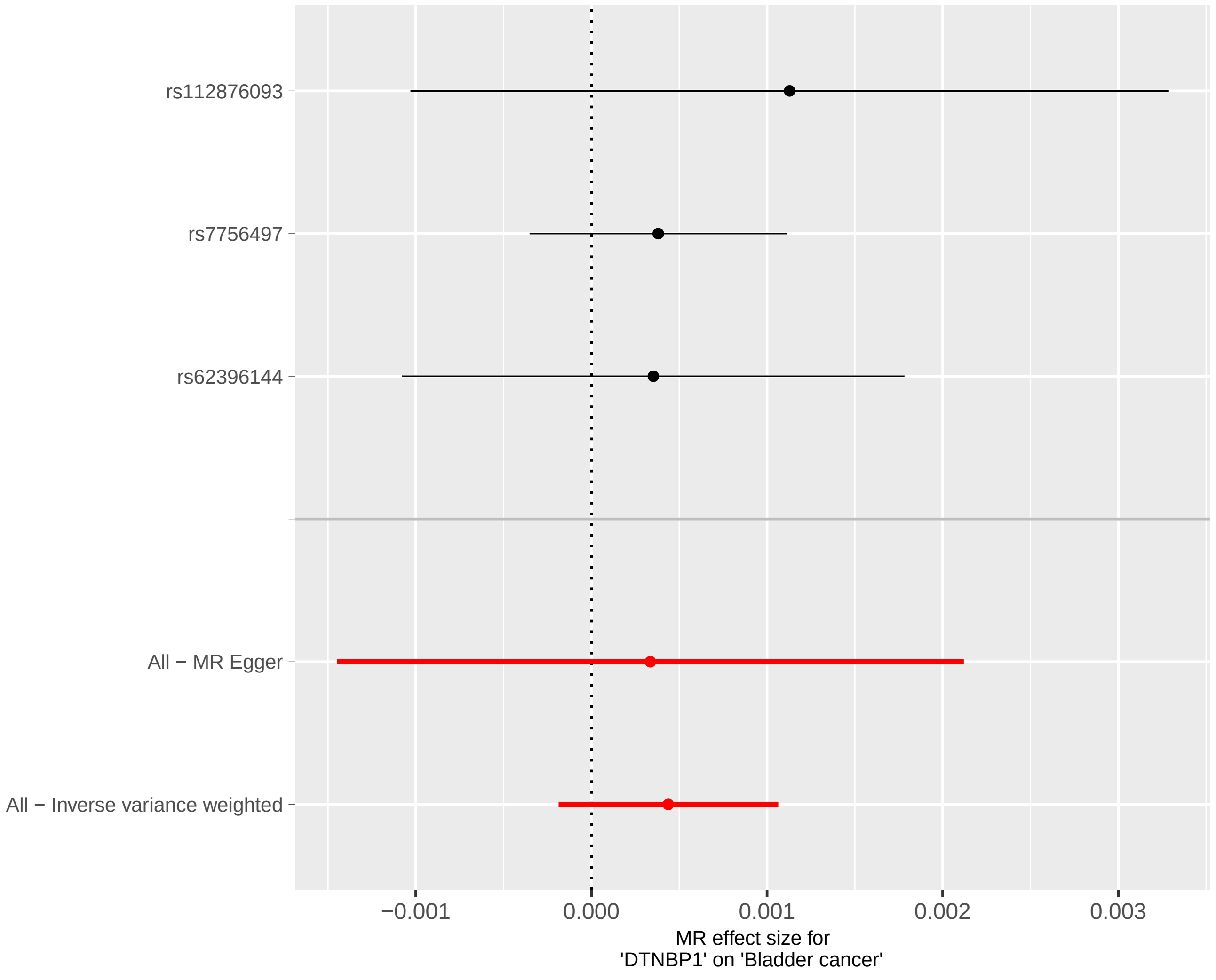
rs112876093
rs7756497
rs62396144
All − MR Egger
All − Inverse variance weighted
−0.001
0.000
0.001
0.002
0.003
MR effect size for
'DTNBP1' on 'Bladder cancer'

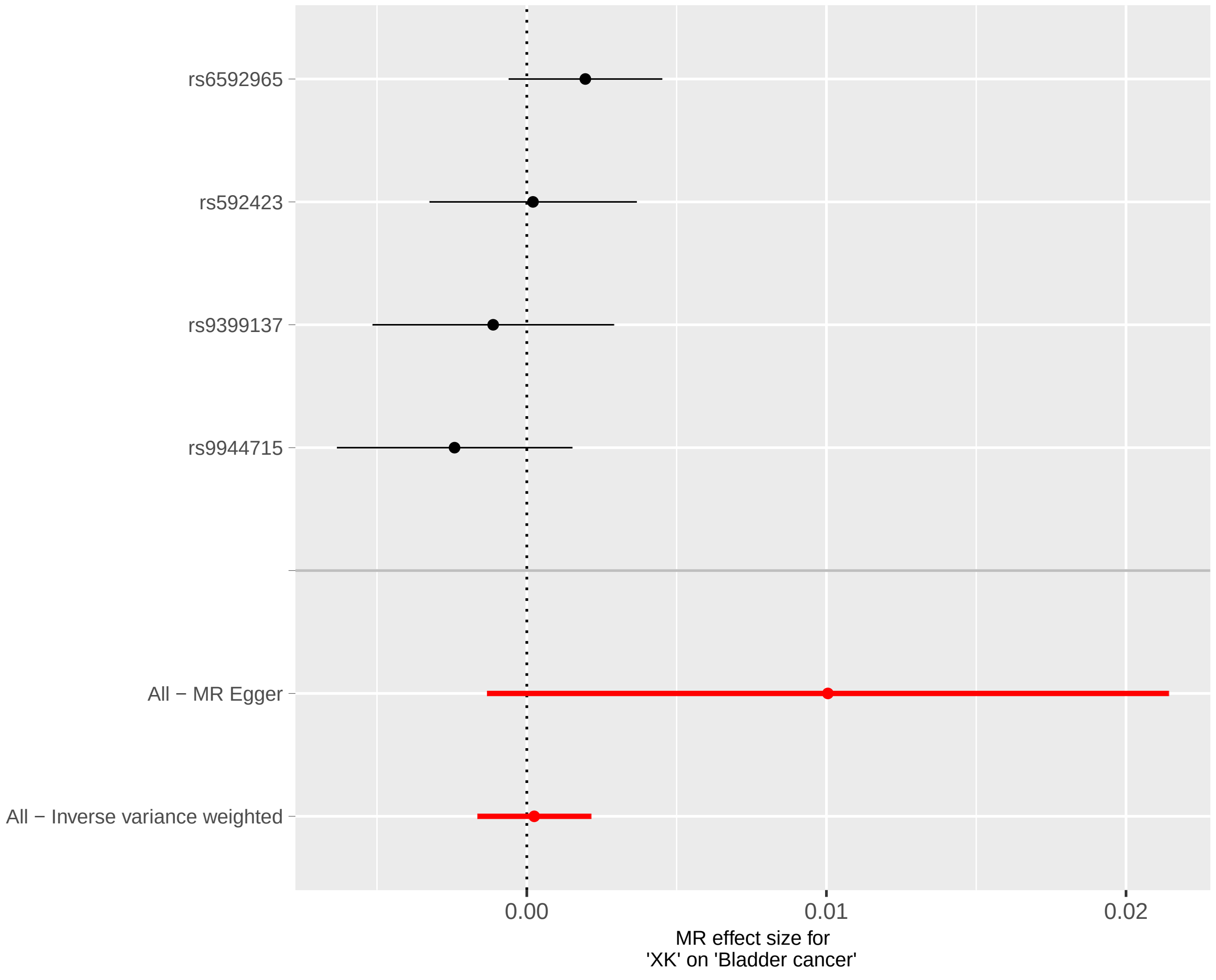
rs6592965
rs592423
rs9399137
rs9944715
All − MR Egger
All − Inverse variance weighted
0.00
0.01
0.02
MR effect size for
'XK' on 'Bladder cancer'

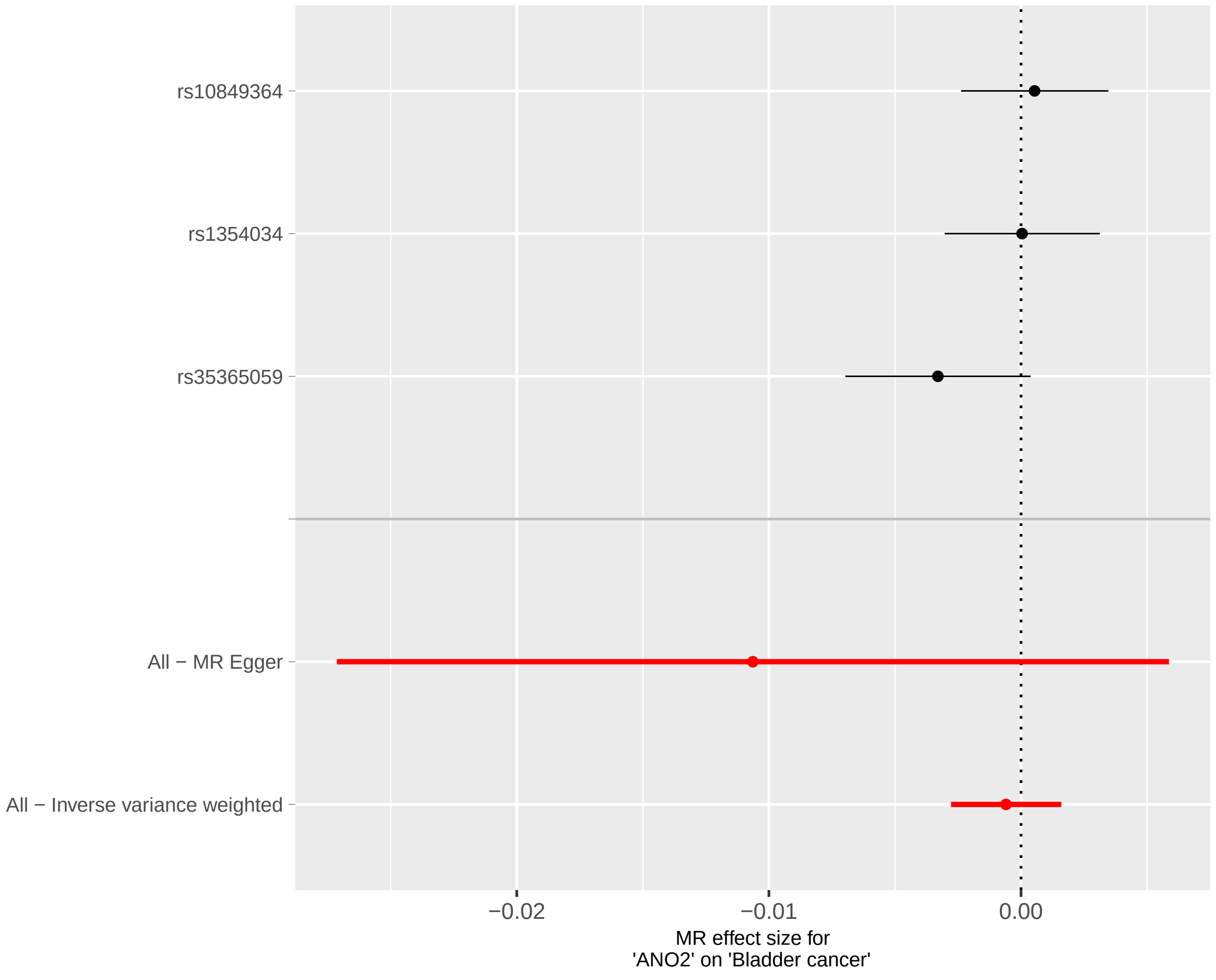
rs10849364
rs1354034
rs35365059
All − MR Egger
All − Inverse variance weighted
−0.02
−0.01
0.00
MR effect size for
'ANO2' on 'Bladder cancer'

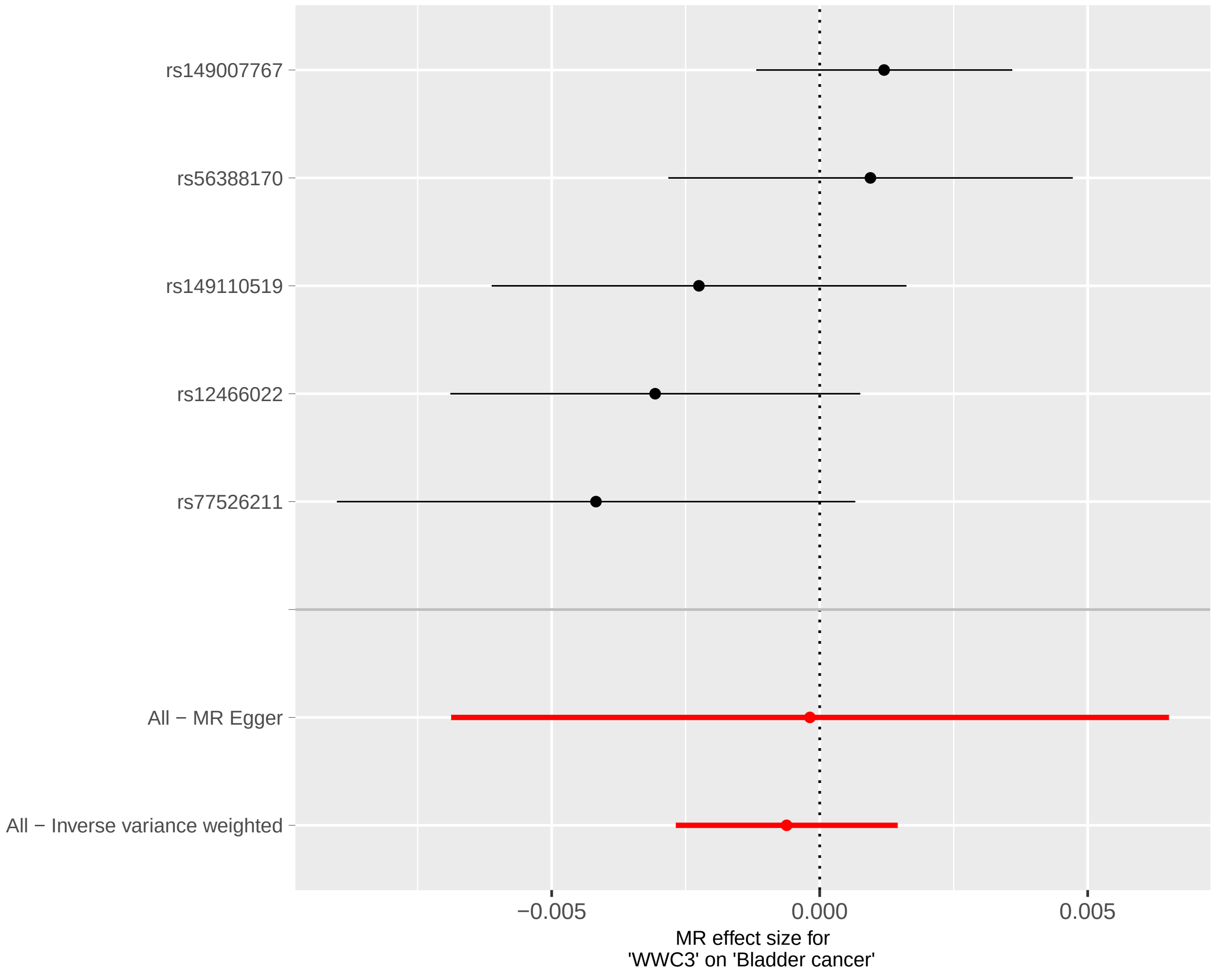
rs149007767
rs56388170
rs149110519
rs12466022
rs77526211
All − MR Egger
All − Inverse variance weighted
−0.005
0.000
0.005
MR effect size for
'WWC3' on 'Bladder cancer'

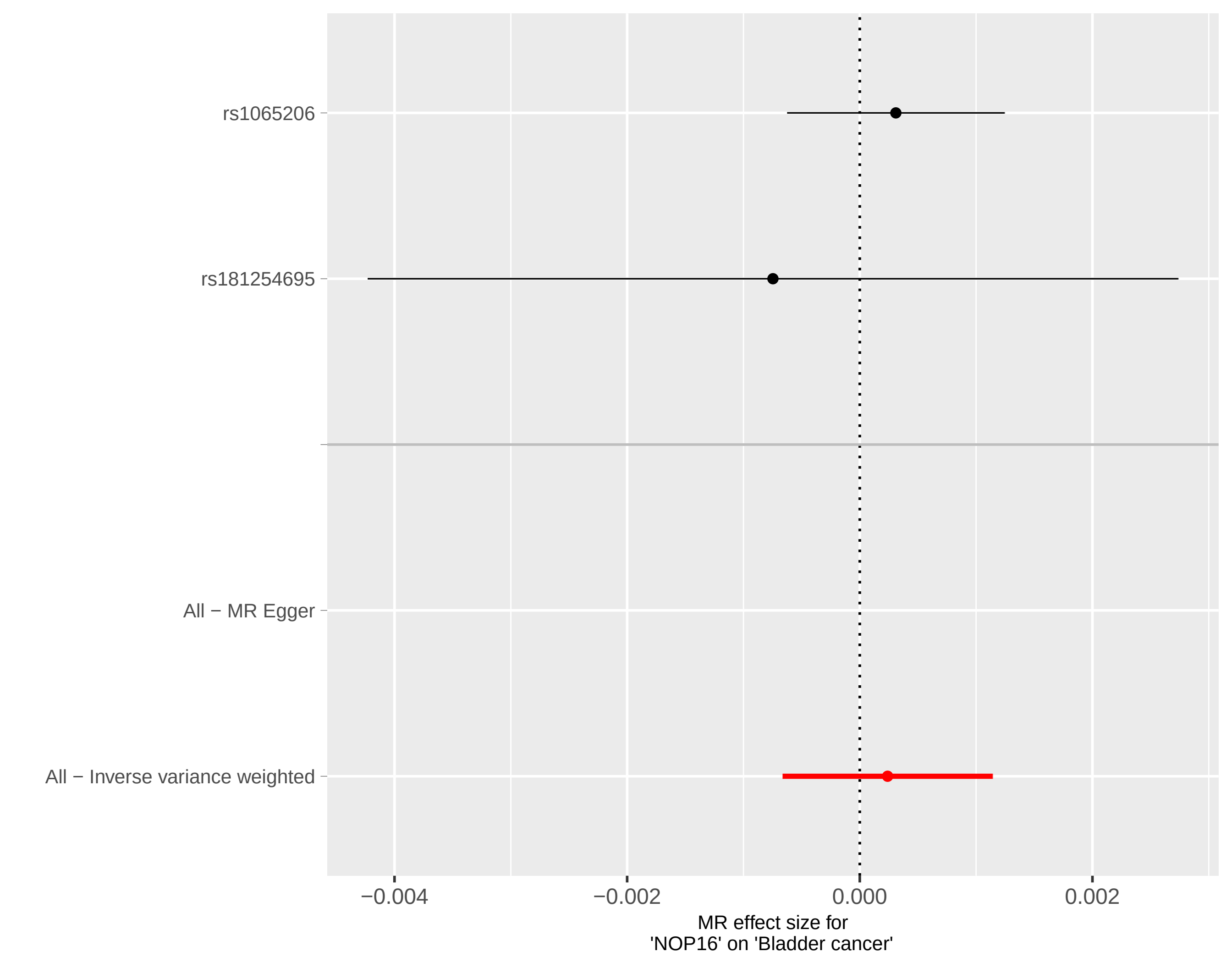

rs1065206
rs181254695
All − MR Egger
All − Inverse variance weighted
−0.004
−0.002
0.000
0.002
MR effect size for
'NOP16' on 'Bladder cancer'

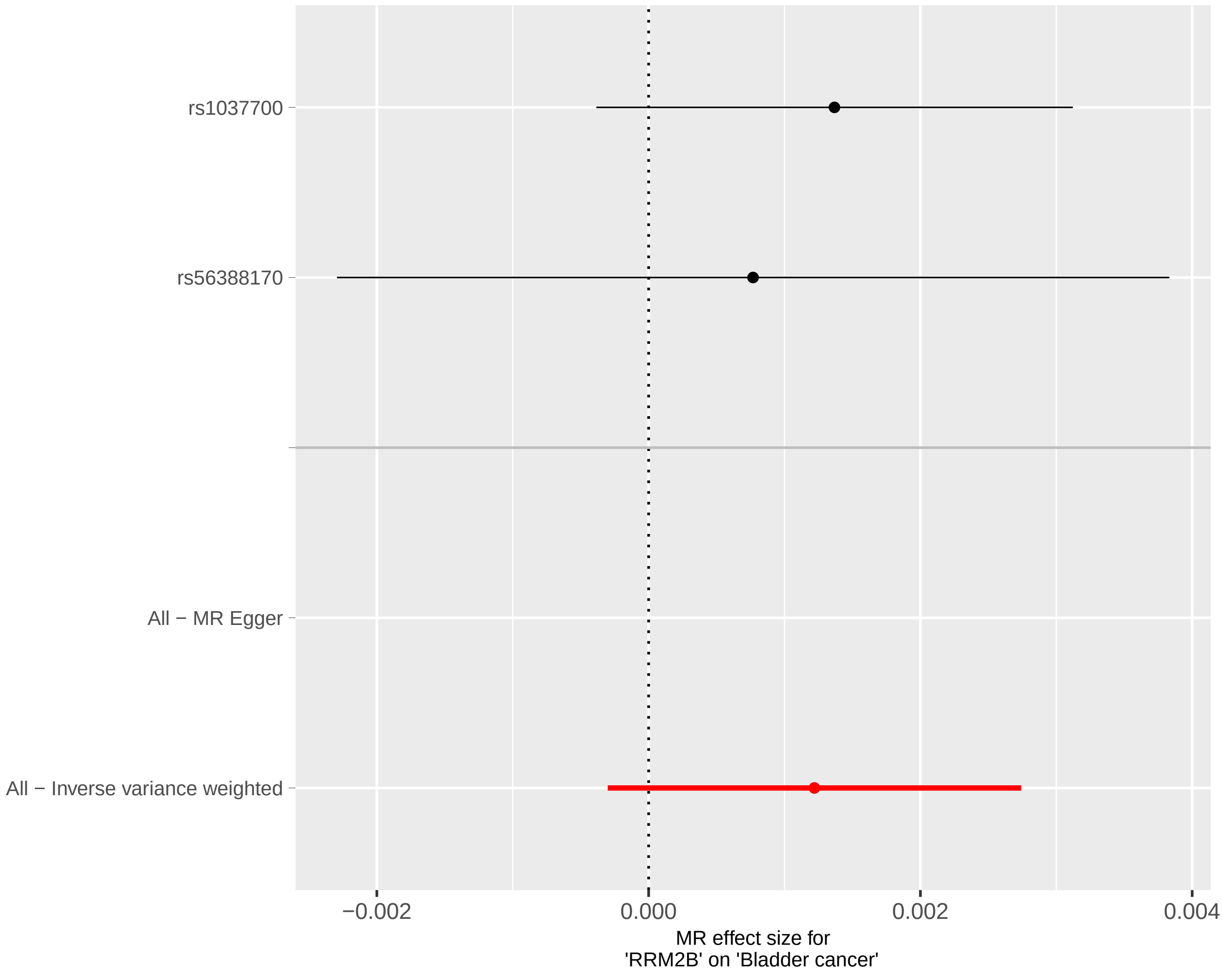
rs1037700
rs56388170
All − MR Egger
All − Inverse variance weighted
−0.002
0.000
0.002
0.004
MR effect size for
'RRM2B' on 'Bladder cancer'

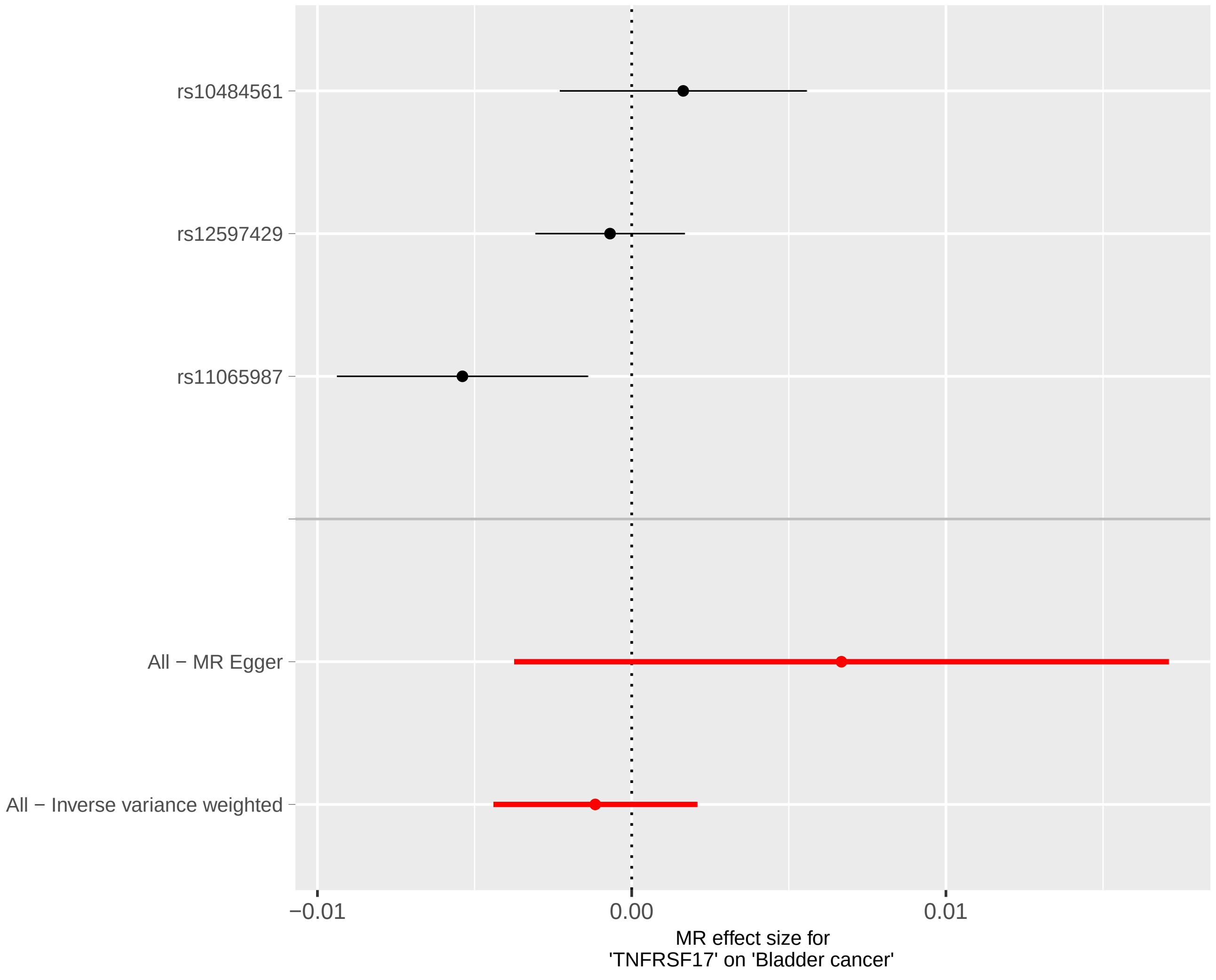

rs10484561
rs12597429
rs11065987
All − MR Egger
All − Inverse variance weighted
−0.01
0.00
0.01
MR effect size for
'TNFRSF17' on 'Bladder cancer'

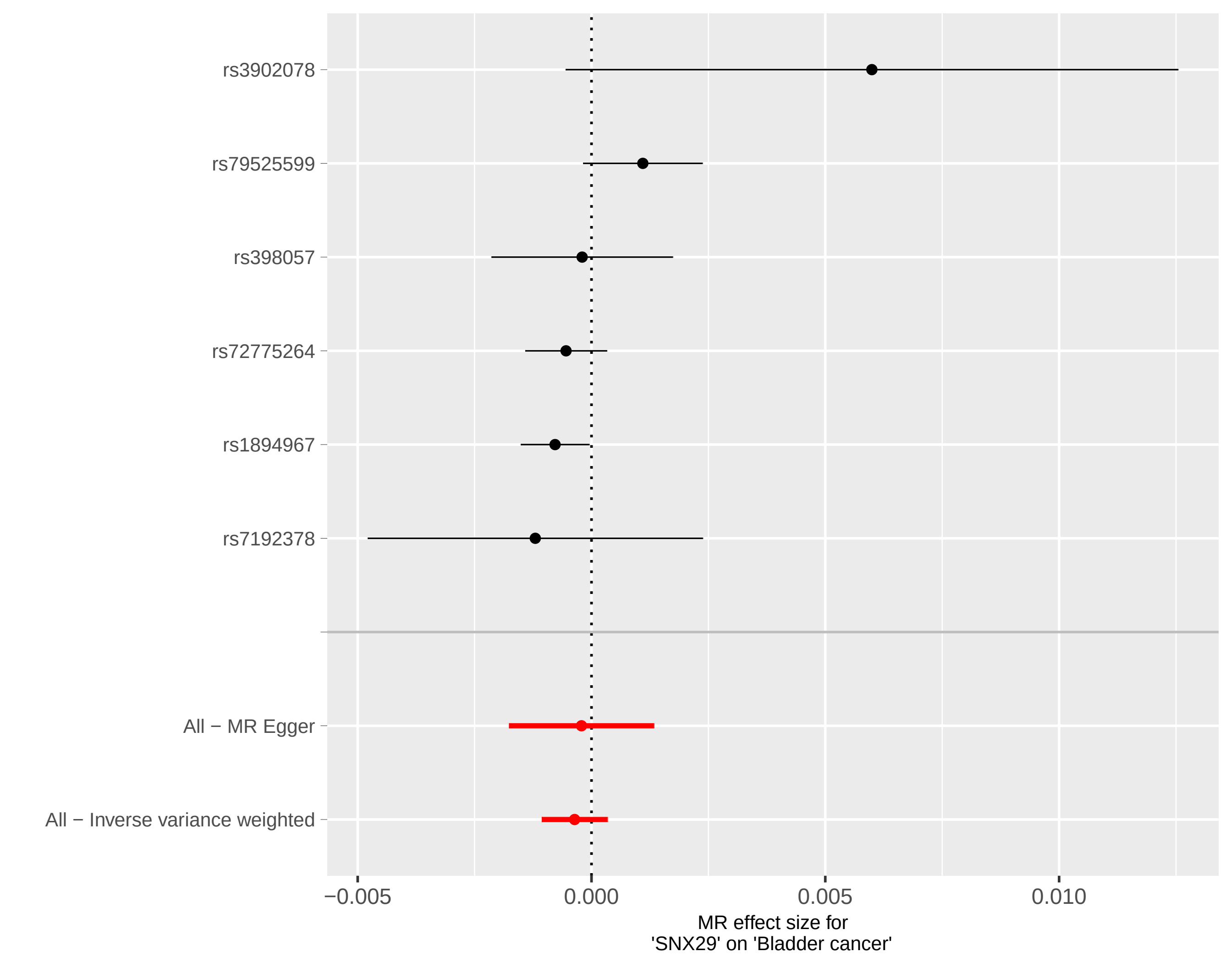

rs3902078
rs79525599
rs398057
rs72775264
rs1894967
rs7192378
All − MR Egger
All − Inverse variance weighted
−0.005
0.000
0.005
0.010
MR effect size for
'SNX29' on 'Bladder cancer'

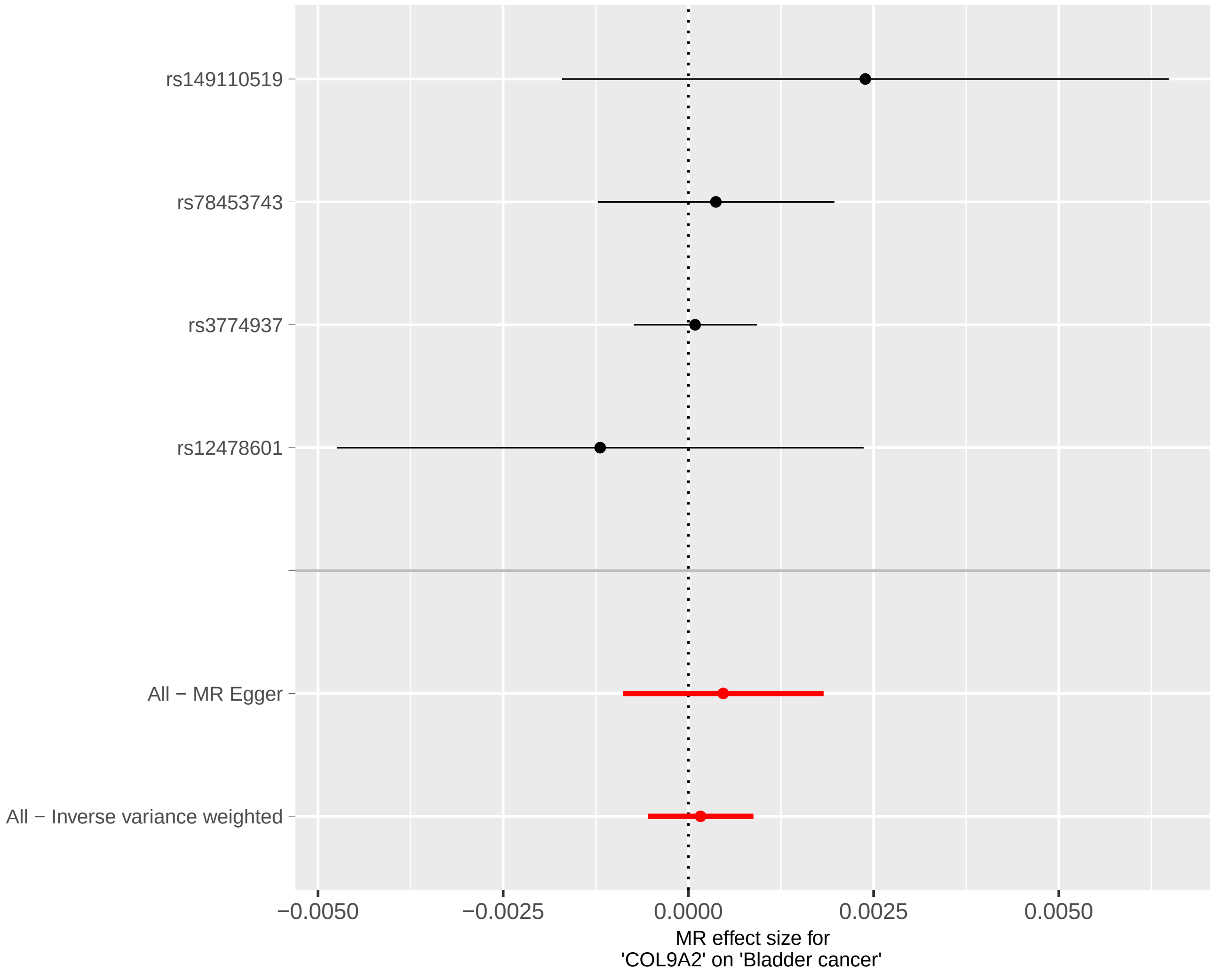
rs149110519
rs78453743
rs3774937
rs12478601
All − MR Egger
All − Inverse variance weighted
−0.0050
−0.0025
0.0000
0.0025
0.0050
MR effect size for
'COL9A2' on 'Bladder cancer'

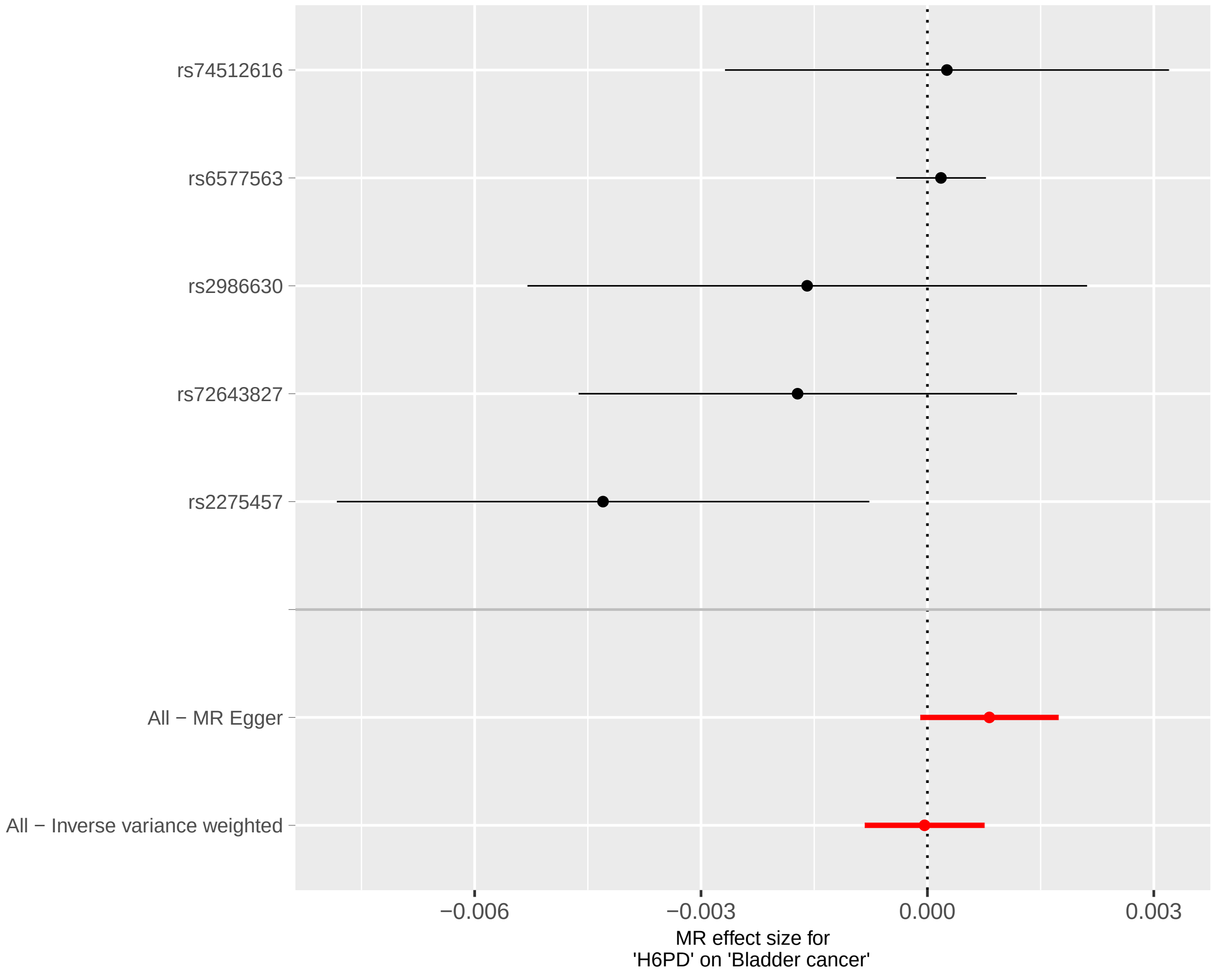

rs74512616
rs6577563
rs2986630
rs72643827
rs2275457
All − MR Egger
All − Inverse variance weighted
−0.006
−0.003
0.000
0.003
MR effect size for
'H6PD' on 'Bladder cancer'

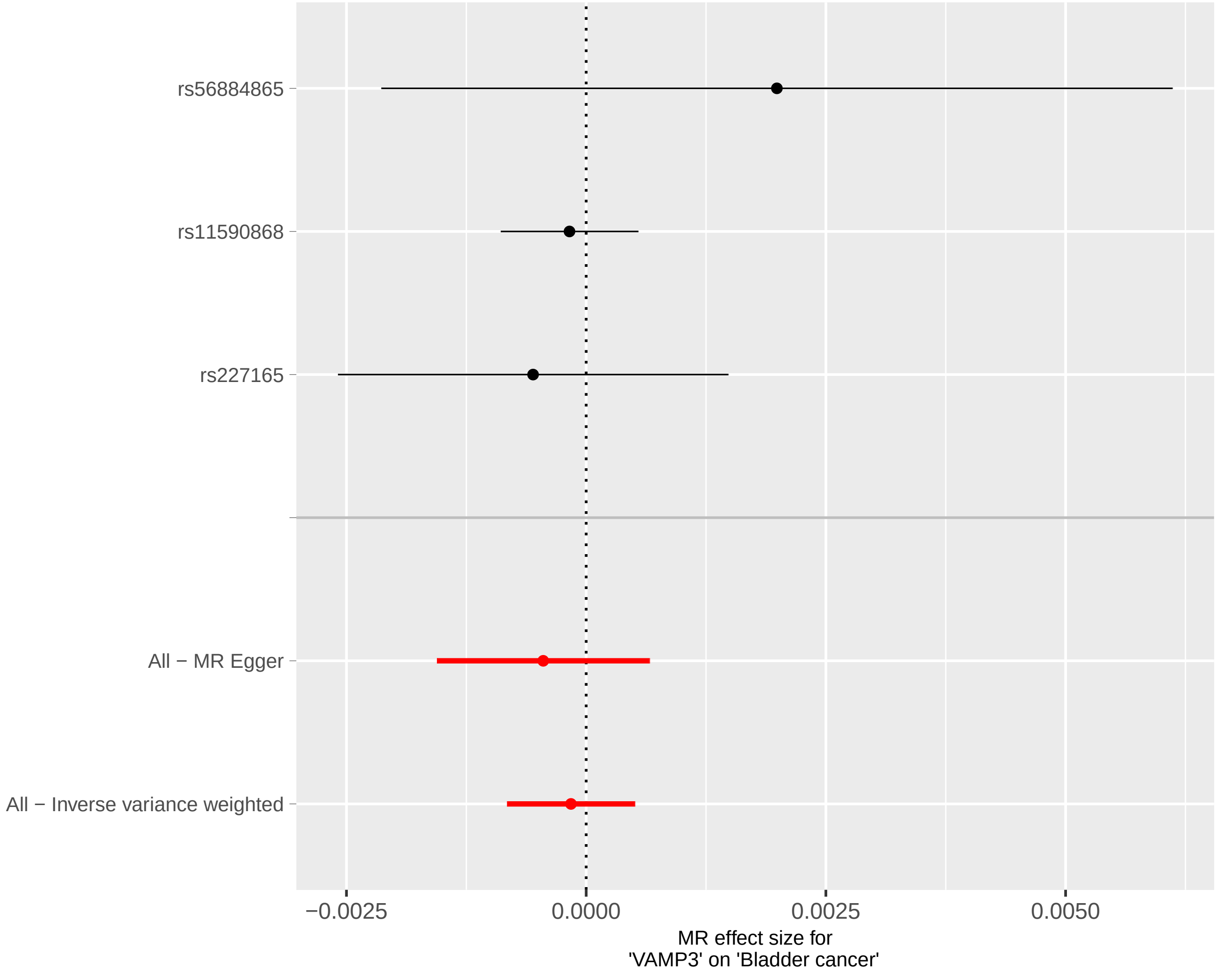

rs56884865
rs11590868
rs227165
All − MR Egger
All − Inverse variance weighted
−0.0025
0.0000
0.0025
0.0050
MR effect size for
'VAMP3' on 'Bladder cancer'

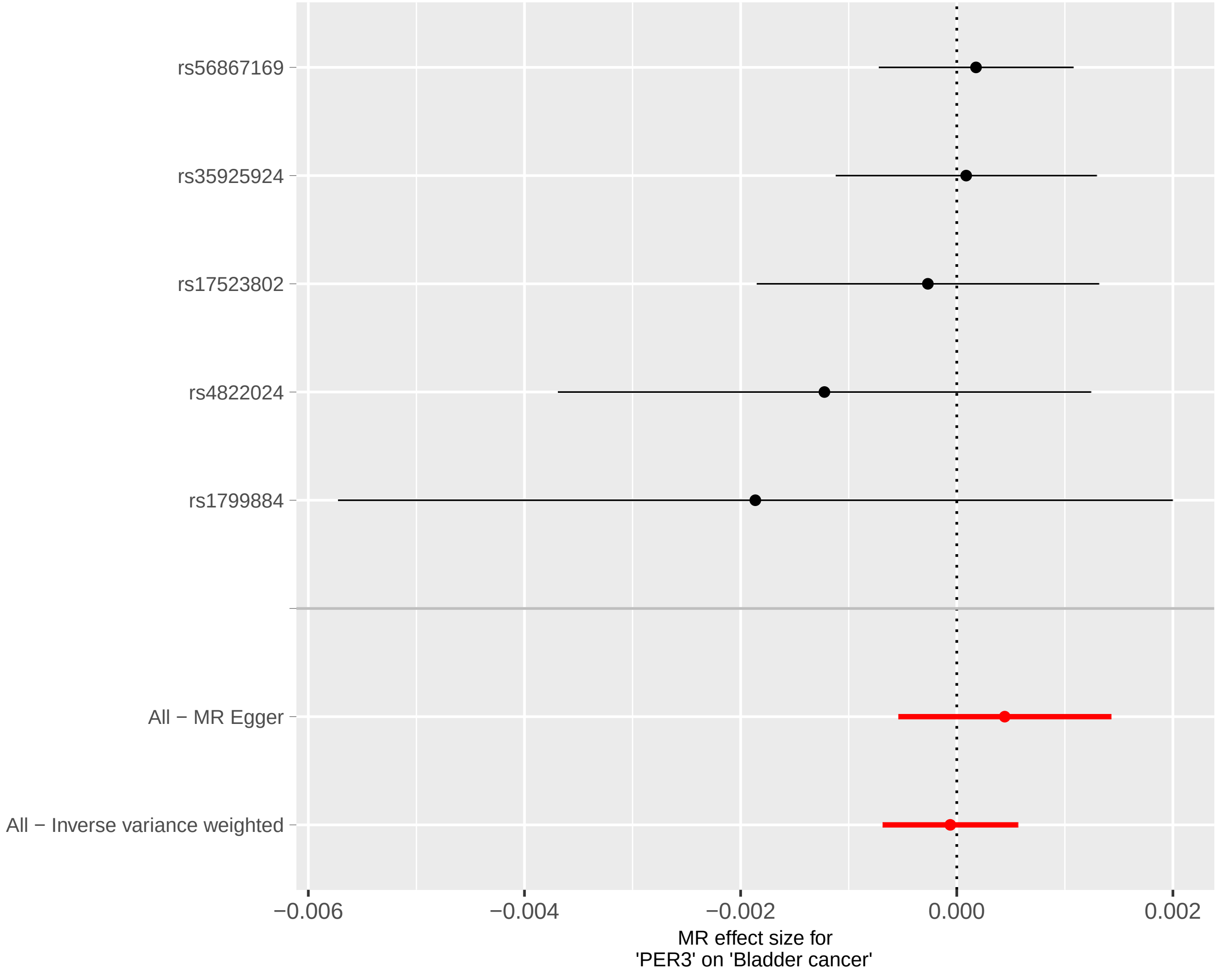
rs56867169
rs35925924
rs17523802
rs4822024
rs1799884
All − MR Egger
All − Inverse variance weighted
−0.006
−0.004
−0.002
0.000
0.002
MR effect size for
'PER3' on 'Bladder cancer'

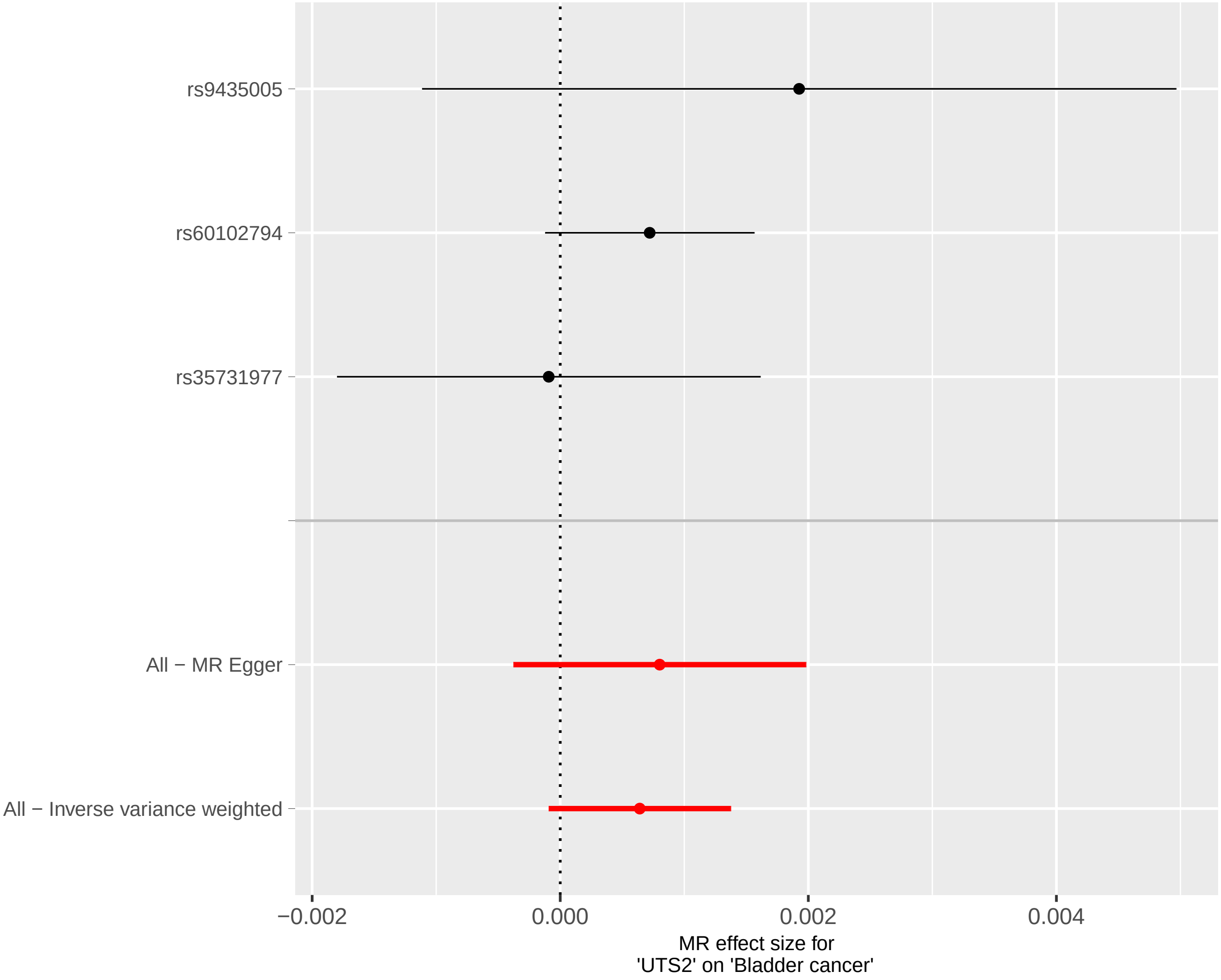
rs9435005
rs60102794
rs35731977
All − MR Egger
All − Inverse variance weighted
−0.002
0.000
0.002
0.004
MR effect size for
'UTS2' on 'Bladder cancer'

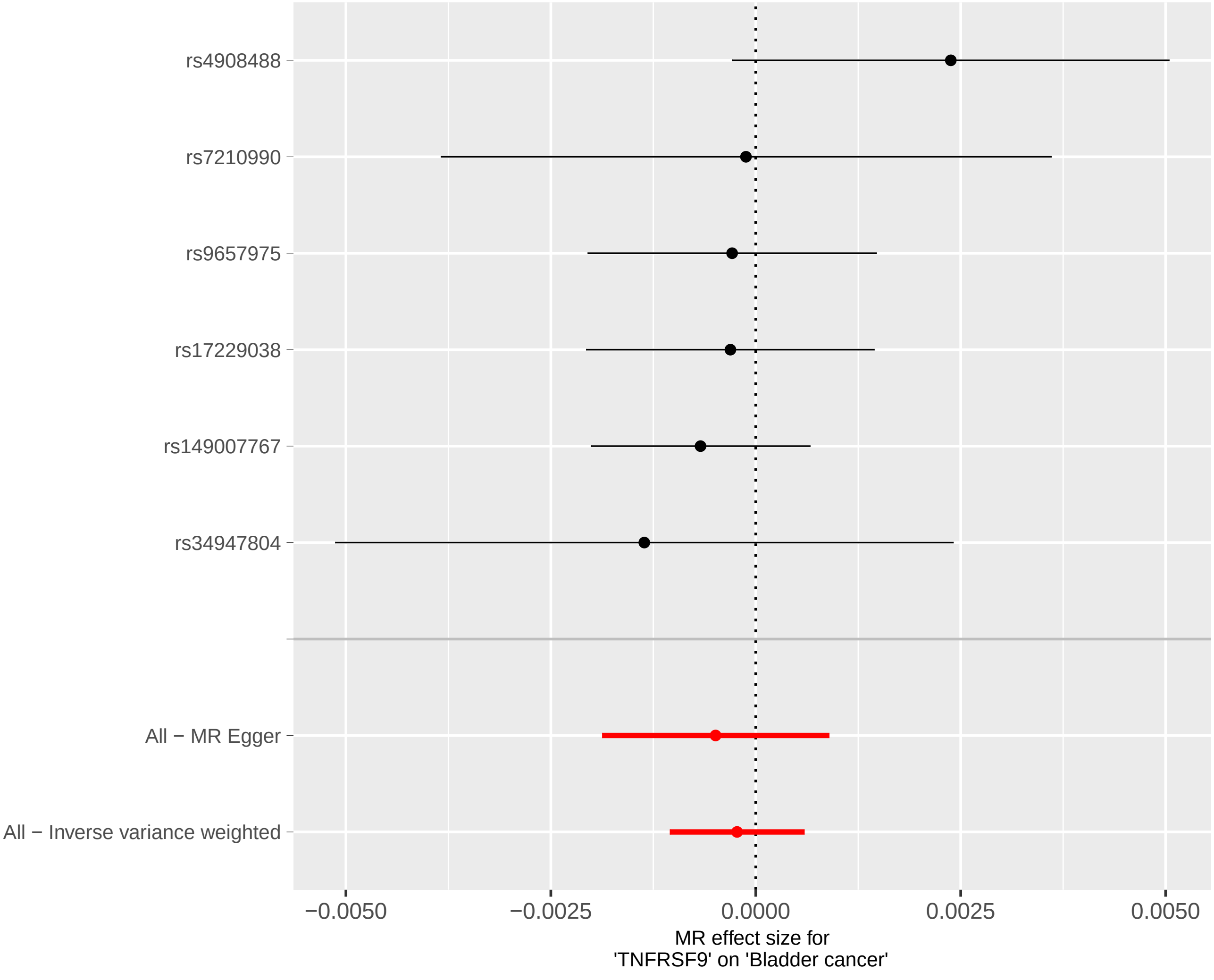

rs4908488
rs7210990
rs9657975
rs17229038
rs149007767
rs34947804
All − MR Egger
All − Inverse variance weighted
−0.0050
−0.0025
0.0000
0.0025
0.0050
MR effect size for
'TNFRSF9' on 'Bladder cancer'

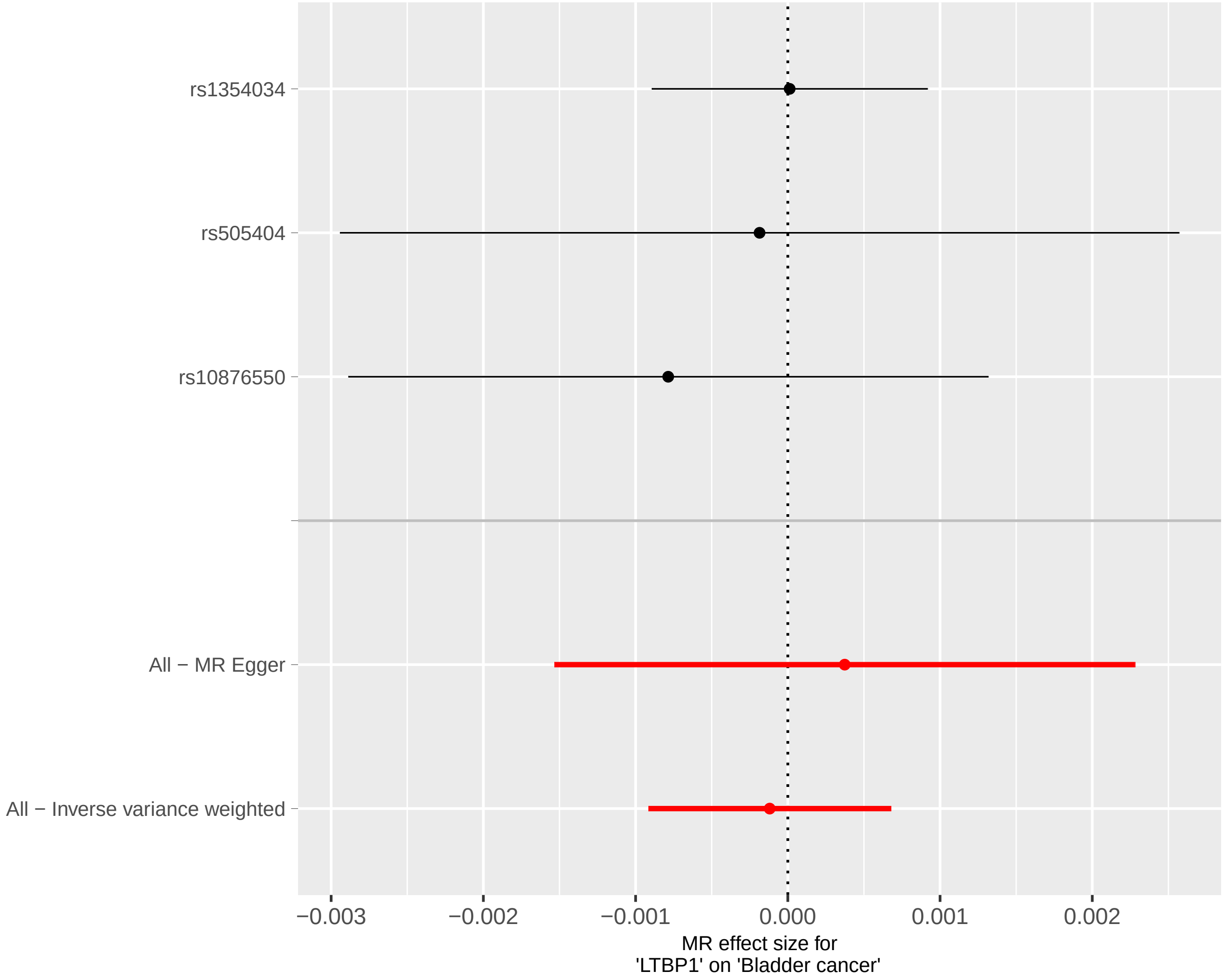

rs1354034
rs505404
rs10876550
All − MR Egger
All − Inverse variance weighted
−0.003
−0.002
−0.001
0.000
0.001
0.002
MR effect size for
'LTBP1' on 'Bladder cancer'

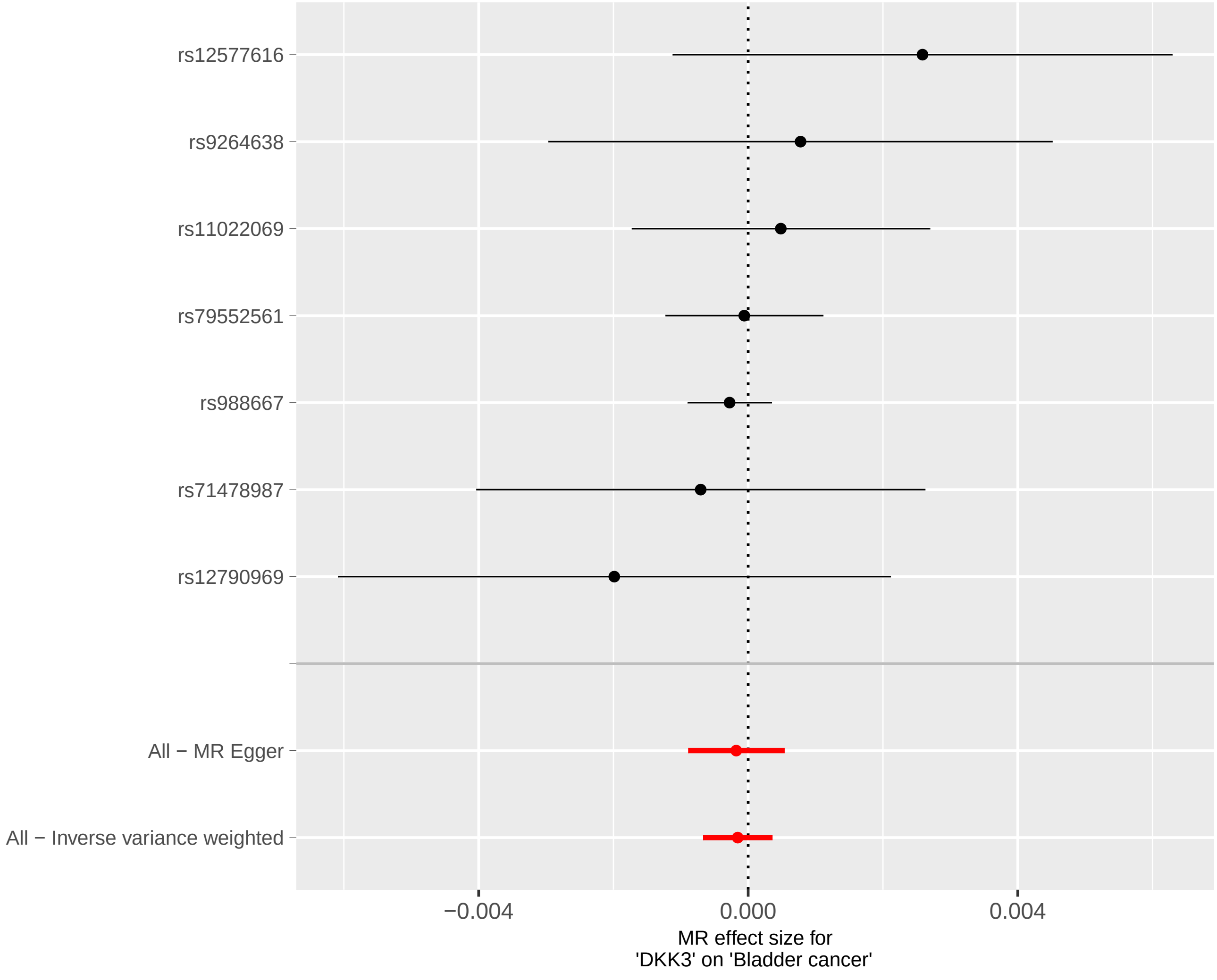

rs12577616
rs9264638
rs11022069
rs79552561
rs988667
rs71478987
rs12790969
All − MR Egger
All − Inverse variance weighted
−0.004
0.000
0.004
MR effect size for
'DKK3' on 'Bladder cancer'

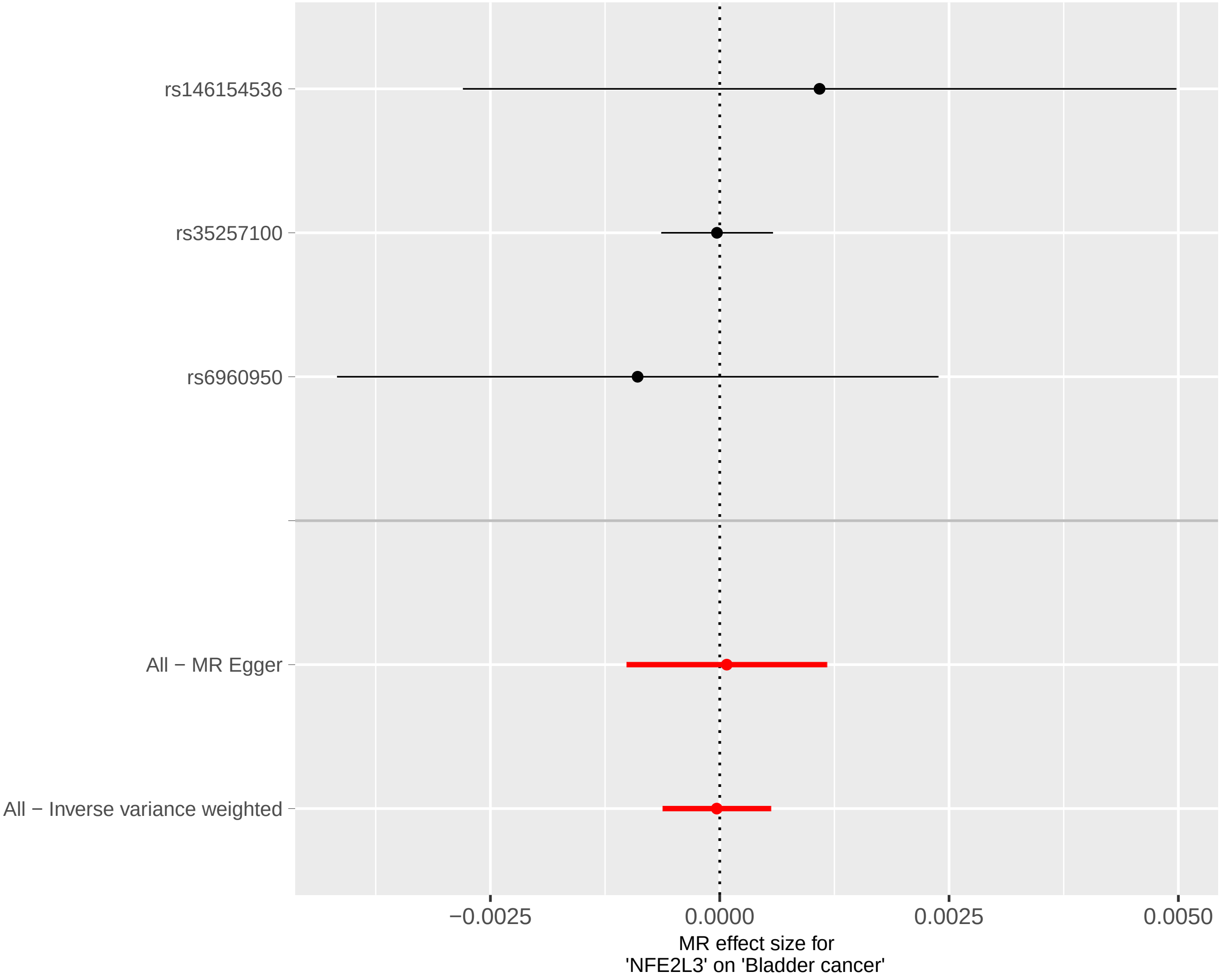

rs146154536
rs35257100
rs6960950
All − MR Egger
All − Inverse variance weighted
−0.0025
0.0000
0.0025
0.0050
MR effect size for
'NFE2L3' on 'Bladder cancer'

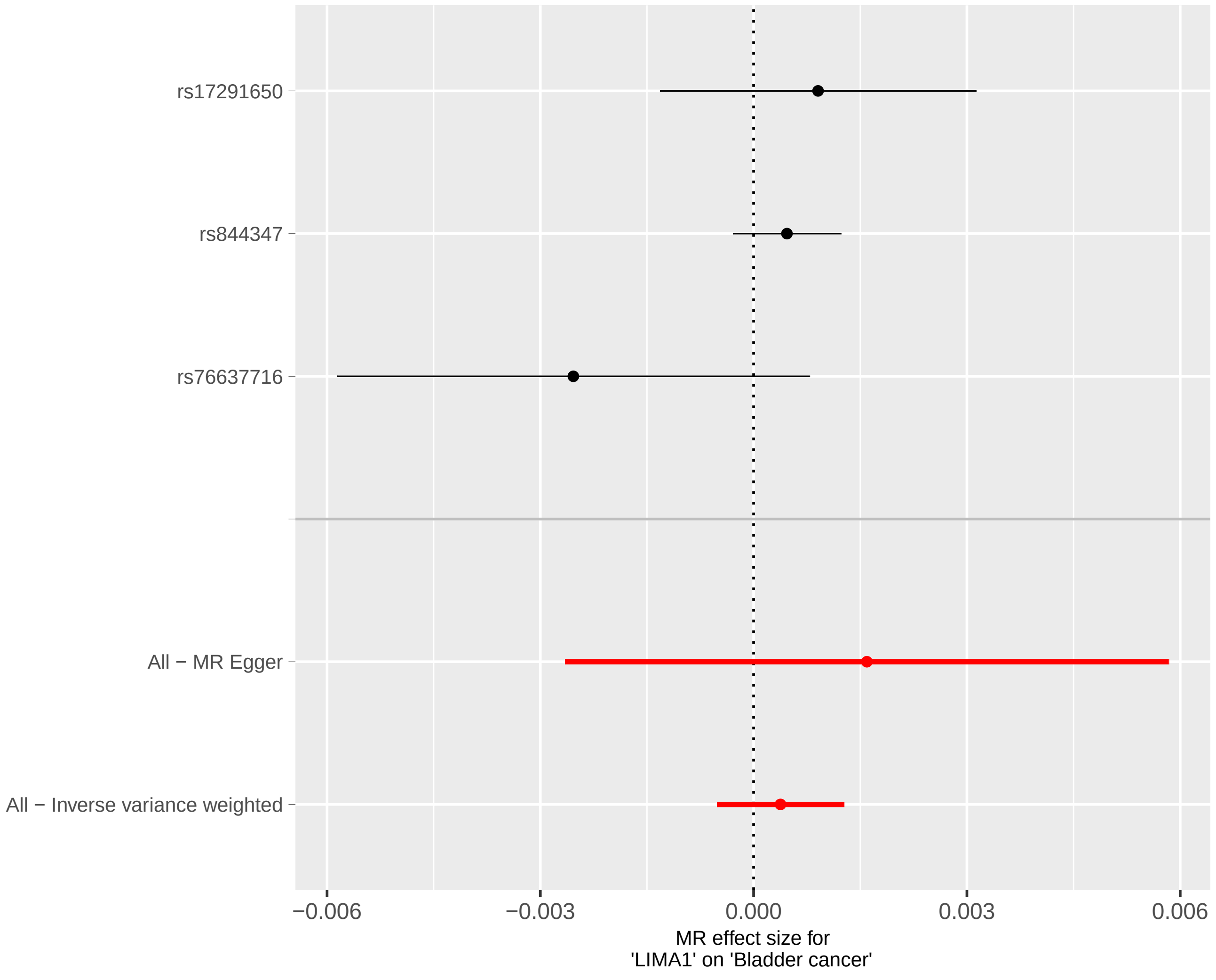

rs17291650
rs844347
rs76637716
All − MR Egger
All − Inverse variance weighted
−0.006
−0.003
0.000
0.003
0.006
MR effect size for
'LIMA1' on 'Bladder cancer'

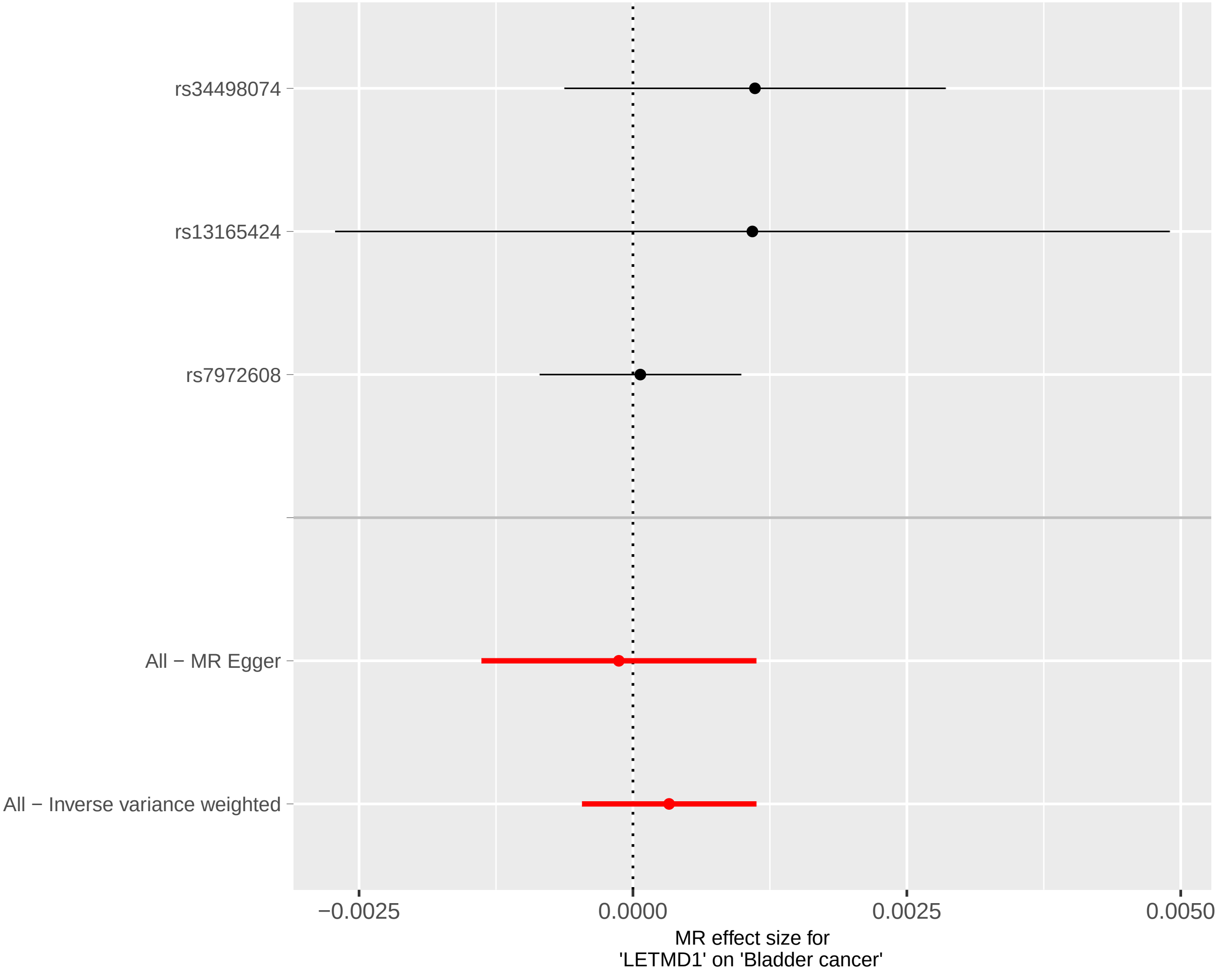

rs34498074
rs13165424
rs7972608
All − MR Egger
All − Inverse variance weighted
−0.0025
0.0000
0.0025
0.0050
MR effect size for
'LETMD1' on 'Bladder cancer'

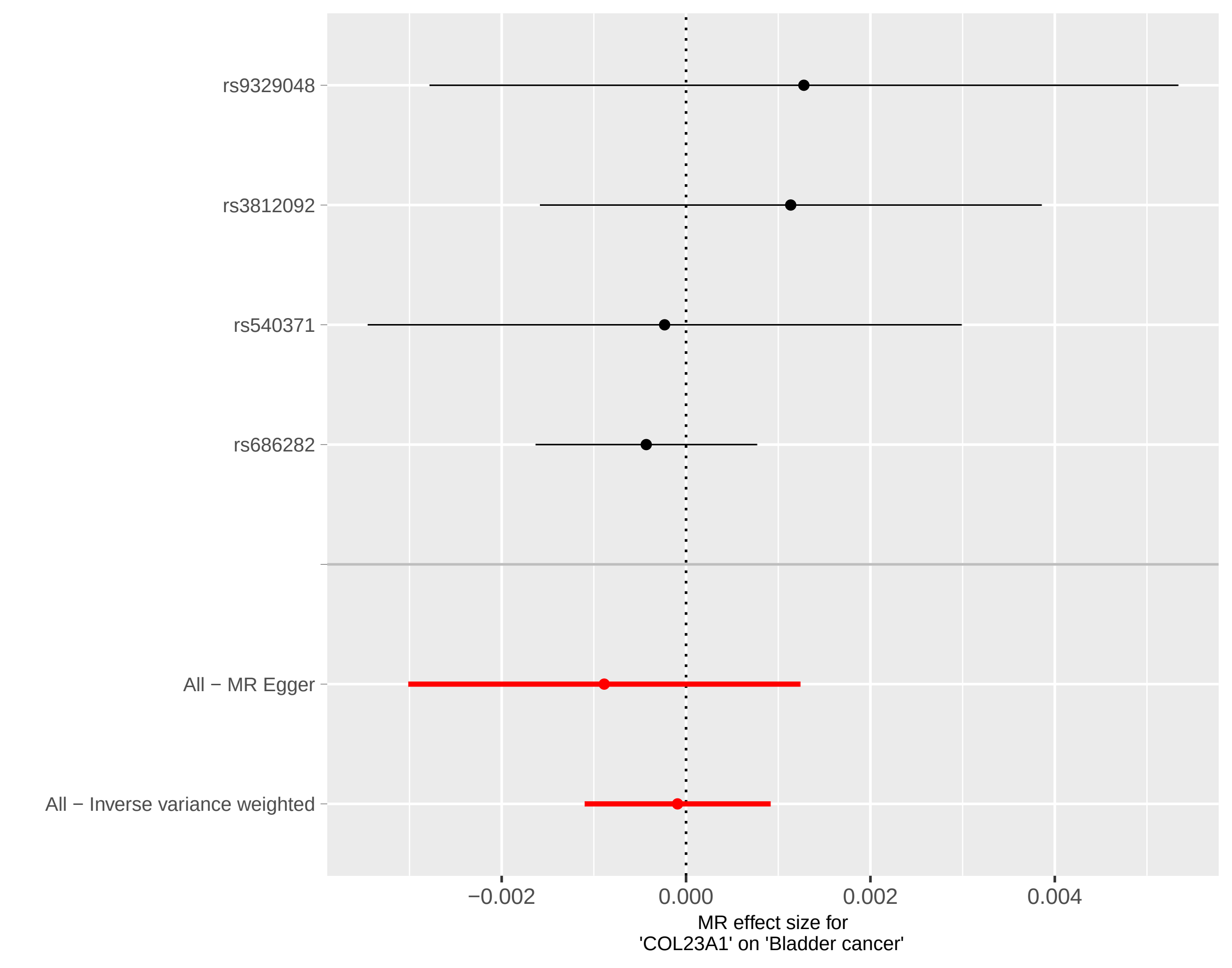

rs9329048
rs3812092
rs540371
rs686282
All − MR Egger
All − Inverse variance weighted
−0.002
0.000
0.002
0.004
MR effect size for
'COL23A1' on 'Bladder cancer'

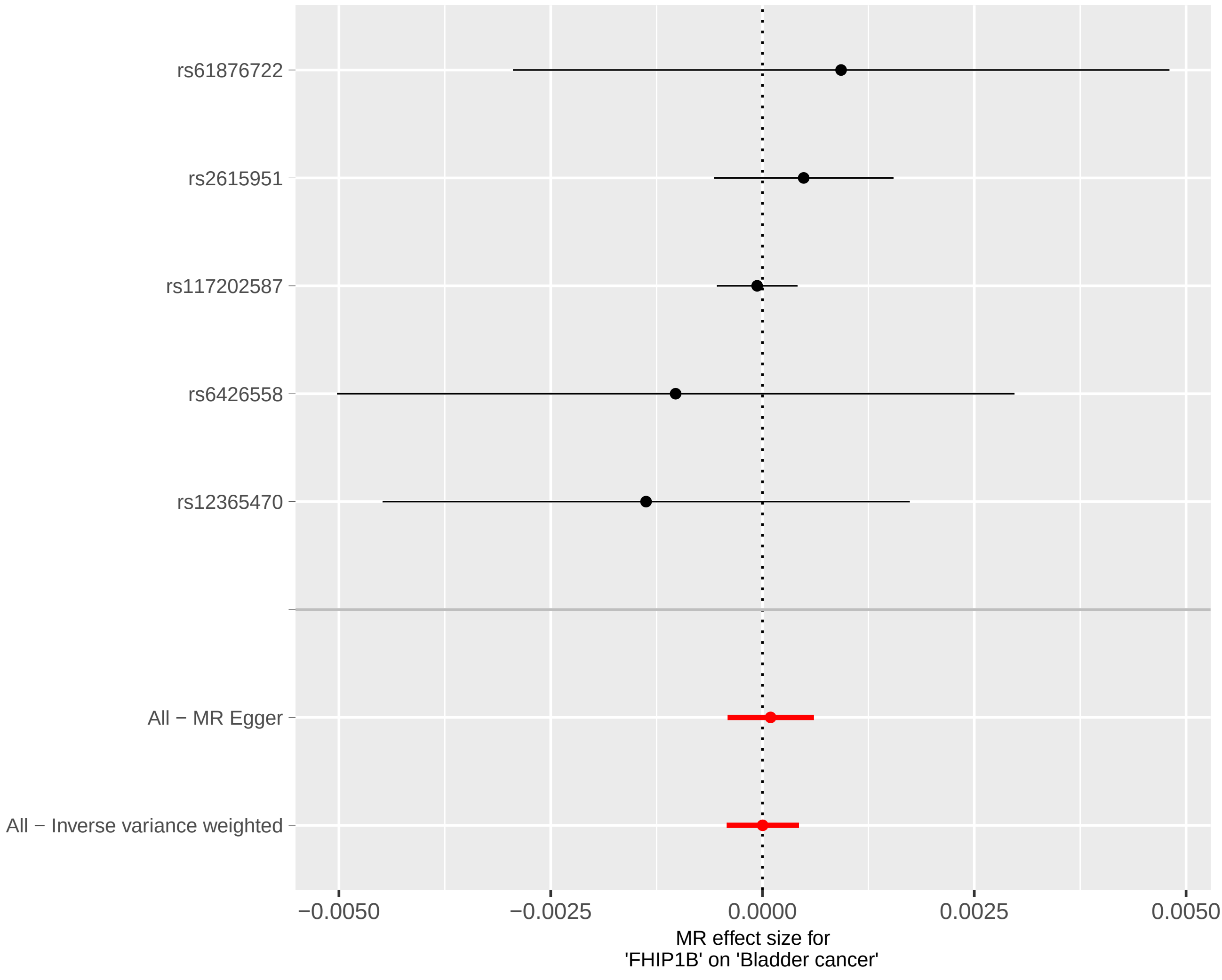

rs61876722
rs2615951
rs117202587
rs6426558
rs12365470
All − MR Egger
All − Inverse variance weighted
−0.0050
−0.0025
0.0000
0.0025
0.0050
MR effect size for
'FHIP1B' on 'Bladder cancer'

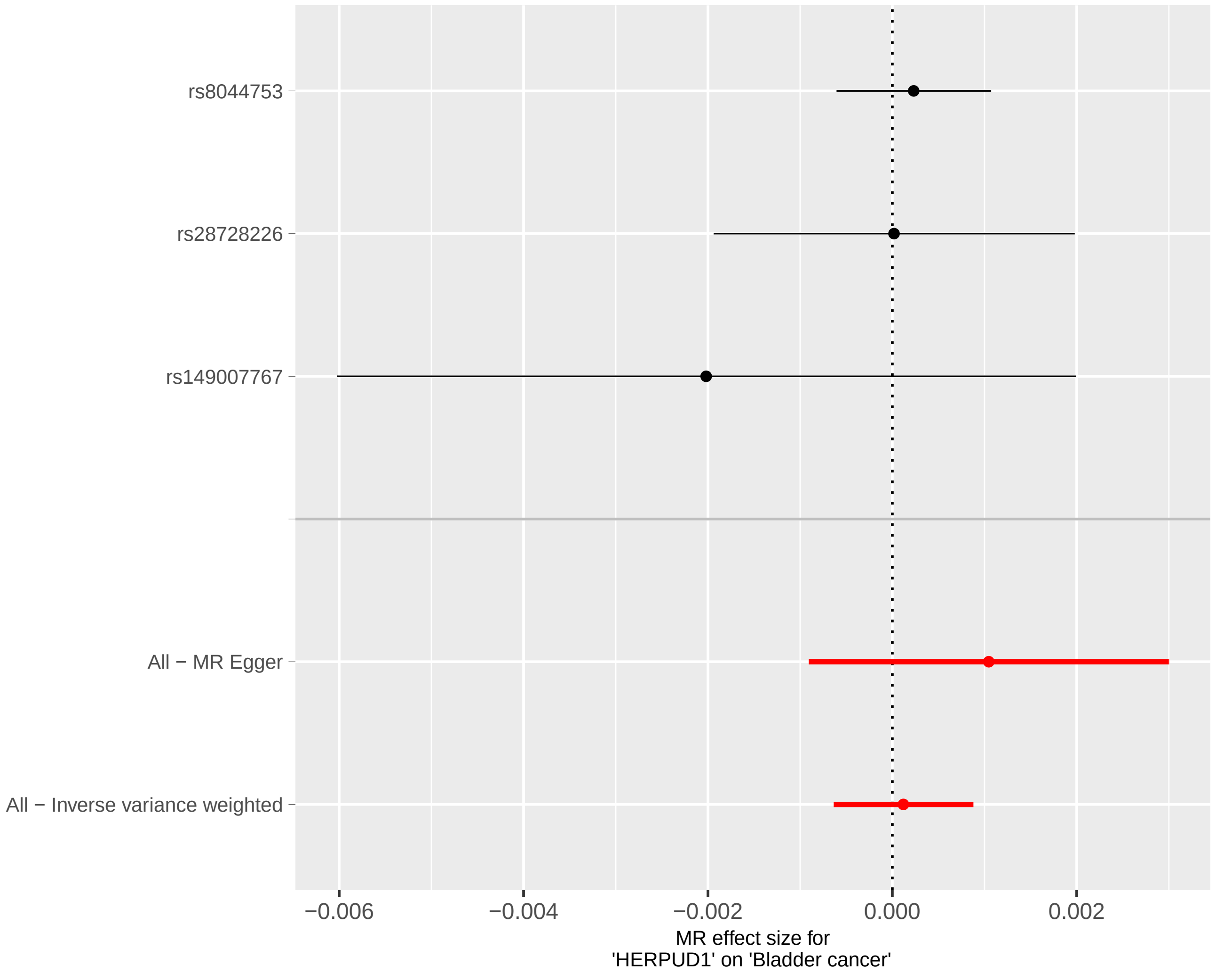

rs8044753
rs28728226
rs149007767
All − MR Egger
All − Inverse variance weighted
−0.006
−0.004
−0.002
0.000
0.002
MR effect size for
'HERPUD1' on 'Bladder cancer'

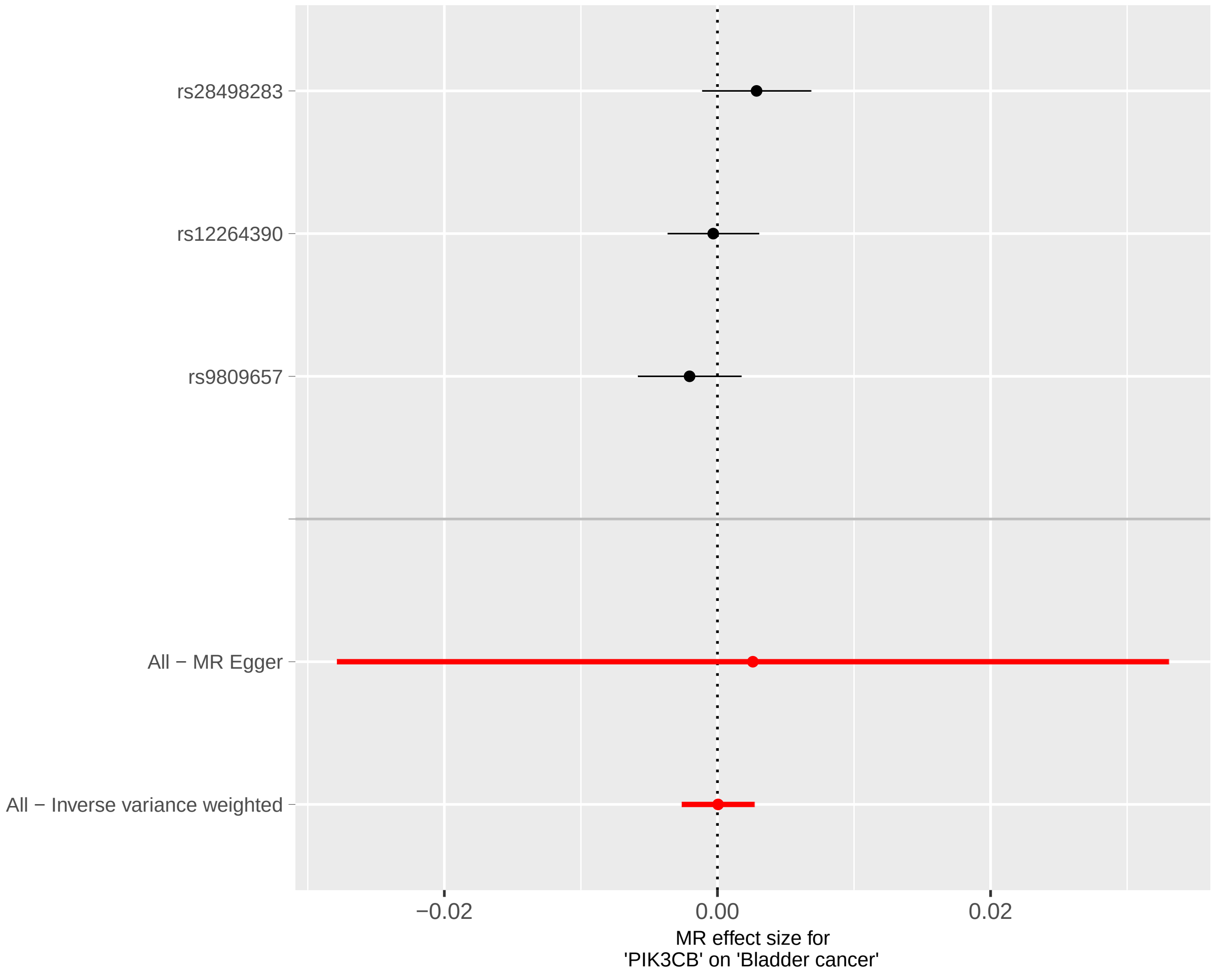
rs28498283
rs12264390
rs9809657
All − MR Egger
All − Inverse variance weighted
−0.02
0.00
0.02
MR effect size for
'PIK3CB' on 'Bladder cancer'

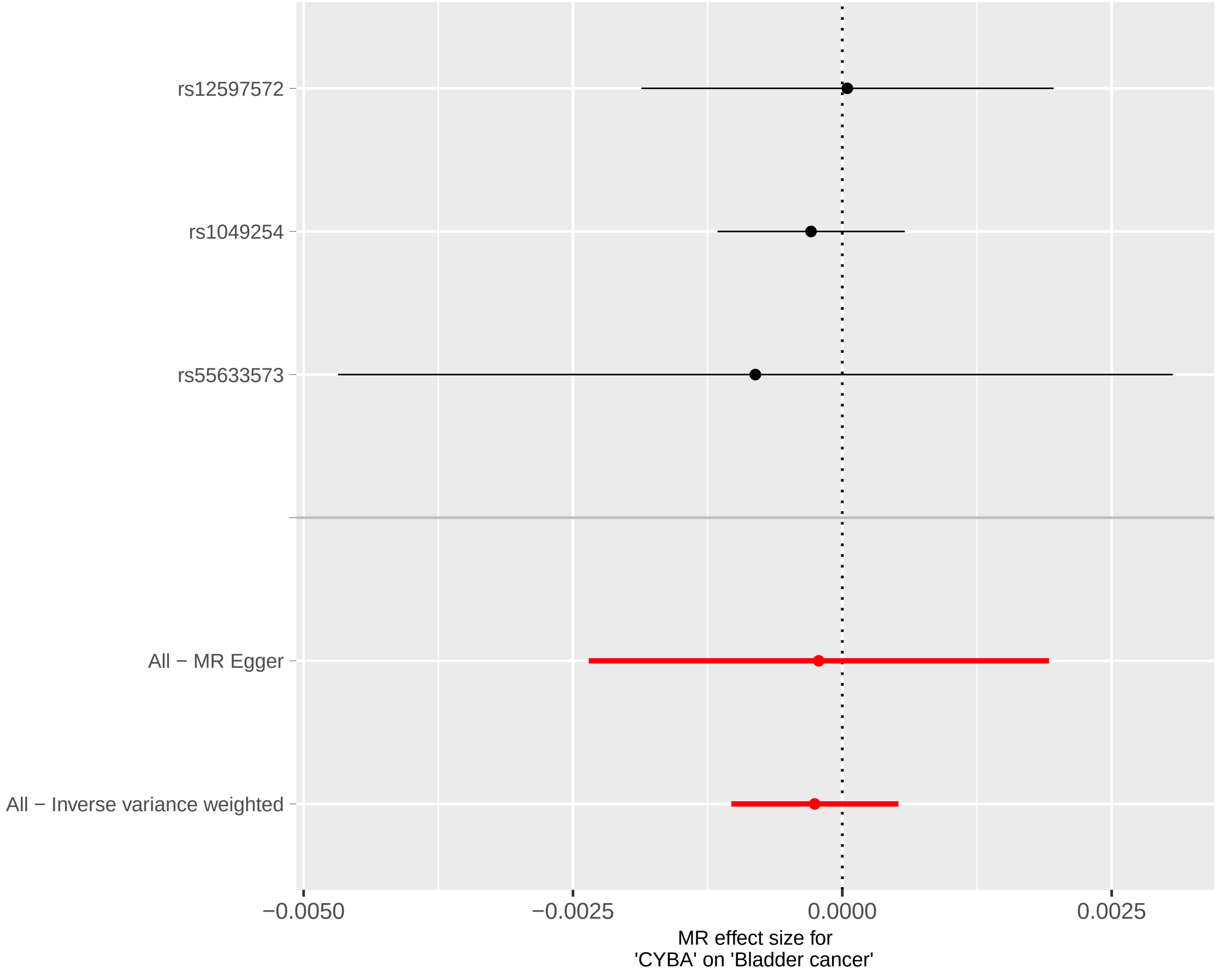

rs12597572
rs1049254
rs55633573
All − MR Egger
All − Inverse variance weighted
−0.0050
−0.0025
0.0000
0.0025
MR effect size for
'CYBA' on 'Bladder cancer'

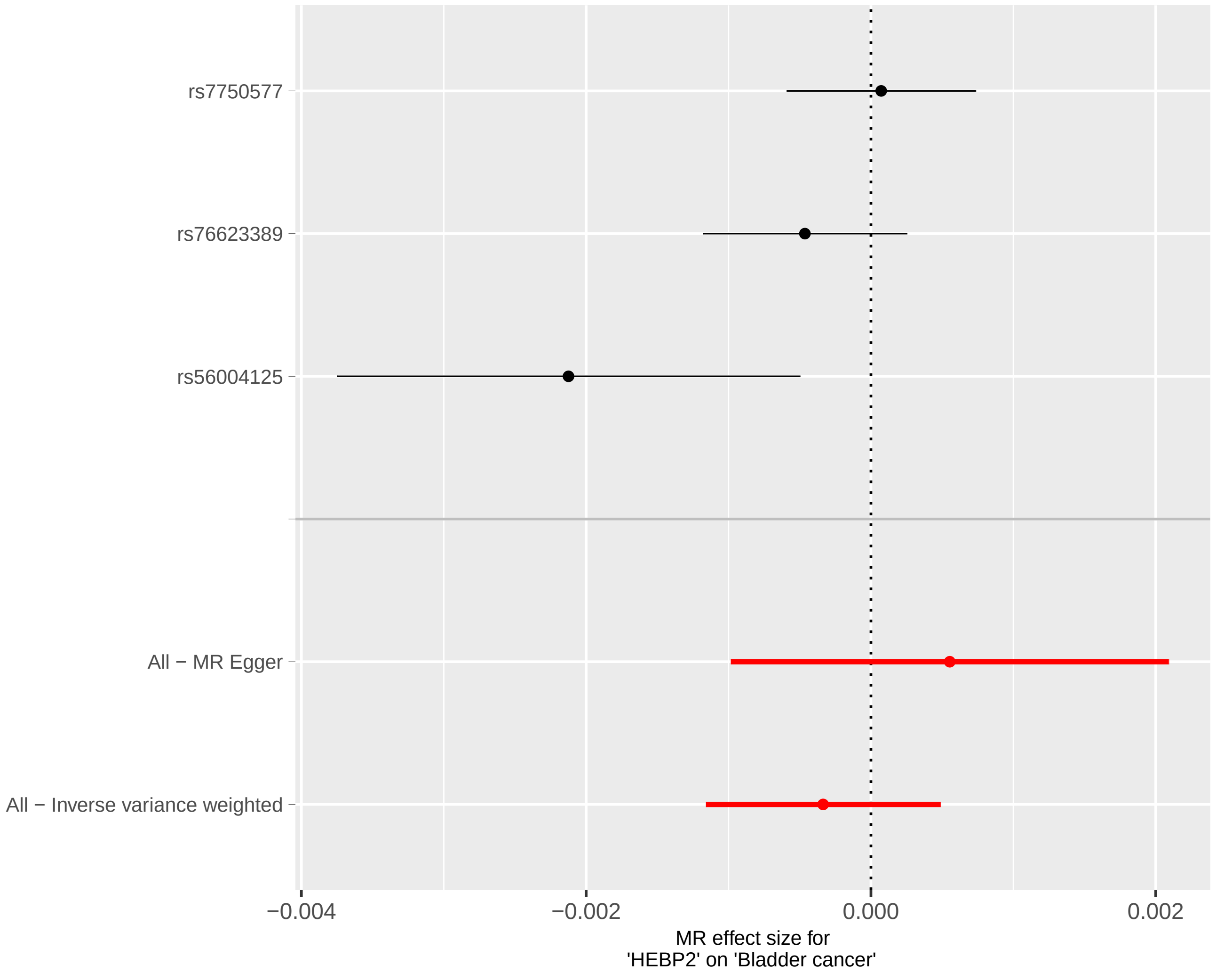
rs7750577
rs76623389
rs56004125
All − MR Egger
All − Inverse variance weighted
−0.004
−0.002
0.000
0.002
MR effect size for
'HEBP2' on 'Bladder cancer'

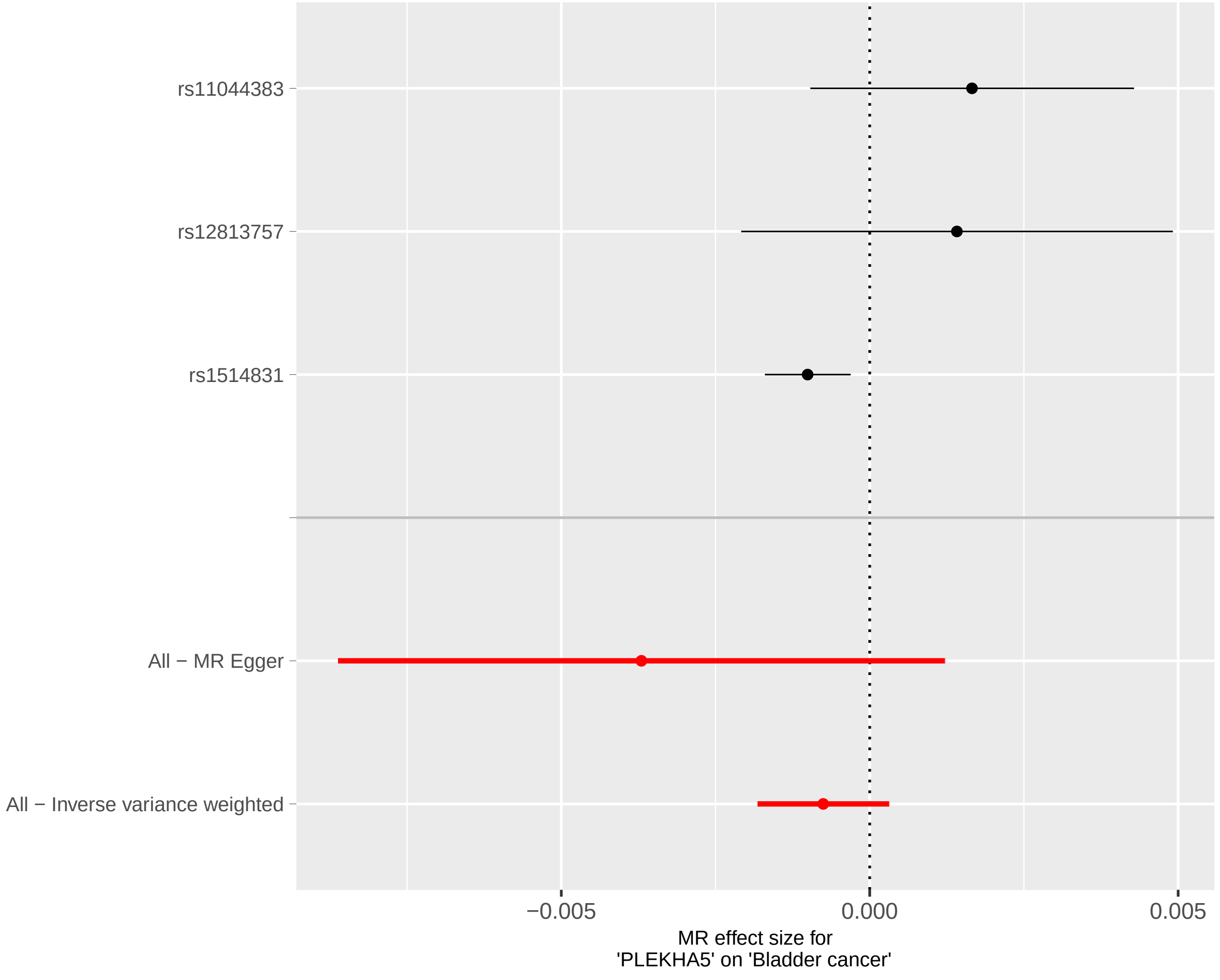
rs11044383
rs12813757
rs1514831
All − MR Egger
All − Inverse variance weighted
−0.005
0.000
0.005
MR effect size for
'PLEKHA5' on 'Bladder cancer'

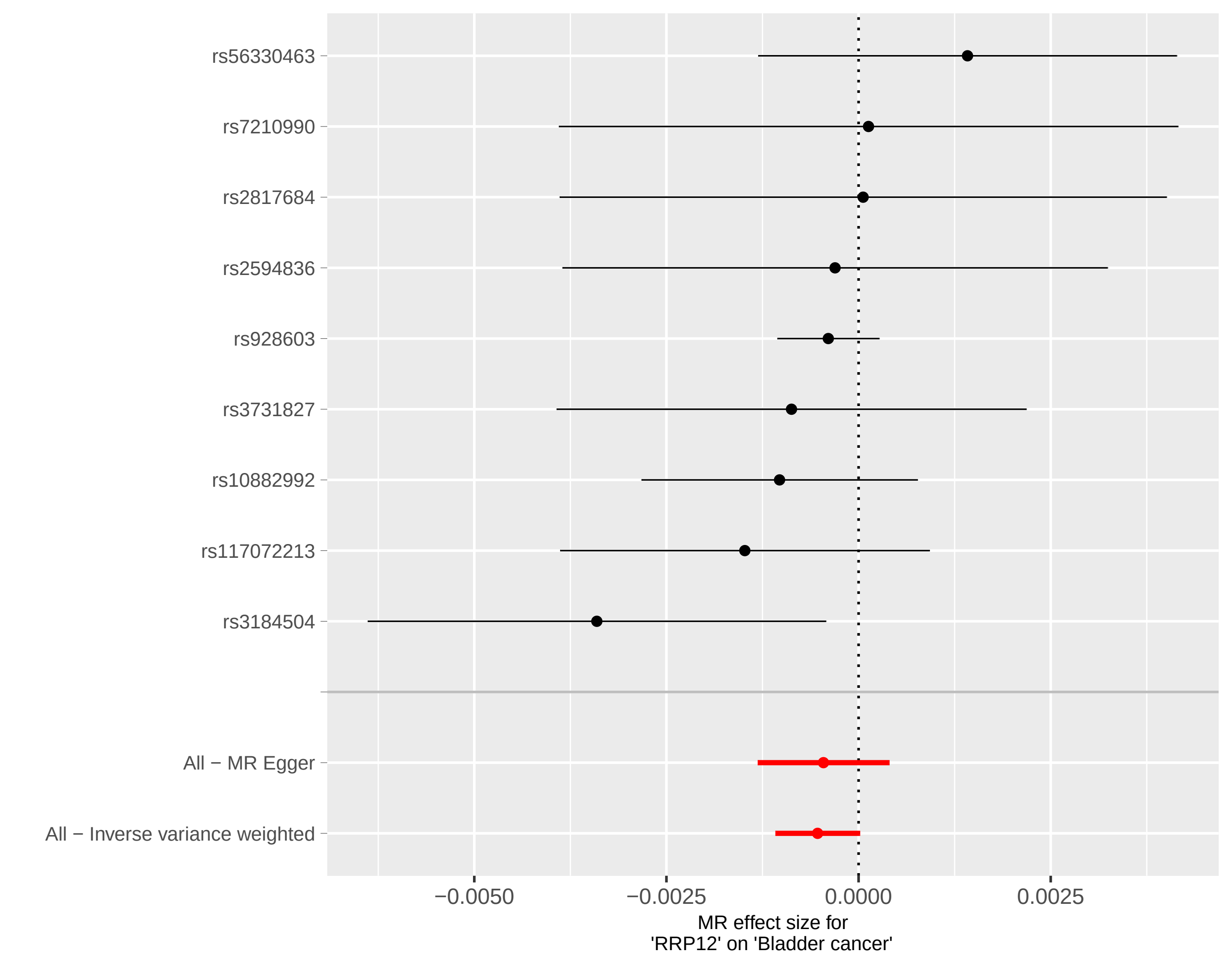
rs56330463
rs7210990
rs2817684
rs2594836
rs928603
rs3731827
rs10882992
rs117072213
rs3184504
All − MR Egger
All − Inverse variance weighted
−0.0050
−0.0025
0.0000
0.0025
MR effect size for
'RRP12' on 'Bladder cancer'

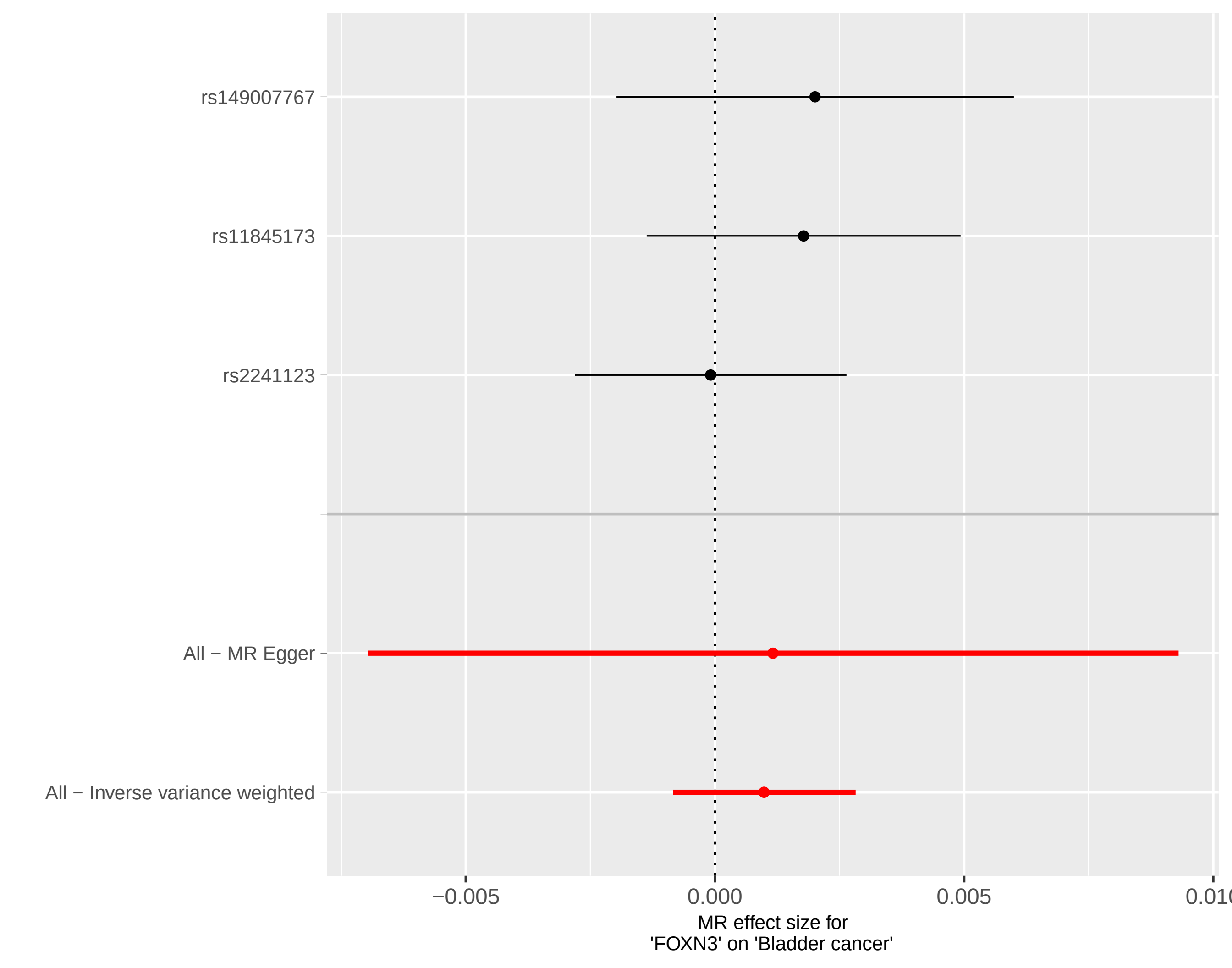

rs149007767
rs11845173
rs2241123
All − MR Egger
All − Inverse variance weighted
−0.005
0.000
0.005
0.010
MR effect size for
'FOXN3' on 'Bladder cancer'

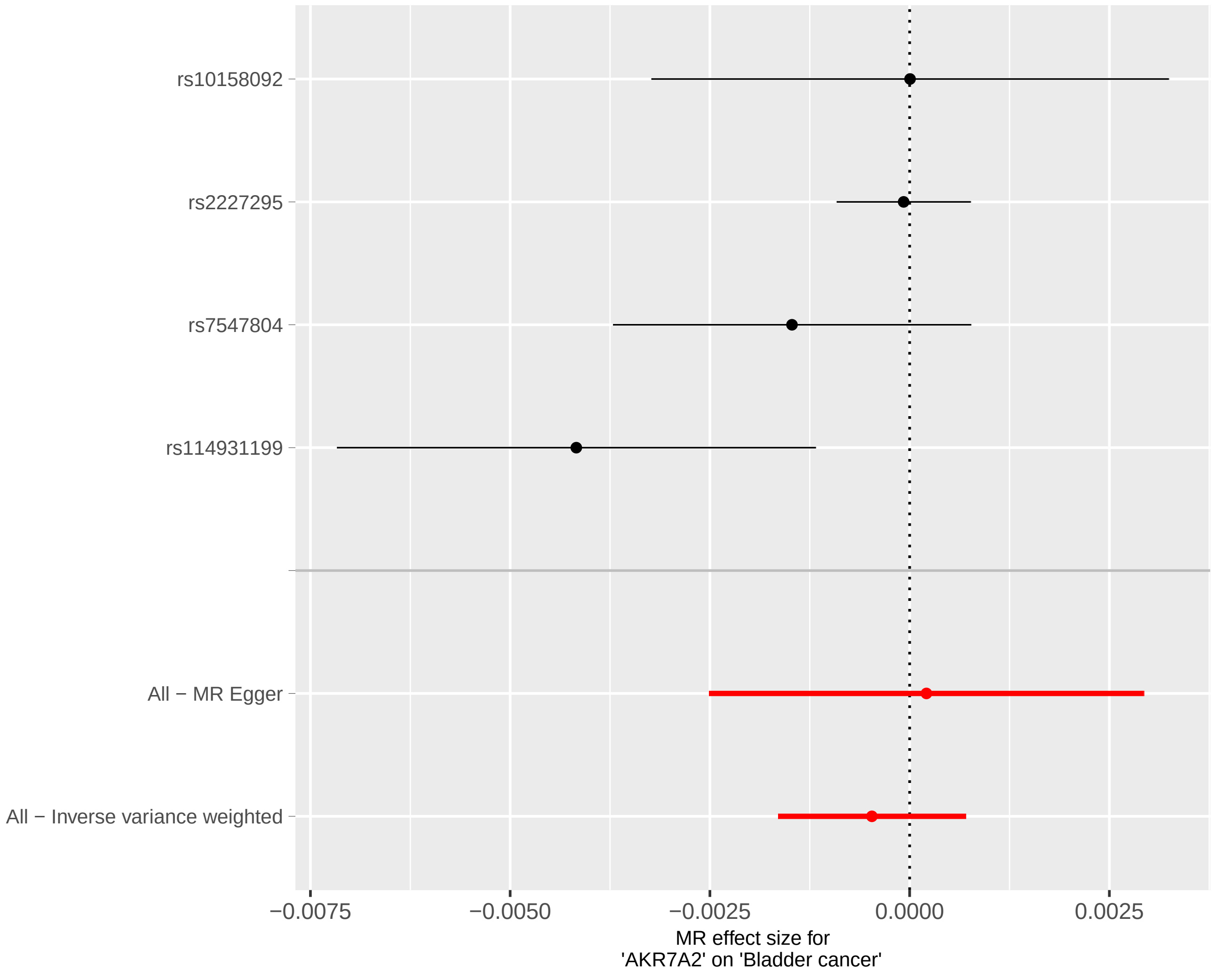

rs10158092
rs2227295
rs7547804
rs114931199
All − MR Egger
All − Inverse variance weighted
−0.0075
−0.0050
−0.0025
0.0000
0.0025
MR effect size for
'AKR7A2' on 'Bladder cancer'

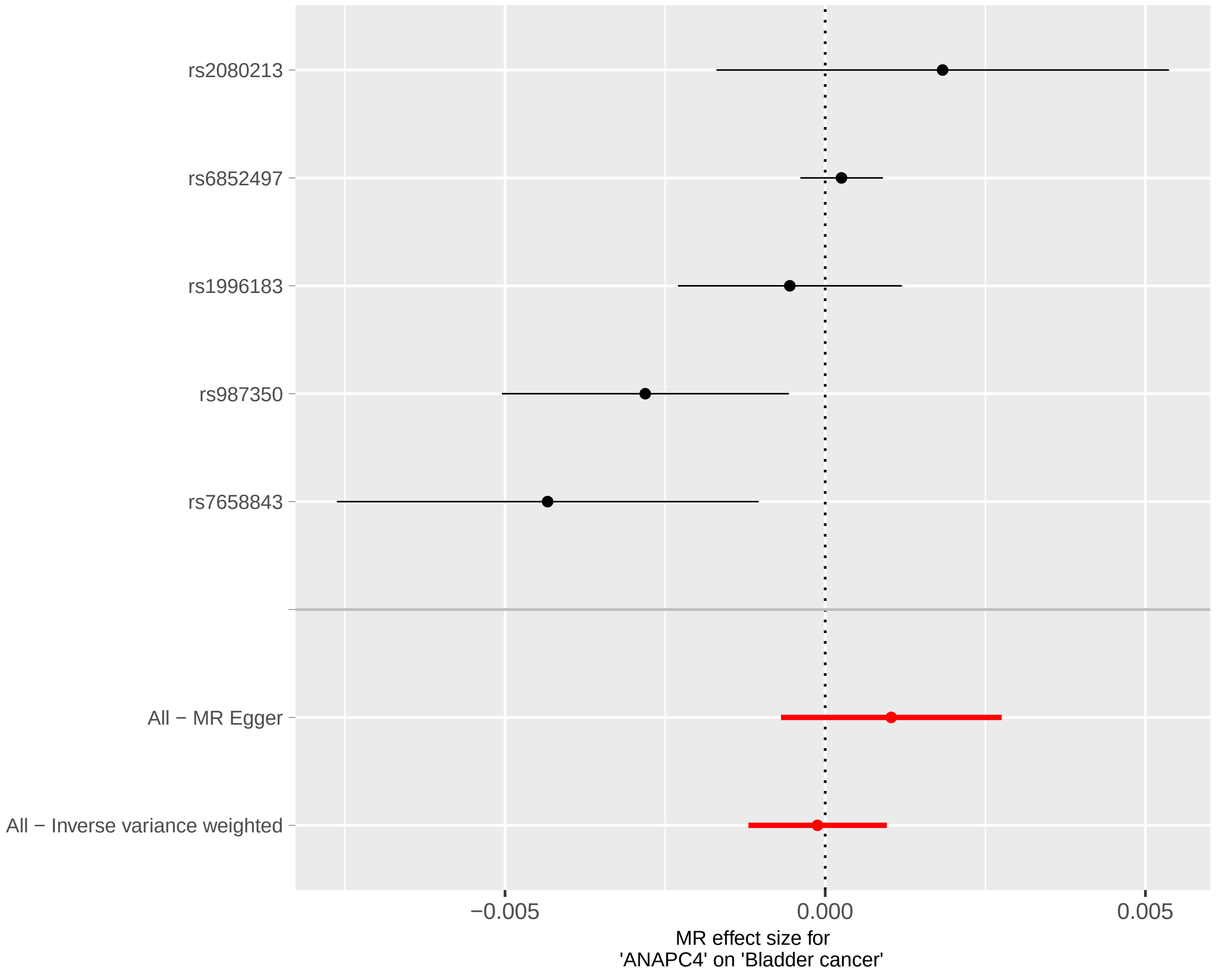

rs2080213
rs6852497
rs1996183
rs987350
rs7658843
All − MR Egger
All − Inverse variance weighted
−0.005
0.000
0.005
MR effect size for
'ANAPC4' on 'Bladder cancer'

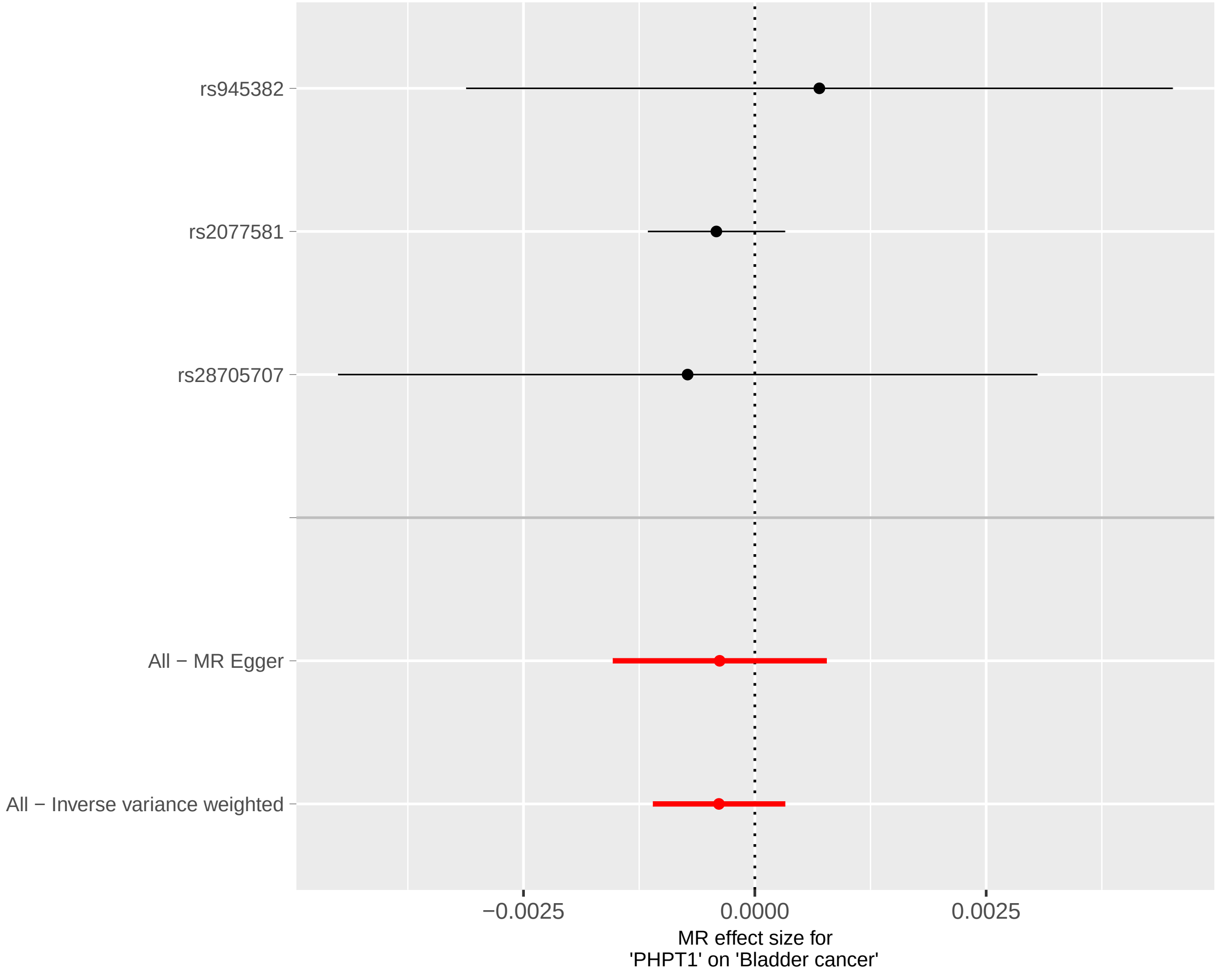

rs945382
rs2077581
rs28705707
All − MR Egger
All − Inverse variance weighted
−0.0025
0.0000
0.0025
MR effect size for
'PHPT1' on 'Bladder cancer'

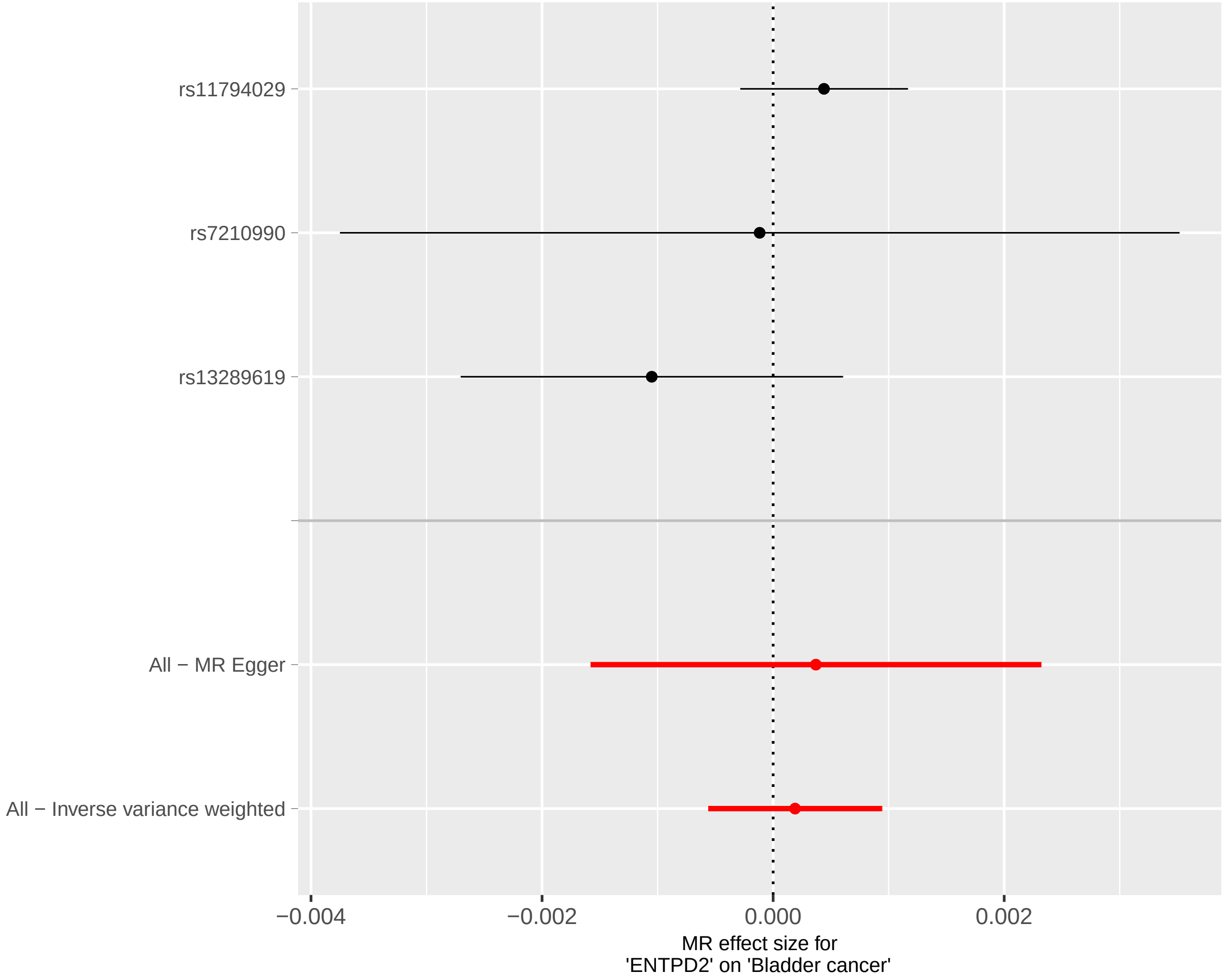

rs11794029
rs7210990
rs13289619
All − MR Egger
All − Inverse variance weighted
−0.004
−0.002
0.000
0.002
MR effect size for
'ENTPD2' on 'Bladder cancer'

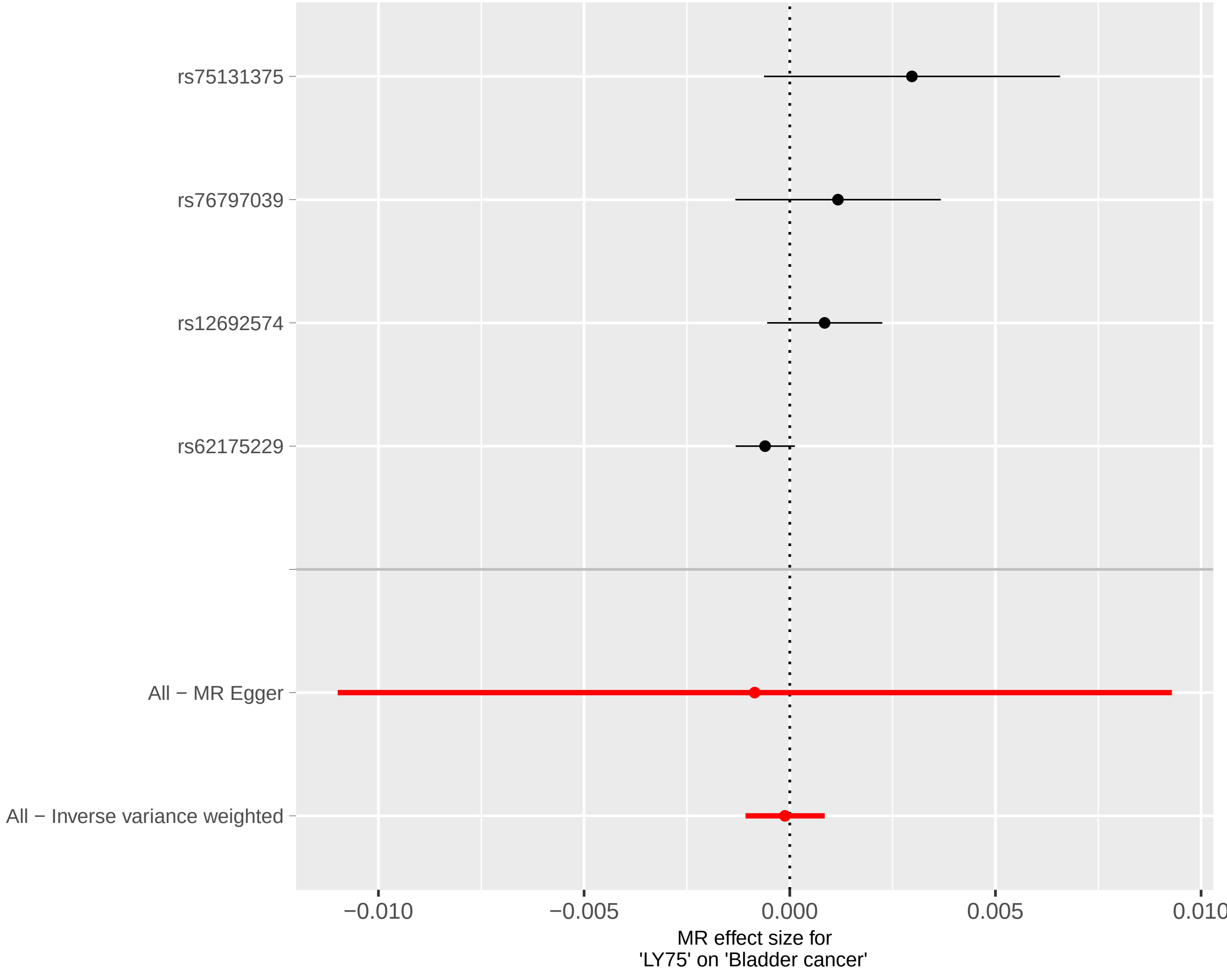

rs75131375
rs76797039
rs12692574
rs62175229
All − MR Egger
All − Inverse variance weighted
−0.010
−0.005
0.000
0.005
0.010
MR effect size for
'LY75' on 'Bladder cancer'

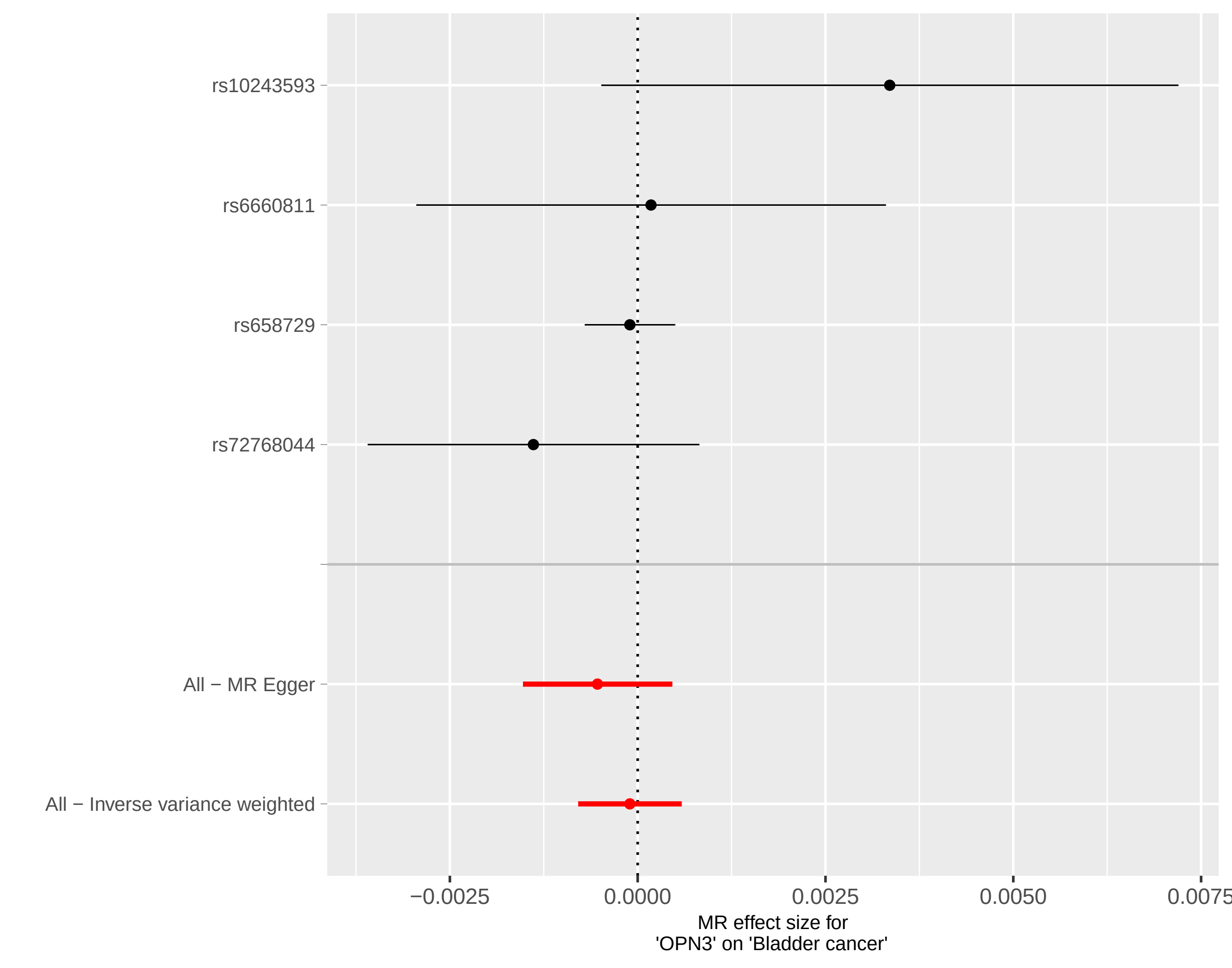
rs10243593
rs6660811
rs658729
rs72768044
All − MR Egger
All − Inverse variance weighted
−0.0025
0.0000
0.0025
0.0050
0.0075
MR effect size for
'OPN3' on 'Bladder cancer'

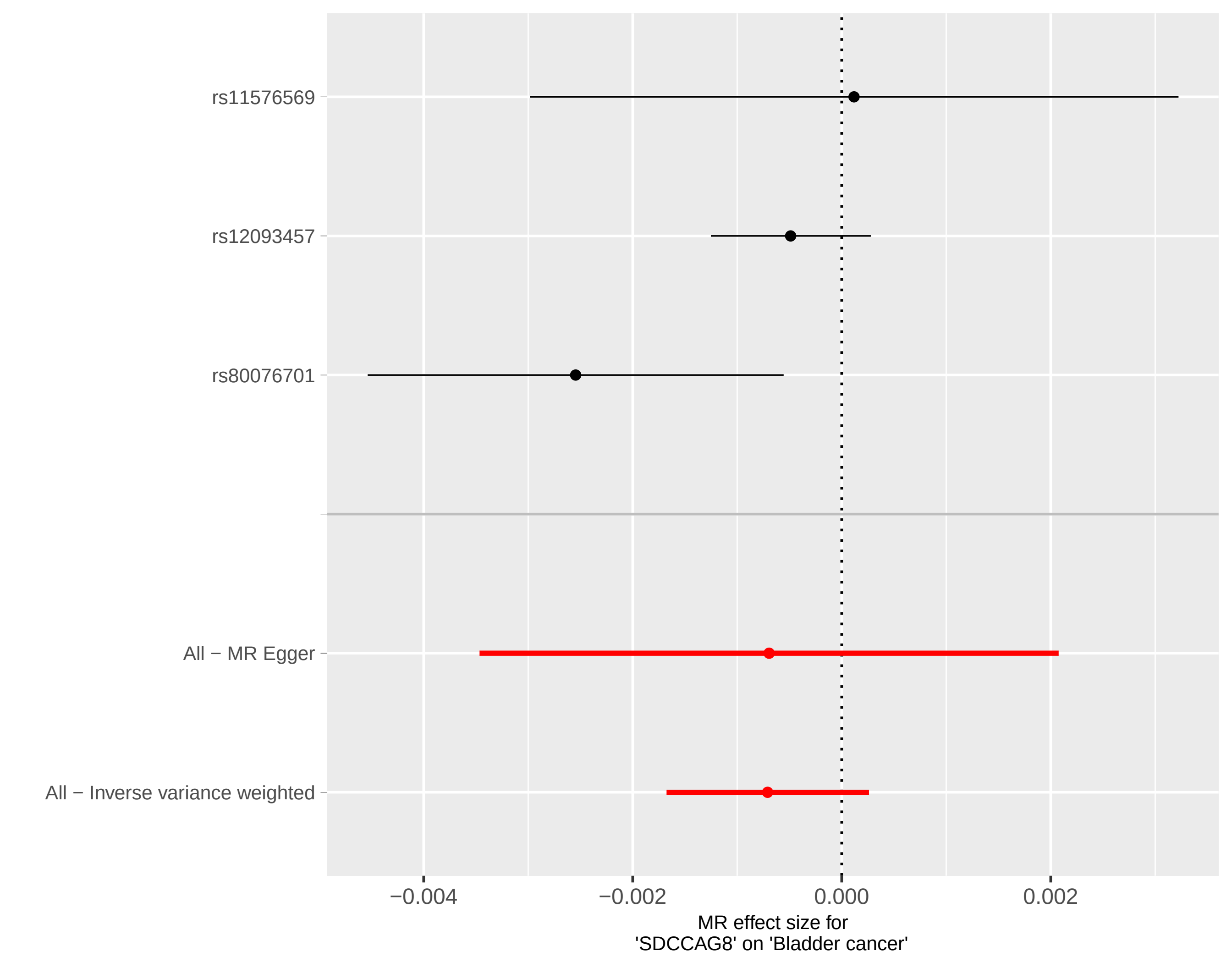
rs11576569
rs12093457
rs80076701
All − MR Egger
All − Inverse variance weighted
−0.004
−0.002
0.000
0.002
MR effect size for
'SDCCAG8' on 'Bladder cancer'

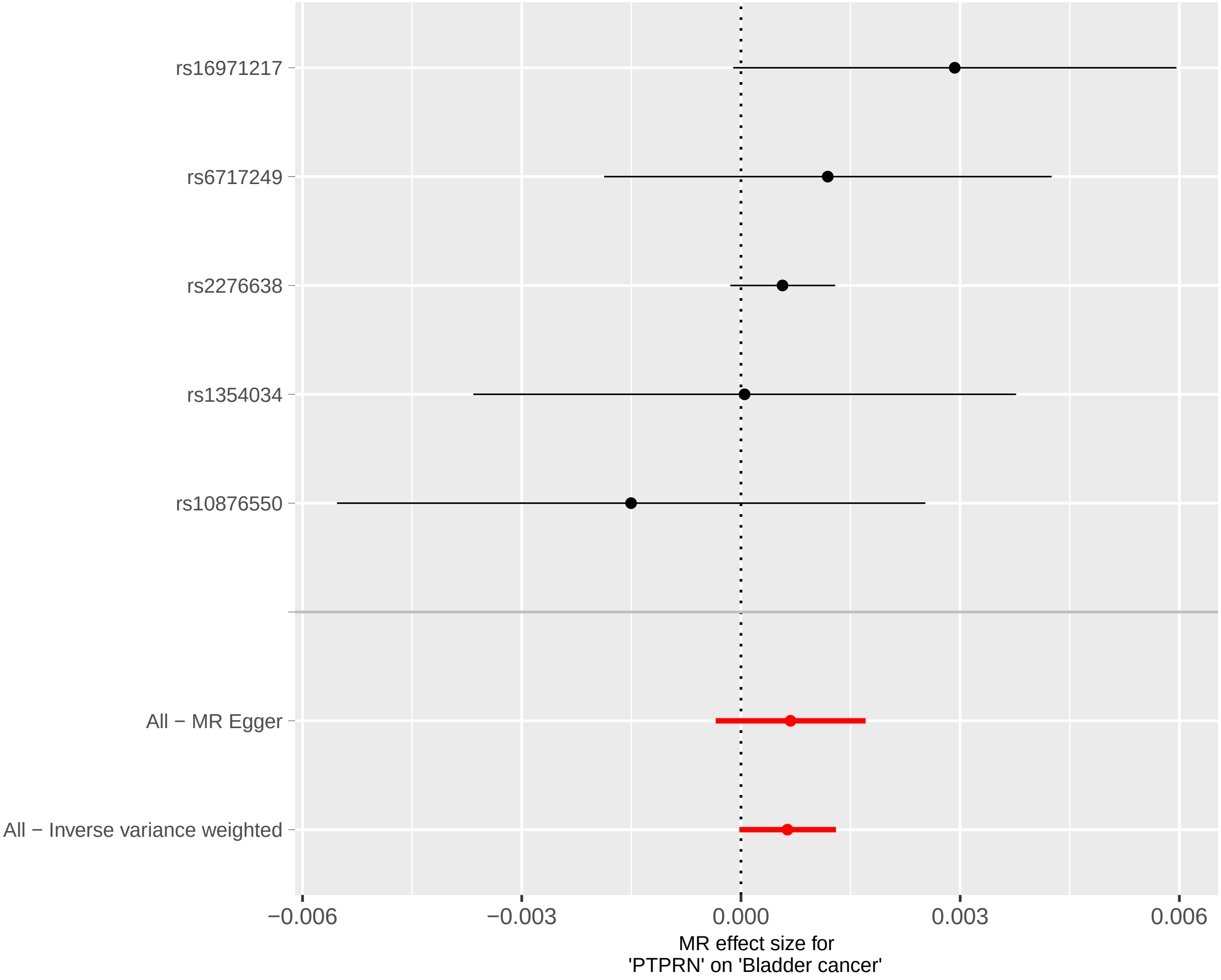

rs16971217
rs6717249
rs2276638
rs1354034
rs10876550
All − MR Egger
All − Inverse variance weighted
−0.006
−0.003
0.000
0.003
0.006
MR effect size for
'PTPRN' on 'Bladder cancer'

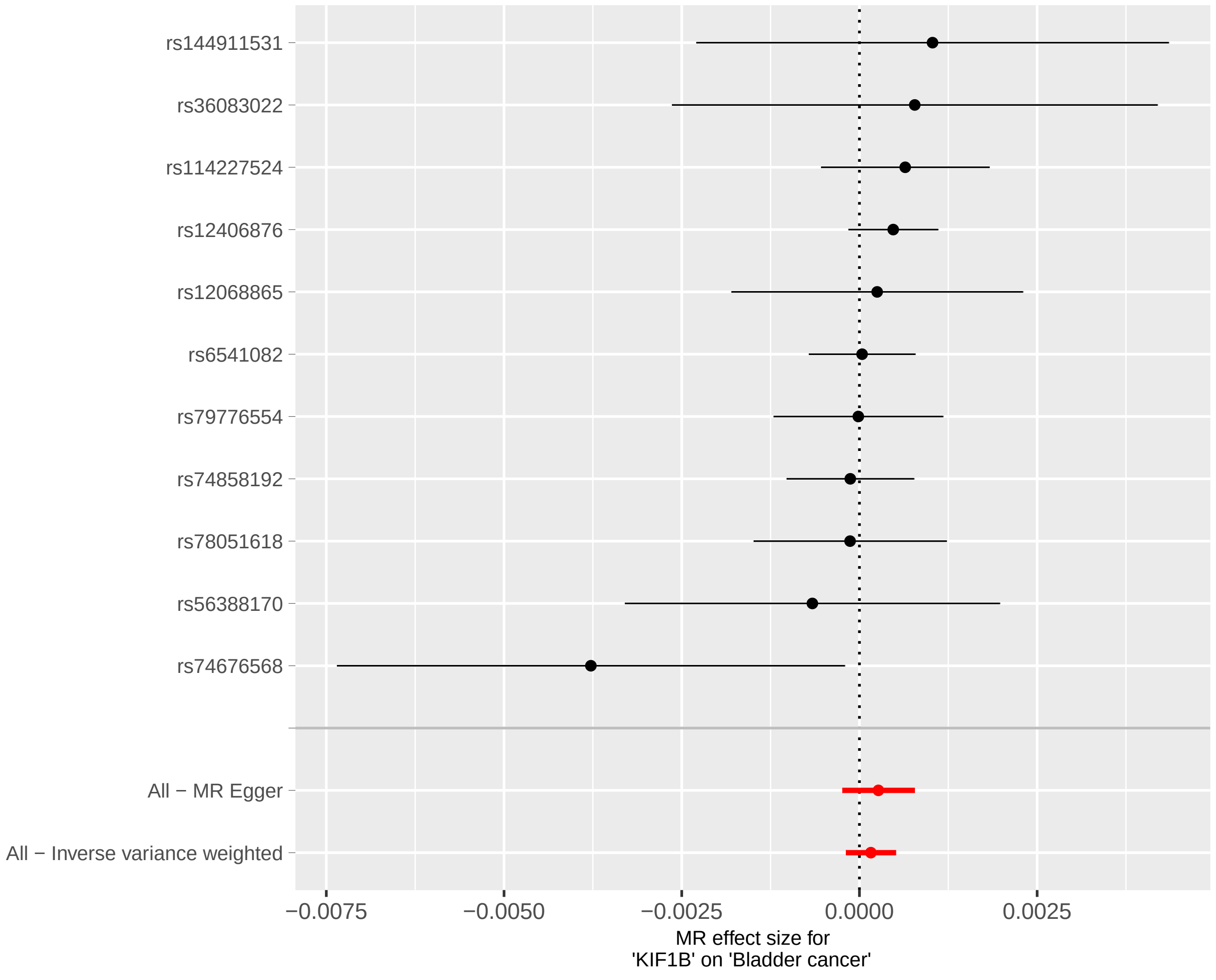
rs144911531
rs36083022
rs114227524
rs12406876
rs12068865
rs6541082
rs79776554
rs74858192
rs78051618
rs56388170
rs74676568
All − MR Egger
All − Inverse variance weighted
−0.0075
−0.0050
−0.0025
0.0000
0.0025
MR effect size for
'KIF1B' on 'Bladder cancer'

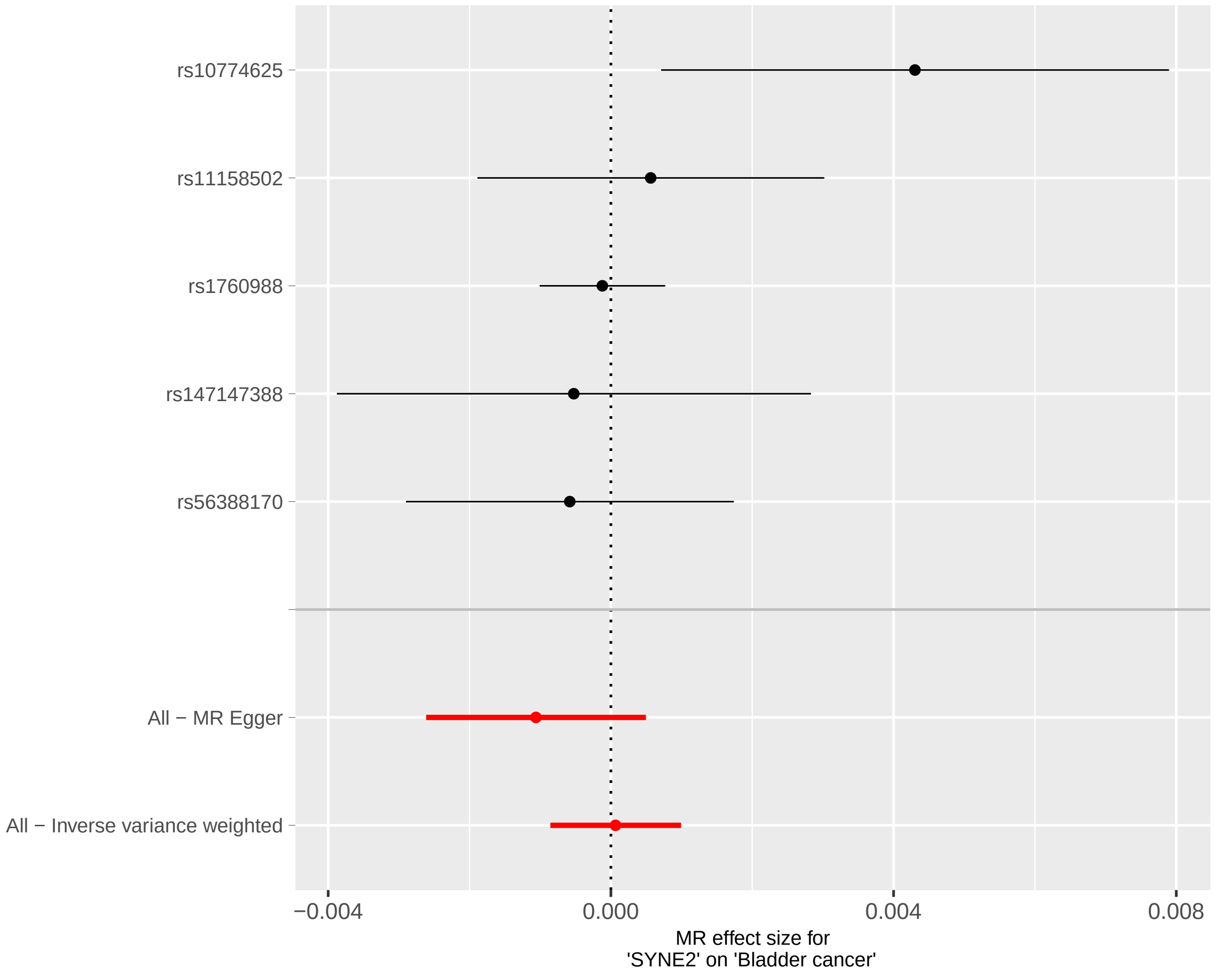

rs10774625
rs11158502
rs1760988
rs147147388
rs56388170
All − MR Egger
All − Inverse variance weighted
−0.004
0.000
0.004
0.008
MR effect size for
'SYNE2' on 'Bladder cancer'

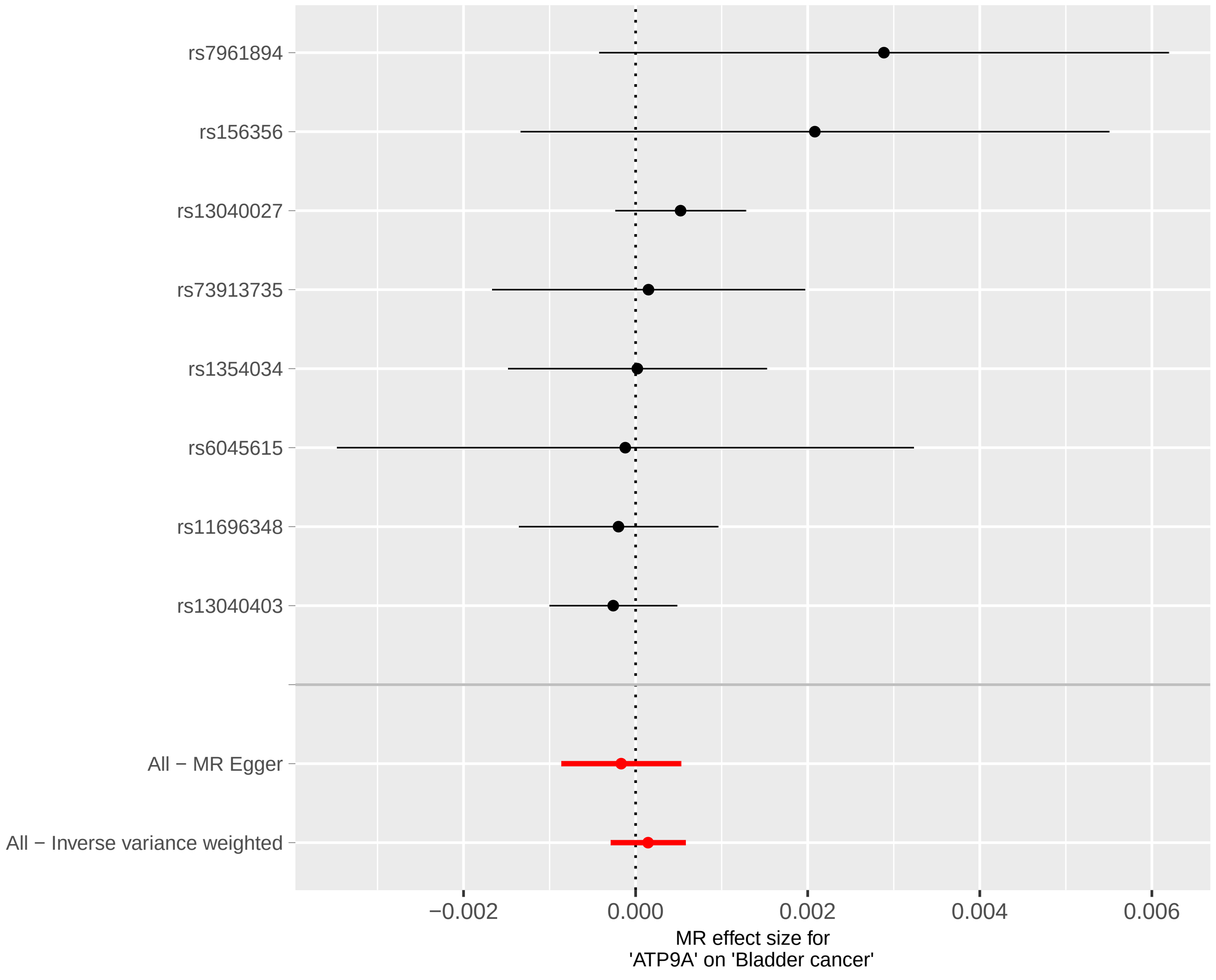

rs7961894
rs156356
rs13040027
rs73913735
rs1354034
rs6045615
rs11696348
rs13040403
All − MR Egger
All − Inverse variance weighted
−0.002
0.000
0.002
0.004
0.006
MR effect size for
'ATP9A' on 'Bladder cancer'

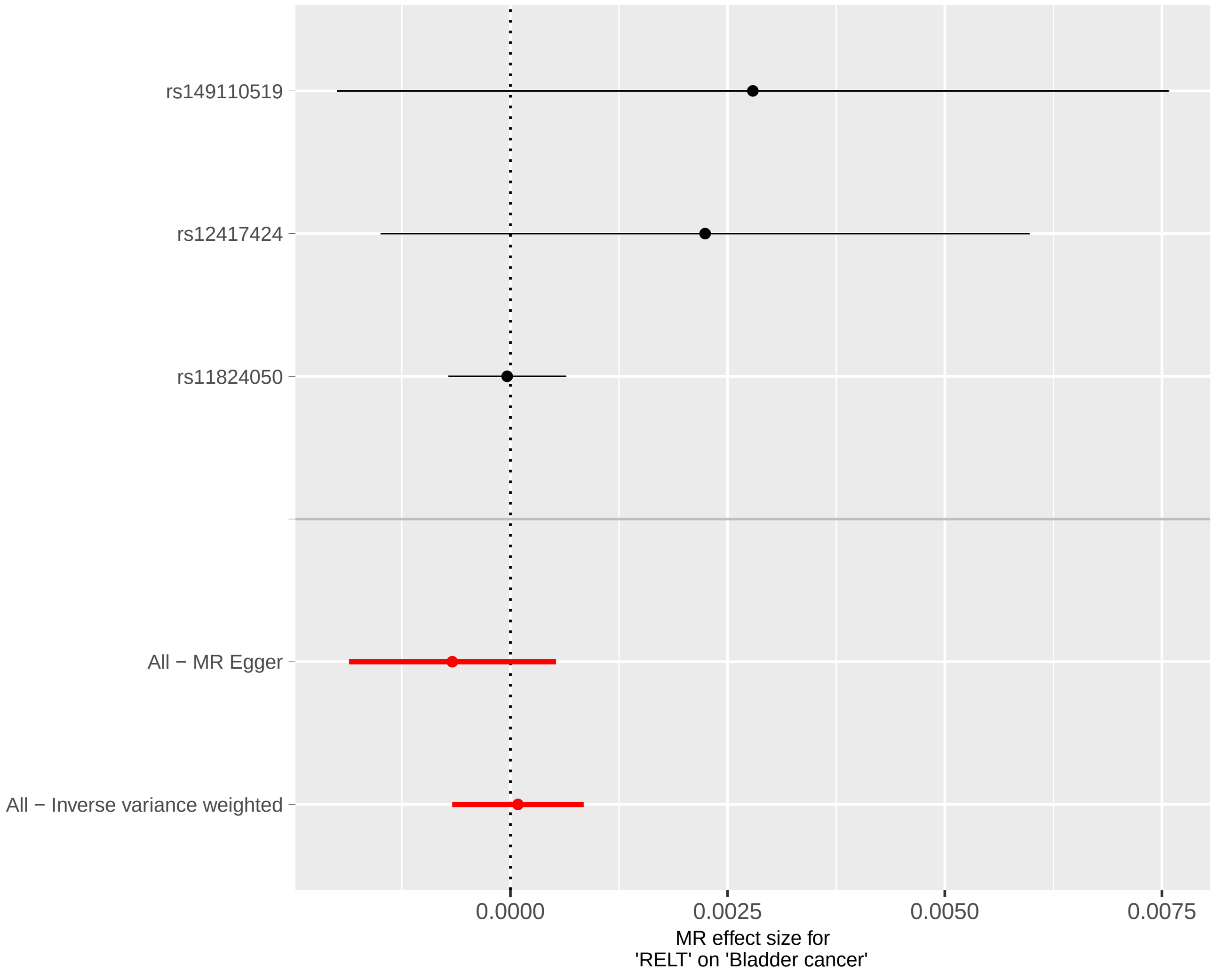
rs149110519
rs12417424
rs11824050
All − MR Egger
All − Inverse variance weighted
0.0000
0.0025
0.0050
0.0075
MR effect size for
'RELT' on 'Bladder cancer'

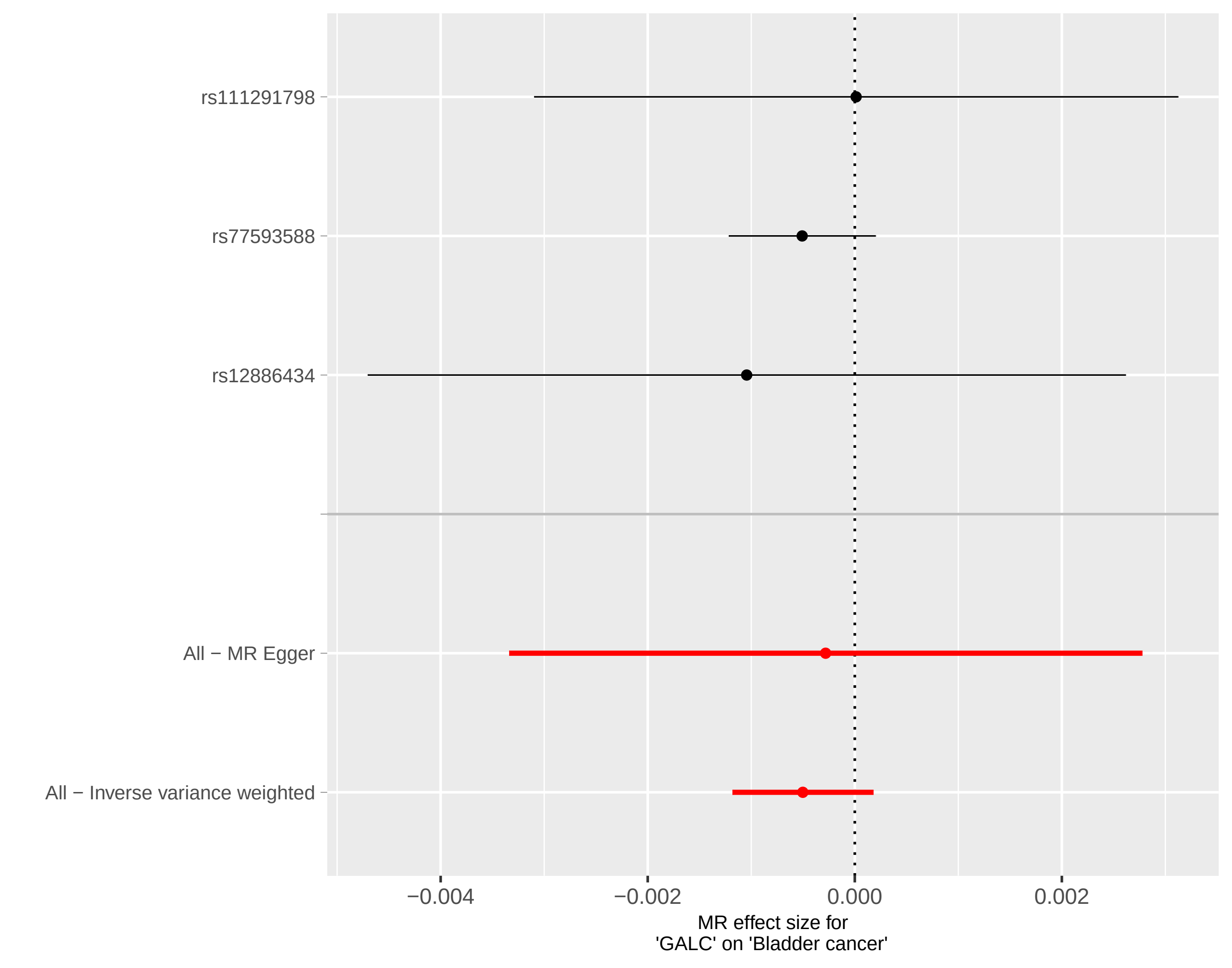

rs111291798
rs77593588
rs12886434
All − MR Egger
All − Inverse variance weighted
−0.004
−0.002
0.000
0.002
MR effect size for
'GALC' on 'Bladder cancer'

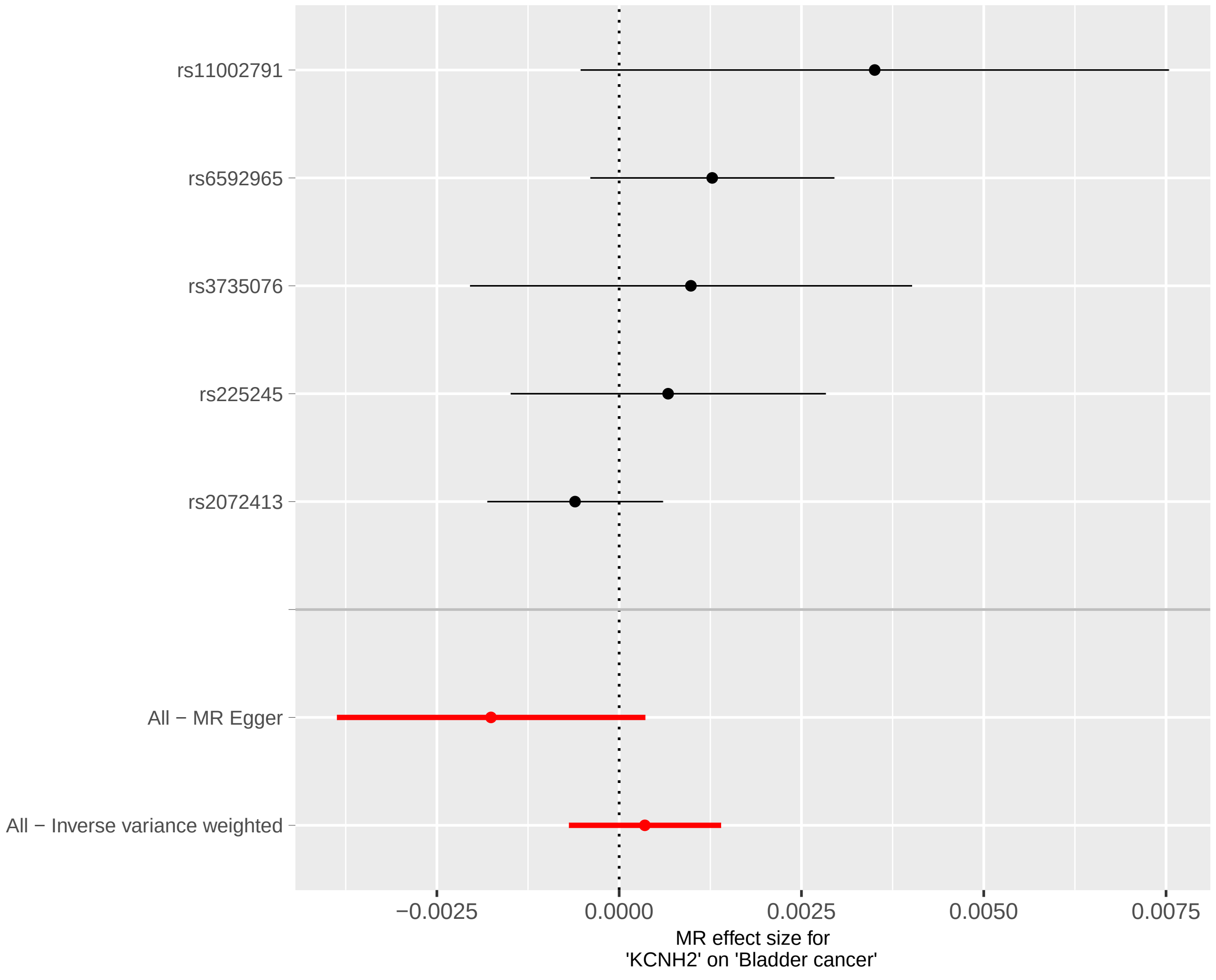

rs11002791
rs6592965
rs3735076
rs225245
rs2072413
All − MR Egger
All − Inverse variance weighted
−0.0025
0.0000
0.0025
0.0050
0.0075
MR effect size for
'KCNH2' on 'Bladder cancer'

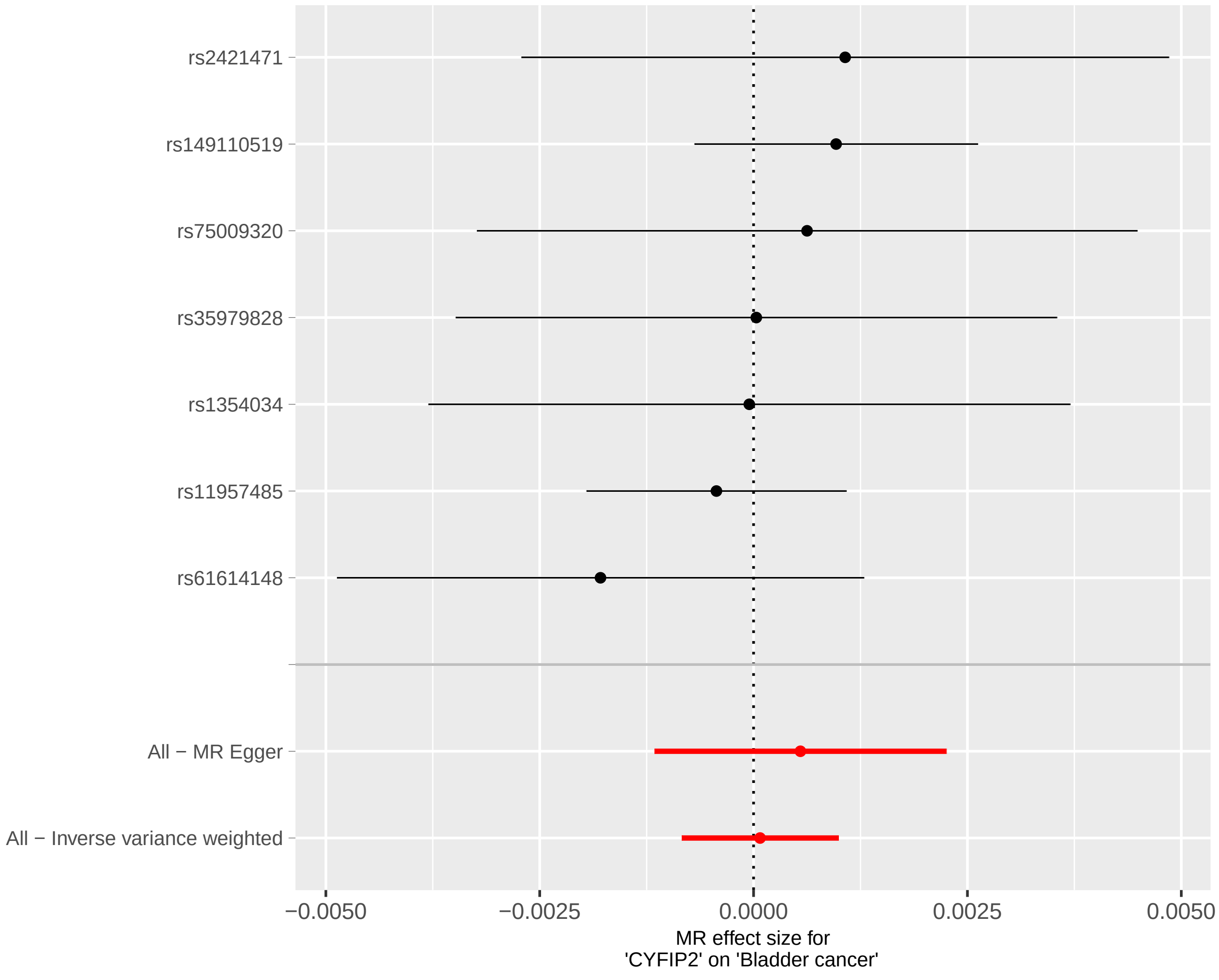

rs2421471
rs149110519
rs75009320
rs35979828
rs1354034
rs11957485
rs61614148
All − MR Egger
All − Inverse variance weighted
−0.0050
−0.0025
0.0000
0.0025
0.0050
MR effect size for
'CYFIP2' on 'Bladder cancer'

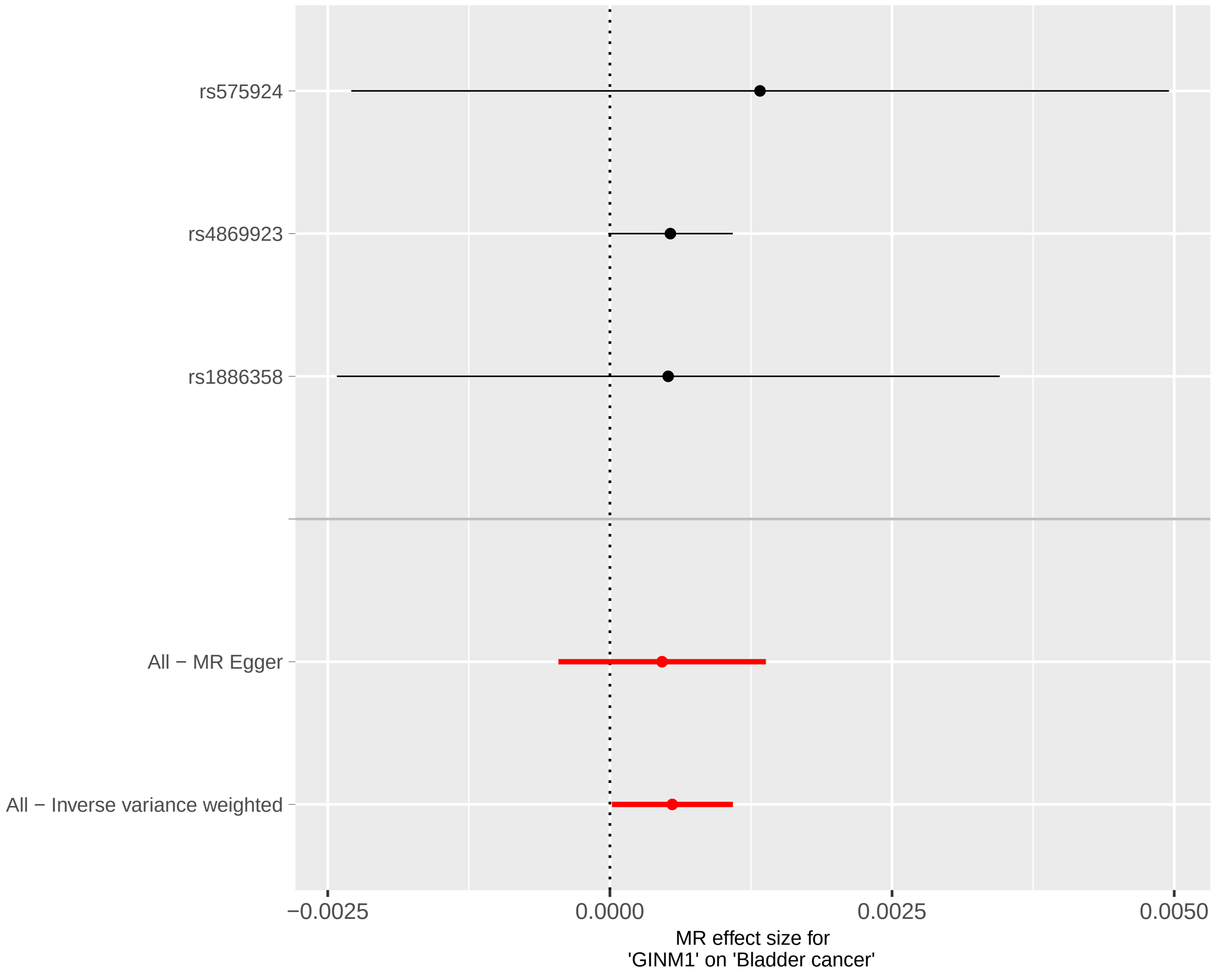

rs575924
rs4869923
rs1886358
All − MR Egger
All − Inverse variance weighted
−0.0025
0.0000
0.0025
0.0050
MR effect size for
'GINM1' on 'Bladder cancer'

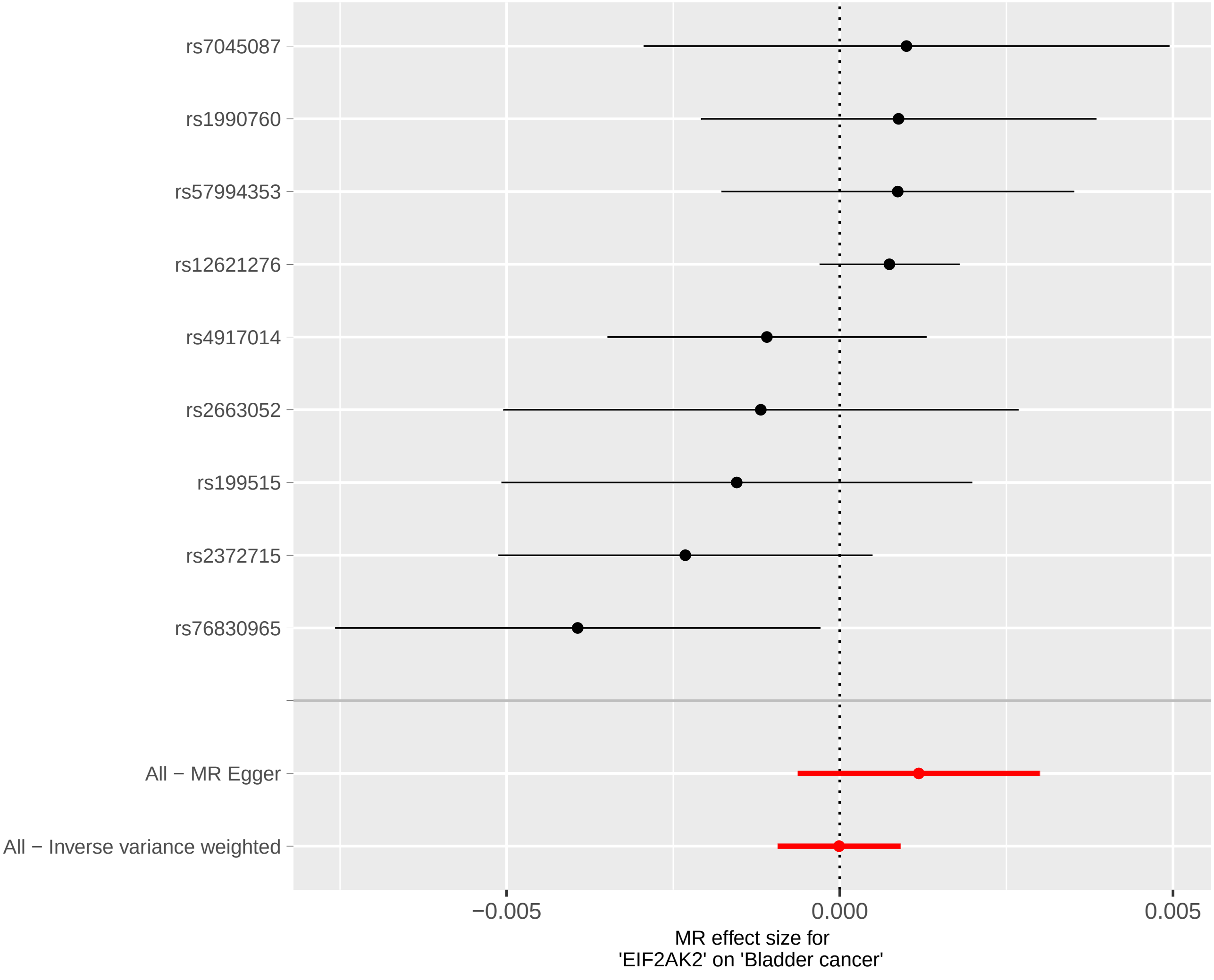
rs7045087
rs1990760
rs57994353
rs12621276
rs4917014
rs2663052
rs199515
rs2372715
rs76830965
All − MR Egger
All − Inverse variance weighted
−0.005
0.000
0.005
MR effect size for
'EIF2AK2' on 'Bladder cancer'

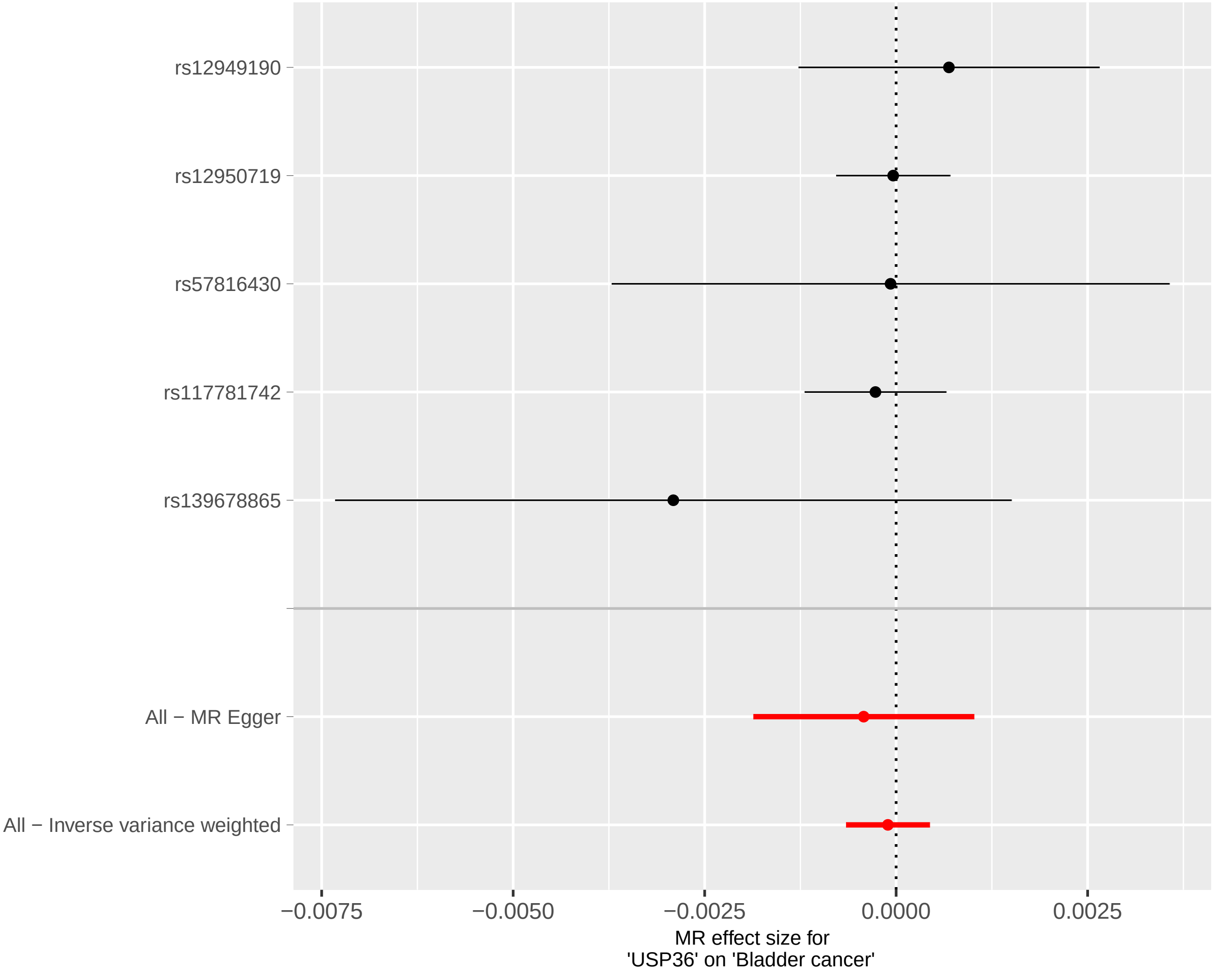
rs12949190
rs12950719
rs57816430
rs117781742
rs139678865
All − MR Egger
All − Inverse variance weighted
−0.0075
−0.0050
−0.0025
0.0000
0.0025
MR effect size for
'USP36' on 'Bladder cancer'

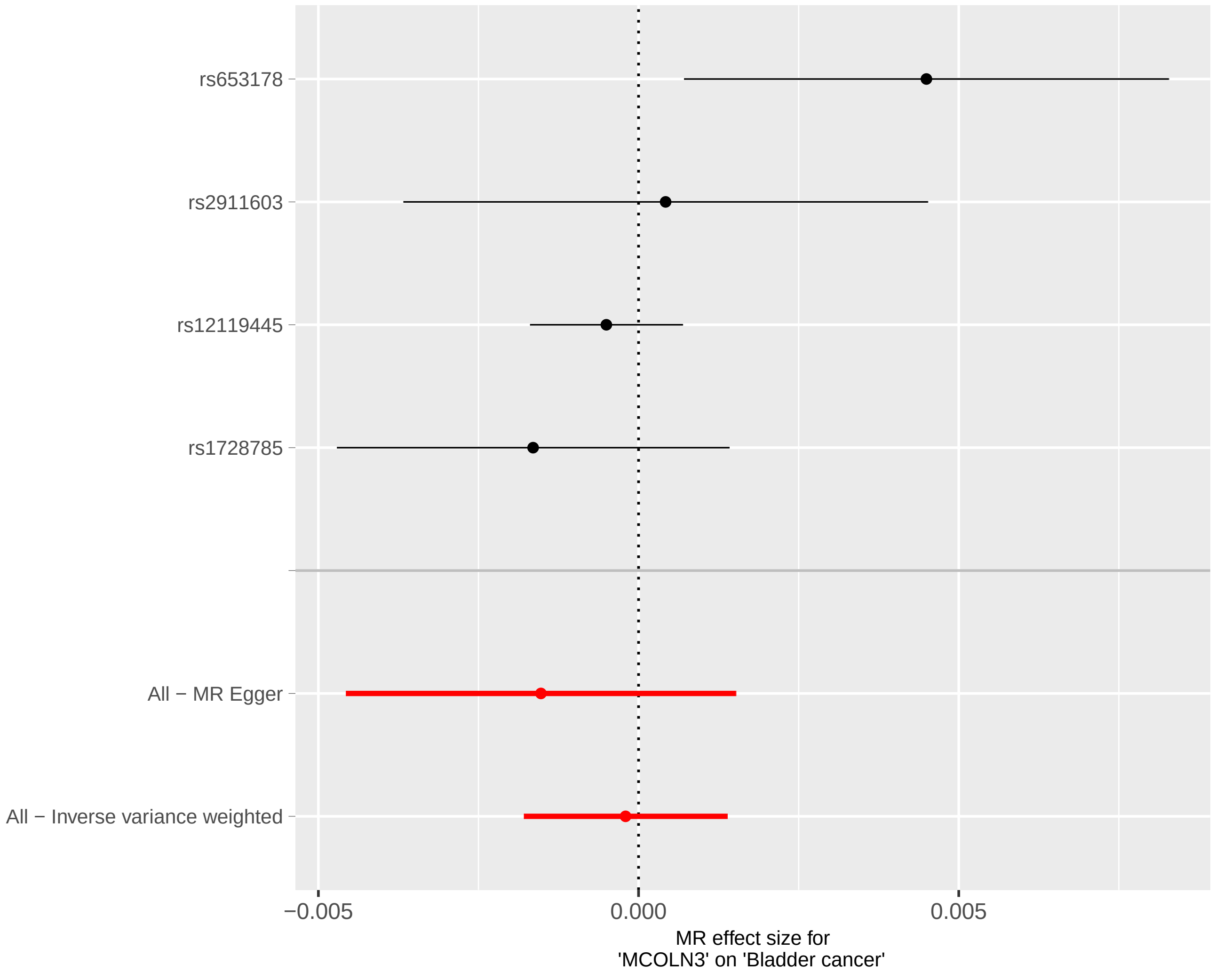
rs653178
rs2911603
rs12119445
rs1728785
All − MR Egger
All − Inverse variance weighted
−0.005
0.000
0.005
MR effect size for
'MCOLN3' on 'Bladder cancer'

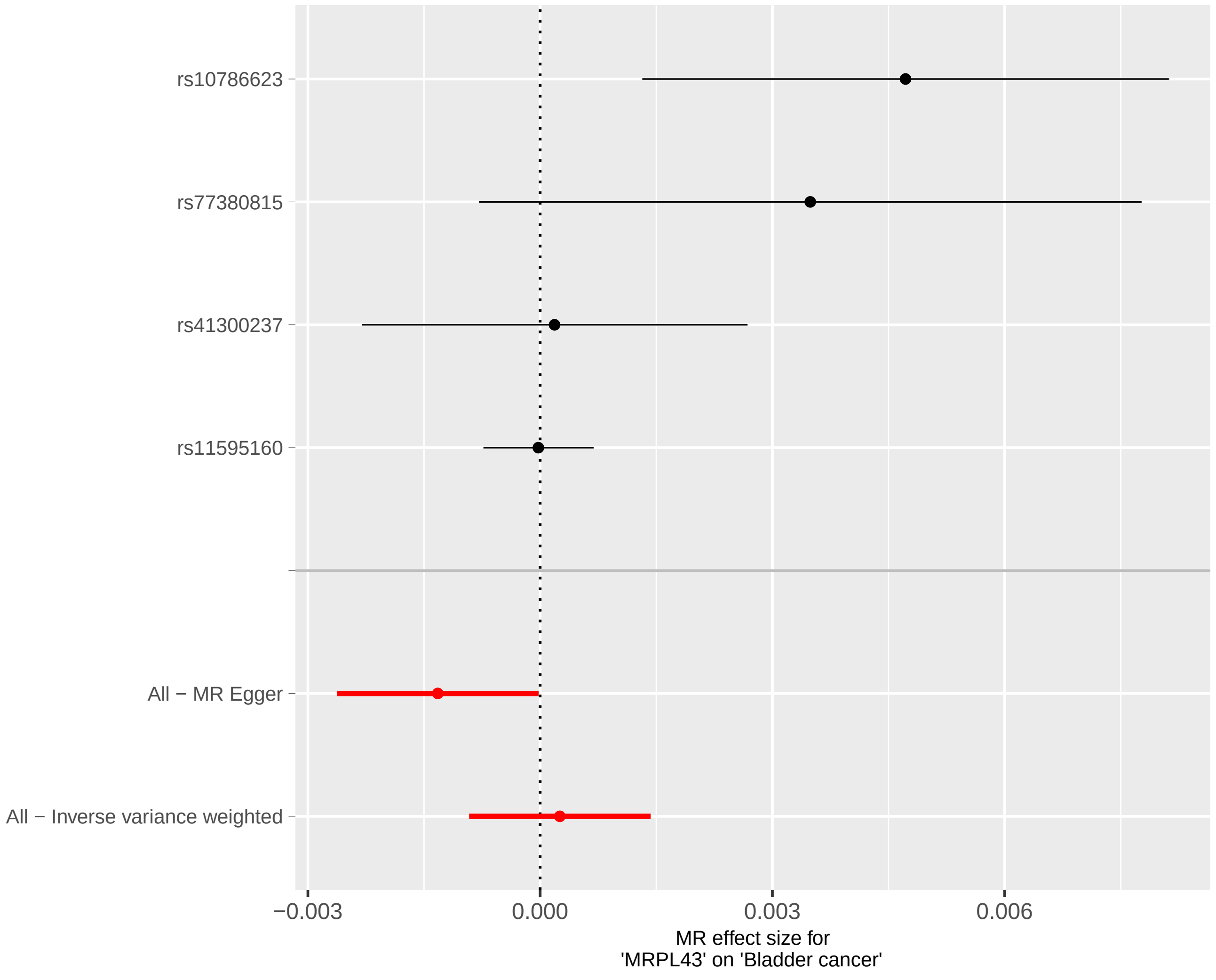

rs10786623
rs77380815
rs41300237
rs11595160
All − MR Egger
All − Inverse variance weighted
−0.003
0.000
0.003
0.006
MR effect size for
'MRPL43' on 'Bladder cancer'

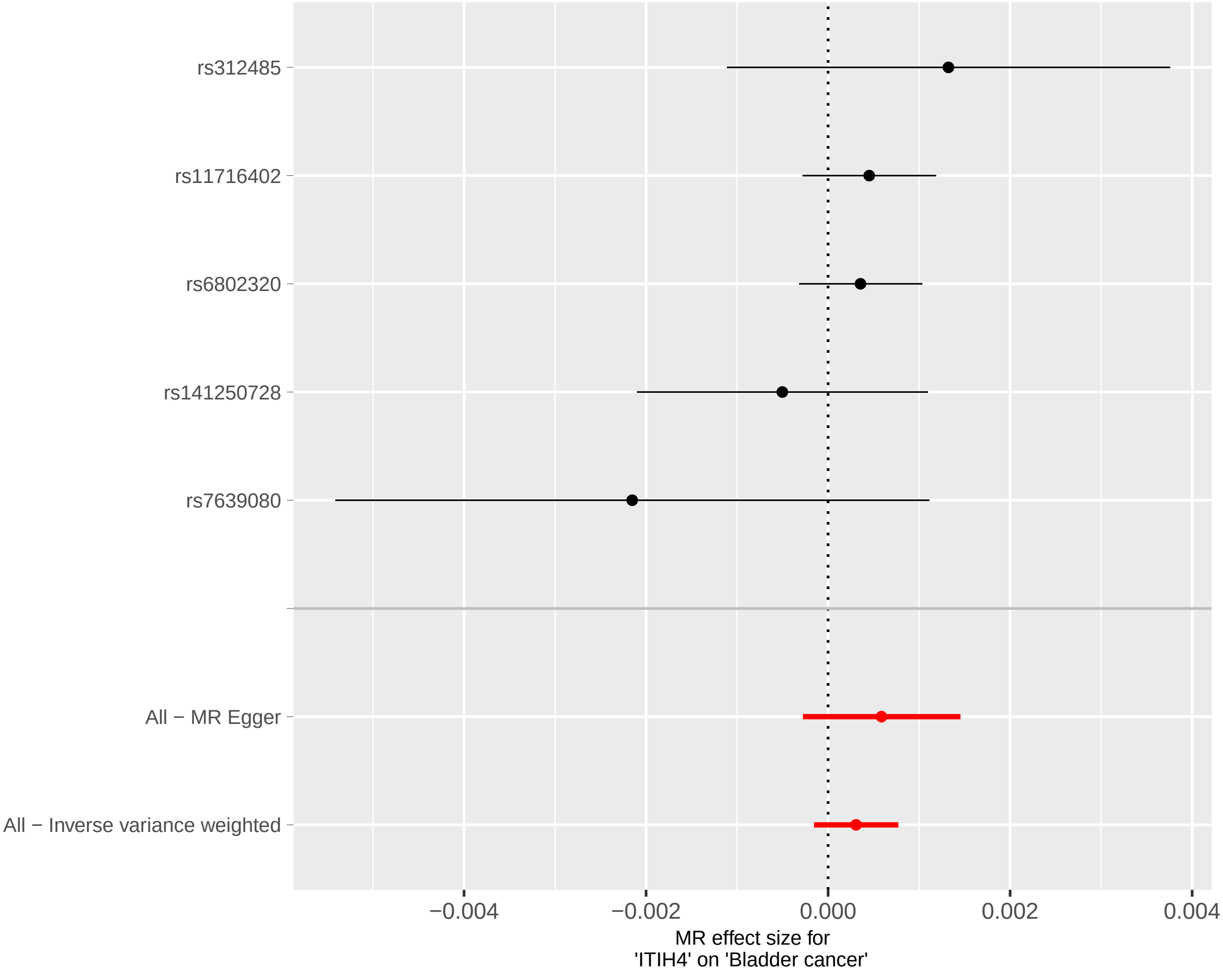

rs312485
rs11716402
rs6802320
rs141250728
rs7639080
All − MR Egger
All − Inverse variance weighted
−0.004
−0.002
0.000
0.002
0.004
MR effect size for
'ITIH4' on 'Bladder cancer'

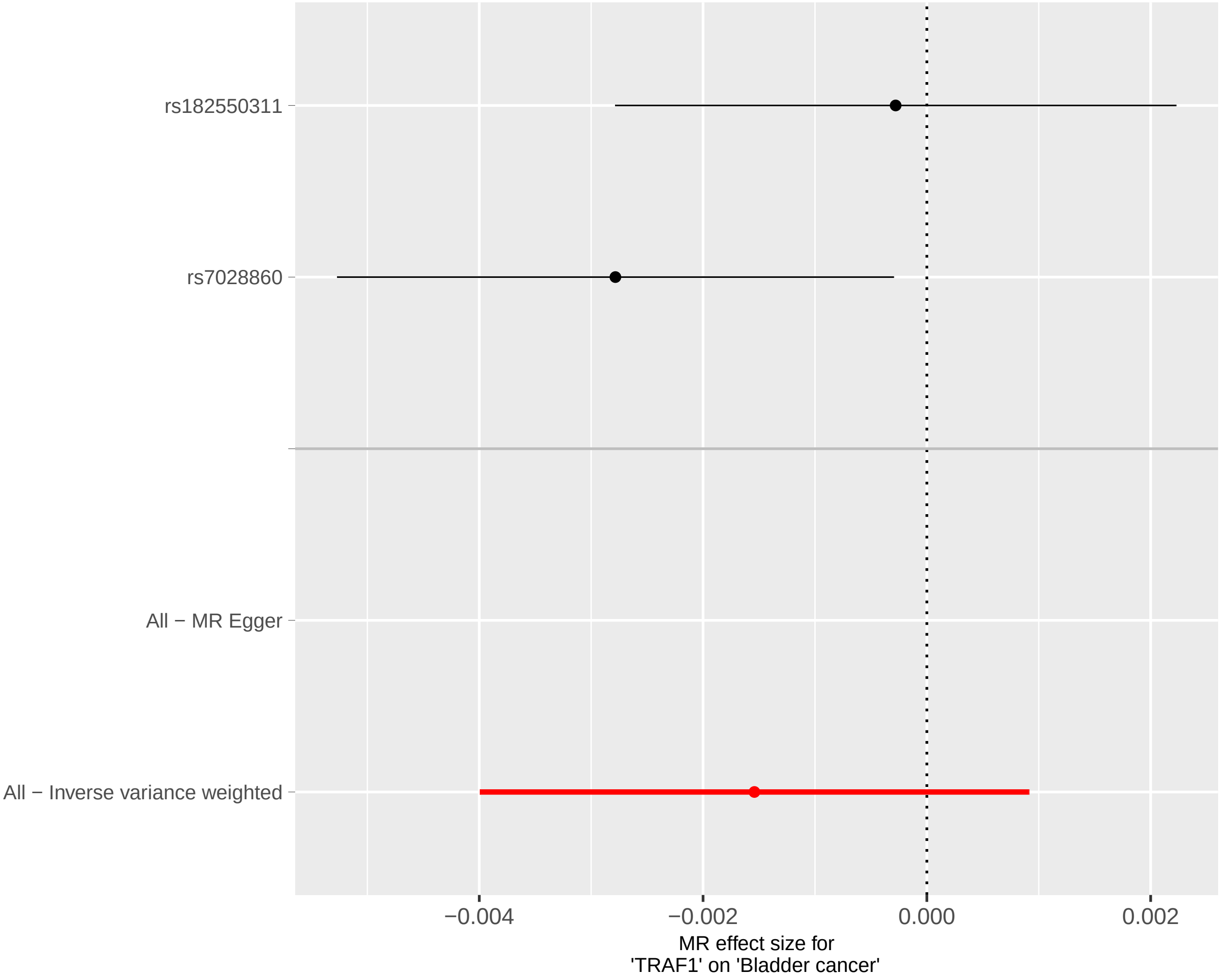

rs182550311
rs7028860
All − MR Egger
All − Inverse variance weighted
−0.004
−0.002
0.000
0.002
MR effect size for
'TRAF1' on 'Bladder cancer'

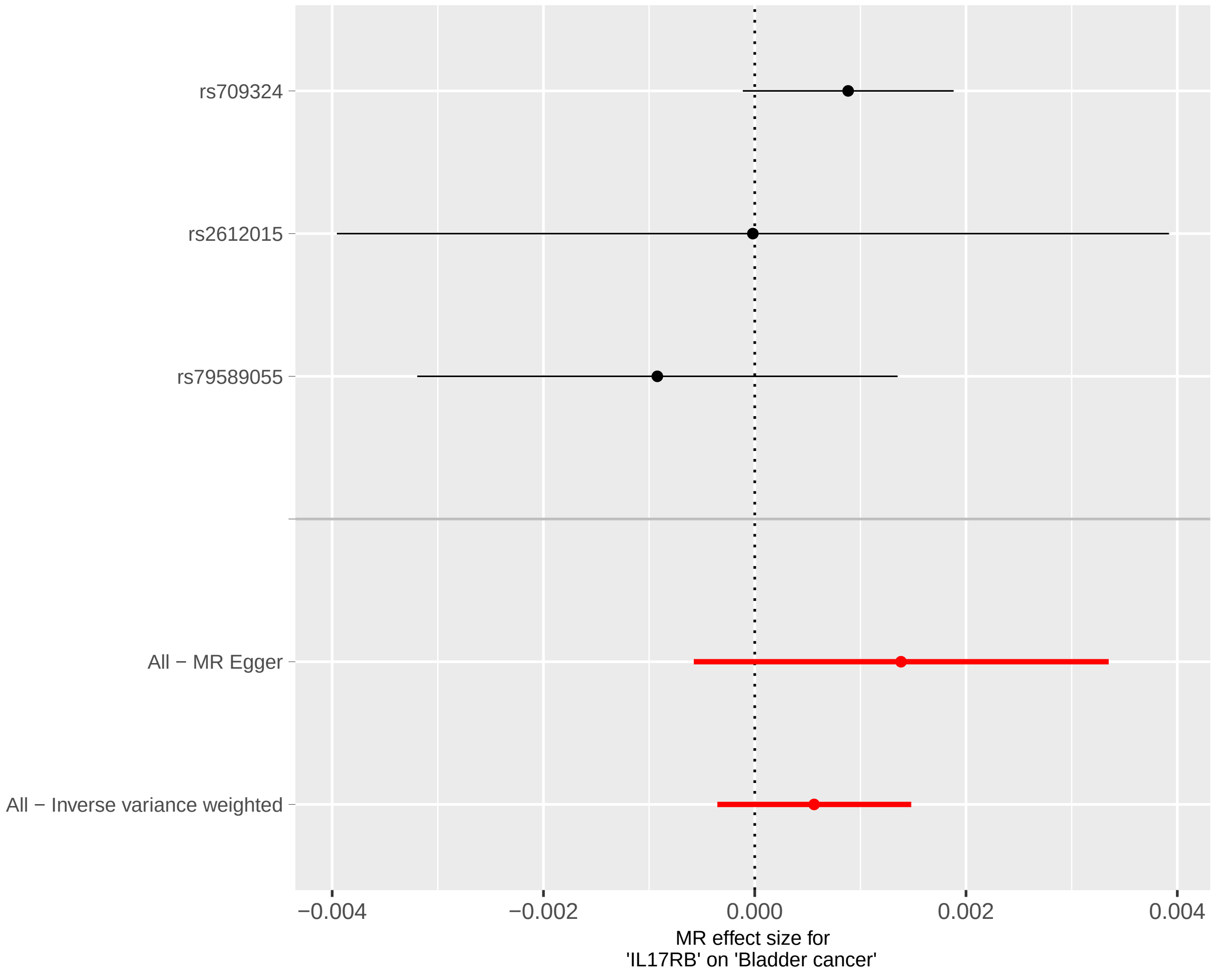
rs709324
rs2612015
rs79589055
All − MR Egger
All − Inverse variance weighted
−0.004
−0.002
0.000
0.002
0.004
MR effect size for
'IL17RB' on 'Bladder cancer'

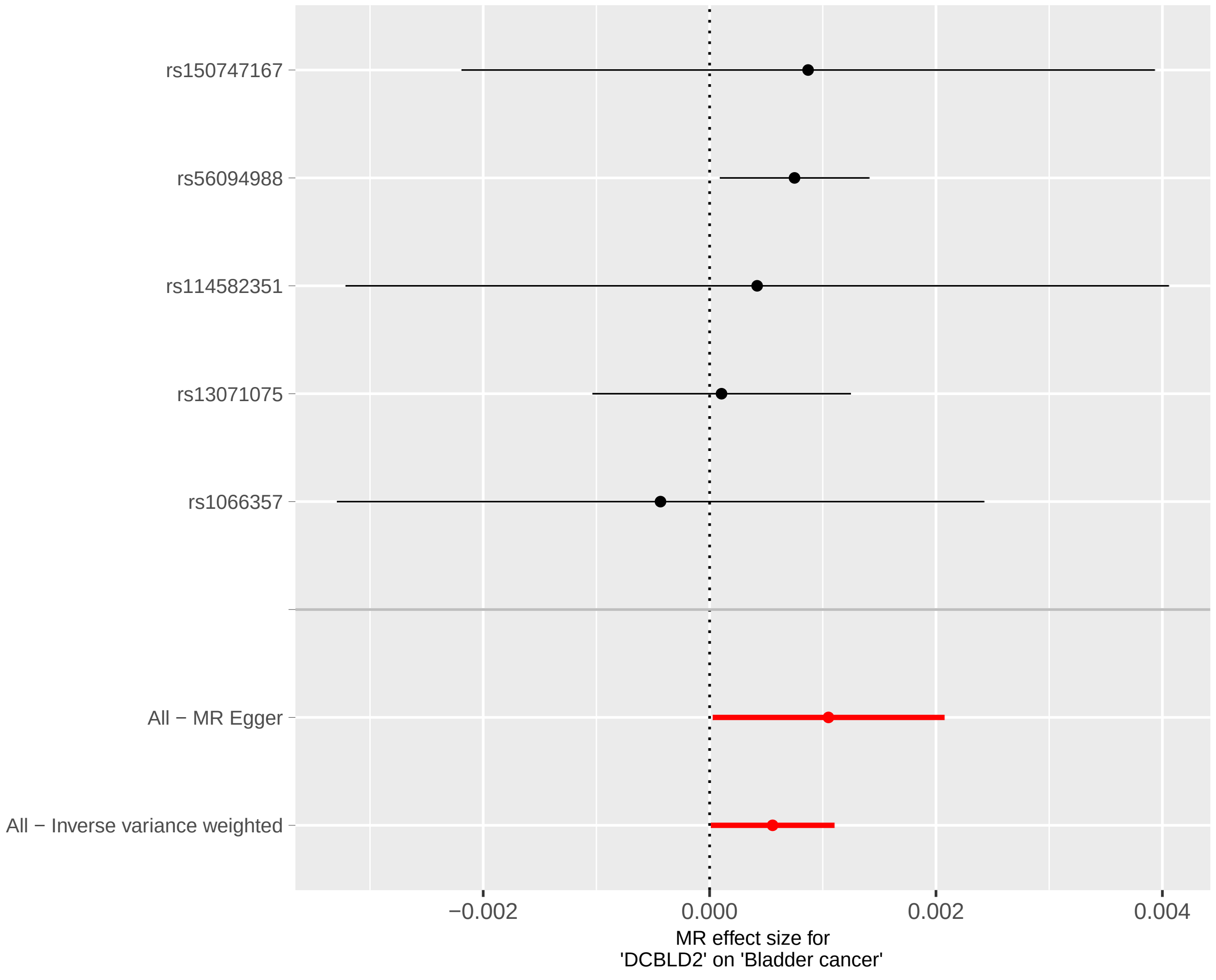

rs150747167
rs56094988
rs114582351
rs13071075
rs1066357
All − MR Egger
All − Inverse variance weighted
−0.002
0.000
0.002
0.004
MR effect size for
'DCBLD2' on 'Bladder cancer'

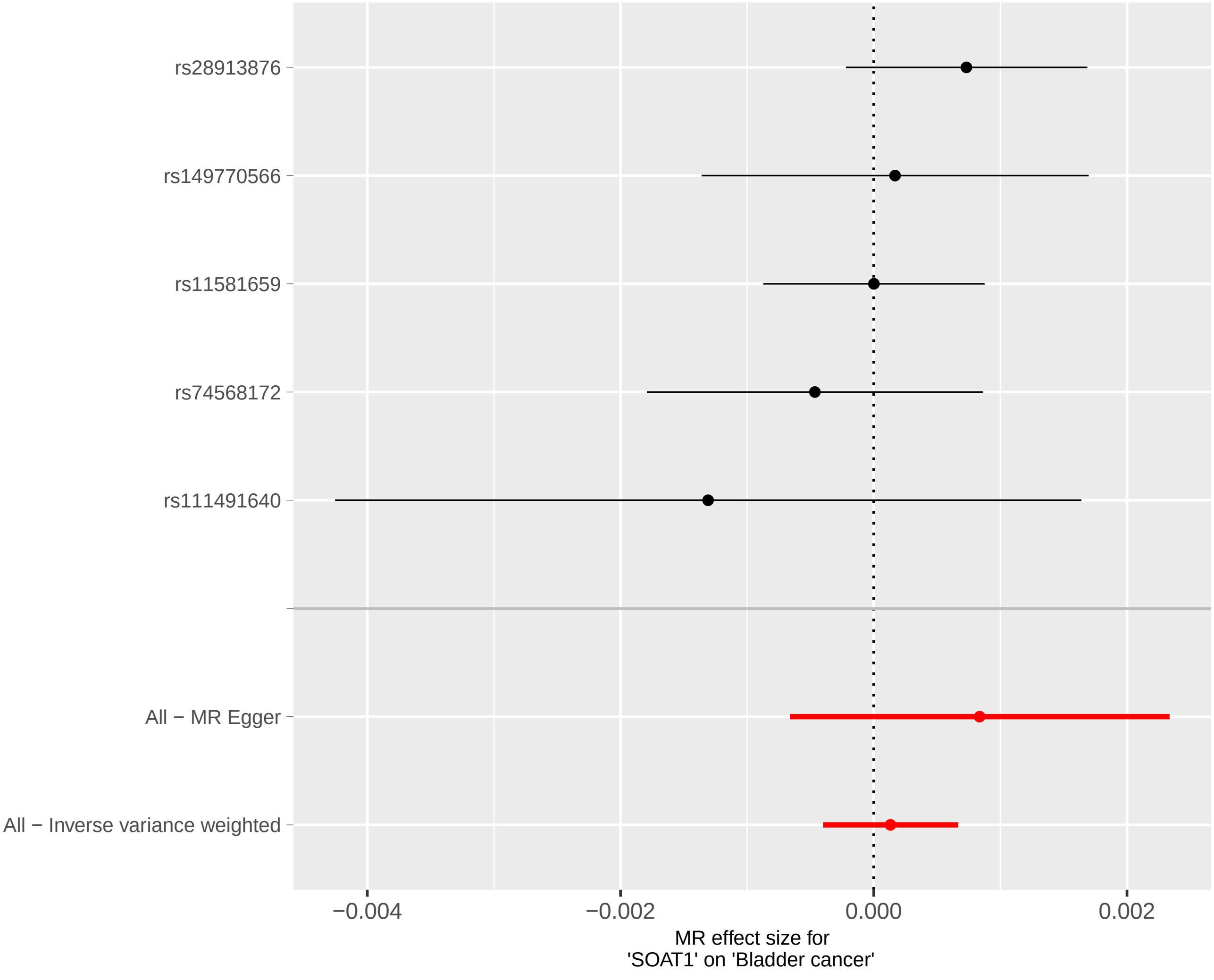
rs28913876
rs149770566
rs11581659
rs74568172
rs111491640
All − MR Egger
All − Inverse variance weighted
−0.004
−0.002
0.000
0.002
MR effect size for
'SOAT1' on 'Bladder cancer'

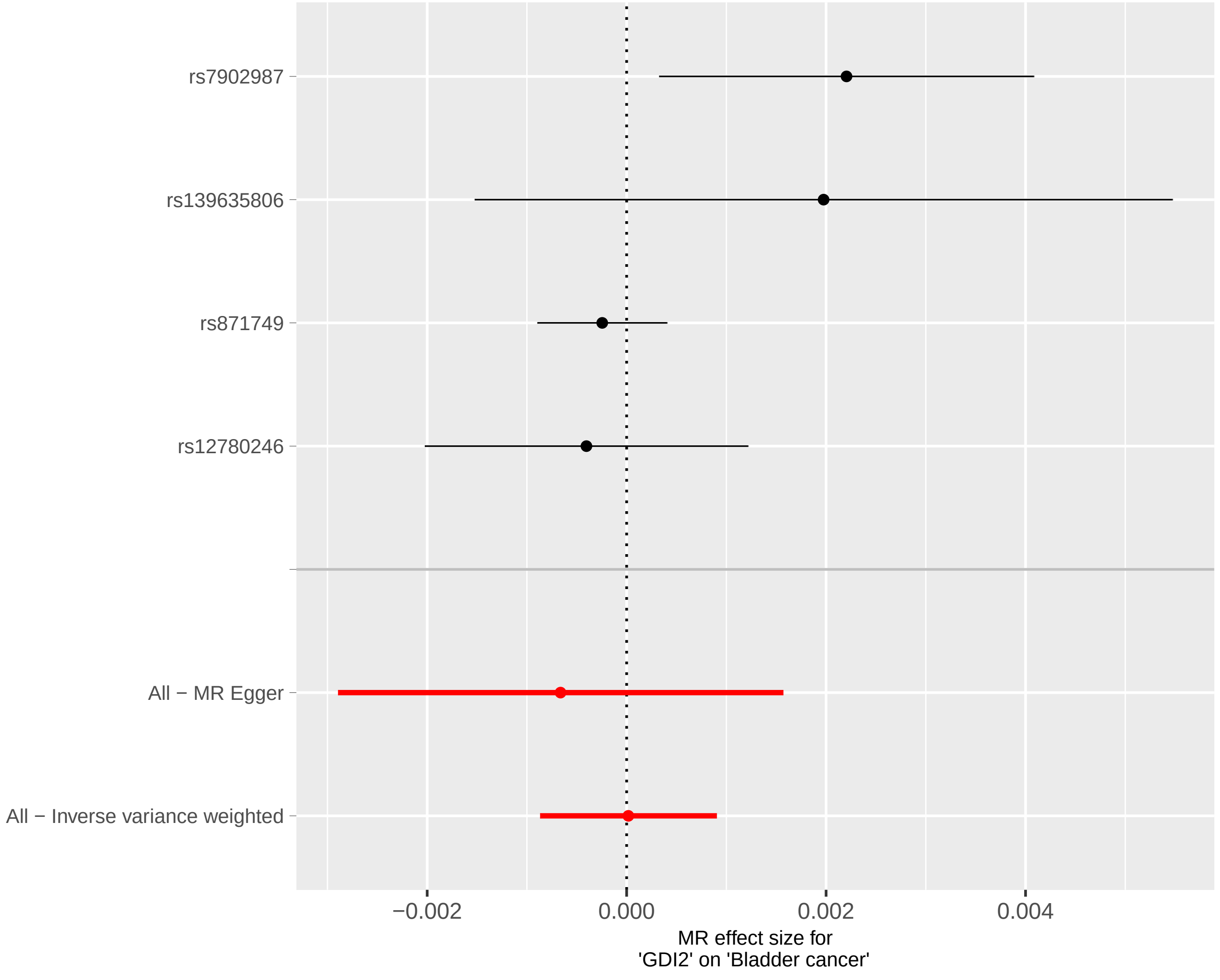
rs7902987
rs139635806
rs871749
rs12780246
All − MR Egger
All − Inverse variance weighted
−0.002
0.000
0.002
0.004
MR effect size for
'GDI2' on 'Bladder cancer'

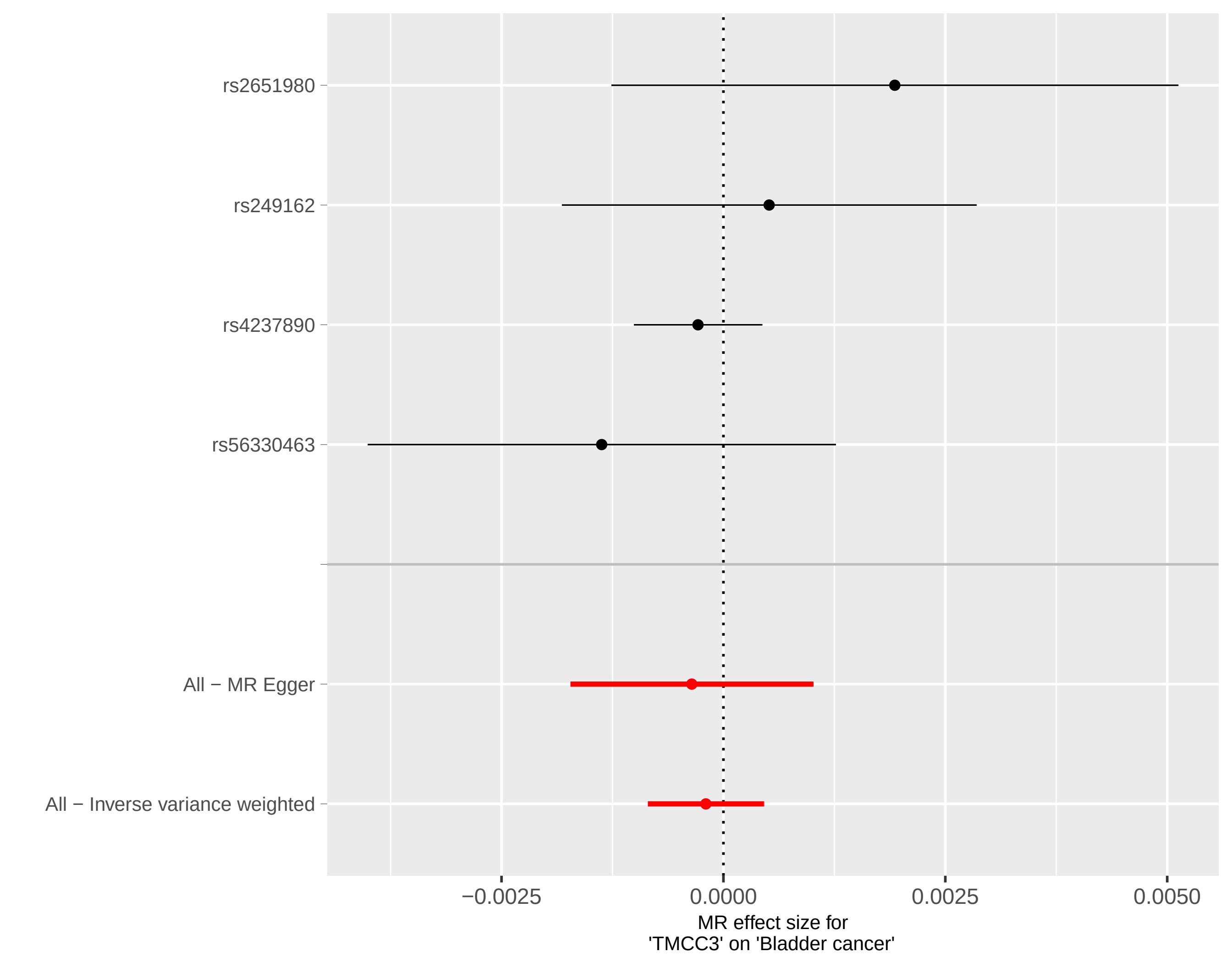

rs2651980
rs249162
rs4237890
rs56330463
All − MR Egger
All − Inverse variance weighted
−0.0025
0.0000
0.0025
0.0050
MR effect size for
'TMCC3' on 'Bladder cancer'

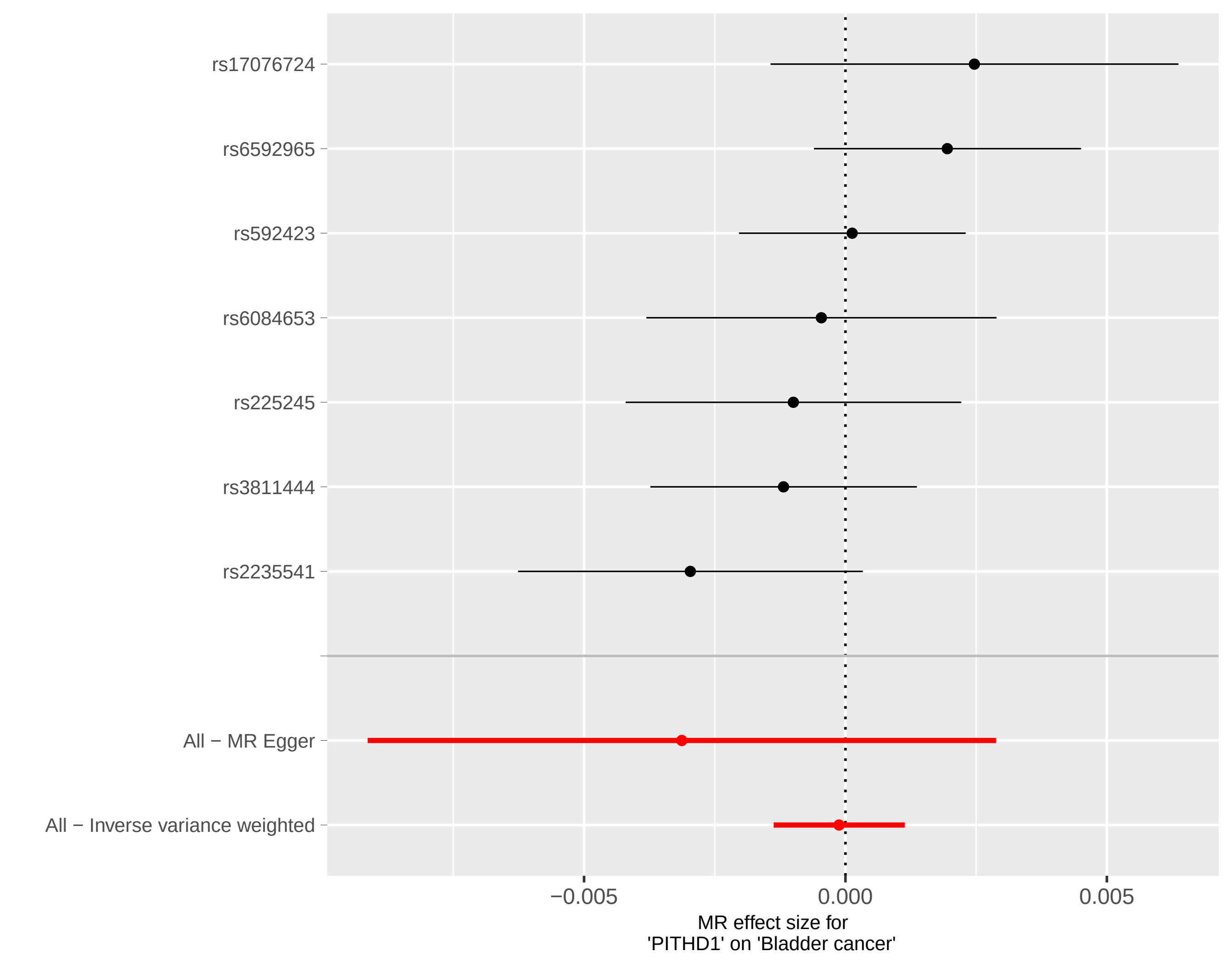

rs17076724
rs6592965
rs592423
rs6084653
rs225245
rs3811444
rs2235541
All − MR Egger
All − Inverse variance weighted
−0.005
0.000
0.005
MR effect size for
'PITHD1' on 'Bladder cancer'

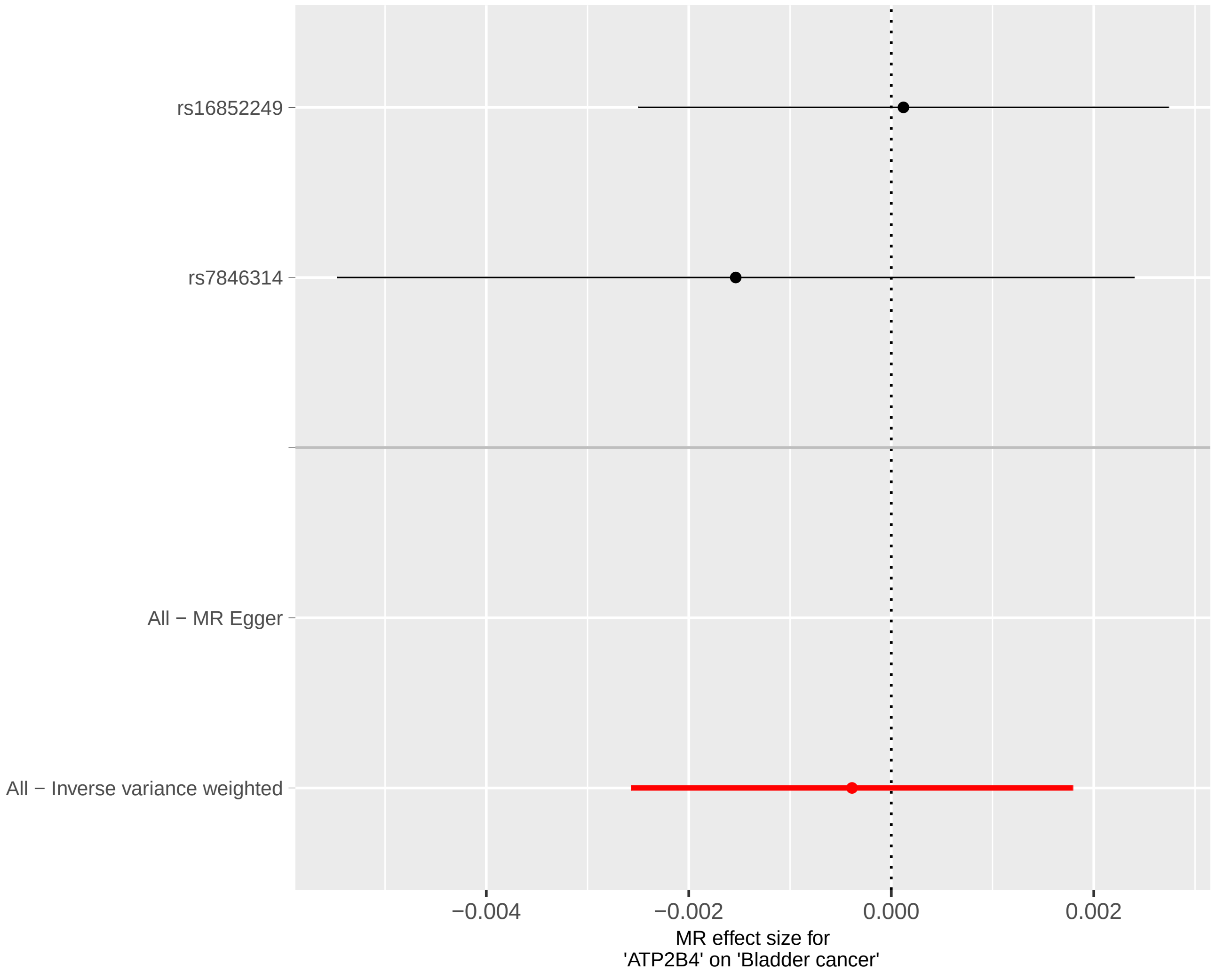

rs16852249
rs7846314
All − MR Egger
All − Inverse variance weighted
−0.004
−0.002
0.000
0.002
MR effect size for
'ATP2B4' on 'Bladder cancer'

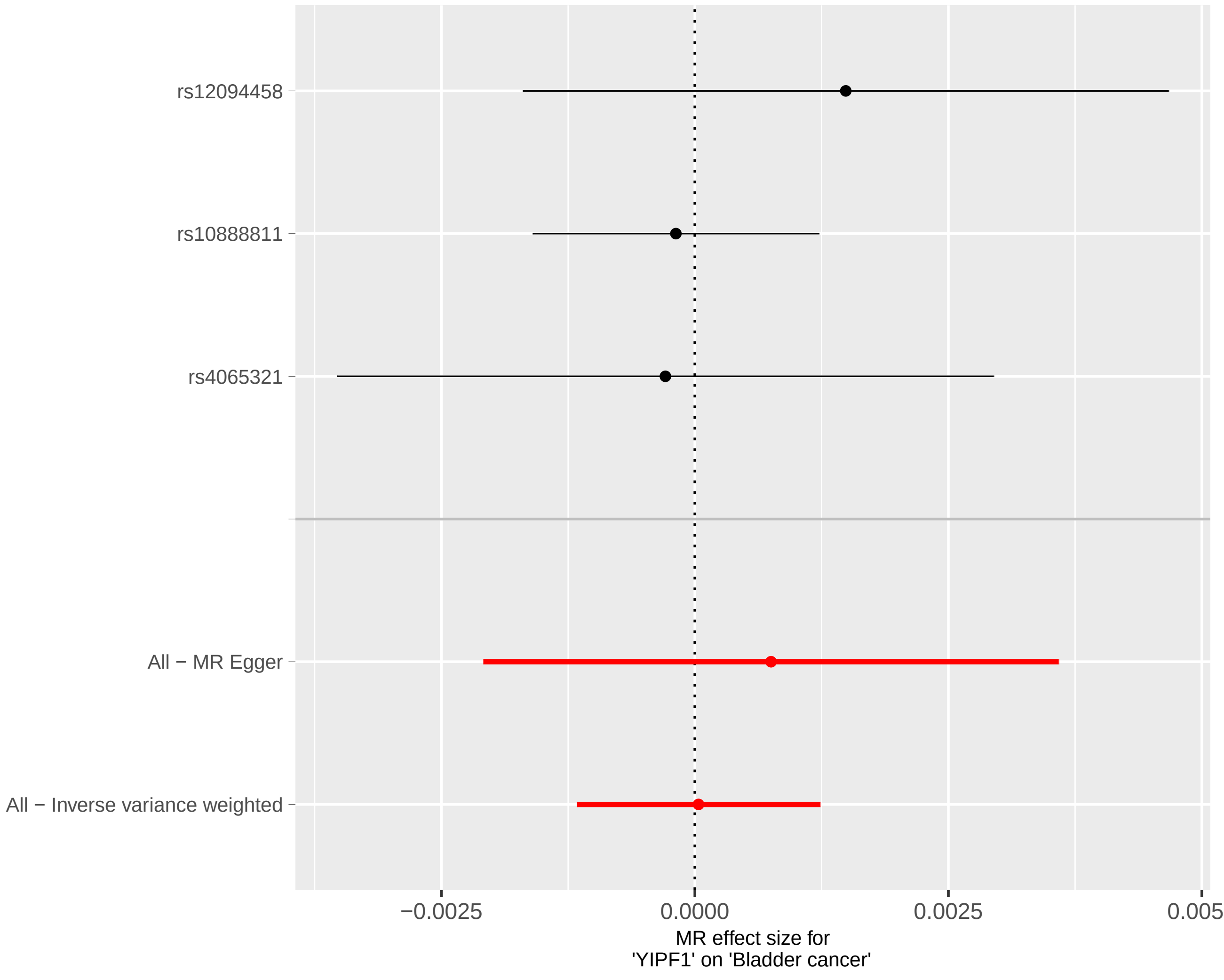

rs12094458
rs10888811
rs4065321
All − MR Egger
All − Inverse variance weighted
−0.0025
0.0000
0.0025
0.005
MR effect size for
'YIPF1' on 'Bladder cancer'

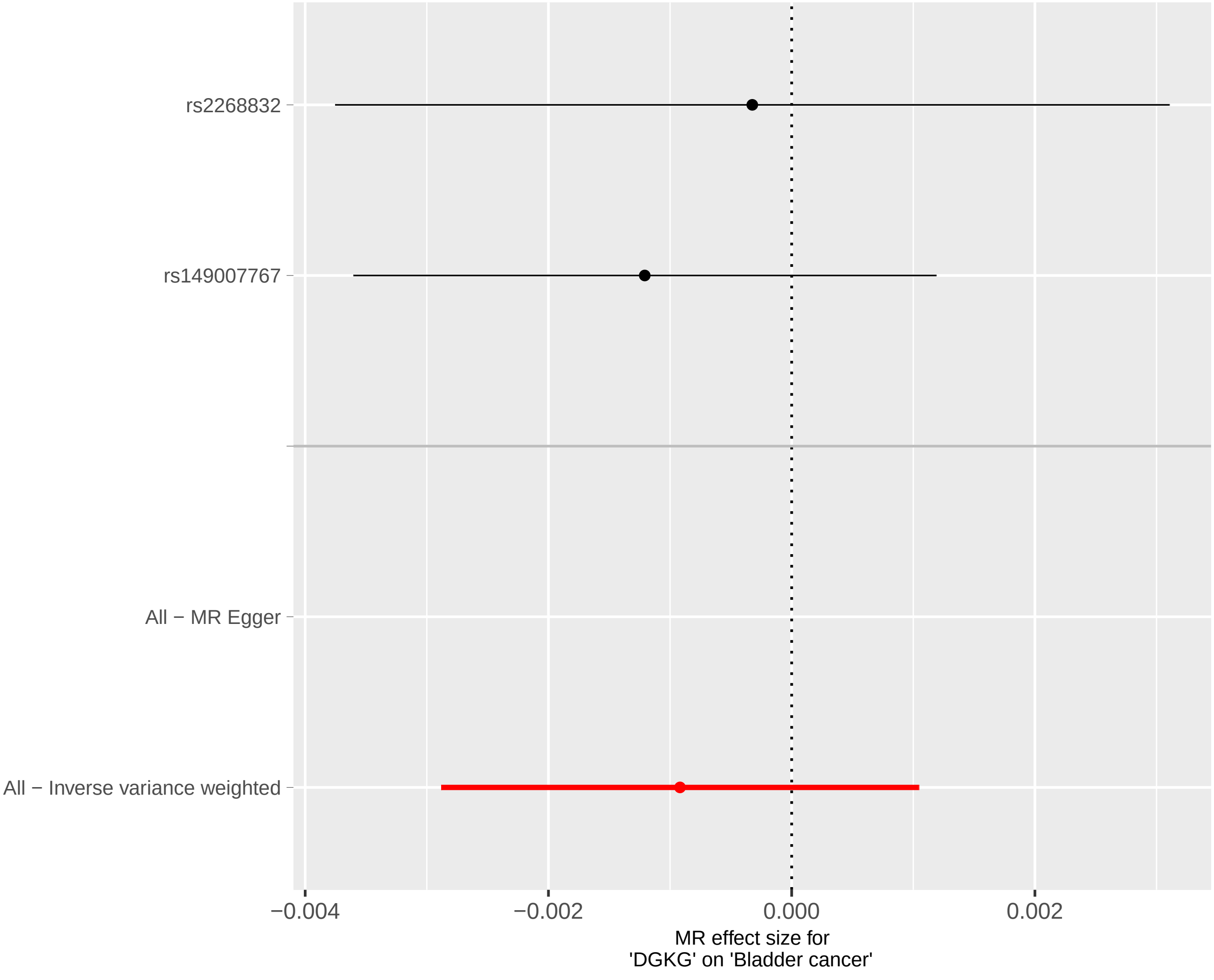

rs2268832
rs149007767
All − MR Egger
All − Inverse variance weighted
−0.004
−0.002
0.000
0.002
MR effect size for
'DGKG' on 'Bladder cancer'

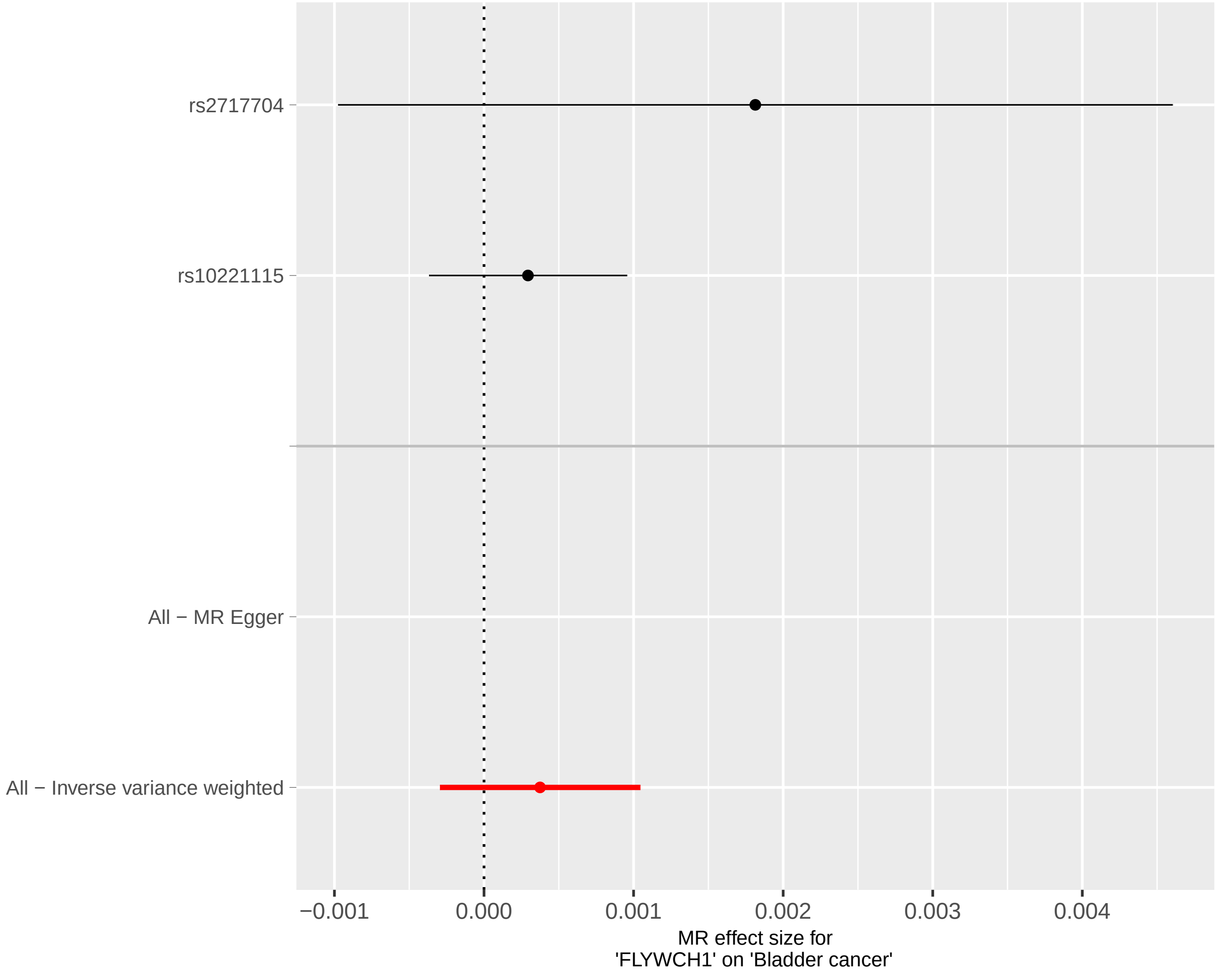
rs2717704
rs10221115
All − MR Egger
All − Inverse variance weighted
−0.001
0.000
0.001
0.002
0.003
0.004
MR effect size for
'FLYWCH1' on 'Bladder cancer'

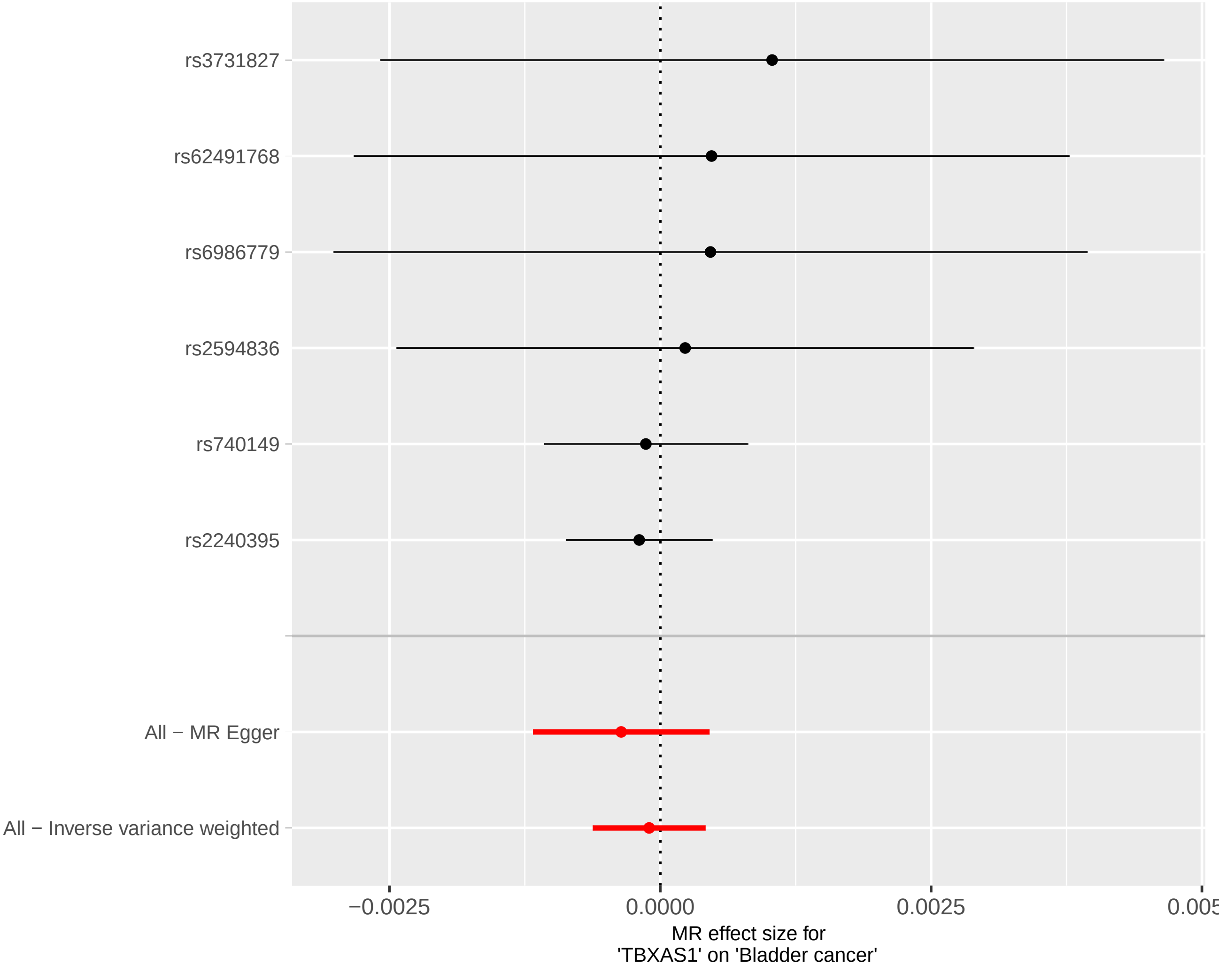

rs3731827
rs62491768
rs6986779
rs2594836
rs740149
rs2240395
All − MR Egger
All − Inverse variance weighted
−0.0025
0.0000
0.0025
0.005
MR effect size for
'TBXAS1' on 'Bladder cancer'

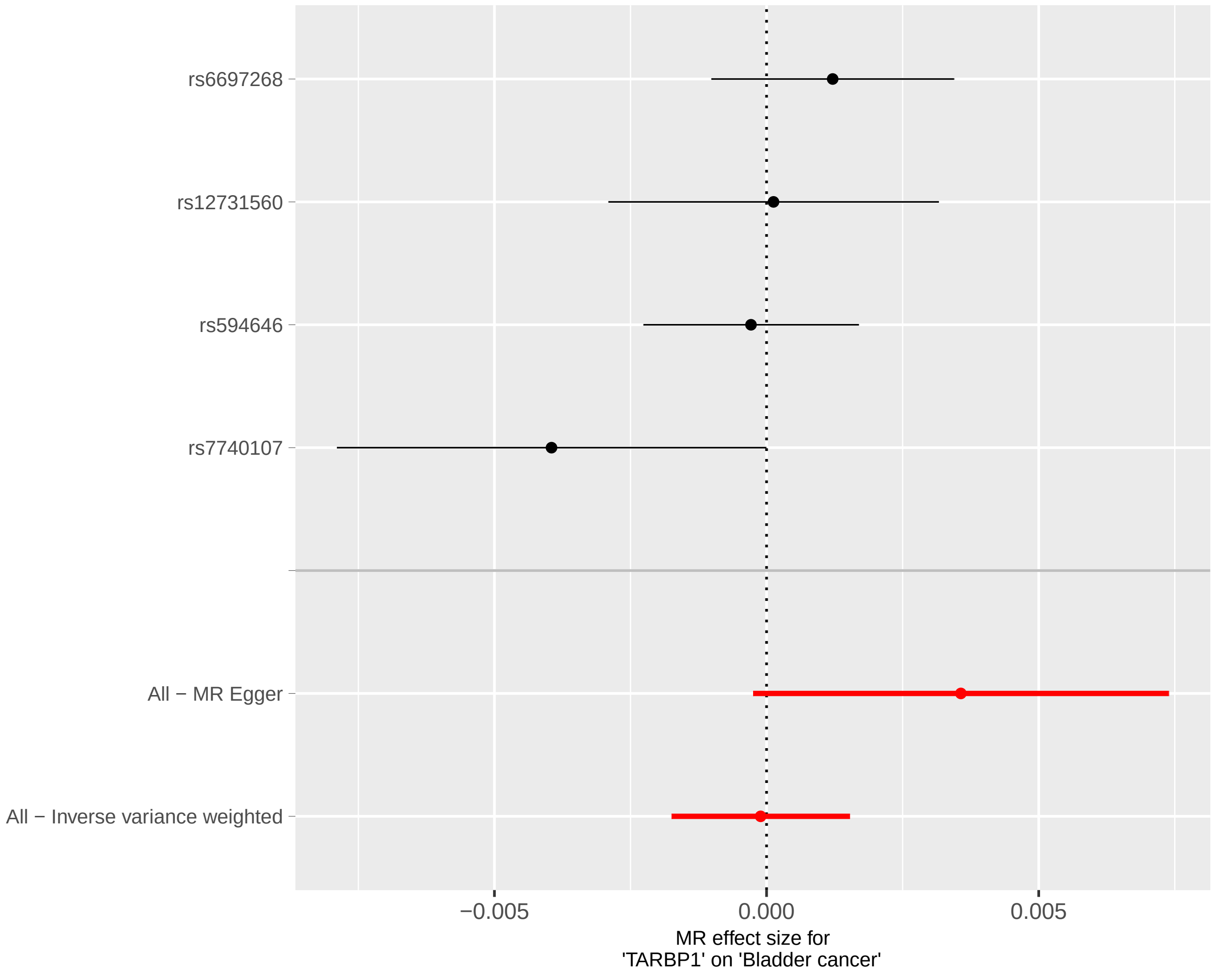

rs6697268
rs12731560
rs594646
rs7740107
All − MR Egger
All − Inverse variance weighted
−0.005
0.000
0.005
MR effect size for
'TARBP1' on 'Bladder cancer'

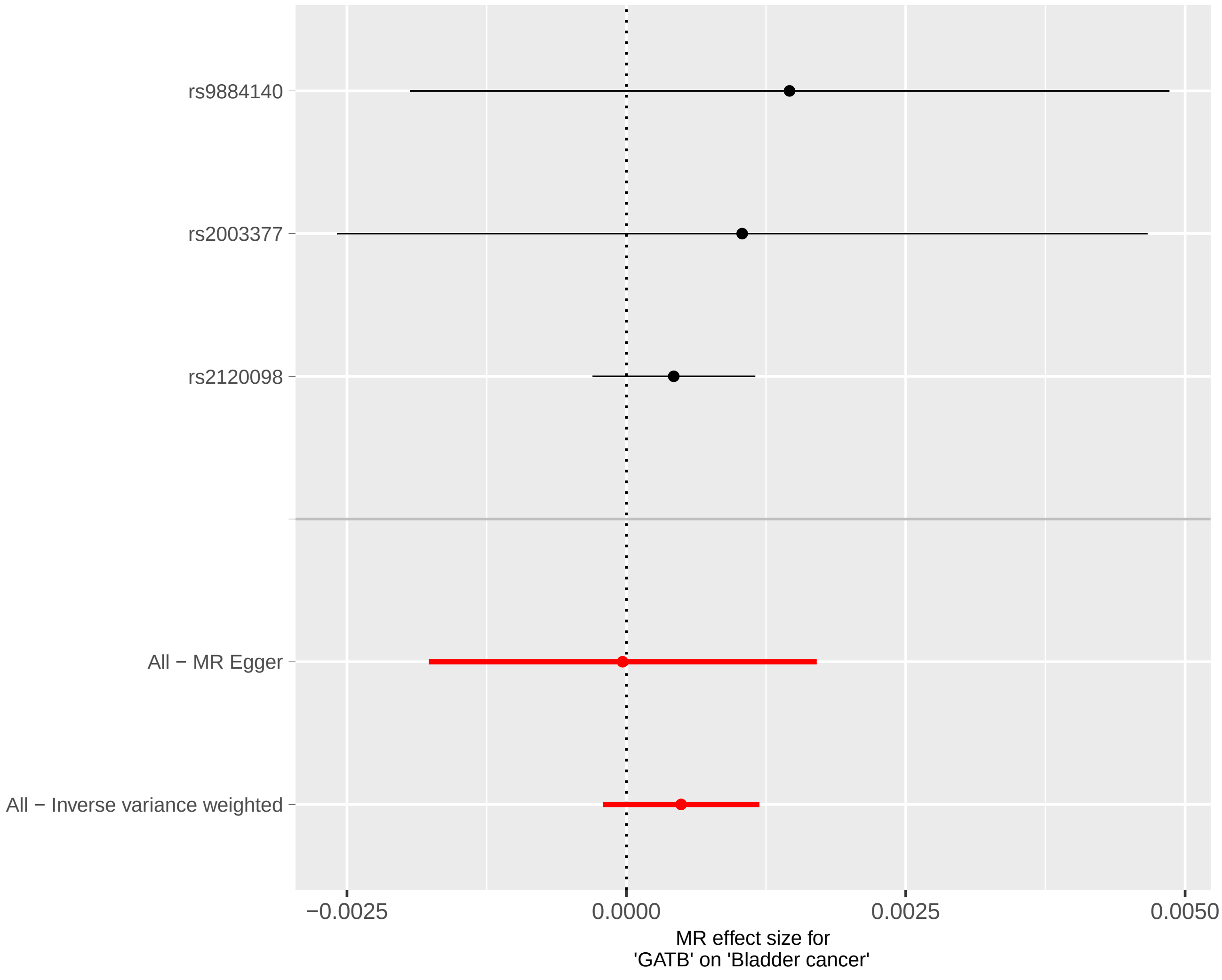

rs9884140
rs2003377
rs2120098
All − MR Egger
All − Inverse variance weighted
−0.0025
0.0000
0.0025
0.0050
MR effect size for
'GATB' on 'Bladder cancer'

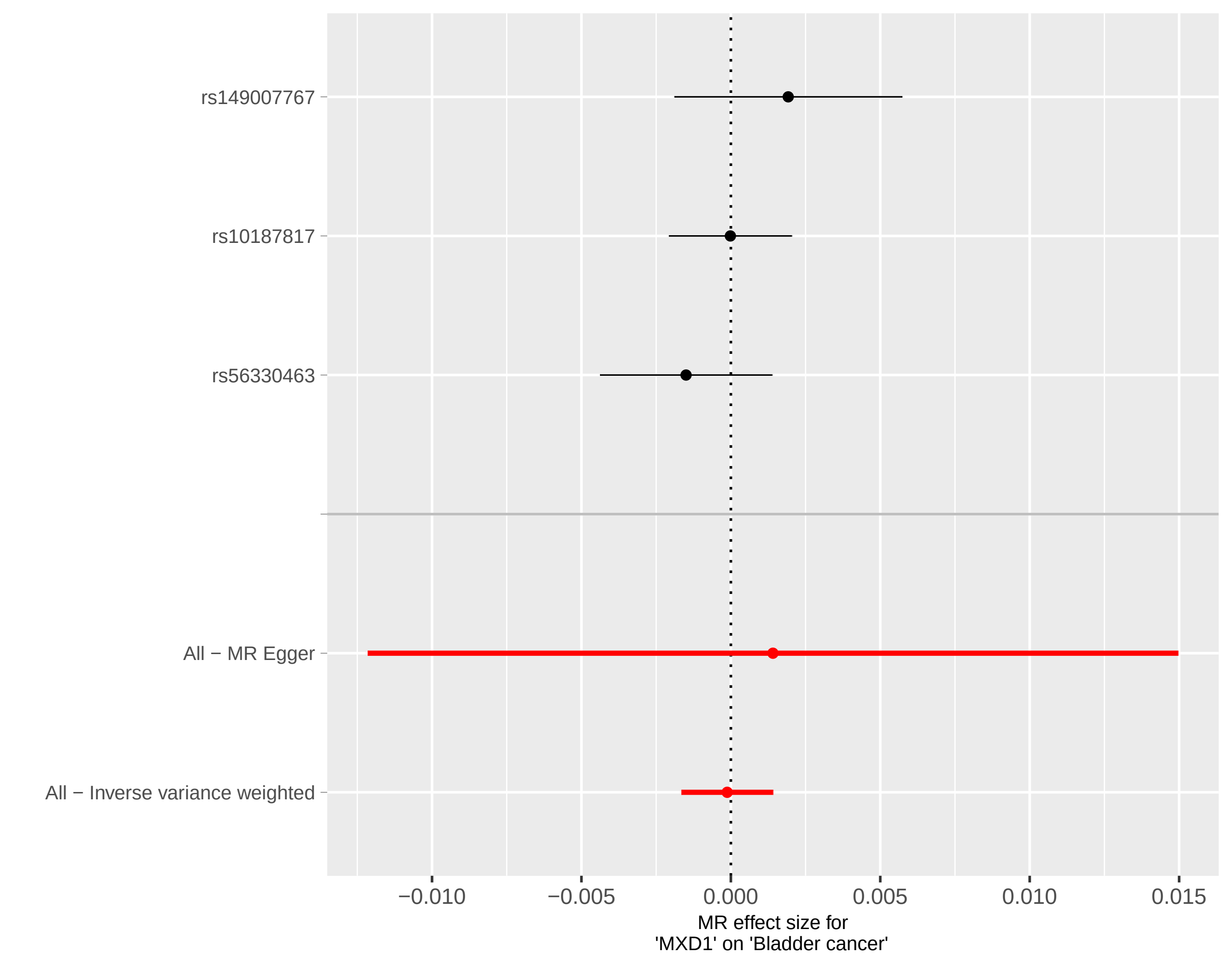

rs149007767
rs10187817
rs56330463
All − MR Egger
All − Inverse variance weighted
−0.010
−0.005
0.000
0.005
0.010
0.015
MR effect size for
'MXD1' on 'Bladder cancer'

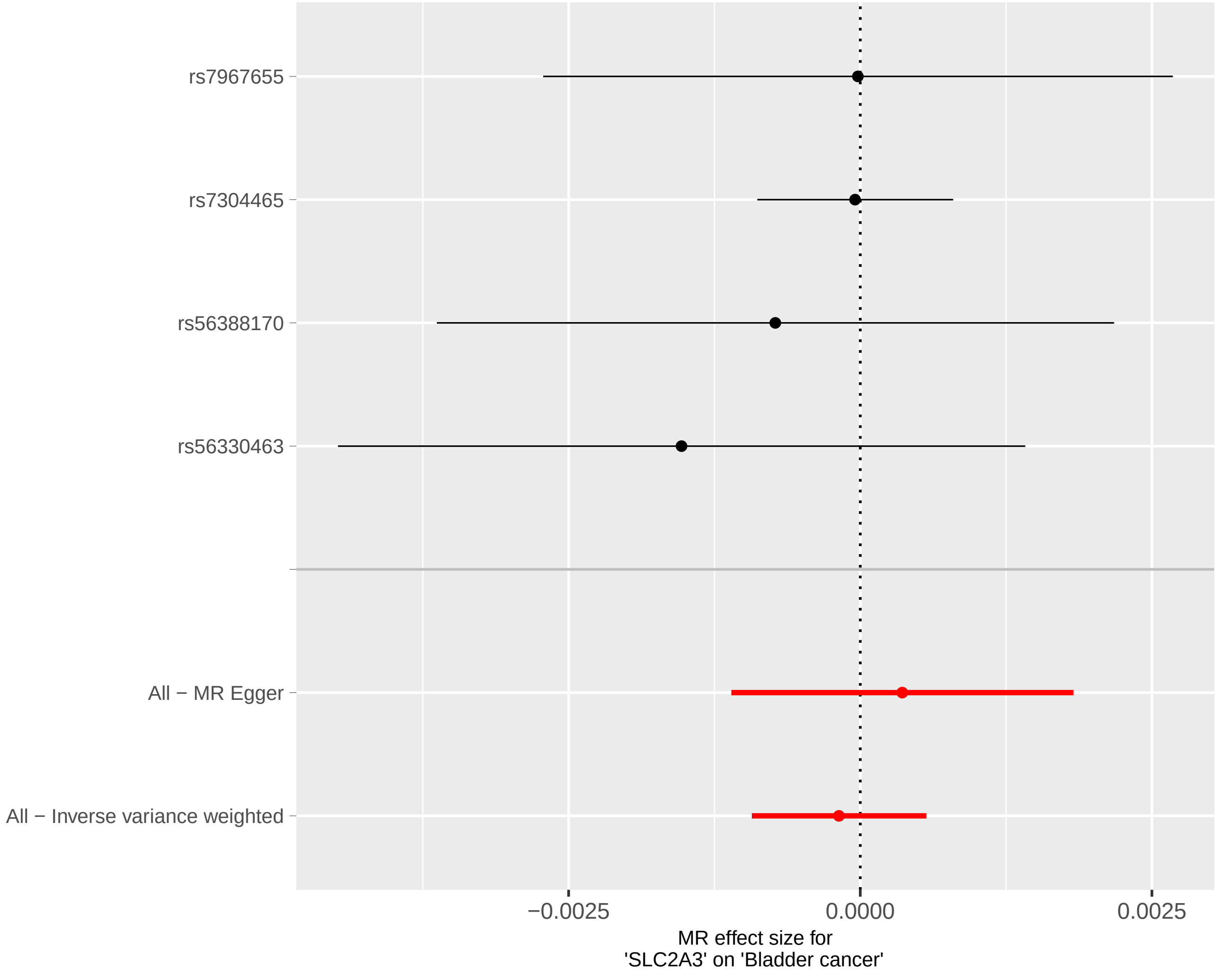

rs7967655
rs7304465
rs56388170
rs56330463
All − MR Egger
All − Inverse variance weighted
−0.0025
0.0000
0.0025
MR effect size for
'SLC2A3' on 'Bladder cancer'

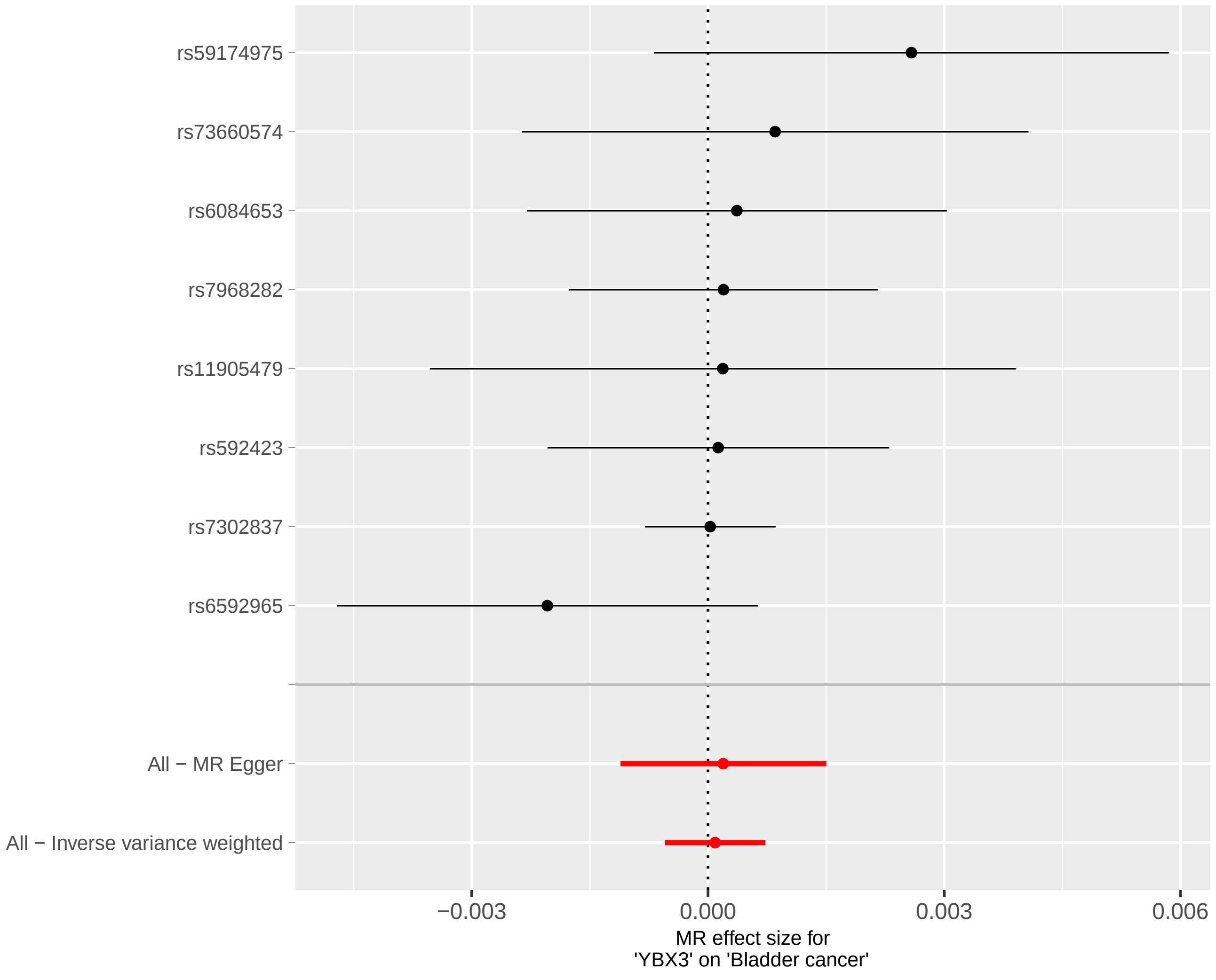
rs59174975
rs73660574
rs6084653
rs7968282
rs11905479
rs592423
rs7302837
rs6592965
All − MR Egger
All − Inverse variance weighted
−0.003
0.000
0.003
0.006
MR effect size for
'YBX3' on 'Bladder cancer'

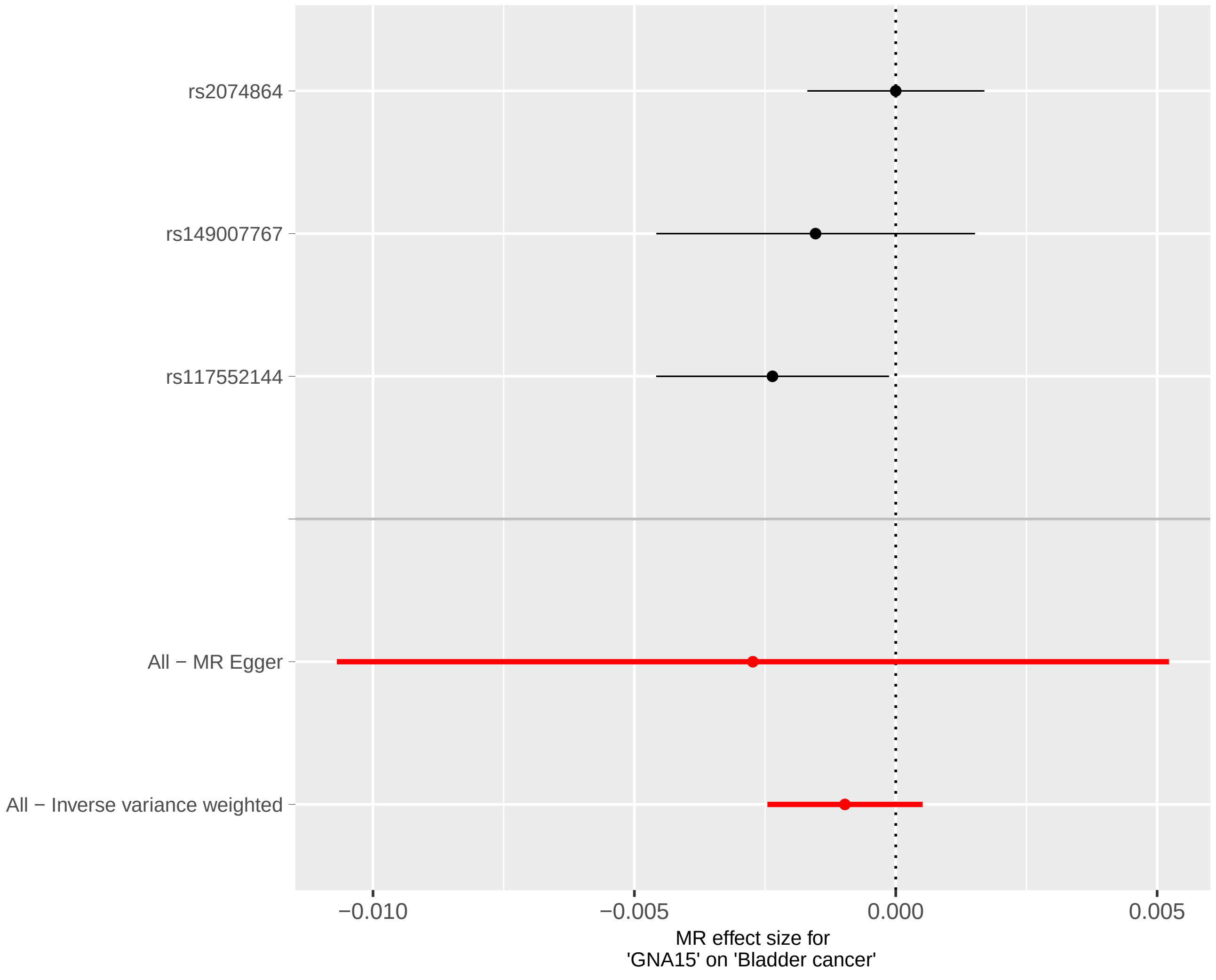
rs2074864
rs149007767
rs117552144
All − MR Egger
All − Inverse variance weighted
−0.010
−0.005
0.000
0.005
MR effect size for
'GNA15' on 'Bladder cancer'

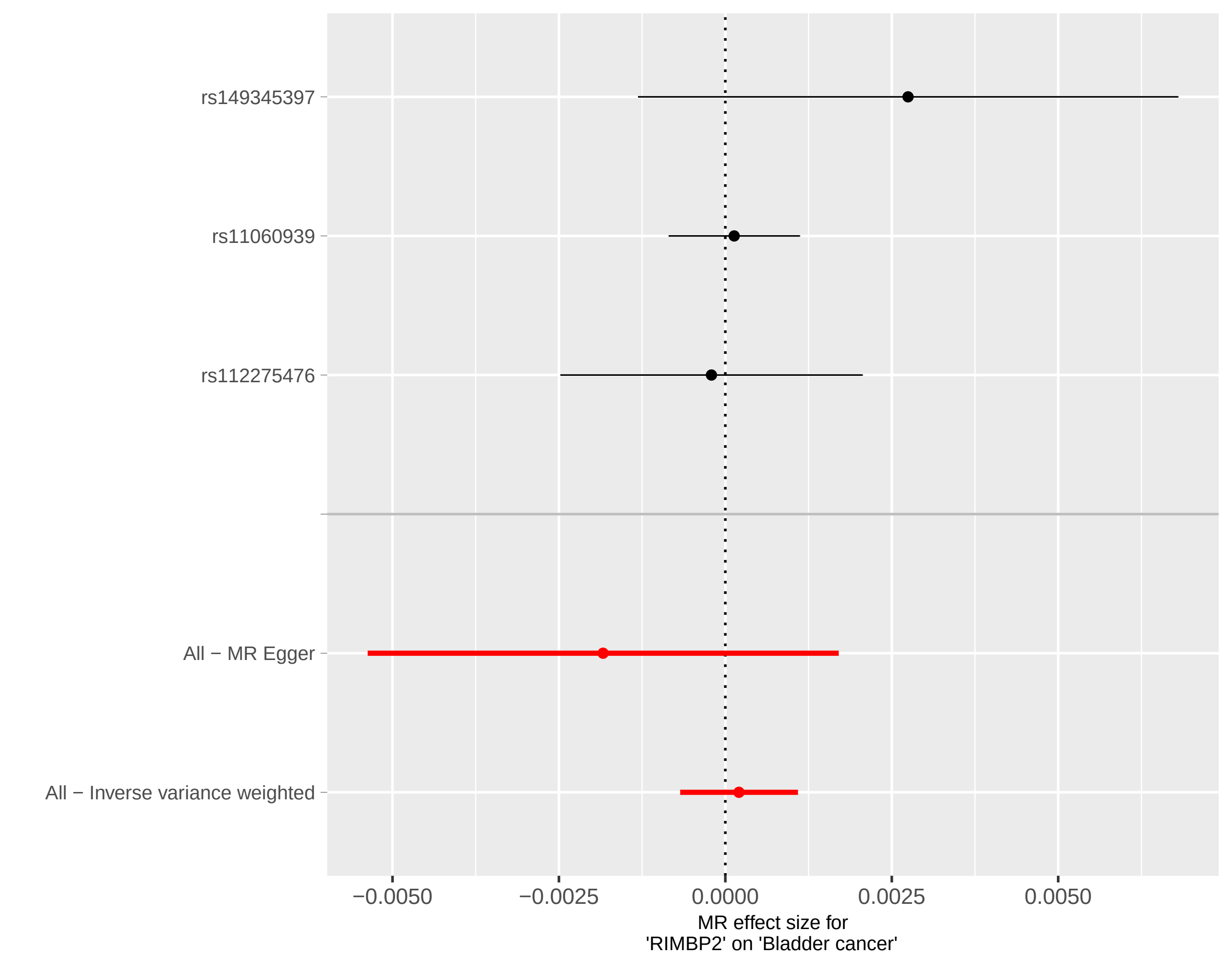
rs149345397
rs11060939
rs112275476
All − MR Egger
All − Inverse variance weighted
−0.0050
−0.0025
0.0000
0.0025
0.0050
MR effect size for
'RIMBP2' on 'Bladder cancer'

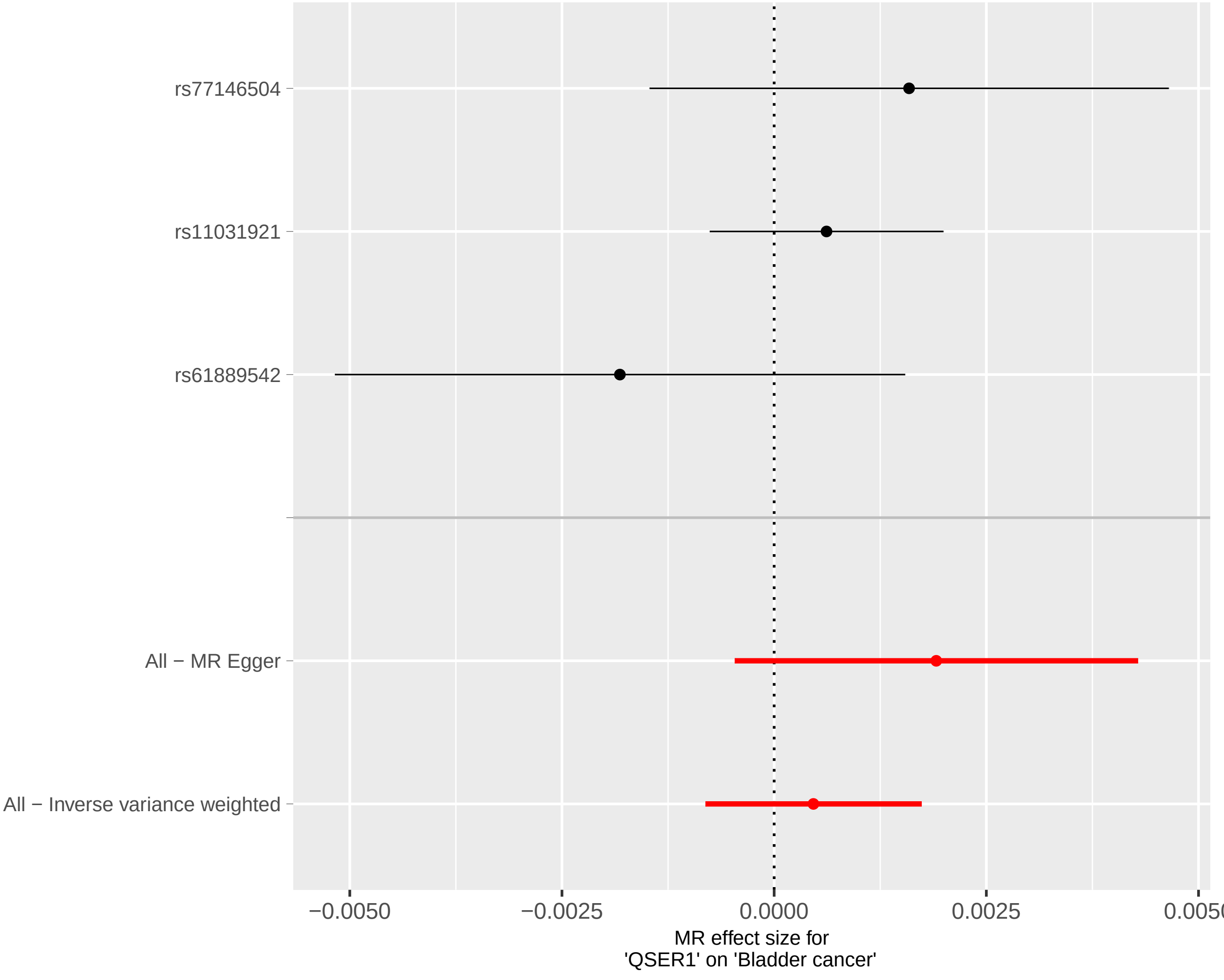

rs77146504
rs11031921
rs61889542
All − MR Egger
All − Inverse variance weighted
−0.0050
−0.0025
0.0000
0.0025
0.005
MR effect size for
'QSER1' on 'Bladder cancer'

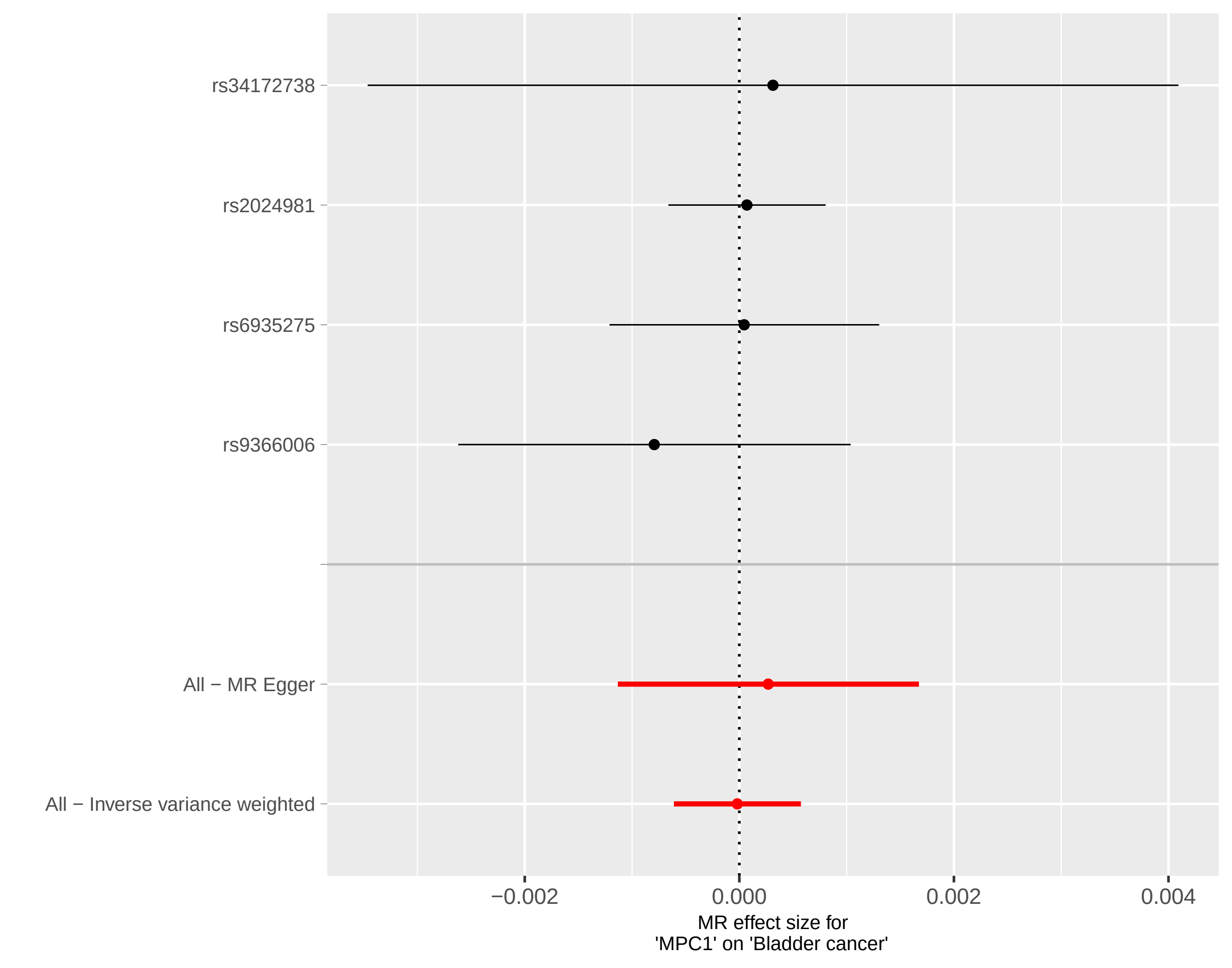

rs34172738
rs2024981
rs6935275
rs9366006
All − MR Egger
All − Inverse variance weighted
−0.002
0.000
0.002
0.004
MR effect size for
'MPC1' on 'Bladder cancer'

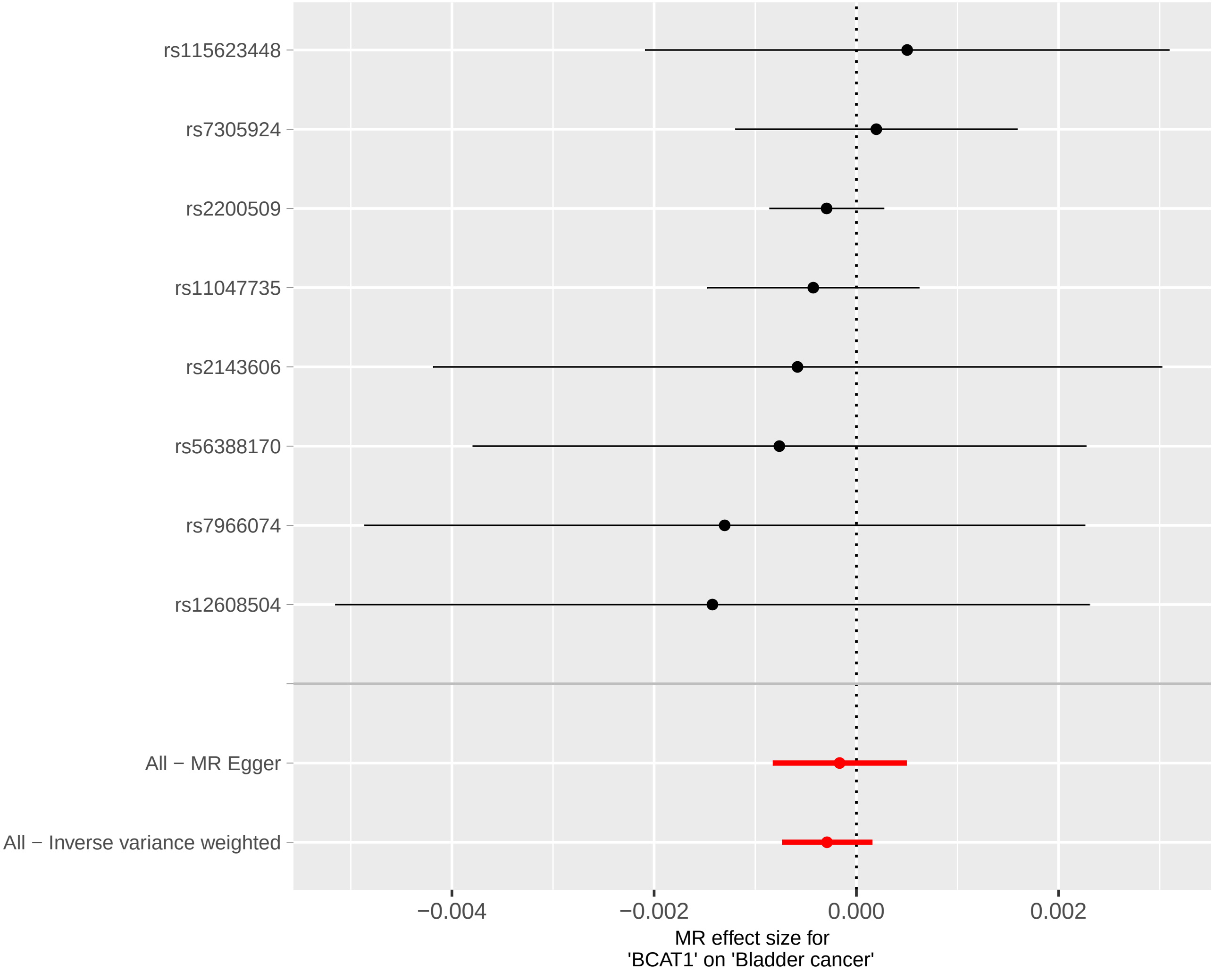
rs115623448
rs7305924
rs2200509
rs11047735
rs2143606
rs56388170
rs7966074
rs12608504
All − MR Egger
All − Inverse variance weighted
−0.004
−0.002
0.000
0.002
MR effect size for
'BCAT1' on 'Bladder cancer'

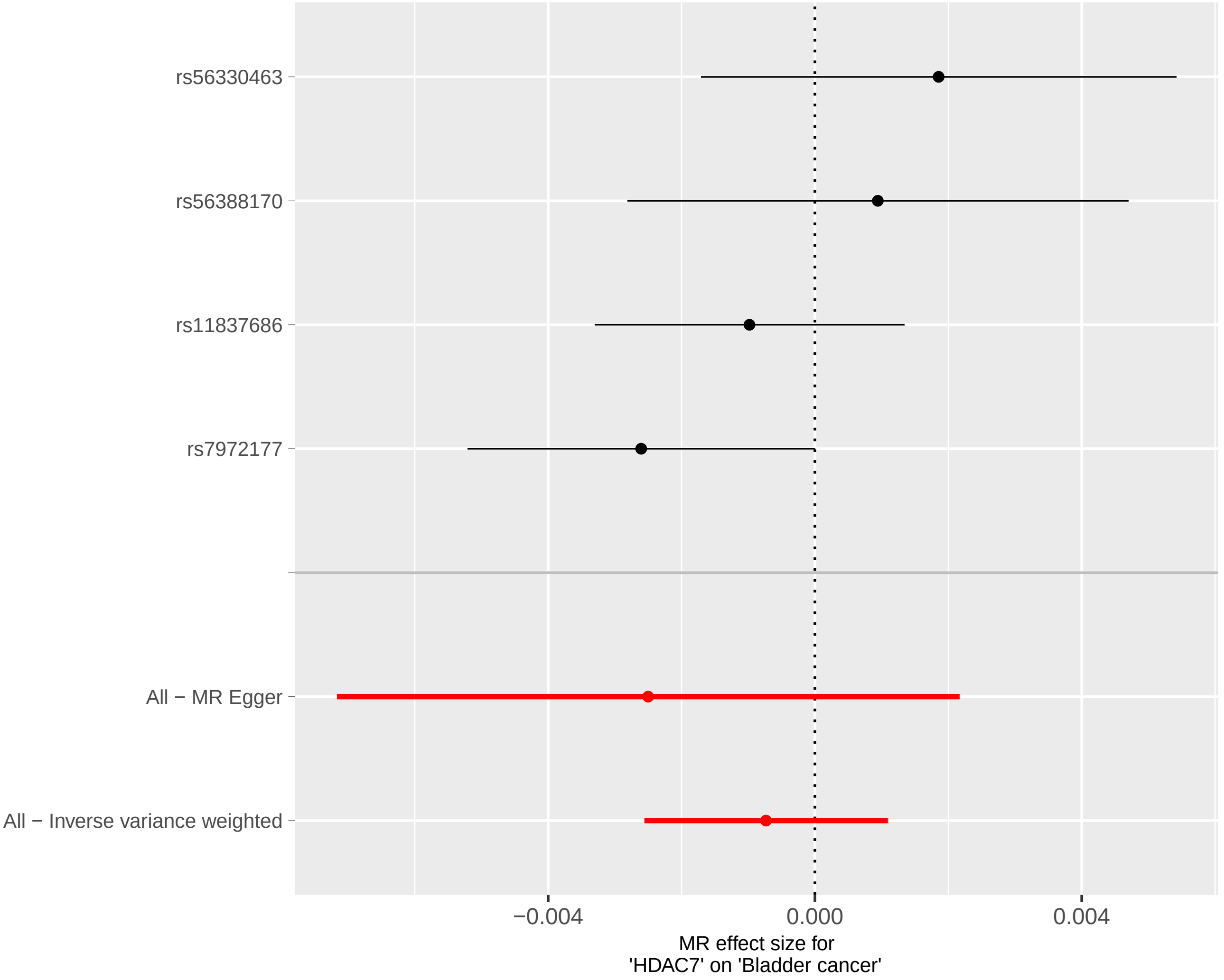
rs56330463
rs56388170
rs11837686
rs7972177
All − MR Egger
All − Inverse variance weighted
−0.004
0.000
0.004
MR effect size for
'HDAC7' on 'Bladder cancer'

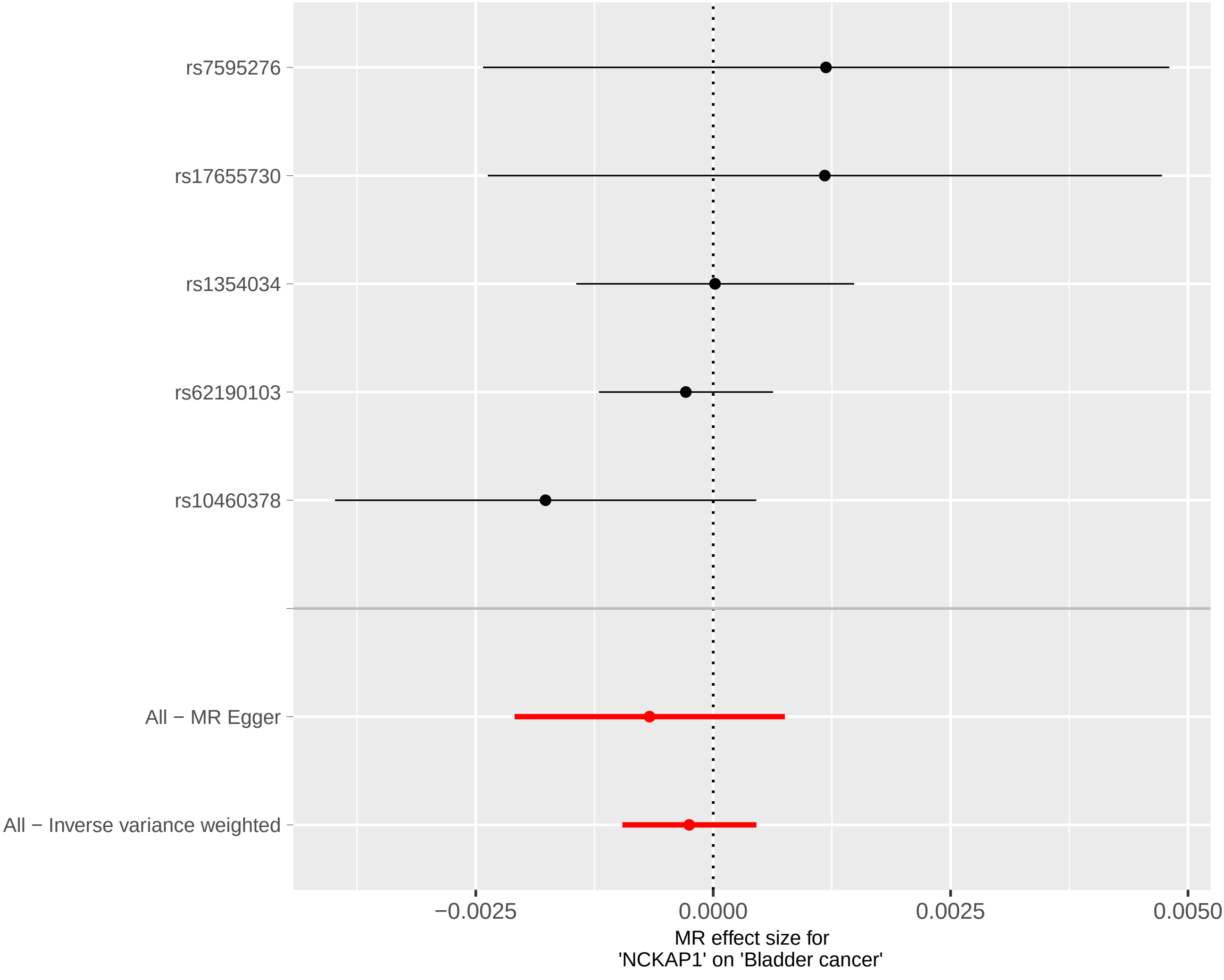

rs7595276
rs17655730
rs1354034
rs62190103
rs10460378
All − MR Egger
All − Inverse variance weighted
−0.0025
0.0000
0.0025
0.0050
MR effect size for
'NCKAP1' on 'Bladder cancer'

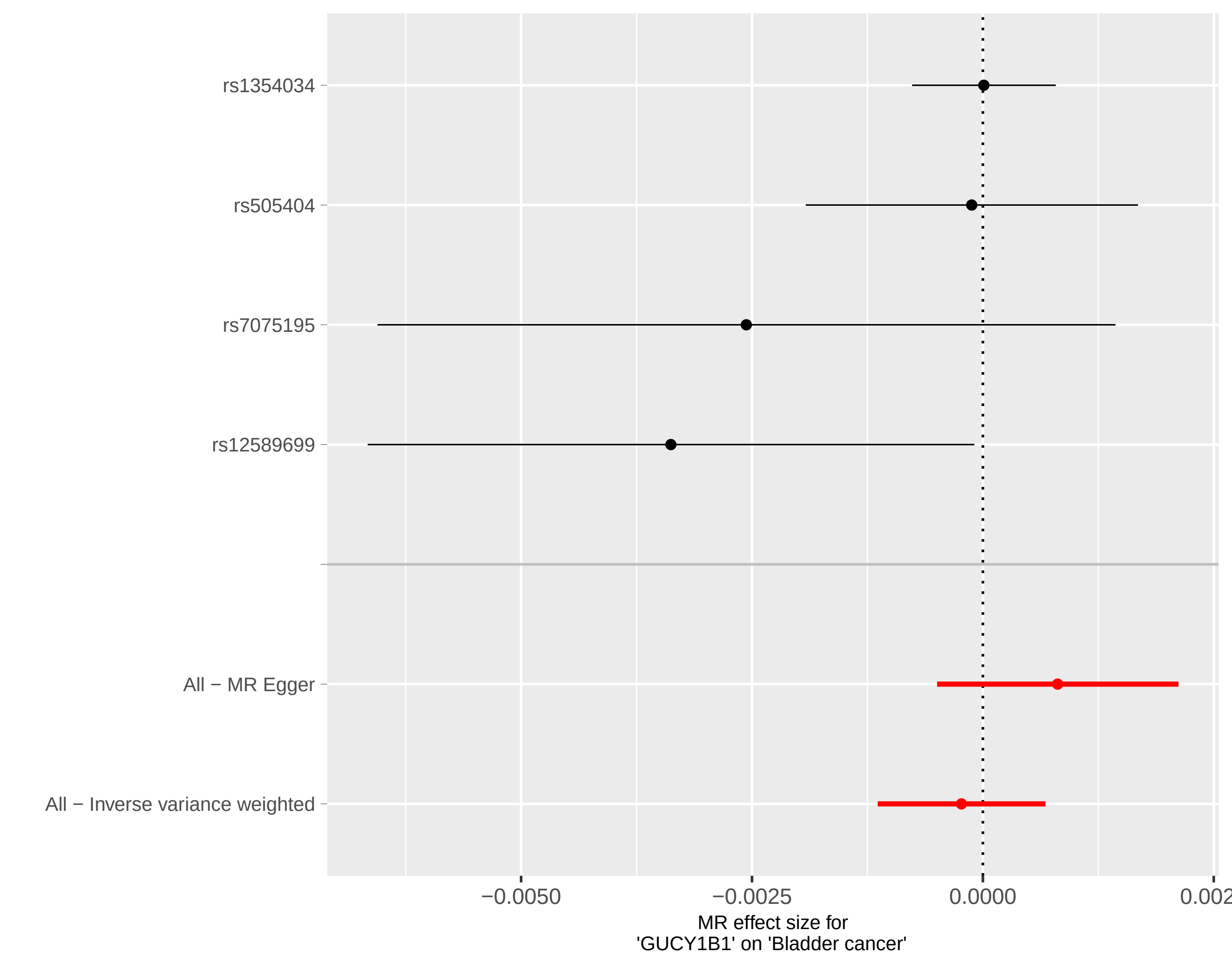

rs1354034
rs505404
rs7075195
rs12589699
All − MR Egger
All − Inverse variance weighted
−0.0050
−0.0025
0.0000
0.002
MR effect size for
'GUCY1B1' on 'Bladder cancer'

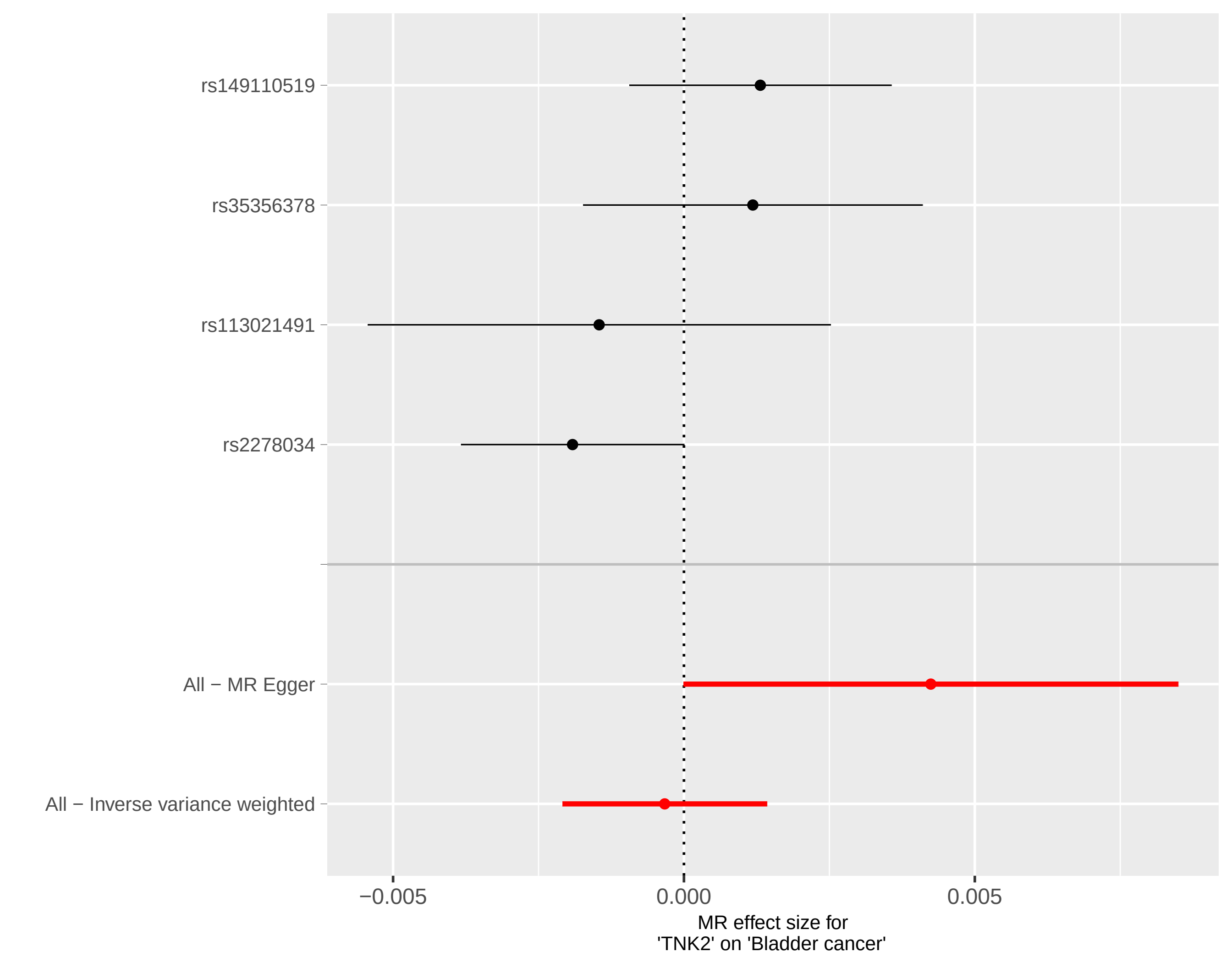
rs149110519
rs35356378
rs113021491
rs2278034
All − MR Egger
All − Inverse variance weighted
−0.005
0.000
0.005
MR effect size for
'TNK2' on 'Bladder cancer'

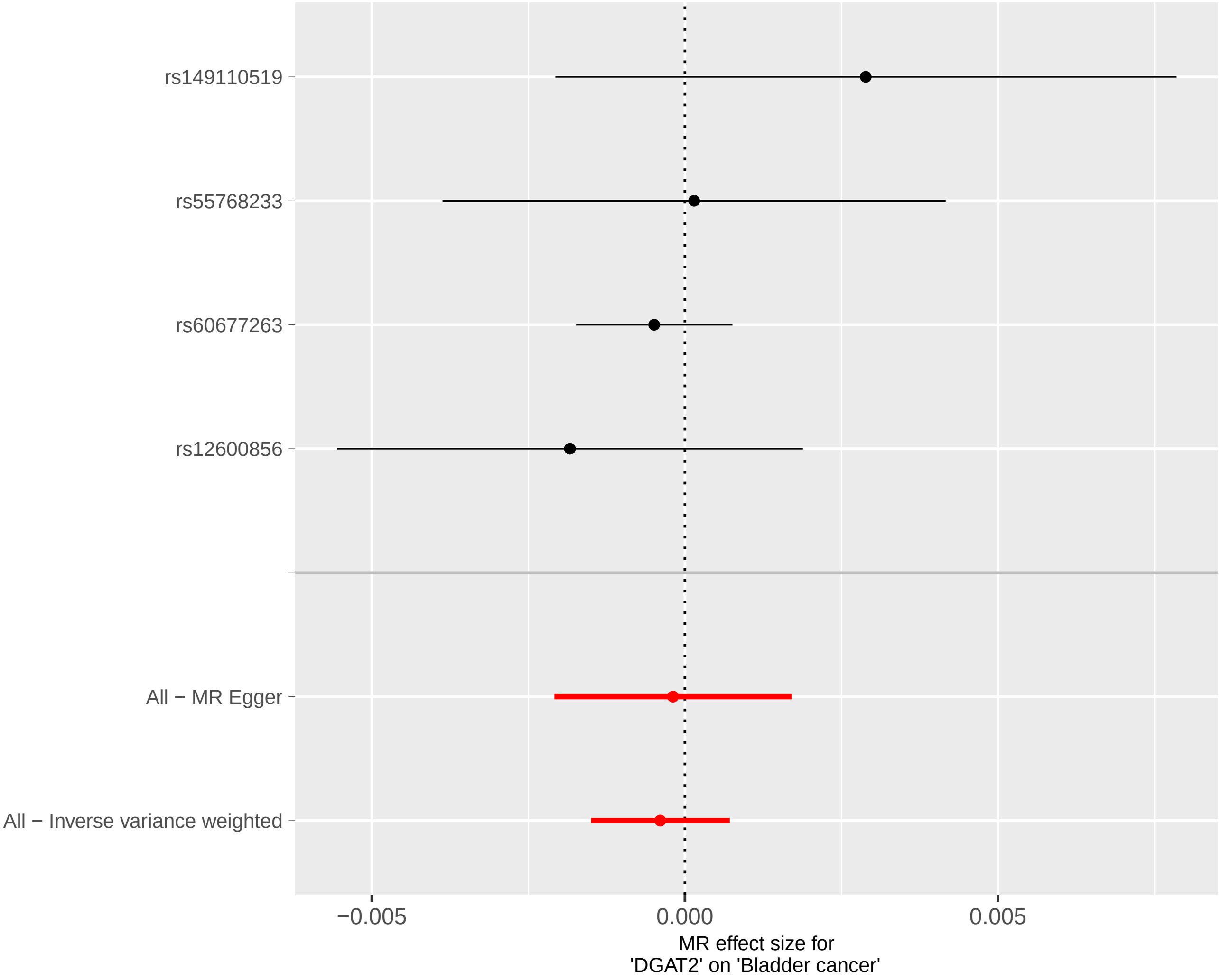

rs149110519
rs55768233
rs60677263
rs12600856
All − MR Egger
All − Inverse variance weighted
−0.005
0.000
0.005
MR effect size for
'DGAT2' on 'Bladder cancer'

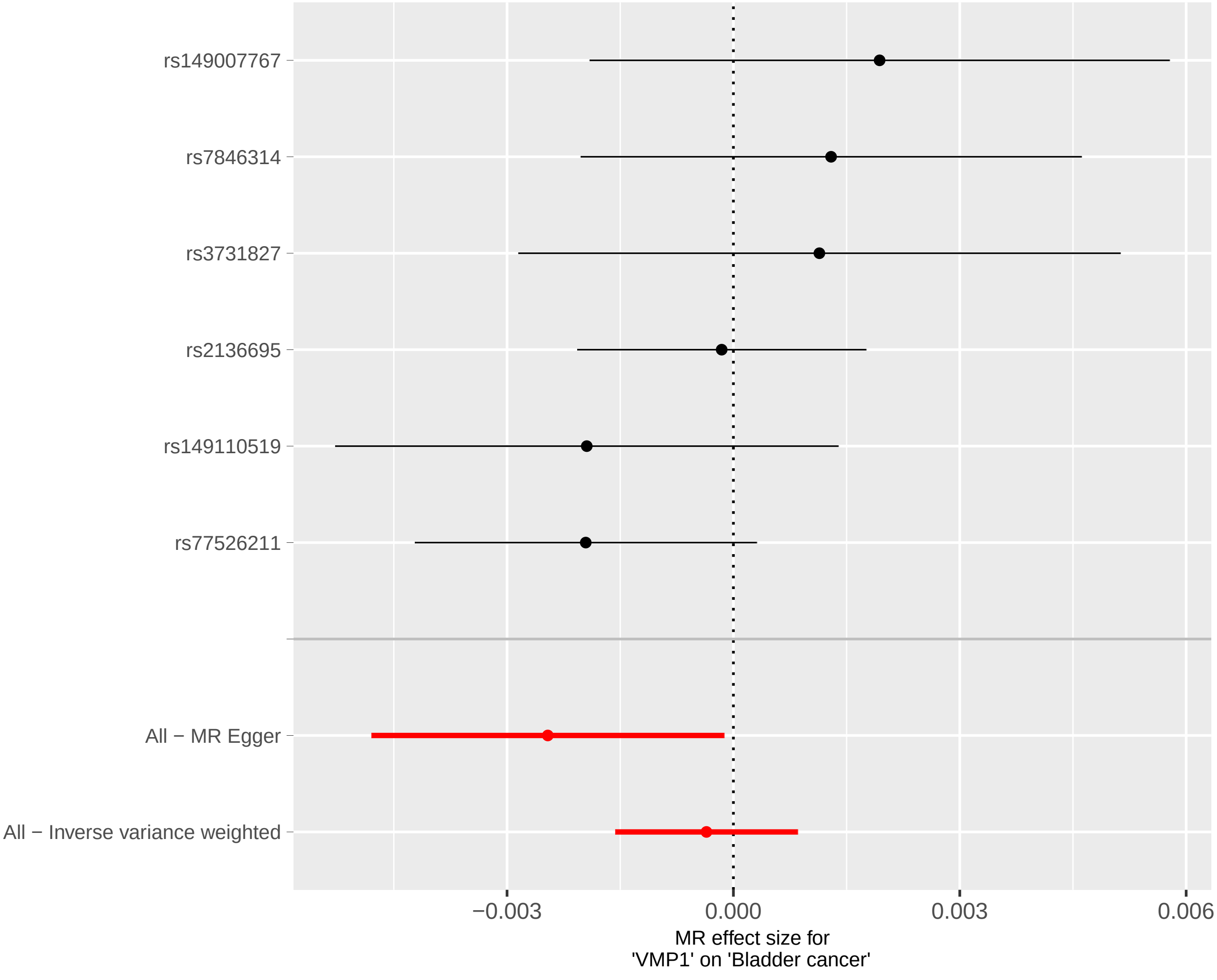

rs149007767
rs7846314
rs3731827
rs2136695
rs149110519
rs77526211
All − MR Egger
All − Inverse variance weighted
−0.003
0.000
0.003
0.006
MR effect size for
'VMP1' on 'Bladder cancer'

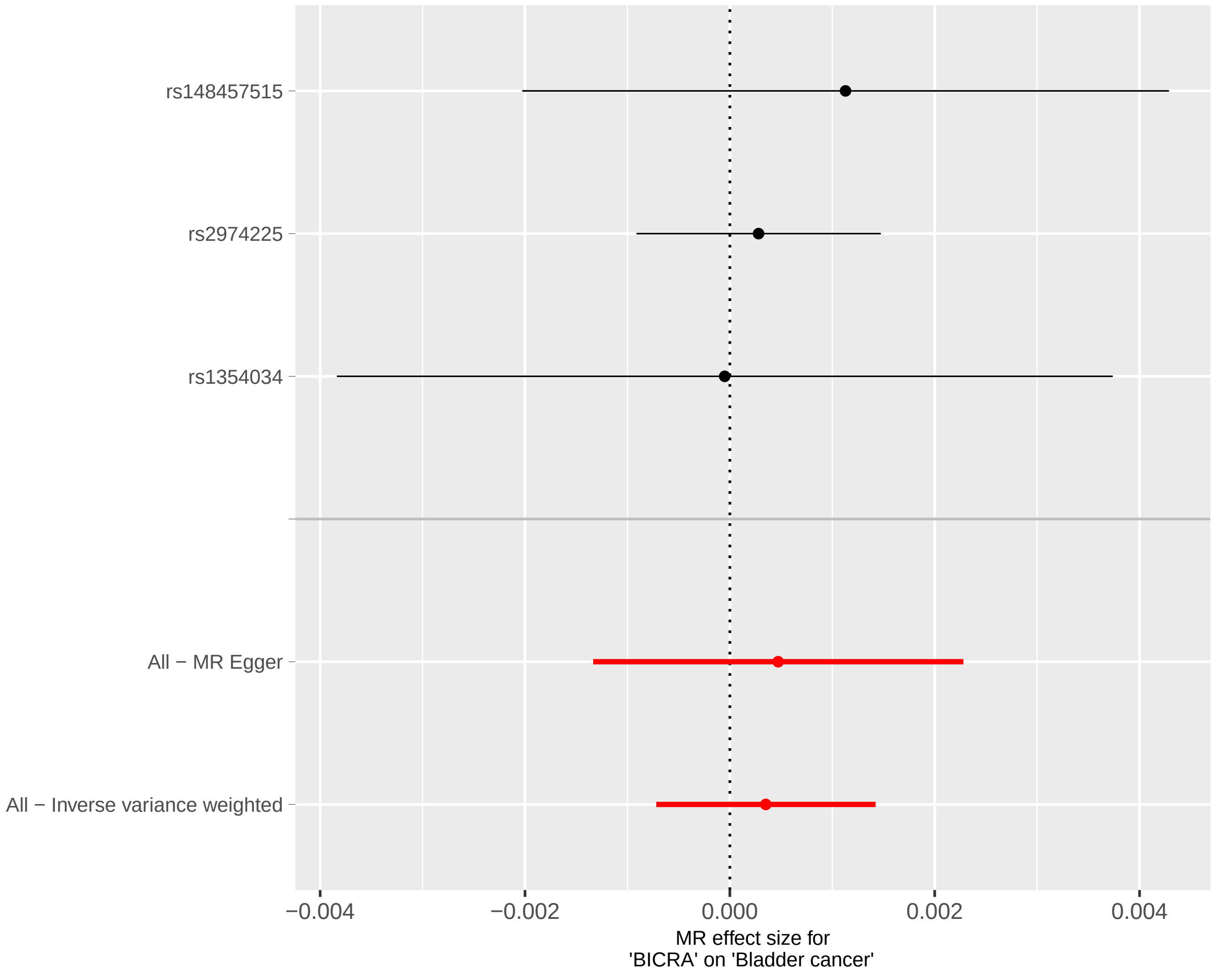
rs148457515
rs2974225
rs1354034
All − MR Egger
All − Inverse variance weighted
−0.004
−0.002
0.000
0.002
0.004
MR effect size for
'BICRA' on 'Bladder cancer'

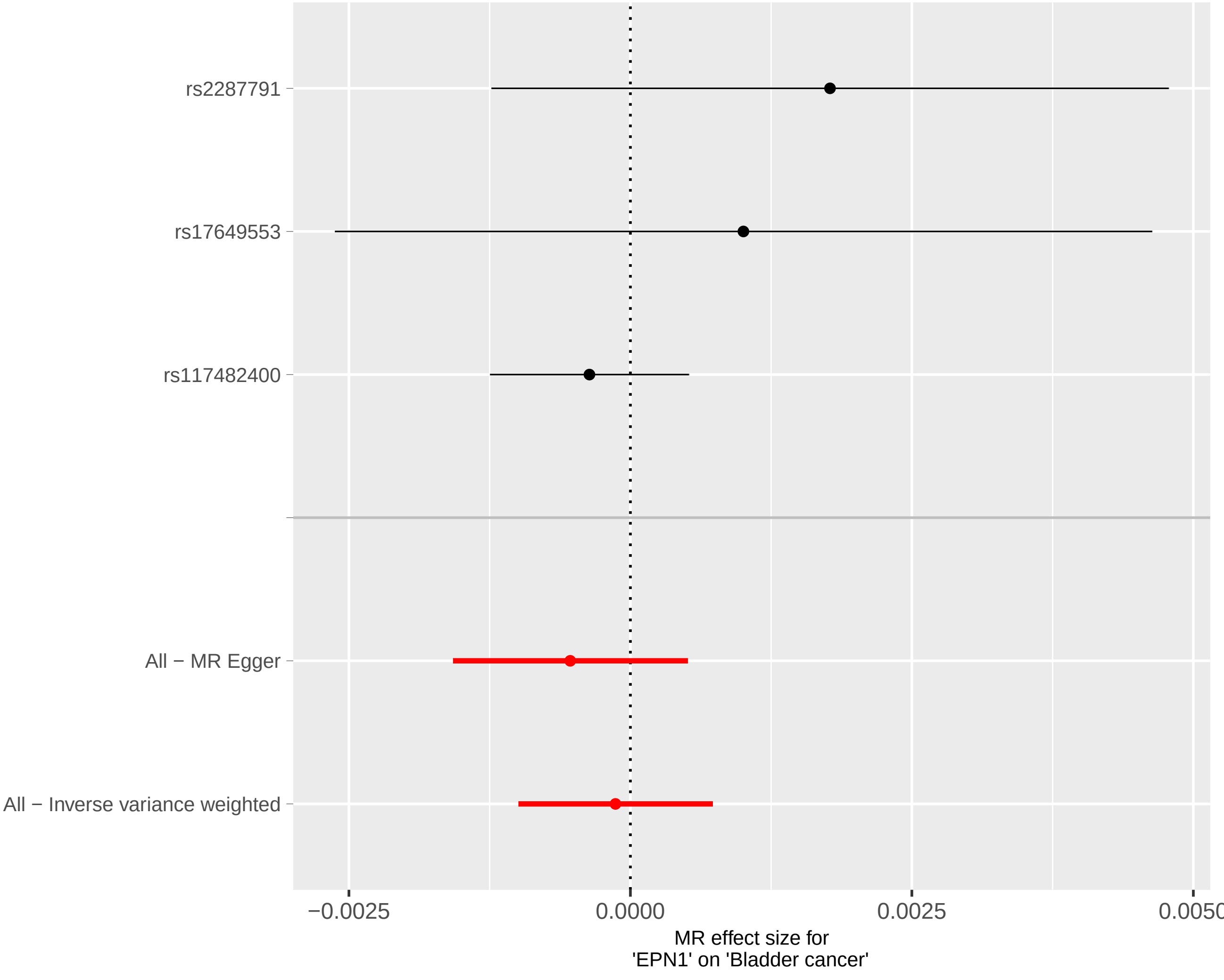
rs2287791
rs17649553
rs117482400
All − MR Egger
All − Inverse variance weighted
−0.0025
0.0000
0.0025
0.0050
MR effect size for
'EPN1' on 'Bladder cancer'

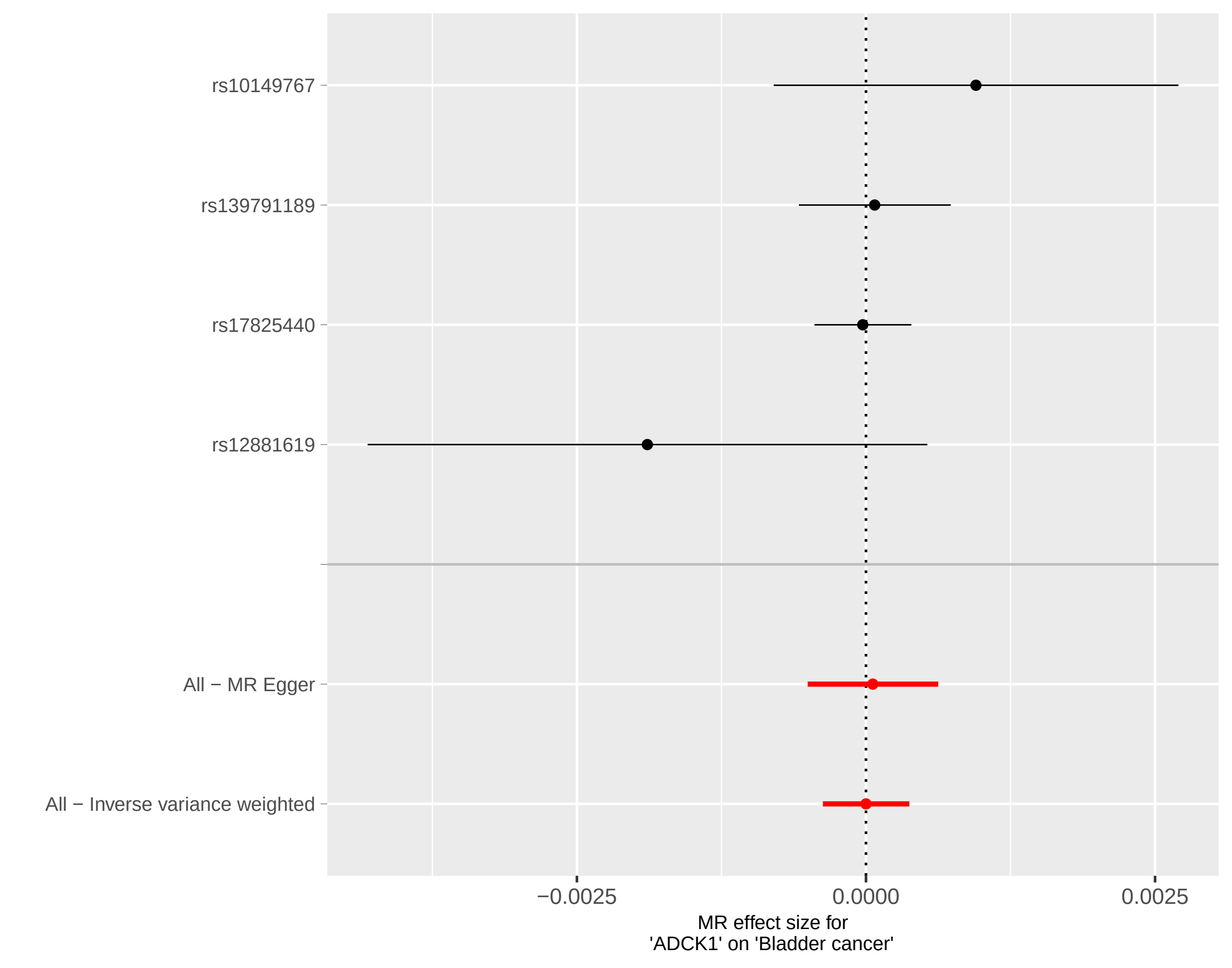
rs10149767
rs139791189
rs17825440
rs12881619
All − MR Egger
All − Inverse variance weighted
−0.0025
0.0000
0.0025
MR effect size for
'ADCK1' on 'Bladder cancer'

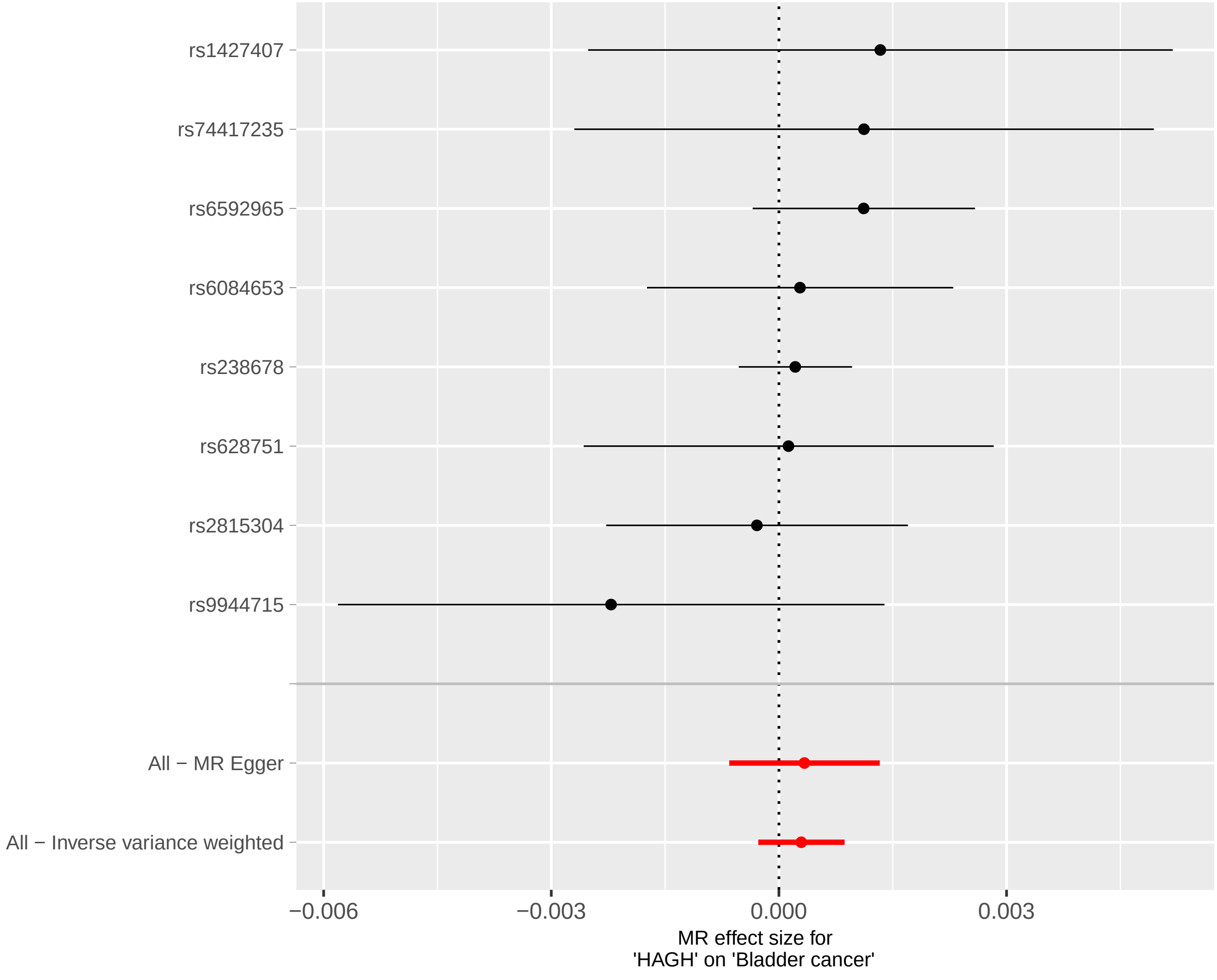
rs1427407
rs74417235
rs6592965
rs6084653
rs238678
rs628751
rs2815304
rs9944715
All − MR Egger
All − Inverse variance weighted
−0.006
−0.003
0.000
0.003
MR effect size for
'HAGH' on 'Bladder cancer'

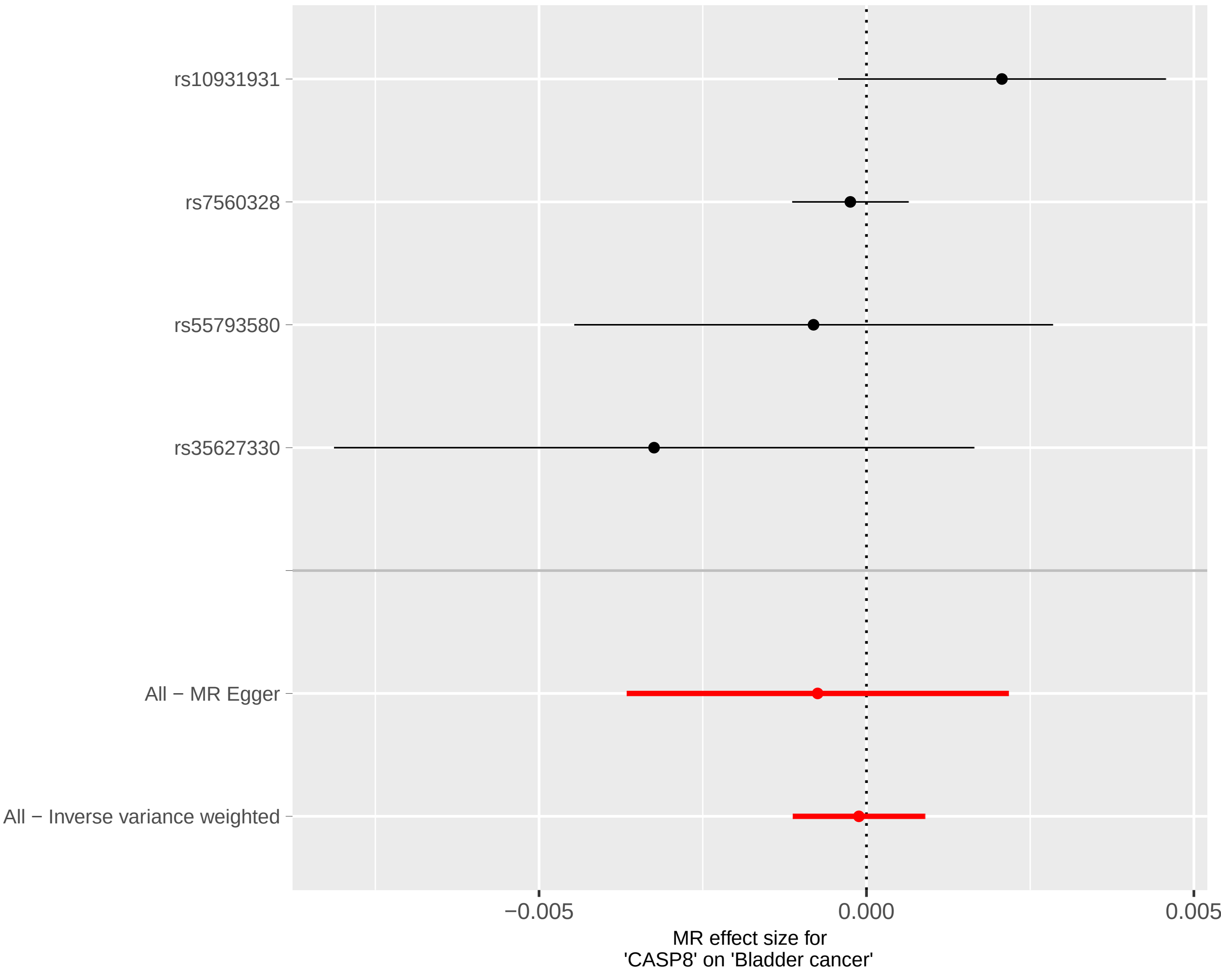
rs10931931
rs7560328
rs55793580
rs35627330
All − MR Egger
All − Inverse variance weighted
−0.005
0.000
0.005
MR effect size for
'CASP8' on 'Bladder cancer'

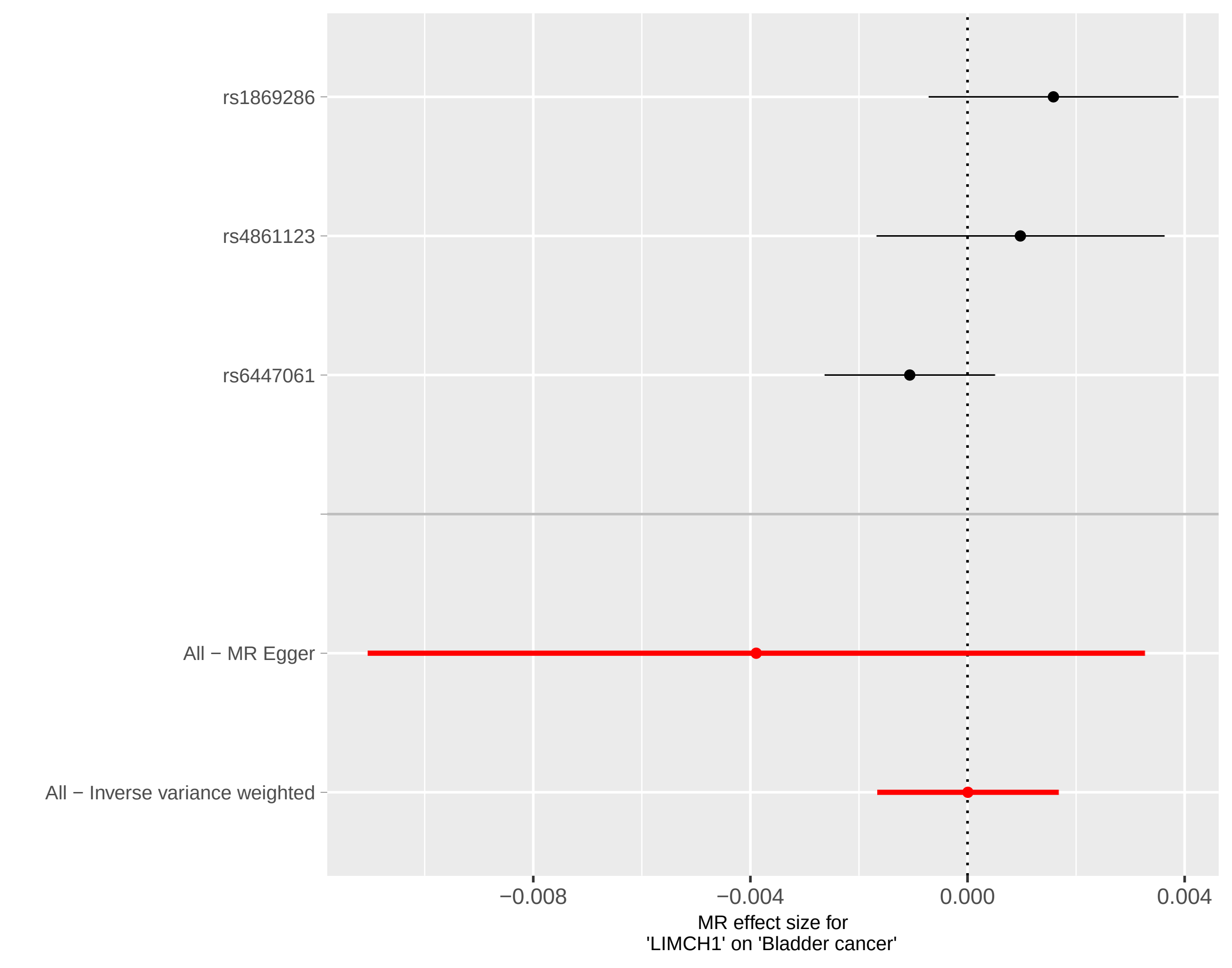
rs1869286
rs4861123
rs6447061
All − MR Egger
All − Inverse variance weighted
−0.008
−0.004
0.000
0.004
MR effect size for
'LIMCH1' on 'Bladder cancer'

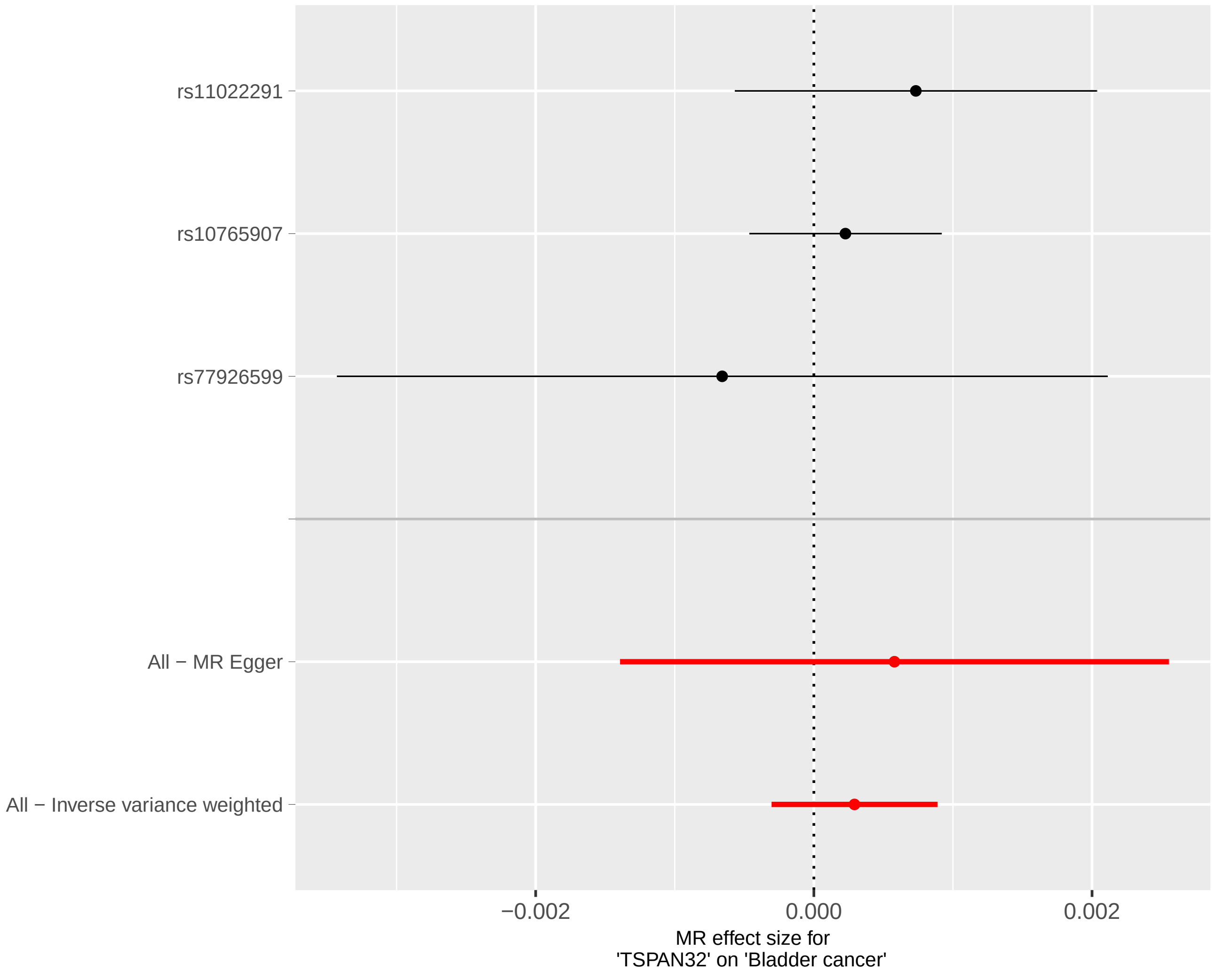
rs11022291
rs10765907
rs77926599
All − MR Egger
All − Inverse variance weighted
−0.002
0.000
0.002
MR effect size for
'TSPAN32' on 'Bladder cancer'

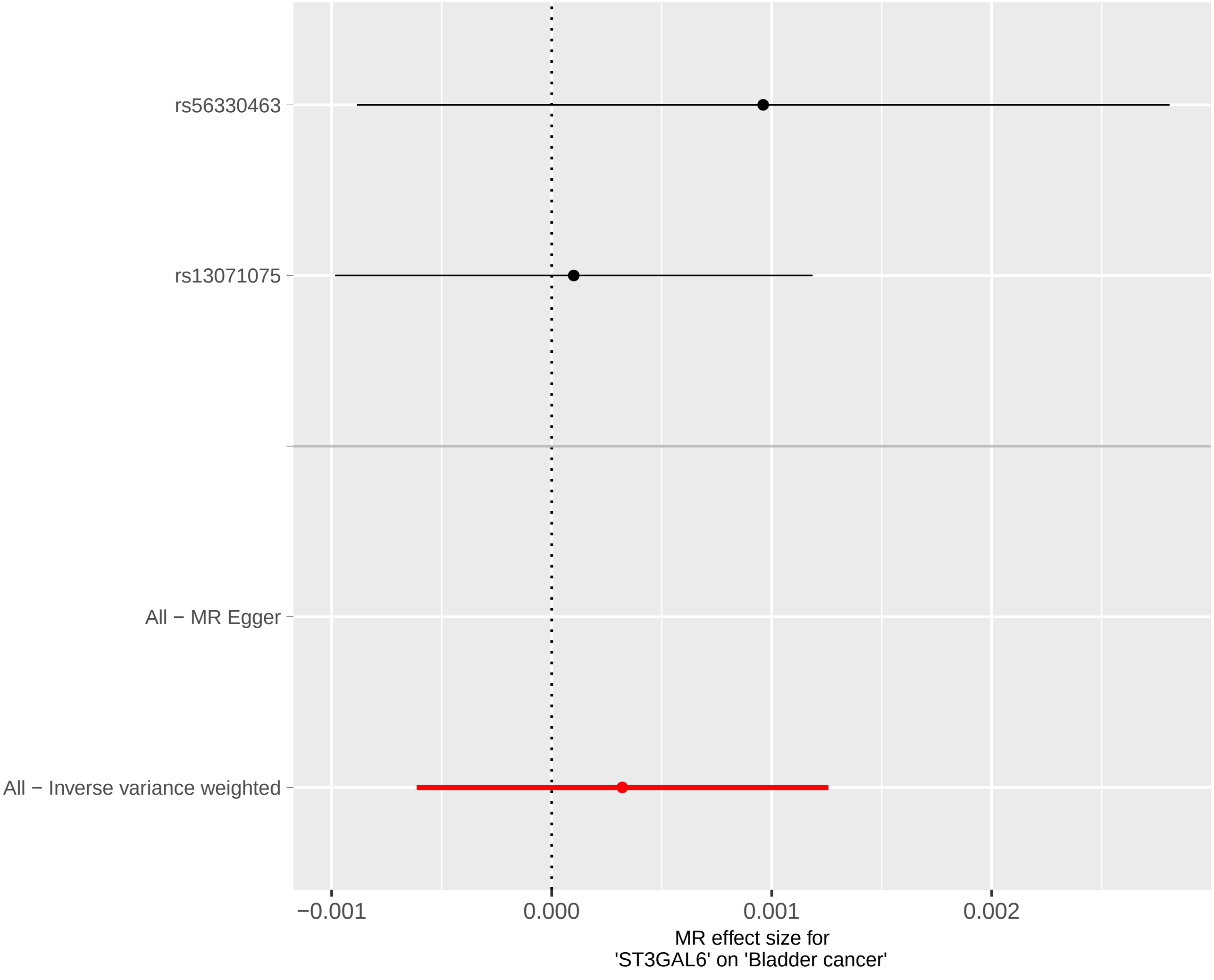

rs56330463
rs13071075
All − MR Egger
All − Inverse variance weighted
−0.001
0.000
0.001
0.002
MR effect size for
'ST3GAL6' on 'Bladder cancer'

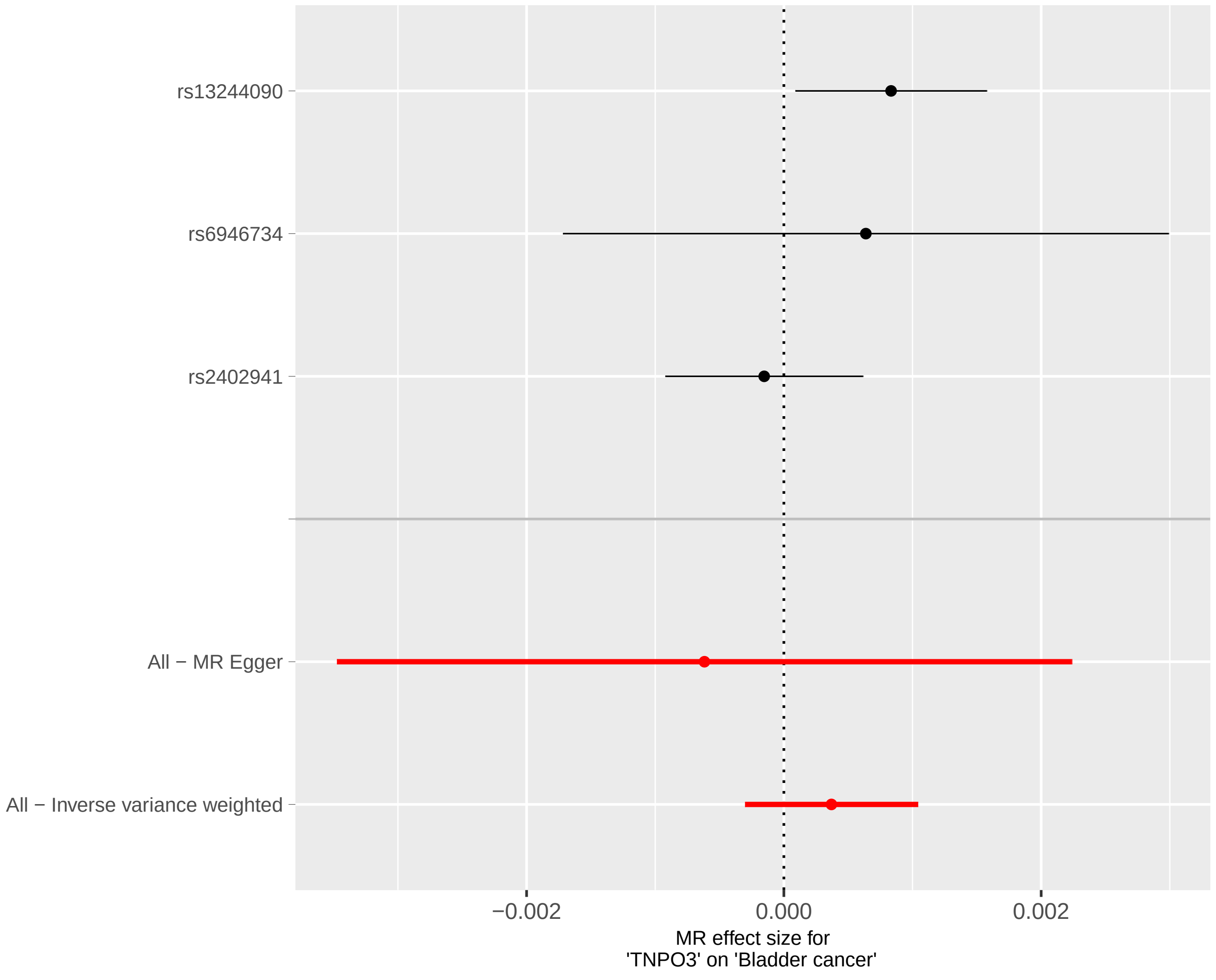
rs13244090
rs6946734
rs2402941
All − MR Egger
All − Inverse variance weighted
−0.002
0.000
0.002
MR effect size for
'TNPO3' on 'Bladder cancer'

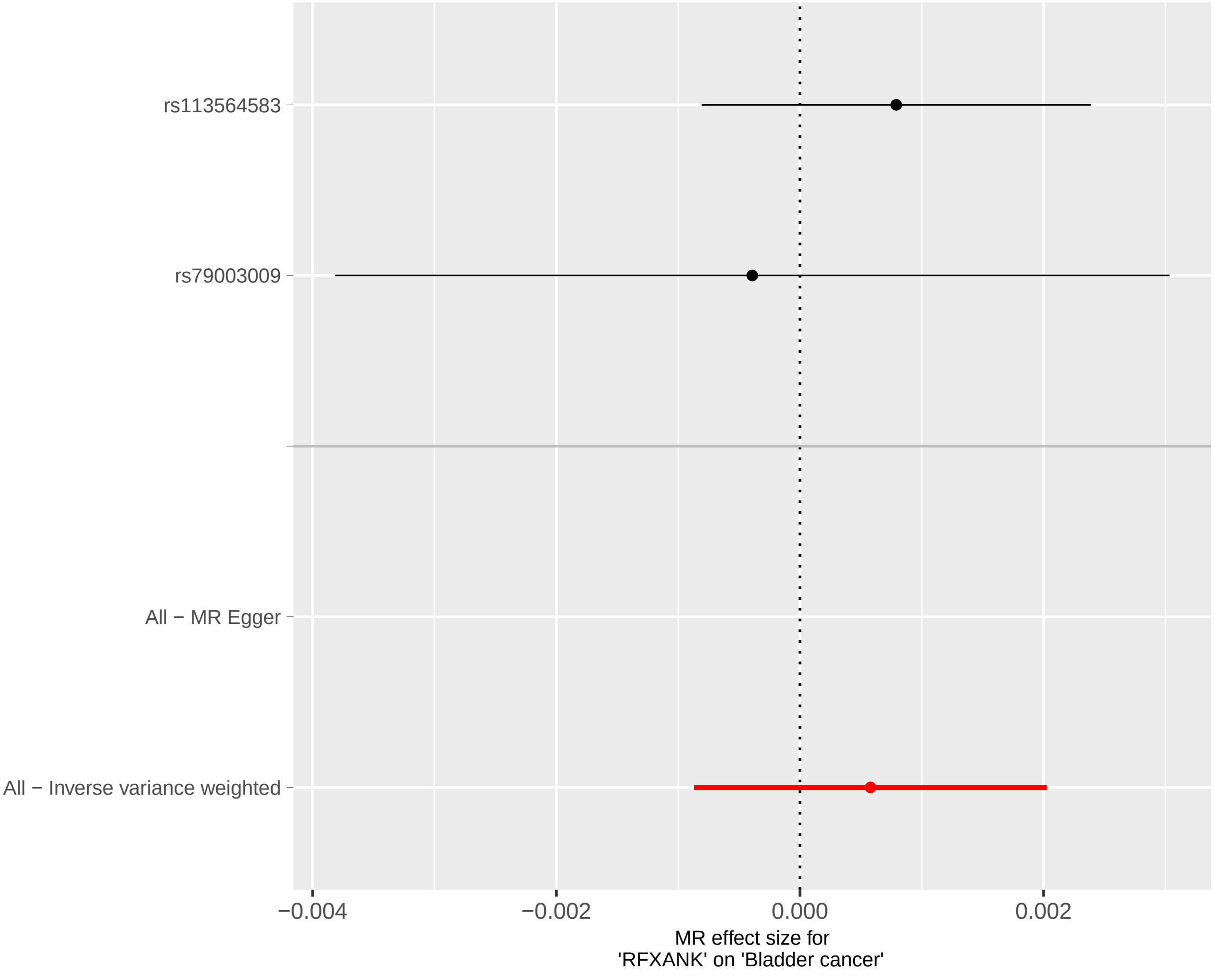
rs113564583
rs79003009
All − MR Egger
All − Inverse variance weighted
−0.004
−0.002
0.000
0.002
MR effect size for
'RFXANK' on 'Bladder cancer'

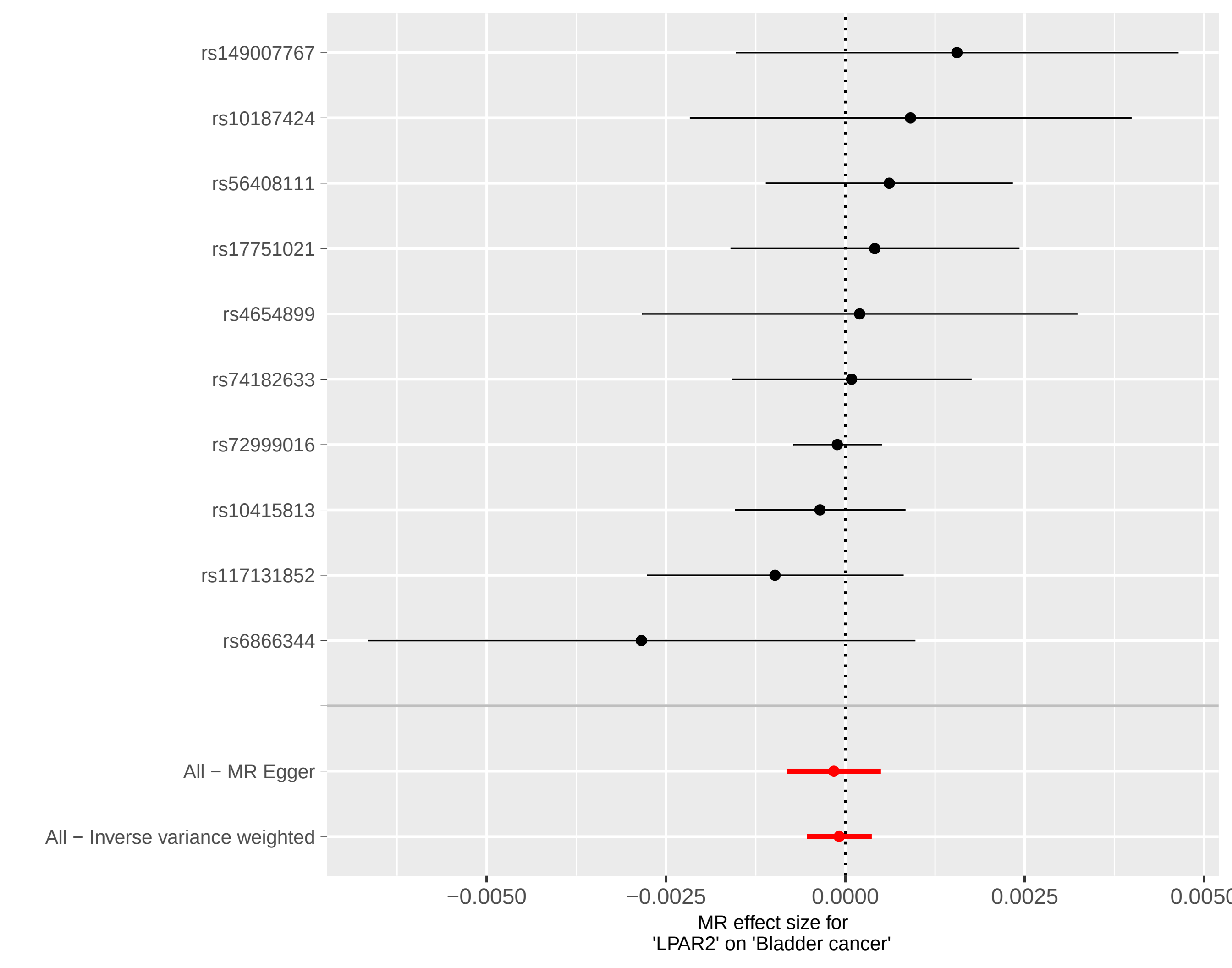
rs149007767
rs10187424
rs56408111
rs17751021
rs4654899
rs74182633
rs72999016
rs10415813
rs117131852
rs6866344
All − MR Egger
All − Inverse variance weighted
−0.0050
−0.0025
0.0000
0.0025
0.0050
MR effect size for
'LPAR2' on 'Bladder cancer'

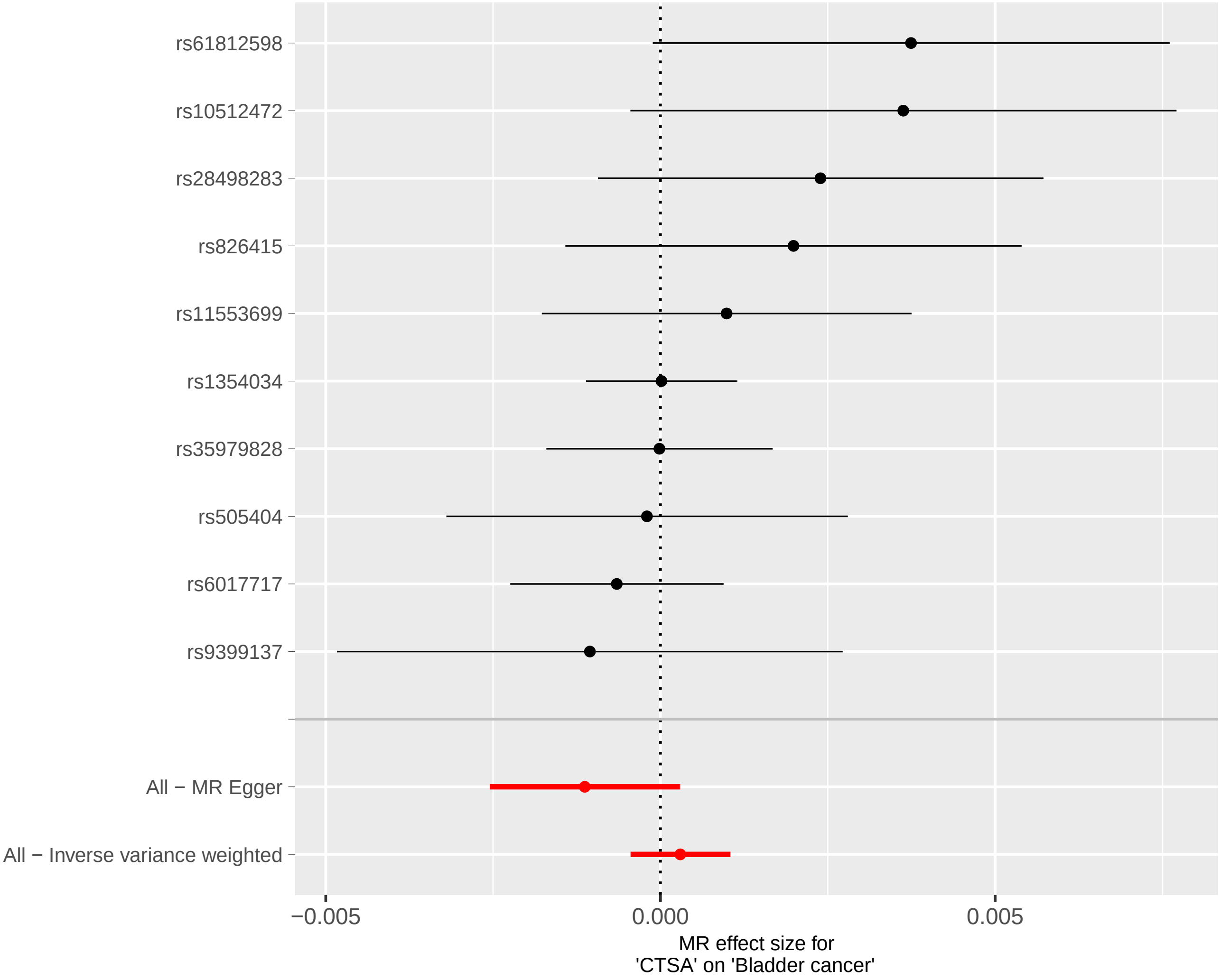

rs61812598
rs10512472
rs28498283
rs826415
rs11553699
rs1354034
rs35979828
rs505404
rs6017717
rs9399137
All − MR Egger
All − Inverse variance weighted
−0.005
0.000
0.005
MR effect size for
'CTSA' on 'Bladder cancer'

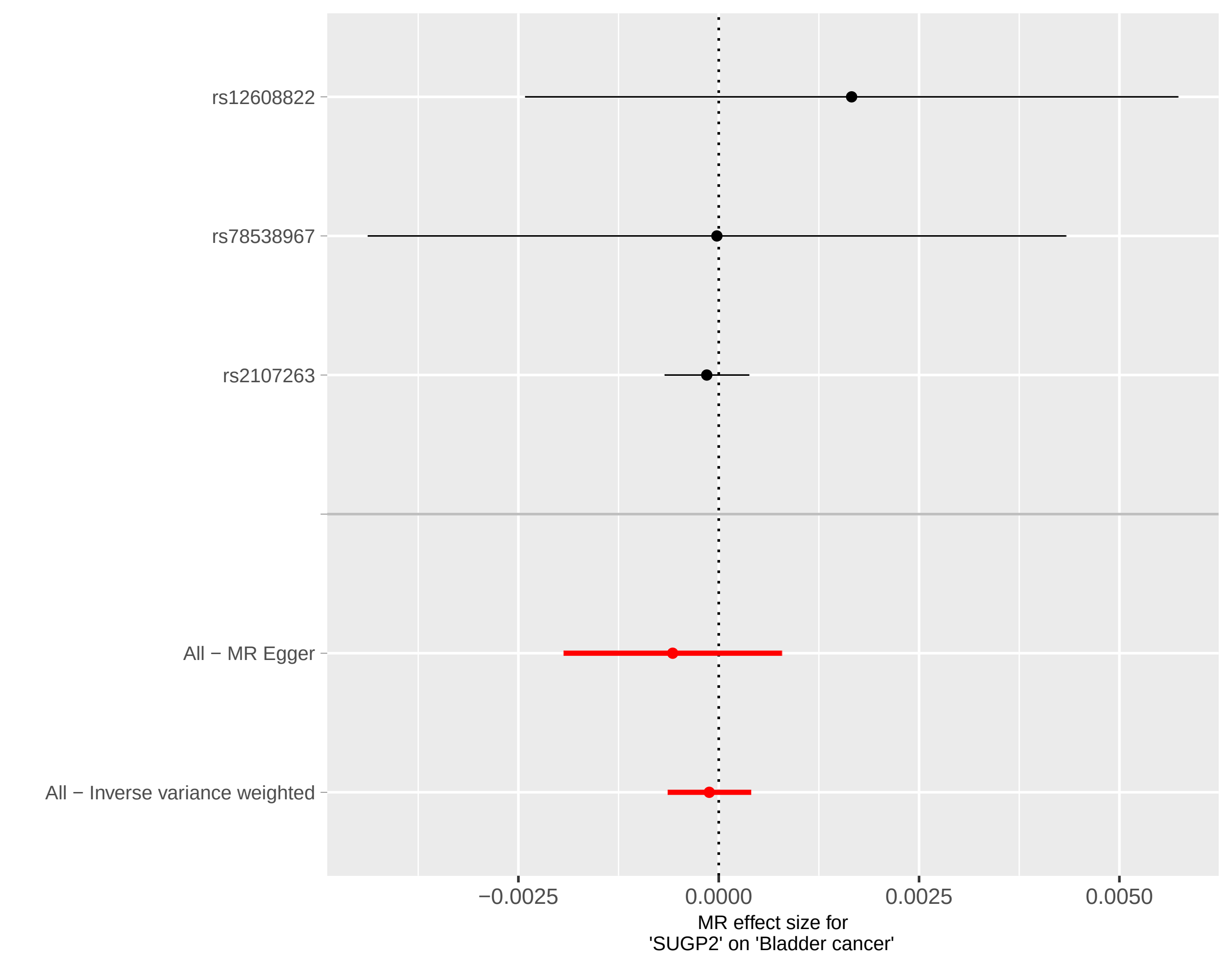

rs12608822
rs78538967
rs2107263
All − MR Egger
All − Inverse variance weighted
−0.0025
0.0000
0.0025
0.0050
MR effect size for
'SUGP2' on 'Bladder cancer'

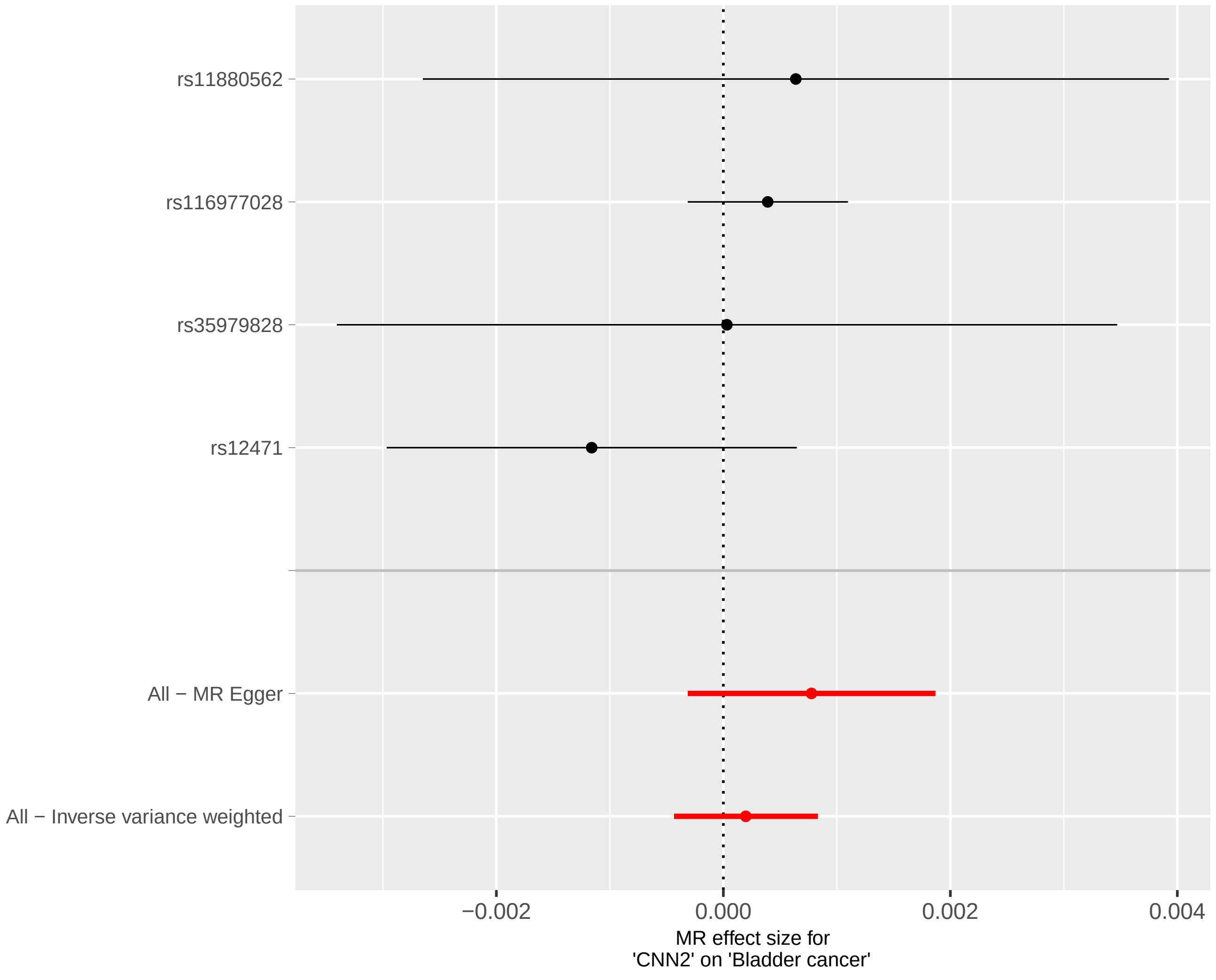

rs11880562
rs116977028
rs35979828
rs12471
All − MR Egger
All − Inverse variance weighted
−0.002
0.000
0.002
0.004
MR effect size for
'CNN2' on 'Bladder cancer'

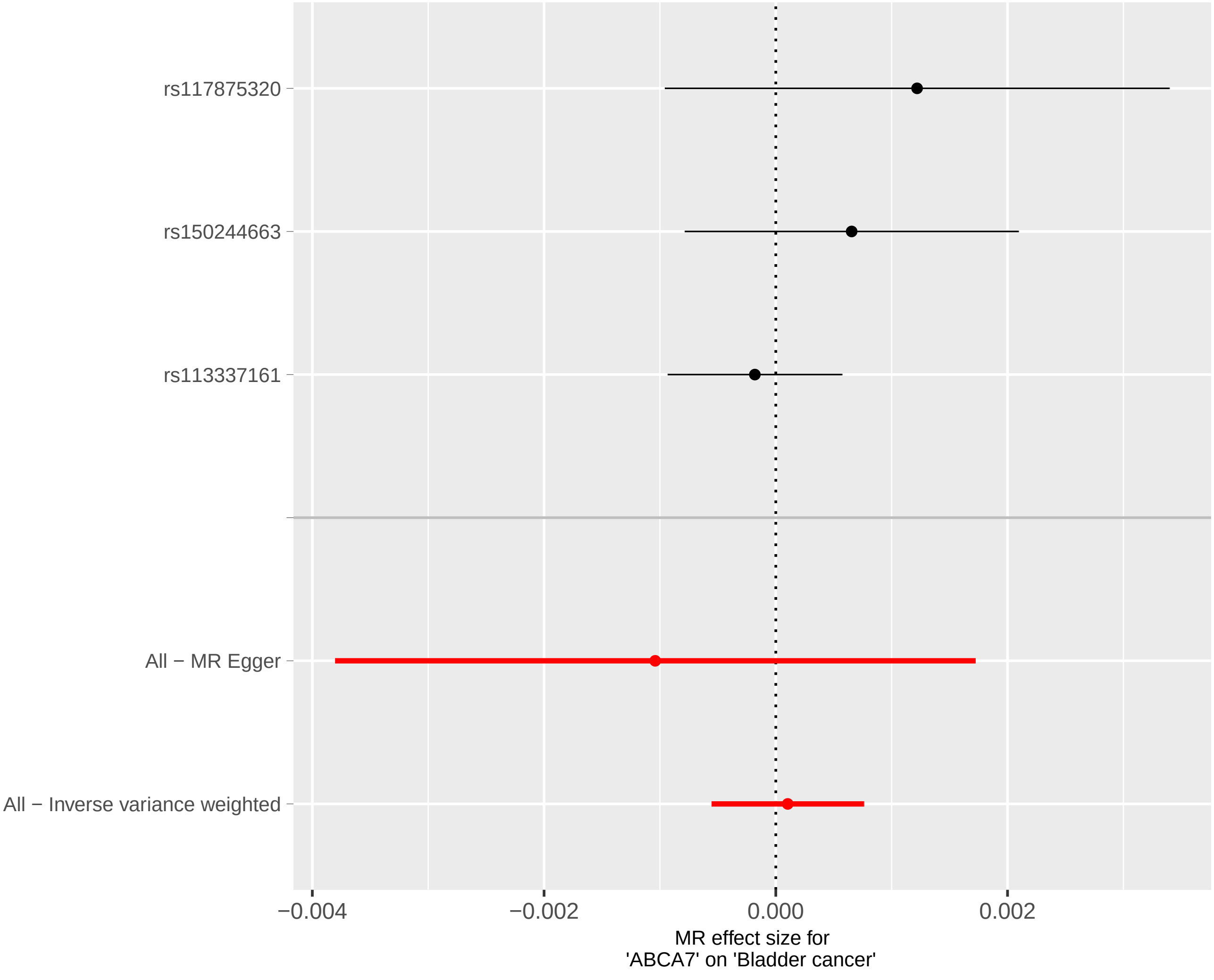

rs117875320
rs150244663
rs113337161
All − MR Egger
All − Inverse variance weighted
−0.004
−0.002
0.000
0.002
MR effect size for
'ABCA7' on 'Bladder cancer'

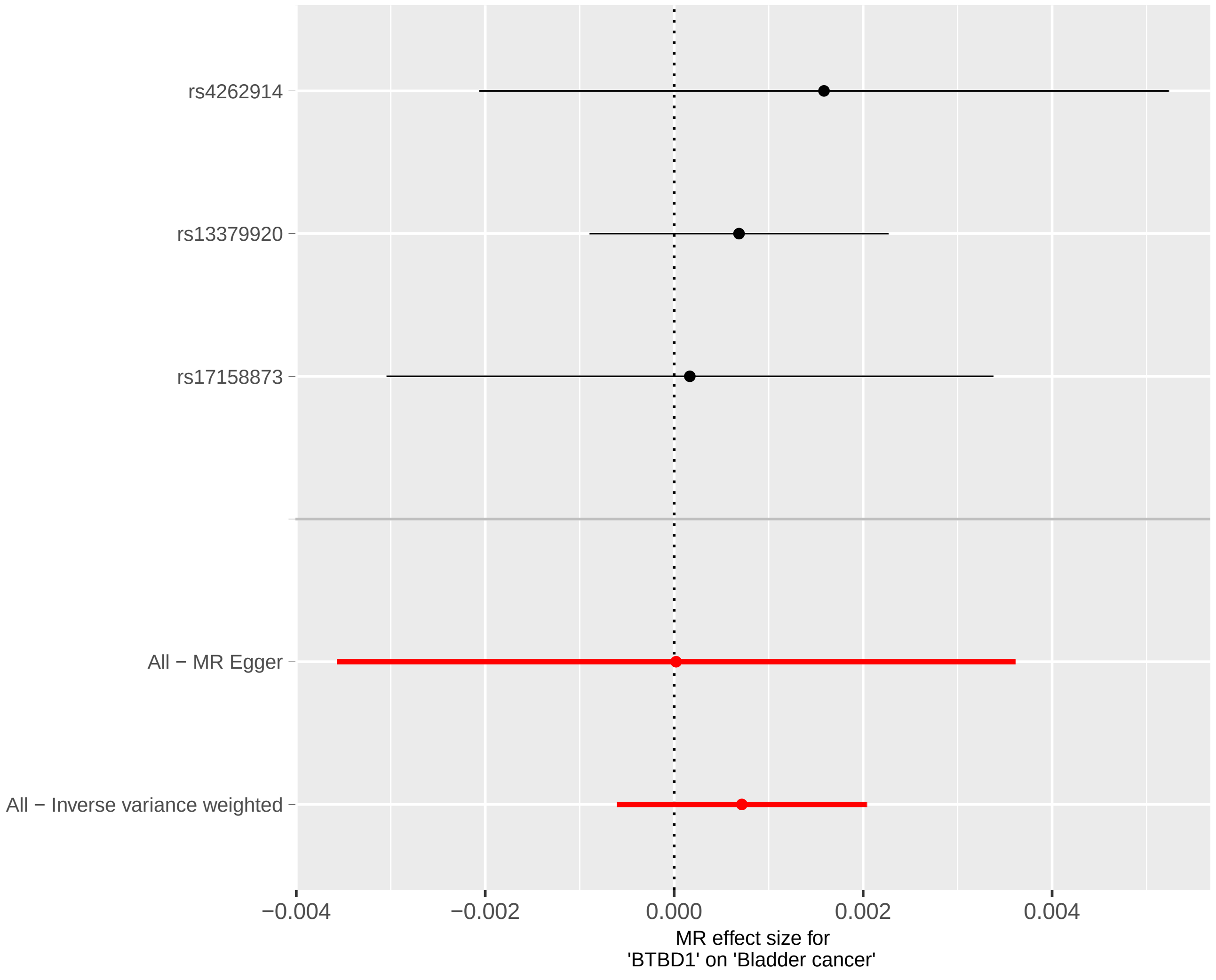
rs4262914
rs13379920
rs17158873
All − MR Egger
All − Inverse variance weighted
−0.004
−0.002
0.000
0.002
0.004
MR effect size for
'BTBD1' on 'Bladder cancer'

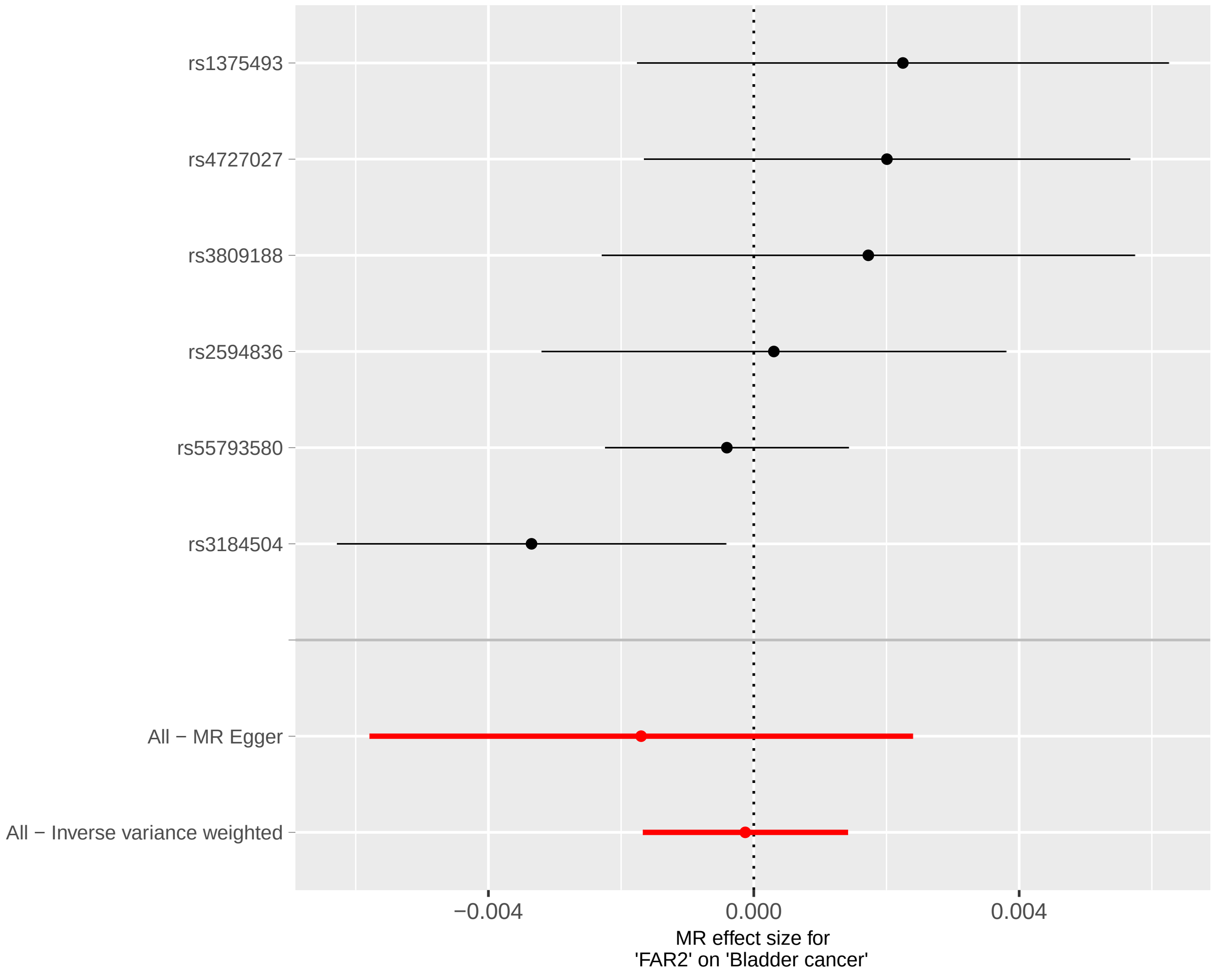
rs1375493
rs4727027
rs3809188
rs2594836
rs55793580
rs3184504
All − MR Egger
All − Inverse variance weighted
−0.004
0.000
0.004
MR effect size for
'FAR2' on 'Bladder cancer'

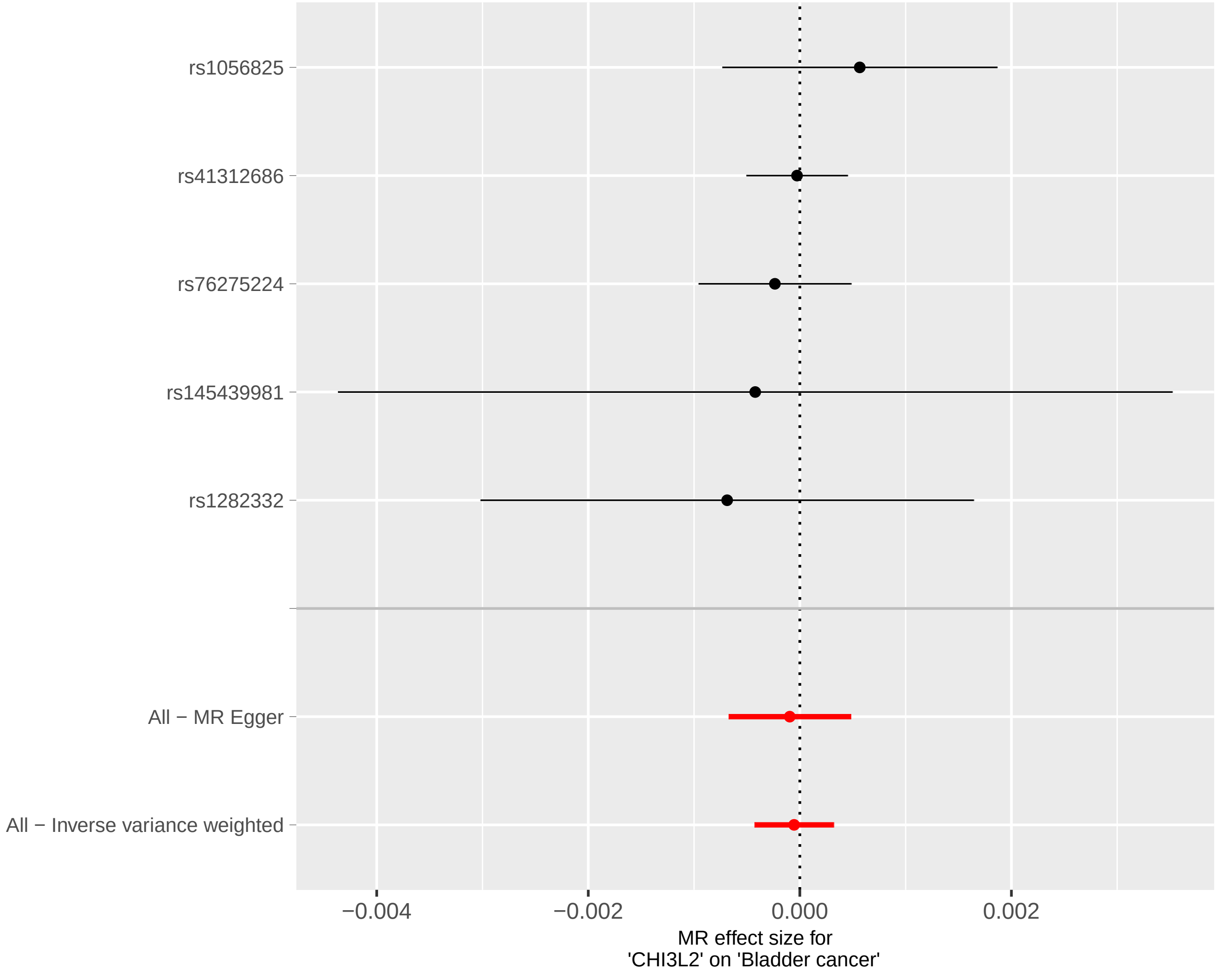
rs1056825
rs41312686
rs76275224
rs145439981
rs1282332
All − MR Egger
All − Inverse variance weighted
−0.004
−0.002
0.000
0.002
MR effect size for
'CHI3L2' on 'Bladder cancer'

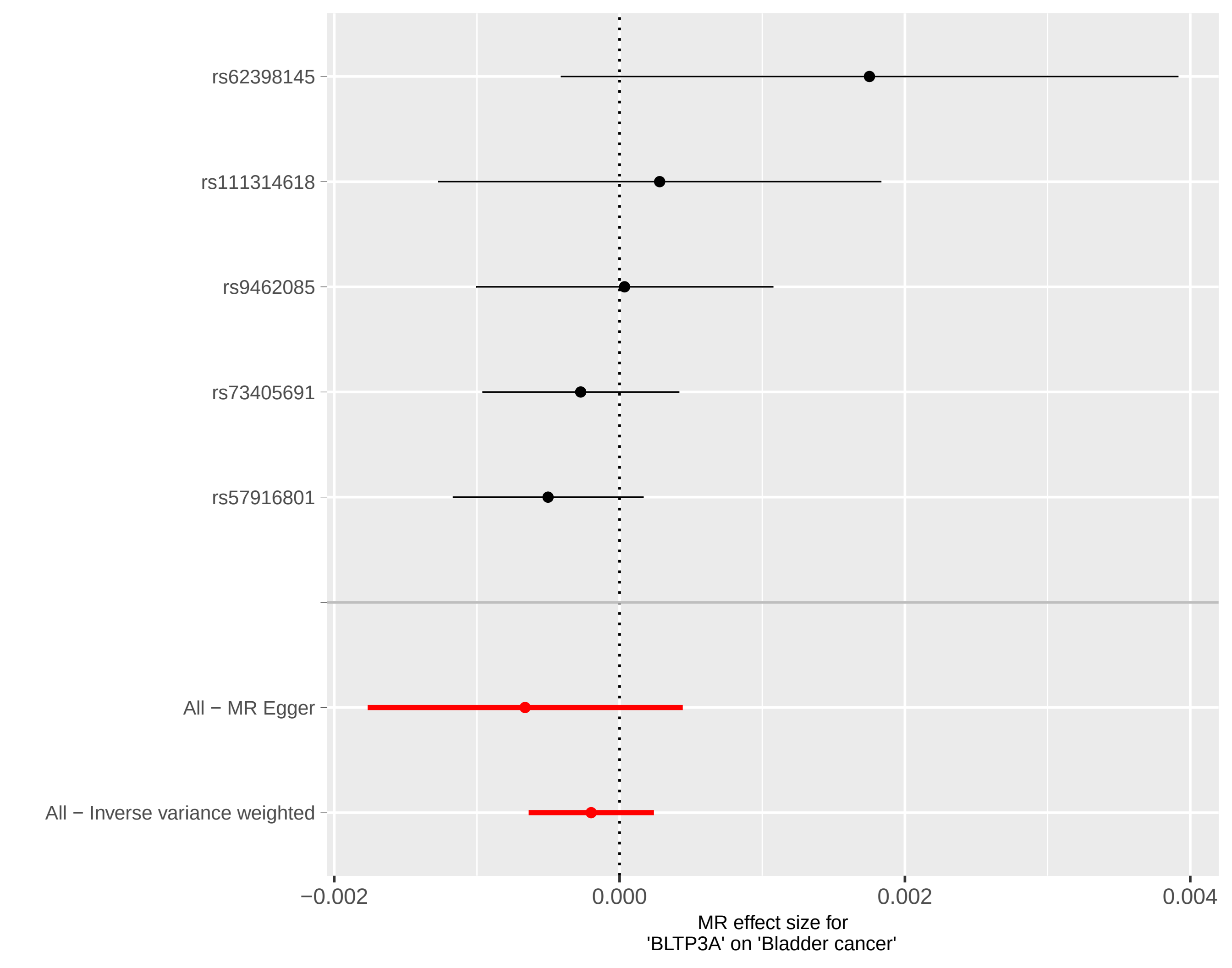

rs62398145
rs111314618
rs9462085
rs73405691
rs57916801
All − MR Egger
All − Inverse variance weighted
−0.002
0.000
0.002
0.004
MR effect size for
'BLTP3A' on 'Bladder cancer'

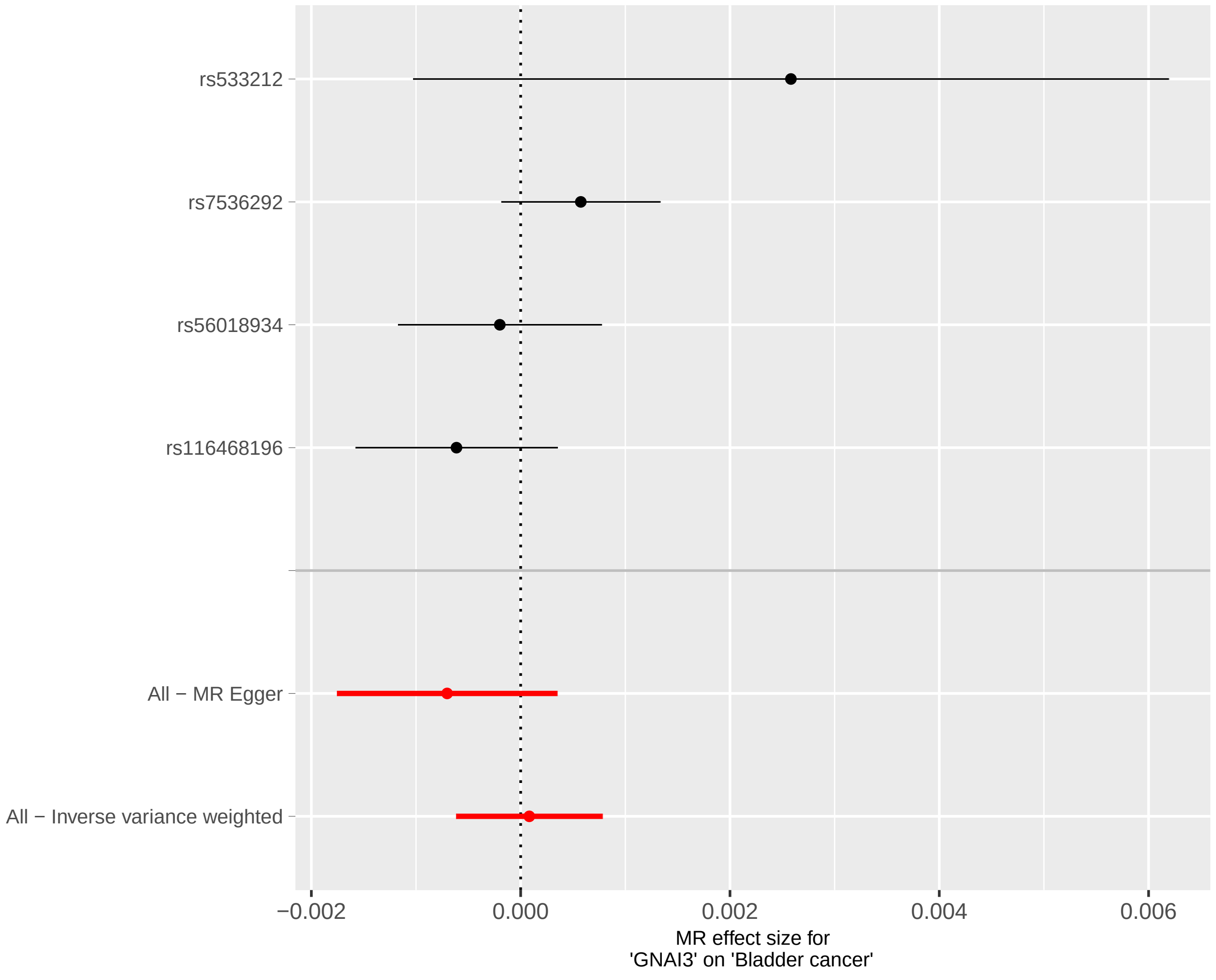

rs533212
rs7536292
rs56018934
rs116468196
All − MR Egger
All − Inverse variance weighted
−0.002
0.000
0.002
0.004
0.006
MR effect size for
'GNAI3' on 'Bladder cancer'

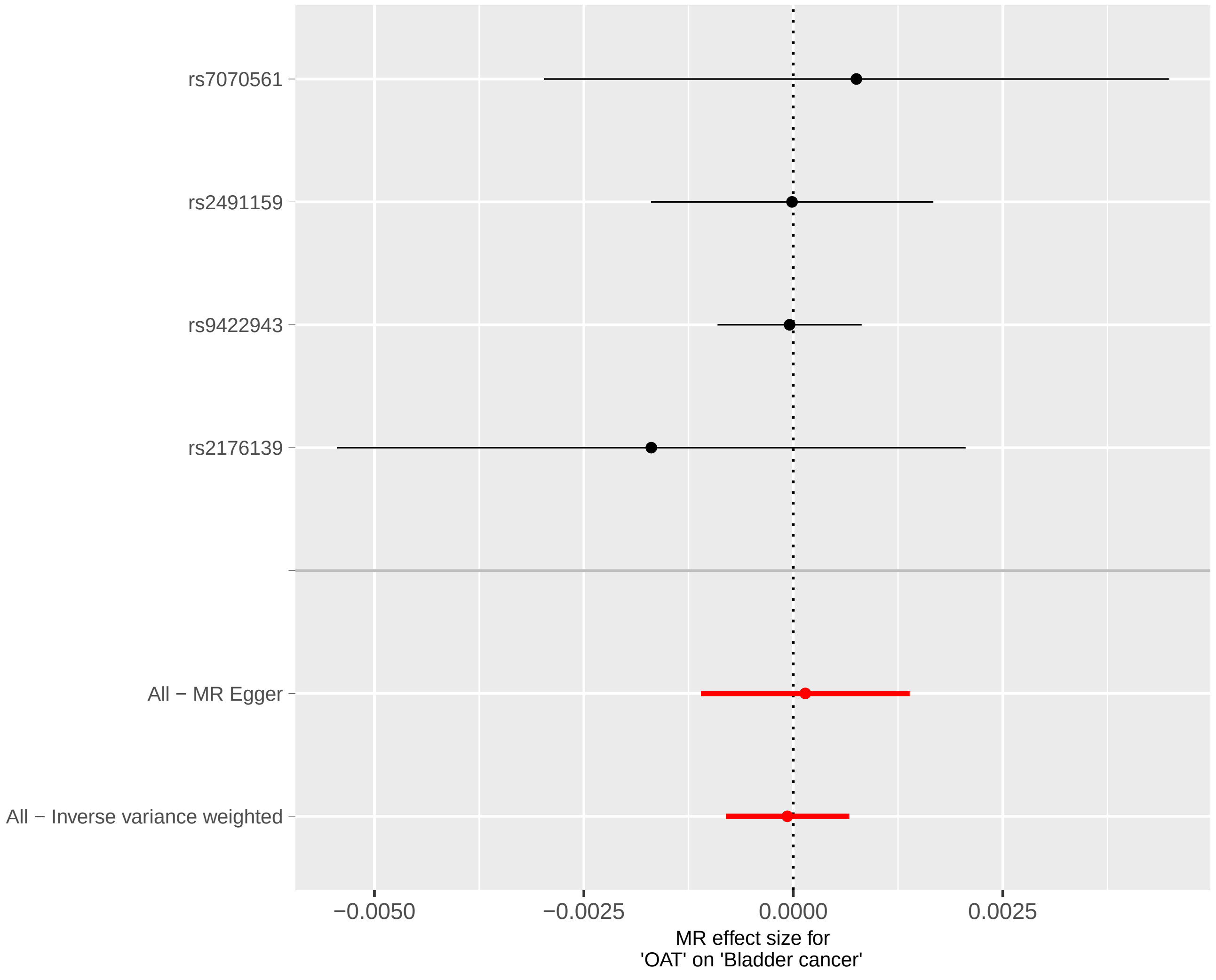

rs7070561
rs2491159
rs9422943
rs2176139
All − MR Egger
All − Inverse variance weighted
−0.0050
−0.0025
0.0000
0.0025
MR effect size for
'OAT' on 'Bladder cancer'

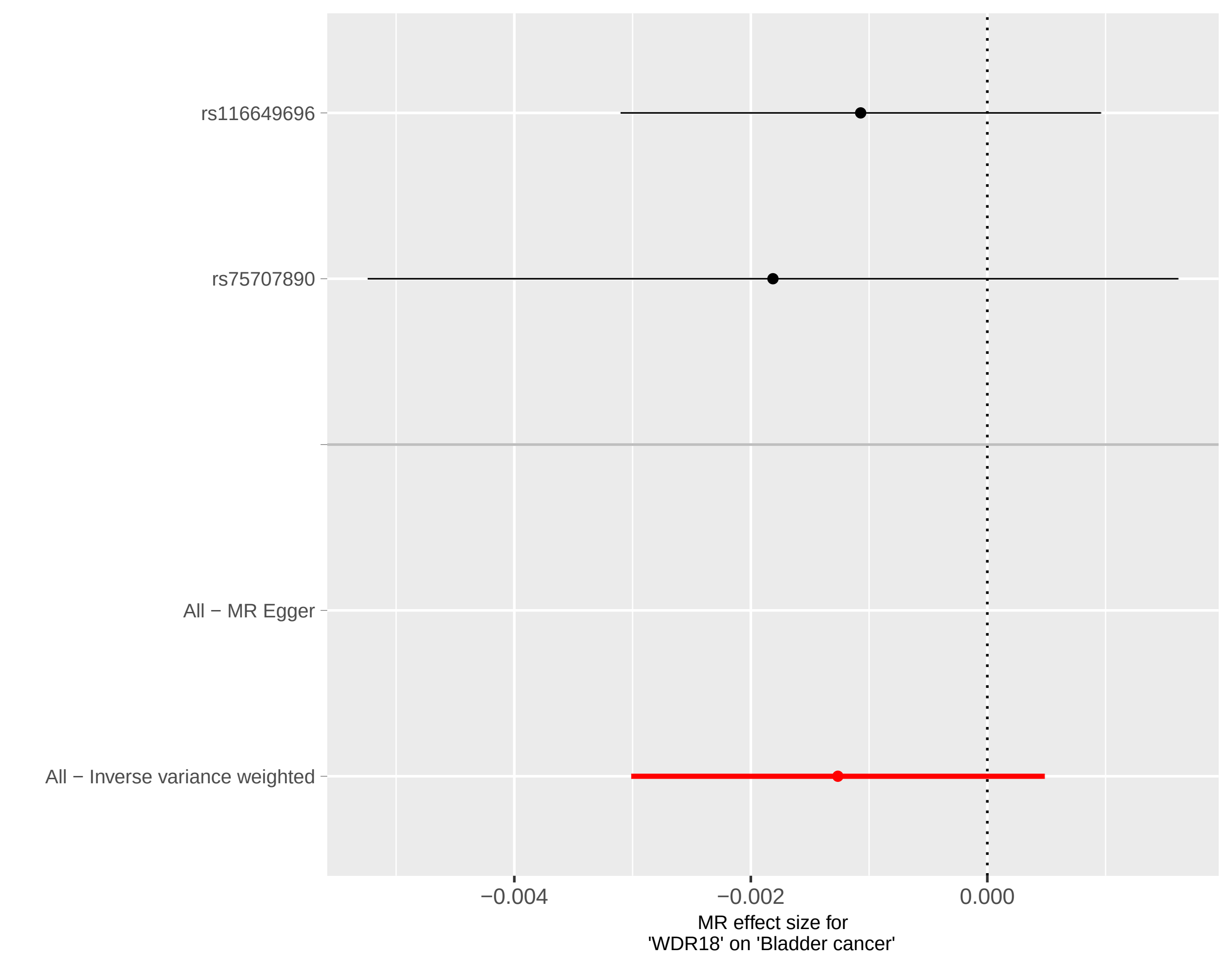

rs116649696
rs75707890
All − MR Egger
All − Inverse variance weighted
−0.004
−0.002
0.000
MR effect size for
'WDR18' on 'Bladder cancer'

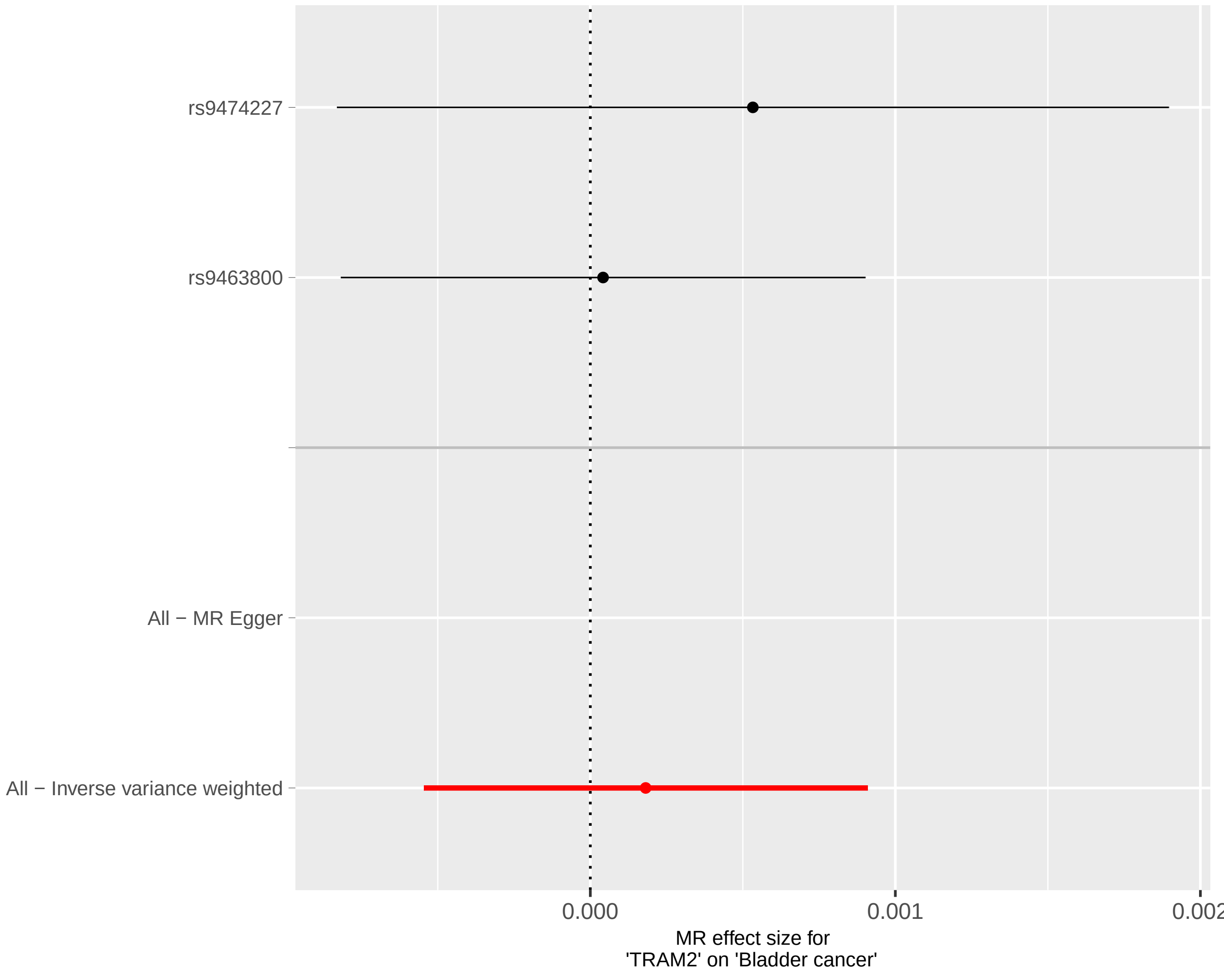
rs9474227
rs9463800
All − MR Egger
All − Inverse variance weighted
0.000
0.001
0.002
MR effect size for
'TRAM2' on 'Bladder cancer'

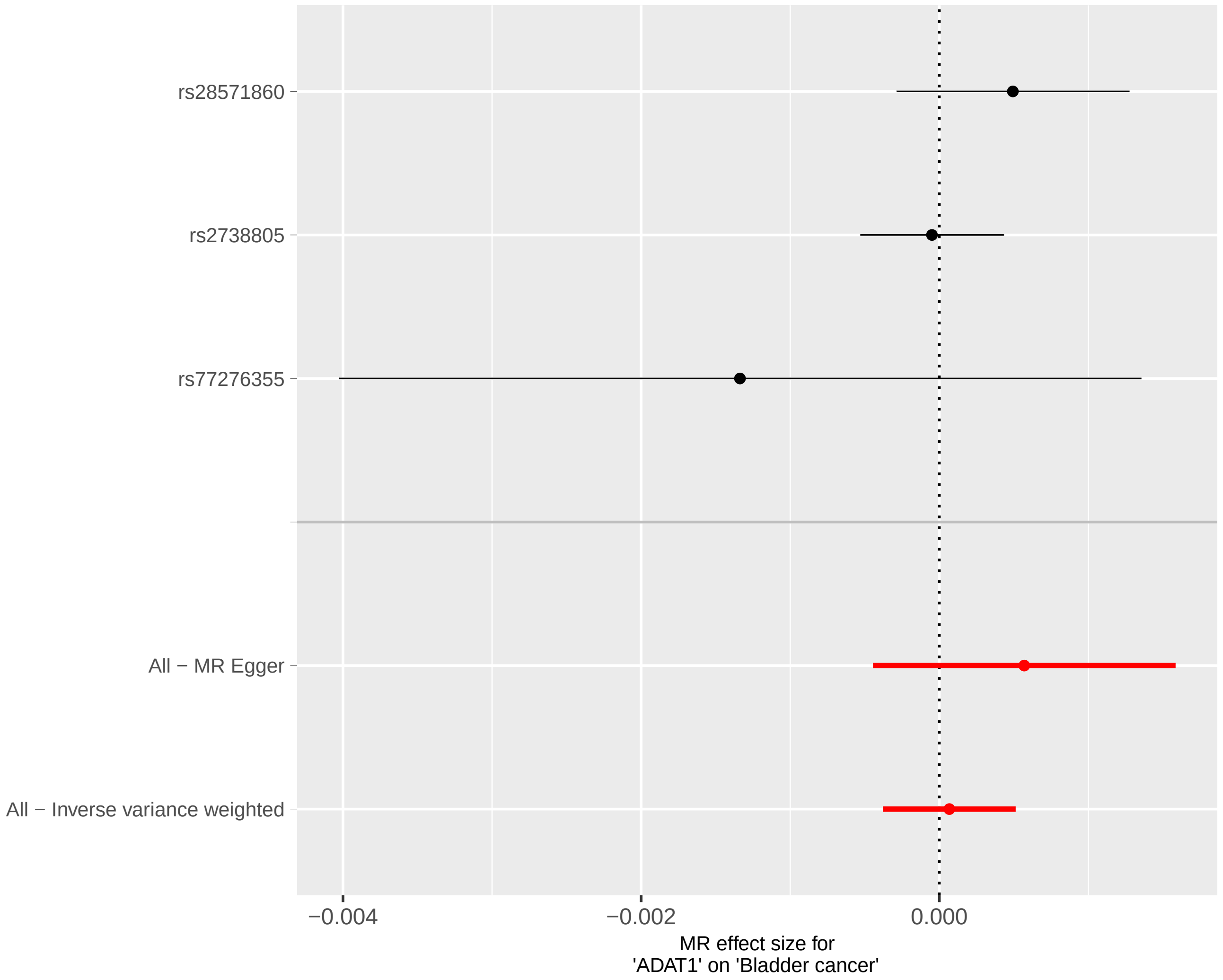
rs28571860
rs2738805
rs77276355
All − MR Egger
All − Inverse variance weighted
−0.004
−0.002
0.000
MR effect size for
'ADAT1' on 'Bladder cancer'

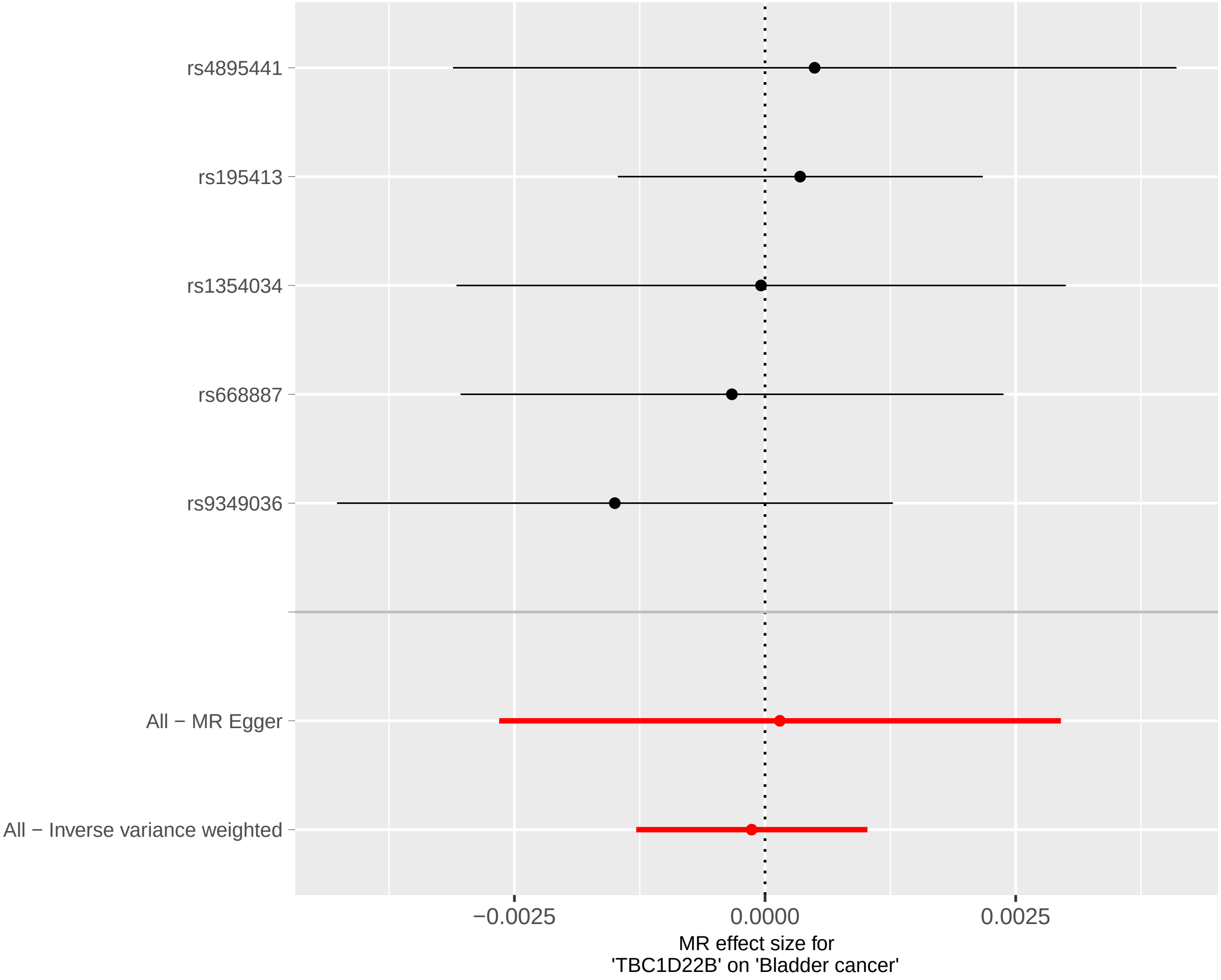
rs4895441
rs195413
rs1354034
rs668887
rs9349036
All − MR Egger
All − Inverse variance weighted
−0.0025
0.0000
0.0025
MR effect size for
'TBC1D22B' on 'Bladder cancer'

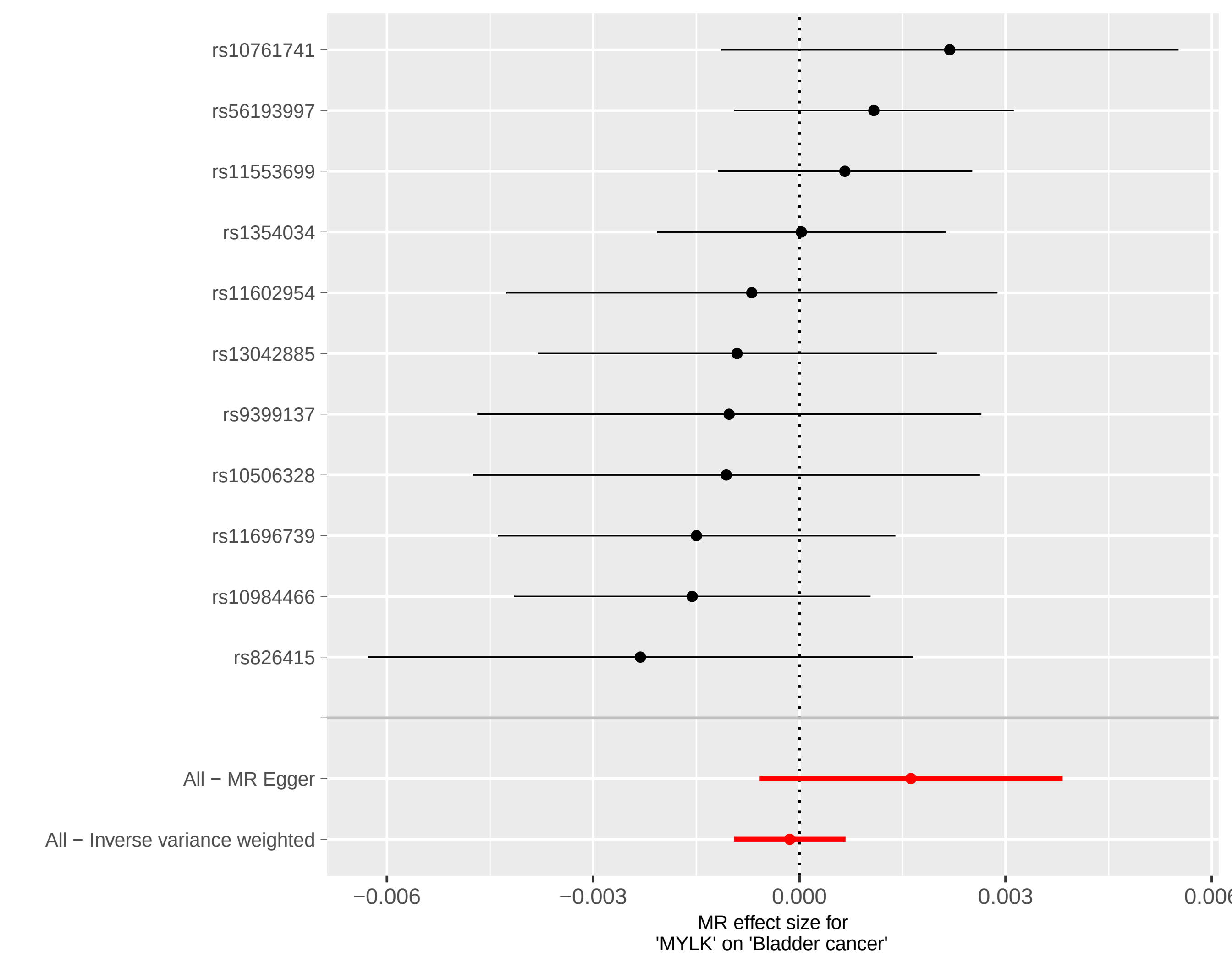

rs10761741
rs56193997
rs11553699
rs1354034
rs11602954
rs13042885
rs9399137
rs10506328
rs11696739
rs10984466
rs826415
All − MR Egger
All − Inverse variance weighted
−0.006
−0.003
0.000
0.003
MR effect size for
'MYLK' on 'Bladder cancer'

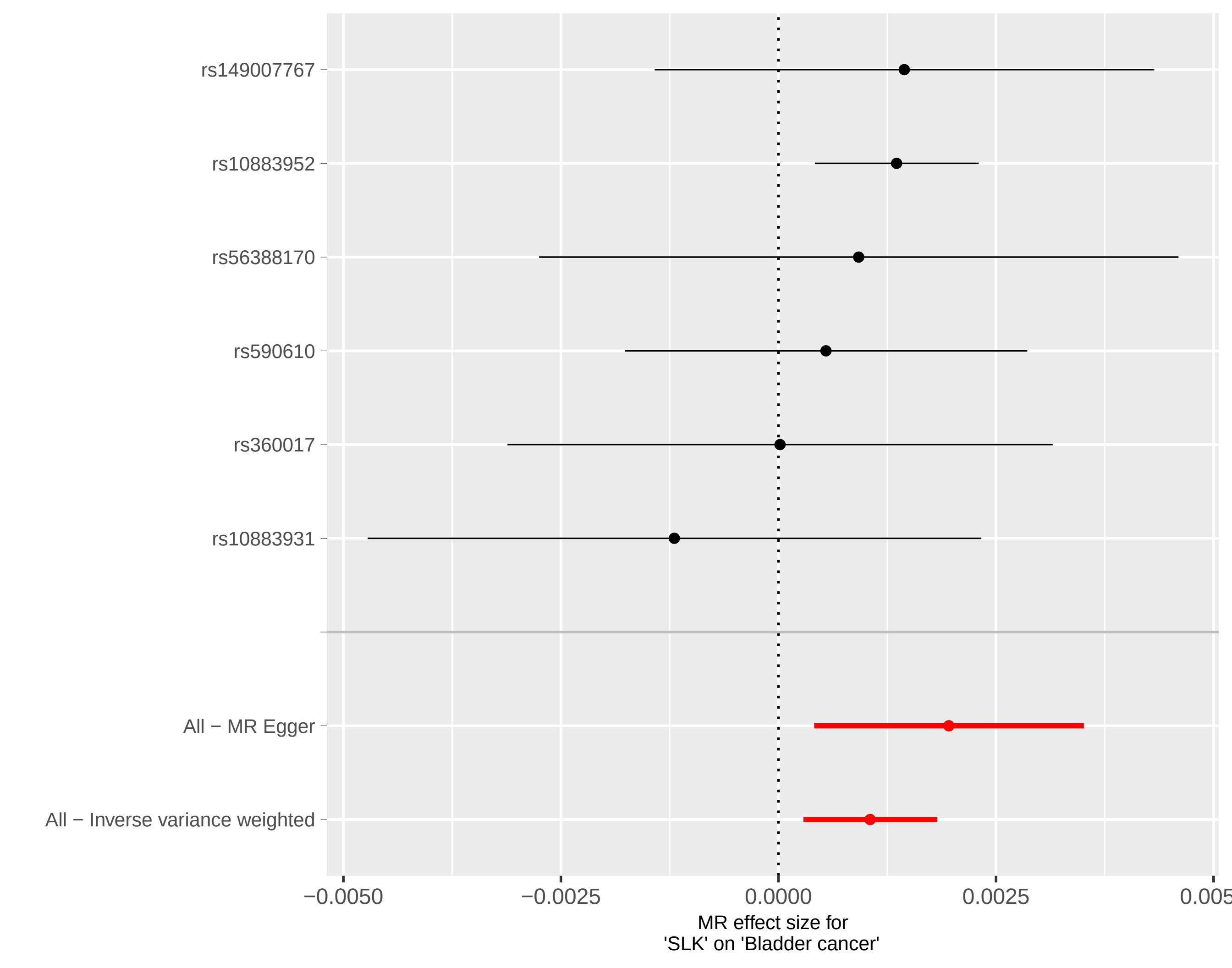
rs149007767
rs10883952
rs56388170
rs590610
rs360017
rs10883931
All − MR Egger
All − Inverse variance weighted
−0.0050
−0.0025
0.0000
0.0025
MR effect size for
'SLK' on 'Bladder cancer'

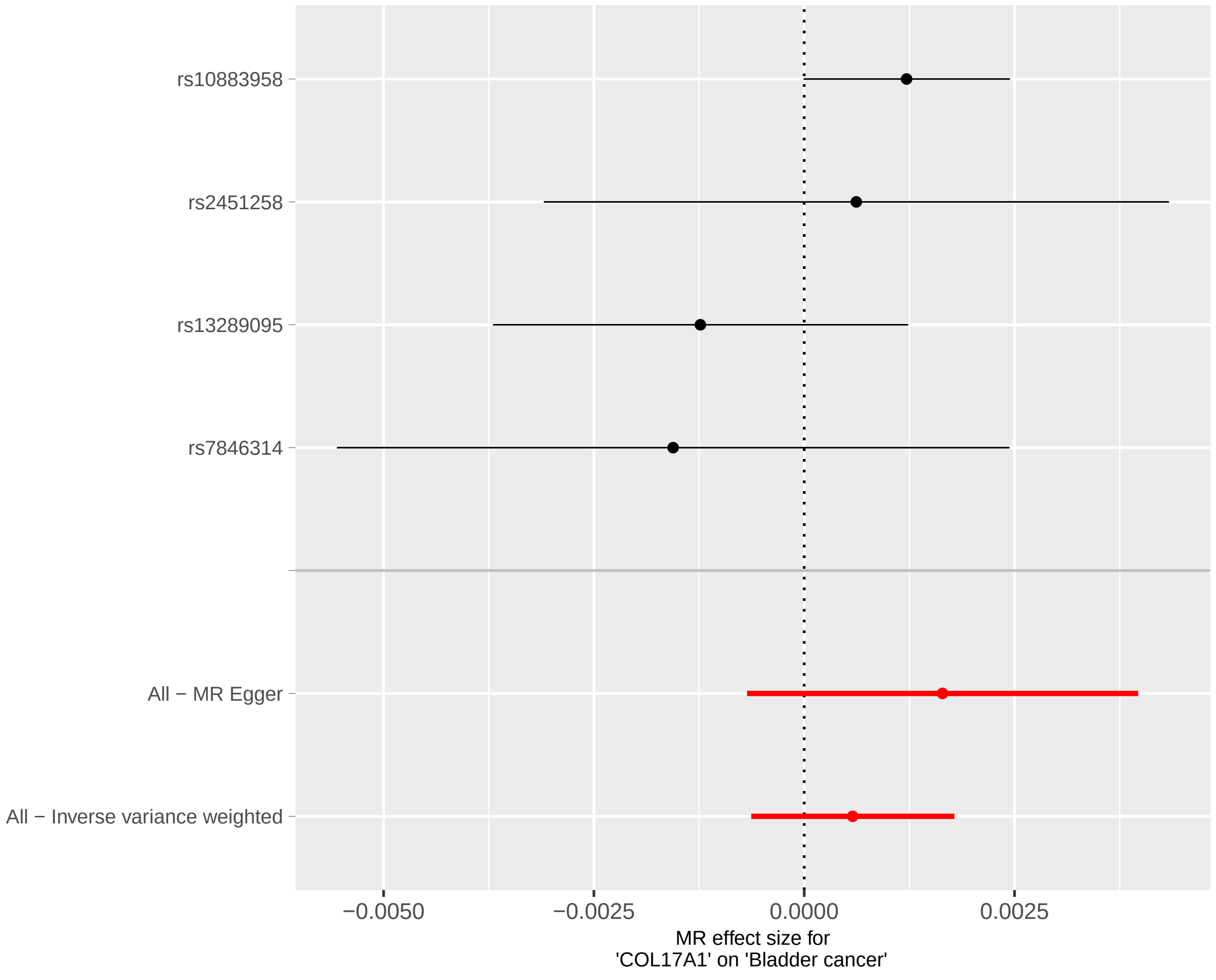

rs10883958
rs2451258
rs13289095
rs7846314
All − MR Egger
All − Inverse variance weighted
−0.0050
−0.0025
0.0000
0.0025
MR effect size for
'COL17A1' on 'Bladder cancer'

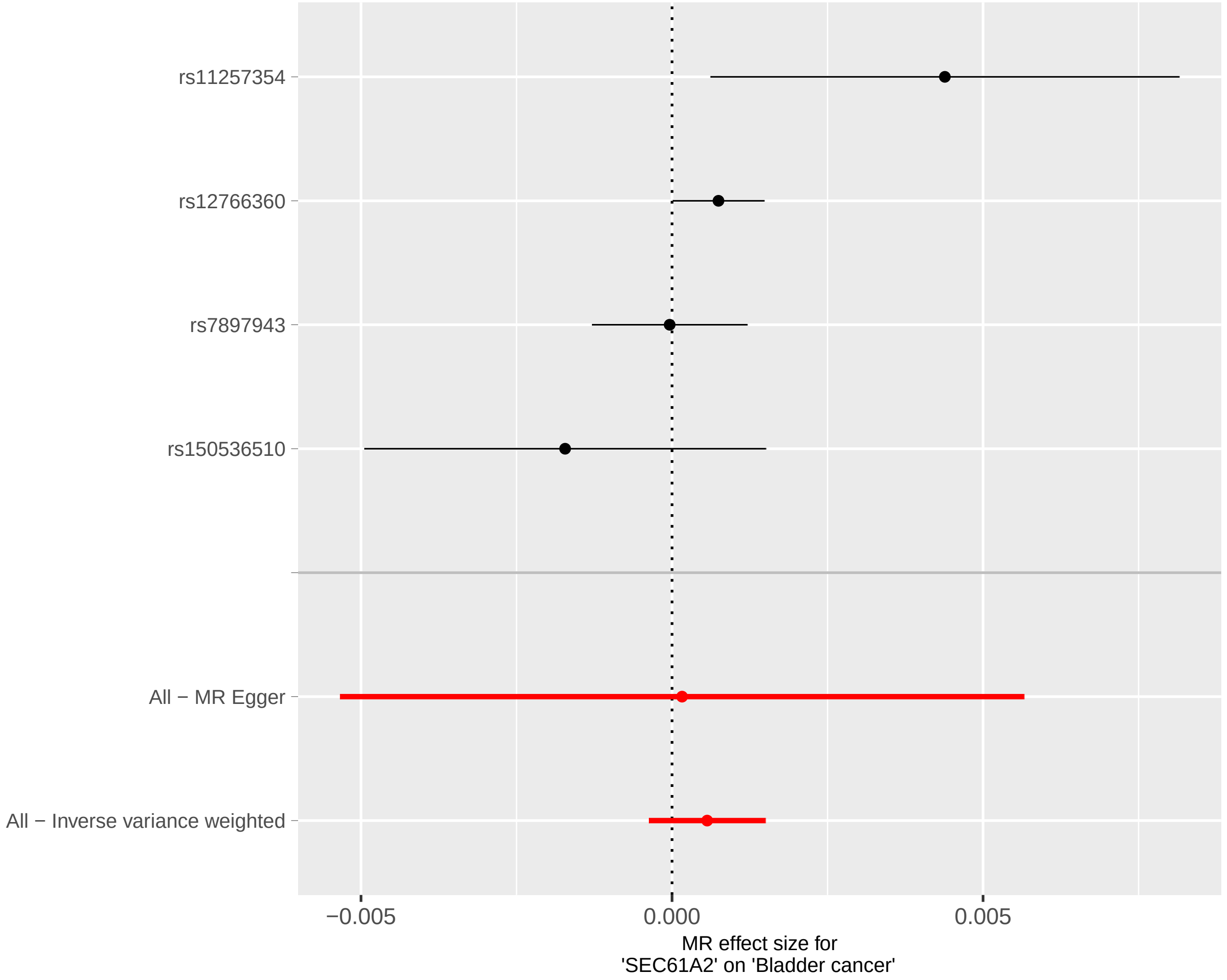
rs11257354
rs12766360
rs7897943
rs150536510
All − MR Egger
All − Inverse variance weighted
−0.005
0.000
0.005
MR effect size for
'SEC61A2' on 'Bladder cancer'

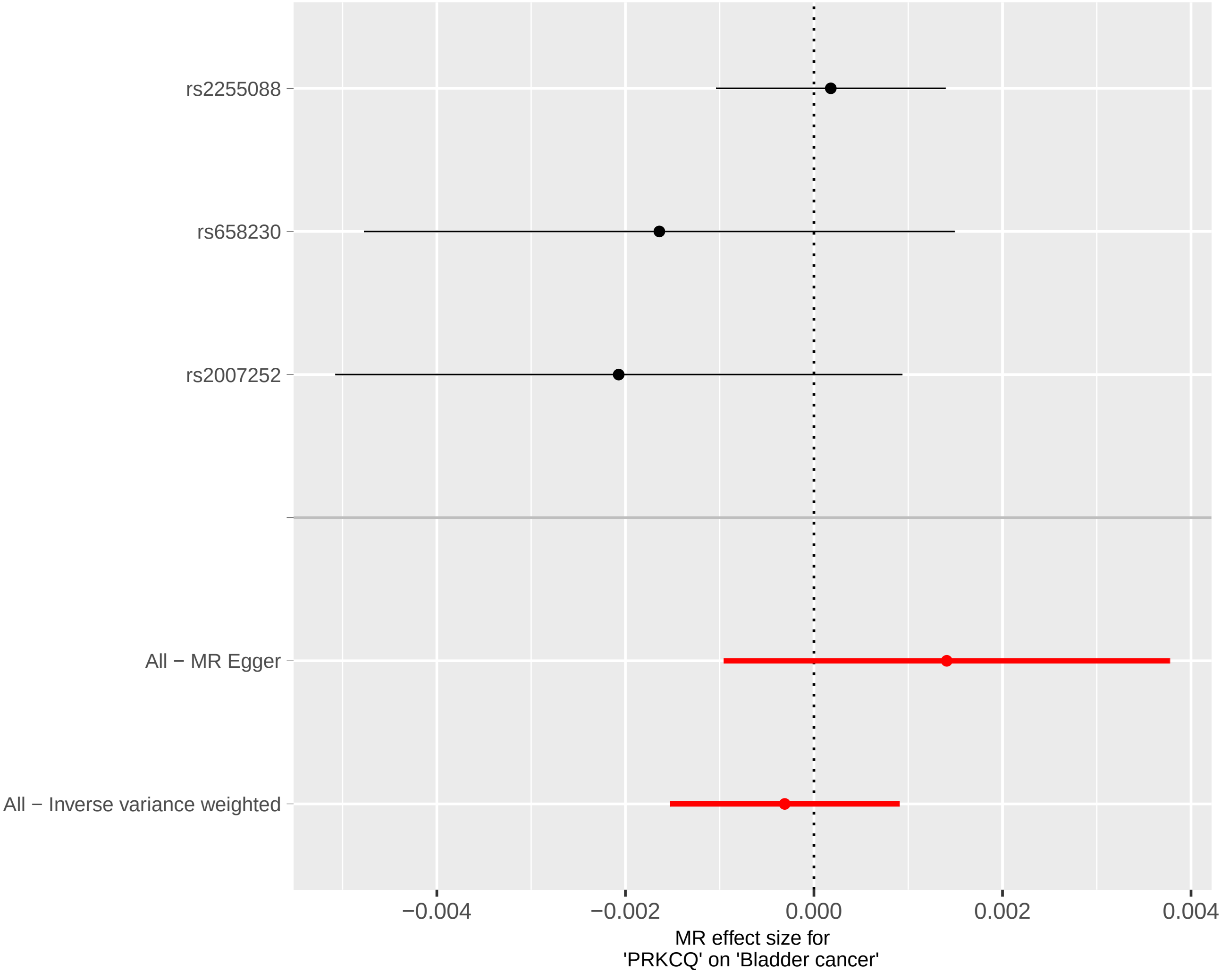

rs2255088
rs658230
rs2007252
All − MR Egger
All − Inverse variance weighted
−0.004
−0.002
0.000
0.002
0.004
MR effect size for
'PRKCQ' on 'Bladder cancer'

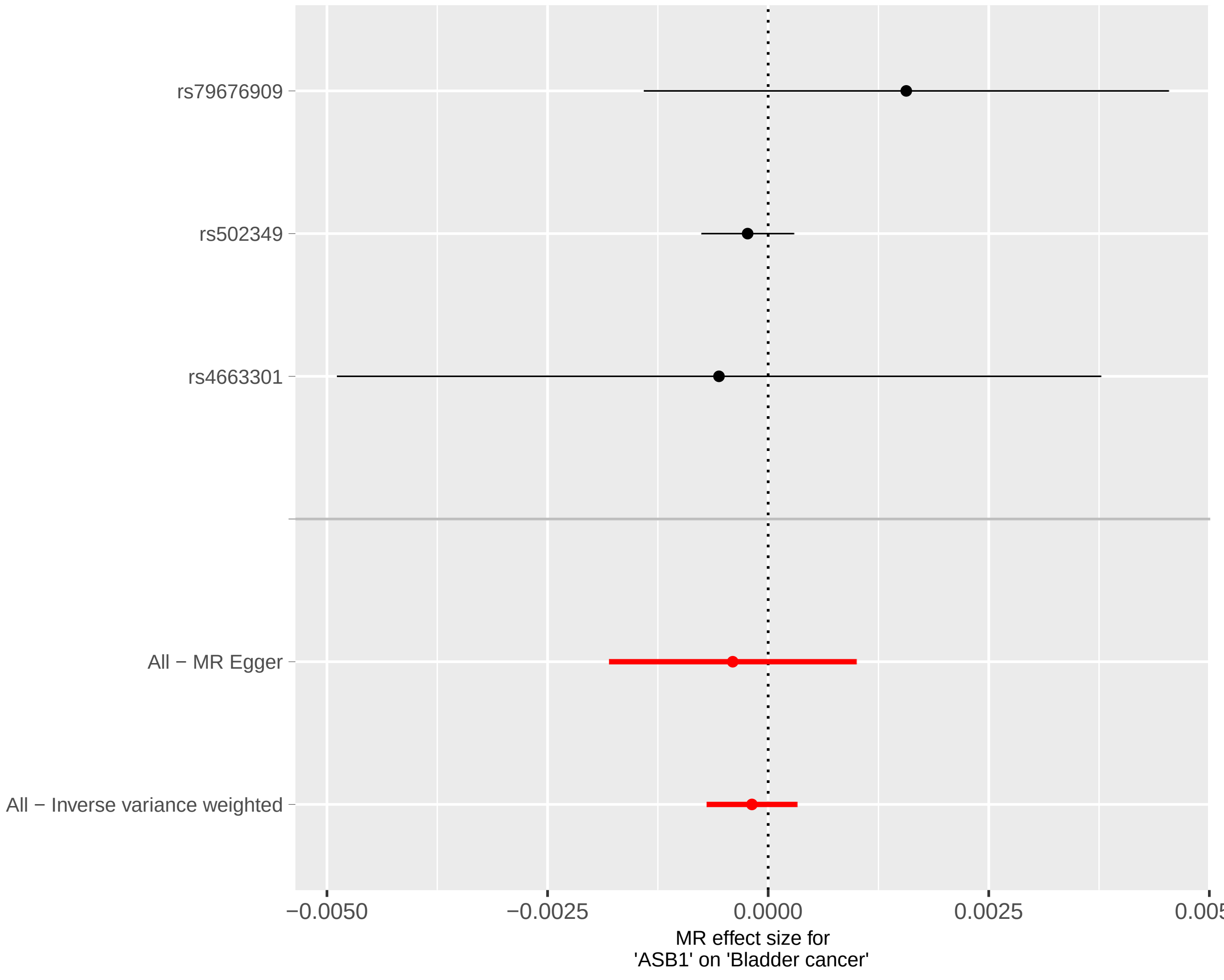
rs79676909
rs502349
rs4663301
All − MR Egger
All − Inverse variance weighted
−0.0050
−0.0025
0.0000
0.0025
MR effect size for
'ASB1' on 'Bladder cancer'

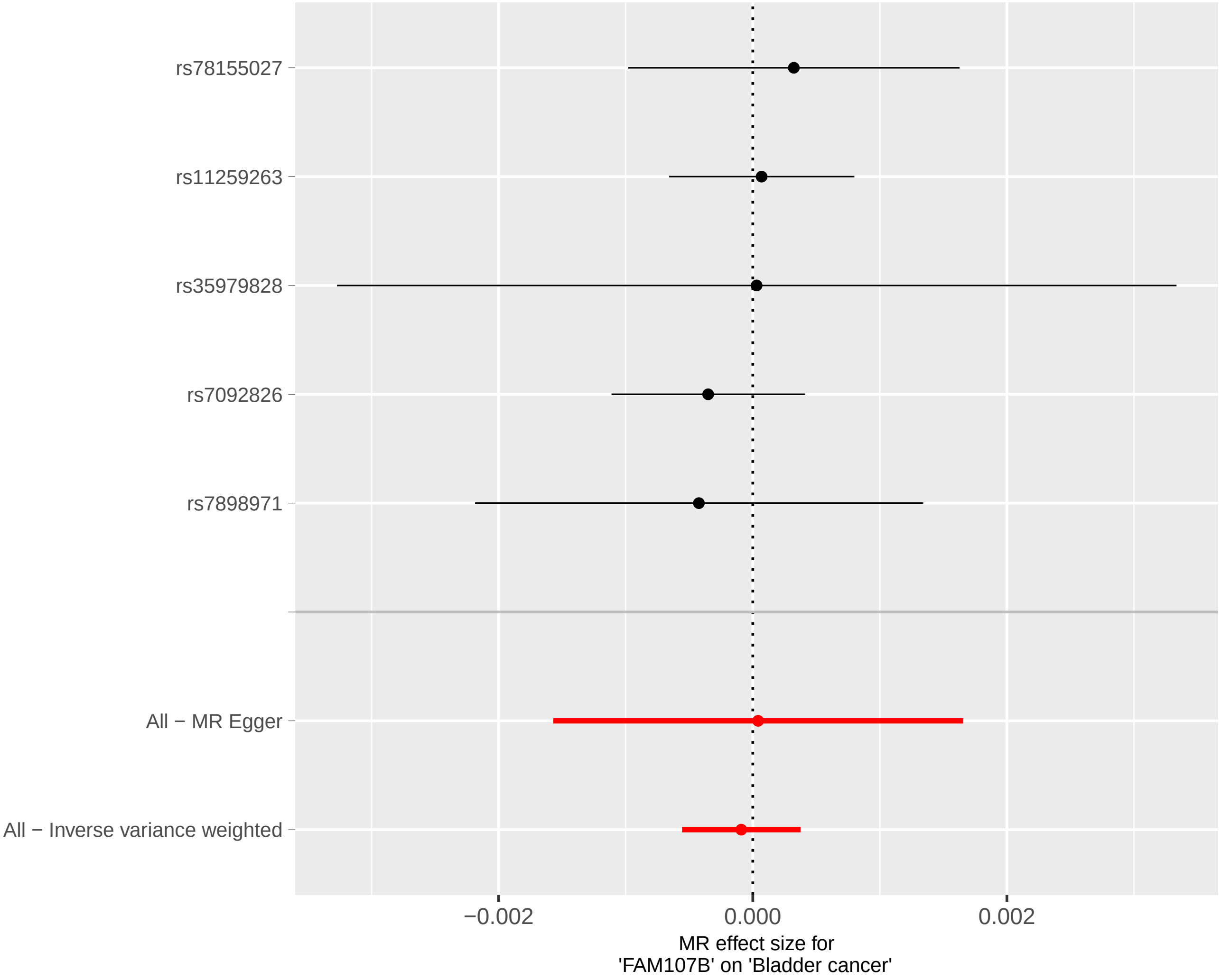
rs78155027
rs11259263
rs35979828
rs7092826
rs7898971
All − MR Egger
All − Inverse variance weighted
−0.002
0.000
0.002
MR effect size for
'FAM107B' on 'Bladder cancer'

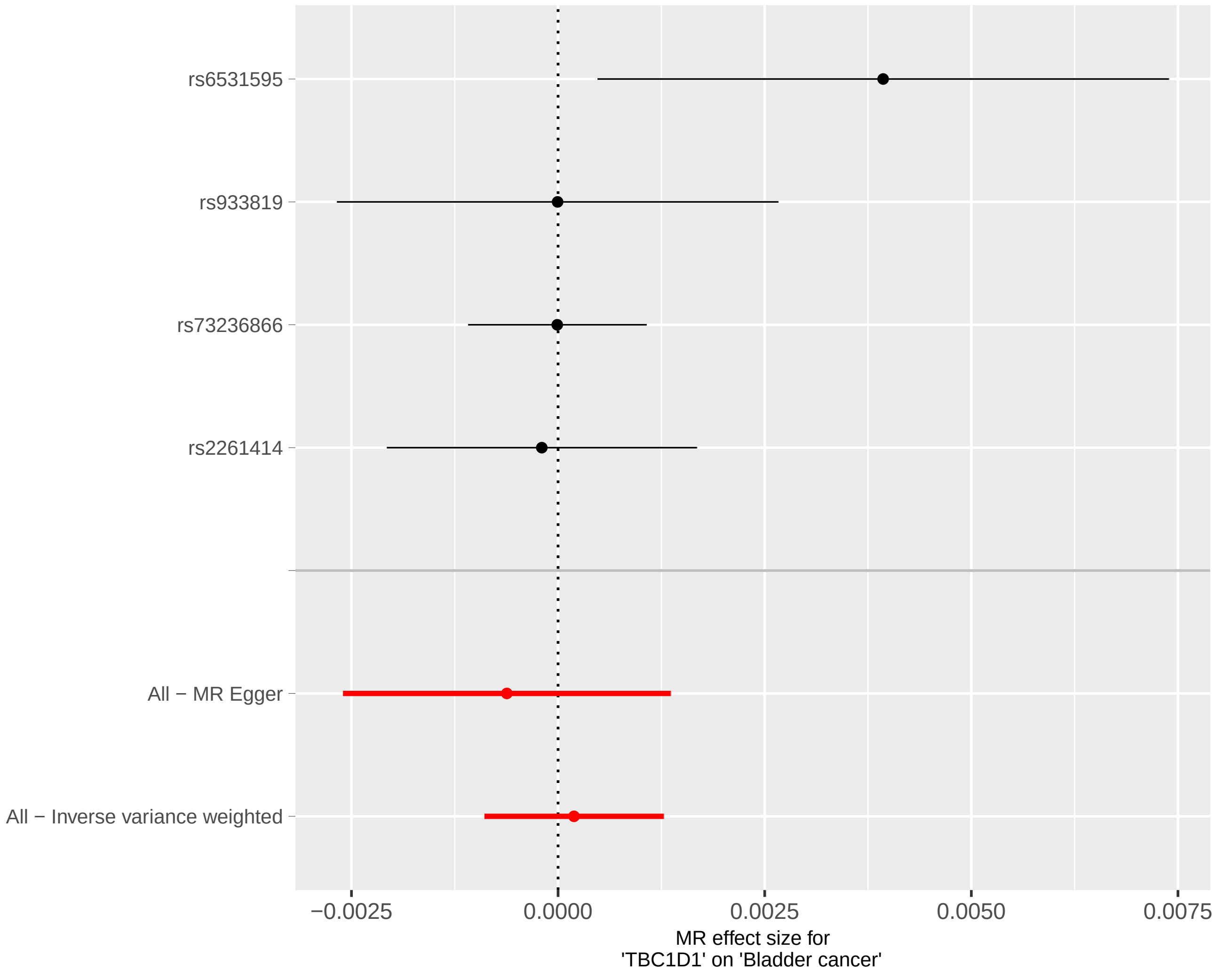
rs6531595
rs933819
rs73236866
rs2261414
All − MR Egger
All − Inverse variance weighted
−0.0025
0.0000
0.0025
0.0050
0.0075
MR effect size for
'TBC1D1' on 'Bladder cancer'

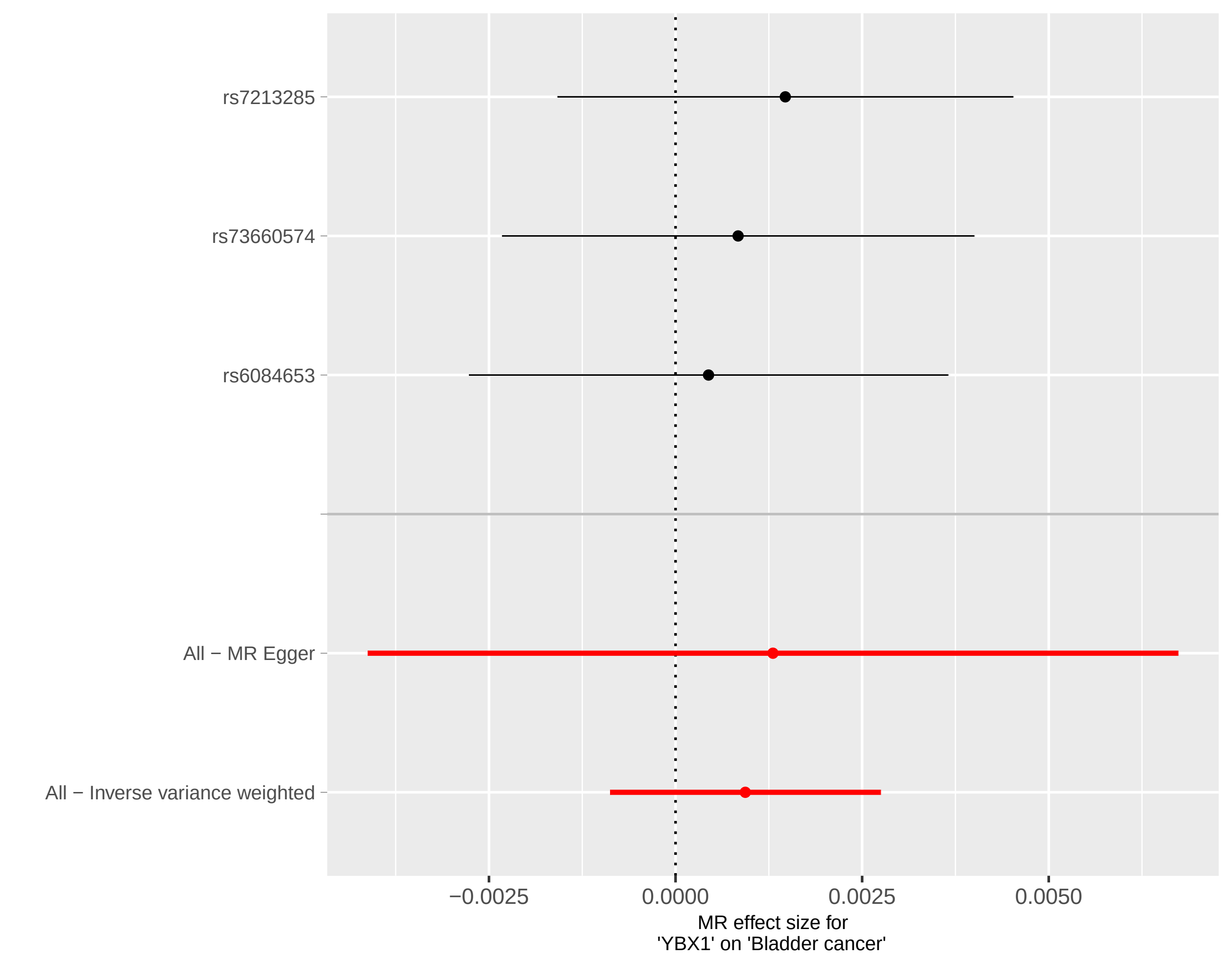
rs7213285
rs73660574
rs6084653
All − MR Egger
All − Inverse variance weighted
−0.0025
0.0000
0.0025
0.0050
MR effect size for
'YBX1' on 'Bladder cancer'

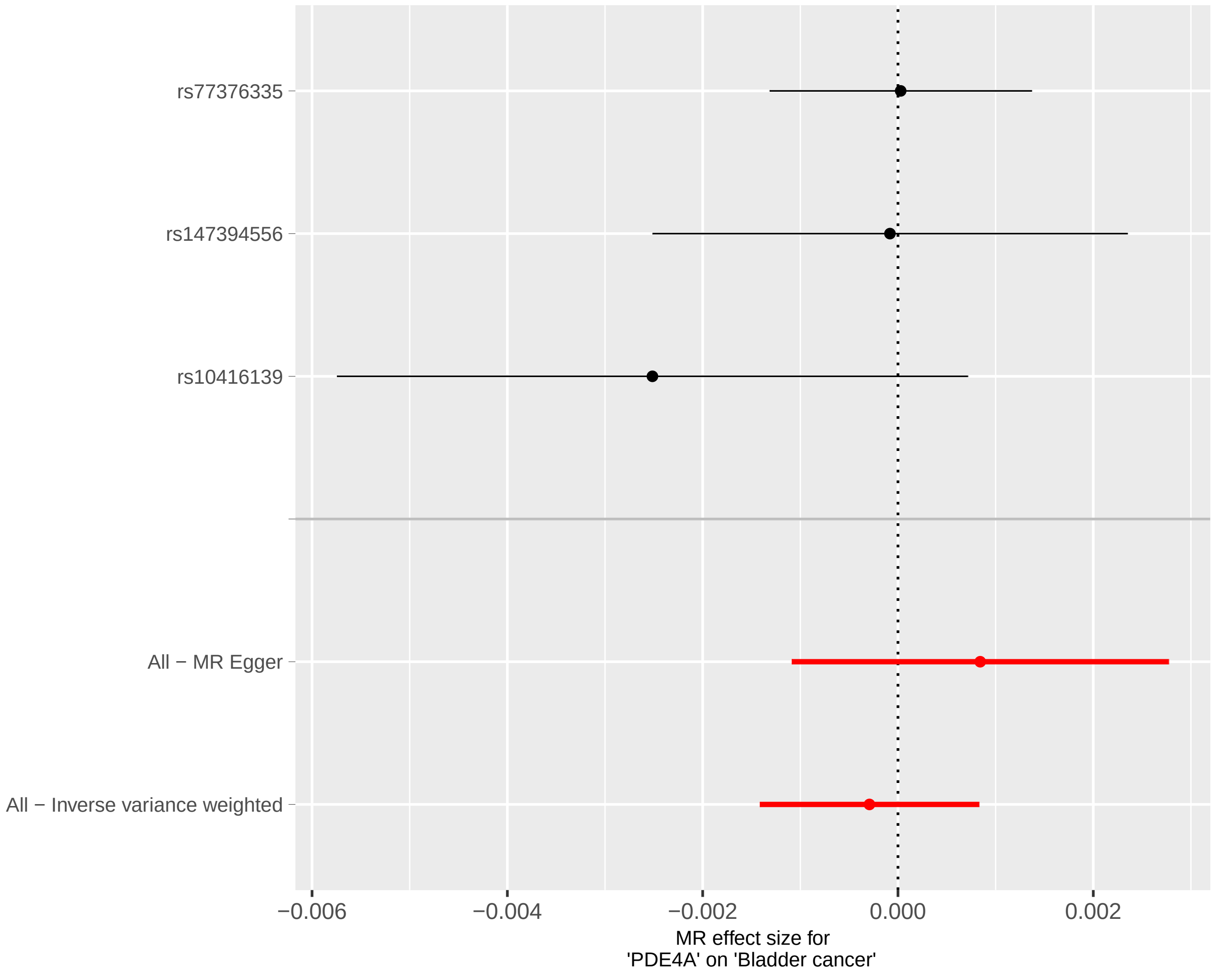

rs77376335
rs147394556
rs10416139
All − MR Egger
All − Inverse variance weighted
−0.006
−0.004
−0.002
0.000
0.002
MR effect size for
'PDE4A' on 'Bladder cancer'

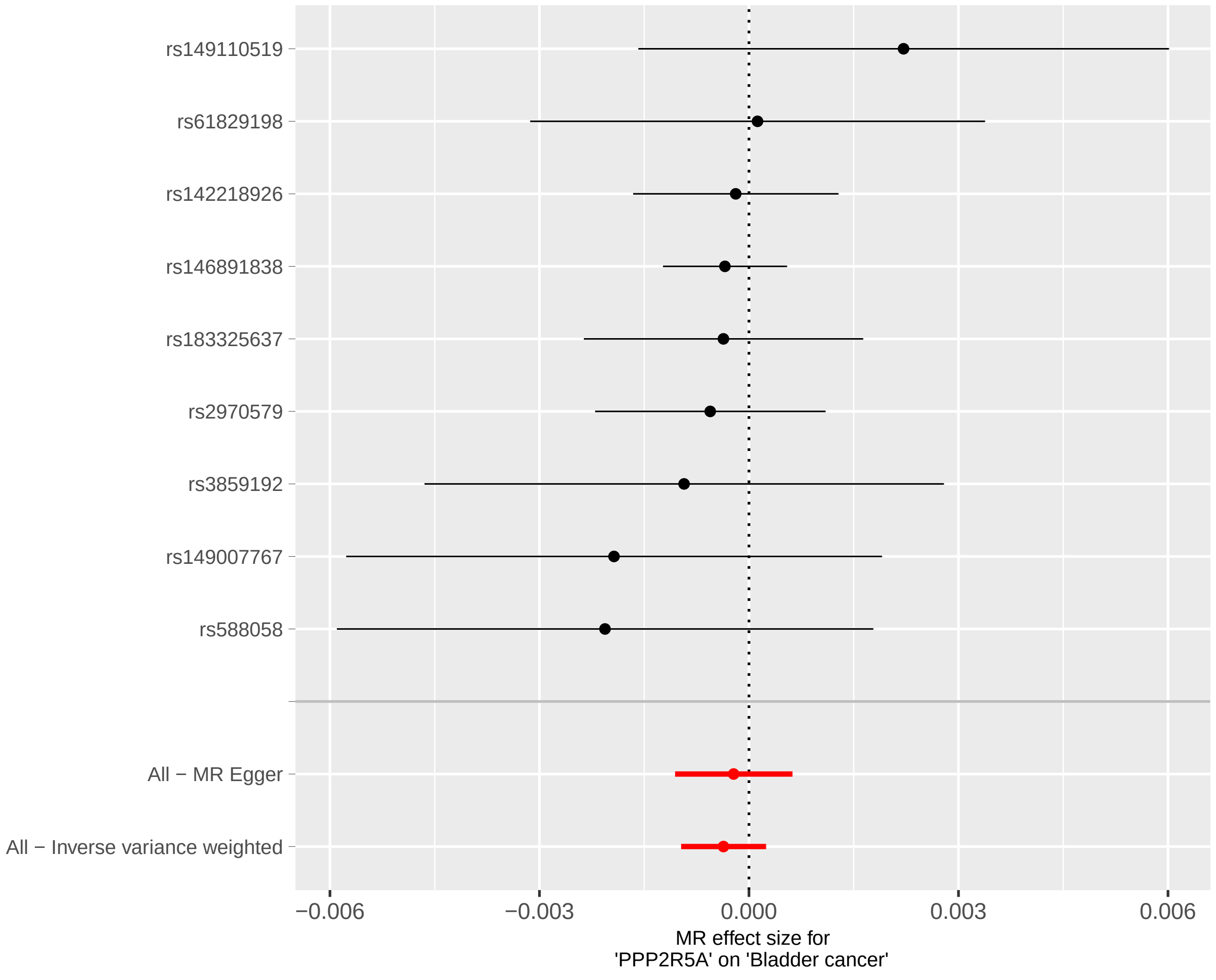

rs149110519
rs61829198
rs142218926
rs146891838
rs183325637
rs2970579
rs3859192
rs149007767
rs588058
All − MR Egger
All − Inverse variance weighted
−0.006
−0.003
0.000
0.003
0.006
MR effect size for
'PPP2R5A' on 'Bladder cancer'

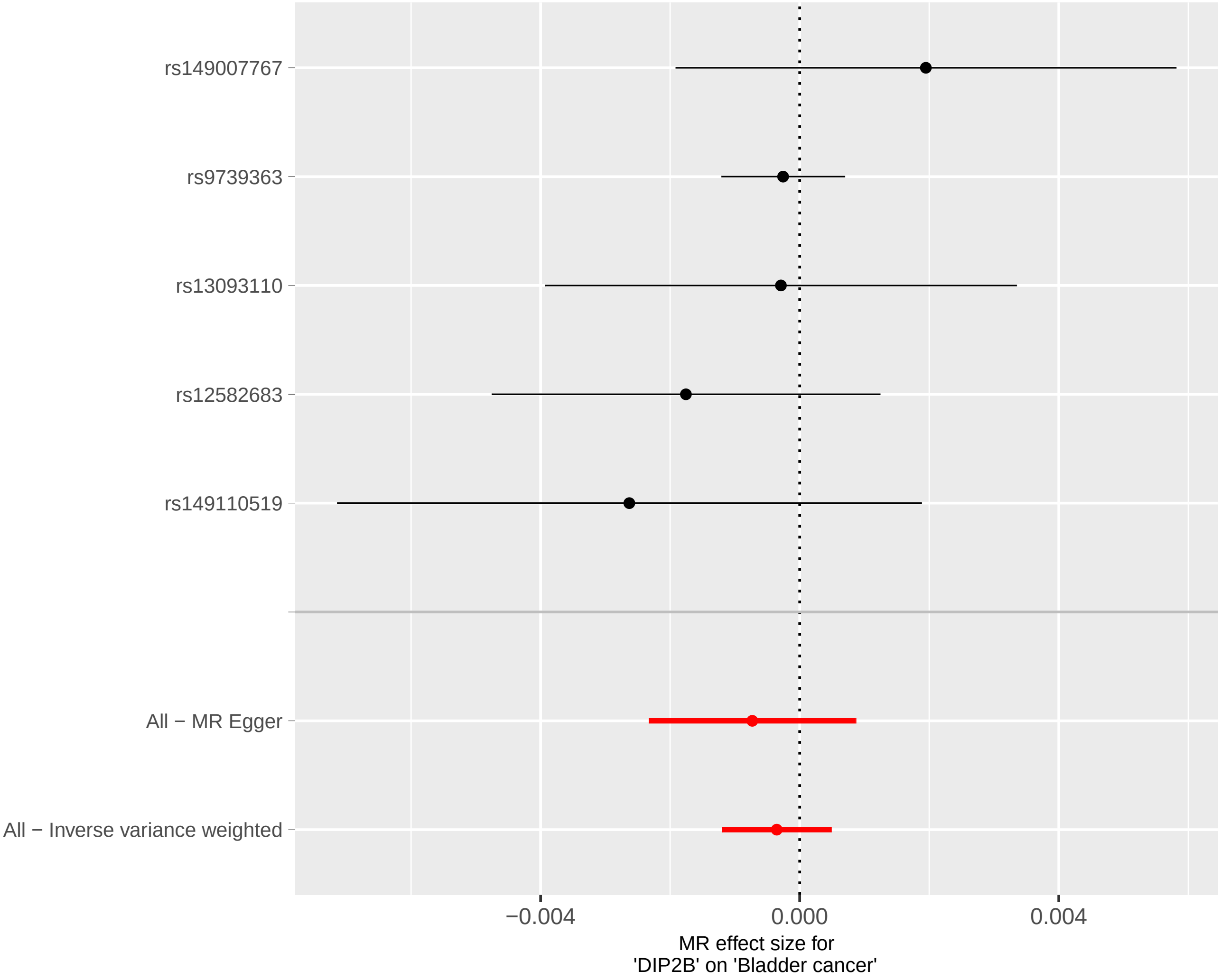
rs149007767
rs9739363
rs13093110
rs12582683
rs149110519
All − MR Egger
All − Inverse variance weighted
−0.004
0.000
0.004
MR effect size for
'DIP2B' on 'Bladder cancer'

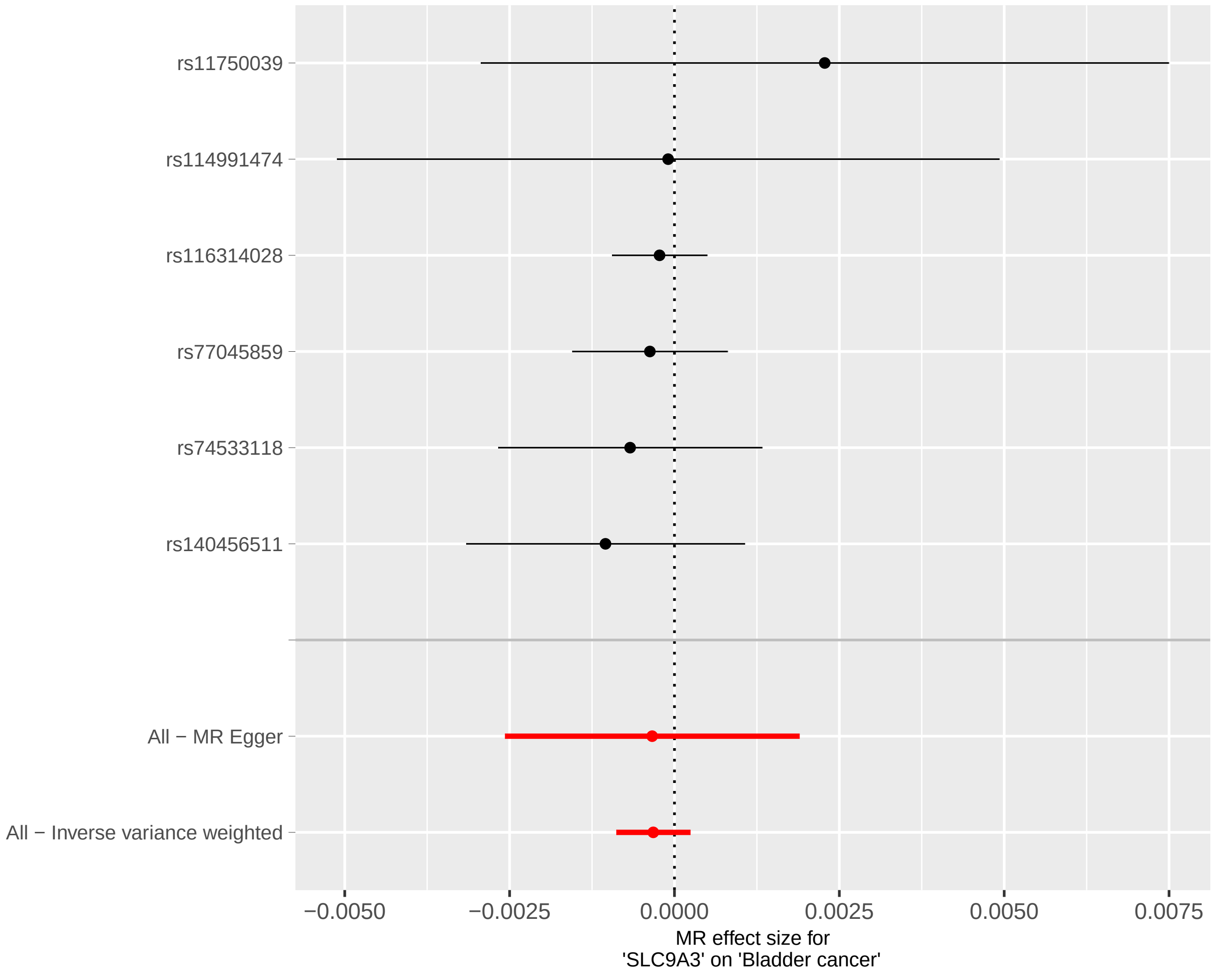

rs11750039
rs114991474
rs116314028
rs77045859
rs74533118
rs140456511
All − MR Egger
All − Inverse variance weighted
−0.0050
−0.0025
0.0000
0.0025
0.0050
0.0075
MR effect size for
'SLC9A3' on 'Bladder cancer'

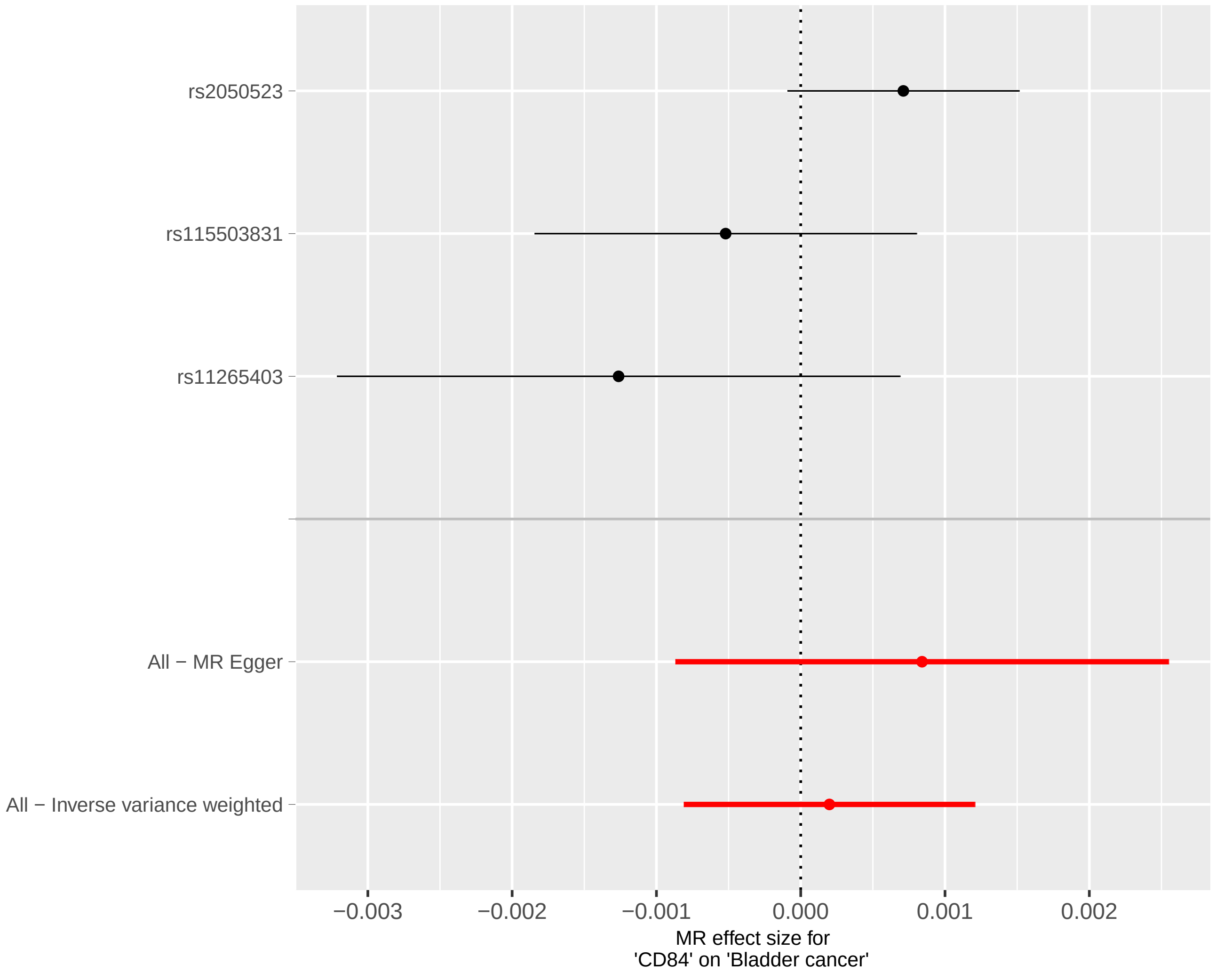

rs2050523
rs115503831
rs11265403
All − MR Egger
All − Inverse variance weighted
−0.003
−0.002
−0.001
0.000
0.001
0.002
MR effect size for
'CD84' on 'Bladder cancer'

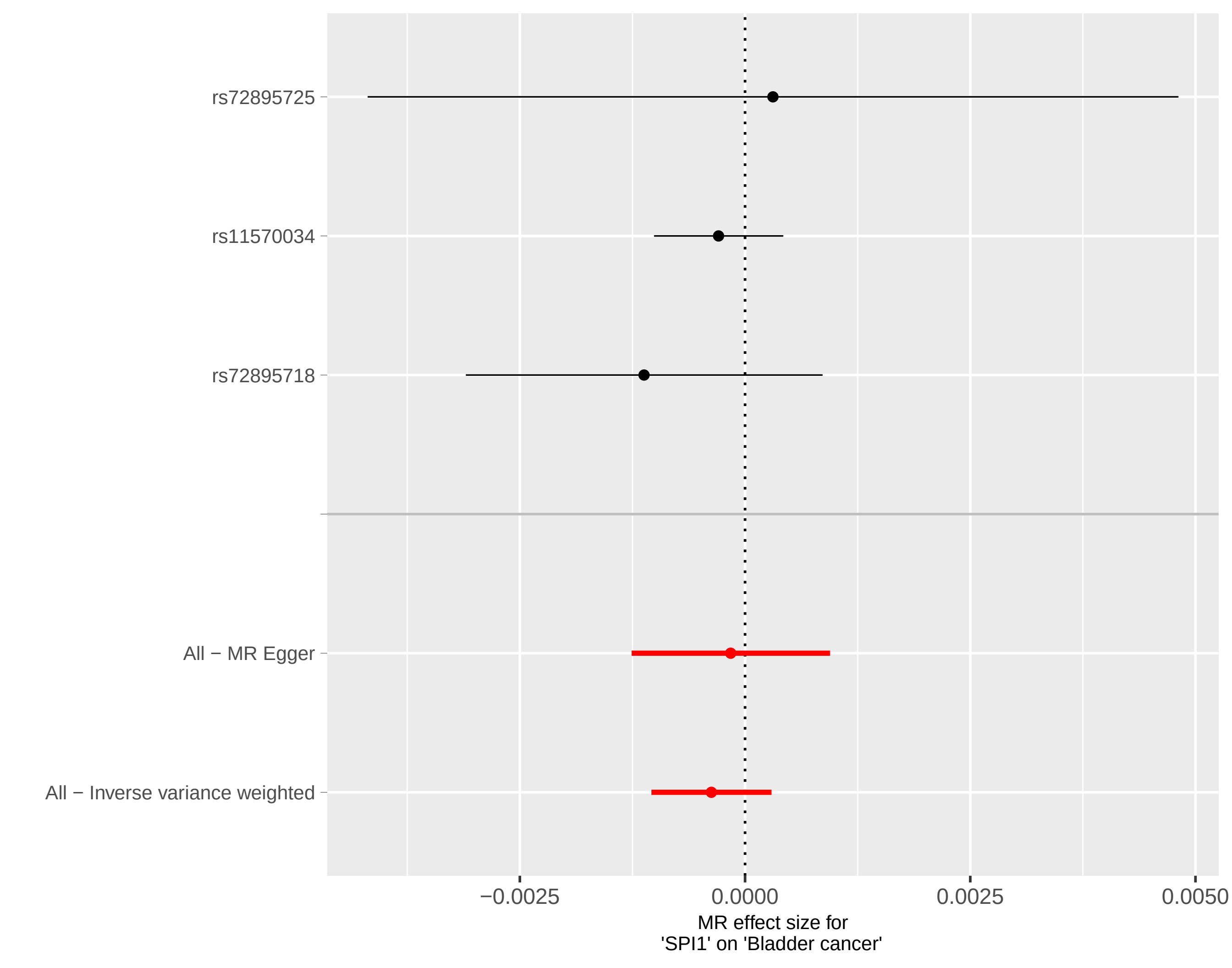

rs72895725
rs11570034
rs72895718
All − MR Egger
All − Inverse variance weighted
−0.0025
0.0000
0.0025
0.0050
MR effect size for
'SPI1' on 'Bladder cancer'

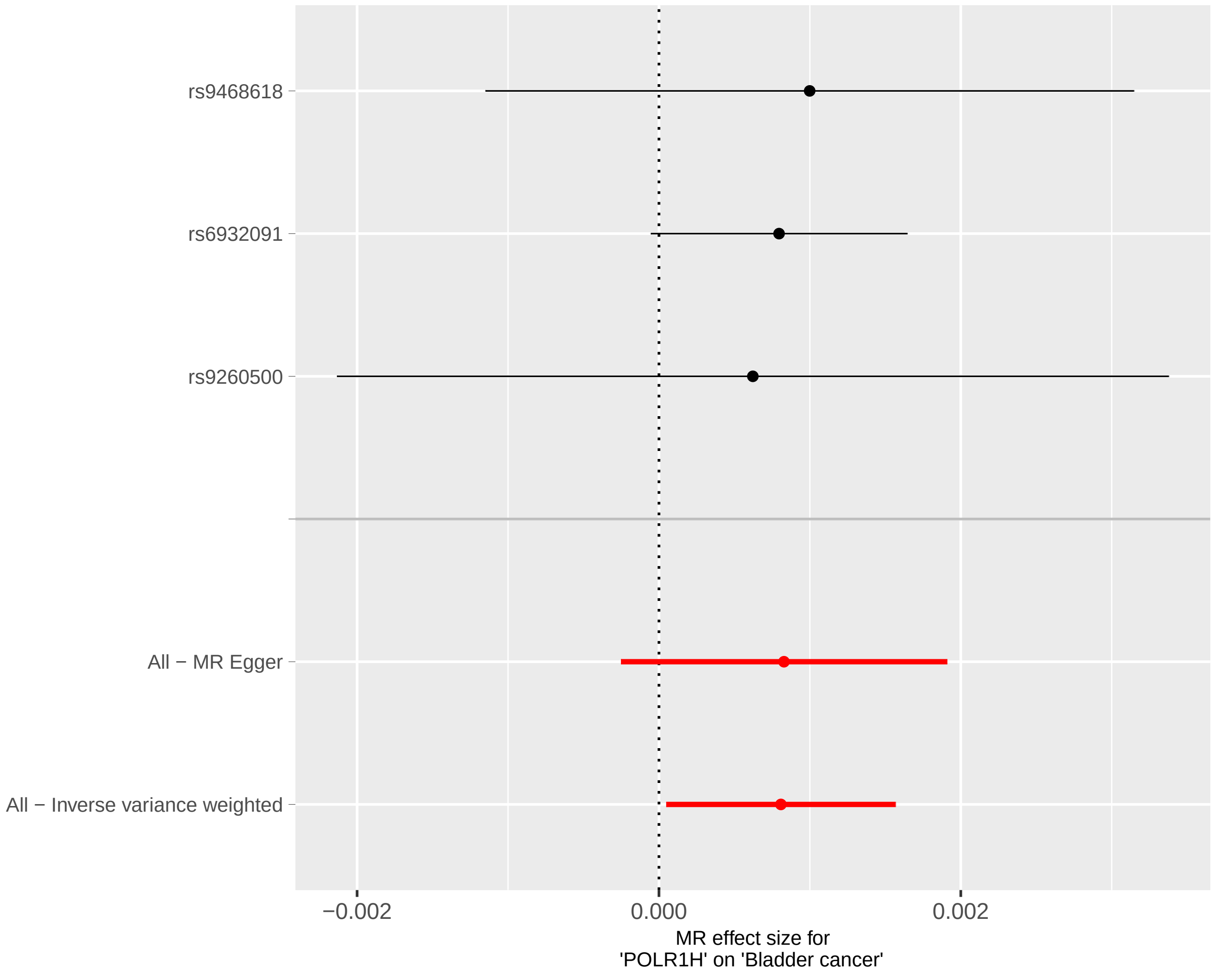
rs9468618
rs6932091
rs9260500
All − MR Egger
All − Inverse variance weighted
−0.002
0.000
0.002
MR effect size for
'POLR1H' on 'Bladder cancer'

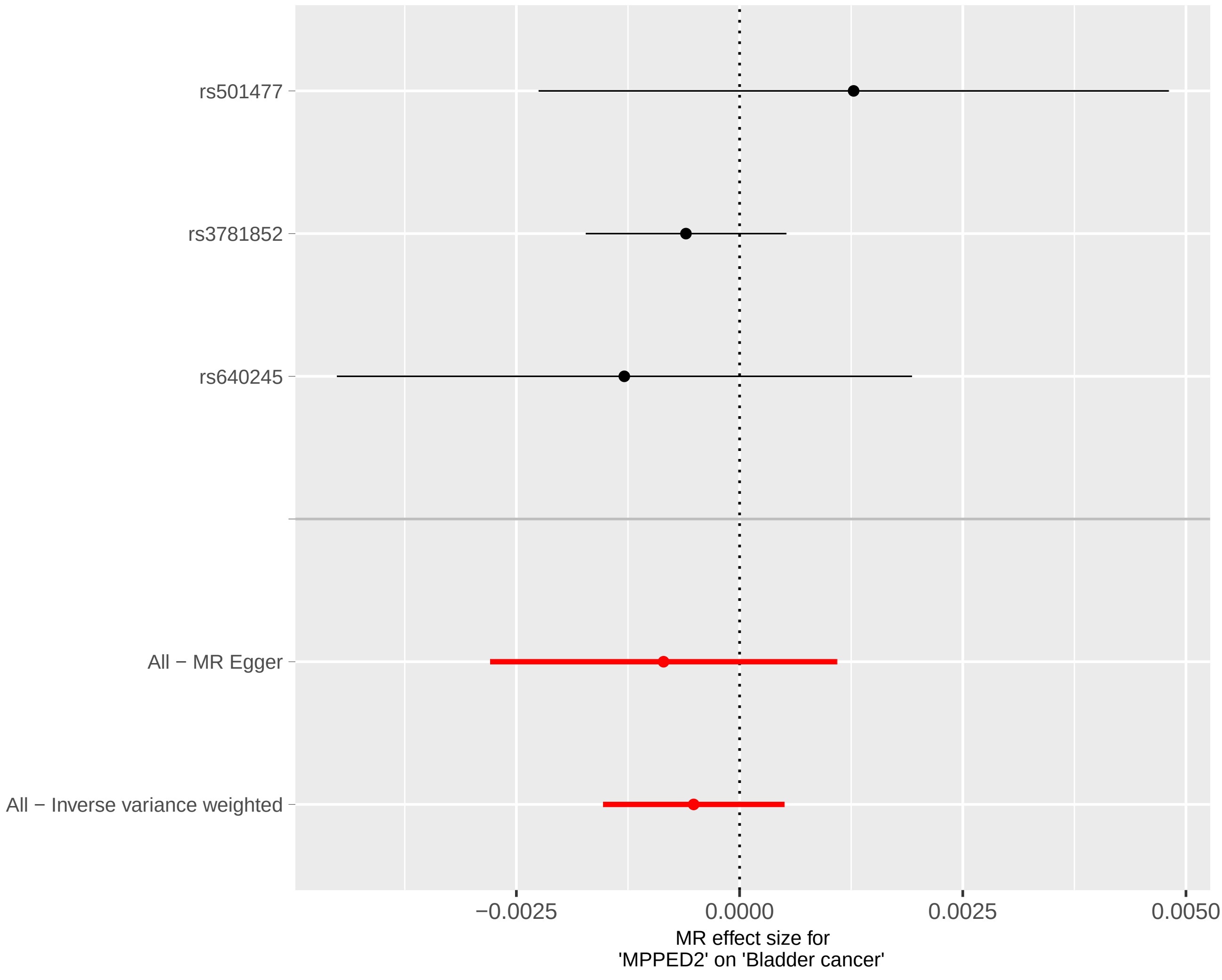
rs501477
rs3781852
rs640245
All − MR Egger
All − Inverse variance weighted
−0.0025
0.0000
0.0025
0.0050
MR effect size for
'MPPED2' on 'Bladder cancer'

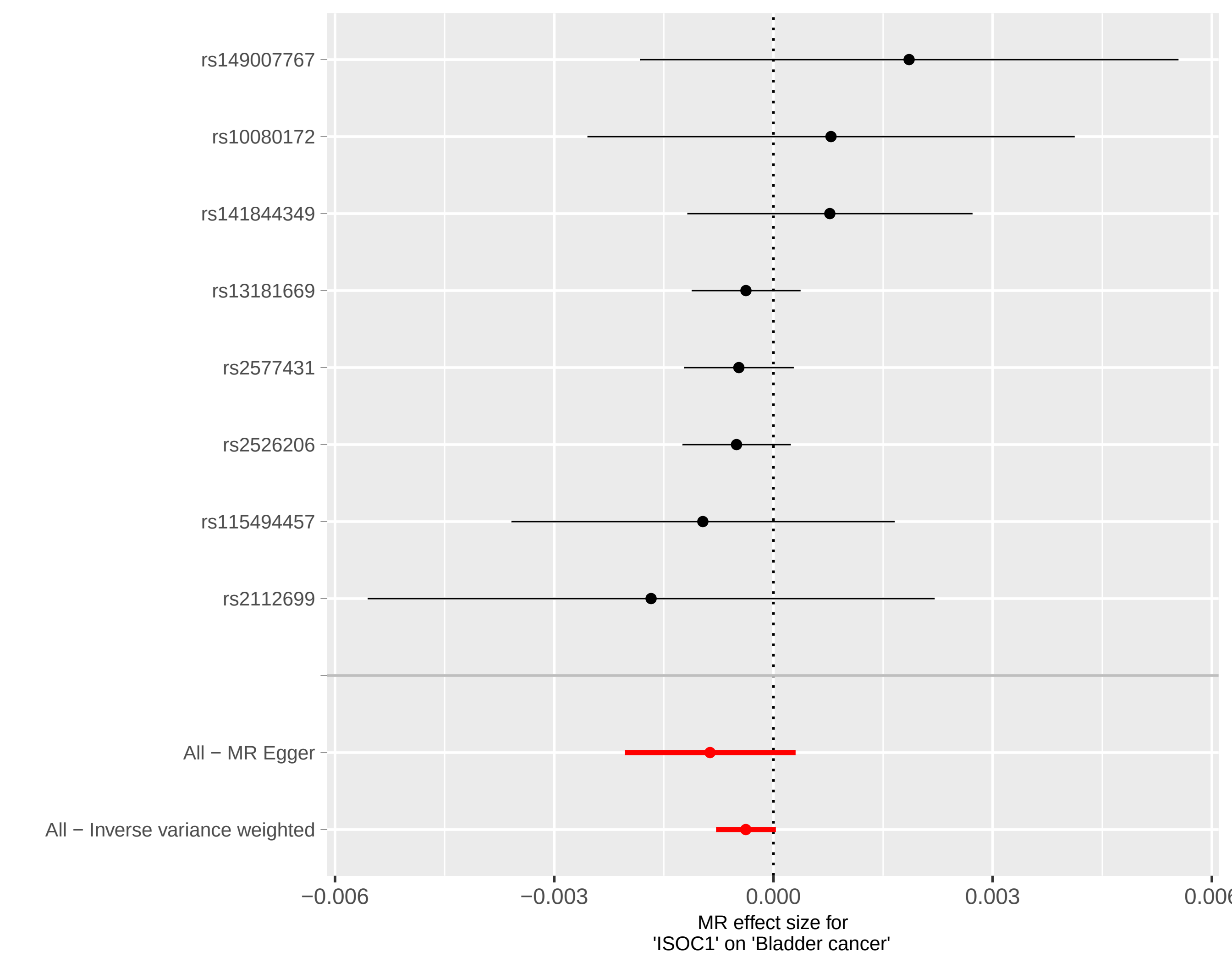

rs149007767
rs10080172
rs141844349
rs13181669
rs2577431
rs2526206
rs115494457
rs2112699
All − MR Egger
All − Inverse variance weighted
−0.006
−0.003
0.000
0.003
MR effect size for
'ISOC1' on 'Bladder cancer'

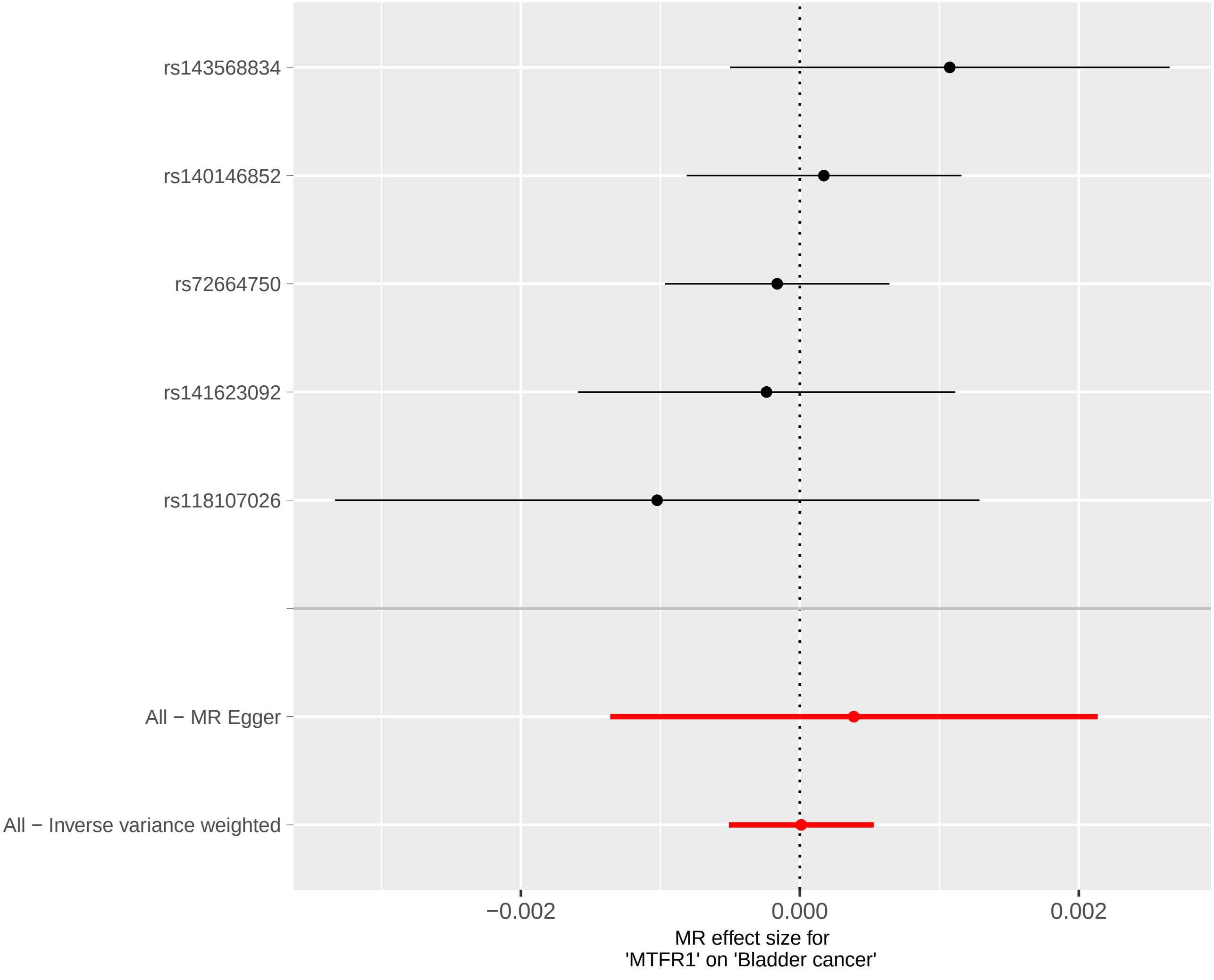
rs143568834
rs140146852
rs72664750
rs141623092
rs118107026
All − MR Egger
All − Inverse variance weighted
−0.002
0.000
0.002
MR effect size for
'MTFR1' on 'Bladder cancer'

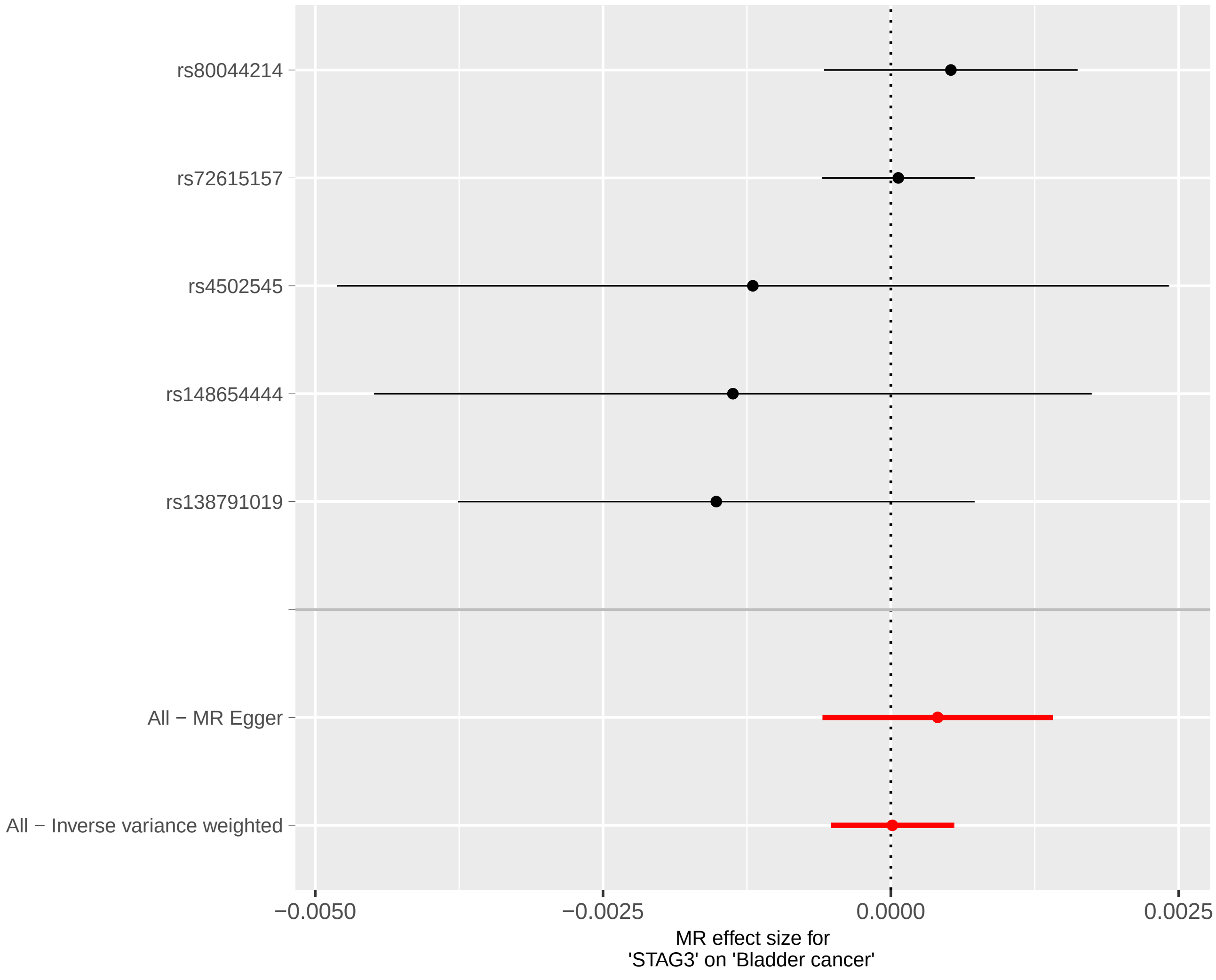

rs80044214
rs72615157
rs4502545
rs148654444
rs138791019
All − MR Egger
All − Inverse variance weighted
−0.0050
−0.0025
0.0000
0.0025
MR effect size for
'STAG3' on 'Bladder cancer'

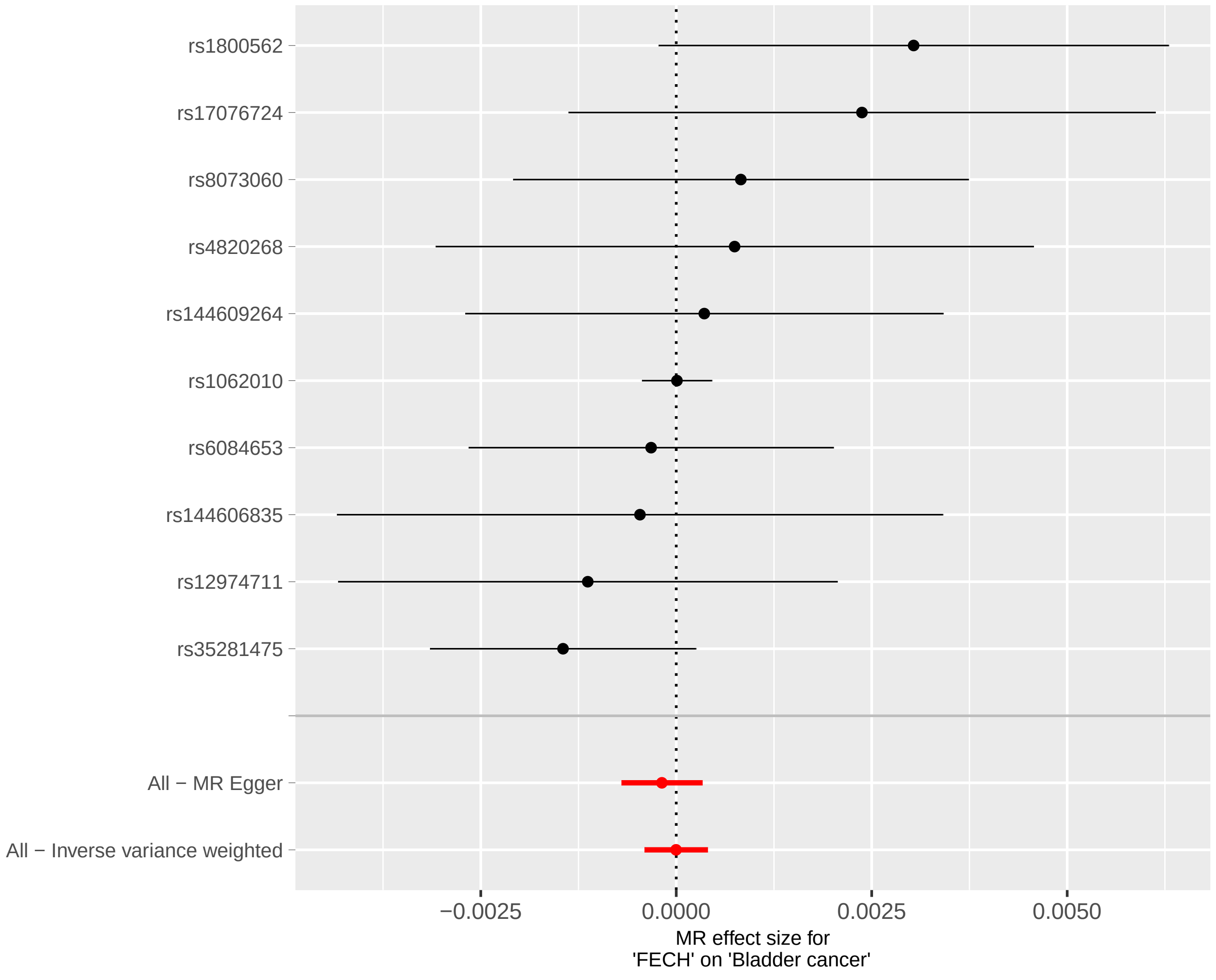

rs1800562
rs17076724
rs8073060
rs4820268
rs144609264
rs1062010
rs6084653
rs144606835
rs12974711
rs35281475
All − MR Egger
All − Inverse variance weighted
−0.0025
0.0000
0.0025
0.0050
MR effect size for
'FECH' on 'Bladder cancer'

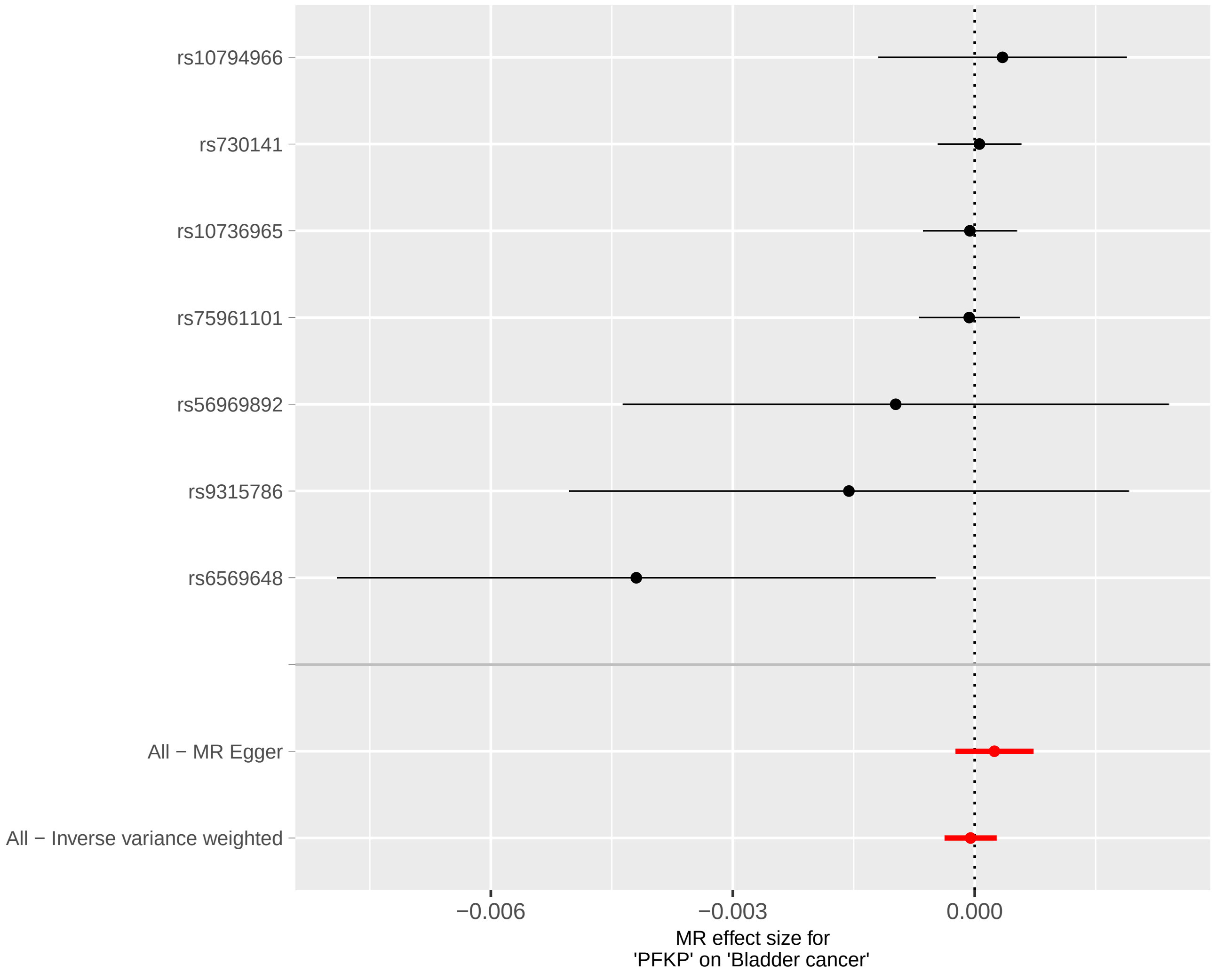

rs10794966
rs730141
rs10736965
rs75961101
rs56969892
rs9315786
rs6569648
All − MR Egger
All − Inverse variance weighted
−0.006
−0.003
0.000
MR effect size for
'PFKP' on 'Bladder cancer'

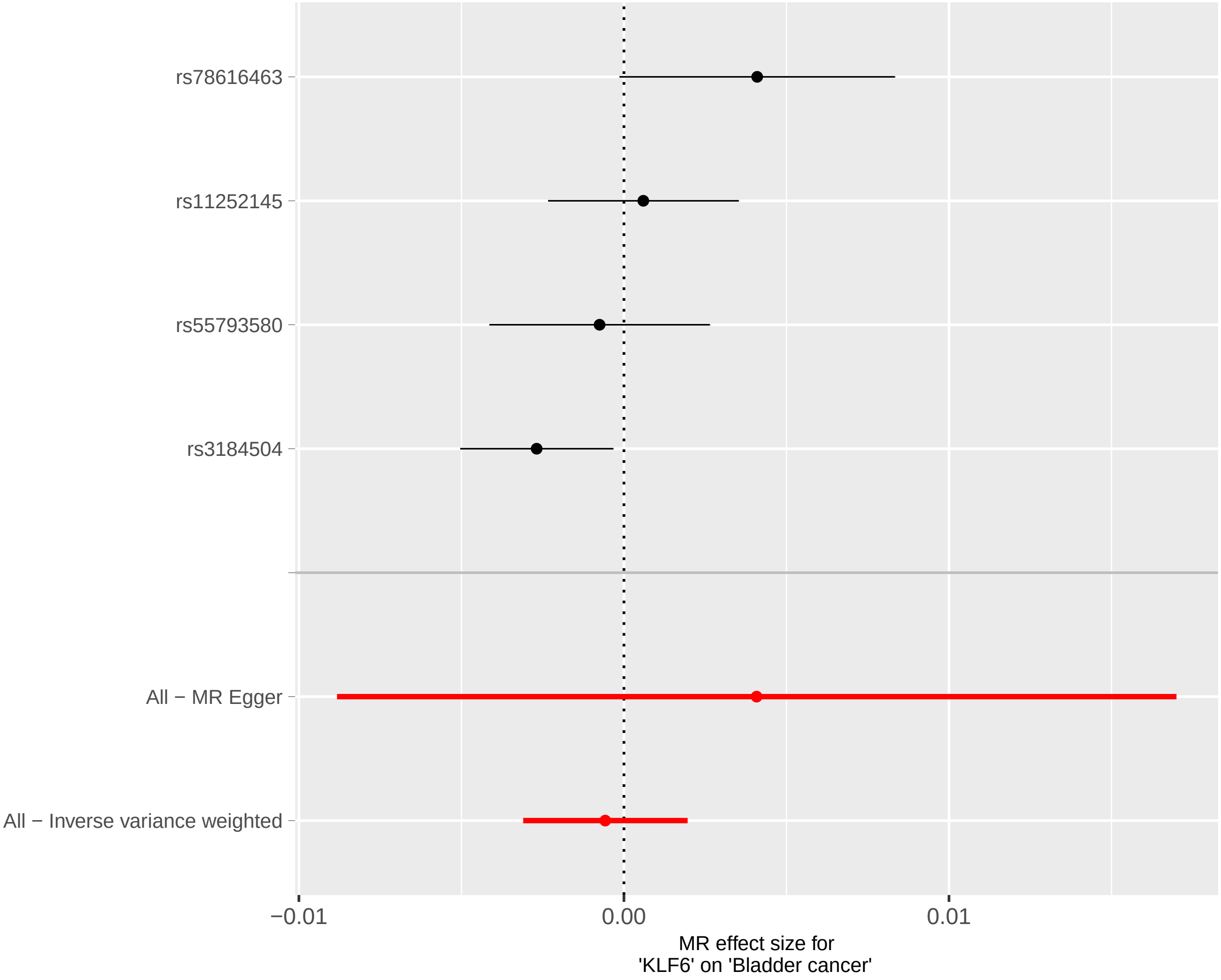
rs78616463
rs11252145
rs55793580
rs3184504
All − MR Egger
All − Inverse variance weighted
−0.01
0.00
0.01
MR effect size for
'KLF6' on 'Bladder cancer'

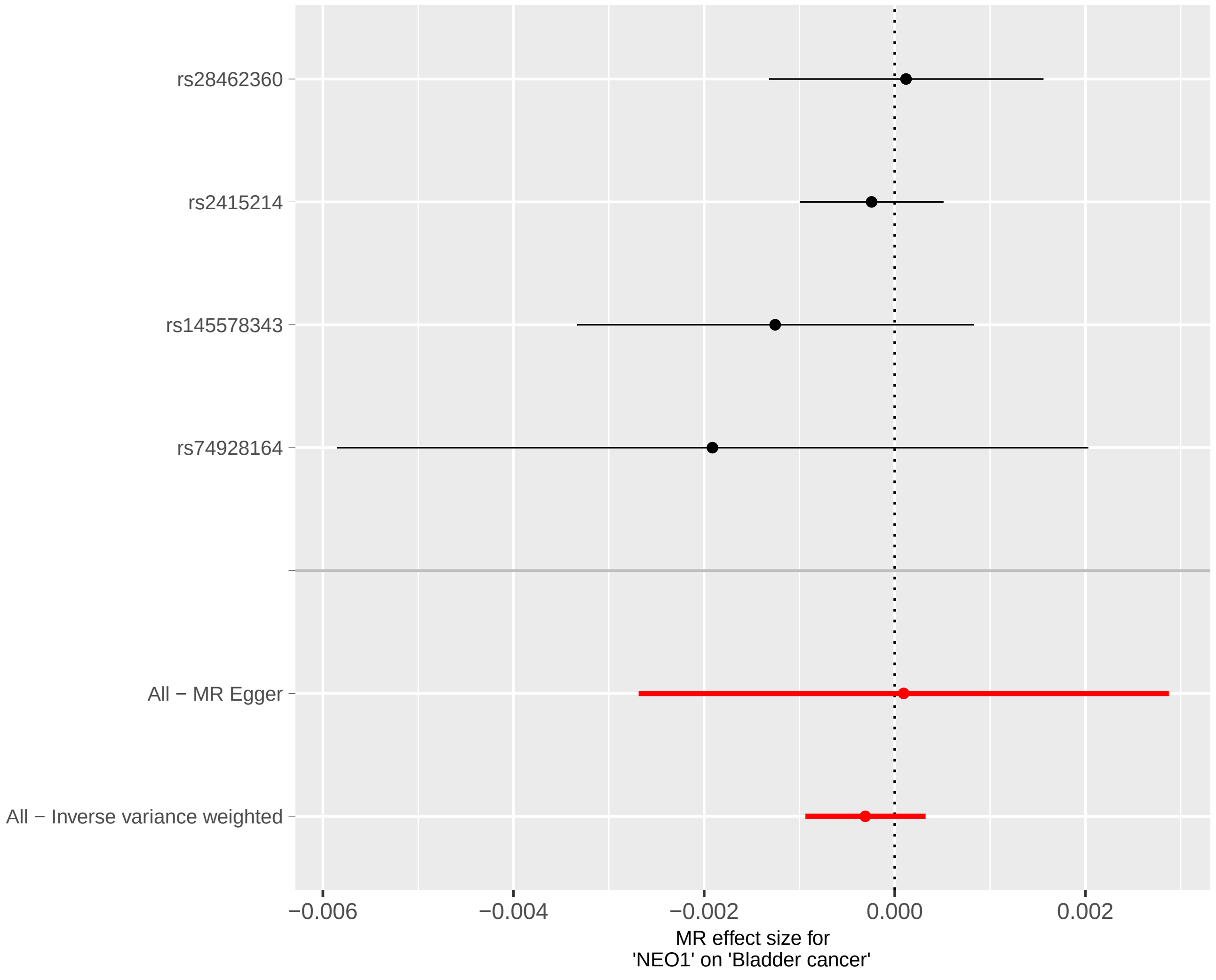
rs28462360
rs2415214
rs145578343
rs74928164
All − MR Egger
All − Inverse variance weighted
−0.006
−0.004
−0.002
0.000
0.002
MR effect size for
'NEO1' on 'Bladder cancer'

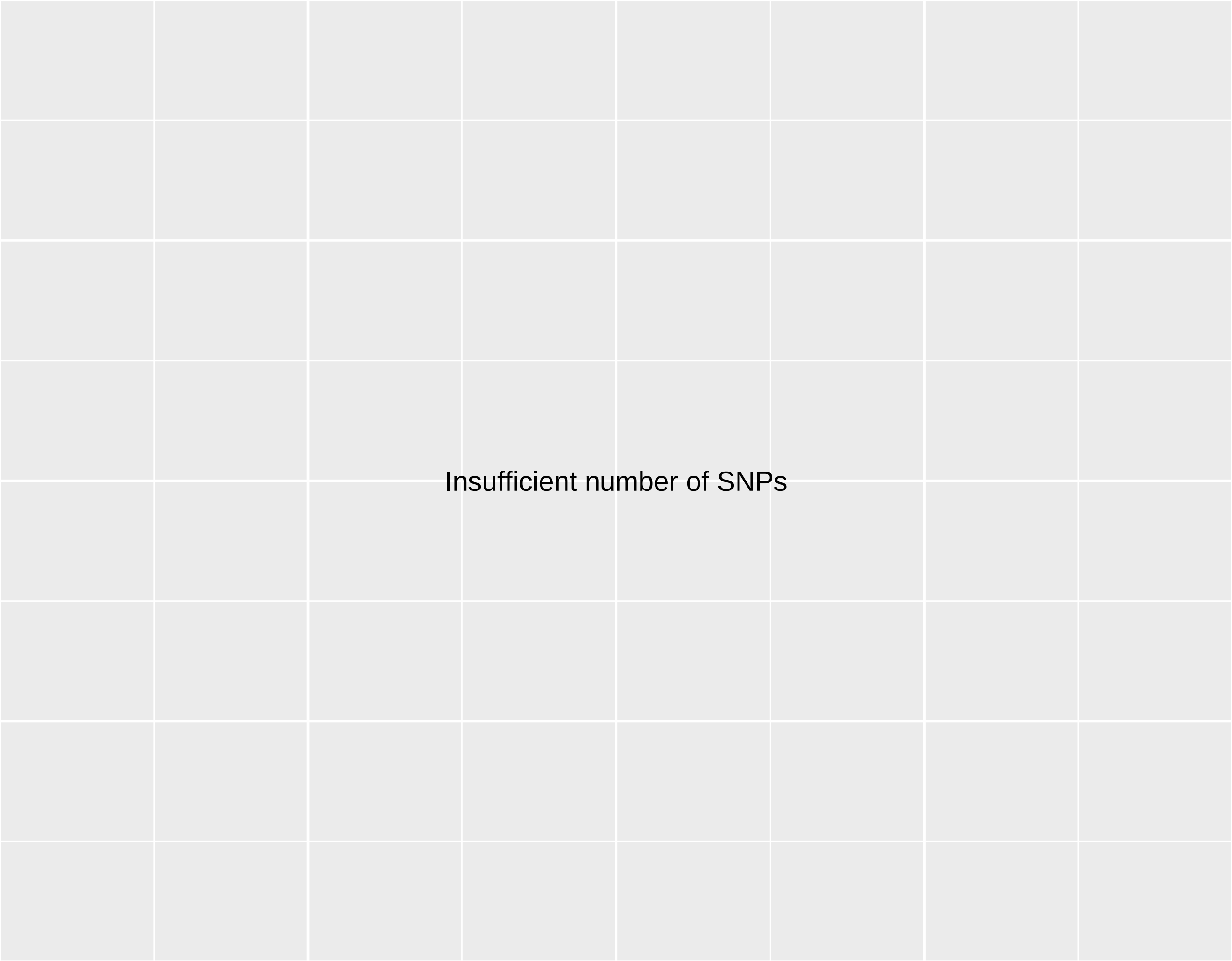
Insufficient number of SNPs

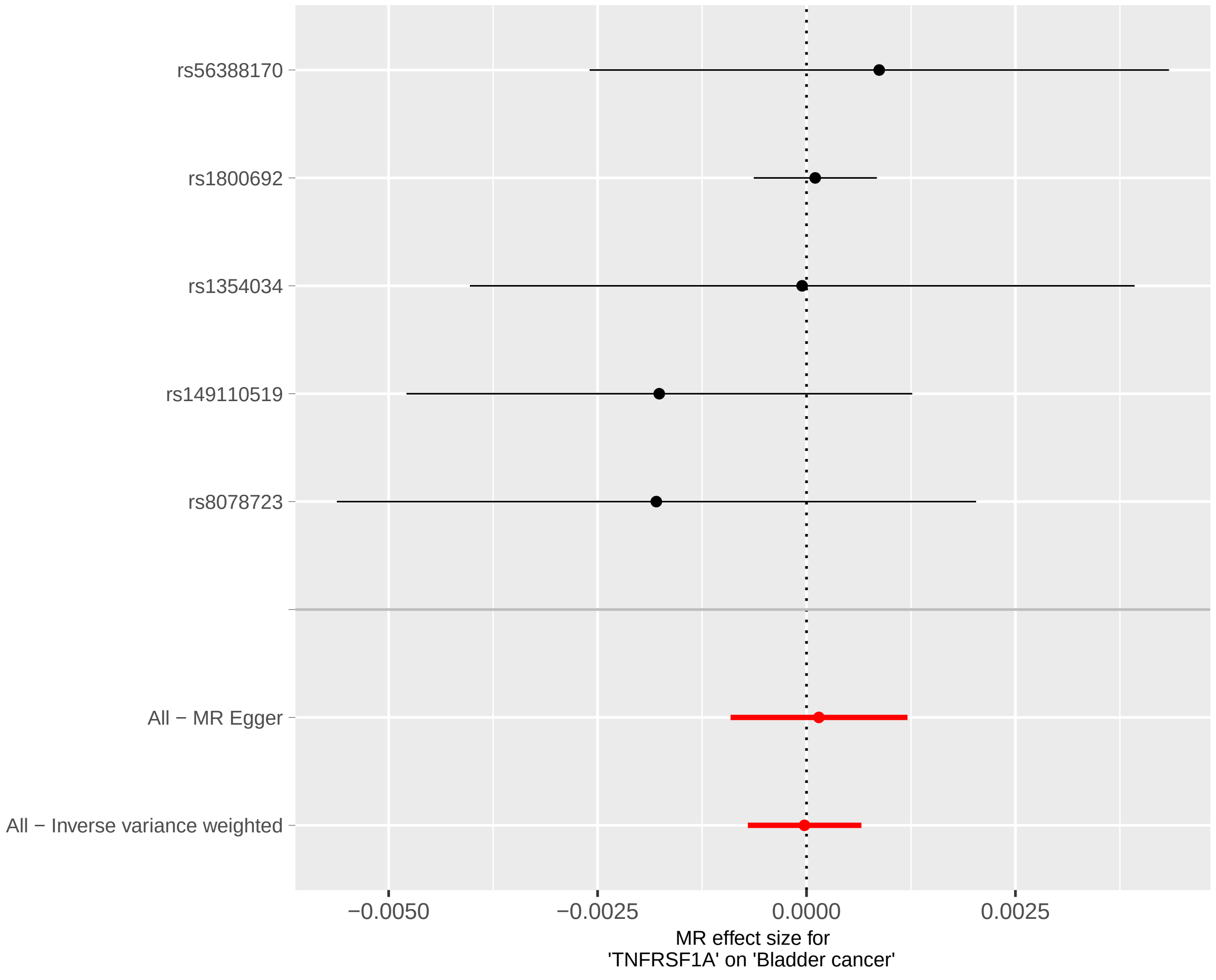
rs56388170
rs1800692
rs1354034
rs149110519
rs8078723
All − MR Egger
All − Inverse variance weighted
−0.0050
−0.0025
0.0000
0.0025
MR effect size for
'TNFRSF1A' on 'Bladder cancer'

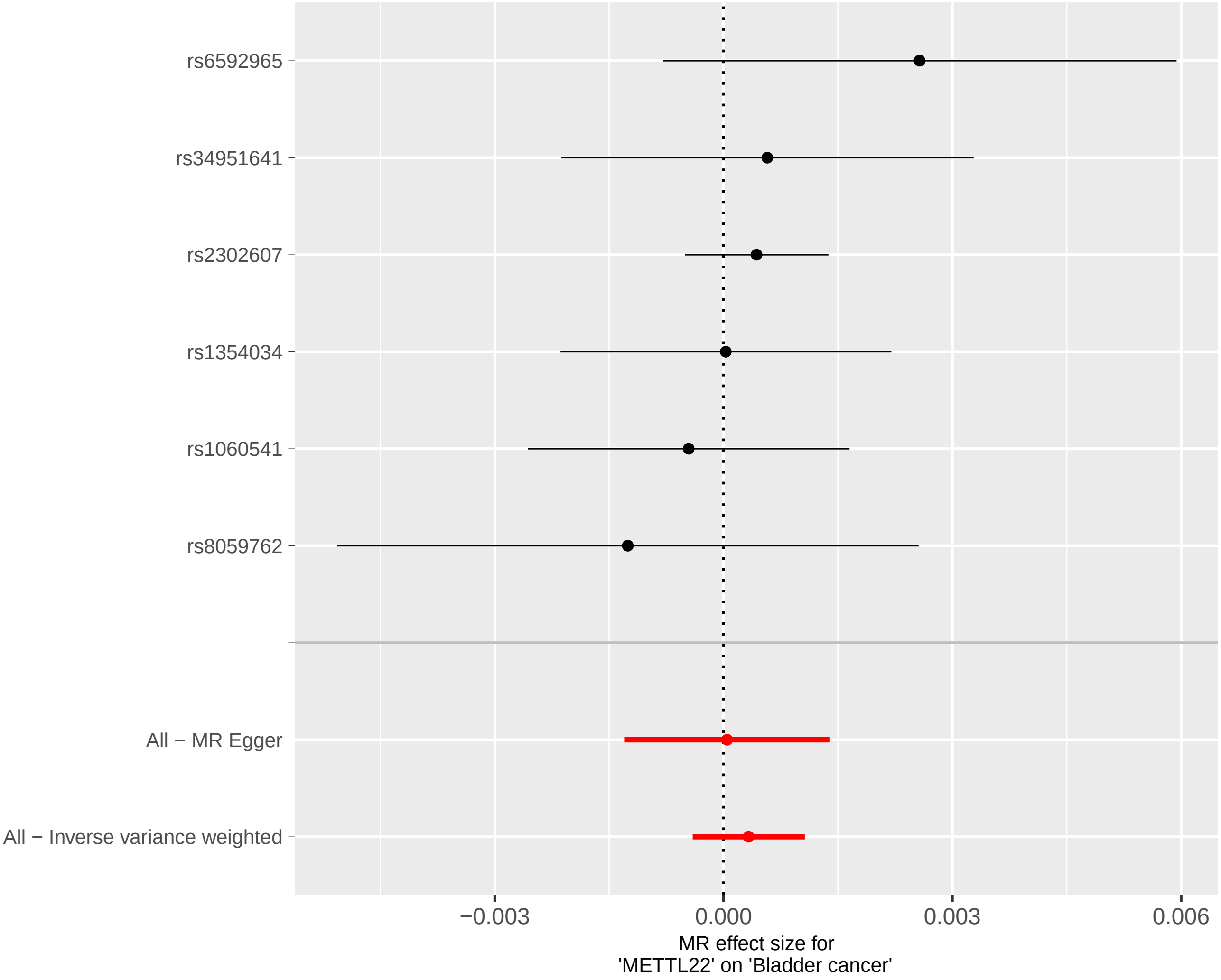

rs6592965
rs34951641
rs2302607
rs1354034
rs1060541
rs8059762
All − MR Egger
All − Inverse variance weighted
−0.003
0.000
0.003
0.006
MR effect size for
'METTL22' on 'Bladder cancer'

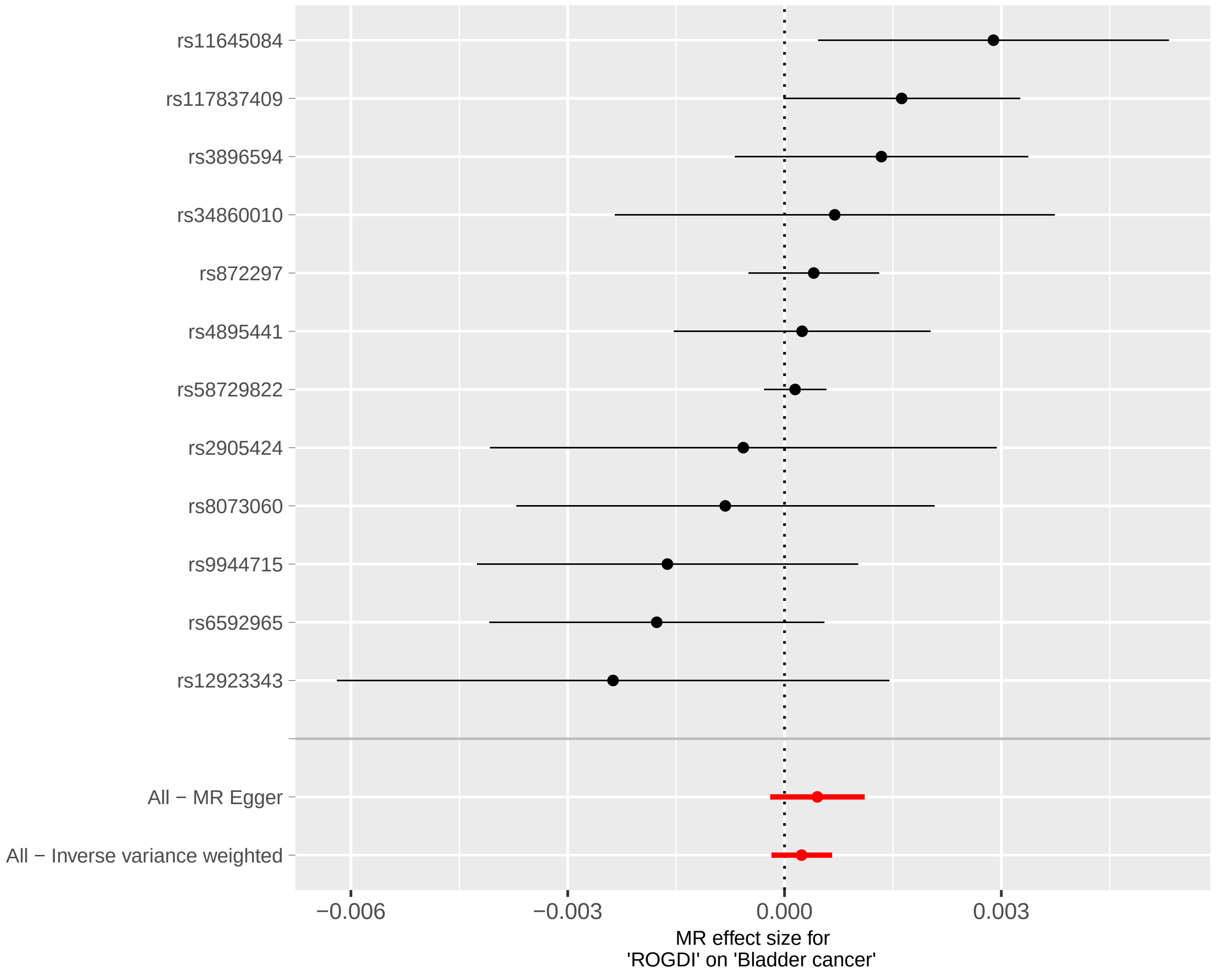
rs11645084
rs117837409
rs3896594
rs34860010
rs872297
rs4895441
rs58729822
rs2905424
rs8073060
rs9944715
rs6592965
rs12923343
All − MR Egger
All − Inverse variance weighted
−0.006
−0.003
0.000
0.003
MR effect size for
'ROGDI' on 'Bladder cancer'

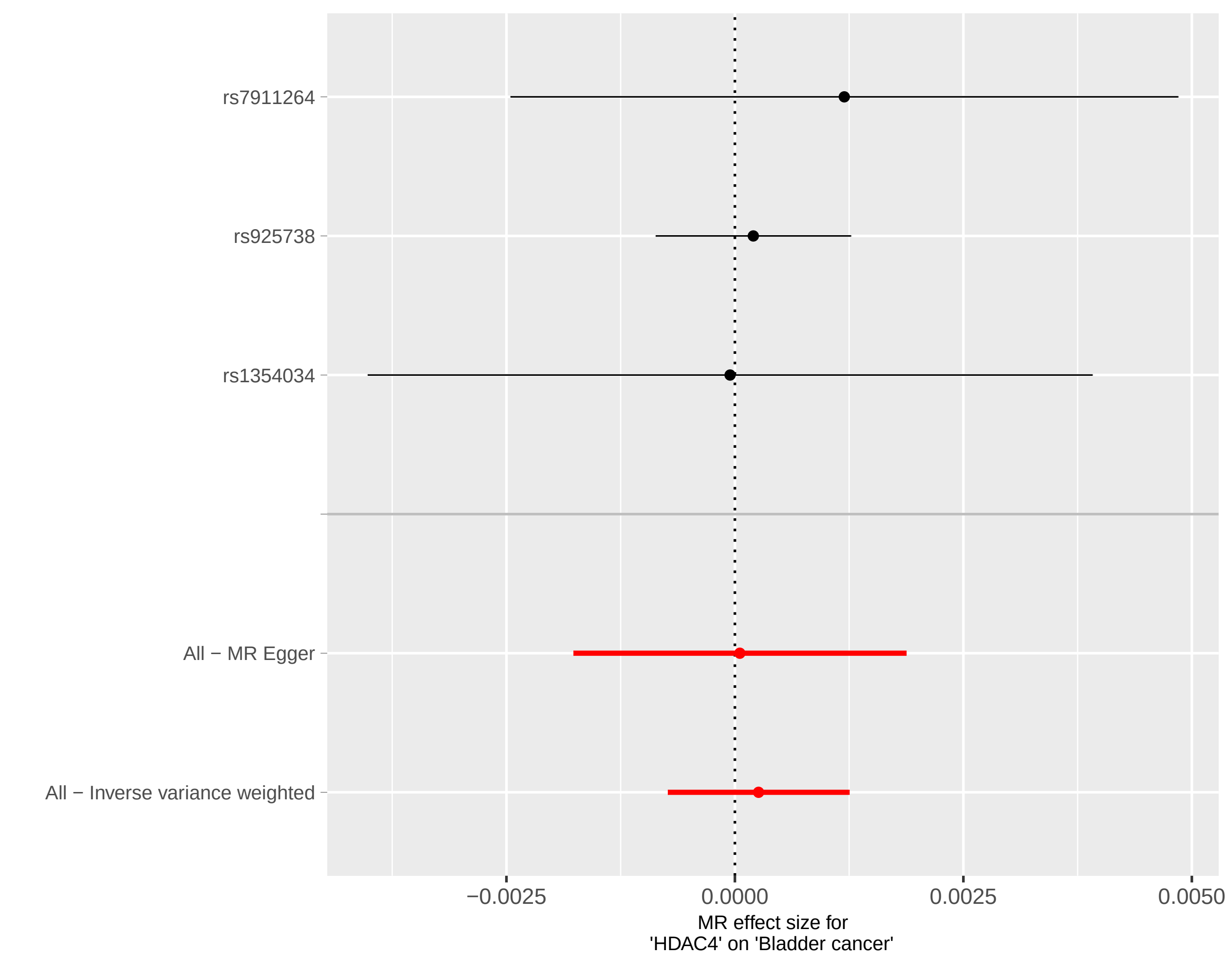

rs7911264
rs925738
rs1354034
All − MR Egger
All − Inverse variance weighted
−0.0025
0.0000
0.0025
0.0050
MR effect size for
'HDAC4' on 'Bladder cancer'

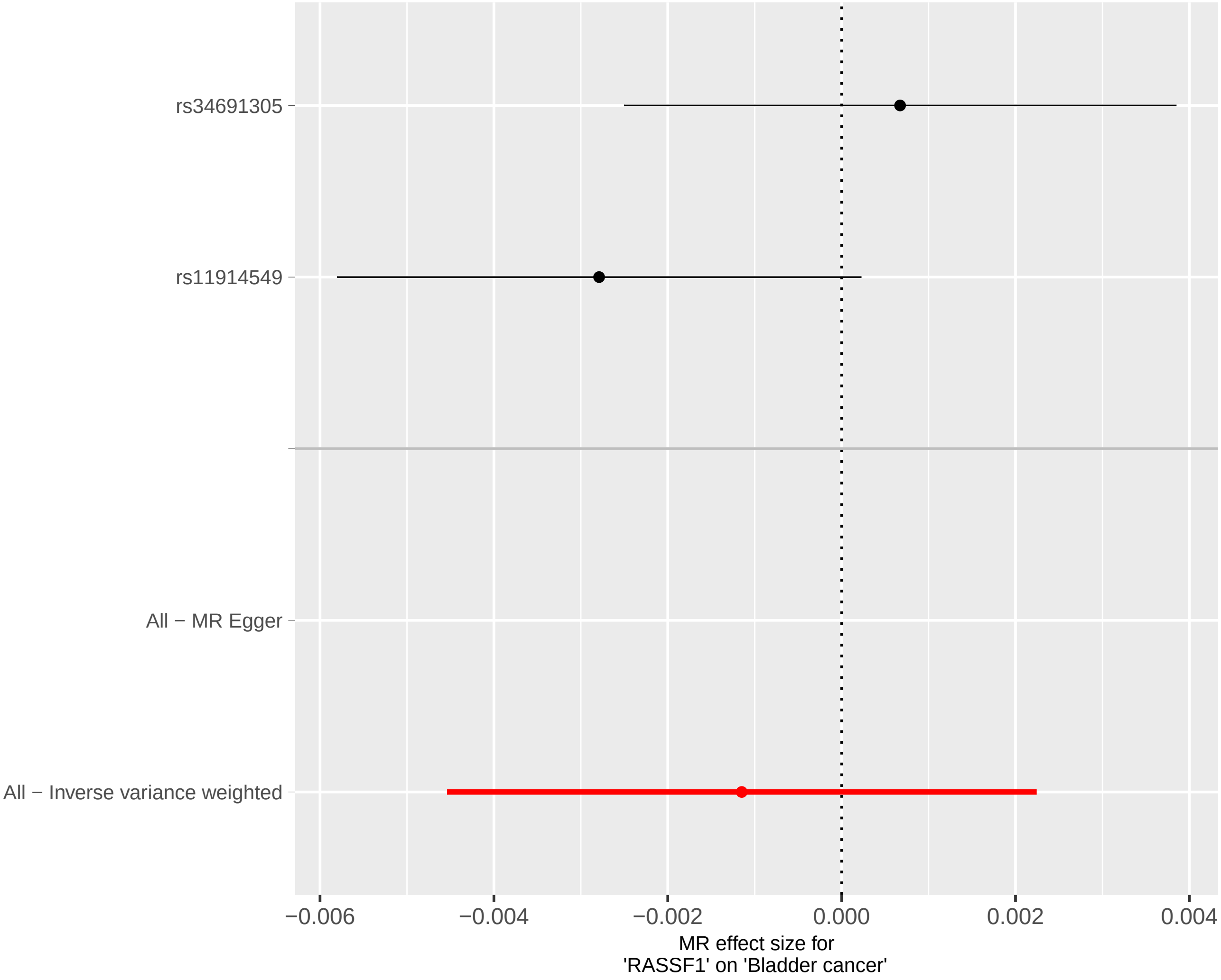

rs34691305
rs11914549
All − MR Egger
All − Inverse variance weighted
−0.006
−0.004
−0.002
0.000
0.002
0.004
MR effect size for
'RASSF1' on 'Bladder cancer'

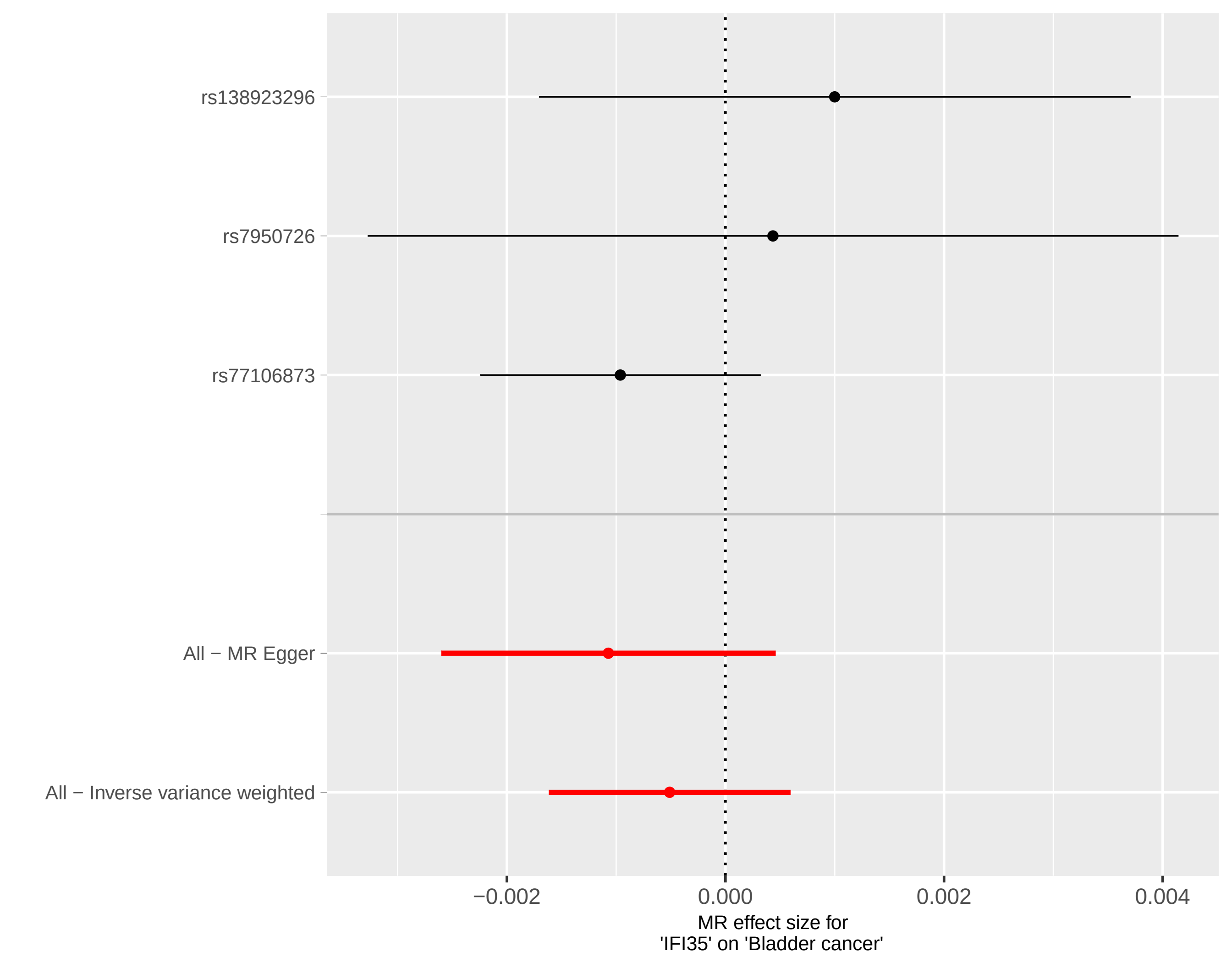
rs138923296
rs7950726
rs77106873
All − MR Egger
All − Inverse variance weighted
−0.002
0.000
0.002
0.004
MR effect size for
'IFI35' on 'Bladder cancer'

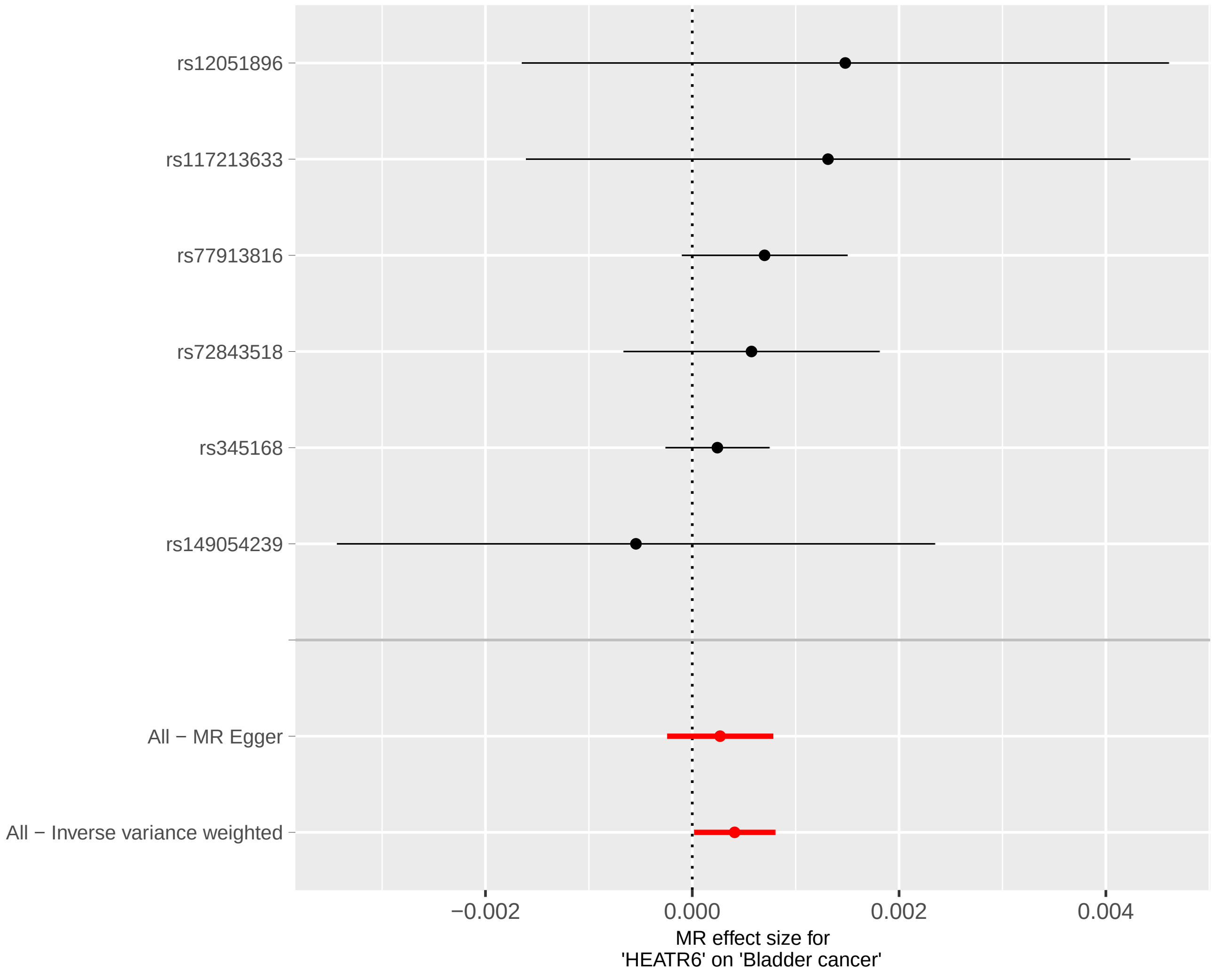
rs12051896
rs117213633
rs77913816
rs72843518
rs345168
rs149054239
All − MR Egger
All − Inverse variance weighted
−0.002
0.000
0.002
0.004
MR effect size for
'HEATR6' on 'Bladder cancer'

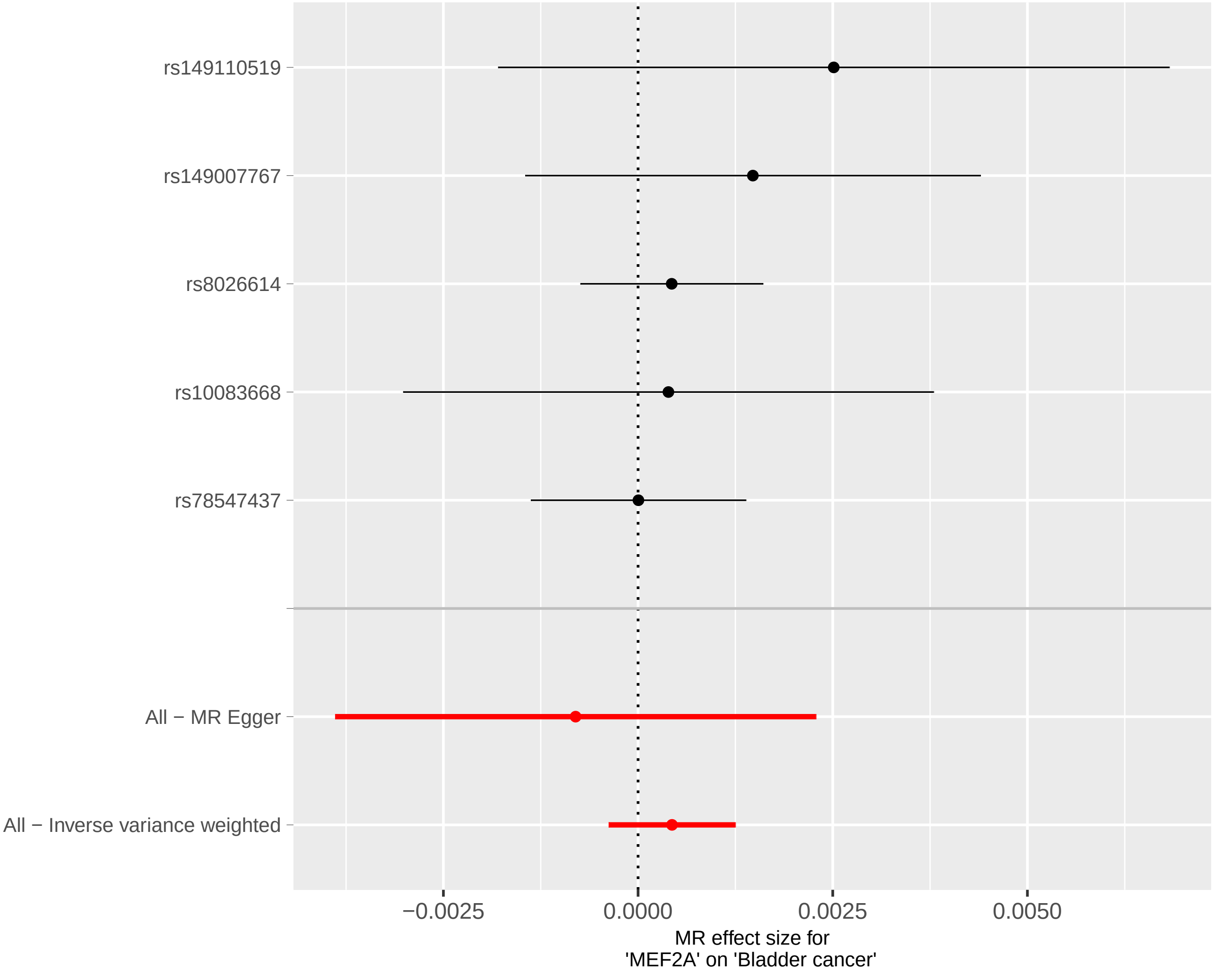

rs149110519
rs149007767
rs8026614
rs10083668
rs78547437
All − MR Egger
All − Inverse variance weighted
−0.0025
0.0000
0.0025
0.0050
MR effect size for
'MEF2A' on 'Bladder cancer'

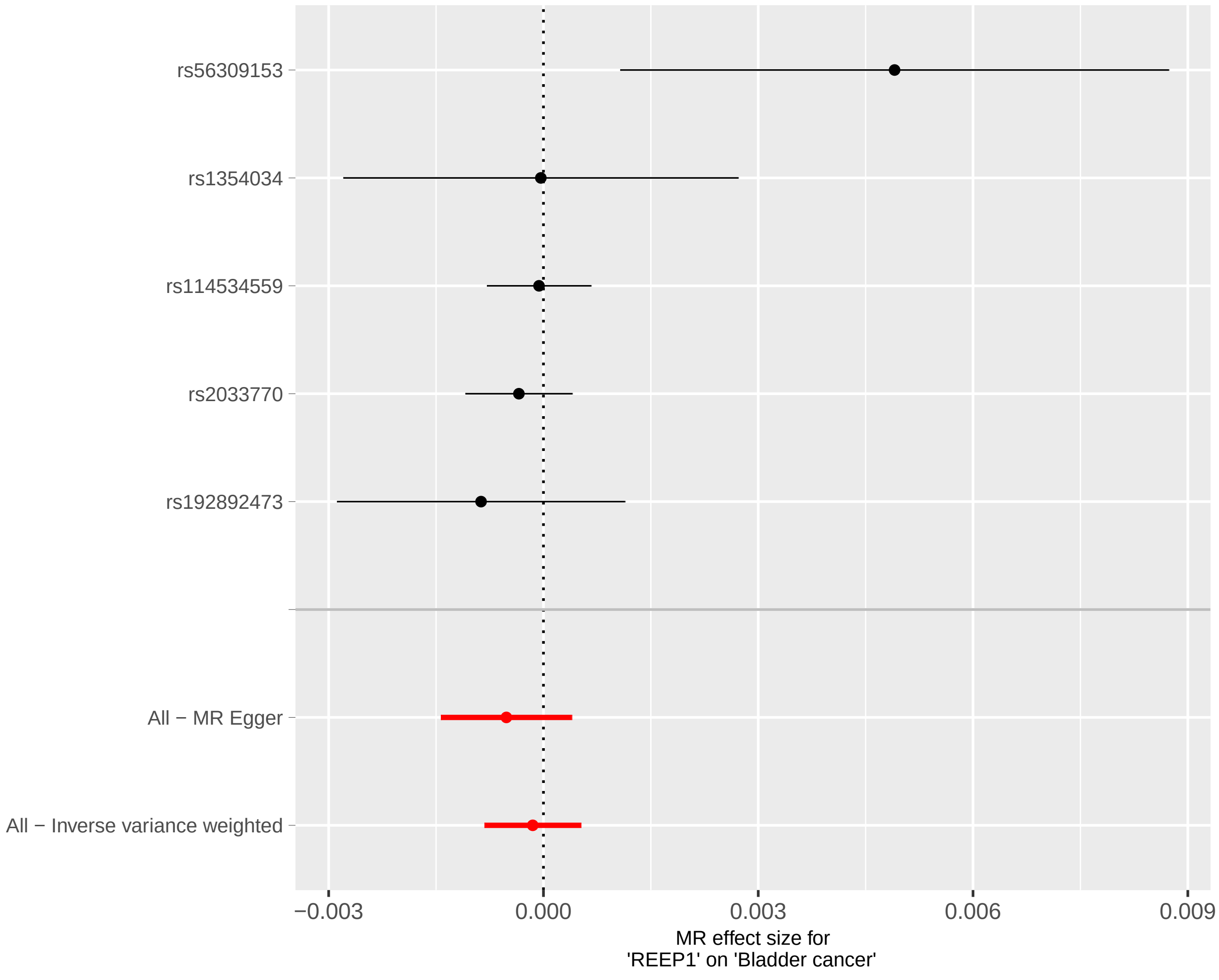

rs56309153
rs1354034
rs114534559
rs2033770
rs192892473
All − MR Egger
All − Inverse variance weighted
−0.003
0.000
0.003
0.006
0.009
MR effect size for
'REEP1' on 'Bladder cancer'

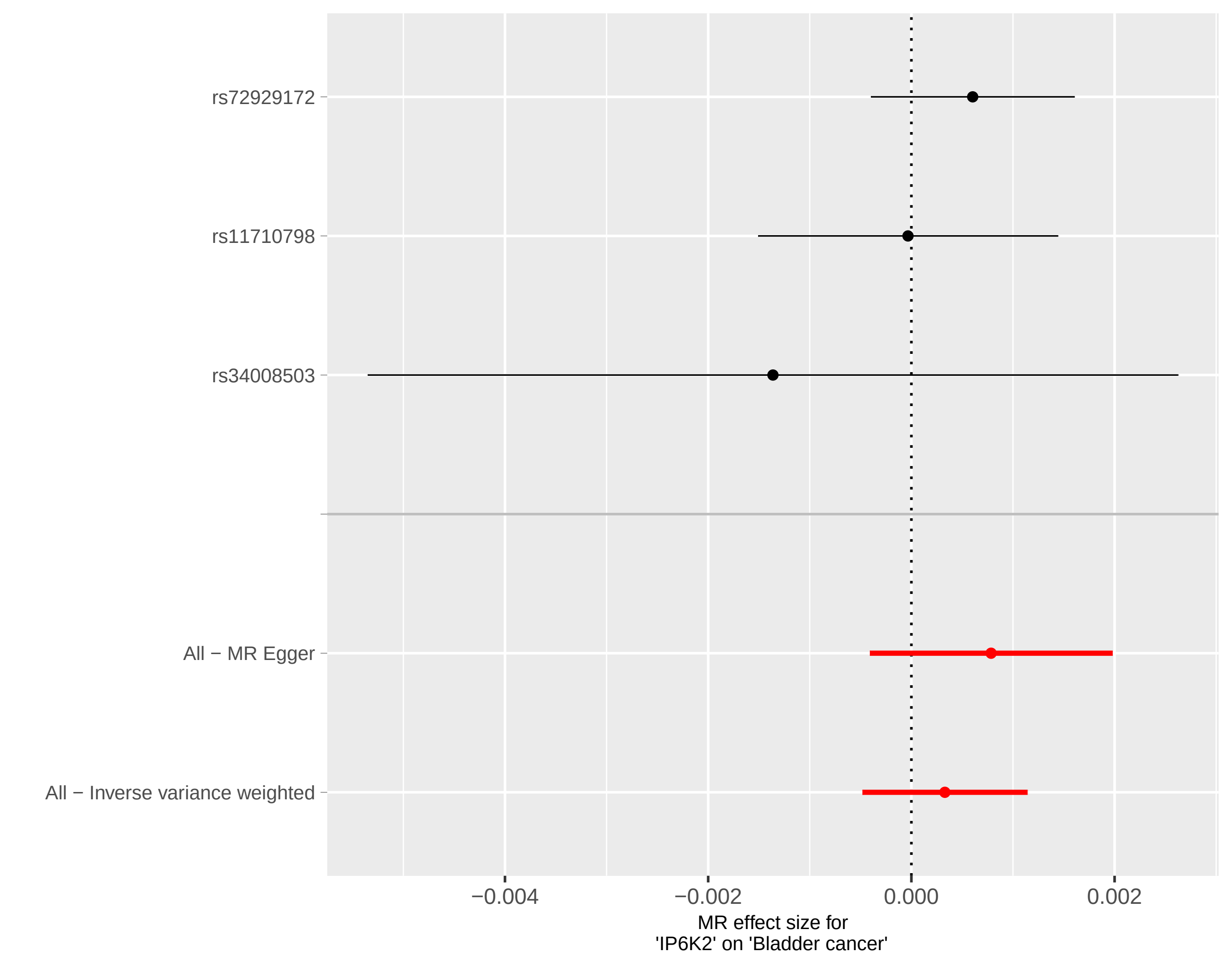

rs72929172
rs11710798
rs34008503
All − MR Egger
All − Inverse variance weighted
−0.004
−0.002
0.000
0.002
MR effect size for
'IP6K2' on 'Bladder cancer'

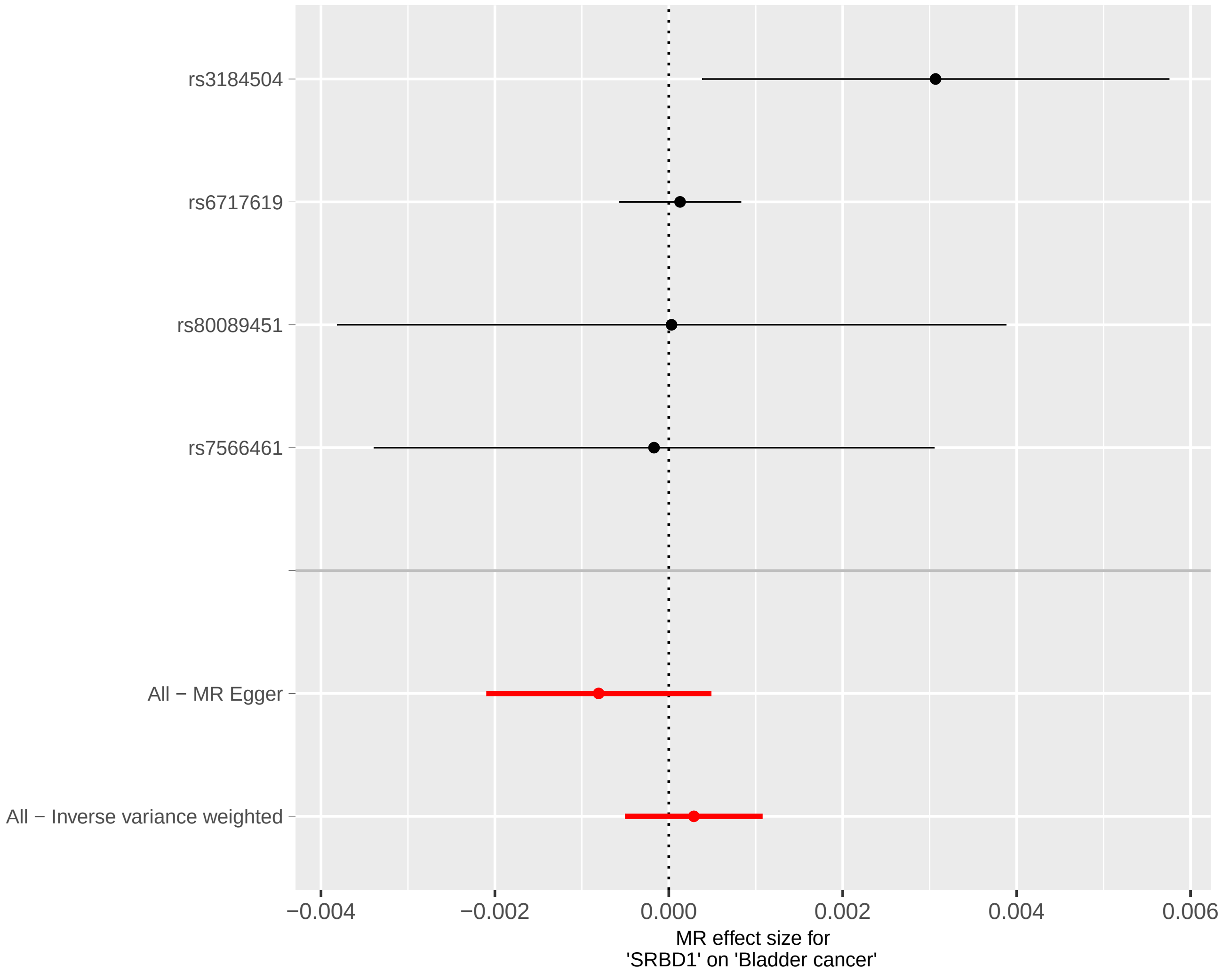
rs3184504
rs6717619
rs80089451
rs7566461
All − MR Egger
All − Inverse variance weighted
−0.004
−0.002
0.000
0.002
0.004
0.006
MR effect size for
'SRBD1' on 'Bladder cancer'

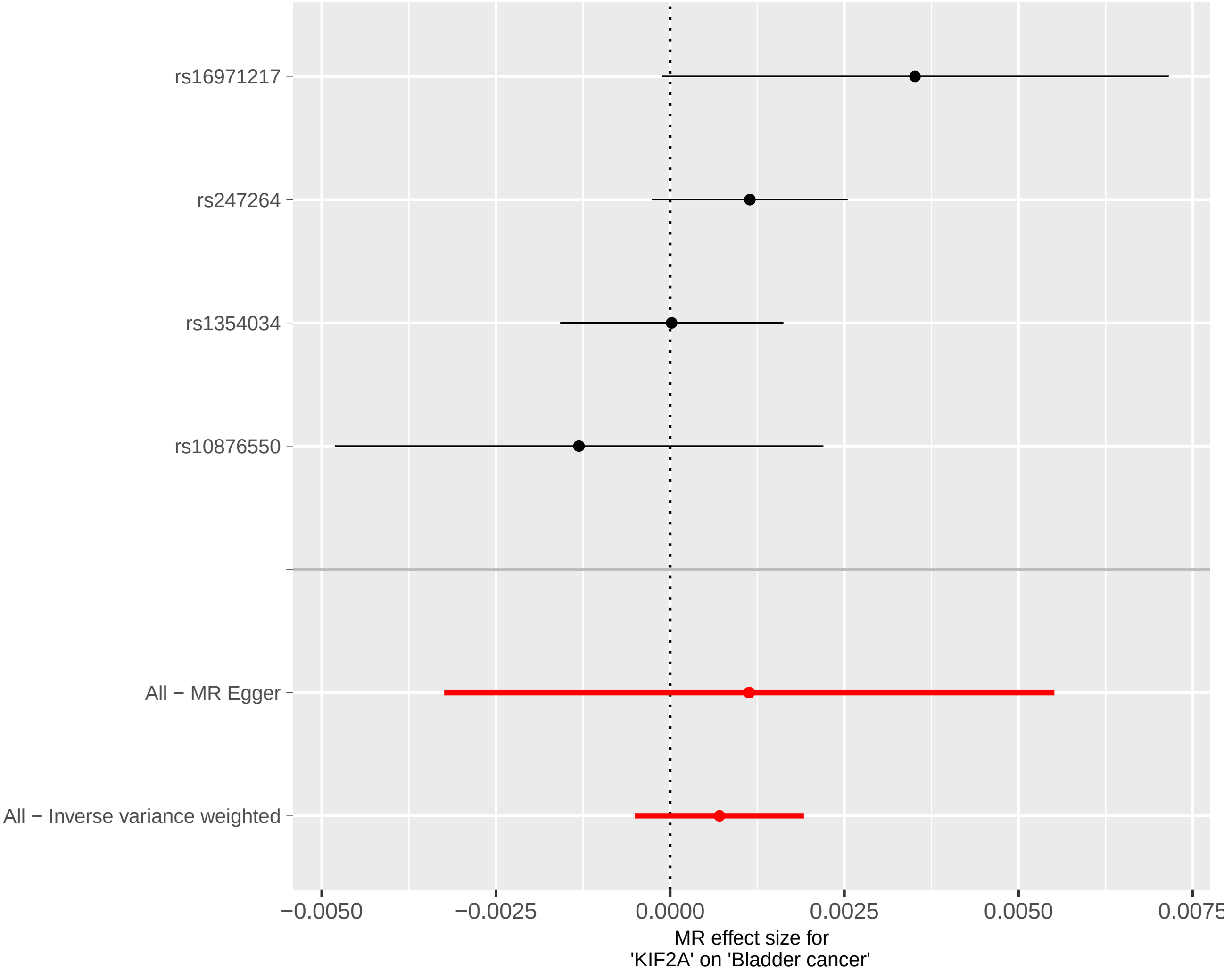
rs16971217
rs247264
rs1354034
rs10876550
All − MR Egger
All − Inverse variance weighted
−0.0050
−0.0025
0.0000
0.0025
0.0050
0.0075
MR effect size for
'KIF2A' on 'Bladder cancer'

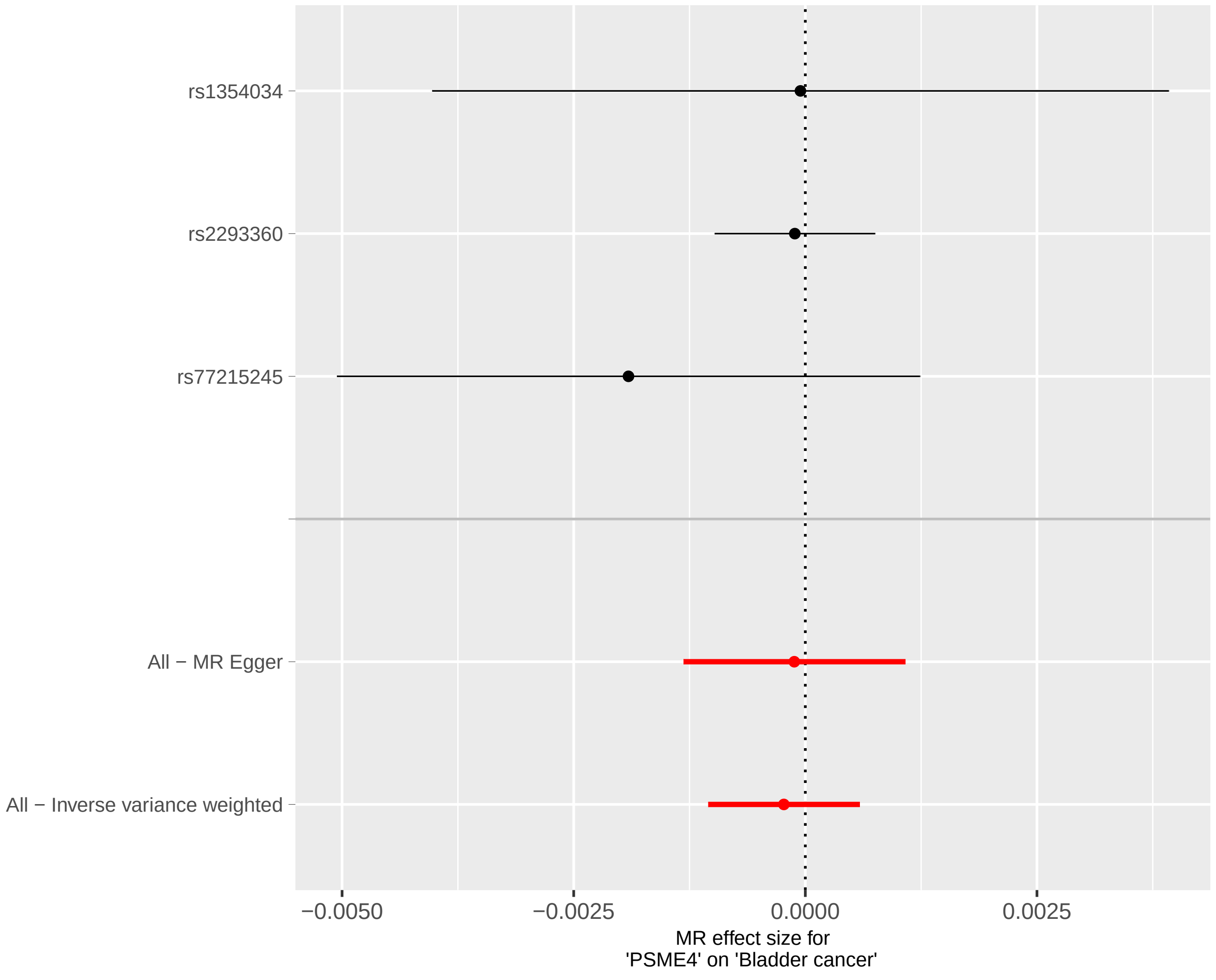
rs1354034
rs2293360
rs77215245
All − MR Egger
All − Inverse variance weighted
−0.0050
−0.0025
0.0000
0.0025
MR effect size for
'PSME4' on 'Bladder cancer'

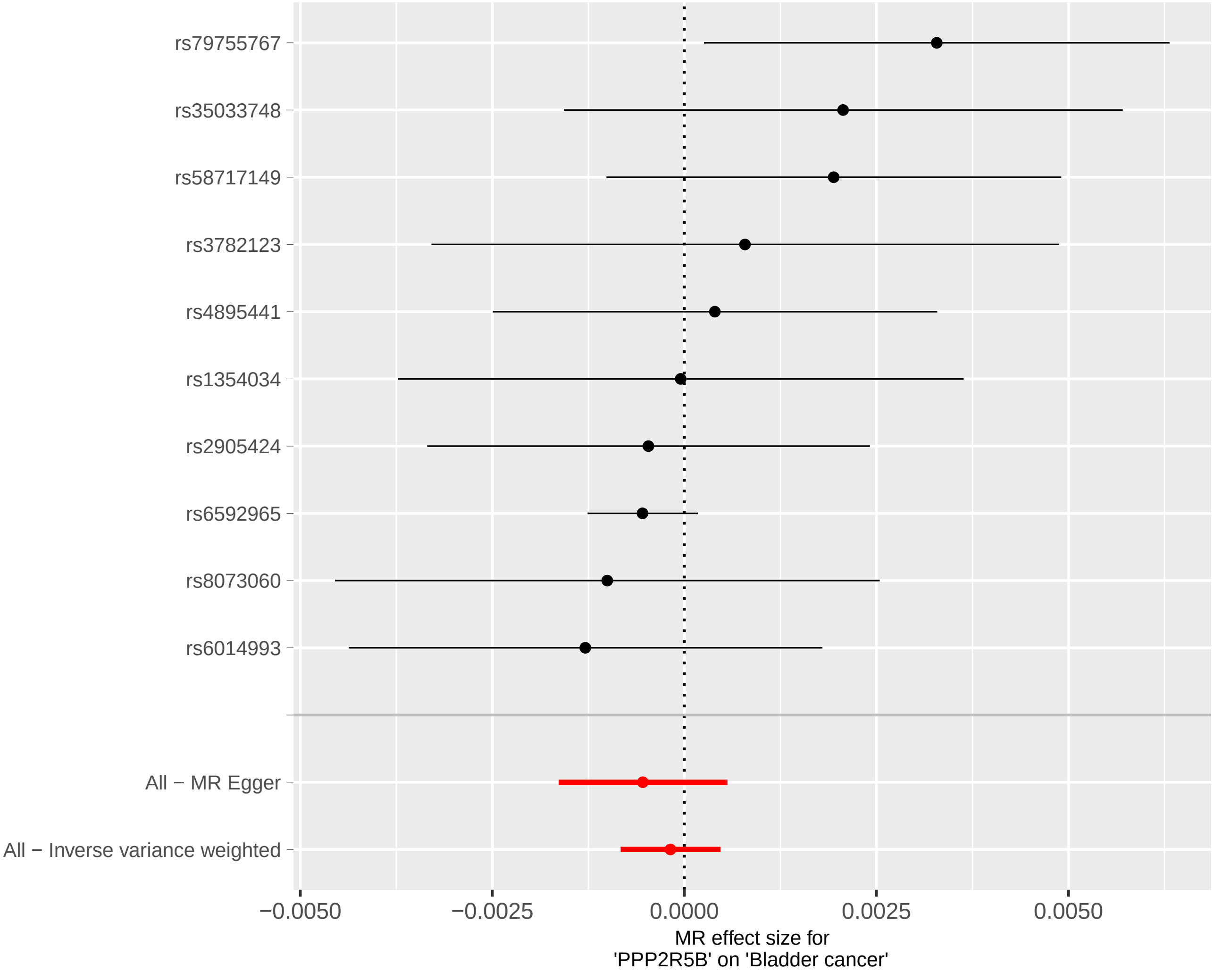

rs79755767
rs35033748
rs58717149
rs3782123
rs4895441
rs1354034
rs2905424
rs6592965
rs8073060
rs6014993
All − MR Egger
All − Inverse variance weighted
−0.0050
−0.0025
0.0000
0.0025
0.0050
MR effect size for
'PPP2R5B' on 'Bladder cancer'

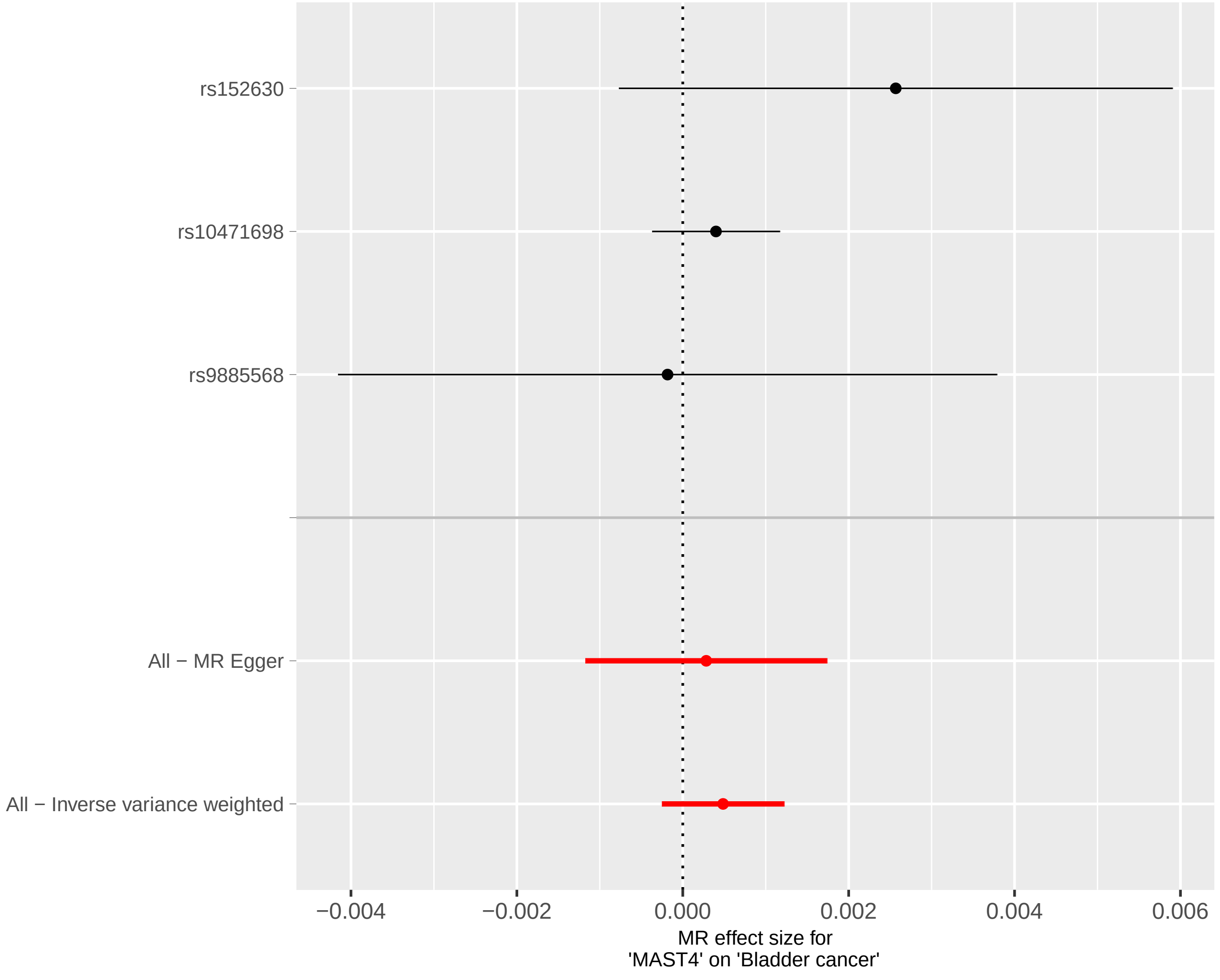

rs152630
rs10471698
rs9885568
All − MR Egger
All − Inverse variance weighted
−0.004
−0.002
0.000
0.002
0.004
0.006
MR effect size for
'MAST4' on 'Bladder cancer'

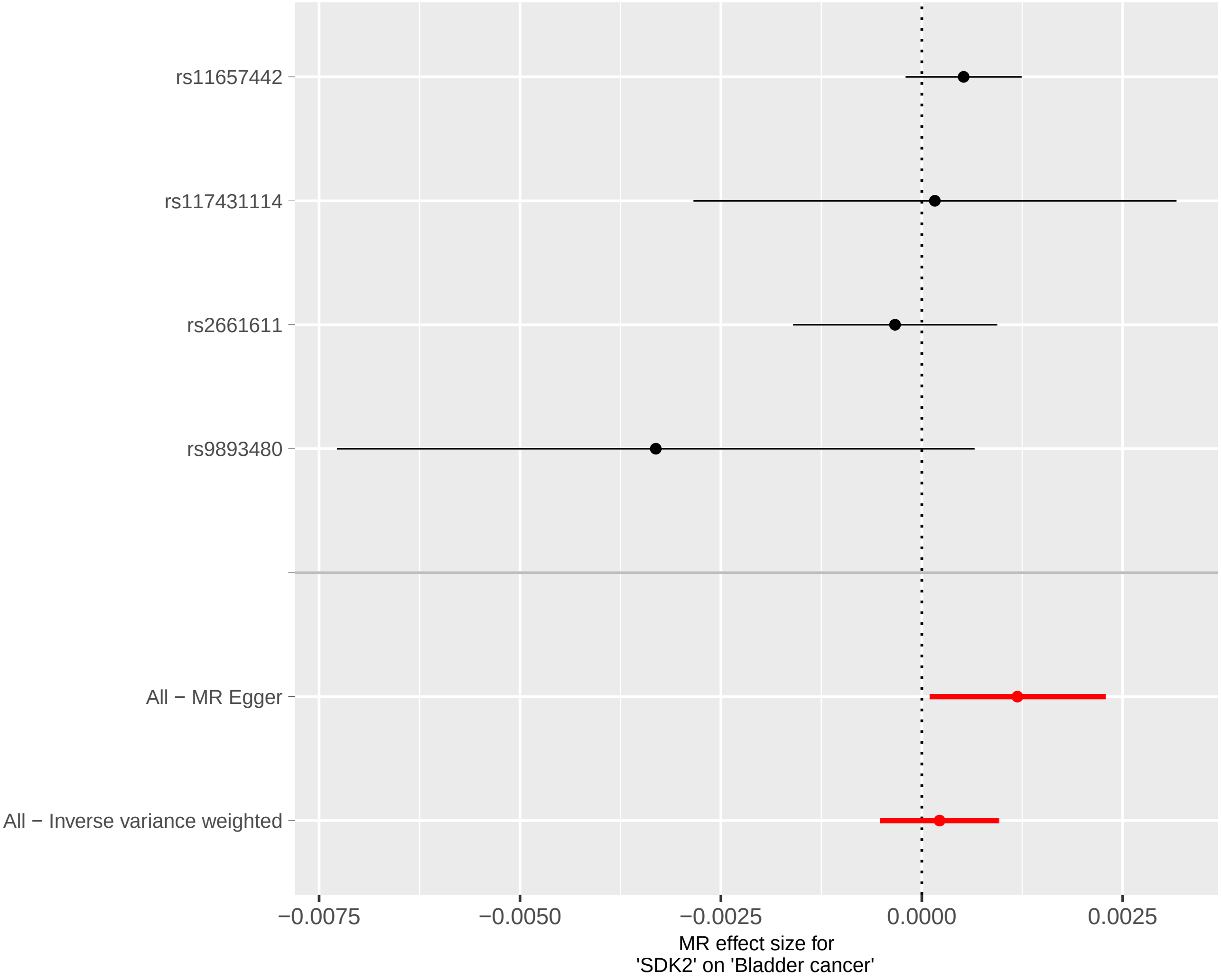
rs11657442
rs117431114
rs2661611
rs9893480
All − MR Egger
All − Inverse variance weighted
−0.0075
−0.0050
−0.0025
0.0000
0.0025
MR effect size for
'SDK2' on 'Bladder cancer'

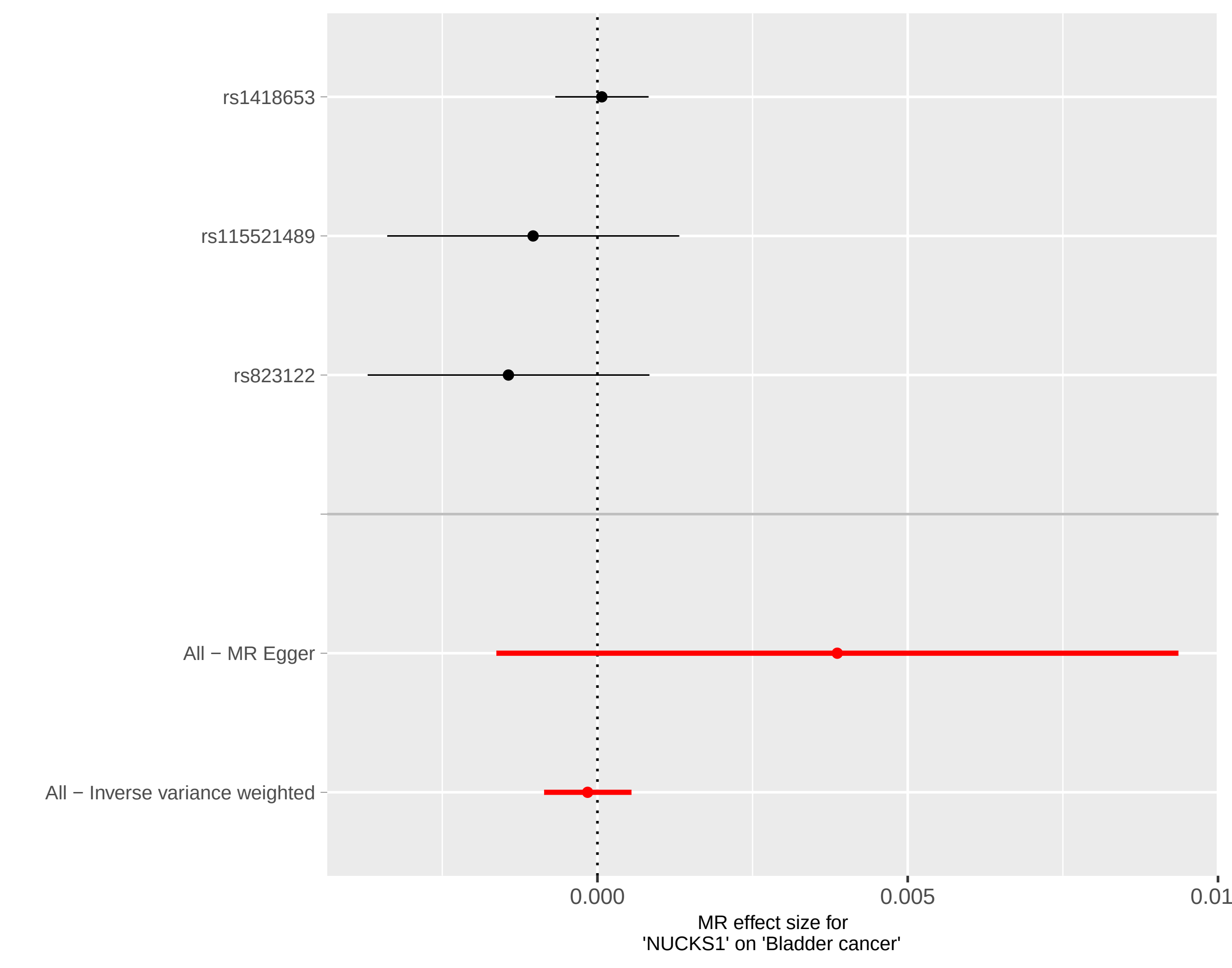
rs1418653
rs115521489
rs823122
All − MR Egger
All − Inverse variance weighted
0.000
0.005
0.01
MR effect size for
'NUCKS1' on 'Bladder cancer'

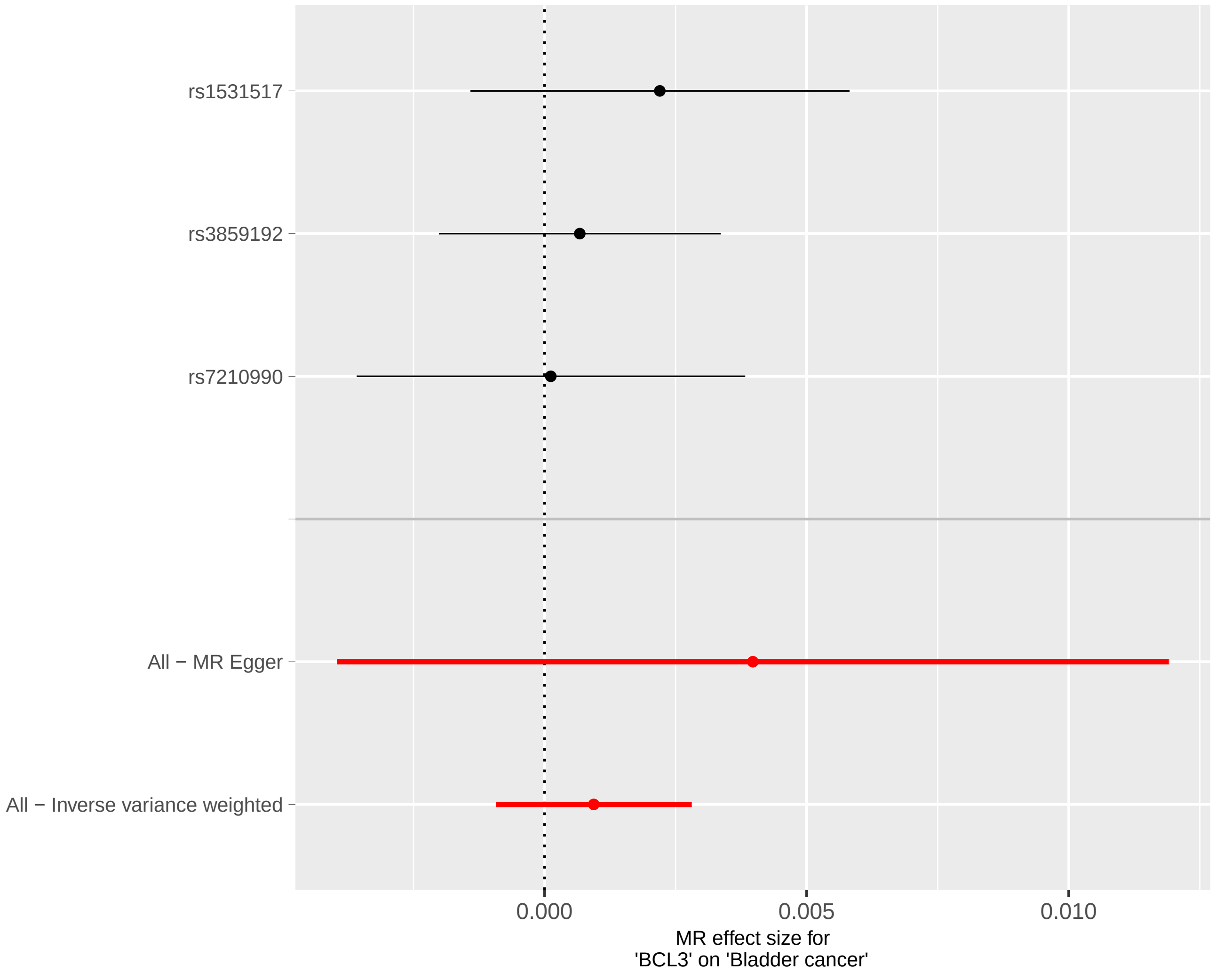
rs1531517
rs3859192
rs7210990
All − MR Egger
All − Inverse variance weighted
0.000
0.005
0.010
MR effect size for
'BCL3' on 'Bladder cancer'

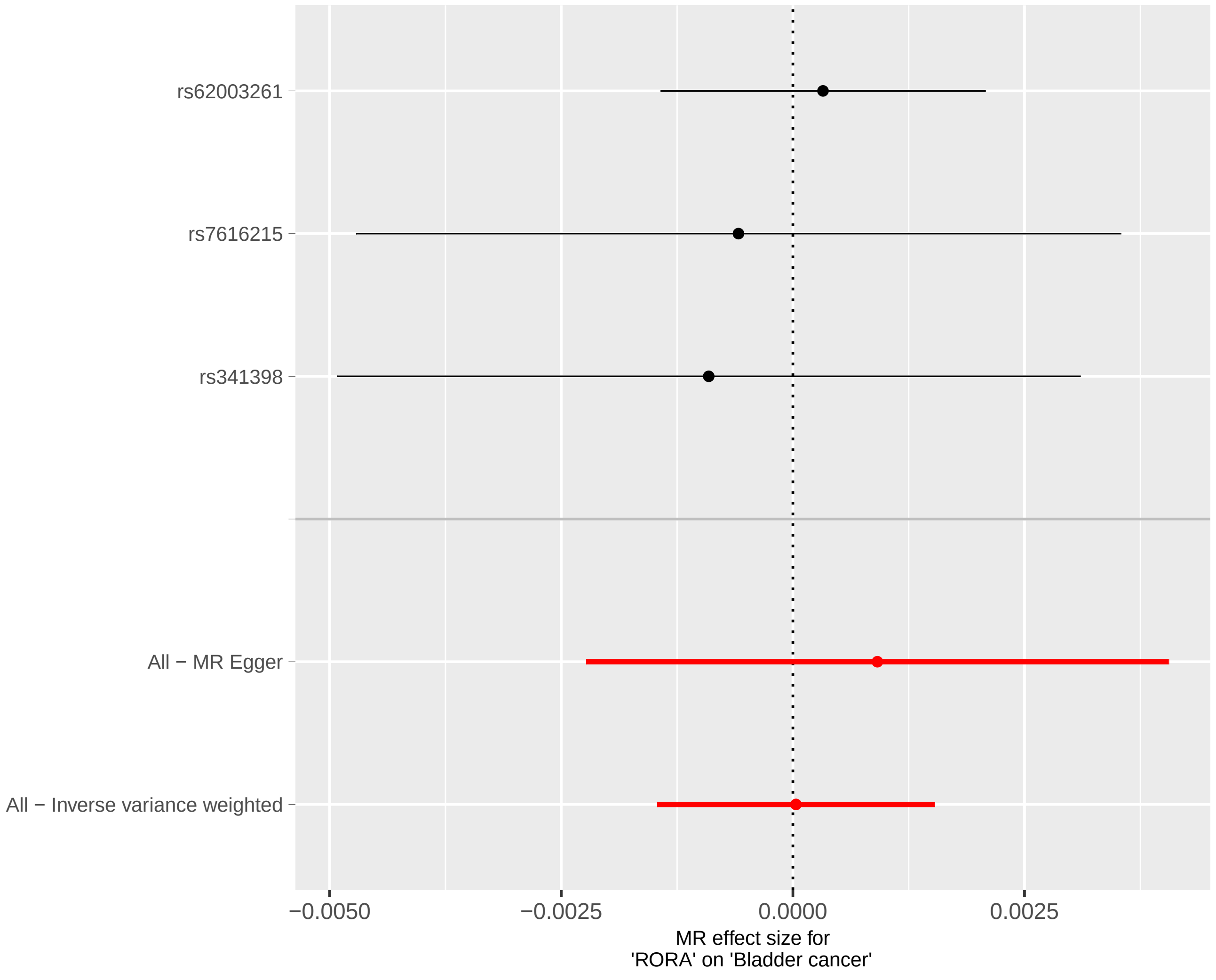
rs62003261
rs7616215
rs341398
All − MR Egger
All − Inverse variance weighted
−0.0050
−0.0025
0.0000
0.0025
MR effect size for
'RORA' on 'Bladder cancer'

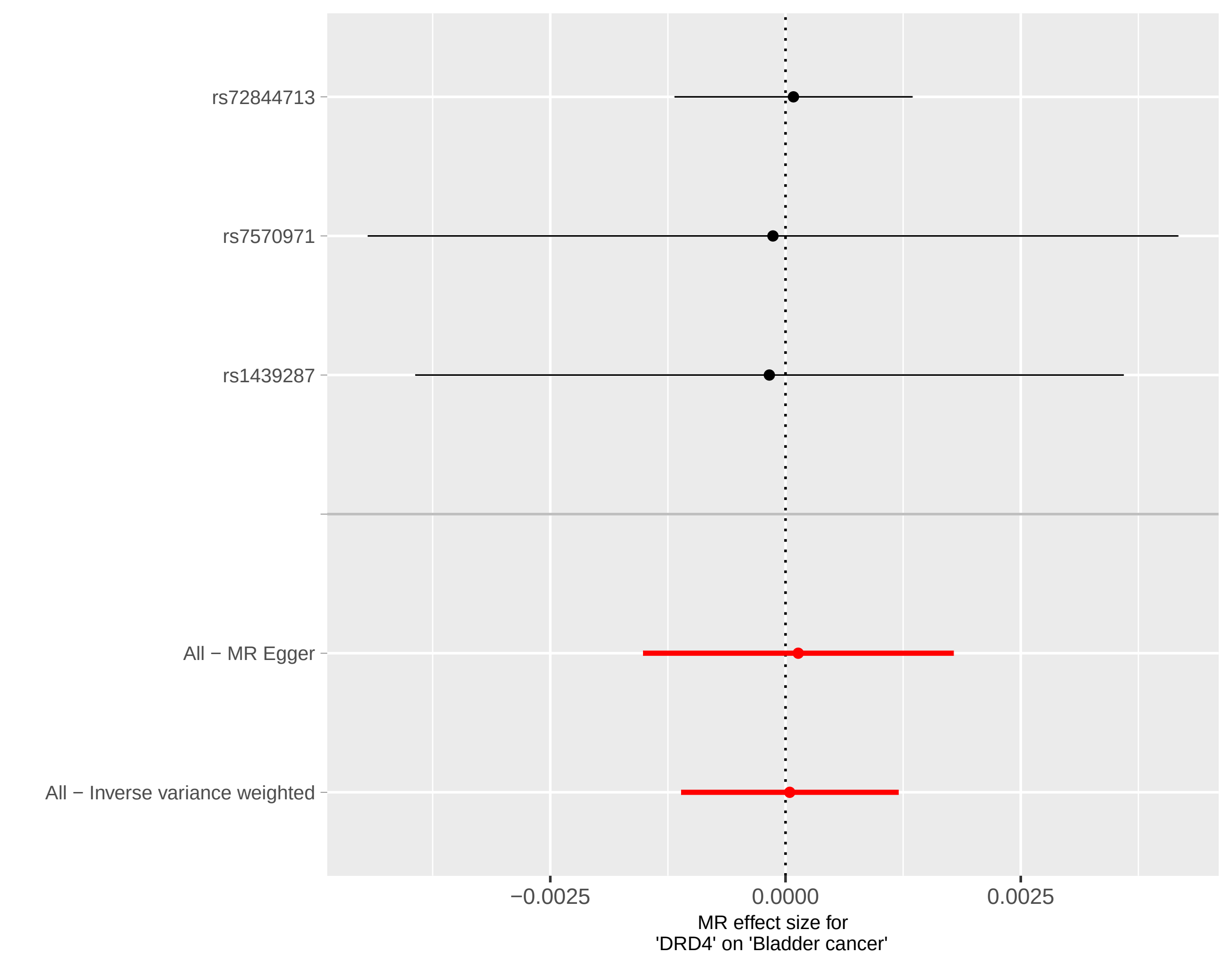
rs72844713
rs7570971
rs1439287
All − MR Egger
All − Inverse variance weighted
−0.0025
0.0000
0.0025
MR effect size for
'DRD4' on 'Bladder cancer'

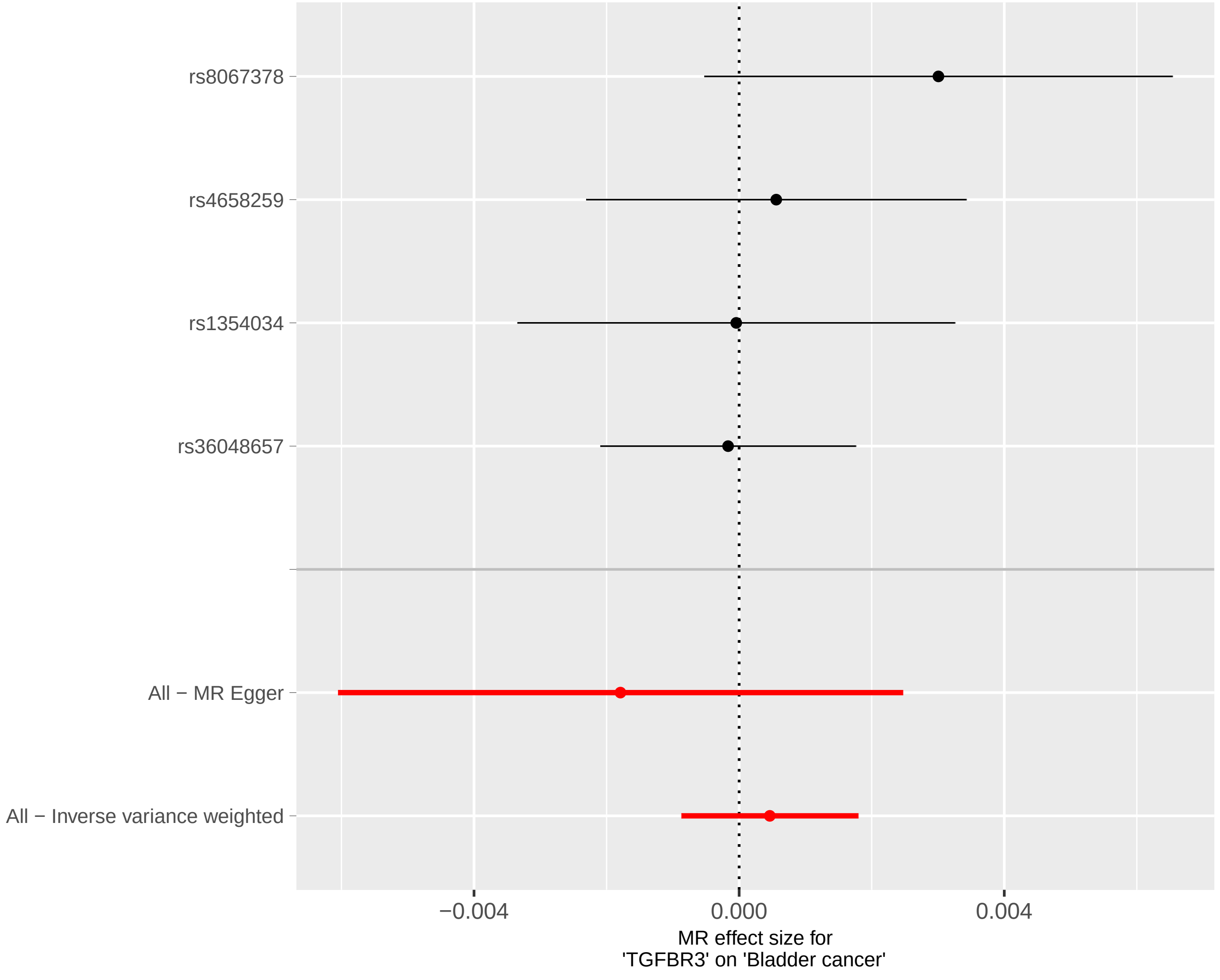
rs8067378
rs4658259
rs1354034
rs36048657
All − MR Egger
All − Inverse variance weighted
−0.004
0.000
0.004
MR effect size for
'TGFBR3' on 'Bladder cancer'

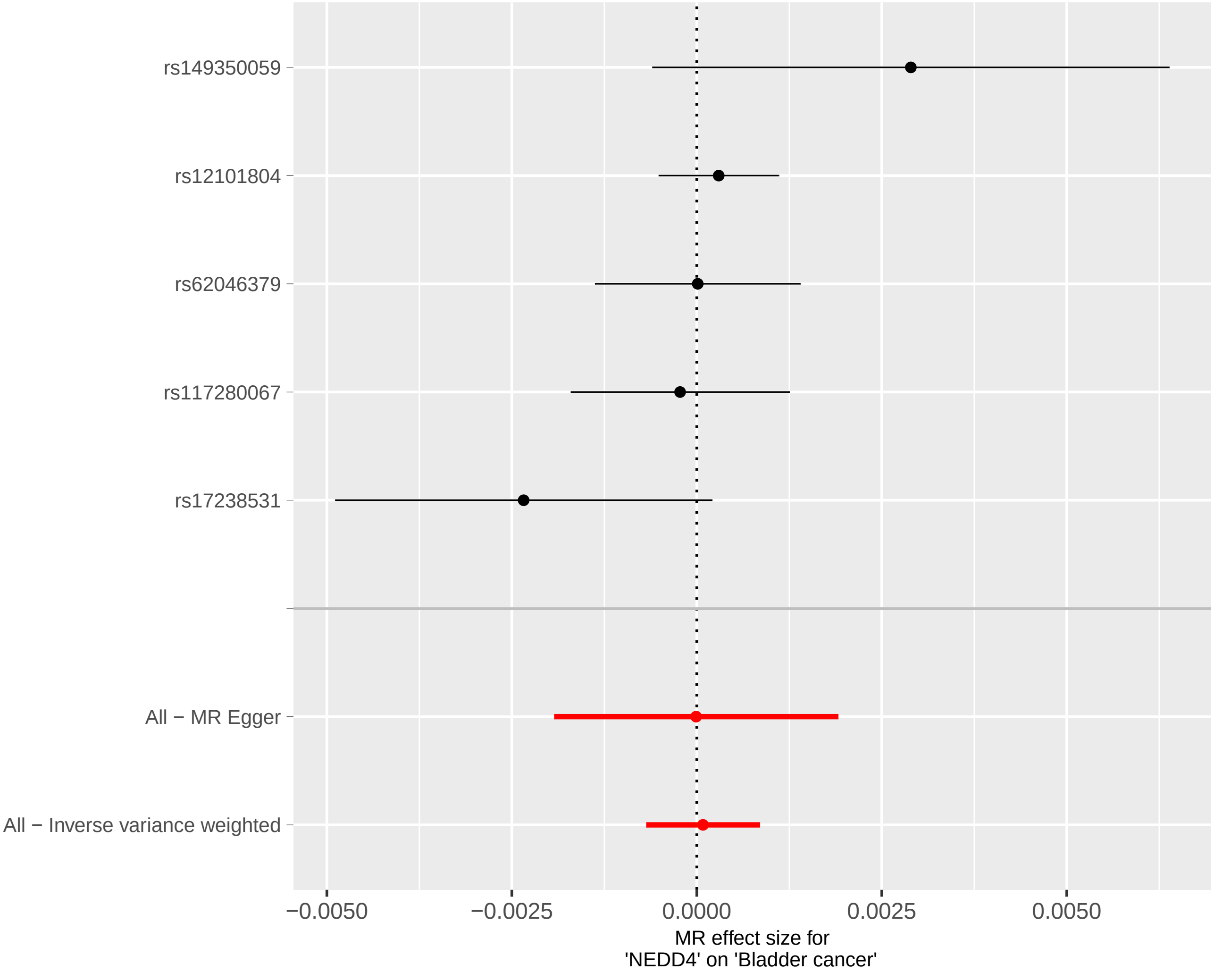
rs149350059
rs12101804
rs62046379
rs117280067
rs17238531
All − MR Egger
All − Inverse variance weighted
−0.0050
−0.0025
0.0000
0.0025
0.0050
MR effect size for
'NEDD4' on 'Bladder cancer'

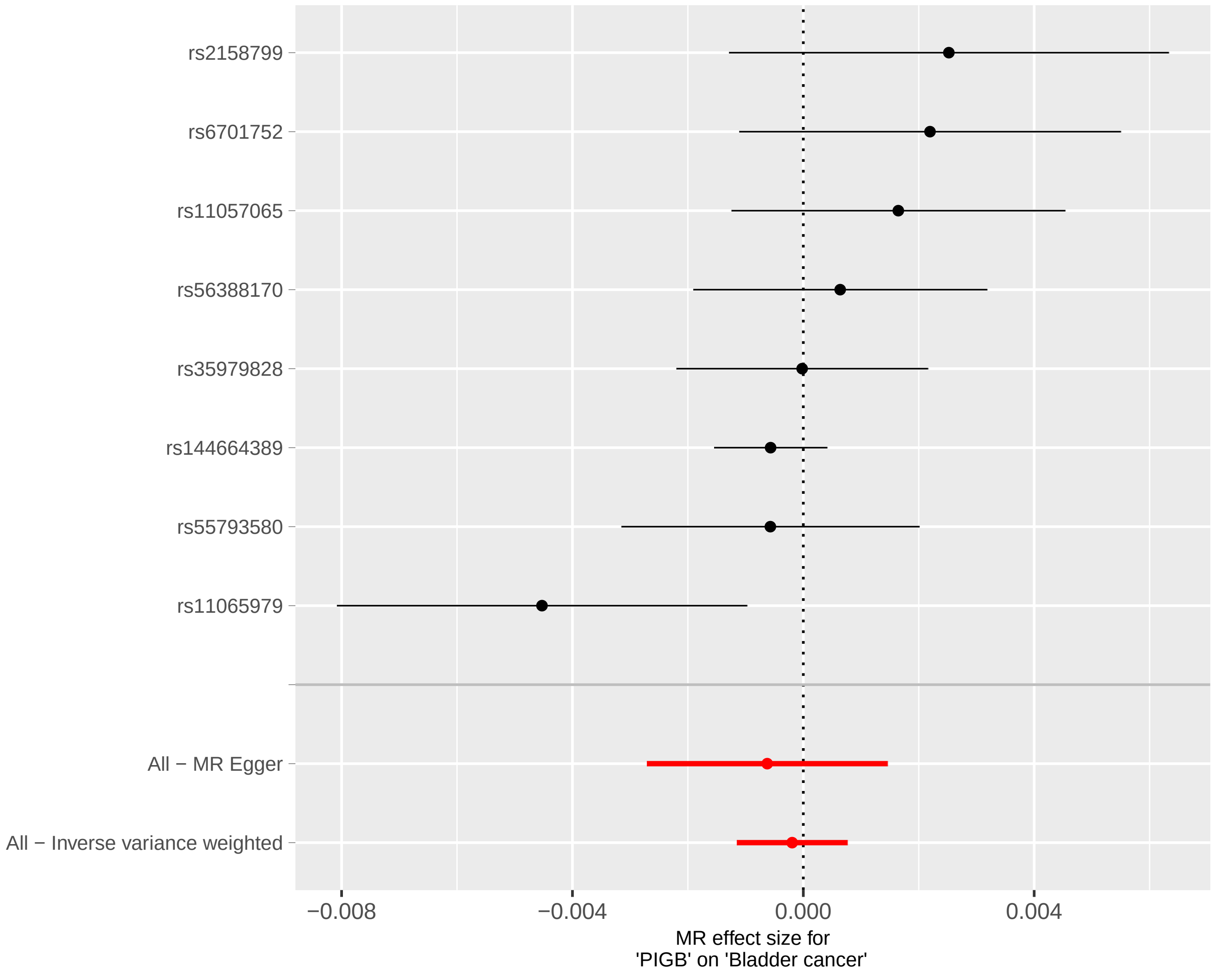

rs2158799
rs6701752
rs11057065
rs56388170
rs35979828
rs144664389
rs55793580
rs11065979
All − MR Egger
All − Inverse variance weighted
−0.008
−0.004
0.000
0.004
MR effect size for
'PIGB' on 'Bladder cancer'

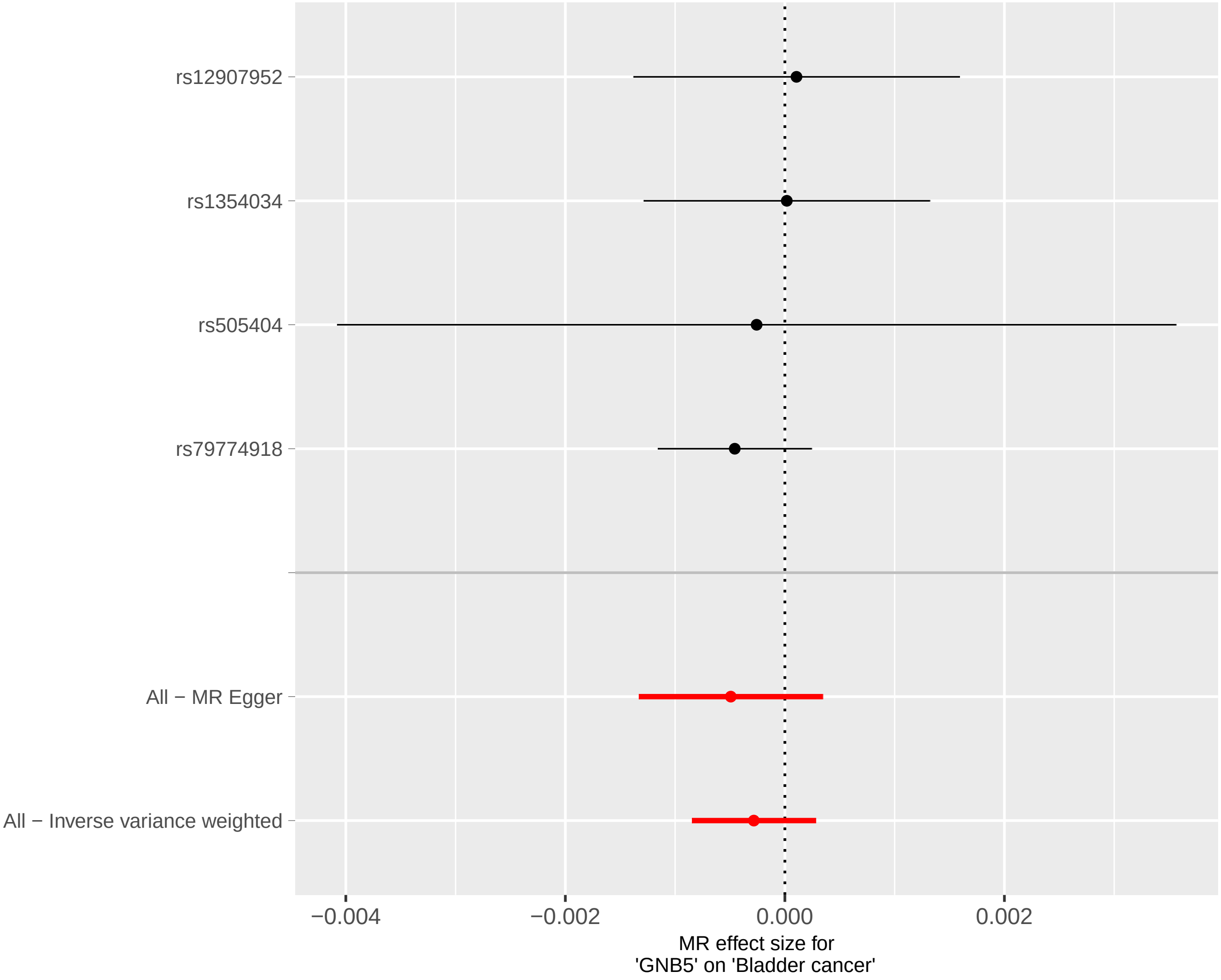

rs12907952
rs1354034
rs505404
rs79774918
All − MR Egger
All − Inverse variance weighted
−0.004
−0.002
0.000
0.002
MR effect size for
'GNB5' on 'Bladder cancer'

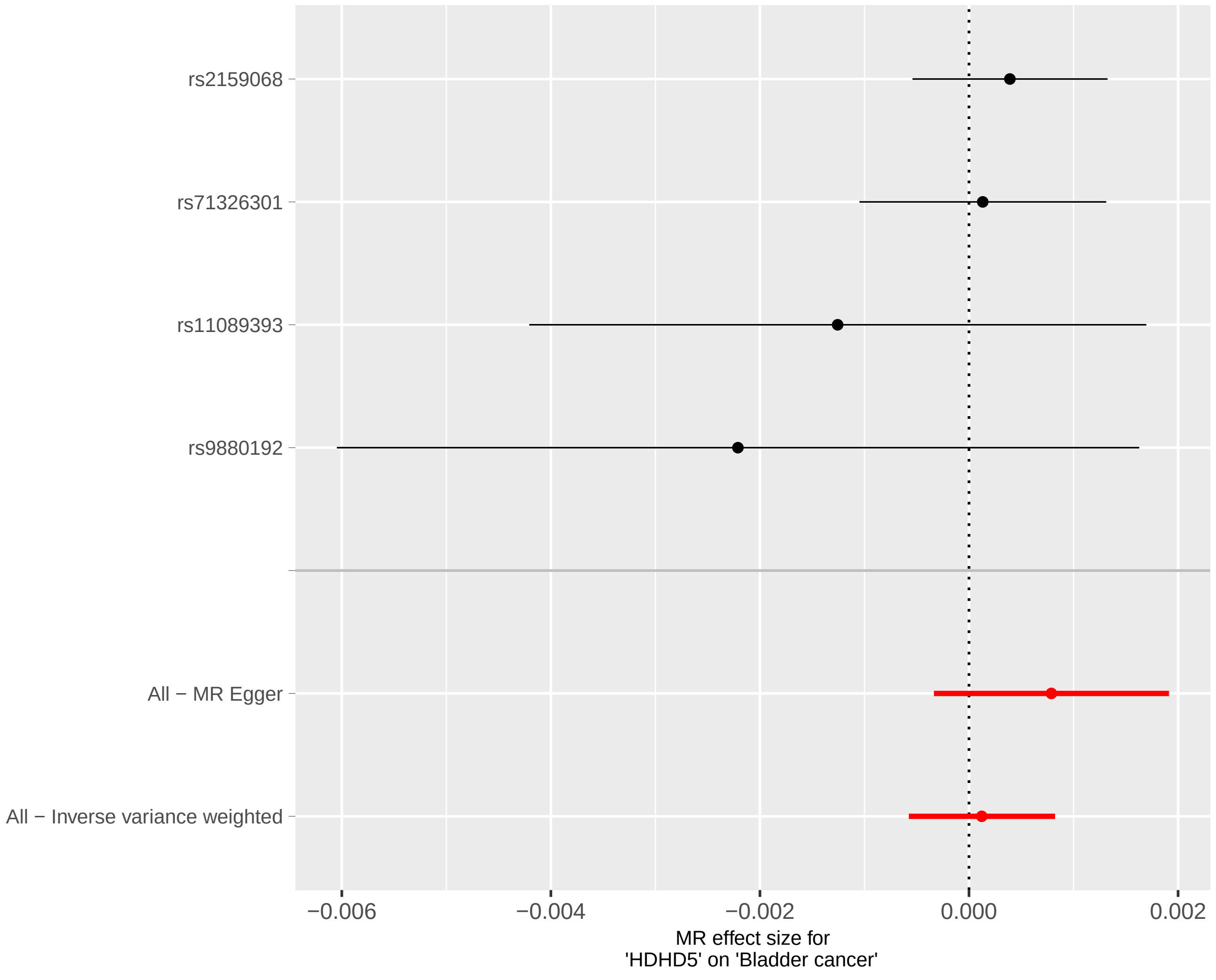
rs2159068
rs71326301
rs11089393
rs9880192
All − MR Egger
All − Inverse variance weighted
−0.006
−0.004
−0.002
0.000
0.002
MR effect size for
'HDHD5' on 'Bladder cancer'

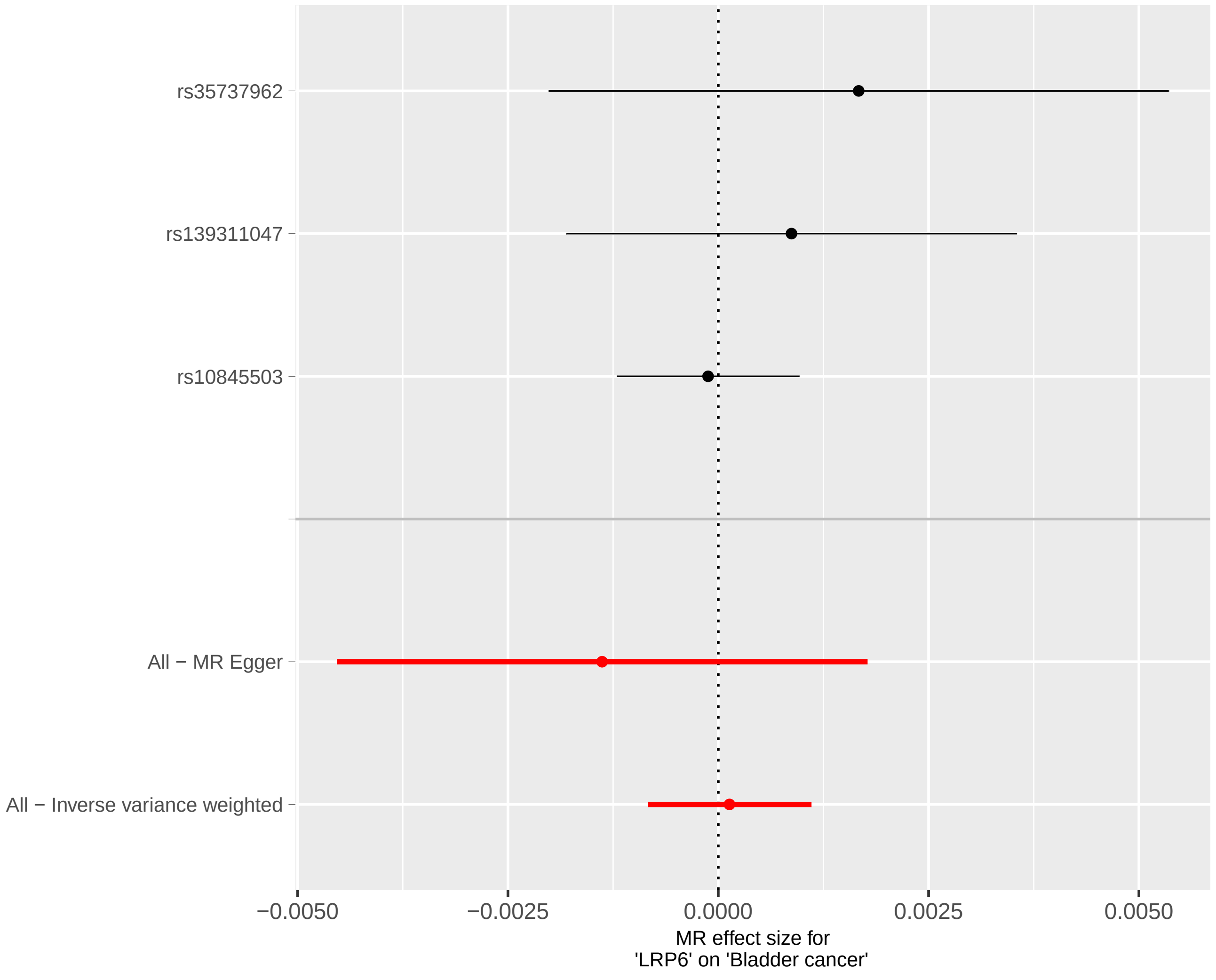

rs35737962
rs139311047
rs10845503
All − MR Egger
All − Inverse variance weighted
−0.0050
−0.0025
0.0000
0.0025
0.0050
MR effect size for
'LRP6' on 'Bladder cancer'

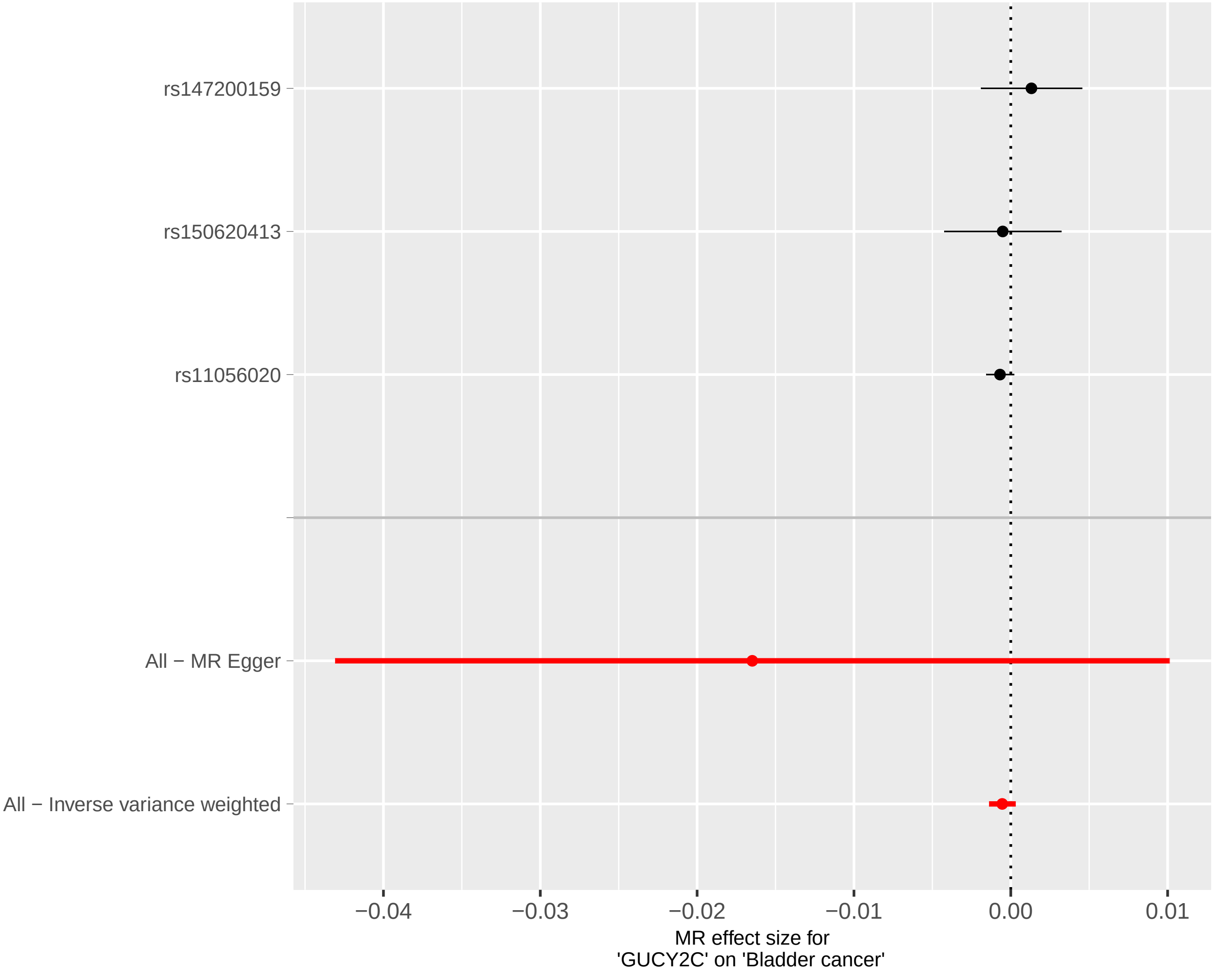
rs147200159
rs150620413
rs11056020
All − MR Egger
All − Inverse variance weighted
−0.04
−0.03
−0.02
−0.01
0.00
0.01
MR effect size for
'GUCY2C' on 'Bladder cancer'

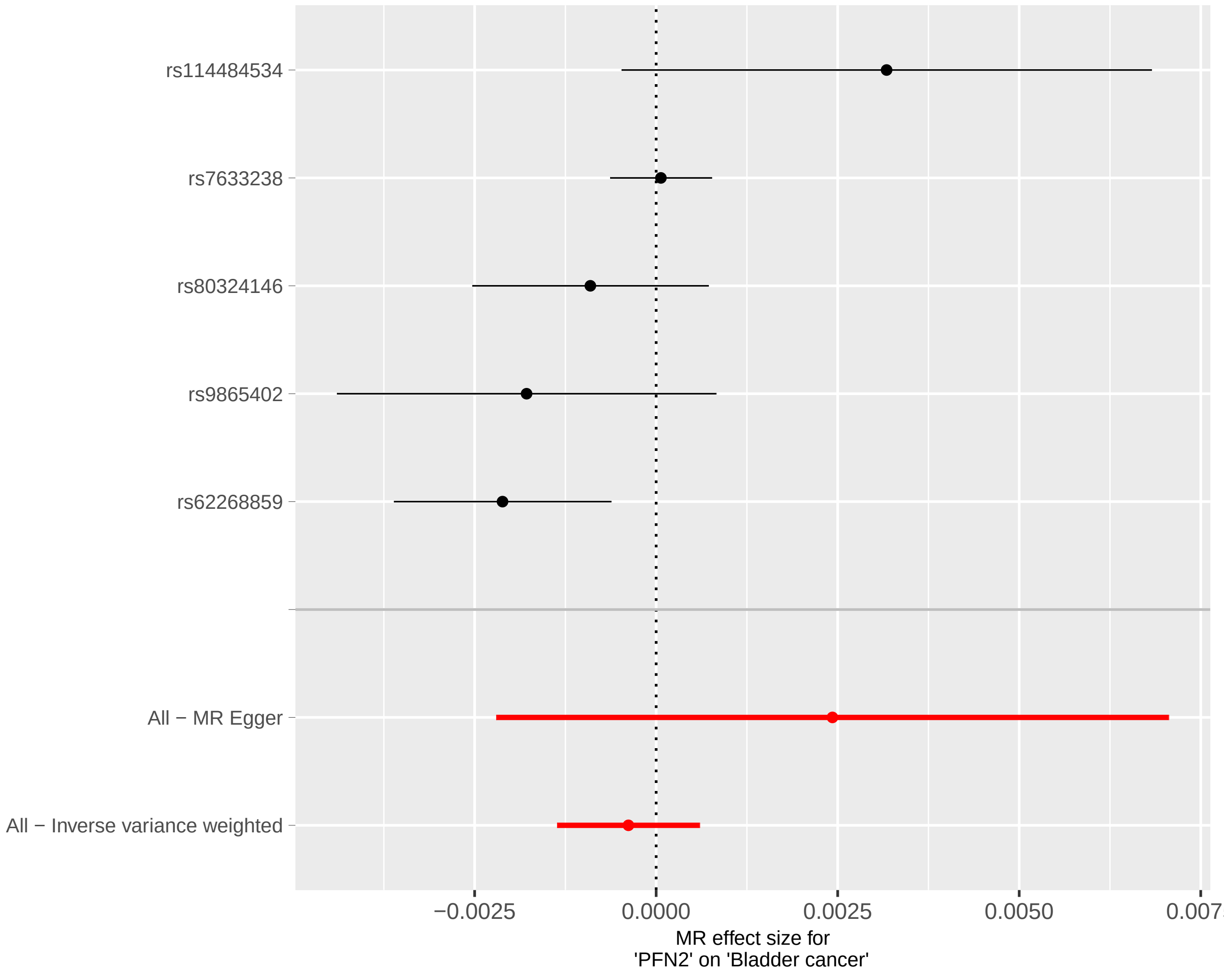

rs114484534
rs7633238
rs80324146
rs9865402
rs62268859
All − MR Egger
All − Inverse variance weighted
−0.0025
0.0000
0.0025
0.0050
MR effect size for
'PFN2' on 'Bladder cancer'

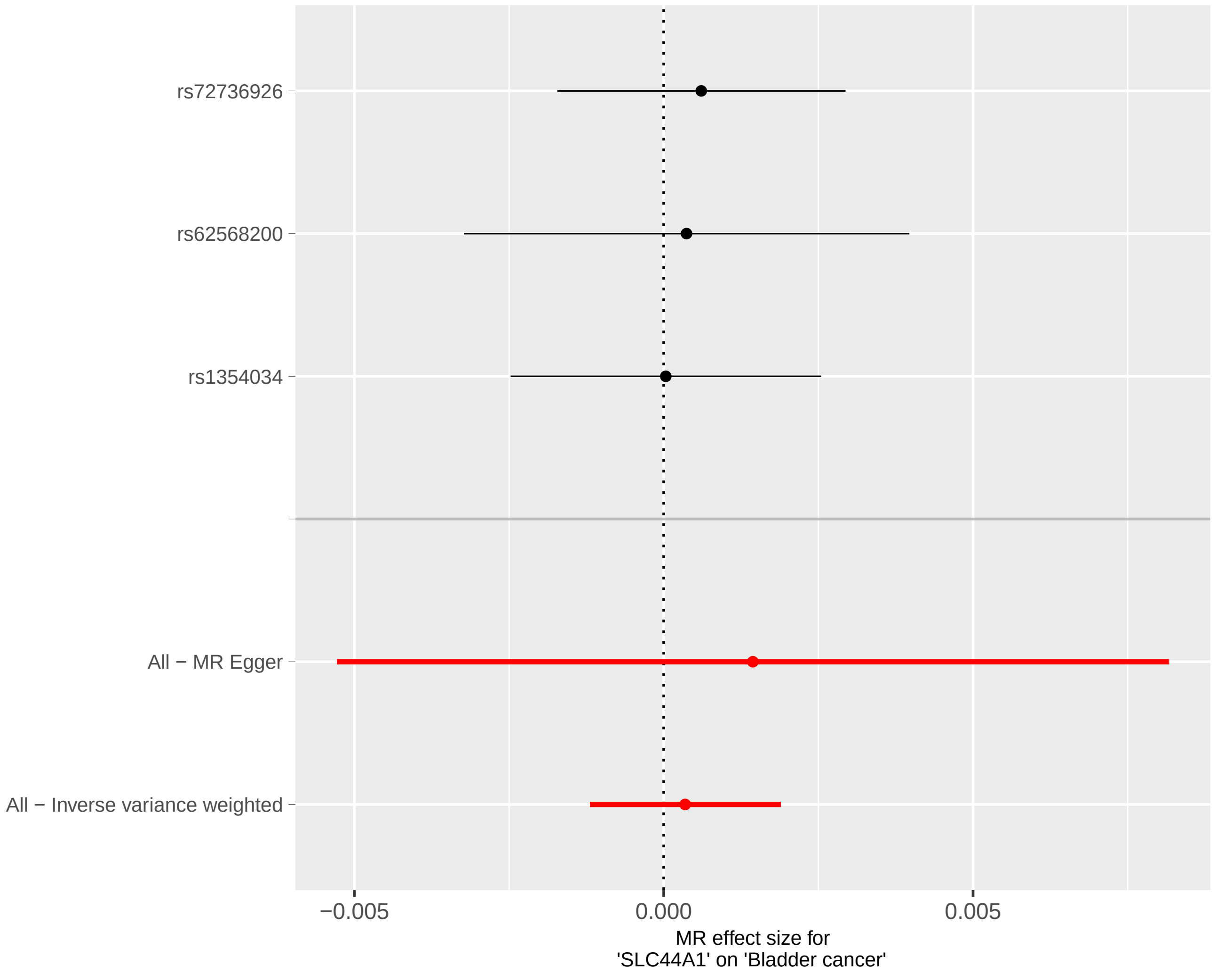

rs72736926
rs62568200
rs1354034
All − MR Egger
All − Inverse variance weighted
−0.005
0.000
0.005
MR effect size for
'SLC44A1' on 'Bladder cancer'

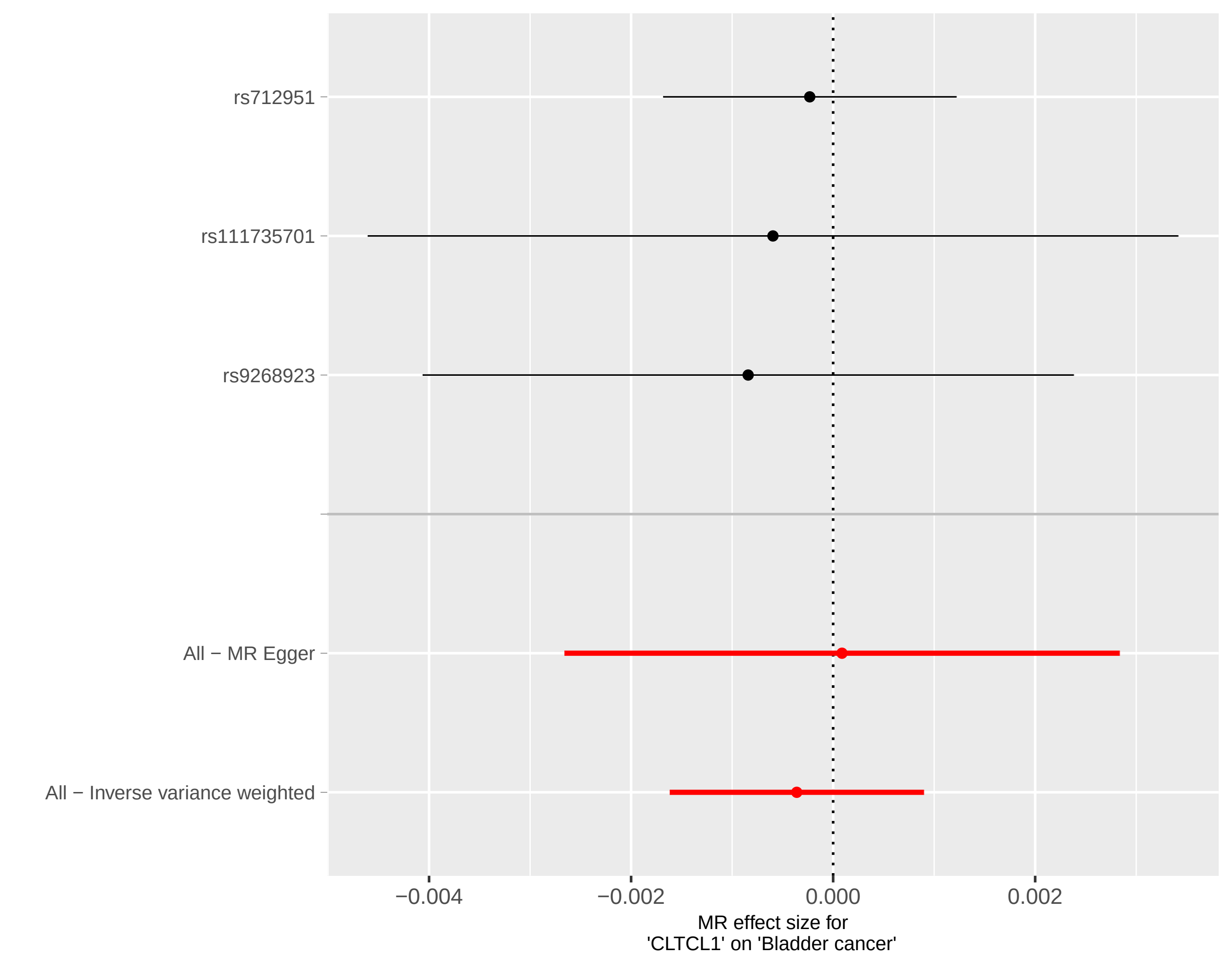
rs712951
rs111735701
rs9268923
All − MR Egger
All − Inverse variance weighted
−0.004
−0.002
0.000
0.002
MR effect size for
'CLTCL1' on 'Bladder cancer'

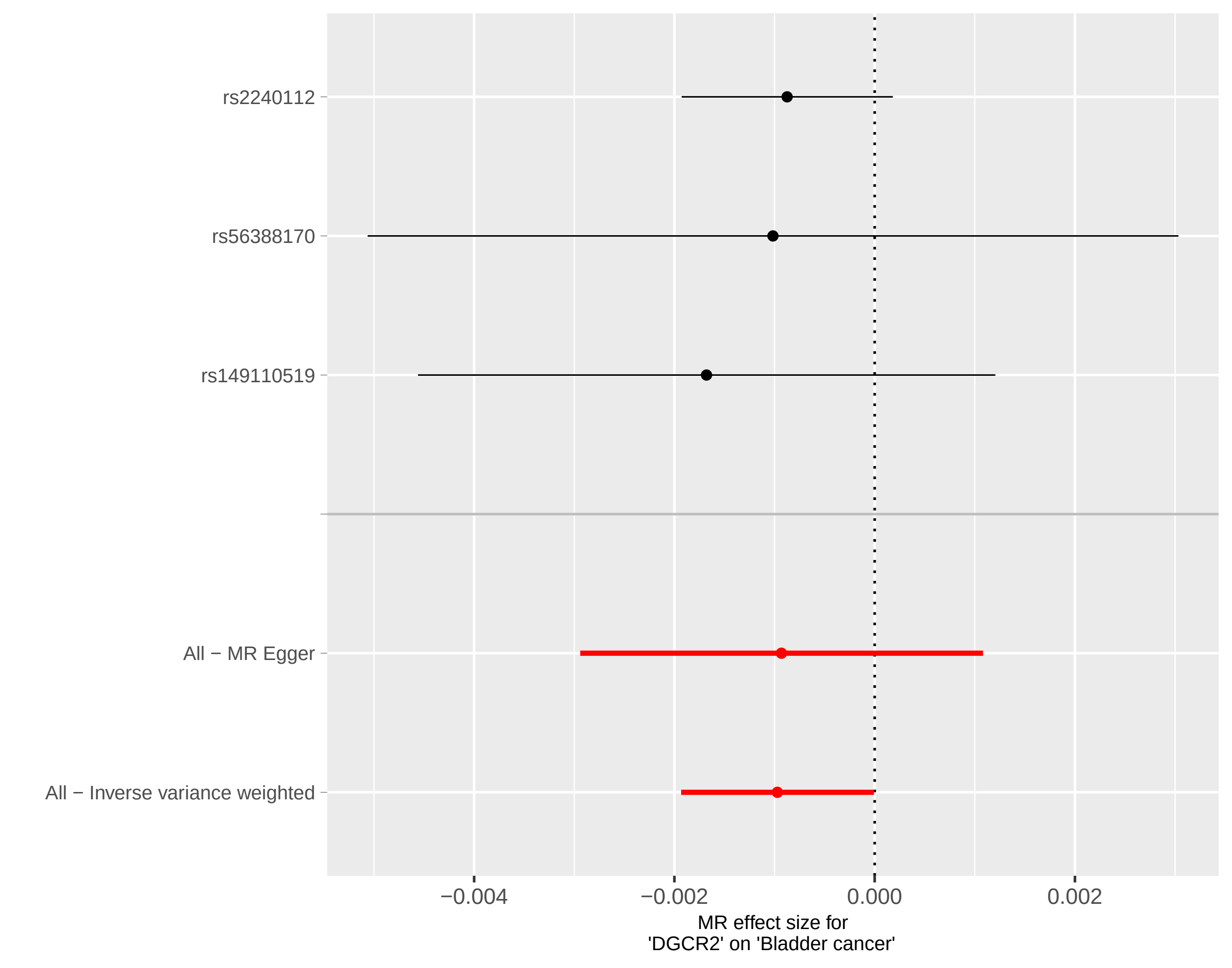

rs2240112
rs56388170
rs149110519
All − MR Egger
All − Inverse variance weighted
−0.004
−0.002
0.000
0.002
MR effect size for
'DGCR2' on 'Bladder cancer'

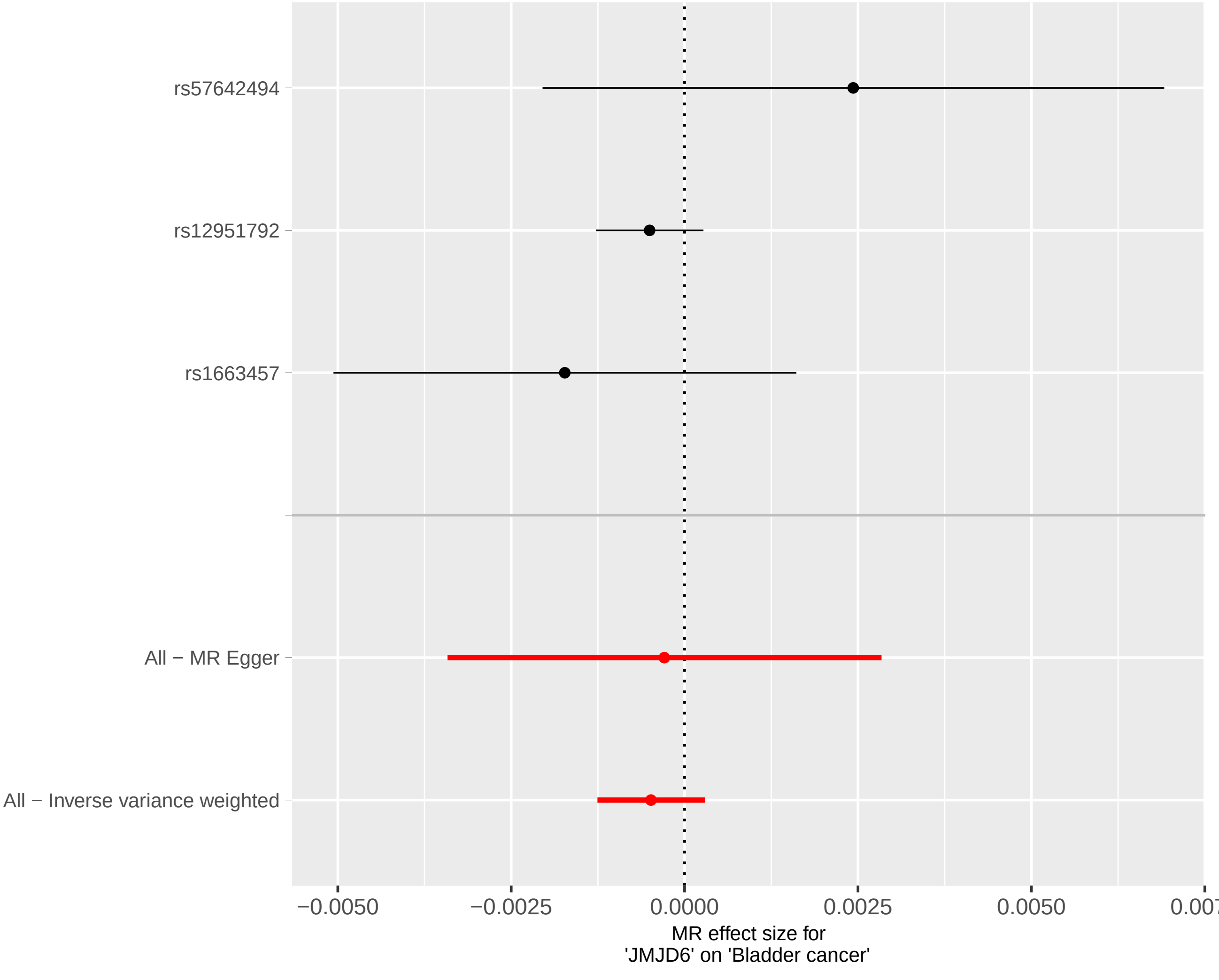

rs57642494
rs12951792
rs1663457
All − MR Egger
All − Inverse variance weighted
−0.0050
−0.0025
0.0000
0.0025
0.0050
MR effect size for
'JMJD6' on 'Bladder cancer'

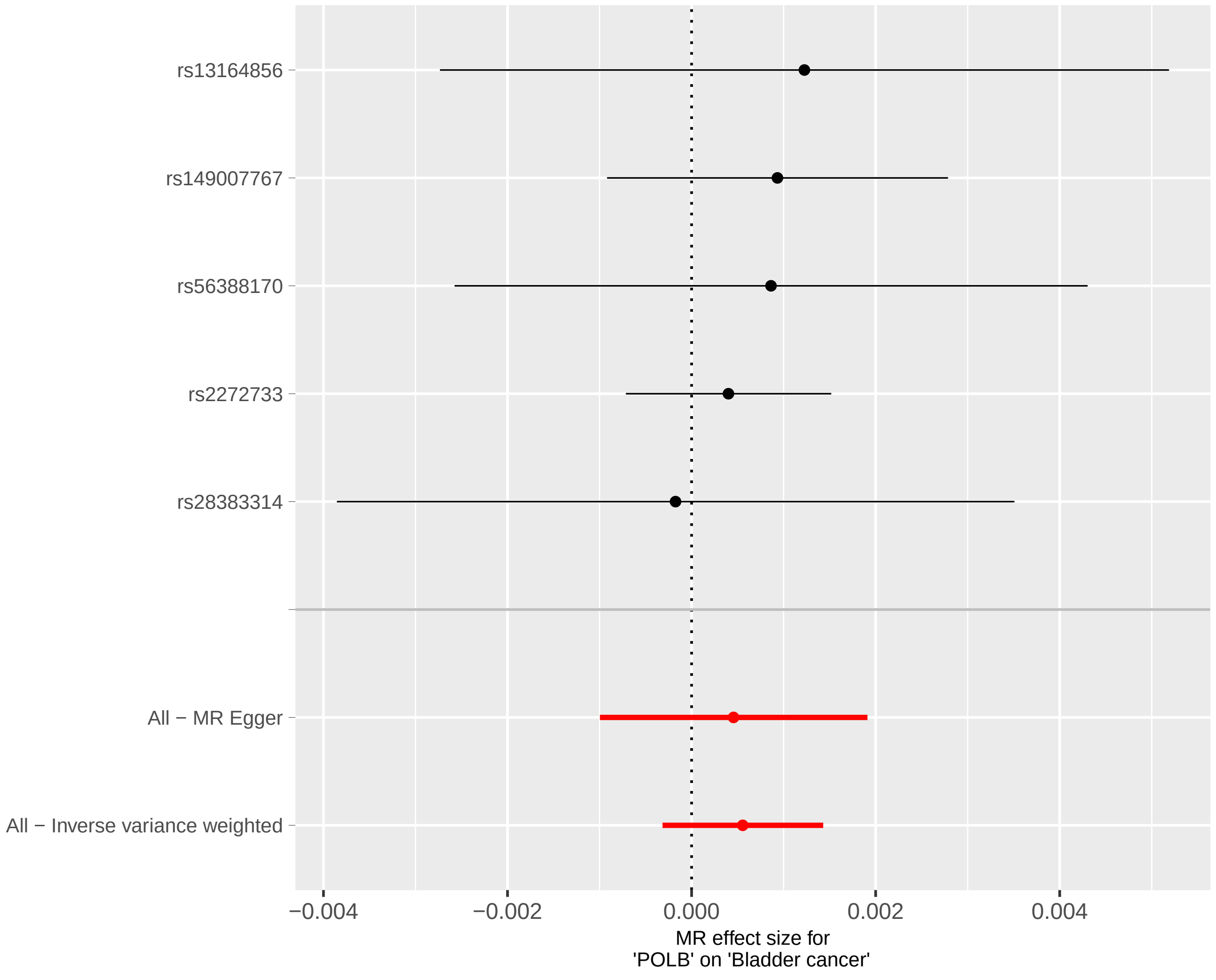
rs13164856
rs149007767
rs56388170
rs2272733
rs28383314
All − MR Egger
All − Inverse variance weighted
−0.004
−0.002
0.000
0.002
0.004
MR effect size for
'POLB' on 'Bladder cancer'

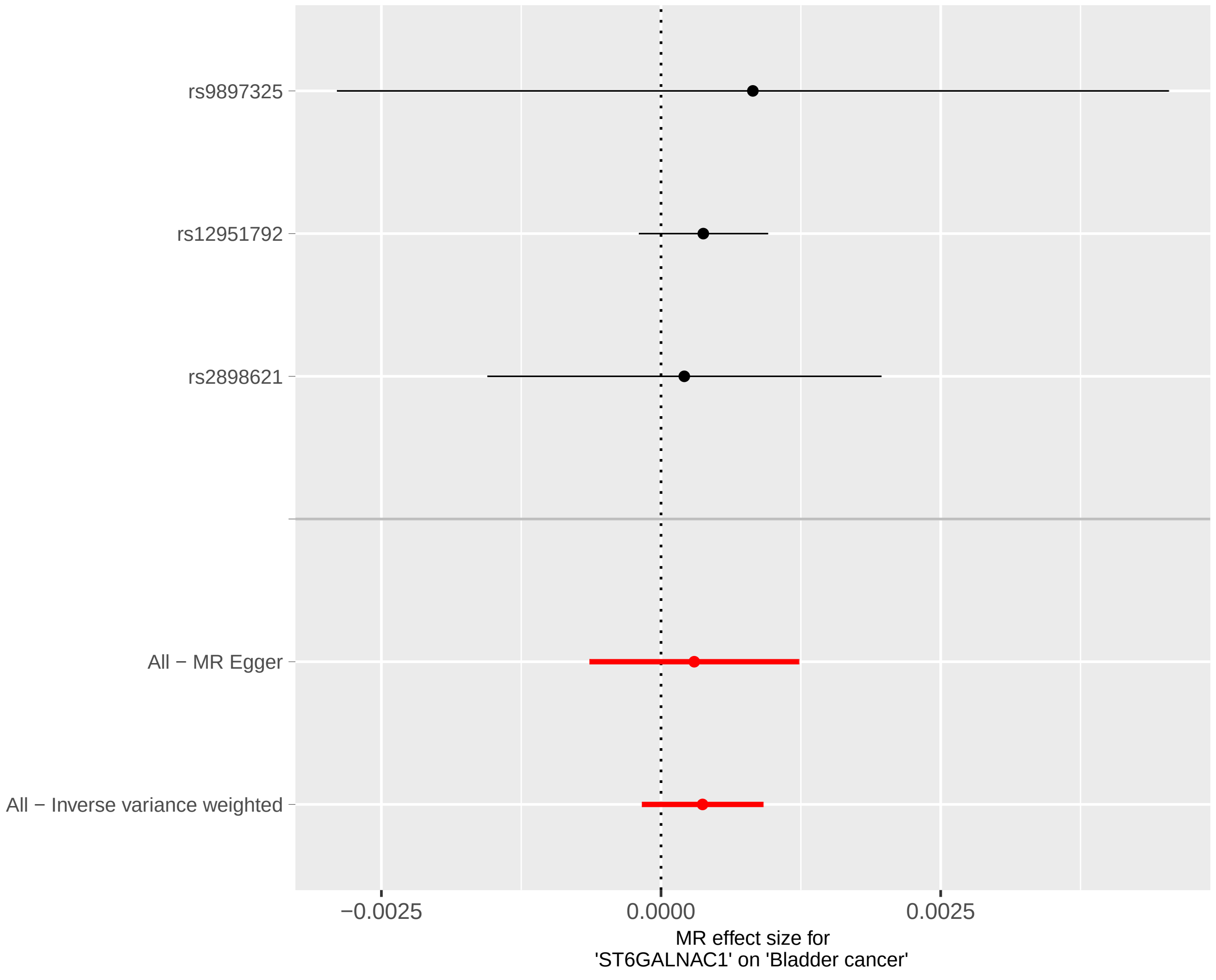

rs9897325
rs12951792
rs2898621
All − MR Egger
All − Inverse variance weighted
−0.0025
0.0000
0.0025
MR effect size for
'ST6GALNAC1' on 'Bladder cancer'

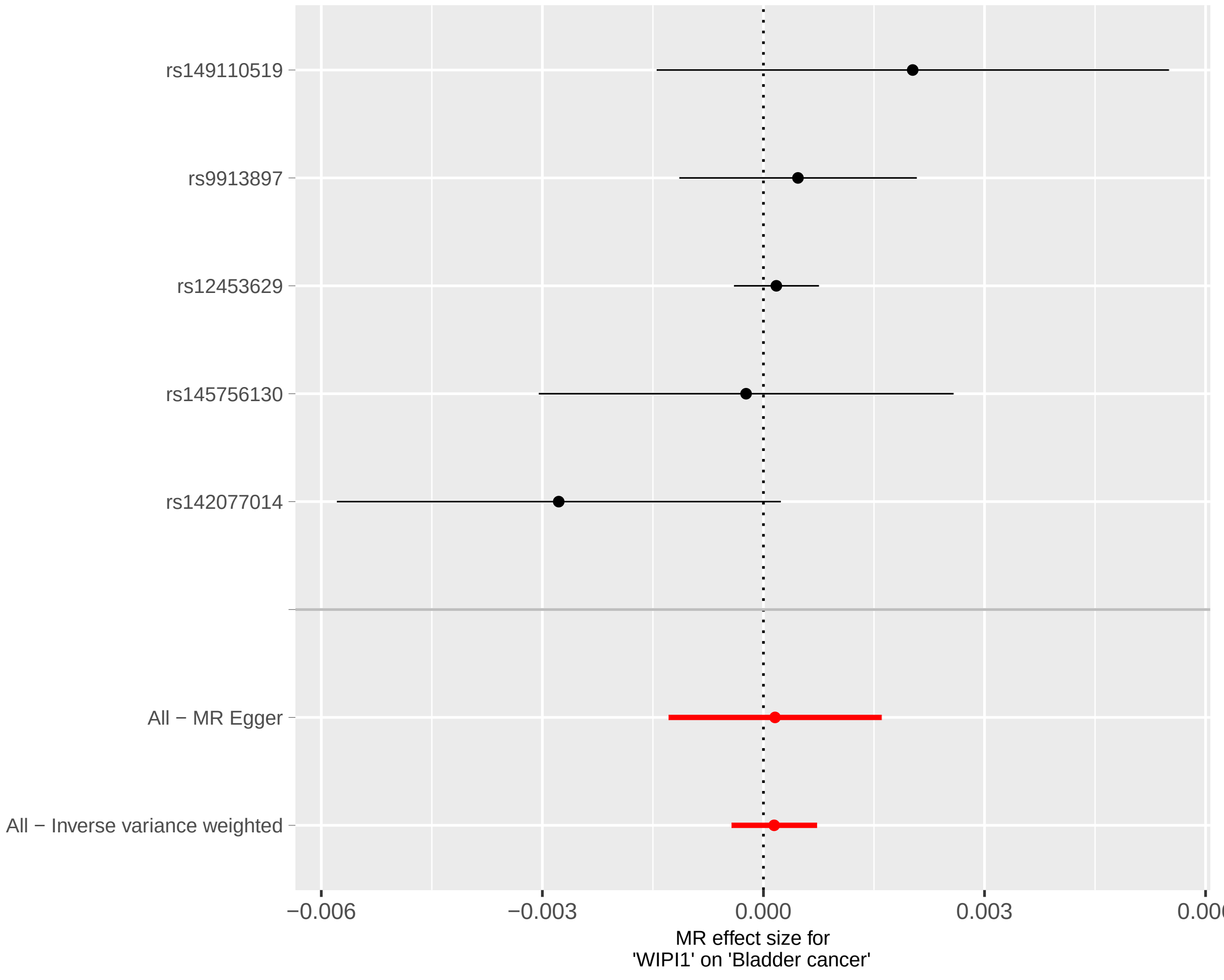
rs149110519
rs9913897
rs12453629
rs145756130
rs142077014
All − MR Egger
All − Inverse variance weighted
−0.006
−0.003
0.000
0.003
MR effect size for
'WIPI1' on 'Bladder cancer'

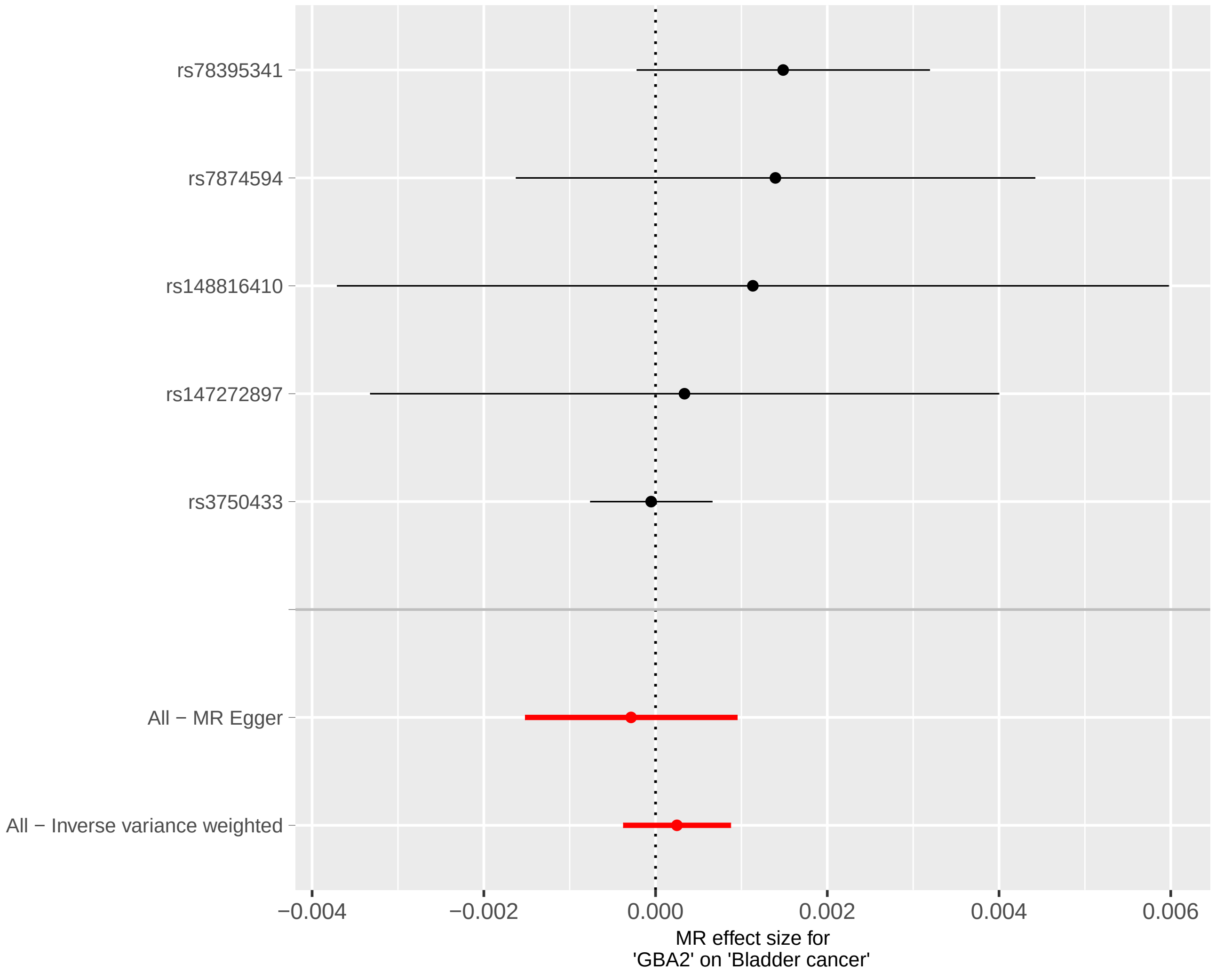
rs78395341
rs7874594
rs148816410
rs147272897
rs3750433
All − MR Egger
All − Inverse variance weighted
−0.004
−0.002
0.000
0.002
0.004
0.006
MR effect size for
'GBA2' on 'Bladder cancer'

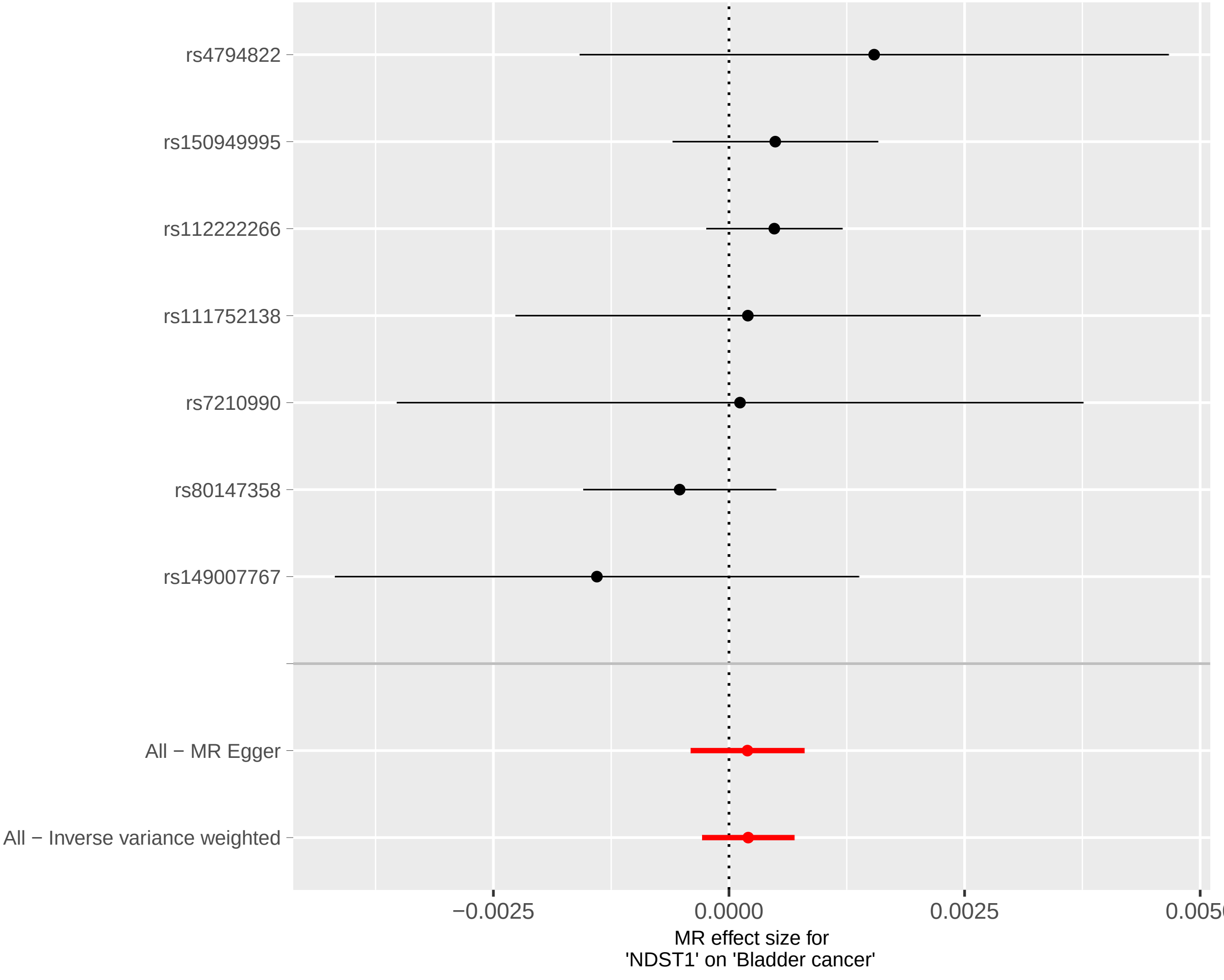

rs4794822
rs150949995
rs112222266
rs111752138
rs7210990
rs80147358
rs149007767
All − MR Egger
All − Inverse variance weighted
−0.0025
0.0000
0.0025
0.005
MR effect size for
'NDST1' on 'Bladder cancer'

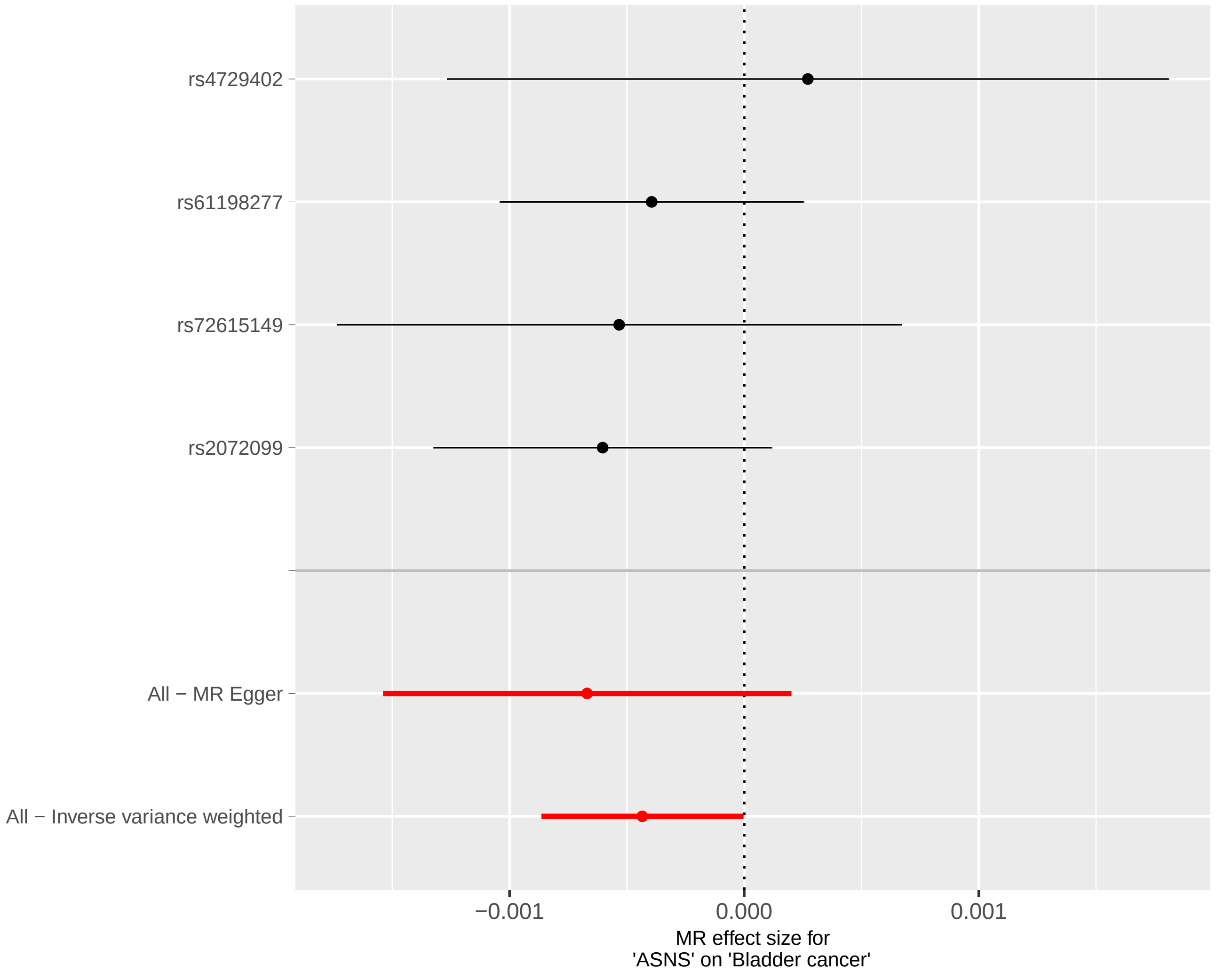

rs4729402
rs61198277
rs72615149
rs2072099
All − MR Egger
All − Inverse variance weighted
−0.001
0.000
0.001
MR effect size for
'ASNS' on 'Bladder cancer'

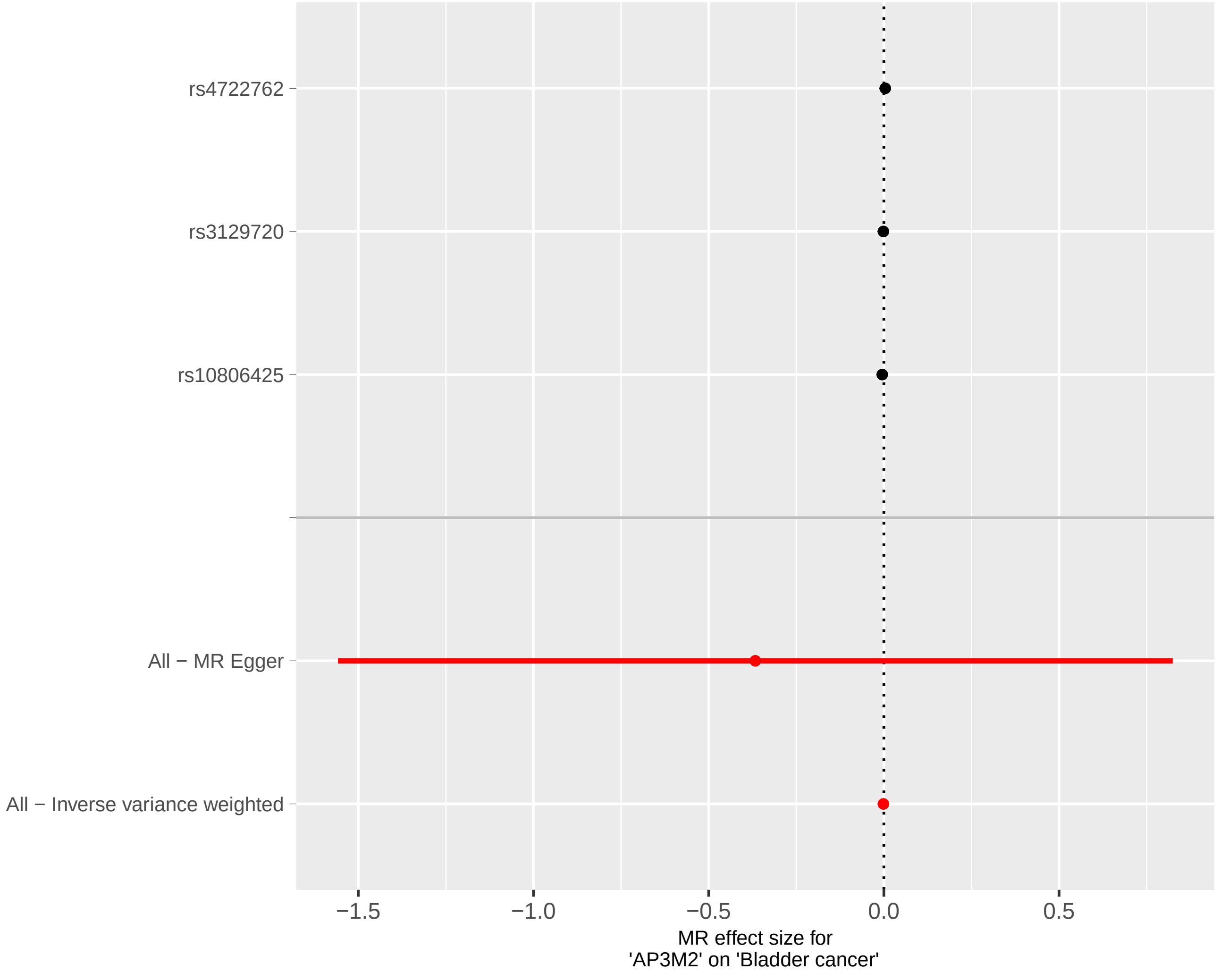

rs4722762
rs3129720
rs10806425
All − MR Egger
All − Inverse variance weighted
−1.5
−1.0
−0.5
0.0
0.5
MR effect size for
'AP3M2' on 'Bladder cancer'

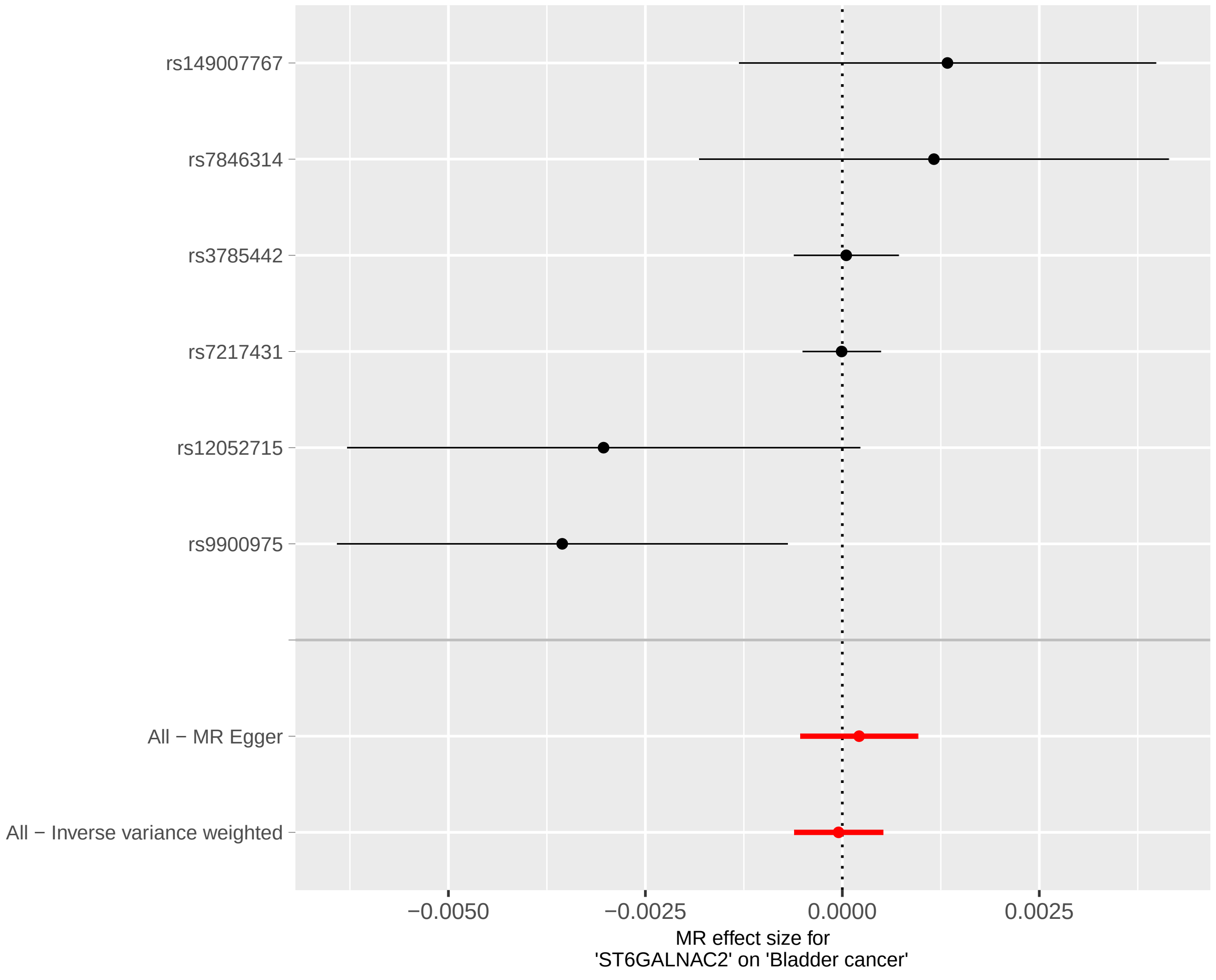

rs149007767
rs7846314
rs3785442
rs7217431
rs12052715
rs9900975
All − MR Egger
All − Inverse variance weighted
−0.0050
−0.0025
0.0000
0.0025
MR effect size for
'ST6GALNAC2' on 'Bladder cancer'

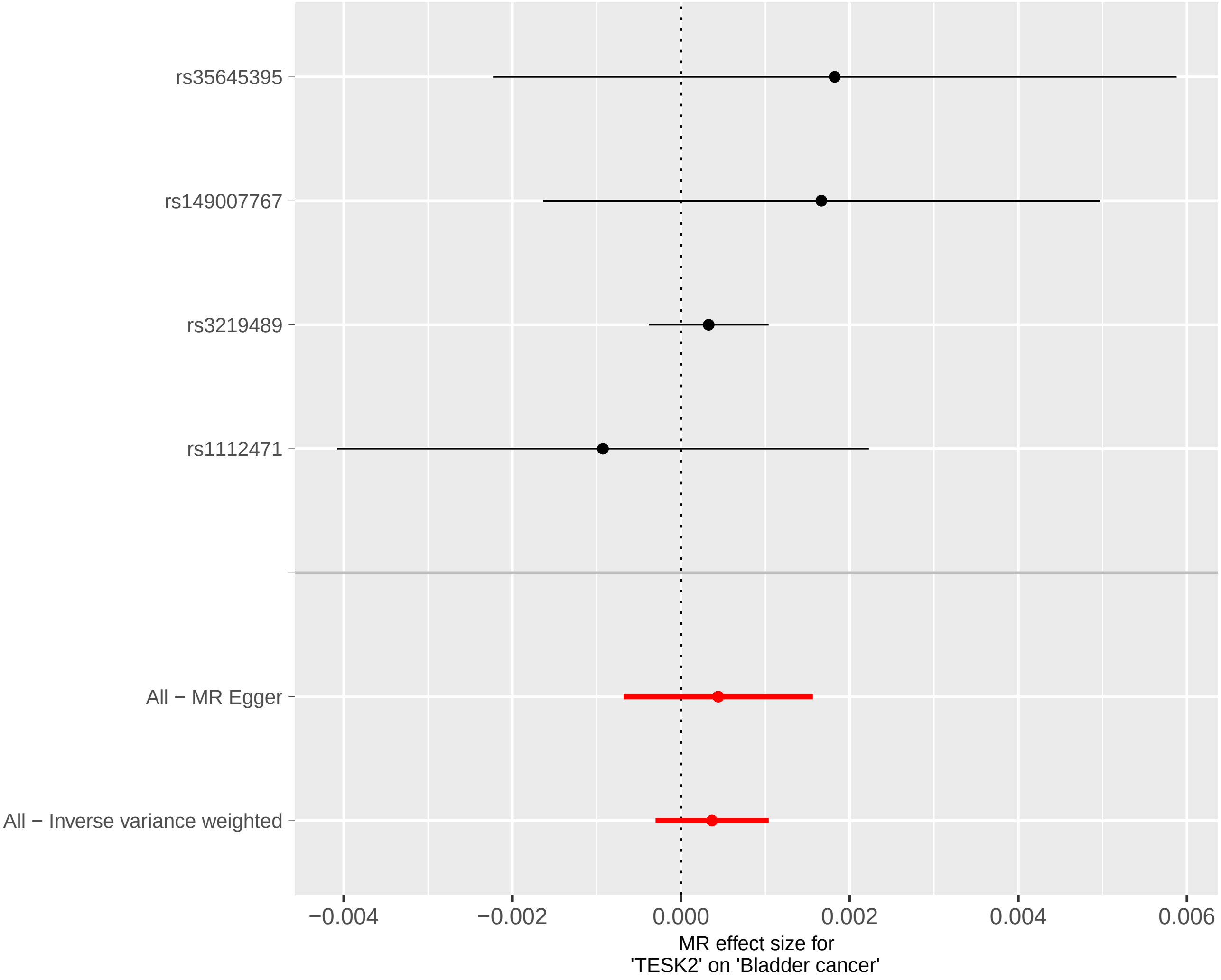
rs35645395
rs149007767
rs3219489
rs1112471
All − MR Egger
All − Inverse variance weighted
−0.004
−0.002
0.000
0.002
0.004
0.006
MR effect size for
'TESK2' on 'Bladder cancer'

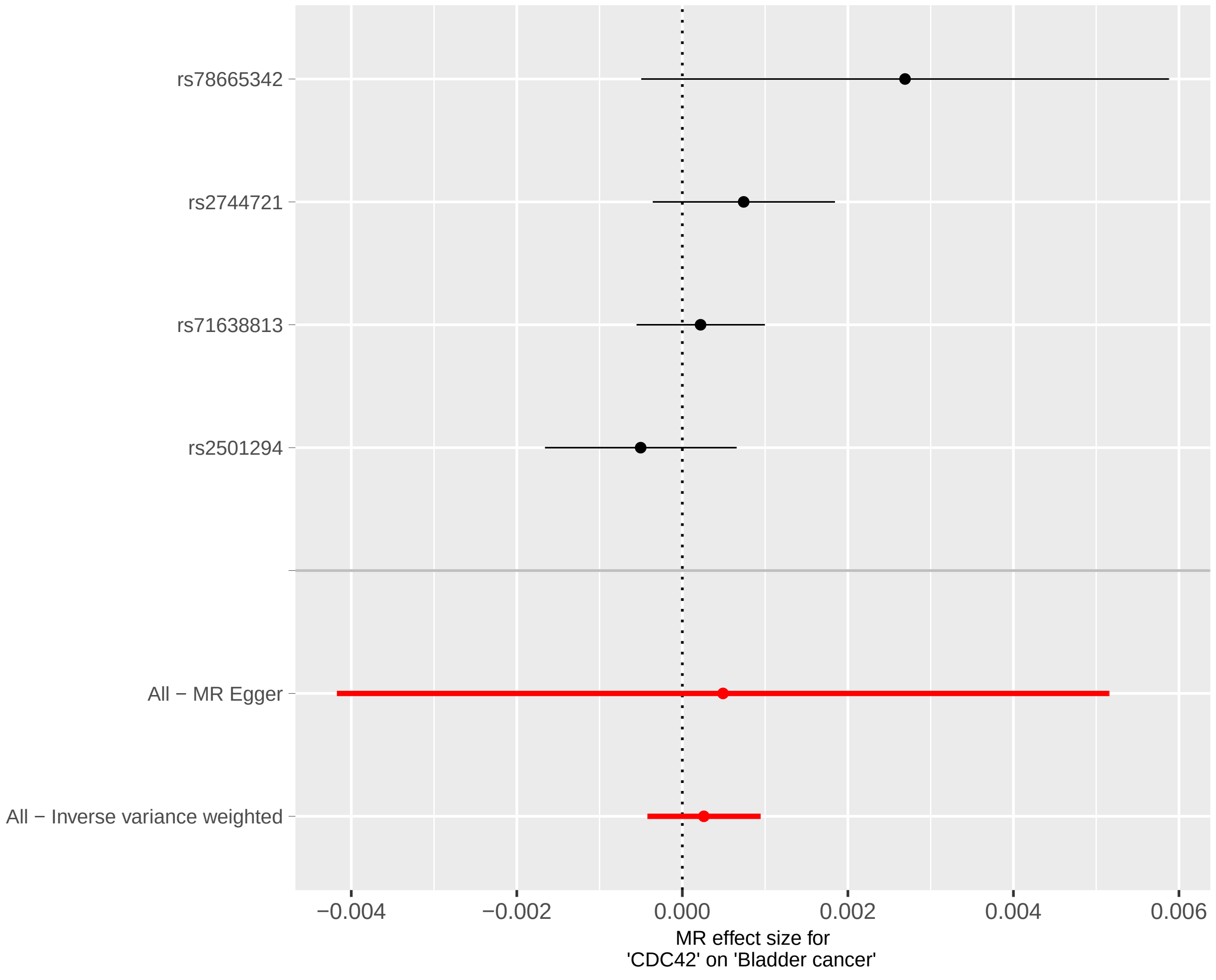
rs78665342
rs2744721
rs71638813
rs2501294
All − MR Egger
All − Inverse variance weighted
−0.004
−0.002
0.000
0.002
0.004
0.006
MR effect size for
'CDC42' on 'Bladder cancer'

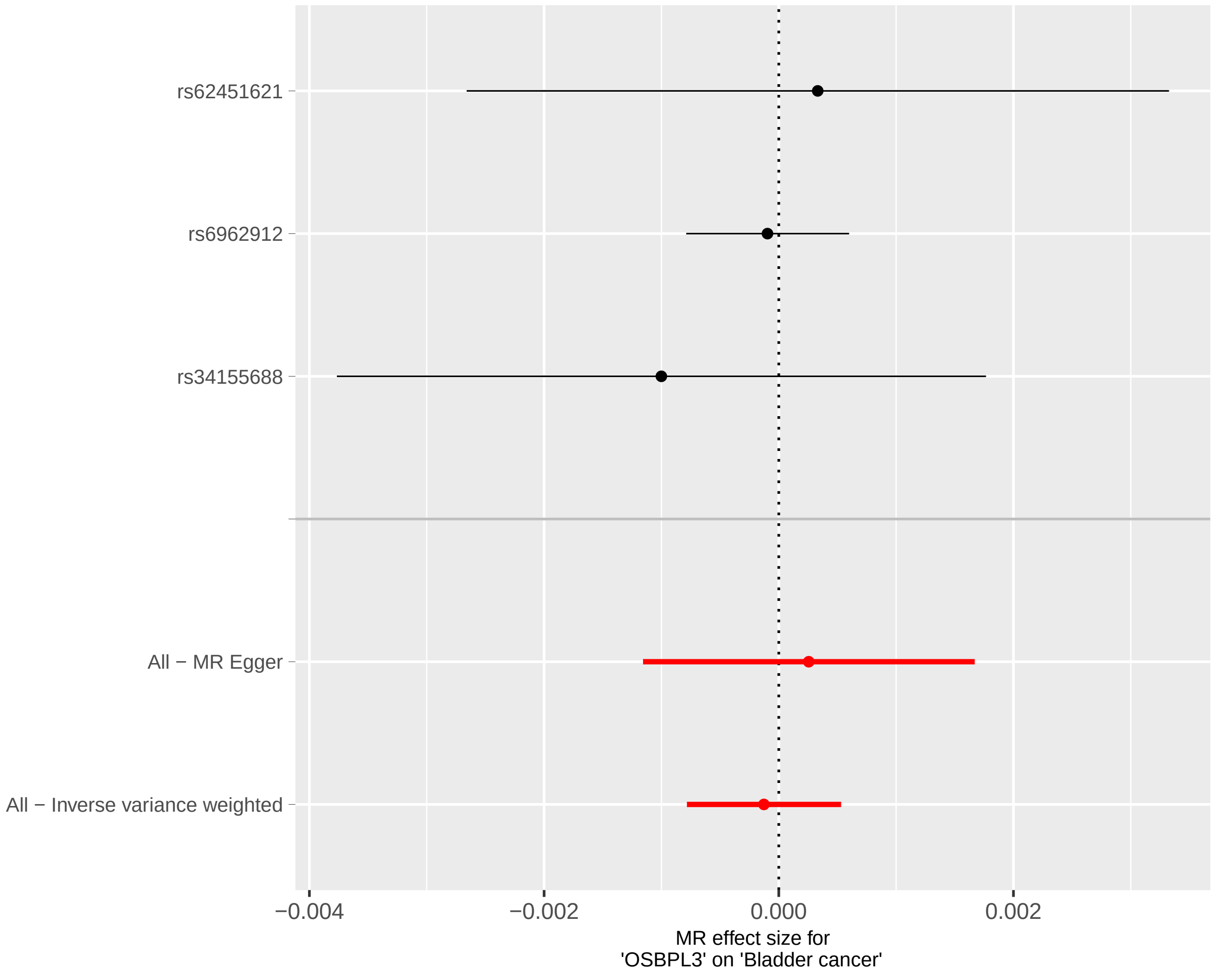

rs62451621
rs6962912
rs34155688
All − MR Egger
All − Inverse variance weighted
−0.004
−0.002
0.000
0.002
MR effect size for
'OSBPL3' on 'Bladder cancer'

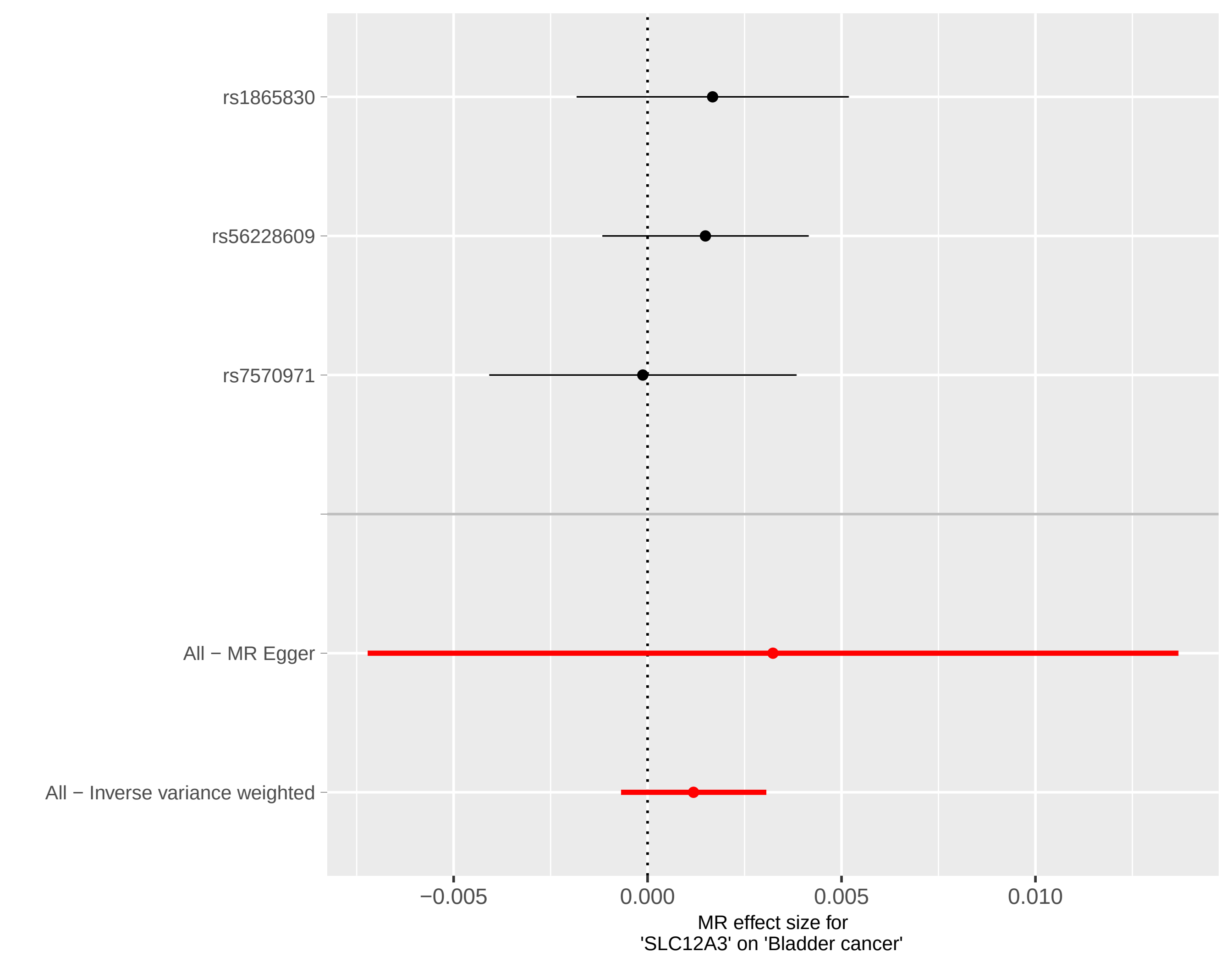
rs1865830
rs56228609
rs7570971
All − MR Egger
All − Inverse variance weighted
−0.005
0.000
0.005
0.010
MR effect size for
'SLC12A3' on 'Bladder cancer'

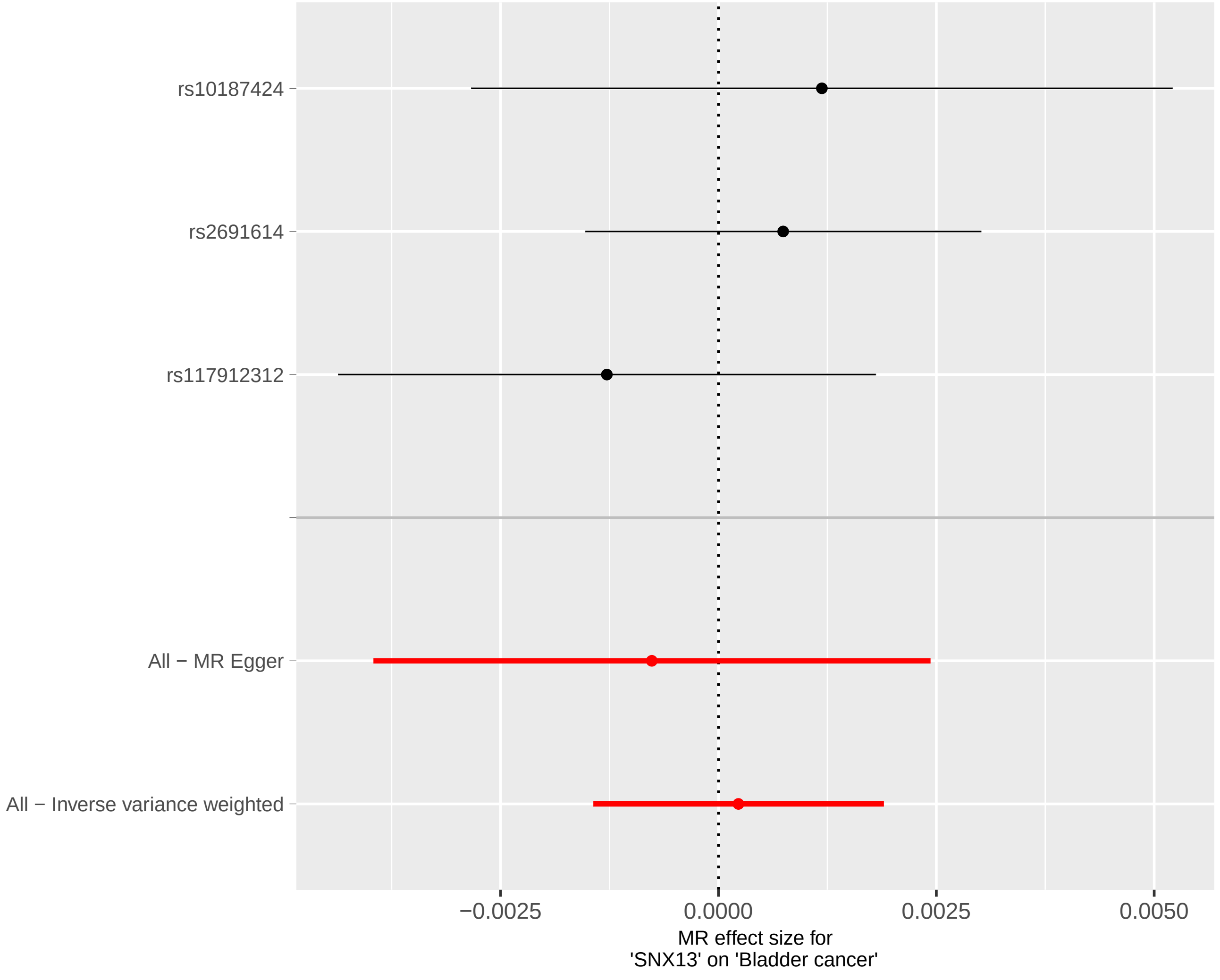
rs10187424
rs2691614
rs117912312
All − MR Egger
All − Inverse variance weighted
−0.0025
0.0000
0.0025
0.0050
MR effect size for
'SNX13' on 'Bladder cancer'

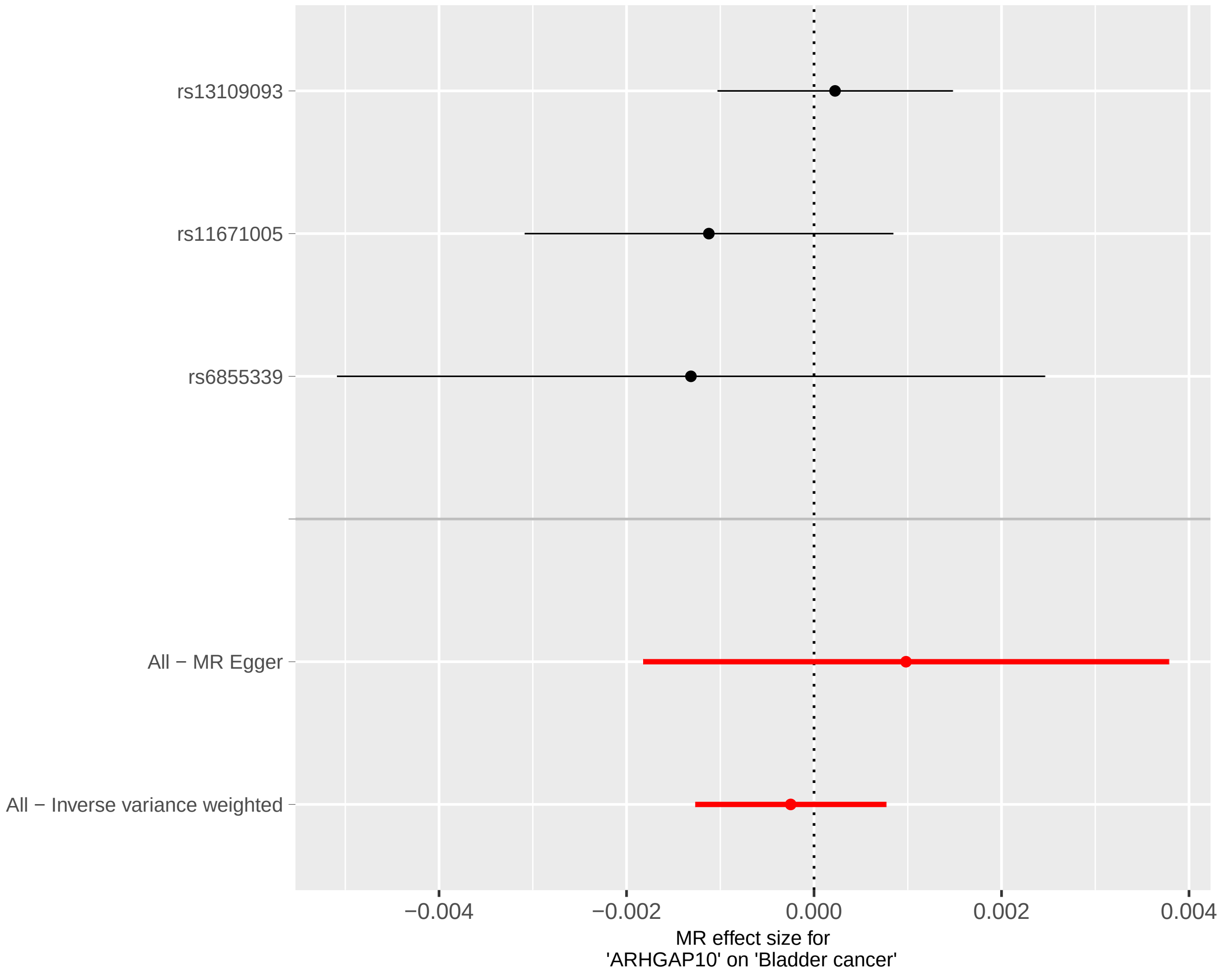
rs13109093
rs11671005
rs6855339
All − MR Egger
All − Inverse variance weighted
−0.004
−0.002
0.000
0.002
0.004
MR effect size for
'ARHGAP10' on 'Bladder cancer'

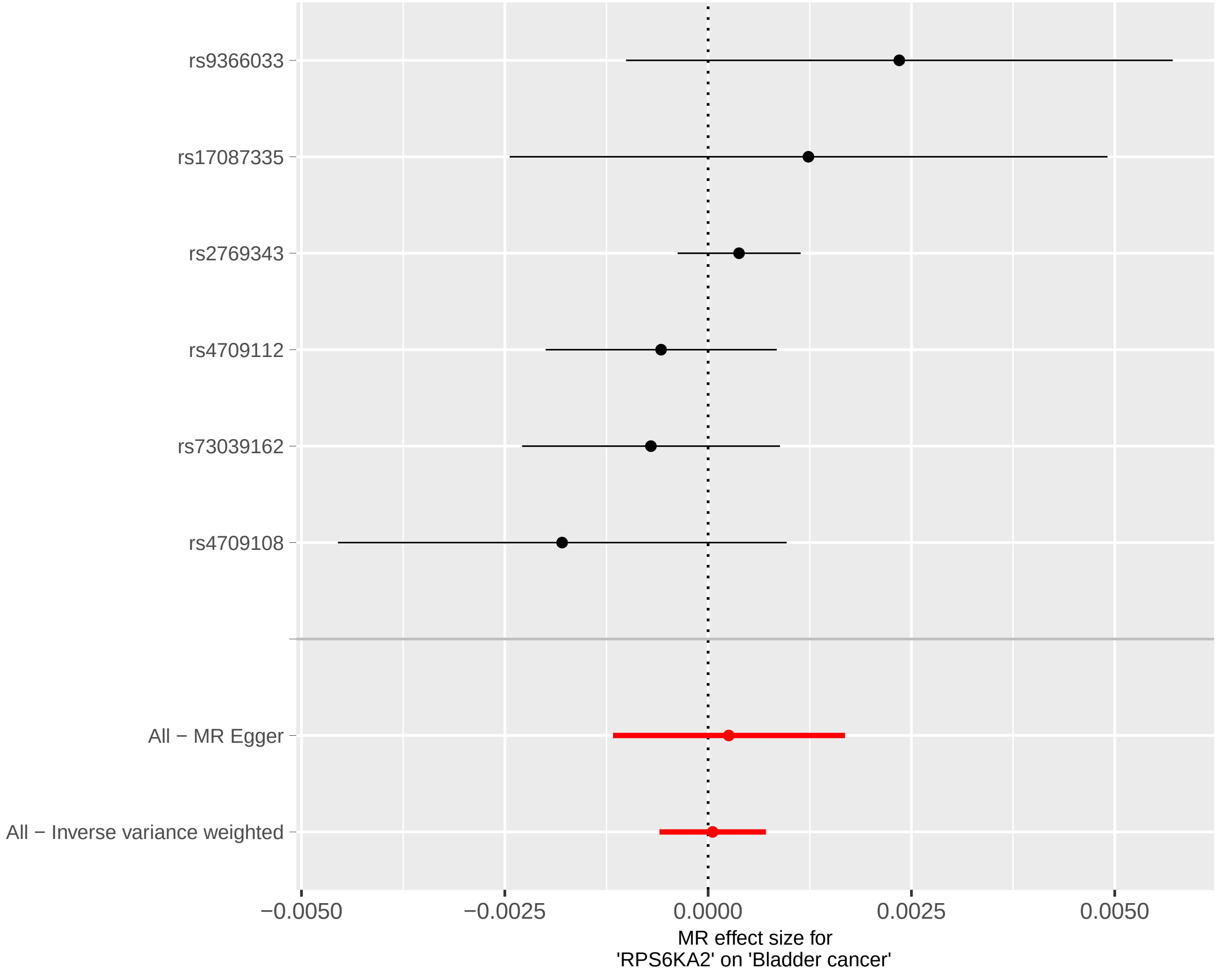

rs9366033
rs17087335
rs2769343
rs4709112
rs73039162
rs4709108
All − MR Egger
All − Inverse variance weighted
−0.0050
−0.0025
0.0000
0.0025
0.0050
MR effect size for
'RPS6KA2' on 'Bladder cancer'

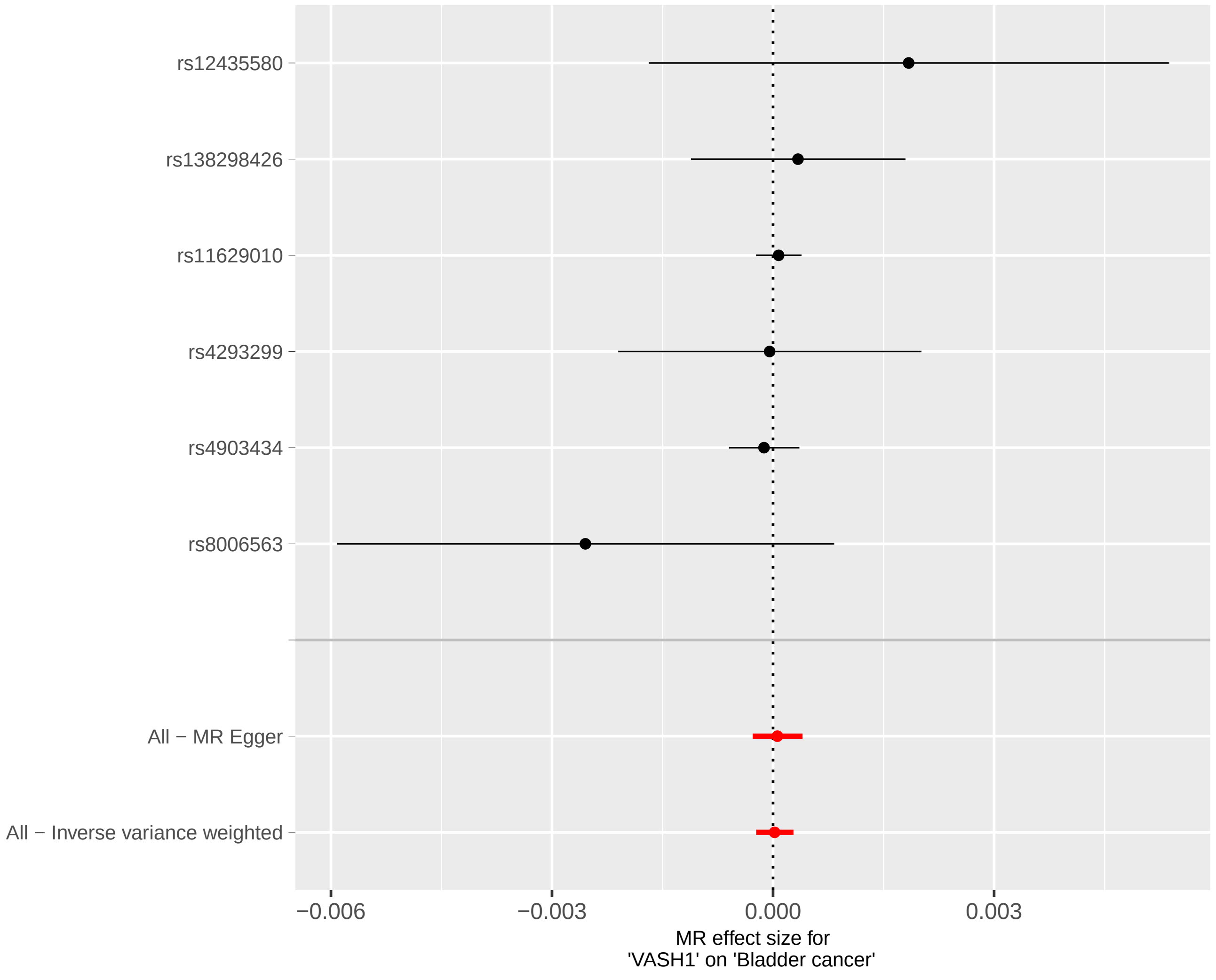
rs12435580
rs138298426
rs11629010
rs4293299
rs4903434
rs8006563
All − MR Egger
All − Inverse variance weighted
−0.006
−0.003
0.000
0.003
MR effect size for
'VASH1' on 'Bladder cancer'

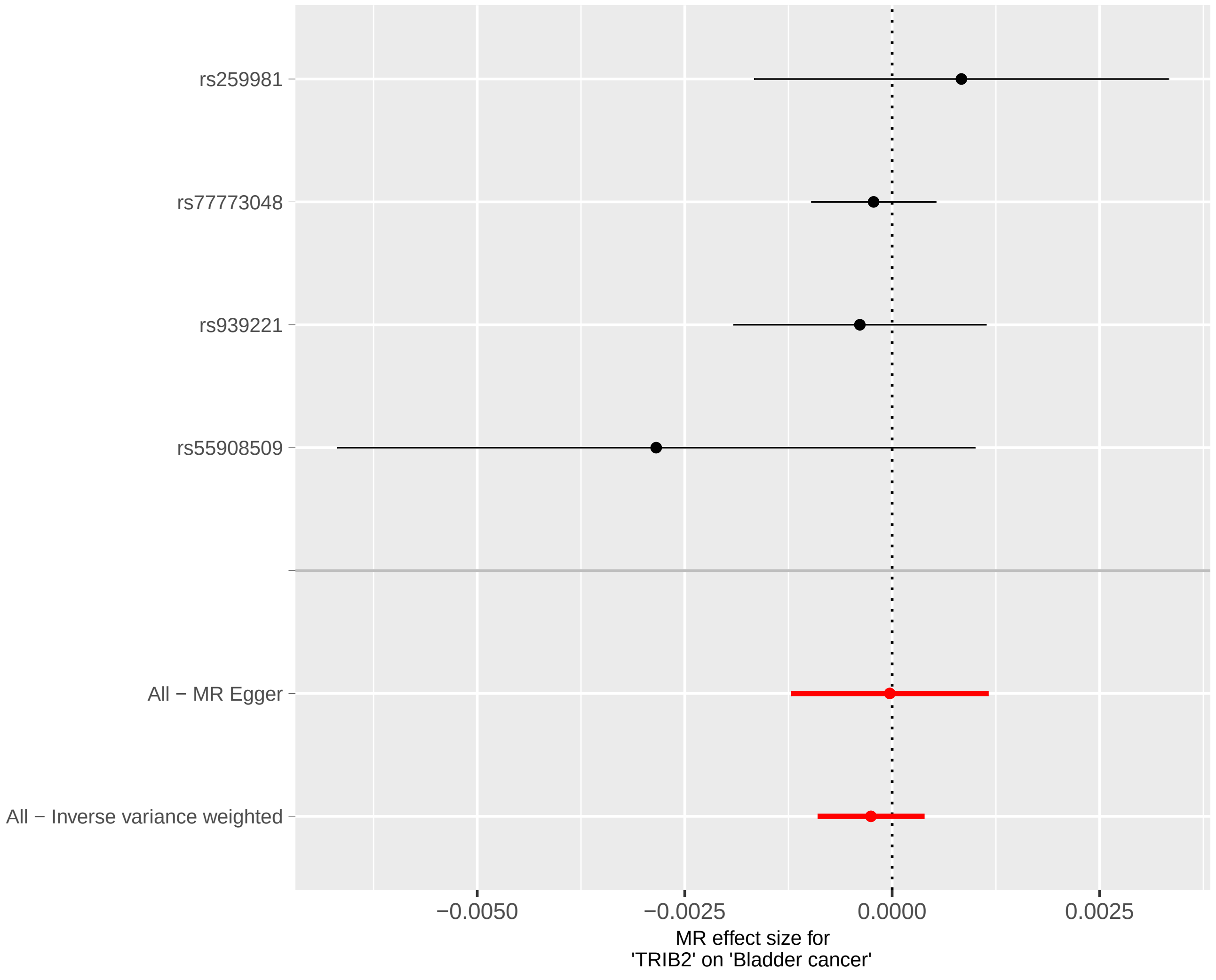
rs259981
rs77773048
rs939221
rs55908509
All − MR Egger
All − Inverse variance weighted
−0.0050
−0.0025
0.0000
0.0025
MR effect size for
'TRIB2' on 'Bladder cancer'

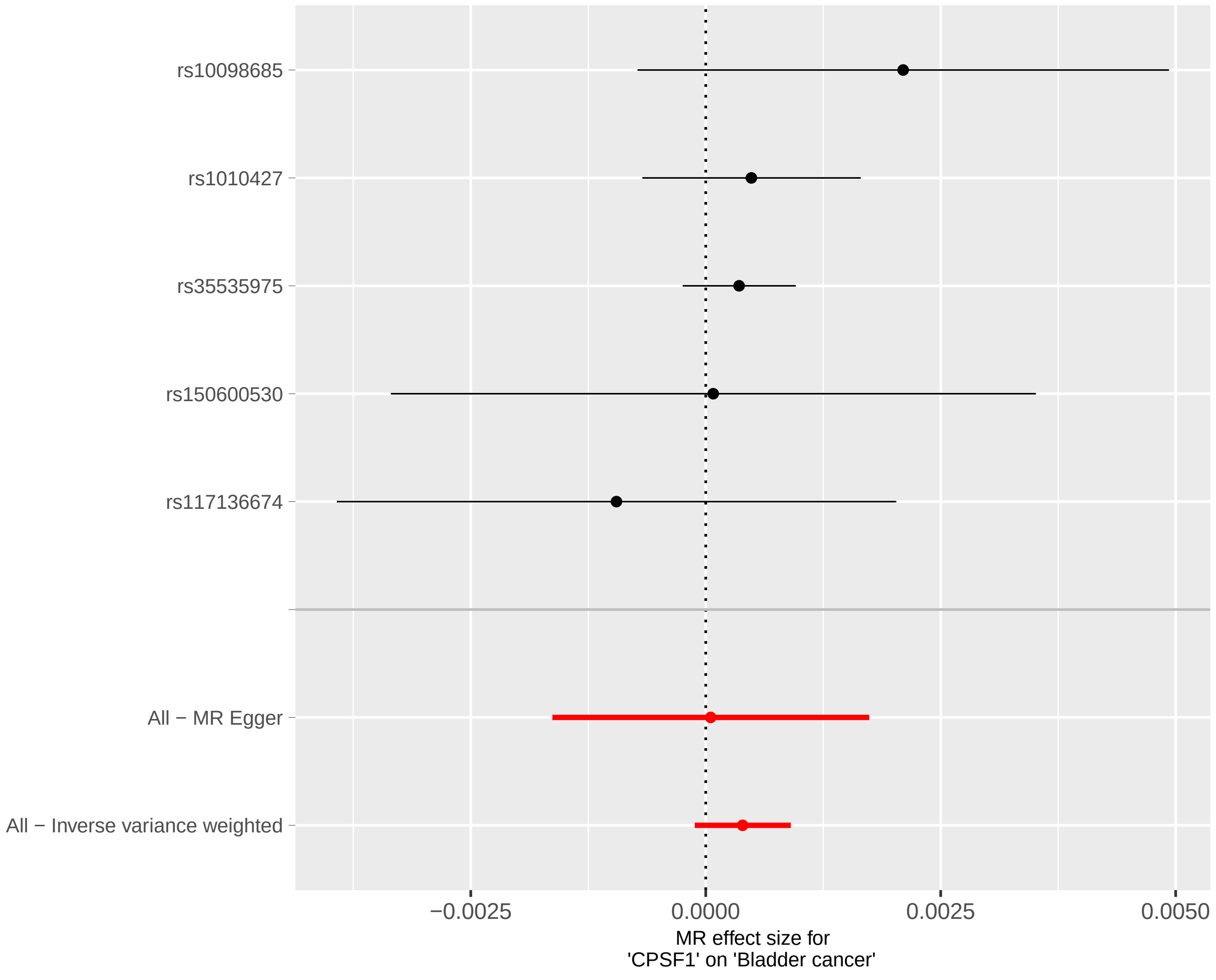
rs10098685
rs1010427
rs35535975
rs150600530
rs117136674
All − MR Egger
All − Inverse variance weighted
−0.0025
0.0000
0.0025
0.0050
MR effect size for
'CPSF1' on 'Bladder cancer'

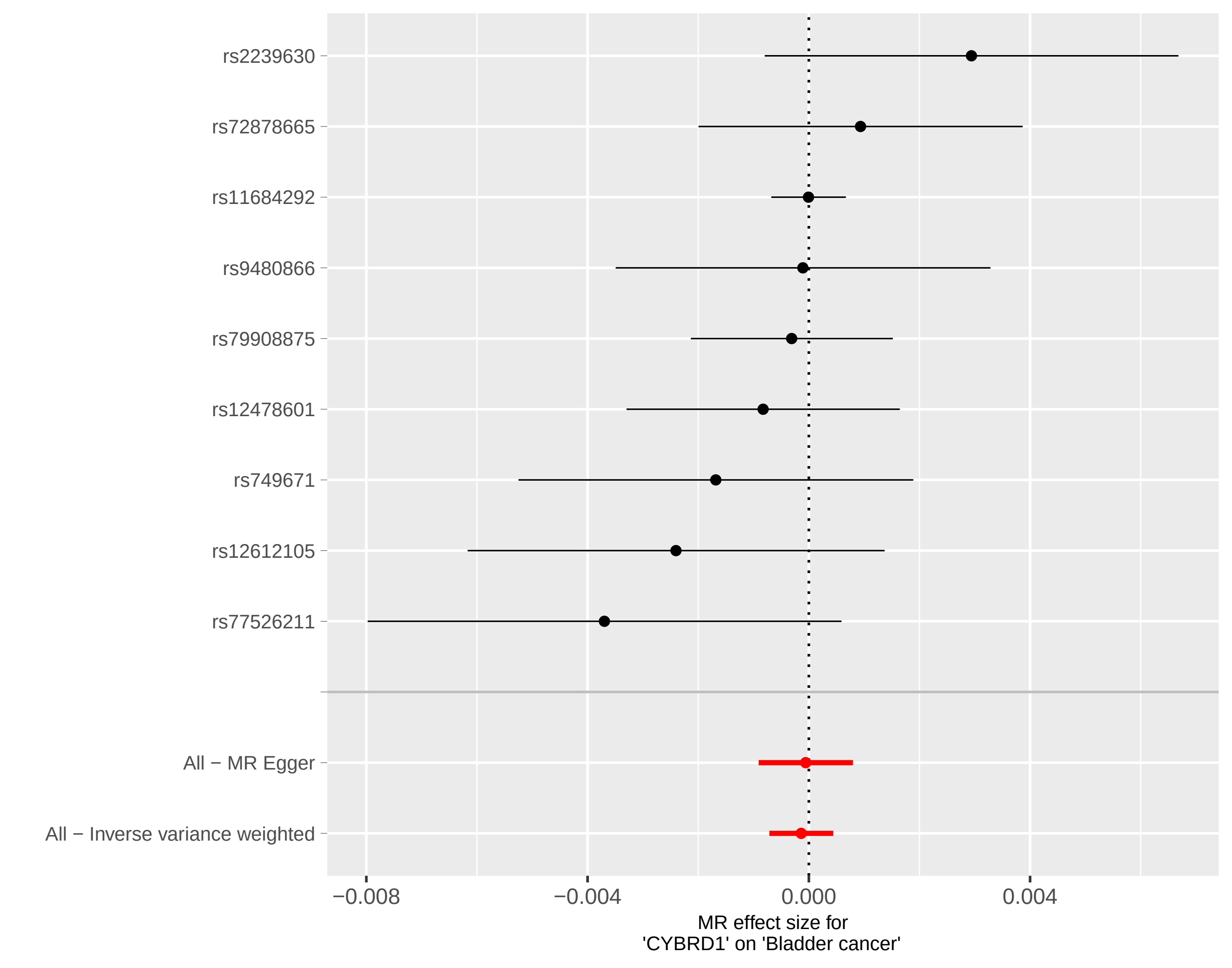

rs2239630
rs72878665
rs11684292
rs9480866
rs79908875
rs12478601
rs749671
rs12612105
rs77526211
All − MR Egger
All − Inverse variance weighted
−0.008
−0.004
0.000
0.004
MR effect size for
'CYBRD1' on 'Bladder cancer'

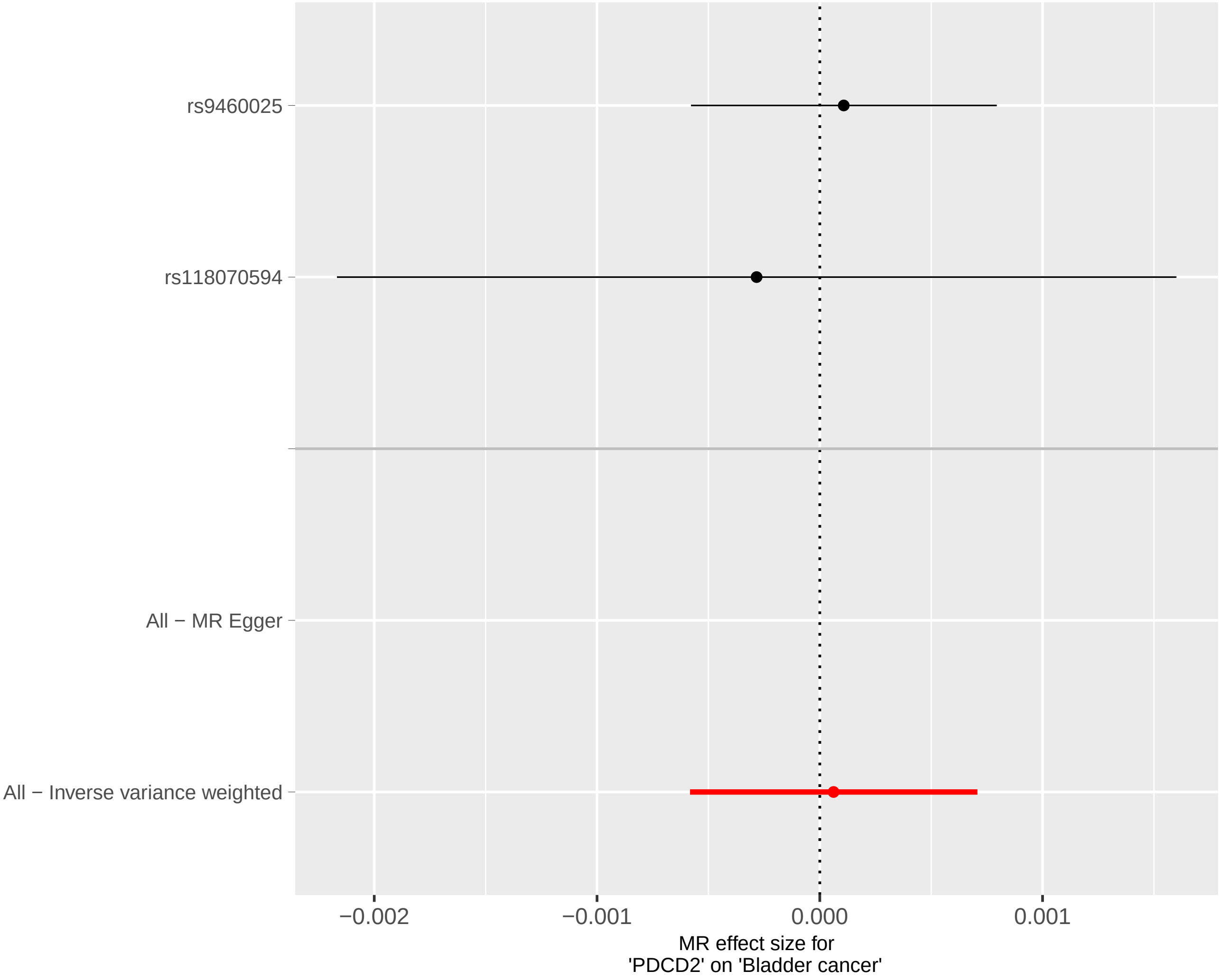
rs9460025
rs118070594
All − MR Egger
All − Inverse variance weighted
−0.002
−0.001
0.000
0.001
MR effect size for
'PDCD2' on 'Bladder cancer'

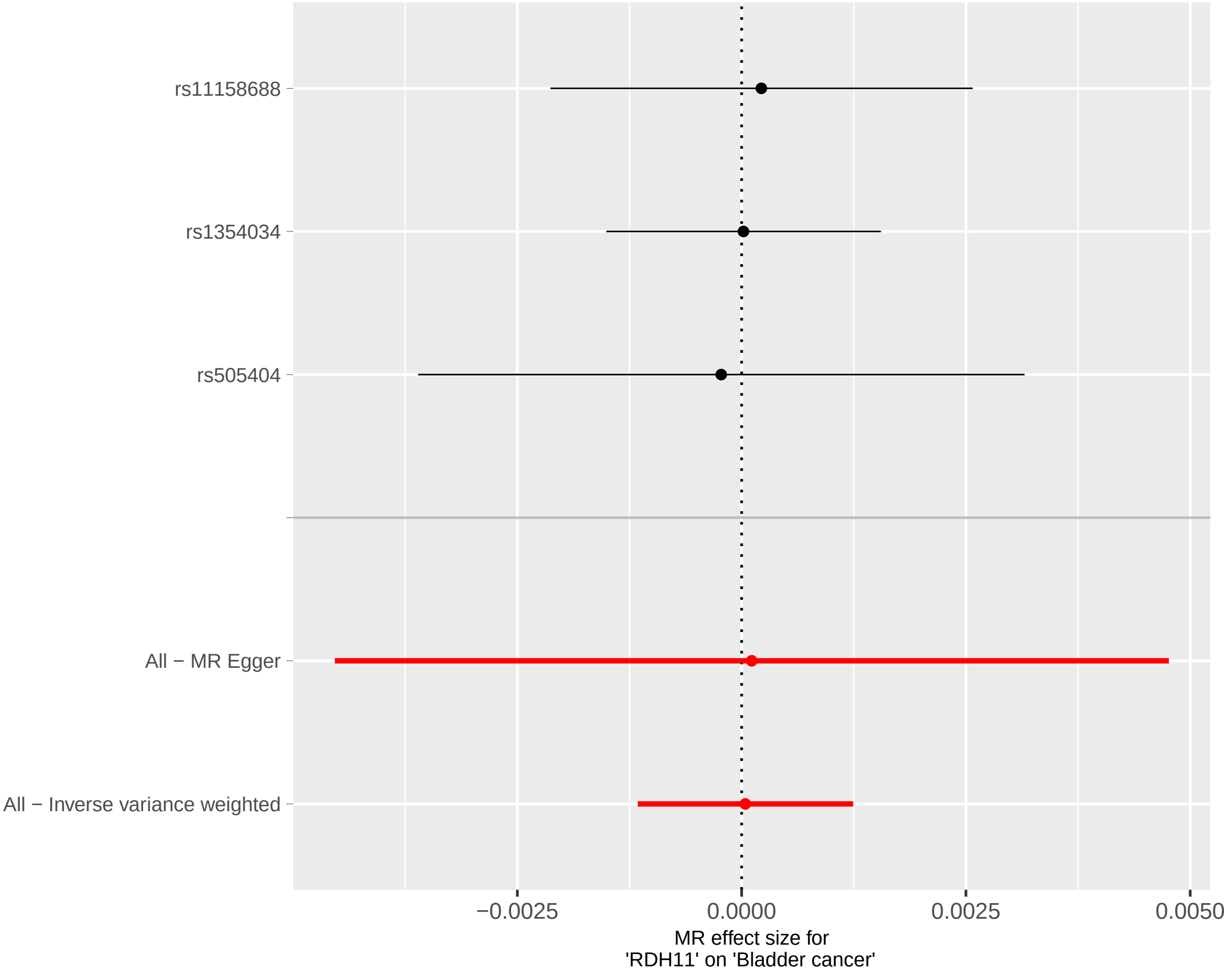
rs11158688
rs1354034
rs505404
All − MR Egger
All − Inverse variance weighted
−0.0025
0.0000
0.0025
0.0050
MR effect size for
'RDH11' on 'Bladder cancer'

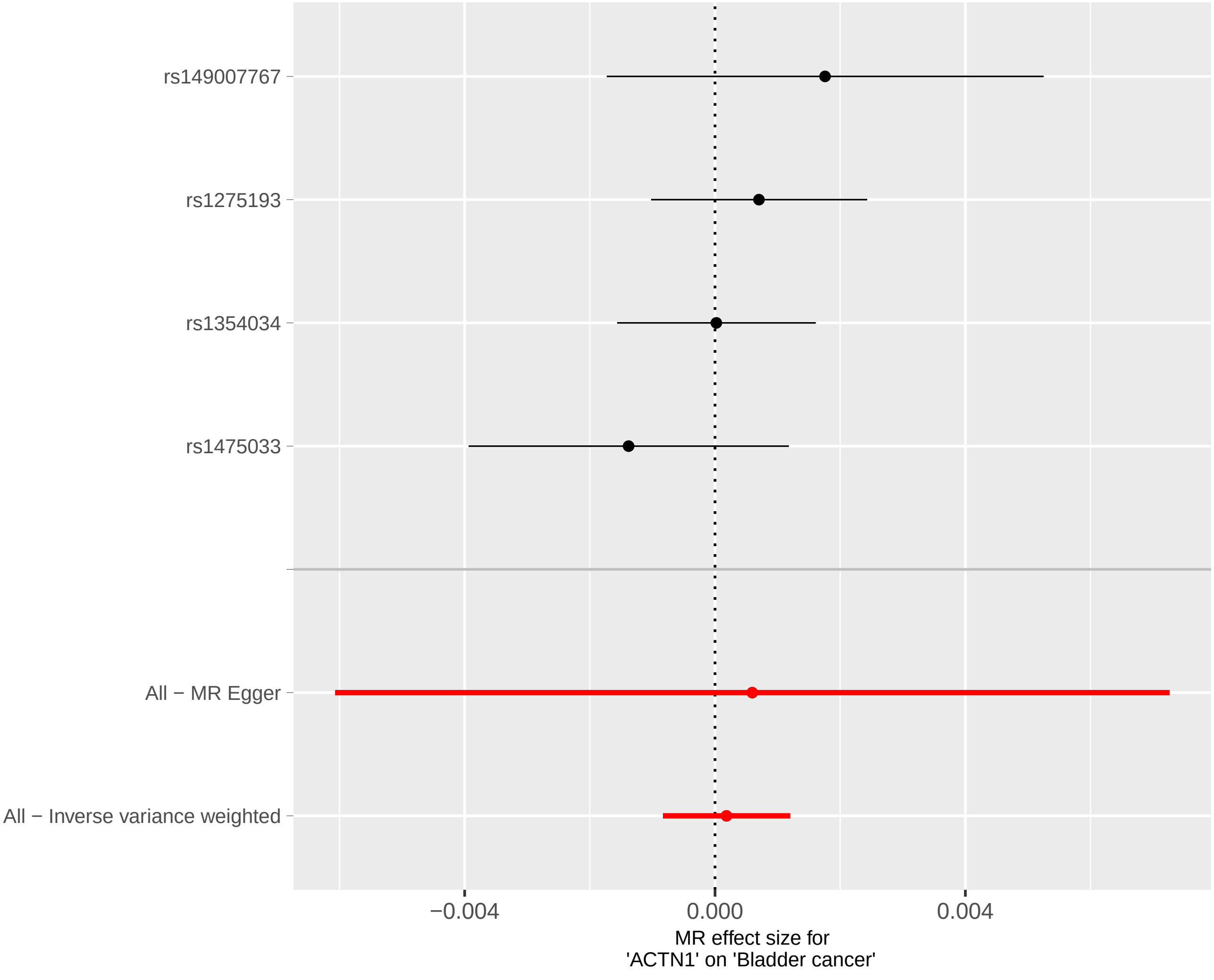
rs149007767
rs1275193
rs1354034
rs1475033
All − MR Egger
All − Inverse variance weighted
−0.004
0.000
0.004
MR effect size for
'ACTN1' on 'Bladder cancer'

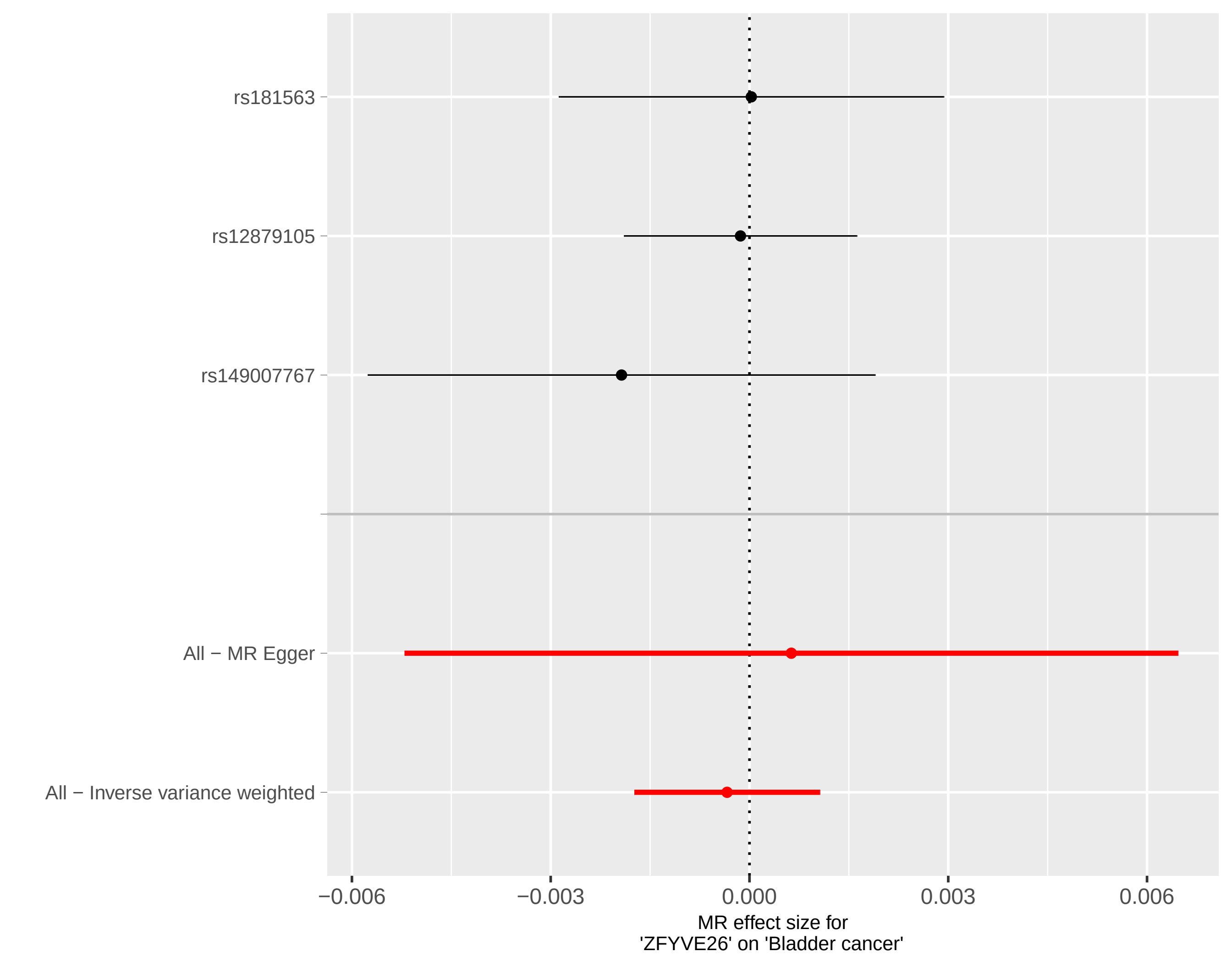
rs181563
rs12879105
rs149007767
All − MR Egger
All − Inverse variance weighted
−0.006
−0.003
0.000
0.003
0.006
MR effect size for
'ZFYVE26' on 'Bladder cancer'

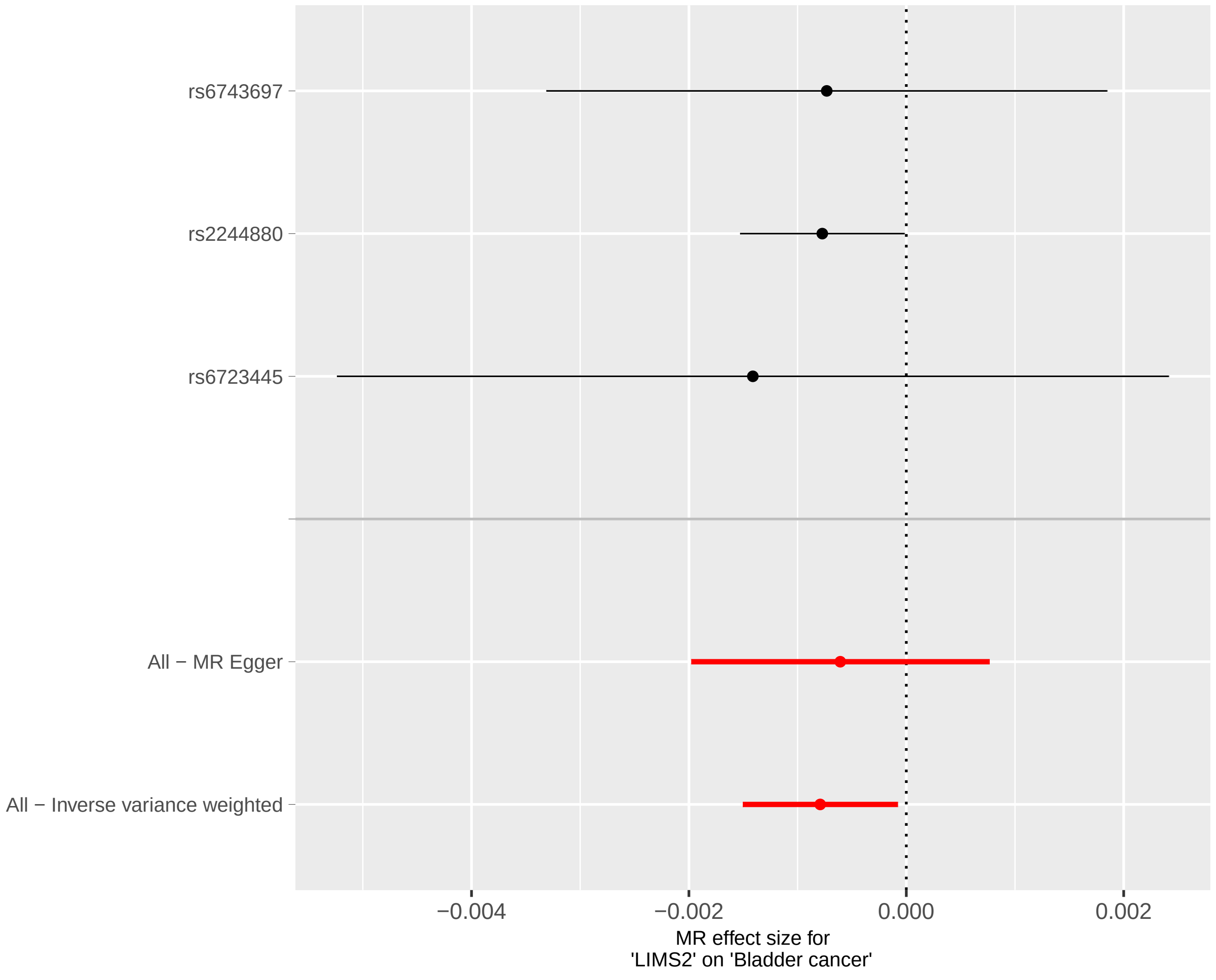

rs6743697
rs2244880
rs6723445
All − MR Egger
All − Inverse variance weighted
−0.004
−0.002
0.000
0.002
MR effect size for
'LIMS2' on 'Bladder cancer'

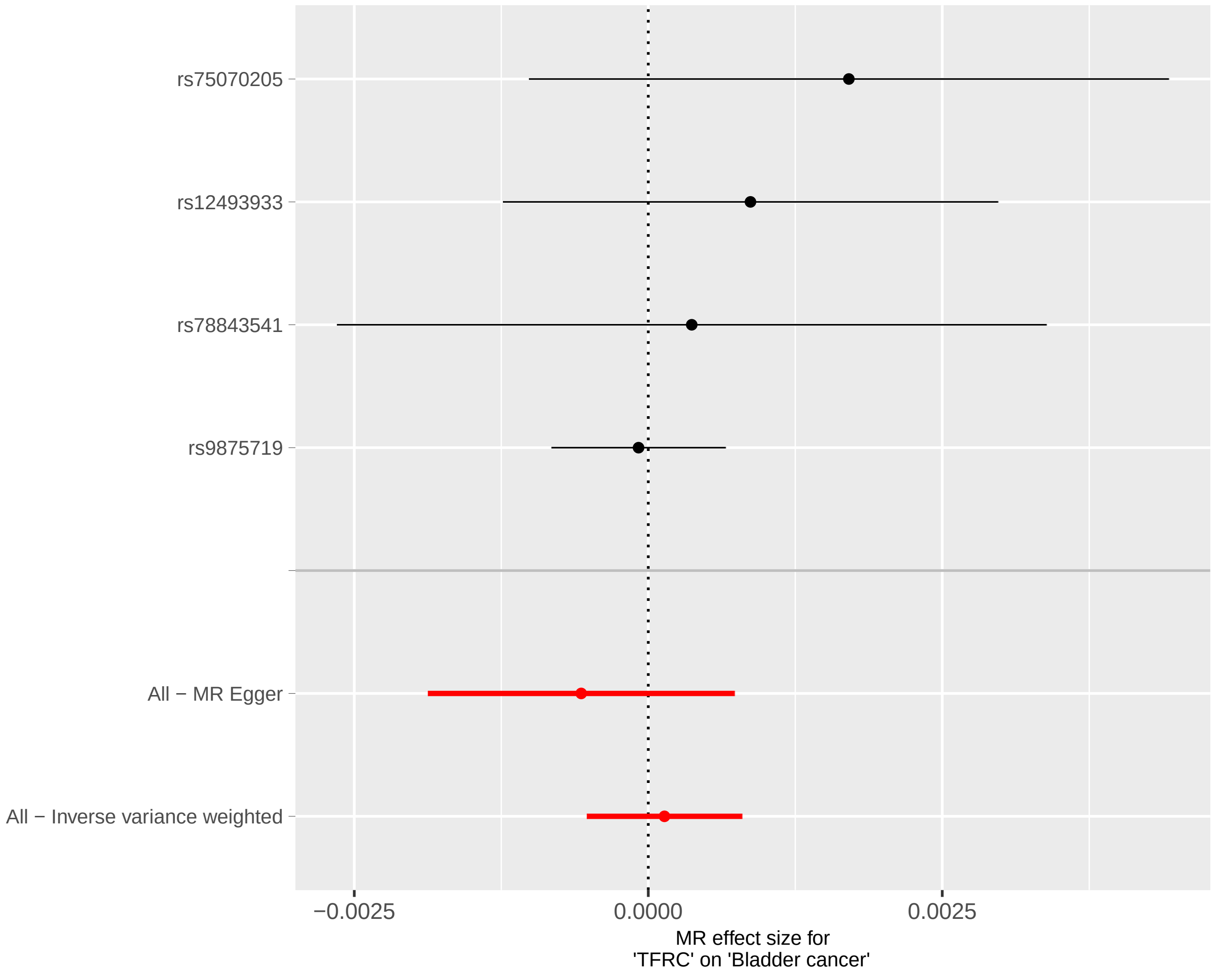
rs75070205
rs12493933
rs78843541
rs9875719
All − MR Egger
All − Inverse variance weighted
−0.0025
0.0000
0.0025
MR effect size for
'TFRC' on 'Bladder cancer'

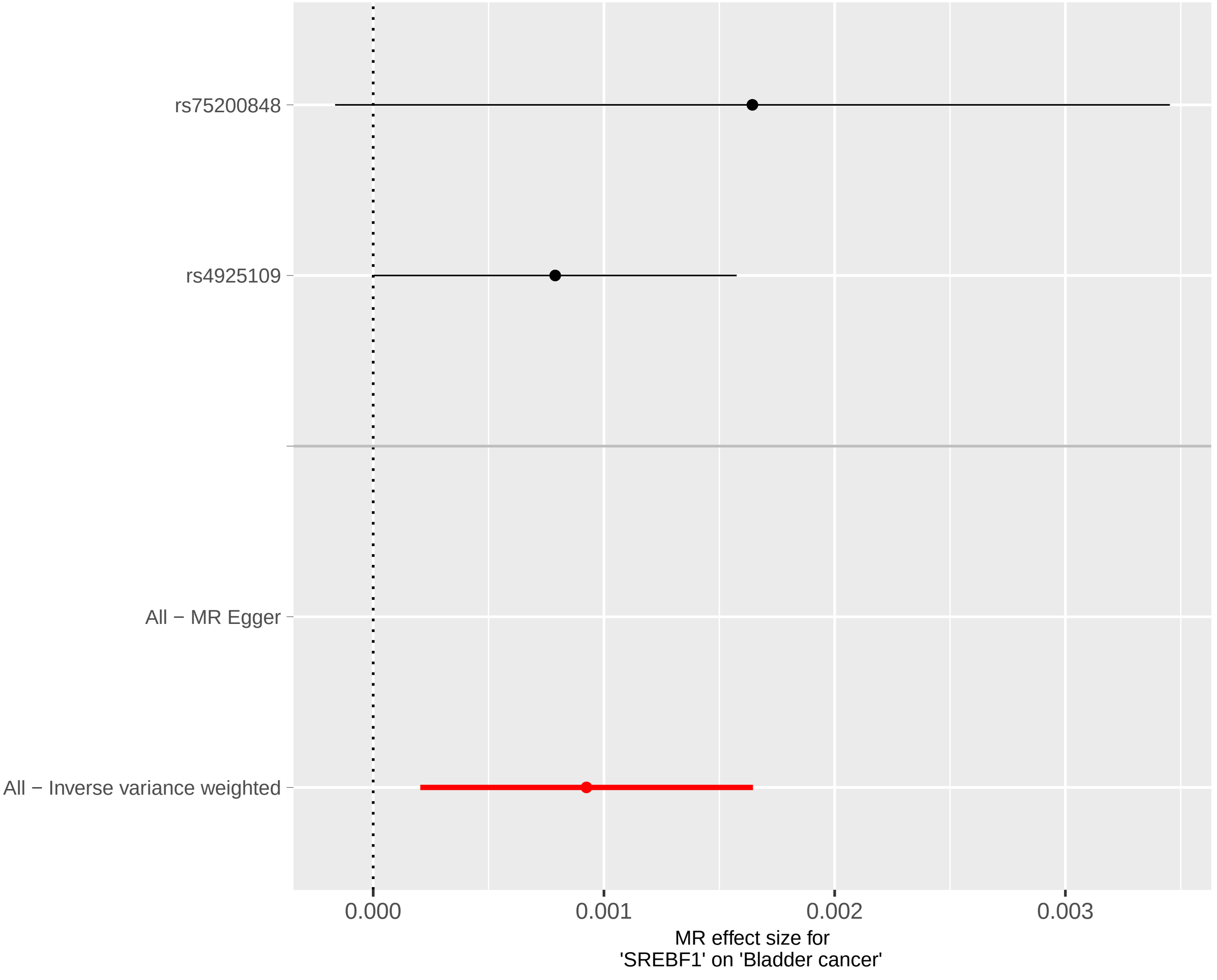
rs75200848
rs4925109
All − MR Egger
All − Inverse variance weighted
0.000
0.001
0.002
0.003
MR effect size for
'SREBF1' on 'Bladder cancer'

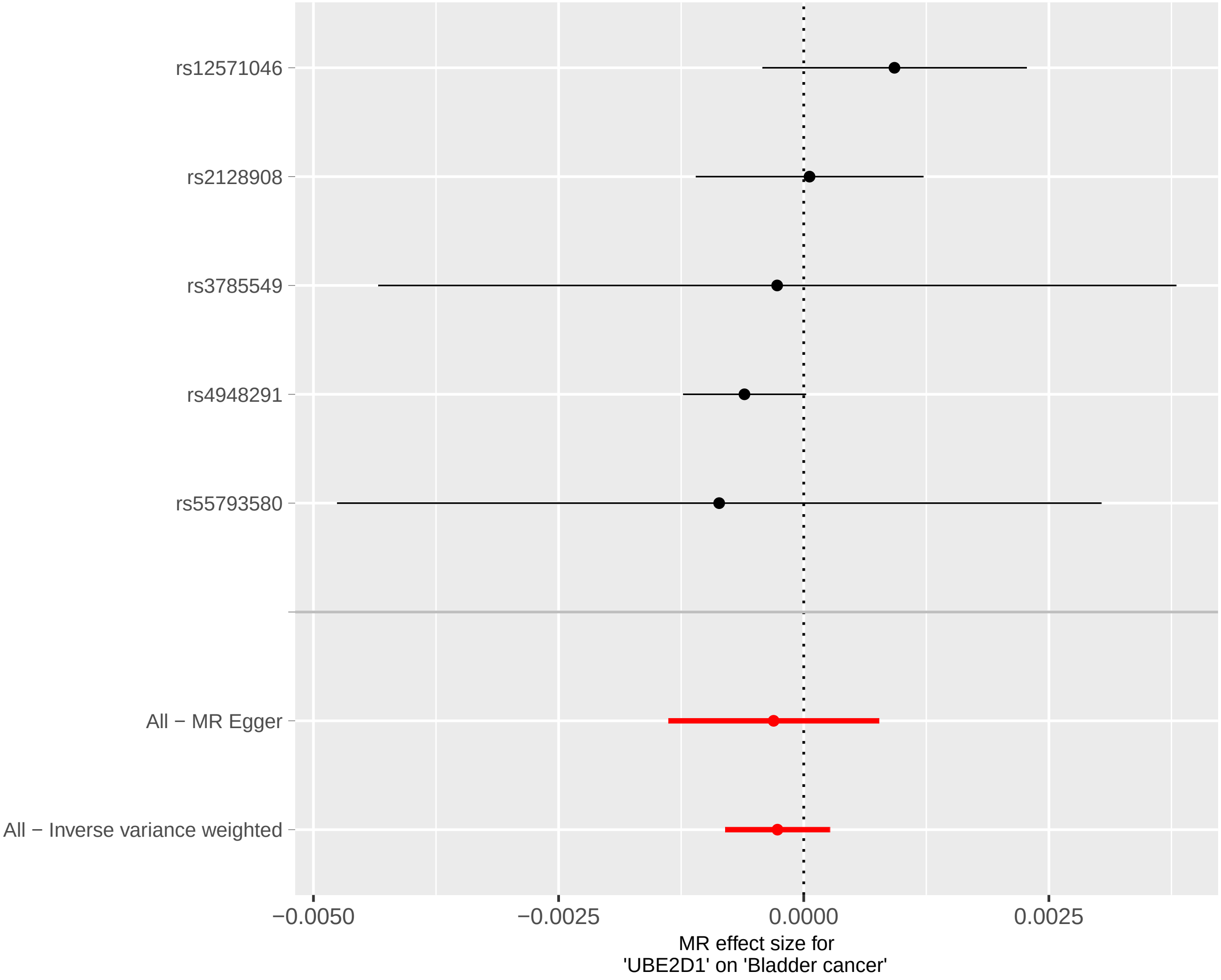
rs12571046
rs2128908
rs3785549
rs4948291
rs55793580
All − MR Egger
All − Inverse variance weighted
−0.0050
−0.0025
0.0000
0.0025
MR effect size for
'UBE2D1' on 'Bladder cancer'

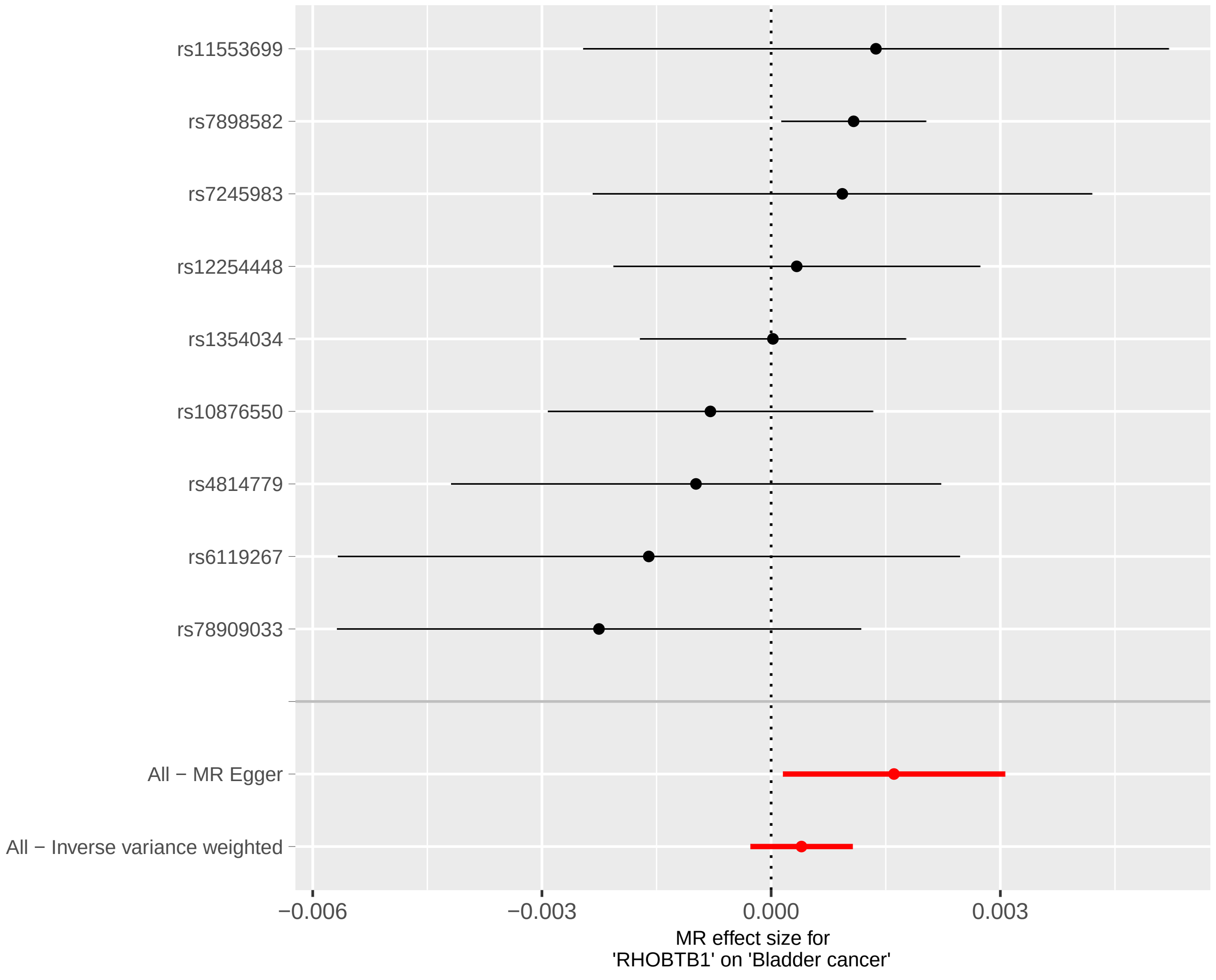
rs11553699
rs7898582
rs7245983
rs12254448
rs1354034
rs10876550
rs4814779
rs6119267
rs78909033
All − MR Egger
All − Inverse variance weighted
−0.006
−0.003
0.000
0.003
MR effect size for
'RHOBTB1' on 'Bladder cancer'

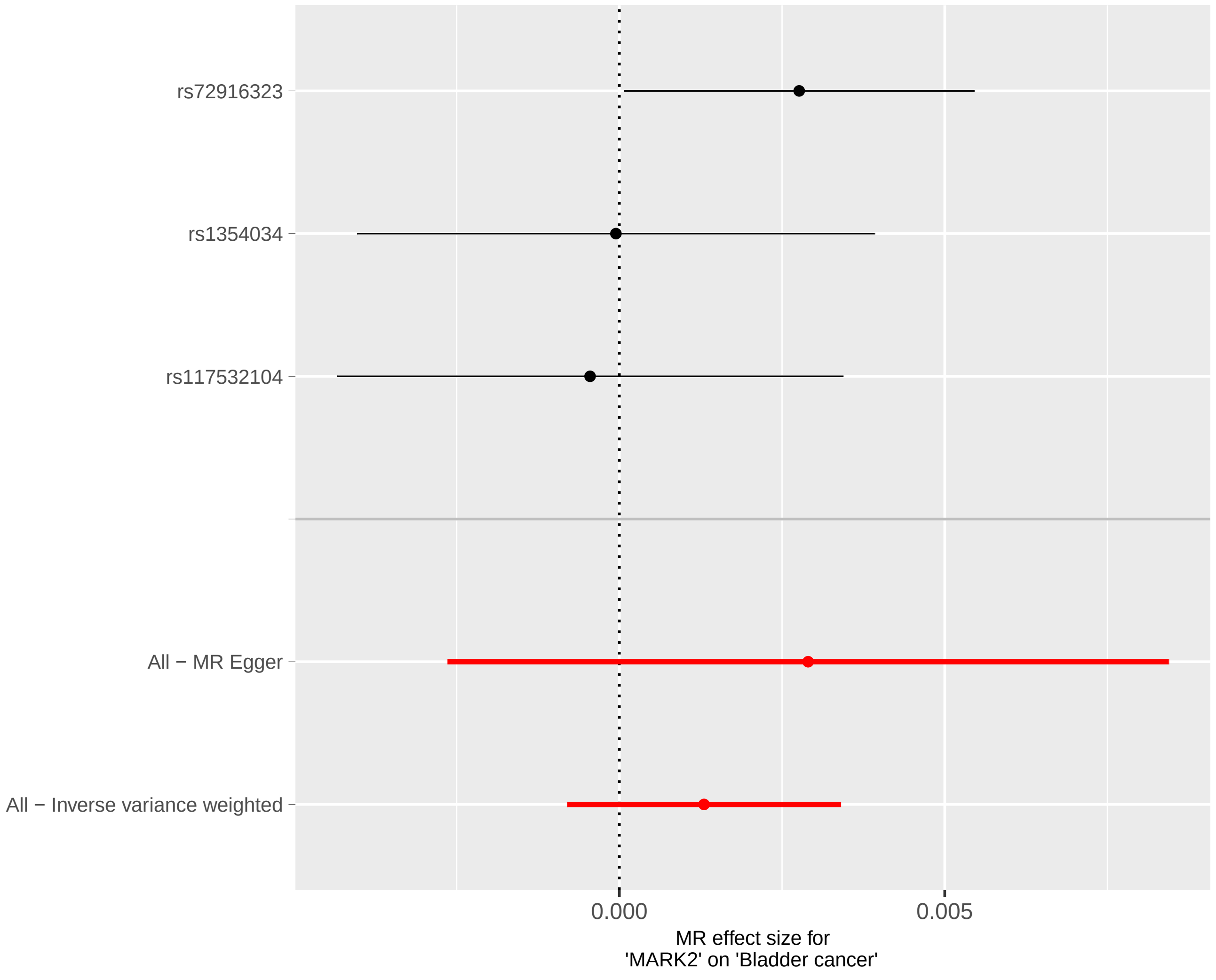

rs72916323
rs1354034
rs117532104
All − MR Egger
All − Inverse variance weighted
0.000
0.005
MR effect size for
'MARK2' on 'Bladder cancer'

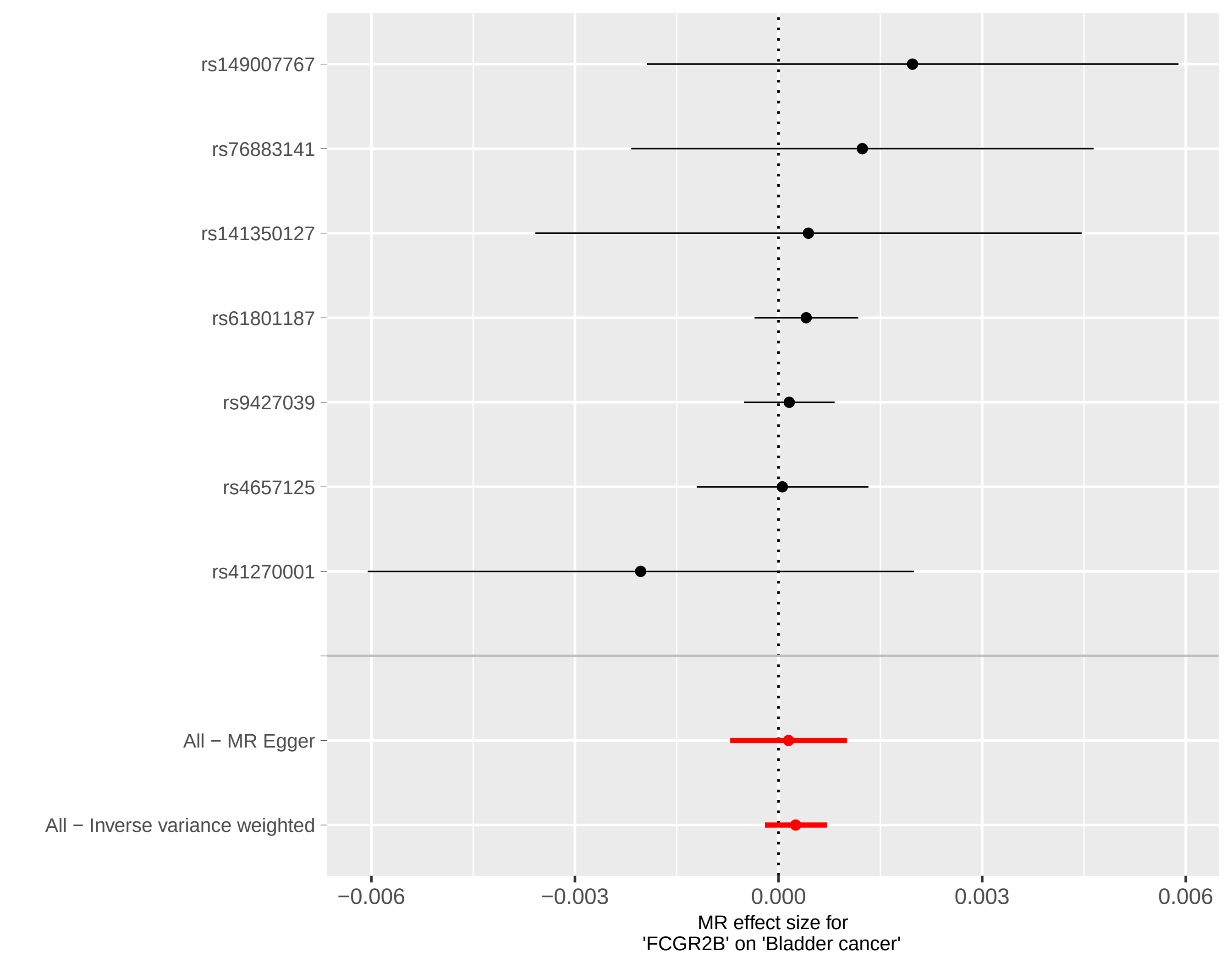
rs149007767
rs76883141
rs141350127
rs61801187
rs9427039
rs4657125
rs41270001
All − MR Egger
All − Inverse variance weighted
−0.006
−0.003
0.000
0.003
0.006
MR effect size for
'FCGR2B' on 'Bladder cancer'

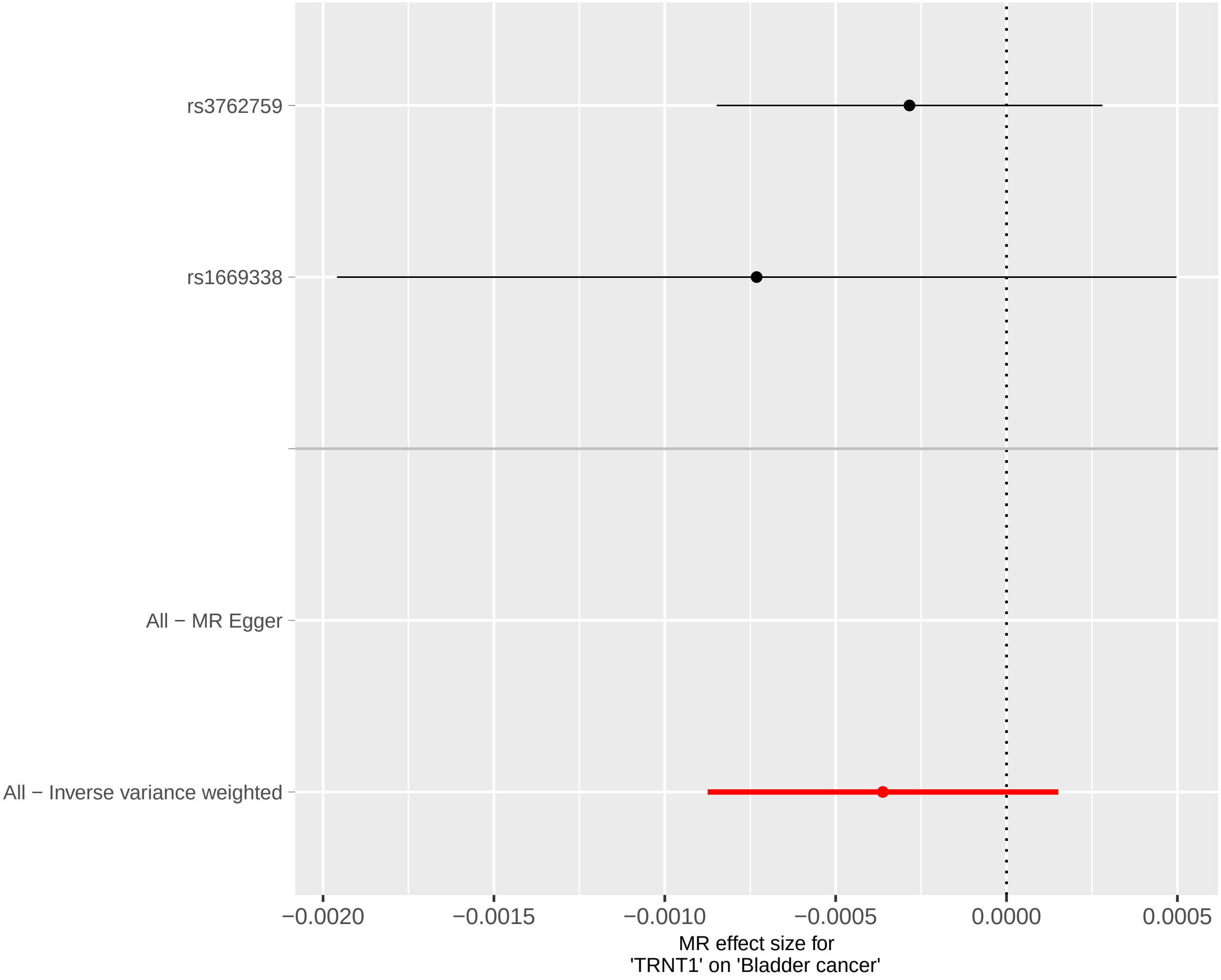
rs3762759
rs1669338
All − MR Egger
All − Inverse variance weighted
−0.0020
−0.0015
−0.0010
−0.0005
0.0000
0.0005
MR effect size for
'TRNT1' on 'Bladder cancer'

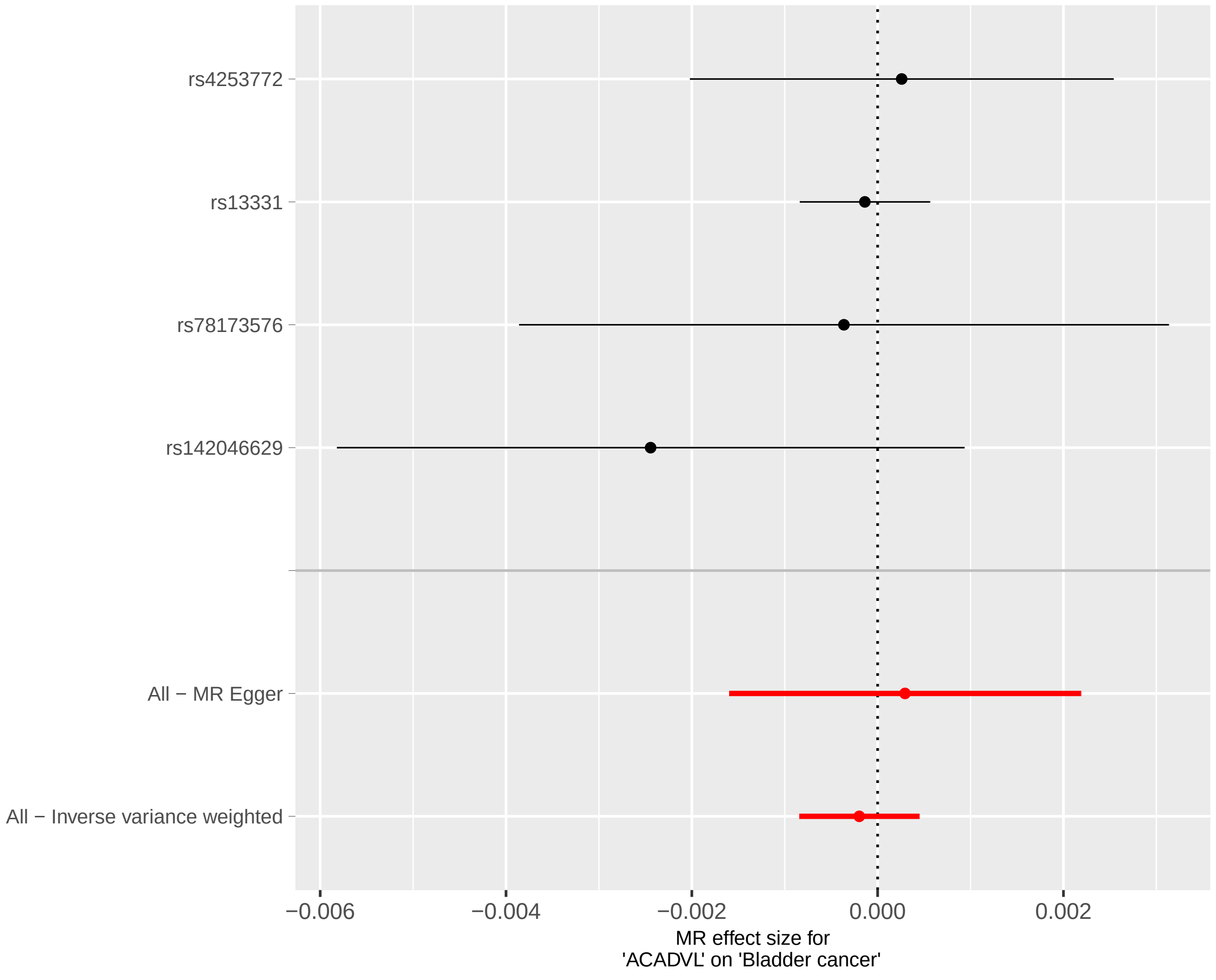

rs4253772
rs13331
rs78173576
rs142046629
All − MR Egger
All − Inverse variance weighted
−0.006
−0.004
−0.002
0.000
0.002
MR effect size for
'ACADVL' on 'Bladder cancer'

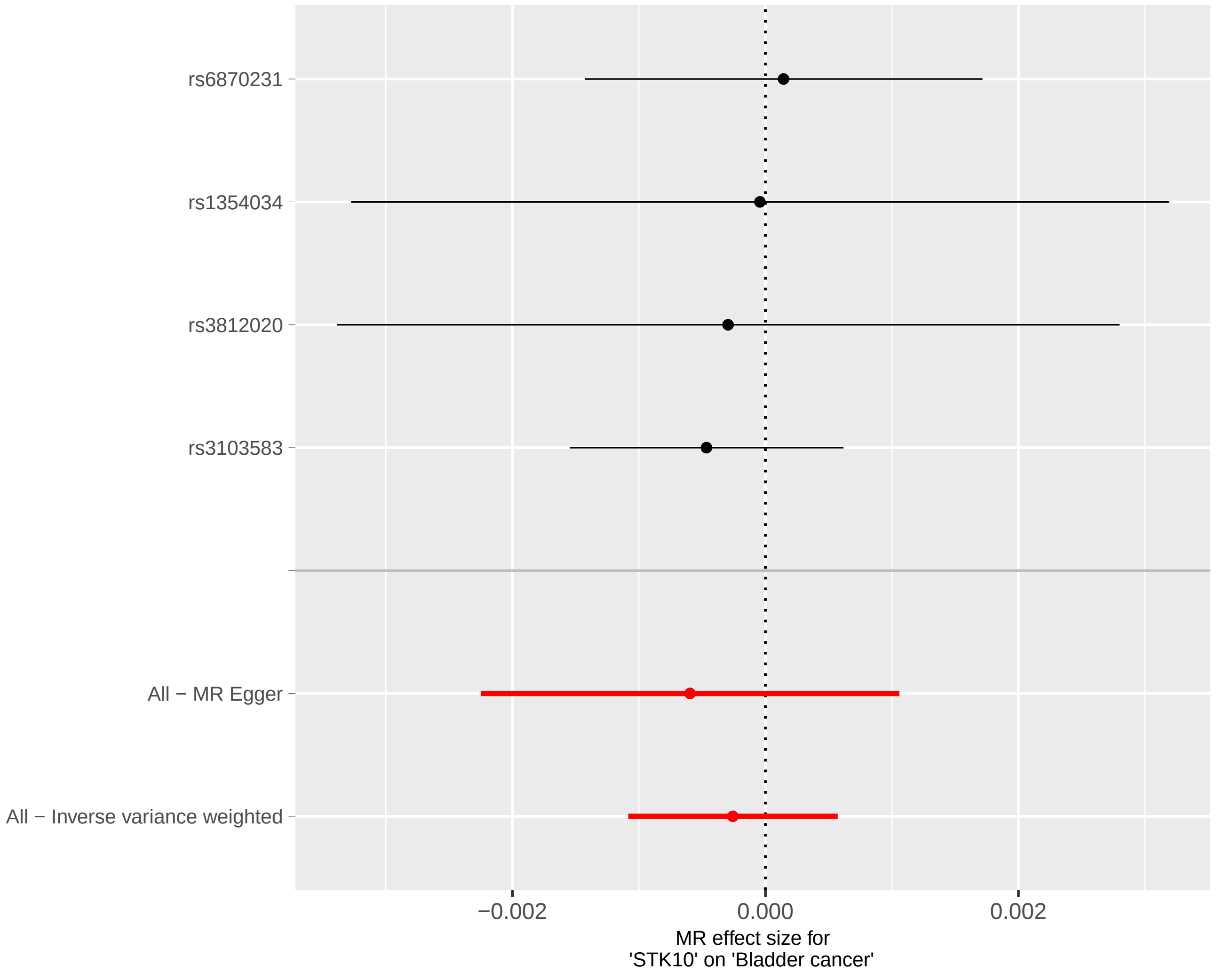
rs6870231
rs1354034
rs3812020
rs3103583
All − MR Egger
All − Inverse variance weighted
−0.002
0.000
0.002
MR effect size for
'STK10' on 'Bladder cancer'

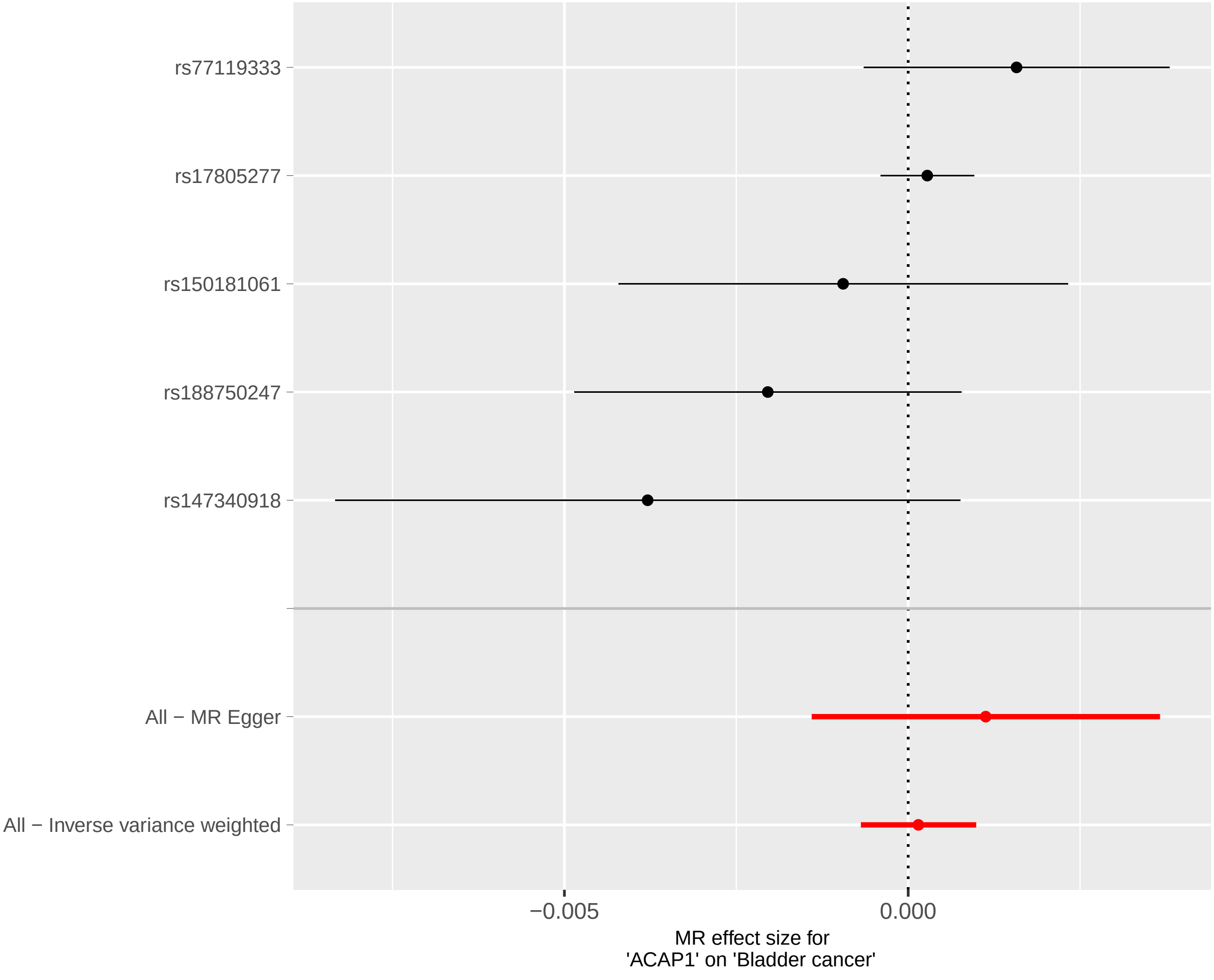
rs77119333
rs17805277
rs150181061
rs188750247
rs147340918
All − MR Egger
All − Inverse variance weighted
−0.005
0.000
MR effect size for
'ACAP1' on 'Bladder cancer'

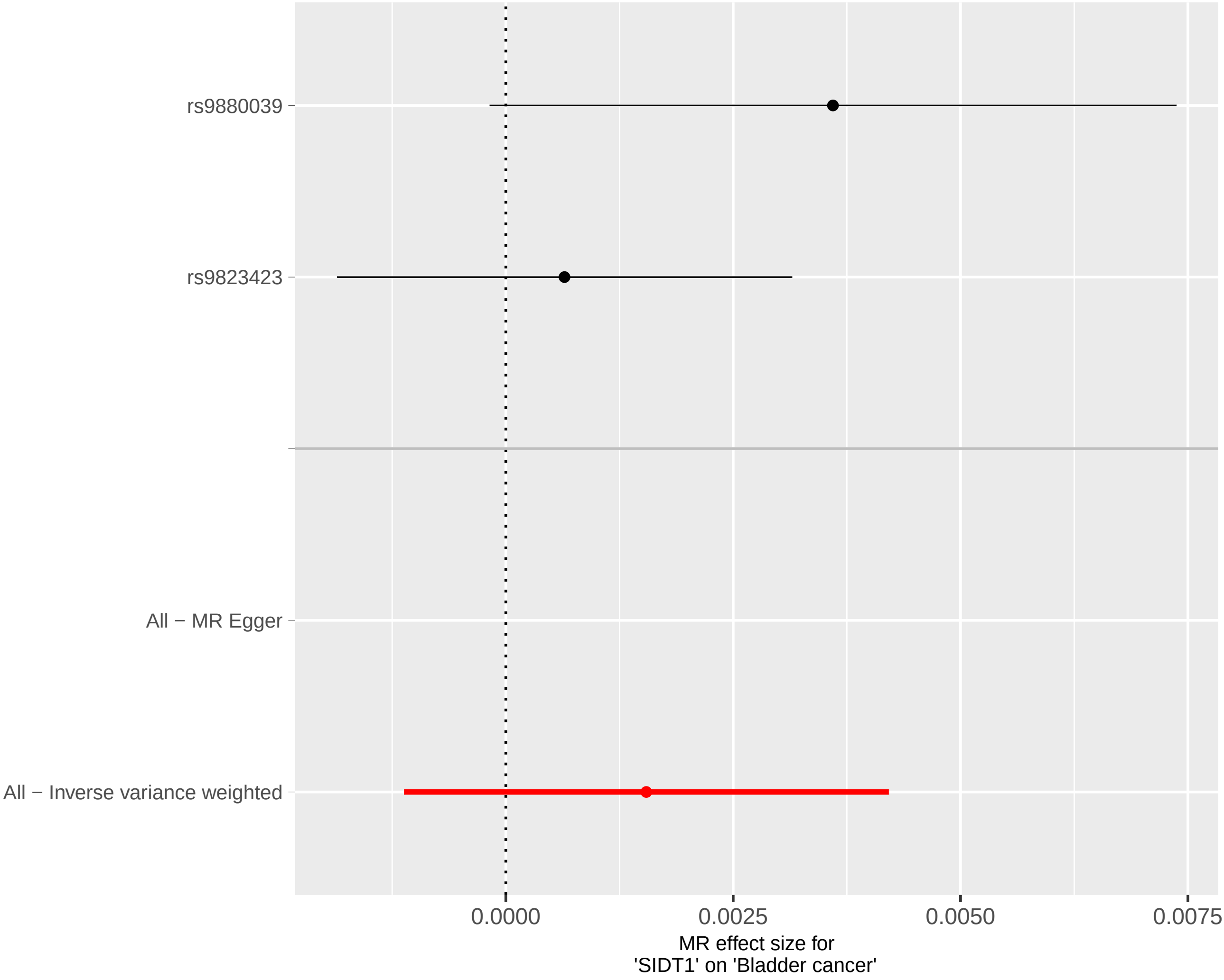
rs9880039
rs9823423
All − MR Egger
All − Inverse variance weighted
0.0000
0.0025
0.0050
0.0075
MR effect size for
'SIDT1' on 'Bladder cancer'

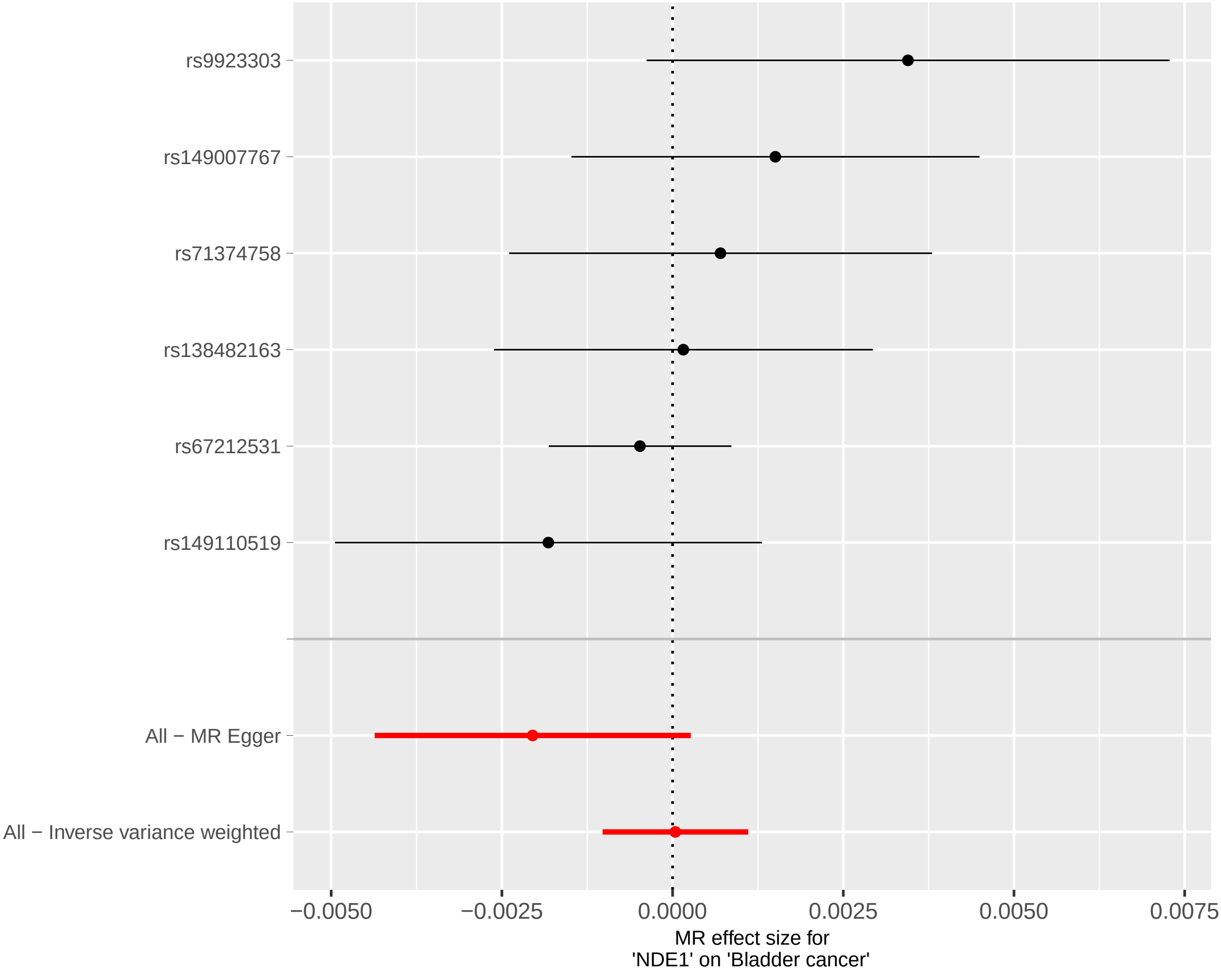

rs9923303
rs149007767
rs71374758
rs138482163
rs67212531
rs149110519
All − MR Egger
All − Inverse variance weighted
−0.0050
−0.0025
0.0000
0.0025
0.0050
0.0075
MR effect size for
'NDE1' on 'Bladder cancer'

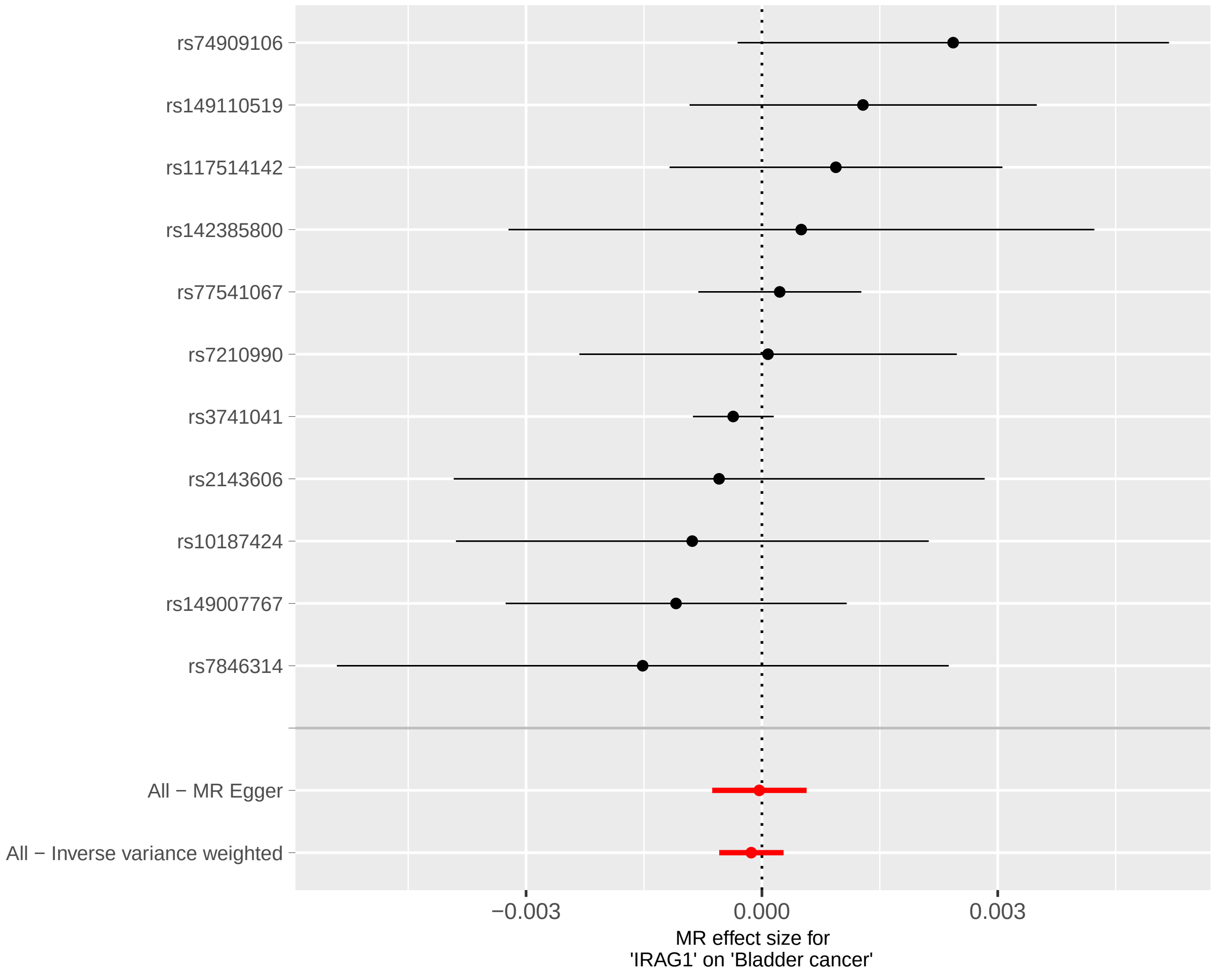
rs74909106
rs149110519
rs117514142
rs142385800
rs77541067
rs7210990
rs3741041
rs2143606
rs10187424
rs149007767
rs7846314
All − MR Egger
All − Inverse variance weighted
−0.003
0.000
0.003
MR effect size for
'IRAG1' on 'Bladder cancer'

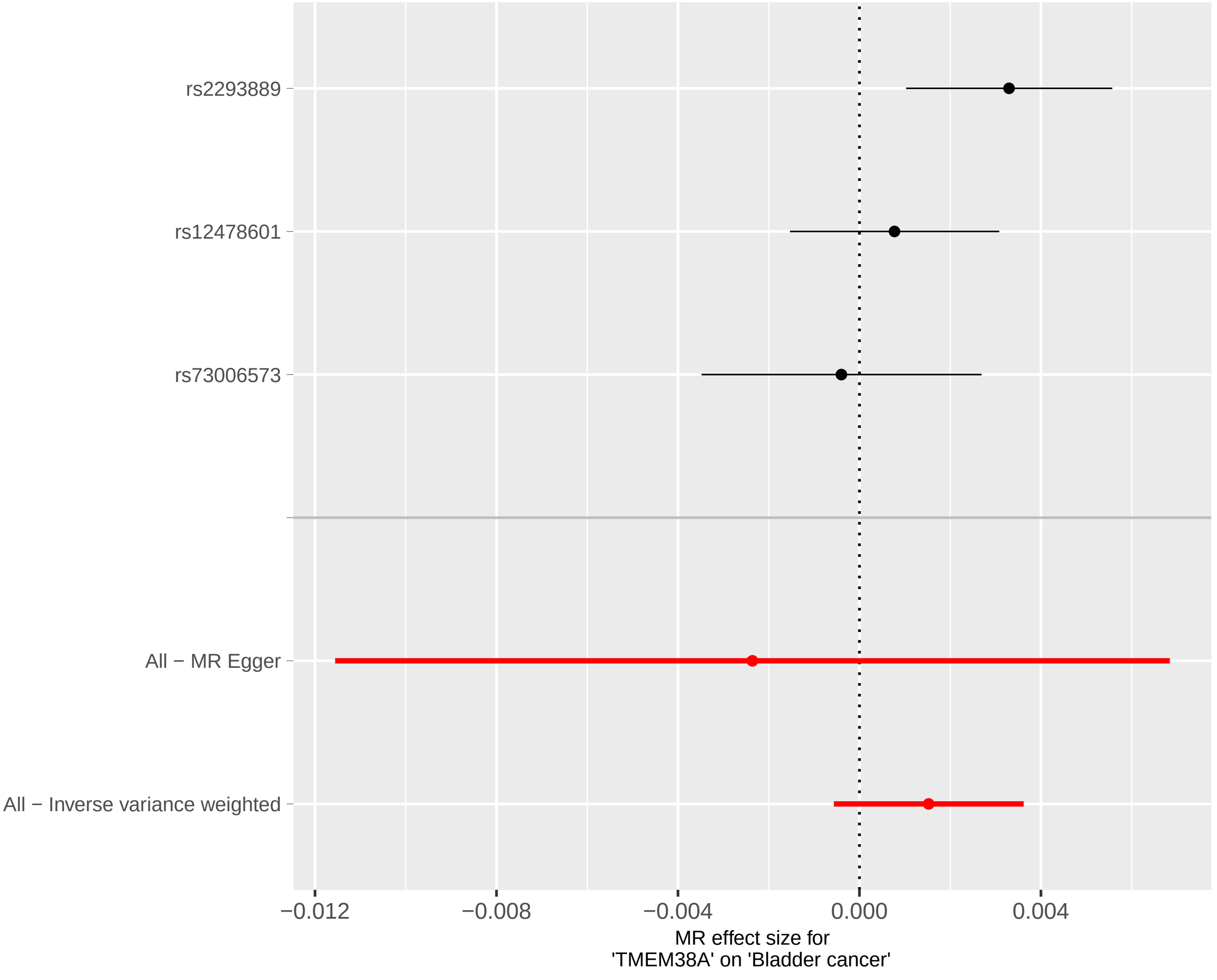
rs2293889
rs12478601
rs73006573
All − MR Egger
All − Inverse variance weighted
−0.012
−0.008
−0.004
0.000
0.004
MR effect size for
'TMEM38A' on 'Bladder cancer'

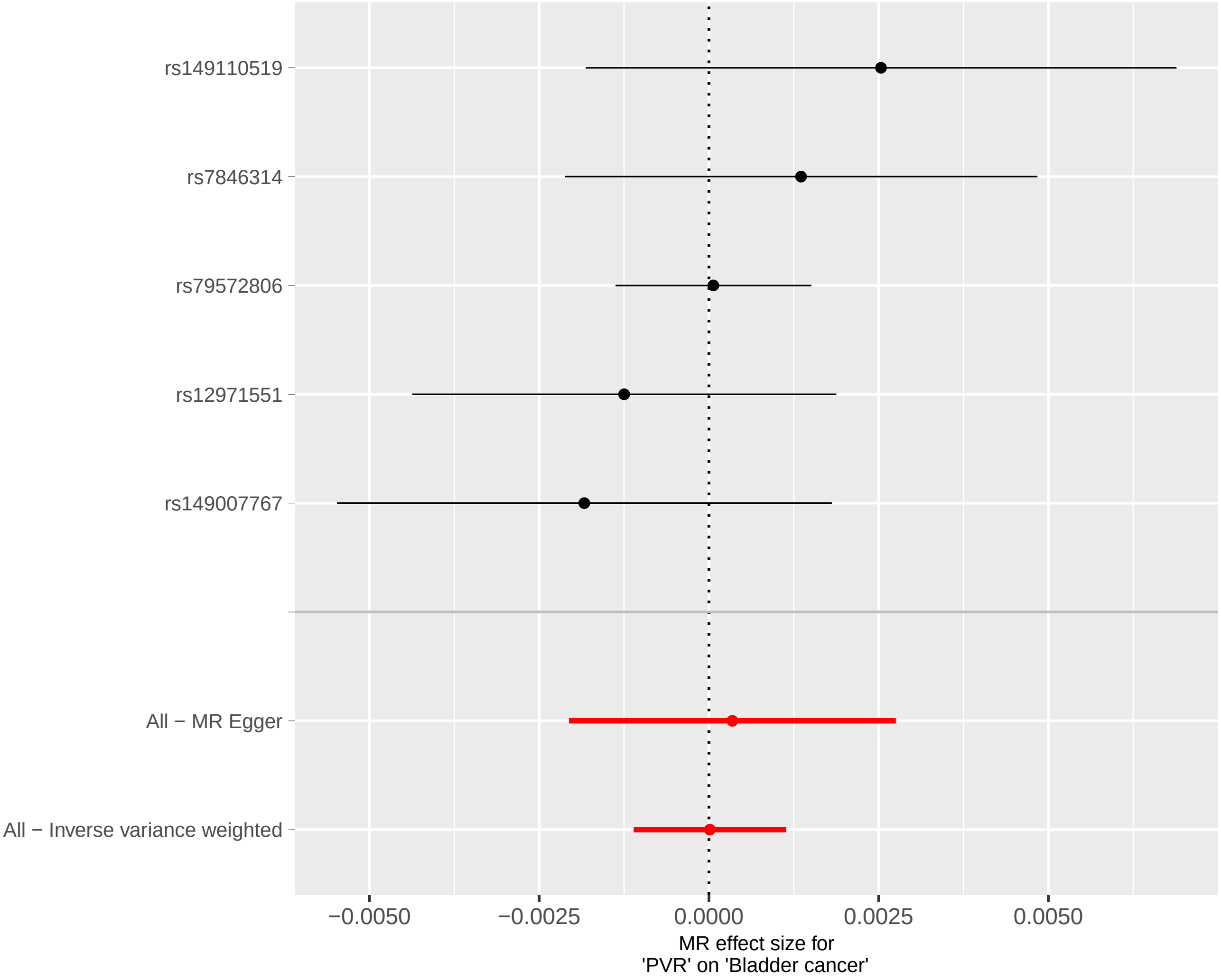
rs149110519
rs7846314
rs79572806
rs12971551
rs149007767
All − MR Egger
All − Inverse variance weighted
−0.0050
−0.0025
0.0000
0.0025
0.0050
MR effect size for
'PVR' on 'Bladder cancer'

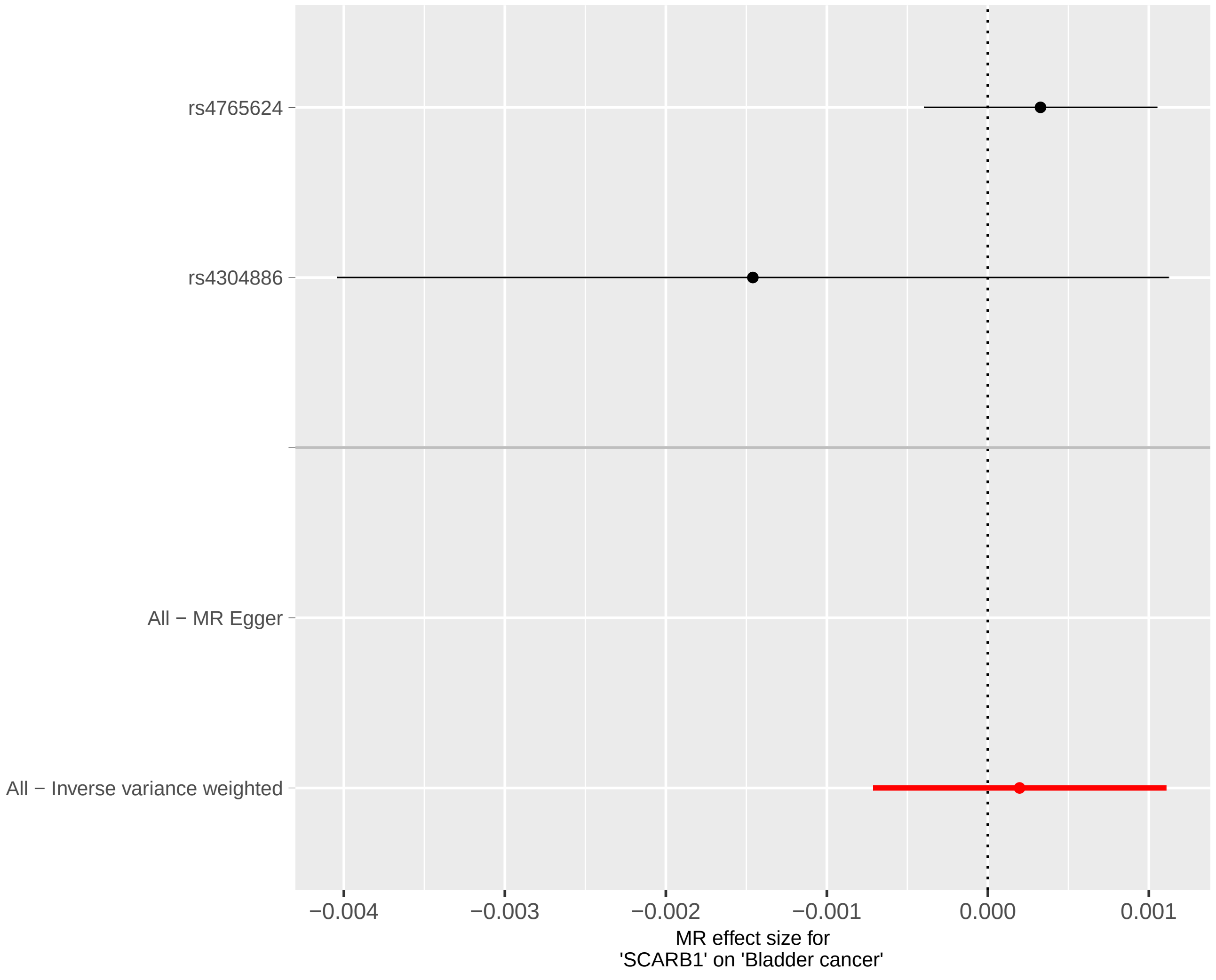
rs4765624
rs4304886
All − MR Egger
All − Inverse variance weighted
−0.004
−0.003
−0.002
−0.001
0.000
0.001
MR effect size for
'SCARB1' on 'Bladder cancer'

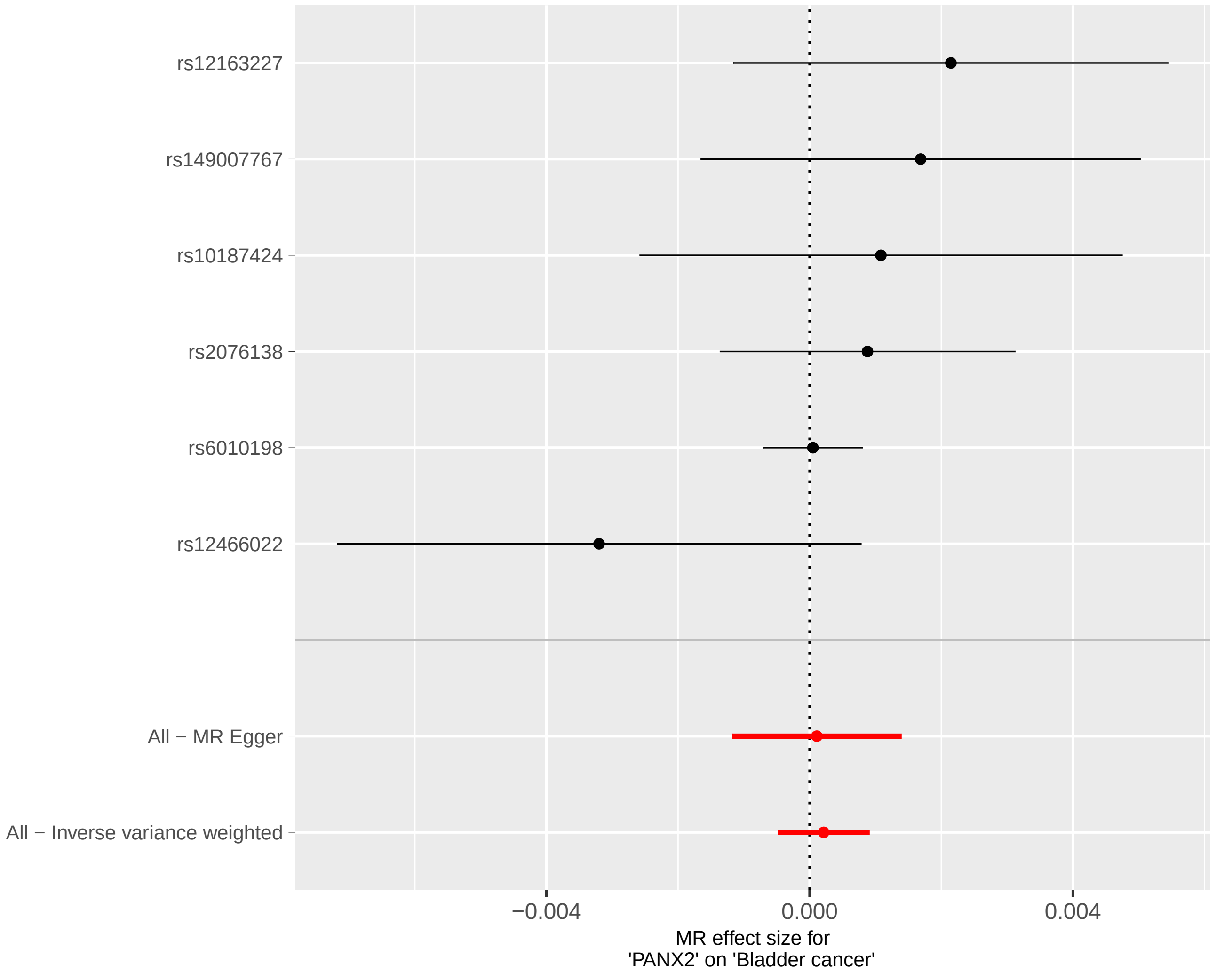
rs12163227
rs149007767
rs10187424
rs2076138
rs6010198
rs12466022
All − MR Egger
All − Inverse variance weighted
−0.004
0.000
0.004
MR effect size for
'PANX2' on 'Bladder cancer'

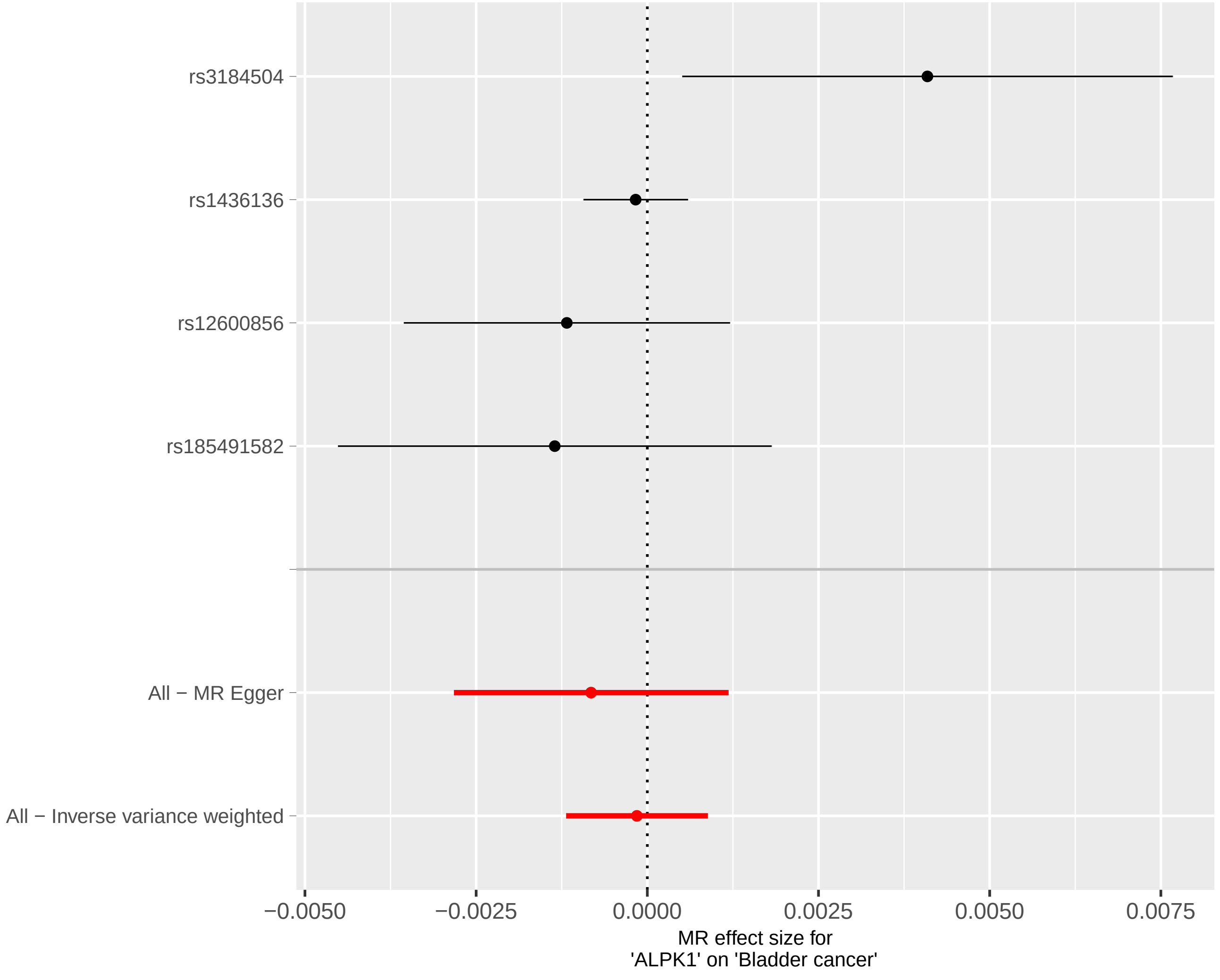
rs3184504
rs1436136
rs12600856
rs185491582
All − MR Egger
All − Inverse variance weighted
−0.0050
−0.0025
0.0000
0.0025
0.0050
0.0075
MR effect size for
'ALPK1' on 'Bladder cancer'

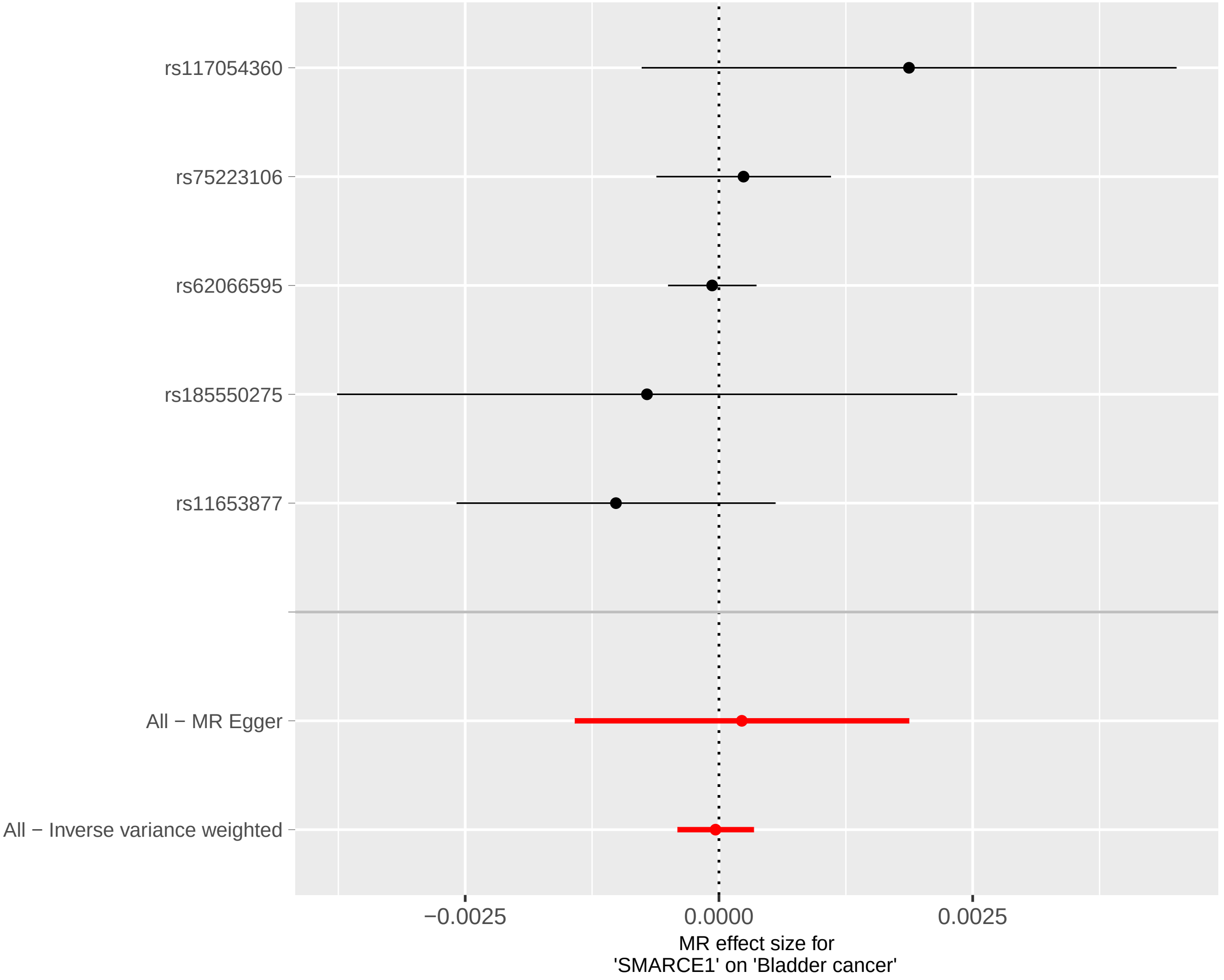
rs117054360
rs75223106
rs62066595
rs185550275
rs11653877
All − MR Egger
All − Inverse variance weighted
−0.0025
0.0000
0.0025
MR effect size for
'SMARCE1' on 'Bladder cancer'

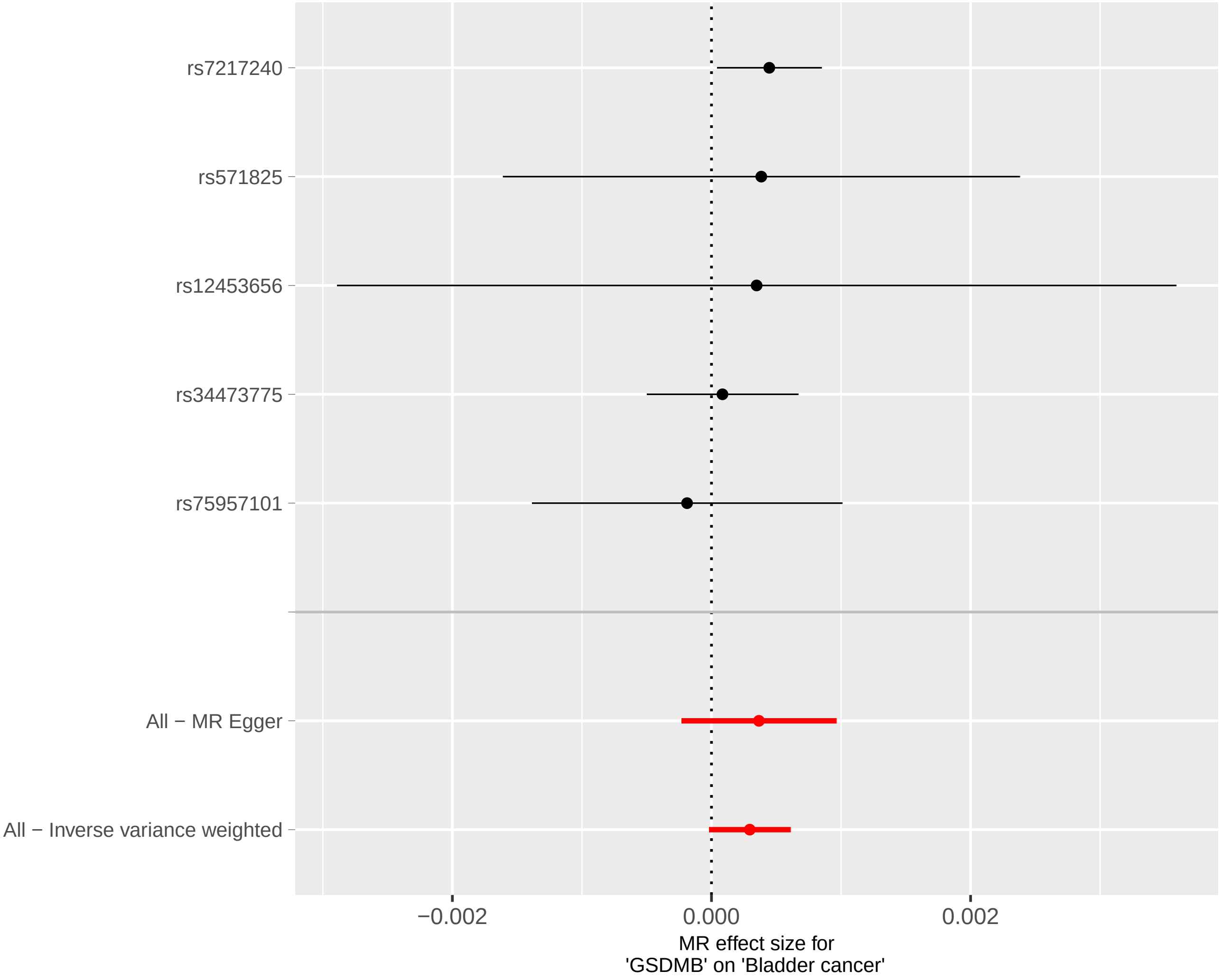

rs7217240
rs571825
rs12453656
rs34473775
rs75957101
All − MR Egger
All − Inverse variance weighted
−0.002
0.000
0.002
MR effect size for
'GSDMB' on 'Bladder cancer'

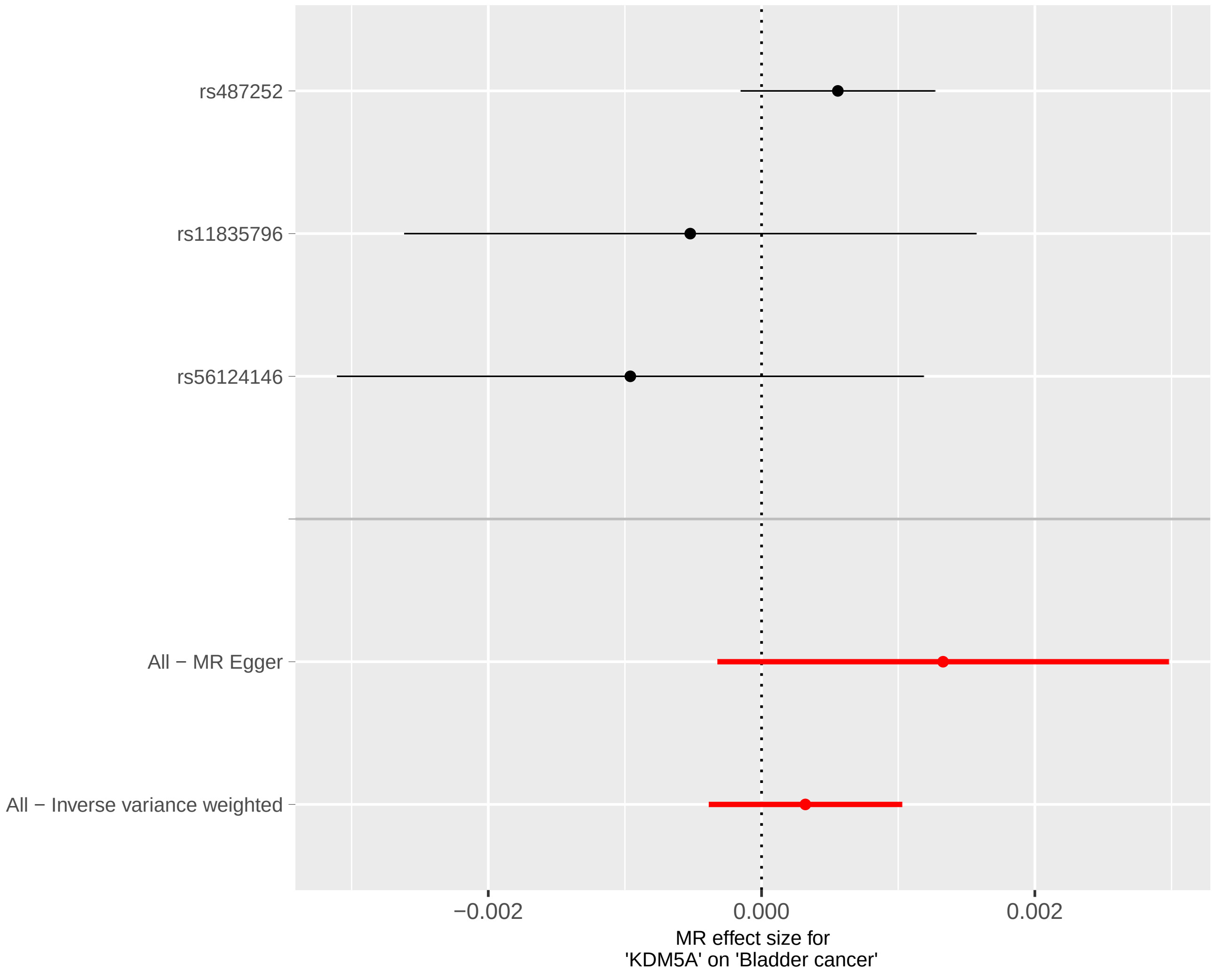

rs487252
rs11835796
rs56124146
All − MR Egger
All − Inverse variance weighted
−0.002
0.000
0.002
MR effect size for
'KDM5A' on 'Bladder cancer'

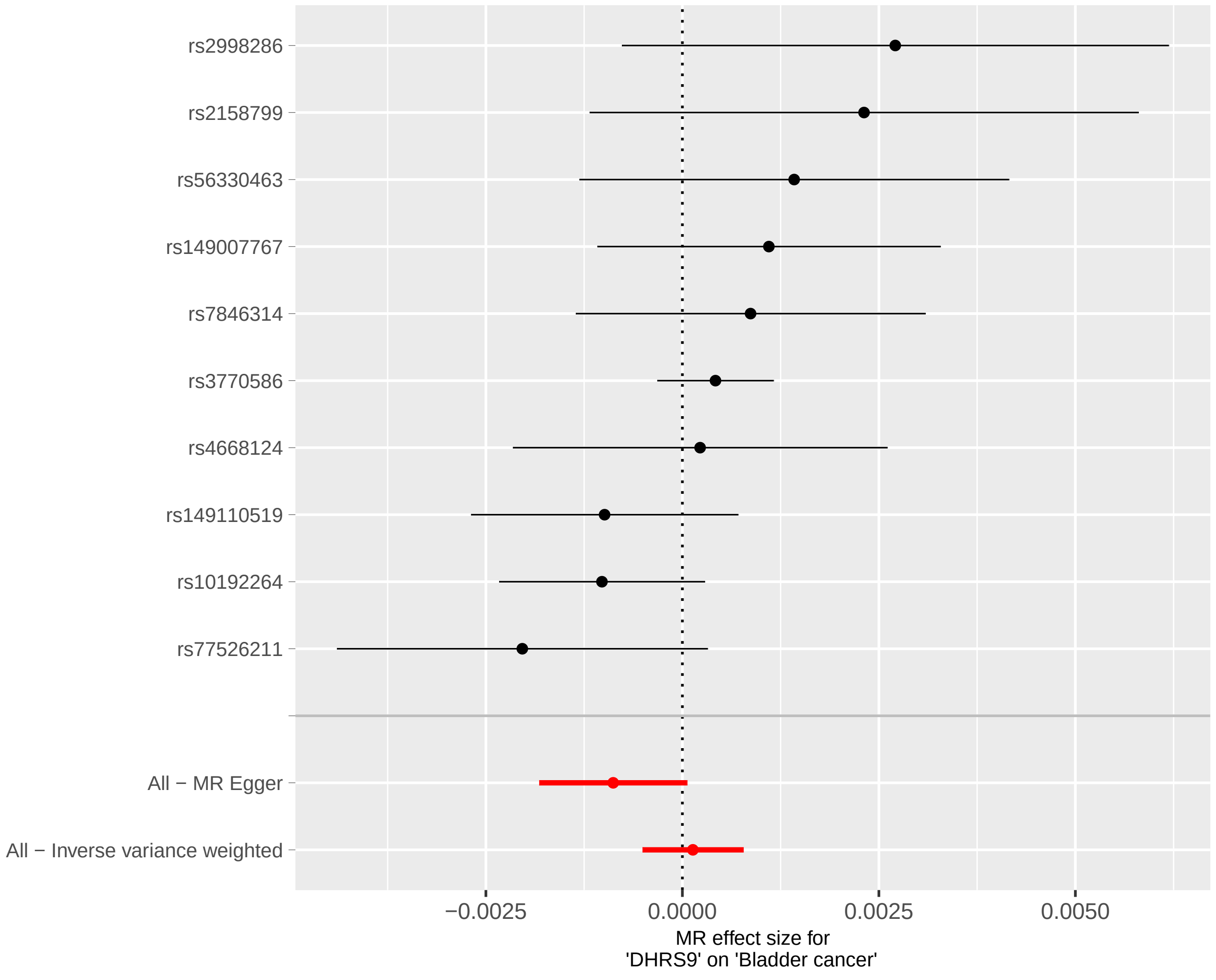

rs2998286
rs2158799
rs56330463
rs149007767
rs7846314
rs3770586
rs4668124
rs149110519
rs10192264
rs77526211
All − MR Egger
All − Inverse variance weighted
−0.0025
0.0000
0.0025
0.0050
MR effect size for
'DHRS9' on 'Bladder cancer'

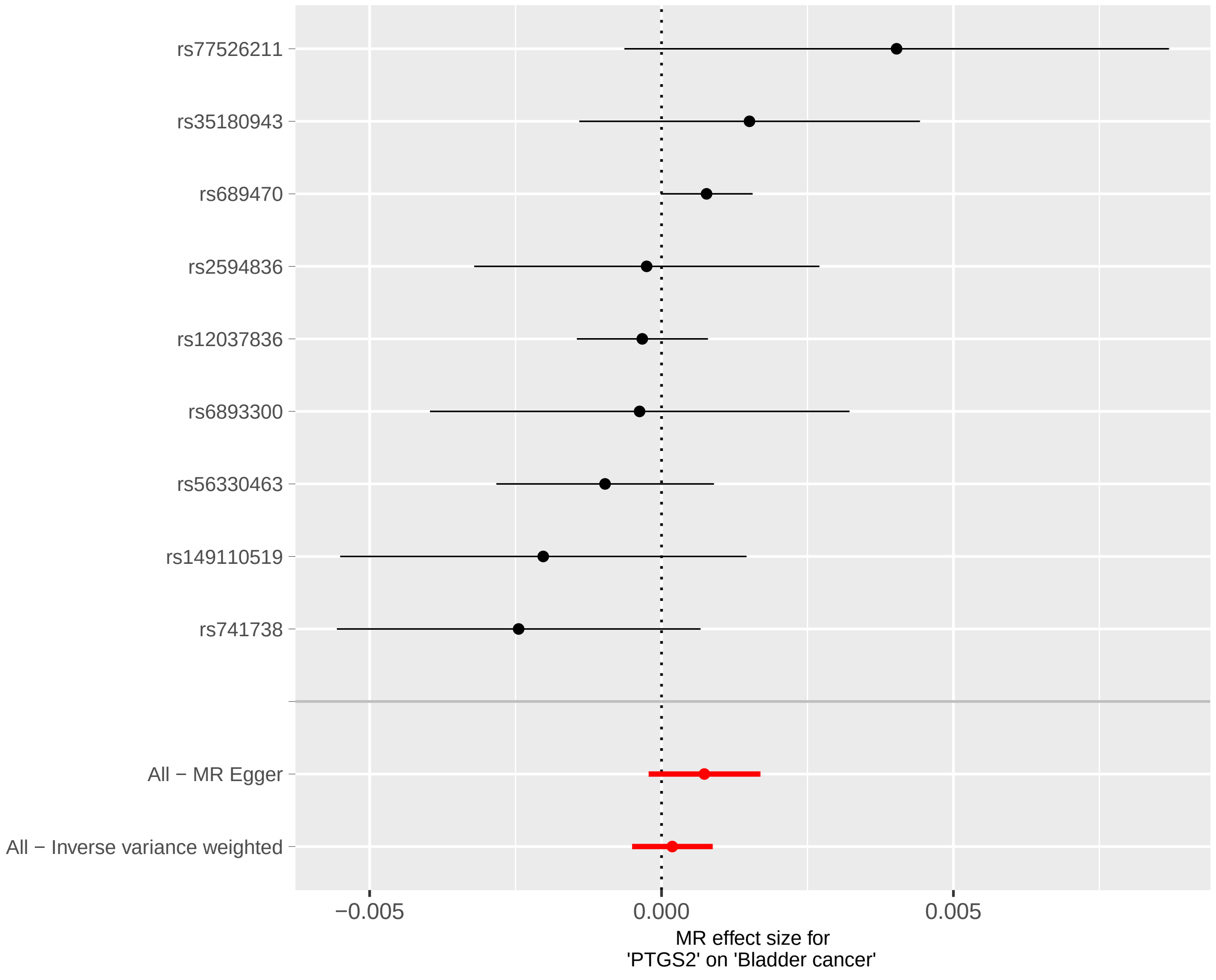
rs77526211
rs35180943
rs689470
rs2594836
rs12037836
rs6893300
rs56330463
rs149110519
rs741738
All − MR Egger
All − Inverse variance weighted
−0.005
0.000
0.005
MR effect size for
'PTGS2' on 'Bladder cancer'

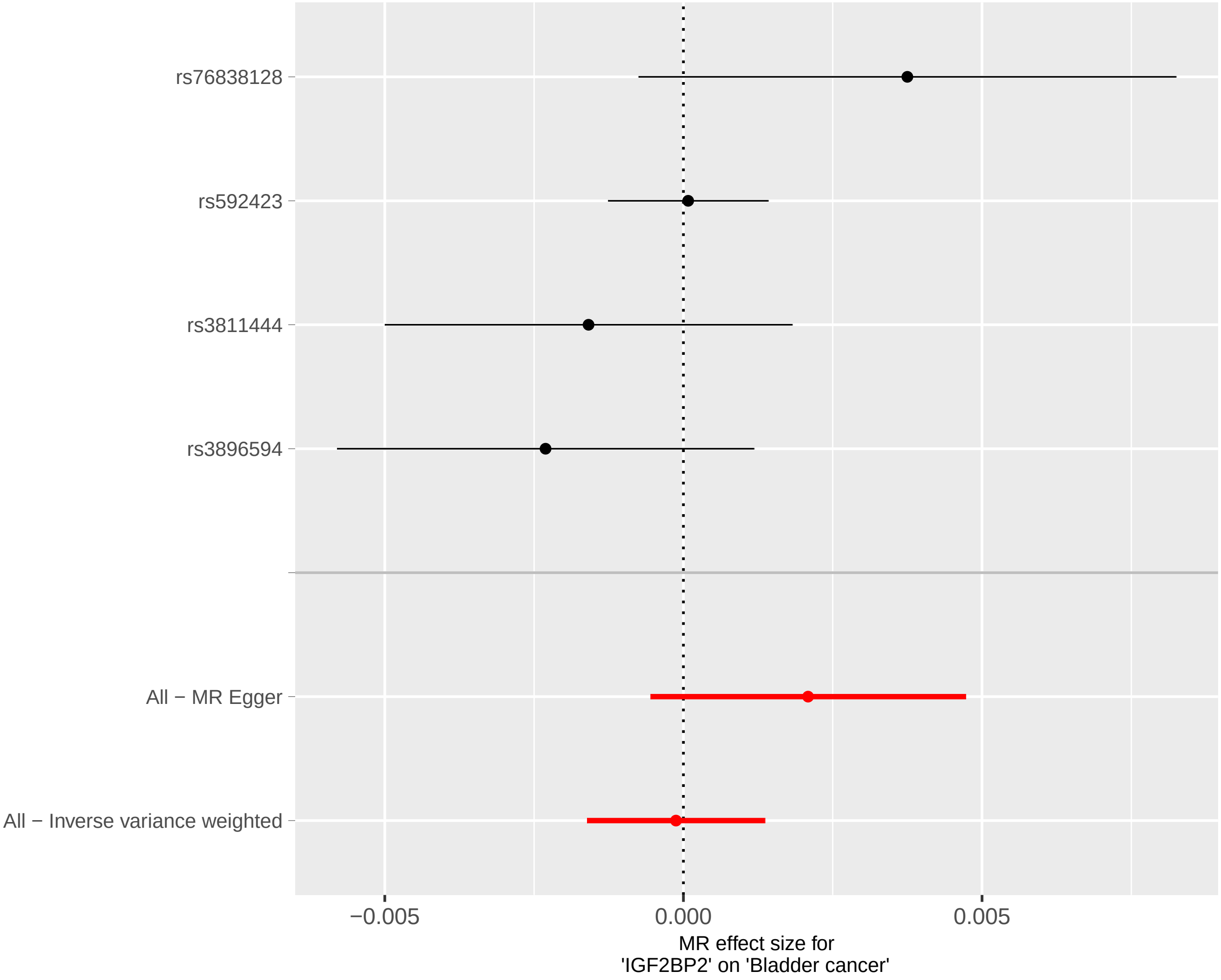

rs76838128
rs592423
rs3811444
rs3896594
All − MR Egger
All − Inverse variance weighted
−0.005
0.000
0.005
MR effect size for
'IGF2BP2' on 'Bladder cancer'

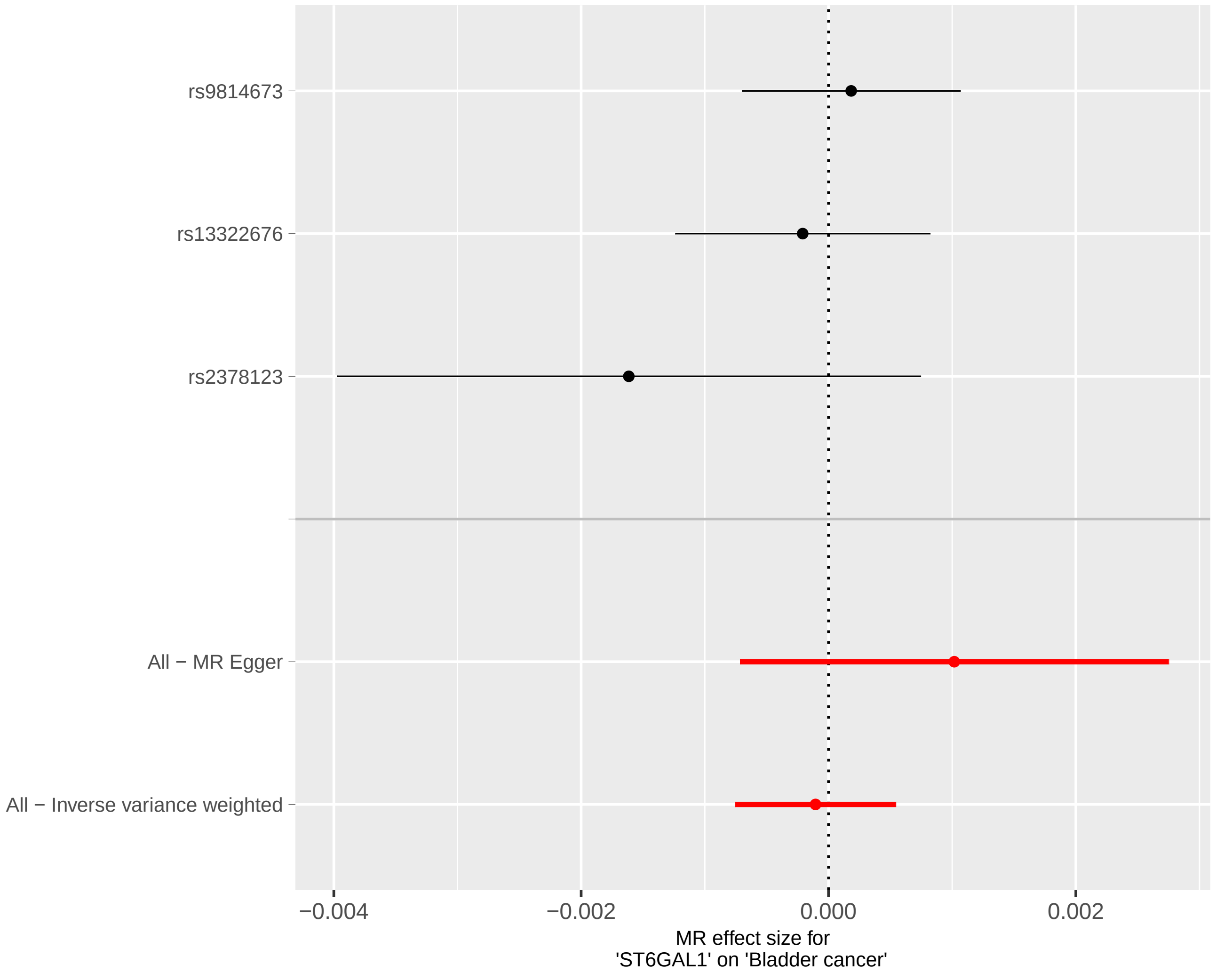
rs9814673
rs13322676
rs2378123
All − MR Egger
All − Inverse variance weighted
−0.004
−0.002
0.000
0.002
MR effect size for
'ST6GAL1' on 'Bladder cancer'

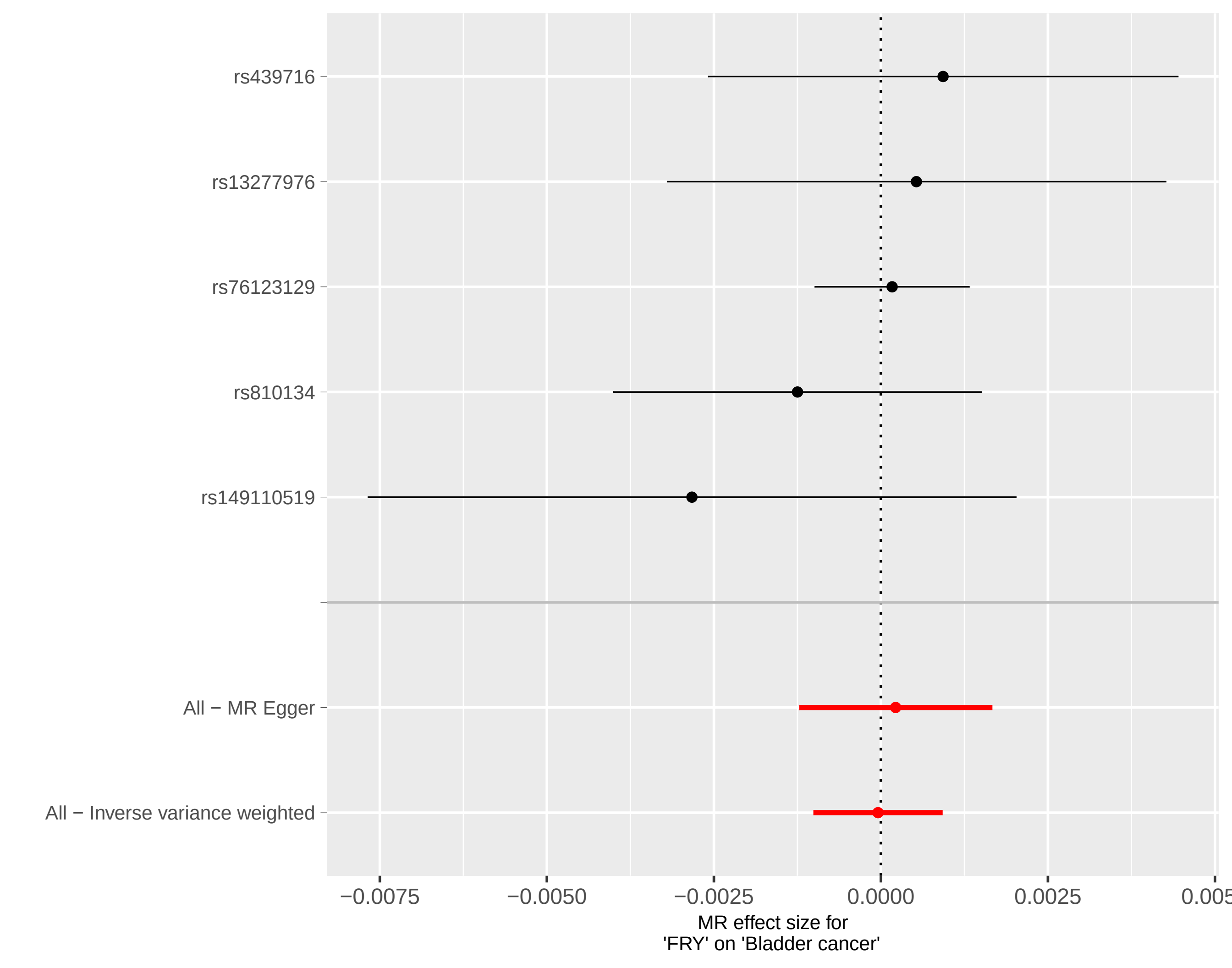

rs439716
rs13277976
rs76123129
rs810134
rs149110519
All − MR Egger
All − Inverse variance weighted
−0.0075
−0.0050
−0.0025
0.0000
0.0025
MR effect size for
'FRY' on 'Bladder cancer'

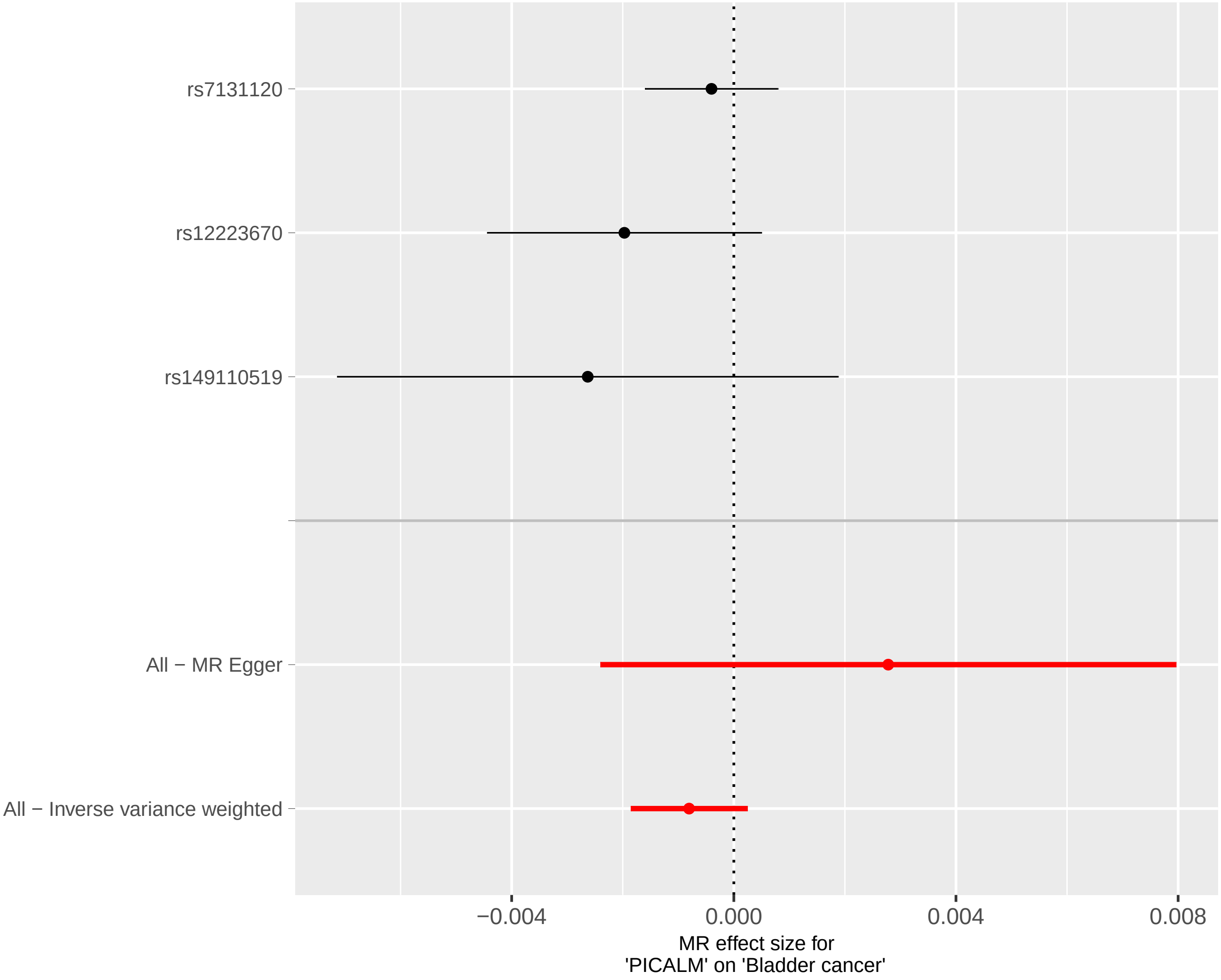

rs7131120
rs12223670
rs149110519
All − MR Egger
All − Inverse variance weighted
−0.004
0.000
0.004
0.008
MR effect size for
'PICALM' on 'Bladder cancer'

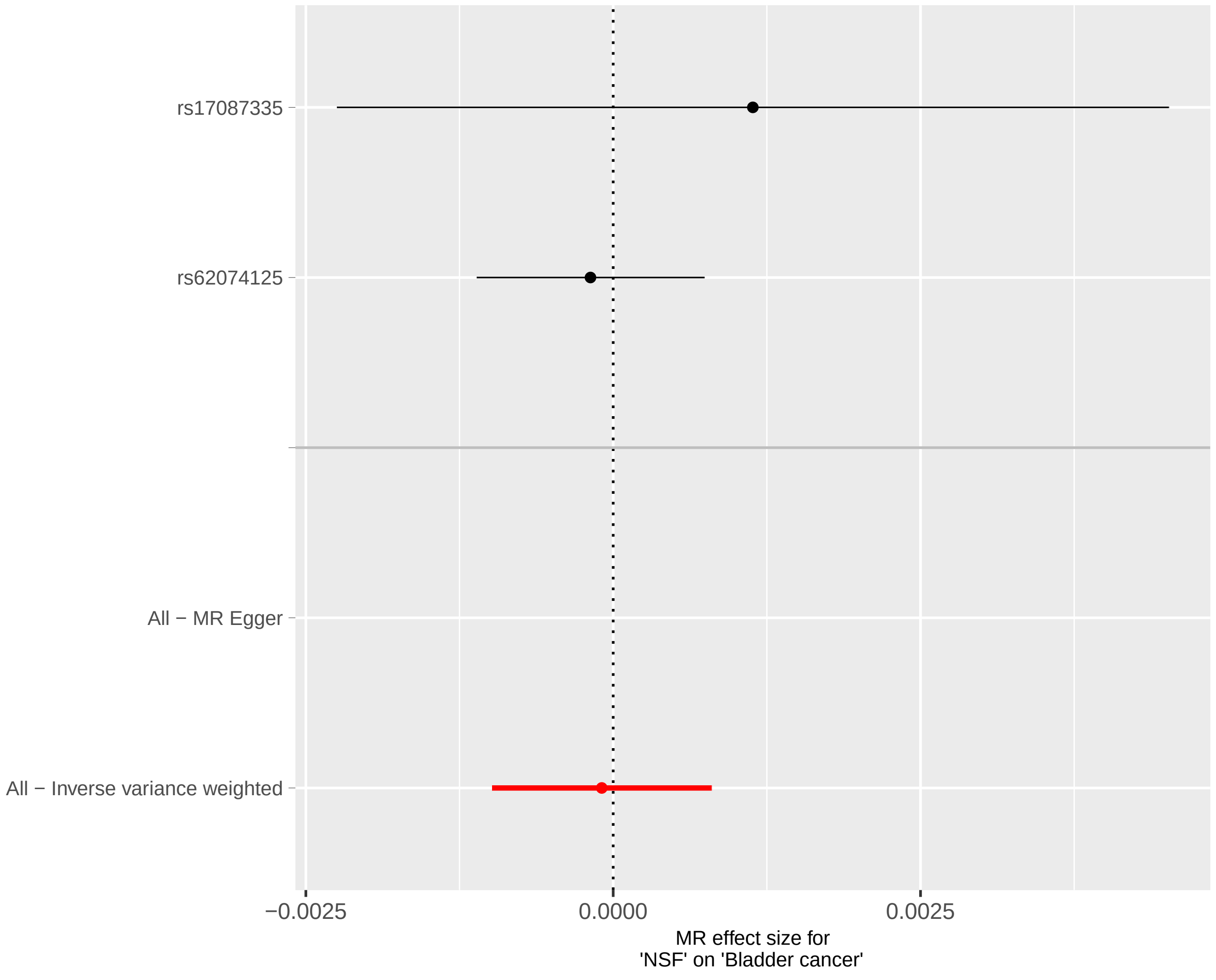

rs17087335
rs62074125
All − MR Egger
All − Inverse variance weighted
−0.0025
0.0000
0.0025
MR effect size for
'NSF' on 'Bladder cancer'

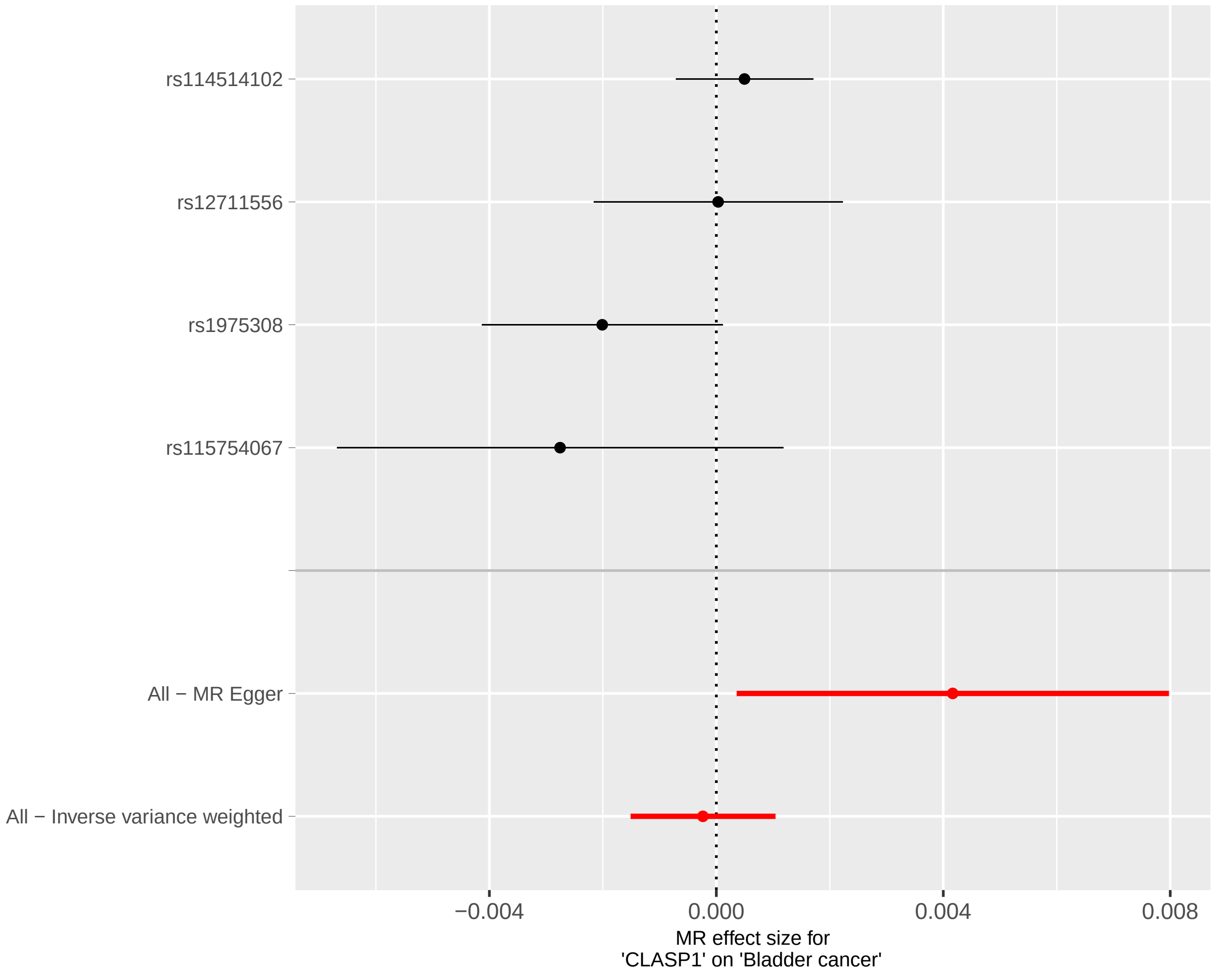
rs114514102
rs12711556
rs1975308
rs115754067
All − MR Egger
All − Inverse variance weighted
−0.004
0.000
0.004
0.008
MR effect size for
'CLASP1' on 'Bladder cancer'

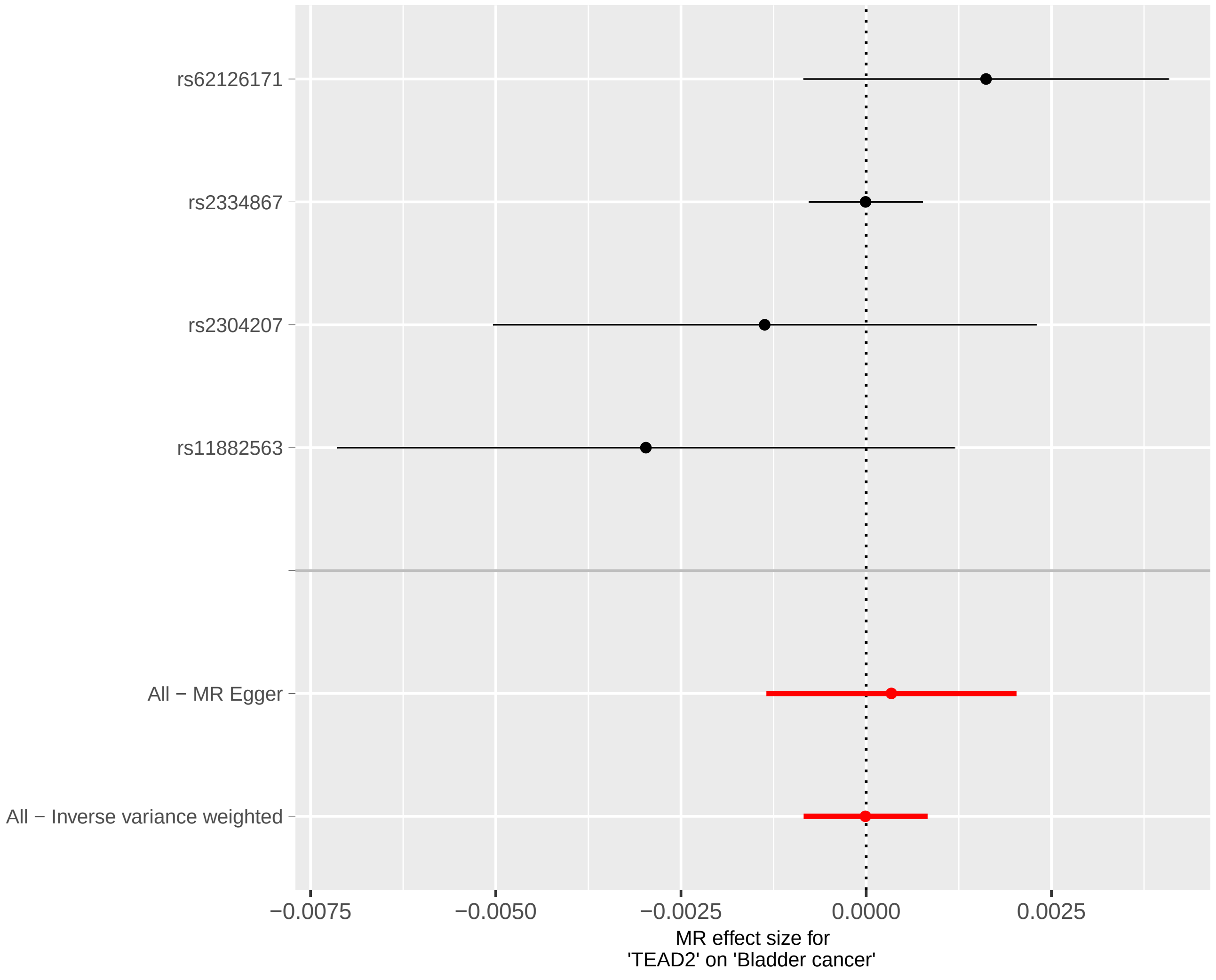
rs62126171
rs2334867
rs2304207
rs11882563
All − MR Egger
All − Inverse variance weighted
−0.0075
−0.0050
−0.0025
0.0000
0.0025
MR effect size for
'TEAD2' on 'Bladder cancer'

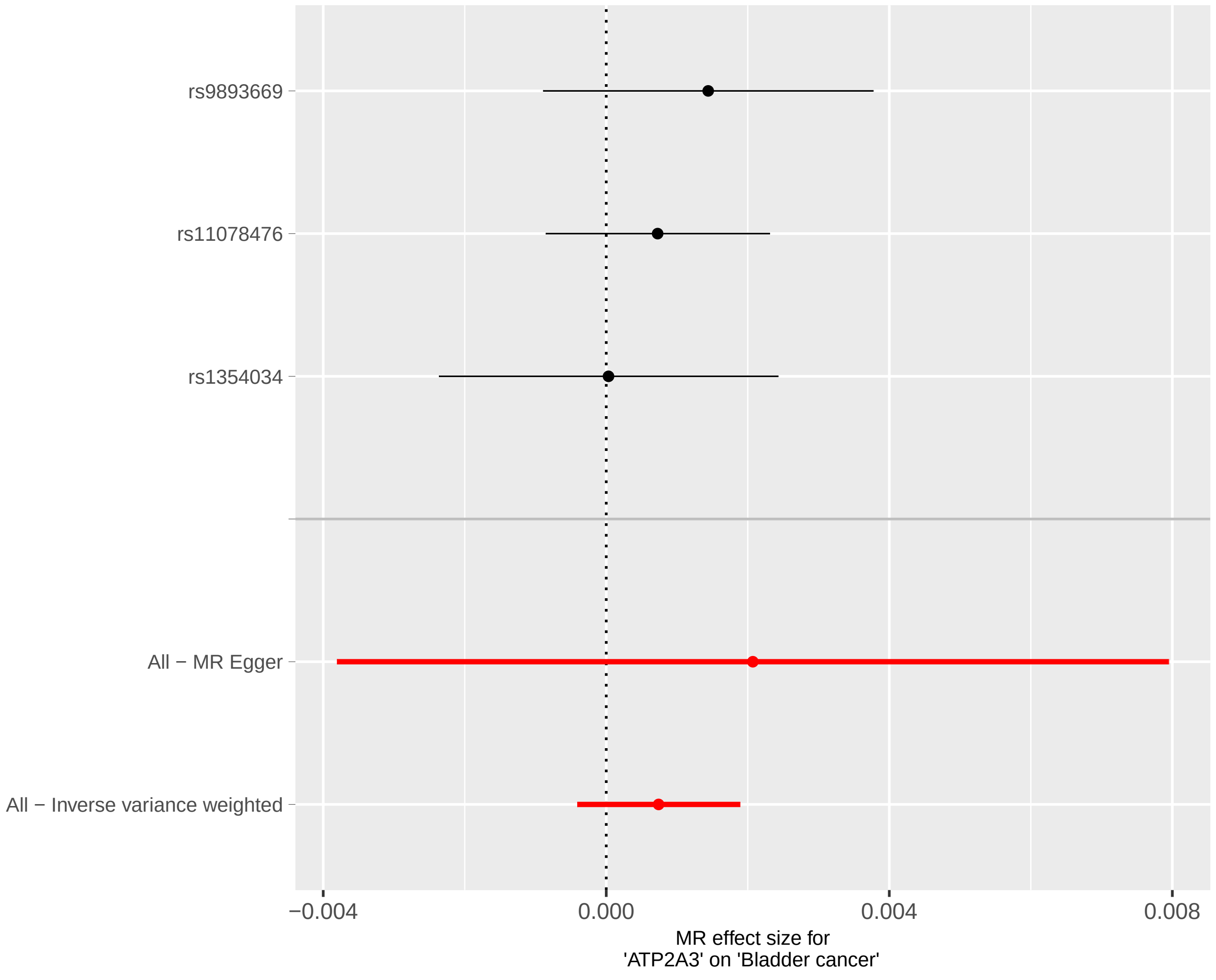

rs9893669
rs11078476
rs1354034
All − MR Egger
All − Inverse variance weighted
−0.004
0.000
0.004
0.008
MR effect size for
'ATP2A3' on 'Bladder cancer'

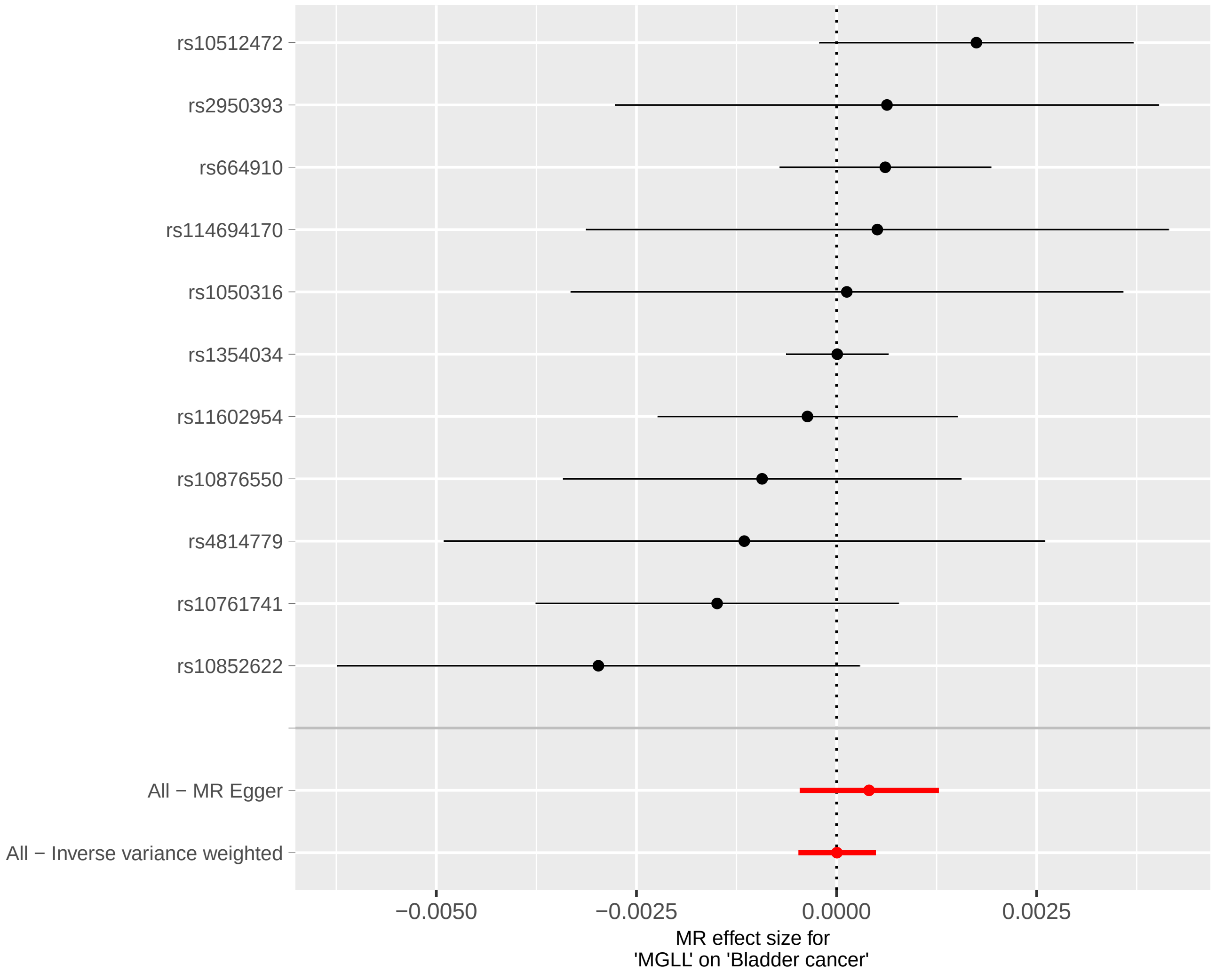

rs10512472
rs2950393
rs664910
rs114694170
rs1050316
rs1354034
rs11602954
rs10876550
rs4814779
rs10761741
rs10852622
All − MR Egger
All − Inverse variance weighted
−0.0050
−0.0025
0.0000
0.0025
MR effect size for
'MGLL' on 'Bladder cancer'

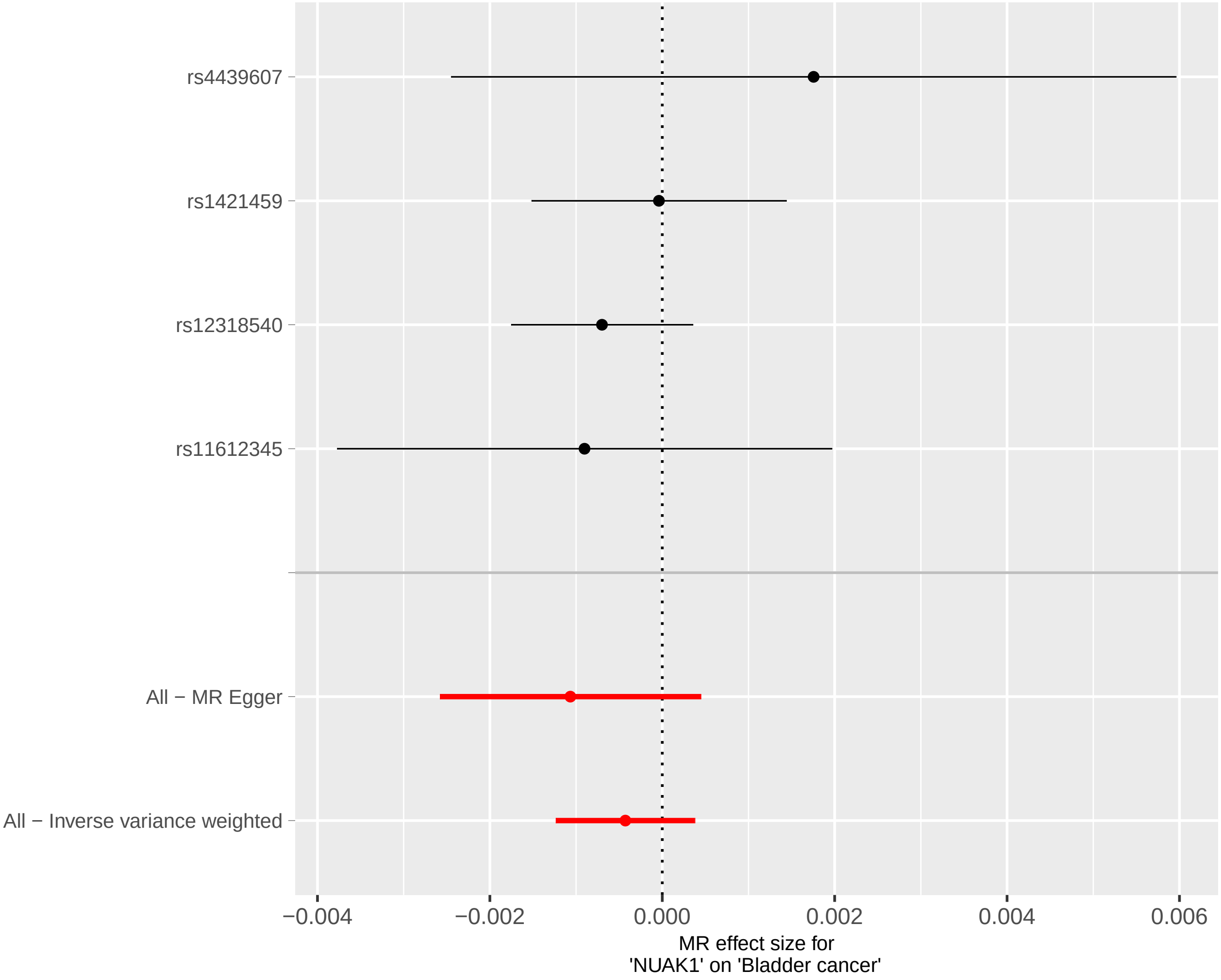

rs4439607
rs1421459
rs12318540
rs11612345
All − MR Egger
All − Inverse variance weighted
−0.004
−0.002
0.000
0.002
0.004
0.006
MR effect size for
'NUAK1' on 'Bladder cancer'

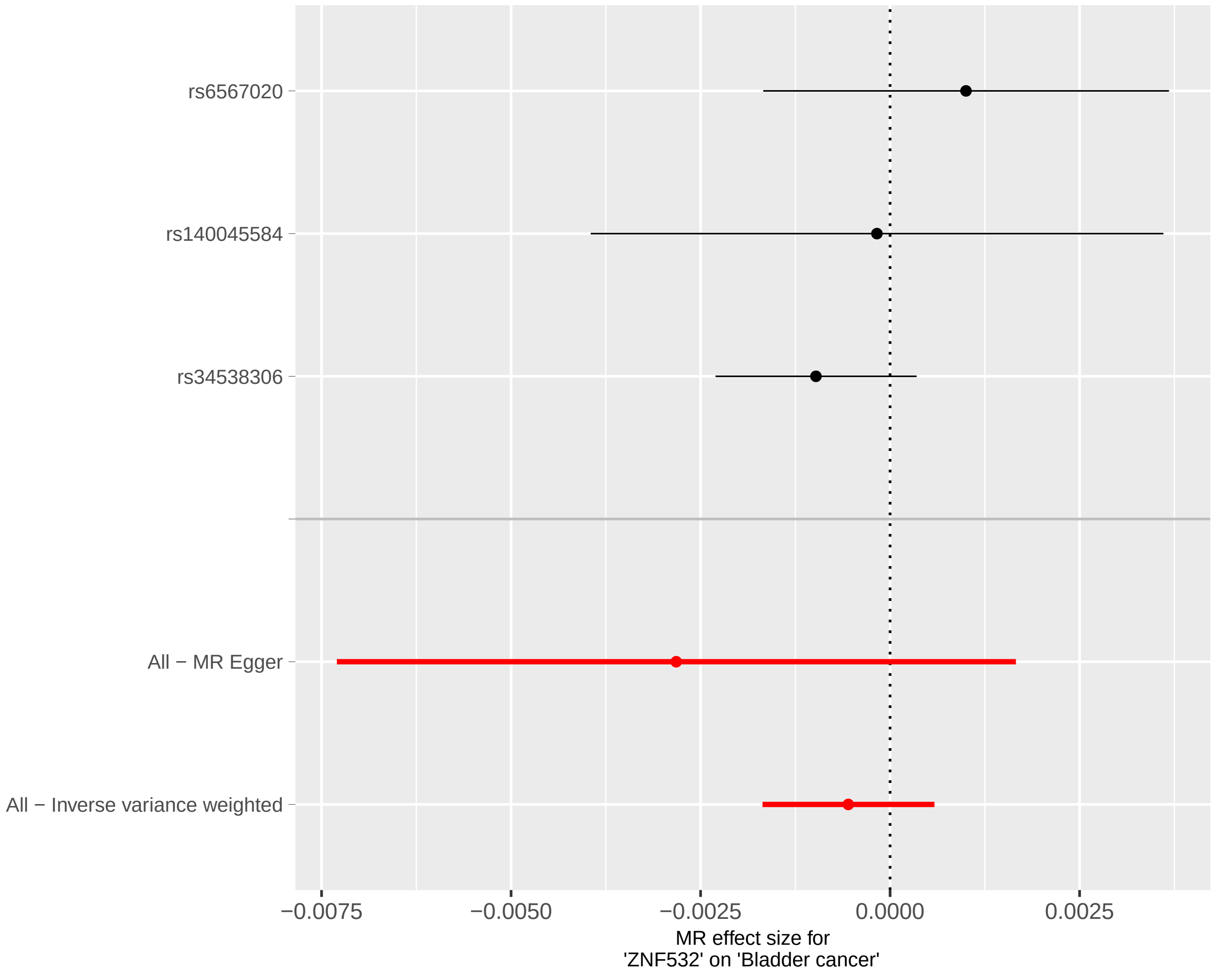

rs6567020
rs140045584
rs34538306
All − MR Egger
All − Inverse variance weighted
−0.0075
−0.0050
−0.0025
0.0000
0.0025
MR effect size for
'ZNF532' on 'Bladder cancer'

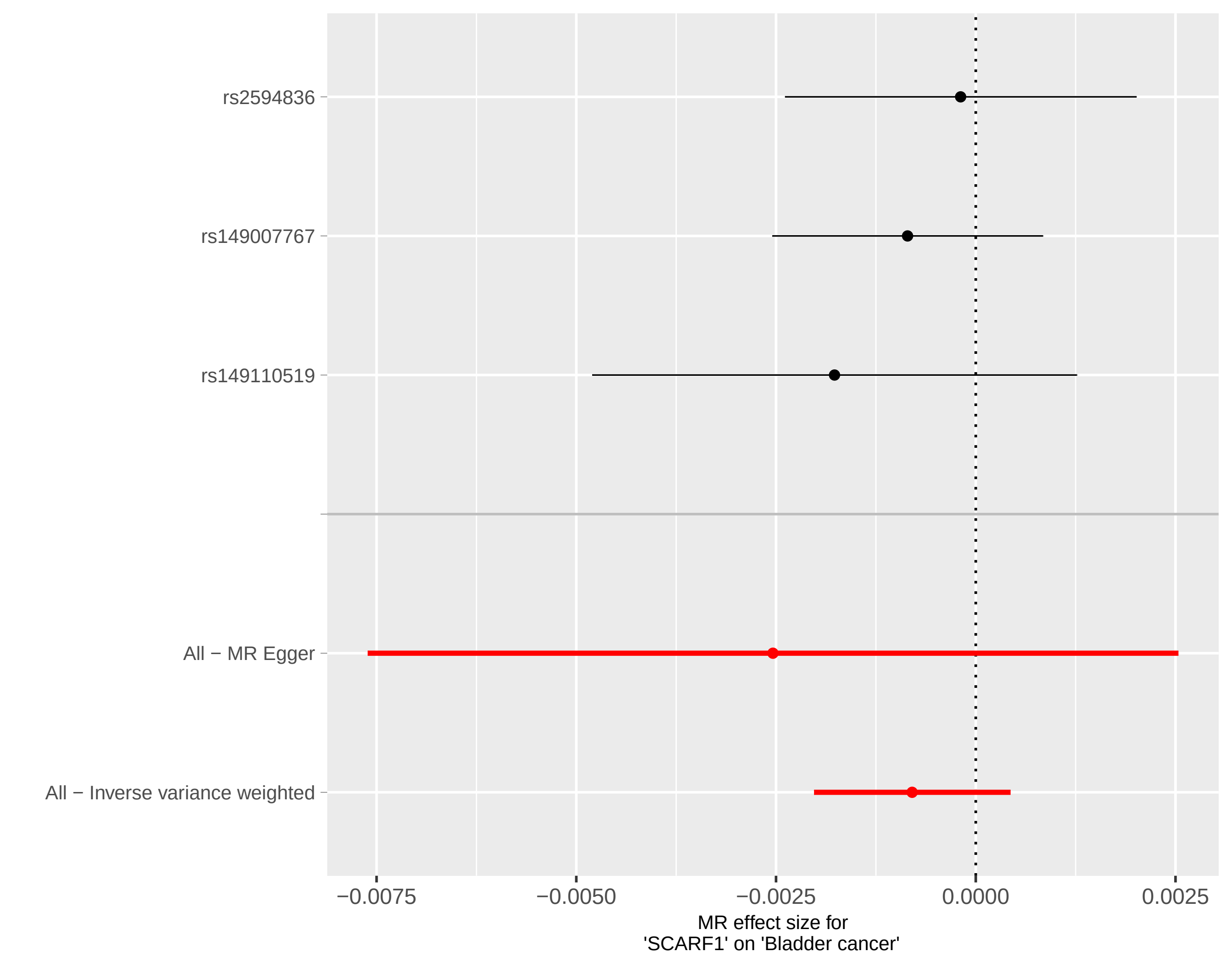
rs2594836
rs149007767
rs149110519
All − MR Egger
All − Inverse variance weighted
−0.0075
−0.0050
−0.0025
0.0000
0.0025
MR effect size for
'SCARF1' on 'Bladder cancer'

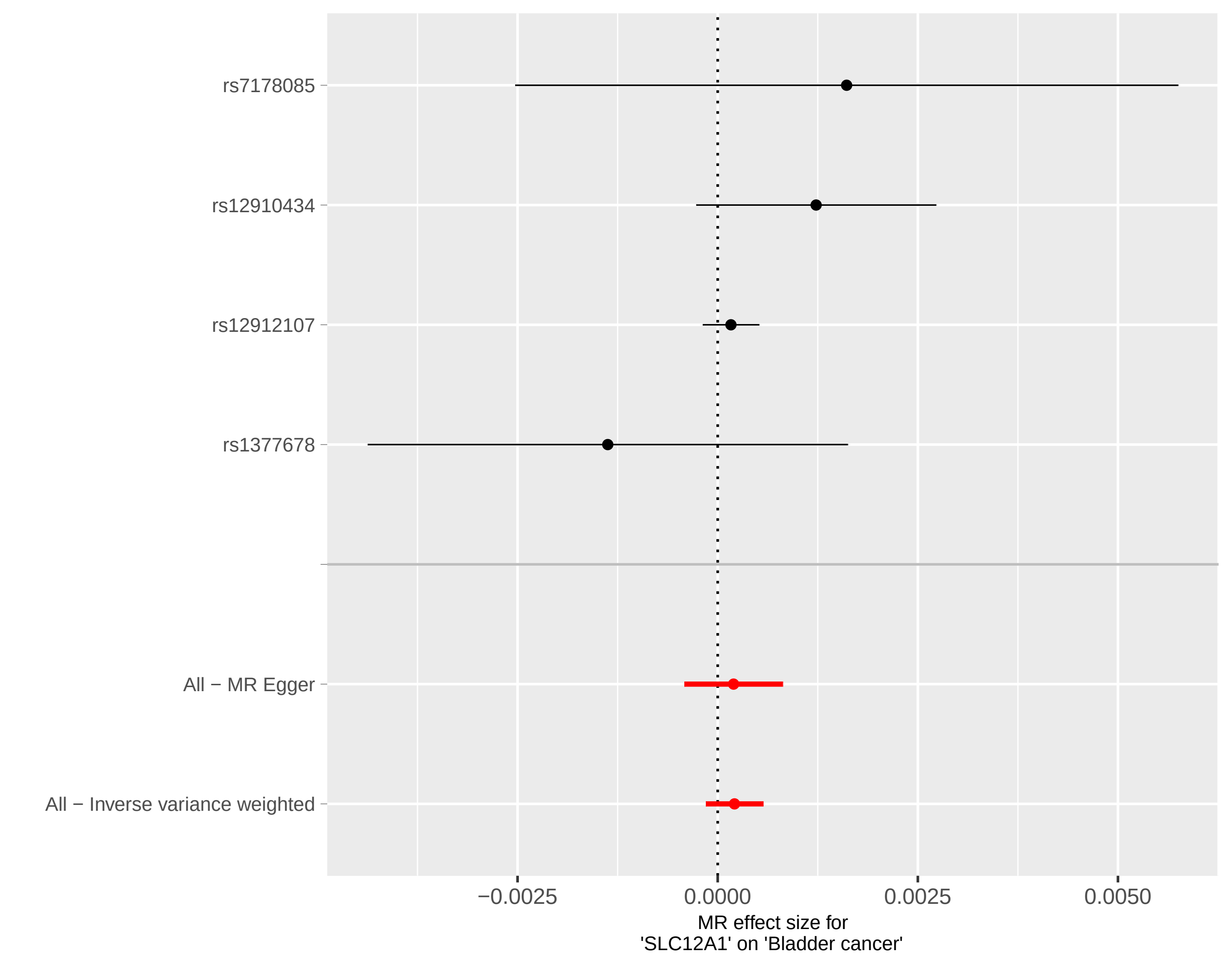

rs7178085
rs12910434
rs12912107
rs1377678
All − MR Egger
All − Inverse variance weighted
−0.0025
0.0000
0.0025
0.0050
MR effect size for
'SLC12A1' on 'Bladder cancer'

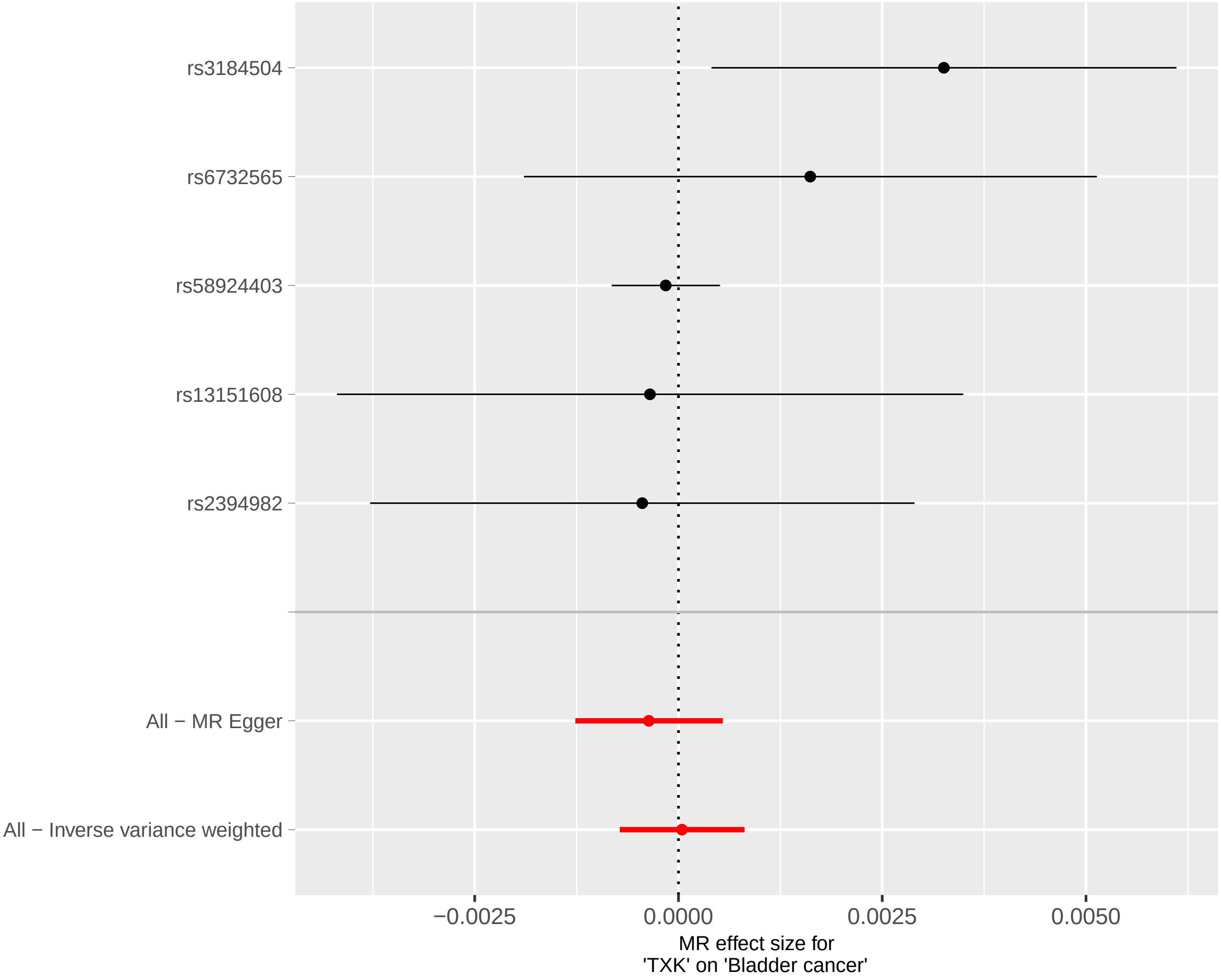
rs3184504
rs6732565
rs58924403
rs13151608
rs2394982
All − MR Egger
All − Inverse variance weighted
−0.0025
0.0000
0.0025
0.0050
MR effect size for
'TXK' on 'Bladder cancer'

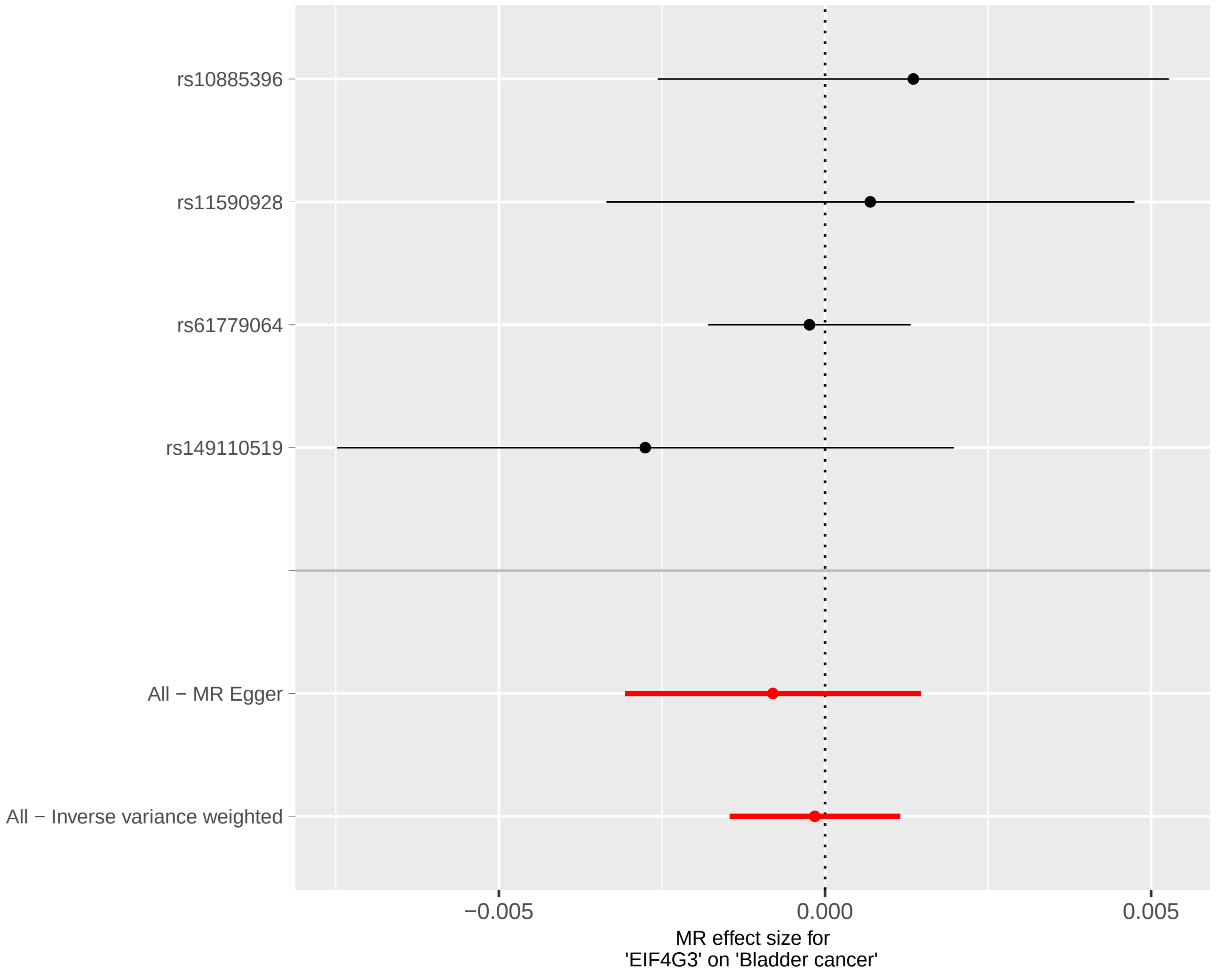

rs10885396
rs11590928
rs61779064
rs149110519
All − MR Egger
All − Inverse variance weighted
−0.005
0.000
0.005
MR effect size for
'EIF4G3' on 'Bladder cancer'

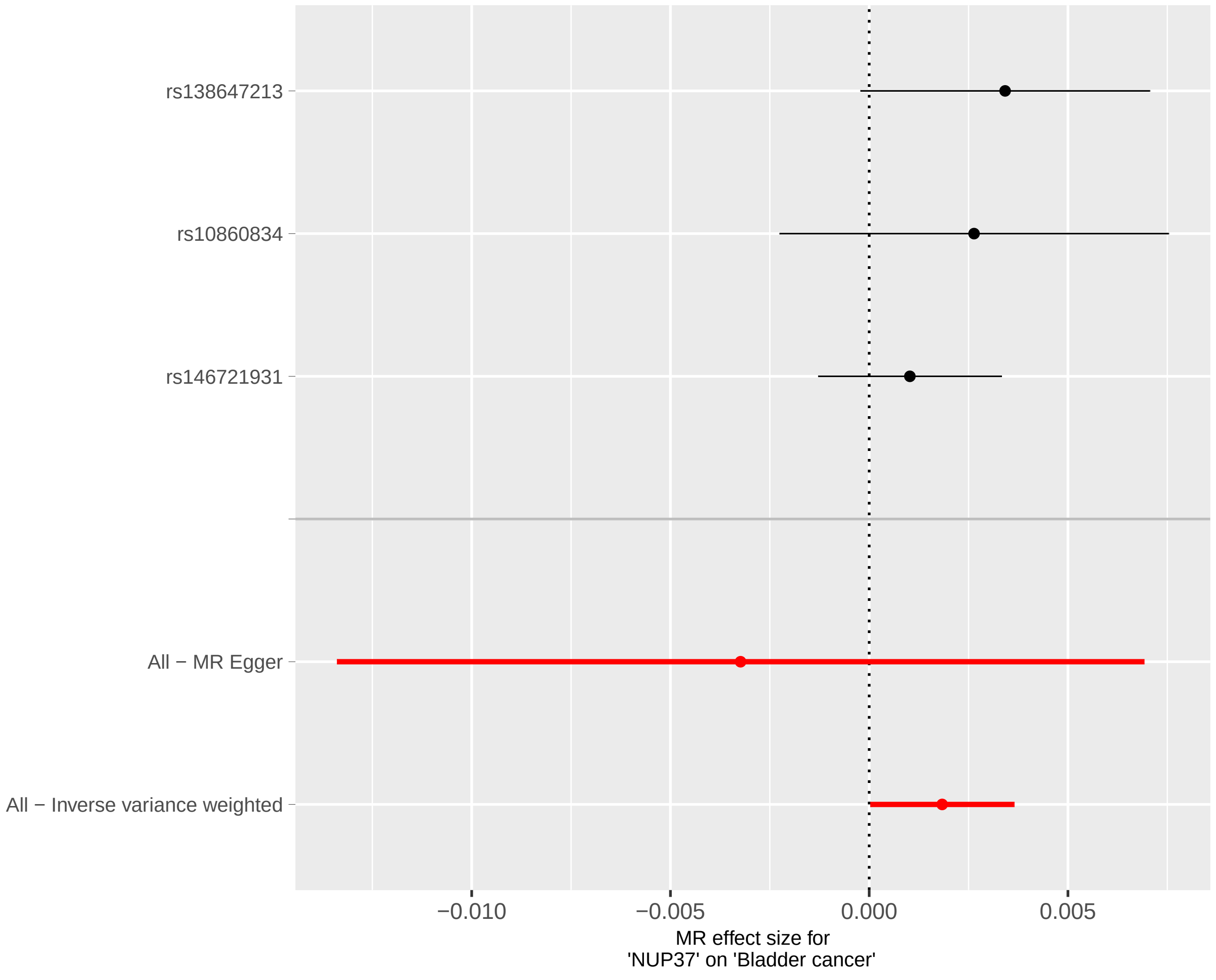
rs138647213
rs10860834
rs146721931
All − MR Egger
All − Inverse variance weighted
−0.010
−0.005
0.000
0.005
MR effect size for
'NUP37' on 'Bladder cancer'

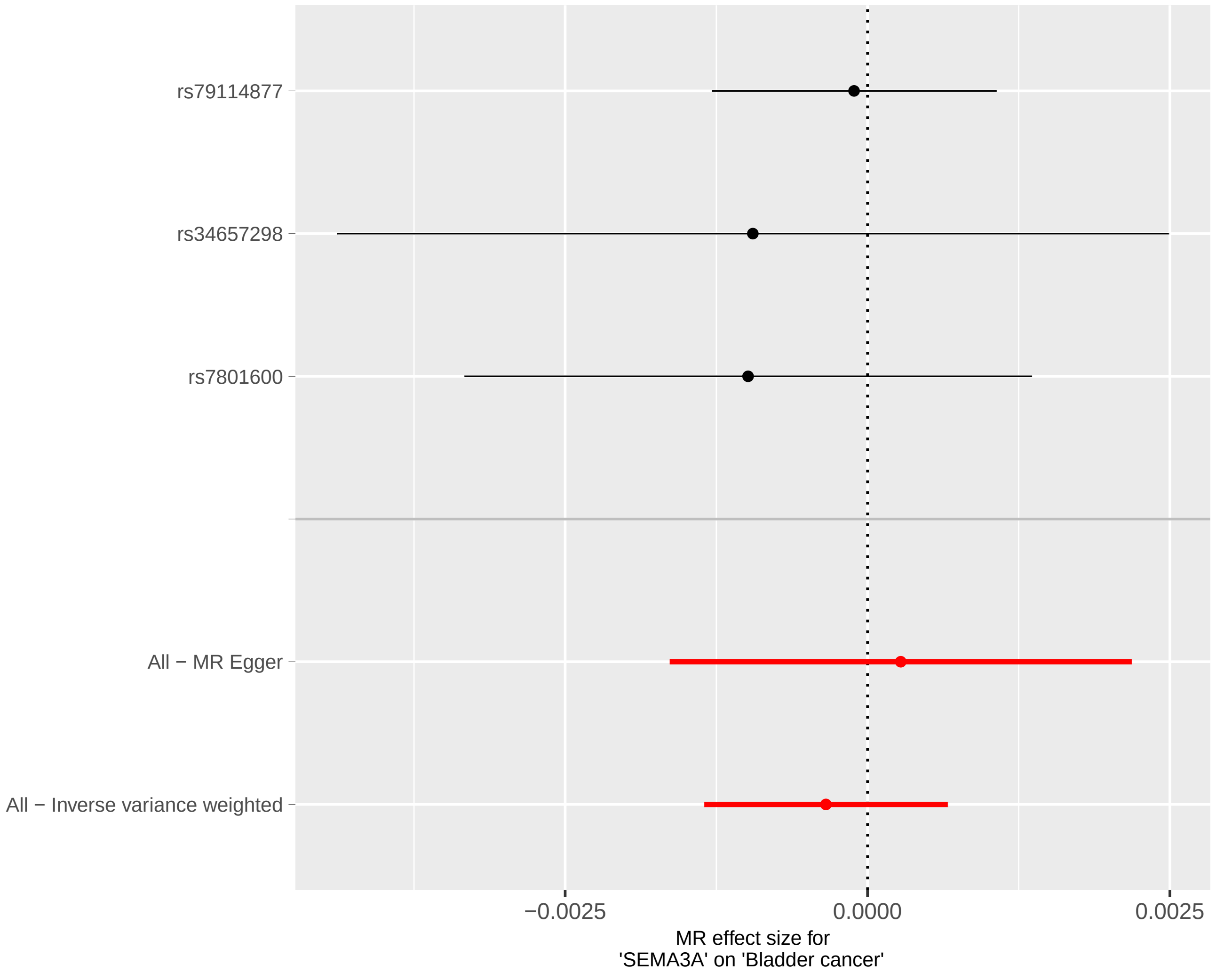
rs79114877
rs34657298
rs7801600
All − MR Egger
All − Inverse variance weighted
−0.0025
0.0000
0.0025
MR effect size for
'SEMA3A' on 'Bladder cancer'

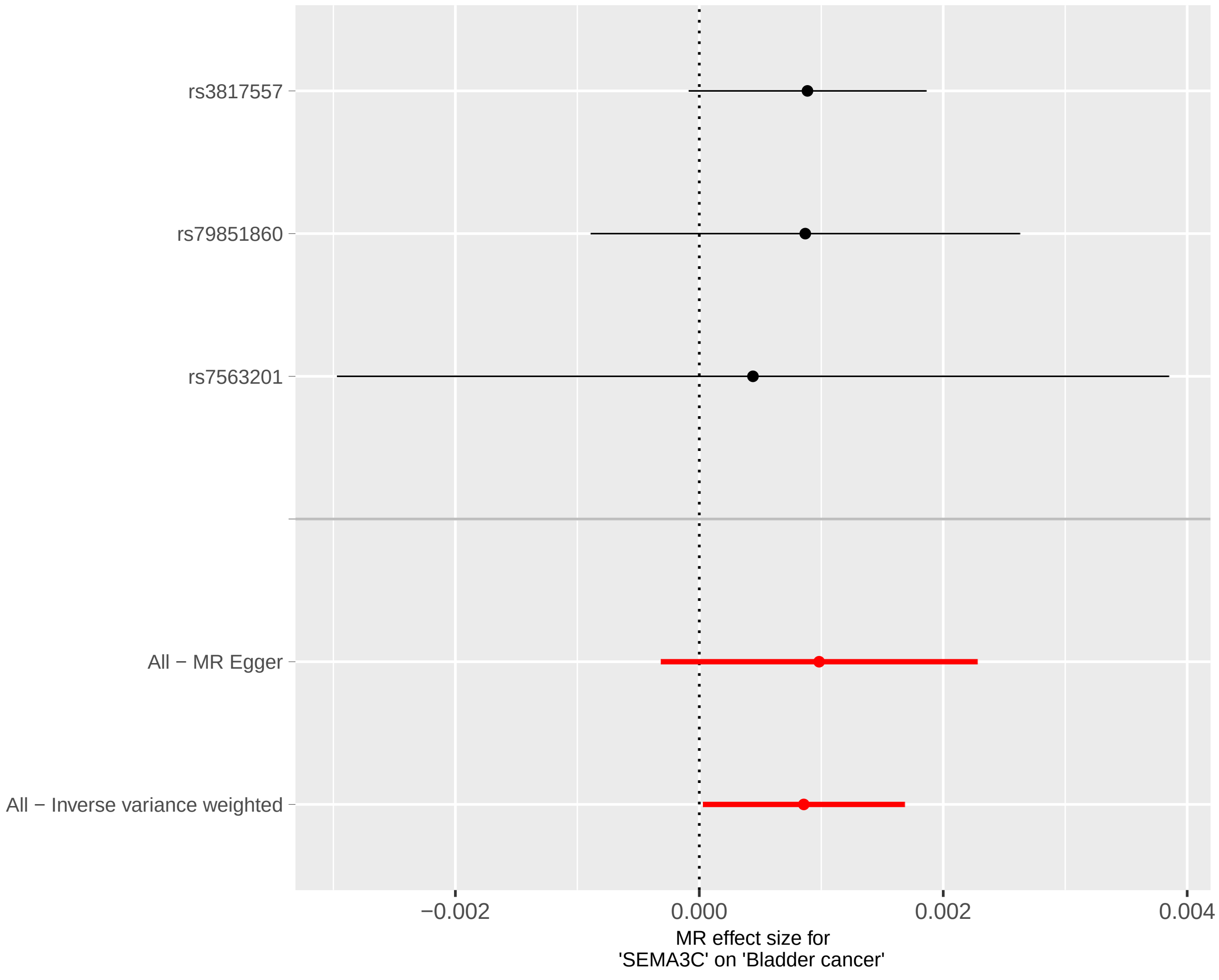

rs3817557
rs79851860
rs7563201
All − MR Egger
All − Inverse variance weighted
−0.002
0.000
0.002
0.004
MR effect size for
'SEMA3C' on 'Bladder cancer'

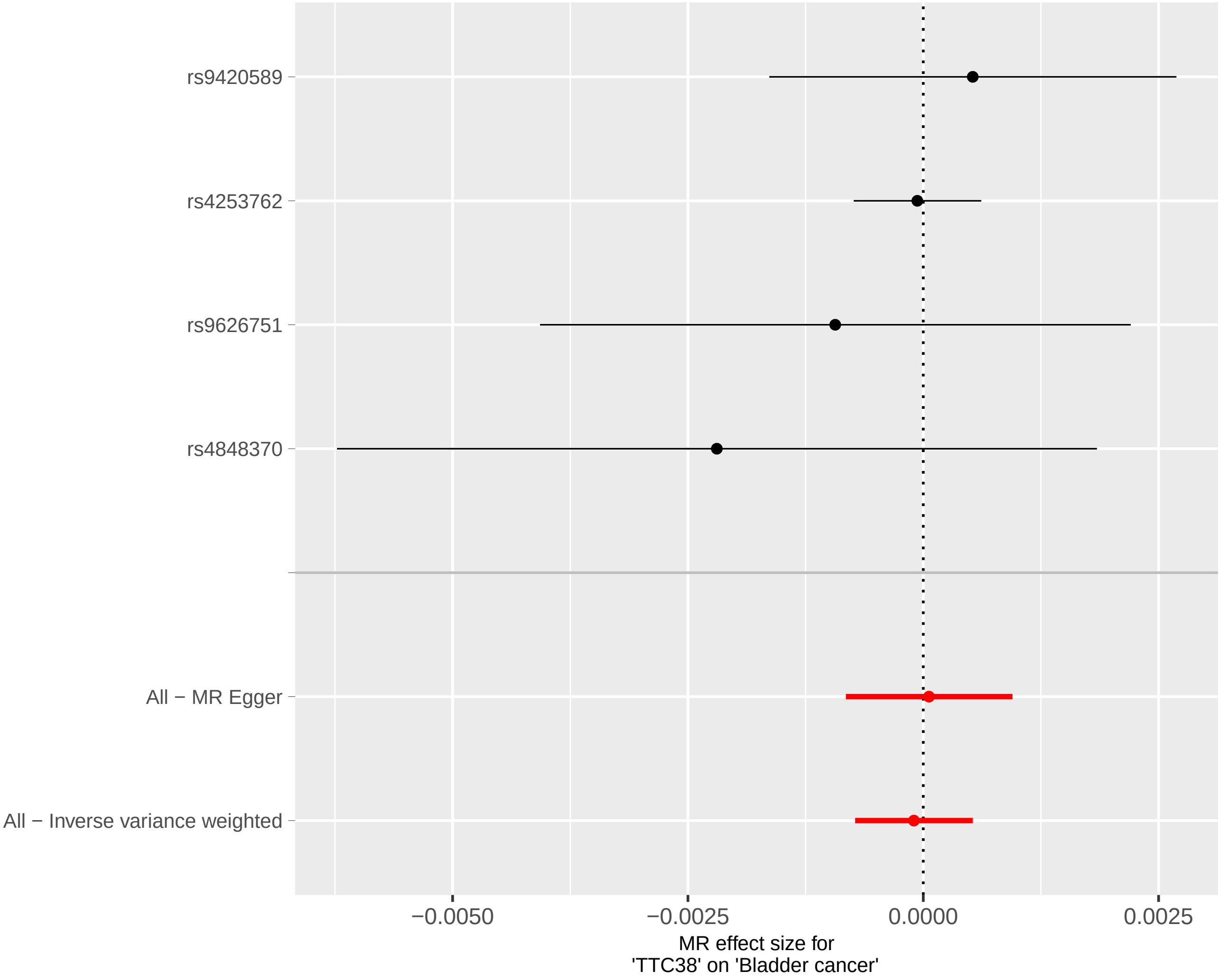
rs9420589
rs4253762
rs9626751
rs4848370
All − MR Egger
All − Inverse variance weighted
−0.0050
−0.0025
0.0000
0.0025
MR effect size for
'TTC38' on 'Bladder cancer'

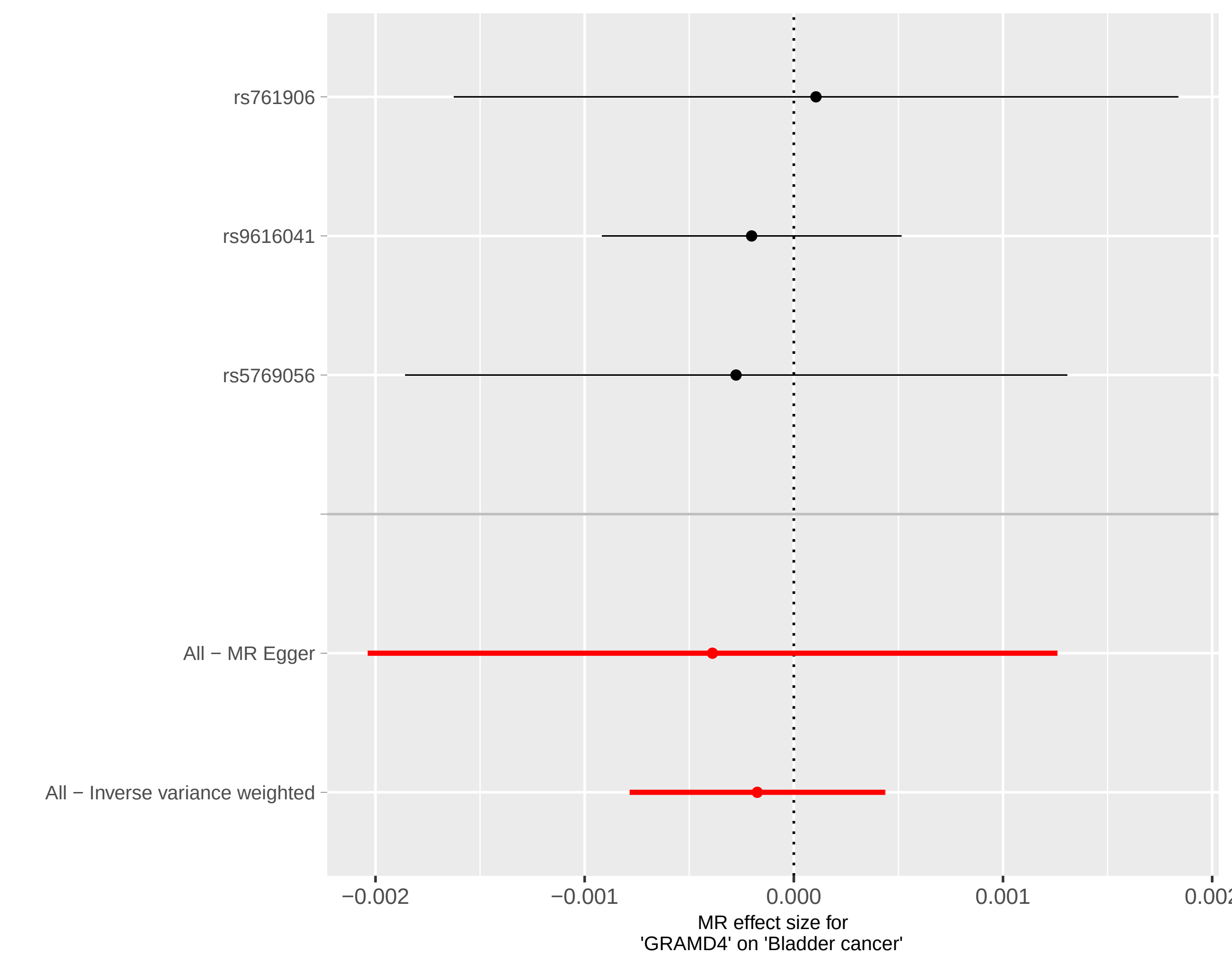

rs761906
rs9616041
rs5769056
All − MR Egger
All − Inverse variance weighted
−0.002
−0.001
0.000
0.001
0.002
MR effect size for
'GRAMD4' on 'Bladder cancer'

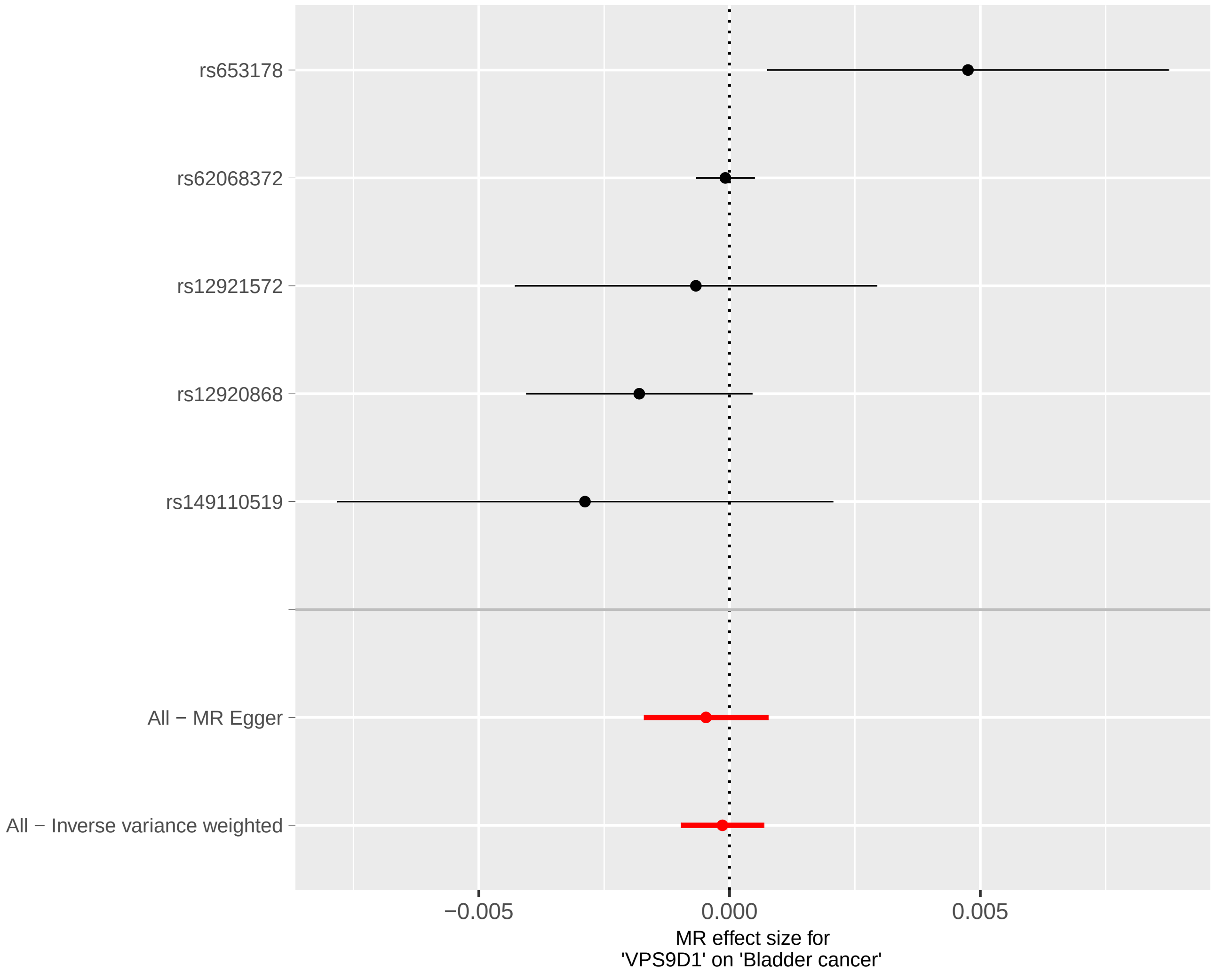
rs653178
rs62068372
rs12921572
rs12920868
rs149110519
All − MR Egger
All − Inverse variance weighted
−0.005
0.000
0.005
MR effect size for
'VPS9D1' on 'Bladder cancer'

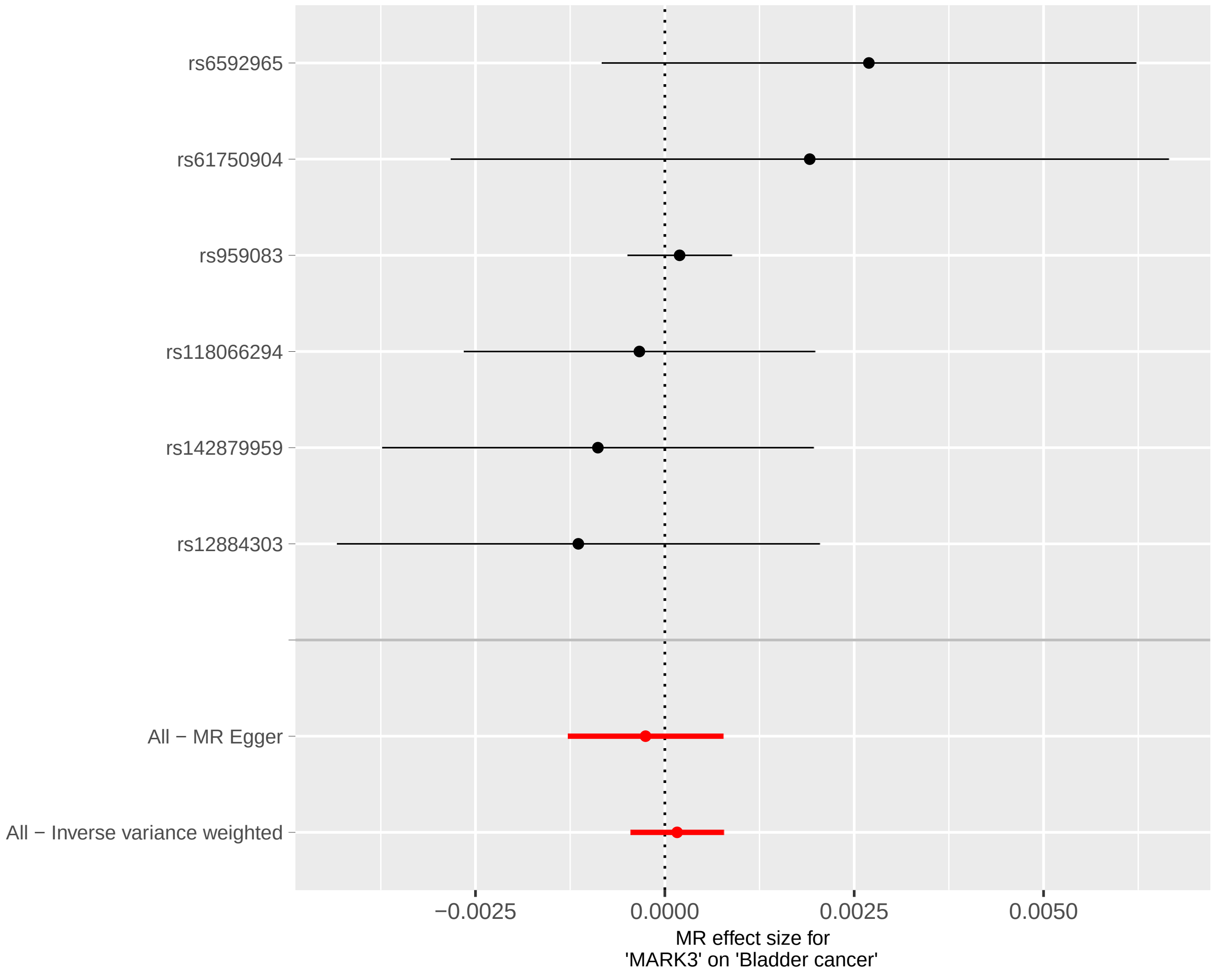
rs6592965
rs61750904
rs959083
rs118066294
rs142879959
rs12884303
All − MR Egger
All − Inverse variance weighted
−0.0025
0.0000
0.0025
0.0050
MR effect size for
'MARK3' on 'Bladder cancer'

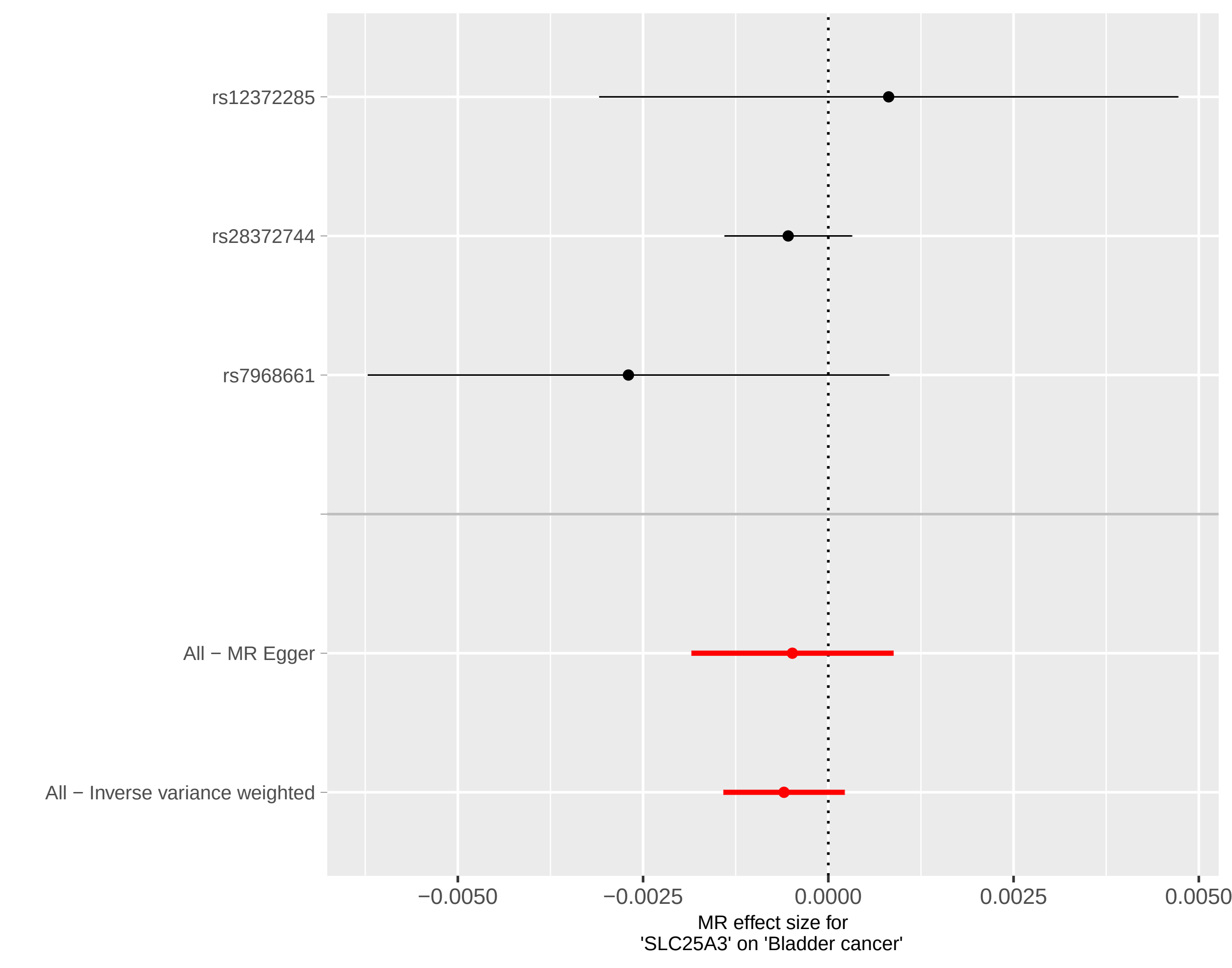
rs12372285
rs28372744
rs7968661
All − MR Egger
All − Inverse variance weighted
−0.0050
−0.0025
0.0000
0.0025
0.0050
MR effect size for
'SLC25A3' on 'Bladder cancer'

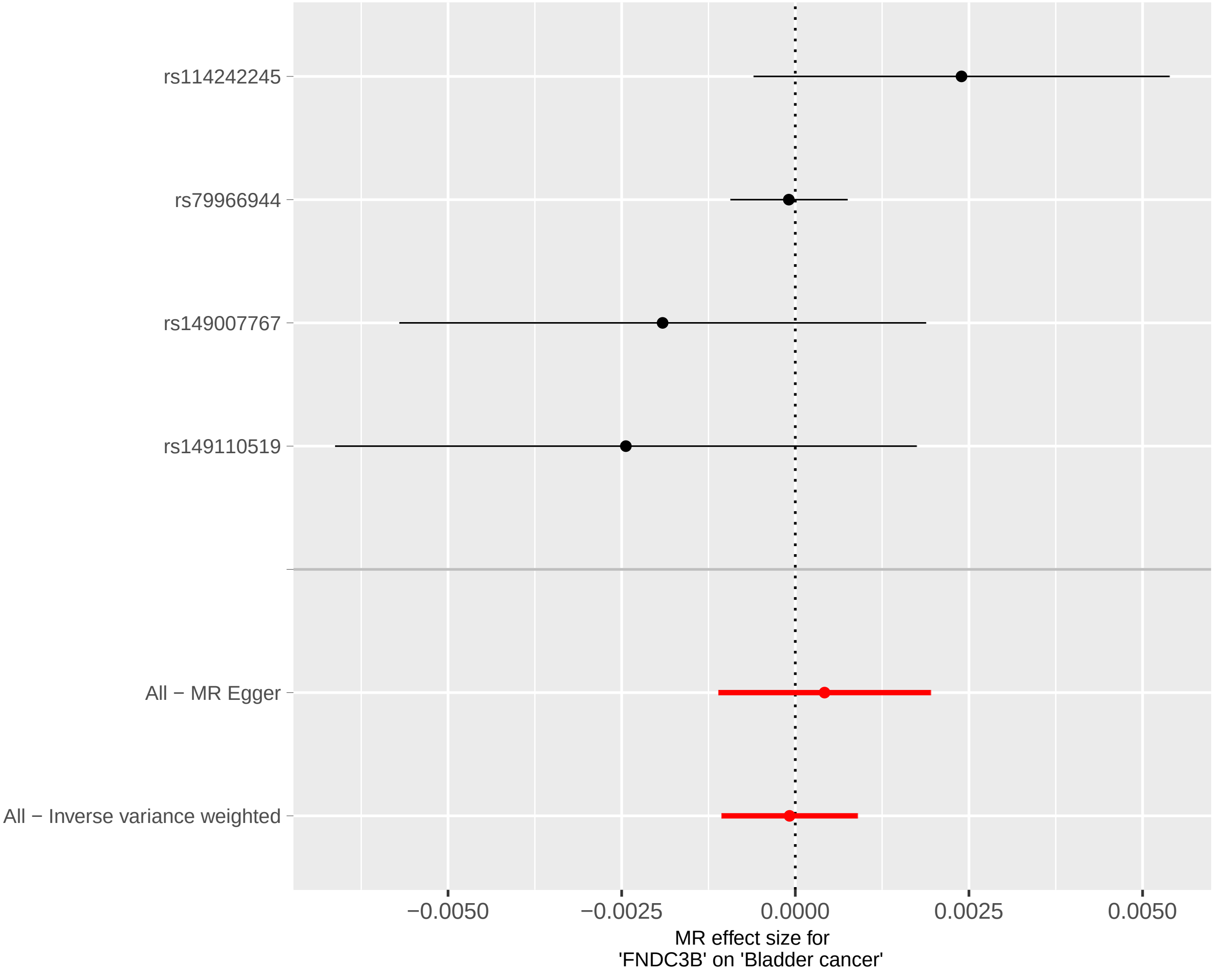
rs114242245
rs79966944
rs149007767
rs149110519
All − MR Egger
All − Inverse variance weighted
−0.0050
−0.0025
0.0000
0.0025
0.0050
MR effect size for
'FNDC3B' on 'Bladder cancer'

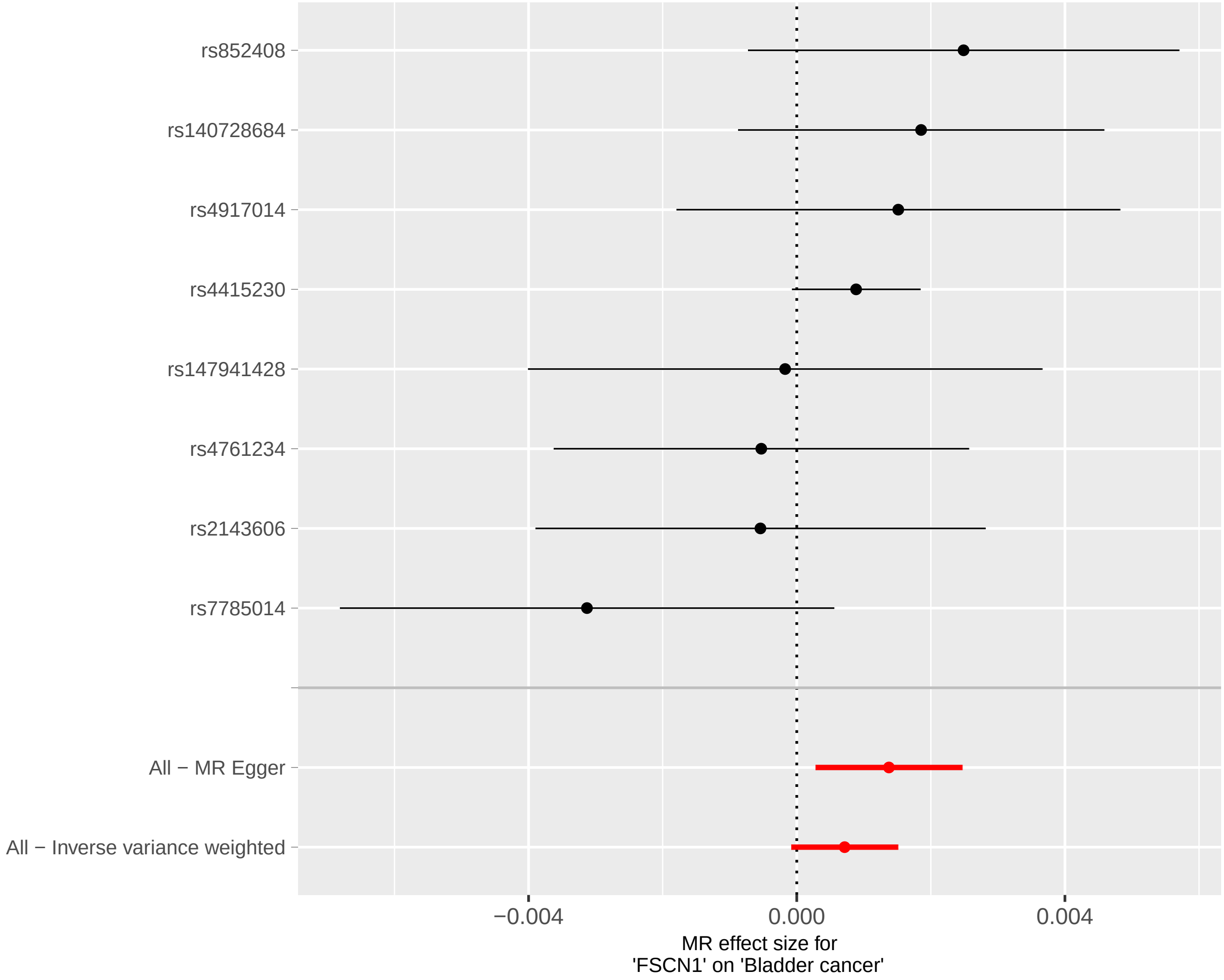
rs852408
rs140728684
rs4917014
rs4415230
rs147941428
rs4761234
rs2143606
rs7785014
All − MR Egger
All − Inverse variance weighted
−0.004
0.000
0.004
MR effect size for
'FSCN1' on 'Bladder cancer'

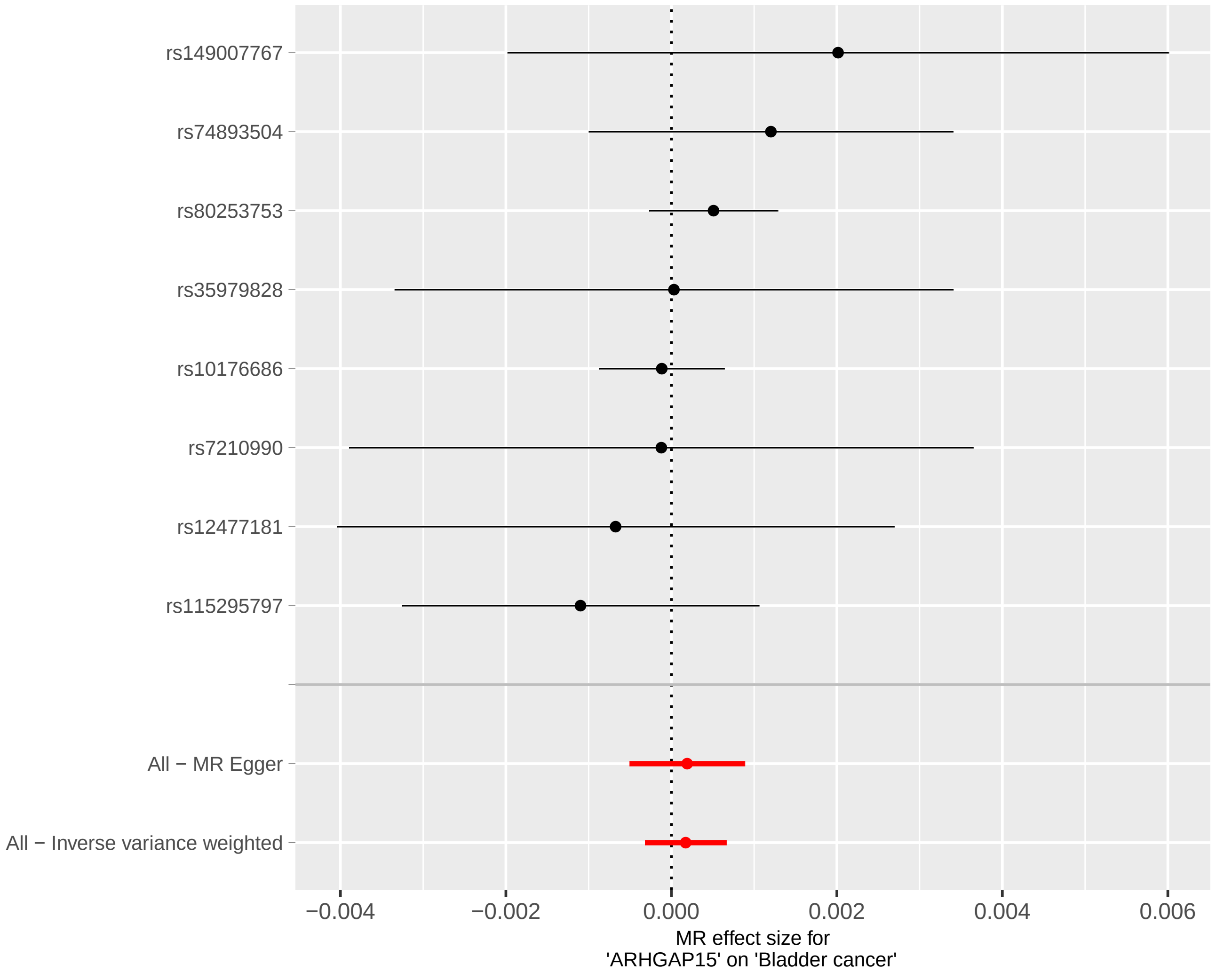

rs149007767
rs74893504
rs80253753
rs35979828
rs10176686
rs7210990
rs12477181
rs115295797
All − MR Egger
All − Inverse variance weighted
−0.004
−0.002
0.000
0.002
0.004
0.006
MR effect size for
'ARHGAP15' on 'Bladder cancer'

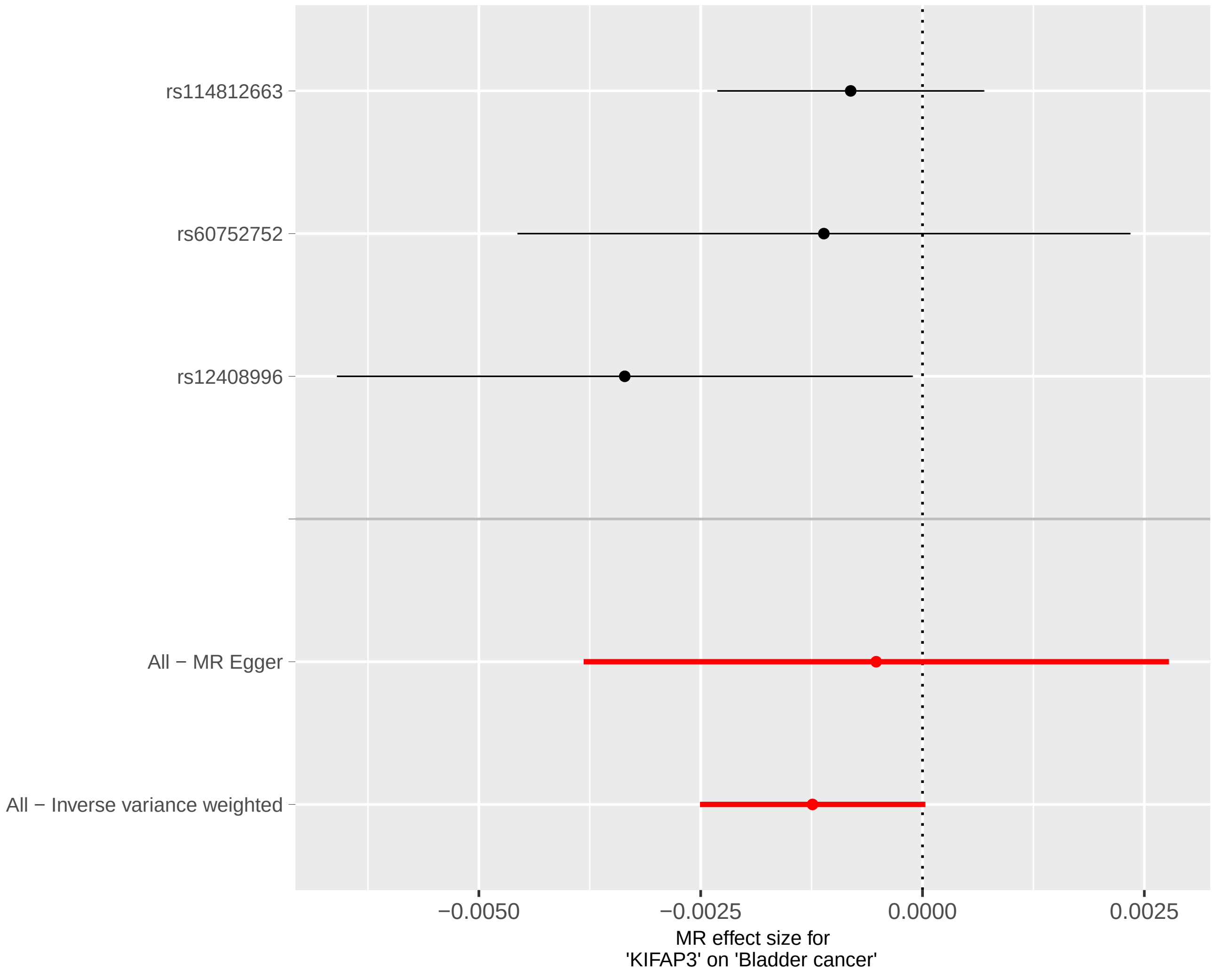
rs114812663
rs60752752
rs12408996
All − MR Egger
All − Inverse variance weighted
−0.0050
−0.0025
0.0000
0.0025
MR effect size for
'KIFAP3' on 'Bladder cancer'

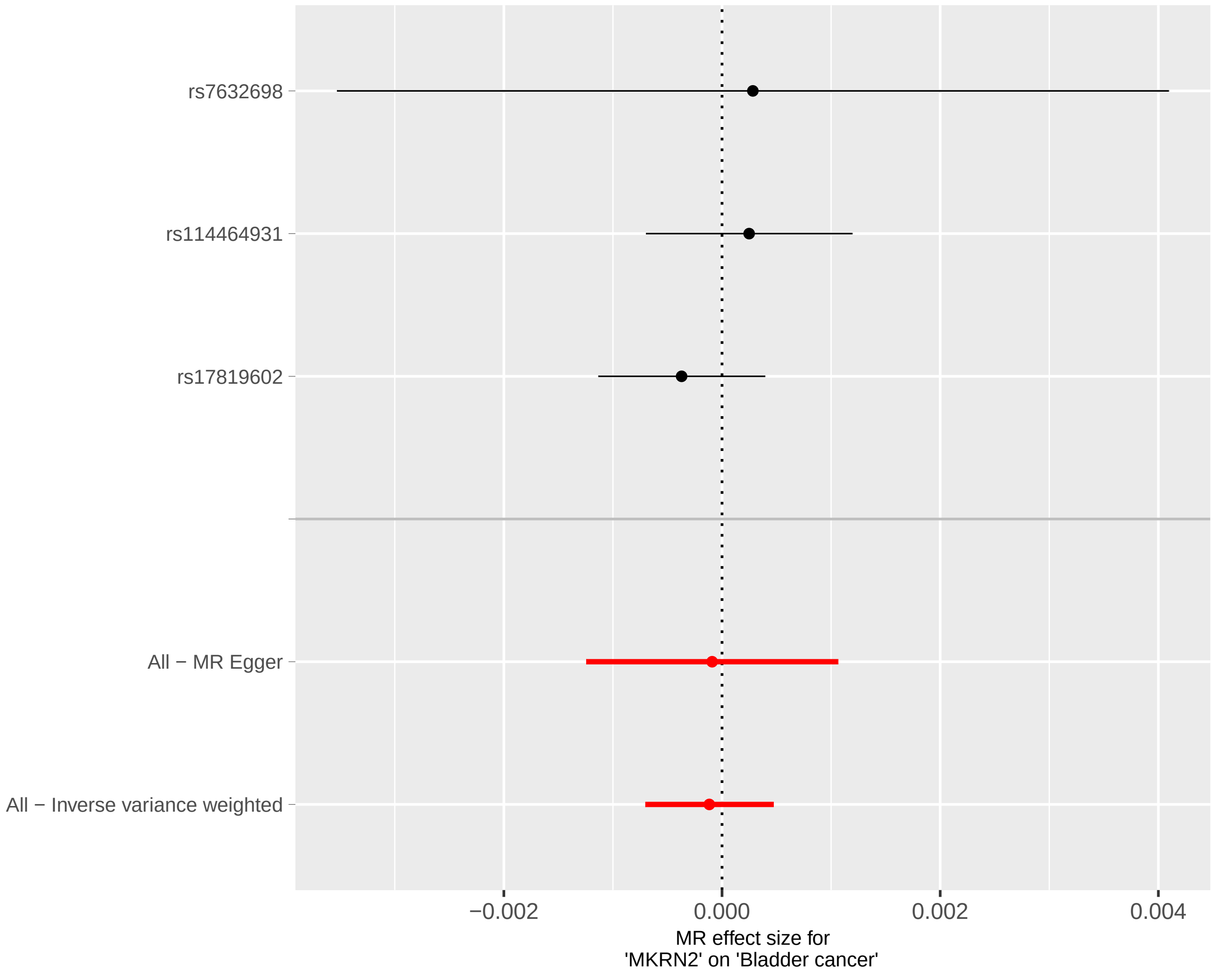
rs7632698
rs114464931
rs17819602
All − MR Egger
All − Inverse variance weighted
−0.002
0.000
0.002
0.004
MR effect size for
'MKRN2' on 'Bladder cancer'

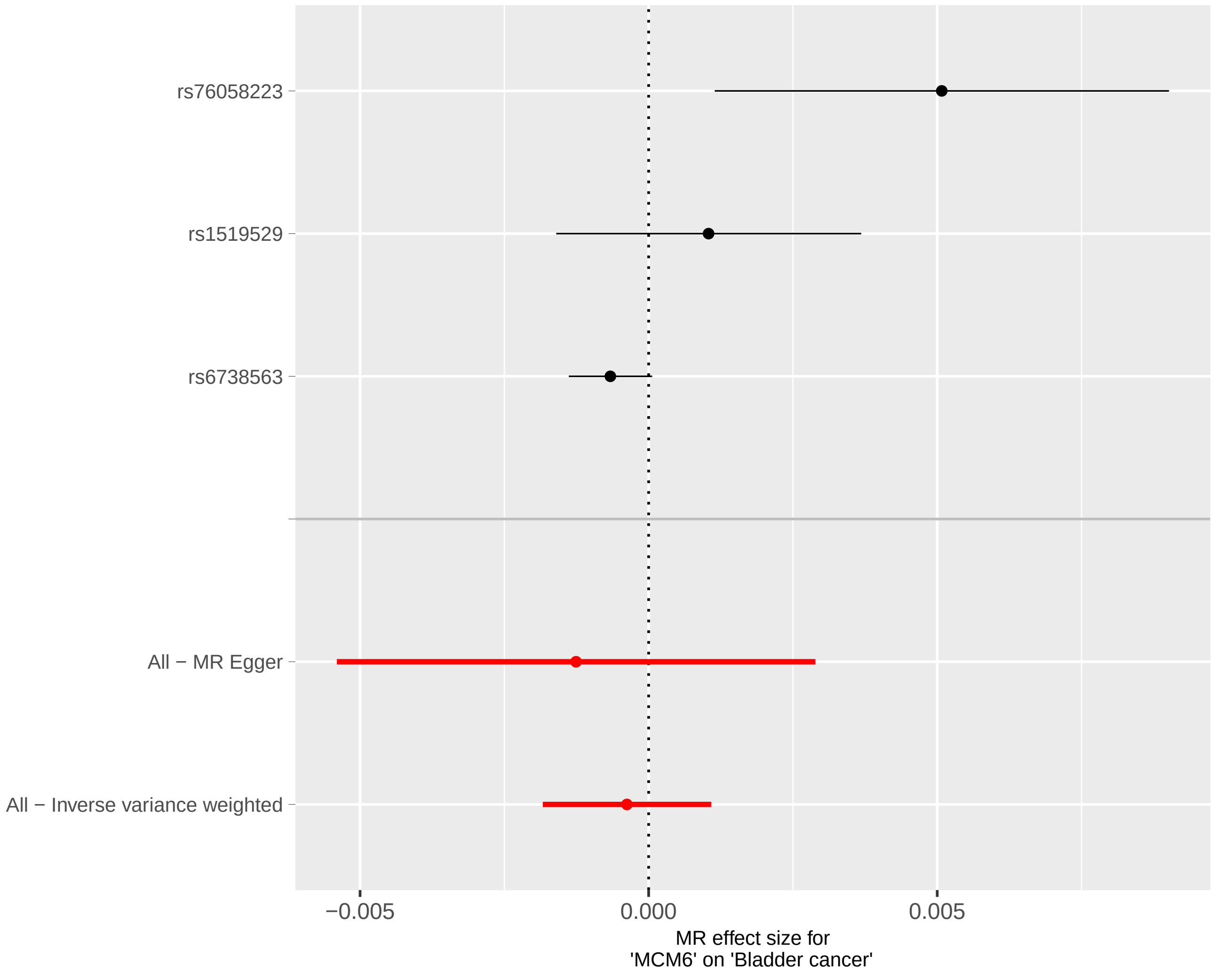

rs76058223
rs1519529
rs6738563
All − MR Egger
All − Inverse variance weighted
−0.005
0.000
0.005
MR effect size for
'MCM6' on 'Bladder cancer'

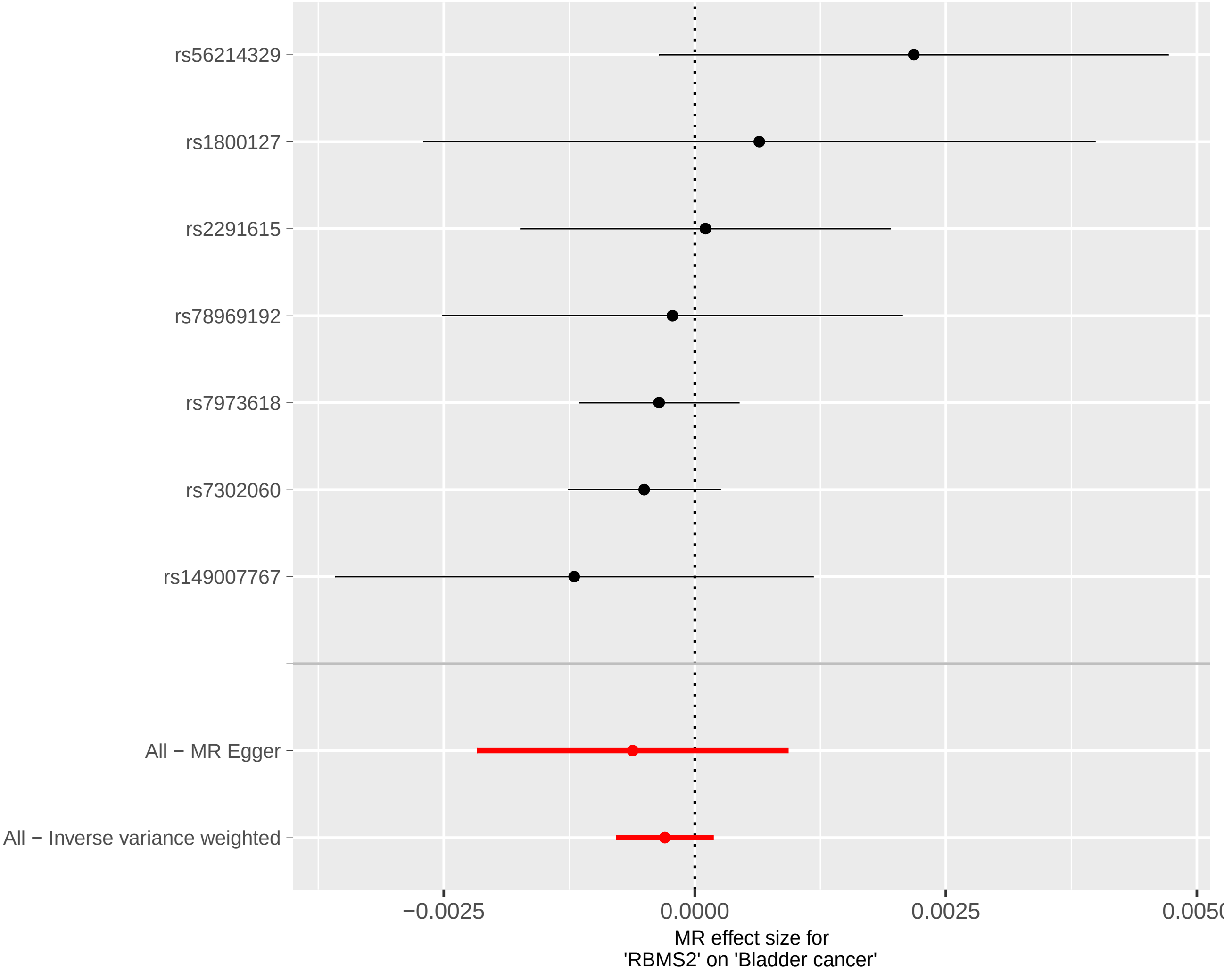

rs56214329
rs1800127
rs2291615
rs78969192
rs7973618
rs7302060
rs149007767
All − MR Egger
All − Inverse variance weighted
−0.0025
0.0000
0.0025
0.0050
MR effect size for
'RBMS2' on 'Bladder cancer'

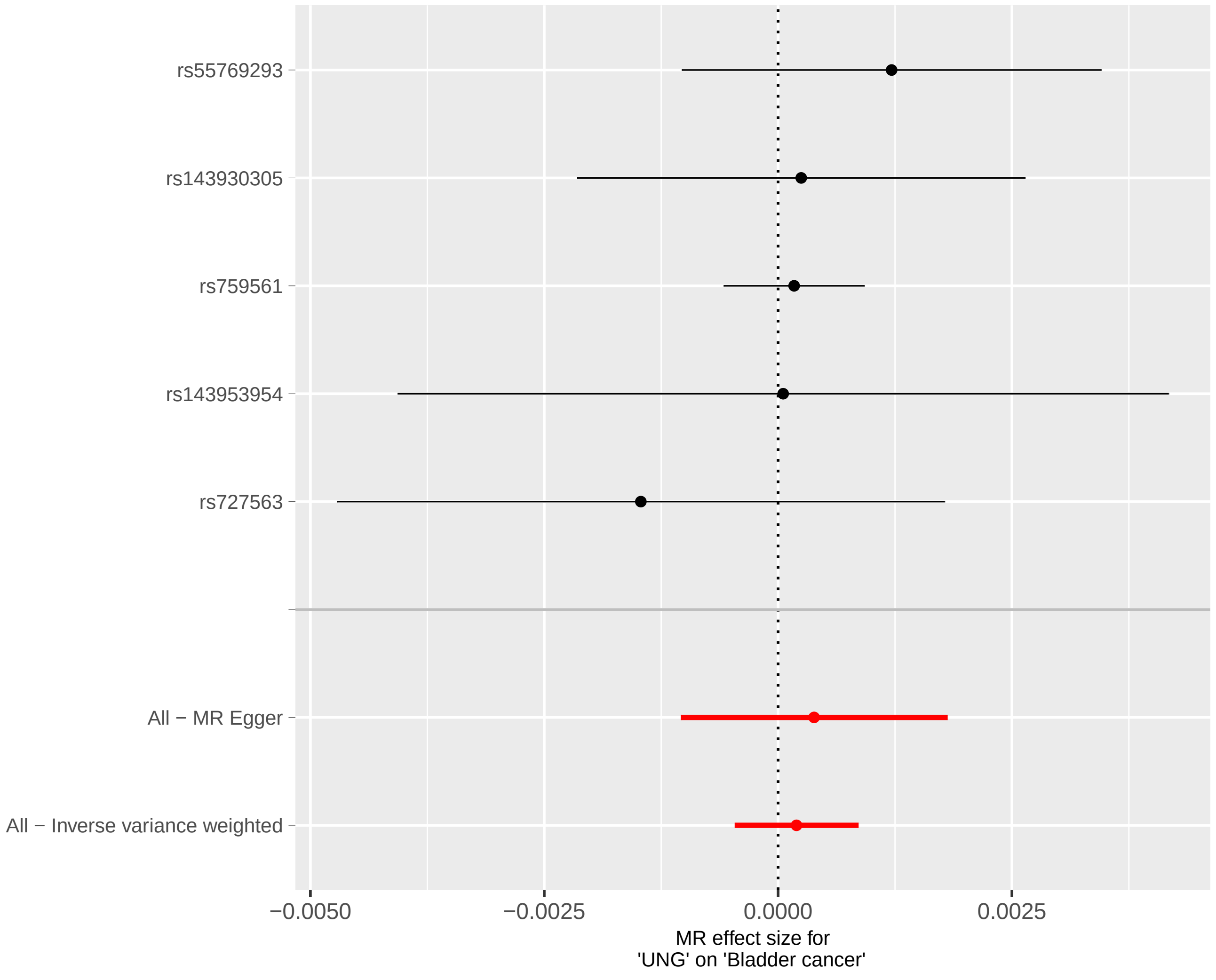
rs55769293
rs143930305
rs759561
rs143953954
rs727563
All − MR Egger
All − Inverse variance weighted
−0.0050
−0.0025
0.0000
0.0025
MR effect size for
'UNG' on 'Bladder cancer'

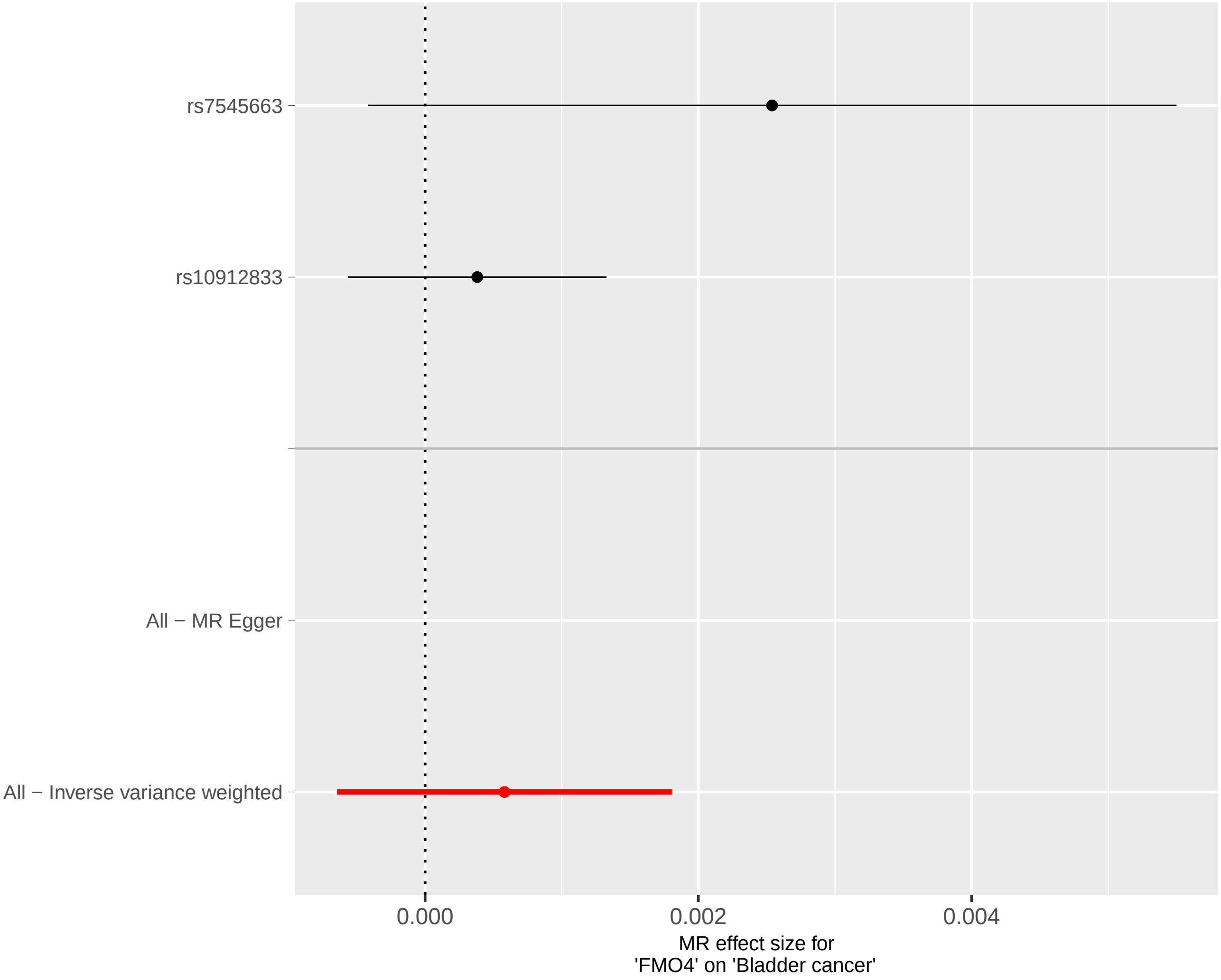

rs7545663
rs10912833
All − MR Egger
All − Inverse variance weighted
0.000
0.002
0.004
MR effect size for
'FMO4' on 'Bladder cancer'

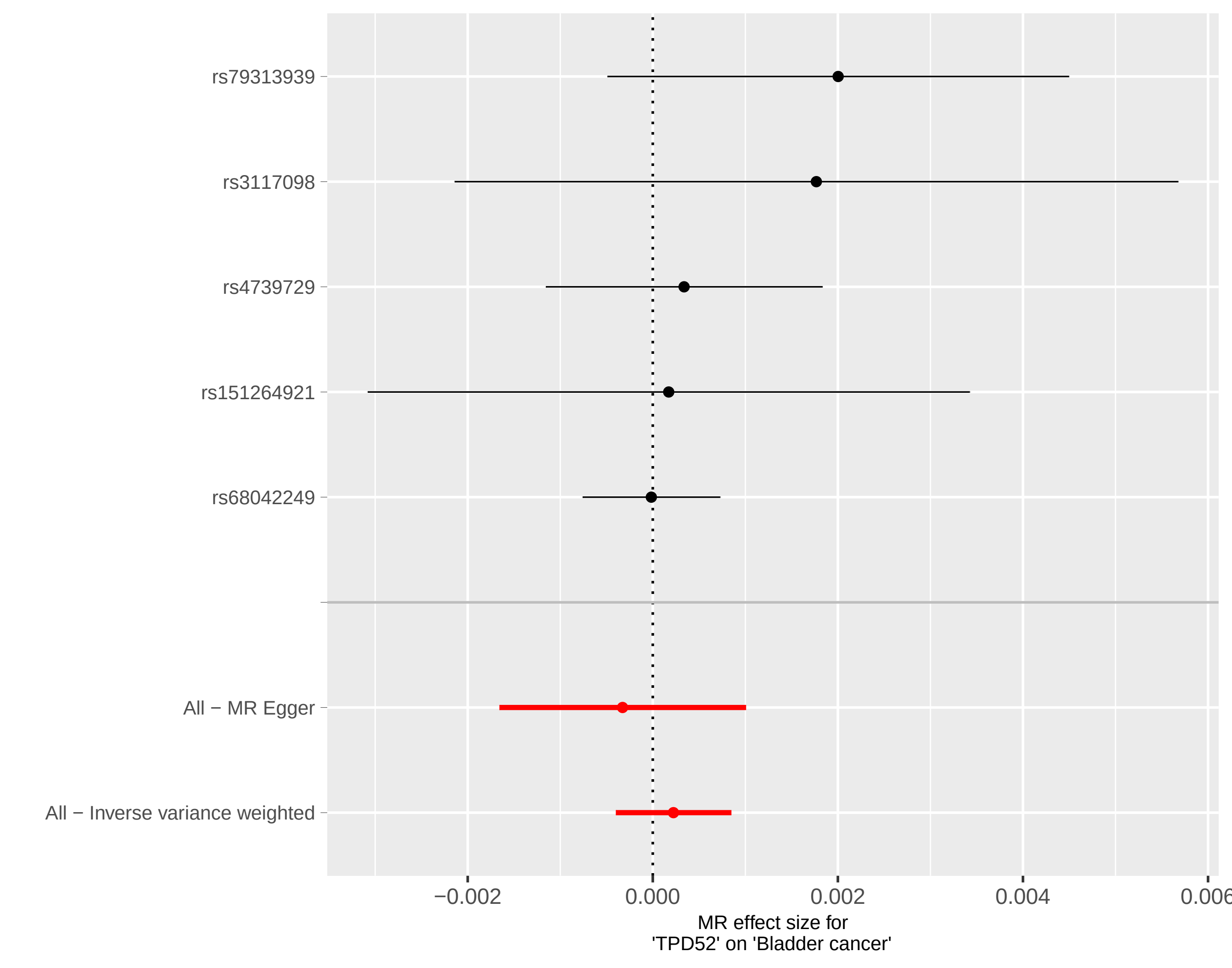

rs79313939
rs3117098
rs4739729
rs151264921
rs68042249
All − MR Egger
All − Inverse variance weighted
−0.002
0.000
0.002
0.004
0.006
MR effect size for
'TPD52' on 'Bladder cancer'

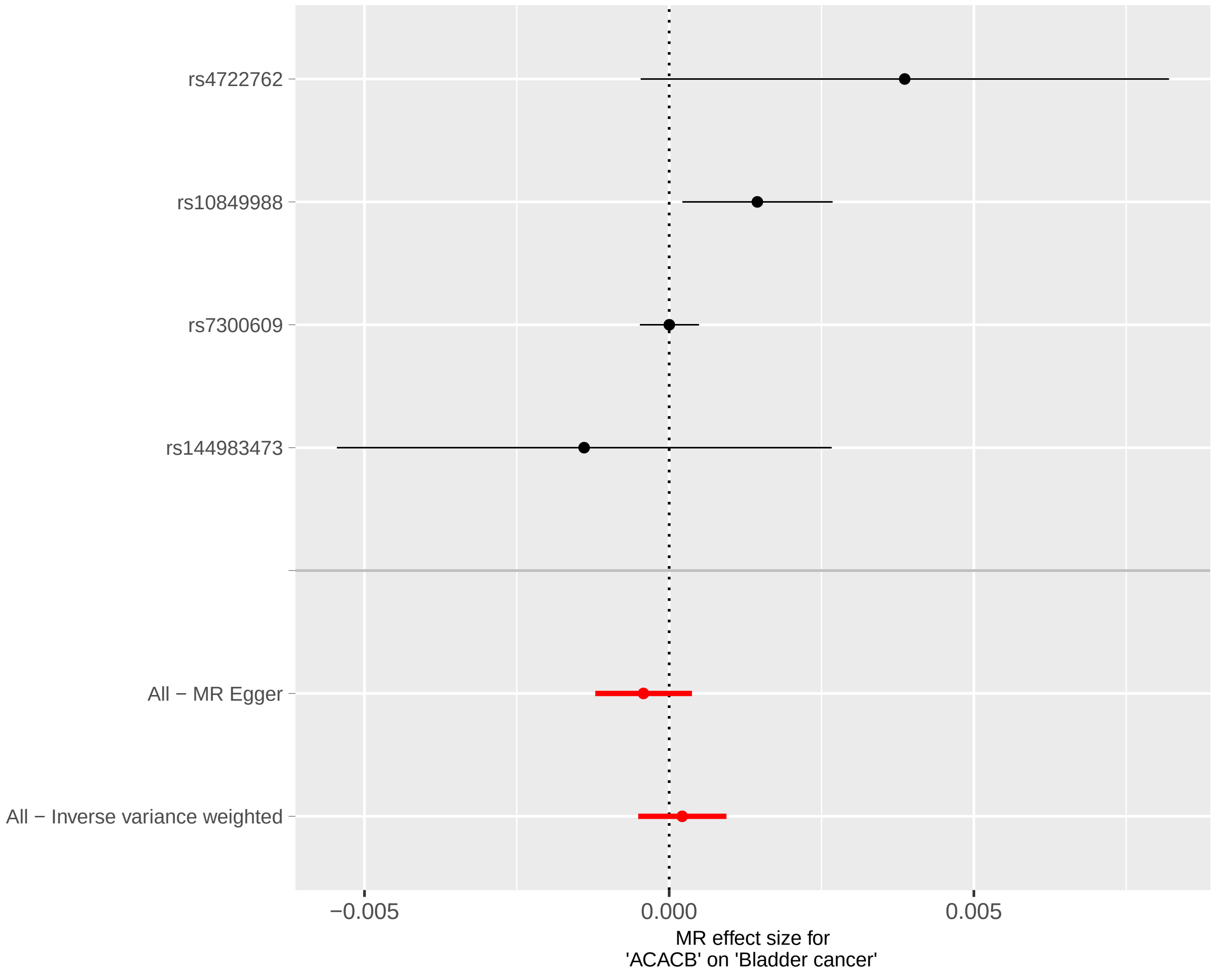
rs4722762
rs10849988
rs7300609
rs144983473
All − MR Egger
All − Inverse variance weighted
−0.005
0.000
0.005
MR effect size for
'ACACB' on 'Bladder cancer'

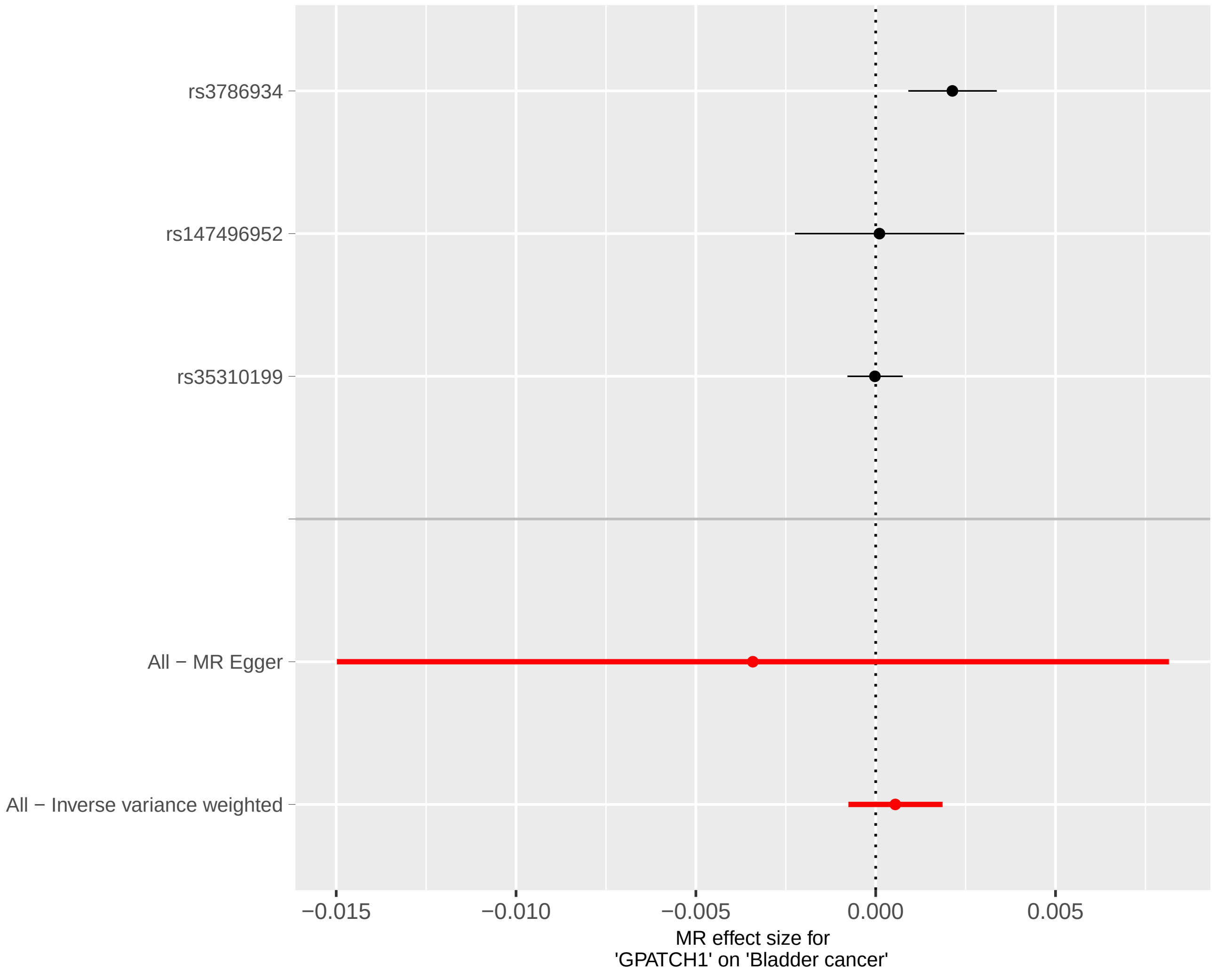
rs3786934
rs147496952
rs35310199
All − MR Egger
All − Inverse variance weighted
−0.015
−0.010
−0.005
0.000
0.005
MR effect size for
'GPATCH1' on 'Bladder cancer'

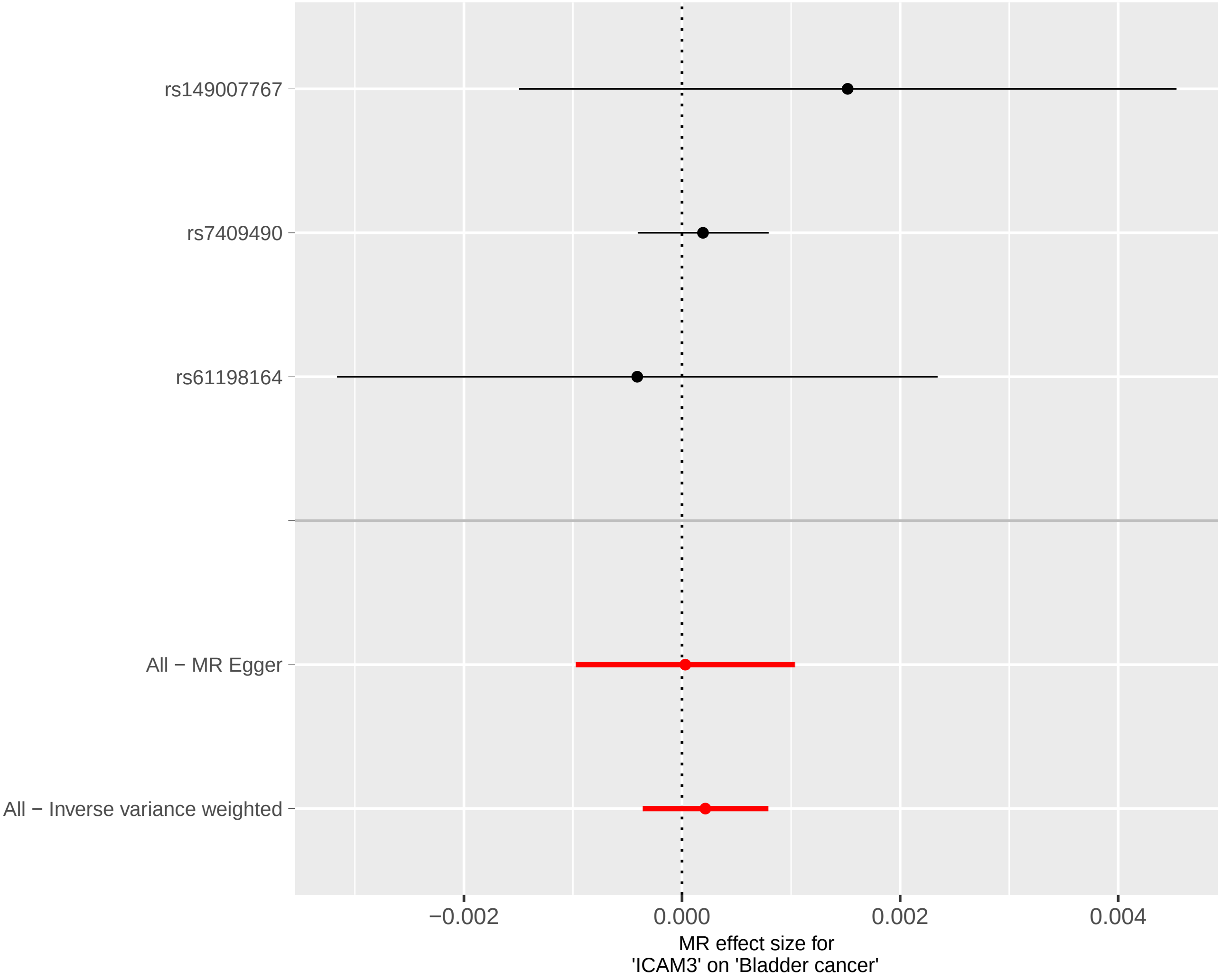
rs149007767
rs7409490
rs61198164
All − MR Egger
All − Inverse variance weighted
−0.002
0.000
0.002
0.004
MR effect size for
'ICAM3' on 'Bladder cancer'

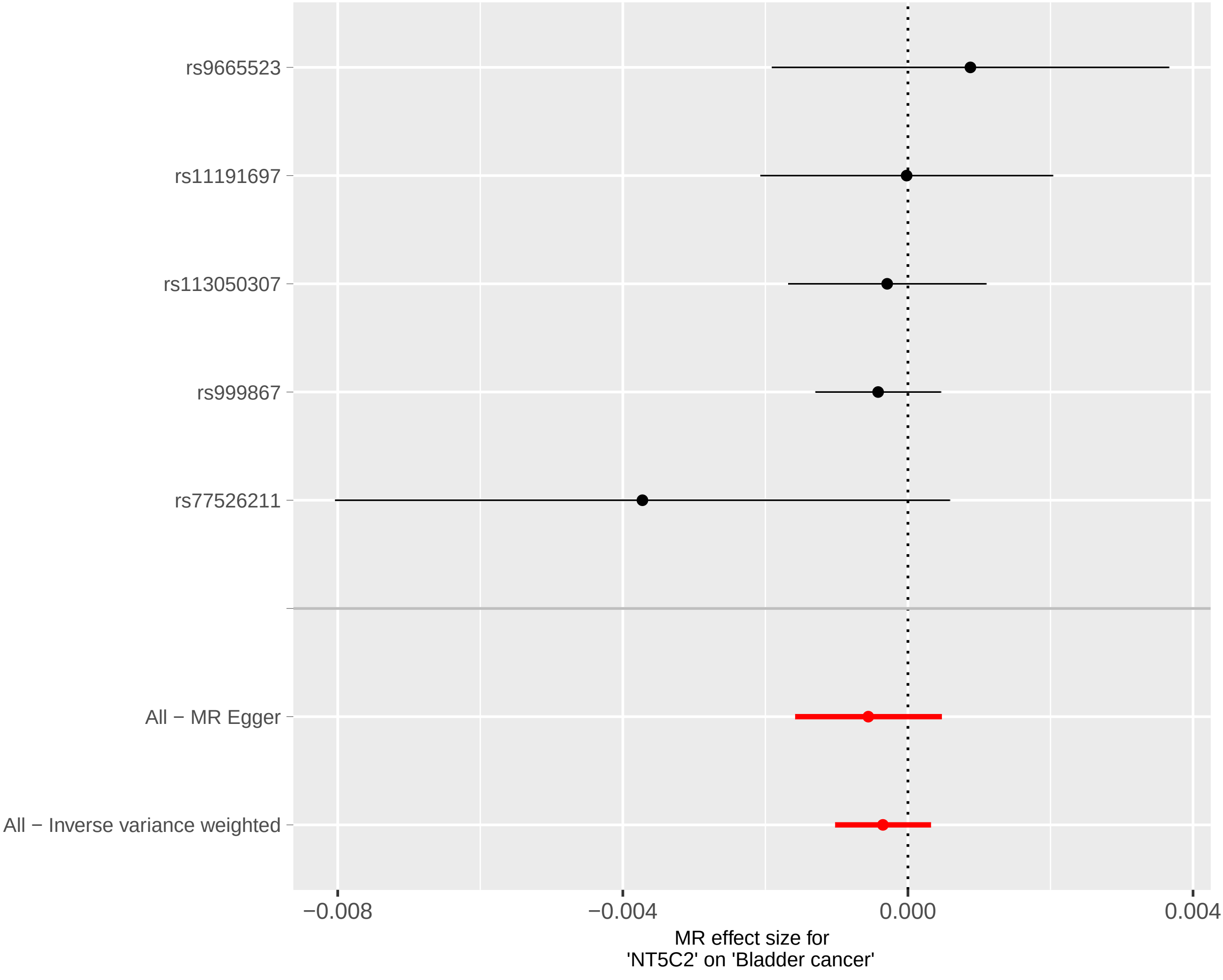

rs9665523
rs11191697
rs113050307
rs999867
rs77526211
All − MR Egger
All − Inverse variance weighted
−0.008
−0.004
0.000
0.004
MR effect size for
'NT5C2' on 'Bladder cancer'

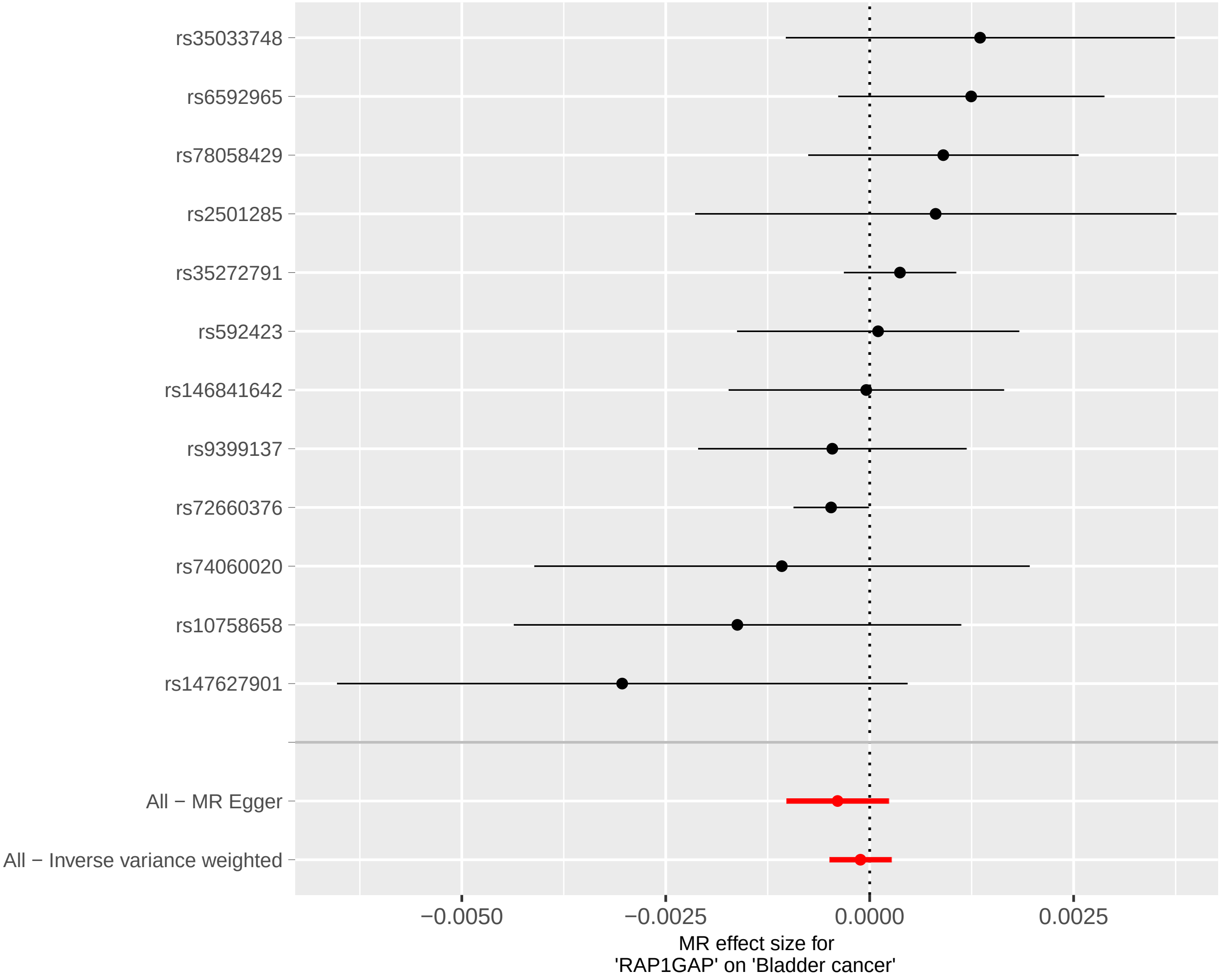
rs35033748
rs6592965
rs78058429
rs2501285
rs35272791
rs592423
rs146841642
rs9399137
rs72660376
rs74060020
rs10758658
rs147627901
All − MR Egger
All − Inverse variance weighted
−0.0050
−0.0025
0.0000
0.0025
MR effect size for
'RAP1GAP' on 'Bladder cancer'

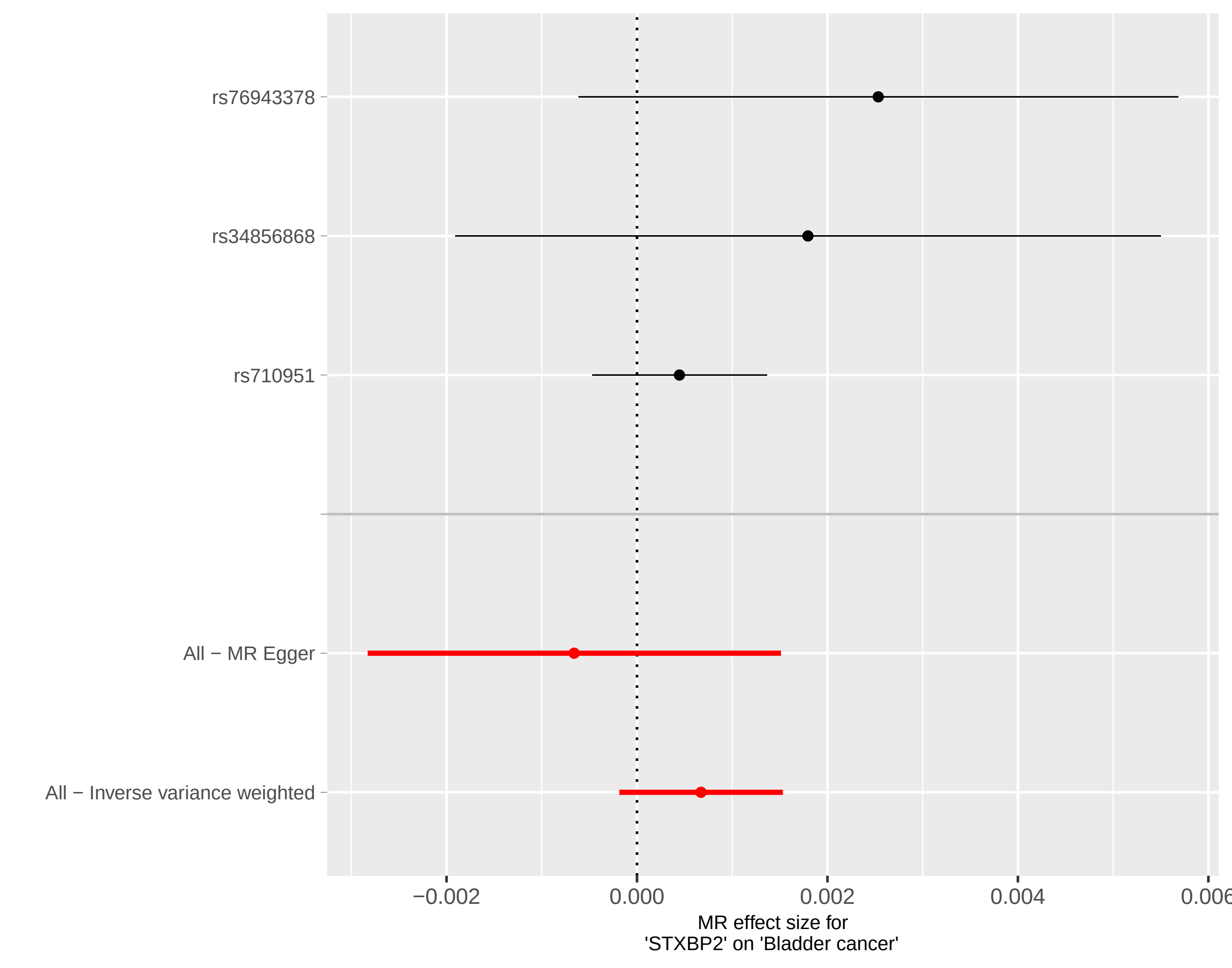
rs76943378
rs34856868
rs710951
All − MR Egger
All − Inverse variance weighted
−0.002
0.000
0.002
0.004
0.006
MR effect size for
'STXBP2' on 'Bladder cancer'

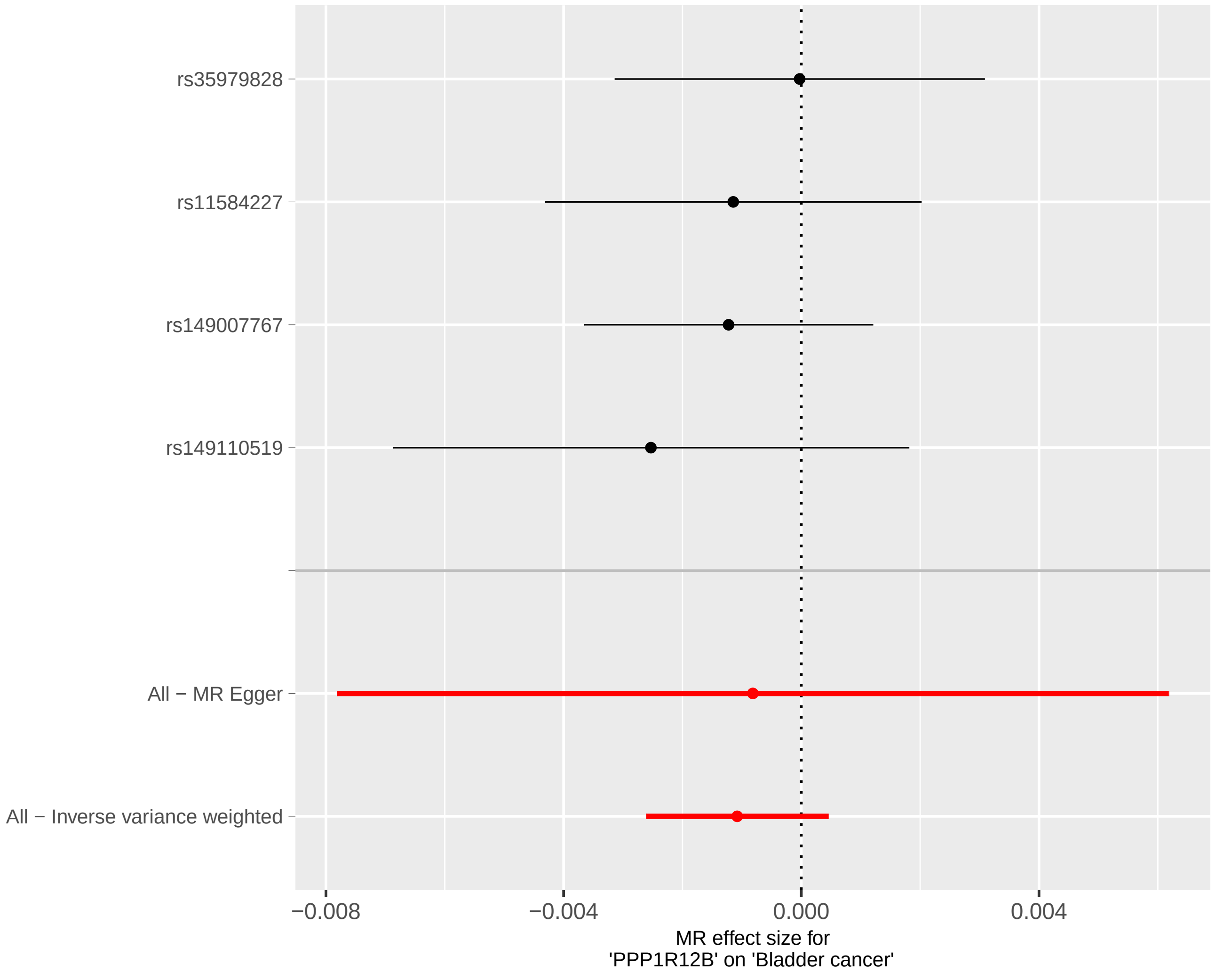

rs35979828
rs11584227
rs149007767
rs149110519
All − MR Egger
All − Inverse variance weighted
−0.008
−0.004
0.000
0.004
MR effect size for
'PPP1R12B' on 'Bladder cancer'

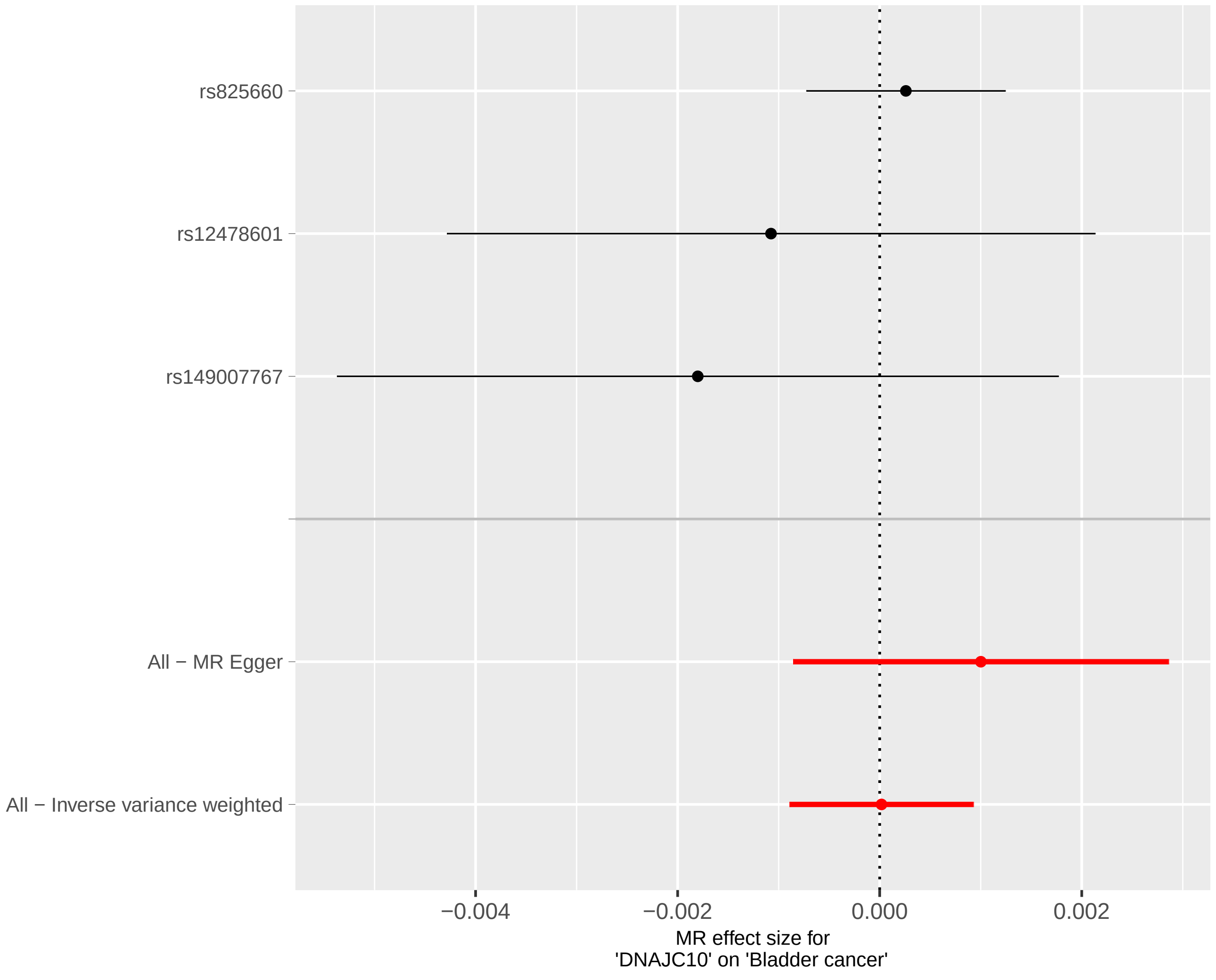

rs825660
rs12478601
rs149007767
All − MR Egger
All − Inverse variance weighted
−0.004
−0.002
0.000
0.002
MR effect size for
'DNAJC10' on 'Bladder cancer'

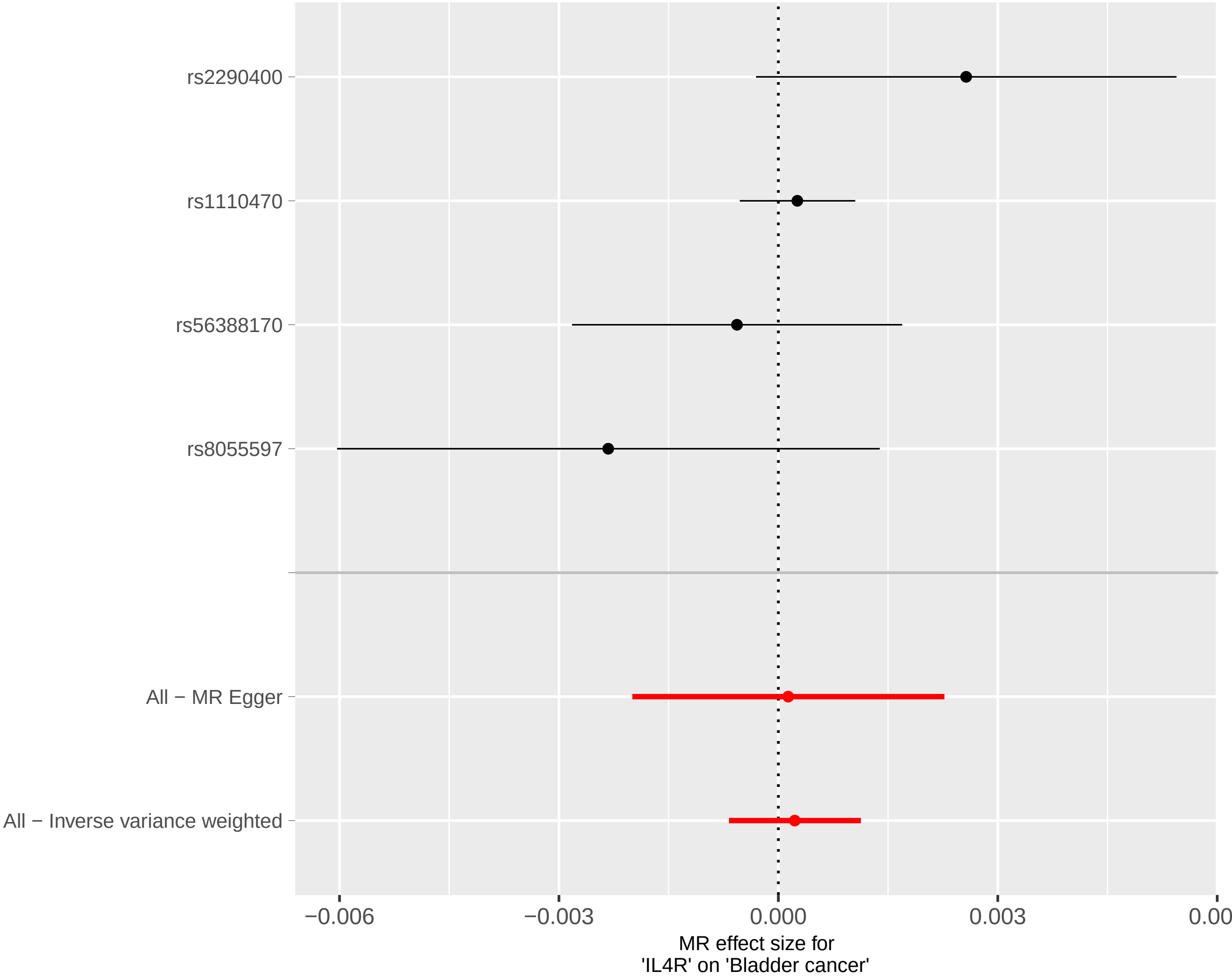
rs2290400
rs1110470
rs56388170
rs8055597
All − MR Egger
All − Inverse variance weighted
−0.006
−0.003
0.000
0.003
0.00
MR effect size for
'IL4R' on 'Bladder cancer'

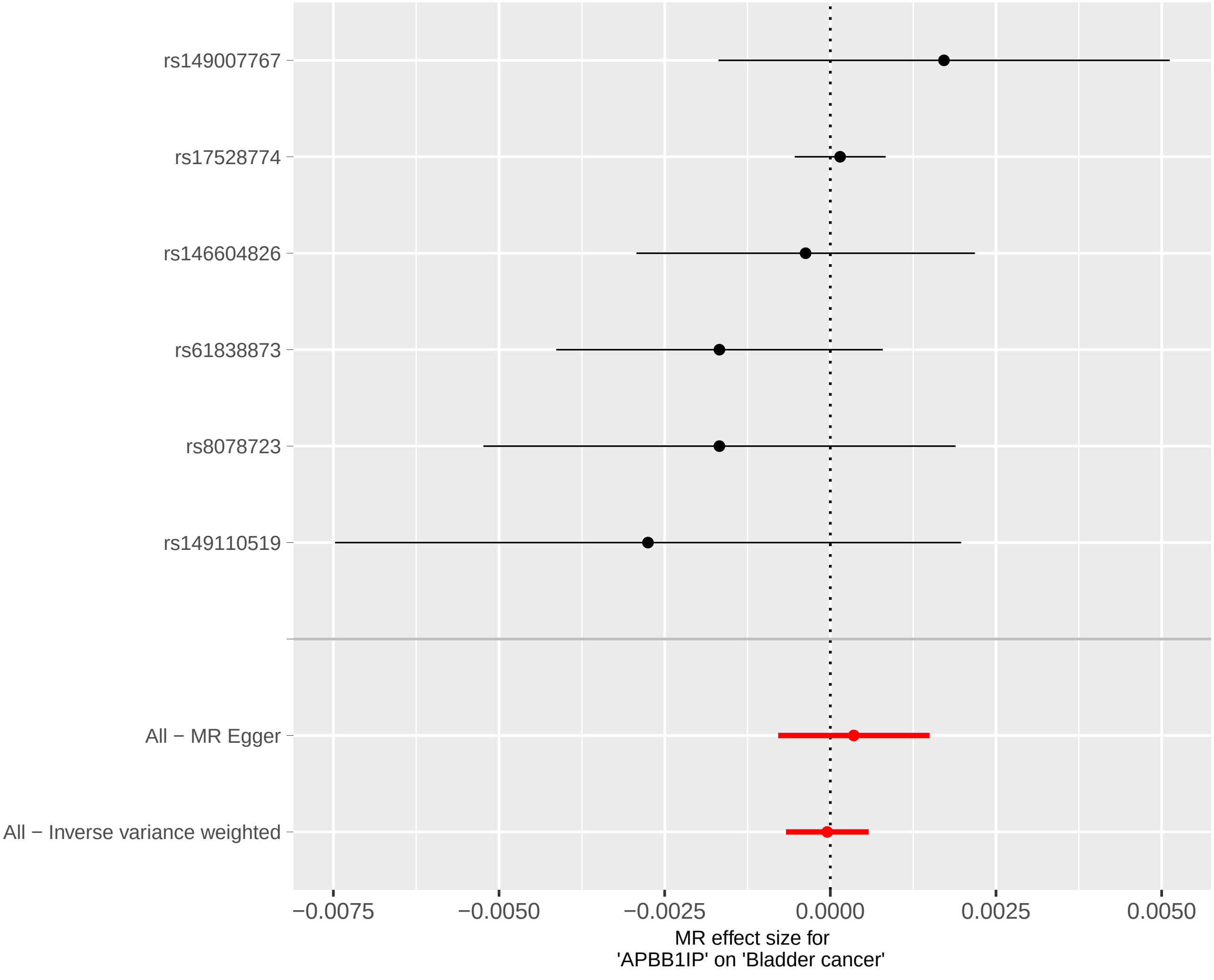
rs149007767
rs17528774
rs146604826
rs61838873
rs8078723
rs149110519
All − MR Egger
All − Inverse variance weighted
−0.0075
−0.0050
−0.0025
0.0000
0.0025
0.0050
MR effect size for
'APBB1IP' on 'Bladder cancer'

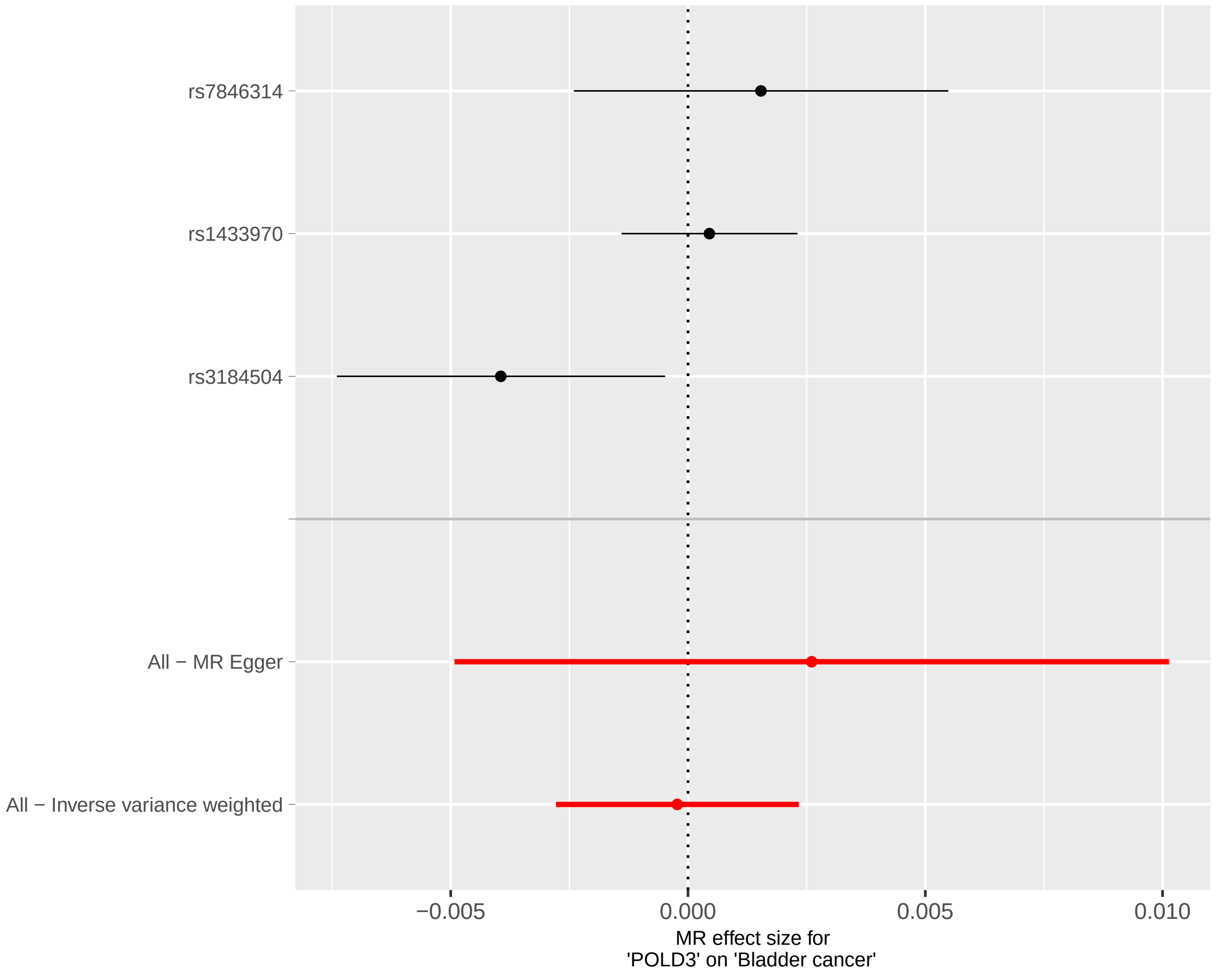
rs7846314
rs1433970
rs3184504
All − MR Egger
All − Inverse variance weighted
−0.005
0.000
0.005
0.010
MR effect size for
'POLD3' on 'Bladder cancer'

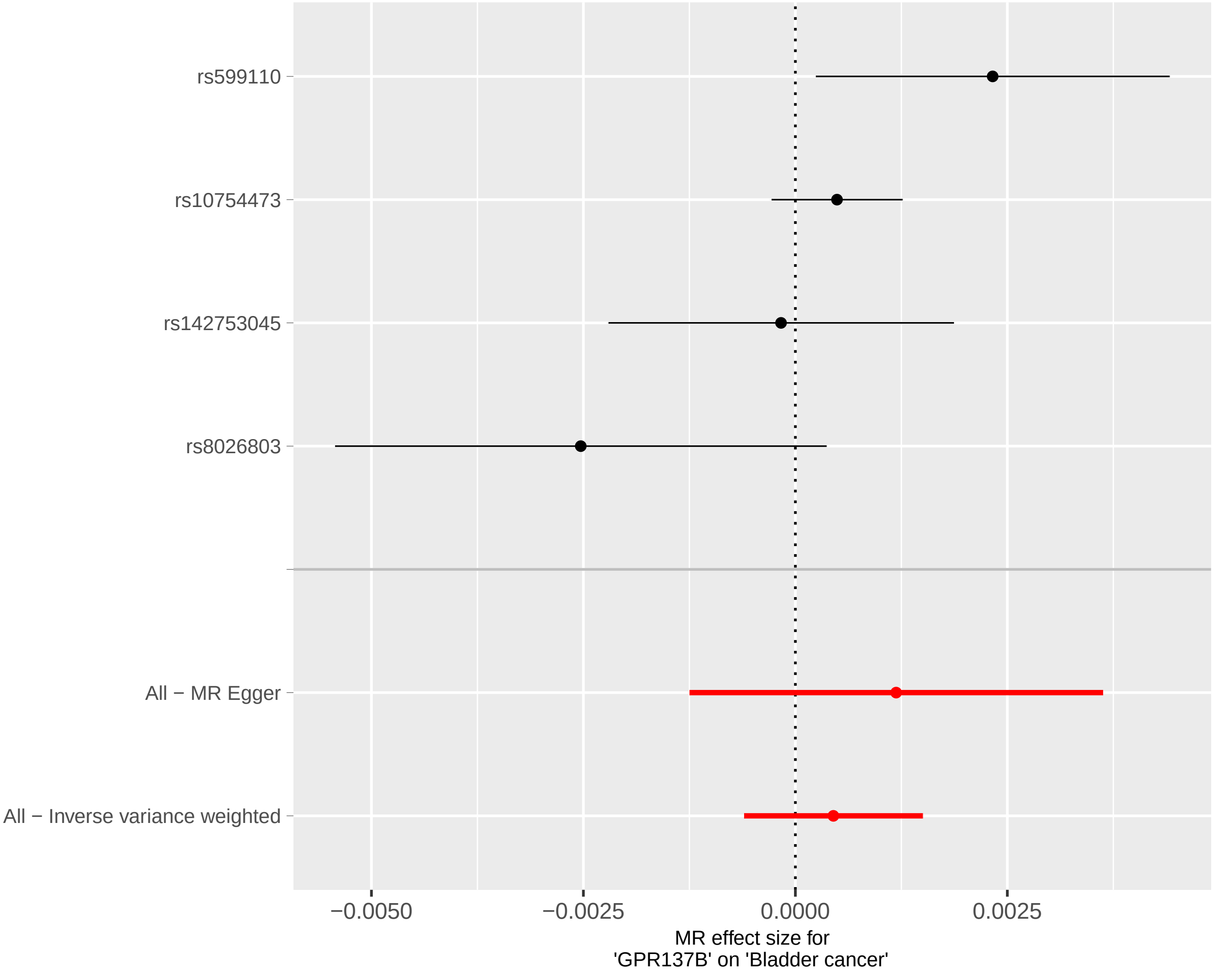

rs599110
rs10754473
rs142753045
rs8026803
All − MR Egger
All − Inverse variance weighted
−0.0050
−0.0025
0.0000
0.0025
MR effect size for
'GPR137B' on 'Bladder cancer'

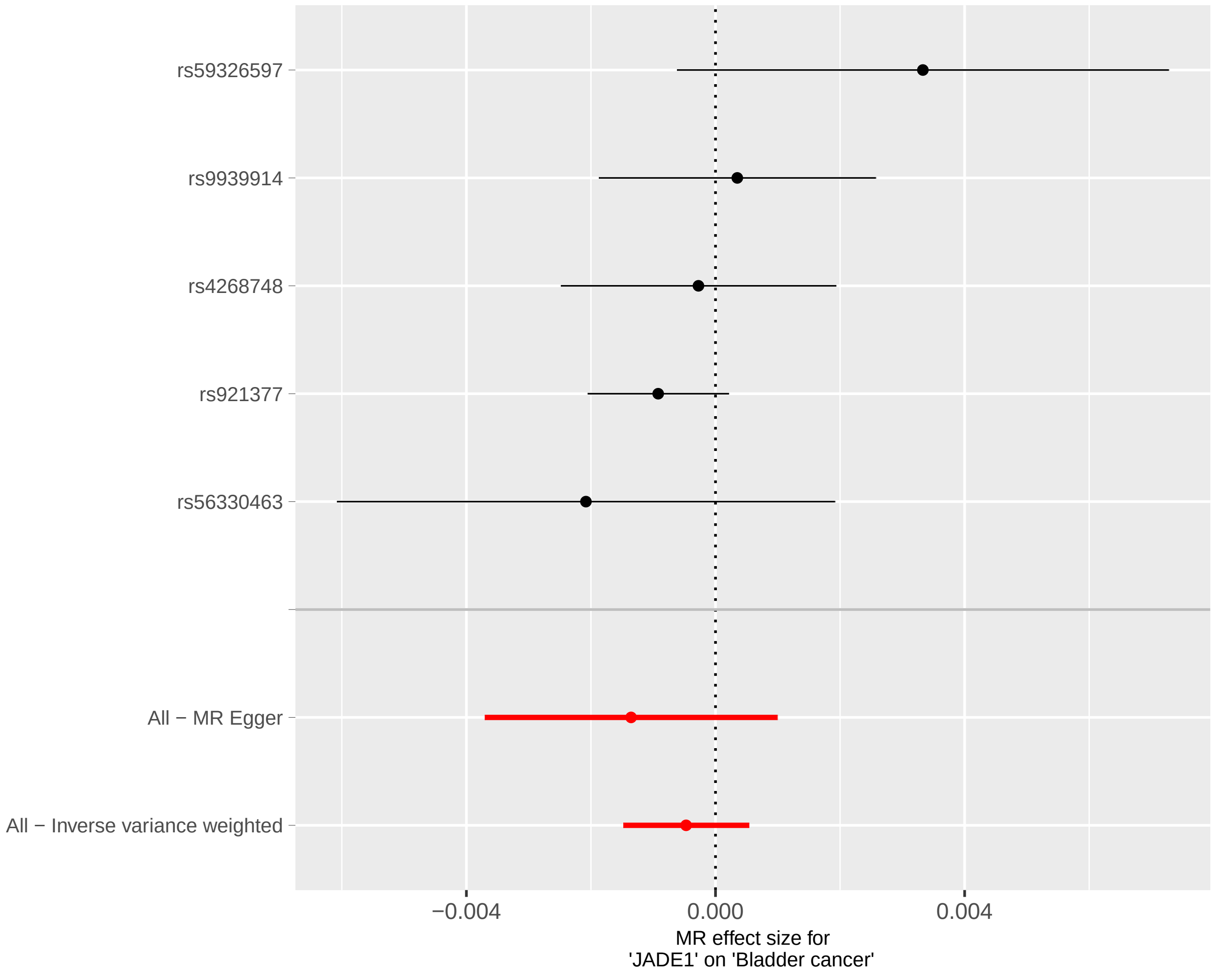

rs59326597
rs9939914
rs4268748
rs921377
rs56330463
All − MR Egger
All − Inverse variance weighted
−0.004
0.000
0.004
MR effect size for
'JADE1' on 'Bladder cancer'

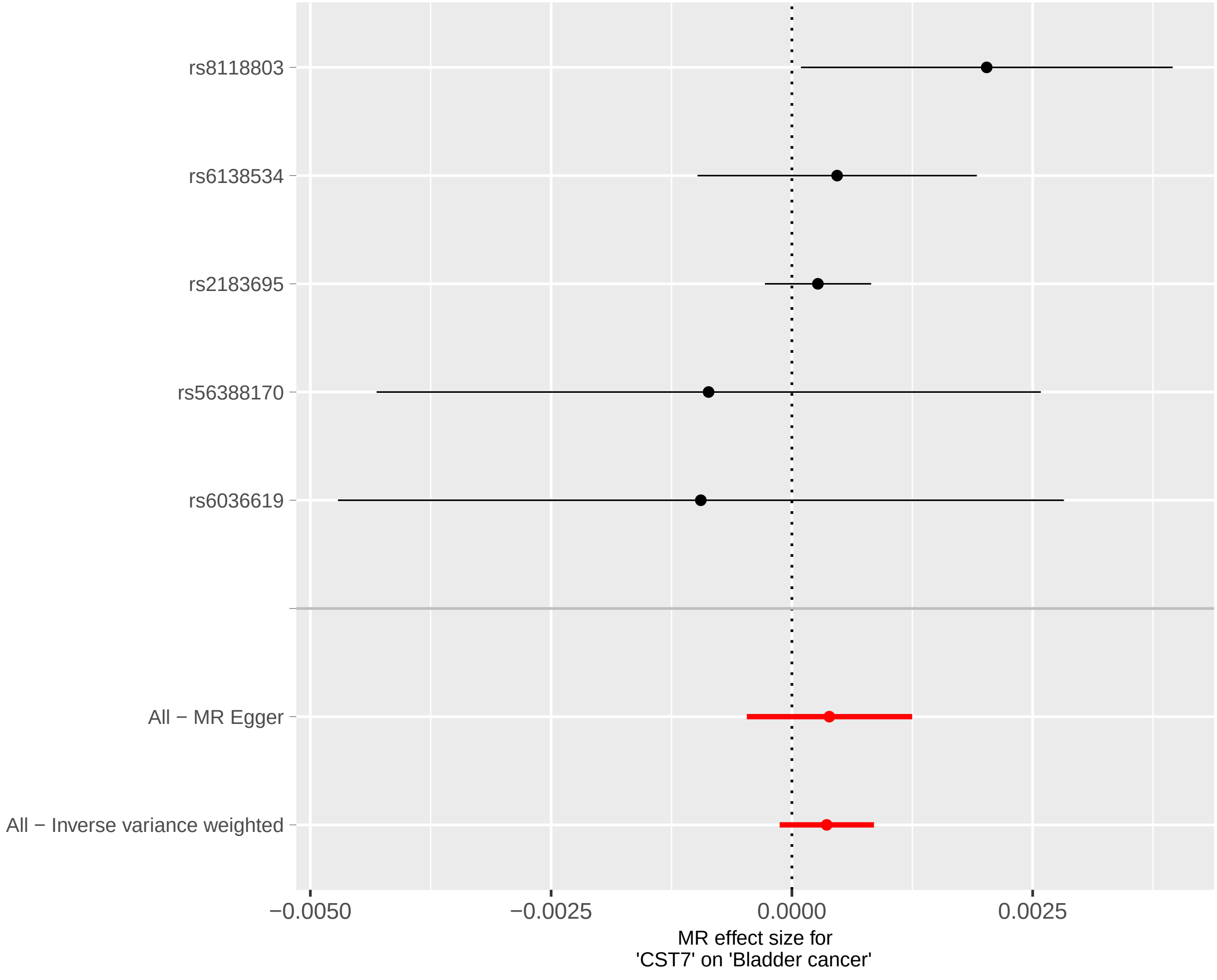
rs8118803
rs6138534
rs2183695
rs56388170
rs6036619
All − MR Egger
All − Inverse variance weighted
−0.0050
−0.0025
0.0000
0.0025
MR effect size for
'CST7' on 'Bladder cancer'

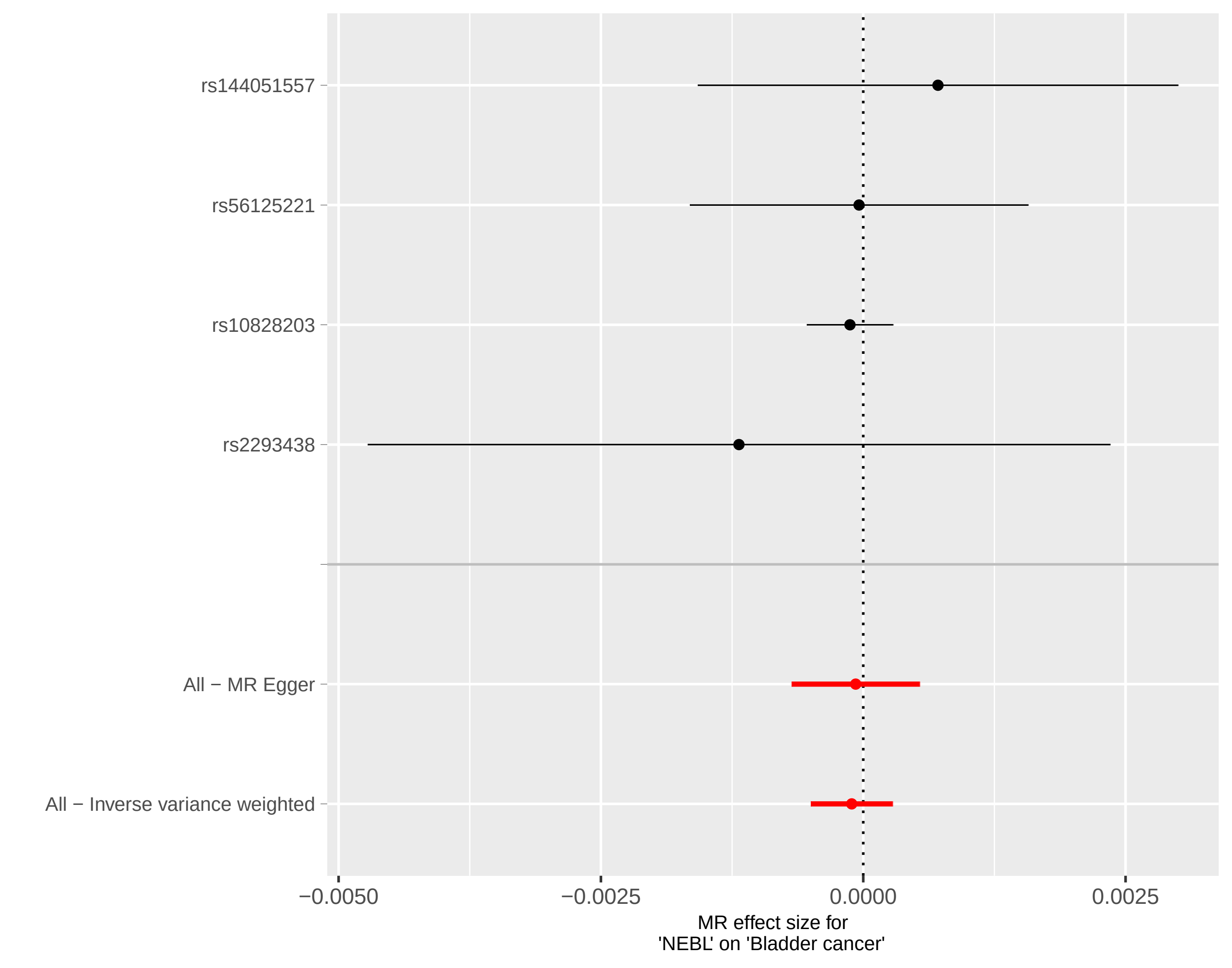

rs144051557
rs56125221
rs10828203
rs2293438
All − MR Egger
All − Inverse variance weighted
−0.0050
−0.0025
0.0000
0.0025
MR effect size for
'NEBL' on 'Bladder cancer'

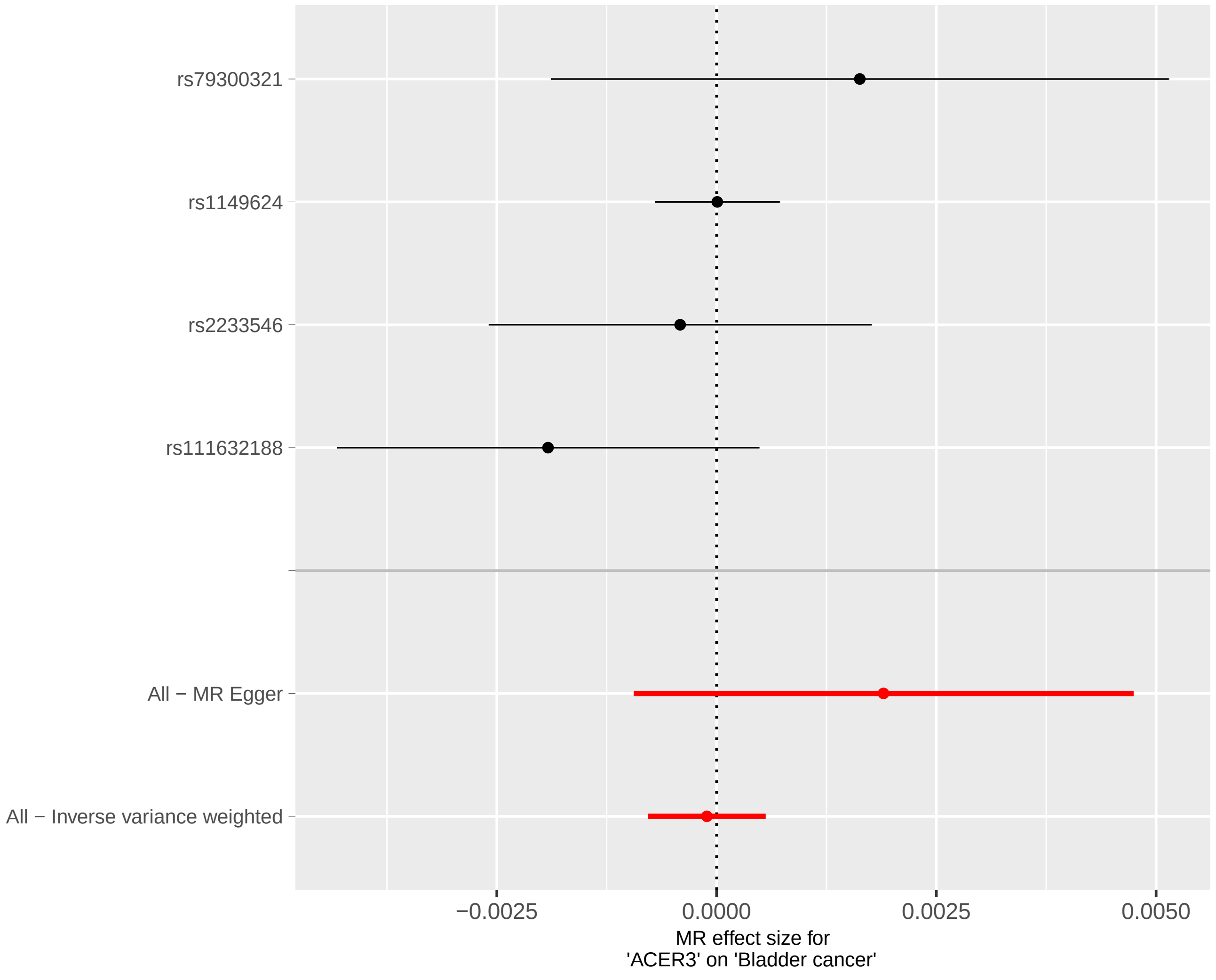

rs79300321
rs1149624
rs2233546
rs111632188
All − MR Egger
All − Inverse variance weighted
−0.0025
0.0000
0.0025
0.0050
MR effect size for
'ACER3' on 'Bladder cancer'

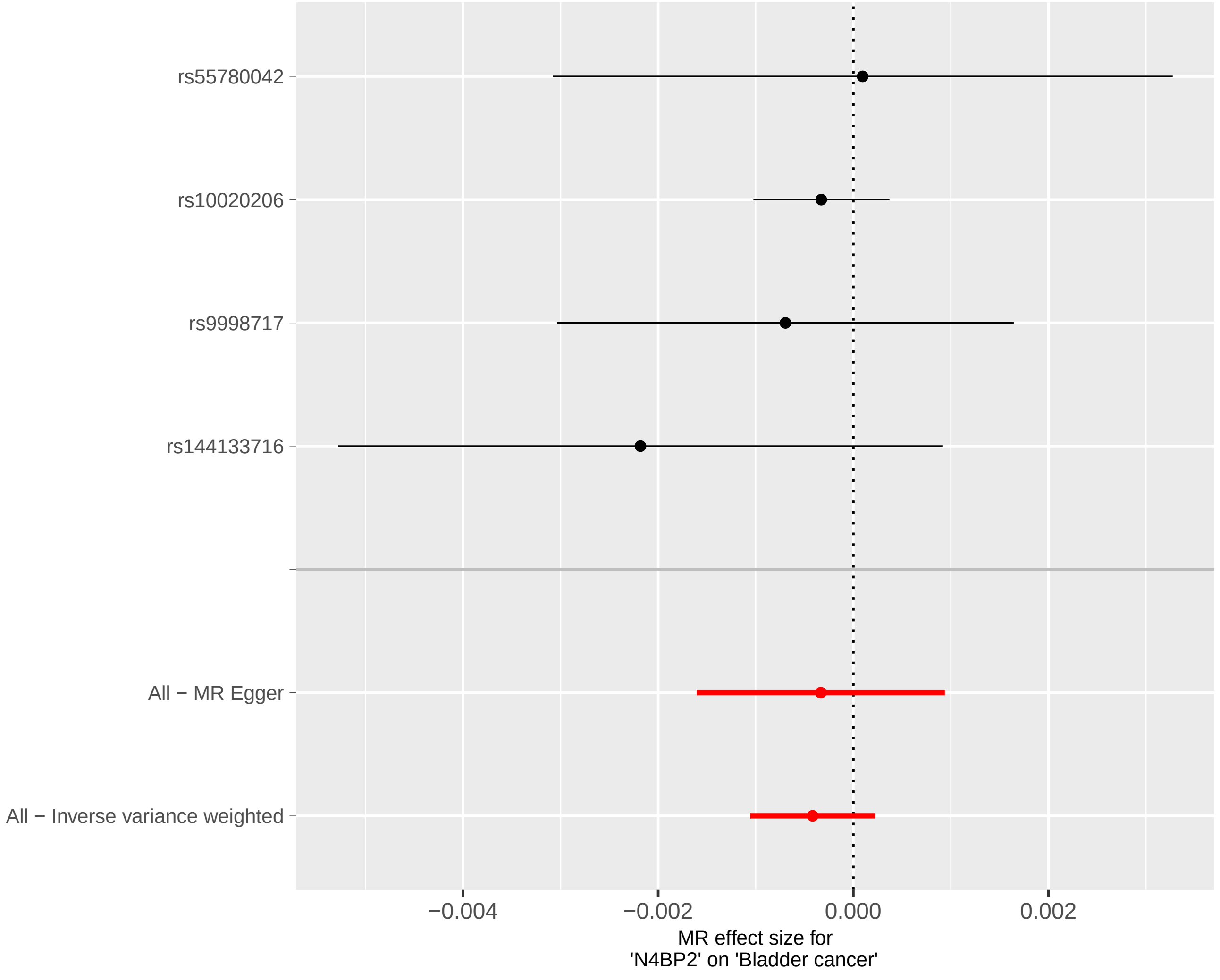
rs55780042
rs10020206
rs9998717
rs144133716
All − MR Egger
All − Inverse variance weighted
−0.004
−0.002
0.000
0.002
MR effect size for
'N4BP2' on 'Bladder cancer'

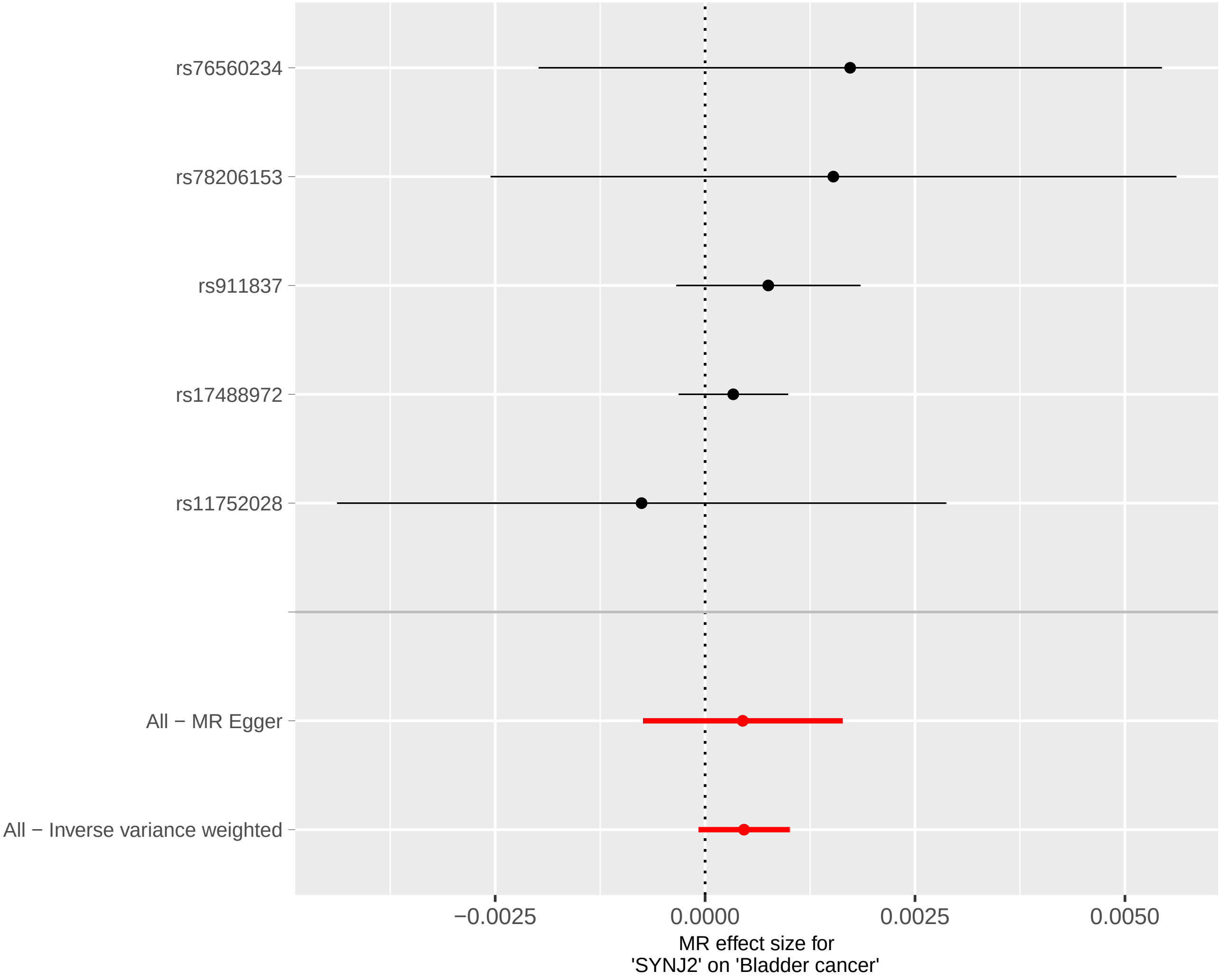
rs76560234
rs78206153
rs911837
rs17488972
rs11752028
All − MR Egger
All − Inverse variance weighted
−0.0025
0.0000
0.0025
0.0050
MR effect size for
'SYNJ2' on 'Bladder cancer'

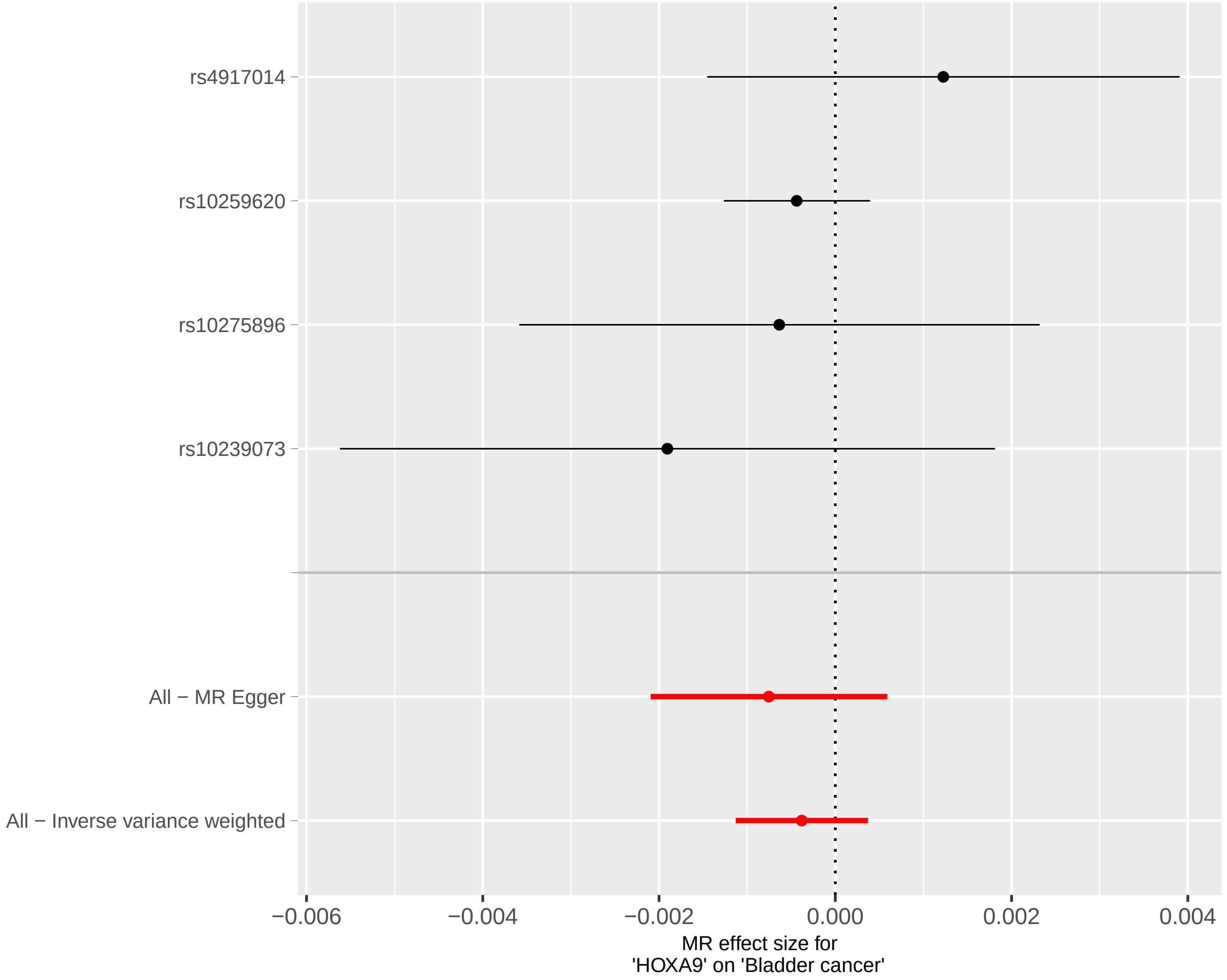

rs4917014
rs10259620
rs10275896
rs10239073
All − MR Egger
All − Inverse variance weighted
−0.006
−0.004
−0.002
0.000
0.002
0.004
MR effect size for
'HOXA9' on 'Bladder cancer'

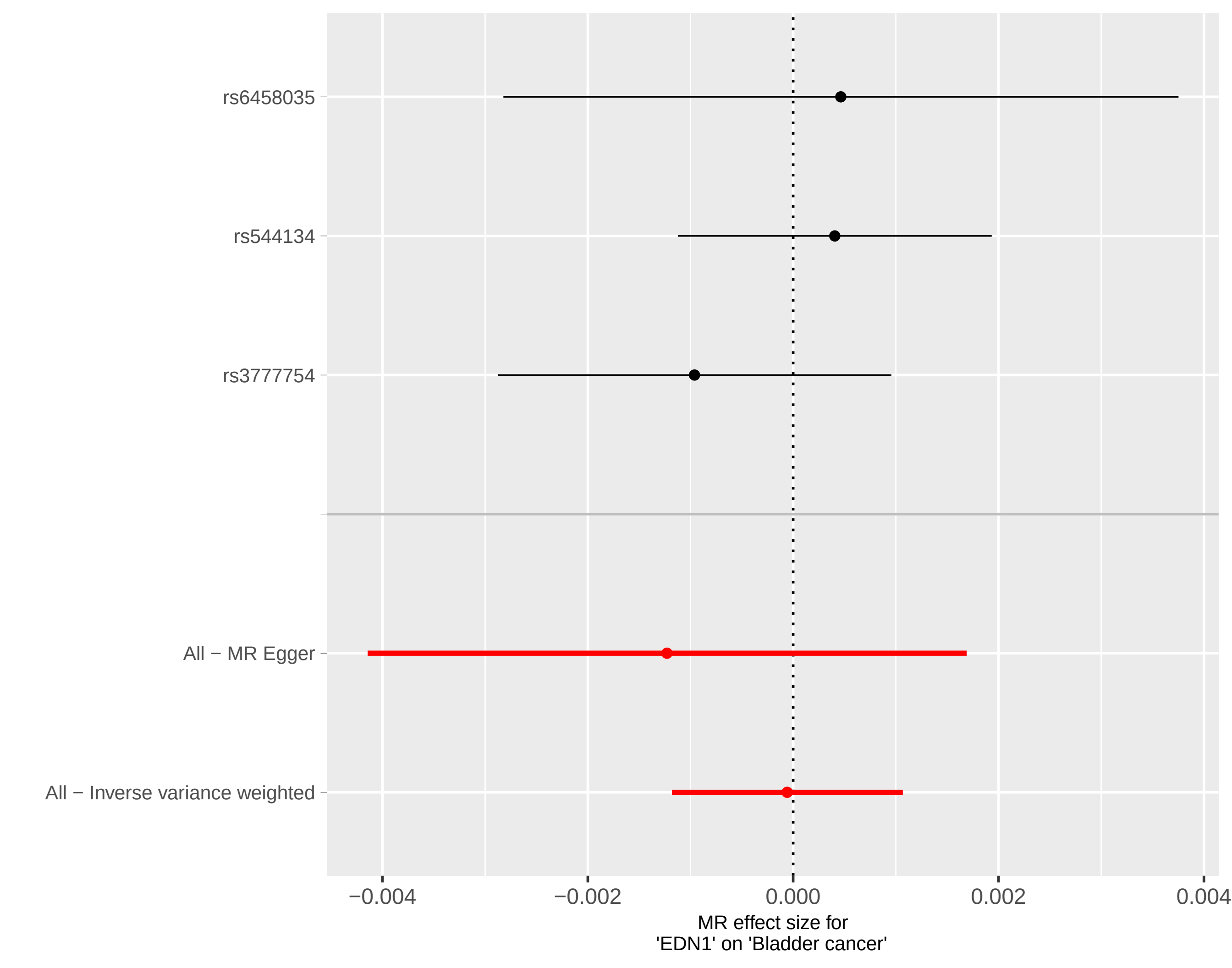

rs6458035
rs544134
rs3777754
All − MR Egger
All − Inverse variance weighted
−0.004
−0.002
0.000
0.002
0.004
MR effect size for
'EDN1' on 'Bladder cancer'

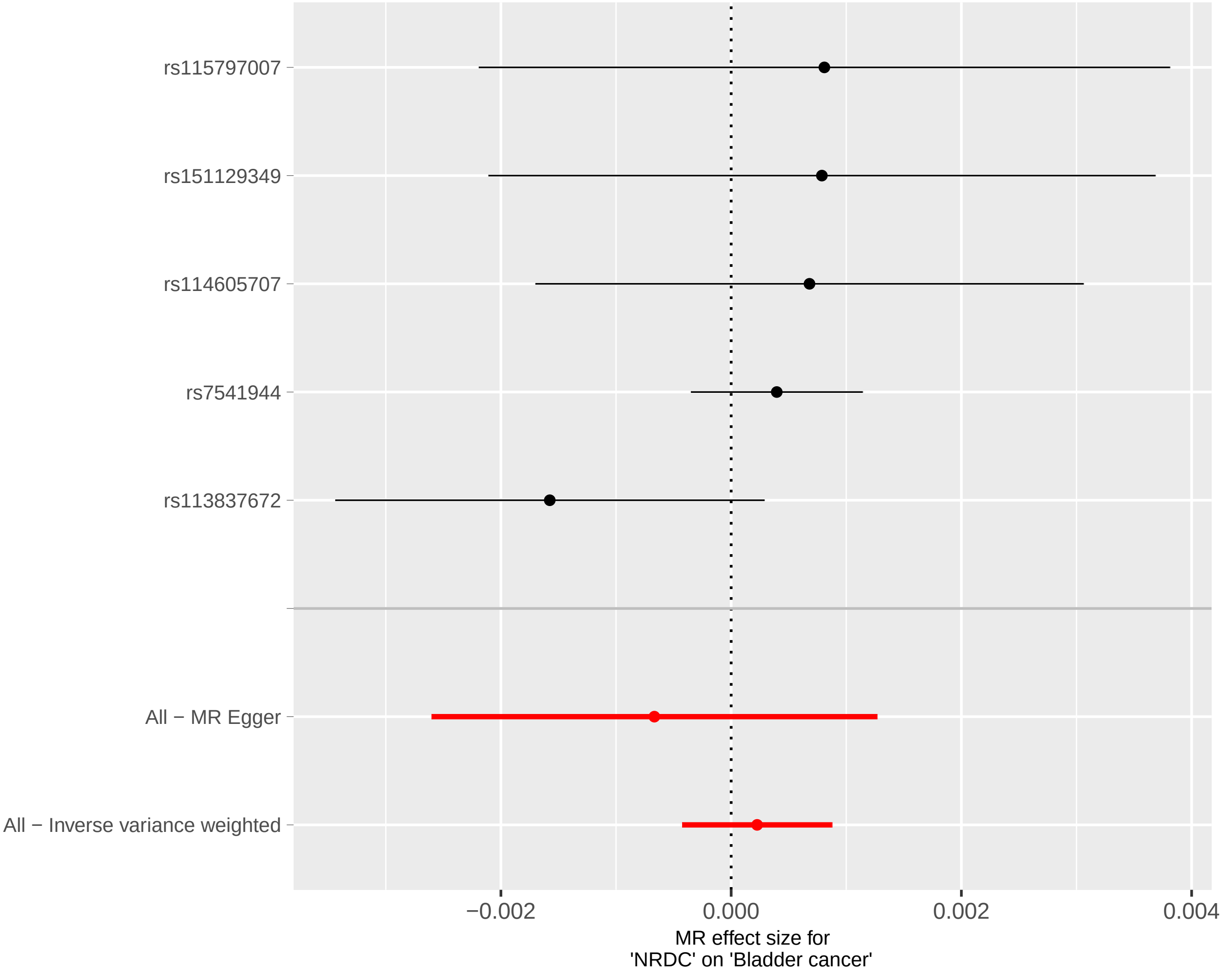
rs115797007
rs151129349
rs114605707
rs7541944
rs113837672
All − MR Egger
All − Inverse variance weighted
−0.002
0.000
0.002
0.004
MR effect size for
'NRDC' on 'Bladder cancer'

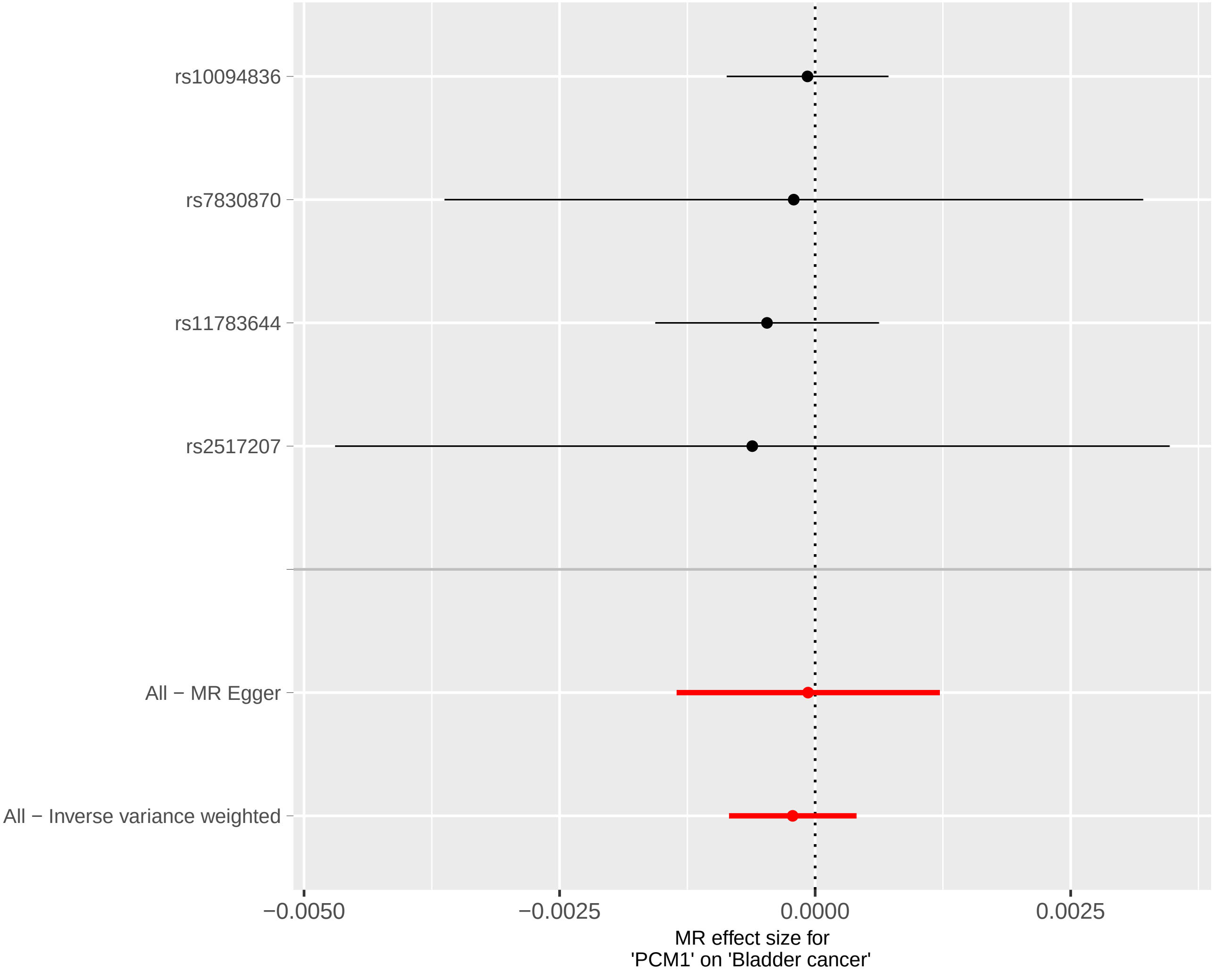
rs10094836
rs7830870
rs11783644
rs2517207
All − MR Egger
All − Inverse variance weighted
−0.0050
−0.0025
0.0000
0.0025
MR effect size for
'PCM1' on 'Bladder cancer'

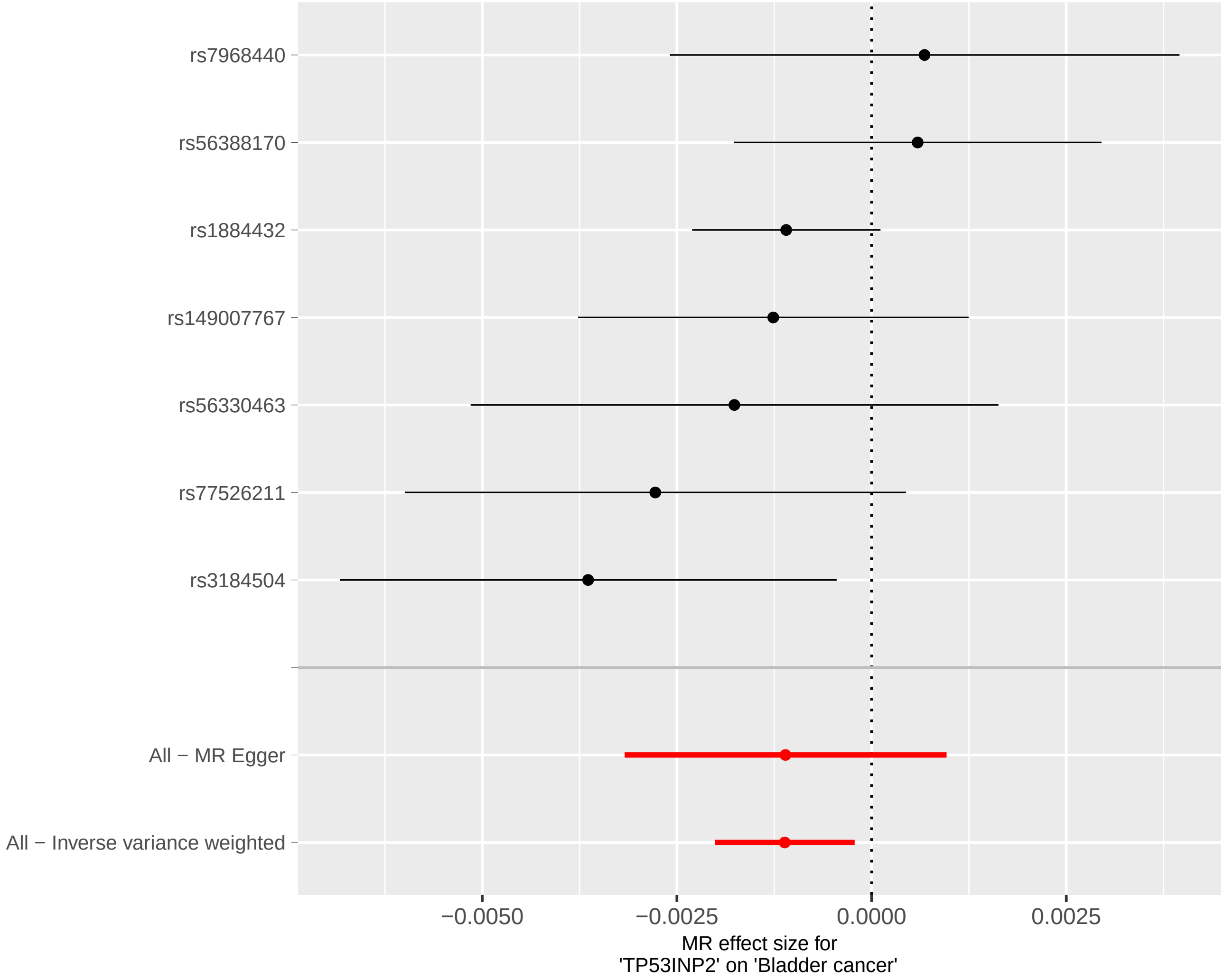

rs7968440
rs56388170
rs1884432
rs149007767
rs56330463
rs77526211
rs3184504
All − MR Egger
All − Inverse variance weighted
−0.0050
−0.0025
0.0000
0.0025
MR effect size for
'TP53INP2' on 'Bladder cancer'

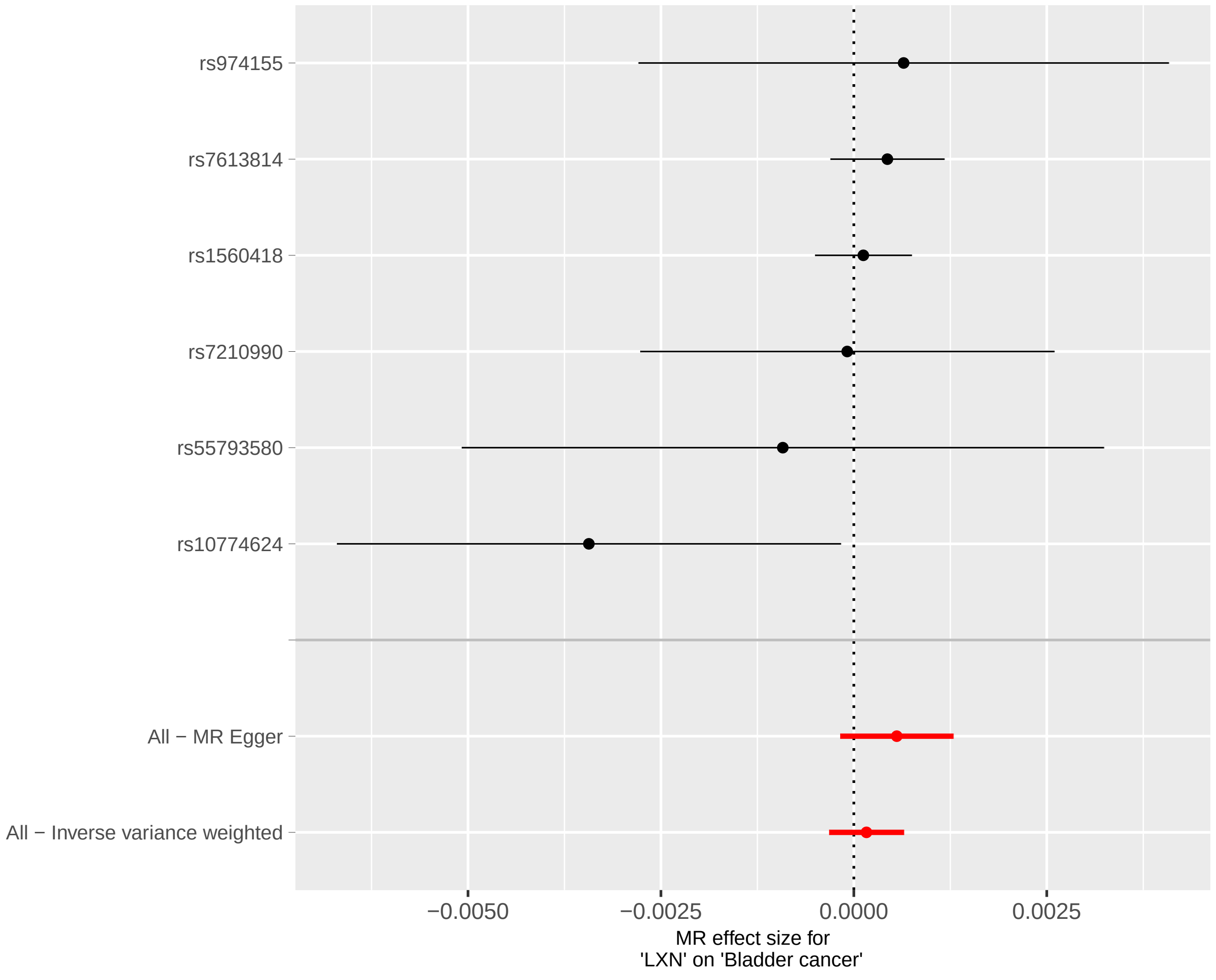
rs974155
rs7613814
rs1560418
rs7210990
rs55793580
rs10774624
All − MR Egger
All − Inverse variance weighted
−0.0050
−0.0025
0.0000
0.0025
MR effect size for
'LXN' on 'Bladder cancer'

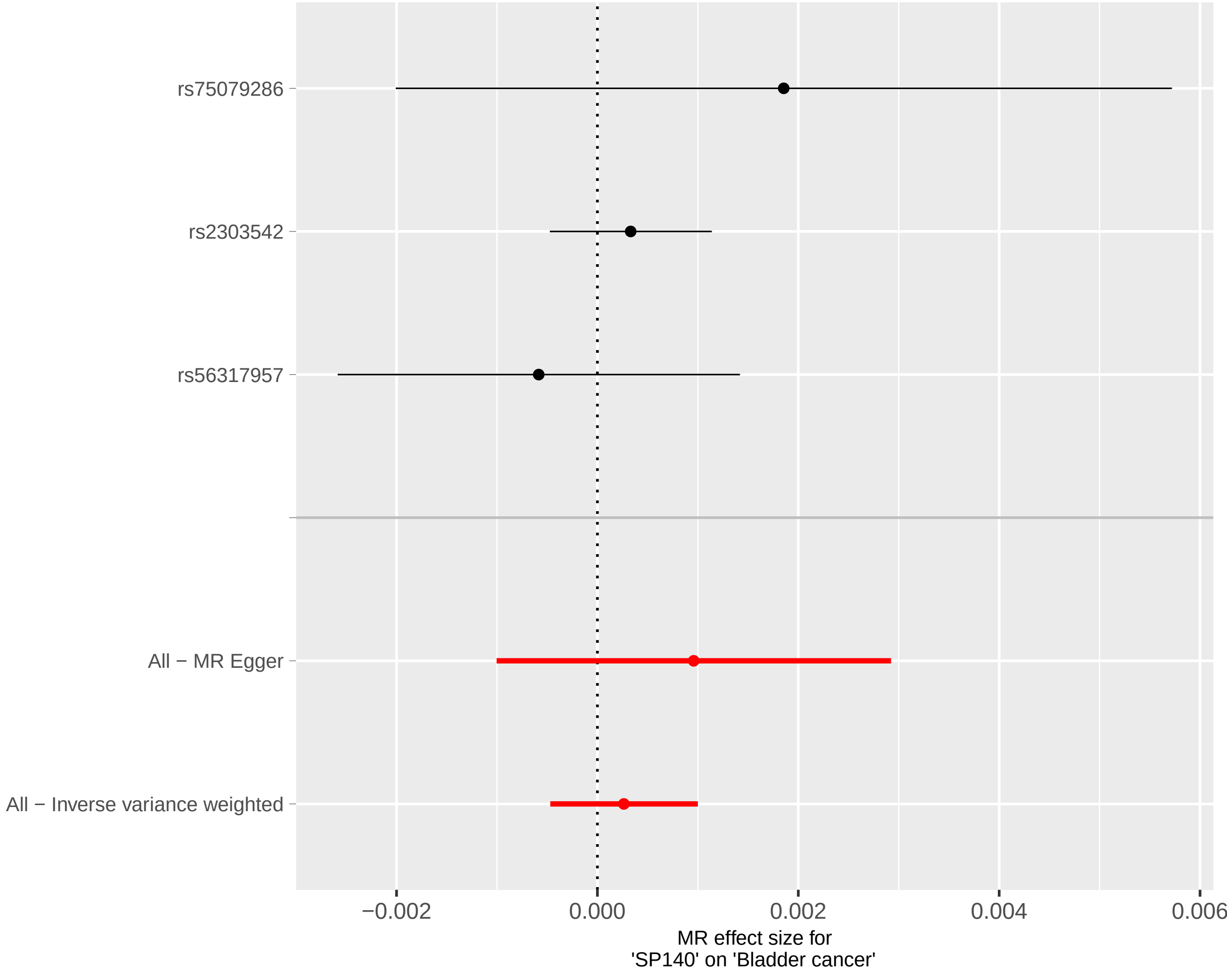

rs75079286
rs2303542
rs56317957
All − MR Egger
All − Inverse variance weighted
−0.002
0.000
0.002
0.004
0.006
MR effect size for
'SP140' on 'Bladder cancer'

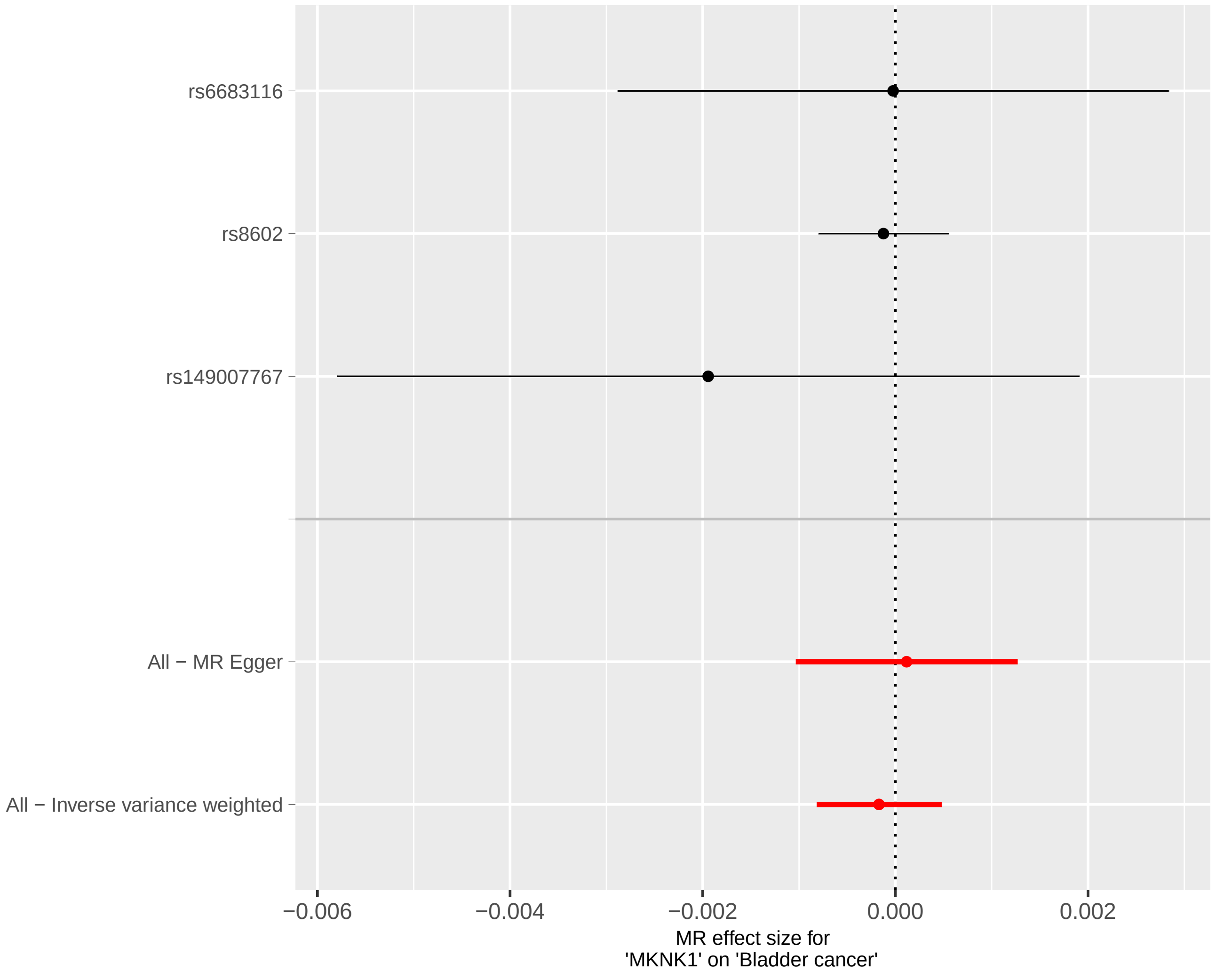
rs6683116
rs8602
rs149007767
All − MR Egger
All − Inverse variance weighted
−0.006
−0.004
−0.002
0.000
0.002
MR effect size for
'MKNK1' on 'Bladder cancer'

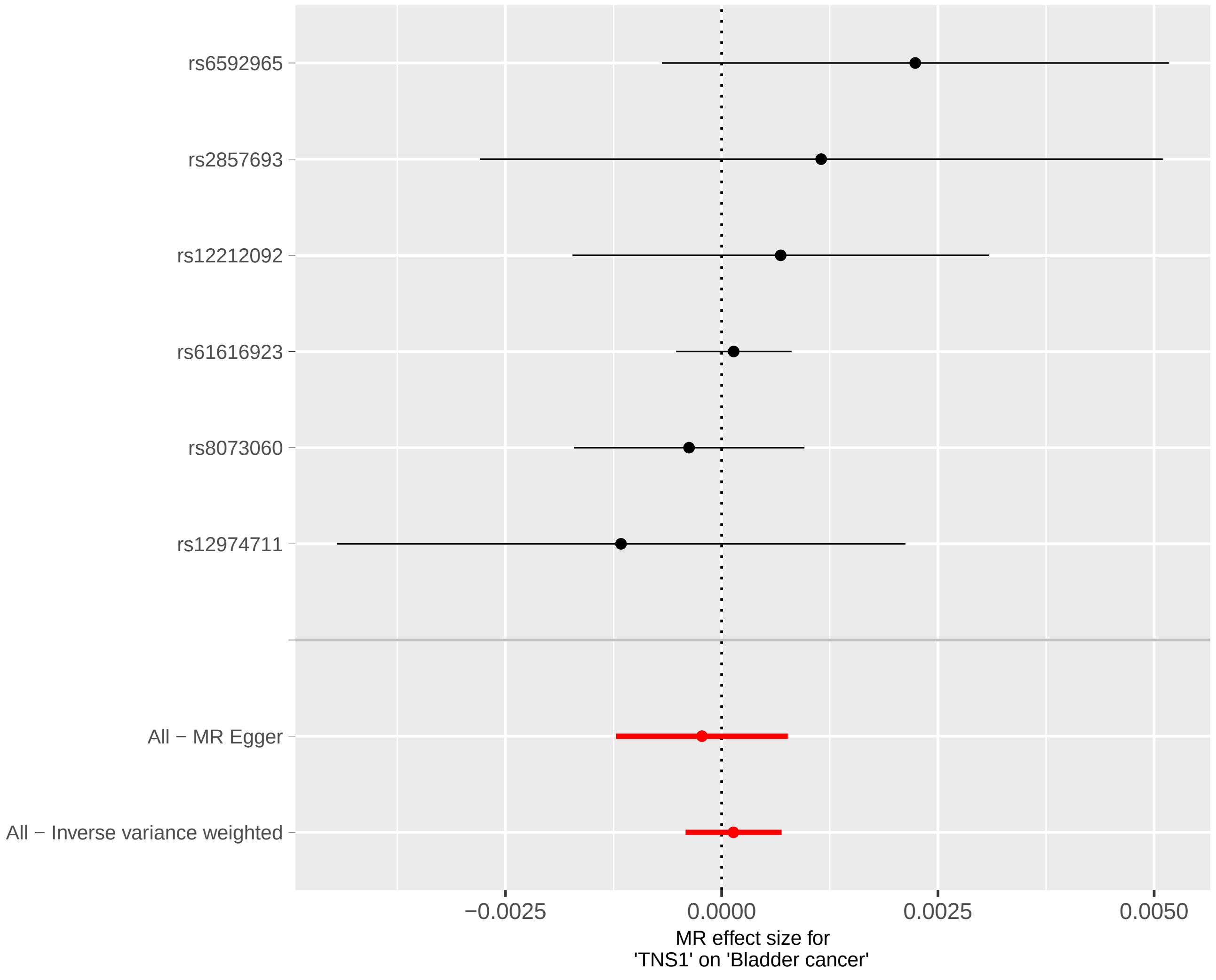

rs6592965
rs2857693
rs12212092
rs61616923
rs8073060
rs12974711
All − MR Egger
All − Inverse variance weighted
−0.0025
0.0000
0.0025
0.0050
MR effect size for
'TNS1' on 'Bladder cancer'

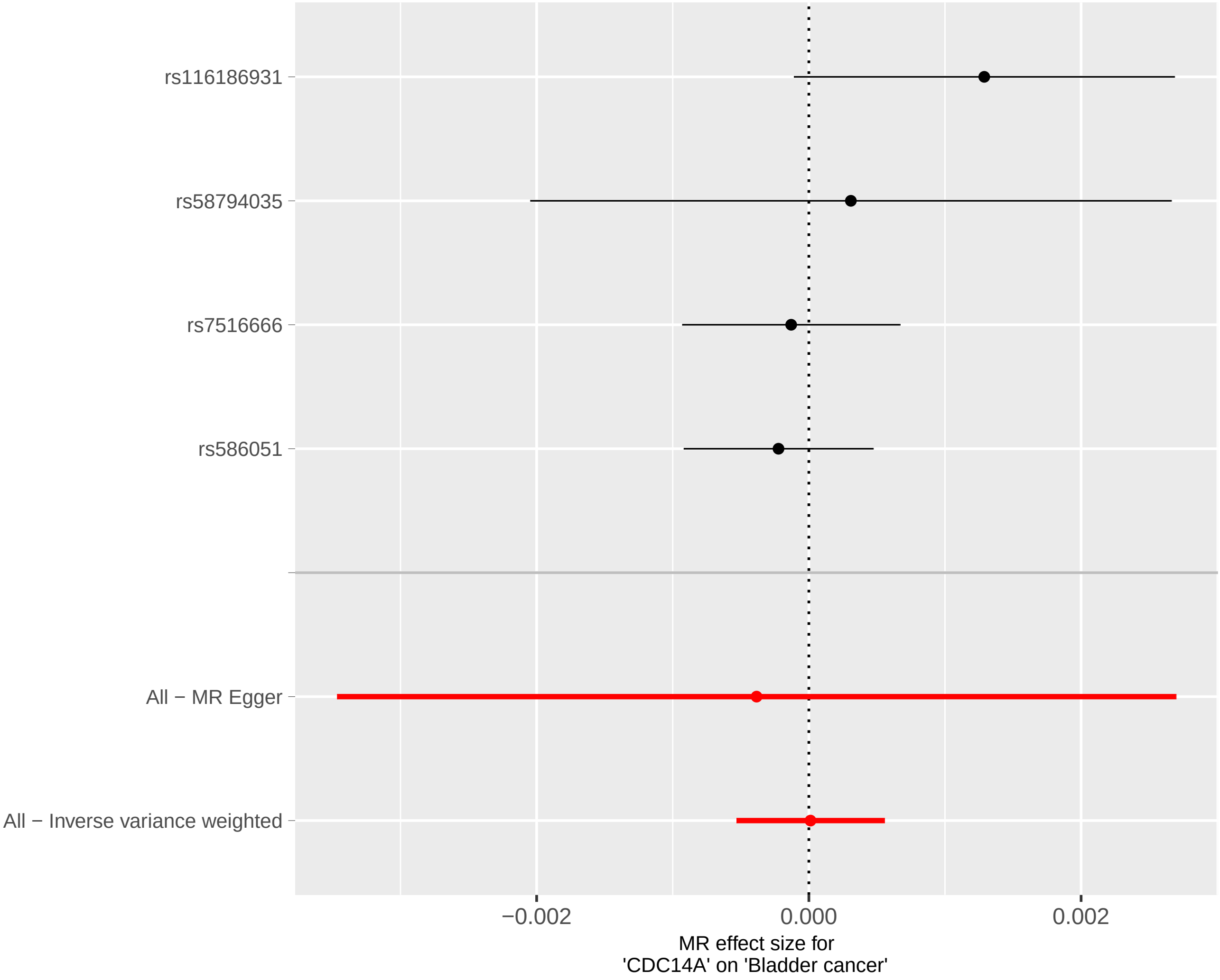
rs116186931
rs58794035
rs7516666
rs586051
All − MR Egger
All − Inverse variance weighted
−0.002
0.000
0.002
MR effect size for
'CDC14A' on 'Bladder cancer'

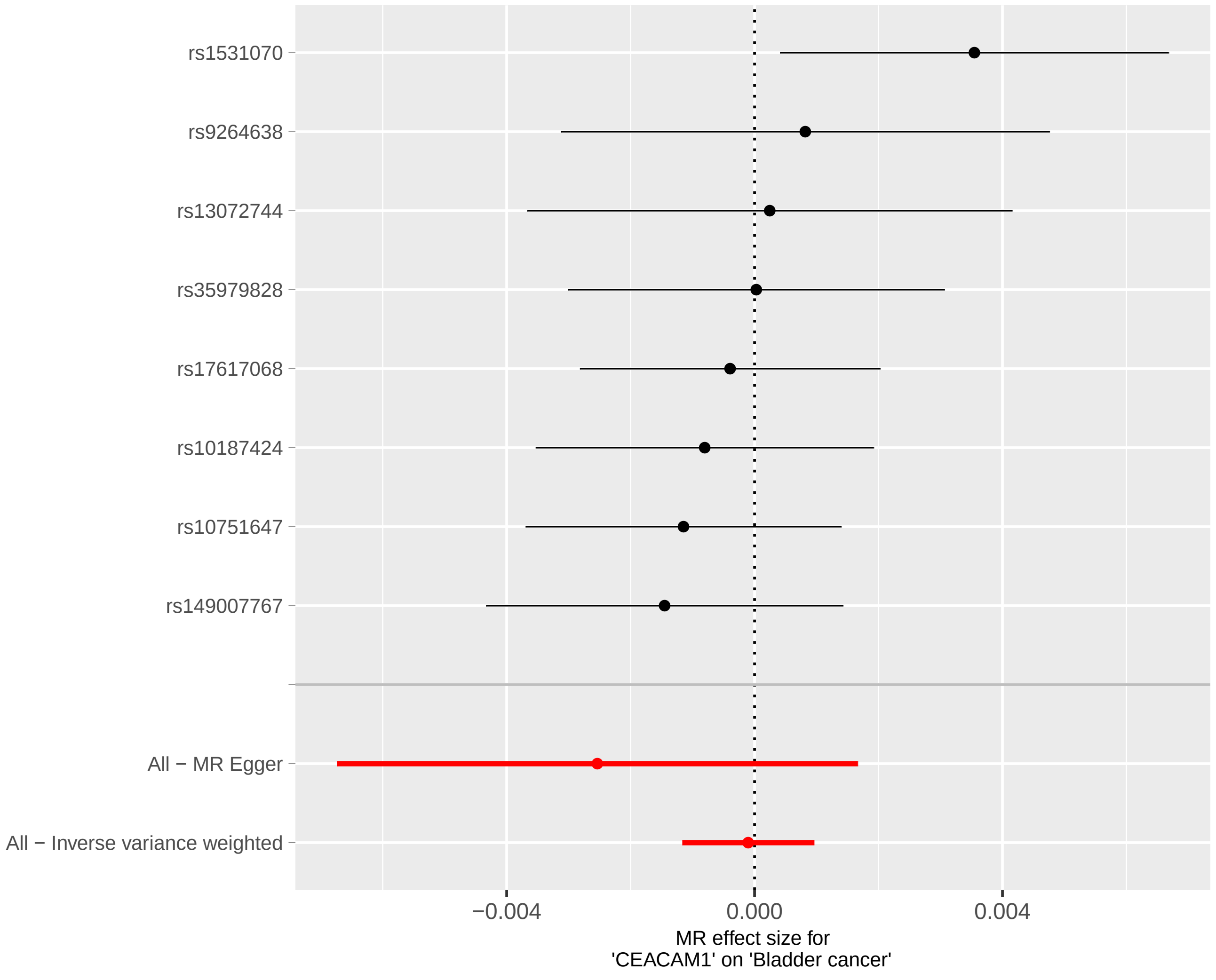

rs1531070
rs9264638
rs13072744
rs35979828
rs17617068
rs10187424
rs10751647
rs149007767
All − MR Egger
All − Inverse variance weighted
−0.004
0.000
0.004
MR effect size for
'CEACAM1' on 'Bladder cancer'

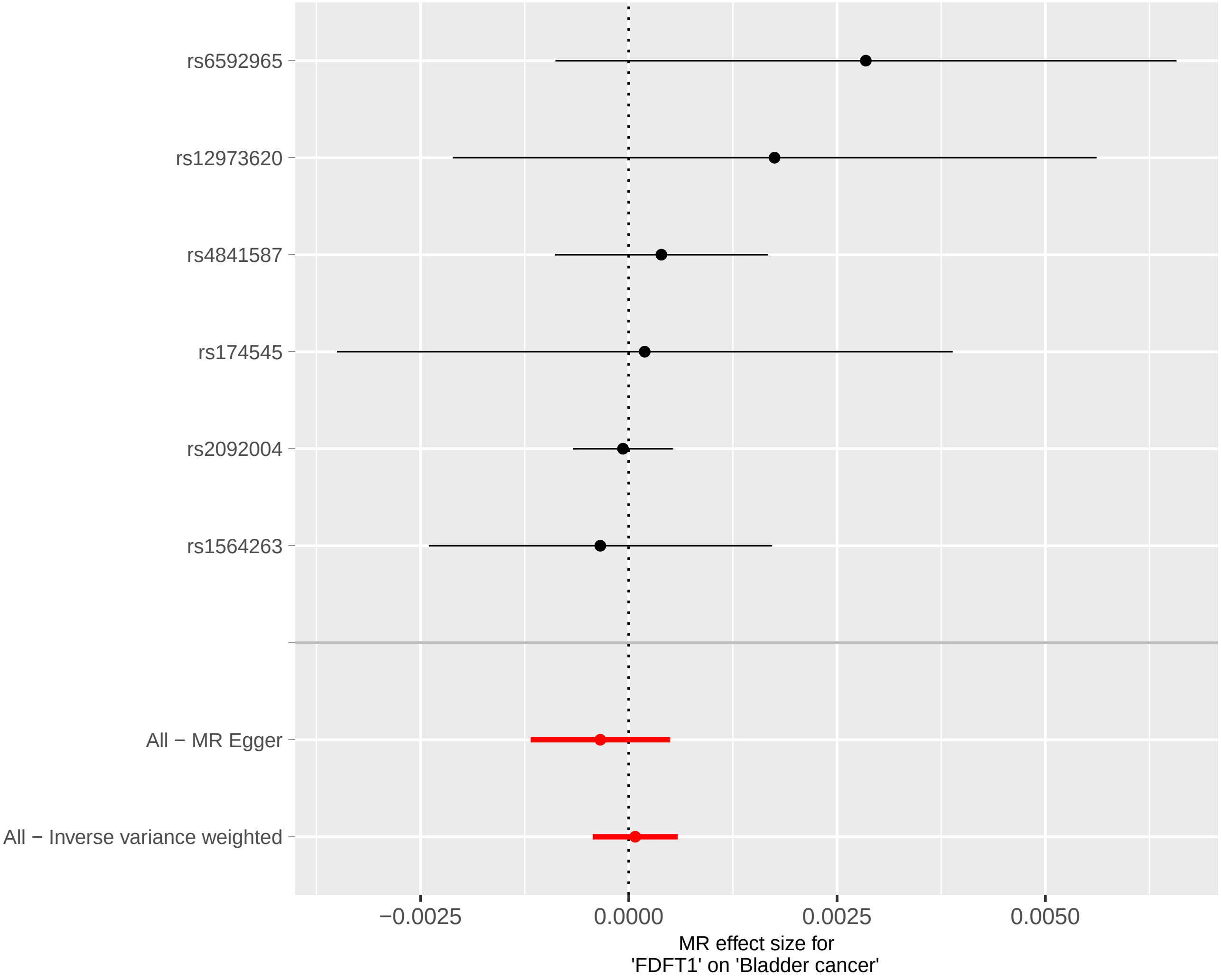

rs6592965
rs12973620
rs4841587
rs174545
rs2092004
rs1564263
All − MR Egger
All − Inverse variance weighted
−0.0025
0.0000
0.0025
0.0050
MR effect size for
'FDFT1' on 'Bladder cancer'

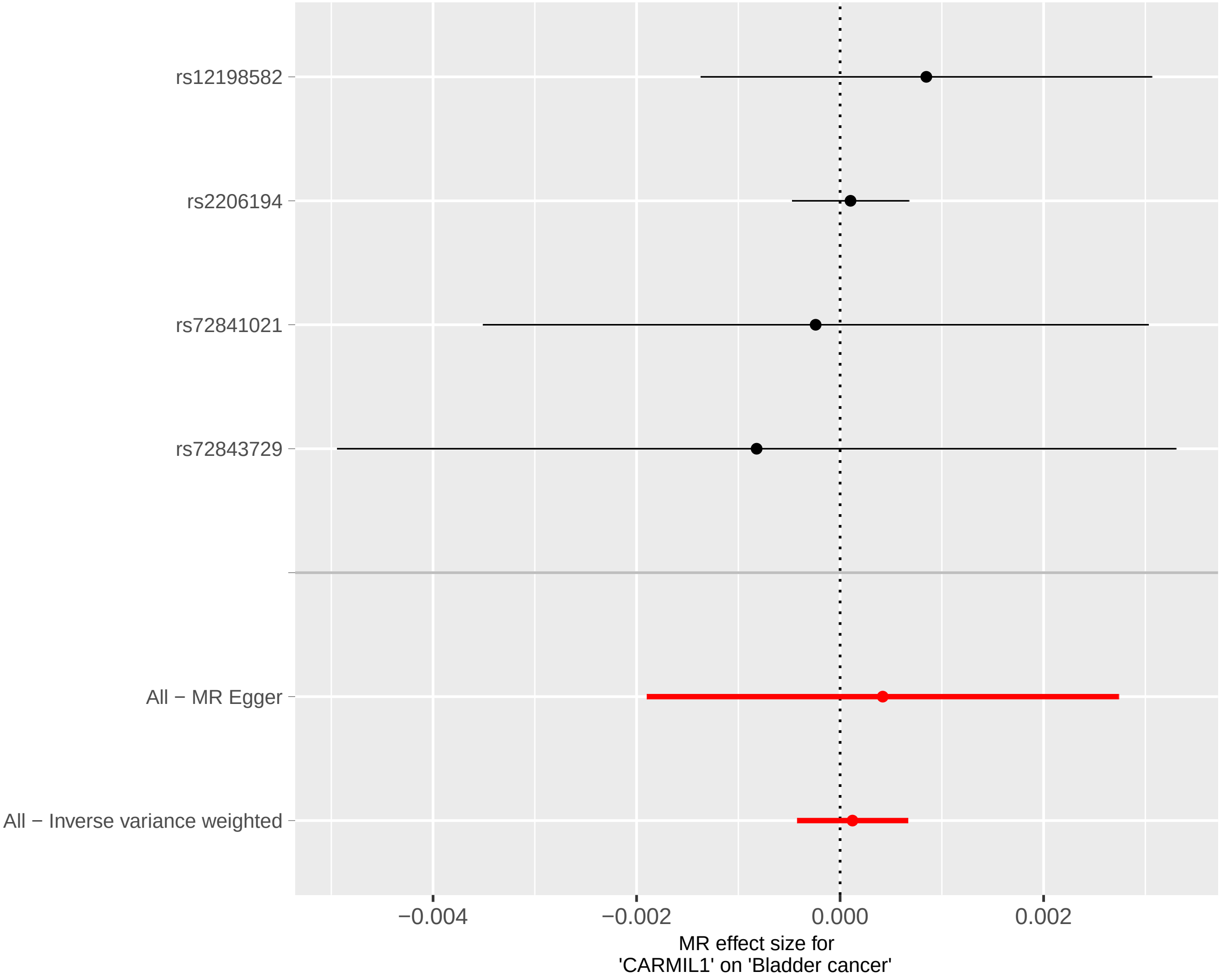

rs12198582
rs2206194
rs72841021
rs72843729
All − MR Egger
All − Inverse variance weighted
−0.004
−0.002
0.000
0.002
MR effect size for
'CARMIL1' on 'Bladder cancer'

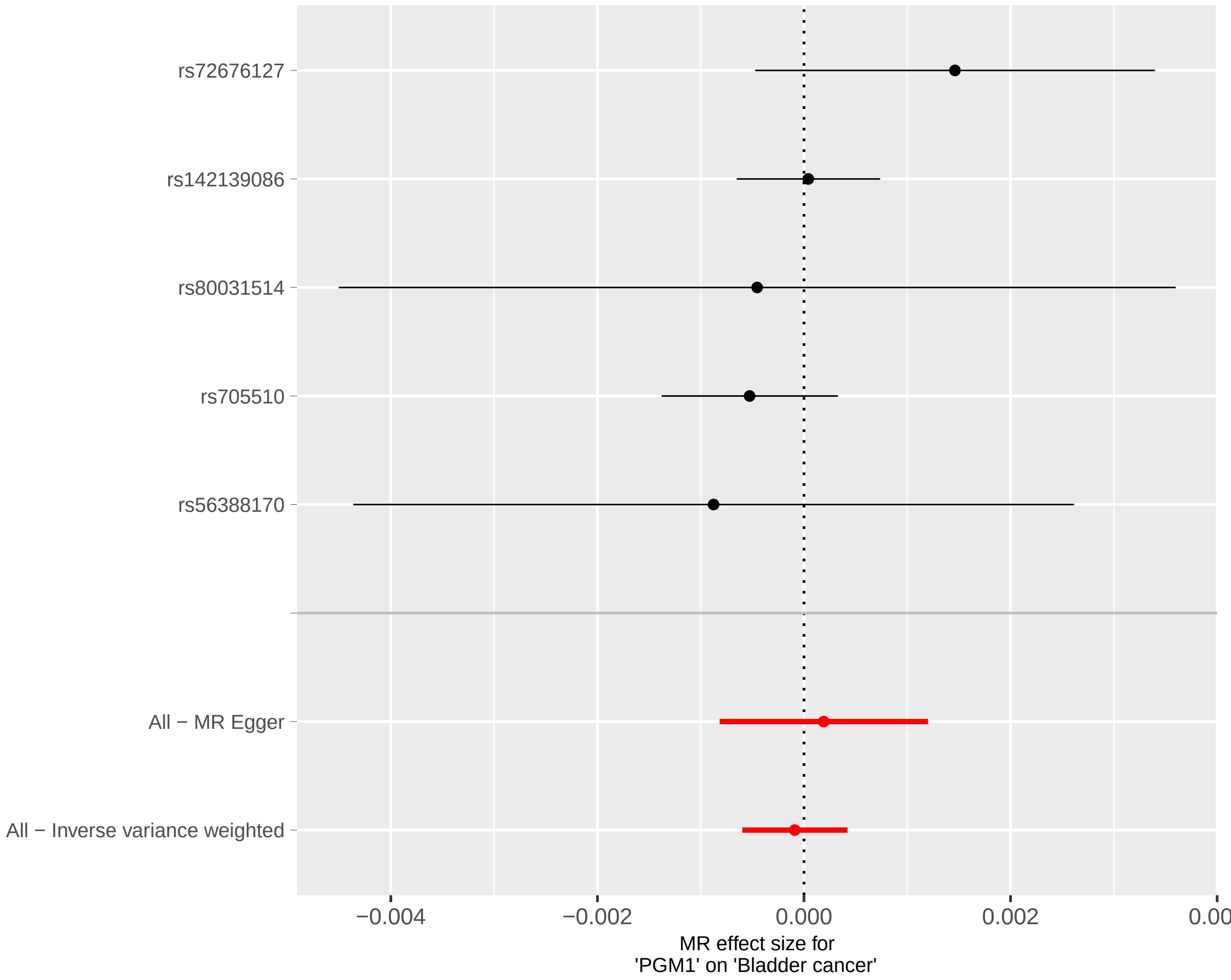

rs72676127
rs142139086
rs80031514
rs705510
rs56388170
All − MR Egger
All − Inverse variance weighted
−0.004
−0.002
0.000
0.002
MR effect size for
'PGM1' on 'Bladder cancer'

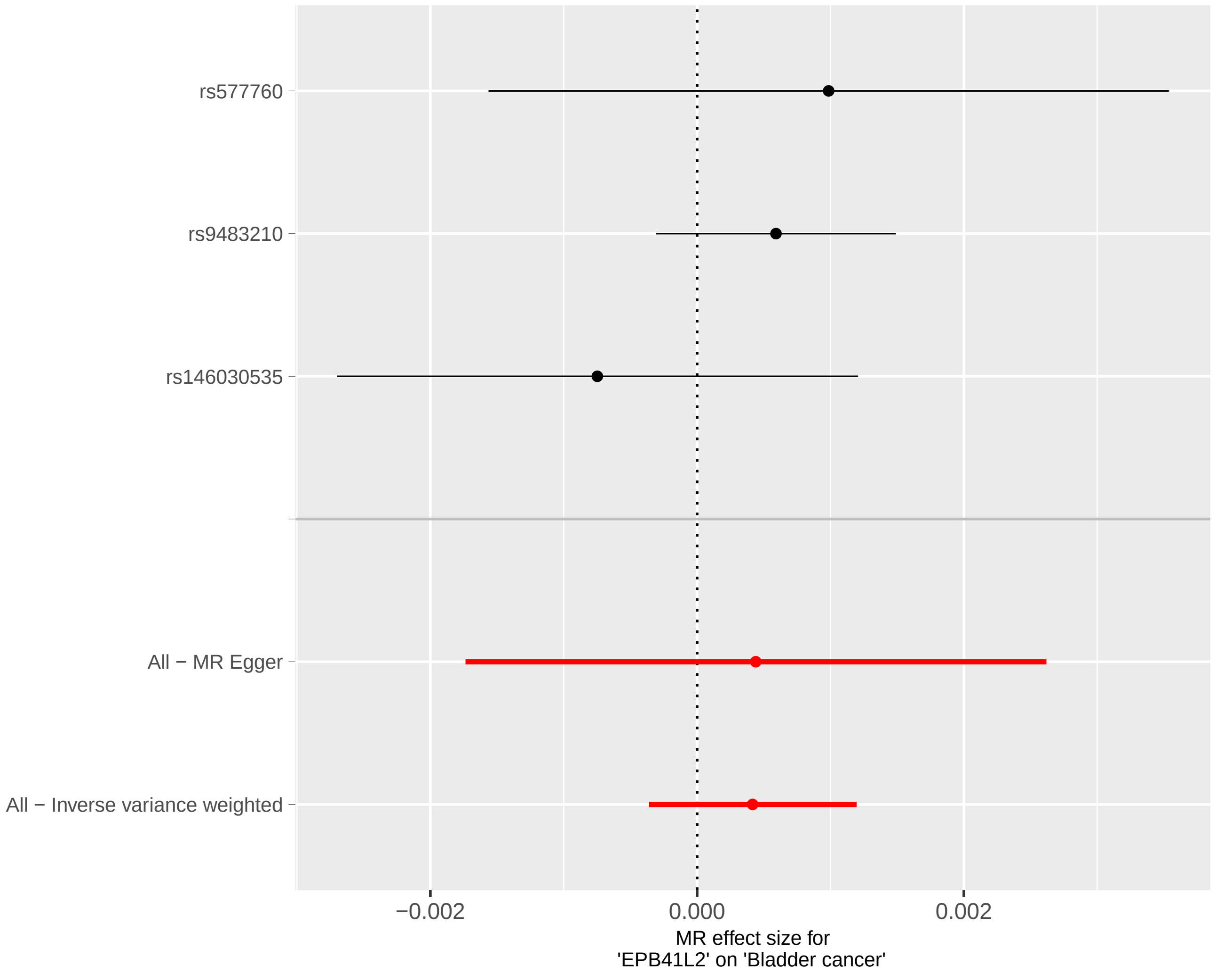

rs577760
rs9483210
rs146030535
All − MR Egger
All − Inverse variance weighted
−0.002
0.000
0.002
MR effect size for
'EPB41L2' on 'Bladder cancer'

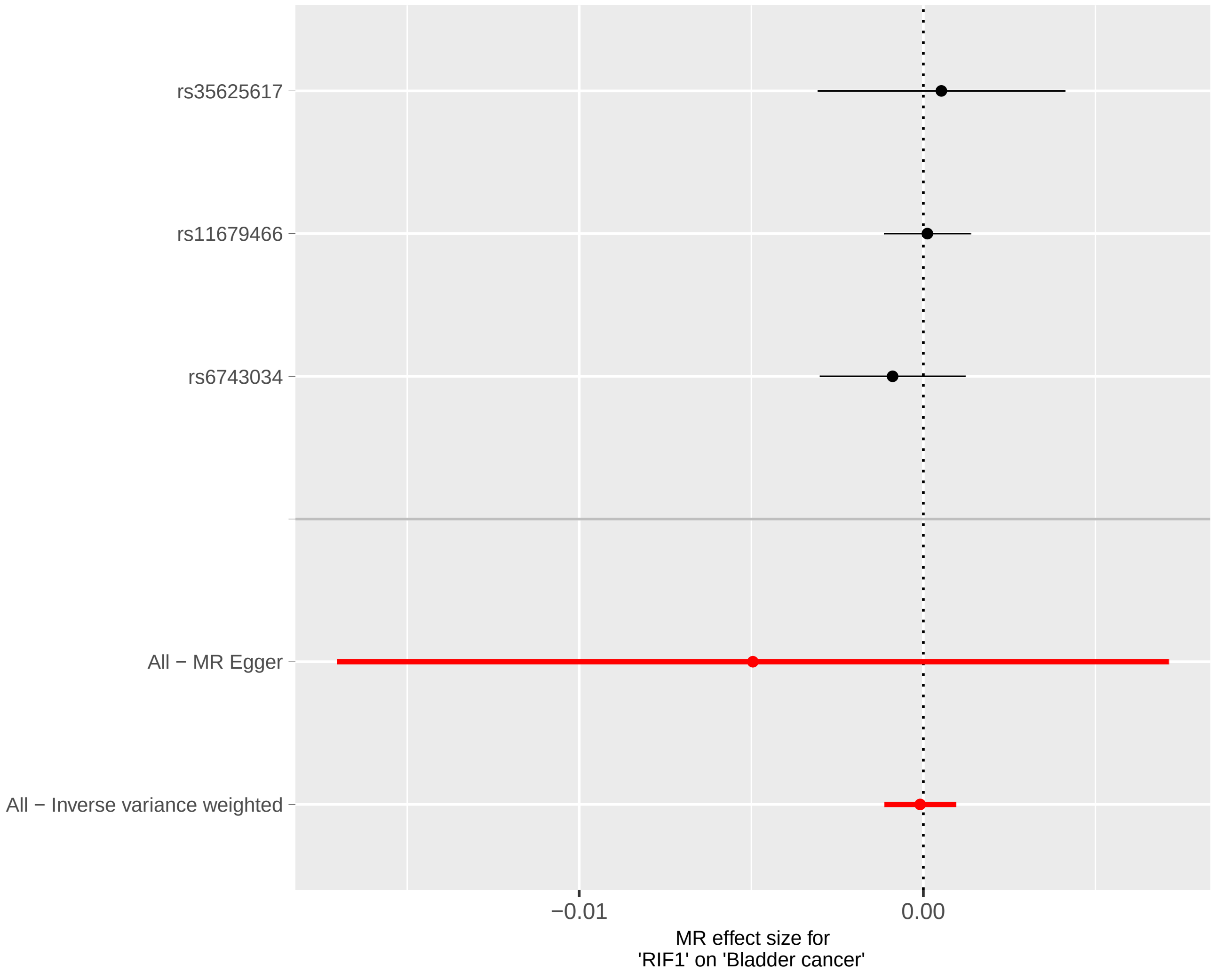

rs35625617
rs11679466
rs6743034
All − MR Egger
All − Inverse variance weighted
−0.01
0.00
MR effect size for
'RIF1' on 'Bladder cancer'

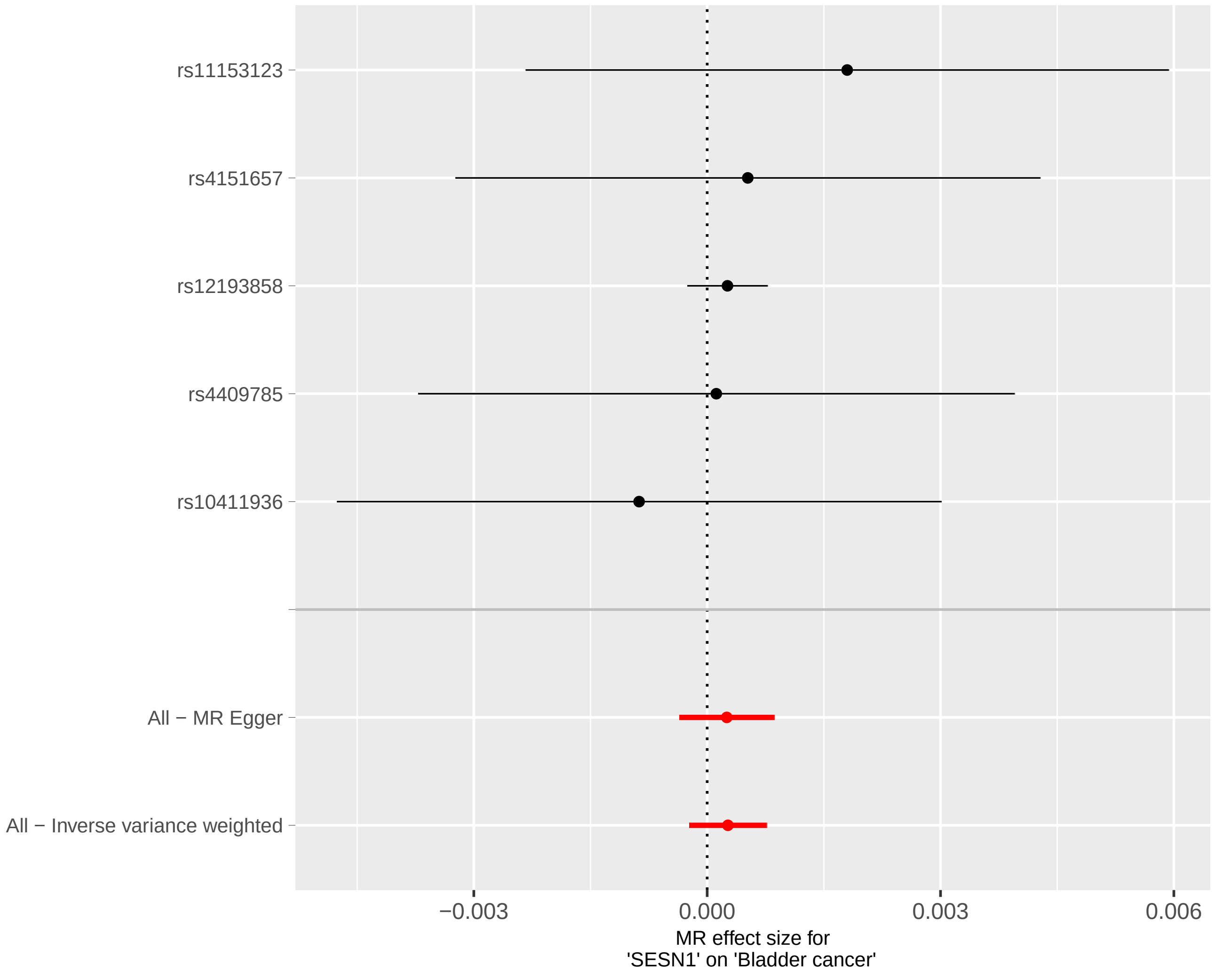

rs11153123
rs4151657
rs12193858
rs4409785
rs10411936
All − MR Egger
All − Inverse variance weighted
−0.003
0.000
0.003
0.006
MR effect size for
'SESN1' on 'Bladder cancer'

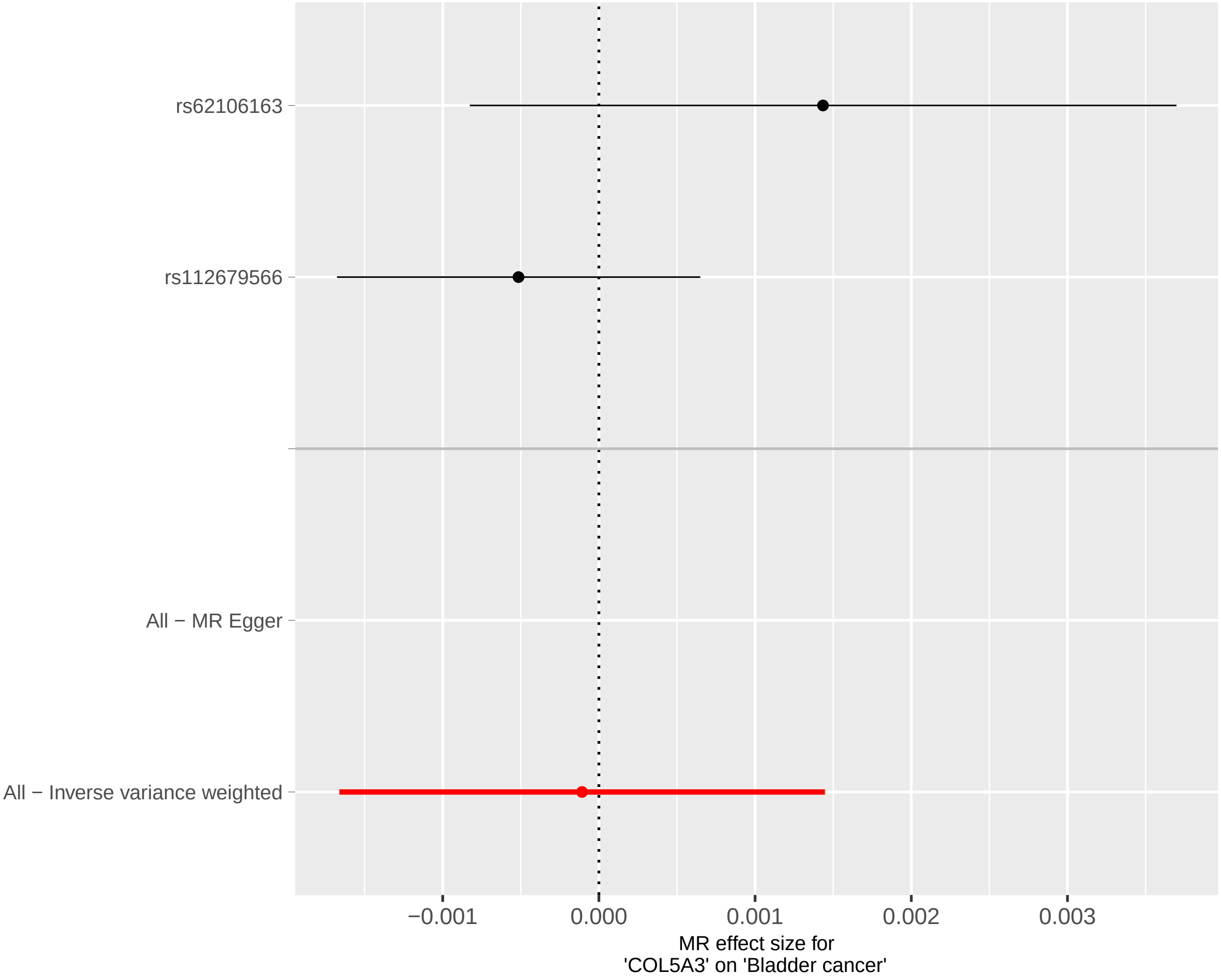

rs62106163
rs112679566
All − MR Egger
All − Inverse variance weighted
−0.001
0.000
0.001
0.002
0.003
MR effect size for
'COL5A3' on 'Bladder cancer'

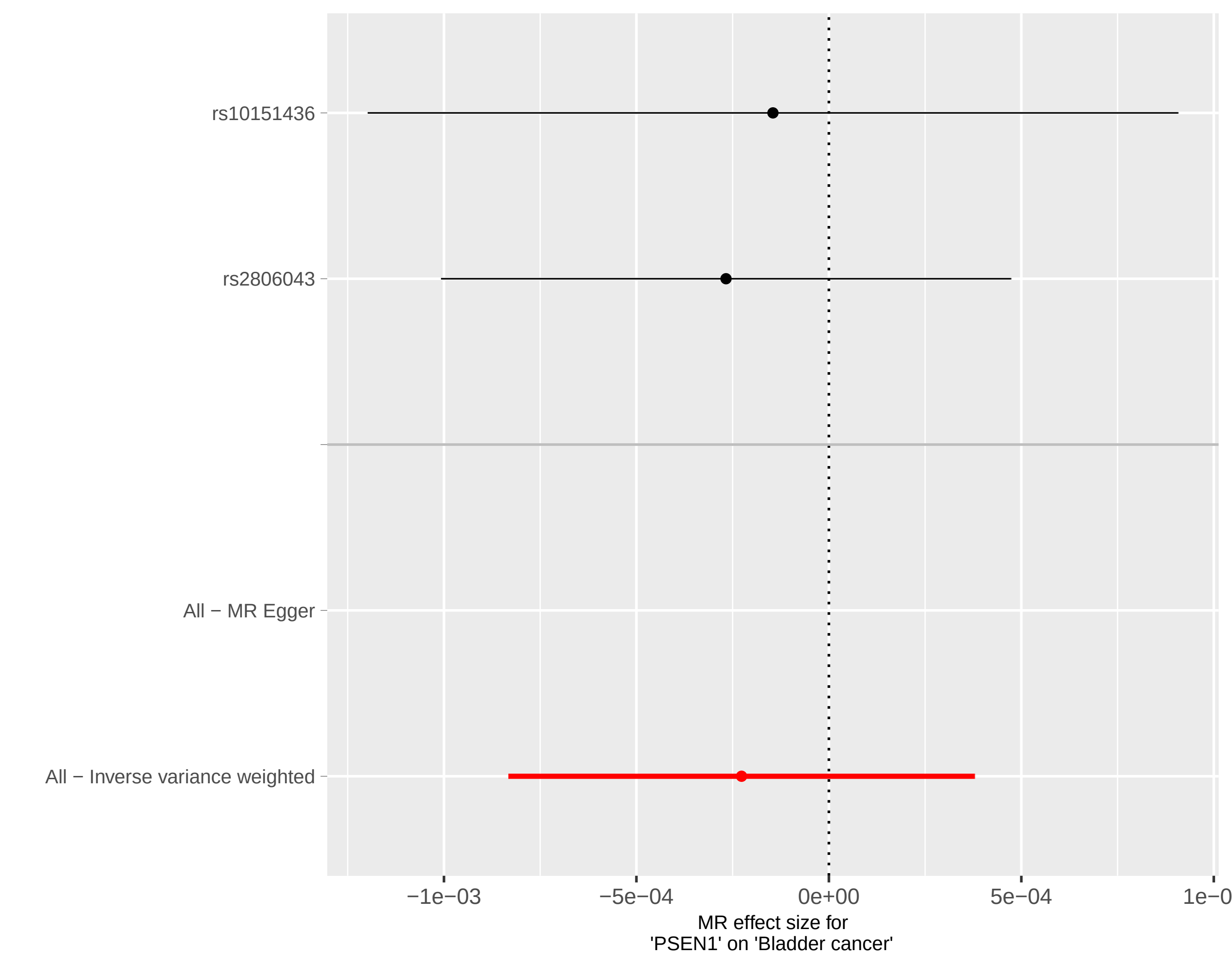
rs10151436
rs2806043
All − MR Egger
All − Inverse variance weighted
−1e−03
−5e−04
0e+00
5e−04
1e−0
MR effect size for
'PSEN1' on 'Bladder cancer'

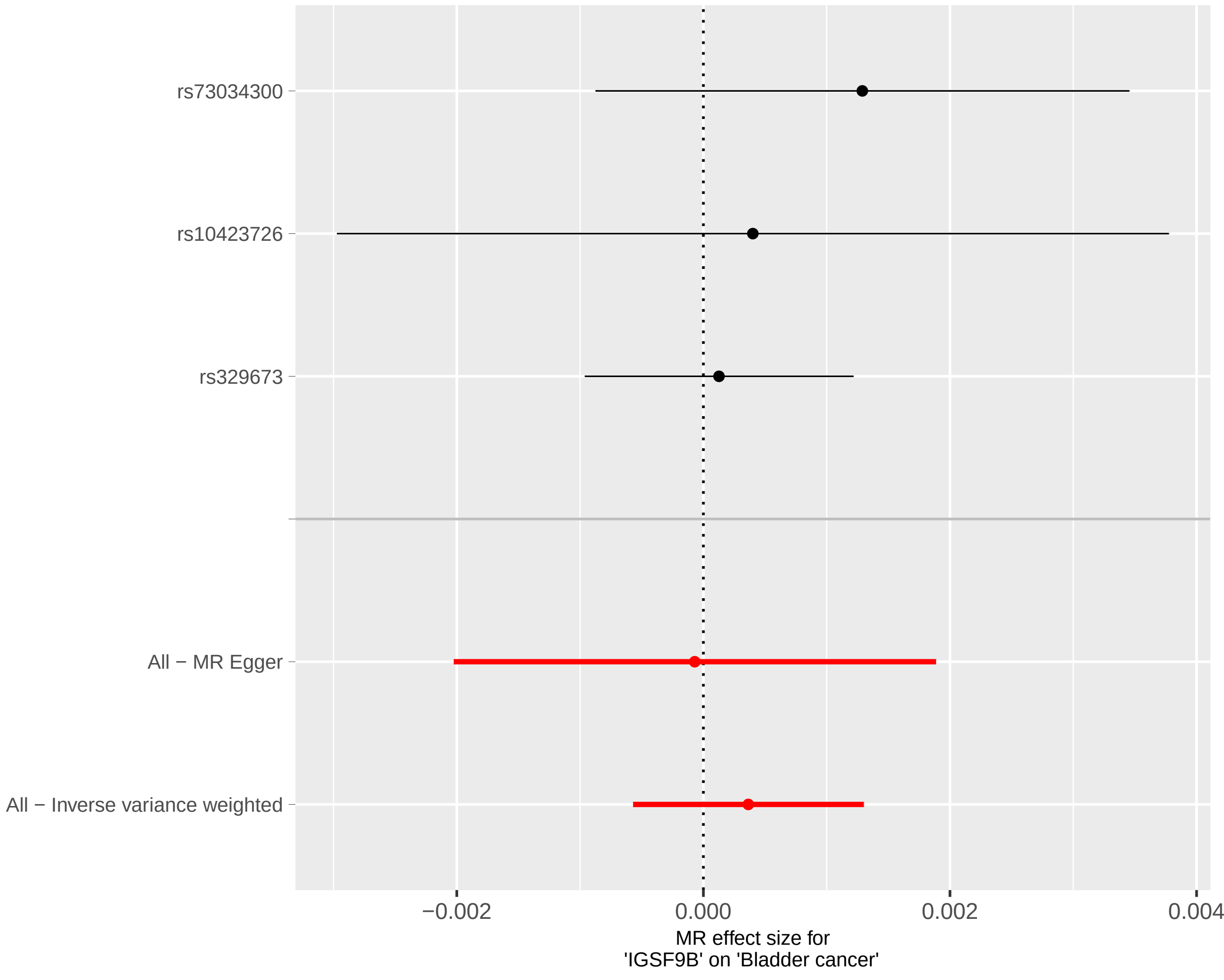
rs73034300
rs10423726
rs329673
All − MR Egger
All − Inverse variance weighted
−0.002
0.000
0.002
0.004
MR effect size for
'IGSF9B' on 'Bladder cancer'

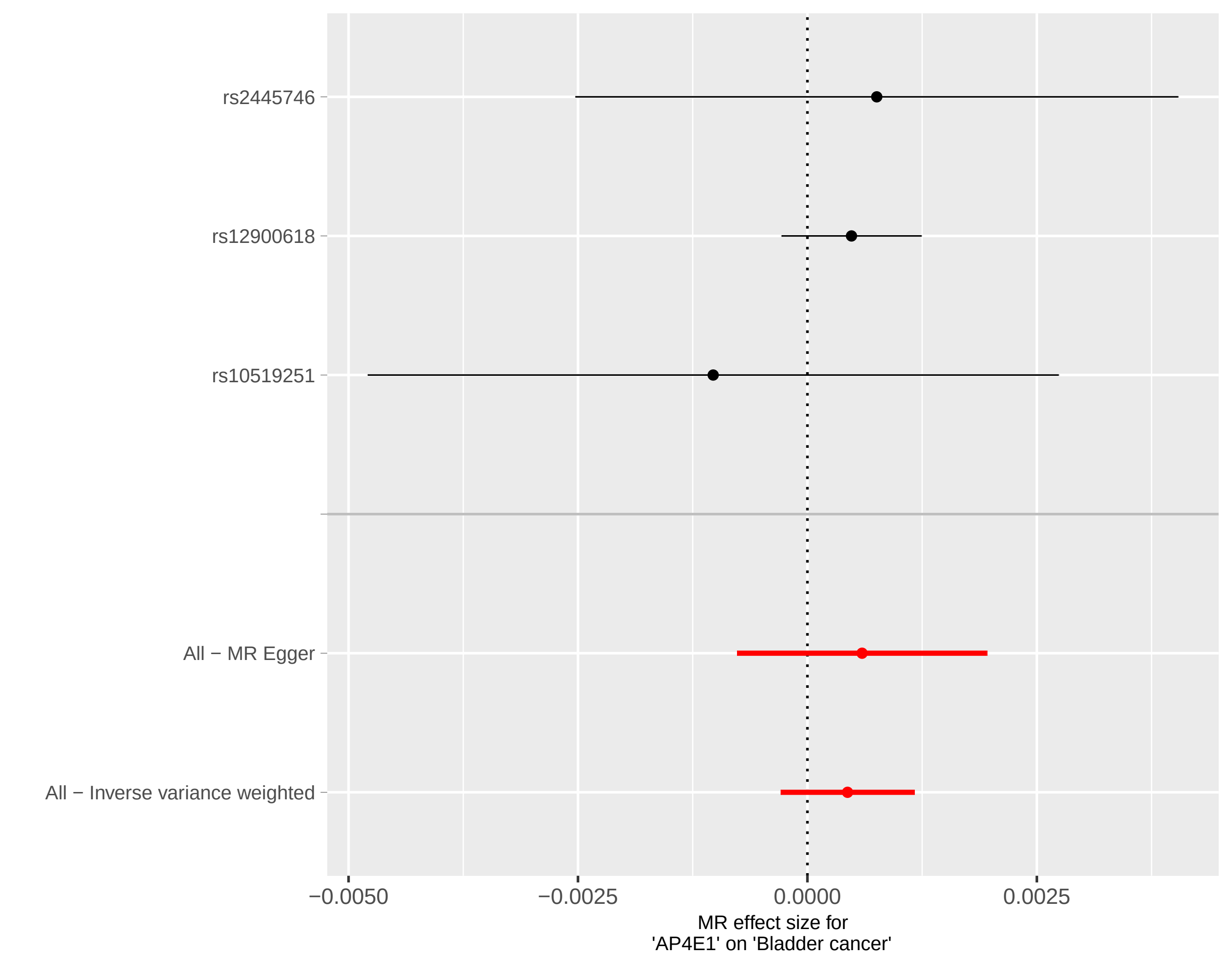

rs2445746
rs12900618
rs10519251
All − MR Egger
All − Inverse variance weighted
−0.0050
−0.0025
0.0000
0.0025
MR effect size for
'AP4E1' on 'Bladder cancer'

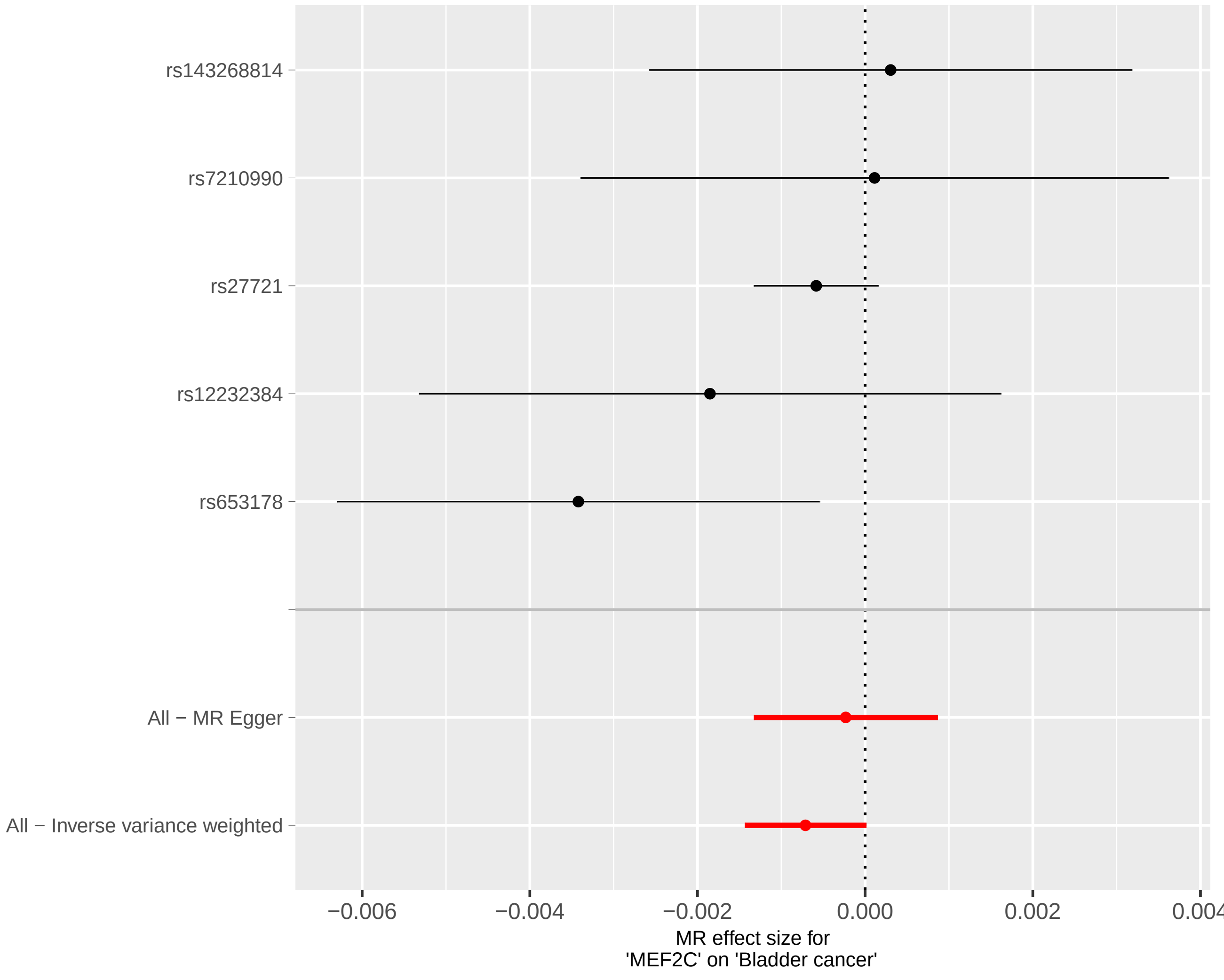

rs143268814
rs7210990
rs27721
rs12232384
rs653178
All − MR Egger
All − Inverse variance weighted
−0.006
−0.004
−0.002
0.000
0.002
0.004
MR effect size for
'MEF2C' on 'Bladder cancer'

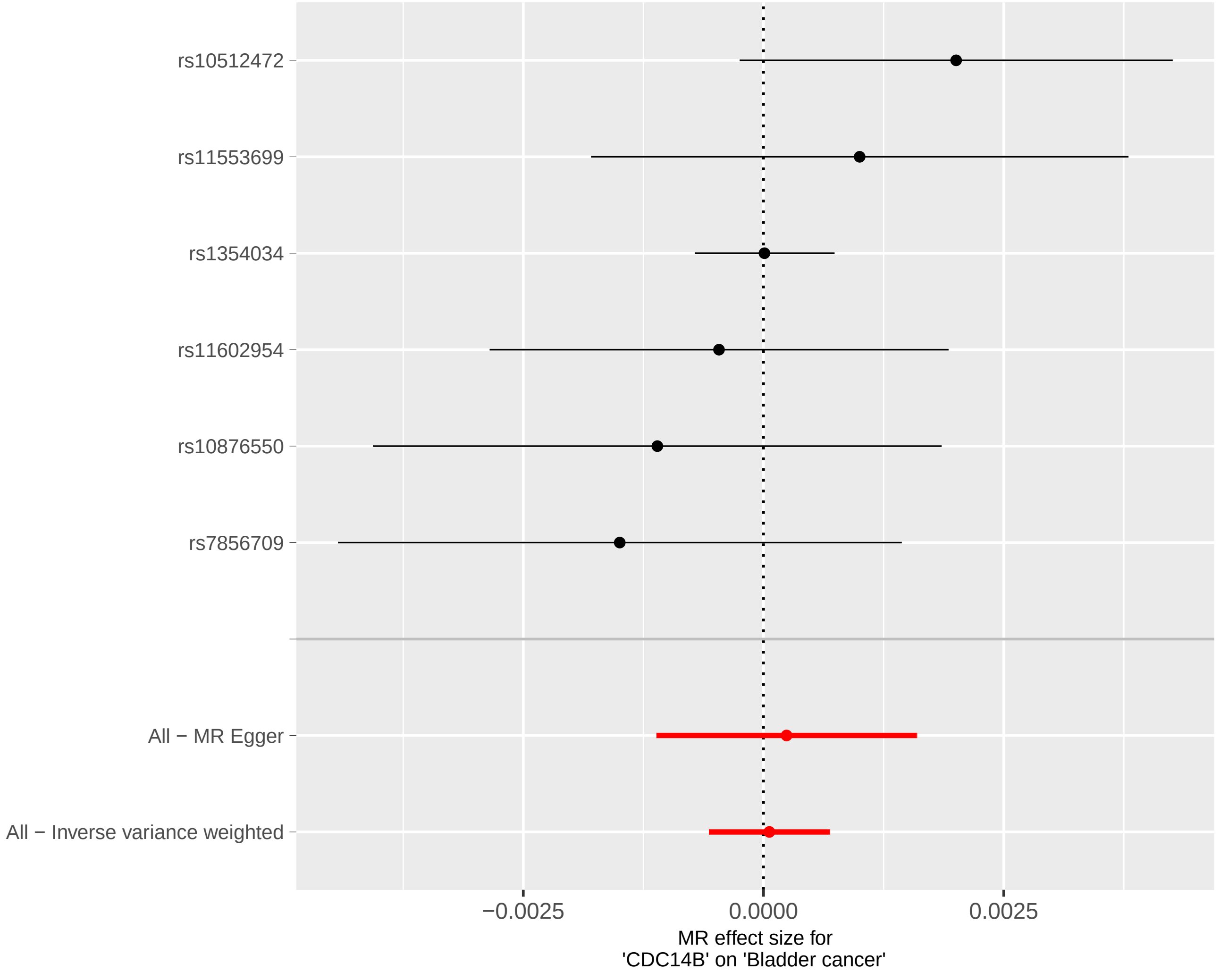

rs10512472
rs11553699
rs1354034
rs11602954
rs10876550
rs7856709
All − MR Egger
All − Inverse variance weighted
−0.0025
0.0000
0.0025
MR effect size for
'CDC14B' on 'Bladder cancer'

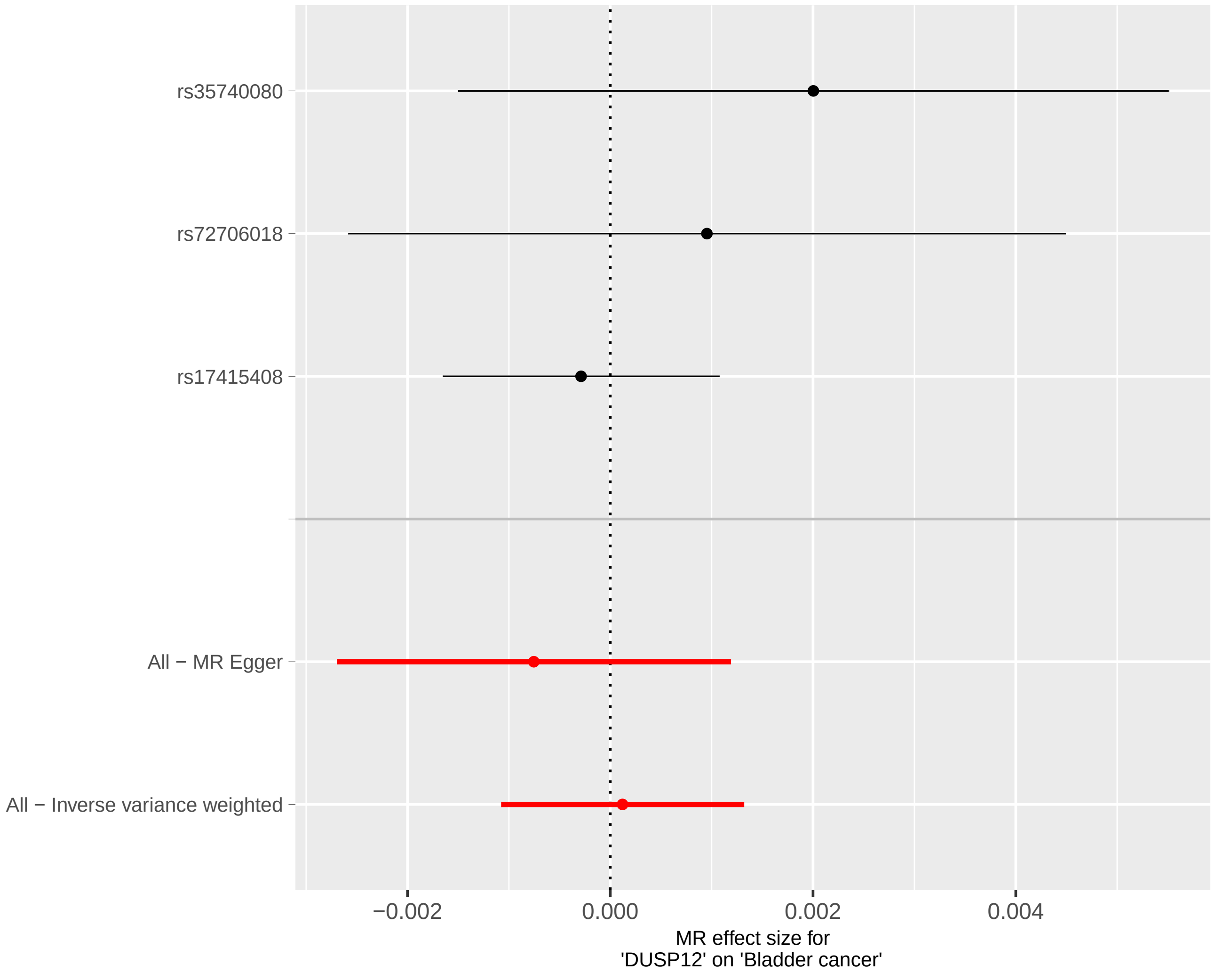
rs35740080
rs72706018
rs17415408
All − MR Egger
All − Inverse variance weighted
−0.002
0.000
0.002
0.004
MR effect size for
'DUSP12' on 'Bladder cancer'

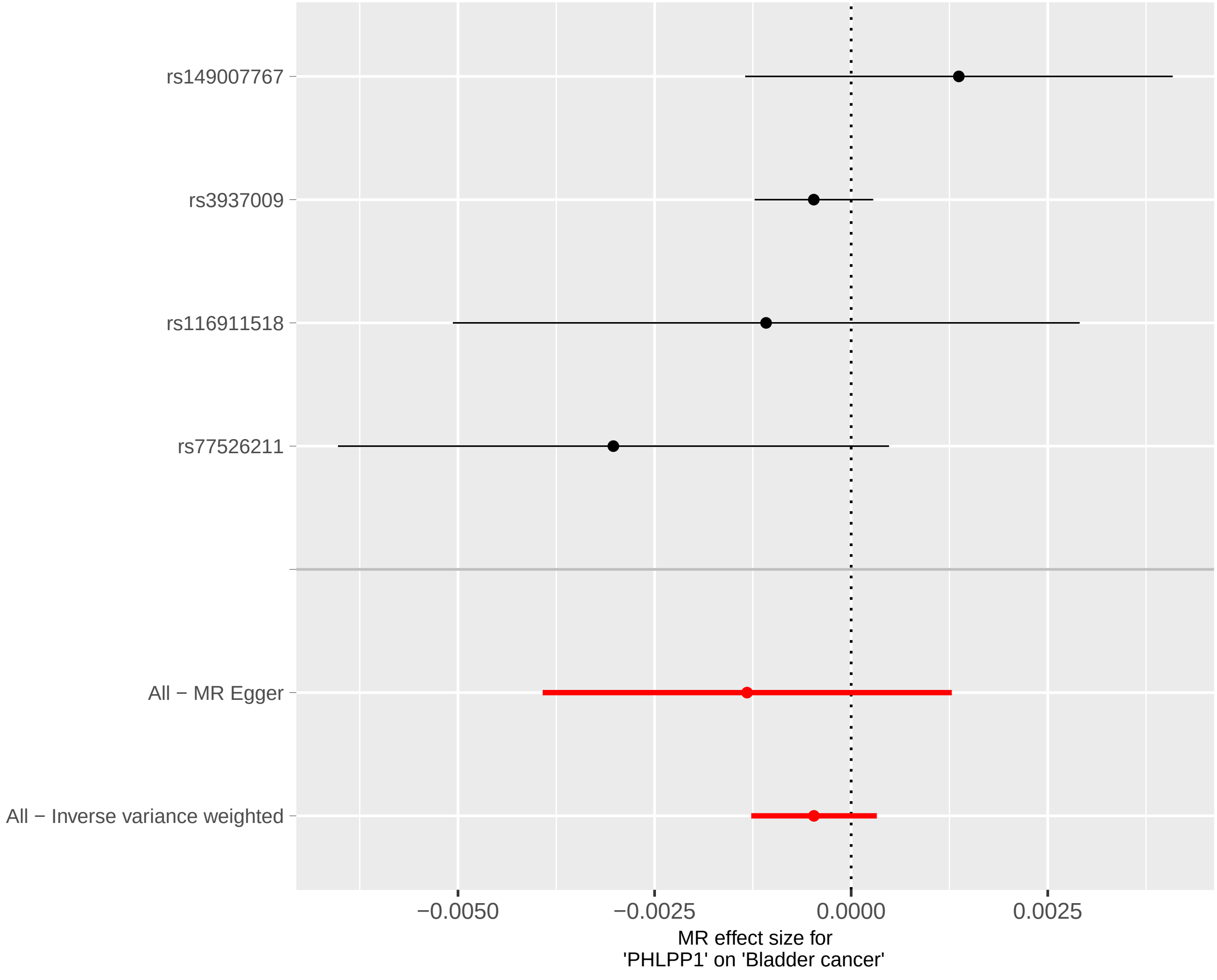
rs149007767
rs3937009
rs116911518
rs77526211
All − MR Egger
All − Inverse variance weighted
−0.0050
−0.0025
0.0000
0.0025
MR effect size for
'PHLPP1' on 'Bladder cancer'

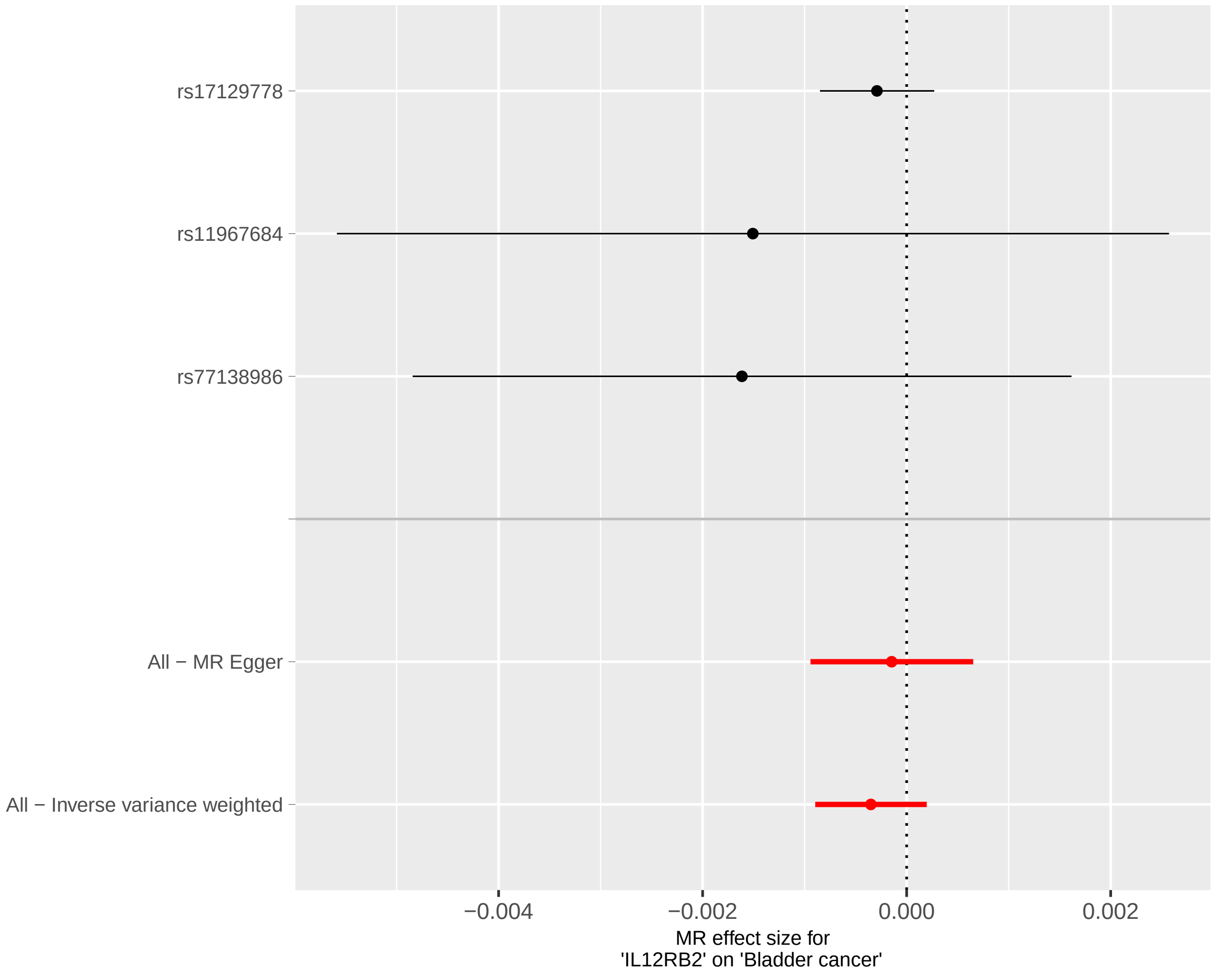
rs17129778
rs11967684
rs77138986
All − MR Egger
All − Inverse variance weighted
−0.004
−0.002
0.000
0.002
MR effect size for
'IL12RB2' on 'Bladder cancer'

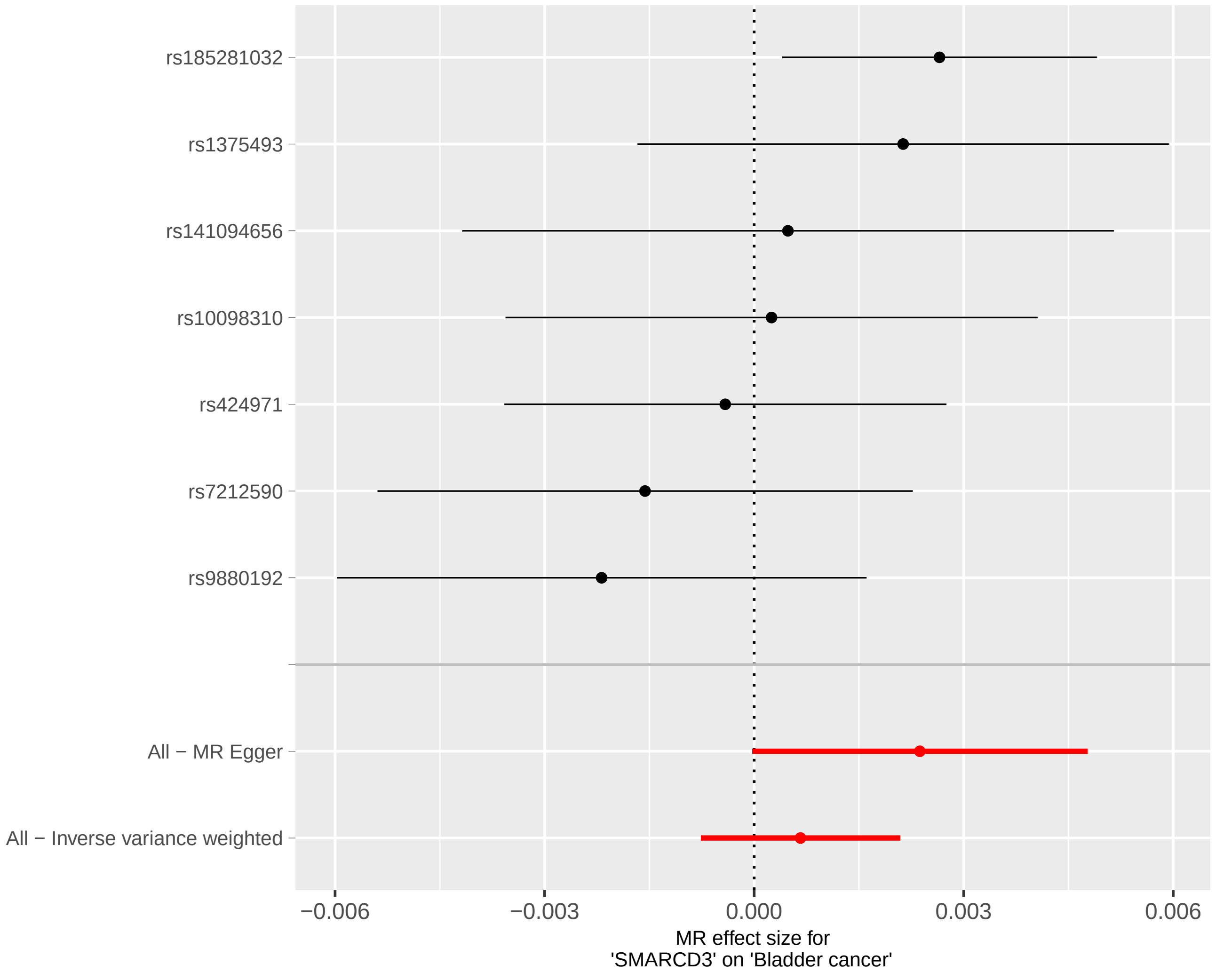

rs185281032
rs1375493
rs141094656
rs10098310
rs424971
rs7212590
rs9880192
All − MR Egger
All − Inverse variance weighted
−0.006
−0.003
0.000
0.003
0.006
MR effect size for
'SMARCD3' on 'Bladder cancer'

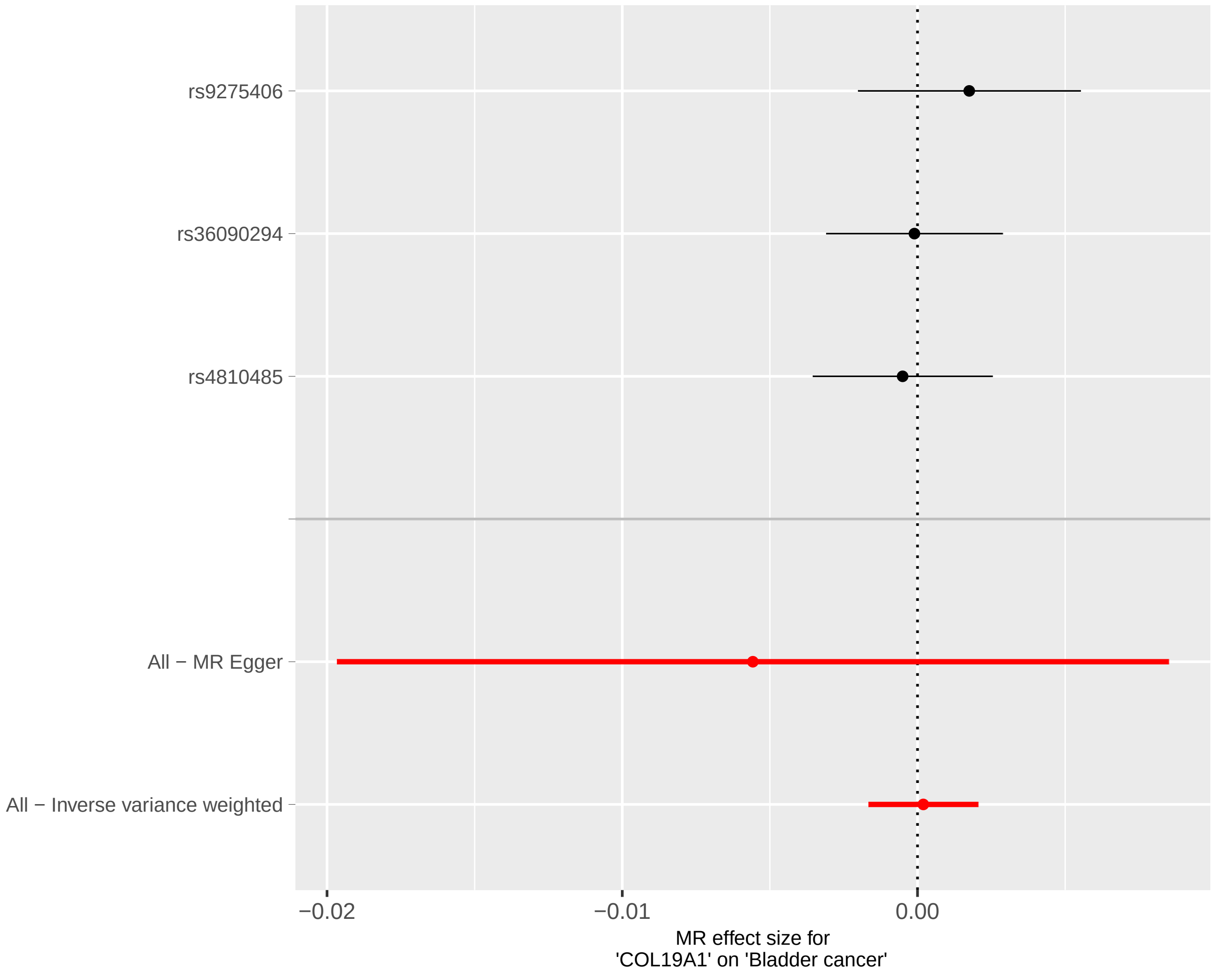
rs9275406
rs36090294
rs4810485
All − MR Egger
All − Inverse variance weighted
−0.02
−0.01
0.00
MR effect size for
'COL19A1' on 'Bladder cancer'

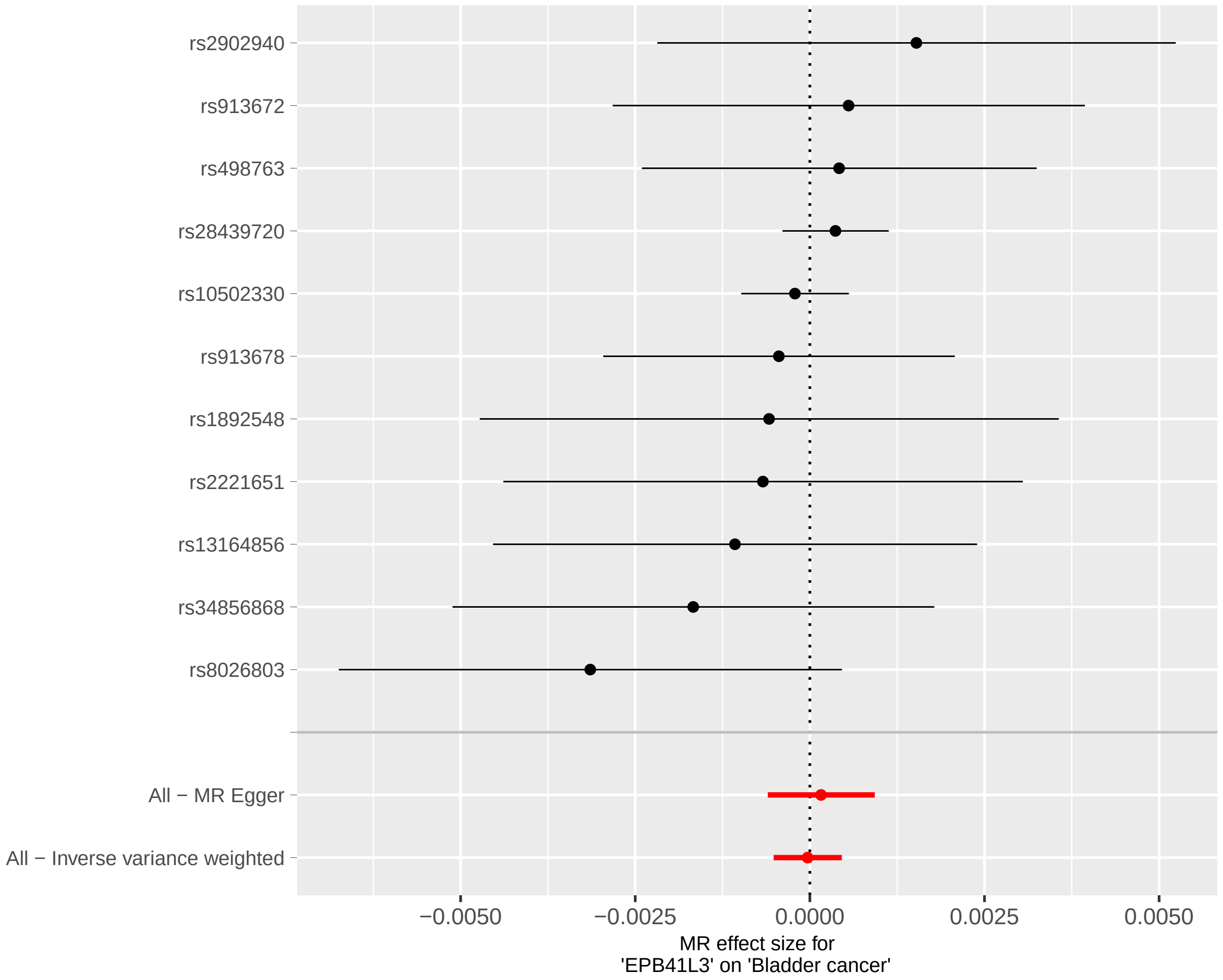
rs2902940
rs913672
rs498763
rs28439720
rs10502330
rs913678
rs1892548
rs2221651
rs13164856
rs34856868
rs8026803
All − MR Egger
All − Inverse variance weighted
−0.0050
−0.0025
0.0000
0.0025
0.0050
MR effect size for
'EPB41L3' on 'Bladder cancer'

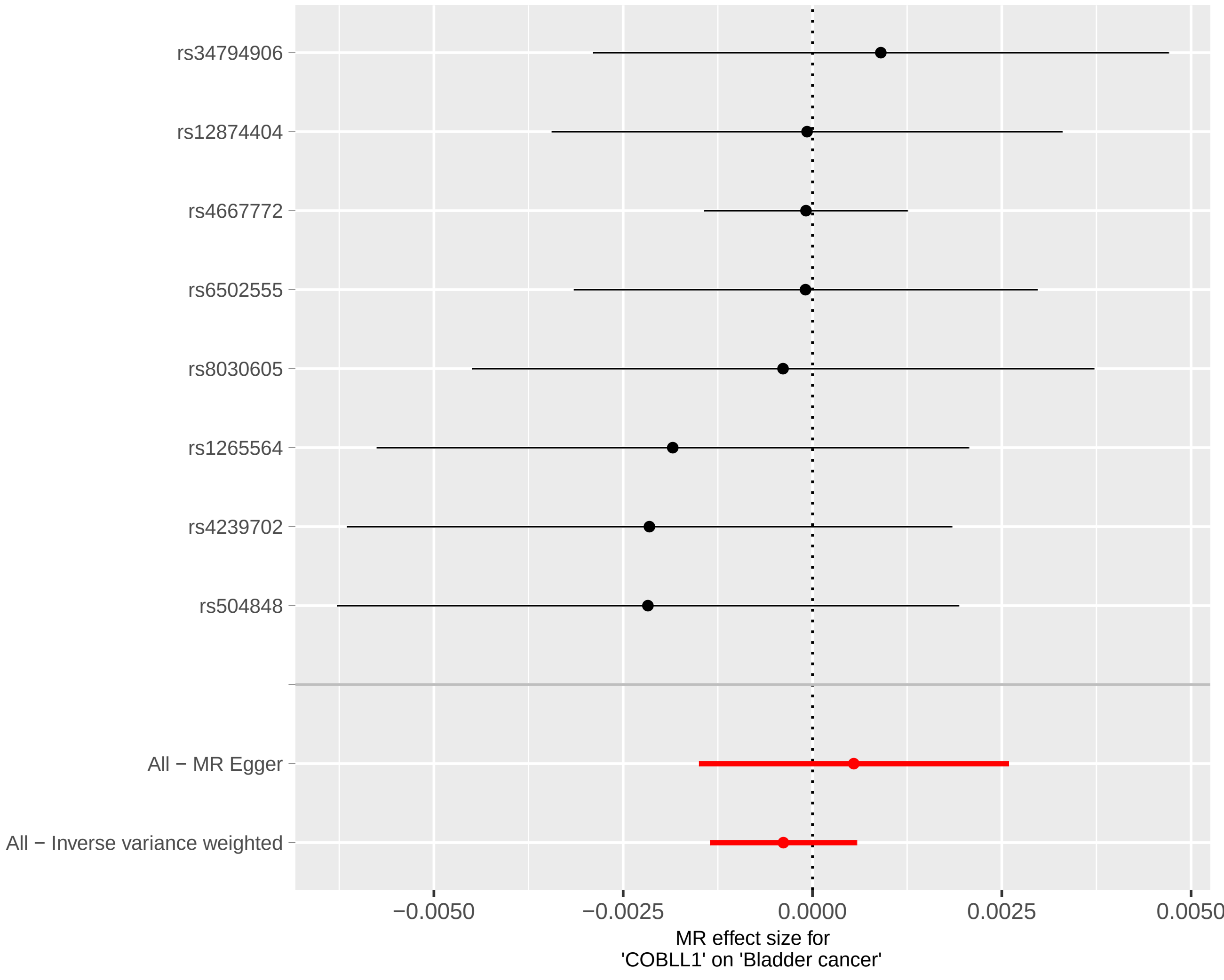

rs34794906
rs12874404
rs4667772
rs6502555
rs8030605
rs1265564
rs4239702
rs504848
All − MR Egger
All − Inverse variance weighted
−0.0050
−0.0025
0.0000
0.0025
0.0050
MR effect size for
'COBLL1' on 'Bladder cancer'

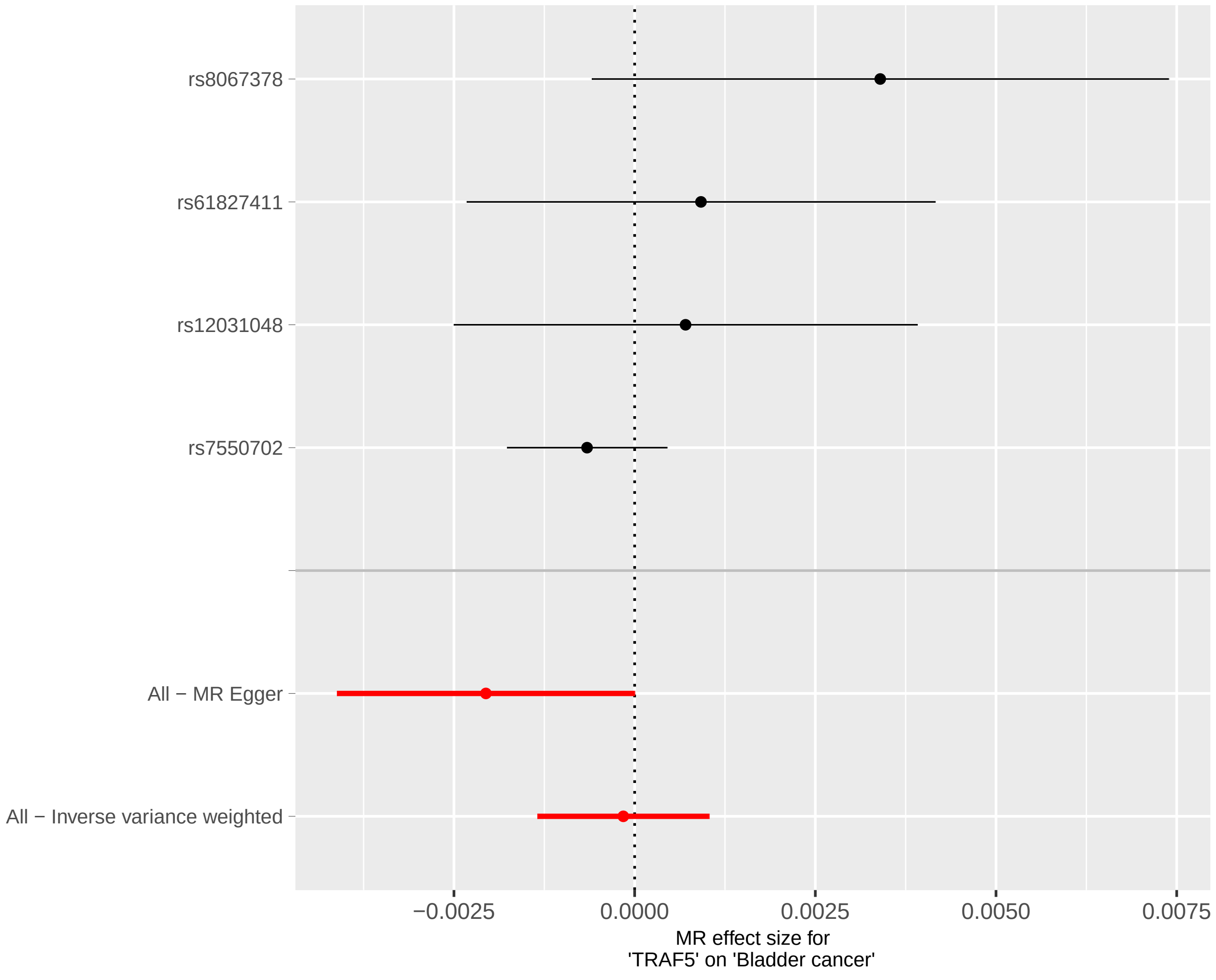

rs8067378
rs61827411
rs12031048
rs7550702
All − MR Egger
All − Inverse variance weighted
−0.0025
0.0000
0.0025
0.0050
0.0075
MR effect size for
'TRAF5' on 'Bladder cancer'

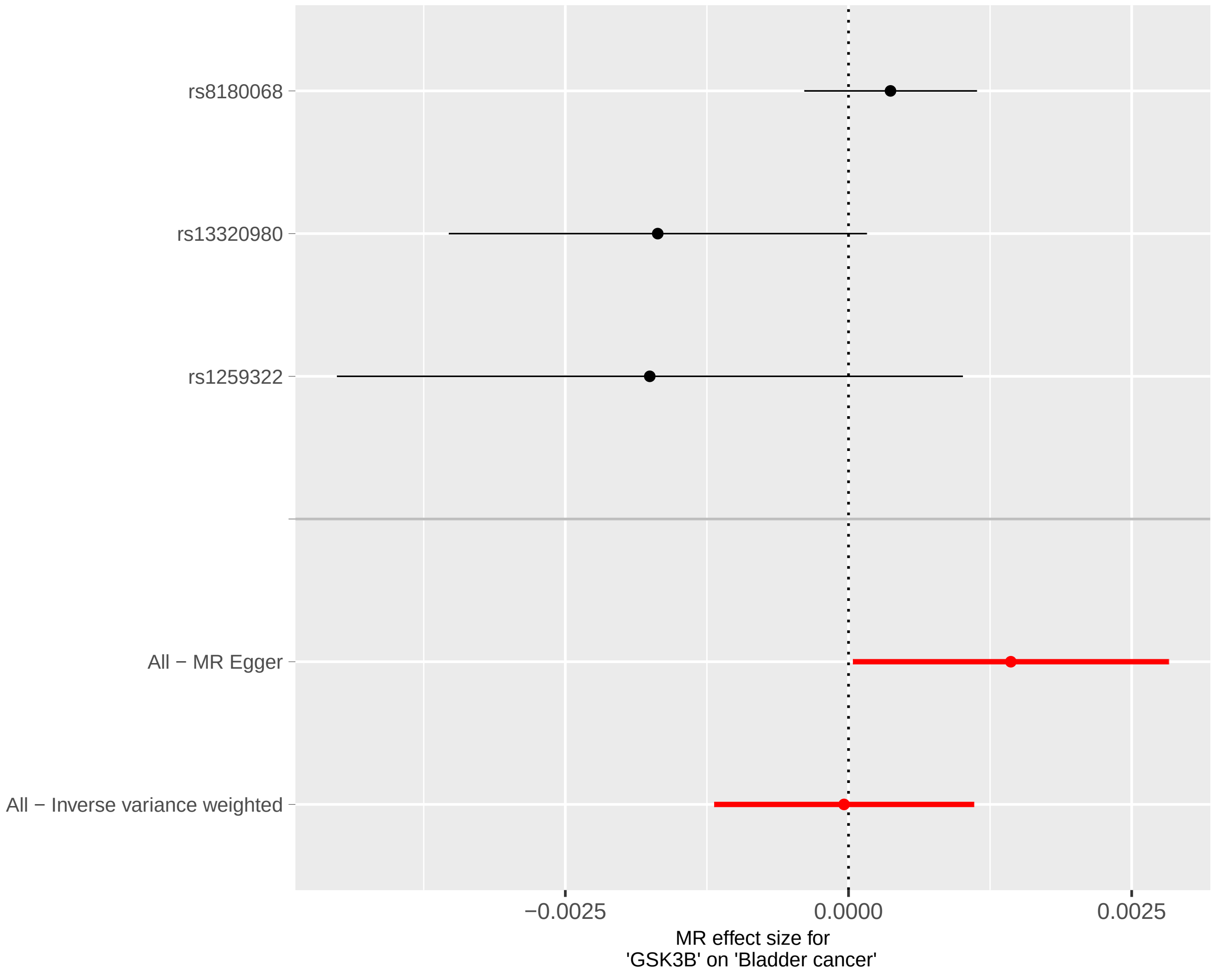

rs8180068
rs13320980
rs1259322
All − MR Egger
All − Inverse variance weighted
−0.0025
0.0000
0.0025
MR effect size for
'GSK3B' on 'Bladder cancer'

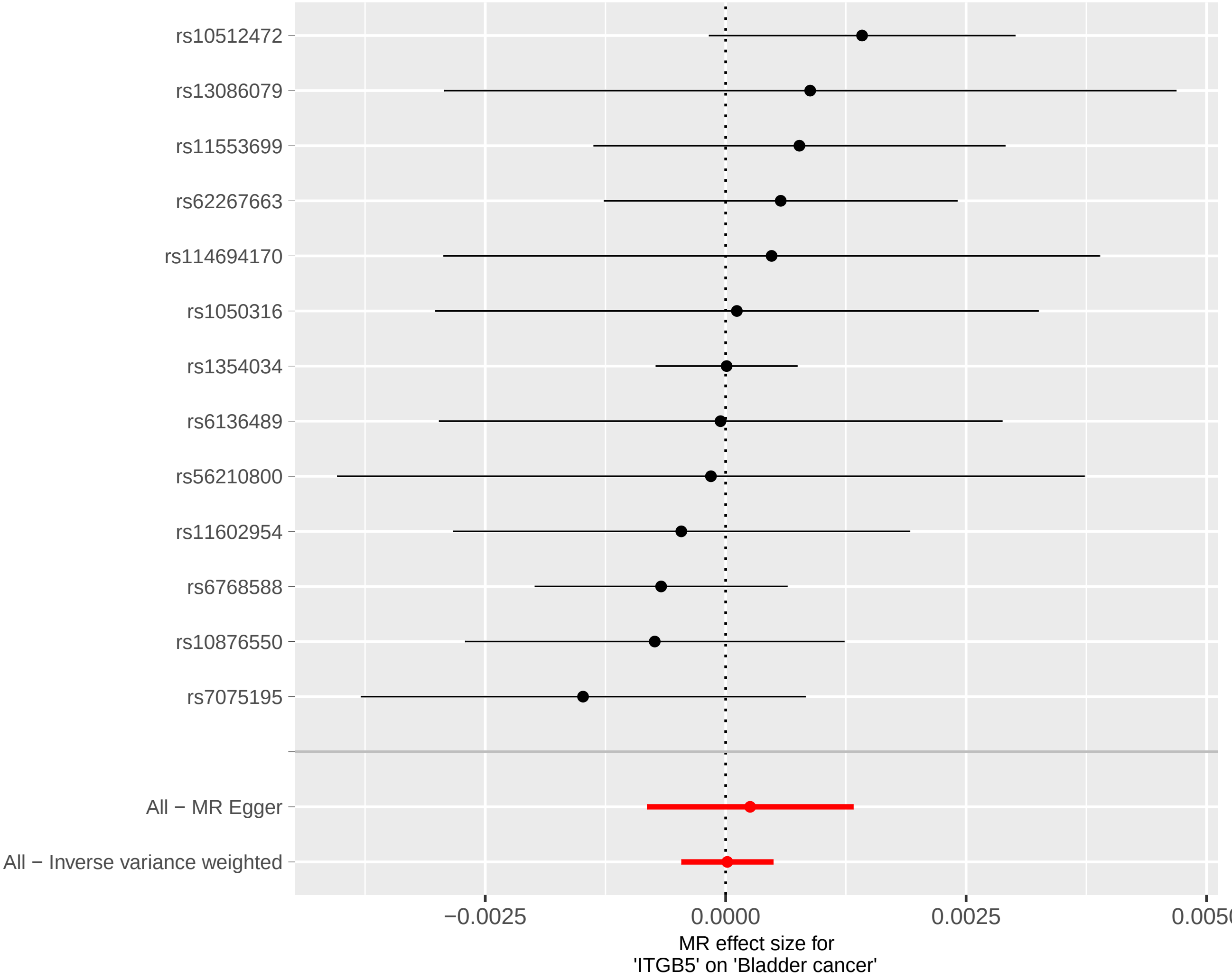
rs10512472
rs13086079
rs11553699
rs62267663
rs114694170
rs1050316
rs1354034
rs6136489
rs56210800
rs11602954
rs6768588
rs10876550
rs7075195
All − MR Egger
All − Inverse variance weighted
−0.0025
0.0000
0.0025
MR effect size for
'ITGB5' on 'Bladder cancer'

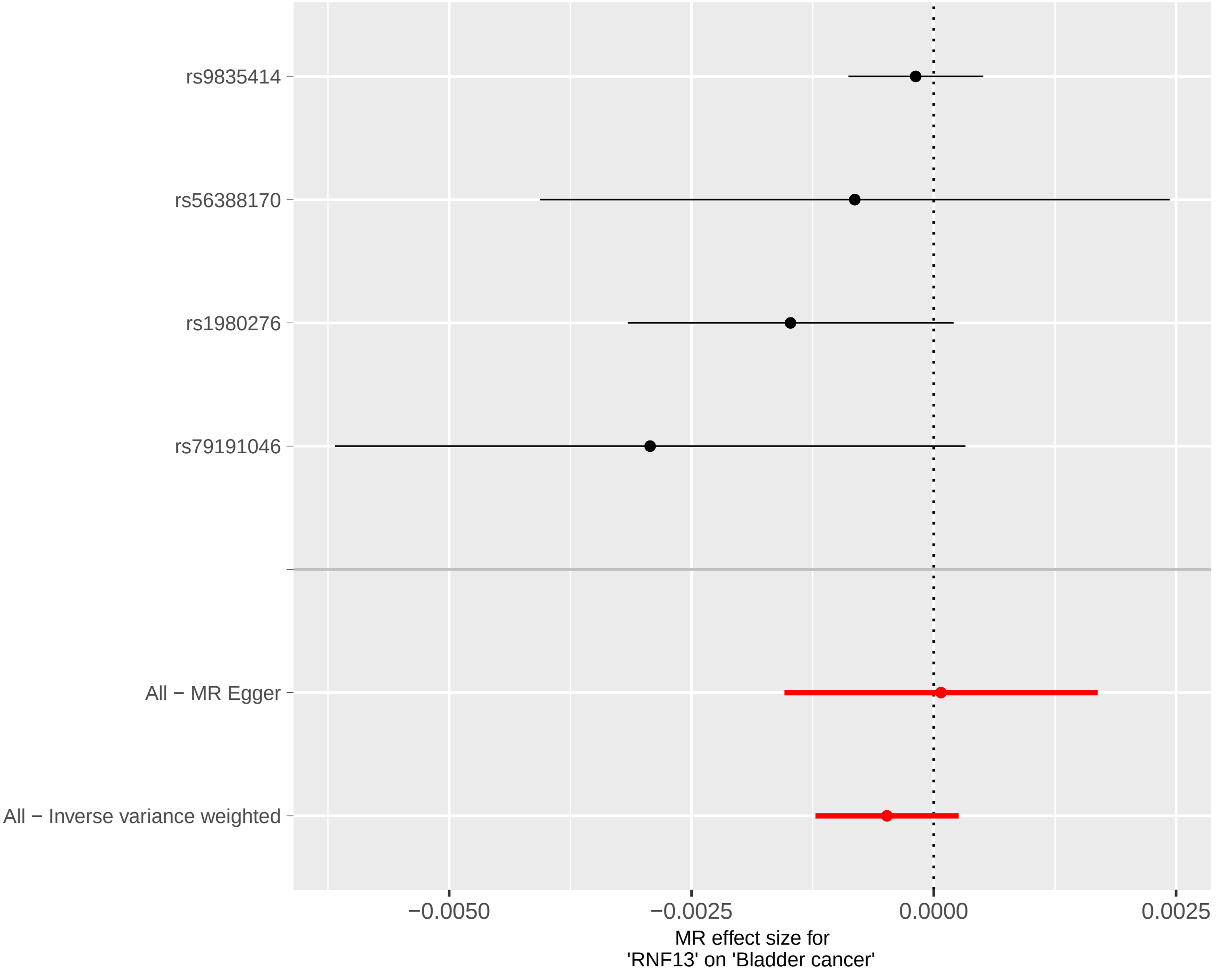
rs9835414
rs56388170
rs1980276
rs79191046
All − MR Egger
All − Inverse variance weighted
−0.0050
−0.0025
0.0000
0.0025
MR effect size for
'RNF13' on 'Bladder cancer'

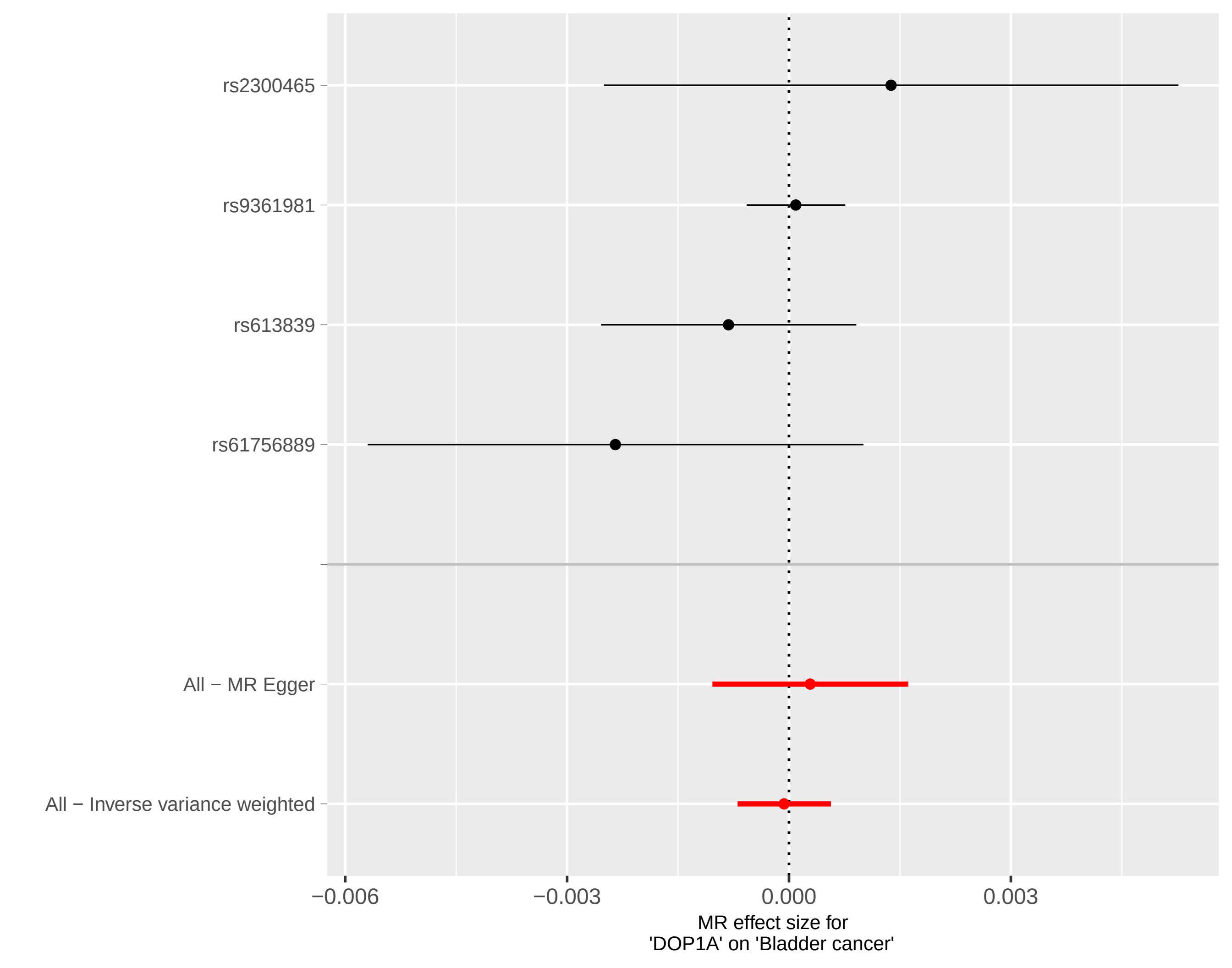

rs2300465
rs9361981
rs613839
rs61756889
All − MR Egger
All − Inverse variance weighted
−0.006
−0.003
0.000
0.003
MR effect size for
'DOP1A' on 'Bladder cancer'

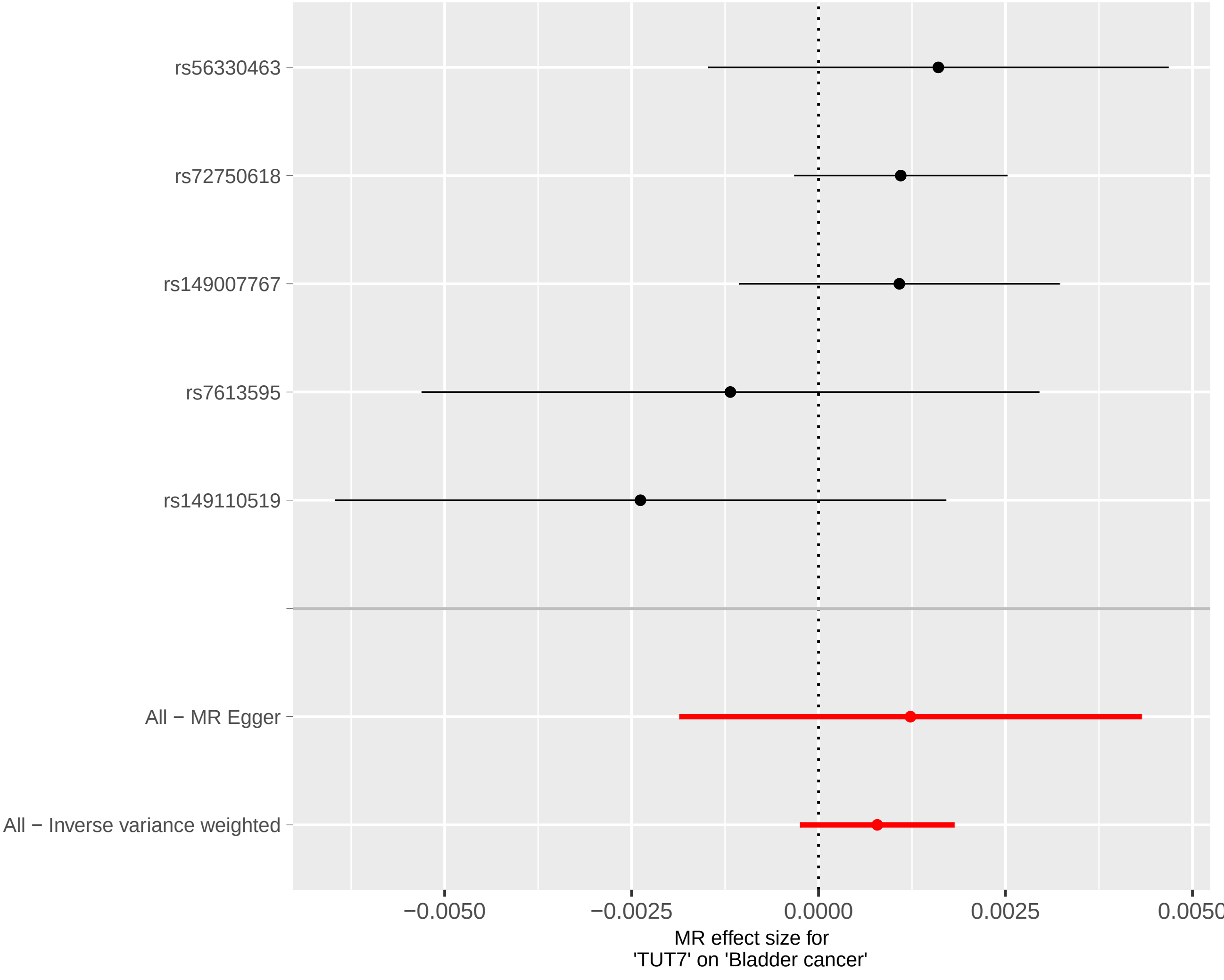

rs56330463
rs72750618
rs149007767
rs7613595
rs149110519
All − MR Egger
All − Inverse variance weighted
−0.0050
−0.0025
0.0000
0.0025
0.0050
MR effect size for
'TUT7' on 'Bladder cancer'

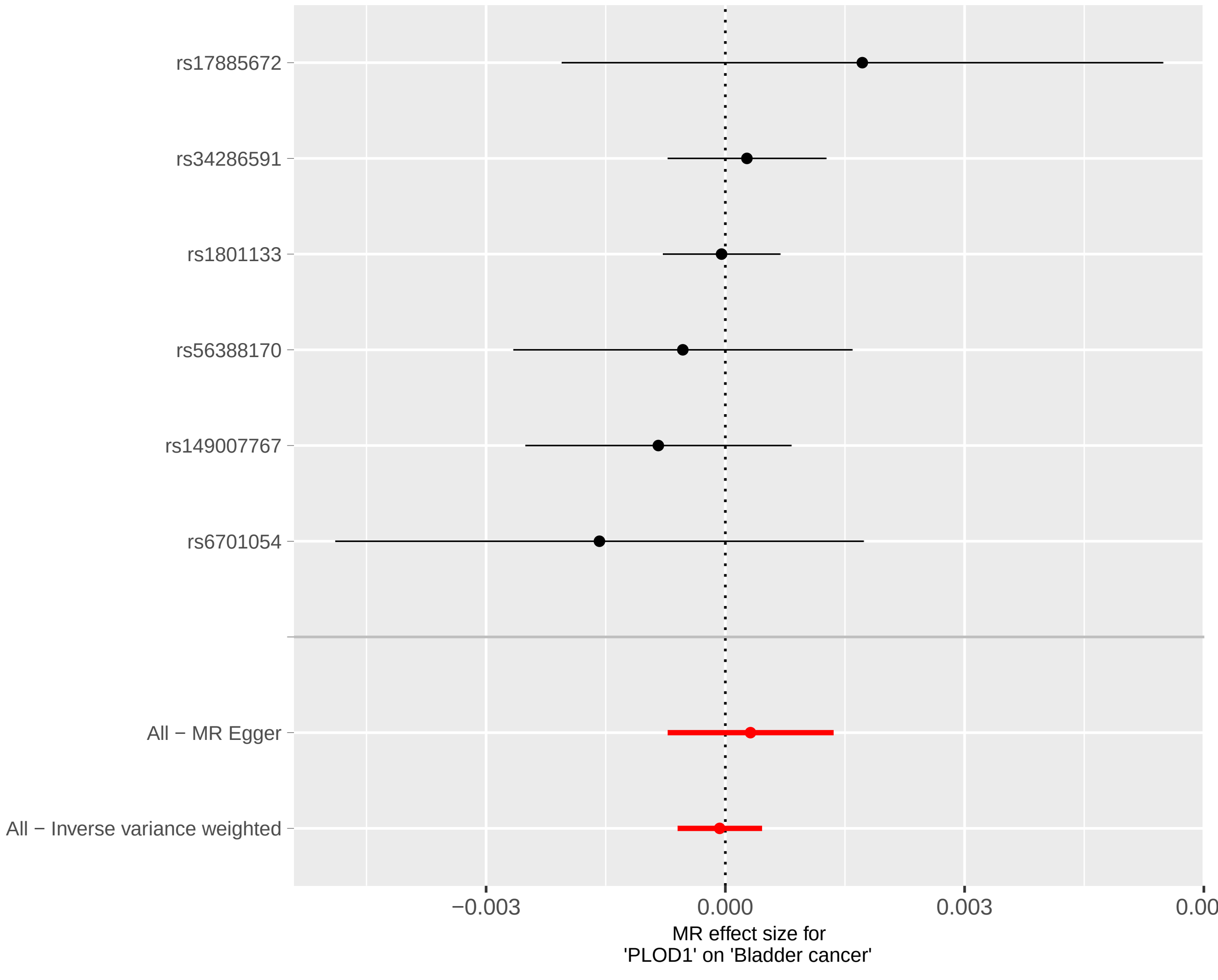

rs17885672
rs34286591
rs1801133
rs56388170
rs149007767
rs6701054
All − MR Egger
All − Inverse variance weighted
−0.003
0.000
0.003
0.00
MR effect size for
'PLOD1' on 'Bladder cancer'

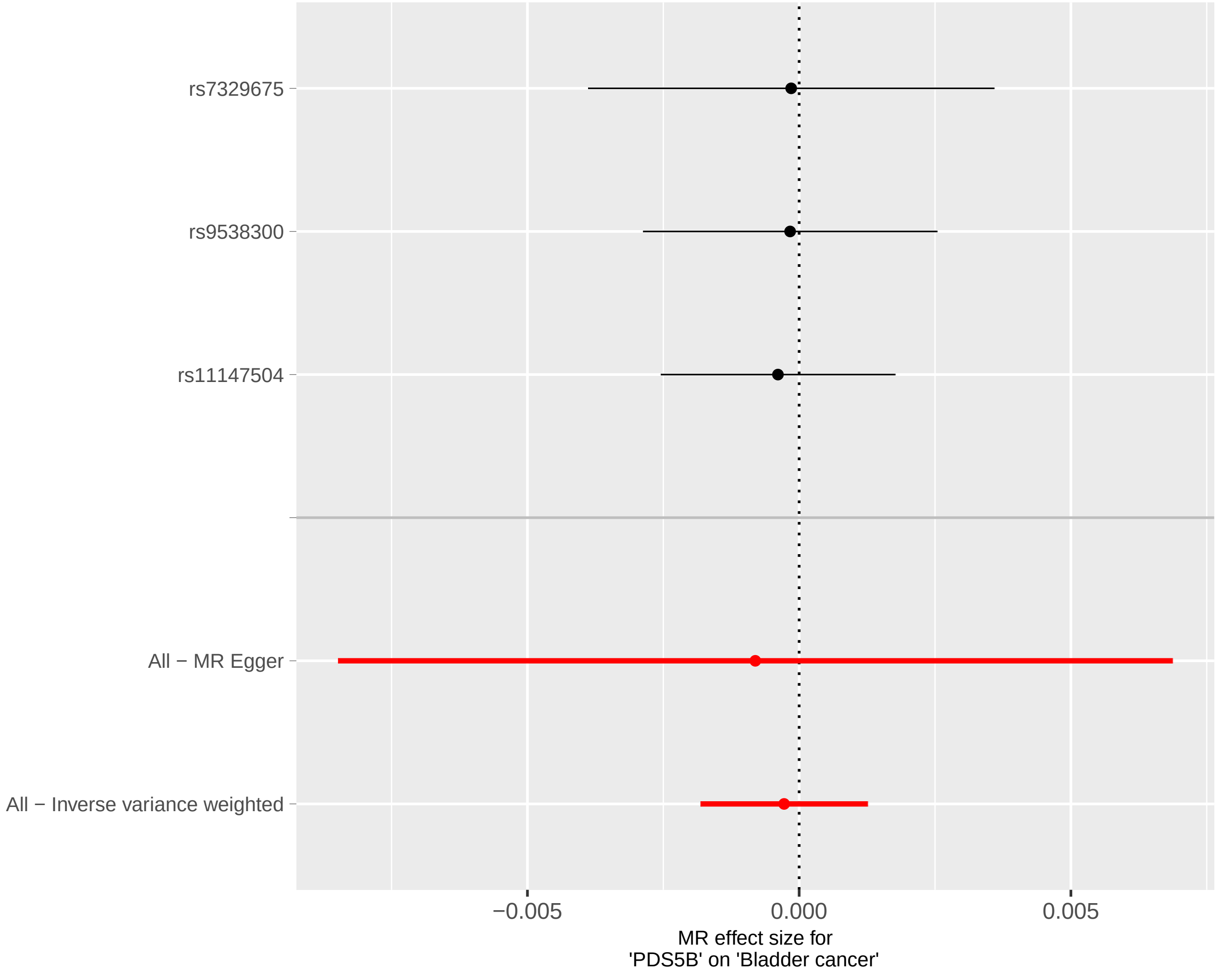

rs7329675
rs9538300
rs11147504
All − MR Egger
All − Inverse variance weighted
−0.005
0.000
0.005
MR effect size for
'PDS5B' on 'Bladder cancer'

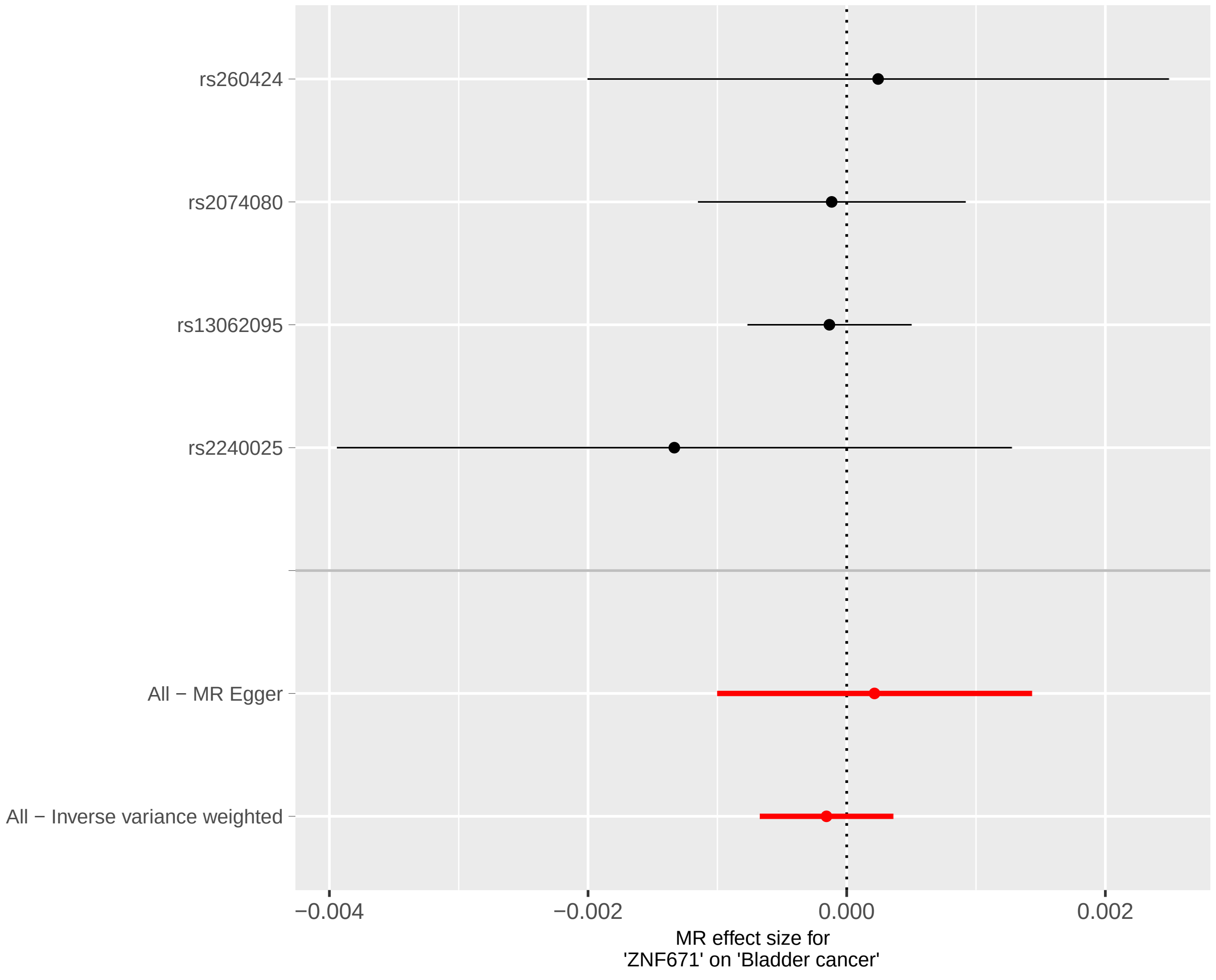
rs260424
rs2074080
rs13062095
rs2240025
All − MR Egger
All − Inverse variance weighted
−0.004
−0.002
0.000
0.002
MR effect size for
'ZNF671' on 'Bladder cancer'

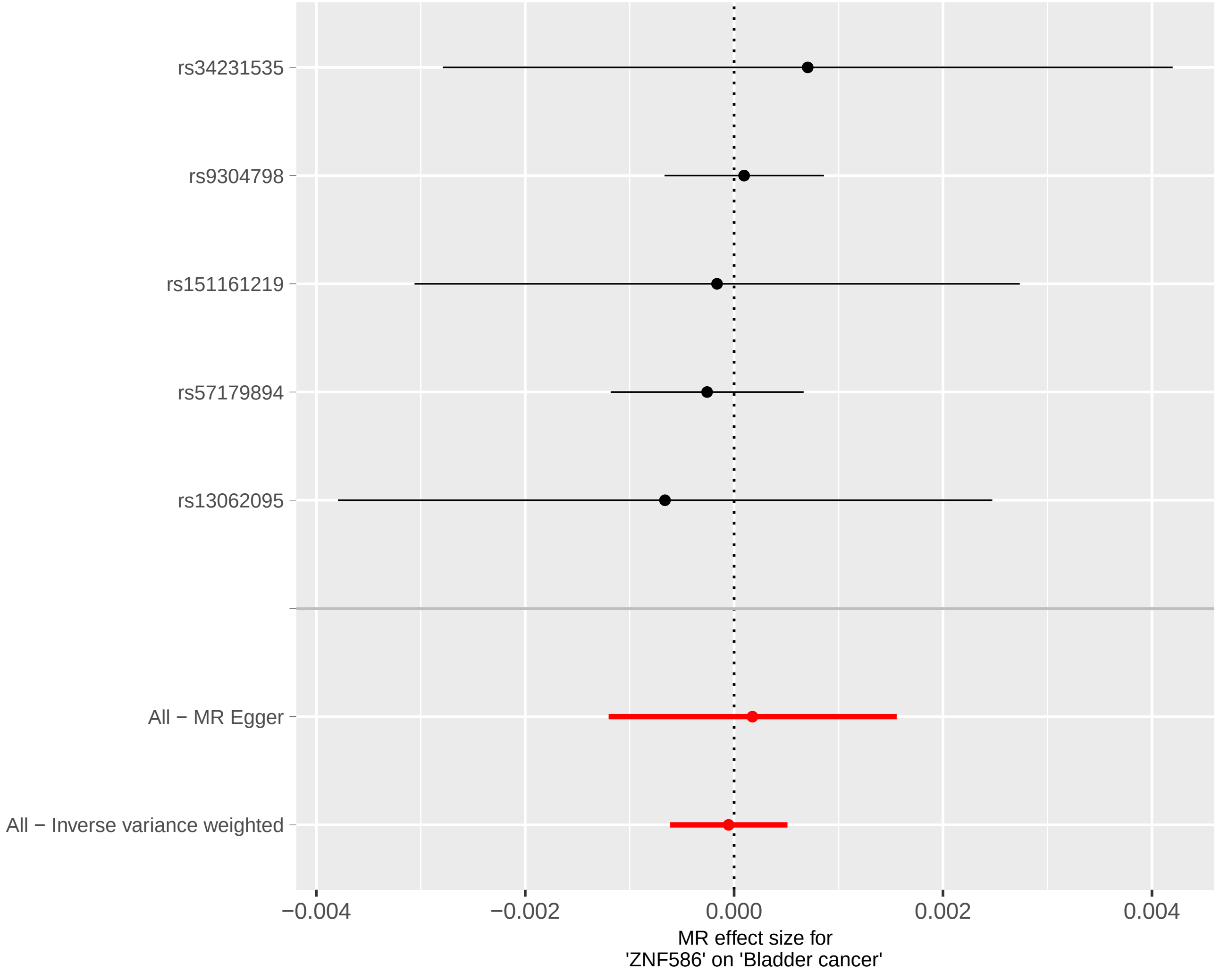
rs34231535
rs9304798
rs151161219
rs57179894
rs13062095
All − MR Egger
All − Inverse variance weighted
−0.004
−0.002
0.000
0.002
0.004
MR effect size for
'ZNF586' on 'Bladder cancer'

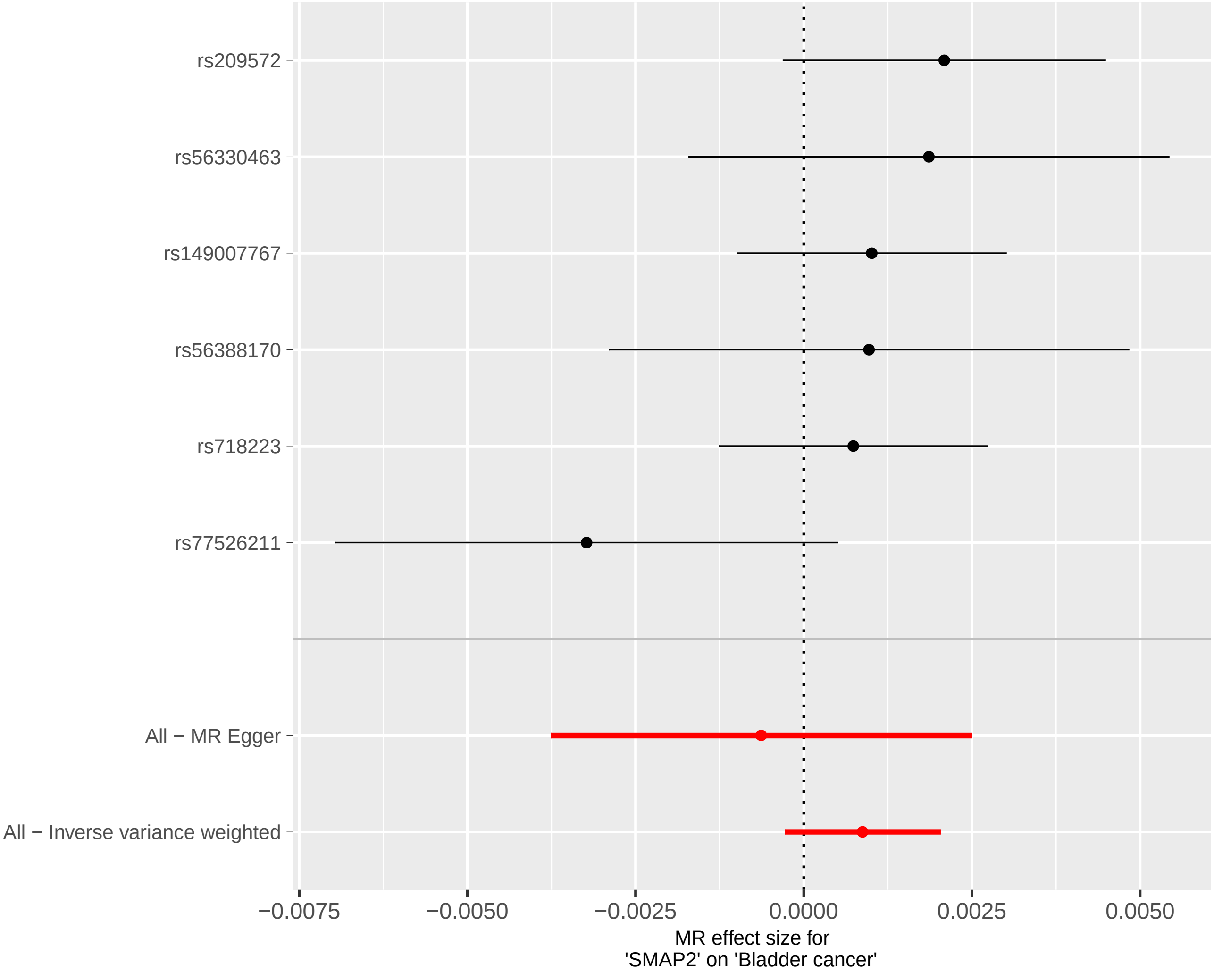

rs209572
rs56330463
rs149007767
rs56388170
rs718223
rs77526211
All − MR Egger
All − Inverse variance weighted
−0.0075
−0.0050
−0.0025
0.0000
0.0025
0.0050
MR effect size for
'SMAP2' on 'Bladder cancer'

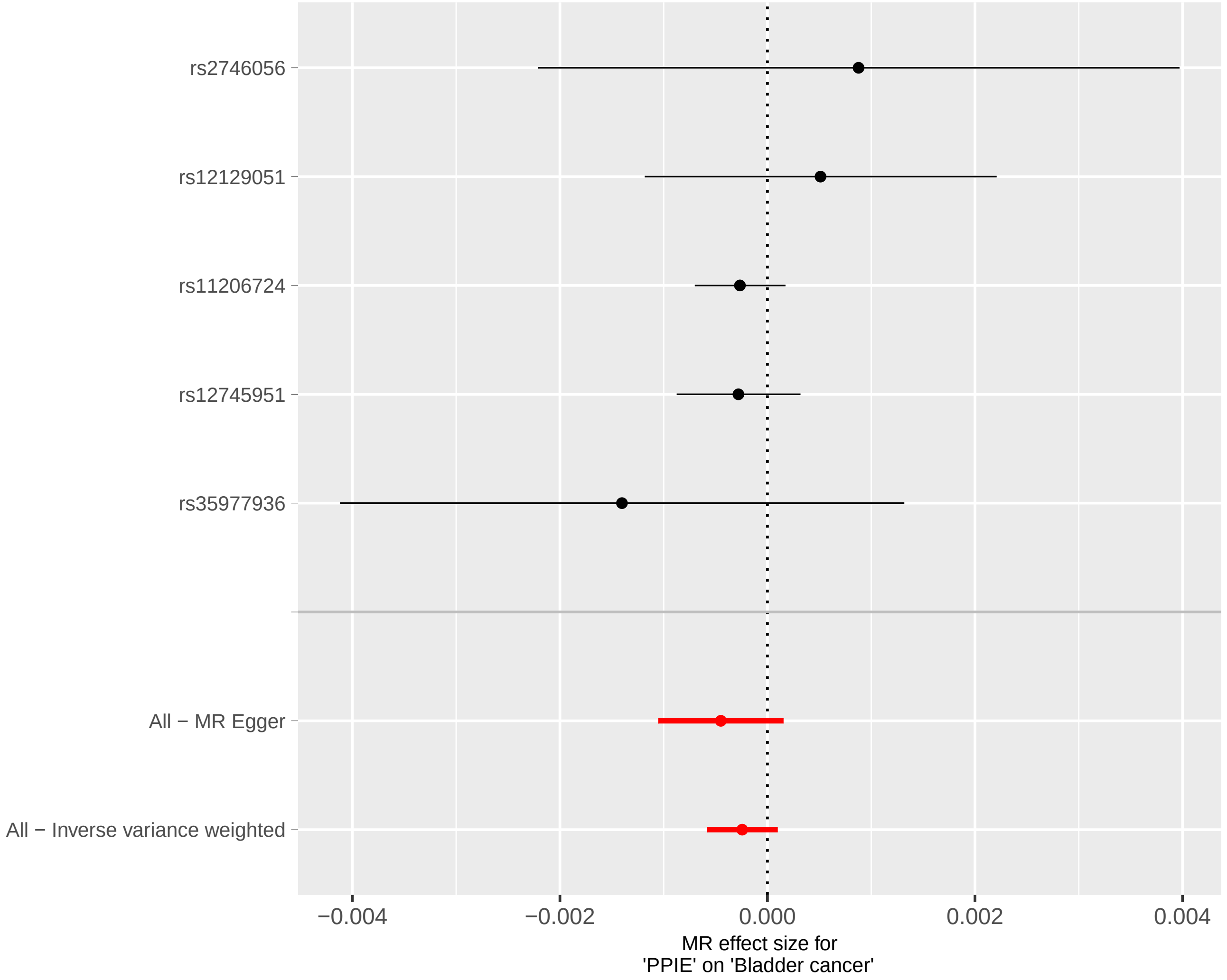
rs2746056
rs12129051
rs11206724
rs12745951
rs35977936
All − MR Egger
All − Inverse variance weighted
−0.004
−0.002
0.000
0.002
0.004
MR effect size for
'PPIE' on 'Bladder cancer'

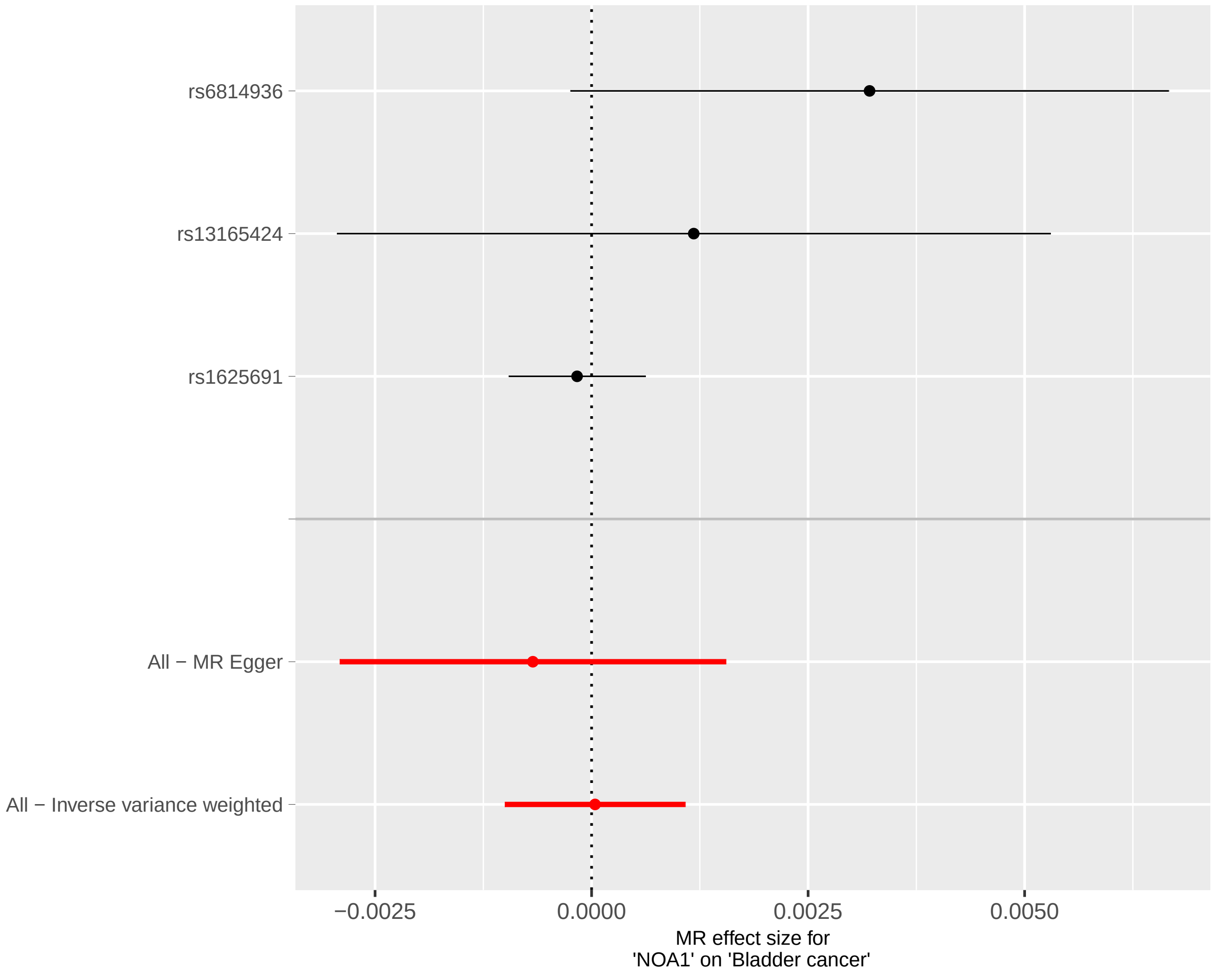
rs6814936
rs13165424
rs1625691
All − MR Egger
All − Inverse variance weighted
−0.0025
0.0000
0.0025
0.0050
MR effect size for
'NOA1' on 'Bladder cancer'

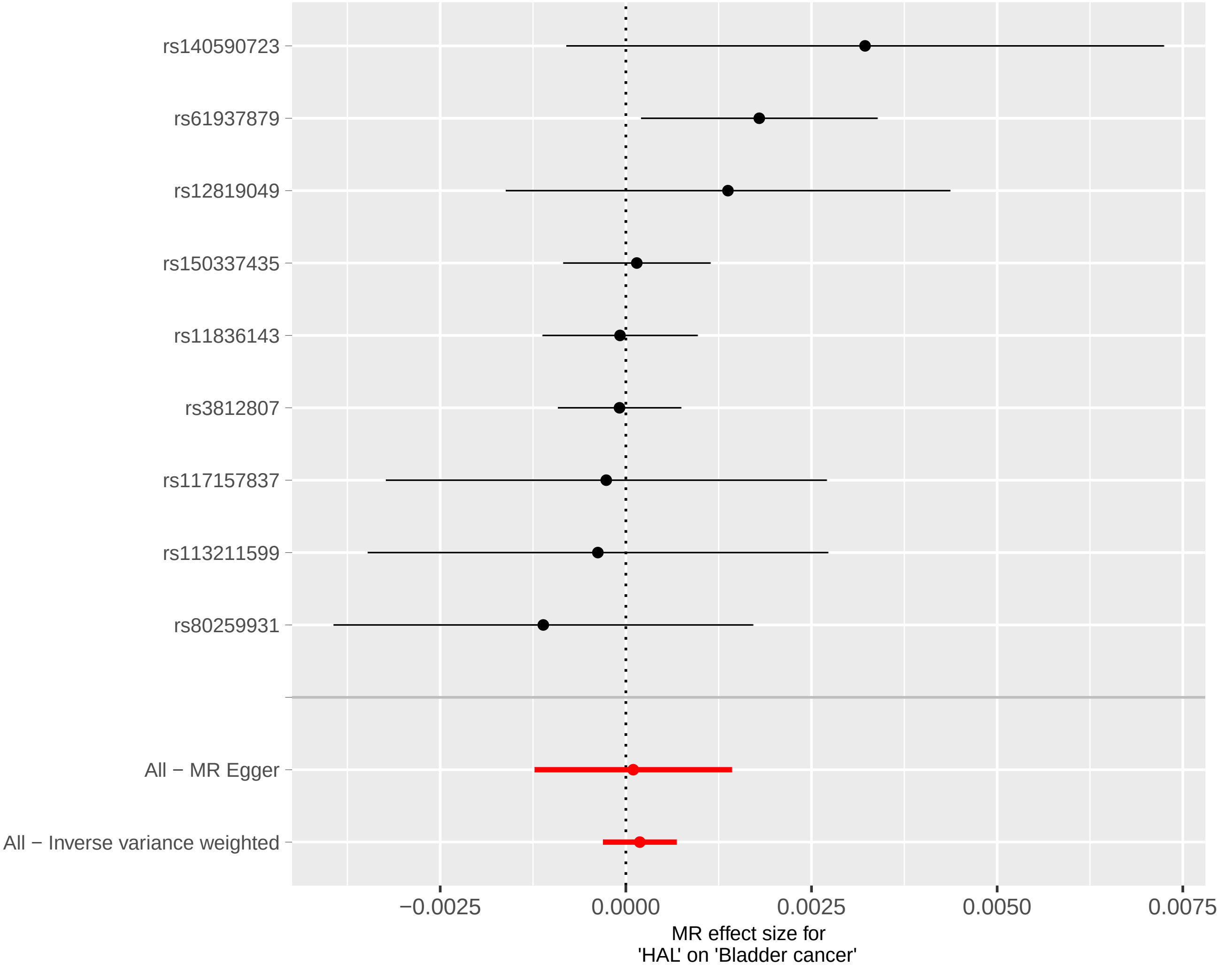

rs140590723
rs61937879
rs12819049
rs150337435
rs11836143
rs3812807
rs117157837
rs113211599
rs80259931
All − MR Egger
All − Inverse variance weighted
−0.0025
0.0000
0.0025
0.0050
0.0075
MR effect size for
'HAL' on 'Bladder cancer'

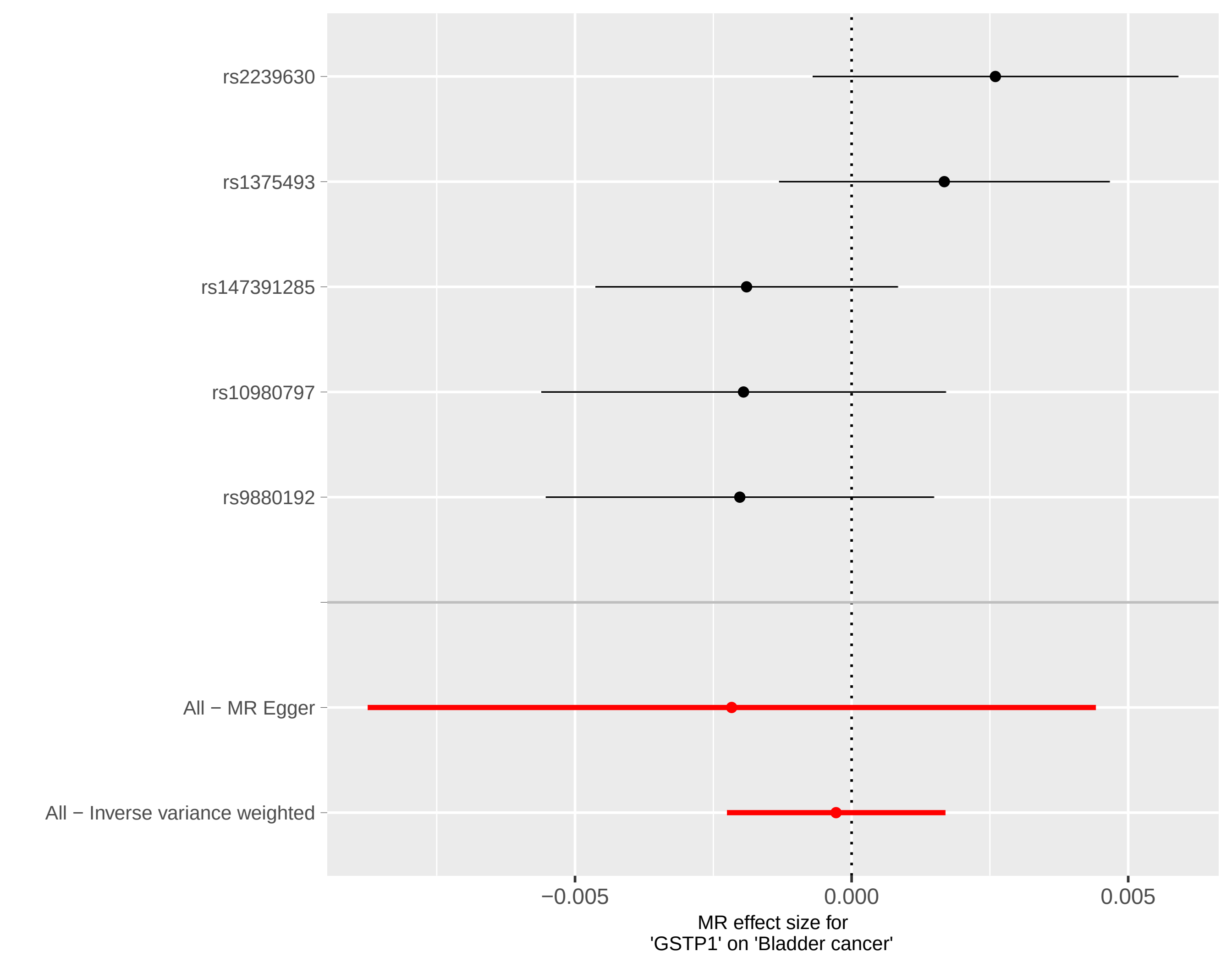
rs2239630
rs1375493
rs147391285
rs10980797
rs9880192
All − MR Egger
All − Inverse variance weighted
−0.005
0.000
0.005
MR effect size for
'GSTP1' on 'Bladder cancer'

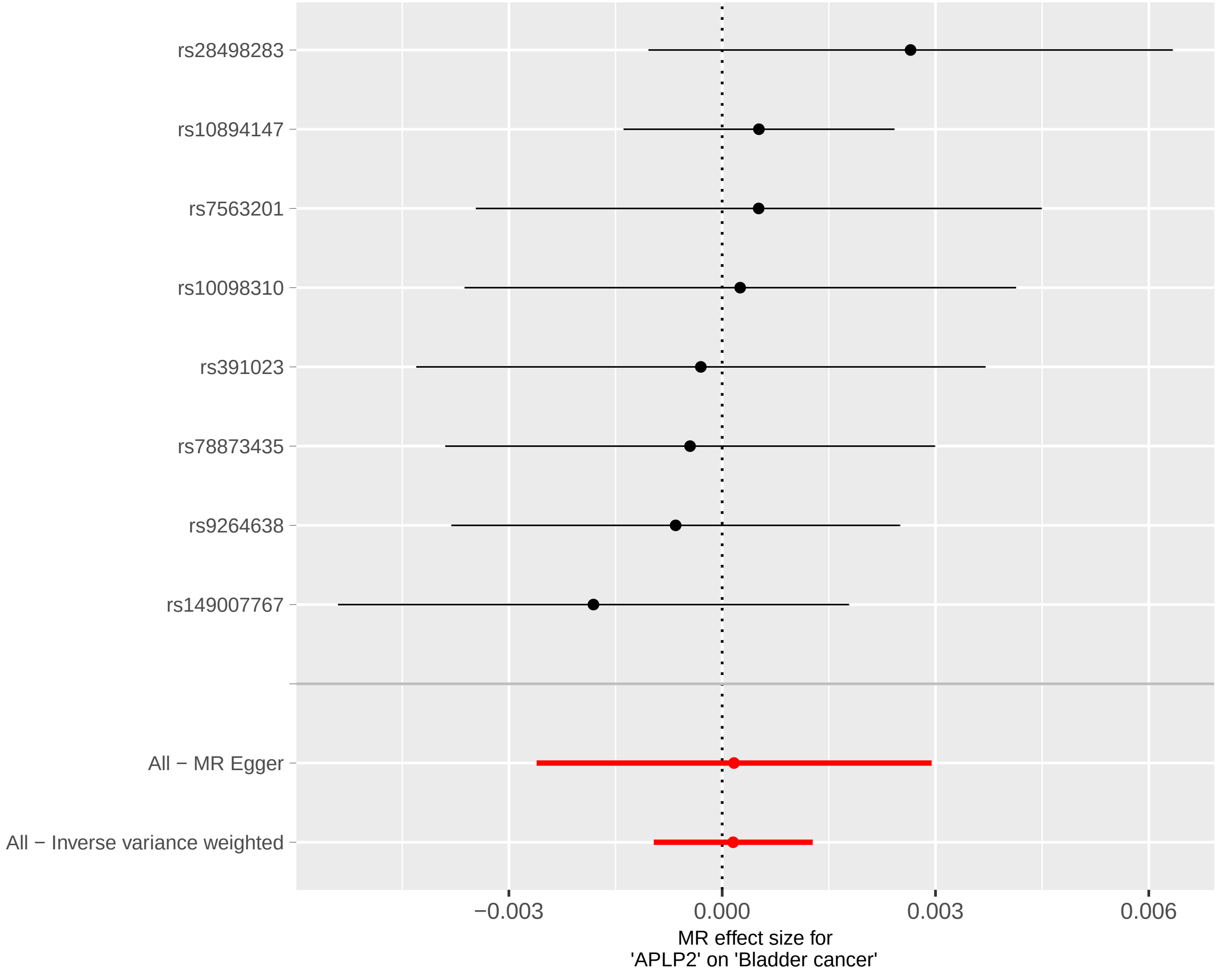
rs28498283
rs10894147
rs7563201
rs10098310
rs391023
rs78873435
rs9264638
rs149007767
All − MR Egger
All − Inverse variance weighted
−0.003
0.000
0.003
0.006
MR effect size for
'APLP2' on 'Bladder cancer'

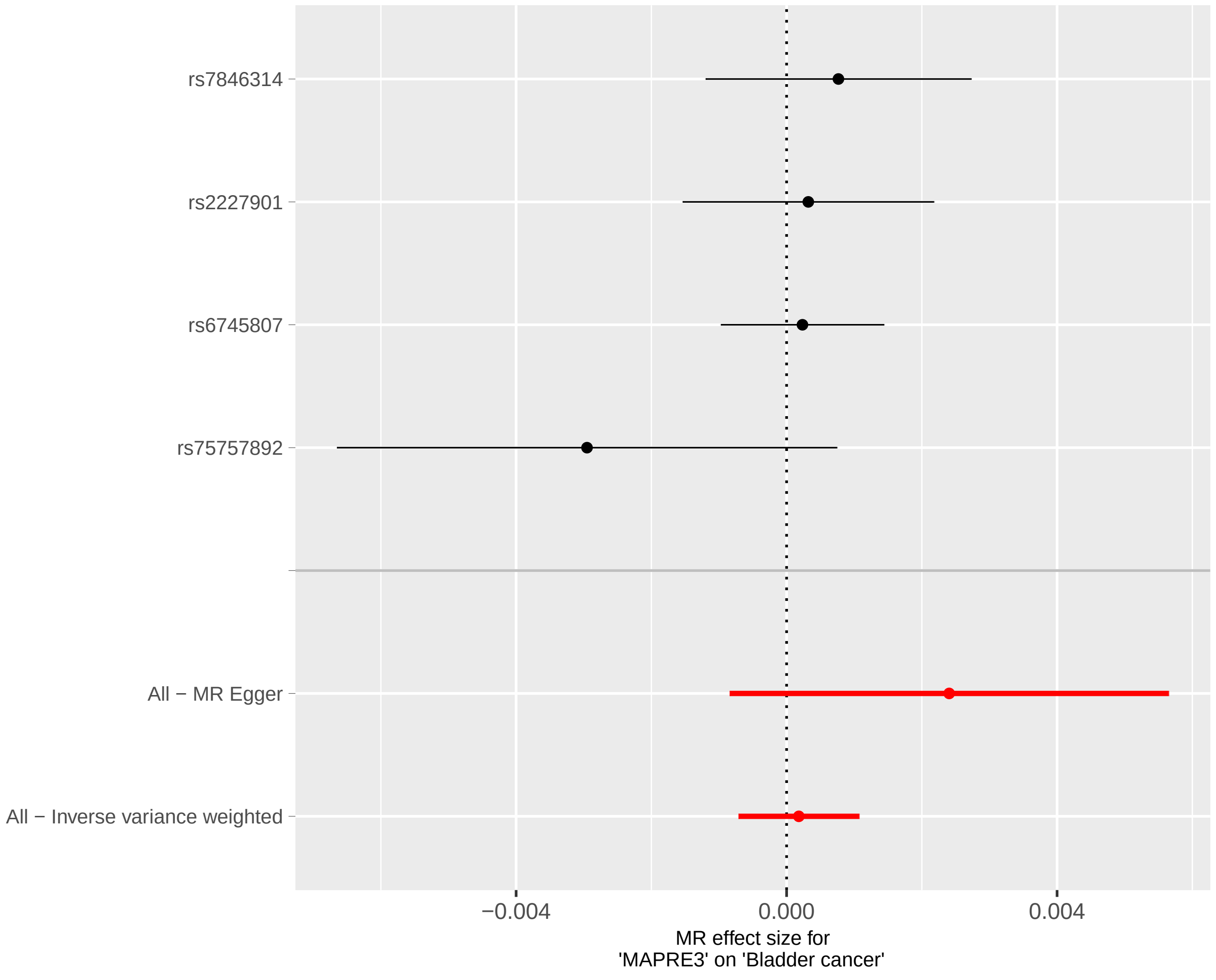

rs7846314
rs2227901
rs6745807
rs75757892
All − MR Egger
All − Inverse variance weighted
−0.004
0.000
0.004
MR effect size for
'MAPRE3' on 'Bladder cancer'

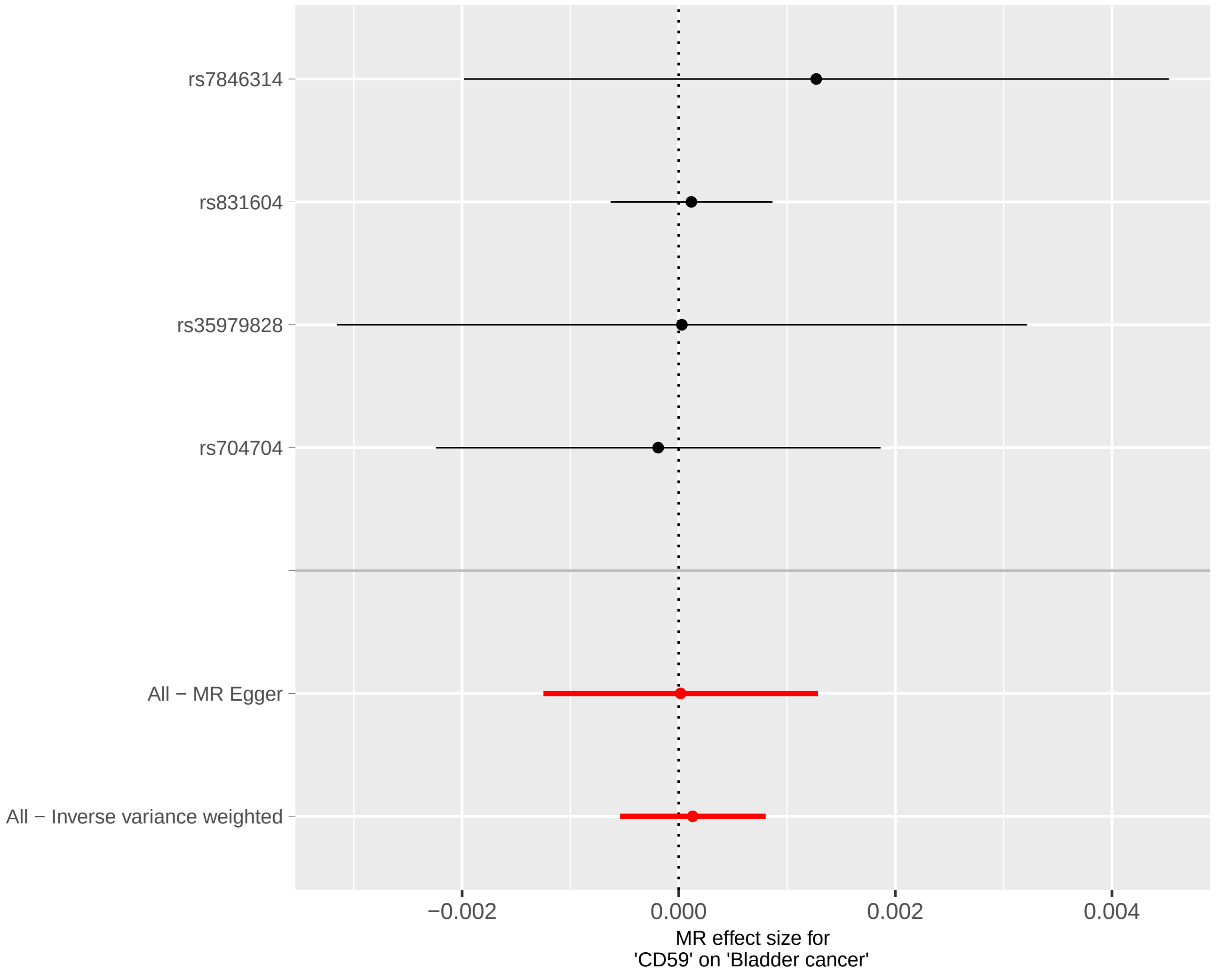

rs7846314
rs831604
rs35979828
rs704704
All − MR Egger
All − Inverse variance weighted
−0.002
0.000
0.002
0.004
MR effect size for
'CD59' on 'Bladder cancer'

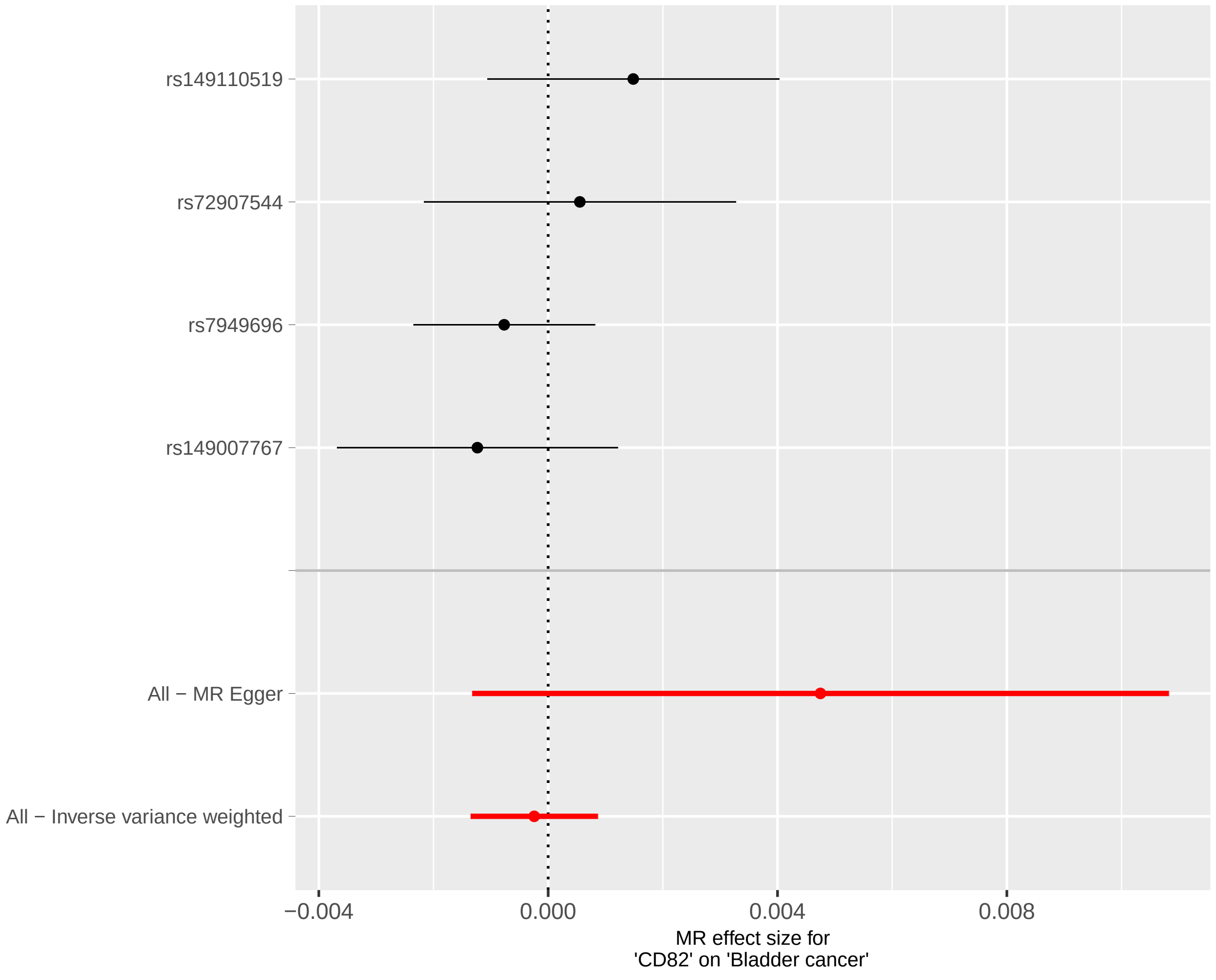
rs149110519
rs72907544
rs7949696
rs149007767
All − MR Egger
All − Inverse variance weighted
−0.004
0.000
0.004
0.008
MR effect size for
'CD82' on 'Bladder cancer'

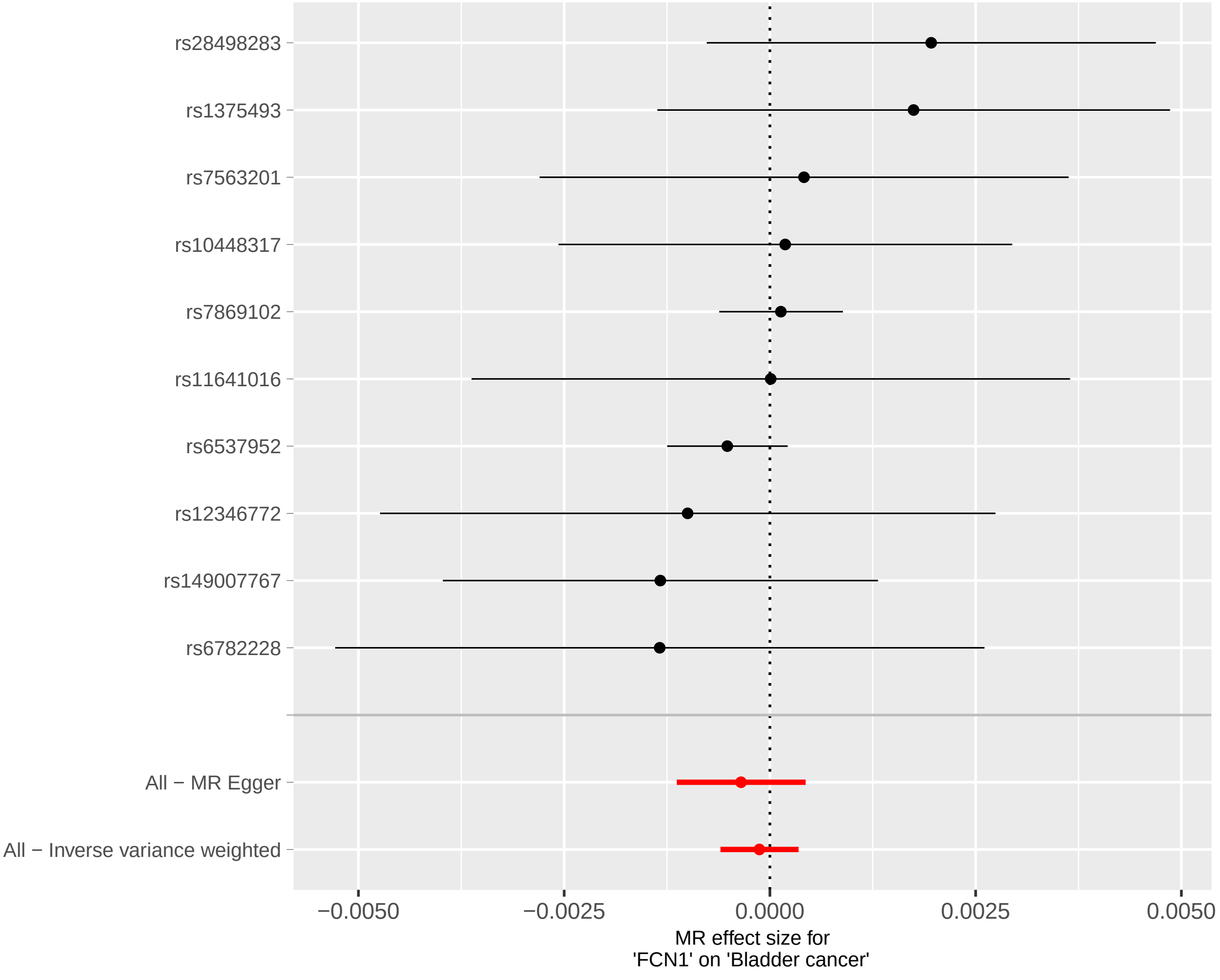

rs28498283
rs1375493
rs7563201
rs10448317
rs7869102
rs11641016
rs6537952
rs12346772
rs149007767
rs6782228
All − MR Egger
All − Inverse variance weighted
−0.0050
−0.0025
0.0000
0.0025
0.0050
MR effect size for
'FCN1' on 'Bladder cancer'

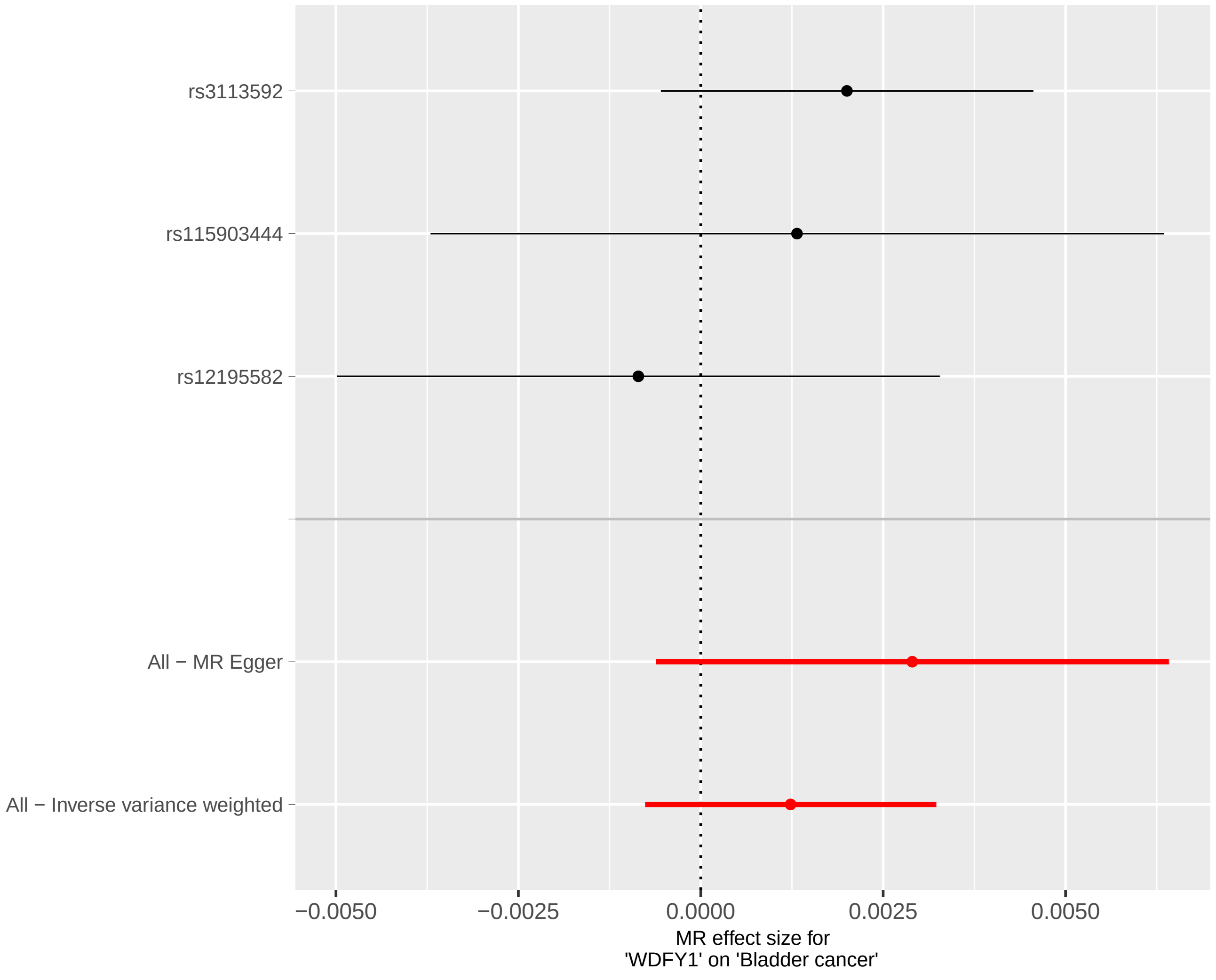
rs3113592
rs115903444
rs12195582
All − MR Egger
All − Inverse variance weighted
−0.0050
−0.0025
0.0000
0.0025
0.0050
MR effect size for
'WDFY1' on 'Bladder cancer'

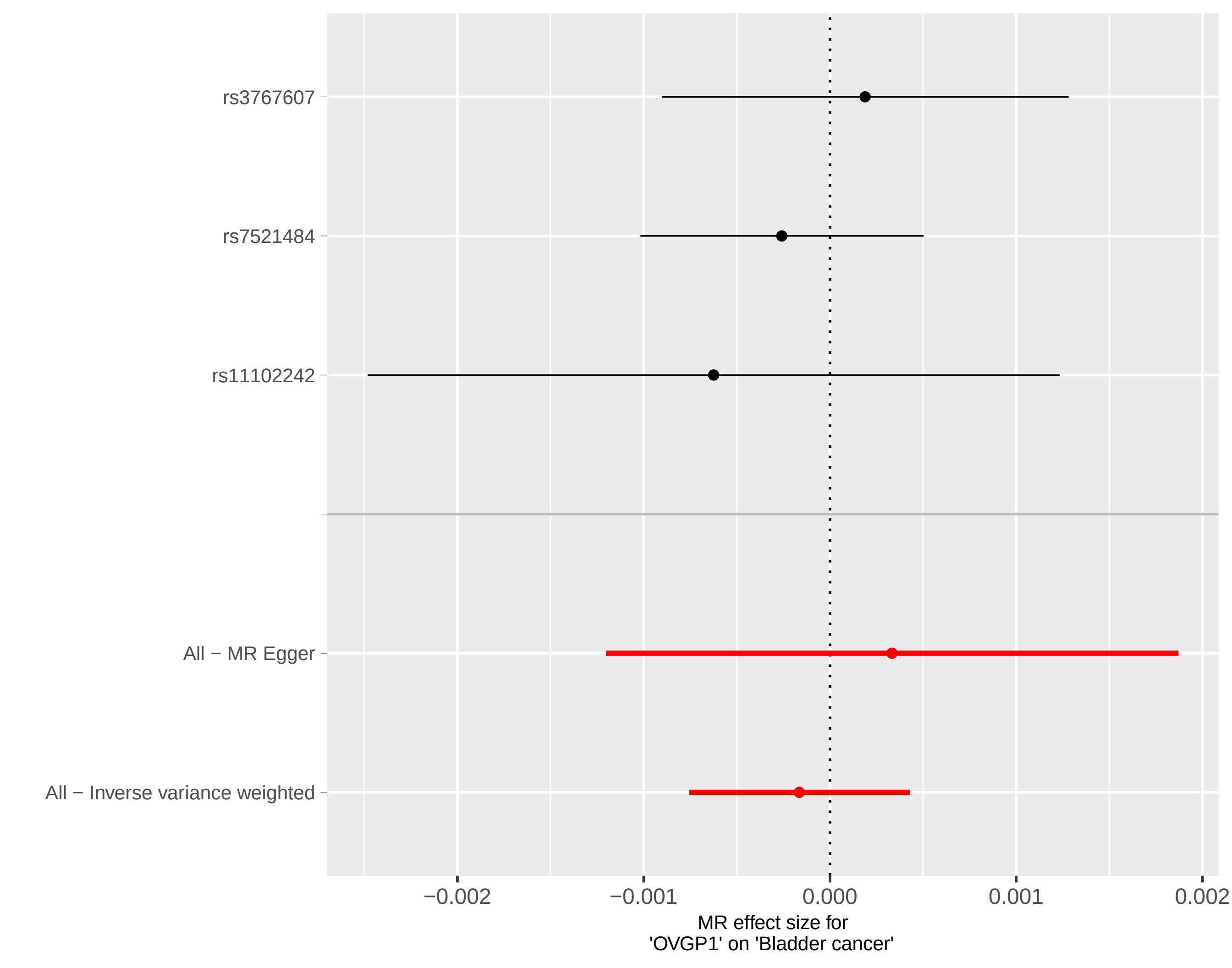

rs3767607
rs7521484
rs11102242
All − MR Egger
All − Inverse variance weighted
−0.002
−0.001
0.000
0.001
0.002
MR effect size for
'OVGP1' on 'Bladder cancer'

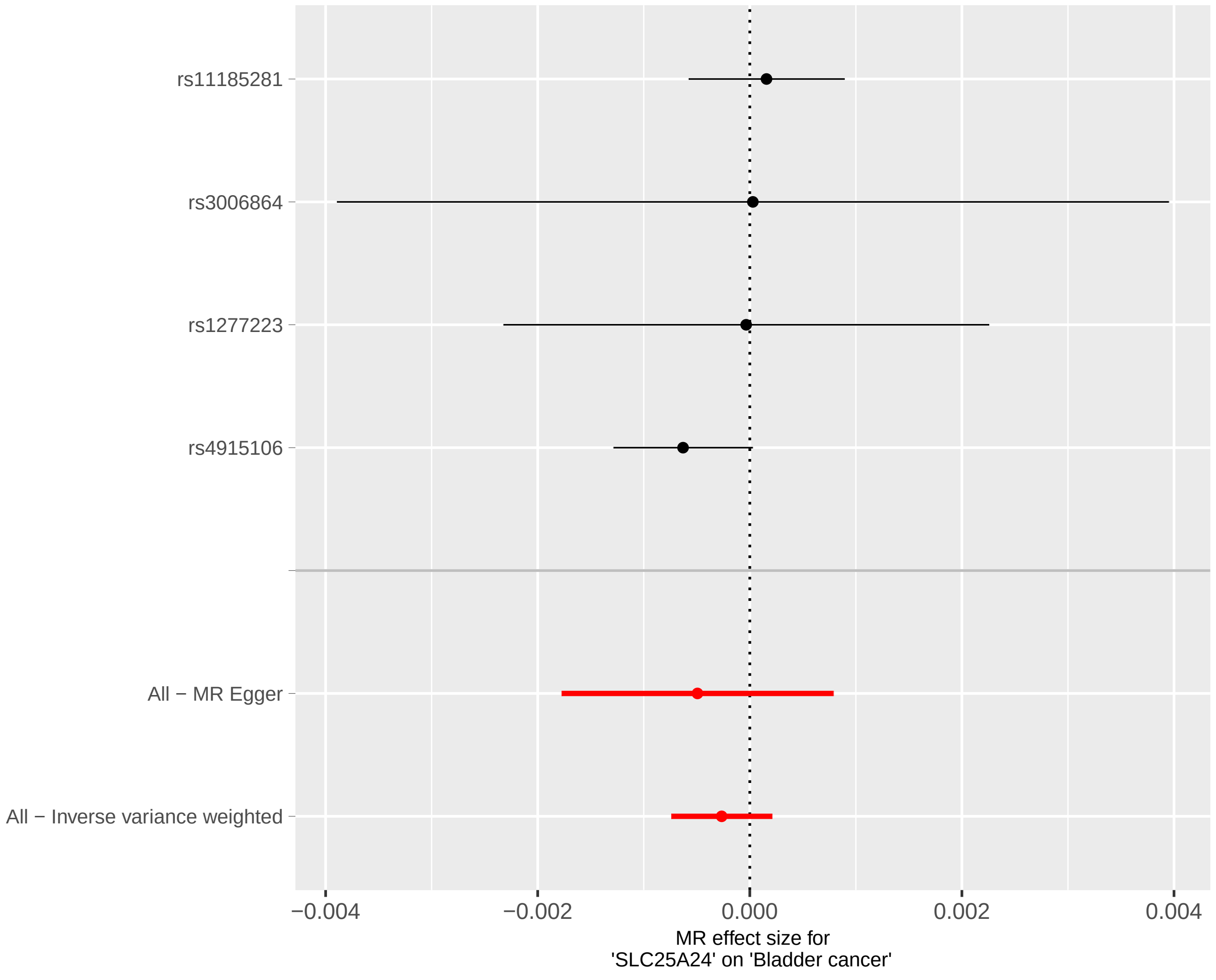

rs11185281
rs3006864
rs1277223
rs4915106
All − MR Egger
All − Inverse variance weighted
−0.004
−0.002
0.000
0.002
0.004
MR effect size for
'SLC25A24' on 'Bladder cancer'

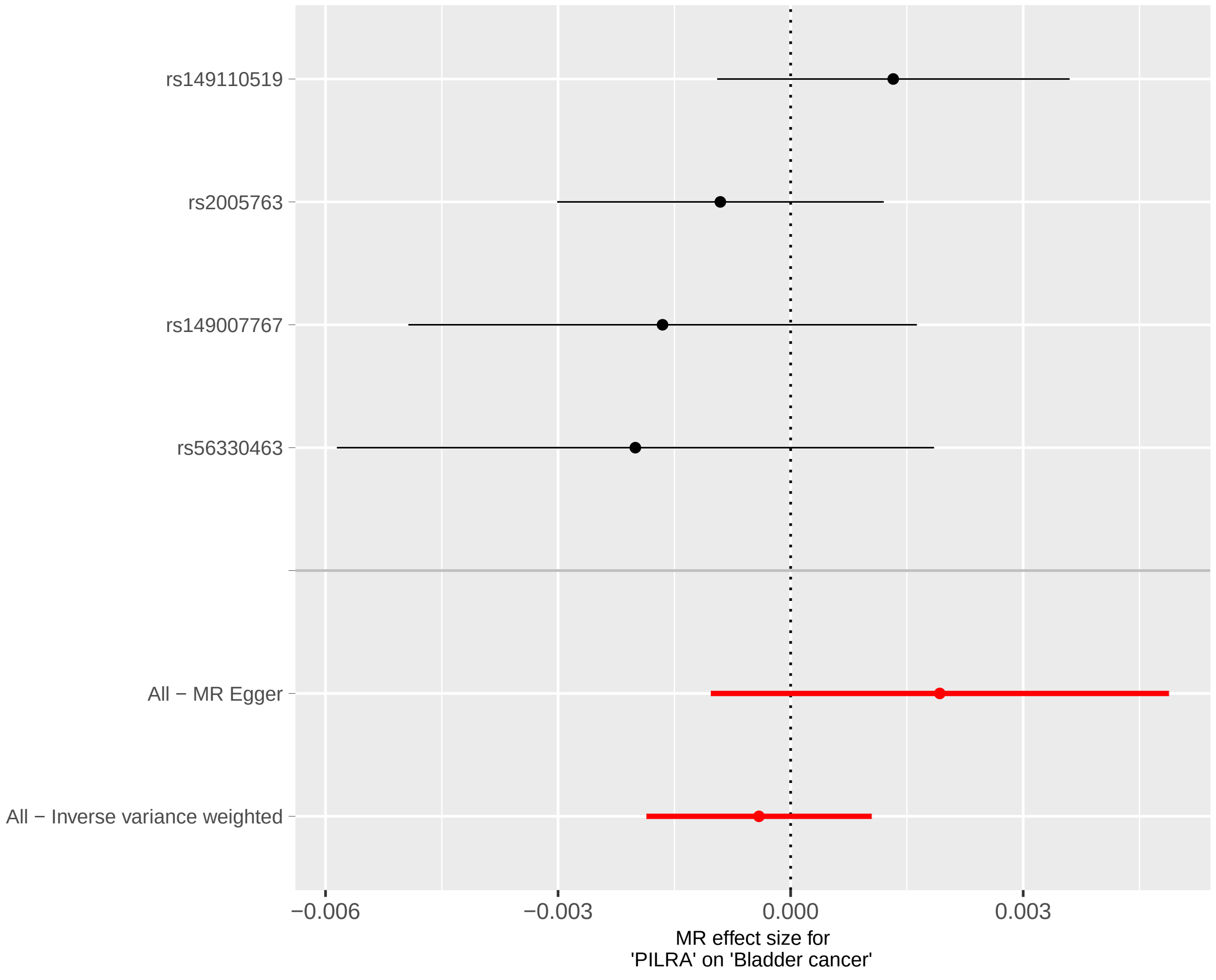
rs149110519
rs2005763
rs149007767
rs56330463
All − MR Egger
All − Inverse variance weighted
−0.006
−0.003
0.000
0.003
MR effect size for
'PILRA' on 'Bladder cancer'

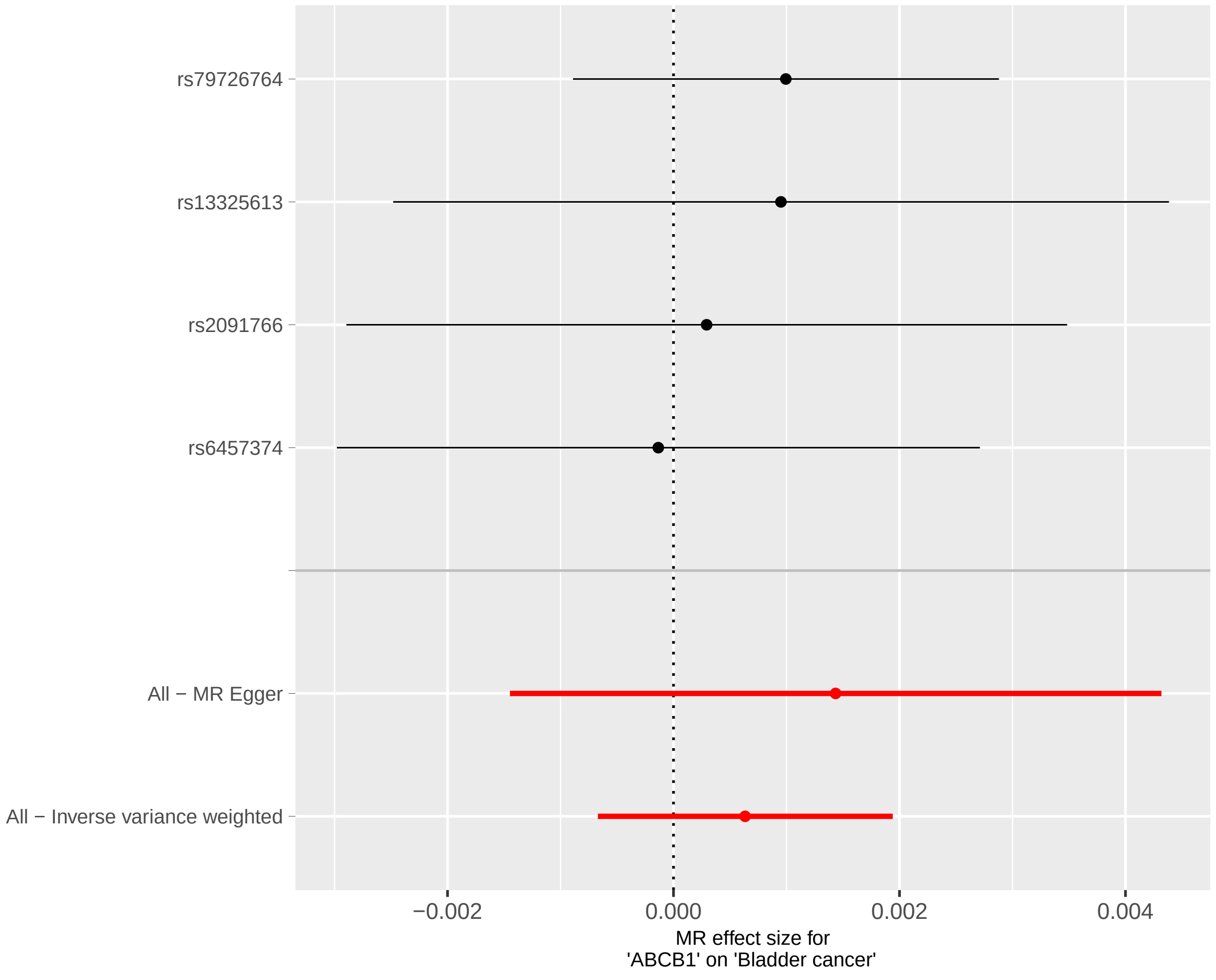
rs79726764
rs13325613
rs2091766
rs6457374
All − MR Egger
All − Inverse variance weighted
−0.002
0.000
0.002
0.004
MR effect size for
'ABCB1' on 'Bladder cancer'

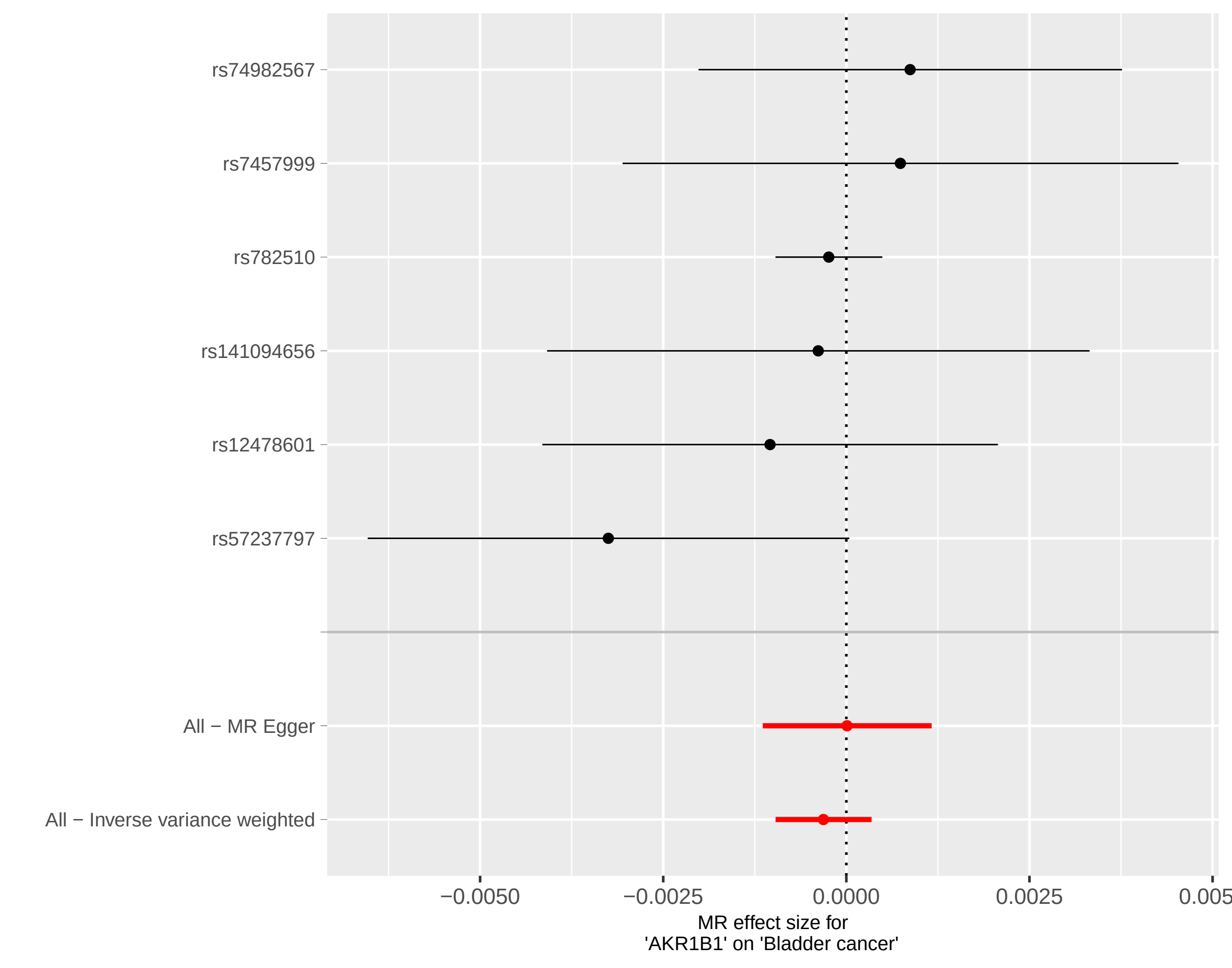

rs74982567
rs7457999
rs782510
rs141094656
rs12478601
rs57237797
All − MR Egger
All − Inverse variance weighted
−0.0050
−0.0025
0.0000
0.0025
0.005
MR effect size for
'AKR1B1' on 'Bladder cancer'

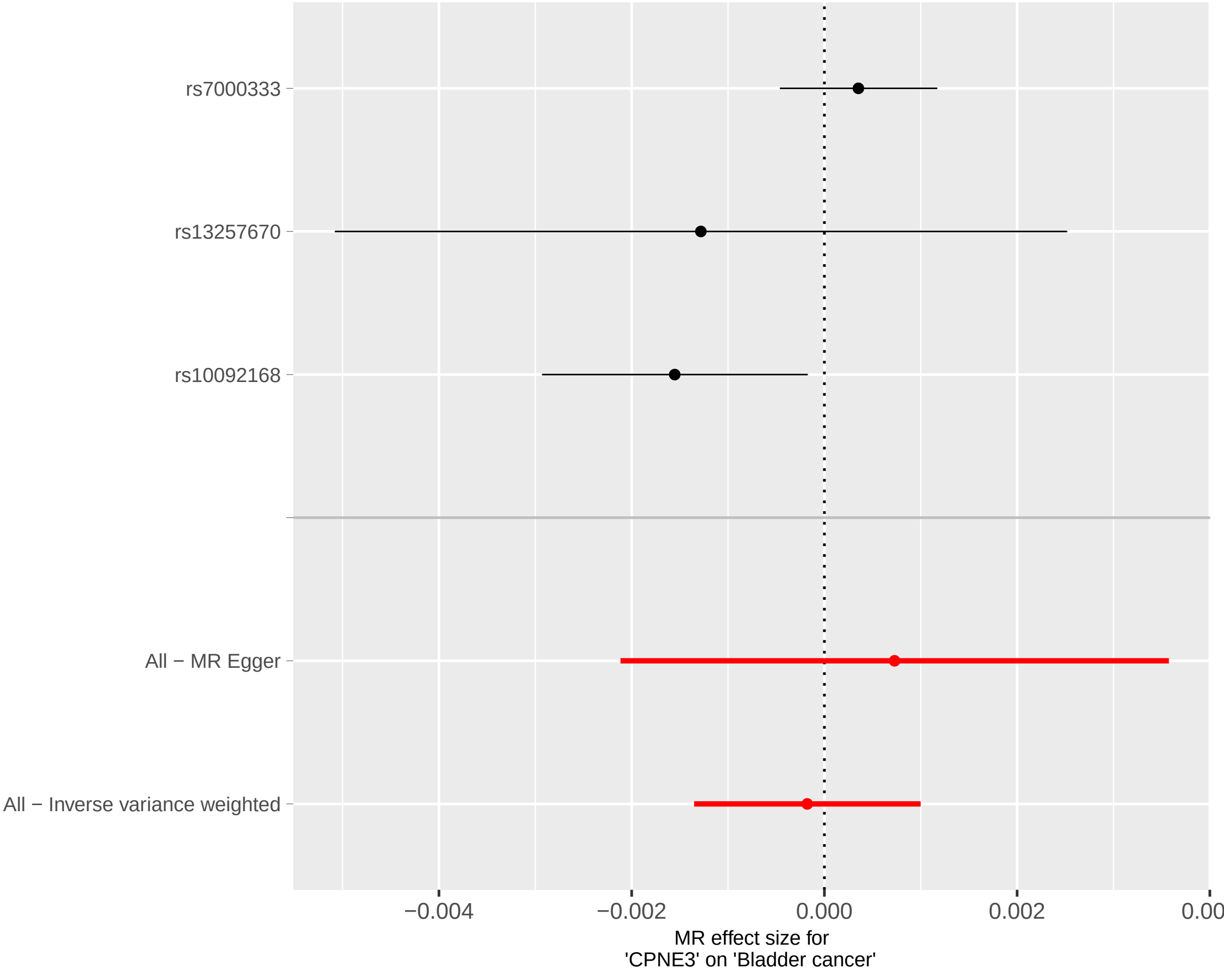

rs7000333
rs13257670
rs10092168
All − MR Egger
All − Inverse variance weighted
−0.004
−0.002
0.000
0.002
MR effect size for
'CPNE3' on 'Bladder cancer'

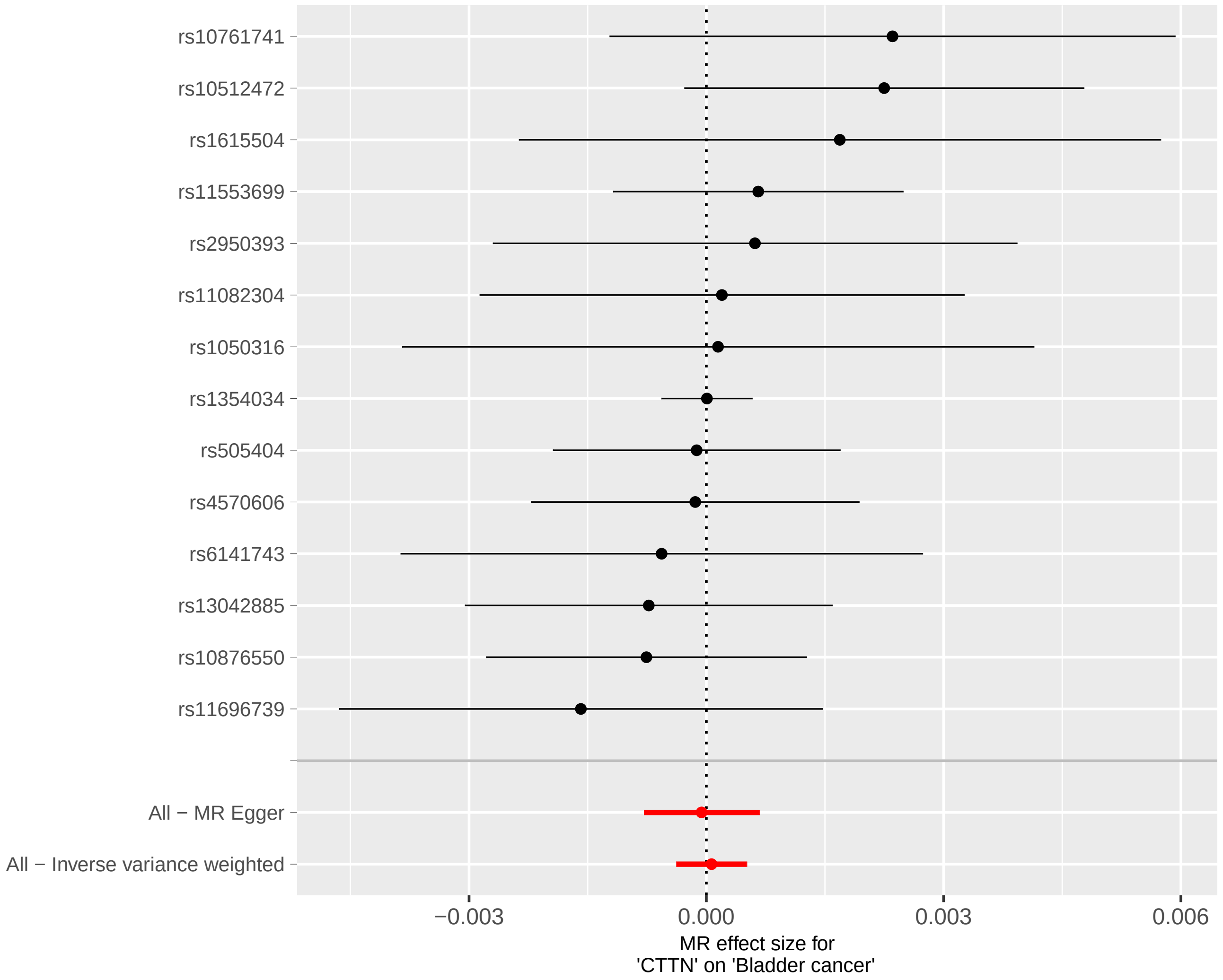
rs10761741
rs10512472
rs1615504
rs11553699
rs2950393
rs11082304
rs1050316
rs1354034
rs505404
rs4570606
rs6141743
rs13042885
rs10876550
rs11696739
All − MR Egger
All − Inverse variance weighted
−0.003
0.000
0.003
0.006
MR effect size for
'CTTN' on 'Bladder cancer'

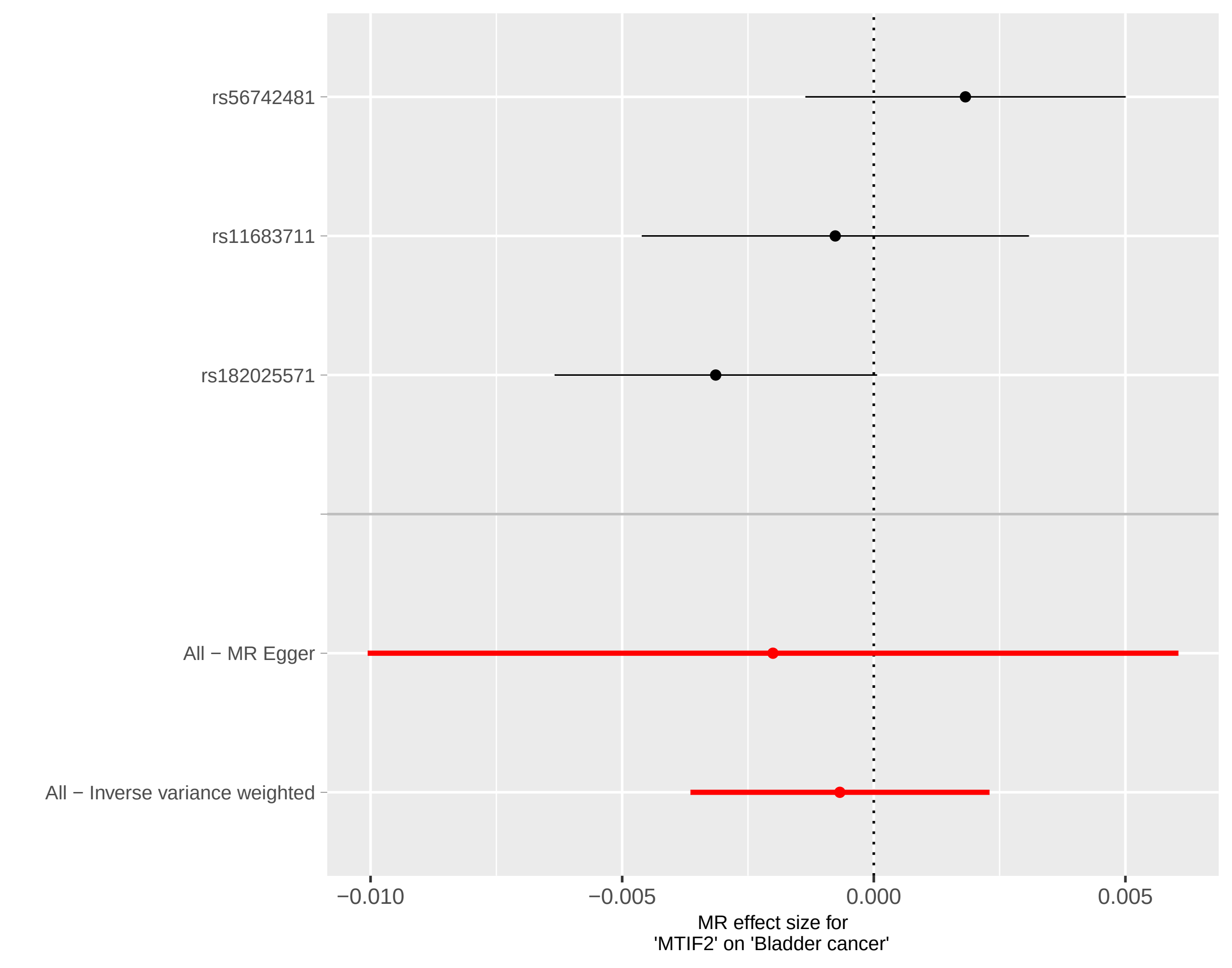
rs56742481
rs11683711
rs182025571
All − MR Egger
All − Inverse variance weighted
−0.010
−0.005
0.000
0.005
MR effect size for
'MTIF2' on 'Bladder cancer'

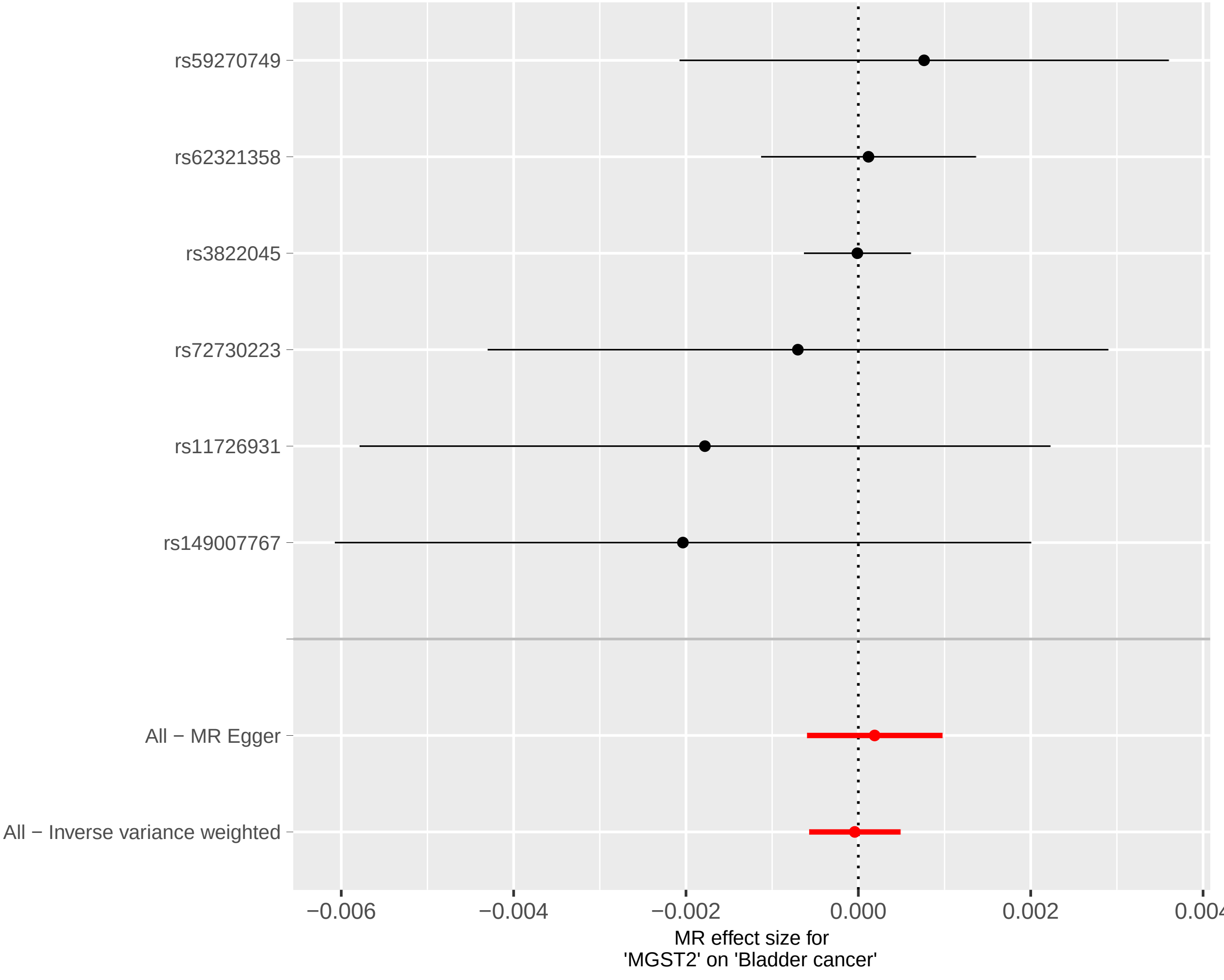
rs59270749
rs62321358
rs3822045
rs72730223
rs11726931
rs149007767
All − MR Egger
All − Inverse variance weighted
−0.006
−0.004
−0.002
0.000
0.002
MR effect size for
'MGST2' on 'Bladder cancer'

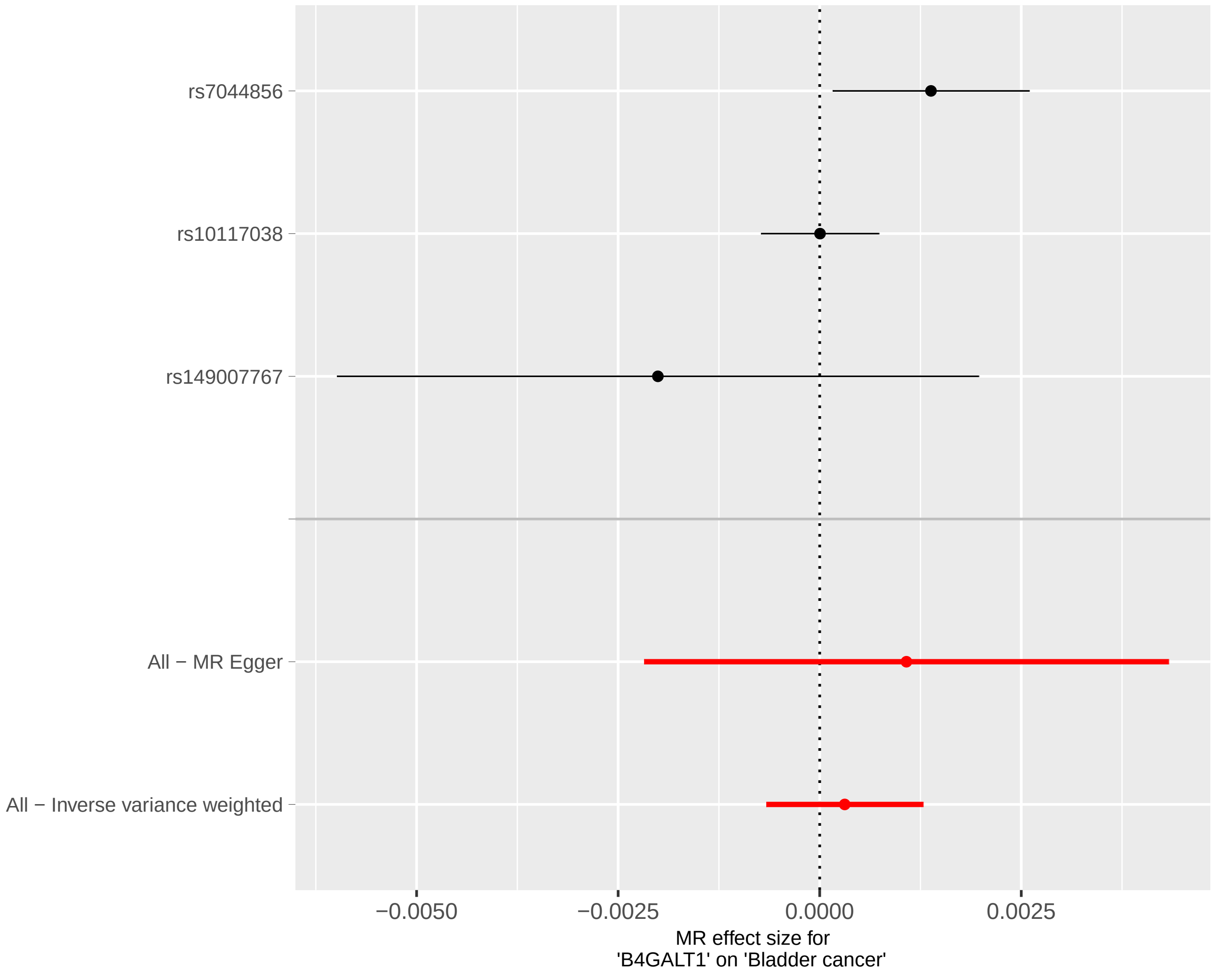
rs7044856
rs10117038
rs149007767
All − MR Egger
All − Inverse variance weighted
−0.0050
−0.0025
0.0000
0.0025
MR effect size for
'B4GALT1' on 'Bladder cancer'

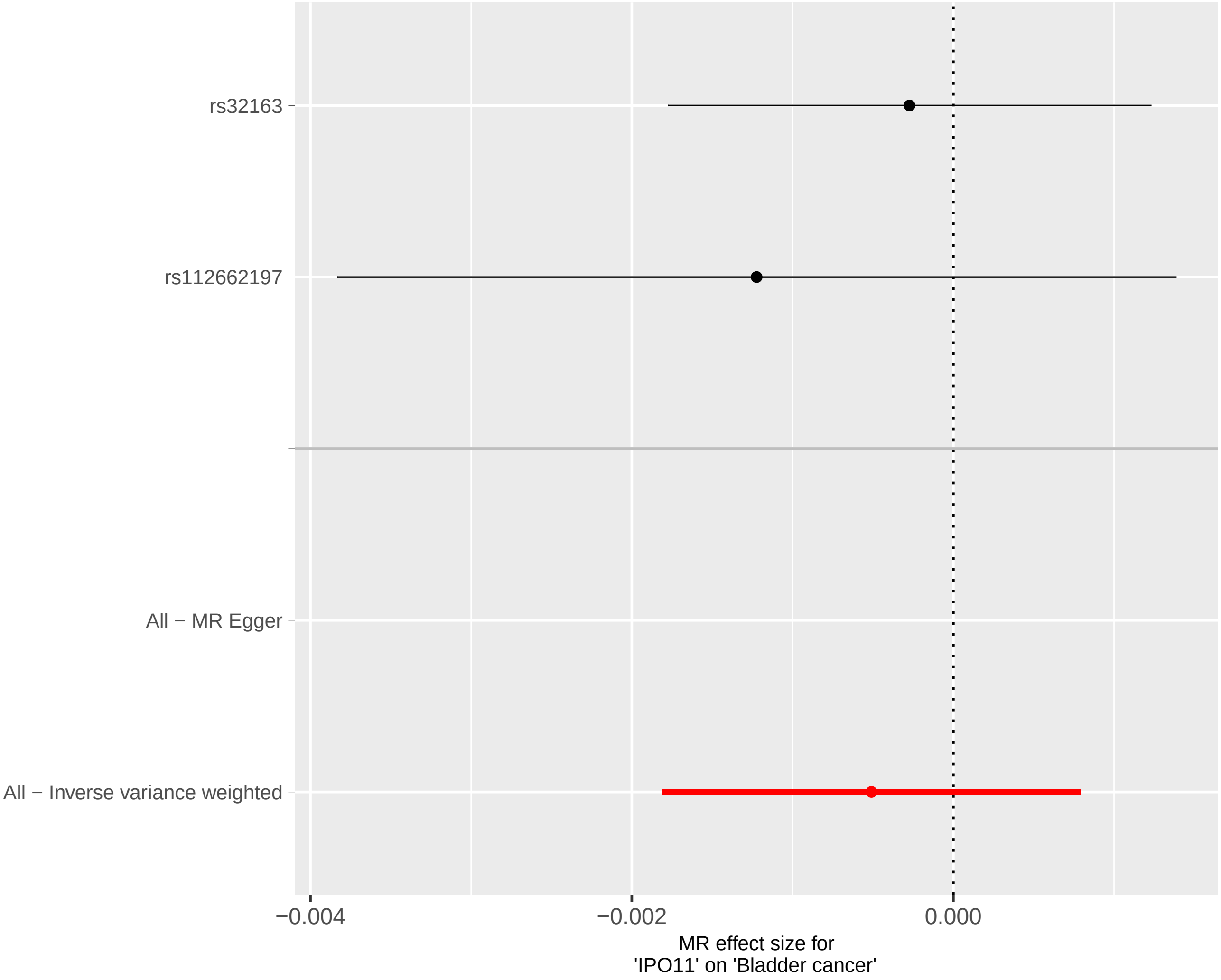

rs32163
rs112662197
All − MR Egger
All − Inverse variance weighted
−0.004
−0.002
0.000
MR effect size for
'IPO11' on 'Bladder cancer'

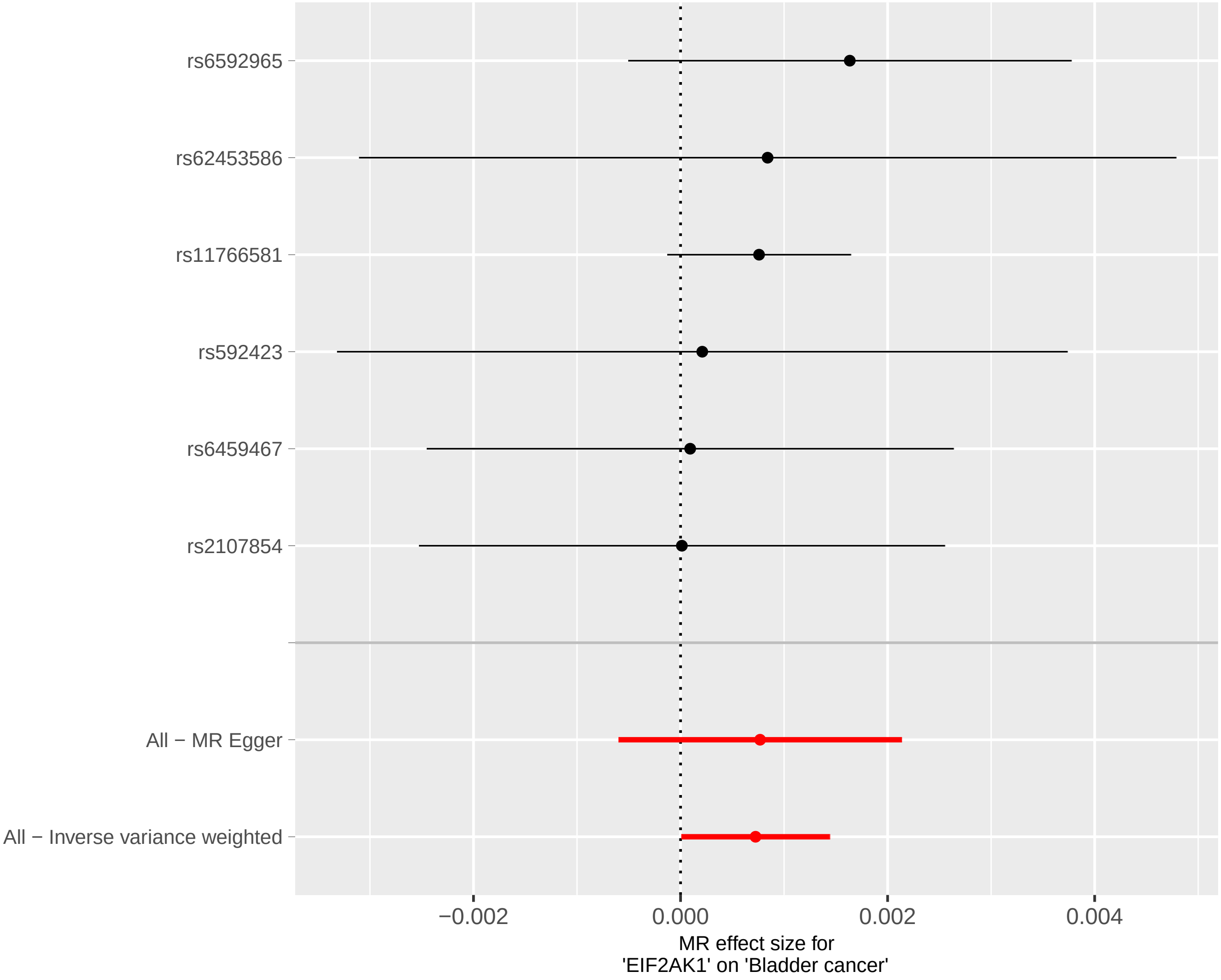
rs6592965
rs62453586
rs11766581
rs592423
rs6459467
rs2107854
All − MR Egger
All − Inverse variance weighted
−0.002
0.000
0.002
0.004
MR effect size for
'EIF2AK1' on 'Bladder cancer'

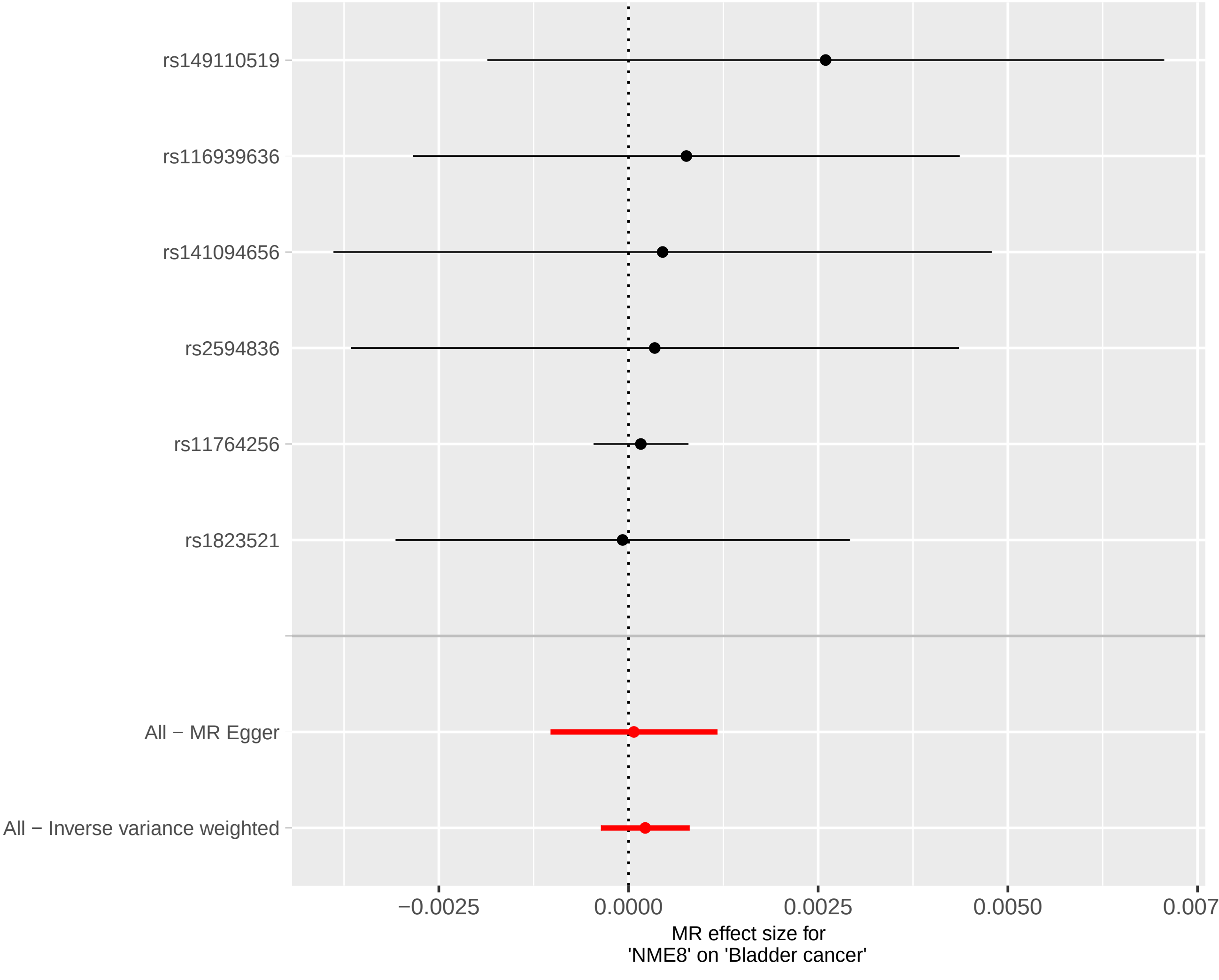
rs149110519
rs116939636
rs141094656
rs2594836
rs11764256
rs1823521
All − MR Egger
All − Inverse variance weighted
−0.0025
0.0000
0.0025
0.0050
0.007
MR effect size for
'NME8' on 'Bladder cancer'

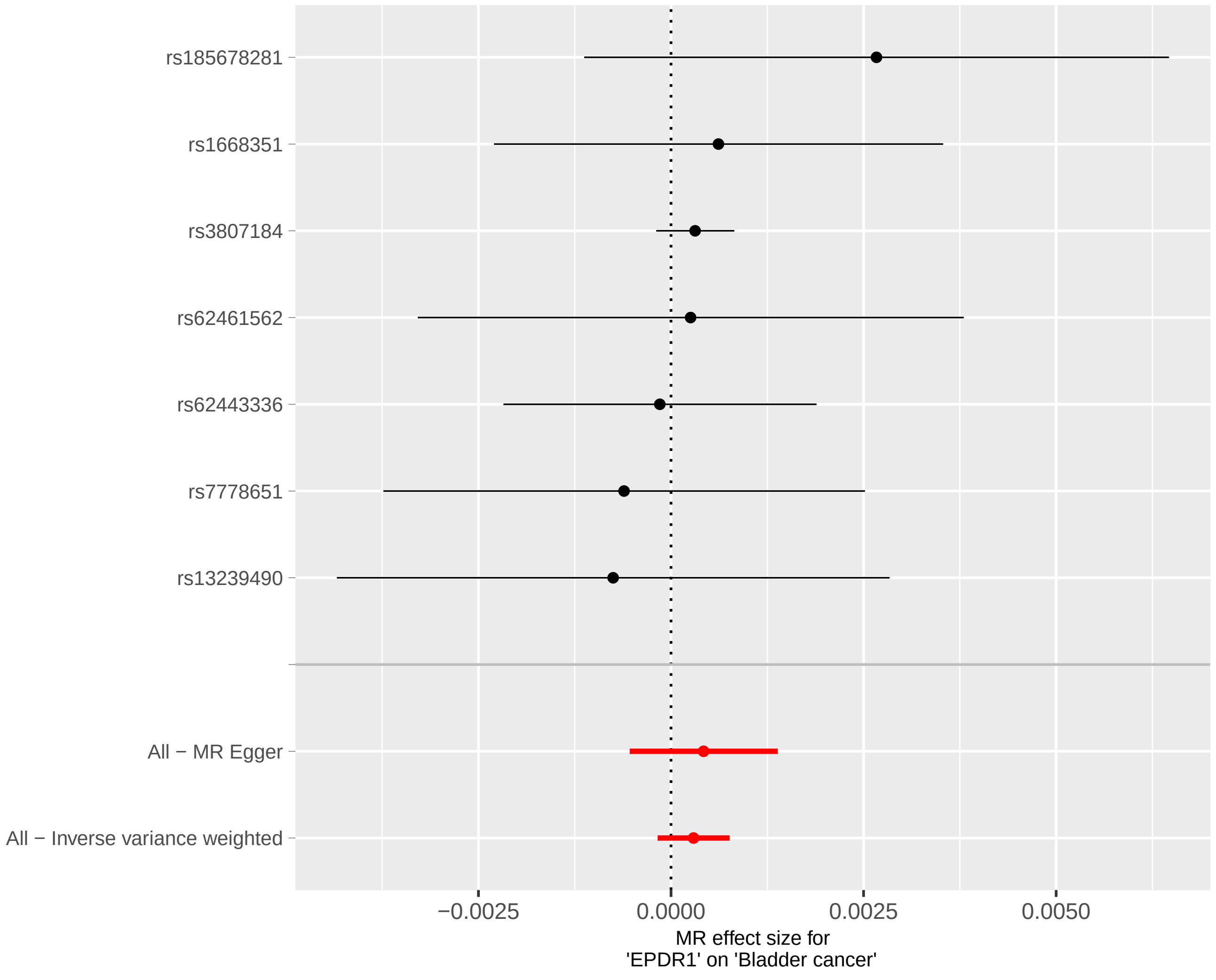

rs185678281
rs1668351
rs3807184
rs62461562
rs62443336
rs7778651
rs13239490
All − MR Egger
All − Inverse variance weighted
−0.0025
0.0000
0.0025
0.0050
MR effect size for
'EPDR1' on 'Bladder cancer'

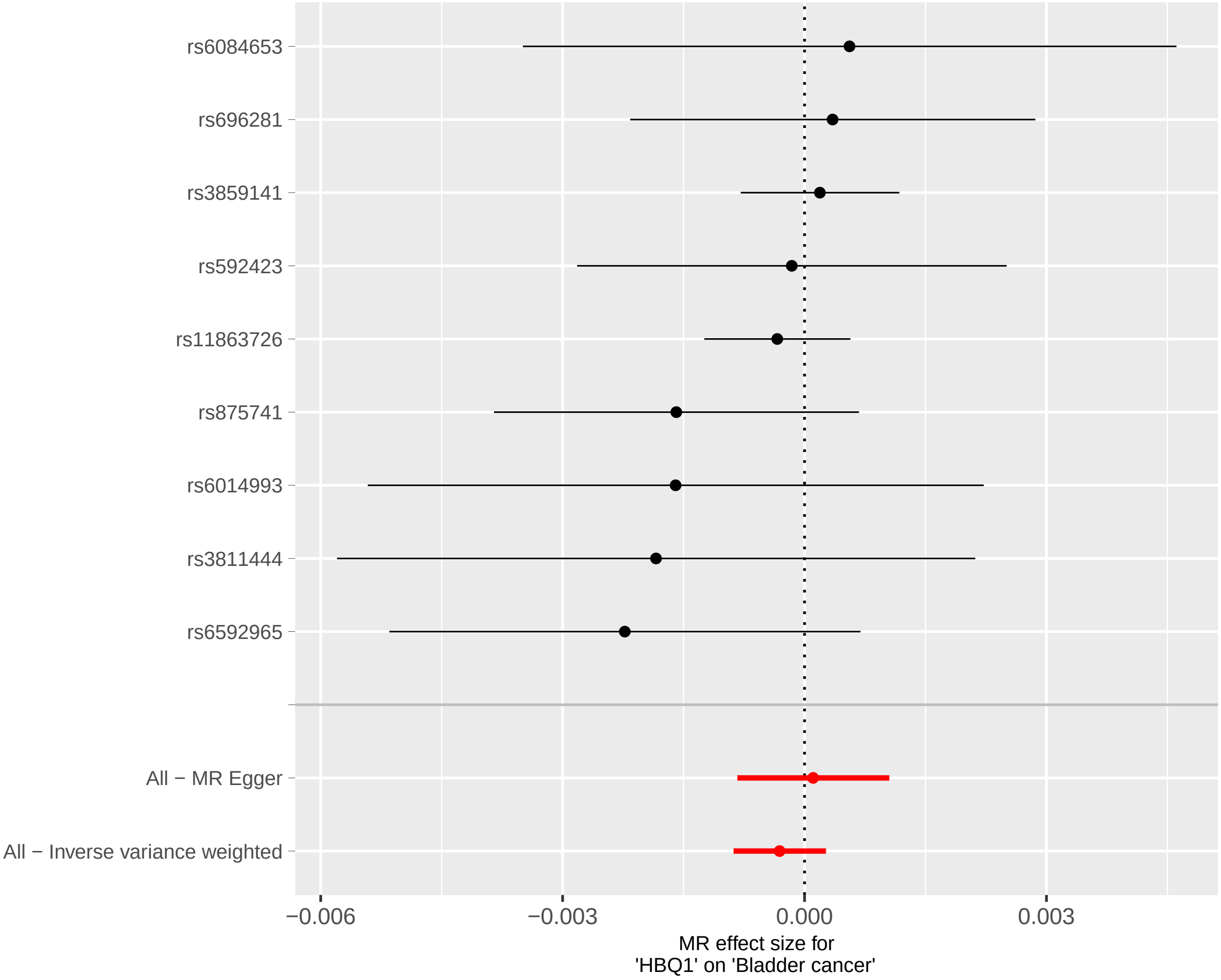
rs6084653
rs696281
rs3859141
rs592423
rs11863726
rs875741
rs6014993
rs3811444
rs6592965
All − MR Egger
All − Inverse variance weighted
−0.006
−0.003
0.000
0.003
MR effect size for
'HBQ1' on 'Bladder cancer'

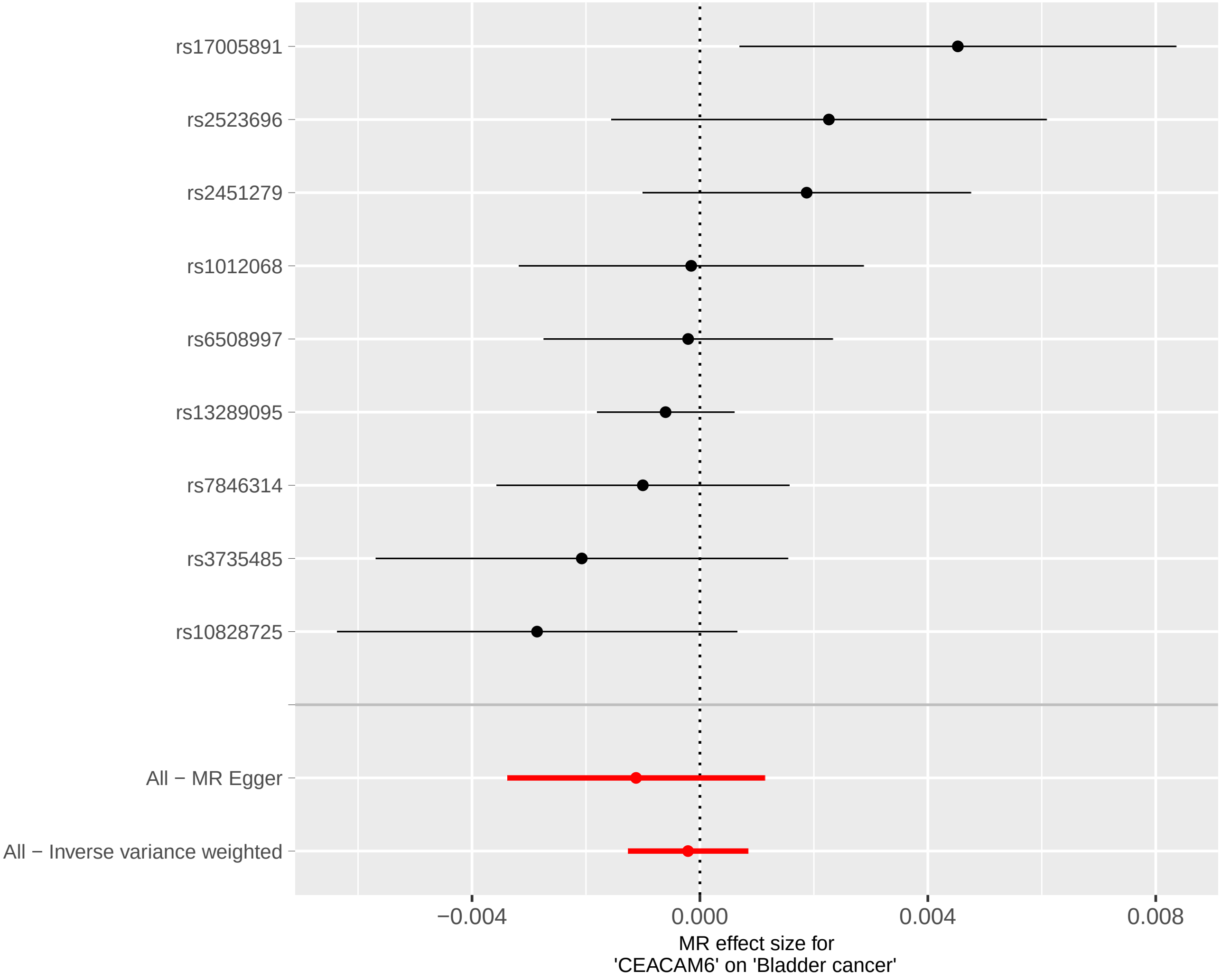
rs17005891
rs2523696
rs2451279
rs1012068
rs6508997
rs13289095
rs7846314
rs3735485
rs10828725
All − MR Egger
All − Inverse variance weighted
−0.004
0.000
0.004
0.008
MR effect size for
'CEACAM6' on 'Bladder cancer'

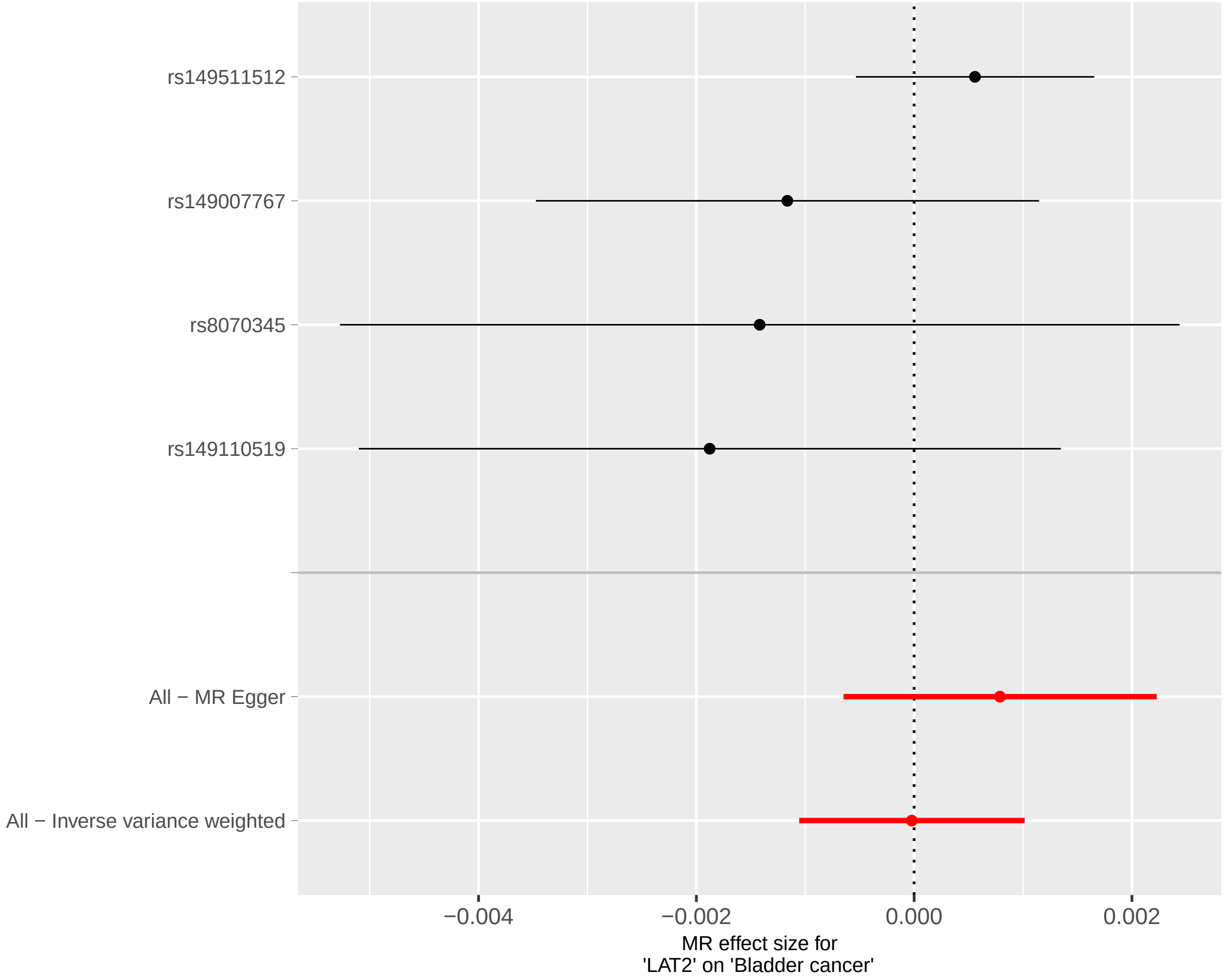

rs149511512
rs149007767
rs8070345
rs149110519
All − MR Egger
All − Inverse variance weighted
−0.004
−0.002
0.000
0.002
MR effect size for
'LAT2' on 'Bladder cancer'

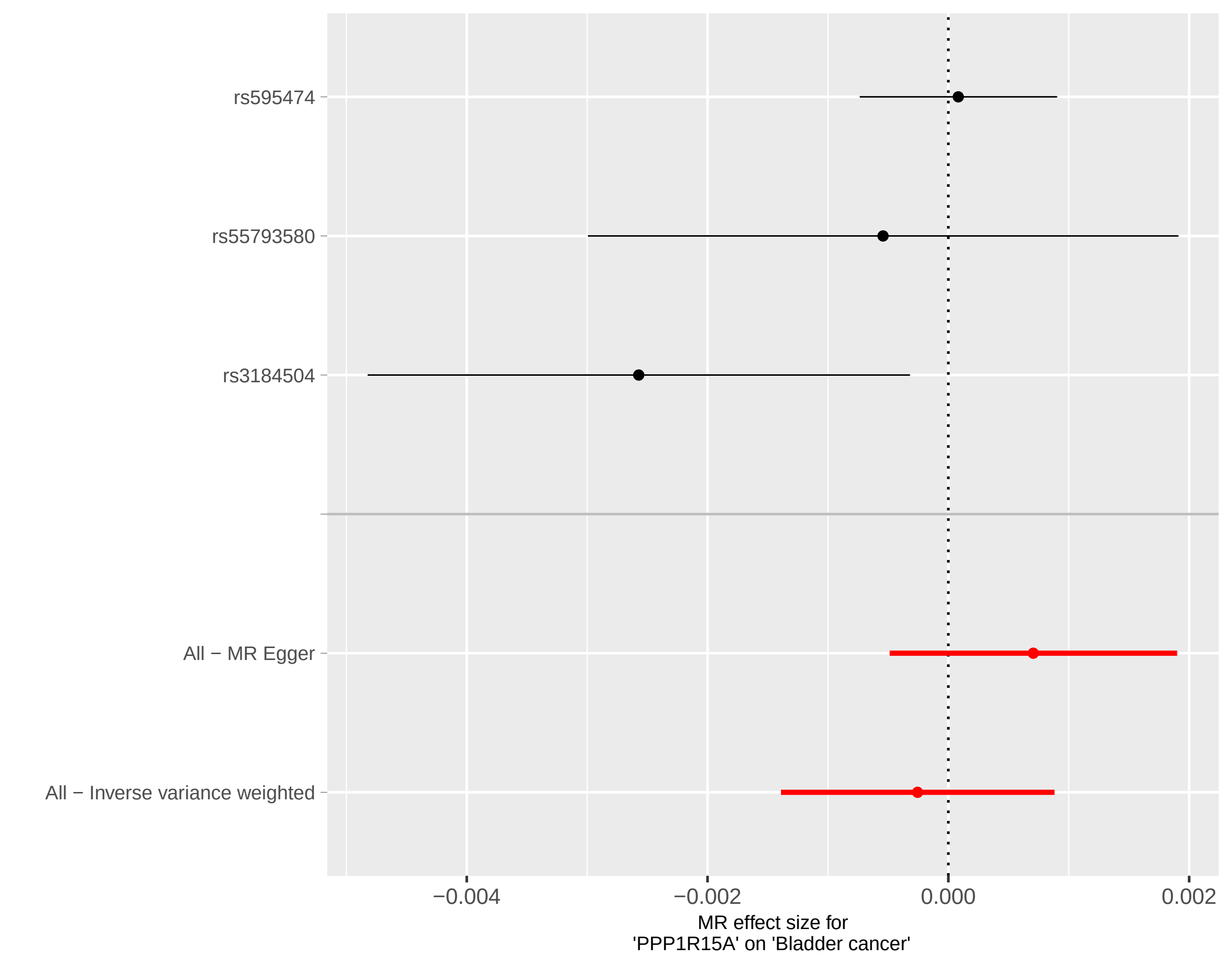

rs595474
rs55793580
rs3184504
All − MR Egger
All − Inverse variance weighted
−0.004
−0.002
0.000
0.002
MR effect size for
'PPP1R15A' on 'Bladder cancer'

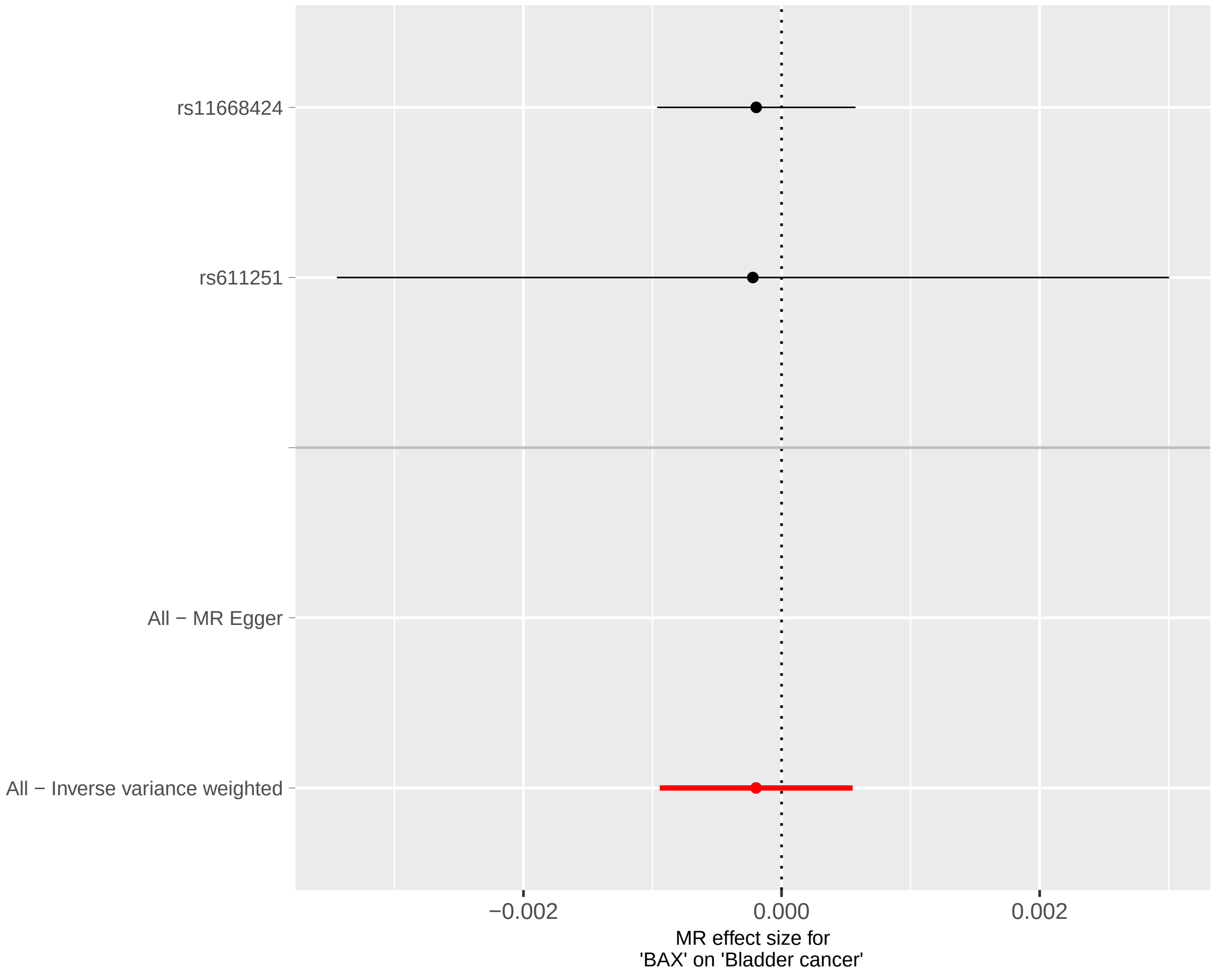
rs11668424
rs611251
All − MR Egger
All − Inverse variance weighted
−0.002
0.000
0.002
MR effect size for
'BAX' on 'Bladder cancer'

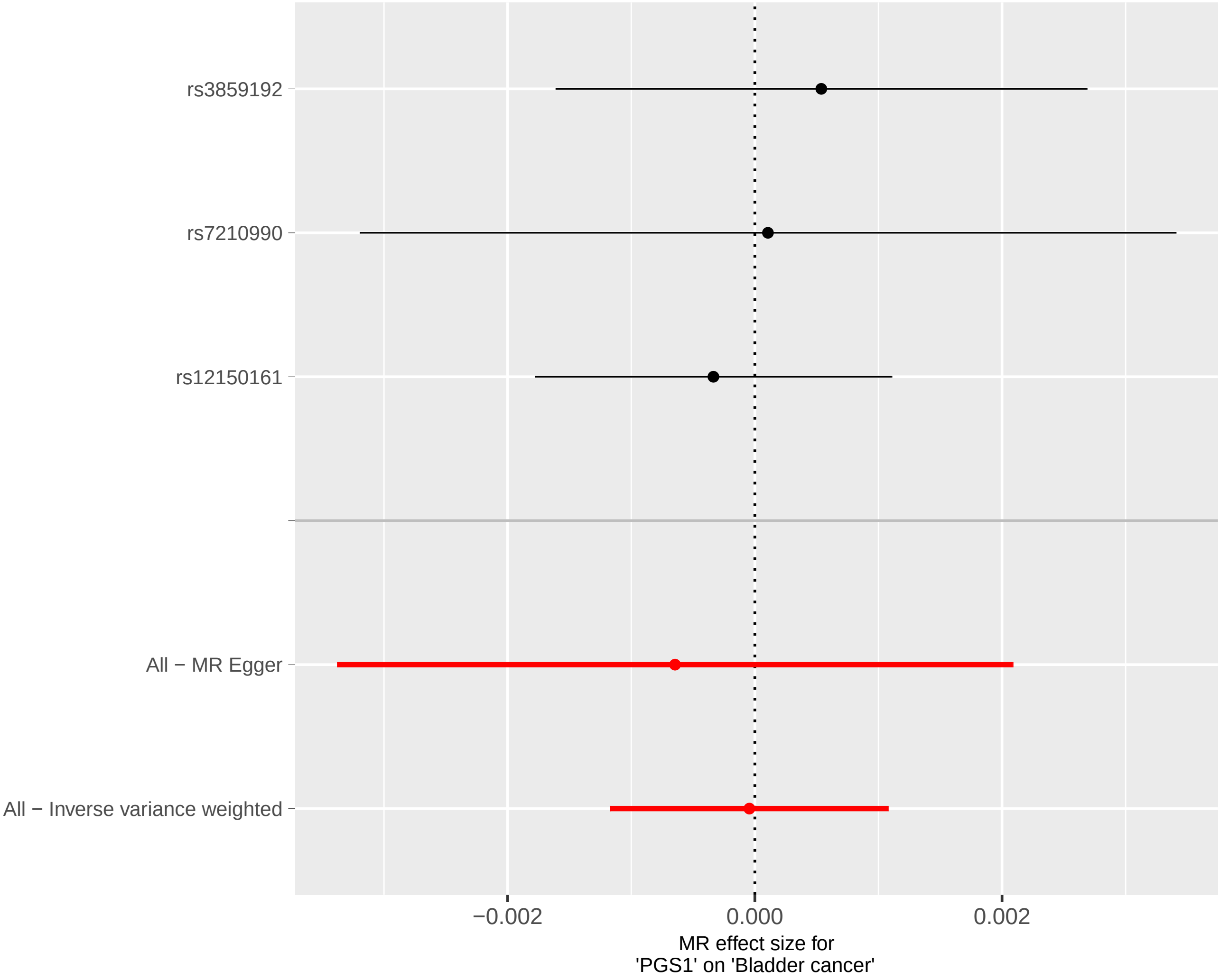
rs3859192
rs7210990
rs12150161
All − MR Egger
All − Inverse variance weighted
−0.002
0.000
0.002
MR effect size for
'PGS1' on 'Bladder cancer'

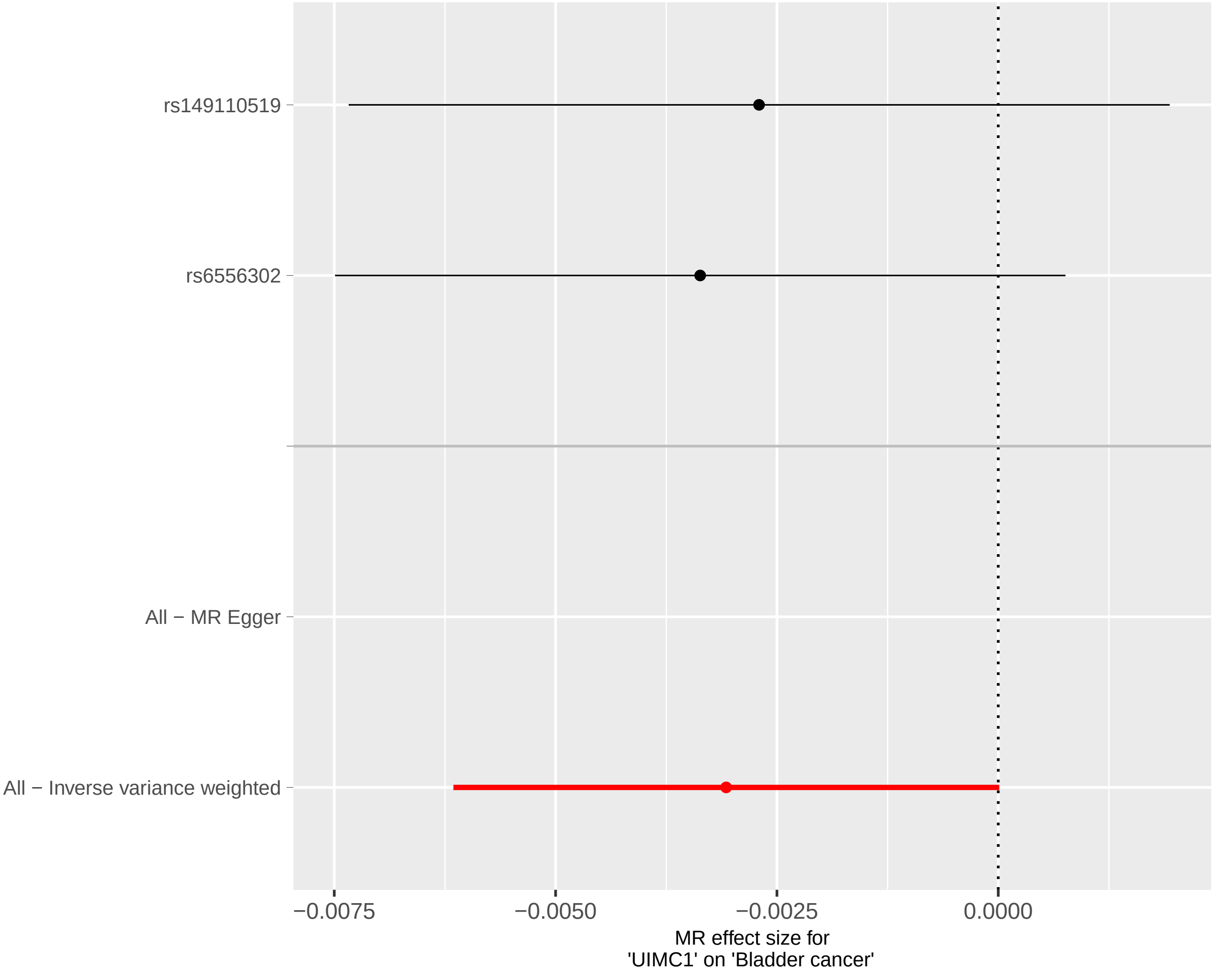
rs149110519
rs6556302
All − MR Egger
All − Inverse variance weighted
−0.0075
−0.0050
−0.0025
0.0000
MR effect size for
'UIMC1' on 'Bladder cancer'

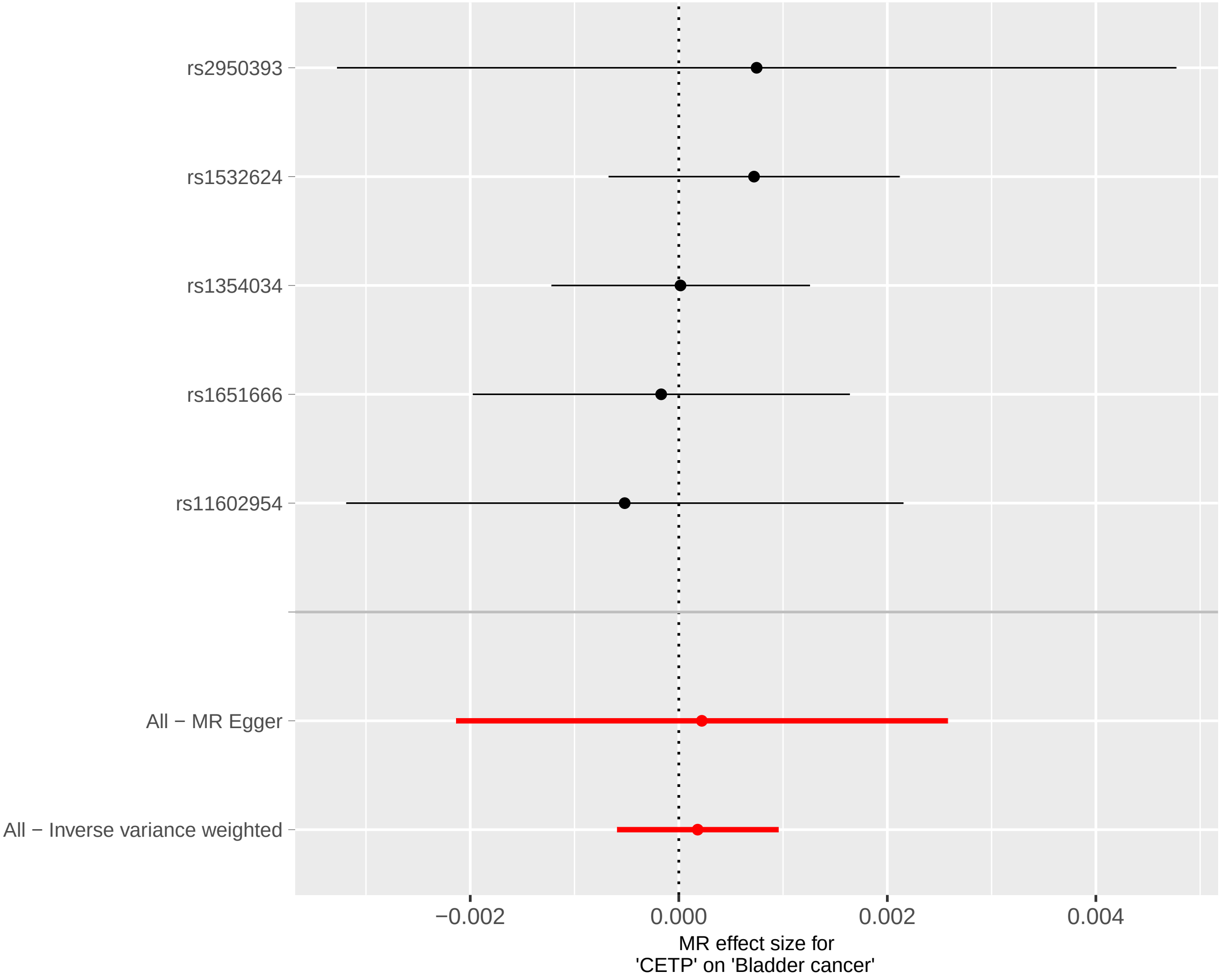
rs2950393
rs1532624
rs1354034
rs1651666
rs11602954
All − MR Egger
All − Inverse variance weighted
−0.002
0.000
0.002
0.004
MR effect size for
'CETP' on 'Bladder cancer'

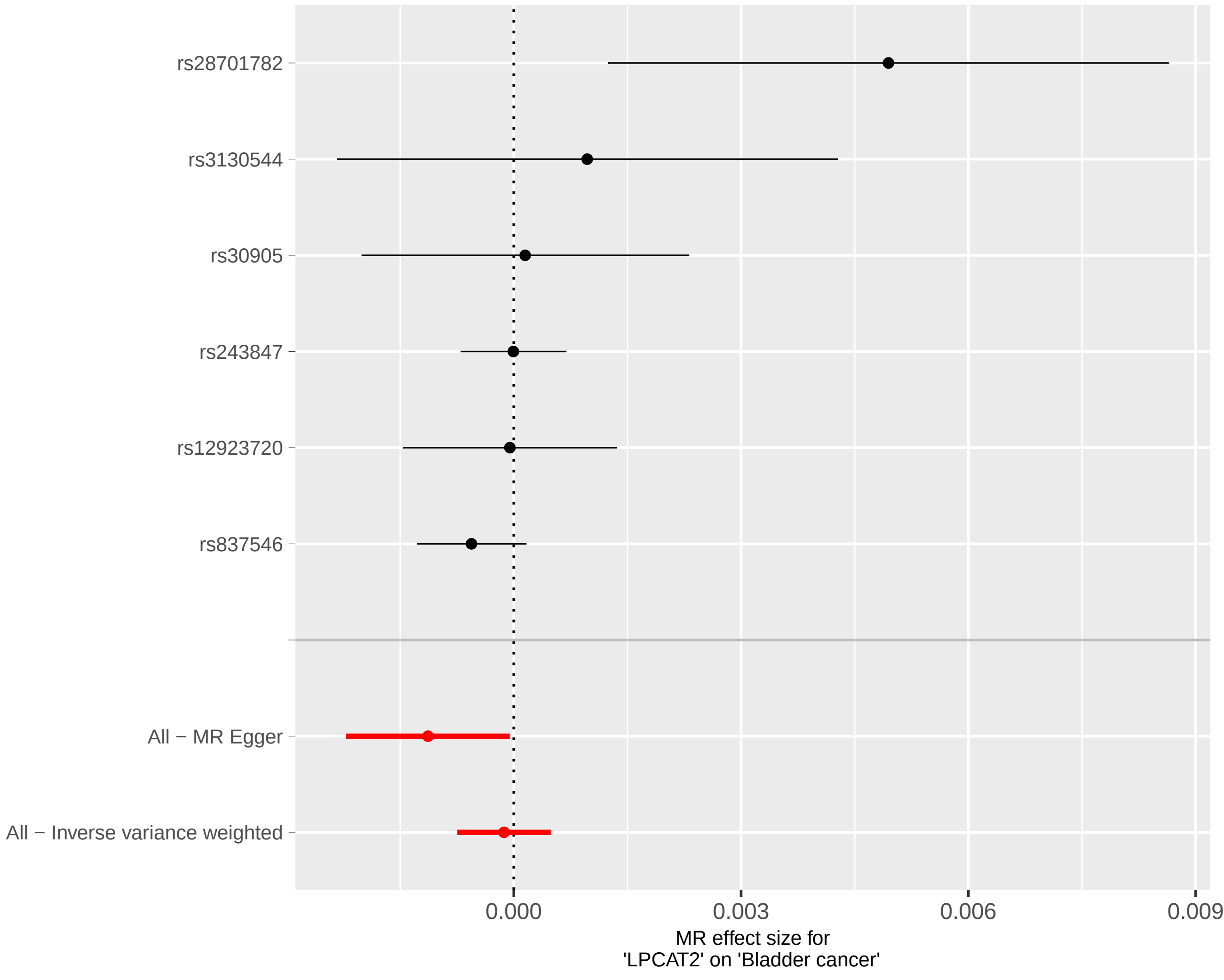
rs28701782
rs3130544
rs30905
rs243847
rs12923720
rs837546
All − MR Egger
All − Inverse variance weighted
0.000
0.003
0.006
0.009
MR effect size for
'LPCAT2' on 'Bladder cancer'

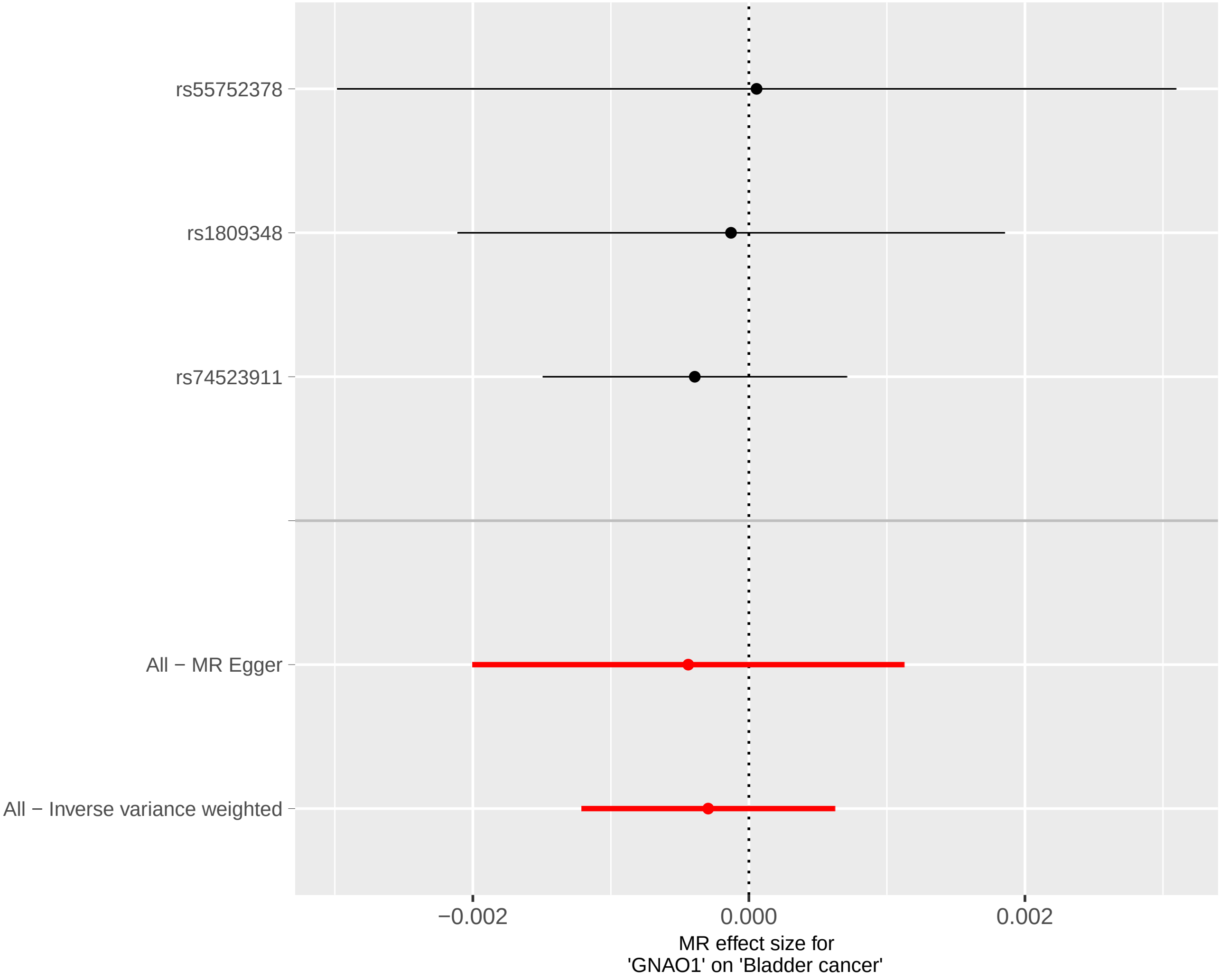
rs55752378
rs1809348
rs74523911
All − MR Egger
All − Inverse variance weighted
−0.002
0.000
0.002
MR effect size for
'GNAO1' on 'Bladder cancer'

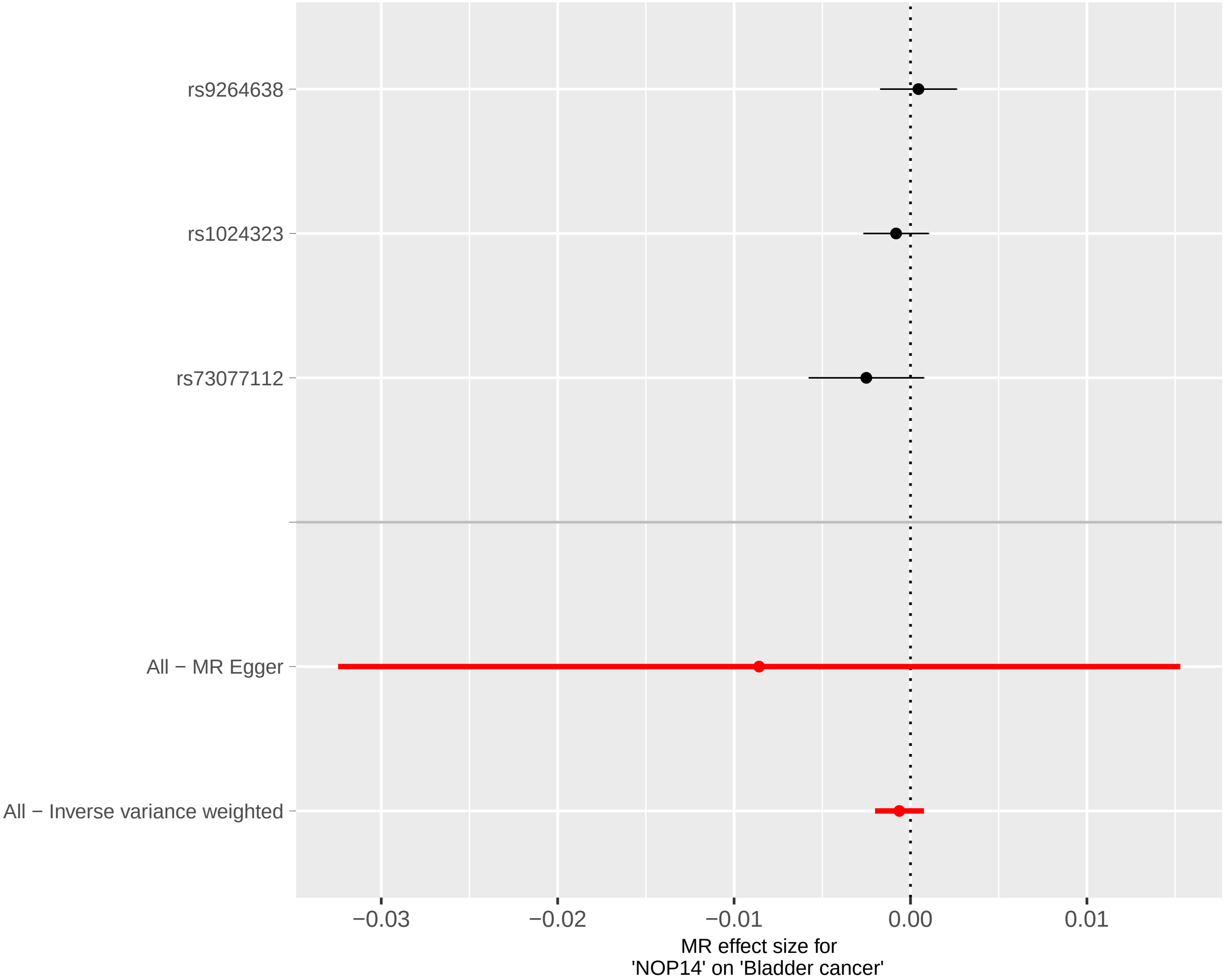
rs9264638
rs1024323
rs73077112
All − MR Egger
All − Inverse variance weighted
−0.03
−0.02
−0.01
0.00
0.01
MR effect size for
'NOP14' on 'Bladder cancer'

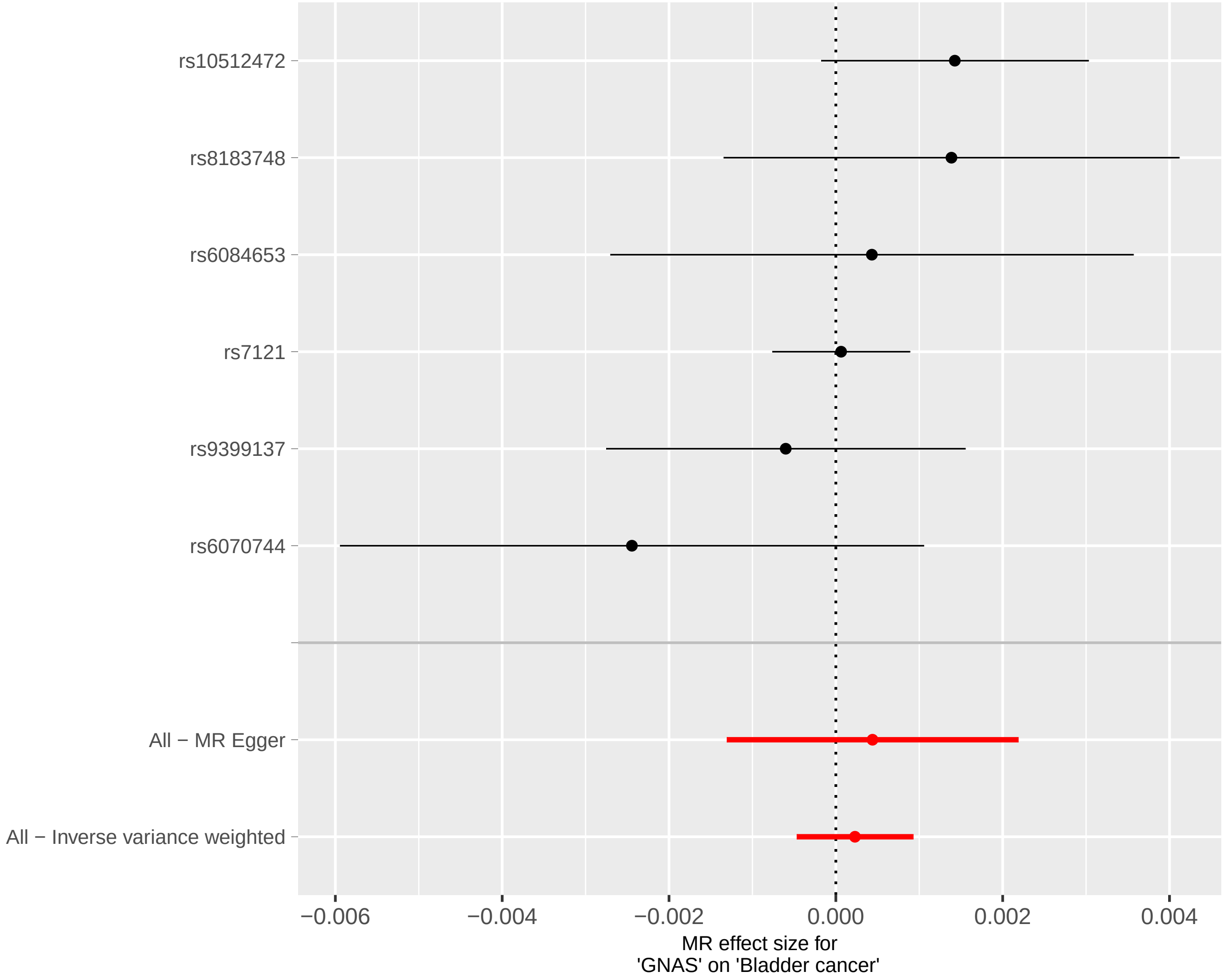

rs10512472
rs8183748
rs6084653
rs7121
rs9399137
rs6070744
All − MR Egger
All − Inverse variance weighted
−0.006
−0.004
−0.002
0.000
0.002
0.004
MR effect size for
'GNAS' on 'Bladder cancer'

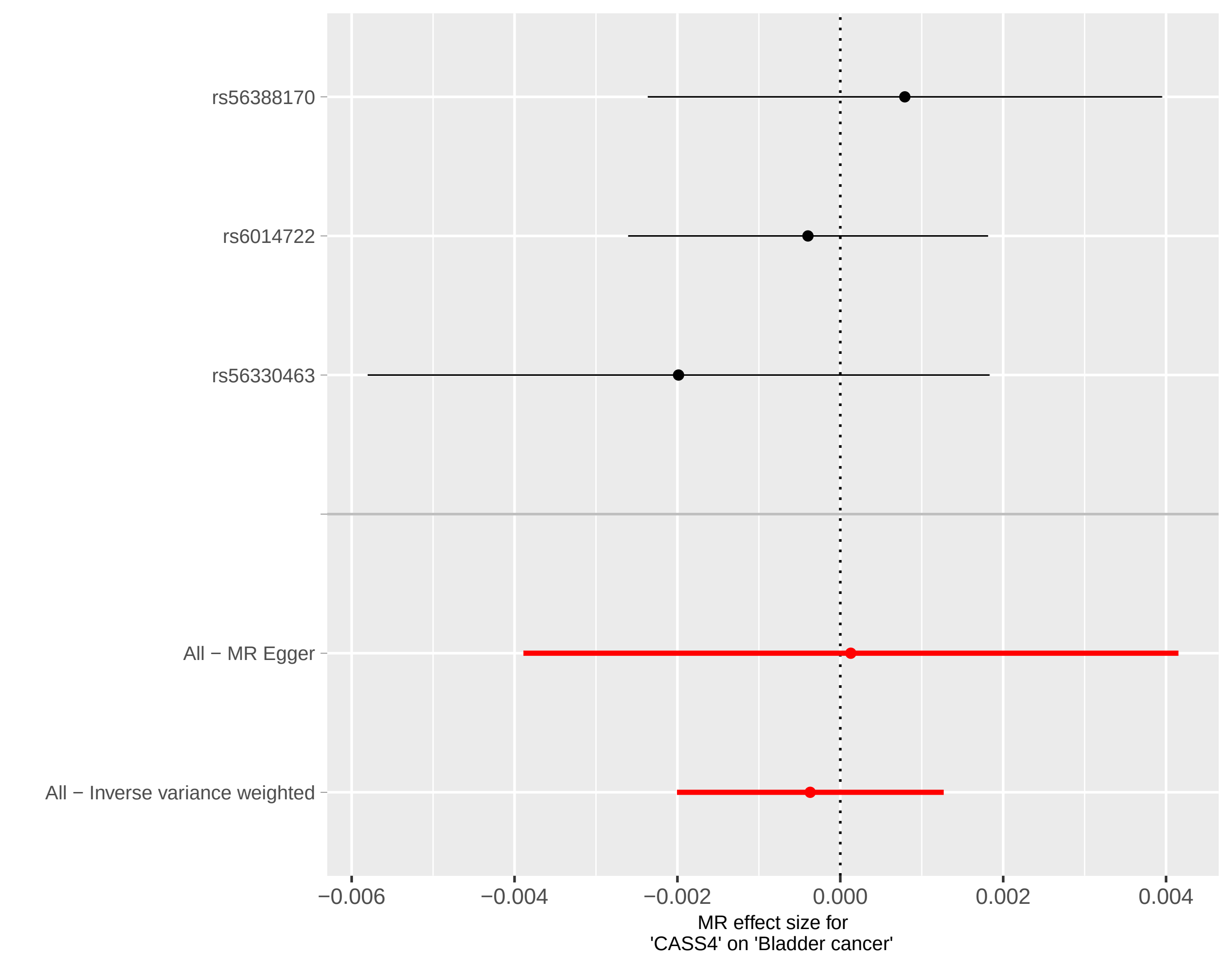
rs56388170
rs6014722
rs56330463
All − MR Egger
All − Inverse variance weighted
−0.006
−0.004
−0.002
0.000
0.002
0.004
MR effect size for
'CASS4' on 'Bladder cancer'

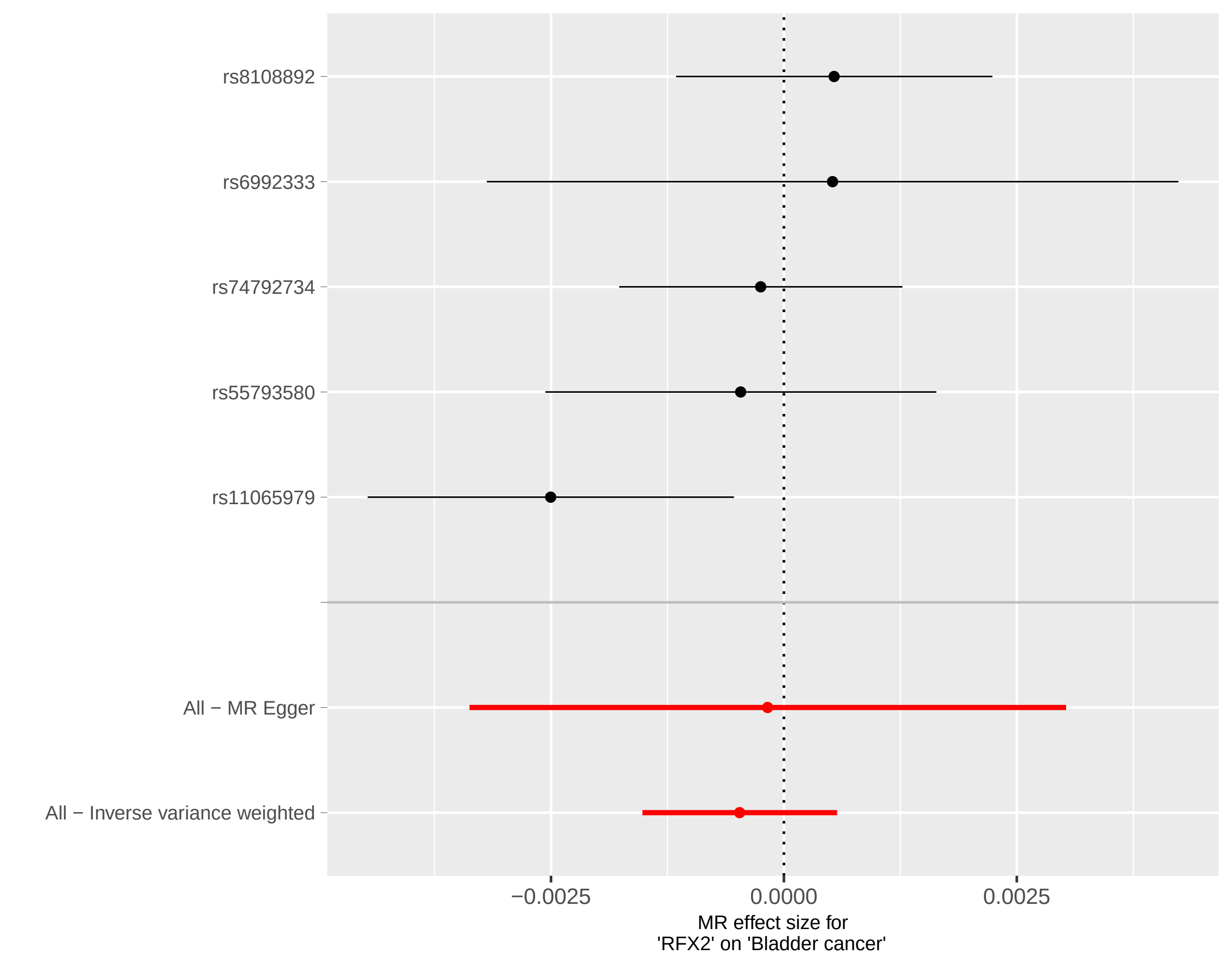
rs8108892
rs6992333
rs74792734
rs55793580
rs11065979
All − MR Egger
All − Inverse variance weighted
−0.0025
0.0000
0.0025
MR effect size for
'RFX2' on 'Bladder cancer'

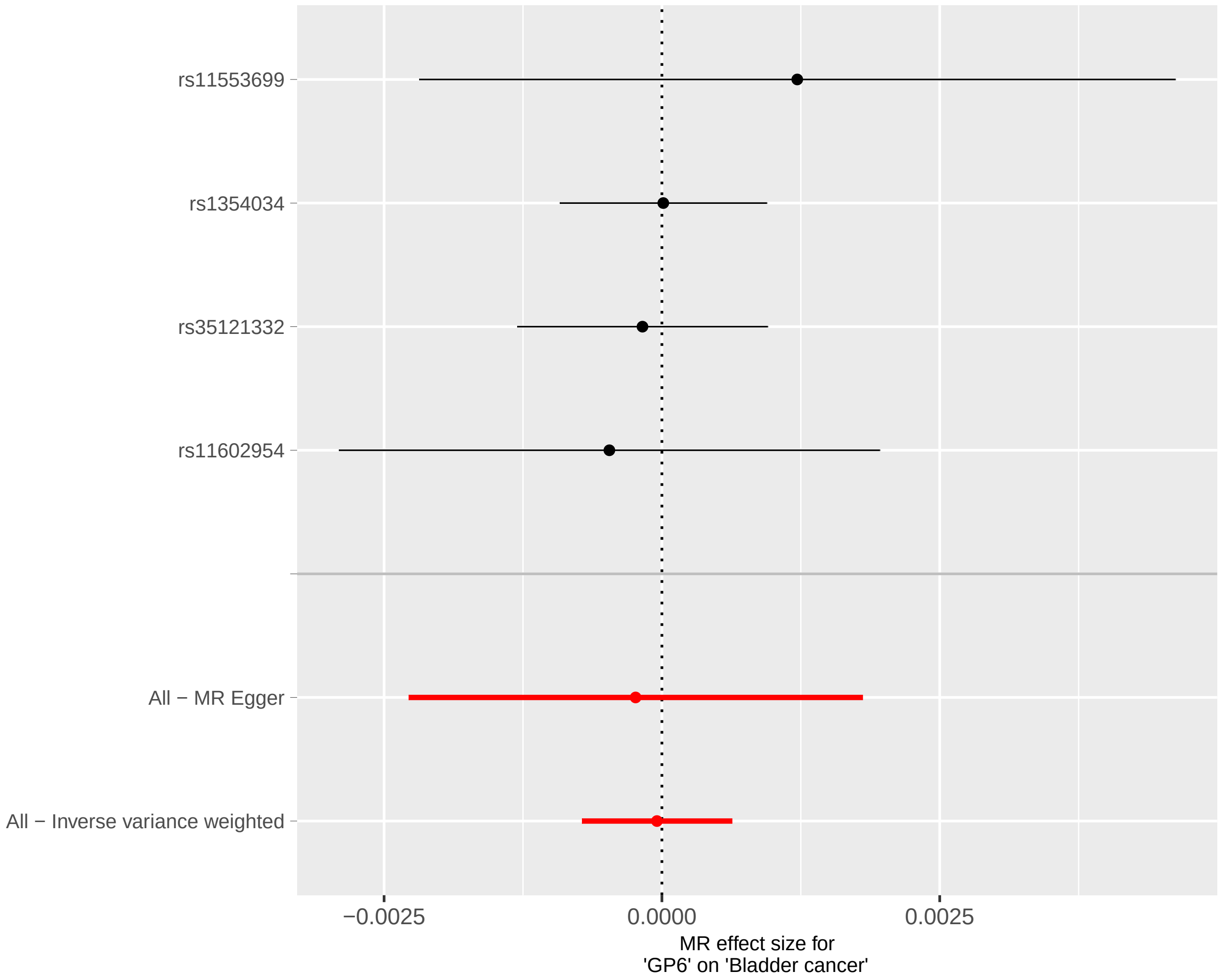
rs11553699
rs1354034
rs35121332
rs11602954
All − MR Egger
All − Inverse variance weighted
−0.0025
0.0000
0.0025
MR effect size for
'GP6' on 'Bladder cancer'

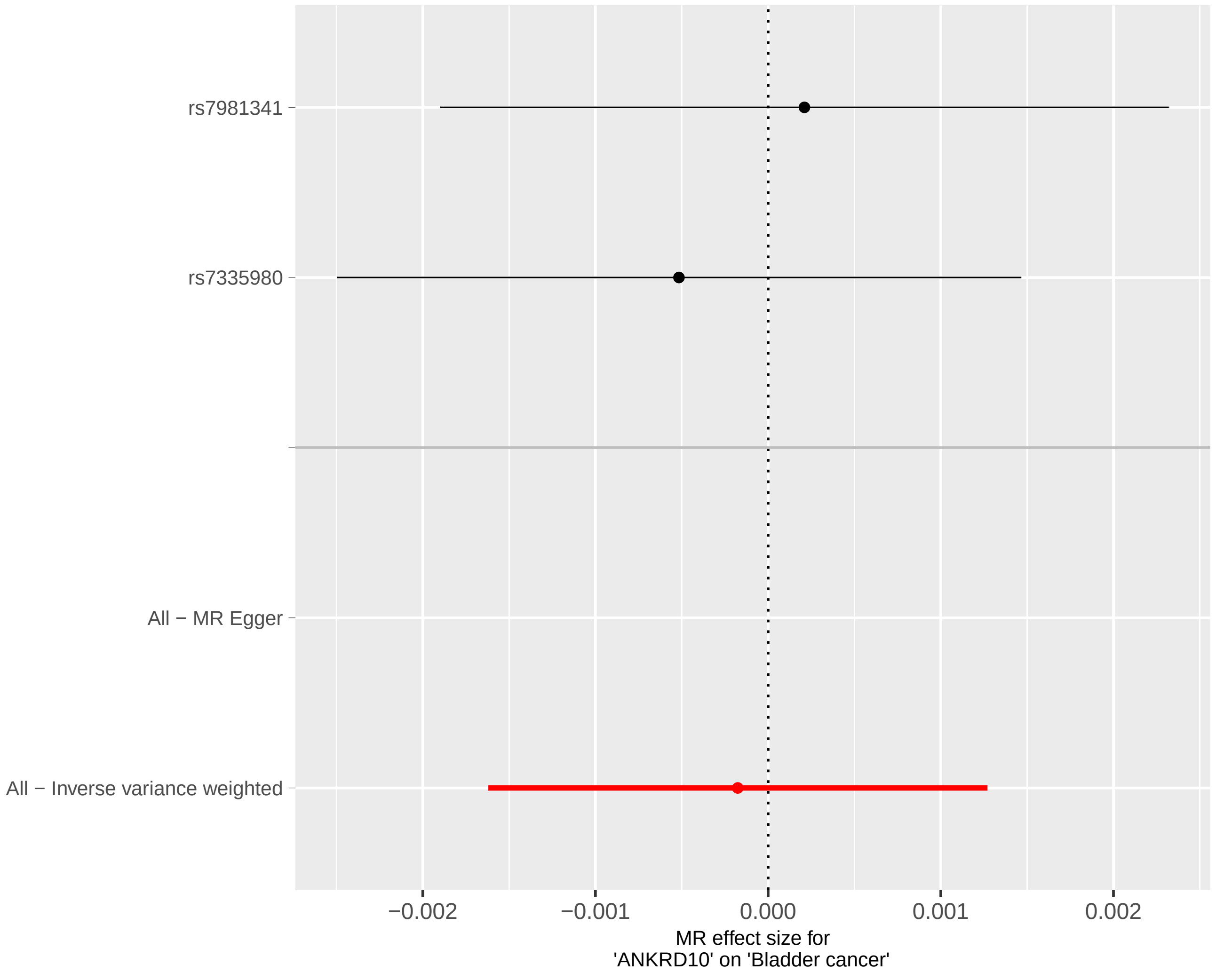
rs7981341
rs7335980
All − MR Egger
All − Inverse variance weighted
−0.002
−0.001
0.000
0.001
0.002
MR effect size for
'ANKRD10' on 'Bladder cancer'

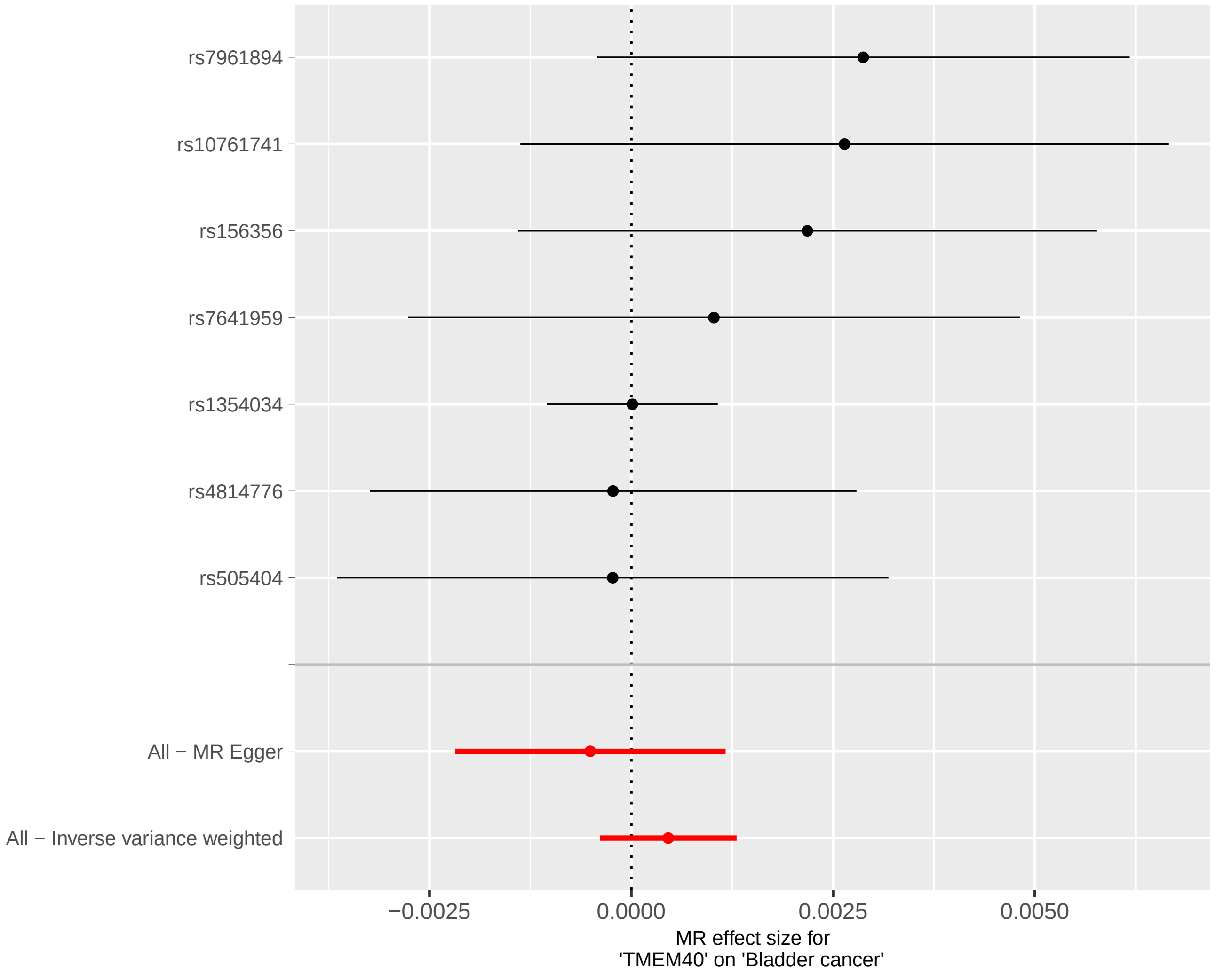
rs7961894
rs10761741
rs156356
rs7641959
rs1354034
rs4814776
rs505404
All − MR Egger
All − Inverse variance weighted
−0.0025
0.0000
0.0025
0.0050
MR effect size for
'TMEM40' on 'Bladder cancer'

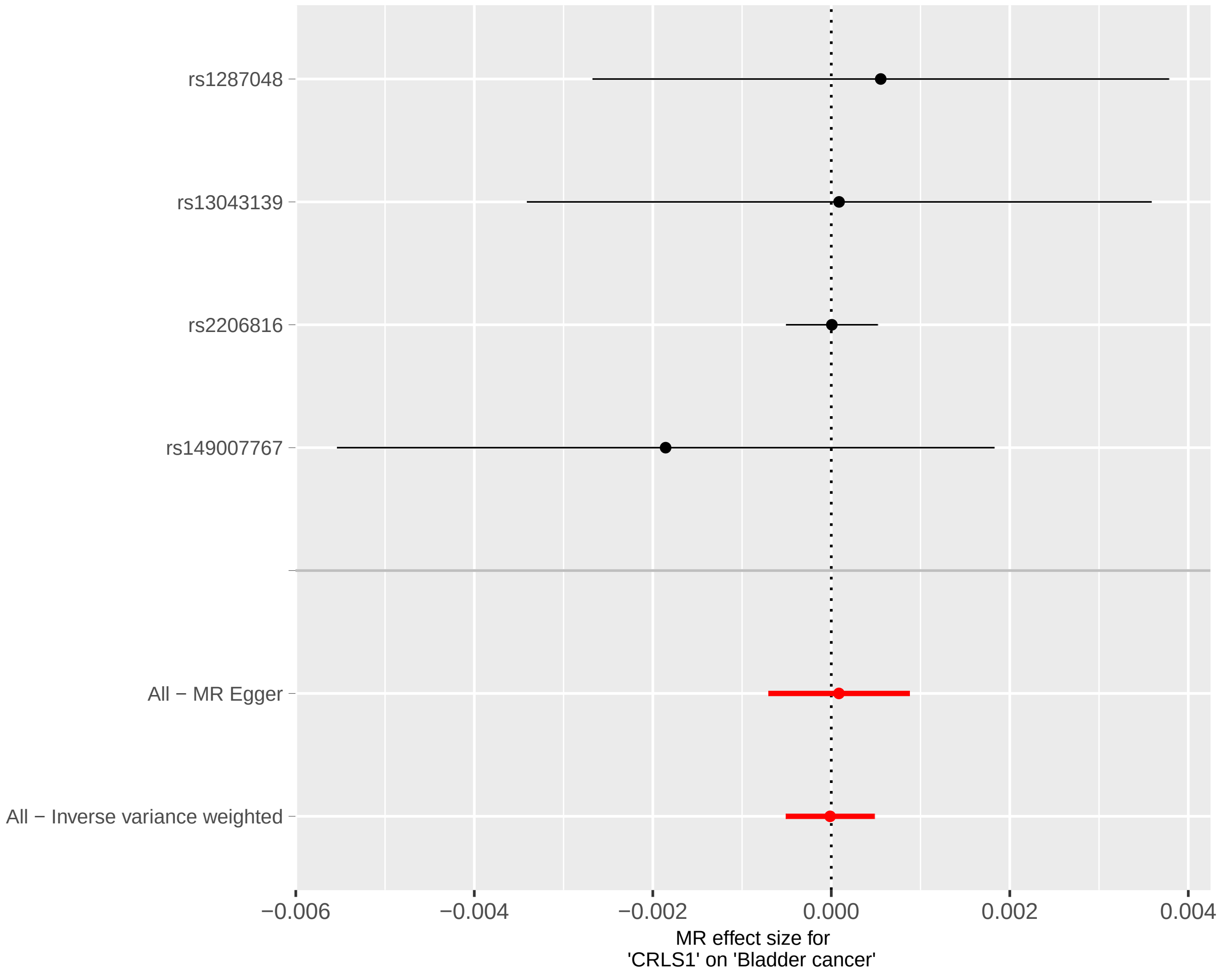

rs1287048
rs13043139
rs2206816
rs149007767
All − MR Egger
All − Inverse variance weighted
−0.006
−0.004
−0.002
0.000
0.002
0.004
MR effect size for
'CRLS1' on 'Bladder cancer'

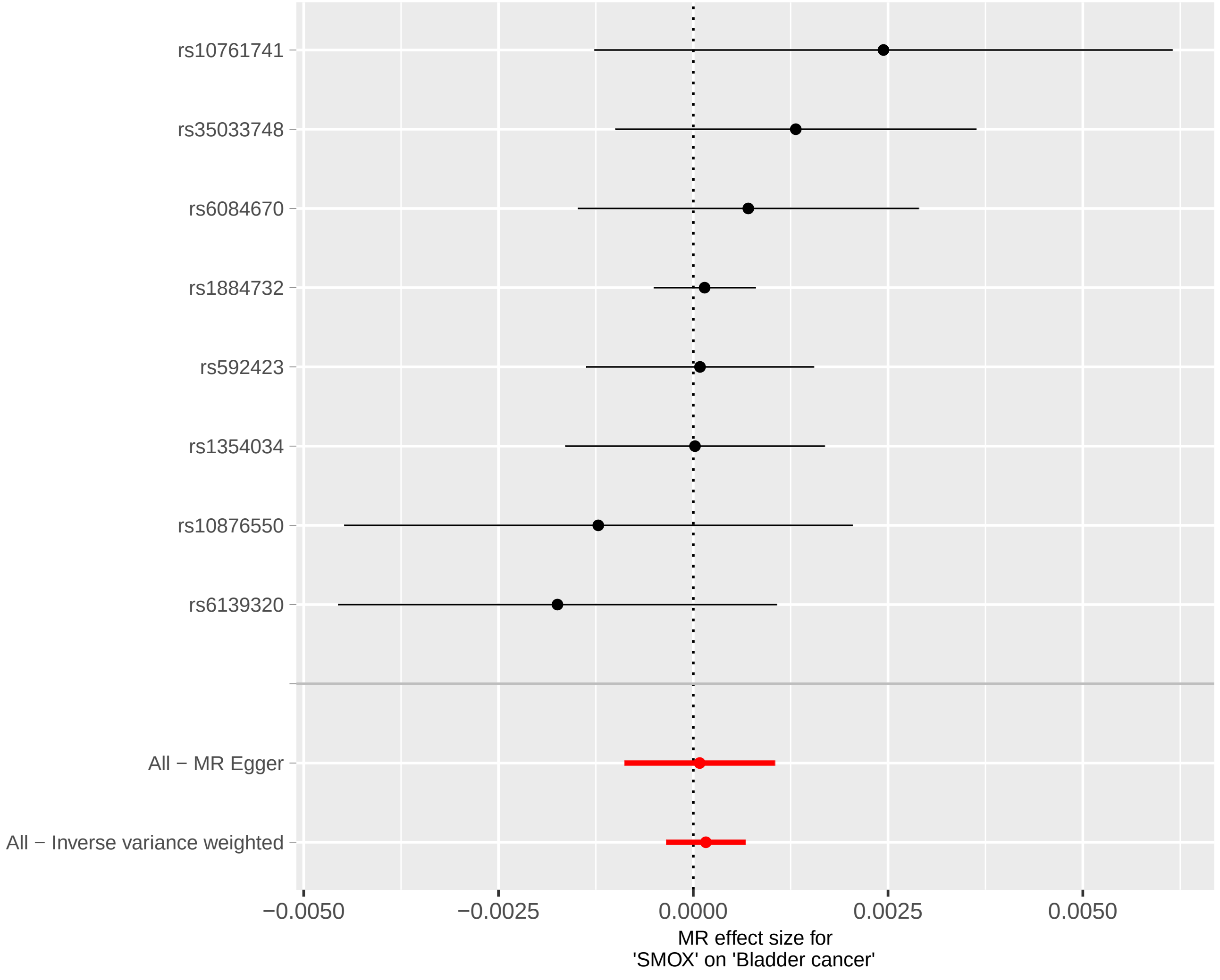
rs10761741
rs35033748
rs6084670
rs1884732
rs592423
rs1354034
rs10876550
rs6139320
All − MR Egger
All − Inverse variance weighted
−0.0050
−0.0025
0.0000
0.0025
0.0050
MR effect size for
'SMOX' on 'Bladder cancer'

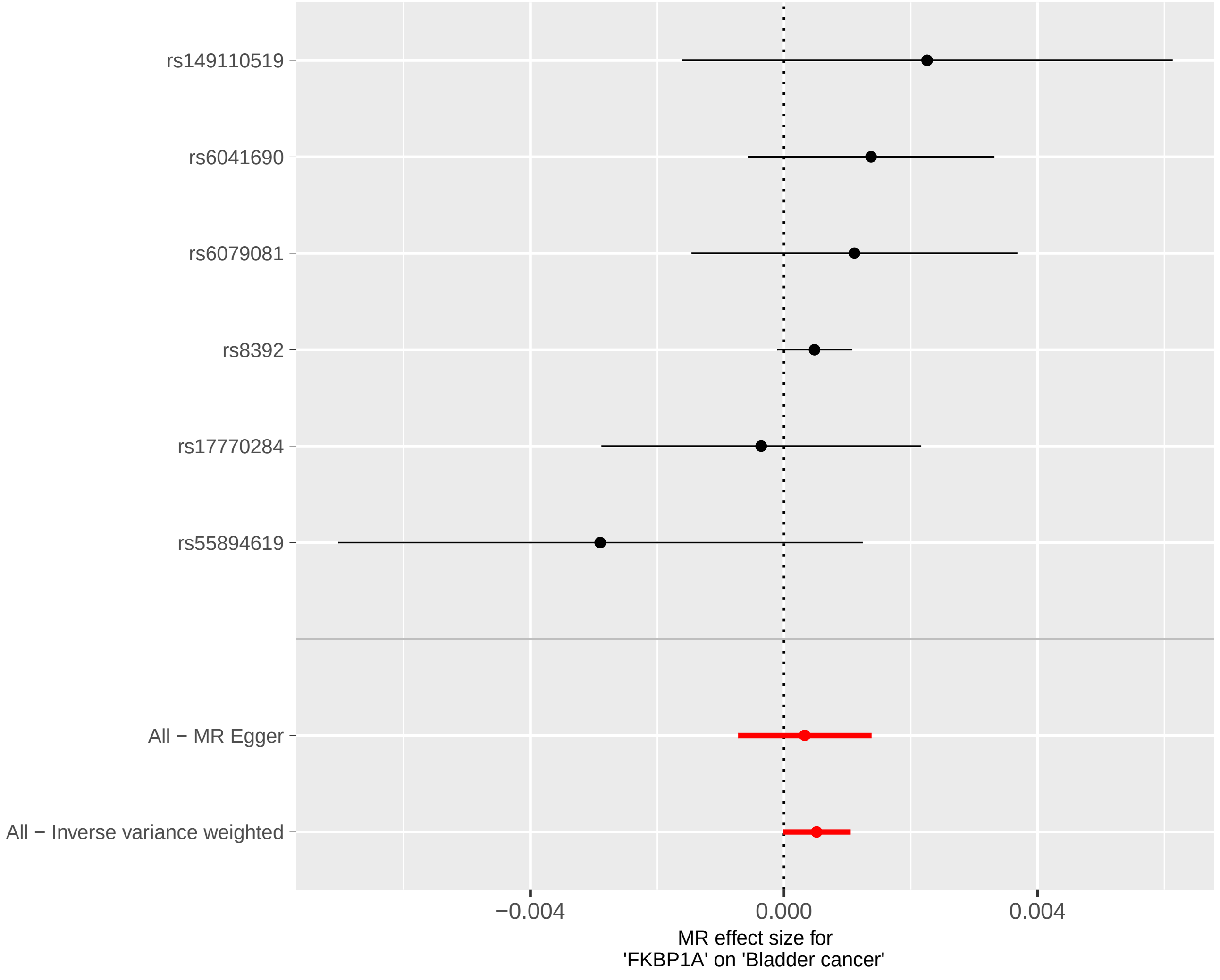

rs149110519
rs6041690
rs6079081
rs8392
rs17770284
rs55894619
All − MR Egger
All − Inverse variance weighted
−0.004
0.000
0.004
MR effect size for
'FKBP1A' on 'Bladder cancer'

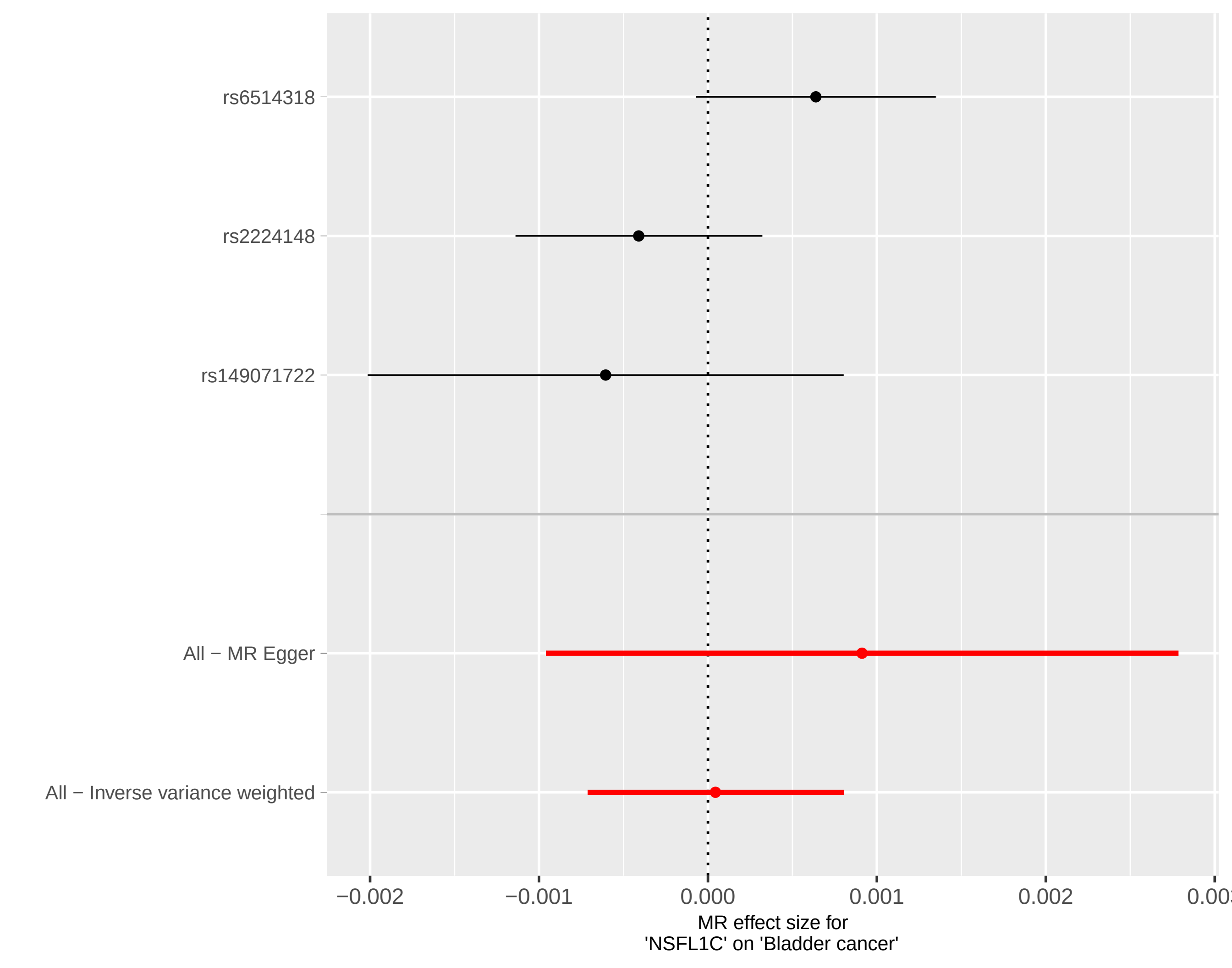
rs6514318
rs2224148
rs149071722
All − MR Egger
All − Inverse variance weighted
−0.002
−0.001
0.000
0.001
0.002
MR effect size for
'NSFL1C' on 'Bladder cancer'

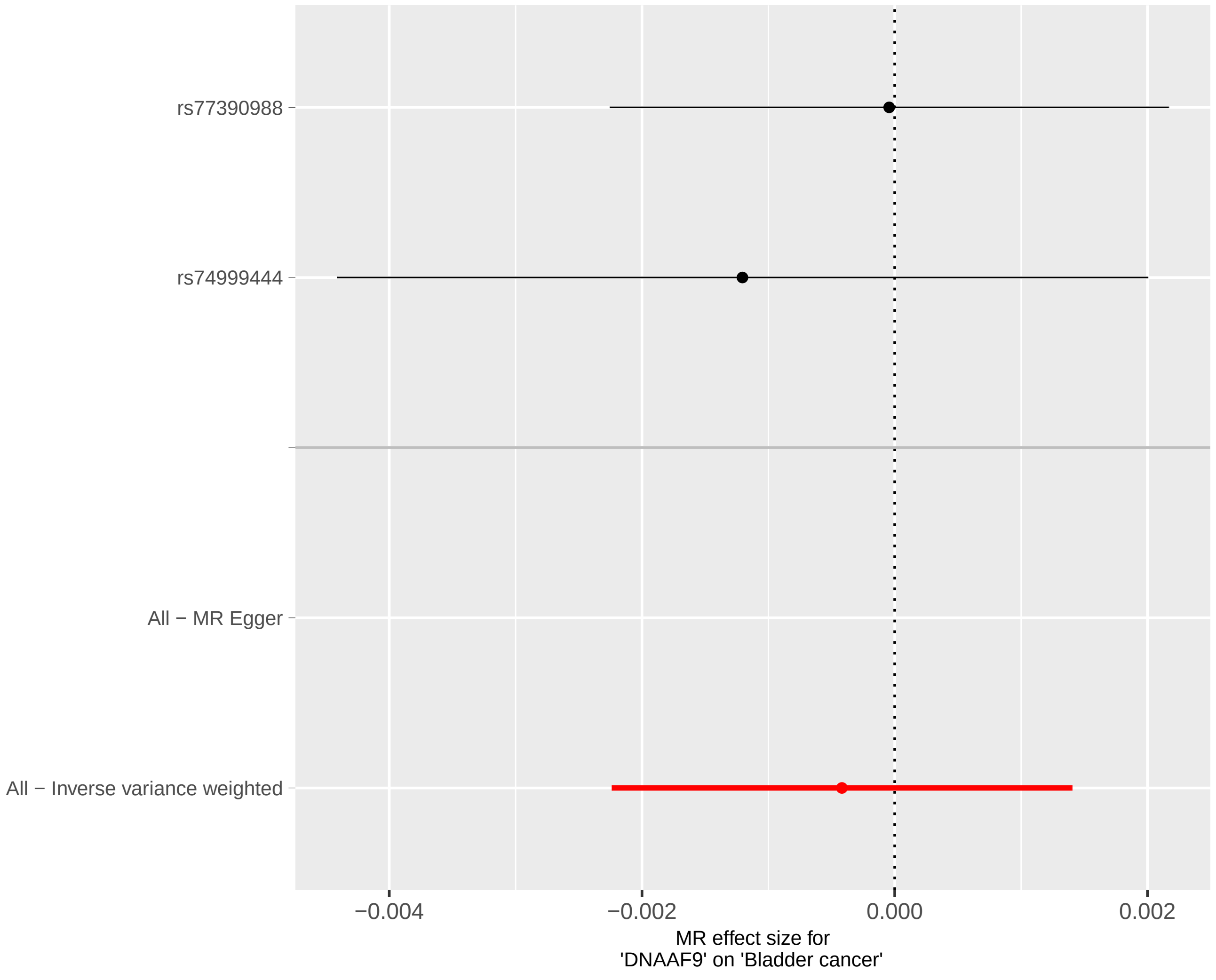
rs77390988
rs74999444
All − MR Egger
All − Inverse variance weighted
−0.004
−0.002
0.000
0.002
MR effect size for
'DNAAF9' on 'Bladder cancer'

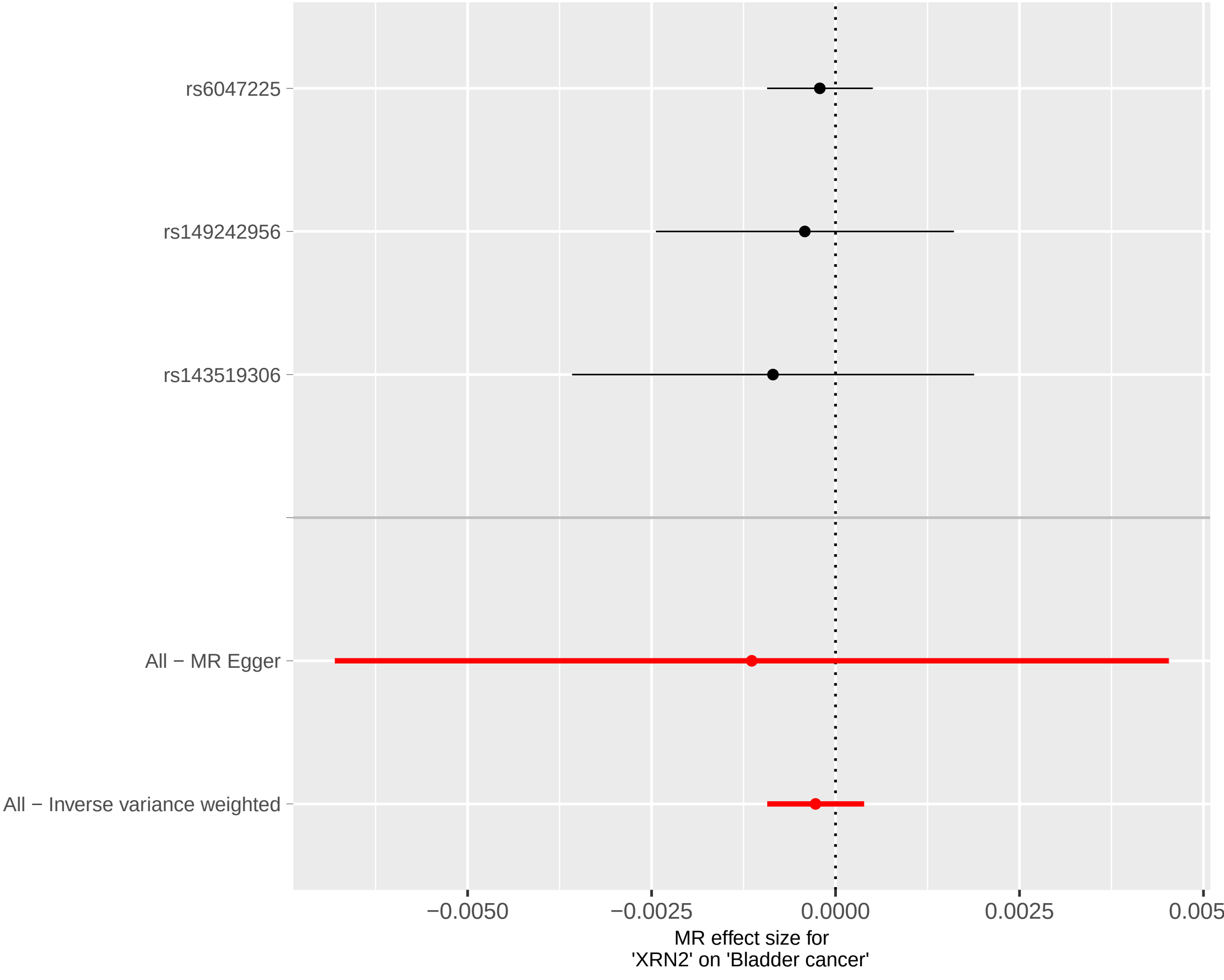
rs6047225
rs149242956
rs143519306
All − MR Egger
All − Inverse variance weighted
−0.0050
−0.0025
0.0000
0.0025
0.005
MR effect size for
'XRN2' on 'Bladder cancer'

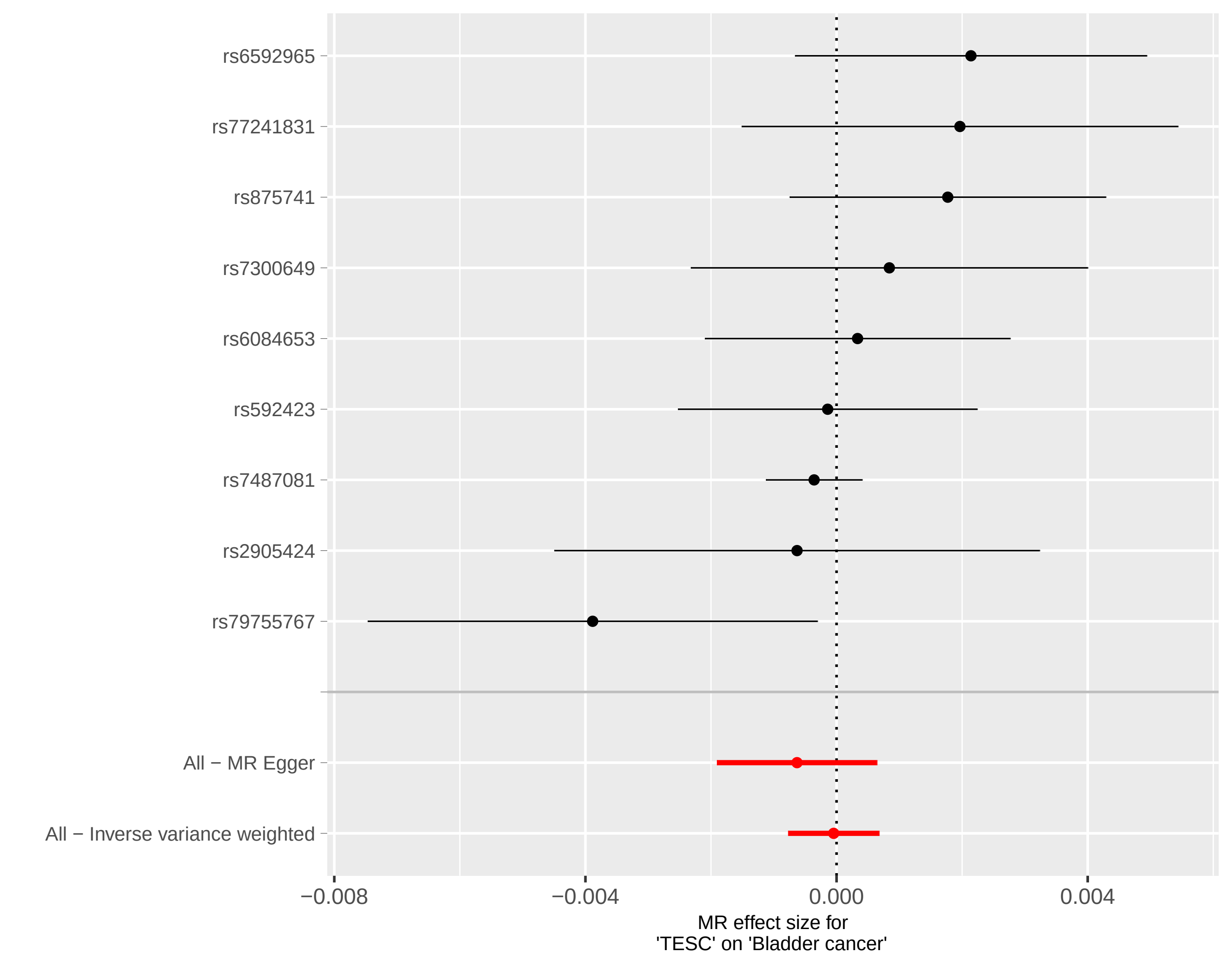

rs6592965
rs77241831
rs875741
rs7300649
rs6084653
rs592423
rs7487081
rs2905424
rs79755767
All − MR Egger
All − Inverse variance weighted
−0.008
−0.004
0.000
0.004
MR effect size for
'TESC' on 'Bladder cancer'

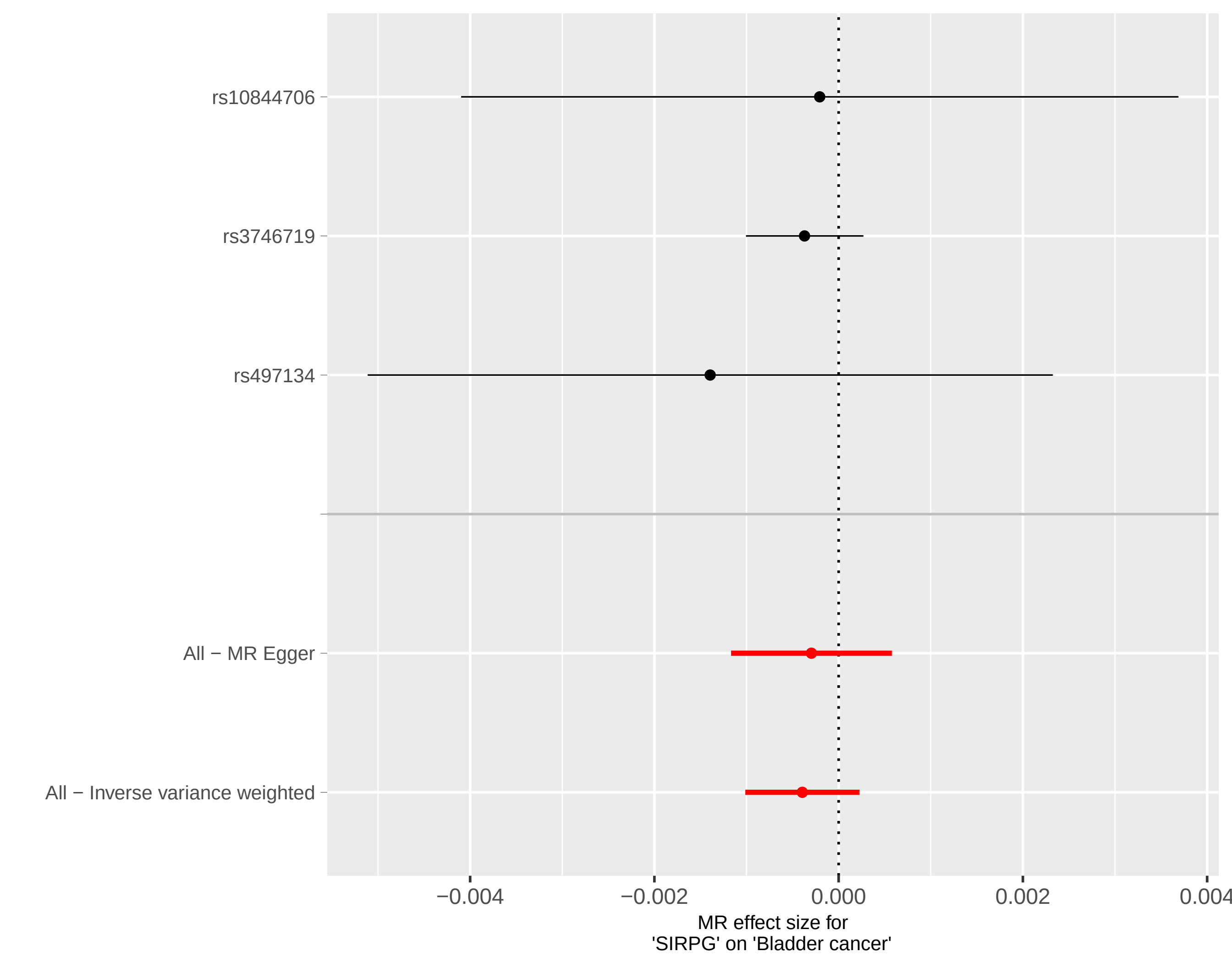

rs10844706
rs3746719
rs497134
All − MR Egger
All − Inverse variance weighted
−0.004
−0.002
0.000
0.002
0.004
MR effect size for
'SIRPG' on 'Bladder cancer'

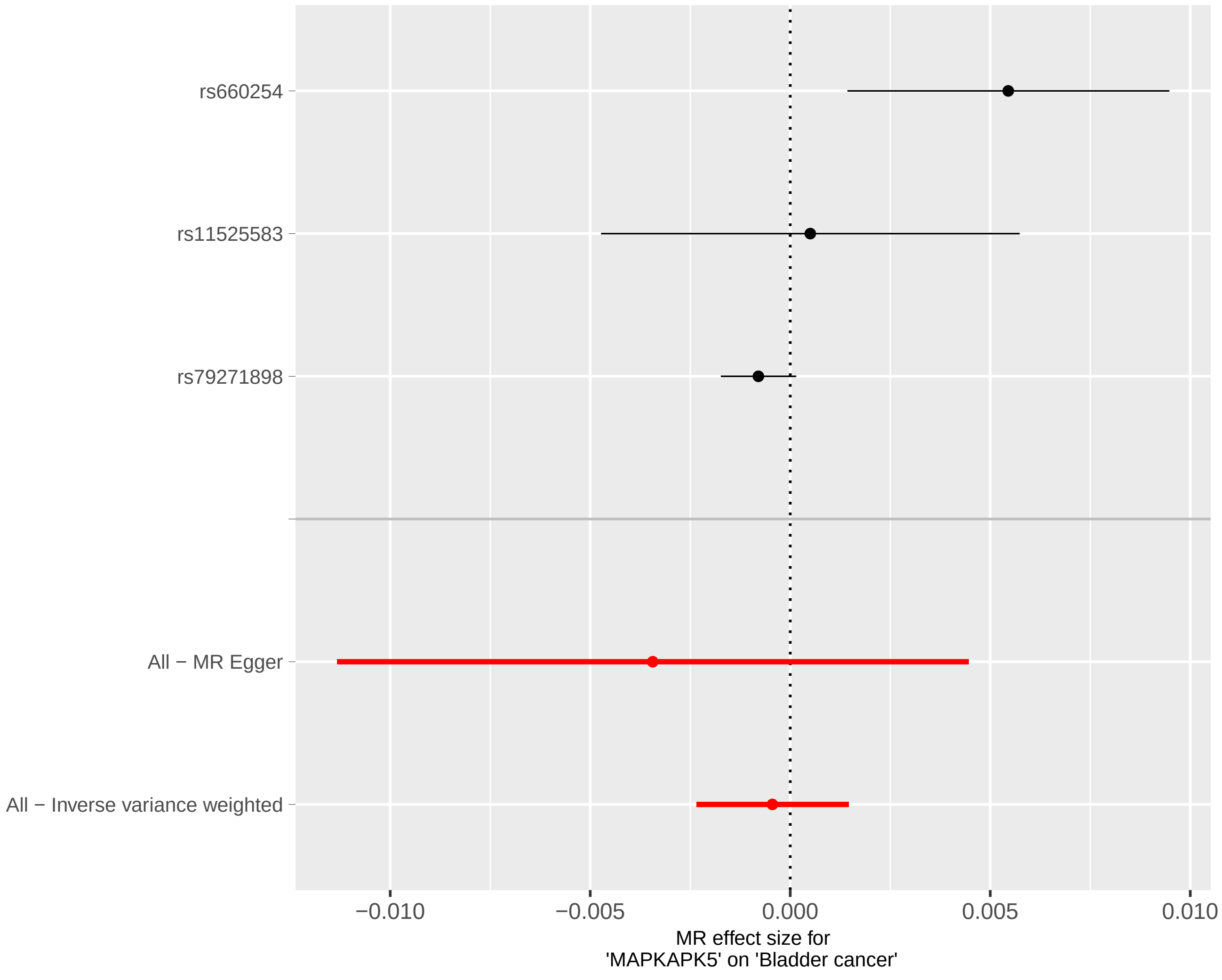
rs660254
rs11525583
rs79271898
All − MR Egger
All − Inverse variance weighted
−0.010
−0.005
0.000
0.005
0.010
MR effect size for
'MAPKAPK5' on 'Bladder cancer'

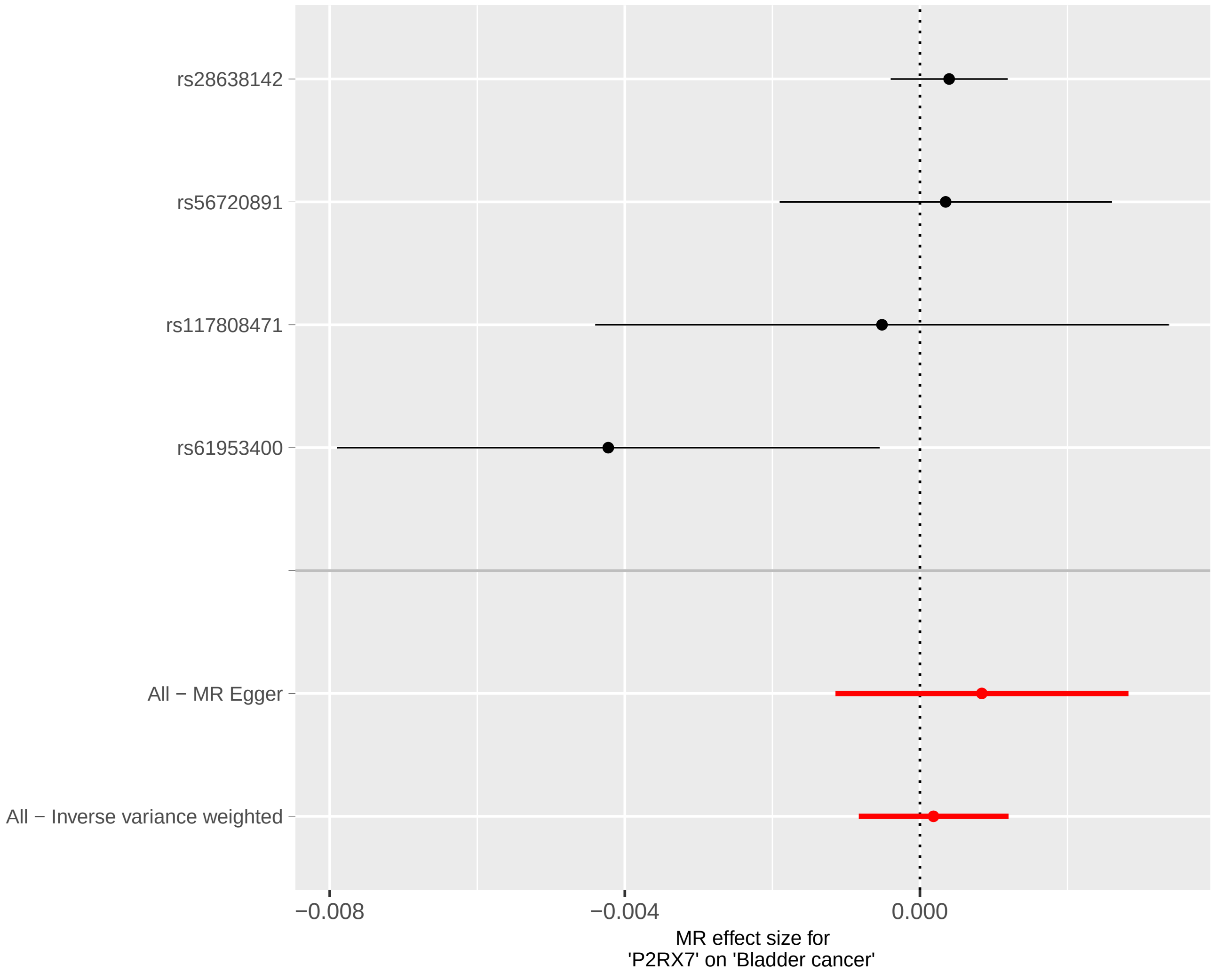

rs28638142
rs56720891
rs117808471
rs61953400
All − MR Egger
All − Inverse variance weighted
−0.008
−0.004
0.000
MR effect size for
'P2RX7' on 'Bladder cancer'

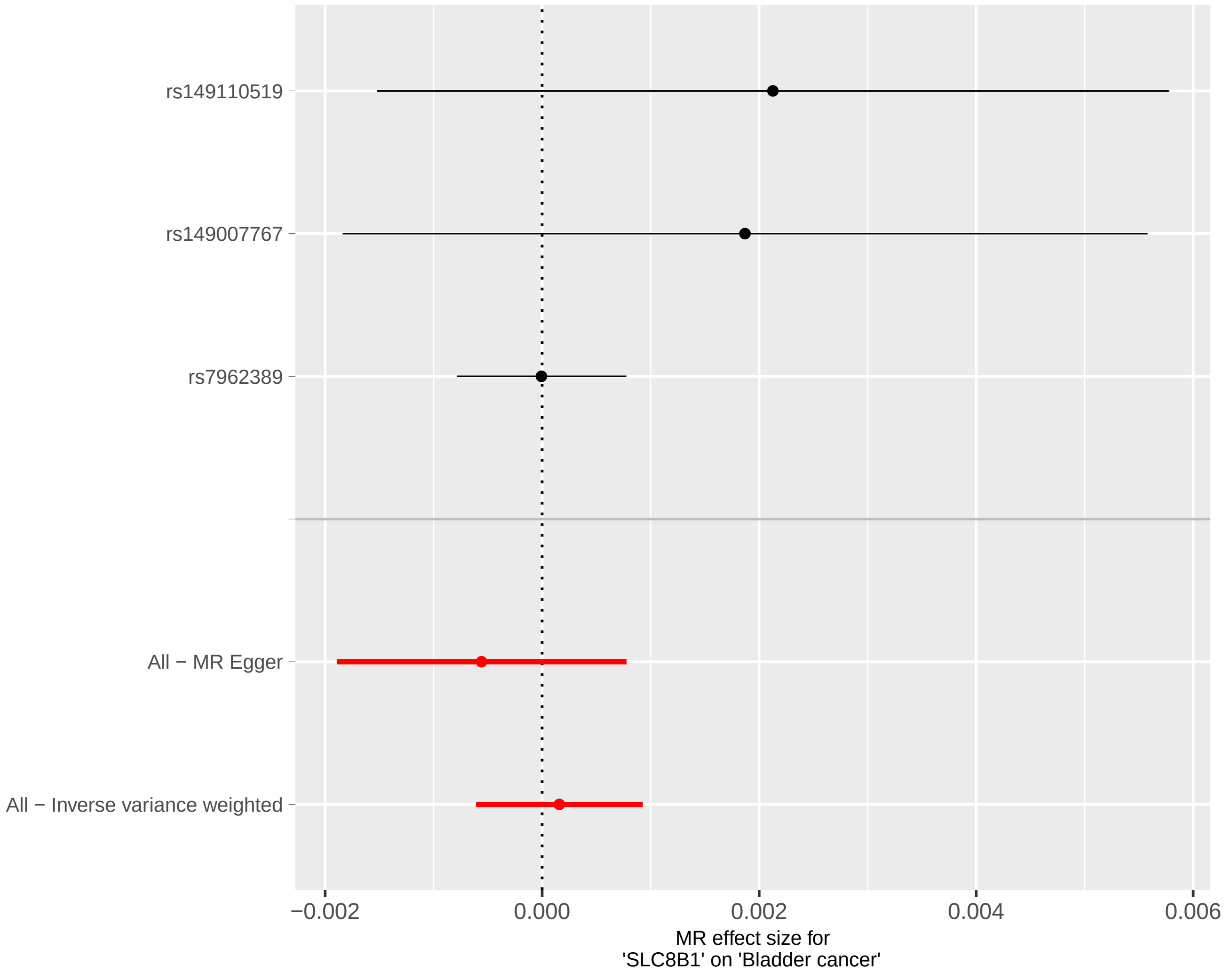
rs149110519
rs149007767
rs7962389
All − MR Egger
All − Inverse variance weighted
−0.002
0.000
0.002
0.004
0.006
MR effect size for
'SLC8B1' on 'Bladder cancer'

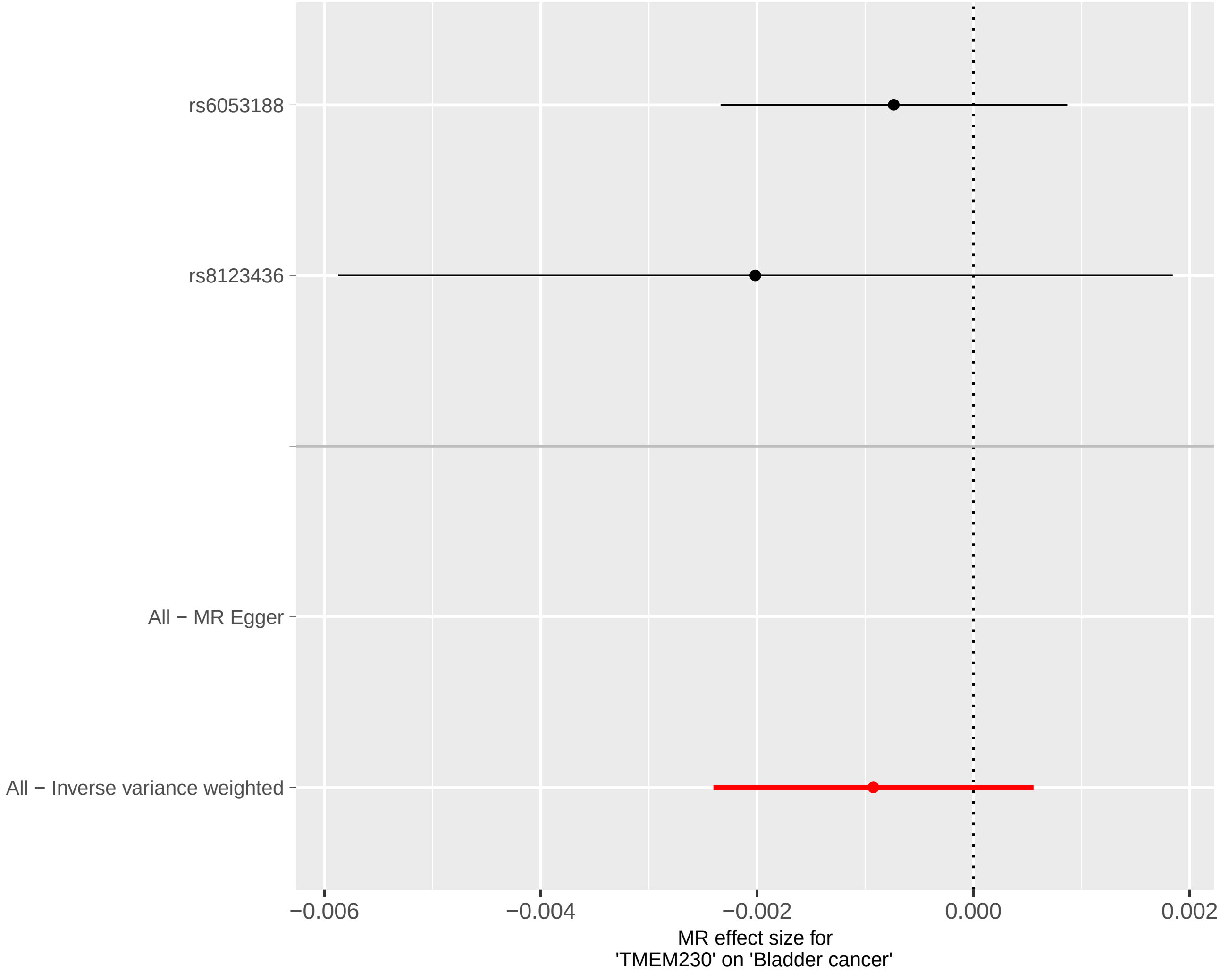

rs6053188
rs8123436
All − MR Egger
All − Inverse variance weighted
−0.006
−0.004
−0.002
0.000
0.002
MR effect size for
'TMEM230' on 'Bladder cancer'

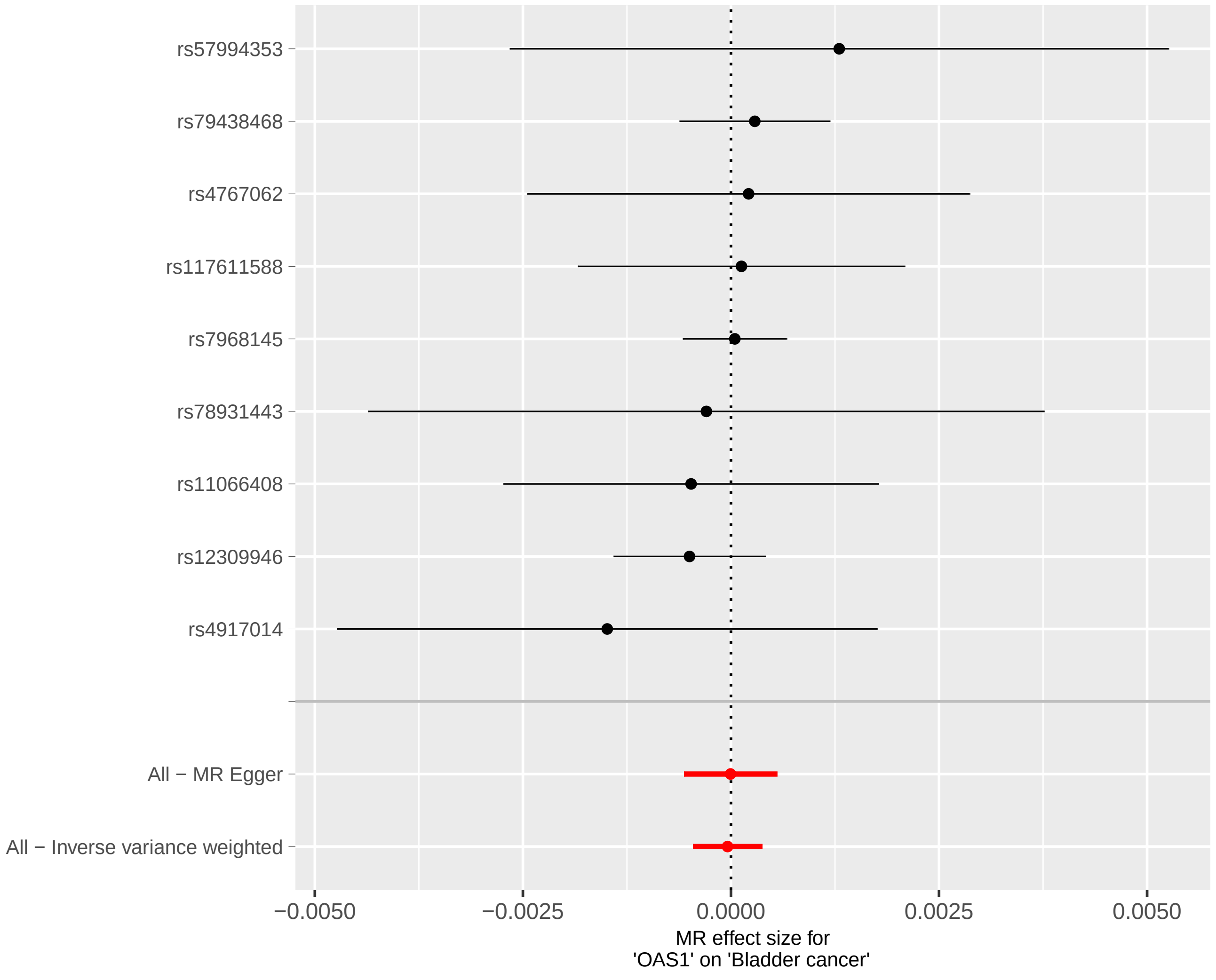
rs57994353
rs79438468
rs4767062
rs117611588
rs7968145
rs78931443
rs11066408
rs12309946
rs4917014
All − MR Egger
All − Inverse variance weighted
−0.0050
−0.0025
0.0000
0.0025
0.0050
MR effect size for
'OAS1' on 'Bladder cancer'

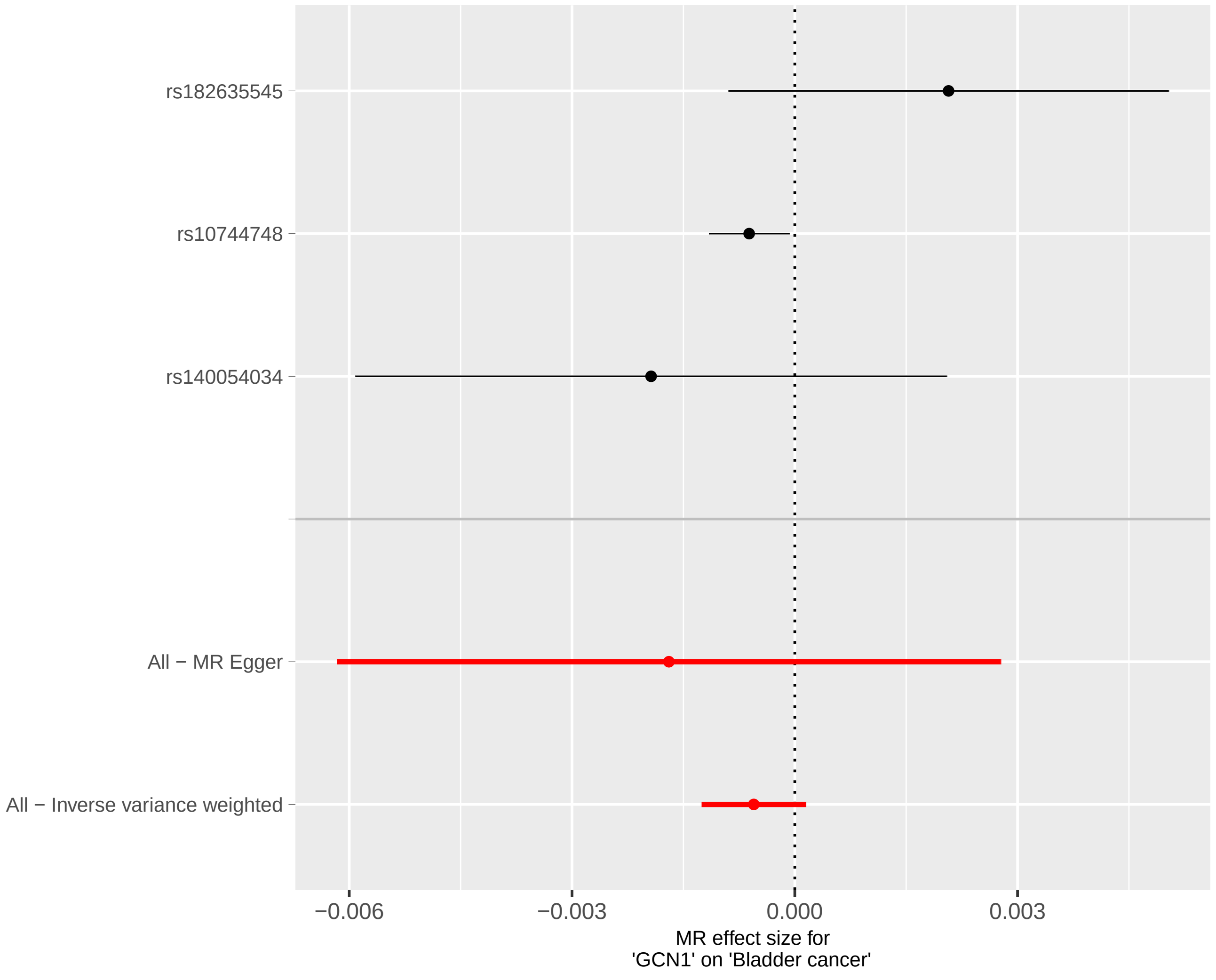
rs182635545
rs10744748
rs140054034
All − MR Egger
All − Inverse variance weighted
−0.006
−0.003
0.000
0.003
MR effect size for
'GCN1' on 'Bladder cancer'

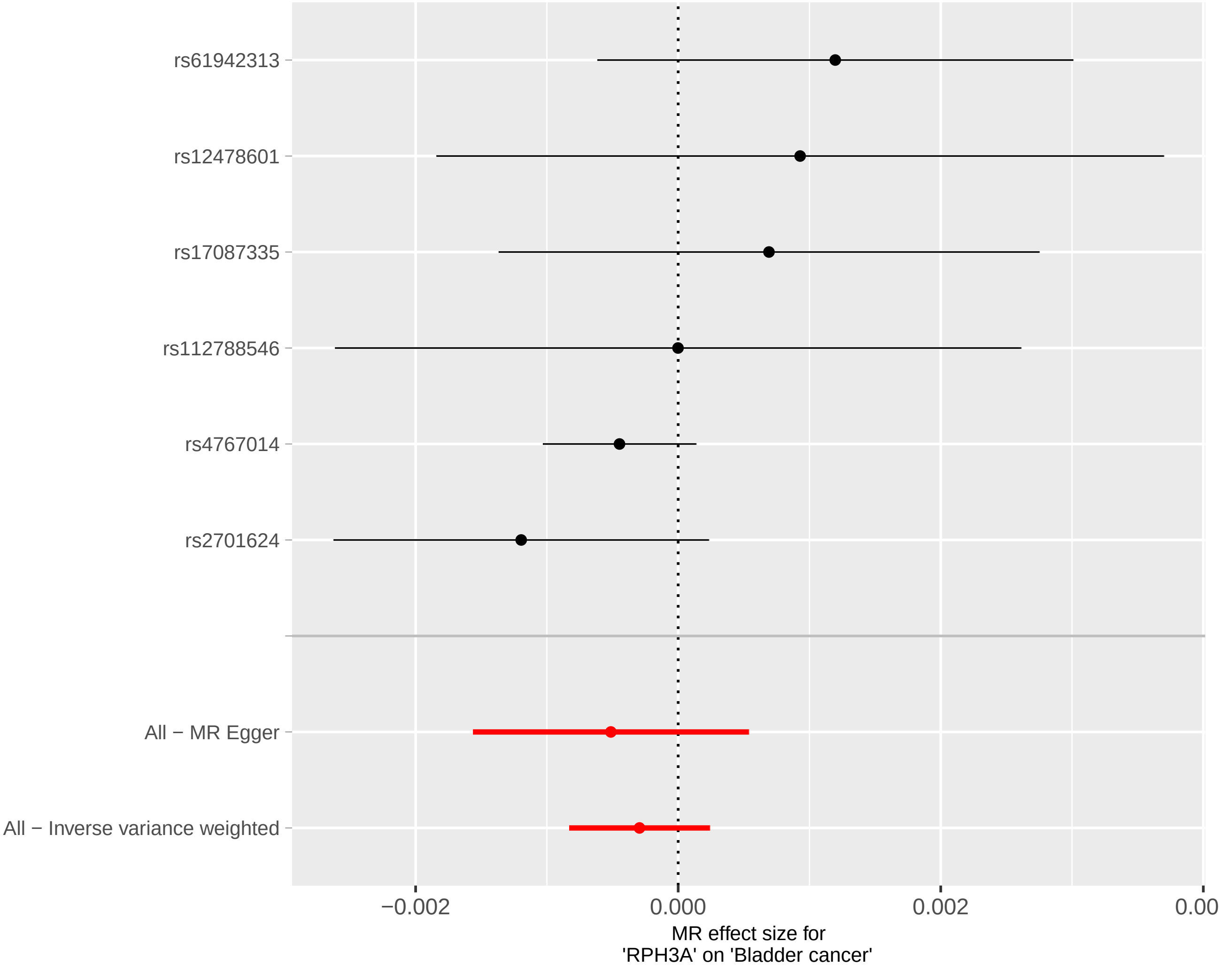
rs61942313
rs12478601
rs17087335
rs112788546
rs4767014
rs2701624
All − MR Egger
All − Inverse variance weighted
−0.002
0.000
0.002
0.00
MR effect size for
'RPH3A' on 'Bladder cancer'

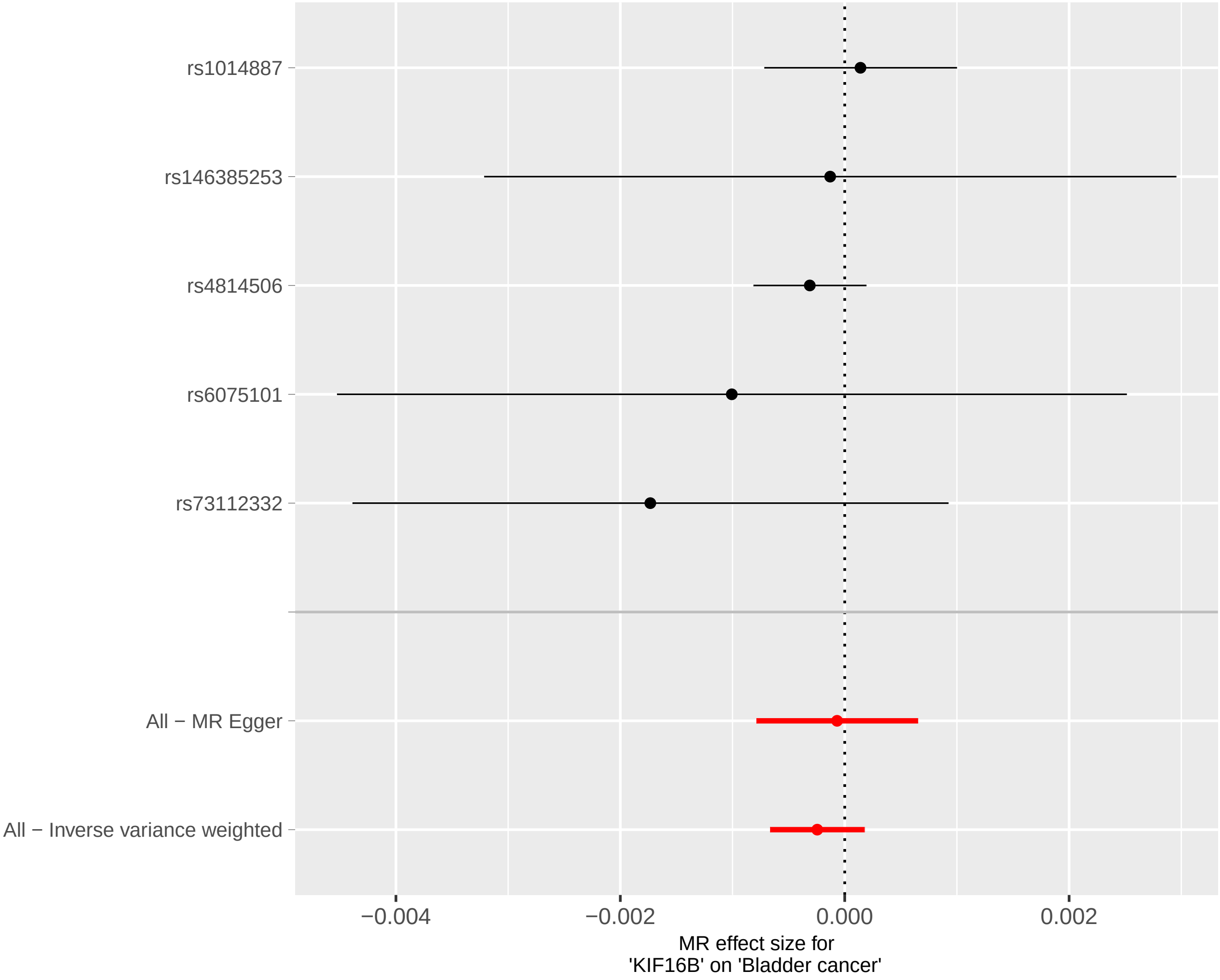
rs1014887
rs146385253
rs4814506
rs6075101
rs73112332
All − MR Egger
All − Inverse variance weighted
−0.004
−0.002
0.000
0.002
MR effect size for
'KIF16B' on 'Bladder cancer'

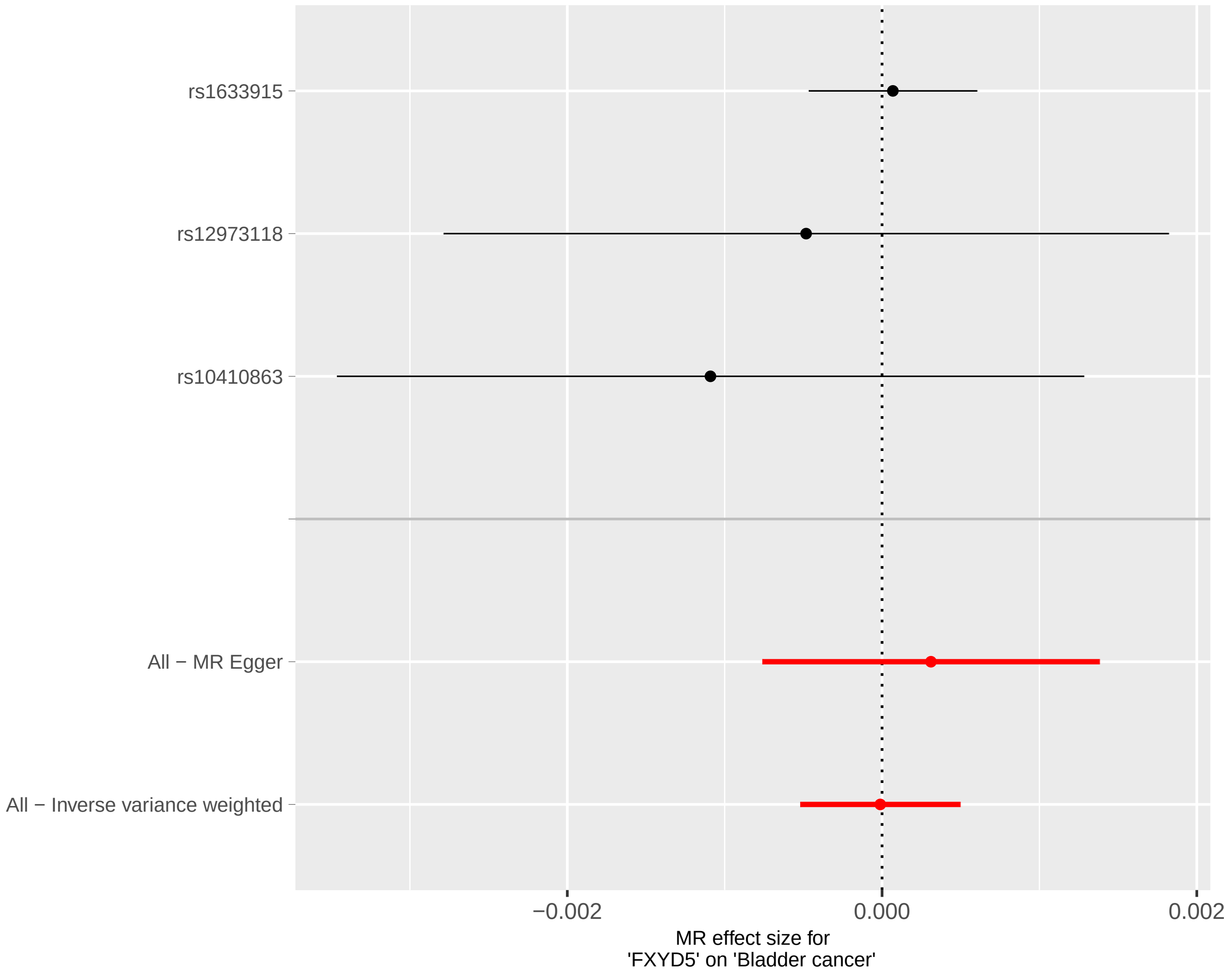
rs1633915
rs12973118
rs10410863
All − MR Egger
All − Inverse variance weighted
−0.002
0.000
0.002
MR effect size for
'FXYD5' on 'Bladder cancer'

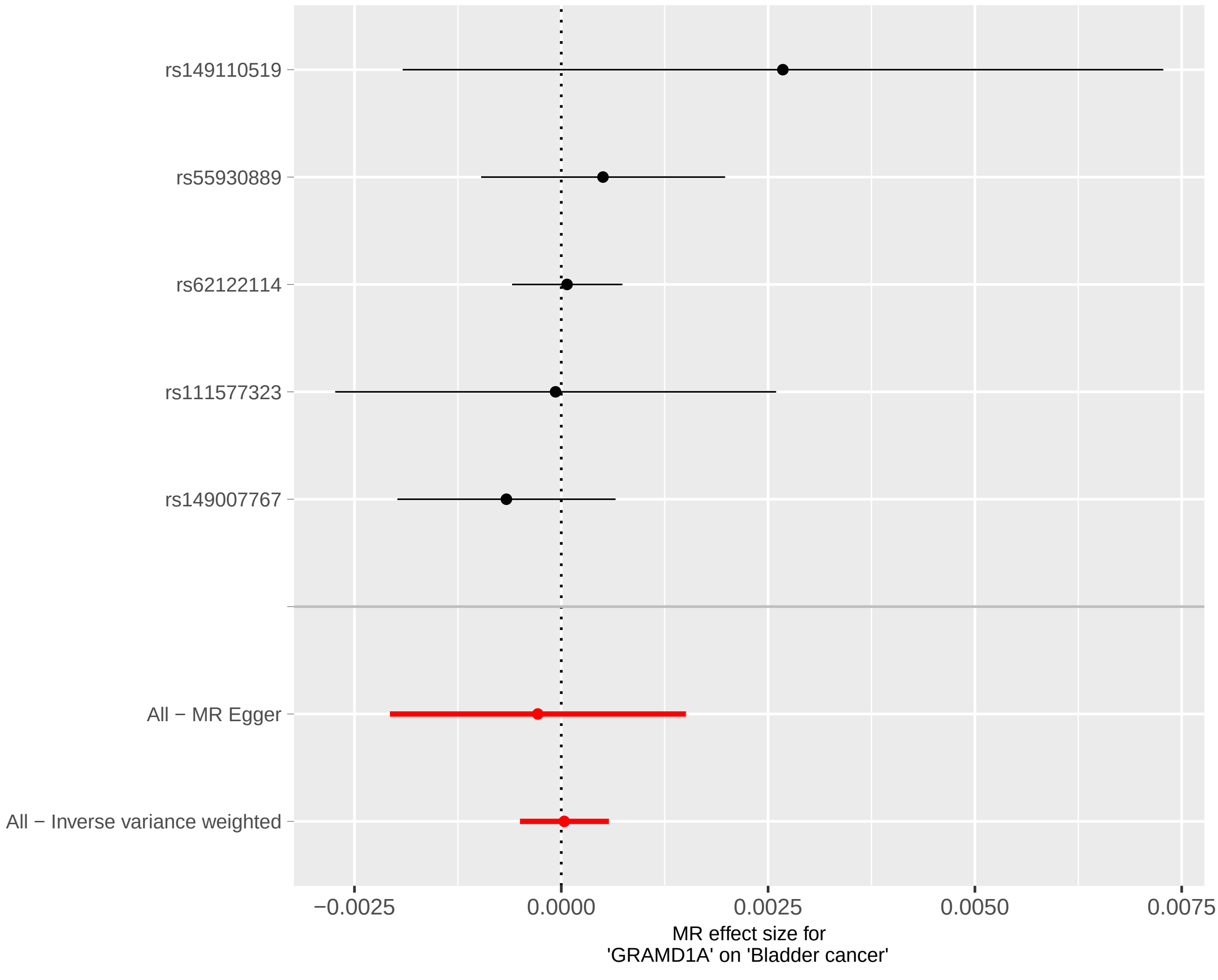

rs149110519
rs55930889
rs62122114
rs111577323
rs149007767
All − MR Egger
All − Inverse variance weighted
−0.0025
0.0000
0.0025
0.0050
0.0075
MR effect size for
'GRAMD1A' on 'Bladder cancer'

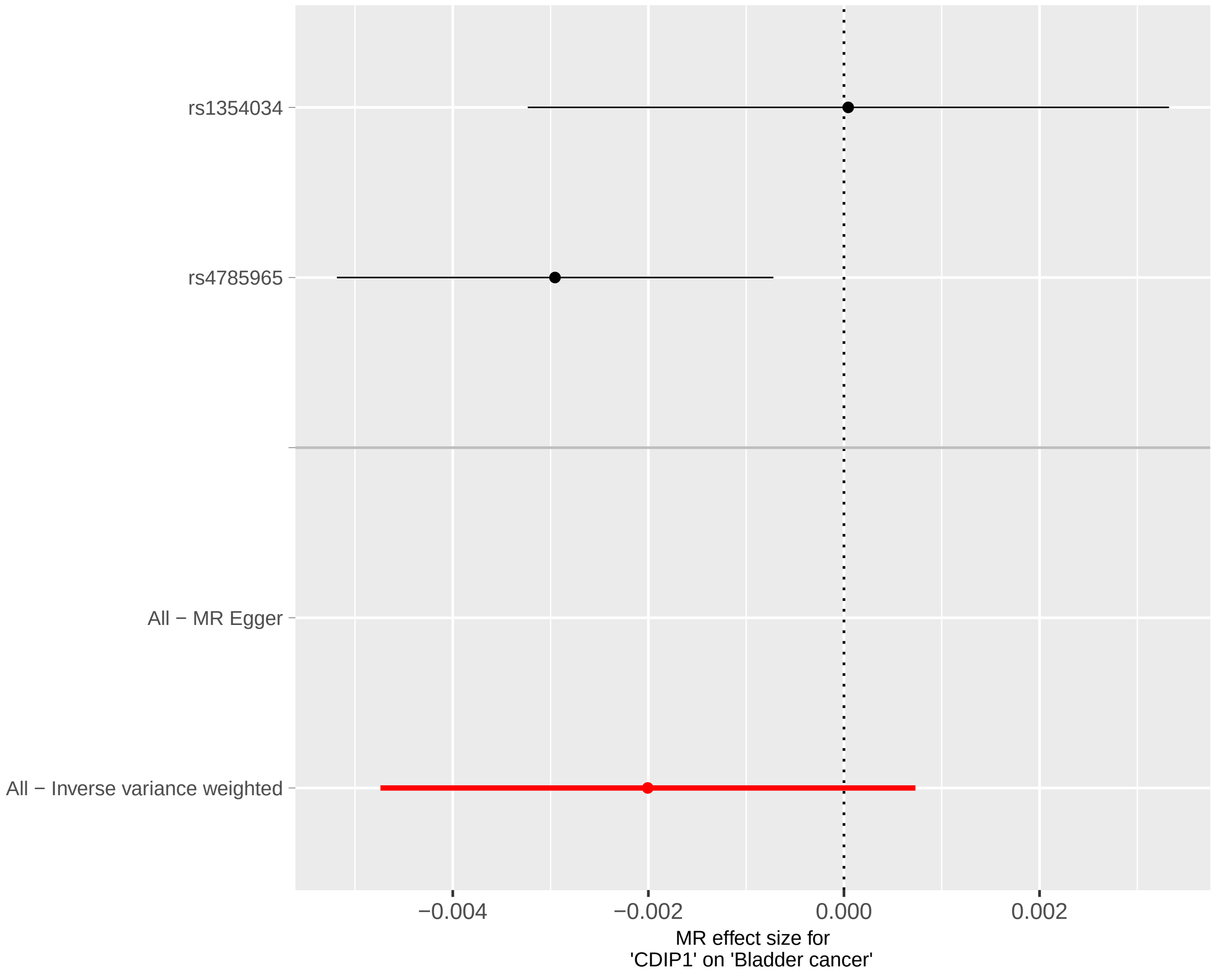

rs1354034
rs4785965
All − MR Egger
All − Inverse variance weighted
−0.004
−0.002
0.000
0.002
MR effect size for
'CDIP1' on 'Bladder cancer'

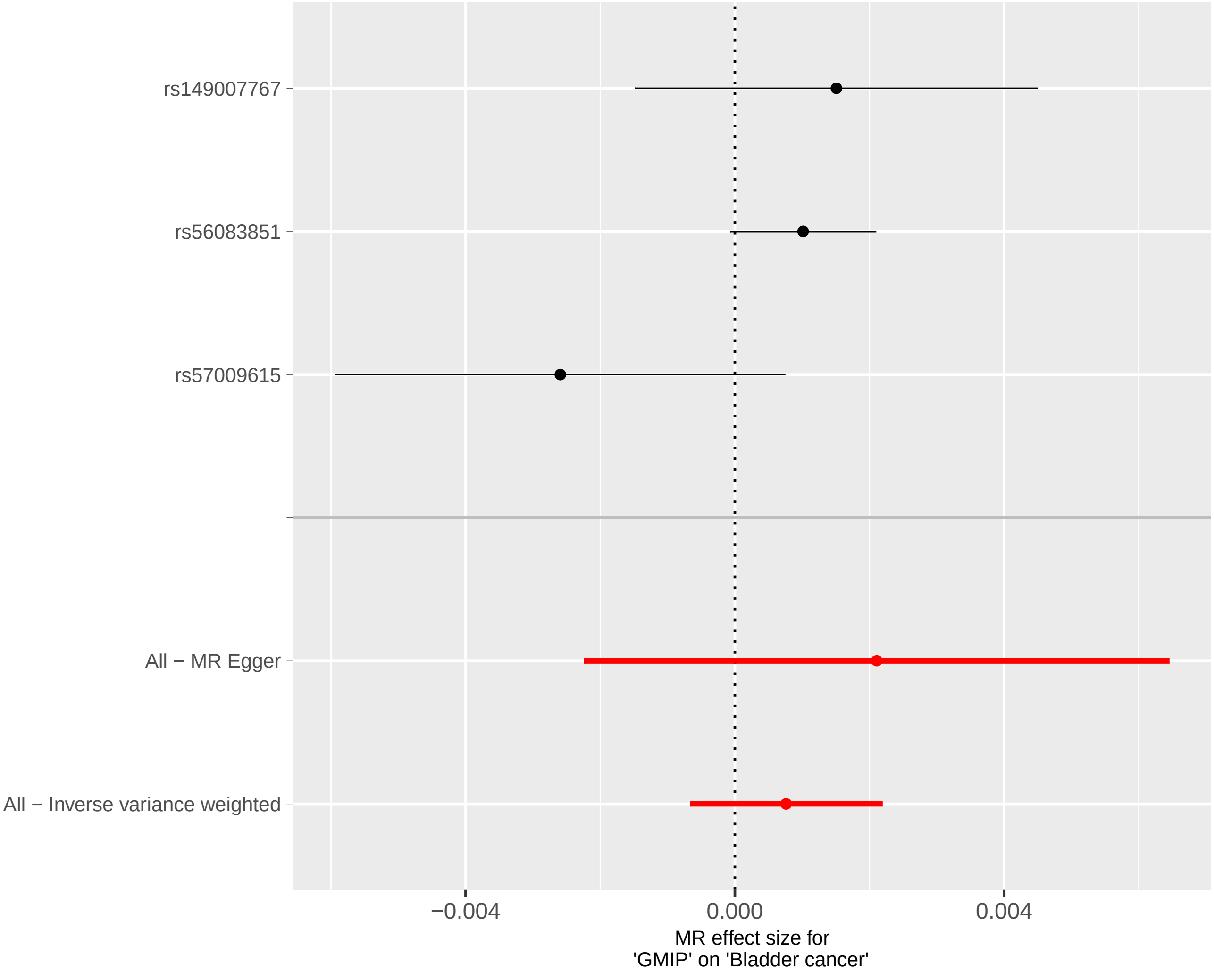

rs149007767
rs56083851
rs57009615
All − MR Egger
All − Inverse variance weighted
−0.004
0.000
0.004
MR effect size for
'GMIP' on 'Bladder cancer'

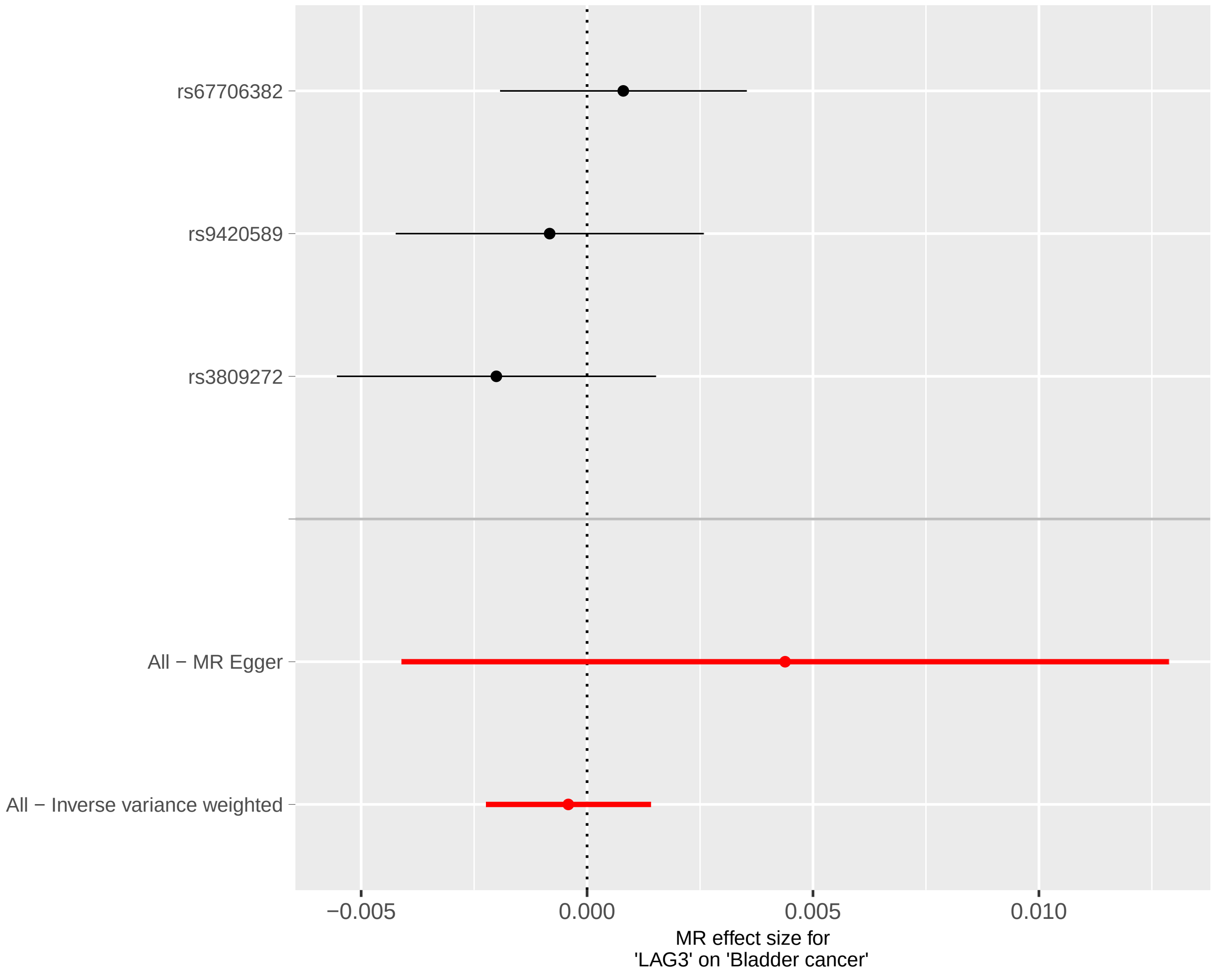
rs67706382
rs9420589
rs3809272
All − MR Egger
All − Inverse variance weighted
−0.005
0.000
0.005
0.010
MR effect size for
'LAG3' on 'Bladder cancer'

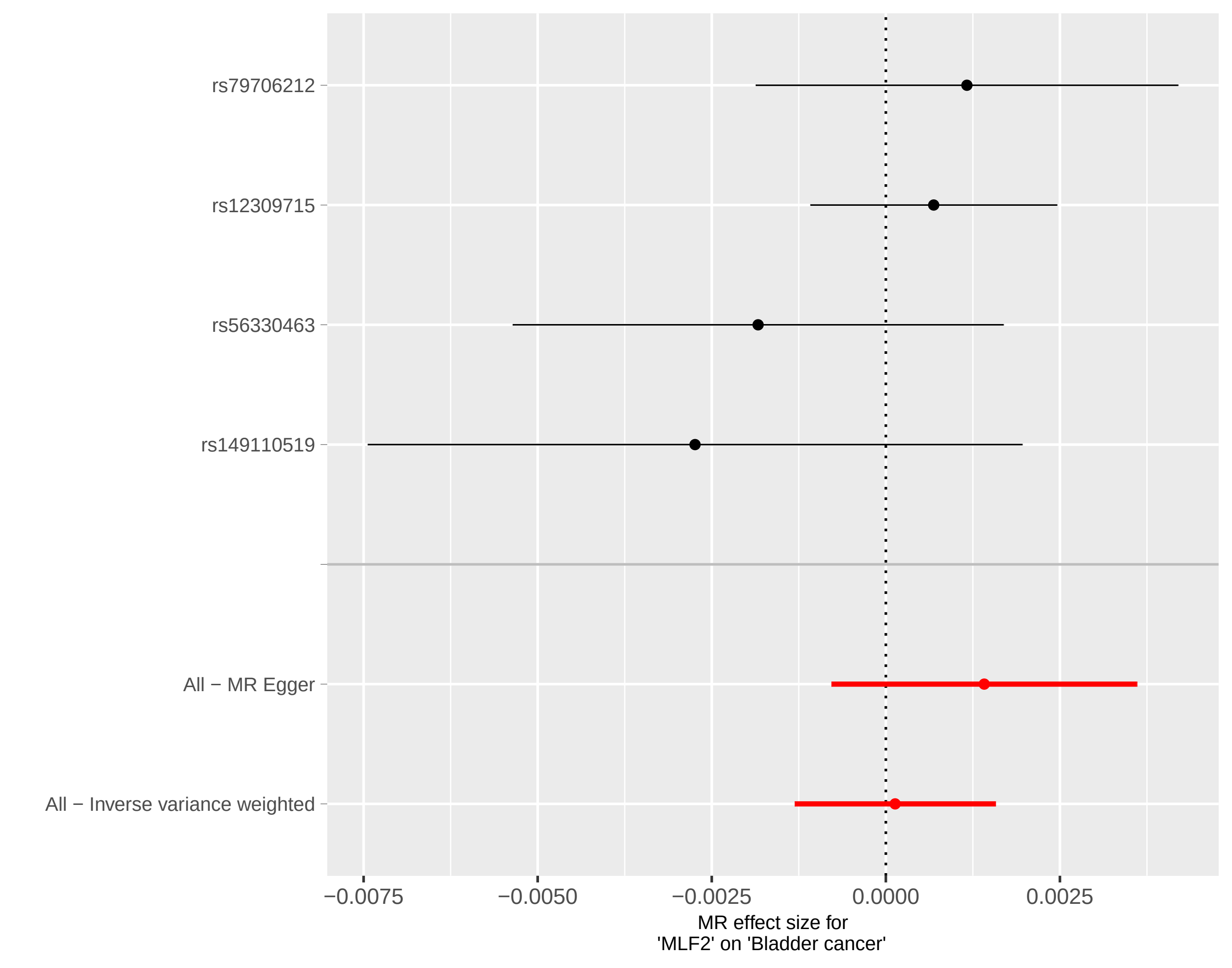

rs79706212
rs12309715
rs56330463
rs149110519
All − MR Egger
All − Inverse variance weighted
−0.0075
−0.0050
−0.0025
0.0000
0.0025
MR effect size for
'MLF2' on 'Bladder cancer'

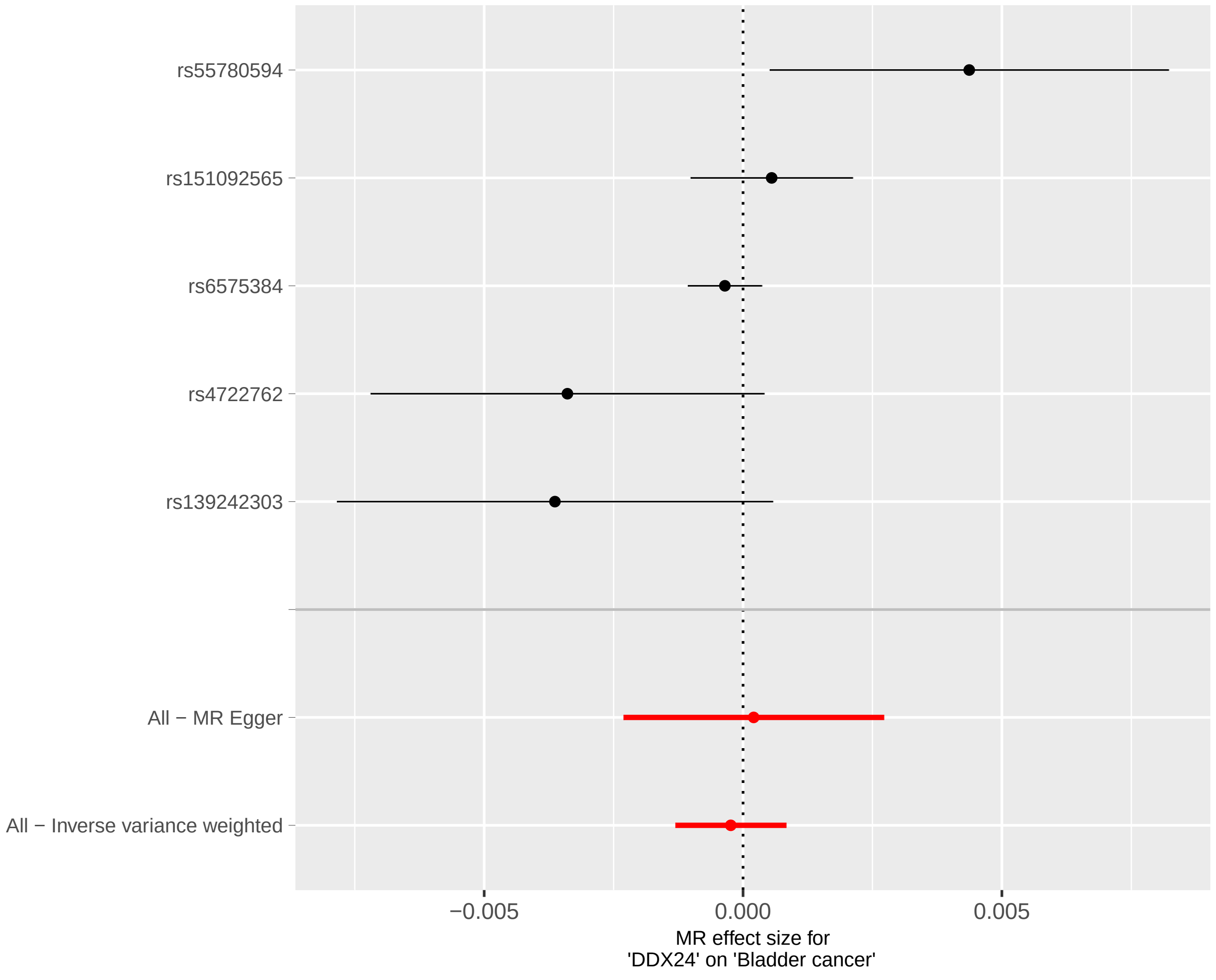
rs55780594
rs151092565
rs6575384
rs4722762
rs139242303
All − MR Egger
All − Inverse variance weighted
−0.005
0.000
0.005
MR effect size for
'DDX24' on 'Bladder cancer'

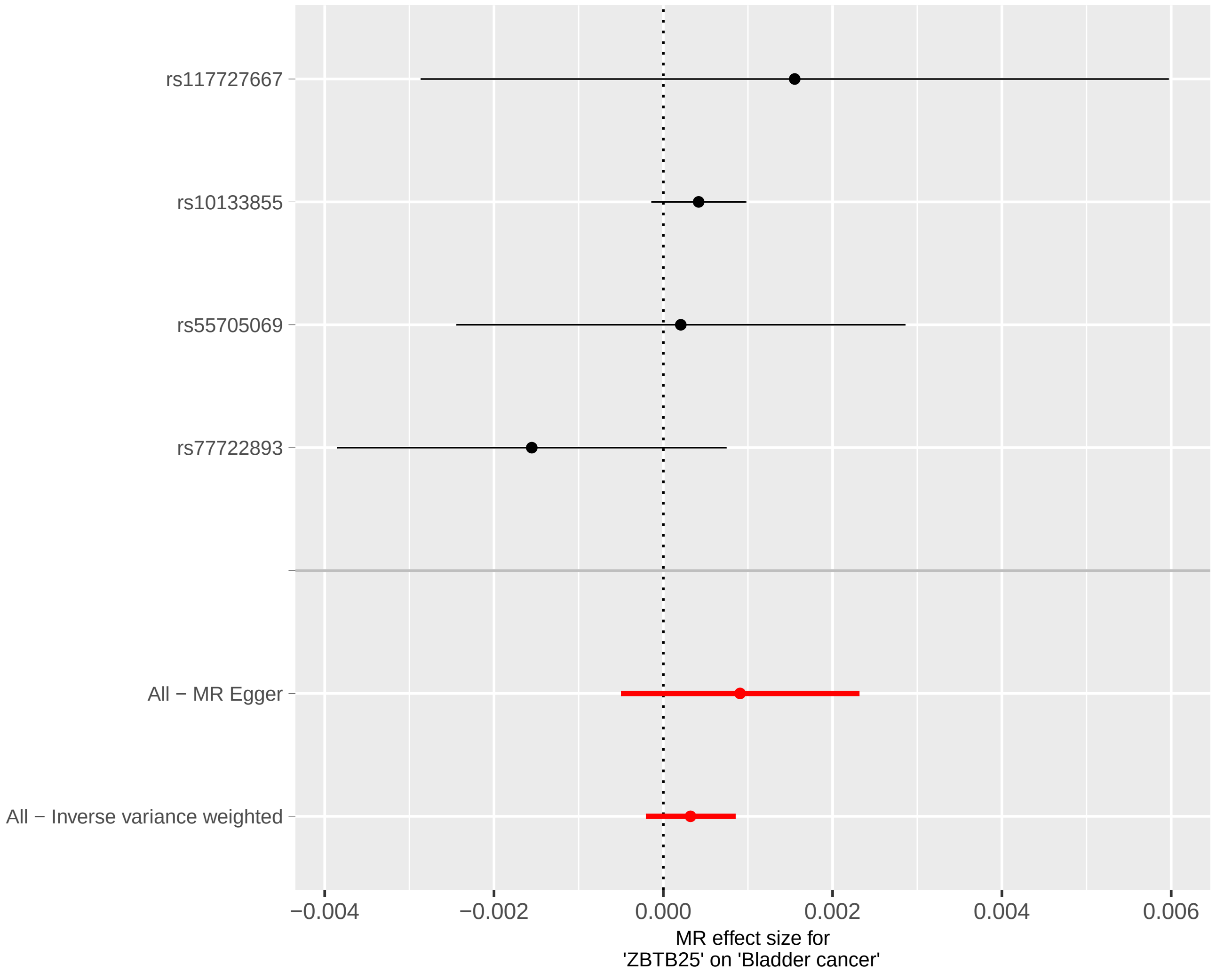

rs117727667
rs10133855
rs55705069
rs77722893
All − MR Egger
All − Inverse variance weighted
−0.004
−0.002
0.000
0.002
0.004
0.006
MR effect size for
'ZBTB25' on 'Bladder cancer'

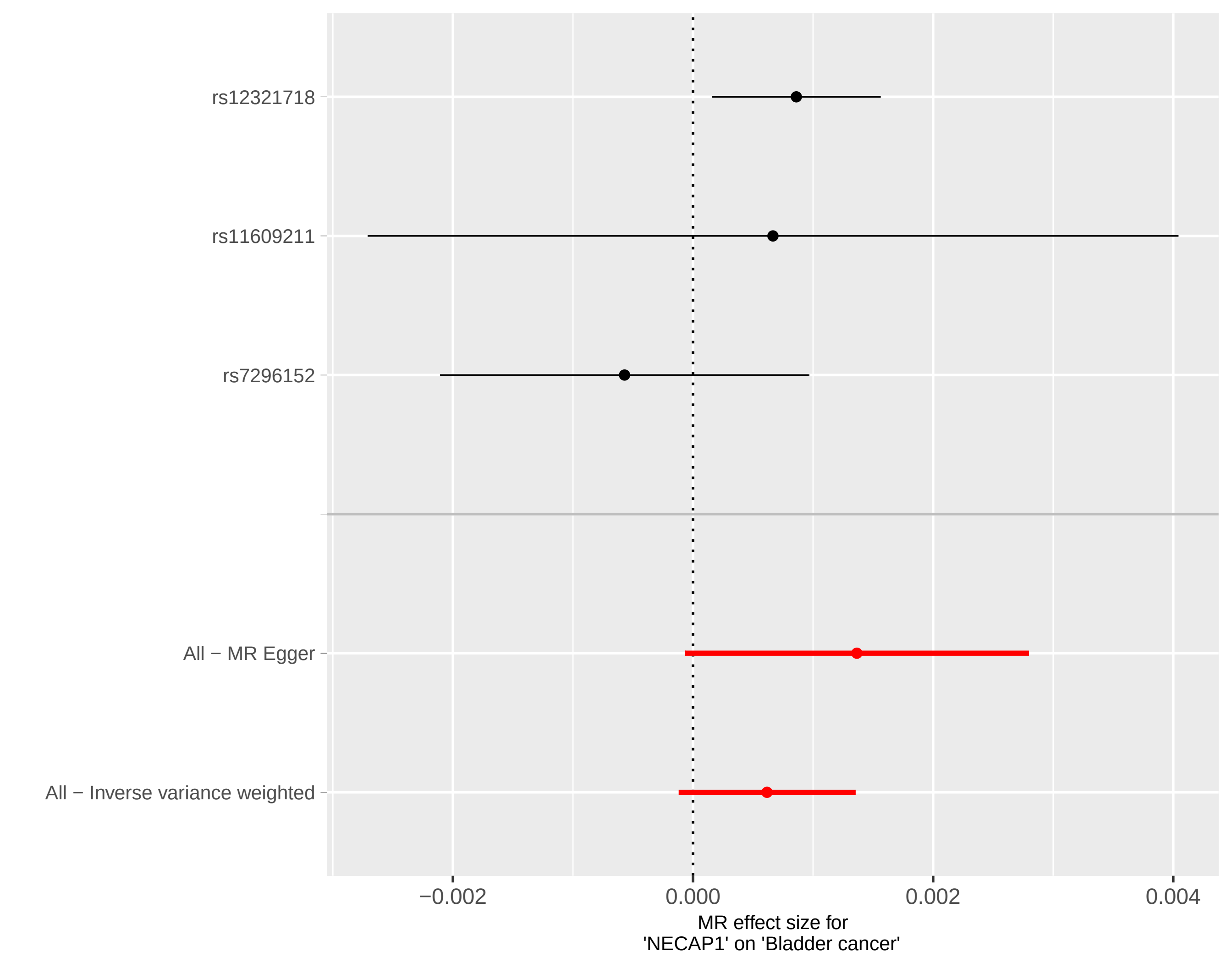
rs12321718
rs11609211
rs7296152
All − MR Egger
All − Inverse variance weighted
−0.002
0.000
0.002
0.004
MR effect size for
'NECAP1' on 'Bladder cancer'

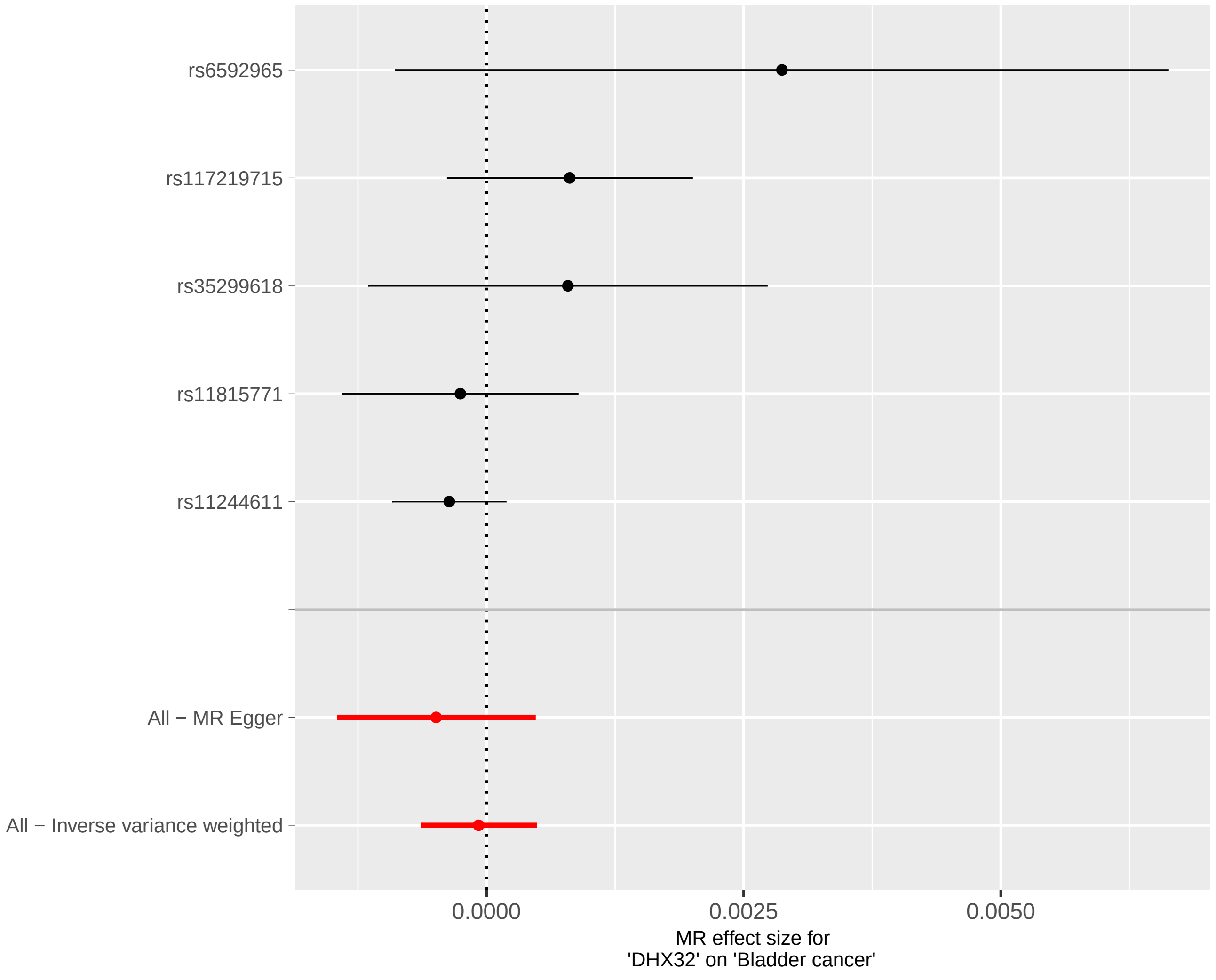

rs6592965
rs117219715
rs35299618
rs11815771
rs11244611
All − MR Egger
All − Inverse variance weighted
0.0000
0.0025
0.0050
MR effect size for
'DHX32' on 'Bladder cancer'

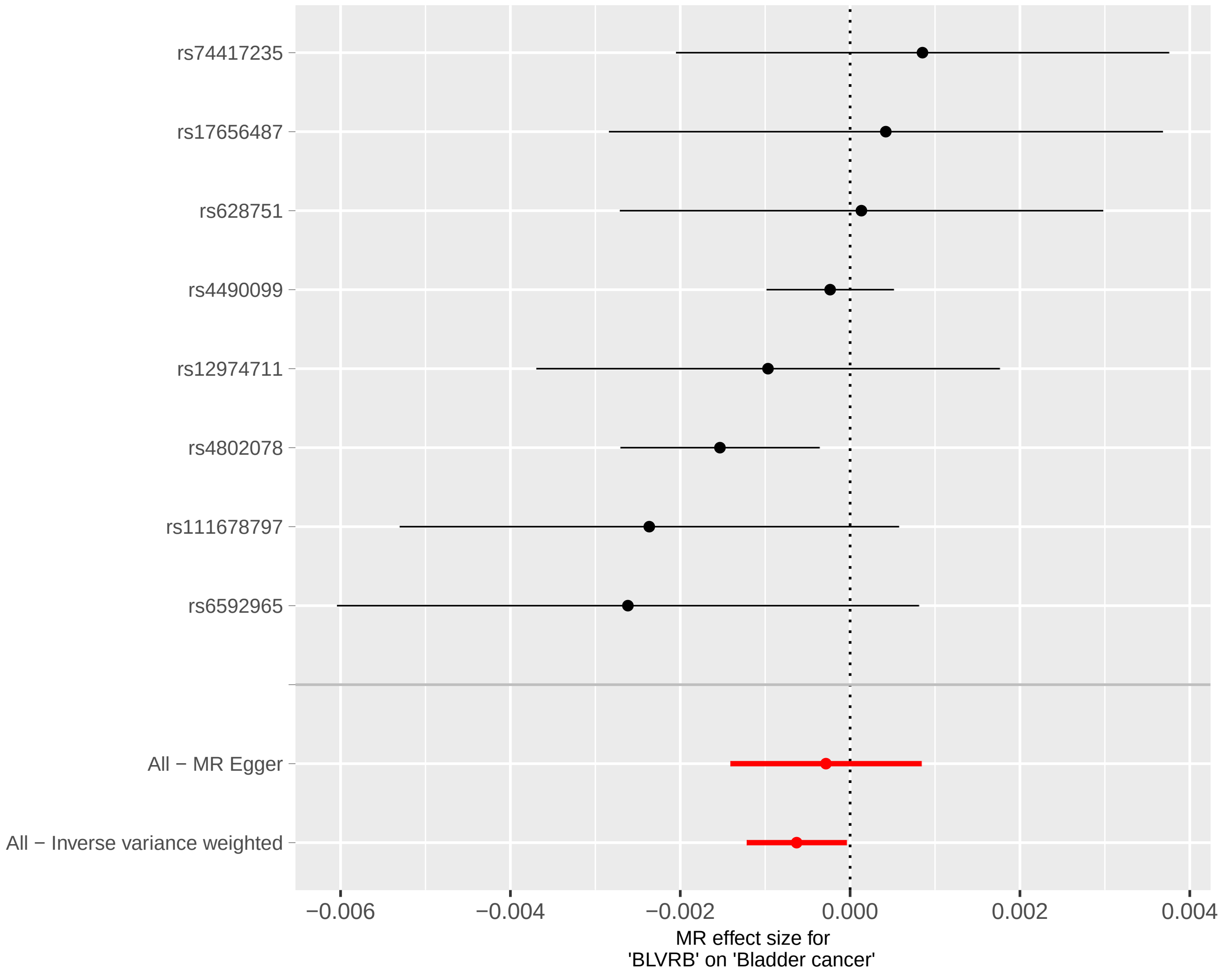

rs74417235
rs17656487
rs628751
rs4490099
rs12974711
rs4802078
rs111678797
rs6592965
All − MR Egger
All − Inverse variance weighted
−0.006
−0.004
−0.002
0.000
0.002
0.004
MR effect size for
'BLVRB' on 'Bladder cancer'

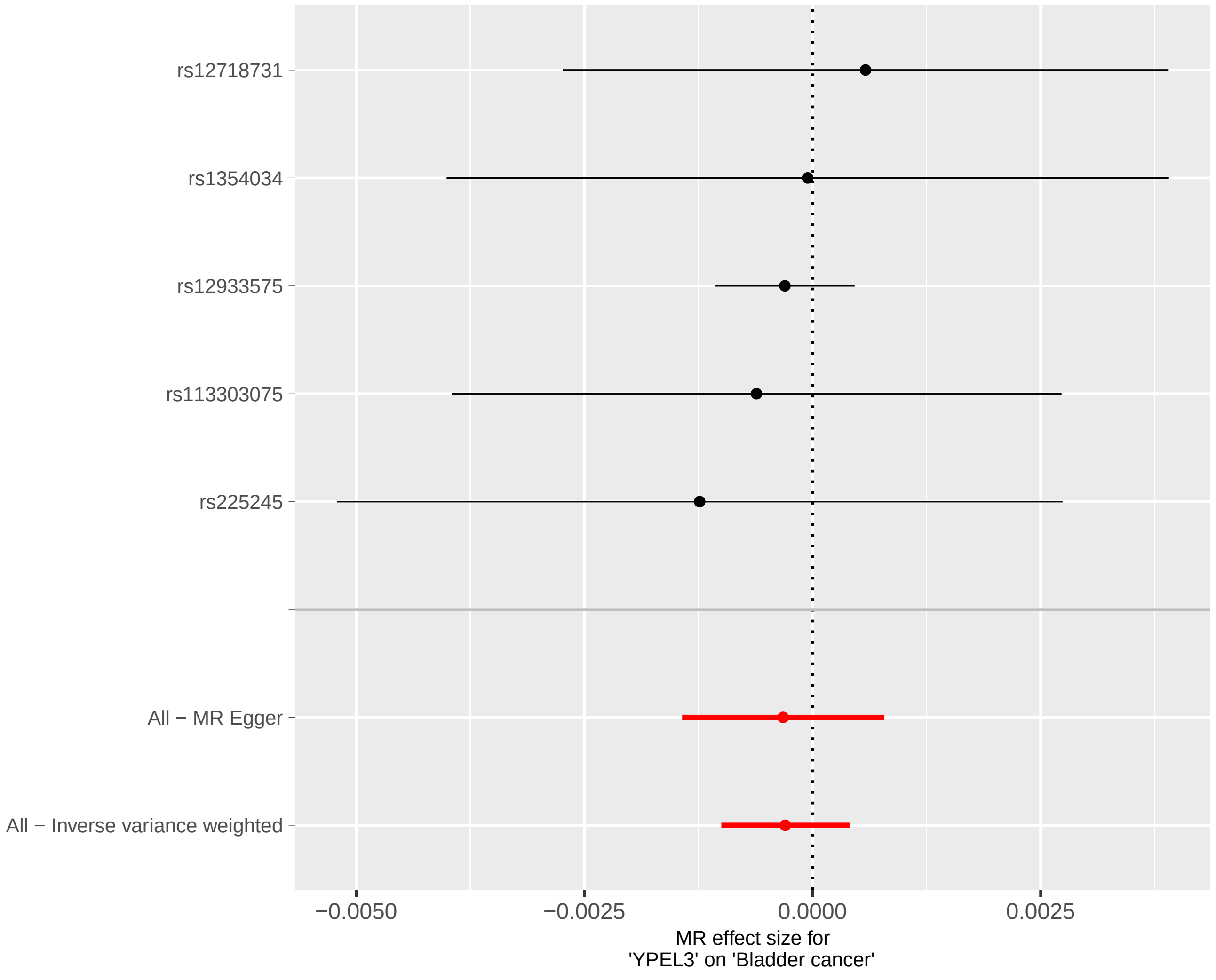
rs12718731
rs1354034
rs12933575
rs113303075
rs225245
All − MR Egger
All − Inverse variance weighted
−0.0050
−0.0025
0.0000
0.0025
MR effect size for
'YPEL3' on 'Bladder cancer'

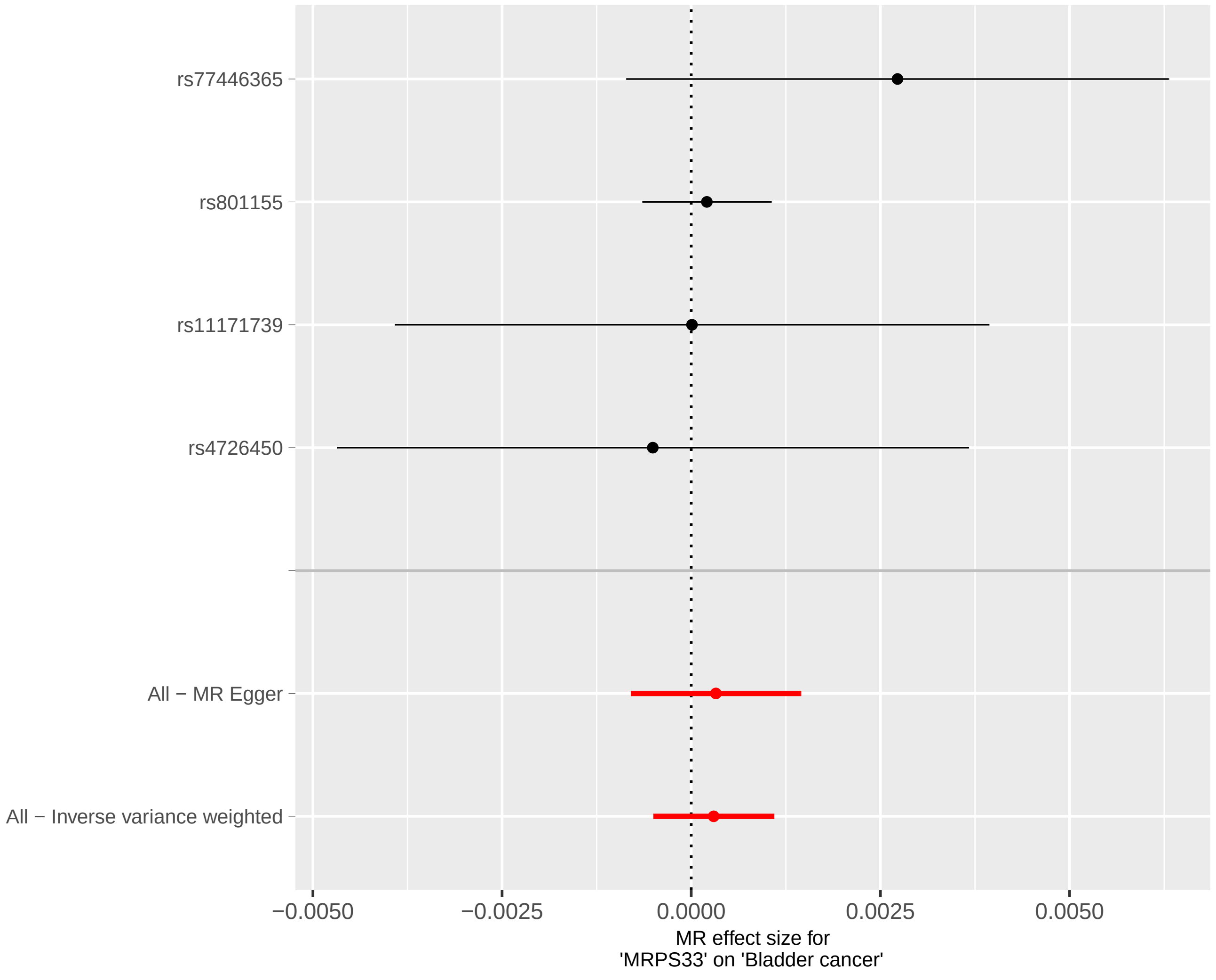

rs77446365
rs801155
rs11171739
rs4726450
All − MR Egger
All − Inverse variance weighted
−0.0050
−0.0025
0.0000
0.0025
0.0050
MR effect size for
'MRPS33' on 'Bladder cancer'

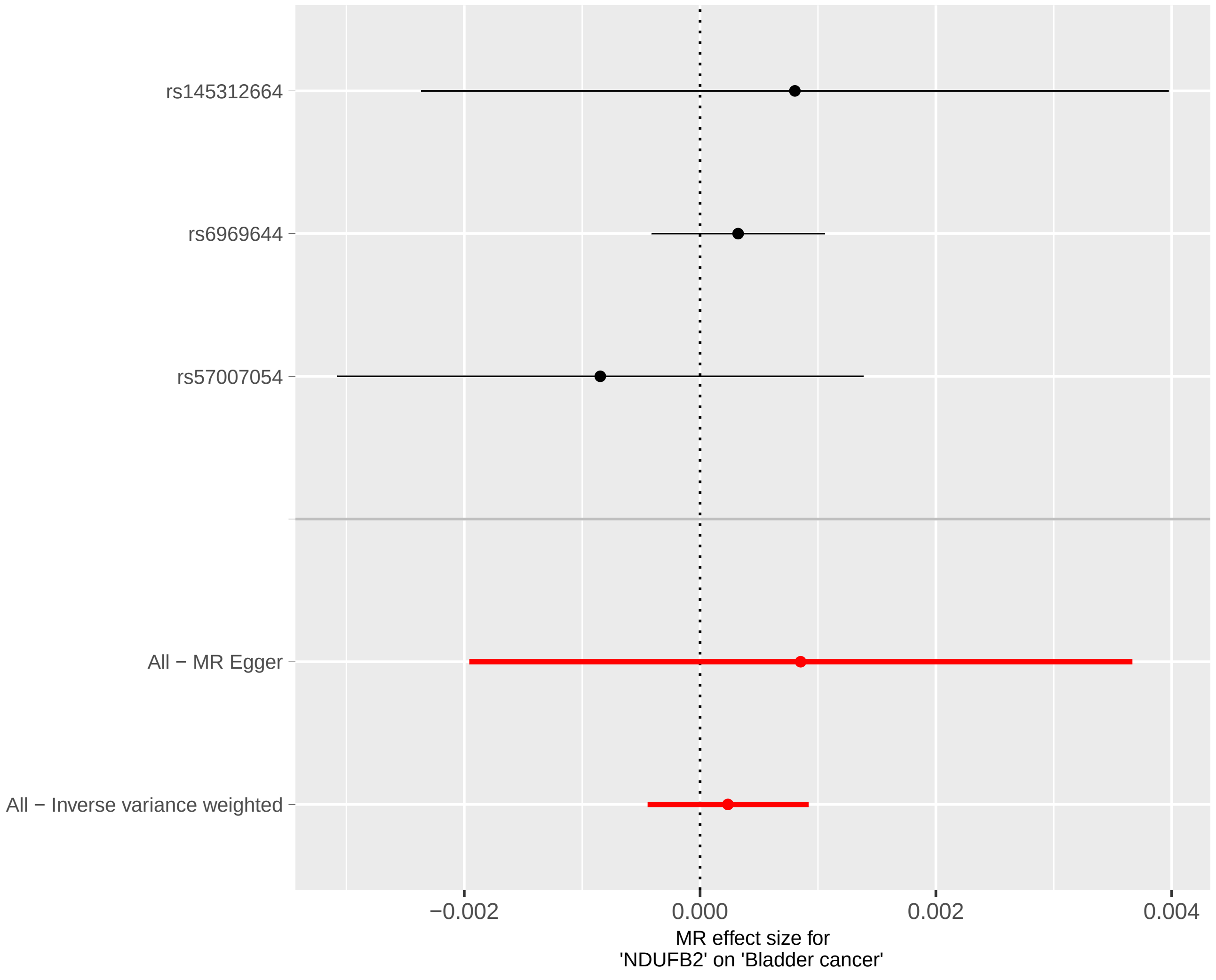

rs145312664
rs6969644
rs57007054
All − MR Egger
All − Inverse variance weighted
−0.002
0.000
0.002
0.004
MR effect size for
'NDUFB2' on 'Bladder cancer'

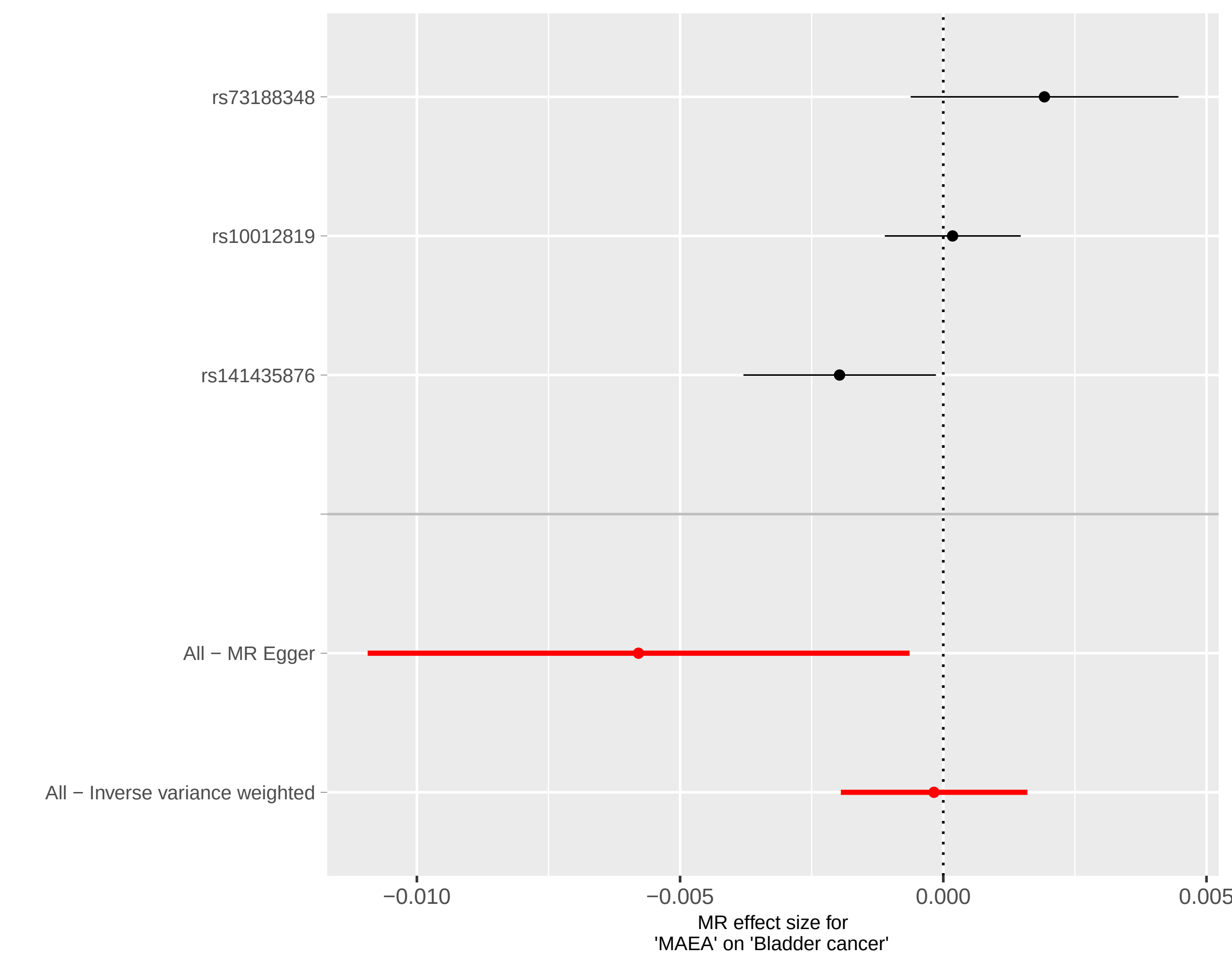

rs73188348
rs10012819
rs141435876
All − MR Egger
All − Inverse variance weighted
−0.010
−0.005
0.000
0.005
MR effect size for
'MAEA' on 'Bladder cancer'

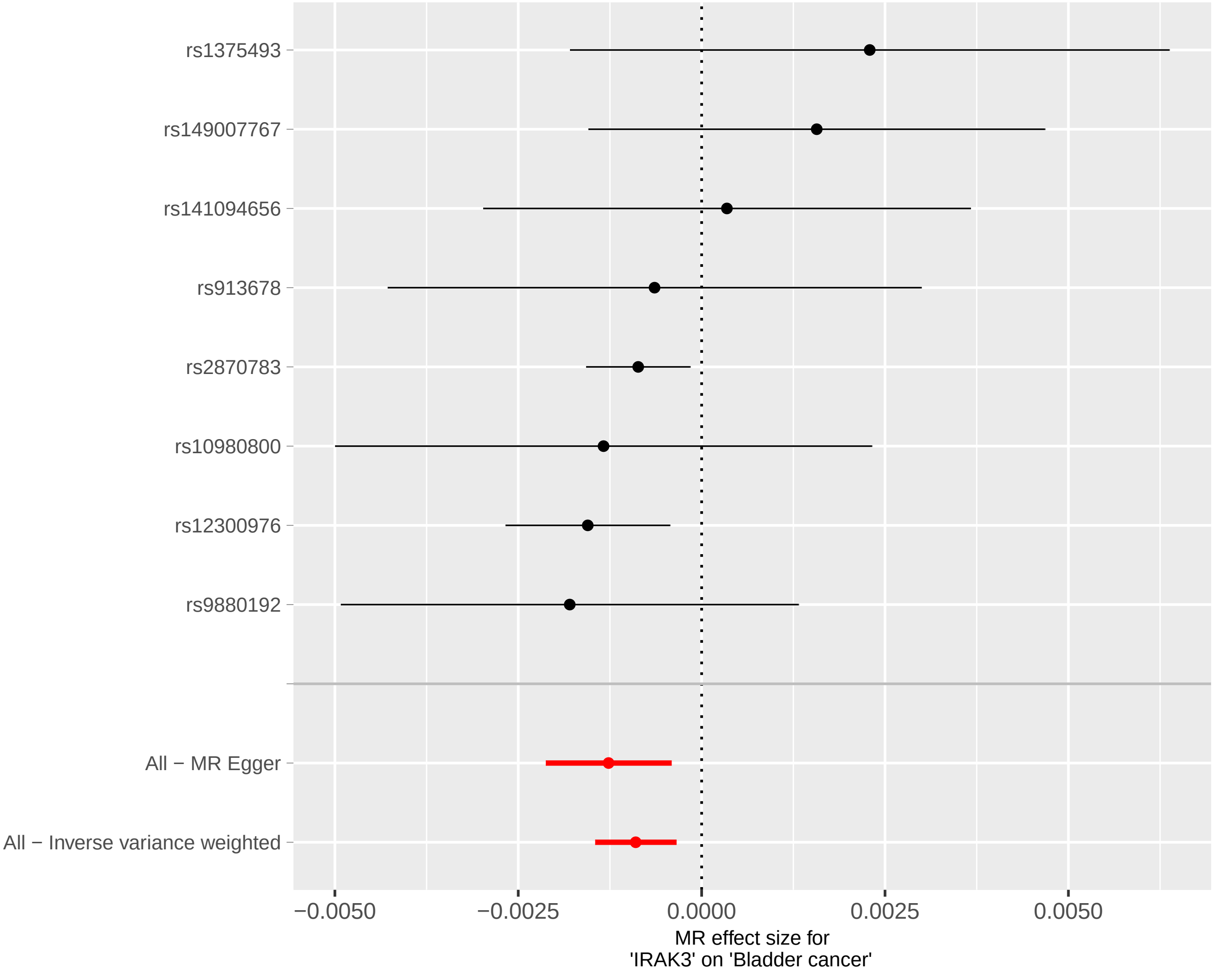
rs1375493
rs149007767
rs141094656
rs913678
rs2870783
rs10980800
rs12300976
rs9880192
All − MR Egger
All − Inverse variance weighted
−0.0050
−0.0025
0.0000
0.0025
0.0050
MR effect size for
'IRAK3' on 'Bladder cancer'

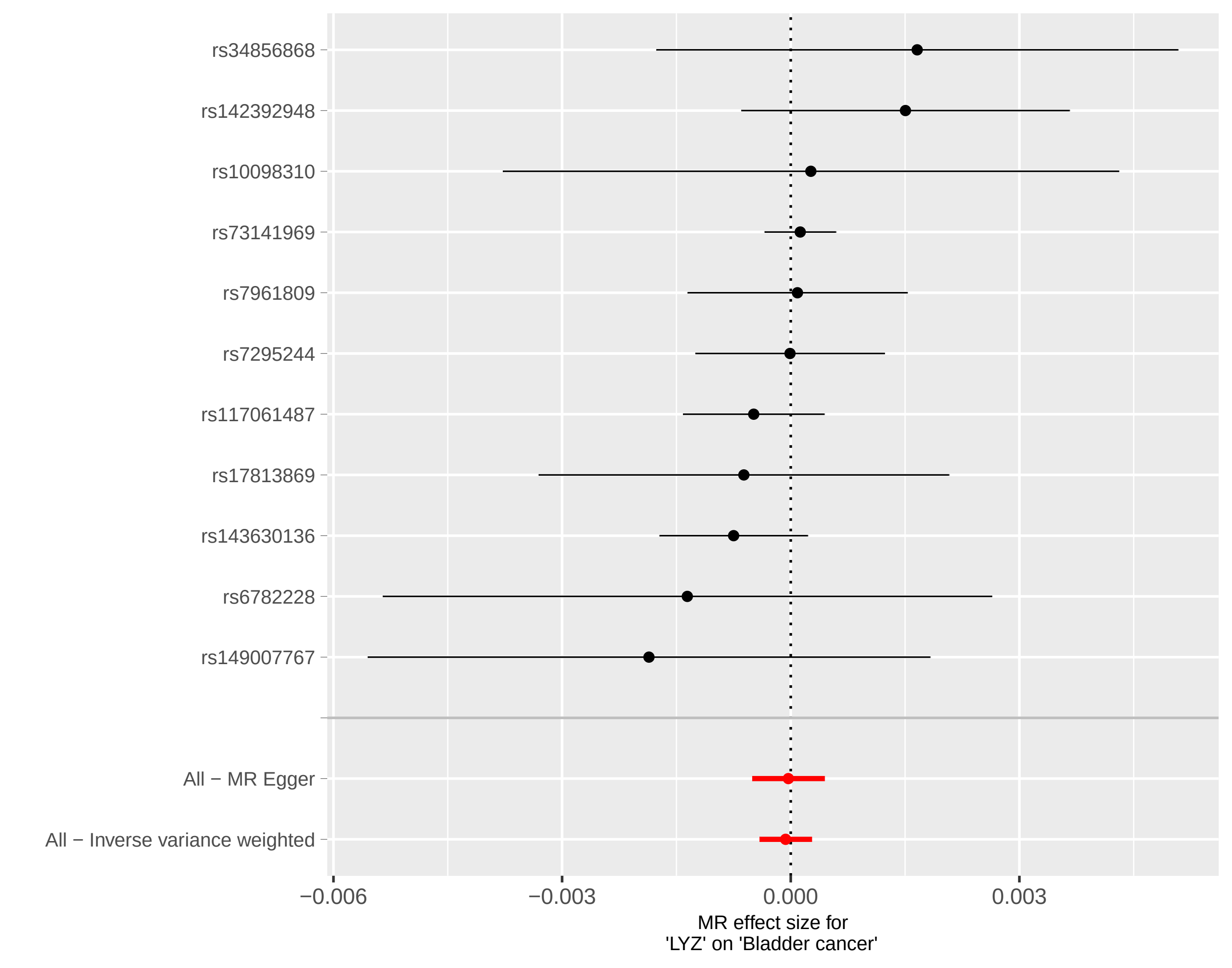
rs34856868
rs142392948
rs10098310
rs73141969
rs7961809
rs7295244
rs117061487
rs17813869
rs143630136
rs6782228
rs149007767
All − MR Egger
All − Inverse variance weighted
−0.006
−0.003
0.000
0.003
MR effect size for
'LYZ' on 'Bladder cancer'

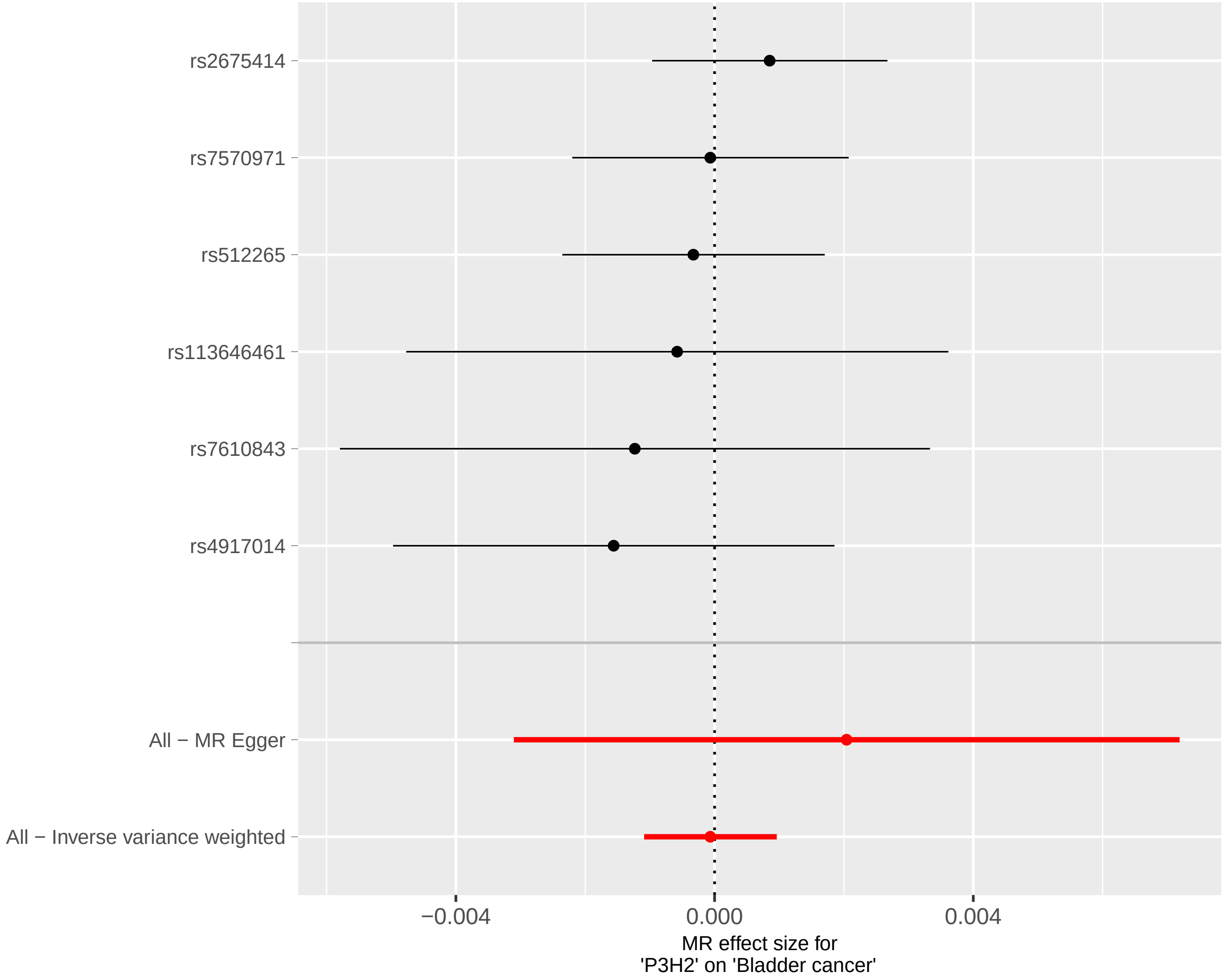

rs2675414
rs7570971
rs512265
rs113646461
rs7610843
rs4917014
All − MR Egger
All − Inverse variance weighted
−0.004
0.000
0.004
MR effect size for
'P3H2' on 'Bladder cancer'

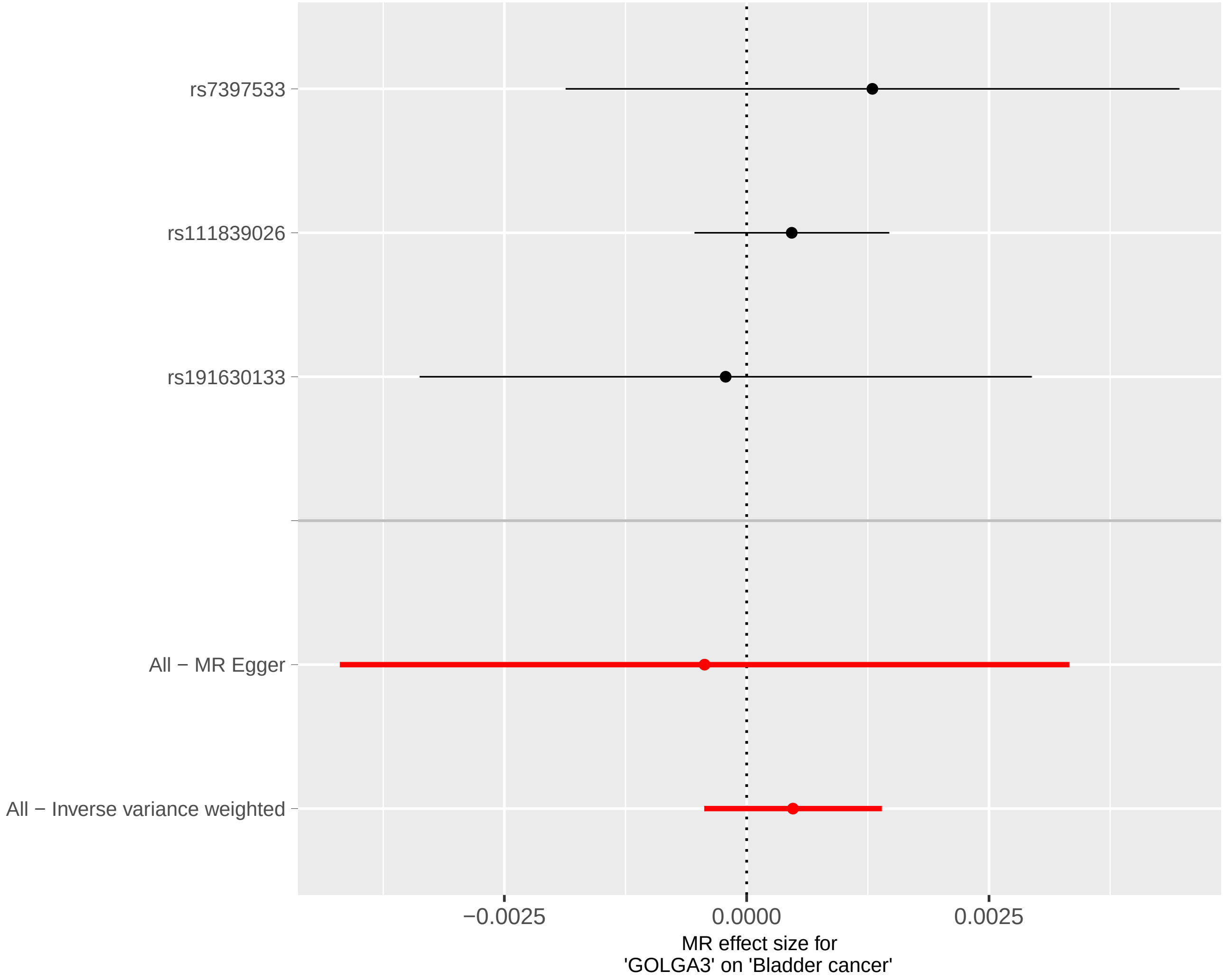
rs7397533
rs111839026
rs191630133
All − MR Egger
All − Inverse variance weighted
−0.0025
0.0000
0.0025
MR effect size for
'GOLGA3' on 'Bladder cancer'

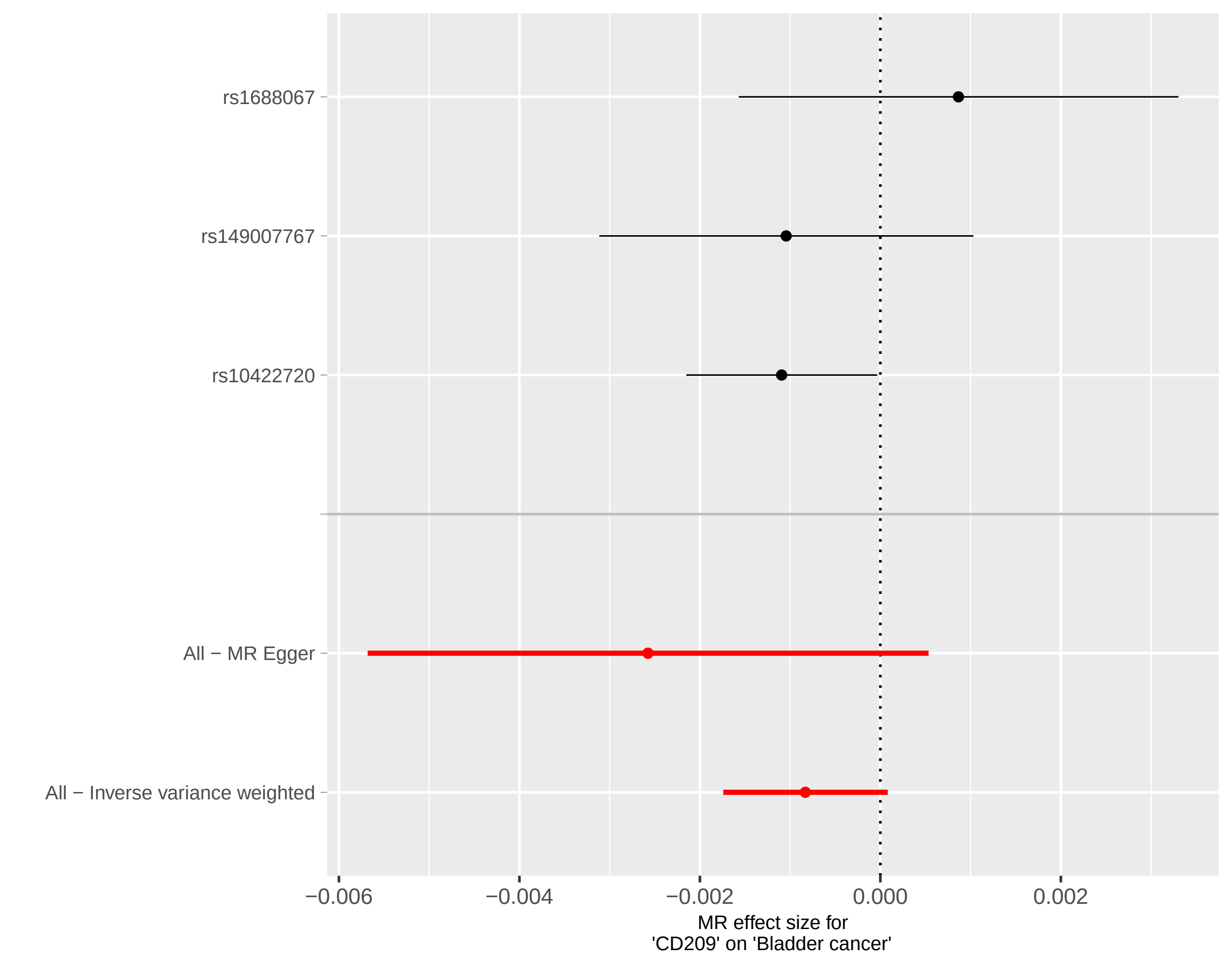

rs1688067
rs149007767
rs10422720
All − MR Egger
All − Inverse variance weighted
−0.006
−0.004
−0.002
0.000
0.002
MR effect size for
'CD209' on 'Bladder cancer'

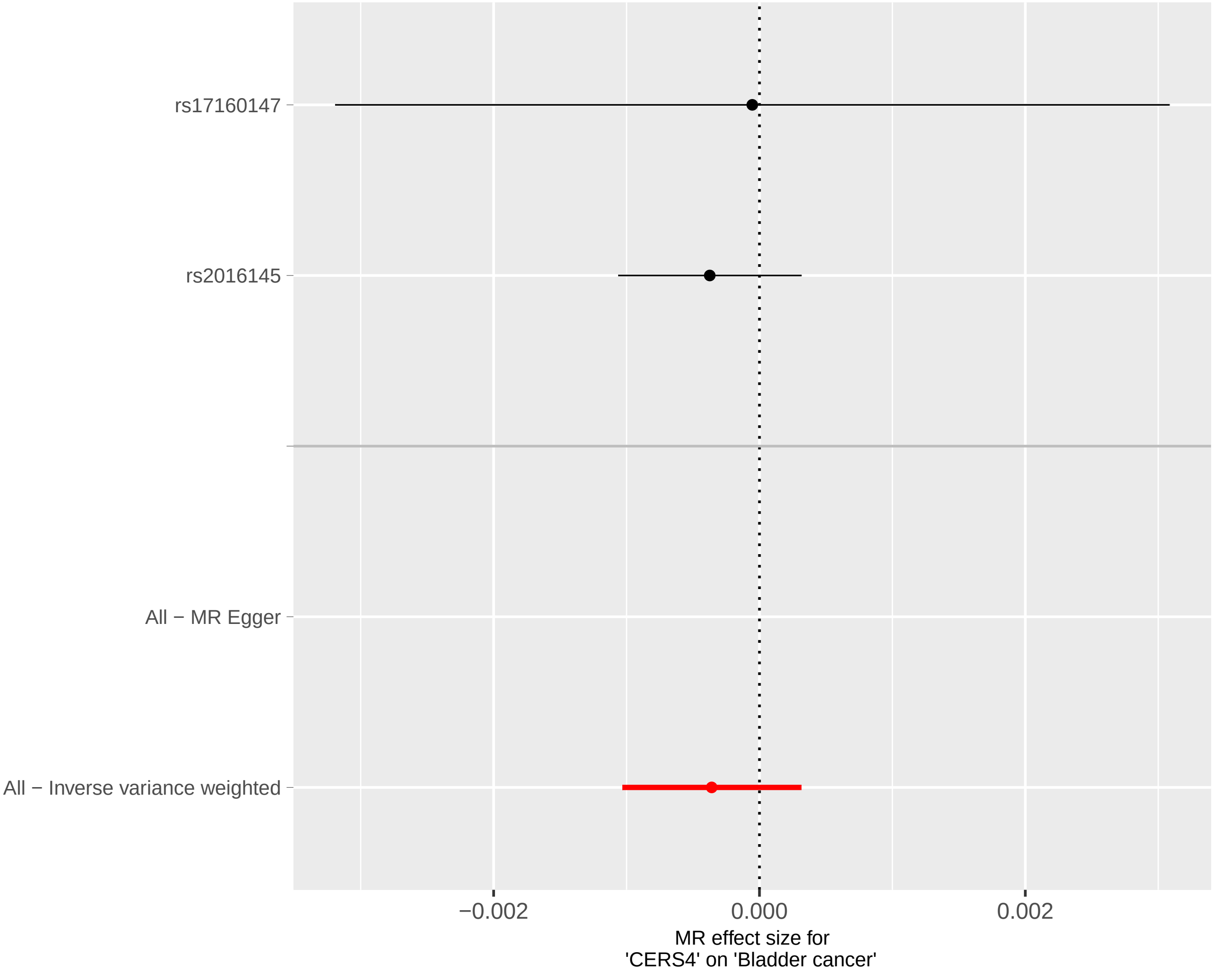

rs17160147
rs2016145
All − MR Egger
All − Inverse variance weighted
−0.002
0.000
0.002
MR effect size for
'CERS4' on 'Bladder cancer'

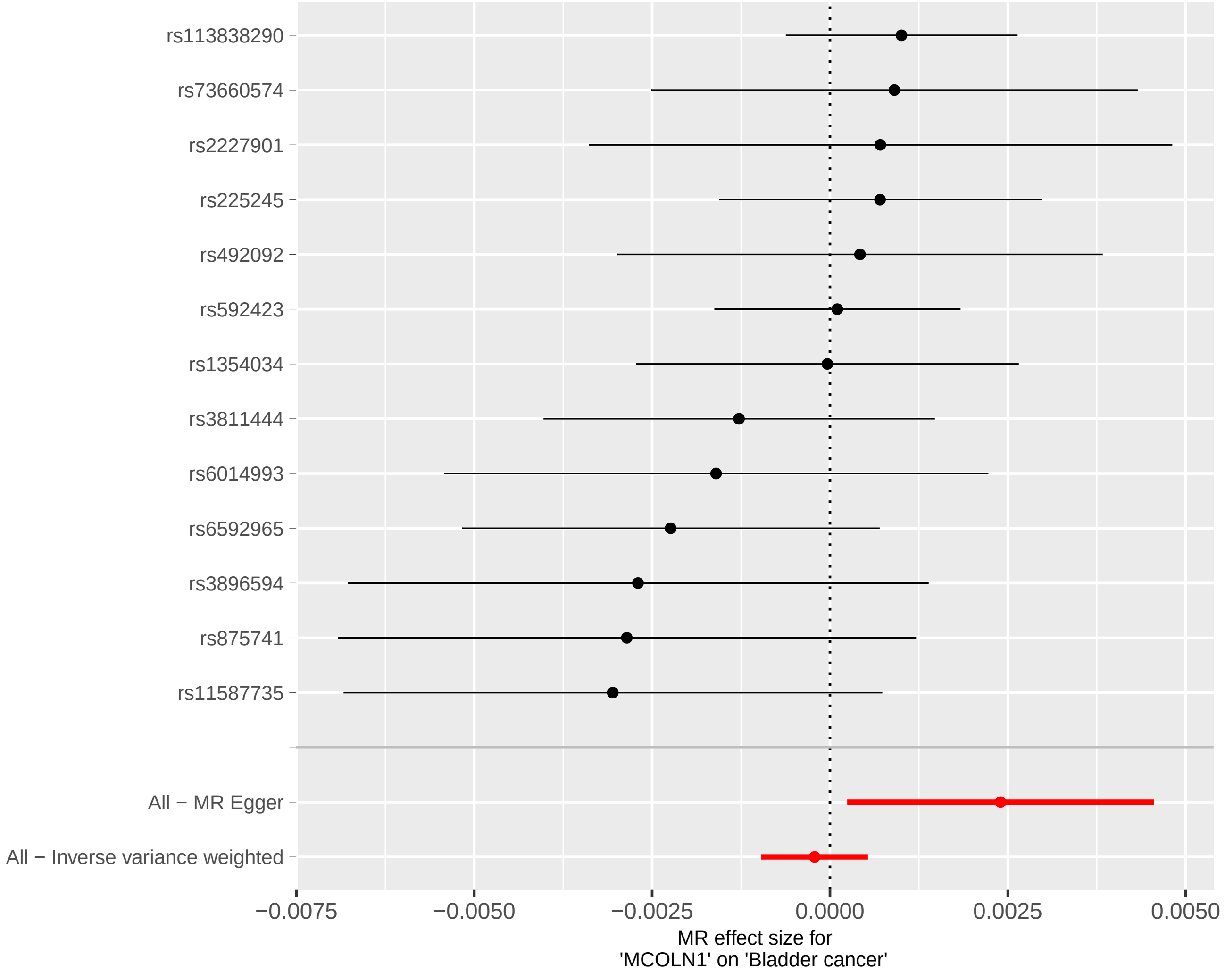
rs113838290
rs73660574
rs2227901
rs225245
rs492092
rs592423
rs1354034
rs3811444
rs6014993
rs6592965
rs3896594
rs875741
rs11587735
All − MR Egger
All − Inverse variance weighted
−0.0075
−0.0050
−0.0025
0.0000
0.0025
0.0050
MR effect size for
'MCOLN1' on 'Bladder cancer'

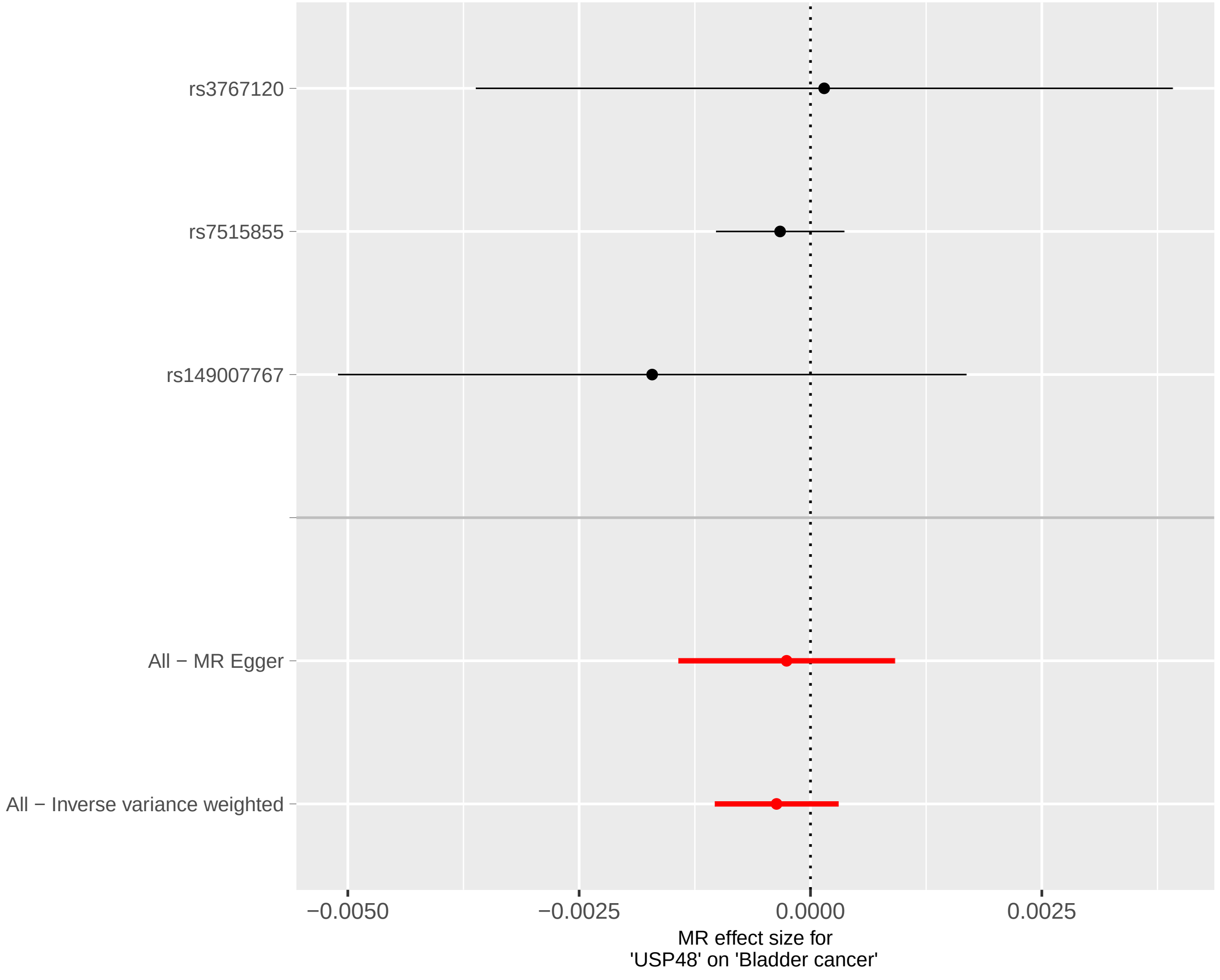
rs3767120
rs7515855
rs149007767
All − MR Egger
All − Inverse variance weighted
−0.0050
−0.0025
0.0000
0.0025
MR effect size for
'USP48' on 'Bladder cancer'

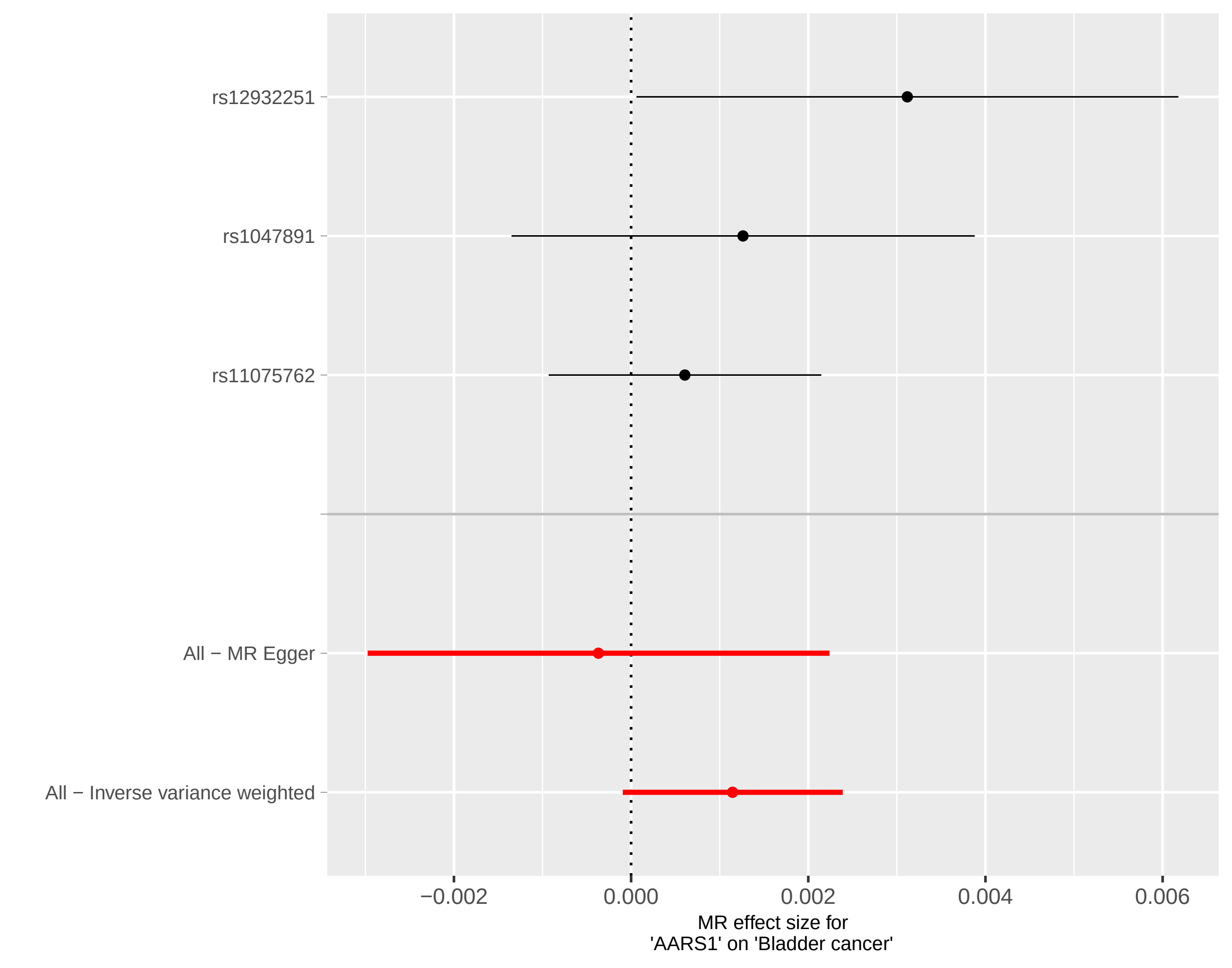
rs12932251
rs1047891
rs11075762
All − MR Egger
All − Inverse variance weighted
−0.002
0.000
0.002
0.004
0.006
MR effect size for
'AARS1' on 'Bladder cancer'

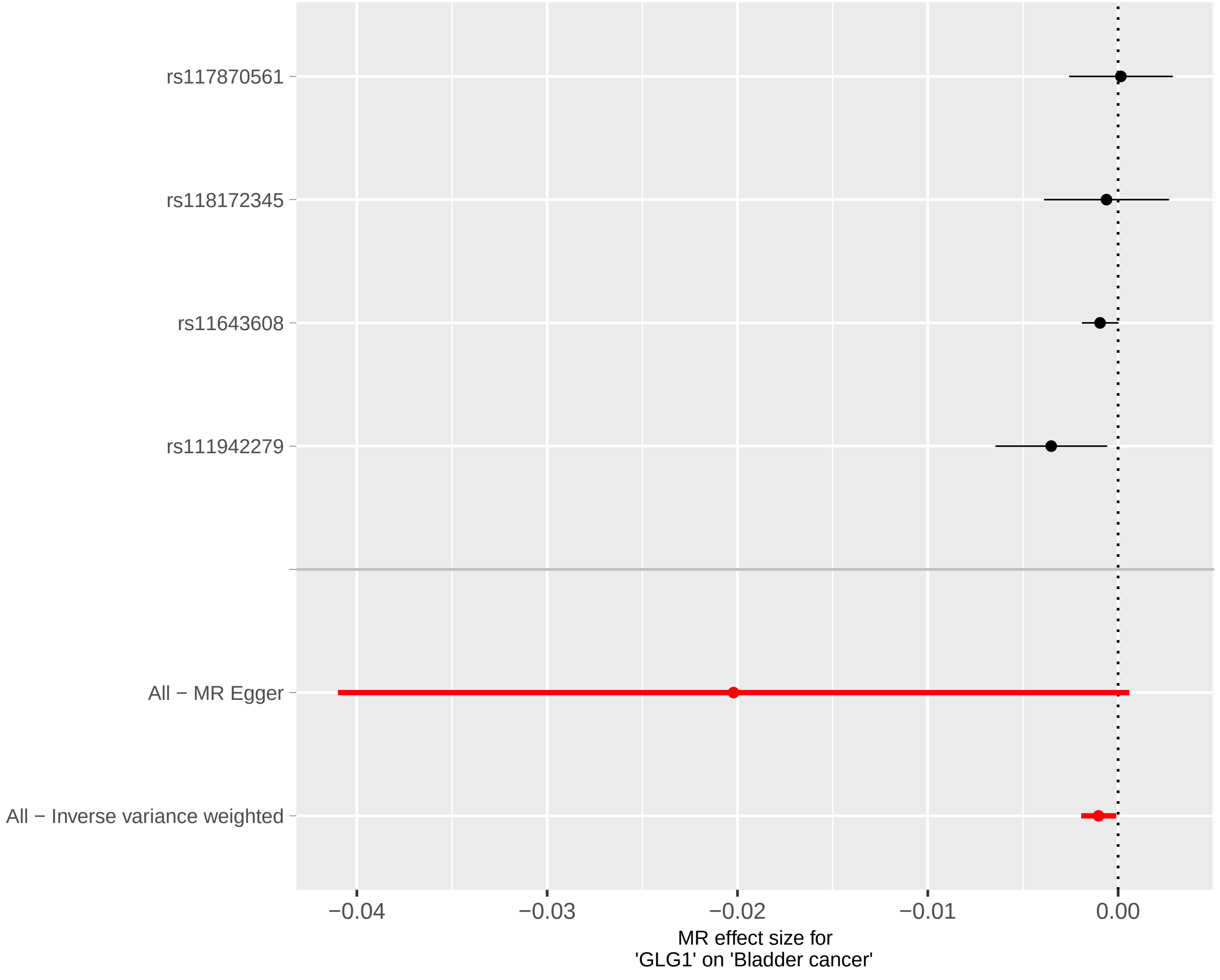
rs117870561
rs118172345
rs11643608
rs111942279
All − MR Egger
All − Inverse variance weighted
−0.04
−0.03
−0.02
−0.01
0.00
MR effect size for
'GLG1' on 'Bladder cancer'

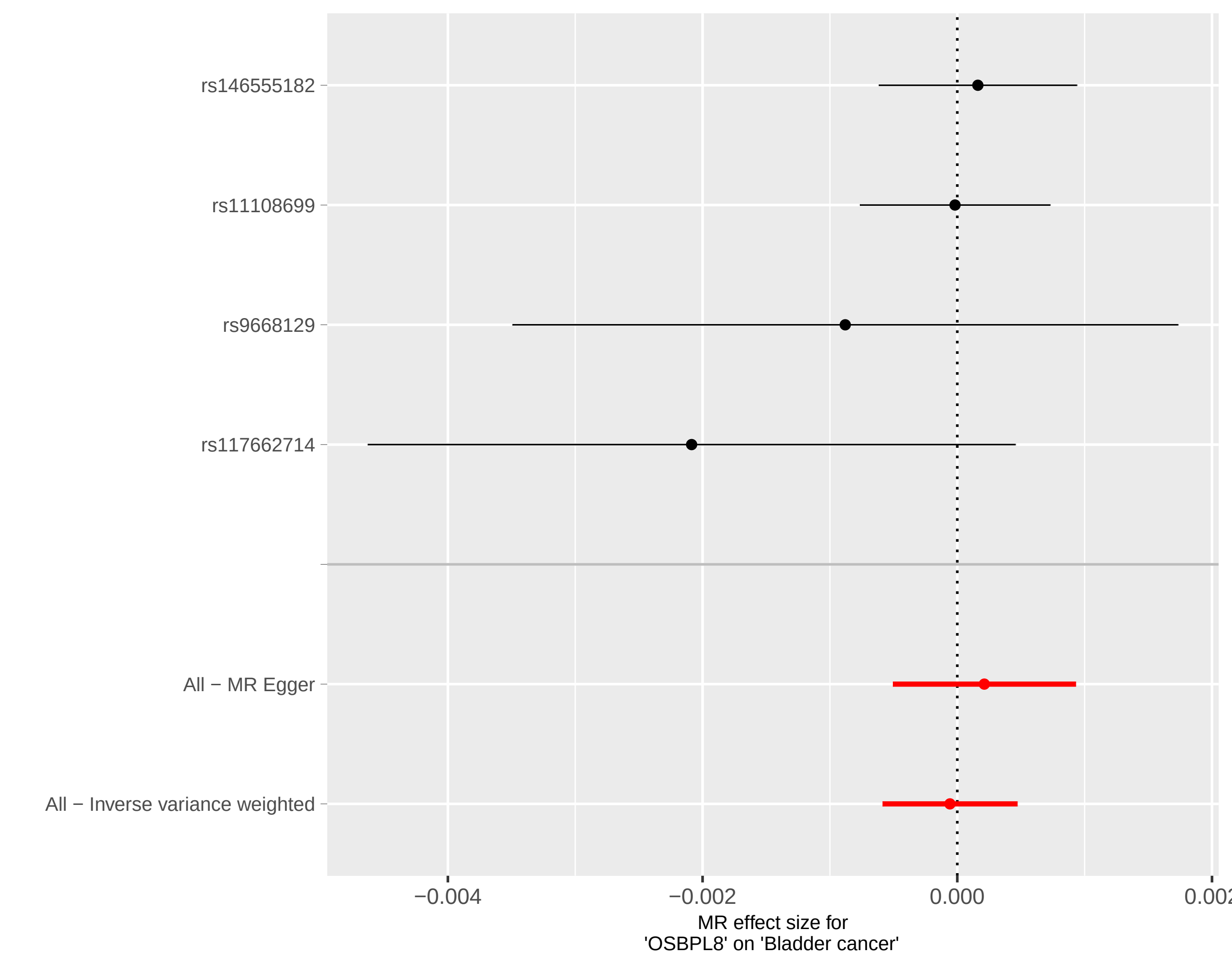
rs146555182
rs11108699
rs9668129
rs117662714
All − MR Egger
All − Inverse variance weighted
−0.004
−0.002
0.000
MR effect size for
'OSBPL8' on 'Bladder cancer'

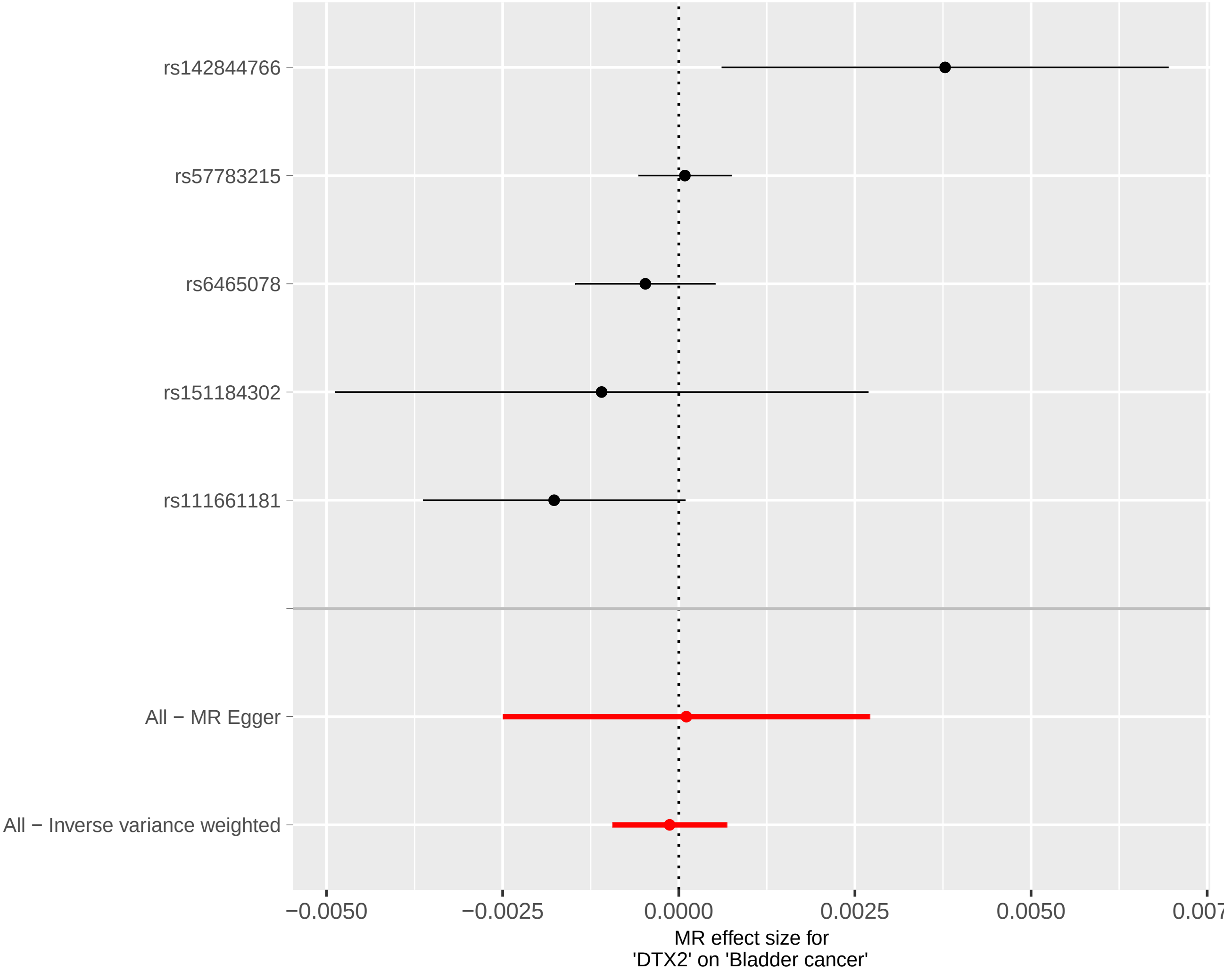
rs142844766
rs57783215
rs6465078
rs151184302
rs111661181
All − MR Egger
All − Inverse variance weighted
−0.0050
−0.0025
0.0000
0.0025
0.0050
MR effect size for
'DTX2' on 'Bladder cancer'

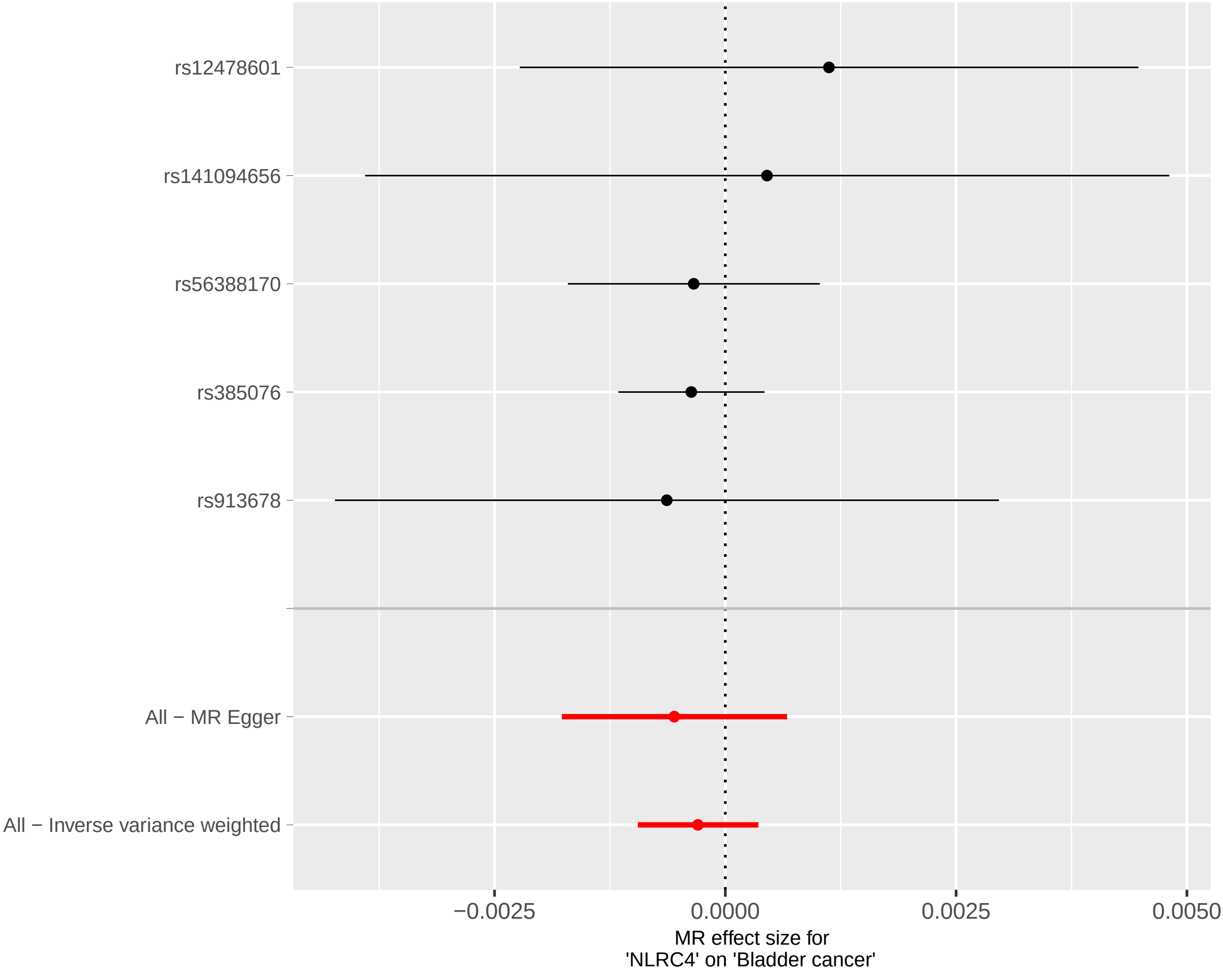

rs12478601
rs141094656
rs56388170
rs385076
rs913678
All − MR Egger
All − Inverse variance weighted
−0.0025
0.0000
0.0025
0.0050
MR effect size for
'NLRC4' on 'Bladder cancer'

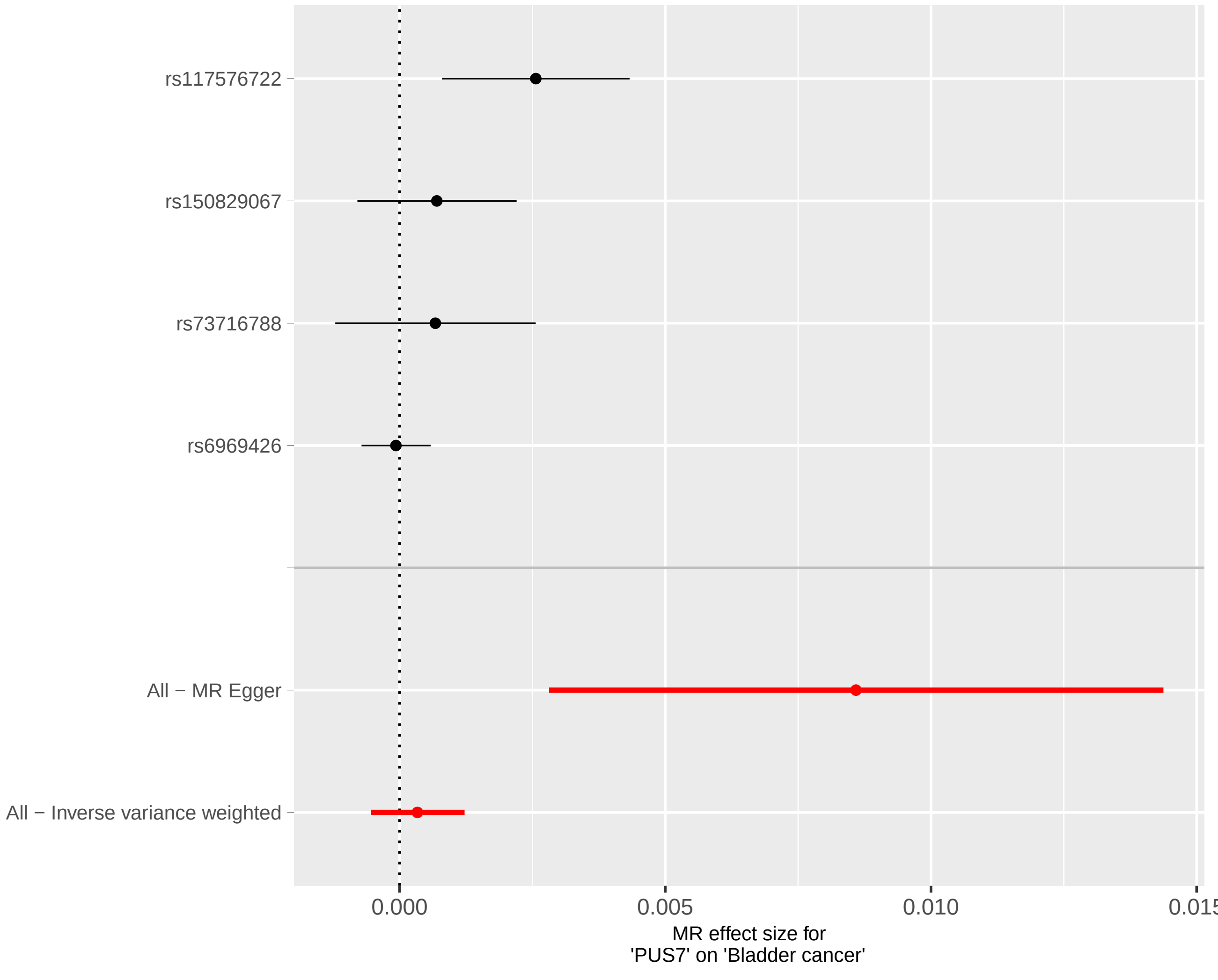

rs117576722
rs150829067
rs73716788
rs6969426
All − MR Egger
All − Inverse variance weighted
0.000
0.005
0.010
0.015
MR effect size for
'PUS7' on 'Bladder cancer'

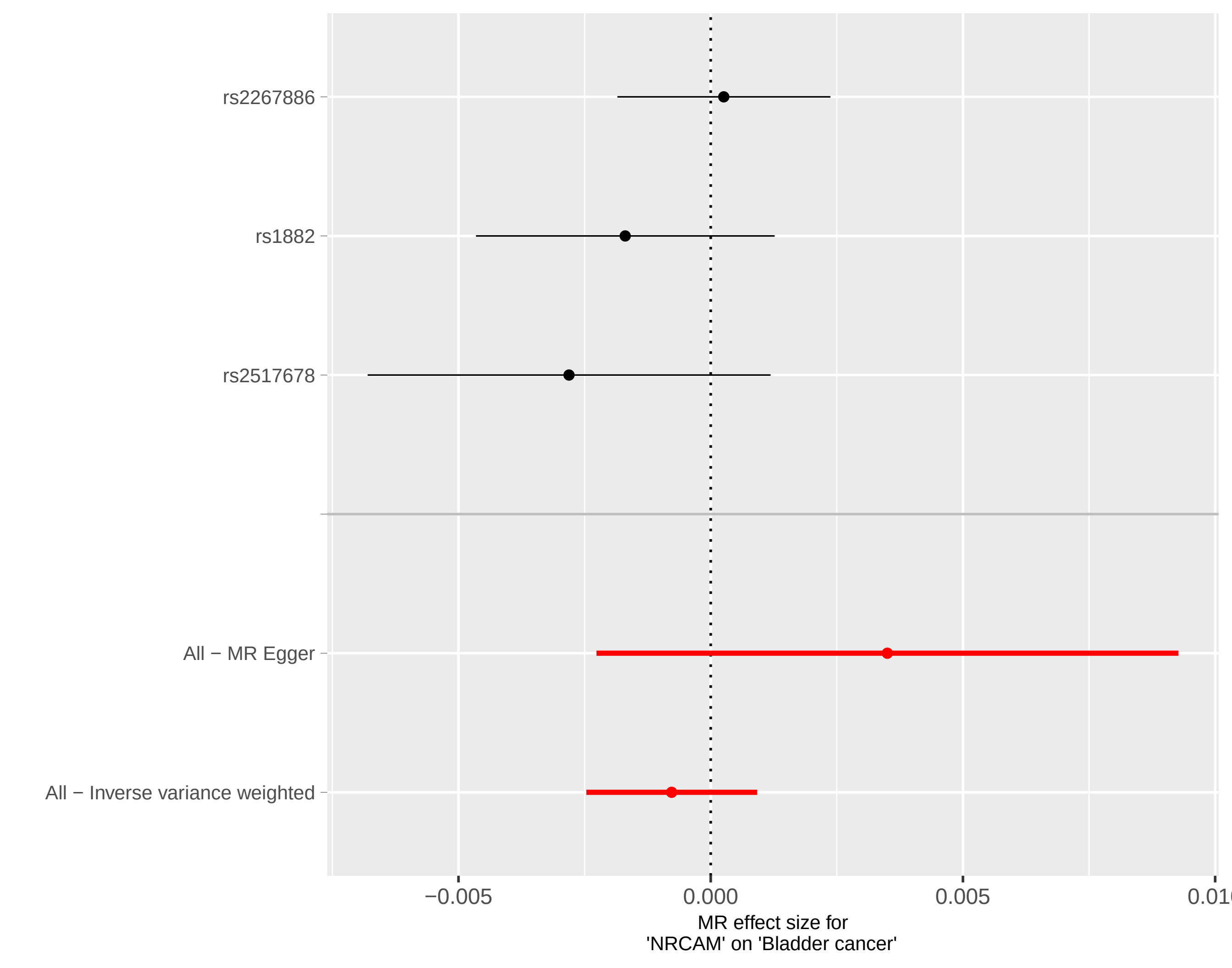
rs2267886
rs1882
rs2517678
All − MR Egger
All − Inverse variance weighted
−0.005
0.000
0.005
0.010
MR effect size for
'NRCAM' on 'Bladder cancer'

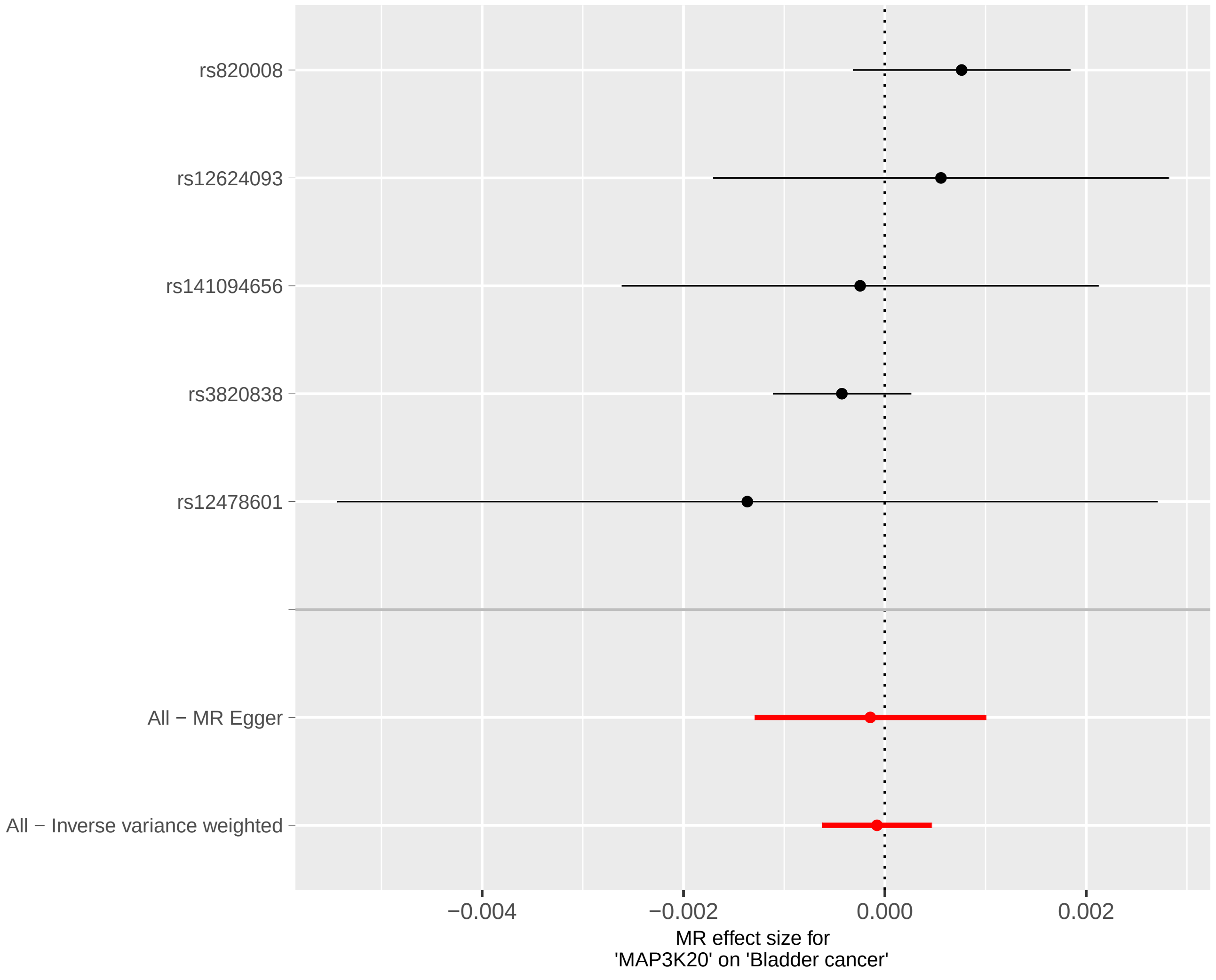
rs820008
rs12624093
rs141094656
rs3820838
rs12478601
All − MR Egger
All − Inverse variance weighted
−0.004
−0.002
0.000
0.002
MR effect size for
'MAP3K20' on 'Bladder cancer'

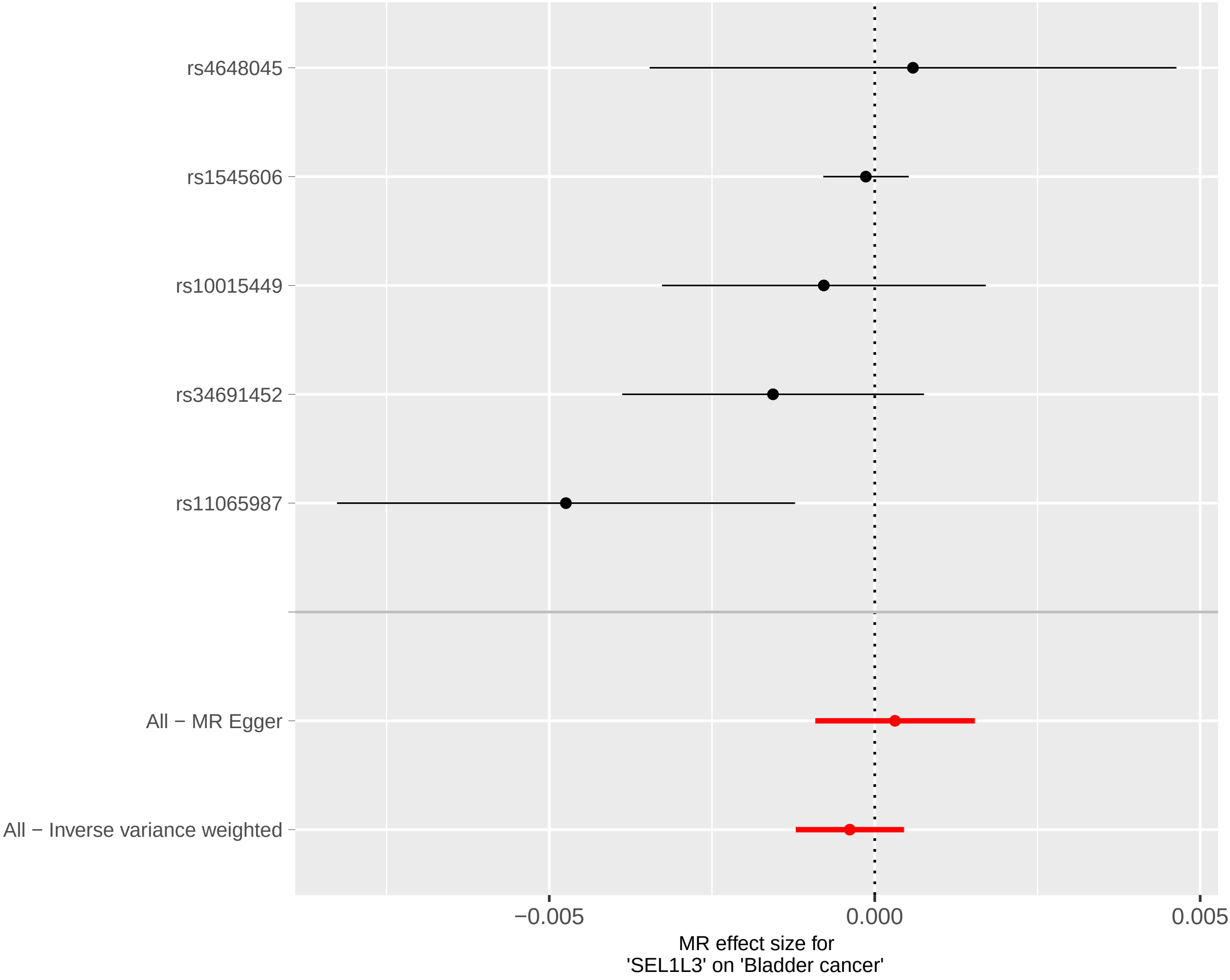

rs4648045
rs1545606
rs10015449
rs34691452
rs11065987
All − MR Egger
All − Inverse variance weighted
−0.005
0.000
0.005
MR effect size for
'SEL1L3' on 'Bladder cancer'

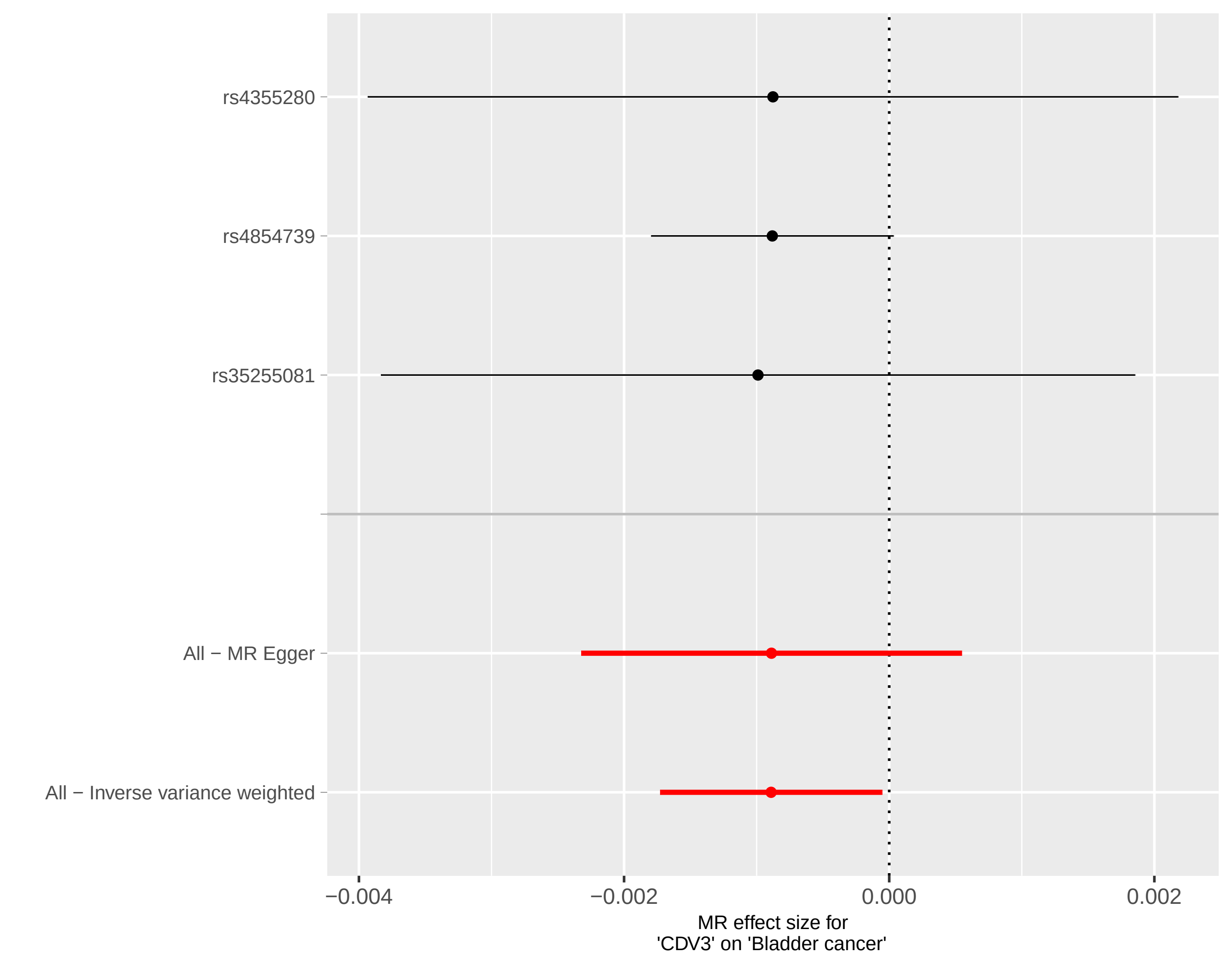

rs4355280
rs4854739
rs35255081
All − MR Egger
All − Inverse variance weighted
−0.004
−0.002
0.000
0.002
MR effect size for
'CDV3' on 'Bladder cancer'

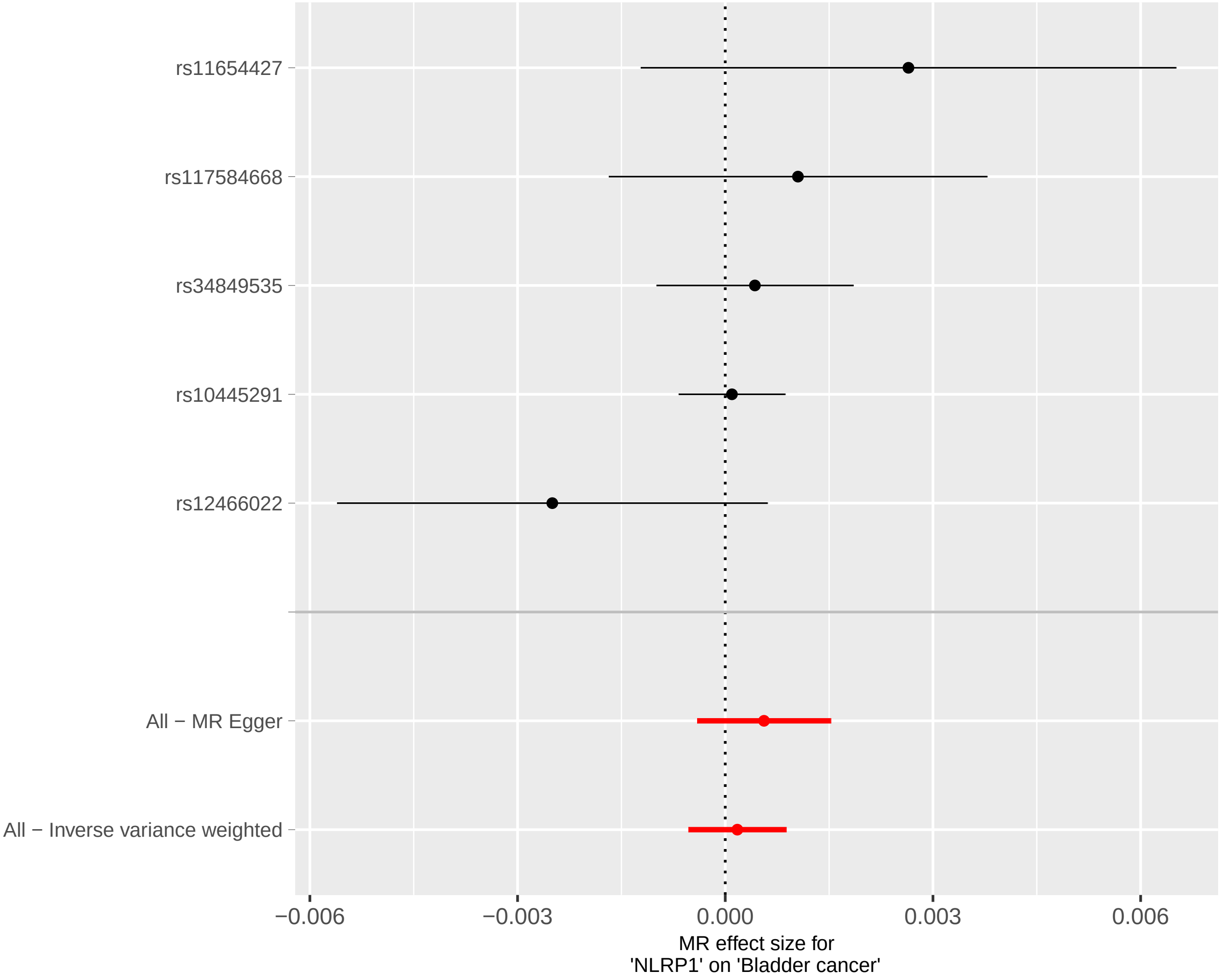
rs11654427
rs117584668
rs34849535
rs10445291
rs12466022
All − MR Egger
All − Inverse variance weighted
−0.006
−0.003
0.000
0.003
0.006
MR effect size for
'NLRP1' on 'Bladder cancer'

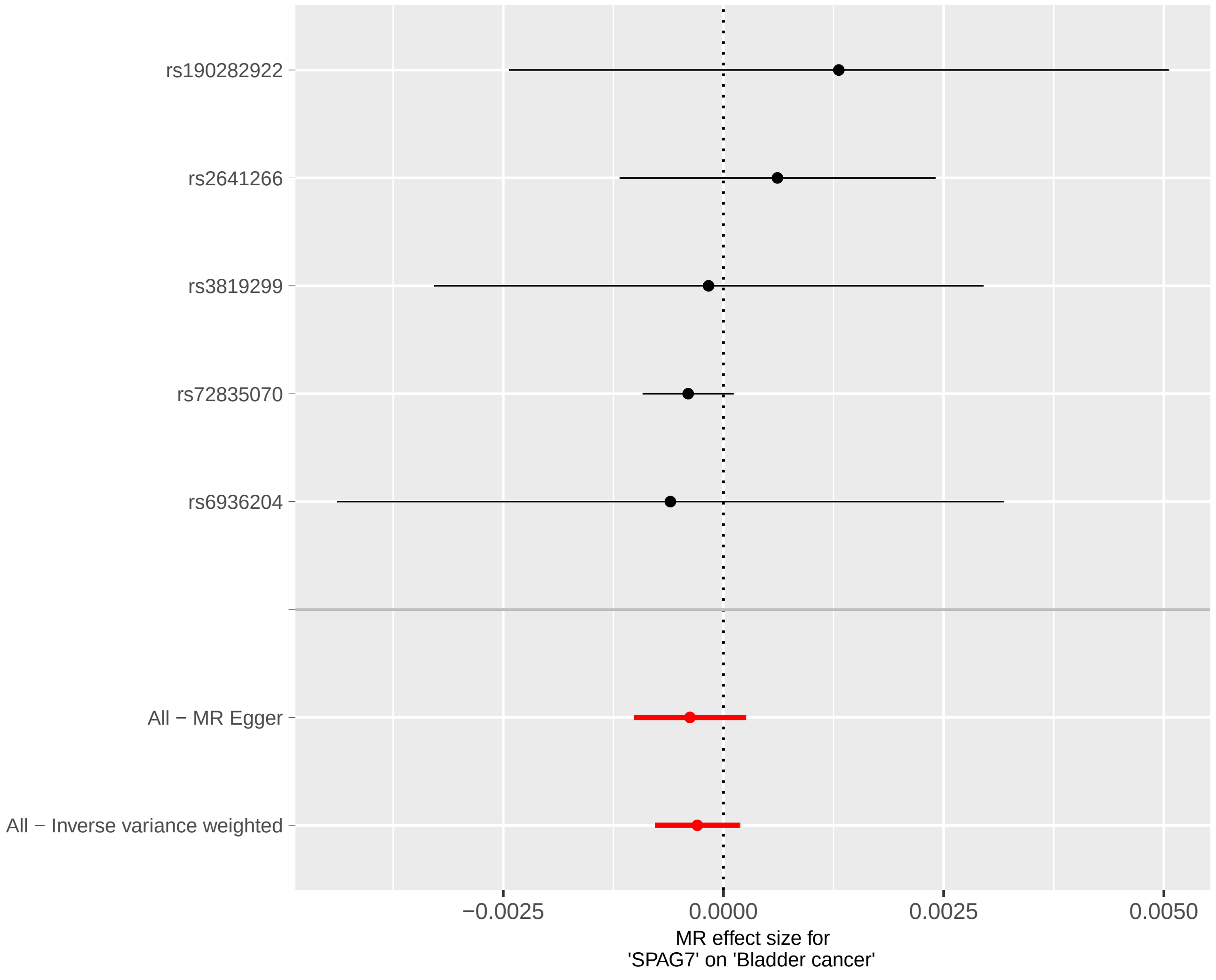

rs190282922
rs2641266
rs3819299
rs72835070
rs6936204
All − MR Egger
All − Inverse variance weighted
−0.0025
0.0000
0.0025
0.0050
MR effect size for
'SPAG7' on 'Bladder cancer'

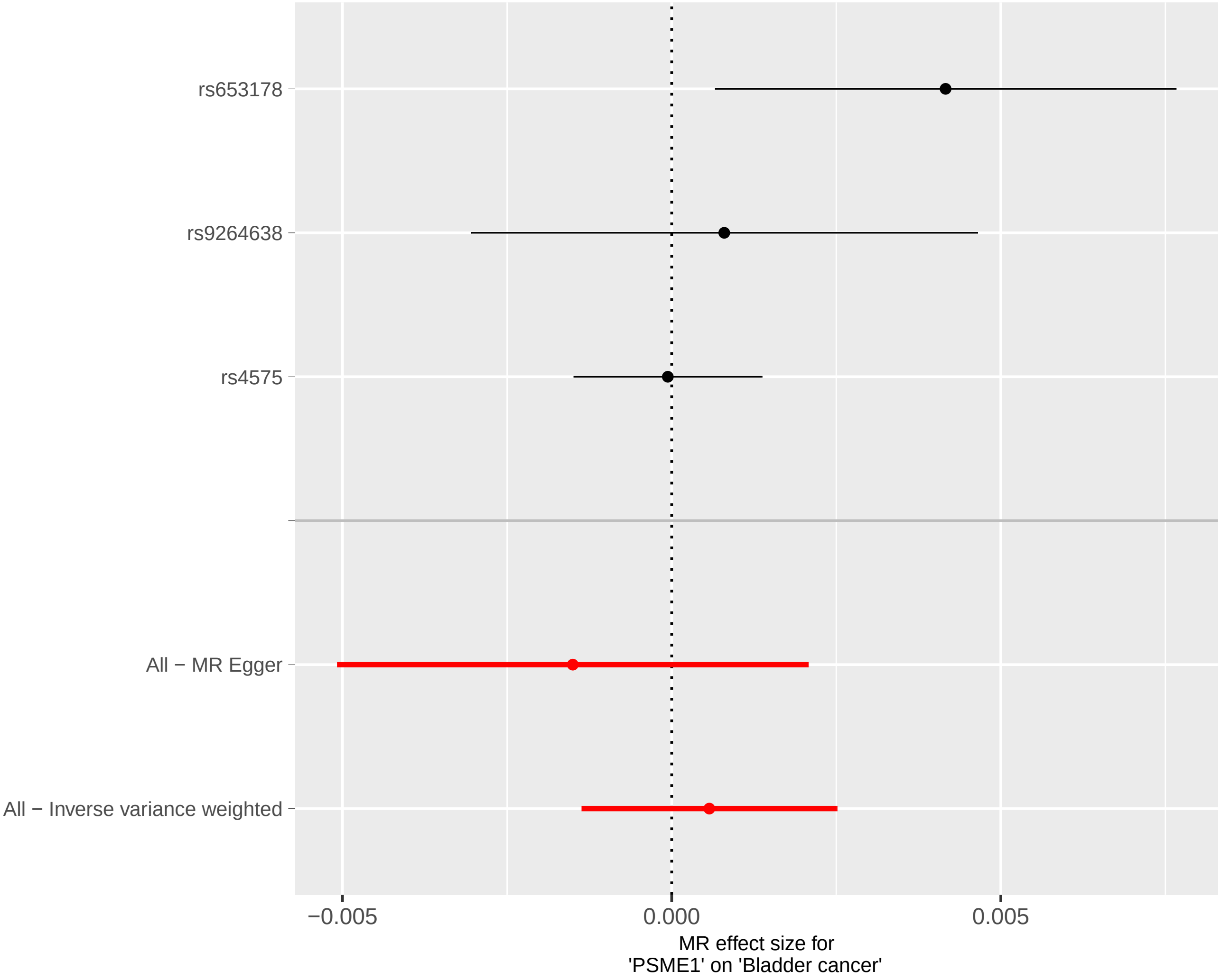
rs653178
rs9264638
rs4575
All − MR Egger
All − Inverse variance weighted
−0.005
0.000
0.005
MR effect size for
'PSME1' on 'Bladder cancer'

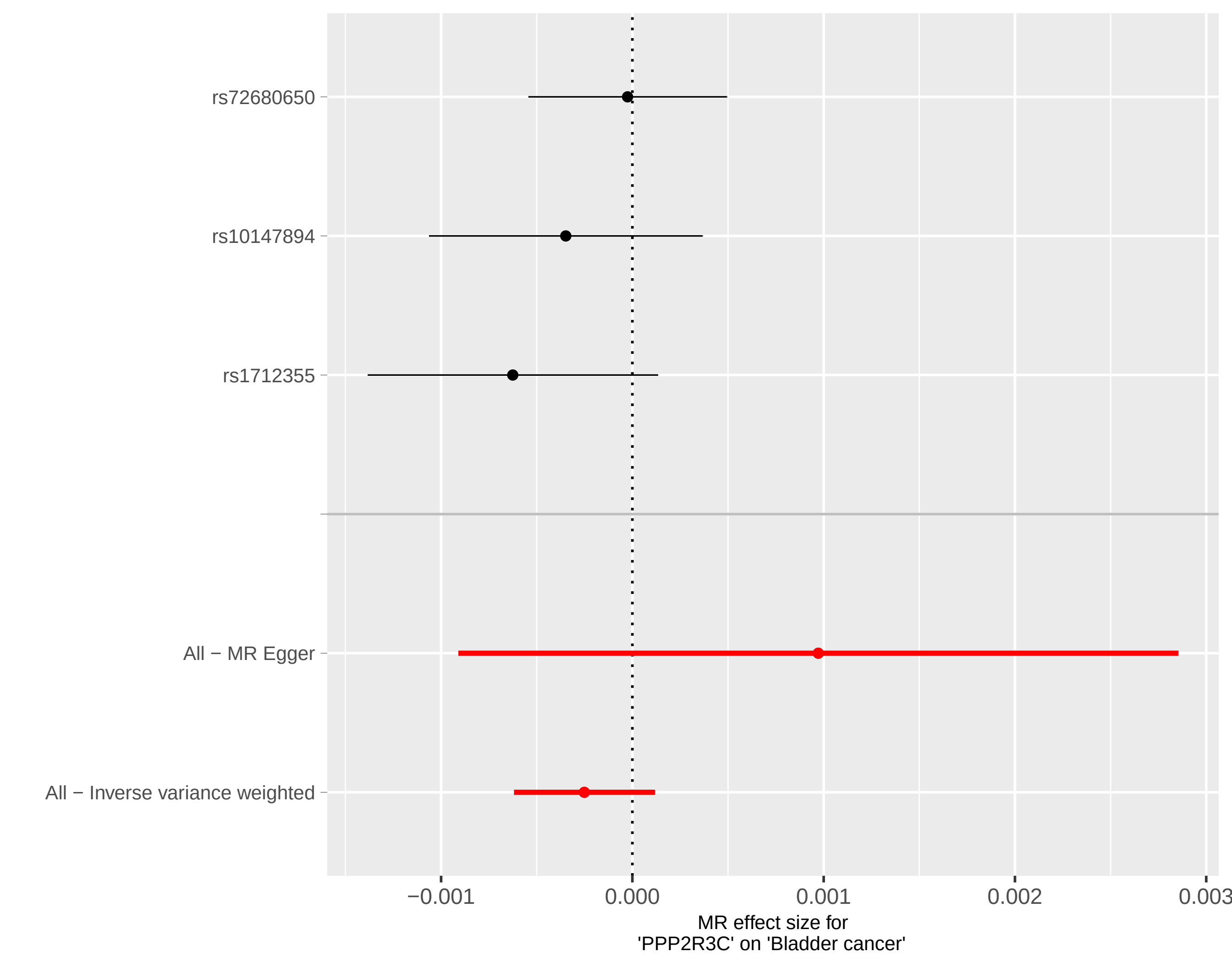
rs72680650
rs10147894
rs1712355
All − MR Egger
All − Inverse variance weighted
−0.001
0.000
0.001
0.002
0.003
MR effect size for
'PPP2R3C' on 'Bladder cancer'

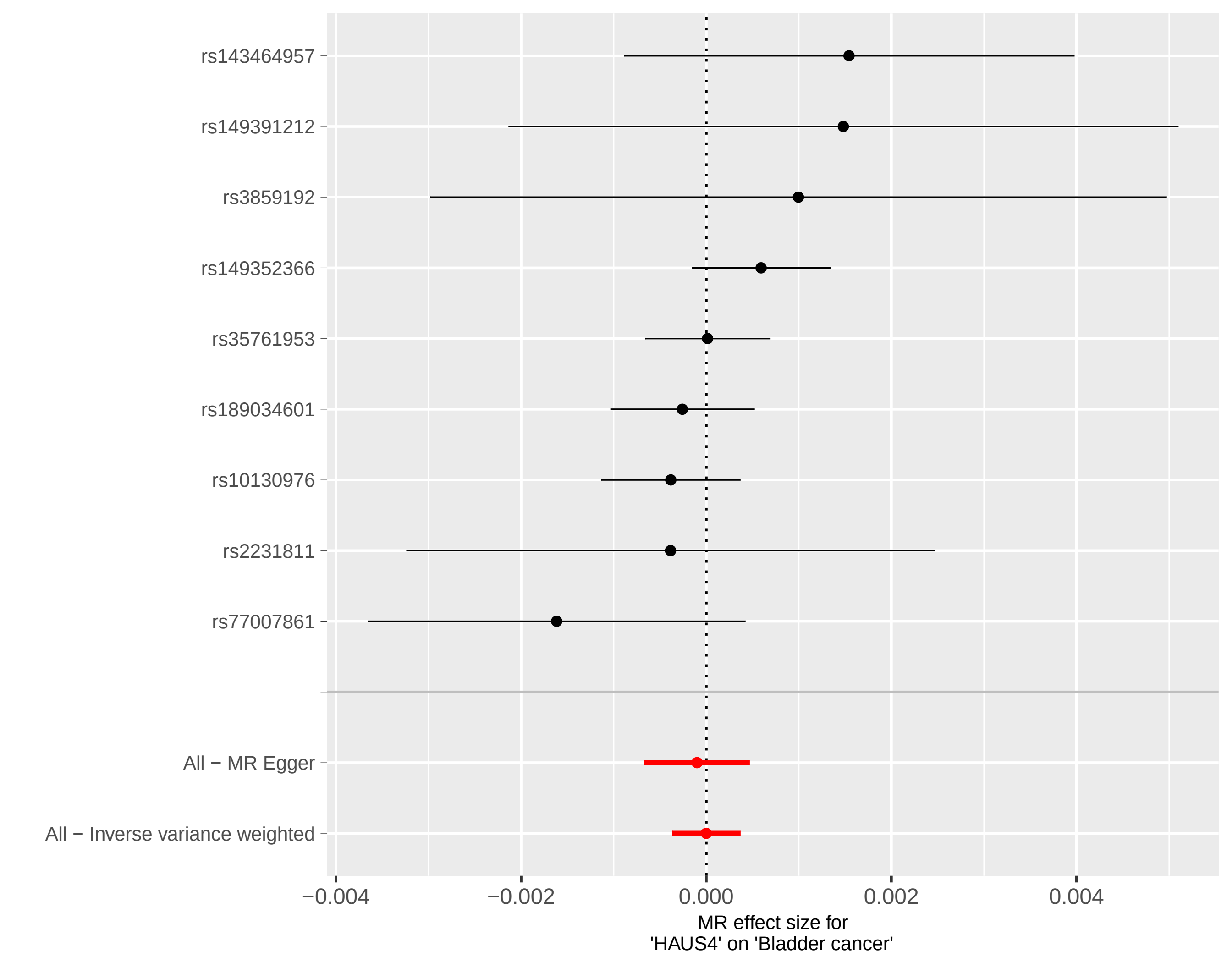

rs143464957
rs149391212
rs3859192
rs149352366
rs35761953
rs189034601
rs10130976
rs2231811
rs77007861
All − MR Egger
All − Inverse variance weighted
−0.004
−0.002
0.000
0.002
0.004
MR effect size for
'HAUS4' on 'Bladder cancer'

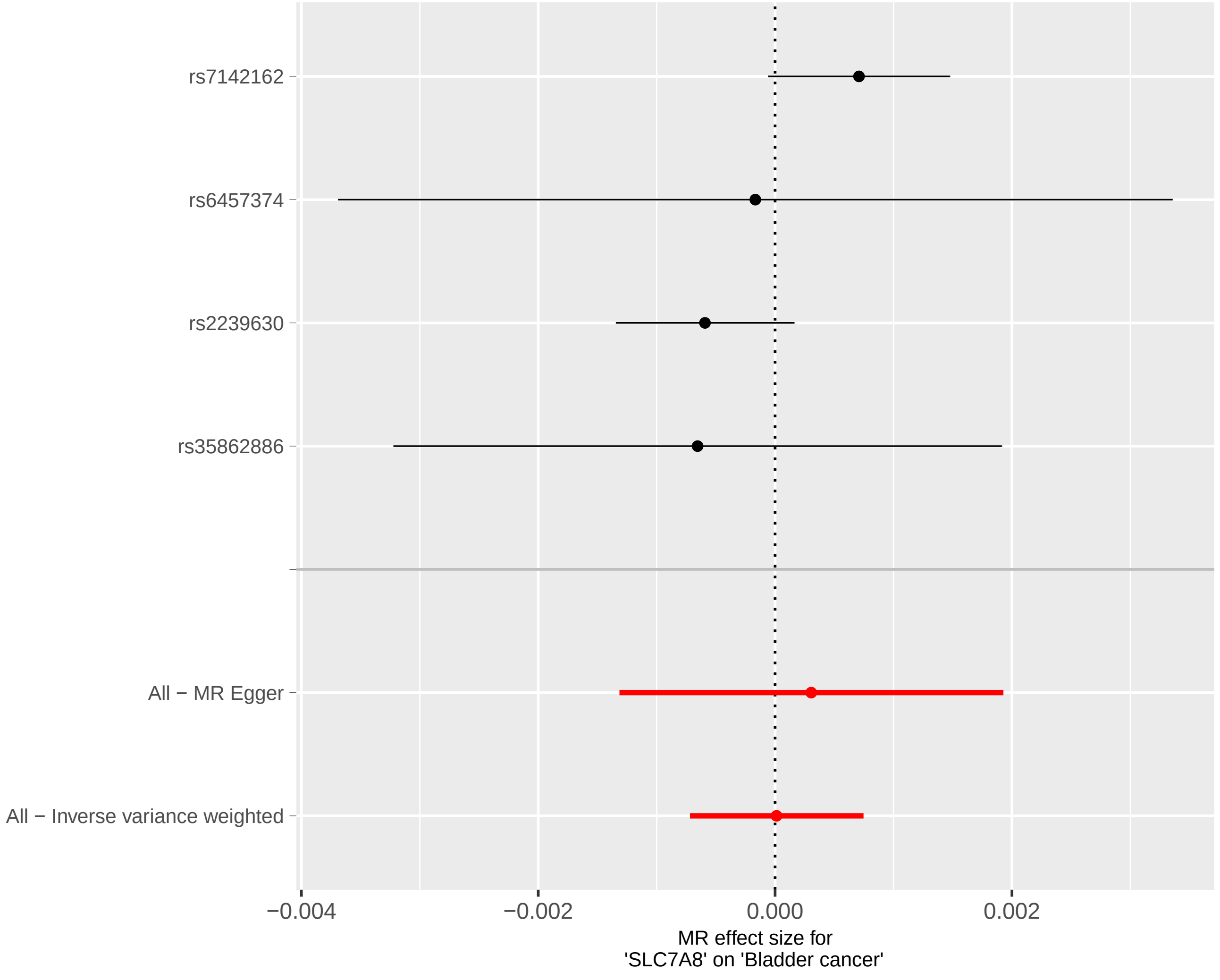

rs7142162
rs6457374
rs2239630
rs35862886
All − MR Egger
All − Inverse variance weighted
−0.004
−0.002
0.000
0.002
MR effect size for
'SLC7A8' on 'Bladder cancer'

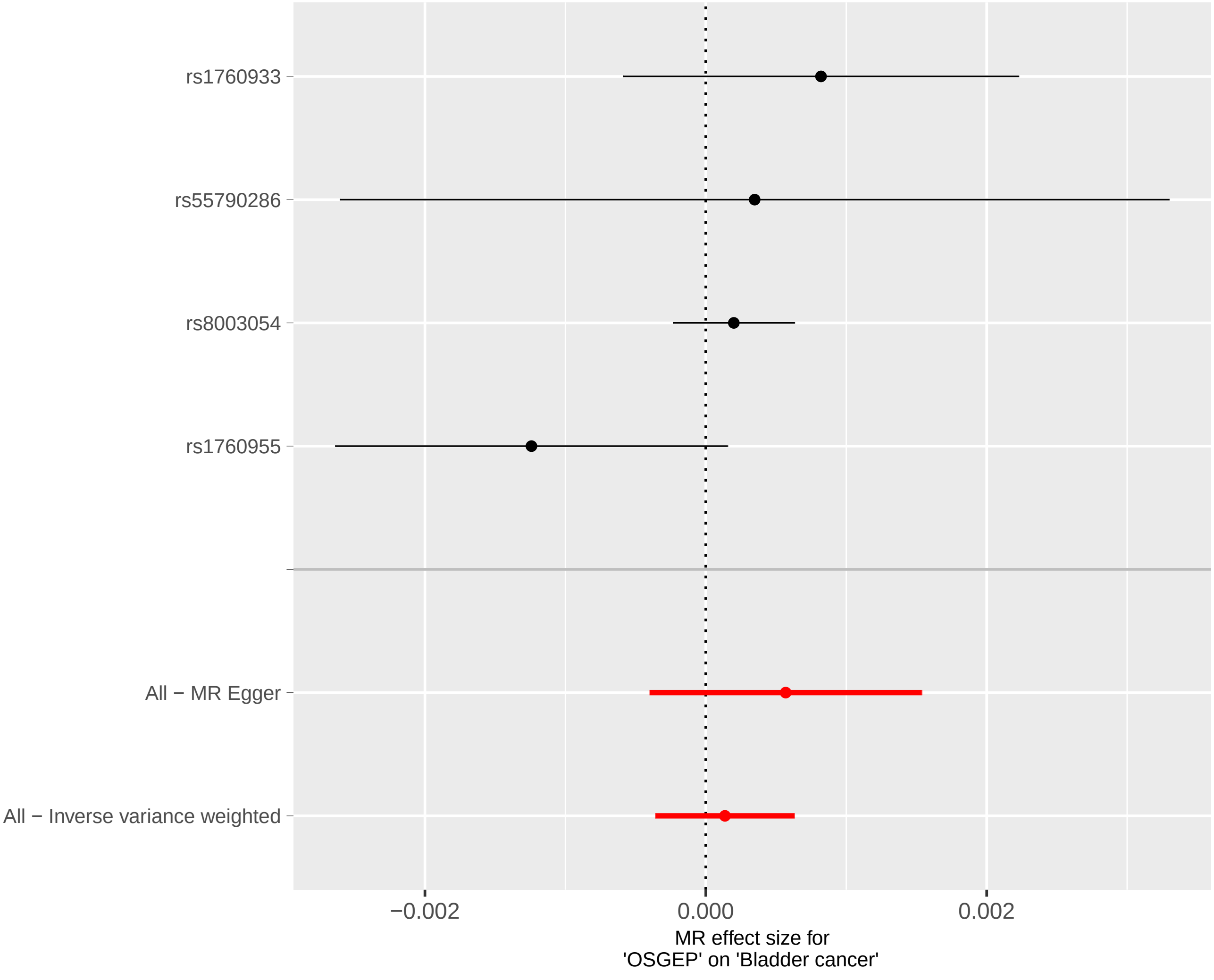
rs1760933
rs55790286
rs8003054
rs1760955
All − MR Egger
All − Inverse variance weighted
−0.002
0.000
0.002
MR effect size for
'OSGEP' on 'Bladder cancer'

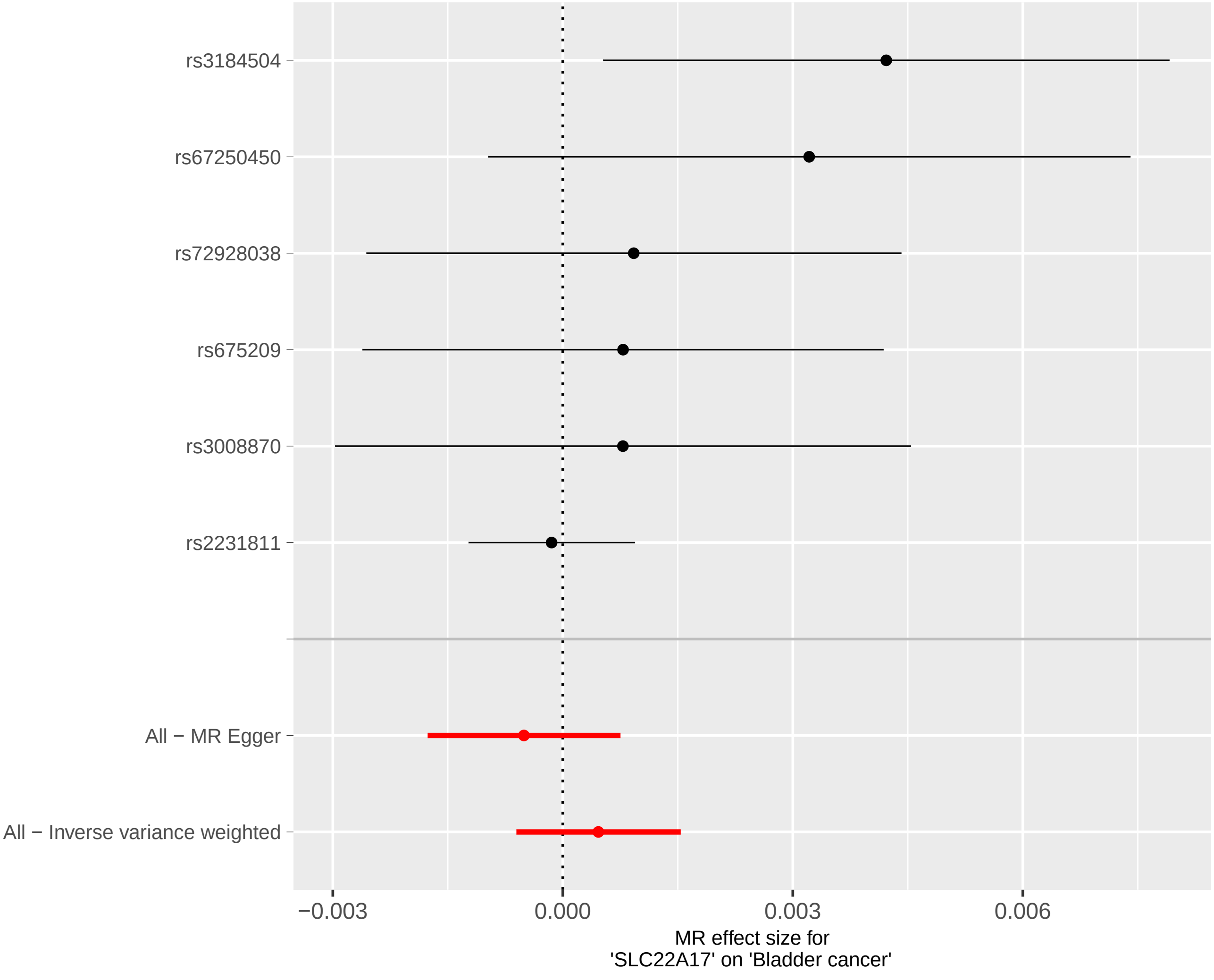

rs3184504
rs67250450
rs72928038
rs675209
rs3008870
rs2231811
All − MR Egger
All − Inverse variance weighted
−0.003
0.000
0.003
0.006
MR effect size for
'SLC22A17' on 'Bladder cancer'

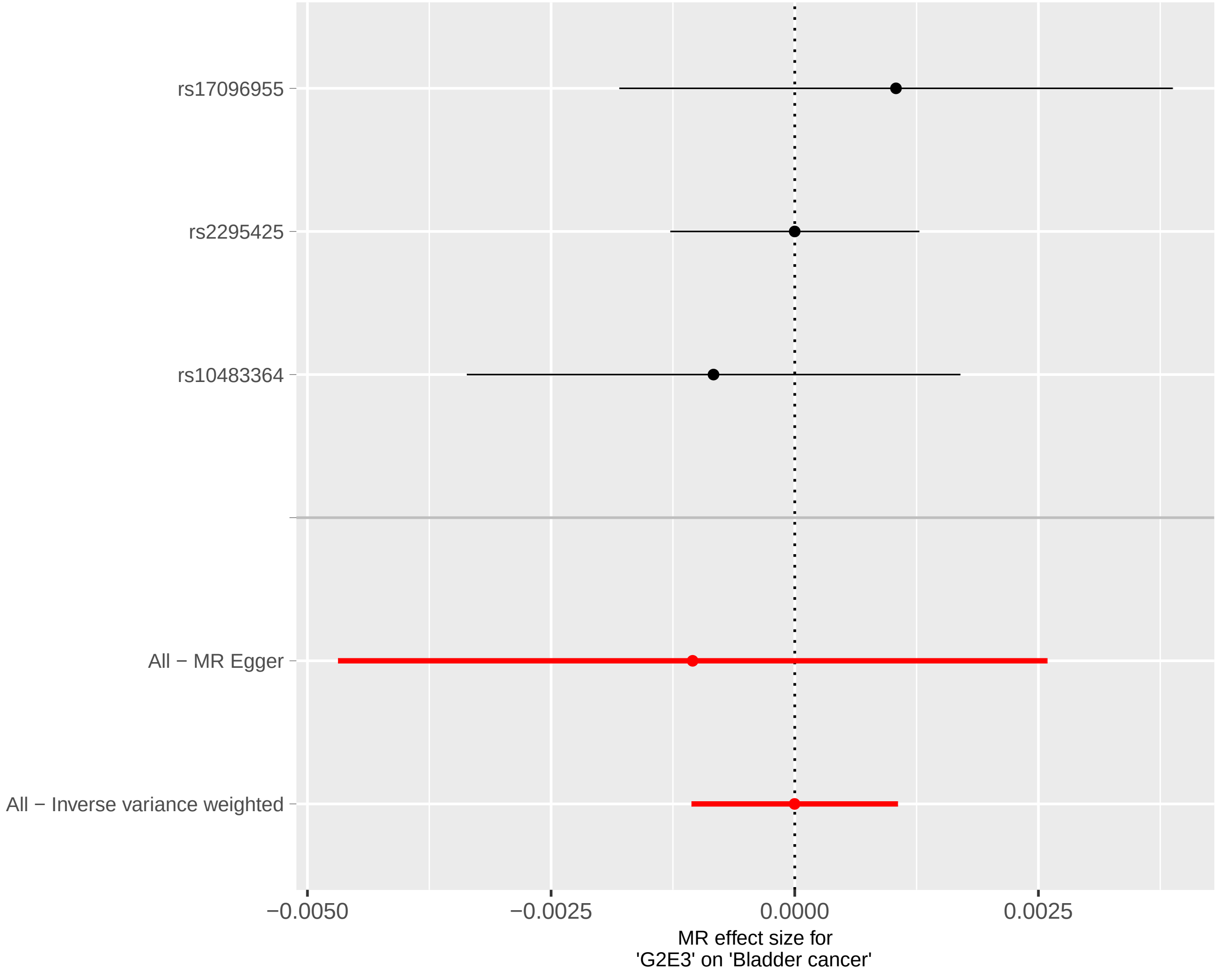

rs17096955
rs2295425
rs10483364
All − MR Egger
All − Inverse variance weighted
−0.0050
−0.0025
0.0000
0.0025
MR effect size for
'G2E3' on 'Bladder cancer'

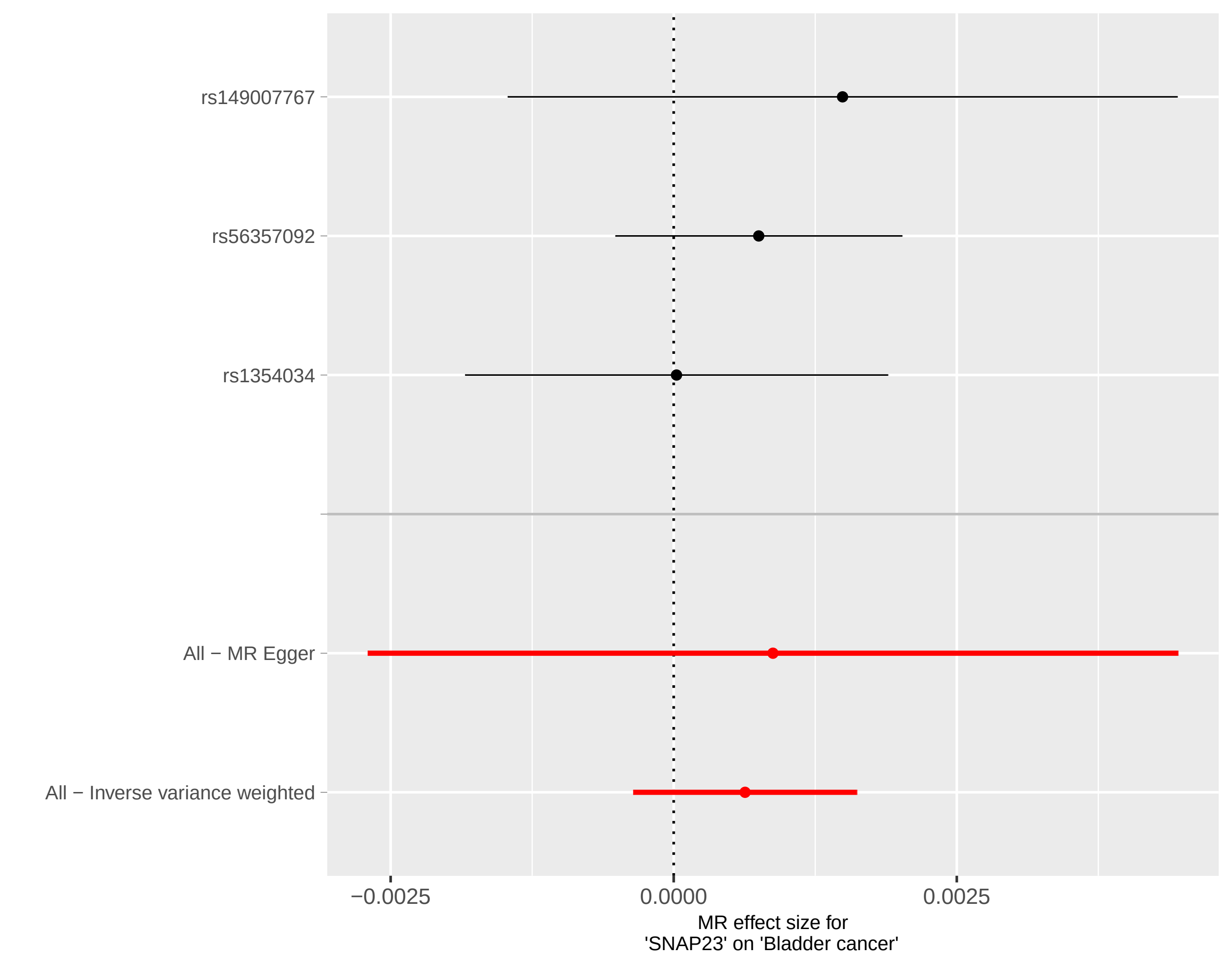

rs149007767
rs56357092
rs1354034
All − MR Egger
All − Inverse variance weighted
−0.0025
0.0000
0.0025
MR effect size for
'SNAP23' on 'Bladder cancer'

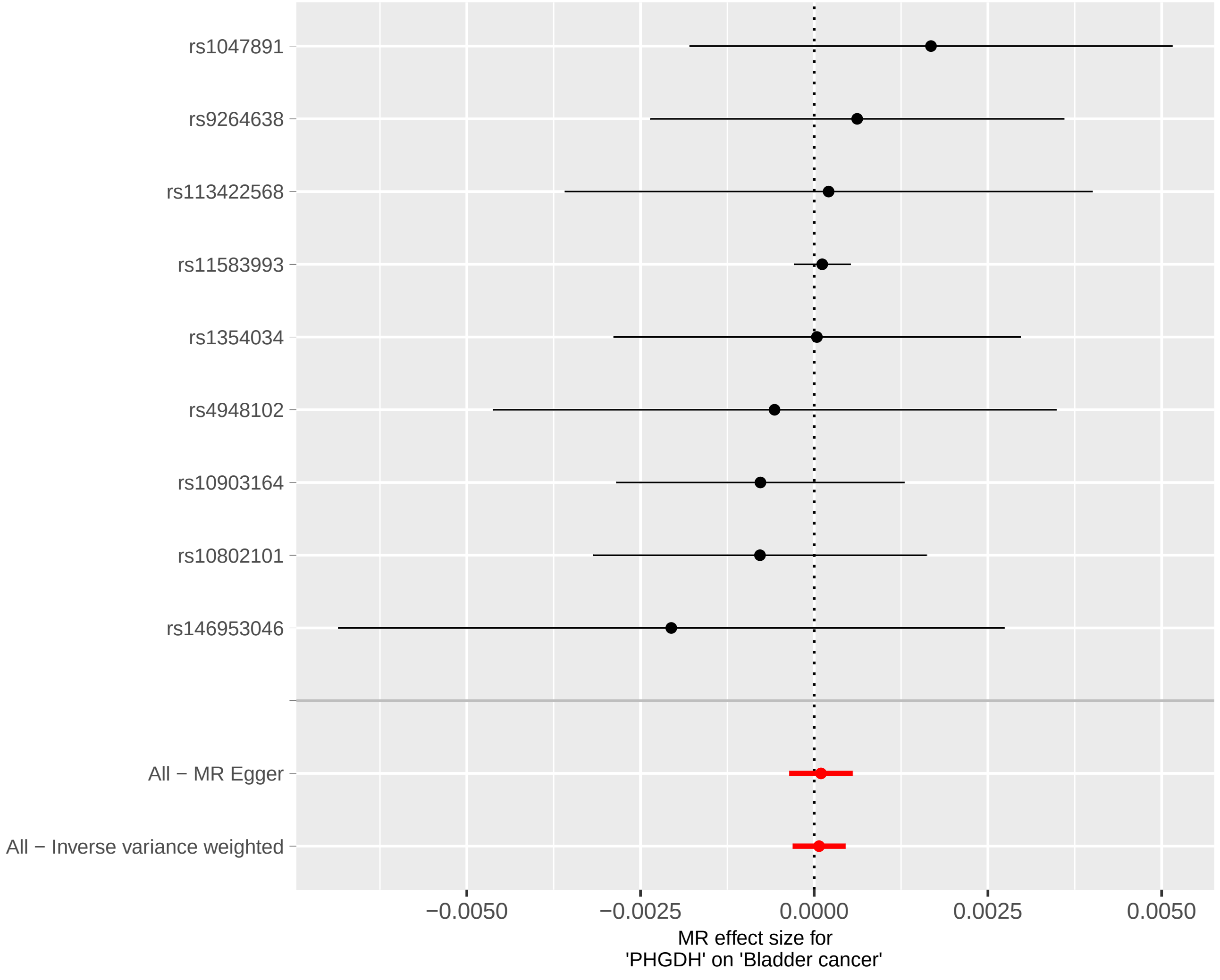

rs1047891
rs9264638
rs113422568
rs11583993
rs1354034
rs4948102
rs10903164
rs10802101
rs146953046
All − MR Egger
All − Inverse variance weighted
−0.0050
−0.0025
0.0000
0.0025
0.0050
MR effect size for
'PHGDH' on 'Bladder cancer'

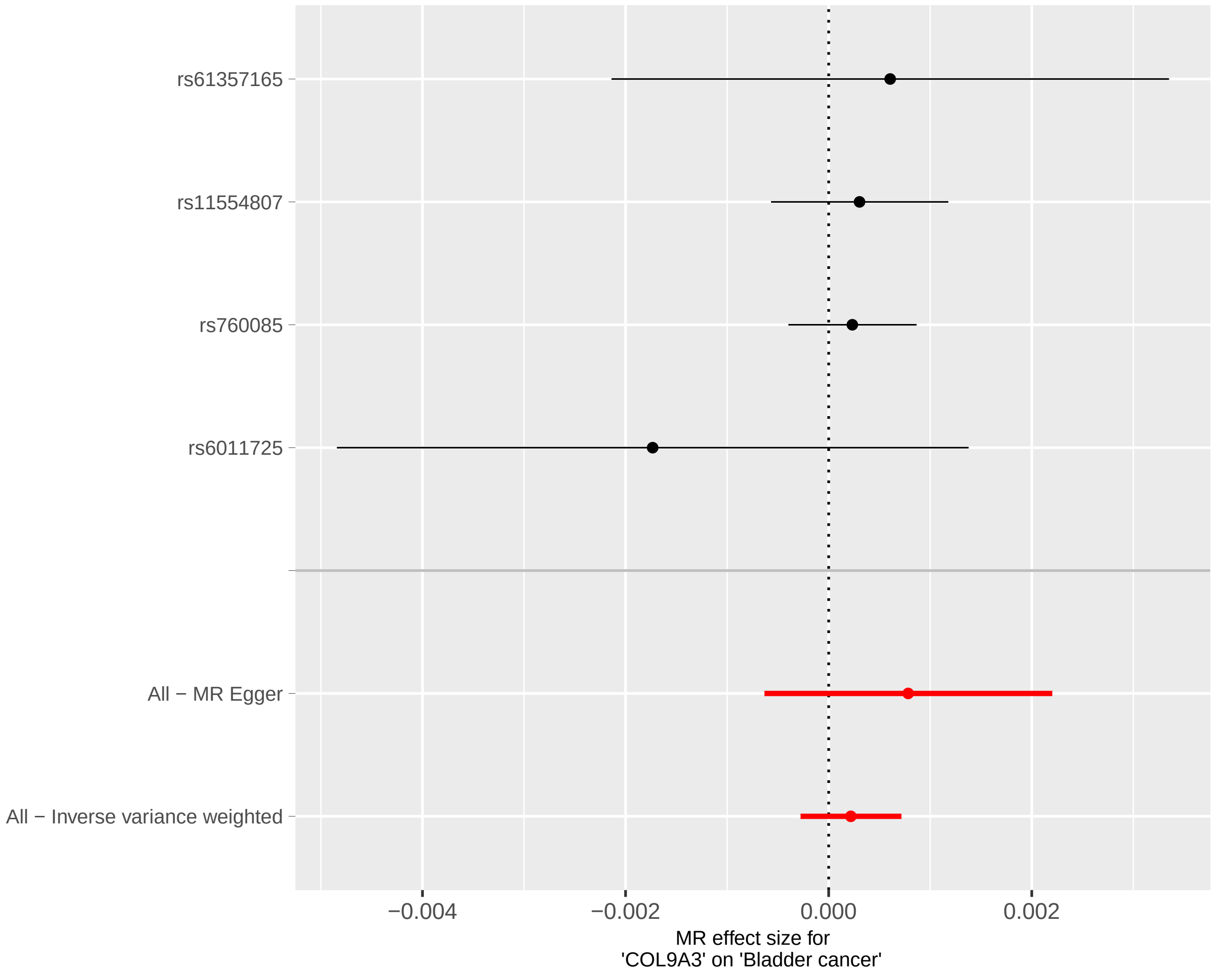
rs61357165
rs11554807
rs760085
rs6011725
All − MR Egger
All − Inverse variance weighted
−0.004
−0.002
0.000
0.002
MR effect size for
'COL9A3' on 'Bladder cancer'

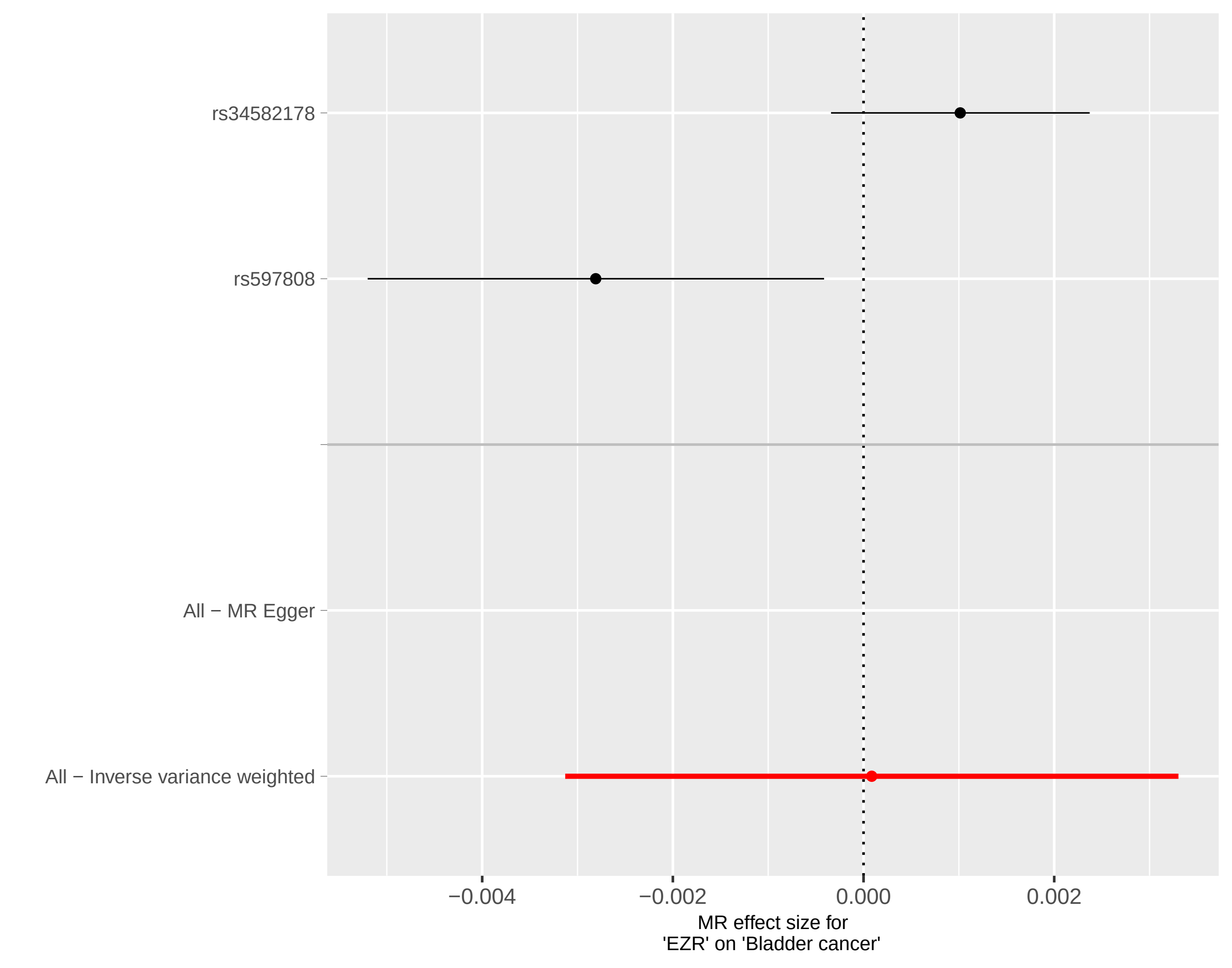
rs34582178
rs597808
All − MR Egger
All − Inverse variance weighted
−0.004
−0.002
0.000
0.002
MR effect size for
'EZR' on 'Bladder cancer'

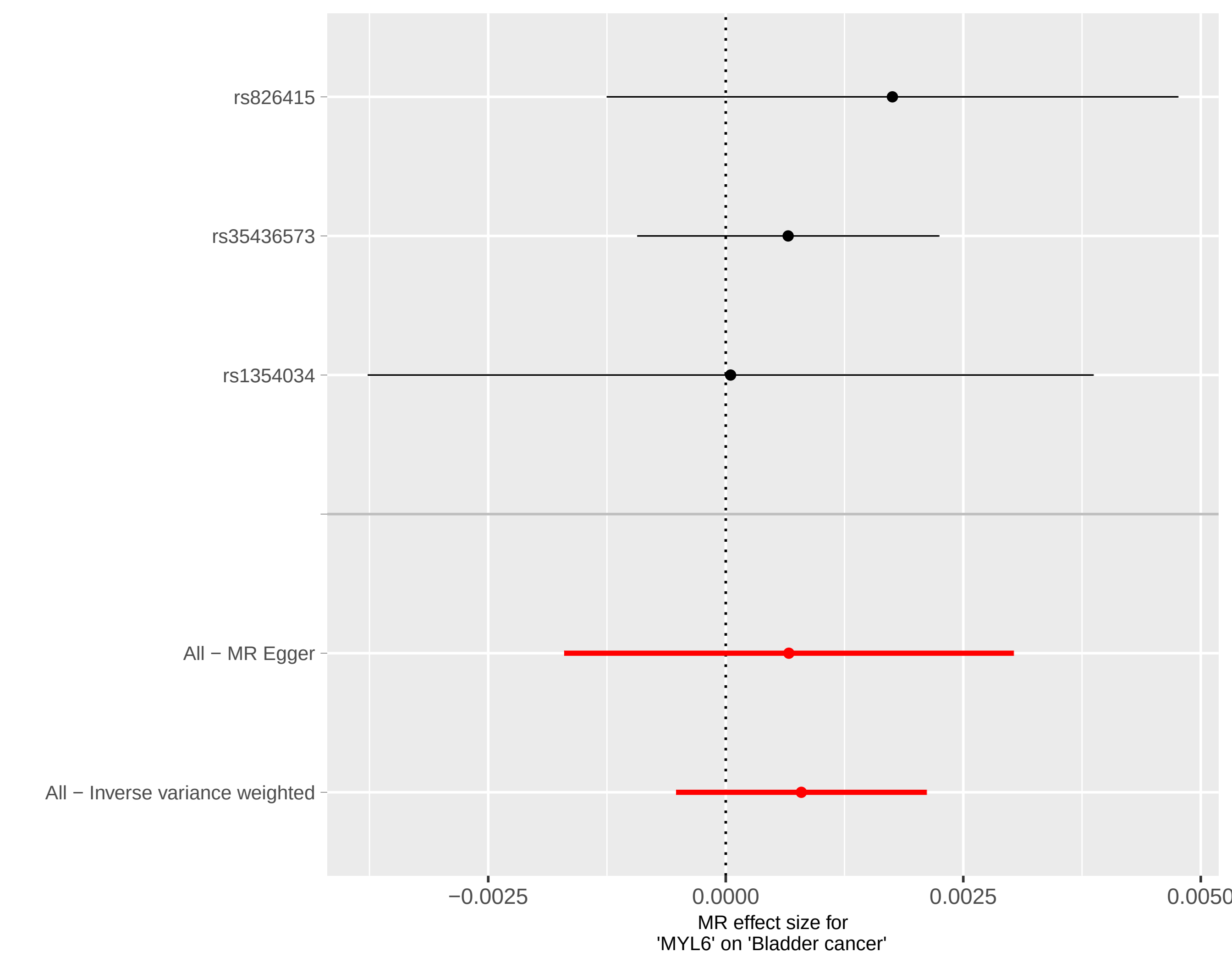
rs826415
rs35436573
rs1354034
All − MR Egger
All − Inverse variance weighted
−0.0025
0.0000
0.0025
0.0050
MR effect size for
'MYL6' on 'Bladder cancer'

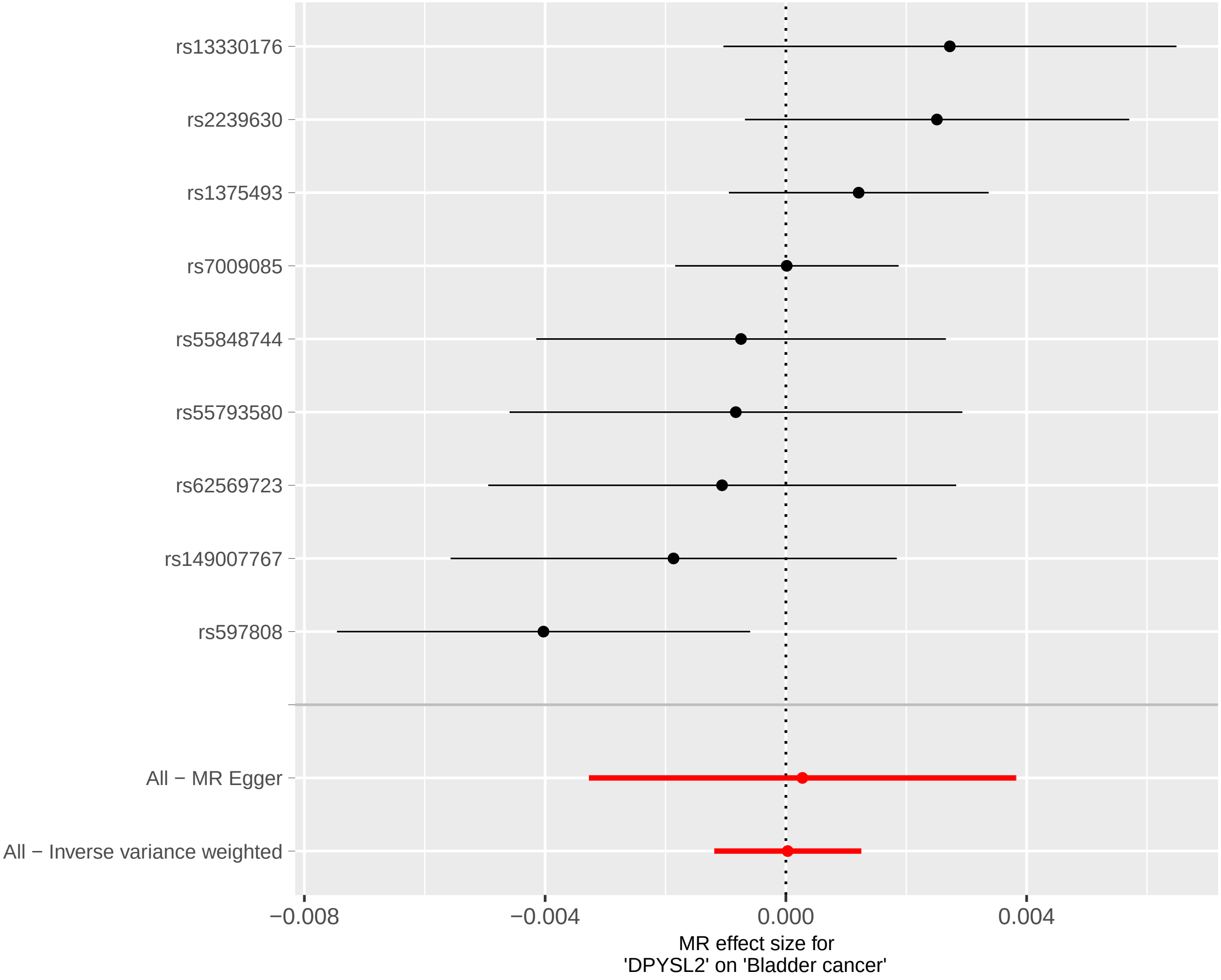
rs13330176
rs2239630
rs1375493
rs7009085
rs55848744
rs55793580
rs62569723
rs149007767
rs597808
All − MR Egger
All − Inverse variance weighted
−0.008
−0.004
0.000
0.004
MR effect size for
'DPYSL2' on 'Bladder cancer'

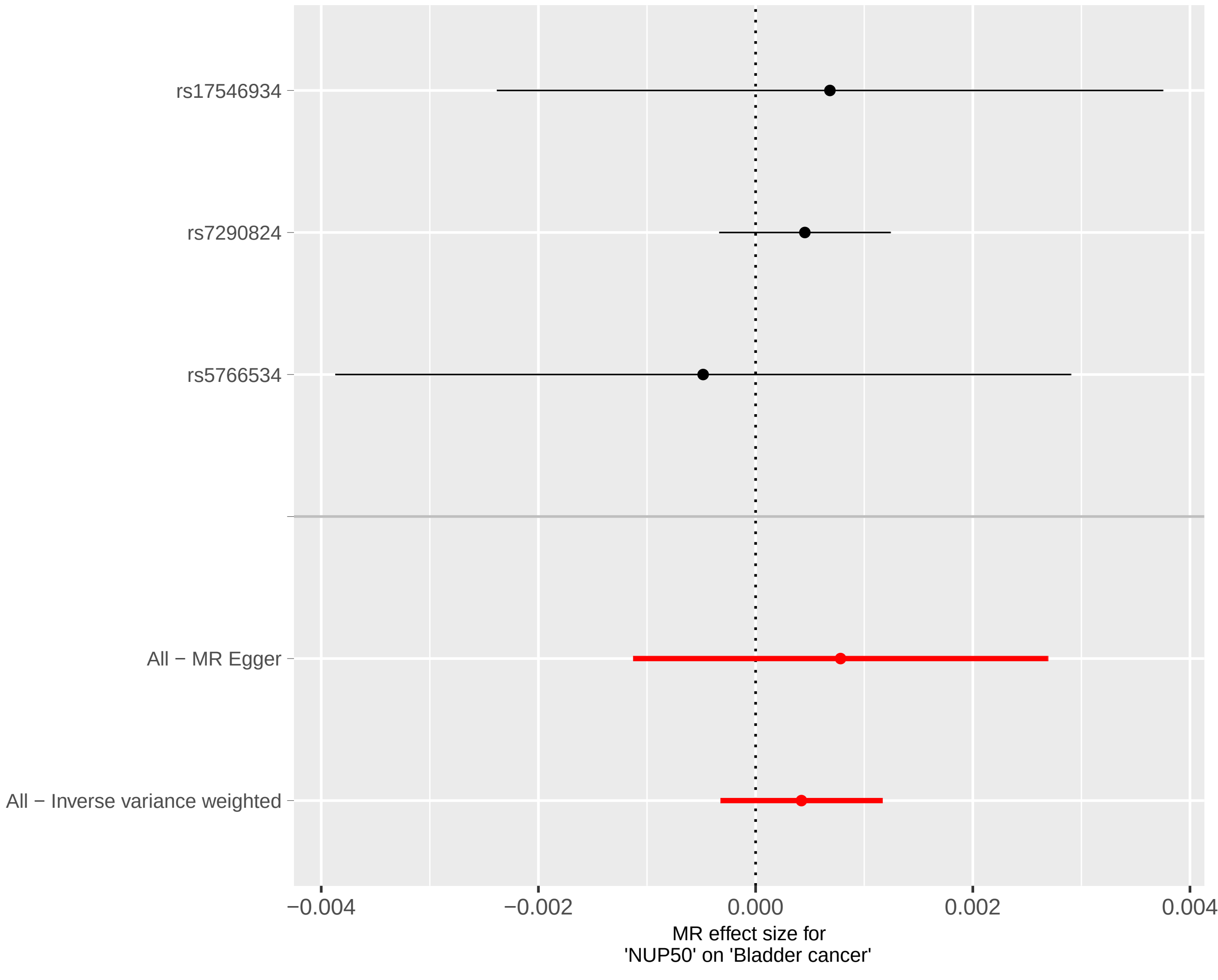

rs17546934
rs7290824
rs5766534
All − MR Egger
All − Inverse variance weighted
−0.004
−0.002
0.000
0.002
0.004
MR effect size for
'NUP50' on 'Bladder cancer'

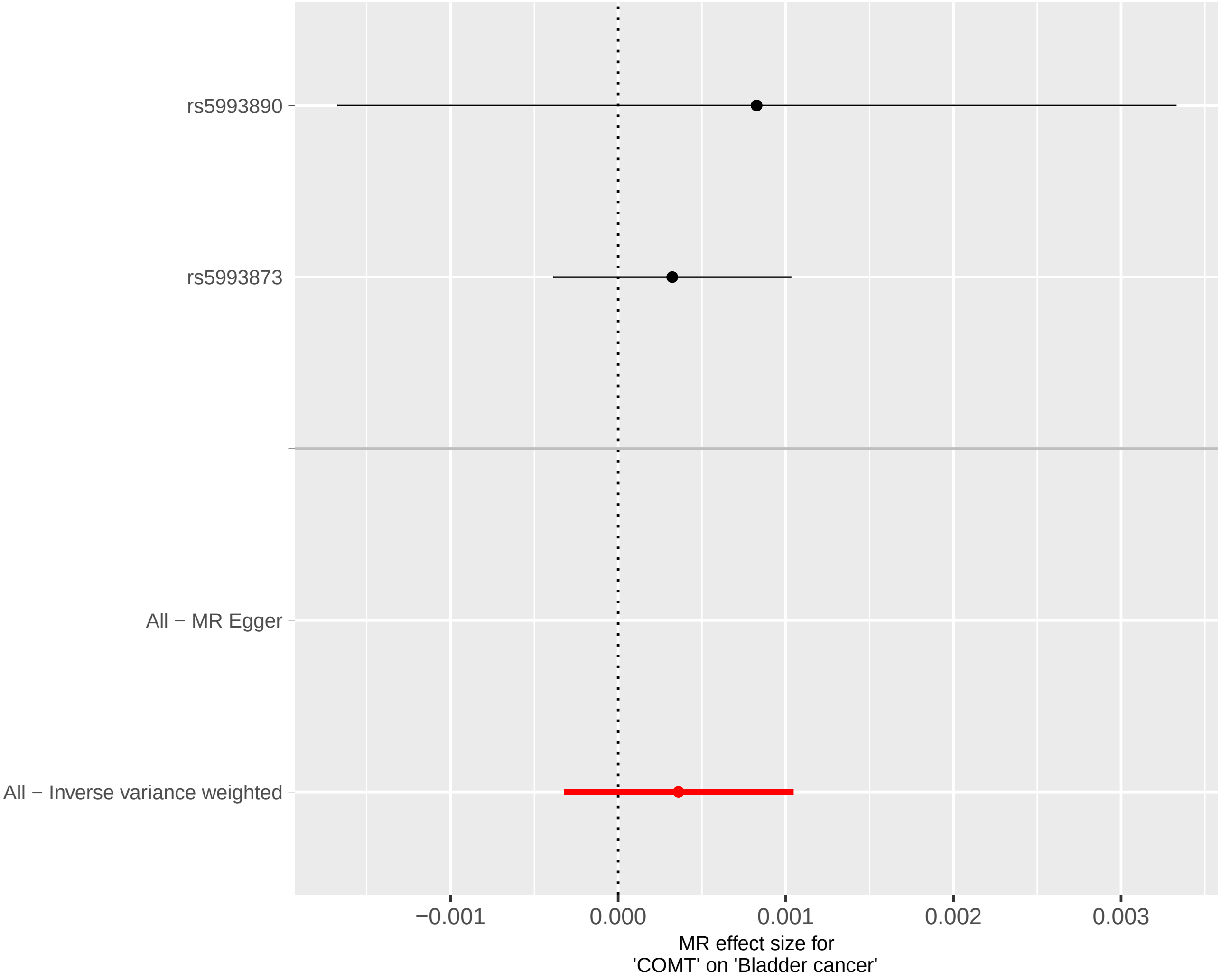
rs5993890
rs5993873
All − MR Egger
All − Inverse variance weighted
−0.001
0.000
0.001
0.002
0.003
MR effect size for
'COMT' on 'Bladder cancer'

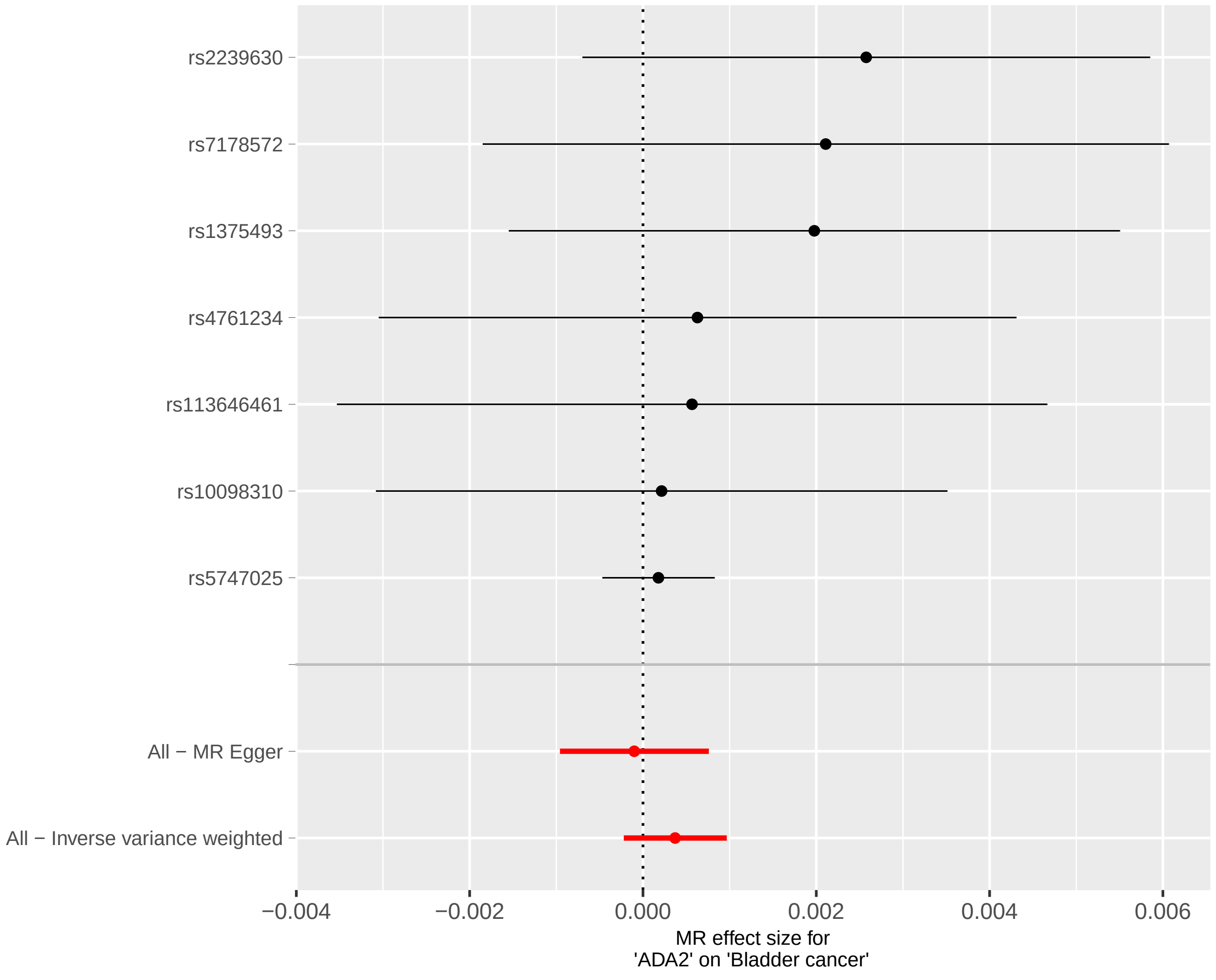

rs2239630
rs7178572
rs1375493
rs4761234
rs113646461
rs10098310
rs5747025
All − MR Egger
All − Inverse variance weighted
−0.004
−0.002
0.000
0.002
0.004
0.006
MR effect size for
'ADA2' on 'Bladder cancer'

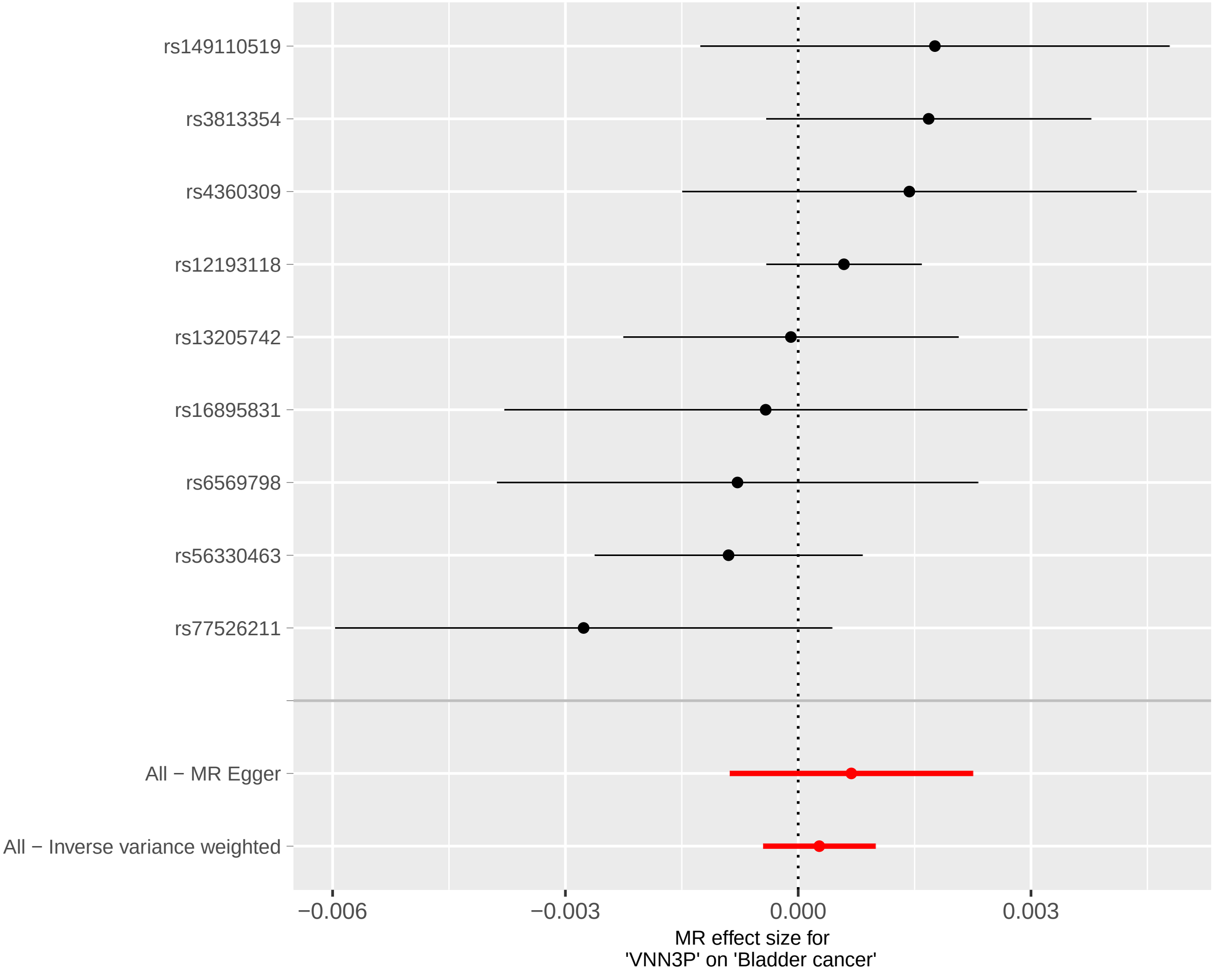
rs149110519
rs3813354
rs4360309
rs12193118
rs13205742
rs16895831
rs6569798
rs56330463
rs77526211
All − MR Egger
All − Inverse variance weighted
−0.006
−0.003
0.000
0.003
MR effect size for
'VNN3P' on 'Bladder cancer'

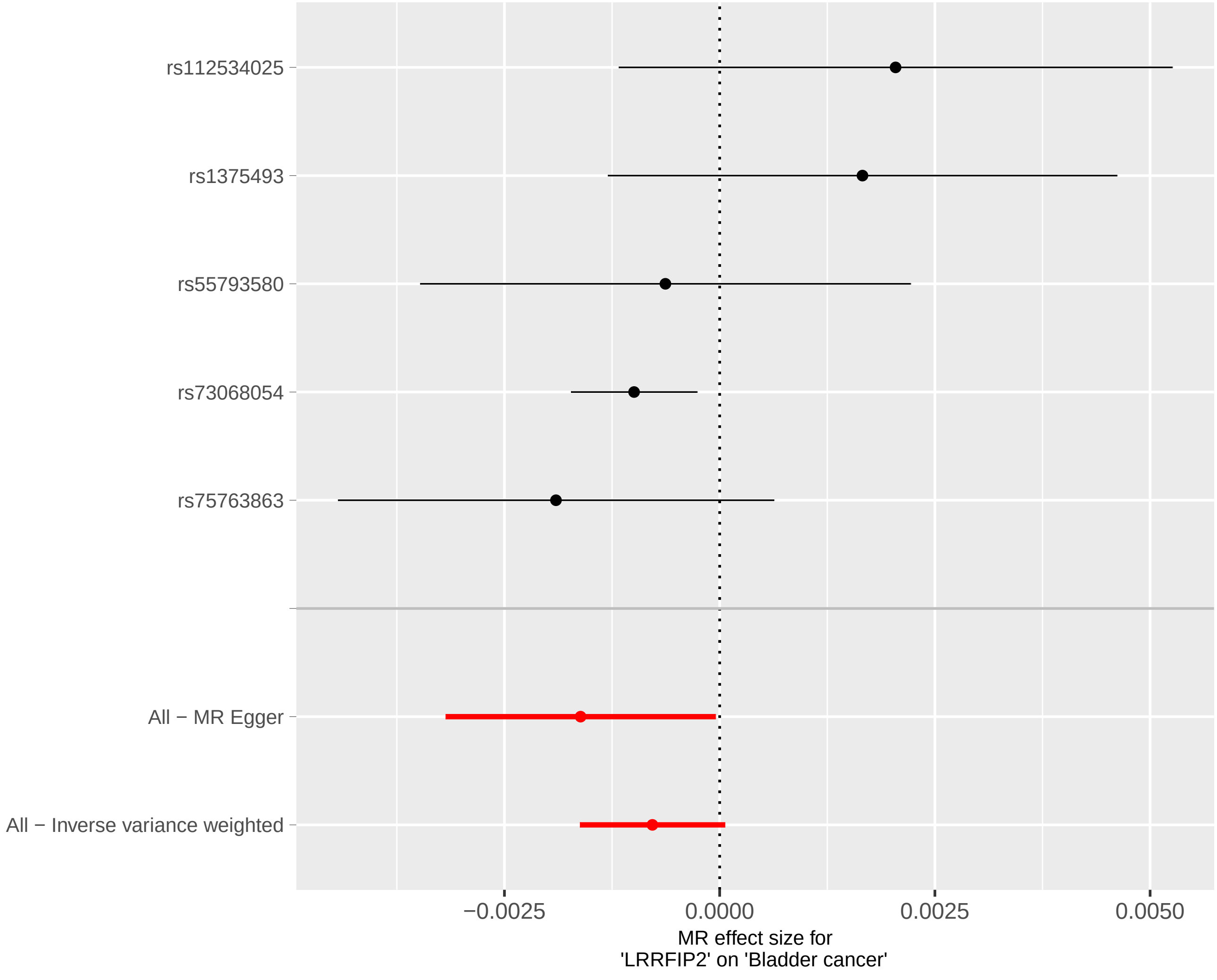
rs112534025
rs1375493
rs55793580
rs73068054
rs75763863
All − MR Egger
All − Inverse variance weighted
−0.0025
0.0000
0.0025
0.0050
MR effect size for
'LRRFIP2' on 'Bladder cancer'

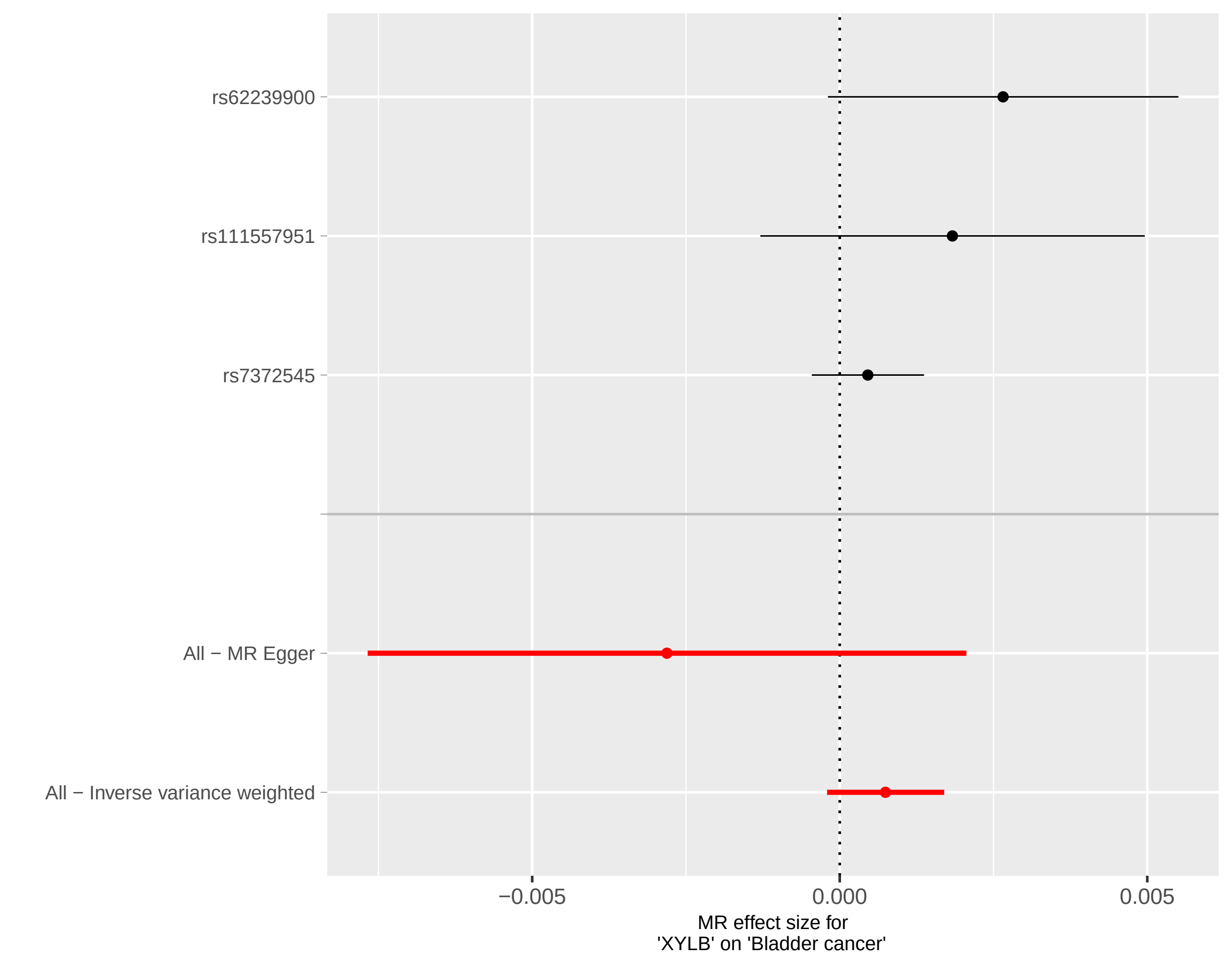

rs62239900
rs111557951
rs7372545
All − MR Egger
All − Inverse variance weighted
−0.005
0.000
0.005
MR effect size for
'XYLB' on 'Bladder cancer'

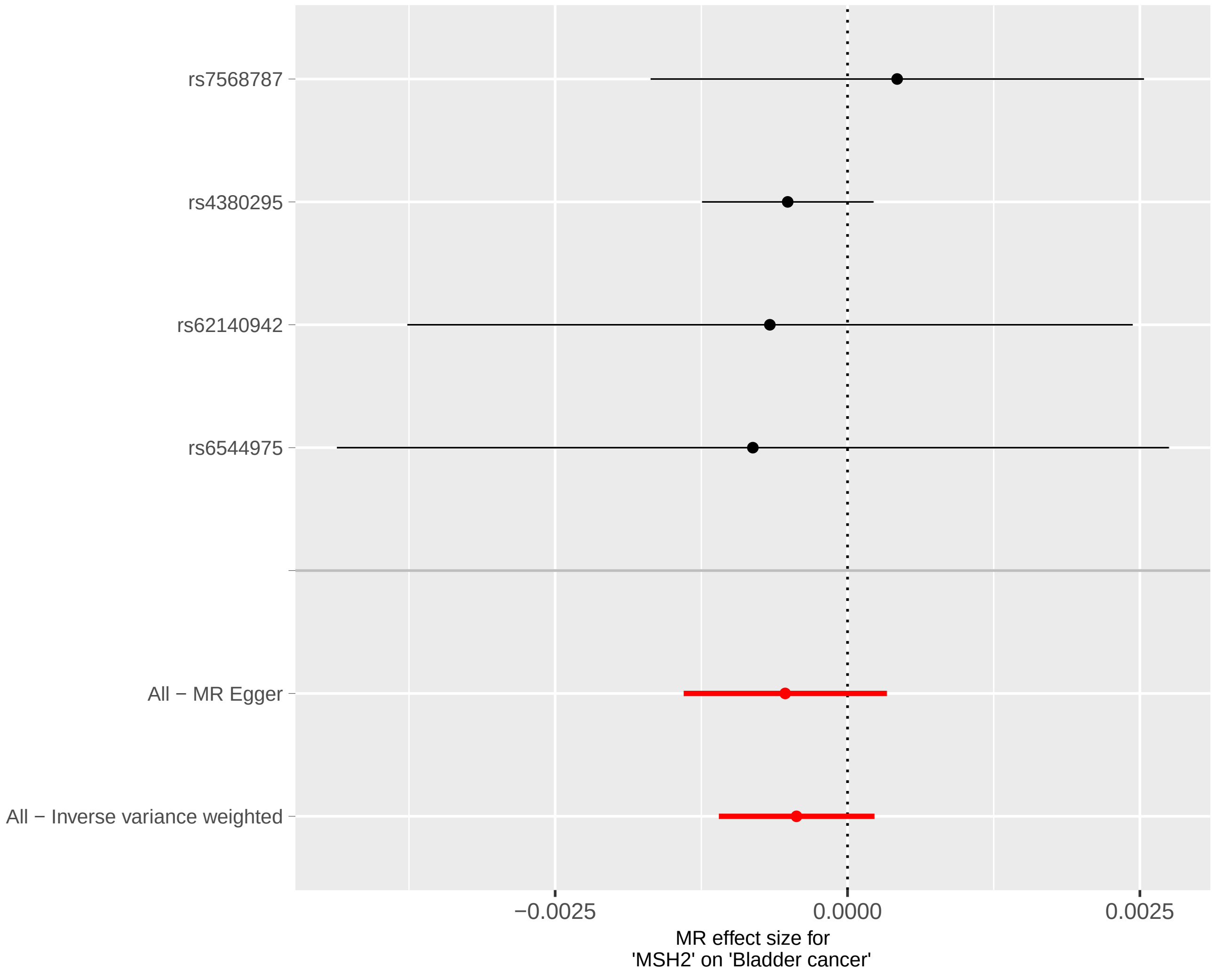
rs7568787
rs4380295
rs62140942
rs6544975
All − MR Egger
All − Inverse variance weighted
−0.0025
0.0000
0.0025
MR effect size for
'MSH2' on 'Bladder cancer'

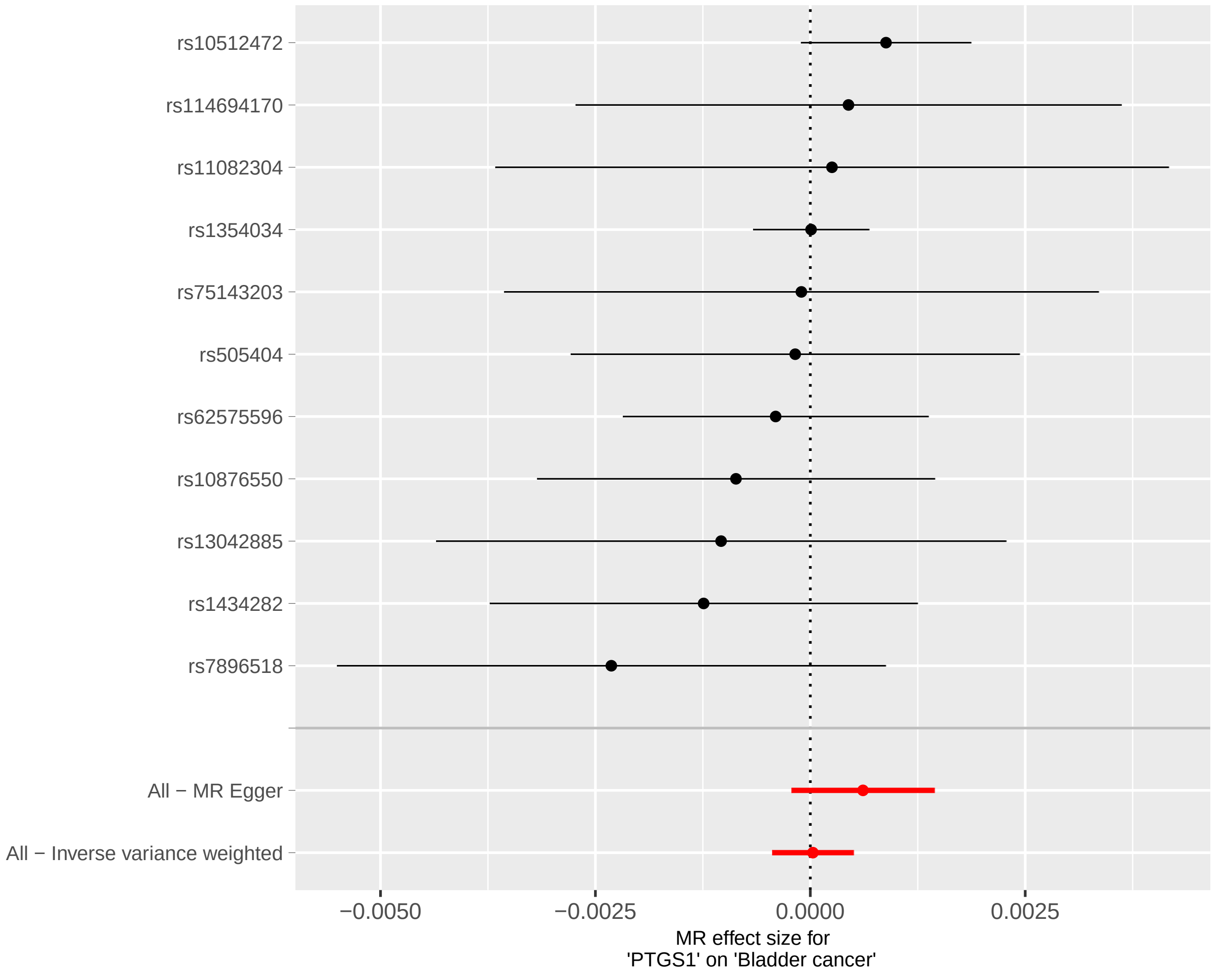
rs10512472
rs114694170
rs11082304
rs1354034
rs75143203
rs505404
rs62575596
rs10876550
rs13042885
rs1434282
rs7896518
All − MR Egger
All − Inverse variance weighted
−0.0050
−0.0025
0.0000
0.0025
MR effect size for
'PTGS1' on 'Bladder cancer'

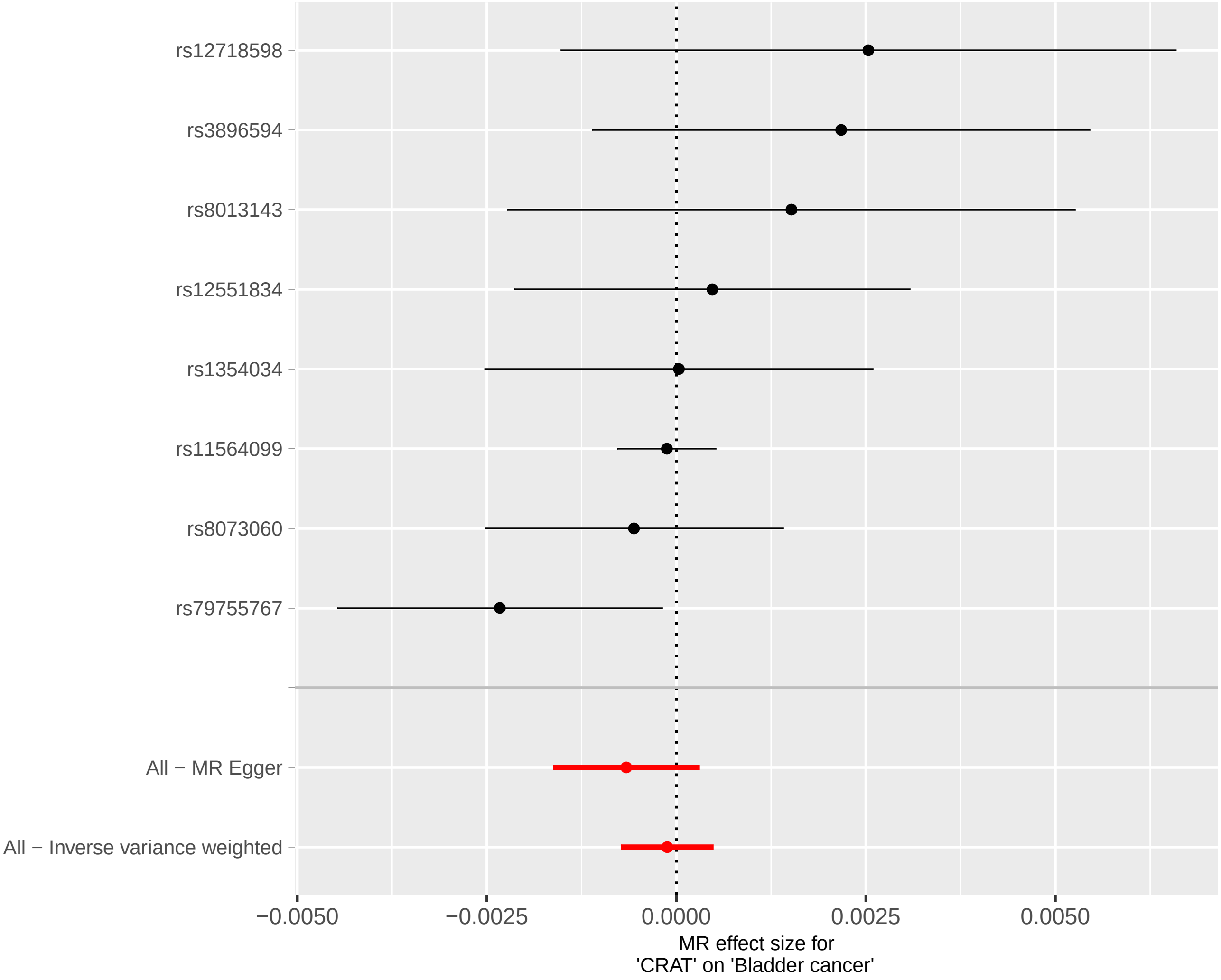
rs12718598
rs3896594
rs8013143
rs12551834
rs1354034
rs11564099
rs8073060
rs79755767
All − MR Egger
All − Inverse variance weighted
−0.0050
−0.0025
0.0000
0.0025
0.0050
MR effect size for
'CRAT' on 'Bladder cancer'

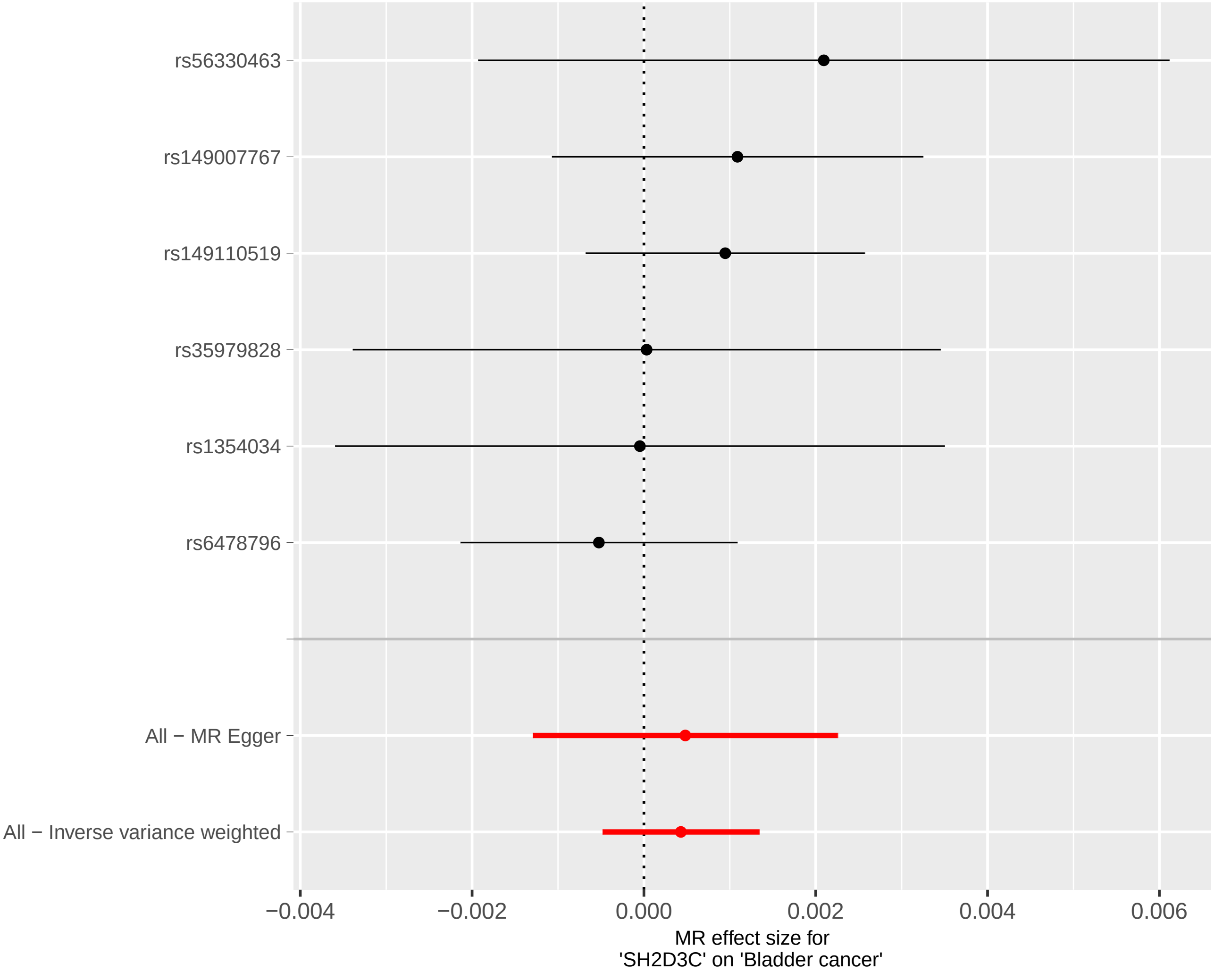

rs56330463
rs149007767
rs149110519
rs35979828
rs1354034
rs6478796
All − MR Egger
All − Inverse variance weighted
−0.004
−0.002
0.000
0.002
0.004
0.006
MR effect size for
'SH2D3C' on 'Bladder cancer'

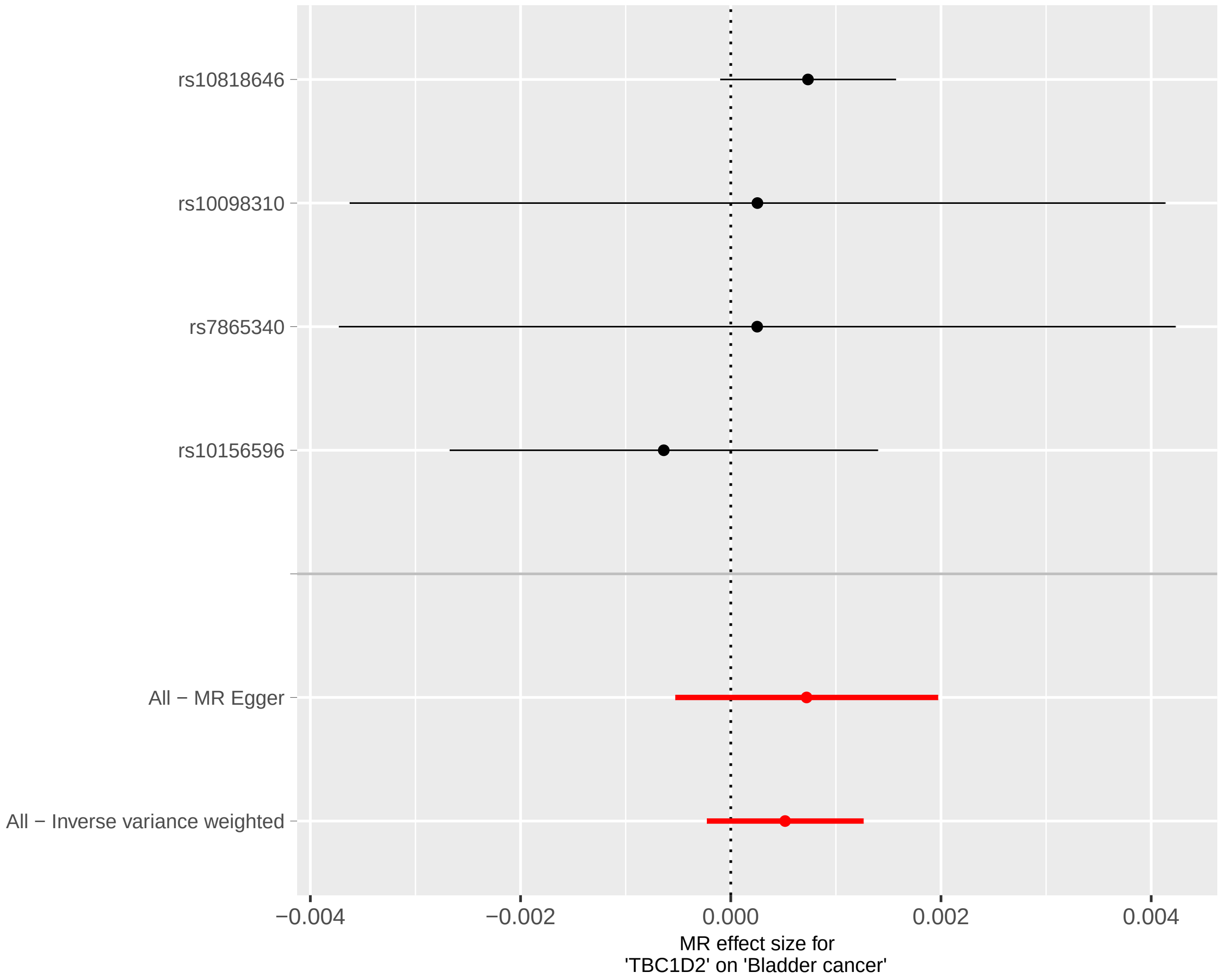

rs10818646
rs10098310
rs7865340
rs10156596
All − MR Egger
All − Inverse variance weighted
−0.004
−0.002
0.000
0.002
0.004
MR effect size for
'TBC1D2' on 'Bladder cancer'

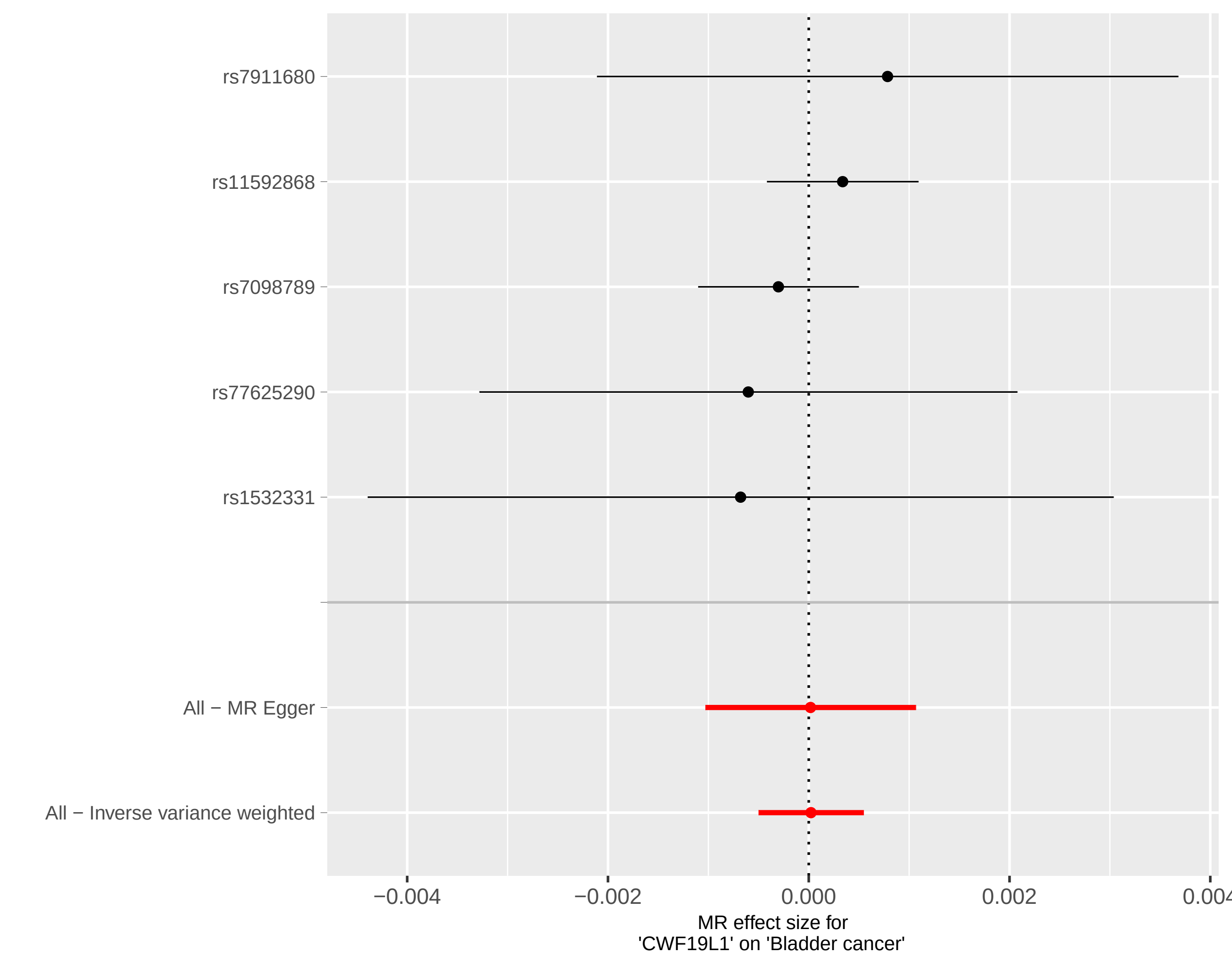

rs7911680
rs11592868
rs7098789
rs77625290
rs1532331
All − MR Egger
All − Inverse variance weighted
−0.004
−0.002
0.000
0.002
MR effect size for
'CWF19L1' on 'Bladder cancer'

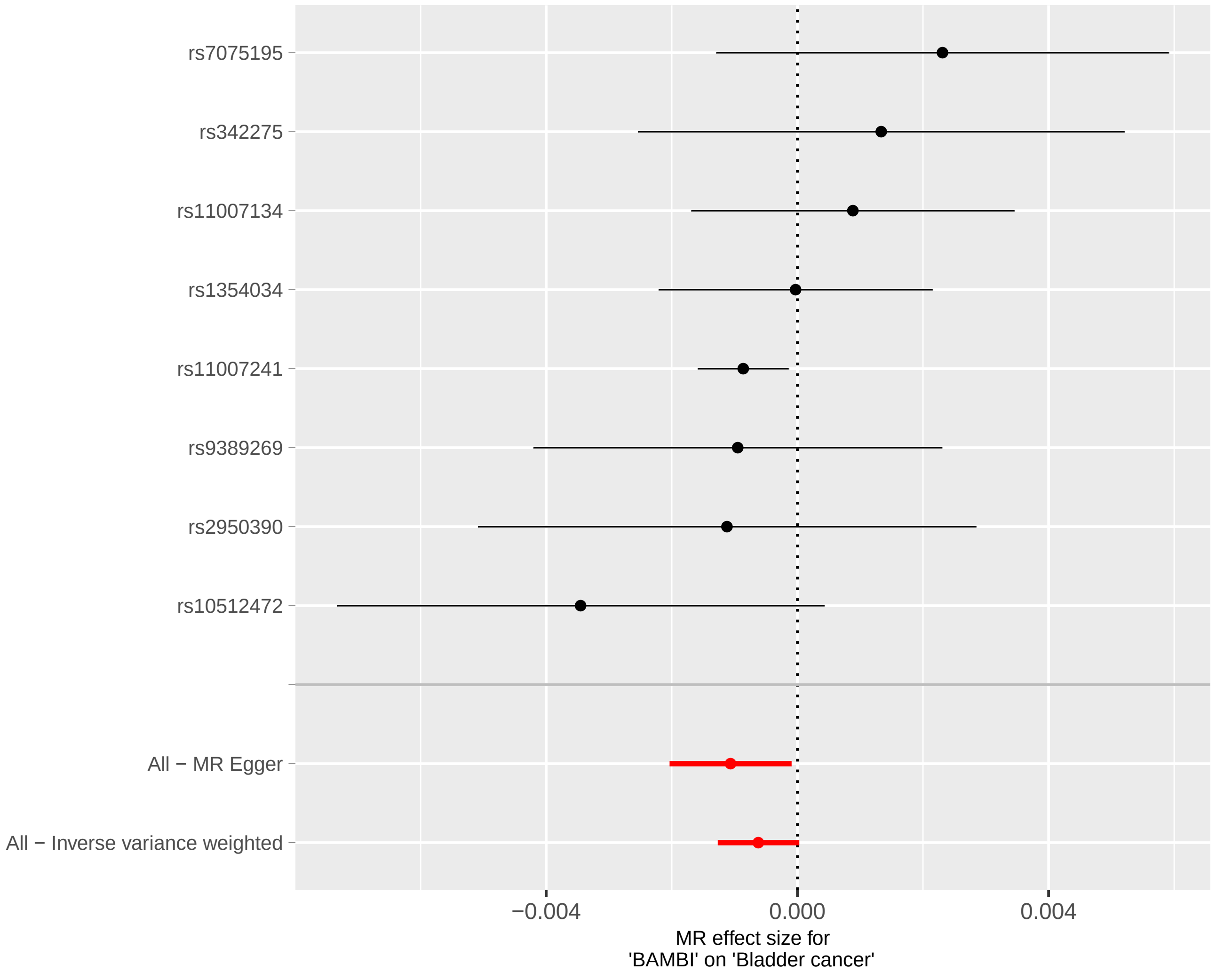
rs7075195
rs342275
rs11007134
rs1354034
rs11007241
rs9389269
rs2950390
rs10512472
All − MR Egger
All − Inverse variance weighted
−0.004
0.000
0.004
MR effect size for
'BAMBI' on 'Bladder cancer'

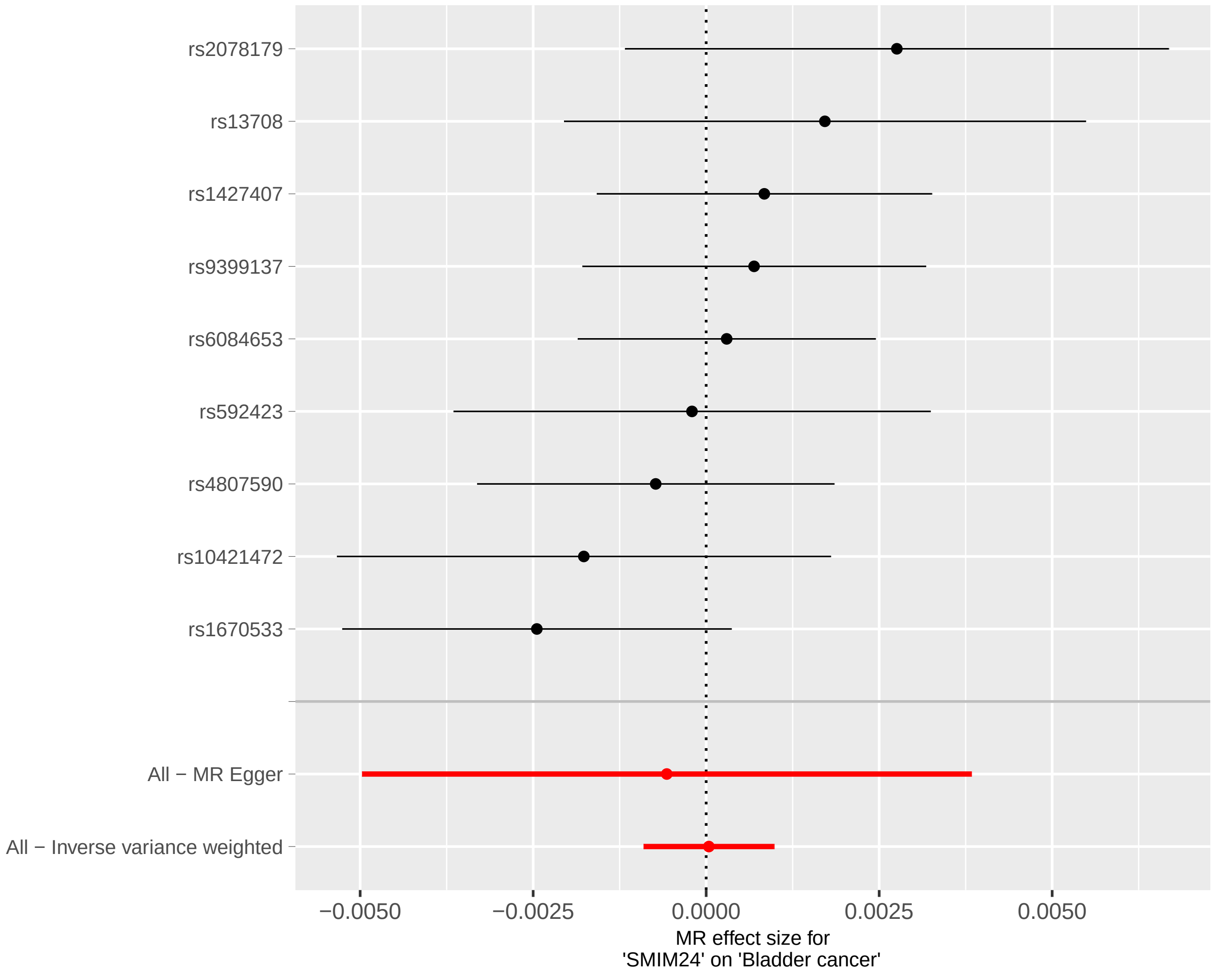

rs2078179
rs13708
rs1427407
rs9399137
rs6084653
rs592423
rs4807590
rs10421472
rs1670533
All − MR Egger
All − Inverse variance weighted
−0.0050
−0.0025
0.0000
0.0025
0.0050
MR effect size for
'SMIM24' on 'Bladder cancer'

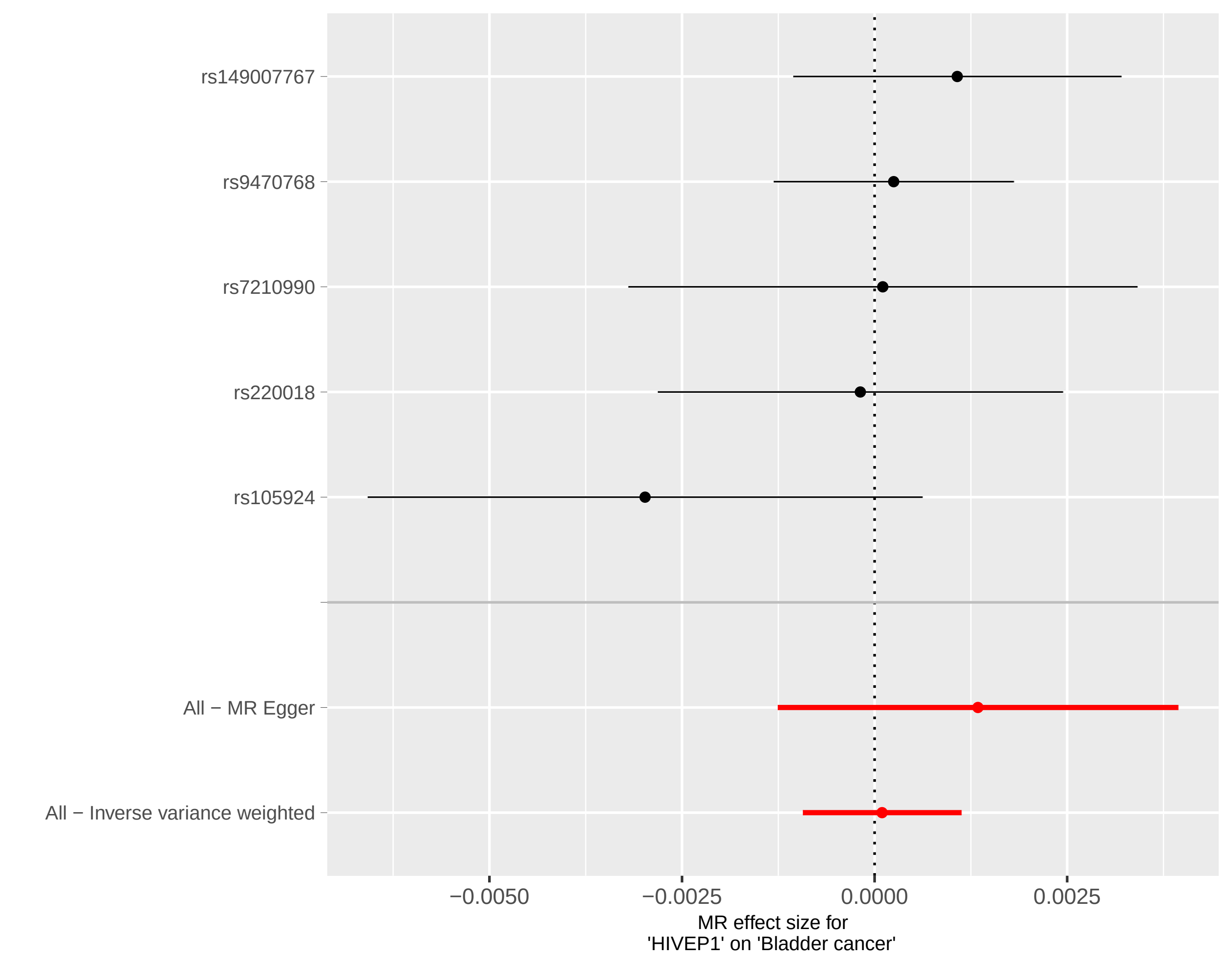
rs149007767
rs9470768
rs7210990
rs220018
rs105924
All − MR Egger
All − Inverse variance weighted
−0.0050
−0.0025
0.0000
0.0025
MR effect size for
'HIVEP1' on 'Bladder cancer'

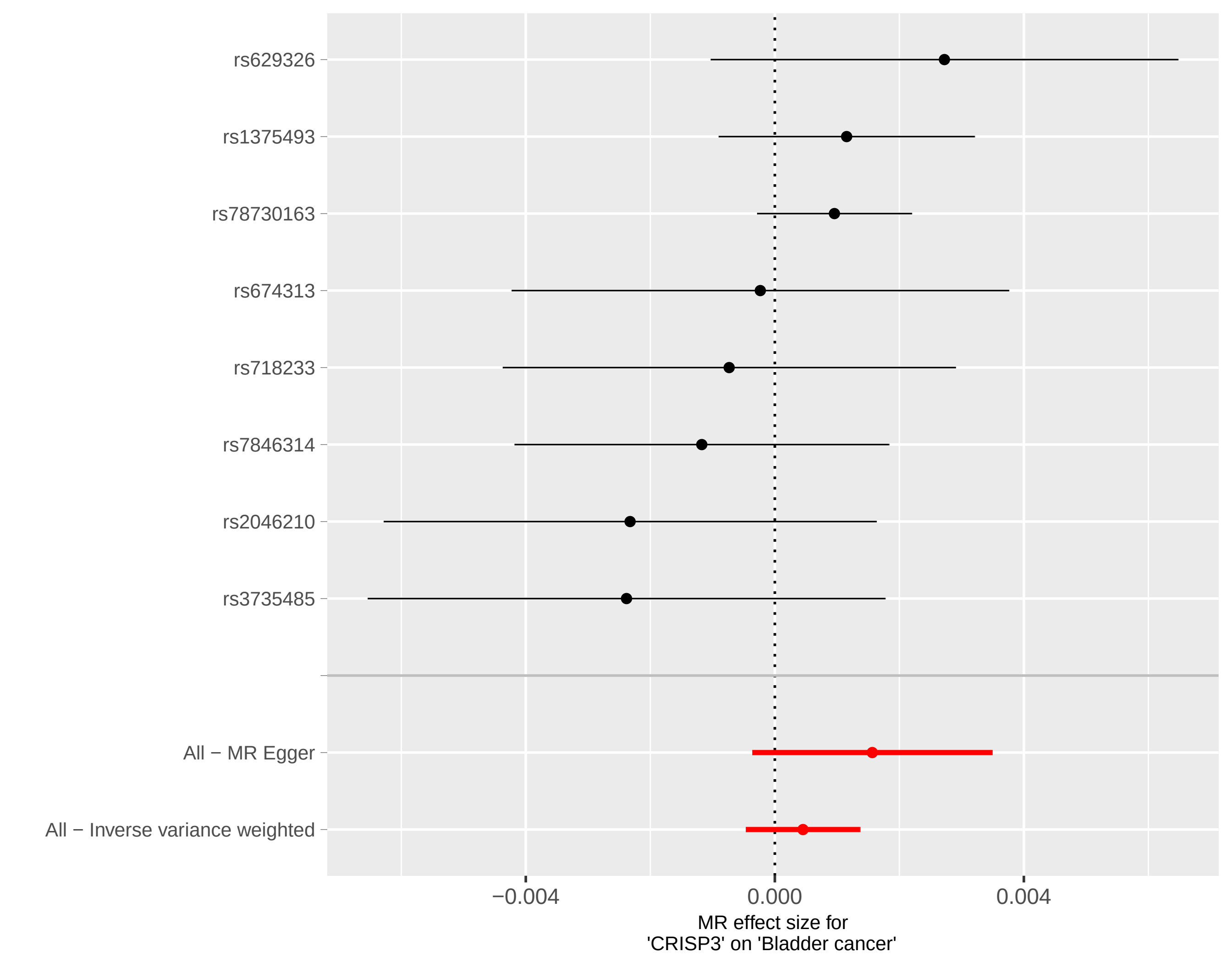
rs629326
rs1375493
rs78730163
rs674313
rs718233
rs7846314
rs2046210
rs3735485
All − MR Egger
All − Inverse variance weighted
−0.004
0.000
0.004
MR effect size for
'CRISP3' on 'Bladder cancer'

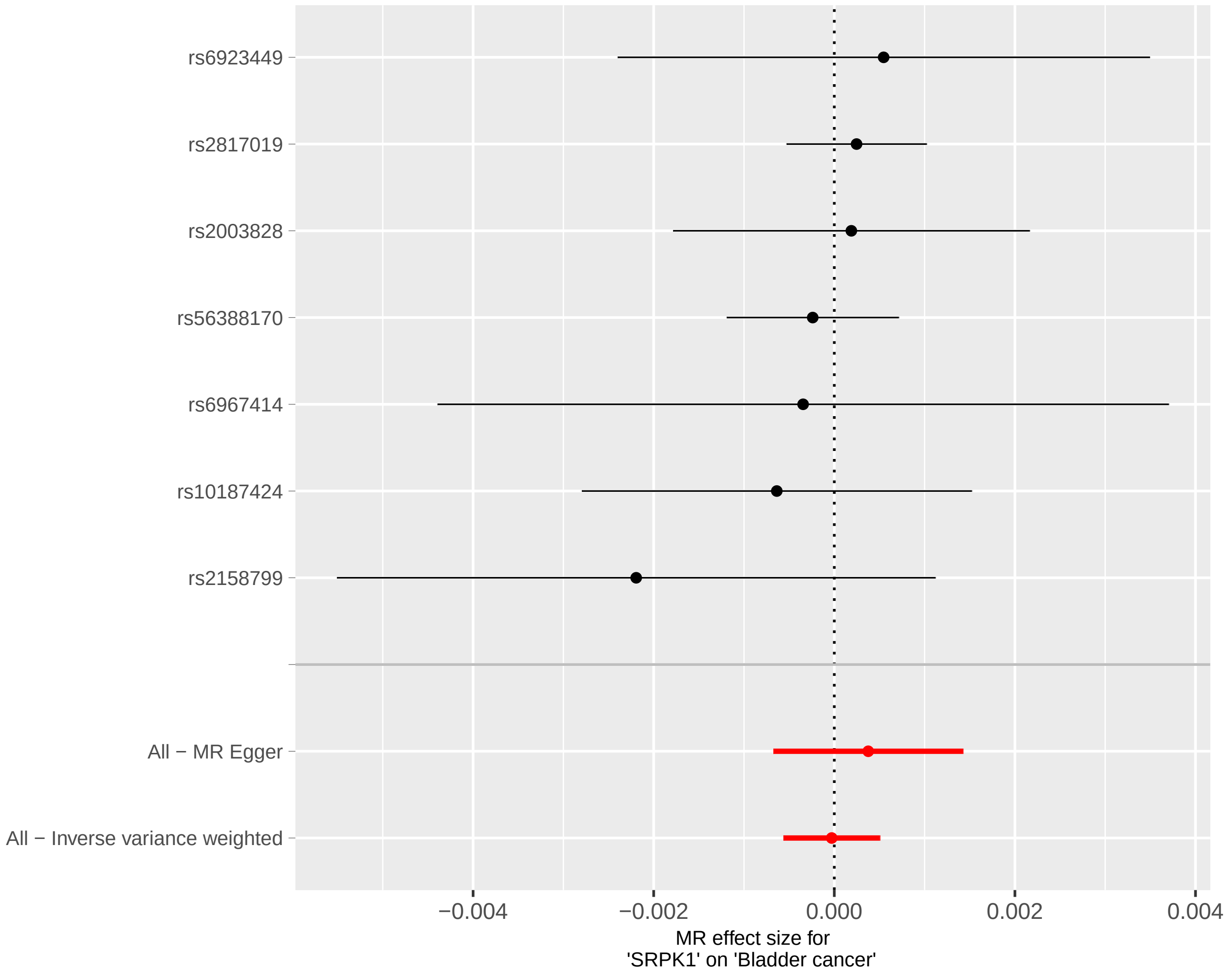

rs6923449
rs2817019
rs2003828
rs56388170
rs6967414
rs10187424
rs2158799
All − MR Egger
All − Inverse variance weighted
−0.004
−0.002
0.000
0.002
0.004
MR effect size for
'SRPK1' on 'Bladder cancer'

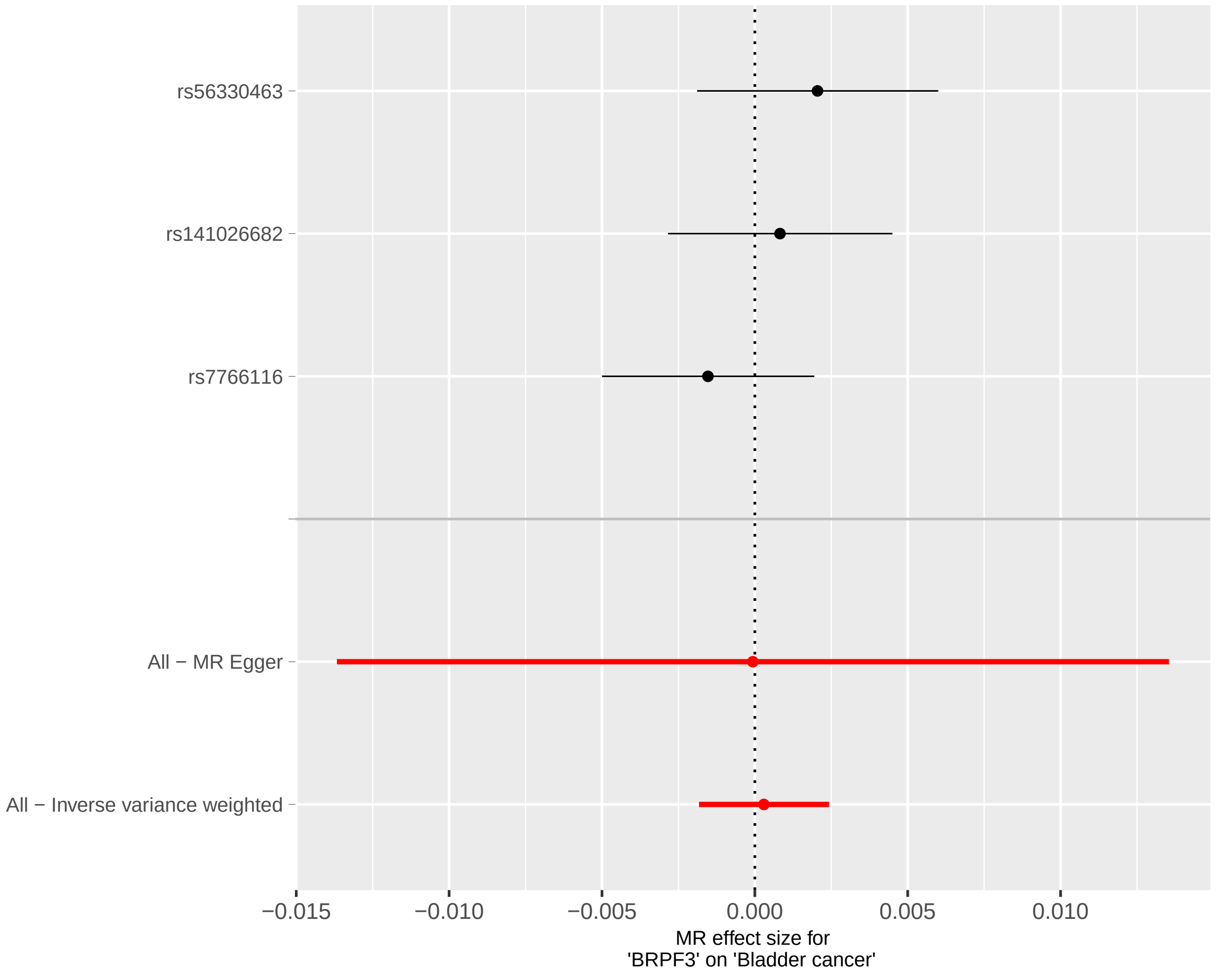

rs56330463
rs141026682
rs7766116
All − MR Egger
All − Inverse variance weighted
−0.015
−0.010
−0.005
0.000
0.005
0.010
MR effect size for
'BRPF3' on 'Bladder cancer'

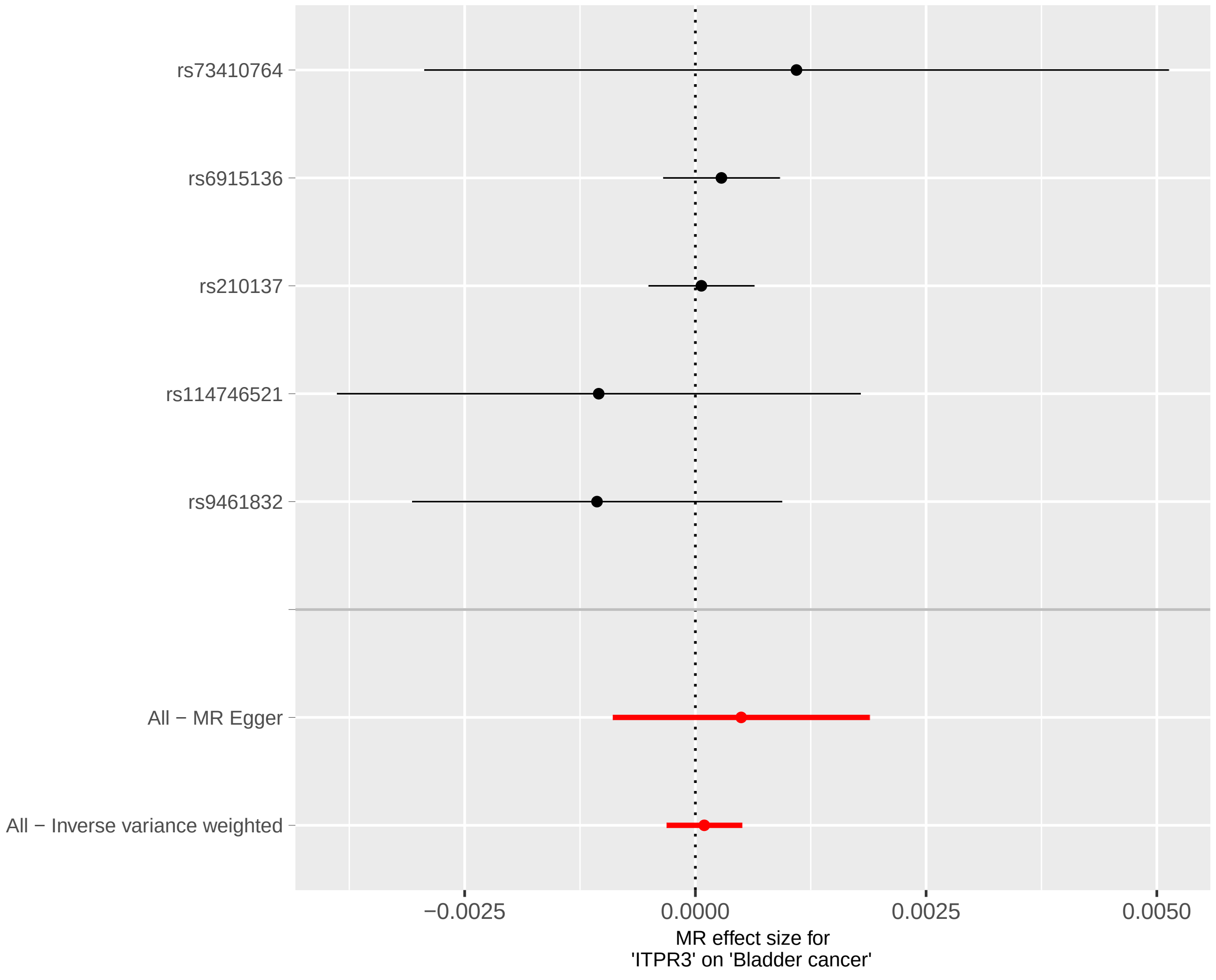

rs73410764
rs6915136
rs210137
rs114746521
rs9461832
All − MR Egger
All − Inverse variance weighted
−0.0025
0.0000
0.0025
0.0050
MR effect size for
'ITPR3' on 'Bladder cancer'

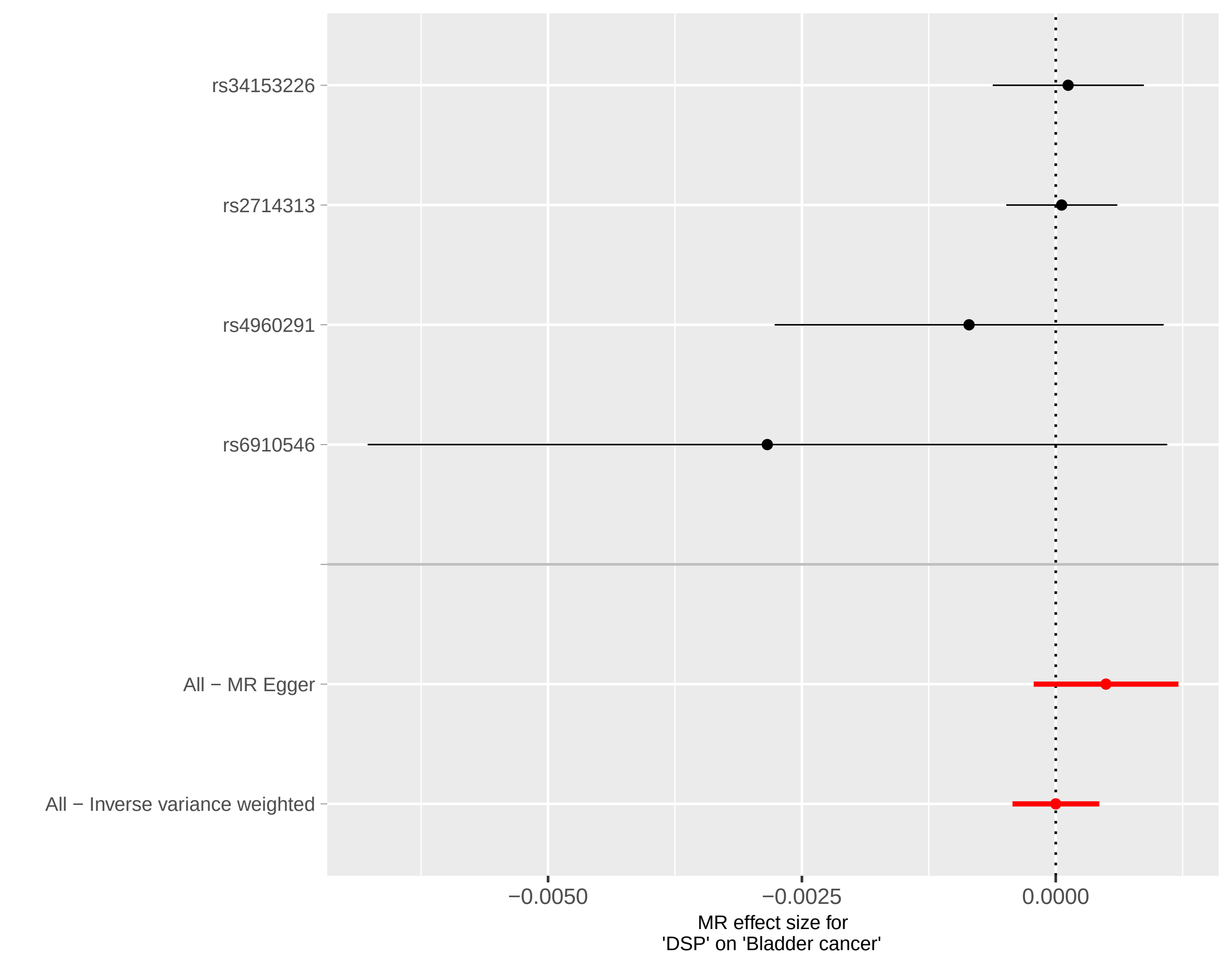
rs34153226
rs2714313
rs4960291
rs6910546
All − MR Egger
All − Inverse variance weighted
−0.0050
−0.0025
0.0000
MR effect size for
'DSP' on 'Bladder cancer'

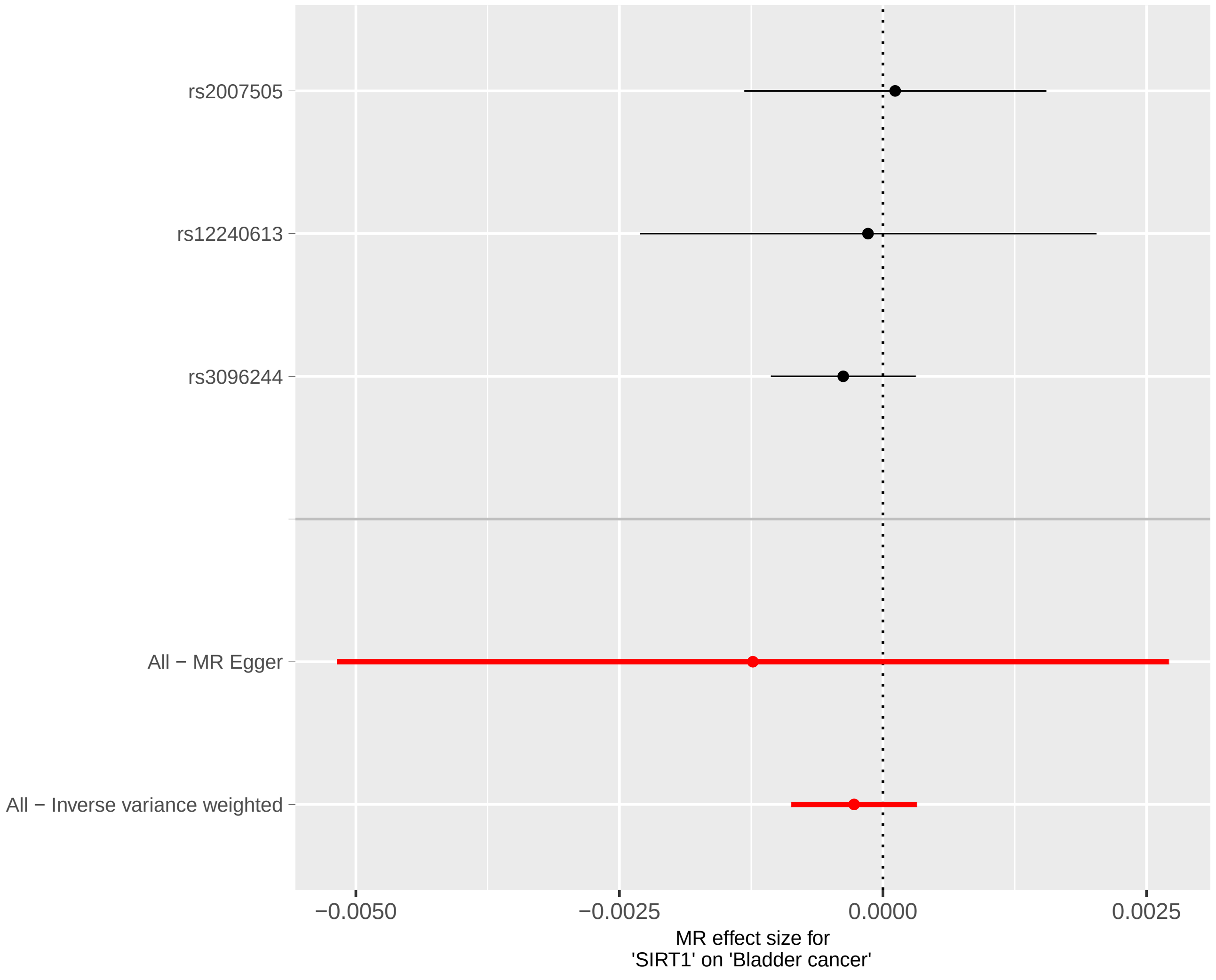
rs2007505
rs12240613
rs3096244
All − MR Egger
All − Inverse variance weighted
−0.0050
−0.0025
0.0000
0.0025
MR effect size for
'SIRT1' on 'Bladder cancer'

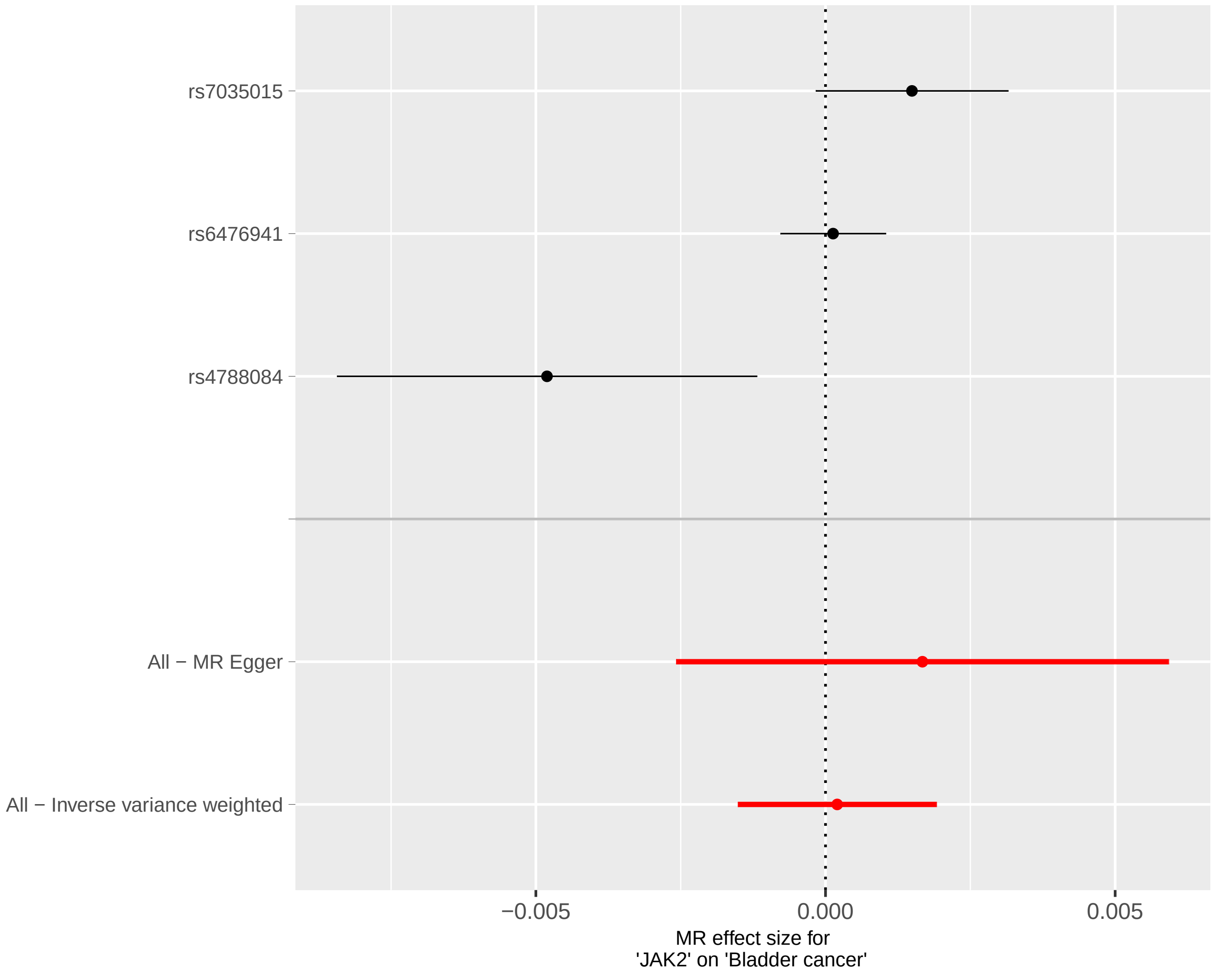

rs7035015
rs6476941
rs4788084
All − MR Egger
All − Inverse variance weighted
−0.005
0.000
0.005
MR effect size for
'JAK2' on 'Bladder cancer'

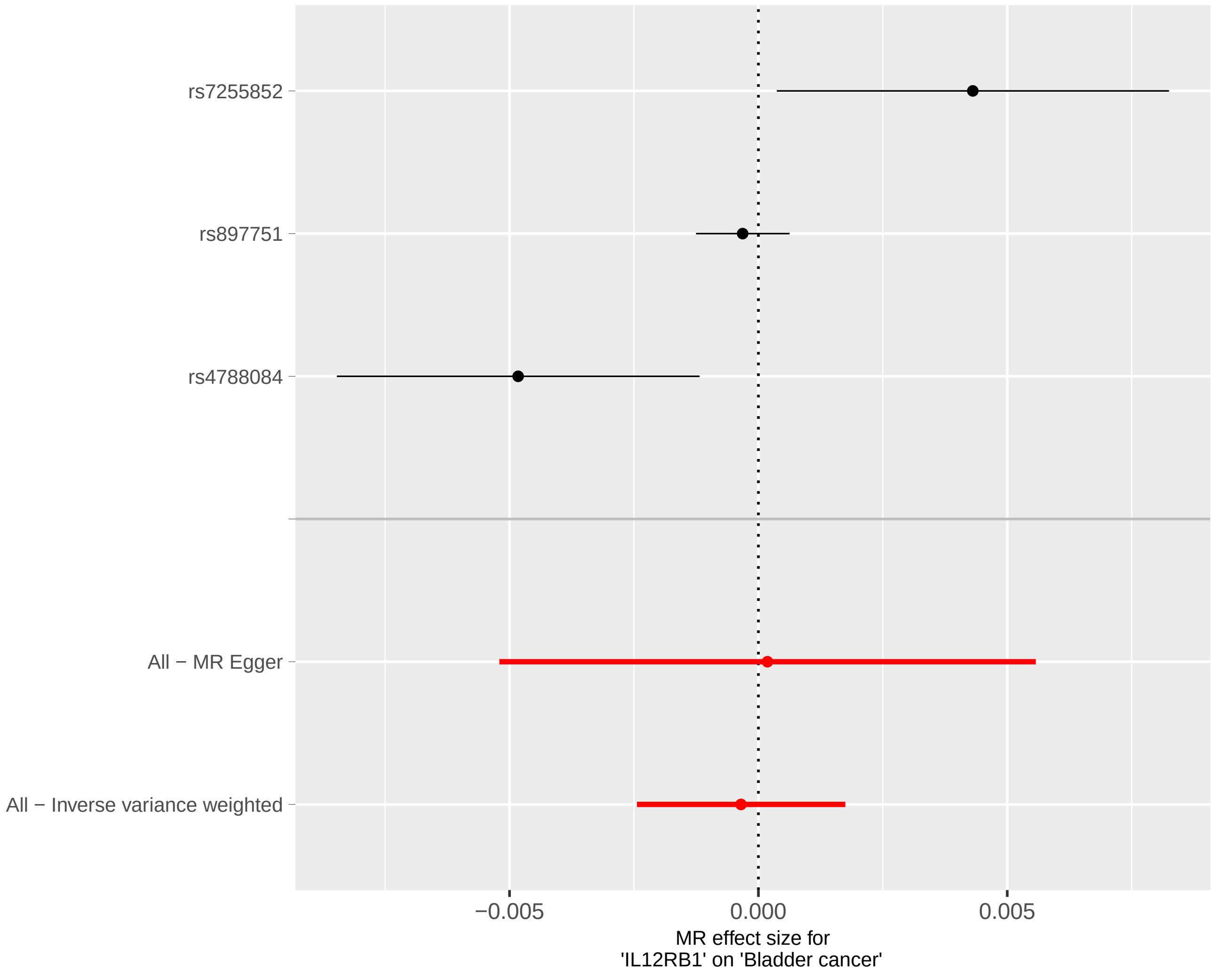
rs7255852
rs897751
rs4788084
All − MR Egger
All − Inverse variance weighted
−0.005
0.000
0.005
MR effect size for
'IL12RB1' on 'Bladder cancer'

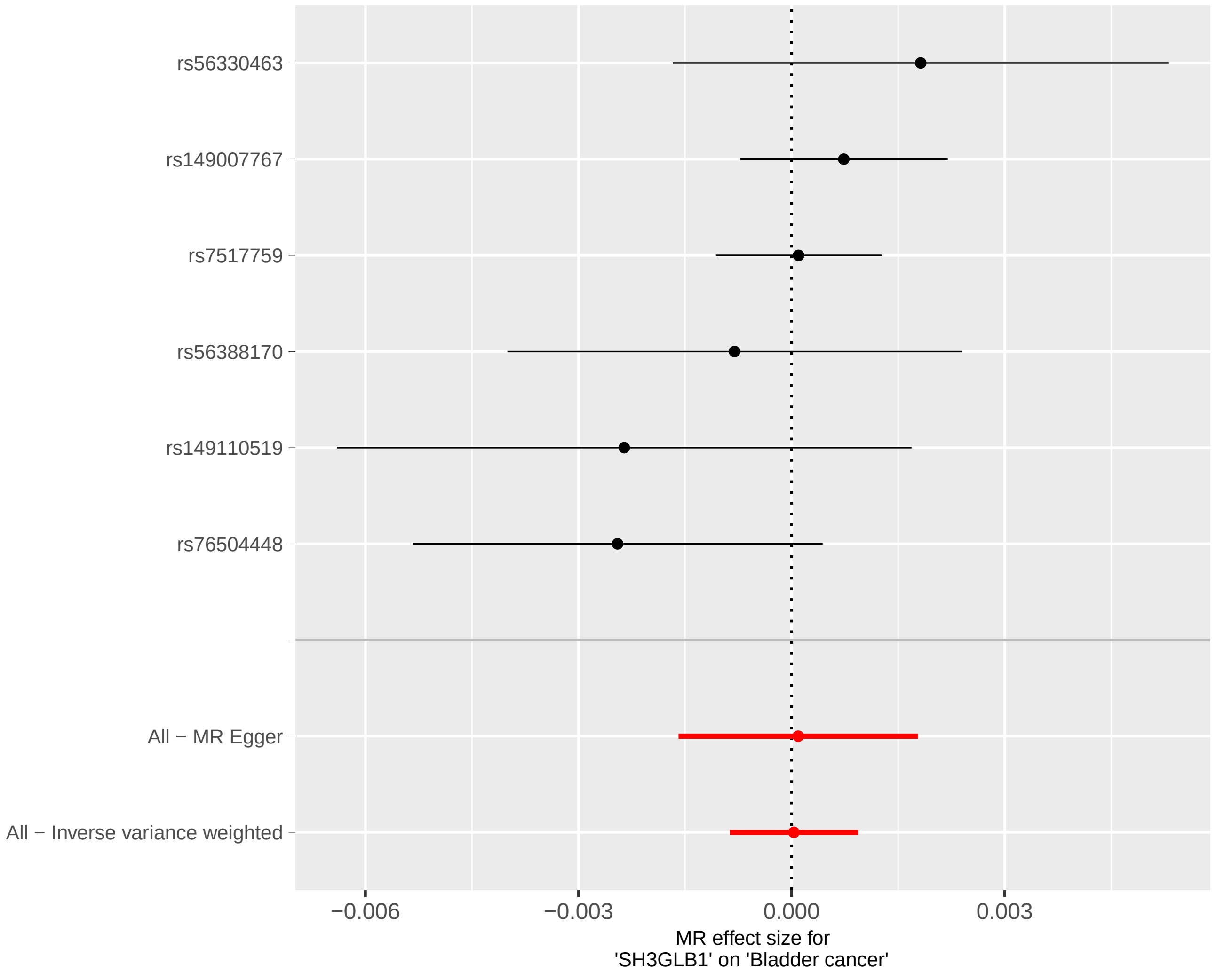

rs56330463
rs149007767
rs7517759
rs56388170
rs149110519
rs76504448
All − MR Egger
All − Inverse variance weighted
−0.006
−0.003
0.000
0.003
MR effect size for
'SH3GLB1' on 'Bladder cancer'

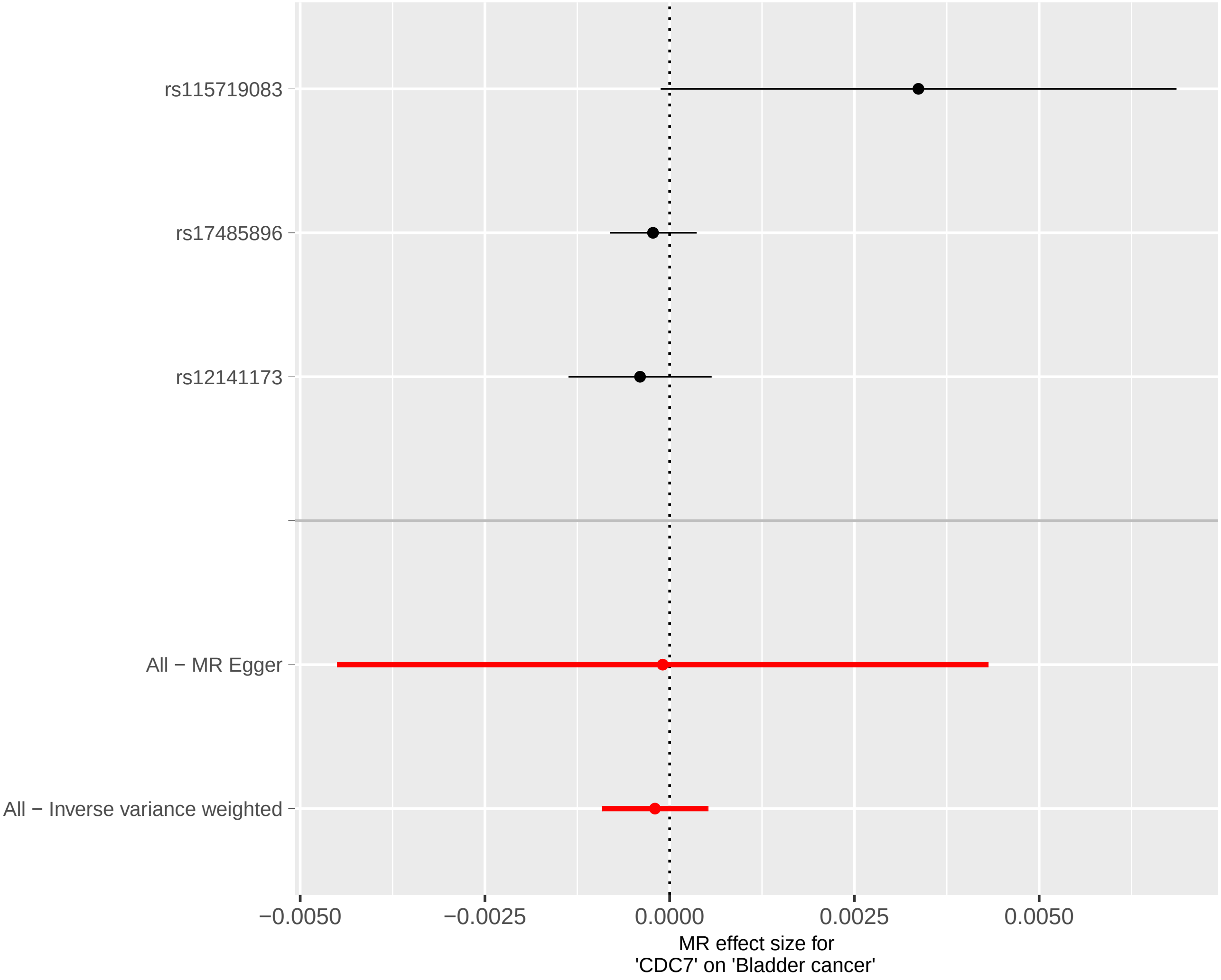
rs115719083
rs17485896
rs12141173
All − MR Egger
All − Inverse variance weighted
−0.0050
−0.0025
0.0000
0.0025
0.0050
MR effect size for
'CDC7' on 'Bladder cancer'

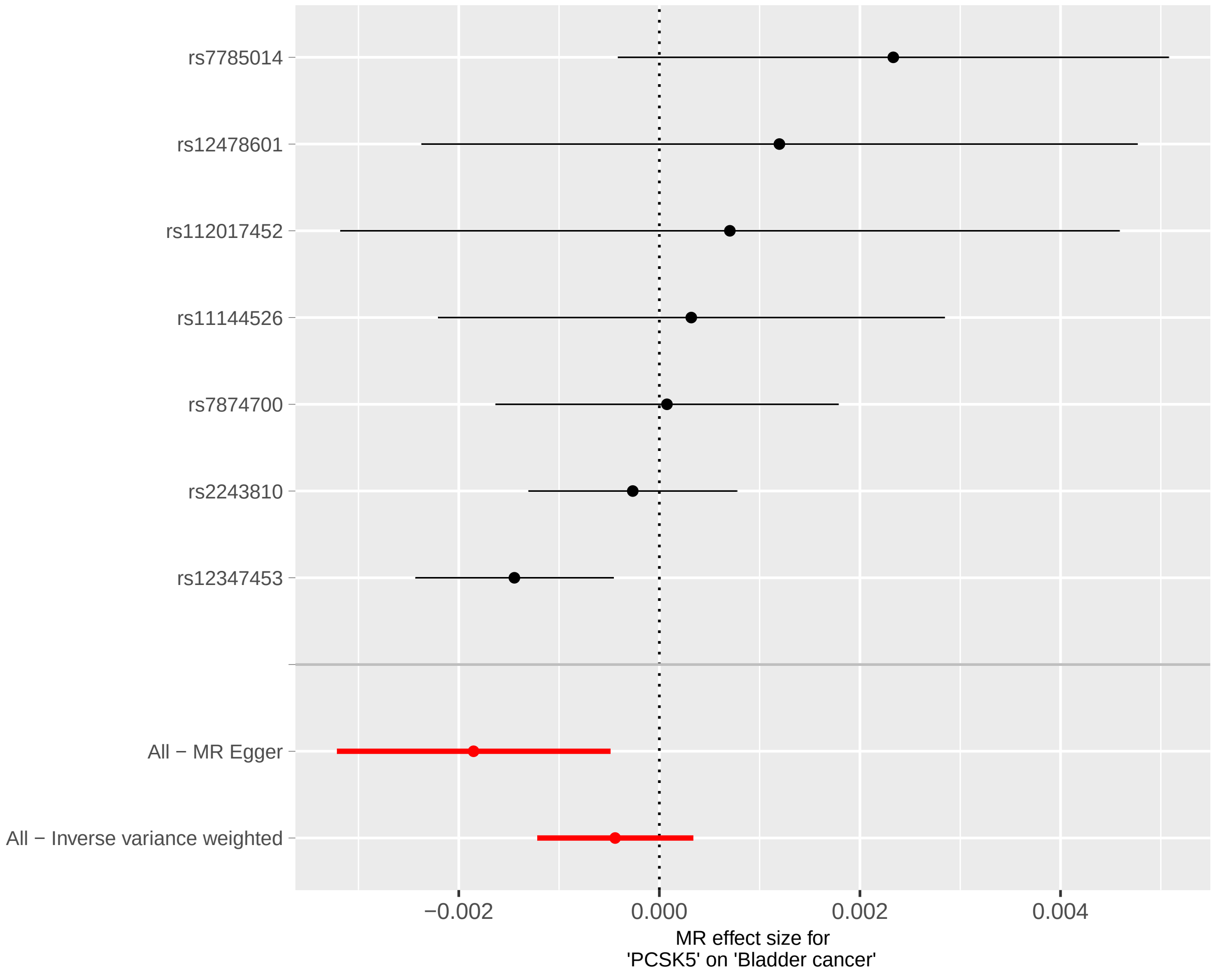

rs7785014
rs12478601
rs112017452
rs11144526
rs7874700
rs2243810
rs12347453
All − MR Egger
All − Inverse variance weighted
−0.002
0.000
0.002
0.004
MR effect size for
'PCSK5' on 'Bladder cancer'

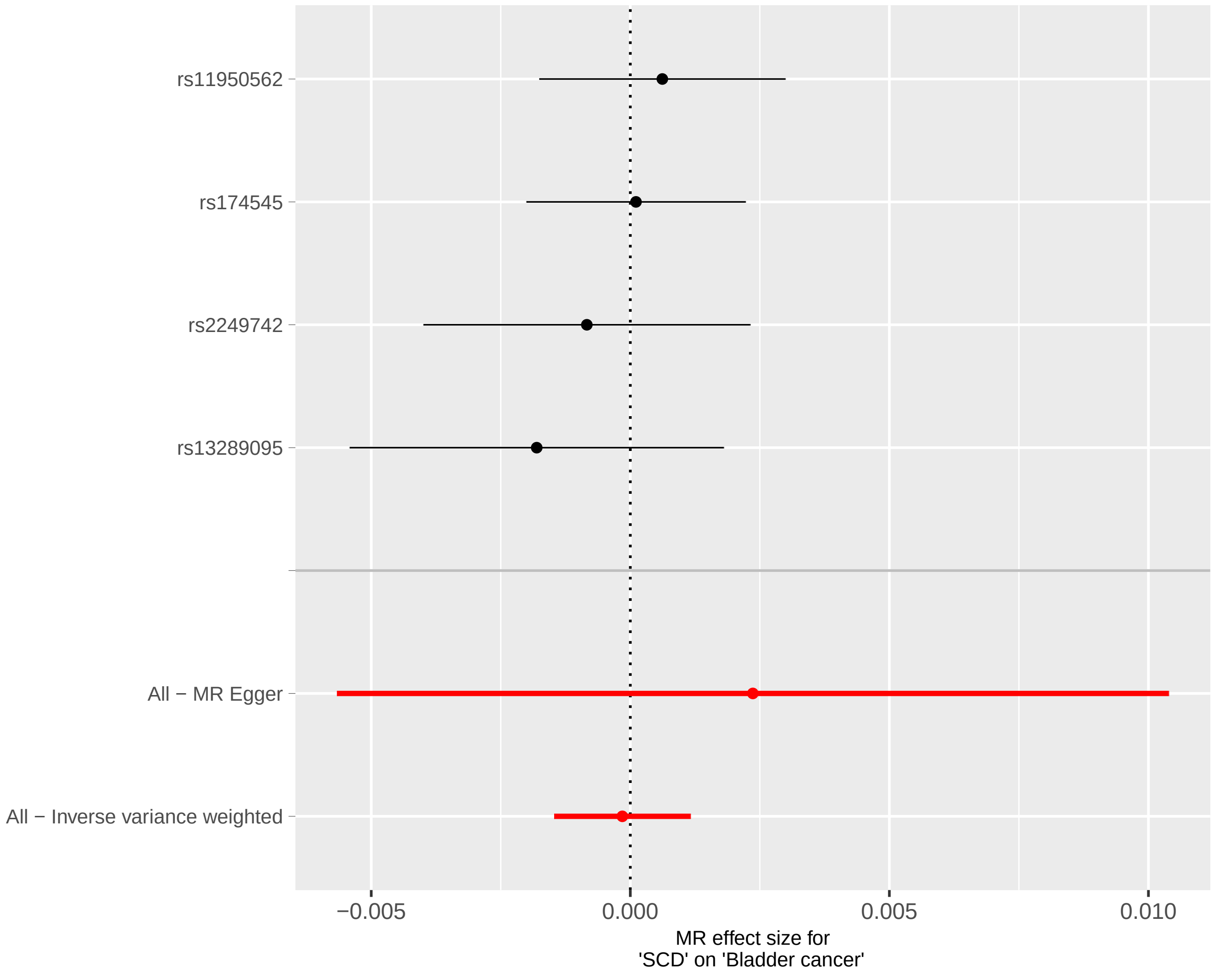
rs11950562
rs174545
rs2249742
rs13289095
All − MR Egger
All − Inverse variance weighted
−0.005
0.000
0.005
0.010
MR effect size for
'SCD' on 'Bladder cancer'

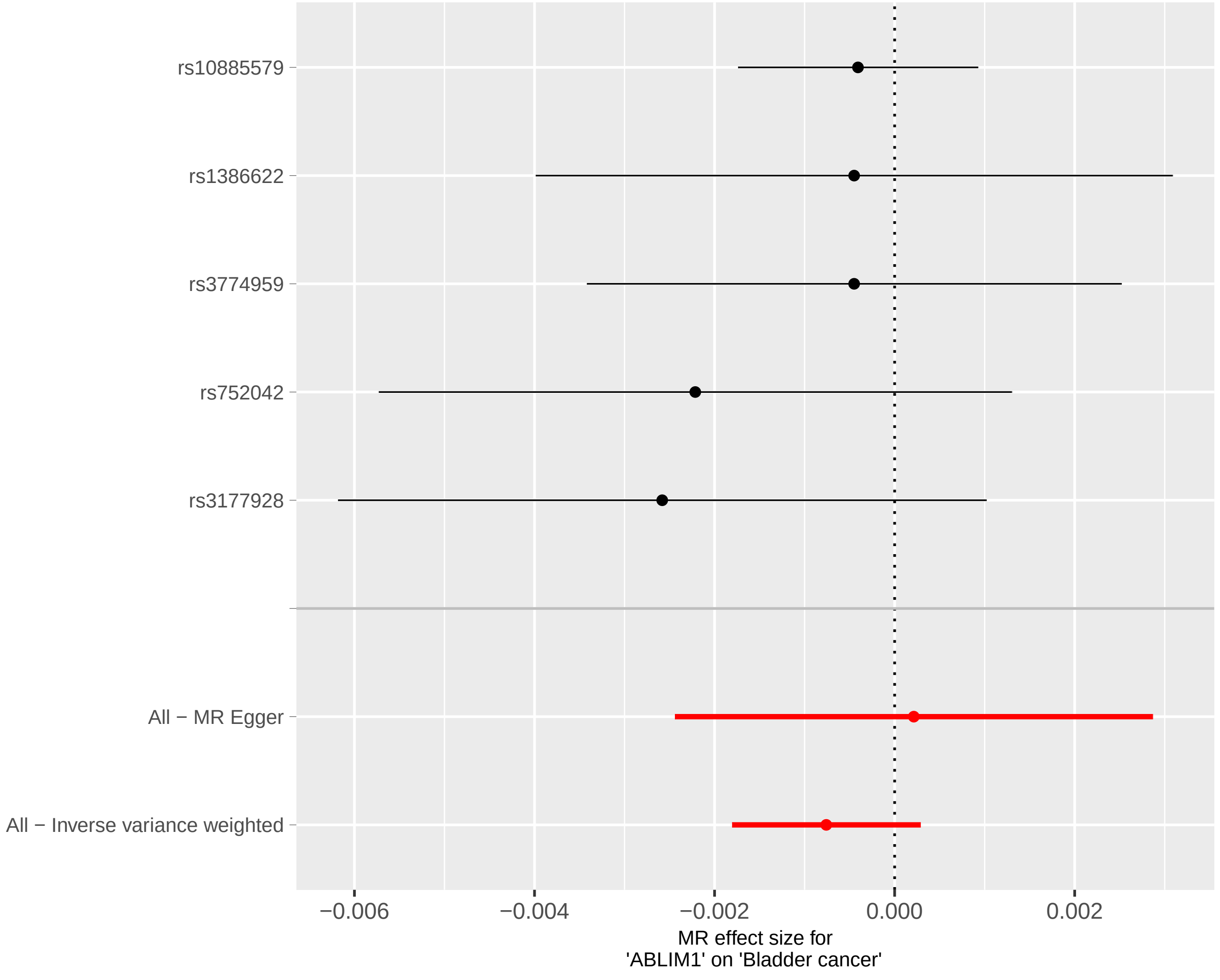

rs10885579
rs1386622
rs3774959
rs752042
rs3177928
All − MR Egger
All − Inverse variance weighted
−0.006
−0.004
−0.002
0.000
0.002
MR effect size for
'ABLIM1' on 'Bladder cancer'

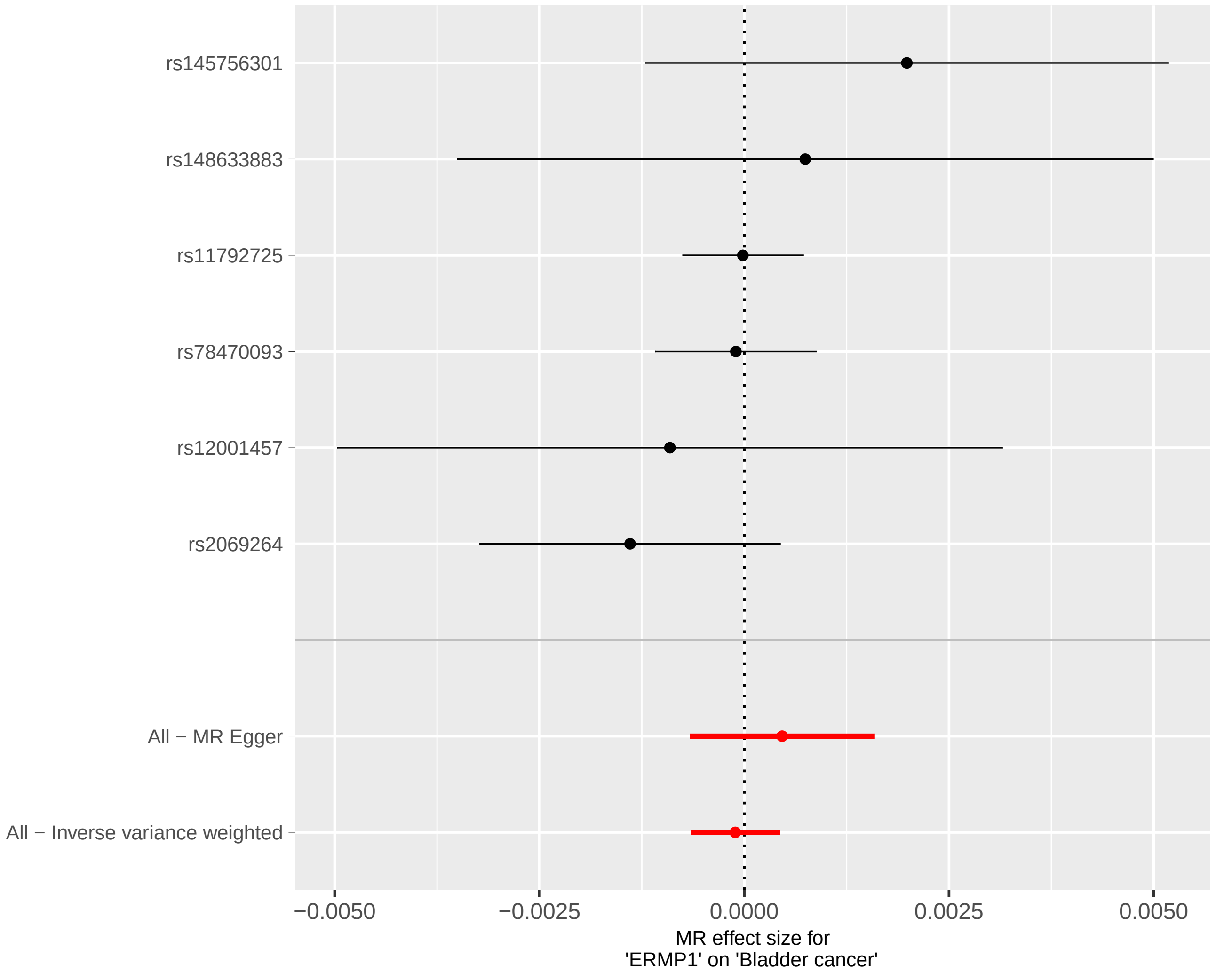
rs145756301
rs148633883
rs11792725
rs78470093
rs12001457
rs2069264
All − MR Egger
All − Inverse variance weighted
−0.0050
−0.0025
0.0000
0.0025
0.0050
MR effect size for
'ERMP1' on 'Bladder cancer'

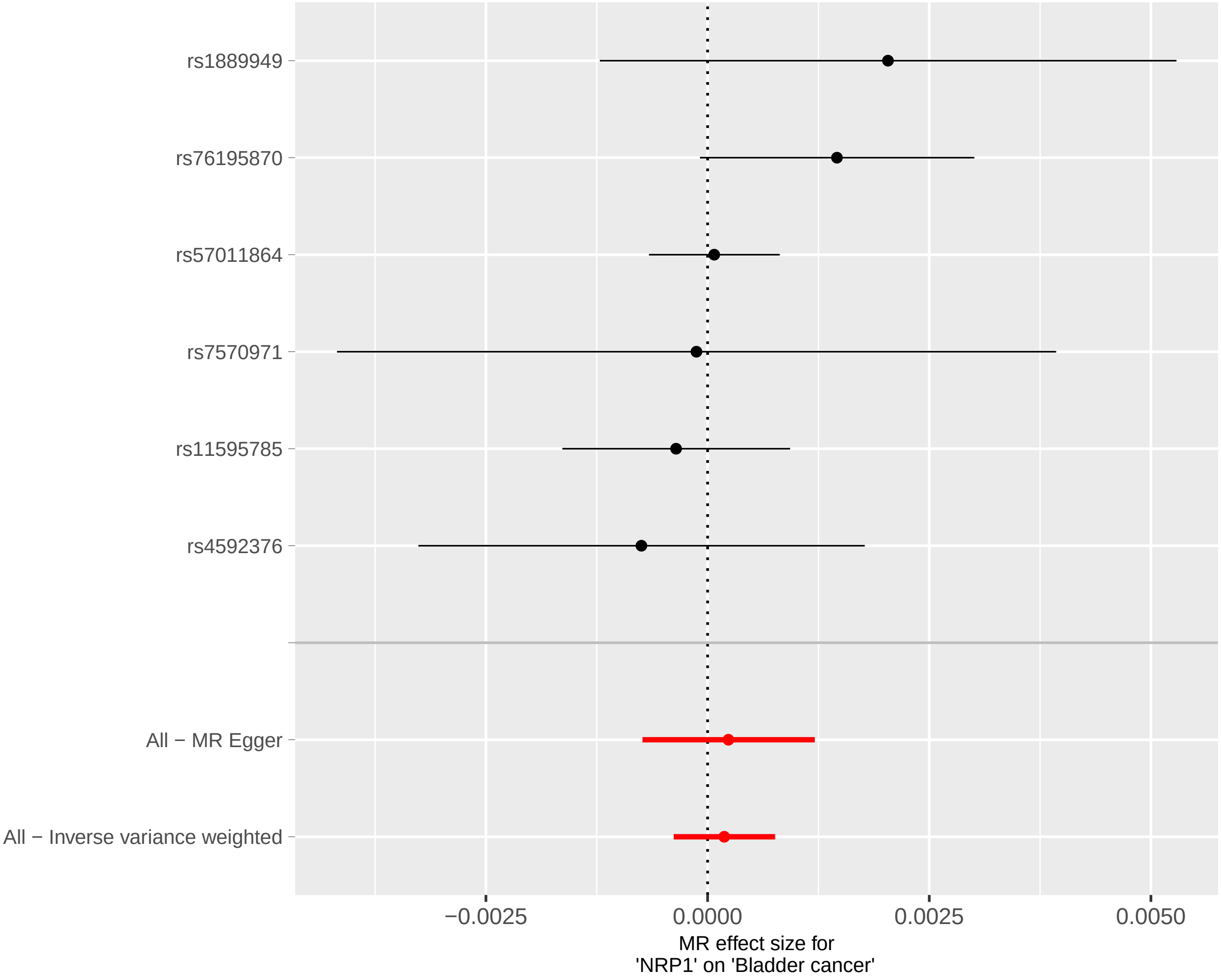

rs1889949
rs76195870
rs57011864
rs7570971
rs11595785
rs4592376
All − MR Egger
All − Inverse variance weighted
−0.0025
0.0000
0.0025
0.0050
MR effect size for
'NRP1' on 'Bladder cancer'

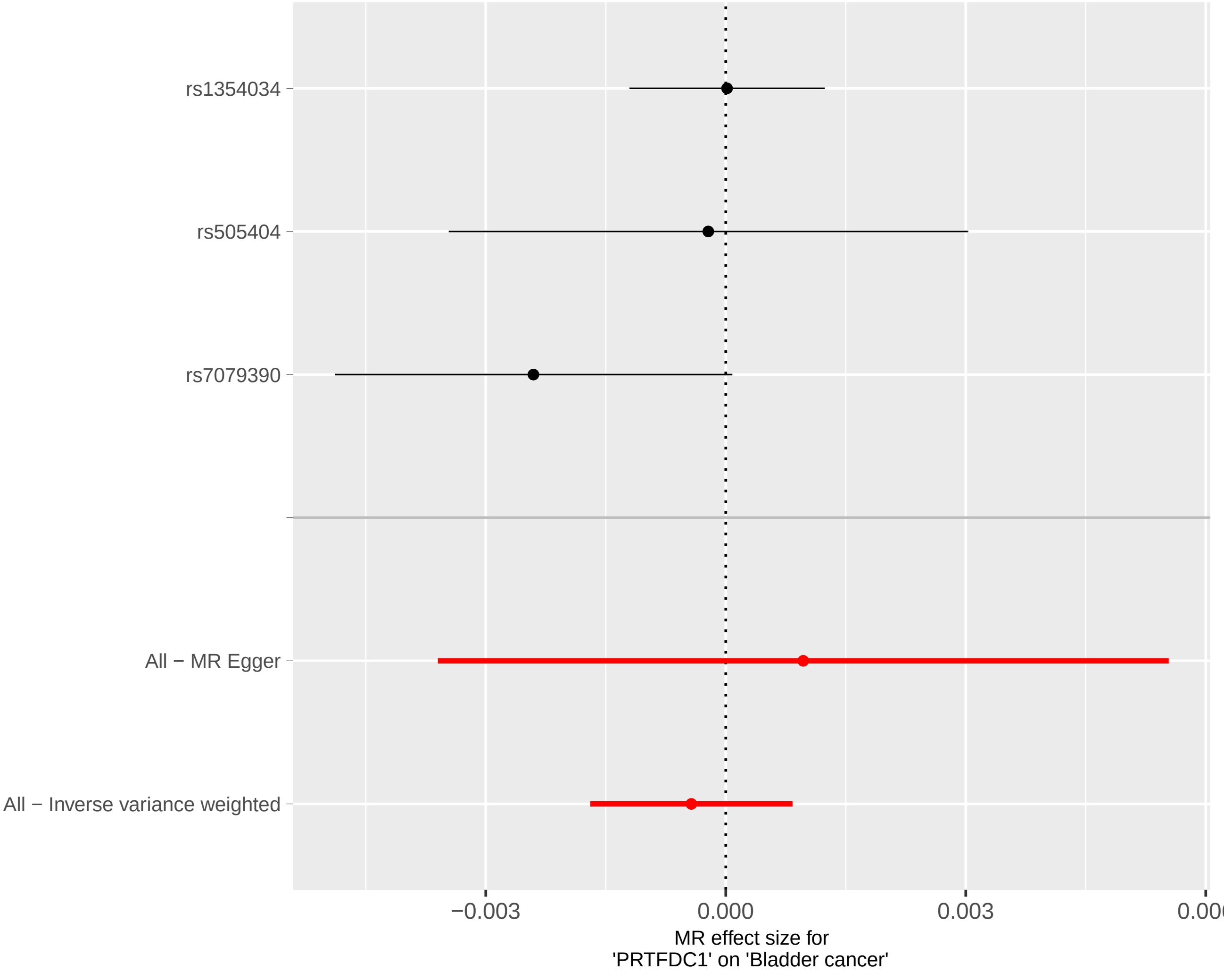

rs1354034
rs505404
rs7079390
All − MR Egger
All − Inverse variance weighted
−0.003
0.000
0.003
MR effect size for
'PRTFDC1' on 'Bladder cancer'

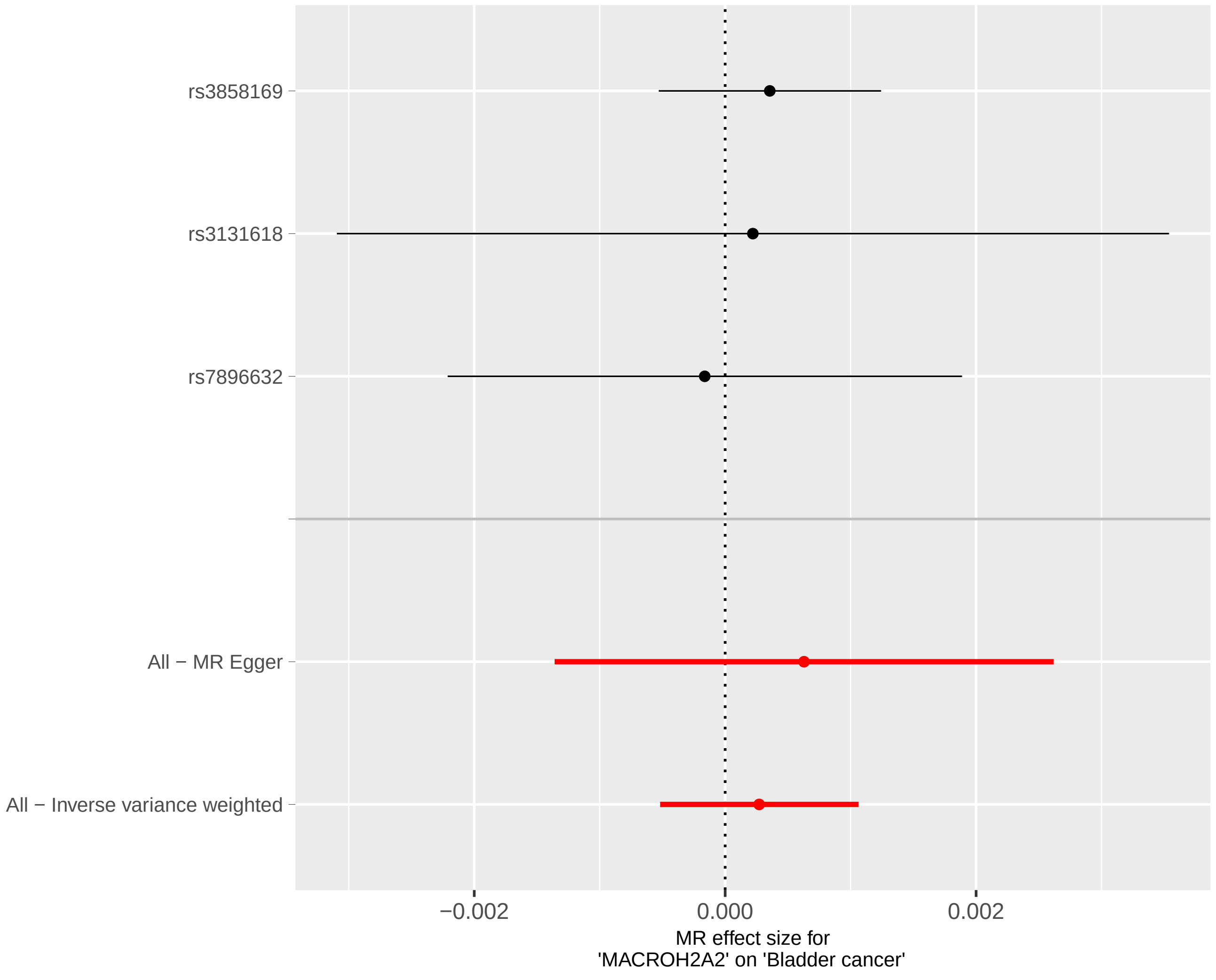

rs3858169
rs3131618
rs7896632
All − MR Egger
All − Inverse variance weighted
−0.002
0.000
0.002
MR effect size for
'MACROH2A2' on 'Bladder cancer'

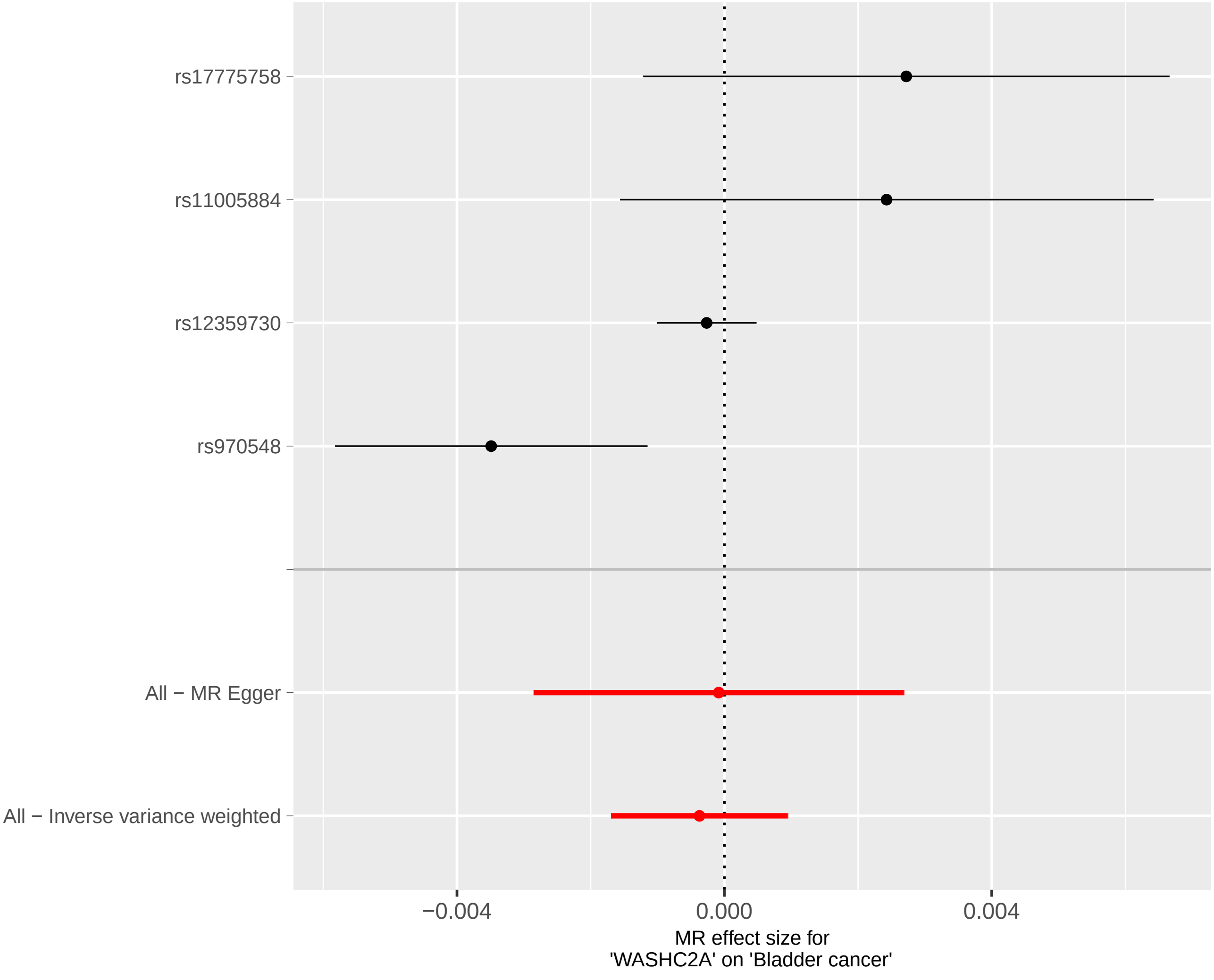

rs17775758
rs11005884
rs12359730
rs970548
All − MR Egger
All − Inverse variance weighted
−0.004
0.000
0.004
MR effect size for
'WASHC2A' on 'Bladder cancer'

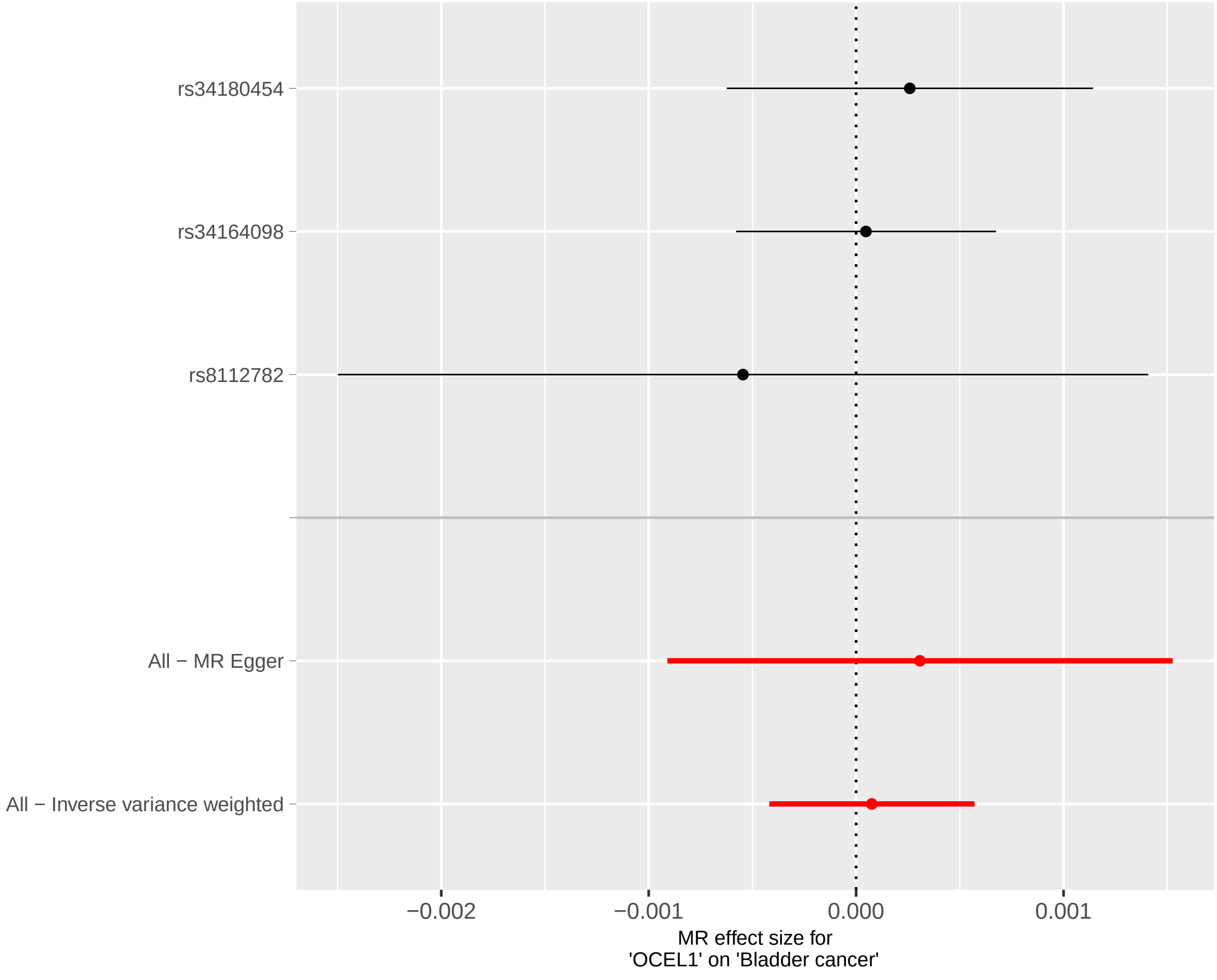

rs34180454
rs34164098
rs8112782
All − MR Egger
All − Inverse variance weighted
−0.002
−0.001
0.000
0.001
MR effect size for
'OCEL1' on 'Bladder cancer'

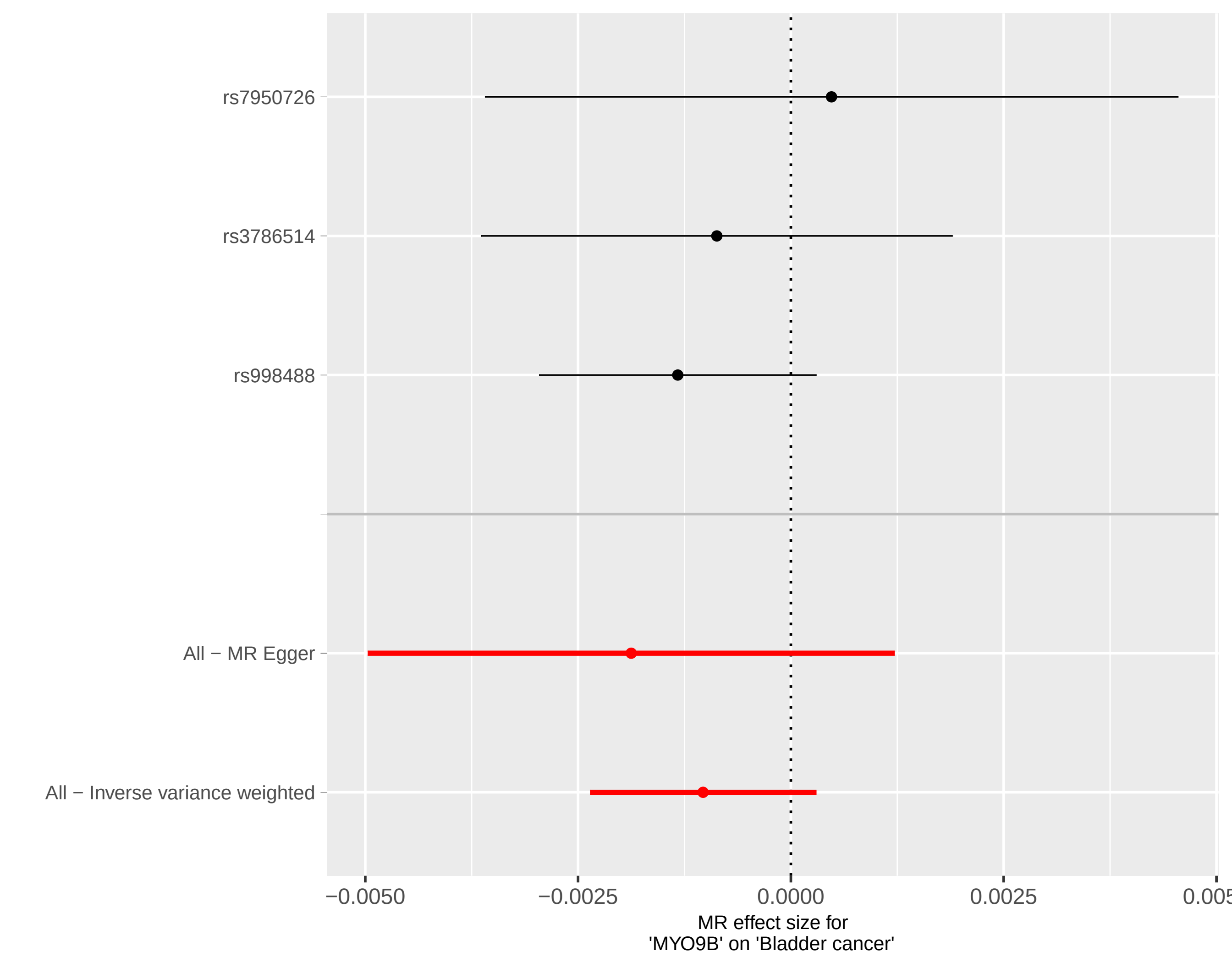

rs7950726
rs3786514
rs998488
All − MR Egger
All − Inverse variance weighted
−0.0050
−0.0025
0.0000
0.0025
0.005
MR effect size for
'MYO9B' on 'Bladder cancer'

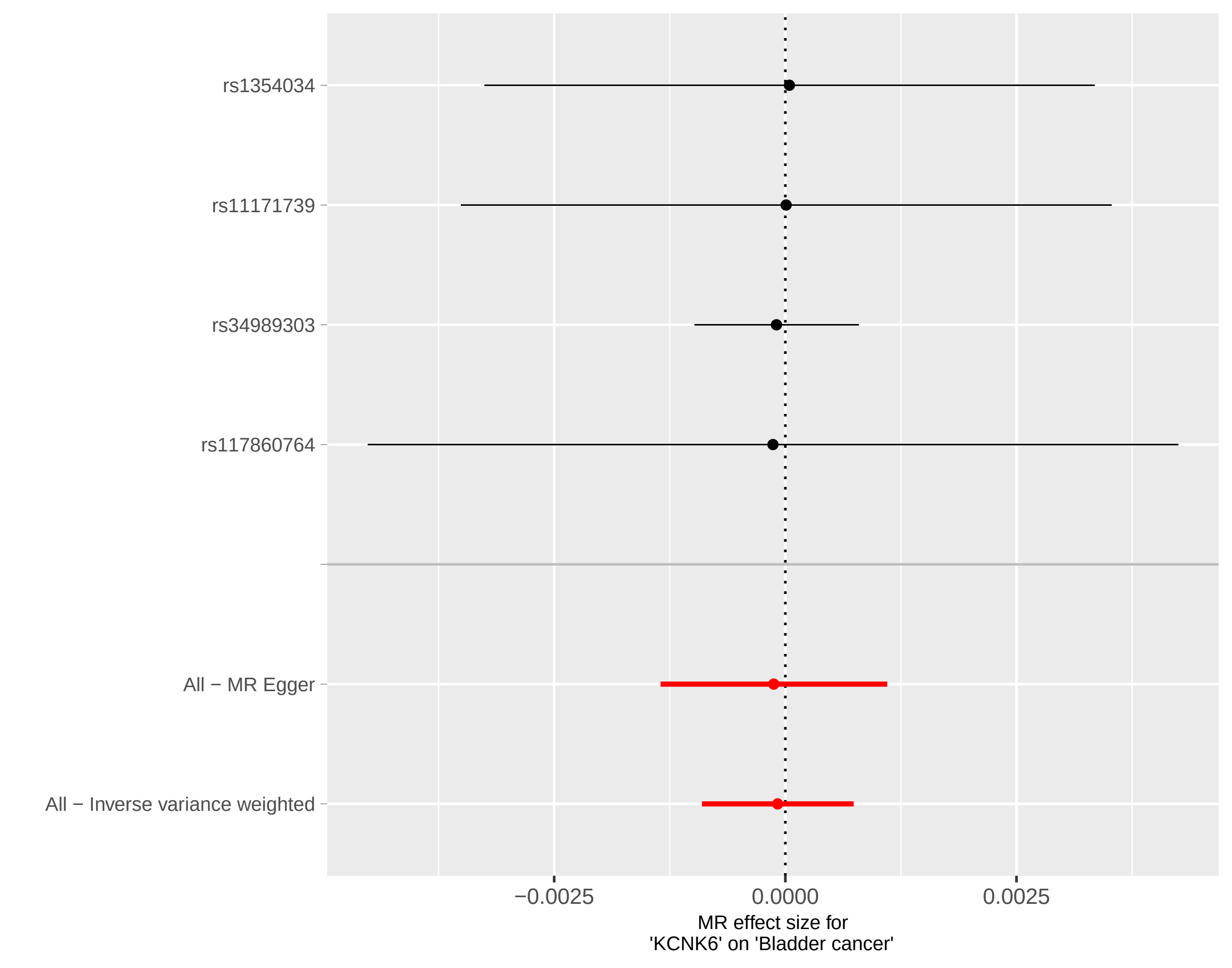
rs1354034
rs11171739
rs34989303
rs117860764
All − MR Egger
All − Inverse variance weighted
−0.0025
0.0000
0.0025
MR effect size for
'KCNK6' on 'Bladder cancer'

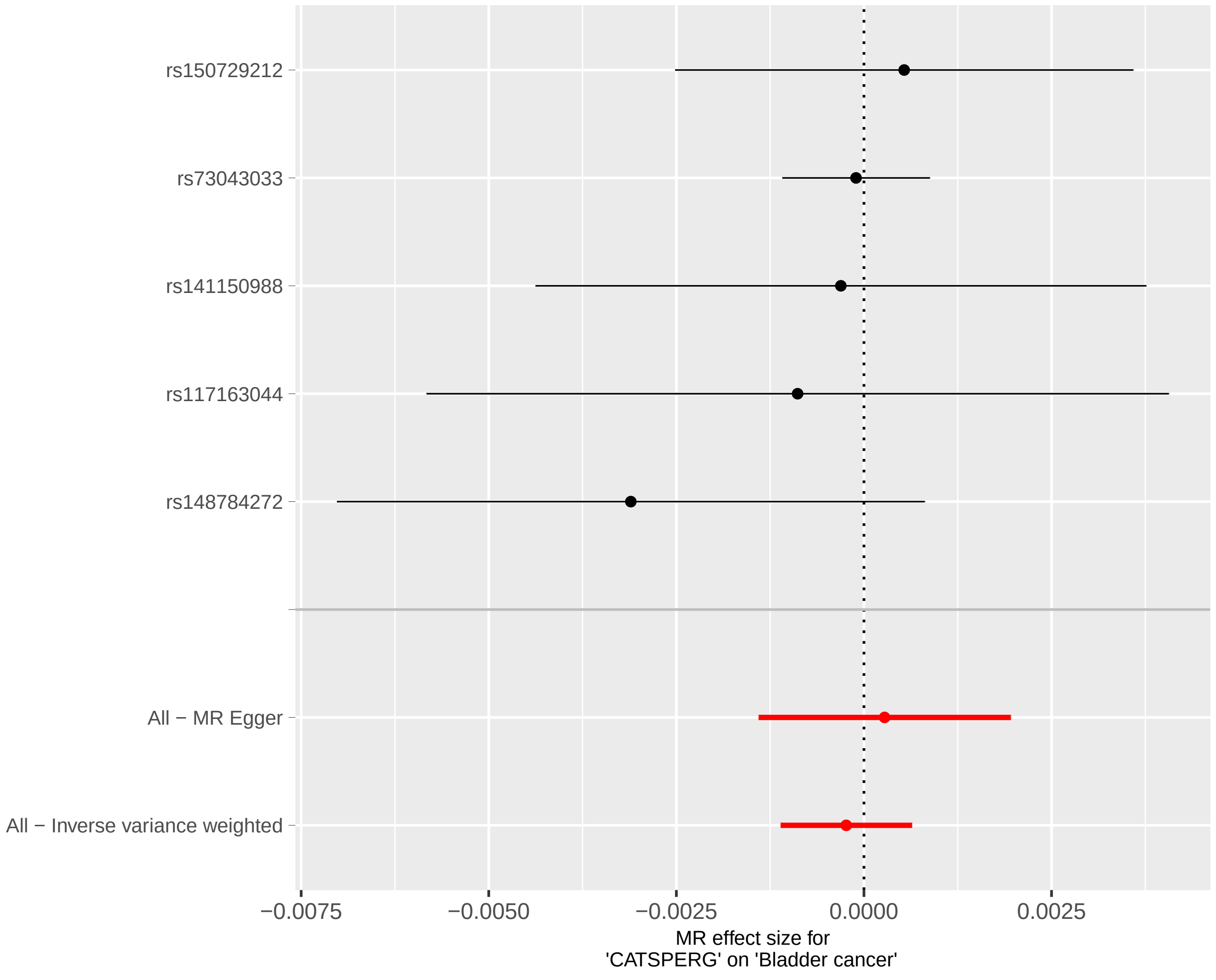

rs150729212
rs73043033
rs141150988
rs117163044
rs148784272
All − MR Egger
All − Inverse variance weighted
−0.0075
−0.0050
−0.0025
0.0000
0.0025
MR effect size for
'CATSPERG' on 'Bladder cancer'

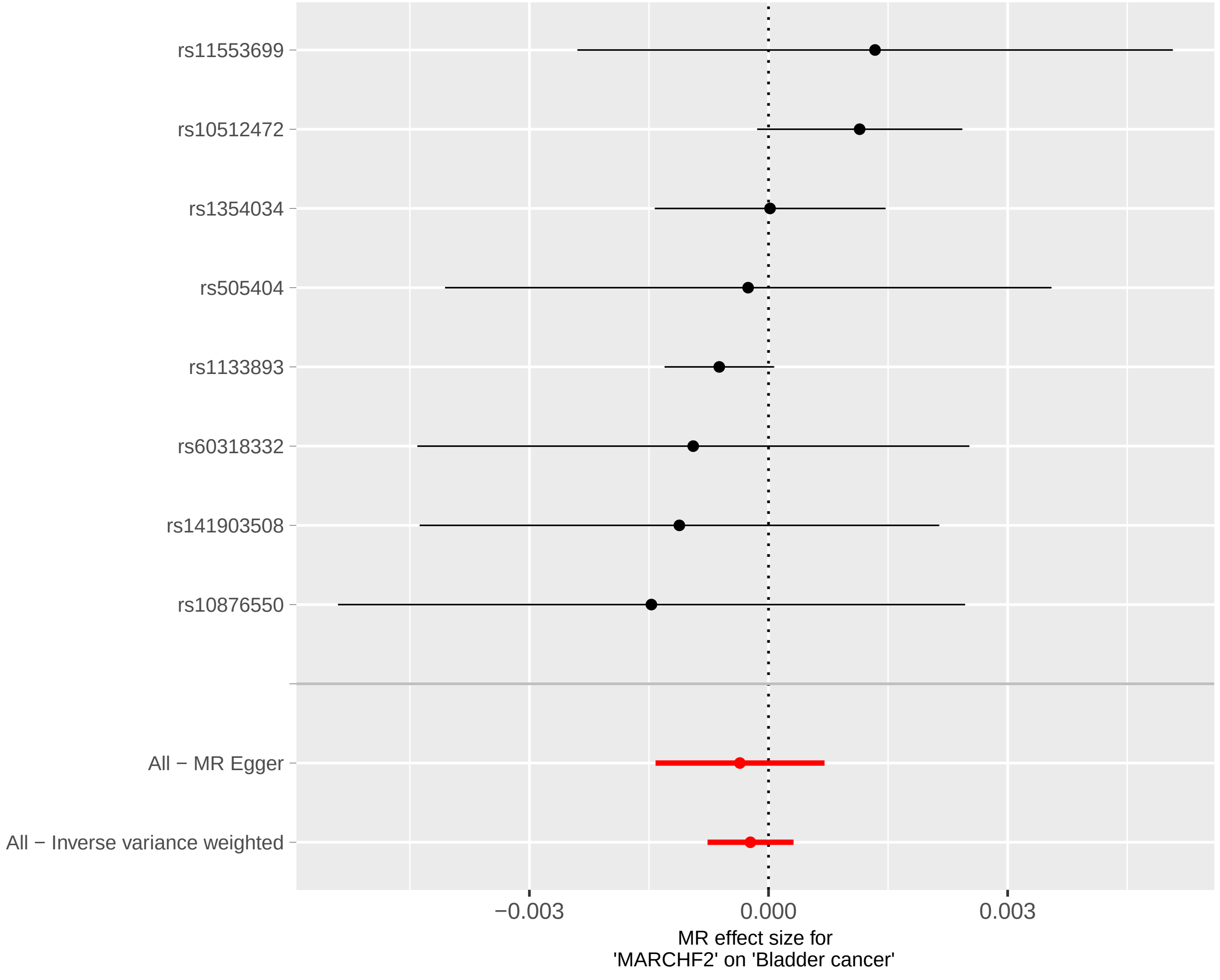

rs11553699
rs10512472
rs1354034
rs505404
rs1133893
rs60318332
rs141903508
rs10876550
All − MR Egger
All − Inverse variance weighted
−0.003
0.000
0.003
MR effect size for
'MARCHF2' on 'Bladder cancer'

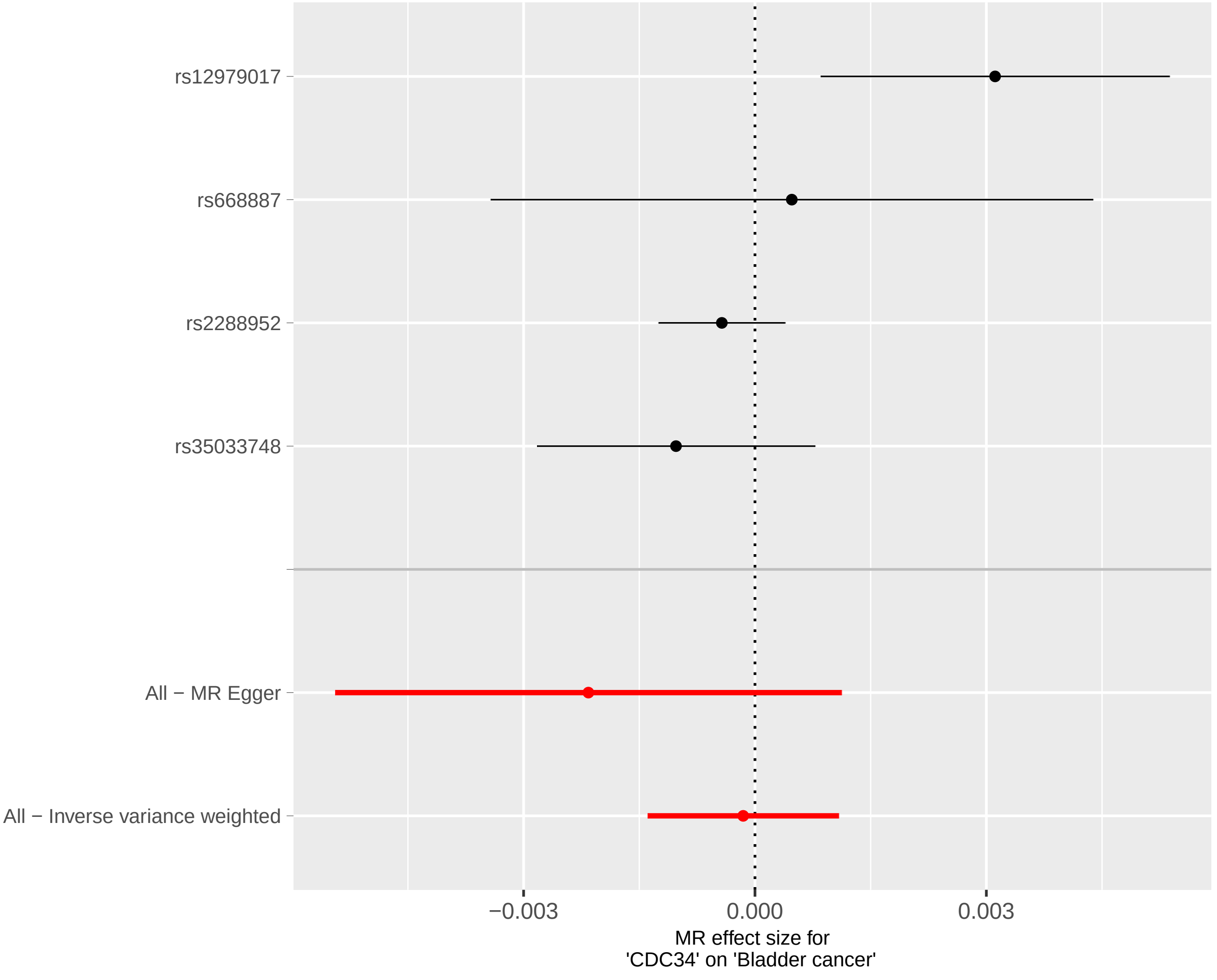

rs12979017
rs668887
rs2288952
rs35033748
All − MR Egger
All − Inverse variance weighted
−0.003
0.000
0.003
MR effect size for
'CDC34' on 'Bladder cancer'

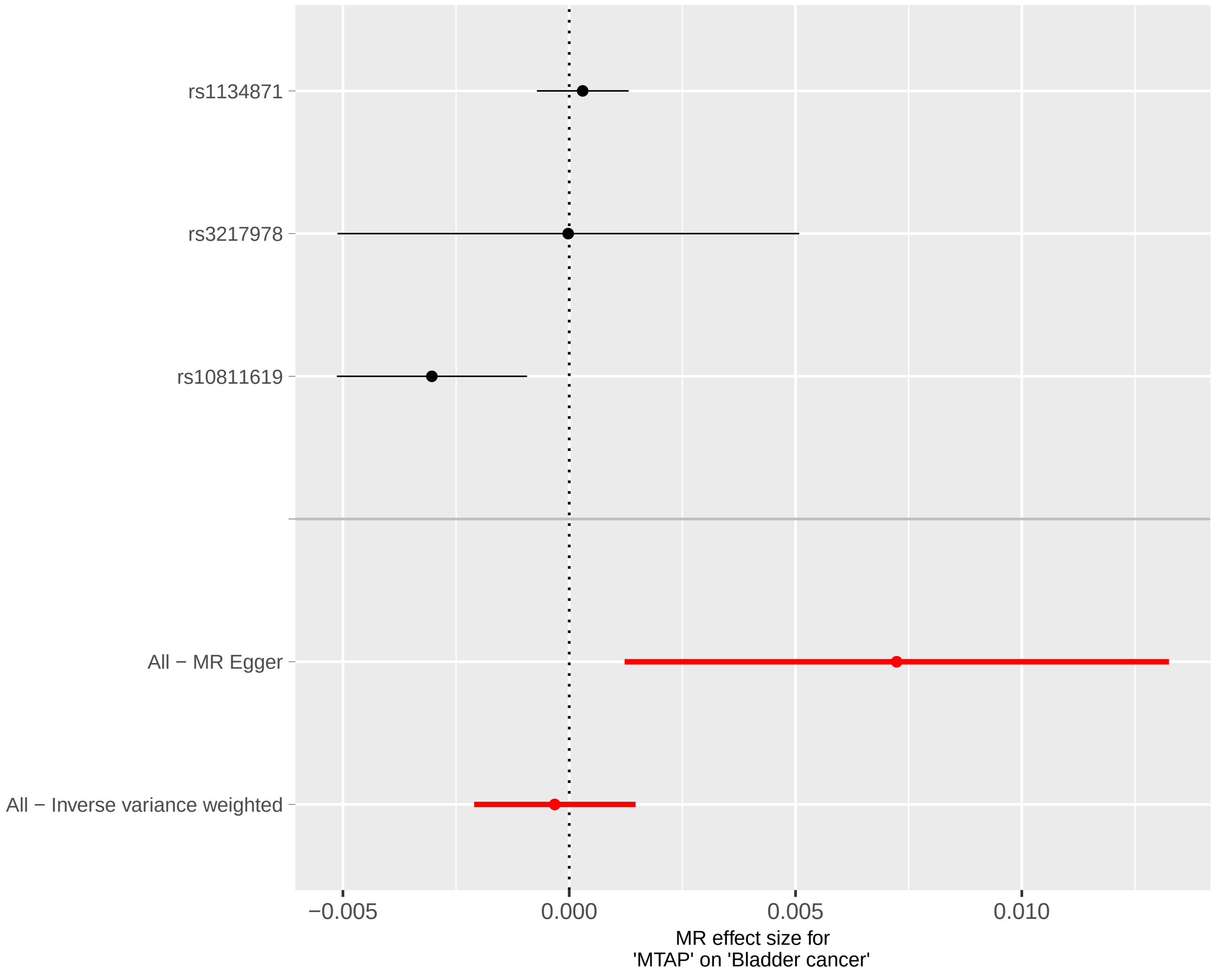
rs1134871
rs3217978
rs10811619
All − MR Egger
All − Inverse variance weighted
−0.005
0.000
0.005
0.010
MR effect size for
'MTAP' on 'Bladder cancer'

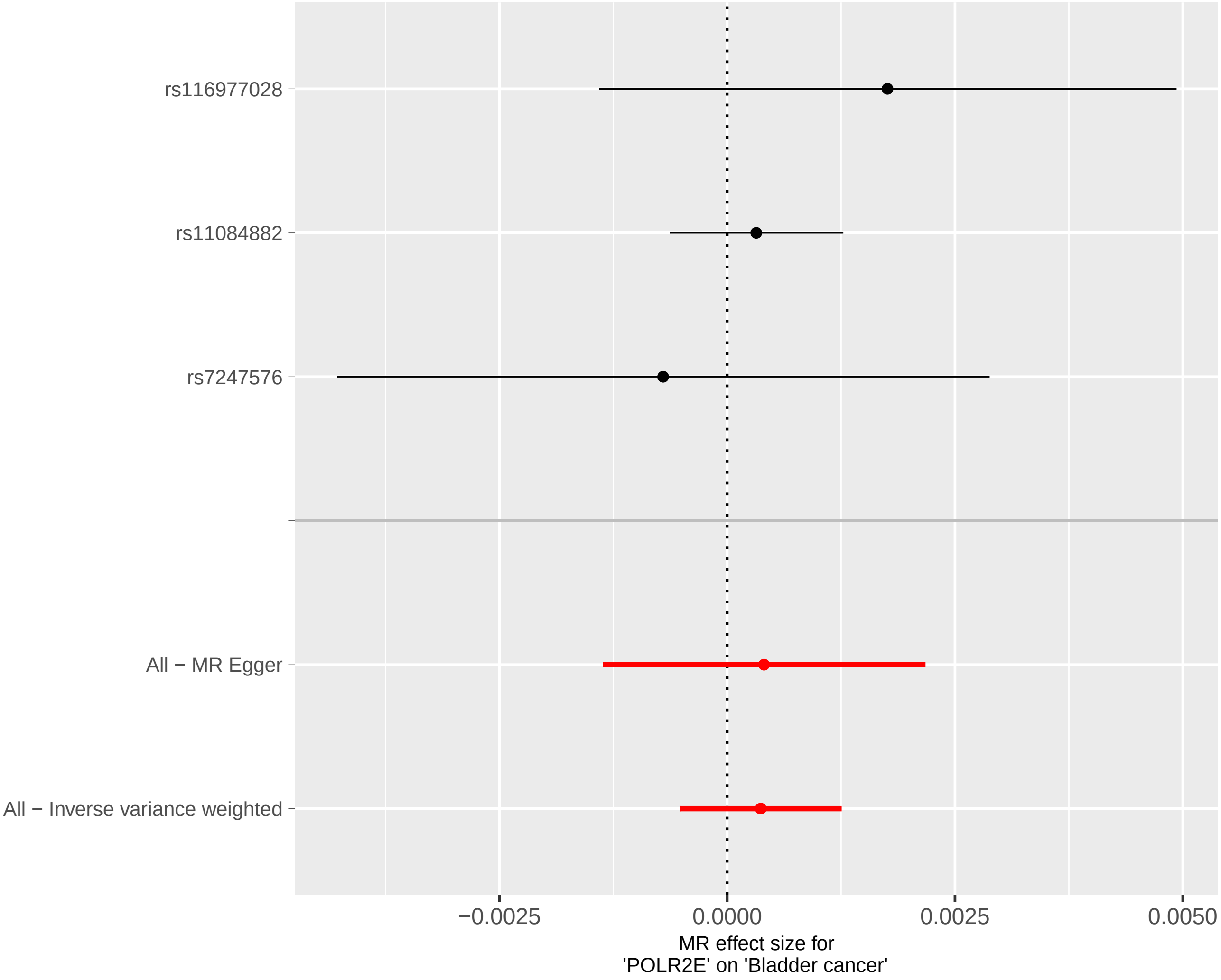
rs116977028
rs11084882
rs7247576
All − MR Egger
All − Inverse variance weighted
−0.0025
0.0000
0.0025
0.0050
MR effect size for
'POLR2E' on 'Bladder cancer'

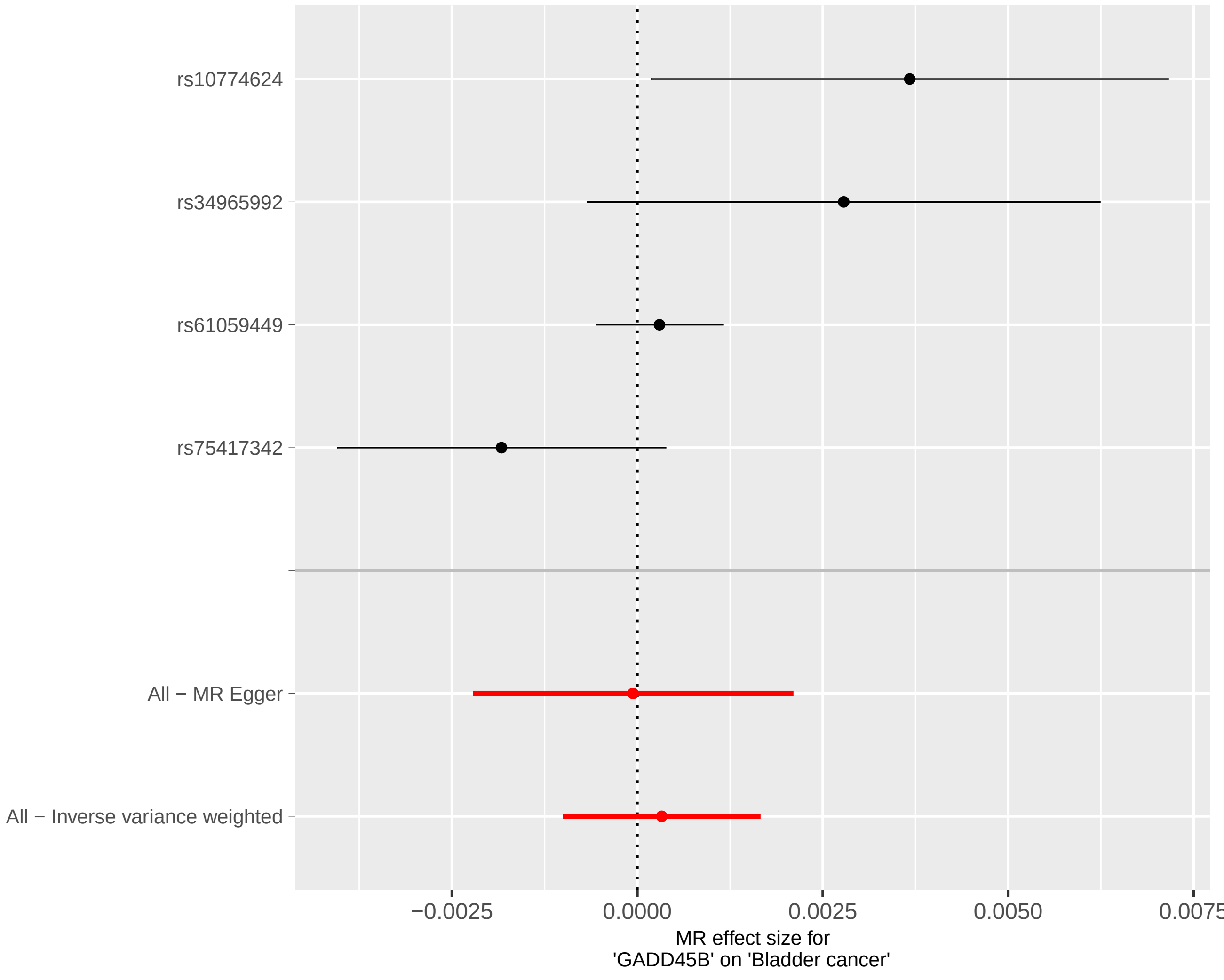
rs10774624
rs34965992
rs61059449
rs75417342
All − MR Egger
All − Inverse variance weighted
−0.0025
0.0000
0.0025
0.0050
0.0075
MR effect size for
'GADD45B' on 'Bladder cancer'

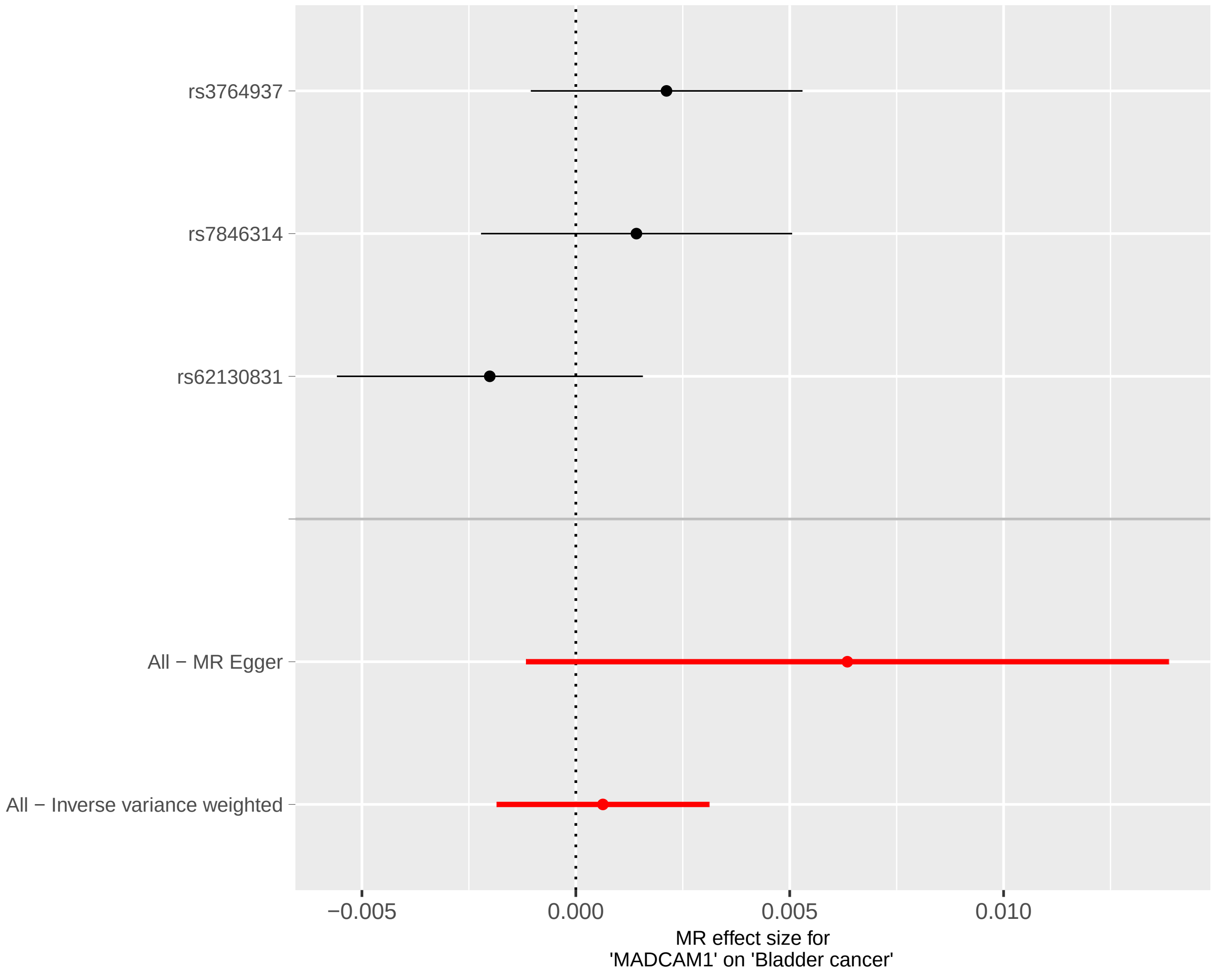
rs3764937
rs7846314
rs62130831
All − MR Egger
All − Inverse variance weighted
−0.005
0.000
0.005
0.010
MR effect size for
'MADCAM1' on 'Bladder cancer'

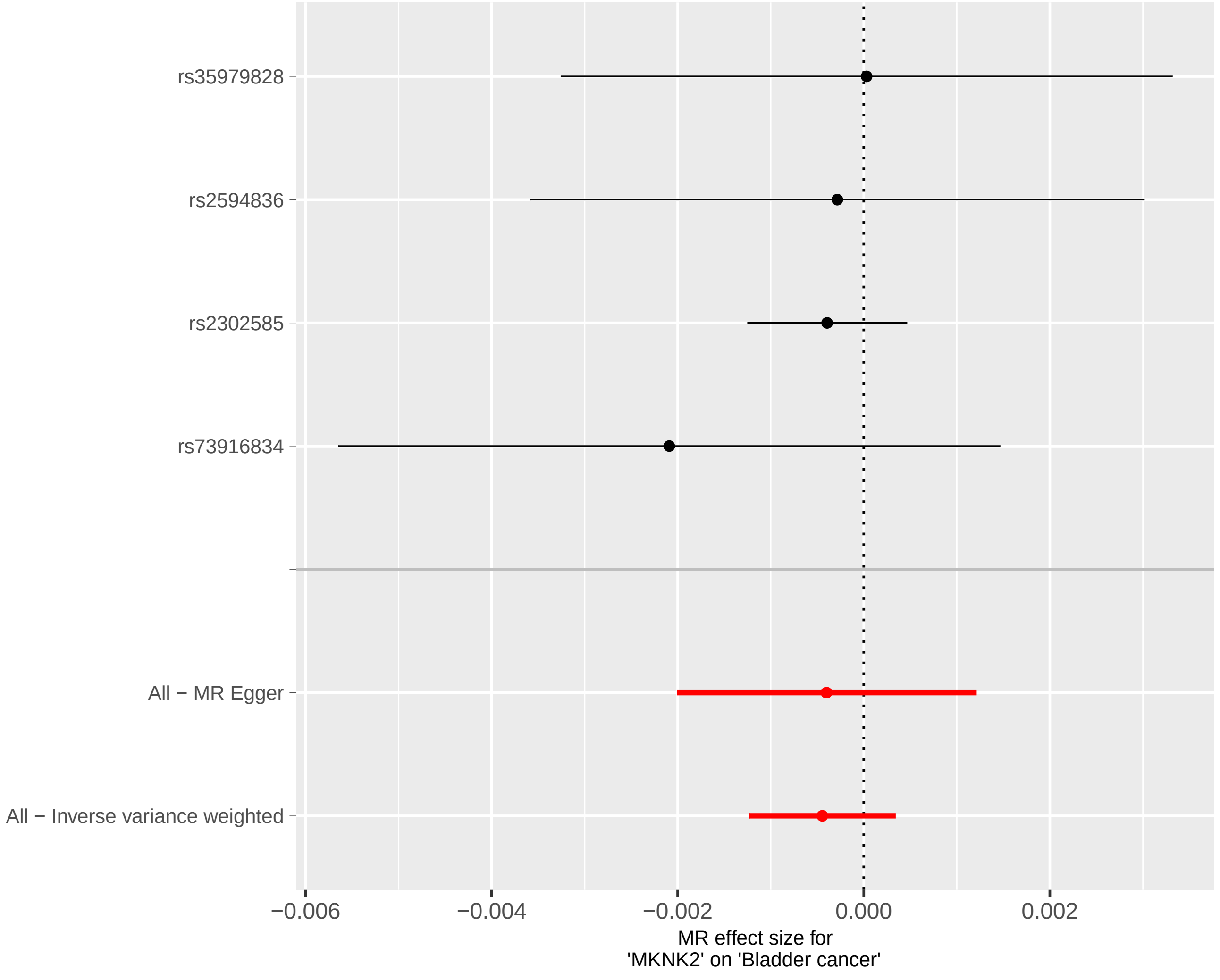

rs35979828
rs2594836
rs2302585
rs73916834
All − MR Egger
All − Inverse variance weighted
−0.006
−0.004
−0.002
0.000
0.002
MR effect size for
'MKNK2' on 'Bladder cancer'

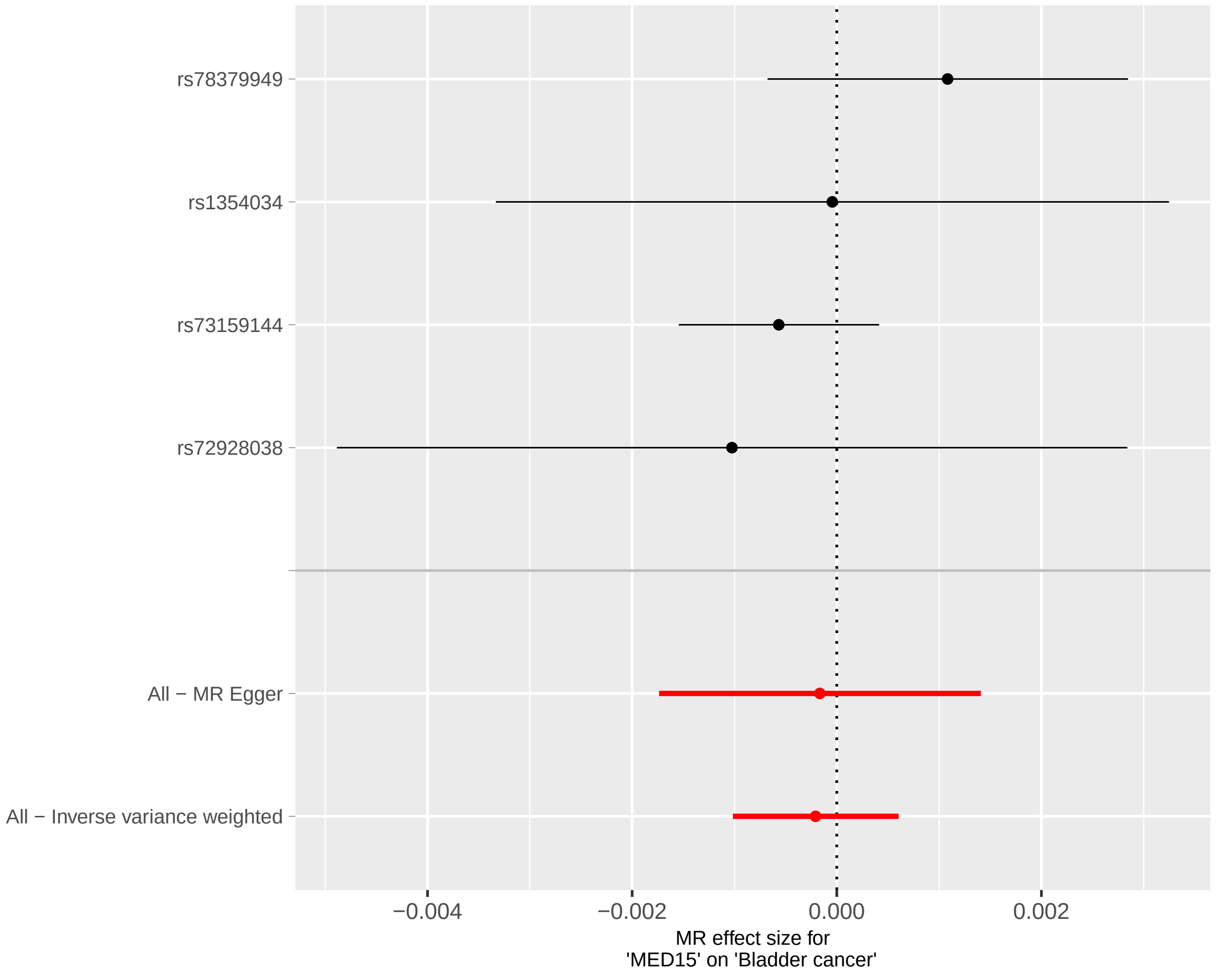
rs78379949
rs1354034
rs73159144
rs72928038
All − MR Egger
All − Inverse variance weighted
−0.004
−0.002
0.000
0.002
MR effect size for
'MED15' on 'Bladder cancer'

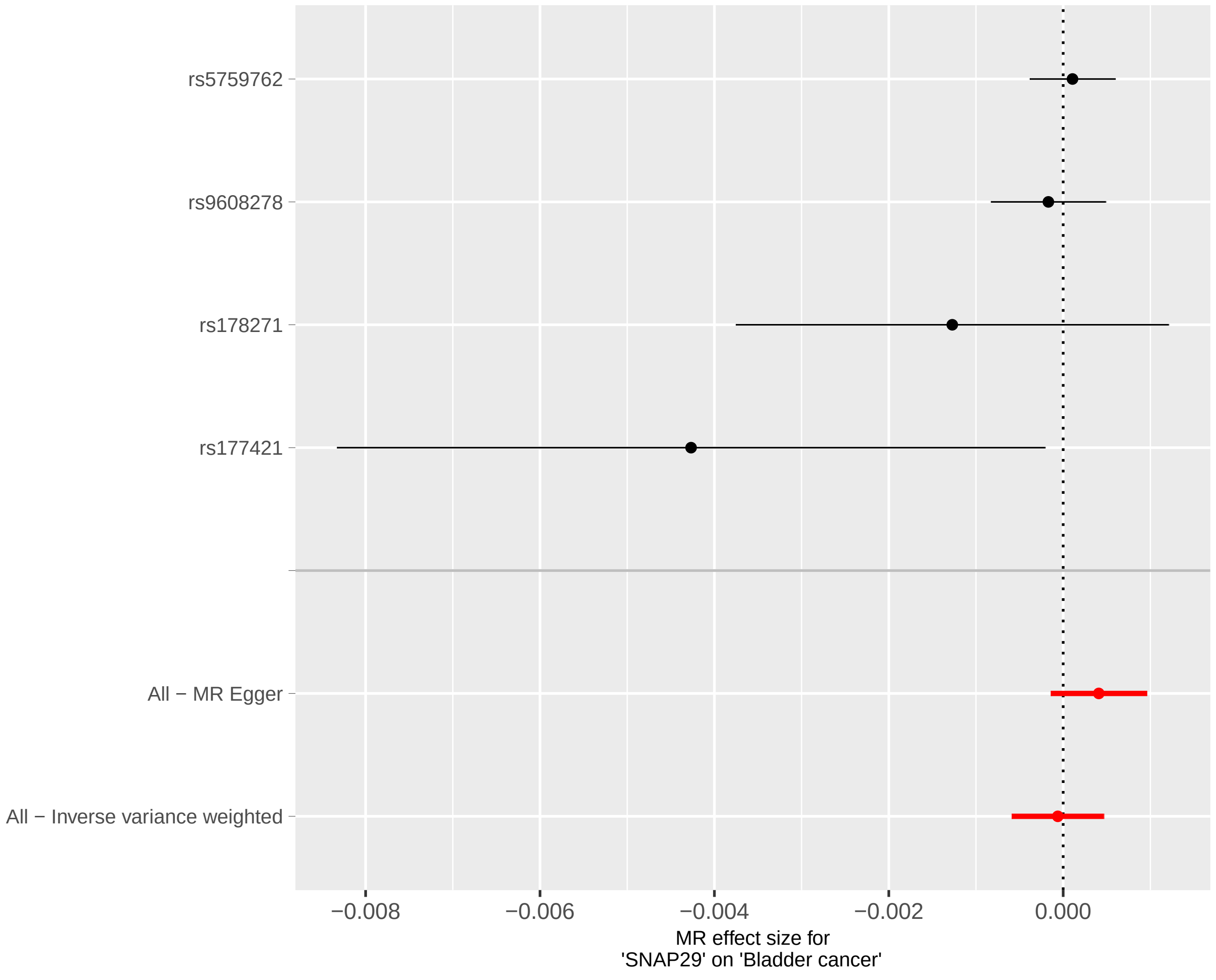

rs5759762
rs9608278
rs178271
rs177421
All − MR Egger
All − Inverse variance weighted
−0.008
−0.006
−0.004
−0.002
0.000
MR effect size for
'SNAP29' on 'Bladder cancer'

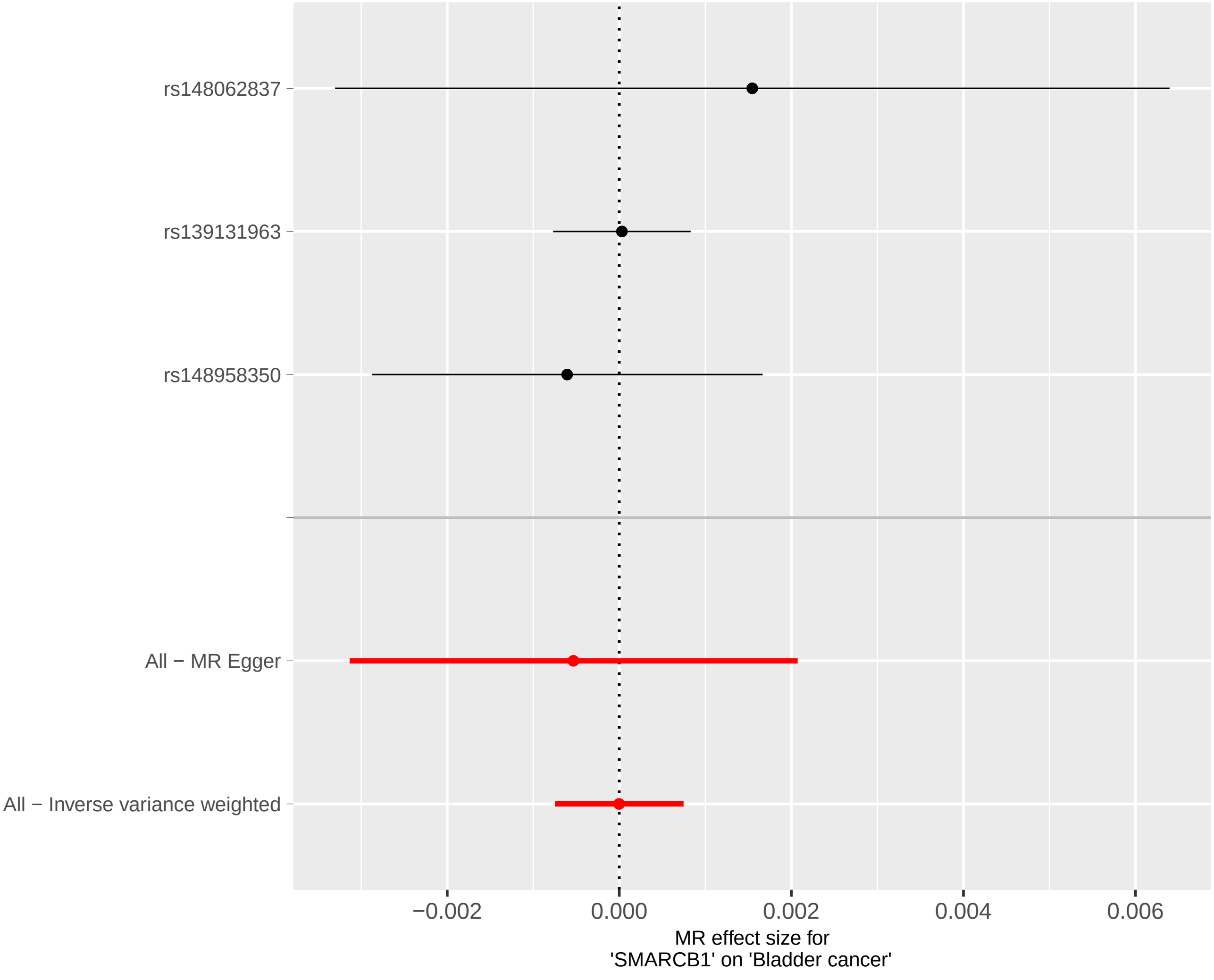
rs148062837
rs139131963
rs148958350
All − MR Egger
All − Inverse variance weighted
−0.002
0.000
0.002
0.004
0.006
MR effect size for
'SMARCB1' on 'Bladder cancer'

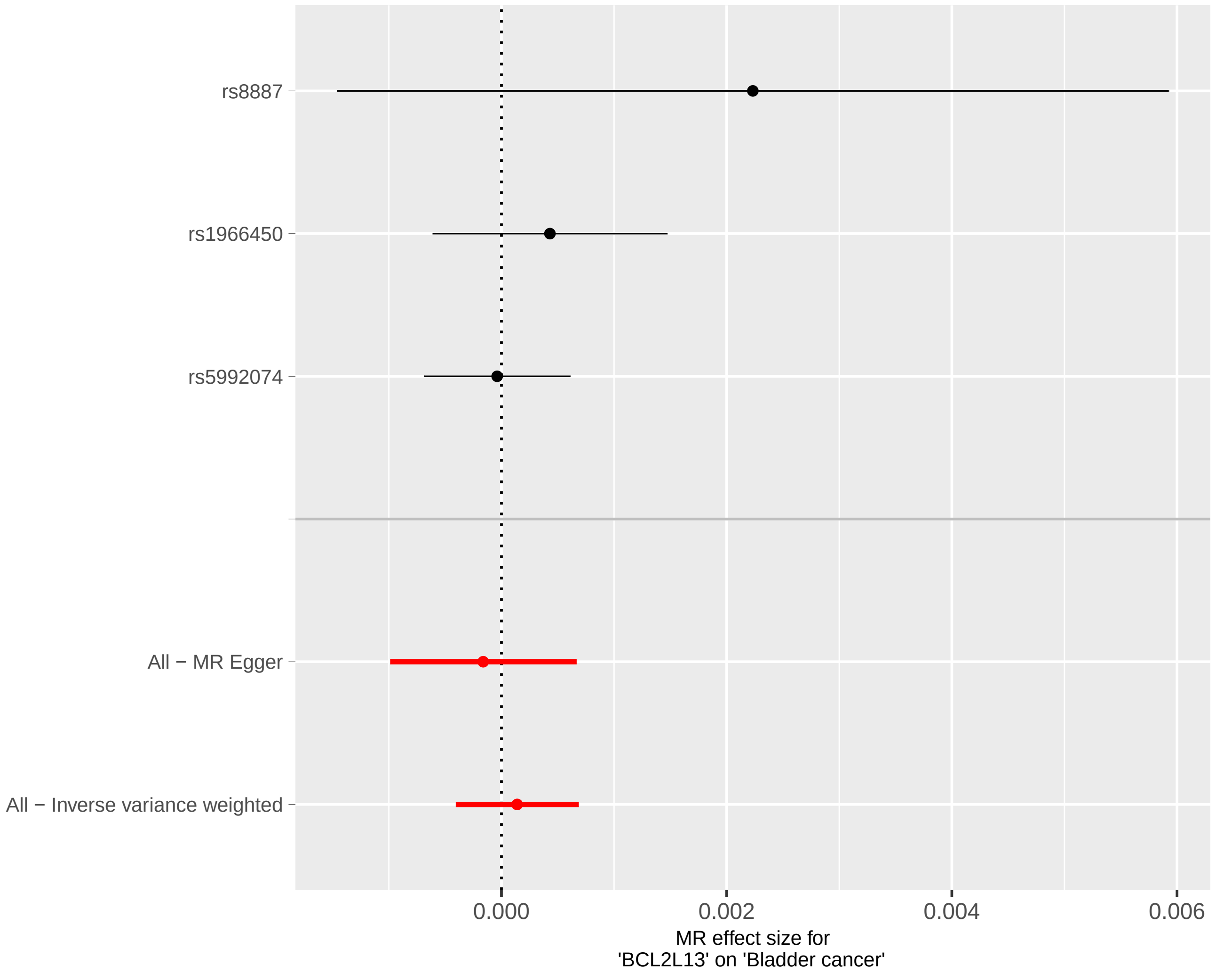

rs8887
rs1966450
rs5992074
All − MR Egger
All − Inverse variance weighted
0.000
0.002
0.004
0.006
MR effect size for
'BCL2L13' on 'Bladder cancer'

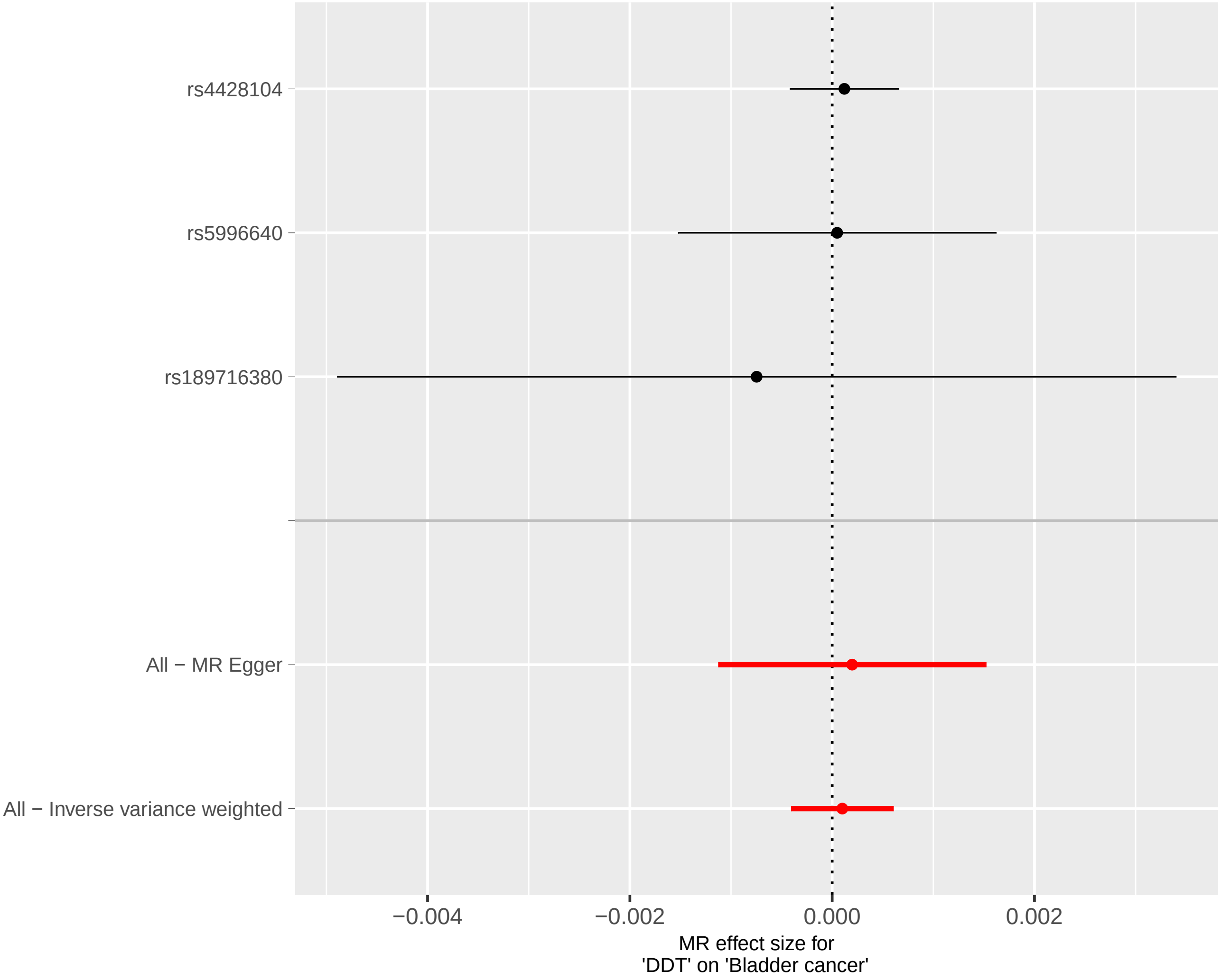

rs4428104
rs5996640
rs189716380
All − MR Egger
All − Inverse variance weighted
−0.004
−0.002
0.000
0.002
MR effect size for
'DDT' on 'Bladder cancer'

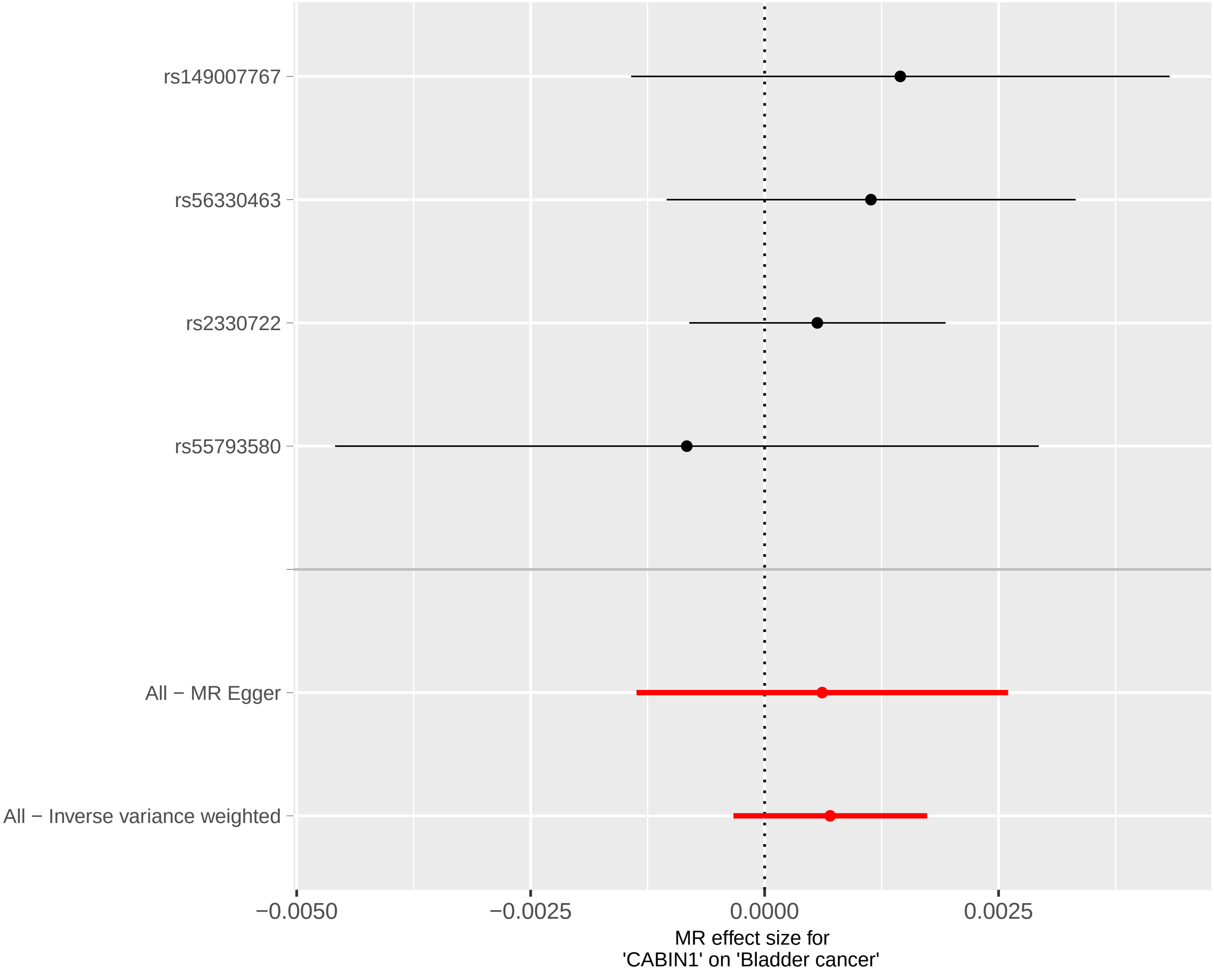

rs149007767
rs56330463
rs2330722
rs55793580
All − MR Egger
All − Inverse variance weighted
−0.0050
−0.0025
0.0000
0.0025
MR effect size for
'CABIN1' on 'Bladder cancer'

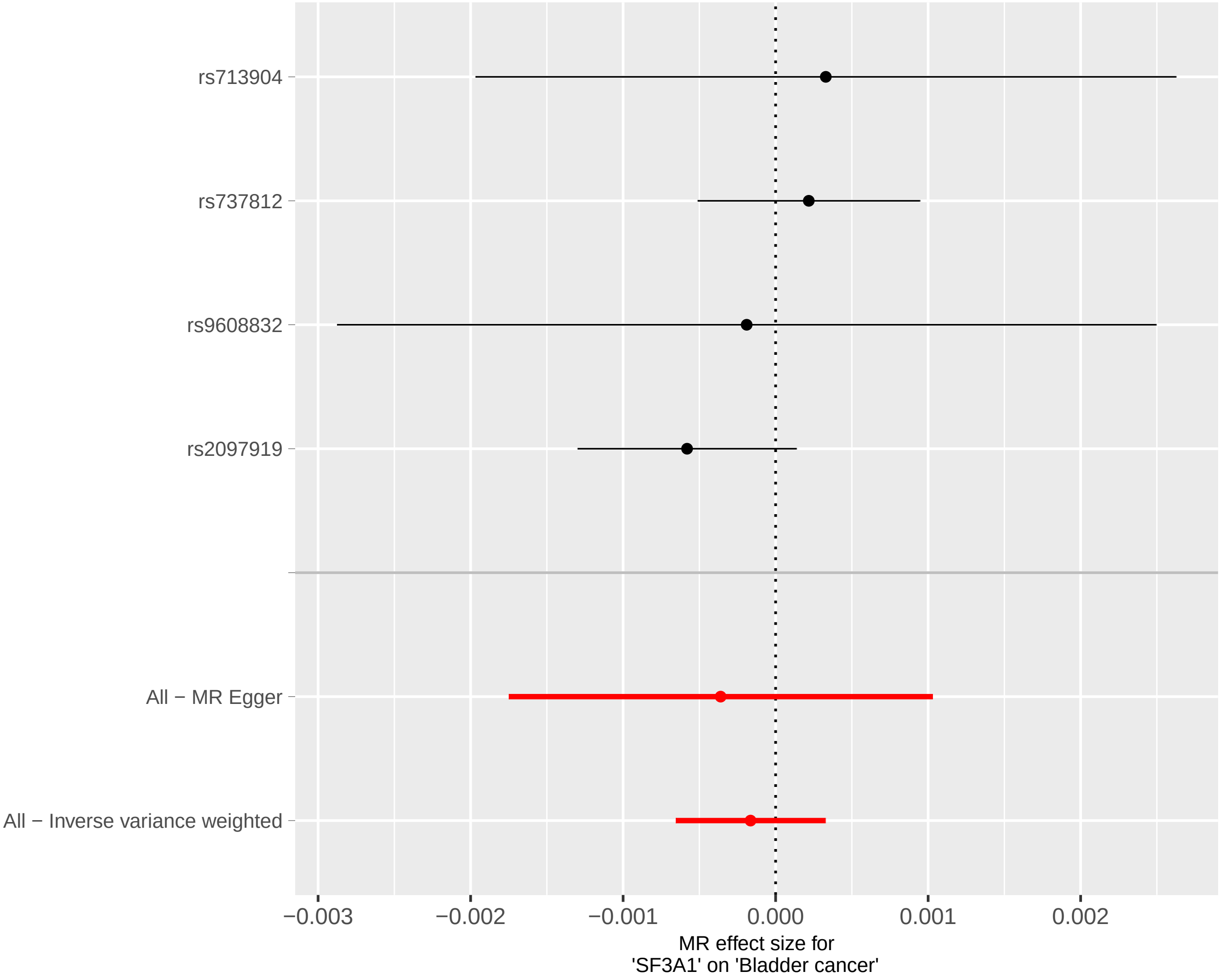

rs713904
rs737812
rs9608832
rs2097919
All − MR Egger
All − Inverse variance weighted
−0.003
−0.002
−0.001
0.000
0.001
0.002
MR effect size for
'SF3A1' on 'Bladder cancer'

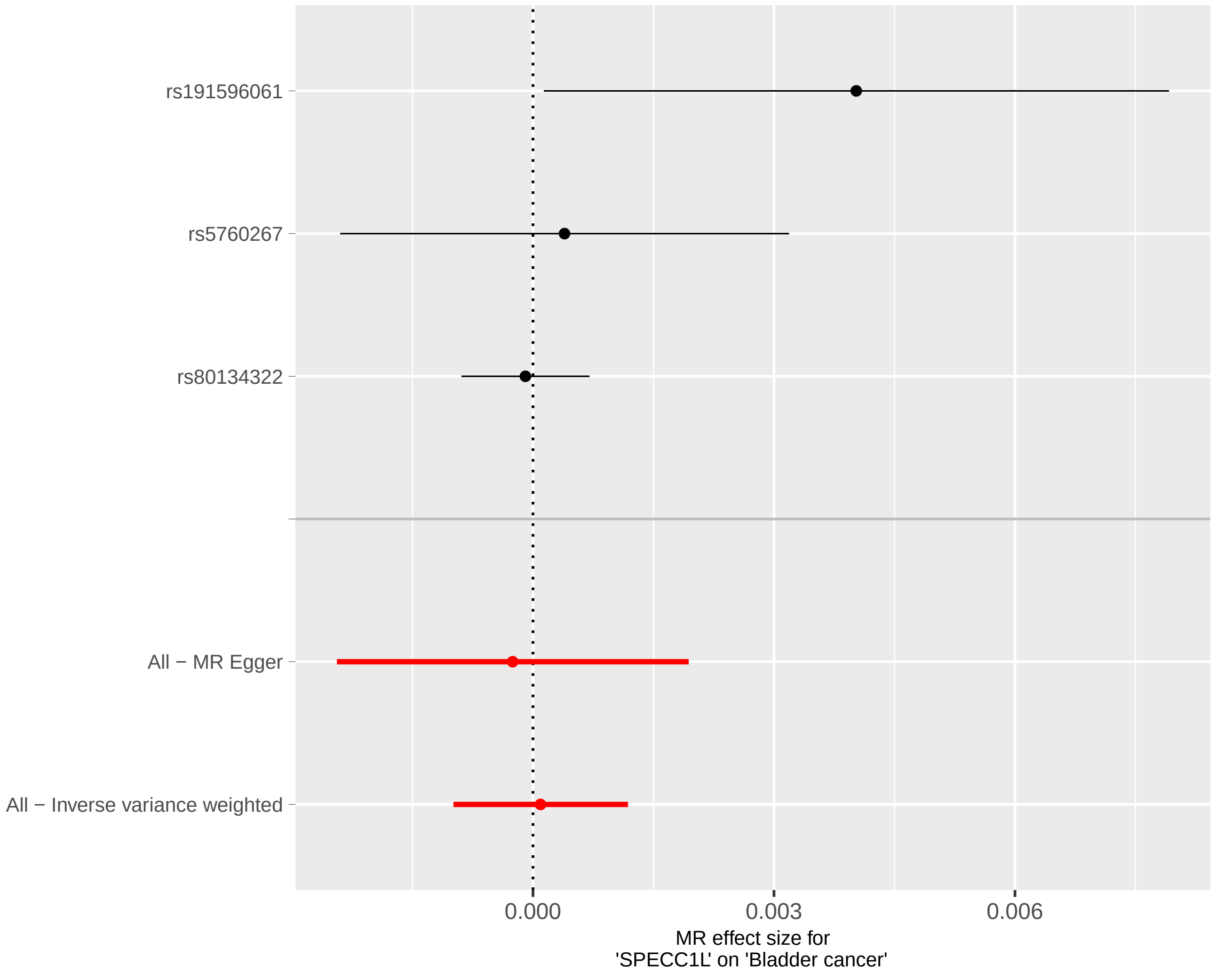

rs191596061
rs5760267
rs80134322
All − MR Egger
All − Inverse variance weighted
0.000
0.003
0.006
MR effect size for
'SPECC1L' on 'Bladder cancer'

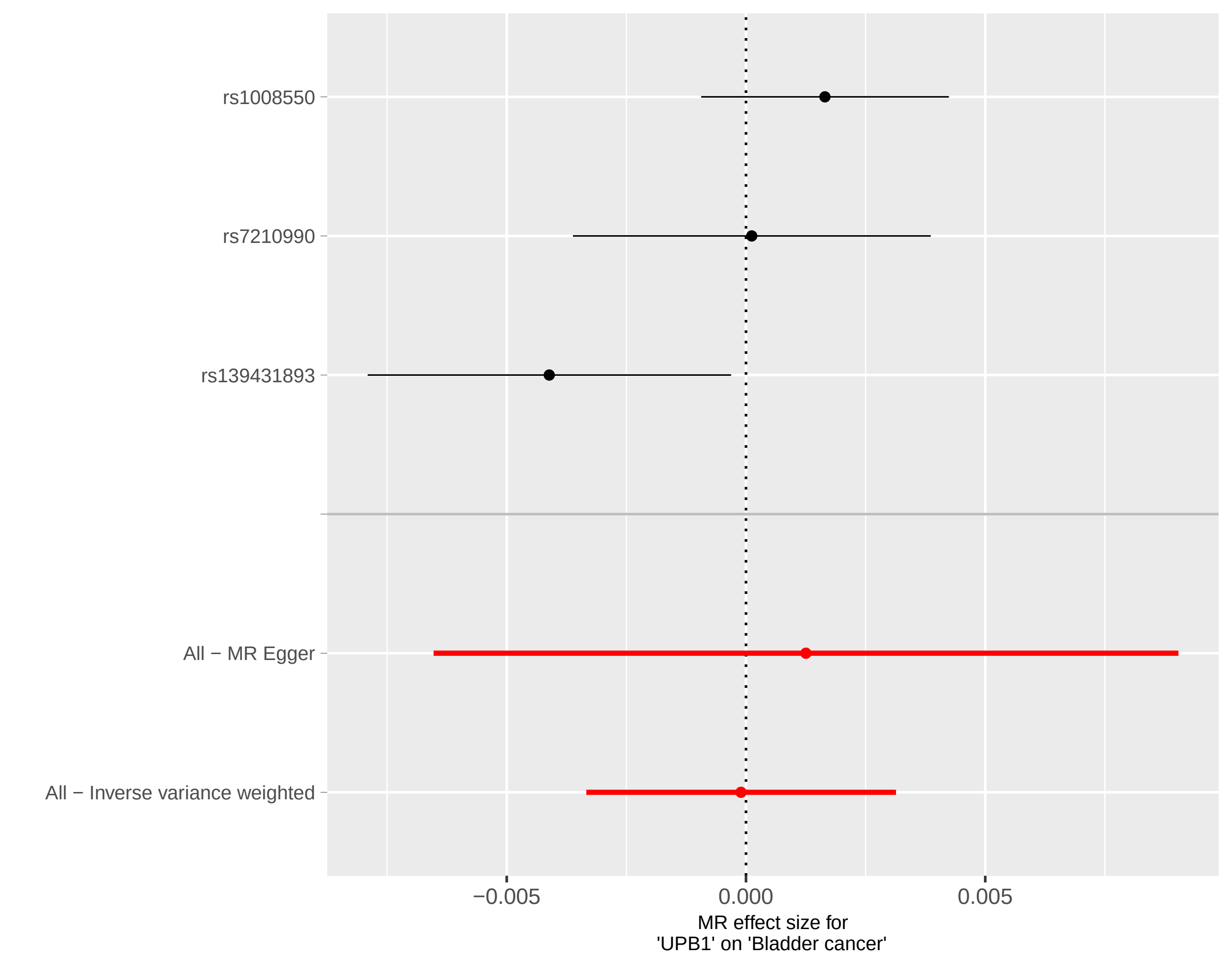

rs1008550
rs7210990
rs139431893
All − MR Egger
All − Inverse variance weighted
−0.005
0.000
0.005
MR effect size for
'UPB1' on 'Bladder cancer'

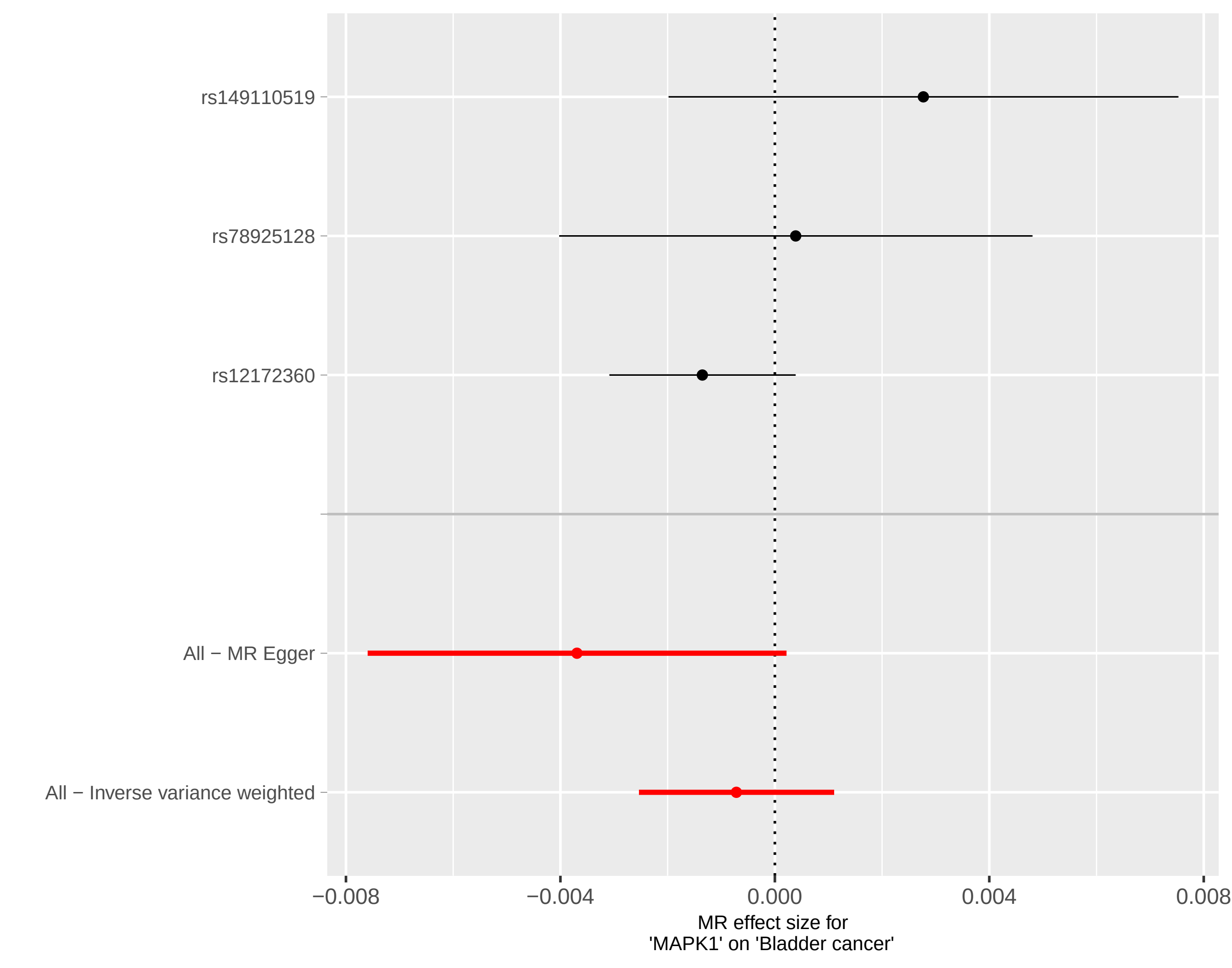

rs149110519
rs78925128
rs12172360
All − MR Egger
All − Inverse variance weighted
−0.008
−0.004
0.000
0.004
0.008
MR effect size for
'MAPK1' on 'Bladder cancer'

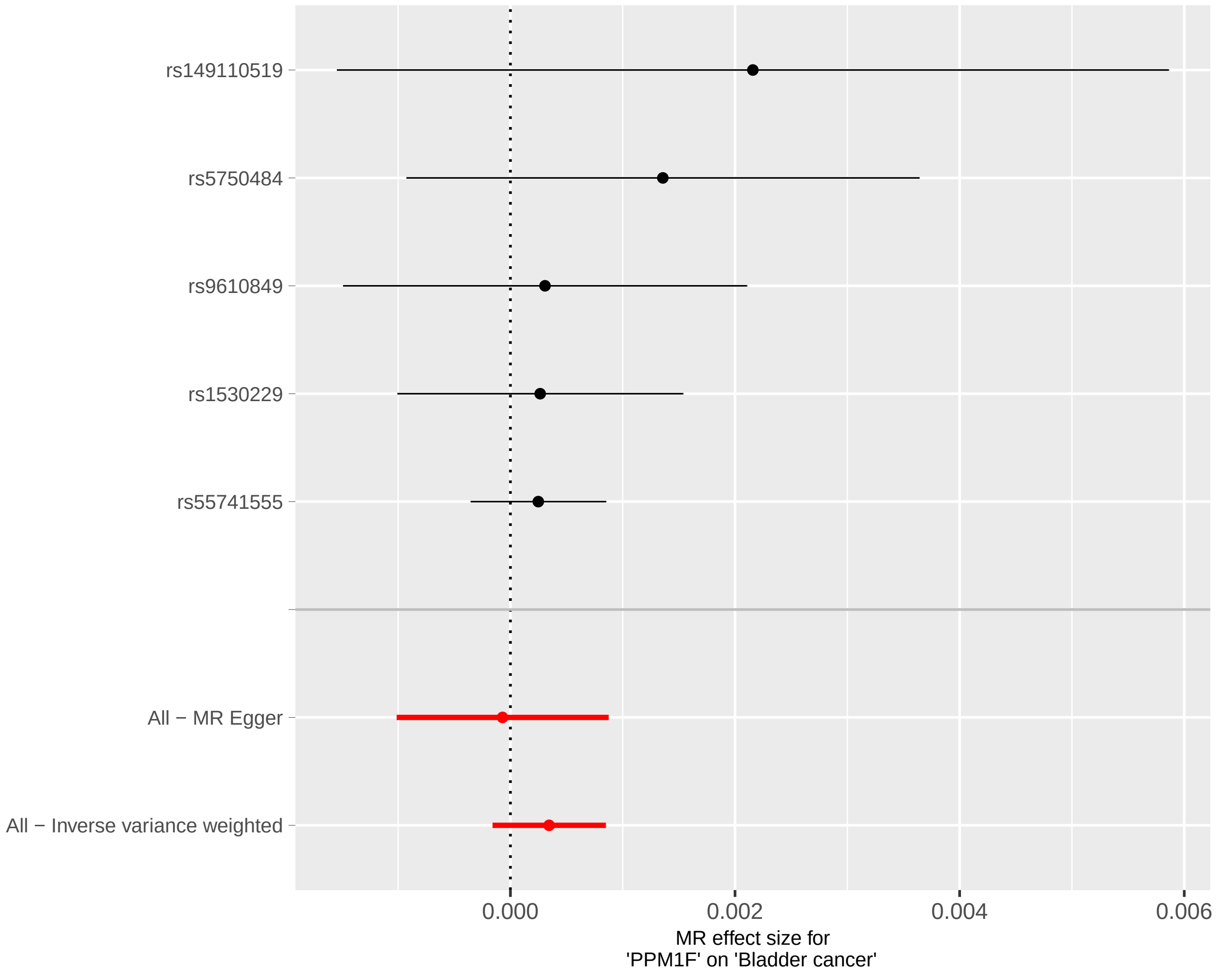
rs149110519
rs5750484
rs9610849
rs1530229
rs55741555
All − MR Egger
All − Inverse variance weighted
0.000
0.002
0.004
0.006
MR effect size for
'PPM1F' on 'Bladder cancer'

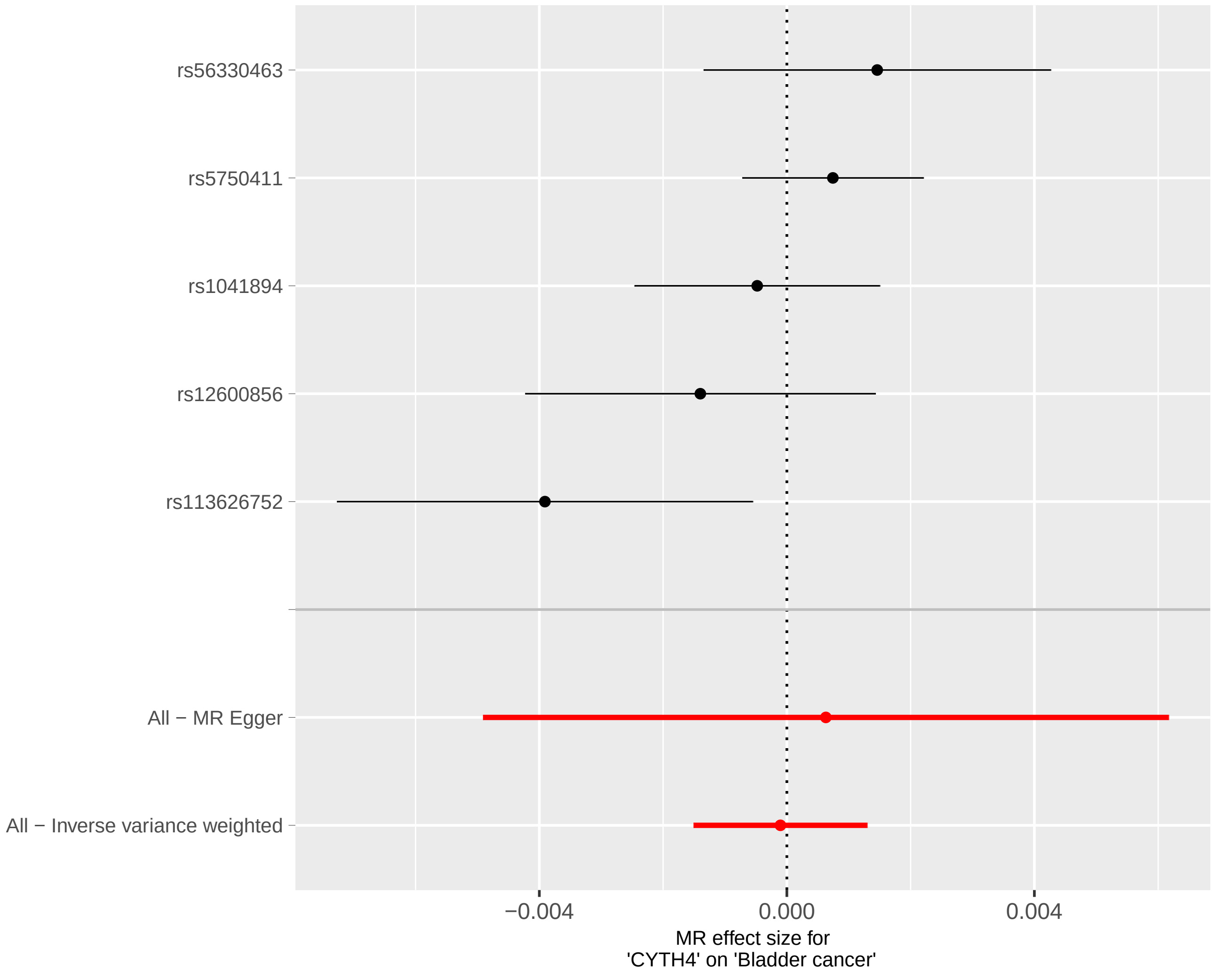

rs56330463
rs5750411
rs1041894
rs12600856
rs113626752
All − MR Egger
All − Inverse variance weighted
−0.004
0.000
0.004
MR effect size for
'CYTH4' on 'Bladder cancer'

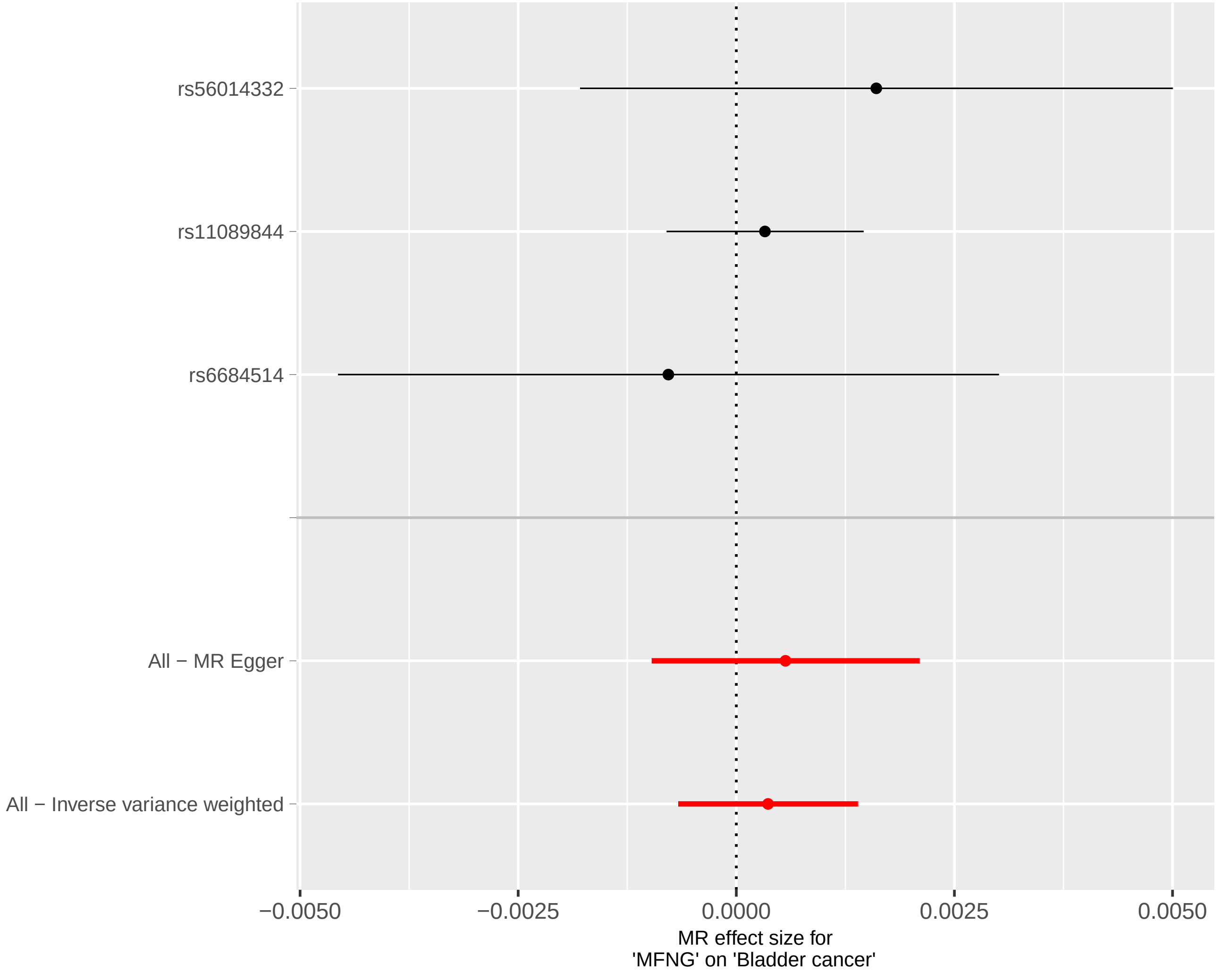

rs56014332
rs11089844
rs6684514
All − MR Egger
All − Inverse variance weighted
−0.0050
−0.0025
0.0000
0.0025
0.0050
MR effect size for
'MFNG' on 'Bladder cancer'

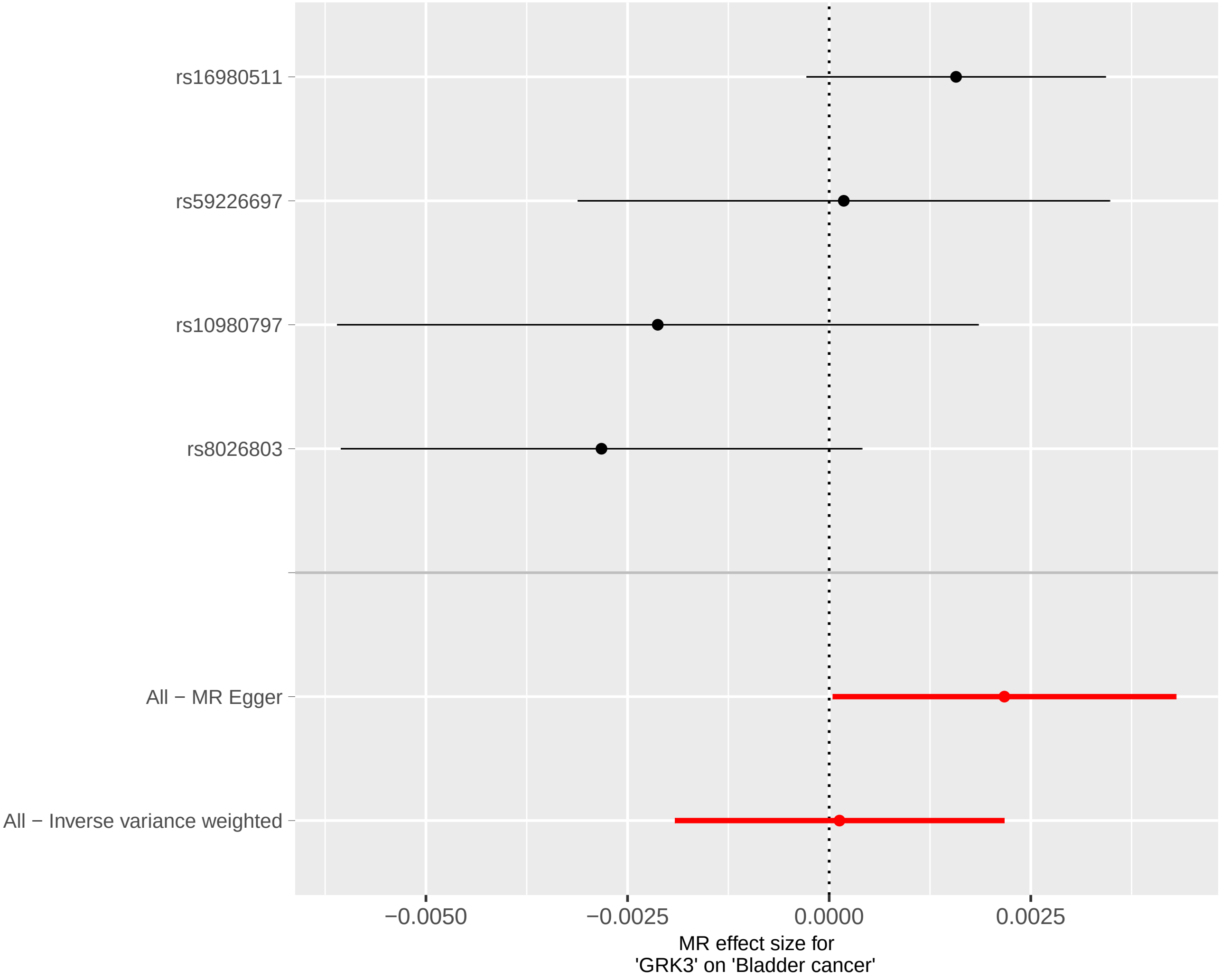
rs16980511
rs59226697
rs10980797
rs8026803
All − MR Egger
All − Inverse variance weighted
−0.0050
−0.0025
0.0000
0.0025
MR effect size for
'GRK3' on 'Bladder cancer'

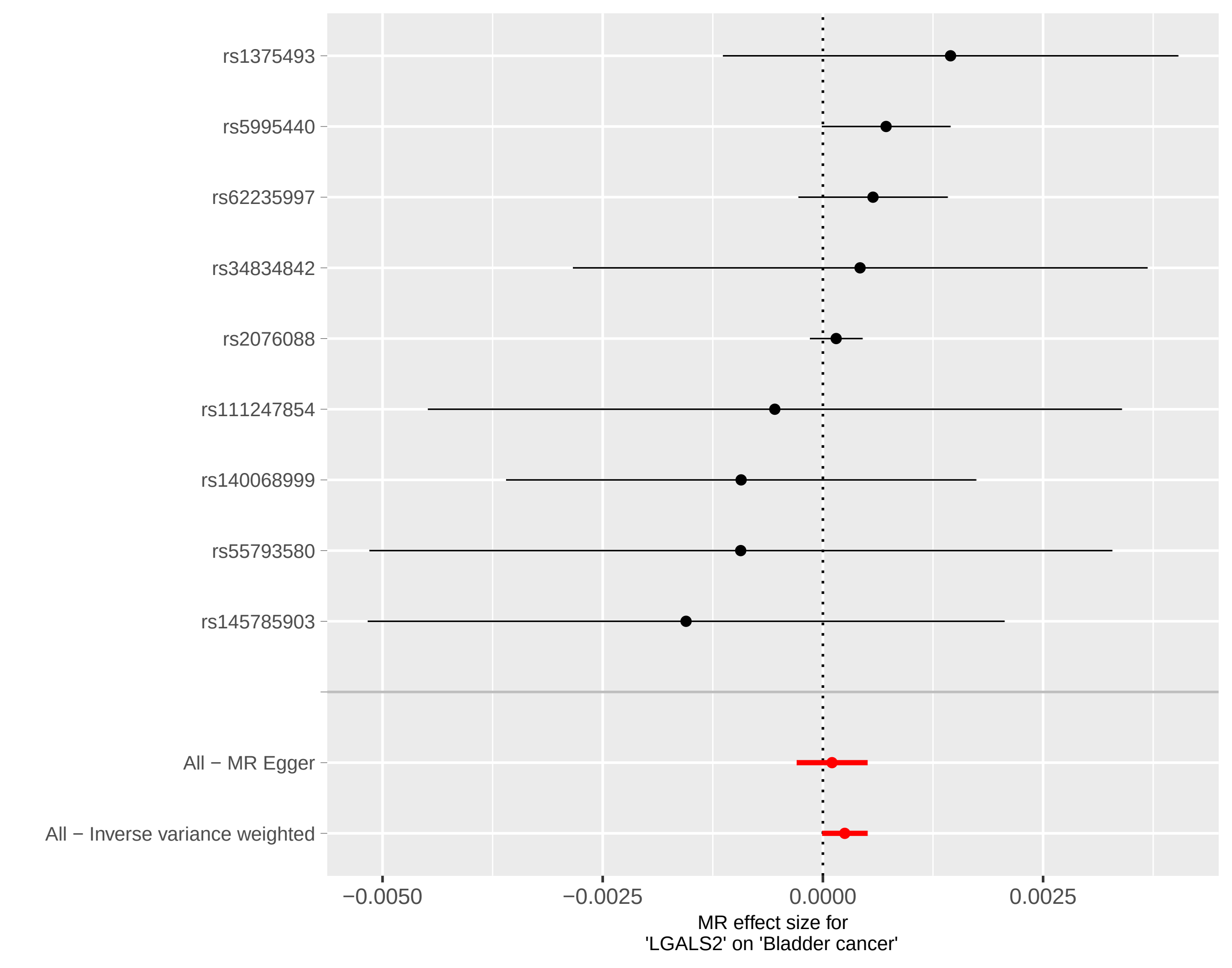

rs1375493
rs5995440
rs62235997
rs34834842
rs2076088
rs111247854
rs140068999
rs55793580
rs145785903
All − MR Egger
All − Inverse variance weighted
−0.0050
−0.0025
0.0000
0.0025
MR effect size for
'LGALS2' on 'Bladder cancer'

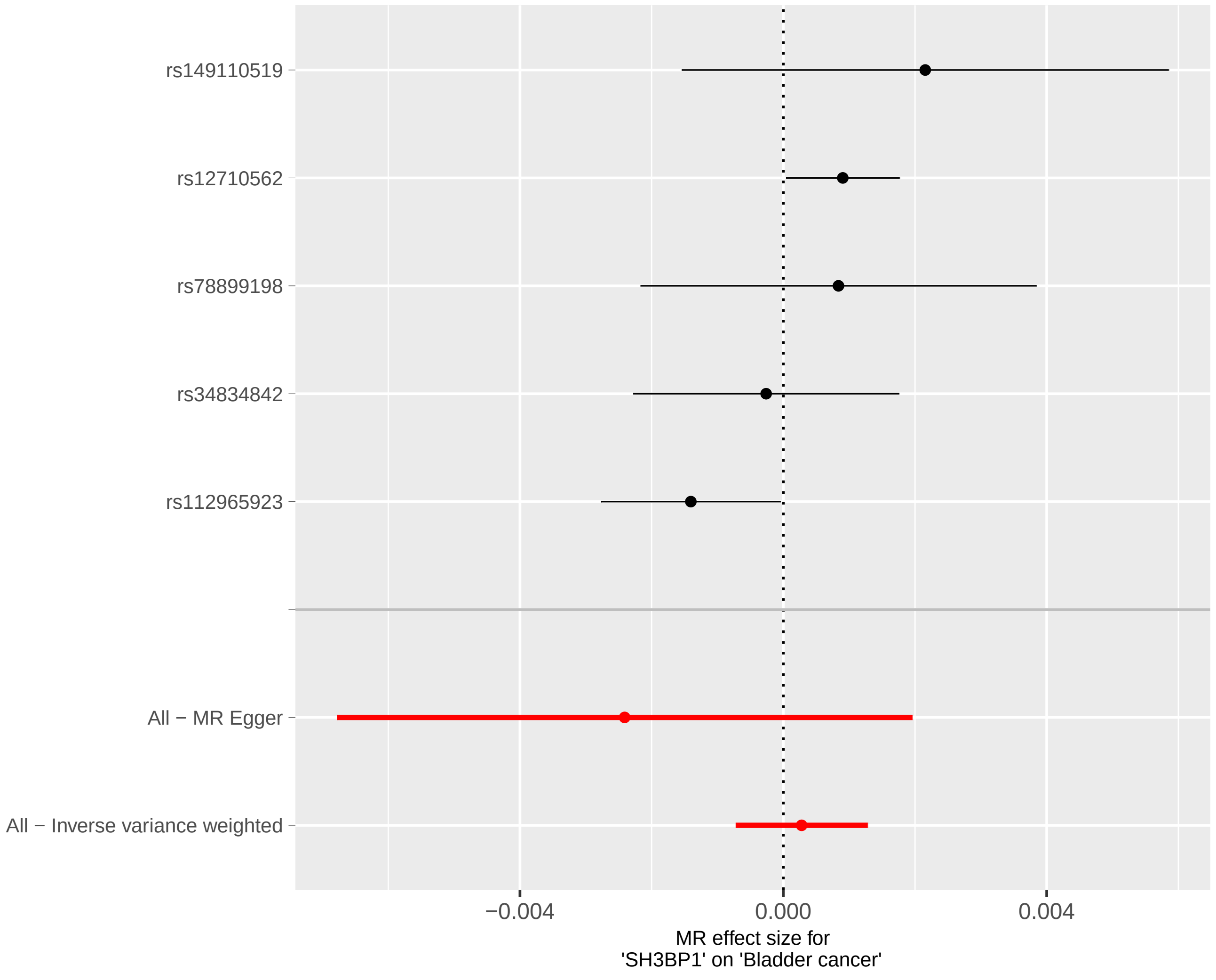
rs149110519
rs12710562
rs78899198
rs34834842
rs112965923
All − MR Egger
All − Inverse variance weighted
−0.004
0.000
0.004
MR effect size for
'SH3BP1' on 'Bladder cancer'

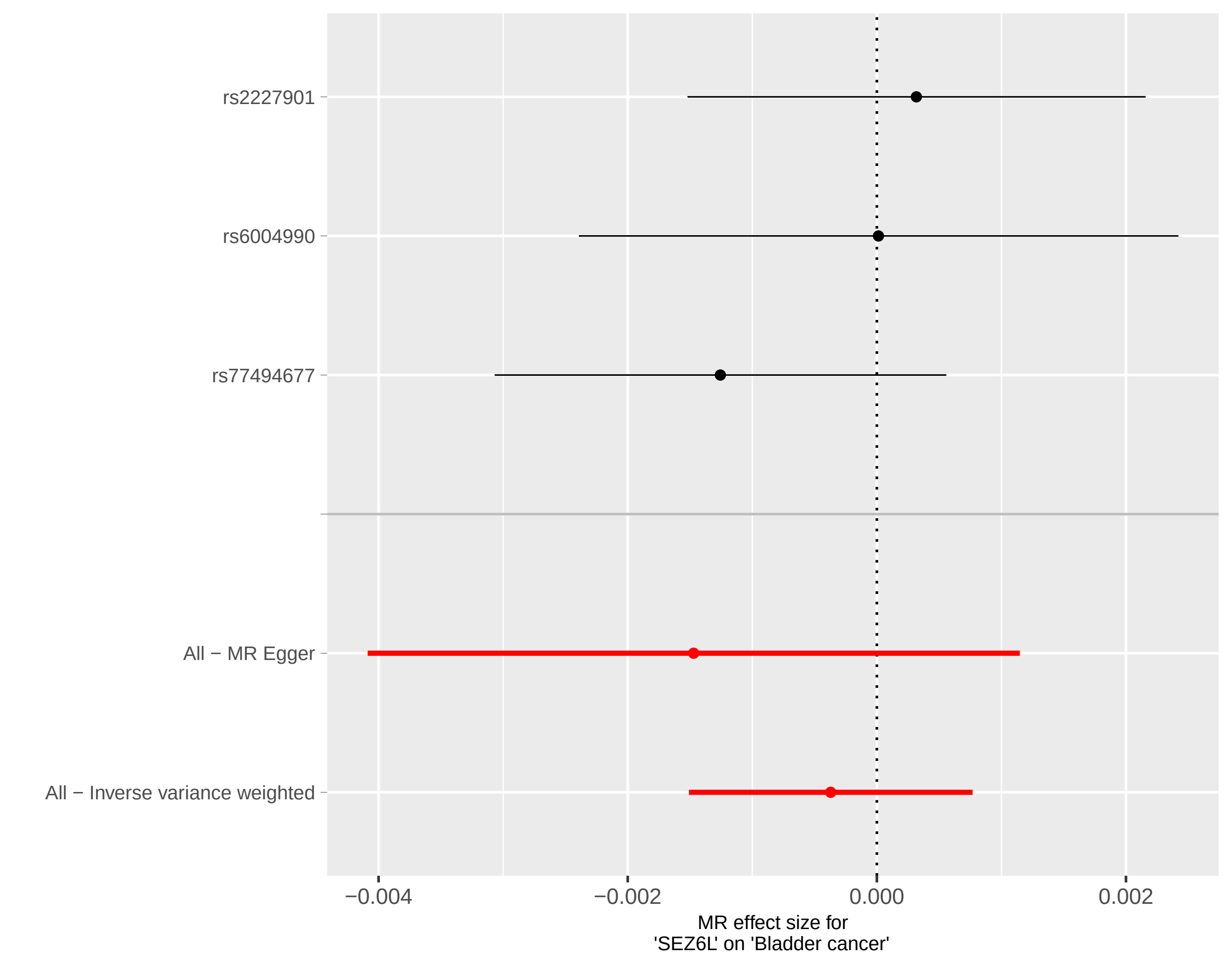
rs2227901
rs6004990
rs77494677
All − MR Egger
All − Inverse variance weighted
−0.004
−0.002
0.000
0.002
MR effect size for
'SEZ6L' on 'Bladder cancer'

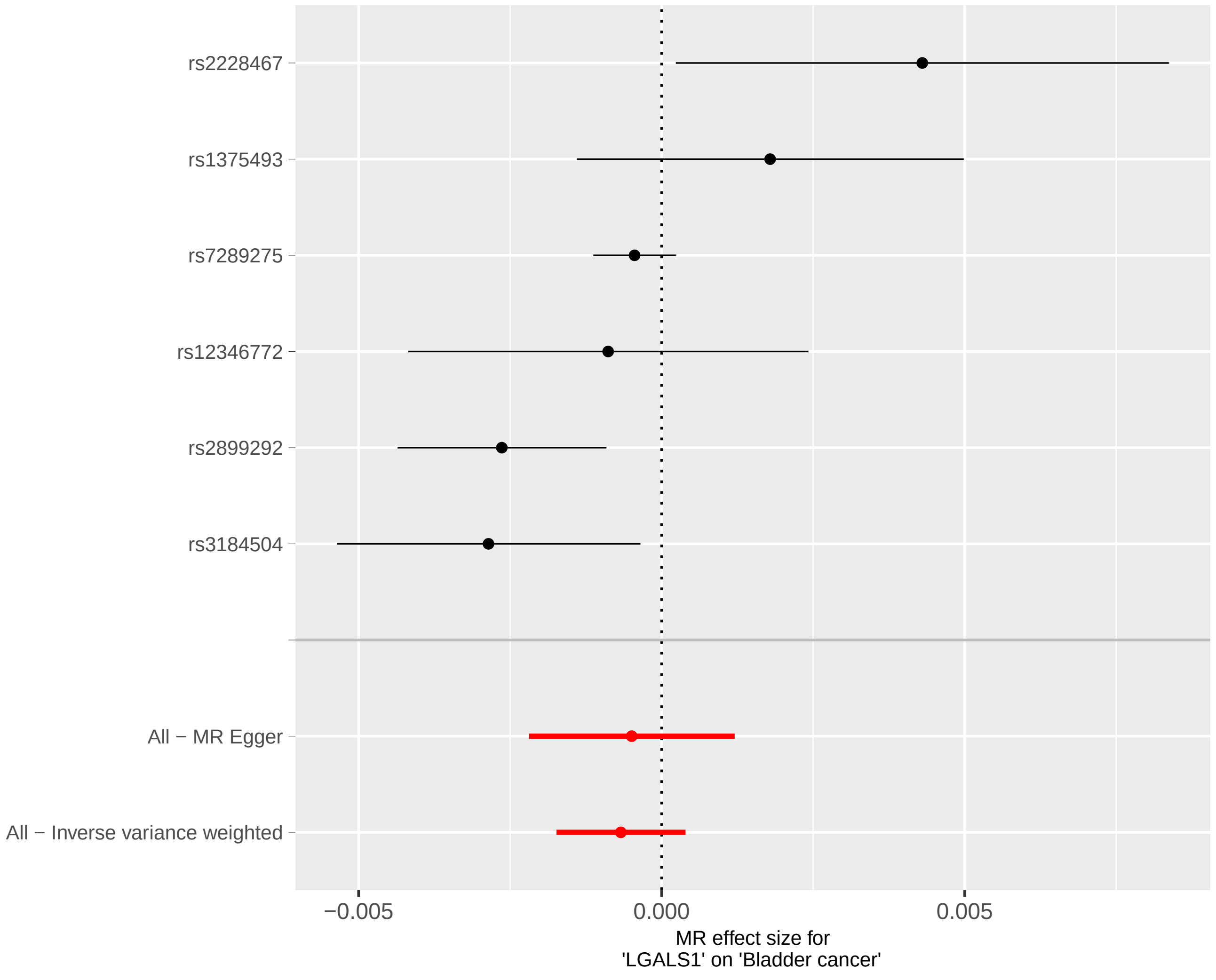

rs2228467
rs1375493
rs7289275
rs12346772
rs2899292
rs3184504
All − MR Egger
All − Inverse variance weighted
−0.005
0.000
0.005
MR effect size for
'LGALS1' on 'Bladder cancer'

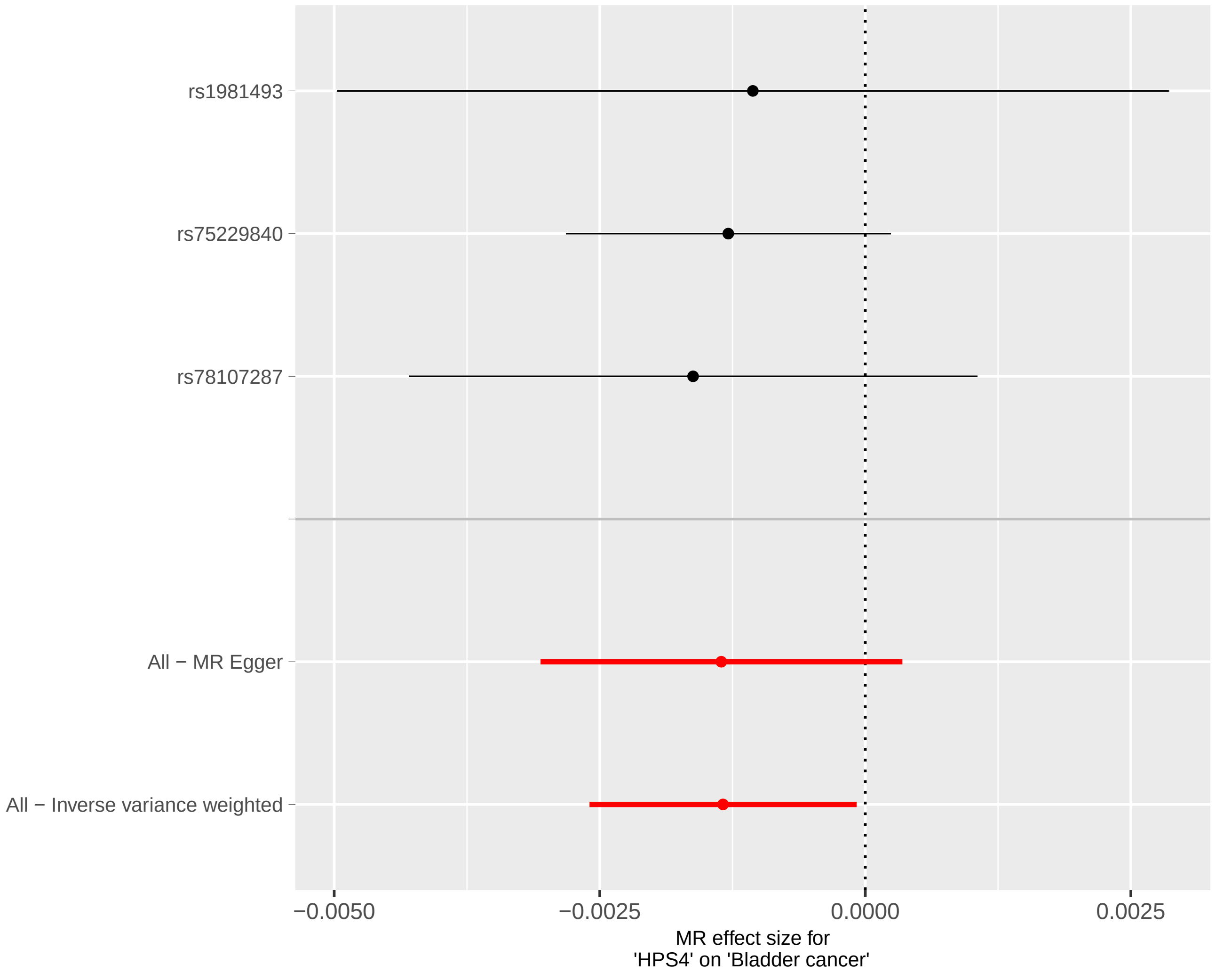
rs1981493
rs75229840
rs78107287
All − MR Egger
All − Inverse variance weighted
−0.0050
−0.0025
0.0000
0.0025
MR effect size for
'HPS4' on 'Bladder cancer'

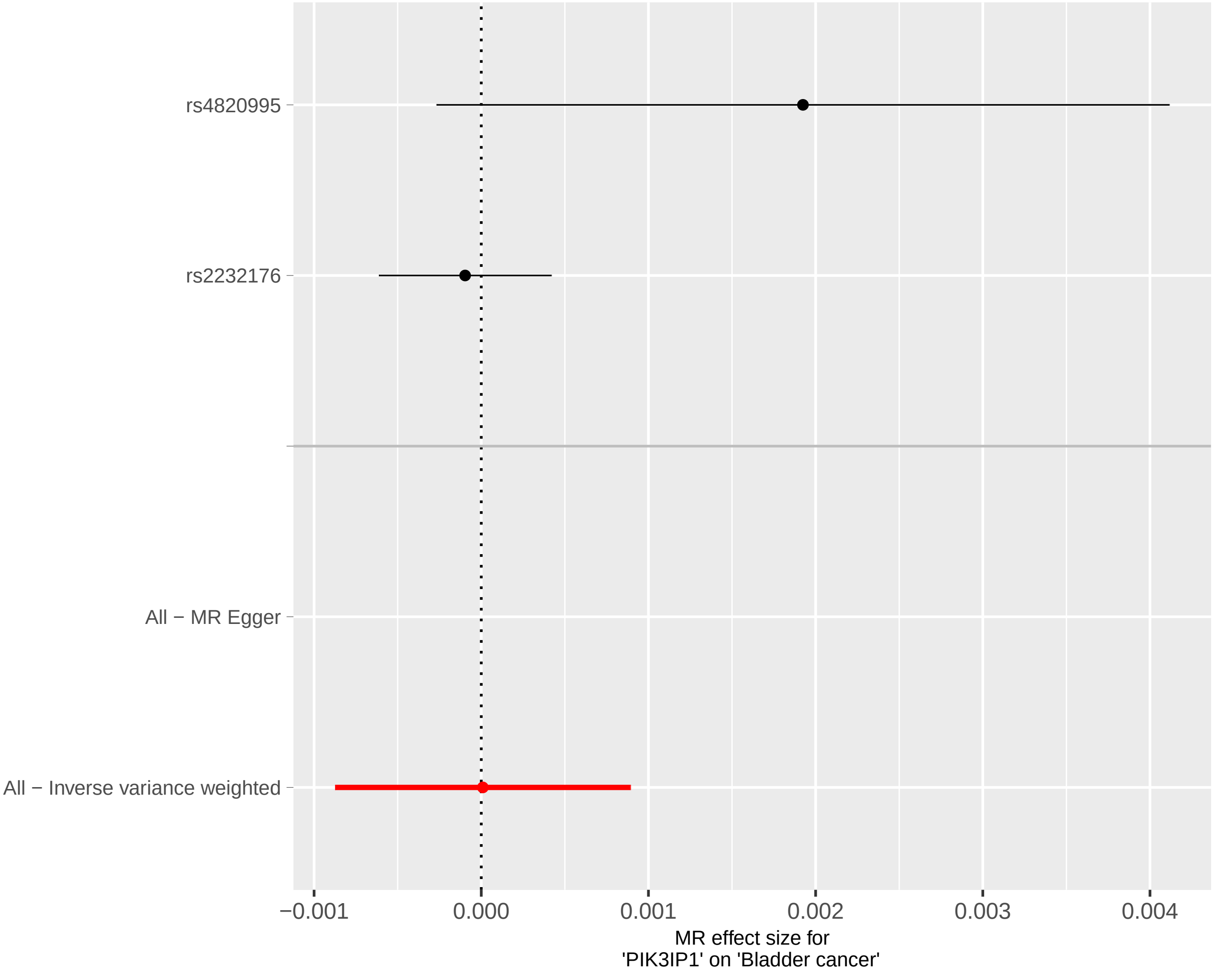

rs4820995
rs2232176
All − MR Egger
All − Inverse variance weighted
−0.001
0.000
0.001
0.002
0.003
0.004
MR effect size for
'PIK3IP1' on 'Bladder cancer'

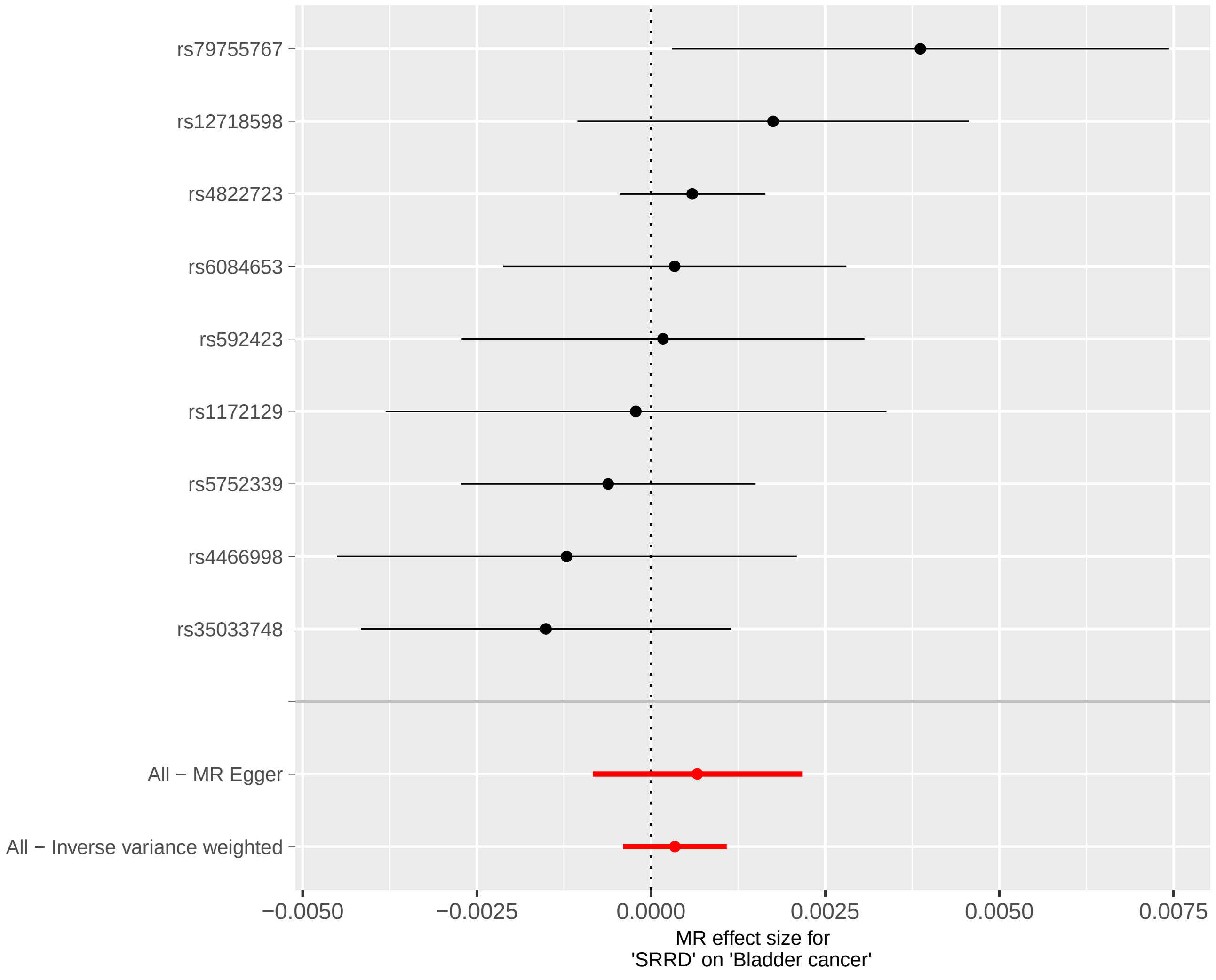
rs79755767
rs12718598
rs4822723
rs6084653
rs592423
rs1172129
rs5752339
rs4466998
rs35033748
All − MR Egger
All − Inverse variance weighted
−0.0050
−0.0025
0.0000
0.0025
0.0050
0.0075
MR effect size for
'SRRD' on 'Bladder cancer'

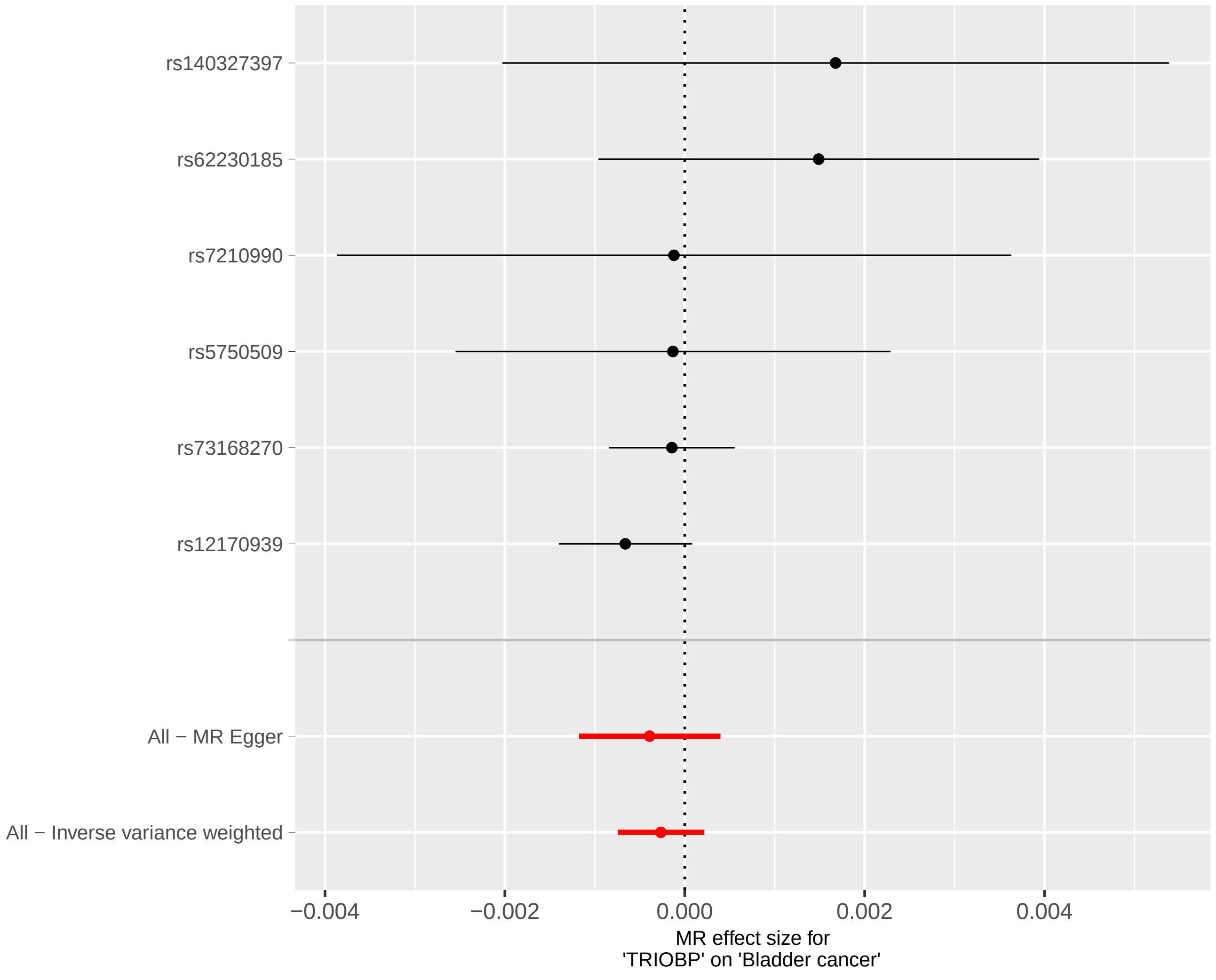
rs140327397
rs62230185
rs7210990
rs5750509
rs73168270
rs12170939
All − MR Egger
All − Inverse variance weighted
−0.004
−0.002
0.000
0.002
0.004
MR effect size for
'TRIOBP' on 'Bladder cancer'

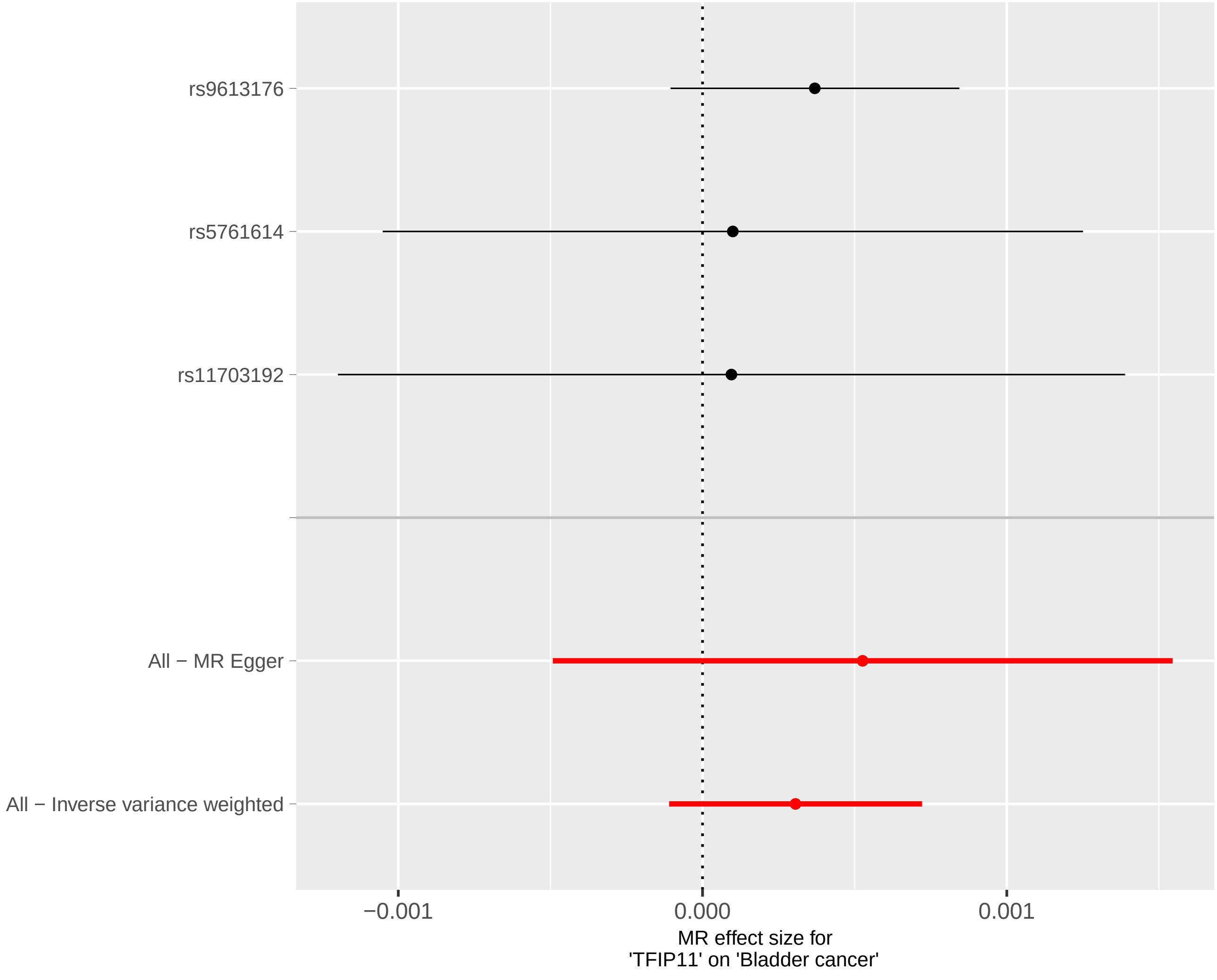

rs9613176
rs5761614
rs11703192
All − MR Egger
All − Inverse variance weighted
−0.001
0.000
0.001
MR effect size for
'TFIP11' on 'Bladder cancer'

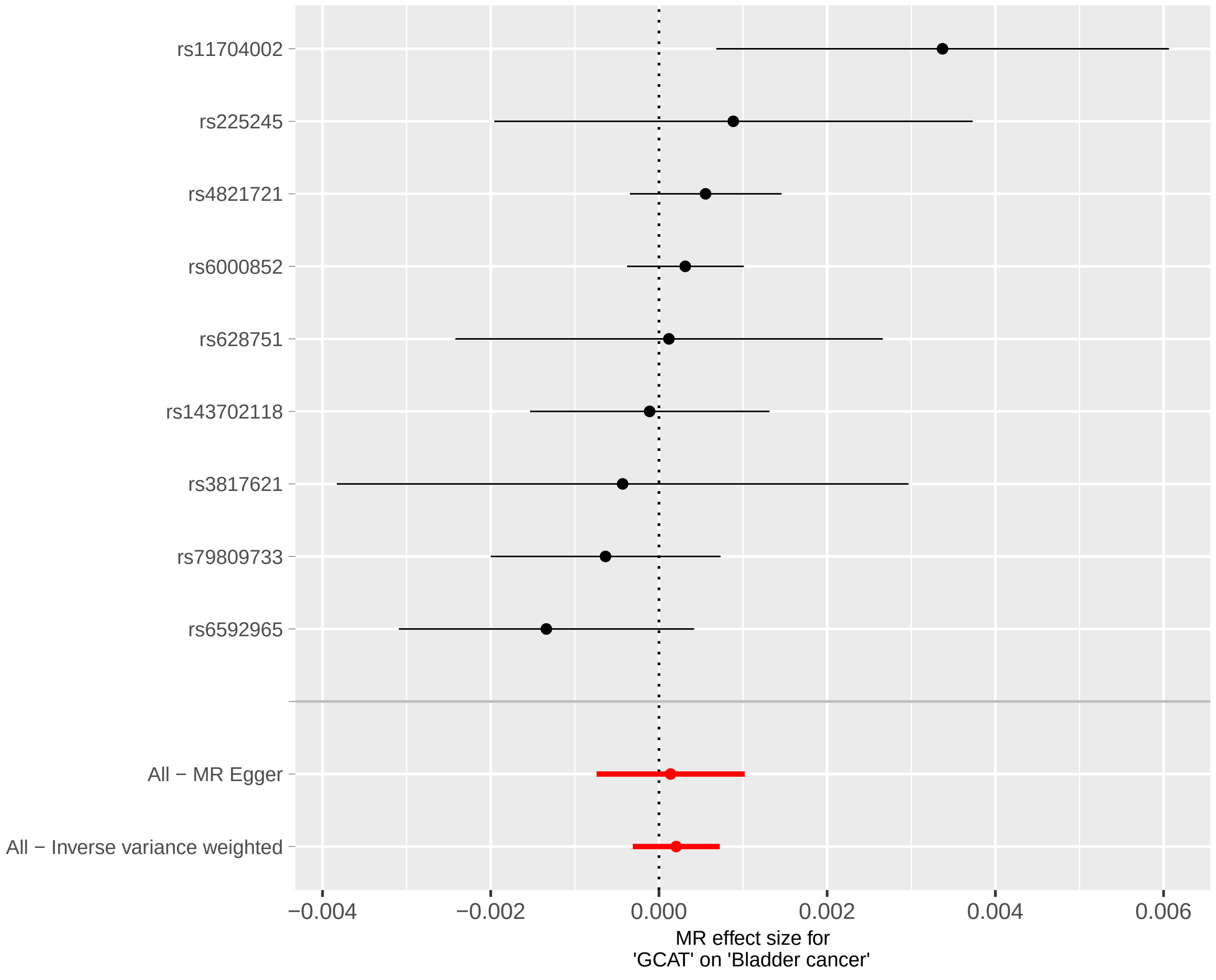

rs11704002
rs225245
rs4821721
rs6000852
rs628751
rs143702118
rs3817621
rs79809733
rs6592965
All − MR Egger
All − Inverse variance weighted
−0.004
−0.002
0.000
0.002
0.004
0.006
MR effect size for
'GCAT' on 'Bladder cancer'

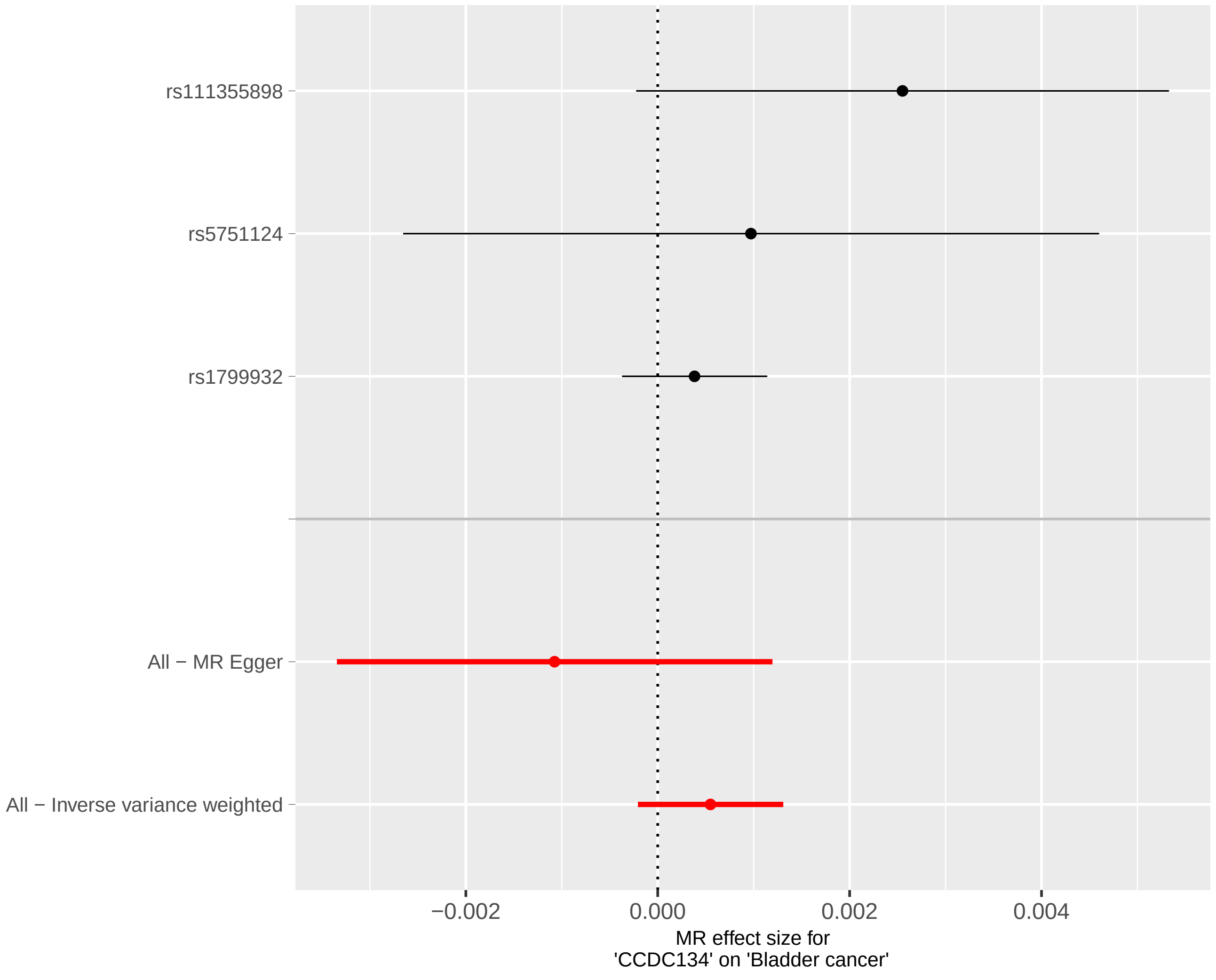

rs111355898
rs5751124
rs1799932
All − MR Egger
All − Inverse variance weighted
−0.002
0.000
0.002
0.004
MR effect size for
'CCDC134' on 'Bladder cancer'

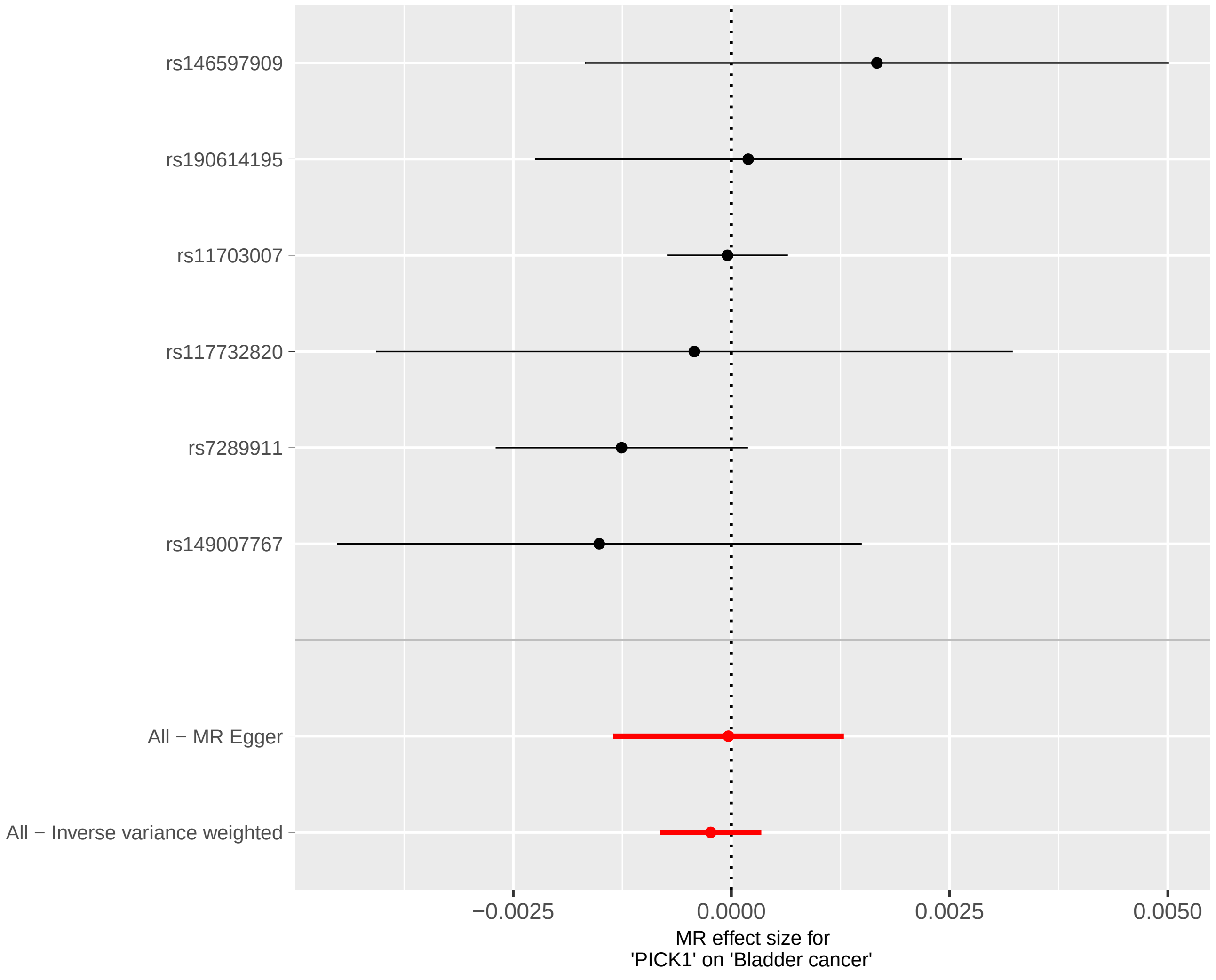

rs146597909
rs190614195
rs11703007
rs117732820
rs7289911
rs149007767
All − MR Egger
All − Inverse variance weighted
−0.0025
0.0000
0.0025
0.0050
MR effect size for
'PICK1' on 'Bladder cancer'

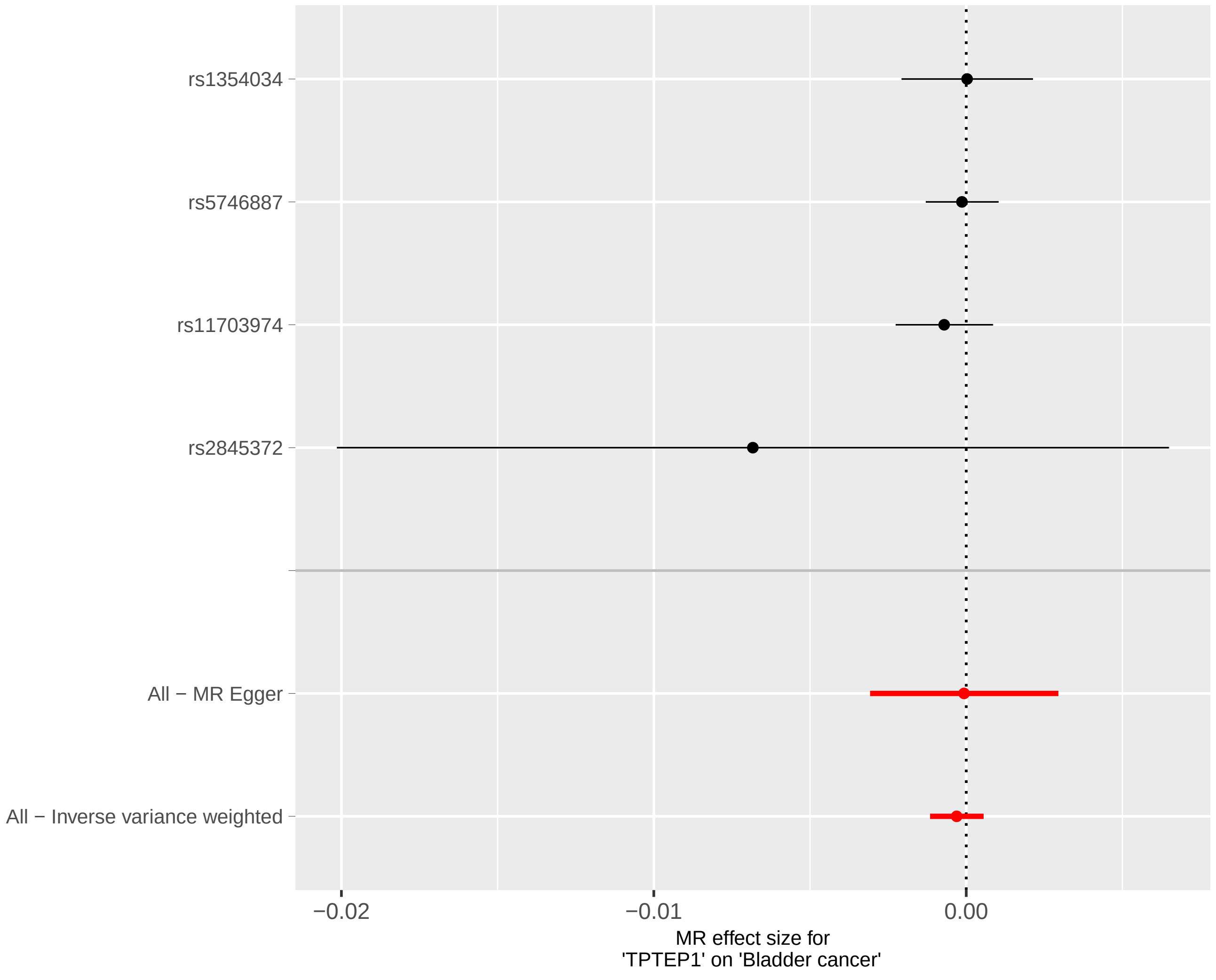
rs1354034
rs5746887
rs11703974
rs2845372
All − MR Egger
All − Inverse variance weighted
−0.02
−0.01
0.00
MR effect size for
'TPTEP1' on 'Bladder cancer'

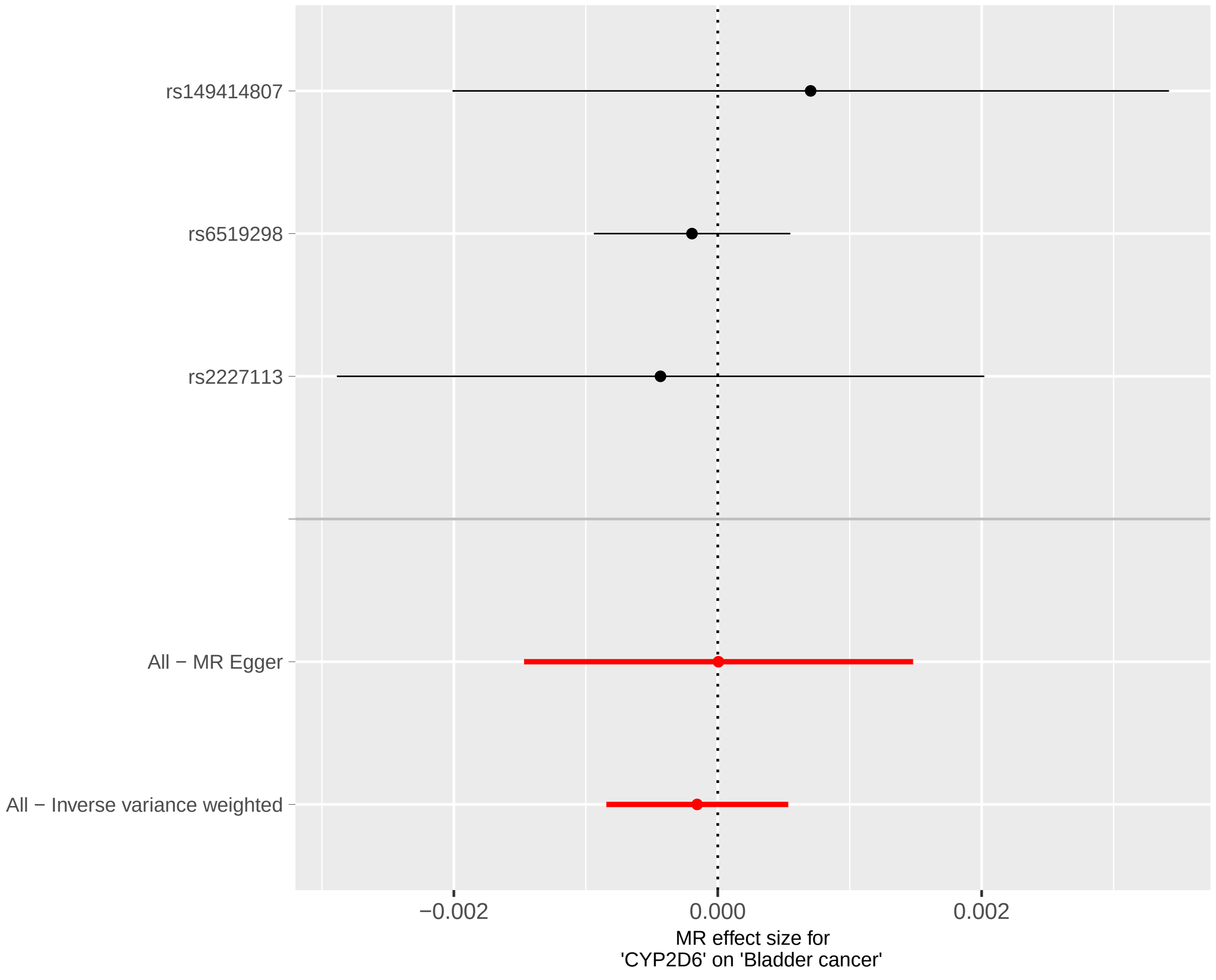

rs149414807
rs6519298
rs2227113
All − MR Egger
All − Inverse variance weighted
−0.002
0.000
0.002
MR effect size for
'CYP2D6' on 'Bladder cancer'

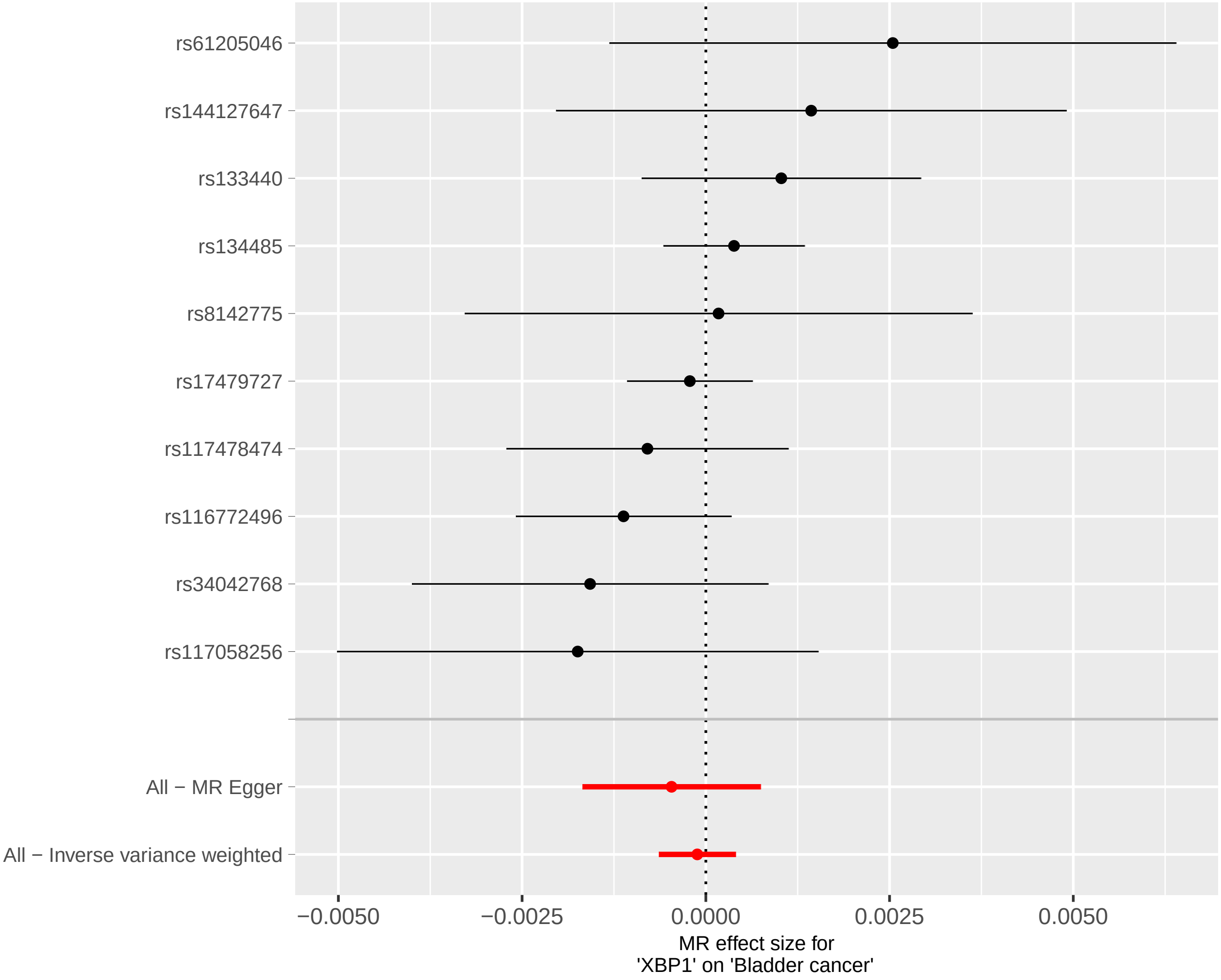
rs61205046
rs144127647
rs133440
rs134485
rs8142775
rs17479727
rs117478474
rs116772496
rs34042768
rs117058256
All − MR Egger
All − Inverse variance weighted
−0.0050
−0.0025
0.0000
0.0025
0.0050
MR effect size for
'XBP1' on 'Bladder cancer'

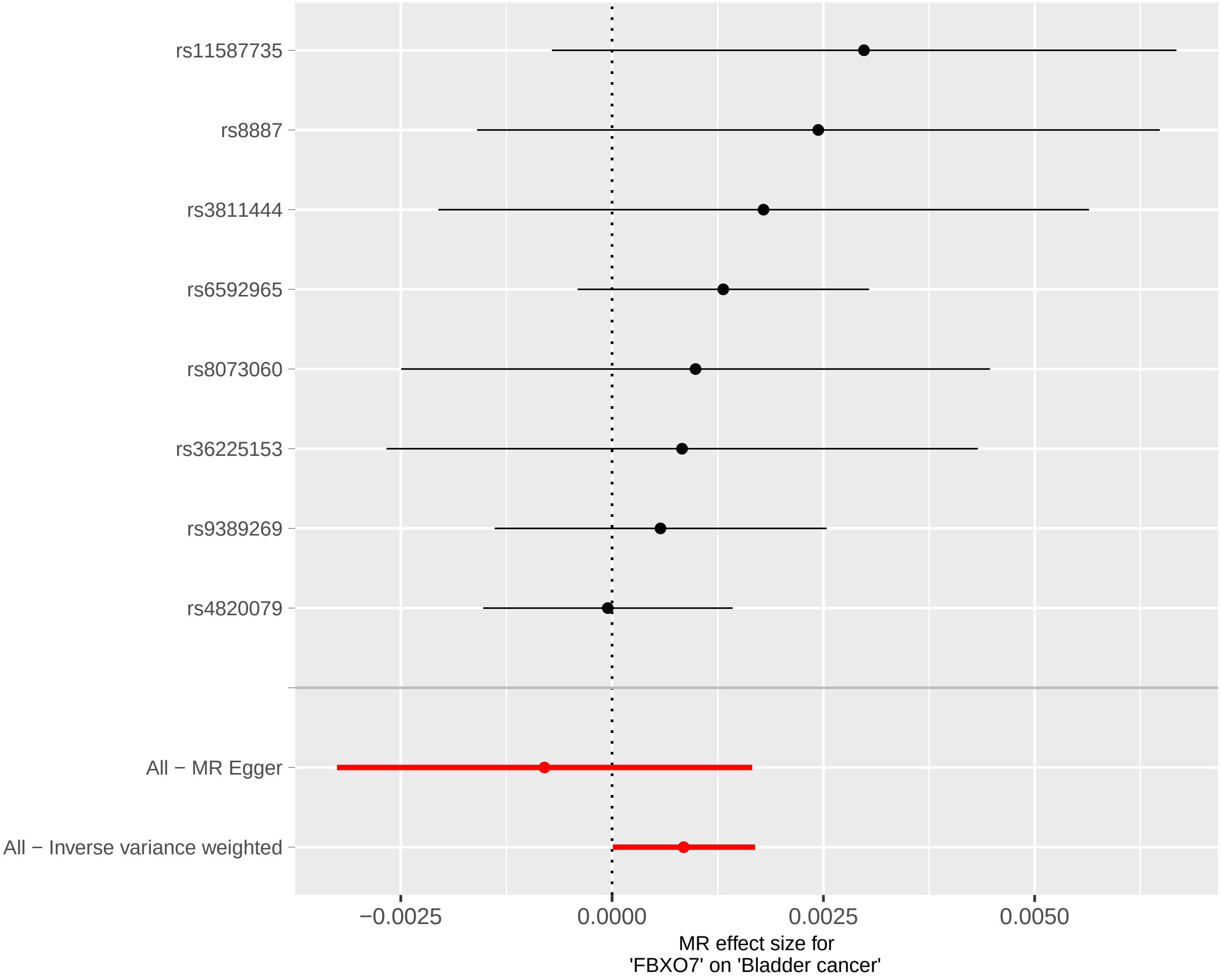

rs11587735
rs8887
rs3811444
rs6592965
rs8073060
rs36225153
rs9389269
rs4820079
All − MR Egger
All − Inverse variance weighted
−0.0025
0.0000
0.0025
0.0050
MR effect size for
'FBXO7' on 'Bladder cancer'

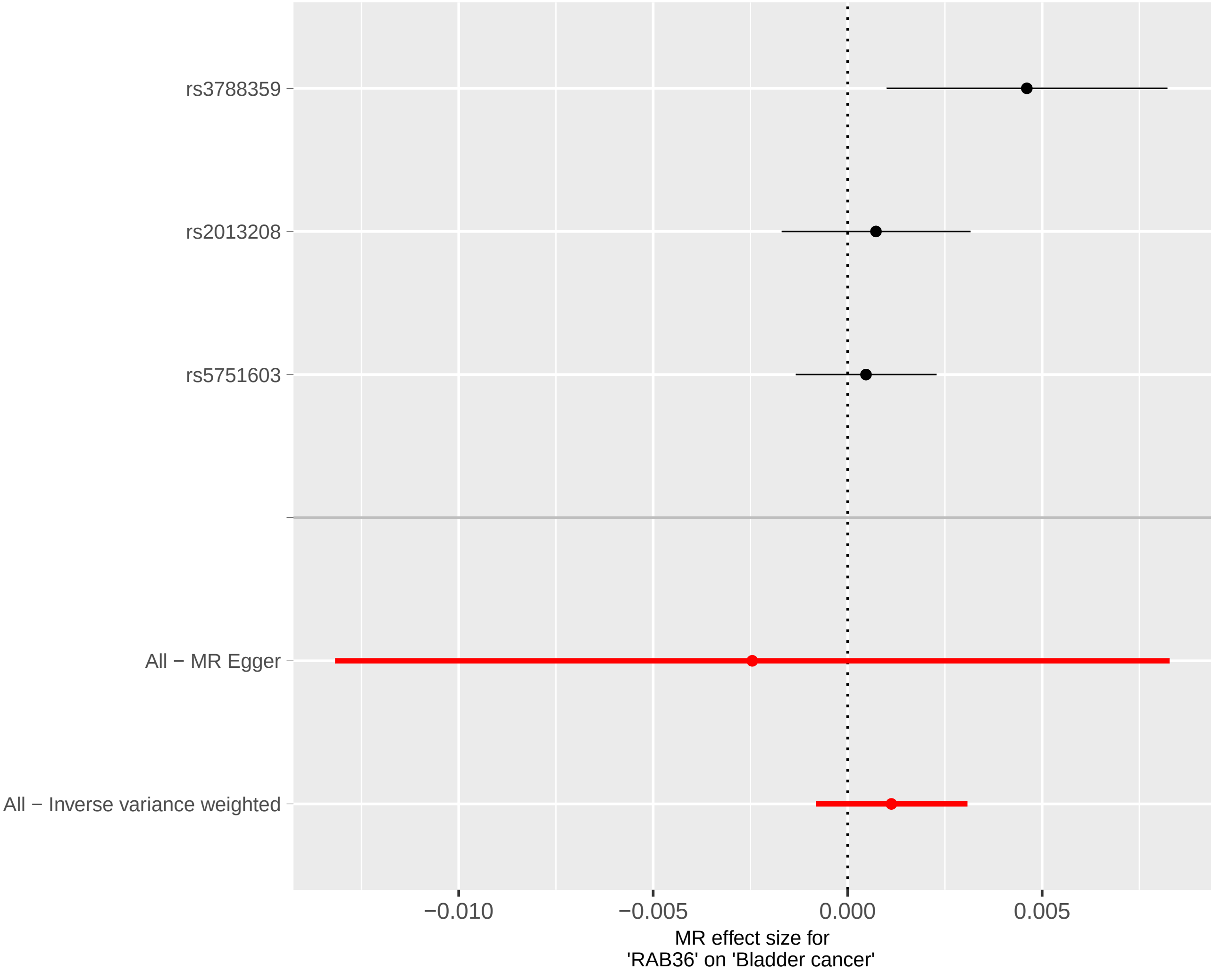
rs3788359
rs2013208
rs5751603
All − MR Egger
All − Inverse variance weighted
−0.010
−0.005
0.000
0.005
MR effect size for
'RAB36' on 'Bladder cancer'

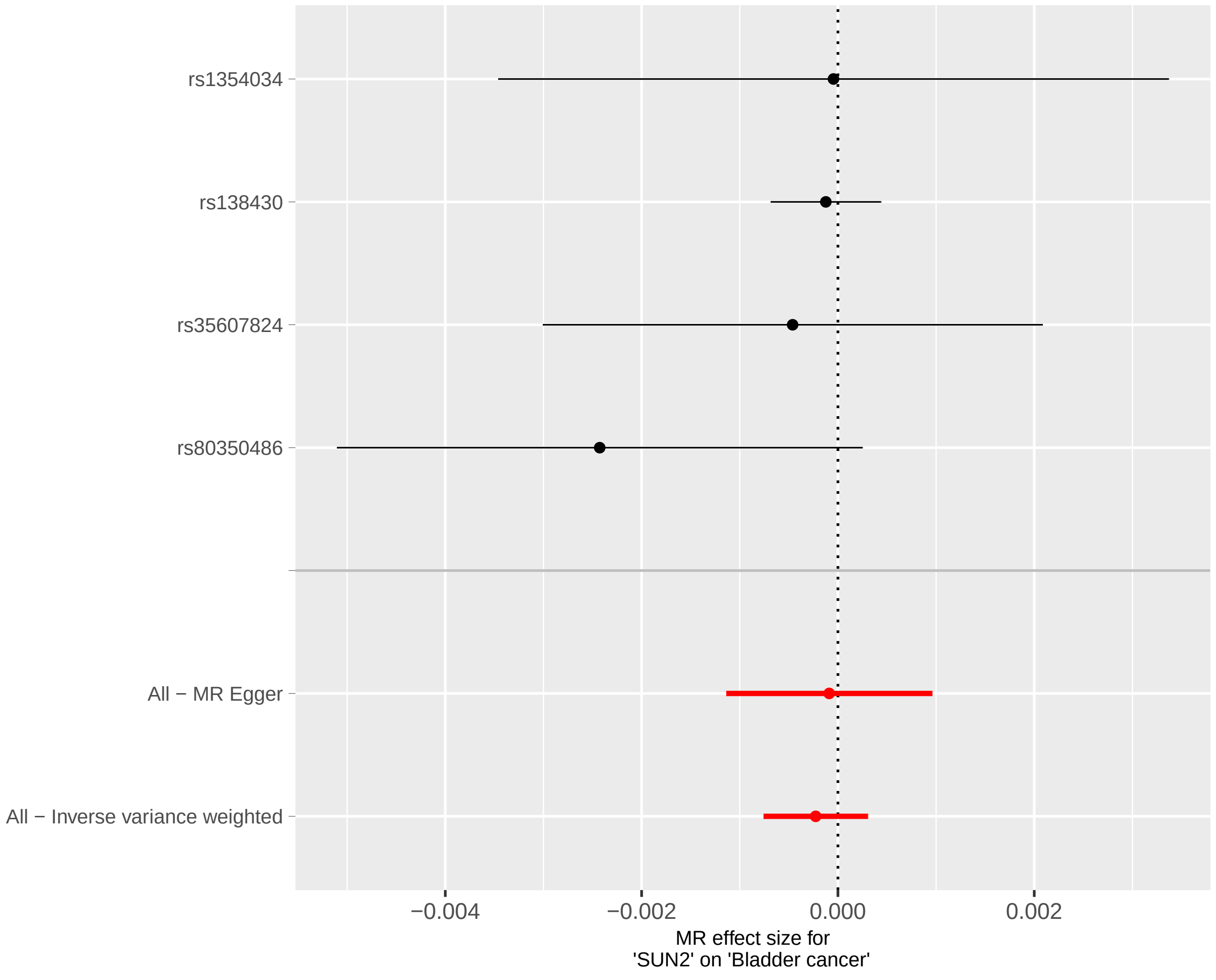

rs1354034
rs138430
rs35607824
rs80350486
All − MR Egger
All − Inverse variance weighted
−0.004
−0.002
0.000
0.002
MR effect size for
'SUN2' on 'Bladder cancer'

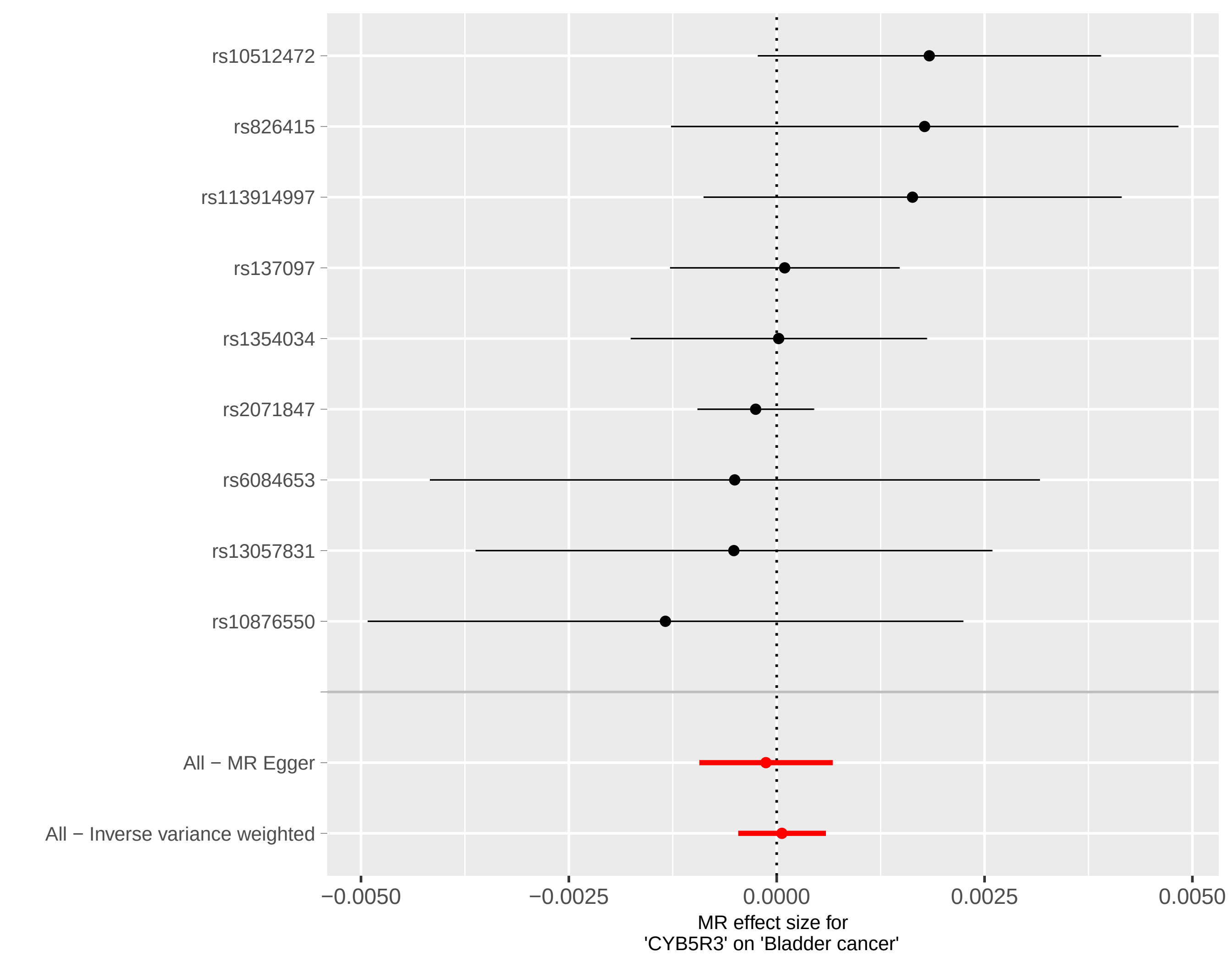
rs10512472
rs826415
rs113914997
rs137097
rs1354034
rs2071847
rs6084653
rs13057831
rs10876550
All − MR Egger
All − Inverse variance weighted
−0.0050
−0.0025
0.0000
0.0025
0.0050
MR effect size for
'CYB5R3' on 'Bladder cancer'

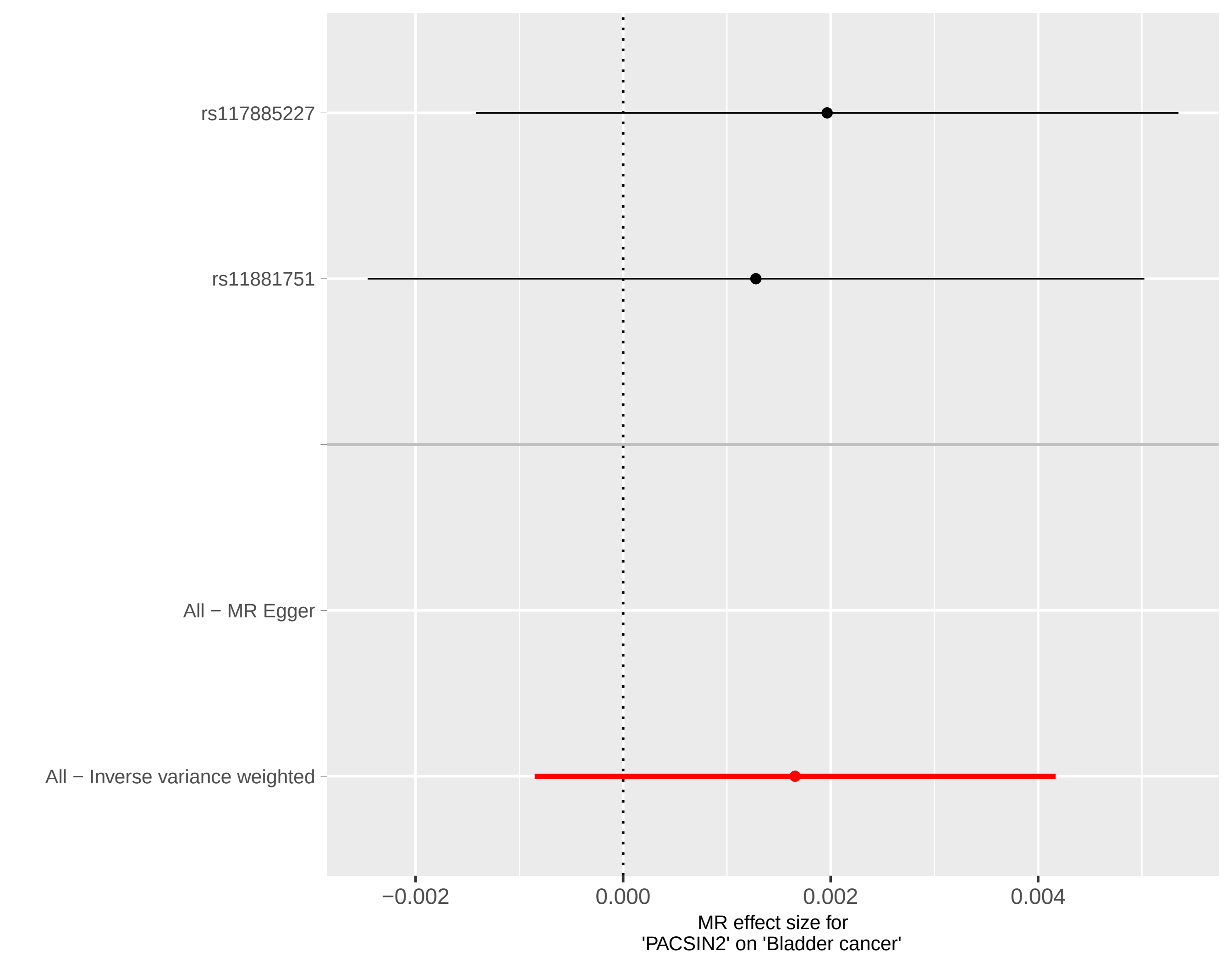
rs117885227
rs11881751
All − MR Egger
All − Inverse variance weighted
−0.002
0.000
0.002
0.004
MR effect size for
'PACSIN2' on 'Bladder cancer'

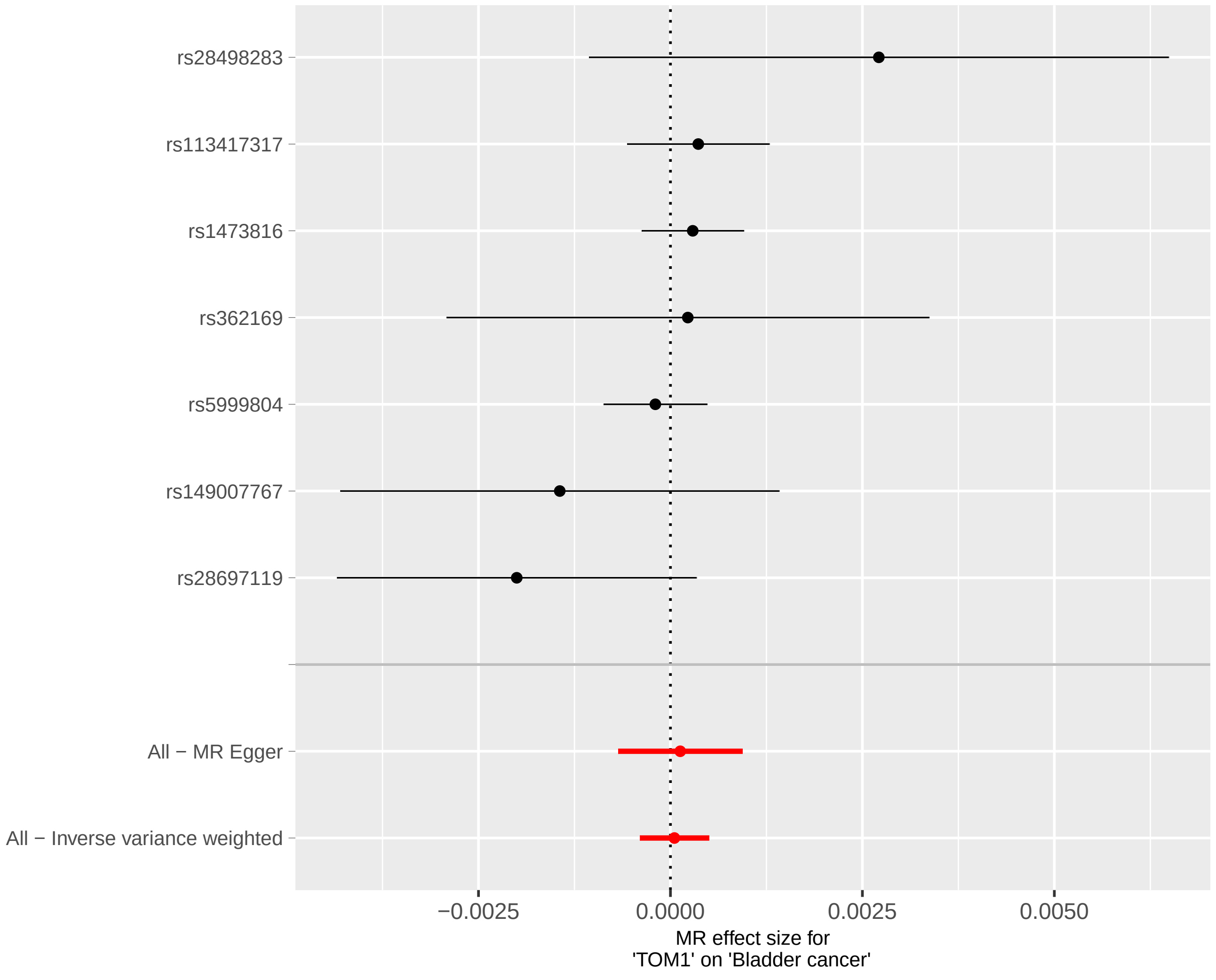
rs28498283
rs113417317
rs1473816
rs362169
rs5999804
rs149007767
rs28697119
All − MR Egger
All − Inverse variance weighted
−0.0025
0.0000
0.0025
0.0050
MR effect size for
'TOM1' on 'Bladder cancer'

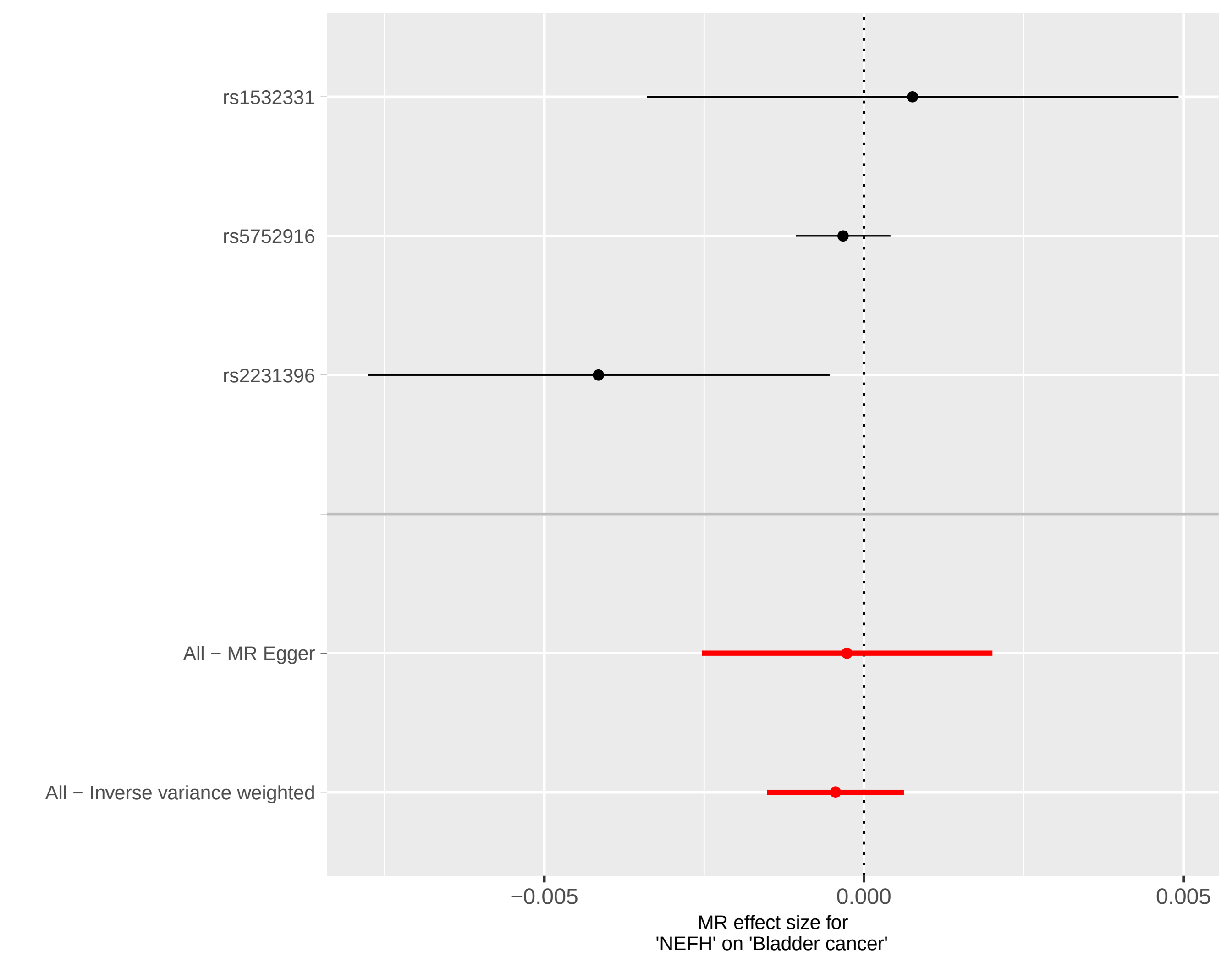
rs1532331
rs5752916
rs2231396
All − MR Egger
All − Inverse variance weighted
−0.005
0.000
0.005
MR effect size for
'NEFH' on 'Bladder cancer'

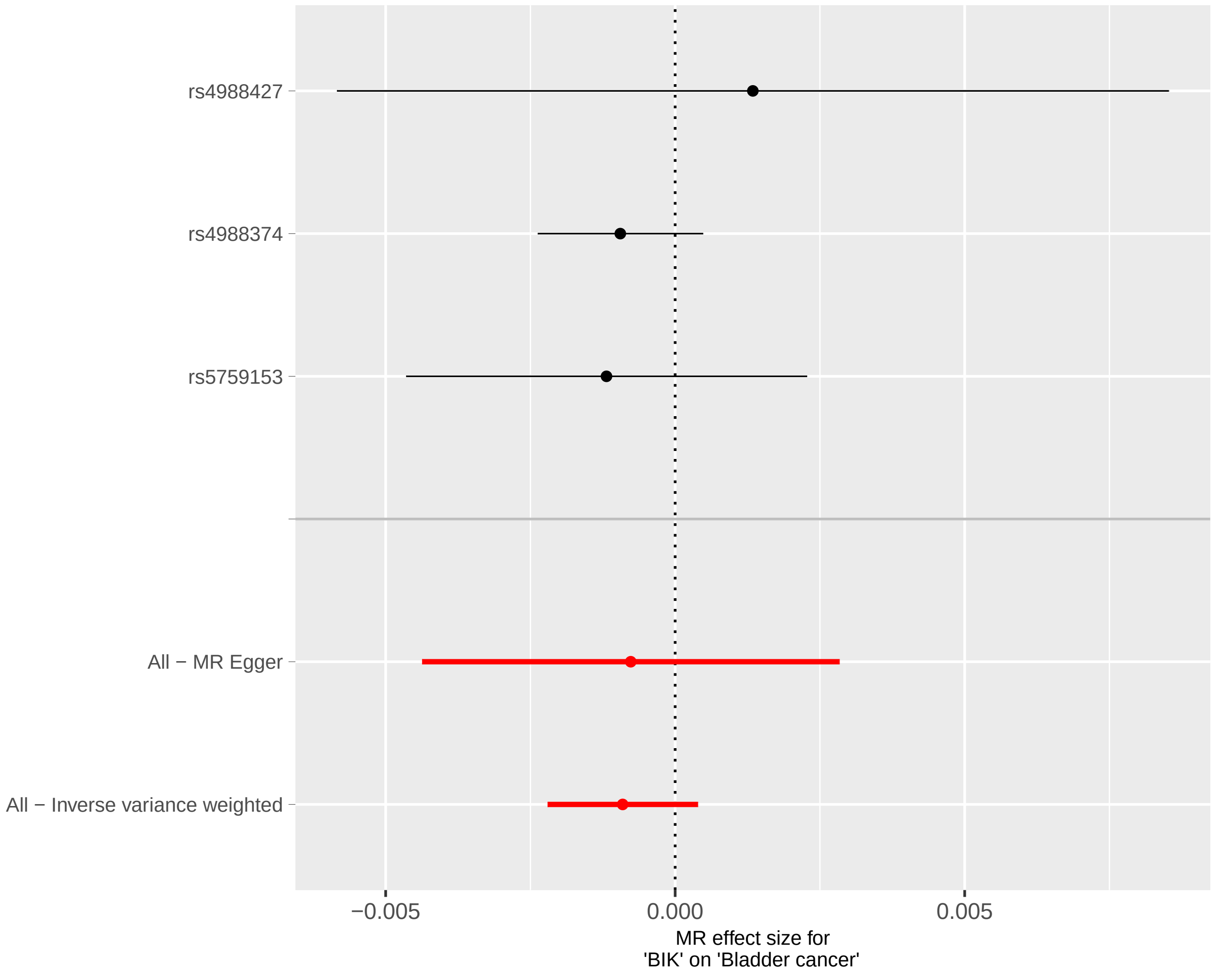

rs4988427
rs4988374
rs5759153
All − MR Egger
All − Inverse variance weighted
−0.005
0.000
0.005
MR effect size for
'BIK' on 'Bladder cancer'

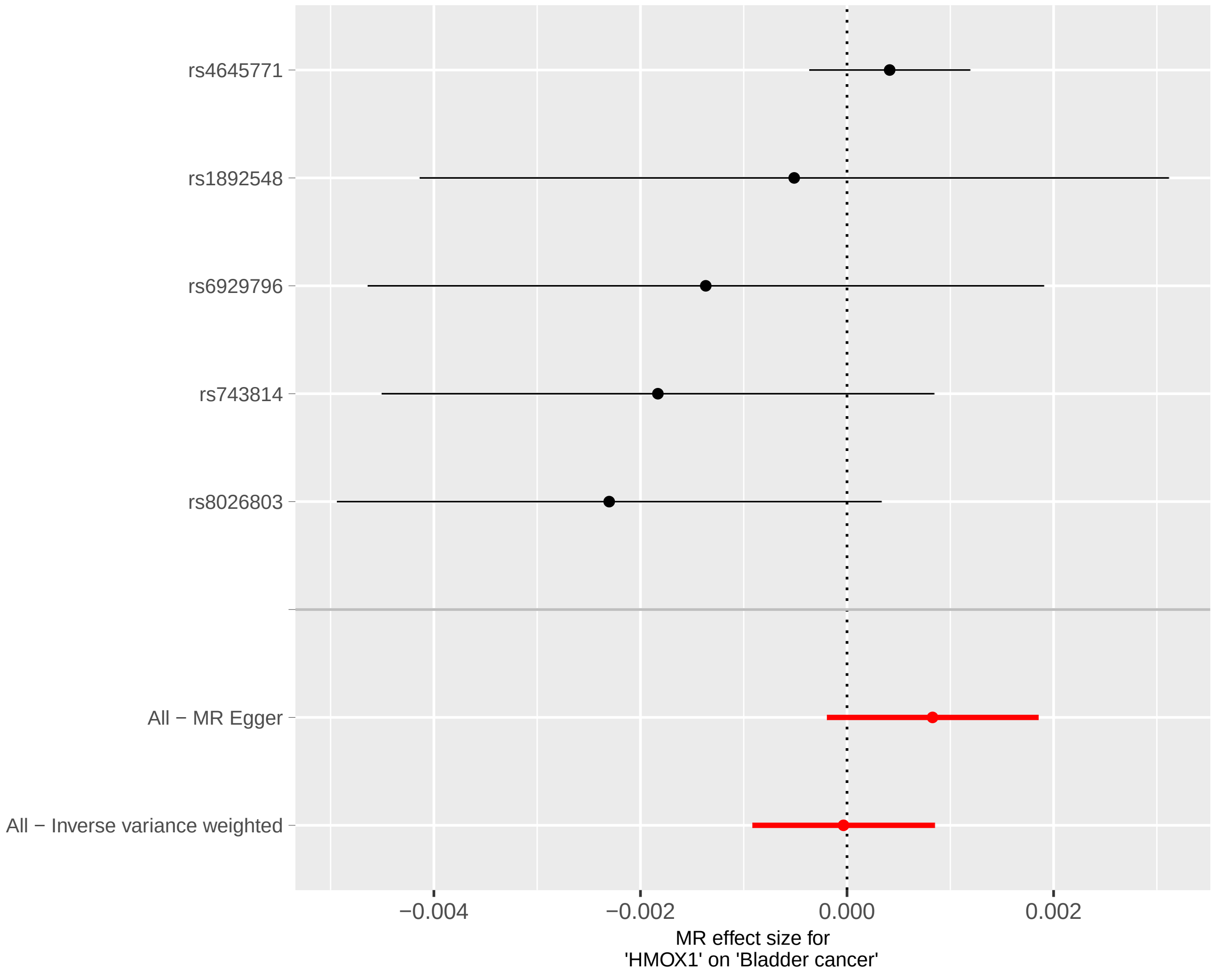

rs4645771
rs1892548
rs6929796
rs743814
rs8026803
All − MR Egger
All − Inverse variance weighted
−0.004
−0.002
0.000
0.002
MR effect size for
'HMOX1' on 'Bladder cancer'

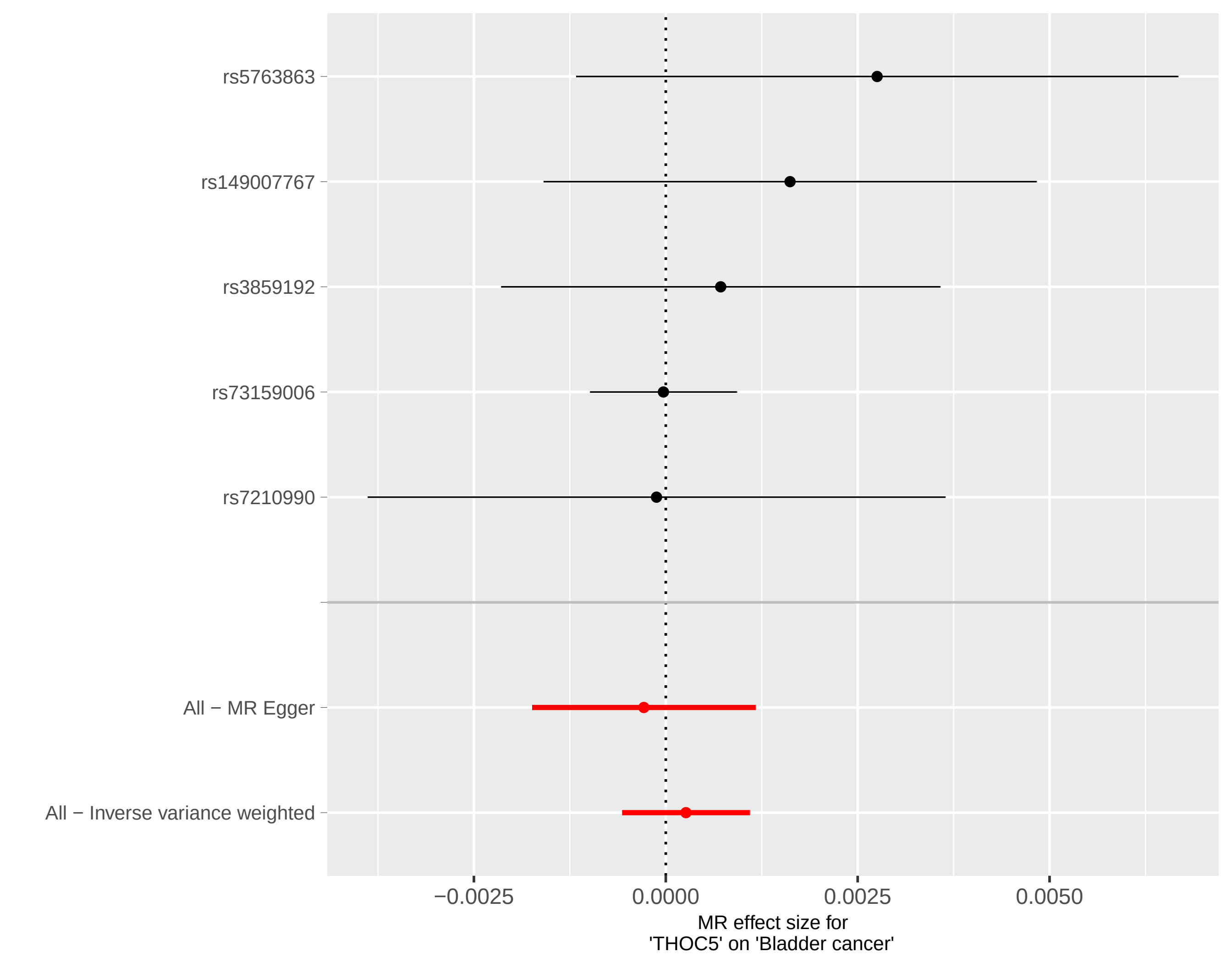

rs5763863
rs149007767
rs3859192
rs73159006
rs7210990
All − MR Egger
All − Inverse variance weighted
−0.0025
0.0000
0.0025
0.0050
MR effect size for
'THOC5' on 'Bladder cancer'

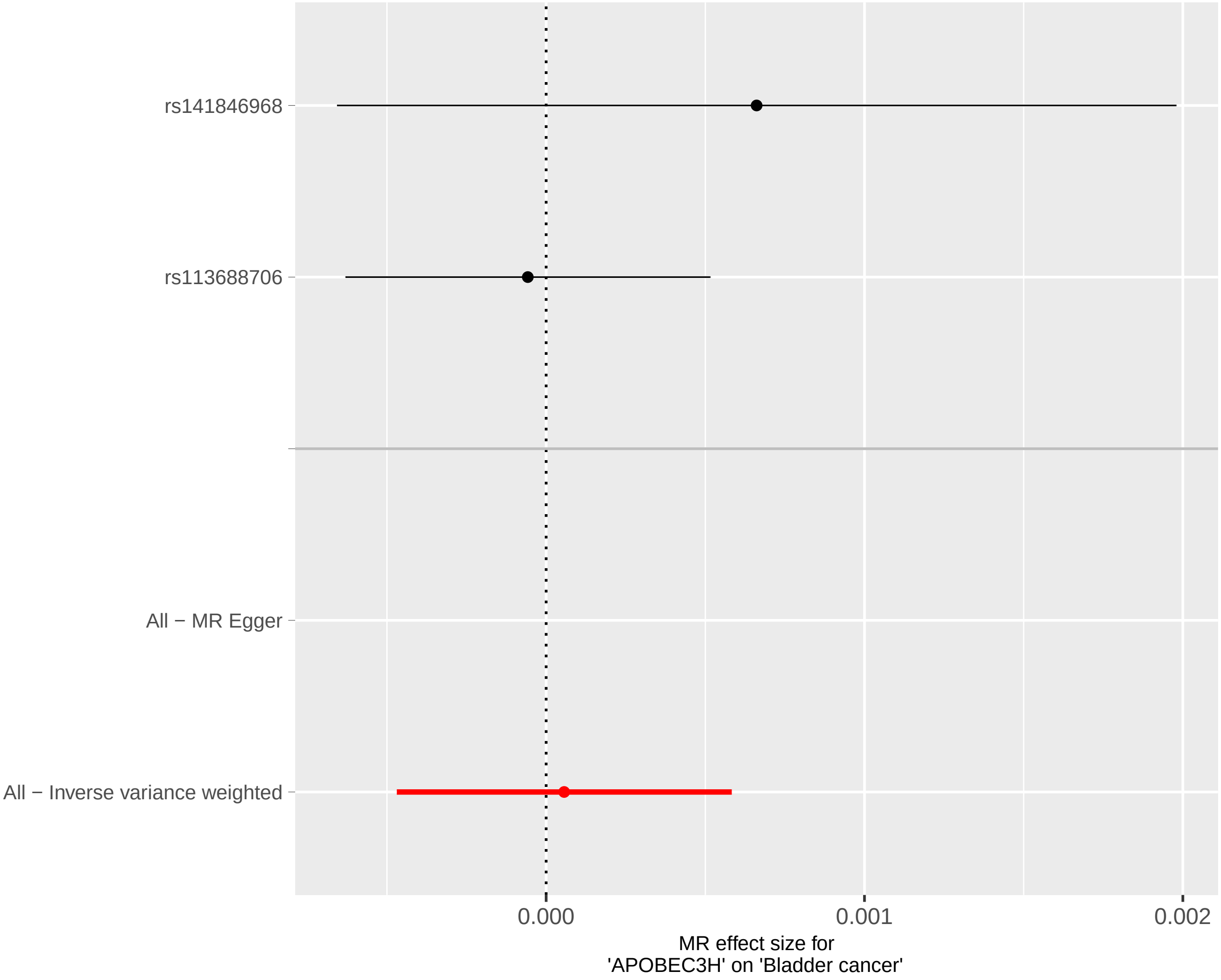

rs141846968
rs113688706
All − MR Egger
All − Inverse variance weighted
0.000
0.001
0.002
MR effect size for
'APOBEC3H' on 'Bladder cancer'

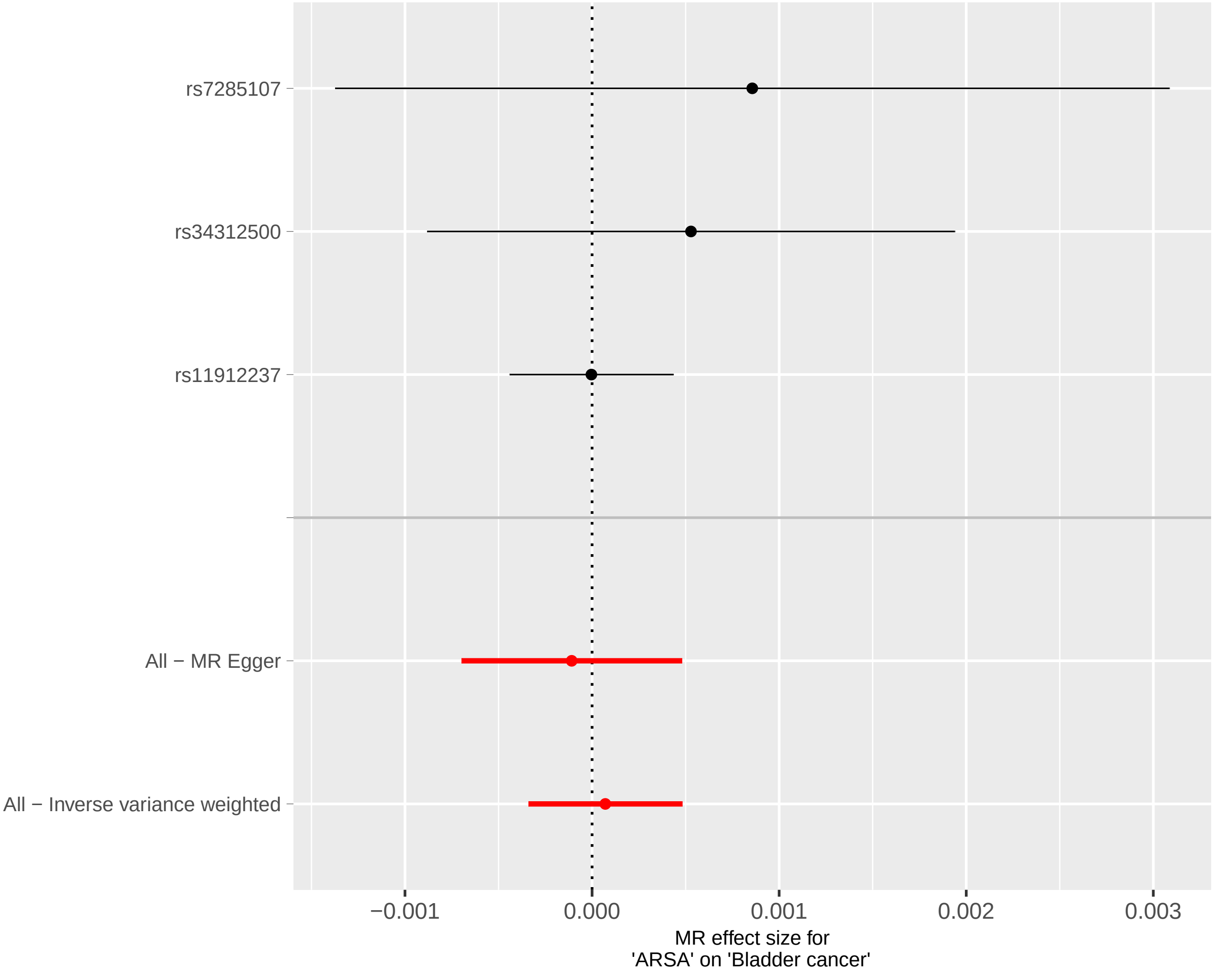

rs7285107
rs34312500
rs11912237
All − MR Egger
All − Inverse variance weighted
−0.001
0.000
0.001
0.002
0.003
MR effect size for
'ARSA' on 'Bladder cancer'

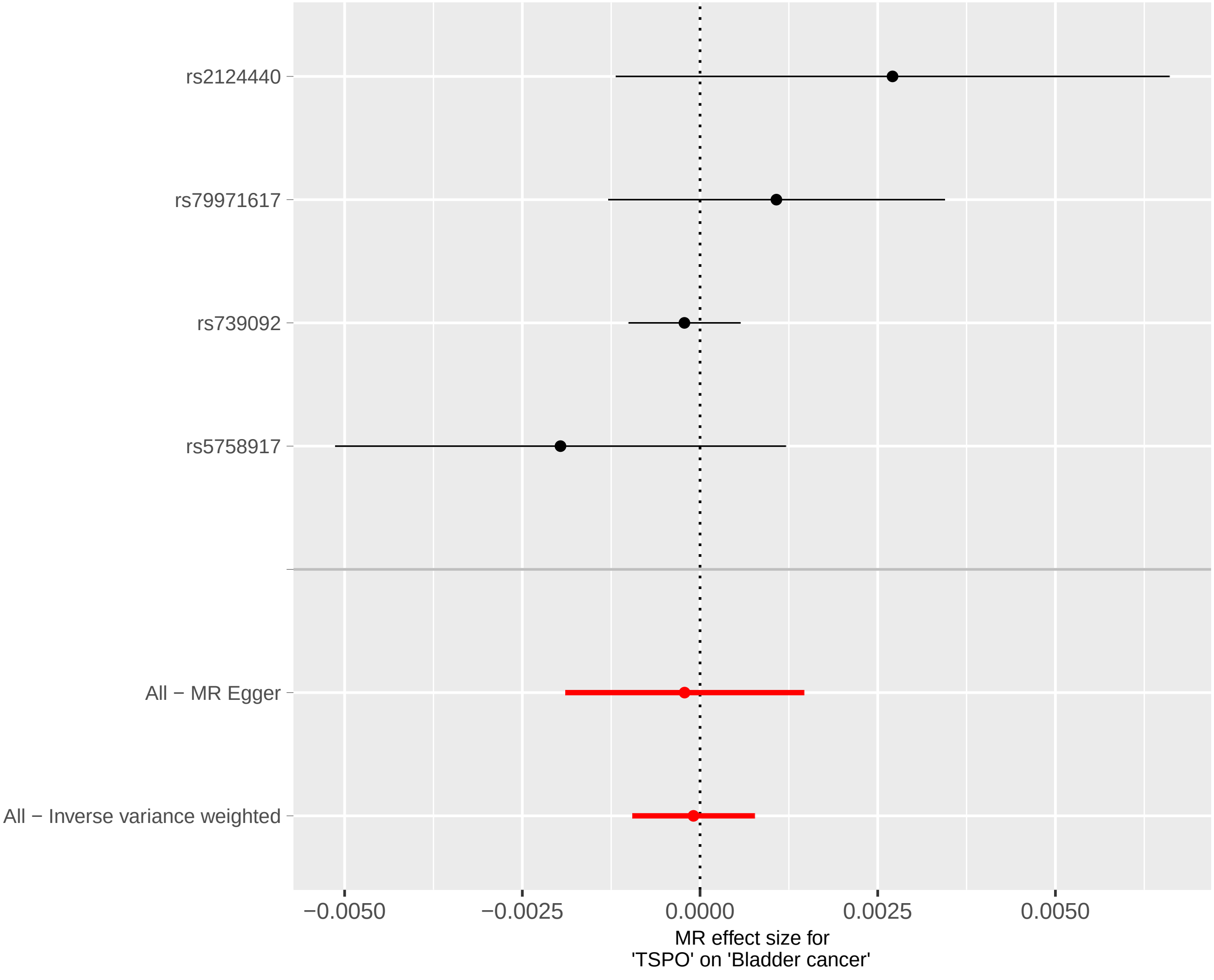
rs2124440
rs79971617
rs739092
rs5758917
All − MR Egger
All − Inverse variance weighted
−0.0050
−0.0025
0.0000
0.0025
0.0050
MR effect size for
'TSPO' on 'Bladder cancer'

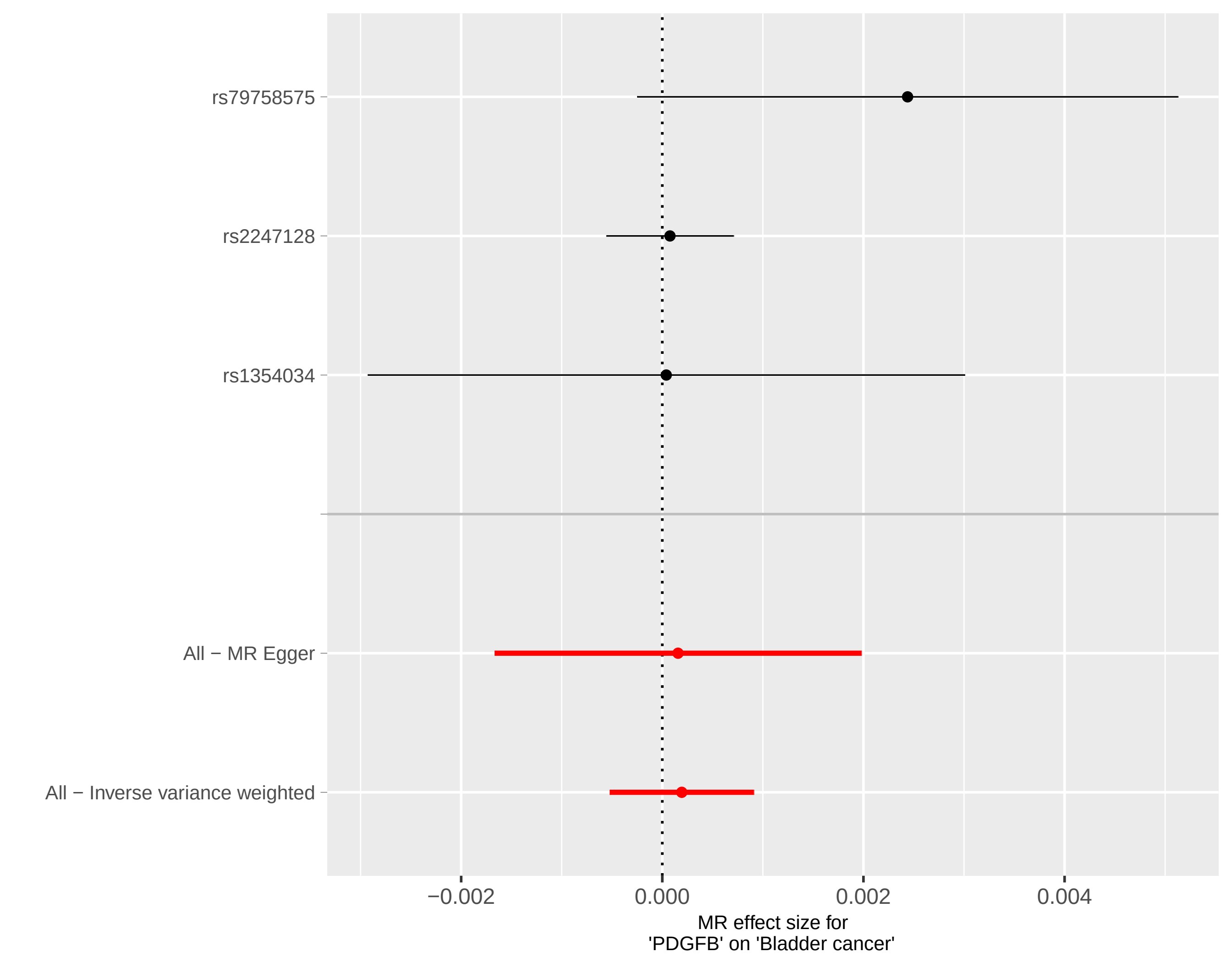
rs79758575
rs2247128
rs1354034
All − MR Egger
All − Inverse variance weighted
−0.002
0.000
0.002
0.004
MR effect size for
'PDGFB' on 'Bladder cancer'

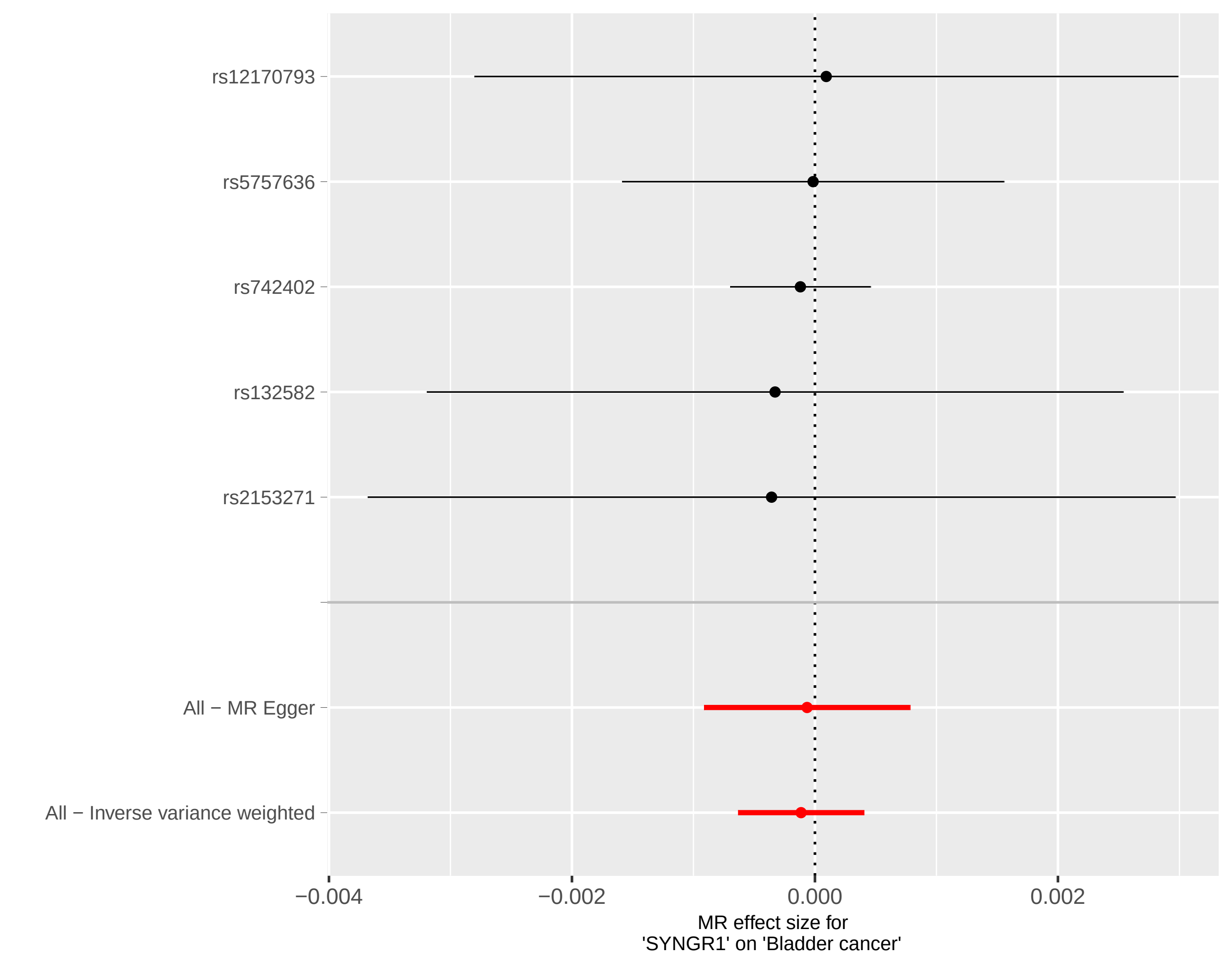
rs12170793
rs5757636
rs742402
rs132582
rs2153271
All − MR Egger
All − Inverse variance weighted
−0.004
−0.002
0.000
0.002
MR effect size for
'SYNGR1' on 'Bladder cancer'

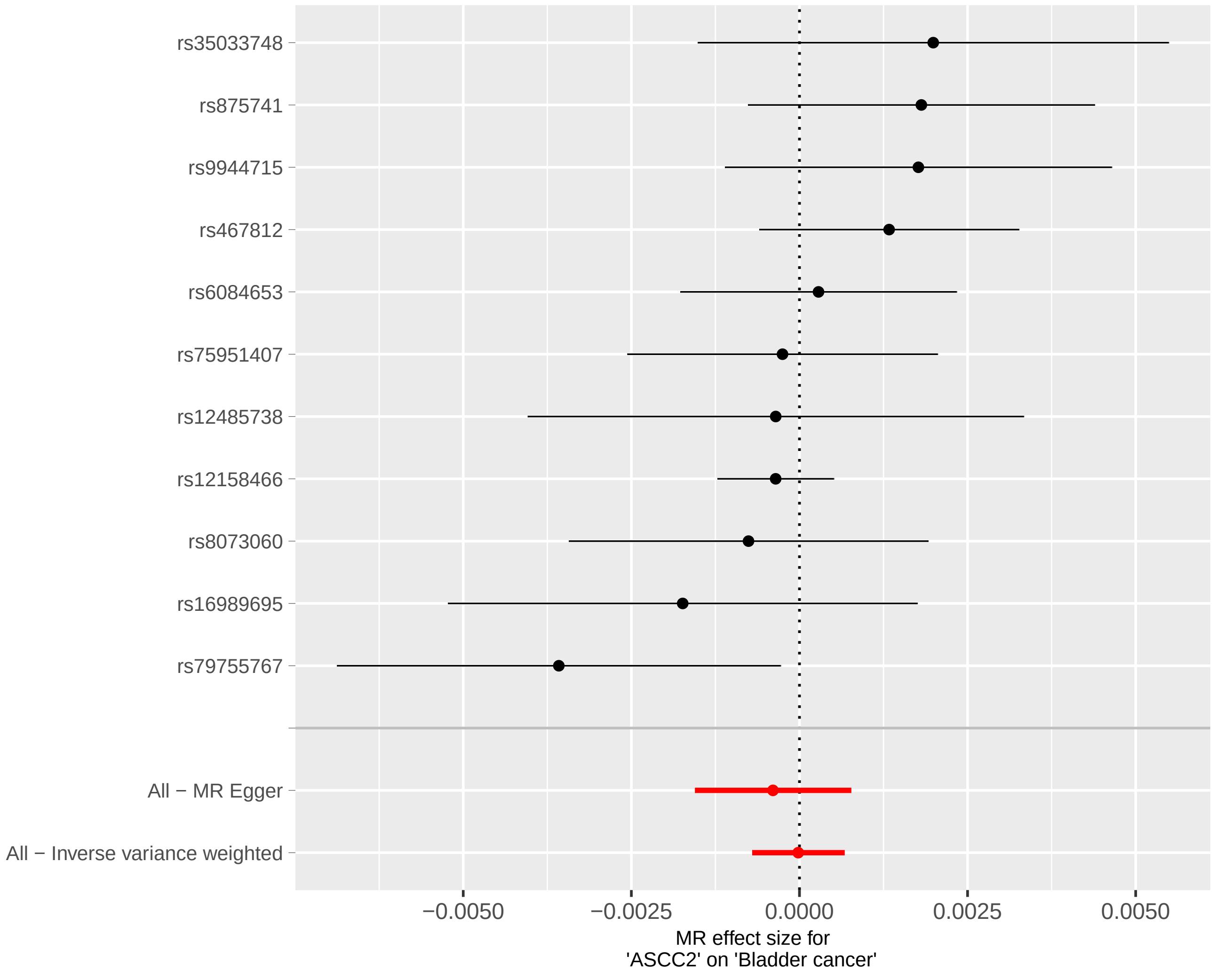
rs35033748
rs875741
rs9944715
rs467812
rs6084653
rs75951407
rs12485738
rs12158466
rs8073060
rs16989695
rs79755767
All − MR Egger
All − Inverse variance weighted
−0.0050
−0.0025
0.0000
0.0025
0.0050
MR effect size for
'ASCC2' on 'Bladder cancer'

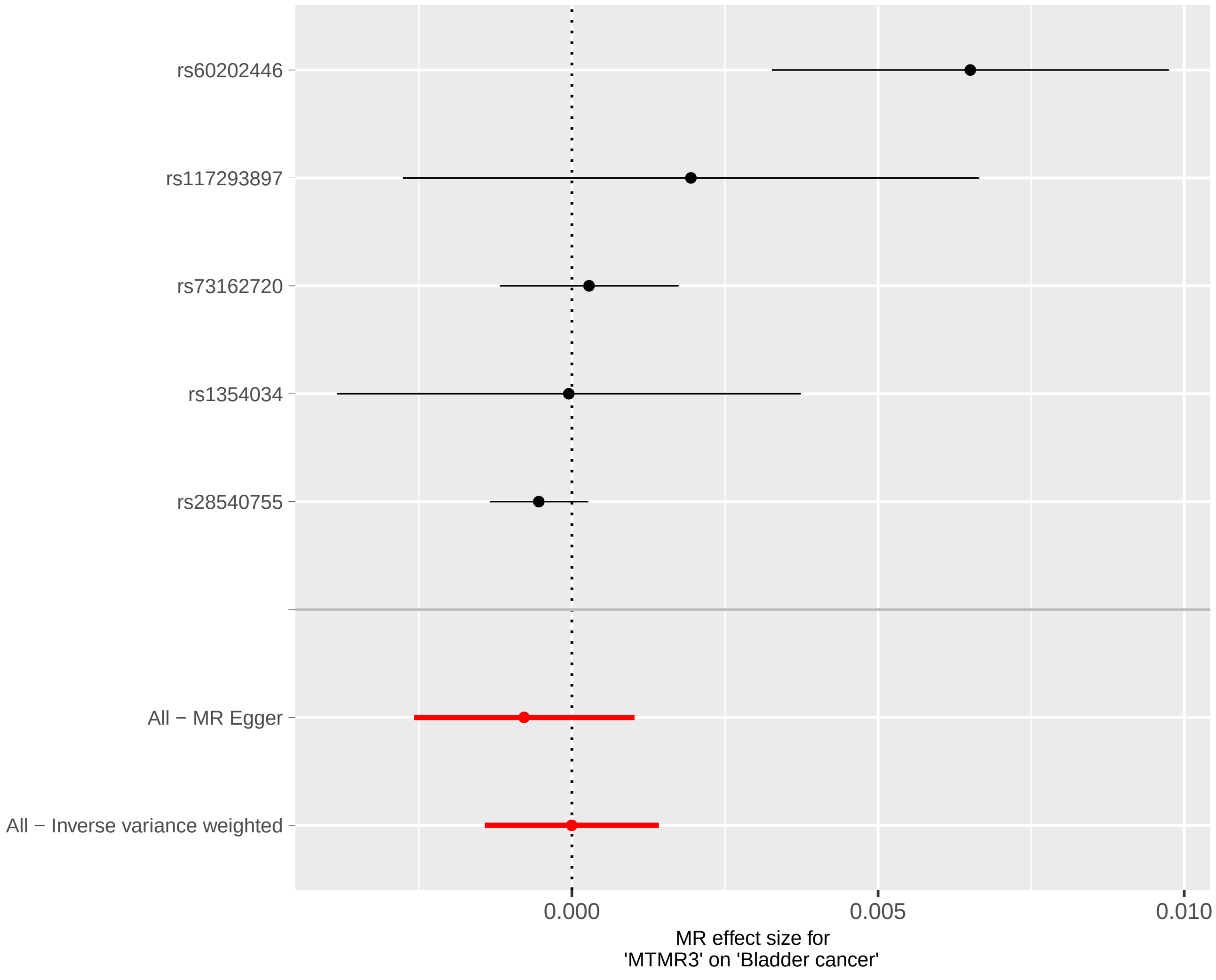

rs60202446
rs117293897
rs73162720
rs1354034
rs28540755
All − MR Egger
All − Inverse variance weighted
0.000
0.005
0.010
MR effect size for
'MTMR3' on 'Bladder cancer'

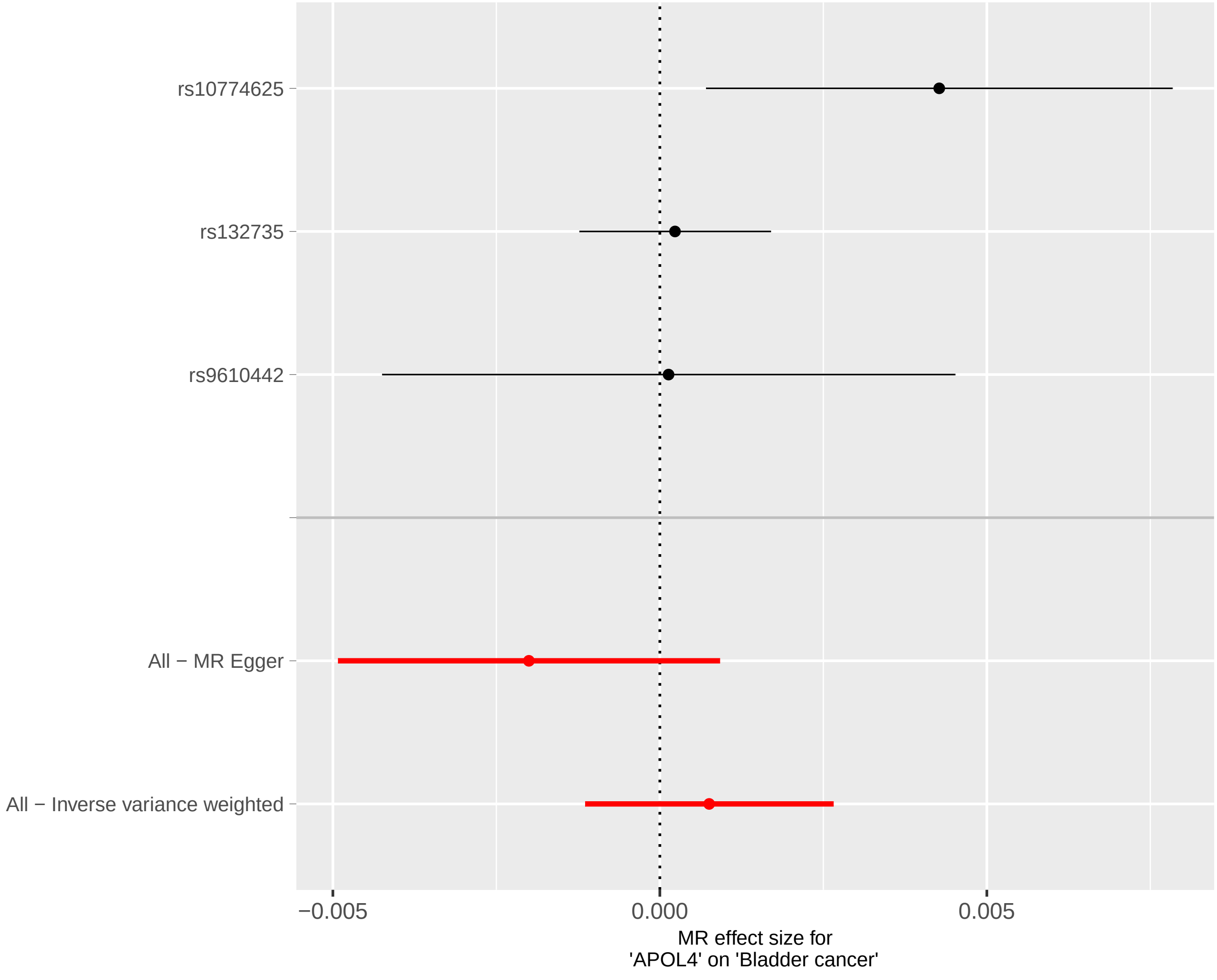
rs10774625
rs132735
rs9610442
All − MR Egger
All − Inverse variance weighted
−0.005
0.000
0.005
MR effect size for
'APOL4' on 'Bladder cancer'

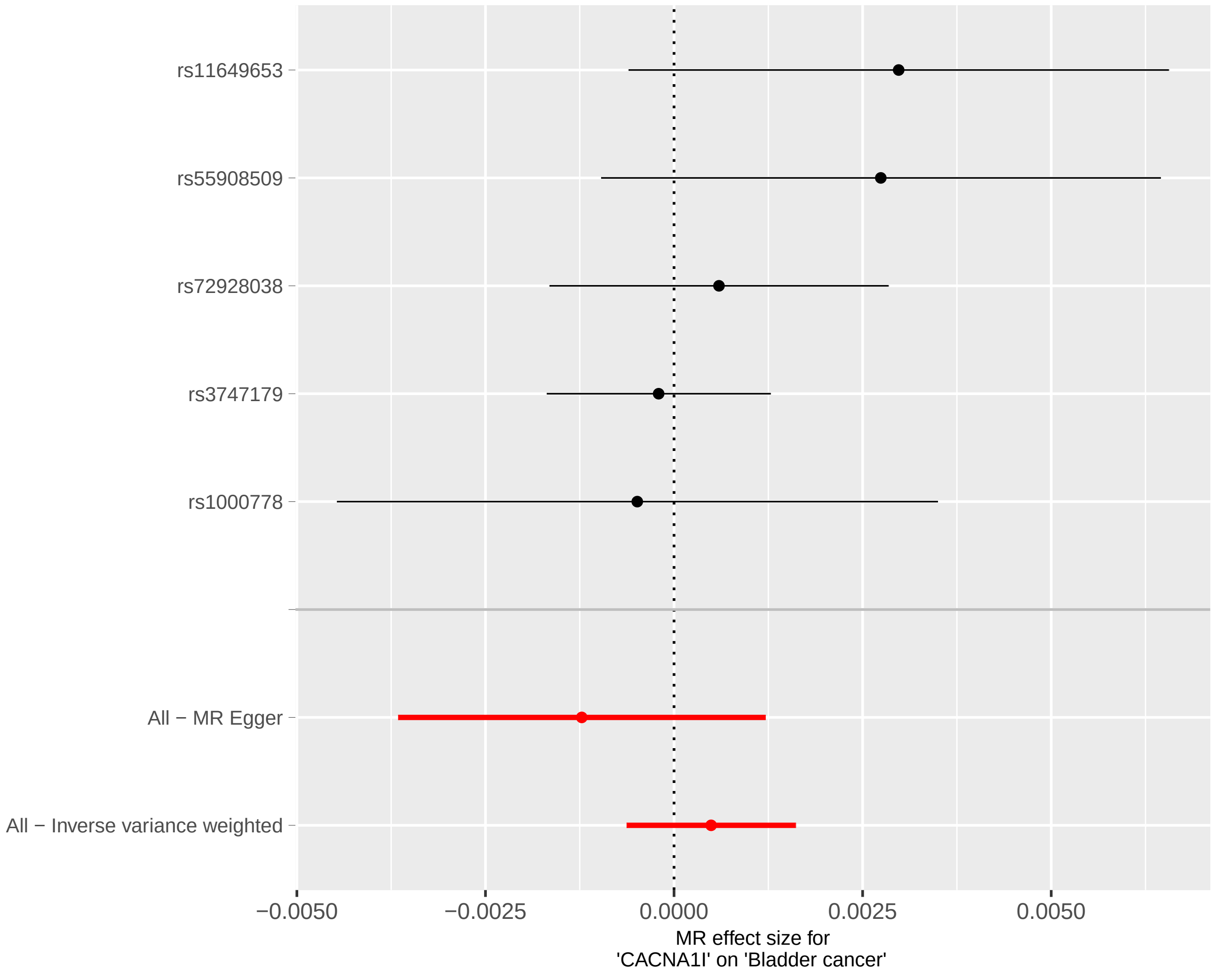
rs11649653
rs55908509
rs72928038
rs3747179
rs1000778
All − MR Egger
All − Inverse variance weighted
−0.0050
−0.0025
0.0000
0.0025
0.0050
MR effect size for
'CACNA1I' on 'Bladder cancer'

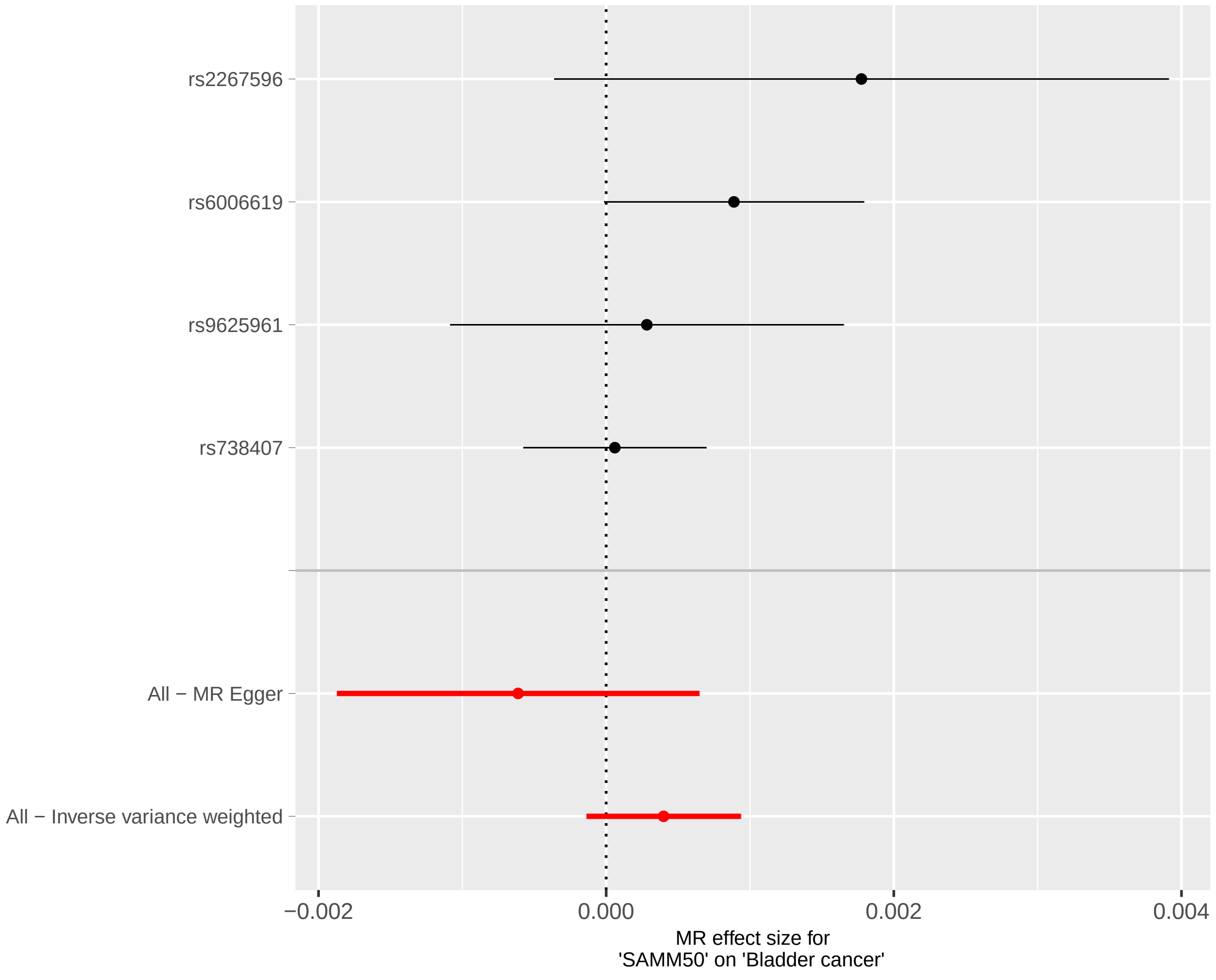

rs2267596
rs6006619
rs9625961
rs738407
All − MR Egger
All − Inverse variance weighted
−0.002
0.000
0.002
0.004
MR effect size for
'SAMM50' on 'Bladder cancer'

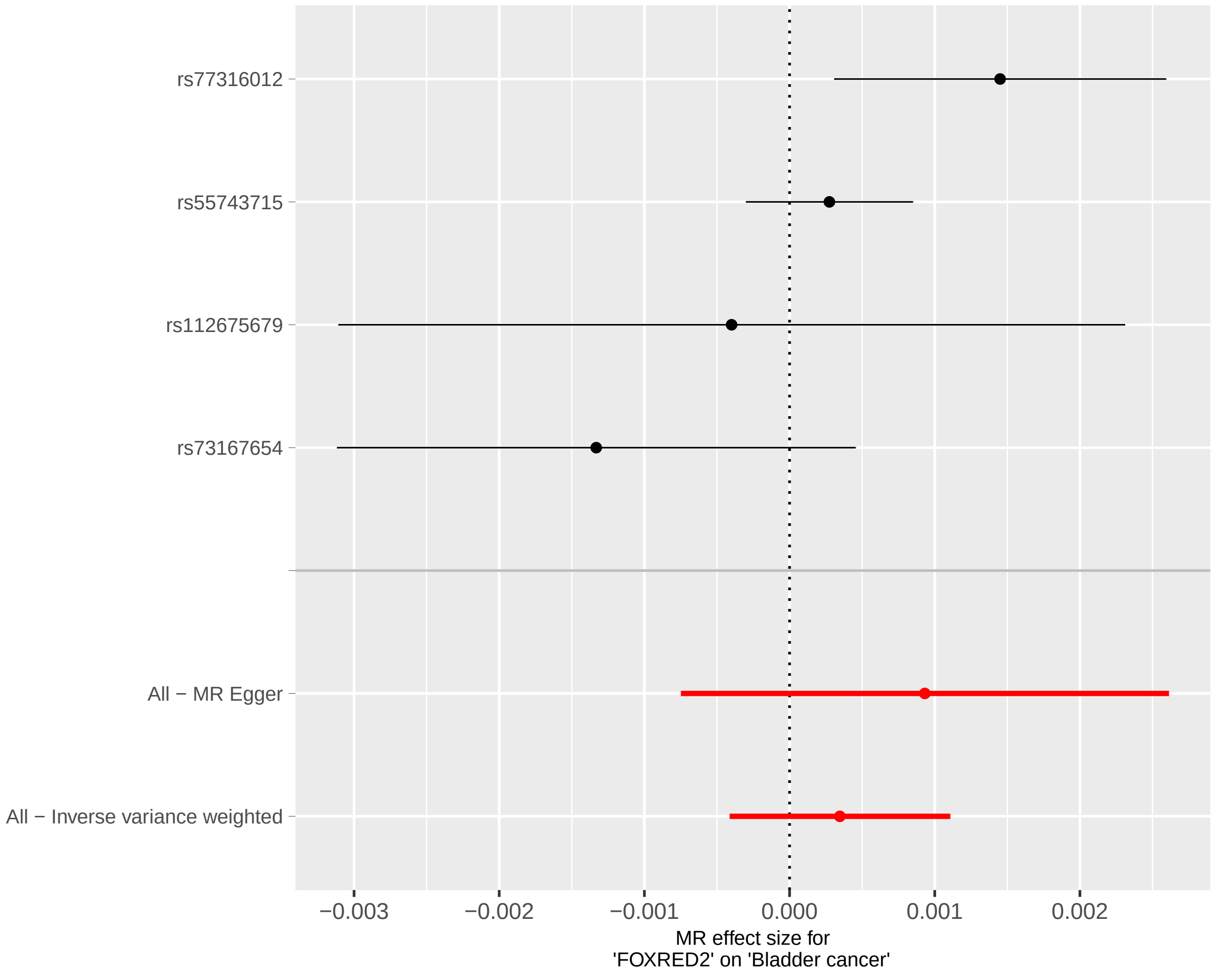
rs77316012
rs55743715
rs112675679
rs73167654
All − MR Egger
All − Inverse variance weighted
−0.003
−0.002
−0.001
0.000
0.001
0.002
MR effect size for
'FOXRED2' on 'Bladder cancer'

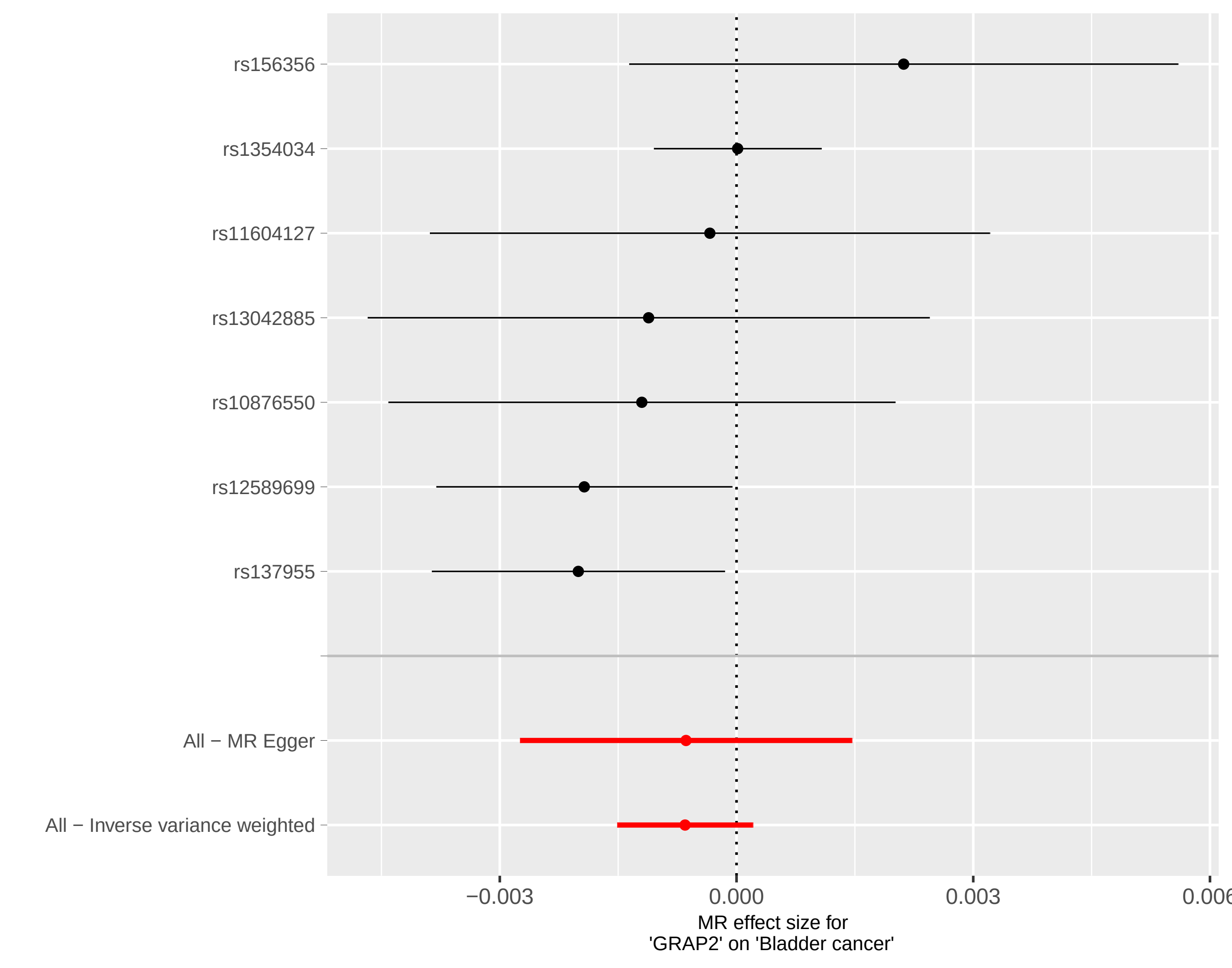
rs156356
rs1354034
rs11604127
rs13042885
rs10876550
rs12589699
rs137955
All − MR Egger
All − Inverse variance weighted
−0.003
0.000
0.003
MR effect size for
'GRAP2' on 'Bladder cancer'

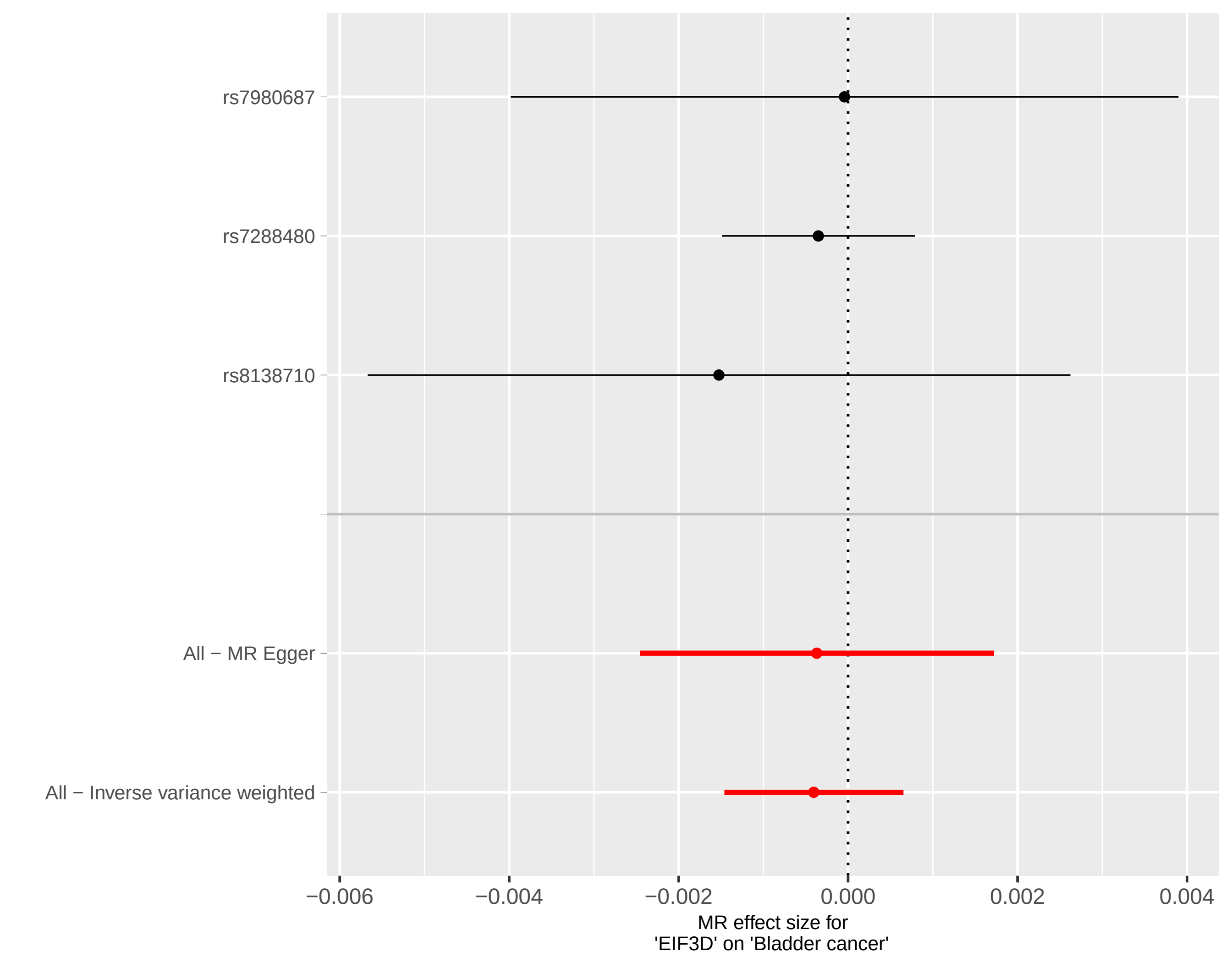

rs7980687
rs7288480
rs8138710
All − MR Egger
All − Inverse variance weighted
−0.006
−0.004
−0.002
0.000
0.002
0.004
MR effect size for
'EIF3D' on 'Bladder cancer'

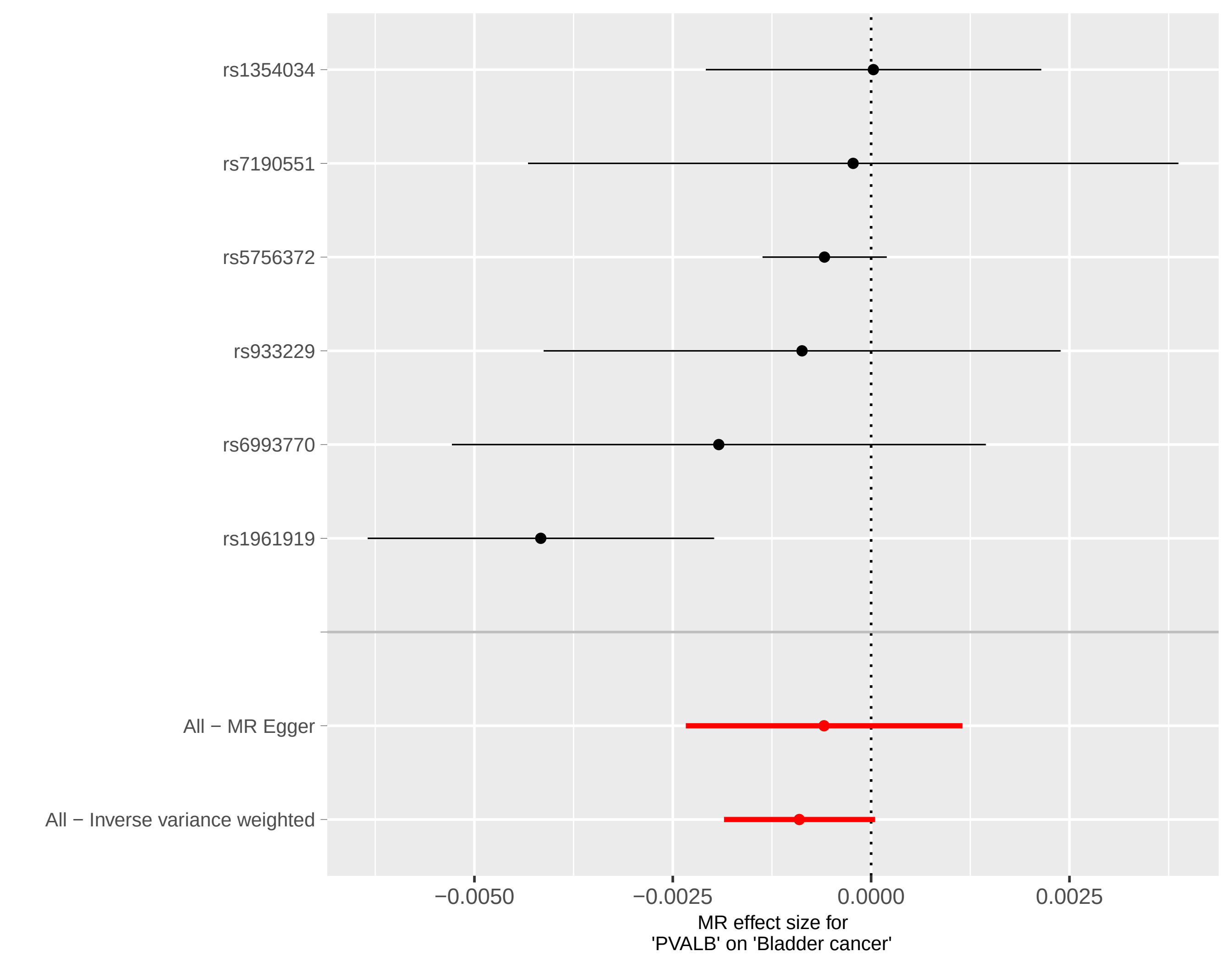

rs1354034
rs7190551
rs5756372
rs933229
rs6993770
rs1961919
All − MR Egger
All − Inverse variance weighted
−0.0050
−0.0025
0.0000
0.0025
MR effect size for
'PVALB' on 'Bladder cancer'

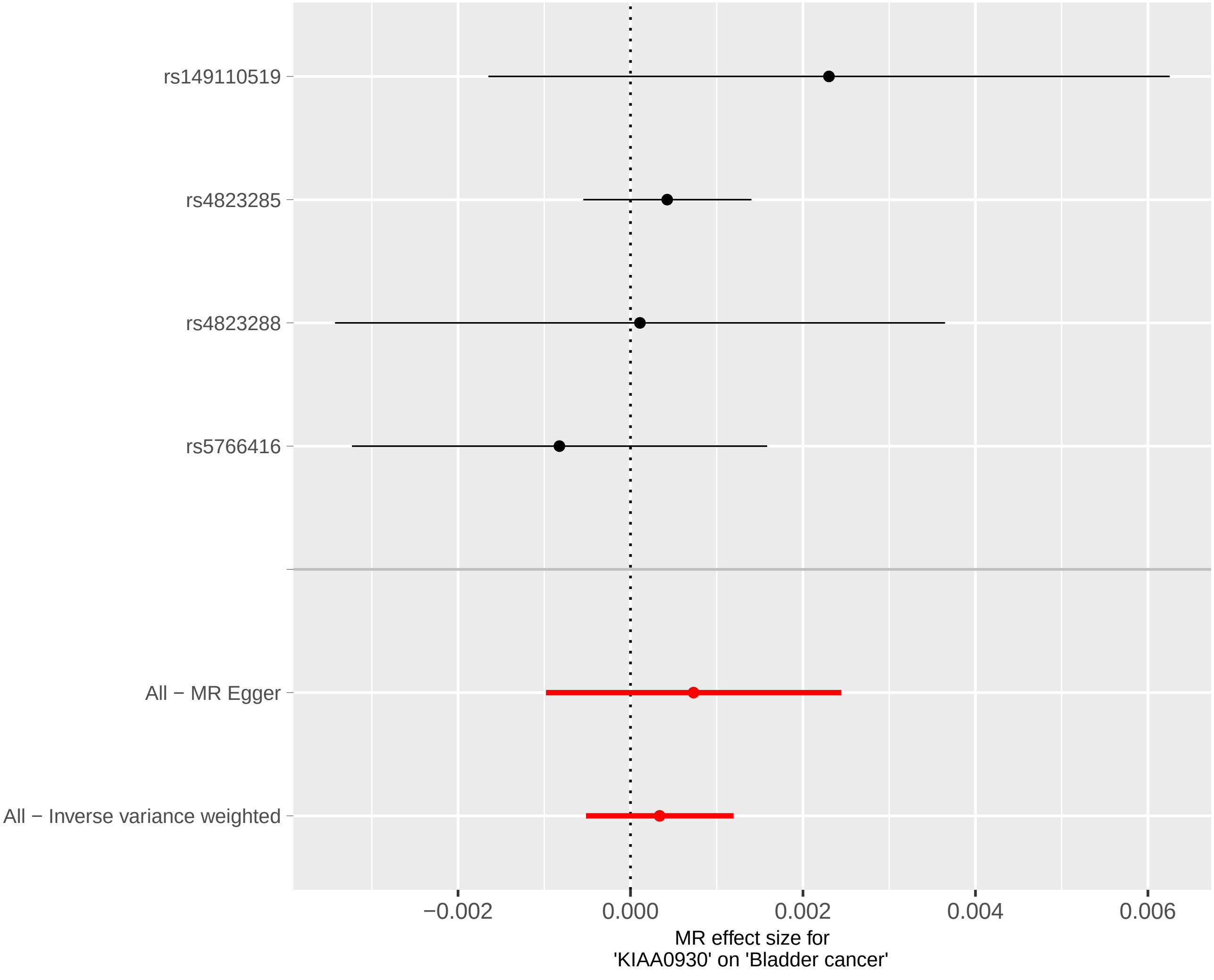

rs149110519
rs4823285
rs4823288
rs5766416
All − MR Egger
All − Inverse variance weighted
−0.002
0.000
0.002
0.004
0.006
MR effect size for
'KIAA0930' on 'Bladder cancer'

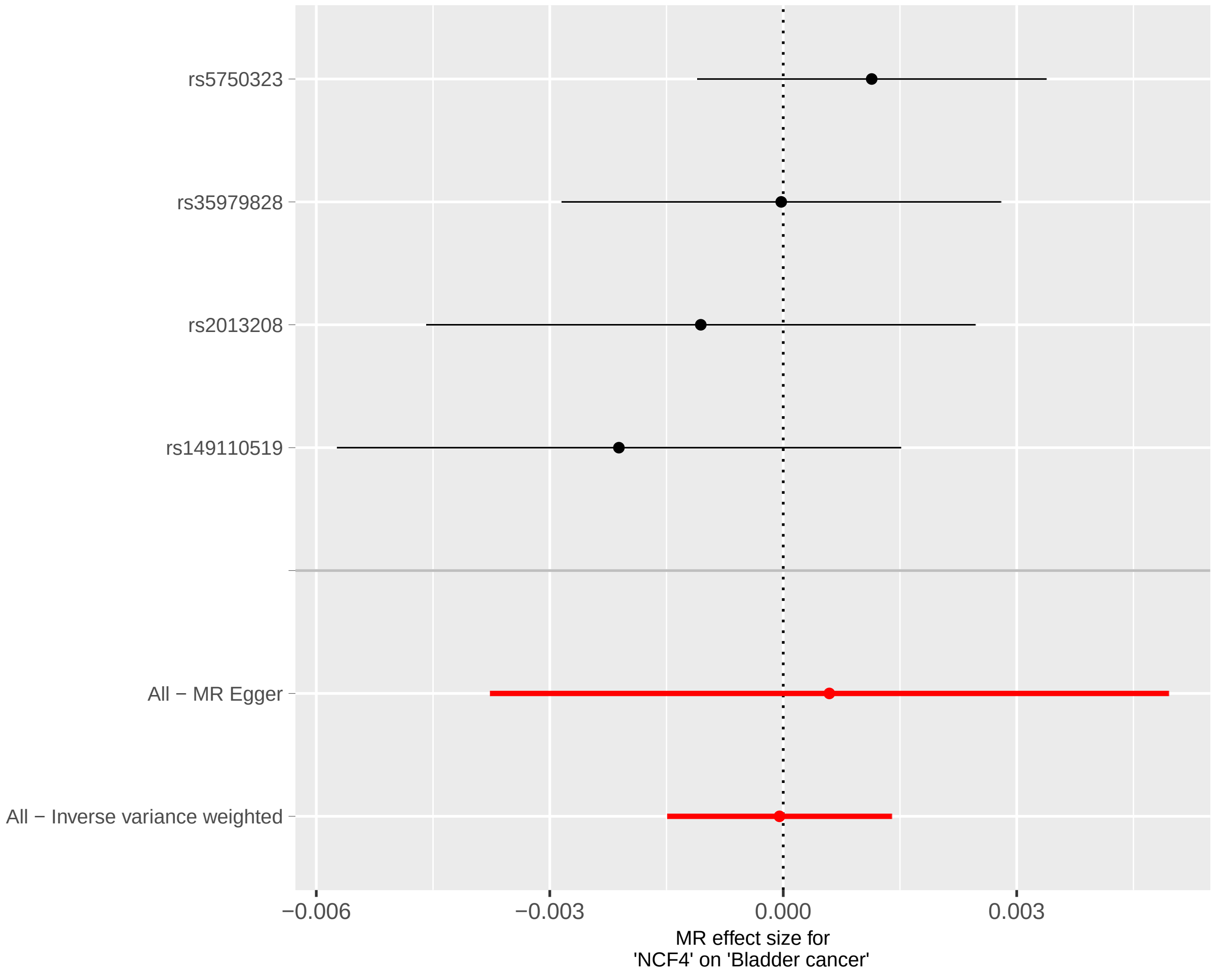
rs5750323
rs35979828
rs2013208
rs149110519
All − MR Egger
All − Inverse variance weighted
−0.006
−0.003
0.000
0.003
MR effect size for
'NCF4' on 'Bladder cancer'

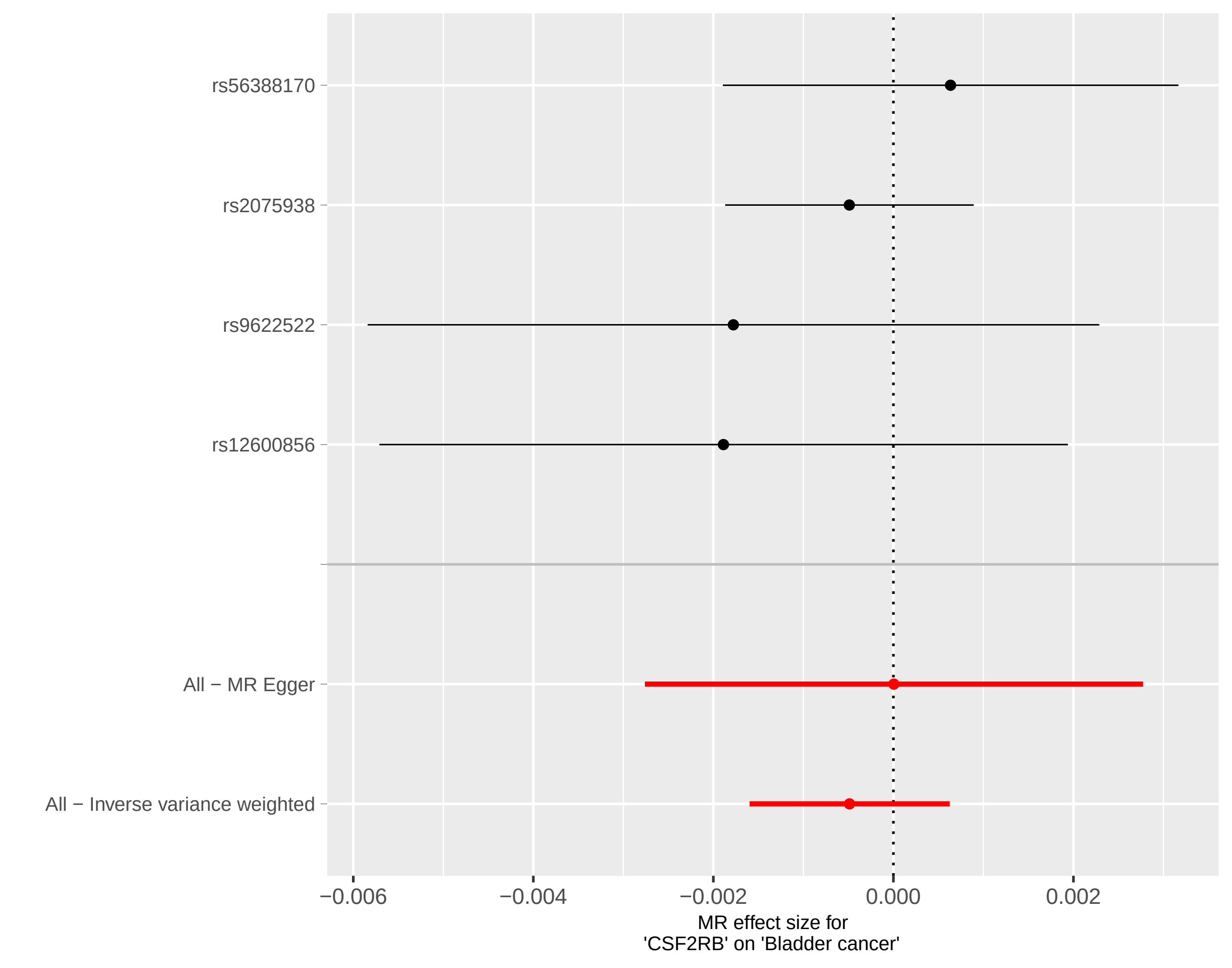

rs56388170
rs2075938
rs9622522
rs12600856
All − MR Egger
All − Inverse variance weighted
−0.006
−0.004
−0.002
0.000
0.002
MR effect size for
'CSF2RB' on 'Bladder cancer'

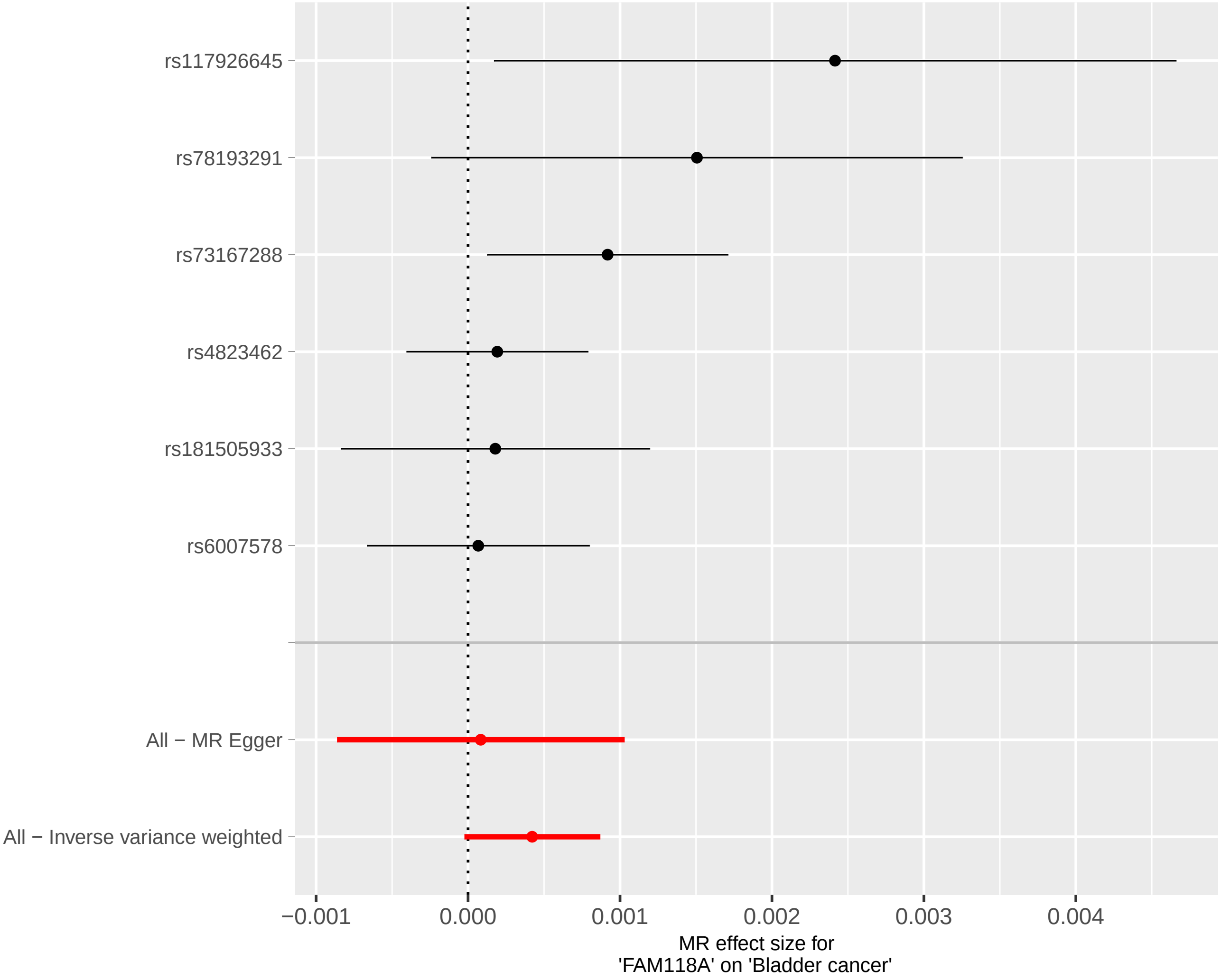
rs117926645
rs78193291
rs73167288
rs4823462
rs181505933
rs6007578
All − MR Egger
All − Inverse variance weighted
−0.001
0.000
0.001
0.002
0.003
0.004
MR effect size for
'FAM118A' on 'Bladder cancer'

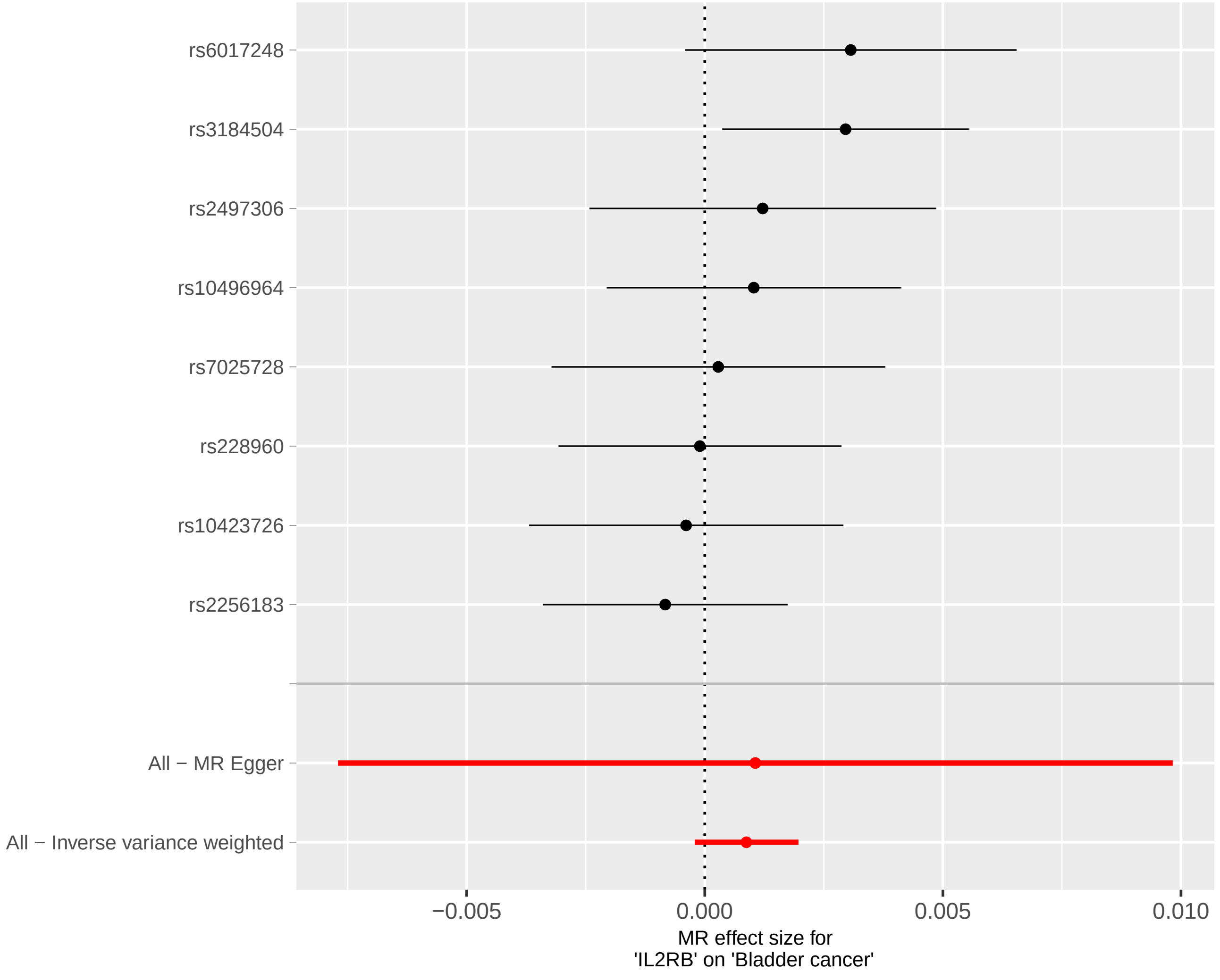

rs6017248
rs3184504
rs2497306
rs10496964
rs7025728
rs228960
rs10423726
rs2256183
All − MR Egger
All − Inverse variance weighted
−0.005
0.000
0.005
0.010
MR effect size for
'IL2RB' on 'Bladder cancer'

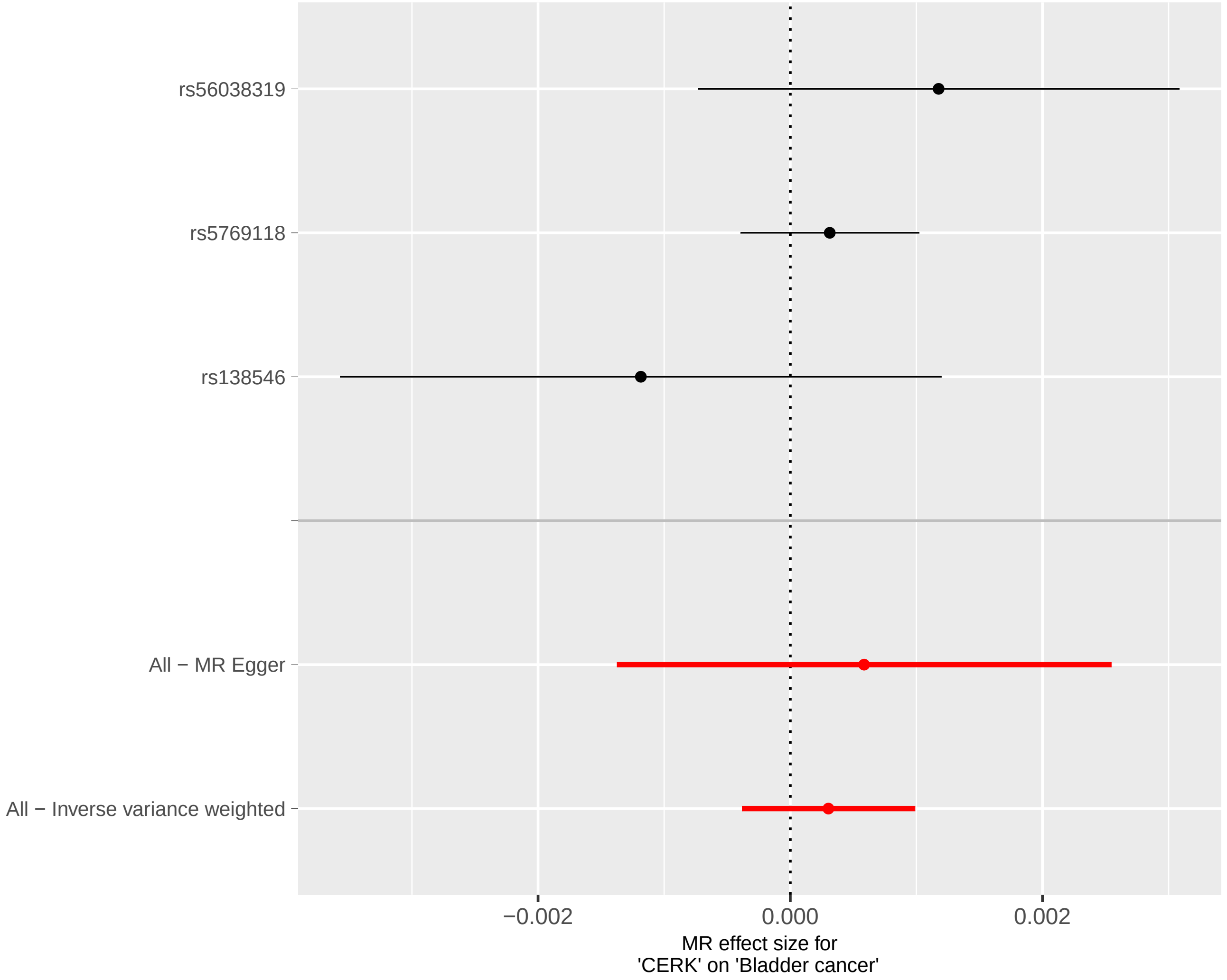
rs56038319
rs5769118
rs138546
All − MR Egger
All − Inverse variance weighted
−0.002
0.000
0.002
MR effect size for
'CERK' on 'Bladder cancer'

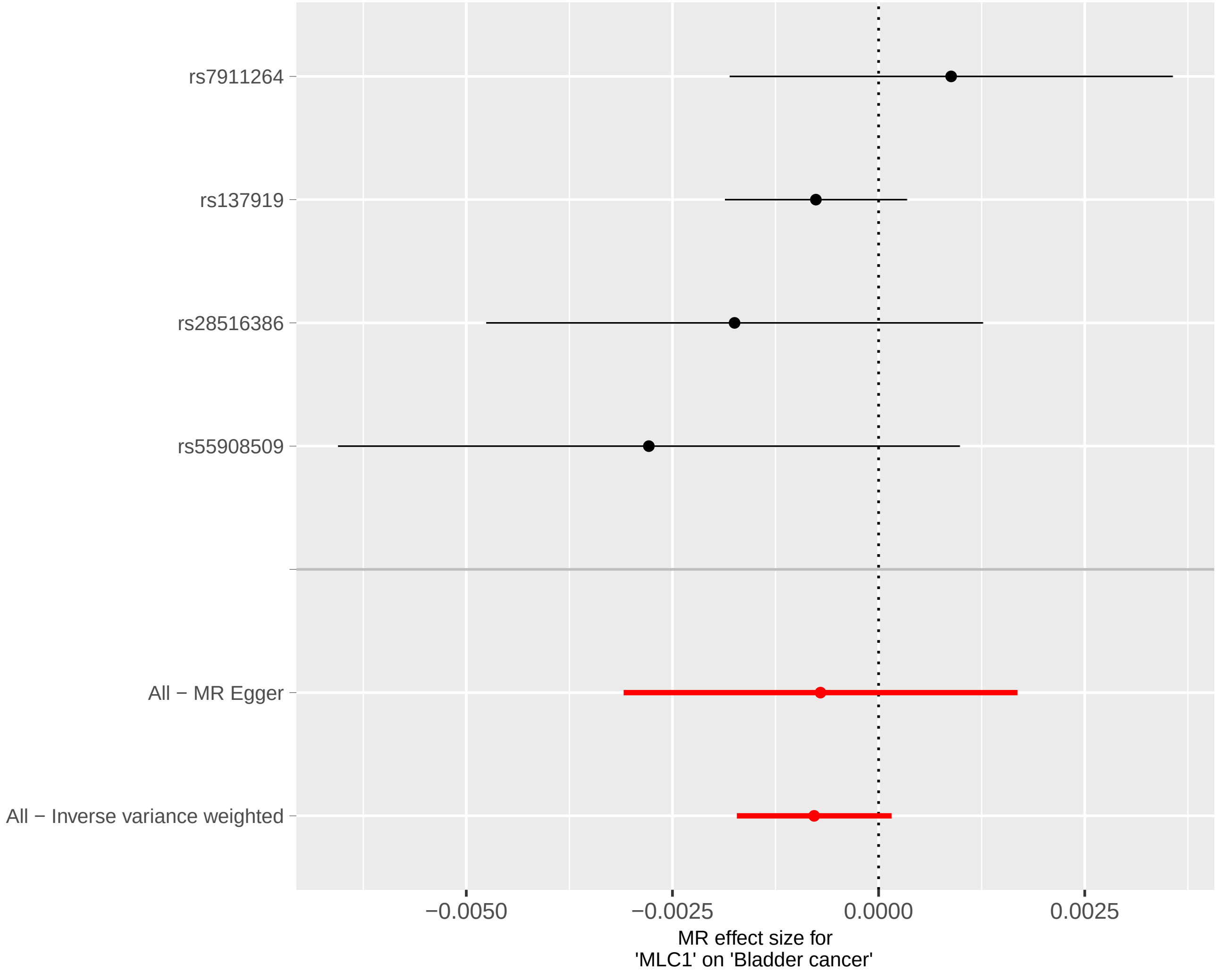
rs7911264
rs137919
rs28516386
rs55908509
All − MR Egger
All − Inverse variance weighted
−0.0050
−0.0025
0.0000
0.0025
MR effect size for
'MLC1' on 'Bladder cancer'

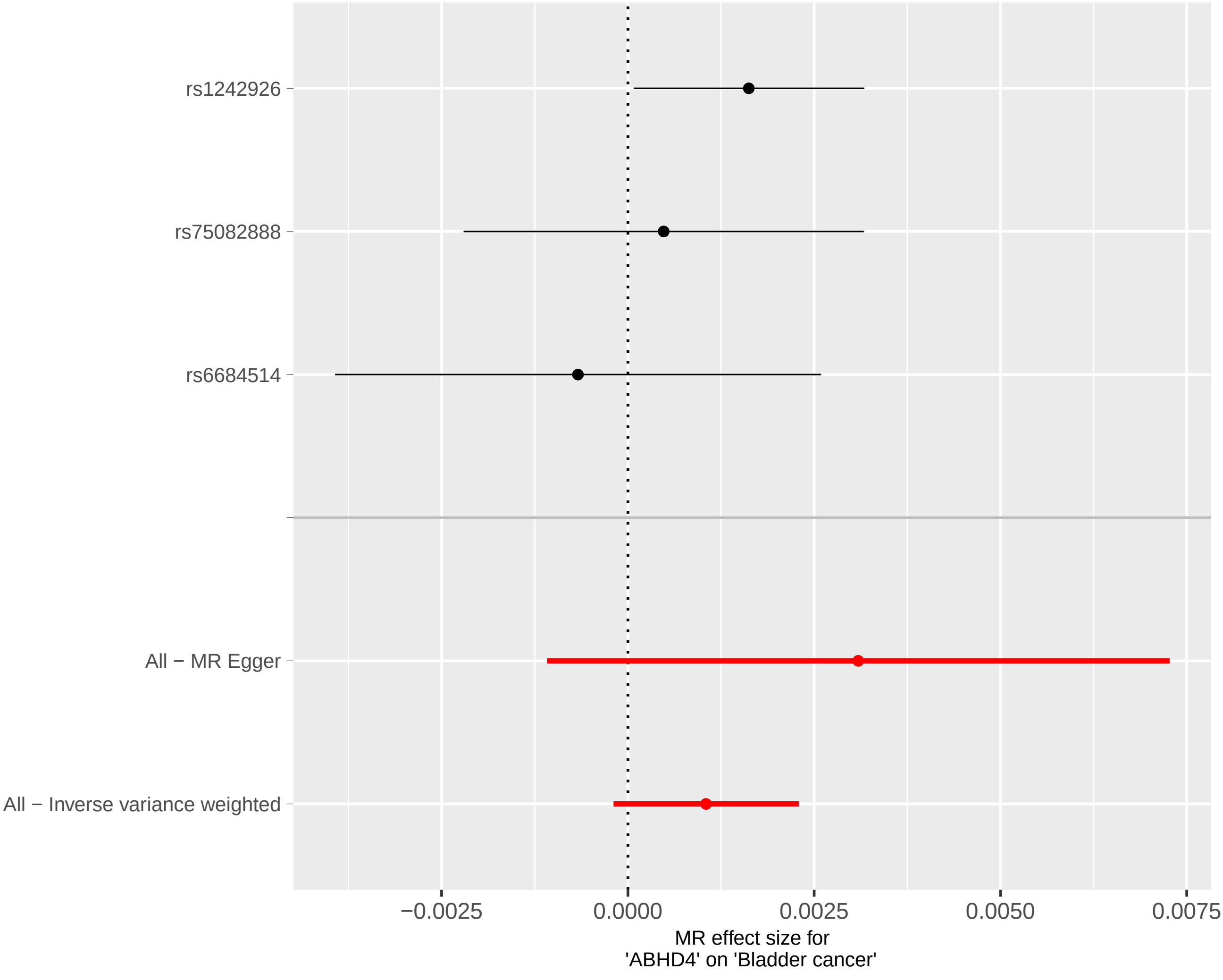
rs1242926
rs75082888
rs6684514
All − MR Egger
All − Inverse variance weighted
−0.0025
0.0000
0.0025
0.0050
0.0075
MR effect size for
'ABHD4' on 'Bladder cancer'

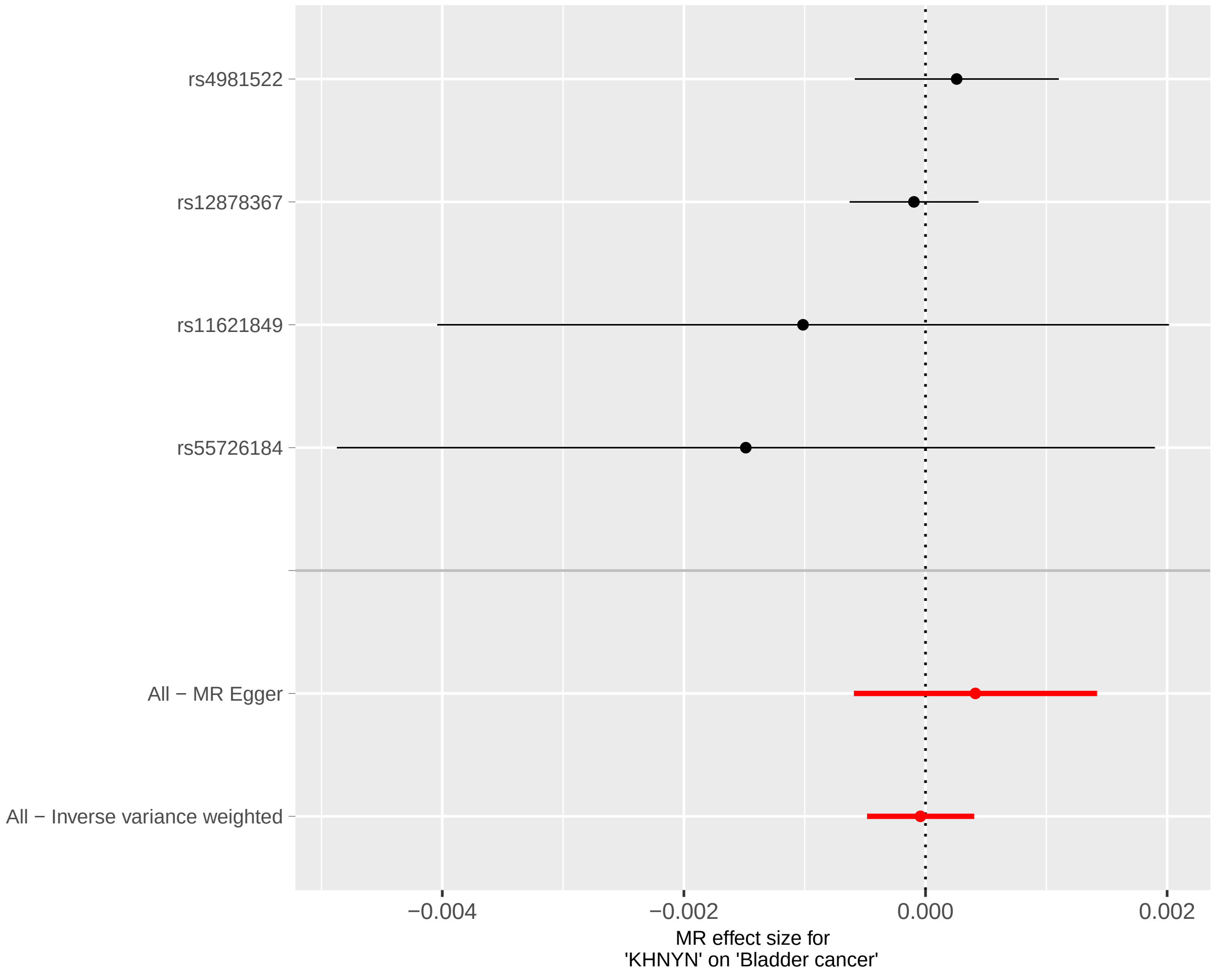
rs4981522
rs12878367
rs11621849
rs55726184
All − MR Egger
All − Inverse variance weighted
−0.004
−0.002
0.000
0.002
MR effect size for
'KHNYN' on 'Bladder cancer'

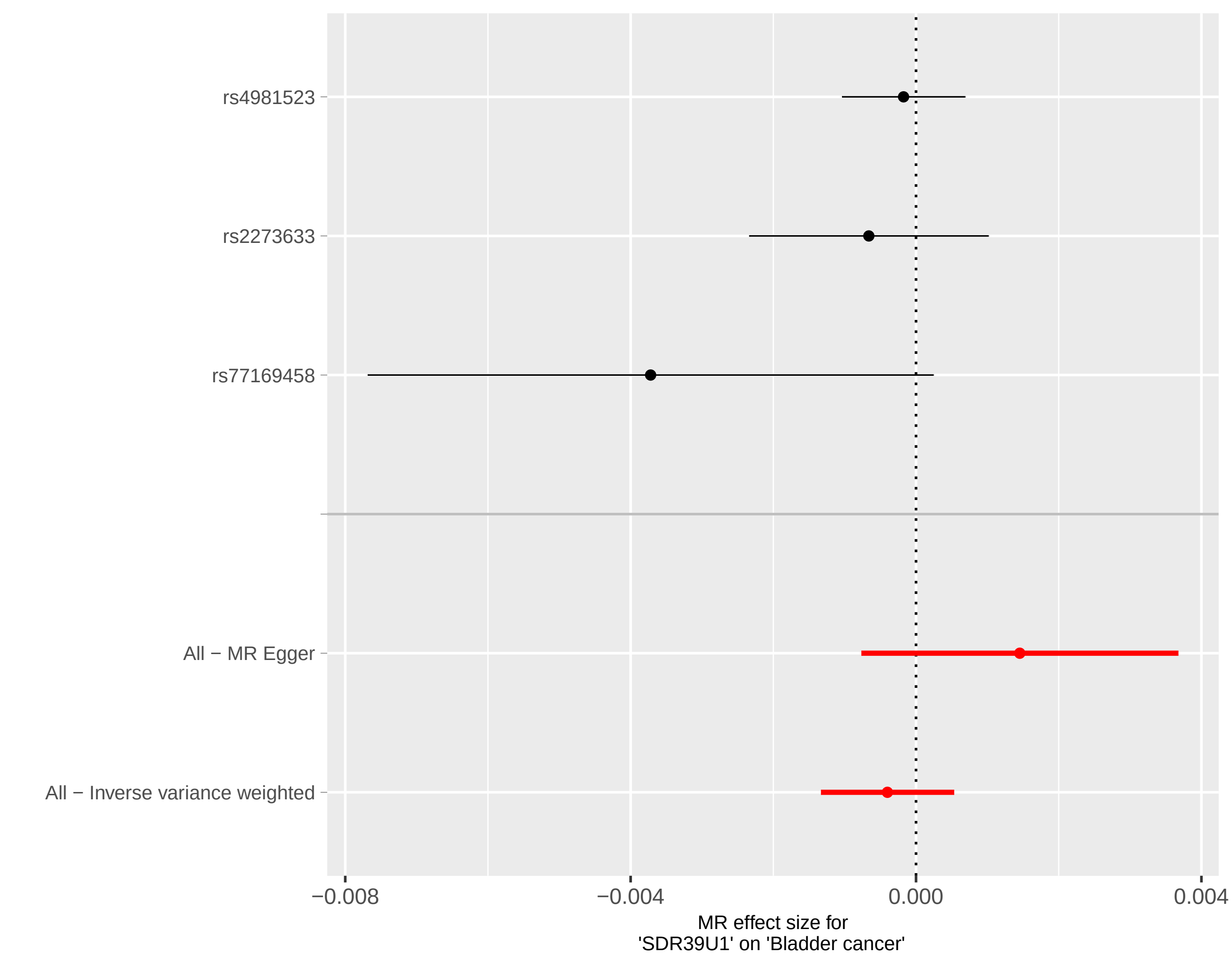

rs4981523
rs2273633
rs77169458
All − MR Egger
All − Inverse variance weighted
−0.008
−0.004
0.000
0.004
MR effect size for
'SDR39U1' on 'Bladder cancer'

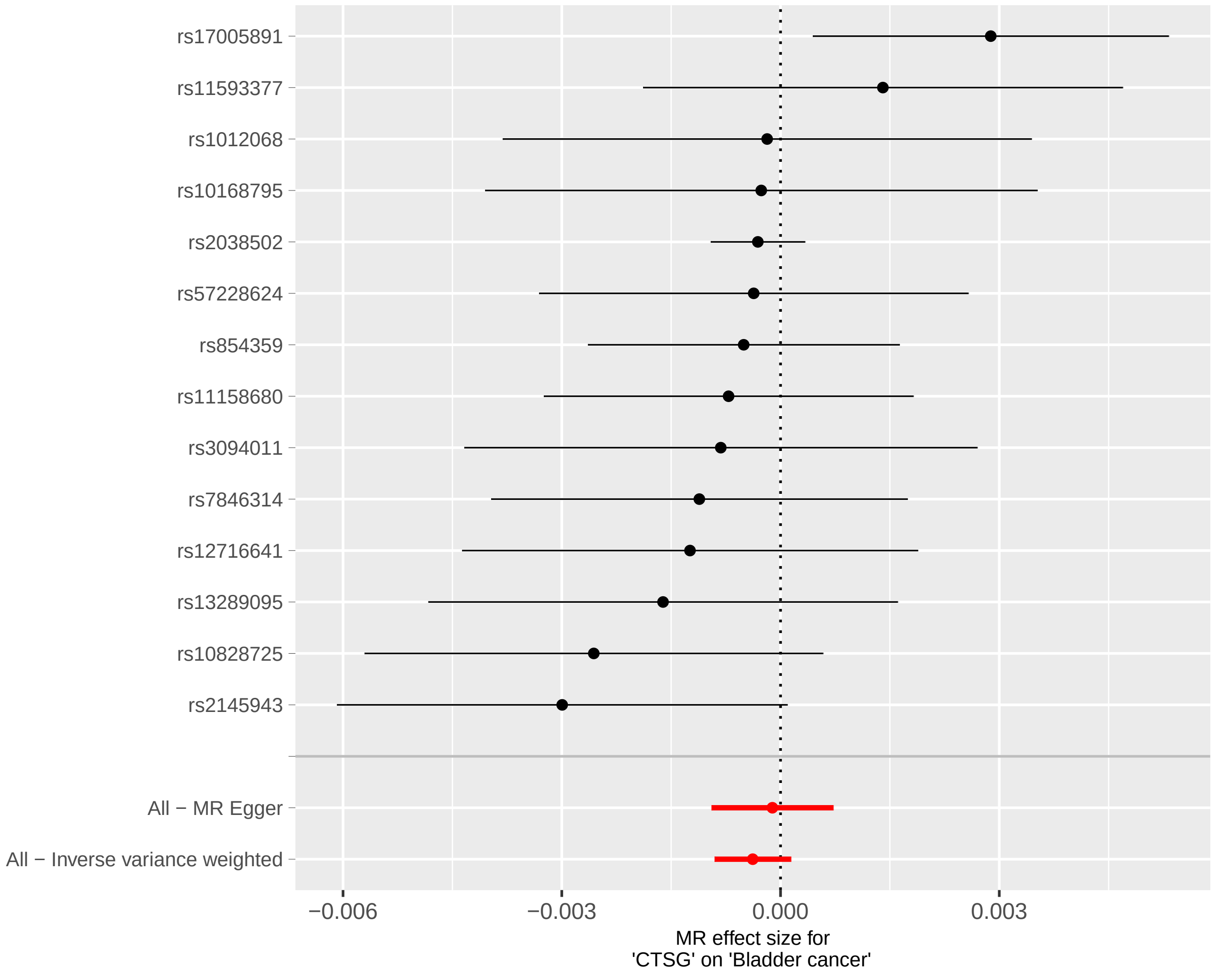

rs17005891
rs11593377
rs1012068
rs10168795
rs2038502
rs57228624
rs854359
rs11158680
rs3094011
rs7846314
rs12716641
rs13289095
rs10828725
rs2145943
All − MR Egger
All − Inverse variance weighted
−0.006
−0.003
0.000
0.003
MR effect size for
'CTSG' on 'Bladder cancer'

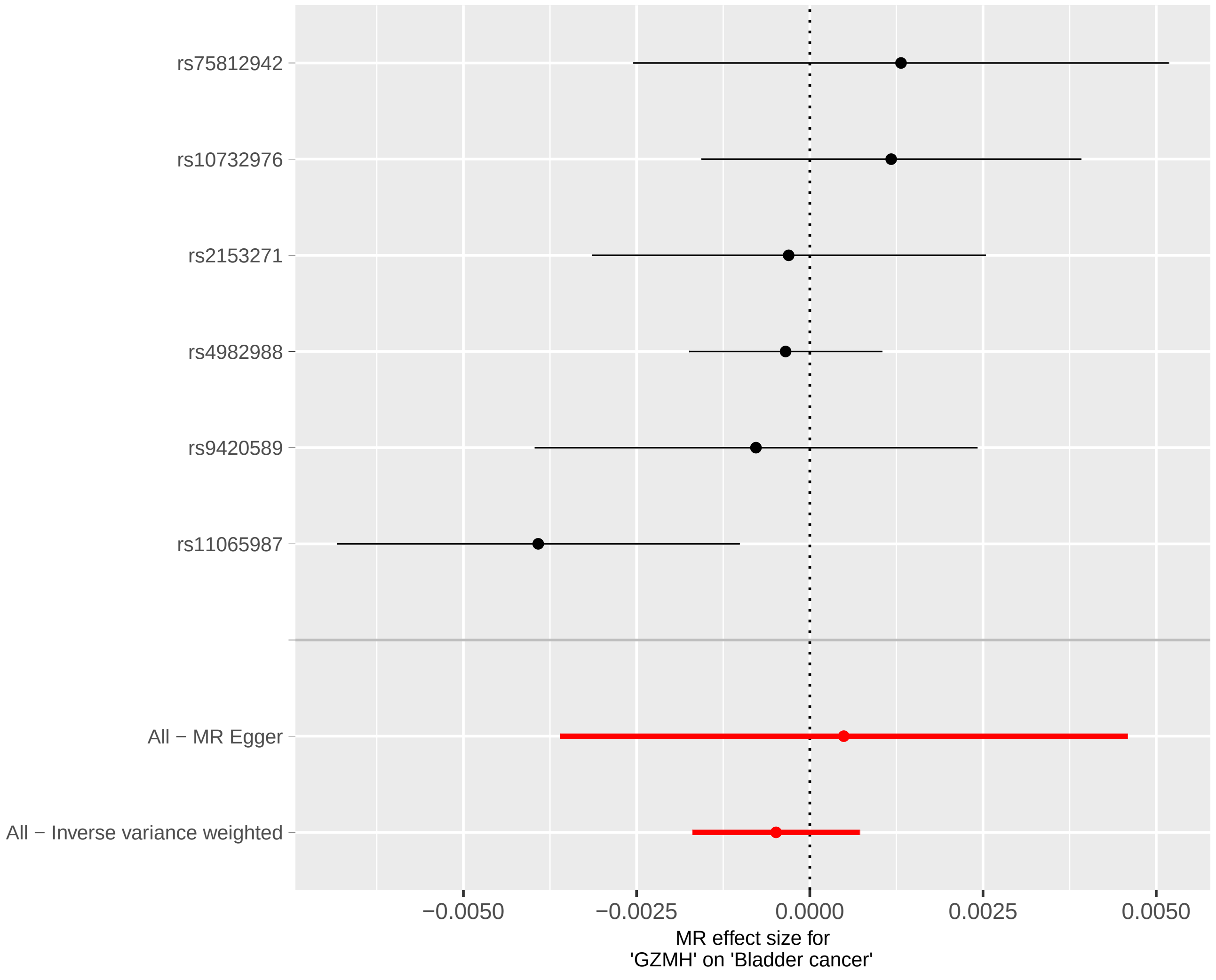
rs75812942
rs10732976
rs2153271
rs4982988
rs9420589
rs11065987
All − MR Egger
All − Inverse variance weighted
−0.0050
−0.0025
0.0000
0.0025
0.0050
MR effect size for
'GZMH' on 'Bladder cancer'

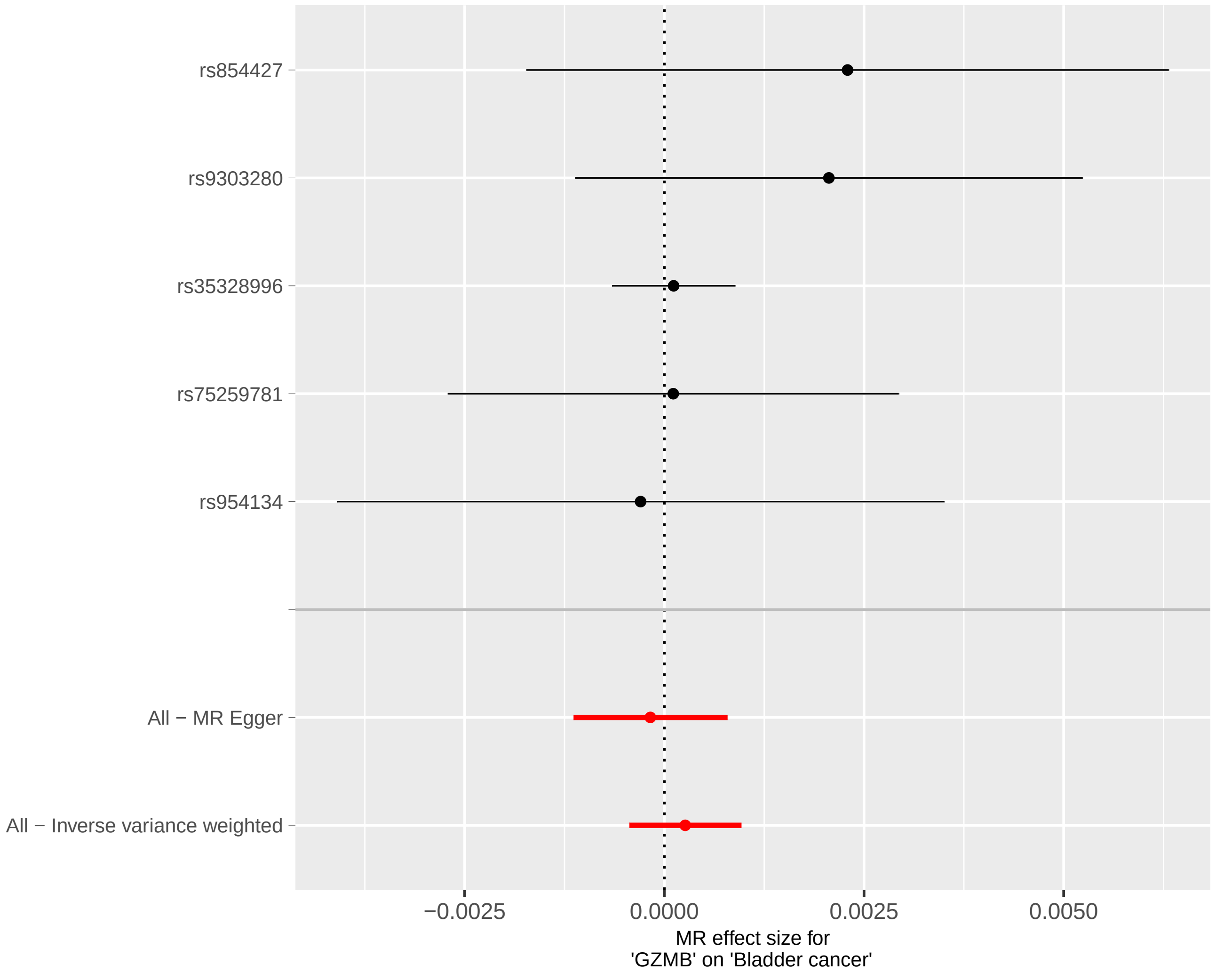

rs854427
rs9303280
rs35328996
rs75259781
rs954134
All − MR Egger
All − Inverse variance weighted
−0.0025
0.0000
0.0025
0.0050
MR effect size for
'GZMB' on 'Bladder cancer'

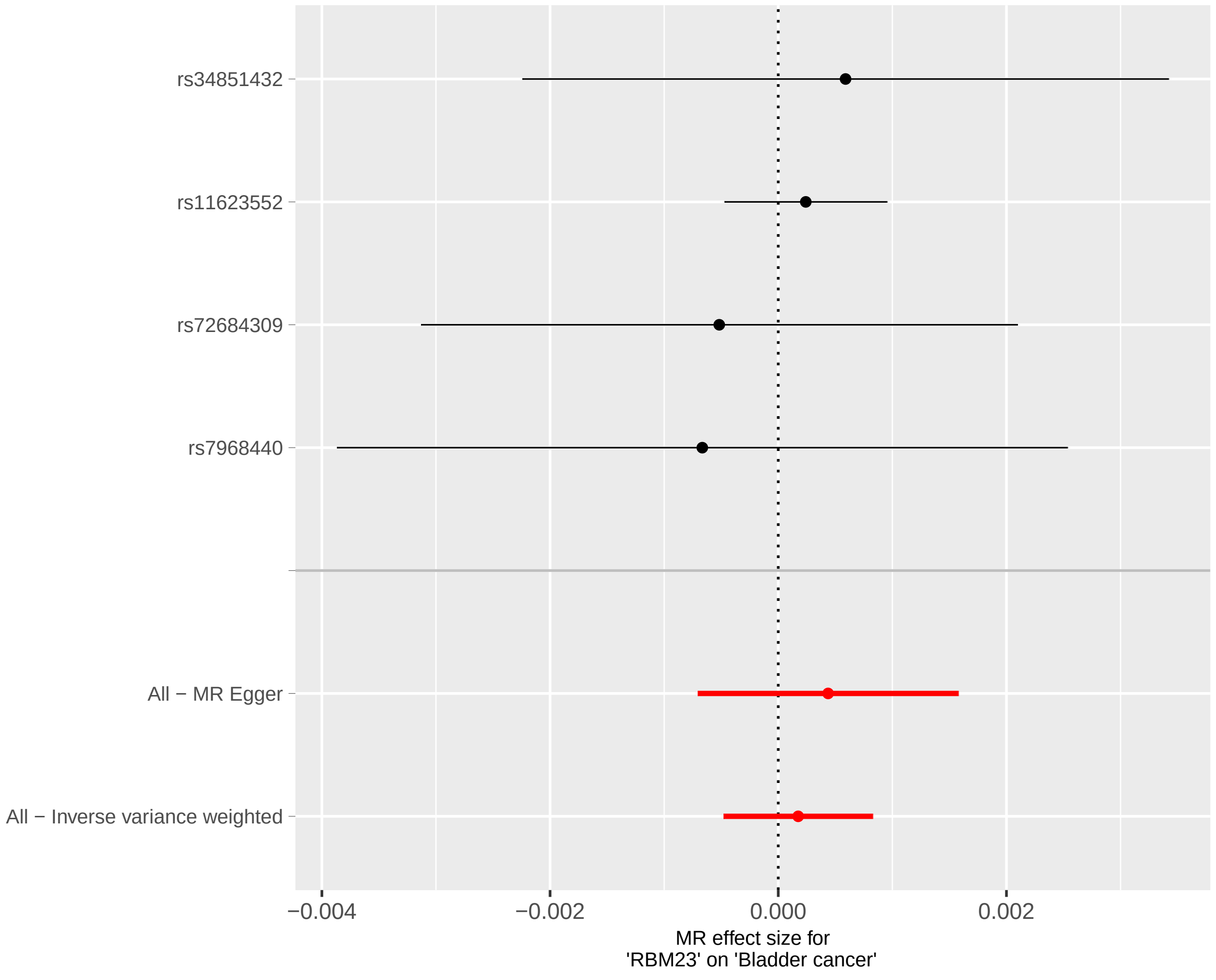
rs34851432
rs11623552
rs72684309
rs7968440
All − MR Egger
All − Inverse variance weighted
−0.004
−0.002
0.000
0.002
MR effect size for
'RBM23' on 'Bladder cancer'

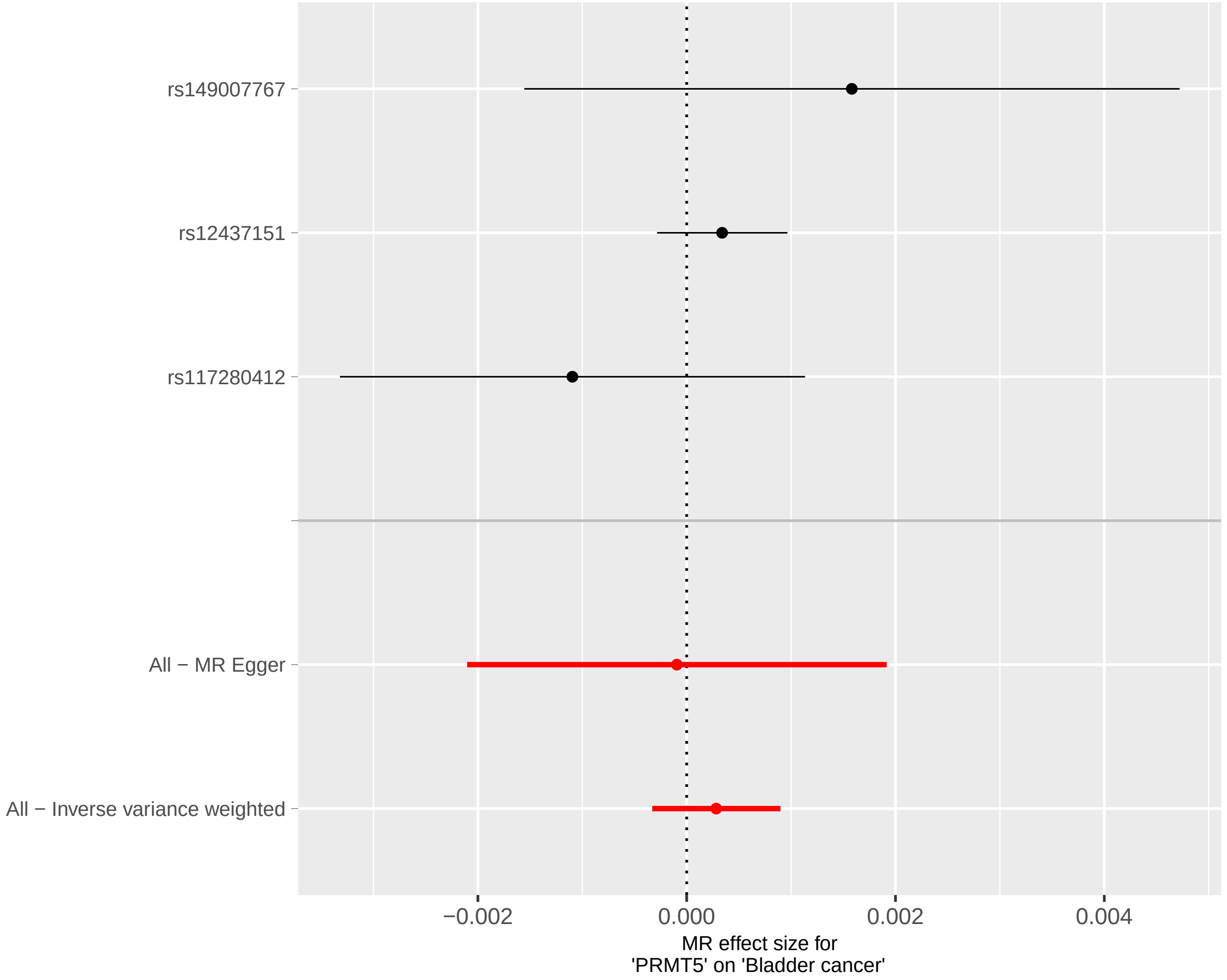
rs149007767
rs12437151
rs117280412
All − MR Egger
All − Inverse variance weighted
−0.002
0.000
0.002
0.004
MR effect size for
'PRMT5' on 'Bladder cancer'

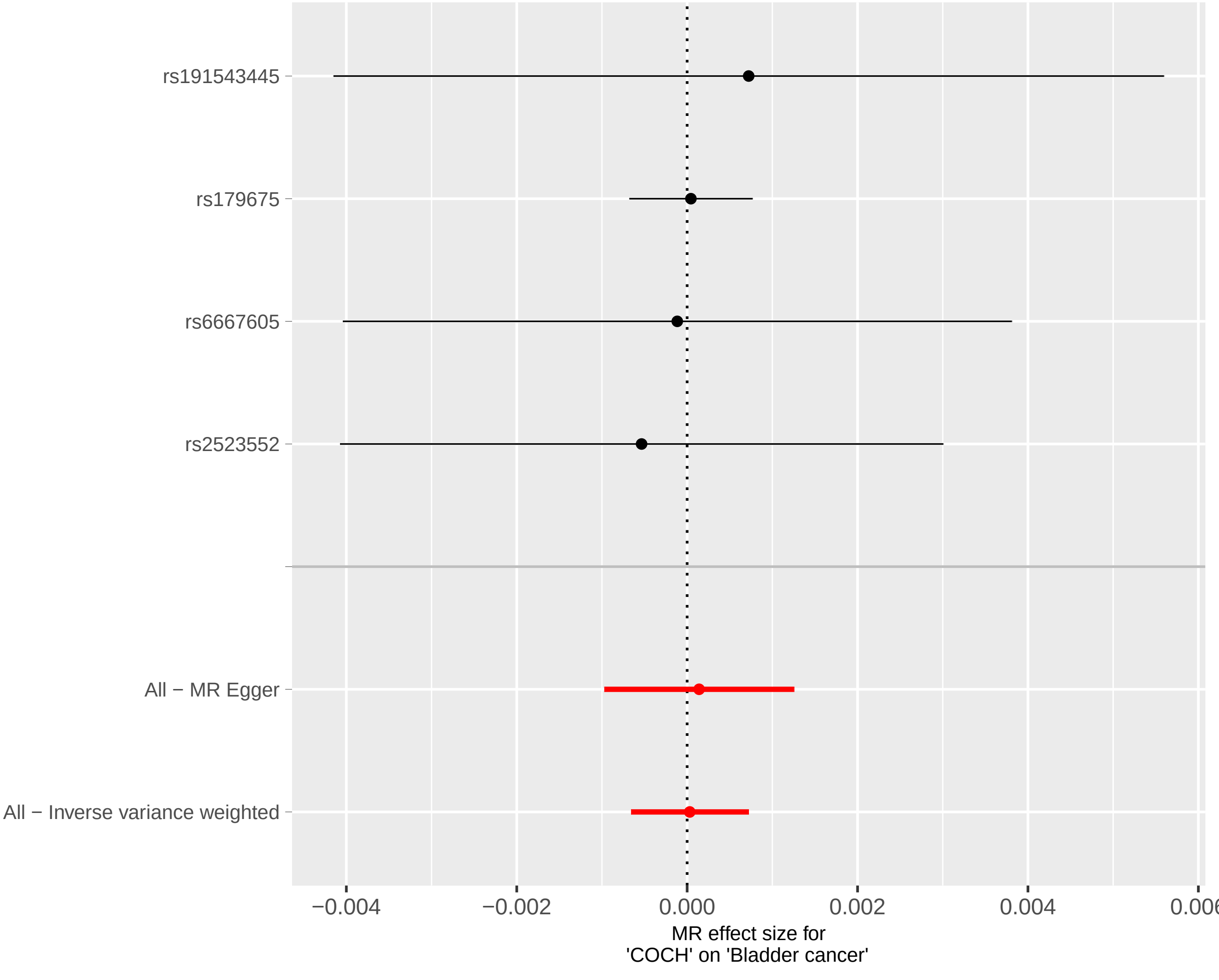
rs191543445
rs179675
rs6667605
rs2523552
All − MR Egger
All − Inverse variance weighted
−0.004
−0.002
0.000
0.002
0.004
MR effect size for
'COCH' on 'Bladder cancer'

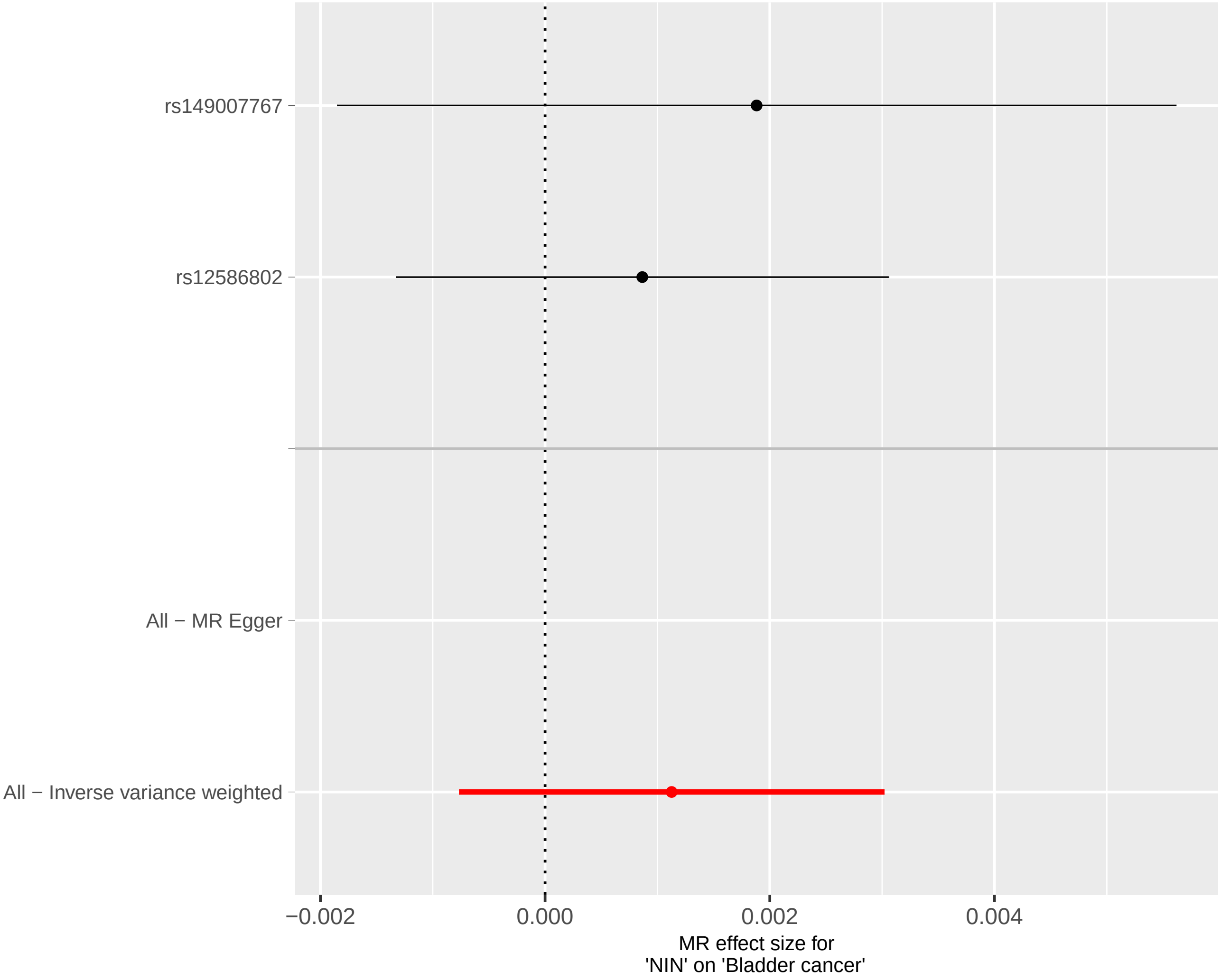
rs149007767
rs12586802
All − MR Egger
All − Inverse variance weighted
−0.002
0.000
0.002
0.004
MR effect size for
'NIN' on 'Bladder cancer'

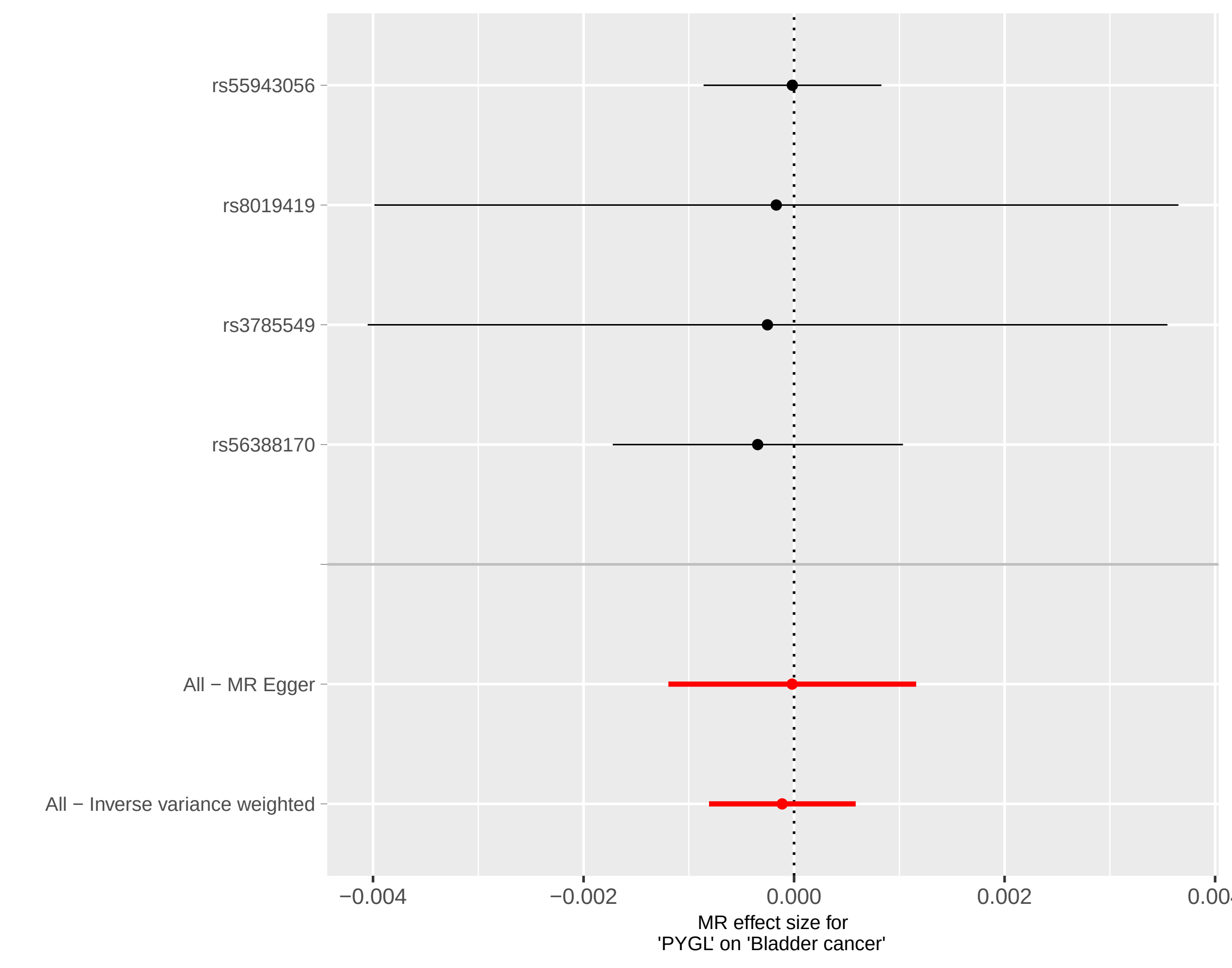
rs55943056
rs8019419
rs3785549
rs56388170
All − MR Egger
All − Inverse variance weighted
−0.004
−0.002
0.000
0.002
MR effect size for
'PYGL' on 'Bladder cancer'

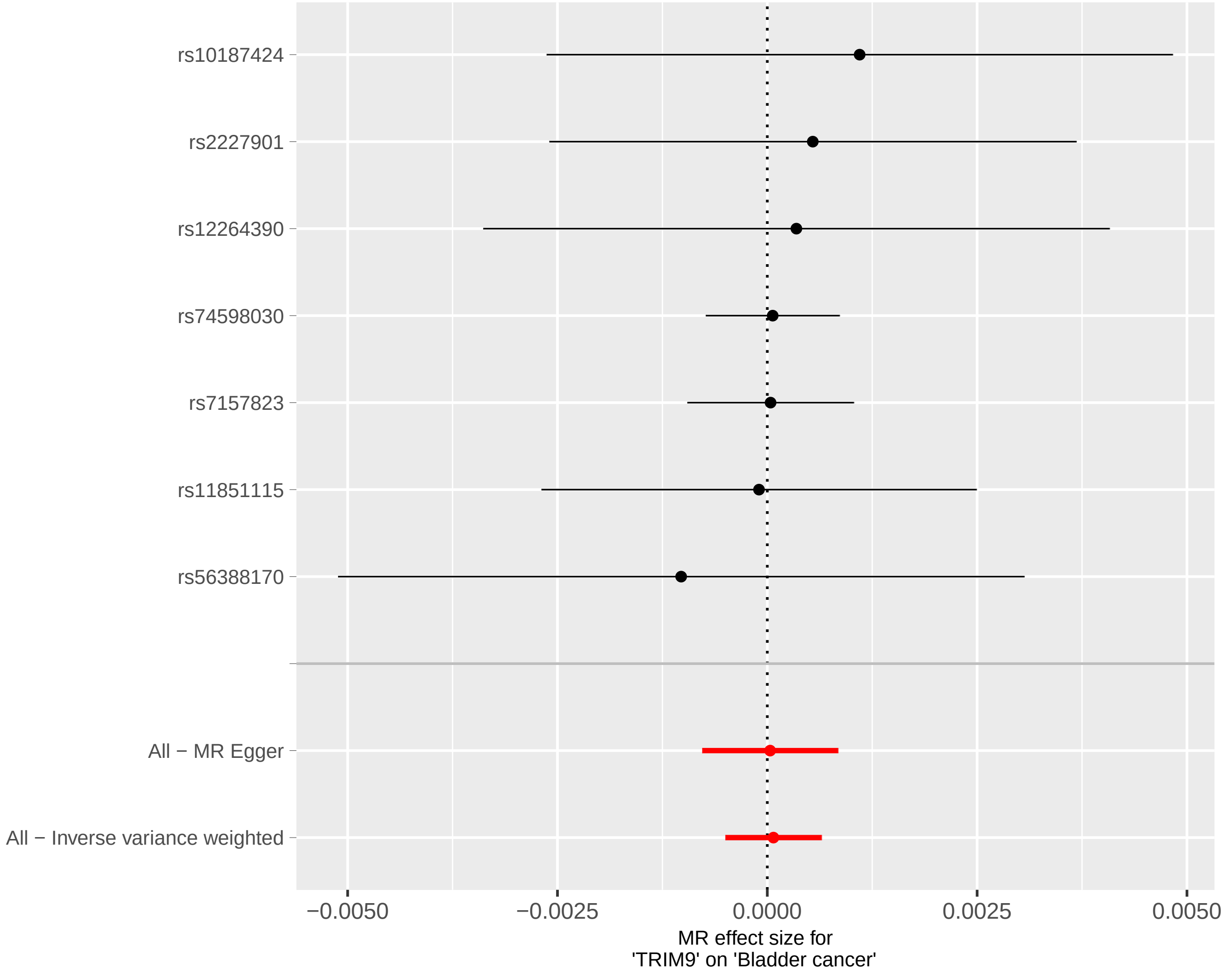

rs10187424
rs2227901
rs12264390
rs74598030
rs7157823
rs11851115
rs56388170
All − MR Egger
All − Inverse variance weighted
−0.0050
−0.0025
0.0000
0.0025
0.0050
MR effect size for
'TRIM9' on 'Bladder cancer'

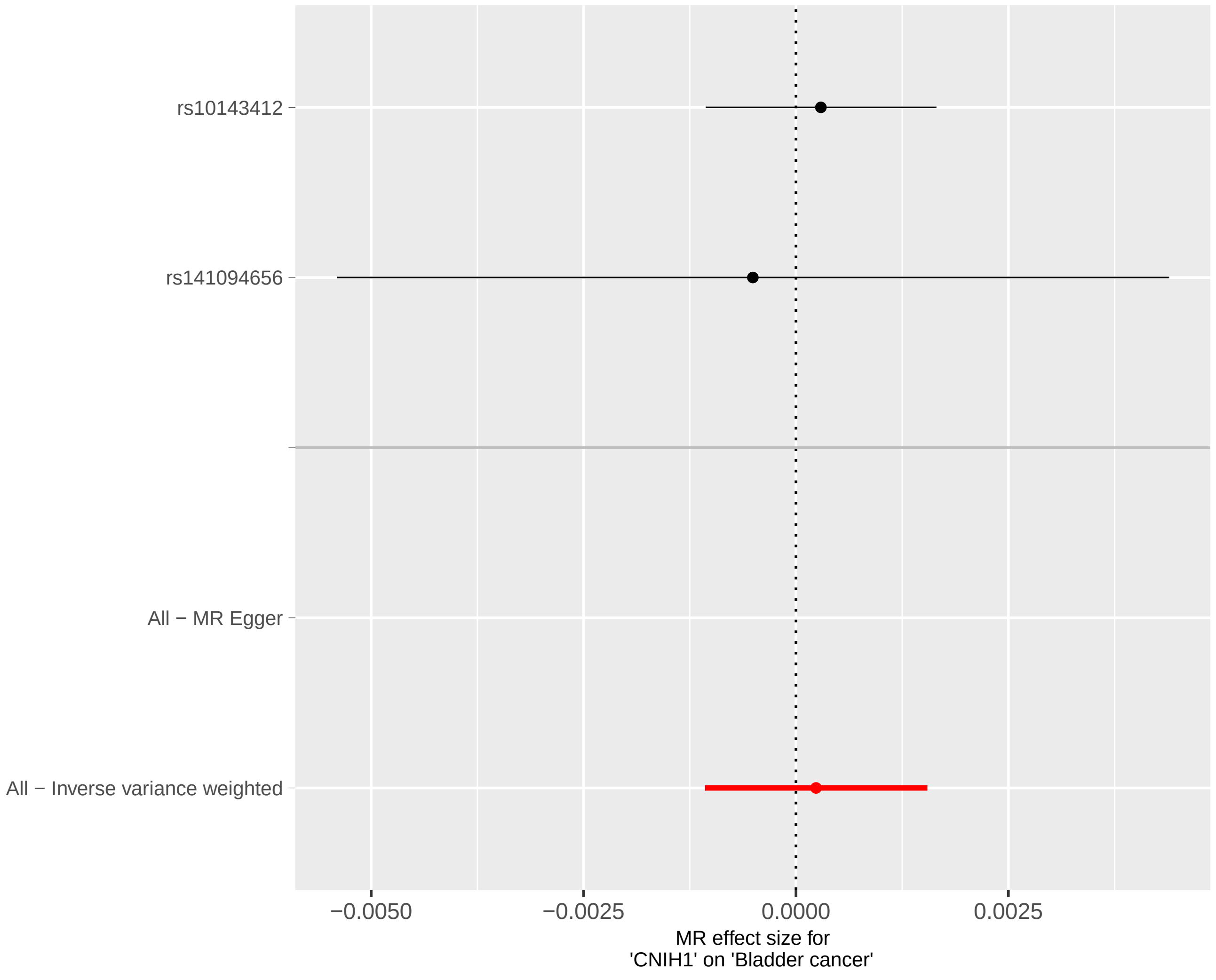

rs10143412
rs141094656
All − MR Egger
All − Inverse variance weighted
−0.0050
−0.0025
0.0000
0.0025
MR effect size for
'CNIH1' on 'Bladder cancer'

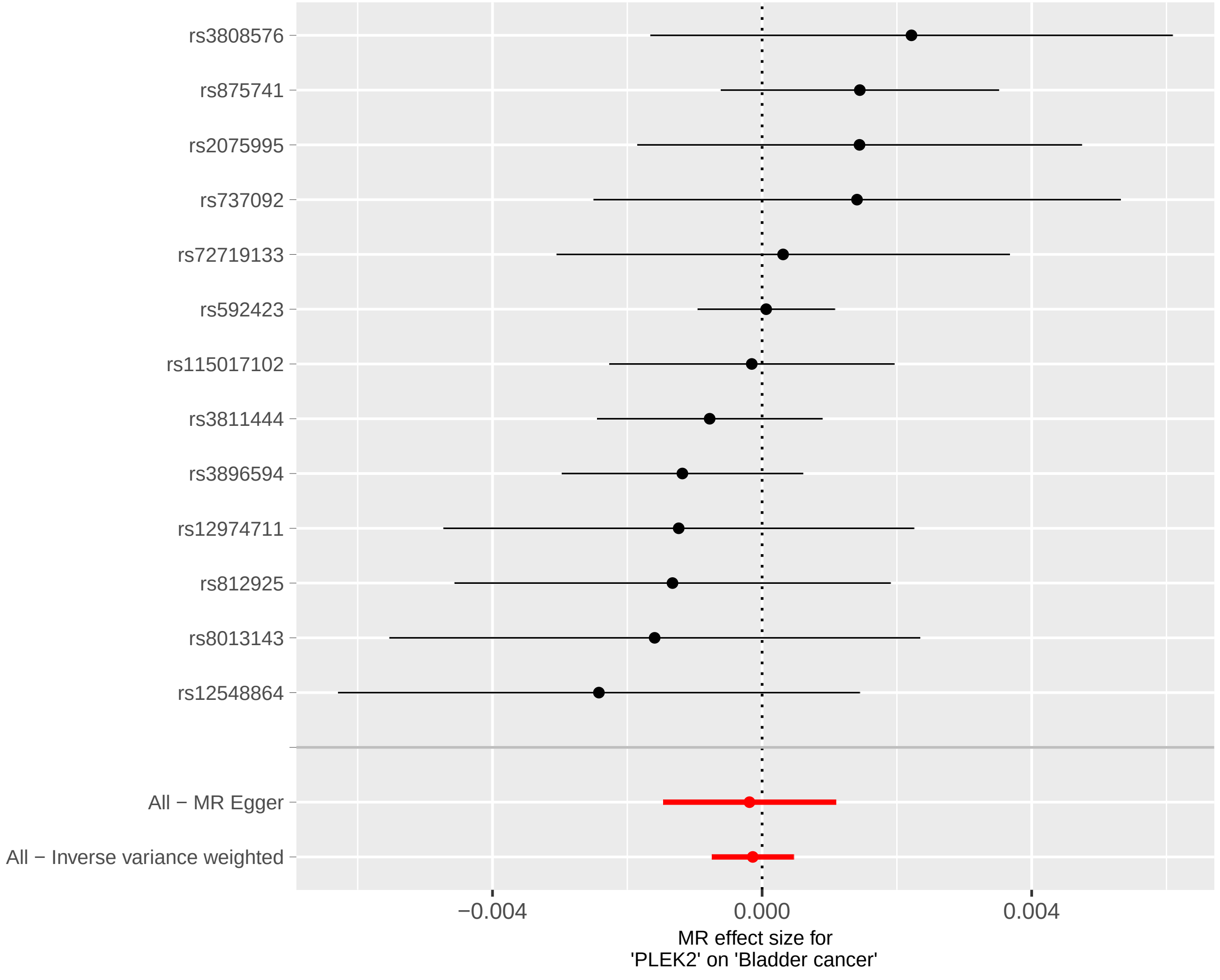
rs3808576
rs875741
rs2075995
rs737092
rs72719133
rs592423
rs115017102
rs3811444
rs3896594
rs12974711
rs812925
rs8013143
rs12548864
All − MR Egger
All − Inverse variance weighted
−0.004
0.000
0.004
MR effect size for
'PLEK2' on 'Bladder cancer'

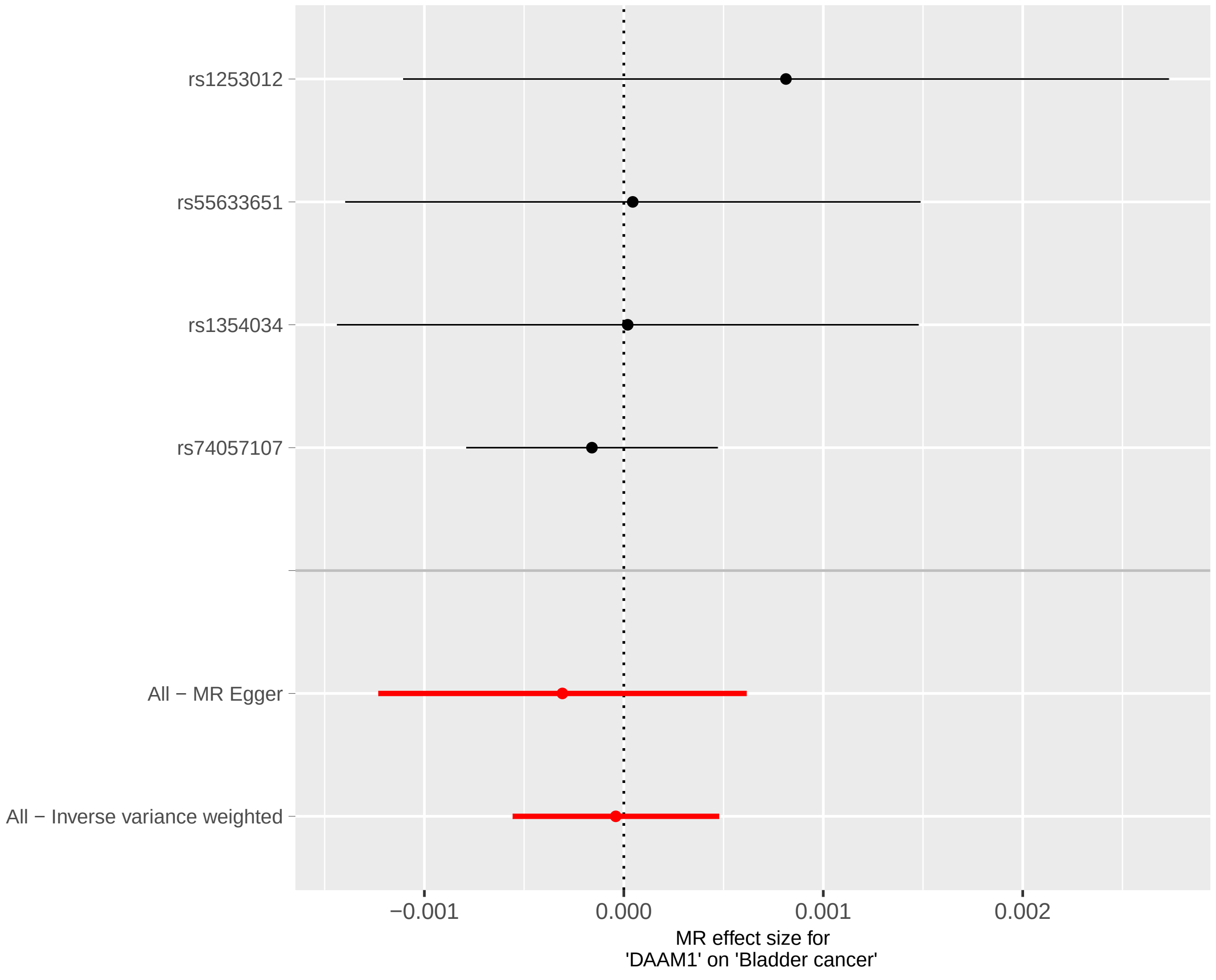

rs1253012
rs55633651
rs1354034
rs74057107
All − MR Egger
All − Inverse variance weighted
−0.001
0.000
0.001
0.002
MR effect size for
'DAAM1' on 'Bladder cancer'

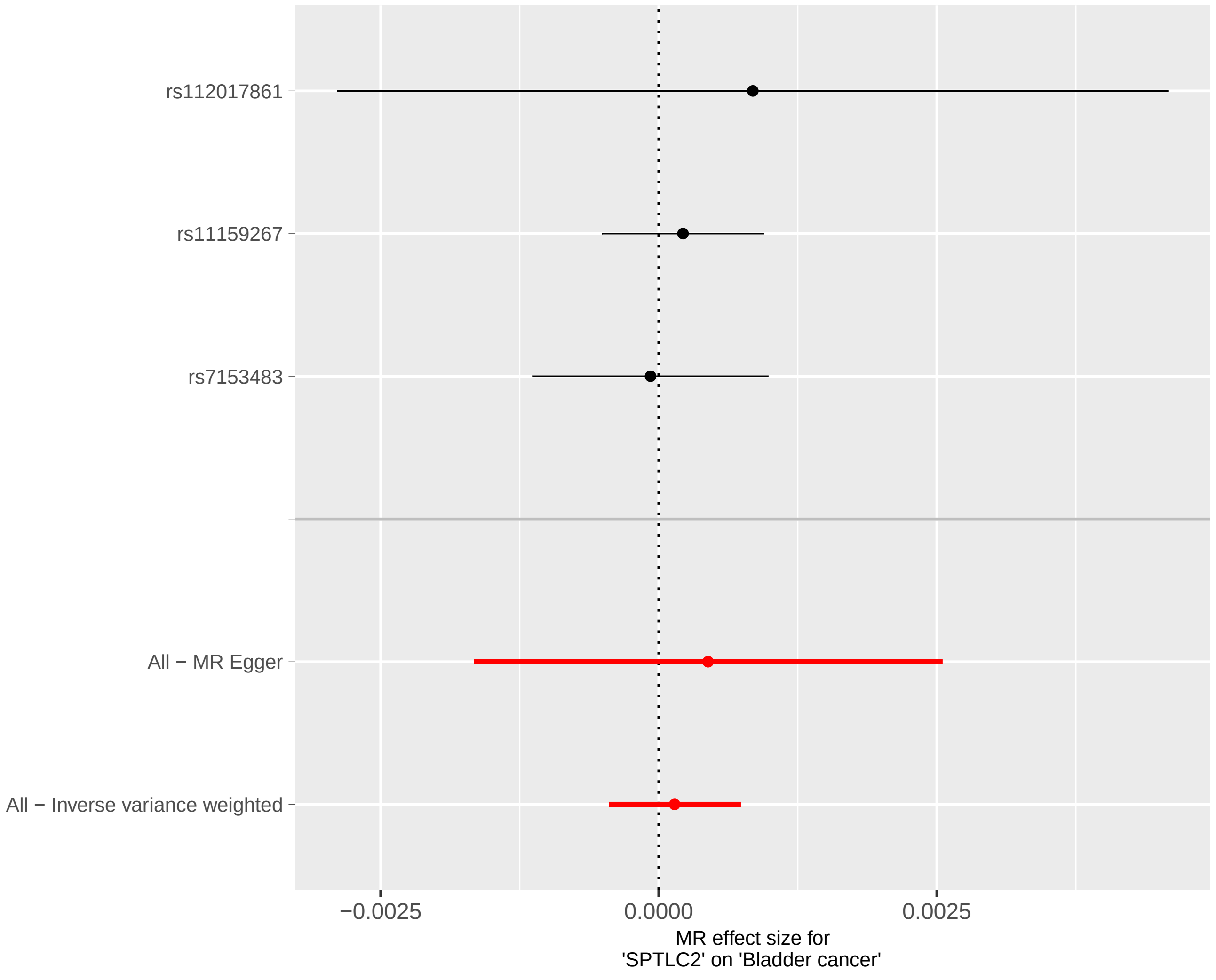
rs112017861
rs11159267
rs7153483
All − MR Egger
All − Inverse variance weighted
−0.0025
0.0000
0.0025
MR effect size for
'SPTLC2' on 'Bladder cancer'

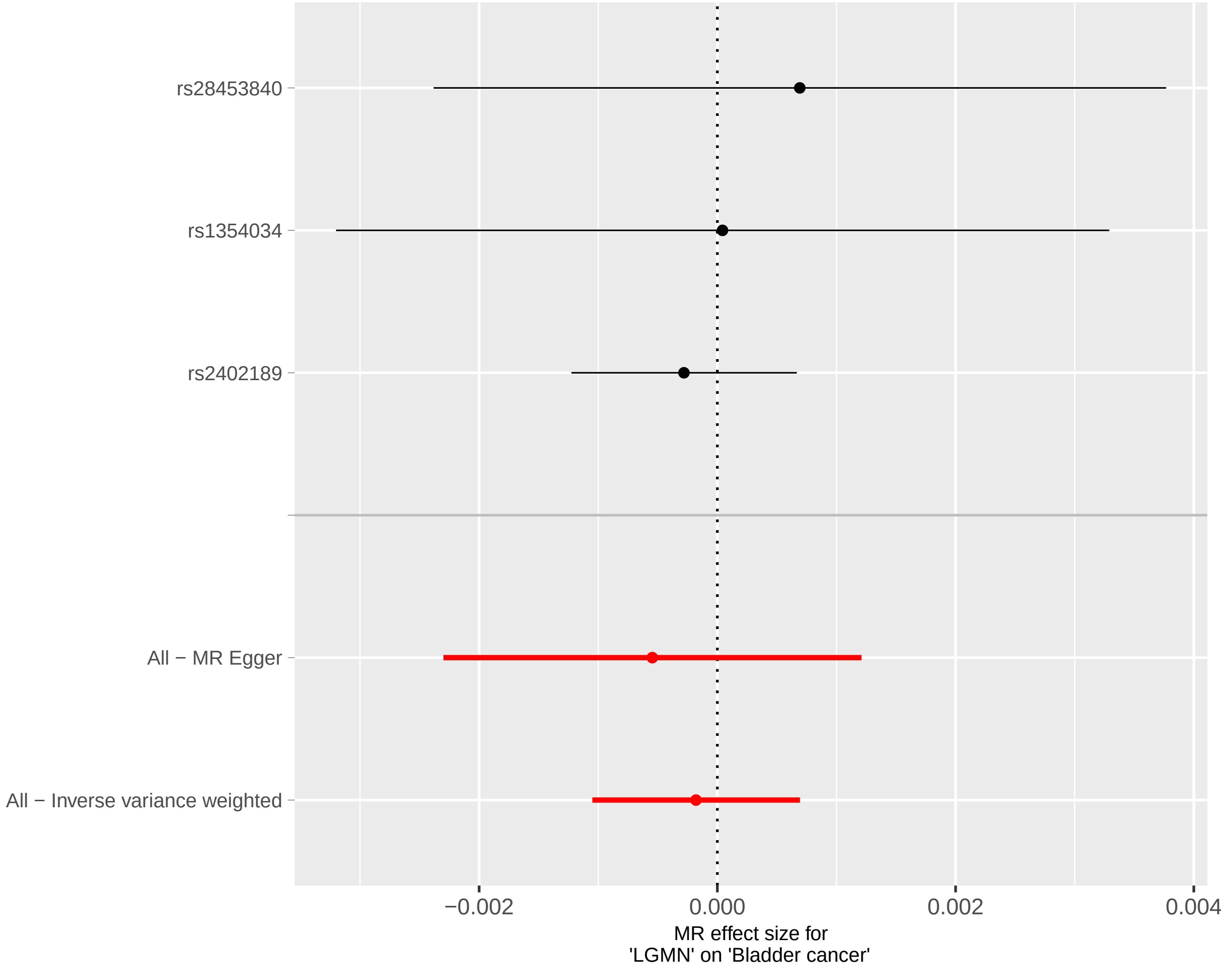
rs28453840
rs1354034
rs2402189
All − MR Egger
All − Inverse variance weighted
−0.002
0.000
0.002
0.004
MR effect size for
'LGMN' on 'Bladder cancer'

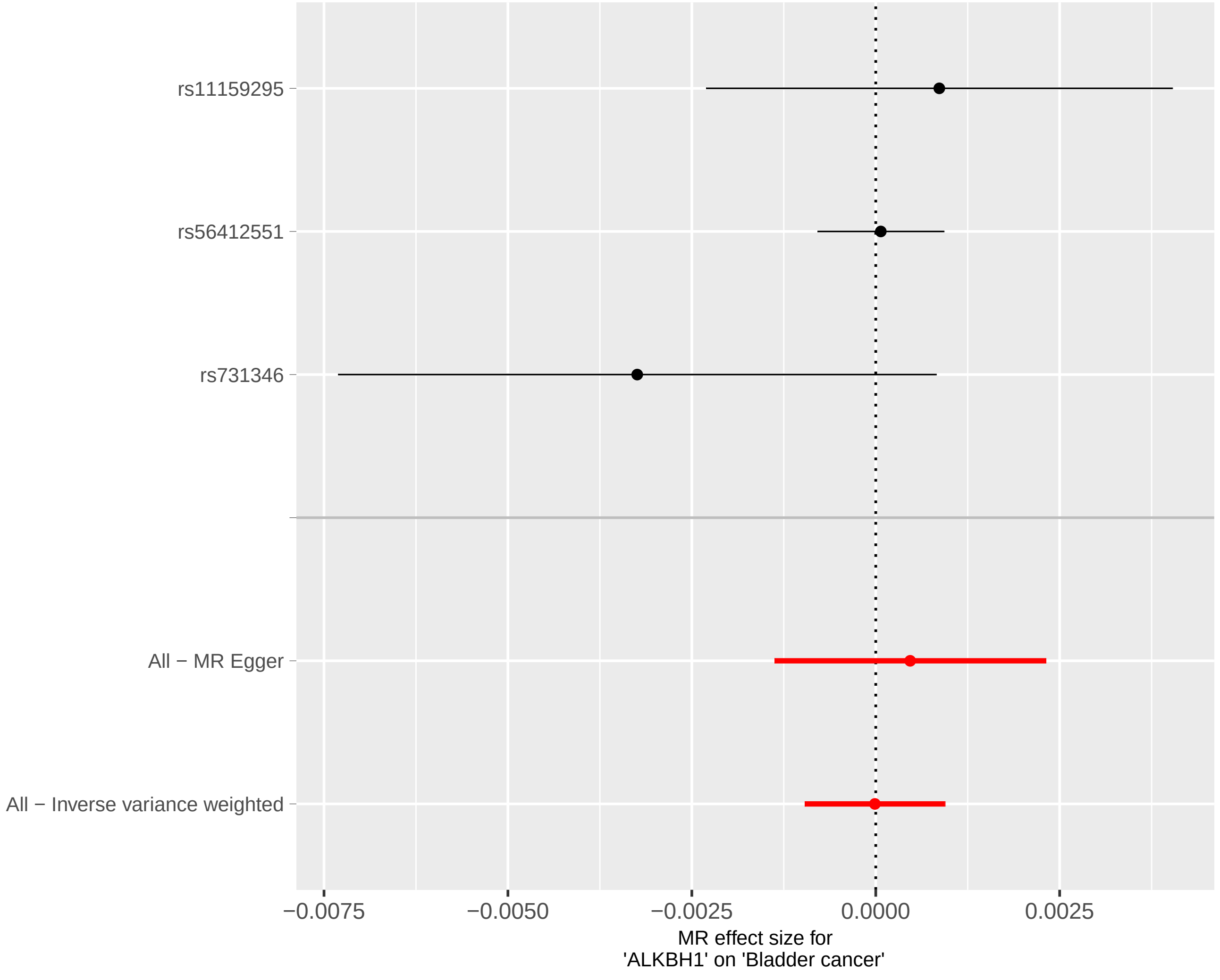
rs11159295
rs56412551
rs731346
All − MR Egger
All − Inverse variance weighted
−0.0075
−0.0050
−0.0025
0.0000
0.0025
MR effect size for
'ALKBH1' on 'Bladder cancer'

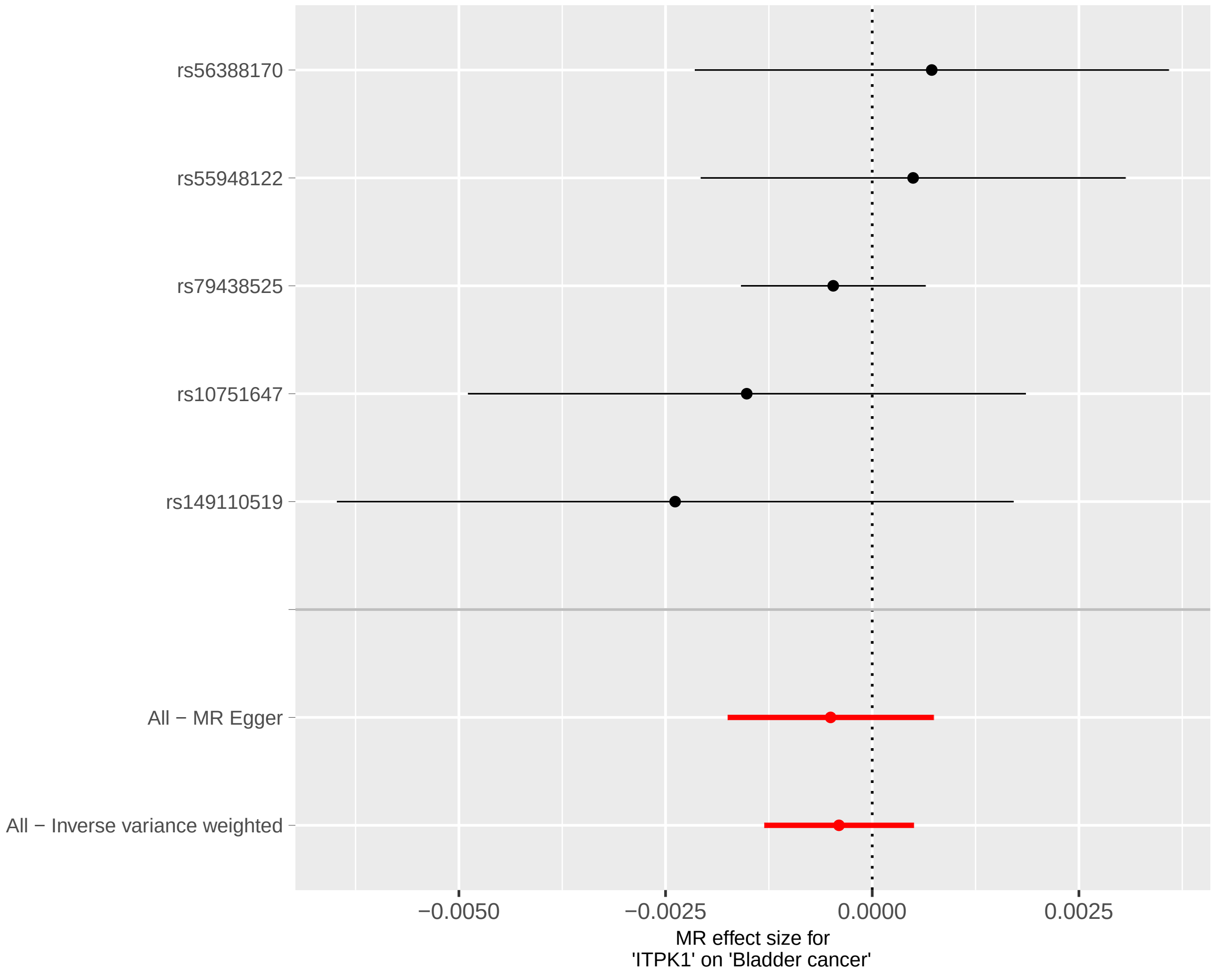

rs56388170
rs55948122
rs79438525
rs10751647
rs149110519
All − MR Egger
All − Inverse variance weighted
−0.0050
−0.0025
0.0000
0.0025
MR effect size for
'ITPK1' on 'Bladder cancer'

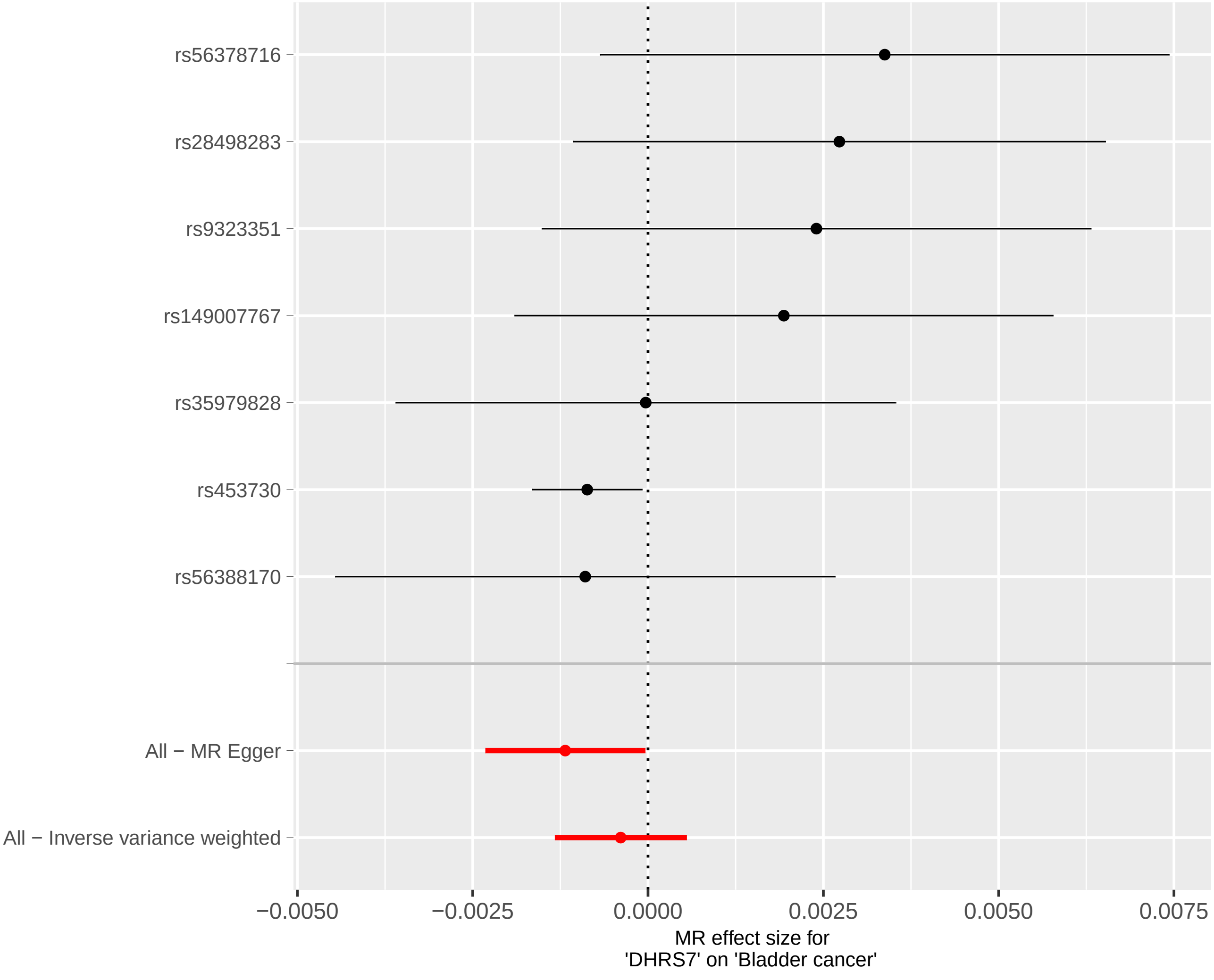

rs56378716
rs28498283
rs9323351
rs149007767
rs35979828
rs453730
rs56388170
All − MR Egger
All − Inverse variance weighted
−0.0050
−0.0025
0.0000
0.0025
0.0050
0.0075
MR effect size for
'DHRS7' on 'Bladder cancer'

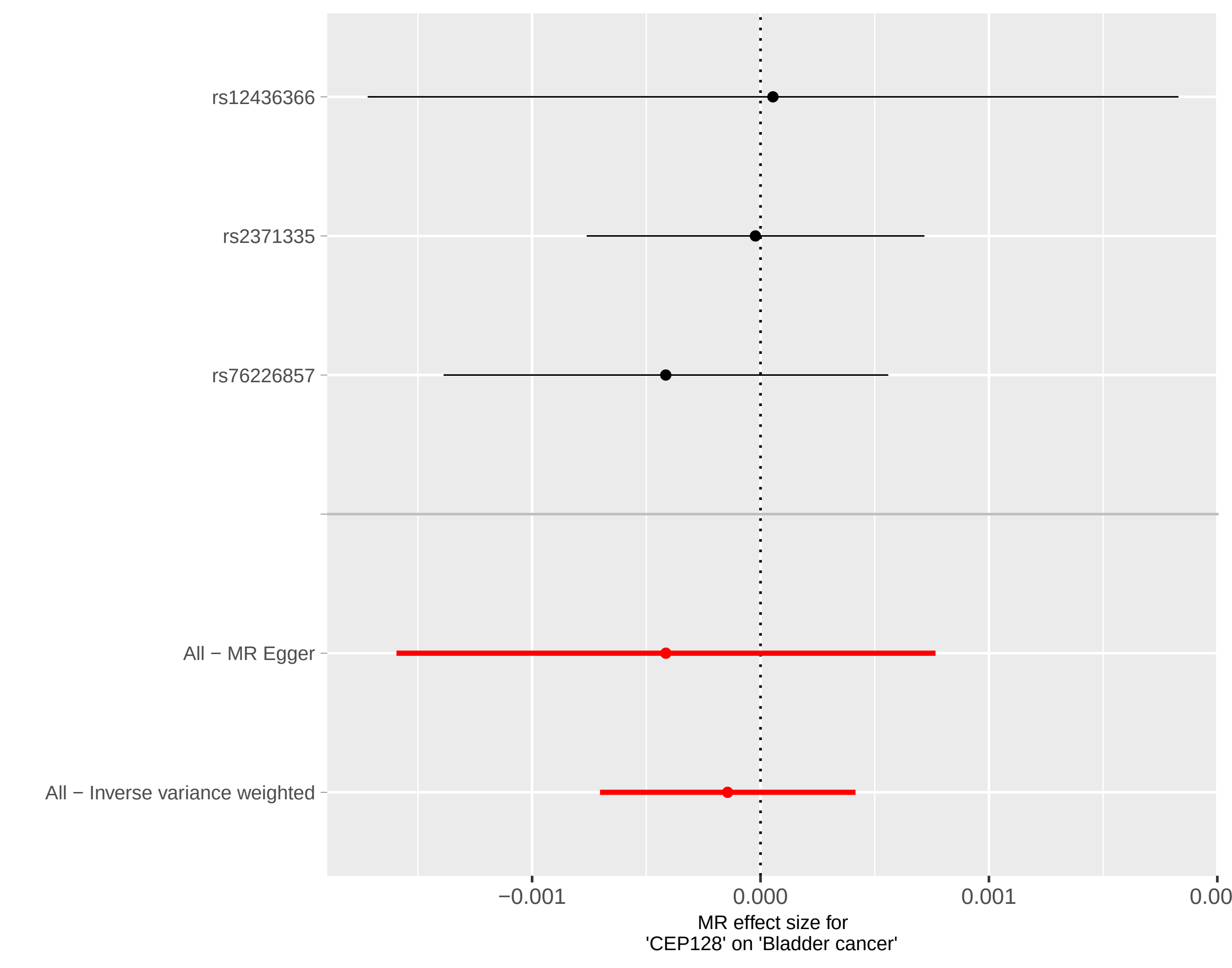
rs12436366
rs2371335
rs76226857
All − MR Egger
All − Inverse variance weighted
−0.001
0.000
0.001
0.00
MR effect size for
'CEP128' on 'Bladder cancer'

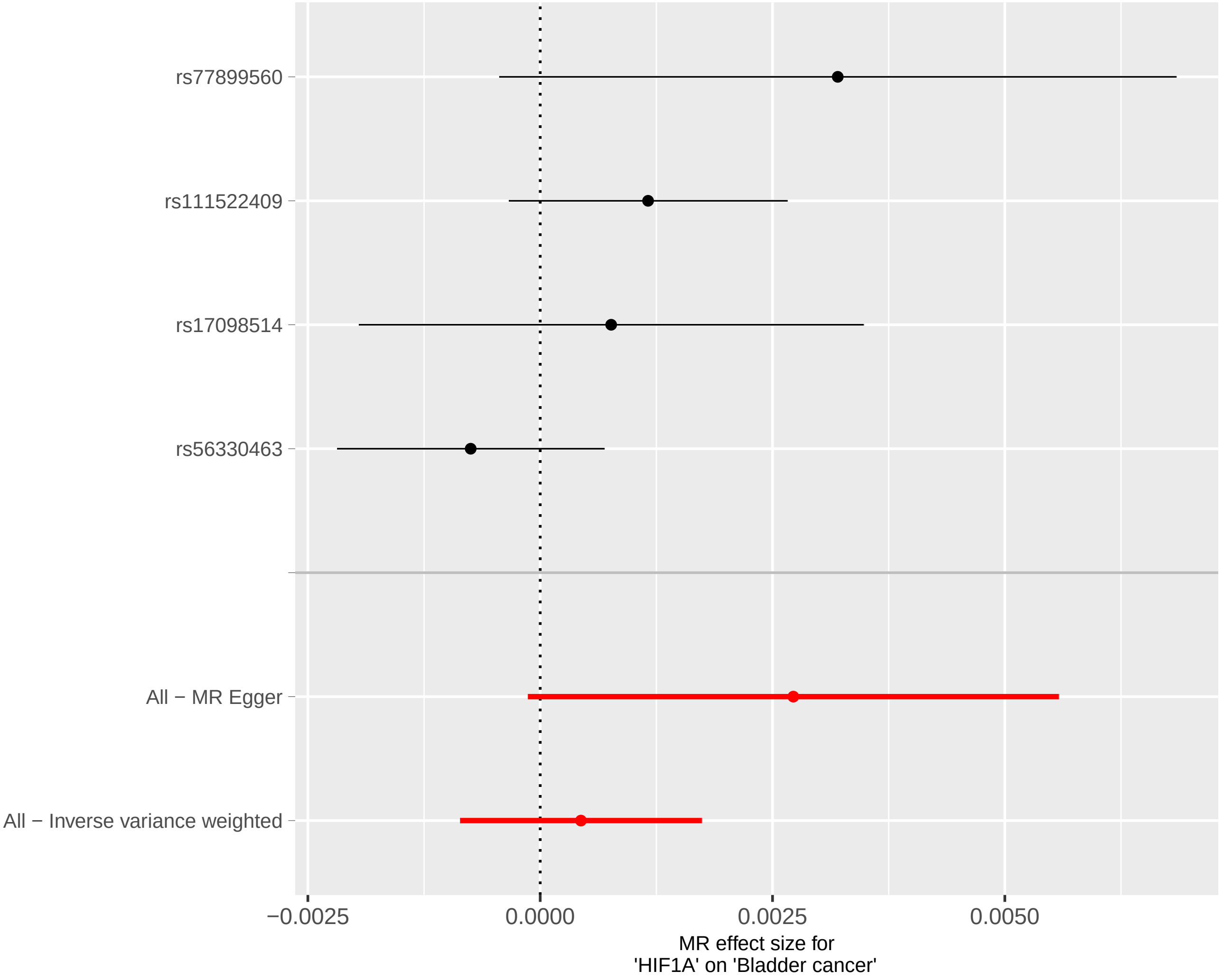
rs77899560
rs111522409
rs17098514
rs56330463
All − MR Egger
All − Inverse variance weighted
−0.0025
0.0000
0.0025
0.0050
MR effect size for
'HIF1A' on 'Bladder cancer'

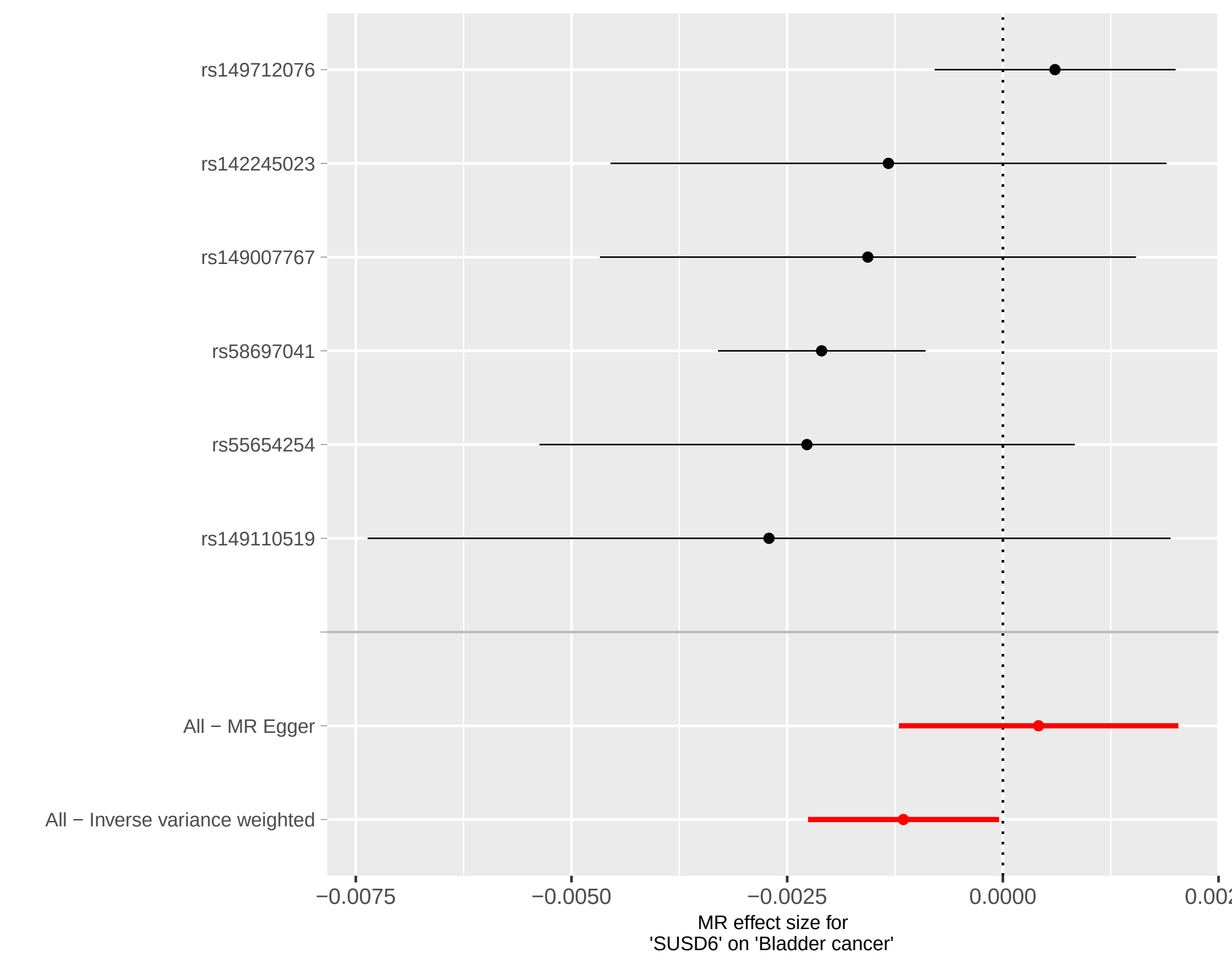
rs149712076
rs142245023
rs149007767
rs58697041
rs55654254
rs149110519
All − MR Egger
All − Inverse variance weighted
−0.0075
−0.0050
−0.0025
0.0000
MR effect size for
'SUSD6' on 'Bladder cancer'

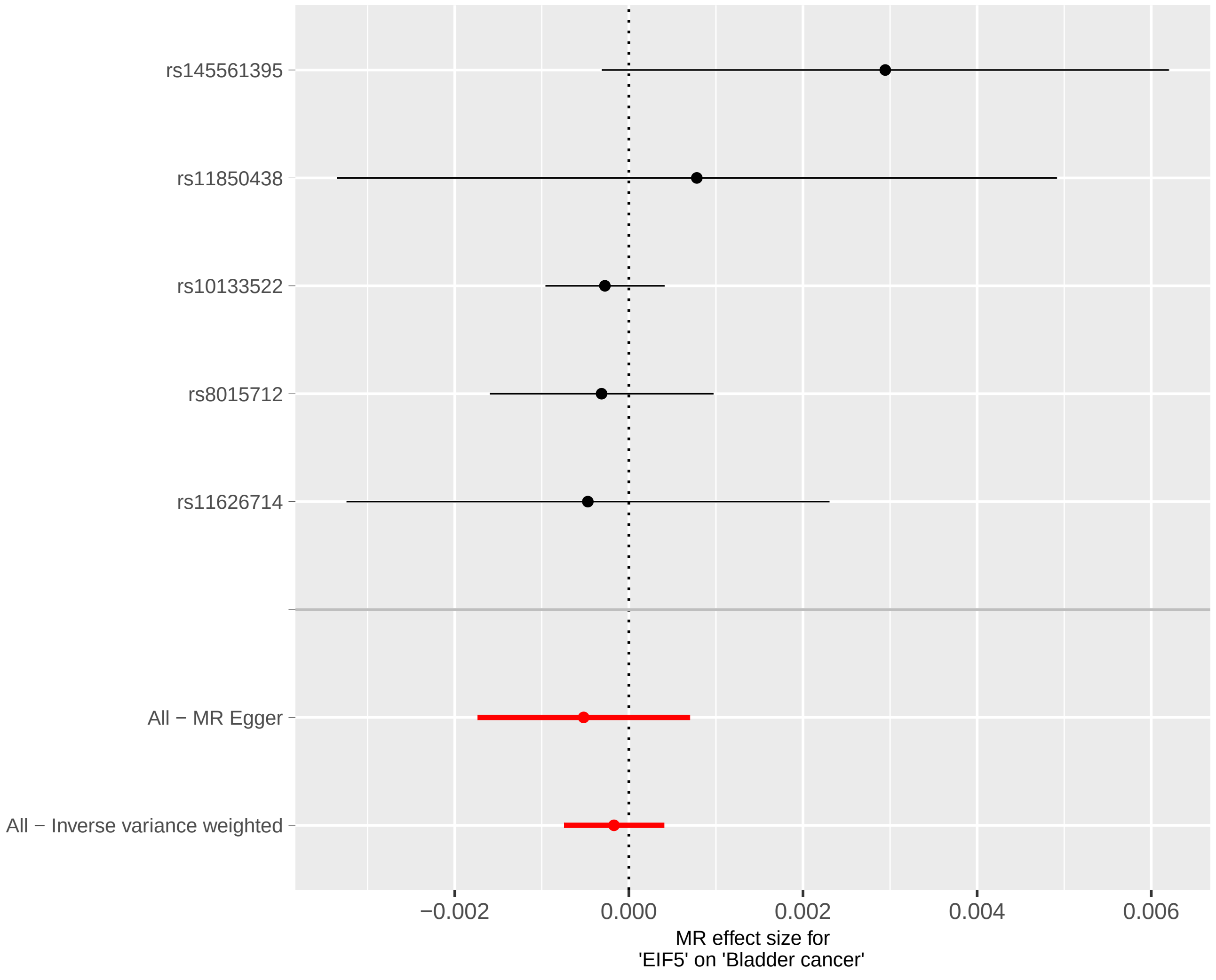

rs145561395
rs11850438
rs10133522
rs8015712
rs11626714
All − MR Egger
All − Inverse variance weighted
−0.002
0.000
0.002
0.004
0.006
MR effect size for
'EIF5' on 'Bladder cancer'

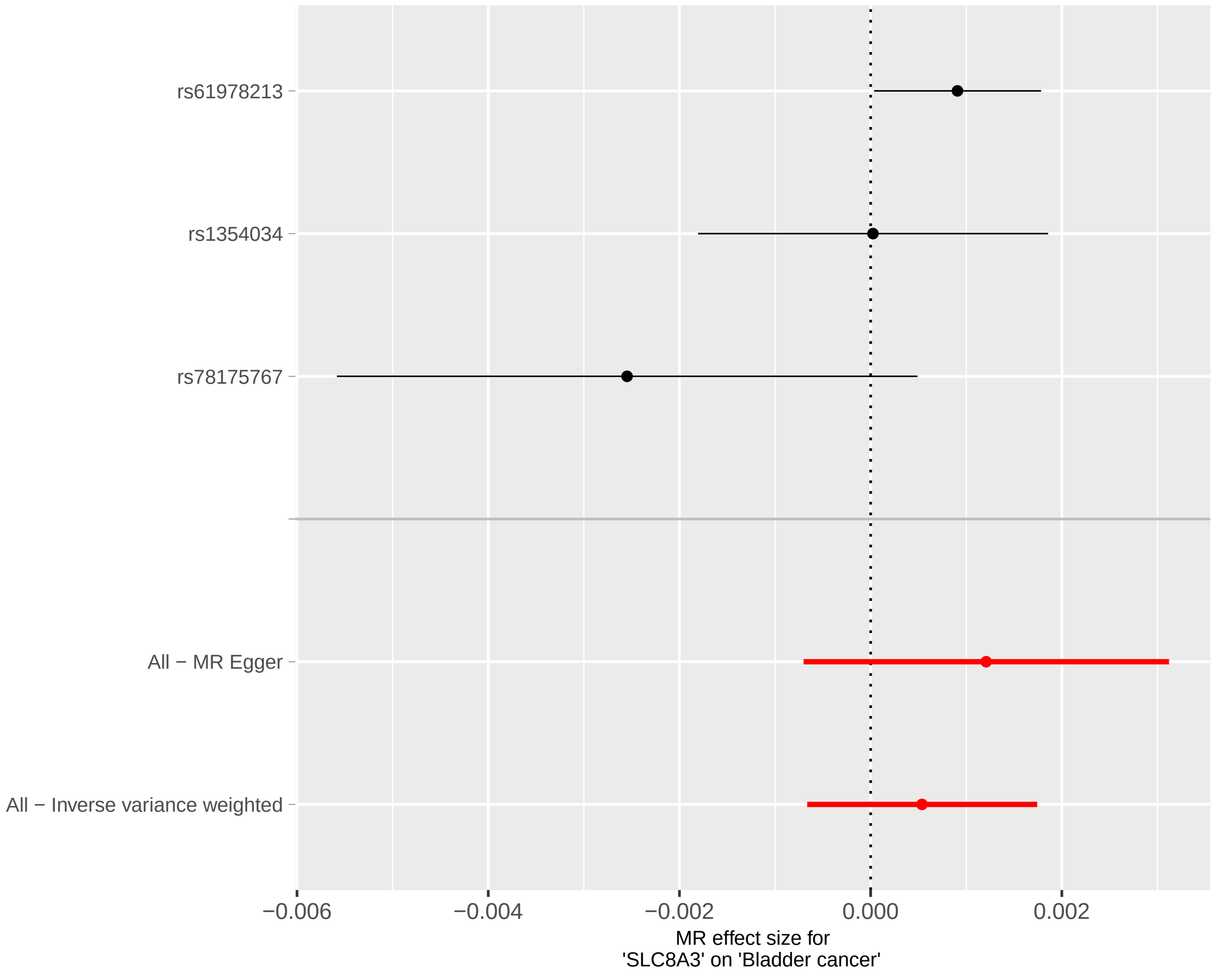
rs61978213
rs1354034
rs78175767
All − MR Egger
All − Inverse variance weighted
−0.006
−0.004
−0.002
0.000
0.002
MR effect size for
'SLC8A3' on 'Bladder cancer'

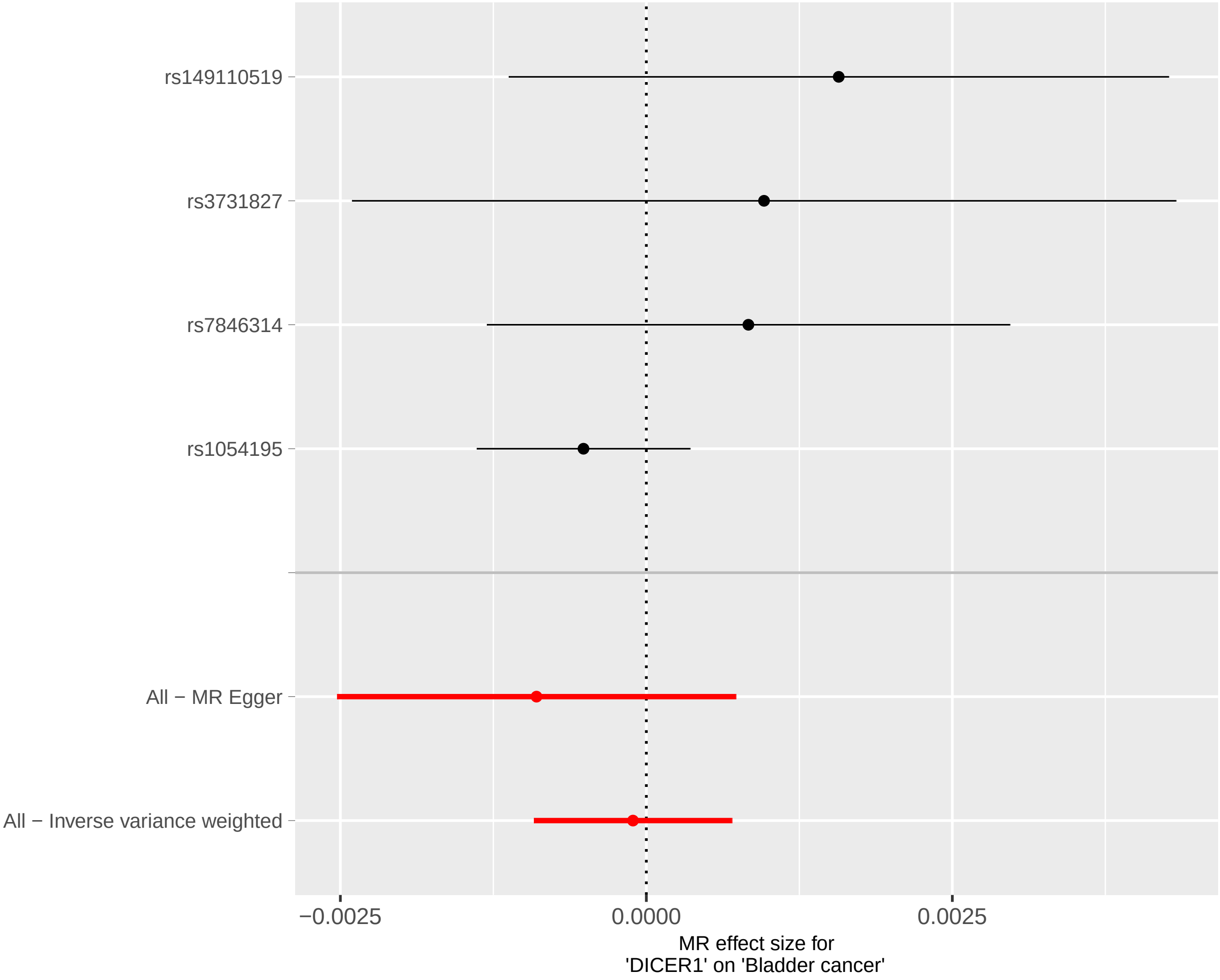
rs149110519
rs3731827
rs7846314
rs1054195
All − MR Egger
All − Inverse variance weighted
−0.0025
0.0000
0.0025
MR effect size for
'DICER1' on 'Bladder cancer'

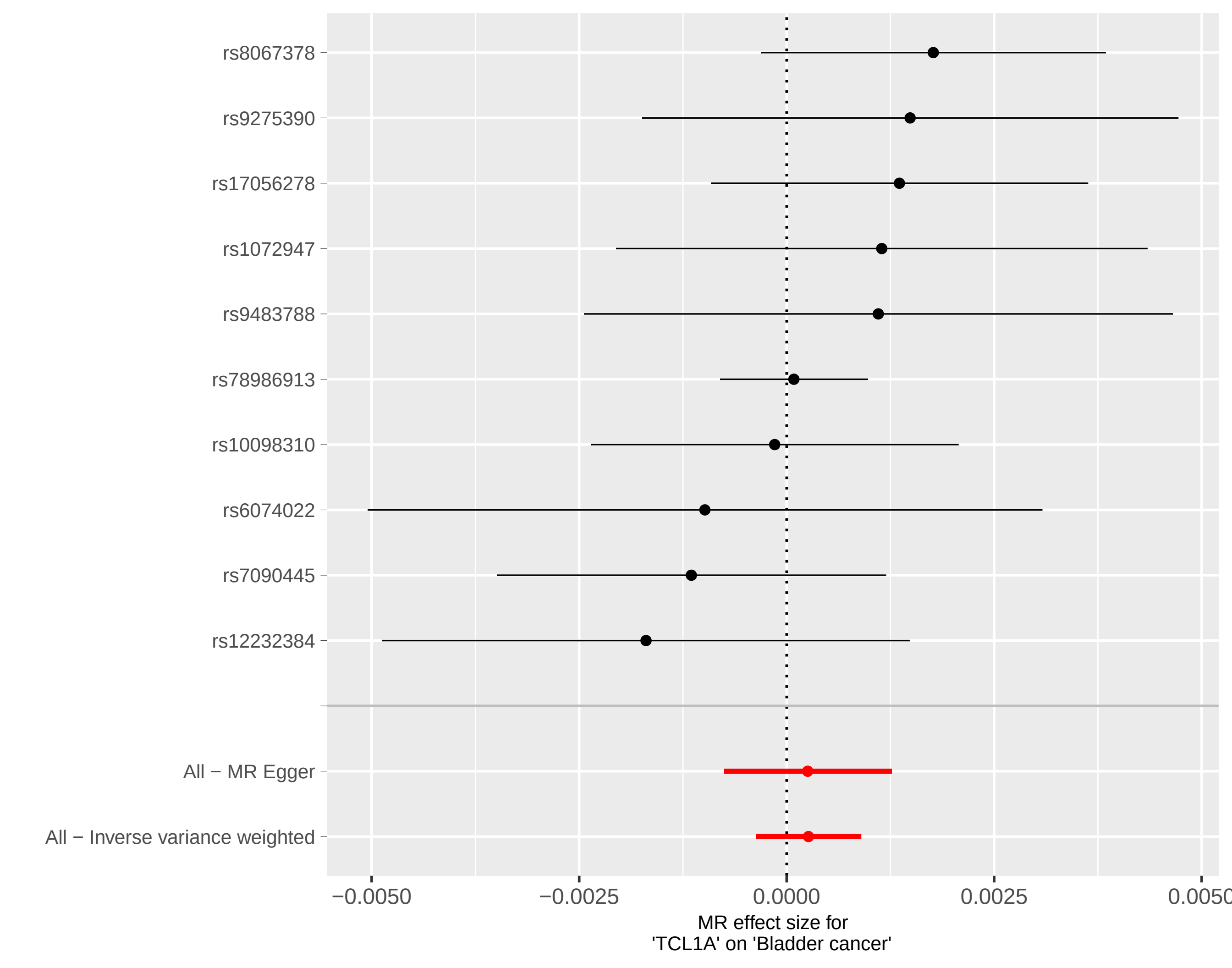
rs8067378
rs9275390
rs17056278
rs1072947
rs9483788
rs78986913
rs10098310
rs6074022
rs7090445
rs12232384
All − MR Egger
All − Inverse variance weighted
−0.0050
−0.0025
0.0000
0.0025
0.0050
MR effect size for
'TCL1A' on 'Bladder cancer'

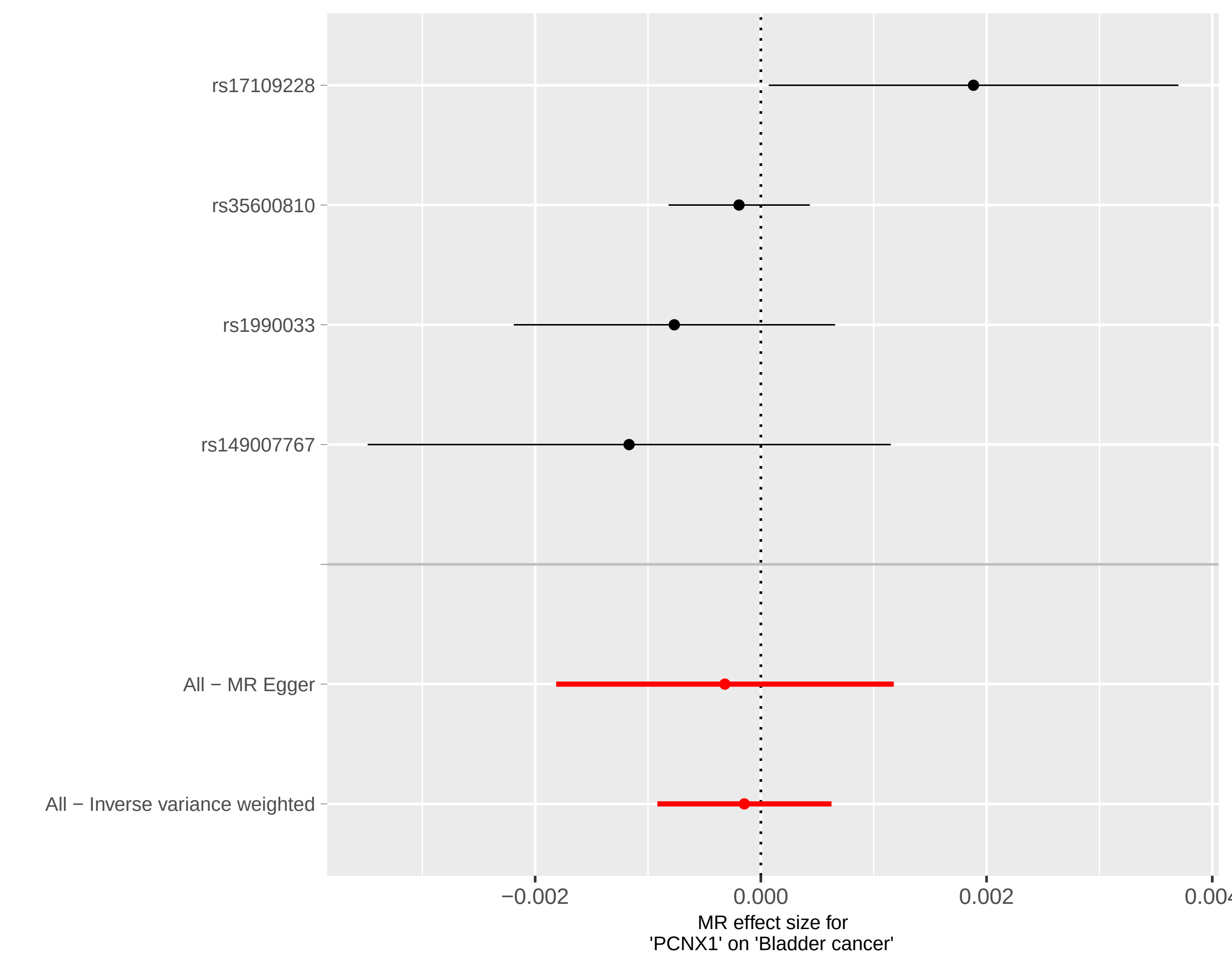

rs17109228
rs35600810
rs1990033
rs149007767
All − MR Egger
All − Inverse variance weighted
−0.002
0.000
0.002
0.004
MR effect size for
'PCNX1' on 'Bladder cancer'

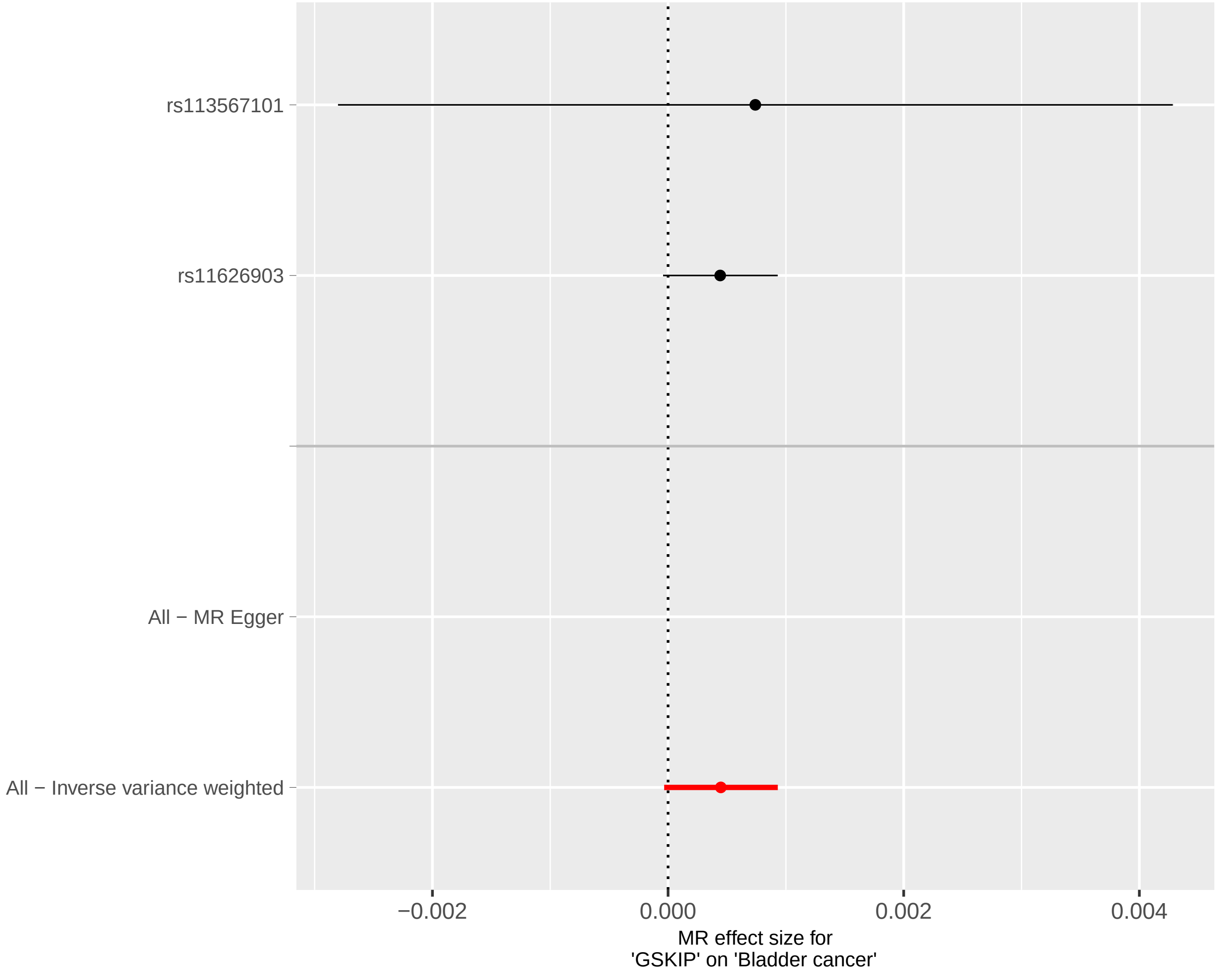

rs113567101
rs11626903
All − MR Egger
All − Inverse variance weighted
−0.002
0.000
0.002
0.004
MR effect size for
'GSKIP' on 'Bladder cancer'

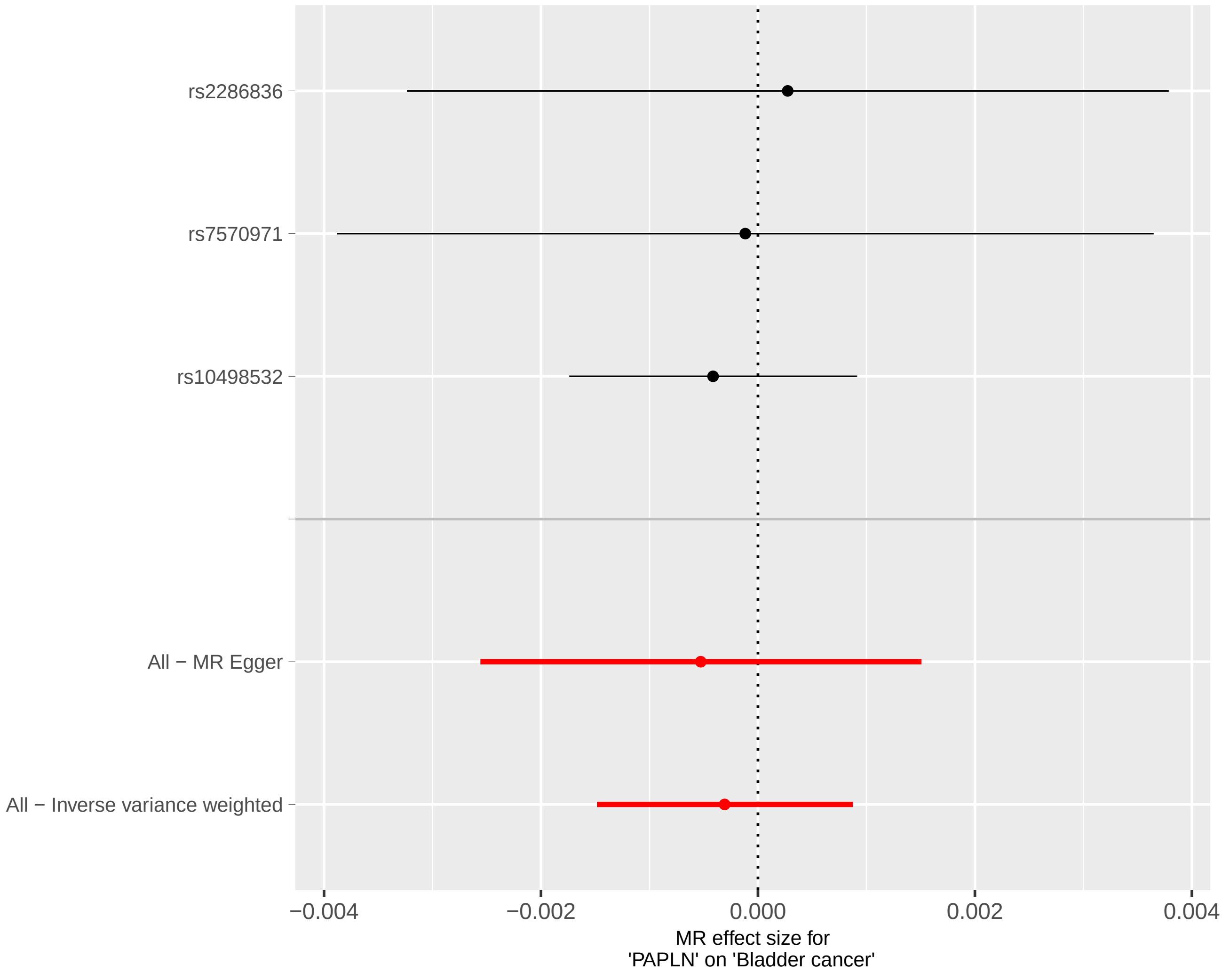

rs2286836
rs7570971
rs10498532
All − MR Egger
All − Inverse variance weighted
−0.004
−0.002
0.000
0.002
0.004
MR effect size for
'PAPLN' on 'Bladder cancer'

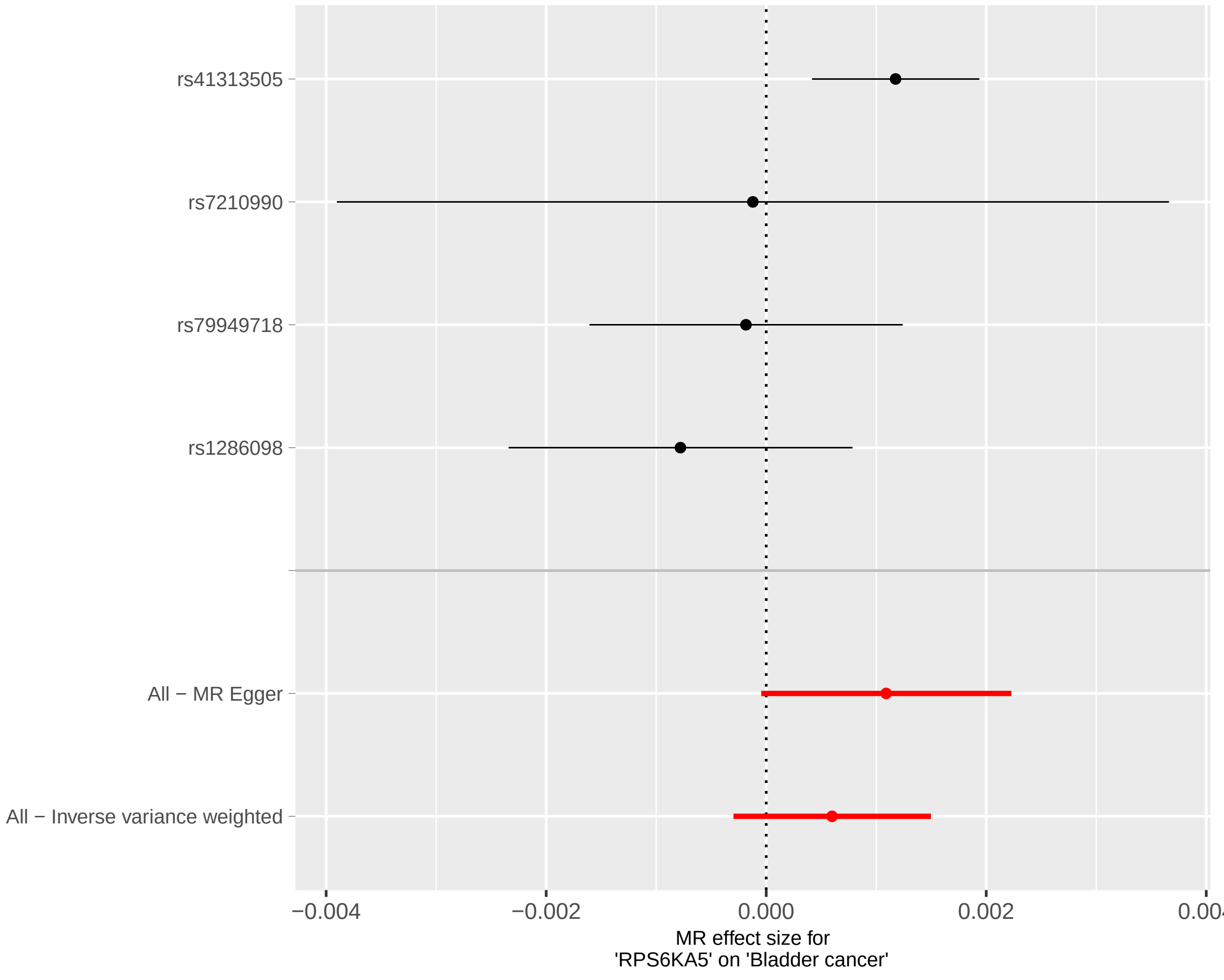

rs41313505
rs7210990
rs79949718
rs1286098
All − MR Egger
All − Inverse variance weighted
−0.004
−0.002
0.000
0.002
MR effect size for
'RPS6KA5' on 'Bladder cancer'

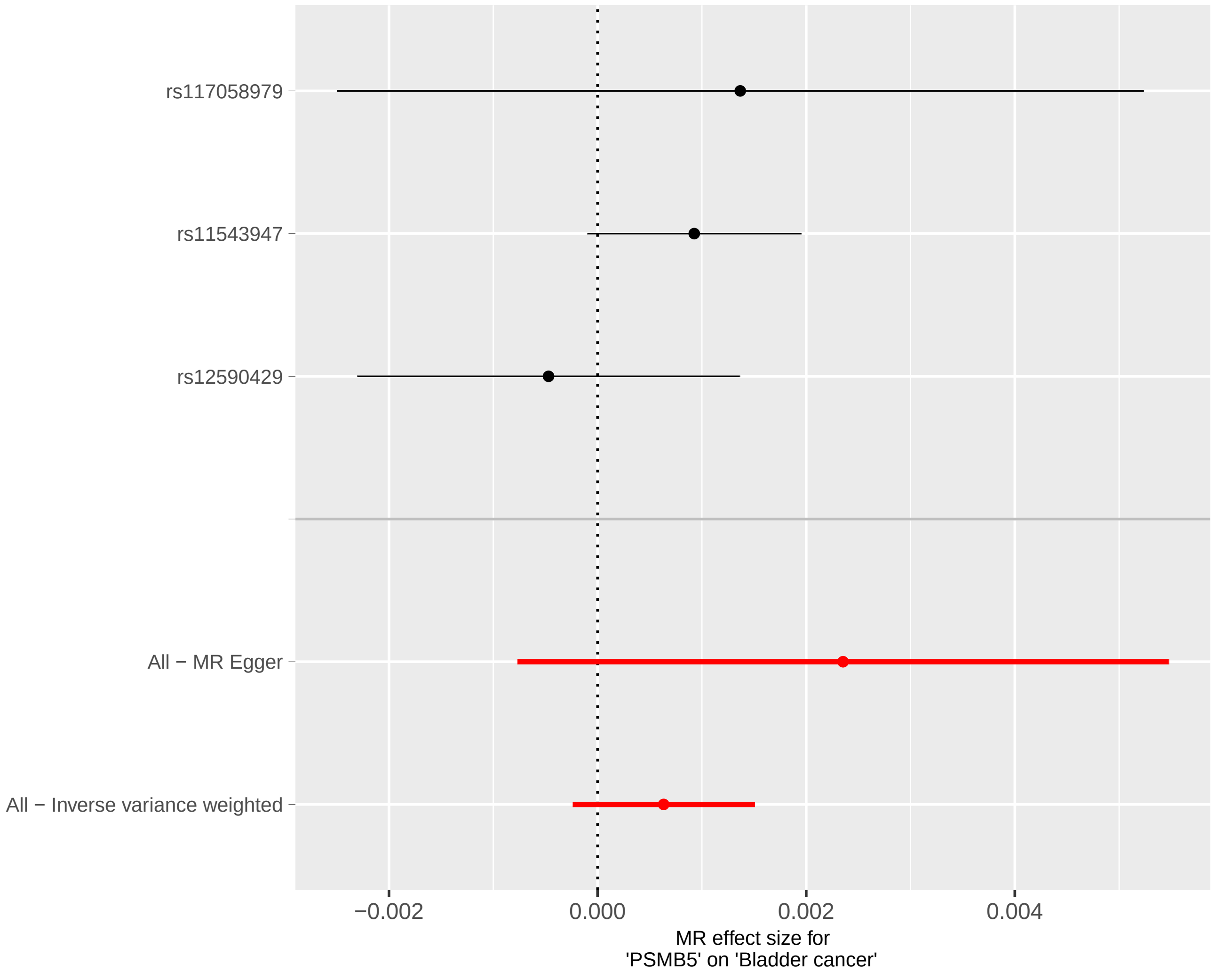

rs117058979
rs11543947
rs12590429
All − MR Egger
All − Inverse variance weighted
−0.002
0.000
0.002
0.004
MR effect size for
'PSMB5' on 'Bladder cancer'

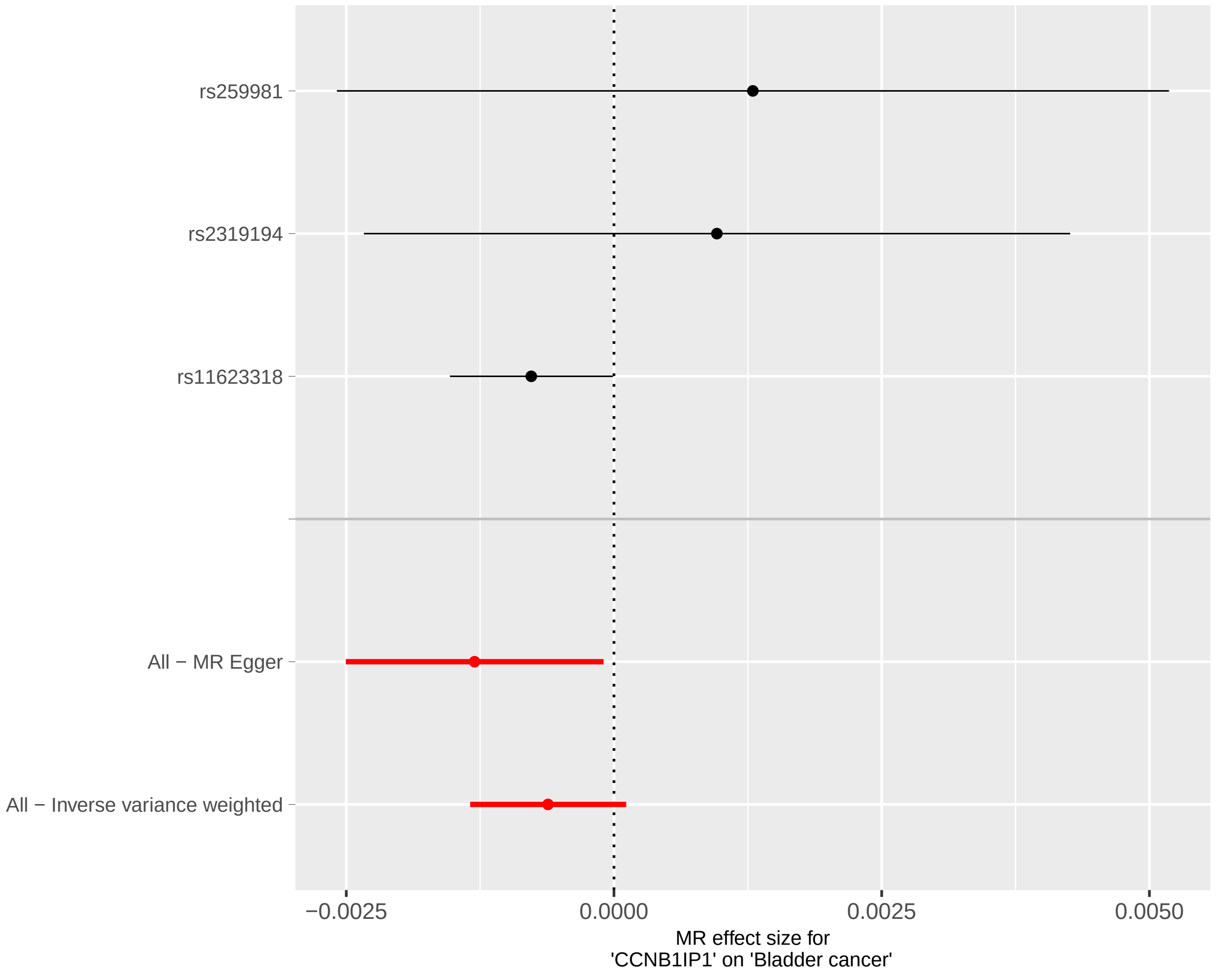
rs259981
rs2319194
rs11623318
All − MR Egger
All − Inverse variance weighted
−0.0025
0.0000
0.0025
0.0050
MR effect size for
'CCNB1IP1' on 'Bladder cancer'

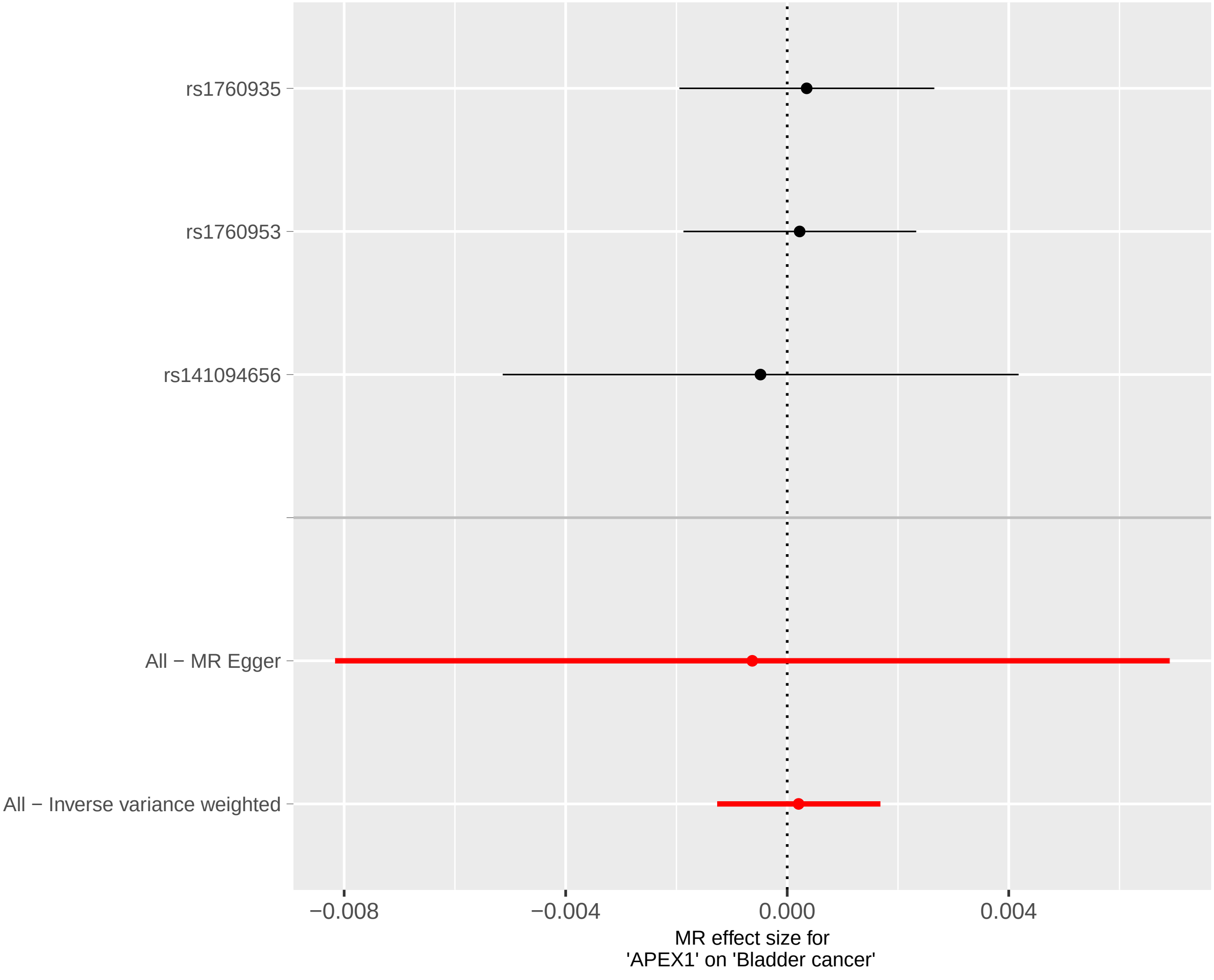

rs1760935
rs1760953
rs141094656
All − MR Egger
All − Inverse variance weighted
−0.008
−0.004
0.000
0.004
MR effect size for
'APEX1' on 'Bladder cancer'

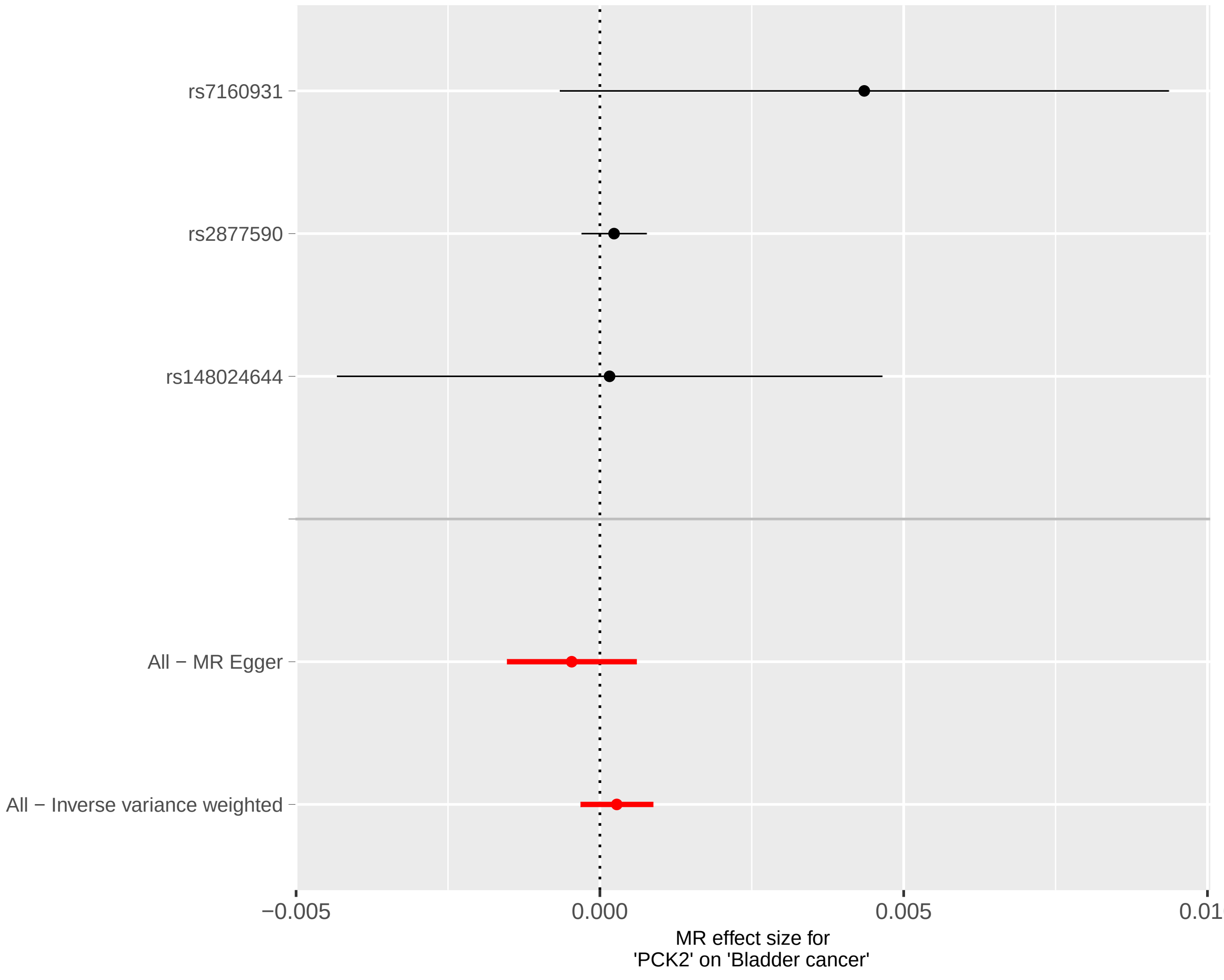
rs7160931
rs2877590
rs148024644
All − MR Egger
All − Inverse variance weighted
−0.005
0.000
0.005
MR effect size for
'PCK2' on 'Bladder cancer'

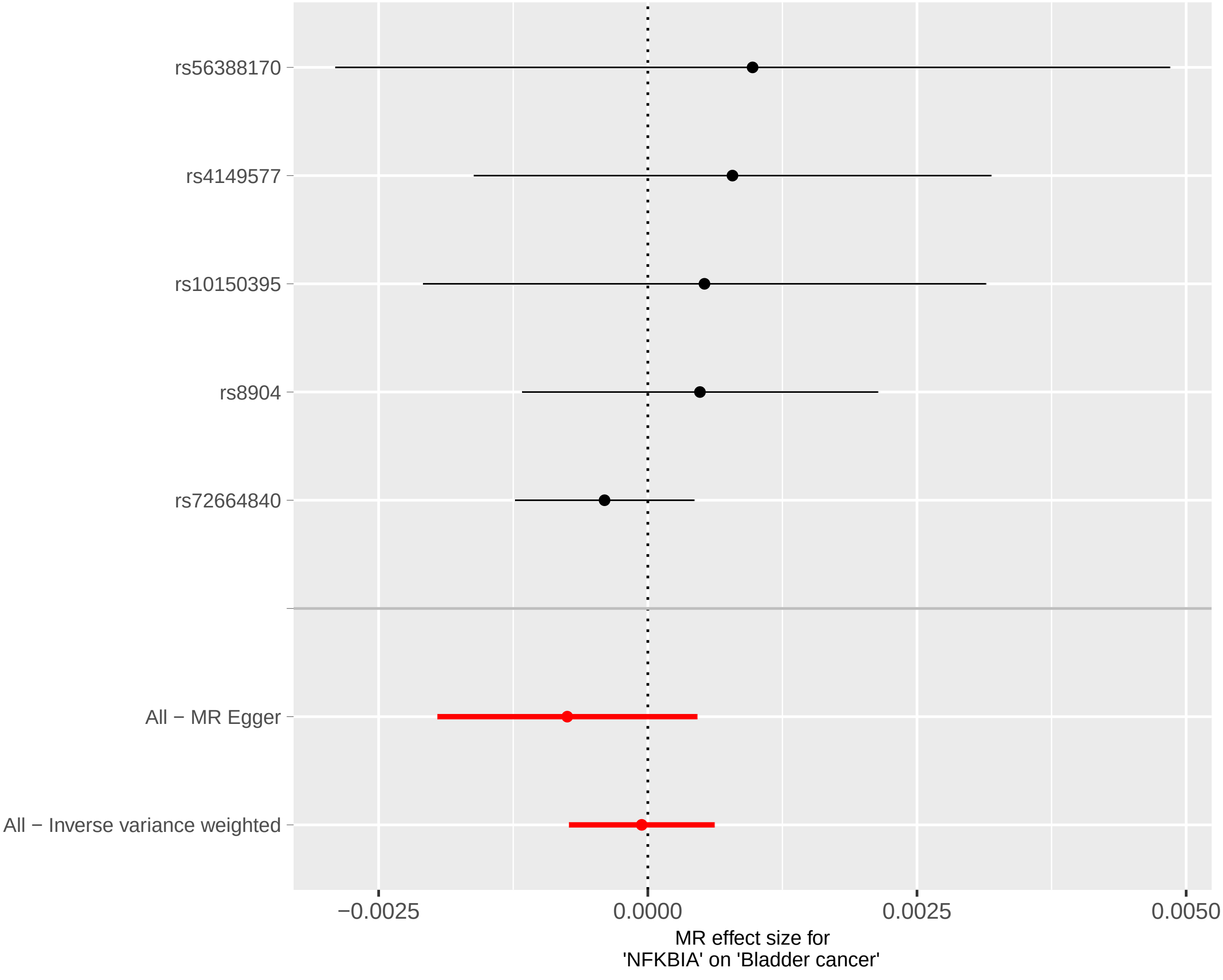
rs56388170
rs4149577
rs10150395
rs8904
rs72664840
All − MR Egger
All − Inverse variance weighted
−0.0025
0.0000
0.0025
0.0050
MR effect size for
'NFKBIA' on 'Bladder cancer'

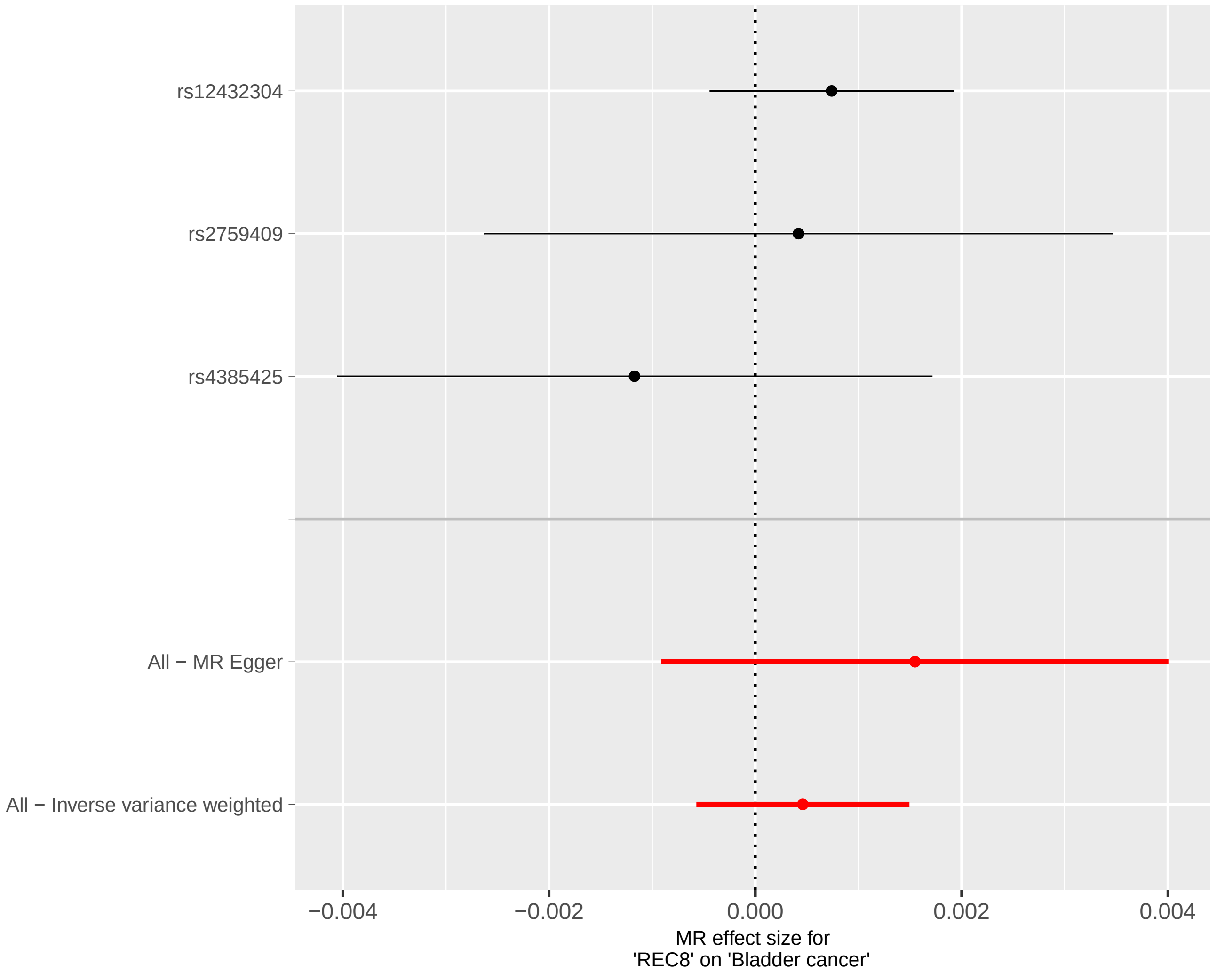
rs12432304
rs2759409
rs4385425
All − MR Egger
All − Inverse variance weighted
−0.004
−0.002
0.000
0.002
0.004
MR effect size for
'REC8' on 'Bladder cancer'

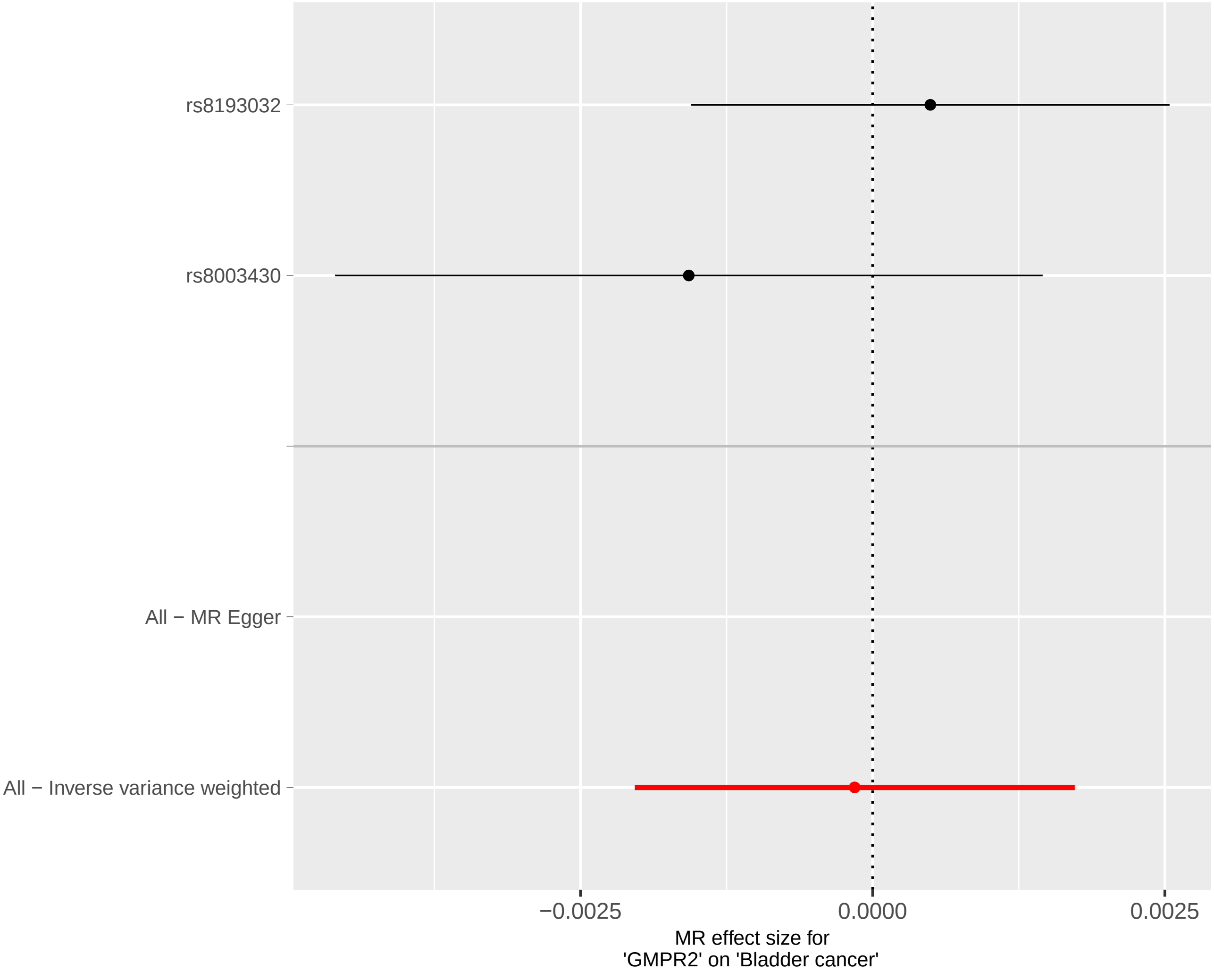
rs8193032
rs8003430
All − MR Egger
All − Inverse variance weighted
−0.0025
0.0000
0.0025
MR effect size for
'GMPR2' on 'Bladder cancer'

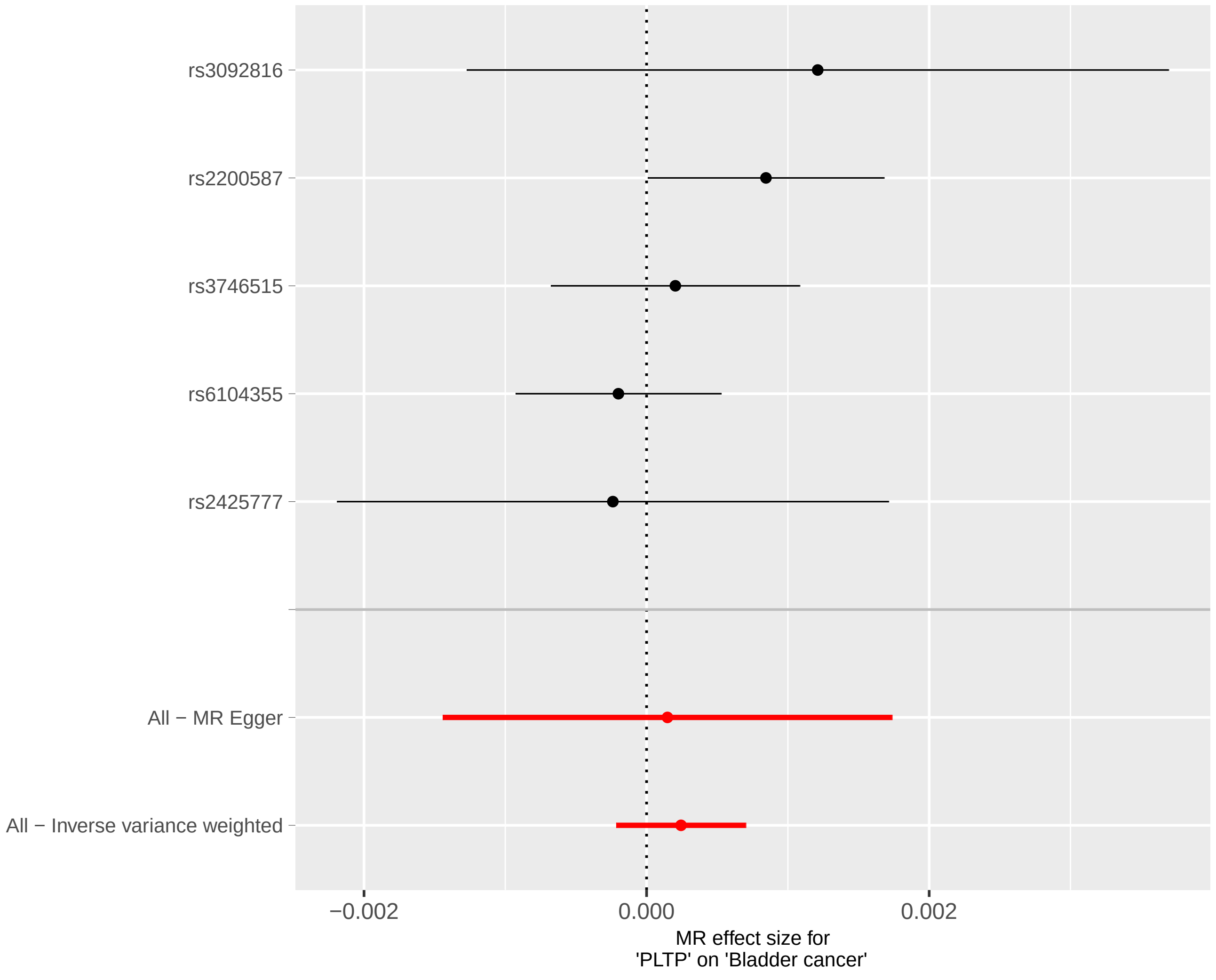
rs3092816
rs2200587
rs3746515
rs6104355
rs2425777
All − MR Egger
All − Inverse variance weighted
−0.002
0.000
0.002
MR effect size for
'PLTP' on 'Bladder cancer'

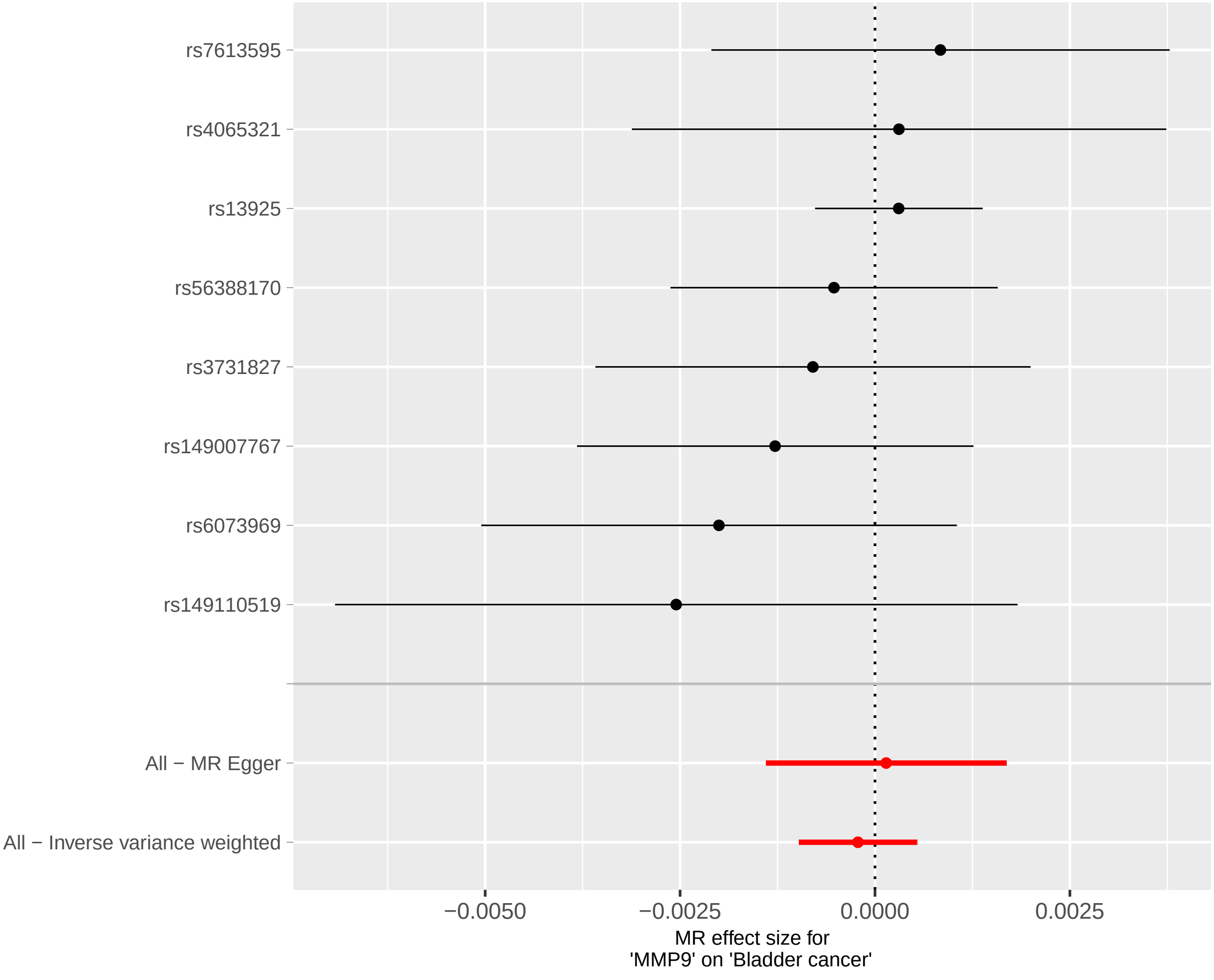
rs7613595
rs4065321
rs13925
rs56388170
rs3731827
rs149007767
rs6073969
rs149110519
All − MR Egger
All − Inverse variance weighted
−0.0050
−0.0025
0.0000
0.0025
MR effect size for
'MMP9' on 'Bladder cancer'

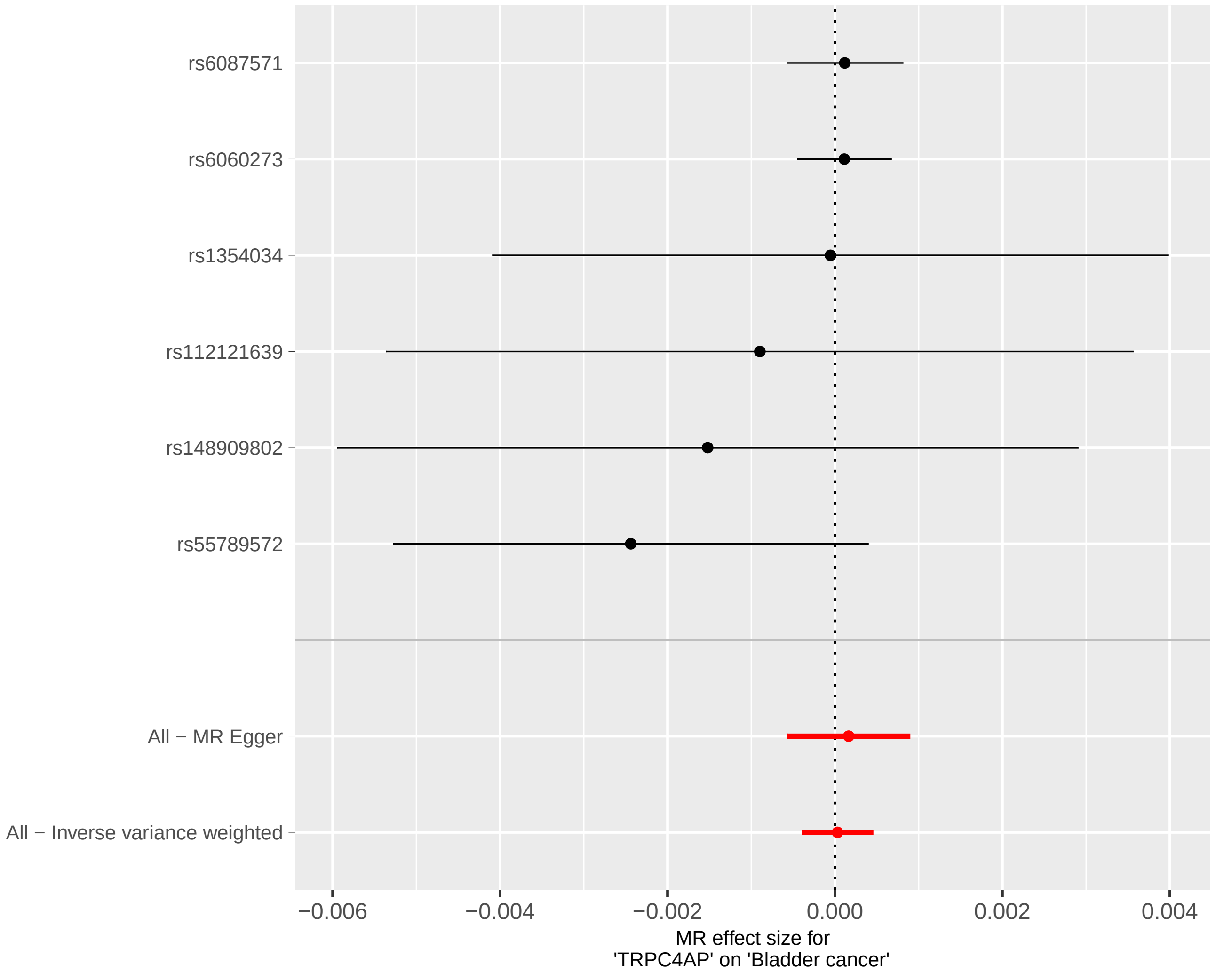
rs6087571
rs6060273
rs1354034
rs112121639
rs148909802
rs55789572
All − MR Egger
All − Inverse variance weighted
−0.006
−0.004
−0.002
0.000
0.002
0.004
MR effect size for
'TRPC4AP' on 'Bladder cancer'

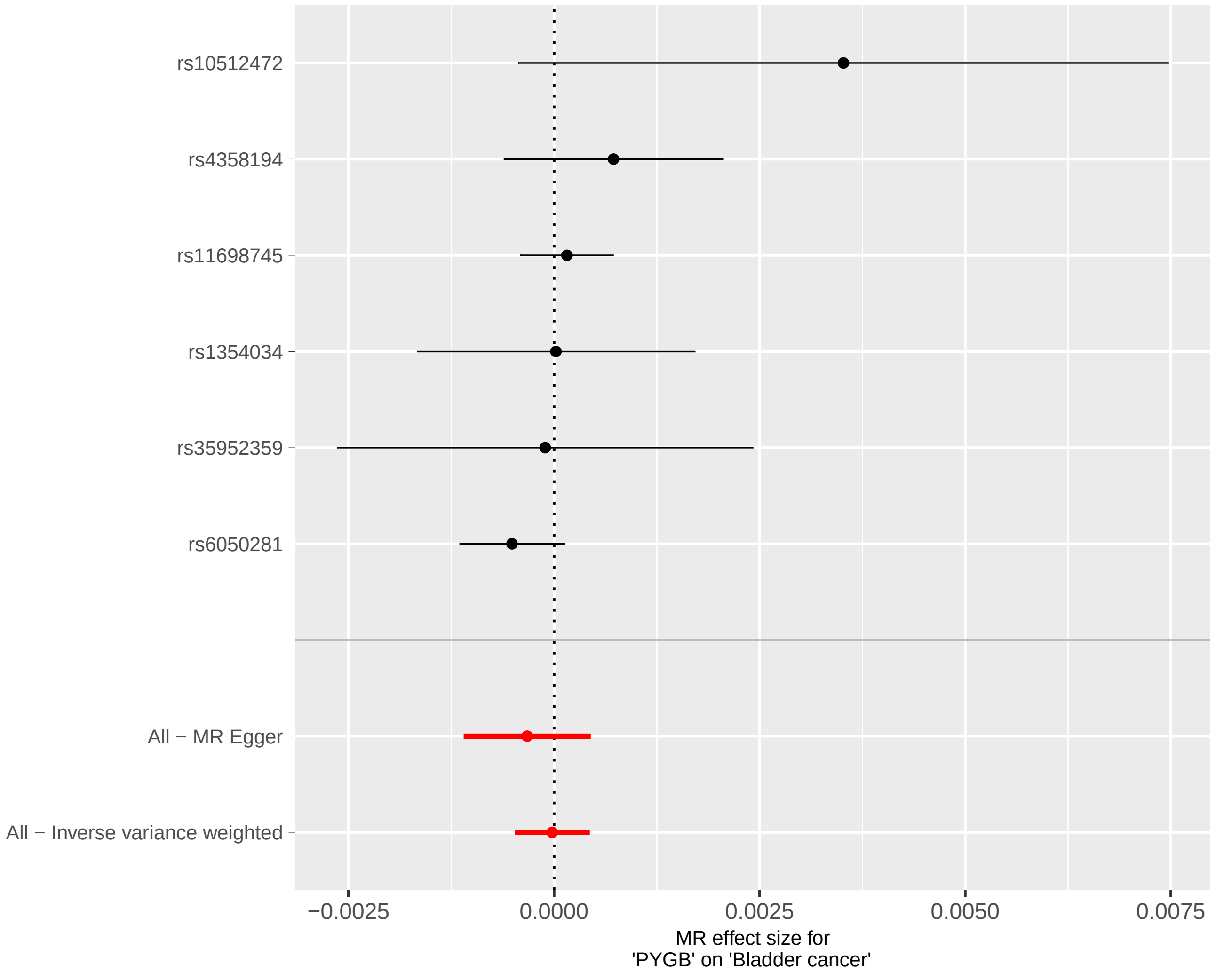

rs10512472
rs4358194
rs11698745
rs1354034
rs35952359
rs6050281
All − MR Egger
All − Inverse variance weighted
−0.0025
0.0000
0.0025
0.0050
0.0075
MR effect size for
'PYGB' on 'Bladder cancer'

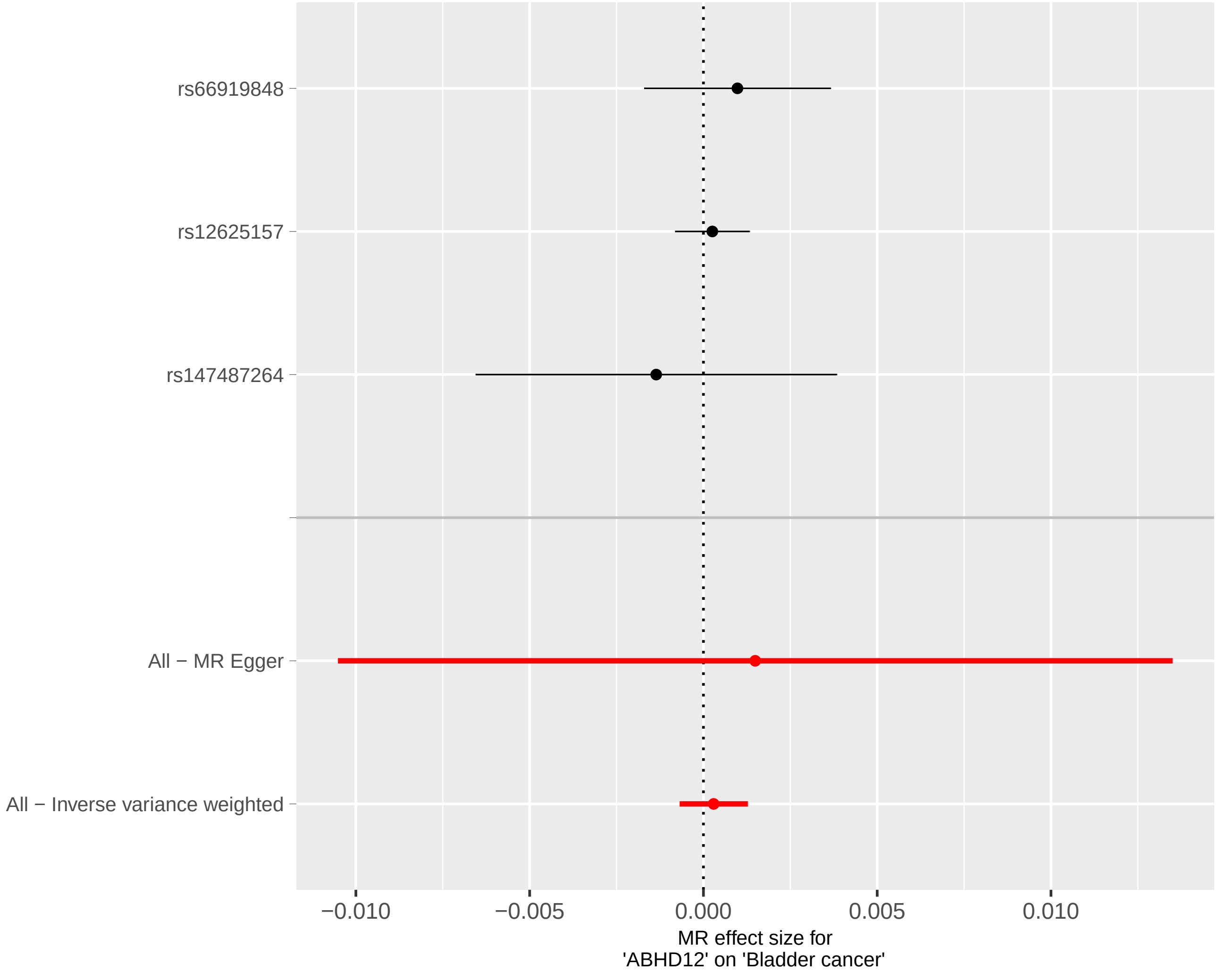

rs66919848
rs12625157
rs147487264
All − MR Egger
All − Inverse variance weighted
−0.010
−0.005
0.000
0.005
0.010
MR effect size for
'ABHD12' on 'Bladder cancer'

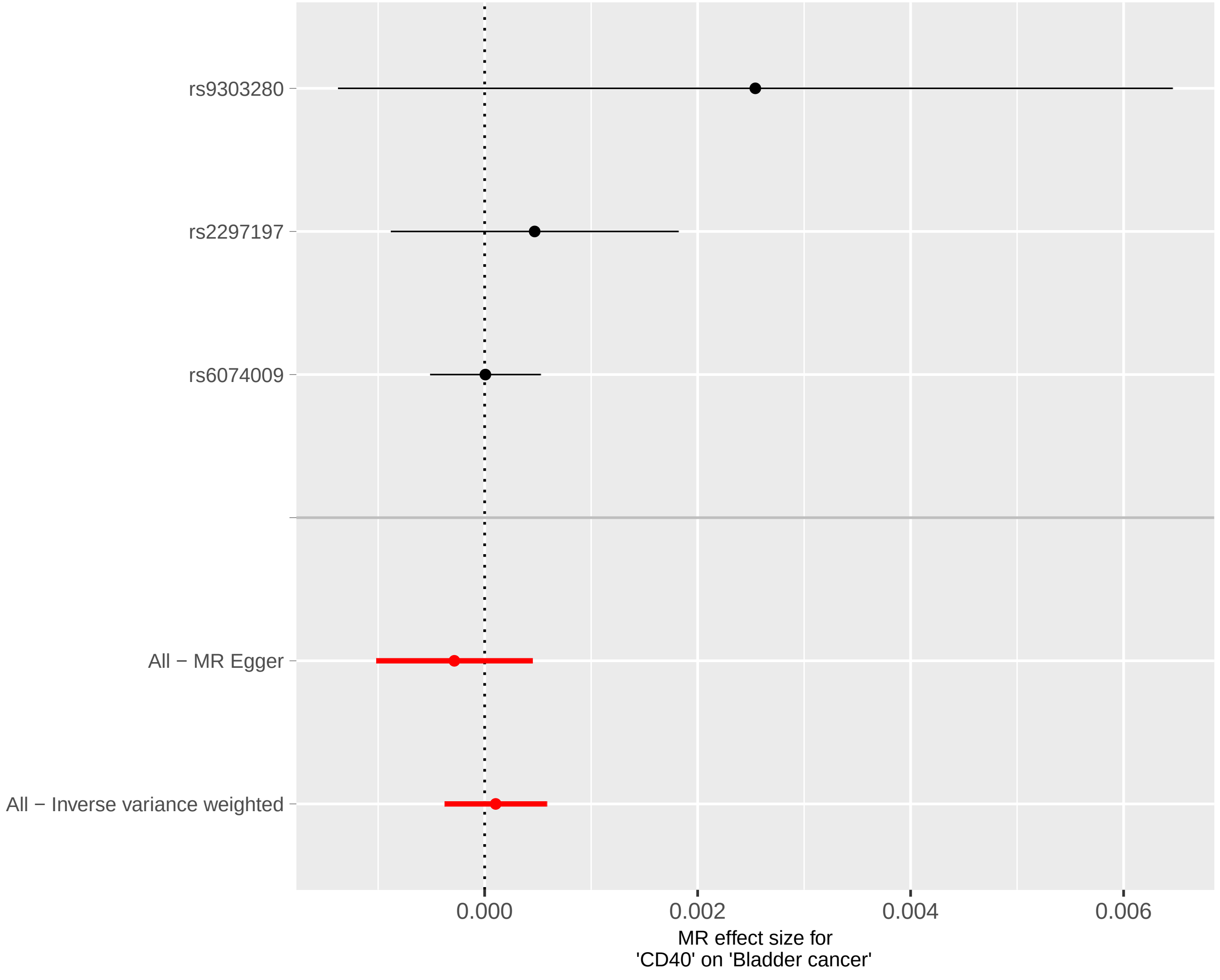

rs9303280
rs2297197
rs6074009
All − MR Egger
All − Inverse variance weighted
0.000
0.002
0.004
0.006
MR effect size for
'CD40' on 'Bladder cancer'

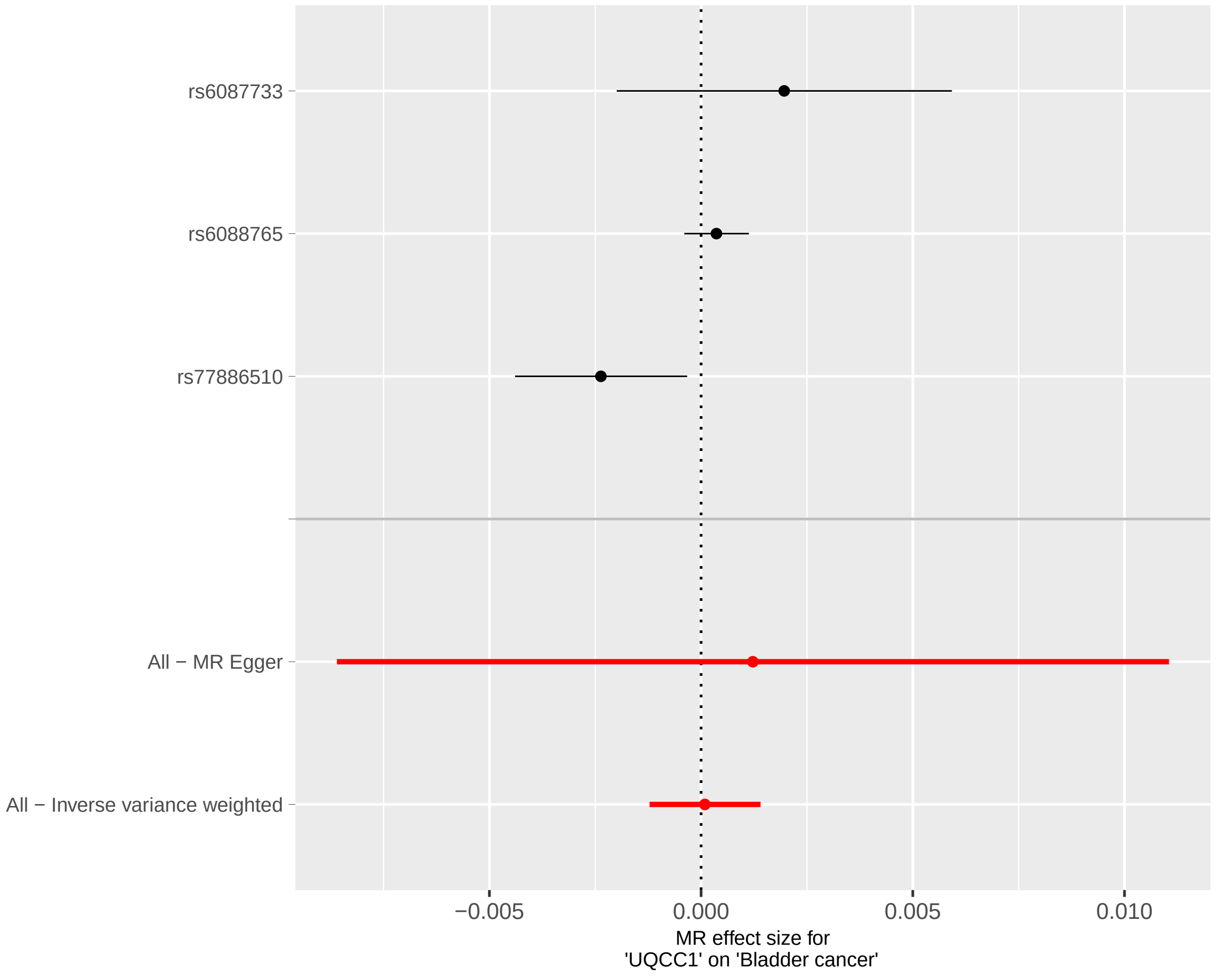
rs6087733
rs6088765
rs77886510
All − MR Egger
All − Inverse variance weighted
−0.005
0.000
0.005
0.010
MR effect size for
'UQCC1' on 'Bladder cancer'

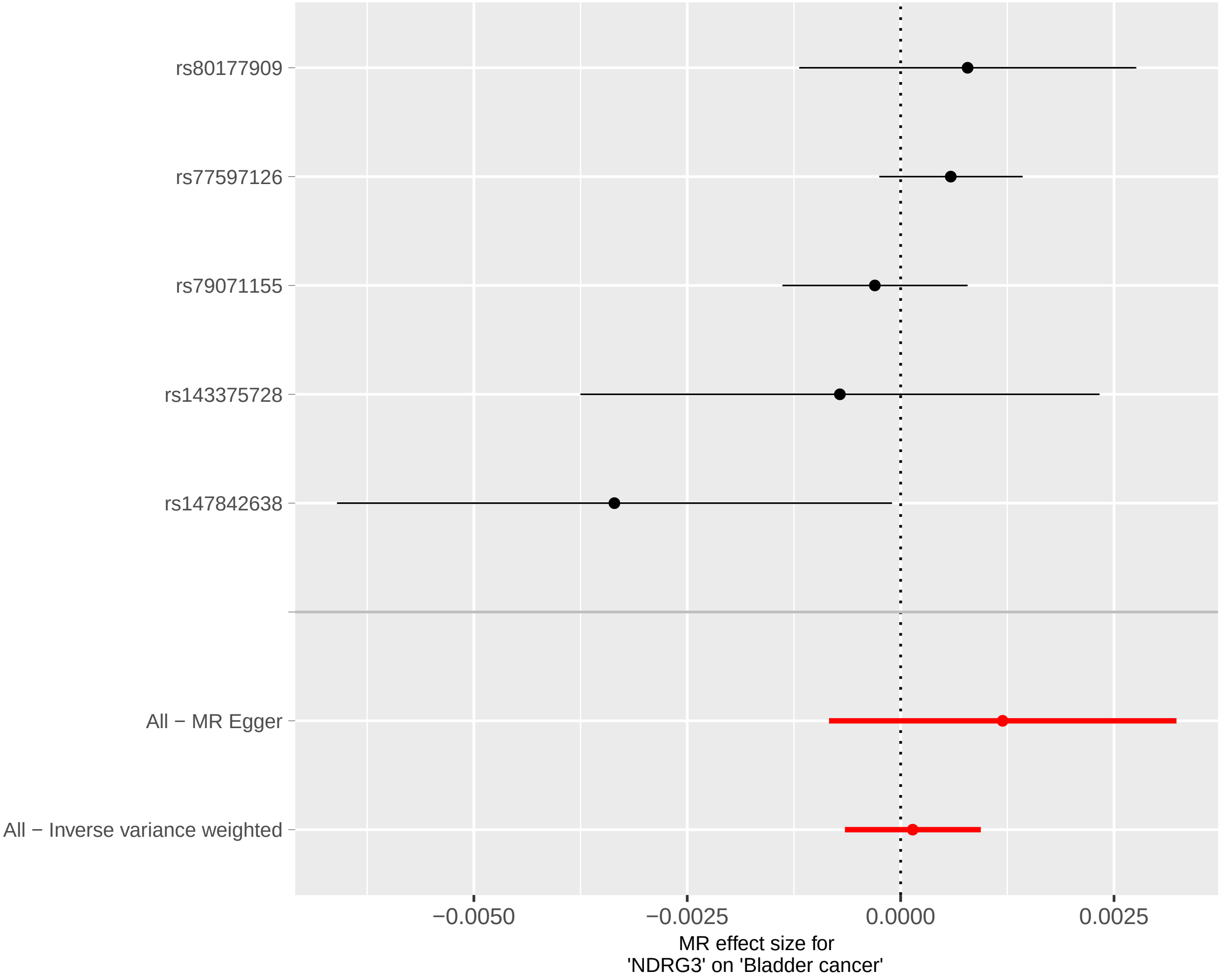

rs80177909
rs77597126
rs79071155
rs143375728
rs147842638
All − MR Egger
All − Inverse variance weighted
−0.0050
−0.0025
0.0000
0.0025
MR effect size for
'NDRG3' on 'Bladder cancer'

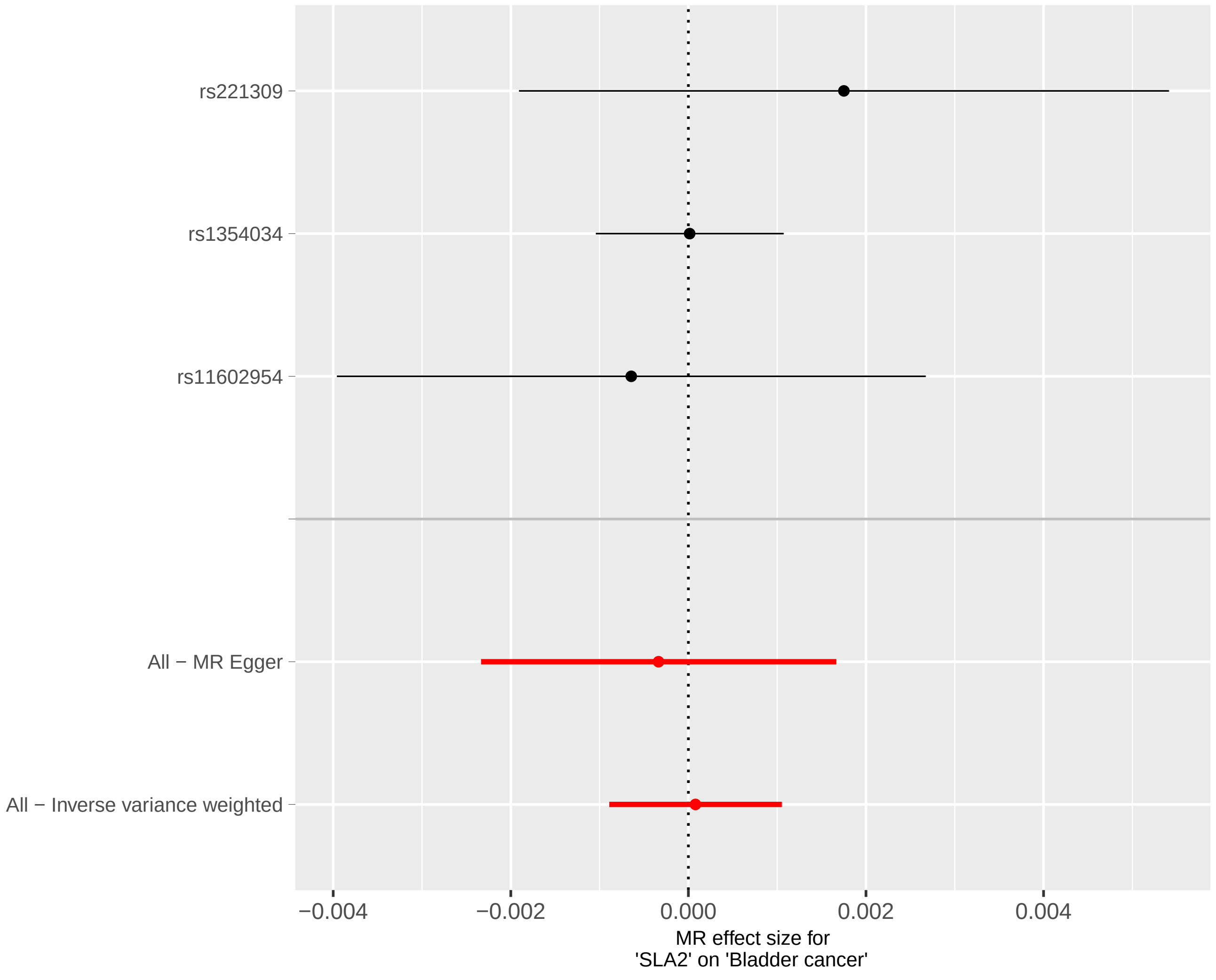

rs221309
rs1354034
rs11602954
All − MR Egger
All − Inverse variance weighted
−0.004
−0.002
0.000
0.002
0.004
MR effect size for
'SLA2' on 'Bladder cancer'

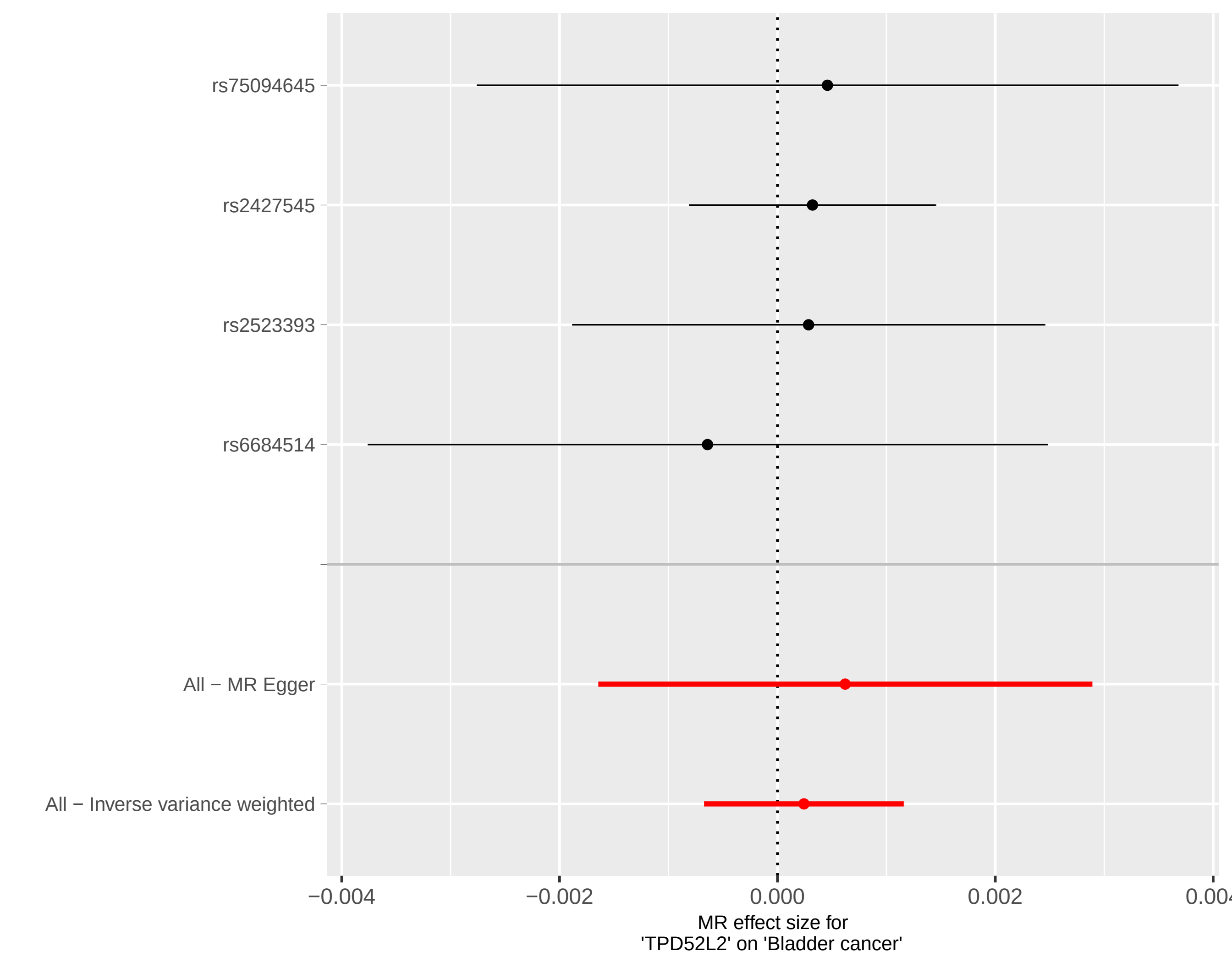

rs75094645
rs2427545
rs2523393
rs6684514
All − MR Egger
All − Inverse variance weighted
−0.004
−0.002
0.000
0.002
0.004
MR effect size for
'TPD52L2' on 'Bladder cancer'

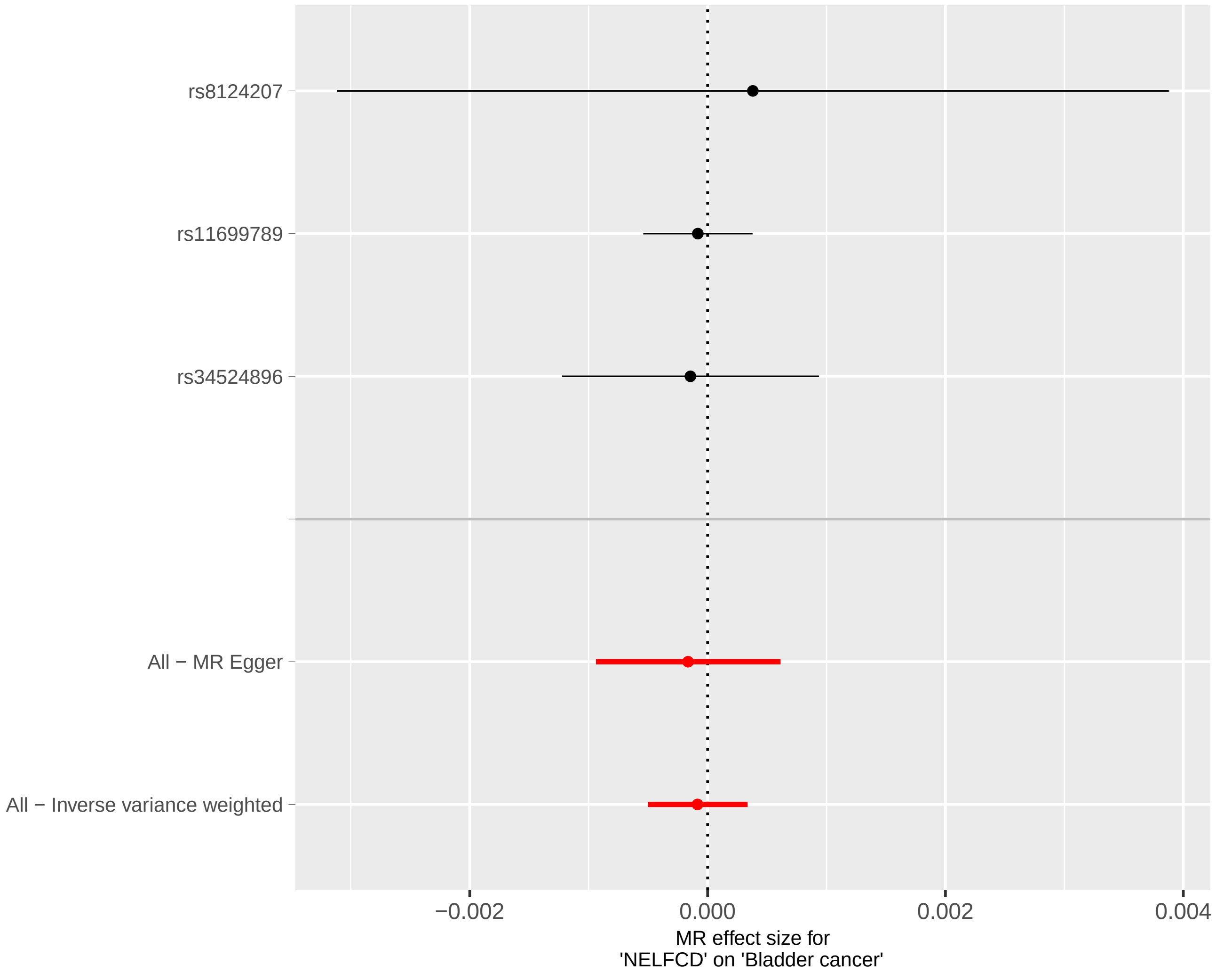

rs8124207
rs11699789
rs34524896
All − MR Egger
All − Inverse variance weighted
−0.002
0.000
0.002
0.004
MR effect size for
'NELFCD' on 'Bladder cancer'

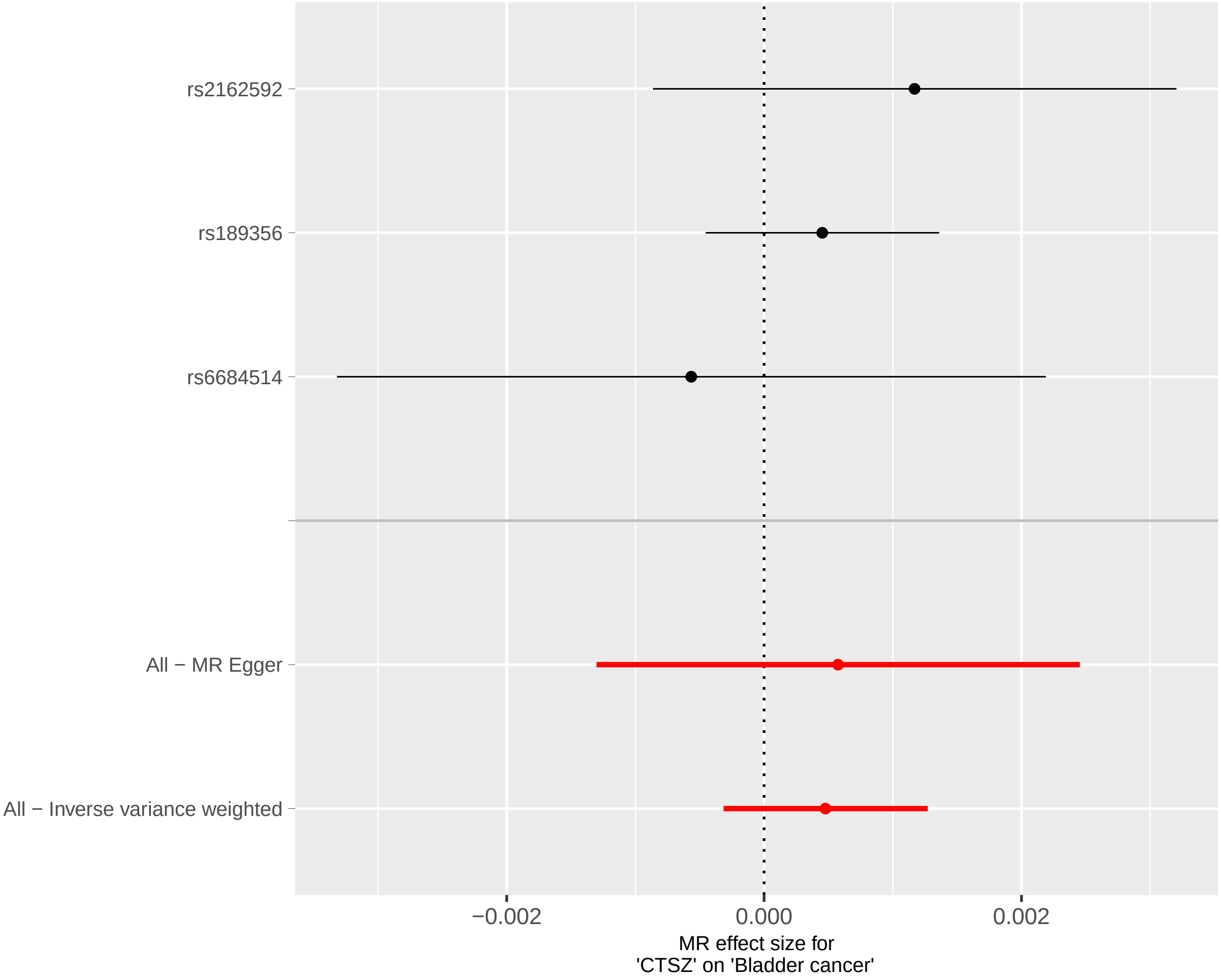

rs2162592
rs189356
rs6684514
All − MR Egger
All − Inverse variance weighted
−0.002
0.000
0.002
MR effect size for
'CTSZ' on 'Bladder cancer'

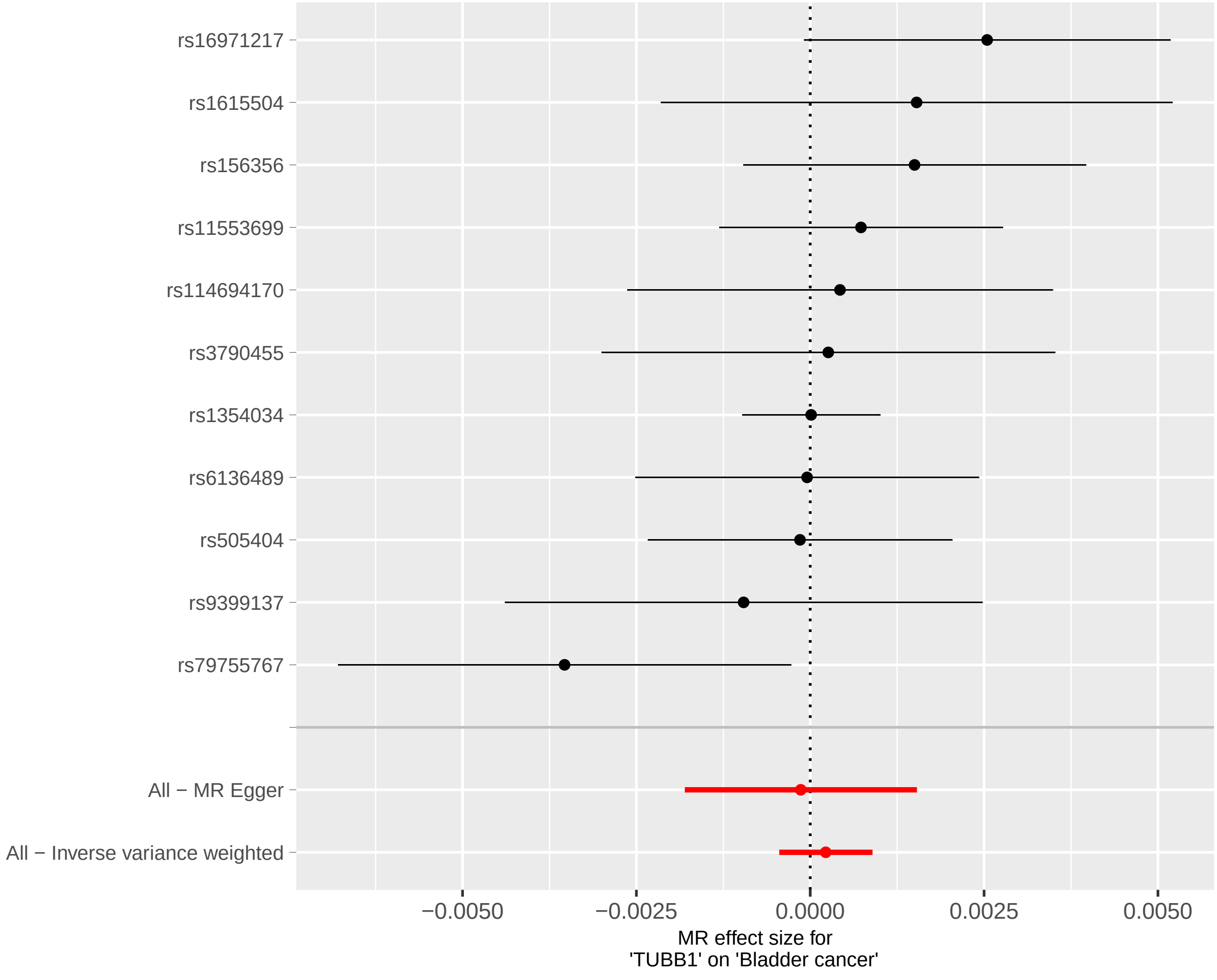
rs16971217
rs1615504
rs156356
rs11553699
rs114694170
rs3790455
rs1354034
rs6136489
rs505404
rs9399137
rs79755767
All − MR Egger
All − Inverse variance weighted
−0.0050
−0.0025
0.0000
0.0025
0.0050
MR effect size for
'TUBB1' on 'Bladder cancer'

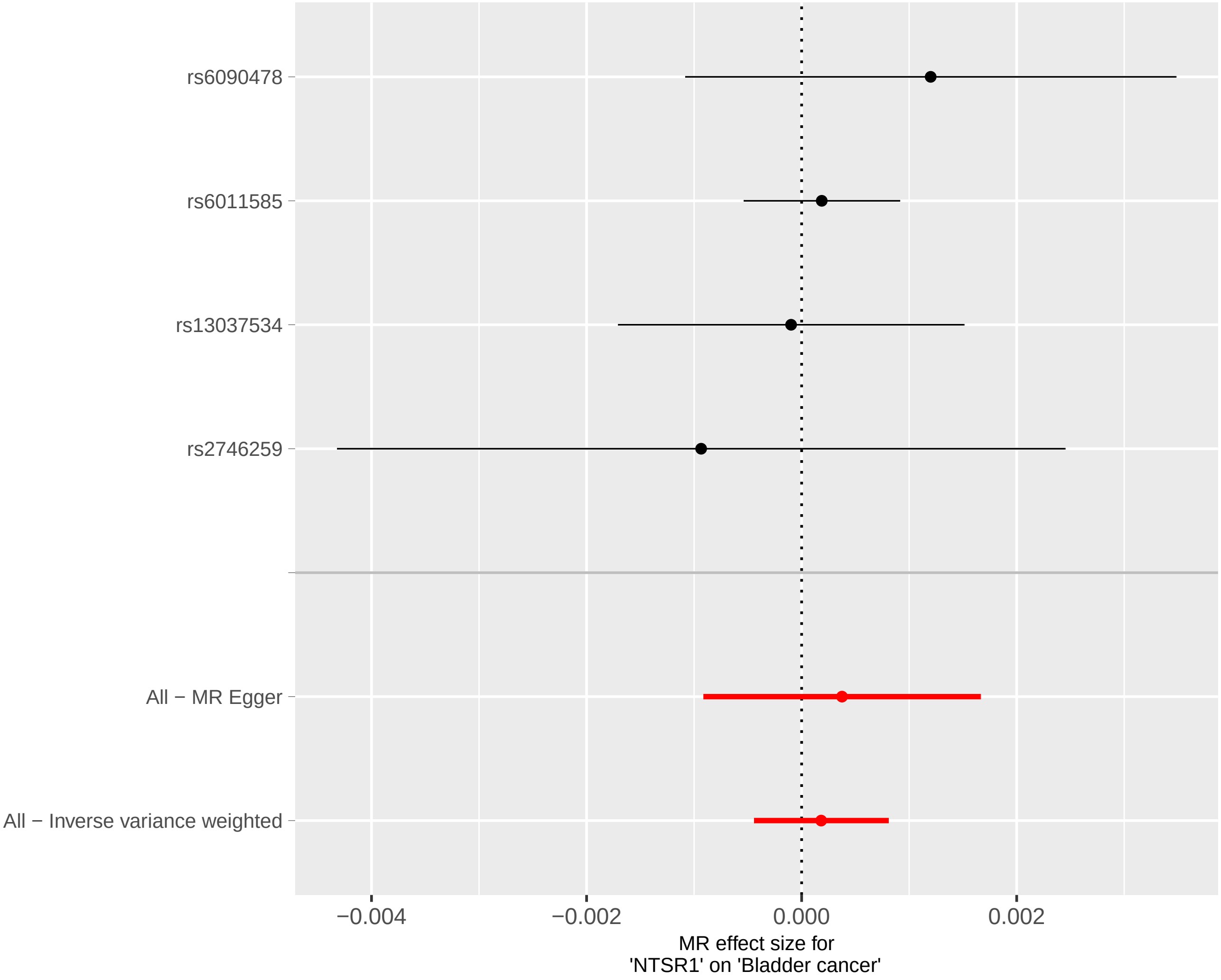

rs6090478
rs6011585
rs13037534
rs2746259
All − MR Egger
All − Inverse variance weighted
−0.004
−0.002
0.000
0.002
MR effect size for
'NTSR1' on 'Bladder cancer'

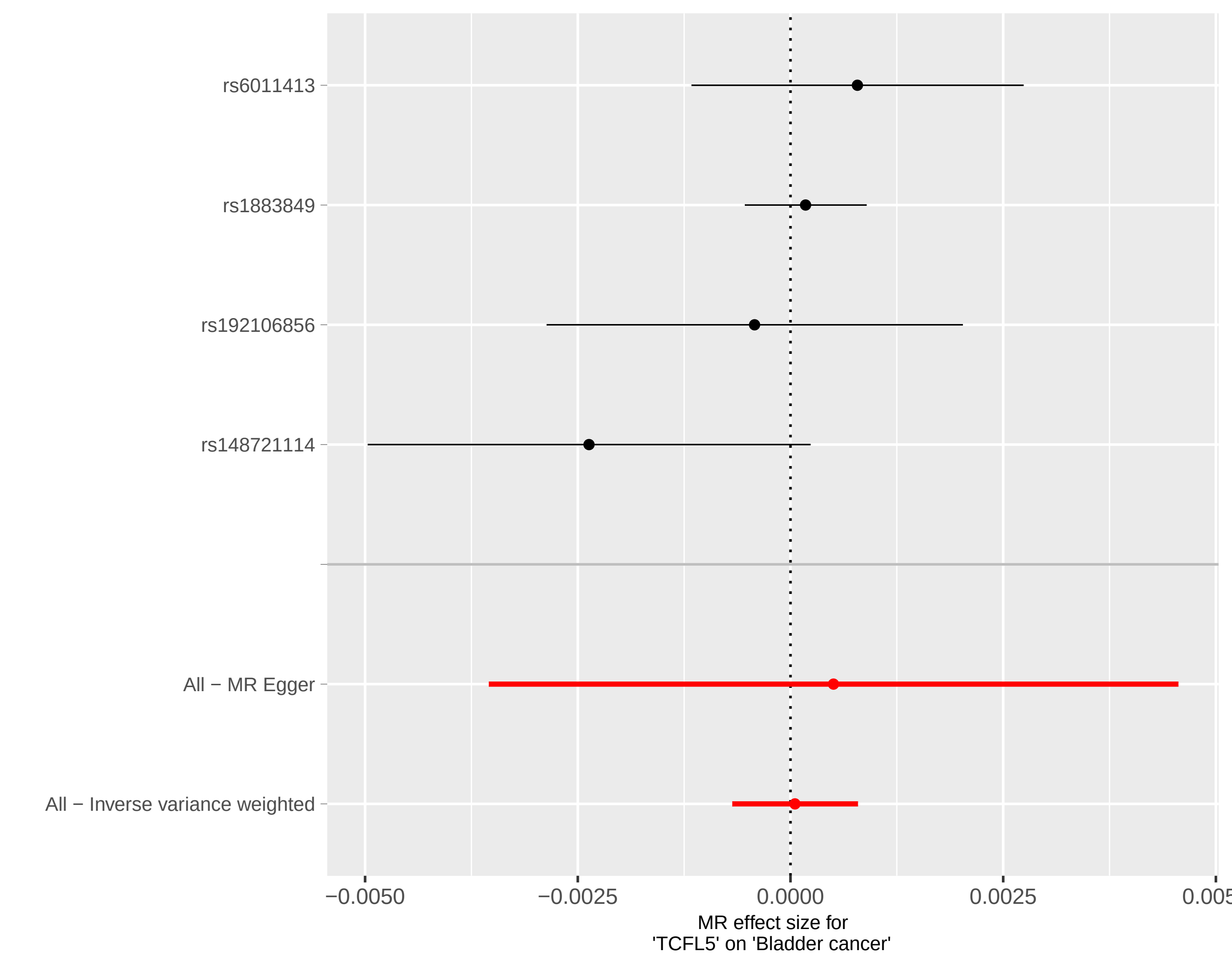
rs6011413
rs1883849
rs192106856
rs148721114
All − MR Egger
All − Inverse variance weighted
−0.0050
−0.0025
0.0000
0.0025
MR effect size for
'TCFL5' on 'Bladder cancer'

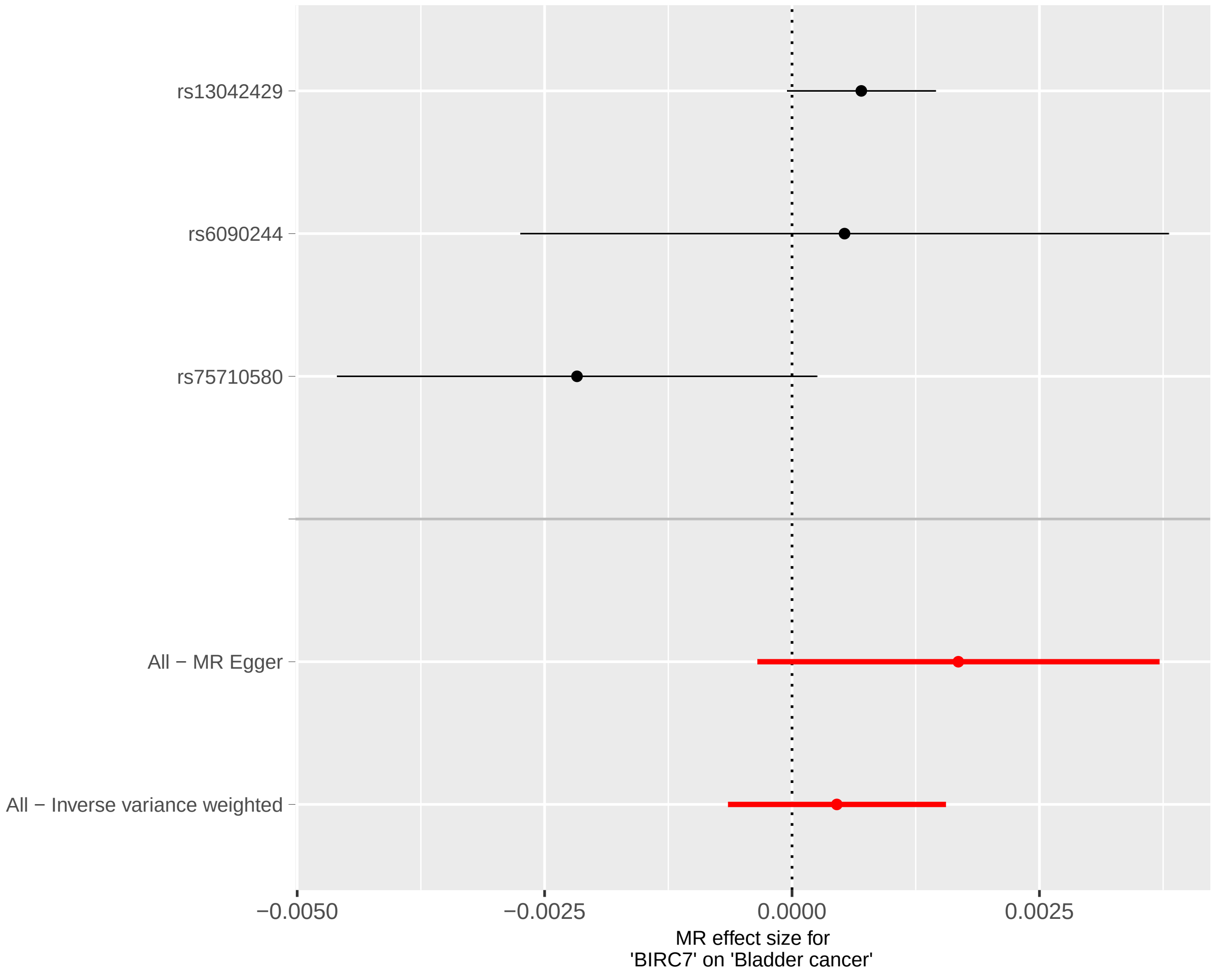

rs13042429
rs6090244
rs75710580
All − MR Egger
All − Inverse variance weighted
−0.0050
−0.0025
0.0000
0.0025
MR effect size for
'BIRC7' on 'Bladder cancer'

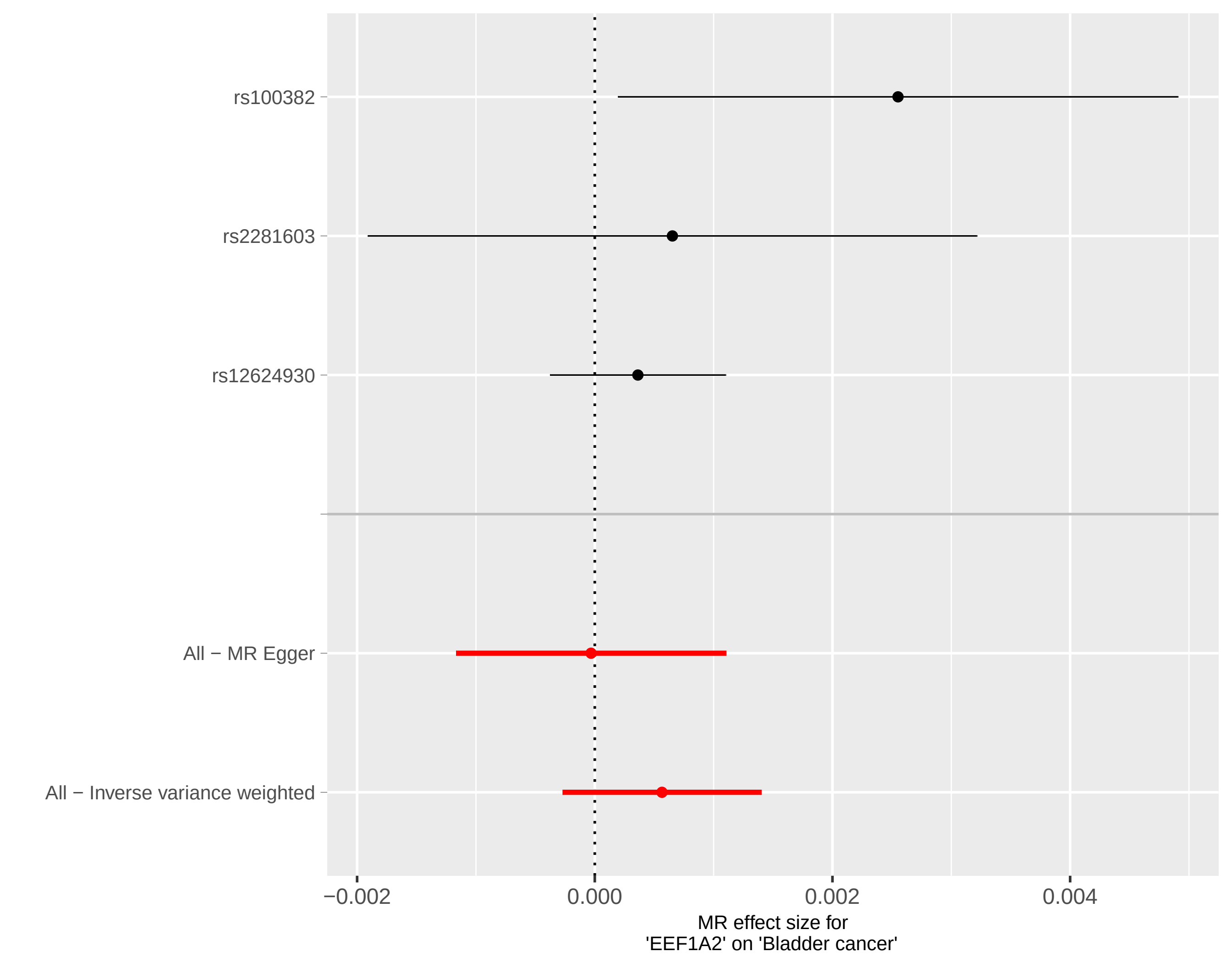
rs100382
rs2281603
rs12624930
All − MR Egger
All − Inverse variance weighted
−0.002
0.000
0.002
0.004
MR effect size for
'EEF1A2' on 'Bladder cancer'

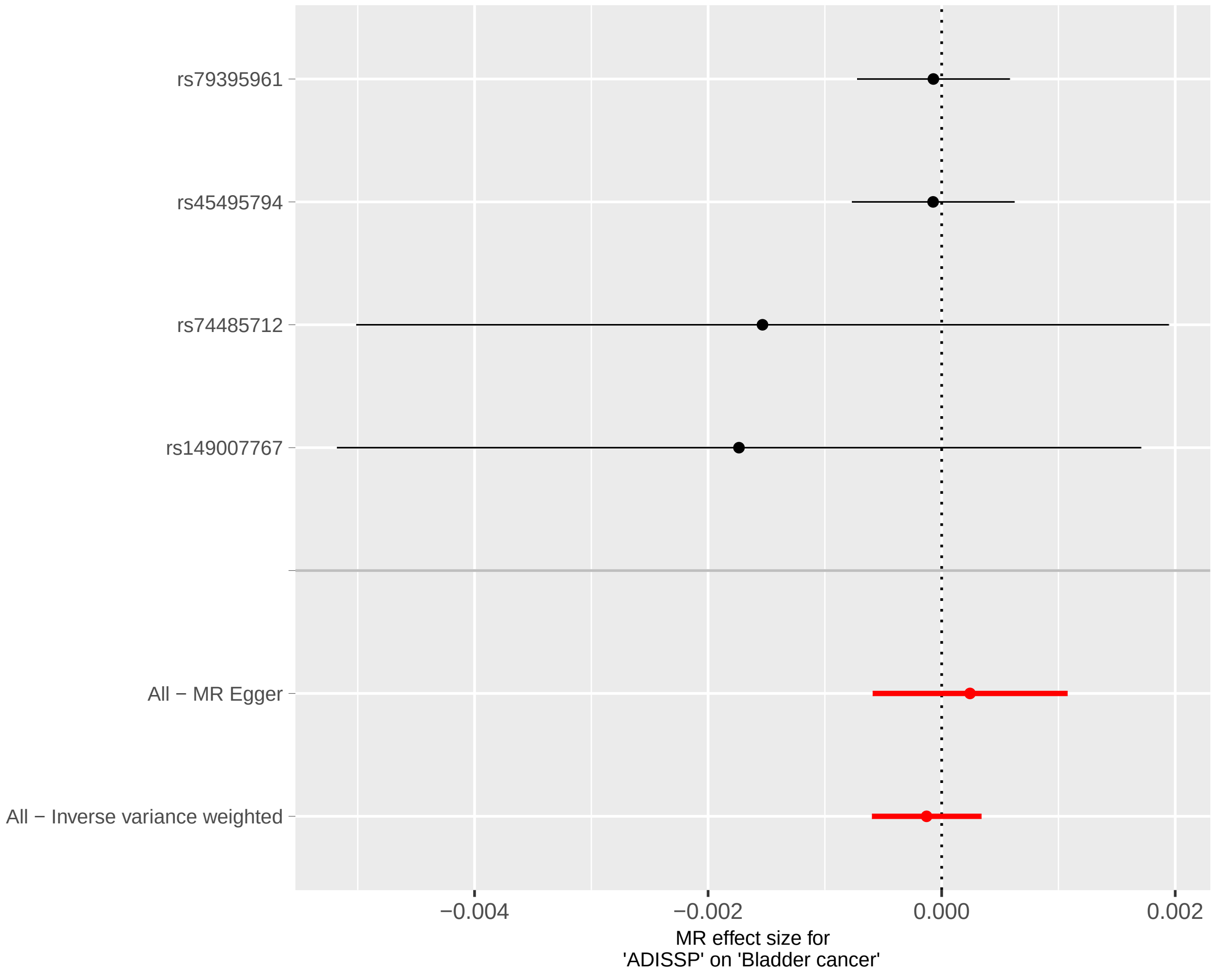

rs79395961
rs45495794
rs74485712
rs149007767
All − MR Egger
All − Inverse variance weighted
−0.004
−0.002
0.000
0.002
MR effect size for
'ADISSP' on 'Bladder cancer'

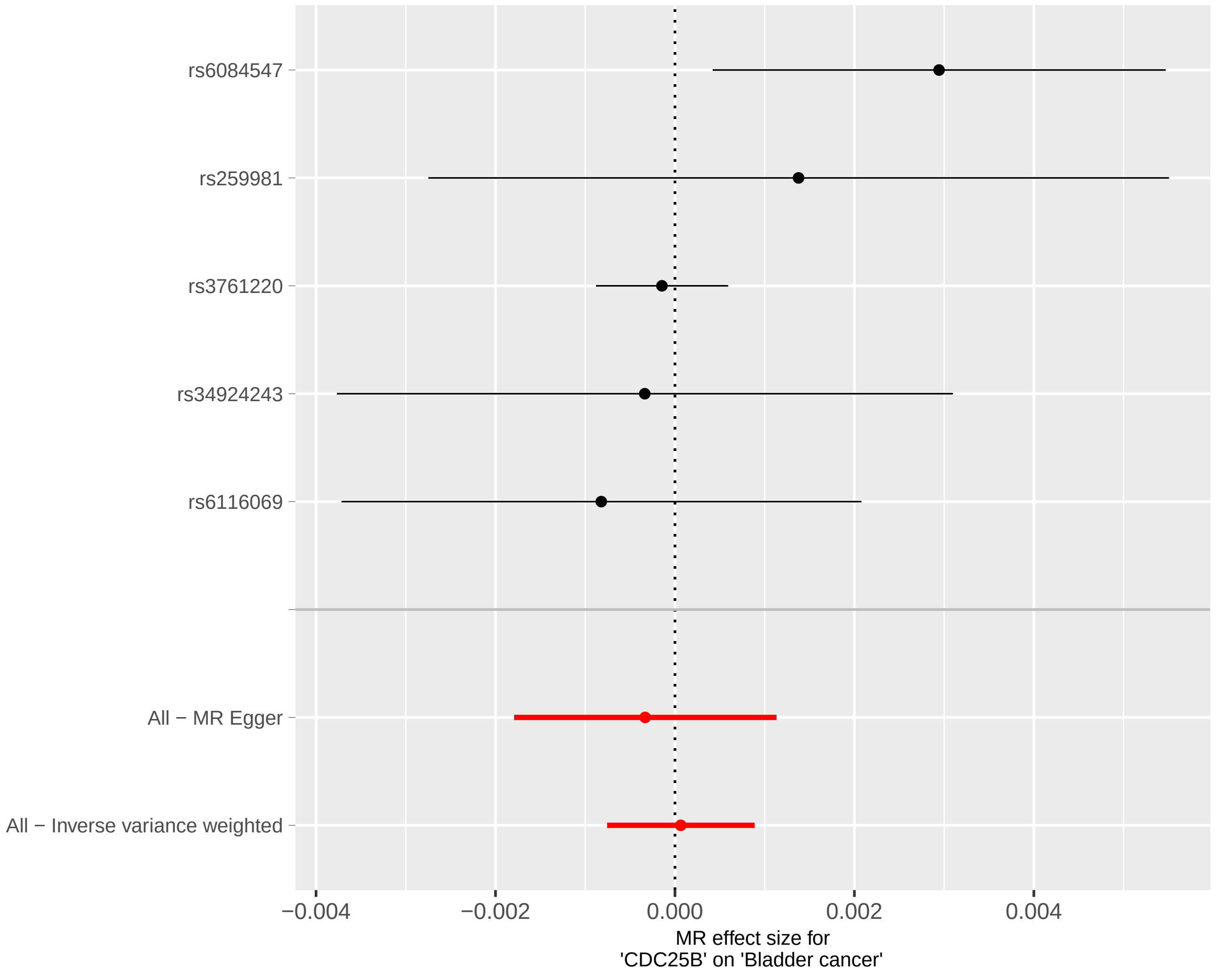
rs6084547
rs259981
rs3761220
rs34924243
rs6116069
All − MR Egger
All − Inverse variance weighted
−0.004
−0.002
0.000
0.002
0.004
MR effect size for
'CDC25B' on 'Bladder cancer'

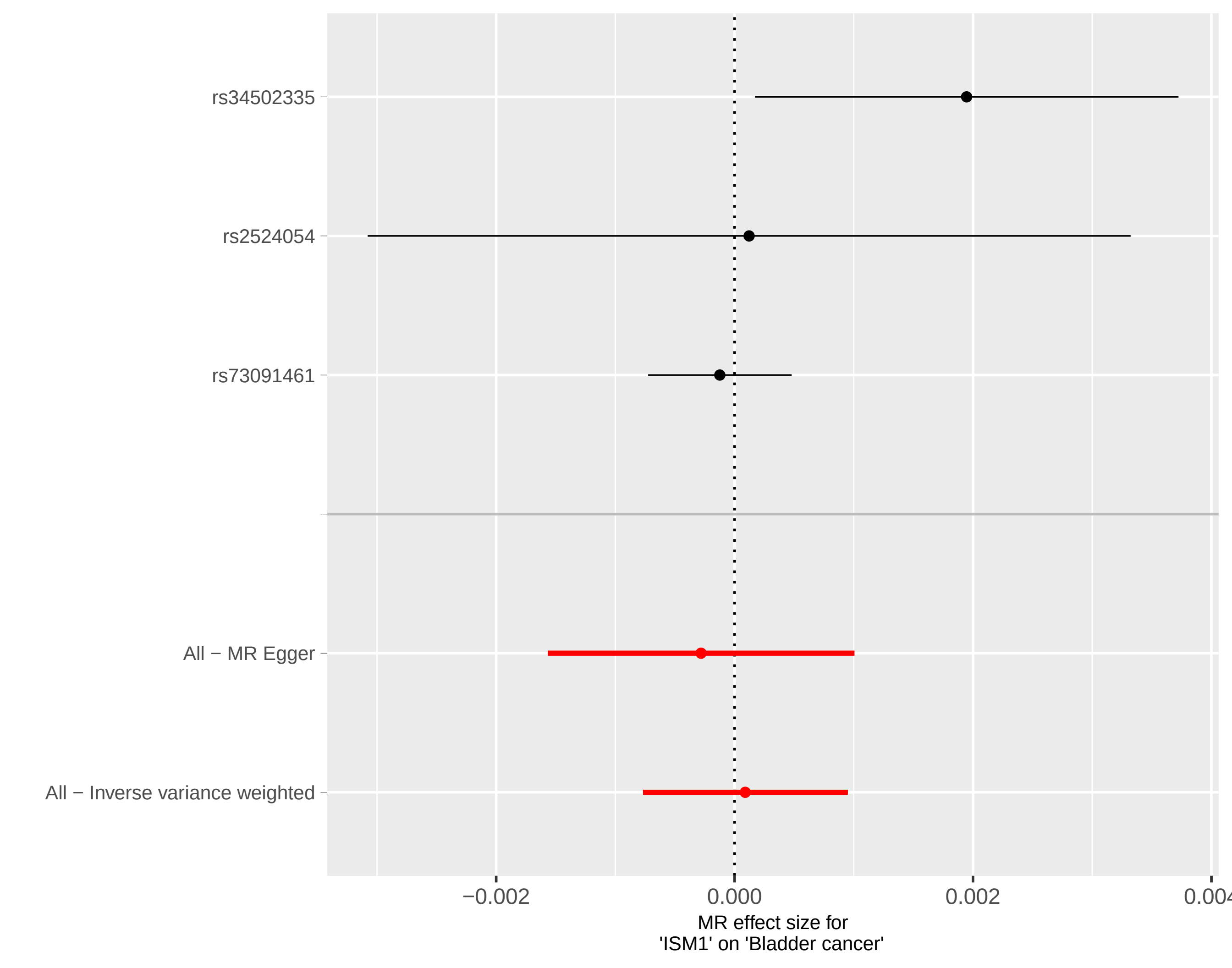
rs34502335
rs2524054
rs73091461
All − MR Egger
All − Inverse variance weighted
−0.002
0.000
0.002
0.004
MR effect size for
'ISM1' on 'Bladder cancer'

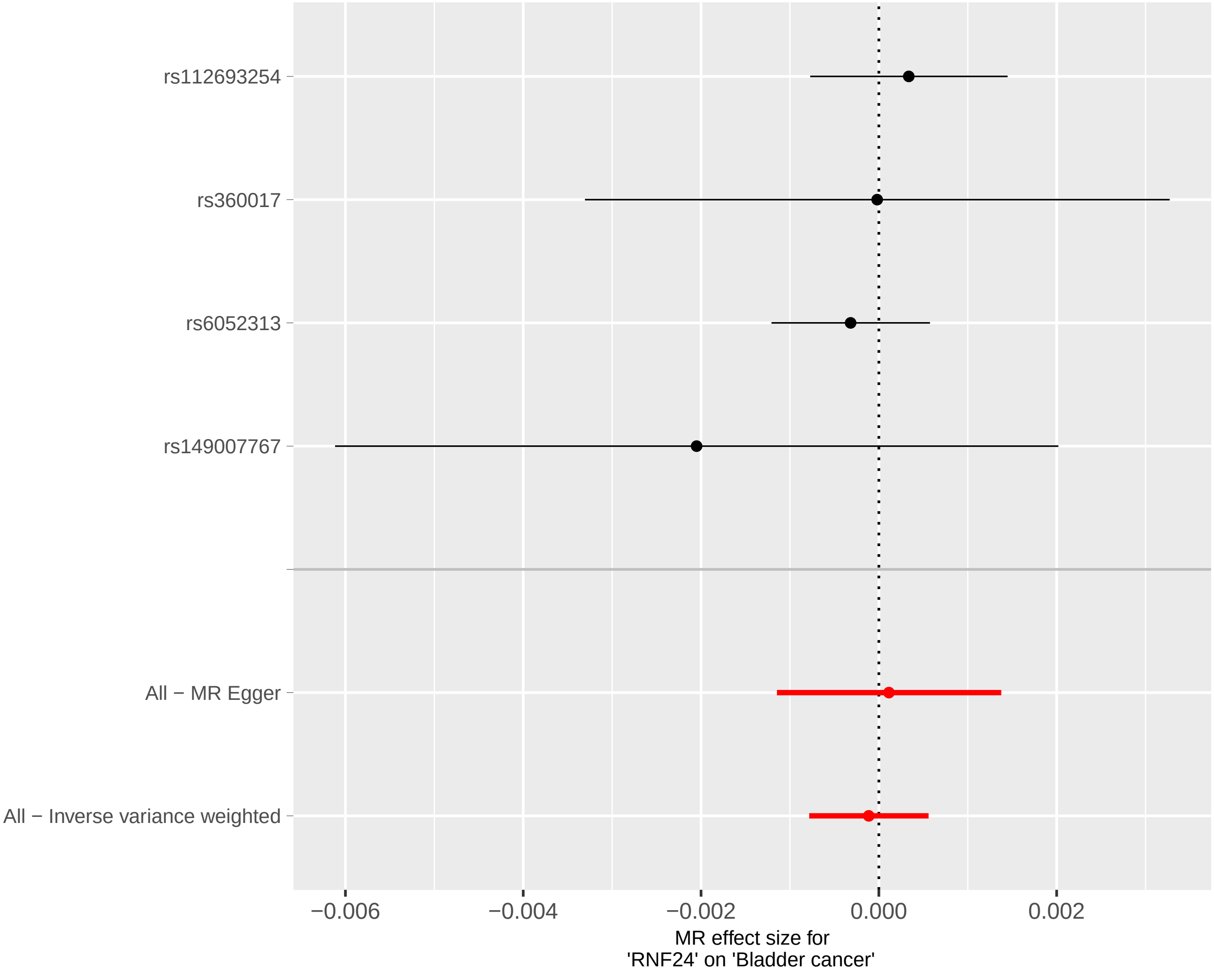

rs112693254
rs360017
rs6052313
rs149007767
All − MR Egger
All − Inverse variance weighted
−0.006
−0.004
−0.002
0.000
0.002
MR effect size for
'RNF24' on 'Bladder cancer'

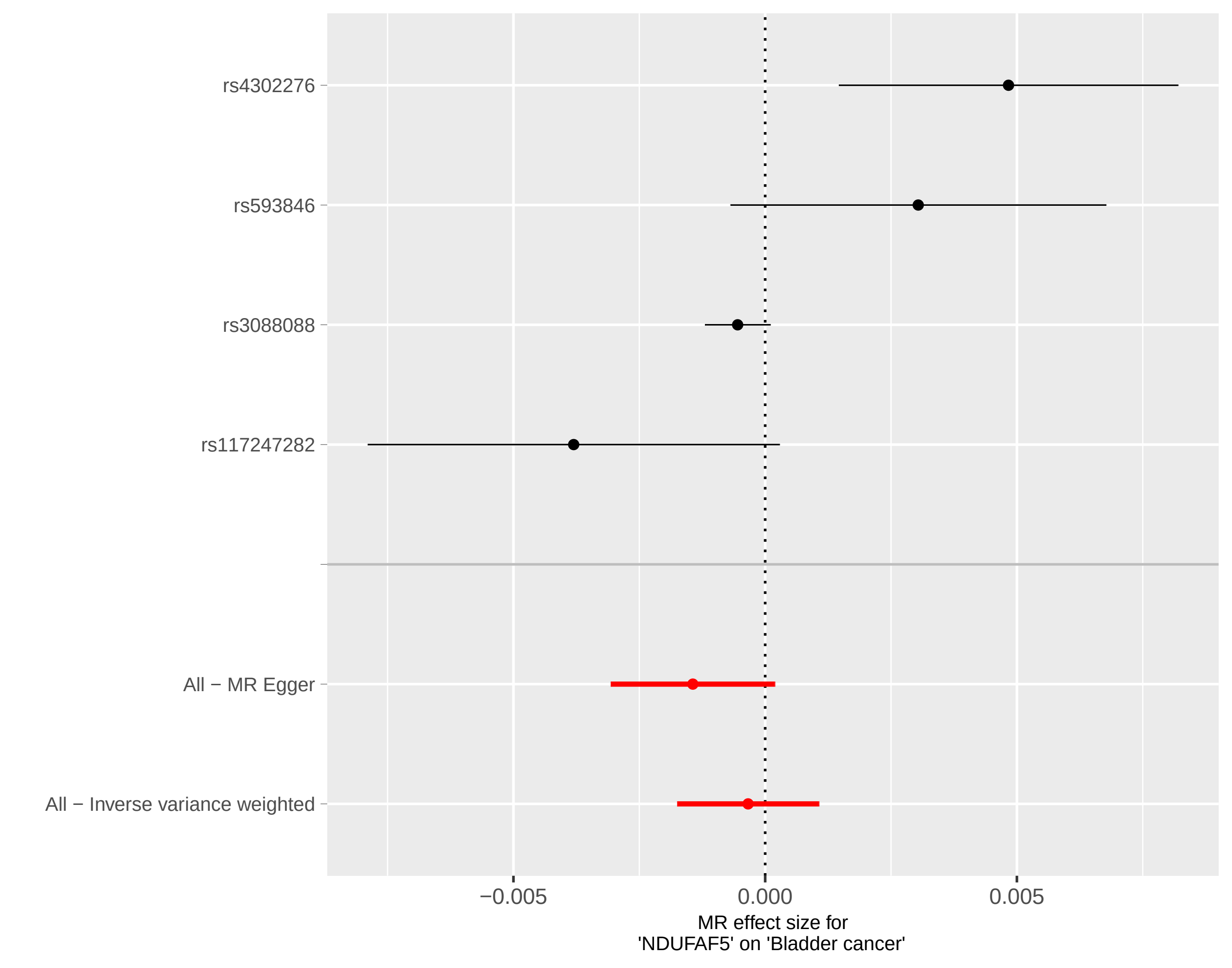
rs4302276
rs593846
rs3088088
rs117247282
All − MR Egger
All − Inverse variance weighted
−0.005
0.000
0.005
MR effect size for
'NDUFAF5' on 'Bladder cancer'

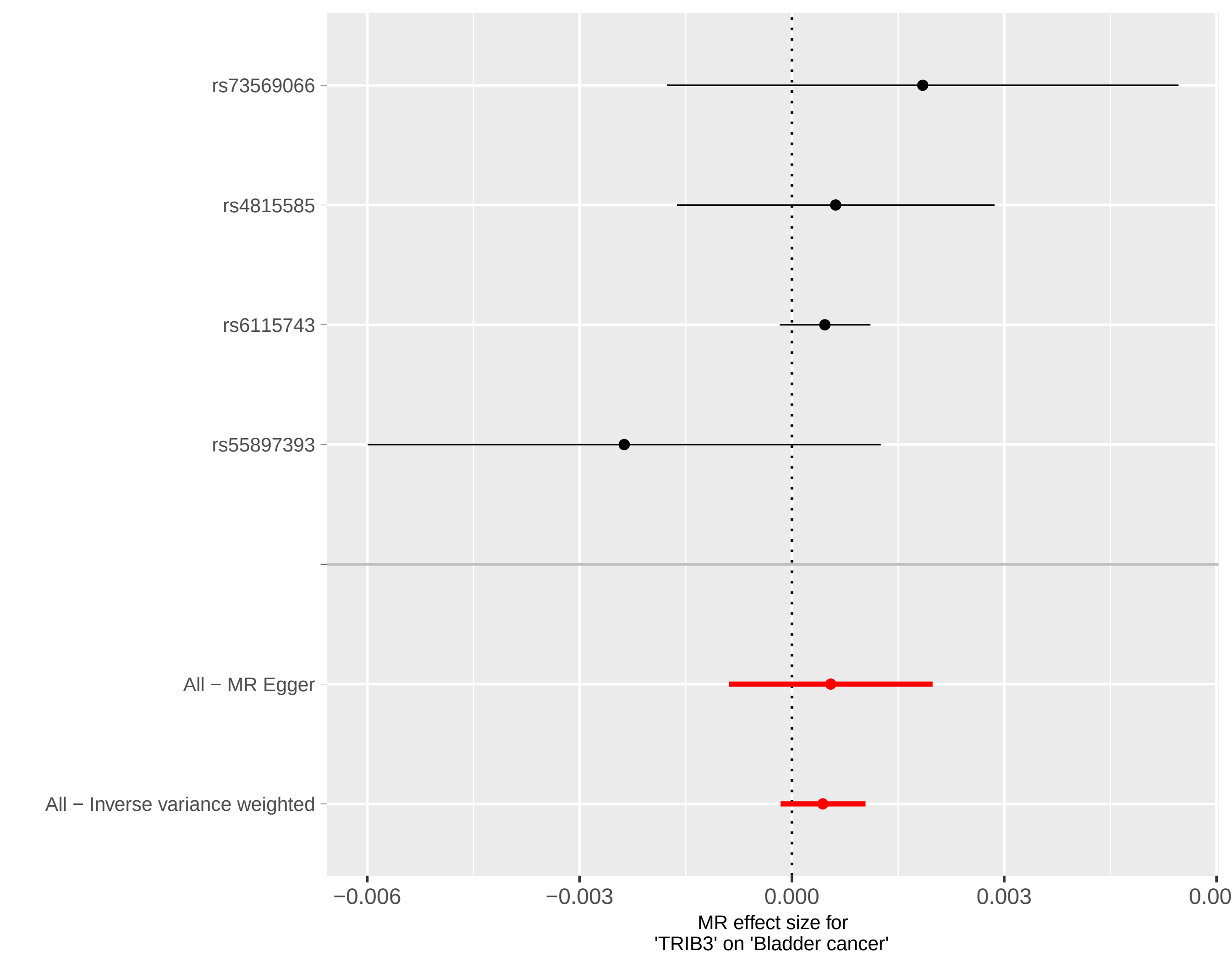

rs73569066
rs4815585
rs6115743
rs55897393
All − MR Egger
All − Inverse variance weighted
−0.006
−0.003
0.000
0.003
0.00
MR effect size for
'TRIB3' on 'Bladder cancer'

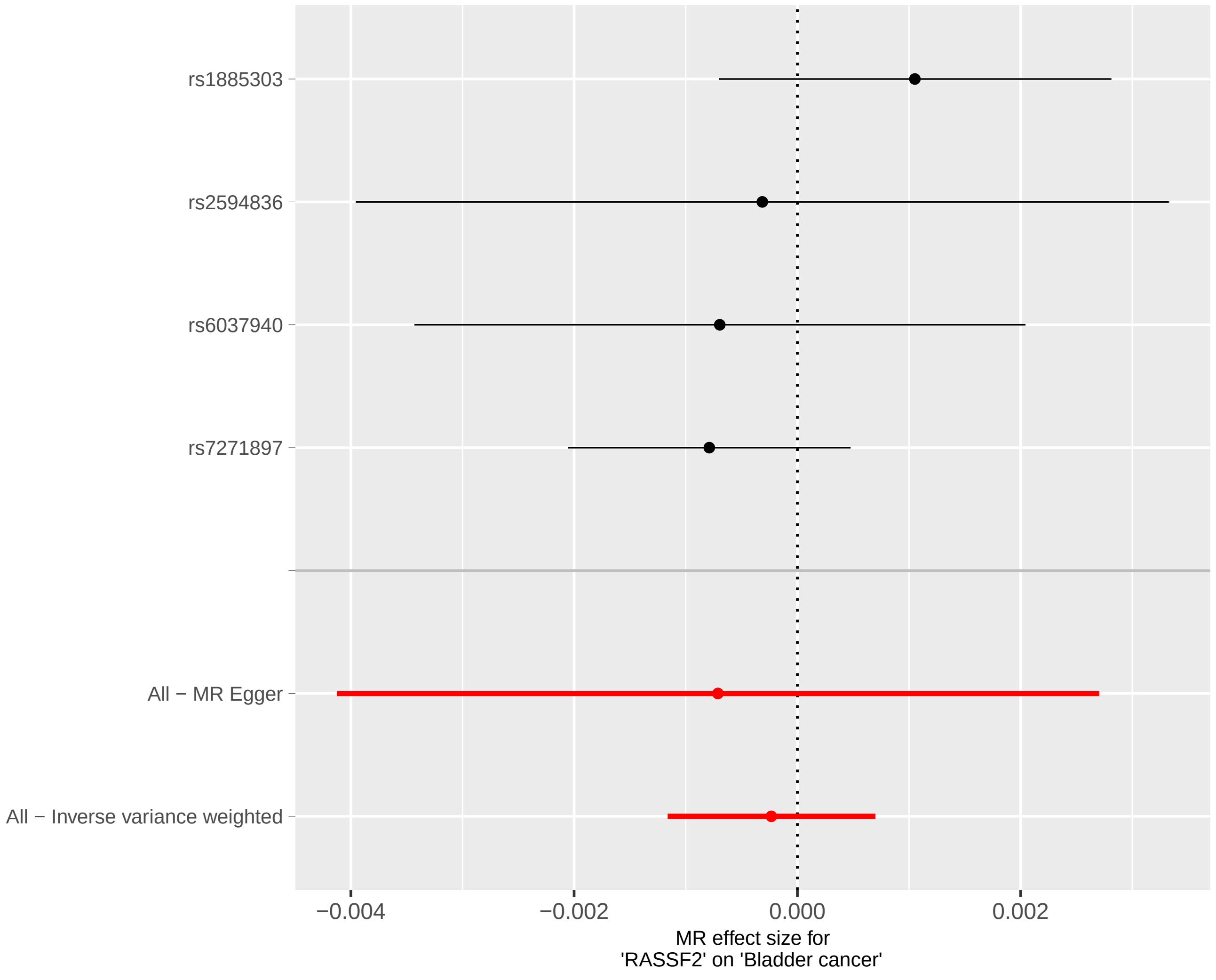

rs1885303
rs2594836
rs6037940
rs7271897
All − MR Egger
All − Inverse variance weighted
−0.004
−0.002
0.000
0.002
MR effect size for
'RASSF2' on 'Bladder cancer'

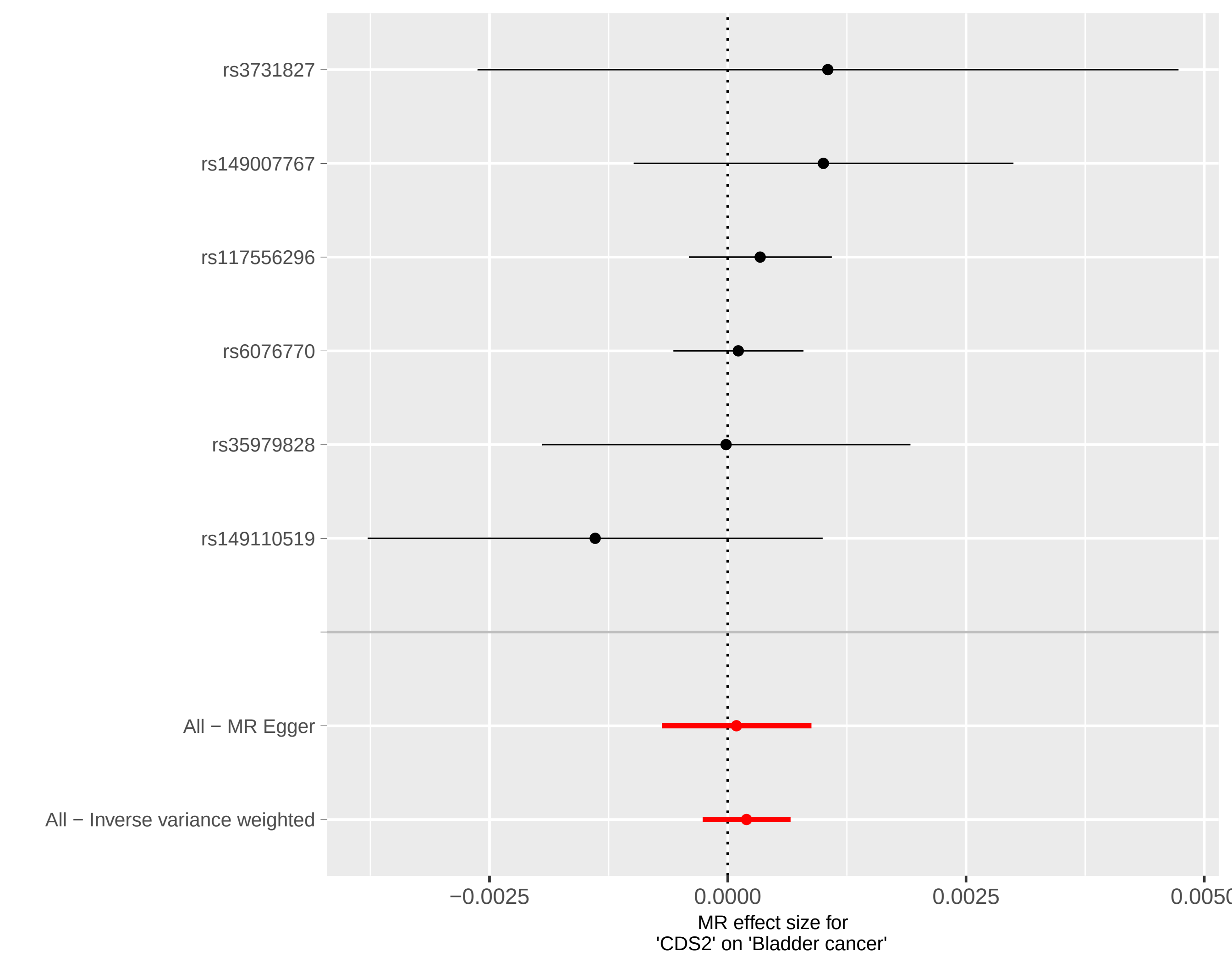

rs3731827
rs149007767
rs117556296
rs6076770
rs35979828
rs149110519
All − MR Egger
All − Inverse variance weighted
−0.0025
0.0000
0.0025
0.0050
MR effect size for
'CDS2' on 'Bladder cancer'

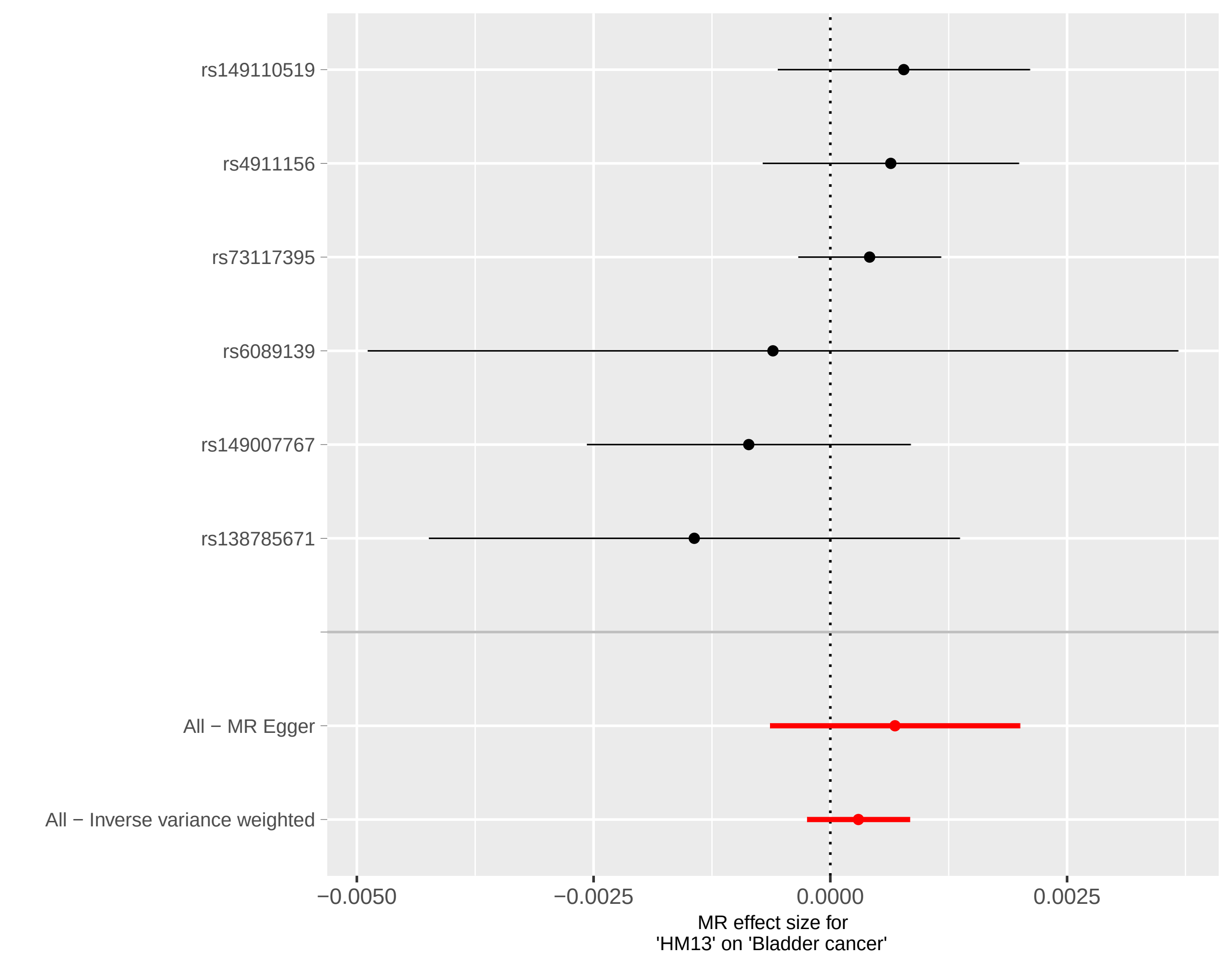
rs149110519
rs4911156
rs73117395
rs6089139
rs149007767
rs138785671
All − MR Egger
All − Inverse variance weighted
−0.0050
−0.0025
0.0000
0.0025
MR effect size for
'HM13' on 'Bladder cancer'

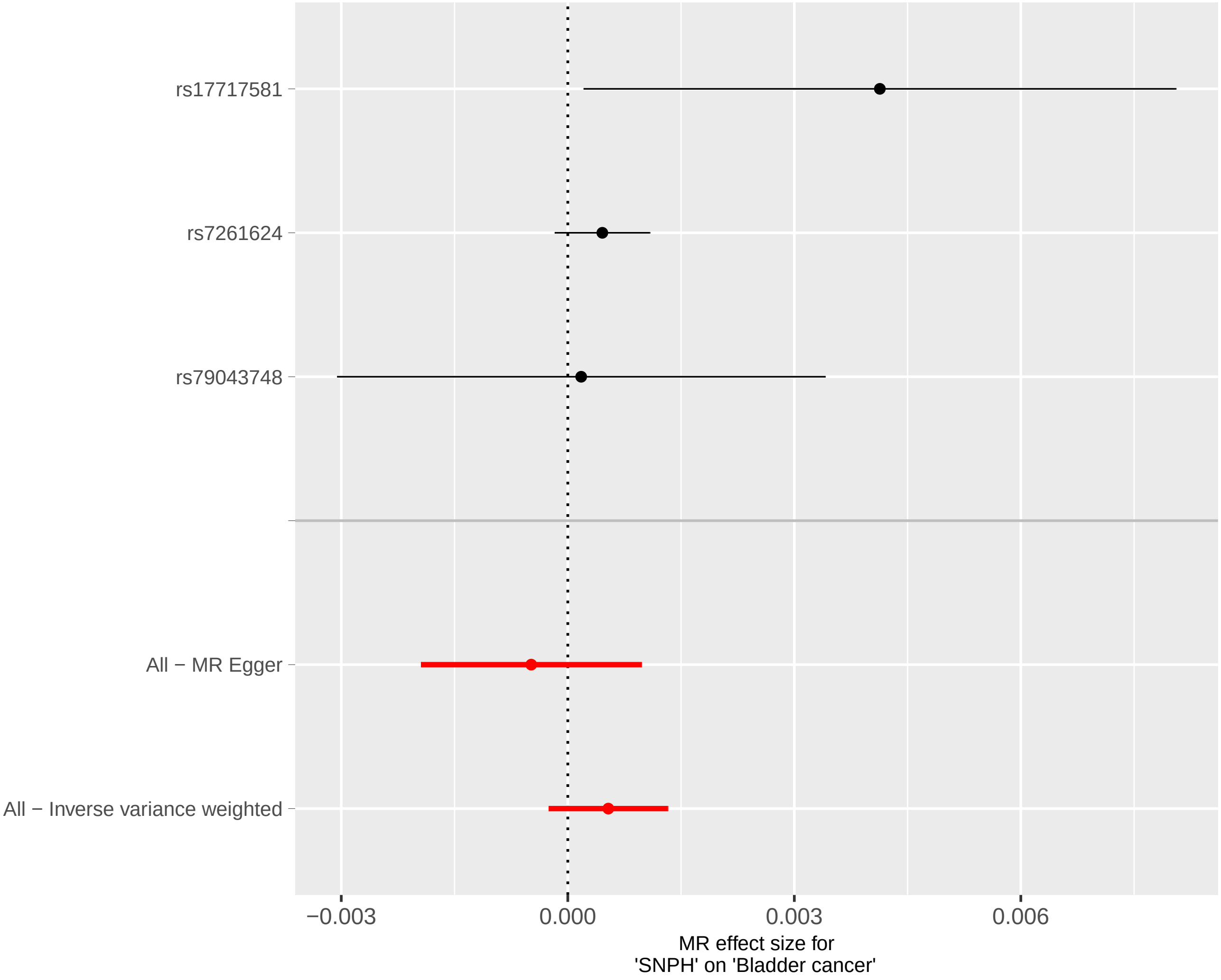

rs17717581
rs7261624
rs79043748
All − MR Egger
All − Inverse variance weighted
−0.003
0.000
0.003
0.006
MR effect size for
'SNPH' on 'Bladder cancer'

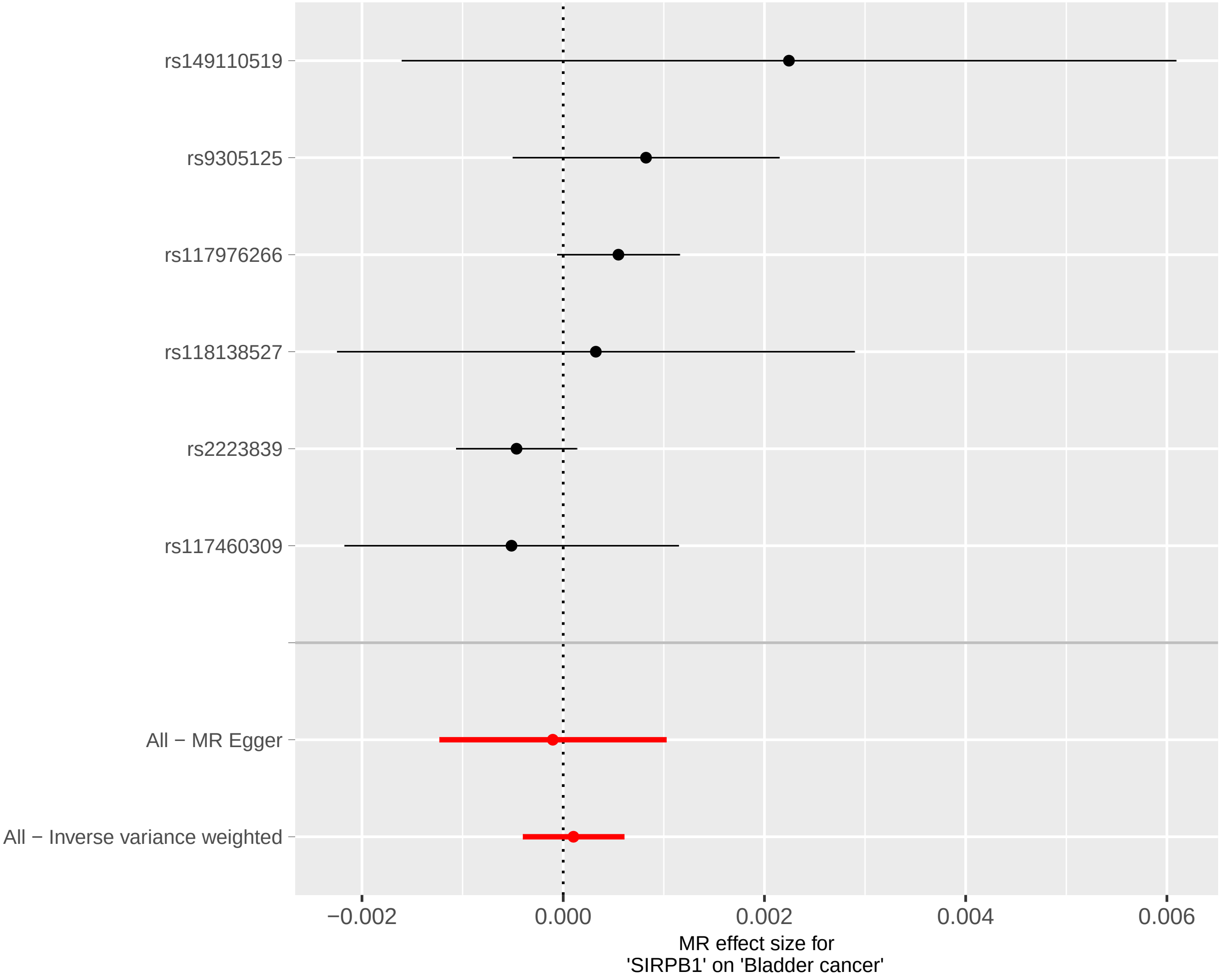

rs149110519
rs9305125
rs117976266
rs118138527
rs2223839
rs117460309
All − MR Egger
All − Inverse variance weighted
−0.002
0.000
0.002
0.004
0.006
MR effect size for
'SIRPB1' on 'Bladder cancer'

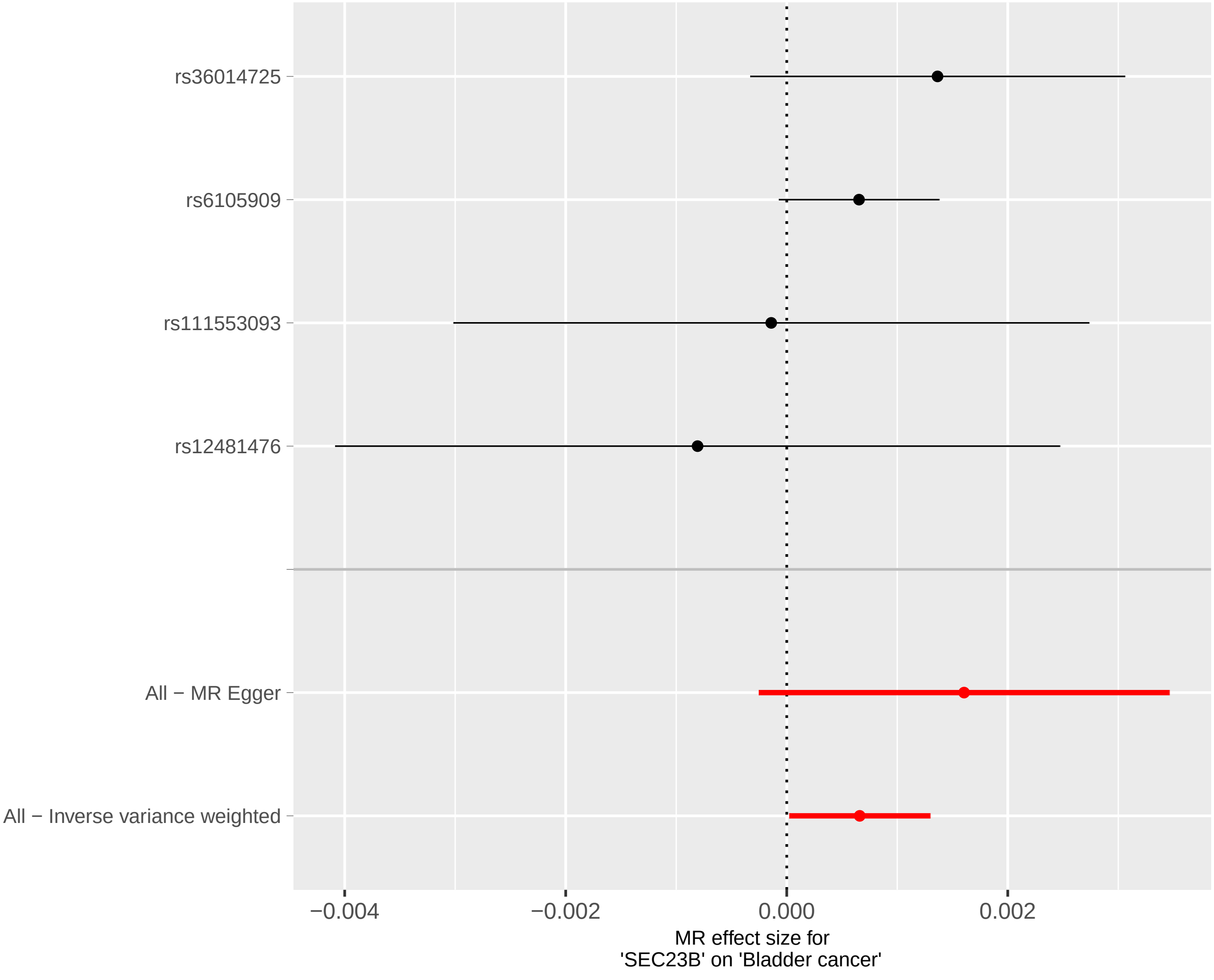

rs36014725
rs6105909
rs111553093
rs12481476
All − MR Egger
All − Inverse variance weighted
−0.004
−0.002
0.000
0.002
MR effect size for
'SEC23B' on 'Bladder cancer'

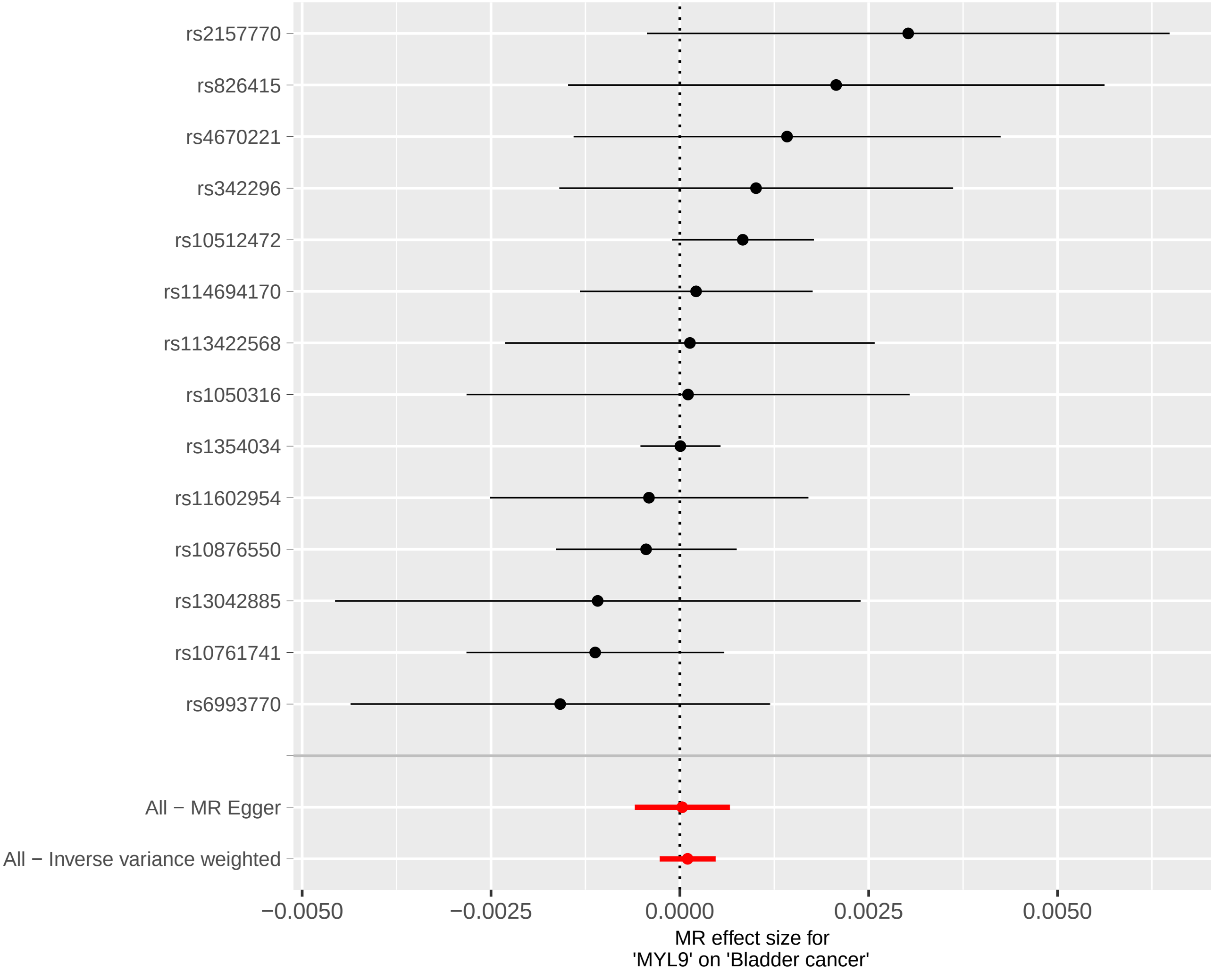
rs2157770
rs826415
rs4670221
rs342296
rs10512472
rs114694170
rs113422568
rs1050316
rs1354034
rs11602954
rs10876550
rs13042885
rs10761741
rs6993770
All − MR Egger
All − Inverse variance weighted
−0.0050
−0.0025
0.0000
0.0025
0.0050
MR effect size for
'MYL9' on 'Bladder cancer'

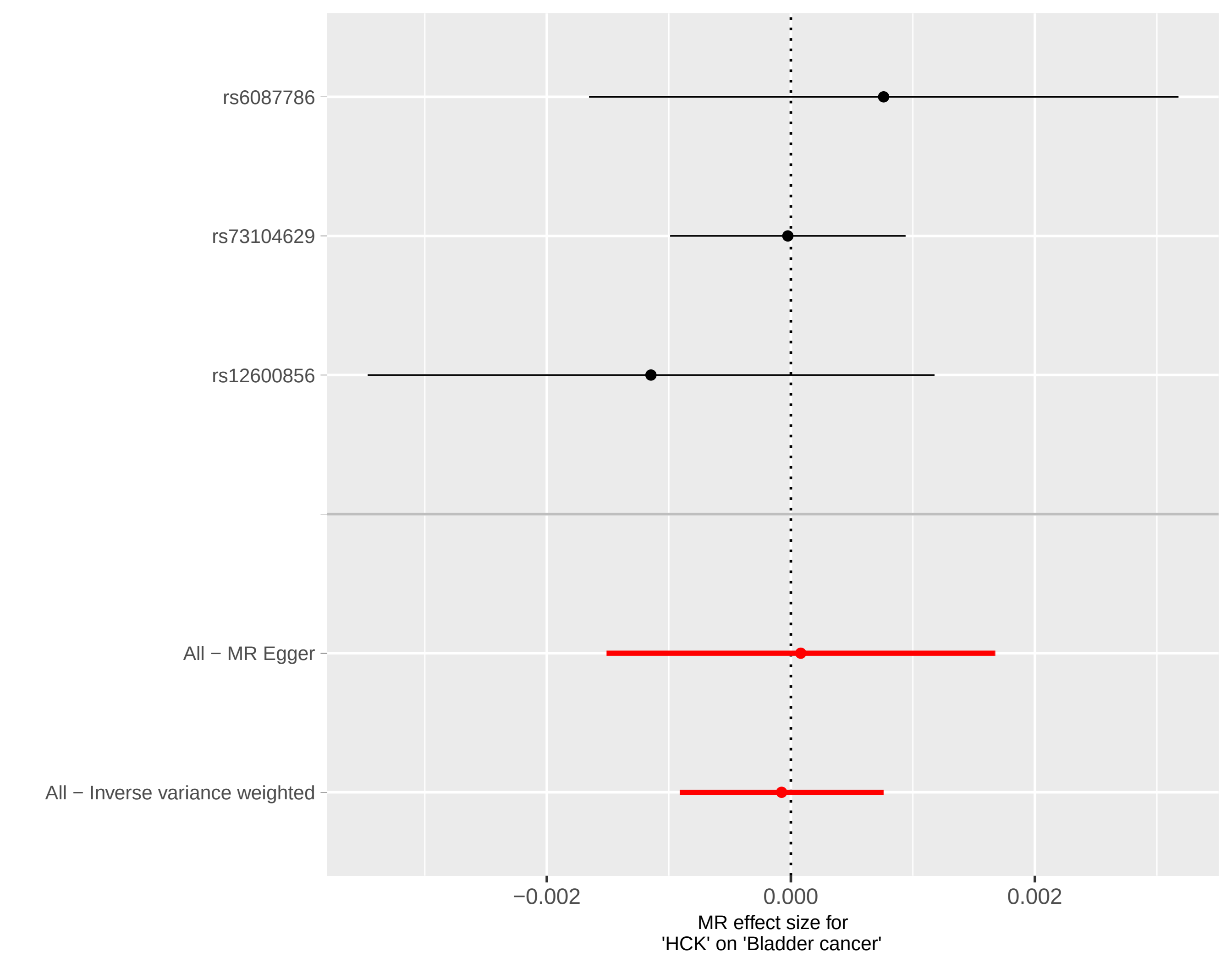
rs6087786
rs73104629
rs12600856
All − MR Egger
All − Inverse variance weighted
−0.002
0.000
0.002
MR effect size for
'HCK' on 'Bladder cancer'

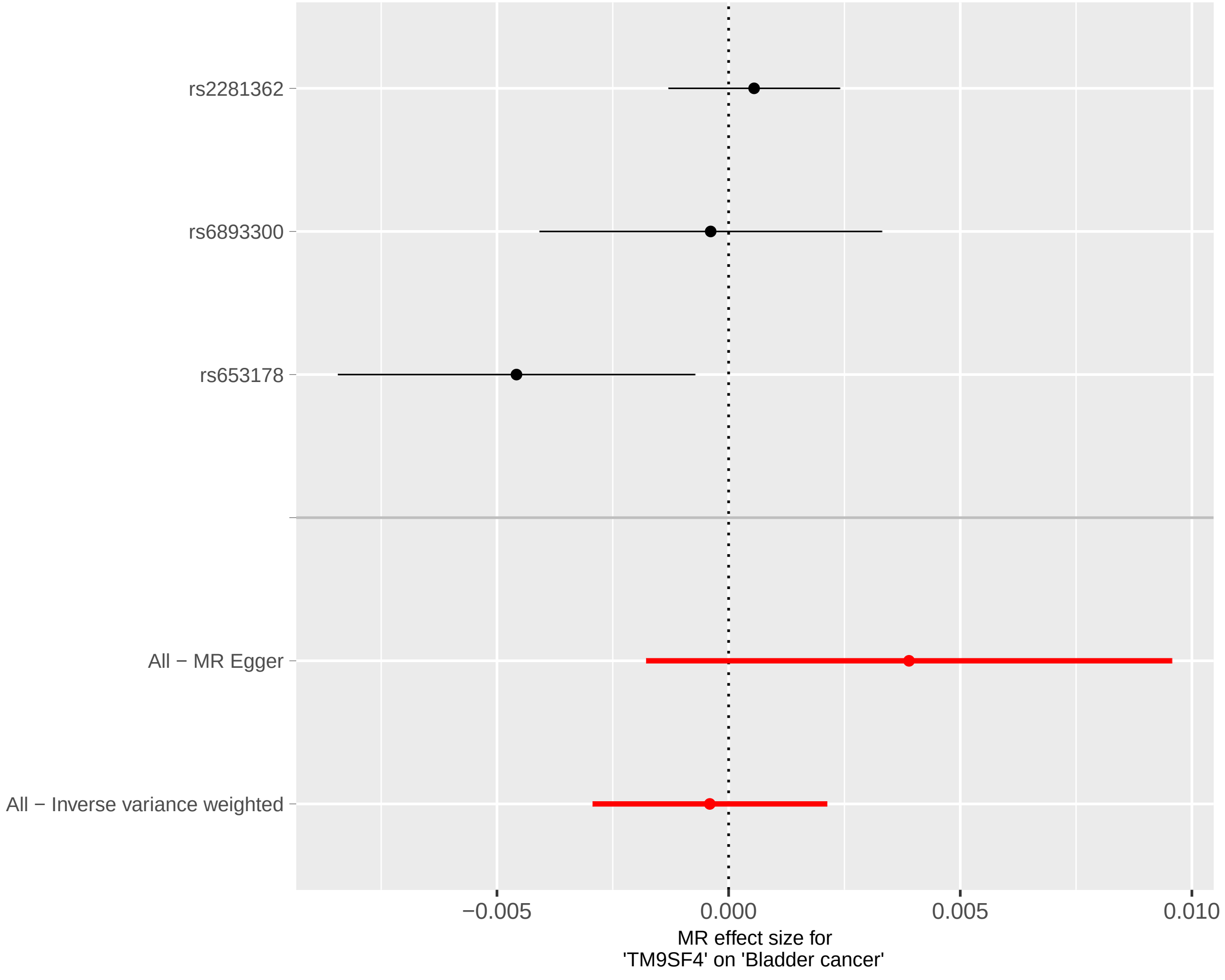
rs2281362
rs6893300
rs653178
All − MR Egger
All − Inverse variance weighted
−0.005
0.000
0.005
0.010
MR effect size for
'TM9SF4' on 'Bladder cancer'

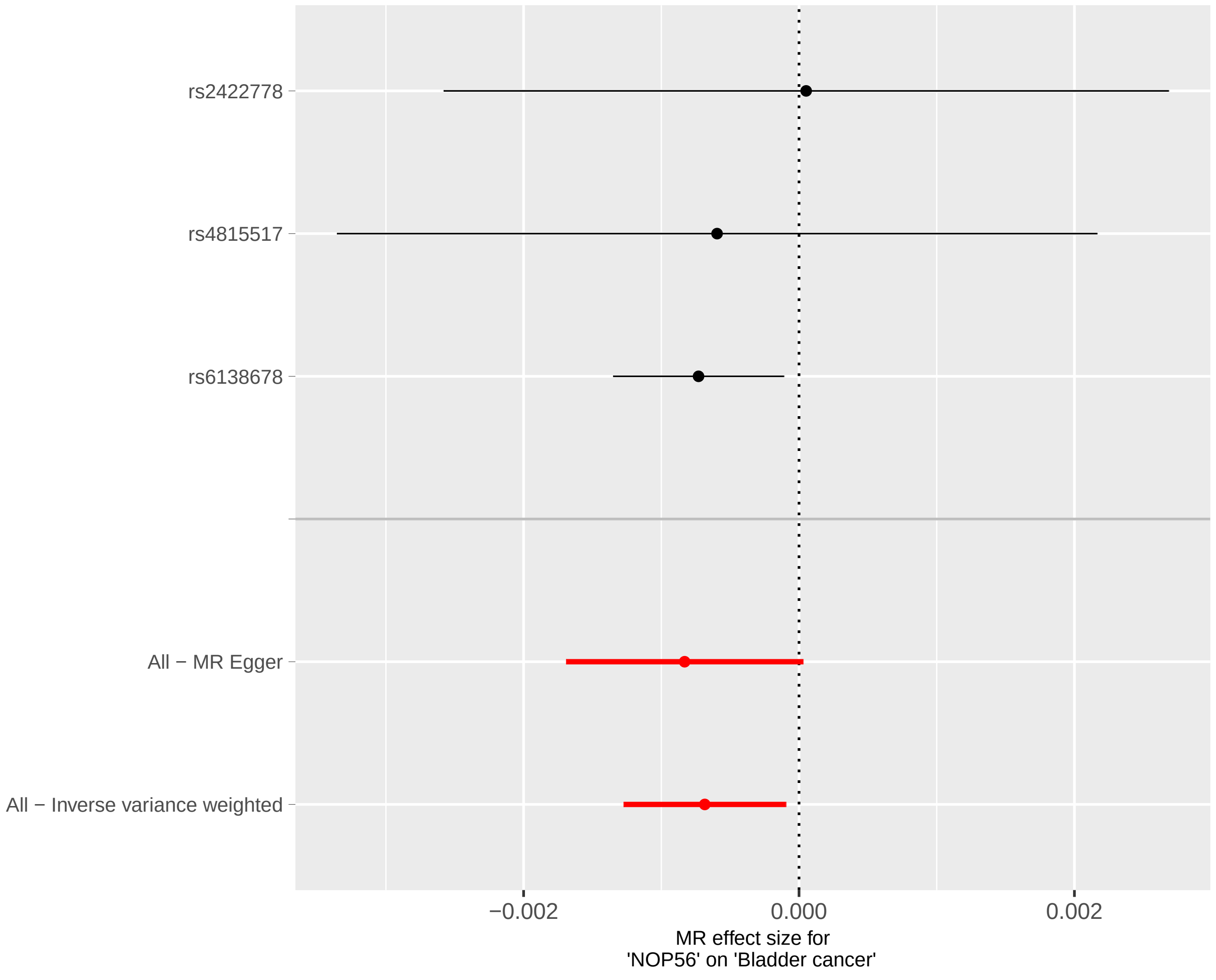

rs2422778
rs4815517
rs6138678
All − MR Egger
All − Inverse variance weighted
−0.002
0.000
0.002
MR effect size for
'NOP56' on 'Bladder cancer'

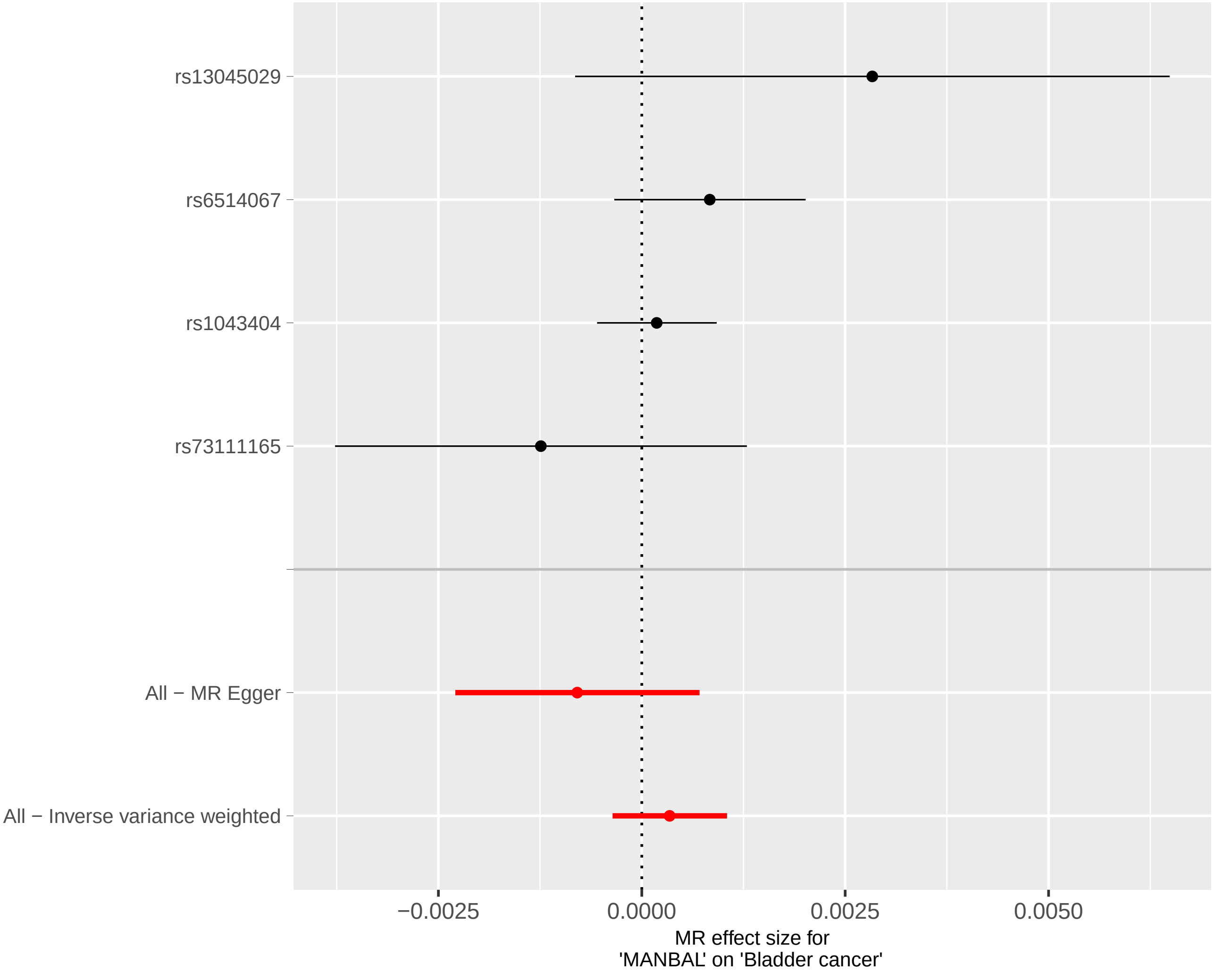
rs13045029
rs6514067
rs1043404
rs73111165
All − MR Egger
All − Inverse variance weighted
−0.0025
0.0000
0.0025
0.0050
MR effect size for
'MANBAL' on 'Bladder cancer'

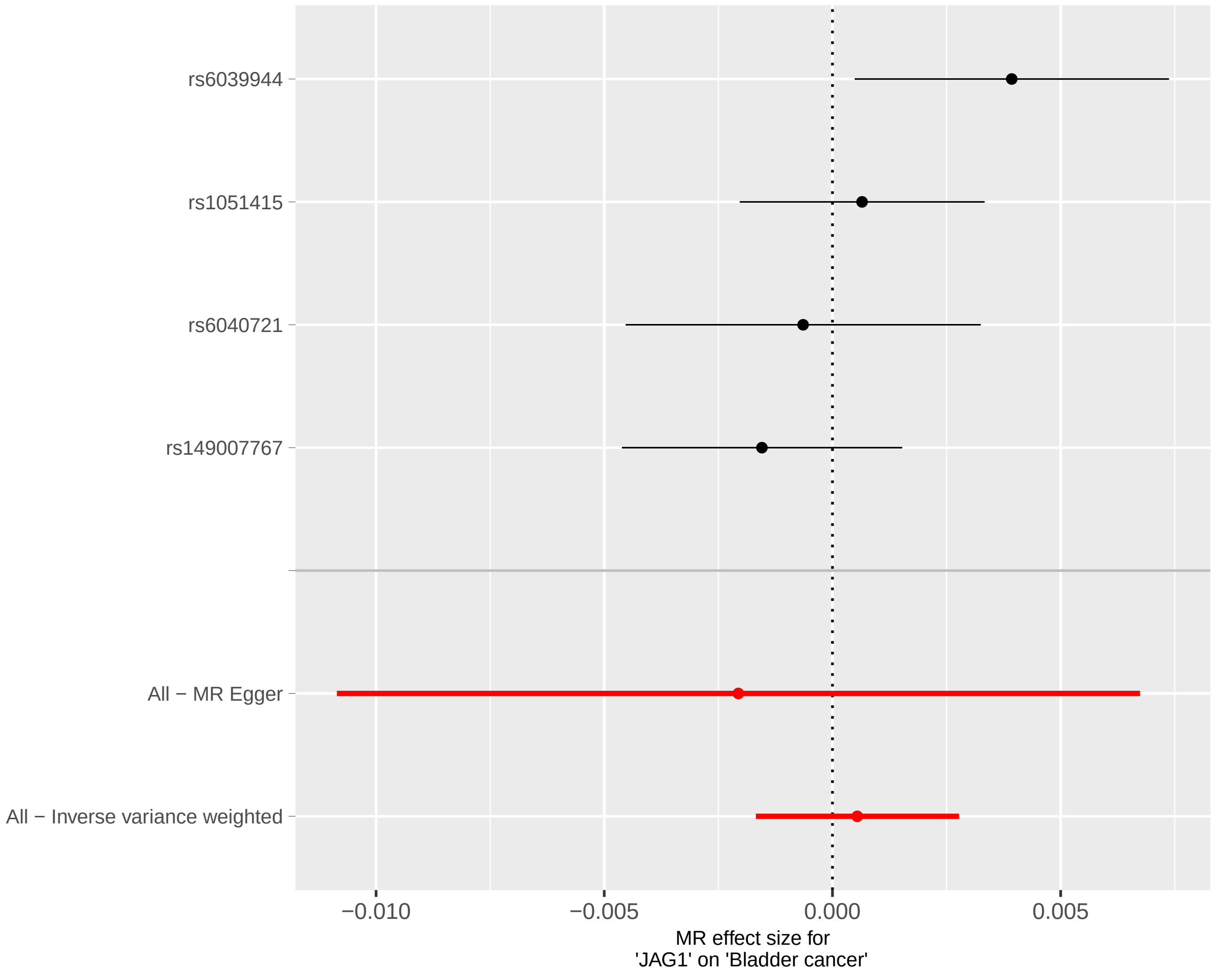

rs6039944
rs1051415
rs6040721
rs149007767
All − MR Egger
All − Inverse variance weighted
−0.010
−0.005
0.000
0.005
MR effect size for
'JAG1' on 'Bladder cancer'

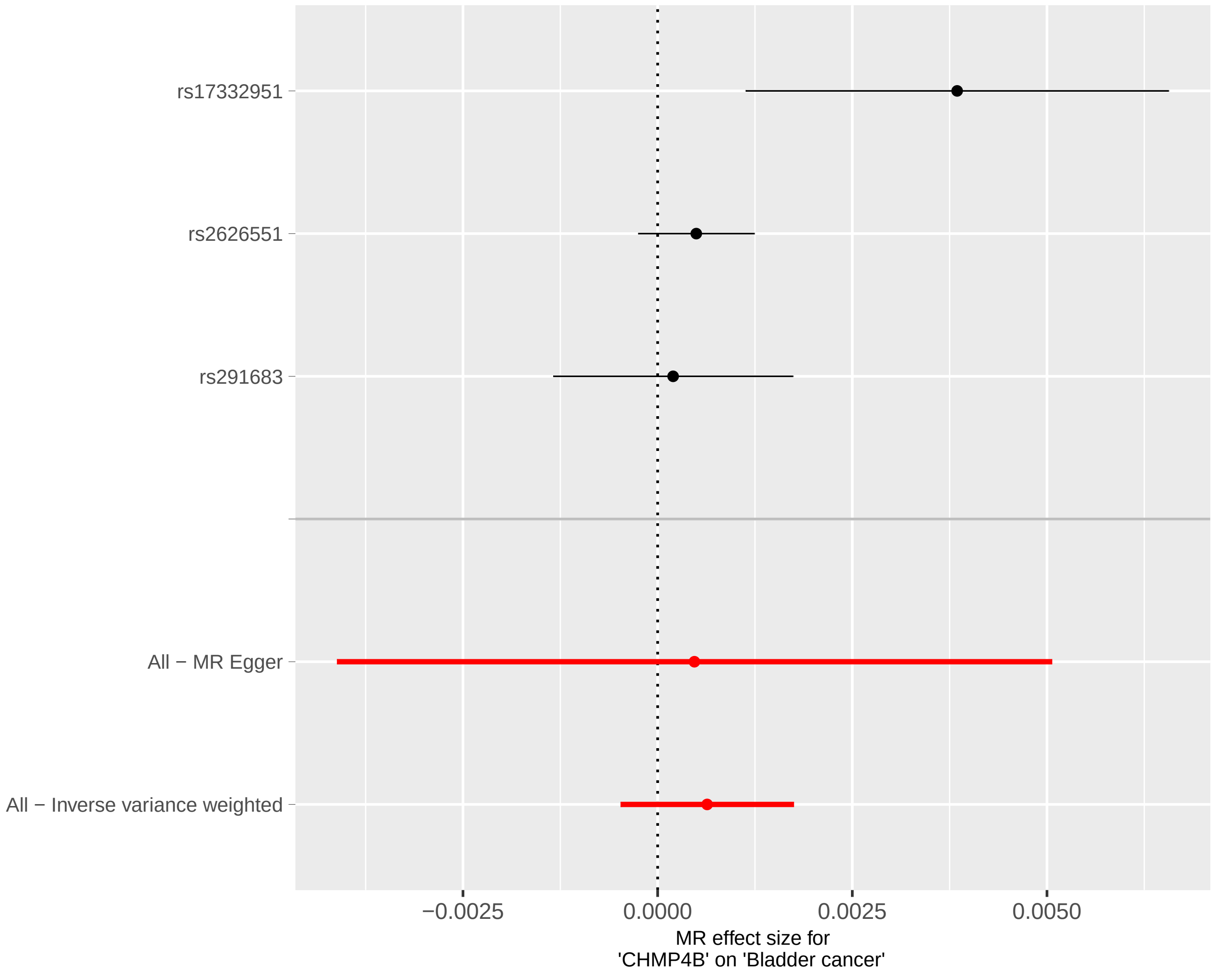

rs17332951
rs2626551
rs291683
All − MR Egger
All − Inverse variance weighted
−0.0025
0.0000
0.0025
0.0050
MR effect size for
'CHMP4B' on 'Bladder cancer'

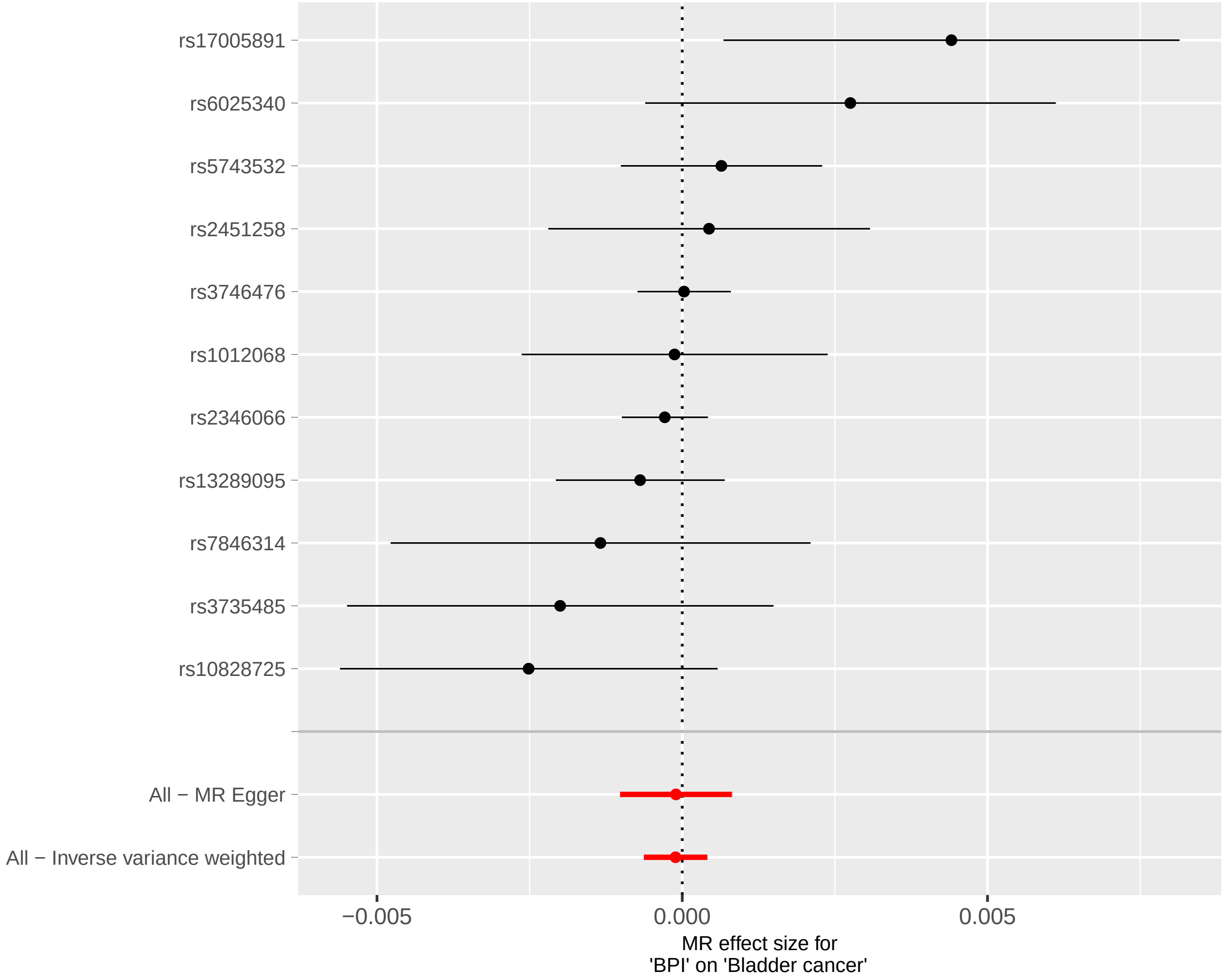
rs17005891
rs6025340
rs5743532
rs2451258
rs3746476
rs1012068
rs2346066
rs13289095
rs7846314
rs3735485
rs10828725
All − MR Egger
All − Inverse variance weighted
−0.005
0.000
0.005
MR effect size for
'BPI' on 'Bladder cancer'

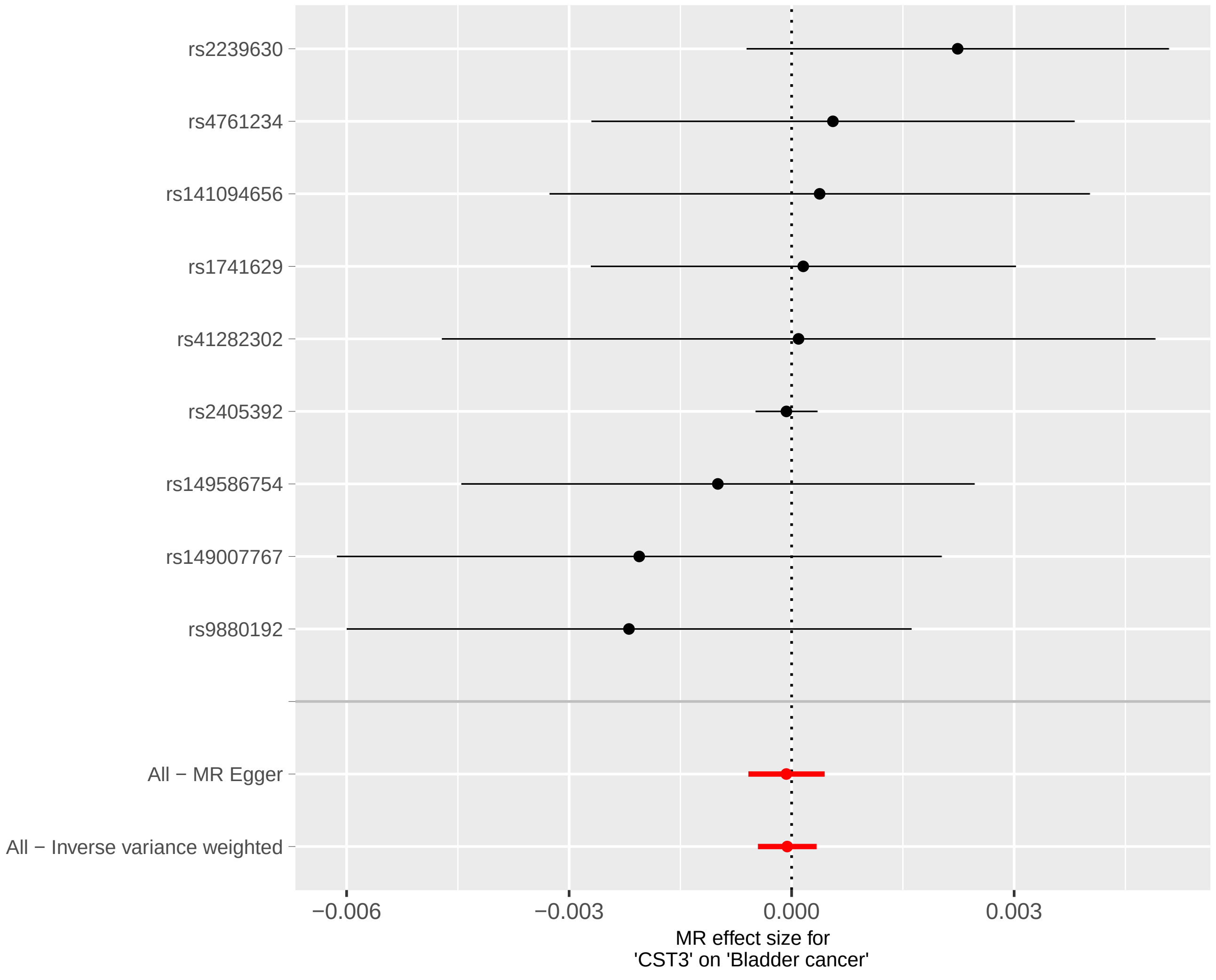
rs2239630
rs4761234
rs141094656
rs1741629
rs41282302
rs2405392
rs149586754
rs149007767
rs9880192
All − MR Egger
All − Inverse variance weighted
−0.006
−0.003
0.000
0.003
MR effect size for
'CST3' on 'Bladder cancer'

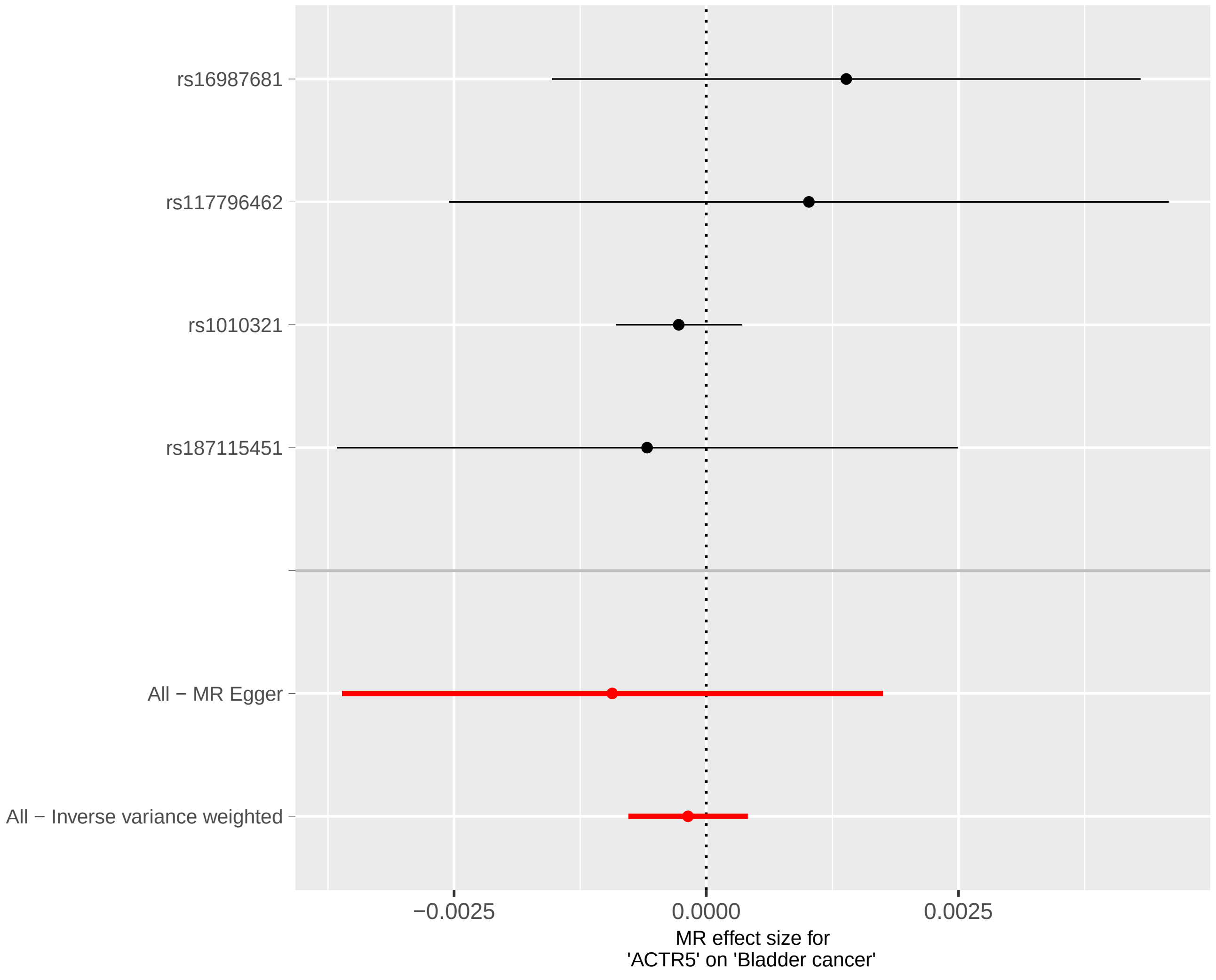
rs16987681
rs117796462
rs1010321
rs187115451
All − MR Egger
All − Inverse variance weighted
−0.0025
0.0000
0.0025
MR effect size for
'ACTR5' on 'Bladder cancer'

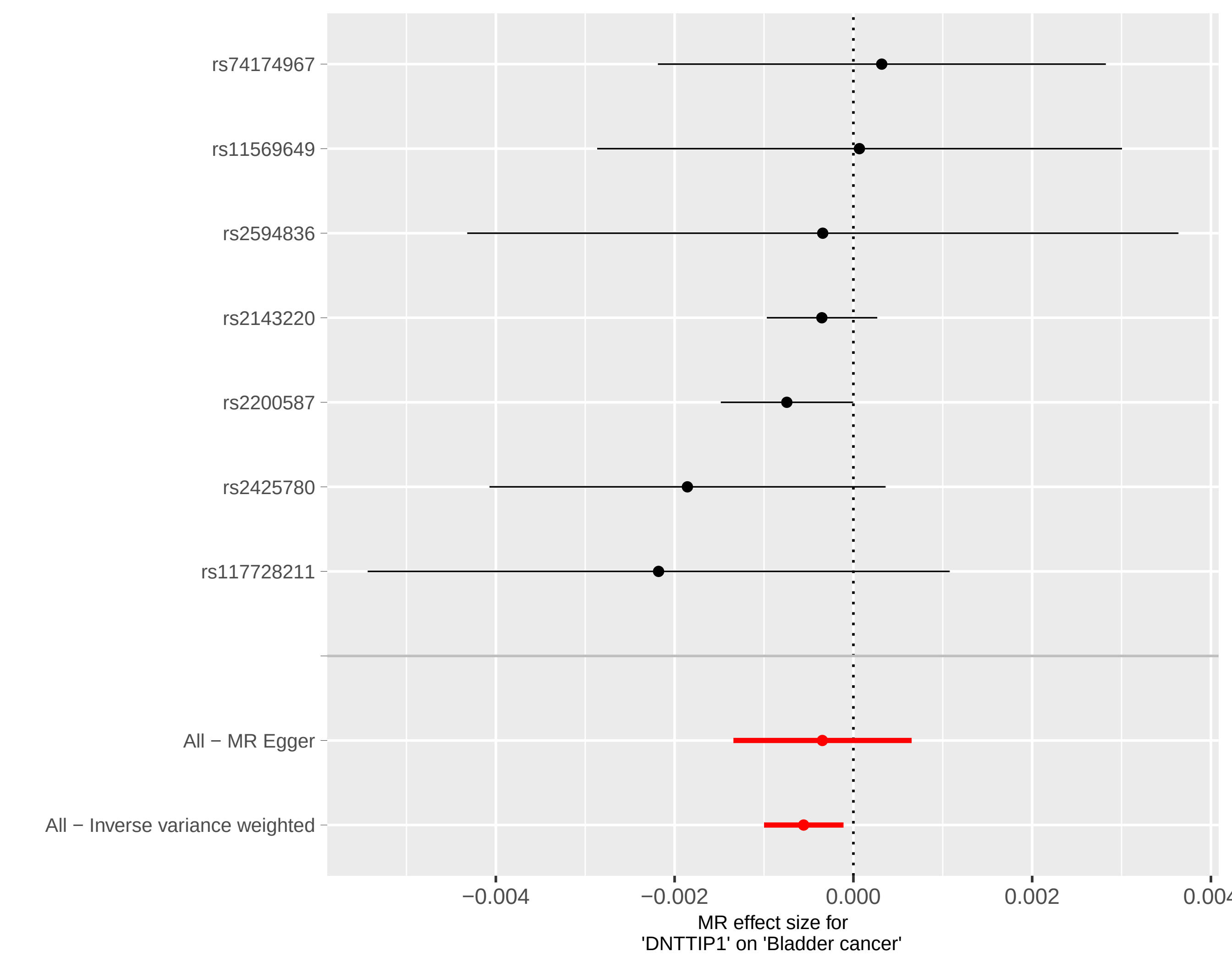
rs74174967
rs11569649
rs2594836
rs2143220
rs2200587
rs2425780
rs117728211
All − MR Egger
All − Inverse variance weighted
−0.004
−0.002
0.000
0.002
0.004
MR effect size for
'DNTTIP1' on 'Bladder cancer'

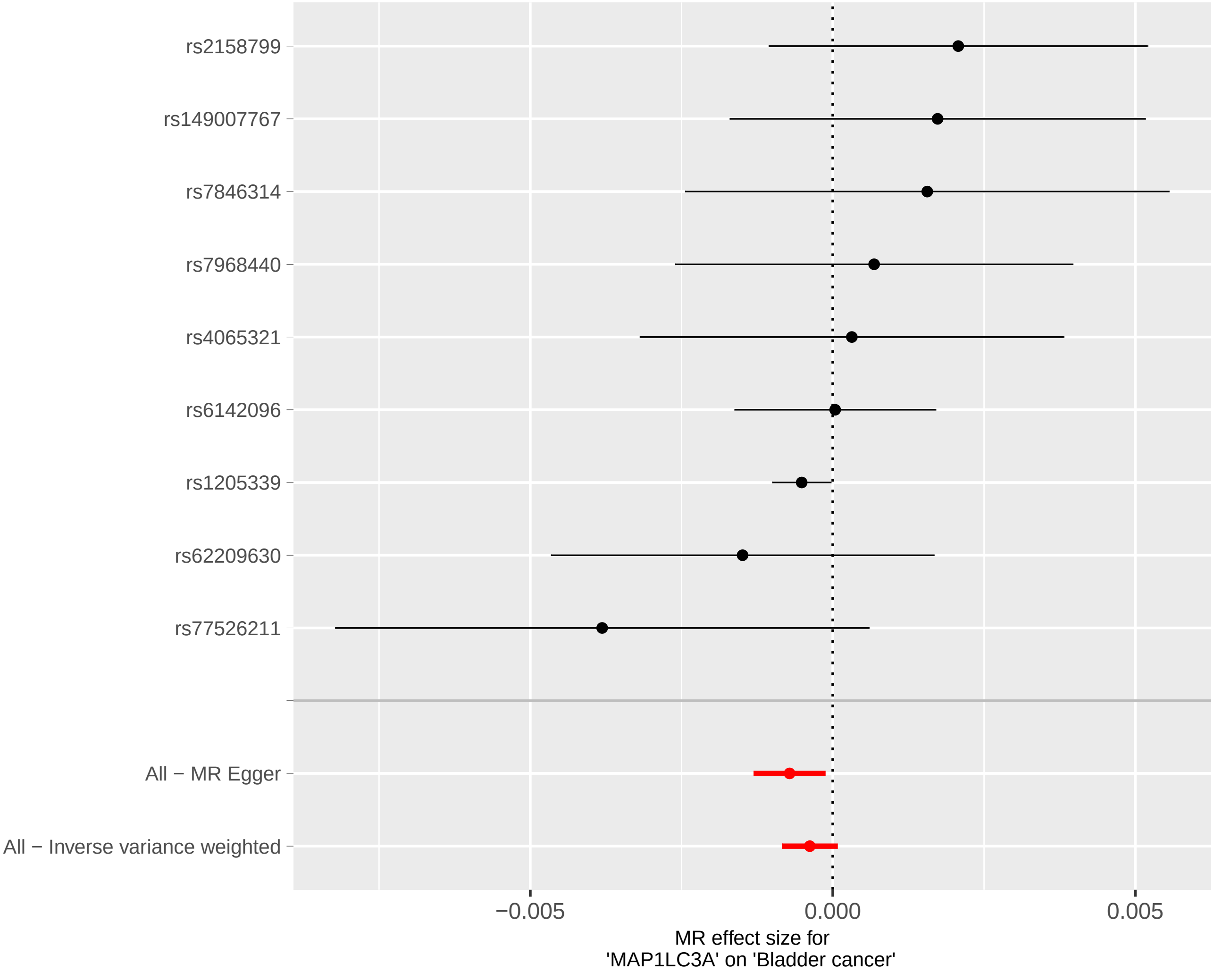

rs2158799
rs149007767
rs7846314
rs7968440
rs4065321
rs6142096
rs1205339
rs62209630
rs77526211
All − MR Egger
All − Inverse variance weighted
−0.005
0.000
0.005
MR effect size for
'MAP1LC3A' on 'Bladder cancer'

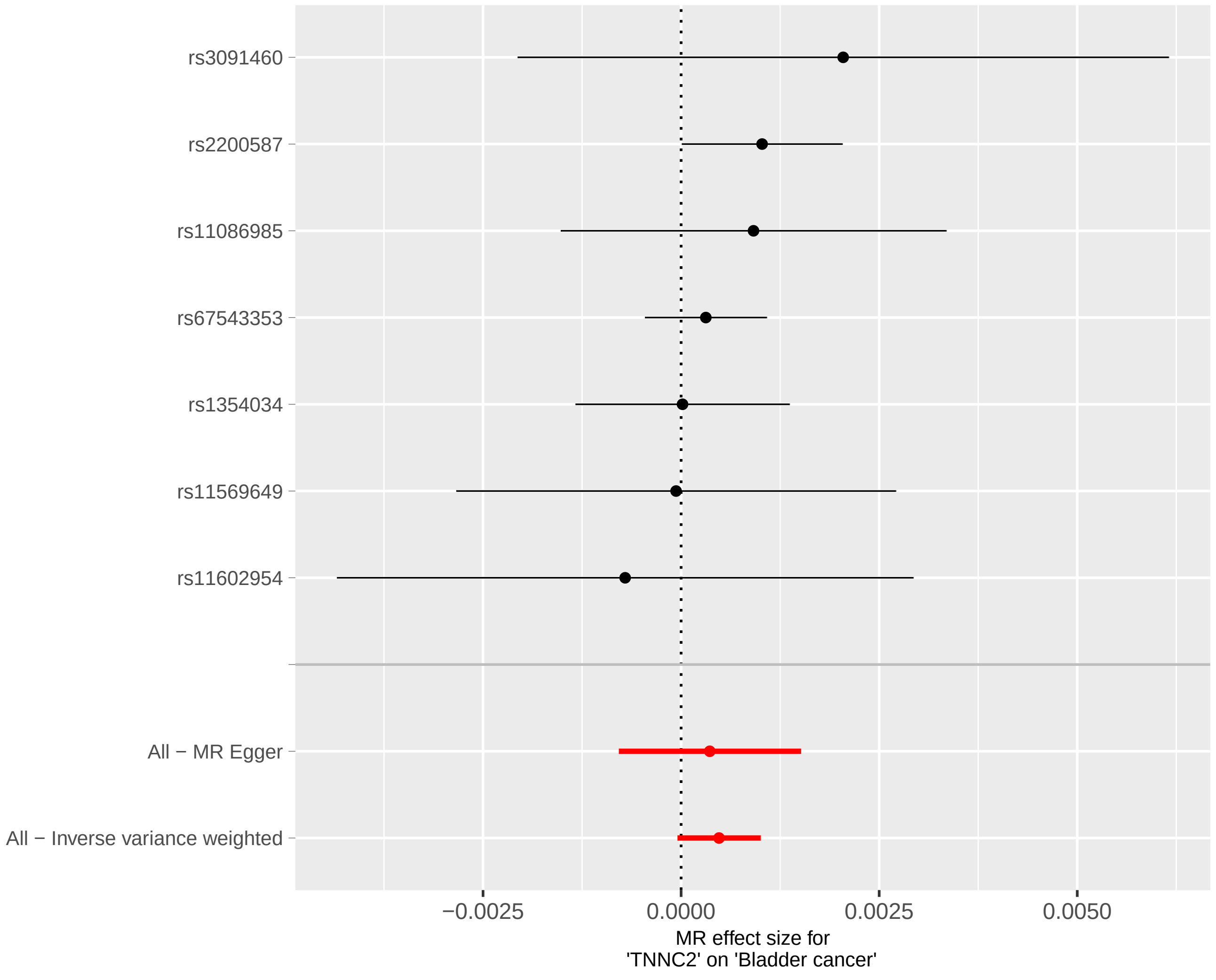
rs3091460
rs2200587
rs11086985
rs67543353
rs1354034
rs11569649
rs11602954
All − MR Egger
All − Inverse variance weighted
−0.0025
0.0000
0.0025
0.0050
MR effect size for
'TNNC2' on 'Bladder cancer'

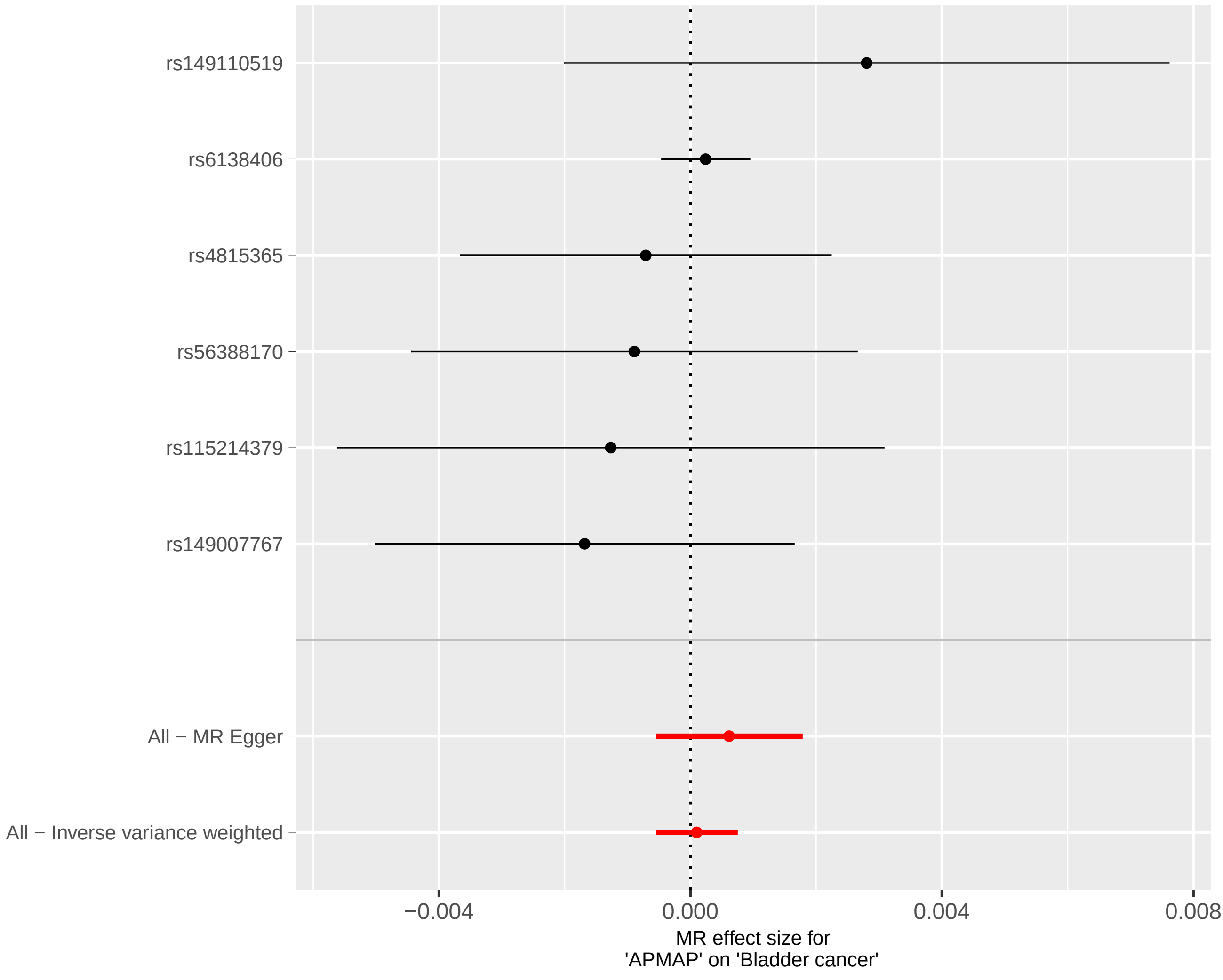
rs149110519
rs6138406
rs4815365
rs56388170
rs115214379
rs149007767
All − MR Egger
All − Inverse variance weighted
−0.004
0.000
0.004
0.008
MR effect size for
'APMAP' on 'Bladder cancer'

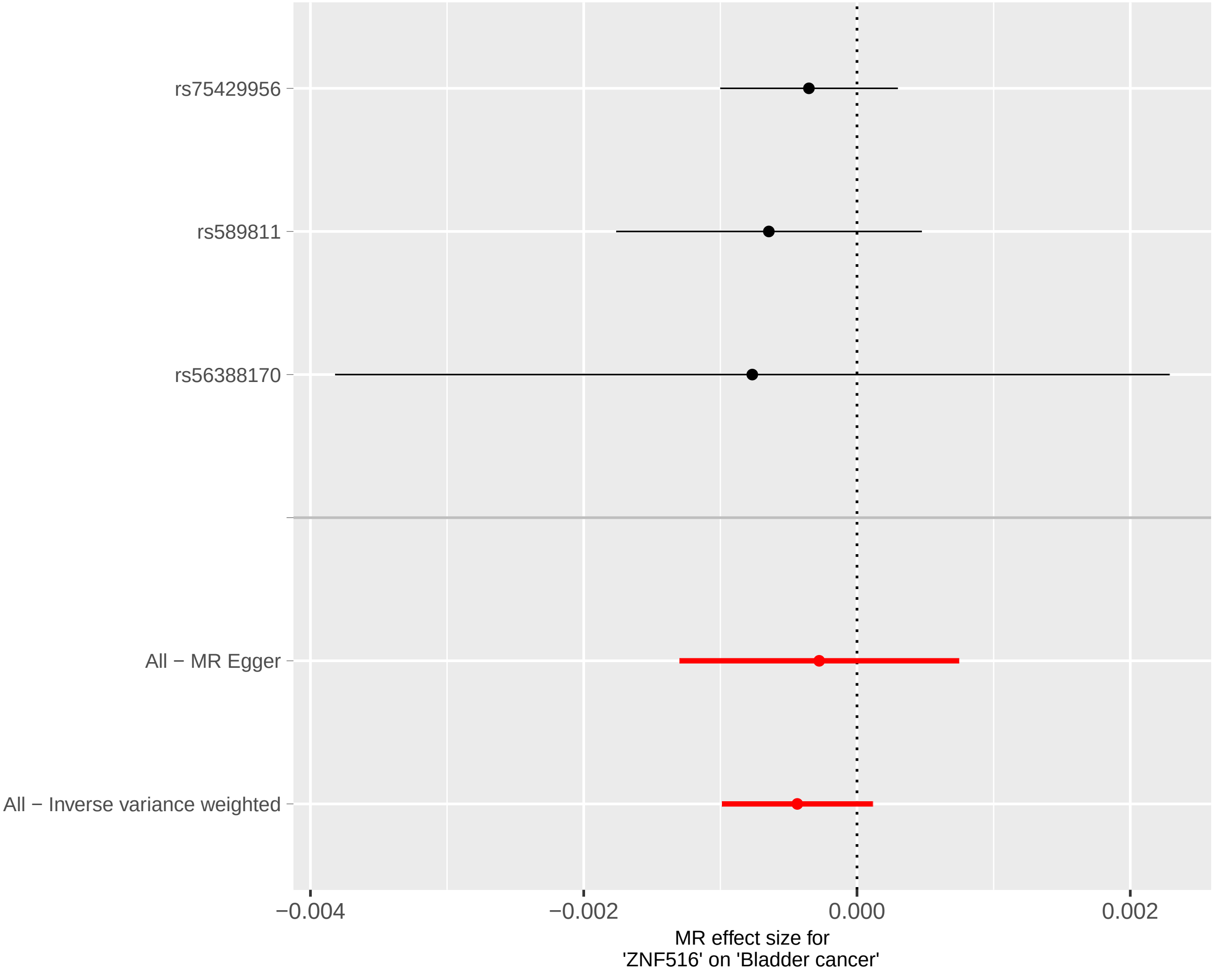

rs75429956
rs589811
rs56388170
All − MR Egger
All − Inverse variance weighted
−0.004
−0.002
0.000
0.002
MR effect size for
'ZNF516' on 'Bladder cancer'

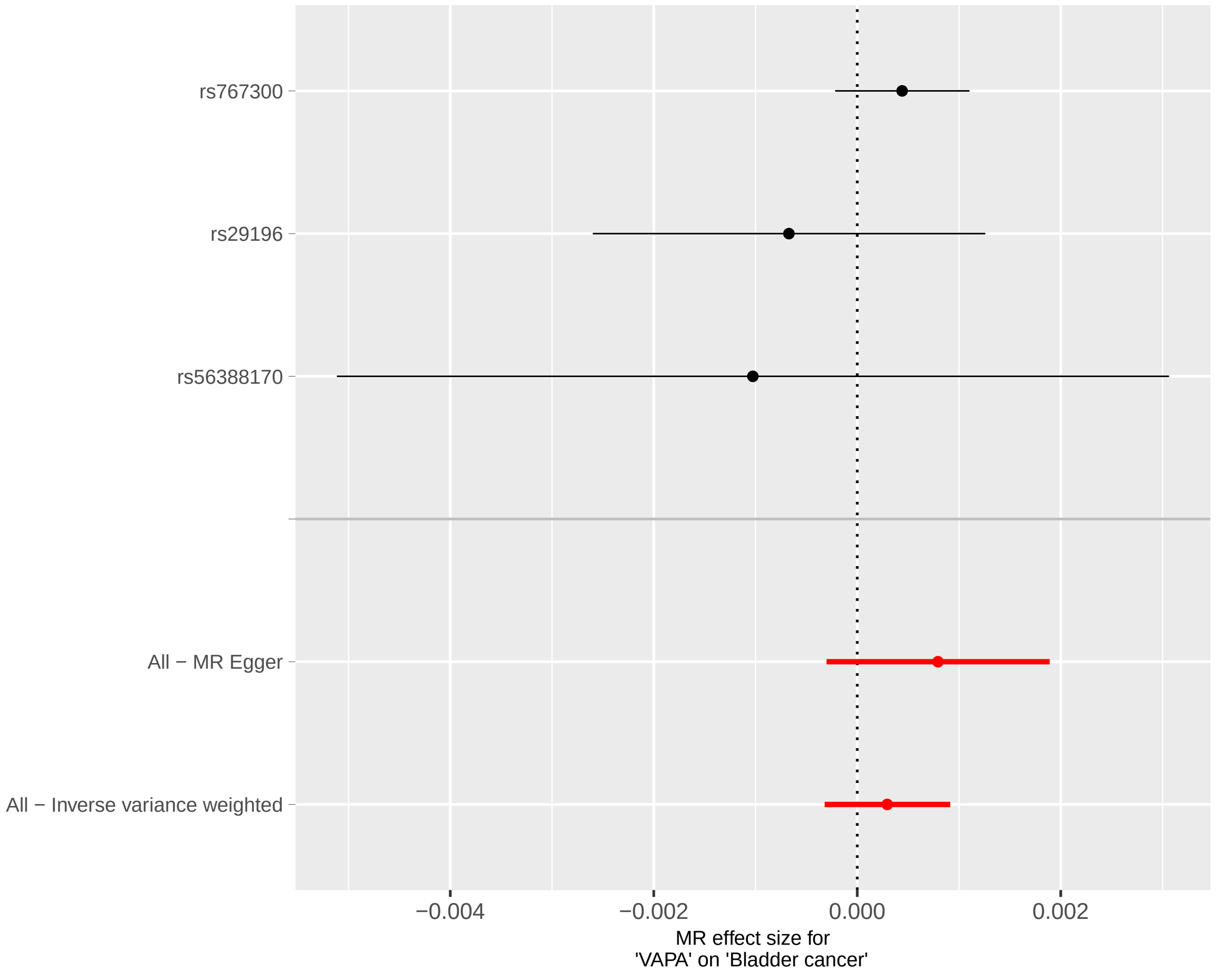
rs767300
rs29196
rs56388170
All − MR Egger
All − Inverse variance weighted
−0.004
−0.002
0.000
0.002
MR effect size for
'VAPA' on 'Bladder cancer'

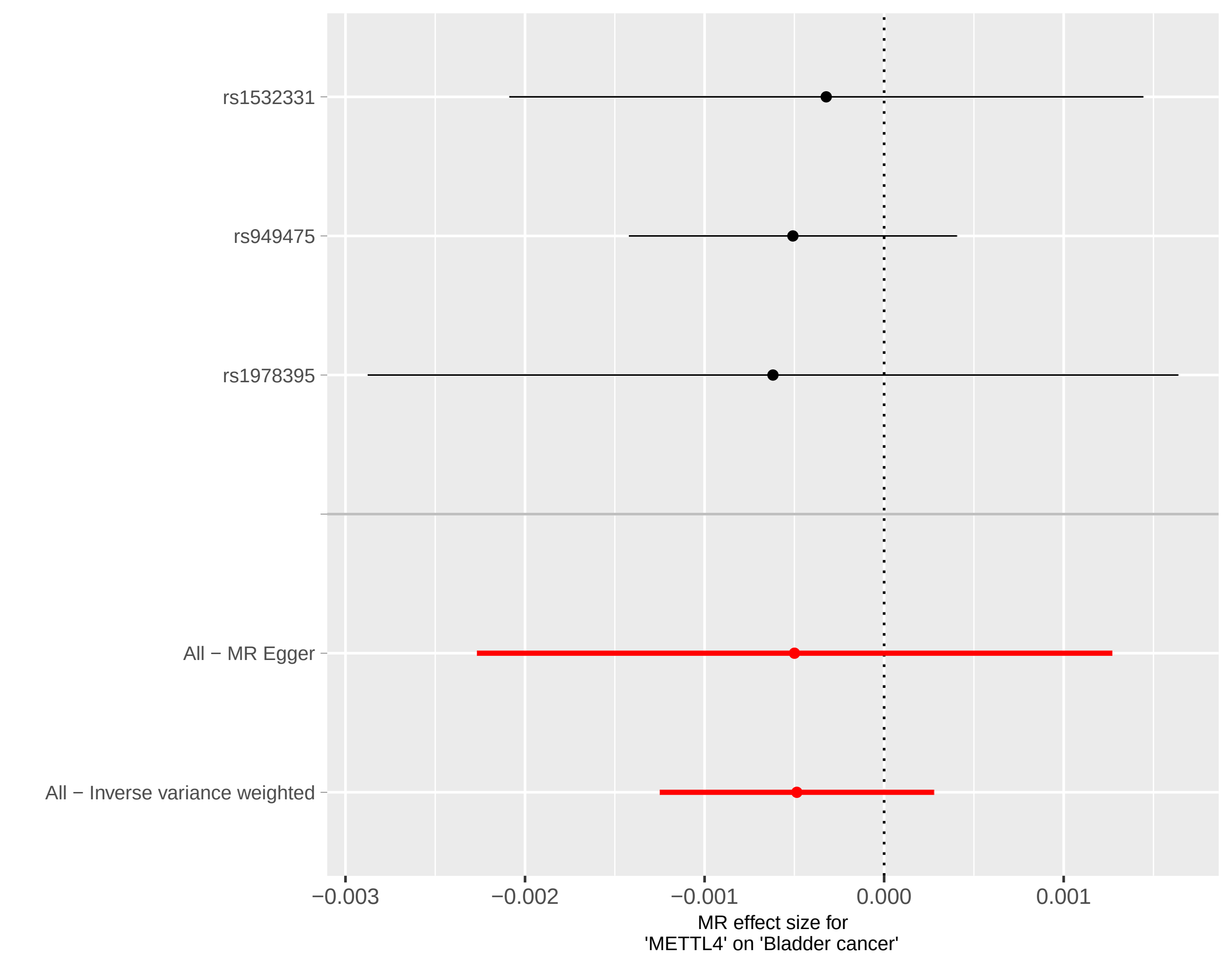
rs1532331
rs949475
rs1978395
All − MR Egger
All − Inverse variance weighted
−0.003
−0.002
−0.001
0.000
0.001
MR effect size for
'METTL4' on 'Bladder cancer'

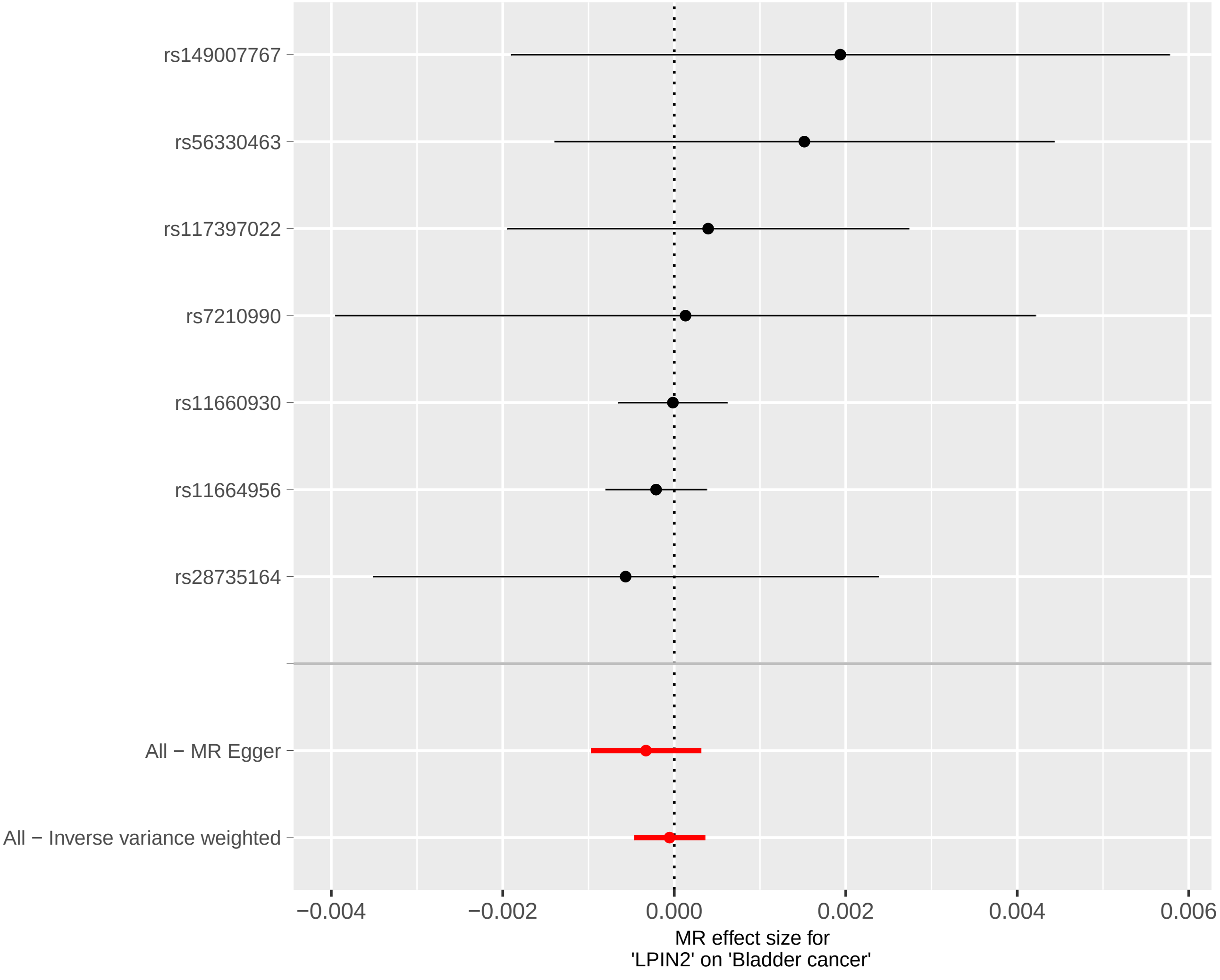
rs149007767
rs56330463
rs117397022
rs7210990
rs11660930
rs11664956
rs28735164
All − MR Egger
All − Inverse variance weighted
−0.004
−0.002
0.000
0.002
0.004
0.006
MR effect size for
'LPIN2' on 'Bladder cancer'

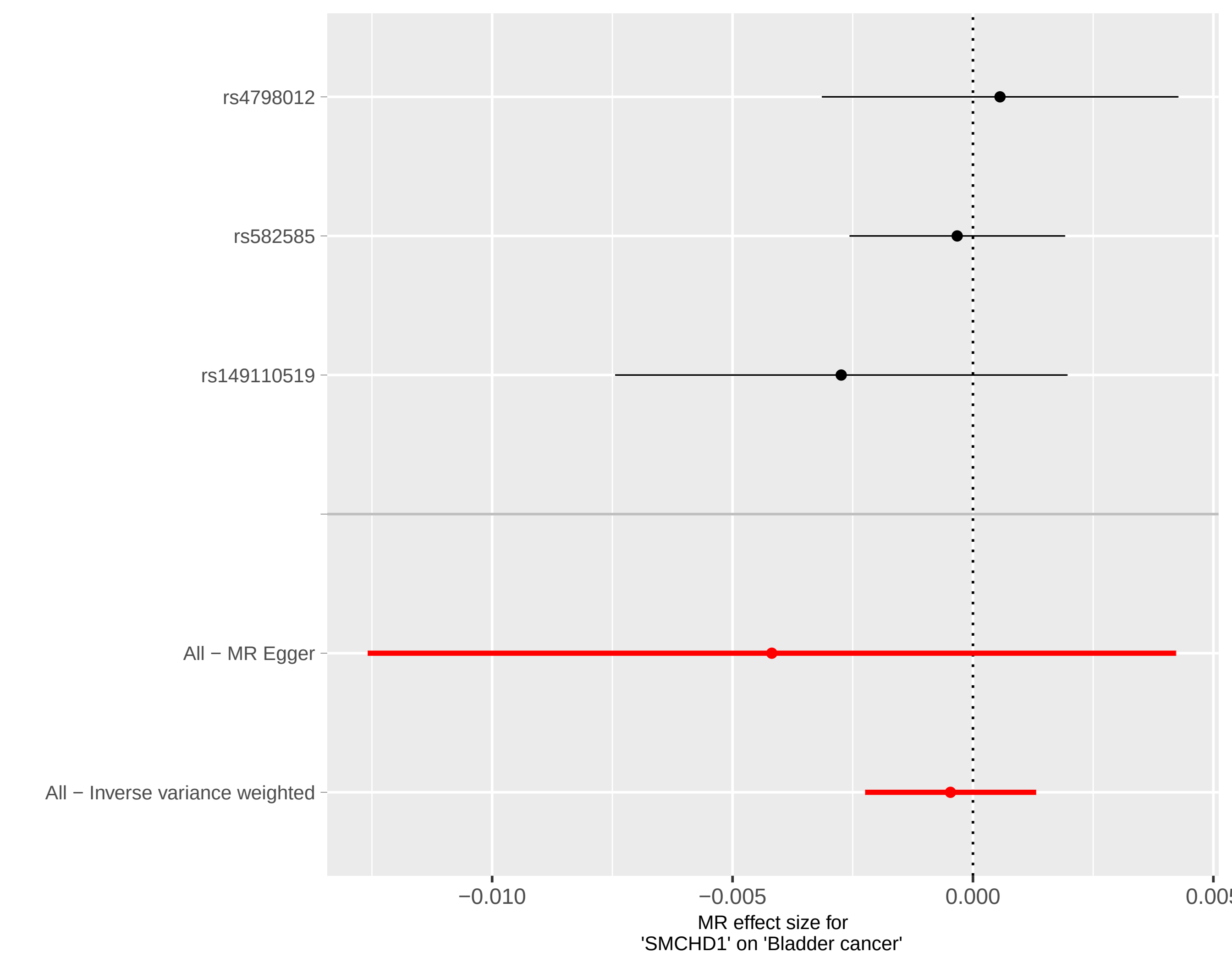

rs4798012
rs582585
rs149110519
All − MR Egger
All − Inverse variance weighted
−0.010
−0.005
0.000
0.005
MR effect size for
'SMCHD1' on 'Bladder cancer'

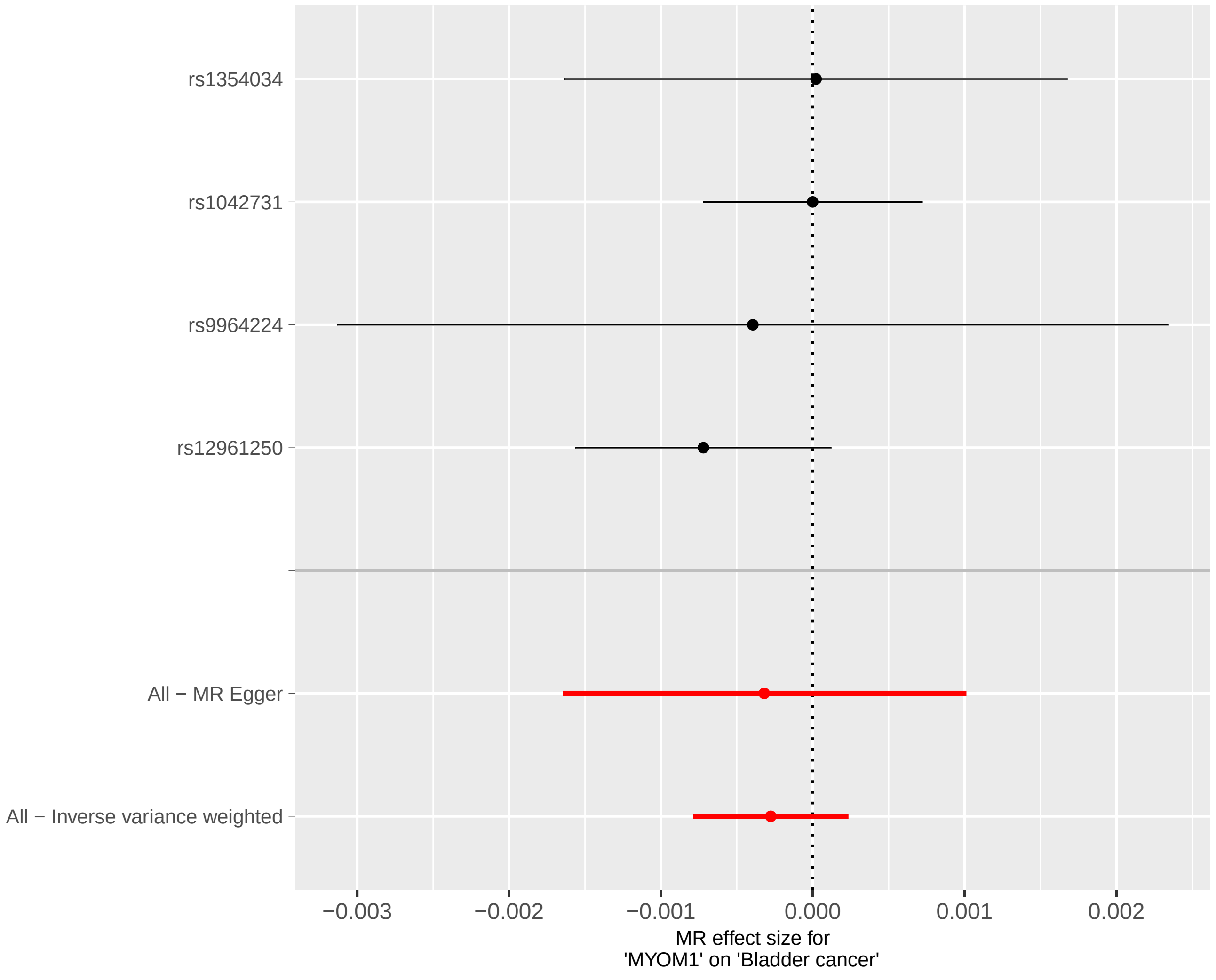

rs1354034
rs1042731
rs9964224
rs12961250
All − MR Egger
All − Inverse variance weighted
−0.003
−0.002
−0.001
0.000
0.001
0.002
MR effect size for
'MYOM1' on 'Bladder cancer'

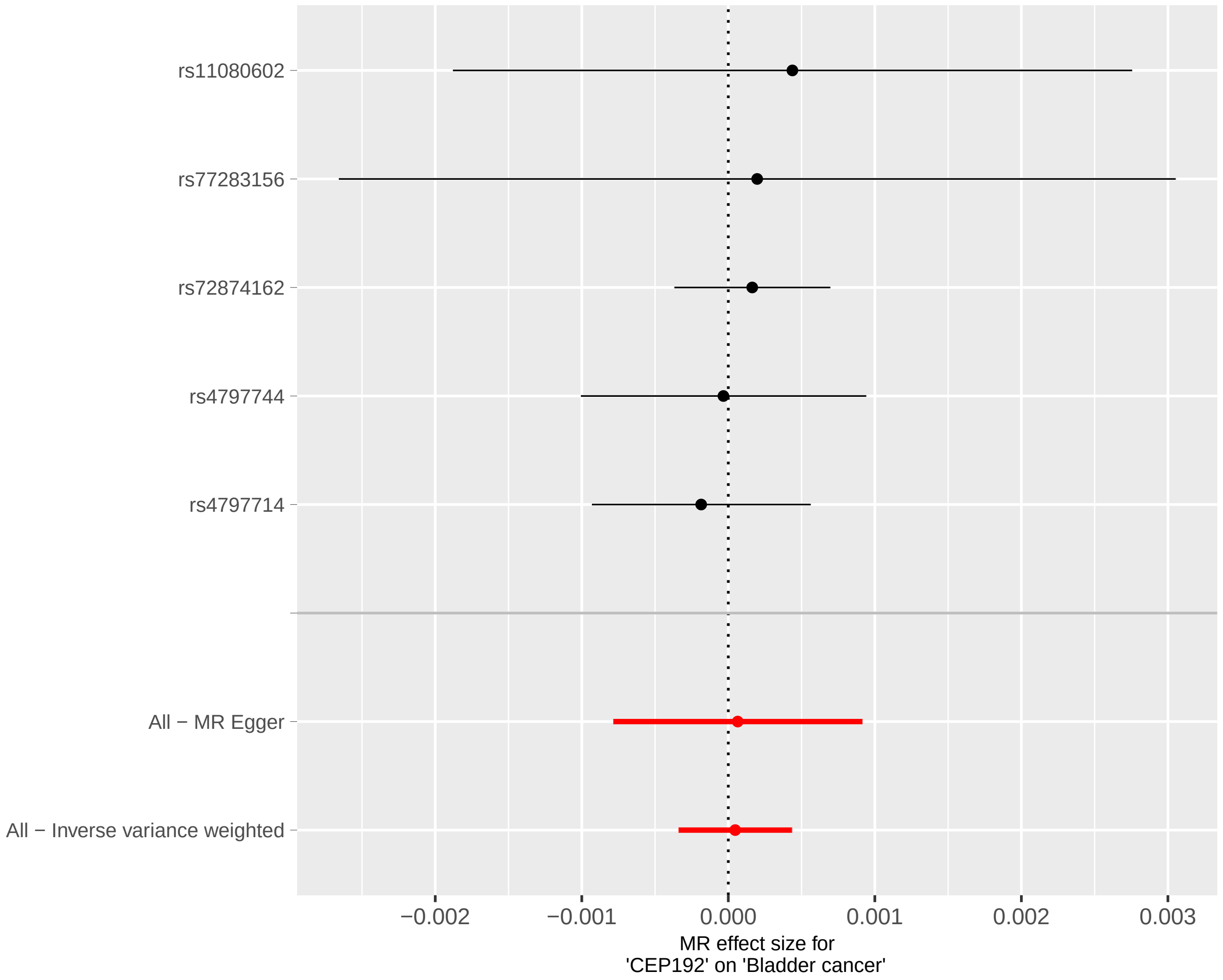

rs11080602
rs77283156
rs72874162
rs4797744
rs4797714
All − MR Egger
All − Inverse variance weighted
−0.002
−0.001
0.000
0.001
0.002
0.003
MR effect size for
'CEP192' on 'Bladder cancer'

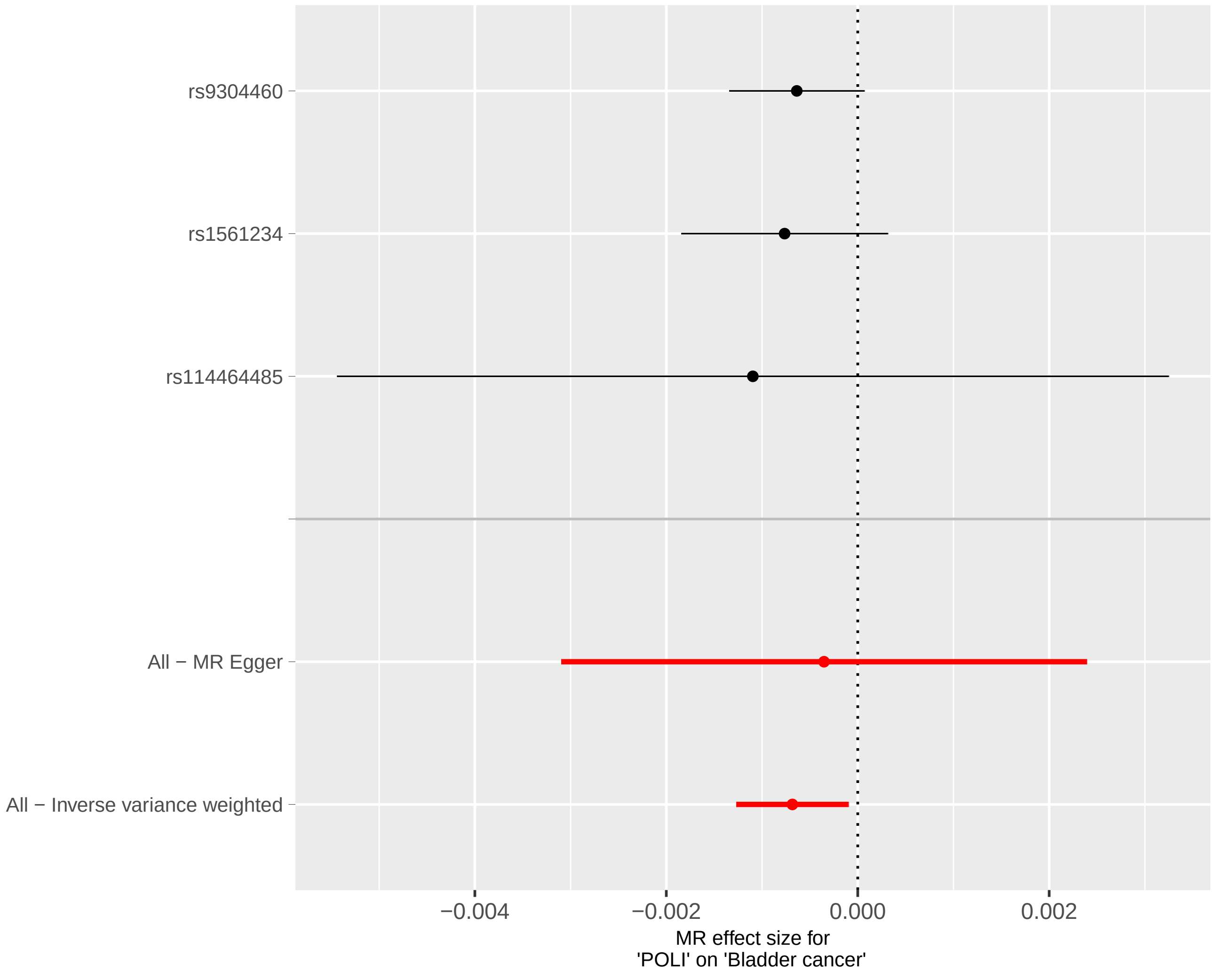
rs9304460
rs1561234
rs114464485
All − MR Egger
All − Inverse variance weighted
−0.004
−0.002
0.000
0.002
MR effect size for
'POLI' on 'Bladder cancer'

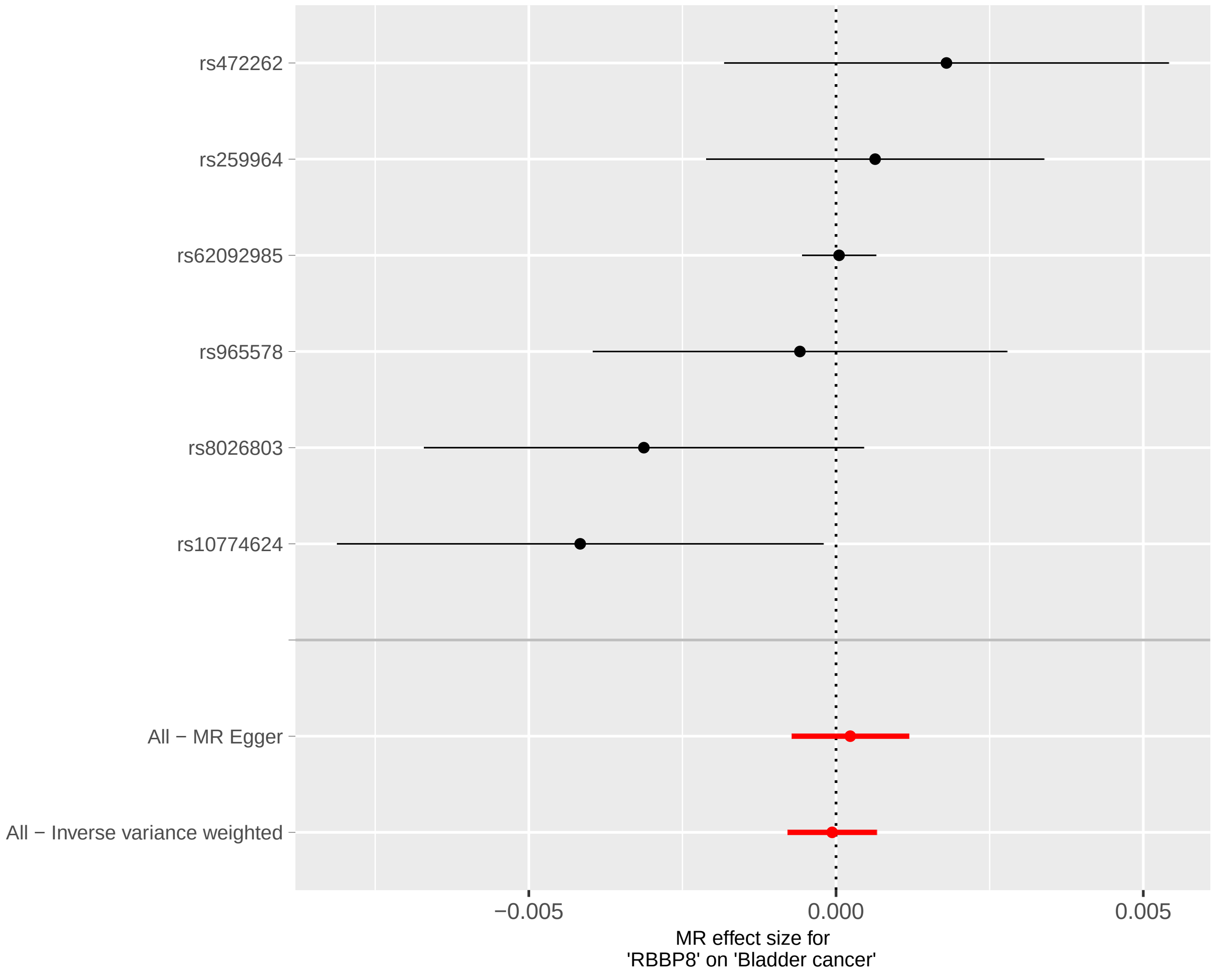

rs472262
rs259964
rs62092985
rs965578
rs8026803
rs10774624
All − MR Egger
All − Inverse variance weighted
−0.005
0.000
0.005
MR effect size for
'RBBP8' on 'Bladder cancer'

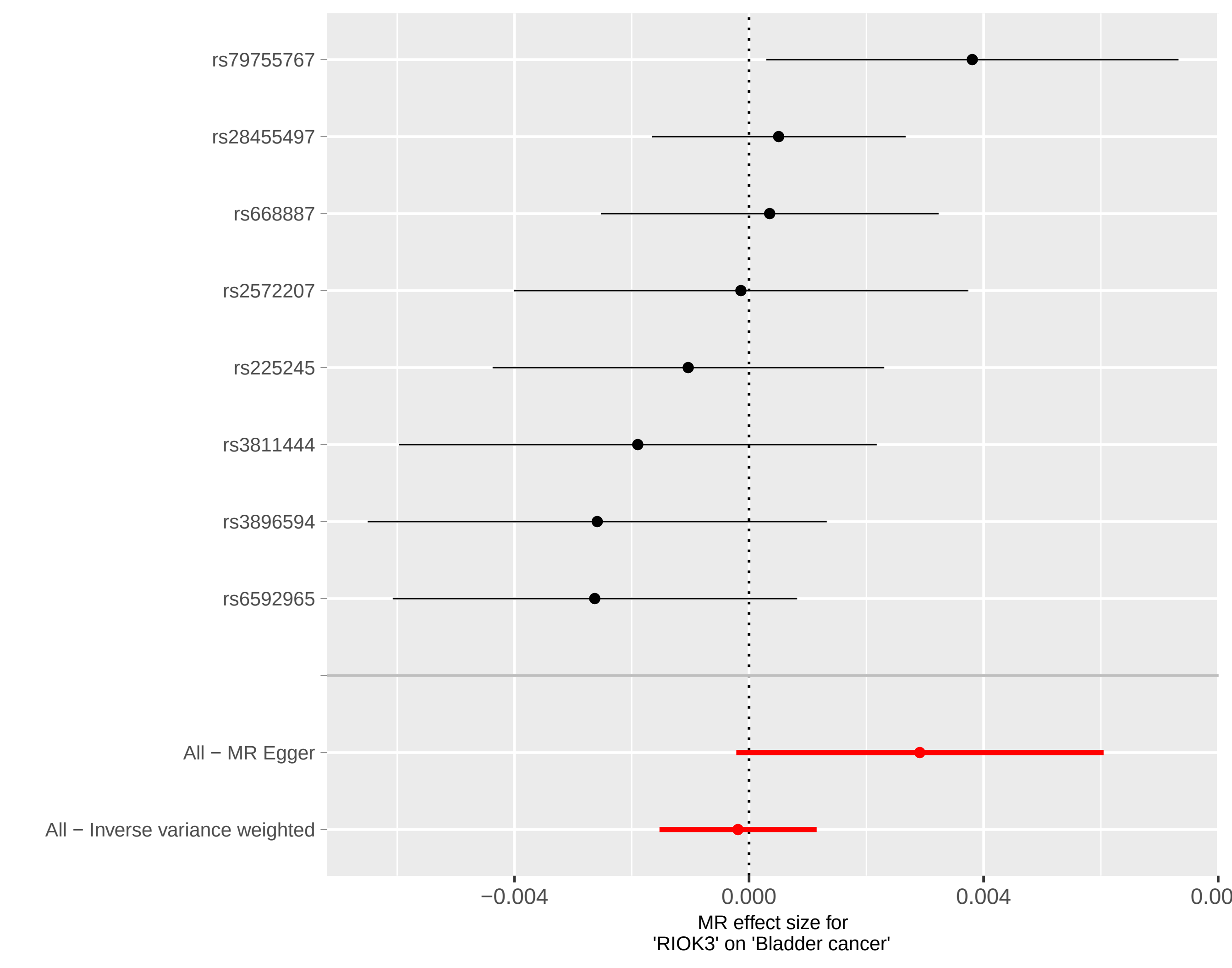

rs79755767
rs28455497
rs668887
rs2572207
rs225245
rs3811444
rs3896594
rs6592965
All − MR Egger
All − Inverse variance weighted
−0.004
0.000
0.004
MR effect size for
'RIOK3' on 'Bladder cancer'

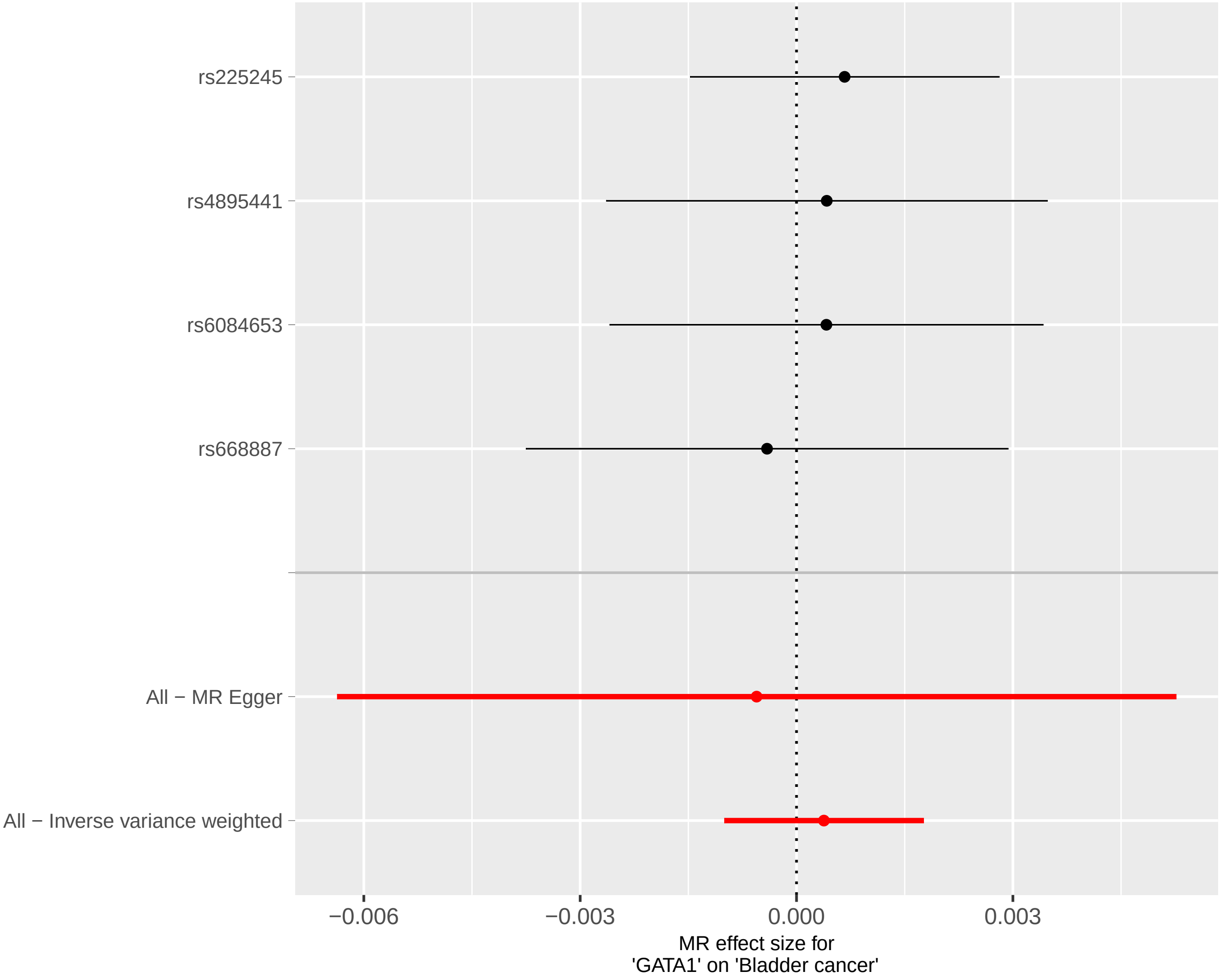
rs225245
rs4895441
rs6084653
rs668887
All − MR Egger
All − Inverse variance weighted
−0.006
−0.003
0.000
0.003
MR effect size for
'GATA1' on 'Bladder cancer'

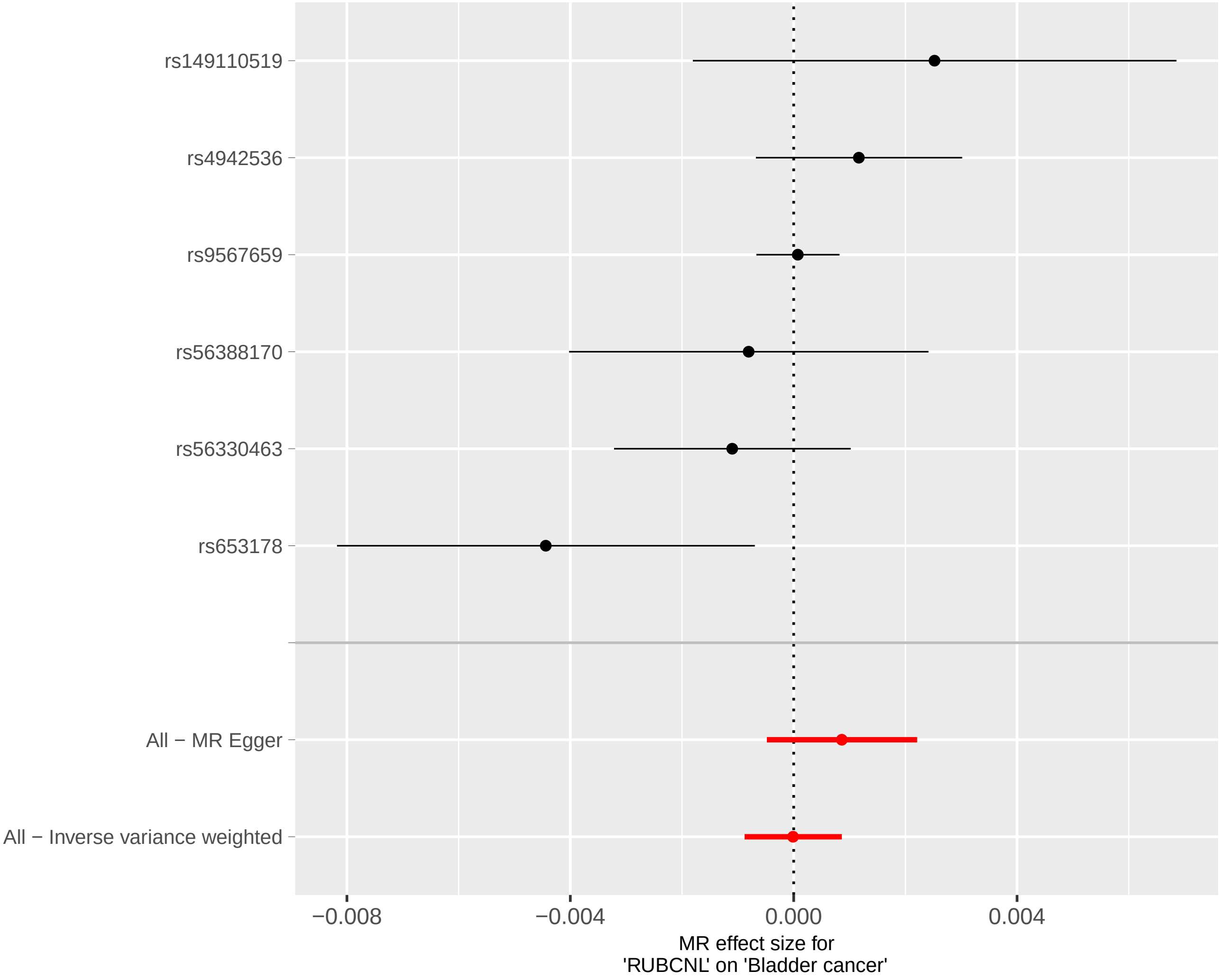

rs149110519
rs4942536
rs9567659
rs56388170
rs56330463
rs653178
All − MR Egger
All − Inverse variance weighted
−0.008
−0.004
0.000
0.004
MR effect size for
'RUBCNL' on 'Bladder cancer'

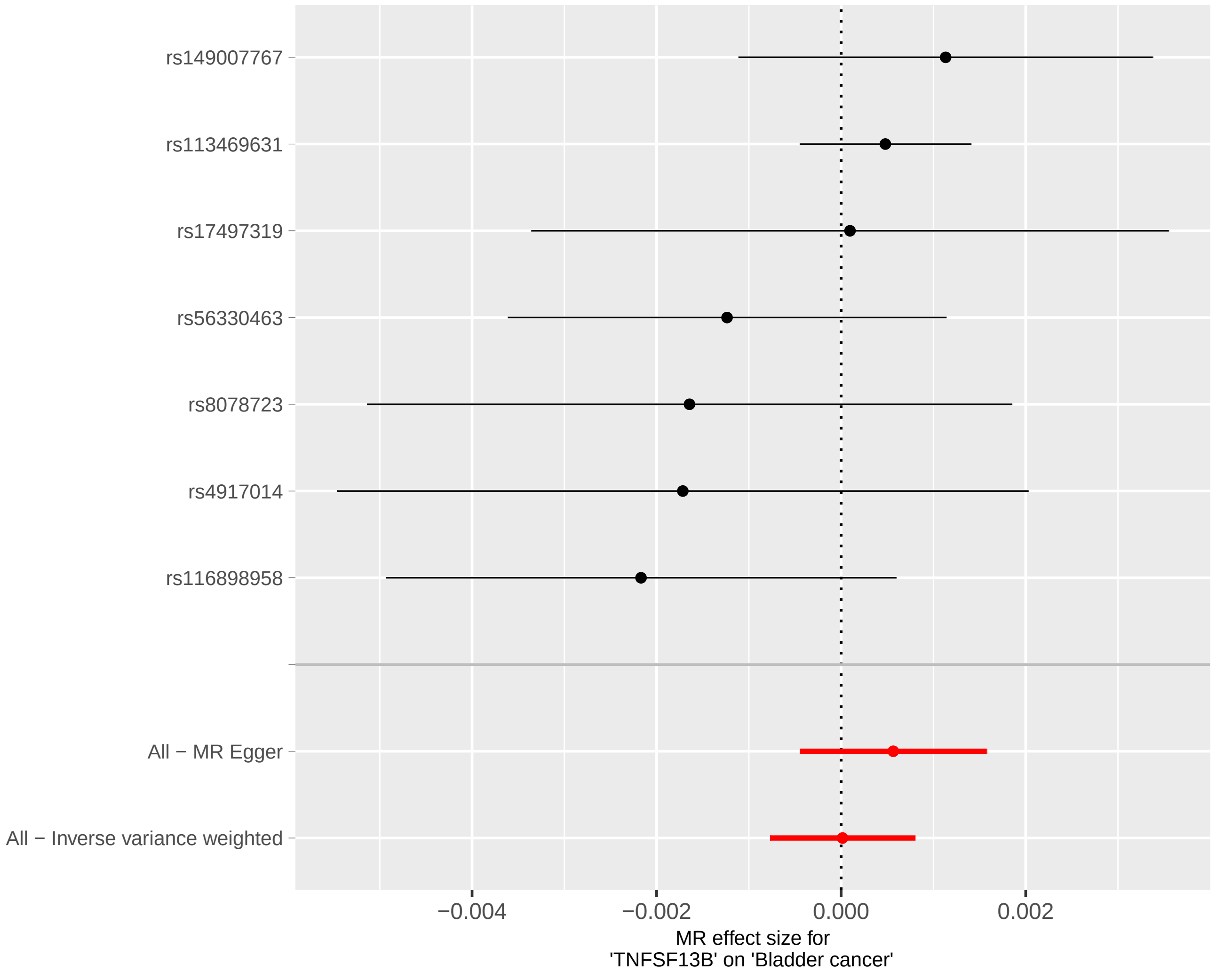

rs149007767
rs113469631
rs17497319
rs56330463
rs8078723
rs4917014
rs116898958
All − MR Egger
All − Inverse variance weighted
−0.004
−0.002
0.000
0.002
MR effect size for
'TNFSF13B' on 'Bladder cancer'

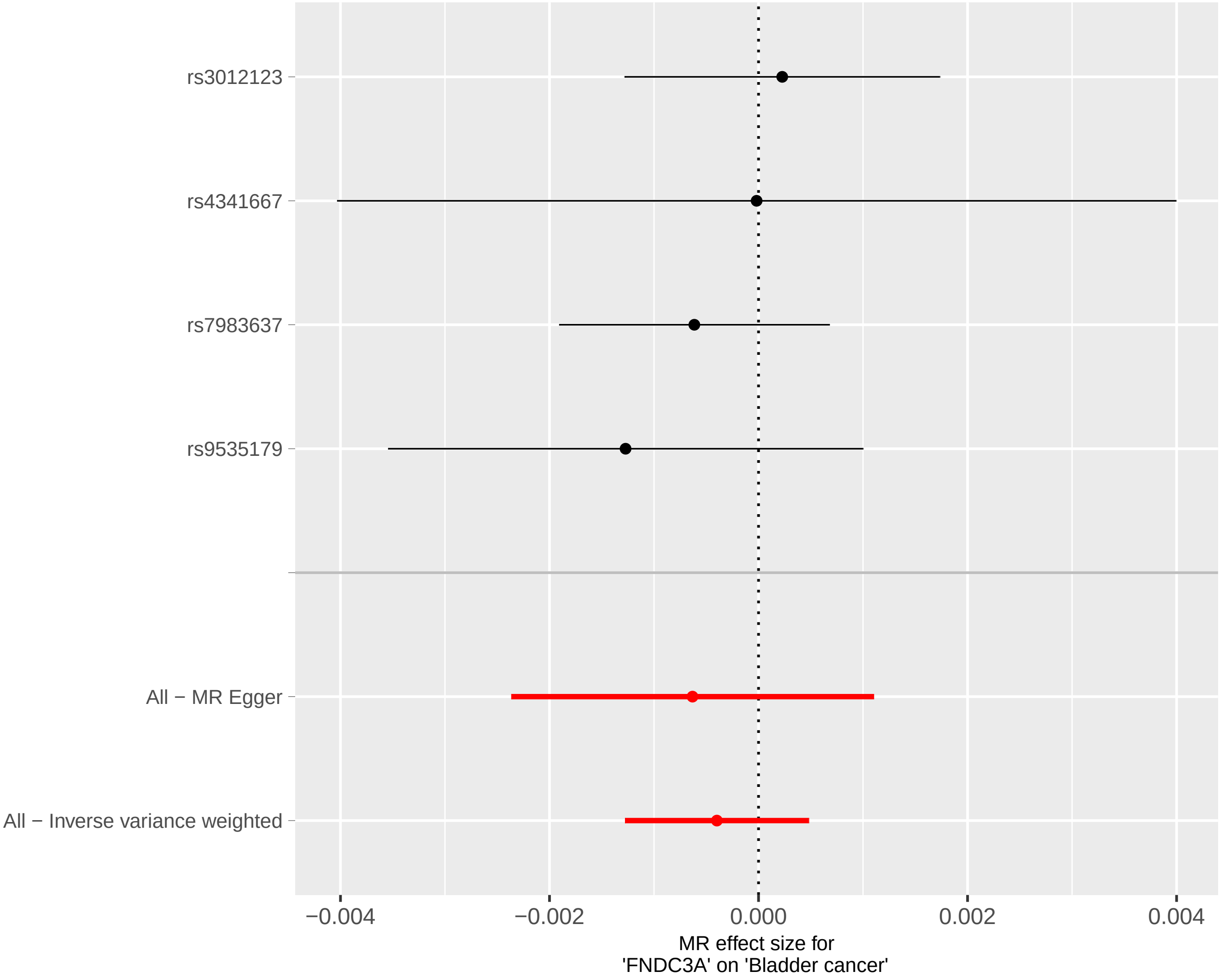

rs3012123
rs4341667
rs7983637
rs9535179
All − MR Egger
All − Inverse variance weighted
−0.004
−0.002
0.000
0.002
0.004
MR effect size for
'FNDC3A' on 'Bladder cancer'

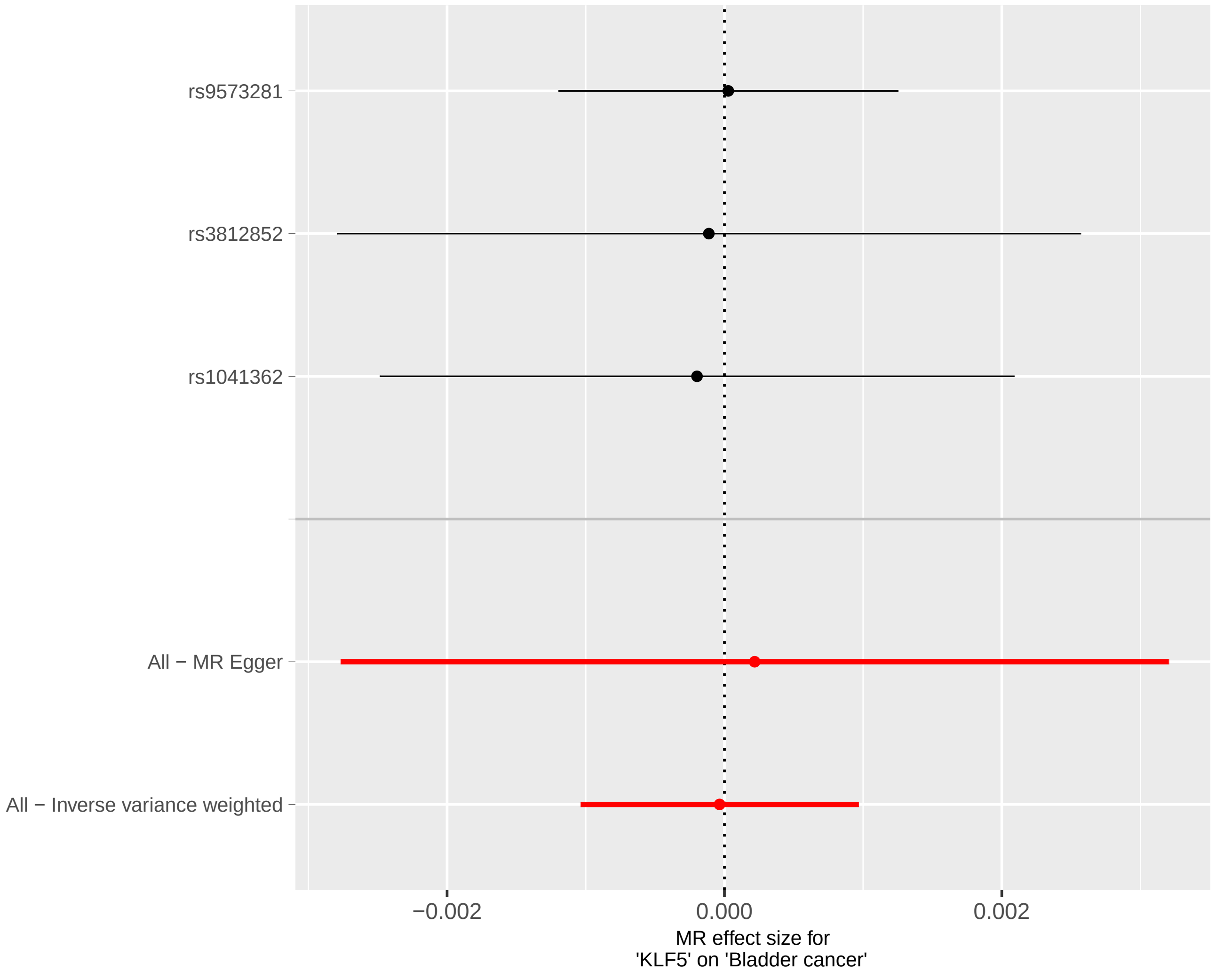

rs9573281
rs3812852
rs1041362
All − MR Egger
All − Inverse variance weighted
−0.002
0.000
0.002
MR effect size for
'KLF5' on 'Bladder cancer'

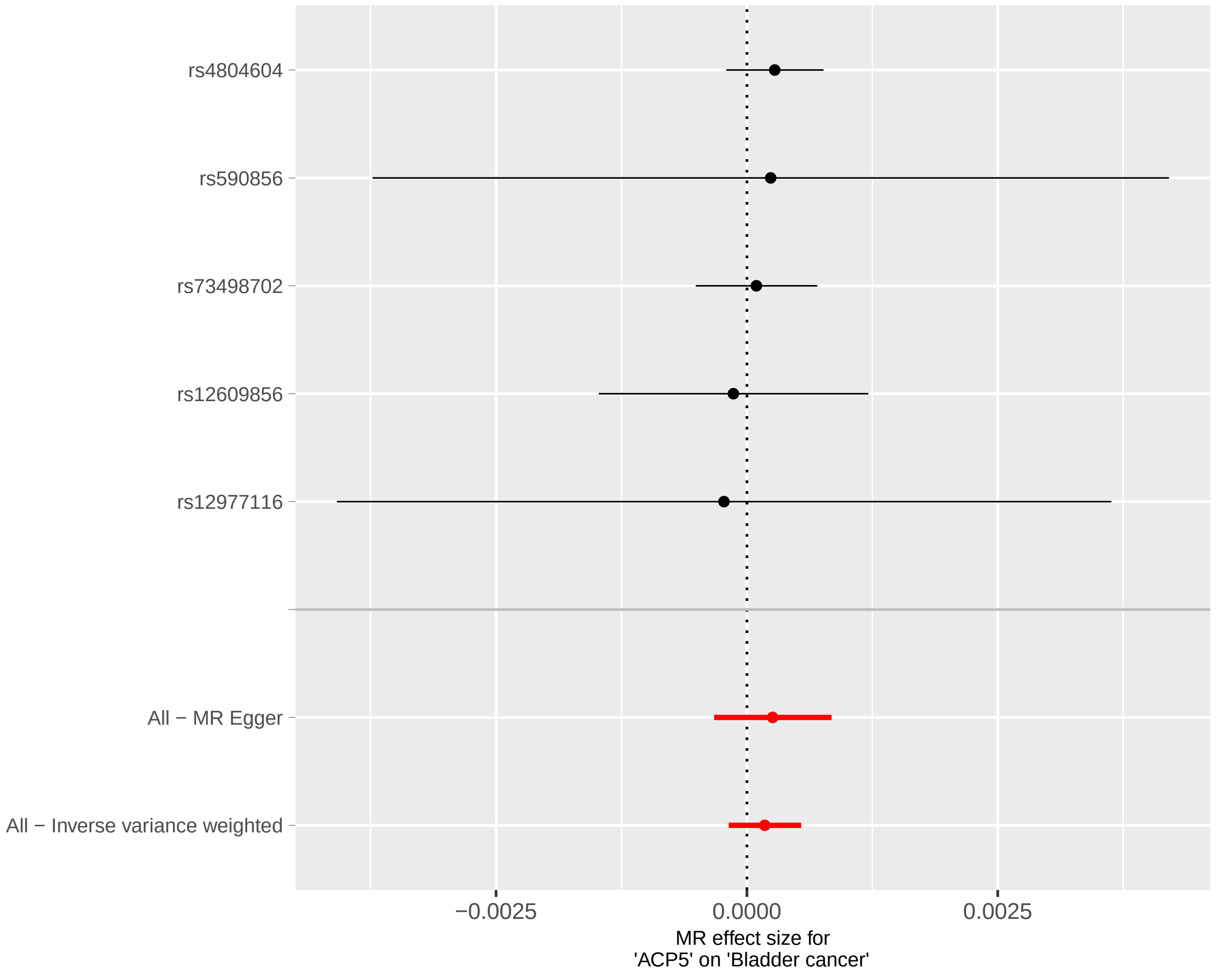
rs4804604
rs590856
rs73498702
rs12609856
rs12977116
All − MR Egger
All − Inverse variance weighted
−0.0025
0.0000
0.0025
MR effect size for
'ACP5' on 'Bladder cancer'

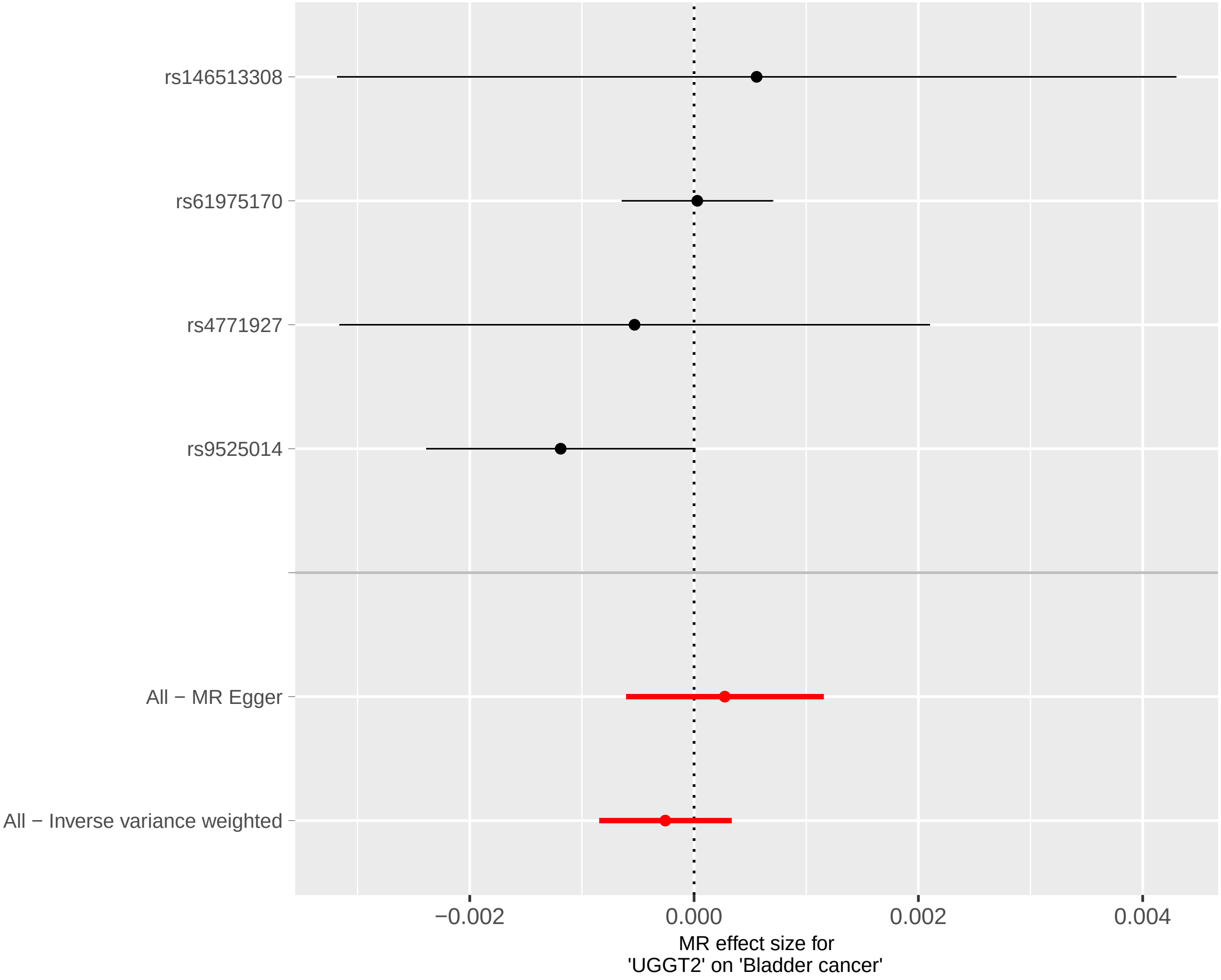

rs146513308
rs61975170
rs4771927
rs9525014
All − MR Egger
All − Inverse variance weighted
−0.002
0.000
0.002
0.004
MR effect size for
'UGGT2' on 'Bladder cancer'

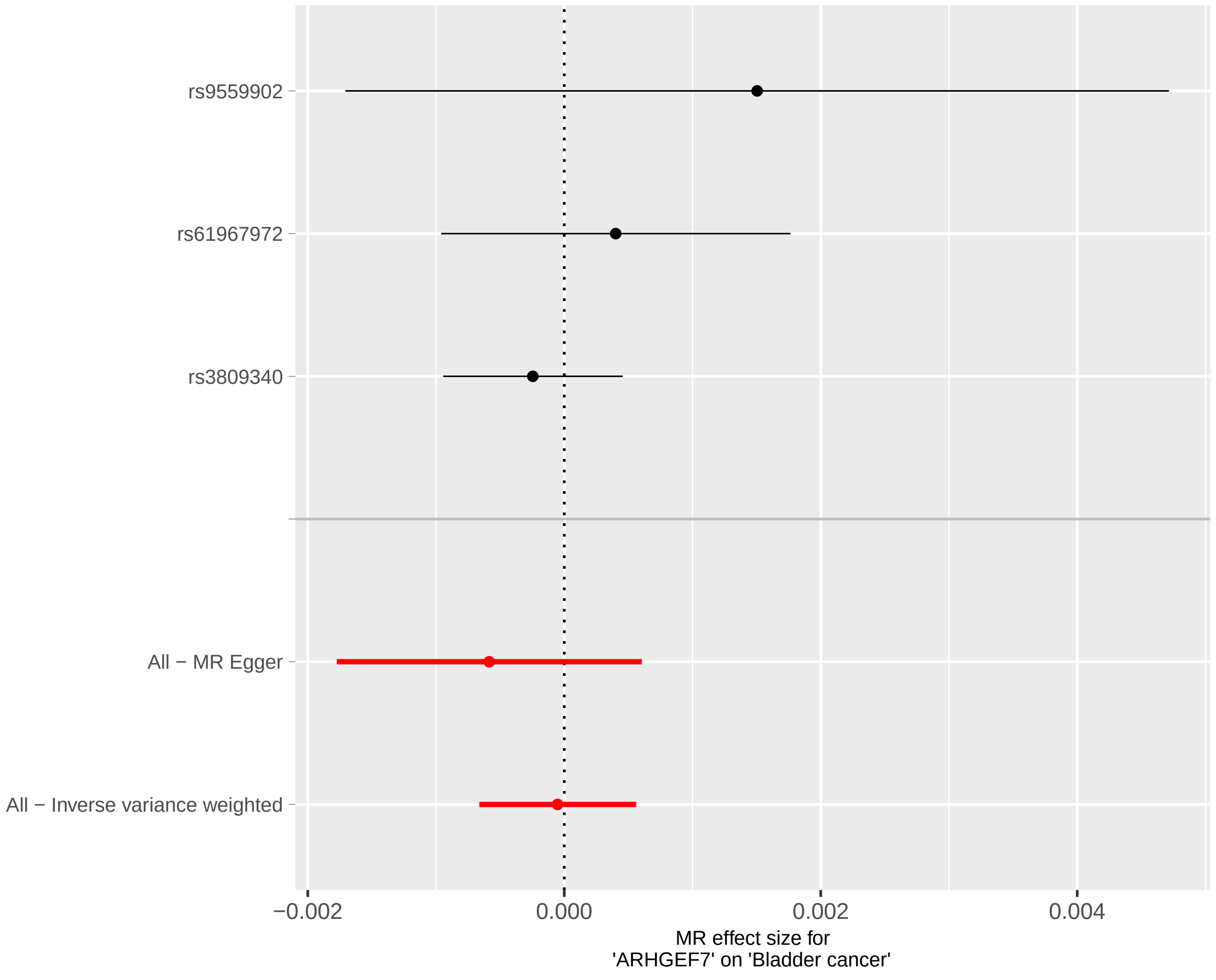

rs9559902
rs61967972
rs3809340
All − MR Egger
All − Inverse variance weighted
−0.002
0.000
0.002
0.004
MR effect size for
'ARHGEF7' on 'Bladder cancer'

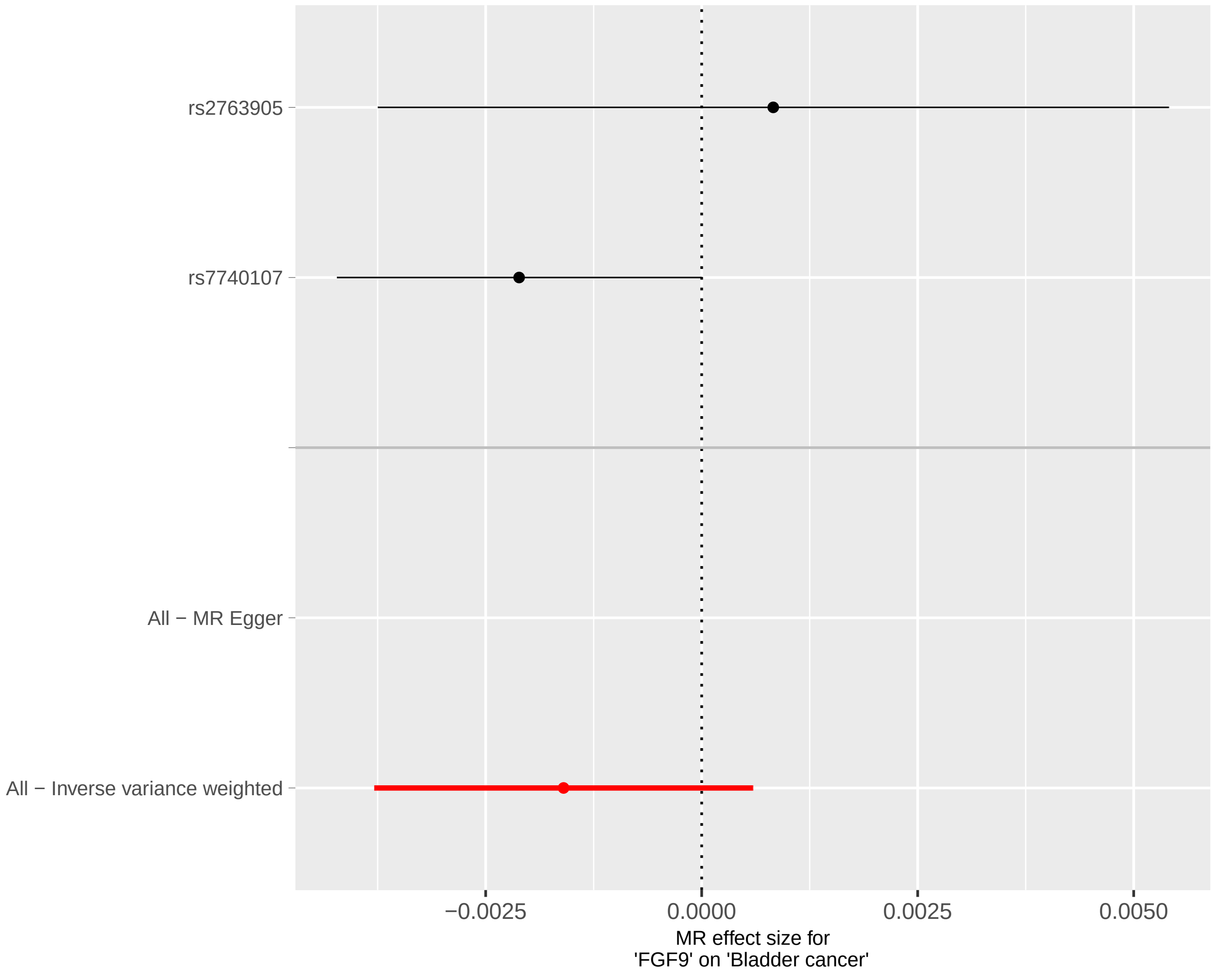

rs2763905
rs7740107
All − MR Egger
All − Inverse variance weighted
−0.0025
0.0000
0.0025
0.0050
MR effect size for
'FGF9' on 'Bladder cancer'

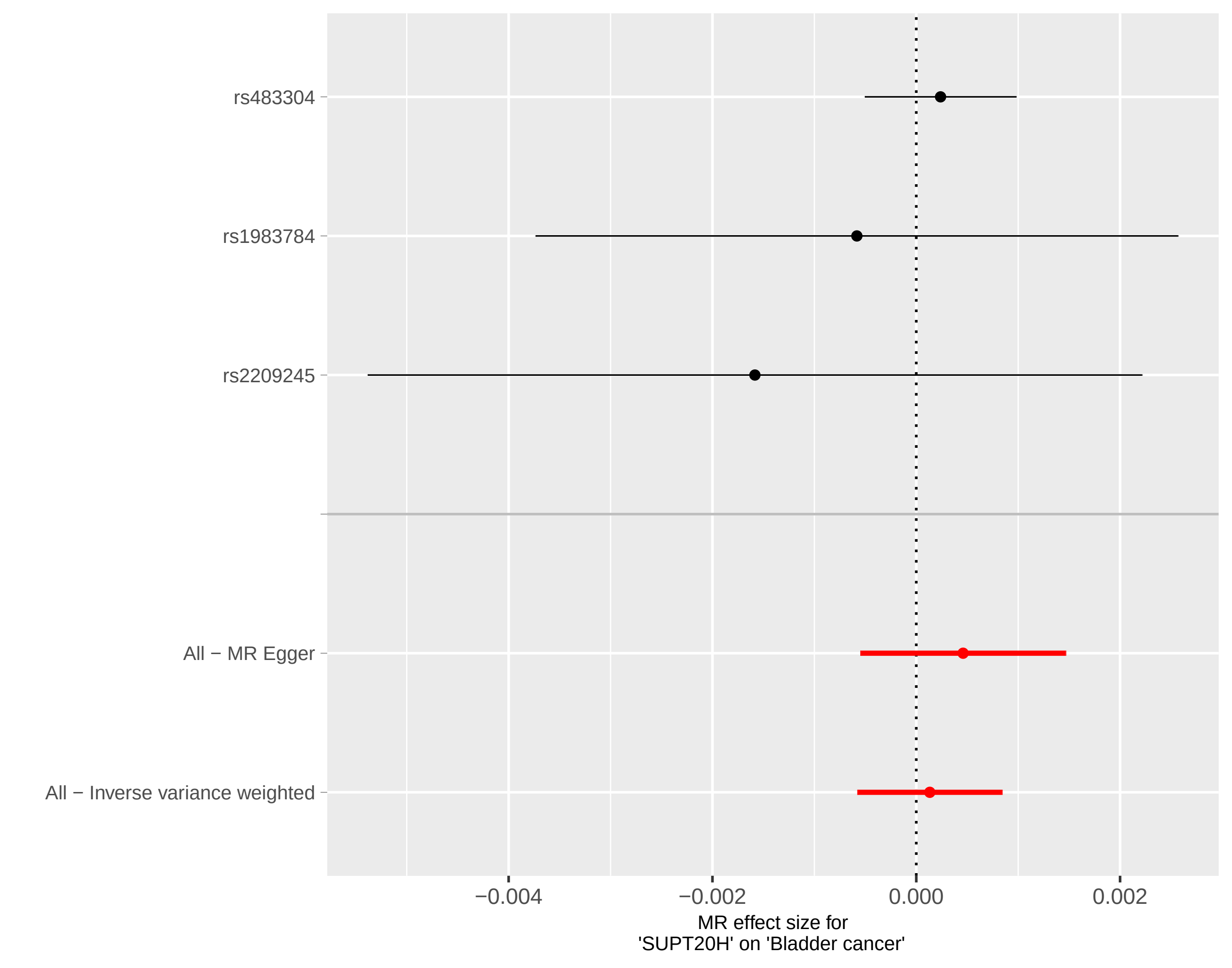
rs483304
rs1983784
rs2209245
All − MR Egger
All − Inverse variance weighted
−0.004
−0.002
0.000
0.002
MR effect size for
'SUPT20H' on 'Bladder cancer'

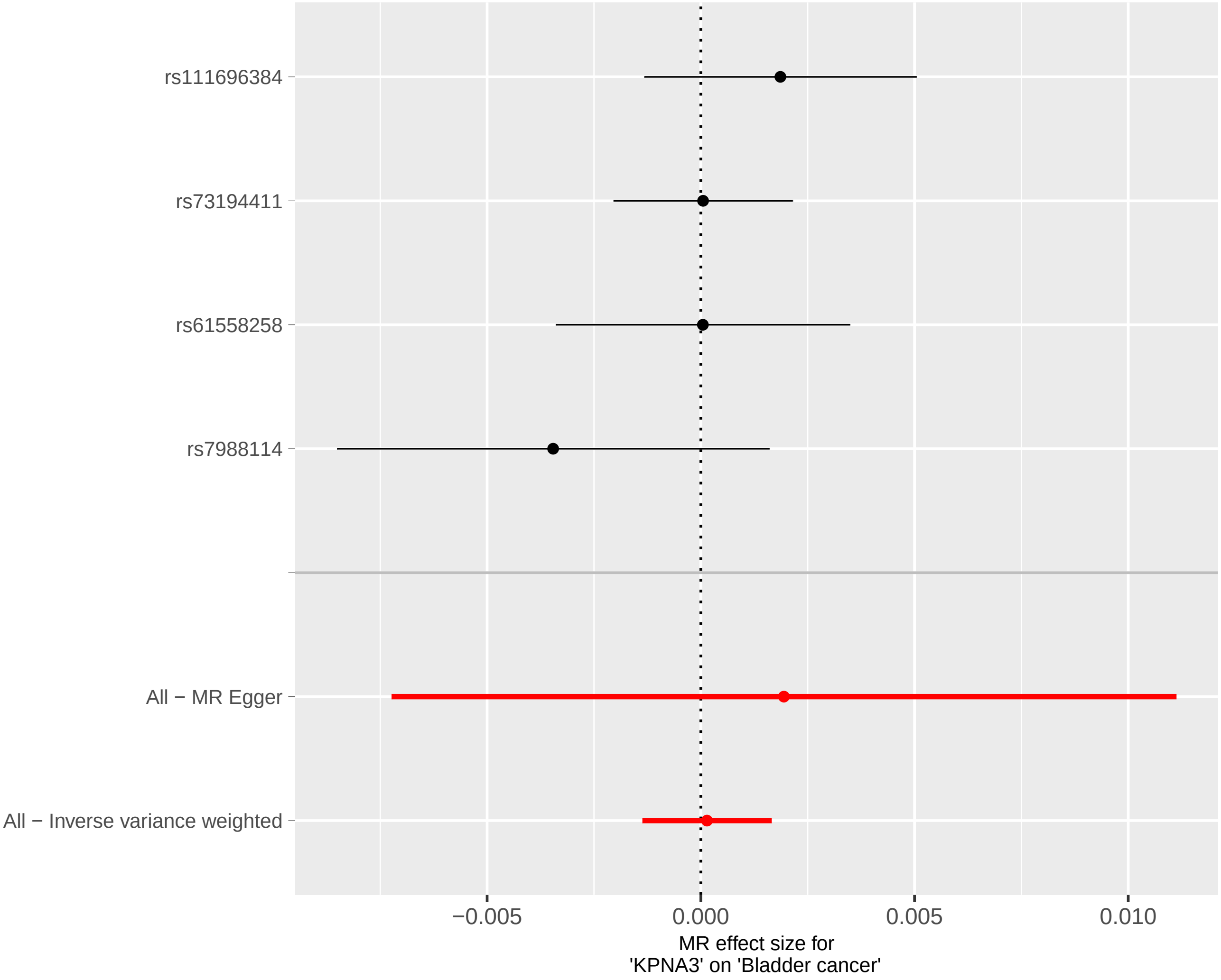

rs111696384
rs73194411
rs61558258
rs7988114
All − MR Egger
All − Inverse variance weighted
−0.005
0.000
0.005
0.010
MR effect size for
'KPNA3' on 'Bladder cancer'

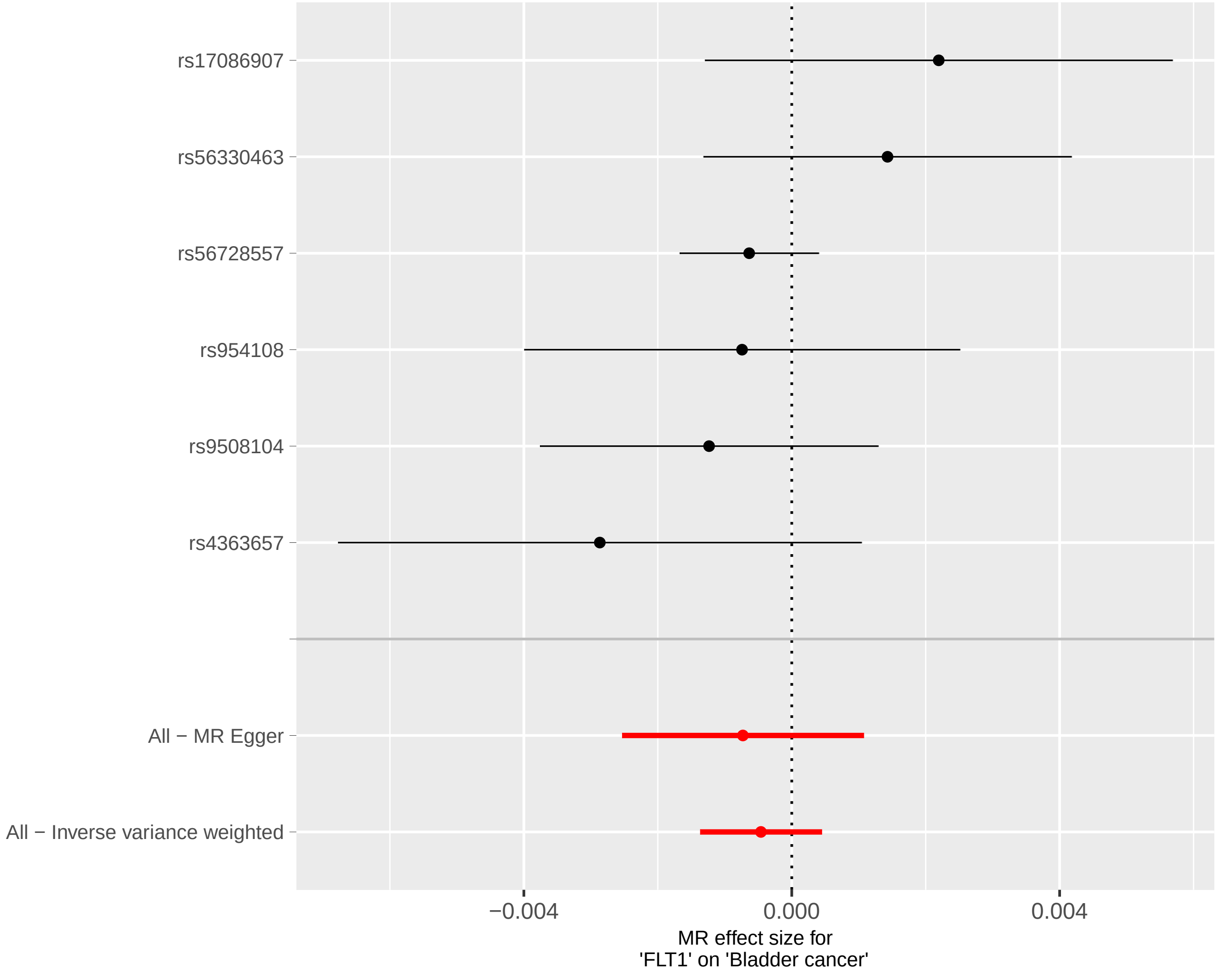
rs17086907
rs56330463
rs56728557
rs954108
rs9508104
rs4363657
All − MR Egger
All − Inverse variance weighted
−0.004
0.000
0.004
MR effect size for
'FLT1' on 'Bladder cancer'

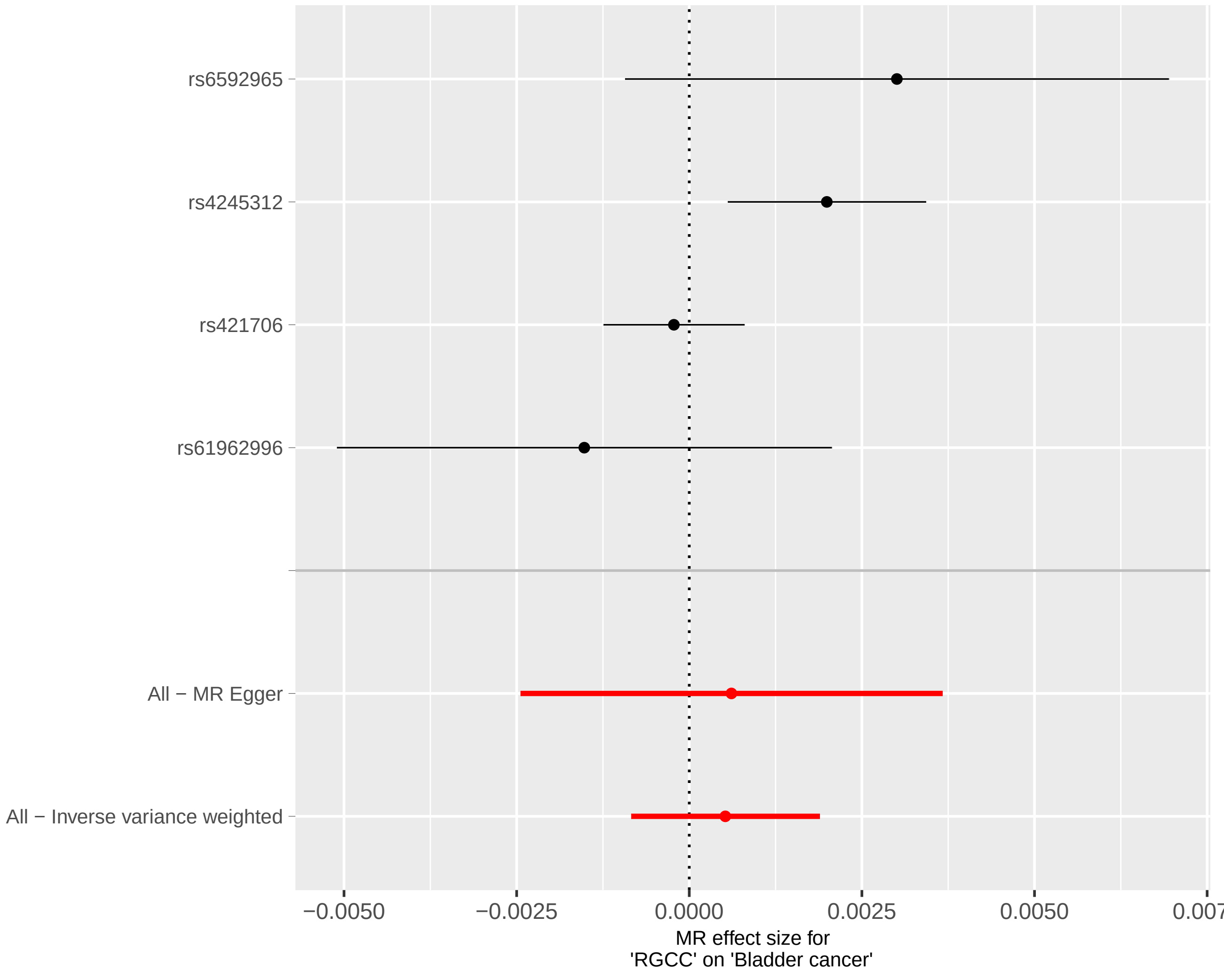
rs6592965
rs4245312
rs421706
rs61962996
All − MR Egger
All − Inverse variance weighted
−0.0050
−0.0025
0.0000
0.0025
0.0050
MR effect size for
'RGCC' on 'Bladder cancer'

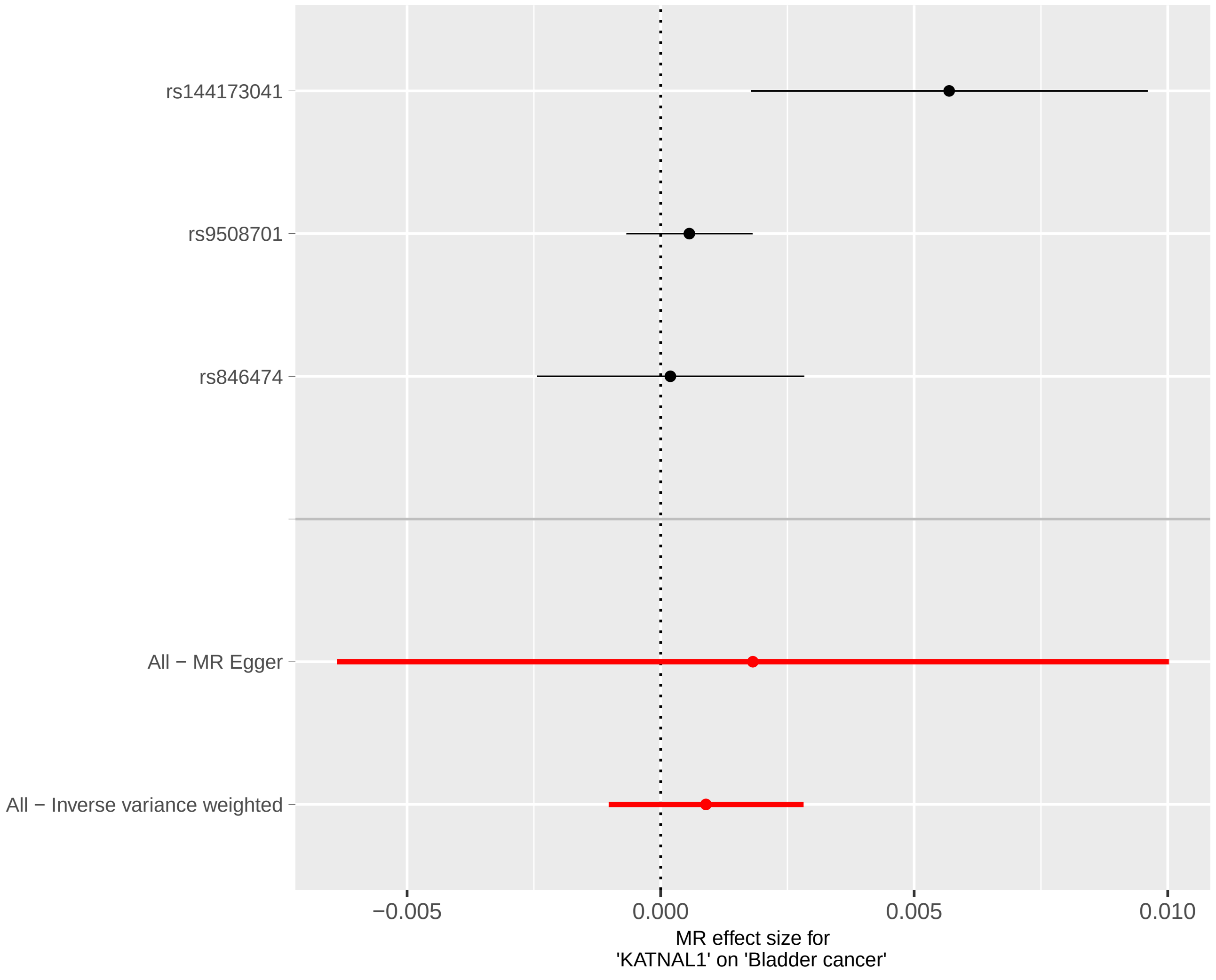
rs144173041
rs9508701
rs846474
All − MR Egger
All − Inverse variance weighted
−0.005
0.000
0.005
0.010
MR effect size for
'KATNAL1' on 'Bladder cancer'

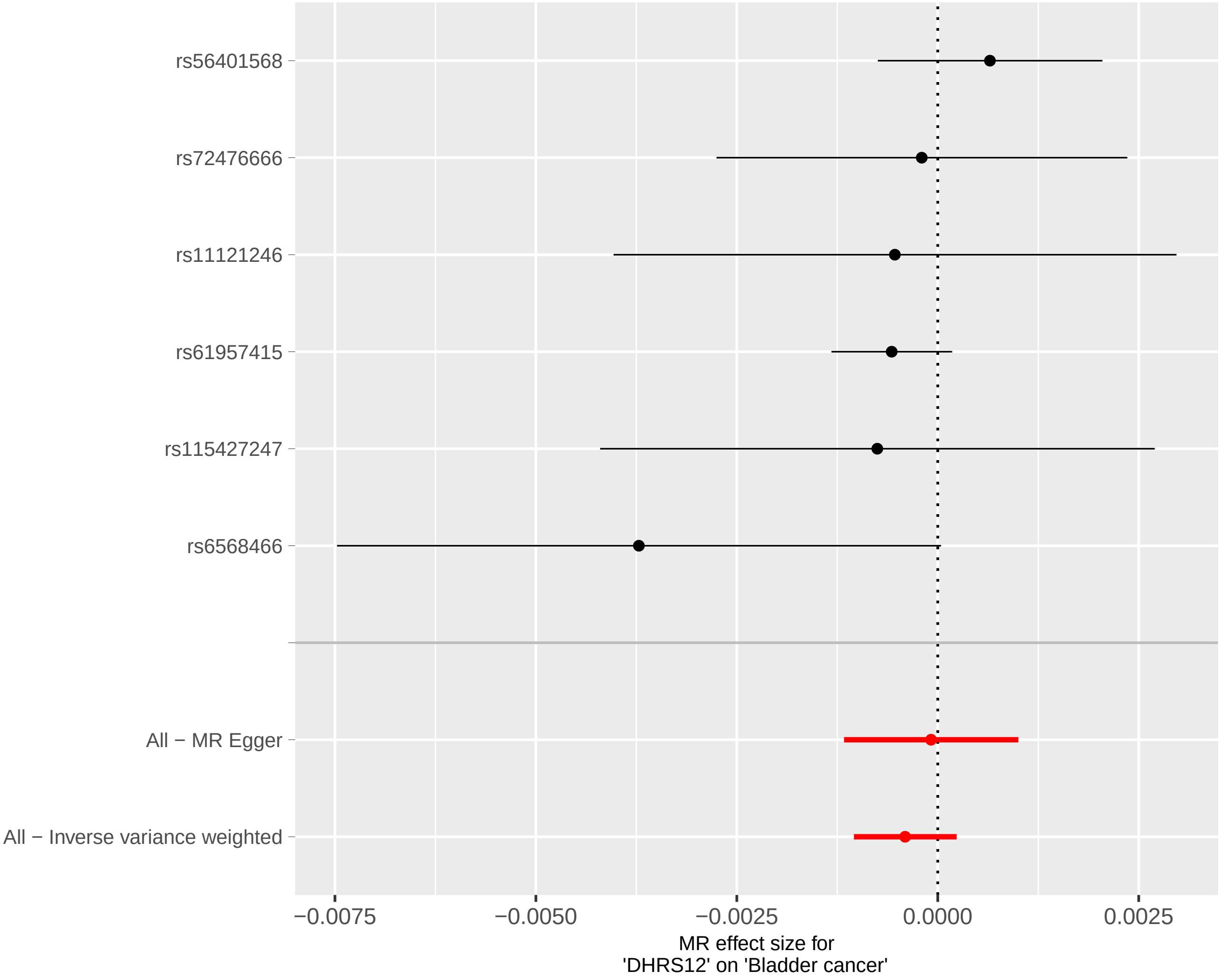

rs56401568
rs72476666
rs11121246
rs61957415
rs115427247
rs6568466
All − MR Egger
All − Inverse variance weighted
−0.0075
−0.0050
−0.0025
0.0000
0.0025
MR effect size for
'DHRS12' on 'Bladder cancer'

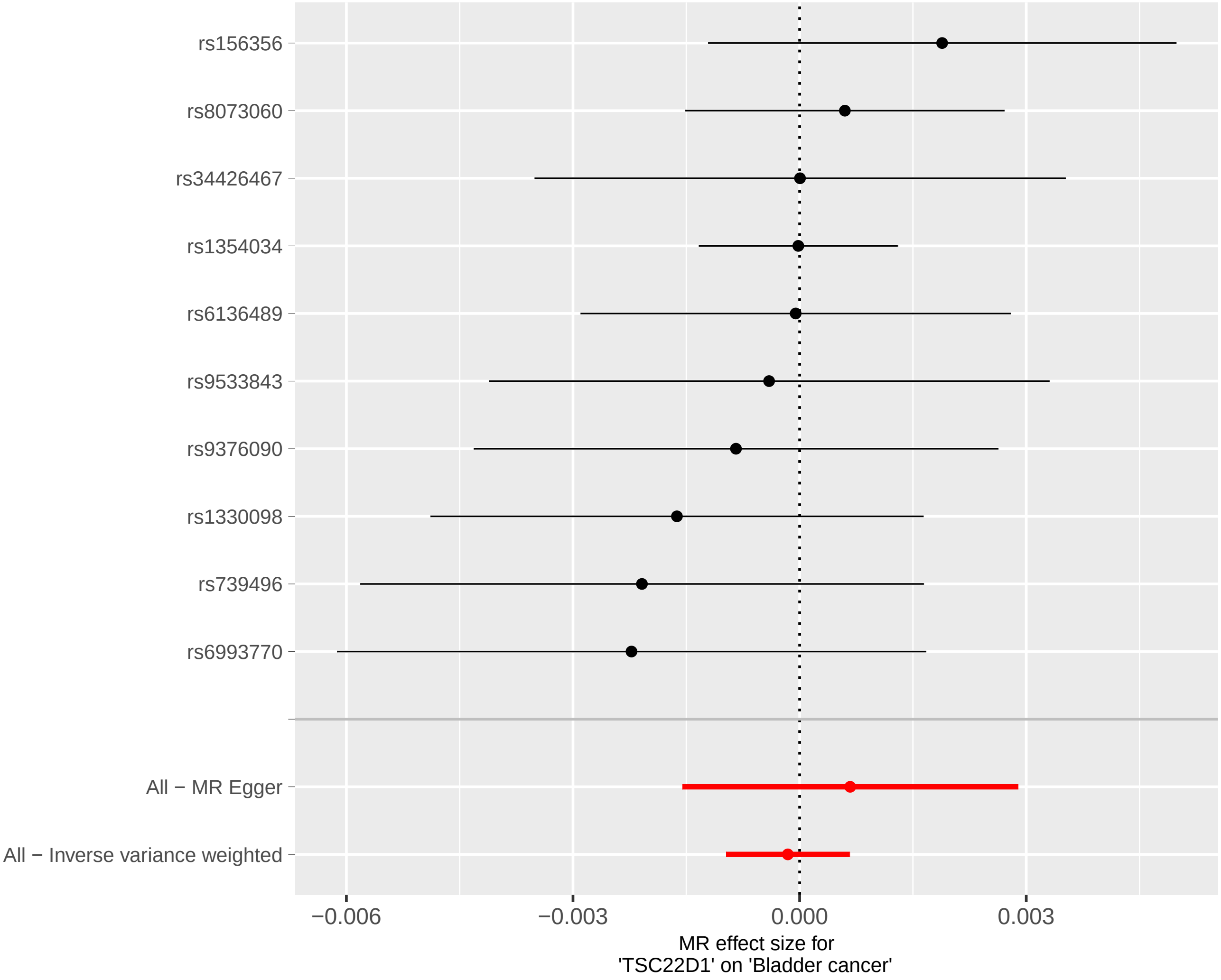
rs156356
rs8073060
rs34426467
rs1354034
rs6136489
rs9533843
rs9376090
rs1330098
rs739496
rs6993770
All − MR Egger
All − Inverse variance weighted
−0.006
−0.003
0.000
0.003
MR effect size for
'TSC22D1' on 'Bladder cancer'

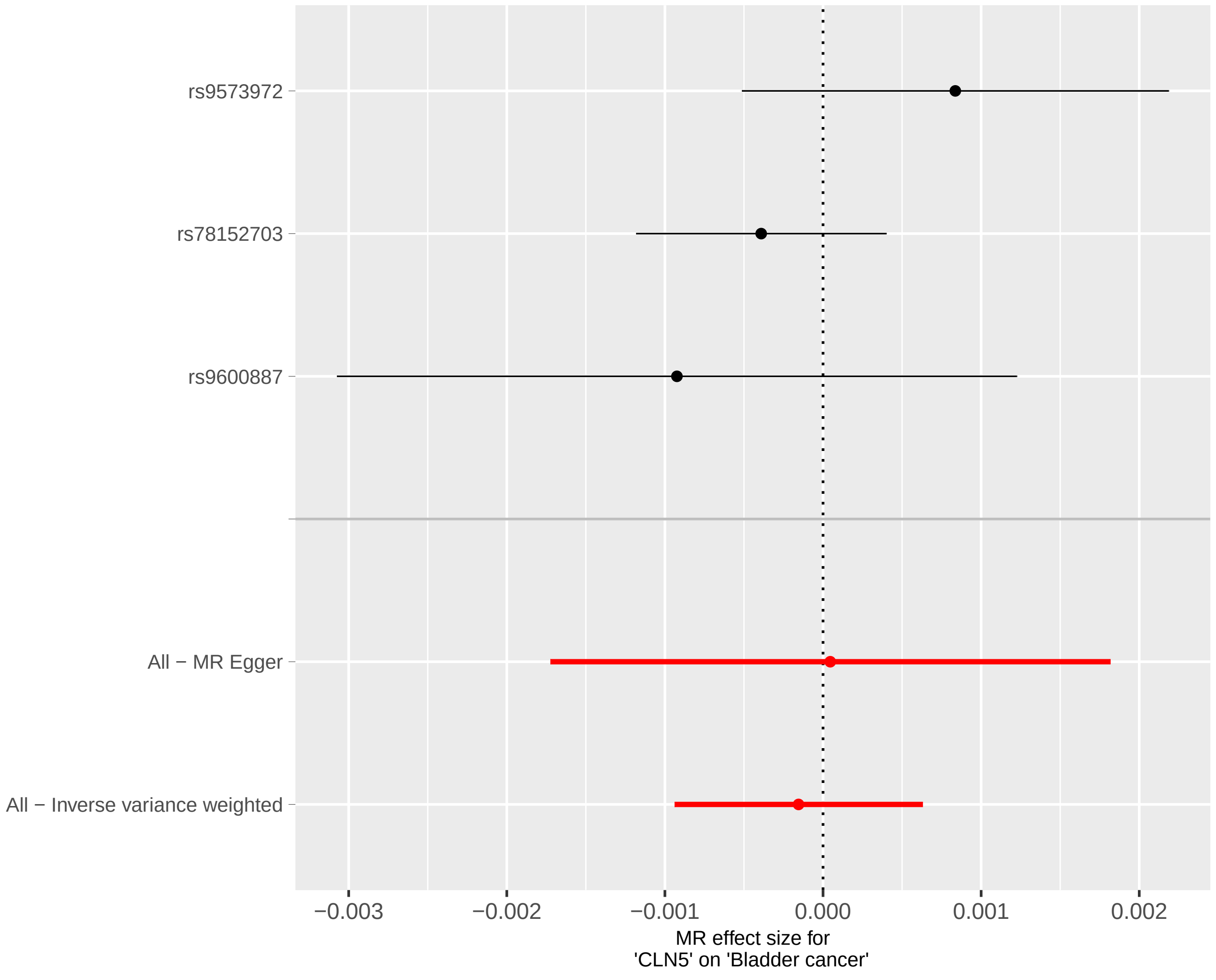
rs9573972
rs78152703
rs9600887
All − MR Egger
All − Inverse variance weighted
−0.003
−0.002
−0.001
0.000
0.001
0.002
MR effect size for
'CLN5' on 'Bladder cancer'

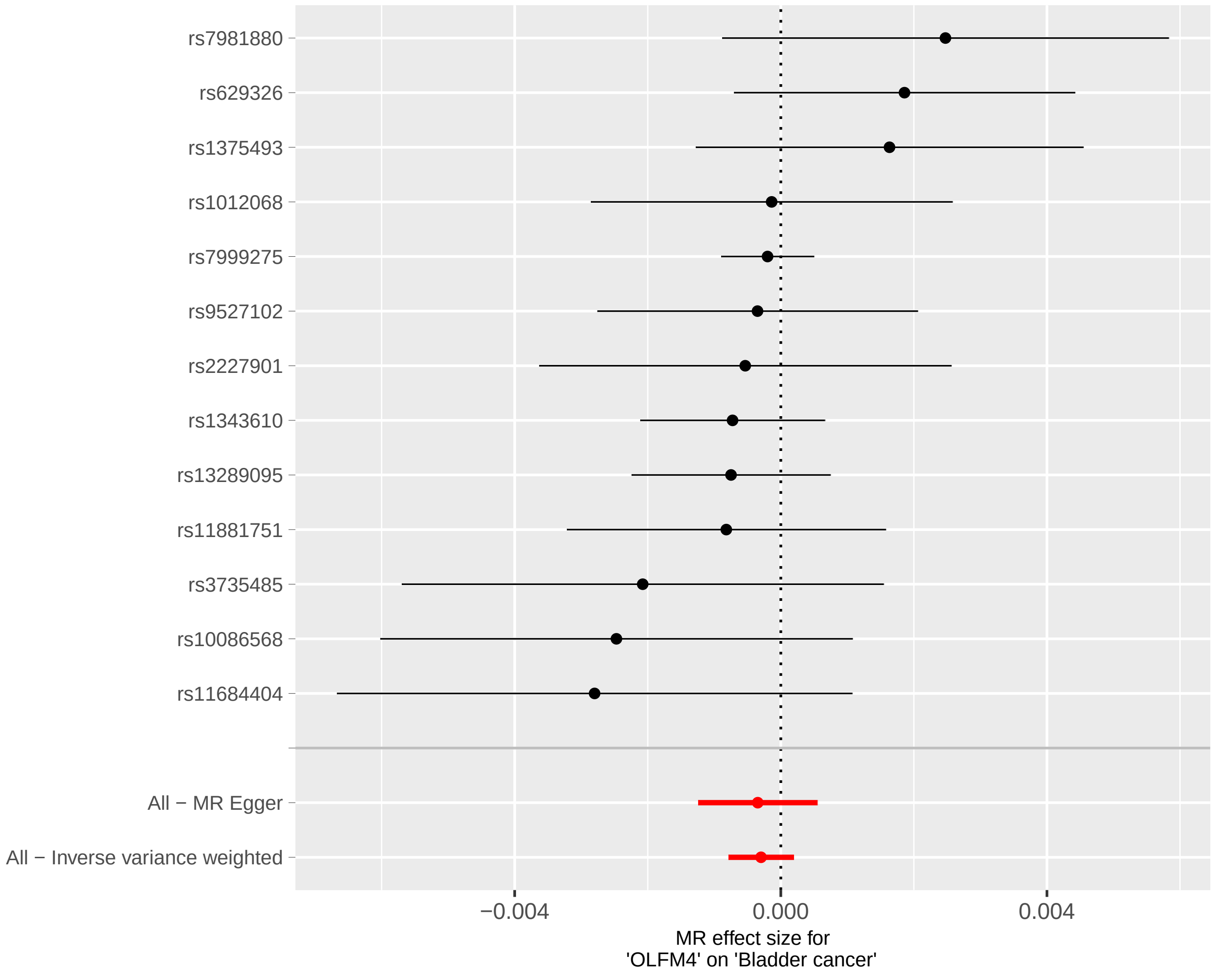

rs7981880
rs629326
rs1375493
rs1012068
rs7999275
rs9527102
rs2227901
rs1343610
rs13289095
rs11881751
rs3735485
rs10086568
rs11684404
All − MR Egger
All − Inverse variance weighted
−0.004
0.000
0.004
MR effect size for
'OLFM4' on 'Bladder cancer'

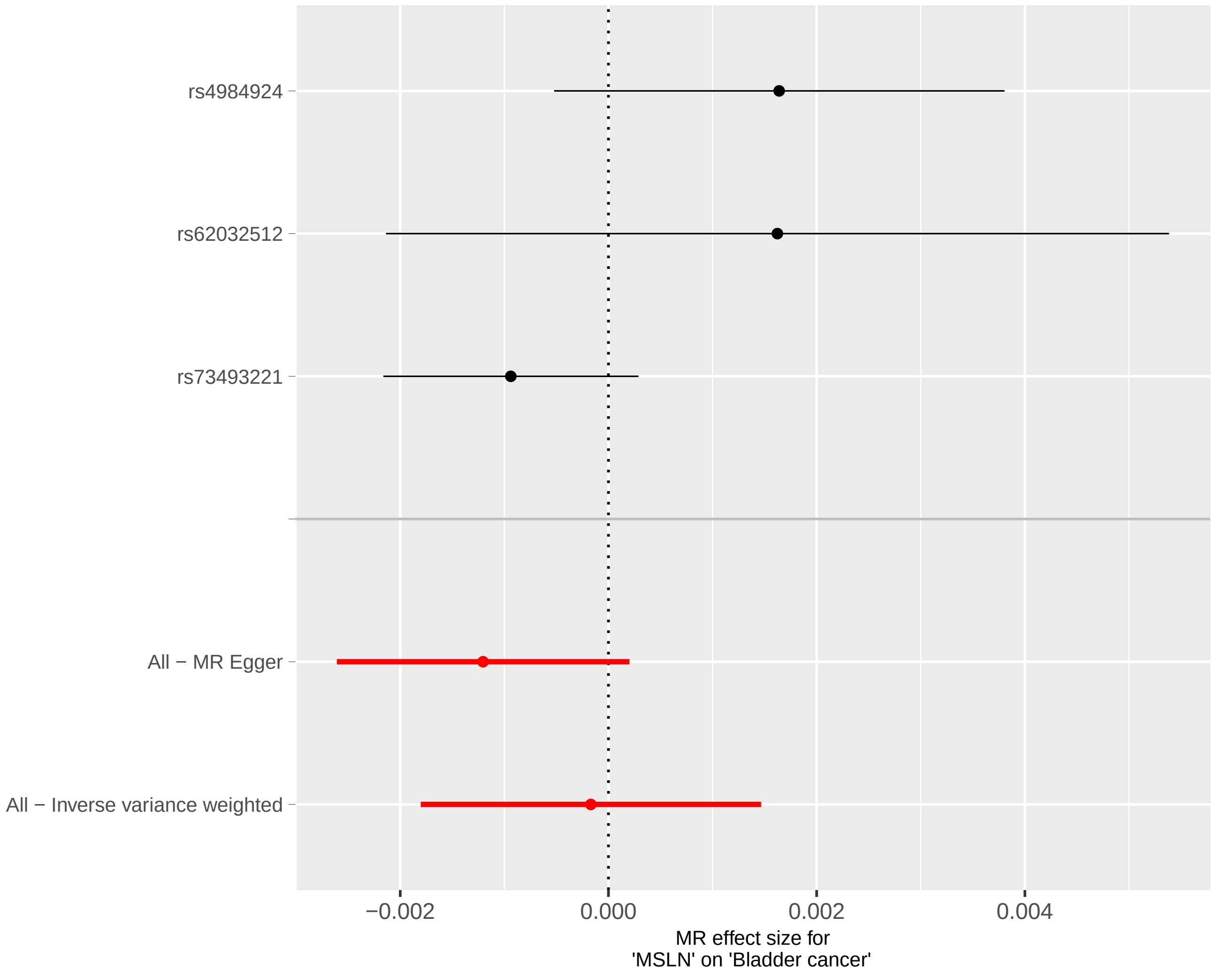

rs4984924
rs62032512
rs73493221
All − MR Egger
All − Inverse variance weighted
−0.002
0.000
0.002
0.004
MR effect size for
'MSLN' on 'Bladder cancer'

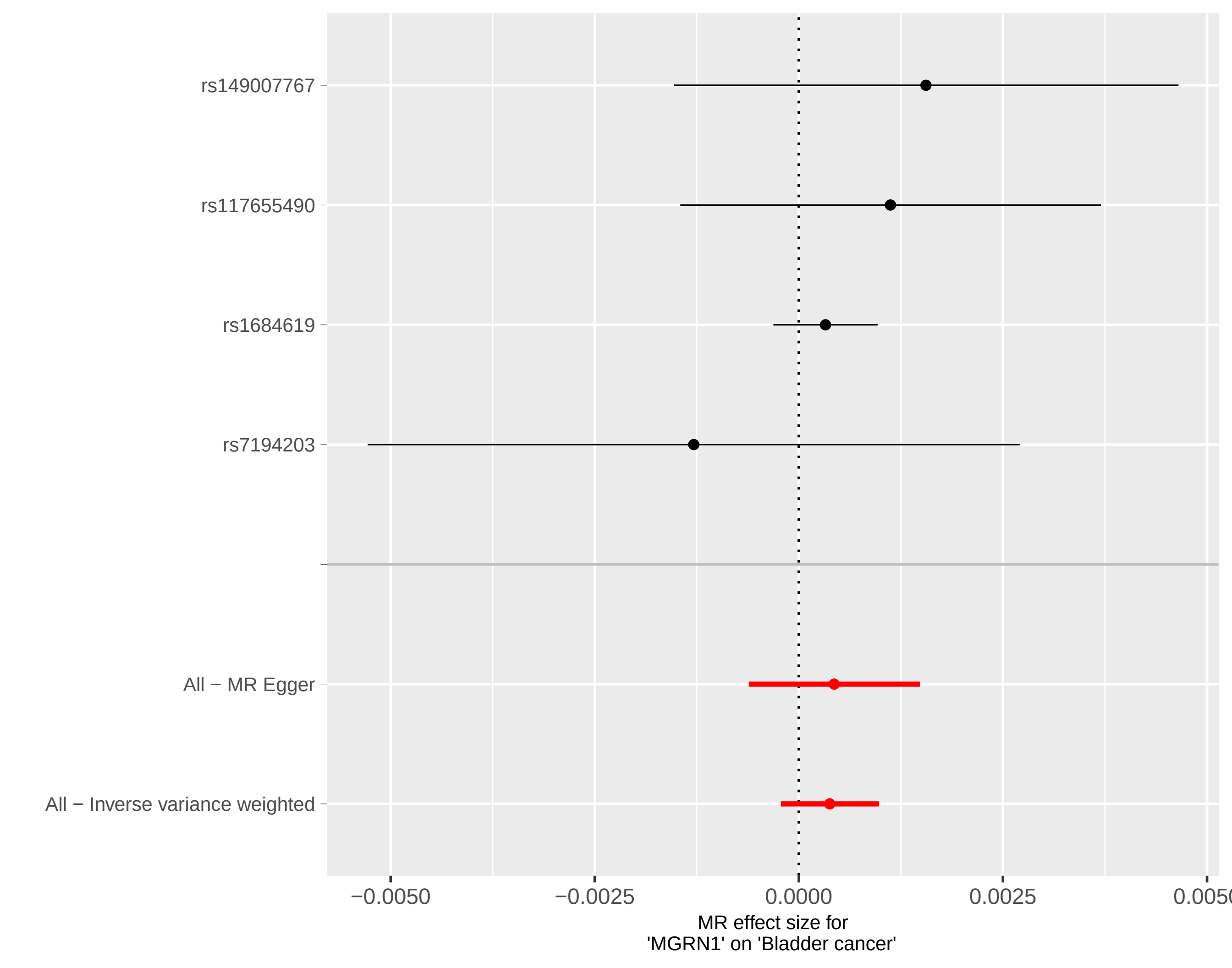

rs149007767
rs117655490
rs1684619
rs7194203
All − MR Egger
All − Inverse variance weighted
−0.0050
−0.0025
0.0000
0.0025
0.0050
MR effect size for
'MGRN1' on 'Bladder cancer'

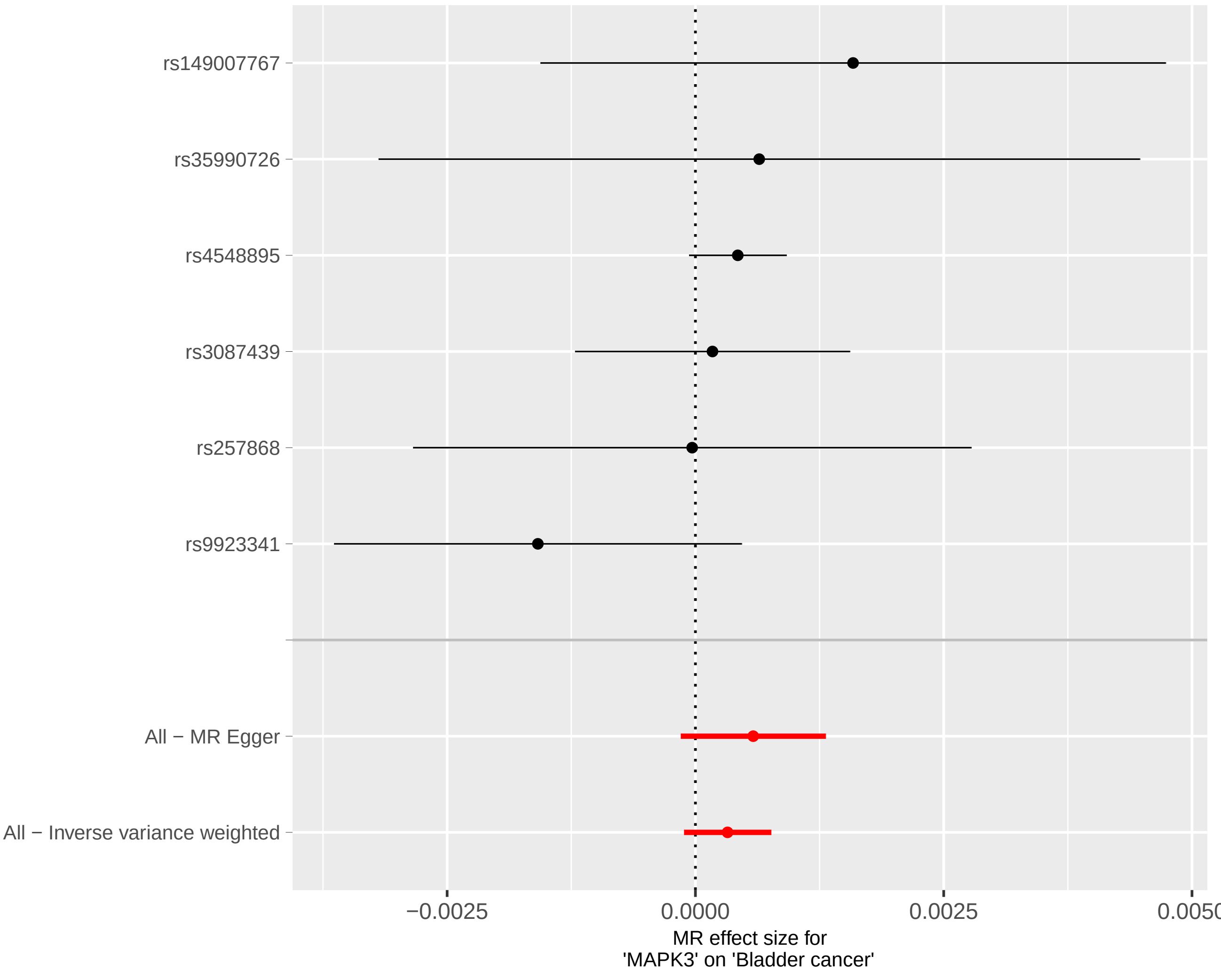

rs149007767
rs35990726
rs4548895
rs3087439
rs257868
rs9923341
All − MR Egger
All − Inverse variance weighted
−0.0025
0.0000
0.0025
0.0050
MR effect size for
'MAPK3' on 'Bladder cancer'

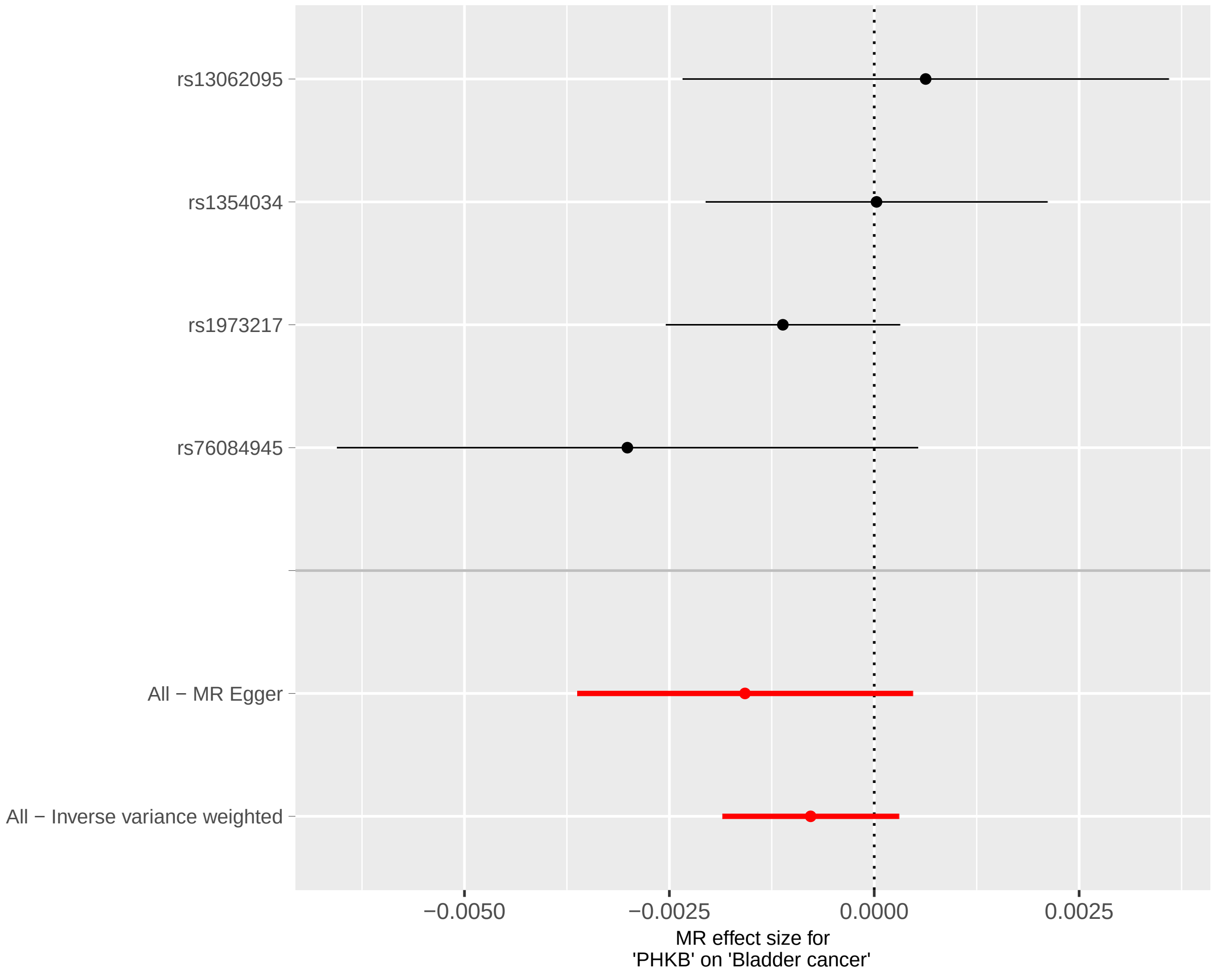
rs13062095
rs1354034
rs1973217
rs76084945
All − MR Egger
All − Inverse variance weighted
−0.0050
−0.0025
0.0000
0.0025
MR effect size for
'PHKB' on 'Bladder cancer'

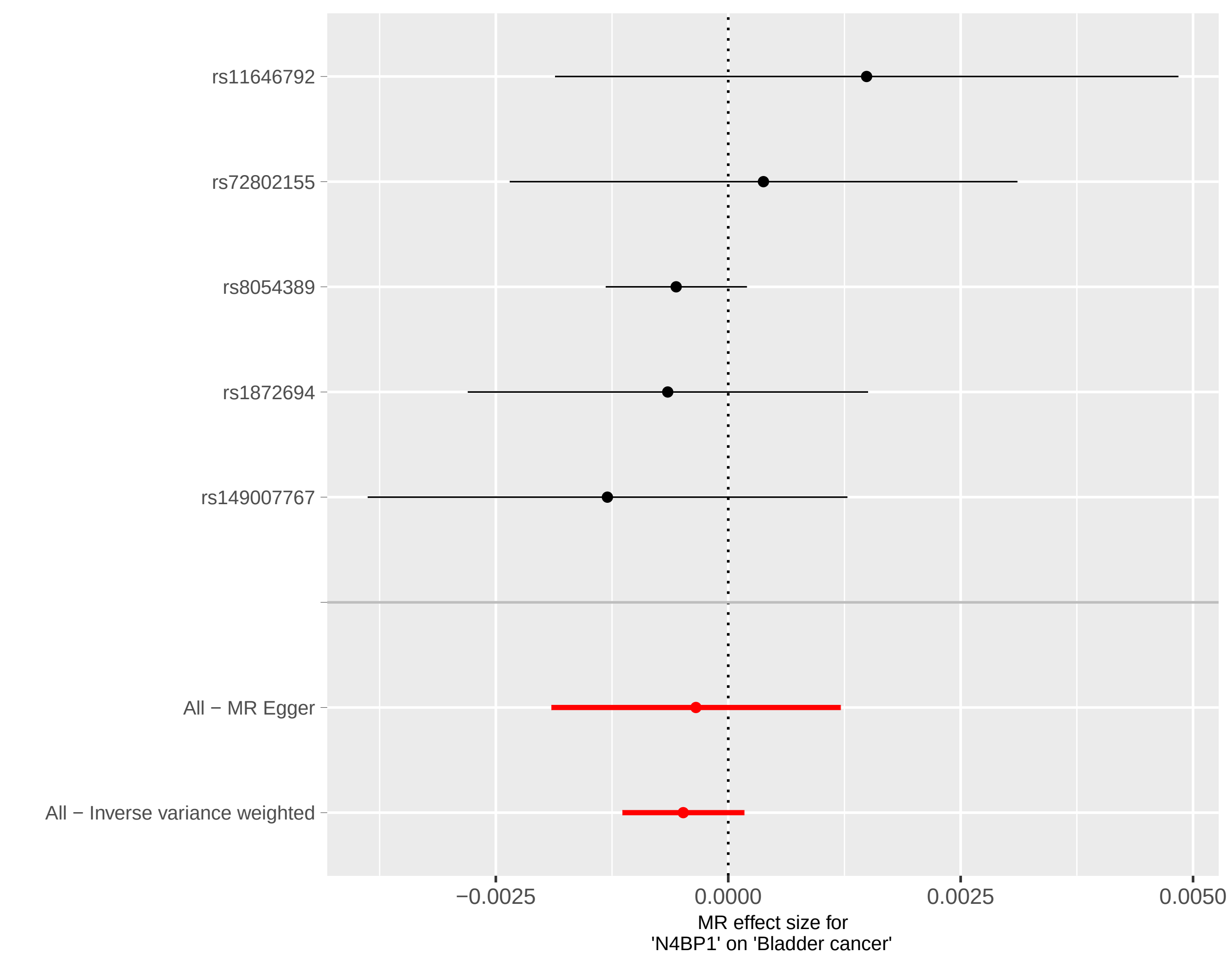

rs11646792
rs72802155
rs8054389
rs1872694
rs149007767
All − MR Egger
All − Inverse variance weighted
−0.0025
0.0000
0.0025
0.0050
MR effect size for
'N4BP1' on 'Bladder cancer'

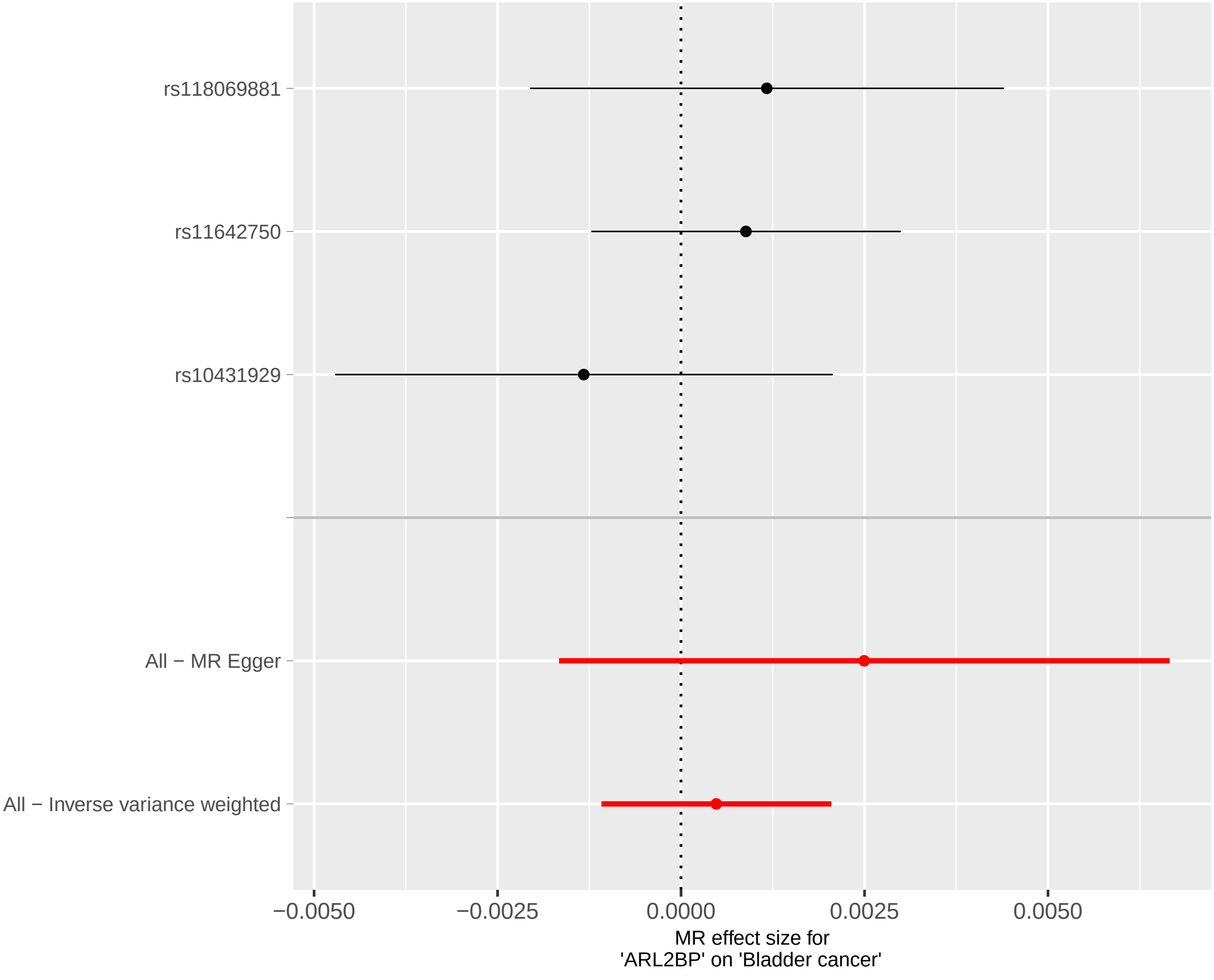
rs118069881
rs11642750
rs10431929
All − MR Egger
All − Inverse variance weighted
−0.0050
−0.0025
0.0000
0.0025
0.0050
MR effect size for
'ARL2BP' on 'Bladder cancer'

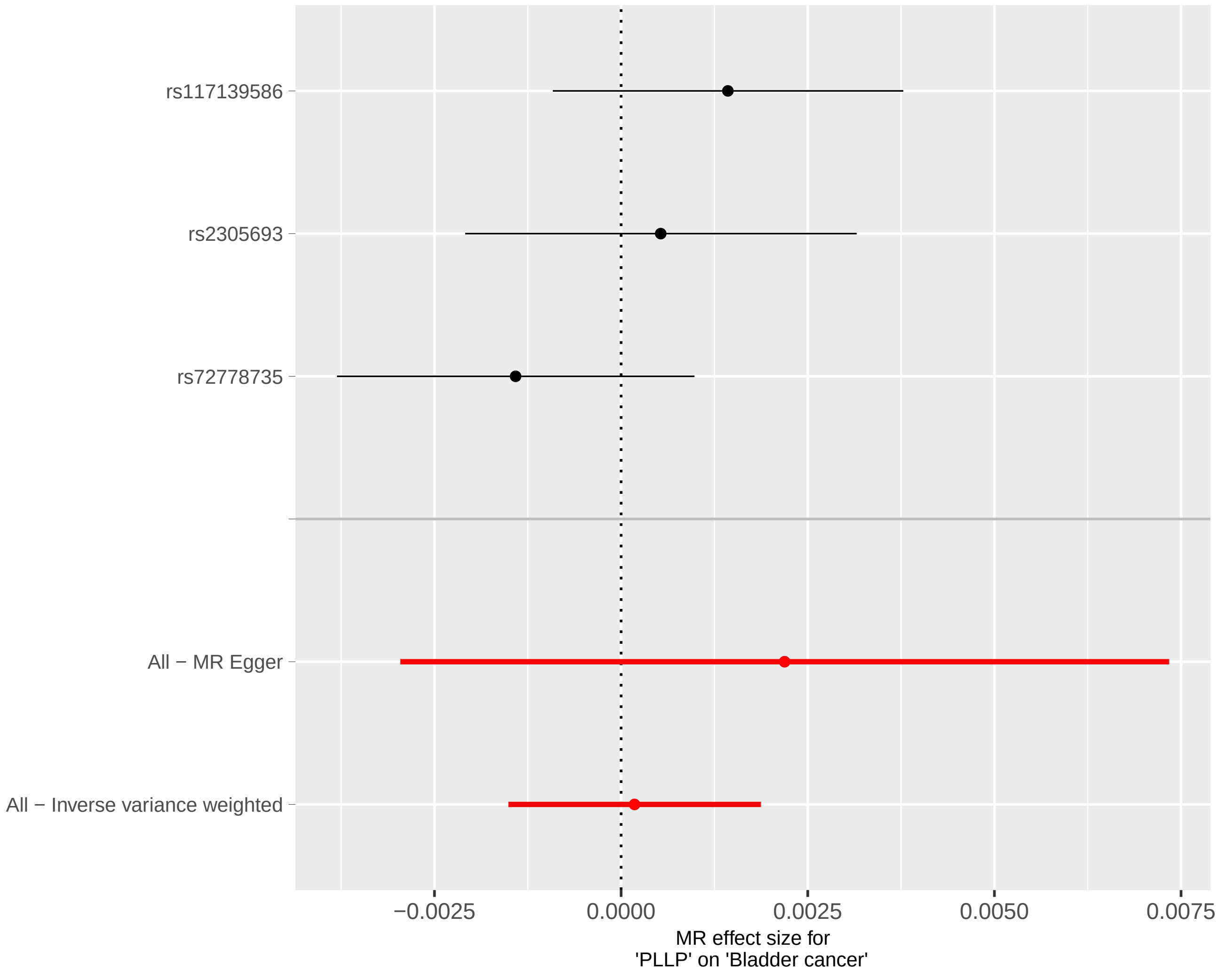
rs117139586
rs2305693
rs72778735
All − MR Egger
All − Inverse variance weighted
−0.0025
0.0000
0.0025
0.0050
0.0075
MR effect size for
'PLLP' on 'Bladder cancer'

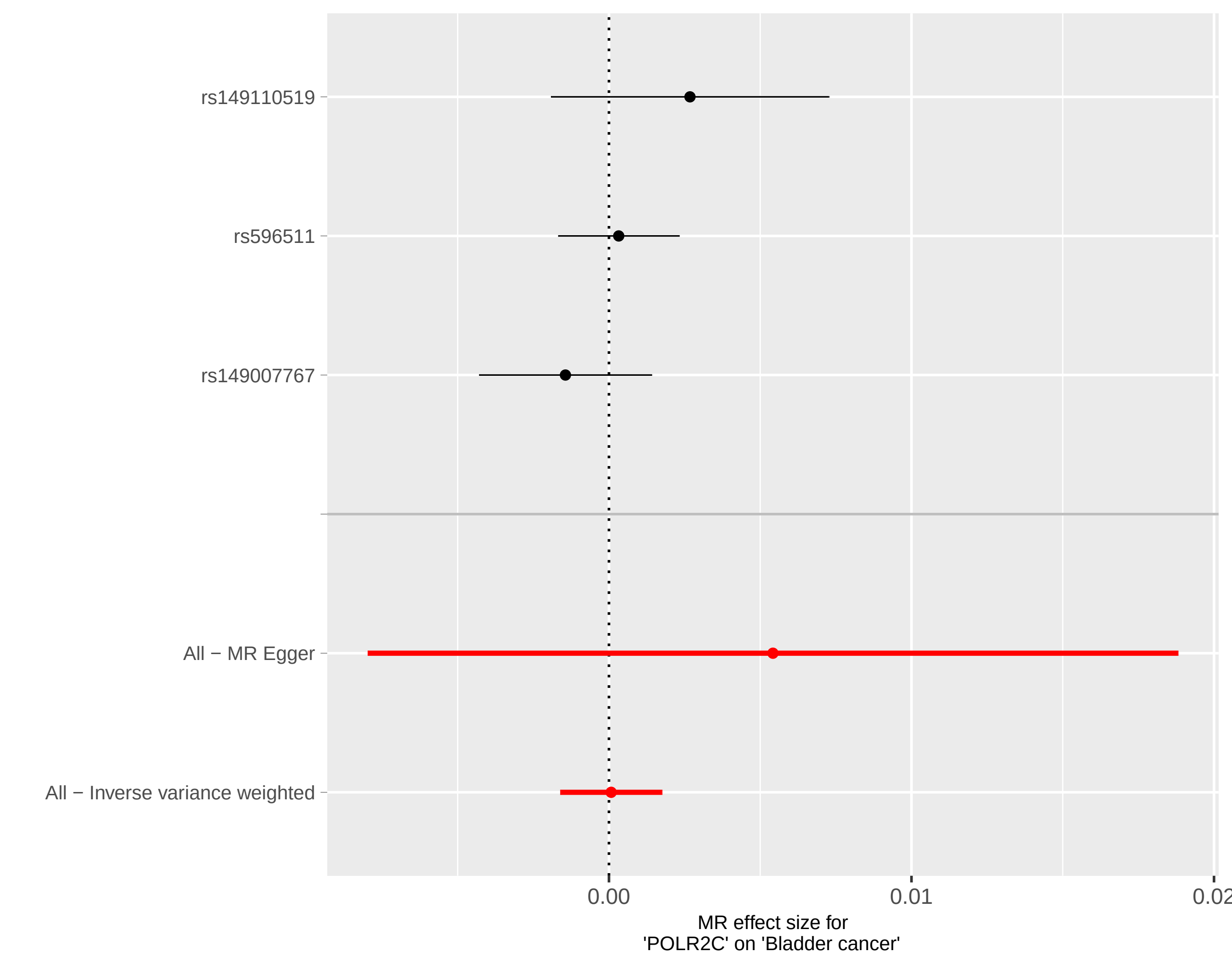

rs149110519
rs596511
rs149007767
All − MR Egger
All − Inverse variance weighted
0.00
0.01
0.02
MR effect size for
'POLR2C' on 'Bladder cancer'

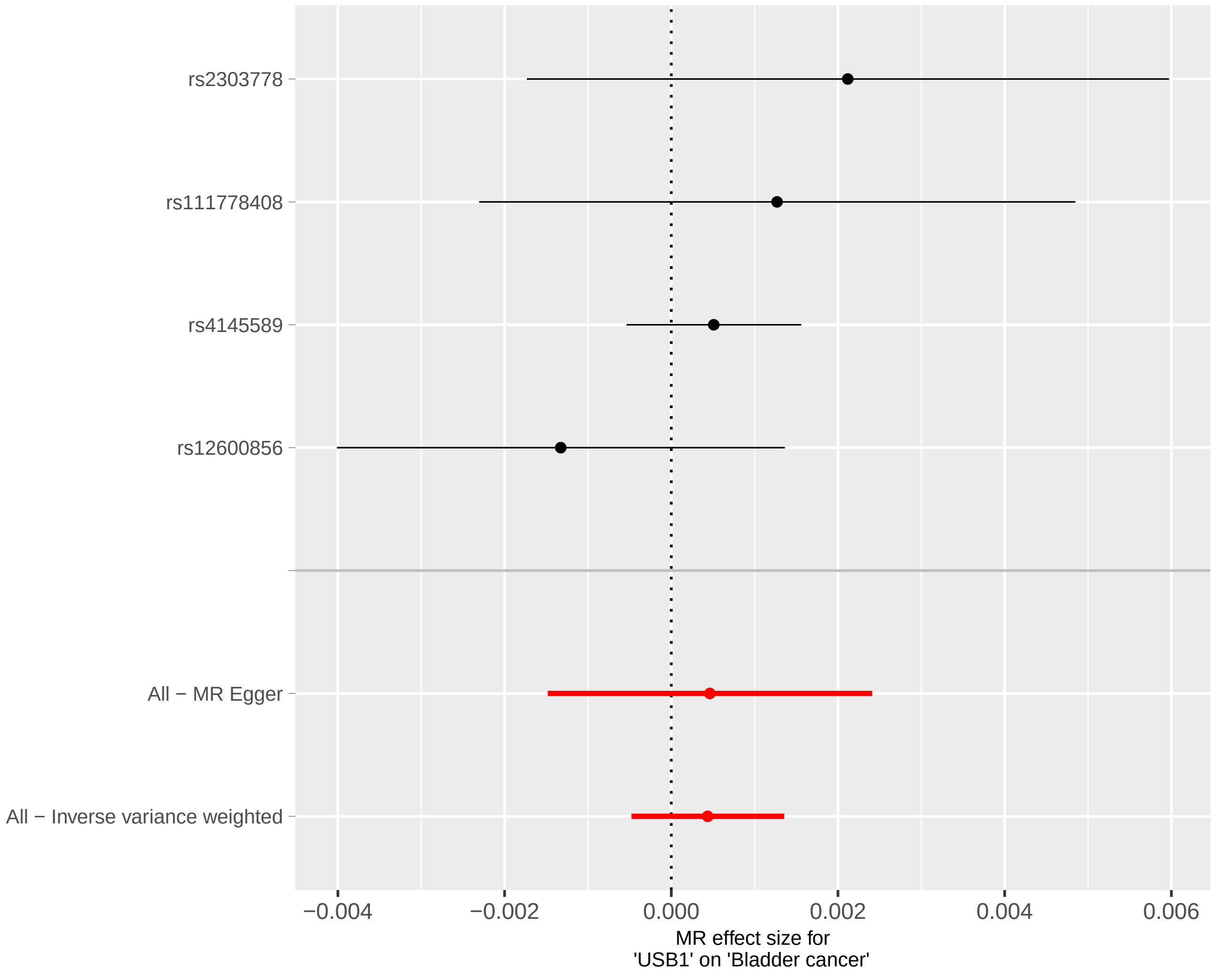

rs2303778
rs111778408
rs4145589
rs12600856
All − MR Egger
All − Inverse variance weighted
−0.004
−0.002
0.000
0.002
0.004
0.006
MR effect size for
'USB1' on 'Bladder cancer'

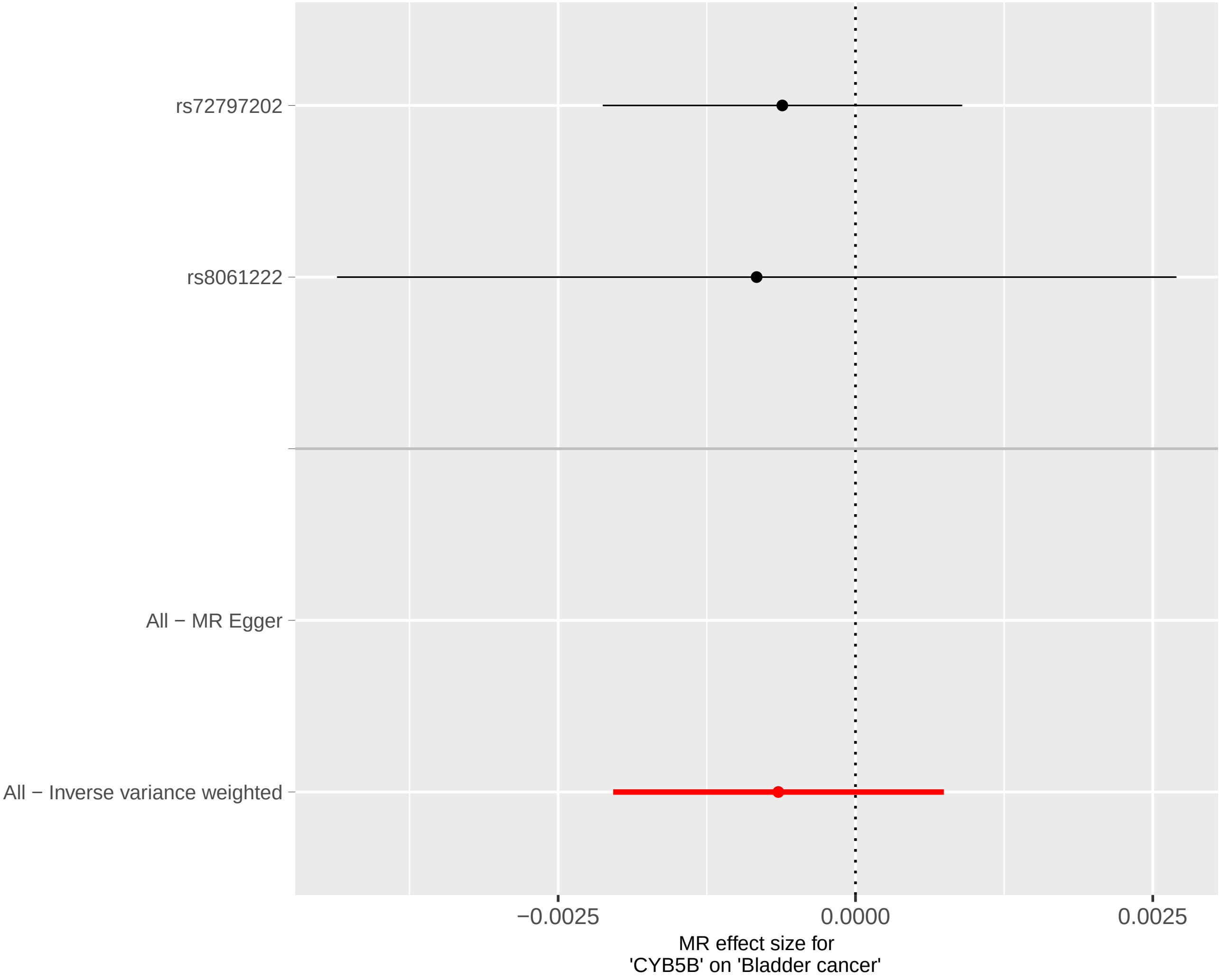
rs72797202
rs8061222
All − MR Egger
All − Inverse variance weighted
−0.0025
0.0000
0.0025
MR effect size for
'CYB5B' on 'Bladder cancer'

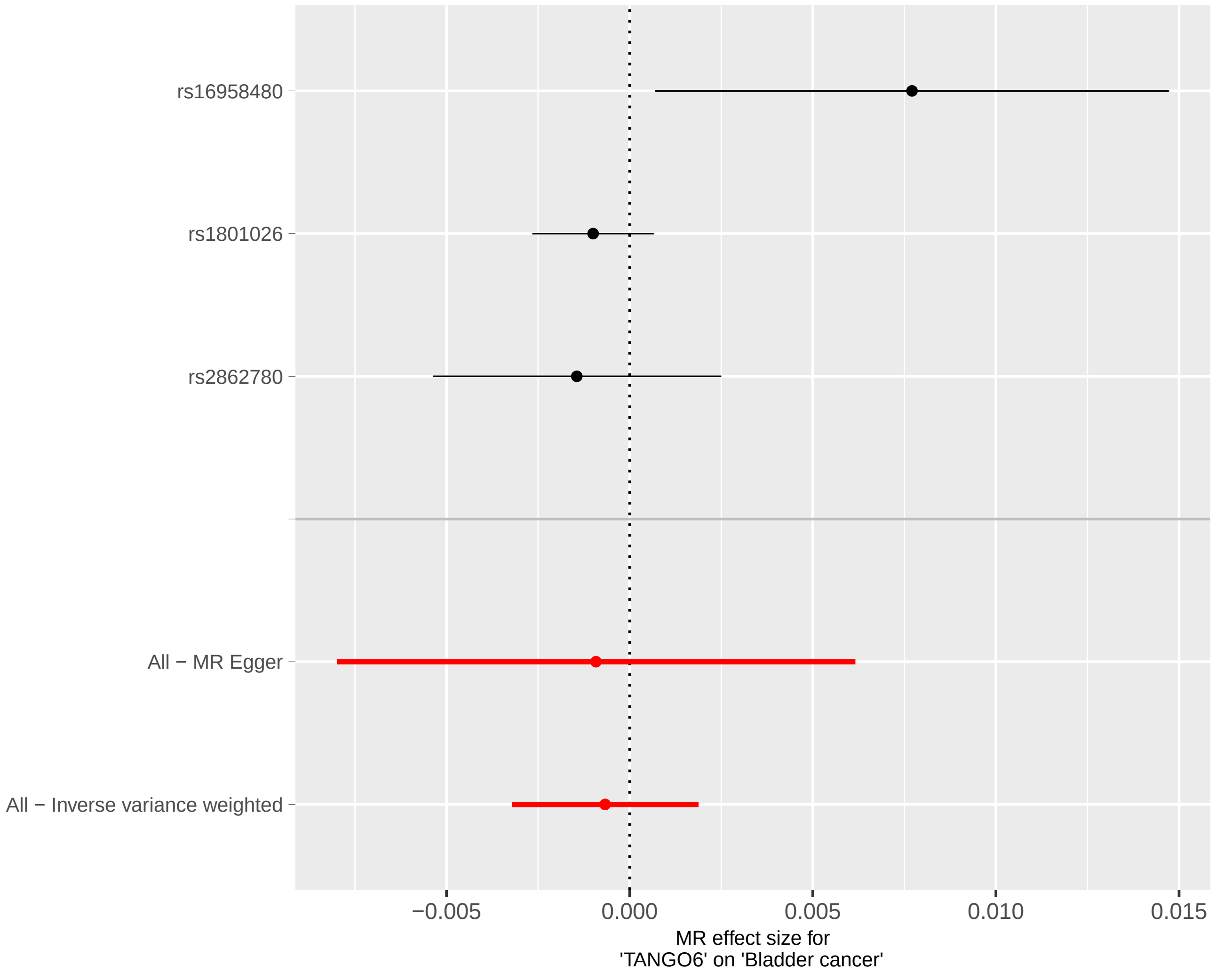
rs16958480
rs1801026
rs2862780
All − MR Egger
All − Inverse variance weighted
−0.005
0.000
0.005
0.010
0.015
MR effect size for
'TANGO6' on 'Bladder cancer'

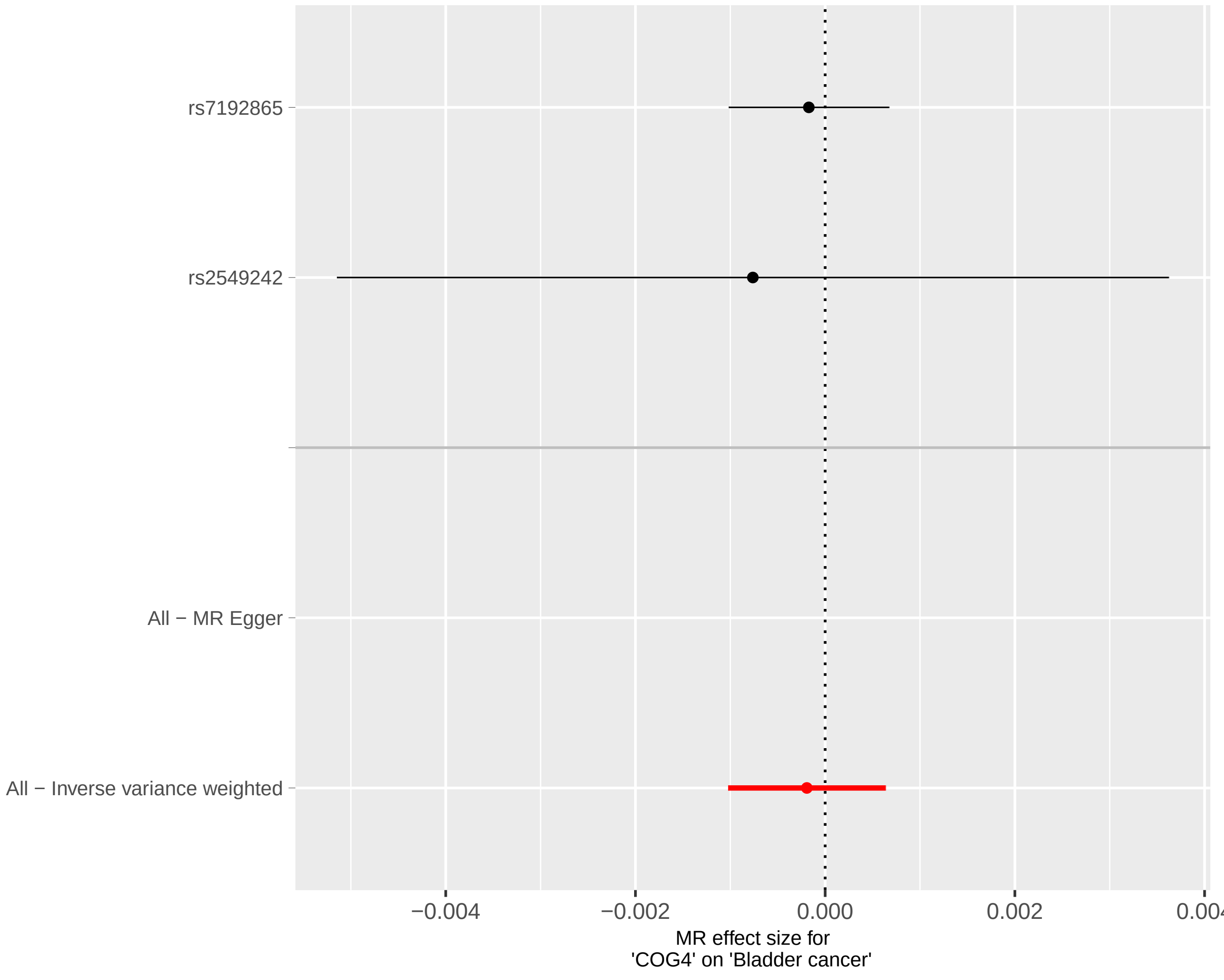

rs7192865
rs2549242
All − MR Egger
All − Inverse variance weighted
−0.004
−0.002
0.000
0.002
0.00
MR effect size for
'COG4' on 'Bladder cancer'

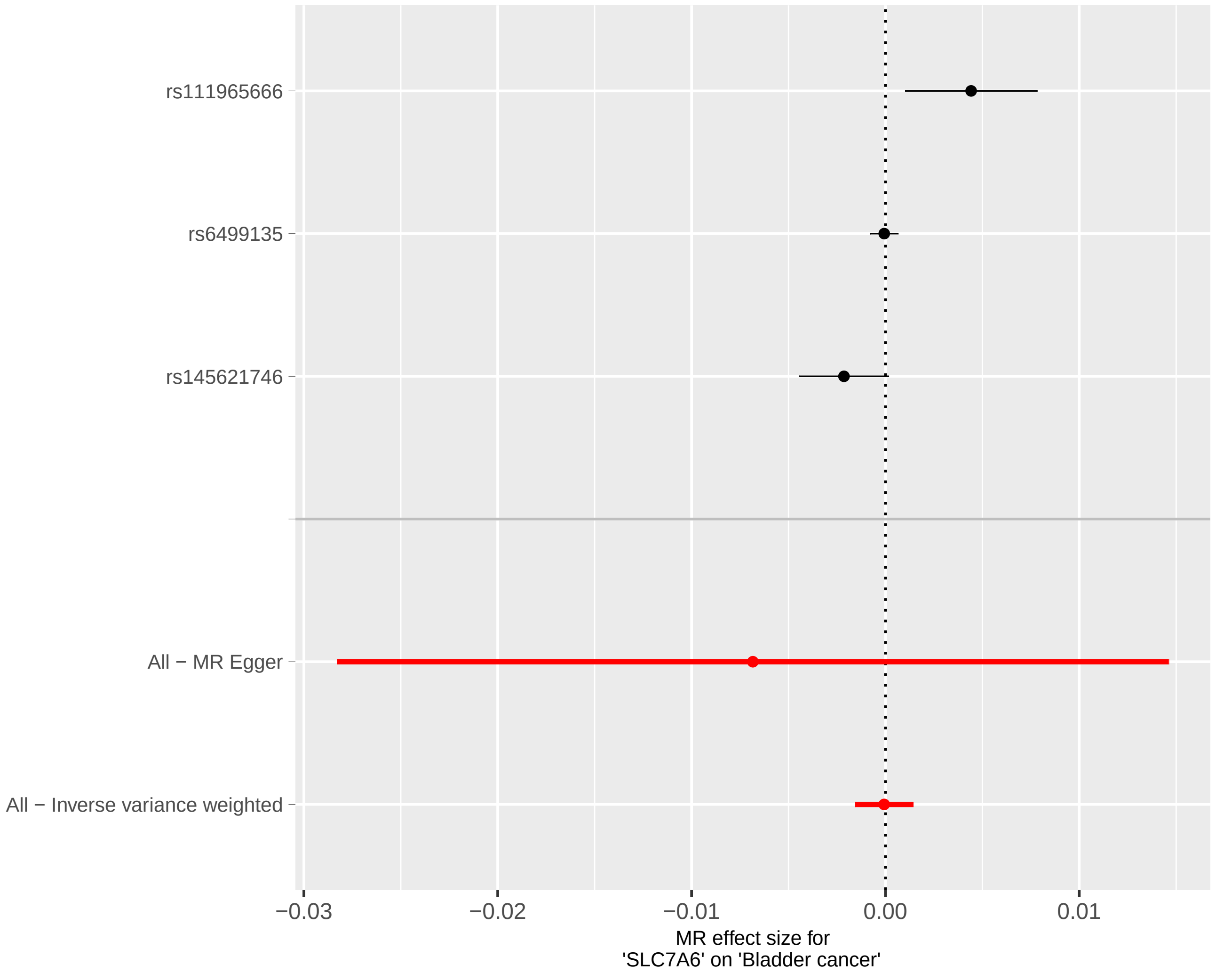

rs111965666
rs6499135
rs145621746
All − MR Egger
All − Inverse variance weighted
−0.03
−0.02
−0.01
0.00
0.01
MR effect size for
'SLC7A6' on 'Bladder cancer'

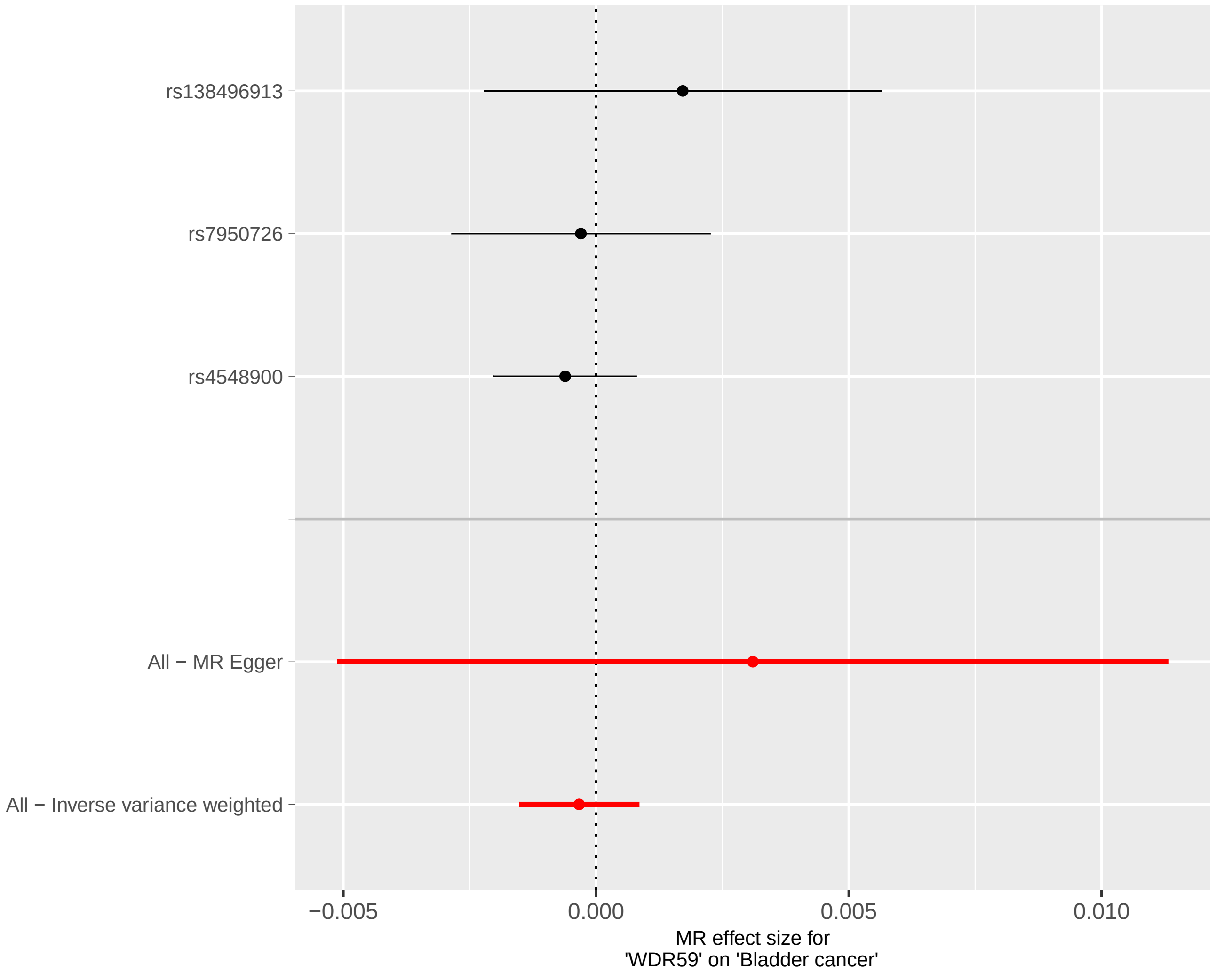

rs138496913
rs7950726
rs4548900
All − MR Egger
All − Inverse variance weighted
−0.005
0.000
0.005
0.010
MR effect size for
'WDR59' on 'Bladder cancer'

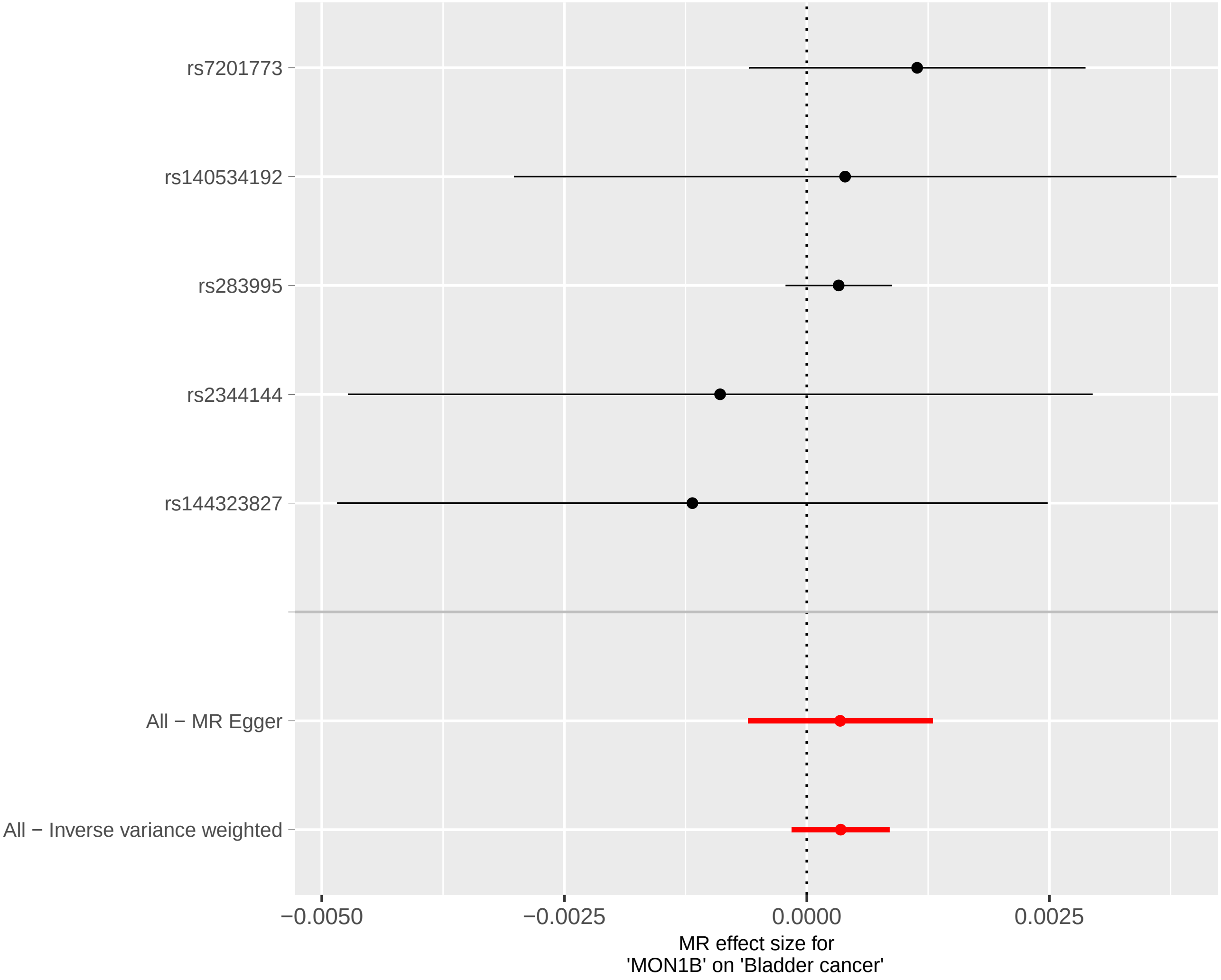
rs7201773
rs140534192
rs283995
rs2344144
rs144323827
All − MR Egger
All − Inverse variance weighted
−0.0050
−0.0025
0.0000
0.0025
MR effect size for
'MON1B' on 'Bladder cancer'

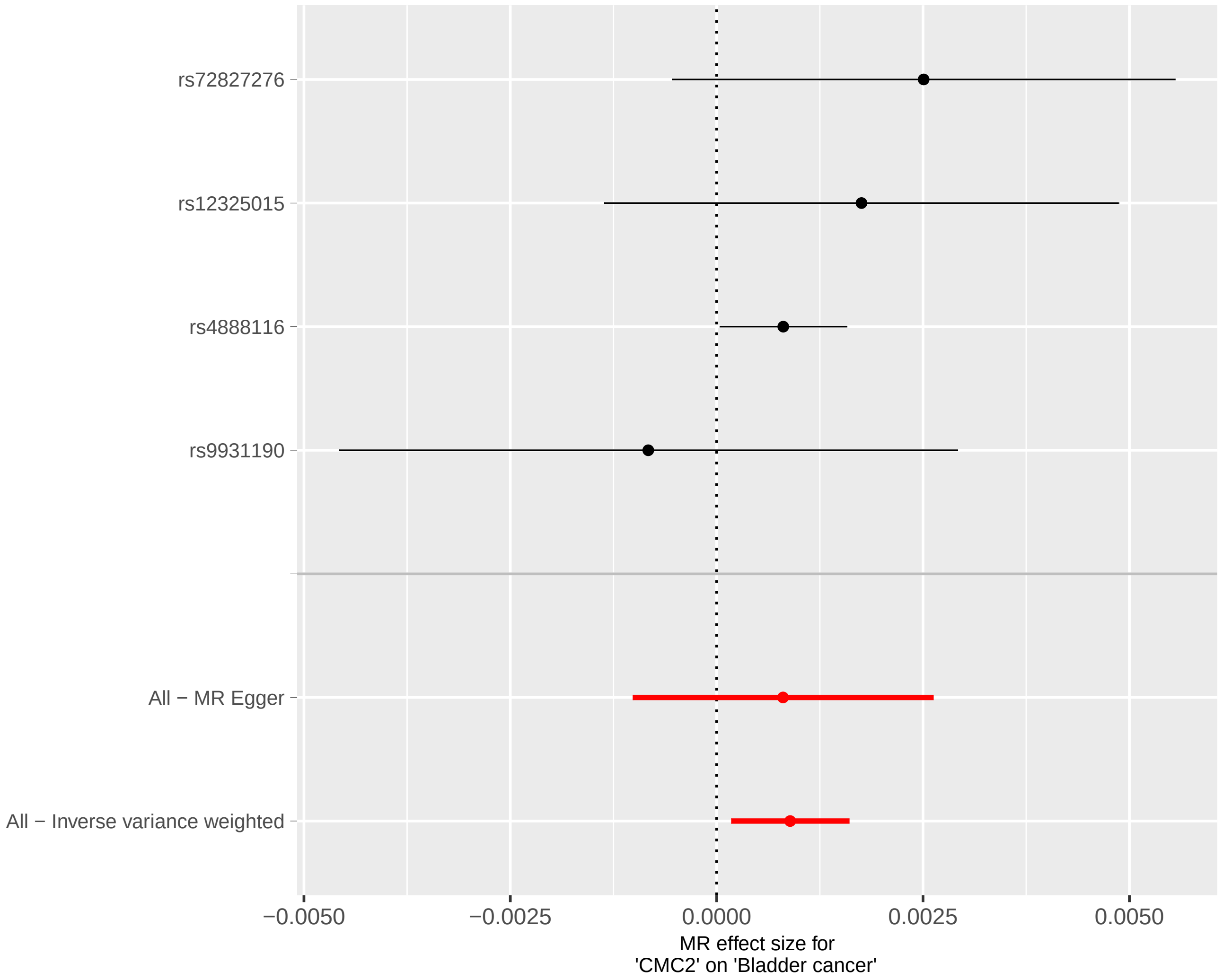
rs72827276
rs12325015
rs4888116
rs9931190
All − MR Egger
All − Inverse variance weighted
−0.0050
−0.0025
0.0000
0.0025
0.0050
MR effect size for
'CMC2' on 'Bladder cancer'

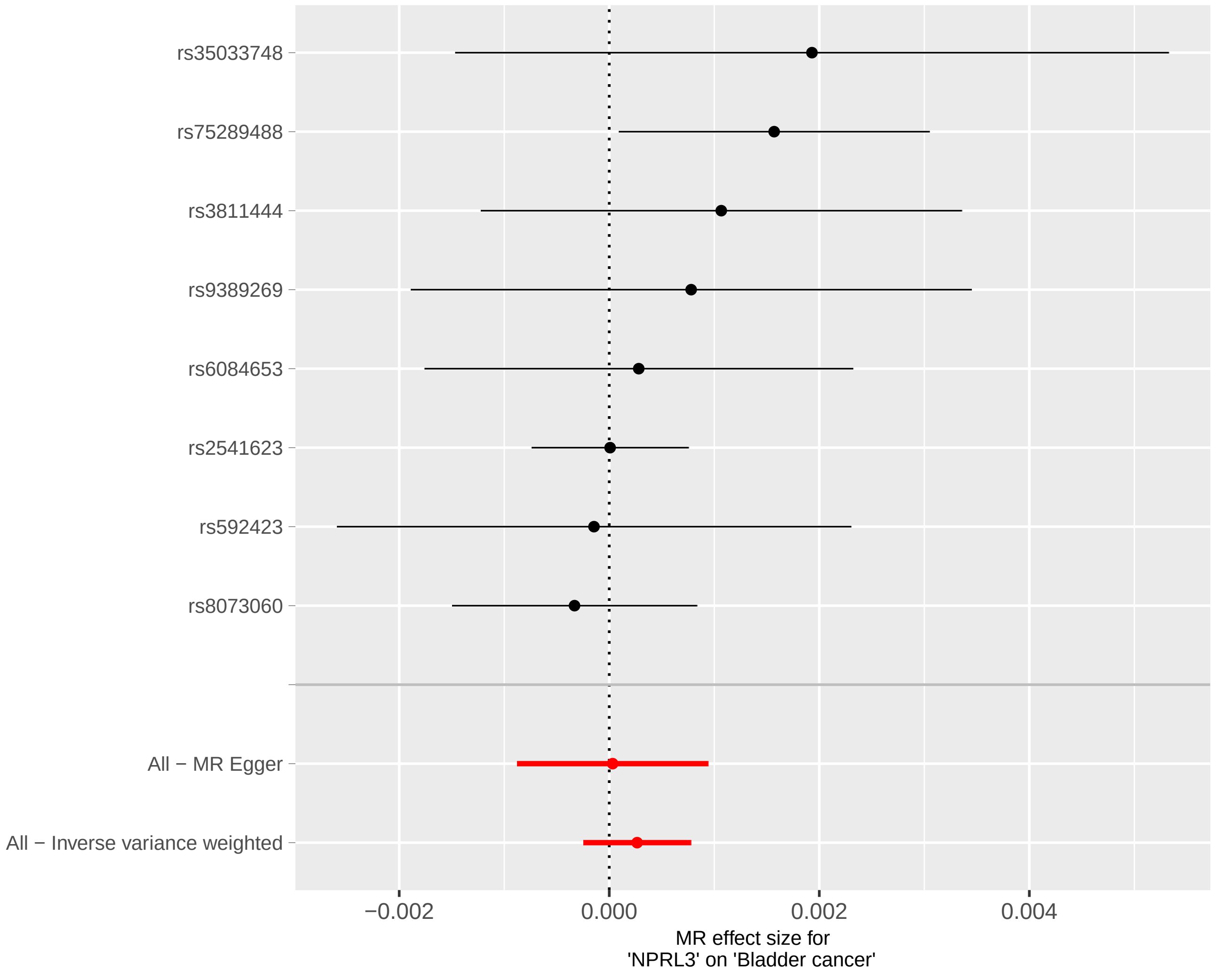

rs35033748
rs75289488
rs3811444
rs9389269
rs6084653
rs2541623
rs592423
rs8073060
All − MR Egger
All − Inverse variance weighted
−0.002
0.000
0.002
0.004
MR effect size for
'NPRL3' on 'Bladder cancer'

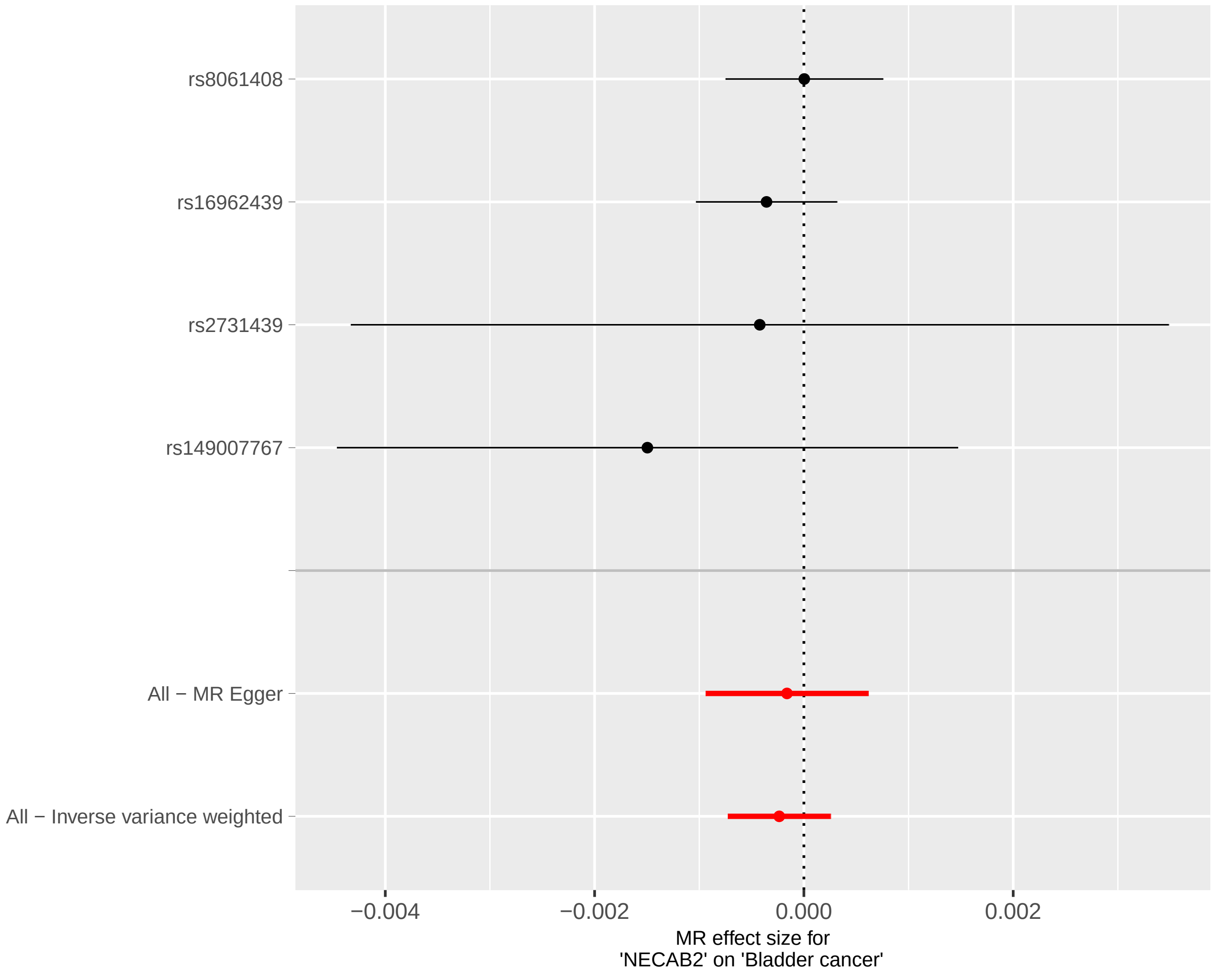
rs8061408
rs16962439
rs2731439
rs149007767
All − MR Egger
All − Inverse variance weighted
−0.004
−0.002
0.000
0.002
MR effect size for
'NECAB2' on 'Bladder cancer'

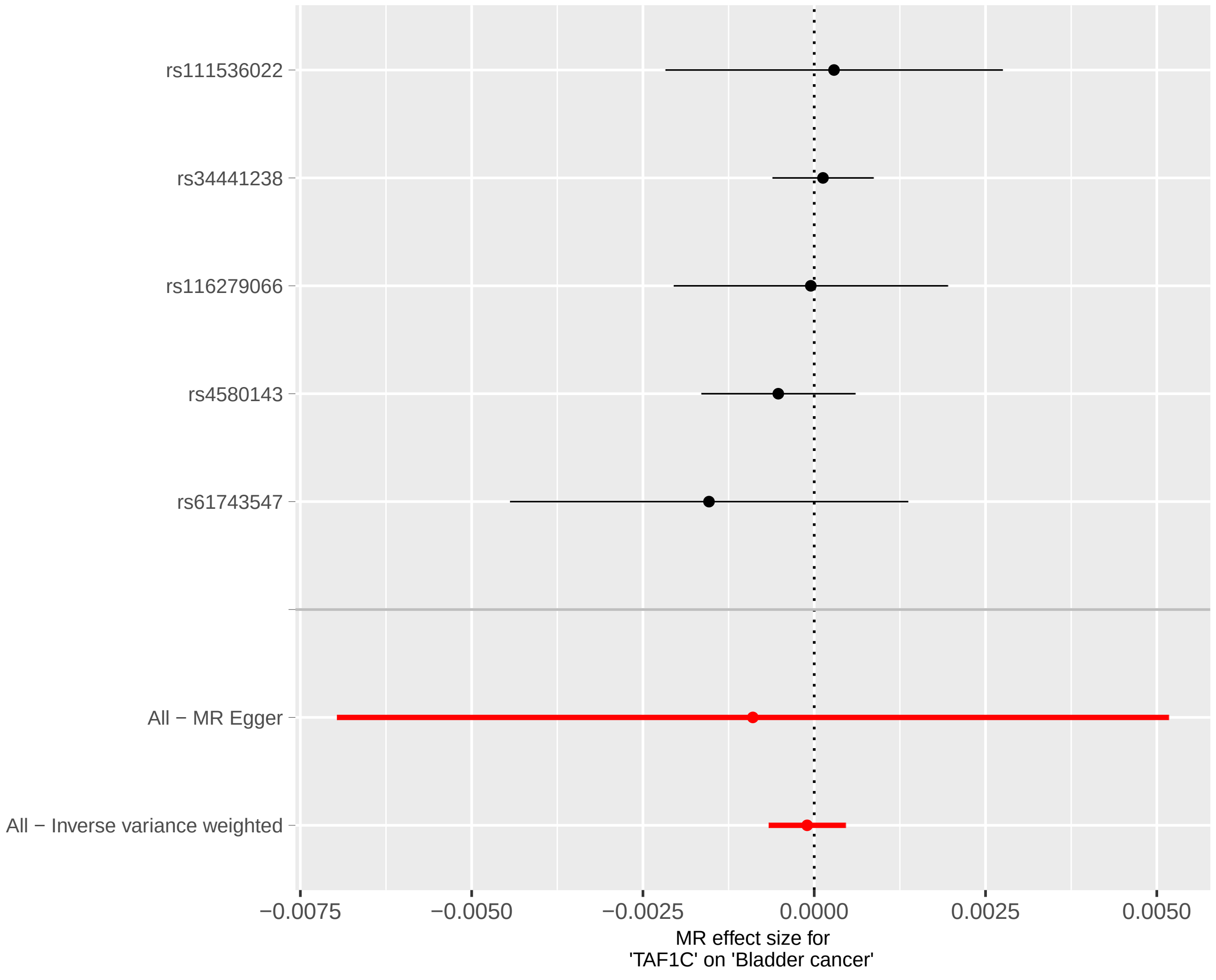

rs111536022
rs34441238
rs116279066
rs4580143
rs61743547
All − MR Egger
All − Inverse variance weighted
−0.0075
−0.0050
−0.0025
0.0000
0.0025
0.0050
MR effect size for
'TAF1C' on 'Bladder cancer'

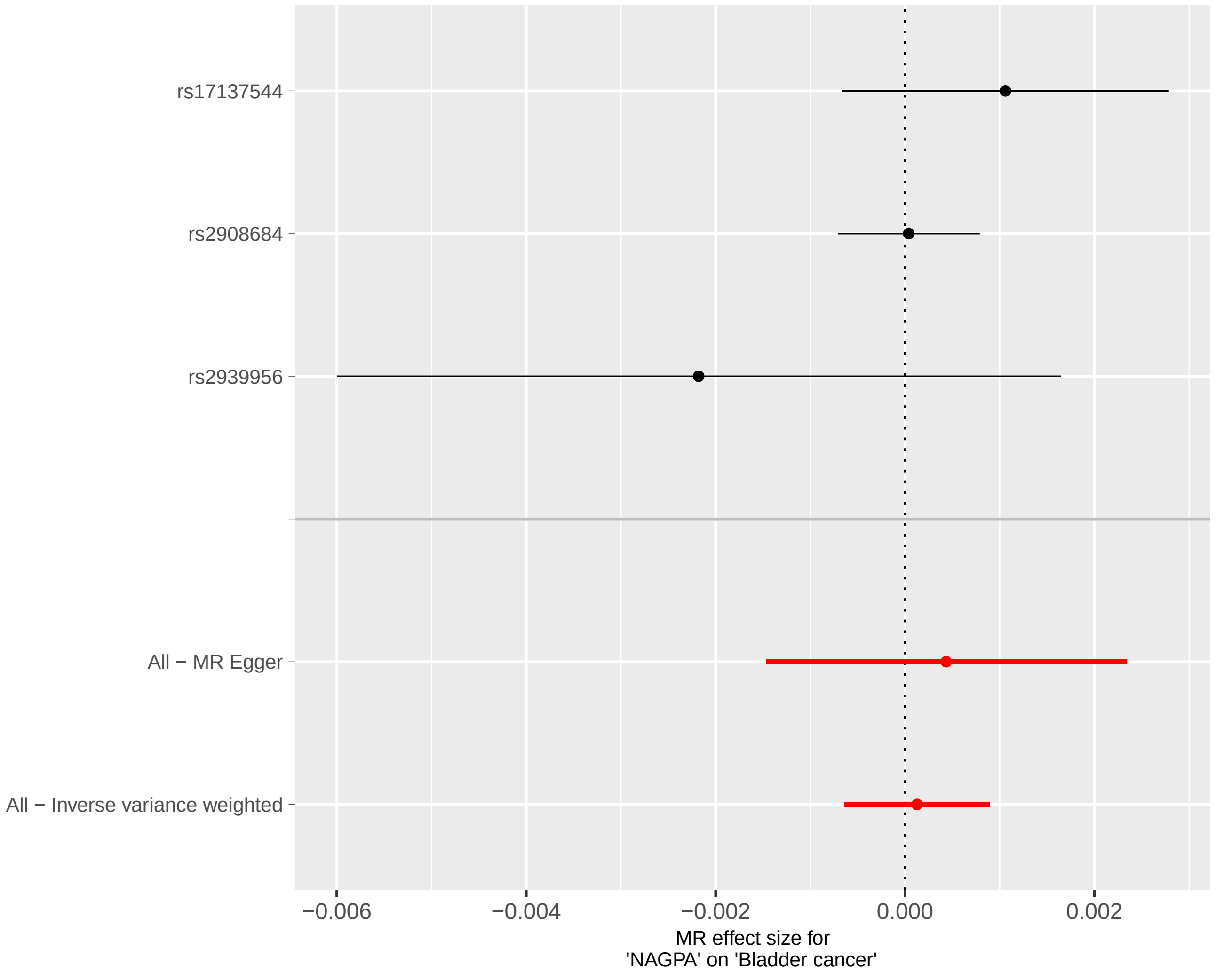

rs17137544
rs2908684
rs2939956
All − MR Egger
All − Inverse variance weighted
−0.006
−0.004
−0.002
0.000
0.002
MR effect size for
'NAGPA' on 'Bladder cancer'

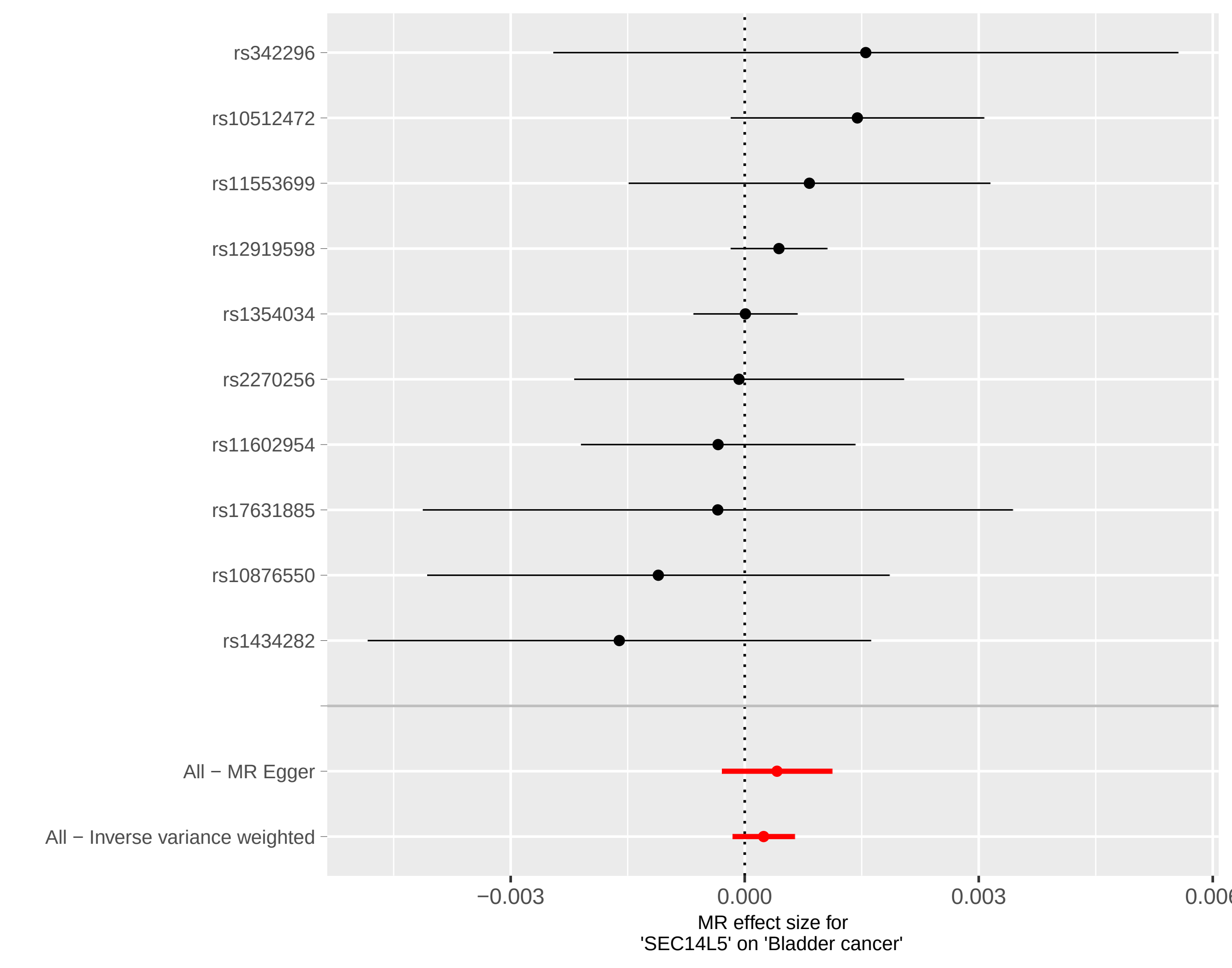

rs342296
rs10512472
rs11553699
rs12919598
rs1354034
rs2270256
rs11602954
rs17631885
rs10876550
rs1434282
All − MR Egger
All − Inverse variance weighted
−0.003
0.000
0.003
MR effect size for
'SEC14L5' on 'Bladder cancer'

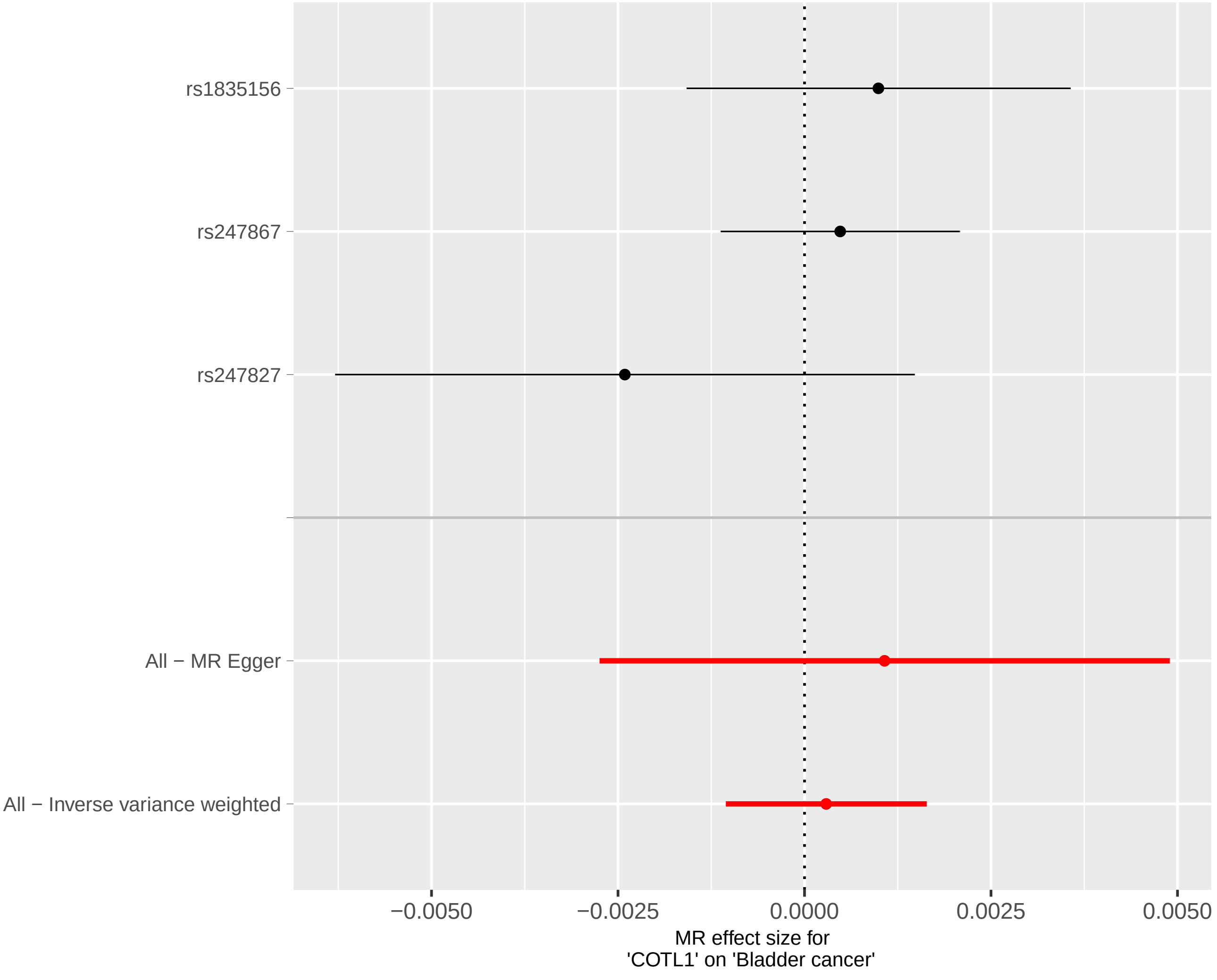

rs1835156
rs247867
rs247827
All − MR Egger
All − Inverse variance weighted
−0.0050
−0.0025
0.0000
0.0025
0.0050
MR effect size for
'COTL1' on 'Bladder cancer'

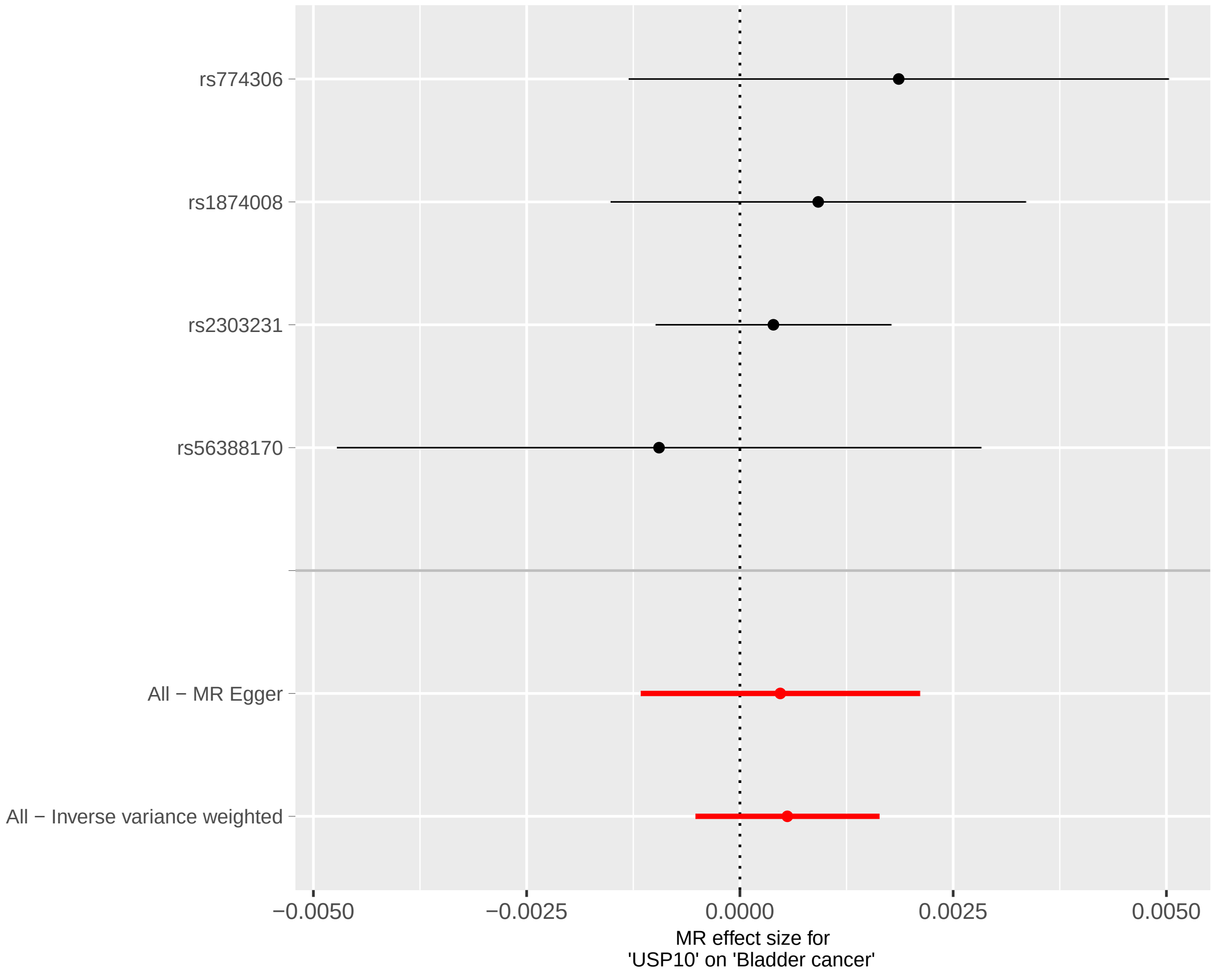
rs774306
rs1874008
rs2303231
rs56388170
All − MR Egger
All − Inverse variance weighted
−0.0050
−0.0025
0.0000
0.0025
0.0050
MR effect size for
'USP10' on 'Bladder cancer'

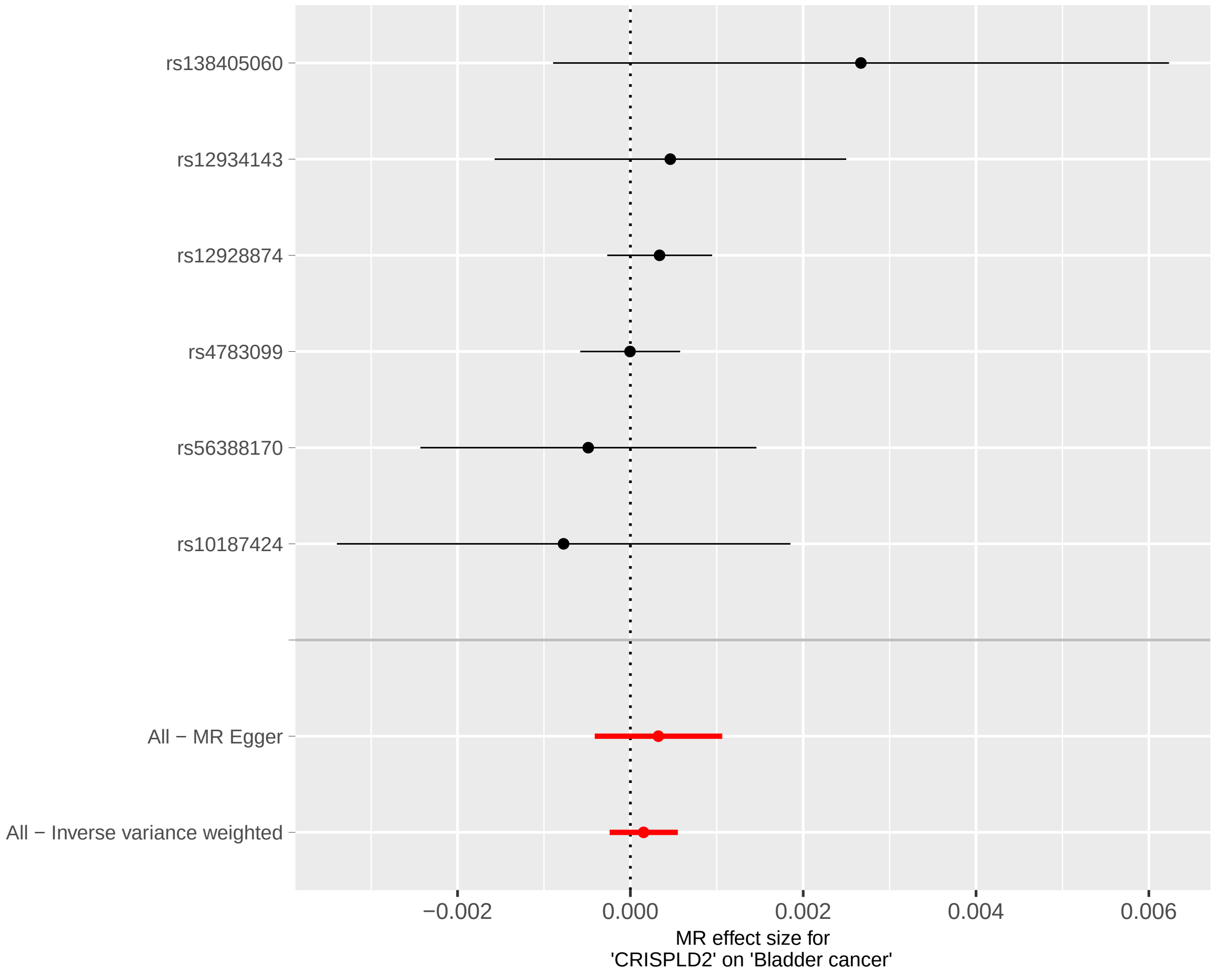
rs138405060
rs12934143
rs12928874
rs4783099
rs56388170
rs10187424
All − MR Egger
All − Inverse variance weighted
−0.002
0.000
0.002
0.004
0.006
MR effect size for
'CRISPLD2' on 'Bladder cancer'

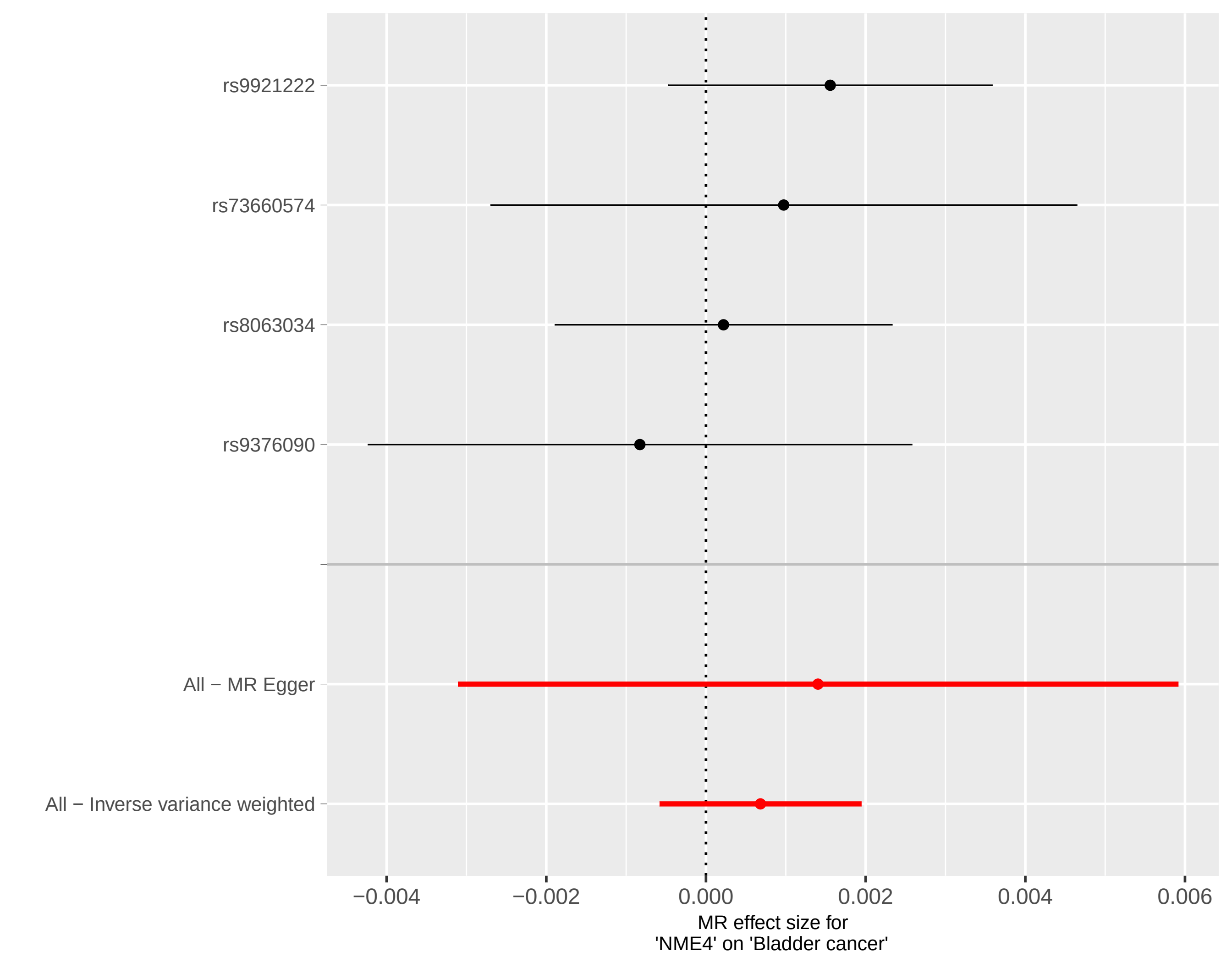

rs9921222
rs73660574
rs8063034
rs9376090
All − MR Egger
All − Inverse variance weighted
−0.004
−0.002
0.000
0.002
0.004
0.006
MR effect size for
'NME4' on 'Bladder cancer'

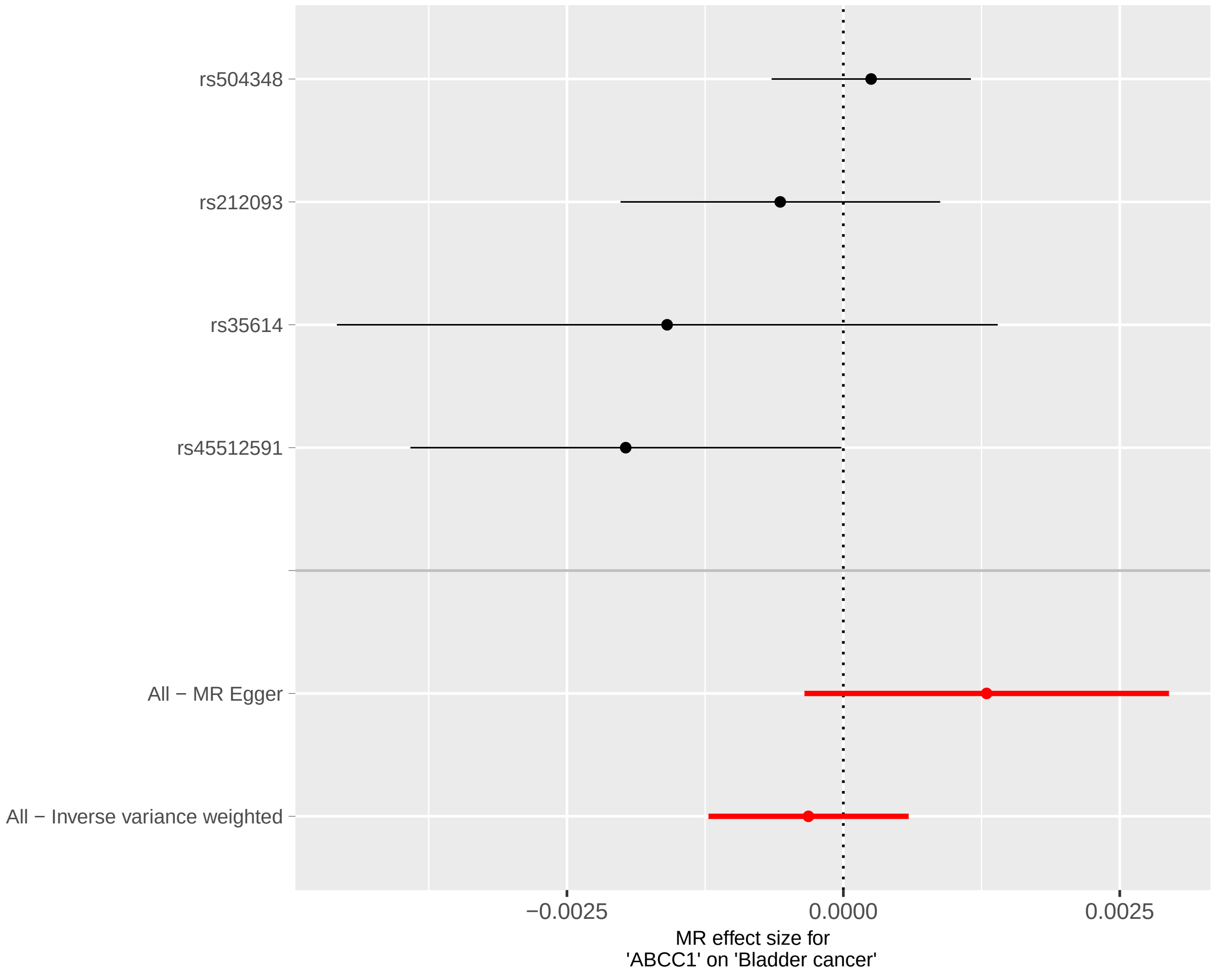
rs504348
rs212093
rs35614
rs45512591
All − MR Egger
All − Inverse variance weighted
−0.0025
0.0000
0.0025
MR effect size for
'ABCC1' on 'Bladder cancer'

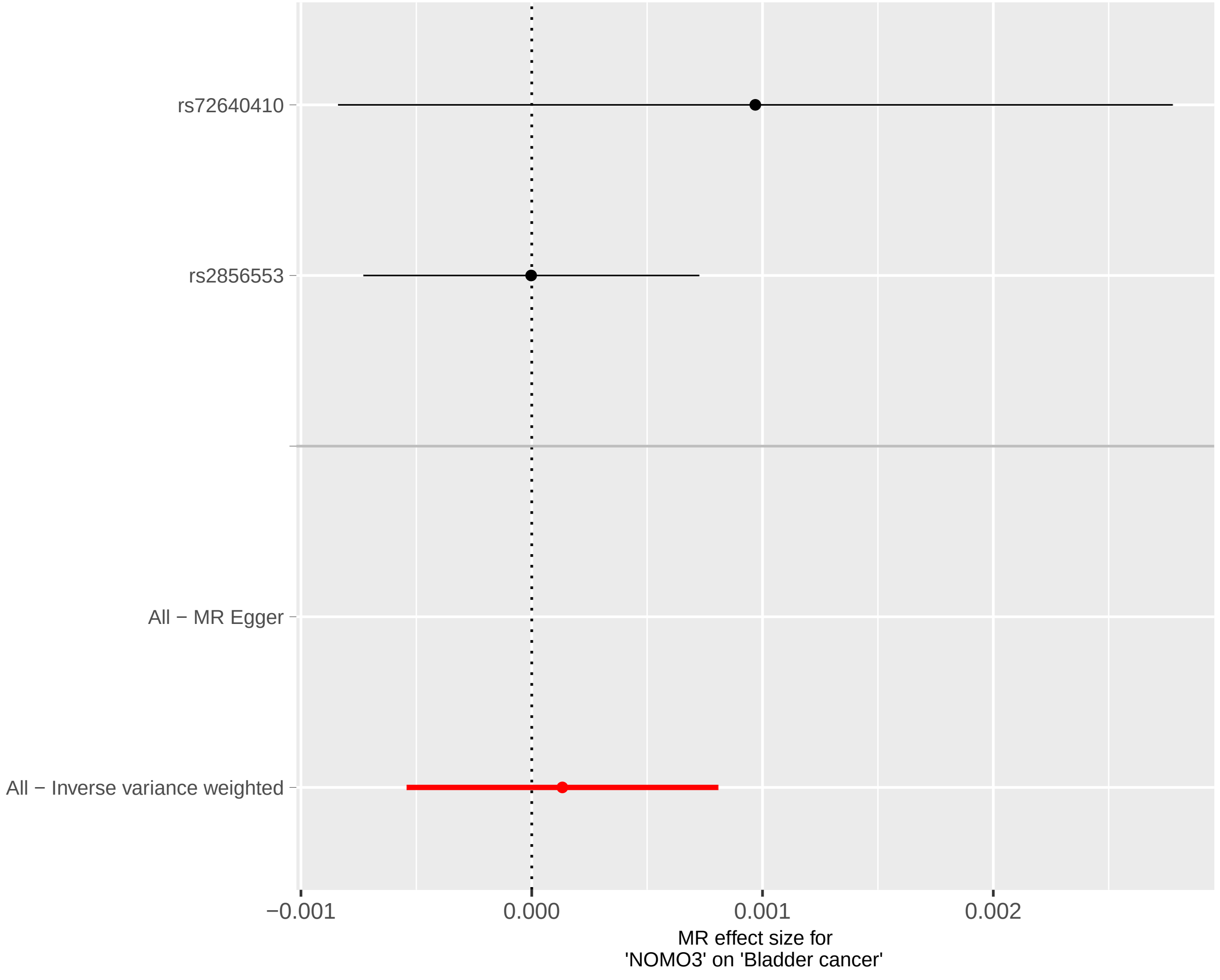
rs72640410
rs2856553
All − MR Egger
All − Inverse variance weighted
−0.001
0.000
0.001
0.002
MR effect size for
'NOMO3' on 'Bladder cancer'

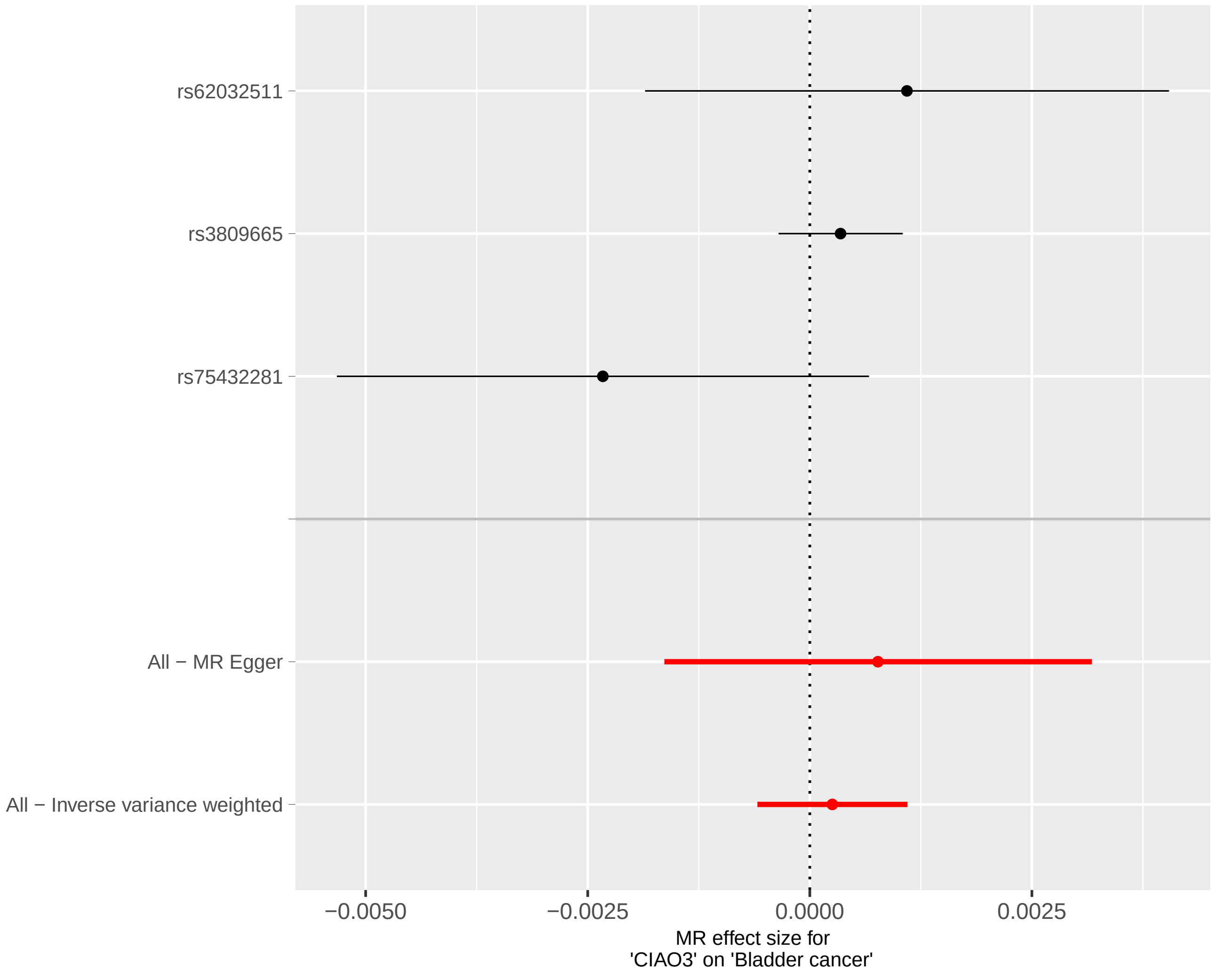

rs62032511
rs3809665
rs75432281
All − MR Egger
All − Inverse variance weighted
−0.0050
−0.0025
0.0000
0.0025
MR effect size for
'CIAO3' on 'Bladder cancer'

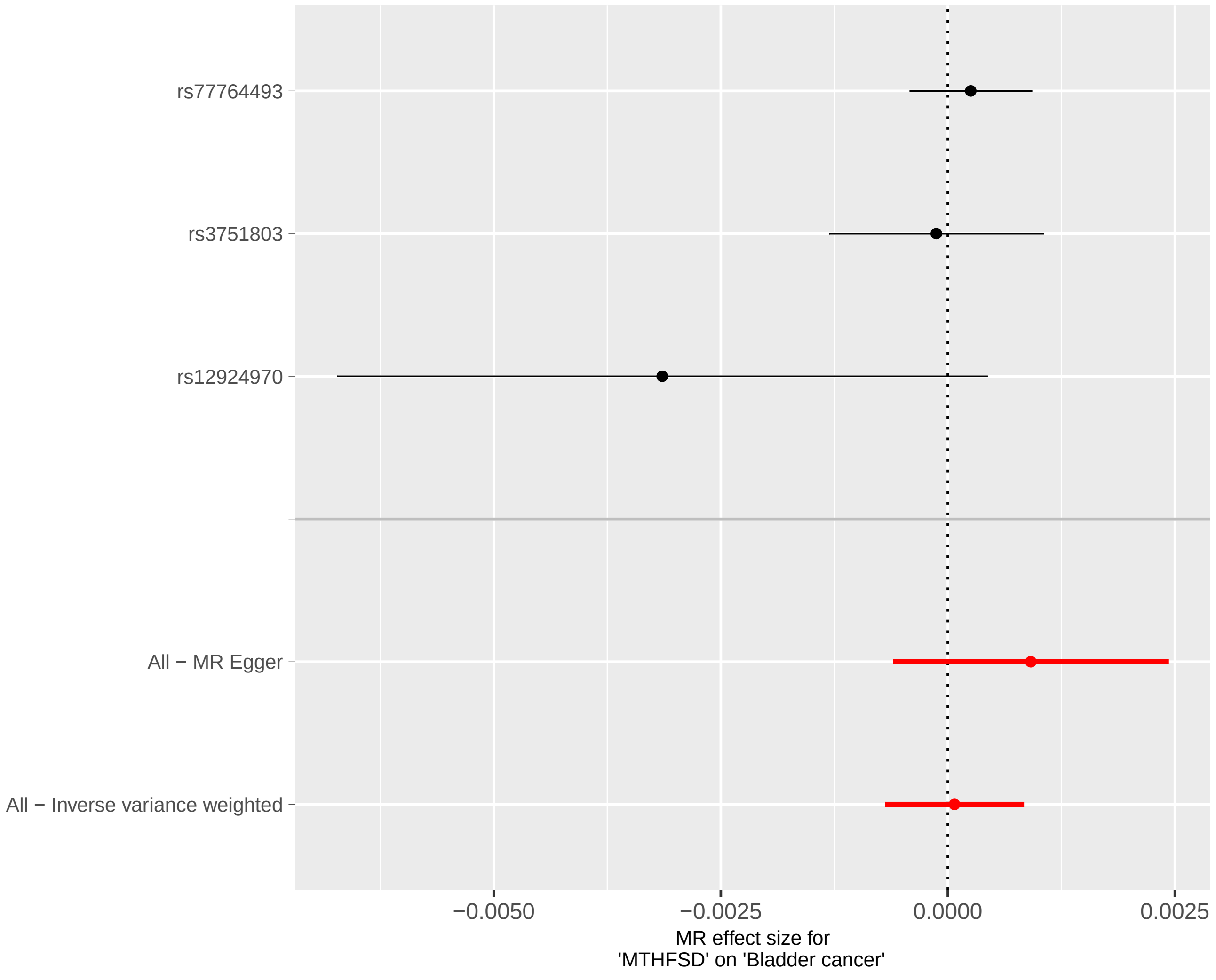
rs77764493
rs3751803
rs12924970
All − MR Egger
All − Inverse variance weighted
−0.0050
−0.0025
0.0000
0.0025
MR effect size for
'MTHFSD' on 'Bladder cancer'

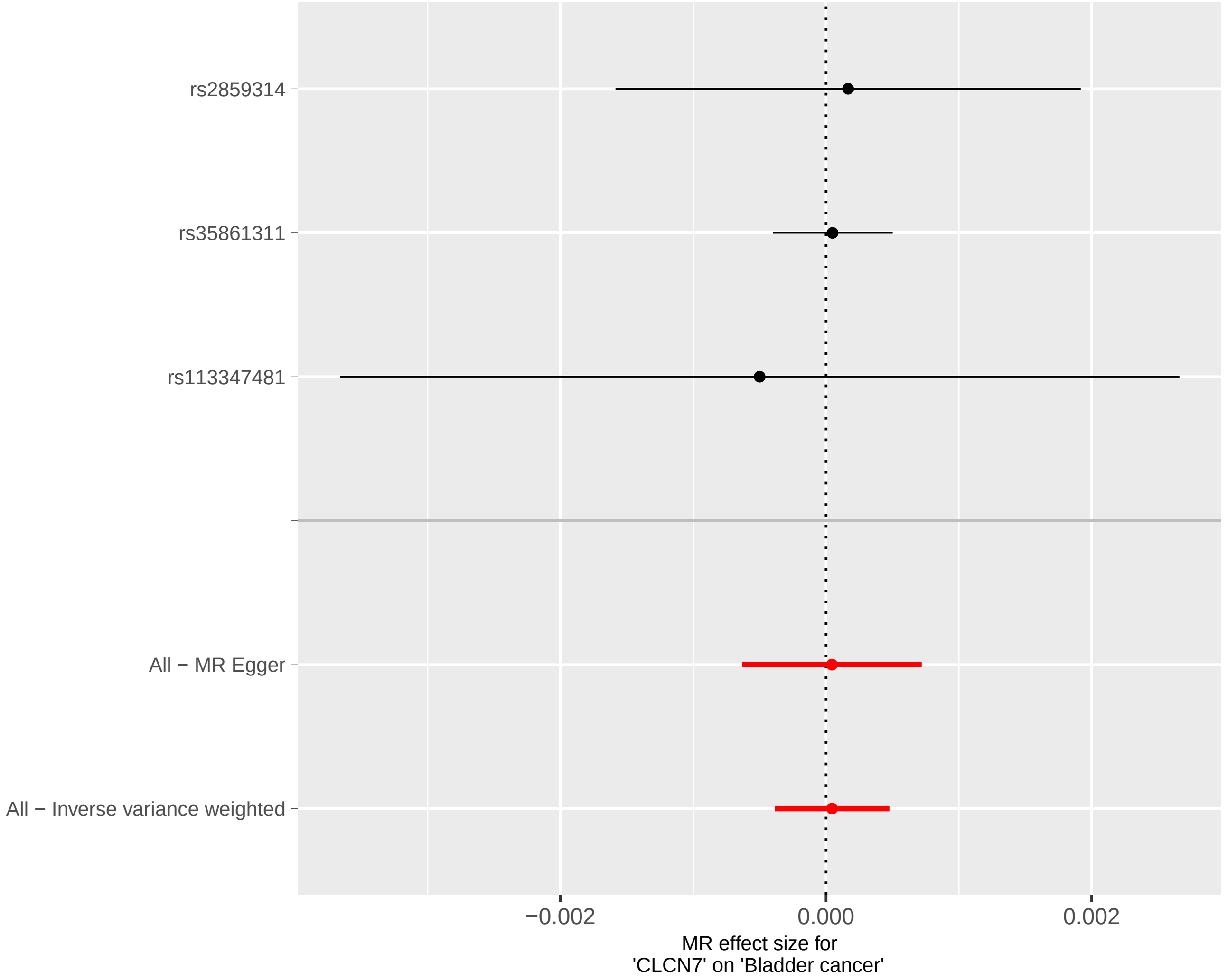

rs2859314
rs35861311
rs113347481
All − MR Egger
All − Inverse variance weighted
−0.002
0.000
0.002
MR effect size for
'CLCN7' on 'Bladder cancer'

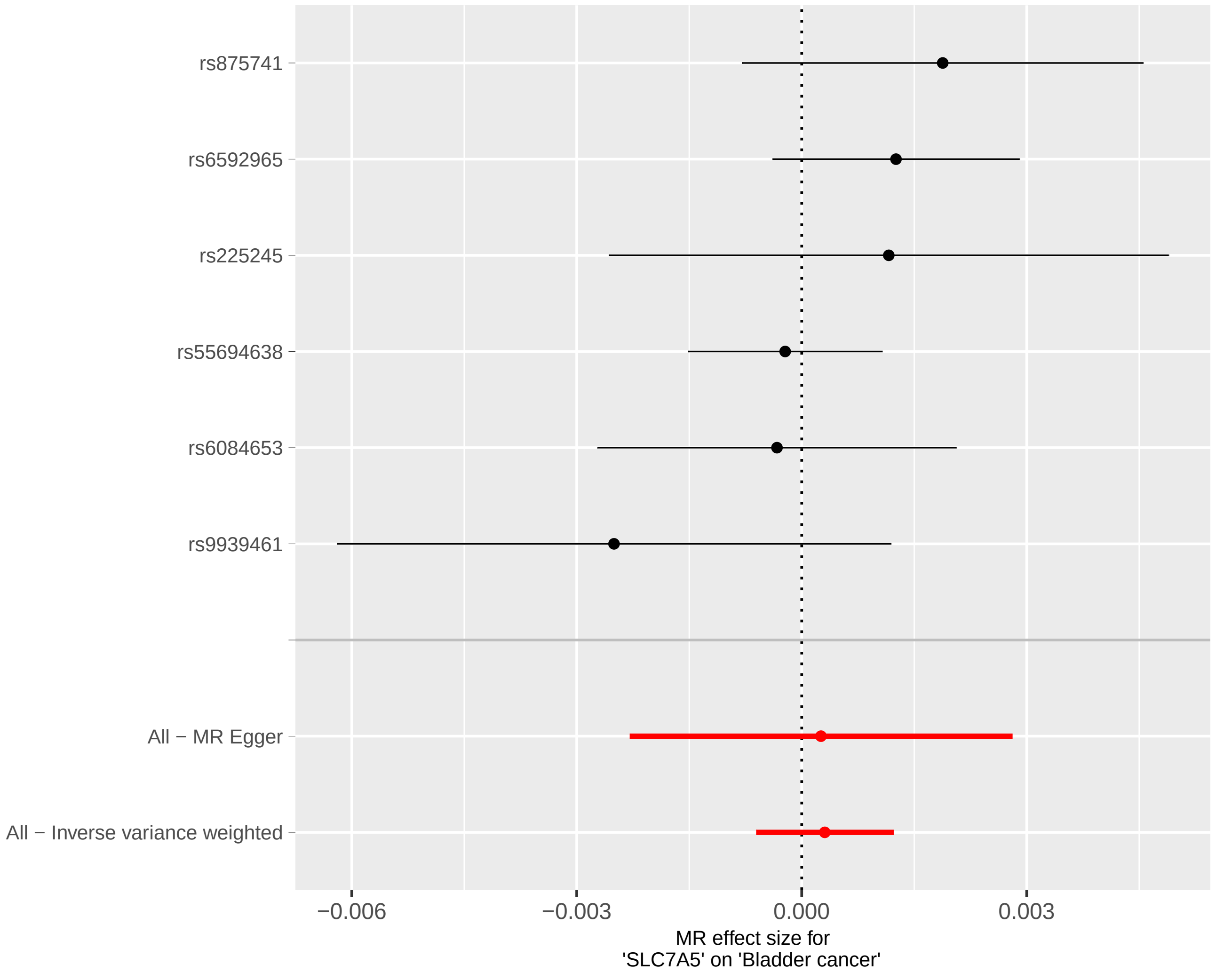
rs875741
rs6592965
rs225245
rs55694638
rs6084653
rs9939461
All − MR Egger
All − Inverse variance weighted
−0.006
−0.003
0.000
0.003
MR effect size for
'SLC7A5' on 'Bladder cancer'

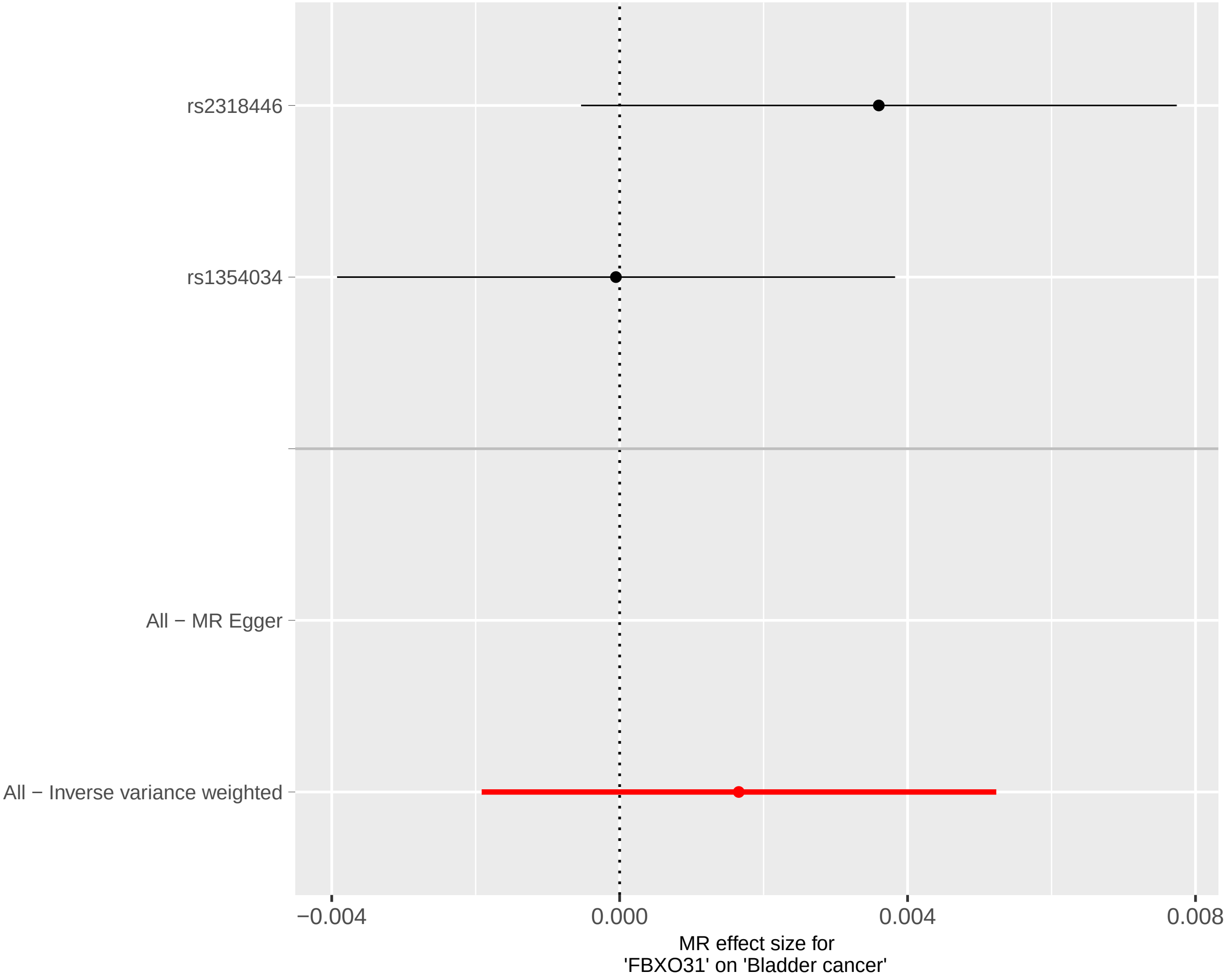

rs2318446
rs1354034
All − MR Egger
All − Inverse variance weighted
−0.004
0.000
0.004
0.008
MR effect size for
'FBXO31' on 'Bladder cancer'

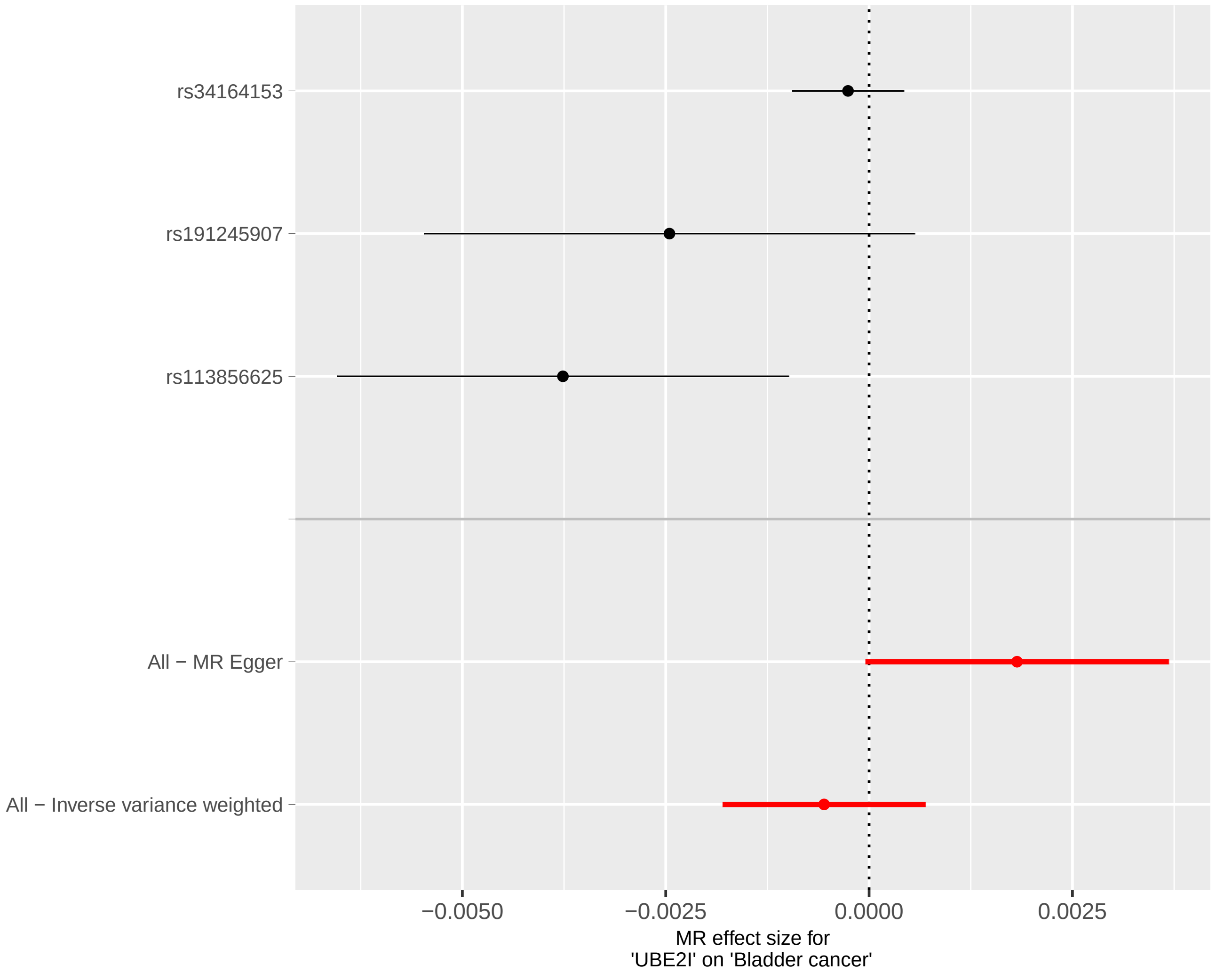
rs34164153
rs191245907
rs113856625
All − MR Egger
All − Inverse variance weighted
−0.0050
−0.0025
0.0000
0.0025
MR effect size for
'UBE2I' on 'Bladder cancer'

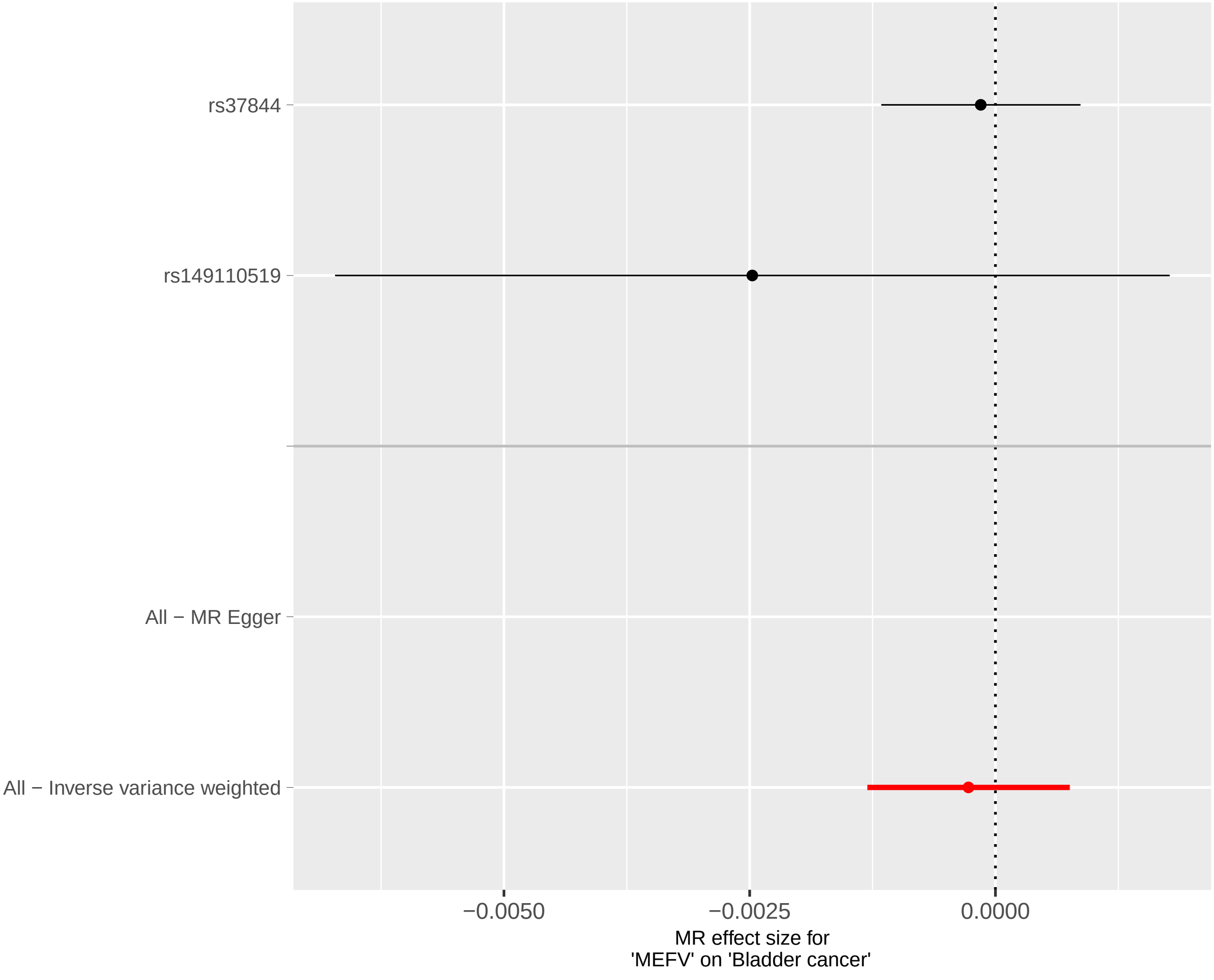
rs37844
rs149110519
All − MR Egger
All − Inverse variance weighted
−0.0050
−0.0025
0.0000
MR effect size for
'MEFV' on 'Bladder cancer'

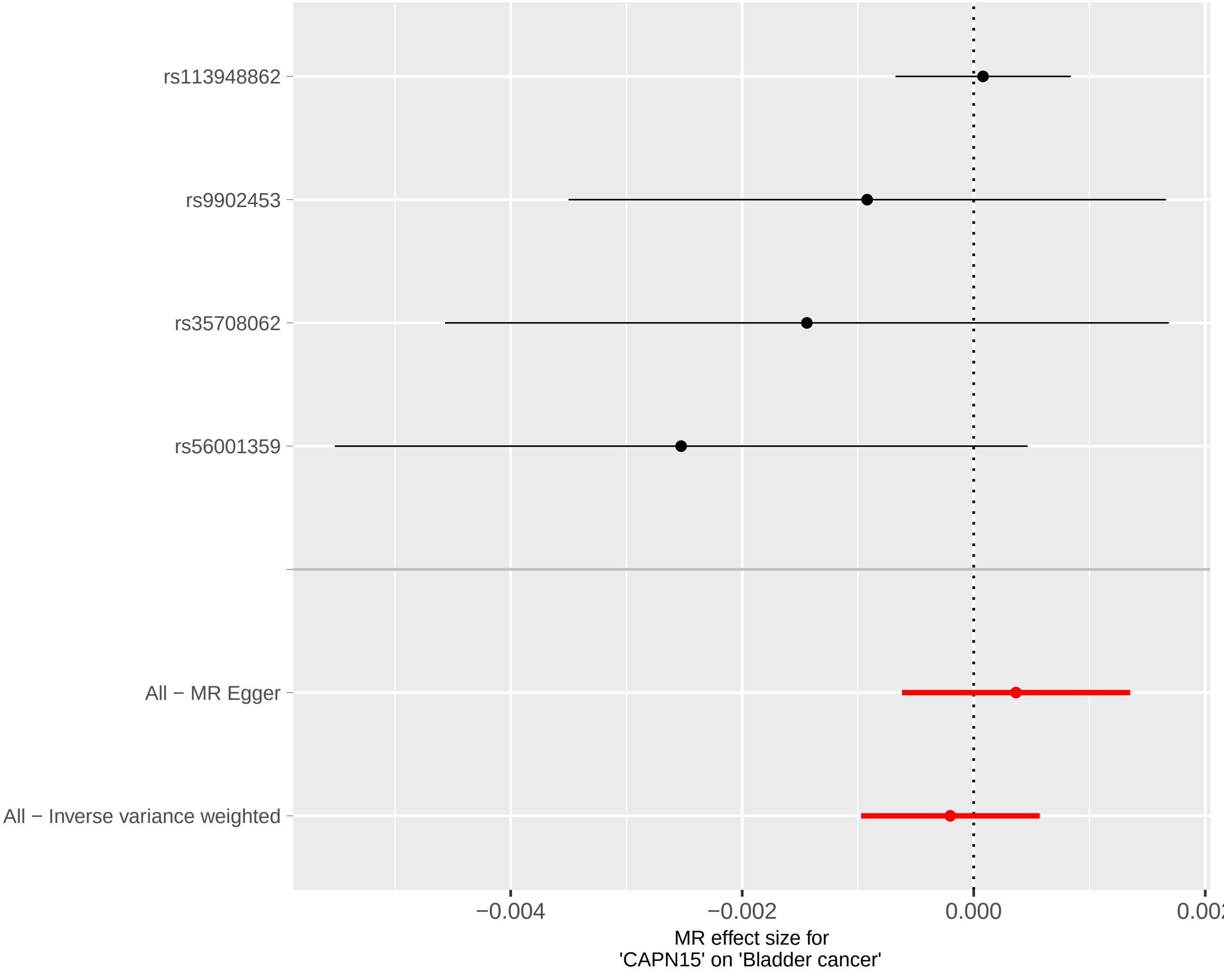

rs113948862
rs9902453
rs35708062
rs56001359
All − MR Egger
All − Inverse variance weighted
−0.004
−0.002
0.000
MR effect size for
'CAPN15' on 'Bladder cancer'

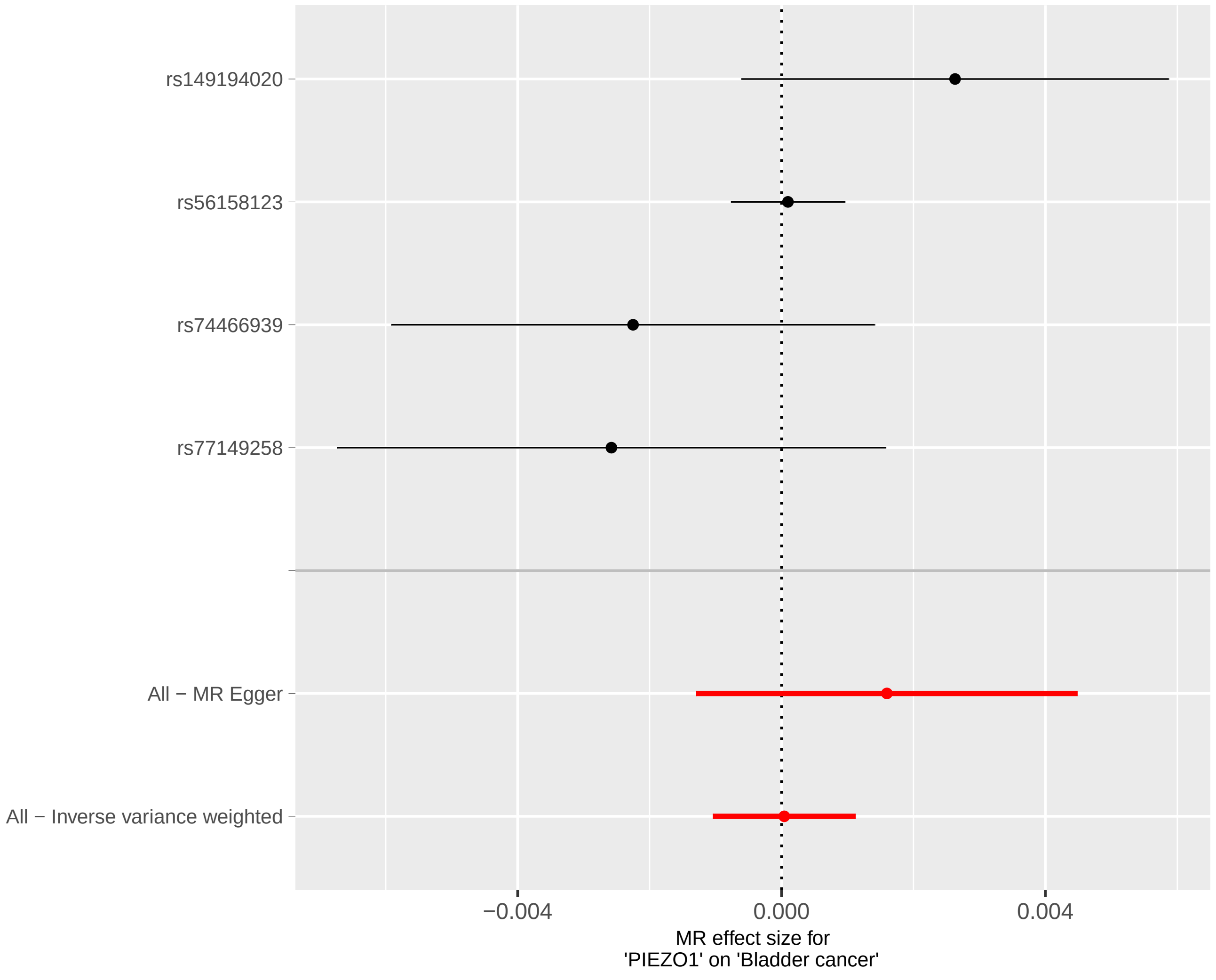
rs149194020
rs56158123
rs74466939
rs77149258
All − MR Egger
All − Inverse variance weighted
−0.004
0.000
0.004
MR effect size for
'PIEZO1' on 'Bladder cancer'

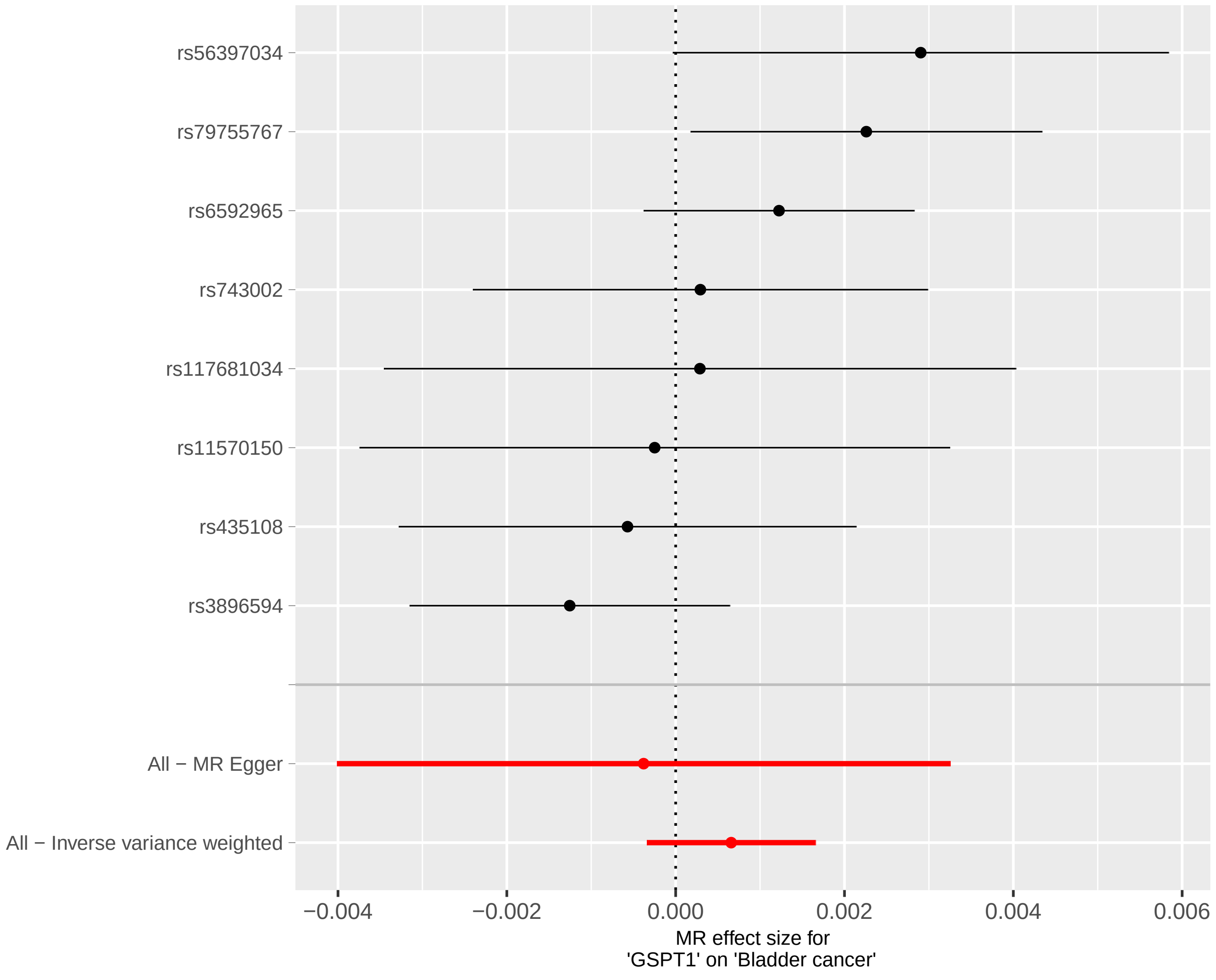

rs56397034
rs79755767
rs6592965
rs743002
rs117681034
rs11570150
rs435108
rs3896594
All − MR Egger
All − Inverse variance weighted
−0.004
−0.002
0.000
0.002
0.004
0.006
MR effect size for
'GSPT1' on 'Bladder cancer'

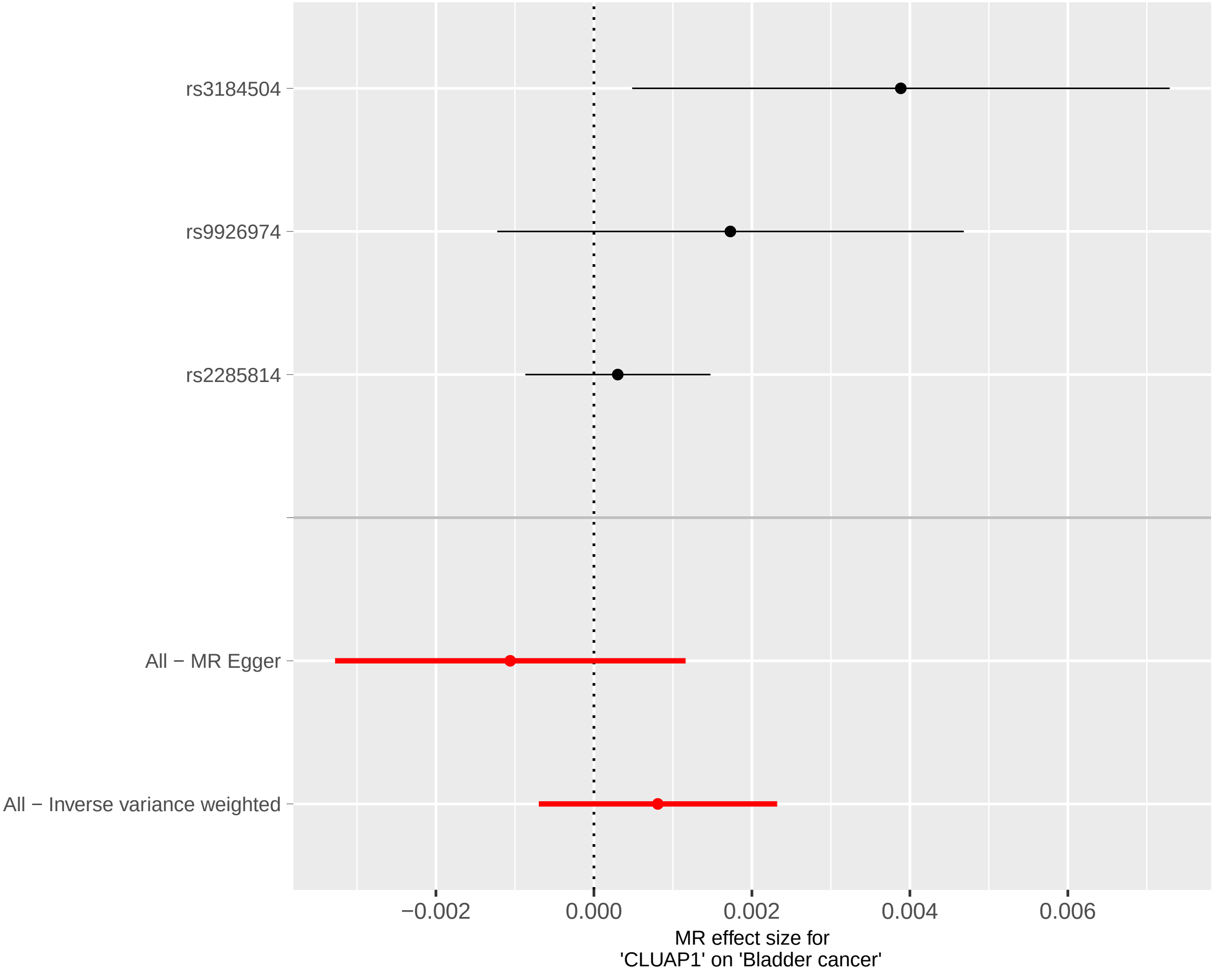

rs3184504
rs9926974
rs2285814
All − MR Egger
All − Inverse variance weighted
−0.002
0.000
0.002
0.004
0.006
MR effect size for
'CLUAP1' on 'Bladder cancer'

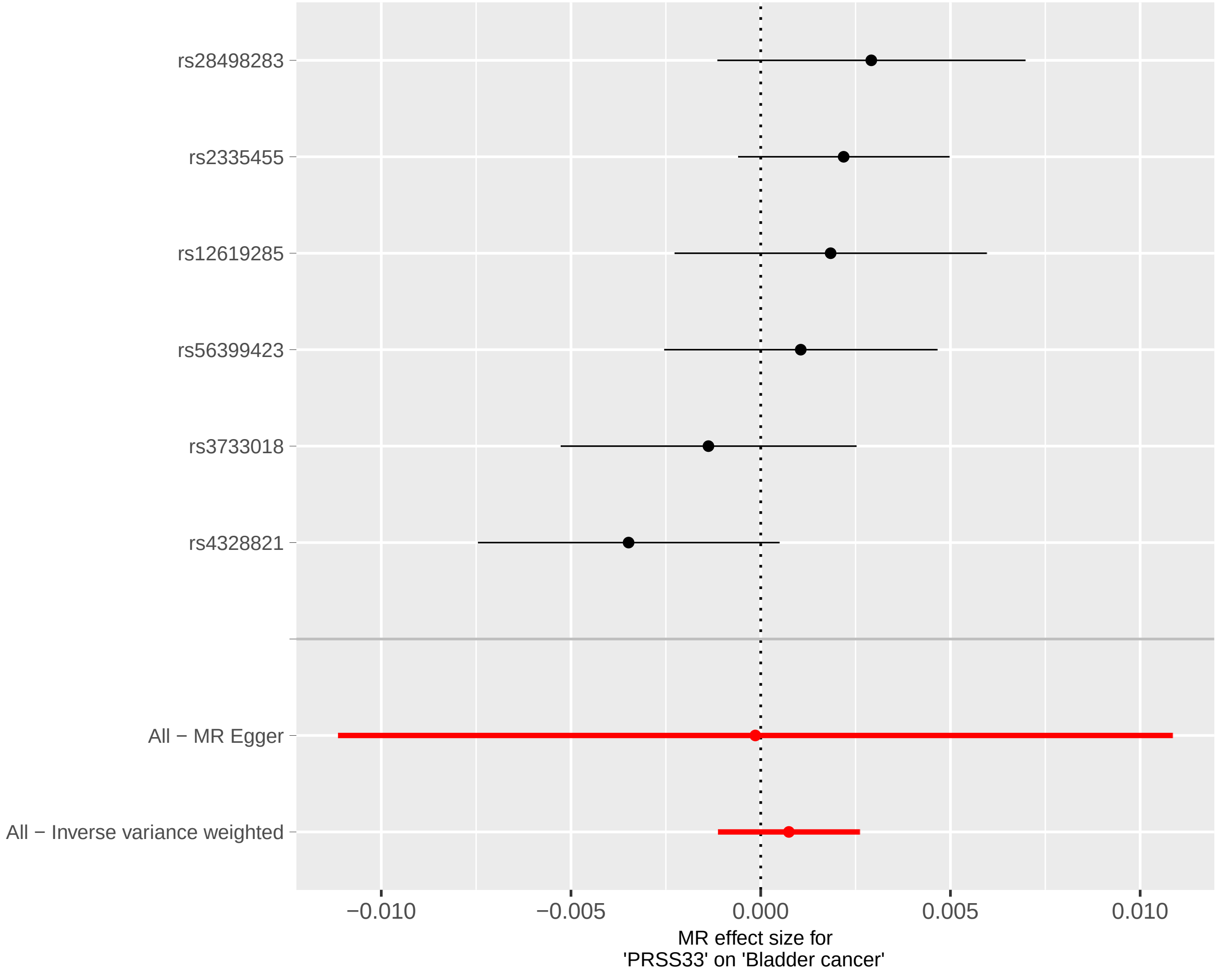
rs28498283
rs2335455
rs12619285
rs56399423
rs3733018
rs4328821
All − MR Egger
All − Inverse variance weighted
−0.010
−0.005
0.000
0.005
0.010
MR effect size for
'PRSS33' on 'Bladder cancer'

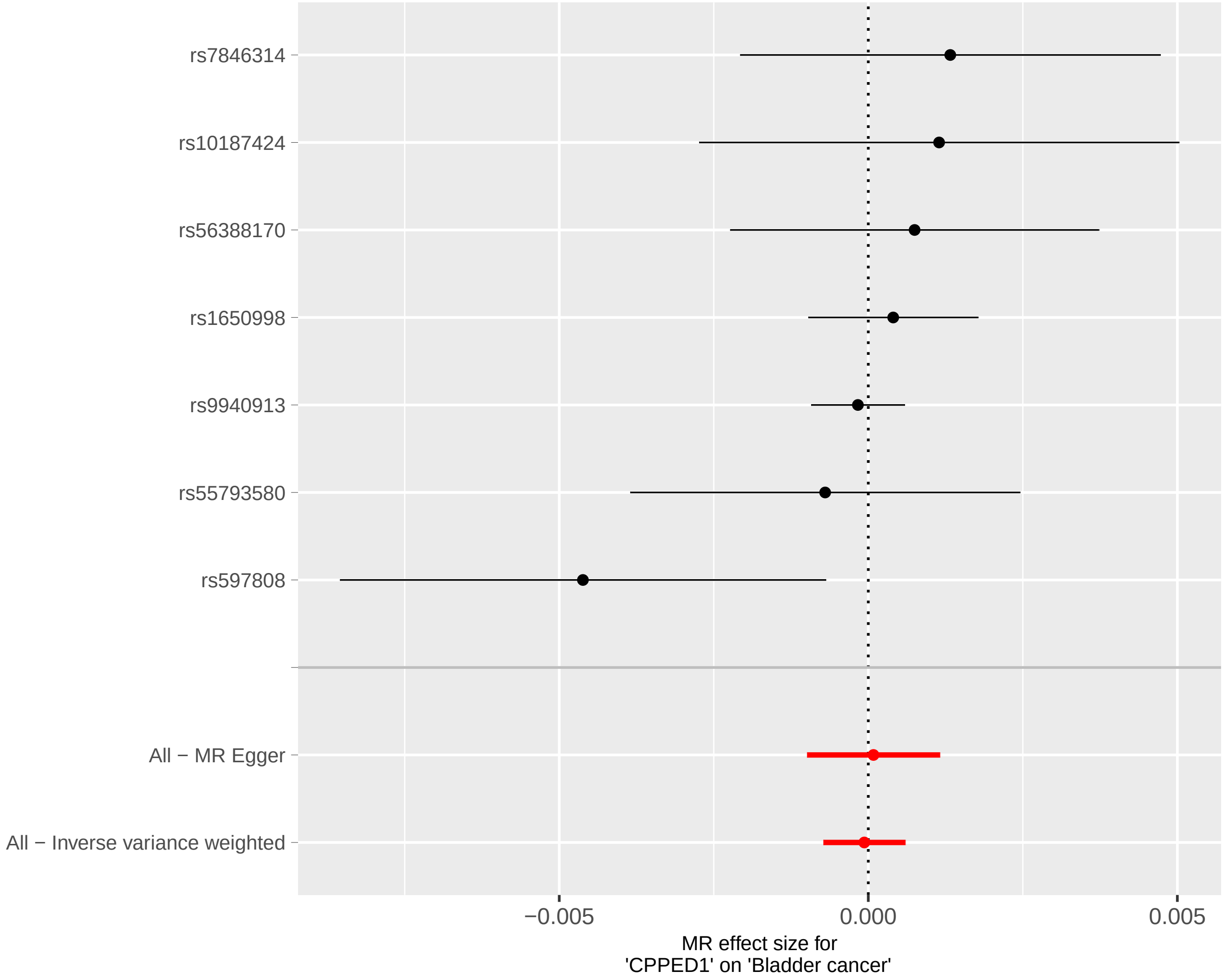
rs7846314
rs10187424
rs56388170
rs1650998
rs9940913
rs55793580
rs597808
All − MR Egger
All − Inverse variance weighted
−0.005
0.000
0.005
MR effect size for
'CPPED1' on 'Bladder cancer'

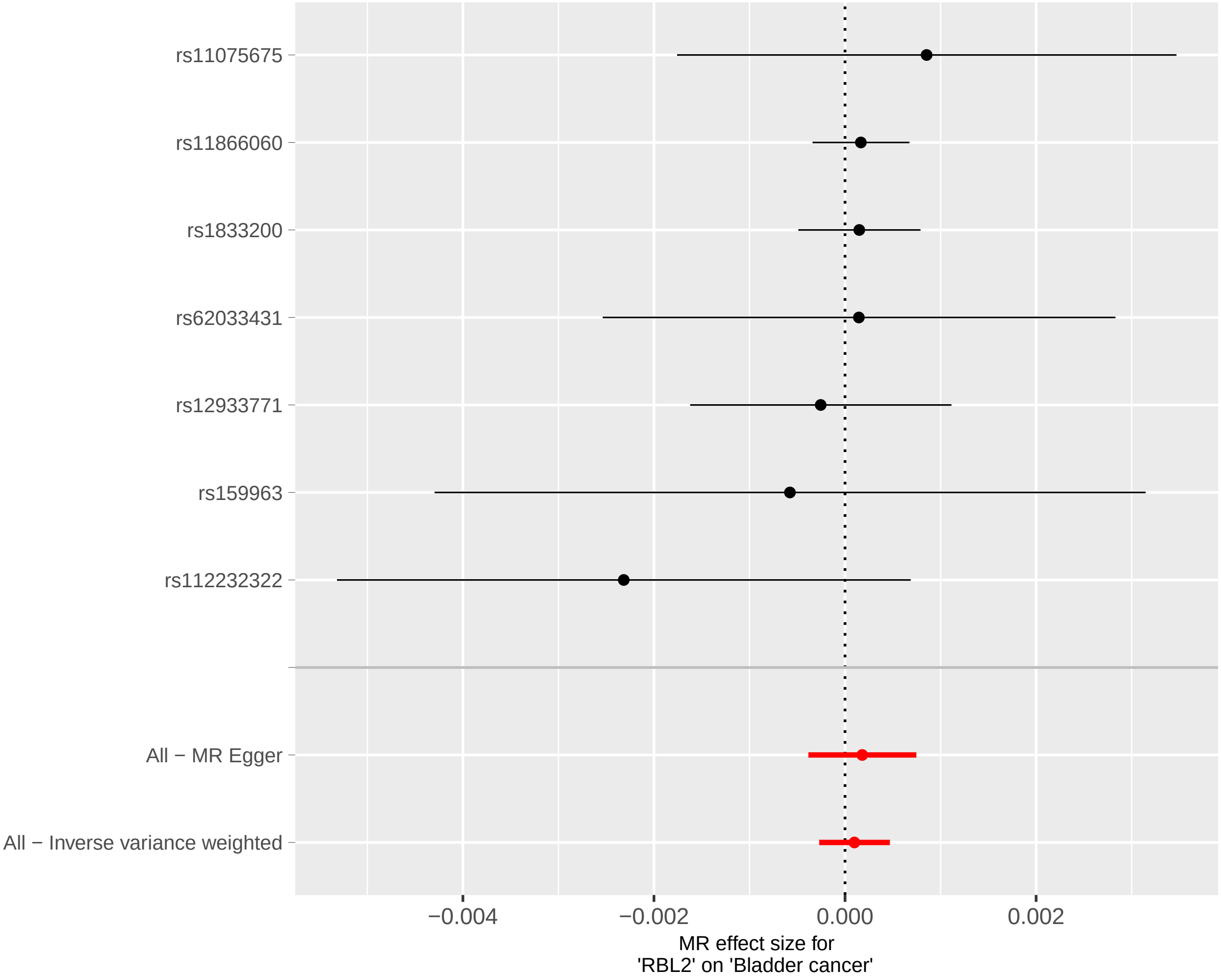
rs11075675
rs11866060
rs1833200
rs62033431
rs12933771
rs159963
rs112232322
All − MR Egger
All − Inverse variance weighted
−0.004
−0.002
0.000
0.002
MR effect size for
'RBL2' on 'Bladder cancer'

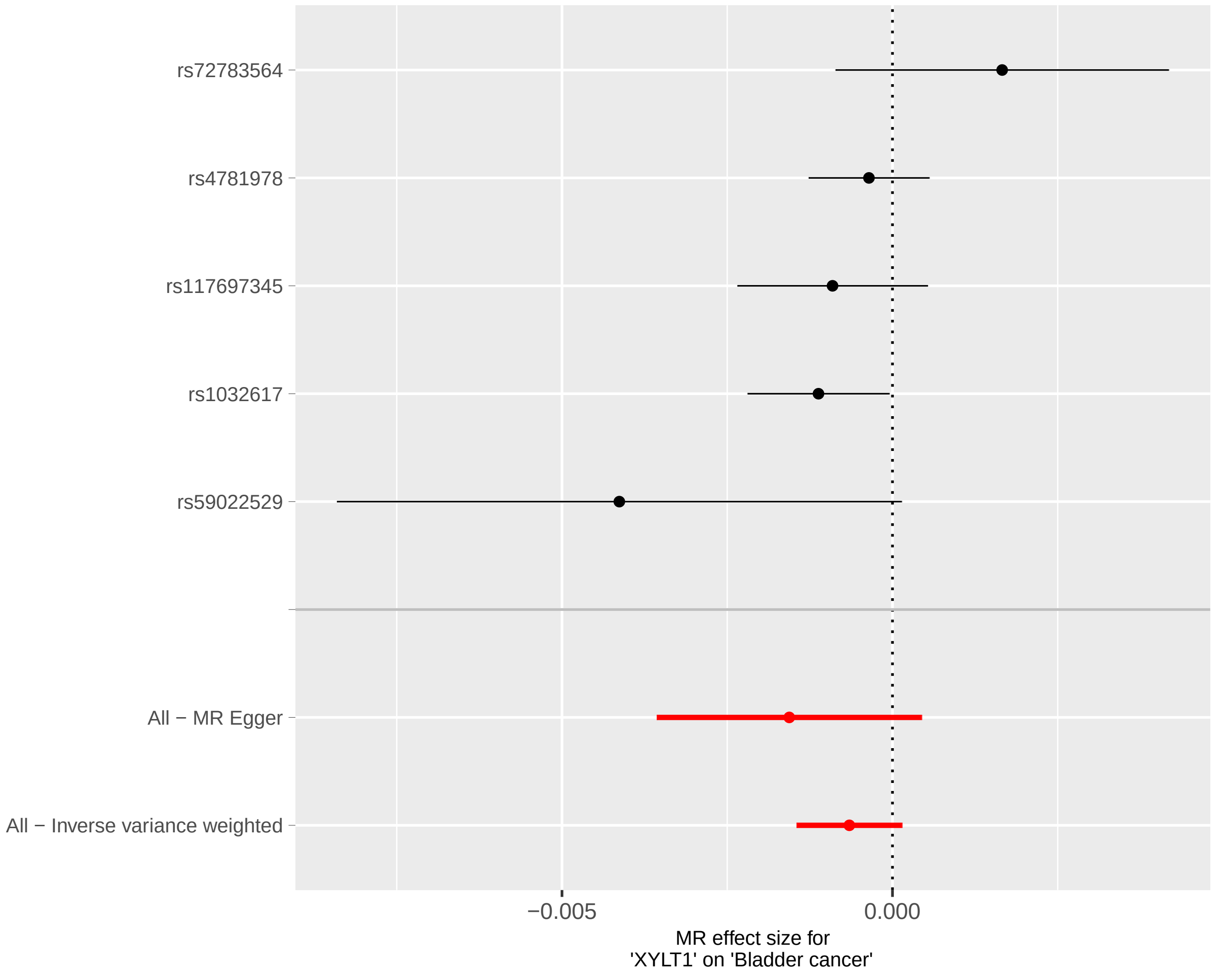

rs72783564
rs4781978
rs117697345
rs1032617
rs59022529
All − MR Egger
All − Inverse variance weighted
−0.005
0.000
MR effect size for
'XYLT1' on 'Bladder cancer'

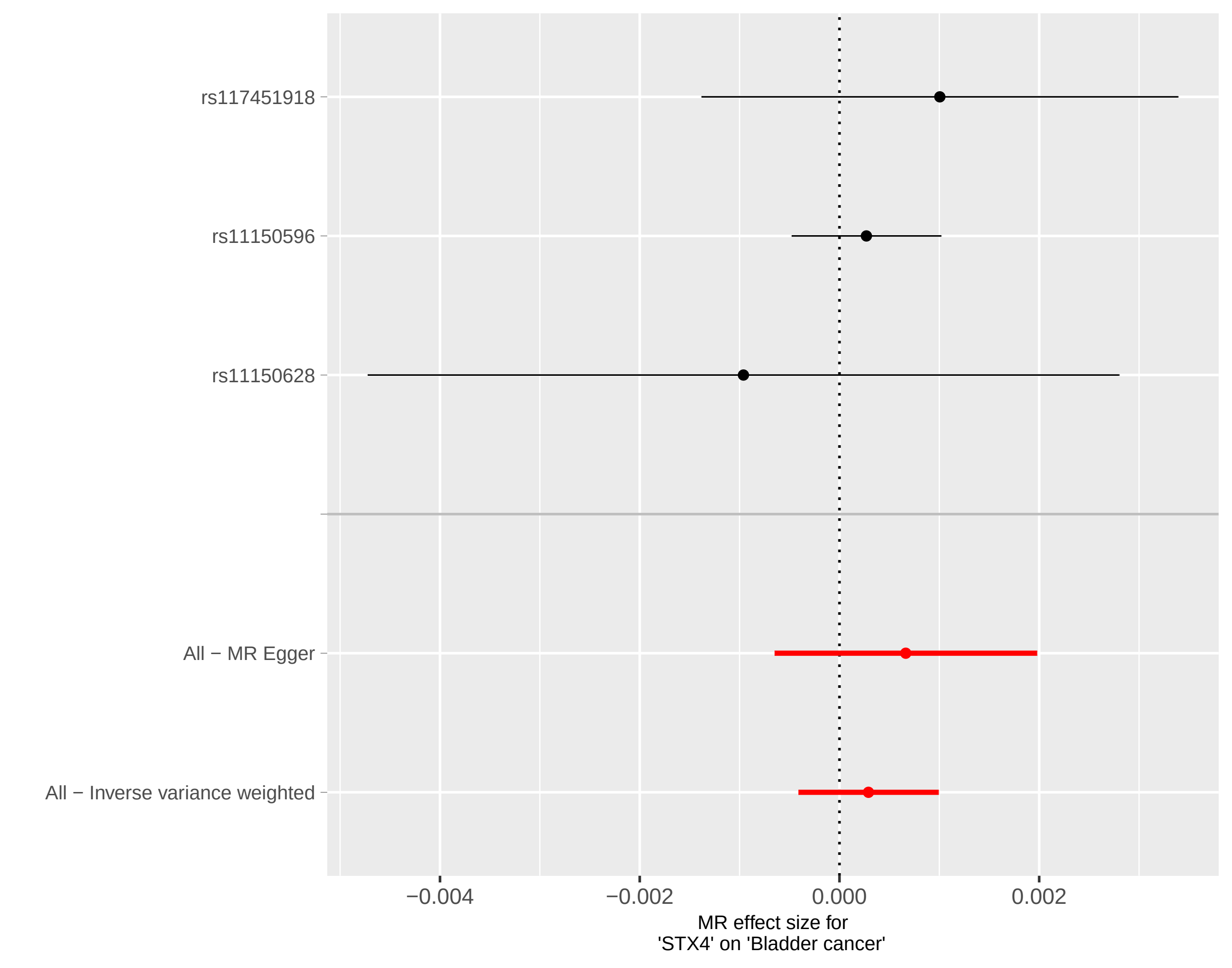

rs117451918
rs11150596
rs11150628
All − MR Egger
All − Inverse variance weighted
−0.004
−0.002
0.000
0.002
MR effect size for
'STX4' on 'Bladder cancer'

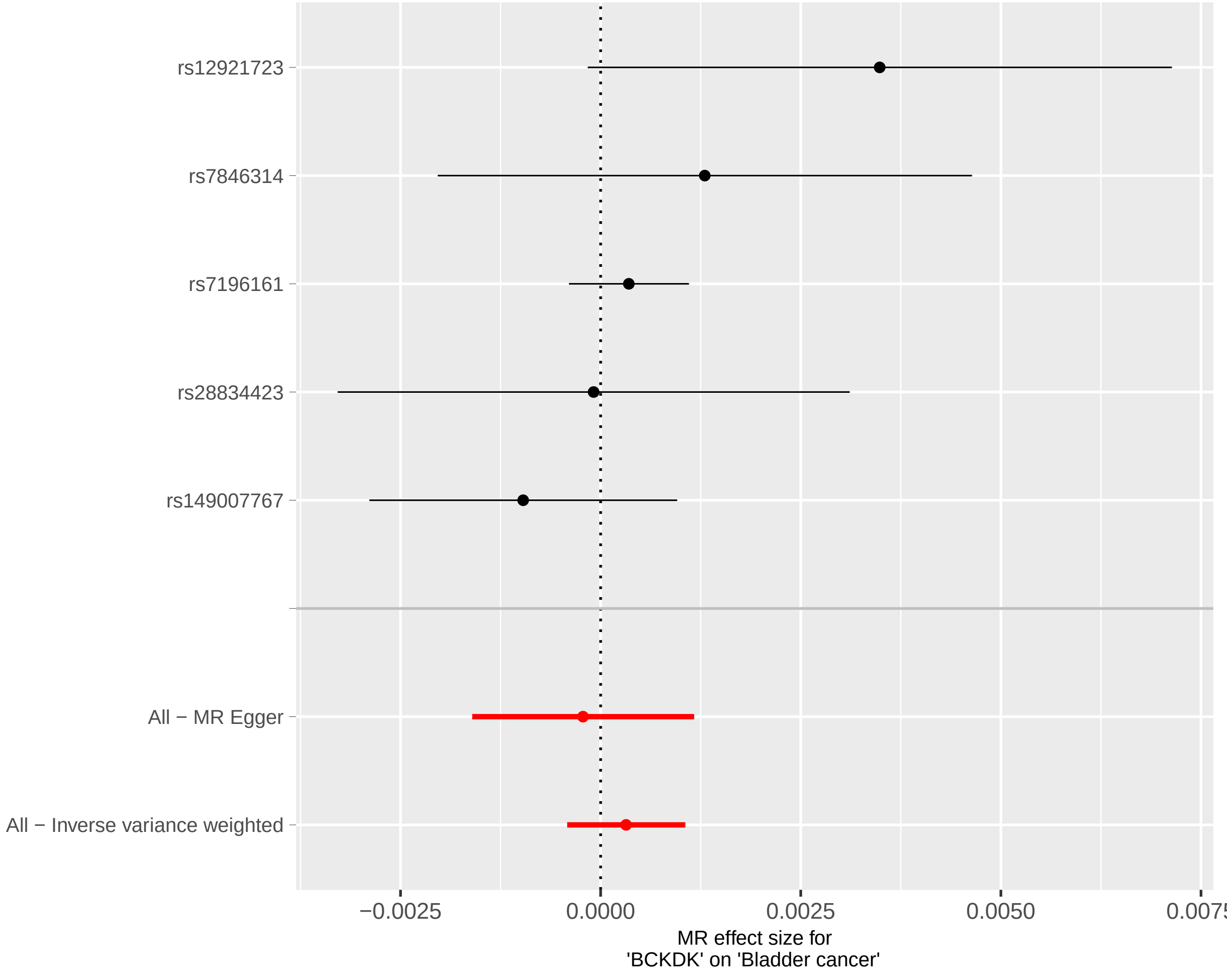
rs12921723
rs7846314
rs7196161
rs28834423
rs149007767
All − MR Egger
All − Inverse variance weighted
−0.0025
0.0000
0.0025
0.0050
MR effect size for
'BCKDK' on 'Bladder cancer'

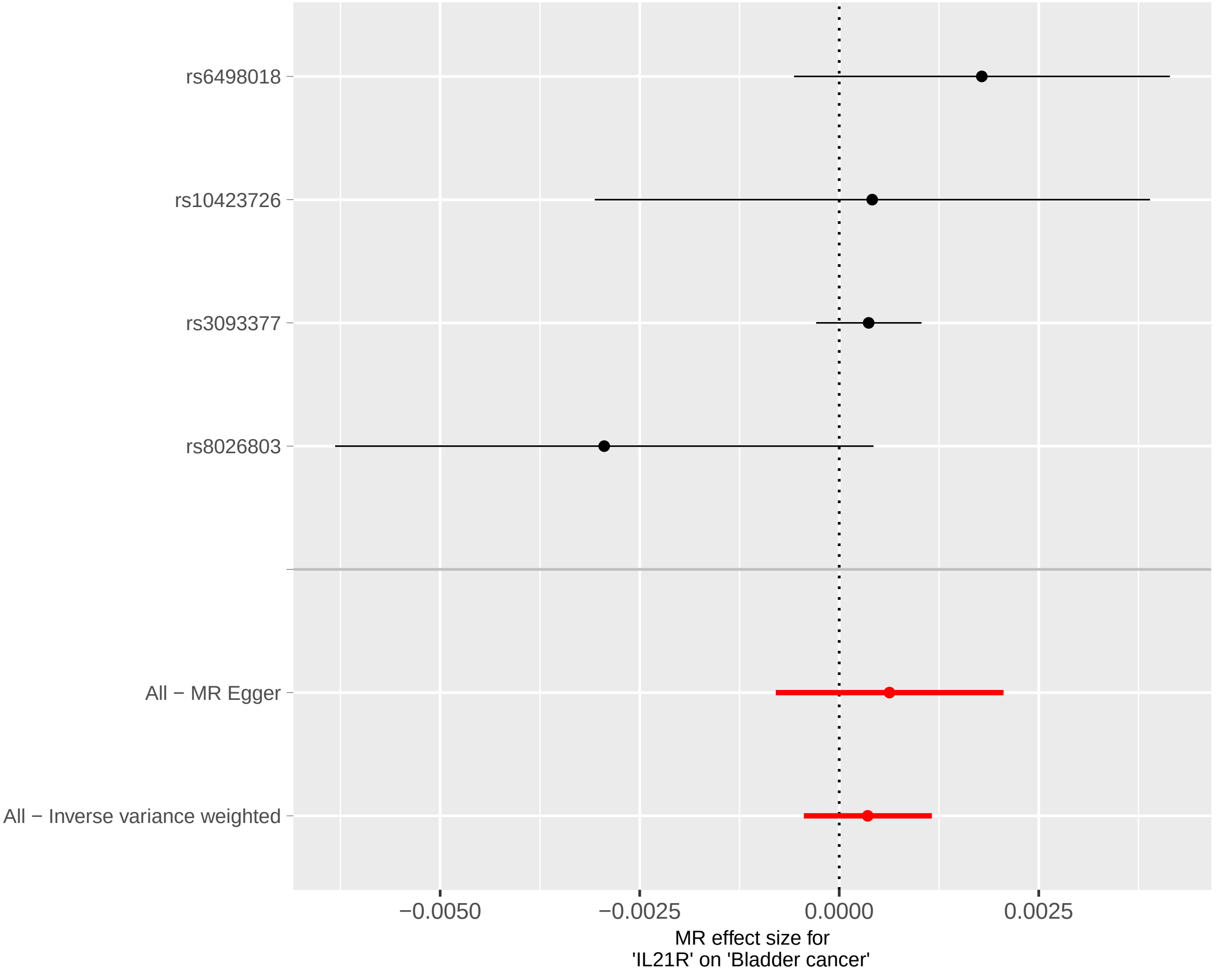

rs6498018
rs10423726
rs3093377
rs8026803
All − MR Egger
All − Inverse variance weighted
−0.0050
−0.0025
0.0000
0.0025
MR effect size for
'IL21R' on 'Bladder cancer'

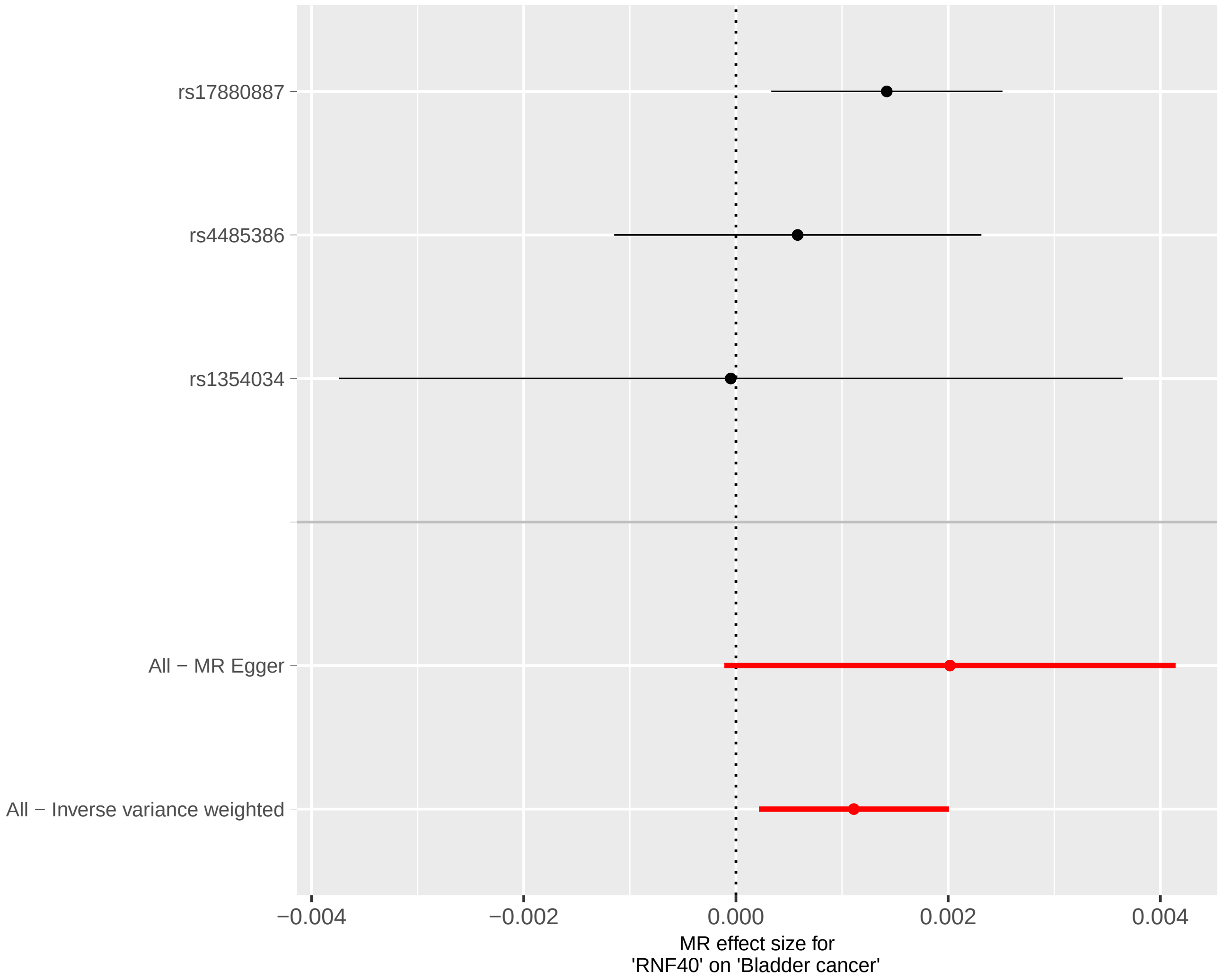
rs17880887
rs4485386
rs1354034
All − MR Egger
All − Inverse variance weighted
−0.004
−0.002
0.000
0.002
0.004
MR effect size for
'RNF40' on 'Bladder cancer'

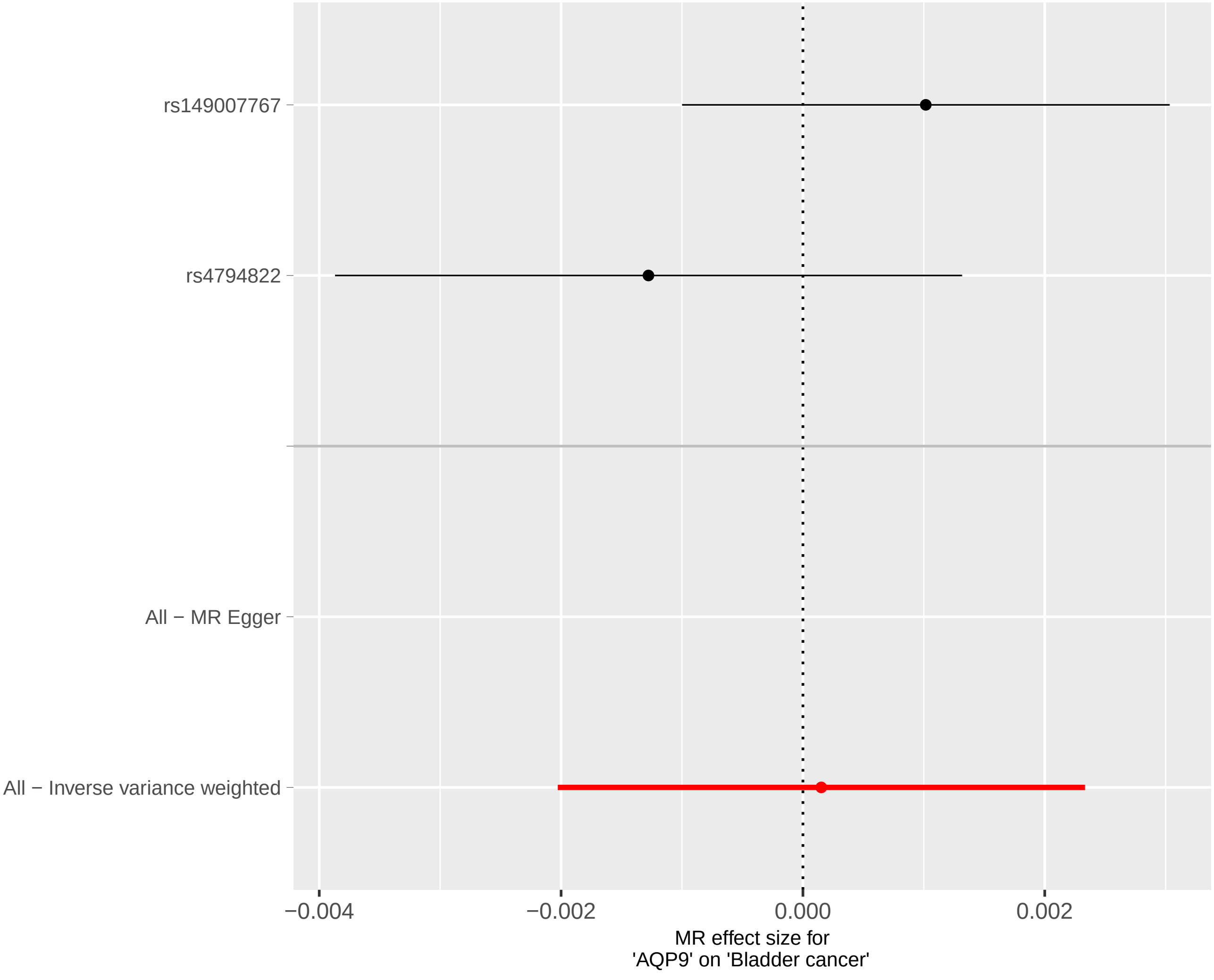
rs149007767
rs4794822
All − MR Egger
All − Inverse variance weighted
−0.004
−0.002
0.000
0.002
MR effect size for
'AQP9' on 'Bladder cancer'

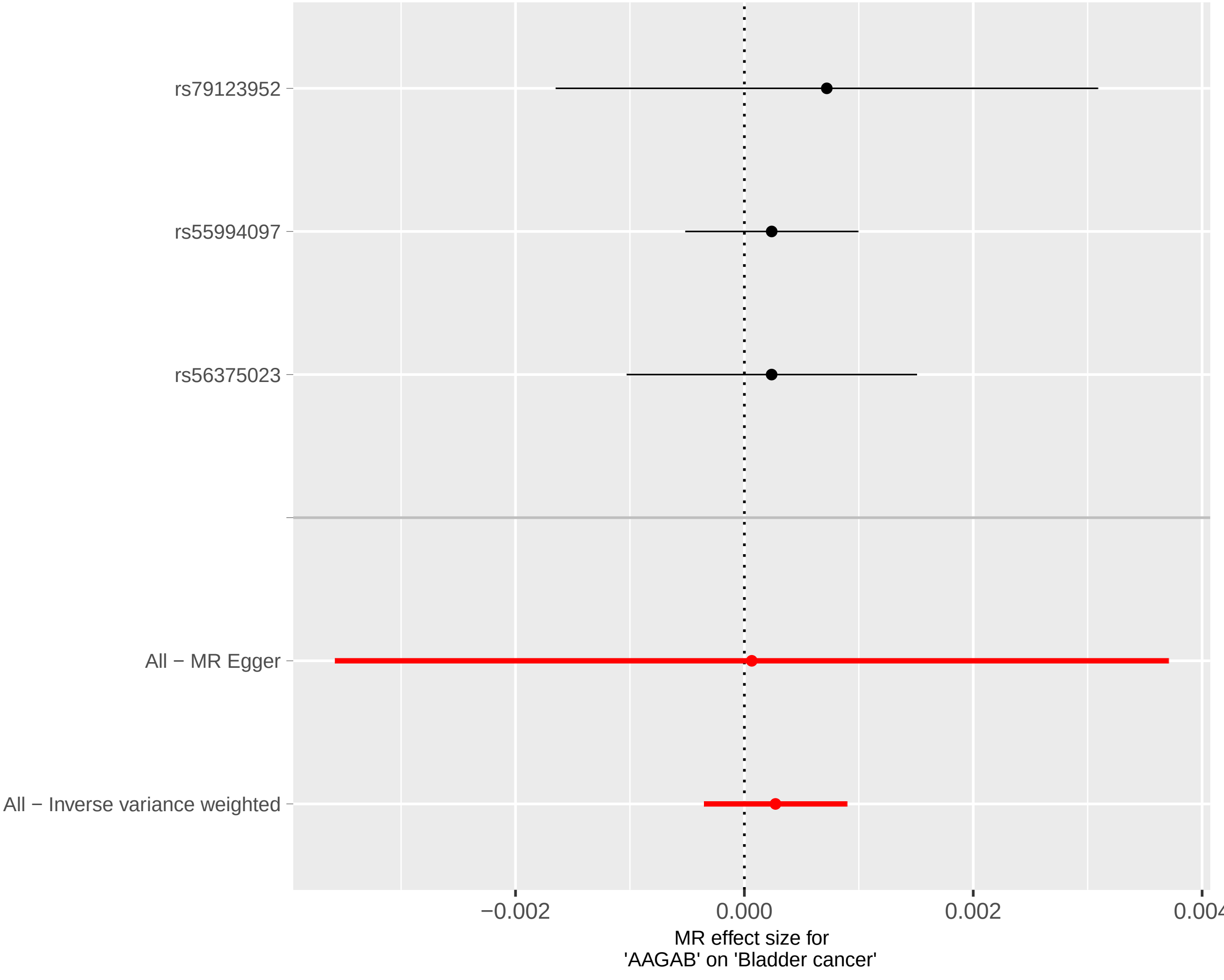
rs79123952
rs55994097
rs56375023
All − MR Egger
All − Inverse variance weighted
−0.002
0.000
0.002
0.004
MR effect size for
'AAGAB' on 'Bladder cancer'

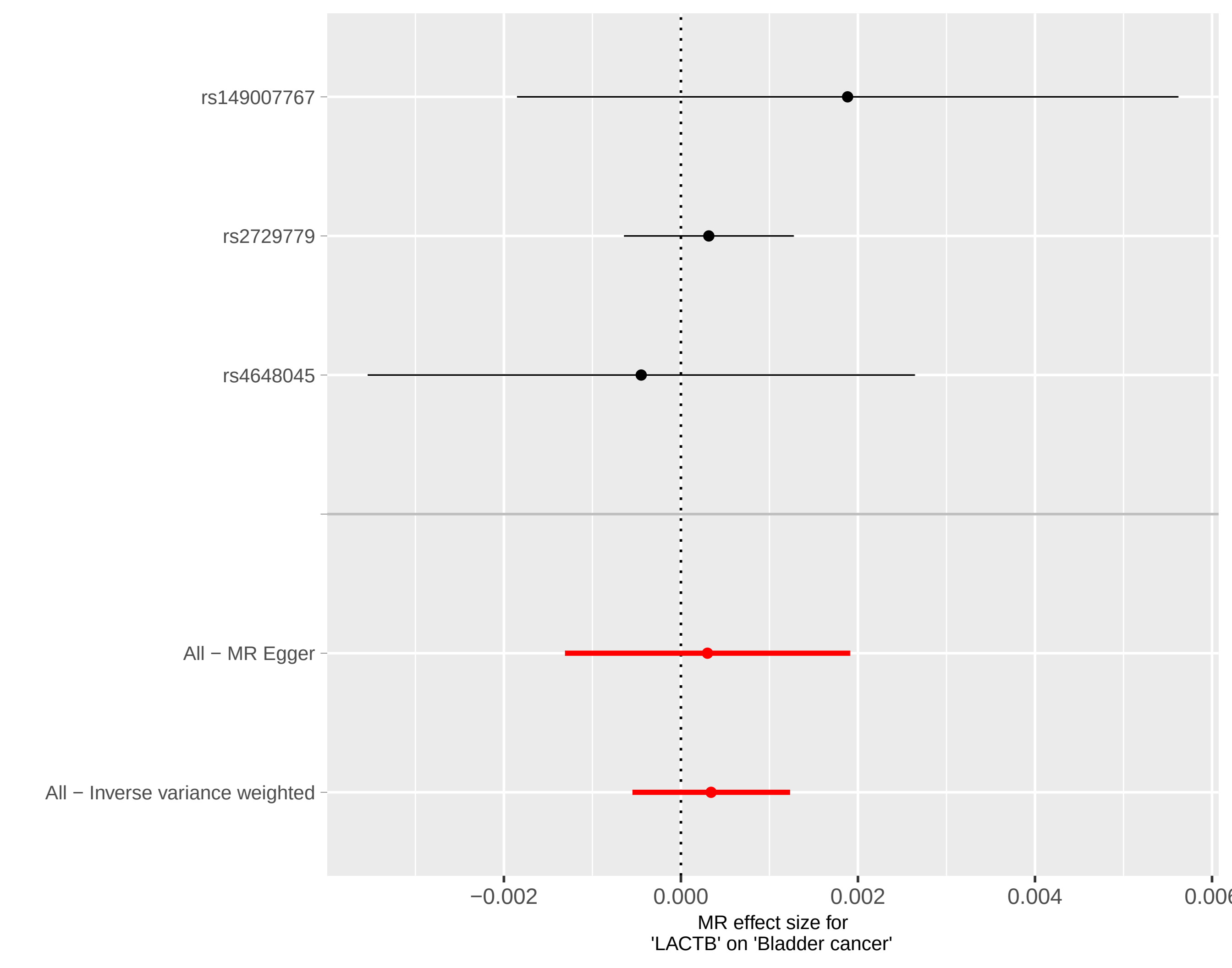
rs149007767
rs2729779
rs4648045
All − MR Egger
All − Inverse variance weighted
−0.002
0.000
0.002
0.004
0.006
MR effect size for
'LACTB' on 'Bladder cancer'

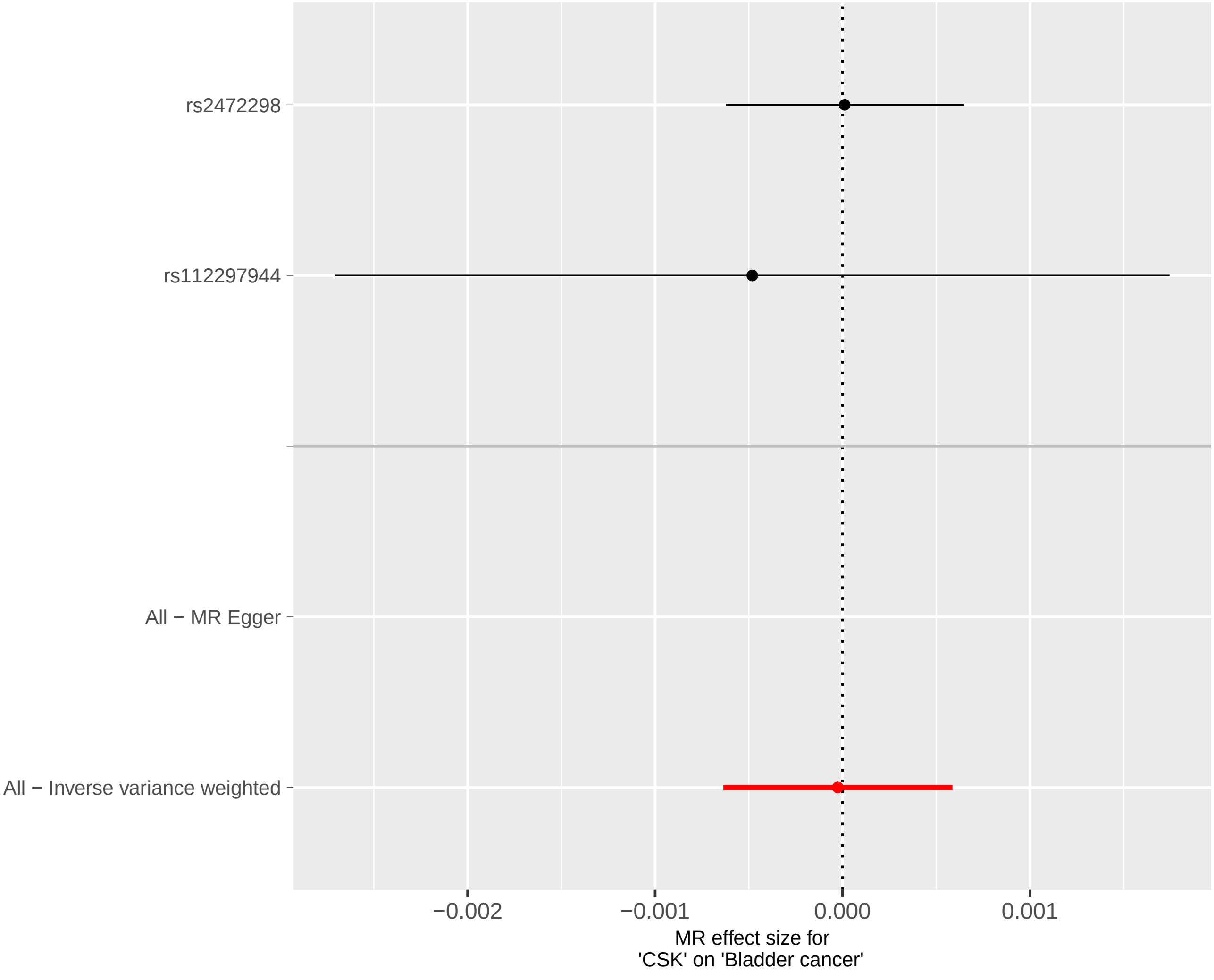

rs2472298
rs112297944
All − MR Egger
All − Inverse variance weighted
−0.002
−0.001
0.000
0.001
MR effect size for
'CSK' on 'Bladder cancer'

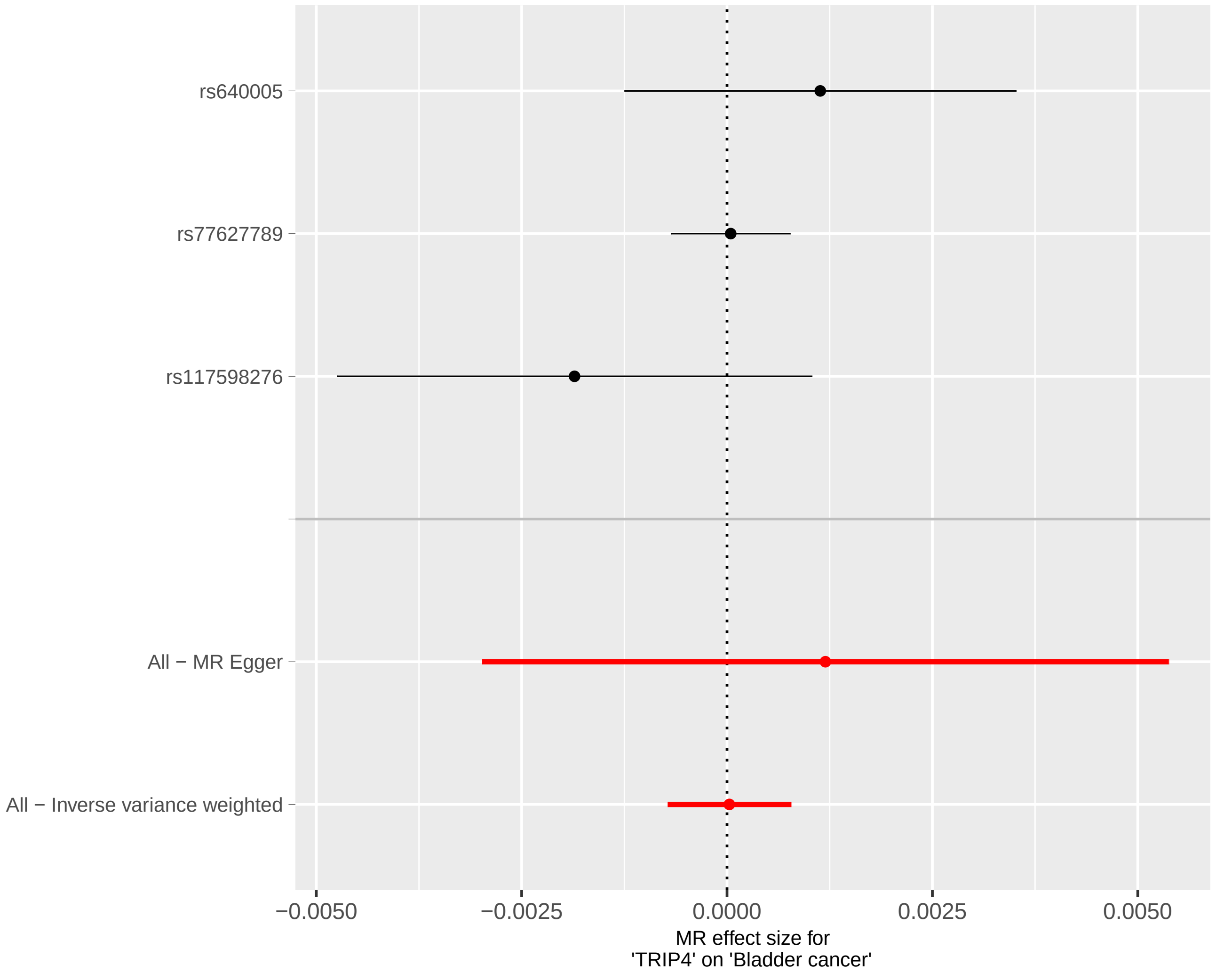
rs640005
rs77627789
rs117598276
All − MR Egger
All − Inverse variance weighted
−0.0050
−0.0025
0.0000
0.0025
0.0050
MR effect size for
'TRIP4' on 'Bladder cancer'

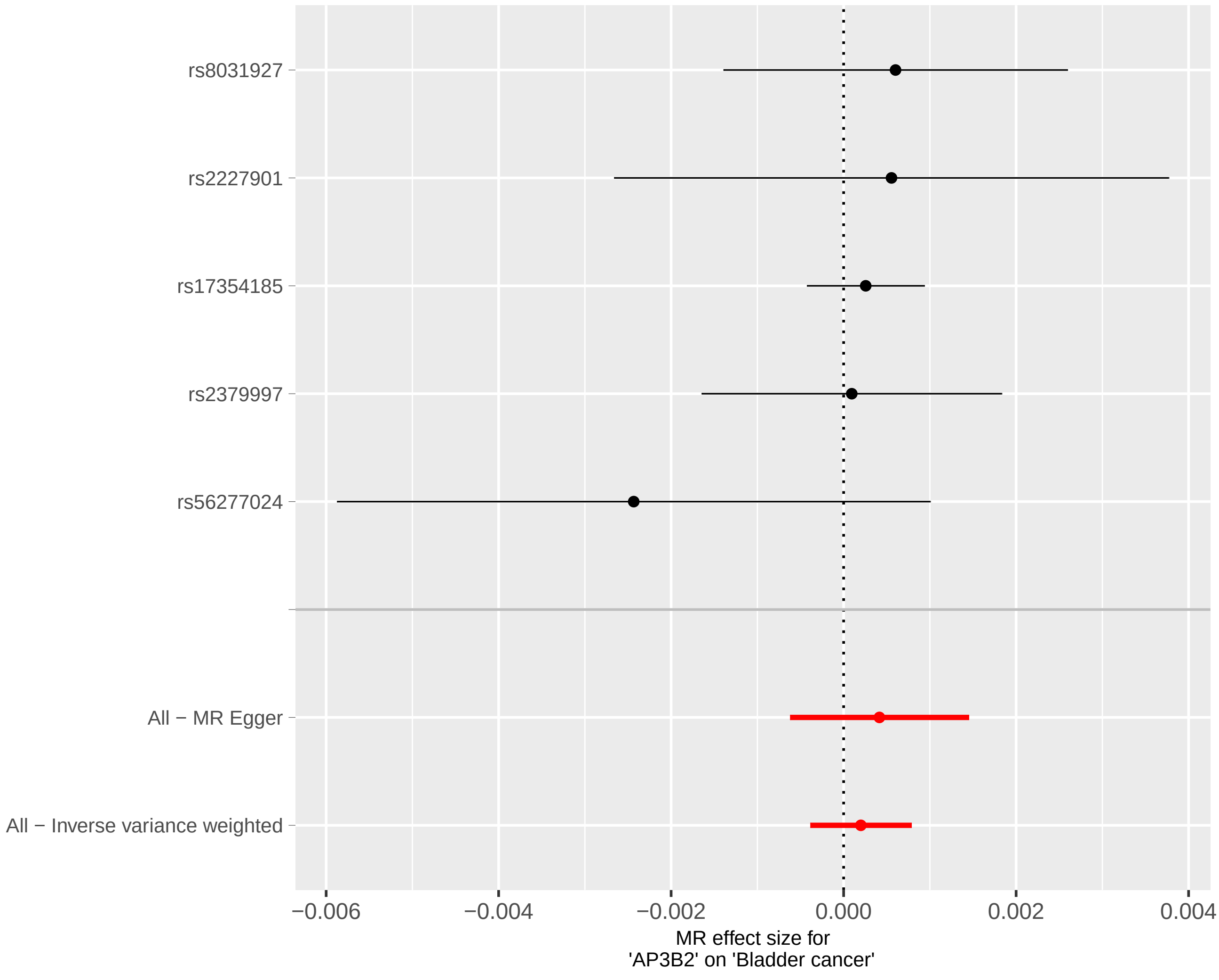

rs8031927
rs2227901
rs17354185
rs2379997
rs56277024
All − MR Egger
All − Inverse variance weighted
−0.006
−0.004
−0.002
0.000
0.002
0.004
MR effect size for
'AP3B2' on 'Bladder cancer'

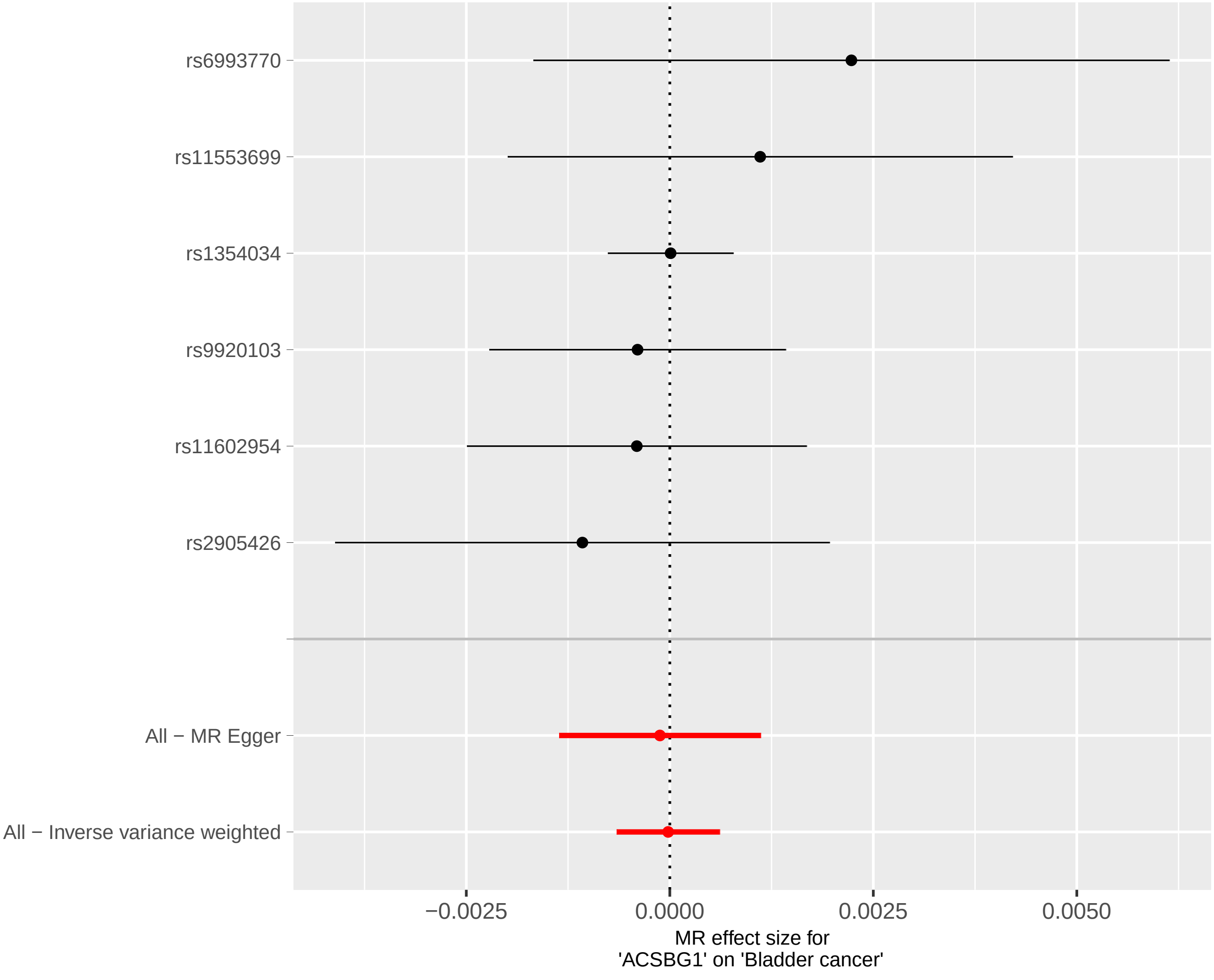

rs6993770
rs11553699
rs1354034
rs9920103
rs11602954
rs2905426
All − MR Egger
All − Inverse variance weighted
−0.0025
0.0000
0.0025
0.0050
MR effect size for
'ACSBG1' on 'Bladder cancer'

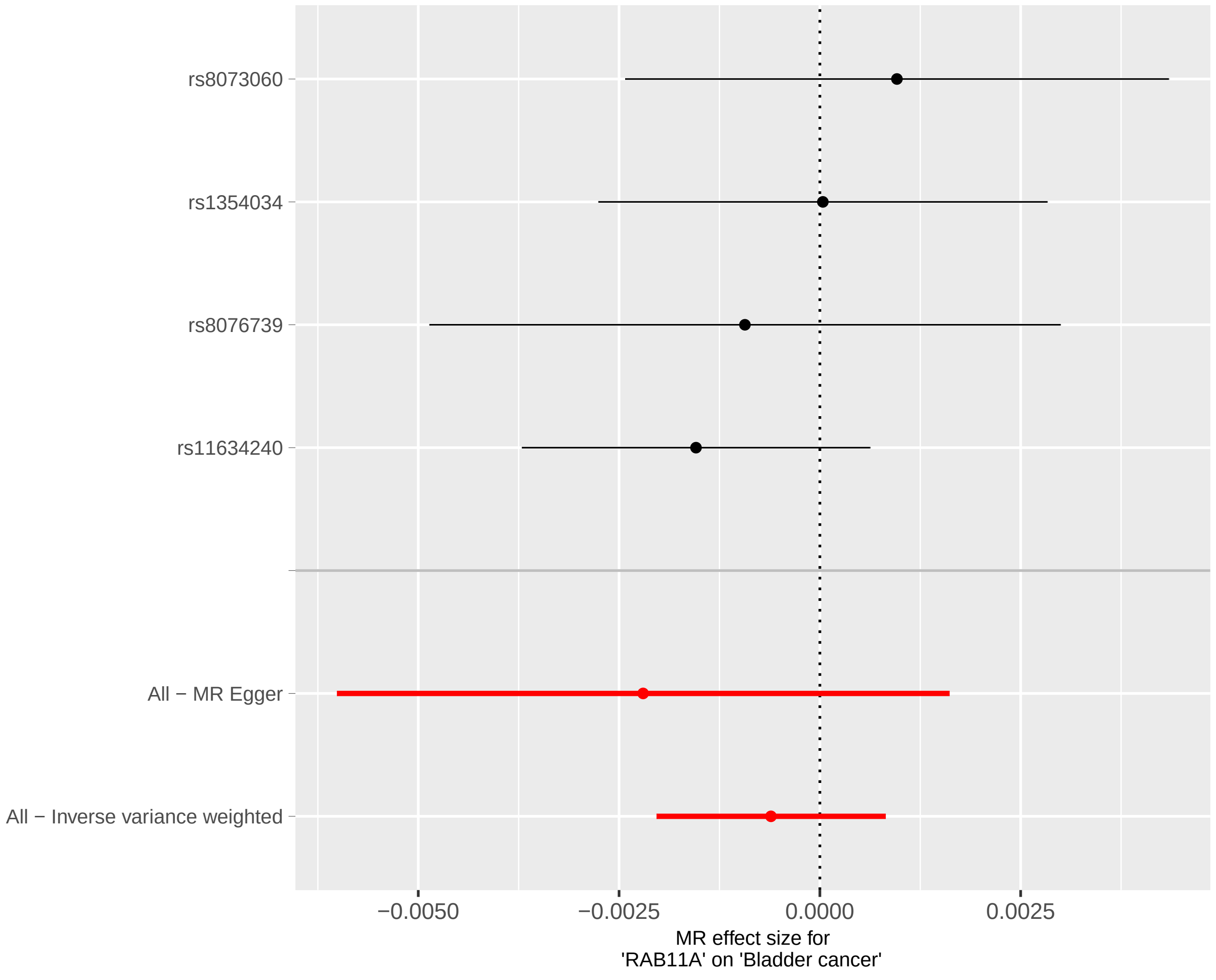
rs8073060
rs1354034
rs8076739
rs11634240
All − MR Egger
All − Inverse variance weighted
−0.0050
−0.0025
0.0000
0.0025
MR effect size for
'RAB11A' on 'Bladder cancer'

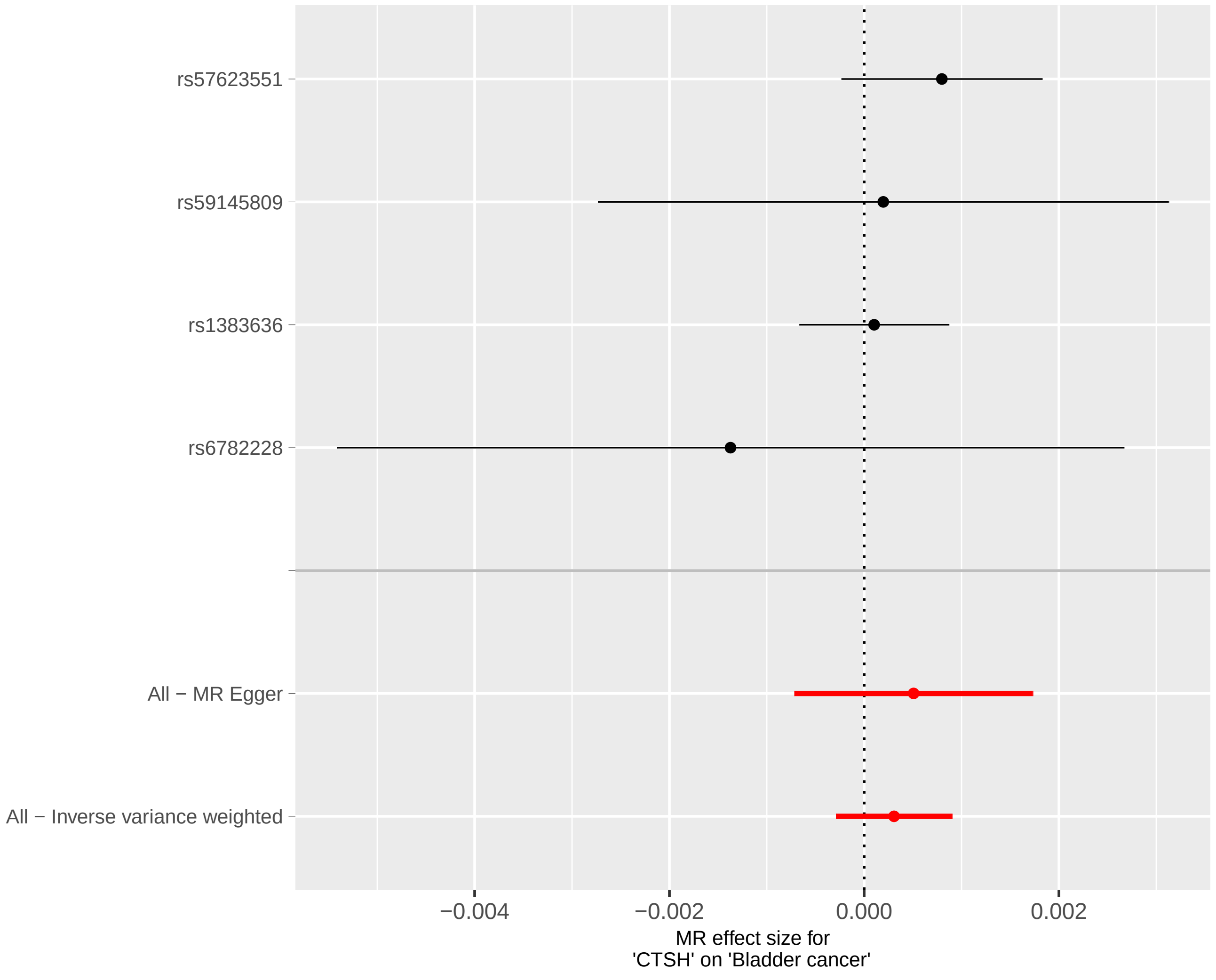

rs57623551
rs59145809
rs1383636
rs6782228
All − MR Egger
All − Inverse variance weighted
−0.004
−0.002
0.000
0.002
MR effect size for
'CTSH' on 'Bladder cancer'

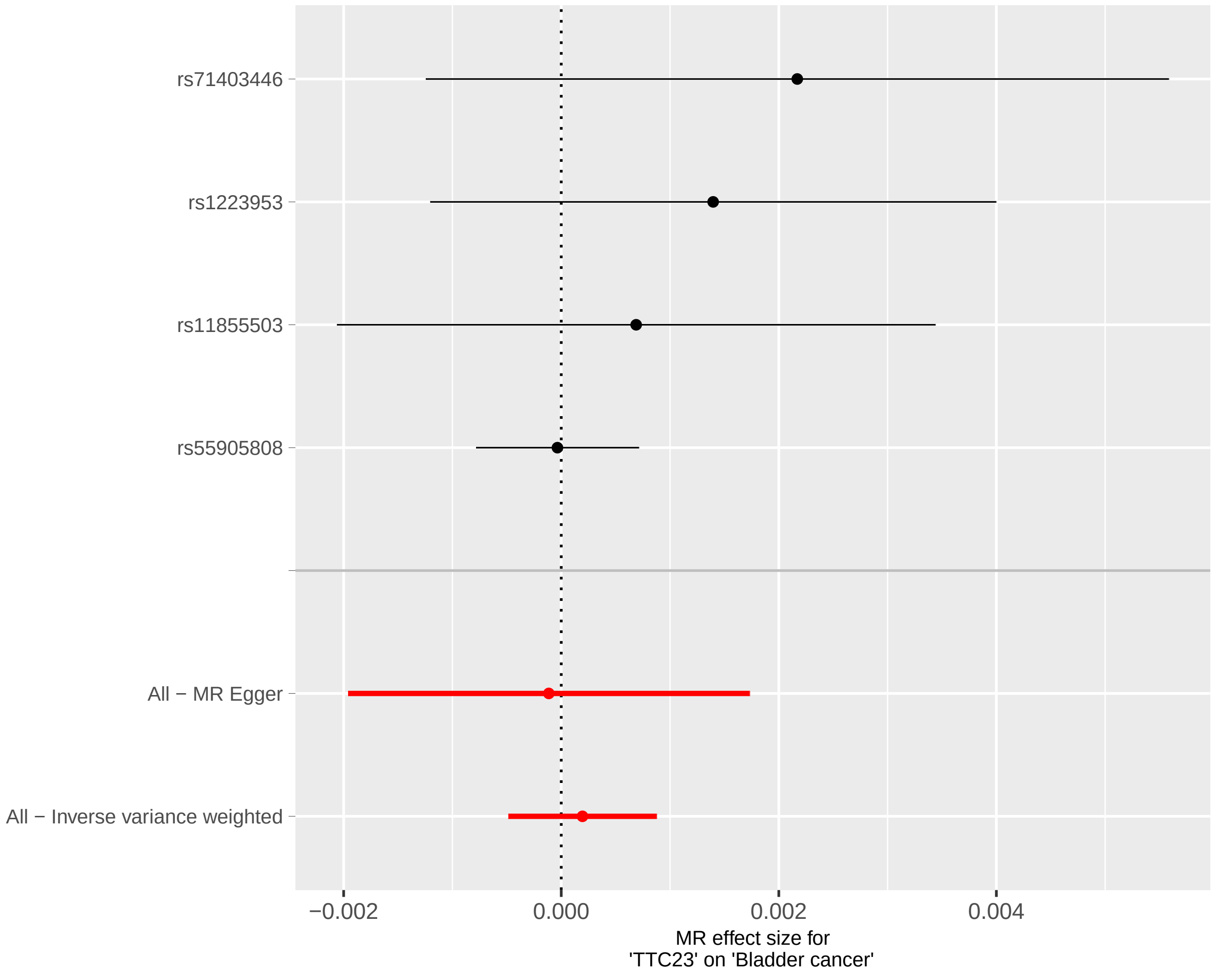

rs71403446
rs1223953
rs11855503
rs55905808
All − MR Egger
All − Inverse variance weighted
−0.002
0.000
0.002
0.004
MR effect size for
'TTC23' on 'Bladder cancer'

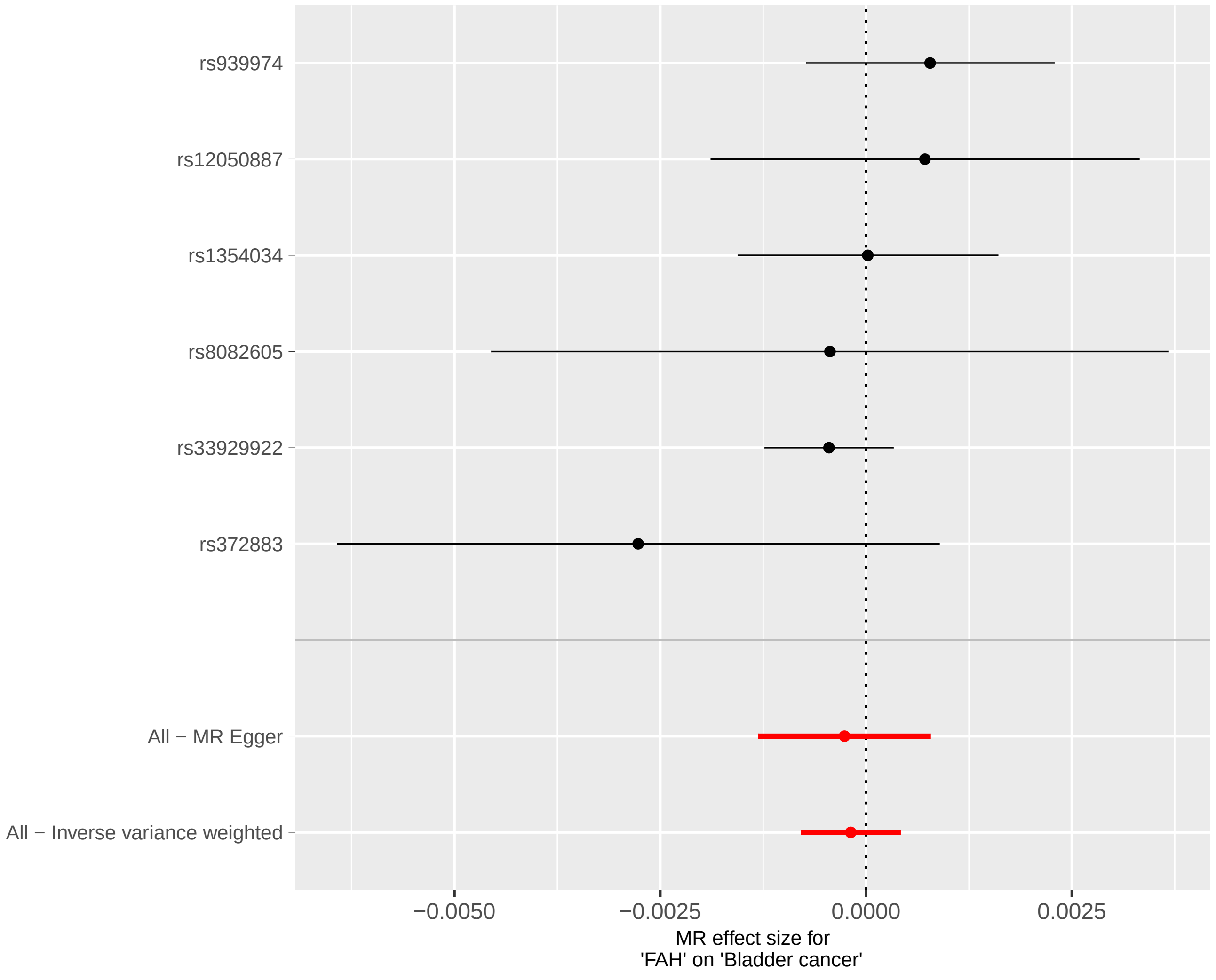
rs939974
rs12050887
rs1354034
rs8082605
rs33929922
rs372883
All − MR Egger
All − Inverse variance weighted
−0.0050
−0.0025
0.0000
0.0025
MR effect size for
'FAH' on 'Bladder cancer'

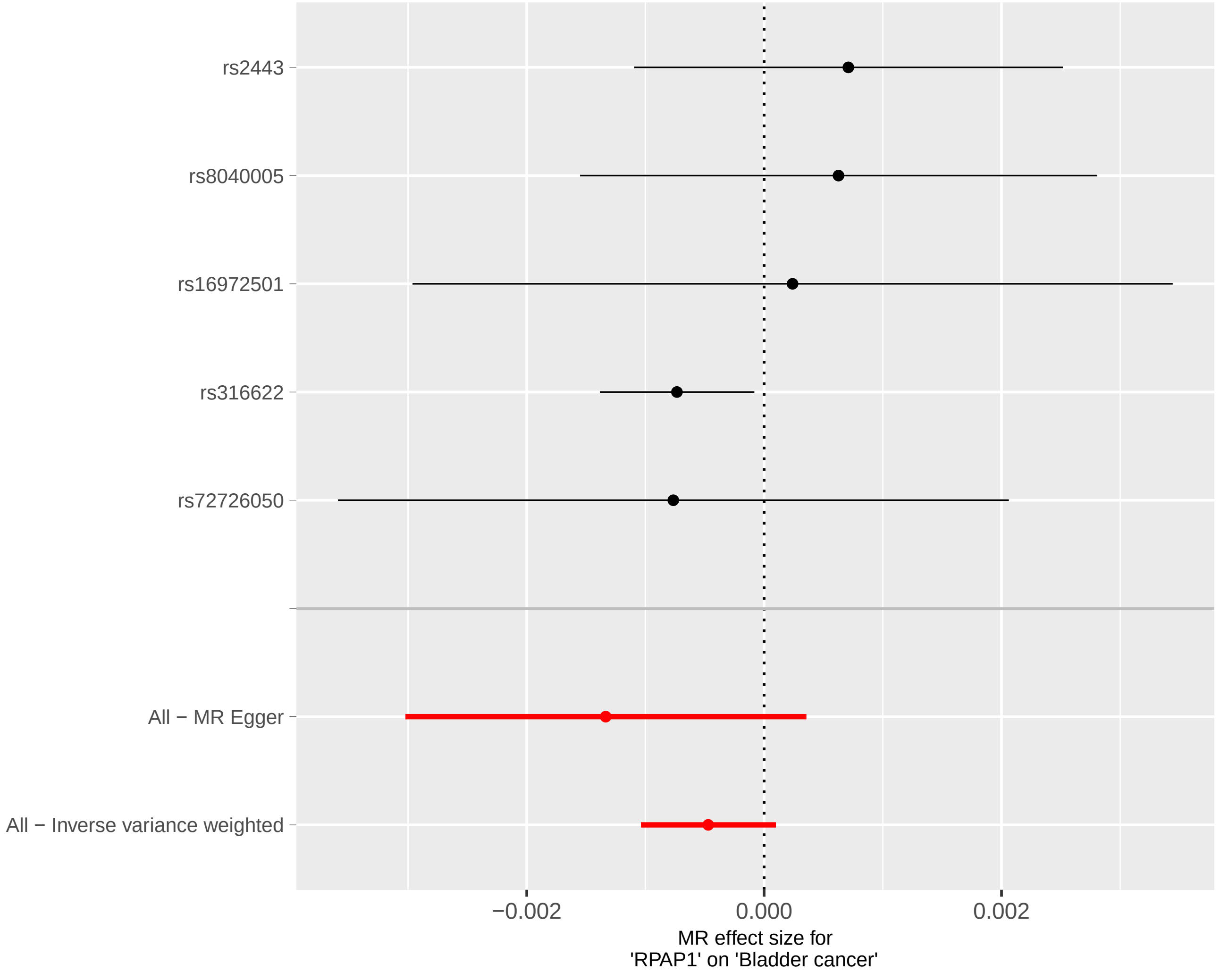

rs2443
rs8040005
rs16972501
rs316622
rs72726050
All − MR Egger
All − Inverse variance weighted
−0.002
0.000
0.002
MR effect size for
'RPAP1' on 'Bladder cancer'

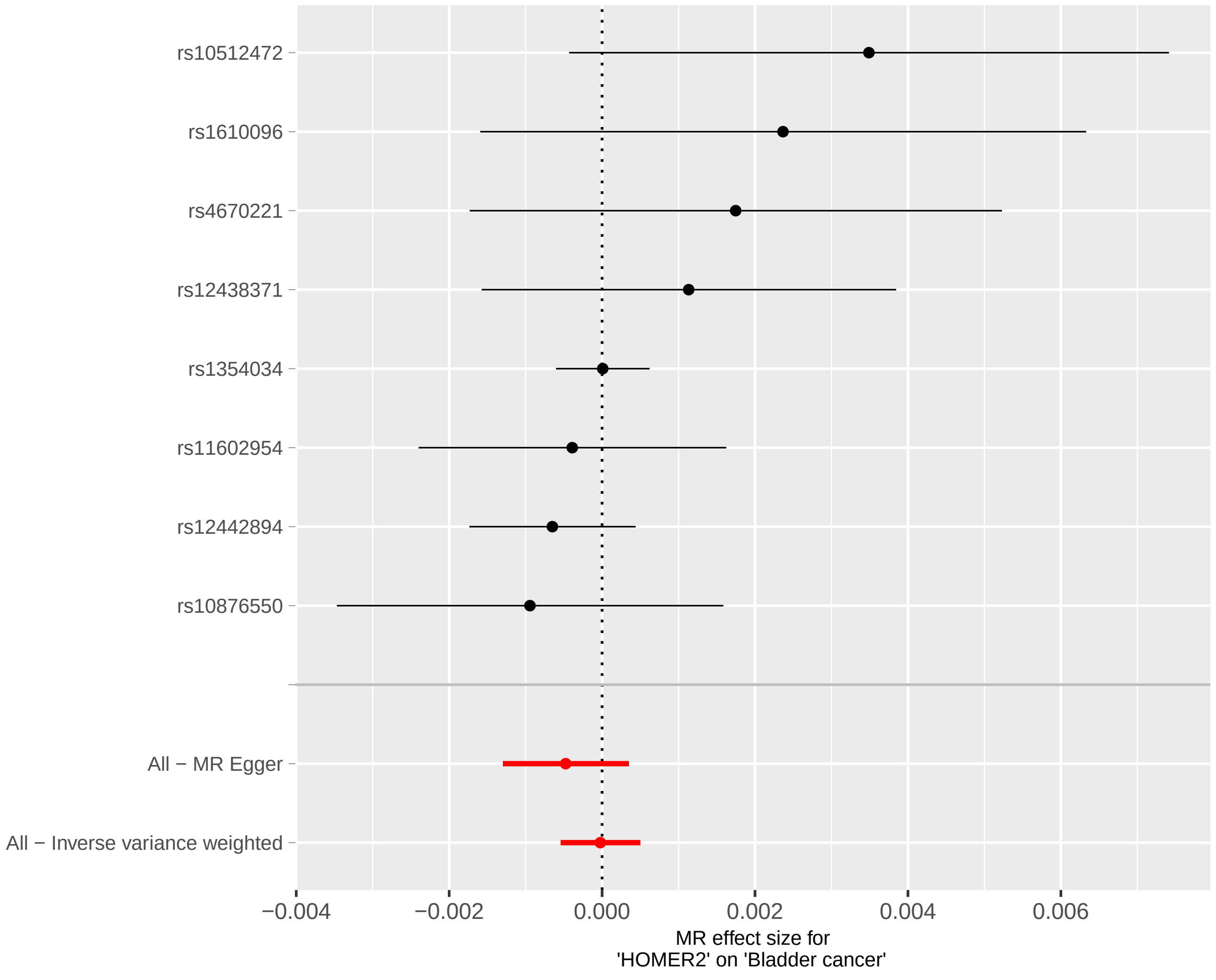

rs10512472
rs1610096
rs4670221
rs12438371
rs1354034
rs11602954
rs12442894
rs10876550
All − MR Egger
All − Inverse variance weighted
−0.004
−0.002
0.000
0.002
0.004
0.006
MR effect size for
'HOMER2' on 'Bladder cancer'

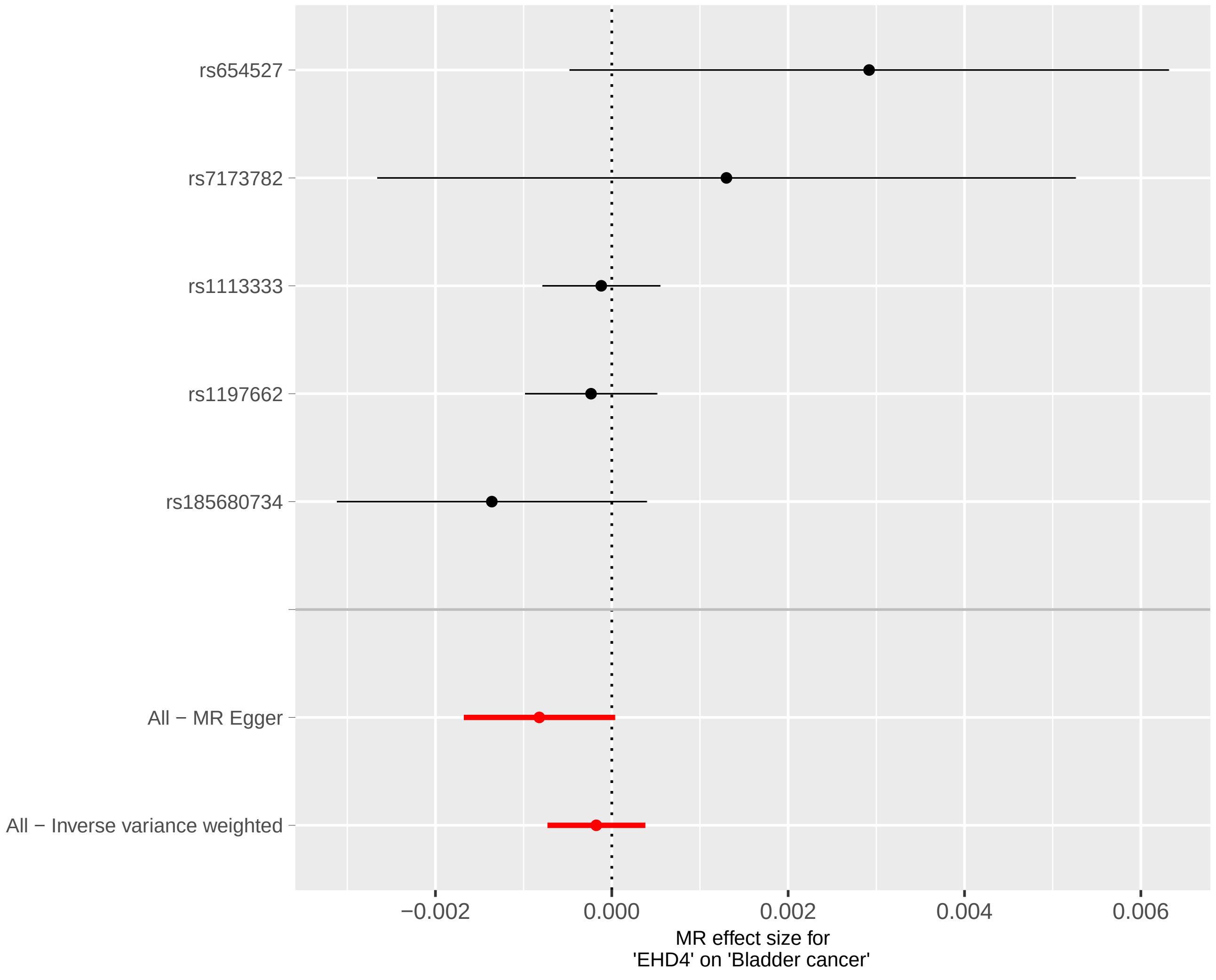

rs654527
rs7173782
rs1113333
rs1197662
rs185680734
All − MR Egger
All − Inverse variance weighted
−0.002
0.000
0.002
0.004
0.006
MR effect size for
'EHD4' on 'Bladder cancer'

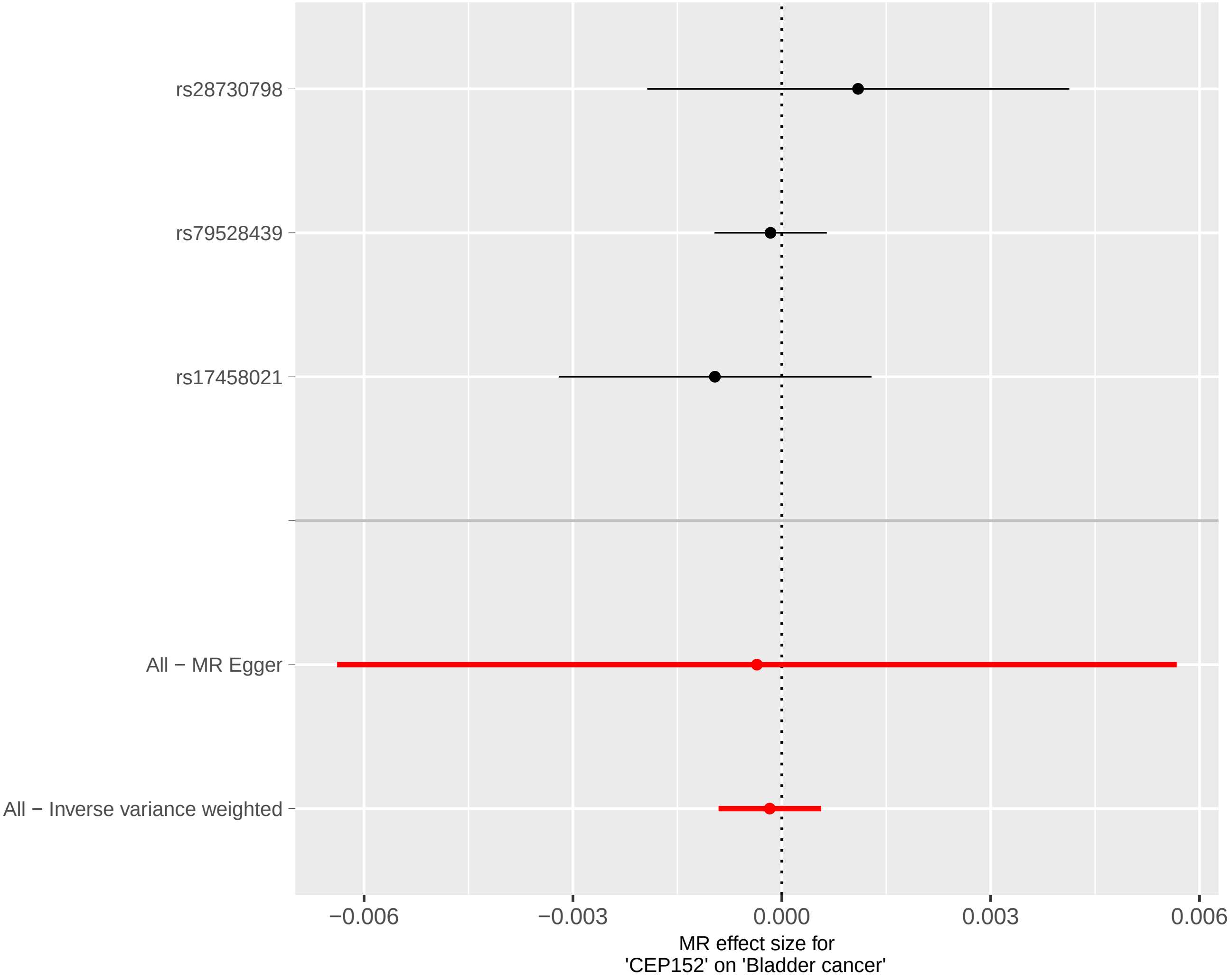
rs28730798
rs79528439
rs17458021
All − MR Egger
All − Inverse variance weighted
−0.006
−0.003
0.000
0.003
0.006
MR effect size for
'CEP152' on 'Bladder cancer'

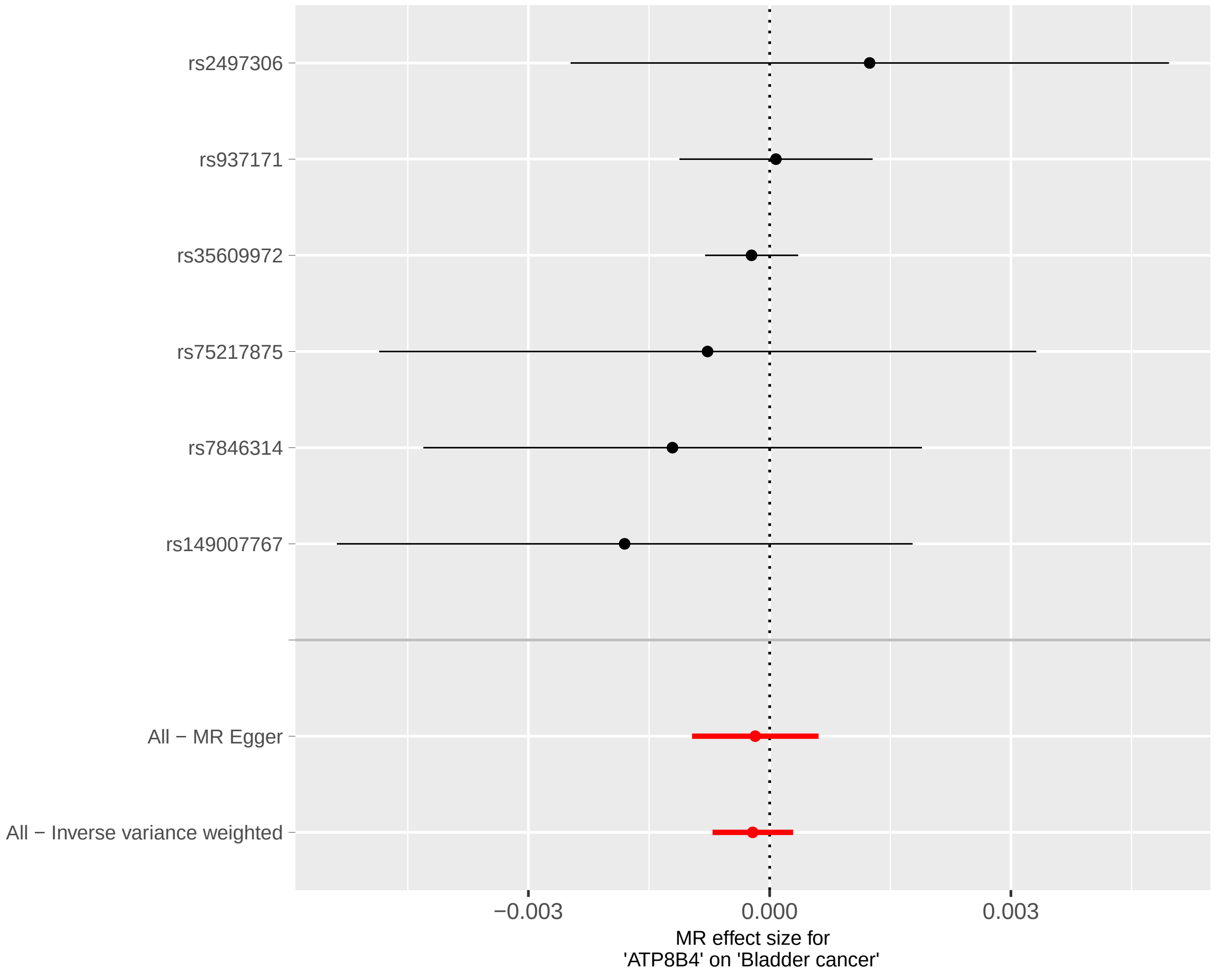

rs2497306
rs937171
rs35609972
rs75217875
rs7846314
rs149007767
All − MR Egger
All − Inverse variance weighted
−0.003
0.000
0.003
MR effect size for
'ATP8B4' on 'Bladder cancer'

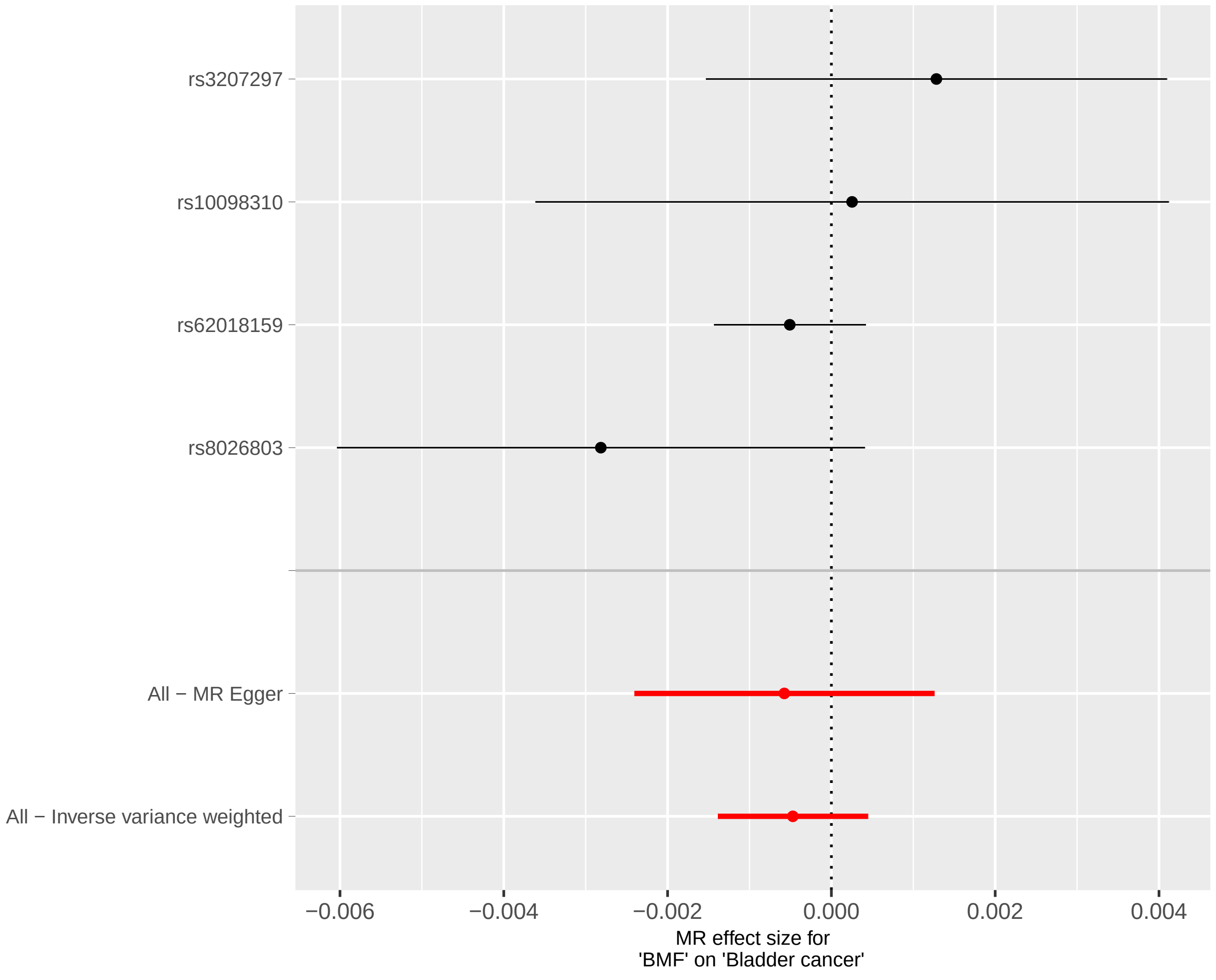
rs3207297
rs10098310
rs62018159
rs8026803
All − MR Egger
All − Inverse variance weighted
−0.006
−0.004
−0.002
0.000
0.002
0.004
MR effect size for
'BMF' on 'Bladder cancer'

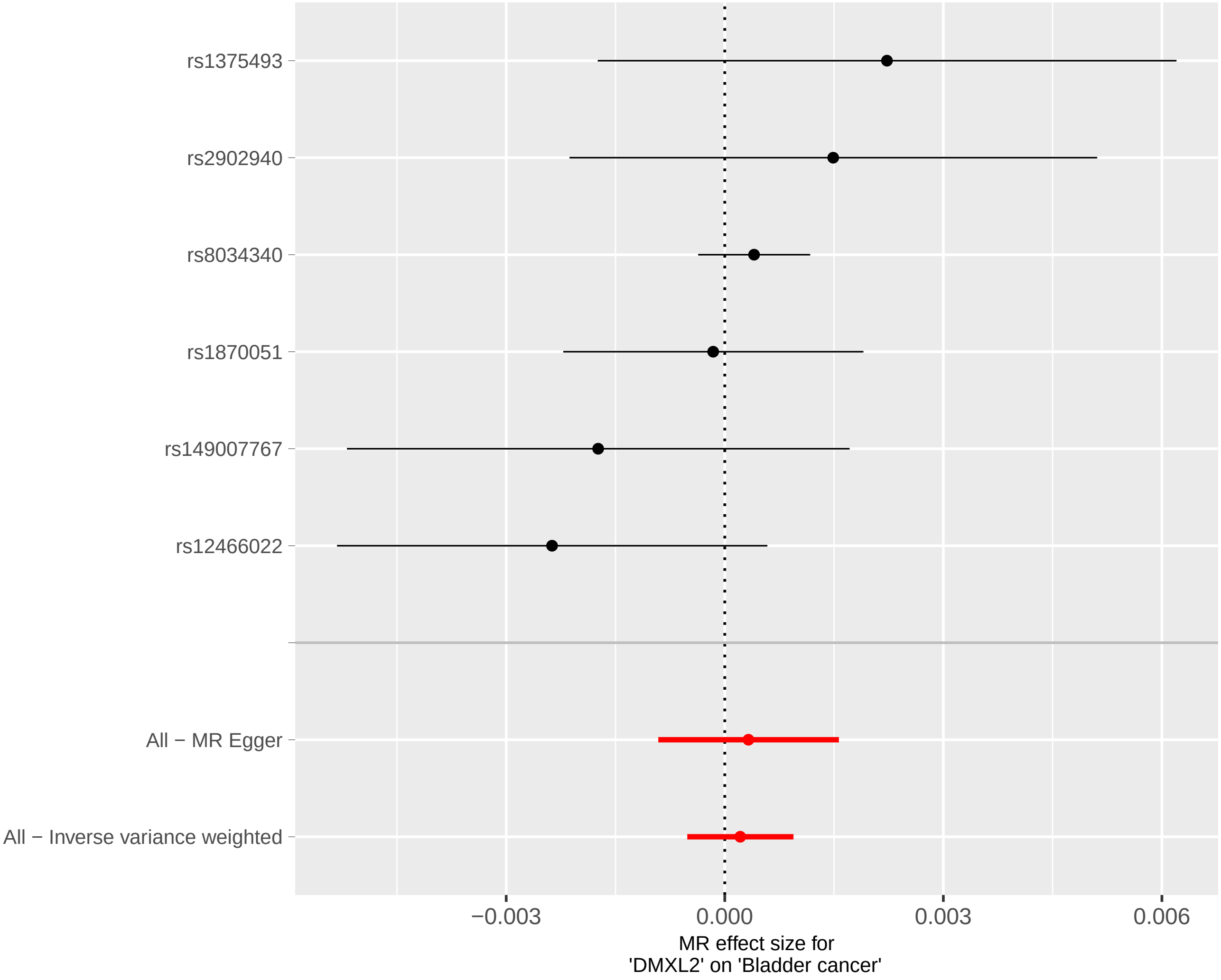

rs1375493
rs2902940
rs8034340
rs1870051
rs149007767
rs12466022
All − MR Egger
All − Inverse variance weighted
−0.003
0.000
0.003
0.006
MR effect size for
'DMXL2' on 'Bladder cancer'

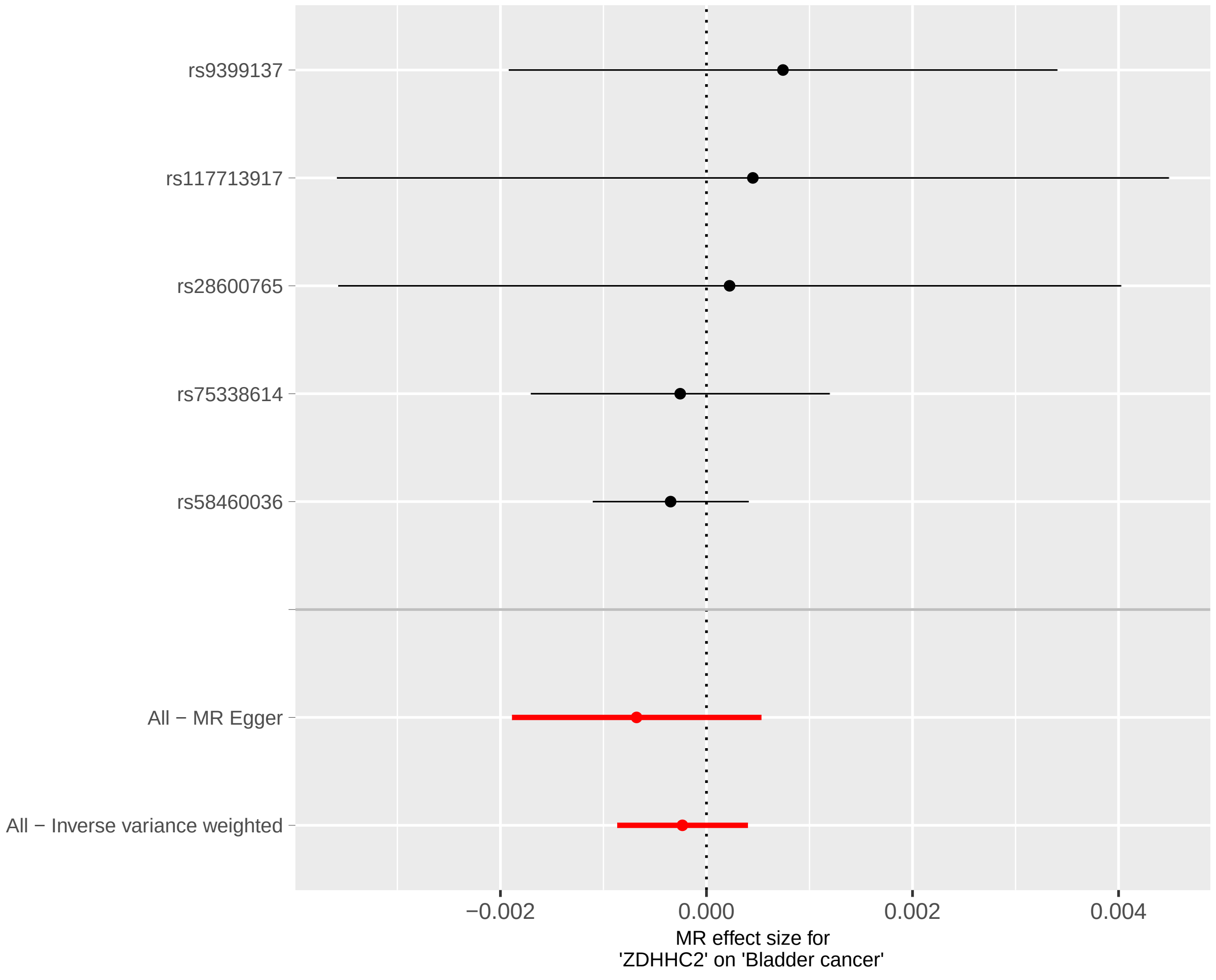
rs9399137
rs117713917
rs28600765
rs75338614
rs58460036
All − MR Egger
All − Inverse variance weighted
−0.002
0.000
0.002
0.004
MR effect size for
'ZDHHC2' on 'Bladder cancer'

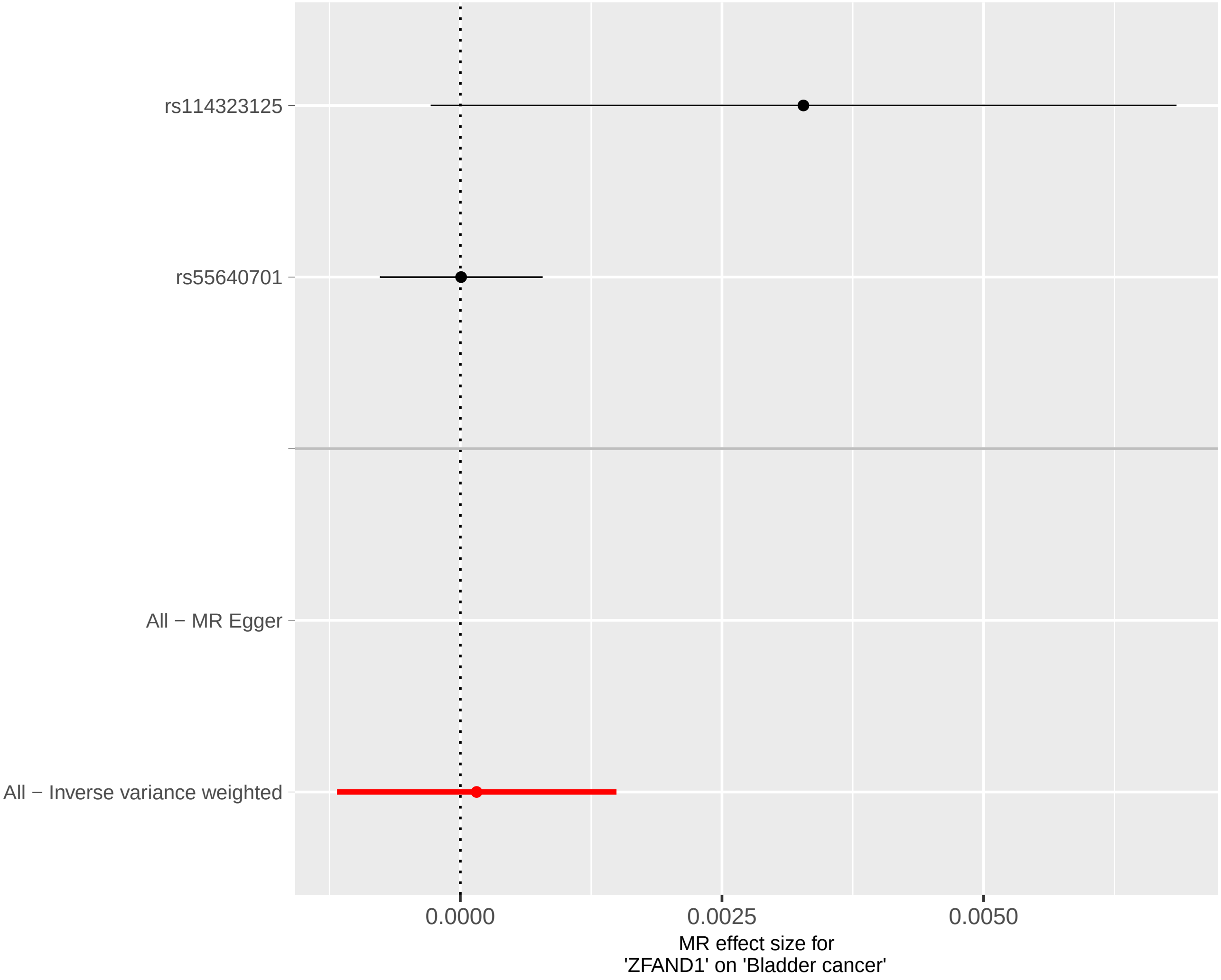
rs114323125
rs55640701
All − MR Egger
All − Inverse variance weighted
0.0000
0.0025
0.0050
MR effect size for
'ZFAND1' on 'Bladder cancer'

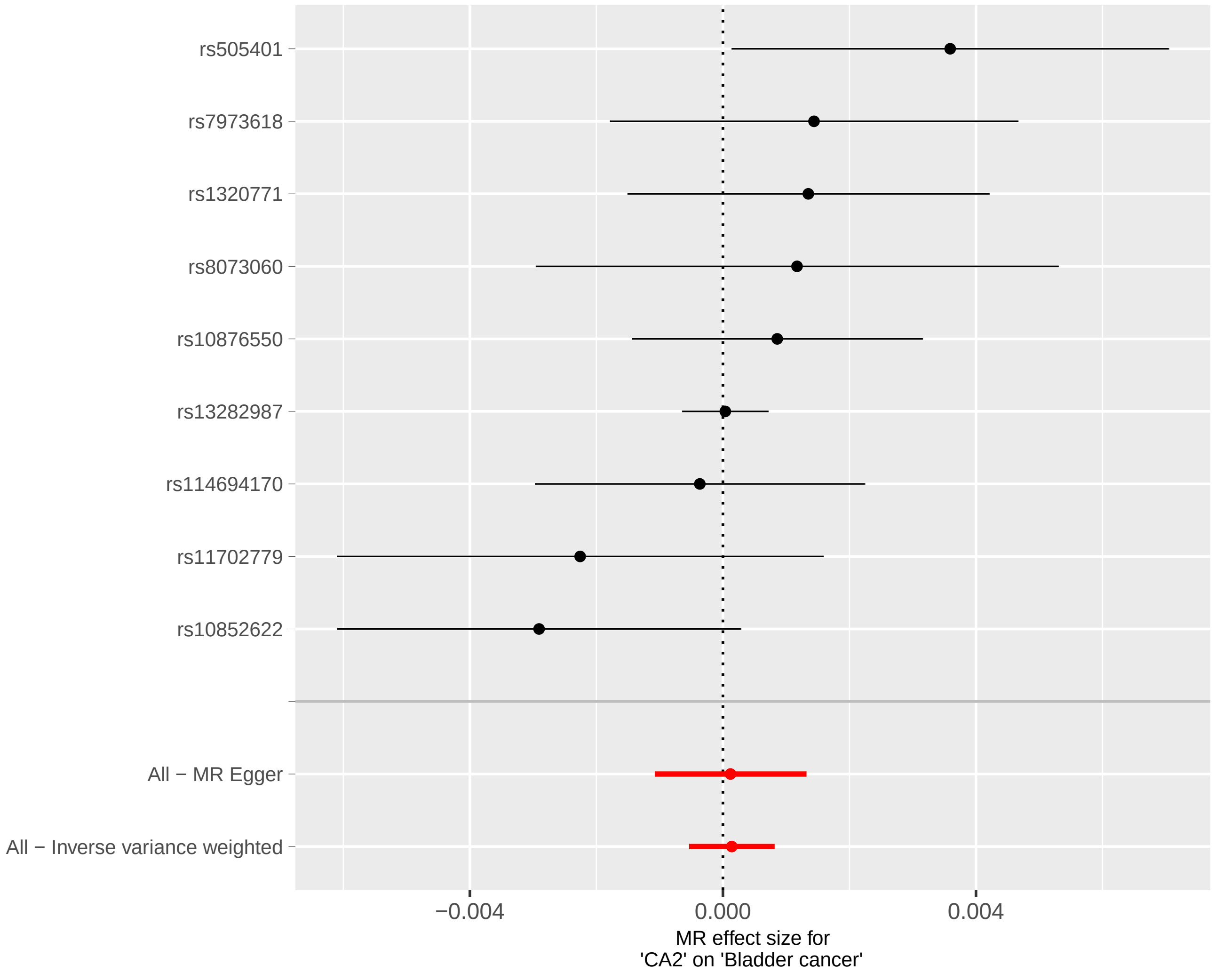

rs505401
rs7973618
rs1320771
rs8073060
rs10876550
rs13282987
rs114694170
rs11702779
rs10852622
All − MR Egger
All − Inverse variance weighted
−0.004
0.000
0.004
MR effect size for
'CA2' on 'Bladder cancer'

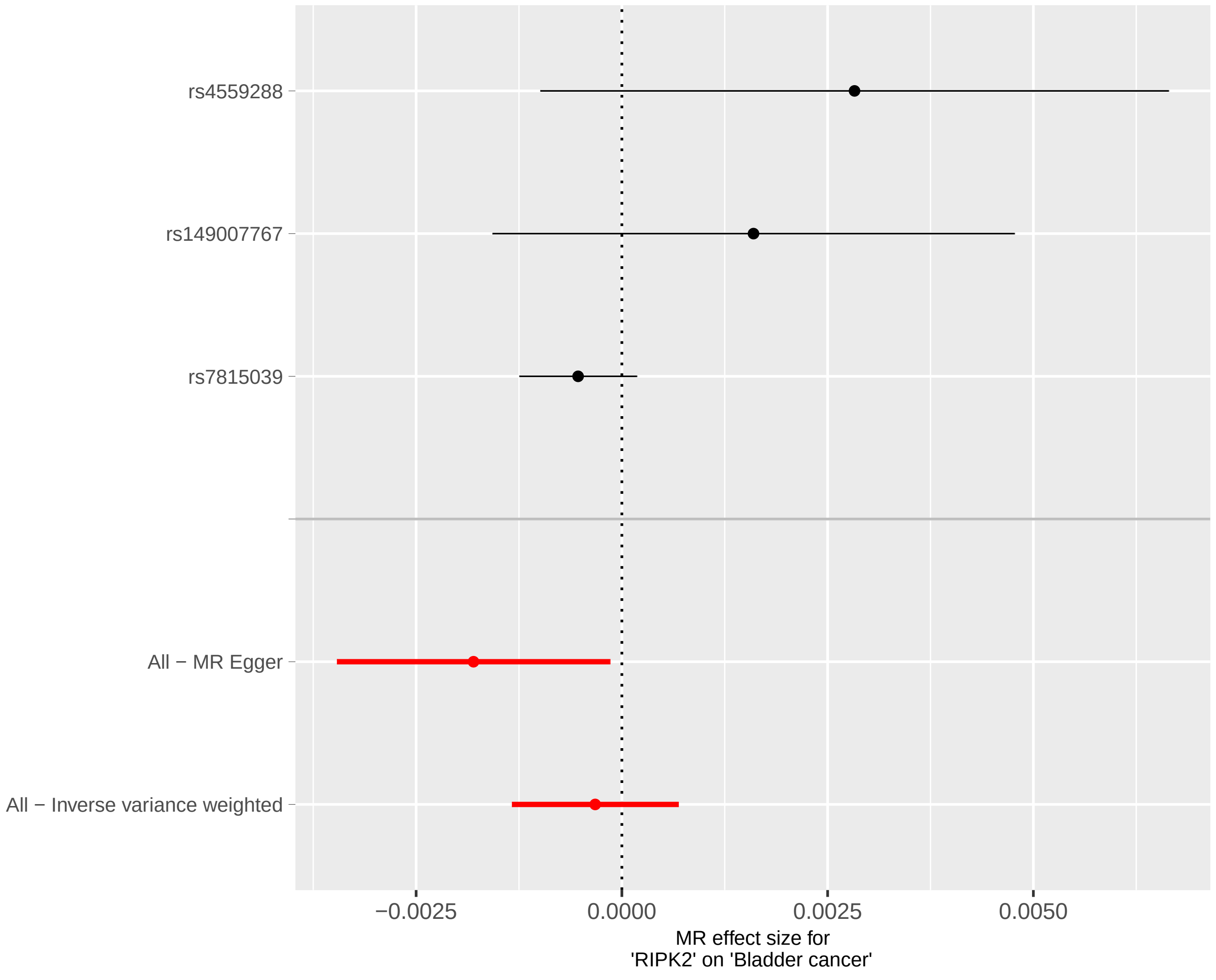

rs4559288
rs149007767
rs7815039
All − MR Egger
All − Inverse variance weighted
−0.0025
0.0000
0.0025
0.0050
MR effect size for
'RIPK2' on 'Bladder cancer'

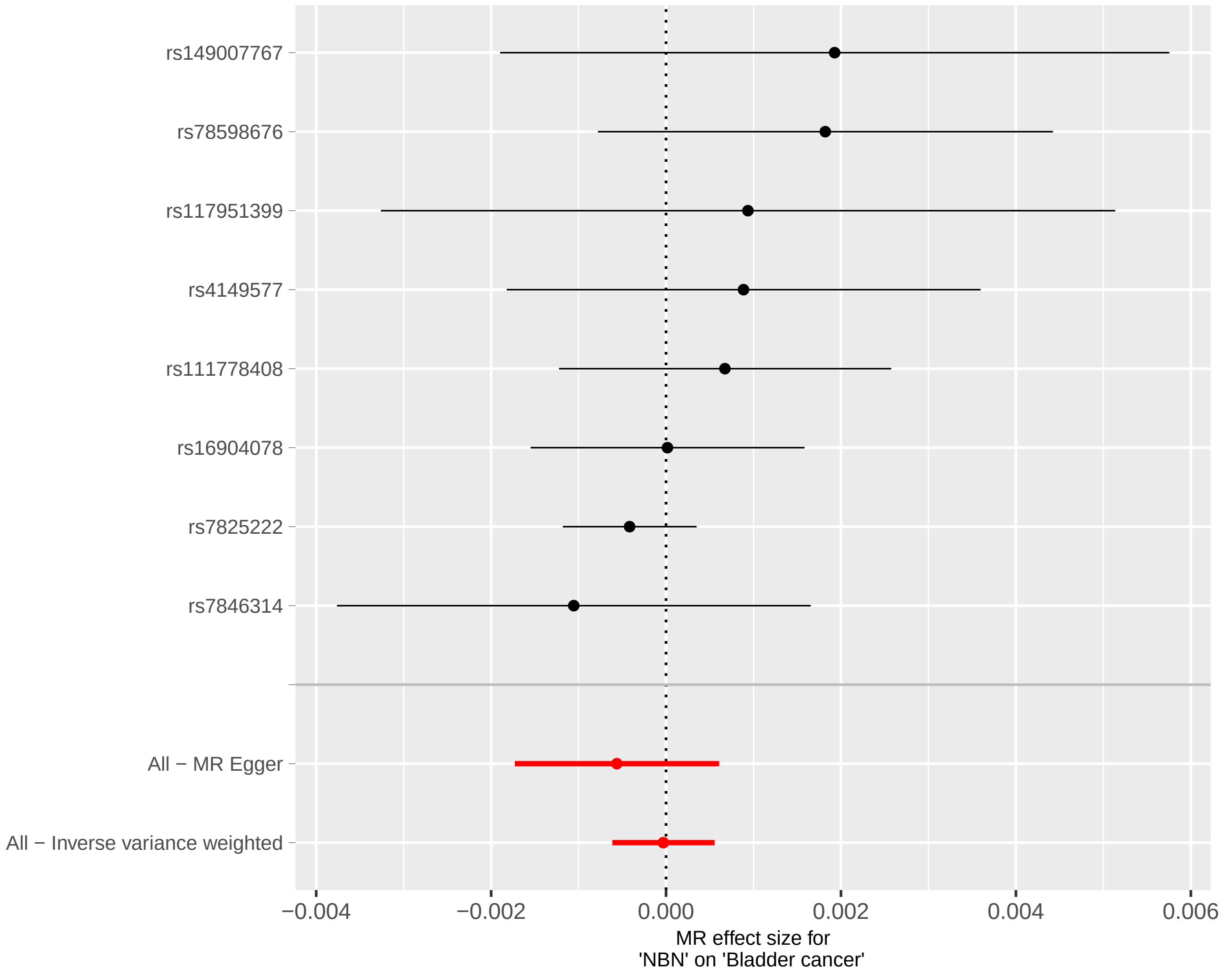

rs149007767
rs78598676
rs117951399
rs4149577
rs111778408
rs16904078
rs7825222
rs7846314
All − MR Egger
All − Inverse variance weighted
−0.004
−0.002
0.000
0.002
0.004
0.006
MR effect size for
'NBN' on 'Bladder cancer'

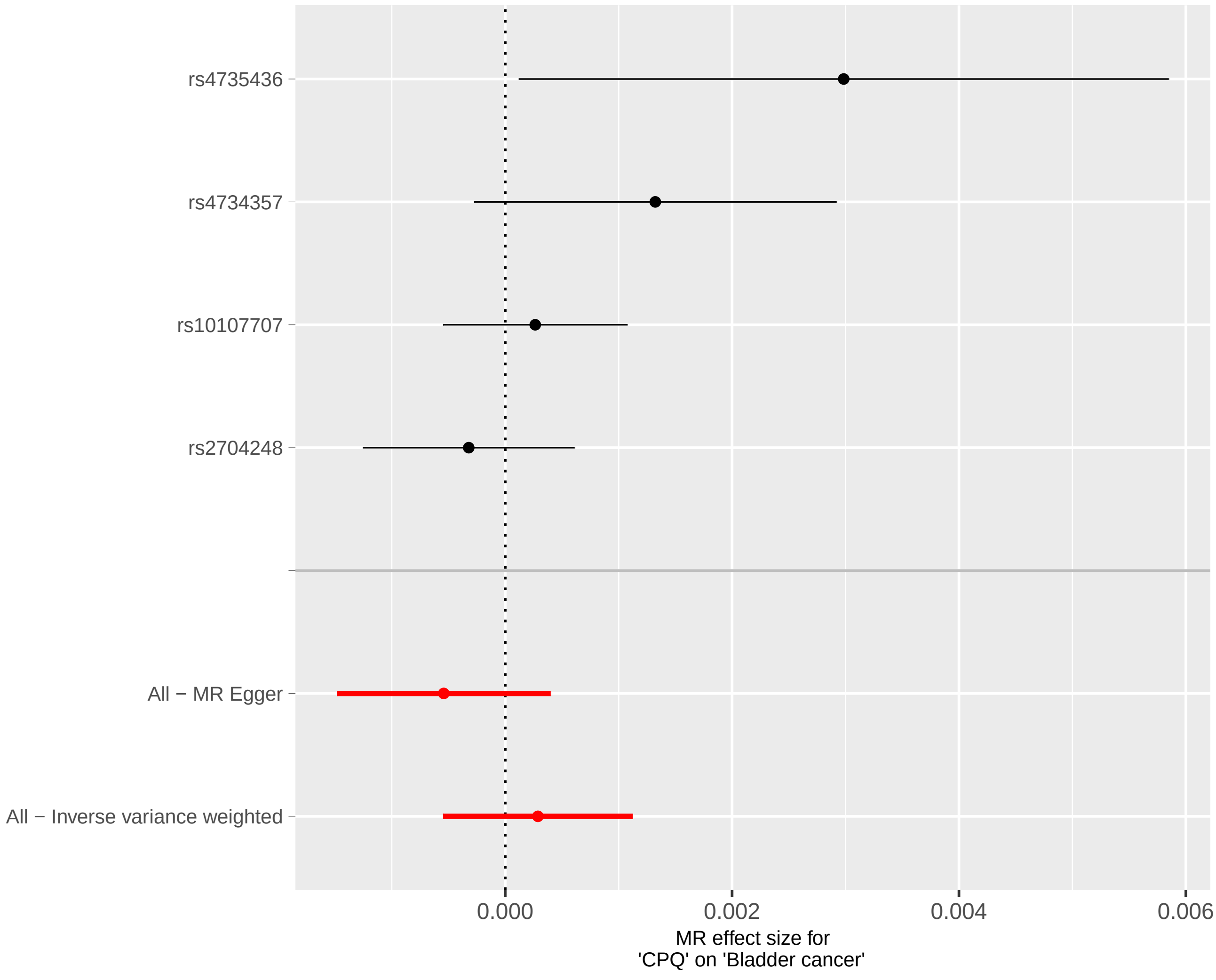
rs4735436
rs4734357
rs10107707
rs2704248
All − MR Egger
All − Inverse variance weighted
0.000
0.002
0.004
0.006
MR effect size for
'CPQ' on 'Bladder cancer'

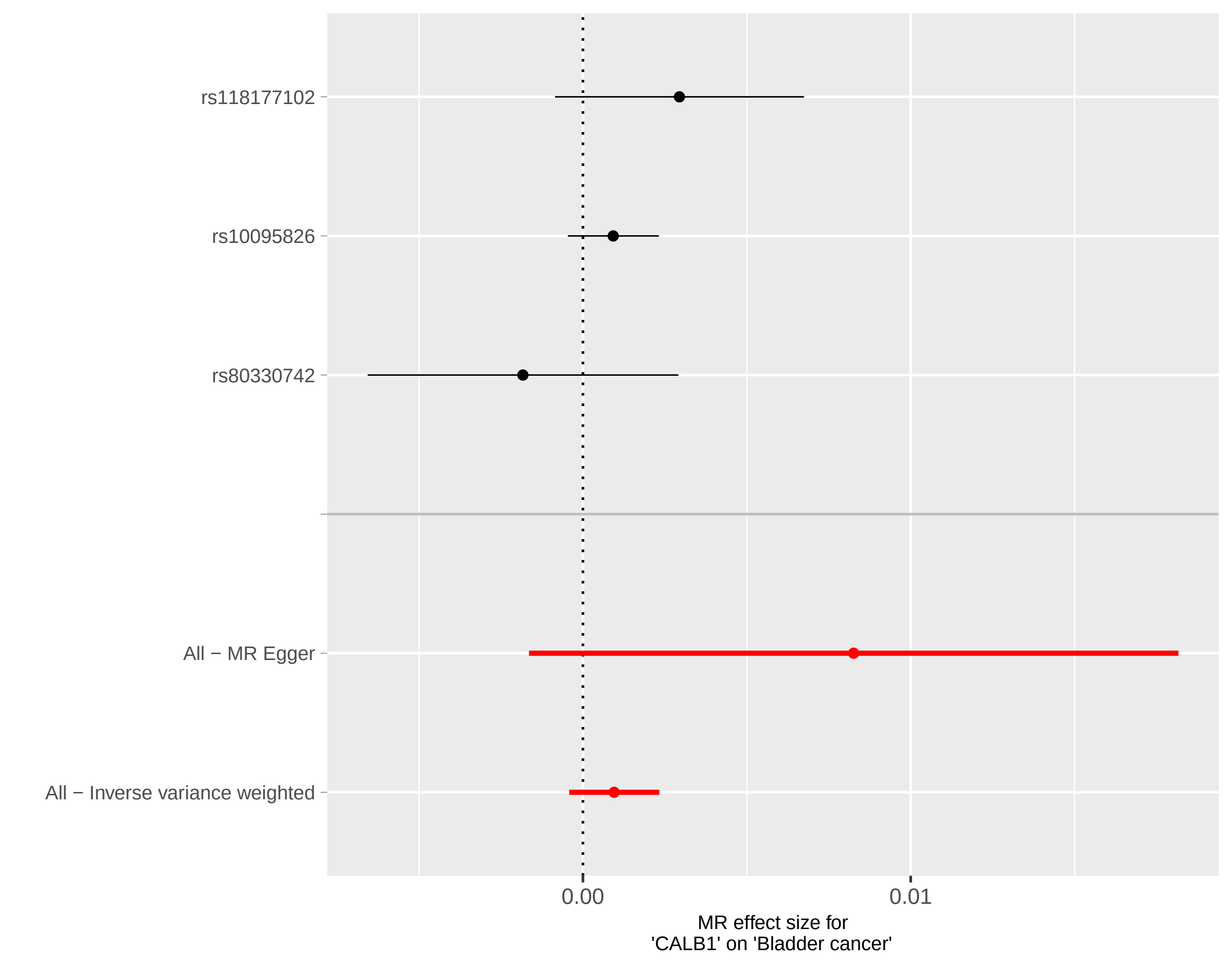

rs118177102
rs10095826
rs80330742
All − MR Egger
All − Inverse variance weighted
0.00
0.01
MR effect size for
'CALB1' on 'Bladder cancer'

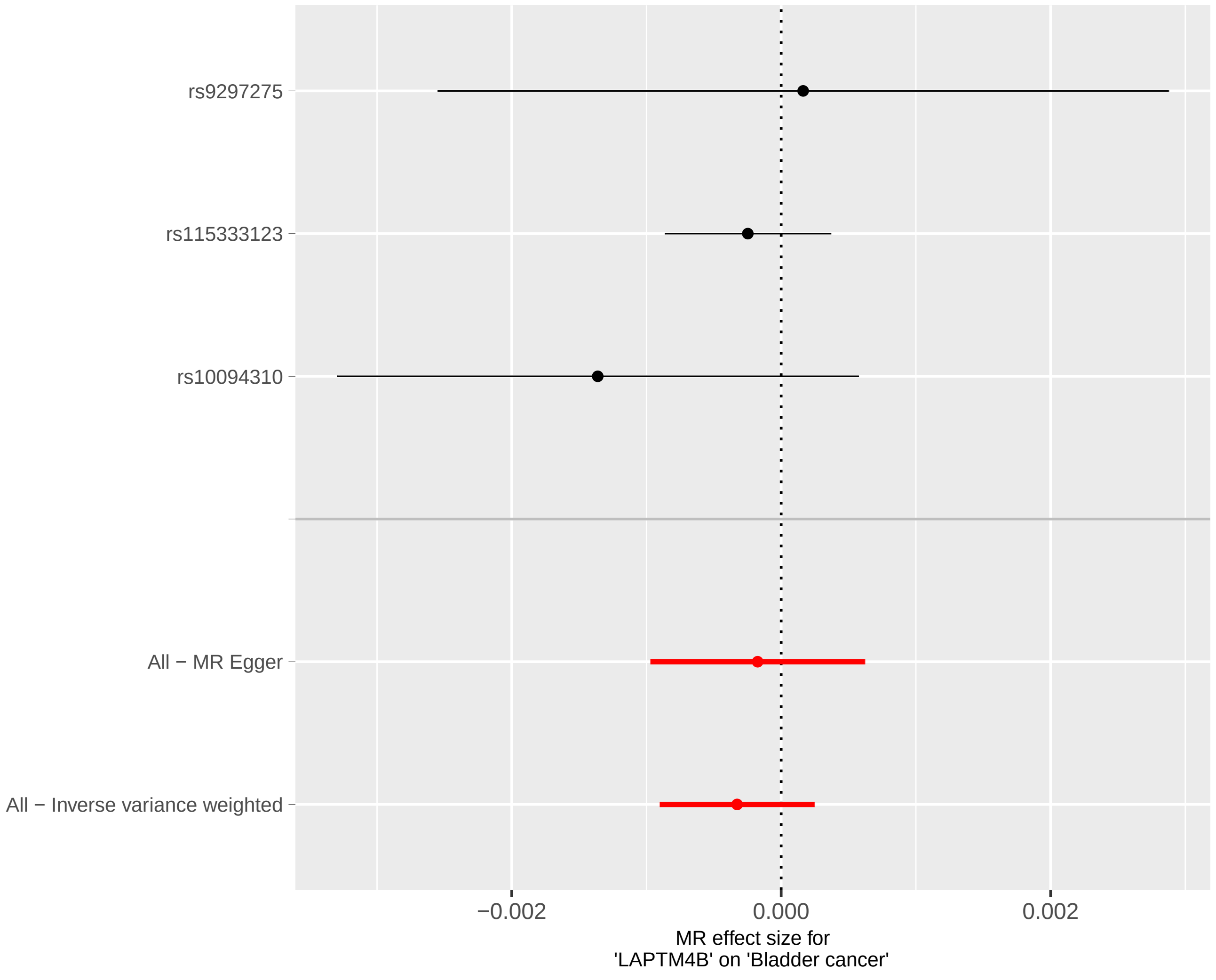
rs9297275
rs115333123
rs10094310
All − MR Egger
All − Inverse variance weighted
−0.002
0.000
0.002
MR effect size for
'LAPTM4B' on 'Bladder cancer'

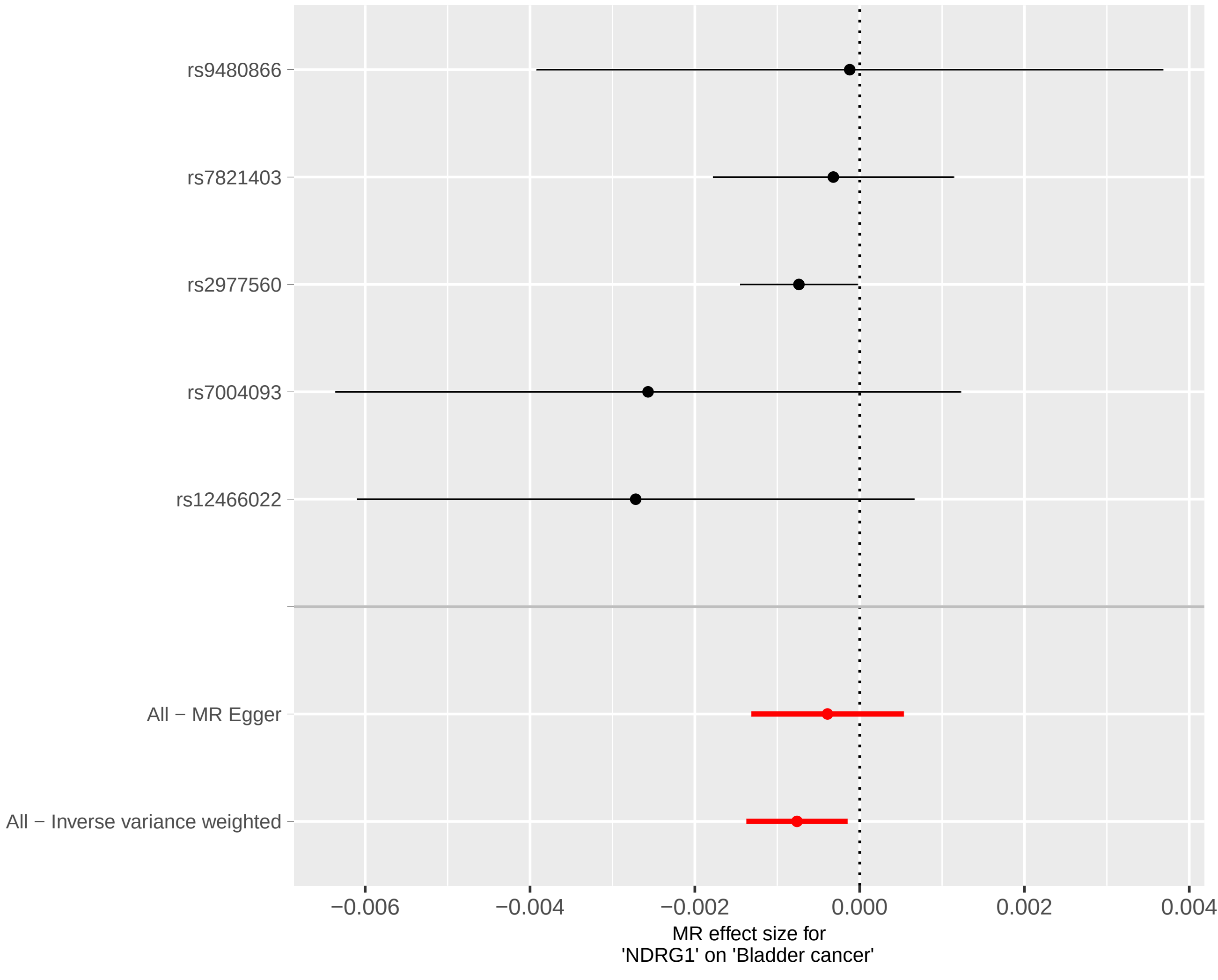

rs9480866
rs7821403
rs2977560
rs7004093
rs12466022
All − MR Egger
All − Inverse variance weighted
−0.006
−0.004
−0.002
0.000
0.002
0.004
MR effect size for
'NDRG1' on 'Bladder cancer'

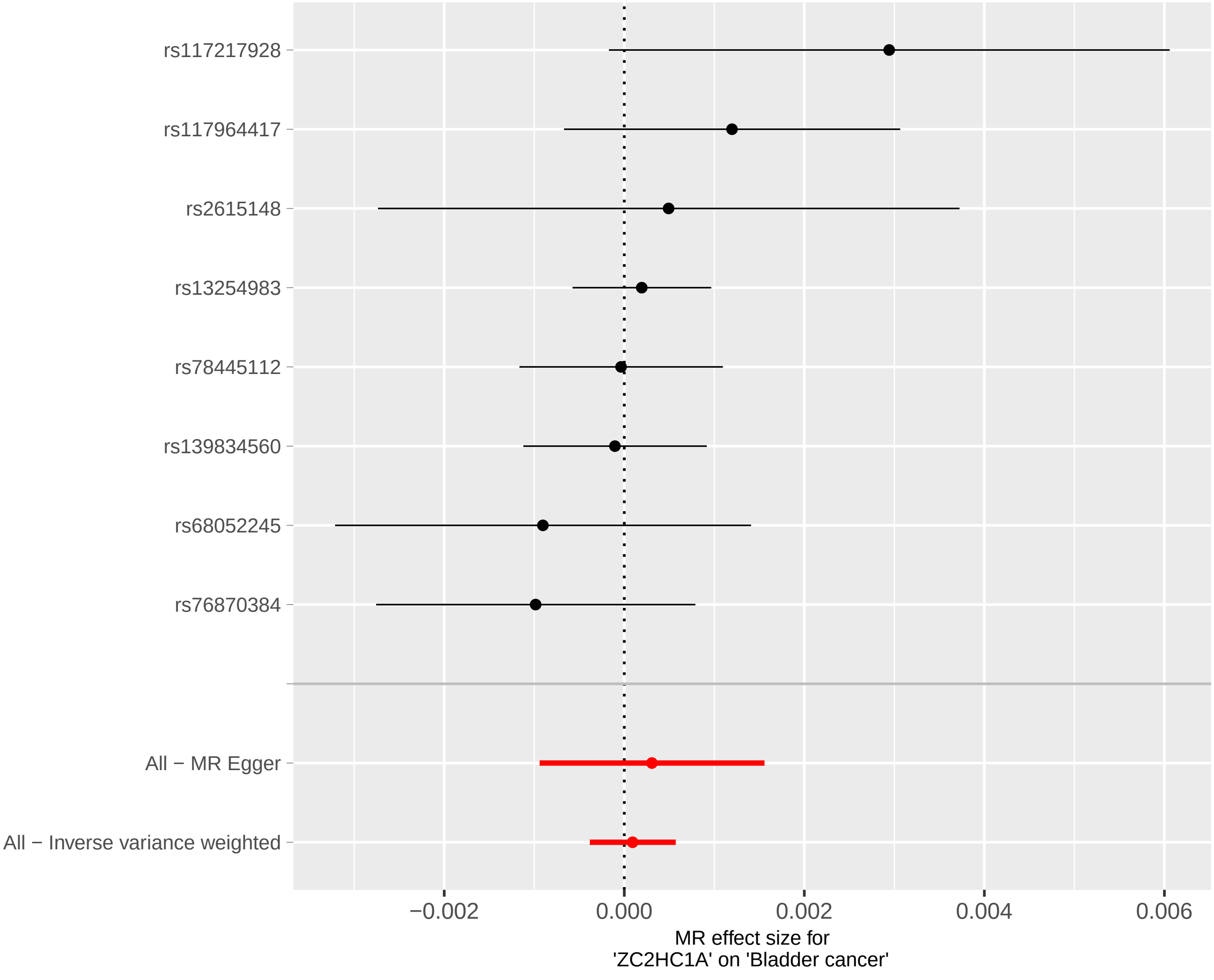
rs117217928
rs117964417
rs2615148
rs13254983
rs78445112
rs139834560
rs68052245
rs76870384
All − MR Egger
All − Inverse variance weighted
−0.002
0.000
0.002
0.004
0.006
MR effect size for
'ZC2HC1A' on 'Bladder cancer'

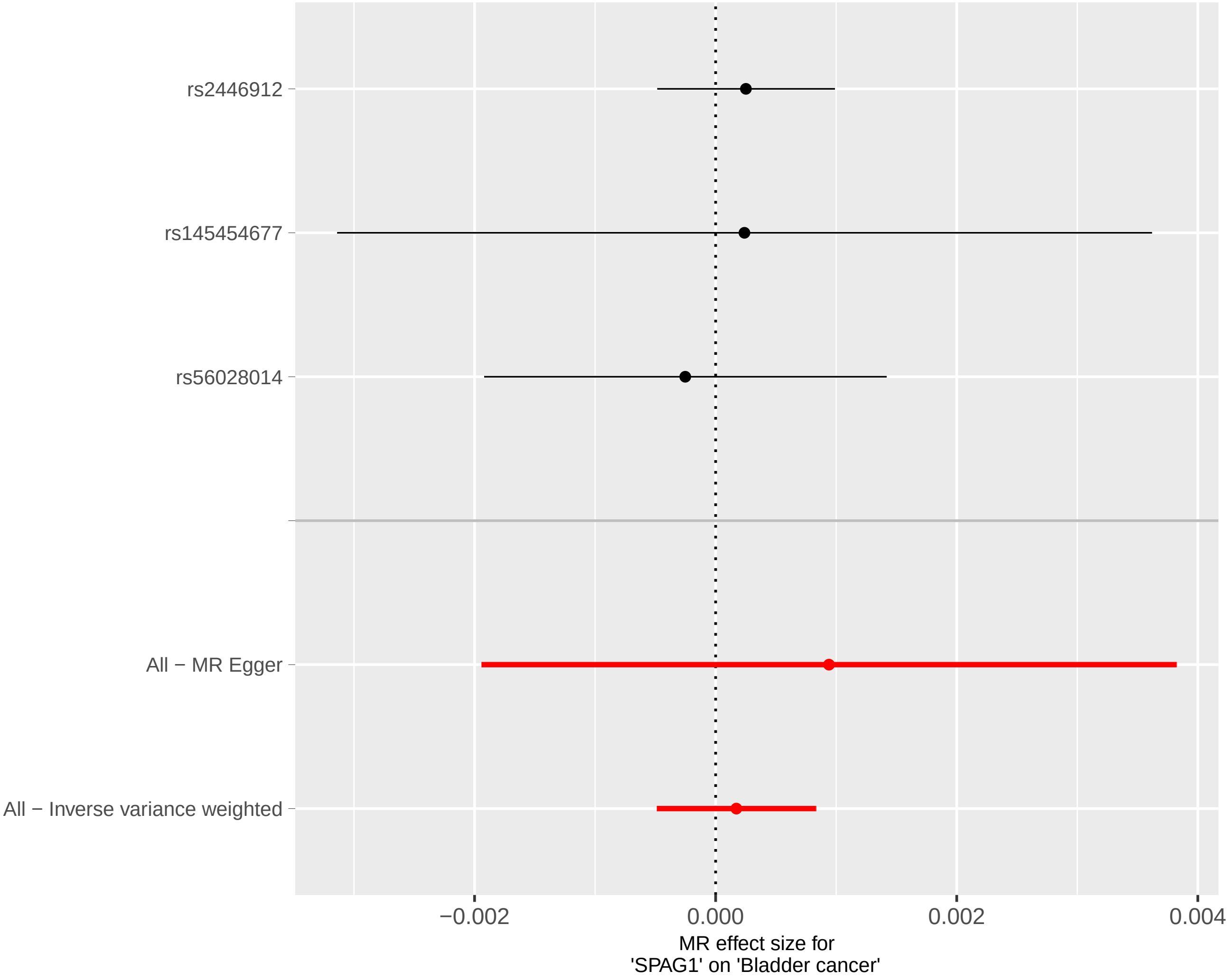
rs2446912
rs145454677
rs56028014
All − MR Egger
All − Inverse variance weighted
−0.002
0.000
0.002
0.004
MR effect size for
'SPAG1' on 'Bladder cancer'

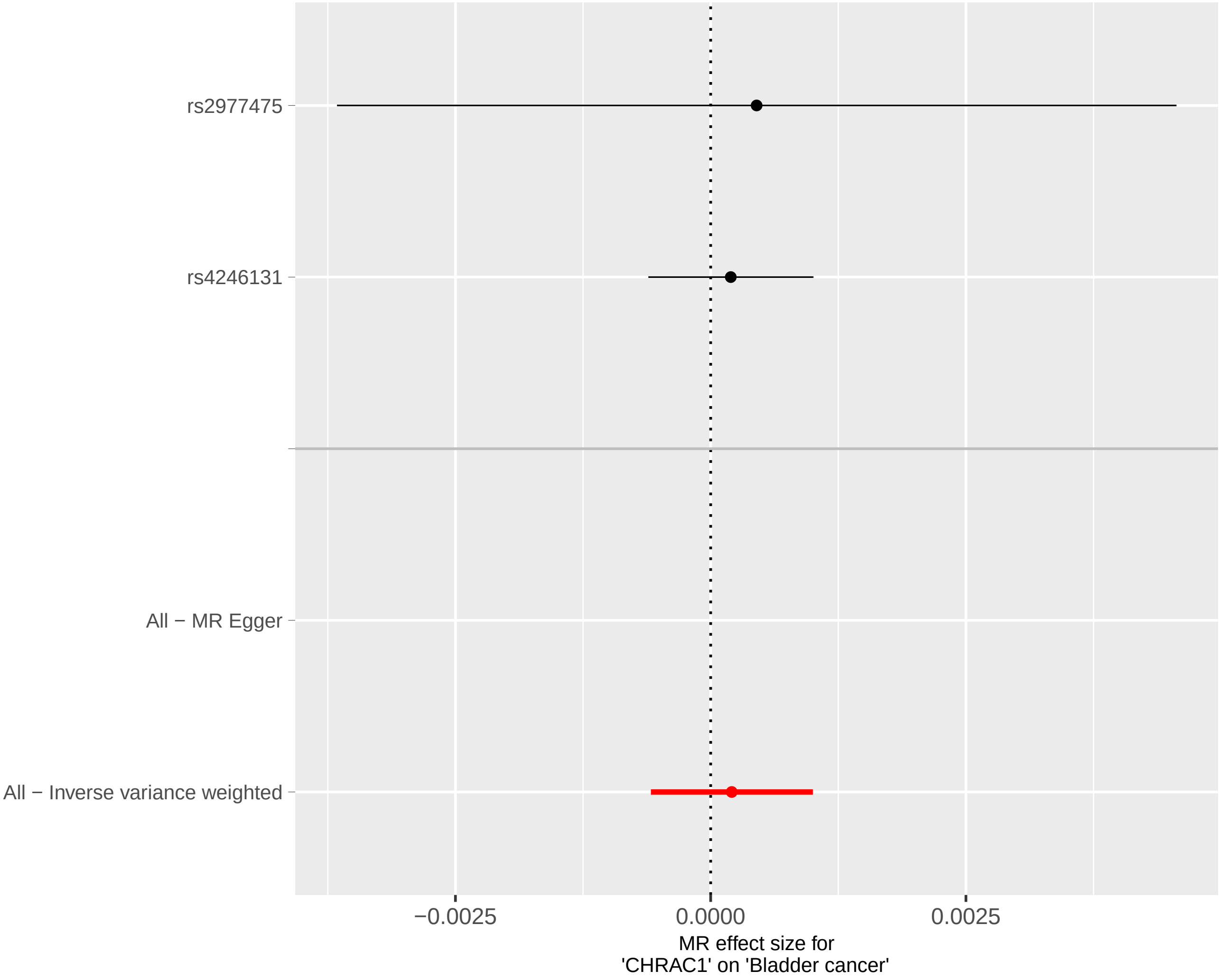

rs2977475
rs4246131
All − MR Egger
All − Inverse variance weighted
−0.0025
0.0000
0.0025
MR effect size for
'CHRAC1' on 'Bladder cancer'

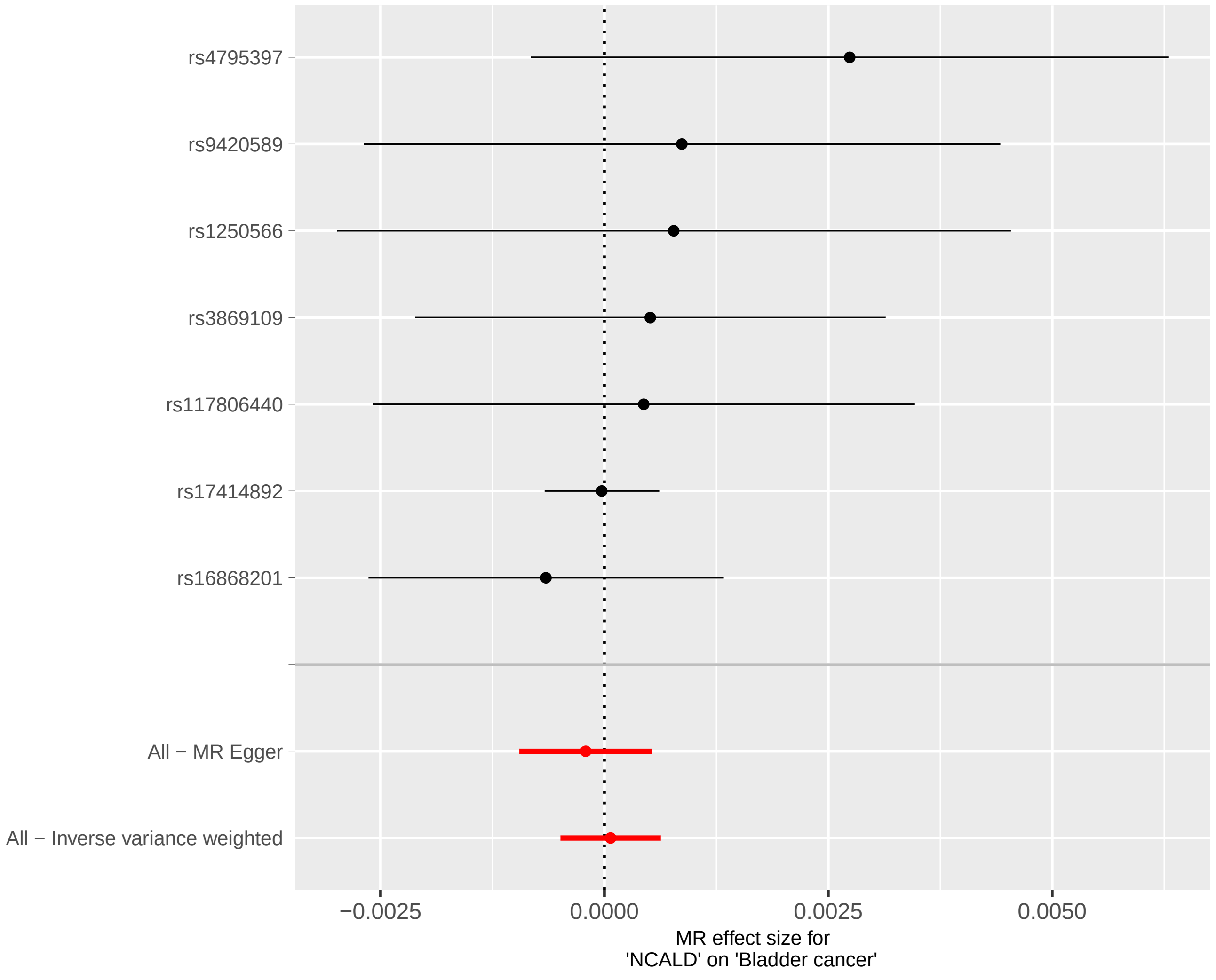

rs4795397
rs9420589
rs1250566
rs3869109
rs117806440
rs17414892
rs16868201
All − MR Egger
All − Inverse variance weighted
−0.0025
0.0000
0.0025
0.0050
MR effect size for
'NCALD' on 'Bladder cancer'

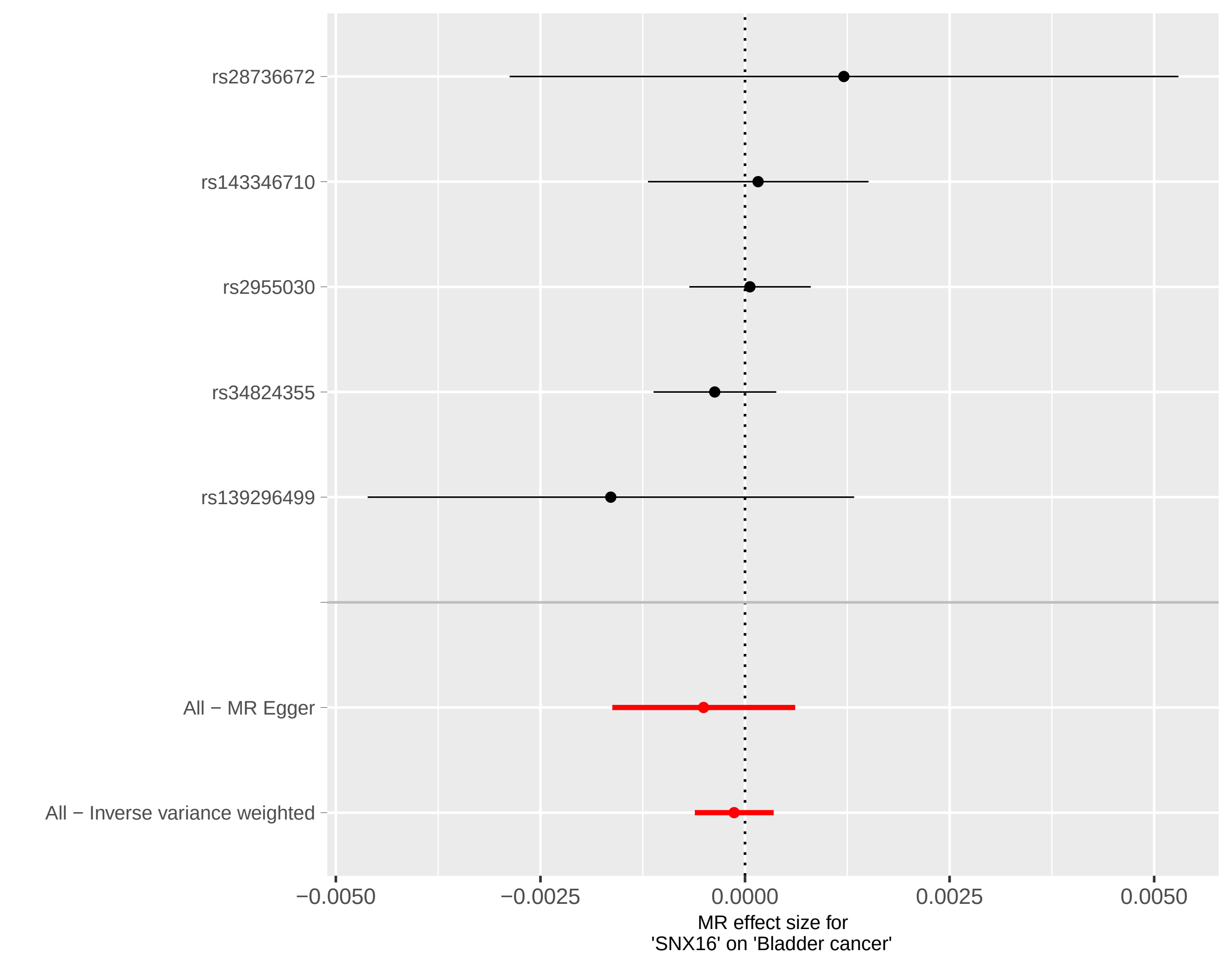
rs28736672
rs143346710
rs2955030
rs34824355
rs139296499
All − MR Egger
All − Inverse variance weighted
−0.0050
−0.0025
0.0000
0.0025
0.0050
MR effect size for
'SNX16' on 'Bladder cancer'

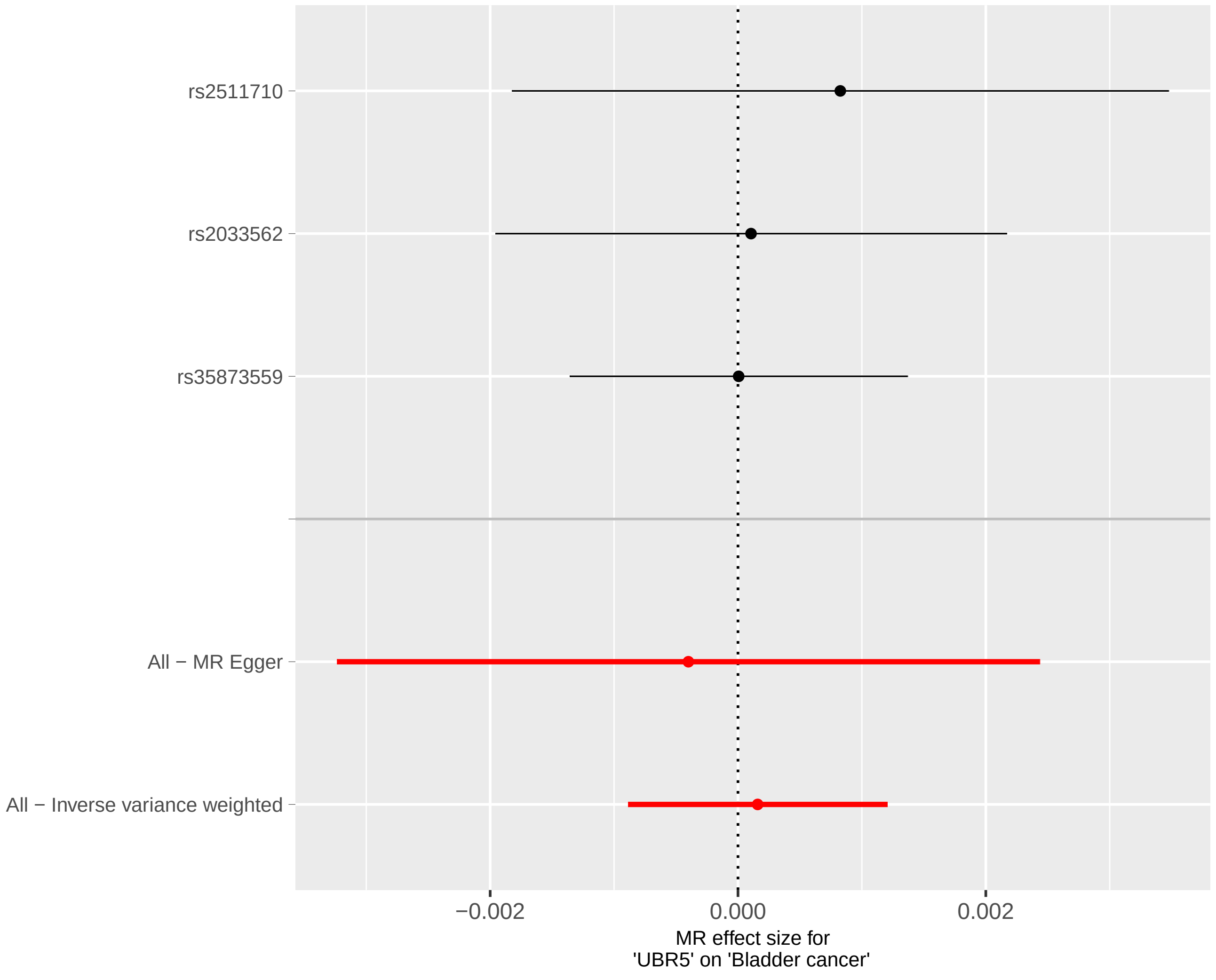

rs2511710
rs2033562
rs35873559
All − MR Egger
All − Inverse variance weighted
−0.002
0.000
0.002
MR effect size for
'UBR5' on 'Bladder cancer'

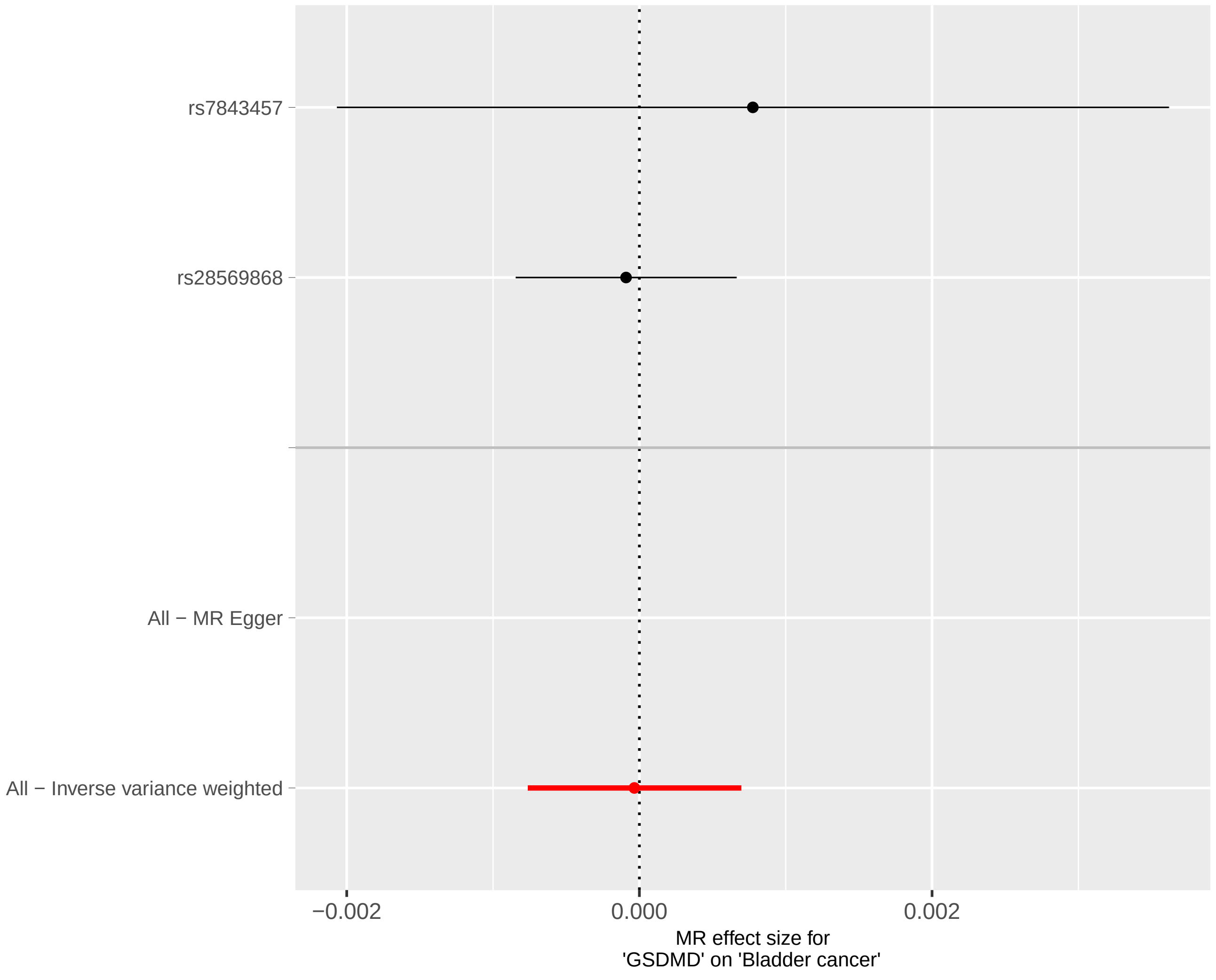

rs7843457
rs28569868
All − MR Egger
All − Inverse variance weighted
−0.002
0.000
0.002
MR effect size for
'GSDMD' on 'Bladder cancer'

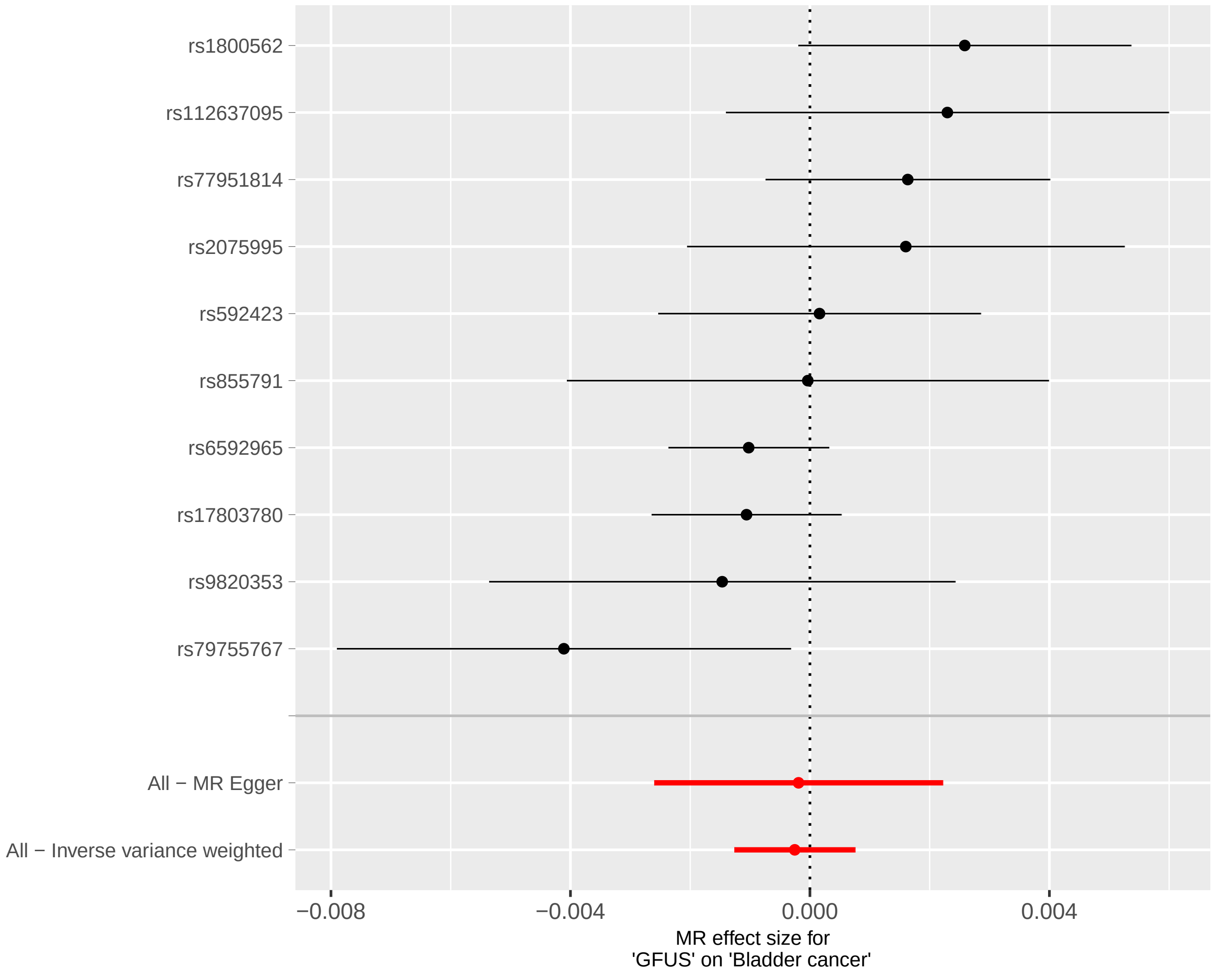

rs1800562
rs112637095
rs77951814
rs2075995
rs592423
rs855791
rs6592965
rs17803780
rs9820353
rs79755767
All − MR Egger
All − Inverse variance weighted
−0.008
−0.004
0.000
0.004
MR effect size for
'GFUS' on 'Bladder cancer'

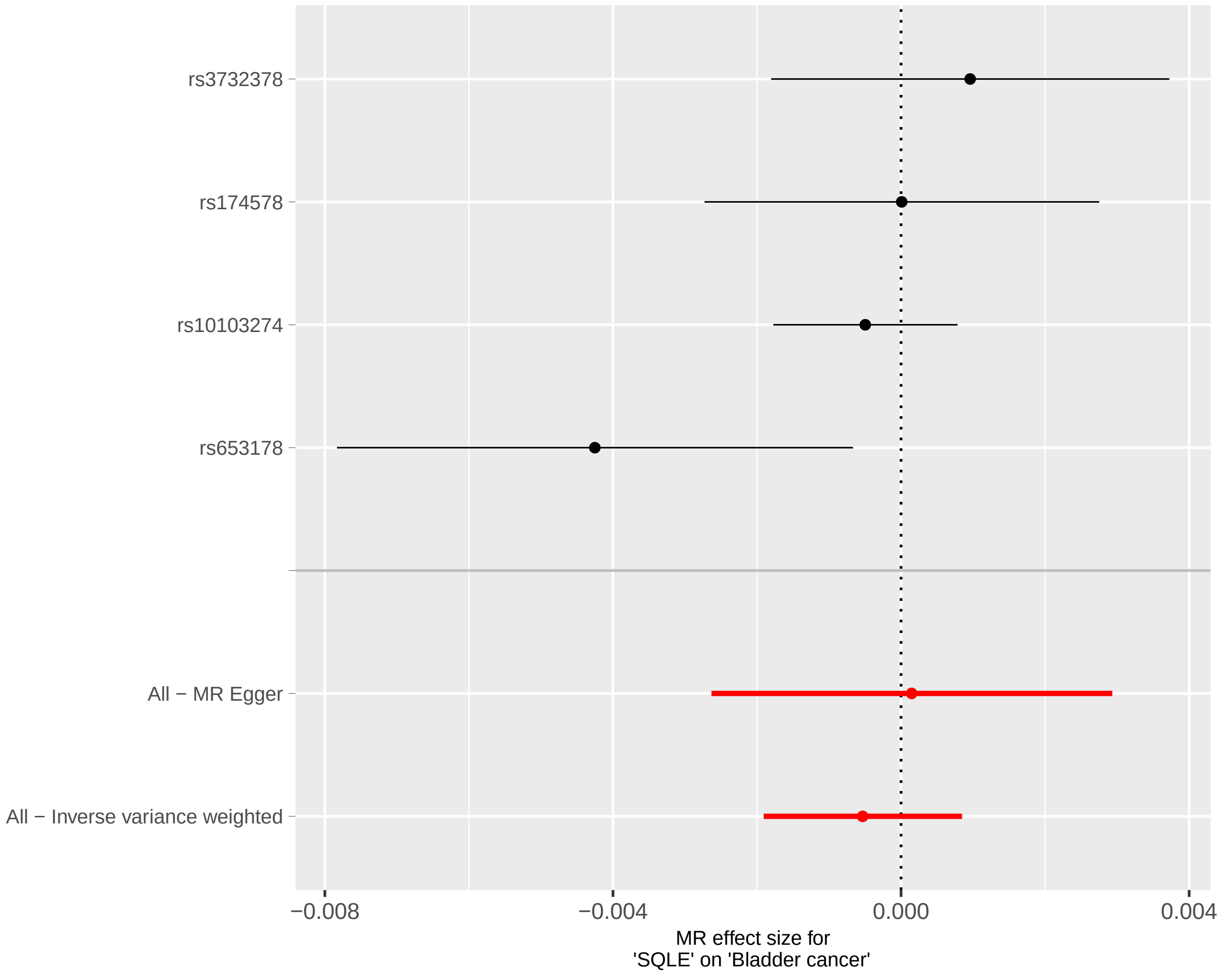
rs3732378
rs174578
rs10103274
rs653178
All − MR Egger
All − Inverse variance weighted
−0.008
−0.004
0.000
0.004
MR effect size for
'SQLE' on 'Bladder cancer'

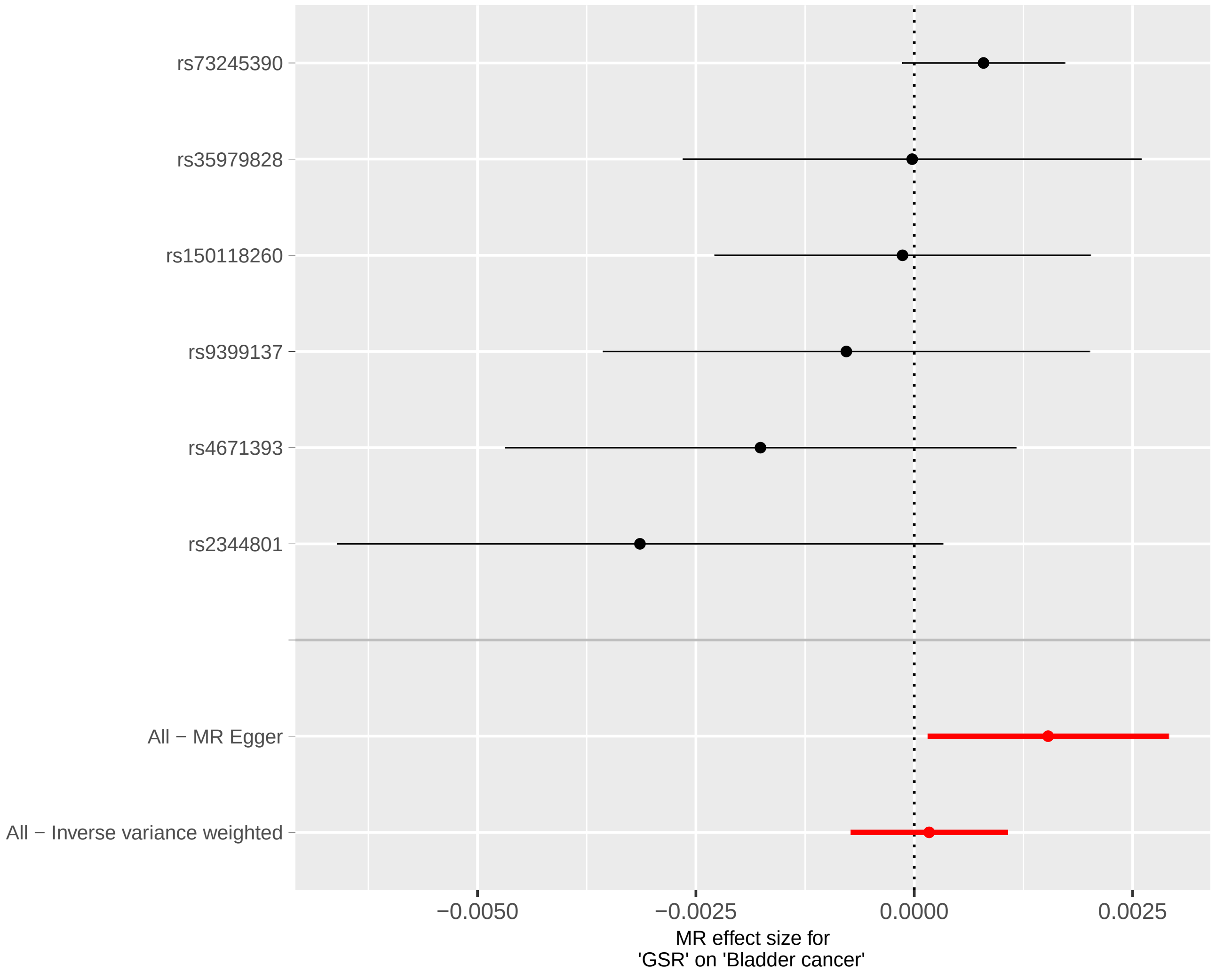
rs73245390
rs35979828
rs150118260
rs9399137
rs4671393
rs2344801
All − MR Egger
All − Inverse variance weighted
−0.0050
−0.0025
0.0000
0.0025
MR effect size for
'GSR' on 'Bladder cancer'

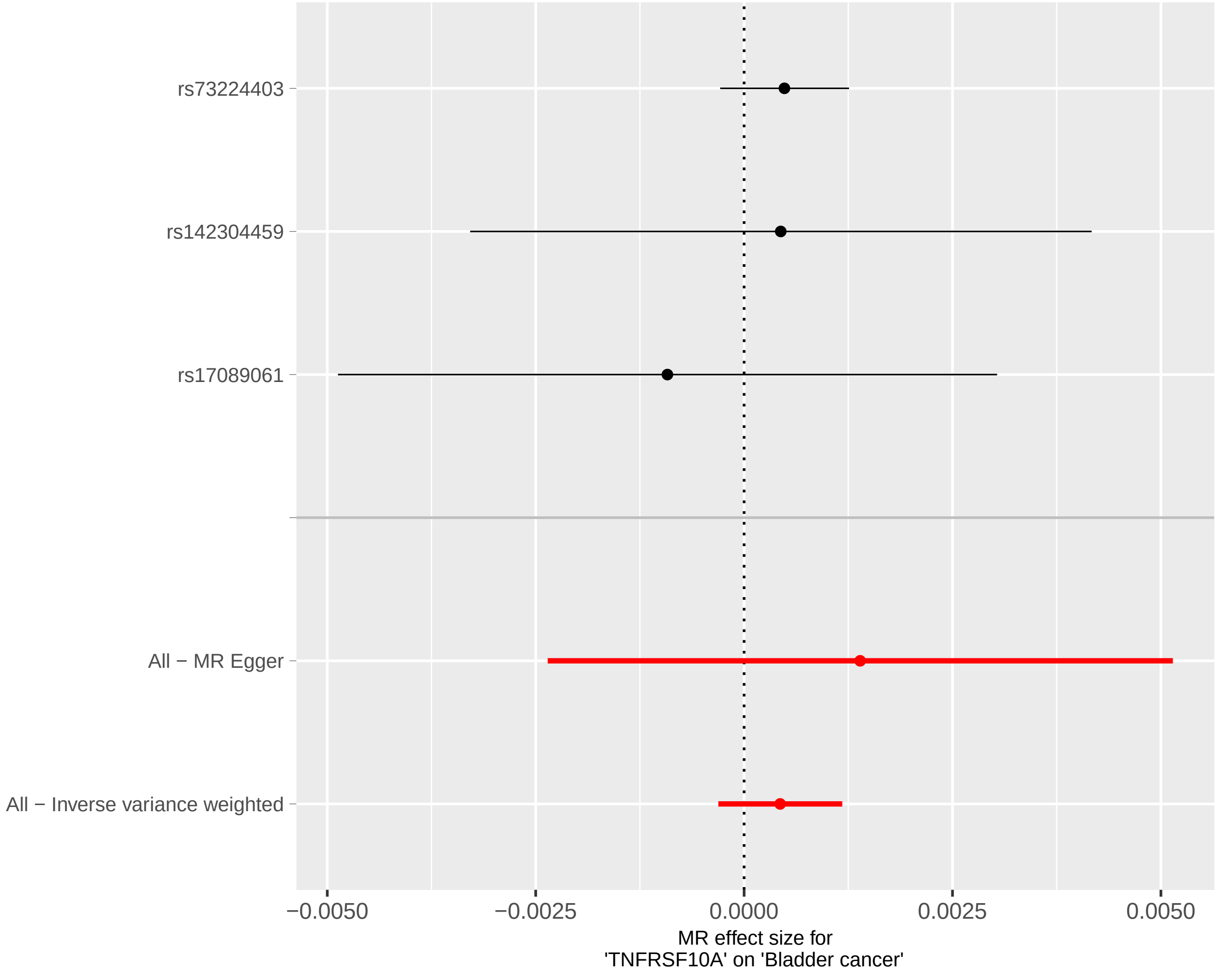

rs73224403
rs142304459
rs17089061
All − MR Egger
All − Inverse variance weighted
−0.0050
−0.0025
0.0000
0.0025
0.0050
MR effect size for
'TNFRSF10A' on 'Bladder cancer'

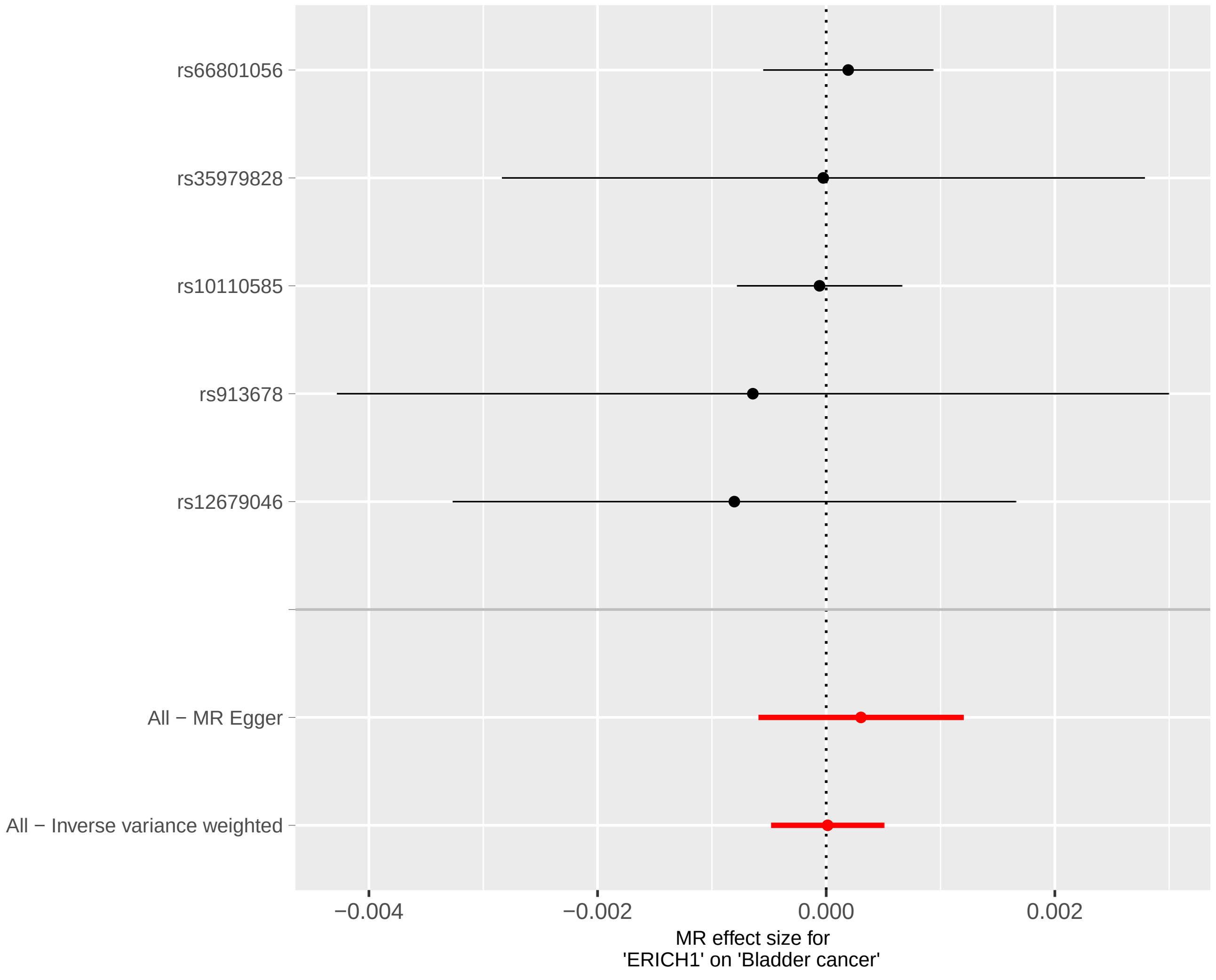

rs66801056
rs35979828
rs10110585
rs913678
rs12679046
All − MR Egger
All − Inverse variance weighted
−0.004
−0.002
0.000
0.002
MR effect size for
'ERICH1' on 'Bladder cancer'

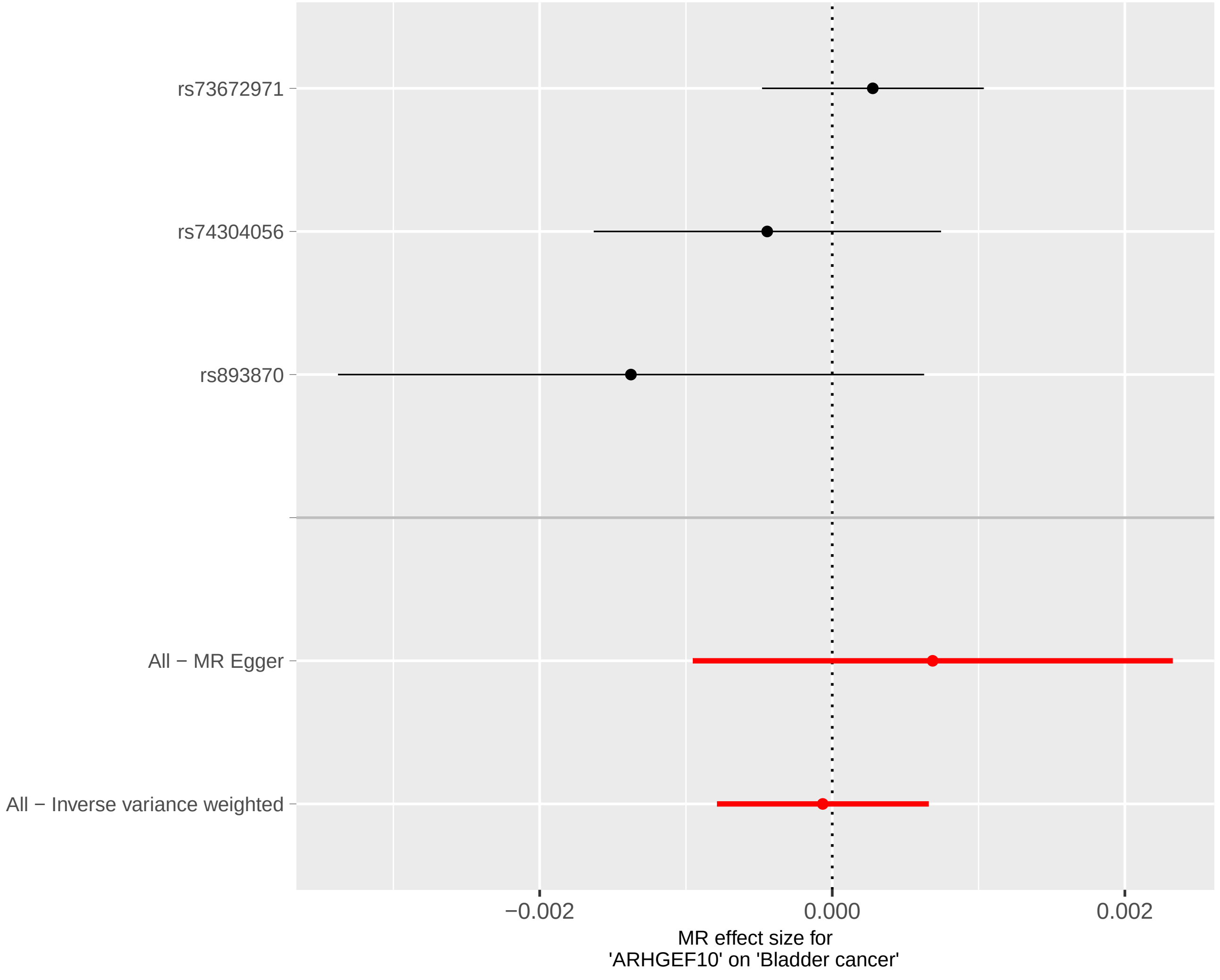
rs73672971
rs74304056
rs893870
All − MR Egger
All − Inverse variance weighted
−0.002
0.000
0.002
MR effect size for
'ARHGEF10' on 'Bladder cancer'

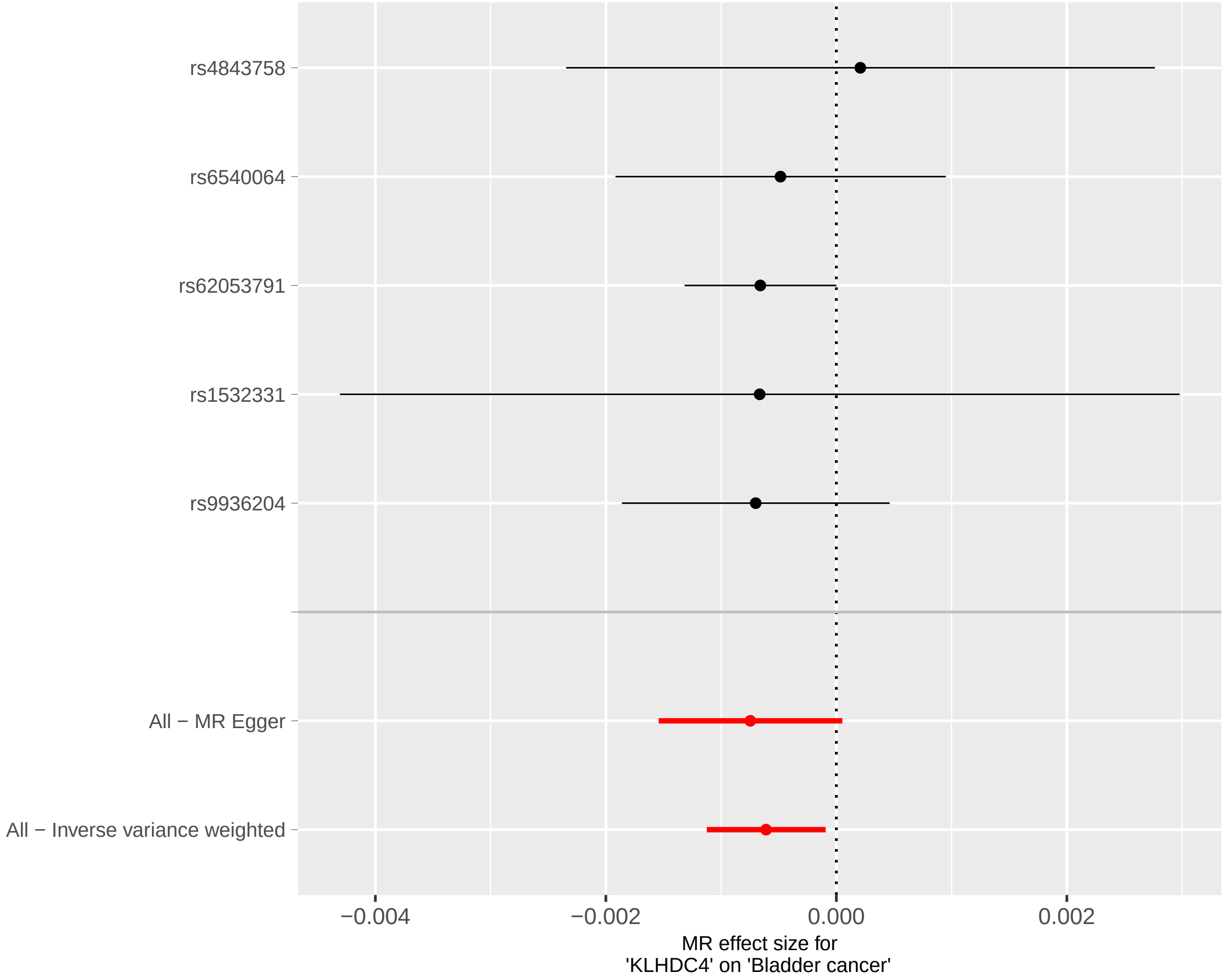
rs4843758
rs6540064
rs62053791
rs1532331
rs9936204
All − MR Egger
All − Inverse variance weighted
−0.004
−0.002
0.000
0.002
MR effect size for
'KLHDC4' on 'Bladder cancer'

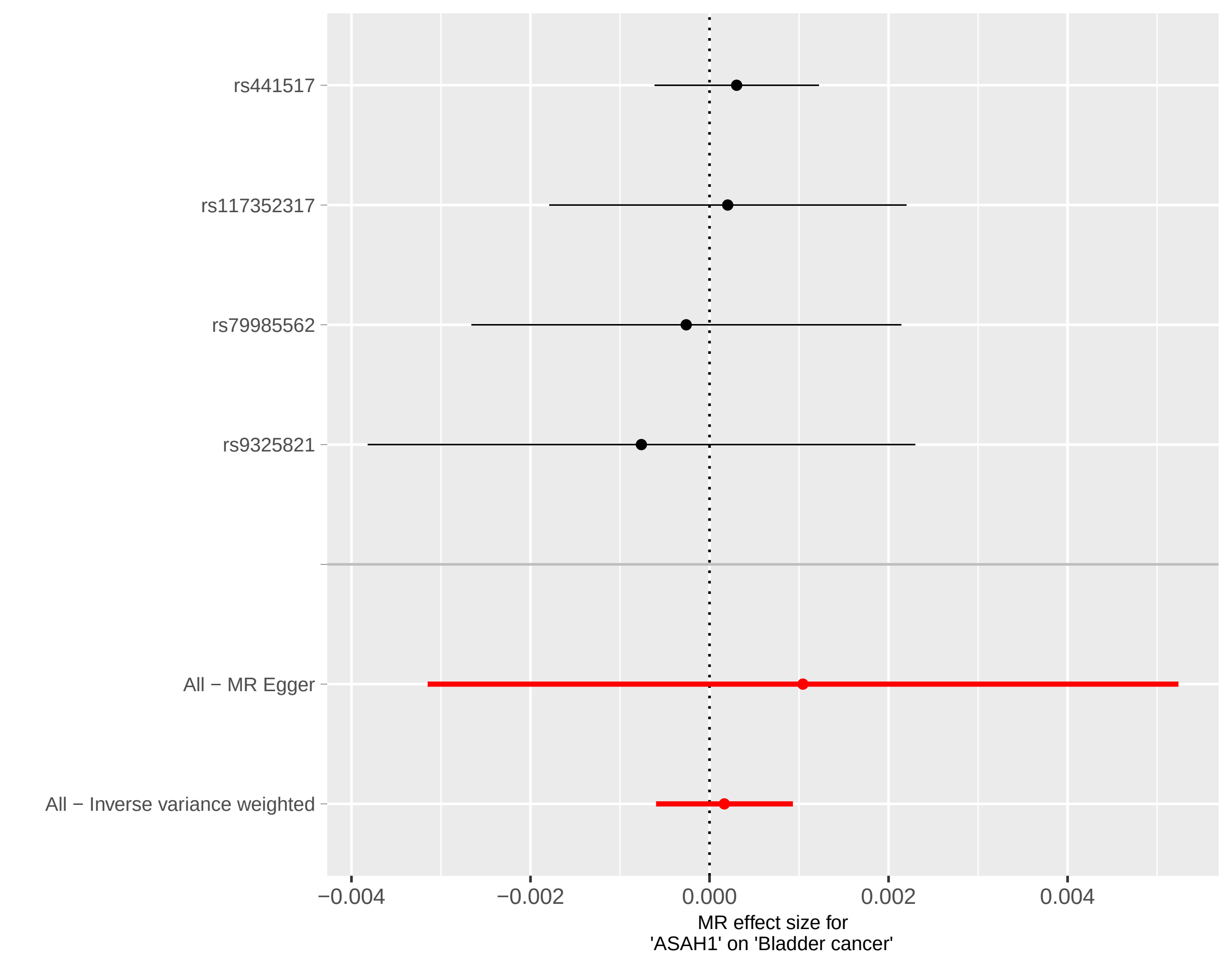

rs441517
rs117352317
rs79985562
rs9325821
All − MR Egger
All − Inverse variance weighted
−0.004
−0.002
0.000
0.002
0.004
MR effect size for
'ASAH1' on 'Bladder cancer'

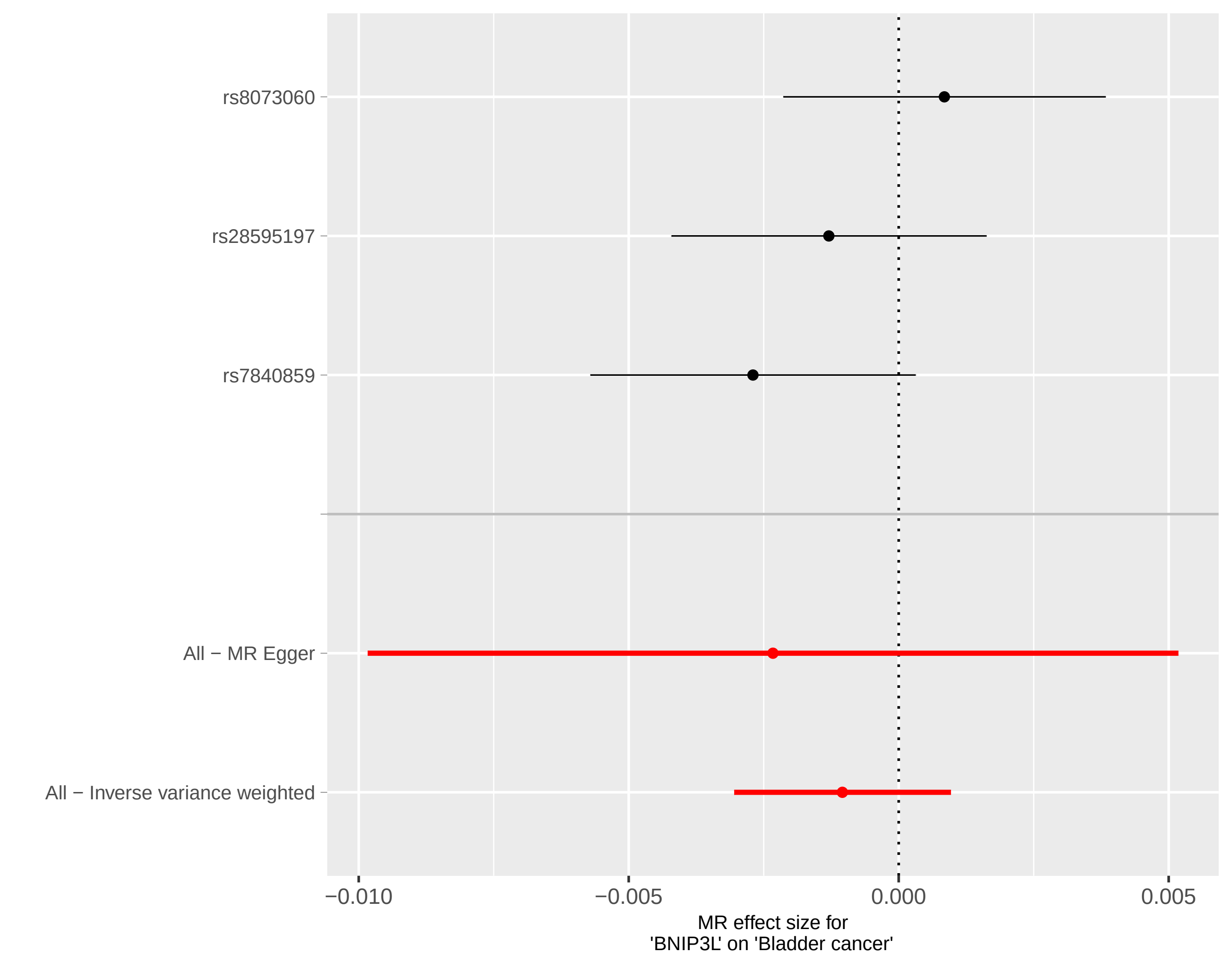
rs8073060
rs28595197
rs7840859
All − MR Egger
All − Inverse variance weighted
−0.010
−0.005
0.000
0.005
MR effect size for
'BNIP3L' on 'Bladder cancer'

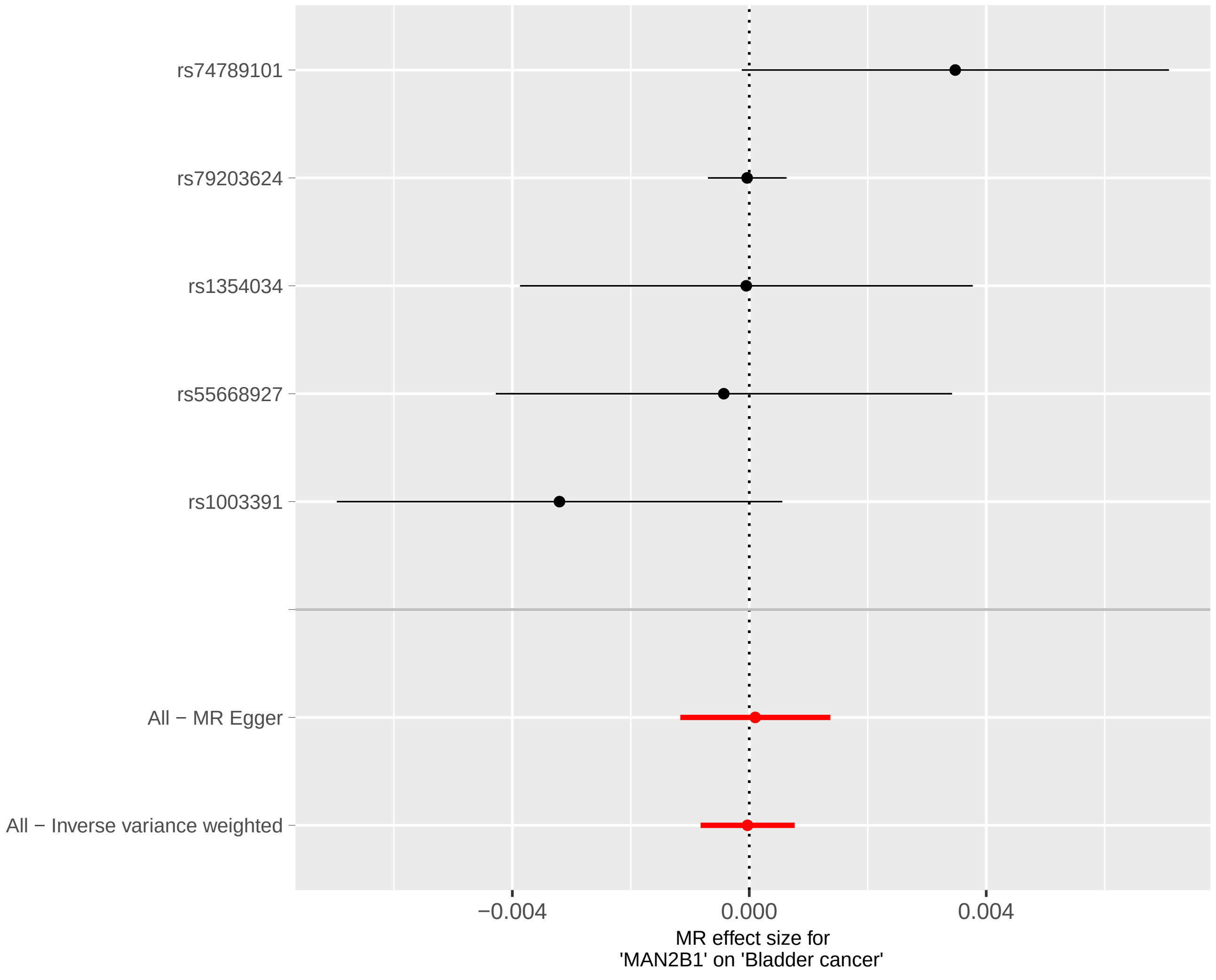
rs74789101
rs79203624
rs1354034
rs55668927
rs1003391
All − MR Egger
All − Inverse variance weighted
−0.004
0.000
0.004
MR effect size for
'MAN2B1' on 'Bladder cancer'

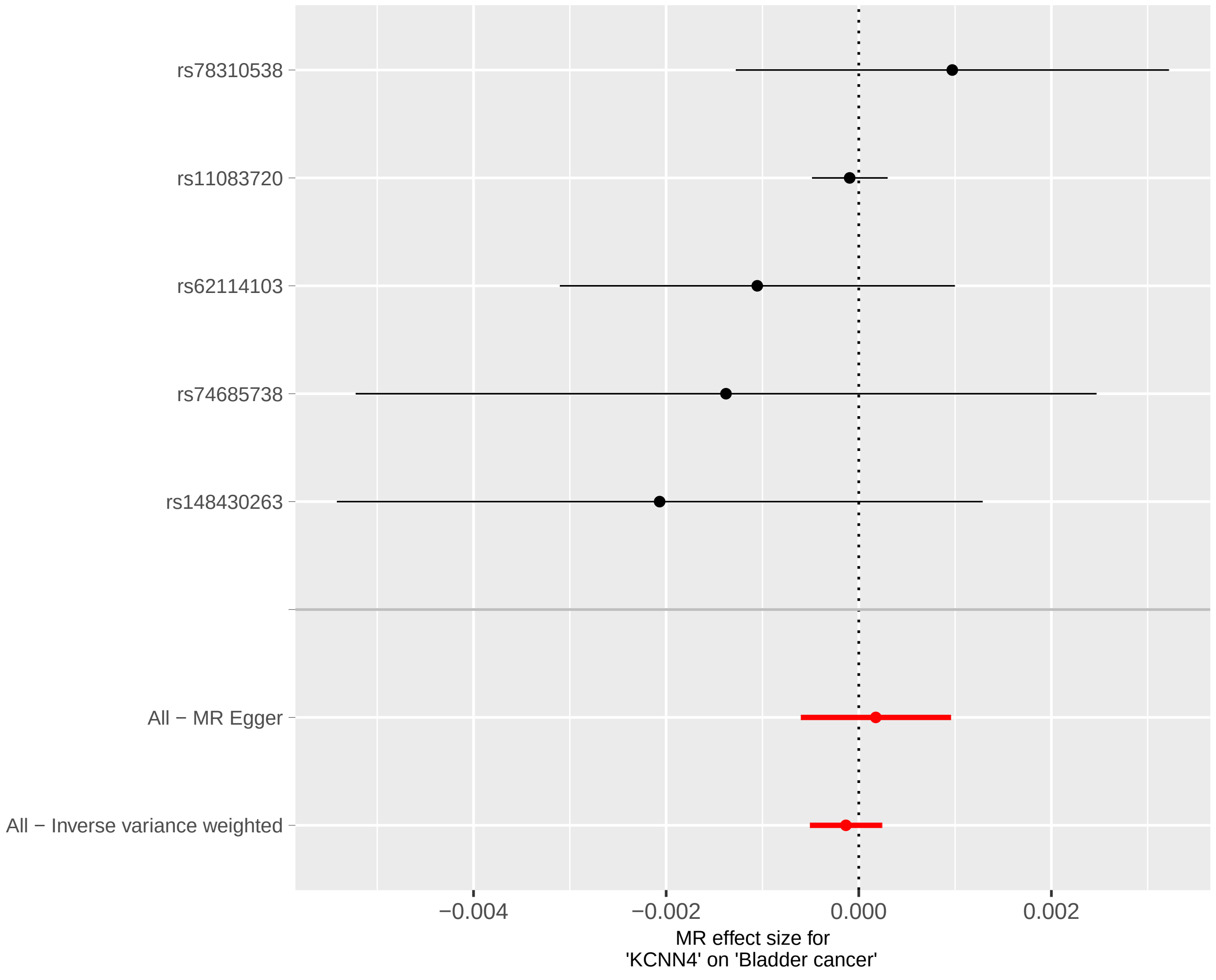
rs78310538
rs11083720
rs62114103
rs74685738
rs148430263
All − MR Egger
All − Inverse variance weighted
−0.004
−0.002
0.000
0.002
MR effect size for
'KCNN4' on 'Bladder cancer'

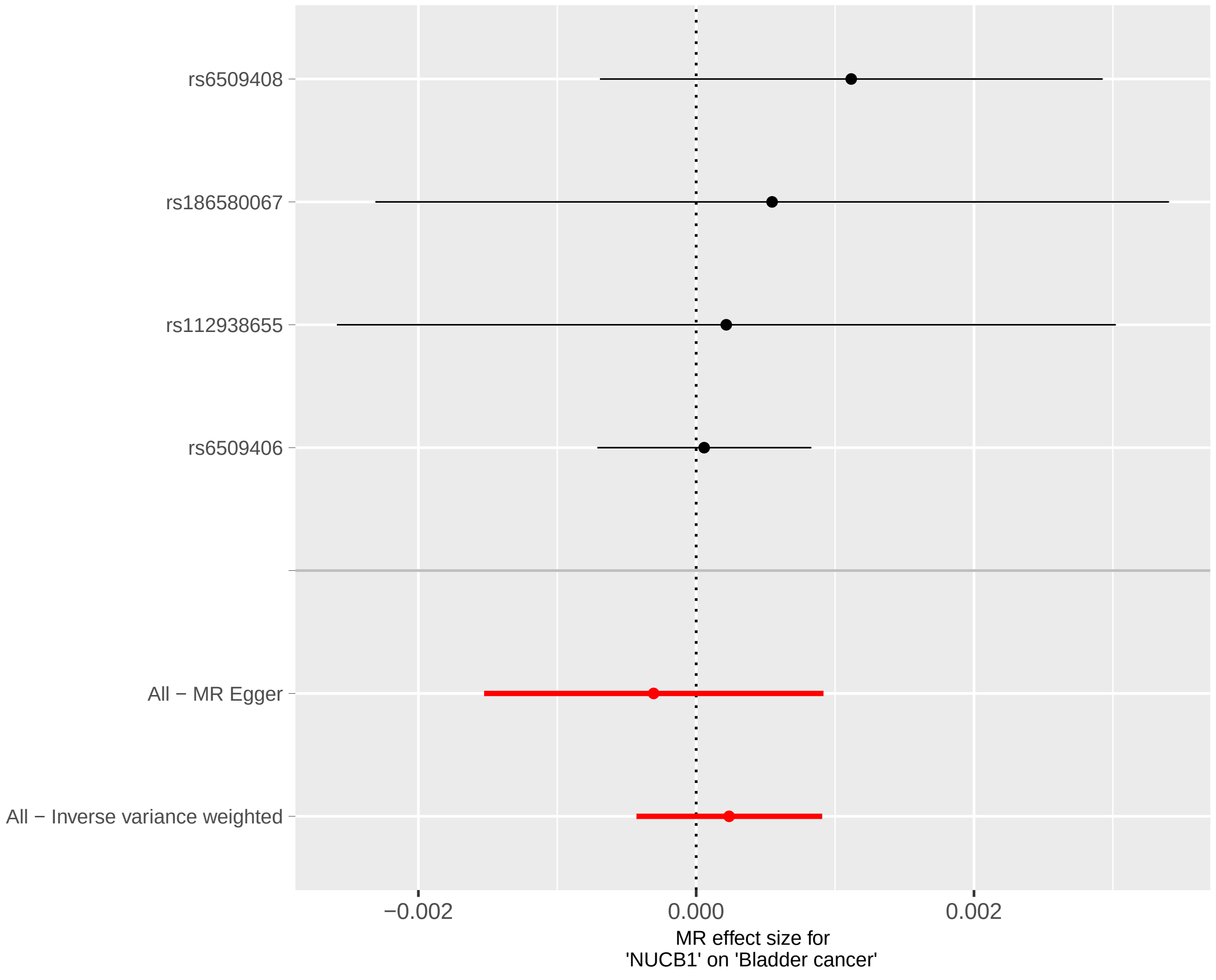
rs6509408
rs186580067
rs112938655
rs6509406
All − MR Egger
All − Inverse variance weighted
−0.002
0.000
0.002
MR effect size for
'NUCB1' on 'Bladder cancer'

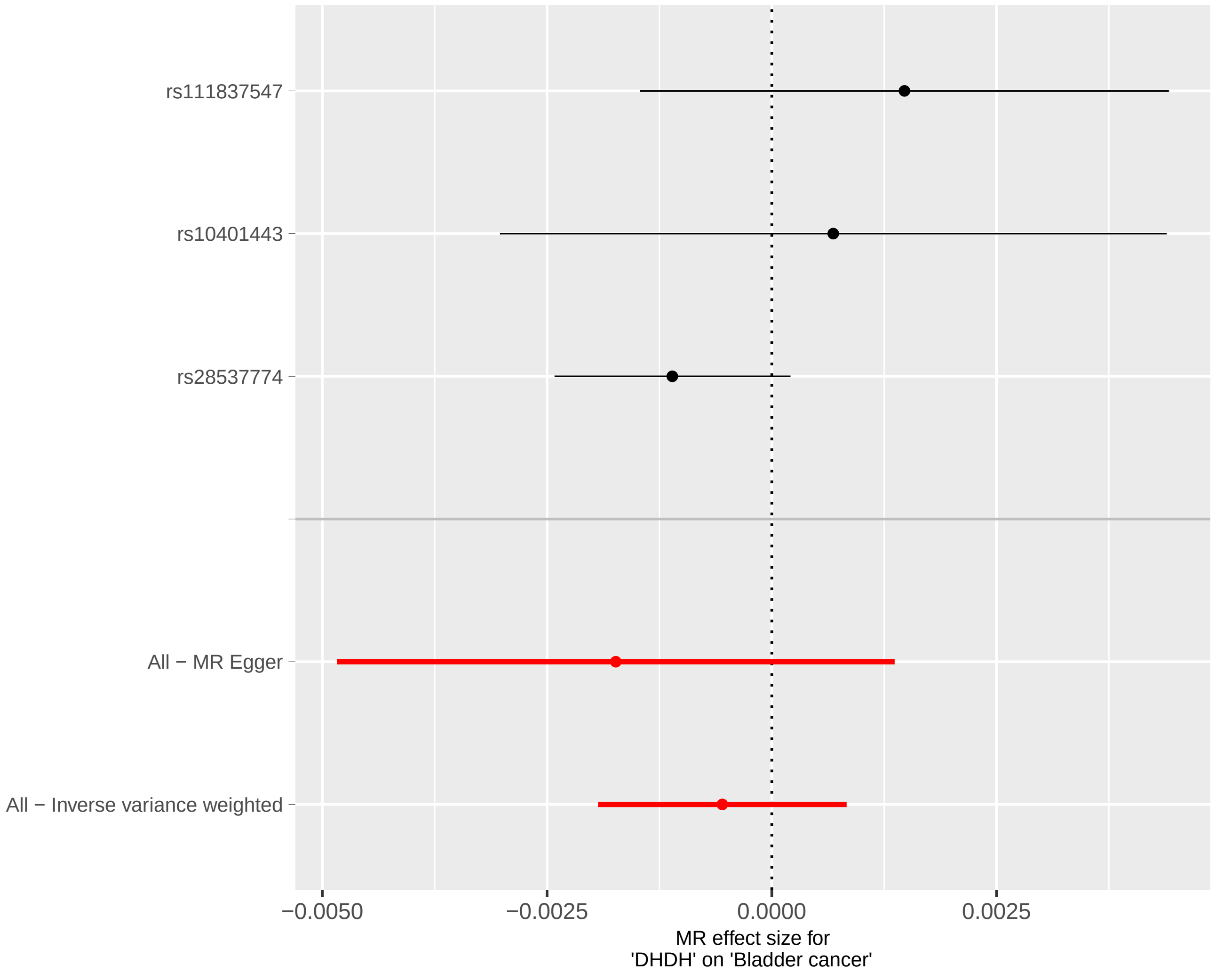

rs111837547
rs10401443
rs28537774
All − MR Egger
All − Inverse variance weighted
−0.0050
−0.0025
0.0000
0.0025
MR effect size for
'DHDH' on 'Bladder cancer'

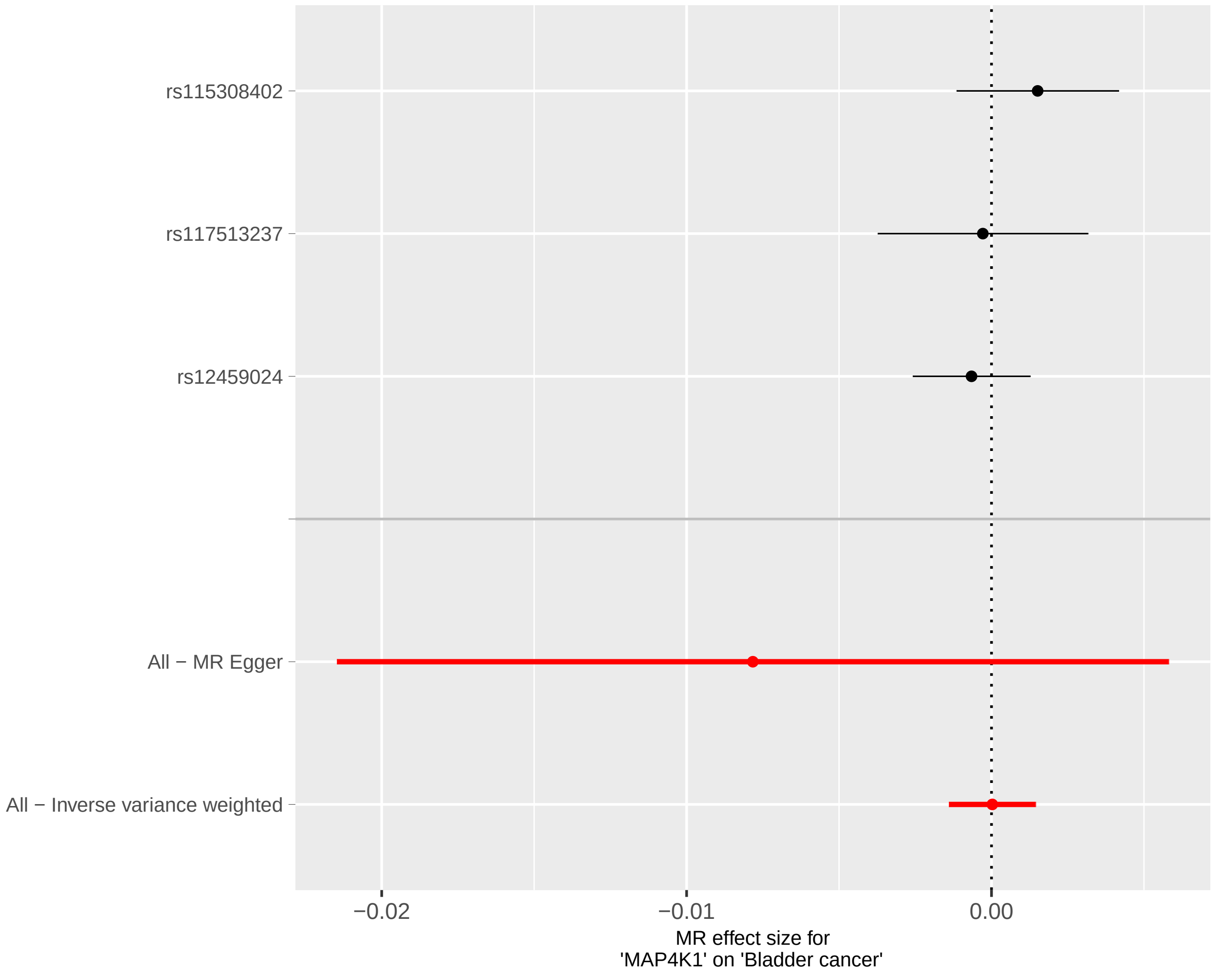
rs115308402
rs117513237
rs12459024
All − MR Egger
All − Inverse variance weighted
−0.02
−0.01
0.00
MR effect size for
'MAP4K1' on 'Bladder cancer'

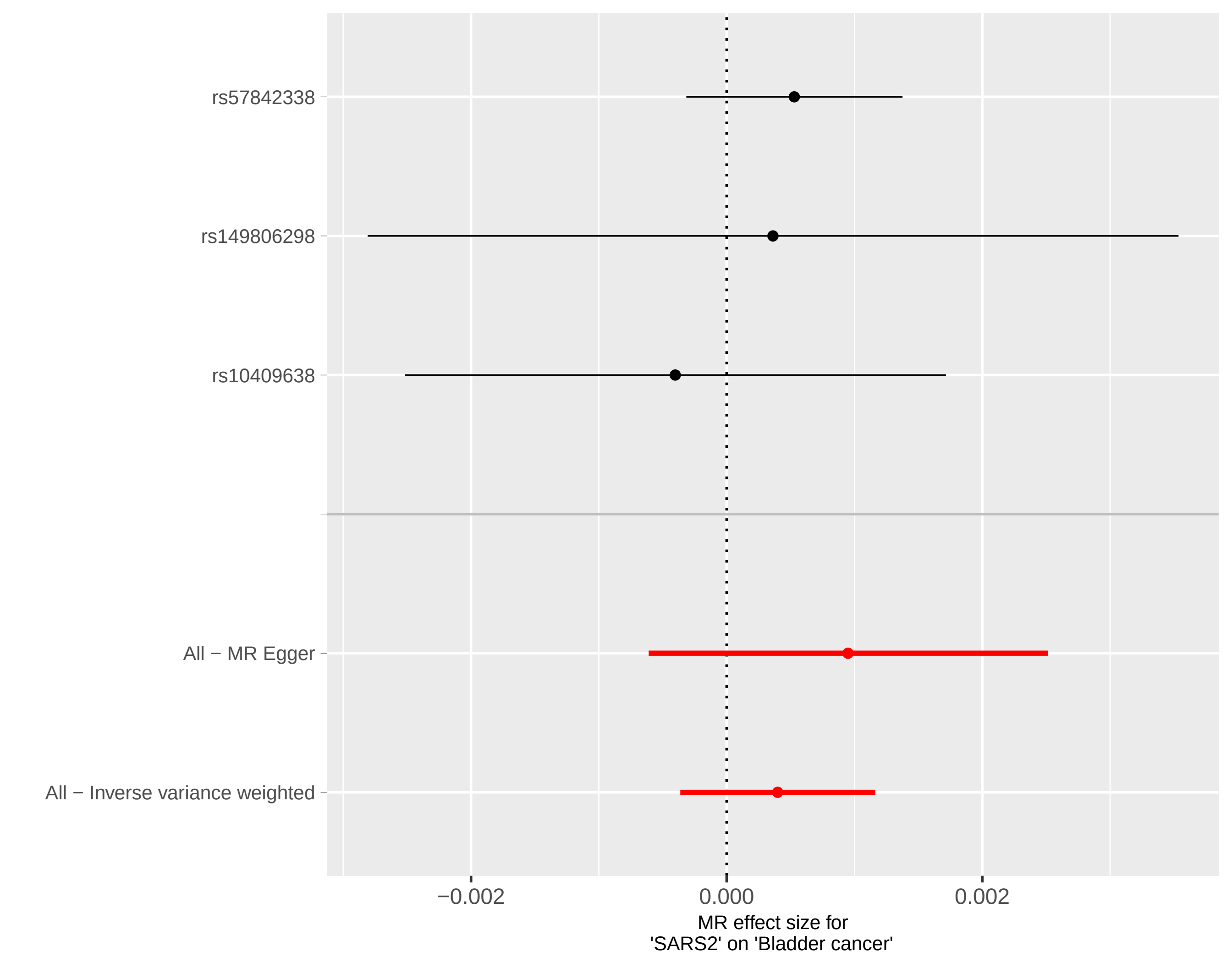

rs57842338
rs149806298
rs10409638
All − MR Egger
All − Inverse variance weighted
−0.002
0.000
0.002
MR effect size for
'SARS2' on 'Bladder cancer'

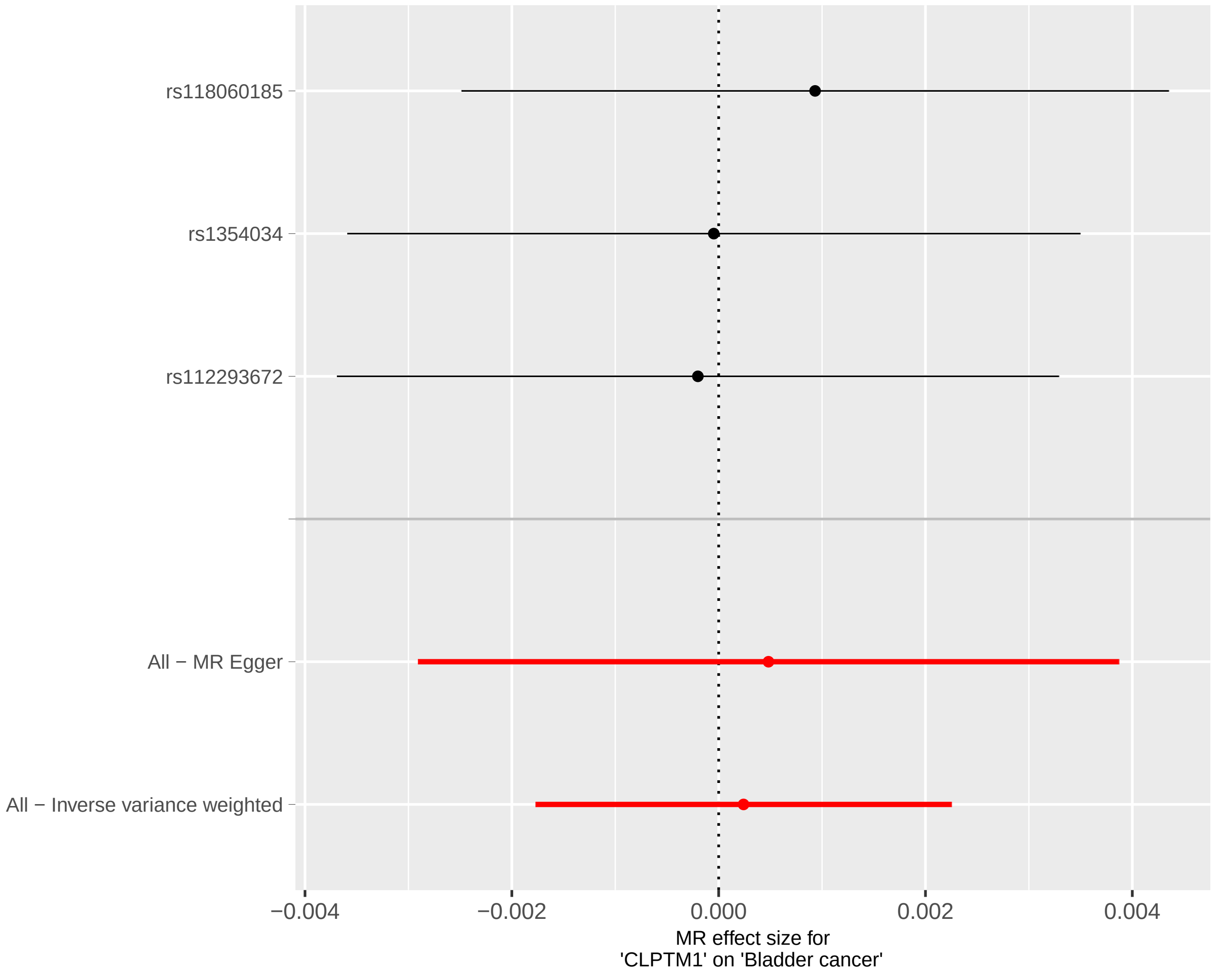
rs118060185
rs1354034
rs112293672
All − MR Egger
All − Inverse variance weighted
−0.004
−0.002
0.000
0.002
0.004
MR effect size for
'CLPTM1' on 'Bladder cancer'

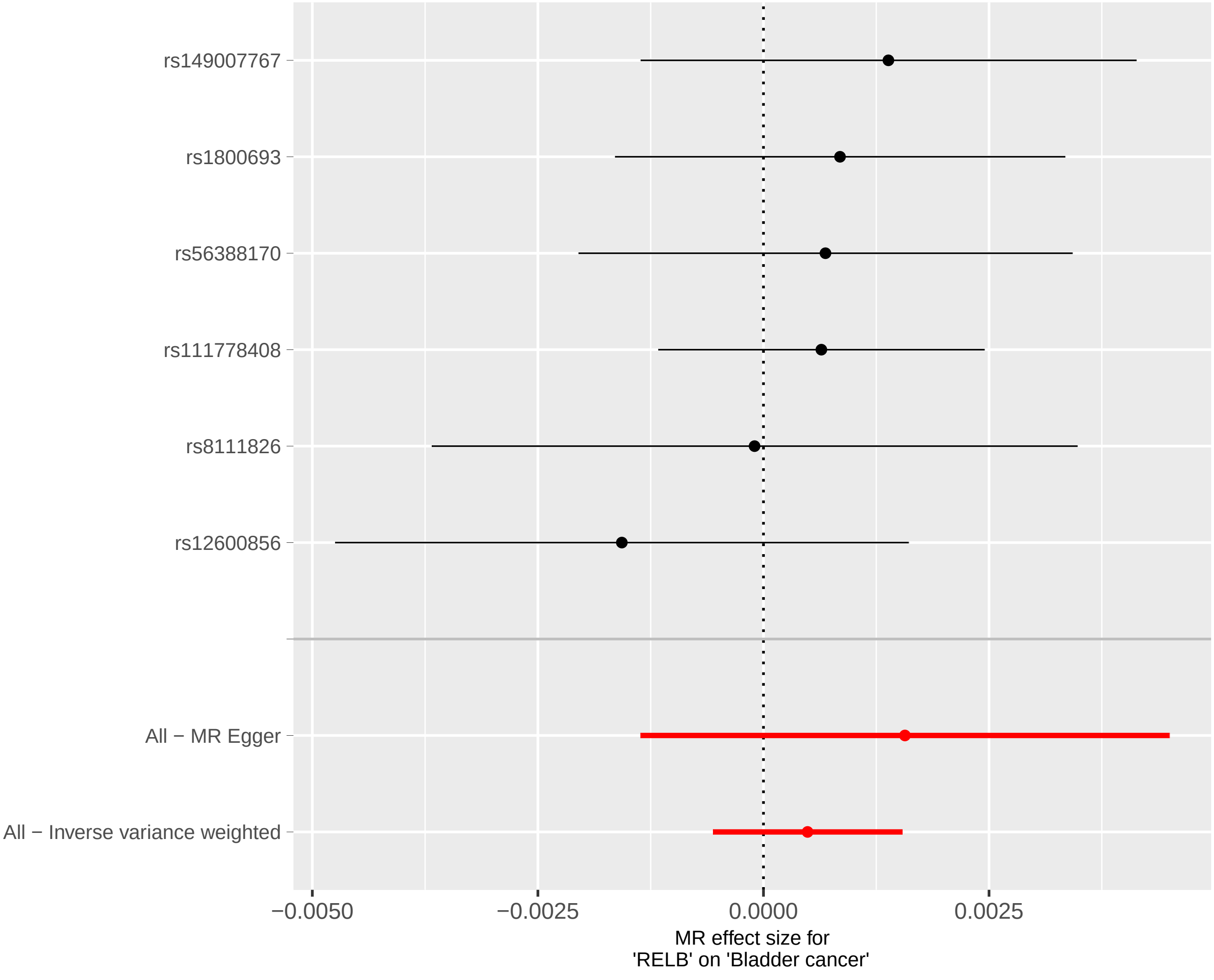
rs149007767
rs1800693
rs56388170
rs111778408
rs8111826
rs12600856
All − MR Egger
All − Inverse variance weighted
−0.0050
−0.0025
0.0000
0.0025
MR effect size for
'RELB' on 'Bladder cancer'

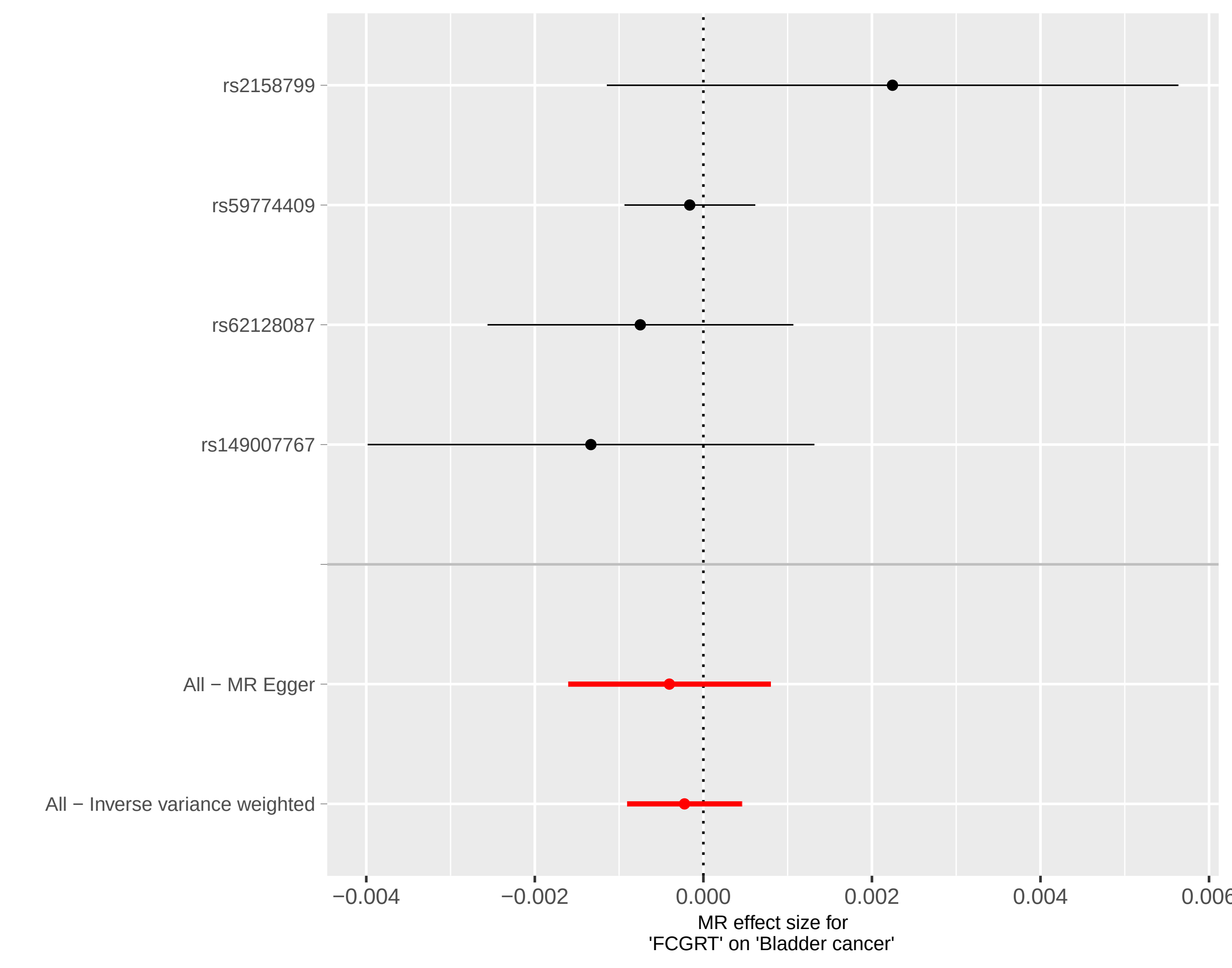

rs2158799
rs59774409
rs62128087
rs149007767
All − MR Egger
All − Inverse variance weighted
−0.004
−0.002
0.000
0.002
0.004
0.006
MR effect size for
'FCGRT' on 'Bladder cancer'

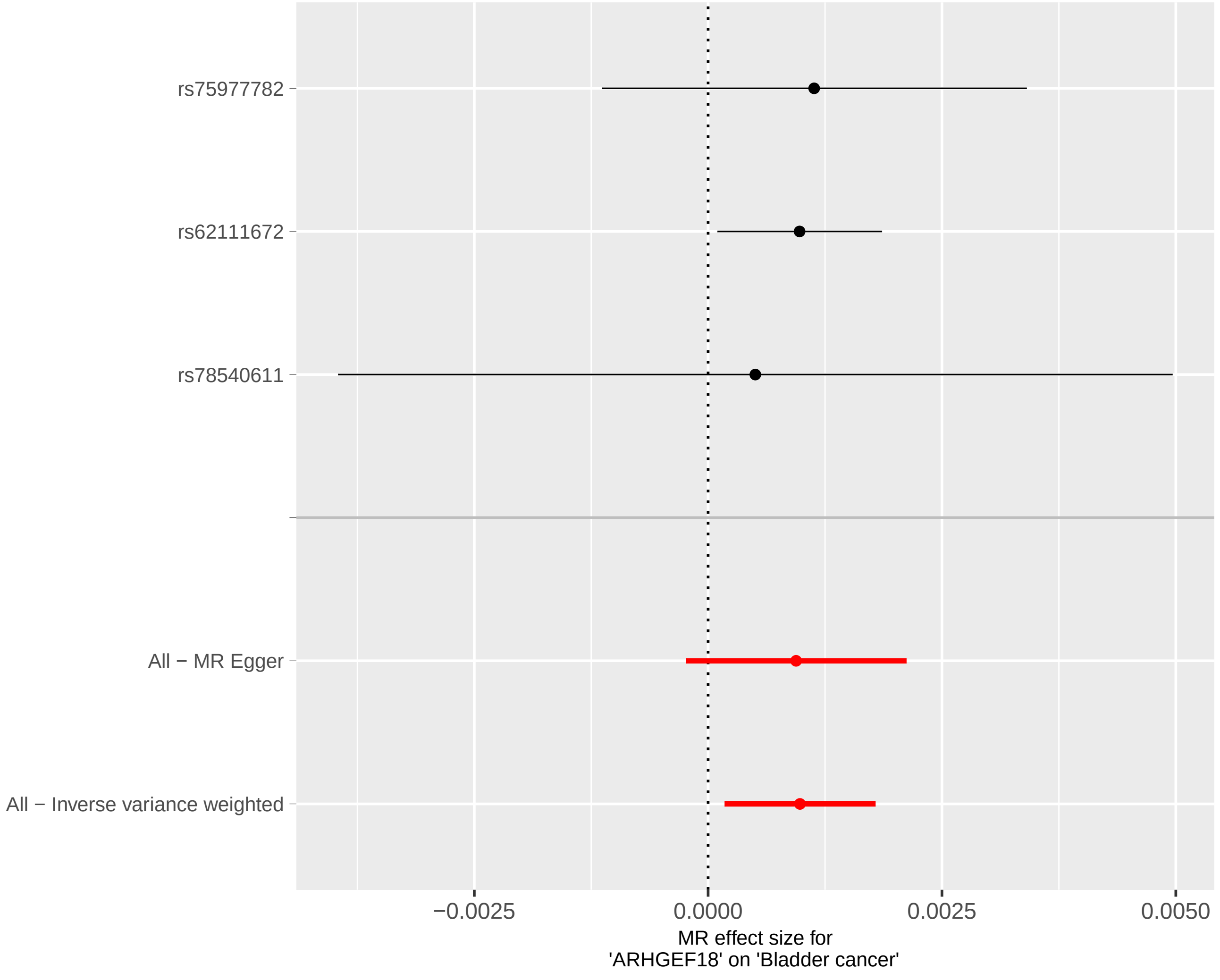

rs75977782
rs62111672
rs78540611
All − MR Egger
All − Inverse variance weighted
−0.0025
0.0000
0.0025
0.0050
MR effect size for
'ARHGEF18' on 'Bladder cancer'

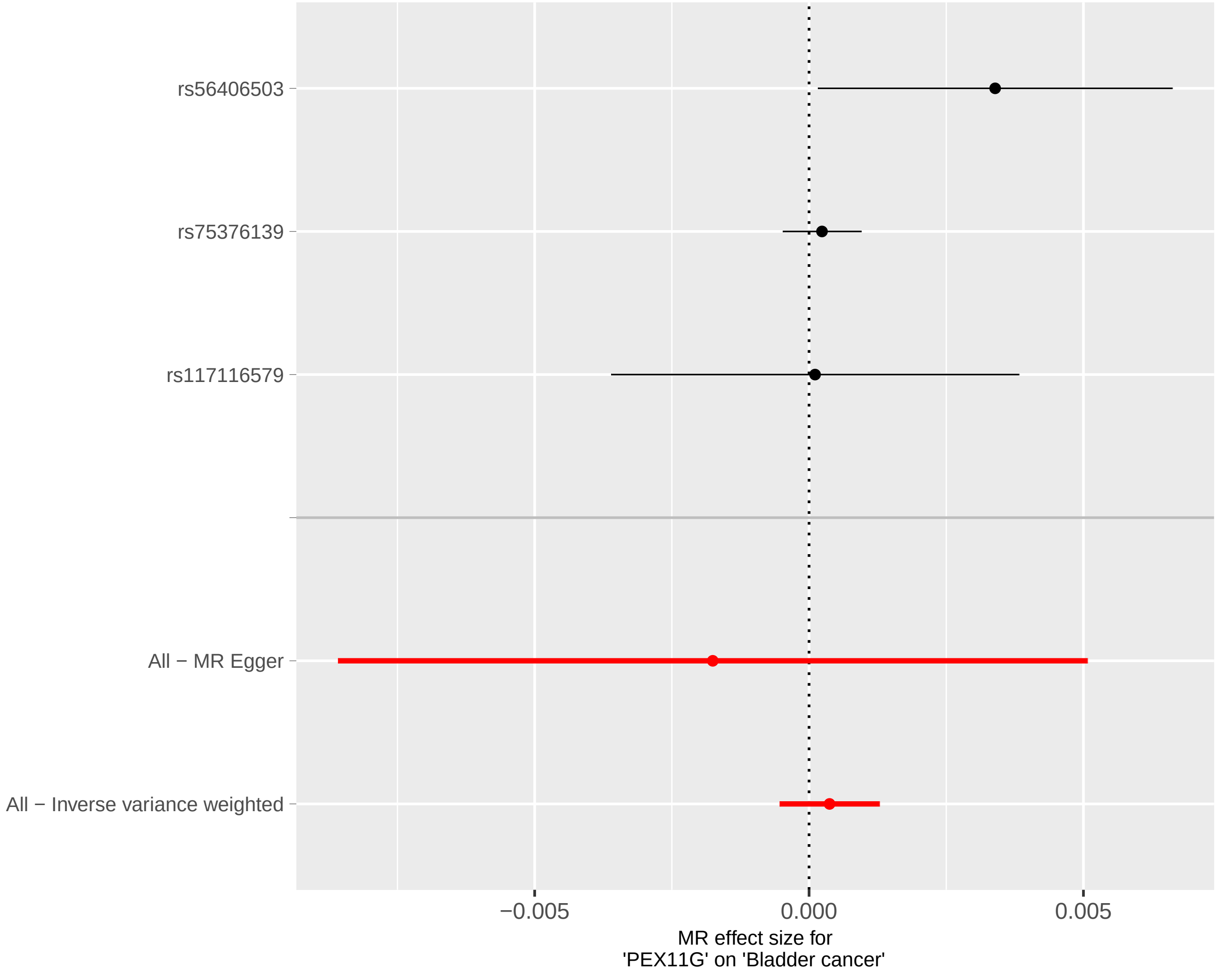

rs56406503
rs75376139
rs117116579
All − MR Egger
All − Inverse variance weighted
−0.005
0.000
0.005
MR effect size for
'PEX11G' on 'Bladder cancer'

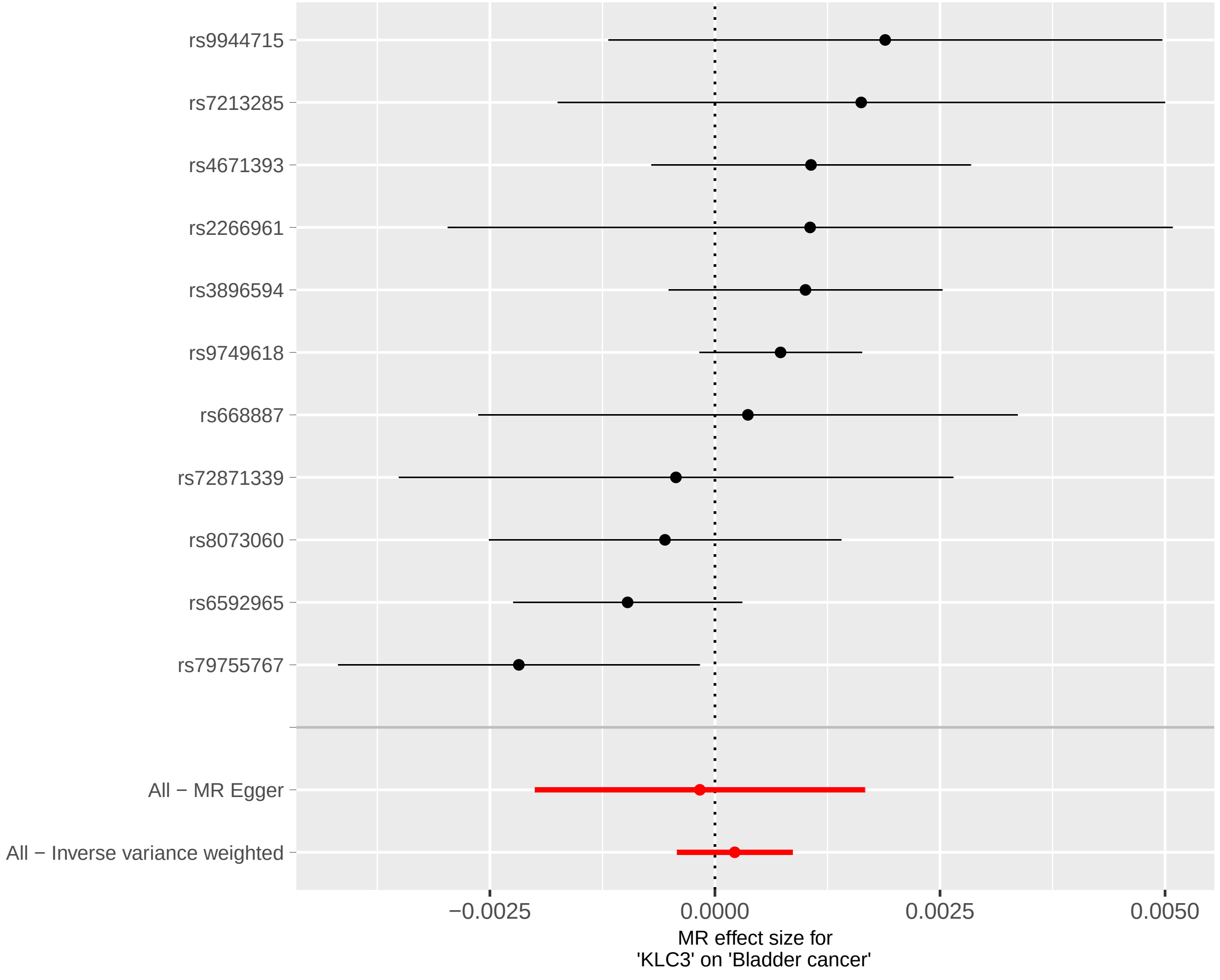

rs9944715
rs7213285
rs4671393
rs2266961
rs3896594
rs9749618
rs668887
rs72871339
rs8073060
rs6592965
rs79755767
All − MR Egger
All − Inverse variance weighted
−0.0025
0.0000
0.0025
0.0050
MR effect size for
'KLC3' on 'Bladder cancer'

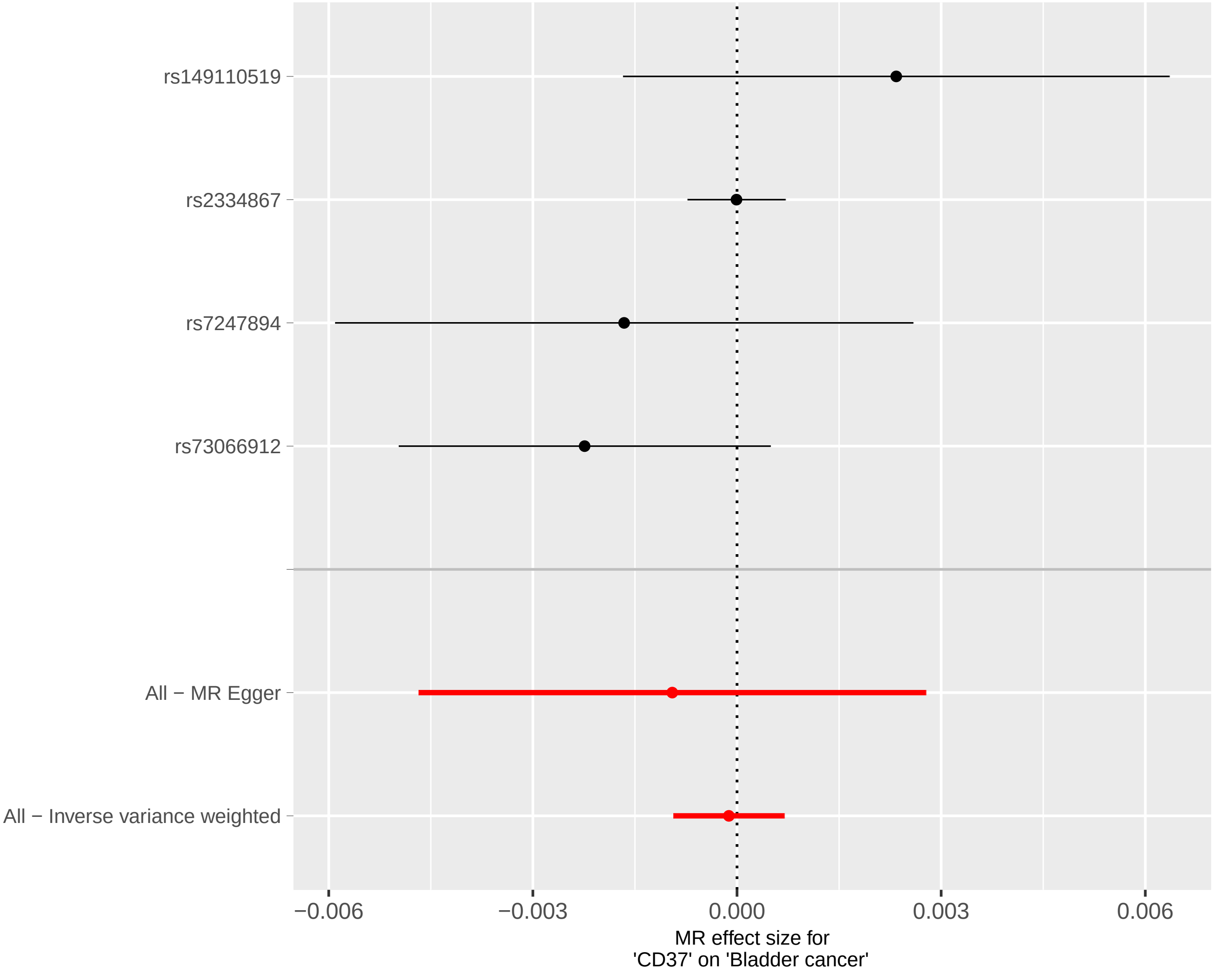

rs149110519
rs2334867
rs7247894
rs73066912
All − MR Egger
All − Inverse variance weighted
−0.006
−0.003
0.000
0.003
0.006
MR effect size for
'CD37' on 'Bladder cancer'

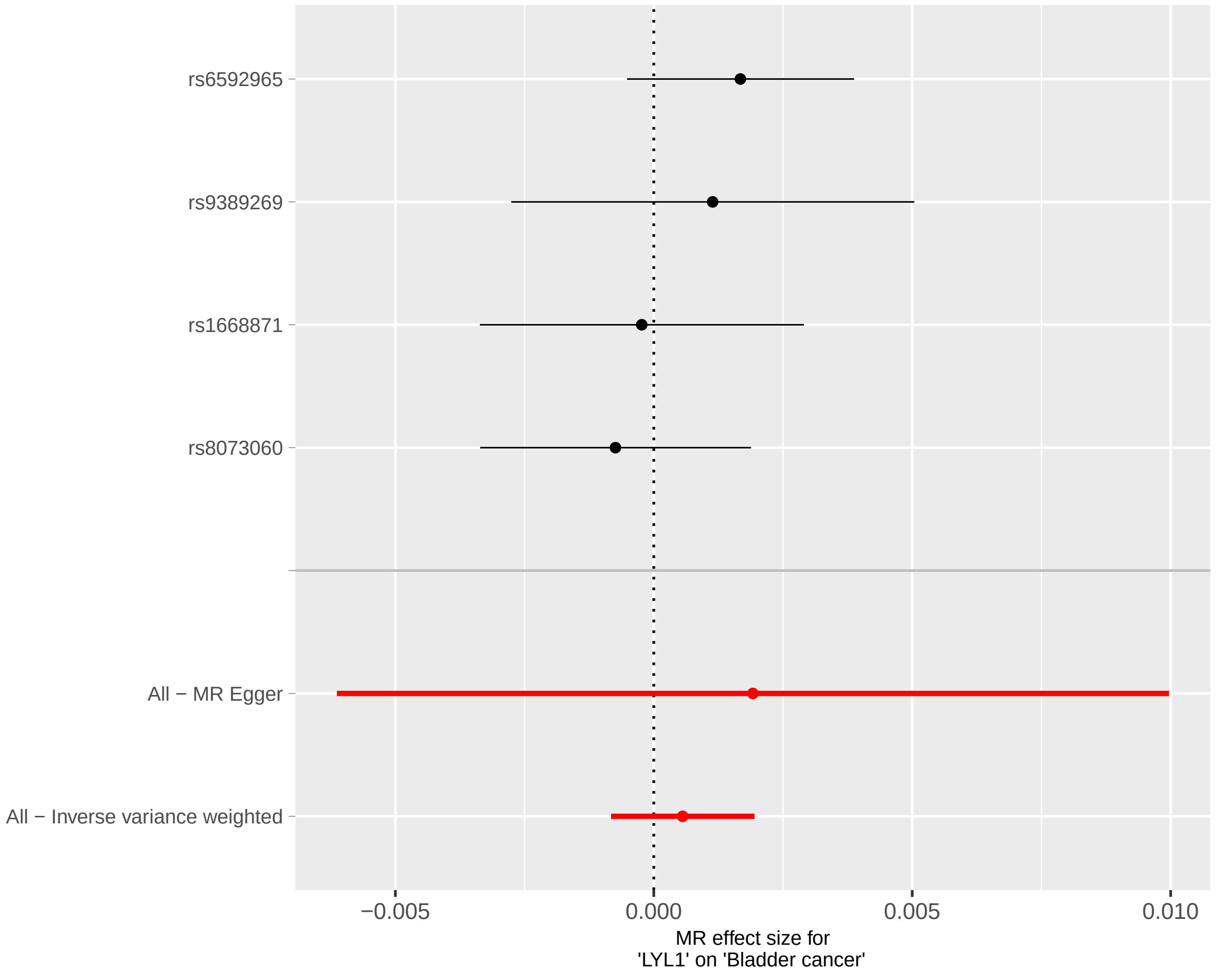
rs6592965
rs9389269
rs1668871
rs8073060
All − MR Egger
All − Inverse variance weighted
−0.005
0.000
0.005
0.010
MR effect size for
'LYL1' on 'Bladder cancer'

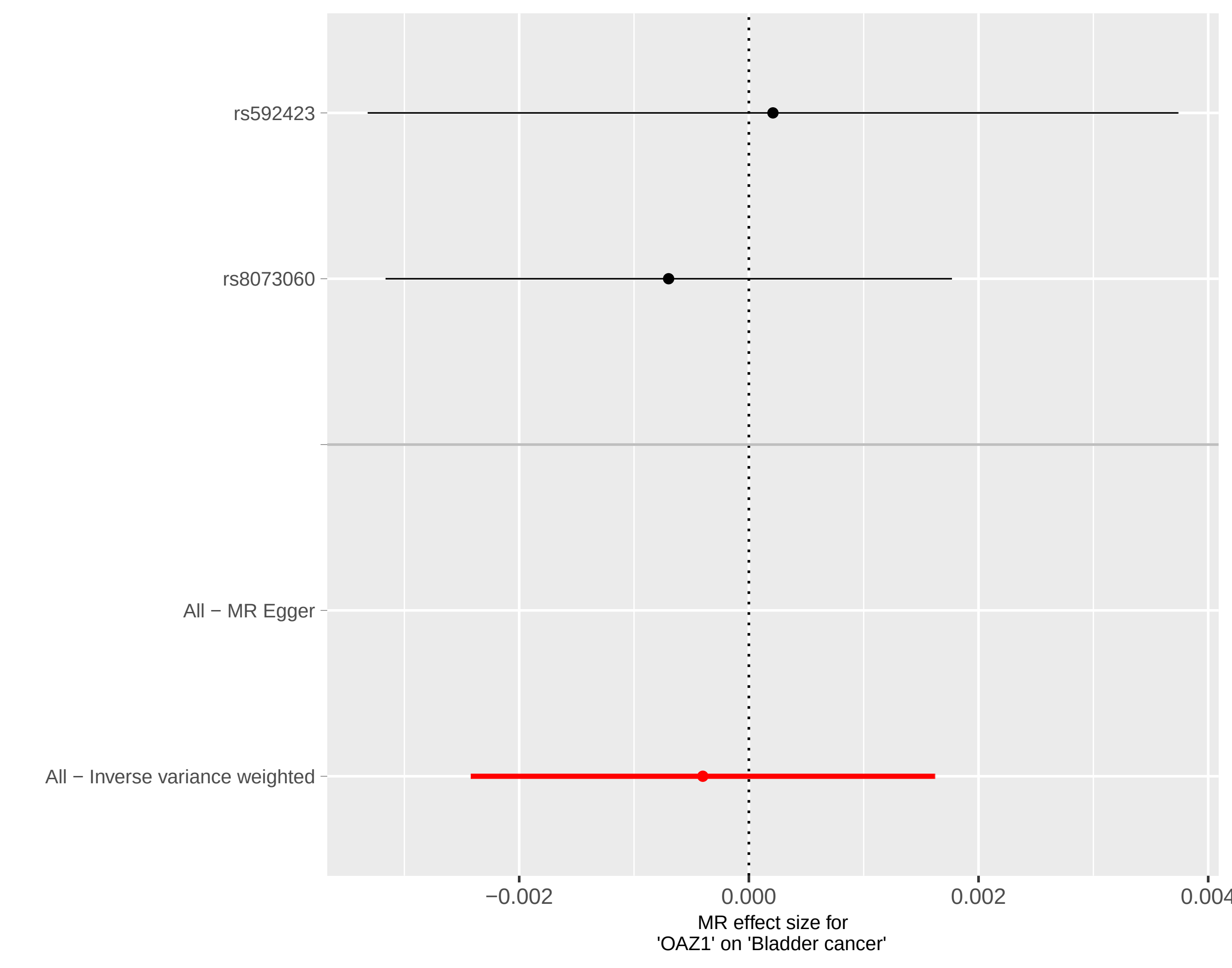

rs592423
rs8073060
All − MR Egger
All − Inverse variance weighted
−0.002
0.000
0.002
MR effect size for
'OAZ1' on 'Bladder cancer'

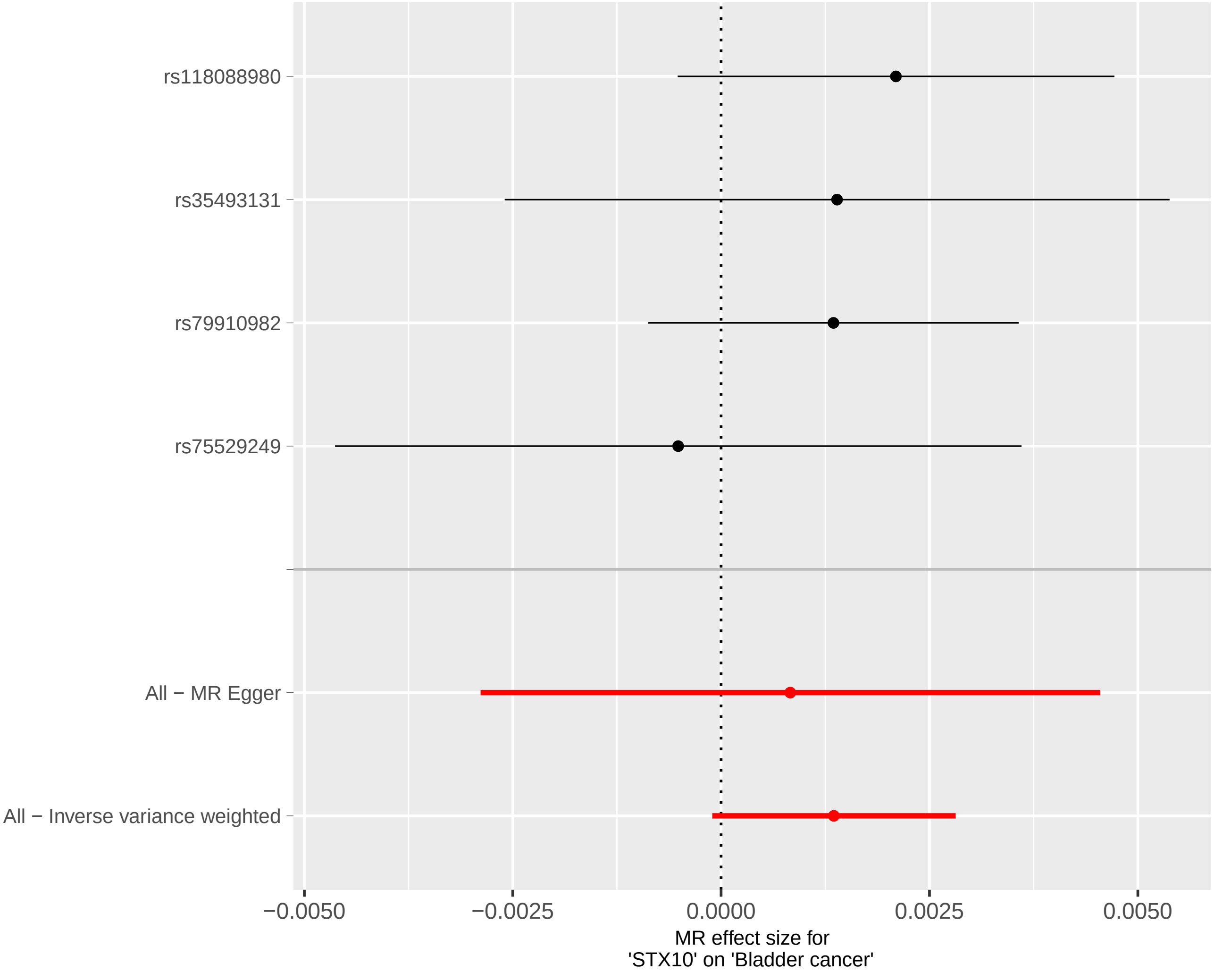

rs118088980
rs35493131
rs79910982
rs75529249
All − MR Egger
All − Inverse variance weighted
−0.0050
−0.0025
0.0000
0.0025
0.0050
MR effect size for
'STX10' on 'Bladder cancer'

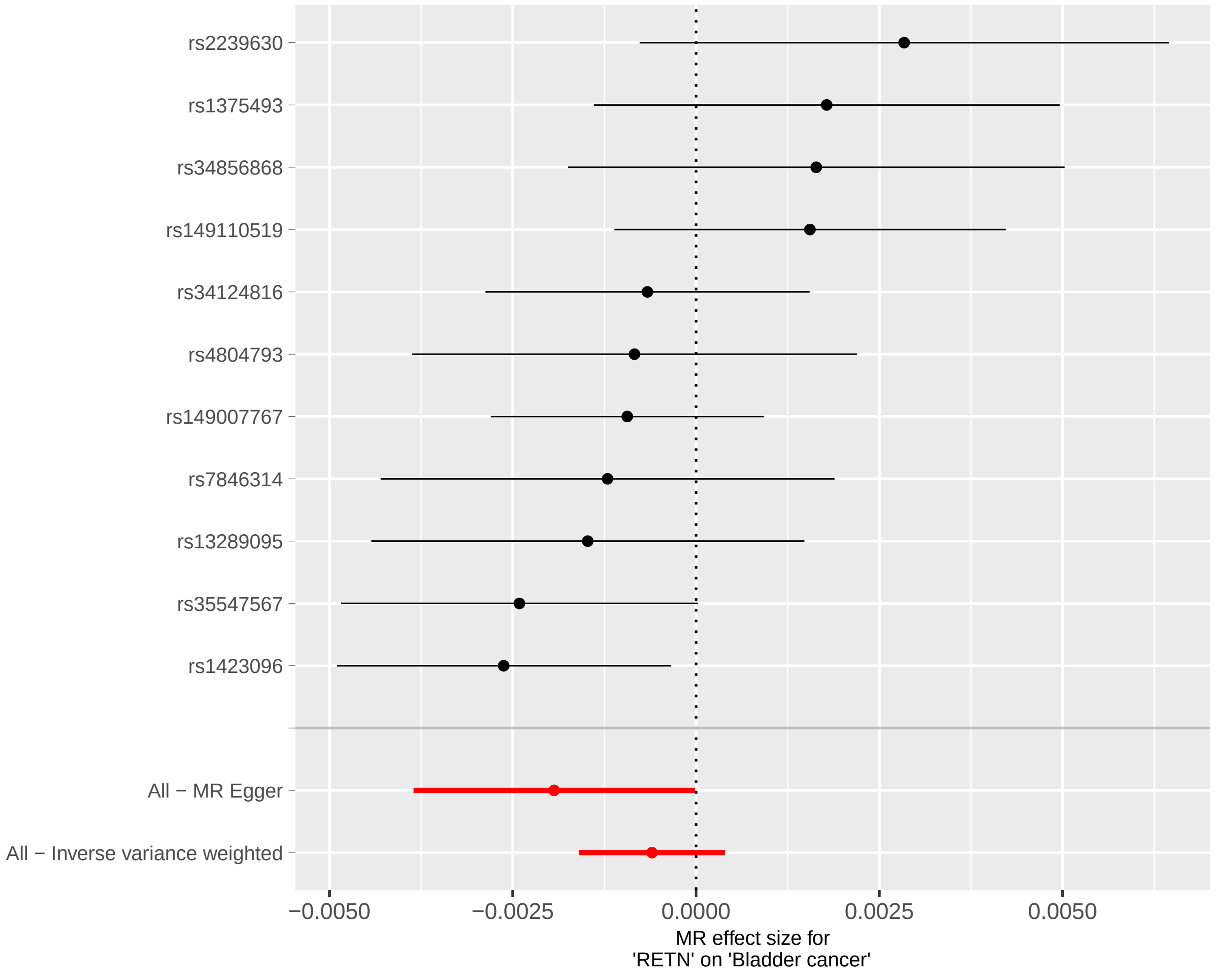
rs2239630
rs1375493
rs34856868
rs149110519
rs34124816
rs4804793
rs149007767
rs7846314
rs13289095
rs35547567
rs1423096
All − MR Egger
All − Inverse variance weighted
−0.0050
−0.0025
0.0000
0.0025
0.0050
MR effect size for
'RETN' on 'Bladder cancer'

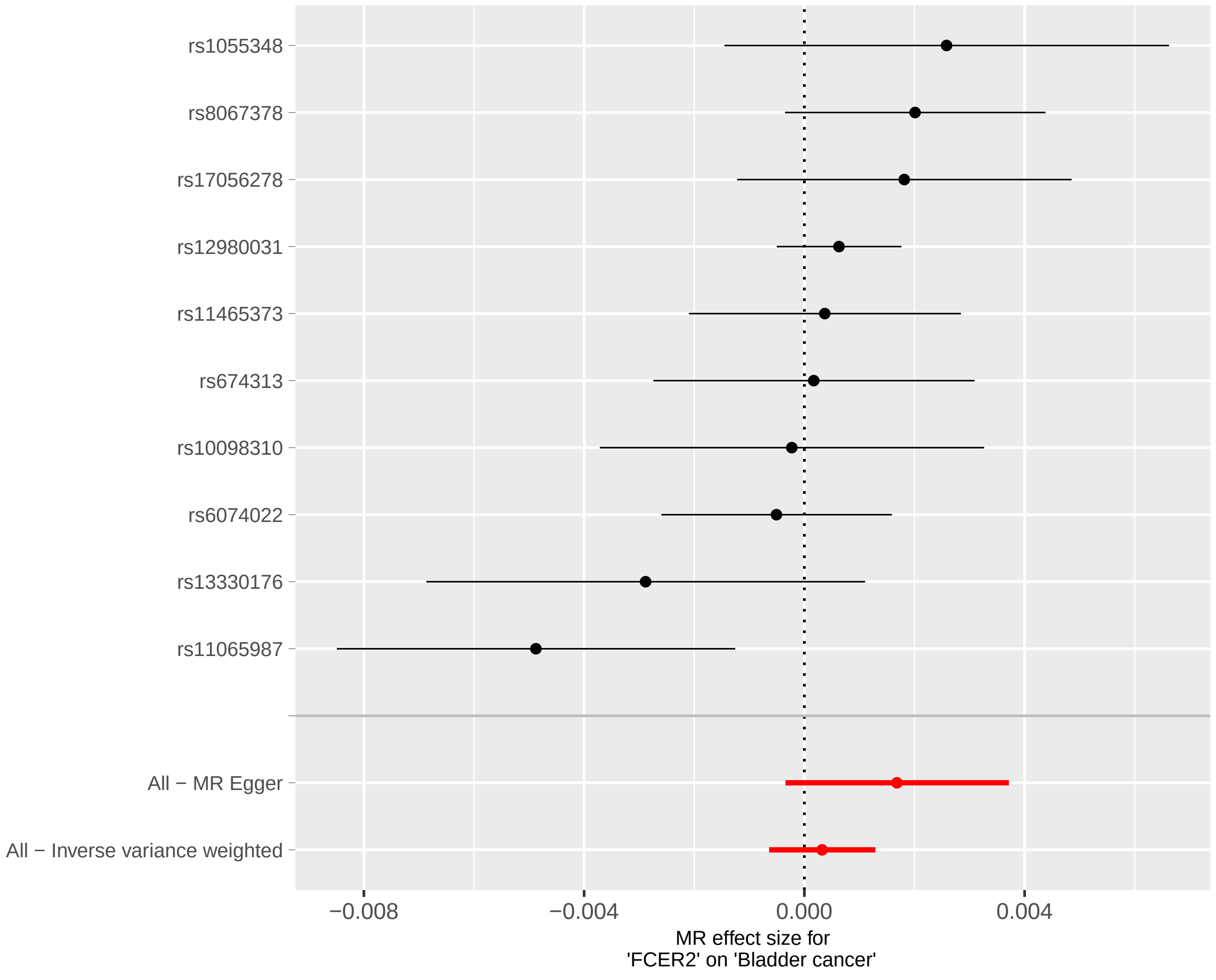
rs1055348
rs8067378
rs17056278
rs12980031
rs11465373
rs674313
rs10098310
rs6074022
rs13330176
rs11065987
All − MR Egger
All − Inverse variance weighted
−0.008
−0.004
0.000
0.004
MR effect size for
'FCER2' on 'Bladder cancer'

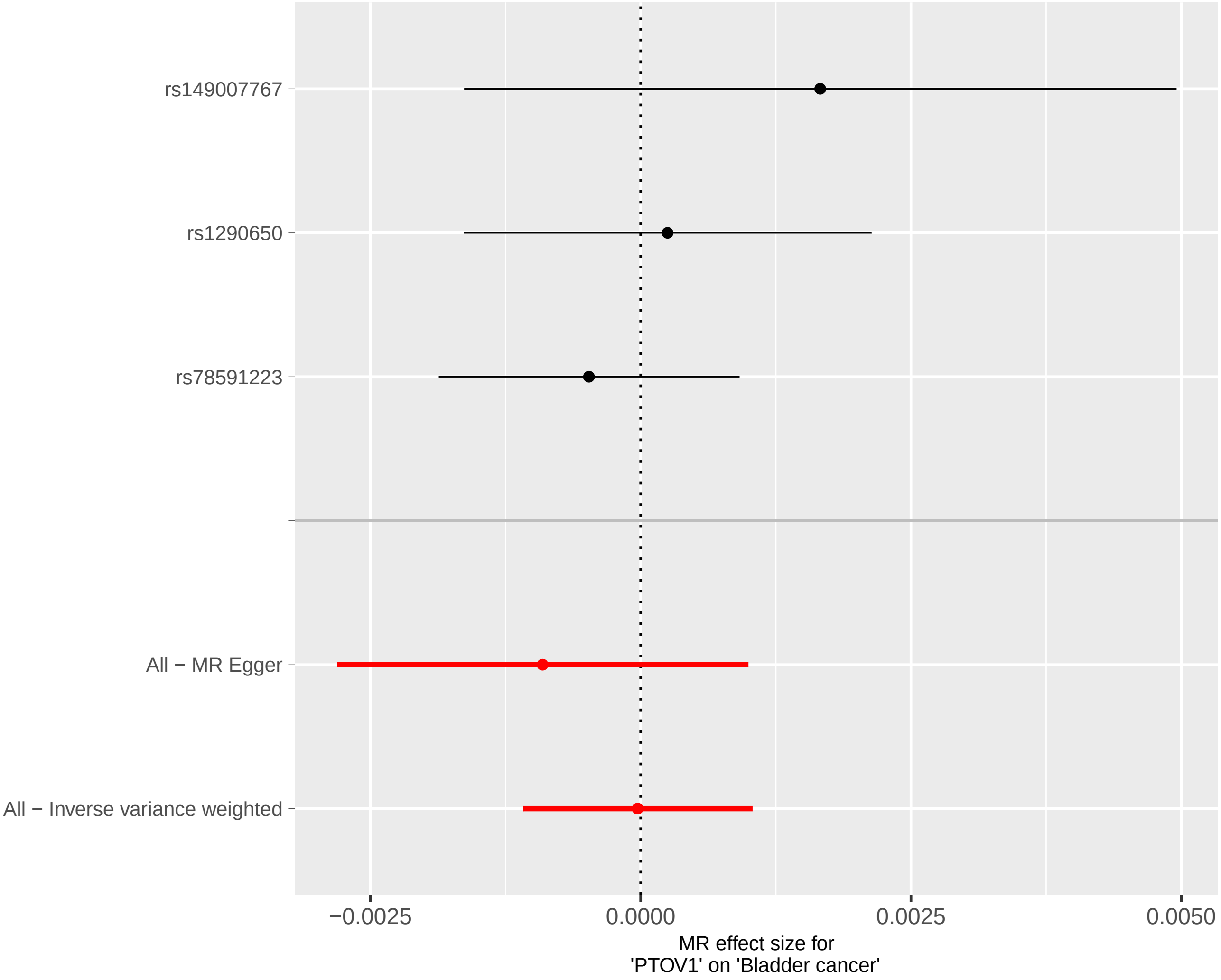
rs149007767
rs1290650
rs78591223
All − MR Egger
All − Inverse variance weighted
−0.0025
0.0000
0.0025
0.0050
MR effect size for
'PTOV1' on 'Bladder cancer'

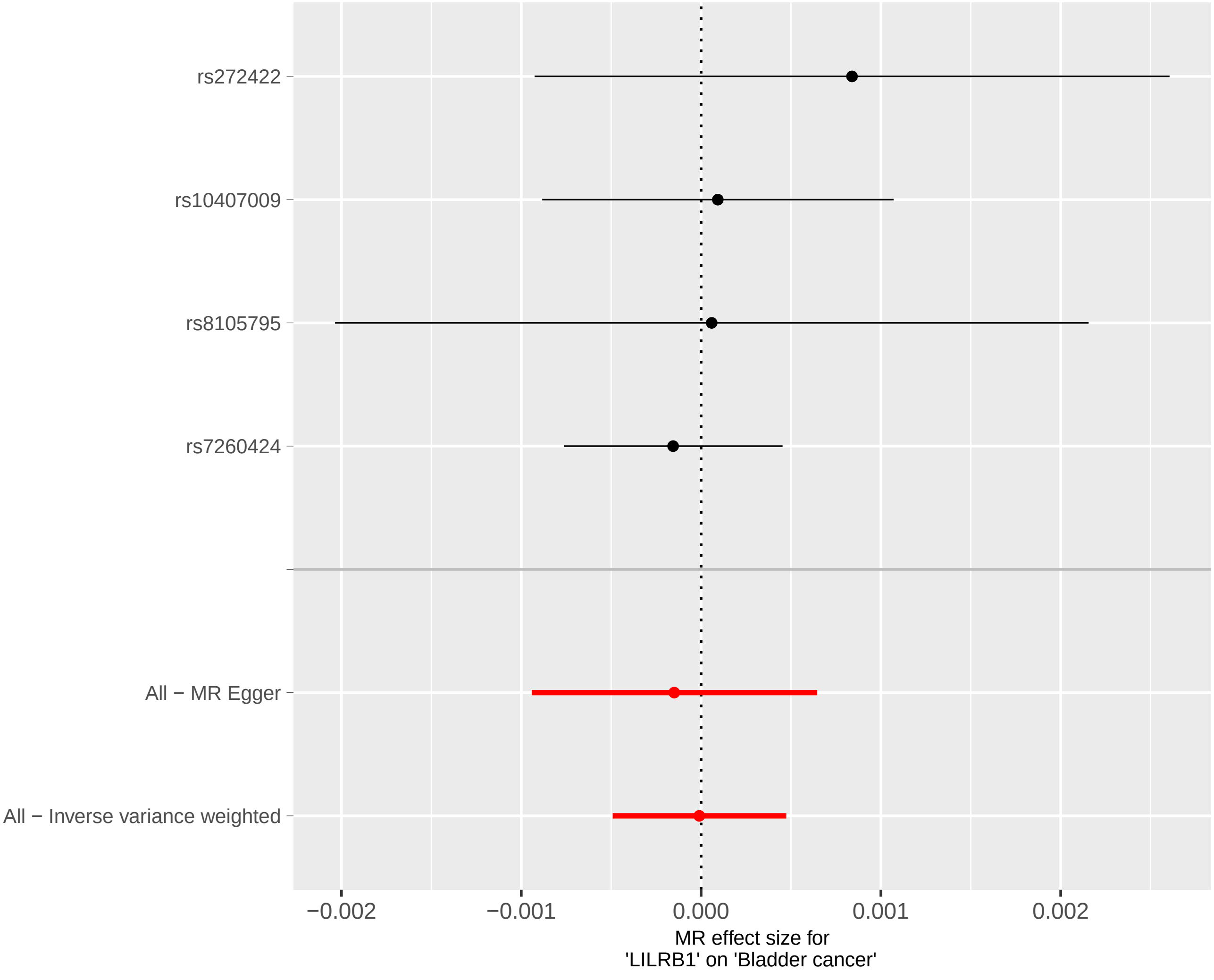

rs272422
rs10407009
rs8105795
rs7260424
All − MR Egger
All − Inverse variance weighted
−0.002
−0.001
0.000
0.001
0.002
MR effect size for
'LILRB1' on 'Bladder cancer'

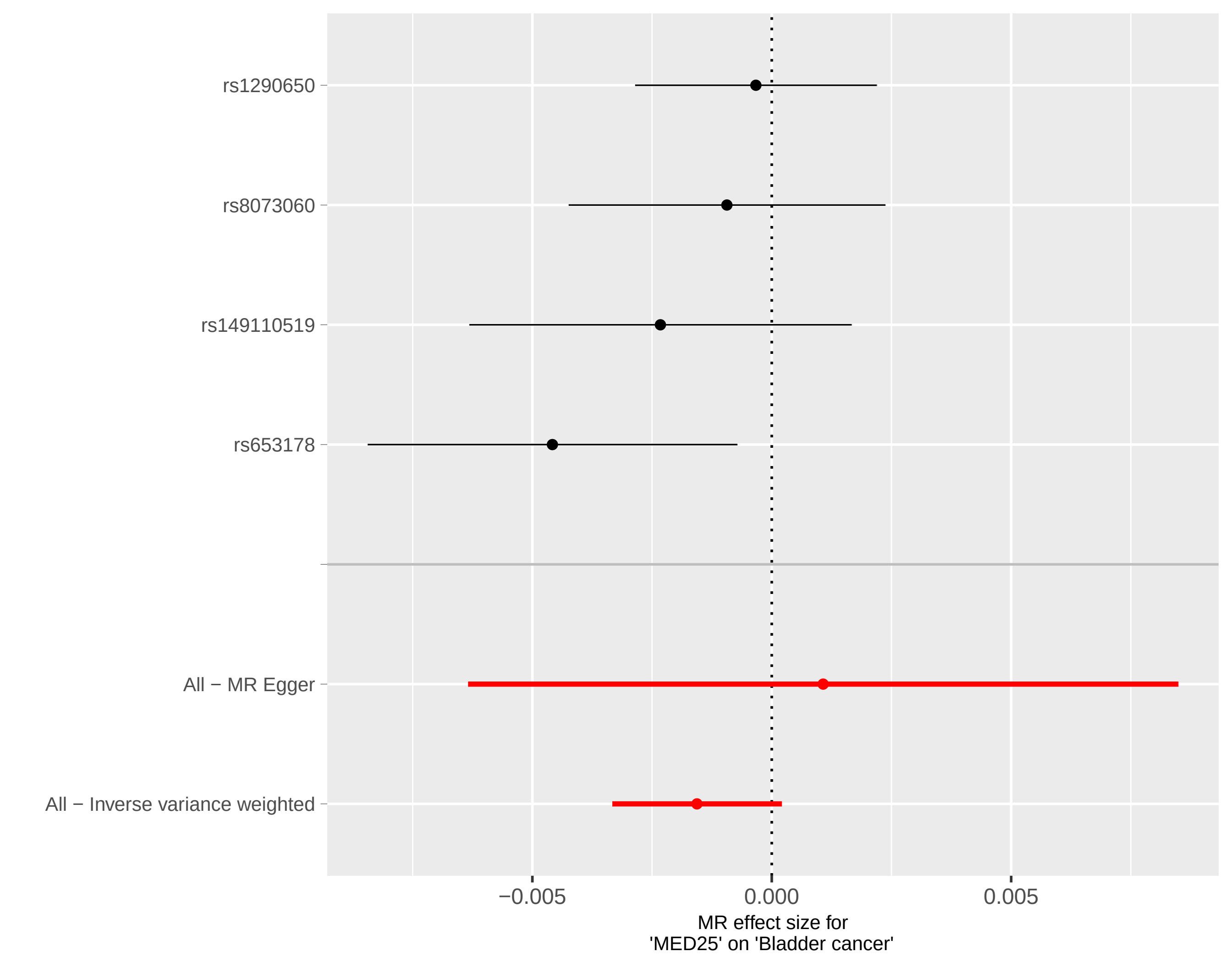

rs1290650
rs8073060
rs149110519
rs653178
All − MR Egger
All − Inverse variance weighted
−0.005
0.000
0.005
MR effect size for
'MED25' on 'Bladder cancer'

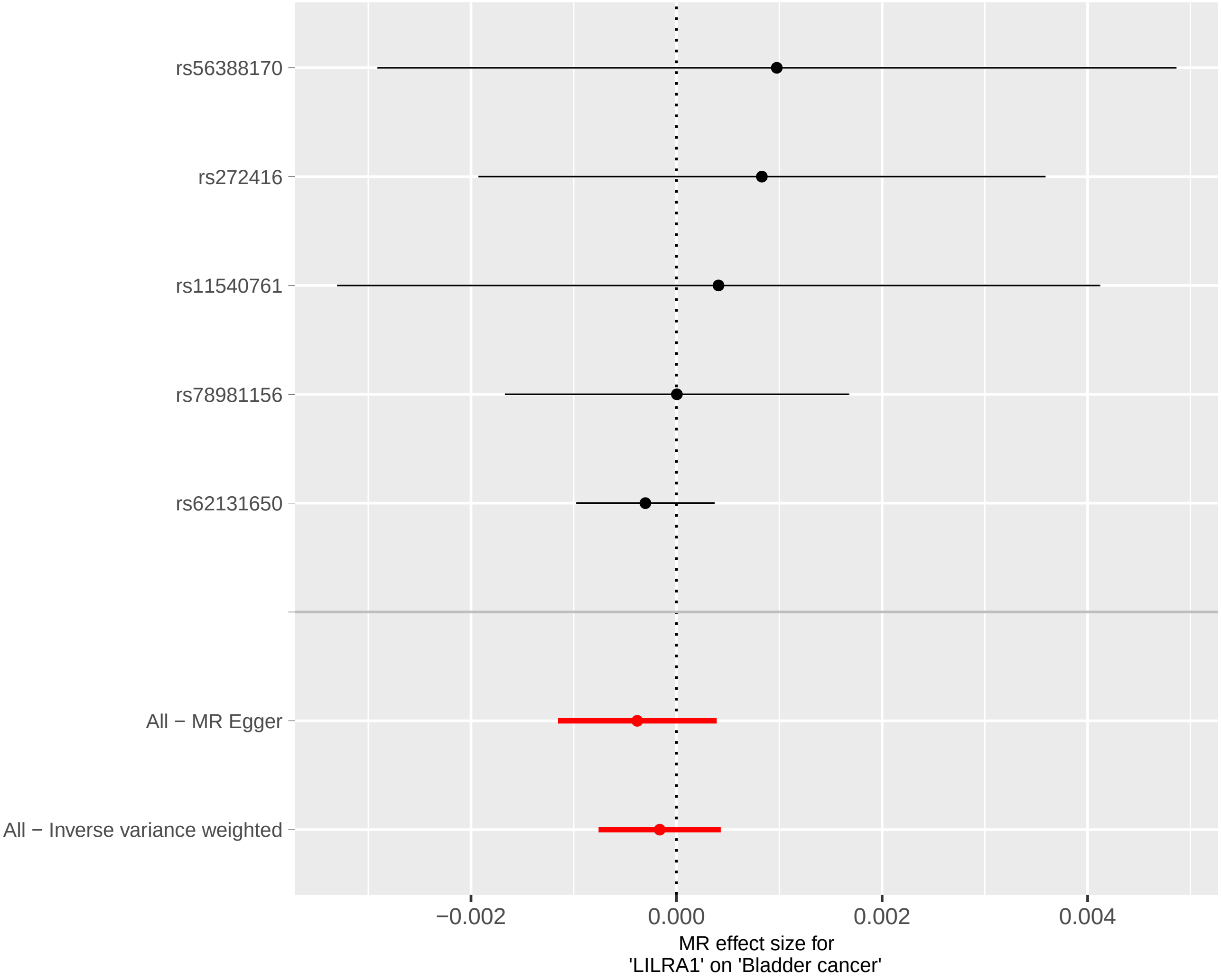

rs56388170
rs272416
rs11540761
rs78981156
rs62131650
All − MR Egger
All − Inverse variance weighted
−0.002
0.000
0.002
0.004
MR effect size for
'LILRA1' on 'Bladder cancer'

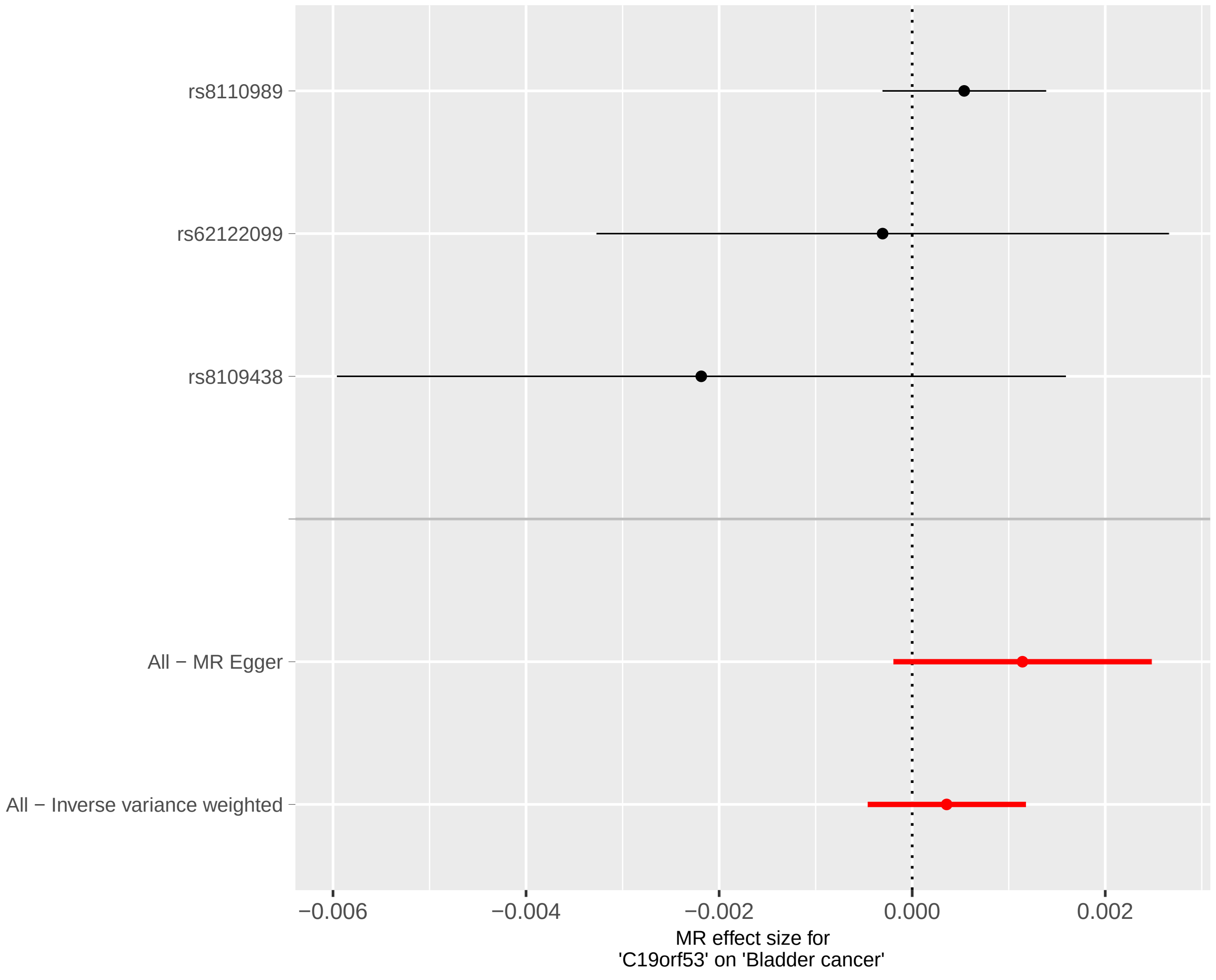

rs8110989
rs62122099
rs8109438
All − MR Egger
All − Inverse variance weighted
−0.006
−0.004
−0.002
0.000
0.002
MR effect size for
'C19orf53' on 'Bladder cancer'

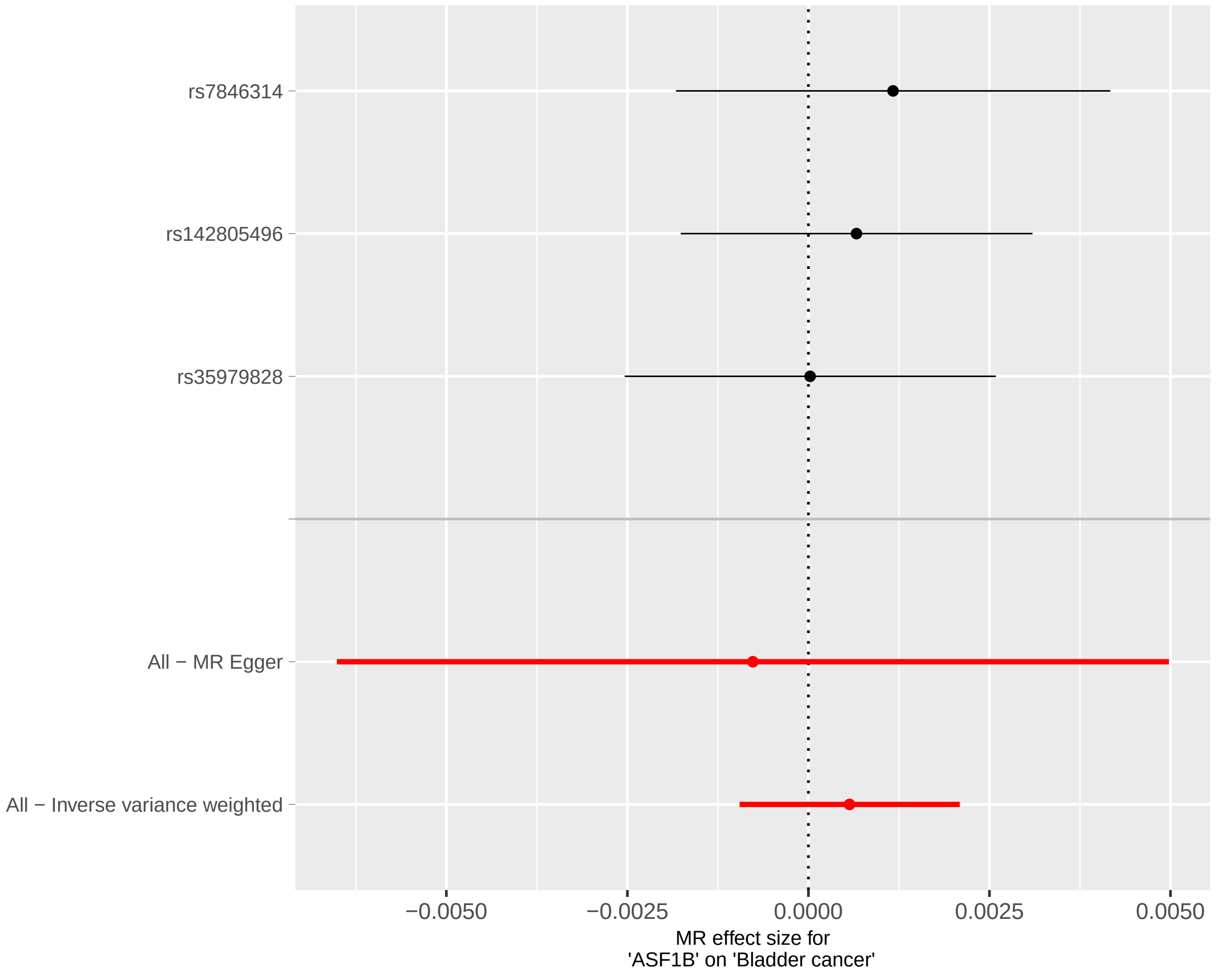
rs7846314
rs142805496
rs35979828
All − MR Egger
All − Inverse variance weighted
−0.0050
−0.0025
0.0000
0.0025
0.0050
MR effect size for
'ASF1B' on 'Bladder cancer'

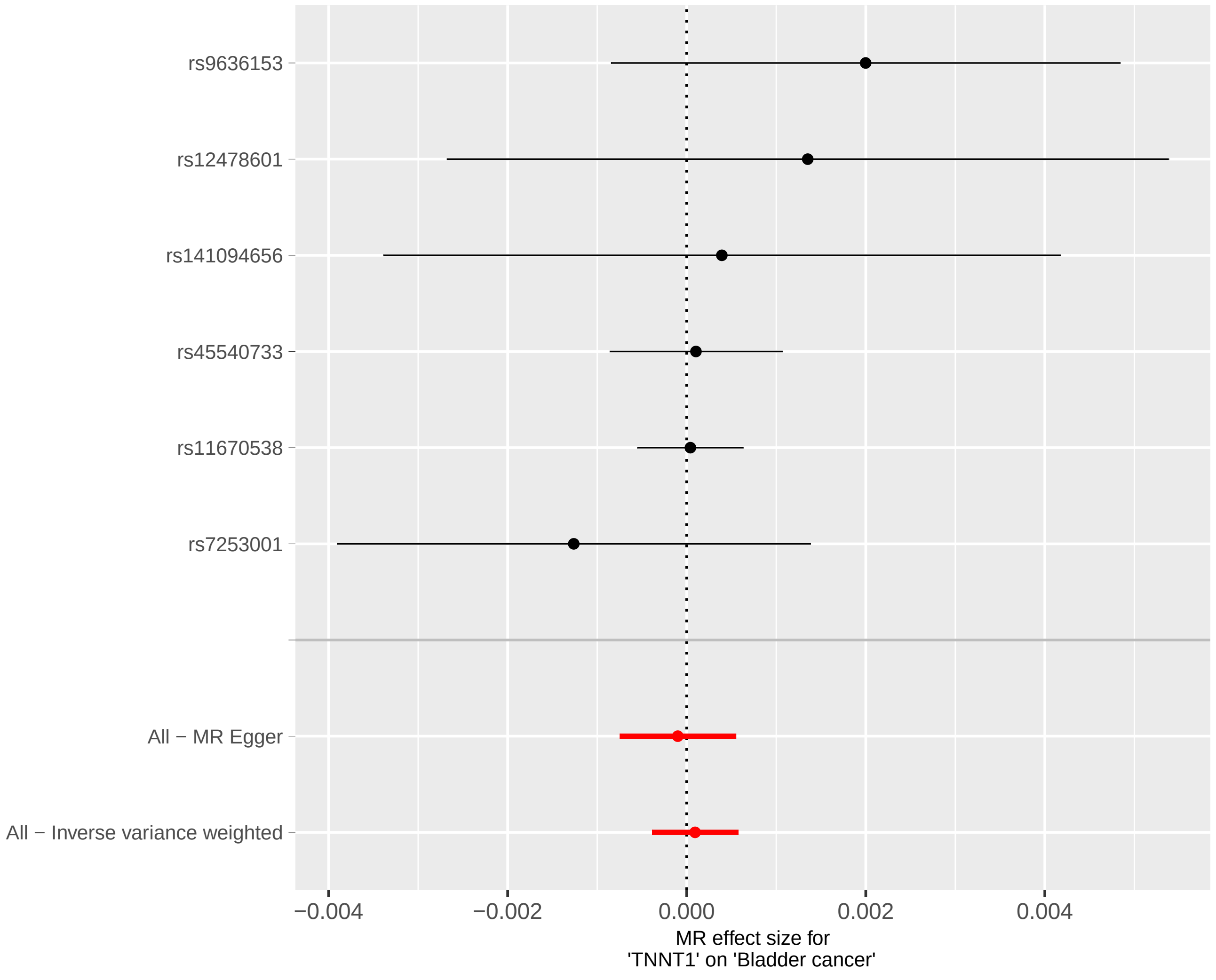
rs9636153
rs12478601
rs141094656
rs45540733
rs11670538
rs7253001
All − MR Egger
All − Inverse variance weighted
−0.004
−0.002
0.000
0.002
0.004
MR effect size for
'TNNT1' on 'Bladder cancer'

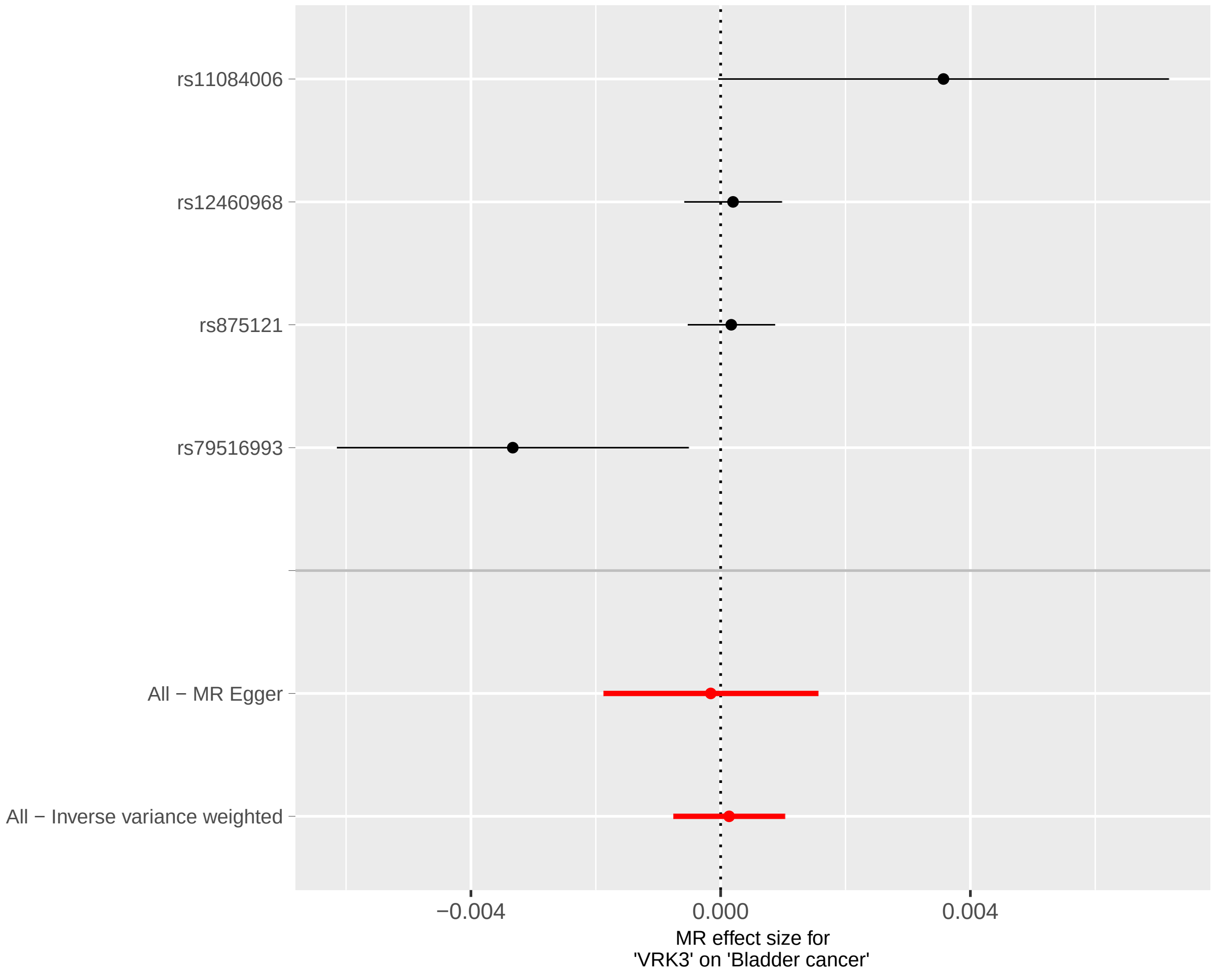

rs11084006
rs12460968
rs875121
rs79516993
All − MR Egger
All − Inverse variance weighted
−0.004
0.000
0.004
MR effect size for
'VRK3' on 'Bladder cancer'

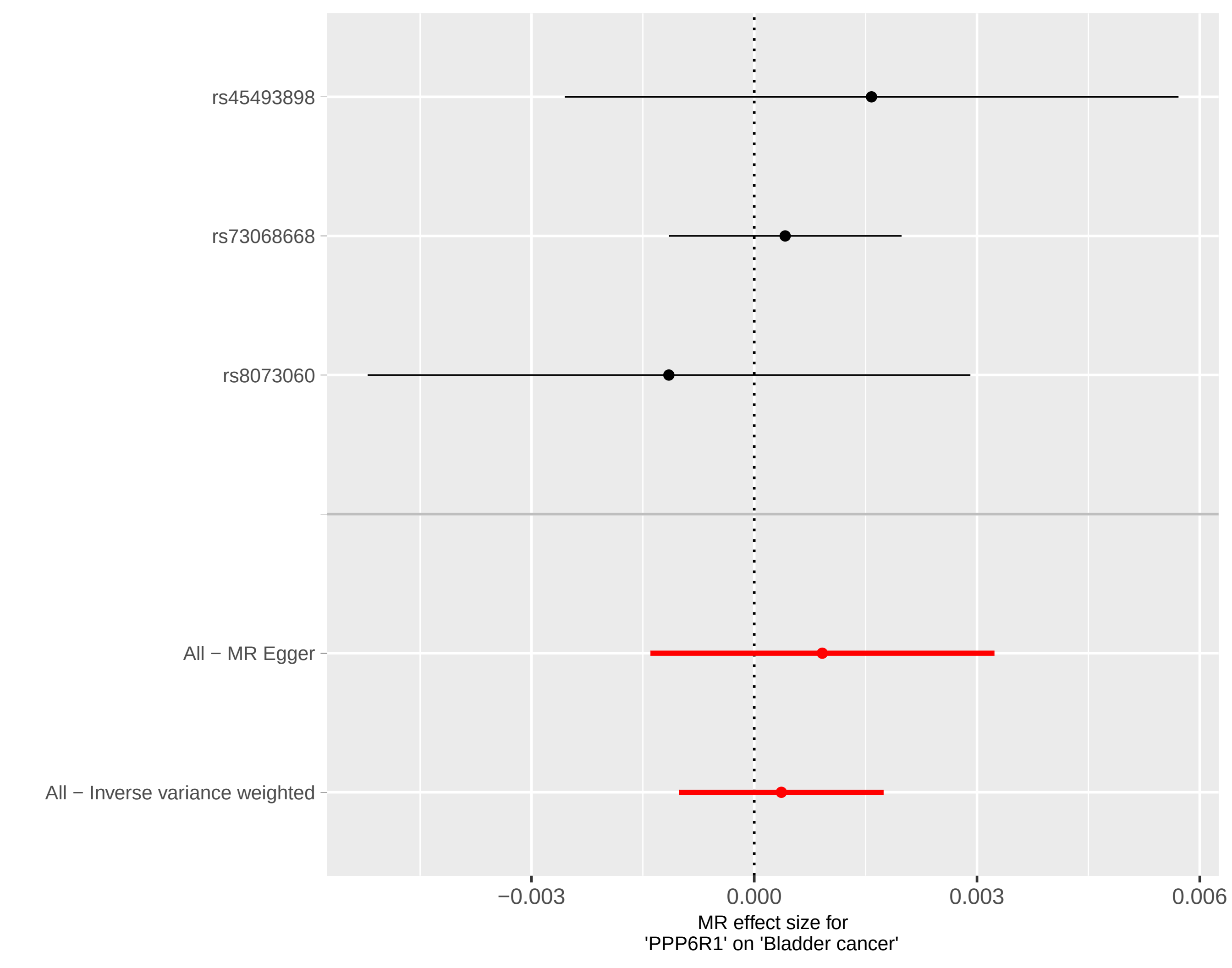

rs45493898
rs73068668
rs8073060
All − MR Egger
All − Inverse variance weighted
−0.003
0.000
0.003
0.006
MR effect size for
'PPP6R1' on 'Bladder cancer'

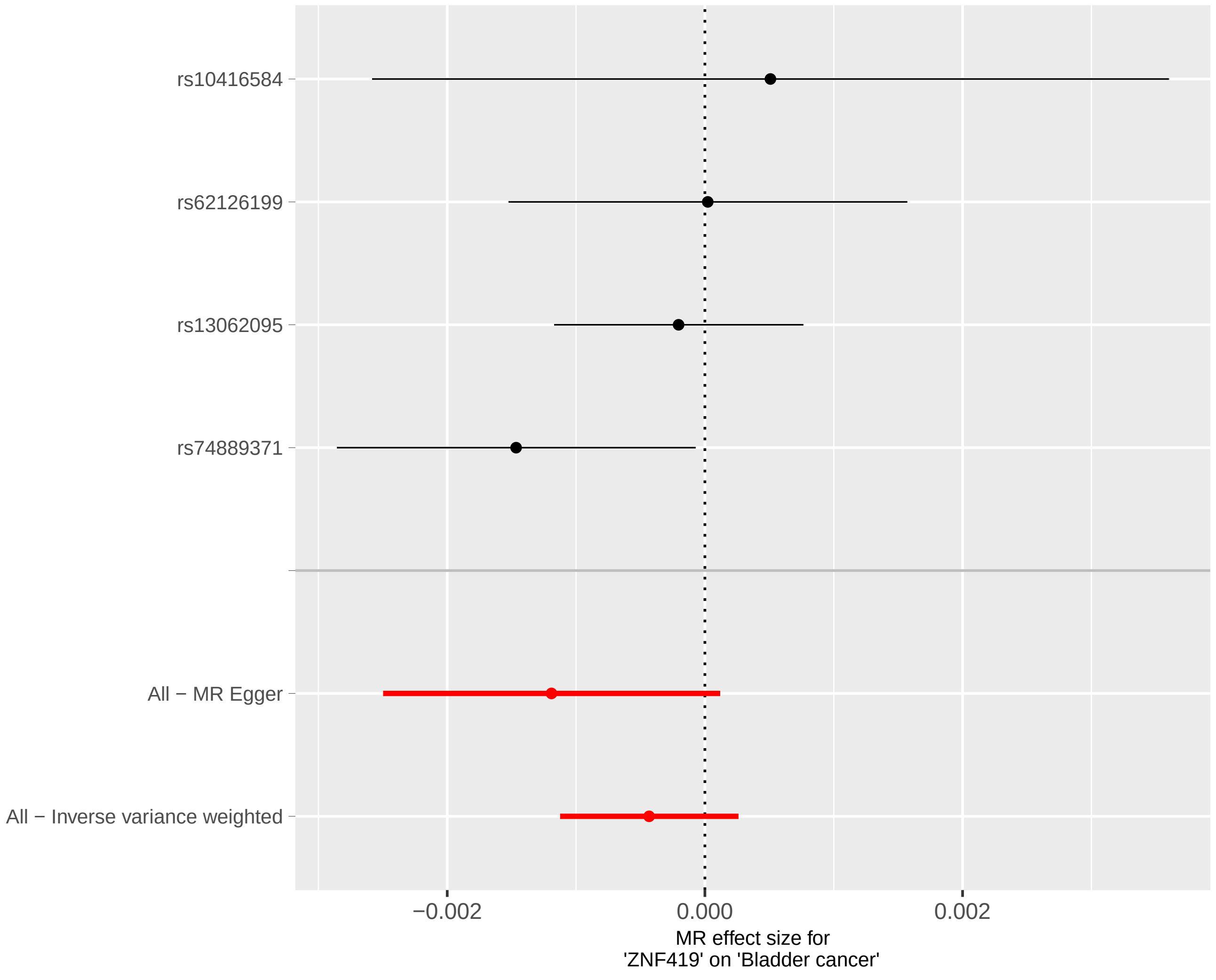
rs10416584
rs62126199
rs13062095
rs74889371
All − MR Egger
All − Inverse variance weighted
−0.002
0.000
0.002
MR effect size for
'ZNF419' on 'Bladder cancer'

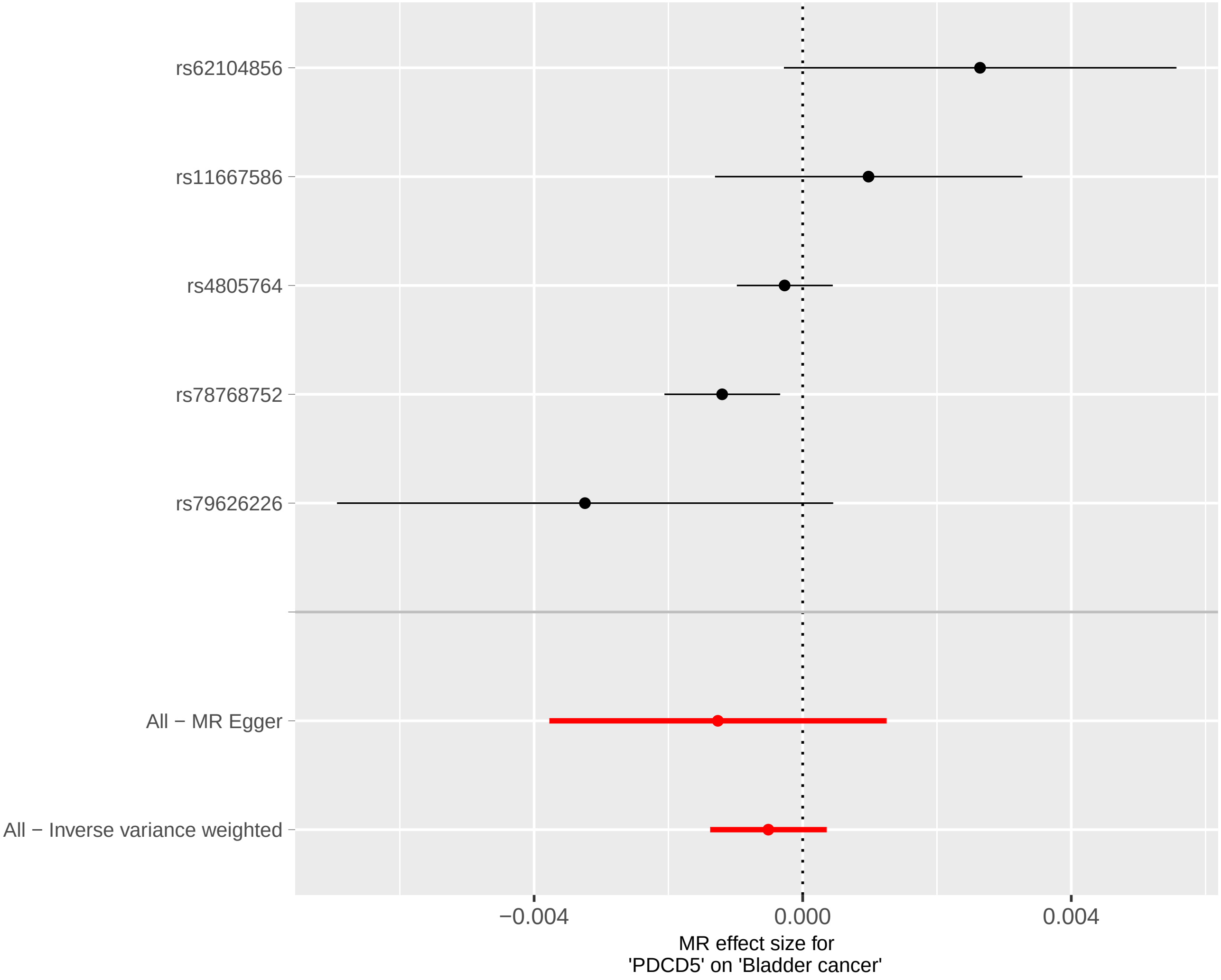
rs62104856
rs11667586
rs4805764
rs78768752
rs79626226
All − MR Egger
All − Inverse variance weighted
−0.004
0.000
0.004
MR effect size for
'PDCD5' on 'Bladder cancer'

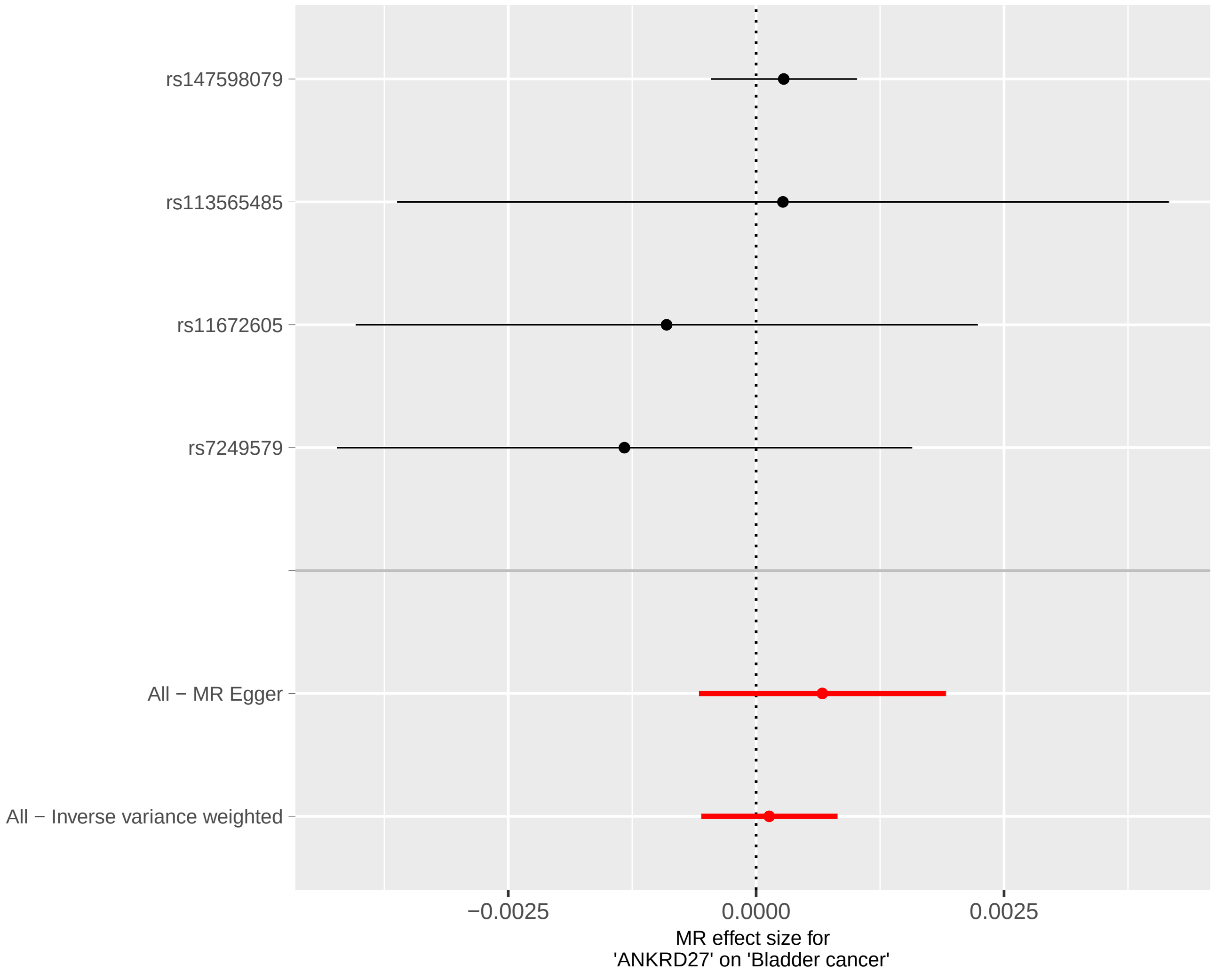
rs147598079
rs113565485
rs11672605
rs7249579
All − MR Egger
All − Inverse variance weighted
−0.0025
0.0000
0.0025
MR effect size for
'ANKRD27' on 'Bladder cancer'

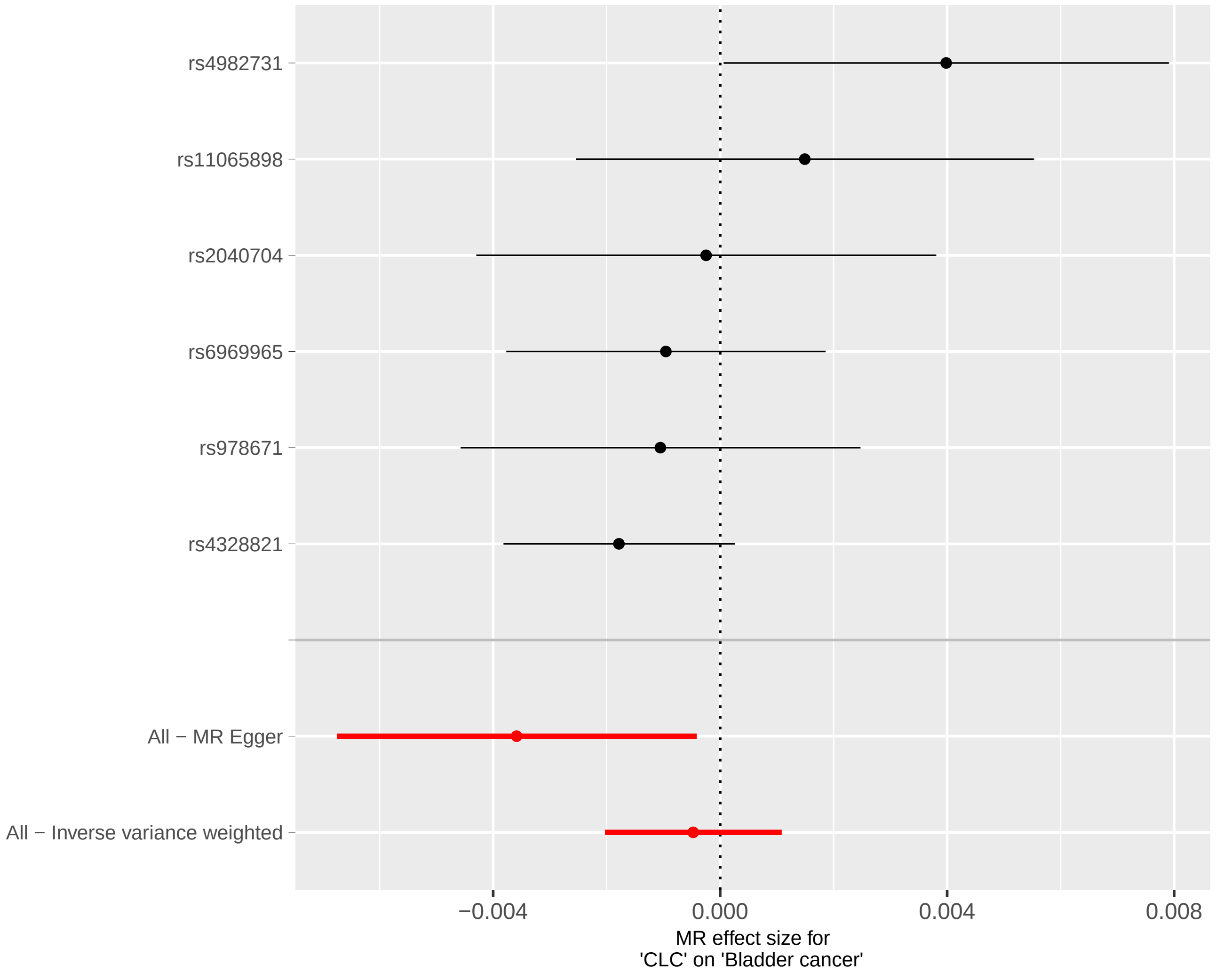
rs4982731
rs11065898
rs2040704
rs6969965
rs978671
rs4328821
All − MR Egger
All − Inverse variance weighted
−0.004
0.000
0.004
0.008
MR effect size for
'CLC' on 'Bladder cancer'

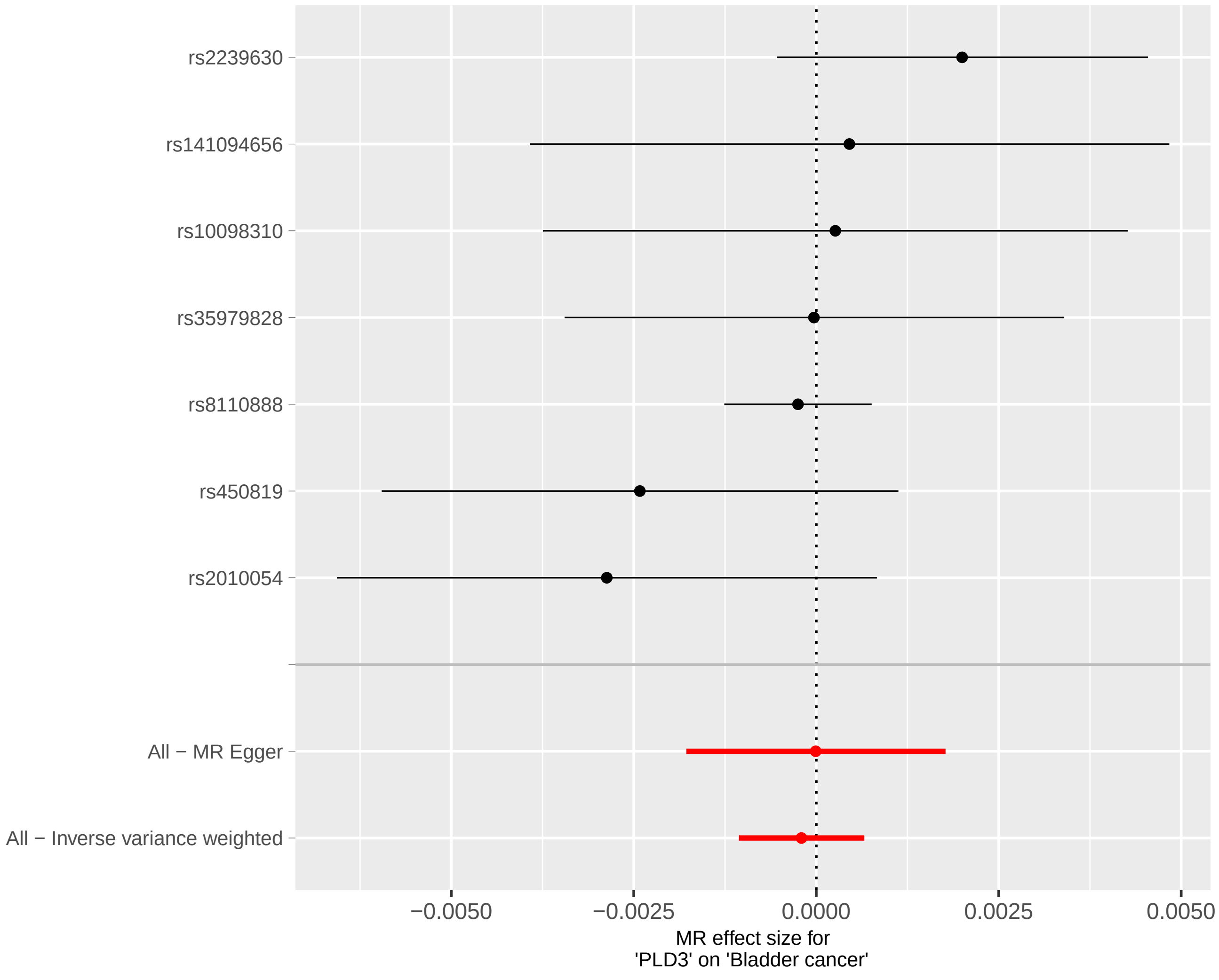
rs2239630
rs141094656
rs10098310
rs35979828
rs8110888
rs450819
rs2010054
All − MR Egger
All − Inverse variance weighted
−0.0050
−0.0025
0.0000
0.0025
0.0050
MR effect size for
'PLD3' on 'Bladder cancer'

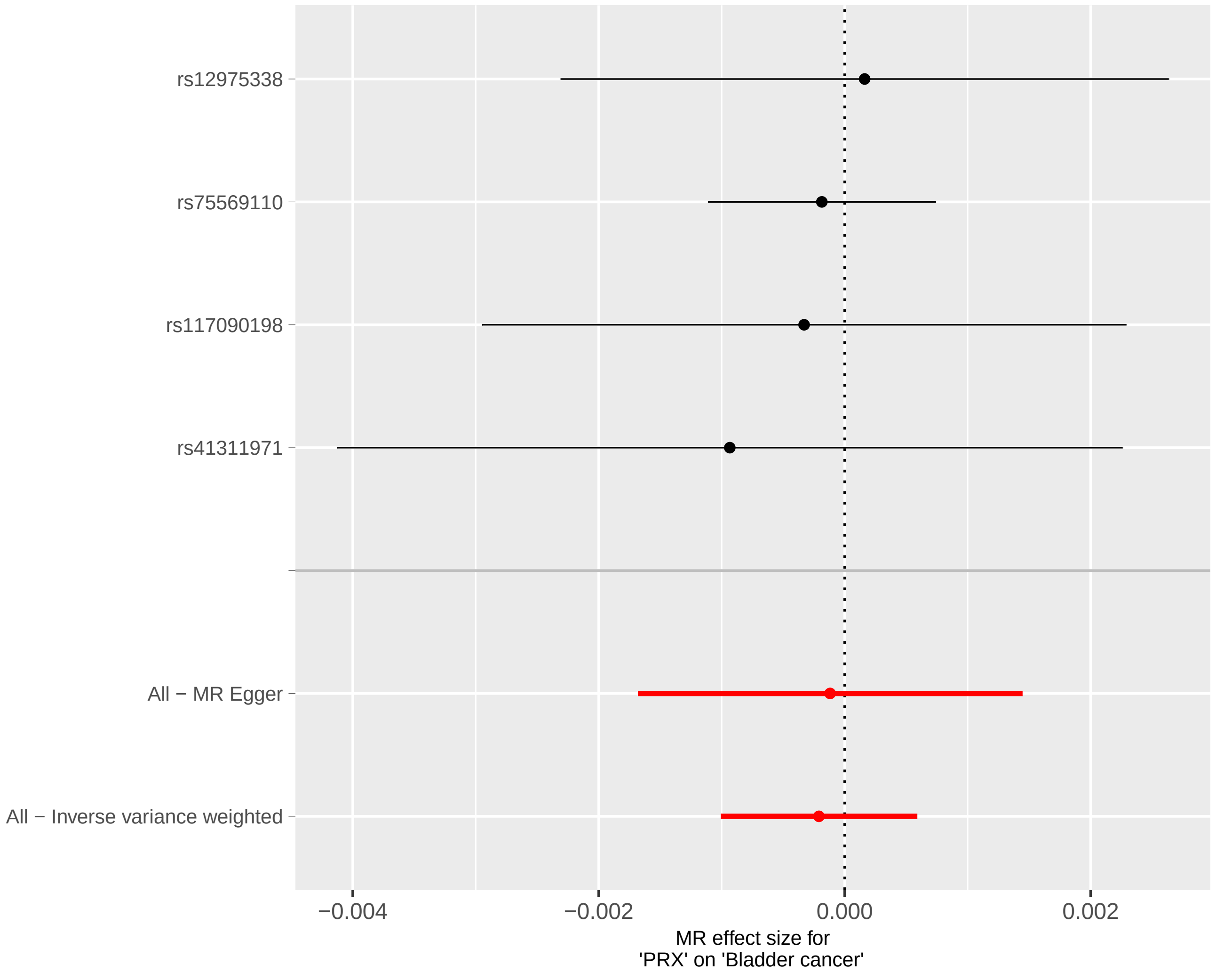
rs12975338
rs75569110
rs117090198
rs41311971
All − MR Egger
All − Inverse variance weighted
−0.004
−0.002
0.000
0.002
MR effect size for
'PRX' on 'Bladder cancer'

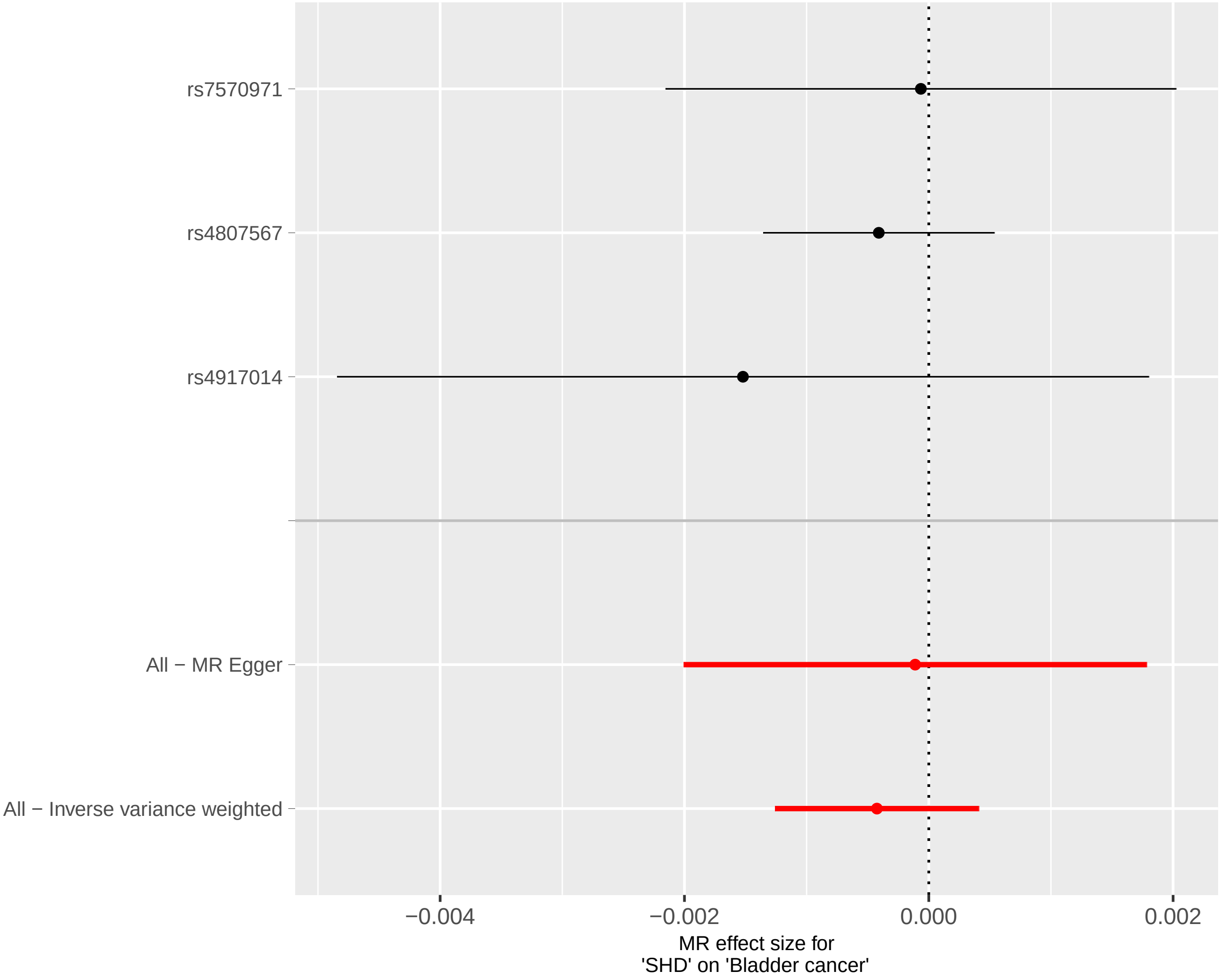

rs7570971
rs4807567
rs4917014
All − MR Egger
All − Inverse variance weighted
−0.004
−0.002
0.000
0.002
MR effect size for
'SHD' on 'Bladder cancer'

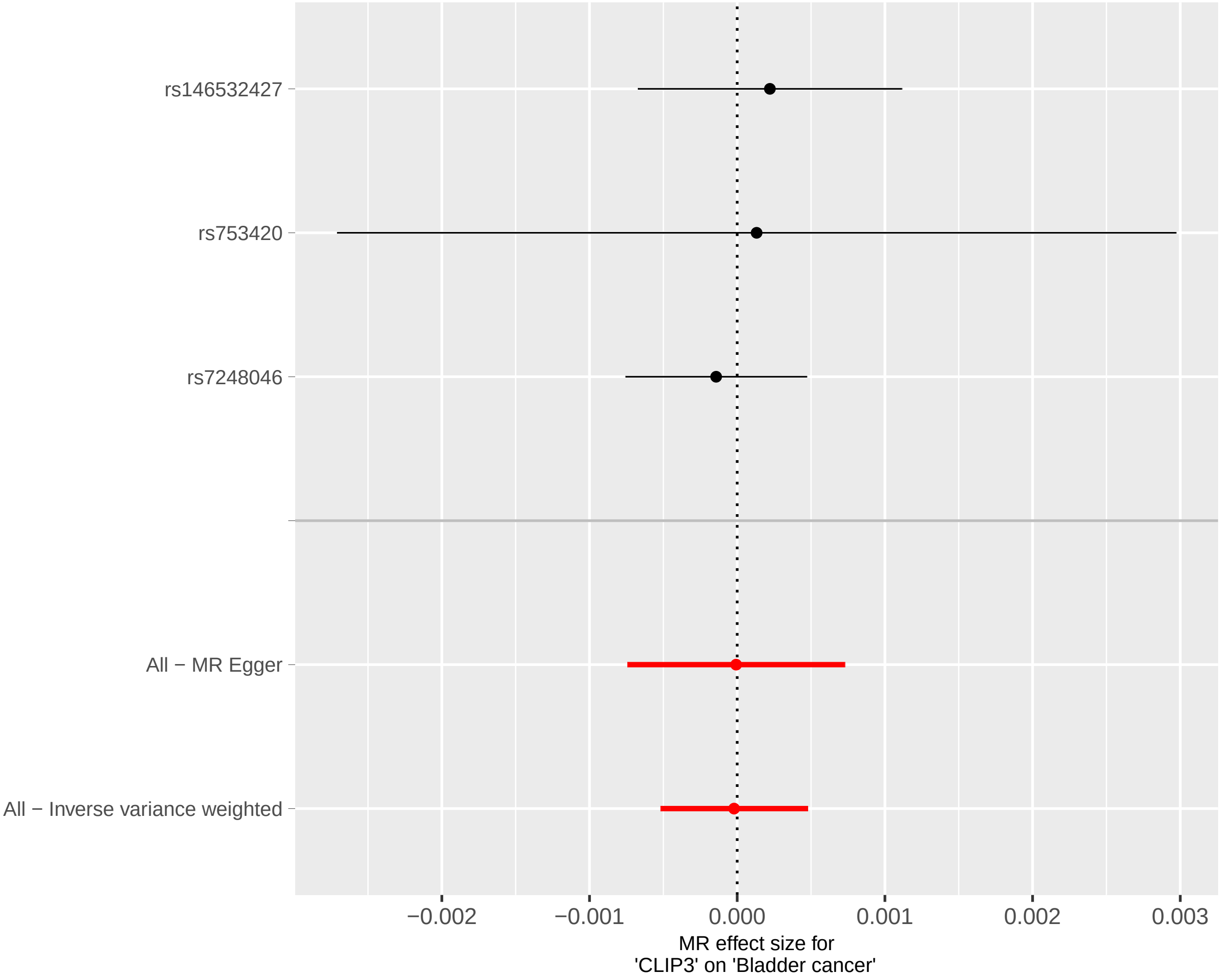
rs146532427
rs753420
rs7248046
All − MR Egger
All − Inverse variance weighted
−0.002
−0.001
0.000
0.001
0.002
0.003
MR effect size for
'CLIP3' on 'Bladder cancer'

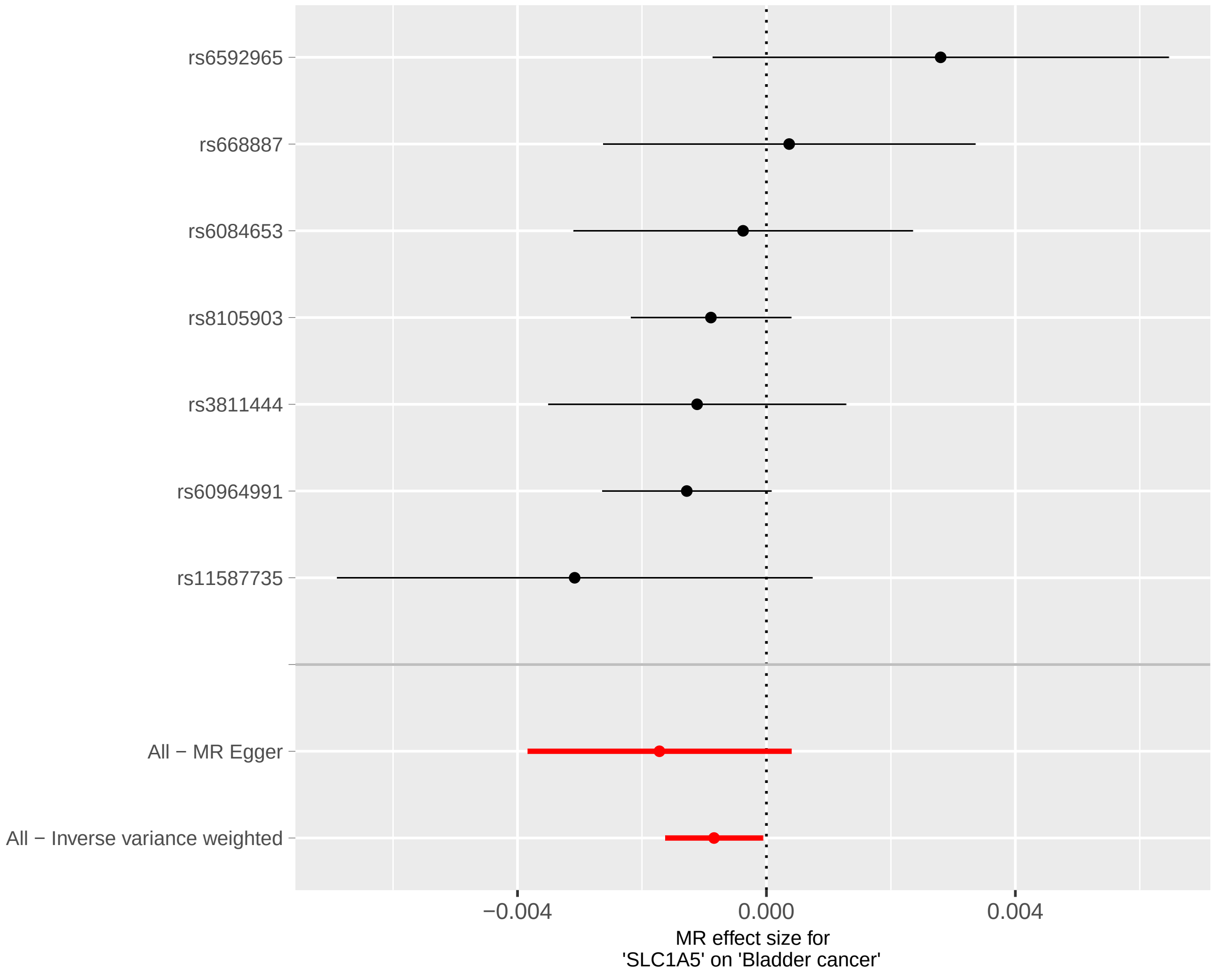
rs6592965
rs668887
rs6084653
rs8105903
rs3811444
rs60964991
rs11587735
All − MR Egger
All − Inverse variance weighted
−0.004
0.000
0.004
MR effect size for
'SLC1A5' on 'Bladder cancer'

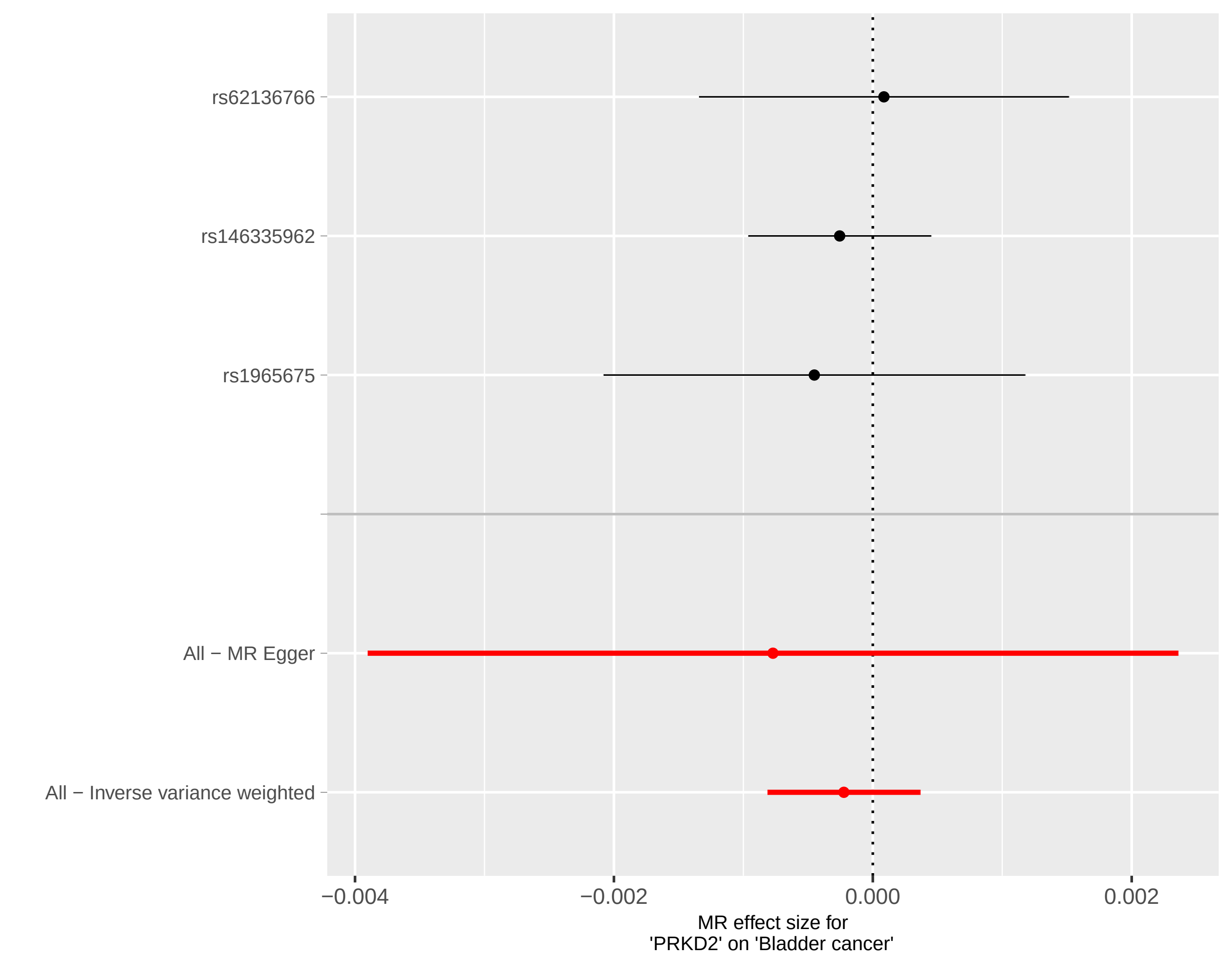
rs62136766
rs146335962
rs1965675
All − MR Egger
All − Inverse variance weighted
−0.004
−0.002
0.000
0.002
MR effect size for
'PRKD2' on 'Bladder cancer'

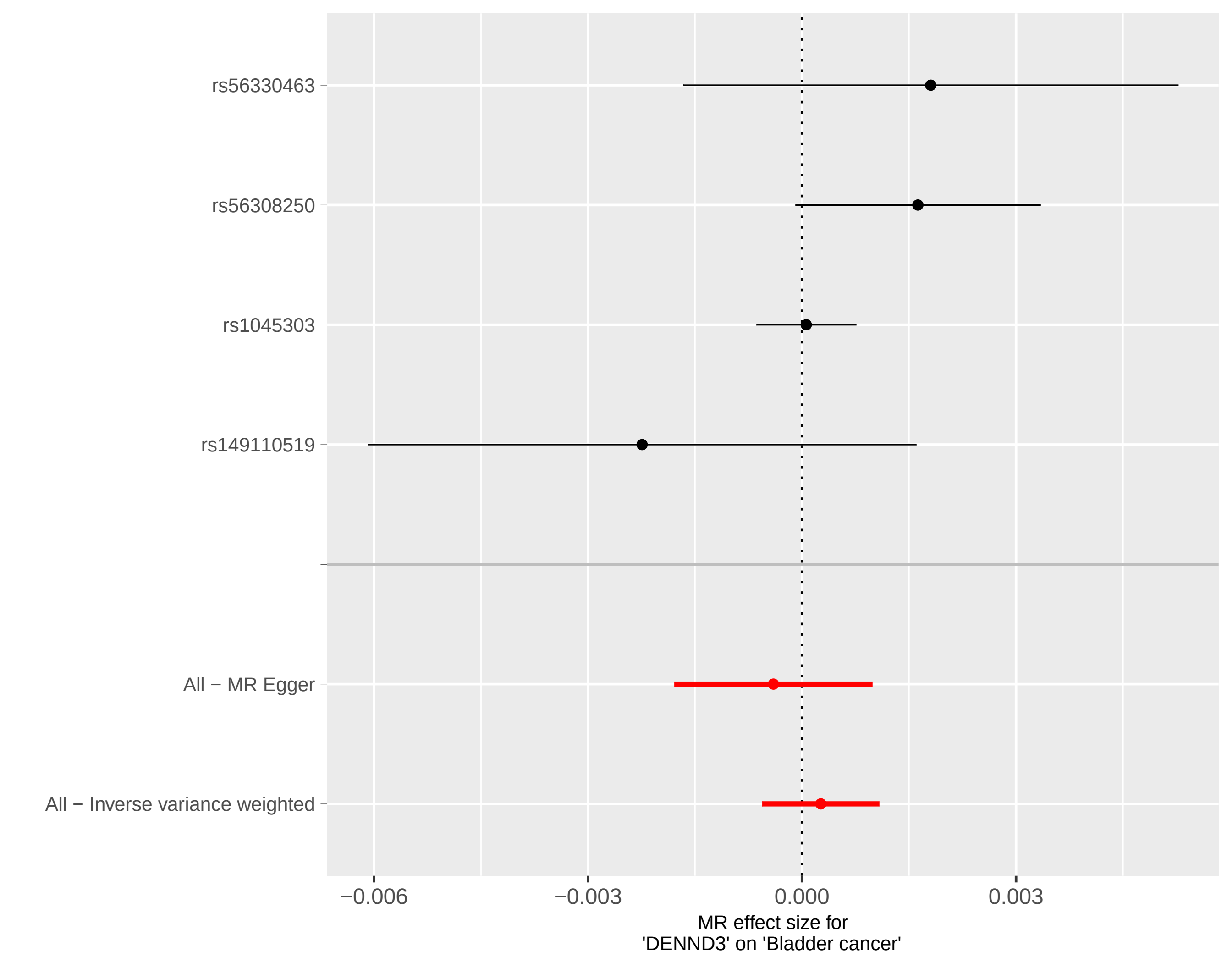

rs56330463
rs56308250
rs1045303
rs149110519
All − MR Egger
All − Inverse variance weighted
−0.006
−0.003
0.000
0.003
MR effect size for
'DENND3' on 'Bladder cancer'

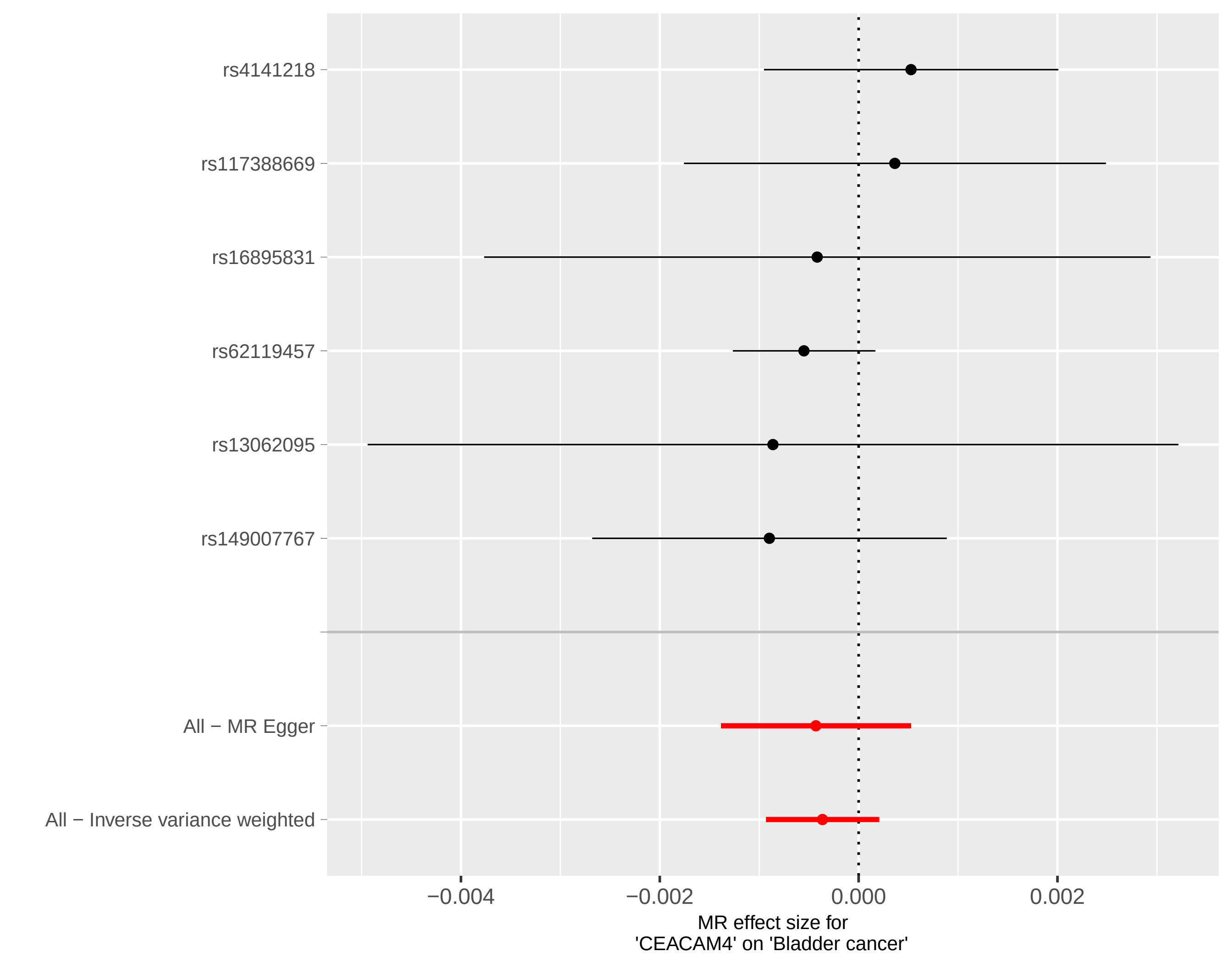

rs4141218
rs117388669
rs16895831
rs62119457
rs13062095
rs149007767
All − MR Egger
All − Inverse variance weighted
−0.004
−0.002
0.000
0.002
MR effect size for
'CEACAM4' on 'Bladder cancer'

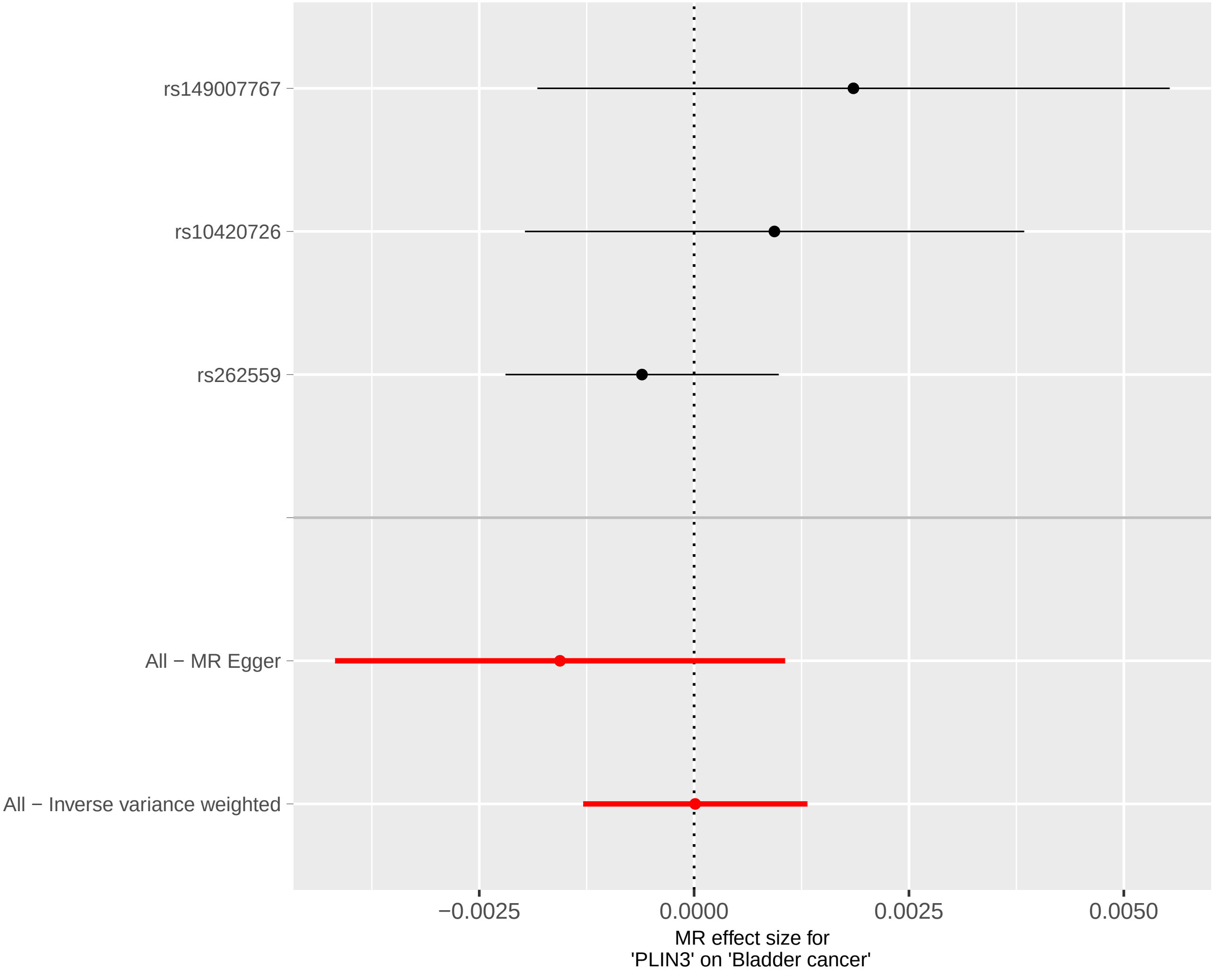

rs149007767
rs10420726
rs262559
All − MR Egger
All − Inverse variance weighted
−0.0025
0.0000
0.0025
0.0050
MR effect size for
'PLIN3' on 'Bladder cancer'

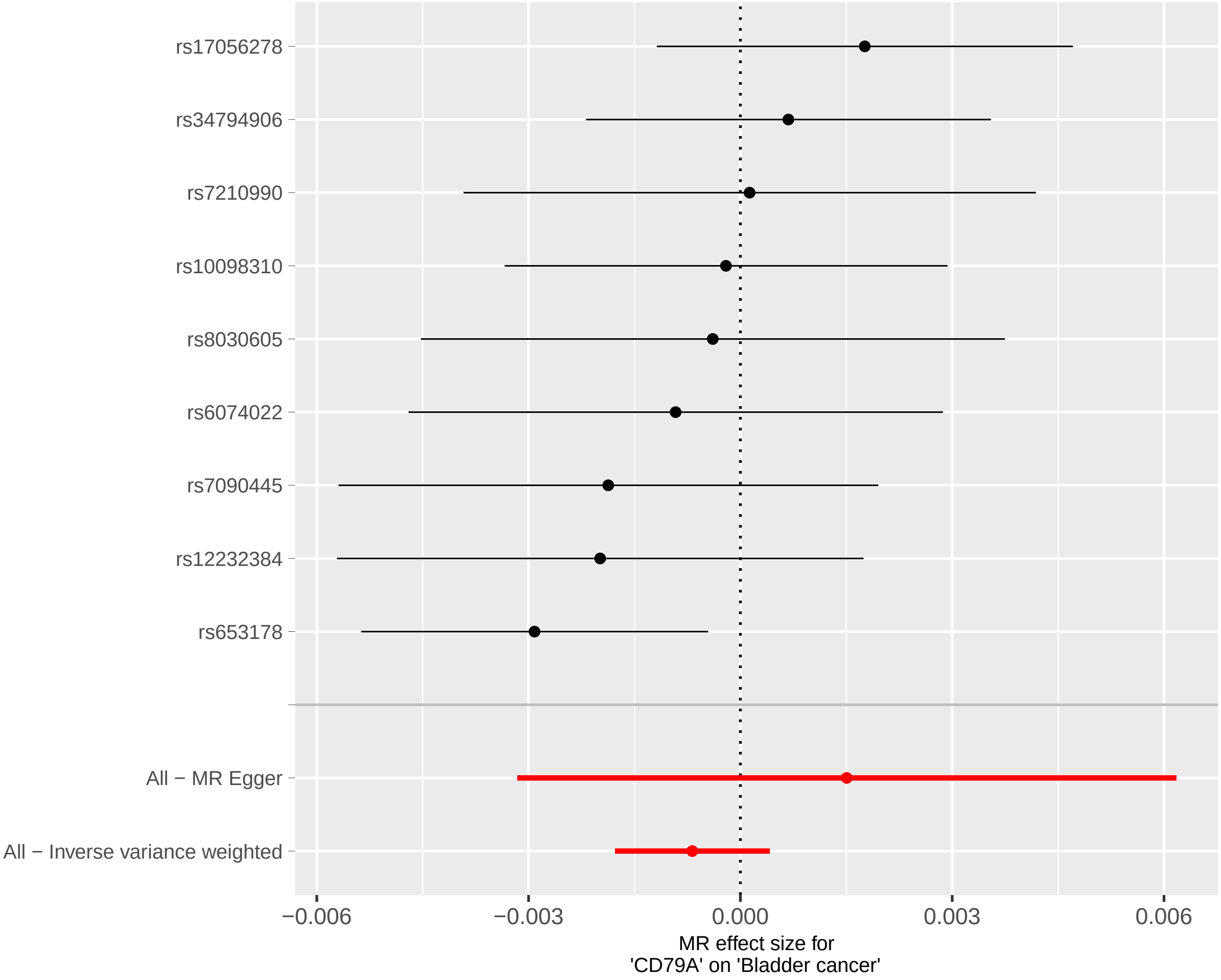
rs17056278
rs34794906
rs7210990
rs10098310
rs8030605
rs6074022
rs7090445
rs12232384
rs653178
All − MR Egger
All − Inverse variance weighted
−0.006
−0.003
0.000
0.003
0.006
MR effect size for
'CD79A' on 'Bladder cancer'

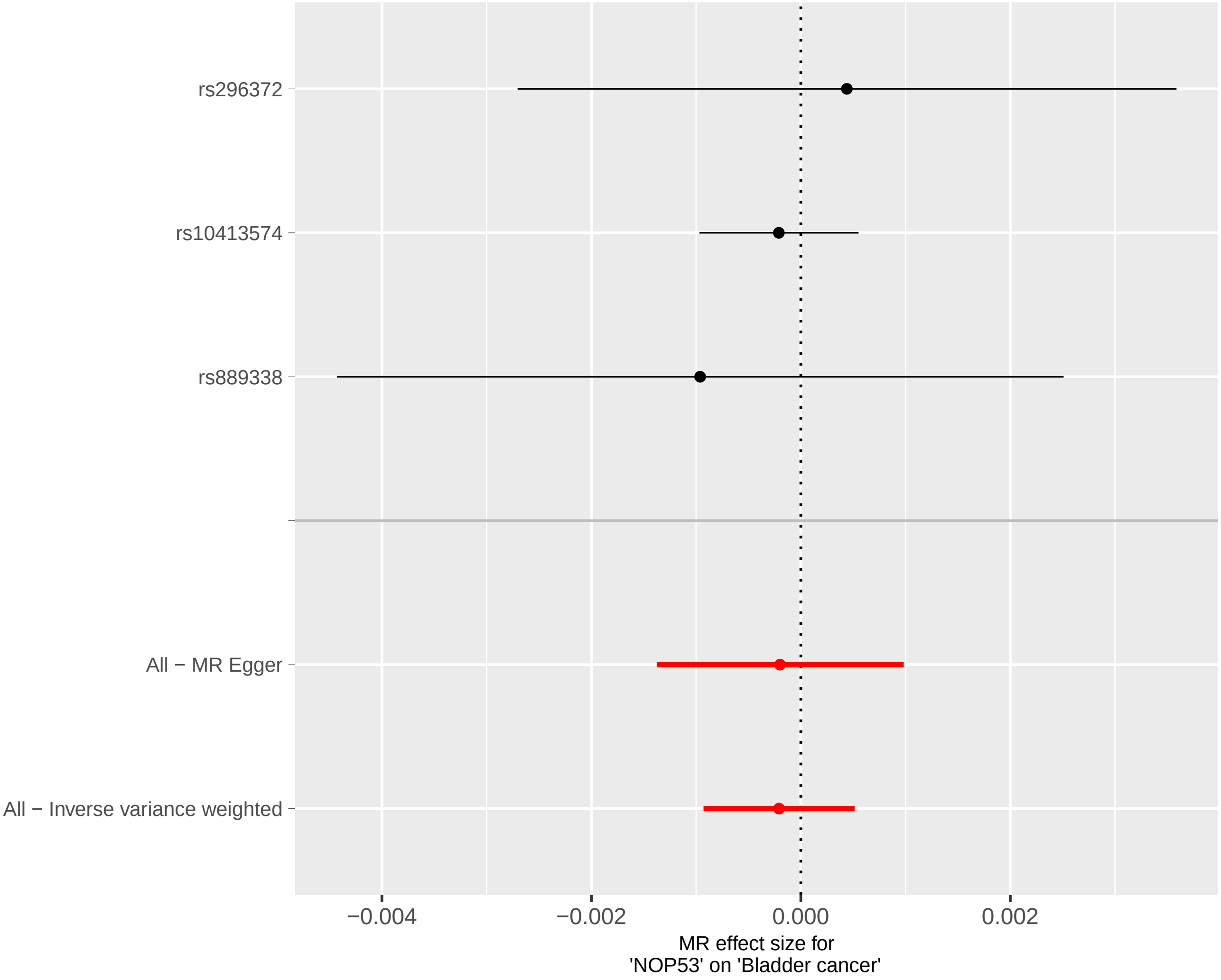
rs296372
rs10413574
rs889338
All − MR Egger
All − Inverse variance weighted
−0.004
−0.002
0.000
0.002
MR effect size for
'NOP53' on 'Bladder cancer'

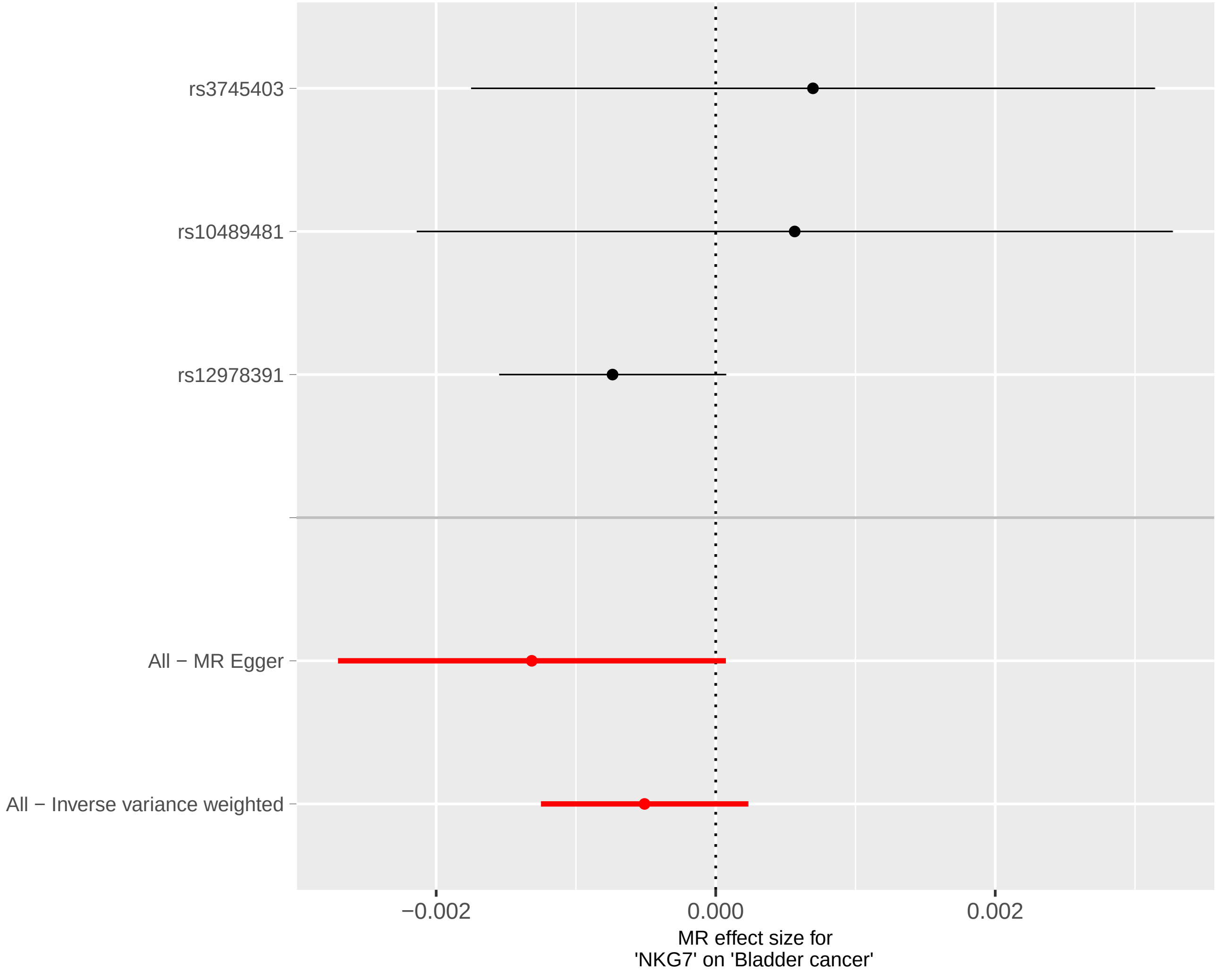

rs3745403
rs10489481
rs12978391
All − MR Egger
All − Inverse variance weighted
−0.002
0.000
0.002
MR effect size for
'NKG7' on 'Bladder cancer'

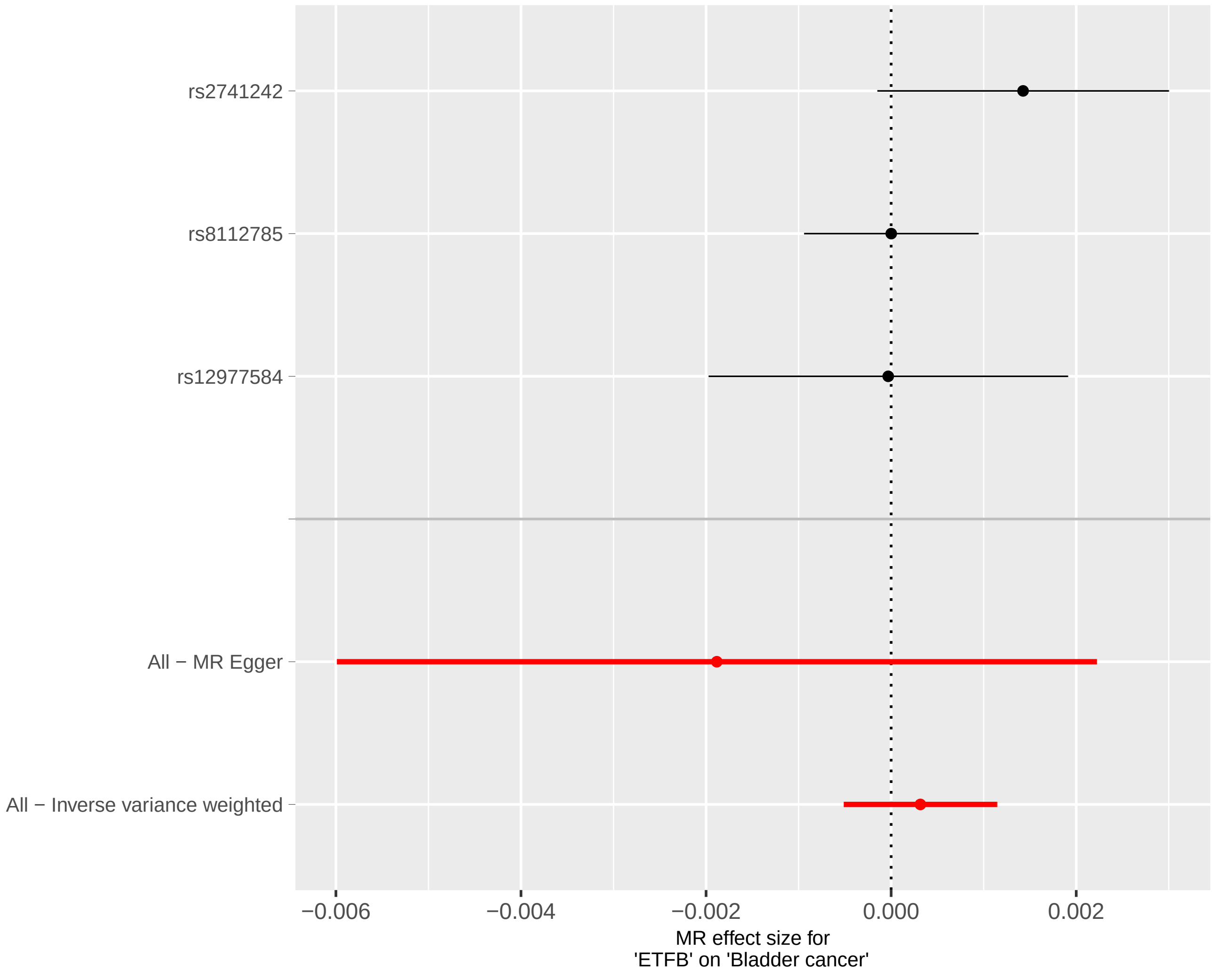
rs2741242
rs8112785
rs12977584
All − MR Egger
All − Inverse variance weighted
−0.006
−0.004
−0.002
0.000
0.002
MR effect size for
'ETFB' on 'Bladder cancer'

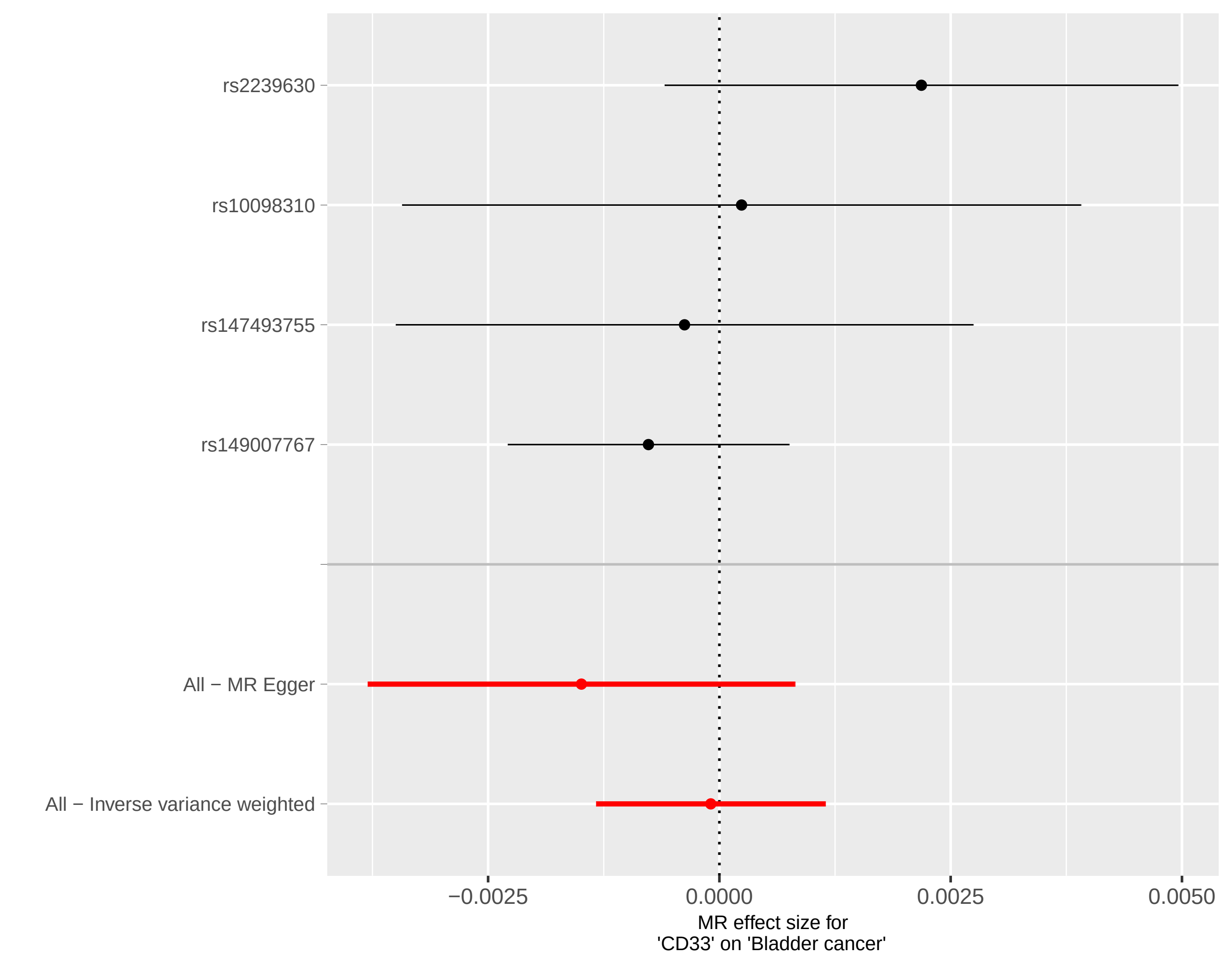
rs2239630
rs10098310
rs147493755
rs149007767
All − MR Egger
All − Inverse variance weighted
−0.0025
0.0000
0.0025
0.0050
MR effect size for
'CD33' on 'Bladder cancer'

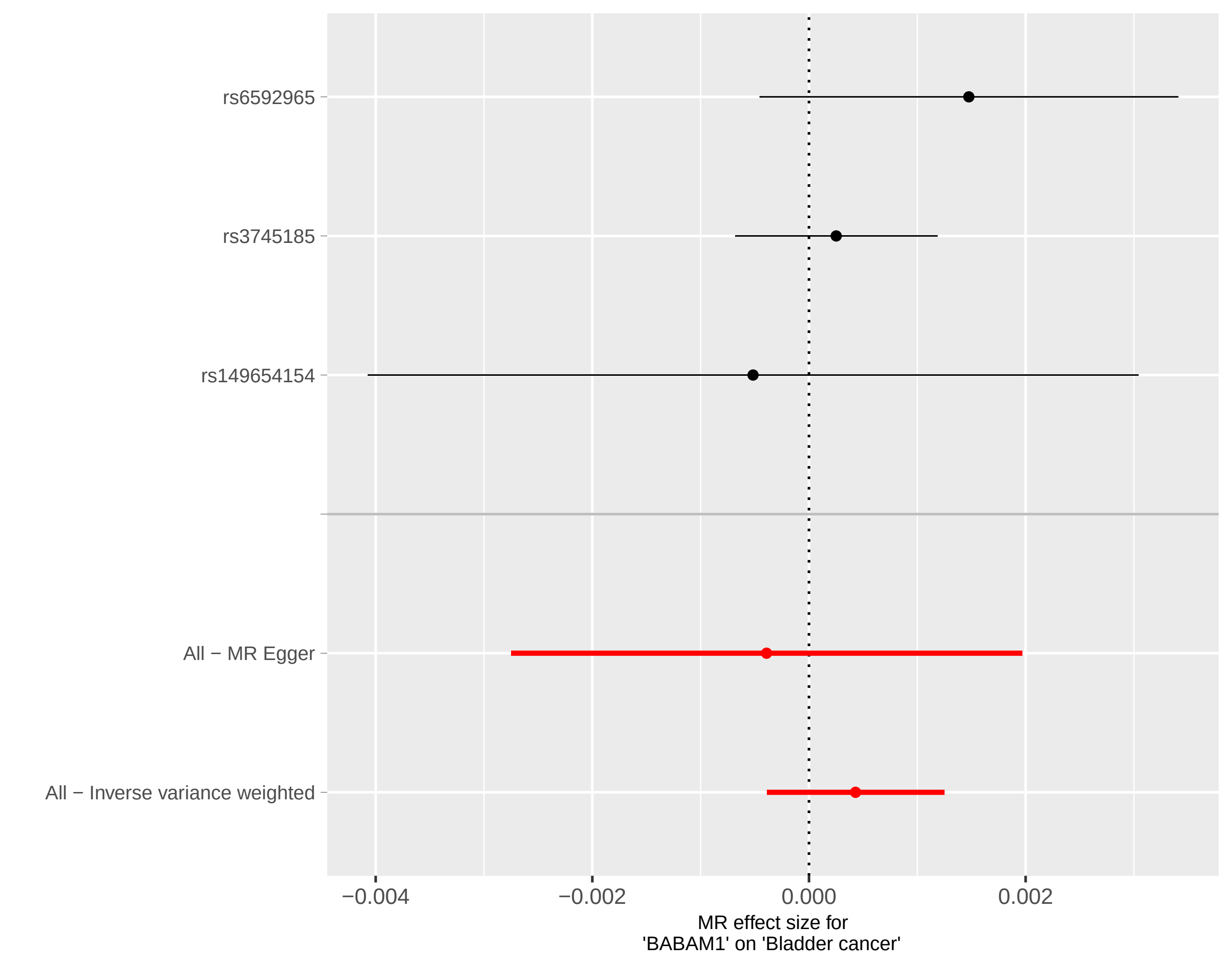
rs6592965
rs3745185
rs149654154
All − MR Egger
All − Inverse variance weighted
−0.004
−0.002
0.000
0.002
MR effect size for
'BABAM1' on 'Bladder cancer'

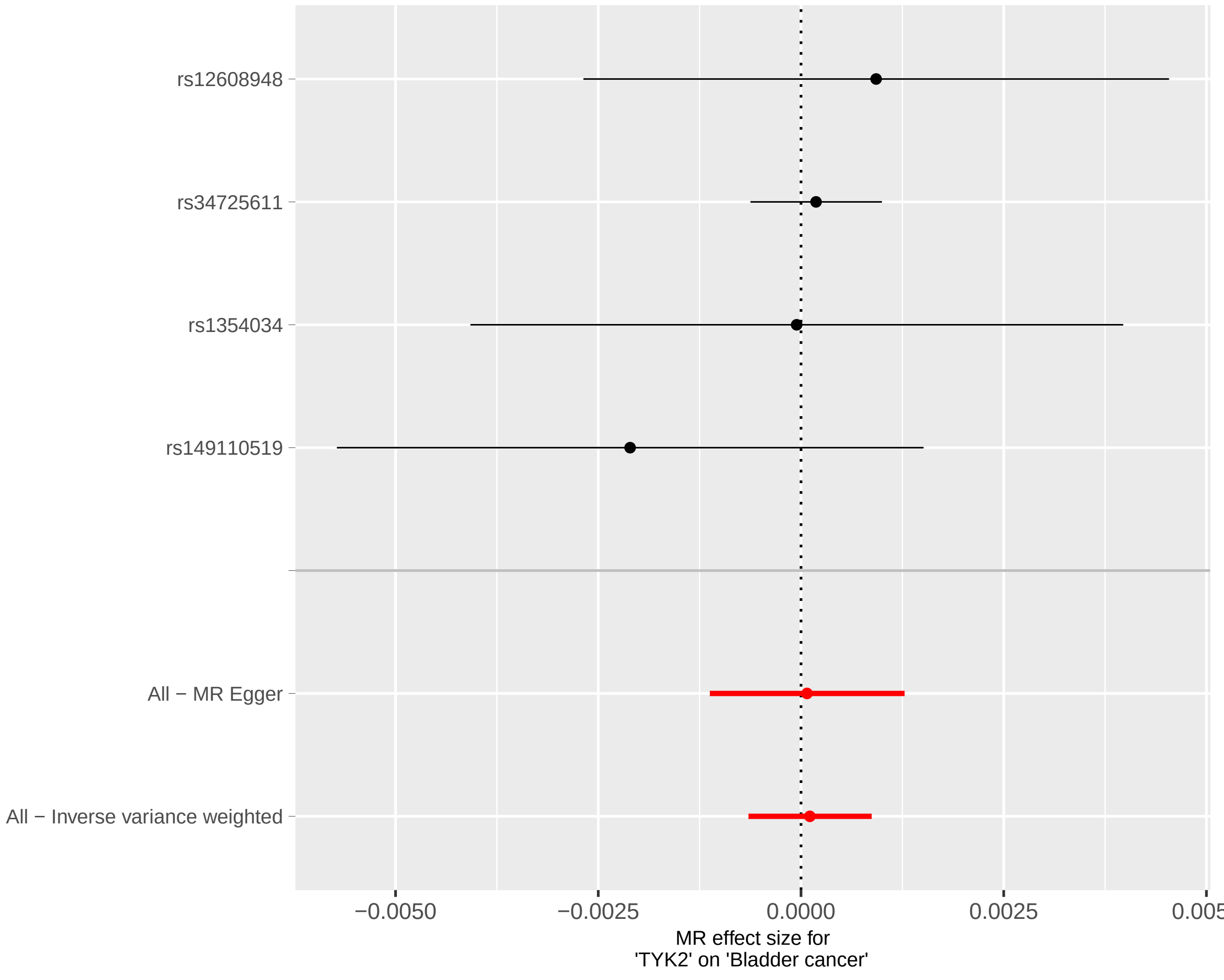
rs12608948
rs34725611
rs1354034
rs149110519
All − MR Egger
All − Inverse variance weighted
−0.0050
−0.0025
0.0000
0.0025
MR effect size for
'TYK2' on 'Bladder cancer'

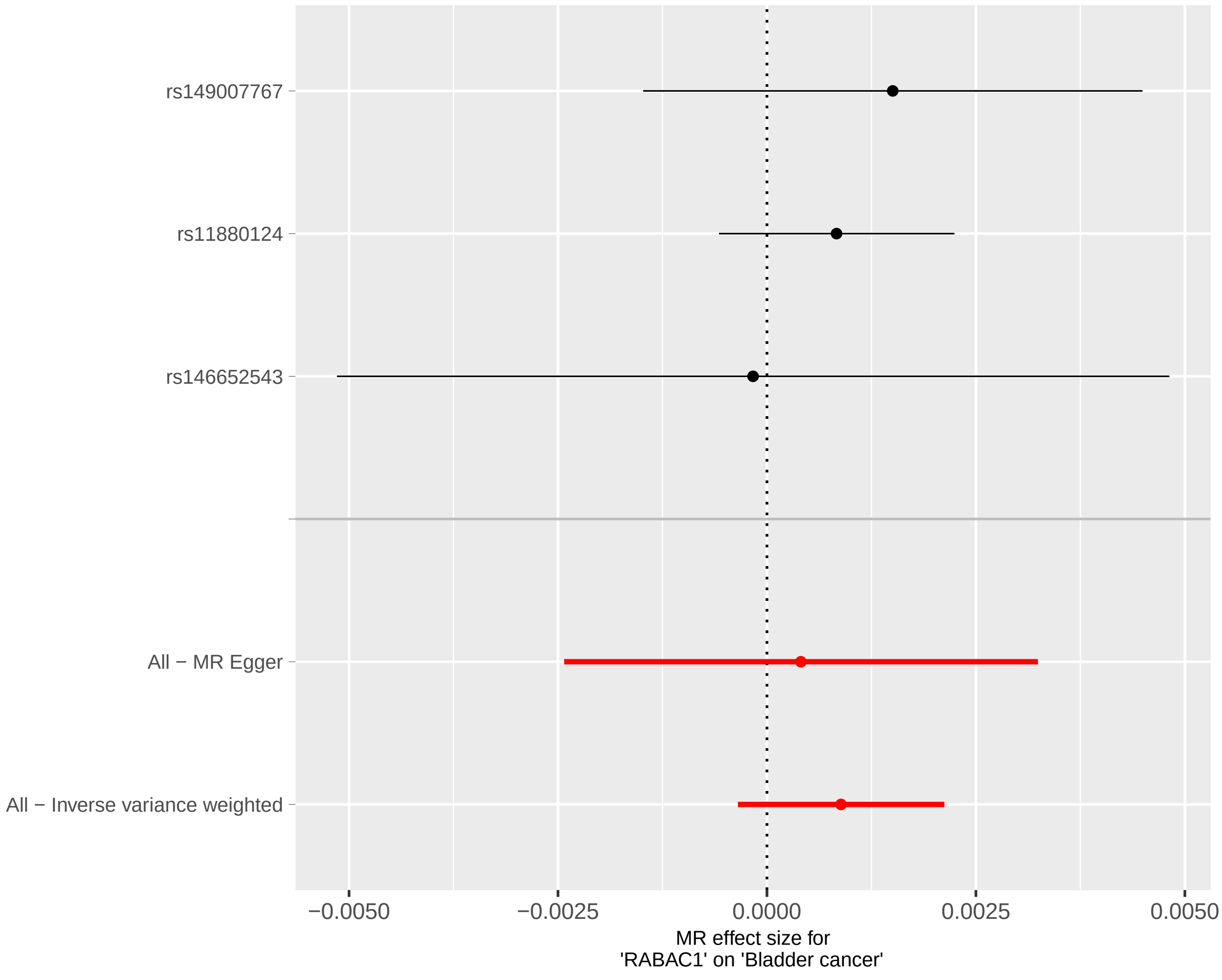

rs149007767
rs11880124
rs146652543
All − MR Egger
All − Inverse variance weighted
−0.0050
−0.0025
0.0000
0.0025
0.0050
MR effect size for
'RABAC1' on 'Bladder cancer'

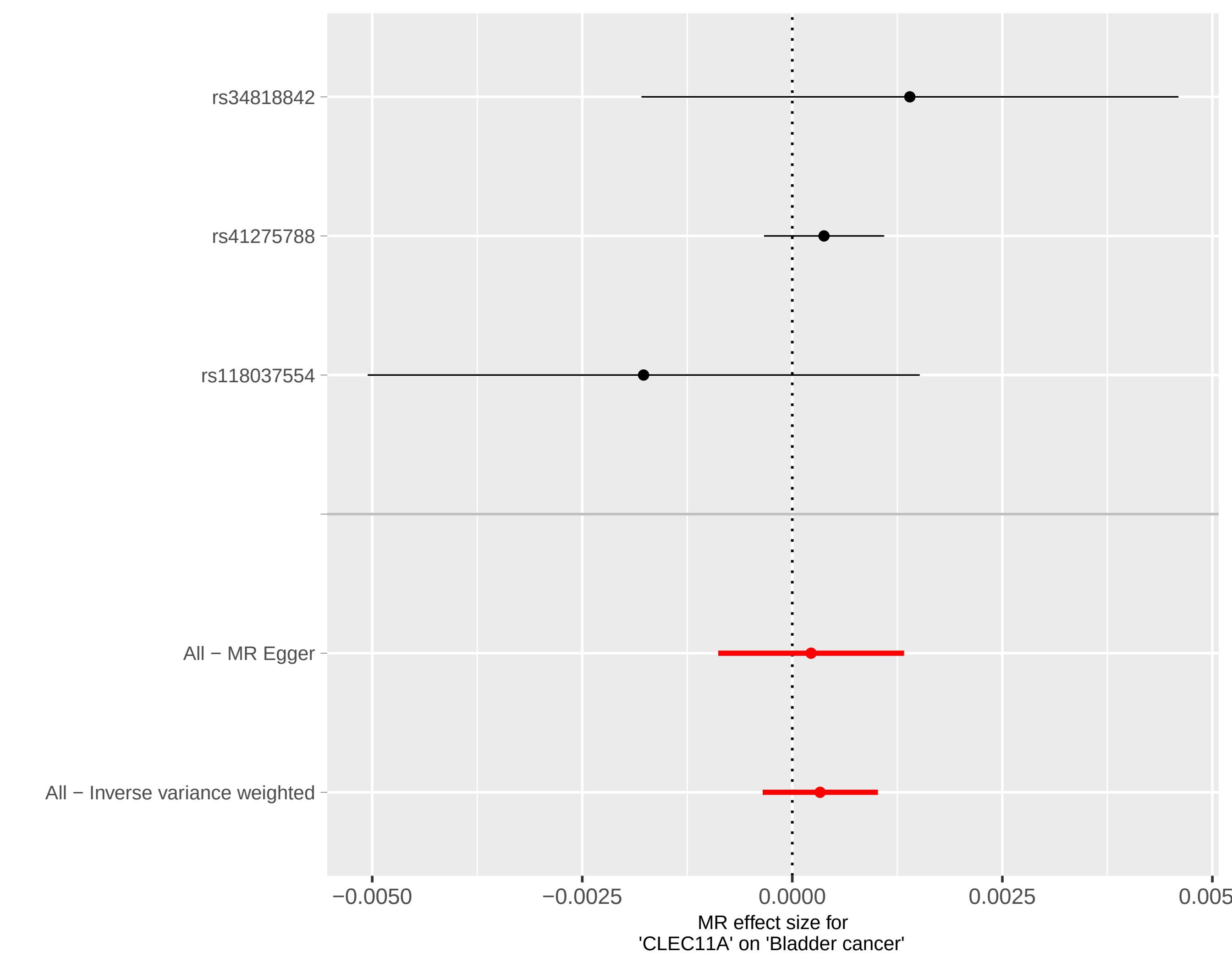

rs34818842
rs41275788
rs118037554
All − MR Egger
All − Inverse variance weighted
−0.0050
−0.0025
0.0000
0.0025
0.005
MR effect size for
'CLEC11A' on 'Bladder cancer'

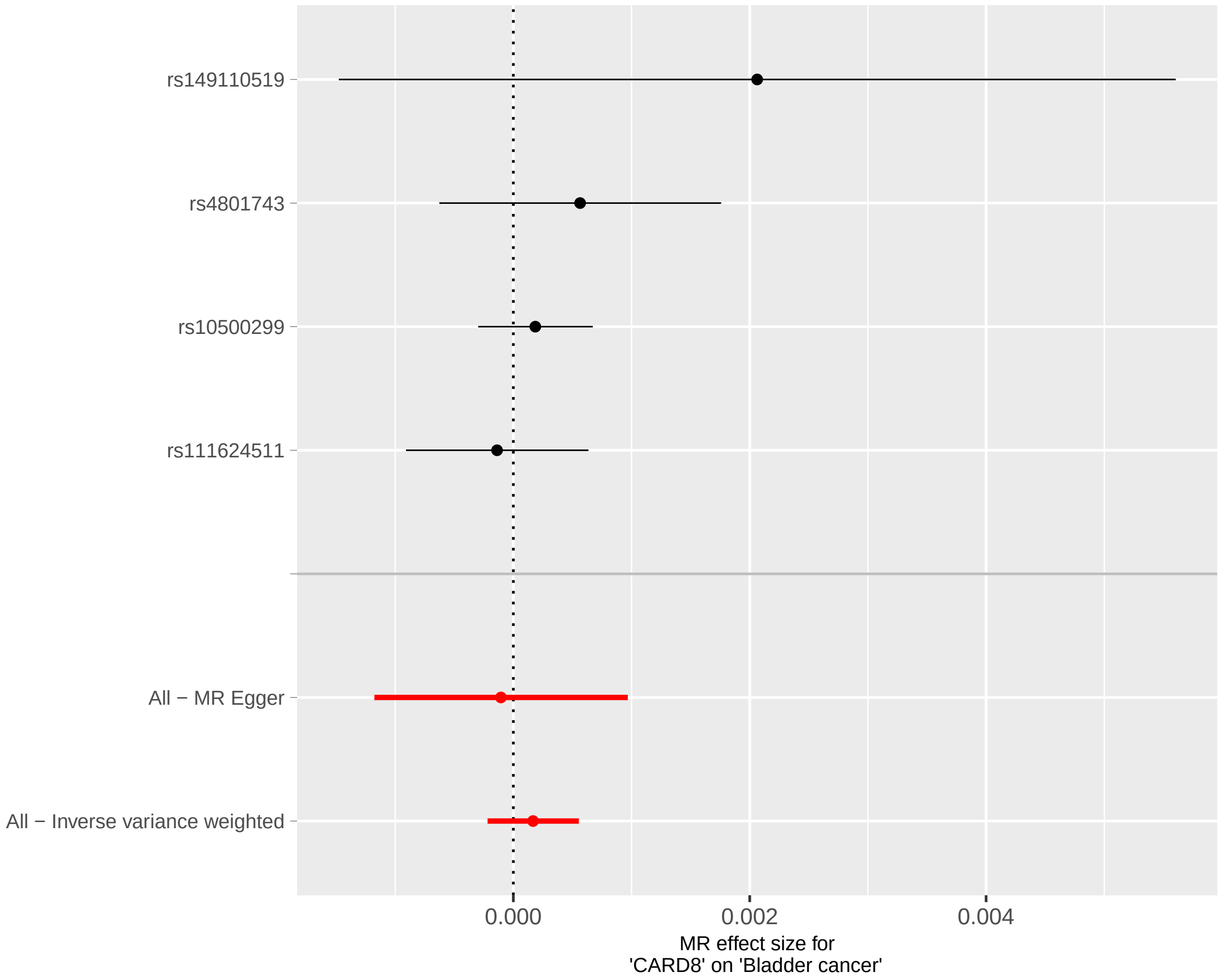

rs149110519
rs4801743
rs10500299
rs111624511
All − MR Egger
All − Inverse variance weighted
0.000
0.002
0.004
MR effect size for
'CARD8' on 'Bladder cancer'

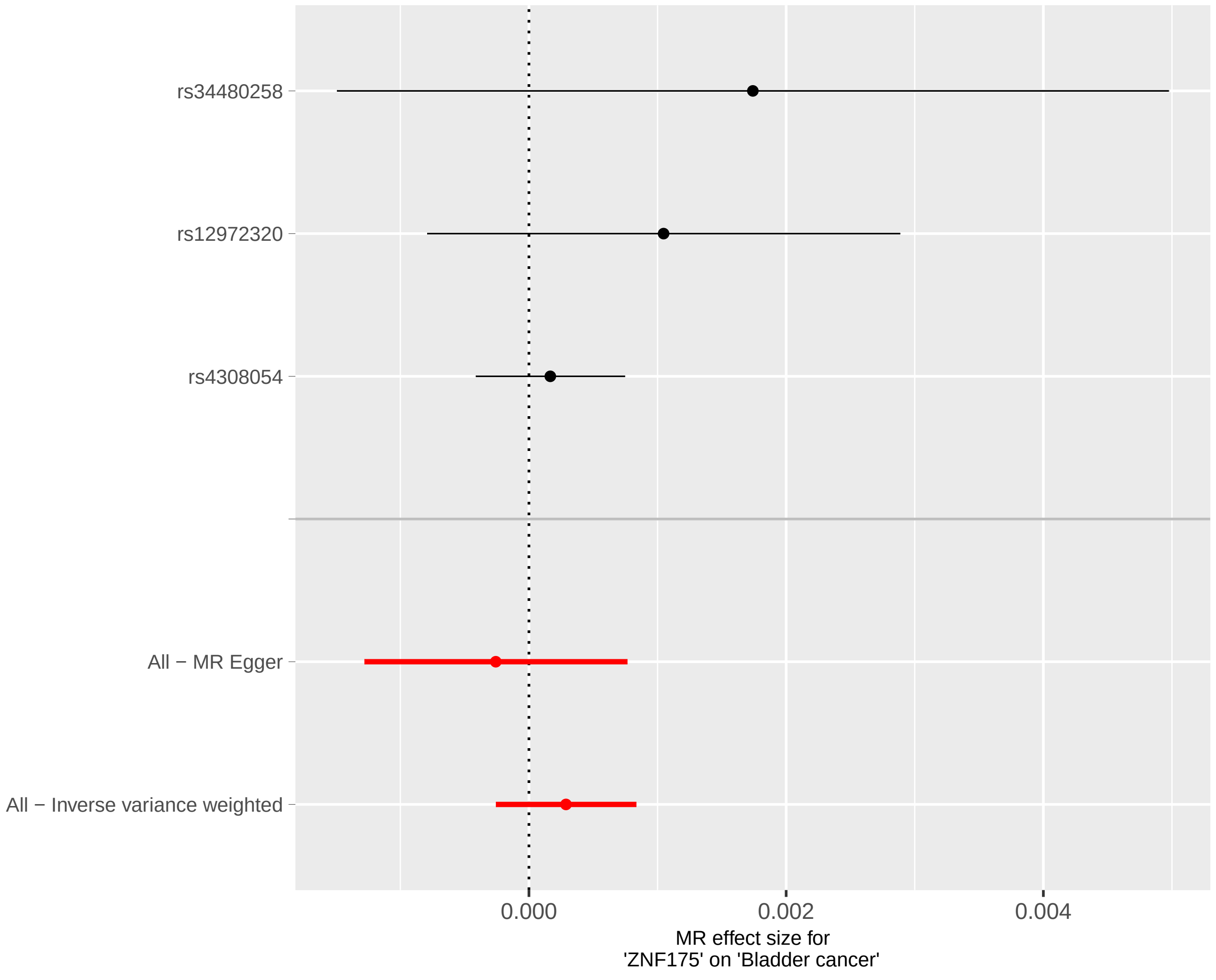
rs34480258
rs12972320
rs4308054
All − MR Egger
All − Inverse variance weighted
0.000
0.002
0.004
MR effect size for
'ZNF175' on 'Bladder cancer'

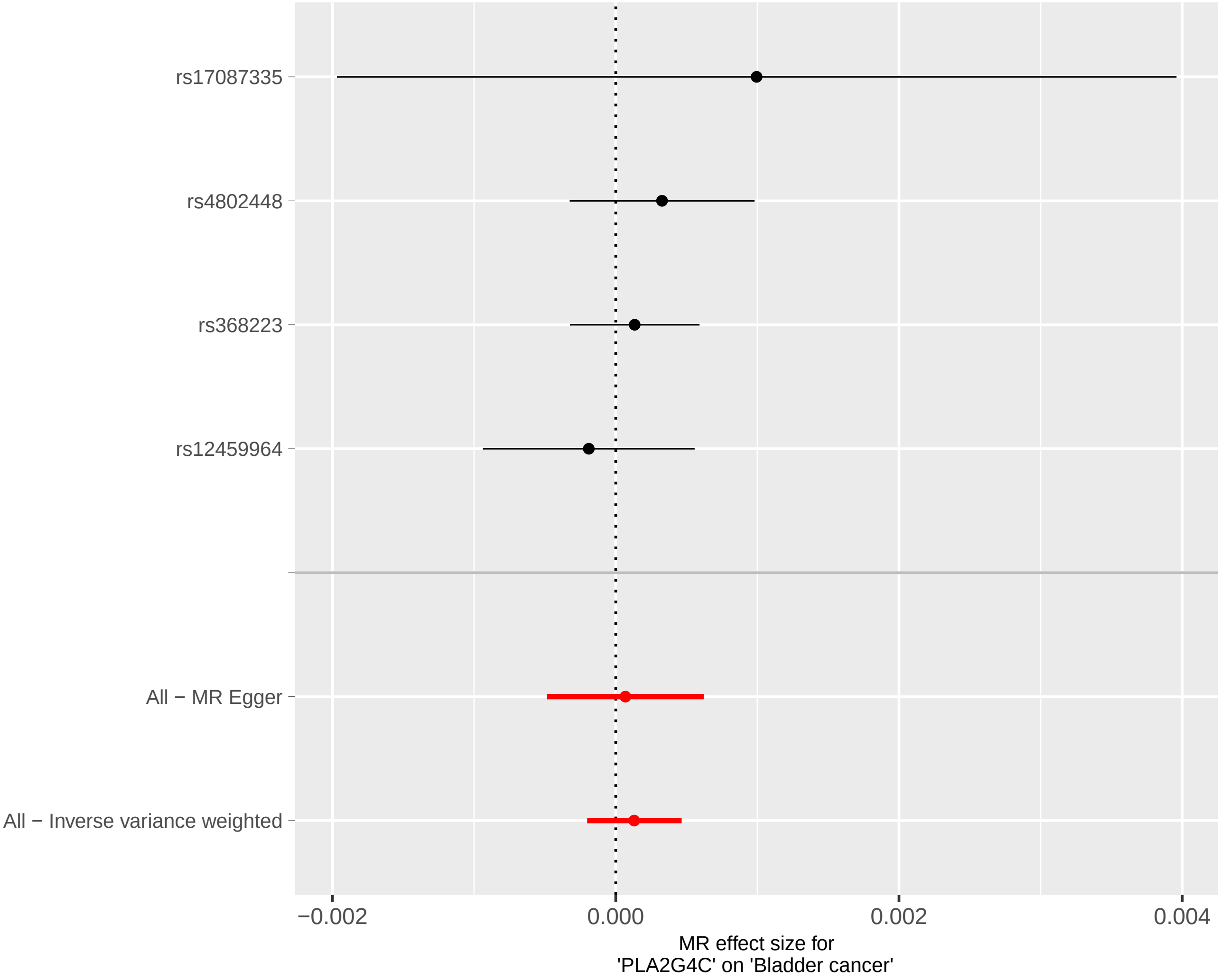
rs17087335
rs4802448
rs368223
rs12459964
All − MR Egger
All − Inverse variance weighted
−0.002
0.000
0.002
0.004
MR effect size for
'PLA2G4C' on 'Bladder cancer'

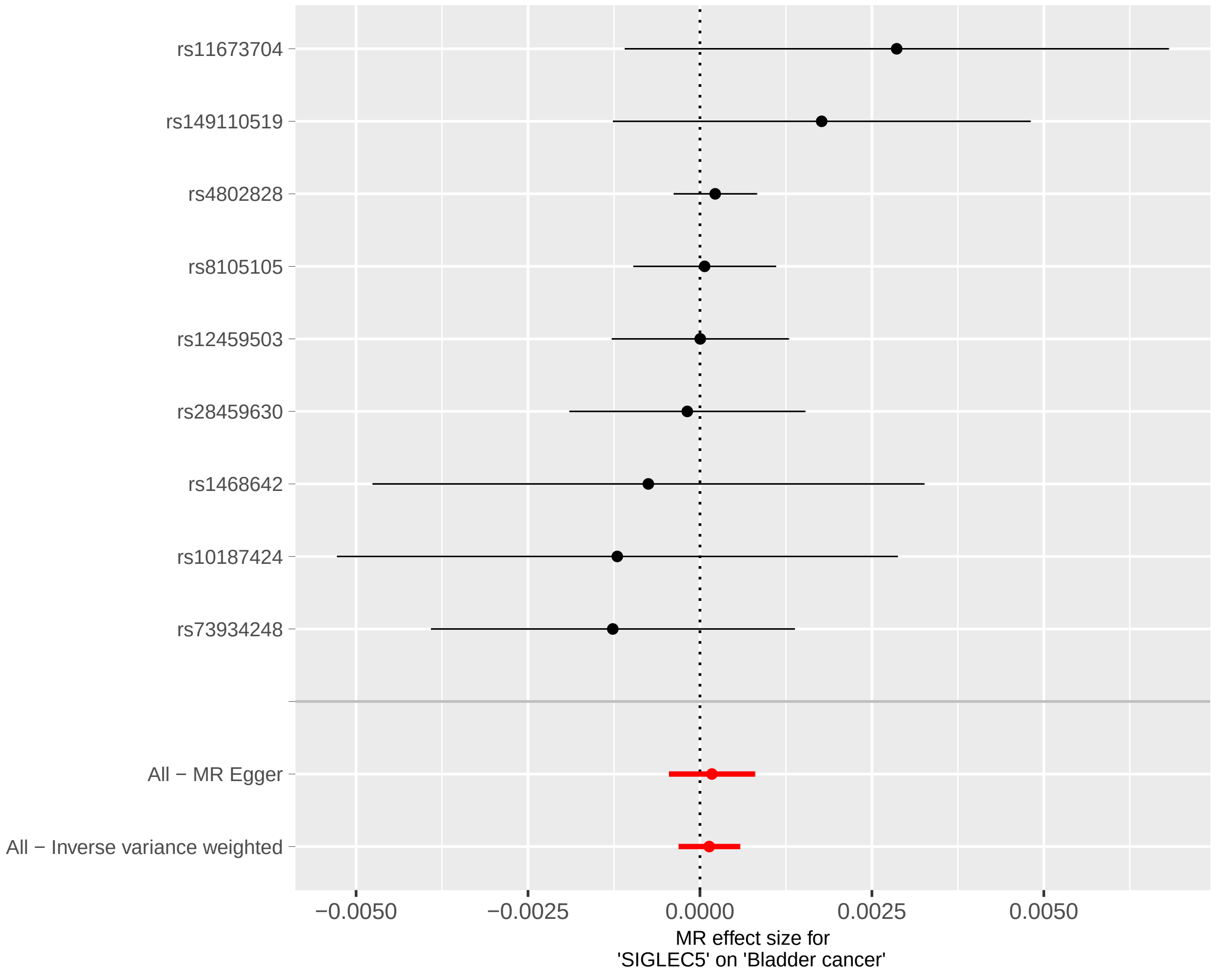
rs11673704
rs149110519
rs4802828
rs8105105
rs12459503
rs28459630
rs1468642
rs10187424
rs73934248
All − MR Egger
All − Inverse variance weighted
−0.0050
−0.0025
0.0000
0.0025
0.0050
MR effect size for
'SIGLEC5' on 'Bladder cancer'

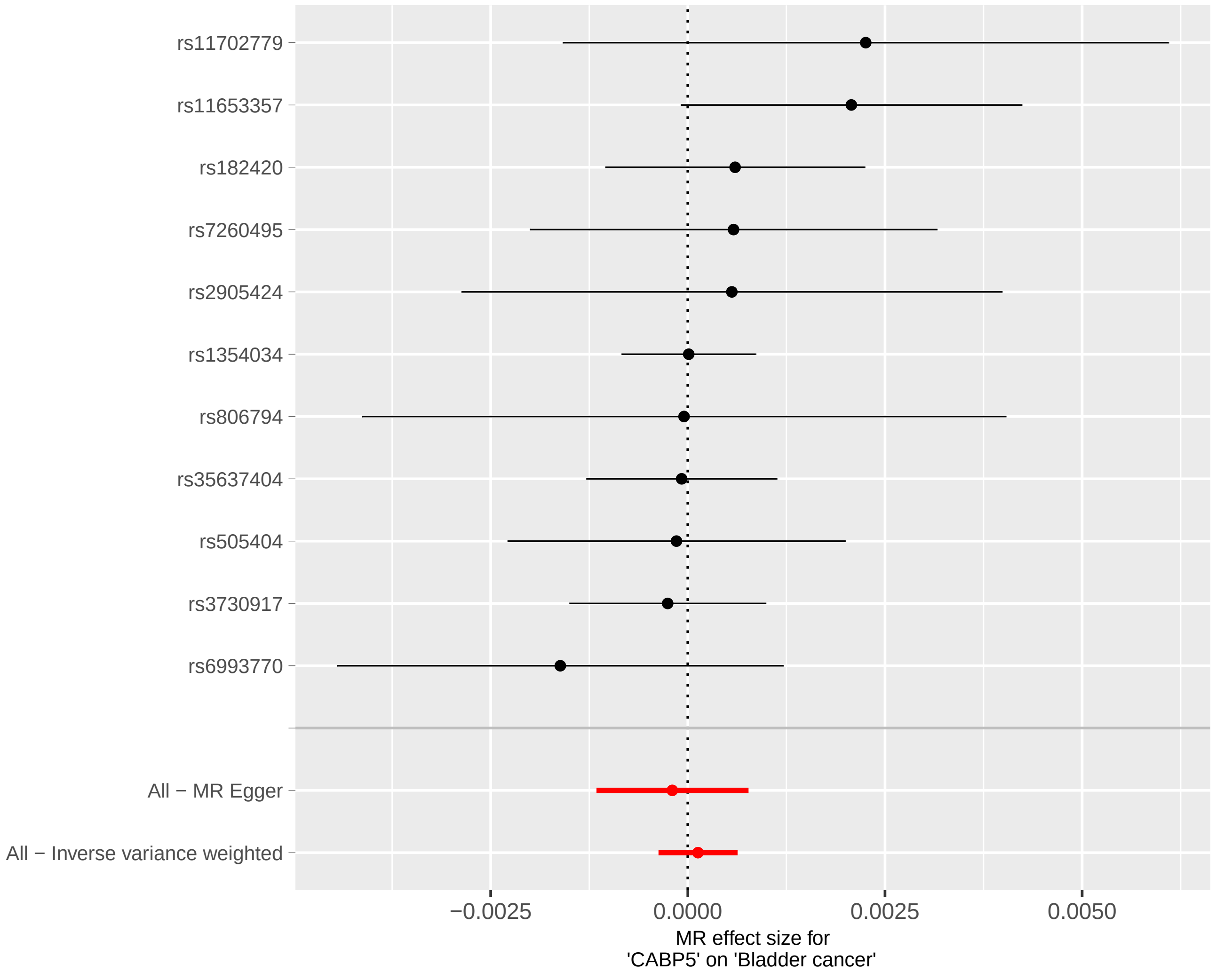

rs11702779
rs11653357
rs182420
rs7260495
rs2905424
rs1354034
rs806794
rs35637404
rs505404
rs3730917
rs6993770
All − MR Egger
All − Inverse variance weighted
−0.0025
0.0000
0.0025
0.0050
MR effect size for
'CABP5' on 'Bladder cancer'

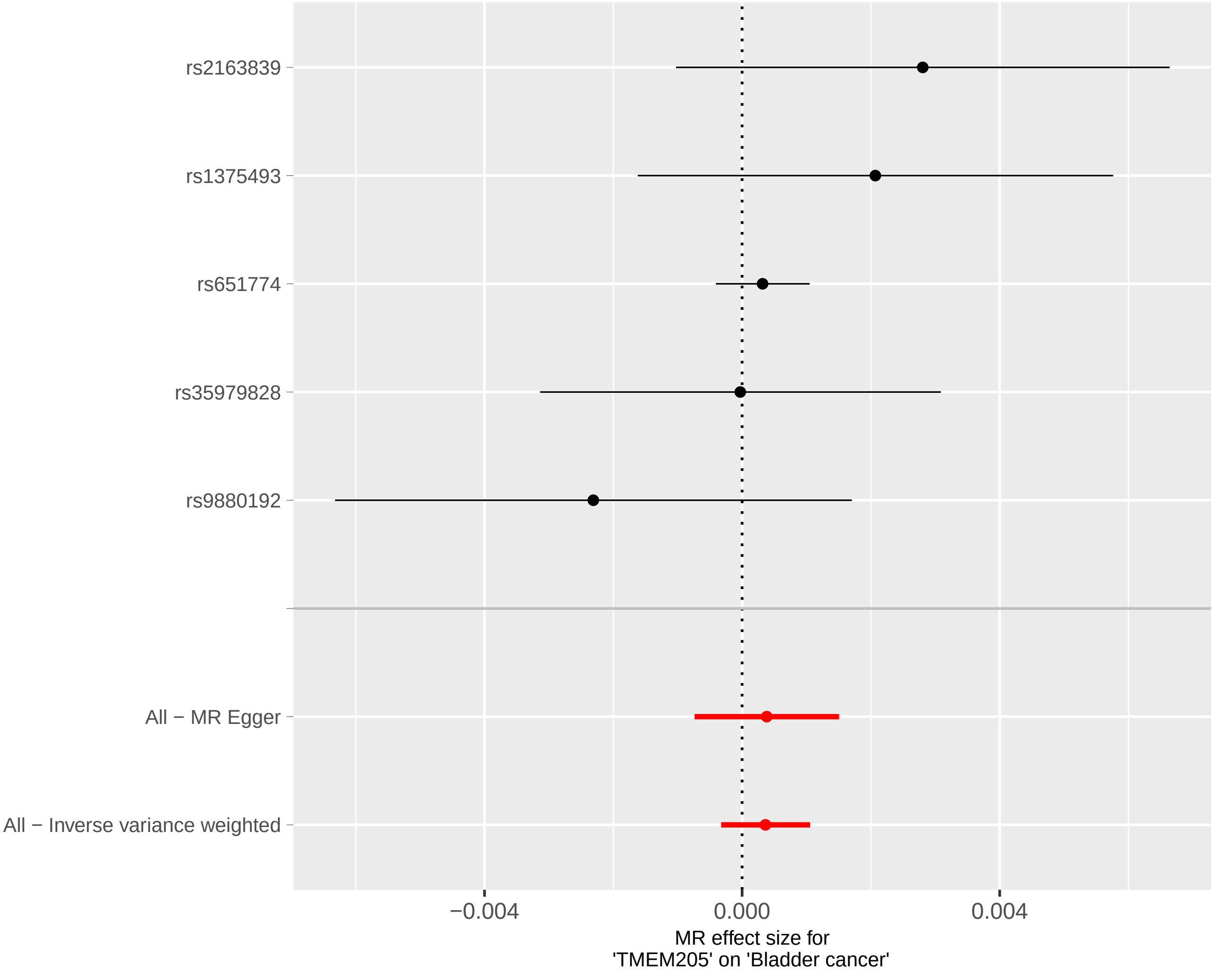
rs2163839
rs1375493
rs651774
rs35979828
rs9880192
All − MR Egger
All − Inverse variance weighted
−0.004
0.000
0.004
MR effect size for
'TMEM205' on 'Bladder cancer'

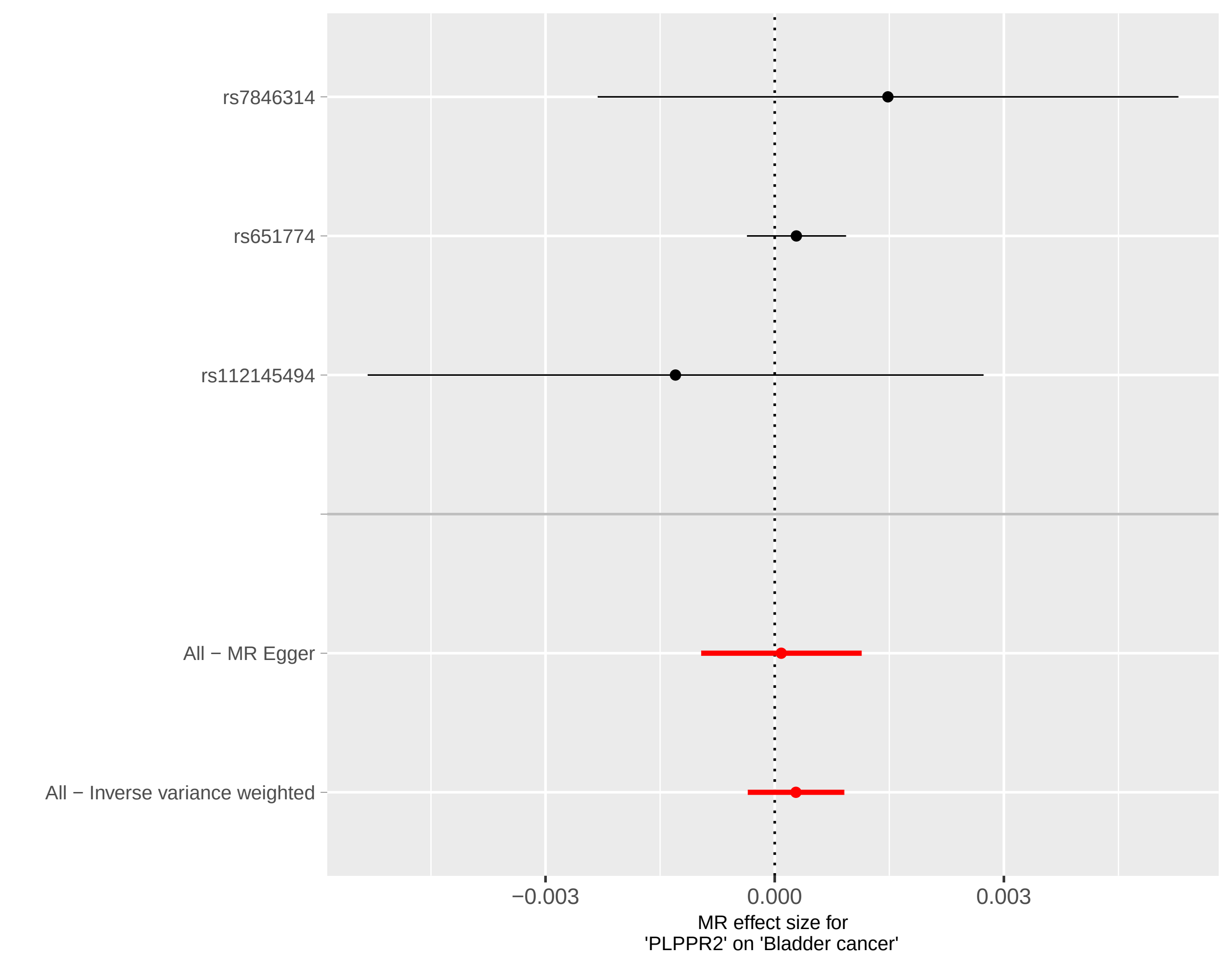

rs7846314
rs651774
rs112145494
All − MR Egger
All − Inverse variance weighted
−0.003
0.000
0.003
MR effect size for
'PLPPR2' on 'Bladder cancer'

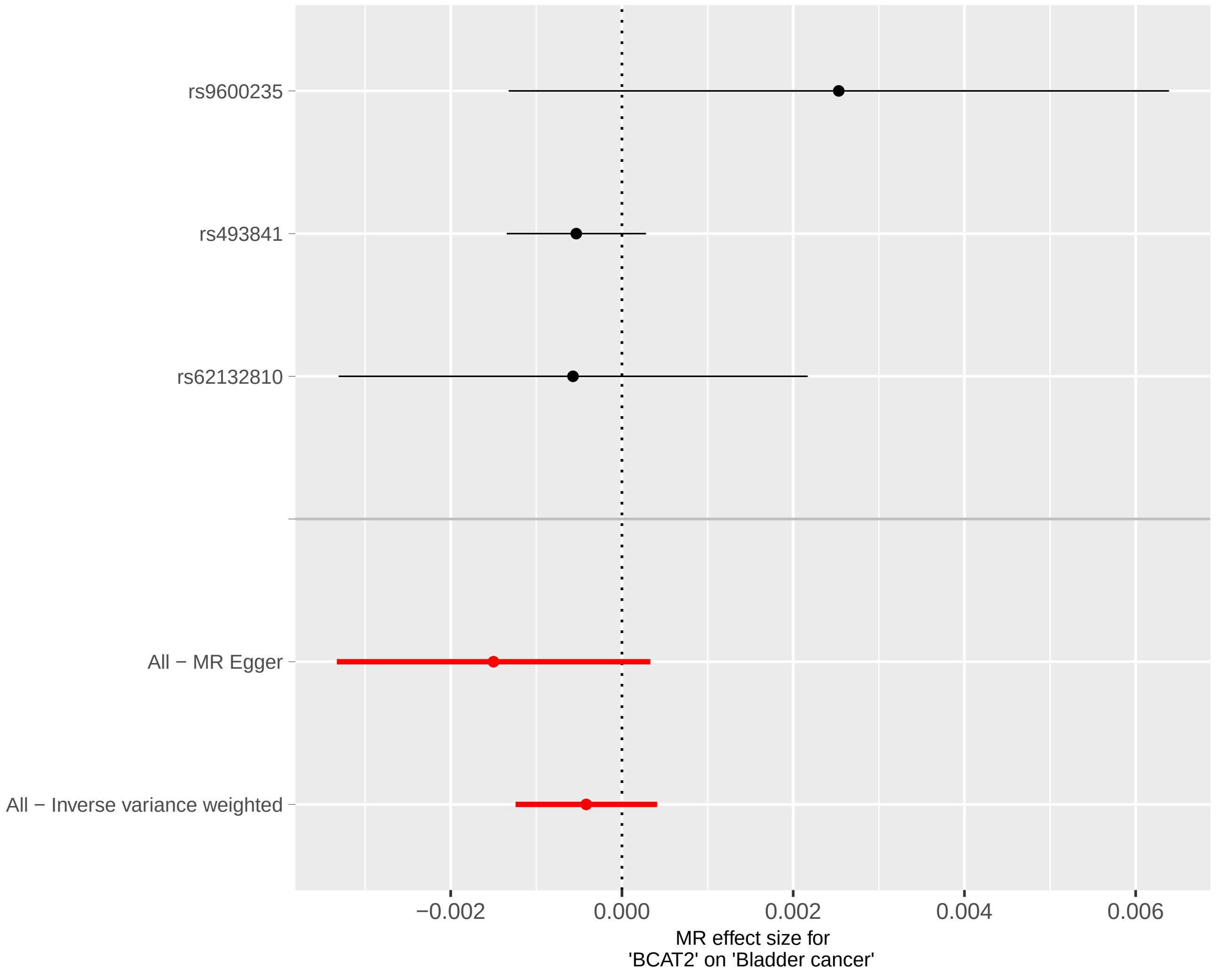
rs9600235
rs493841
rs62132810
All − MR Egger
All − Inverse variance weighted
−0.002
0.000
0.002
0.004
0.006
MR effect size for
'BCAT2' on 'Bladder cancer'

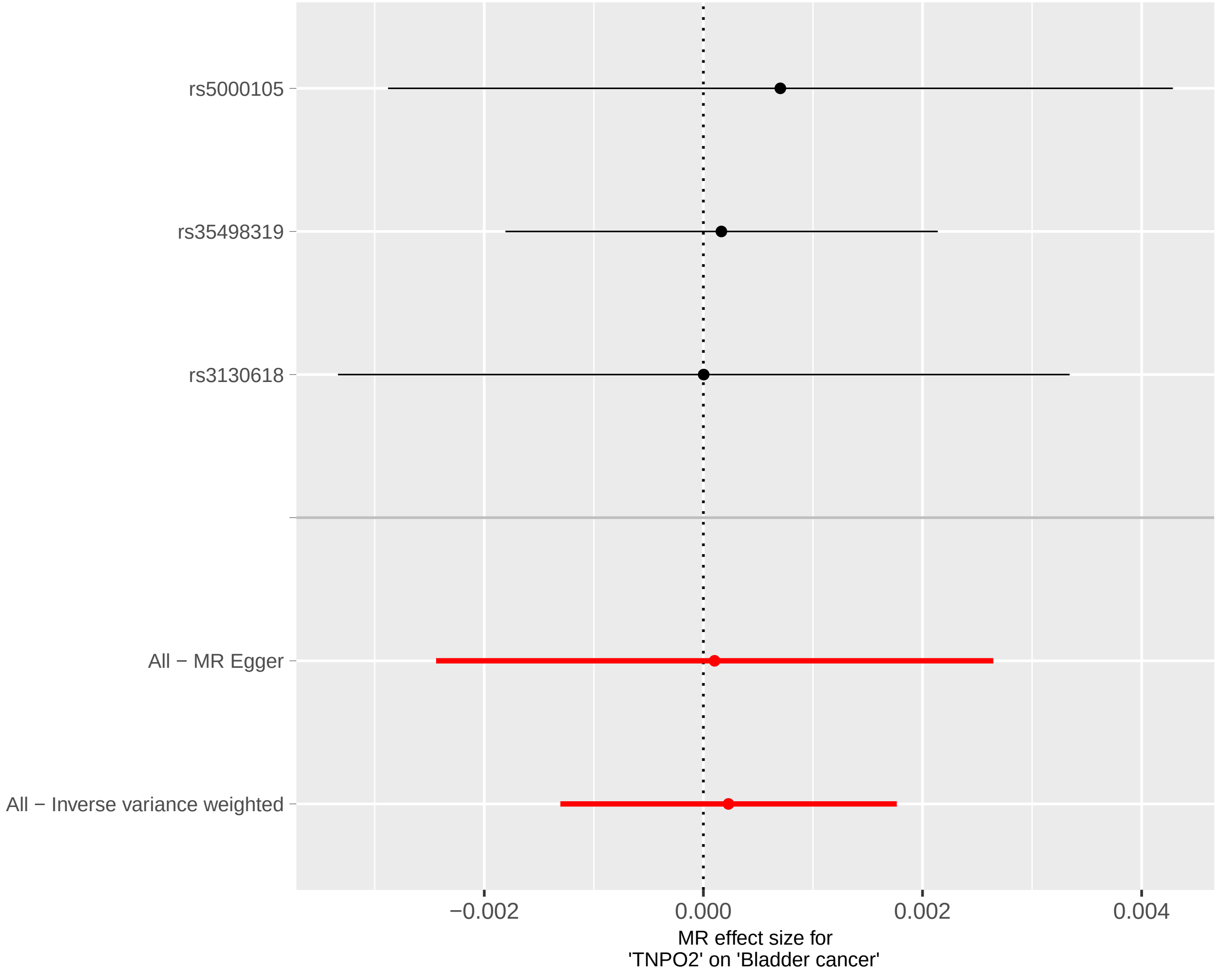
rs5000105
rs35498319
rs3130618
All − MR Egger
All − Inverse variance weighted
−0.002
0.000
0.002
0.004
MR effect size for
'TNPO2' on 'Bladder cancer'

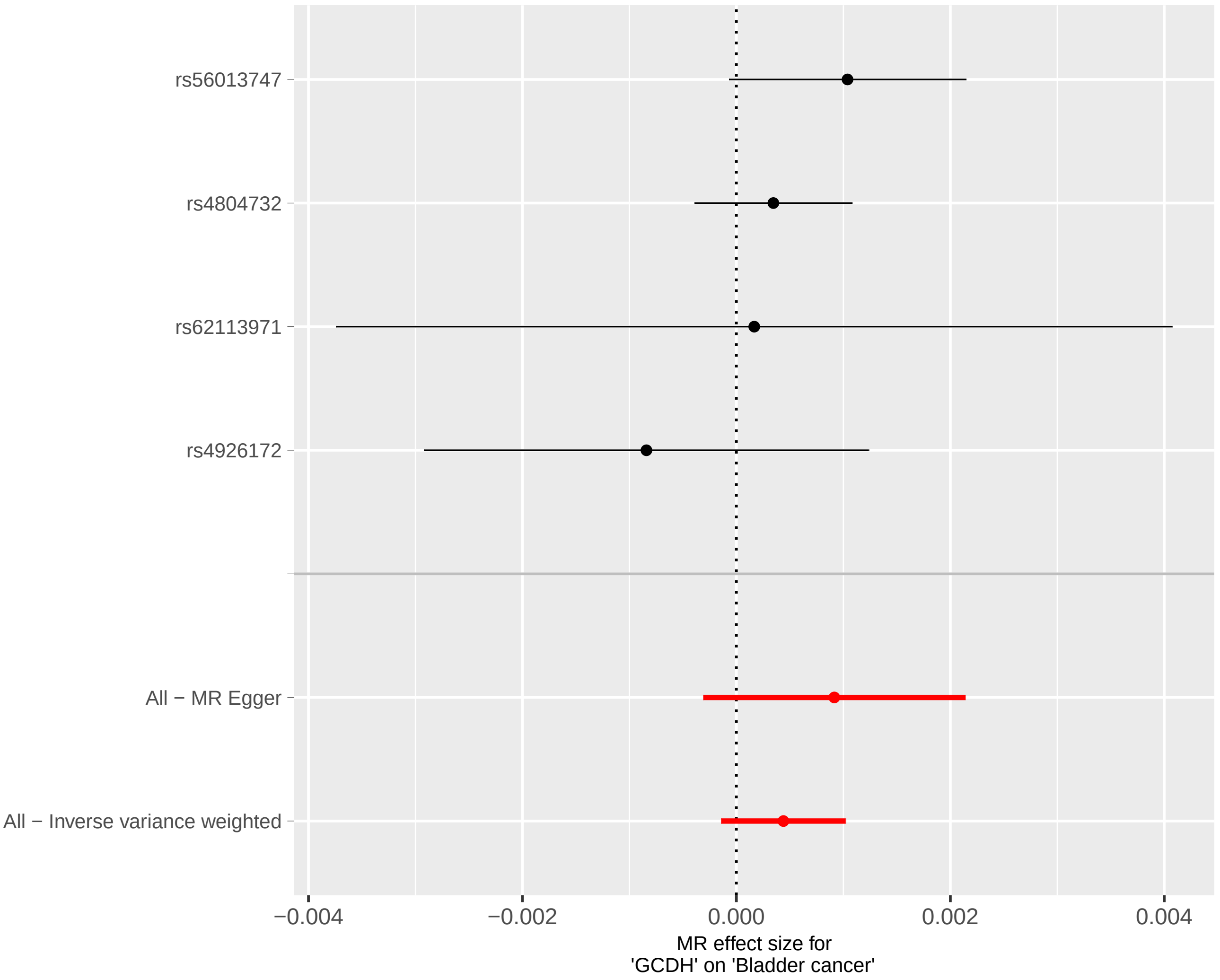

rs56013747
rs4804732
rs62113971
rs4926172
All − MR Egger
All − Inverse variance weighted
−0.004
−0.002
0.000
0.002
0.004
MR effect size for
'GCDH' on 'Bladder cancer'

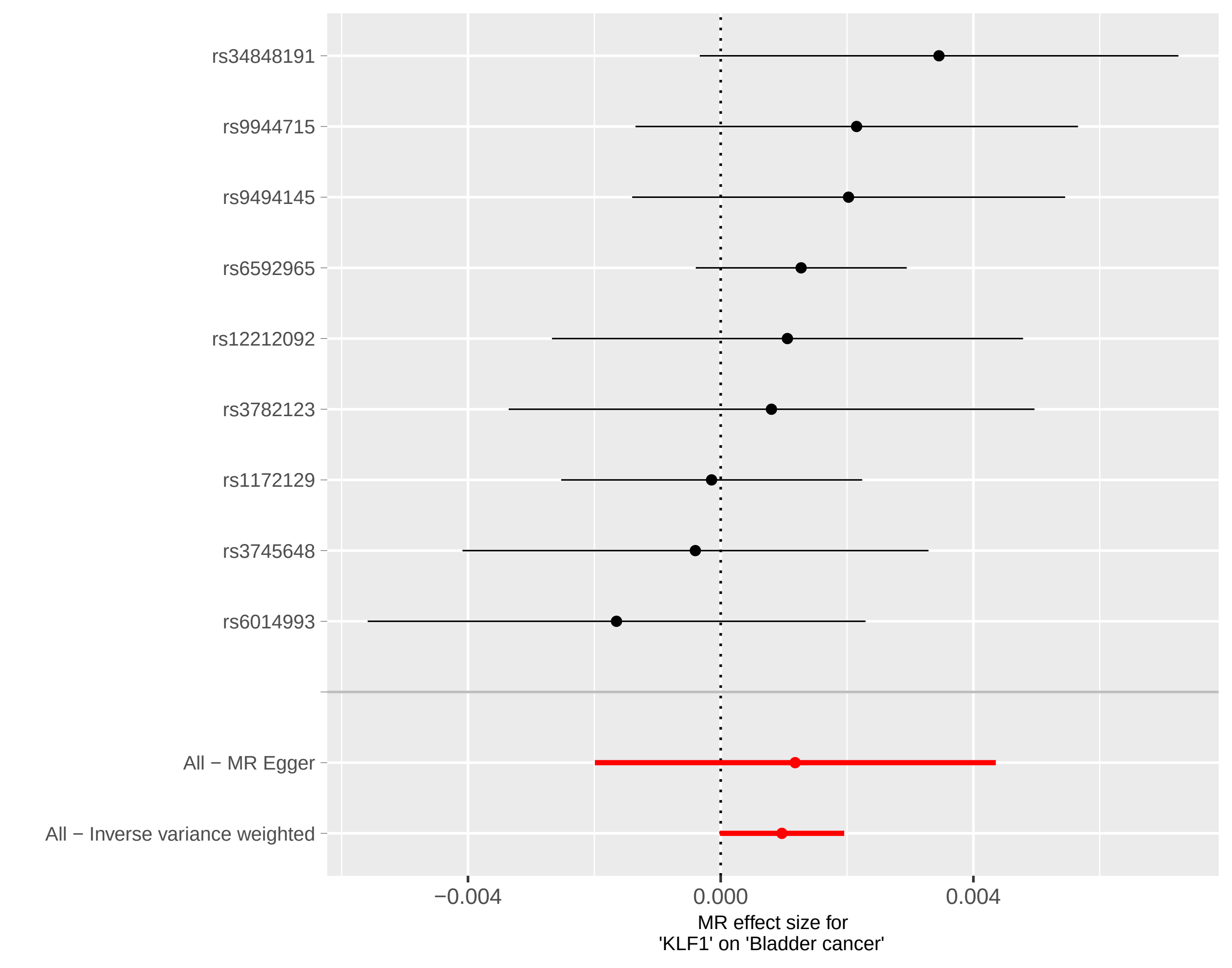
rs34848191
rs9944715
rs9494145
rs6592965
rs12212092
rs3782123
rs1172129
rs3745648
rs6014993
All − MR Egger
All − Inverse variance weighted
−0.004
0.000
0.004
MR effect size for
'KLF1' on 'Bladder cancer'

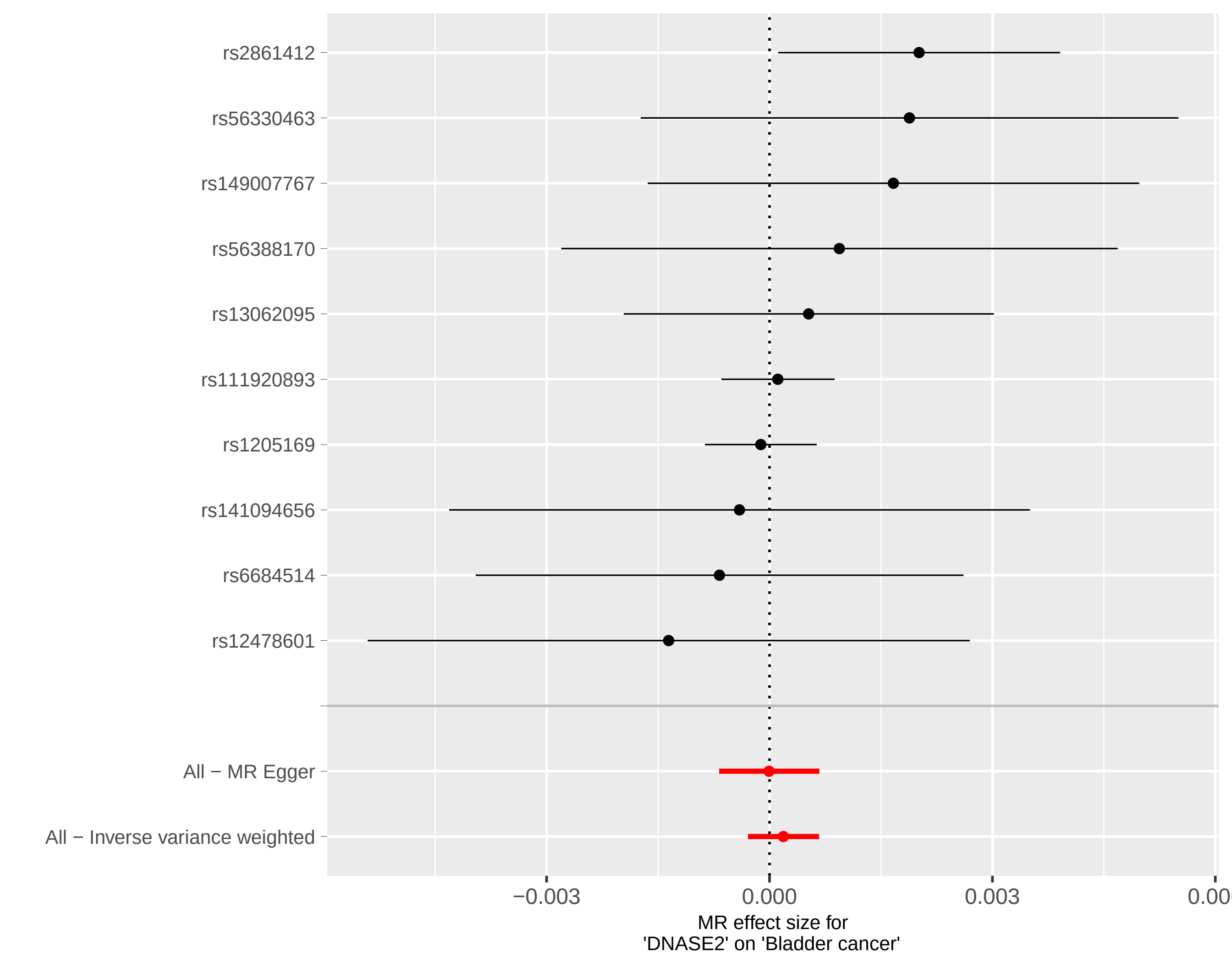
rs2861412
rs56330463
rs149007767
rs56388170
rs13062095
rs111920893
rs1205169
rs141094656
rs6684514
rs12478601
All − MR Egger
All − Inverse variance weighted
−0.003
0.000
0.003
0.00
MR effect size for
'DNASE2' on 'Bladder cancer'

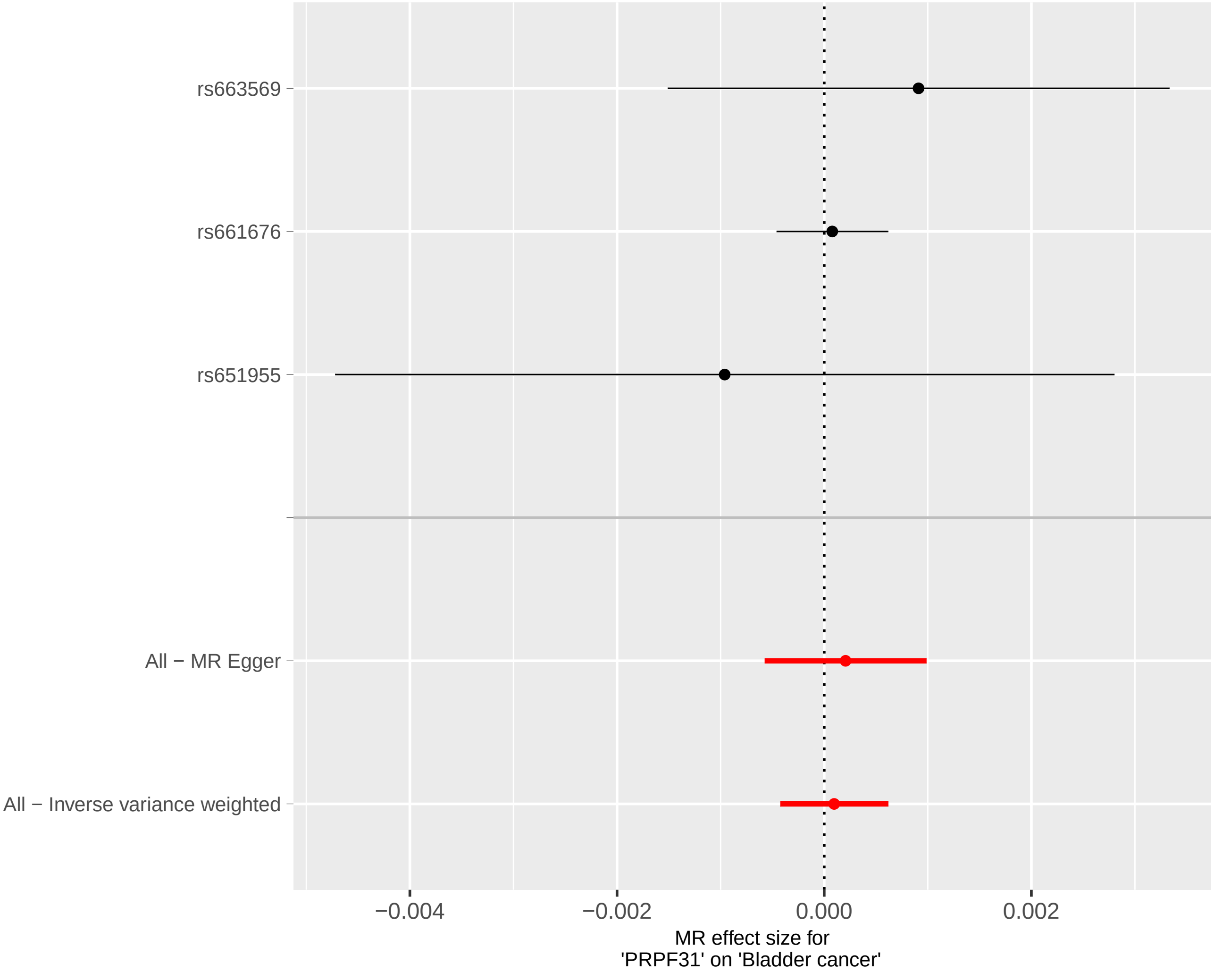

rs663569
rs661676
rs651955
All − MR Egger
All − Inverse variance weighted
−0.004
−0.002
0.000
0.002
MR effect size for
'PRPF31' on 'Bladder cancer'

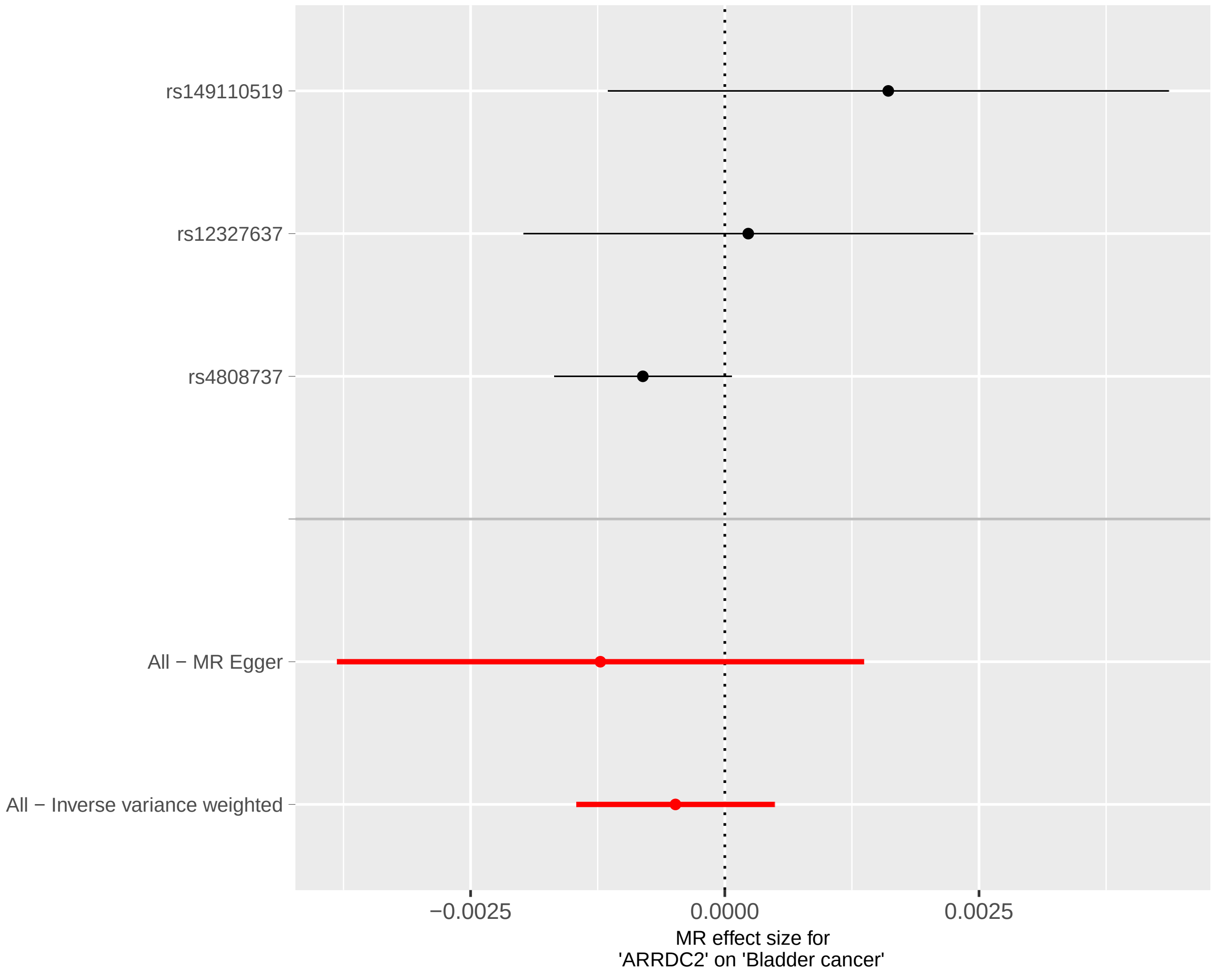

rs149110519
rs12327637
rs4808737
All − MR Egger
All − Inverse variance weighted
−0.0025
0.0000
0.0025
MR effect size for
'ARRDC2' on 'Bladder cancer'

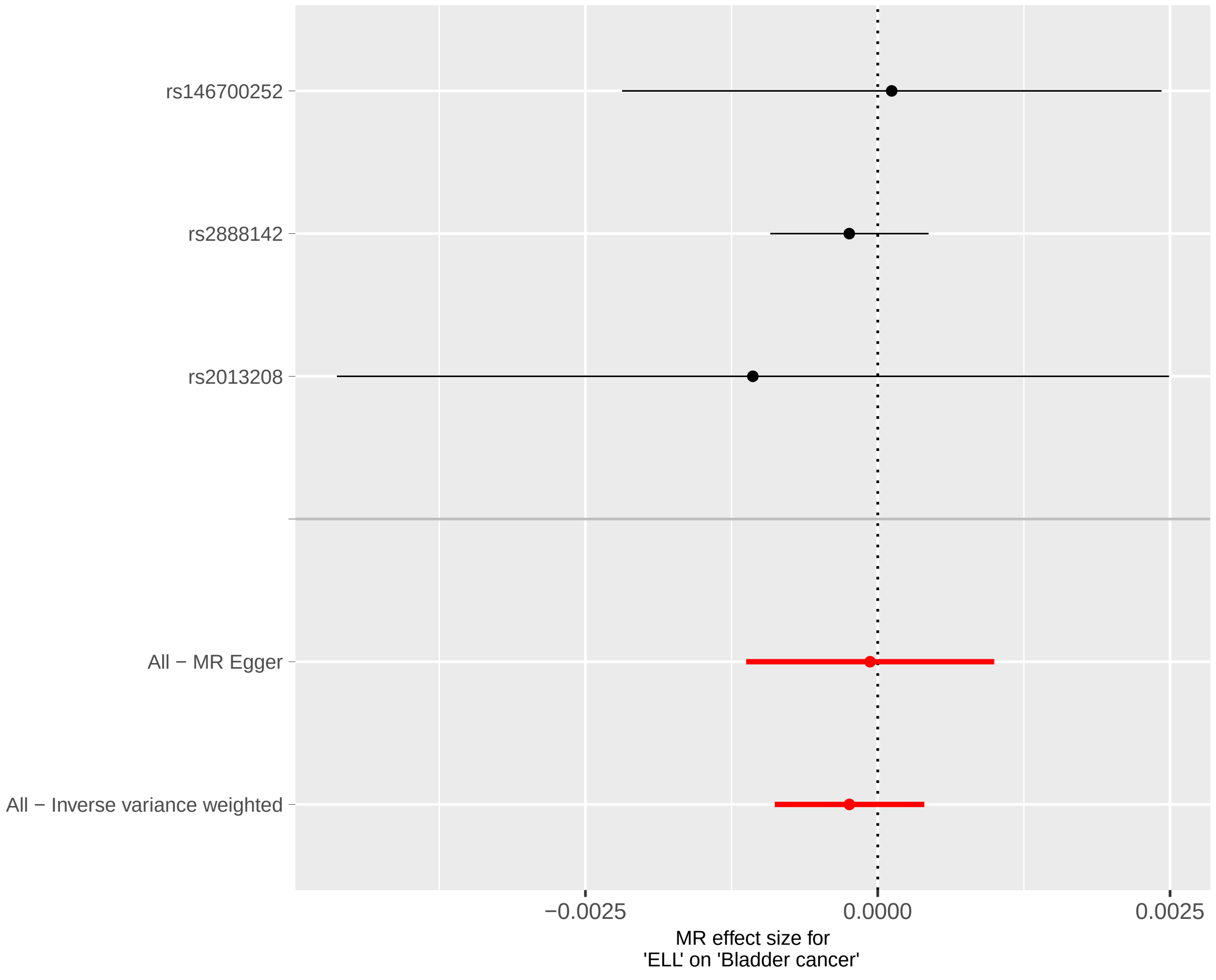
rs146700252
rs2888142
rs2013208
All − MR Egger
All − Inverse variance weighted
−0.0025
0.0000
0.0025
MR effect size for
'ELL' on 'Bladder cancer'

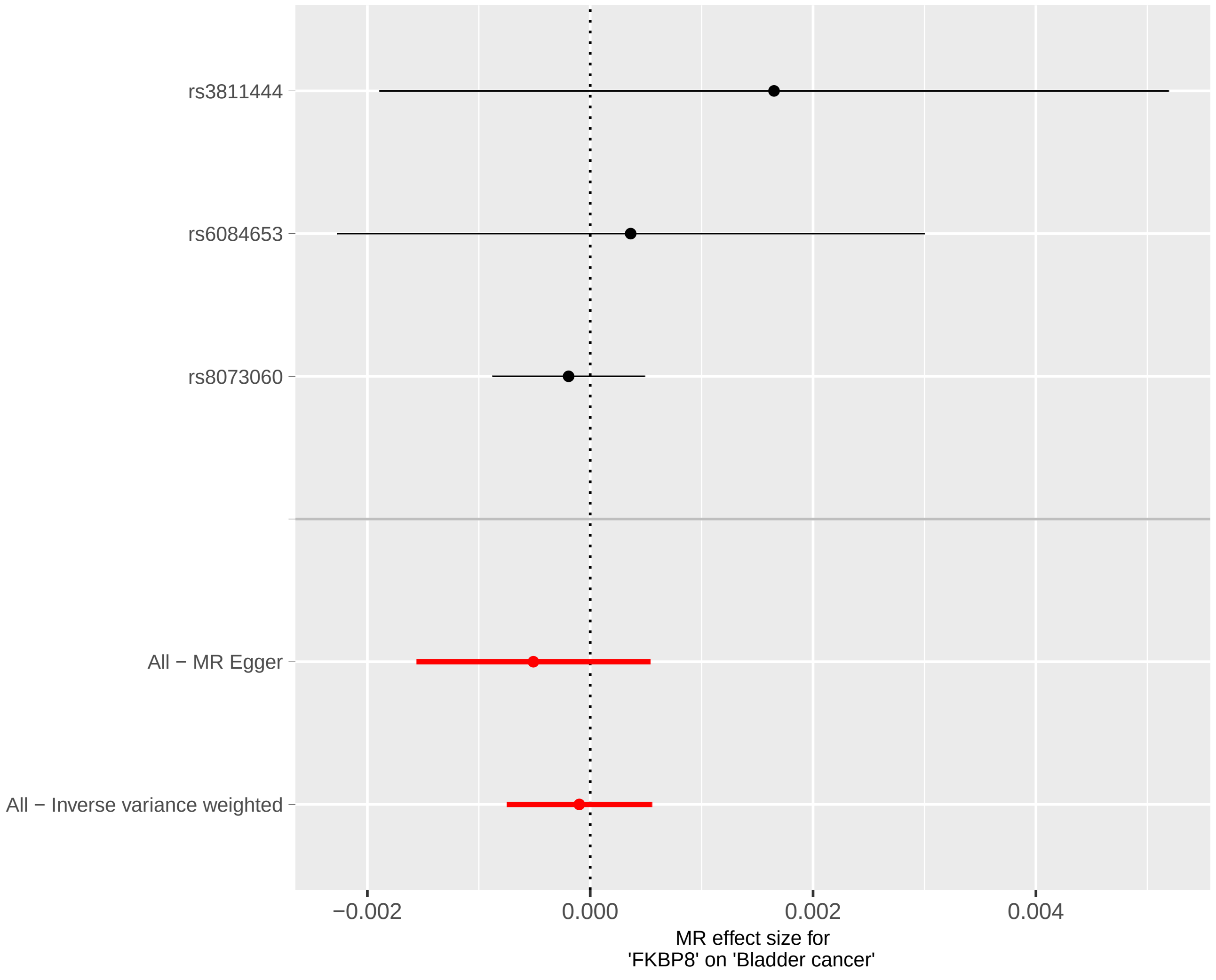
rs3811444
rs6084653
rs8073060
All − MR Egger
All − Inverse variance weighted
−0.002
0.000
0.002
0.004
MR effect size for
'FKBP8' on 'Bladder cancer'

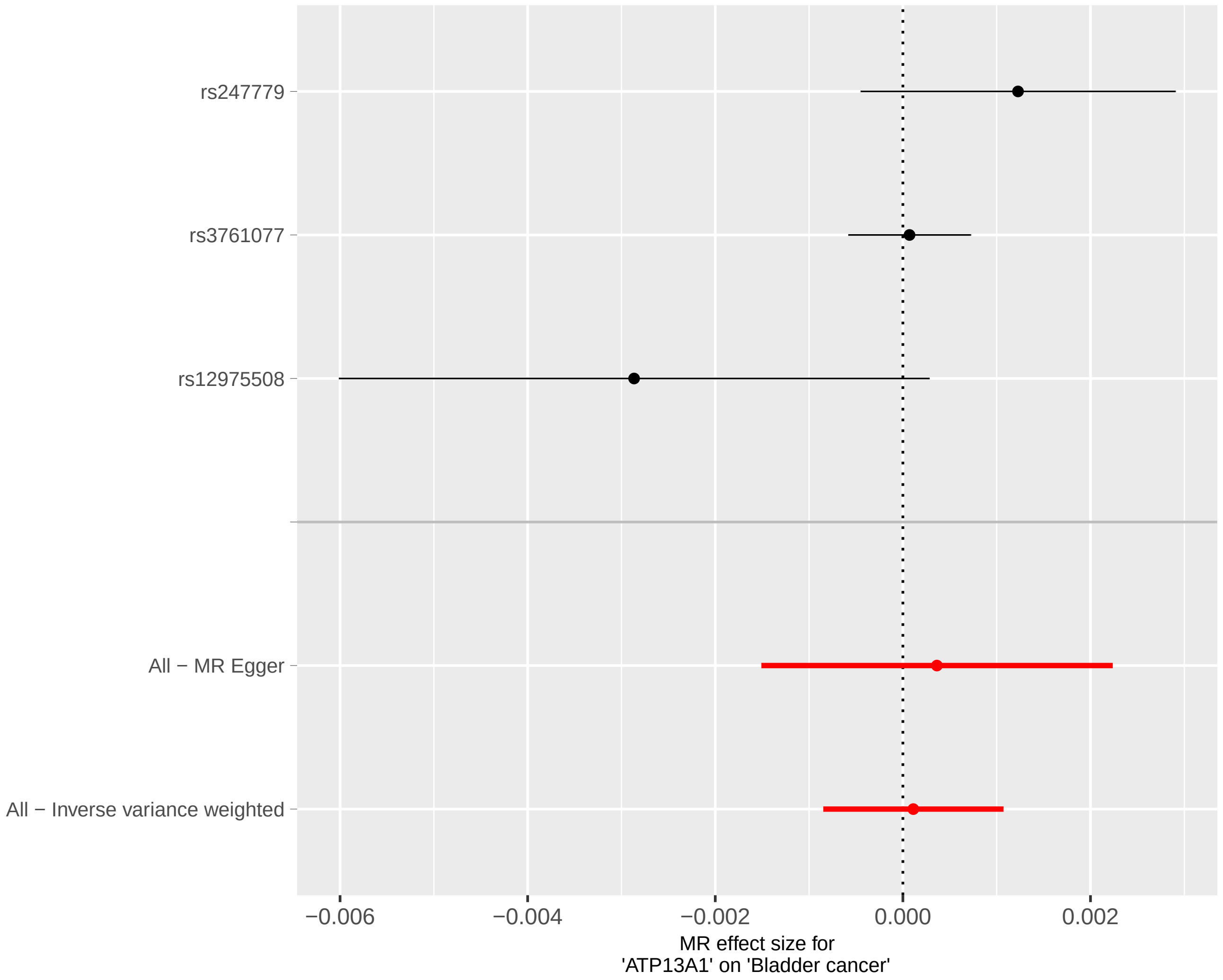
rs247779
rs3761077
rs12975508
All − MR Egger
All − Inverse variance weighted
−0.006
−0.004
−0.002
0.000
0.002
MR effect size for
'ATP13A1' on 'Bladder cancer'

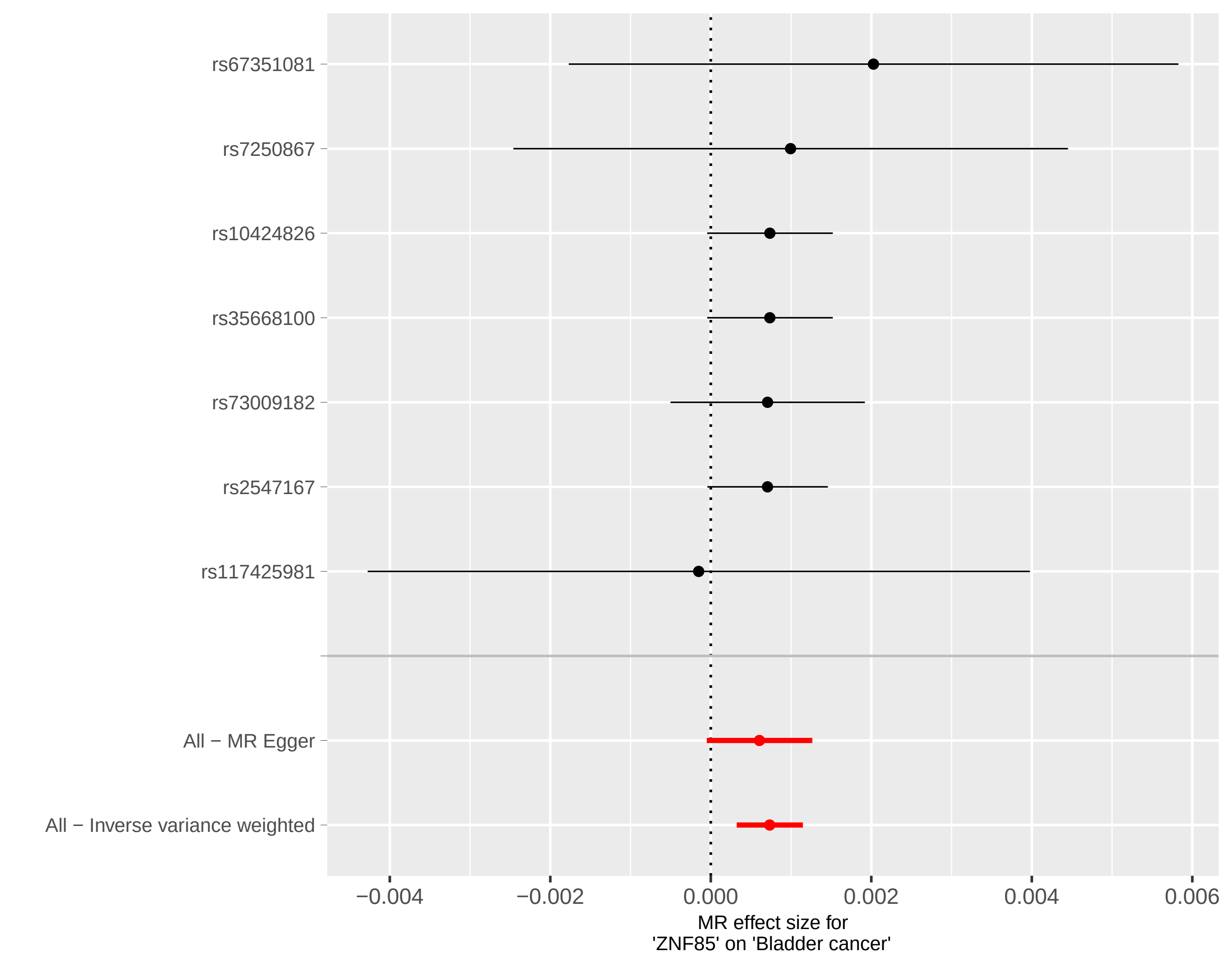

rs67351081
rs7250867
rs10424826
rs35668100
rs73009182
rs2547167
rs117425981
All − MR Egger
All − Inverse variance weighted
−0.004
−0.002
0.000
0.002
0.004
0.006
MR effect size for
'ZNF85' on 'Bladder cancer'

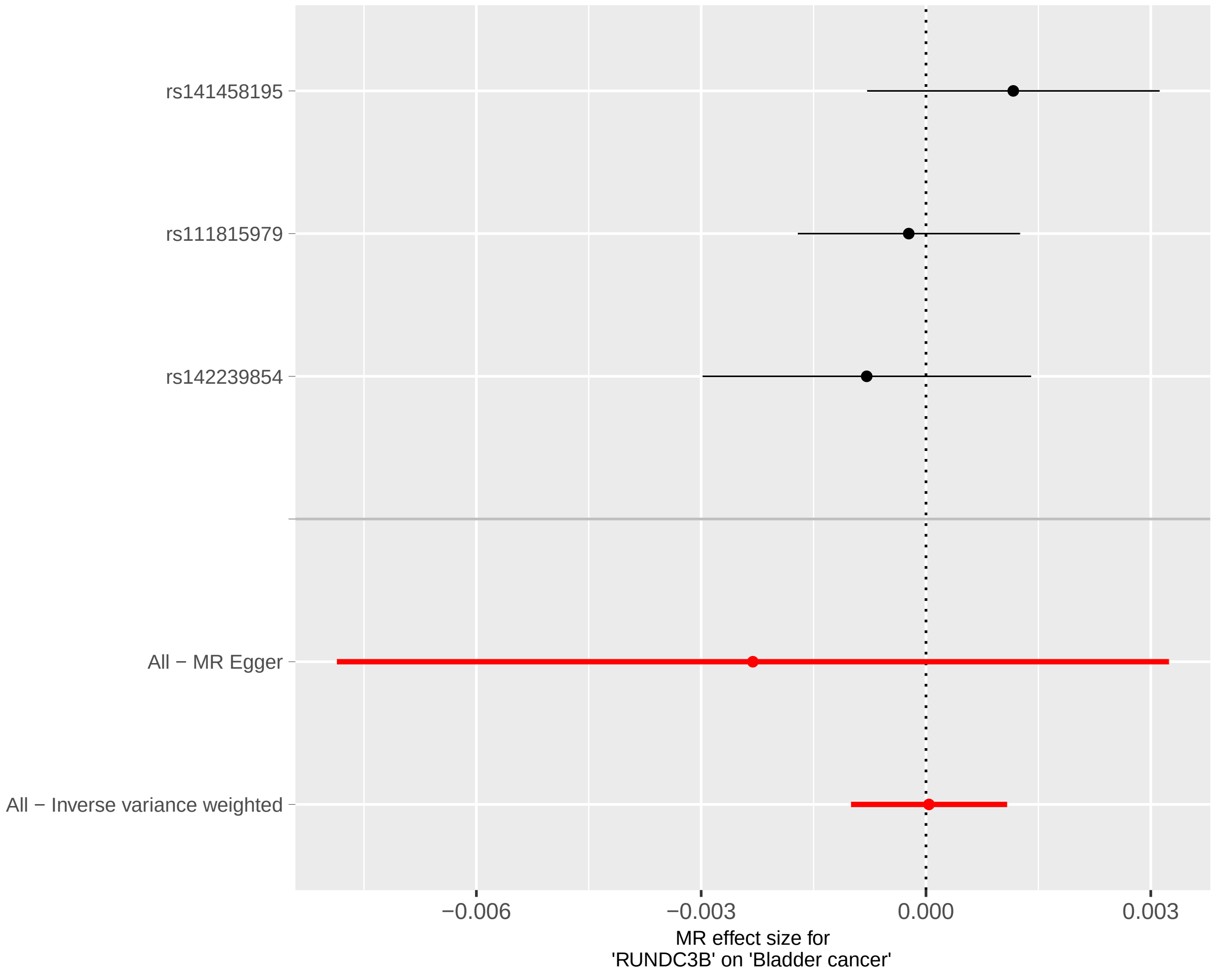
rs141458195
rs111815979
rs142239854
All − MR Egger
All − Inverse variance weighted
−0.006
−0.003
0.000
0.003
MR effect size for
'RUNDC3B' on 'Bladder cancer'

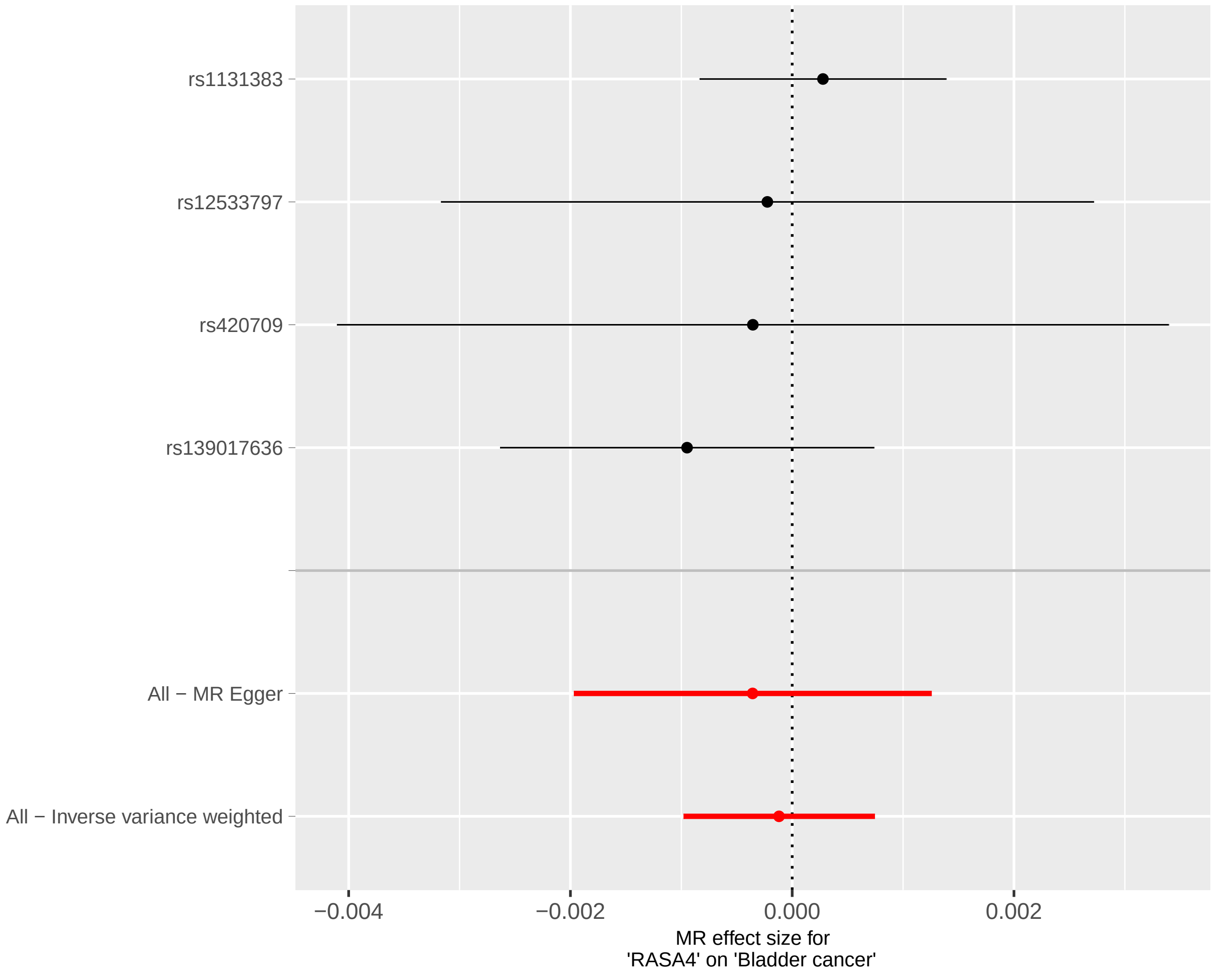
rs1131383
rs12533797
rs420709
rs139017636
All − MR Egger
All − Inverse variance weighted
−0.004
−0.002
0.000
0.002
MR effect size for
'RASA4' on 'Bladder cancer'

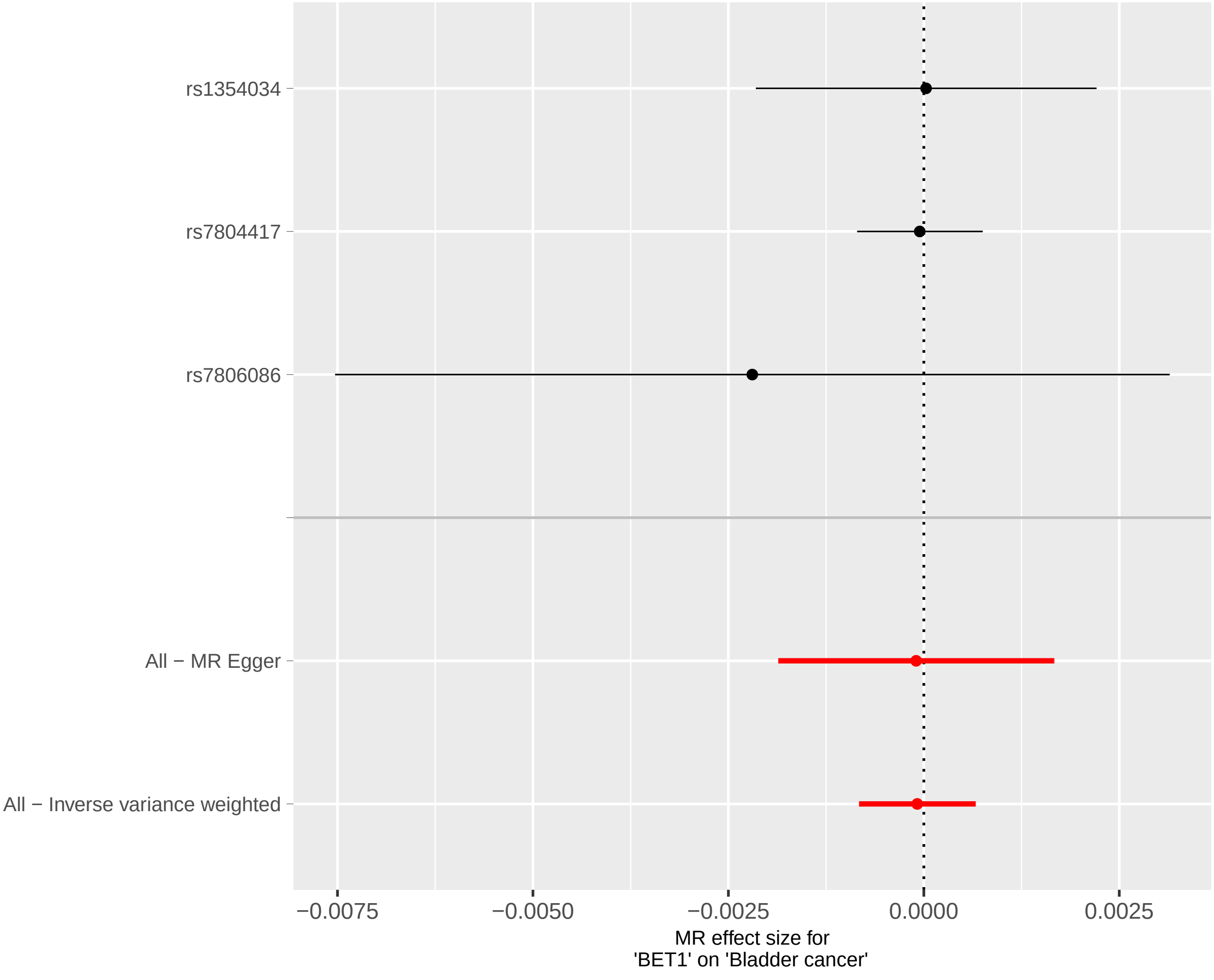

rs1354034
rs7804417
rs7806086
All − MR Egger
All − Inverse variance weighted
−0.0075
−0.0050
−0.0025
0.0000
0.0025
MR effect size for
'BET1' on 'Bladder cancer'

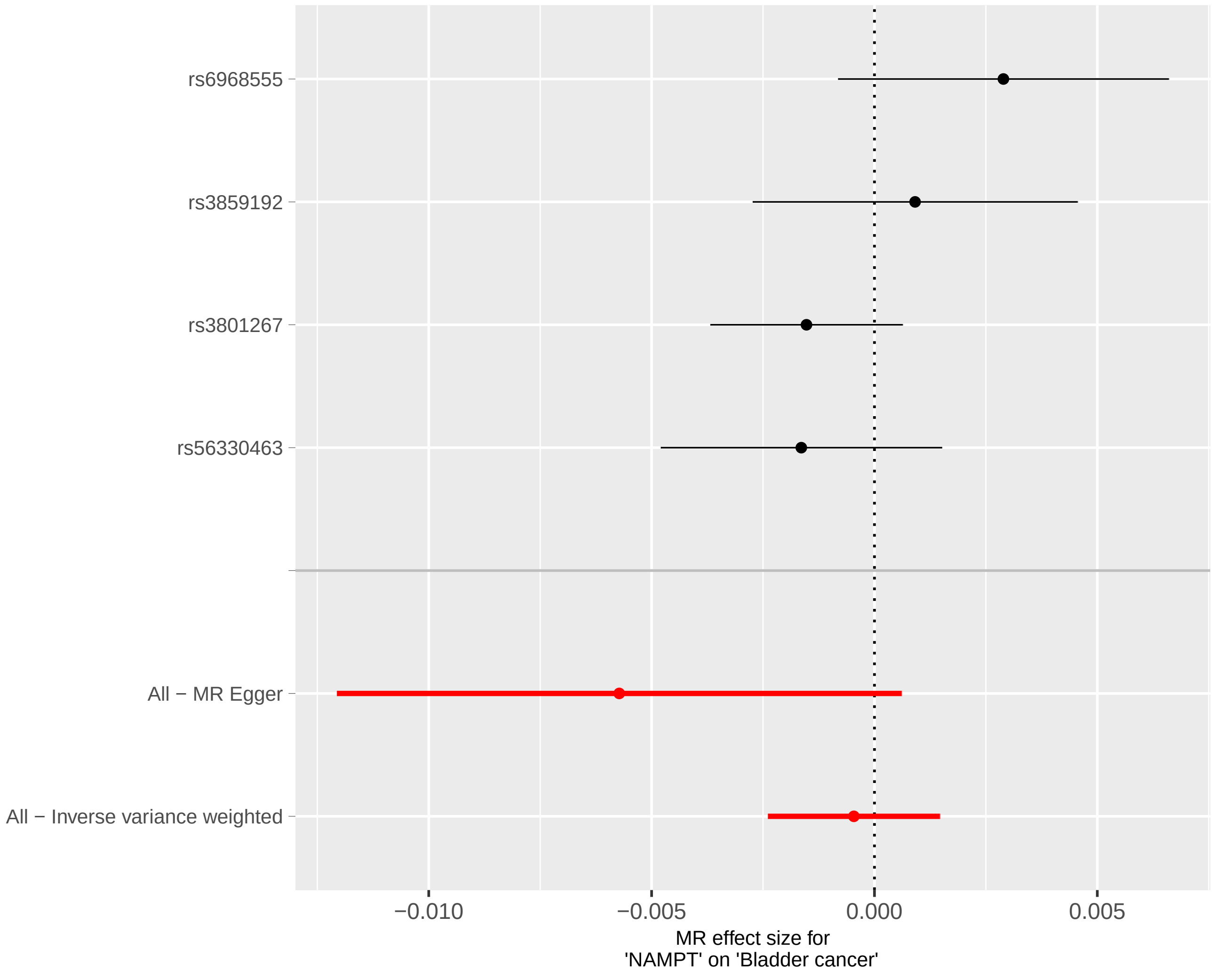
rs6968555
rs3859192
rs3801267
rs56330463
All − MR Egger
All − Inverse variance weighted
−0.010
−0.005
0.000
0.005
MR effect size for
'NAMPT' on 'Bladder cancer'

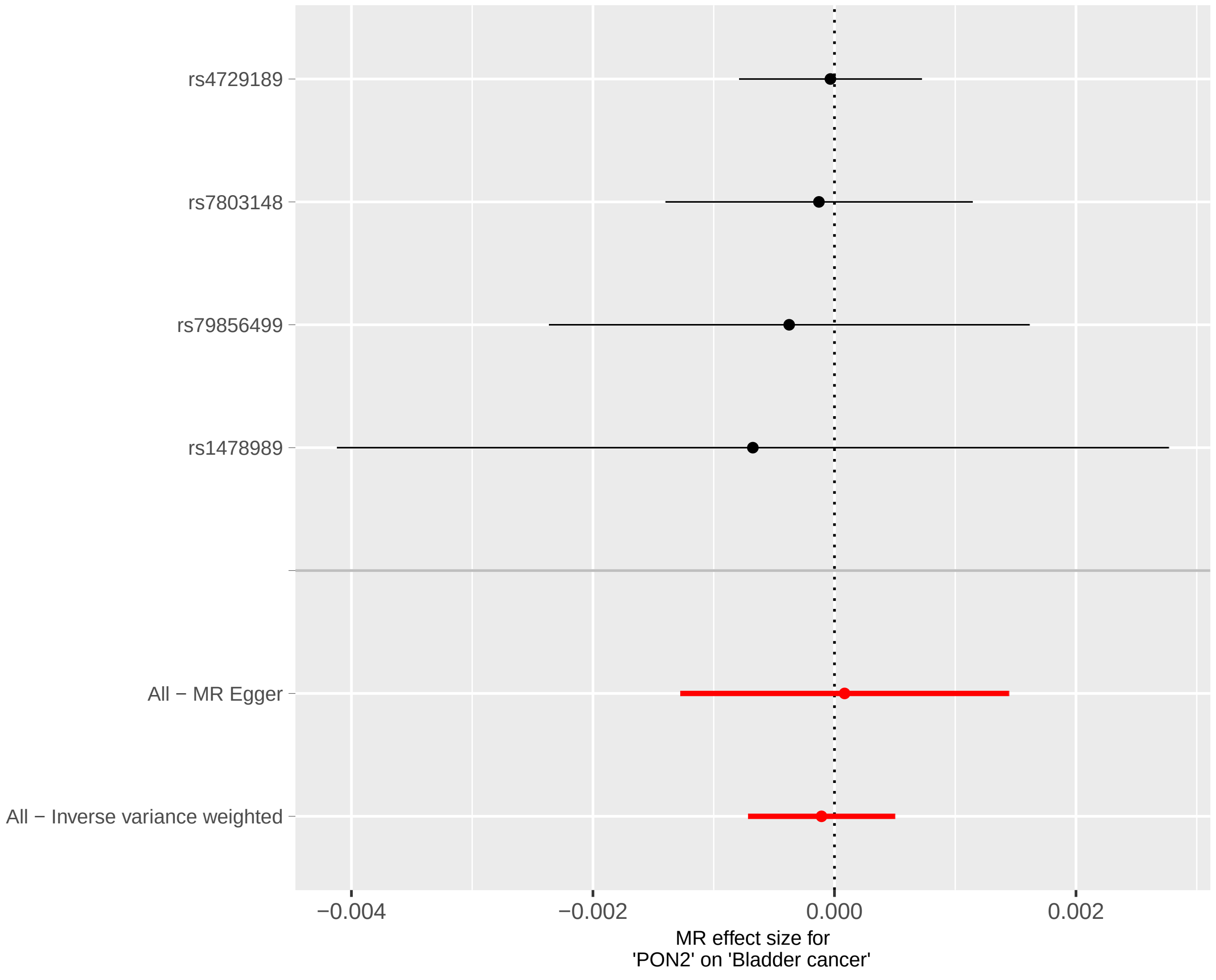

rs4729189
rs7803148
rs79856499
rs1478989
All − MR Egger
All − Inverse variance weighted
−0.004
−0.002
0.000
0.002
MR effect size for
'PON2' on 'Bladder cancer'

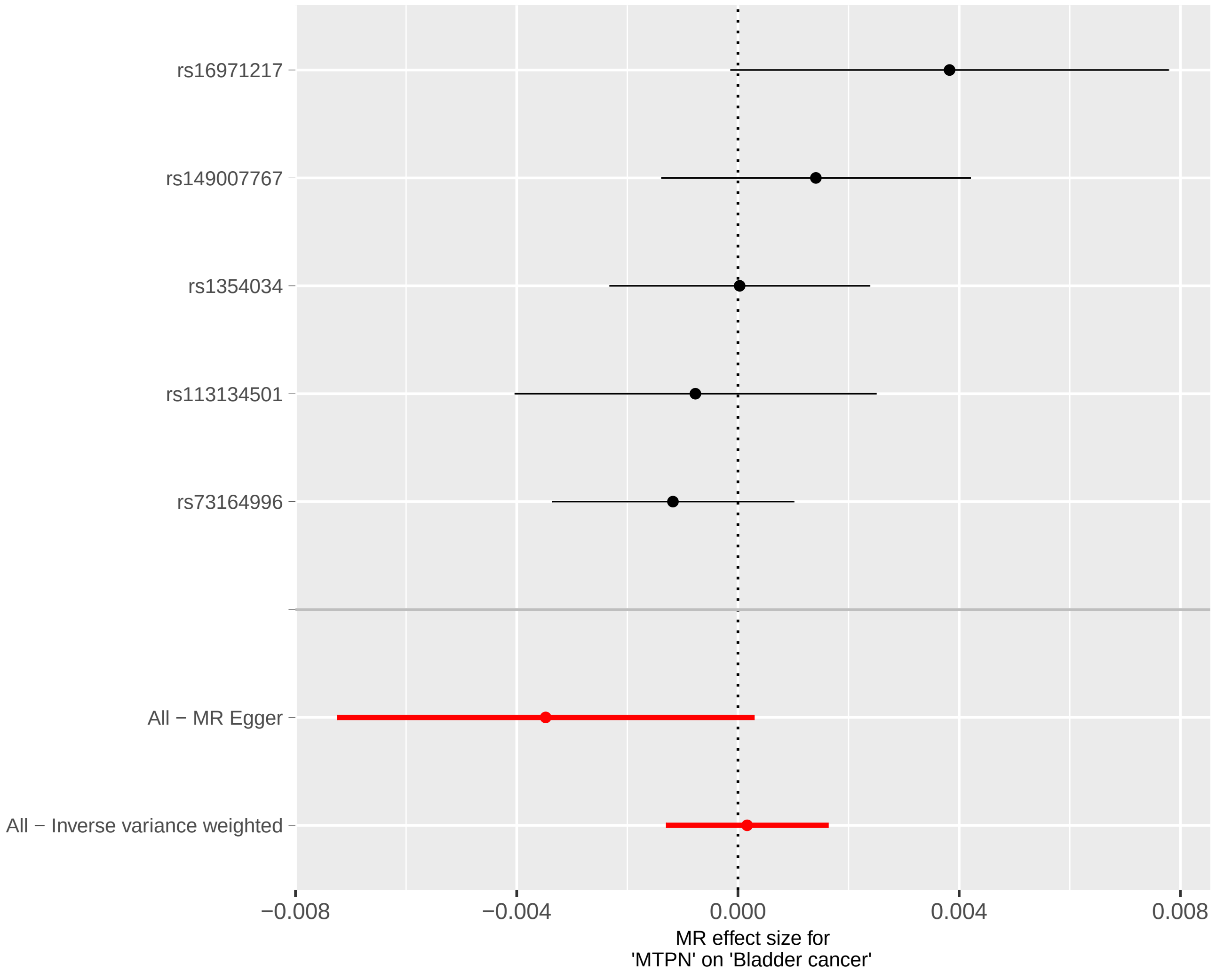

rs16971217
rs149007767
rs1354034
rs113134501
rs73164996
All − MR Egger
All − Inverse variance weighted
−0.008
−0.004
0.000
0.004
0.008
MR effect size for
'MTPN' on 'Bladder cancer'

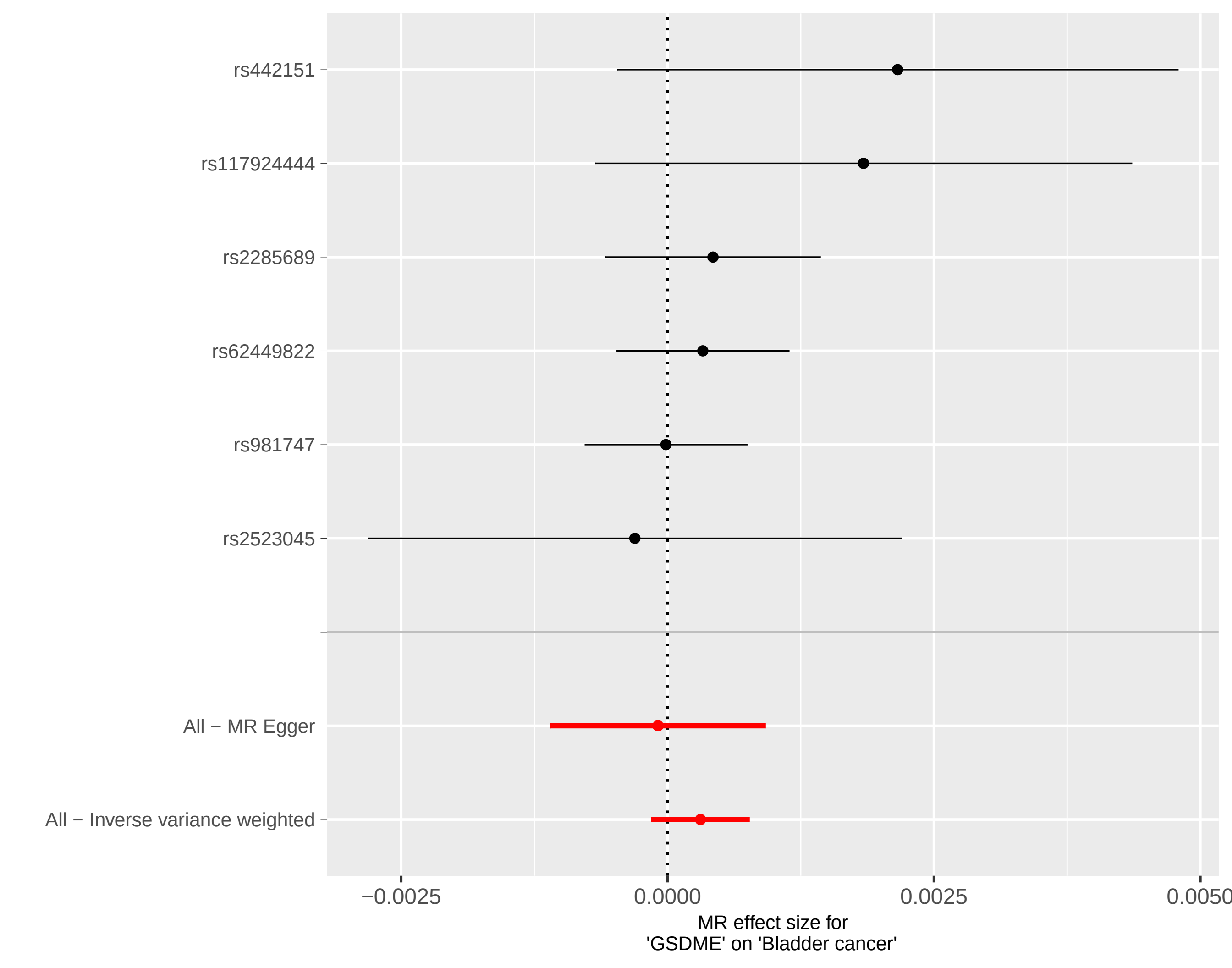
rs442151
rs117924444
rs2285689
rs62449822
rs981747
rs2523045
All − MR Egger
All − Inverse variance weighted
−0.0025
0.0000
0.0025
0.0050
MR effect size for
'GSDME' on 'Bladder cancer'

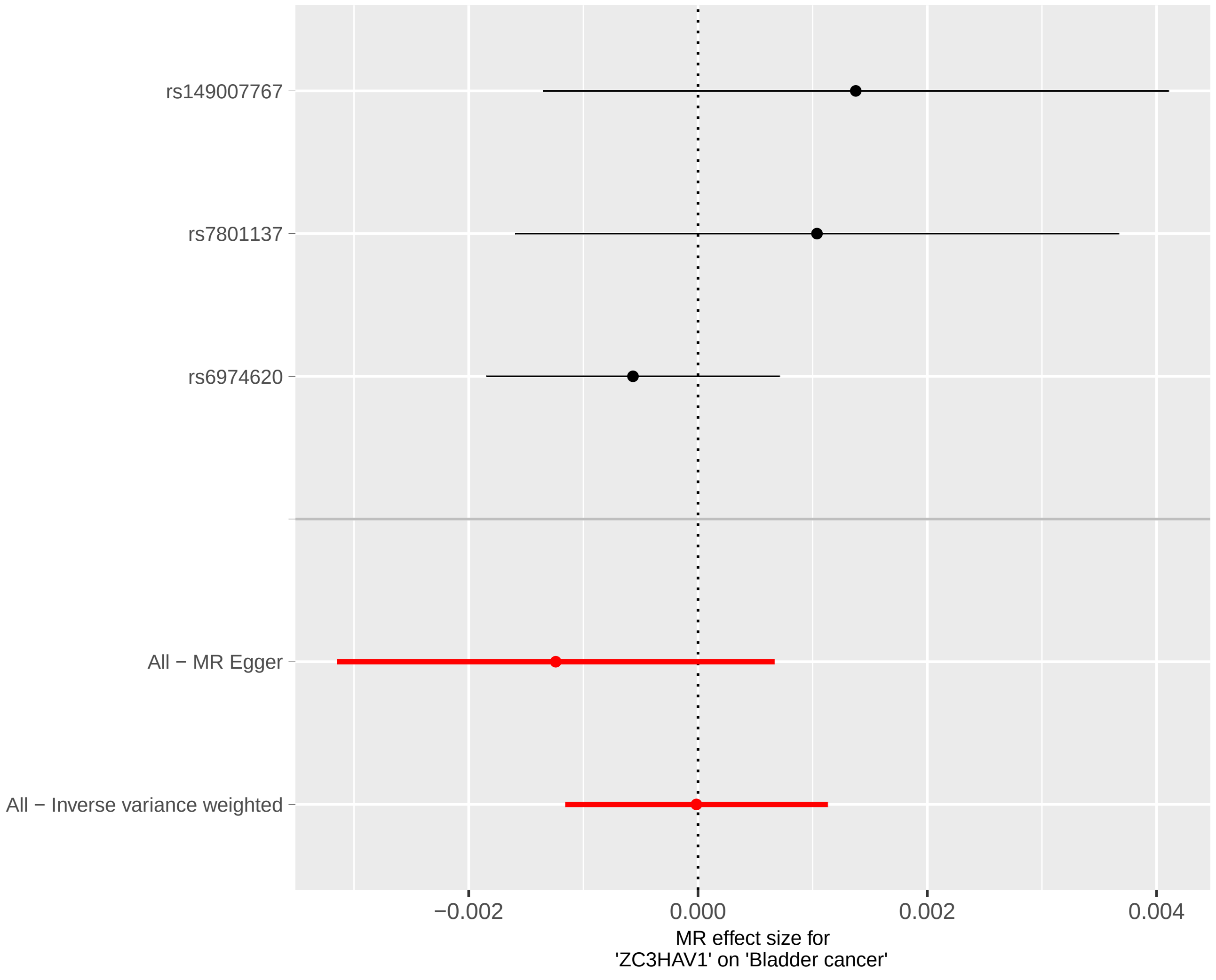

rs149007767
rs7801137
rs6974620
All − MR Egger
All − Inverse variance weighted
−0.002
0.000
0.002
0.004
MR effect size for
'ZC3HAV1' on 'Bladder cancer'

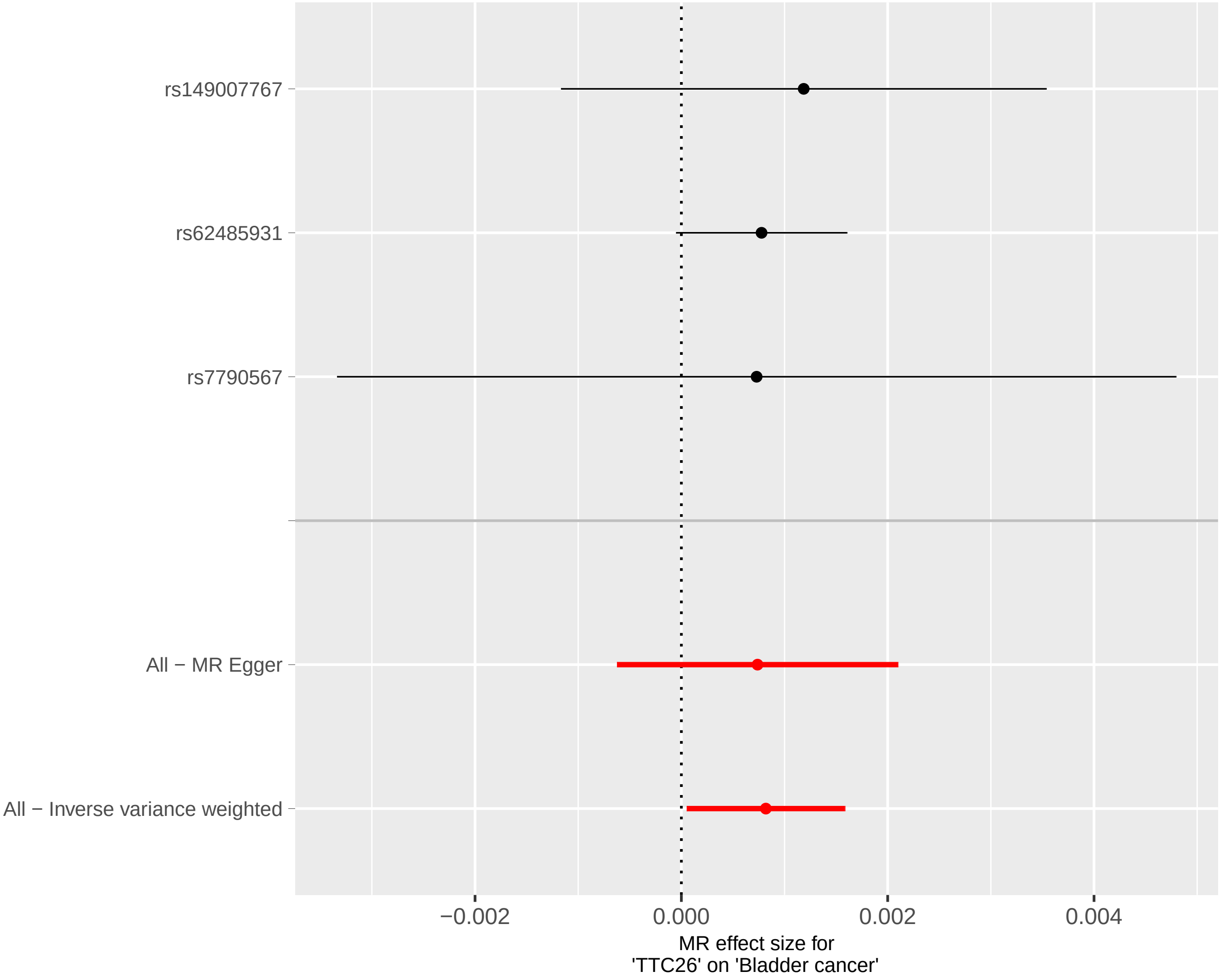
rs149007767
rs62485931
rs7790567
All − MR Egger
All − Inverse variance weighted
−0.002
0.000
0.002
0.004
MR effect size for
'TTC26' on 'Bladder cancer'

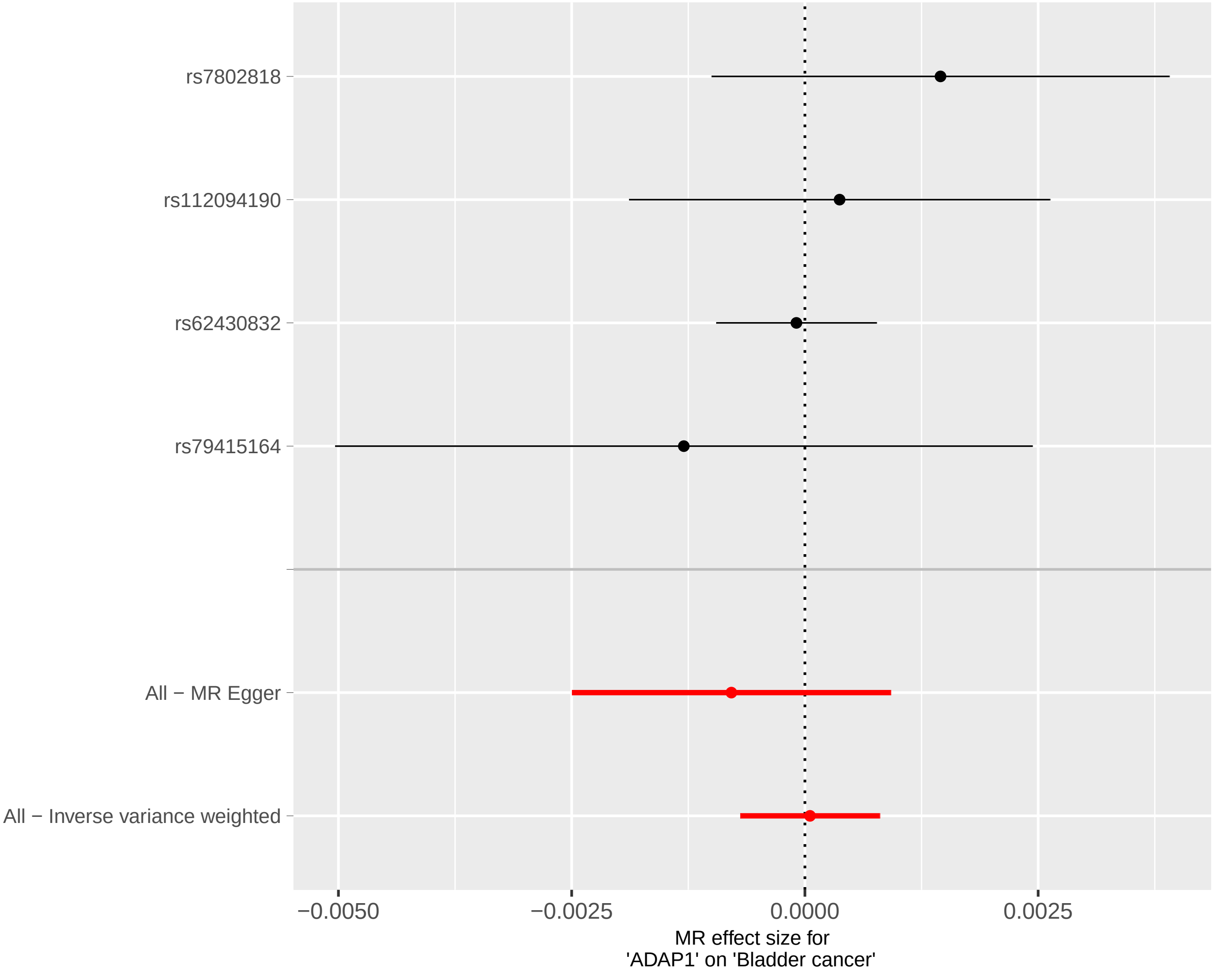
rs7802818
rs112094190
rs62430832
rs79415164
All − MR Egger
All − Inverse variance weighted
−0.0050
−0.0025
0.0000
0.0025
MR effect size for
'ADAP1' on 'Bladder cancer'

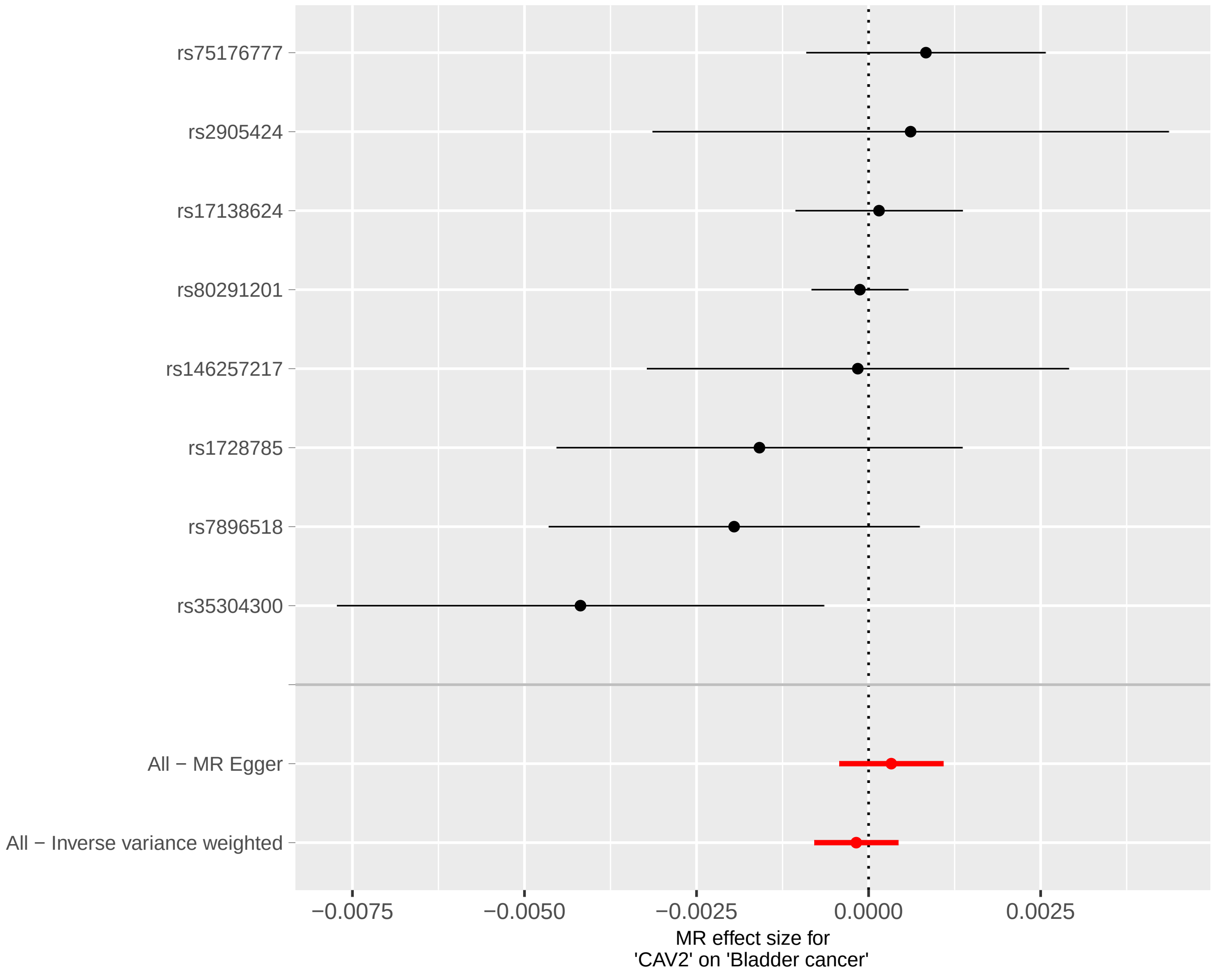

rs75176777
rs2905424
rs17138624
rs80291201
rs146257217
rs1728785
rs7896518
rs35304300
All − MR Egger
All − Inverse variance weighted
−0.0075
−0.0050
−0.0025
0.0000
0.0025
MR effect size for
'CAV2' on 'Bladder cancer'

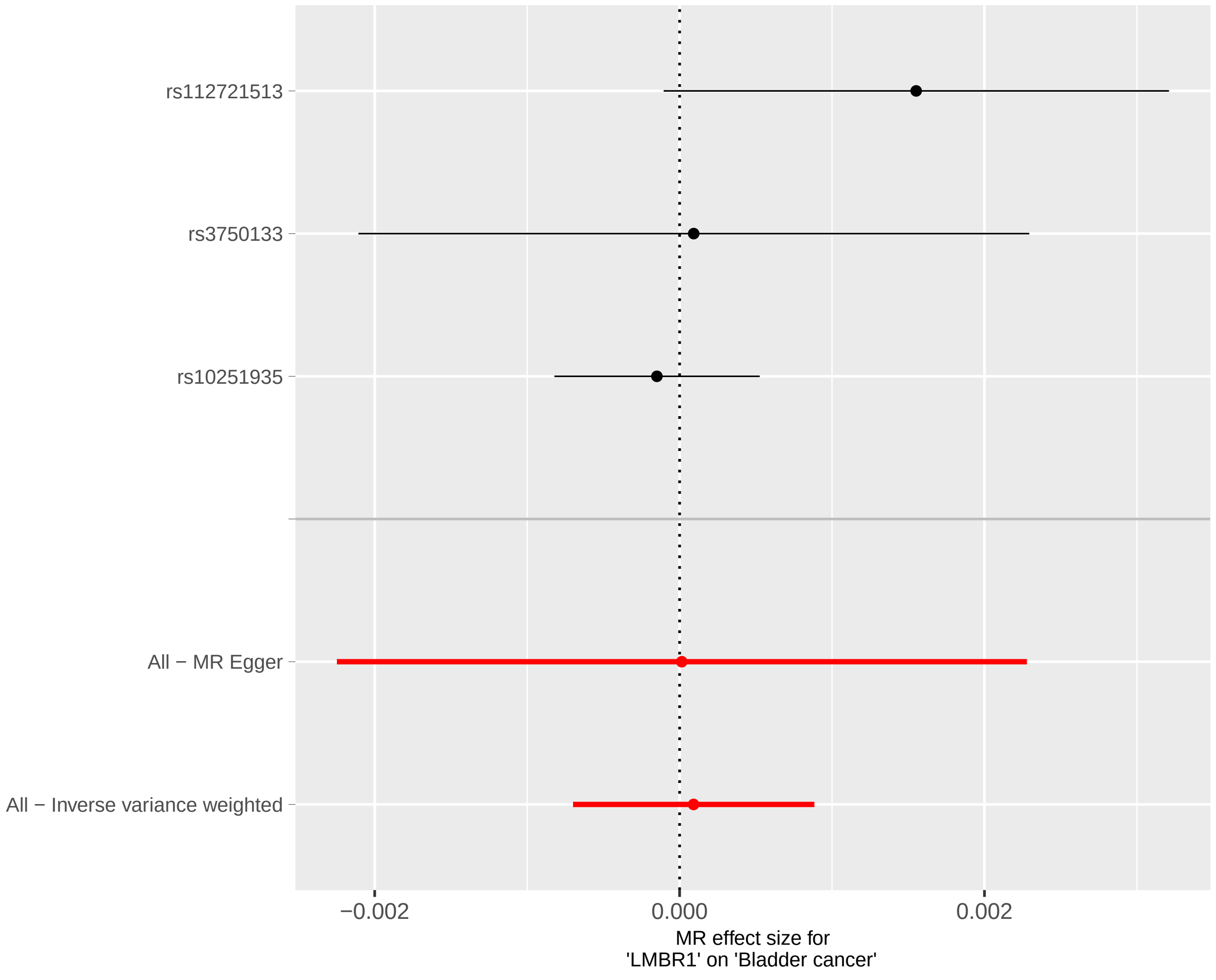

rs112721513
rs3750133
rs10251935
All − MR Egger
All − Inverse variance weighted
−0.002
0.000
0.002
MR effect size for
'LMBR1' on 'Bladder cancer'

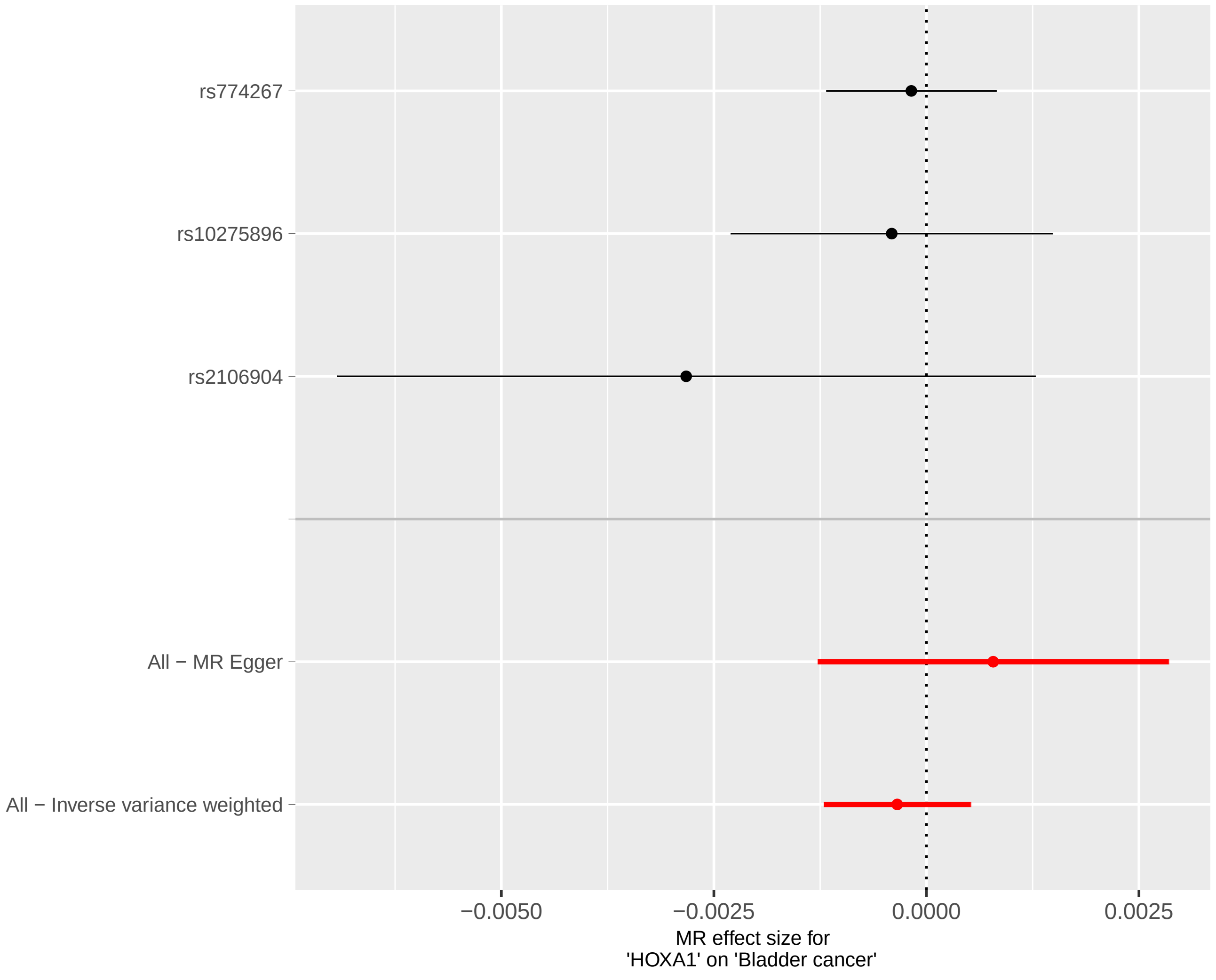
rs774267
rs10275896
rs2106904
All − MR Egger
All − Inverse variance weighted
−0.0050
−0.0025
0.0000
0.0025
MR effect size for
'HOXA1' on 'Bladder cancer'

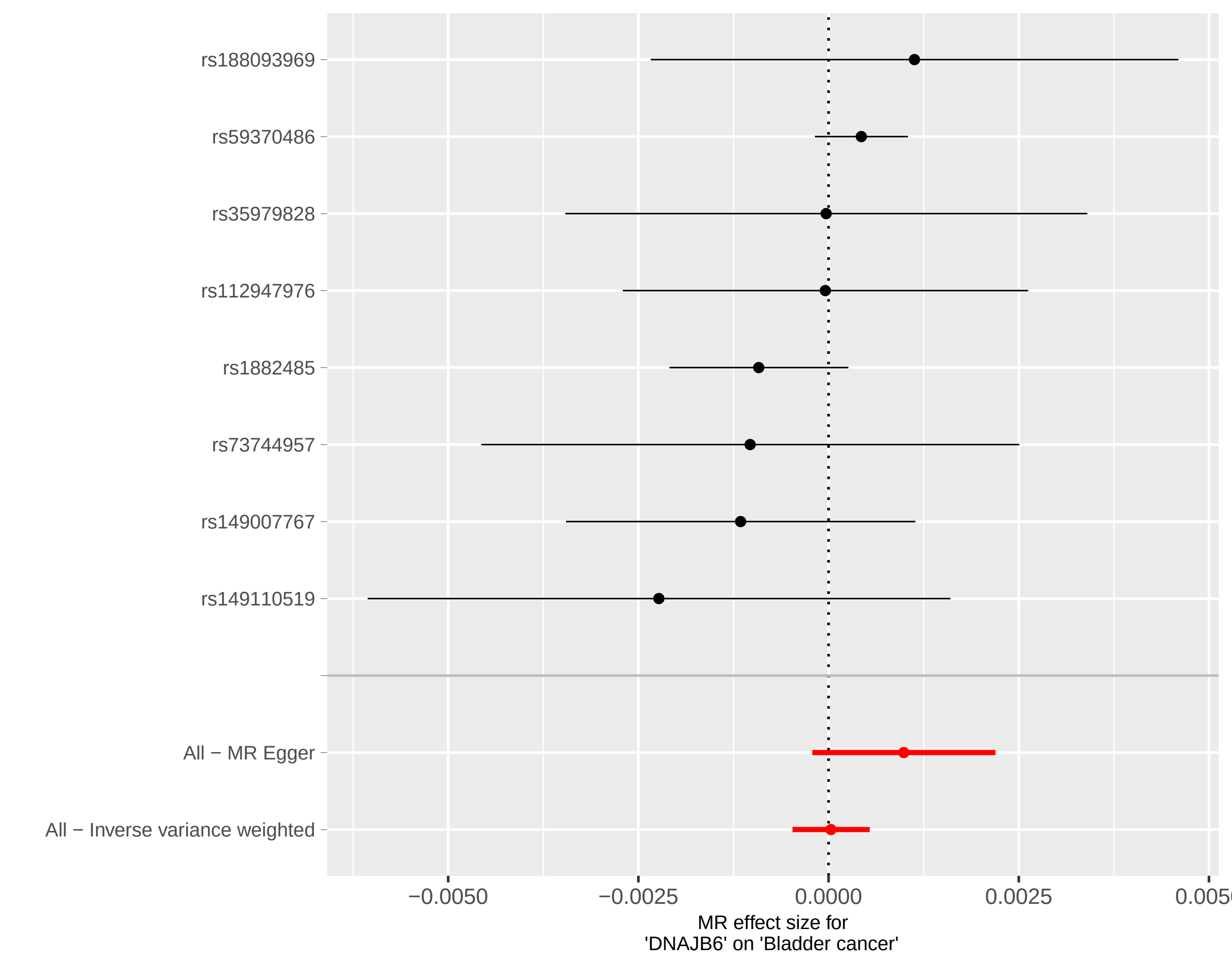

rs188093969
rs59370486
rs35979828
rs112947976
rs1882485
rs73744957
rs149007767
rs149110519
All − MR Egger
All − Inverse variance weighted
−0.0050
−0.0025
0.0000
0.0025
0.005
MR effect size for
'DNAJB6' on 'Bladder cancer'

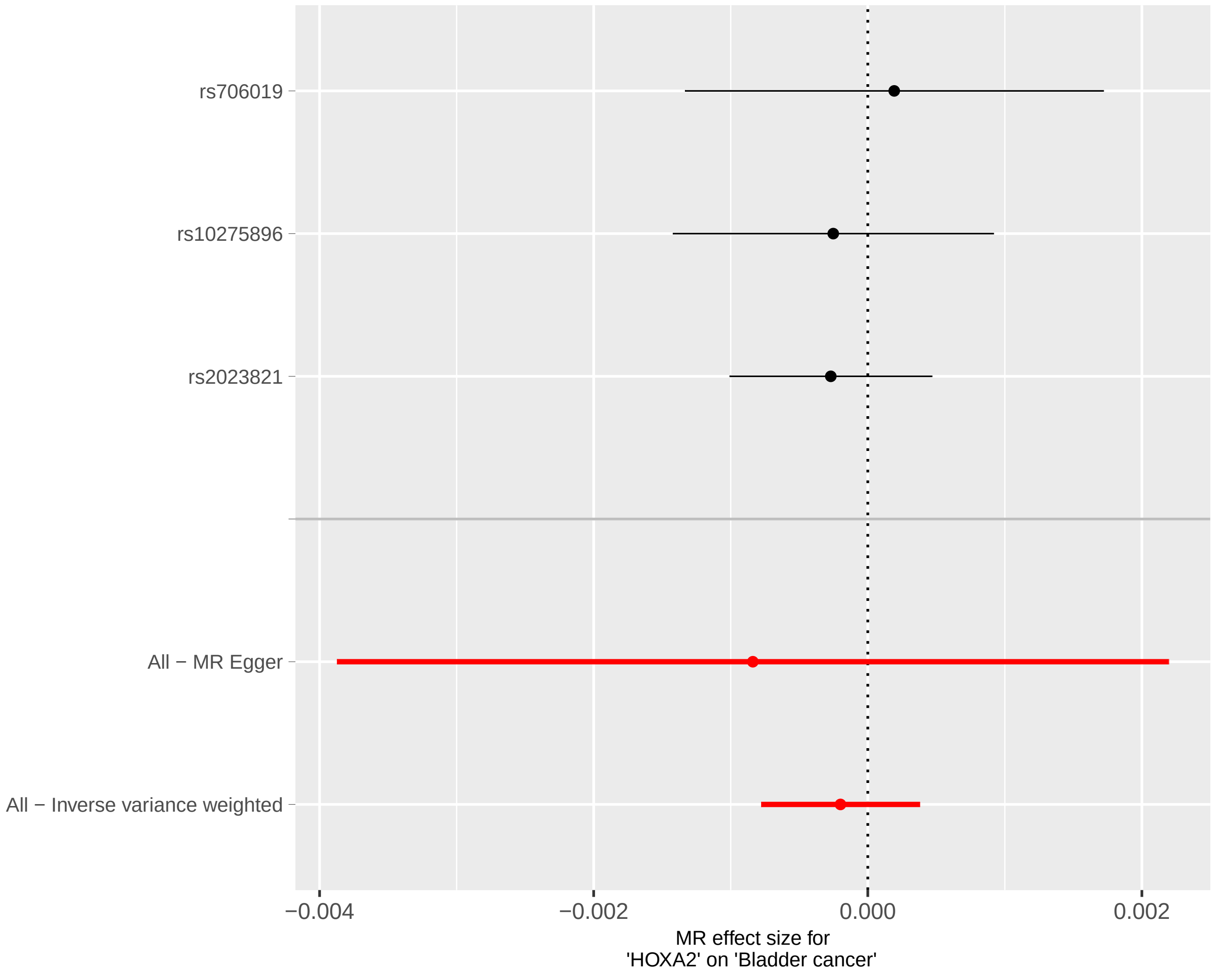
rs706019
rs10275896
rs2023821
All − MR Egger
All − Inverse variance weighted
−0.004
−0.002
0.000
0.002
MR effect size for
'HOXA2' on 'Bladder cancer'

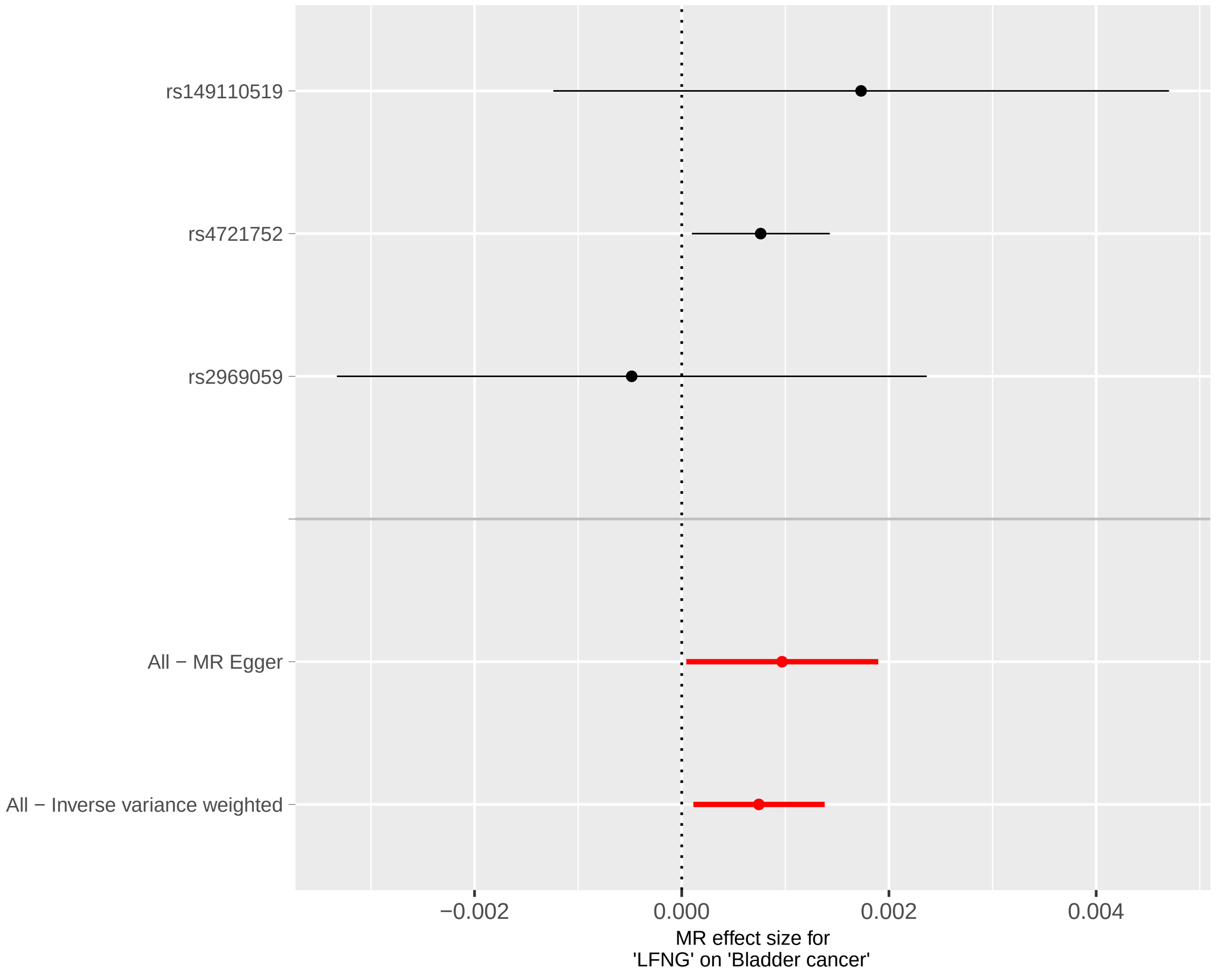

rs149110519
rs4721752
rs2969059
All − MR Egger
All − Inverse variance weighted
−0.002
0.000
0.002
0.004
MR effect size for
'LFNG' on 'Bladder cancer'

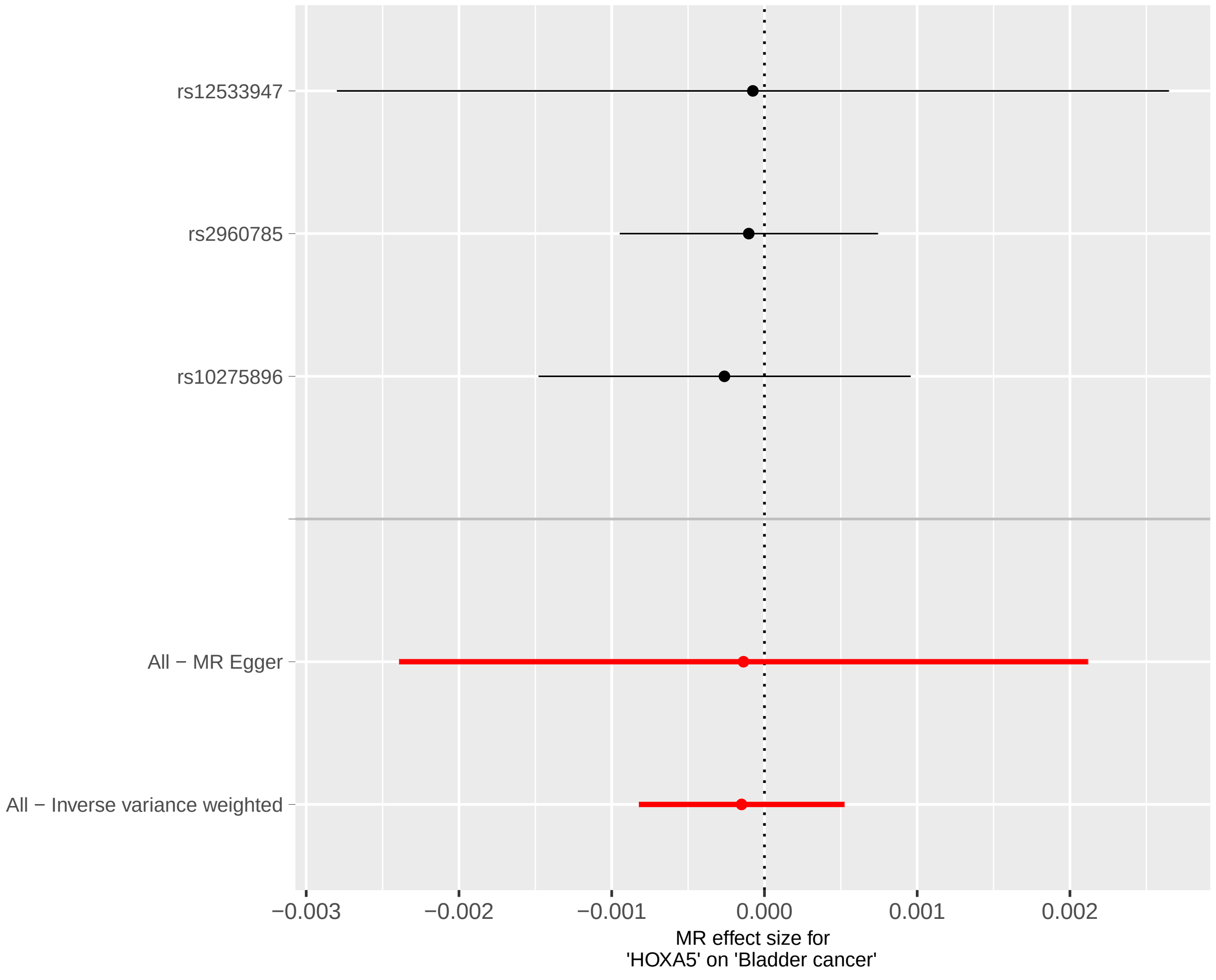
rs12533947
rs2960785
rs10275896
All − MR Egger
All − Inverse variance weighted
−0.003
−0.002
−0.001
0.000
0.001
0.002
MR effect size for
'HOXA5' on 'Bladder cancer'

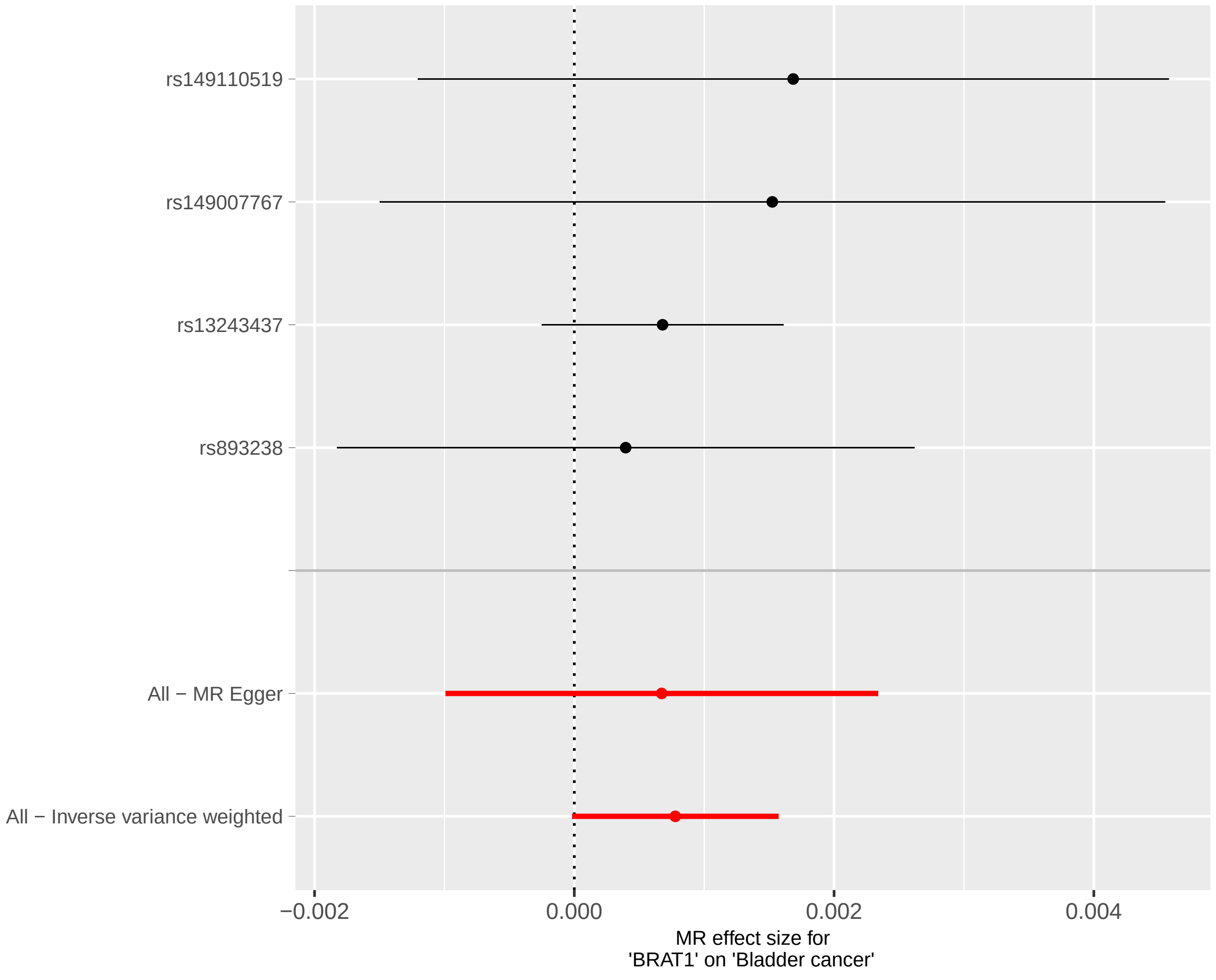
rs149110519
rs149007767
rs13243437
rs893238
All − MR Egger
All − Inverse variance weighted
−0.002
0.000
0.002
0.004
MR effect size for
'BRAT1' on 'Bladder cancer'

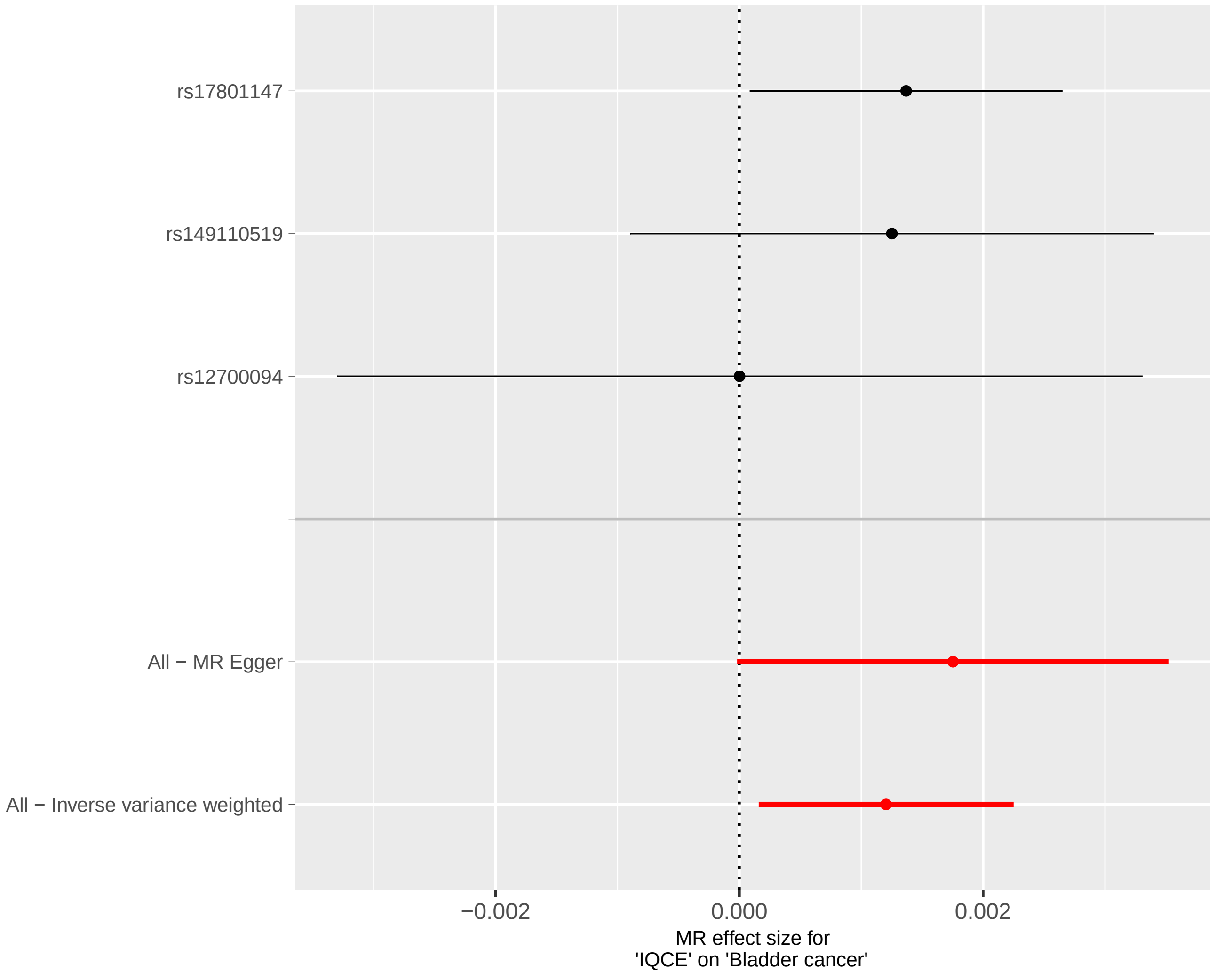

rs17801147
rs149110519
rs12700094
All − MR Egger
All − Inverse variance weighted
−0.002
0.000
0.002
MR effect size for
'IQCE' on 'Bladder cancer'

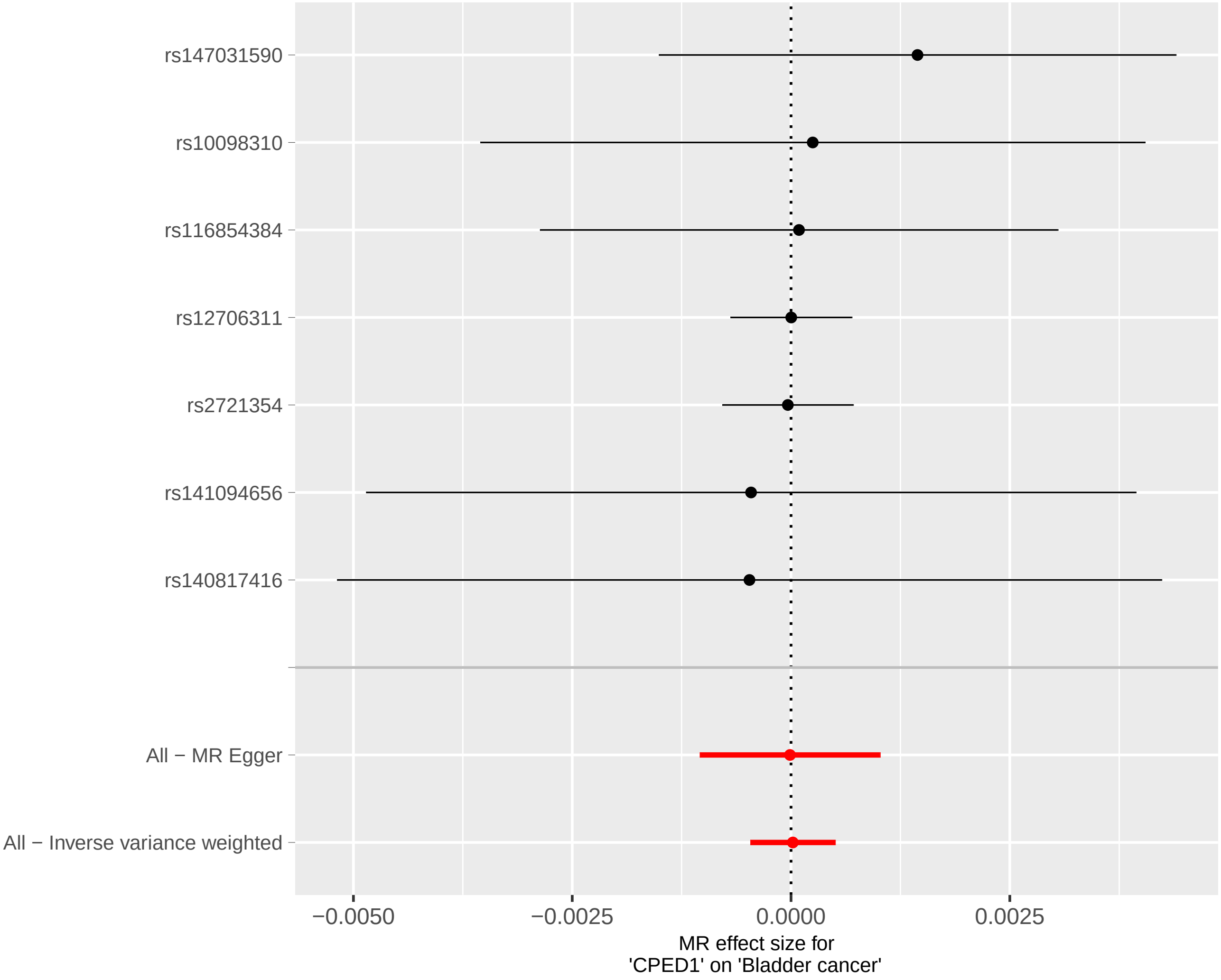

rs147031590
rs10098310
rs116854384
rs12706311
rs2721354
rs141094656
rs140817416
All − MR Egger
All − Inverse variance weighted
−0.0050
−0.0025
0.0000
0.0025
MR effect size for
'CPED1' on 'Bladder cancer'

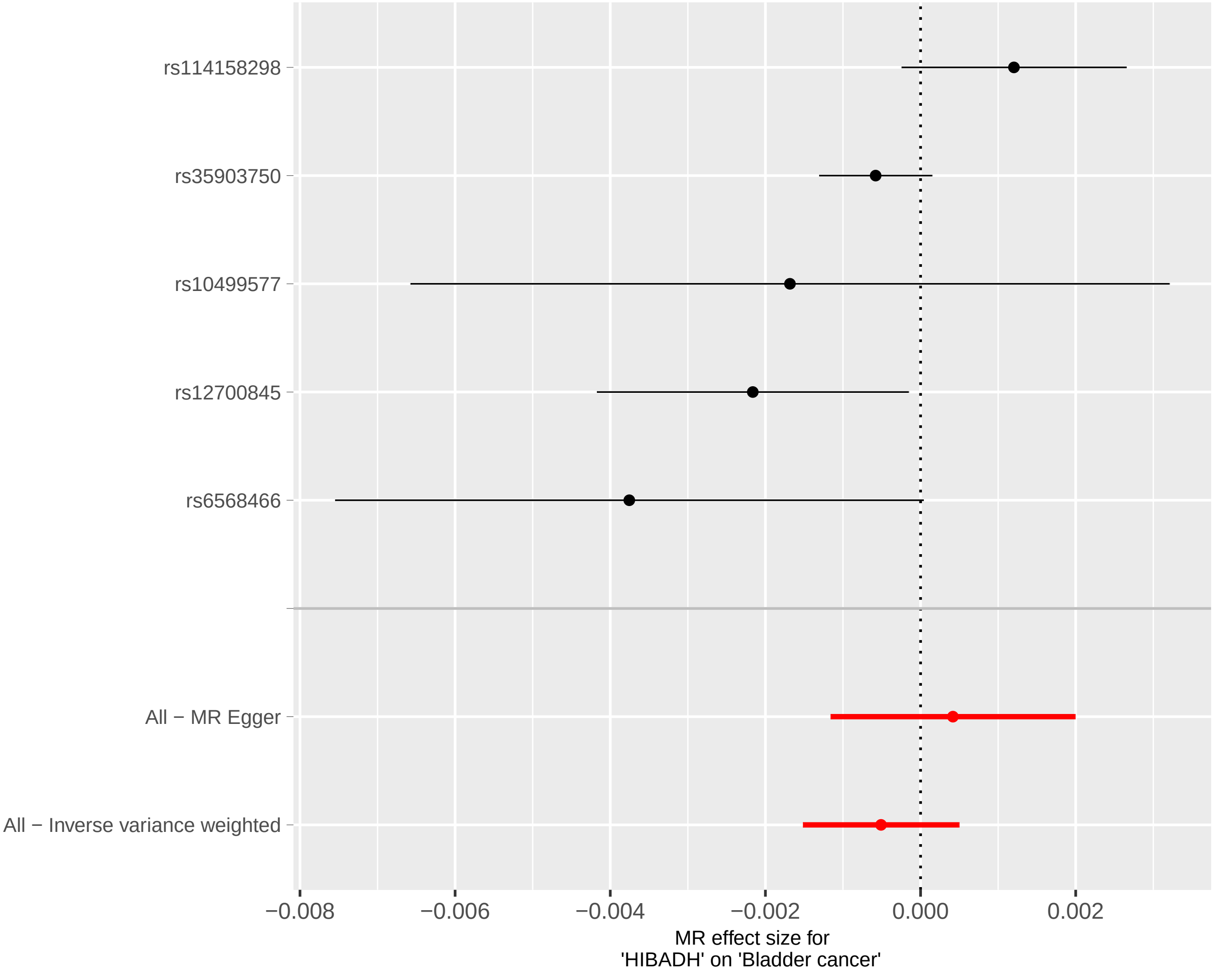
rs114158298
rs35903750
rs10499577
rs12700845
rs6568466
All − MR Egger
All − Inverse variance weighted
−0.008
−0.006
−0.004
−0.002
0.000
0.002
MR effect size for
'HIBADH' on 'Bladder cancer'

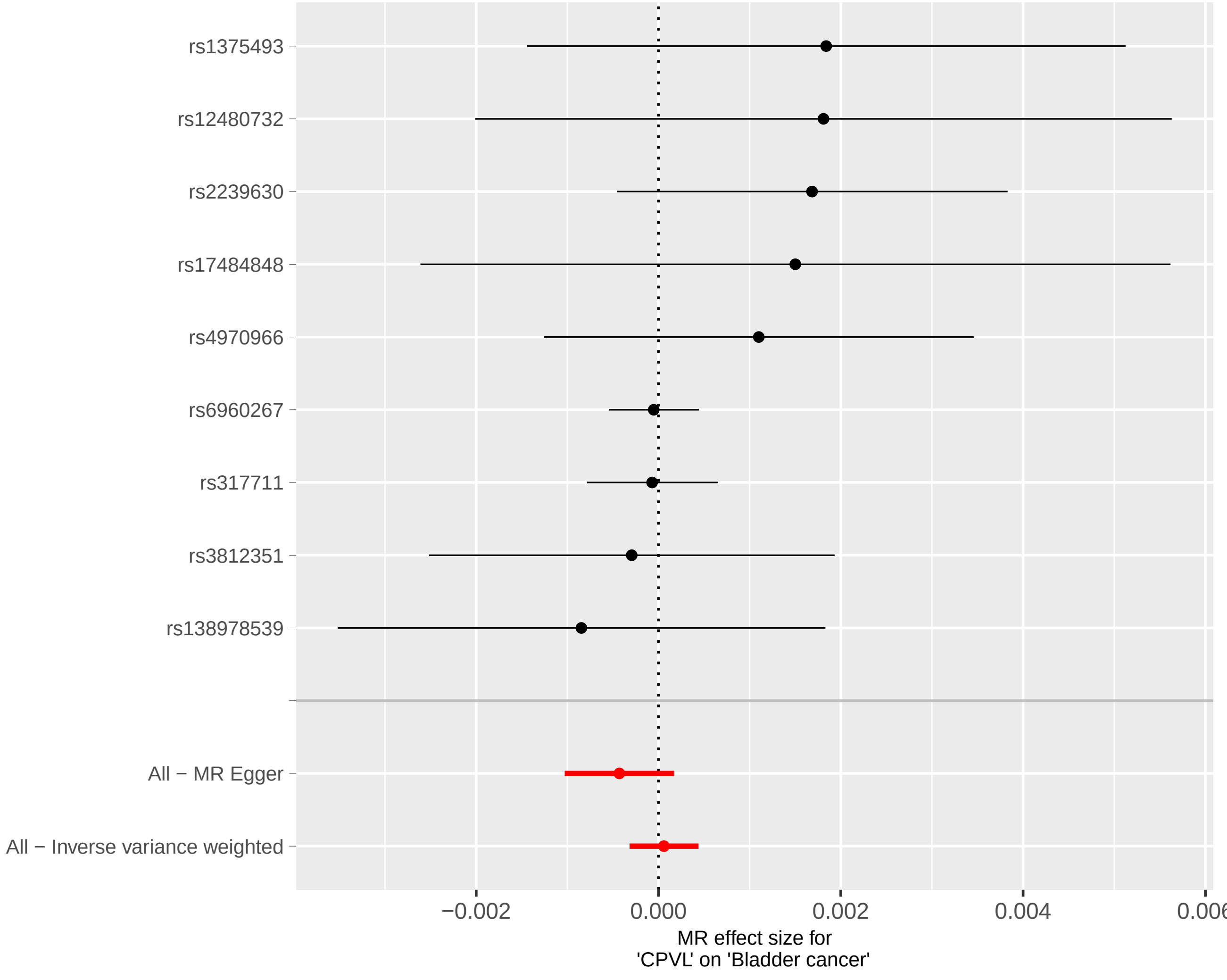

rs1375493
rs12480732
rs2239630
rs17484848
rs4970966
rs6960267
rs317711
rs3812351
rs138978539
All − MR Egger
All − Inverse variance weighted
−0.002
0.000
0.002
0.004
MR effect size for
'CPVL' on 'Bladder cancer'

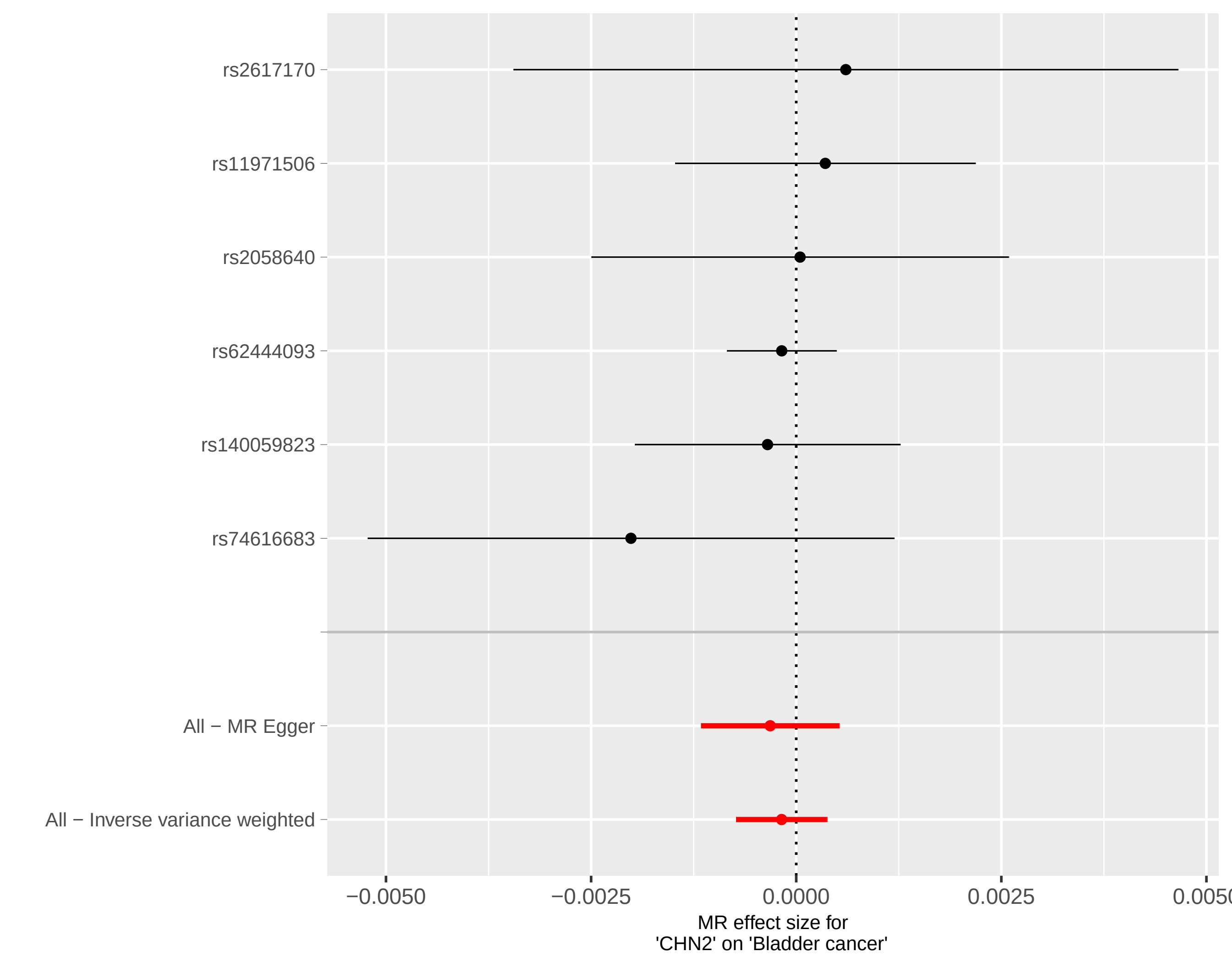

rs2617170
rs11971506
rs2058640
rs62444093
rs140059823
rs74616683
All − MR Egger
All − Inverse variance weighted
−0.0050
−0.0025
0.0000
0.0025
0.0050
MR effect size for
'CHN2' on 'Bladder cancer'
