# Supplementary material for: Integrative multi-omics and single-cell transcriptomics reveal ARHGEF12 driving chemoresistance in bladder cancer
Source: Hereditas. 2025 Nov 27;162:234. doi: 10.1186/s41065-025-00606-1 (PMC12661753; doi:10.1186/s41065-025-00606-1)
Supplement: Supplementary file 1 — Supplementary Material 1. [file 41065_2025_606_MOESM1_ESM.zip › Supplementary1/Supplementary - MR/eQTL-MR/MR/pic.funnel_plot.pdf]

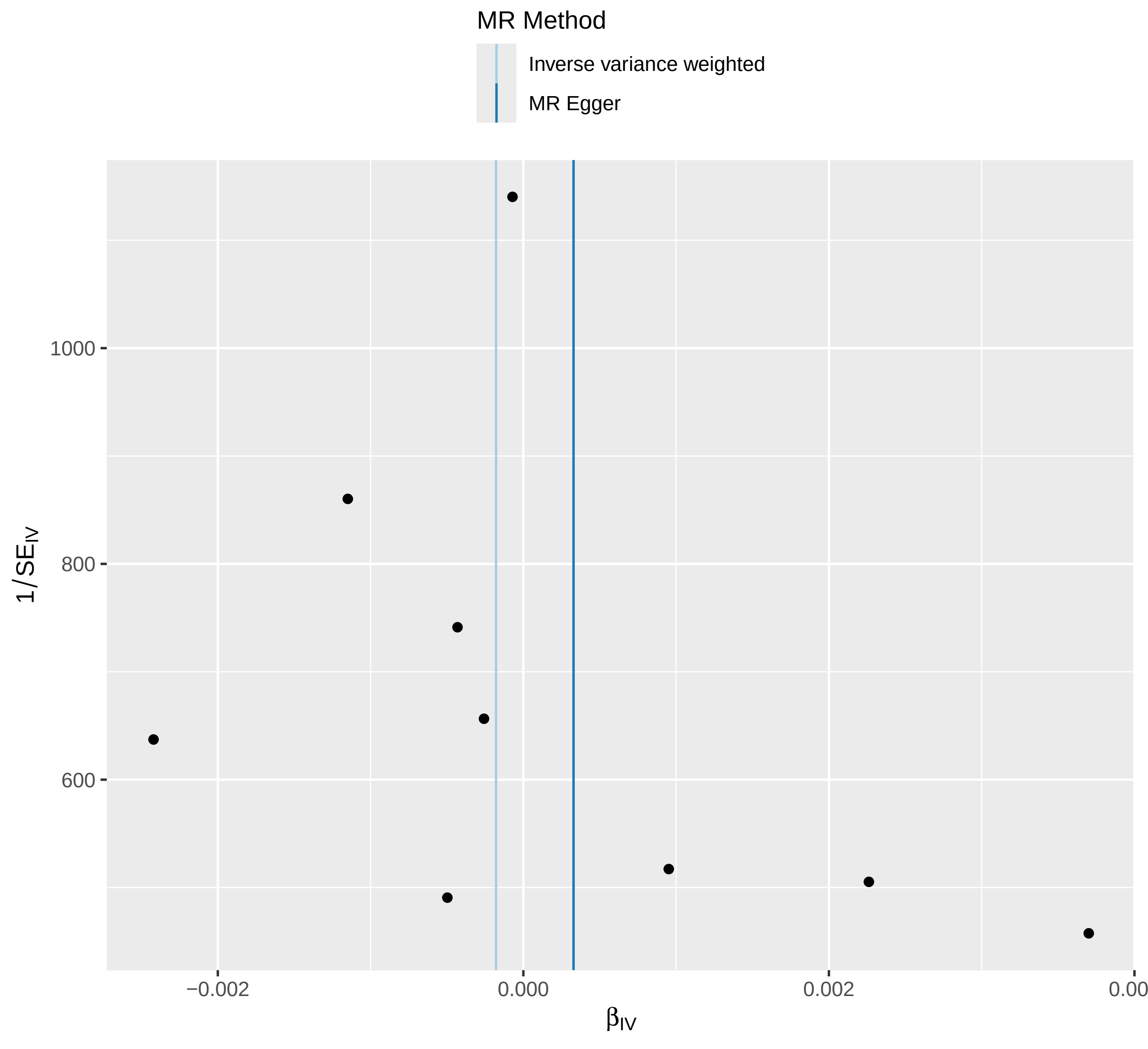
MR Method
Inverse variance weighted
MR Egger
1000
800
600
1/SE$_{IV}$
−0.002
0.000
0.002
0.00
β$_{IV}$

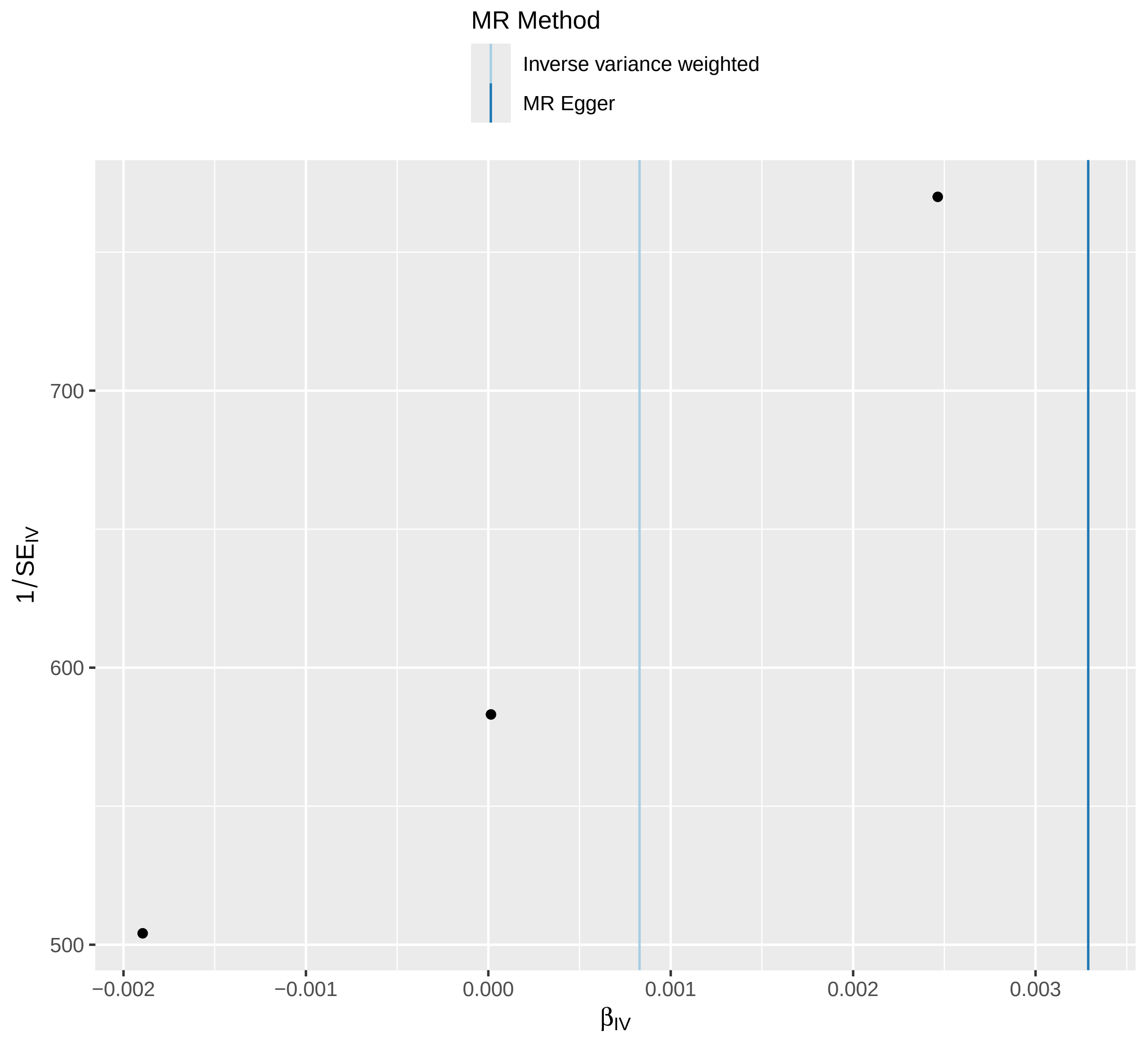
MR Method
Inverse variance weighted
MR Egger
700
600
500
1/SE_IV
−0.002
−0.001
0.000
0.001
0.002
0.003
β_IV

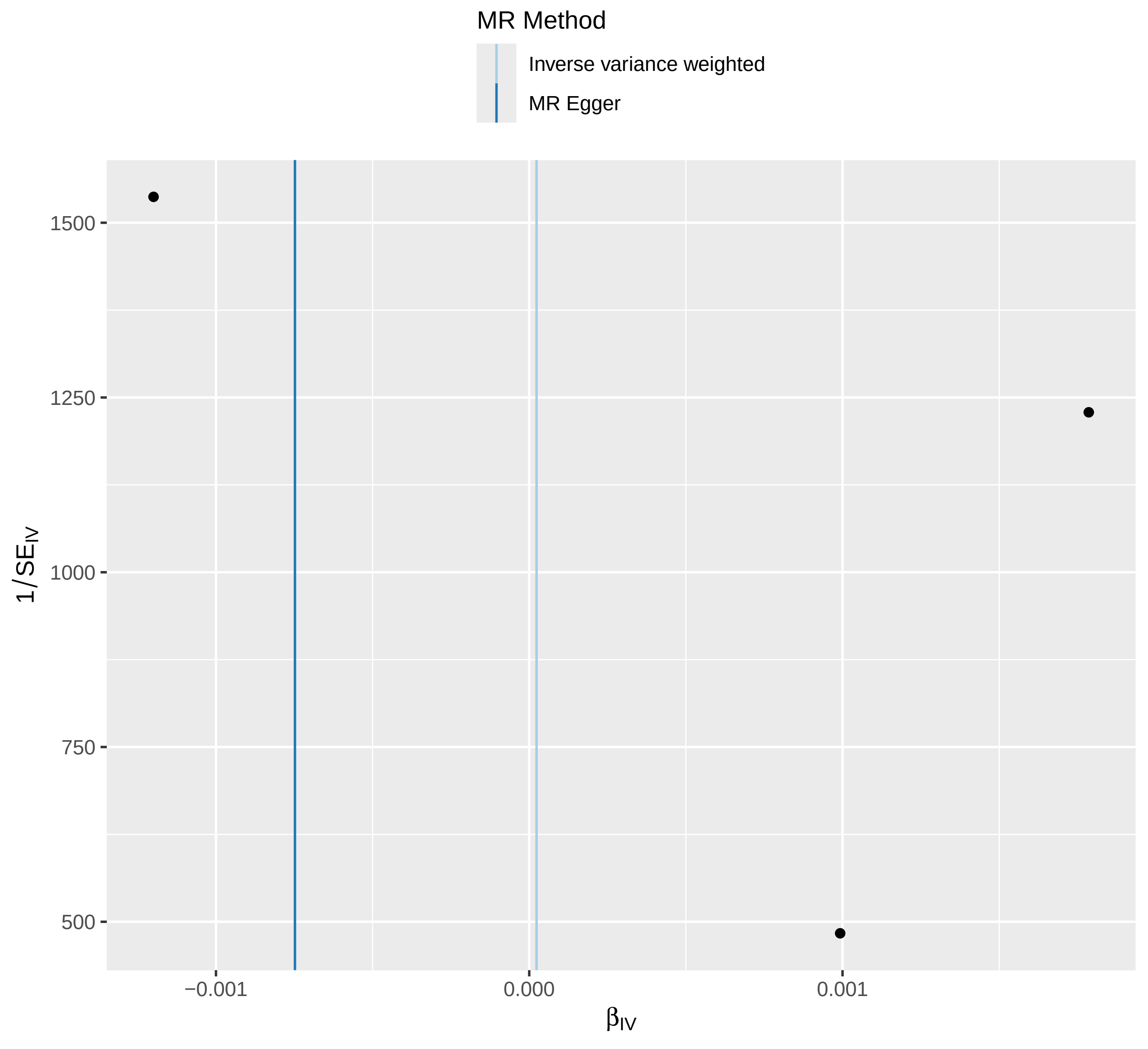
MR Method
Inverse variance weighted
MR Egger
1500
1250
1000
750
500
$1/SE_{IV}$
−0.001
0.000
0.001
β_{IV}

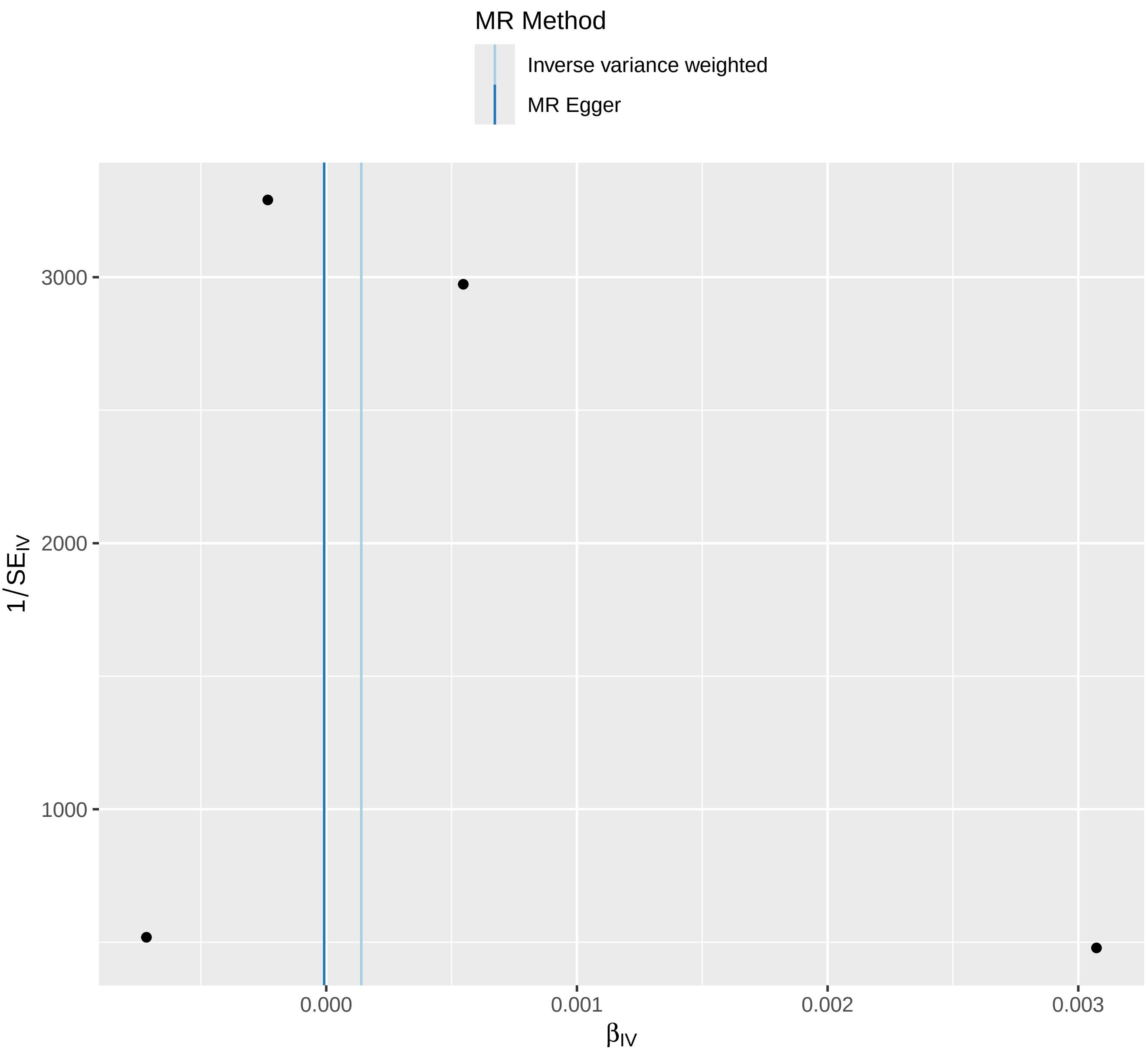

MR Method
Inverse variance weighted
MR Egger
3000
2000
1000
1/SE_IV
0.000
0.001
0.002
0.003
β_IV

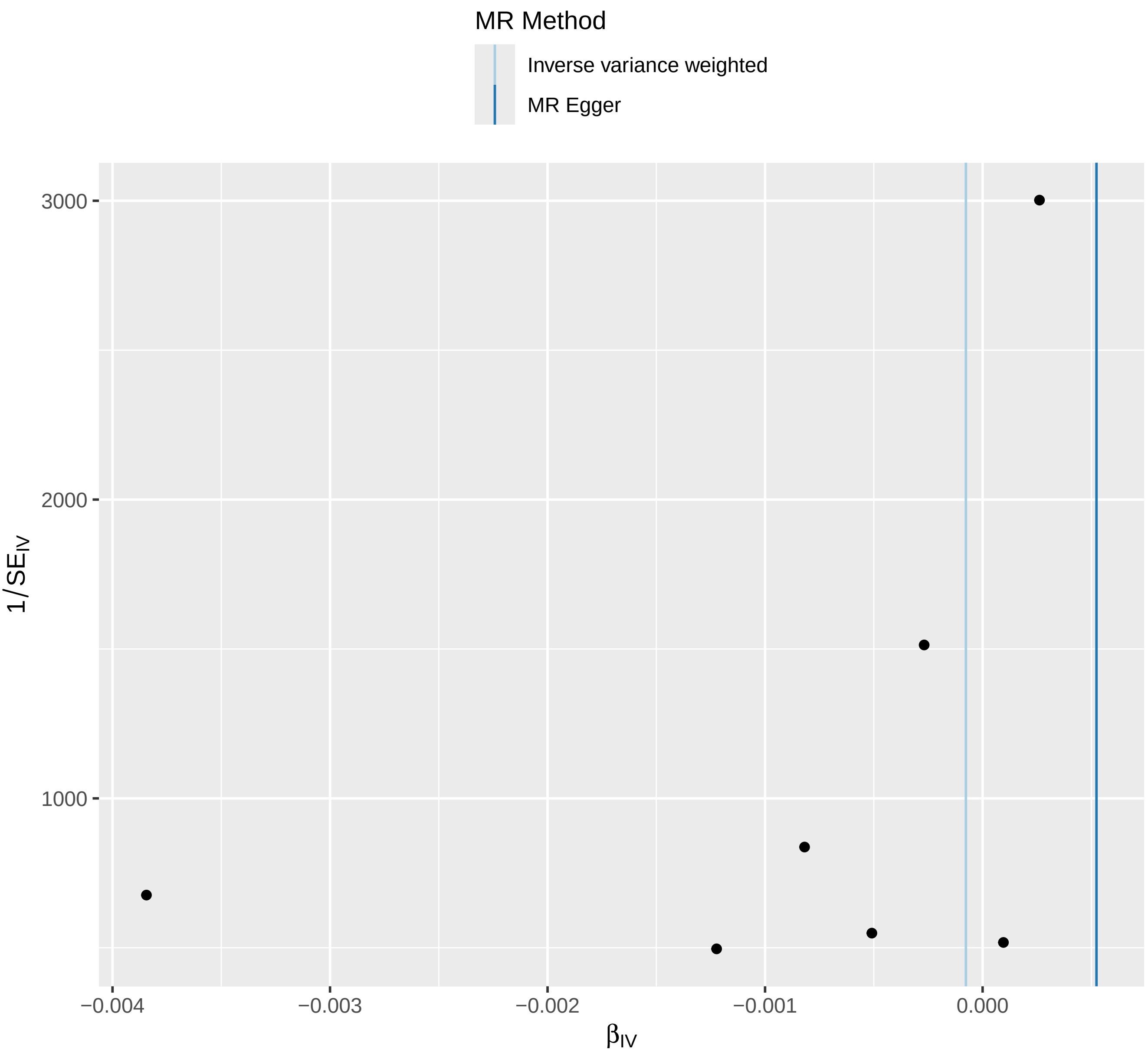
MR Method
Inverse variance weighted
MR Egger
3000
2000
1000
1/SE_IV
−0.004
−0.003
−0.002
−0.001
0.000
β_IV

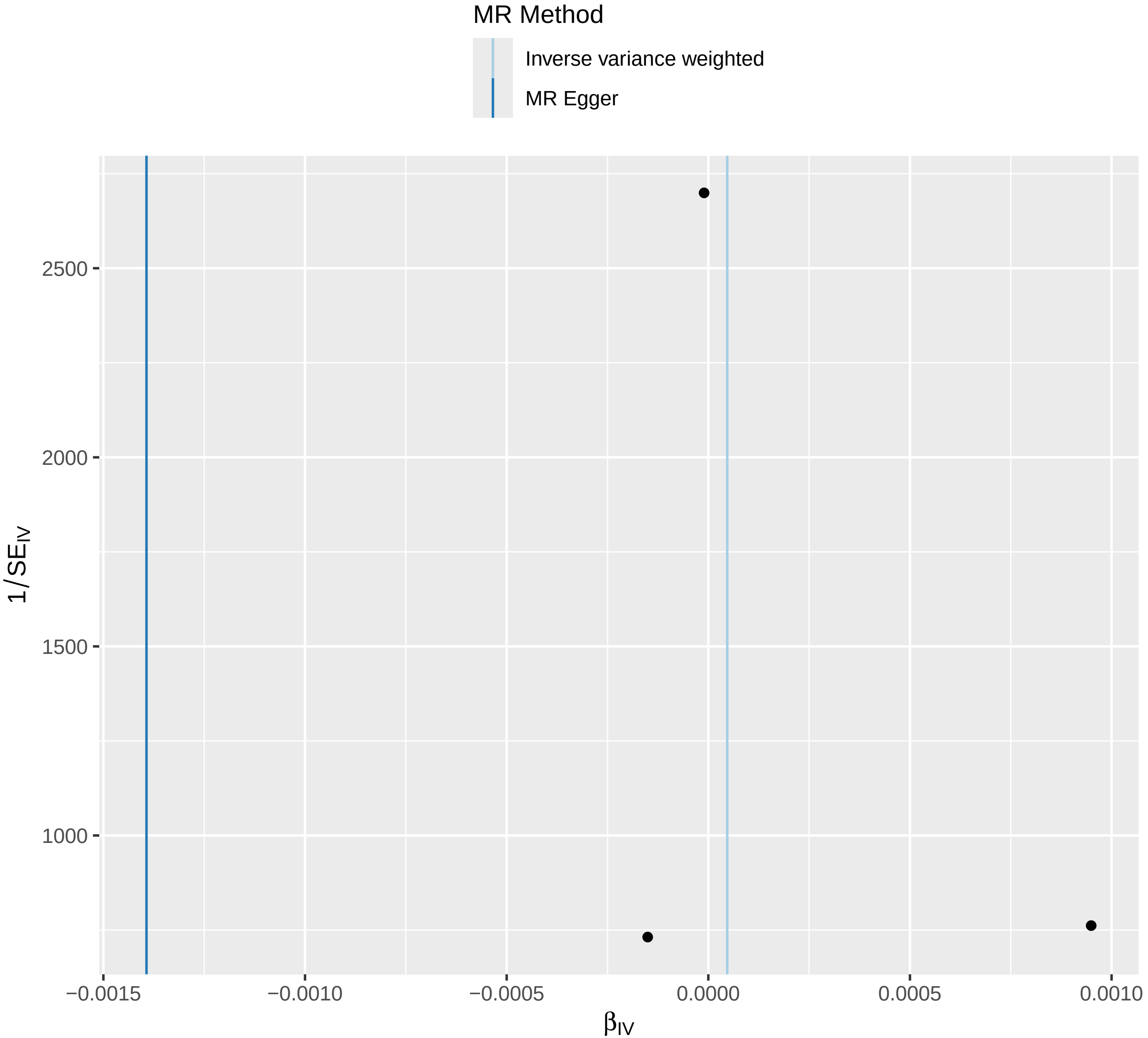
MR Method
Inverse variance weighted
MR Egger
2500
2000
1500
1000
1/SE_IV
−0.0015
−0.0010
−0.0005
0.0000
0.0005
0.0010
β_IV

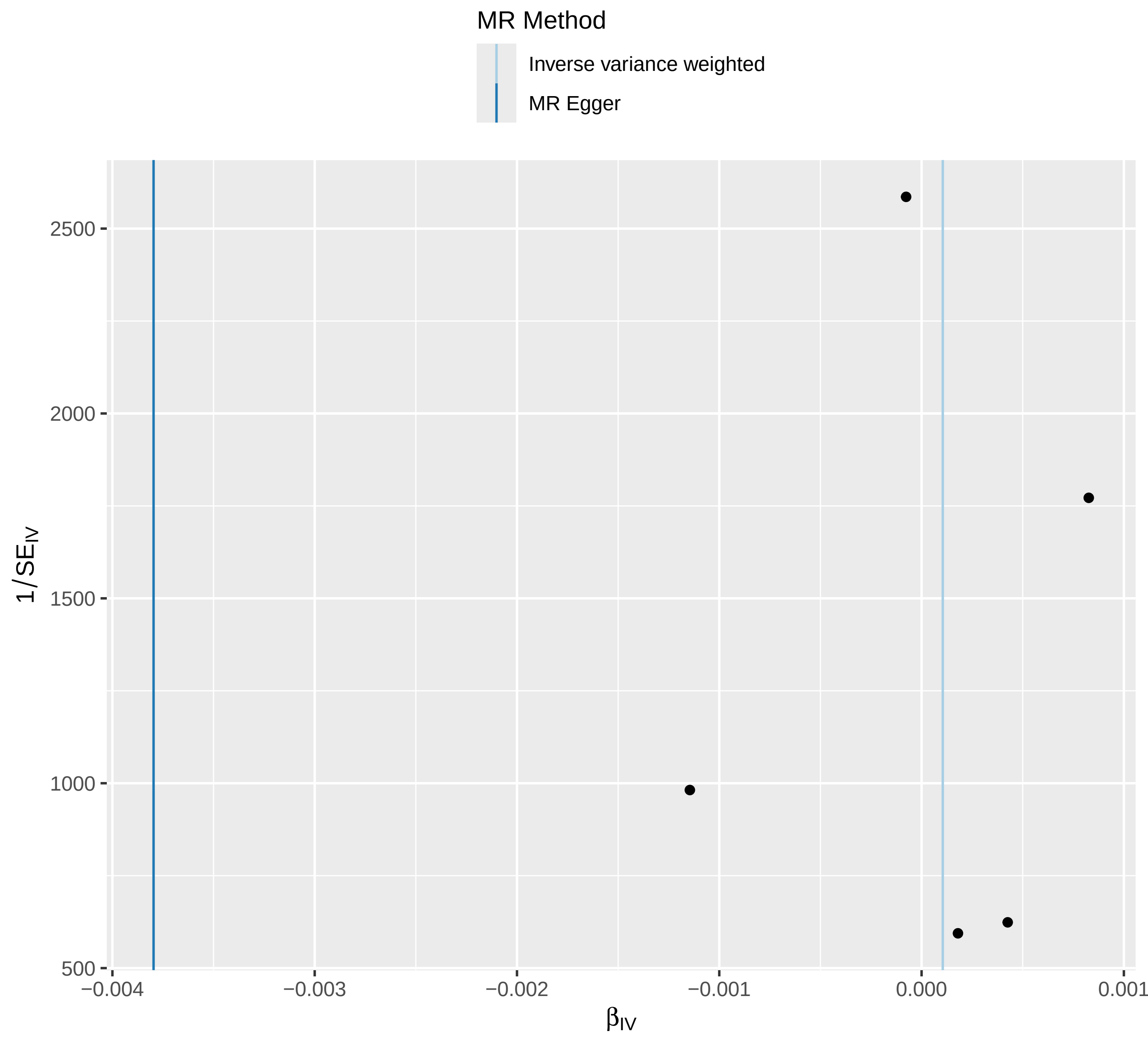
MR Method
Inverse variance weighted
MR Egger
2500
2000
1500
1000
500
1/SE_IV
−0.004
−0.003
−0.002
−0.001
0.000
0.001
β_IV

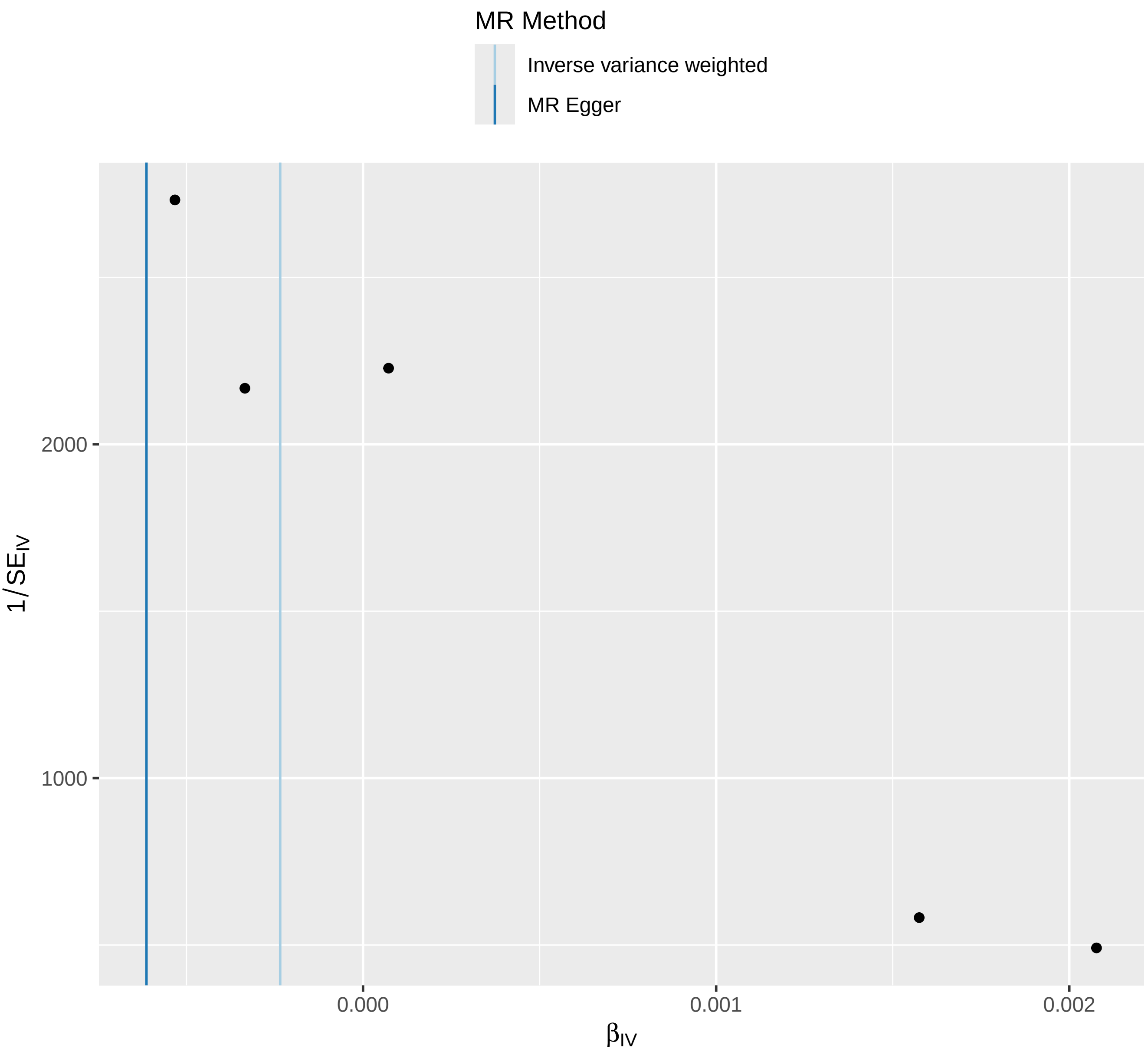

MR Method
Inverse variance weighted
MR Egger
2000
1000
1/SE_IV
0.000
0.001
0.002
β_IV

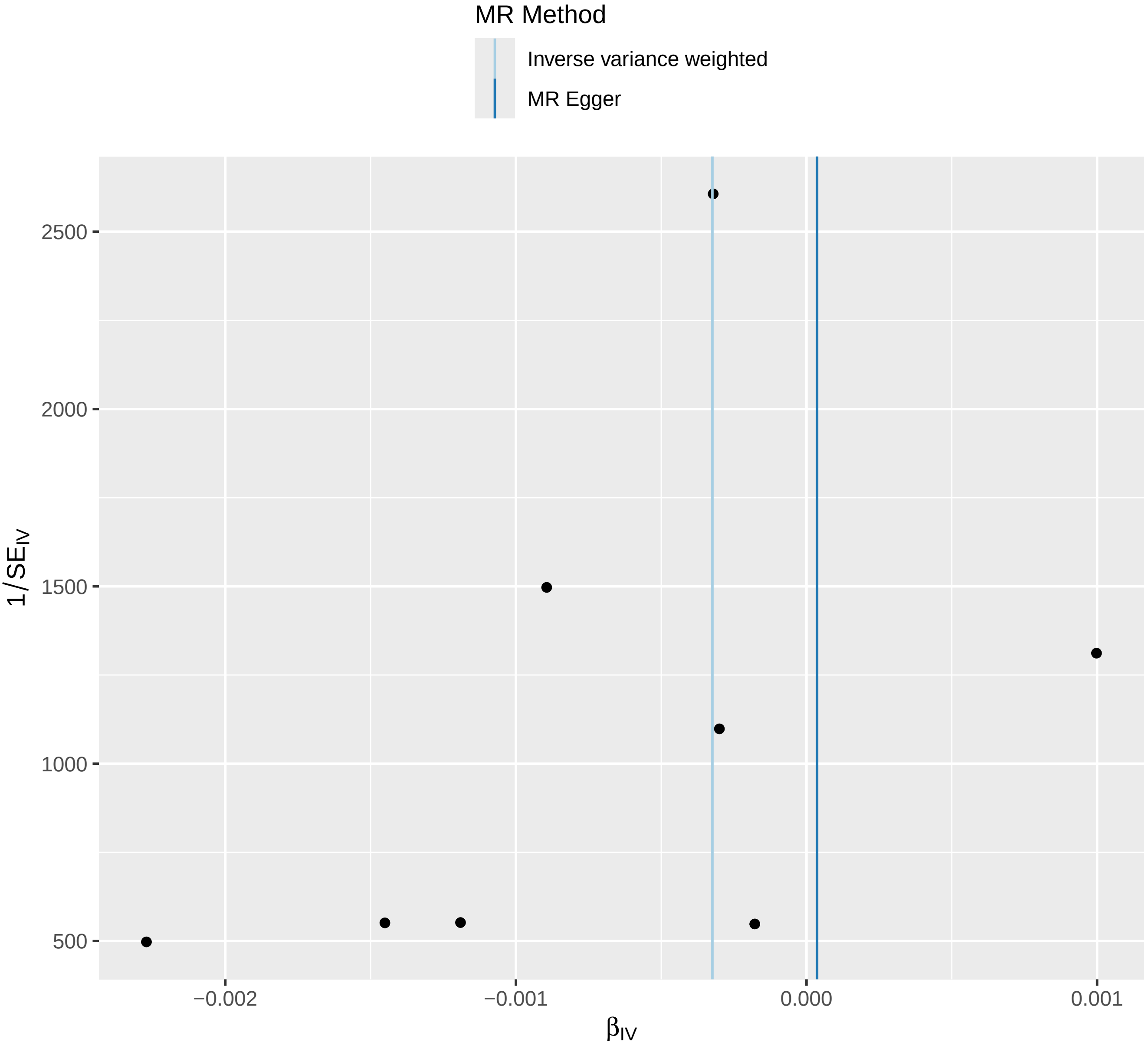
MR Method
Inverse variance weighted
MR Egger
2500
2000
1500
1000
500
1/SE_IV
−0.002
−0.001
0.000
0.001
β_IV

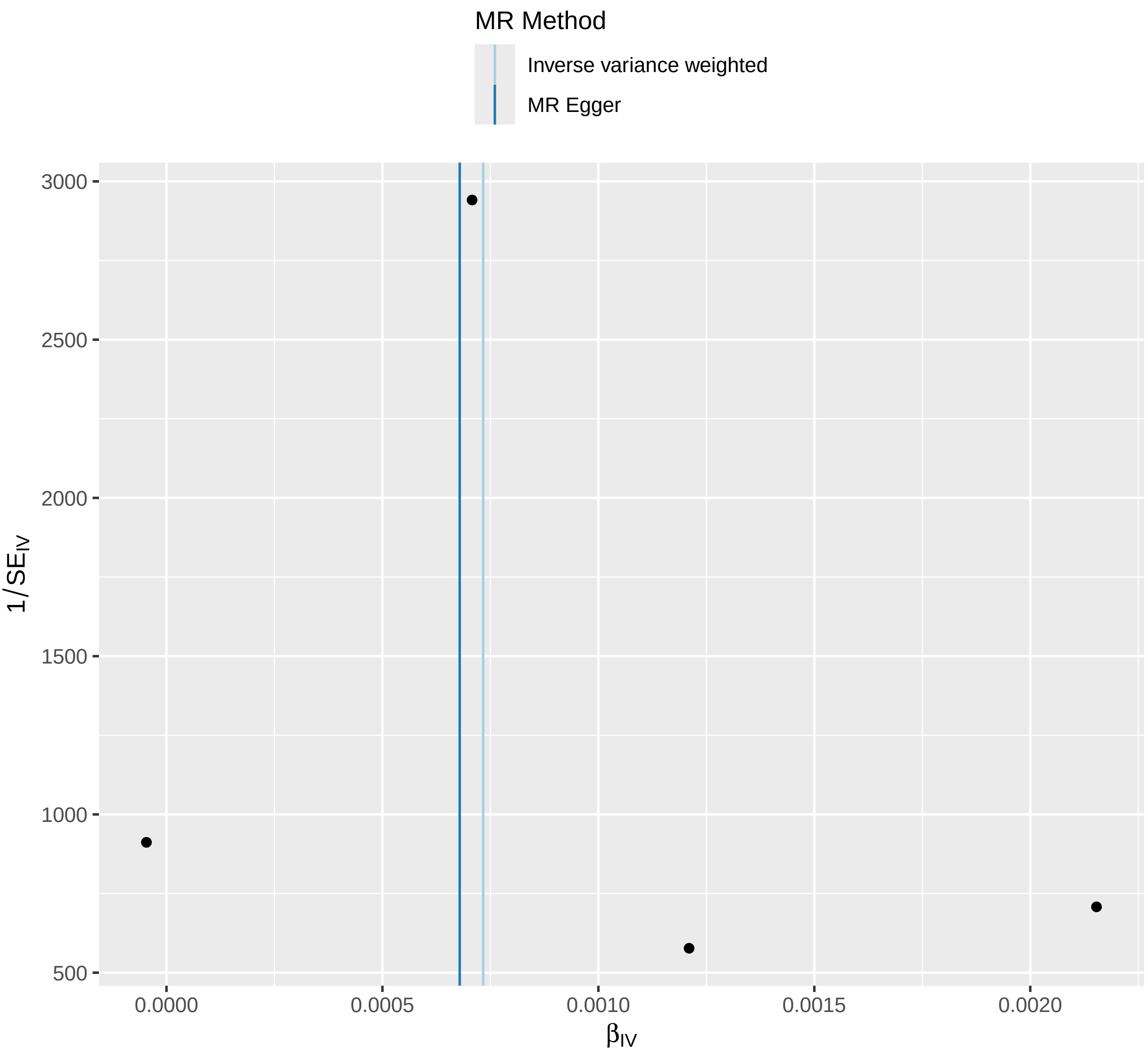
MR Method
Inverse variance weighted
MR Egger
1/SE_IV
3000
2500
2000
1500
1000
500
0.0000
0.0005
0.0010
0.0015
0.0020
β_IV

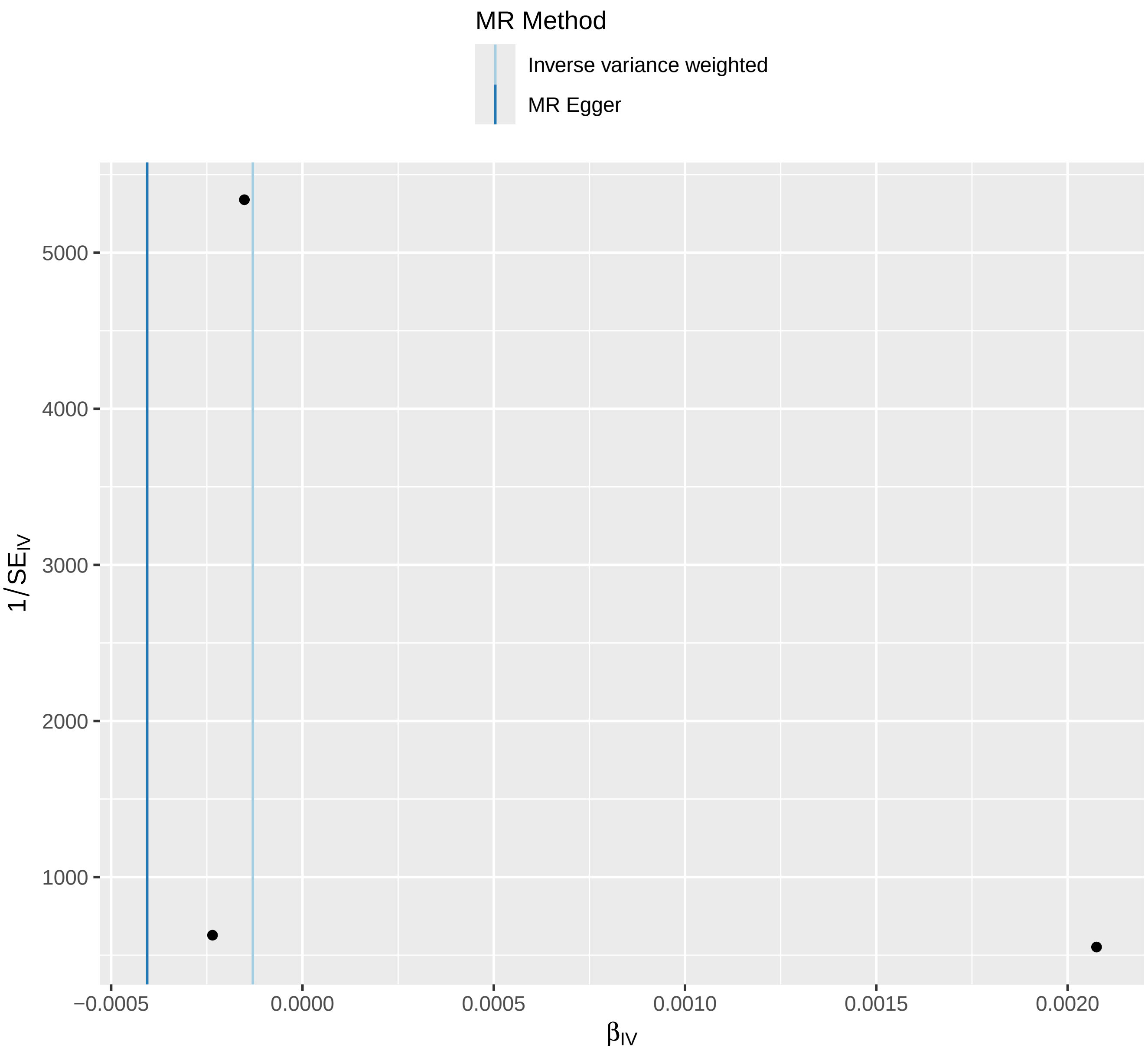

MR Method
Inverse variance weighted
MR Egger
5000
4000
3000
2000
1000
1/SE$_{IV}$
−0.0005
0.0000
0.0005
0.0010
0.0015
0.0020
β$_{IV}$

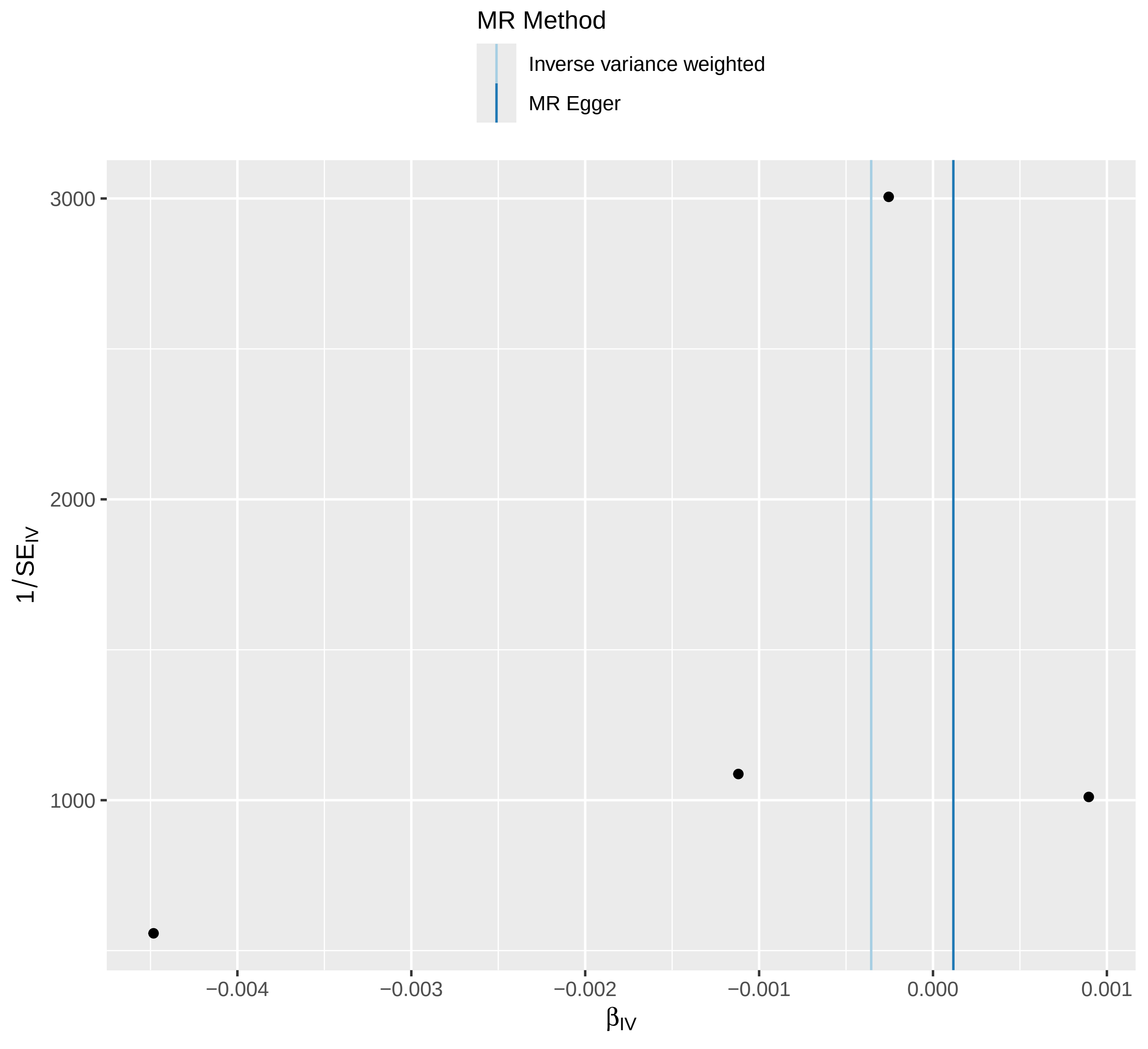

MR Method
Inverse variance weighted
MR Egger
3000
2000
1000
$1/SE_{IV}$
−0.004
−0.003
−0.002
−0.001
0.000
0.001
β_{IV}

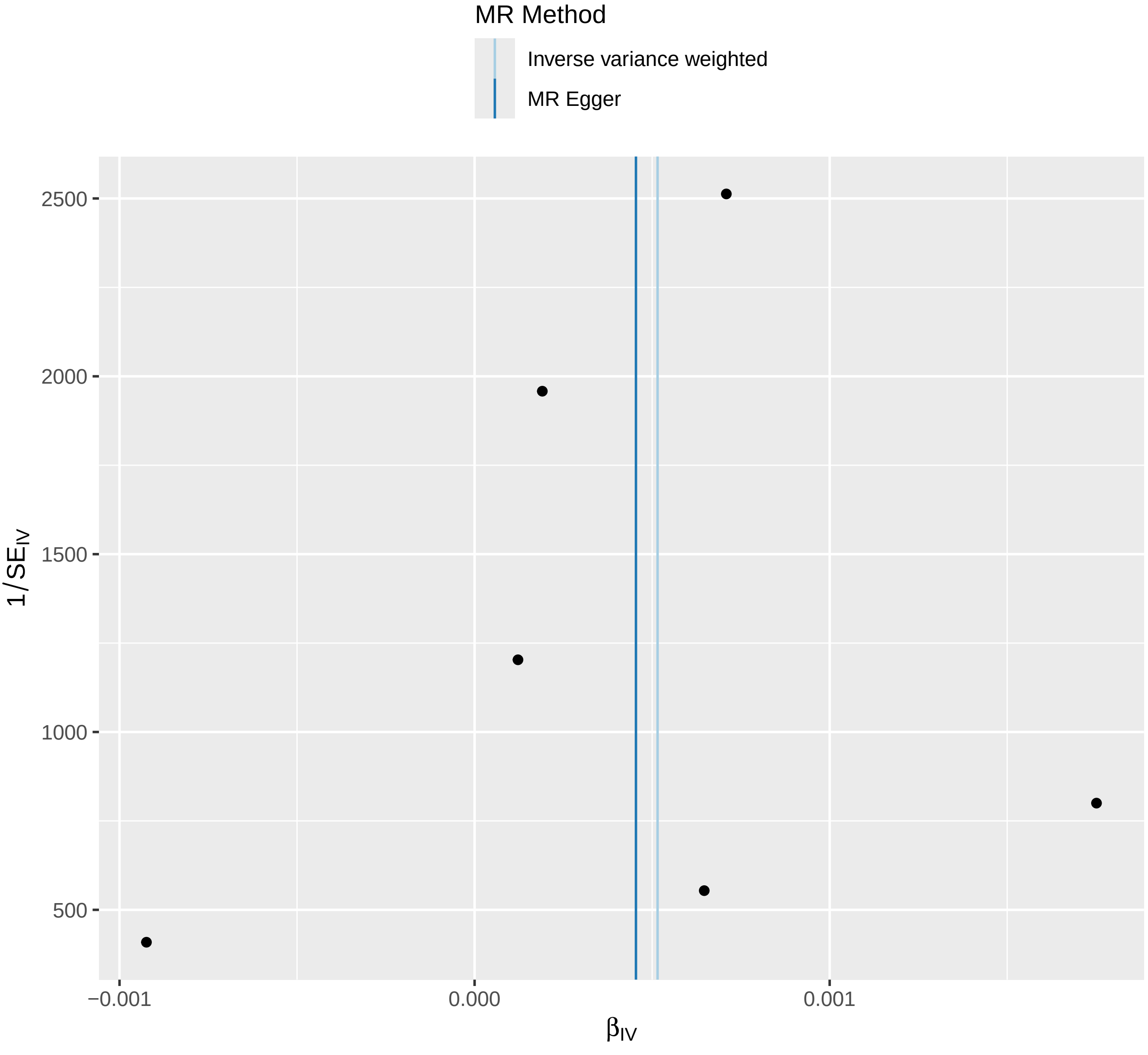
MR Method
Inverse variance weighted
MR Egger
2500
2000
1500
1000
500
1/SE_IV
−0.001
0.000
0.001
β_IV

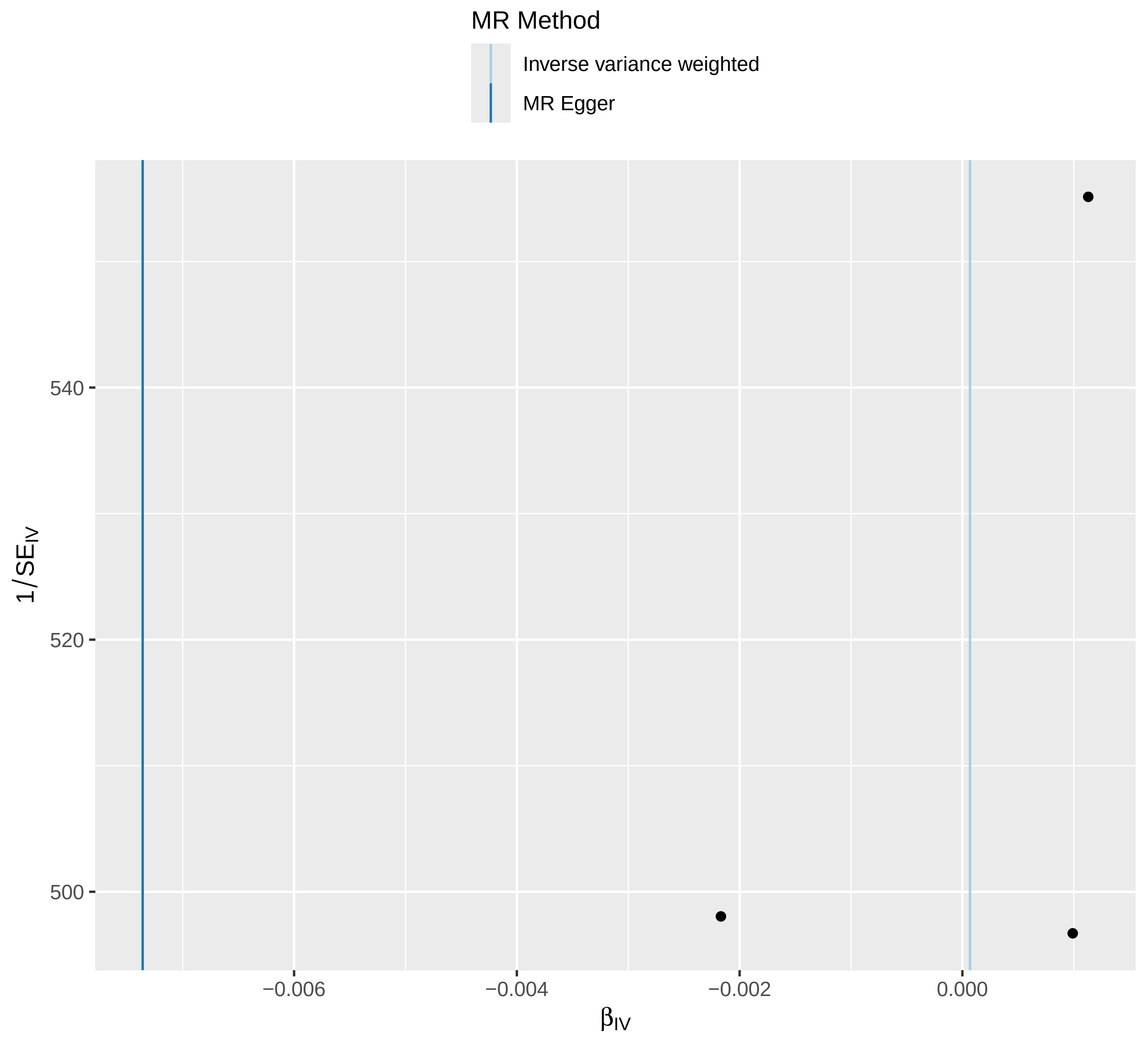

MR Method
Inverse variance weighted
MR Egger
540
520
500
1/SE_IV
−0.006
−0.004
−0.002
0.000
β_IV

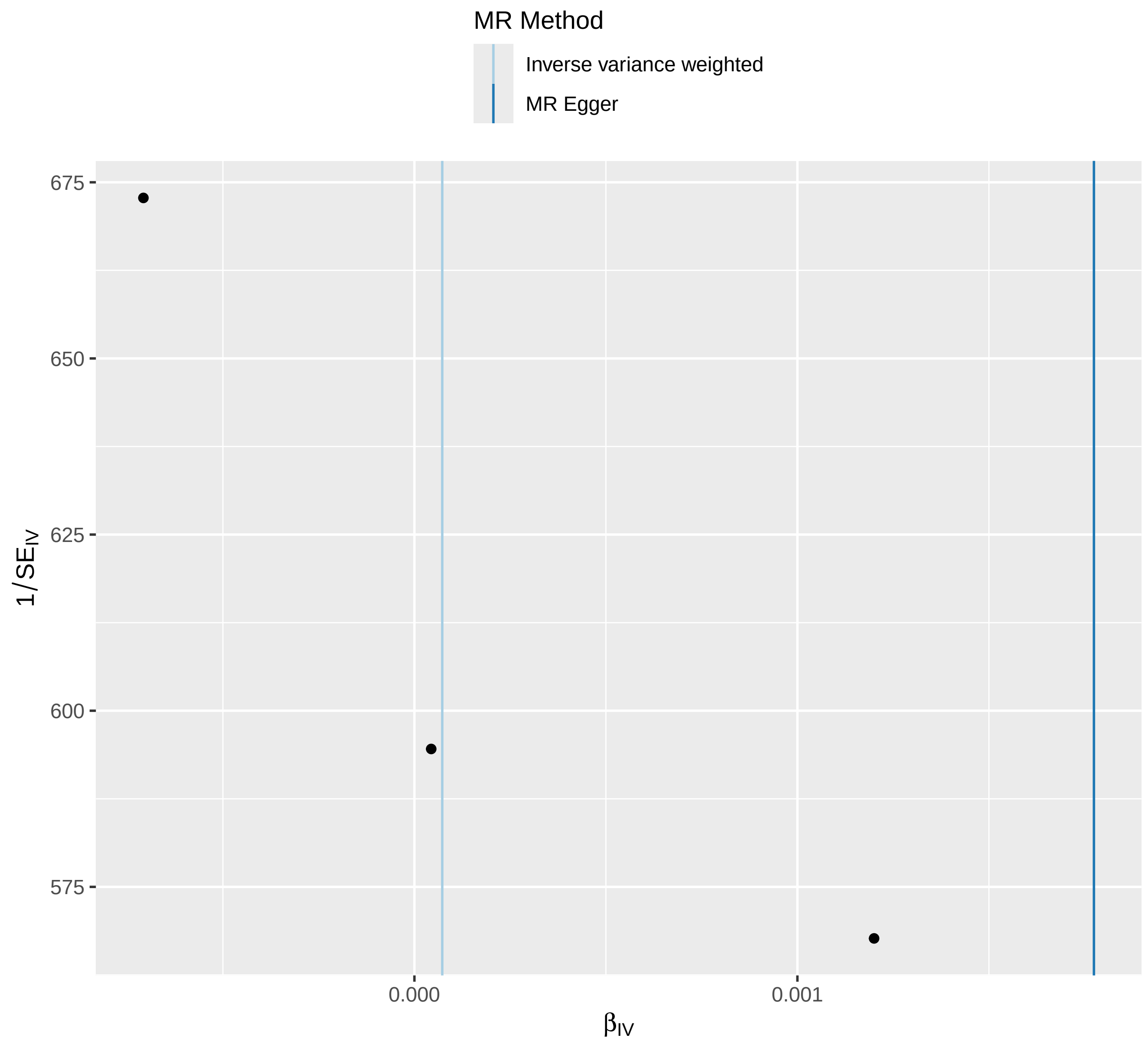
MR Method
Inverse variance weighted
MR Egger
675
650
625
600
575
0.000
0.001
$1/SE_{IV}$
β_{IV}

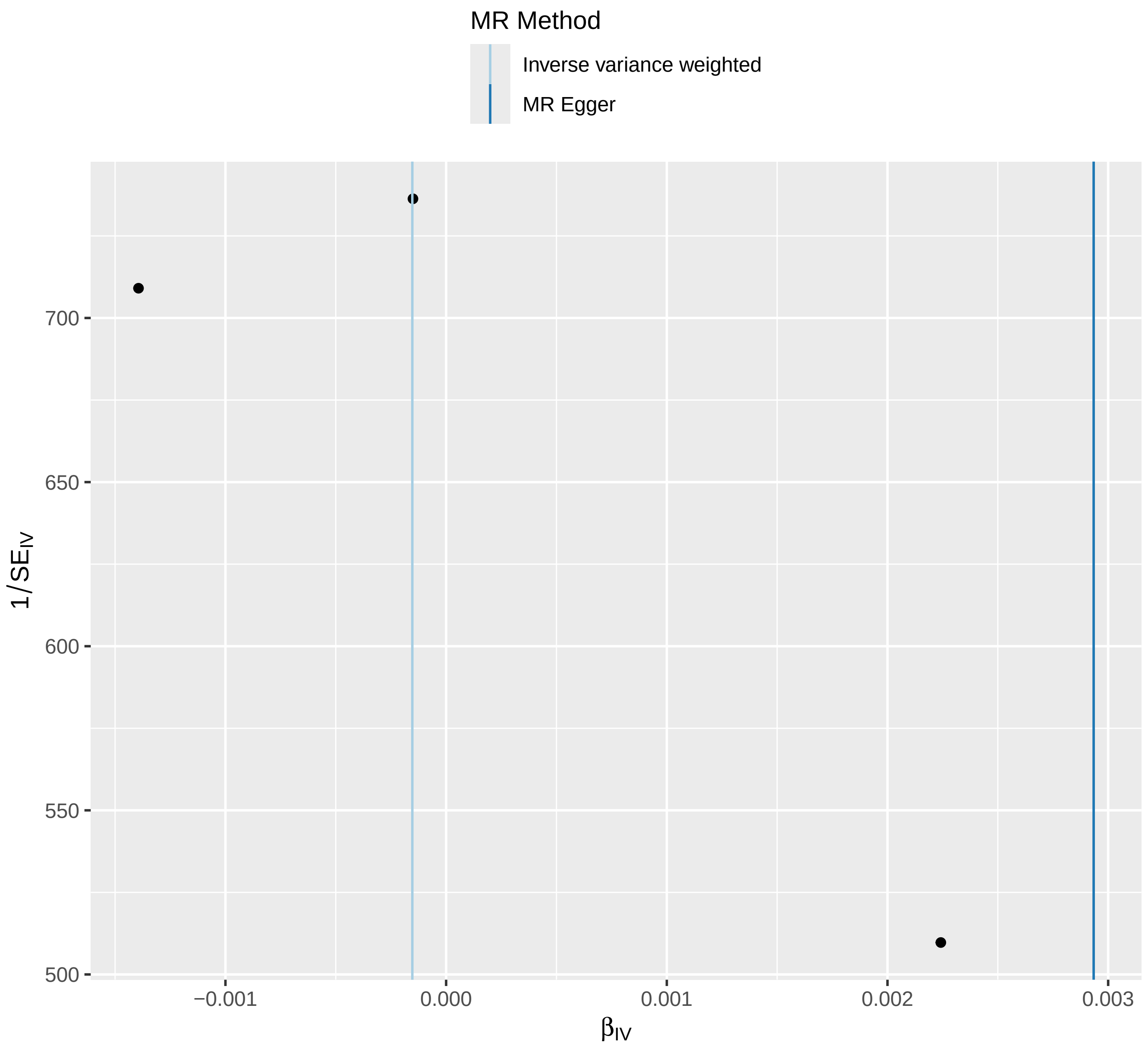

MR Method
Inverse variance weighted
MR Egger
700
650
600
550
500
1/SE_IV
−0.001
0.000
0.001
0.002
0.003
β_IV

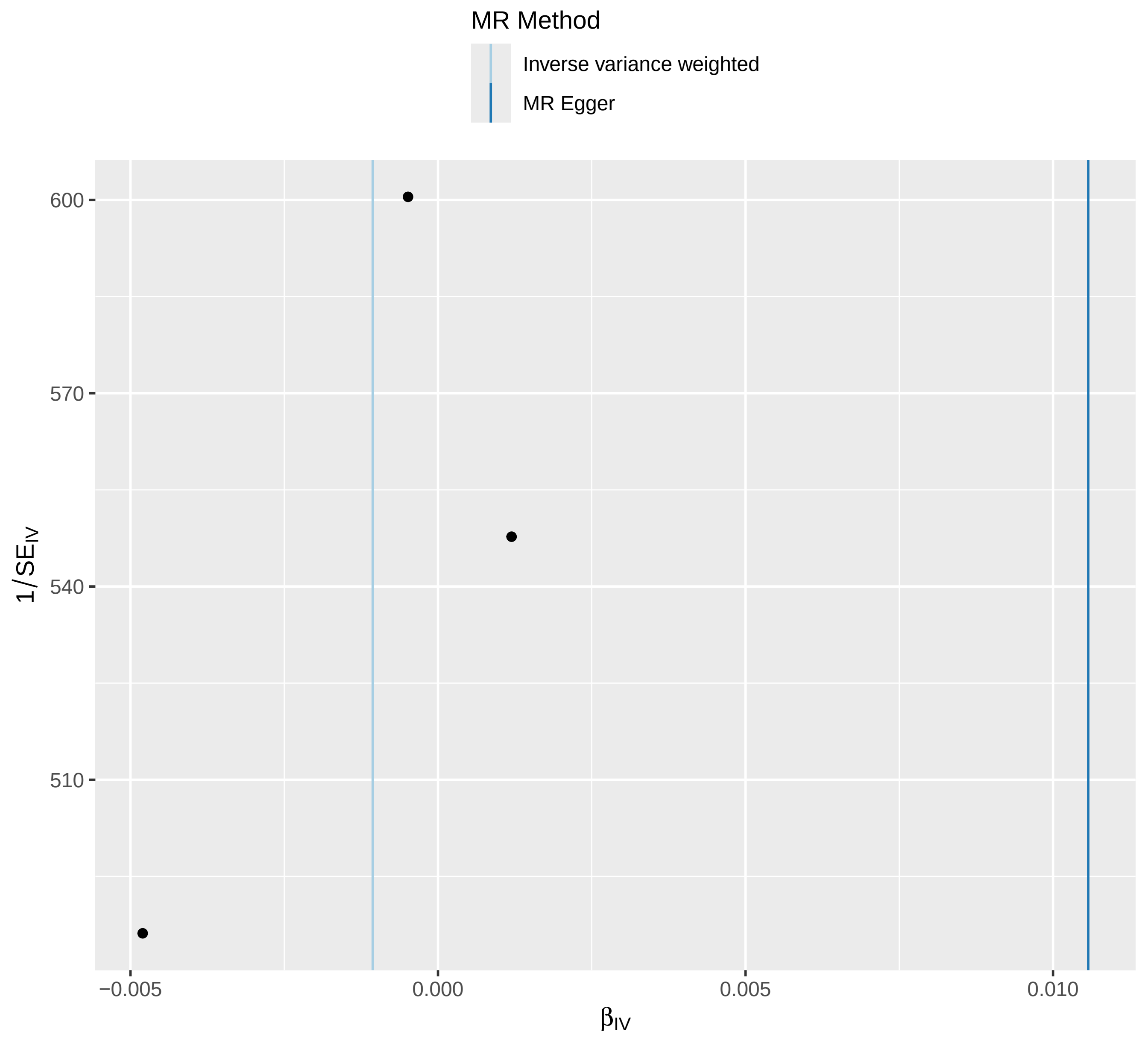
MR Method
Inverse variance weighted
MR Egger
600
570
540
510
1/SE$_{IV}$
−0.005
0.000
0.005
0.010
β$_{IV}$

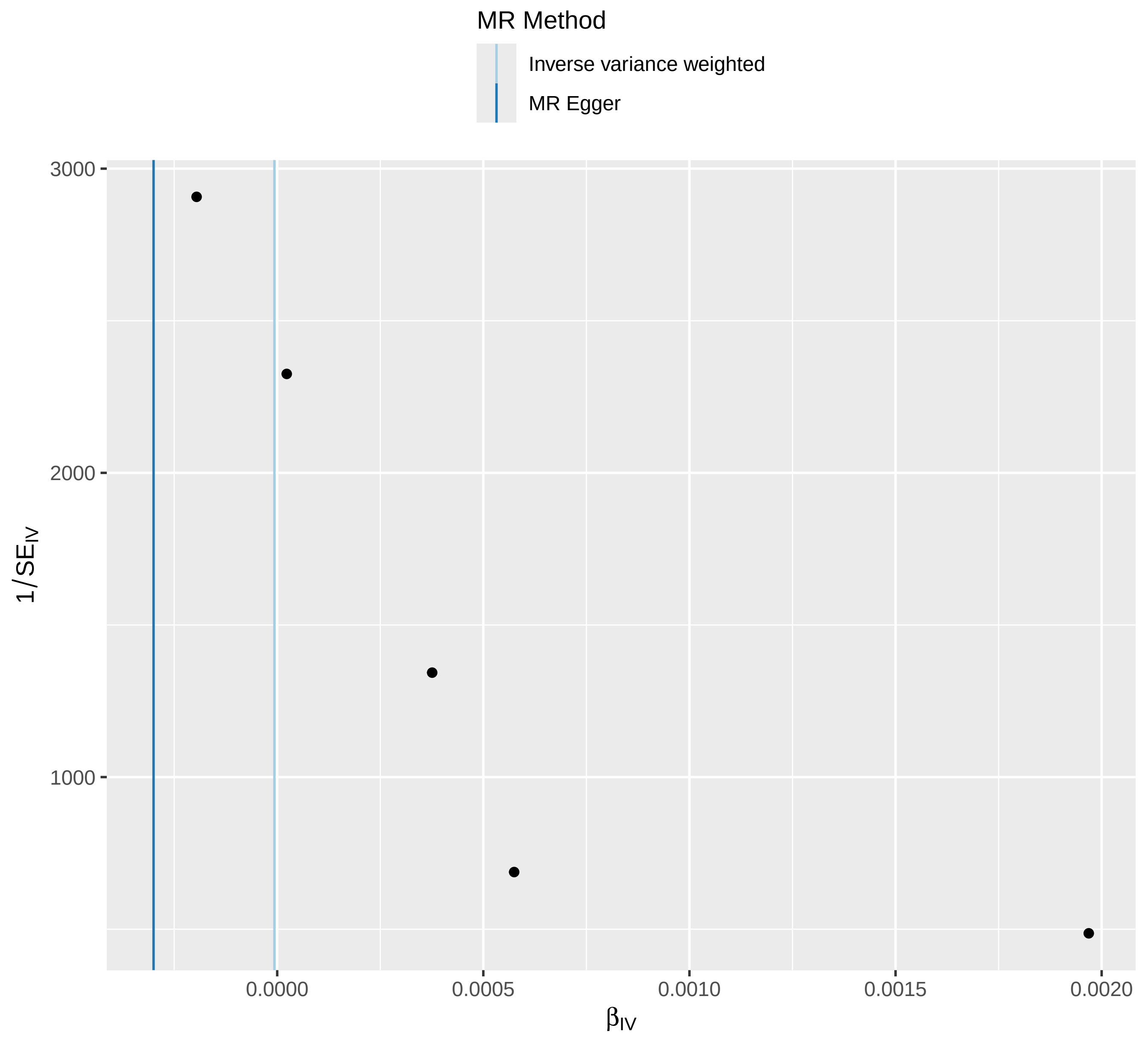

MR Method
Inverse variance weighted
MR Egger
3000
2000
1000
1/SE_IV
0.0000
0.0005
0.0010
0.0015
0.0020
β_IV

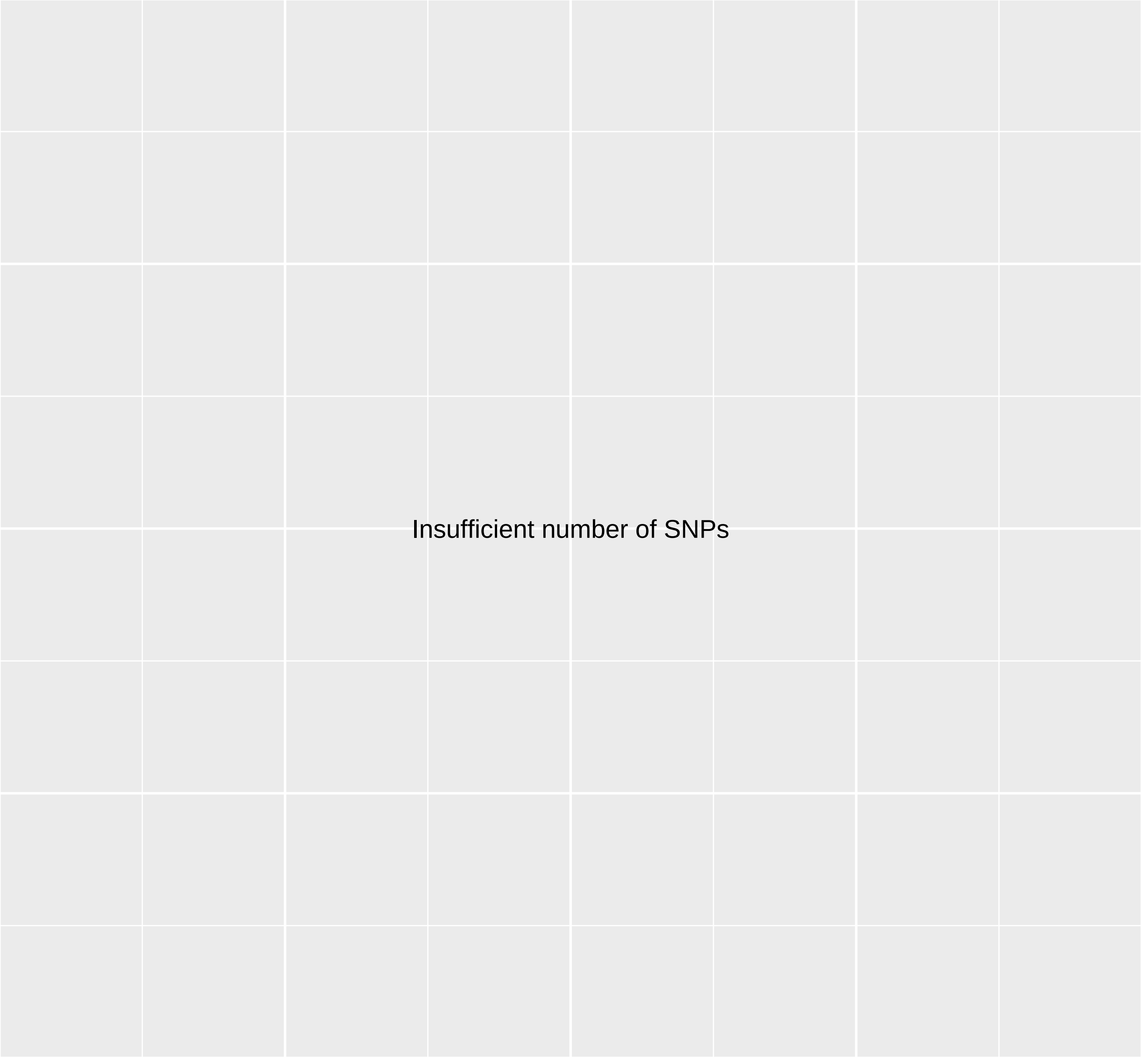
Insufficient number of SNPs

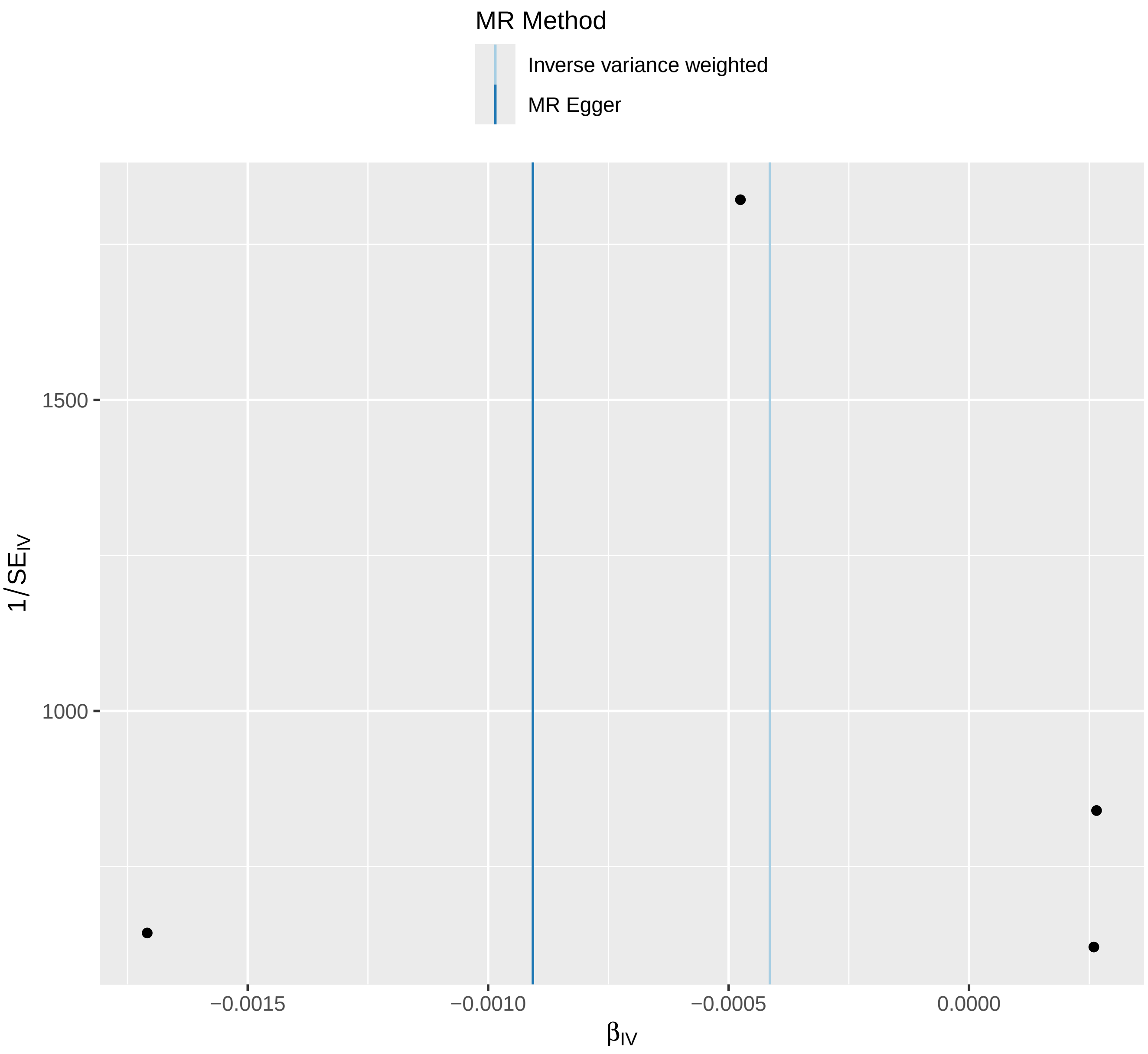
MR Method
Inverse variance weighted
MR Egger
1500
1000
−0.0015
−0.0010
−0.0005
0.0000
$1/SE_{IV}$
β_{IV}

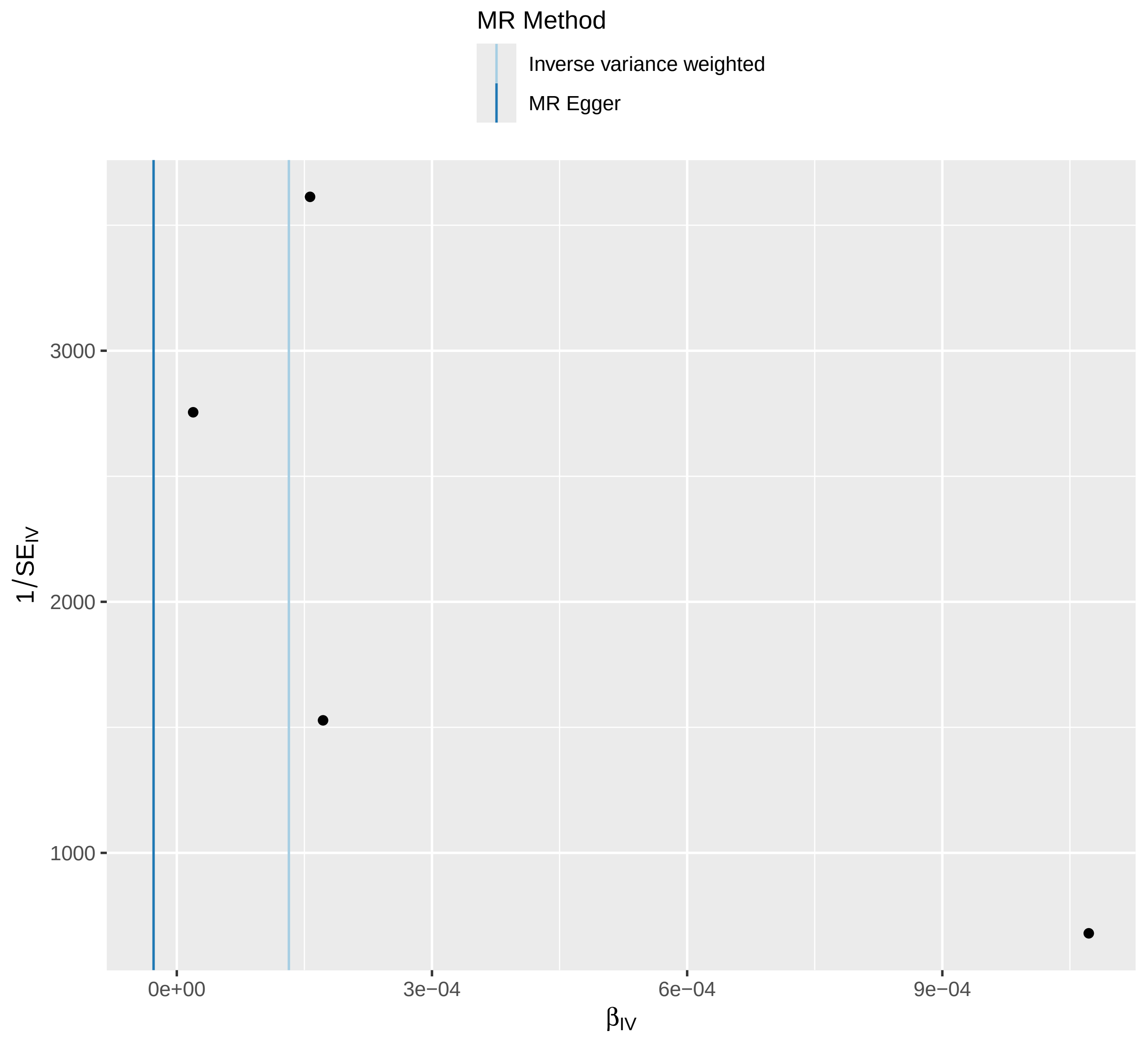

MR Method
Inverse variance weighted
MR Egger
3000
2000
1000
$1/SE_{IV}$
0e+00
3e−04
6e−04
9e−04
β_{IV}

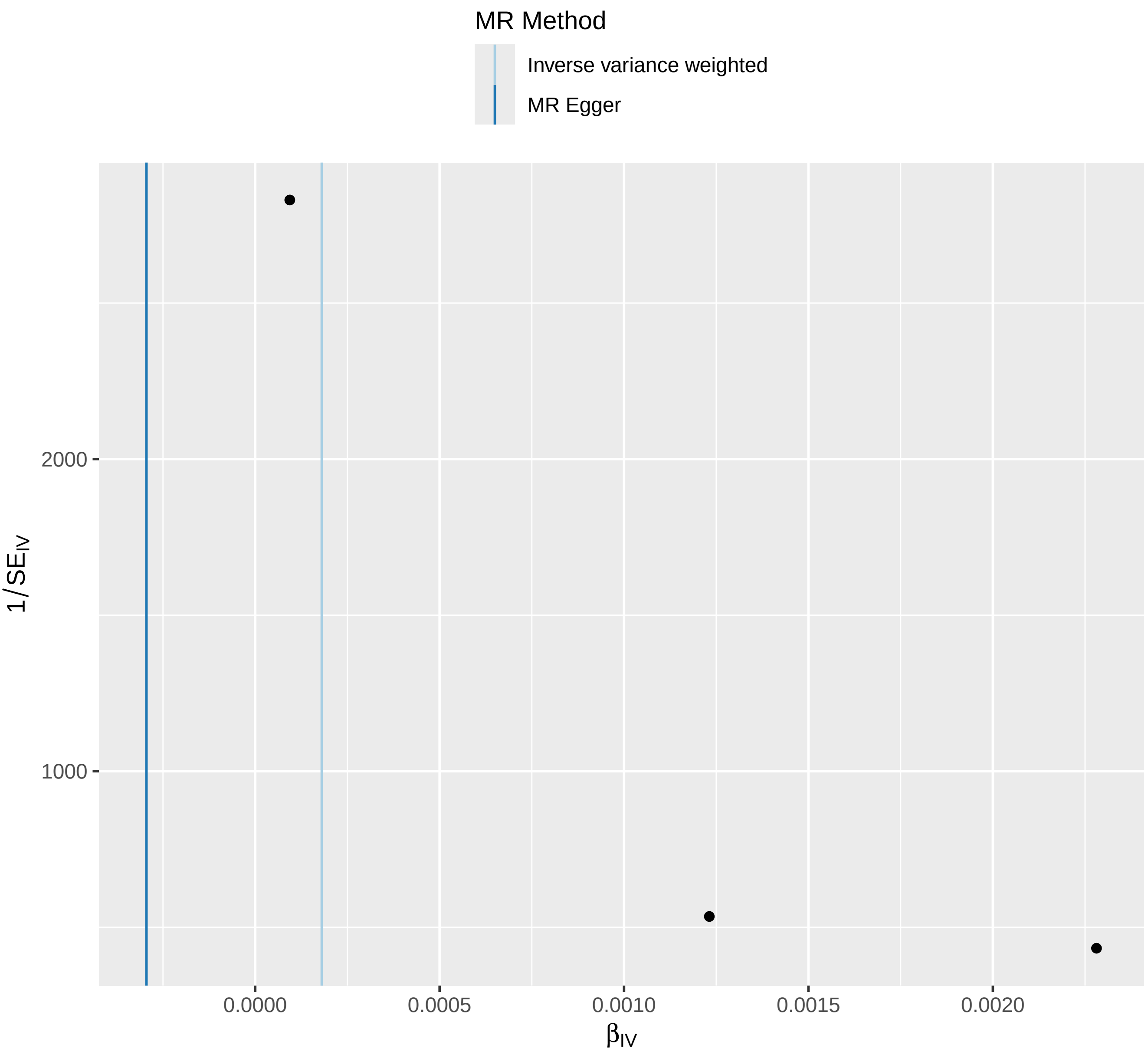

MR Method
Inverse variance weighted
MR Egger
2000
1000
1/SE_IV
0.0000
0.0005
0.0010
0.0015
0.0020
β_IV

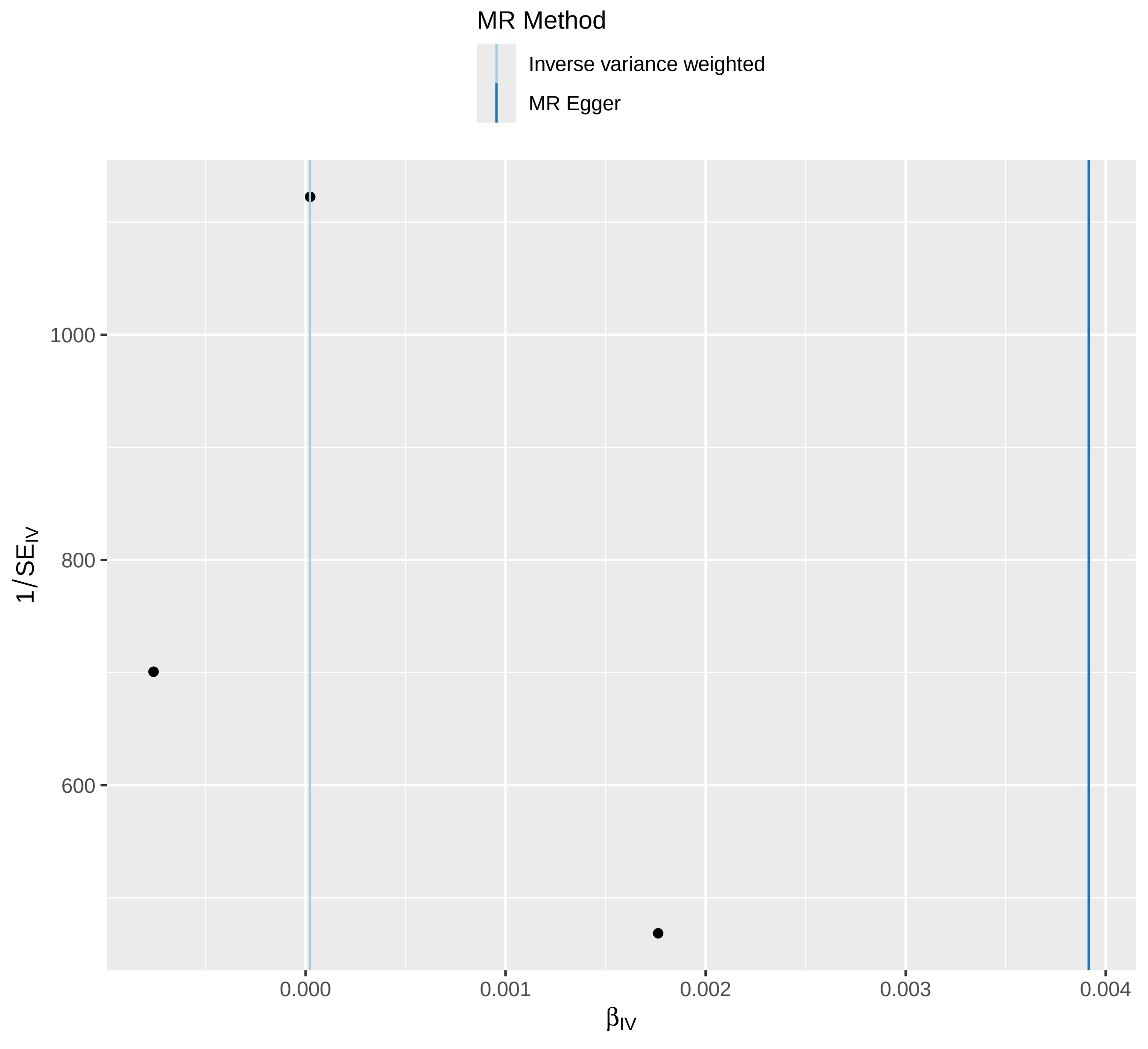
MR Method
Inverse variance weighted
MR Egger
1000
800
600
1/SE_IV
0.000
0.001
0.002
0.003
0.004
β_IV

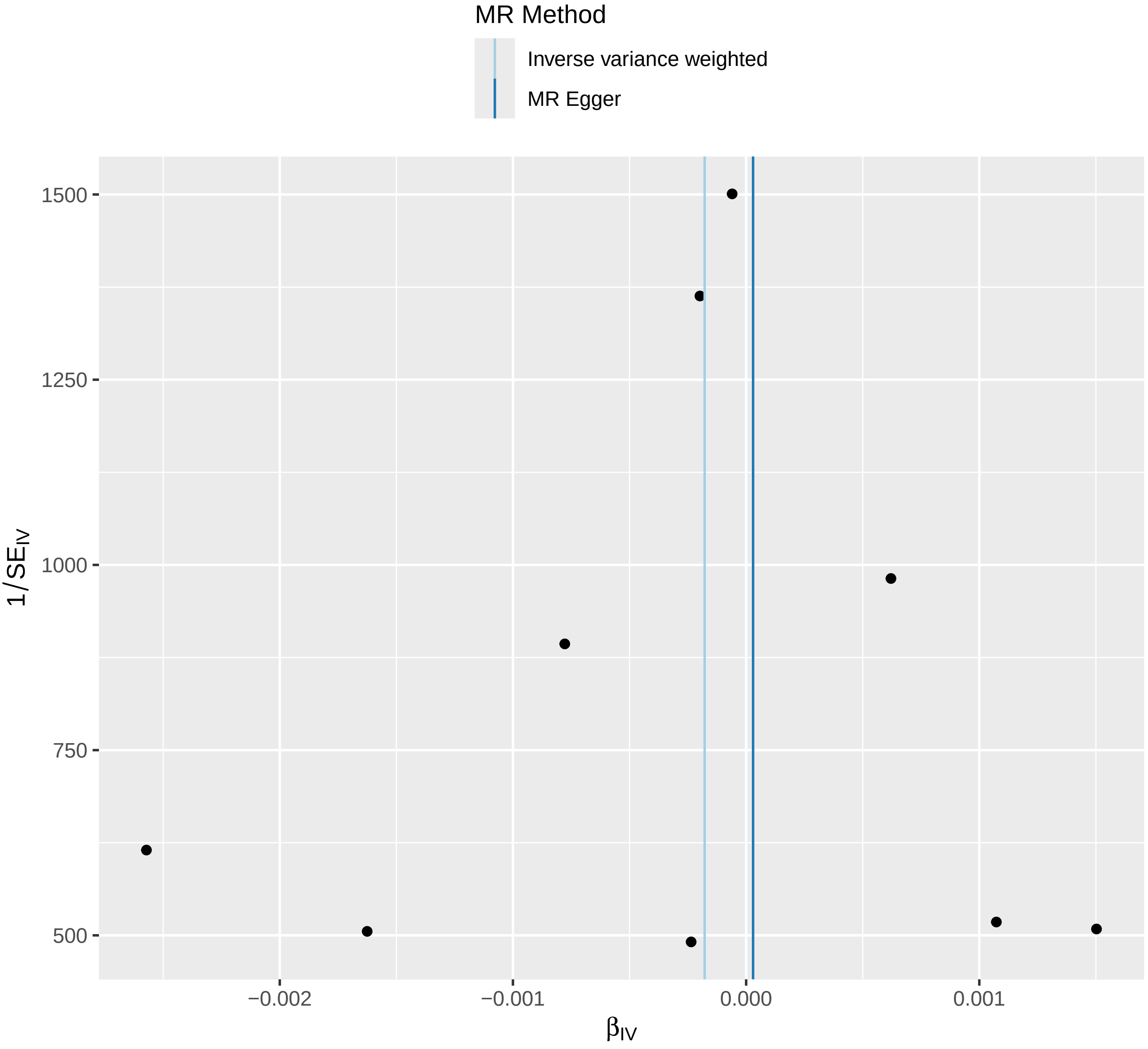
MR Method
Inverse variance weighted
MR Egger
1500
1250
1000
750
500
1/SE_IV
−0.002
−0.001
0.000
0.001
β_IV

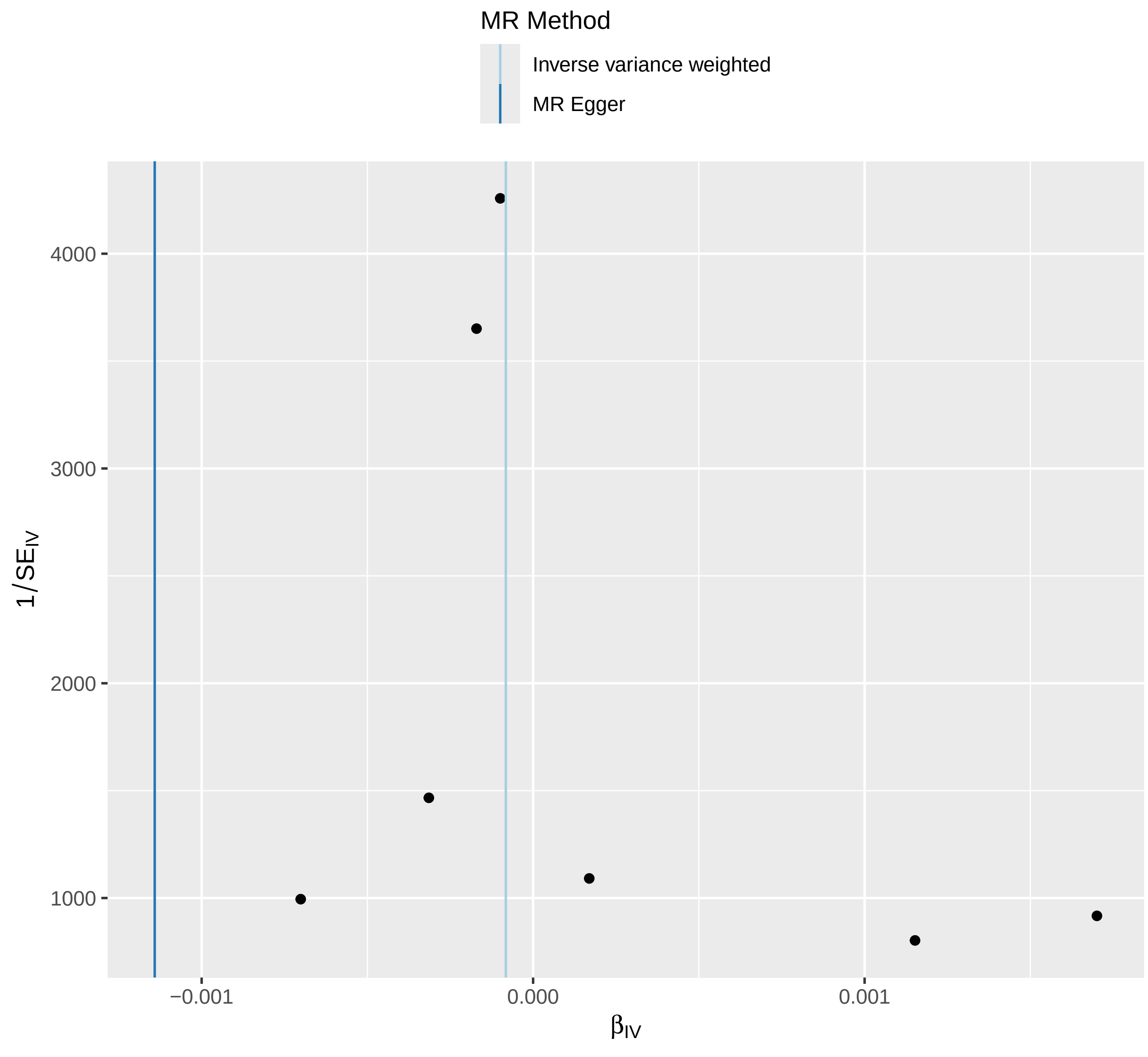
MR Method
Inverse variance weighted
MR Egger
4000
3000
2000
1000
1/SE_IV
−0.001
0.000
0.001
β_IV

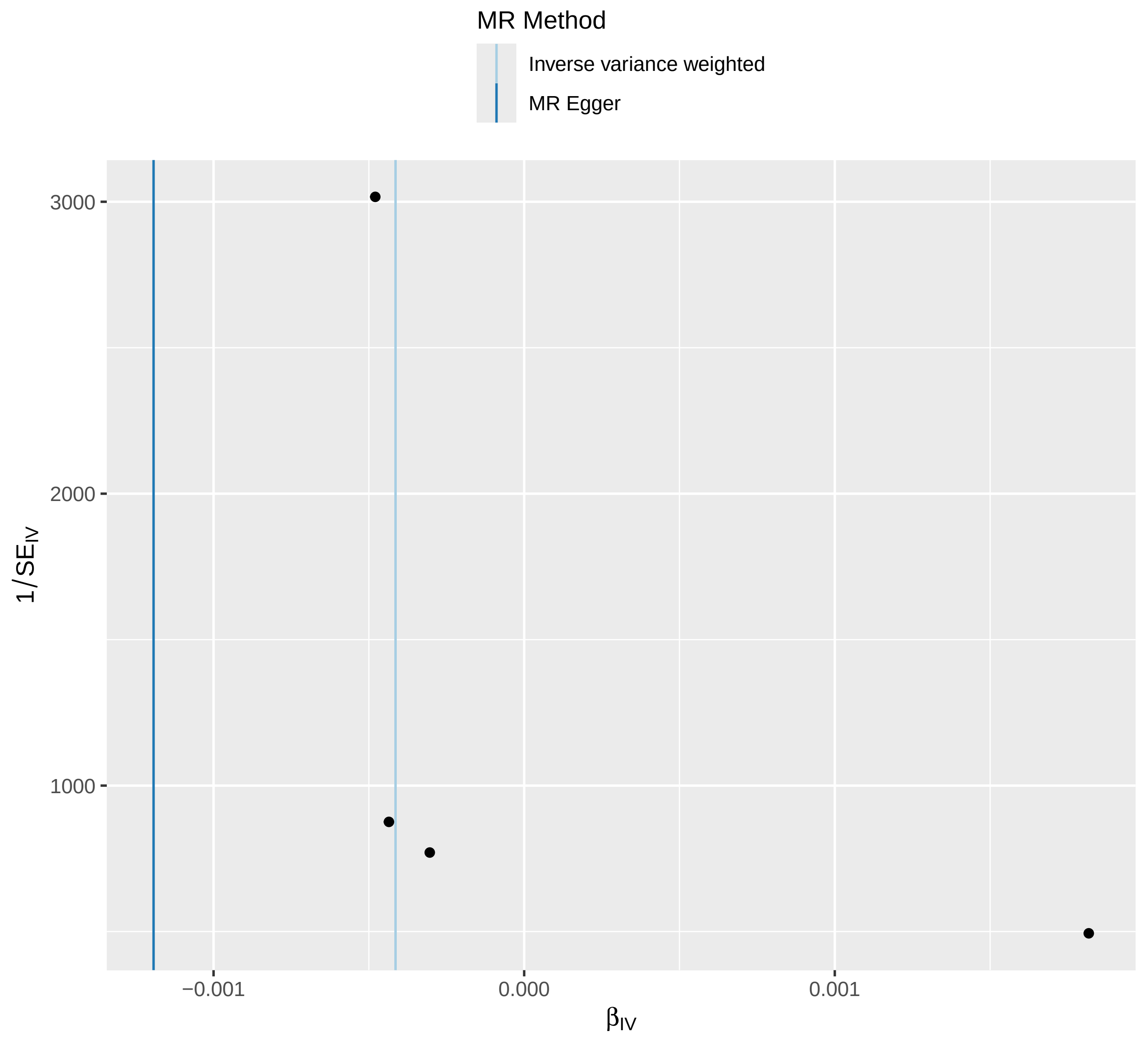
MR Method
Inverse variance weighted
MR Egger
3000
2000
1000
$1/SE_{IV}$
−0.001
0.000
0.001
β_{IV}

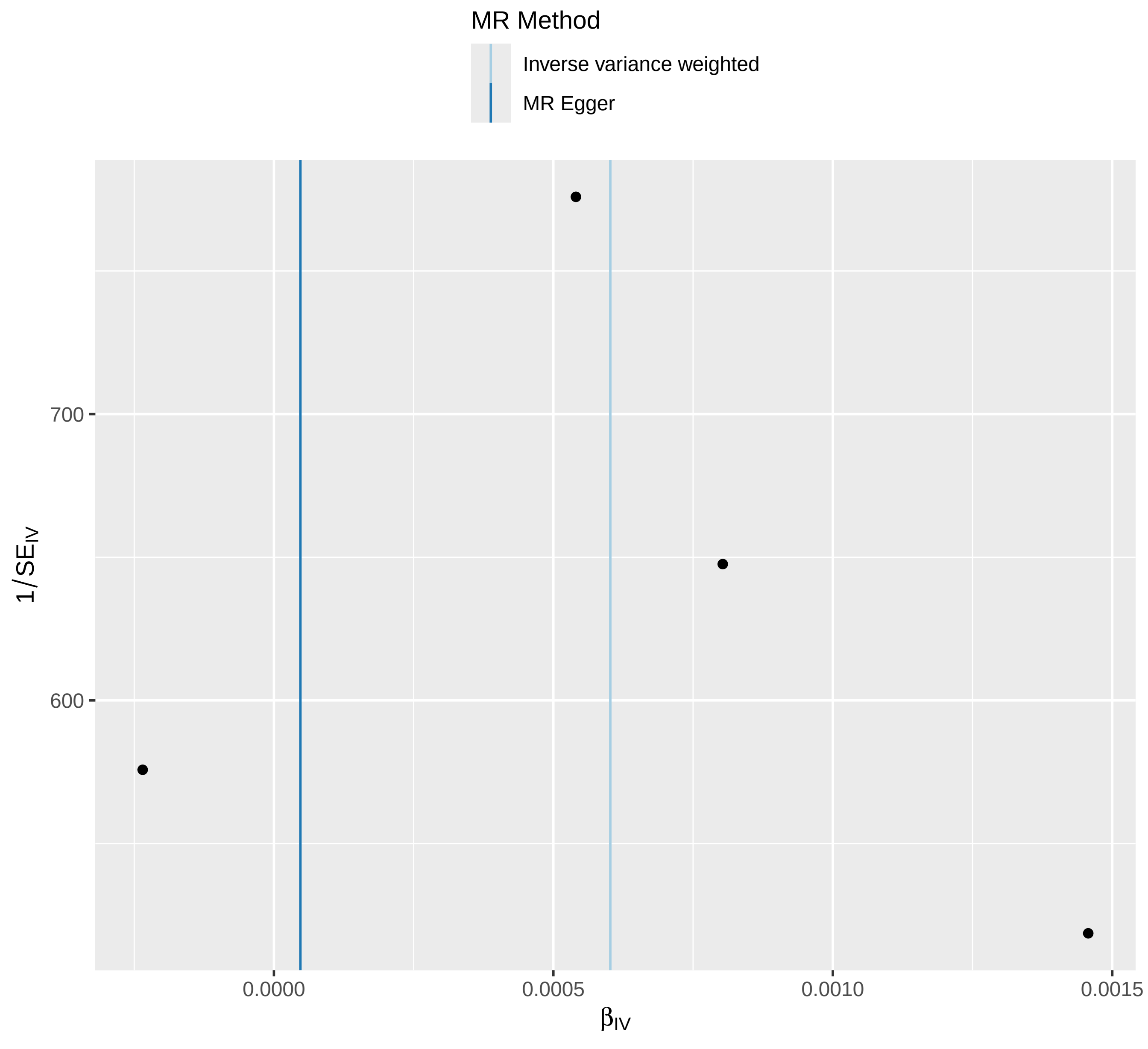
MR Method
Inverse variance weighted
MR Egger
700
600
1/SE_IV
0.0000
0.0005
0.0010
0.0015
β_IV

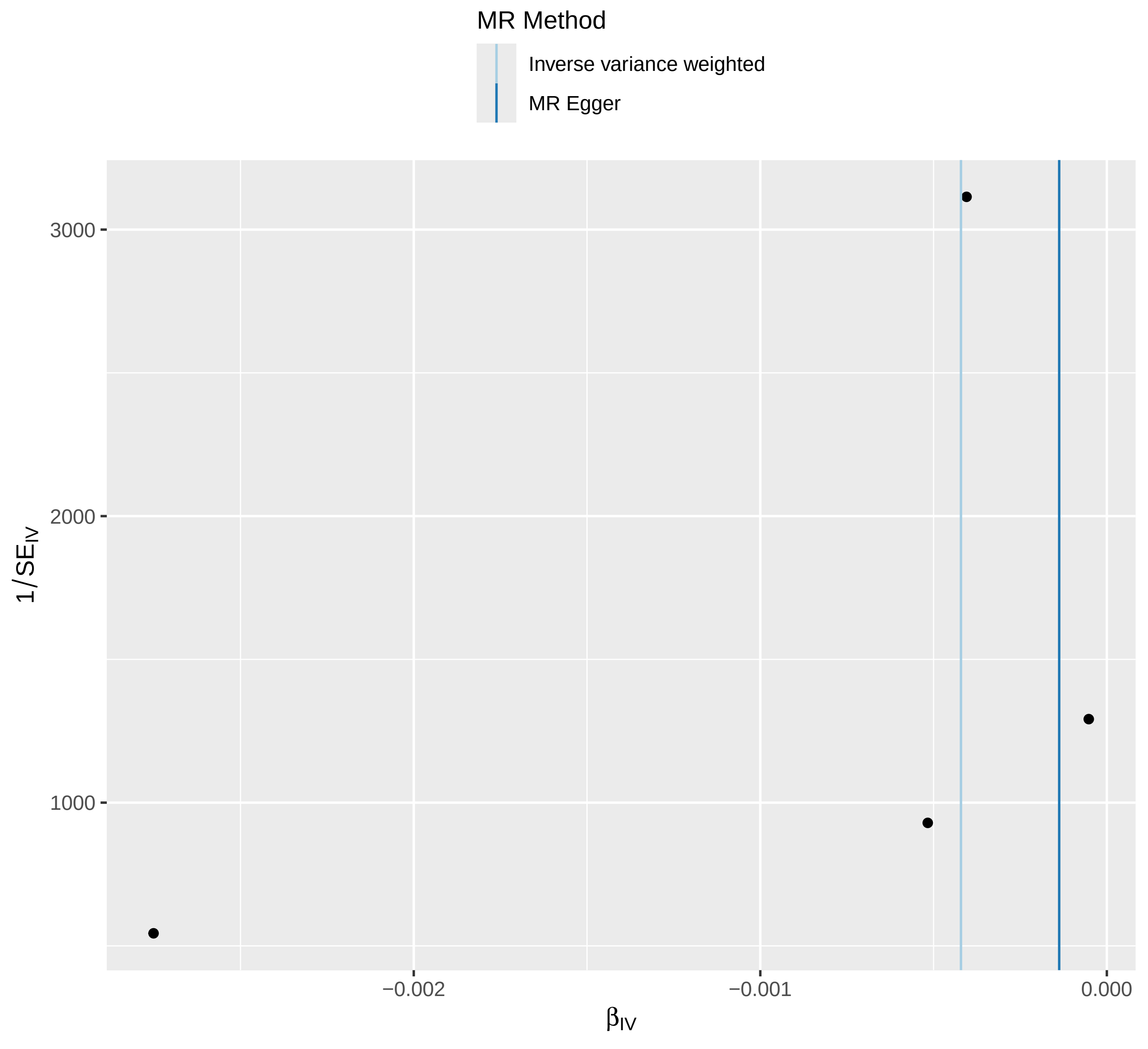
MR Method
Inverse variance weighted
MR Egger
3000
2000
1000
1/SE$_{IV}$
−0.002
−0.001
0.000
β$_{IV}$

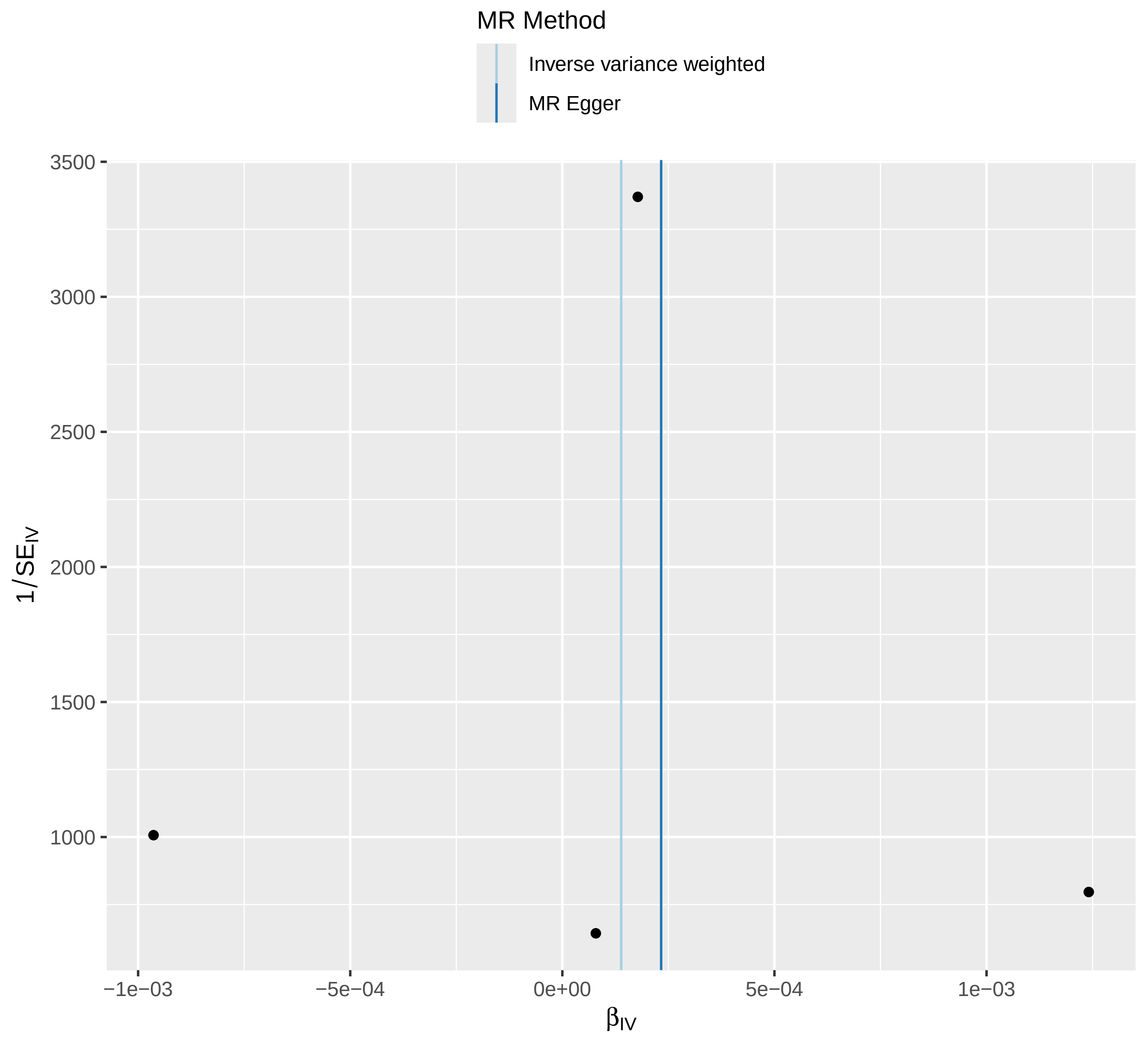
MR Method
Inverse variance weighted
MR Egger
3500
3000
2500
2000
1500
1000
1/SE_IV
−1e−03
−5e−04
0e+00
5e−04
1e−03
β_IV

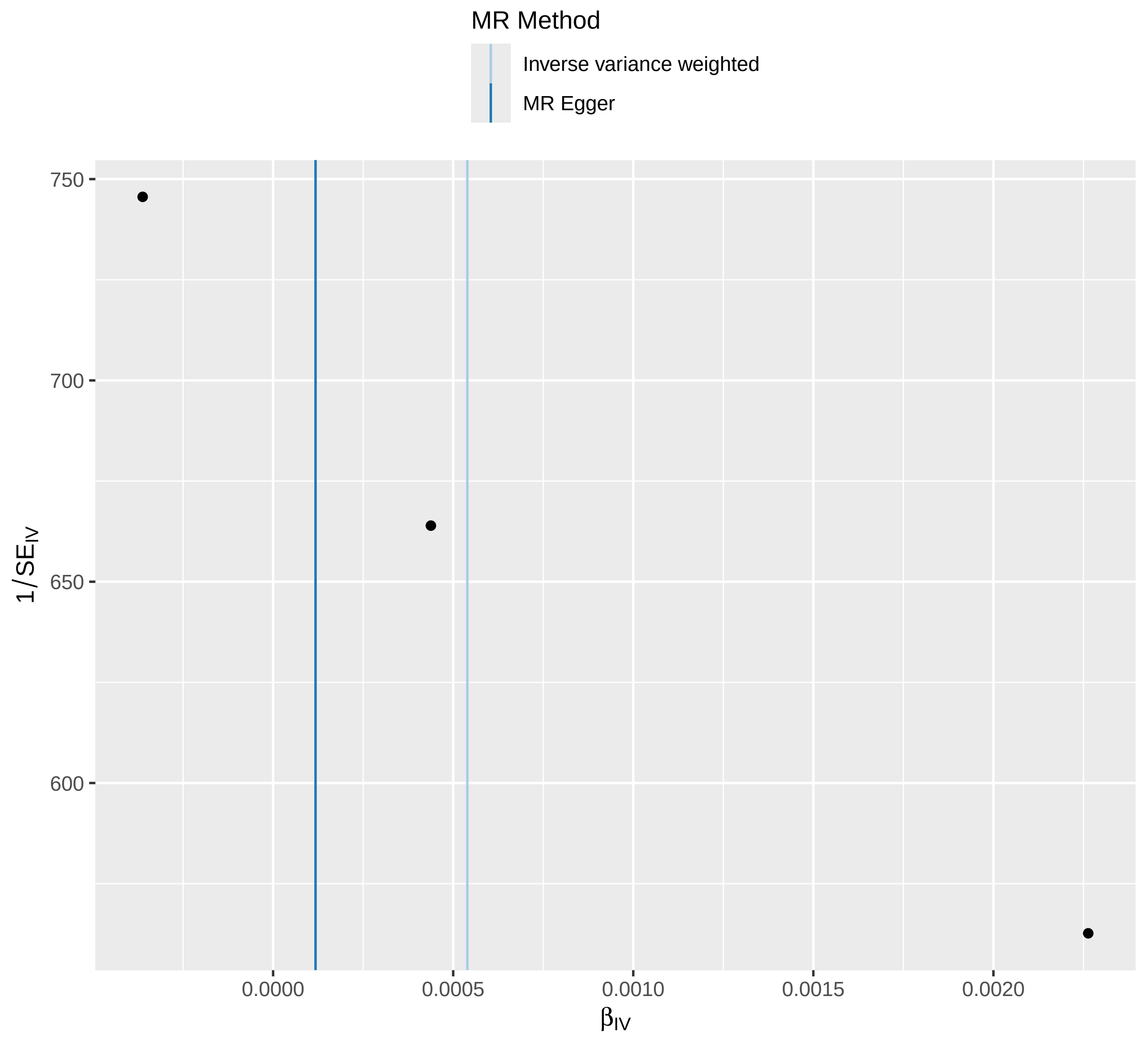
MR Method
Inverse variance weighted
MR Egger
750
700
650
600
1/SE_IV
0.0000
0.0005
0.0010
0.0015
0.0020
β_IV

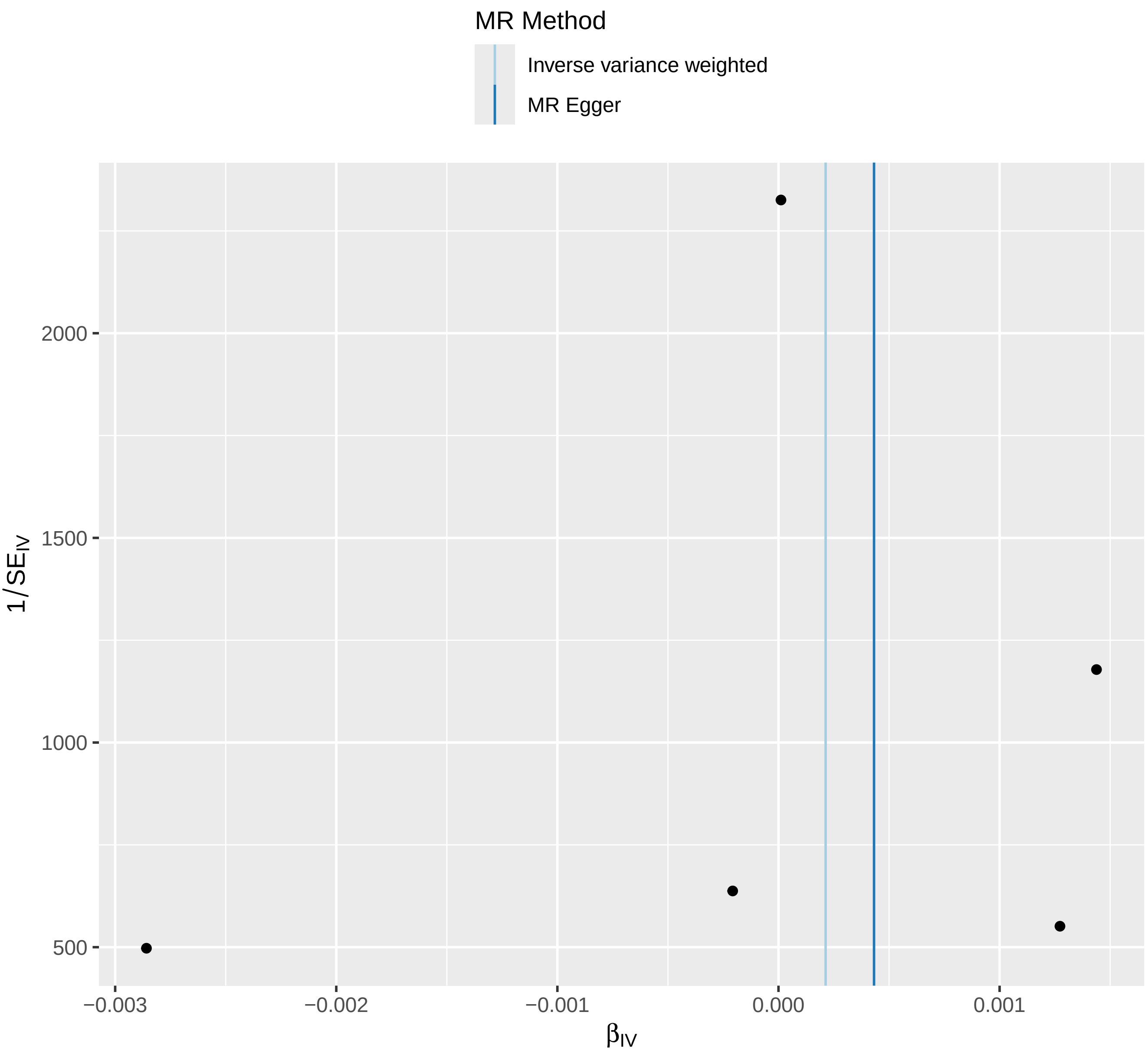
MR Method
Inverse variance weighted
MR Egger
2000
1500
1000
500
1/SE_IV
−0.003
−0.002
−0.001
0.000
0.001
β_IV

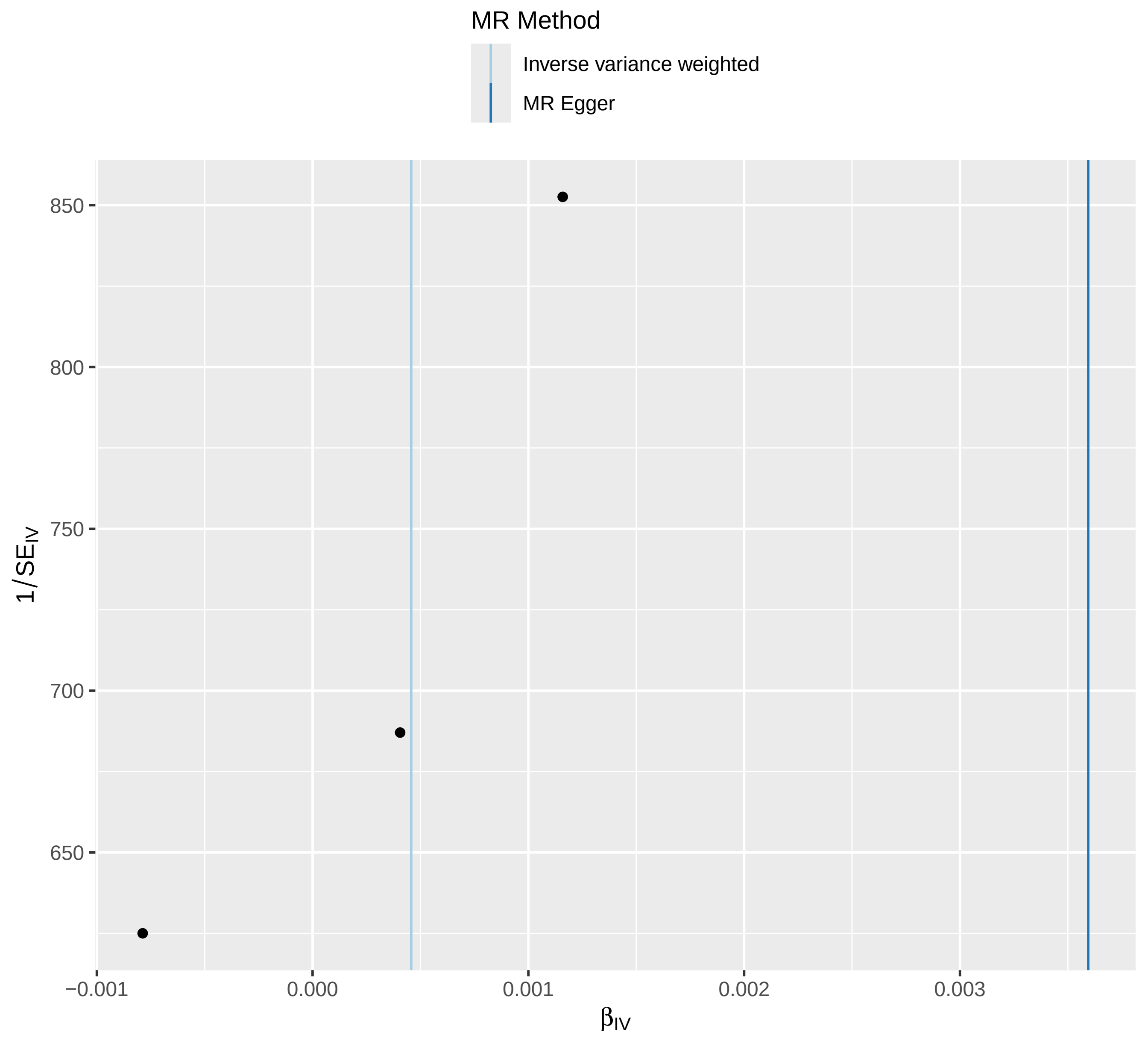
MR Method
Inverse variance weighted
MR Egger
850
800
750
700
650
1/SE_IV
−0.001
0.000
0.001
0.002
0.003
β_IV

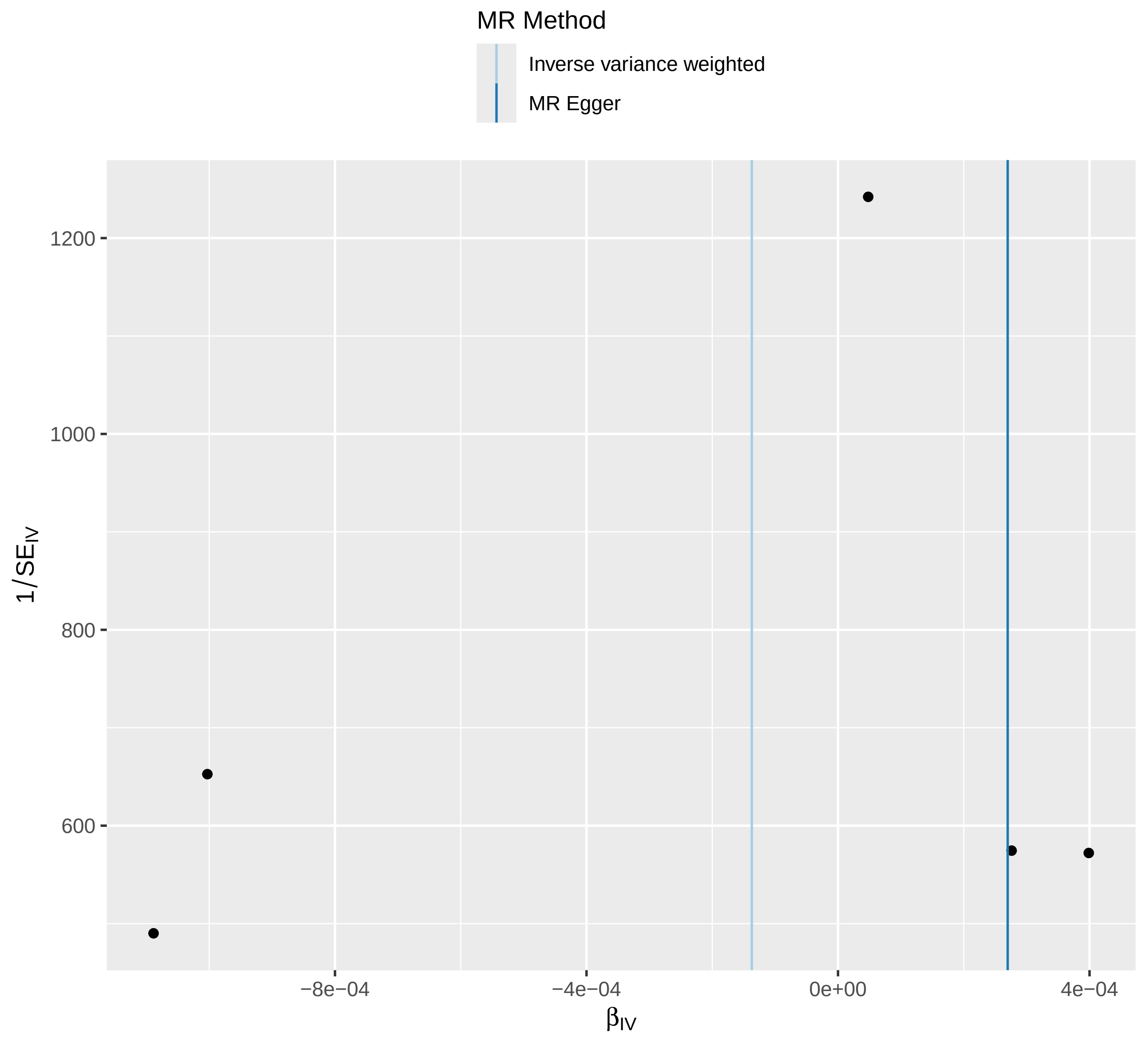

MR Method
Inverse variance weighted
MR Egger
1200
1000
800
600
1/SE_IV
−8e−04
−4e−04
0e+00
4e−04
β_IV

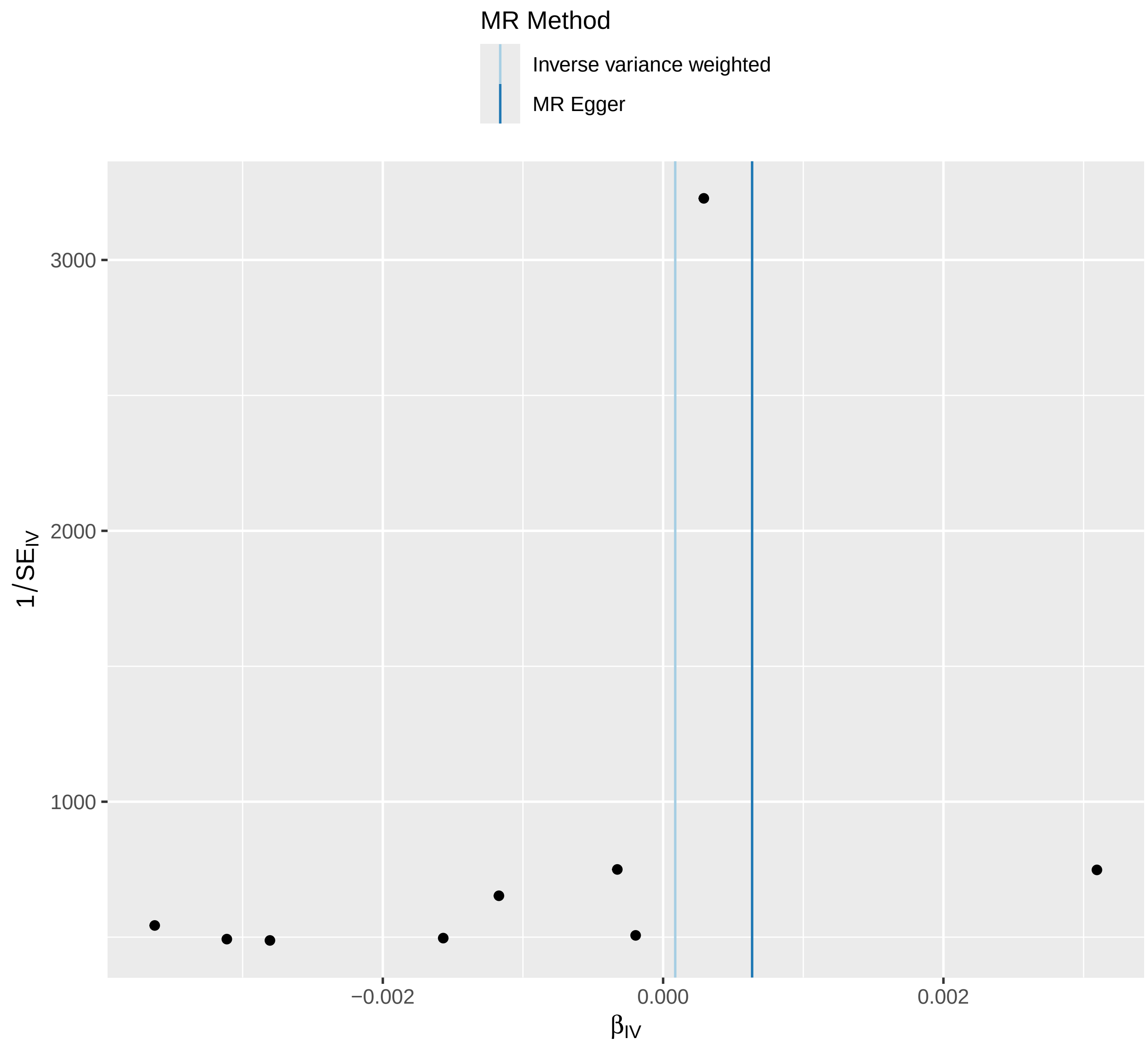
MR Method
Inverse variance weighted
MR Egger
3000
2000
1000
1/SE_{IV}
−0.002
0.000
0.002
β_{IV}

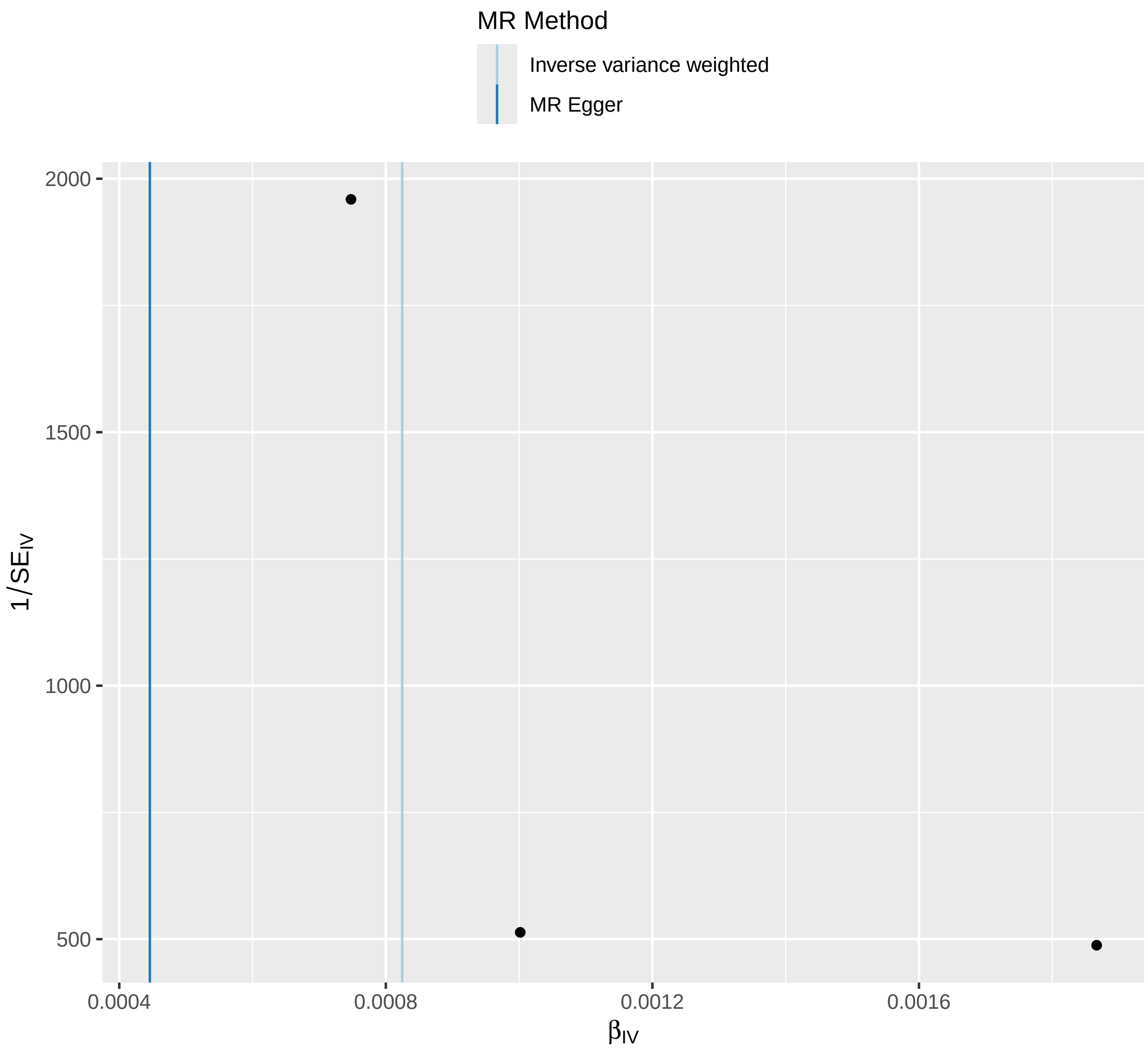

MR Method
Inverse variance weighted
MR Egger
2000
1500
1000
500
1/SE_IV
0.0004
0.0008
0.0012
0.0016
β_IV

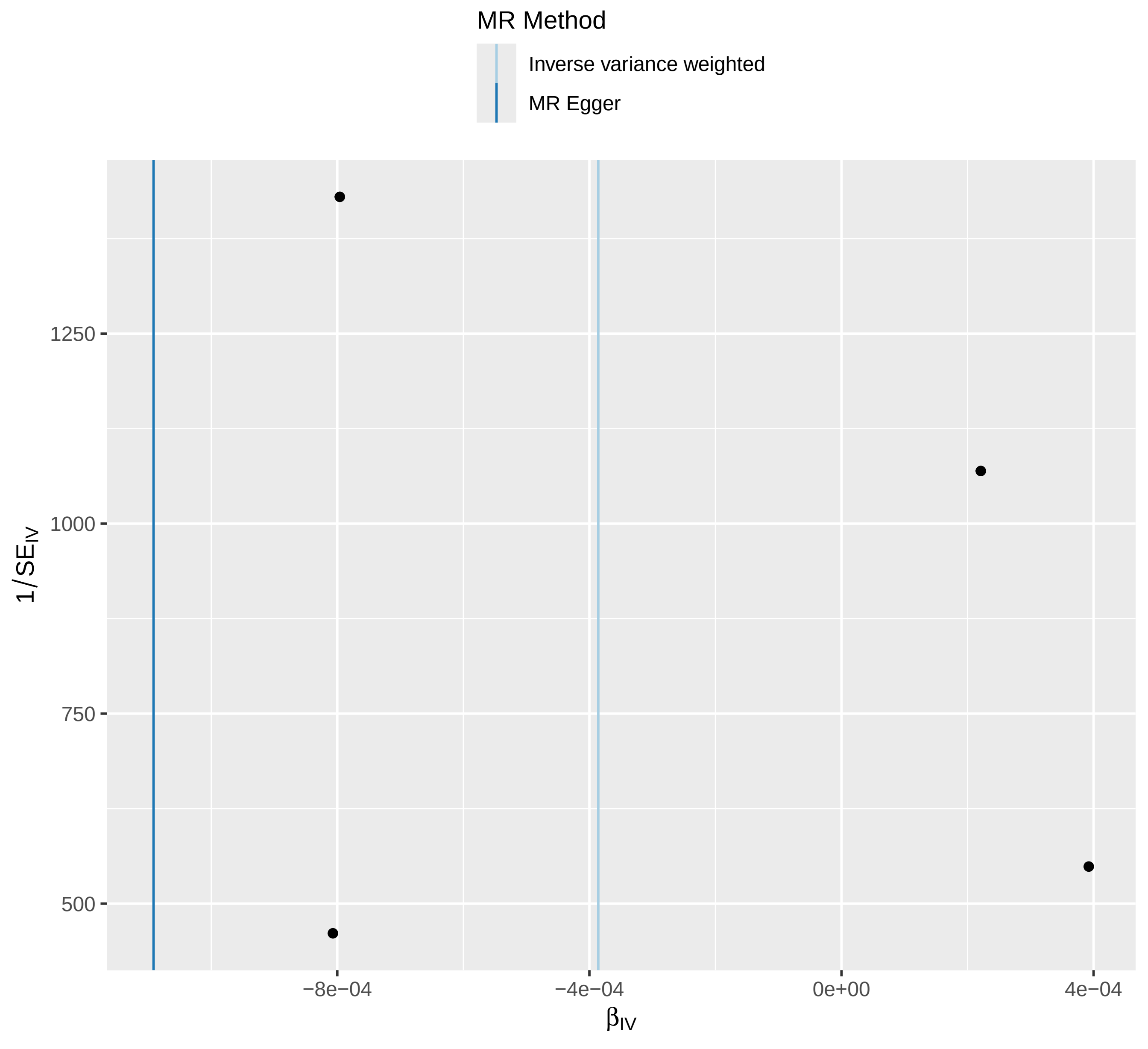
MR Method
Inverse variance weighted
MR Egger
1250
1000
750
500
1/SE$_{IV}$
−8e−04
−4e−04
0e+00
4e−04
β$_{IV}$

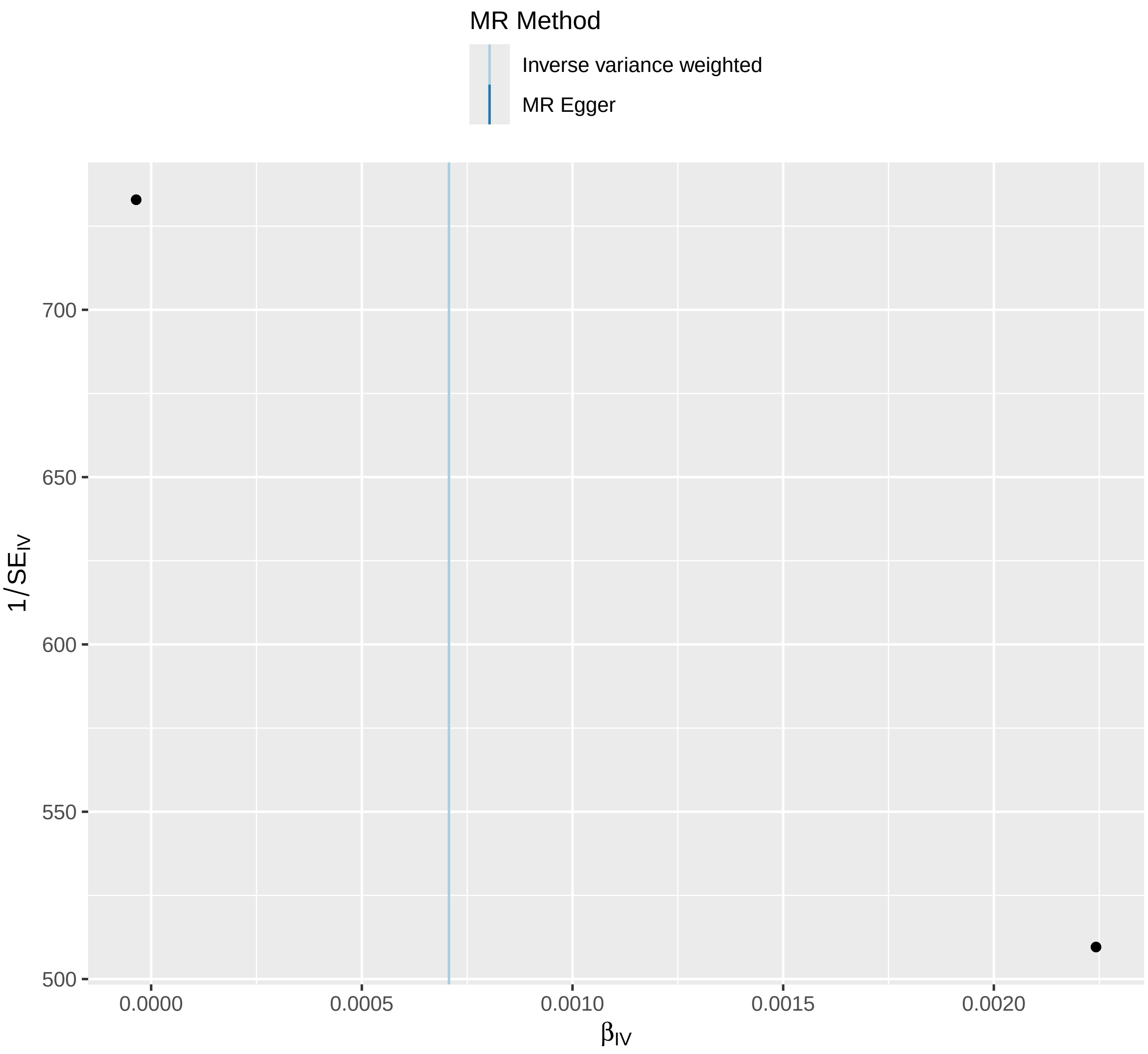

MR Method
Inverse variance weighted
MR Egger
700
650
600
550
500
0.0000
0.0005
0.0010
0.0015
0.0020
$1/SE_{IV}$
β_{IV}

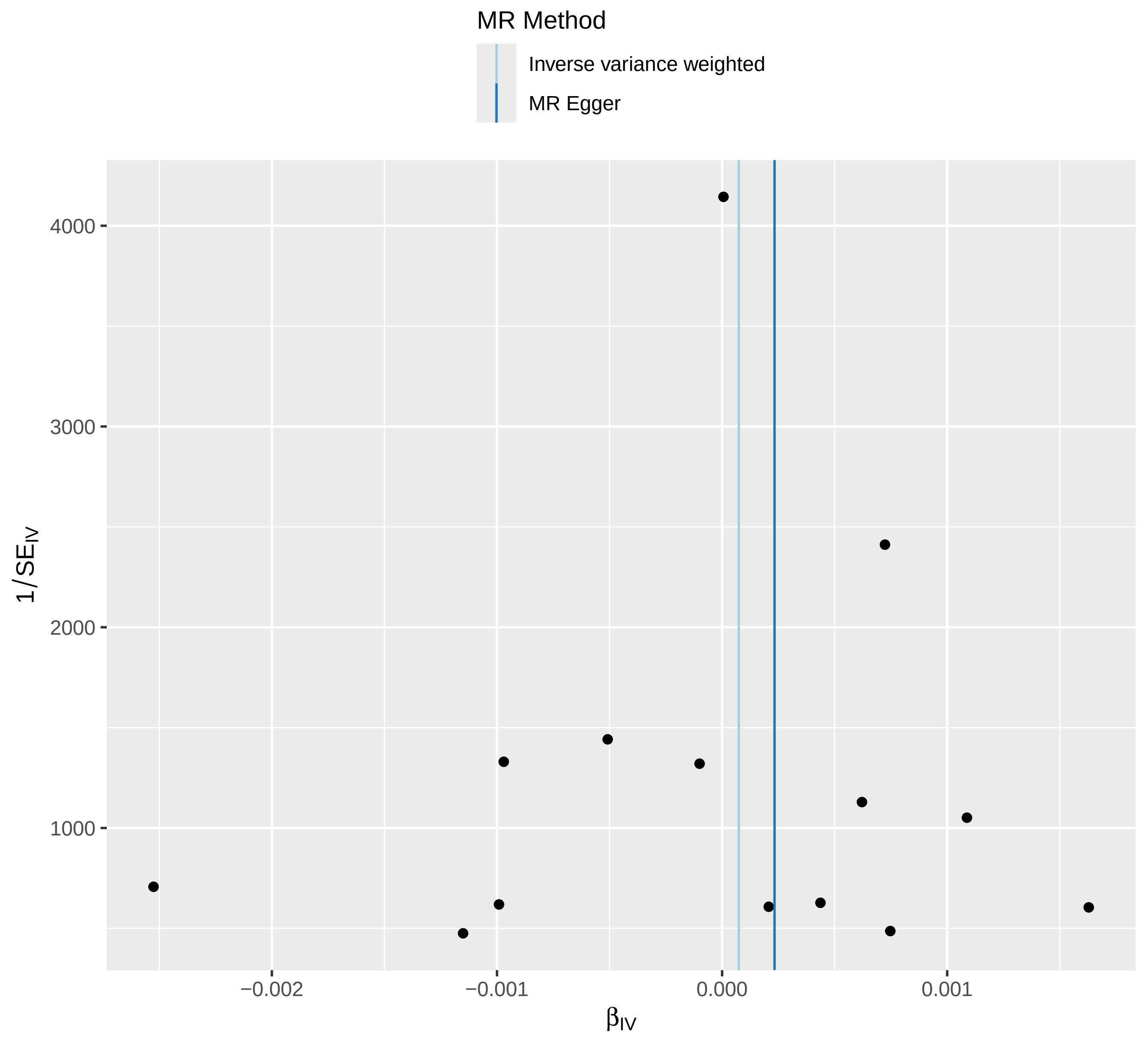
MR Method
Inverse variance weighted
MR Egger
4000
3000
2000
1000
1/SE_IV
−0.002
−0.001
0.000
0.001
β_IV

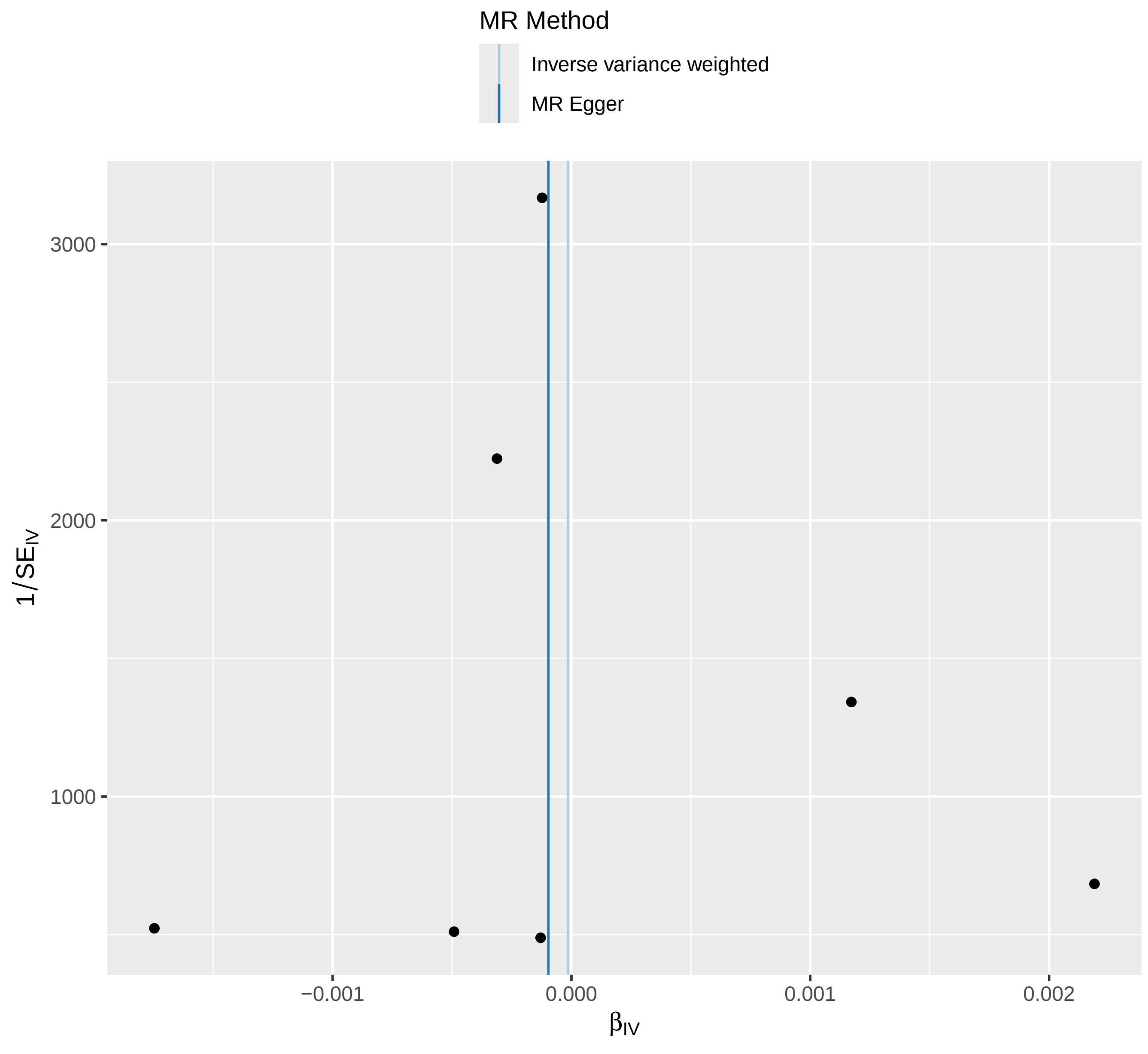
MR Method
Inverse variance weighted
MR Egger
3000
2000
1000
1/SE_IV
−0.001
0.000
0.001
0.002
β_IV

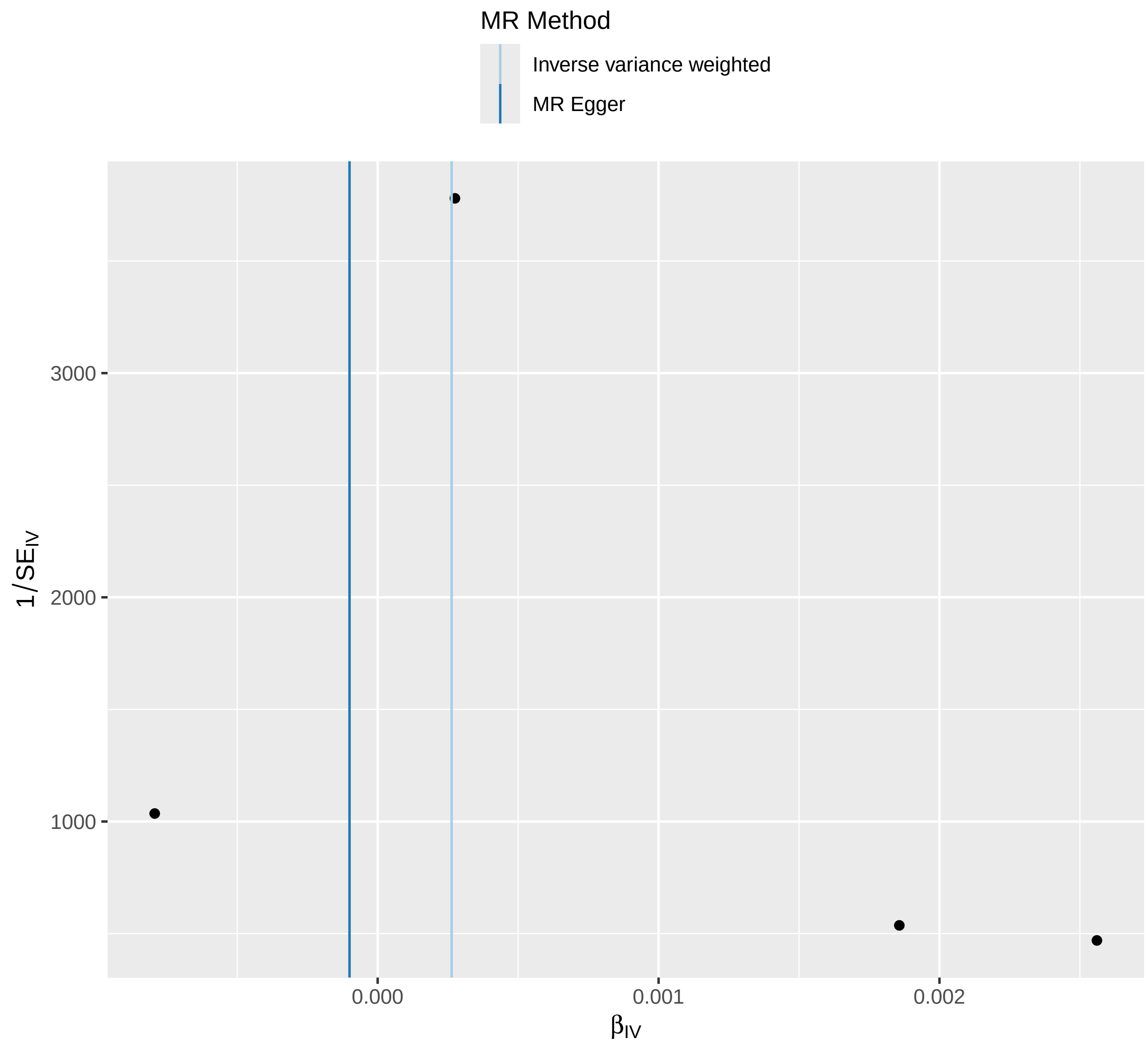
MR Method
Inverse variance weighted
MR Egger
3000
2000
1000
$1/SE_{IV}$
0.000
0.001
0.002
β_{IV}

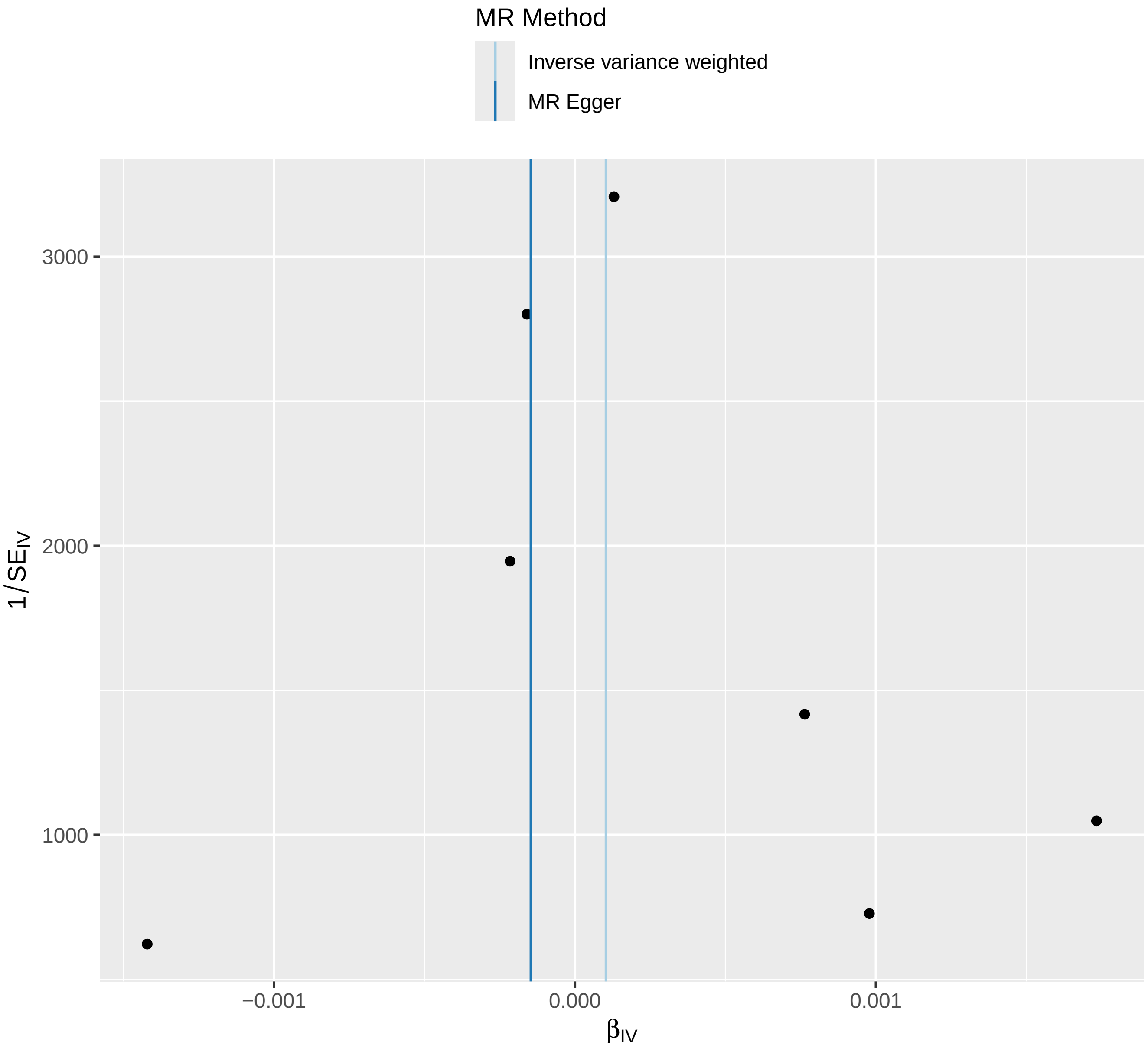

MR Method
Inverse variance weighted
MR Egger
3000
2000
1000
1/SE_IV
−0.001
0.000
0.001
β_IV

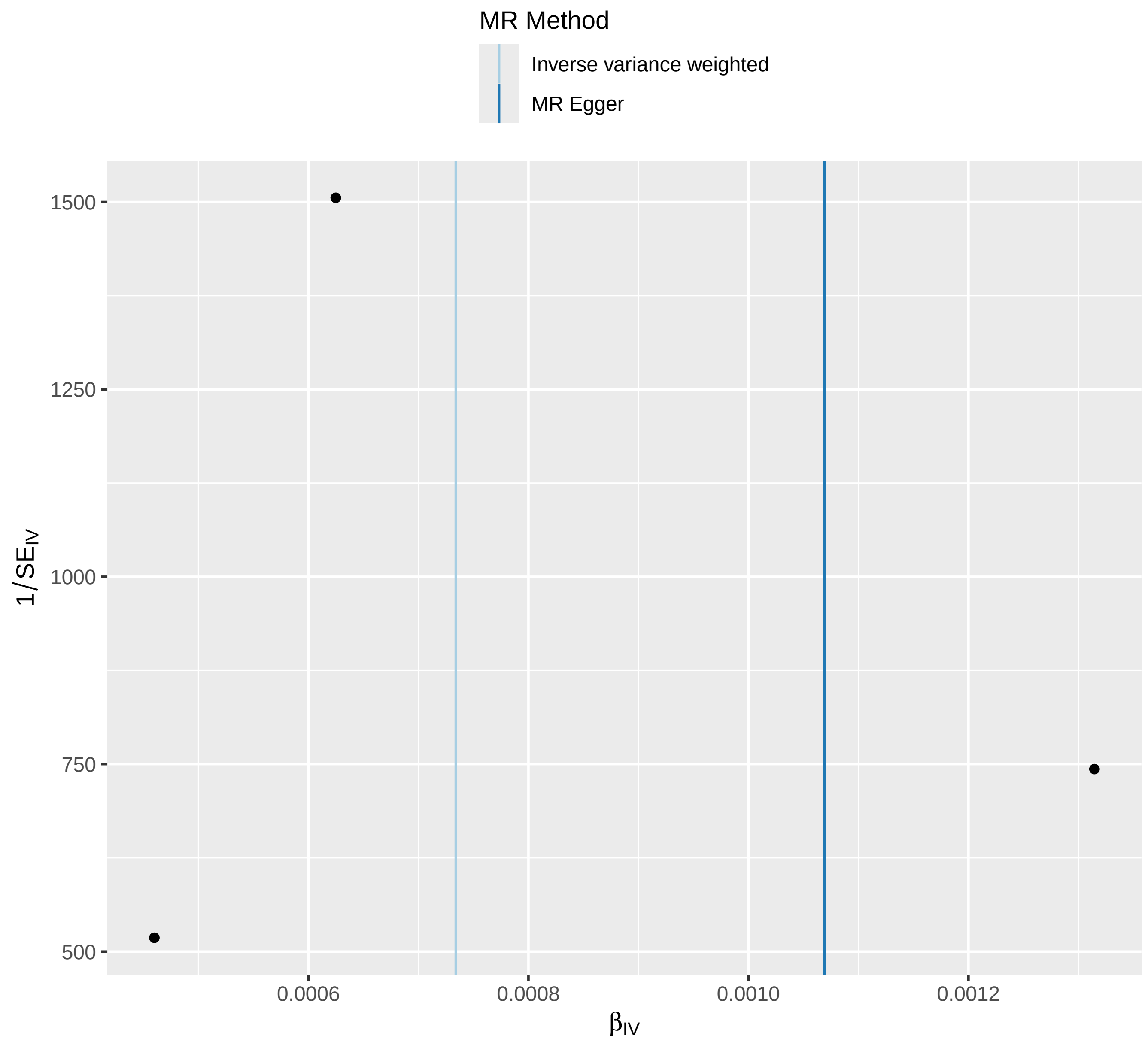

MR Method
Inverse variance weighted
MR Egger
1500
1250
1000
750
500
1/SE$_{IV}$
0.0006
0.0008
0.0010
0.0012
β$_{IV}$

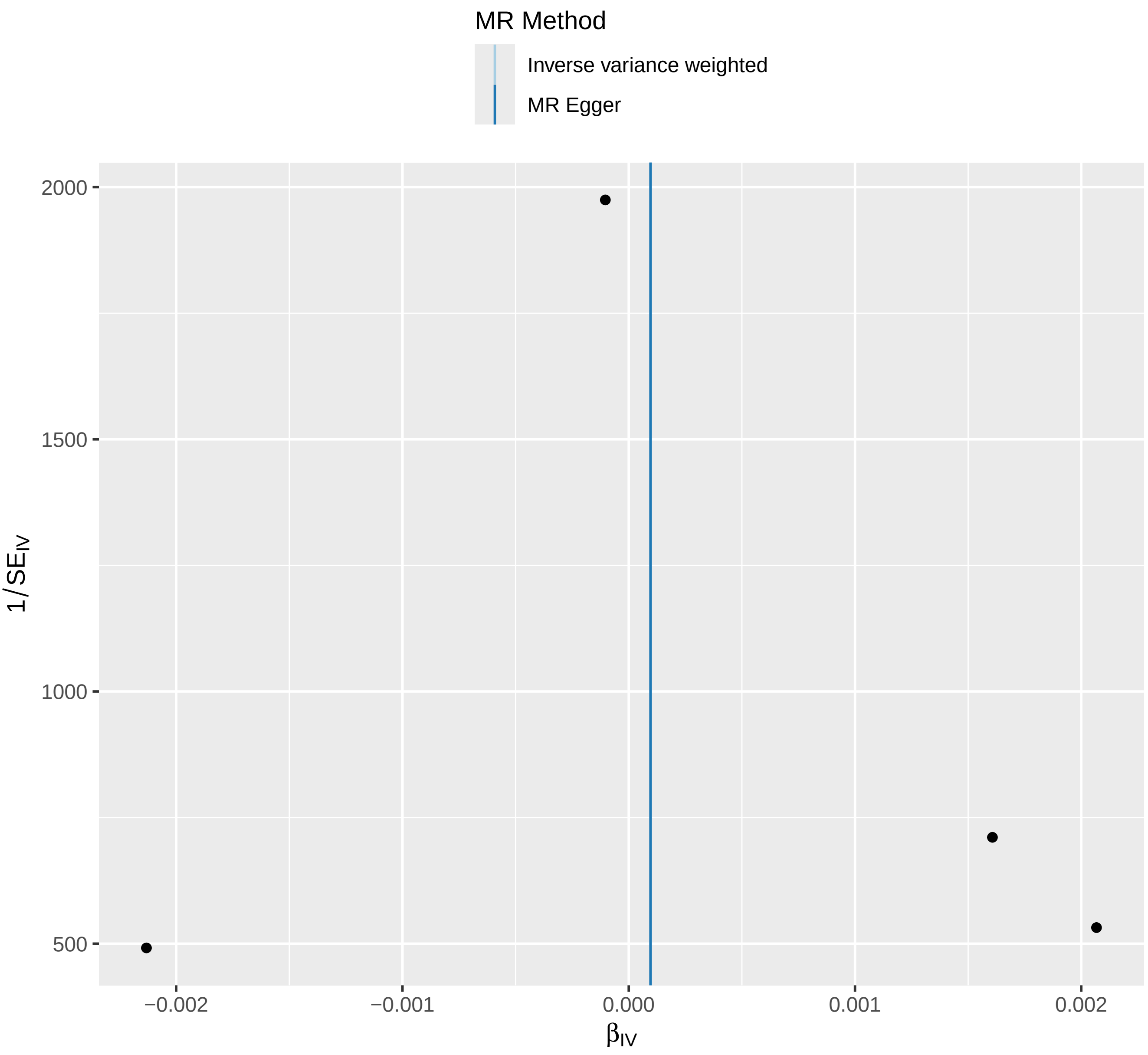
MR Method
Inverse variance weighted
MR Egger
2000
1500
1000
500
1/SE_IV
−0.002
−0.001
0.000
0.001
0.002
β_IV

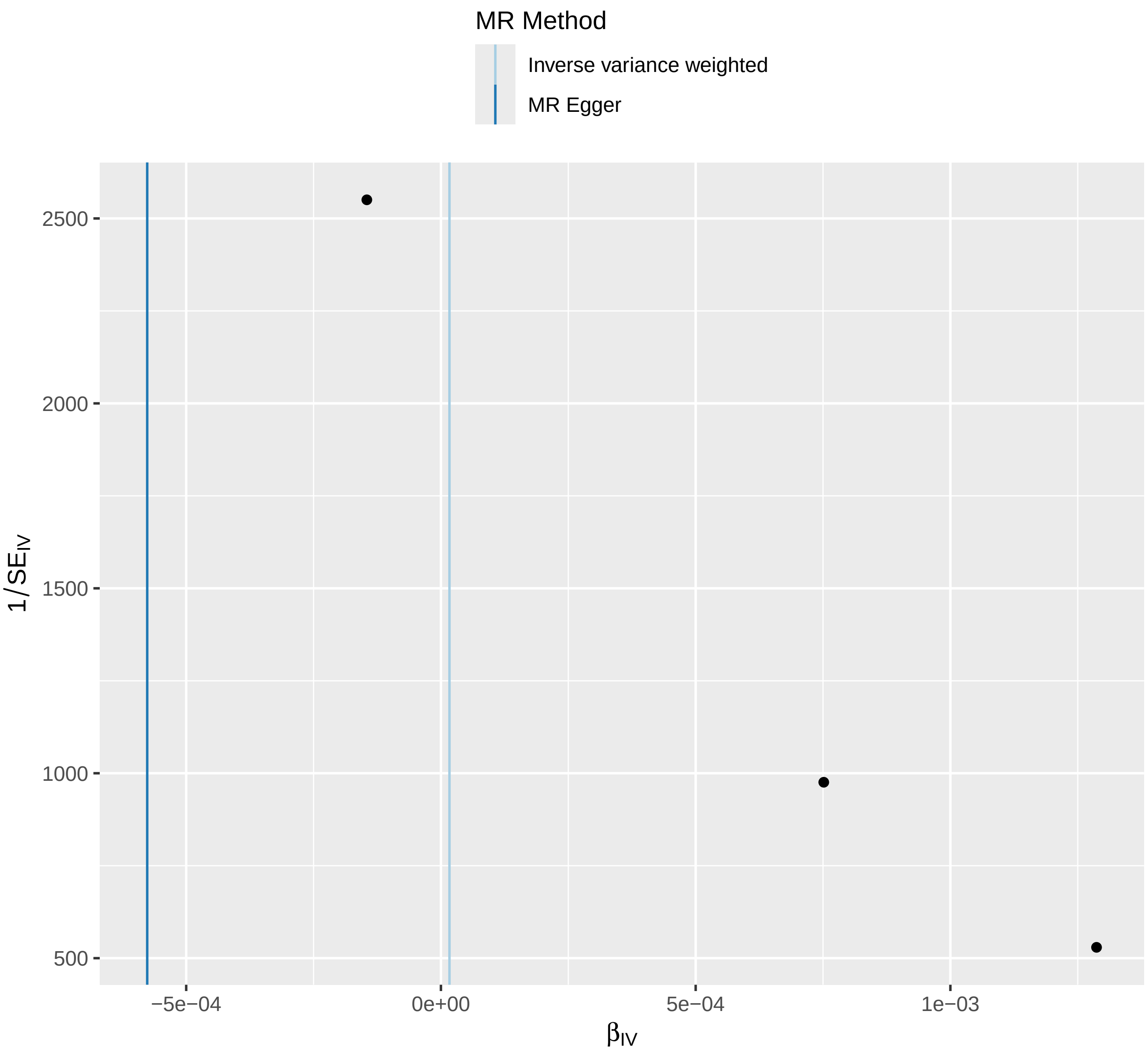
MR Method
Inverse variance weighted
MR Egger
2500
2000
1500
1000
500
$1/SE_{IV}$
−5e−04
0e+00
5e−04
1e−03
β_{IV}

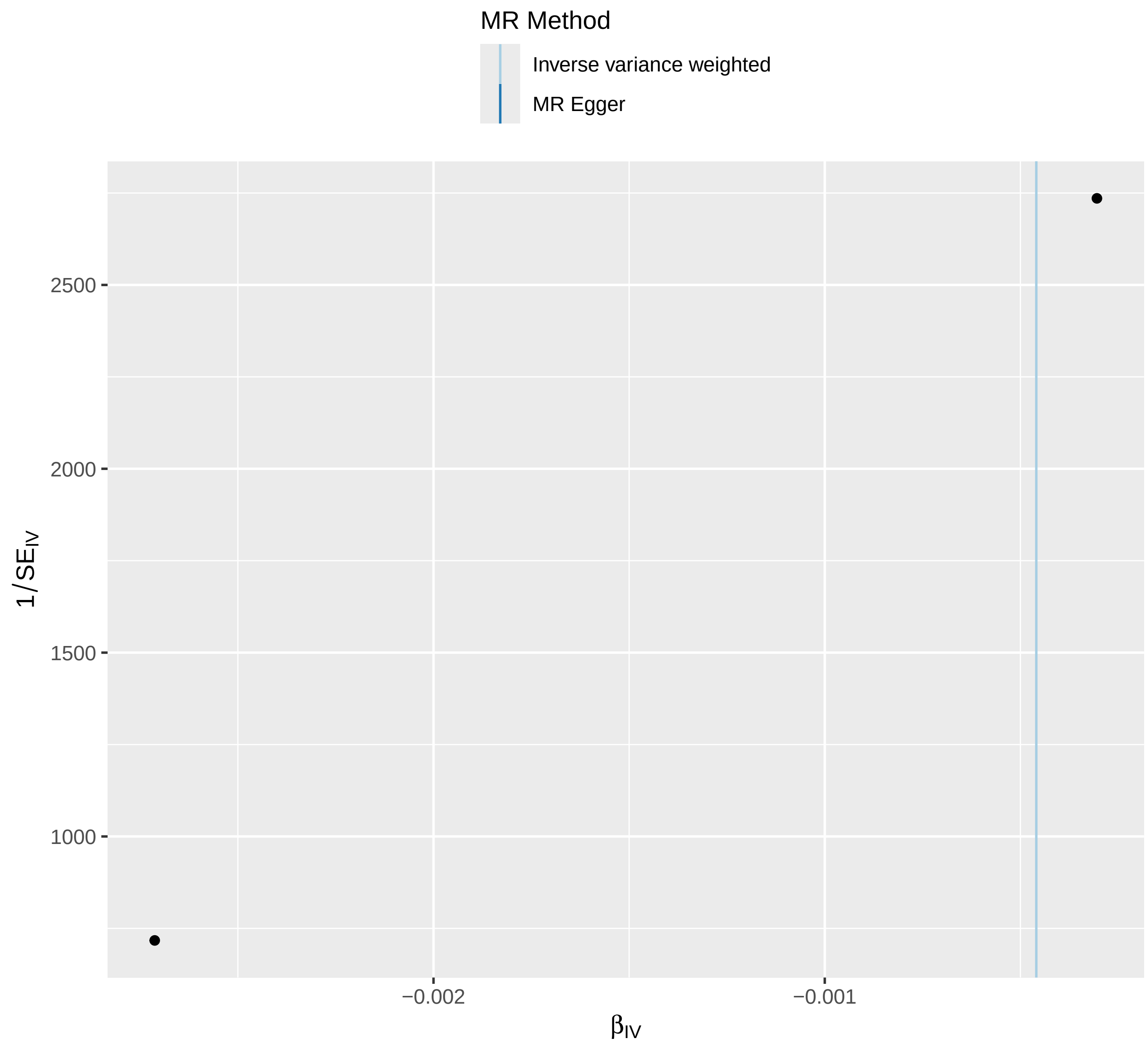
MR Method
Inverse variance weighted
MR Egger
2500
2000
1500
1000
1/SE_IV
−0.002
−0.001
β_IV

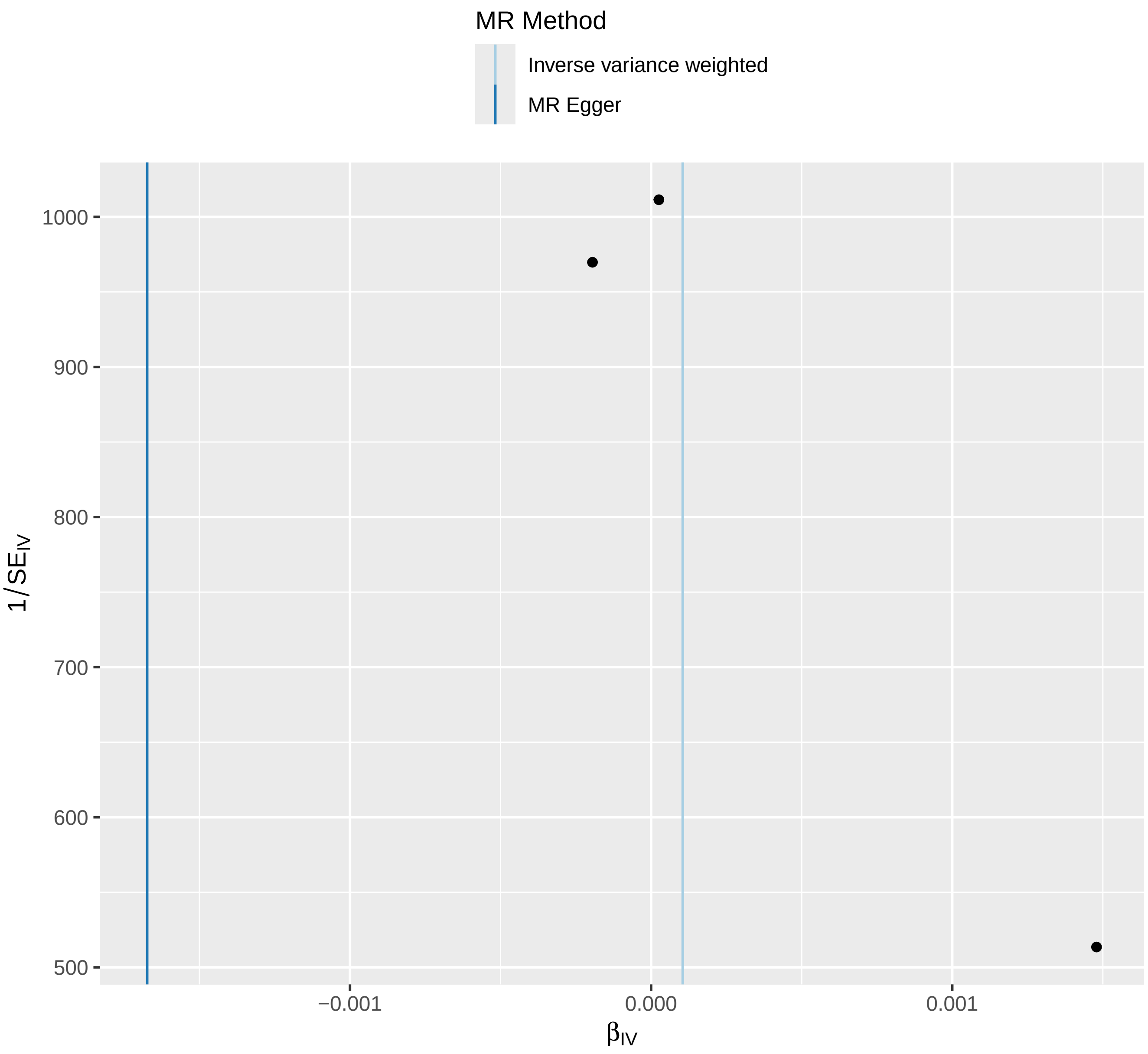
MR Method
Inverse variance weighted
MR Egger
1000
900
800
700
600
500
1/SE_IV
−0.001
0.000
0.001
β_IV

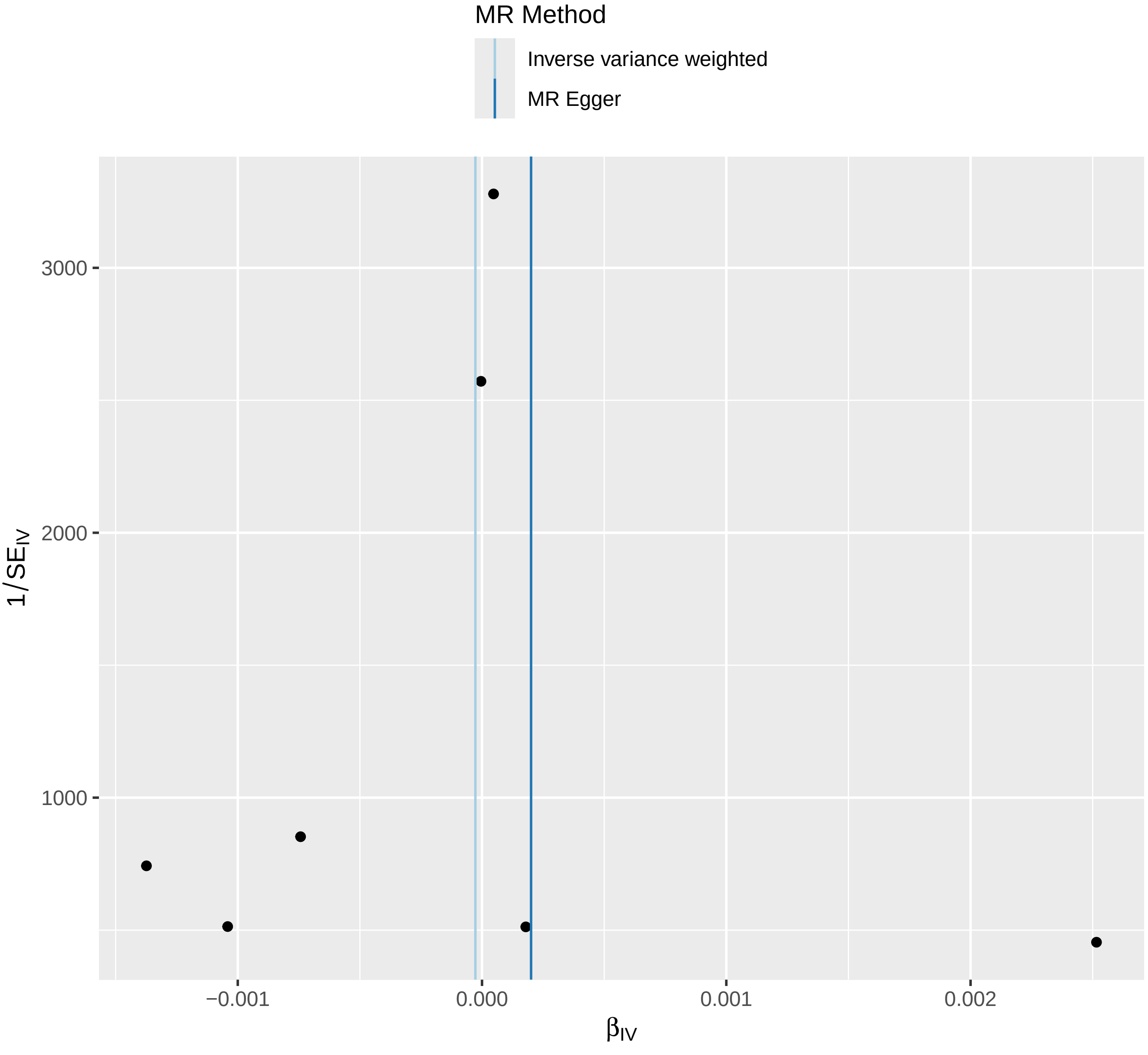

MR Method
Inverse variance weighted
MR Egger
3000
2000
1000
$1/SE_{IV}$
−0.001
0.000
0.001
0.002
β_{IV}

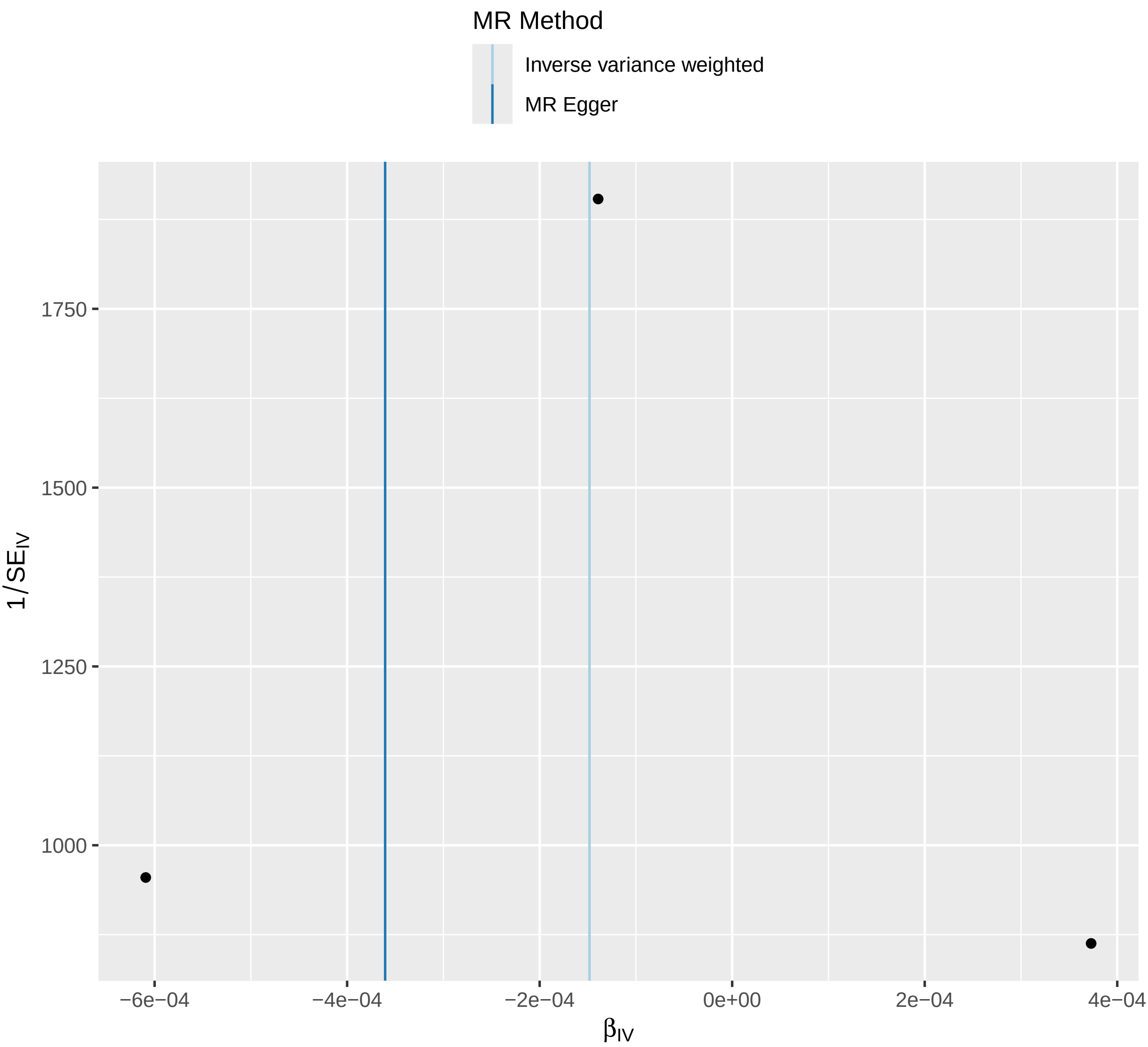

MR Method
Inverse variance weighted
MR Egger
1750
1500
1250
1000
1/SE_IV
−6e−04
−4e−04
−2e−04
0e+00
2e−04
4e−04
β_IV

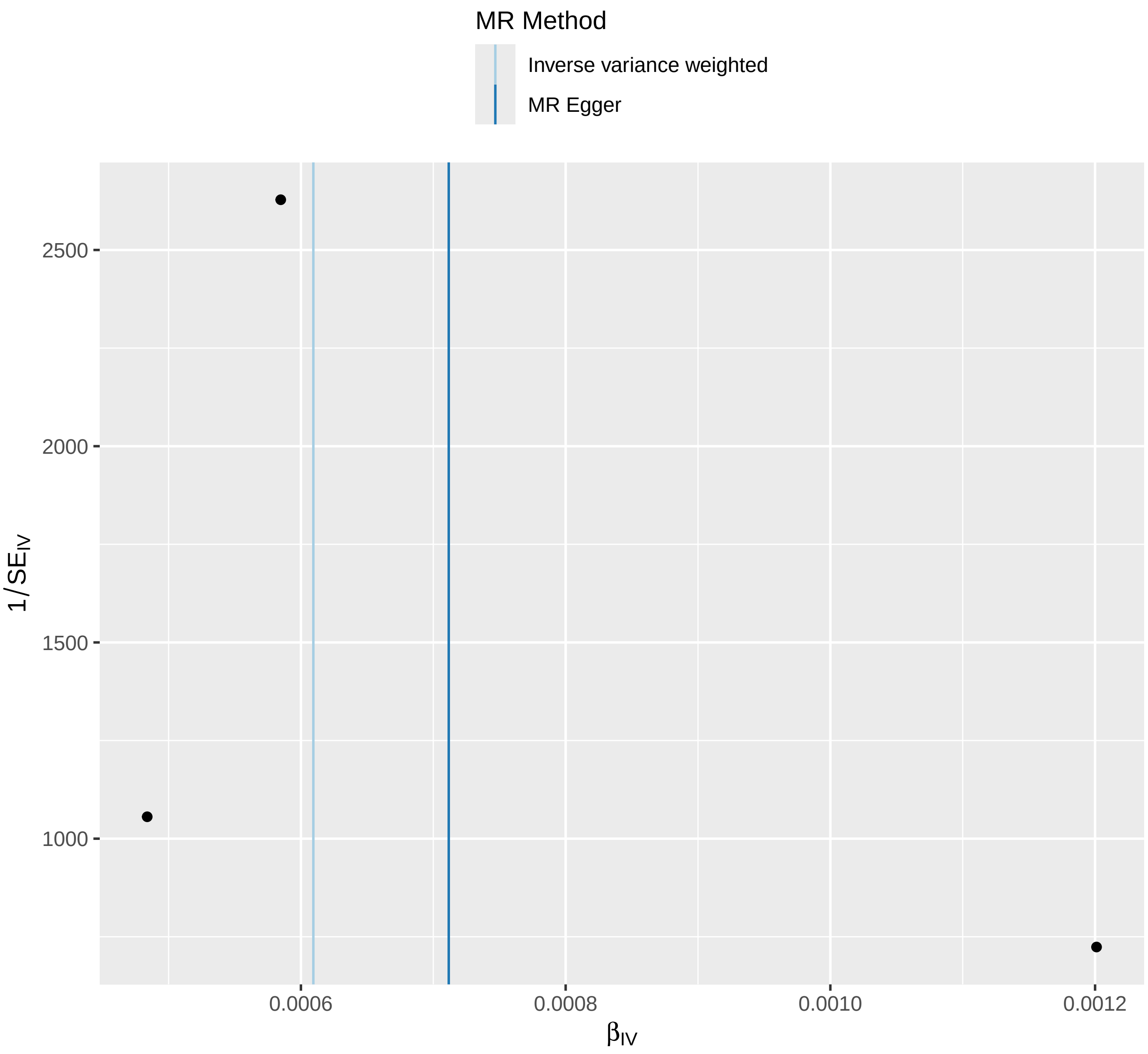

MR Method
Inverse variance weighted
MR Egger
2500
2000
1500
1000
1/SE$_{IV}$
0.0006
0.0008
0.0010
0.0012
β$_{IV}$

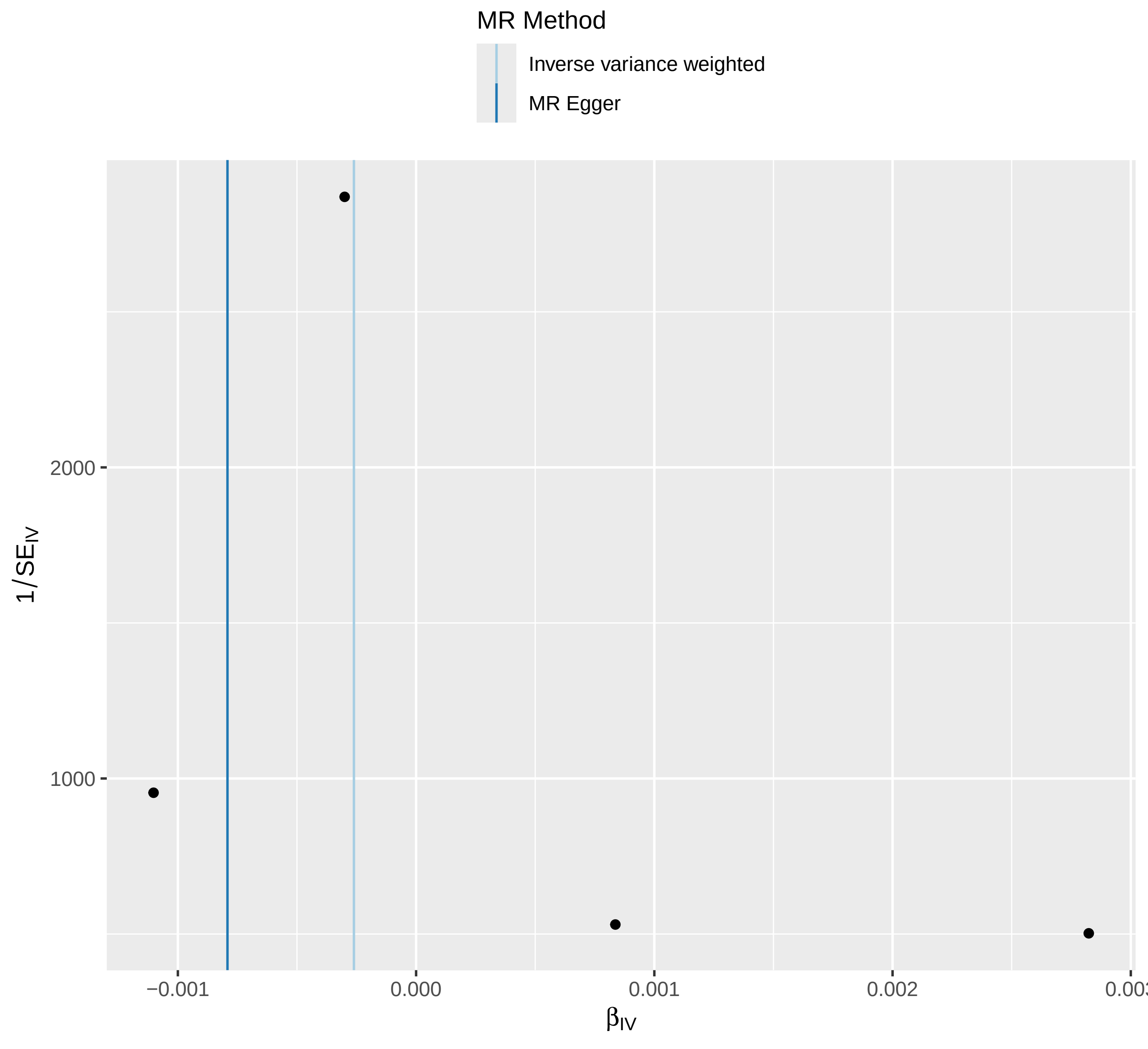

MR Method
Inverse variance weighted
MR Egger
2000
1000
1/SE$_{IV}$
−0.001
0.000
0.001
0.002
β$_{IV}$

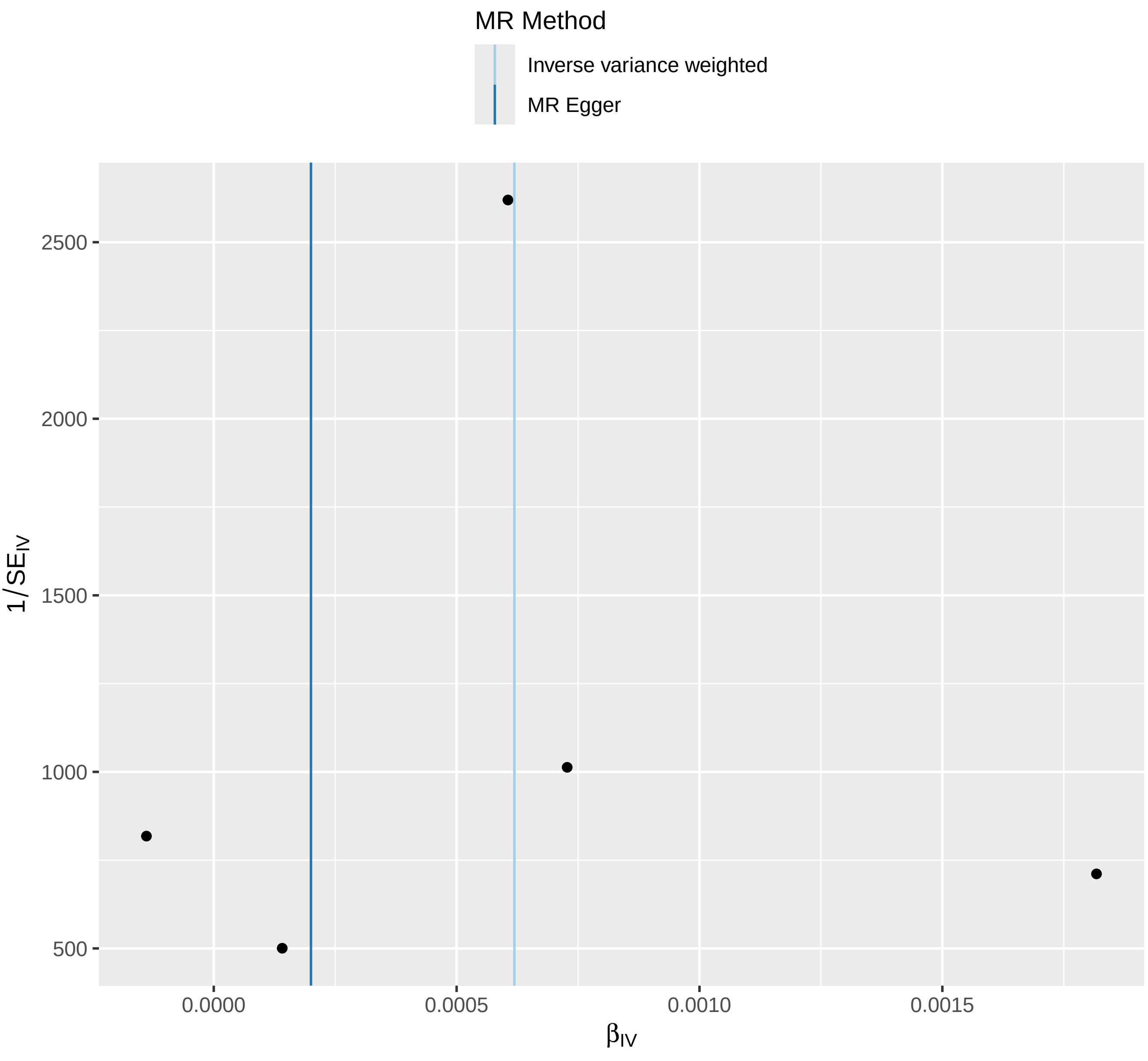
MR Method
Inverse variance weighted
MR Egger
2500
2000
1500
1000
500
$1/SE_{IV}$
0.0000
0.0005
0.0010
0.0015
β_{IV}

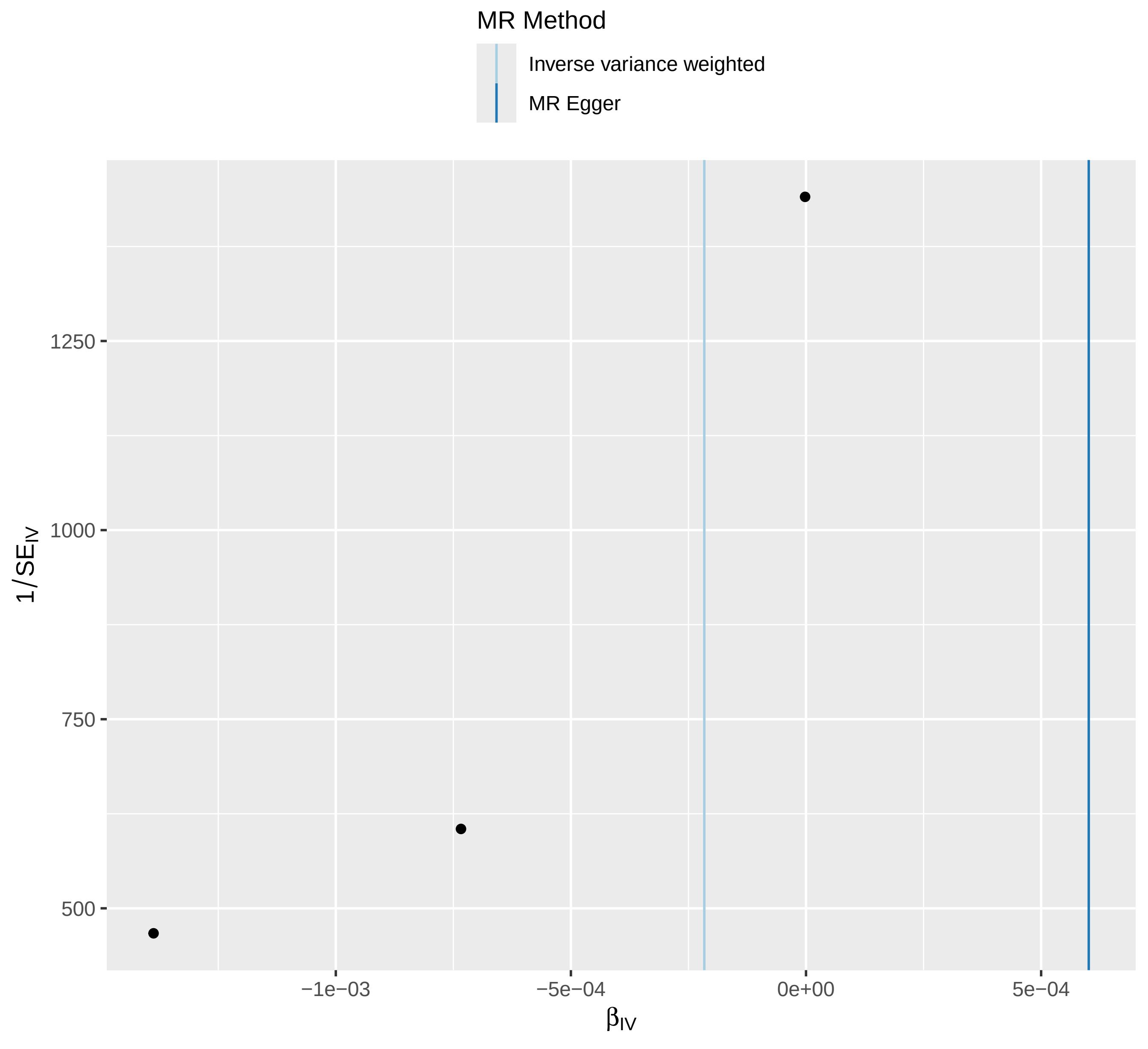

MR Method
Inverse variance weighted
MR Egger
1250
1000
750
500
1/SE$_{IV}$
−1e−03
−5e−04
0e+00
5e−04
β_{IV}

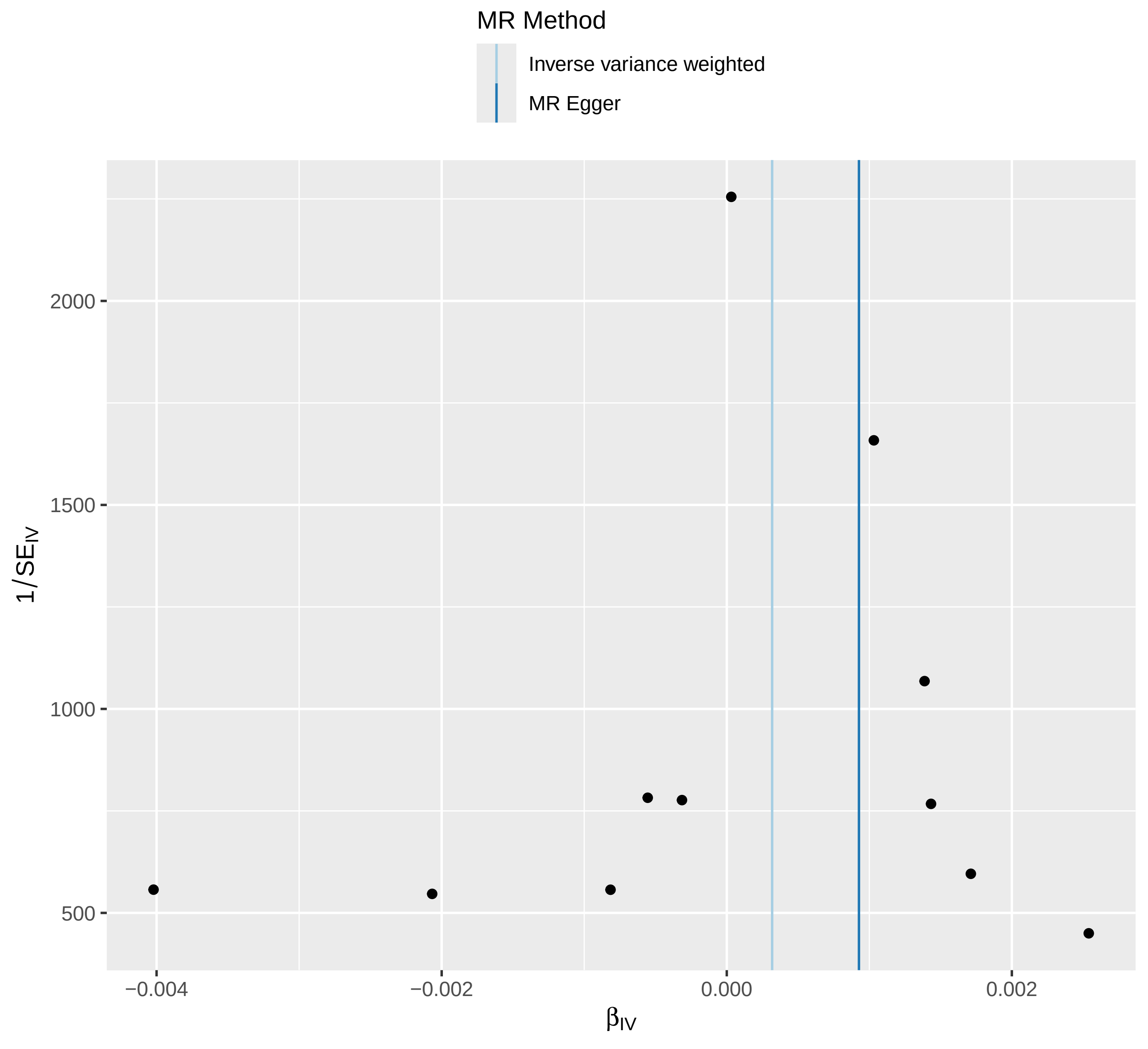

MR Method
Inverse variance weighted
MR Egger
2000
1500
1000
500
1/SE_IV
−0.004
−0.002
0.000
0.002
β_IV

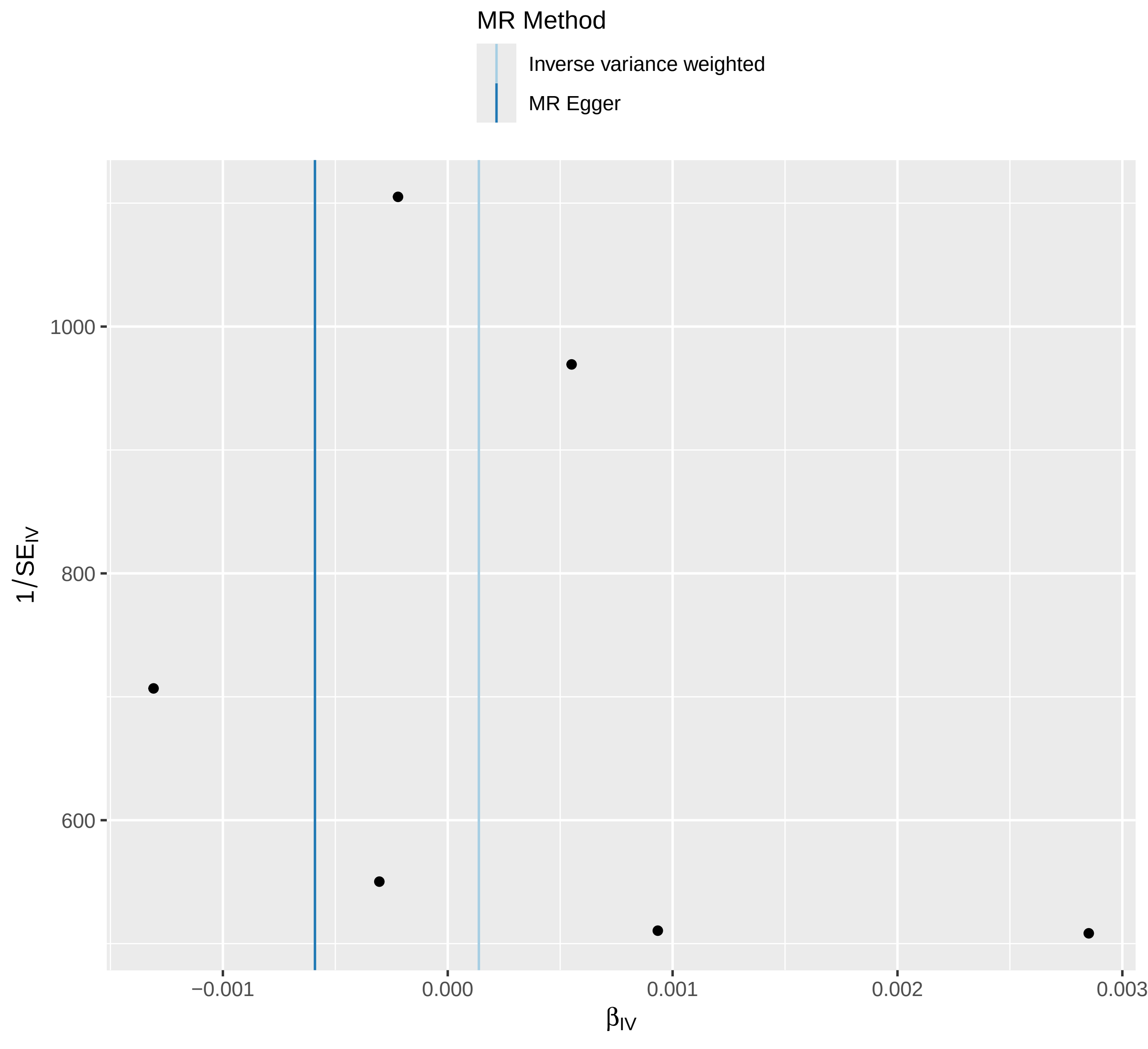
MR Method
Inverse variance weighted
MR Egger
1/SE_IV
1000
800
600
−0.001
0.000
0.001
0.002
0.003
β_IV

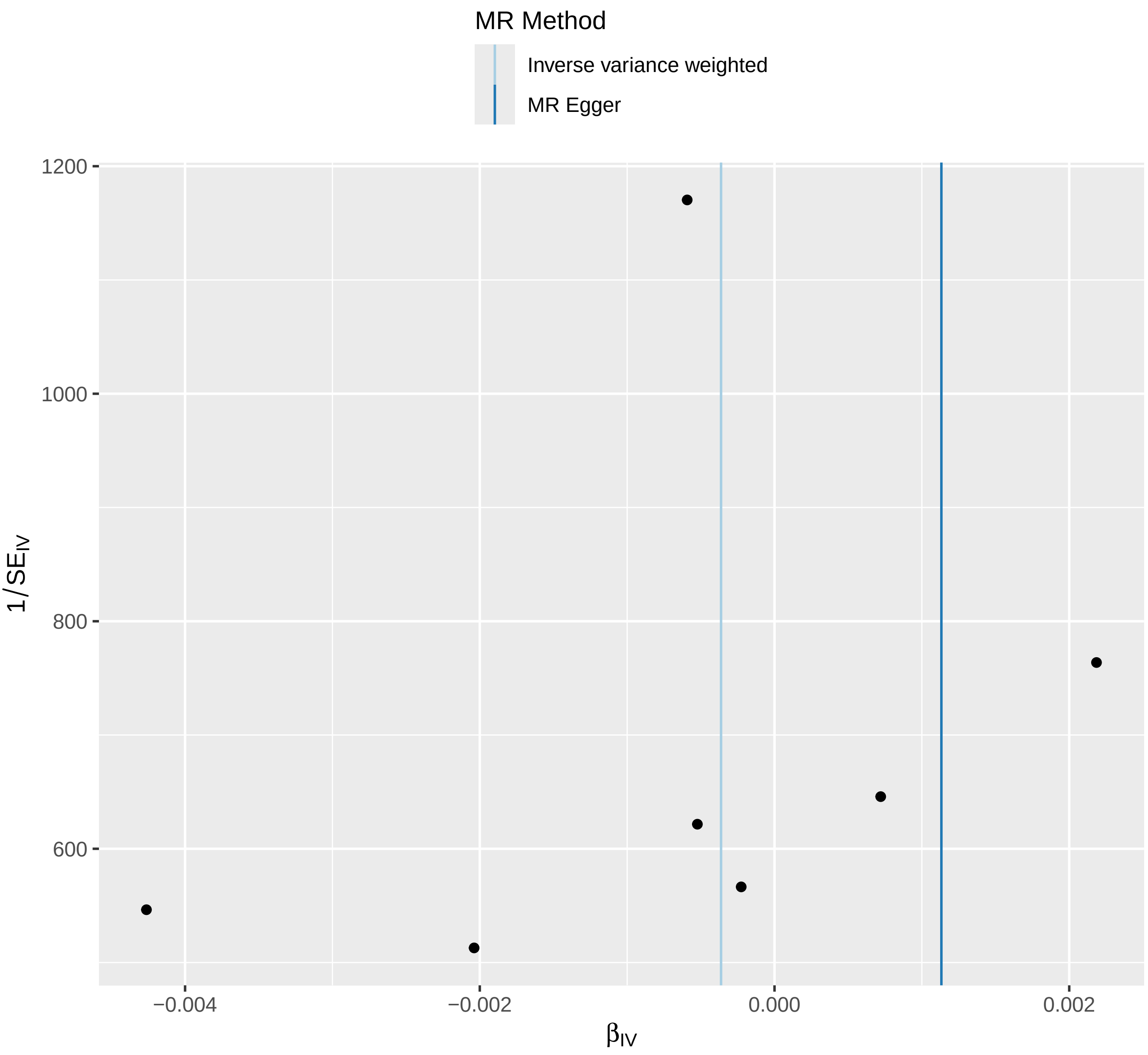
MR Method
Inverse variance weighted
MR Egger
1200
1000
800
600
1/SE_IV
−0.004
−0.002
0.000
0.002
β_IV

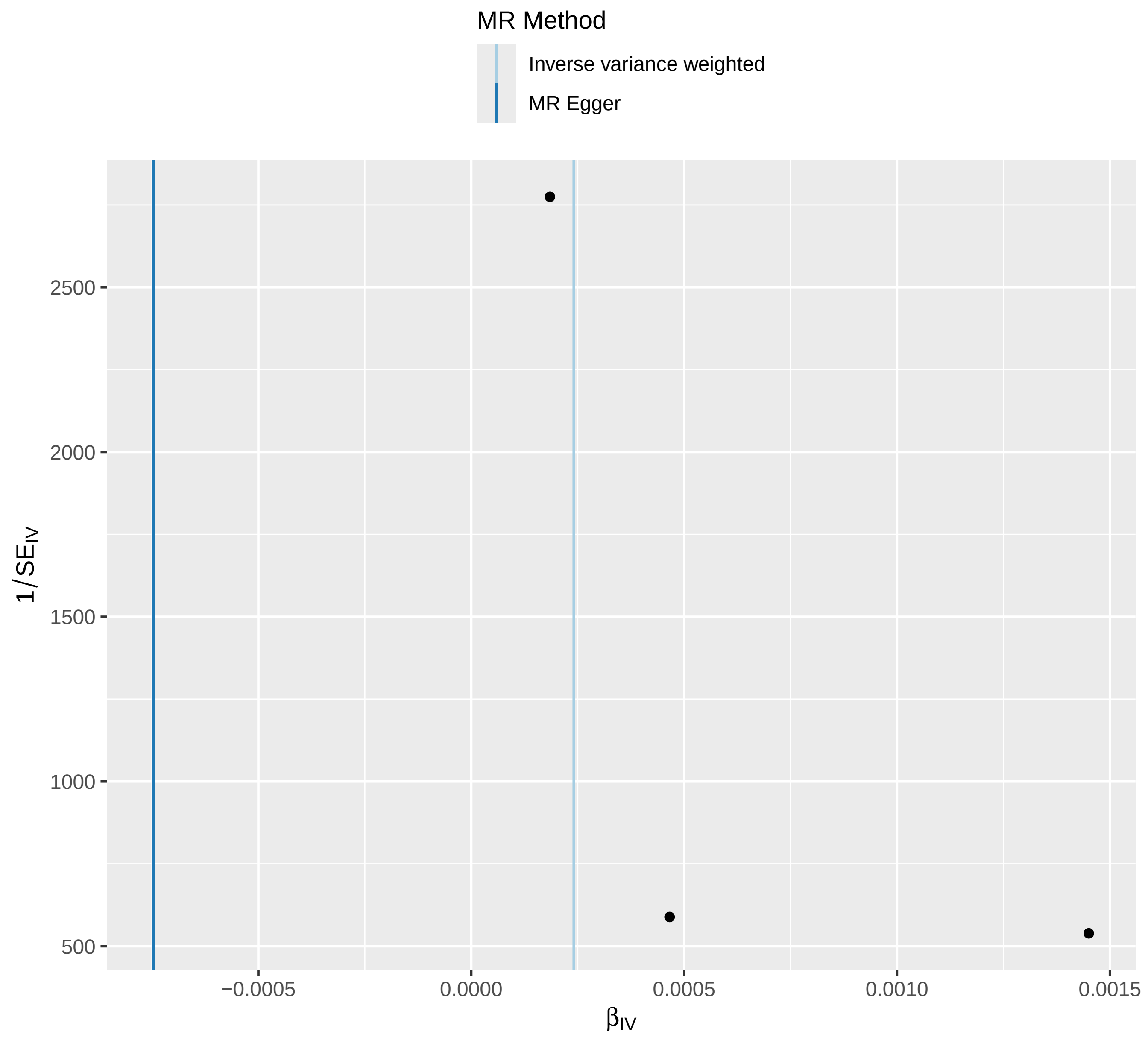
MR Method
Inverse variance weighted
MR Egger
2500
2000
1500
1000
500
1/SE$_{IV}$
−0.0005
0.0000
0.0005
0.0010
0.0015
β$_{IV}$

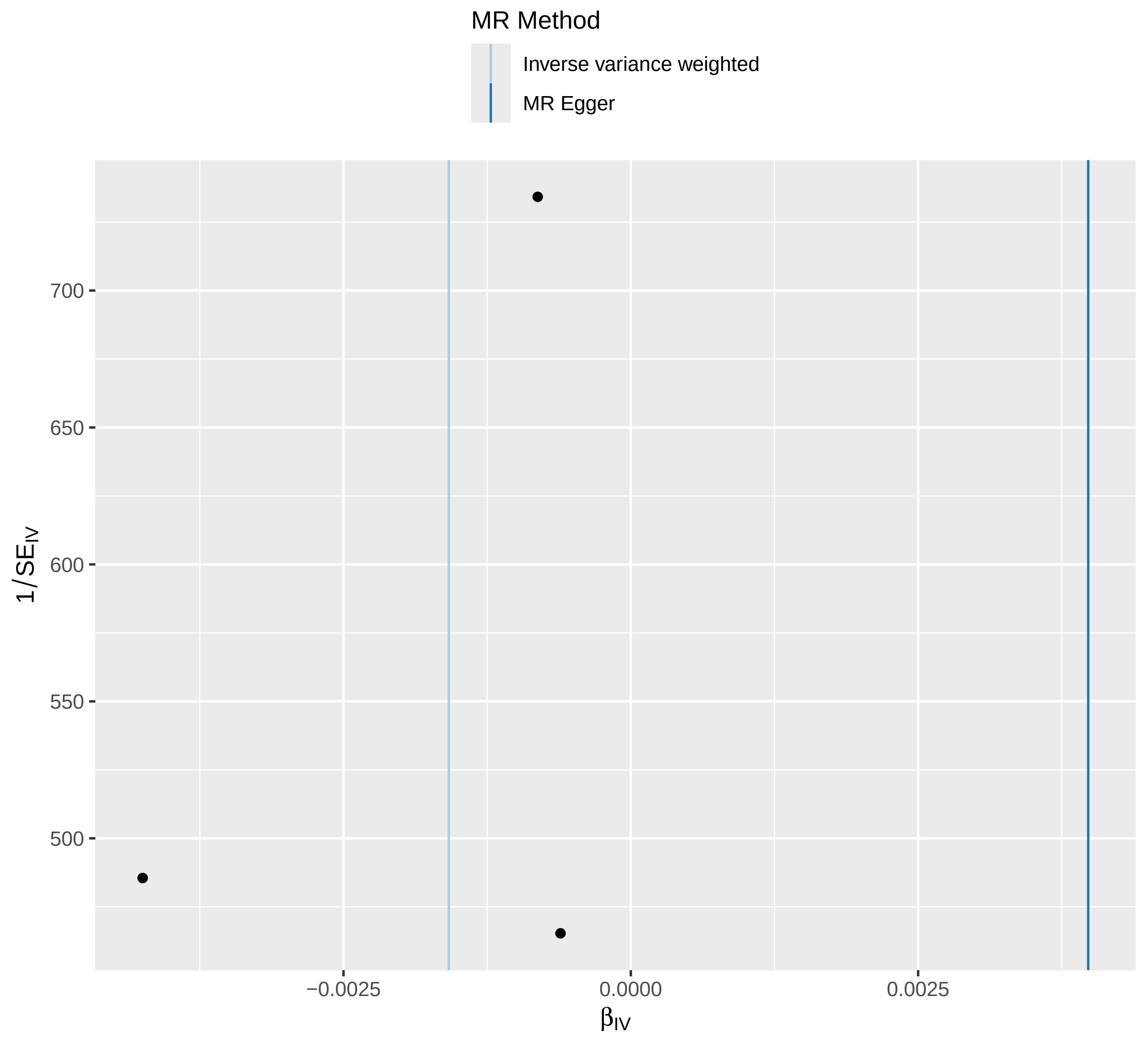

MR Method
Inverse variance weighted
MR Egger
700
650
600
550
500
$1/SE_{IV}$
−0.0025
0.0000
0.0025
β_{IV}

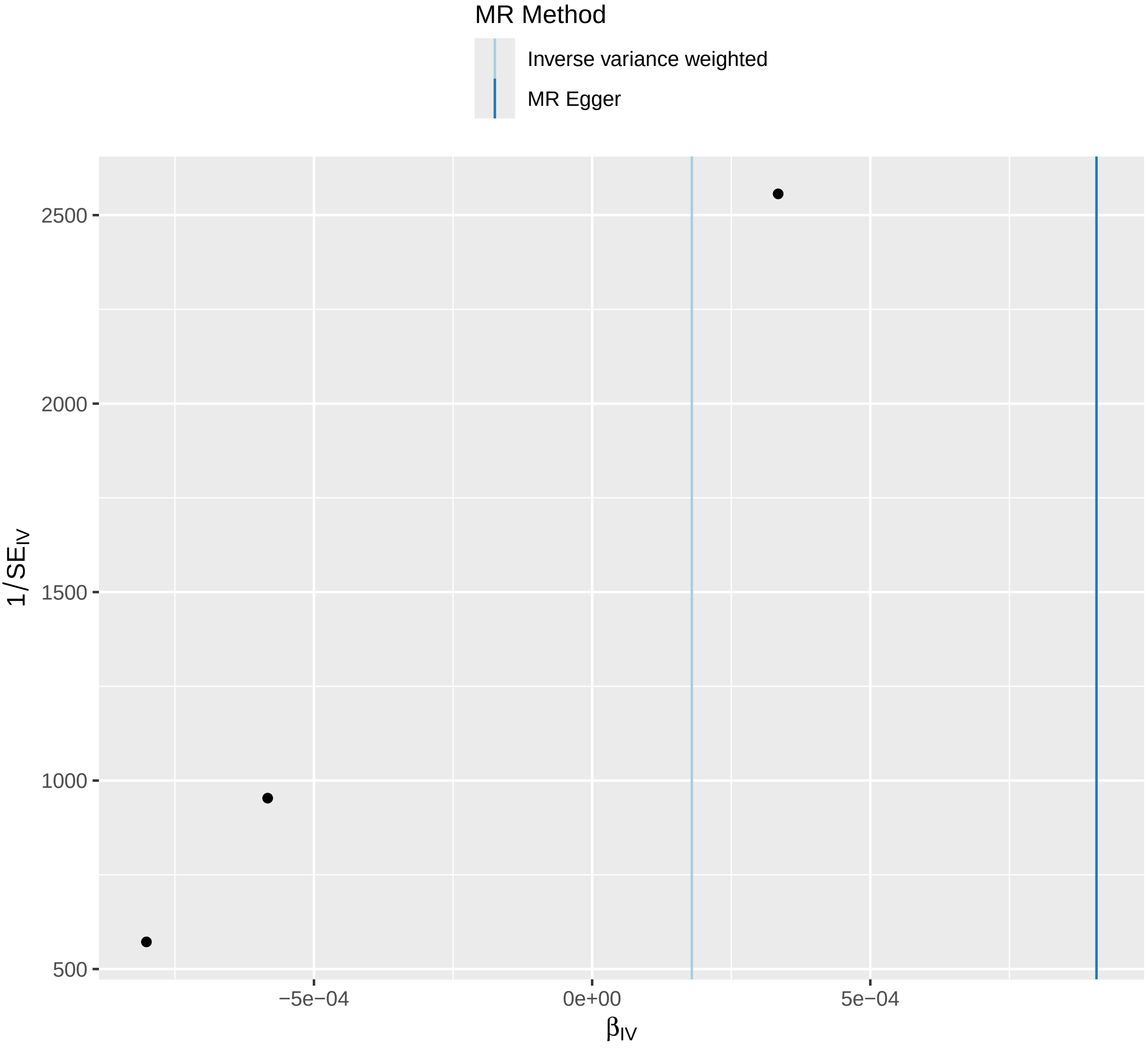
MR Method
Inverse variance weighted
MR Egger
2500
2000
1500
1000
500
1/SE$_{IV}$
−5e−04
0e+00
5e−04
β_{IV}

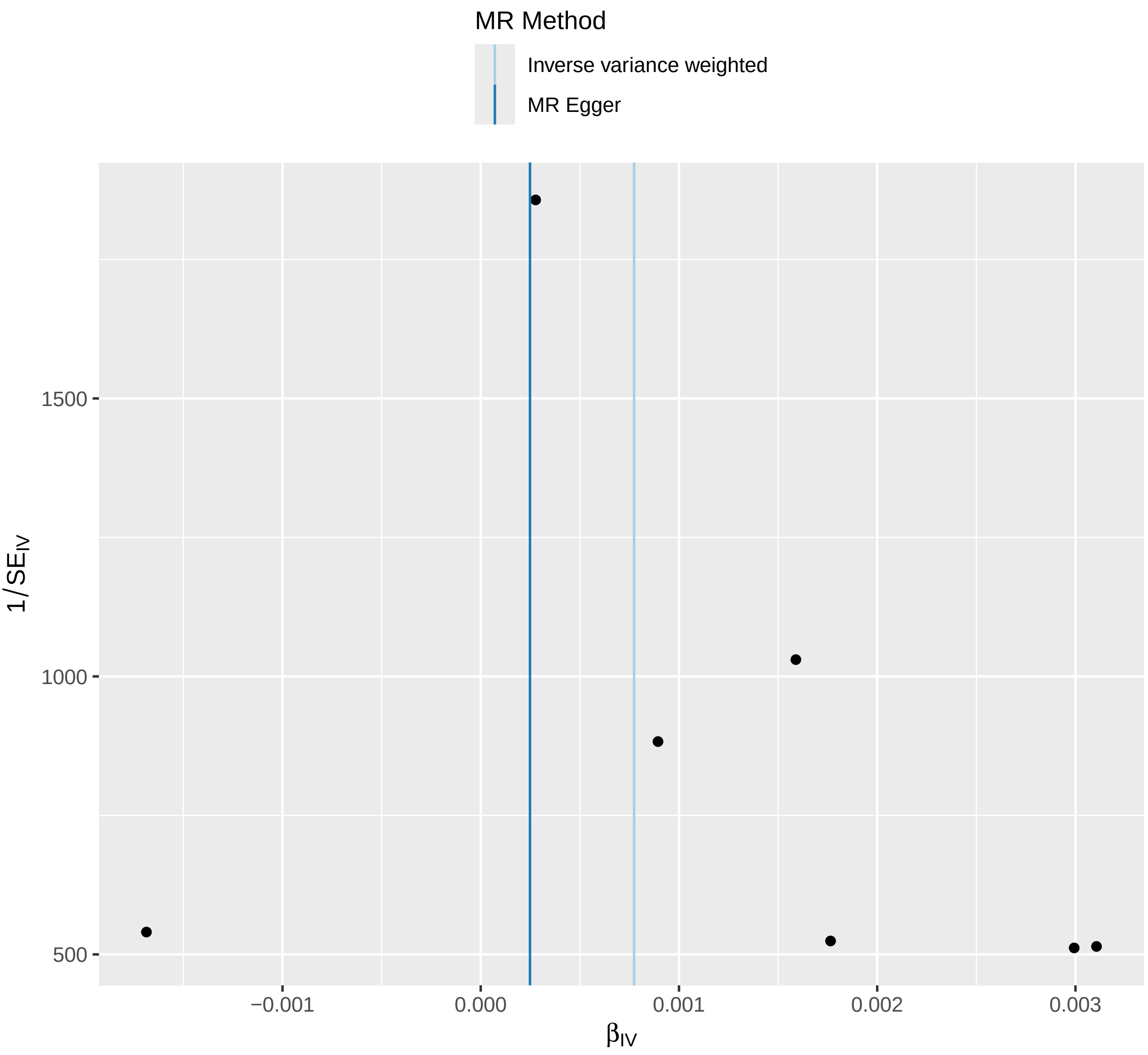

MR Method
Inverse variance weighted
MR Egger
1500
1000
500
1/SE_IV
−0.001
0.000
0.001
0.002
0.003
β_IV

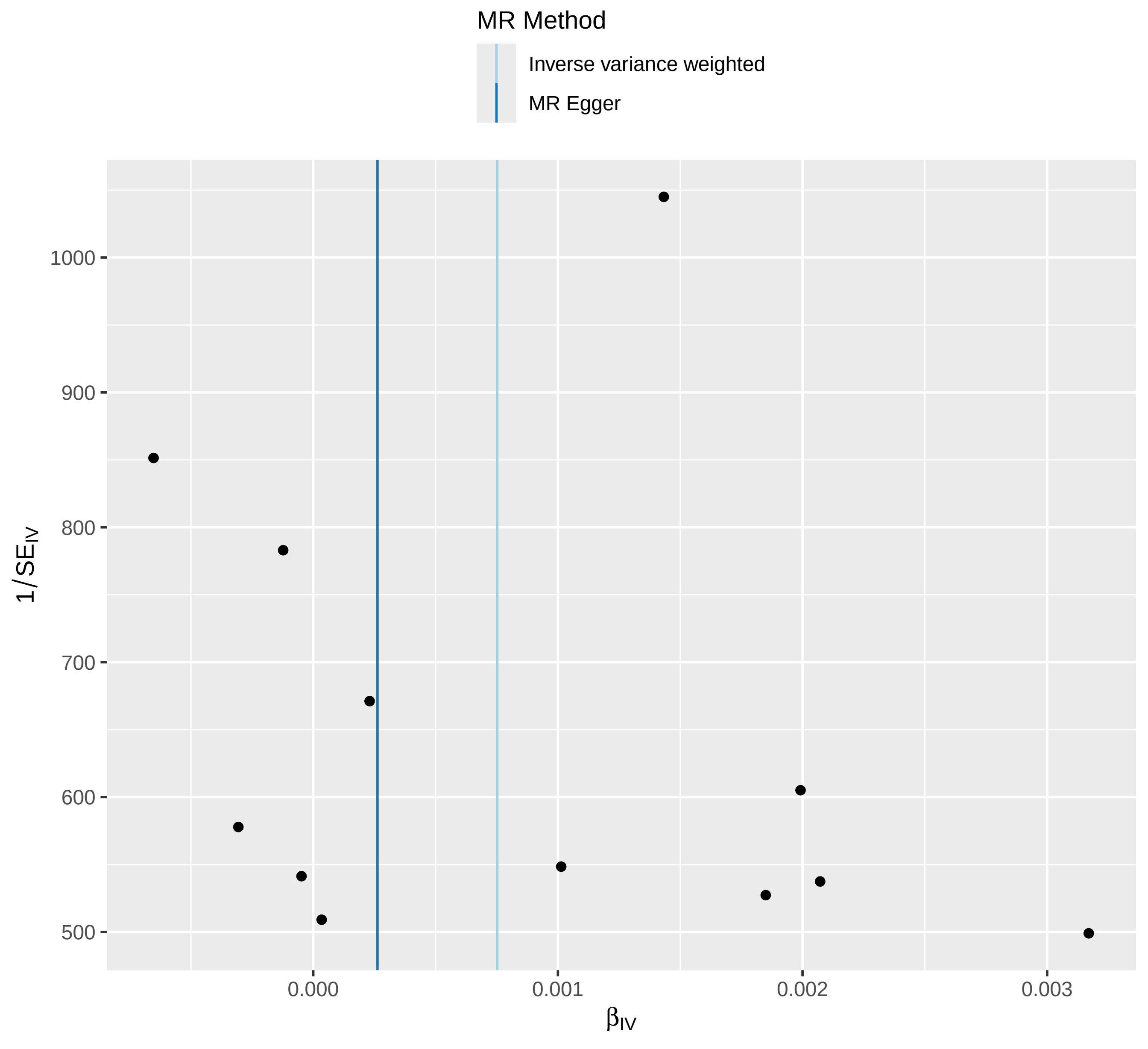
MR Method
Inverse variance weighted
MR Egger
1000
900
800
700
600
500
$1/SE_{IV}$
0.000
0.001
0.002
0.003
β_{IV}

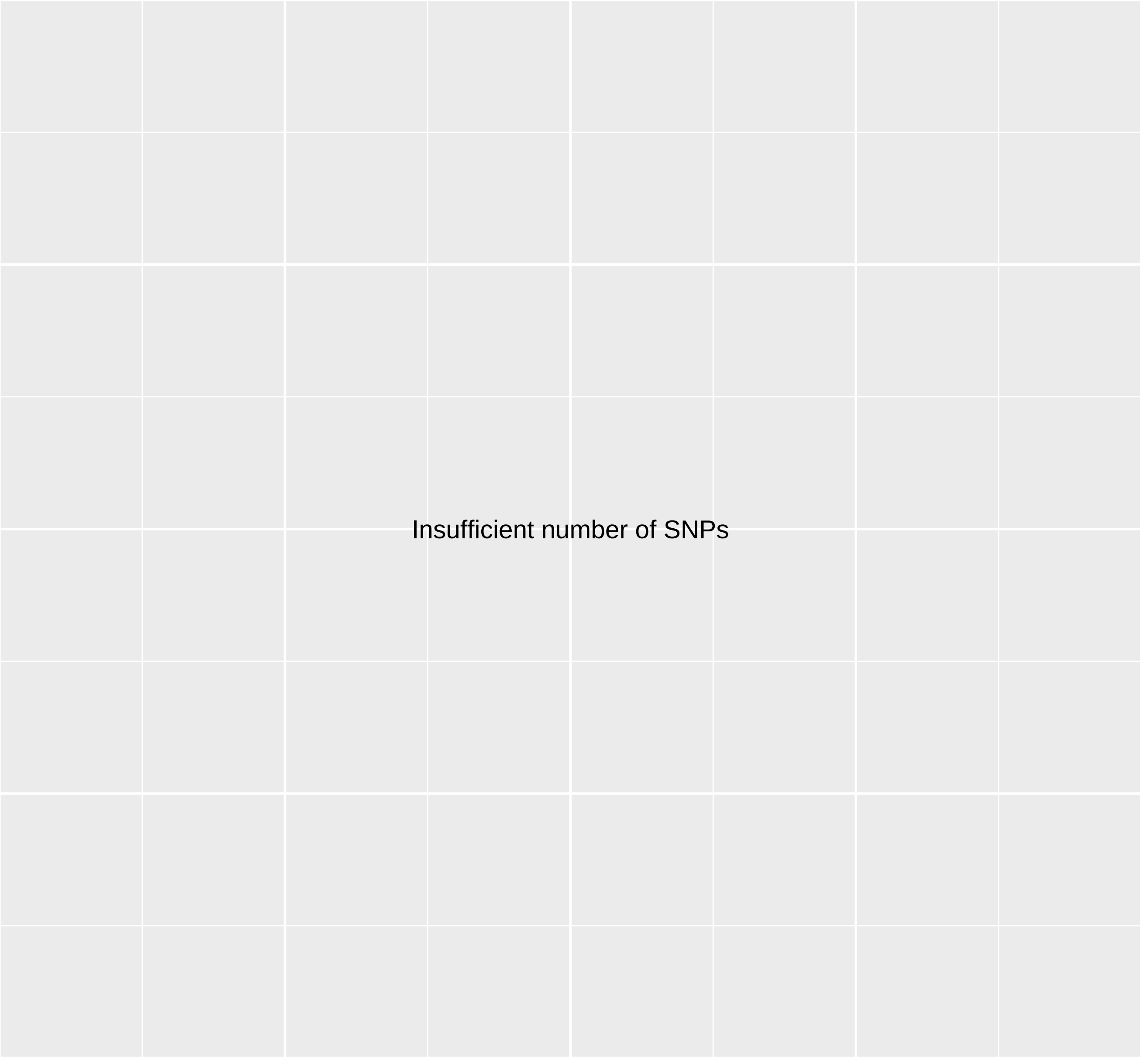
Insufficient number of SNPs

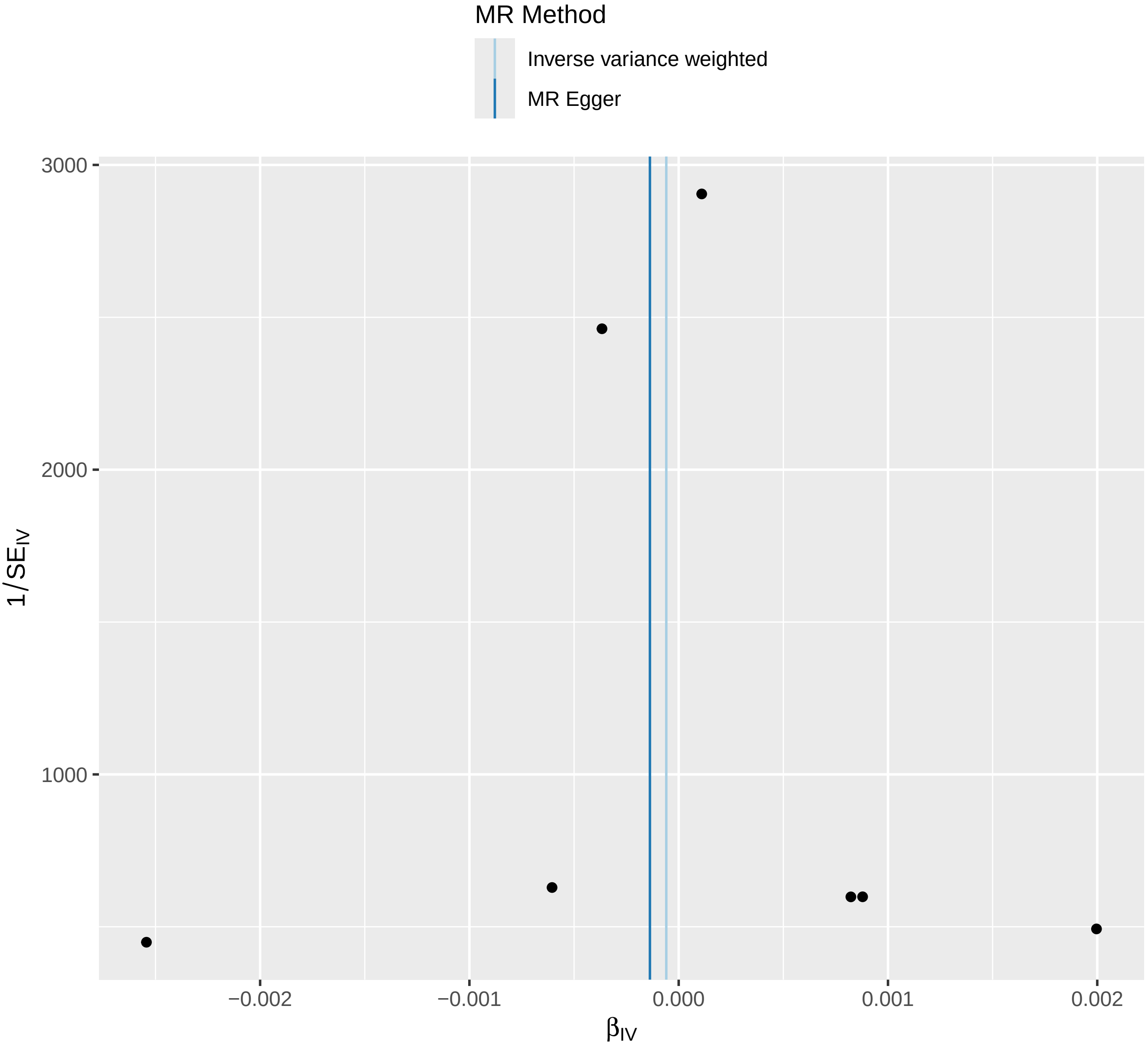

MR Method
Inverse variance weighted
MR Egger
3000
2000
1000
1/SE_IV
−0.002
−0.001
0.000
0.001
0.002
β_IV

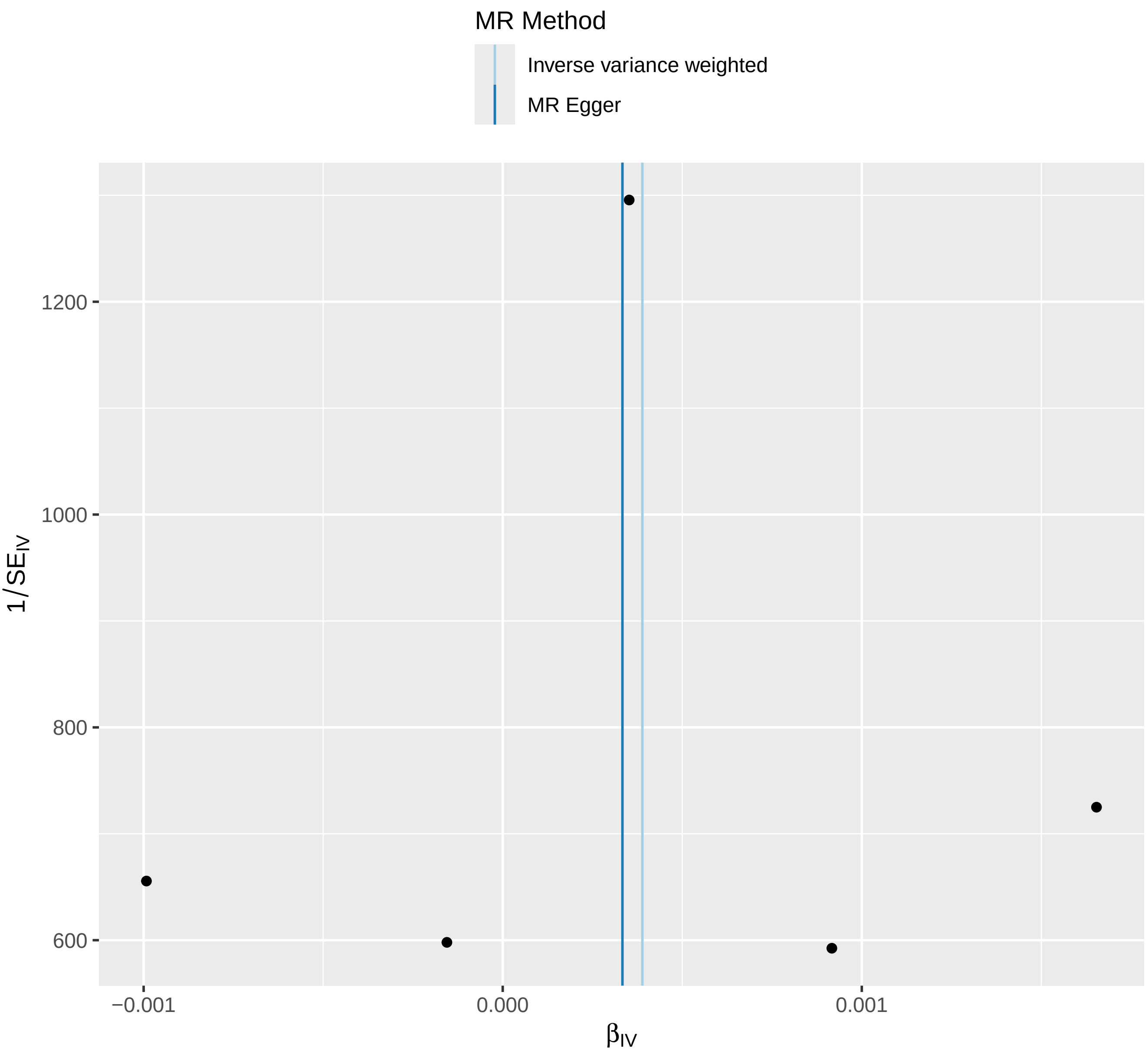

MR Method
Inverse variance weighted
MR Egger
1200
1000
800
600
1/SE$_{IV}$
−0.001
0.000
0.001
β$_{IV}$

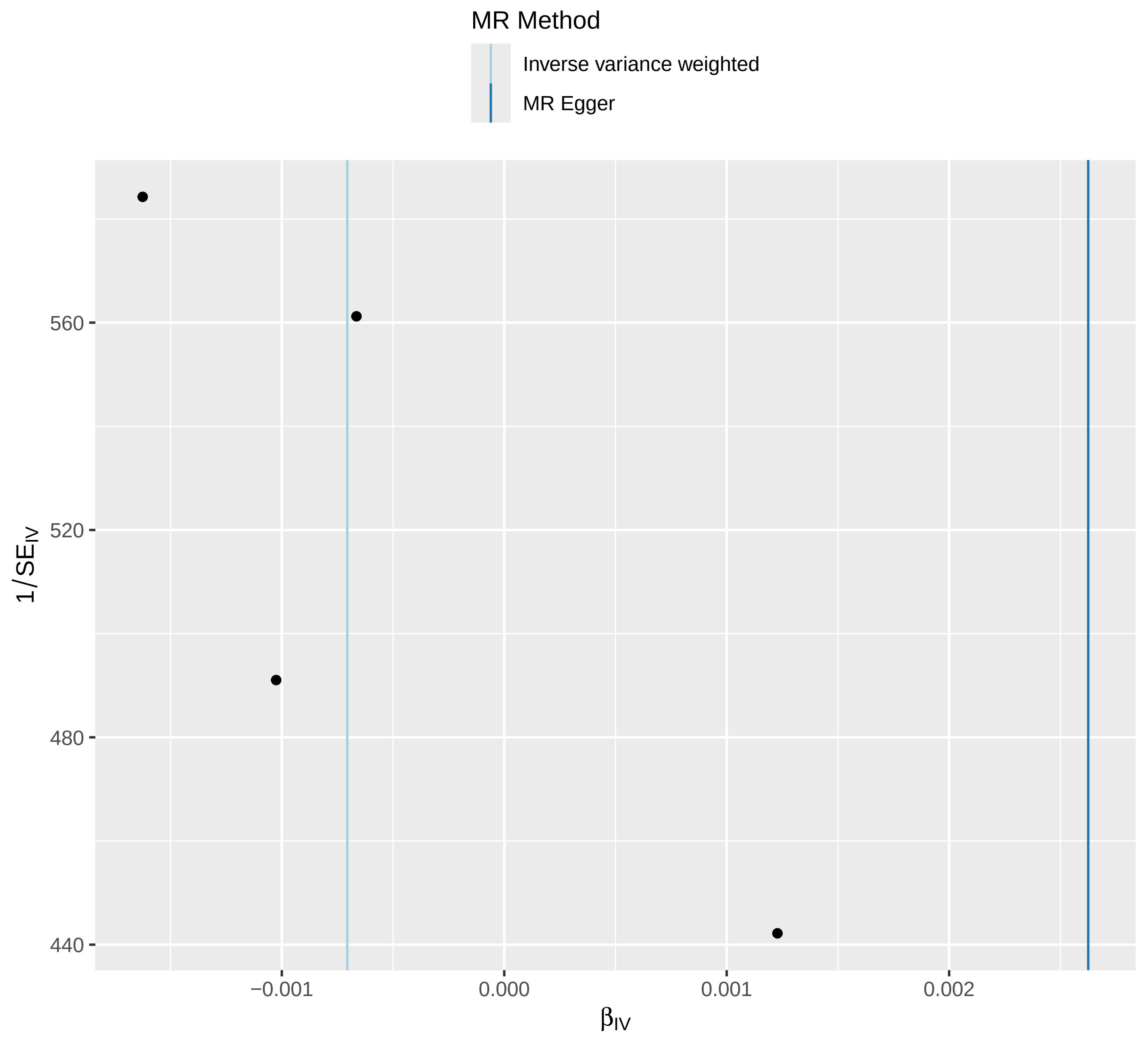

MR Method
Inverse variance weighted
MR Egger
560
520
480
440
1/SE_IV
−0.001
0.000
0.001
0.002
β_IV

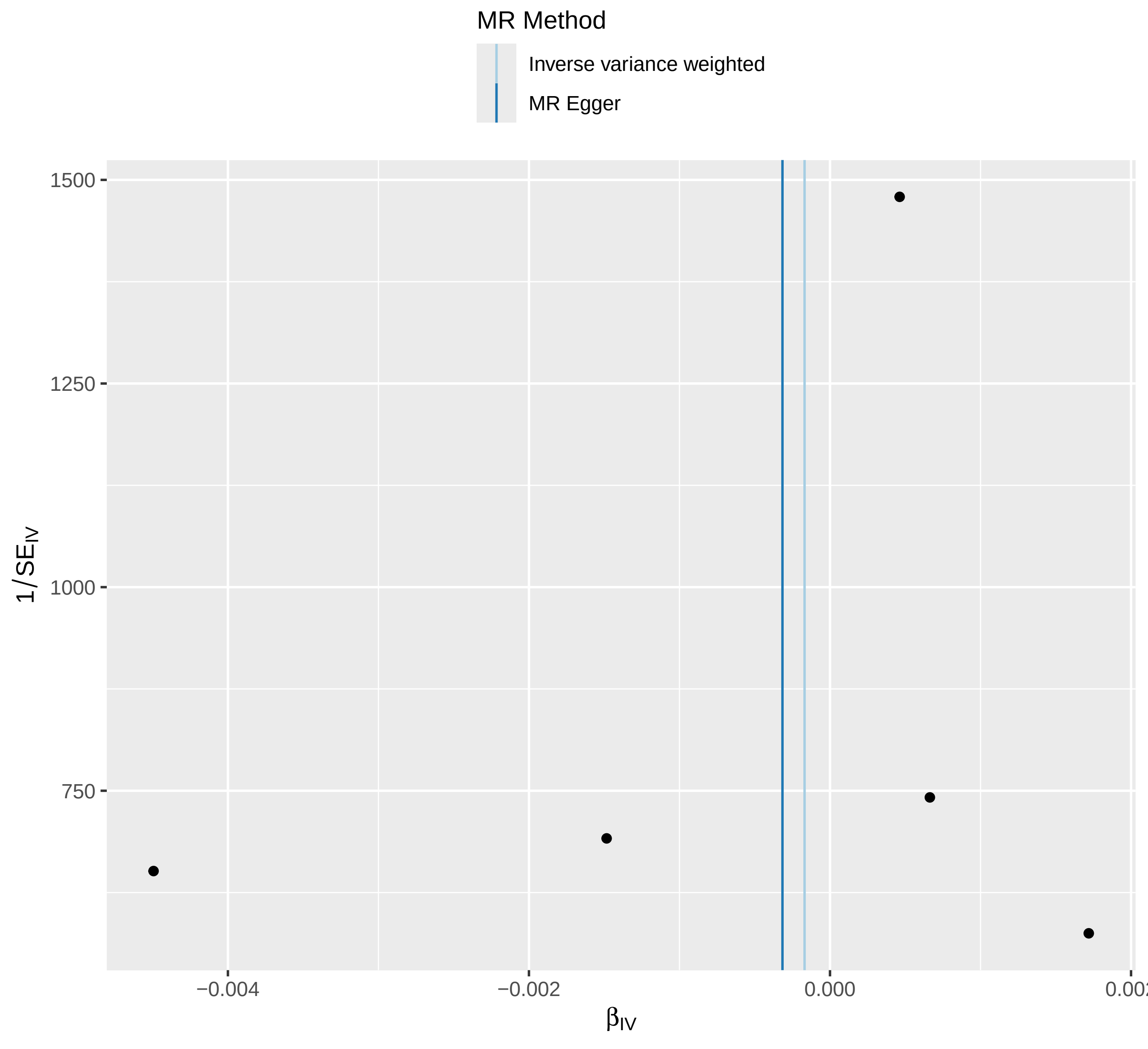

MR Method
Inverse variance weighted
MR Egger
1500
1250
1000
750
1/SE_IV
−0.004
−0.002
0.000
β_IV

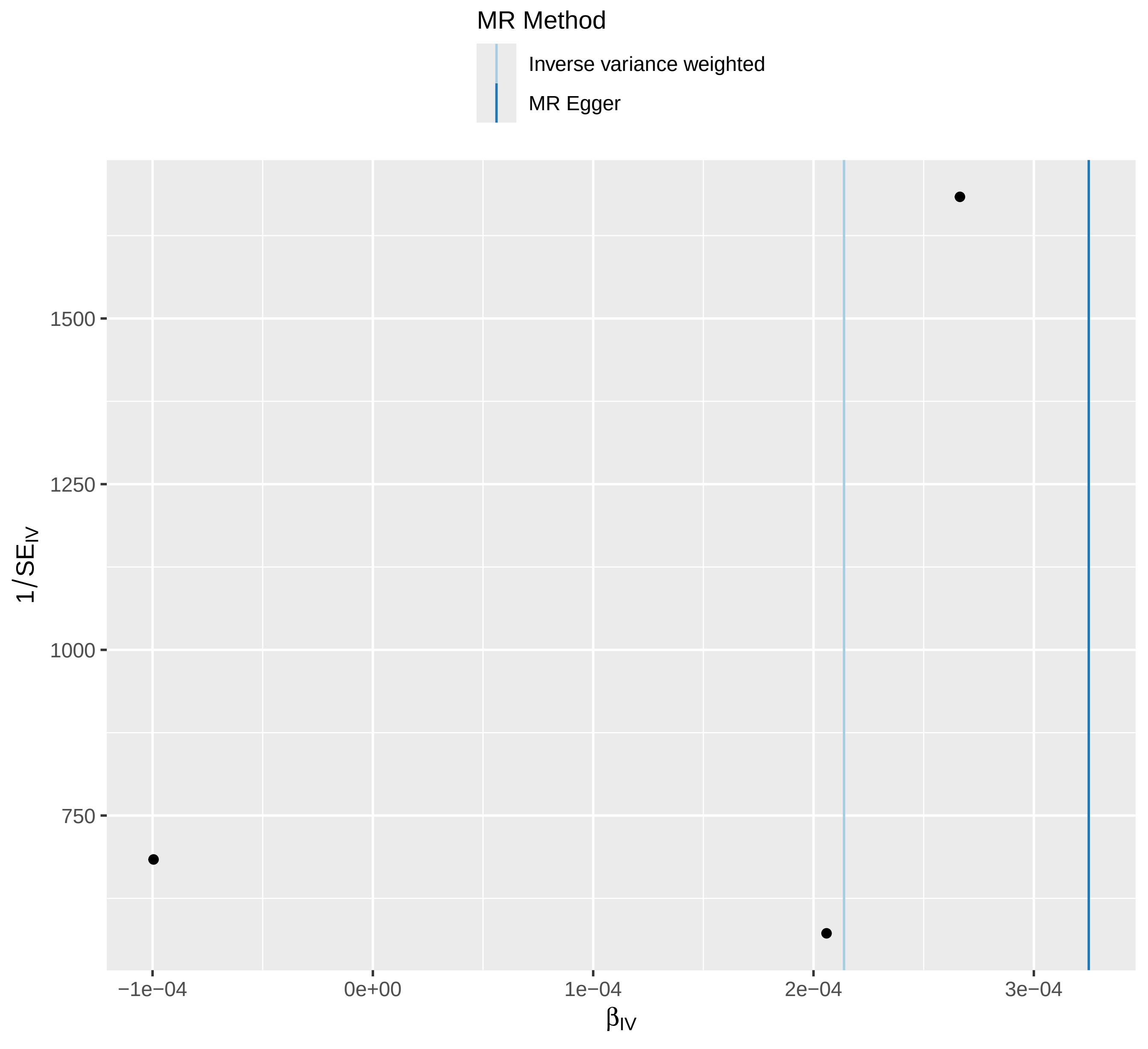

MR Method
Inverse variance weighted
MR Egger
1500
1250
1000
750
1/SE$_{IV}$
−1e−04
0e+00
1e−04
2e−04
3e−04
β_{IV}

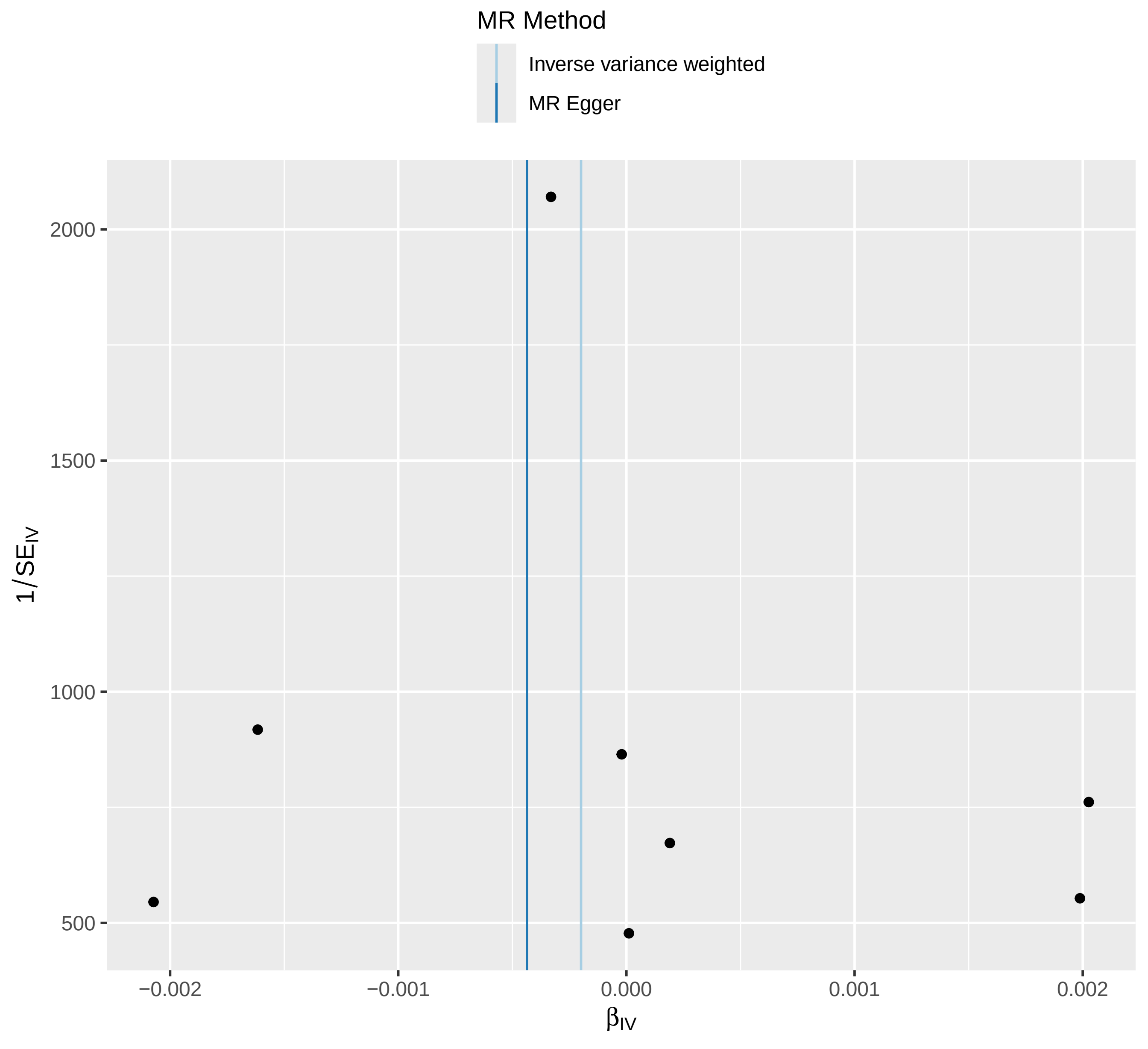
MR Method
Inverse variance weighted
MR Egger
2000
1500
1000
500
1/SE_IV
−0.002
−0.001
0.000
0.001
0.002
β_IV

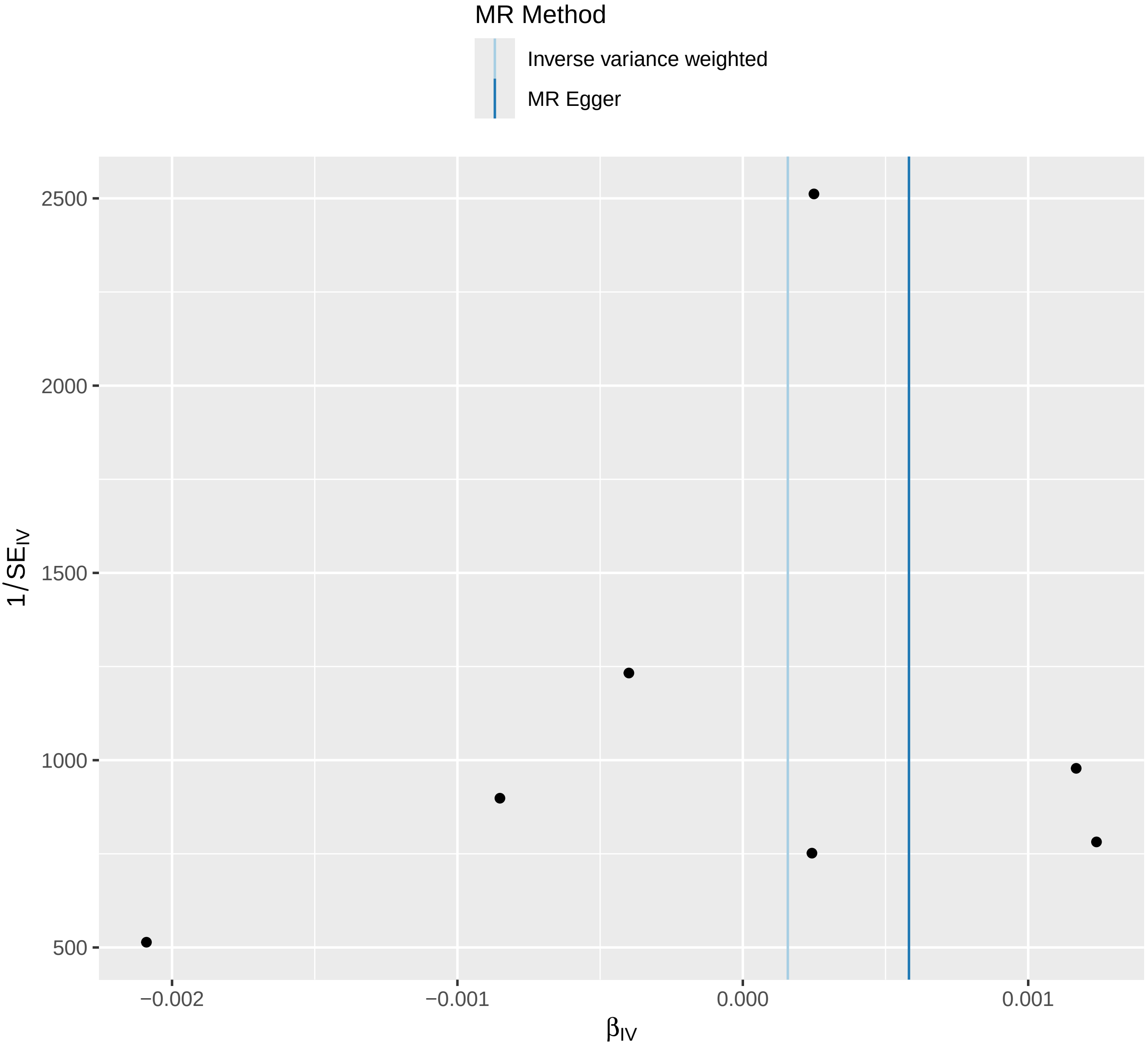
MR Method
Inverse variance weighted
MR Egger
2500
2000
1500
1000
500
1/SE$_{IV}$
−0.002
−0.001
0.000
0.001
β$_{IV}$

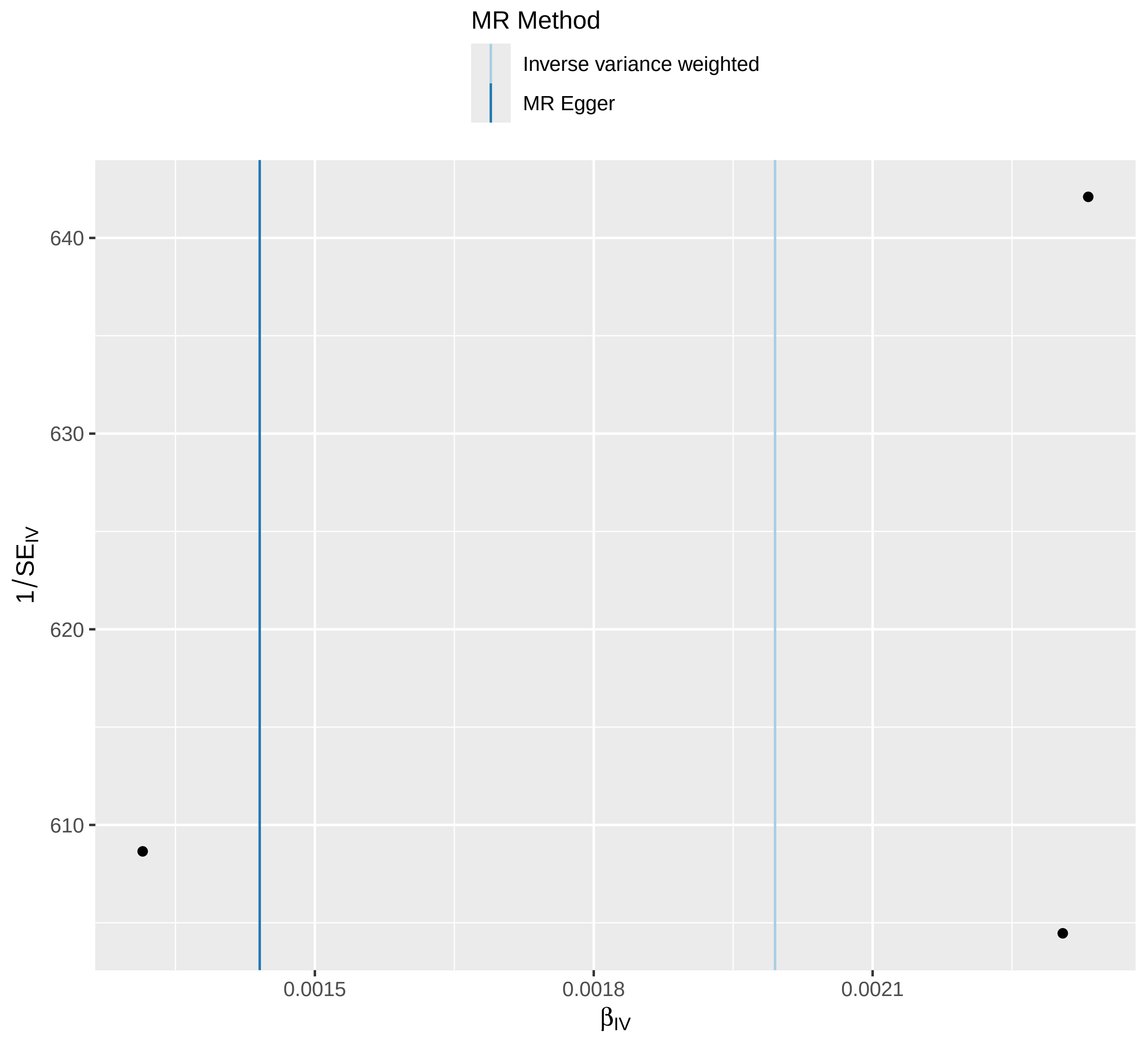

MR Method
Inverse variance weighted
MR Egger
640
630
620
610
0.0015
0.0018
0.0021
1/SE_IV
β_IV

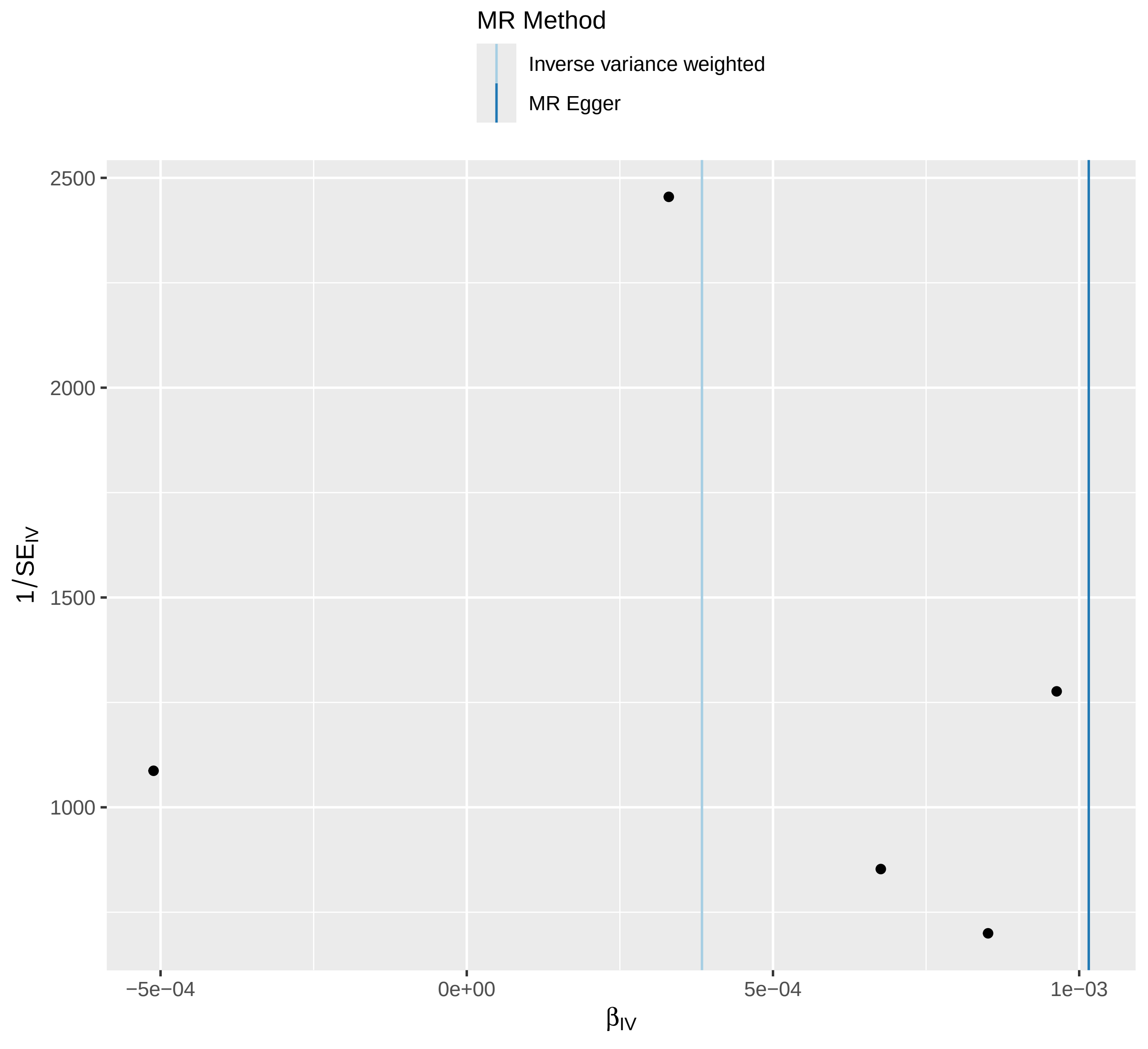

MR Method
Inverse variance weighted
MR Egger
2500
2000
1500
1000
1/SE_IV
−5e−04
0e+00
5e−04
1e−03
β_IV

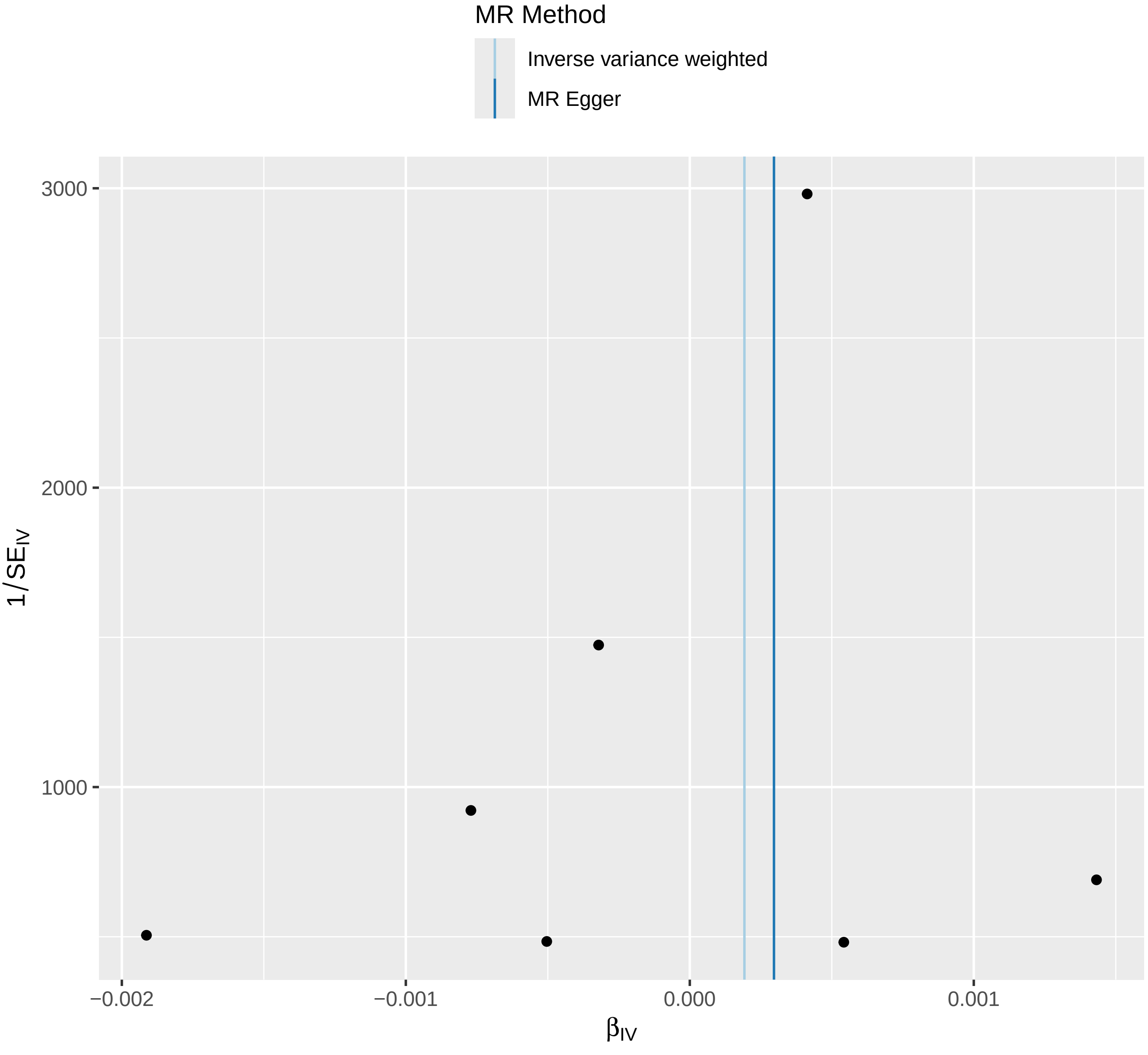

MR Method
Inverse variance weighted
MR Egger
3000
2000
1000
1/SE_IV
−0.002
−0.001
0.000
0.001
β_IV

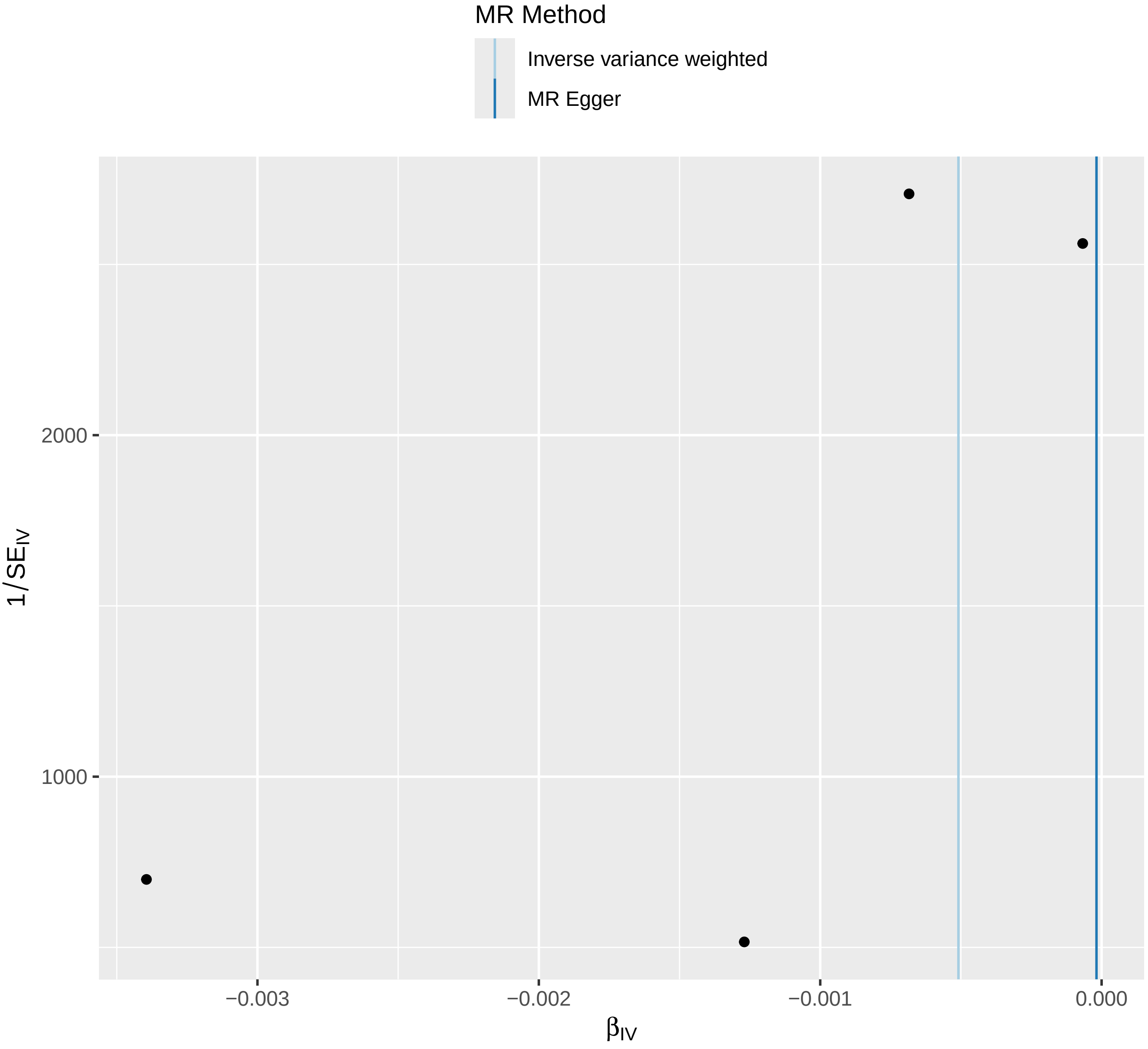

MR Method
Inverse variance weighted
MR Egger
2000
1000
1/SE_IV
−0.003
−0.002
−0.001
0.000
β_IV

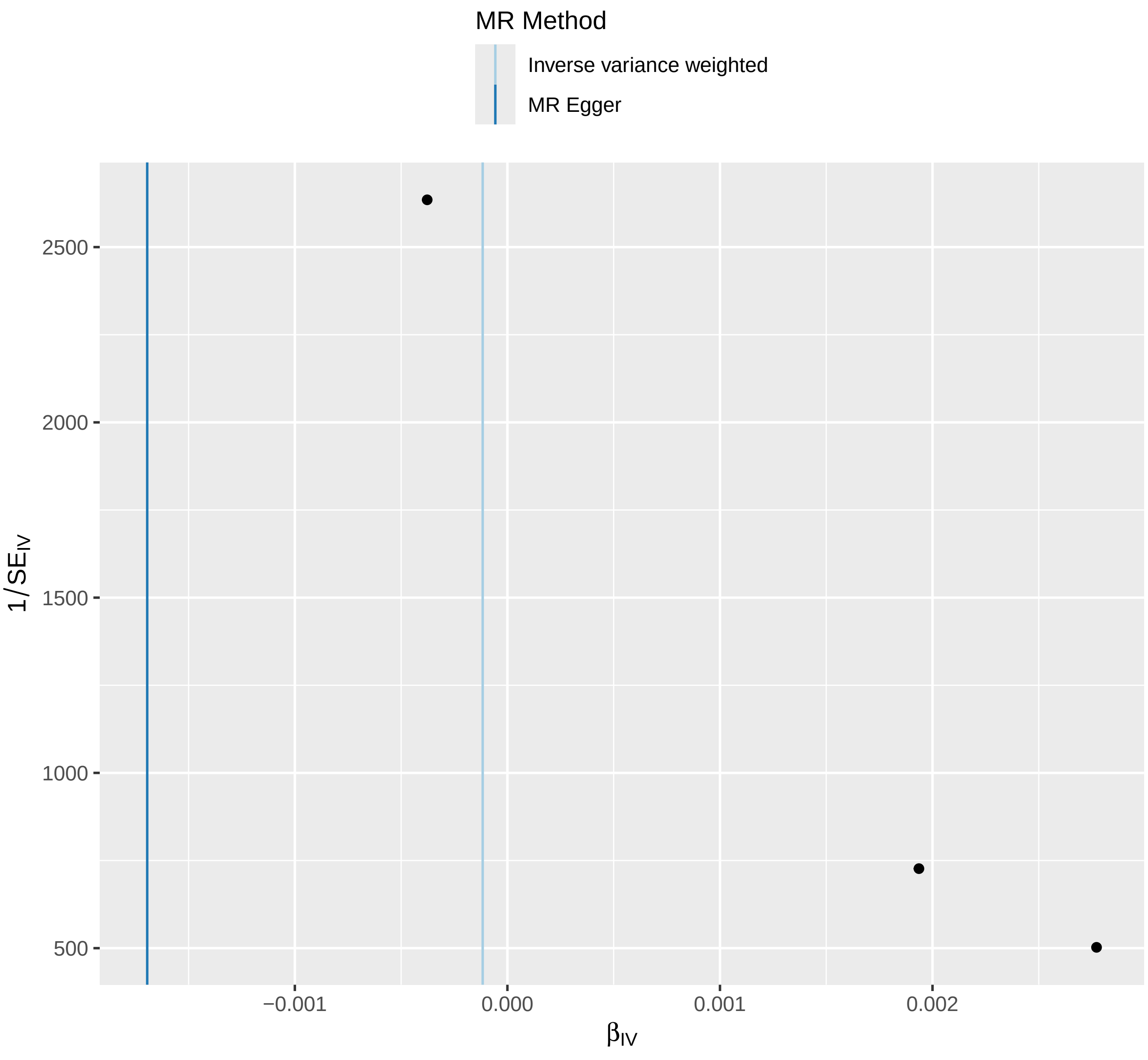
MR Method
Inverse variance weighted
MR Egger
2500
2000
1500
1000
500
$1/SE_{IV}$
−0.001
0.000
0.001
0.002
β_{IV}

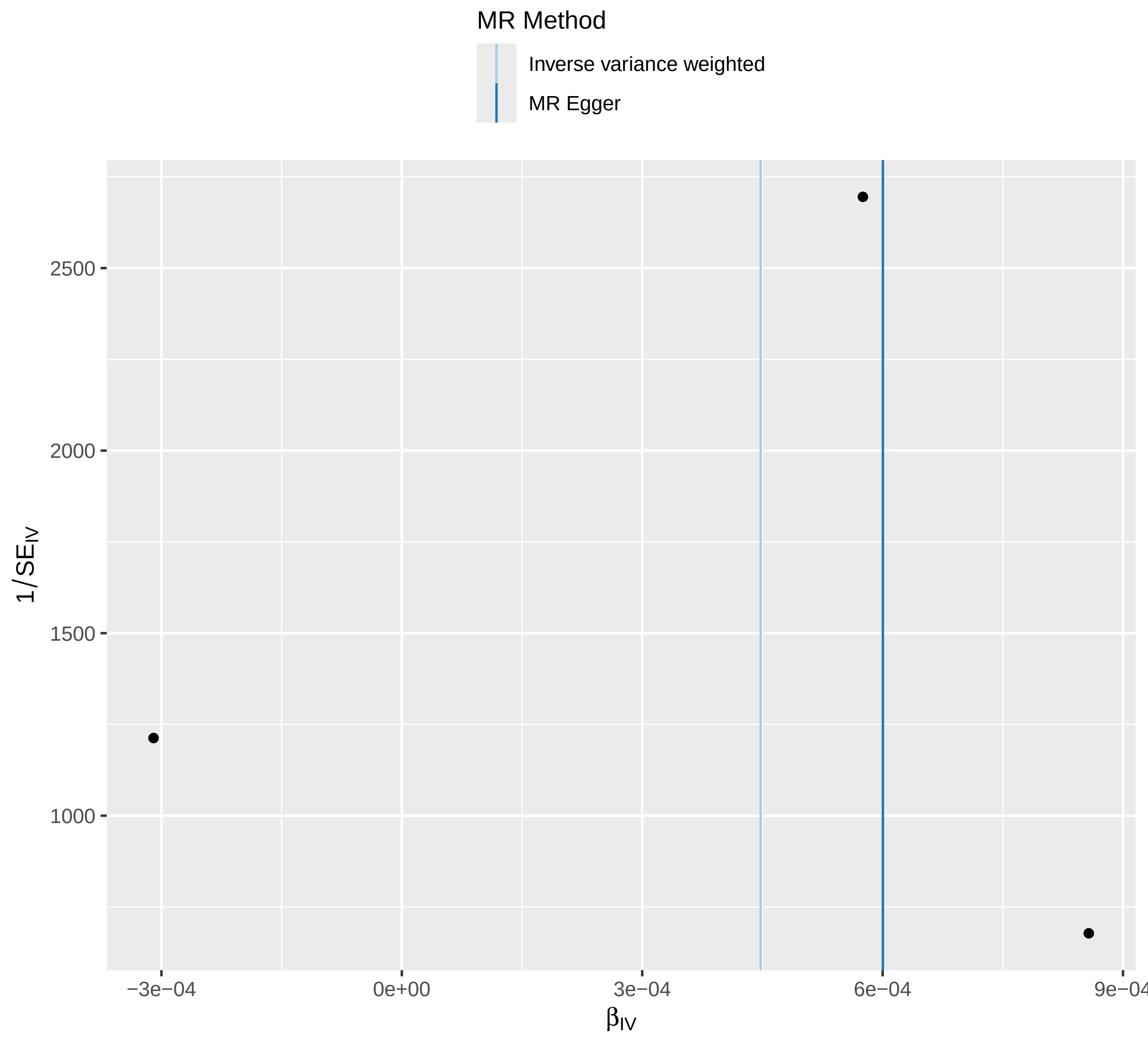
MR Method
Inverse variance weighted
MR Egger
2500
2000
1500
1000
1/SE_IV
−3e−04
0e+00
3e−04
6e−04
9e−04
β_IV

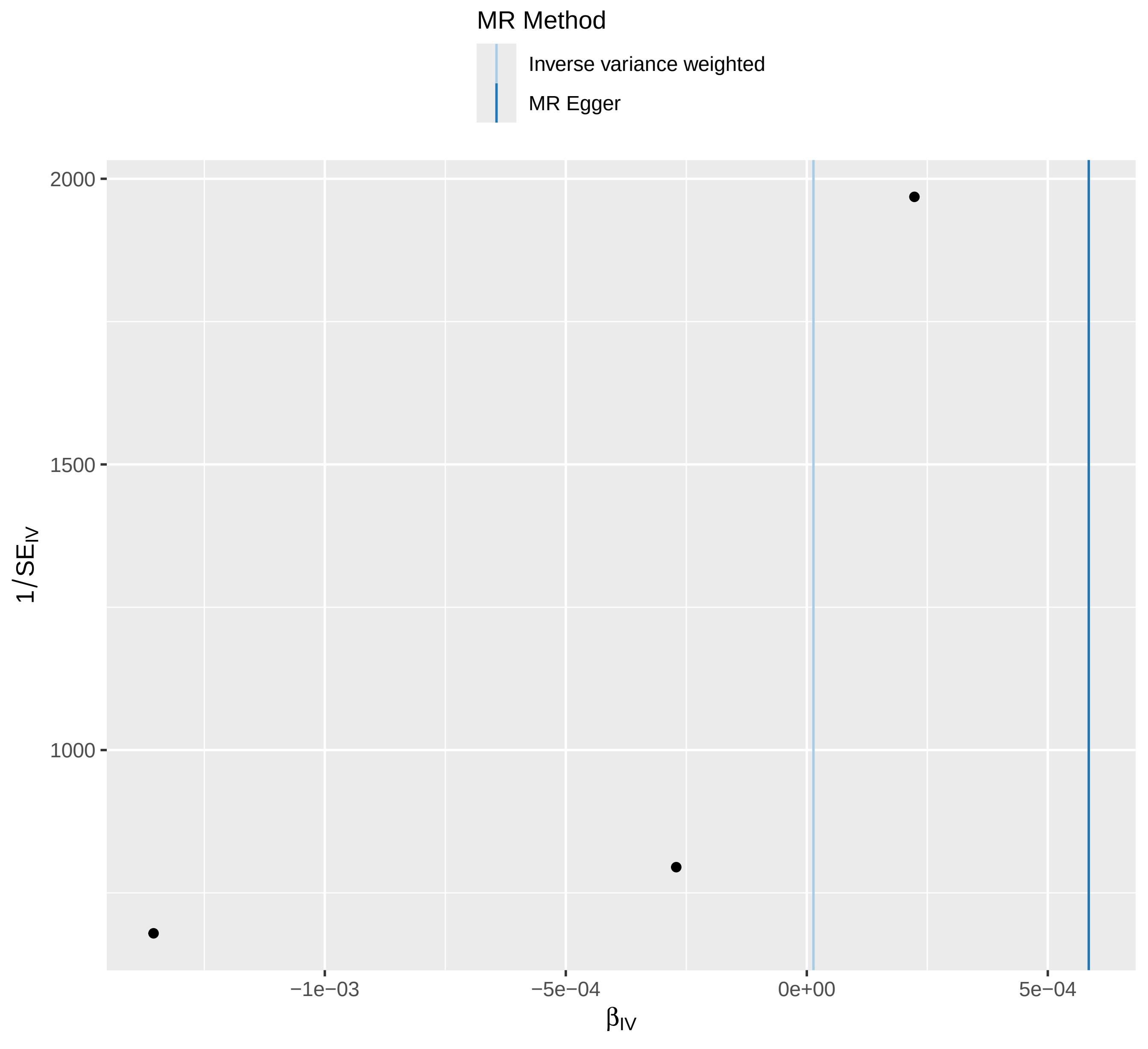

MR Method
Inverse variance weighted
MR Egger
2000
1500
1000
1/SE$_{IV}$
−1e−03
−5e−04
0e+00
5e−04
β$_{IV}$

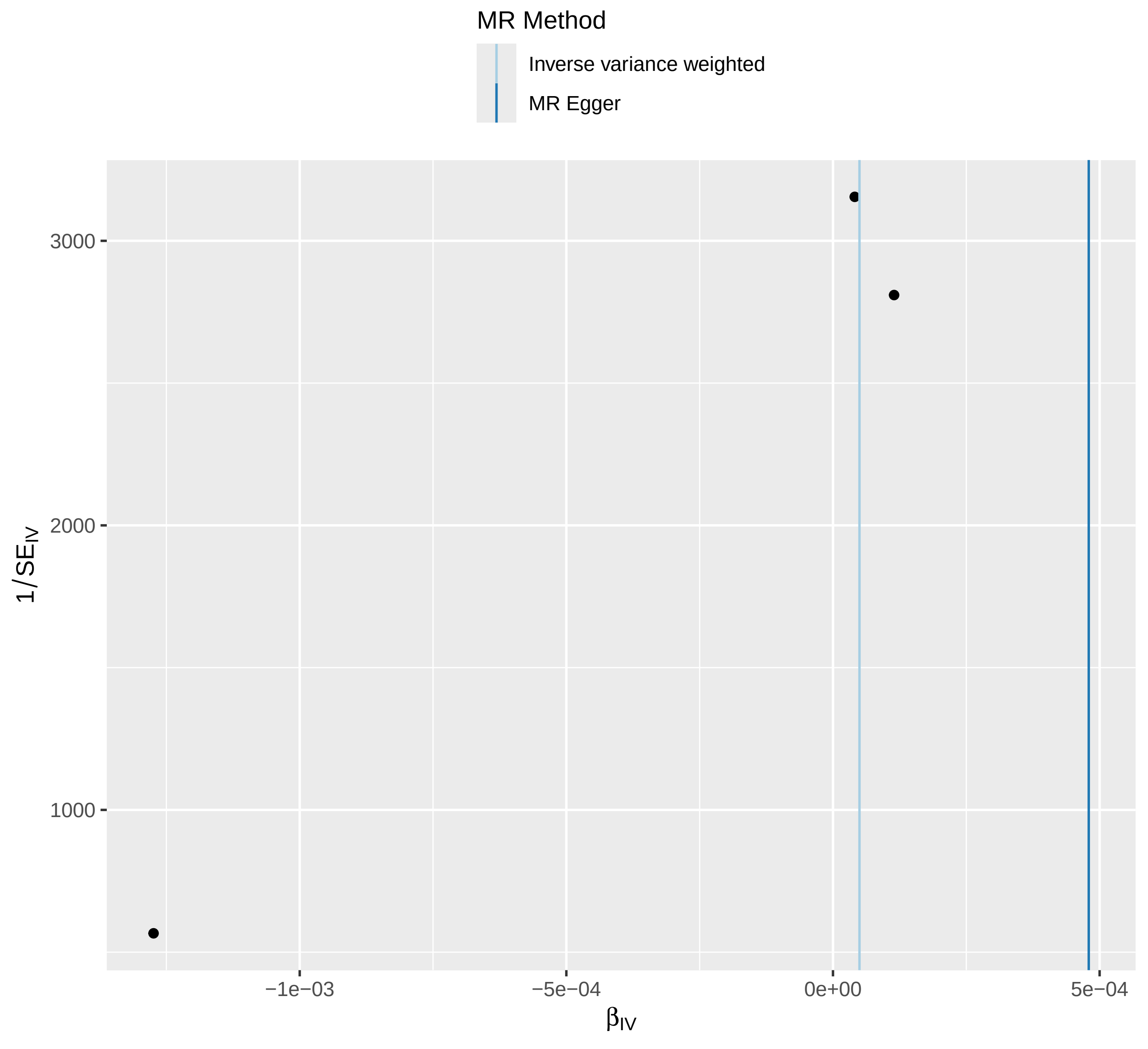

MR Method
Inverse variance weighted
MR Egger
3000
2000
1000
$1/SE_{IV}$
−1e−03
−5e−04
0e+00
5e−04
β_{IV}

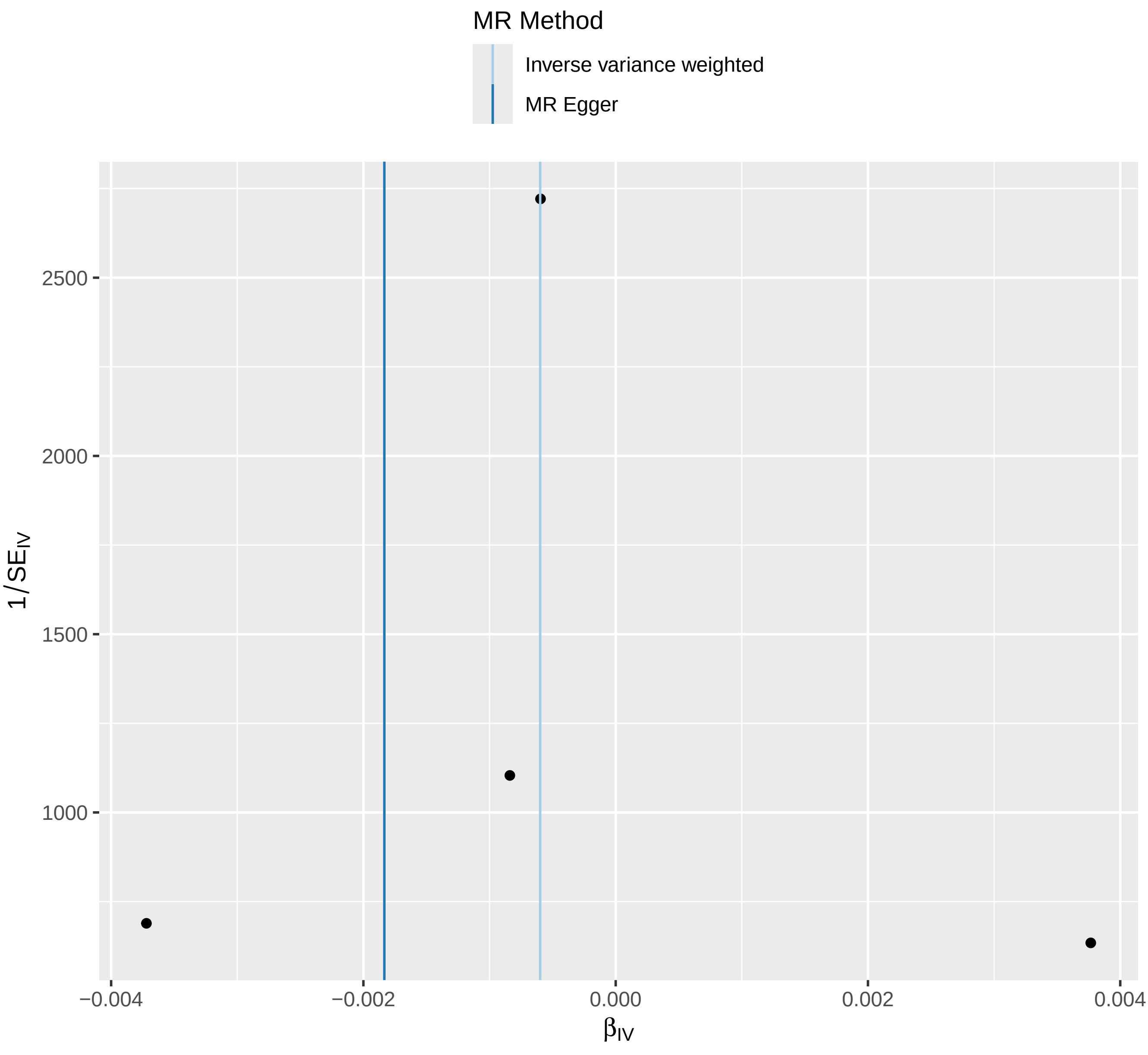
MR Method
Inverse variance weighted
MR Egger
2500
2000
1500
1000
1/SE_IV
−0.004
−0.002
0.000
0.002
0.004
β_IV

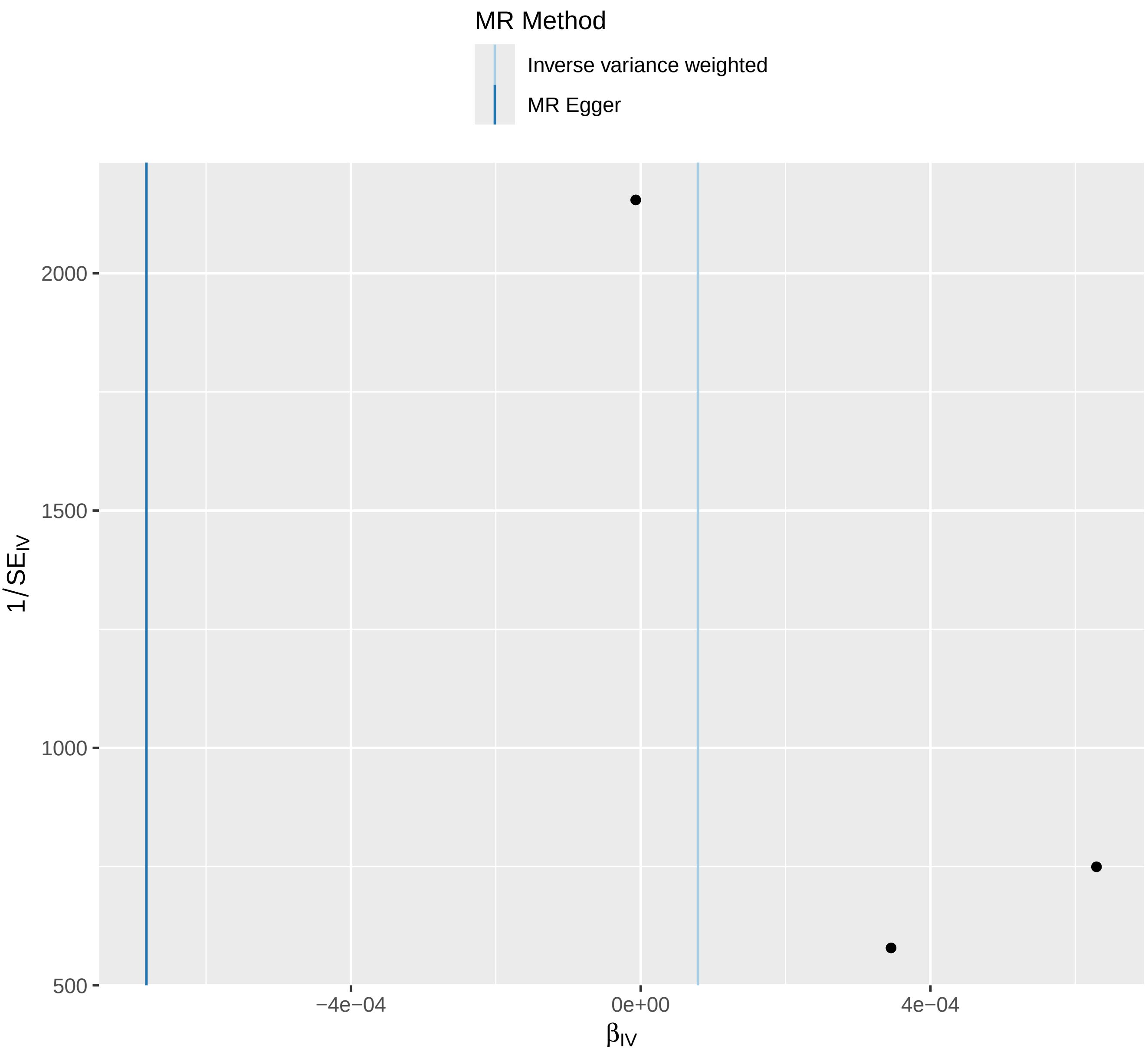
MR Method
Inverse variance weighted
MR Egger
2000
1500
1000
500
1/SE_IV
−4e−04
0e+00
4e−04
β_IV

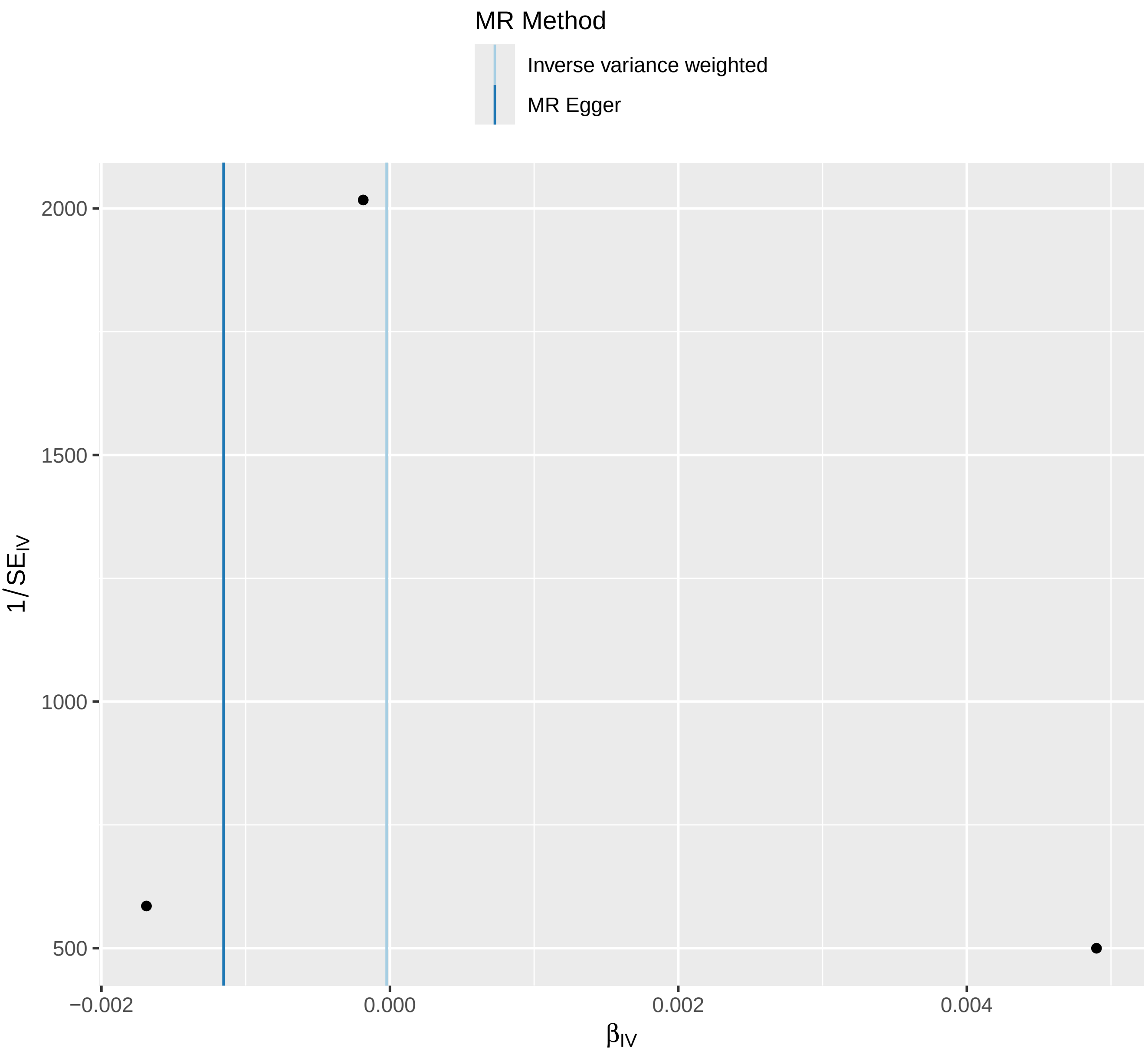
MR Method
Inverse variance weighted
MR Egger
2000
1500
1000
500
1/SE$_{IV}$
−0.002
0.000
0.002
0.004
β$_{IV}$

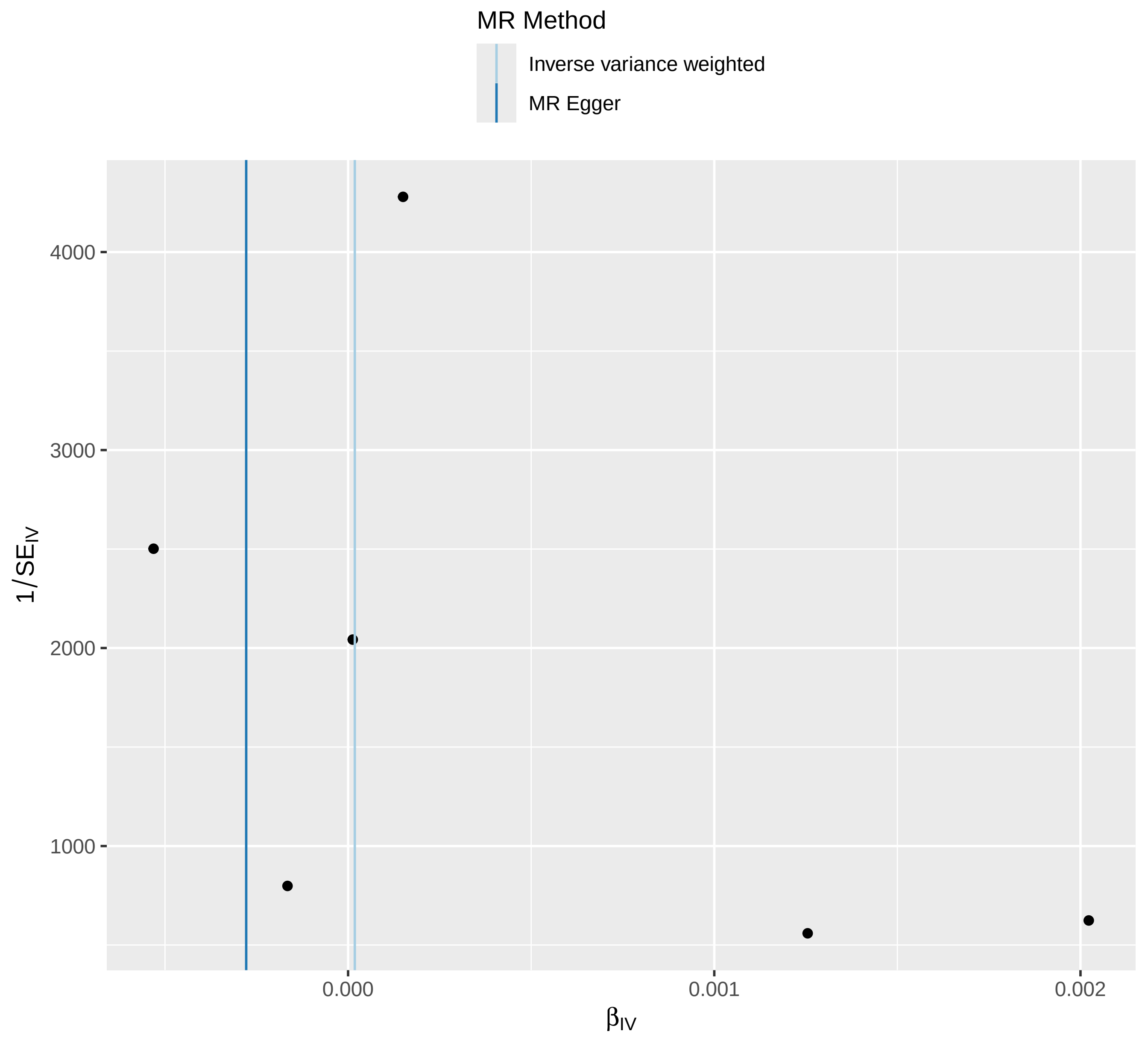
MR Method
Inverse variance weighted
MR Egger
4000
3000
2000
1000
1/SE_IV
0.000
0.001
0.002
β_IV

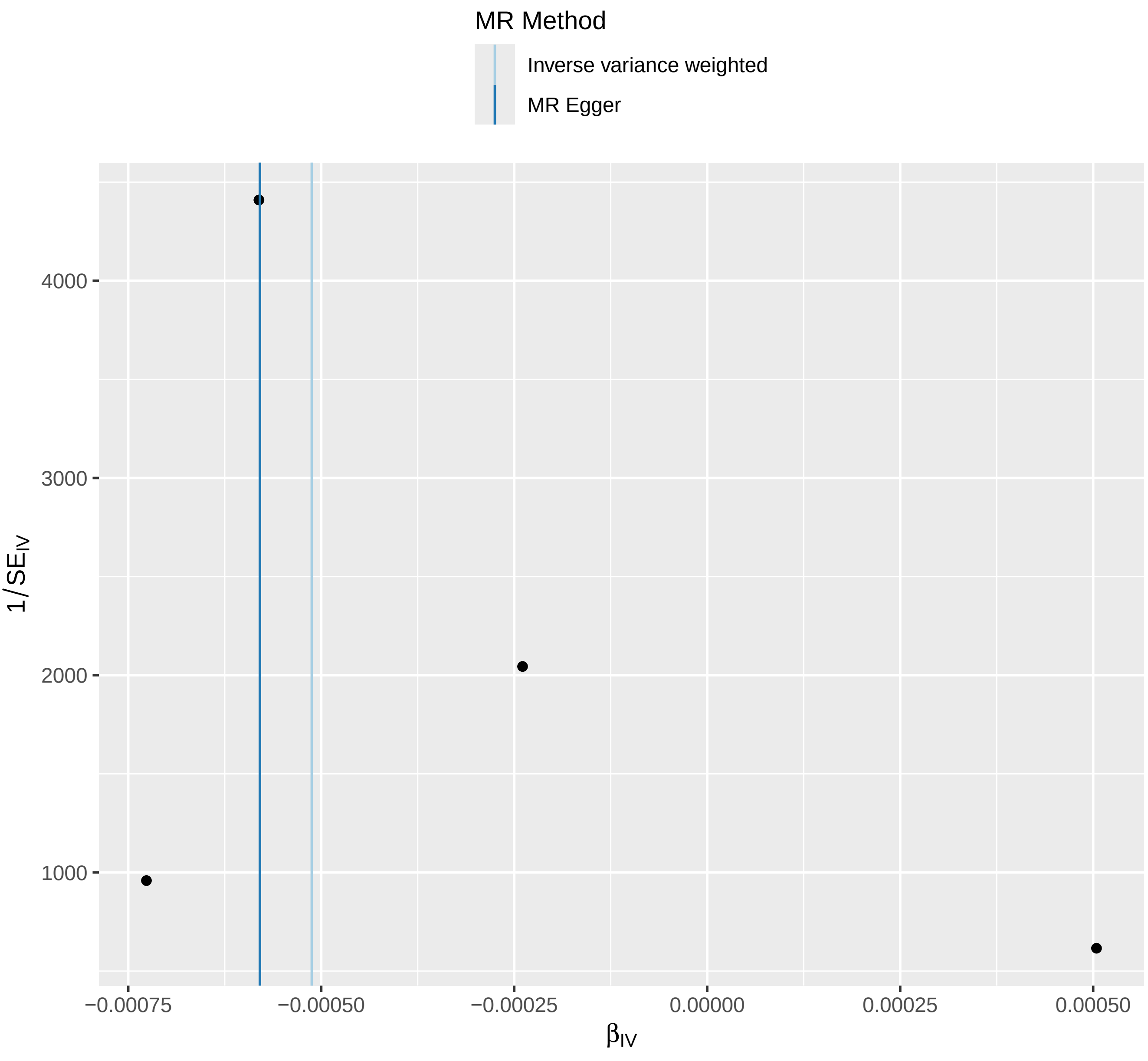
MR Method
Inverse variance weighted
MR Egger
4000
3000
2000
1000
1/SE_IV
−0.00075
−0.00050
−0.00025
0.00000
0.00025
0.00050
β_IV

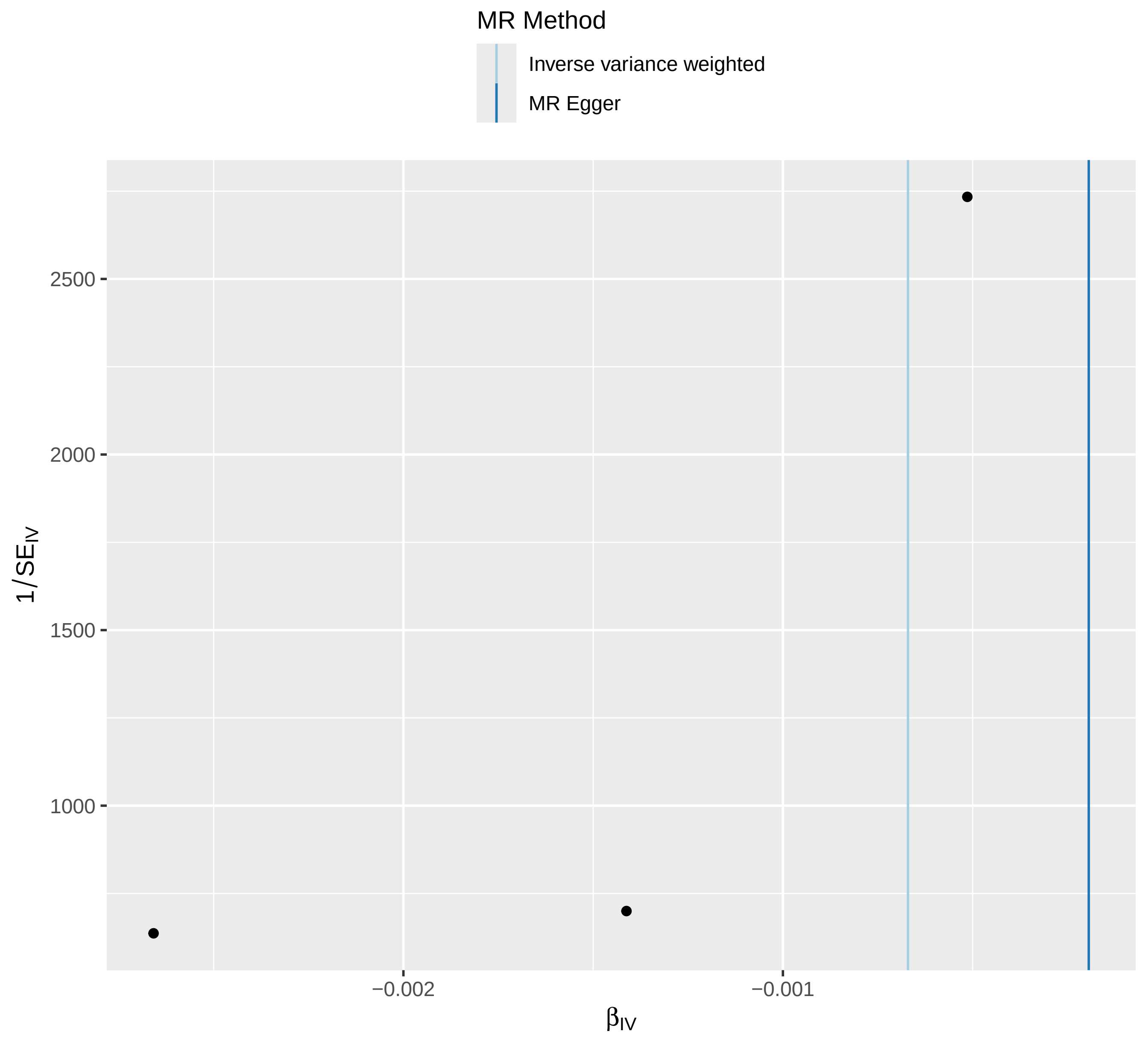
MR Method
Inverse variance weighted
MR Egger
2500
2000
1500
1000
1/SE_IV
−0.002
−0.001
β_IV

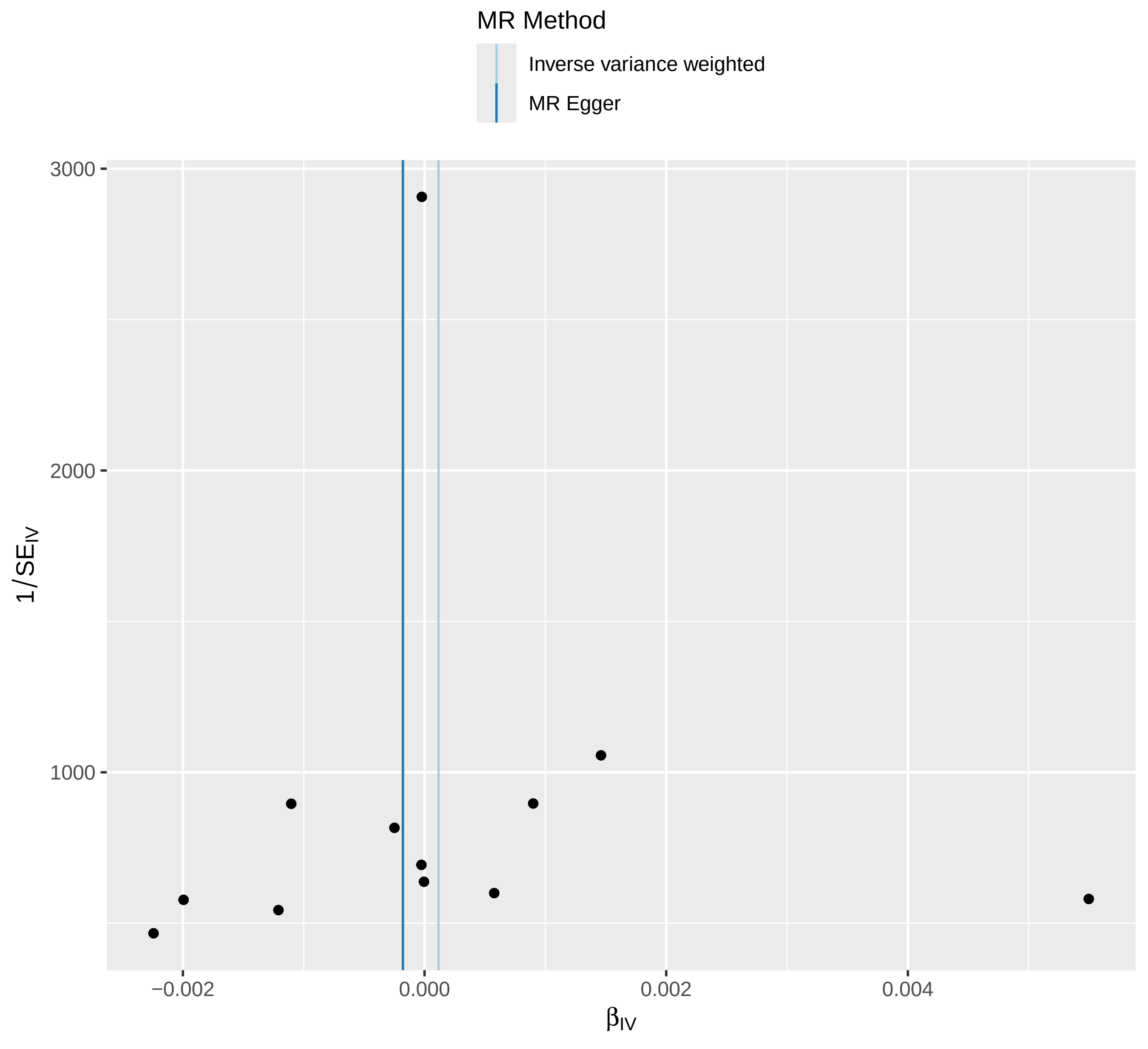
MR Method
Inverse variance weighted
MR Egger
3000
2000
1000
1/SE_IV
−0.002
0.000
0.002
0.004
β_IV

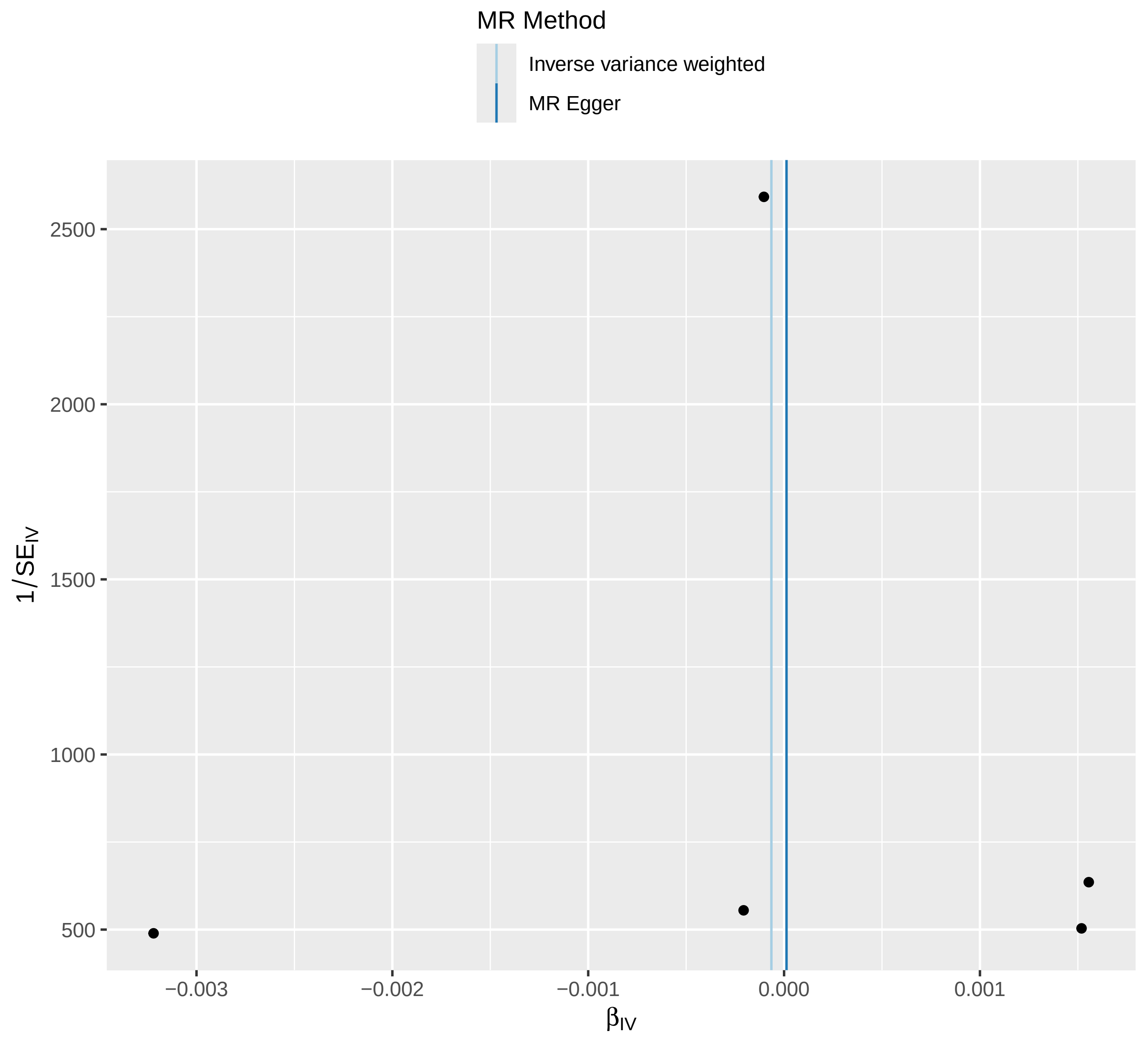

MR Method
Inverse variance weighted
MR Egger
2500
2000
1500
1000
500
1/SE$_{IV}$
−0.003
−0.002
−0.001
0.000
0.001
β$_{IV}$

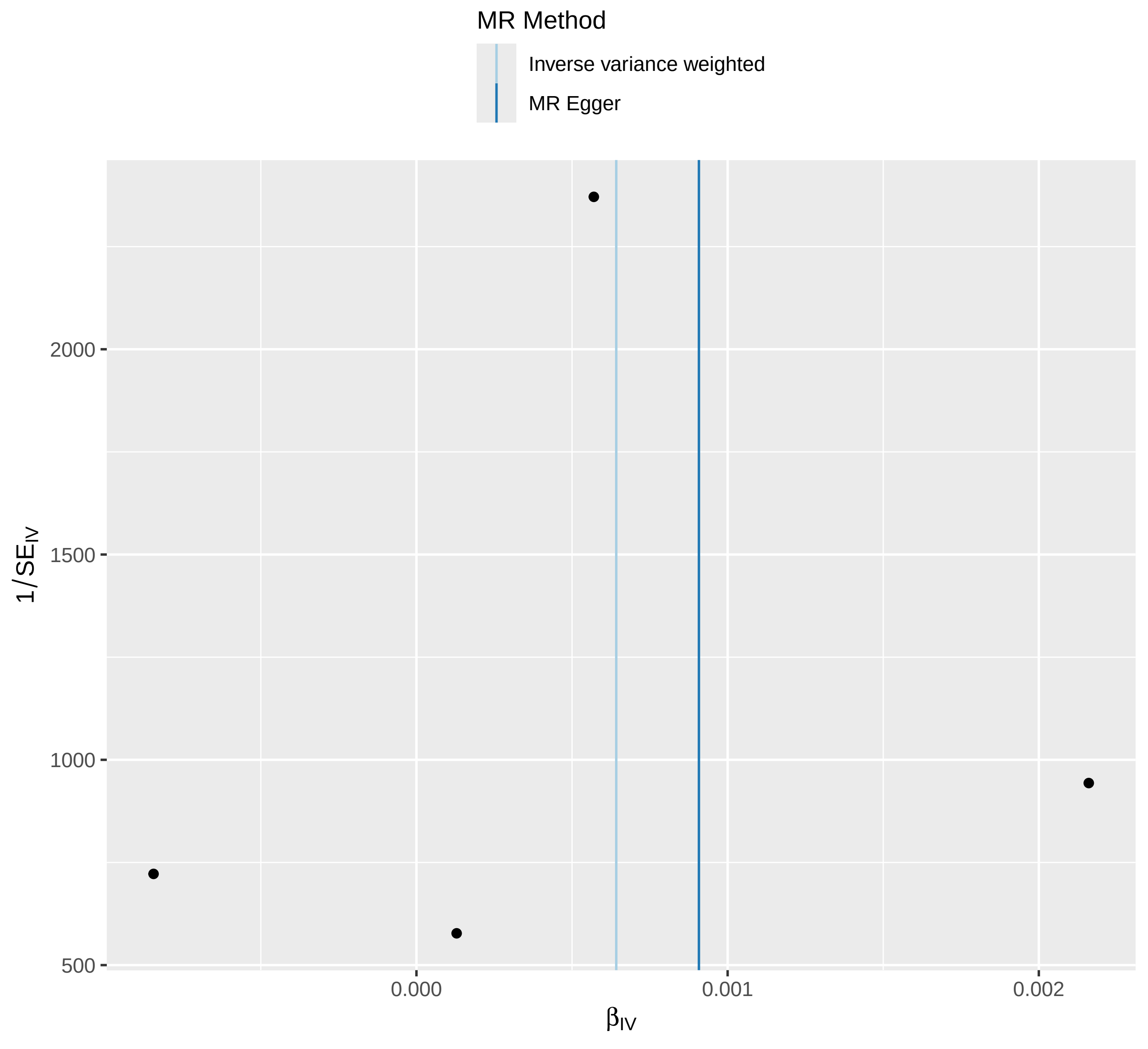
MR Method
Inverse variance weighted
MR Egger
1/SE_IV
2000
1500
1000
500
0.000
0.001
0.002
β_IV

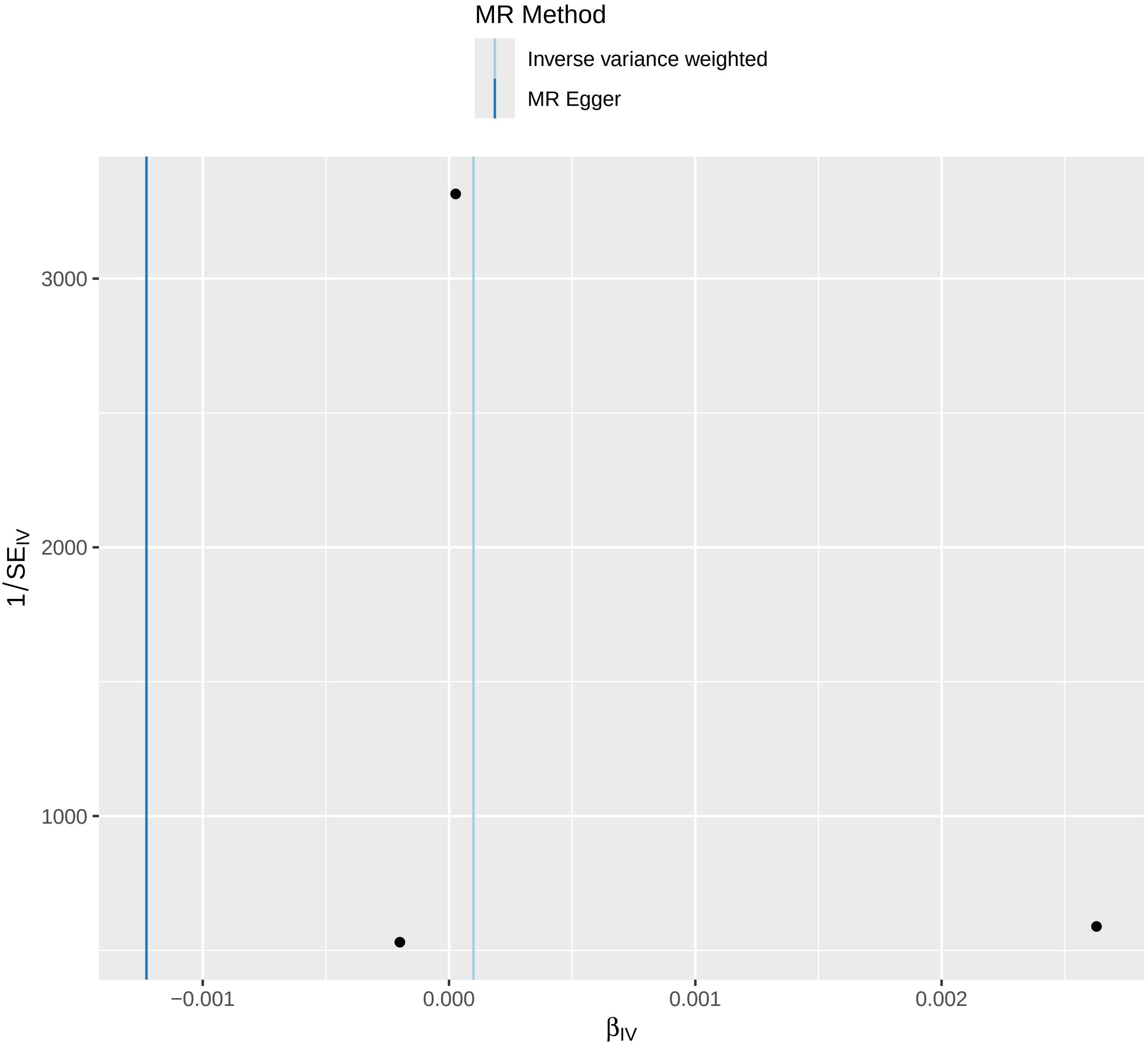

MR Method
Inverse variance weighted
MR Egger
3000
2000
1000
1/SE$_{IV}$
−0.001
0.000
0.001
0.002
β$_{IV}$

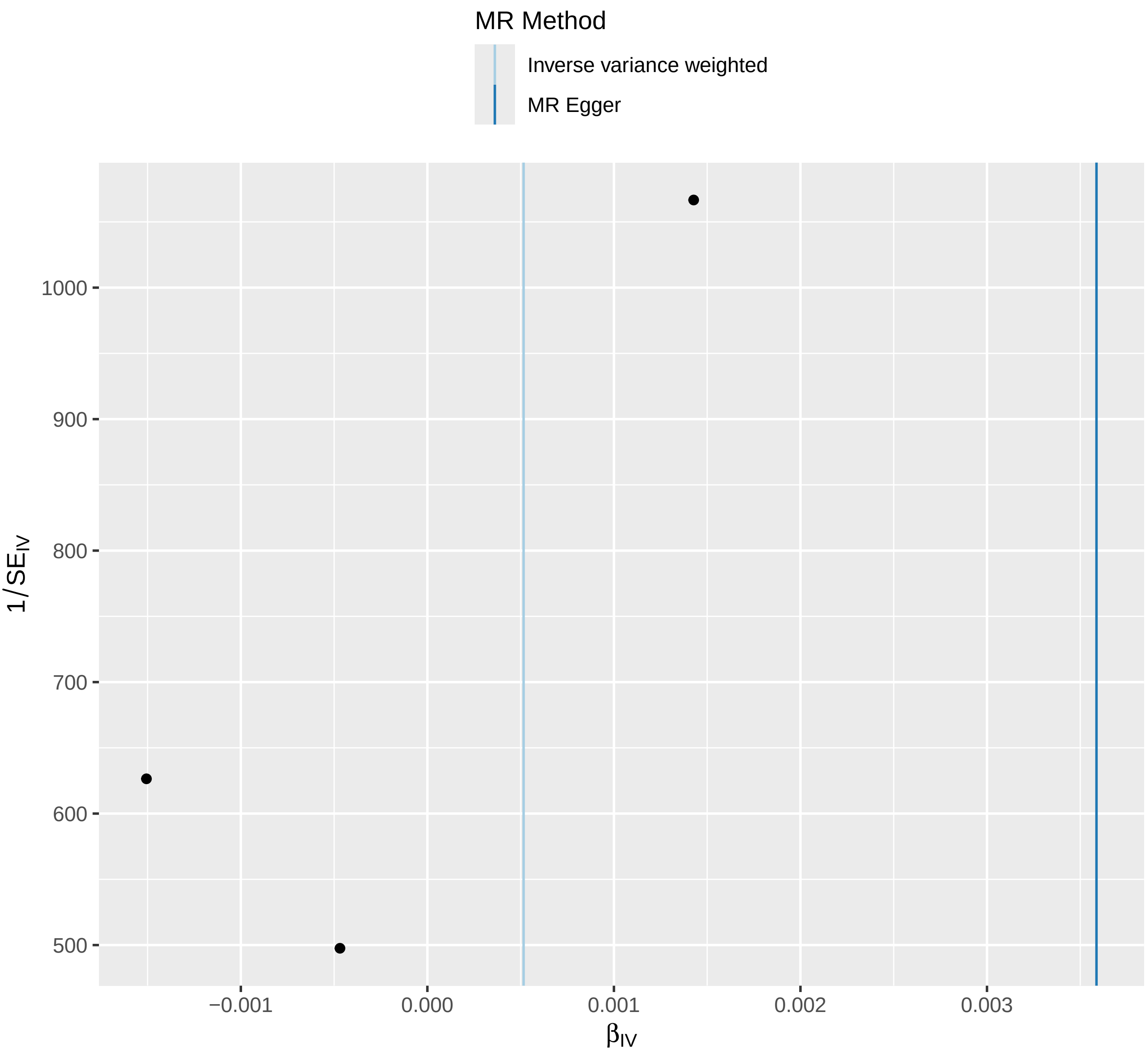
MR Method
Inverse variance weighted
MR Egger
1000
900
800
700
600
500
1/SE_IV
−0.001
0.000
0.001
0.002
0.003
β_IV

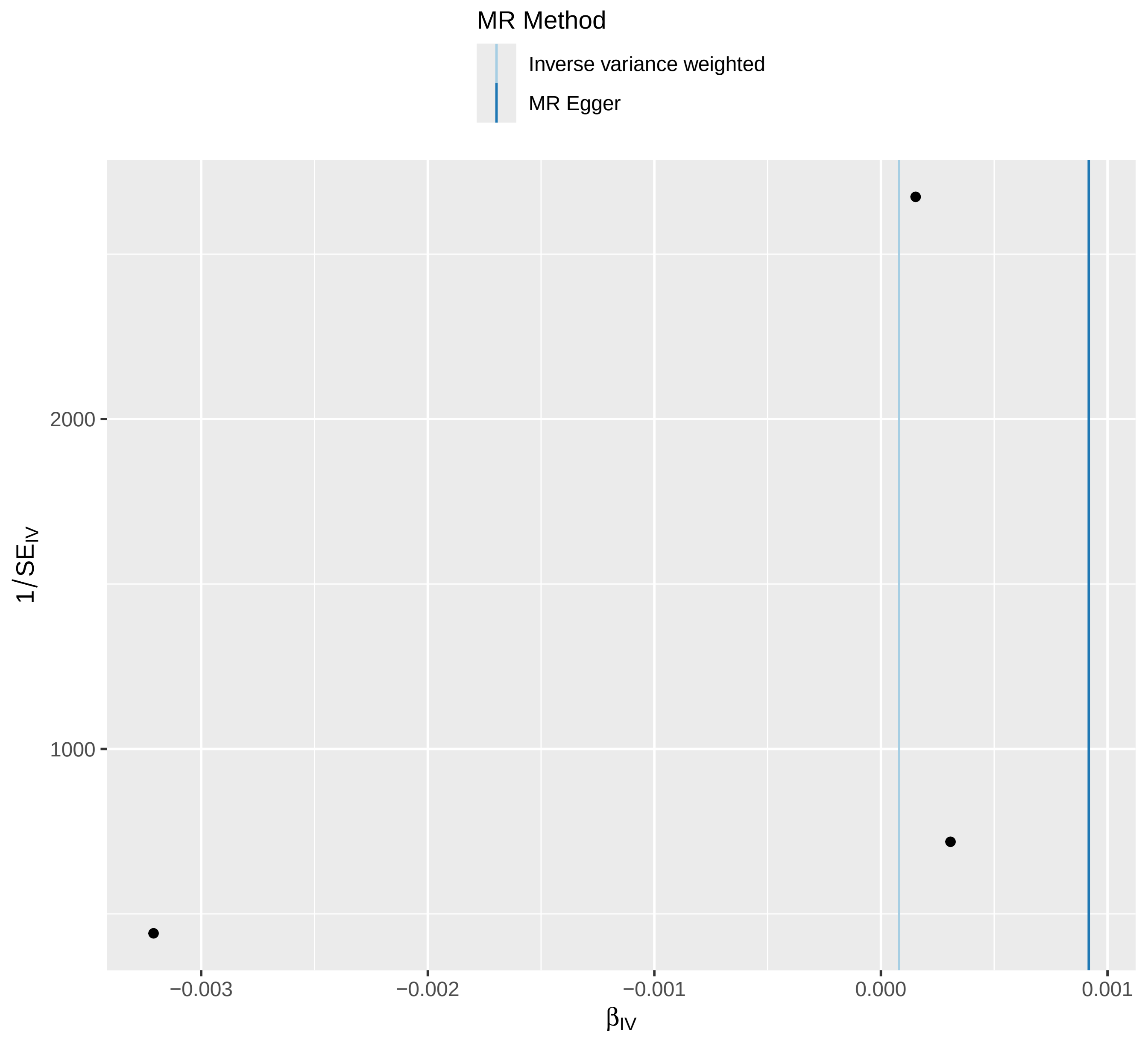

MR Method
Inverse variance weighted
MR Egger
2000
1000
1/SE_IV
−0.003
−0.002
−0.001
0.000
0.001
β_IV

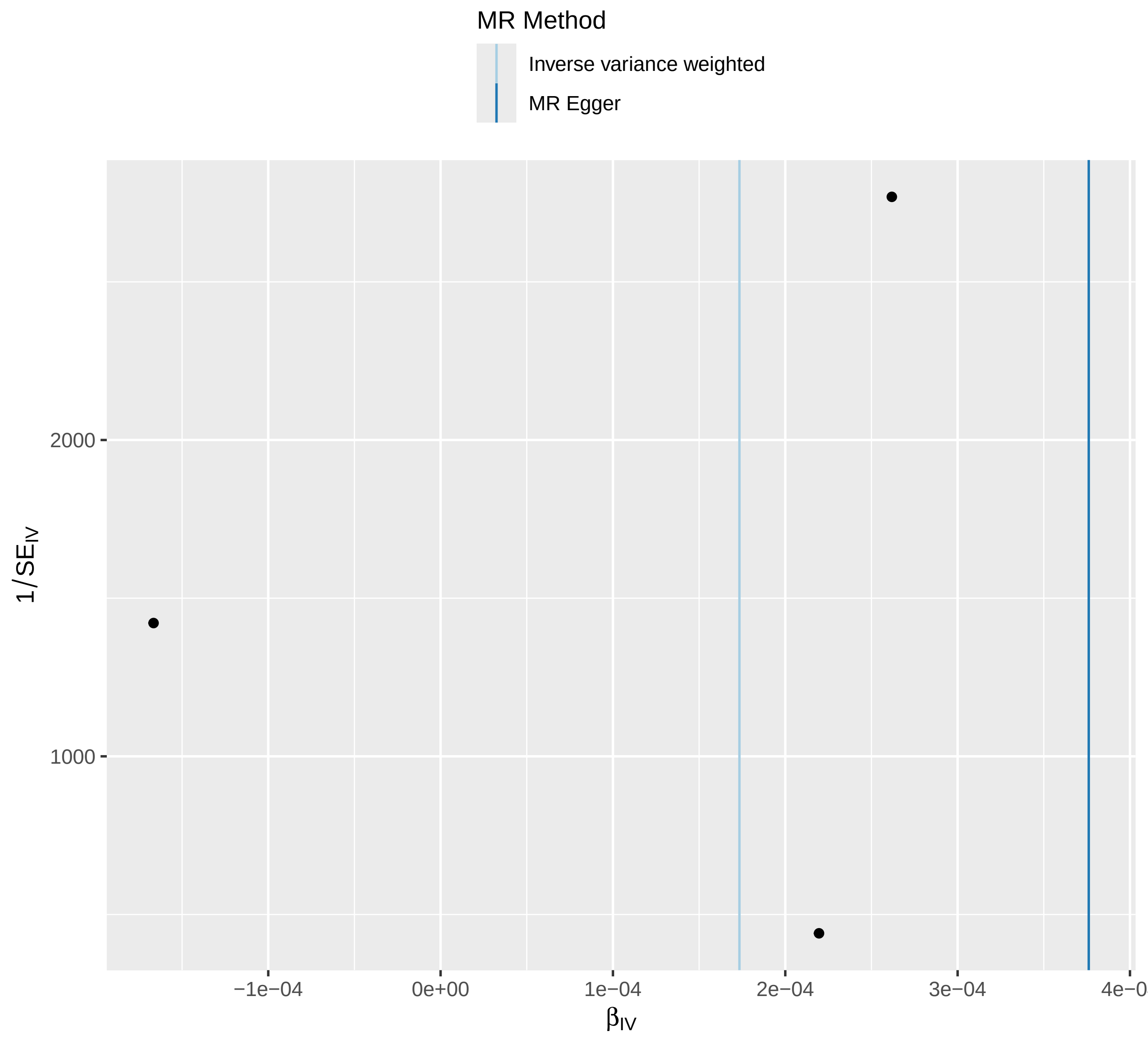

MR Method
Inverse variance weighted
MR Egger
2000
1000
1/SE_IV
−1e−04
0e+00
1e−04
2e−04
3e−04
β_IV

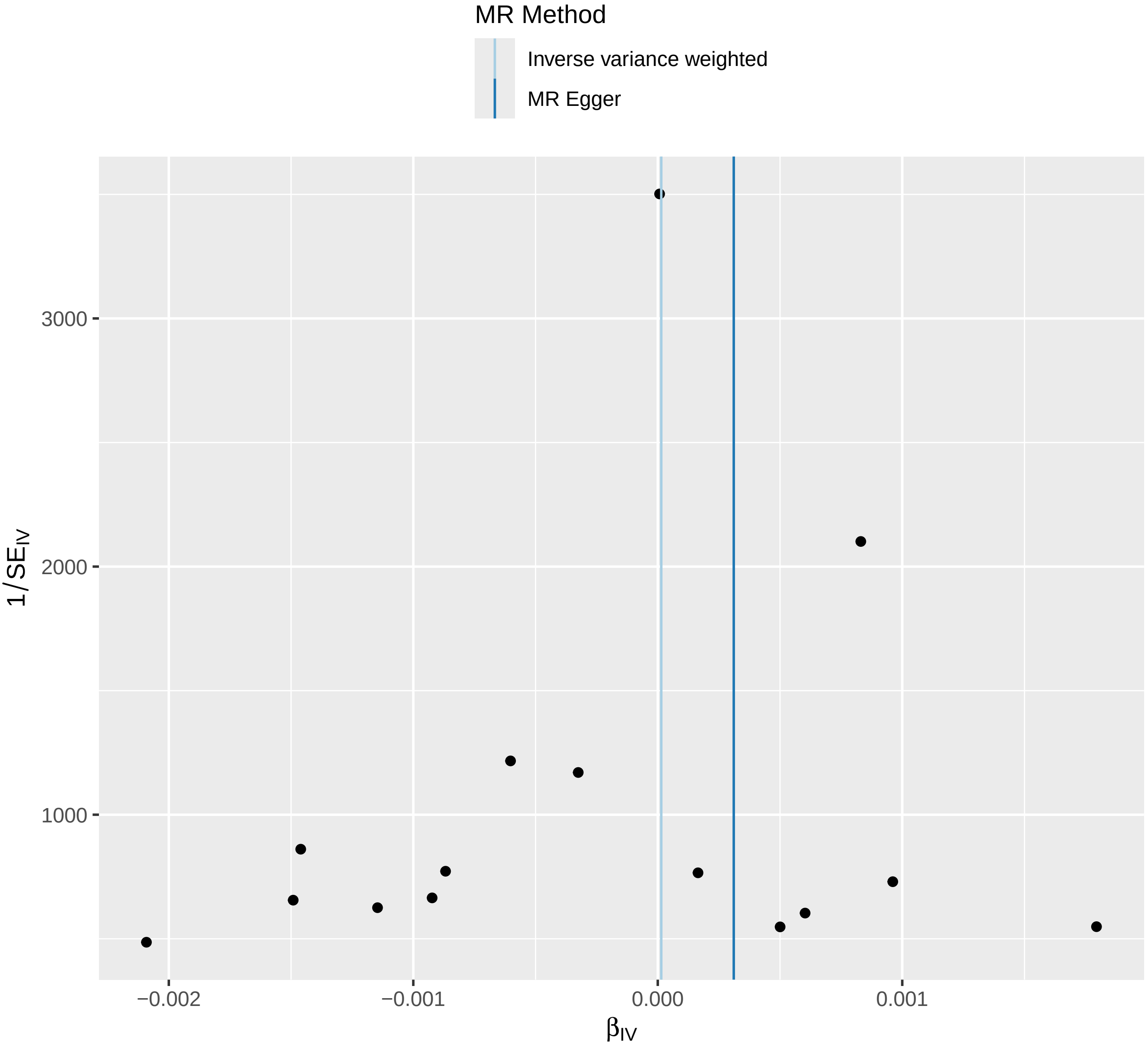
MR Method
Inverse variance weighted
MR Egger
3000
2000
1000
1/SE_IV
−0.002
−0.001
0.000
0.001
β_IV

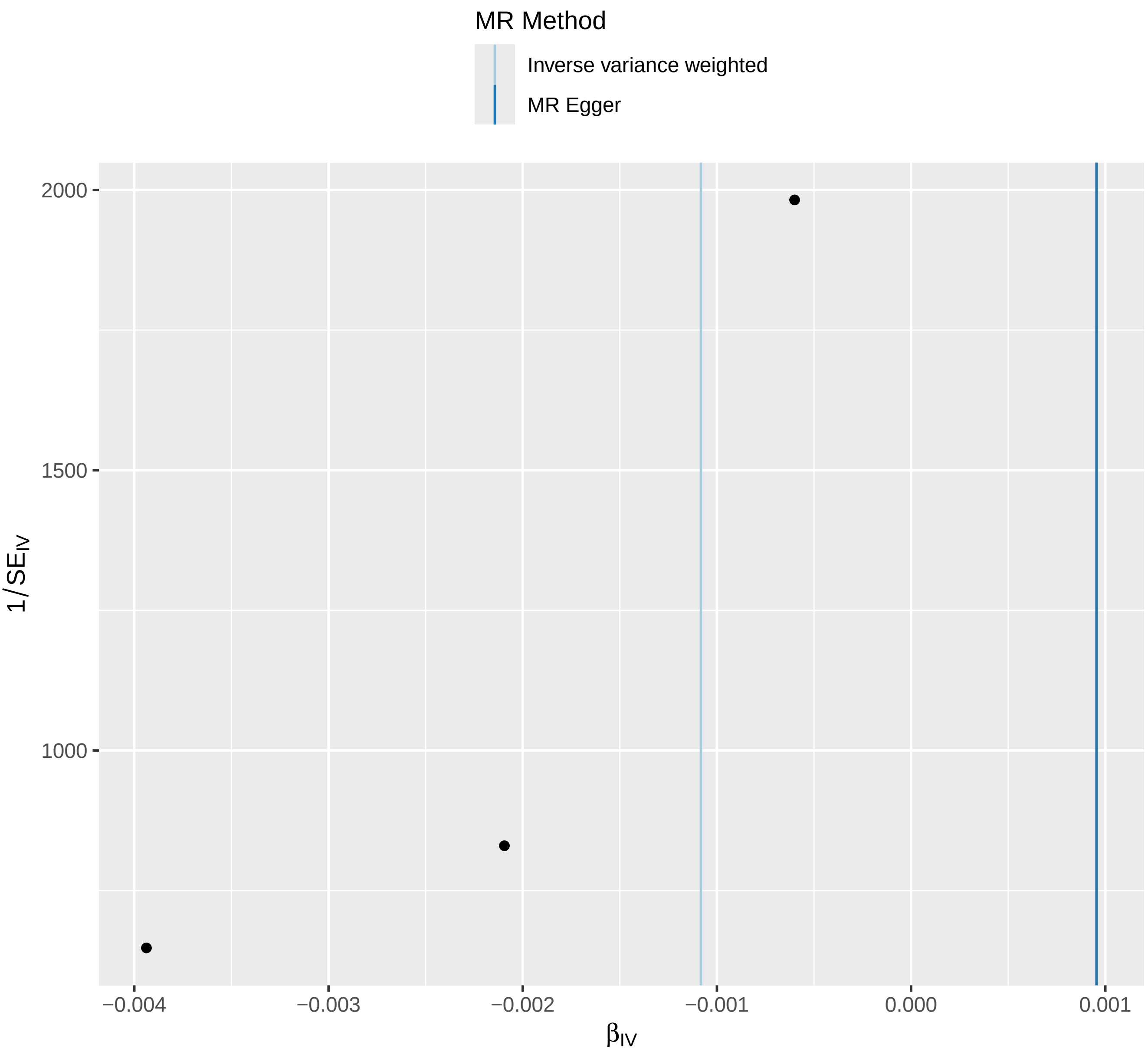

MR Method
Inverse variance weighted
MR Egger
2000
1500
1000
1/SE_IV
−0.004
−0.003
−0.002
−0.001
0.000
0.001
β_IV

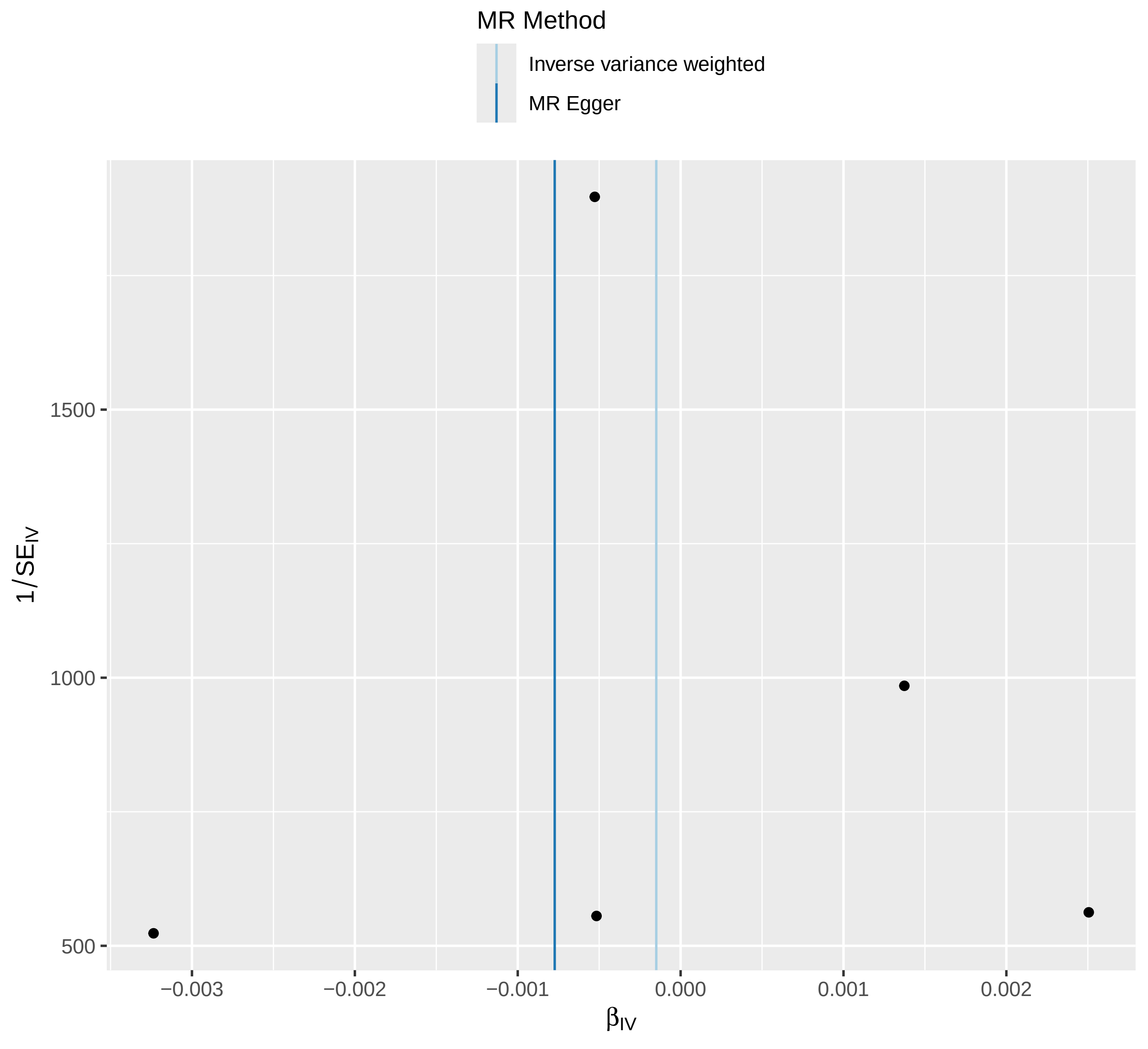
MR Method
Inverse variance weighted
MR Egger
1500
1000
500
1/SE_IV
−0.003
−0.002
−0.001
0.000
0.001
0.002
β_IV

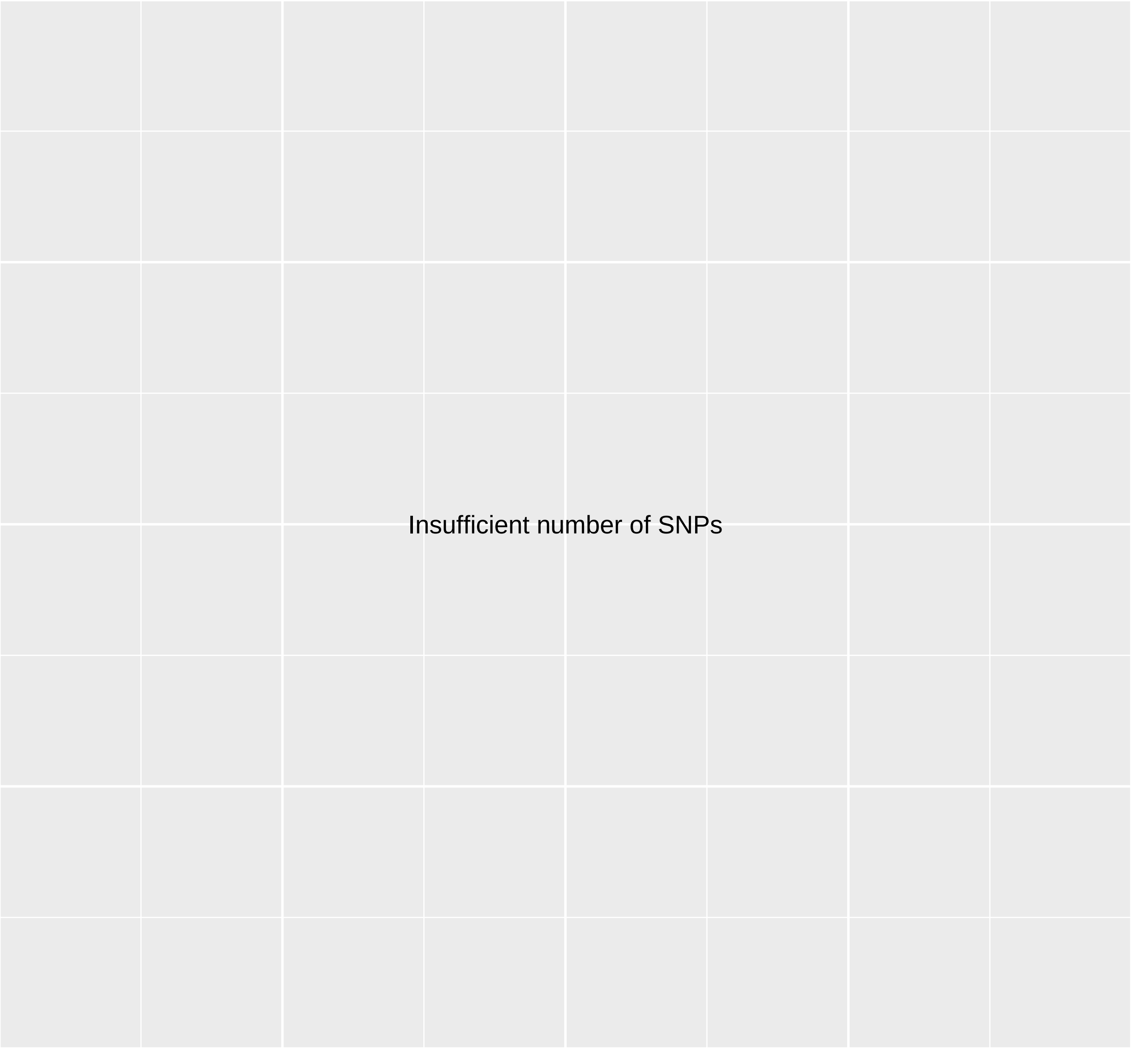
Insufficient number of SNPs

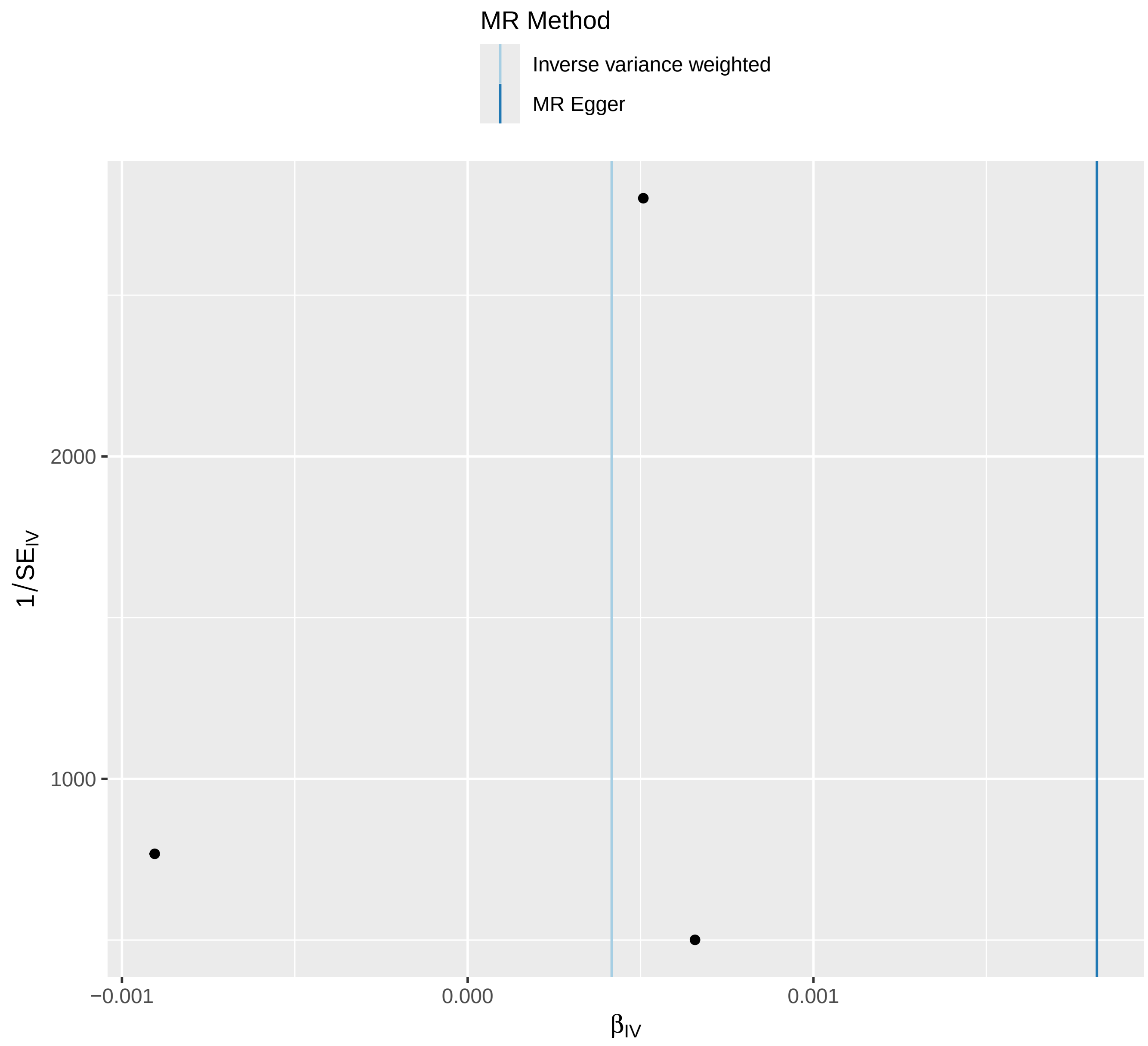

MR Method
Inverse variance weighted
MR Egger
2000
1000
$1/SE_{IV}$
−0.001
0.000
0.001
β_{IV}

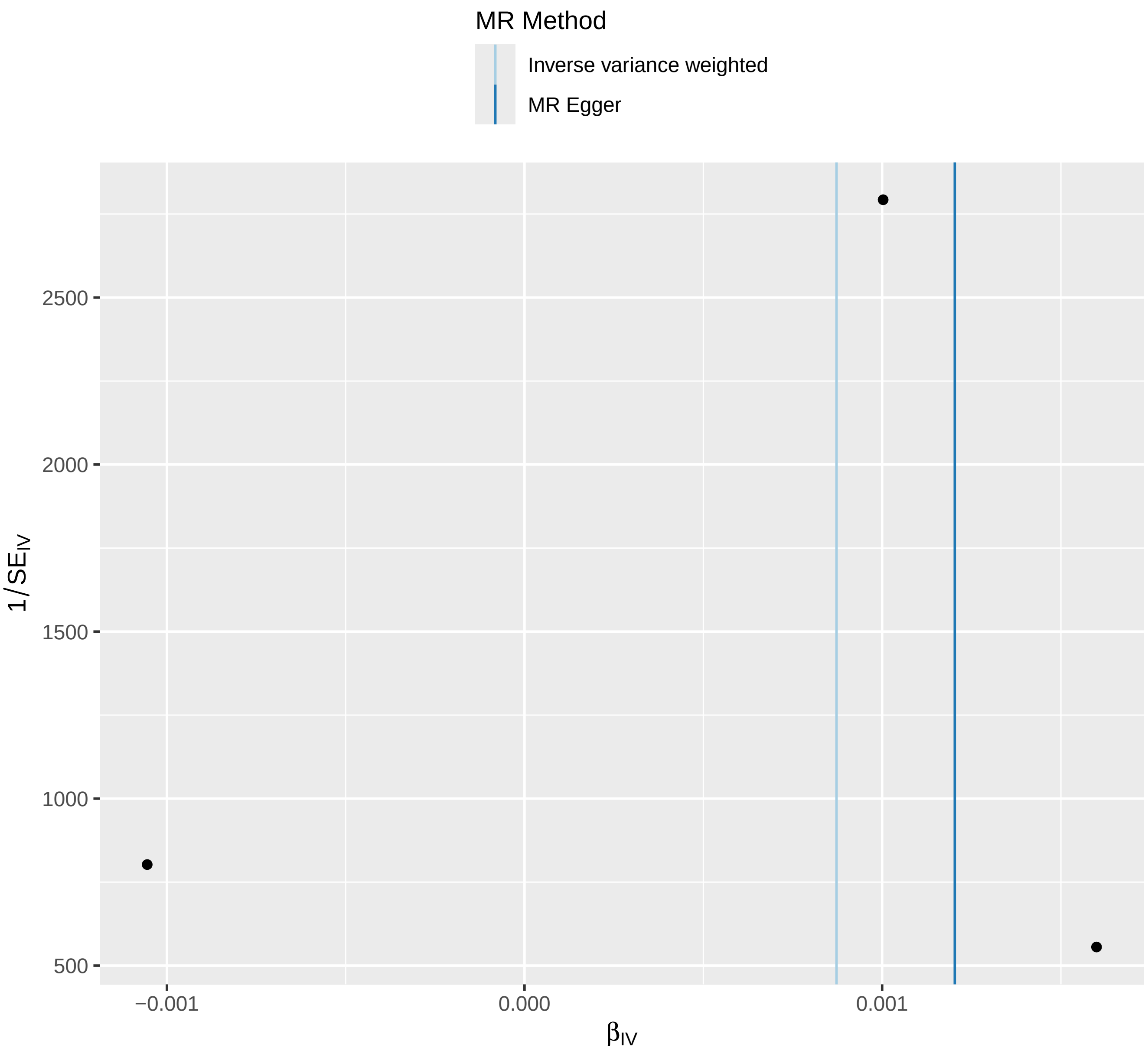

MR Method
Inverse variance weighted
MR Egger
2500
2000
1500
1000
500
1/SE$_{IV}$
−0.001
0.000
0.001
β_{IV}

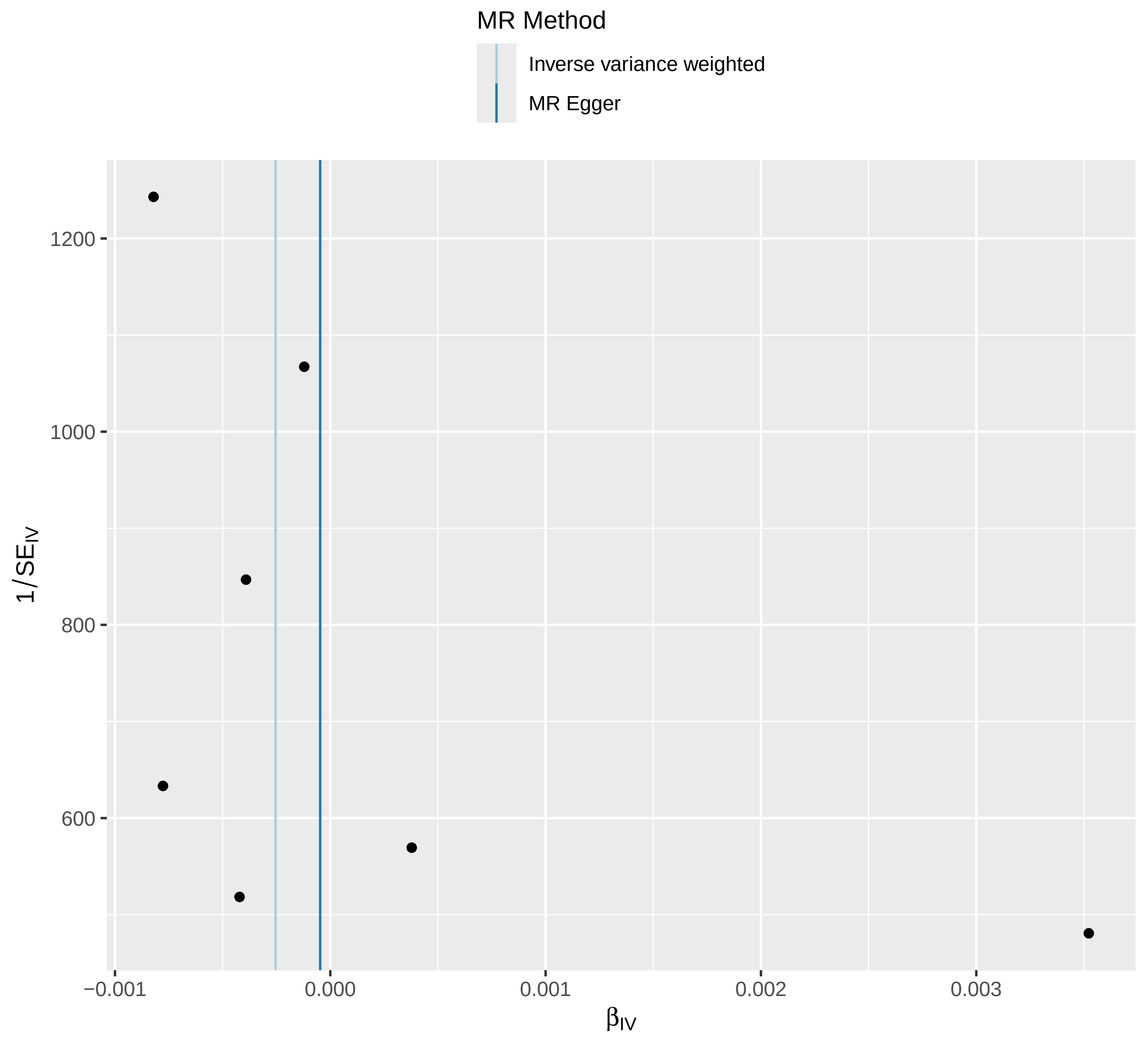

MR Method
Inverse variance weighted
MR Egger
1200
1000
800
600
1/SE_IV
−0.001
0.000
0.001
0.002
0.003
β_IV

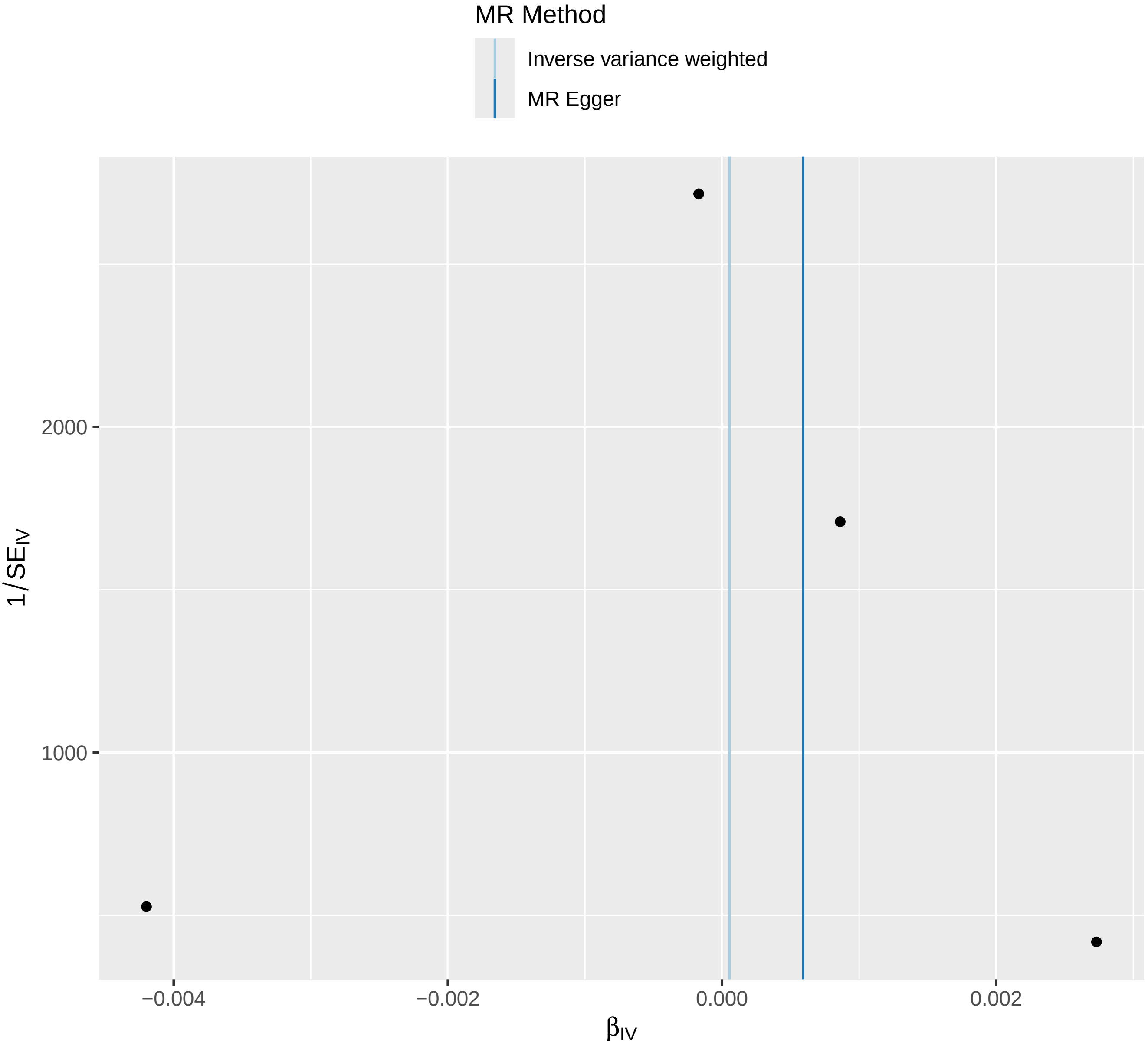
MR Method
Inverse variance weighted
MR Egger
2000
1000
1/SE_IV
−0.004
−0.002
0.000
0.002
β_IV

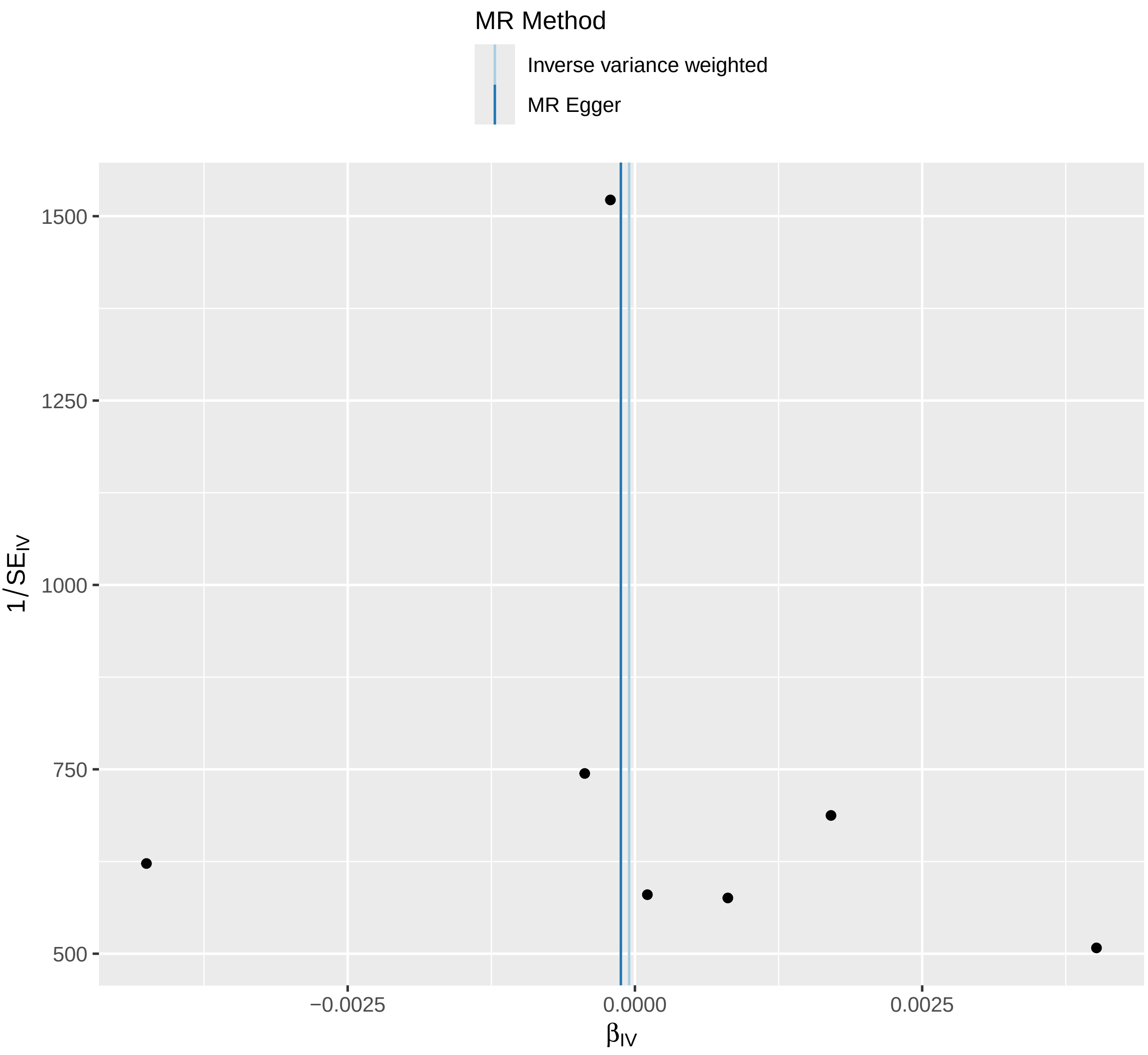
MR Method
Inverse variance weighted
MR Egger
1500
1250
1000
750
500
1/SE_IV
−0.0025
0.0000
0.0025
β_IV

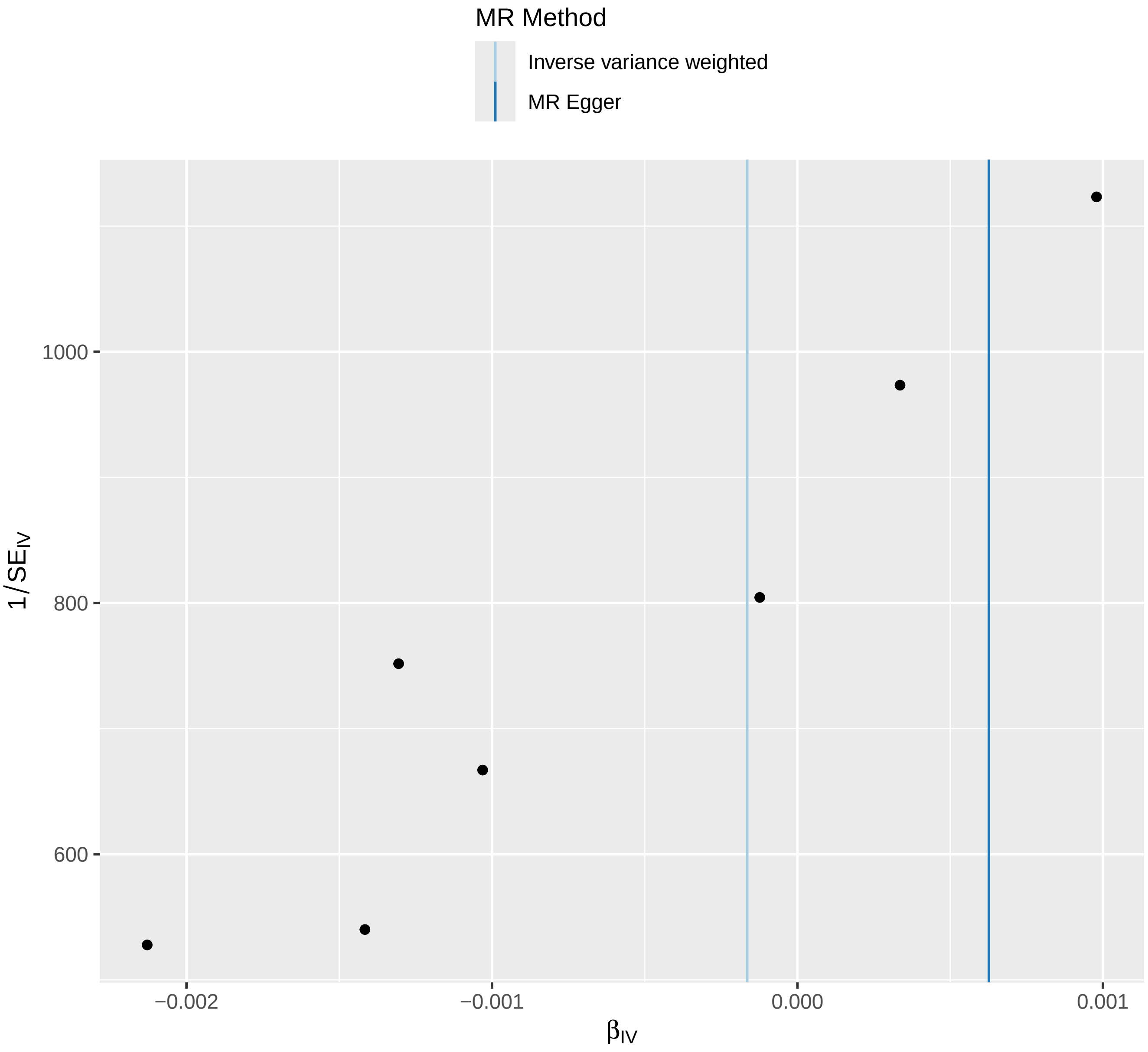
MR Method
Inverse variance weighted
MR Egger
1000
800
600
−0.002
−0.001
0.000
0.001
1/SE$_{IV}$
β$_{IV}$

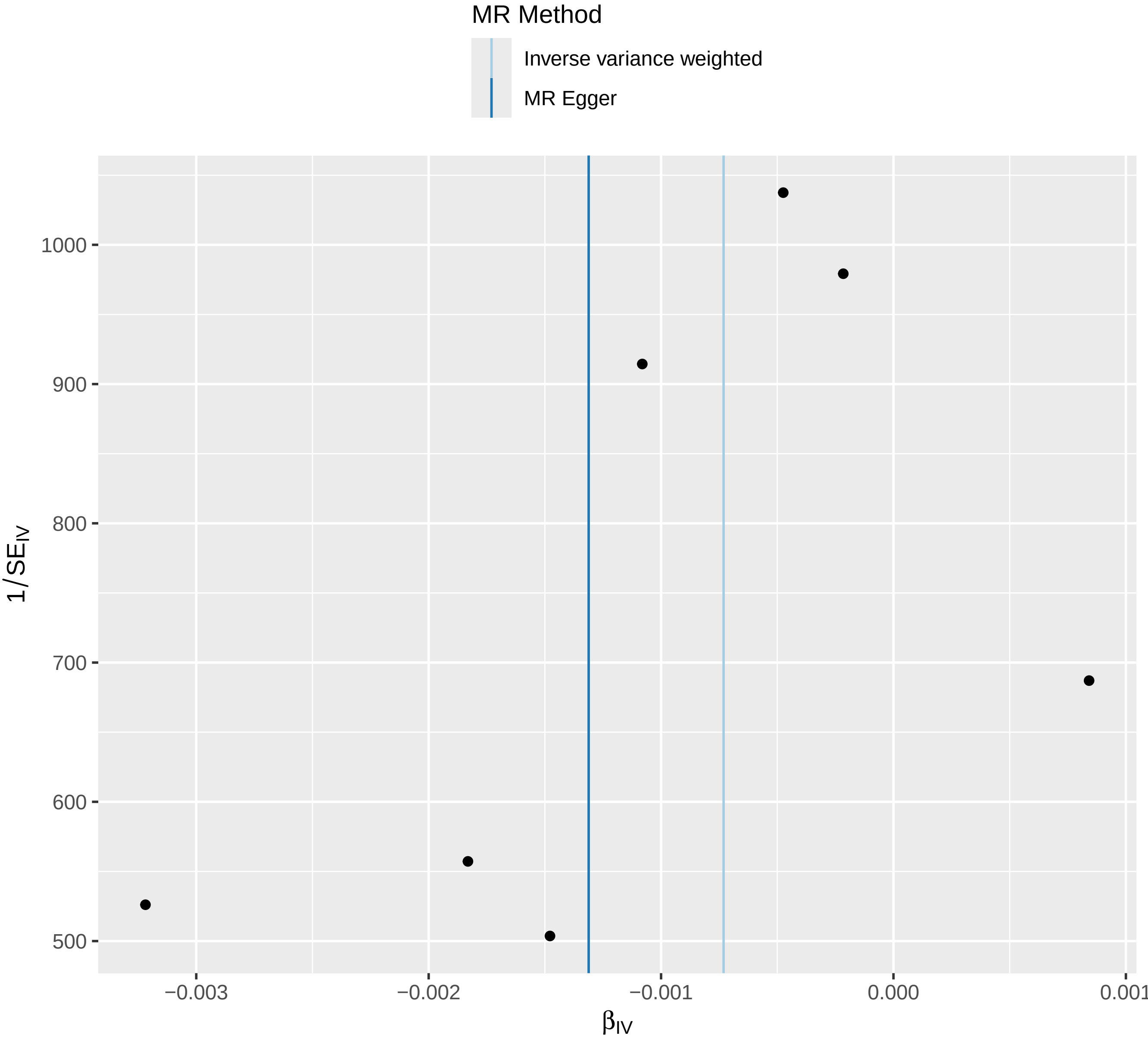
MR Method
Inverse variance weighted
MR Egger
1000
900
800
700
600
500
1/SE$_{IV}$
−0.003
−0.002
−0.001
0.000
0.001
β_{IV}

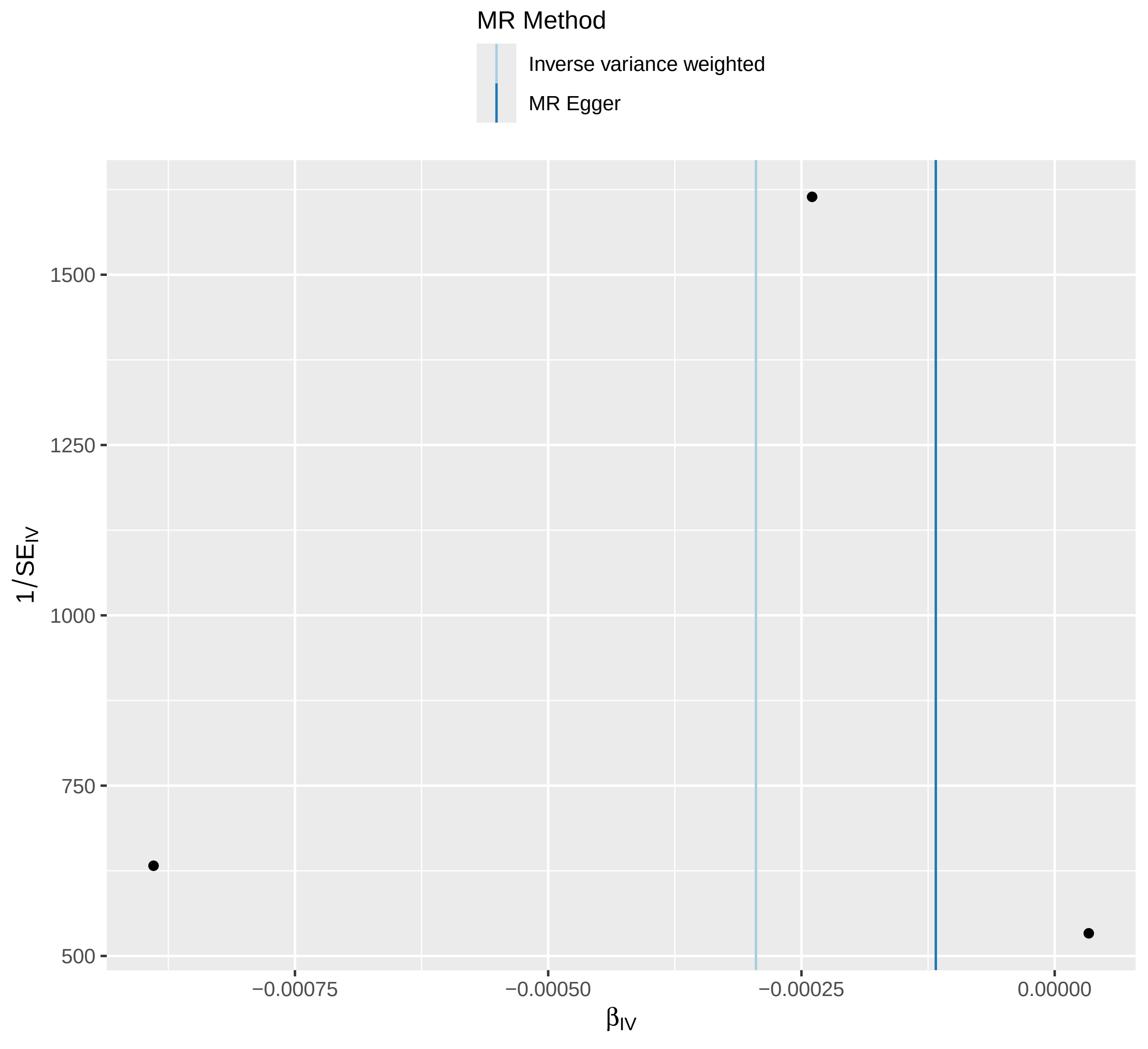
MR Method
Inverse variance weighted
MR Egger
1500
1250
1000
750
500
1/SE_IV
−0.00075
−0.00050
−0.00025
0.00000
β_IV

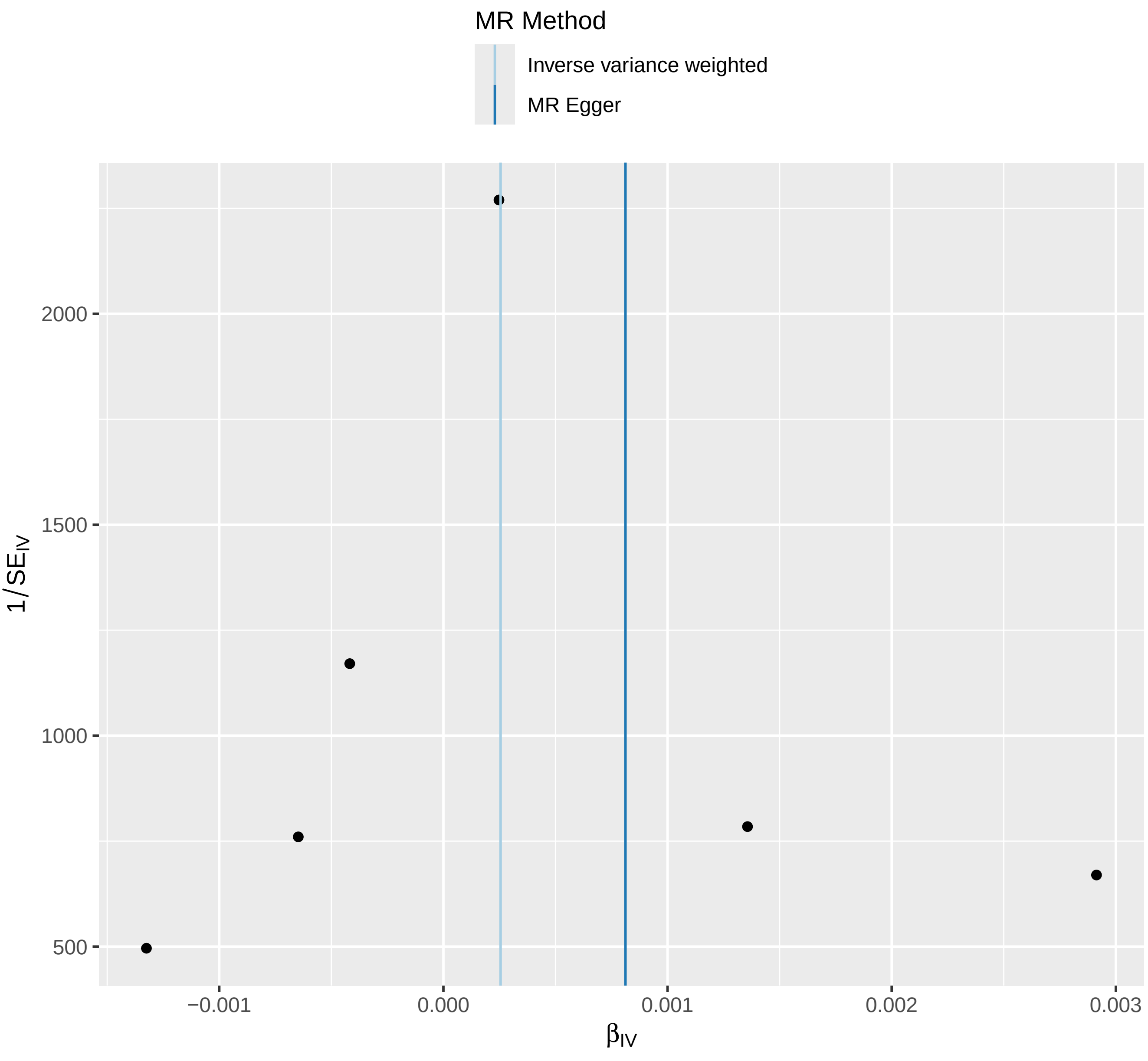
MR Method
Inverse variance weighted
MR Egger
2000
1500
1000
500
1/SE_IV
−0.001
0.000
0.001
0.002
0.003
β_IV

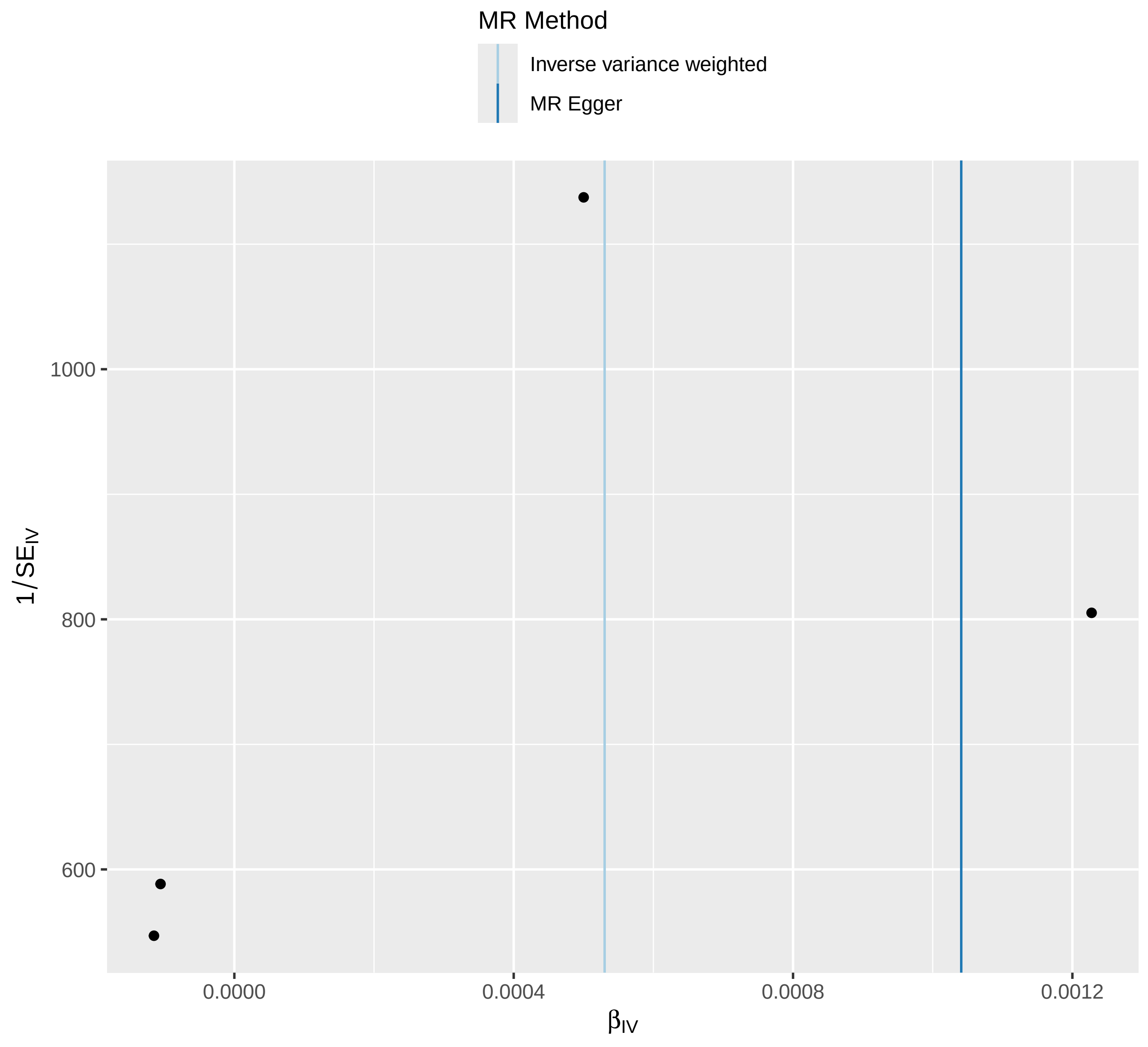
MR Method
Inverse variance weighted
MR Egger
1000
800
600
1/SE_IV
0.0000
0.0004
0.0008
0.0012
β_IV

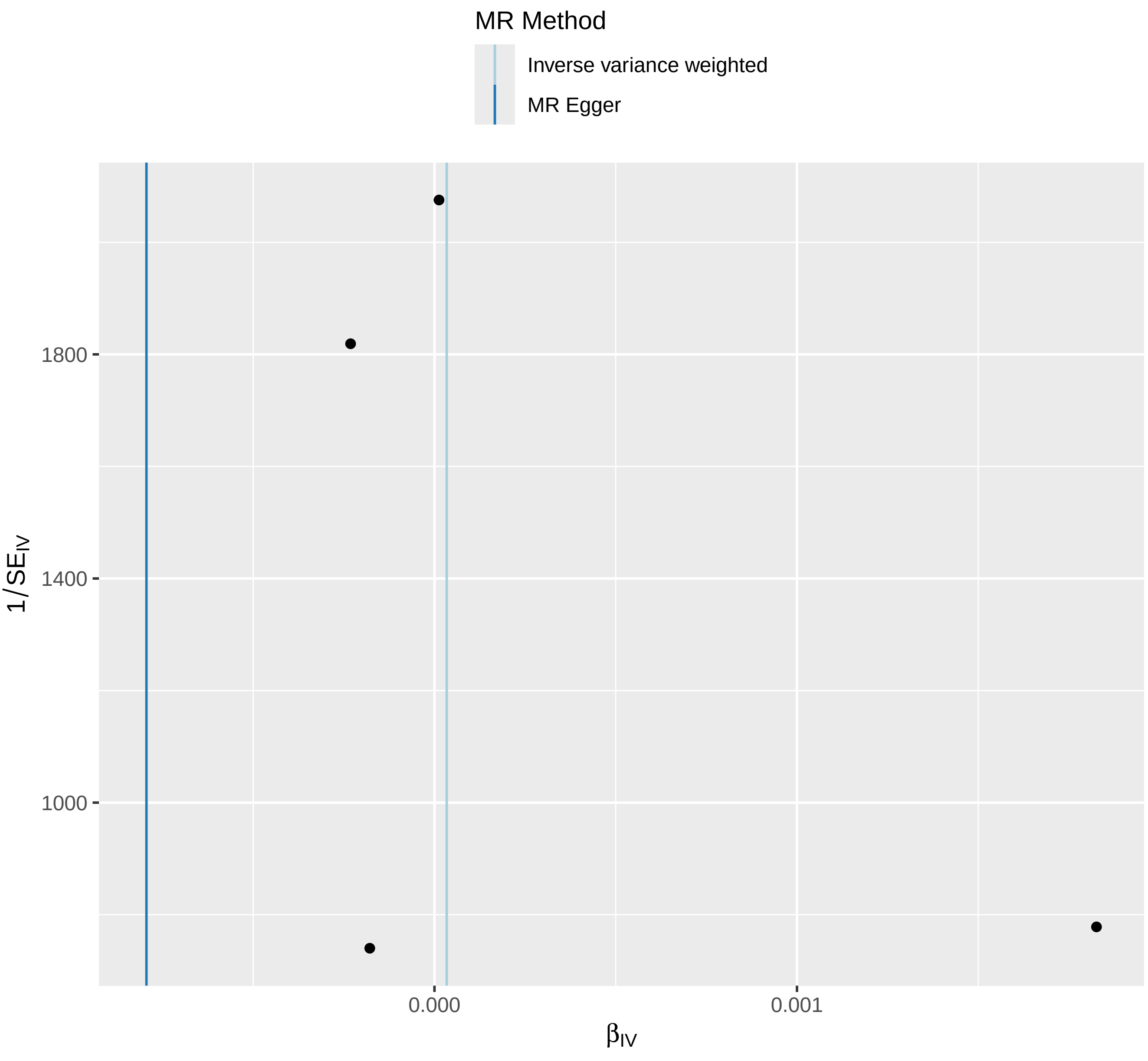
MR Method
Inverse variance weighted
MR Egger
1800
1400
1000
1/SE_IV
0.000
0.001
β_IV

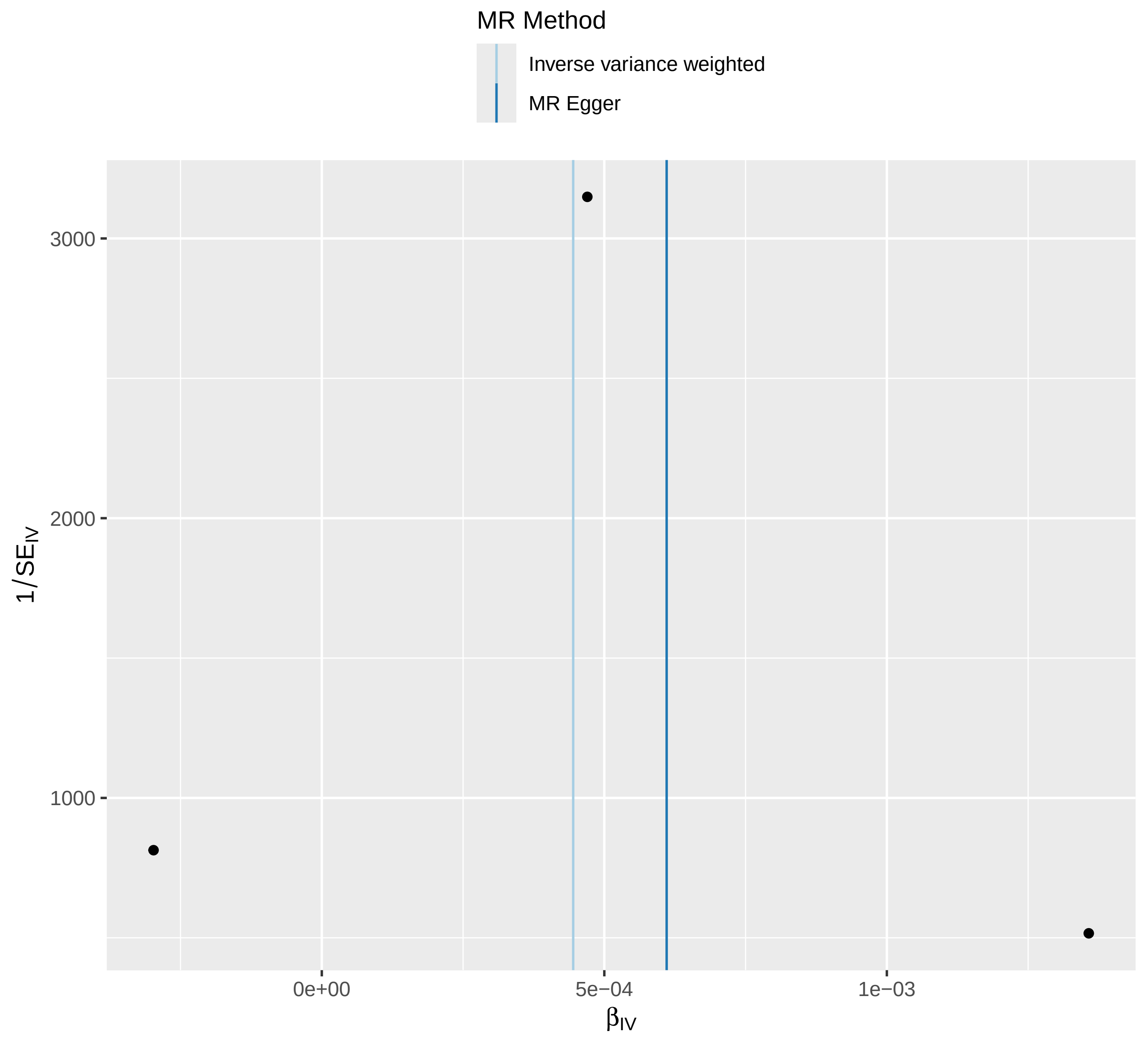

MR Method
Inverse variance weighted
MR Egger
3000
2000
1000
1/SE$_{IV}$
0e+00
5e−04
1e−03
β$_{IV}$

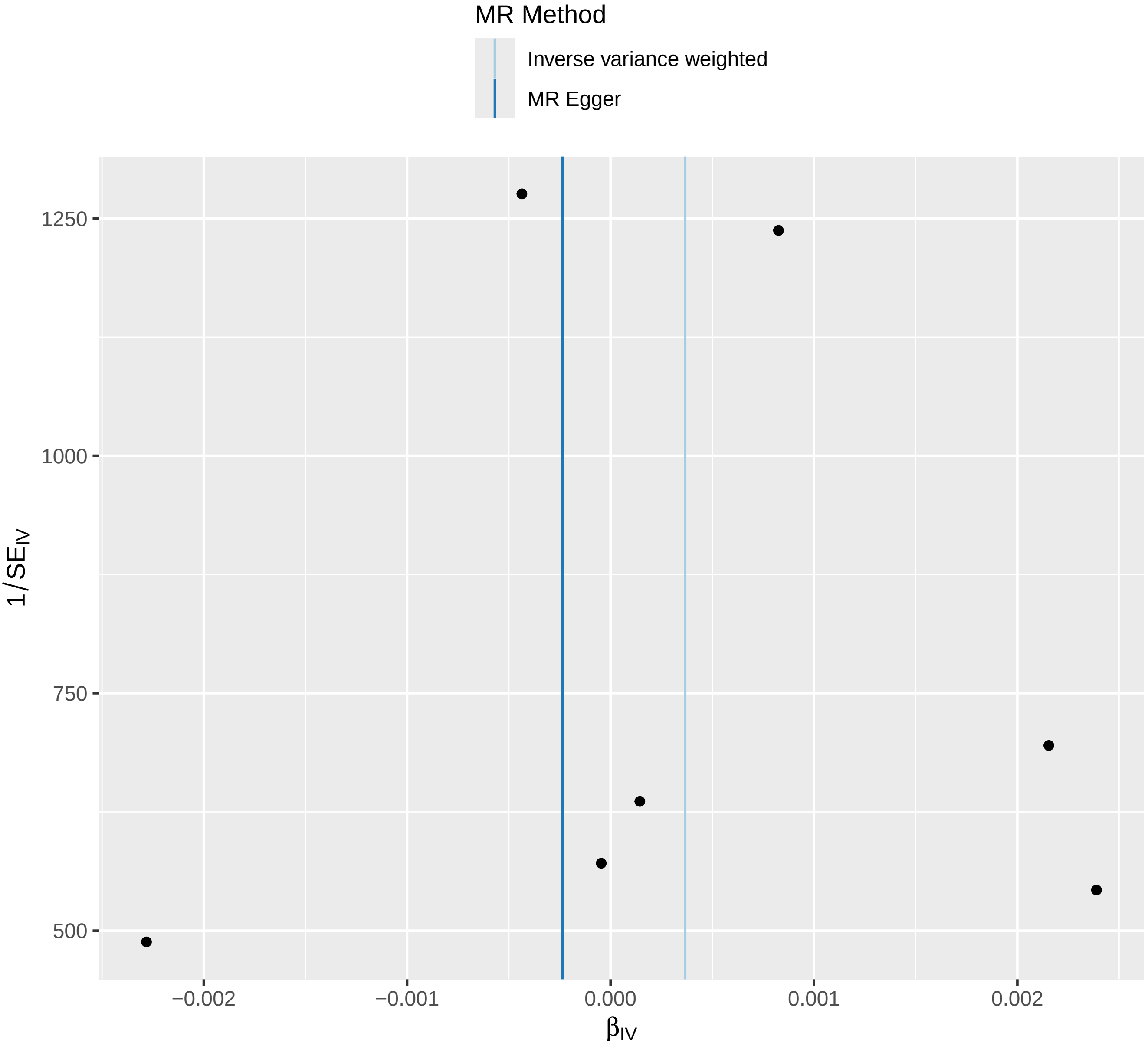
MR Method
Inverse variance weighted
MR Egger
1250
1000
750
500
−0.002
−0.001
0.000
0.001
0.002
β_{IV}
$1/SE_{IV}$

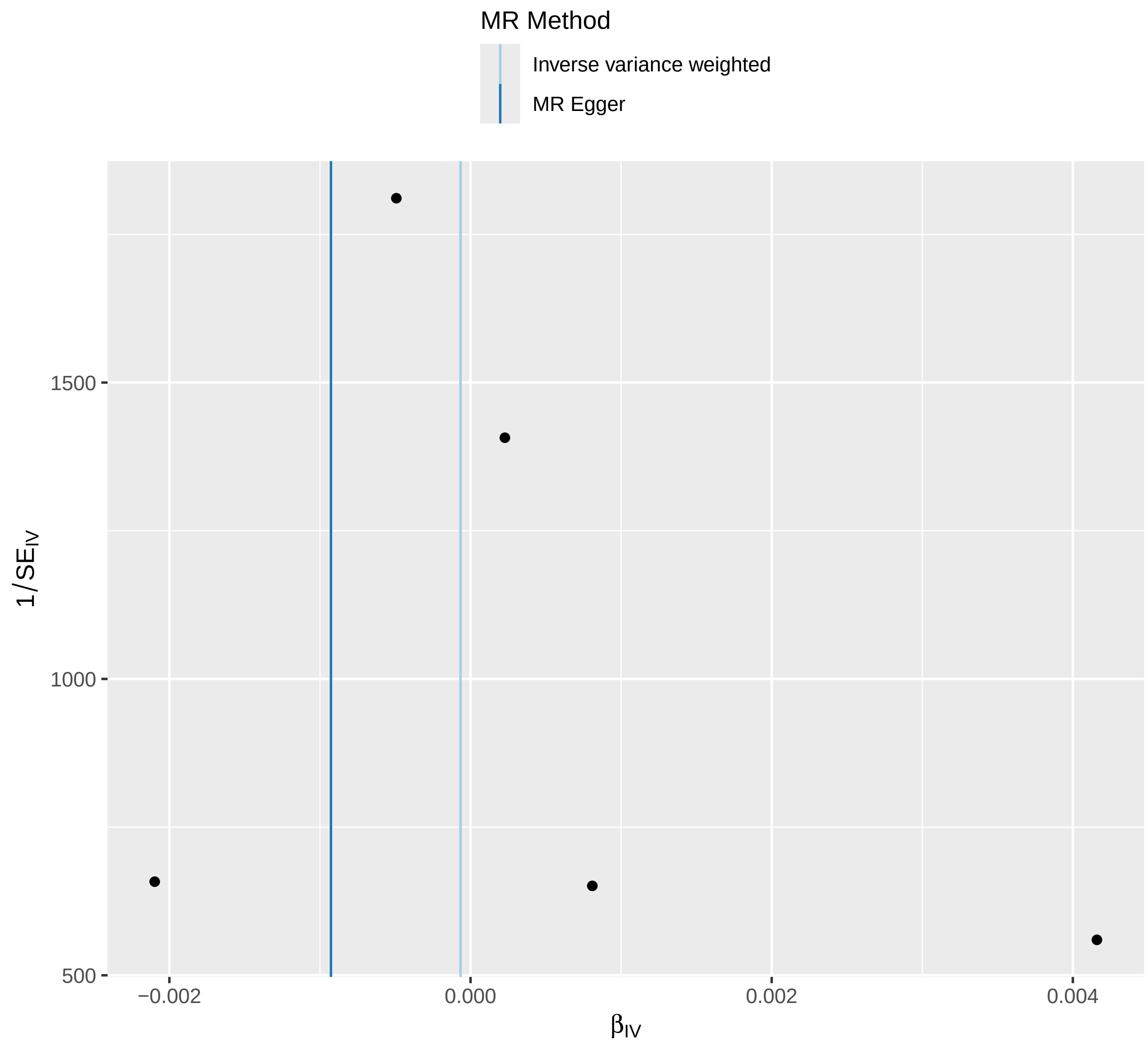
MR Method
Inverse variance weighted
MR Egger
1500
1000
500
1/SE_IV
−0.002
0.000
0.002
0.004
β_IV

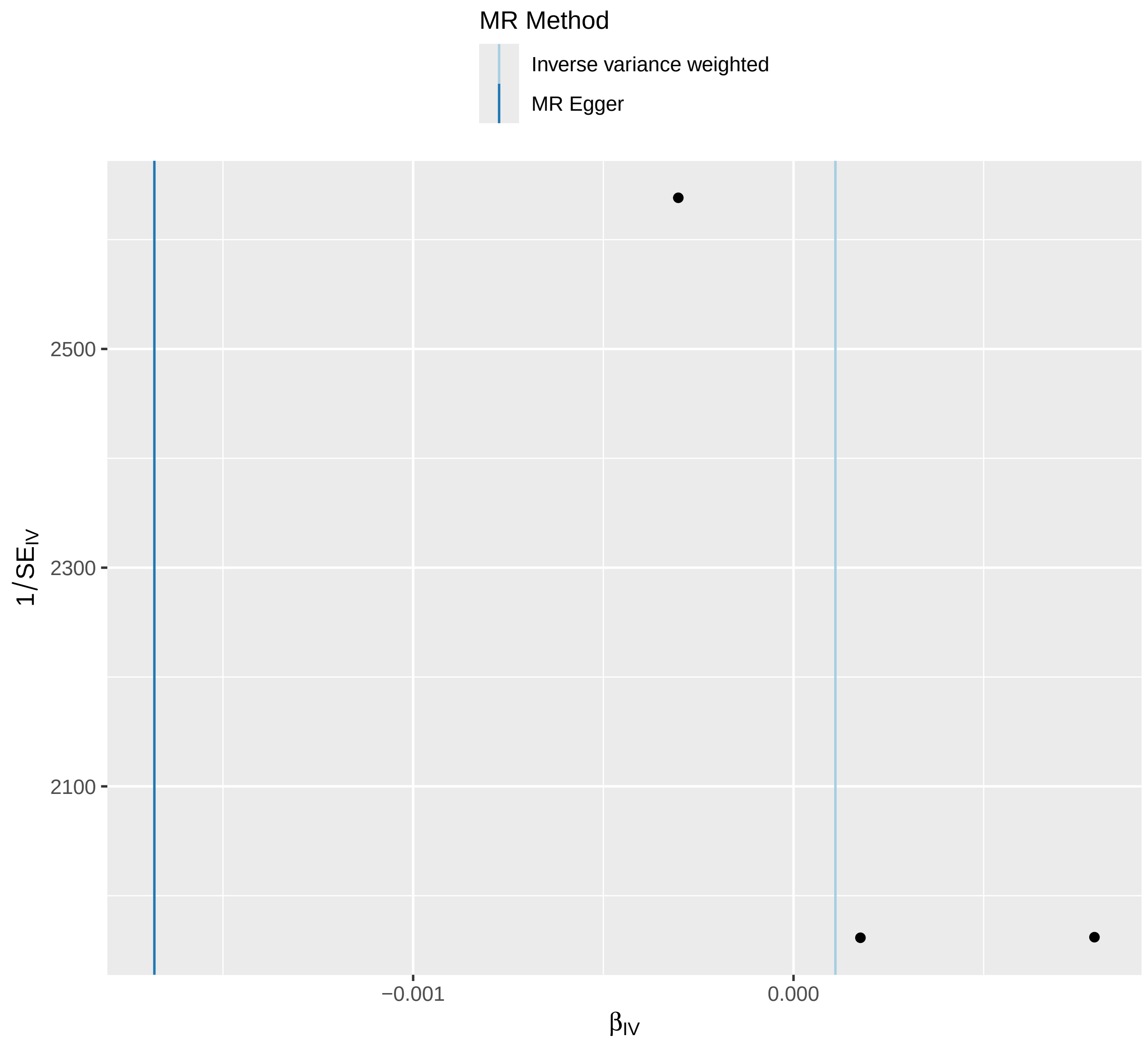

MR Method
Inverse variance weighted
MR Egger
2500
2300
2100
1/SE_IV
−0.001
0.000
β_IV

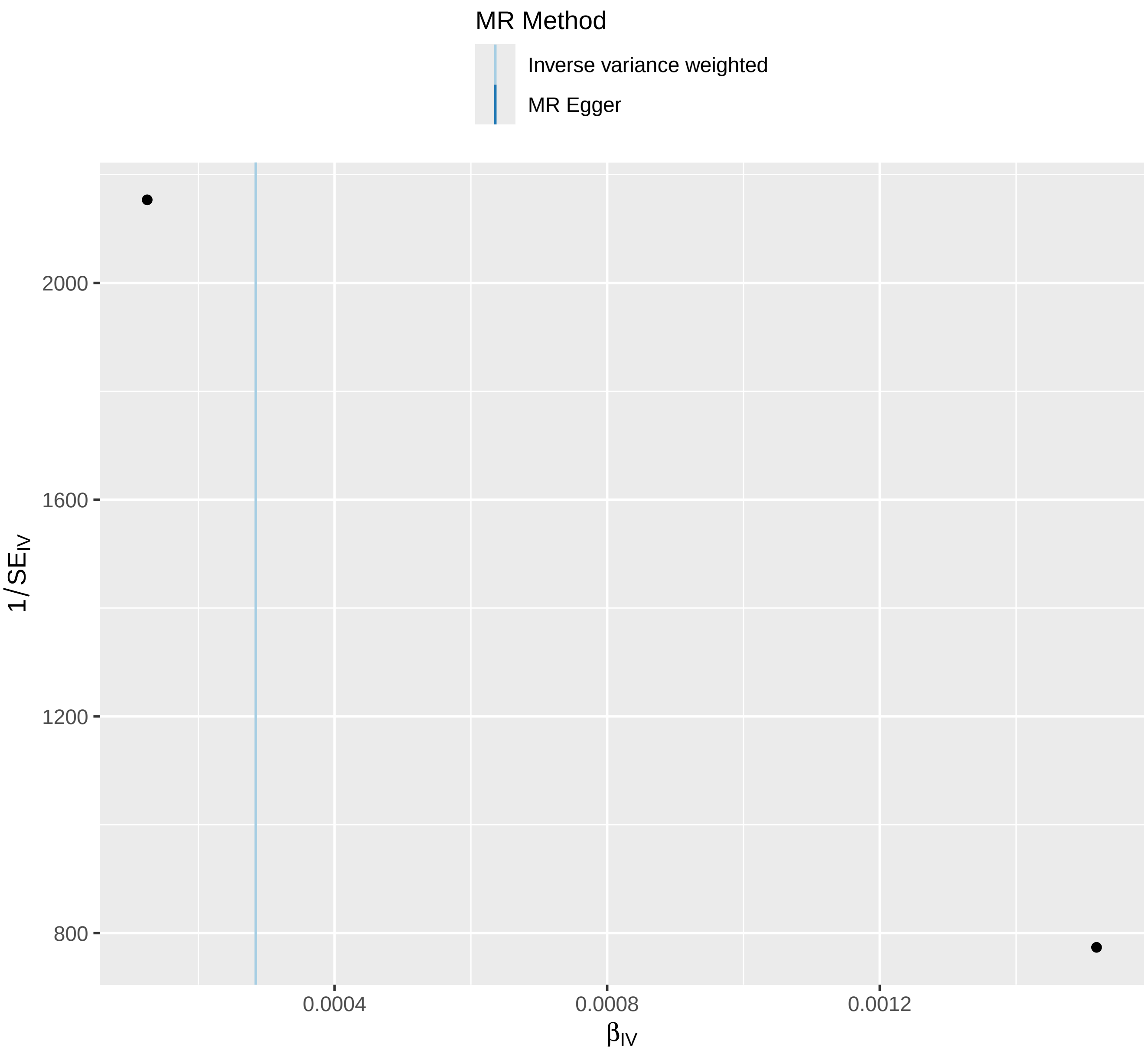
MR Method
Inverse variance weighted
MR Egger
2000
1600
1200
800
1/SE_IV
0.0004
0.0008
0.0012
β_IV

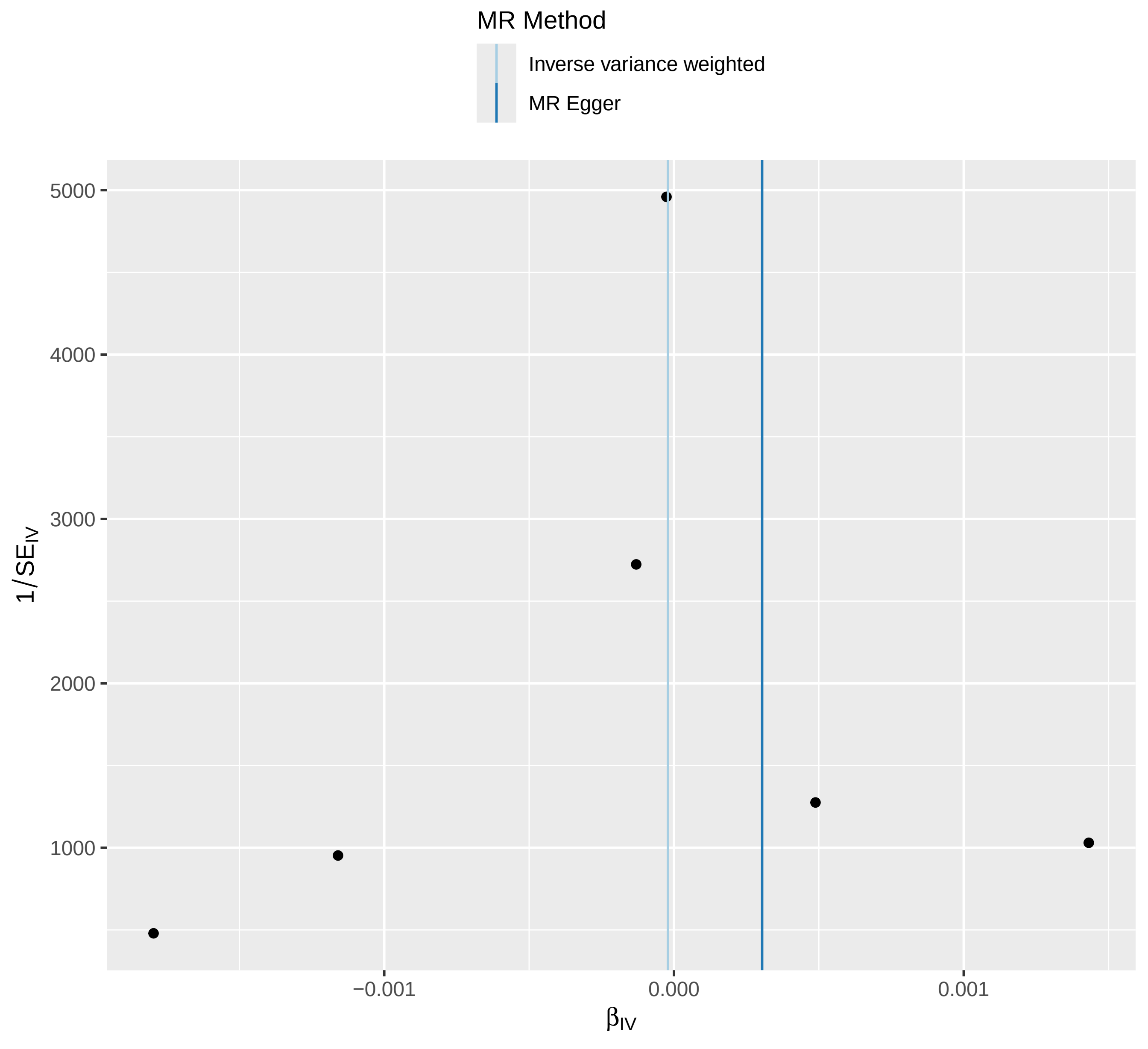
MR Method
Inverse variance weighted
MR Egger
5000
4000
3000
2000
1000
1/SE_IV
−0.001
0.000
0.001
β_IV

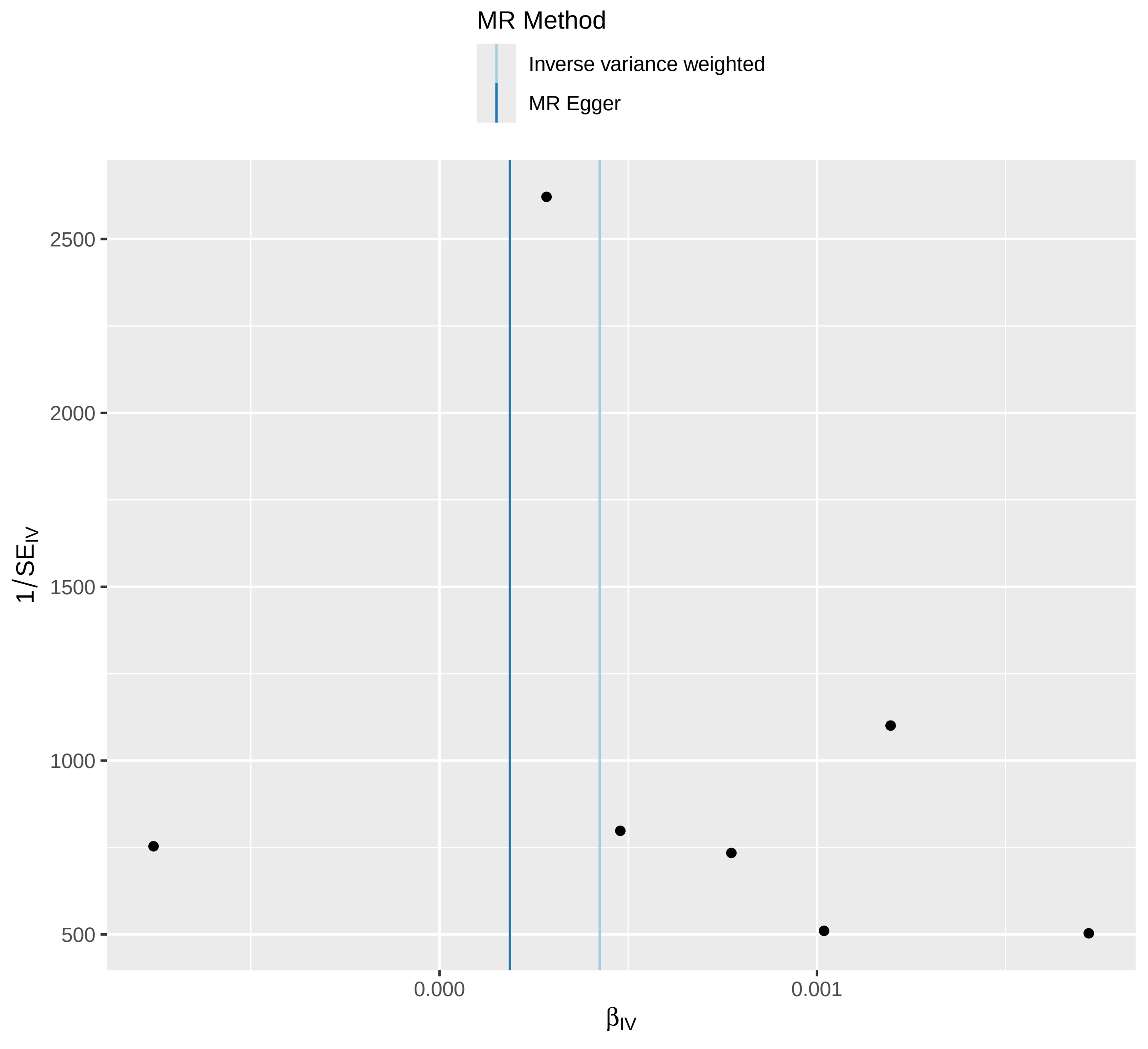
MR Method
Inverse variance weighted
MR Egger
2500
2000
1500
1000
500
$1/SE_{IV}$
0.000
0.001
β_{IV}

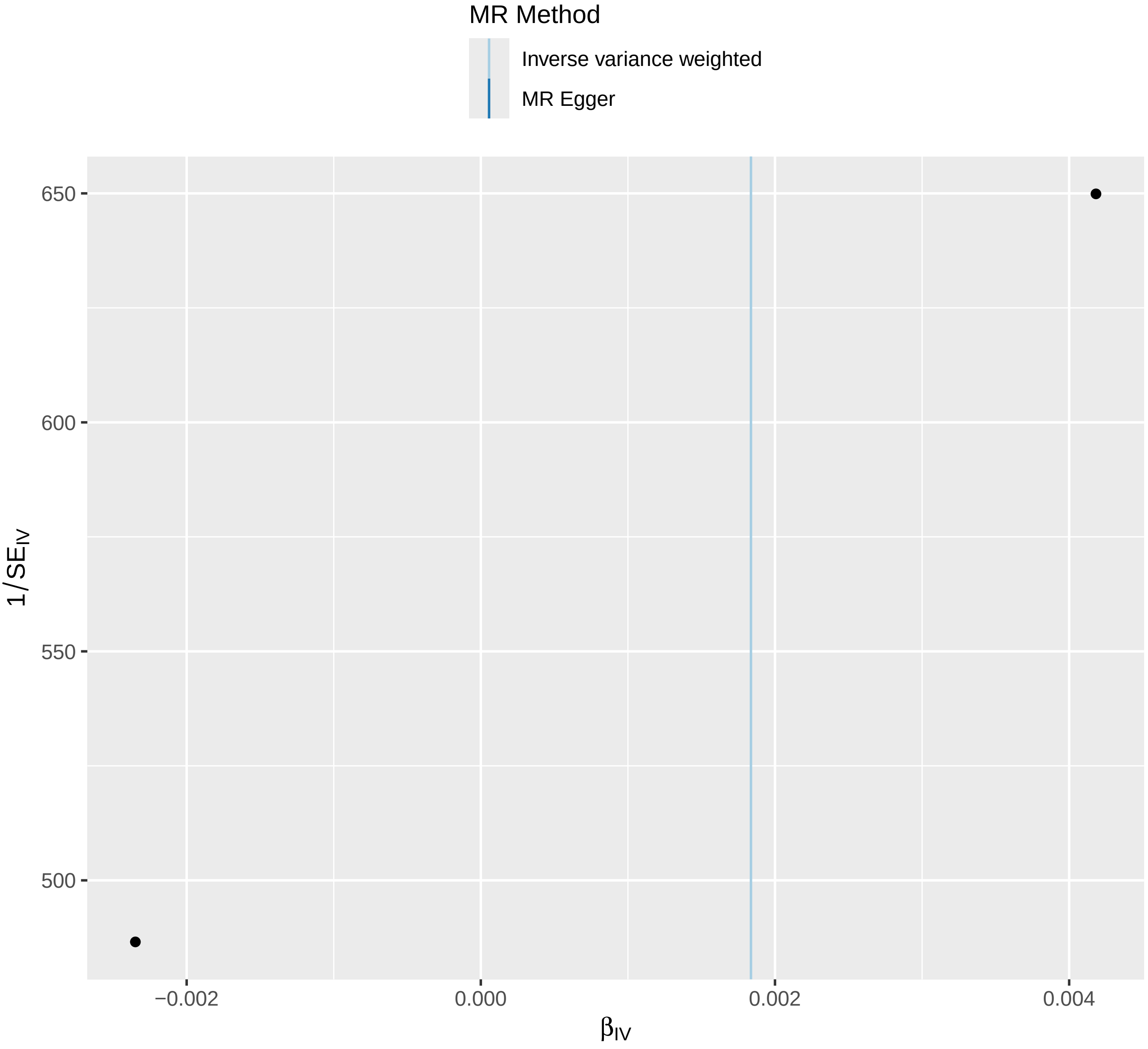

MR Method
Inverse variance weighted
MR Egger
650
600
550
500
1/SE_IV
−0.002
0.000
0.002
0.004
β_IV

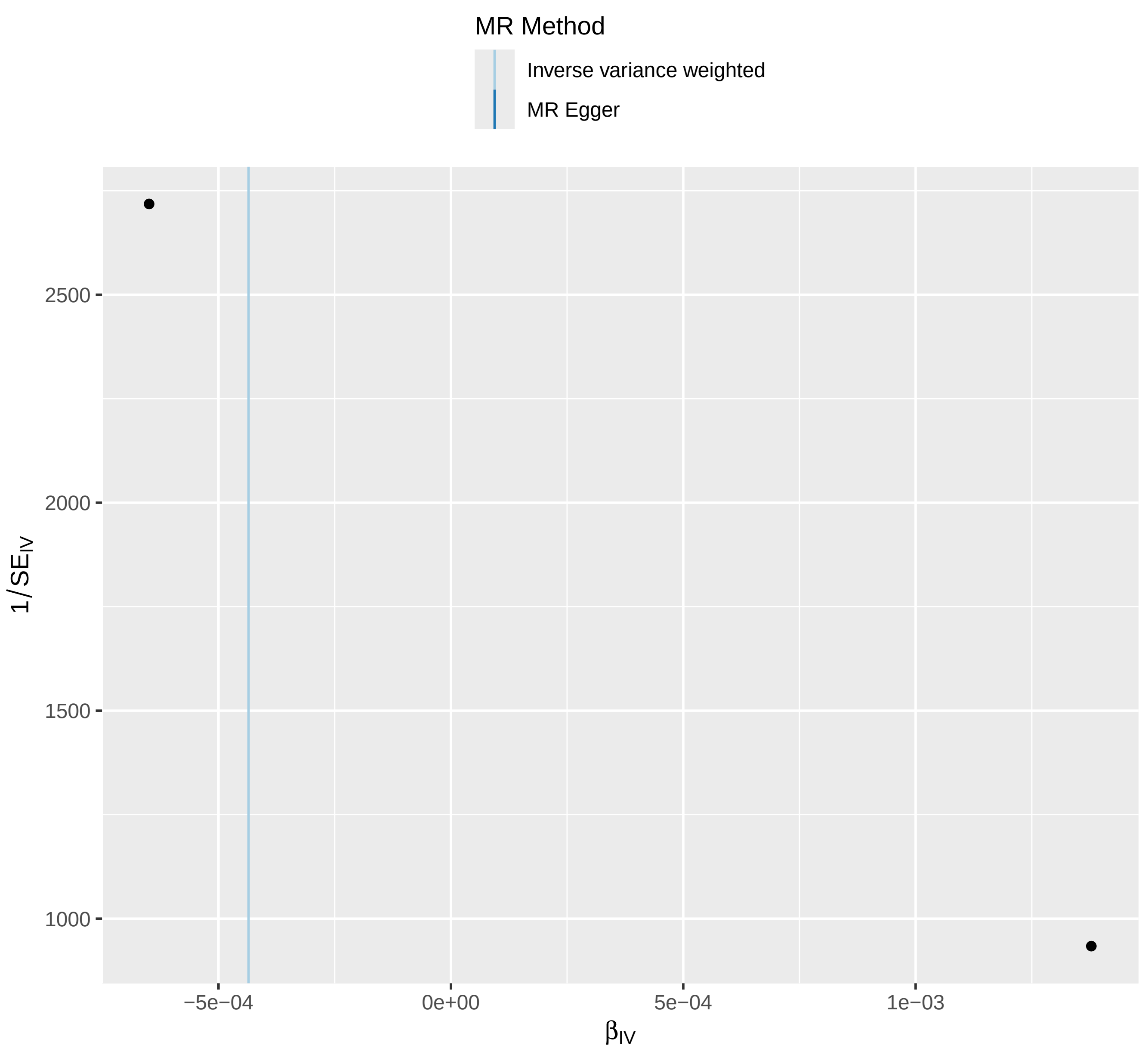
MR Method
Inverse variance weighted
MR Egger
2500
2000
1500
1000
1/SE$_{IV}$
−5e−04
0e+00
5e−04
1e−03
β$_{IV}$

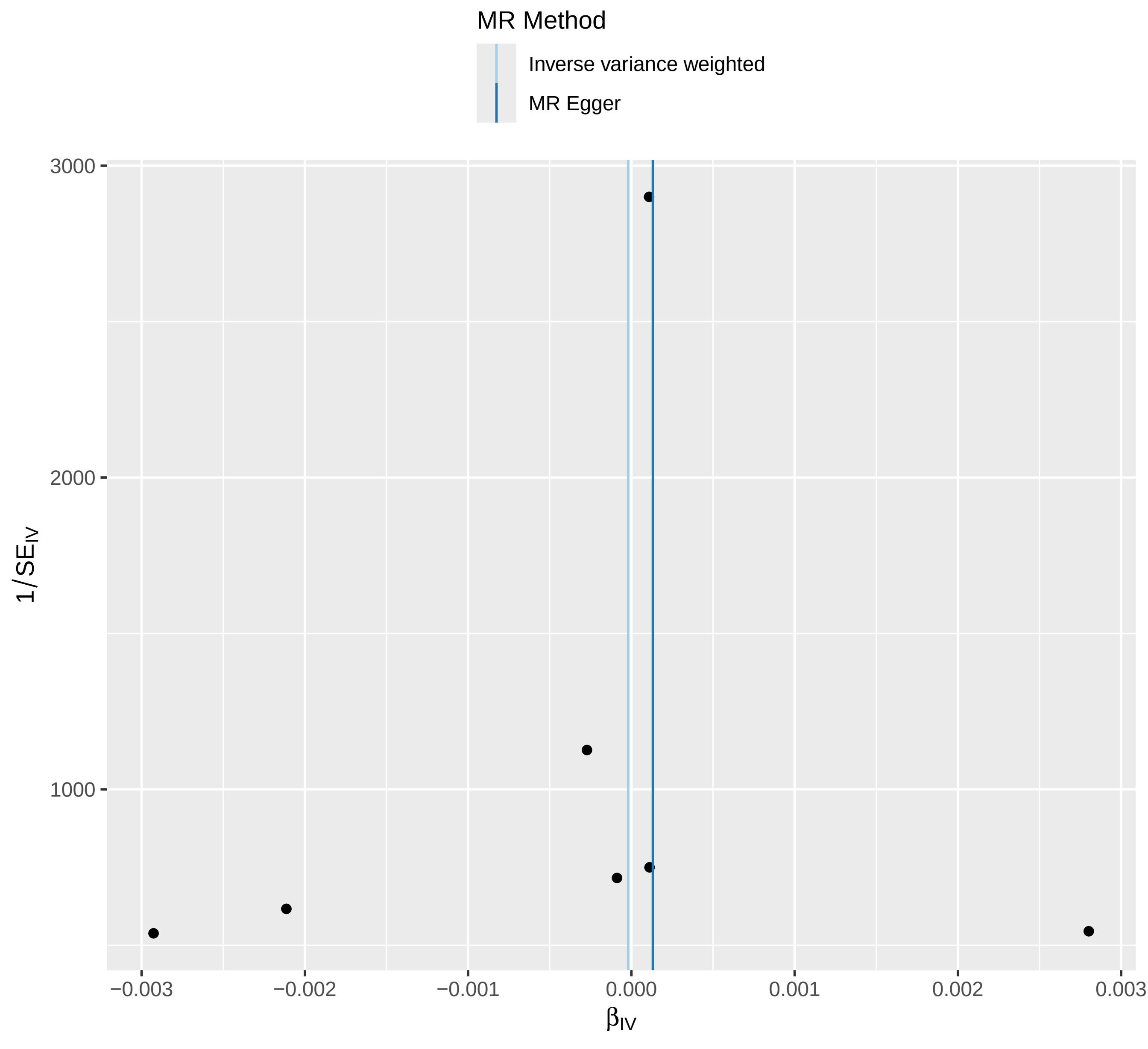
MR Method
Inverse variance weighted
MR Egger
3000
2000
1000
1/SE_IV
−0.003
−0.002
−0.001
0.000
0.001
0.002
0.003
β_IV

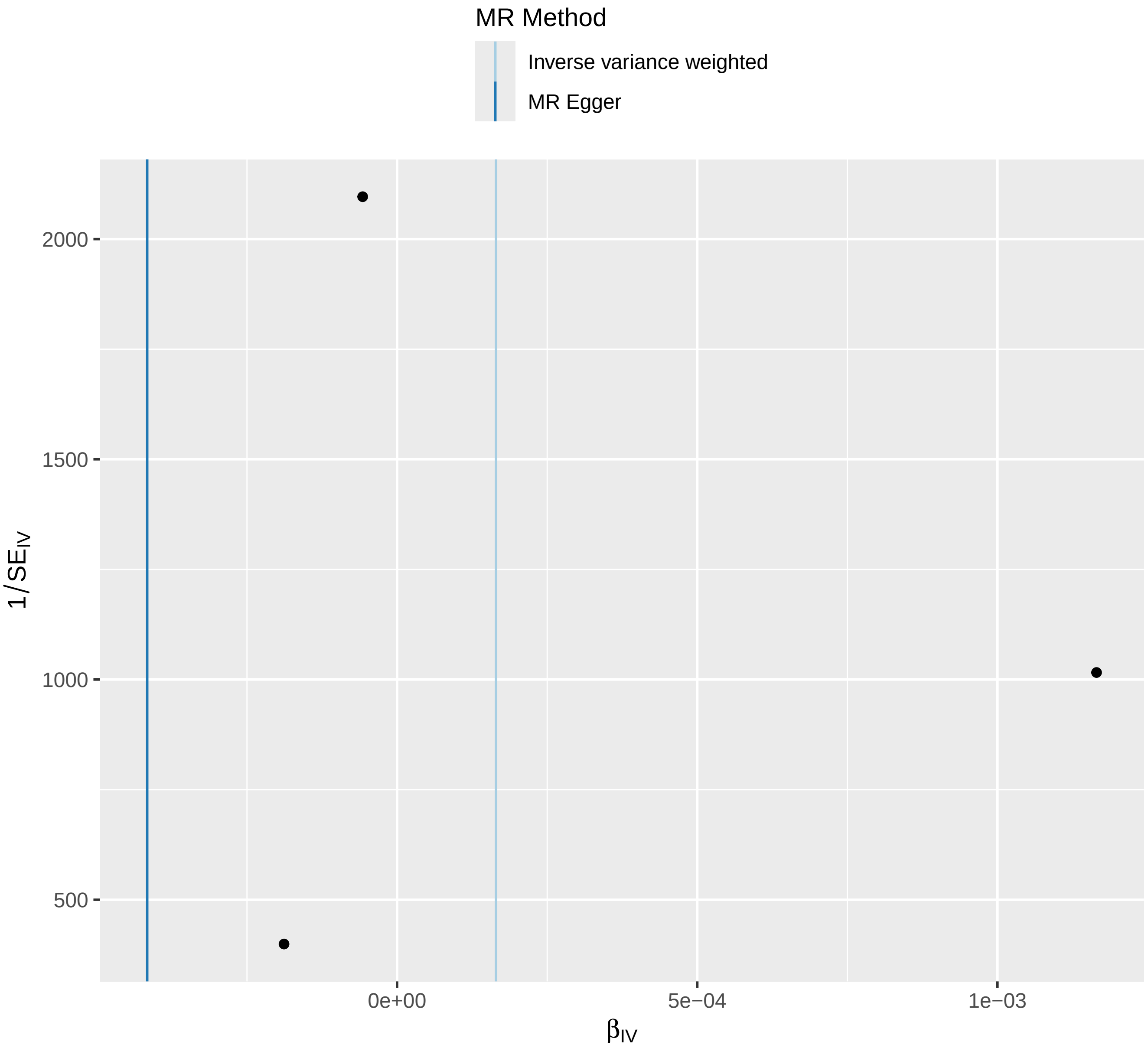

MR Method
Inverse variance weighted
MR Egger
2000
1500
1000
500
$1/SE_{IV}$
0e+00
5e−04
1e−03
β_{IV}

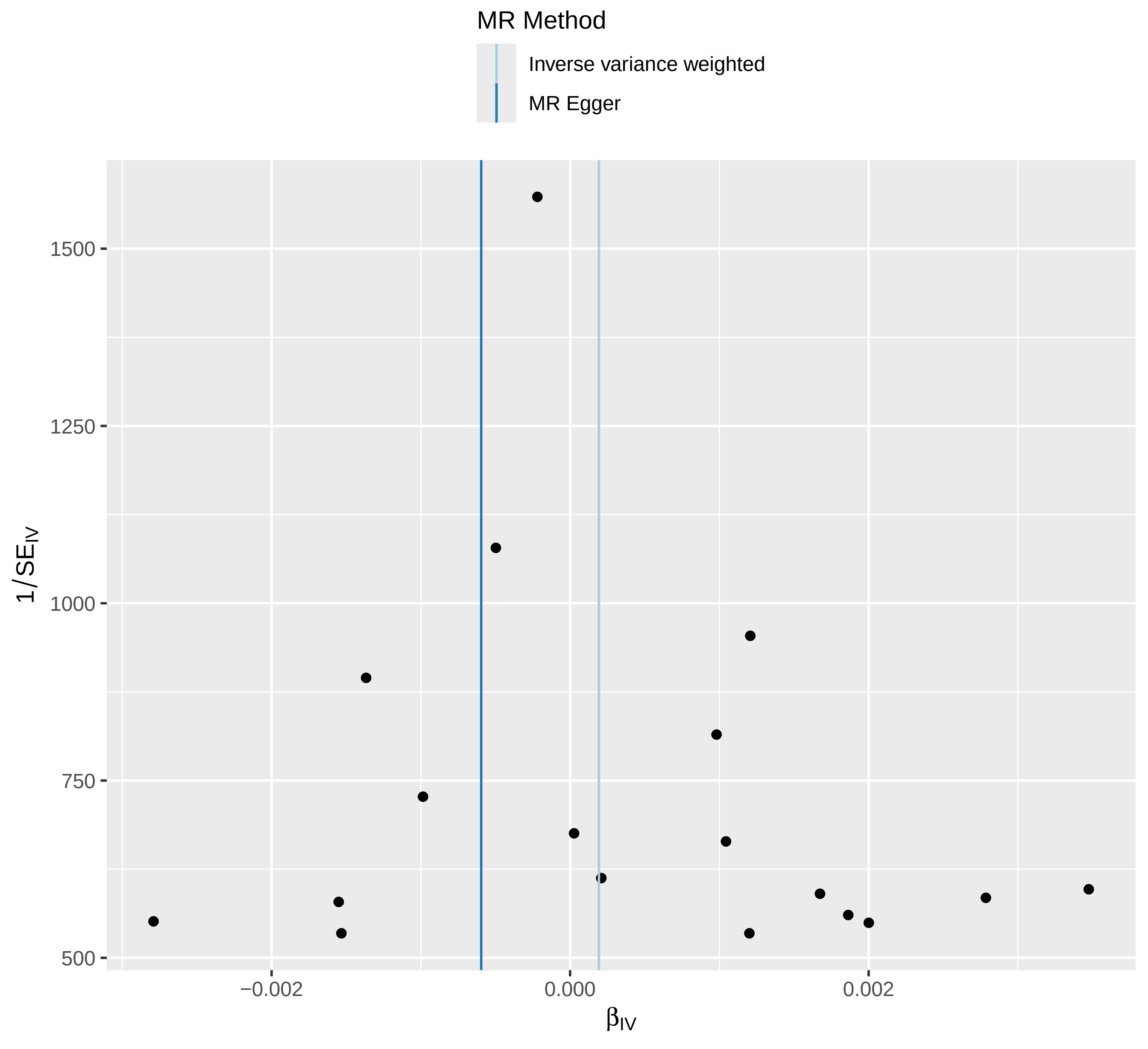
MR Method
Inverse variance weighted
MR Egger
1500
1250
1000
750
500
$1/SE_{IV}$
−0.002
0.000
0.002
β_{IV}

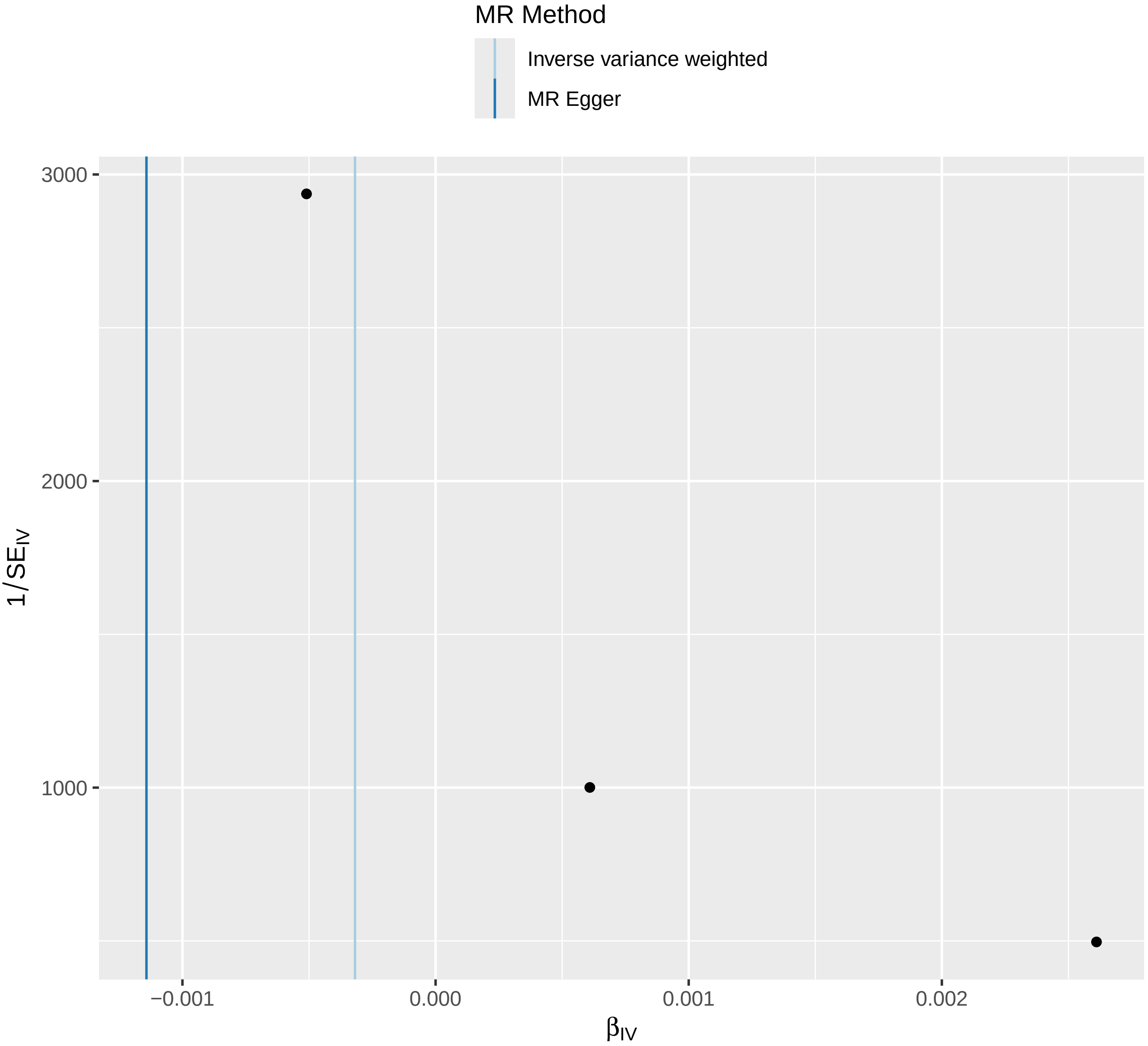
MR Method
Inverse variance weighted
MR Egger
3000
2000
1000
1/SE_IV
−0.001
0.000
0.001
0.002
β_IV

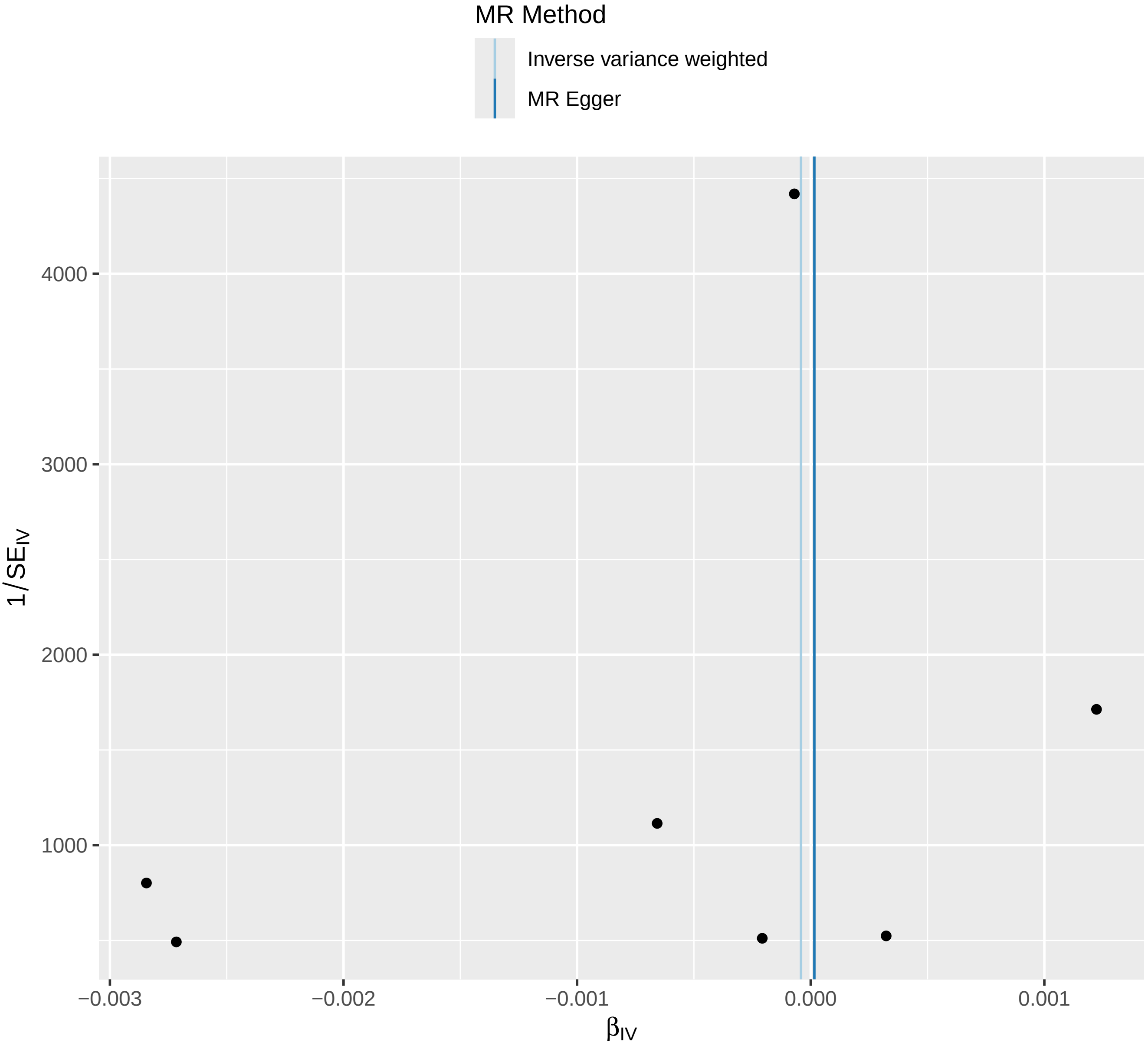
MR Method
Inverse variance weighted
MR Egger
4000
3000
2000
1000
1/SE_IV
−0.003
−0.002
−0.001
0.000
0.001
β_IV

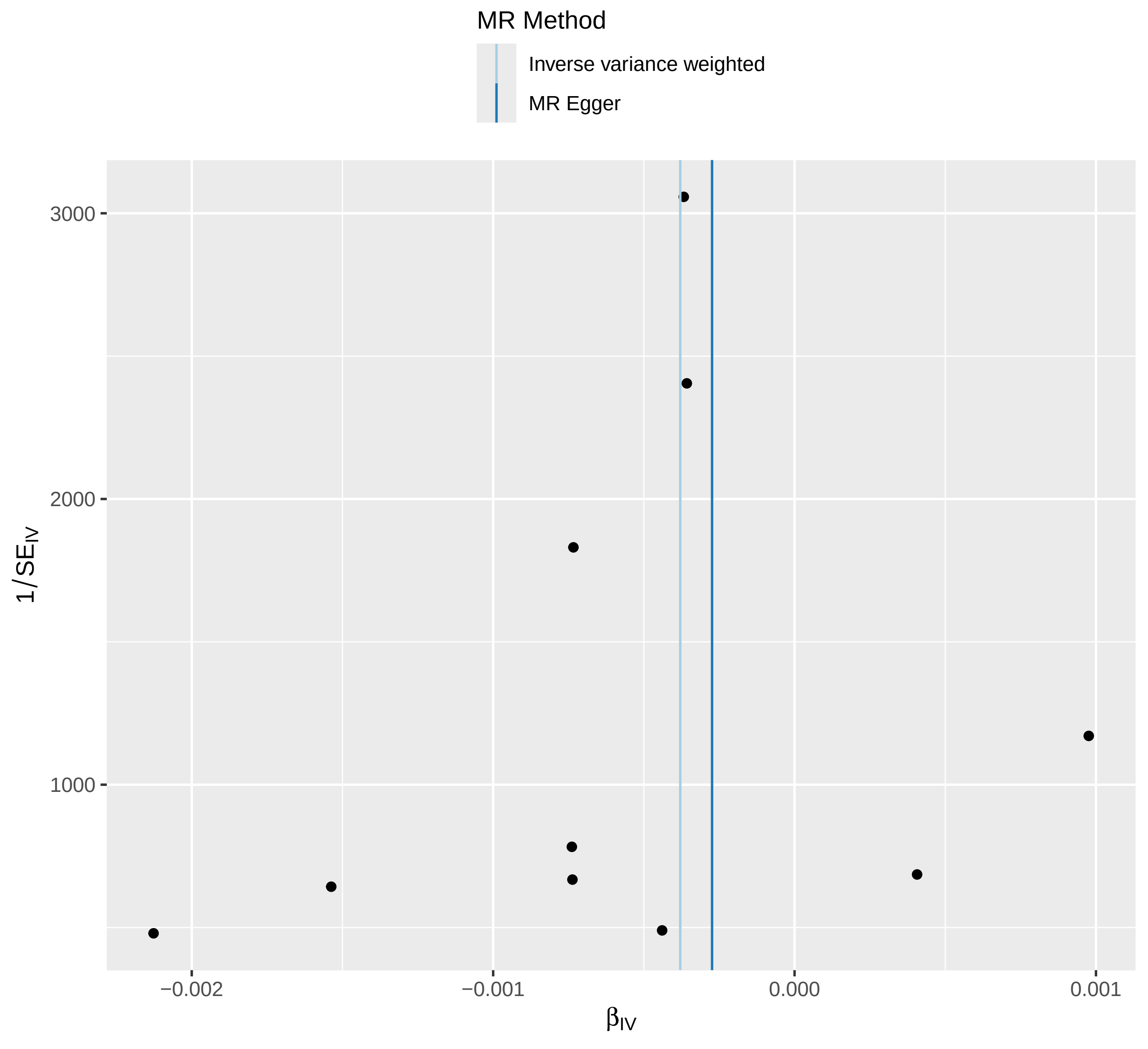

MR Method
Inverse variance weighted
MR Egger
3000
2000
1000
1/SE_IV
−0.002
−0.001
0.000
0.001
β_IV

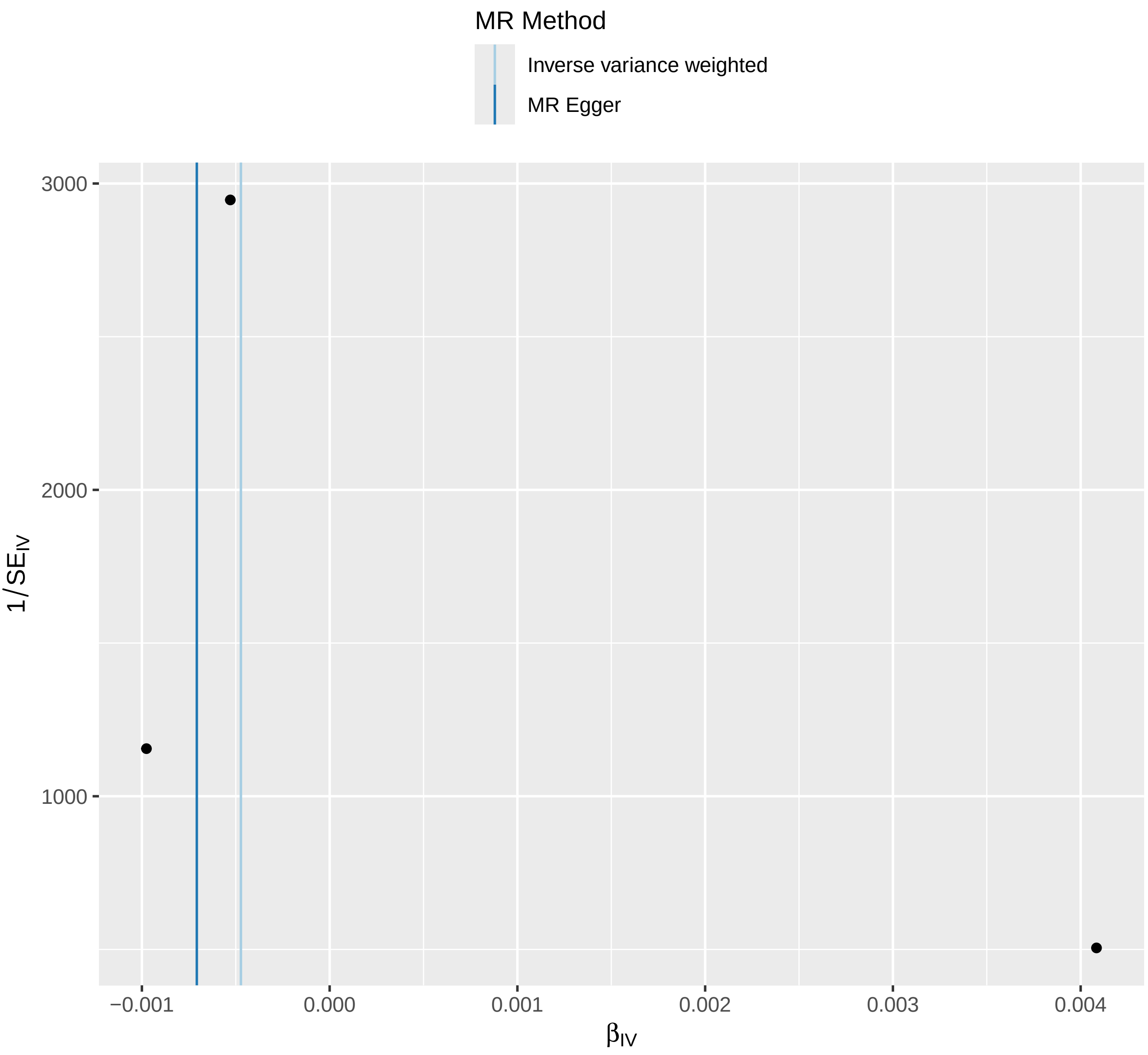
MR Method
Inverse variance weighted
MR Egger
3000
2000
1000
1/SE_IV
−0.001
0.000
0.001
0.002
0.003
0.004
β_IV

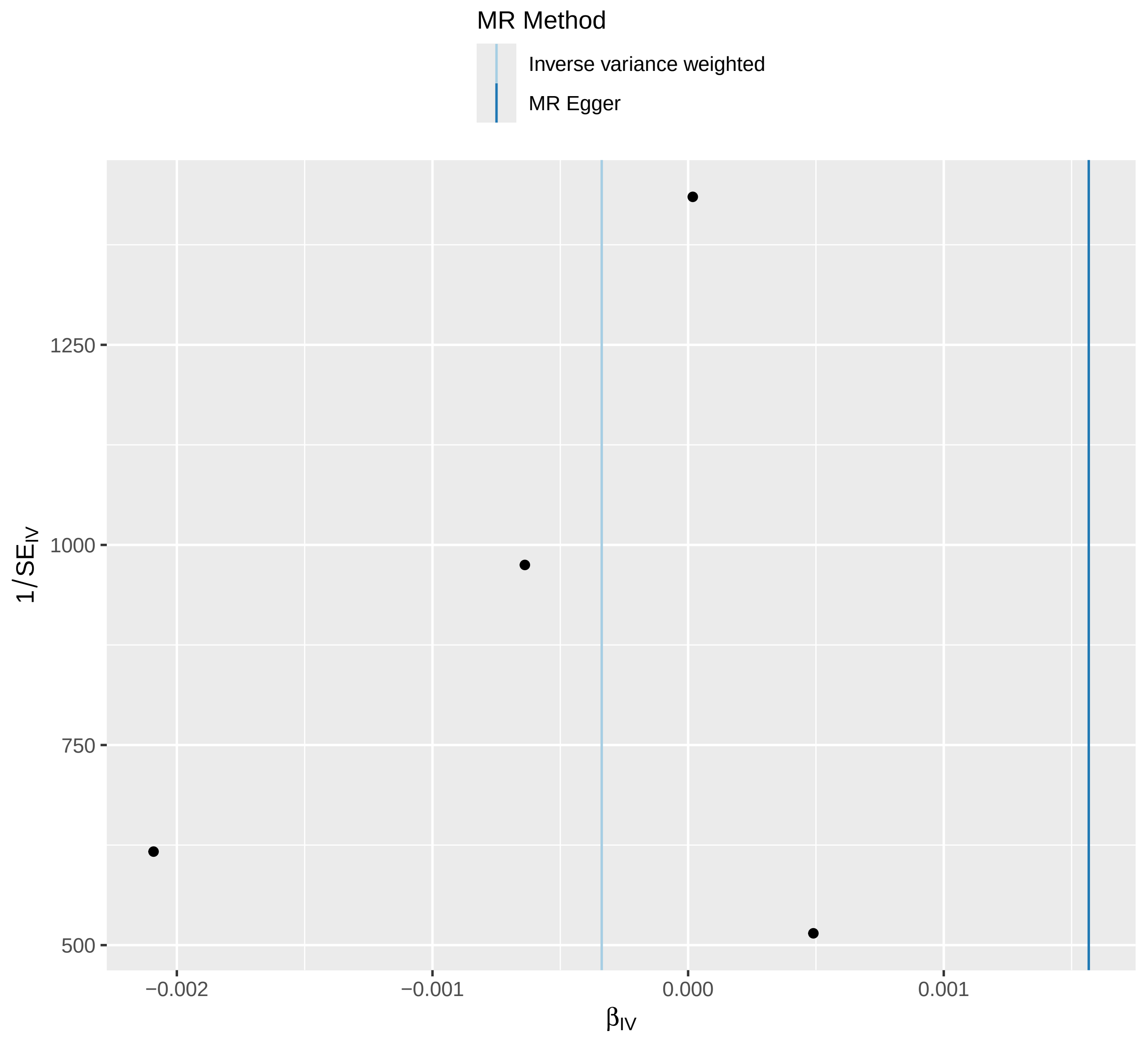
MR Method
Inverse variance weighted
MR Egger
1250
1000
750
500
1/SE_IV
−0.002
−0.001
0.000
0.001
β_IV

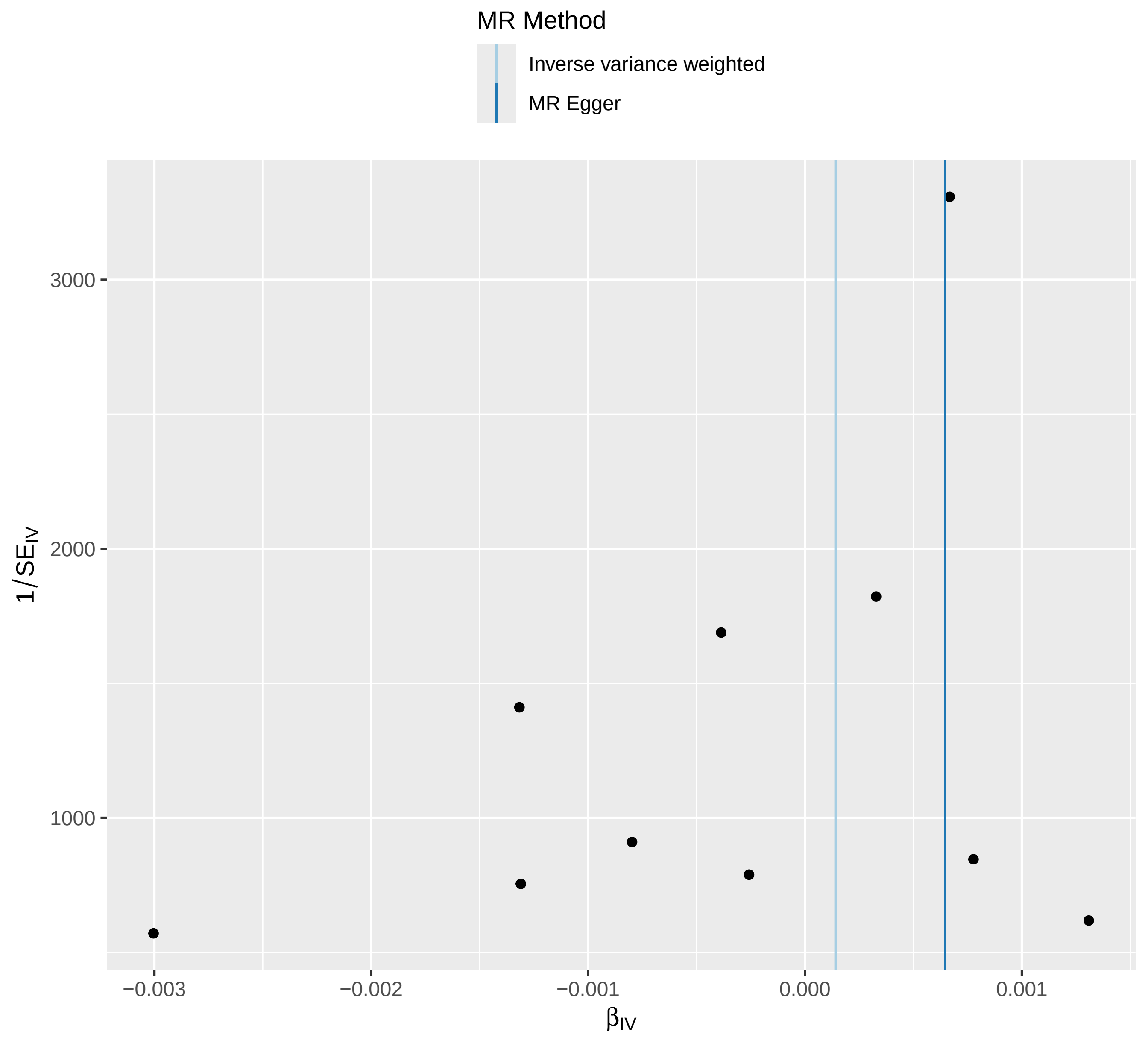
MR Method
Inverse variance weighted
MR Egger
3000
2000
1000
$1/SE_{IV}$
−0.003
−0.002
−0.001
0.000
0.001
β_{IV}

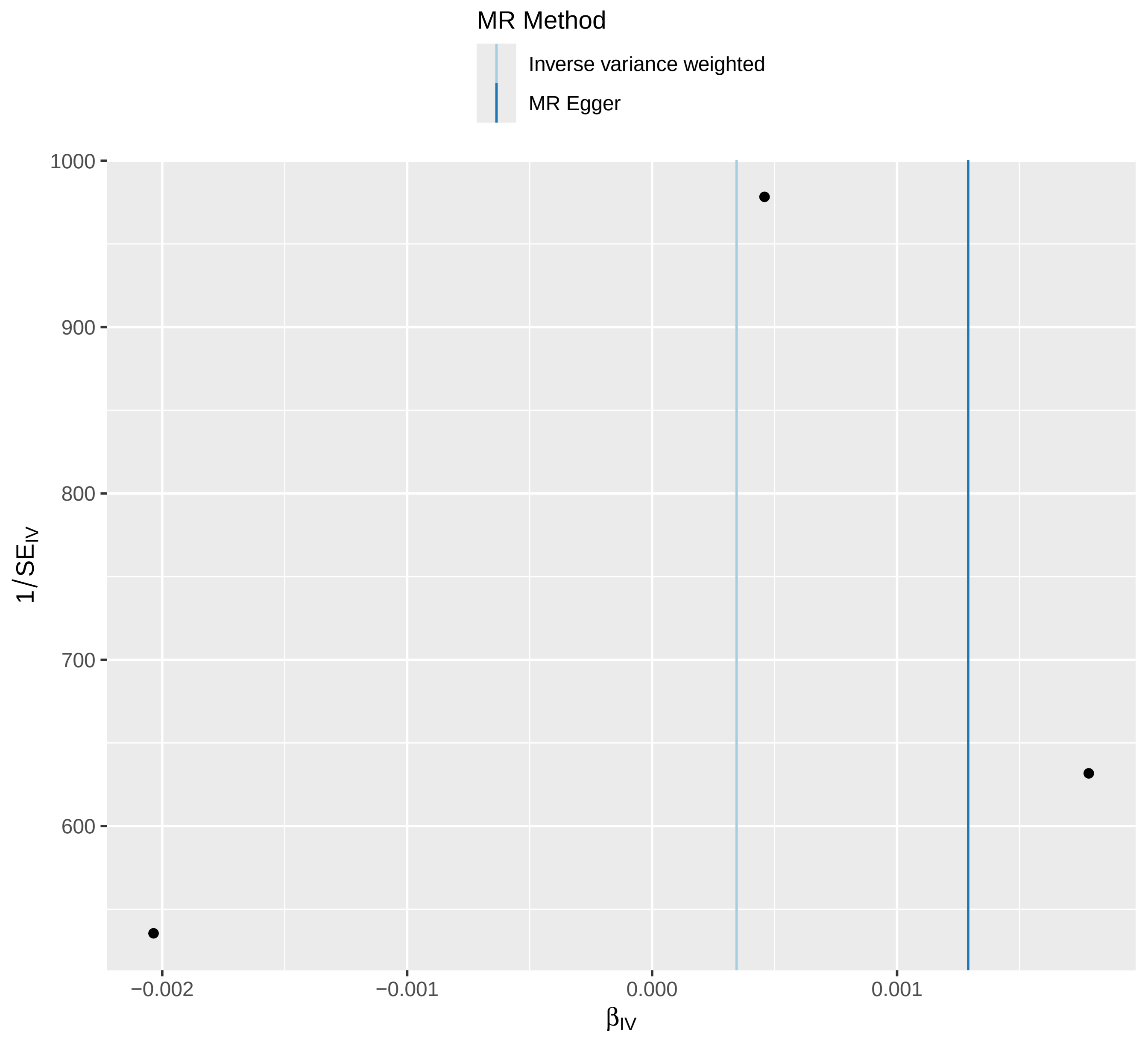
MR Method
Inverse variance weighted
MR Egger
1000
900
800
700
600
1/SE$_{IV}$
−0.002
−0.001
0.000
0.001
β$_{IV}$

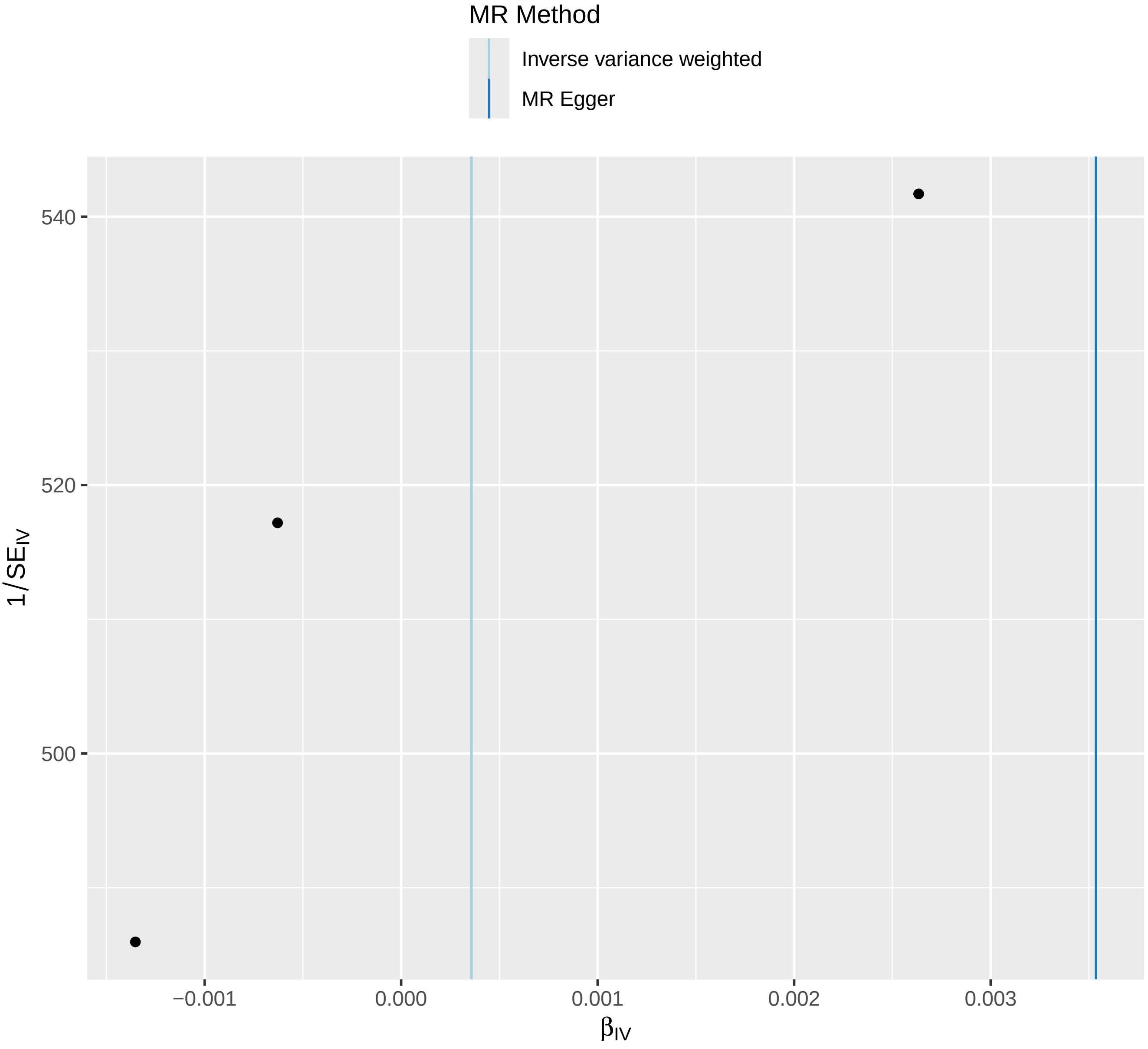
MR Method
Inverse variance weighted
MR Egger
540
520
500
1/SE$_{IV}$
−0.001
0.000
0.001
0.002
0.003
β$_{IV}$

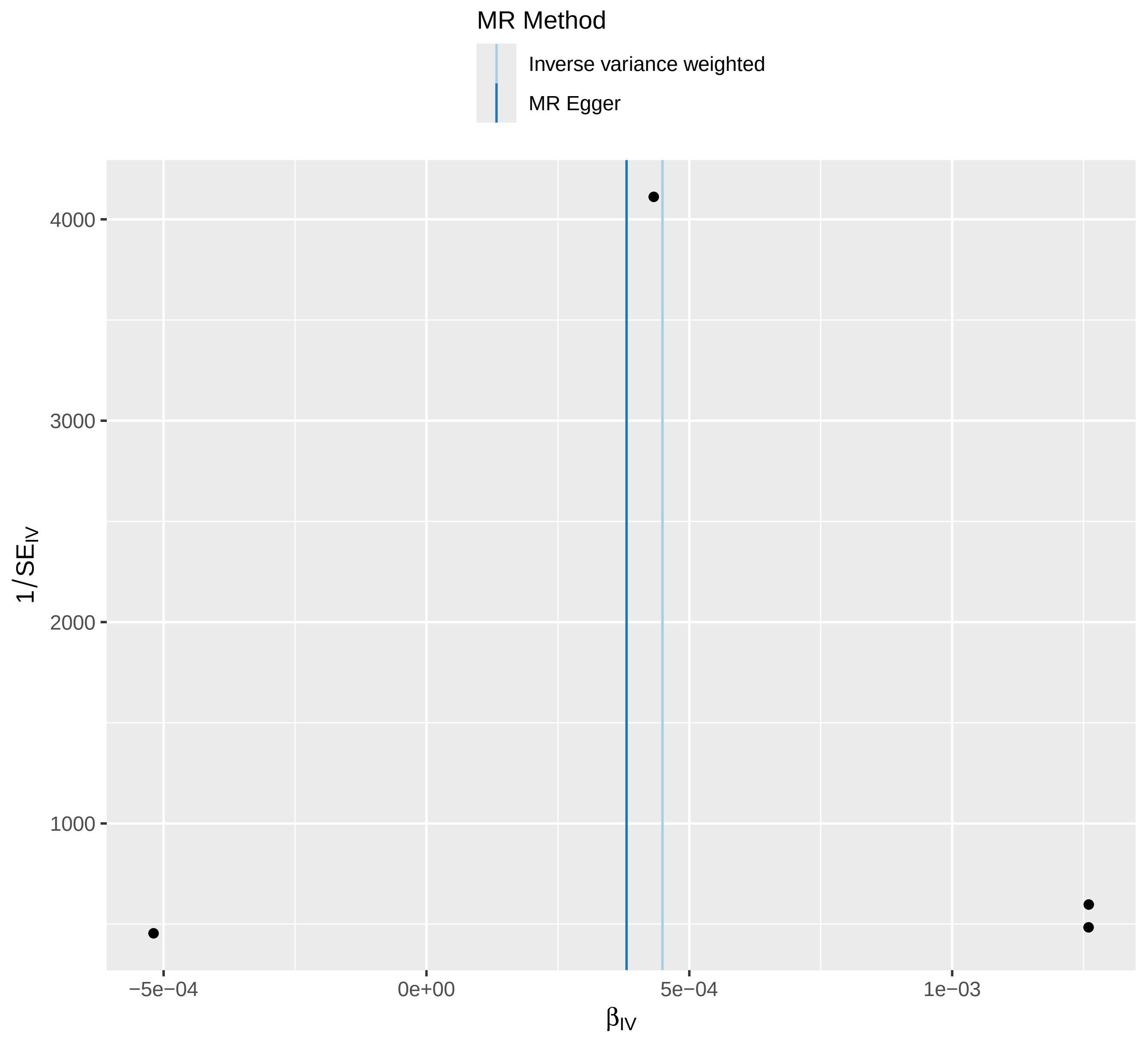
MR Method
Inverse variance weighted
MR Egger
4000
3000
2000
1000
1/SE$_{IV}$
−5e−04
0e+00
5e−04
1e−03
β$_{IV}$

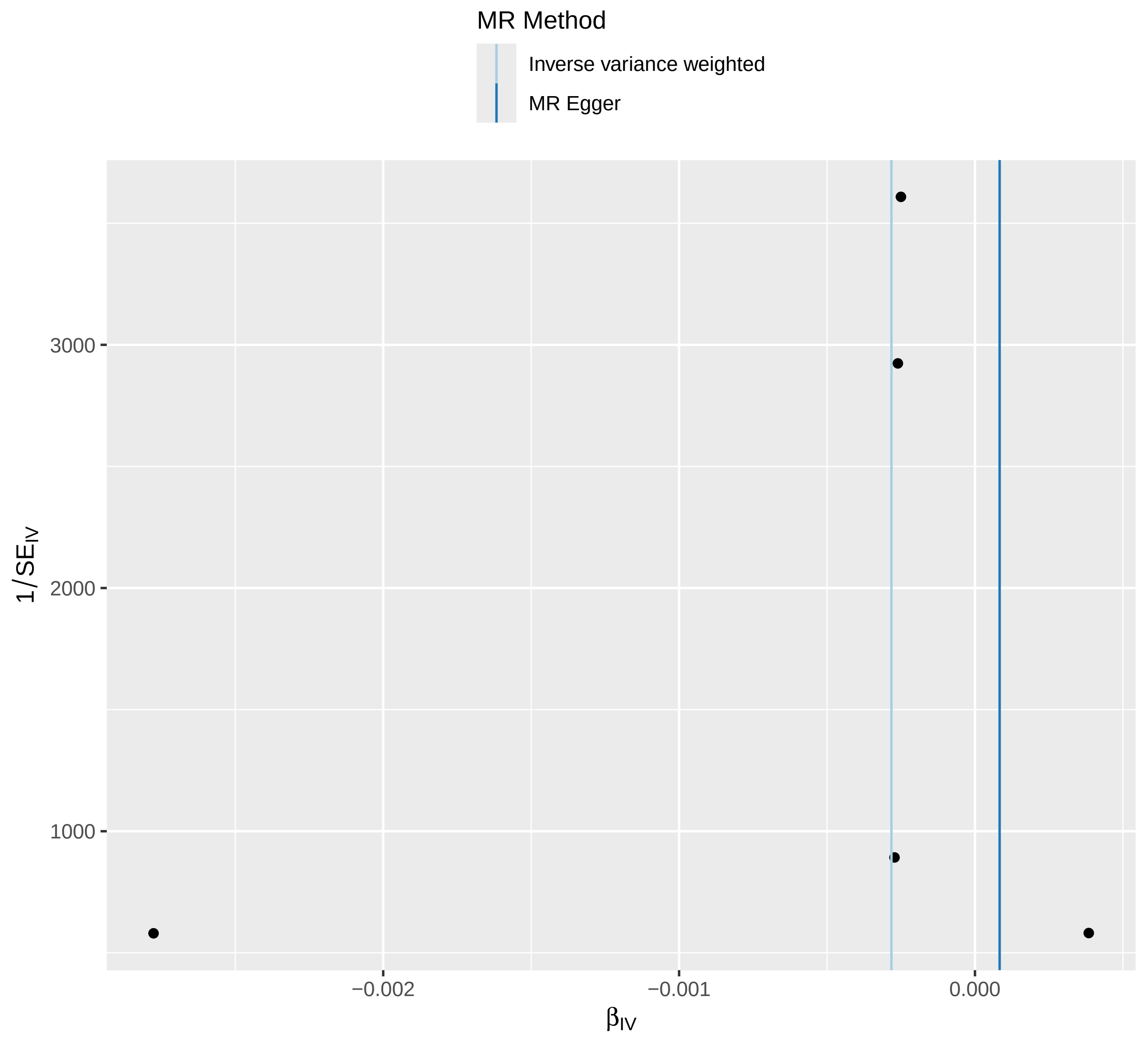
MR Method
Inverse variance weighted
MR Egger
3000
2000
1000
1/SE_IV
−0.002
−0.001
0.000
β_IV

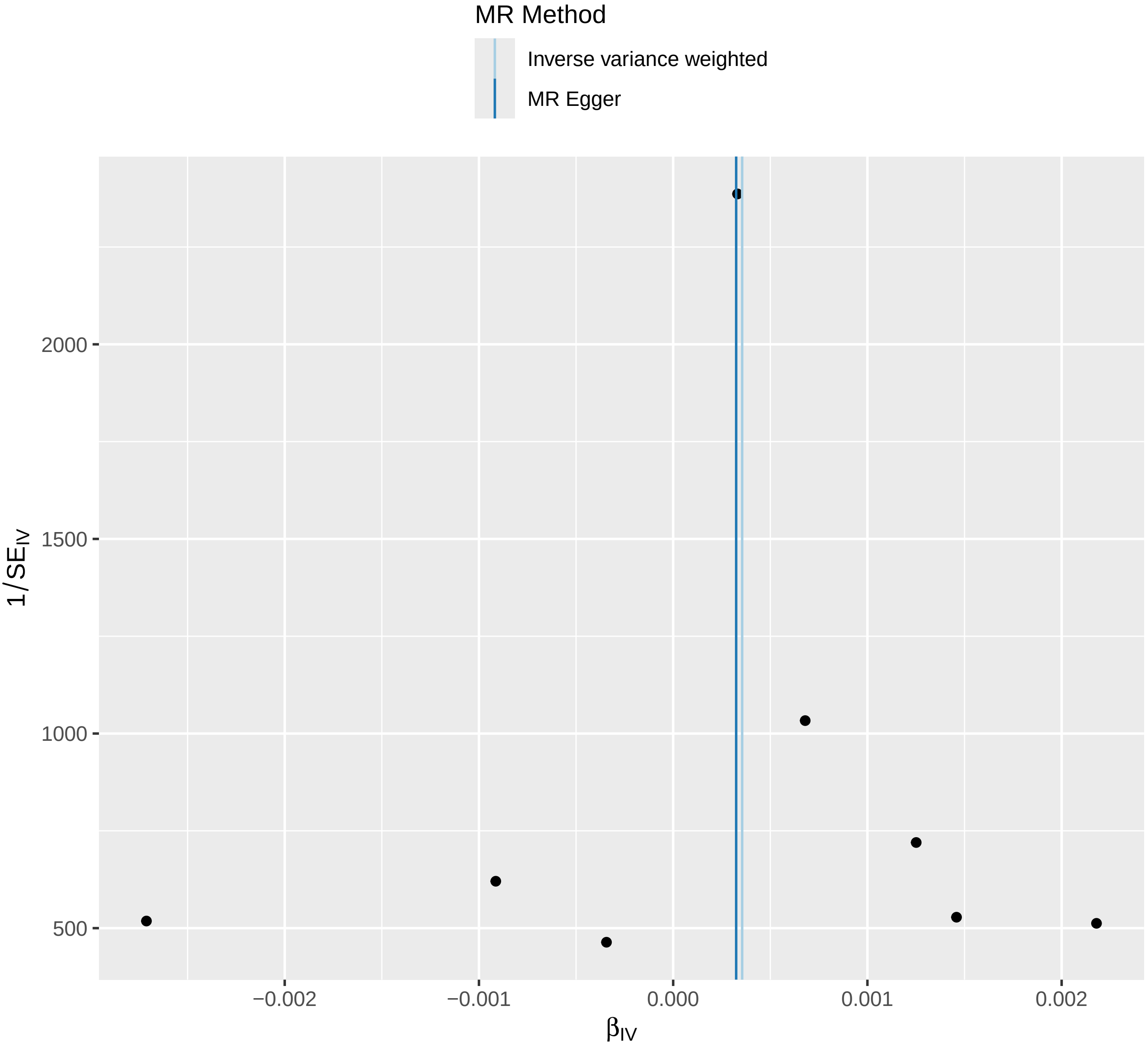

MR Method
Inverse variance weighted
MR Egger
2000
1500
1000
500
$1/SE_{IV}$
−0.002
−0.001
0.000
0.001
0.002
β_{IV}

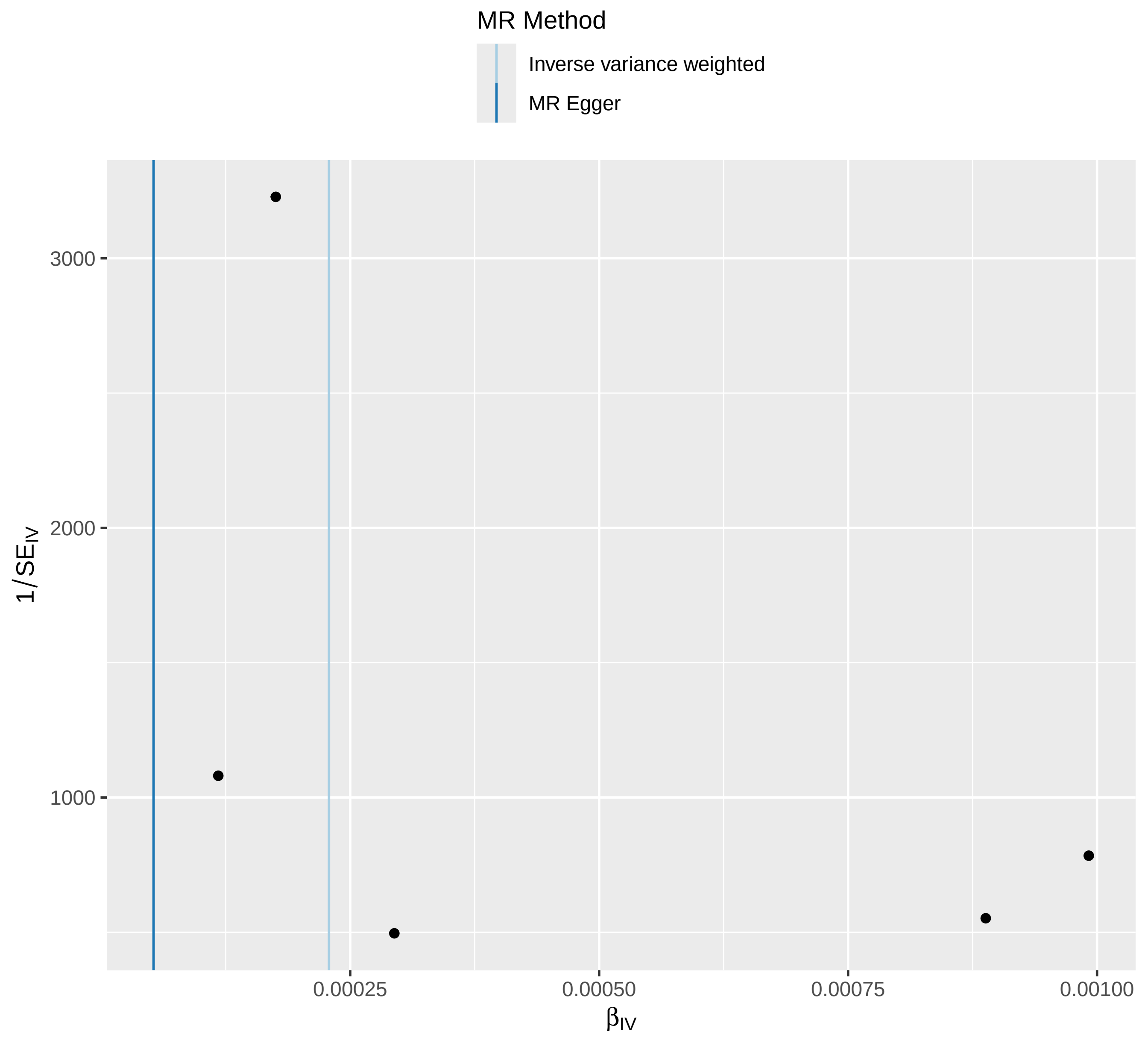
MR Method
Inverse variance weighted
MR Egger
3000
2000
1000
1/SE$_{IV}$
0.00025
0.00050
0.00075
0.00100
β$_{IV}$

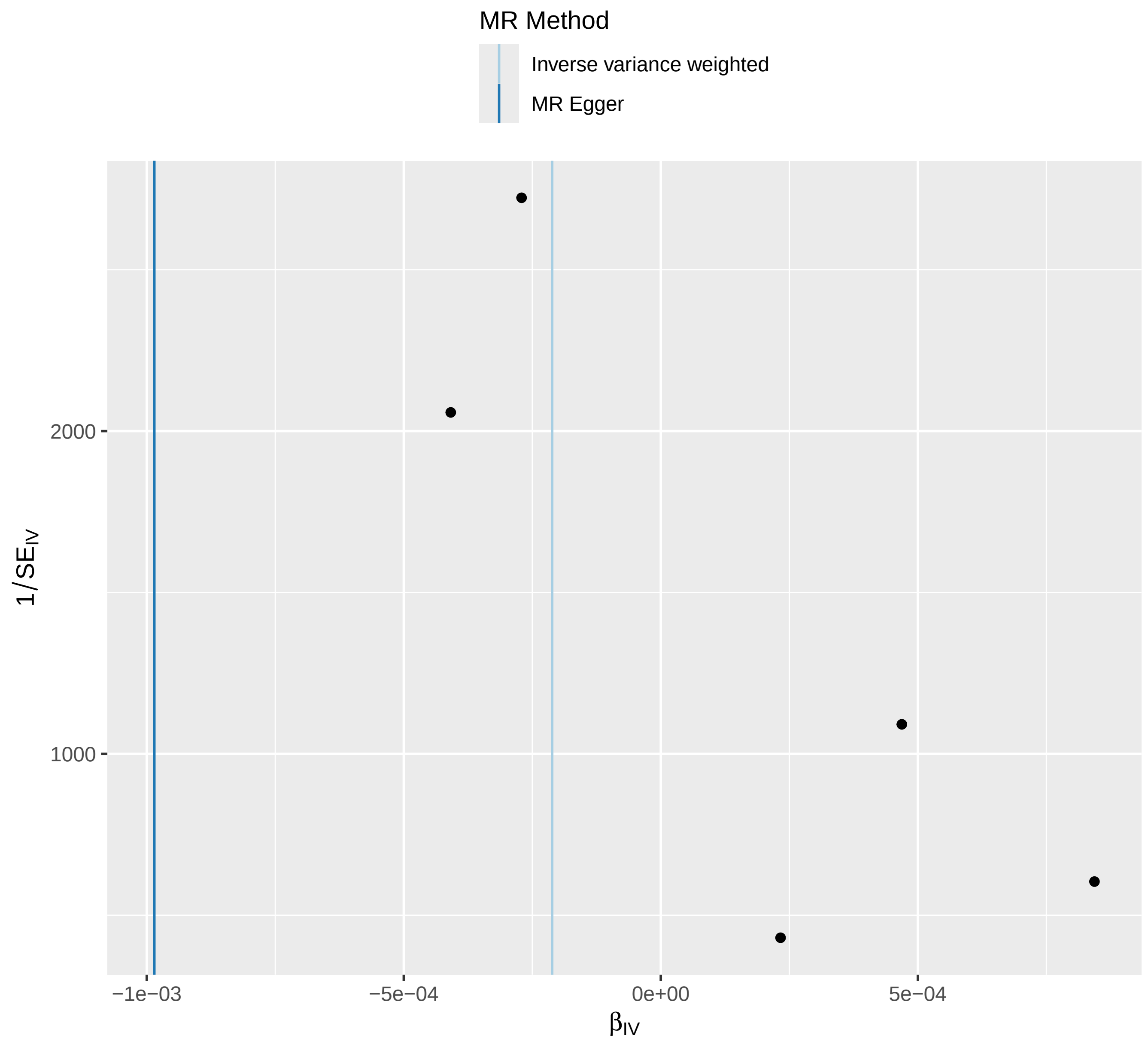
MR Method
Inverse variance weighted
MR Egger
2000
1000
1/SE_IV
−1e−03
−5e−04
0e+00
5e−04
β_IV

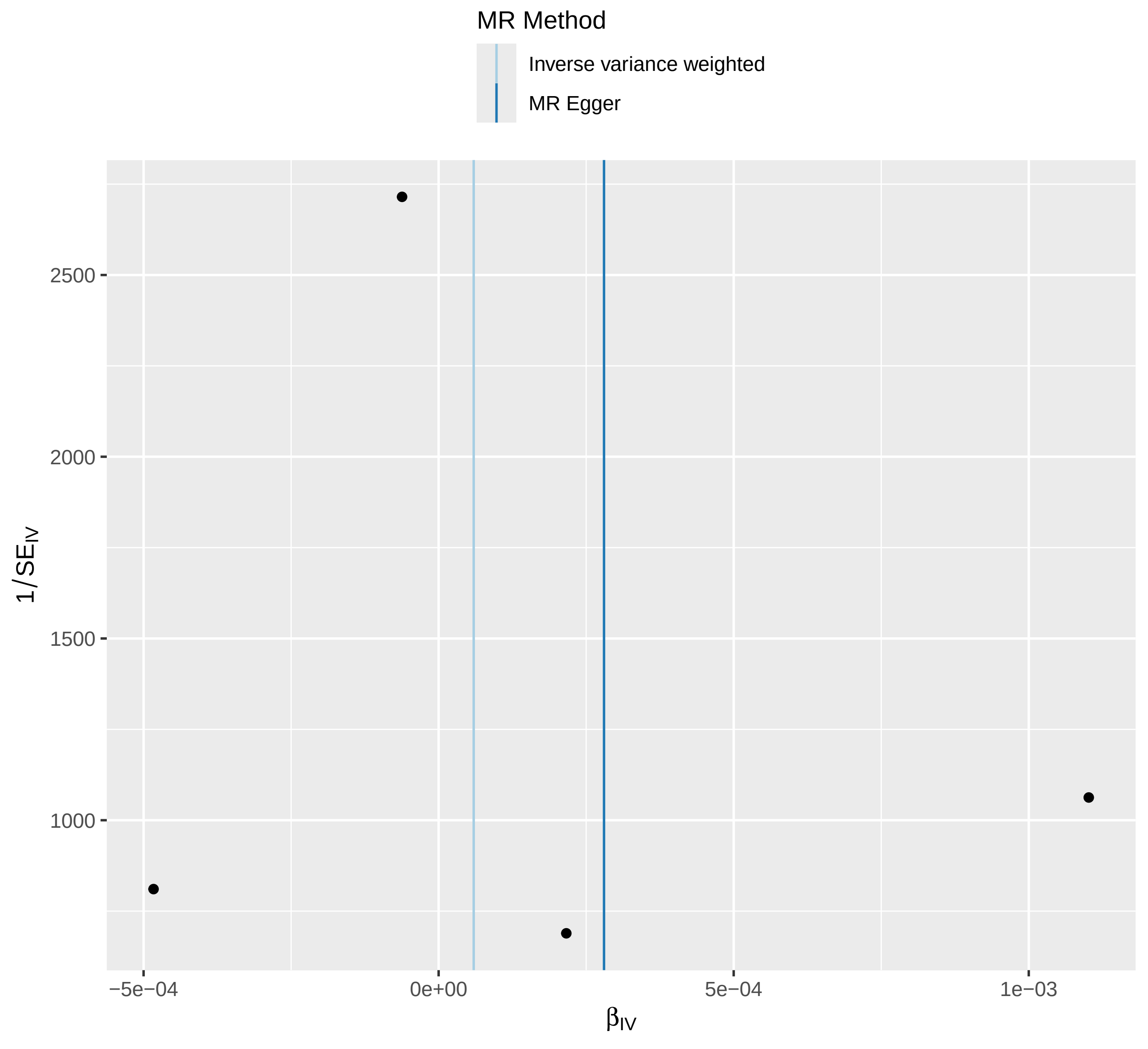

MR Method
Inverse variance weighted
MR Egger
2500
2000
1500
1000
1/SE_IV
−5e−04
0e+00
5e−04
1e−03
β_IV

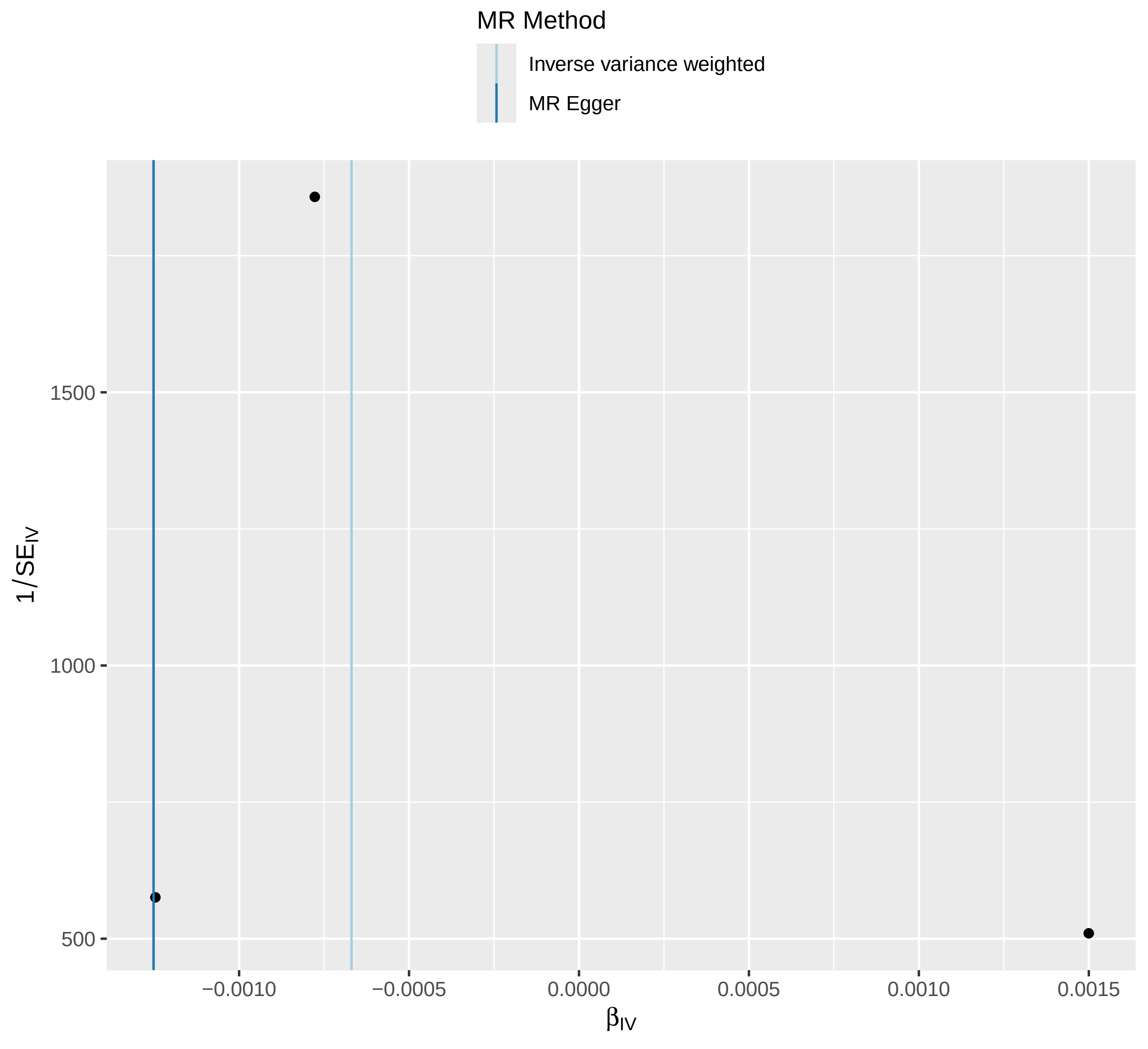
MR Method
Inverse variance weighted
MR Egger
1500
1000
500
1/SE_IV
−0.0010
−0.0005
0.0000
0.0005
0.0010
0.0015
β_IV

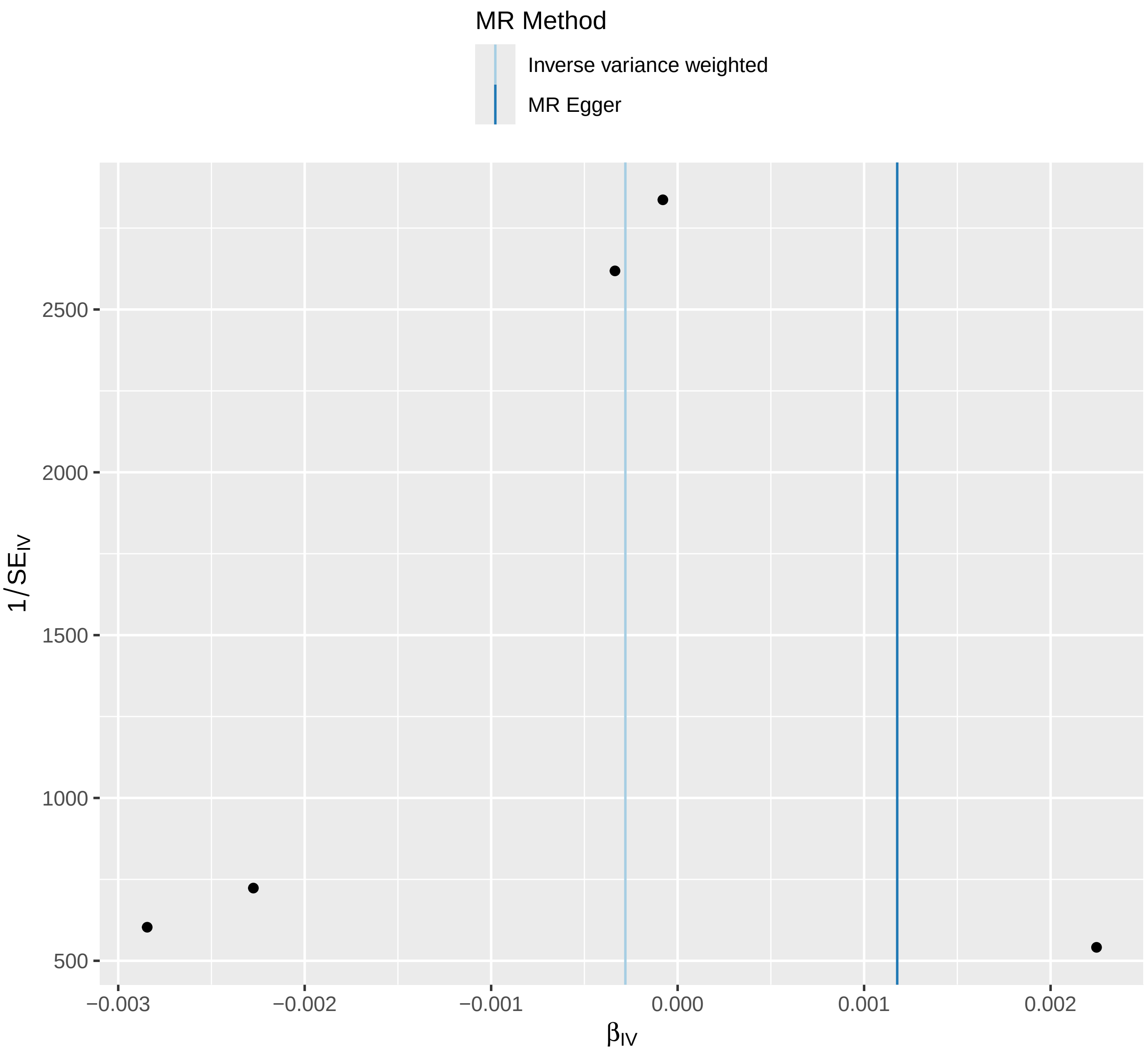

MR Method
Inverse variance weighted
MR Egger
2500
2000
1500
1000
500
1/SE_IV
−0.003
−0.002
−0.001
0.000
0.001
0.002
β_IV

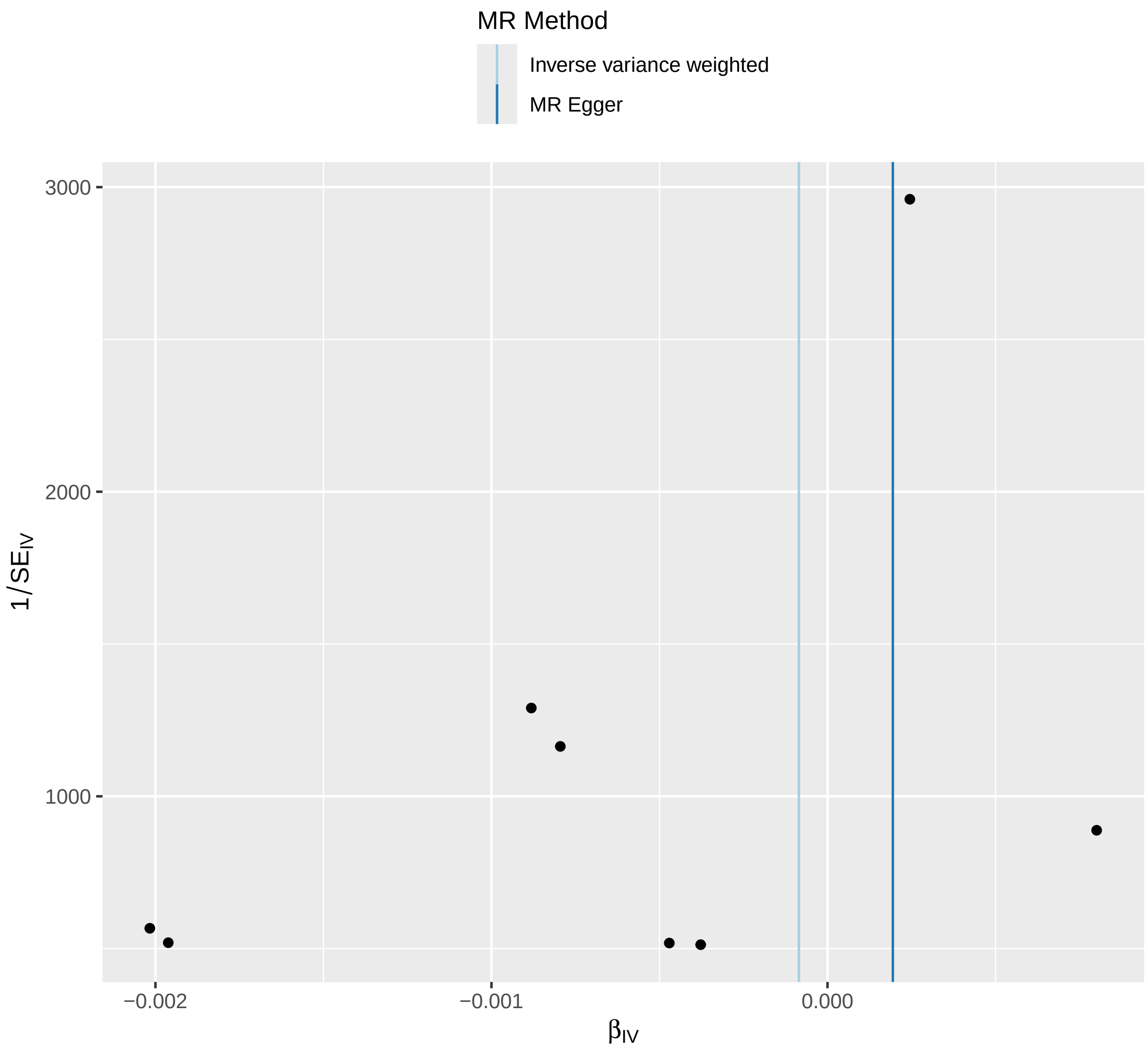

MR Method
Inverse variance weighted
MR Egger
3000
2000
1000
1/SE$_{IV}$
−0.002
−0.001
0.000
β$_{IV}$

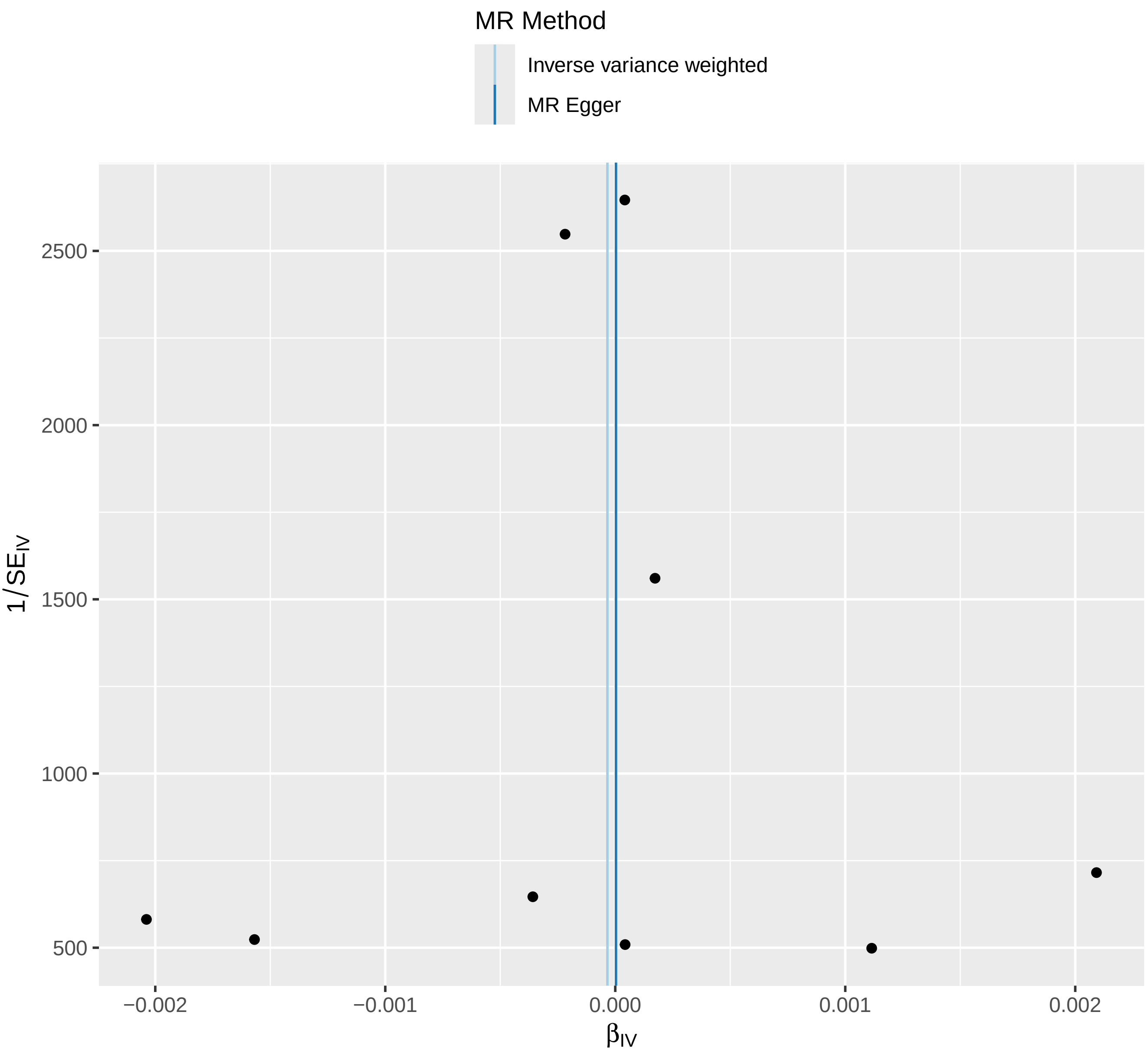
MR Method
Inverse variance weighted
MR Egger
2500
2000
1500
1000
500
1/SE_IV
−0.002
−0.001
0.000
0.001
0.002
β_IV

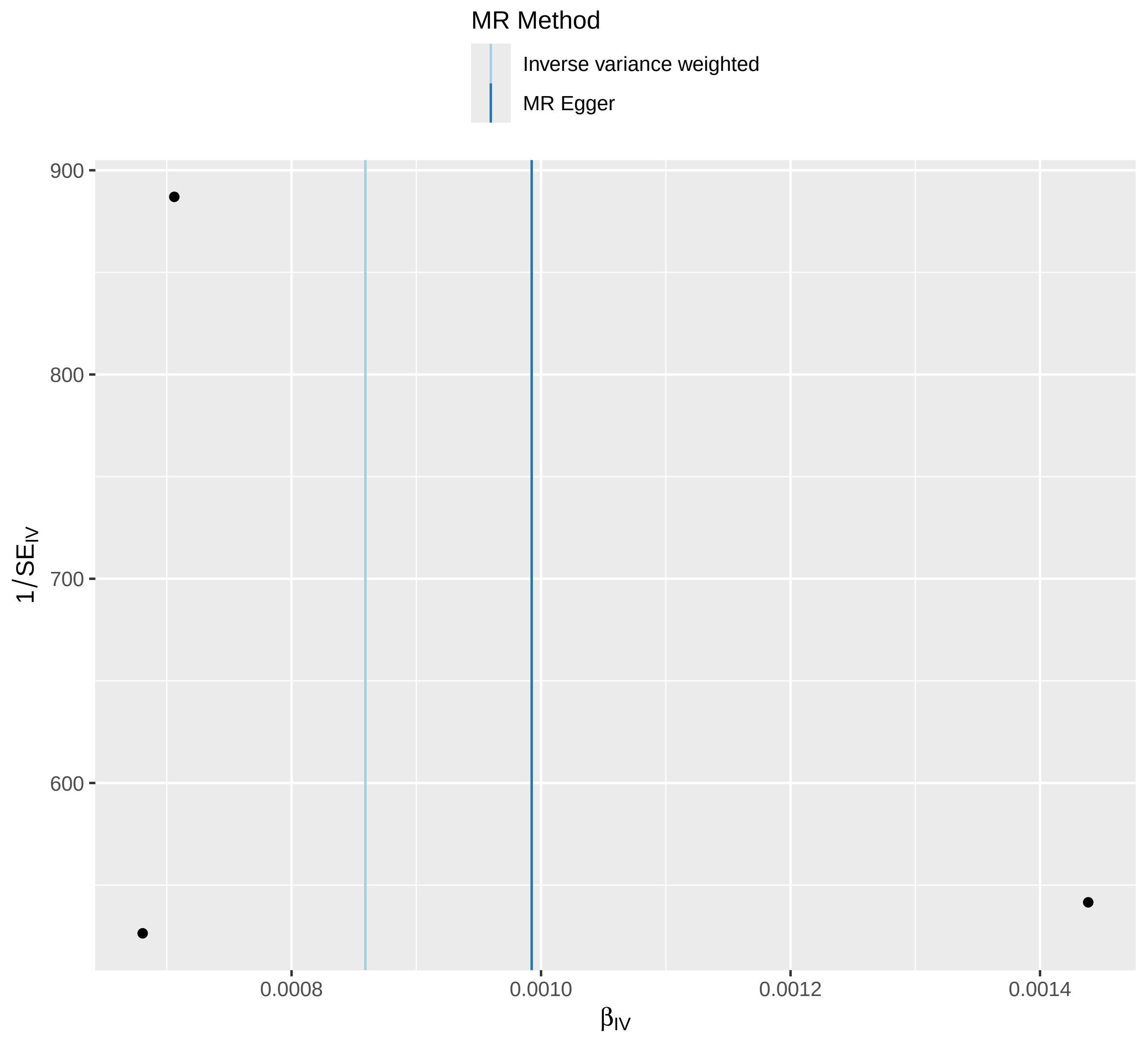
MR Method
Inverse variance weighted
MR Egger
900
800
700
600
1/SE_IV
0.0008
0.0010
0.0012
0.0014
β_IV

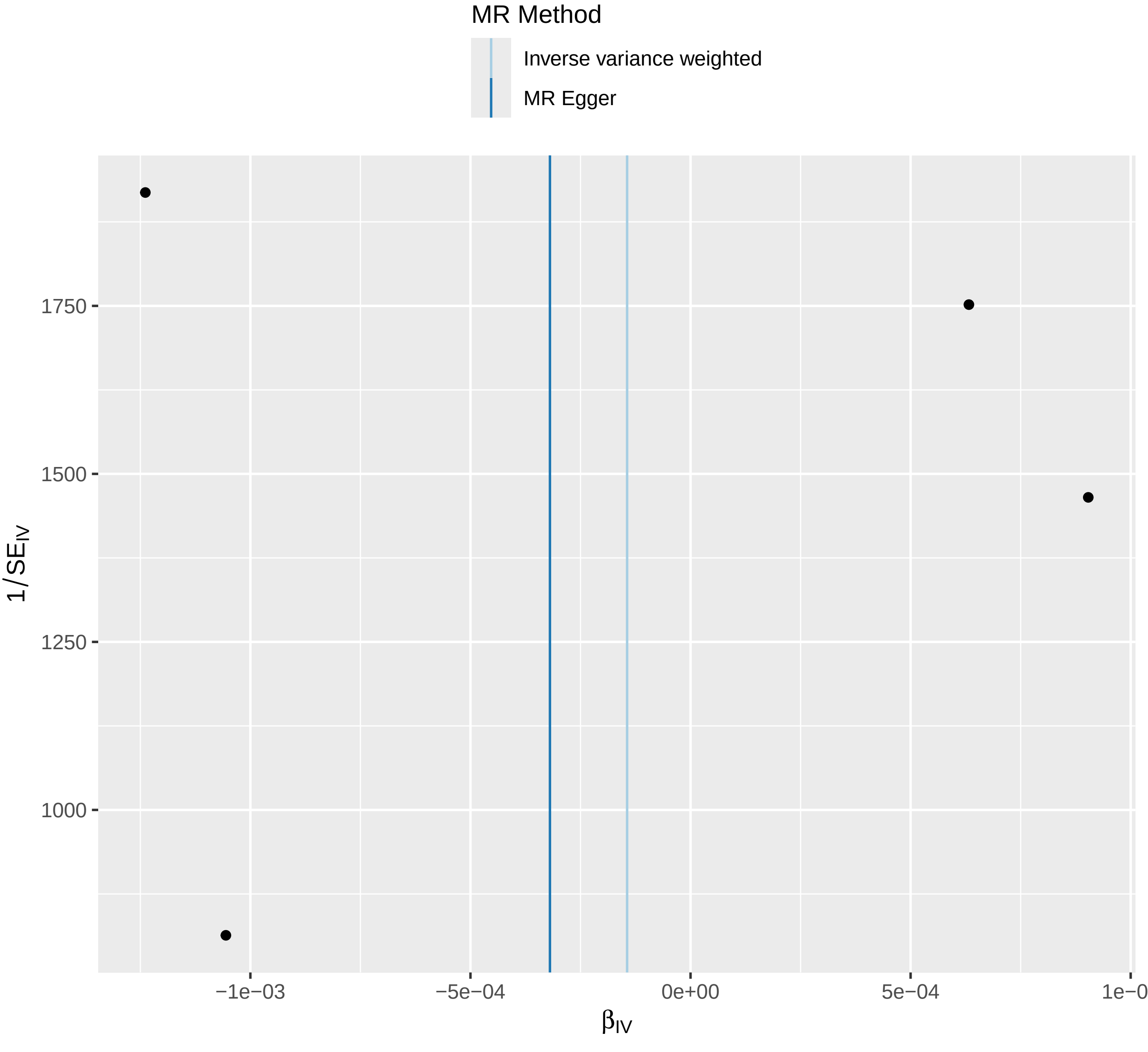

MR Method
Inverse variance weighted
MR Egger
1750
1500
1250
1000
1/SE$_{IV}$
−1e−03
−5e−04
0e+00
5e−04
1e−0
β$_{IV}$

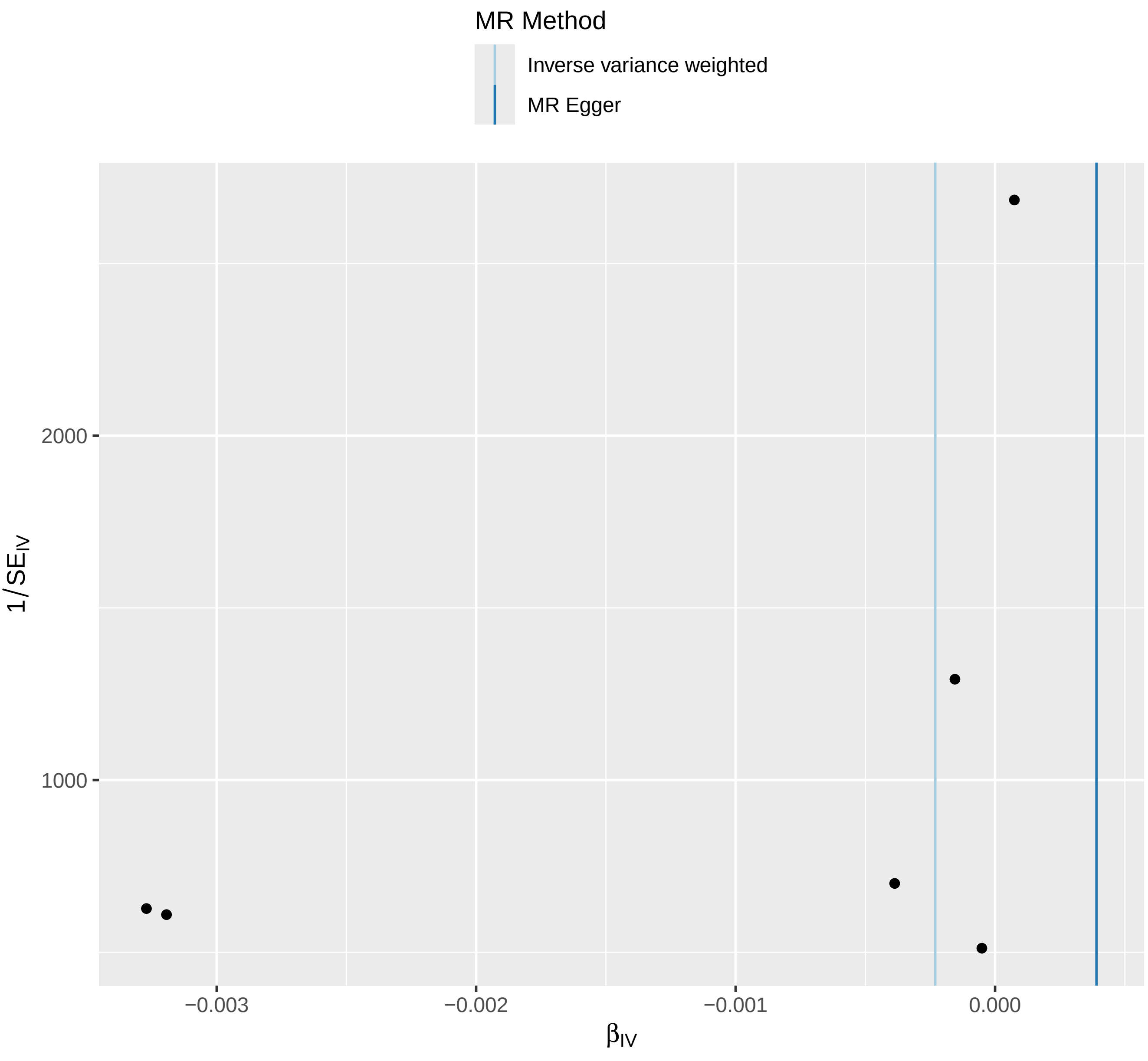
MR Method
Inverse variance weighted
MR Egger
2000
1000
1/SE_IV
−0.003
−0.002
−0.001
0.000
β_IV

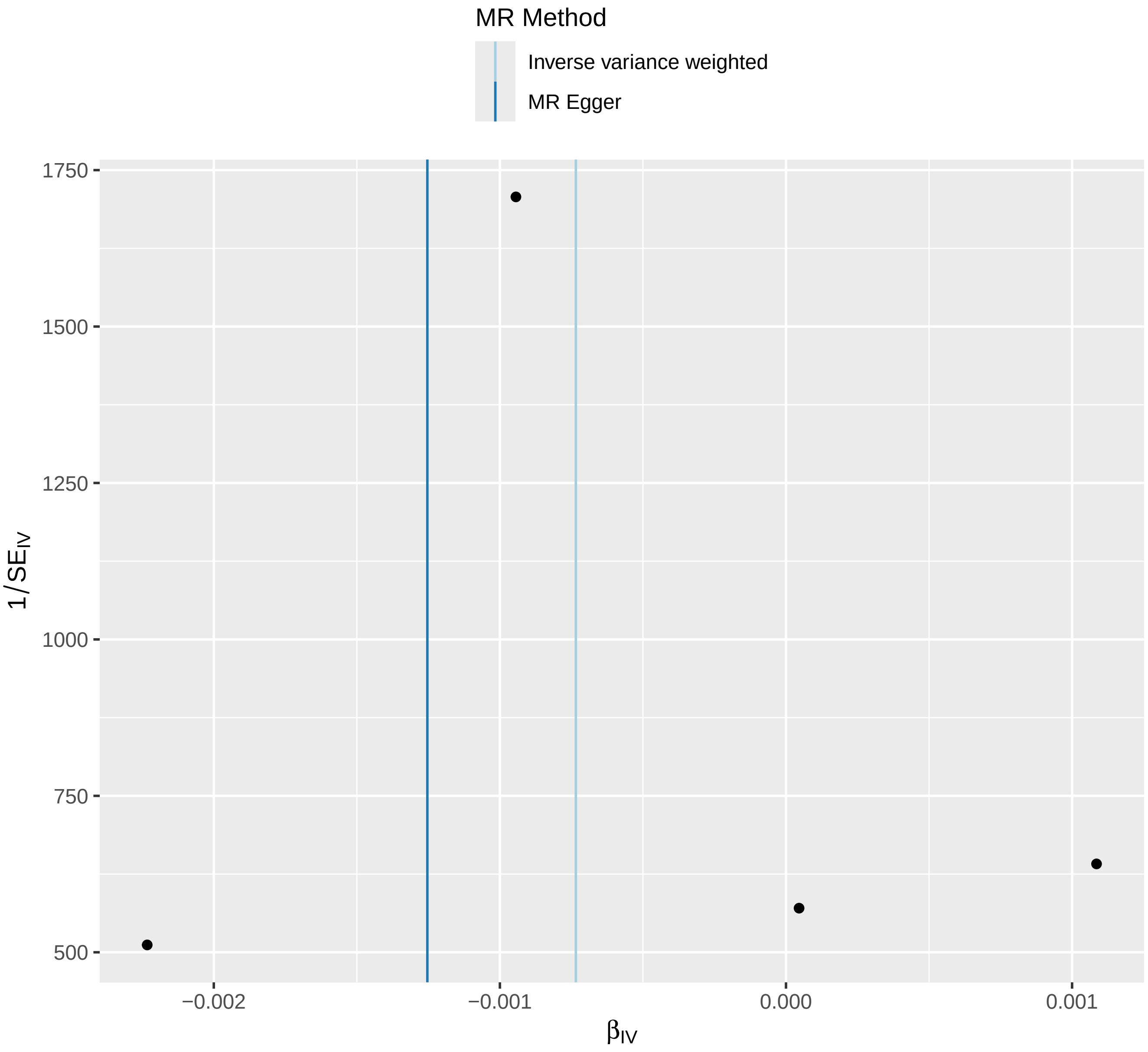

MR Method
Inverse variance weighted
MR Egger
1750
1500
1250
1000
750
500
1/SE_IV
−0.002
−0.001
0.000
0.001
β_IV

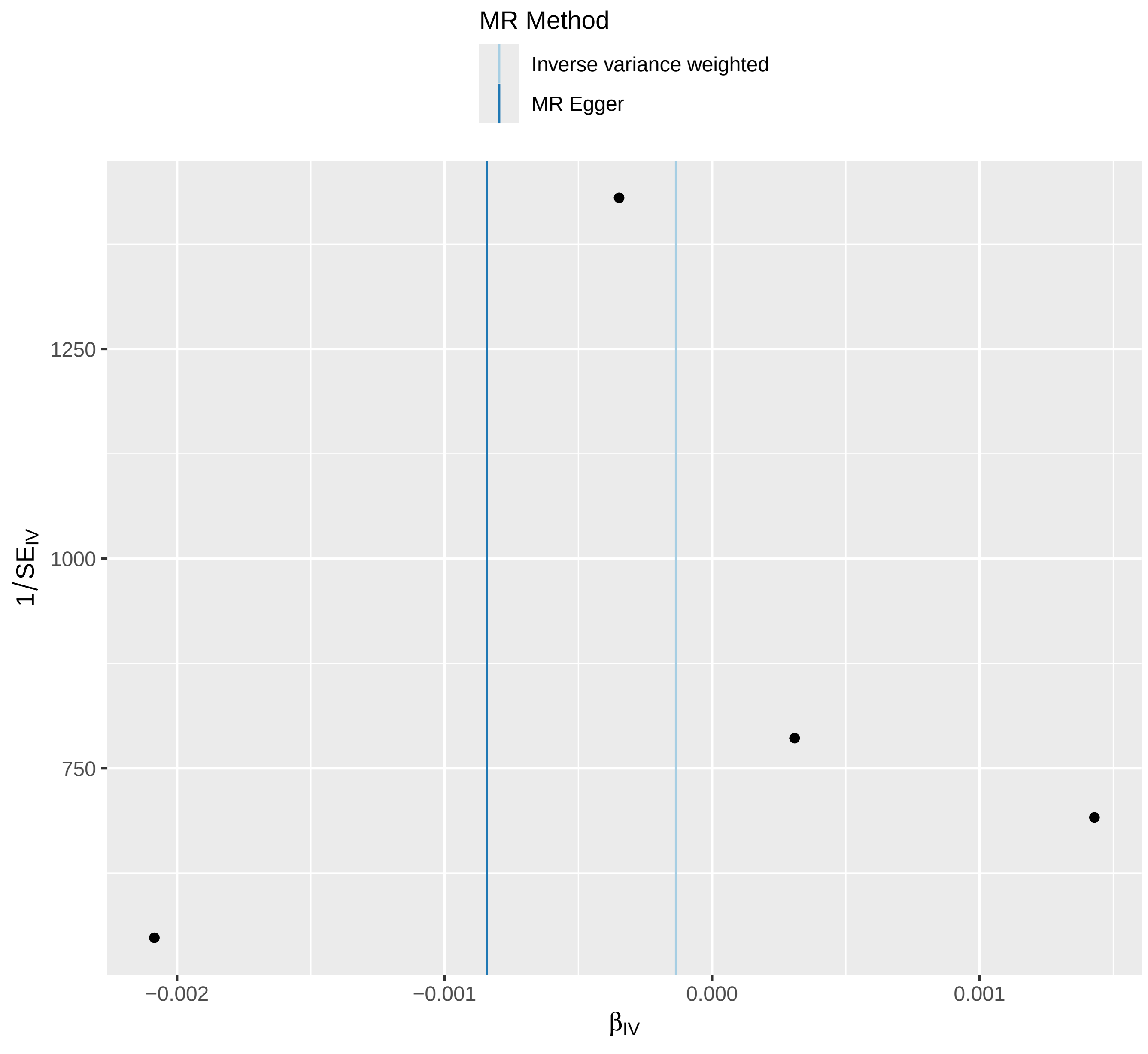

MR Method
Inverse variance weighted
MR Egger
1250
1000
750
1/SE$_{IV}$
−0.002
−0.001
0.000
0.001
β$_{IV}$

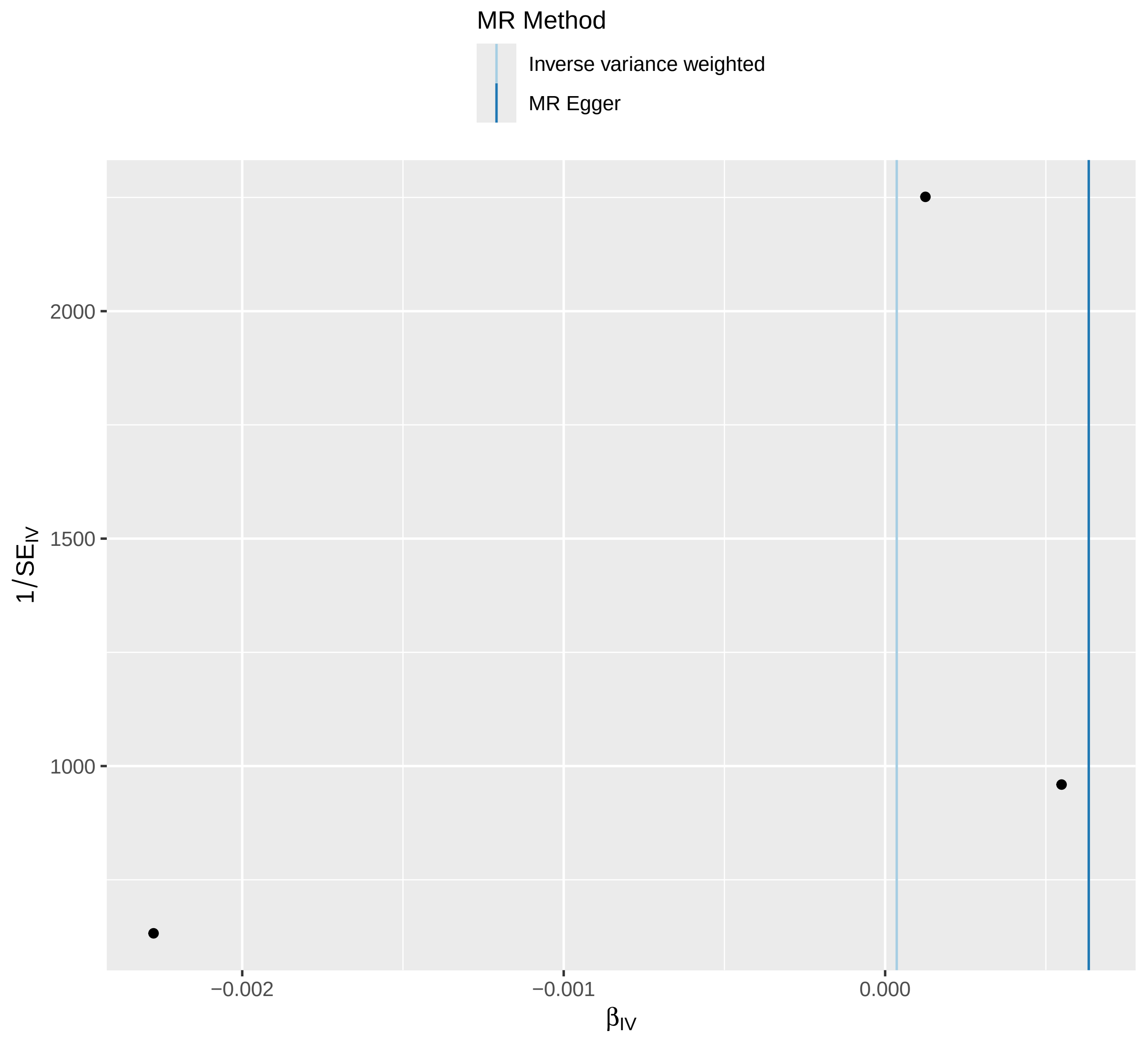
MR Method
Inverse variance weighted
MR Egger
2000
1500
1000
1/SE_IV
−0.002
−0.001
0.000
β_IV

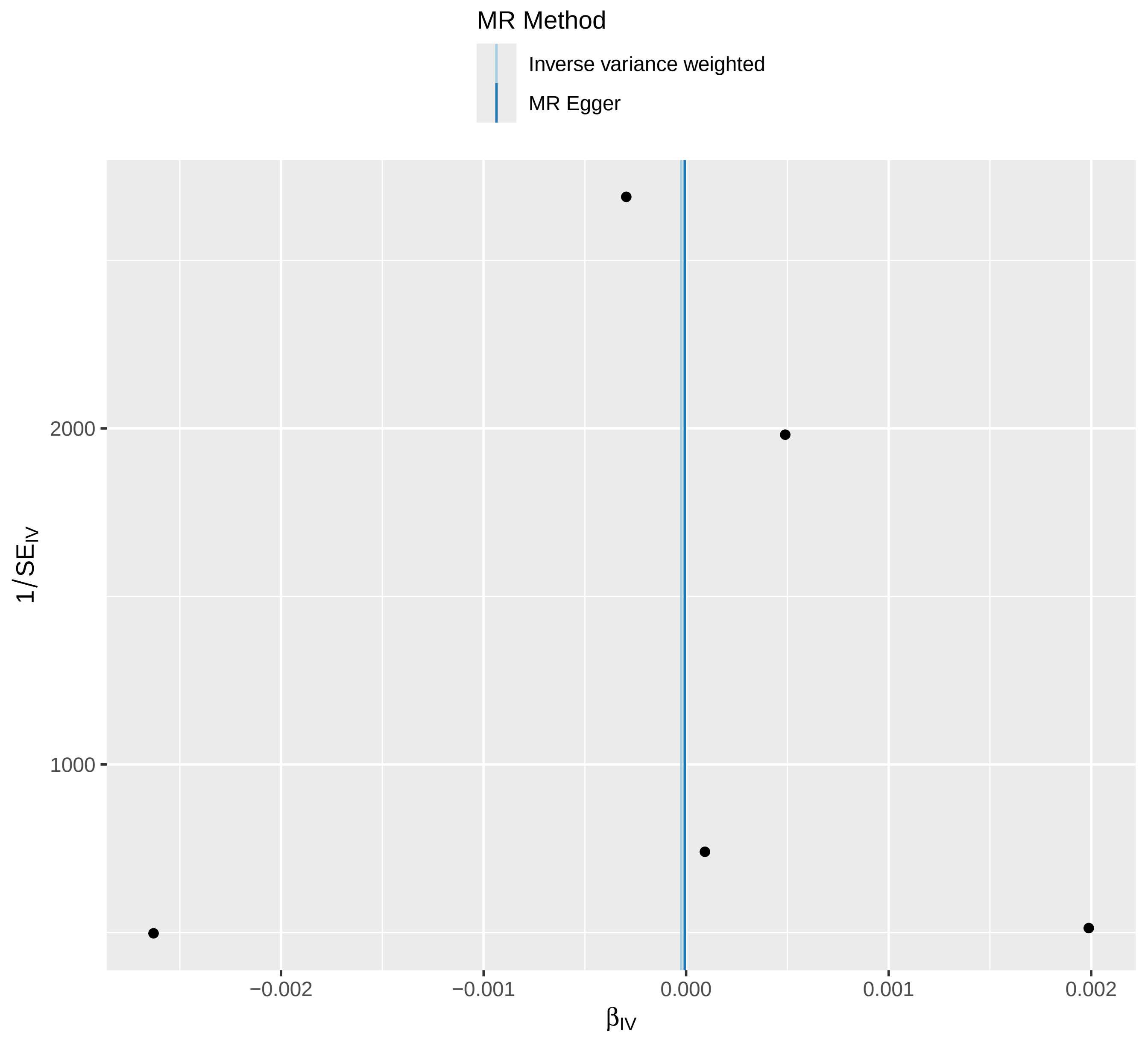

MR Method
Inverse variance weighted
MR Egger
2000
1000
1/SE_IV
−0.002
−0.001
0.000
0.001
0.002
β_IV

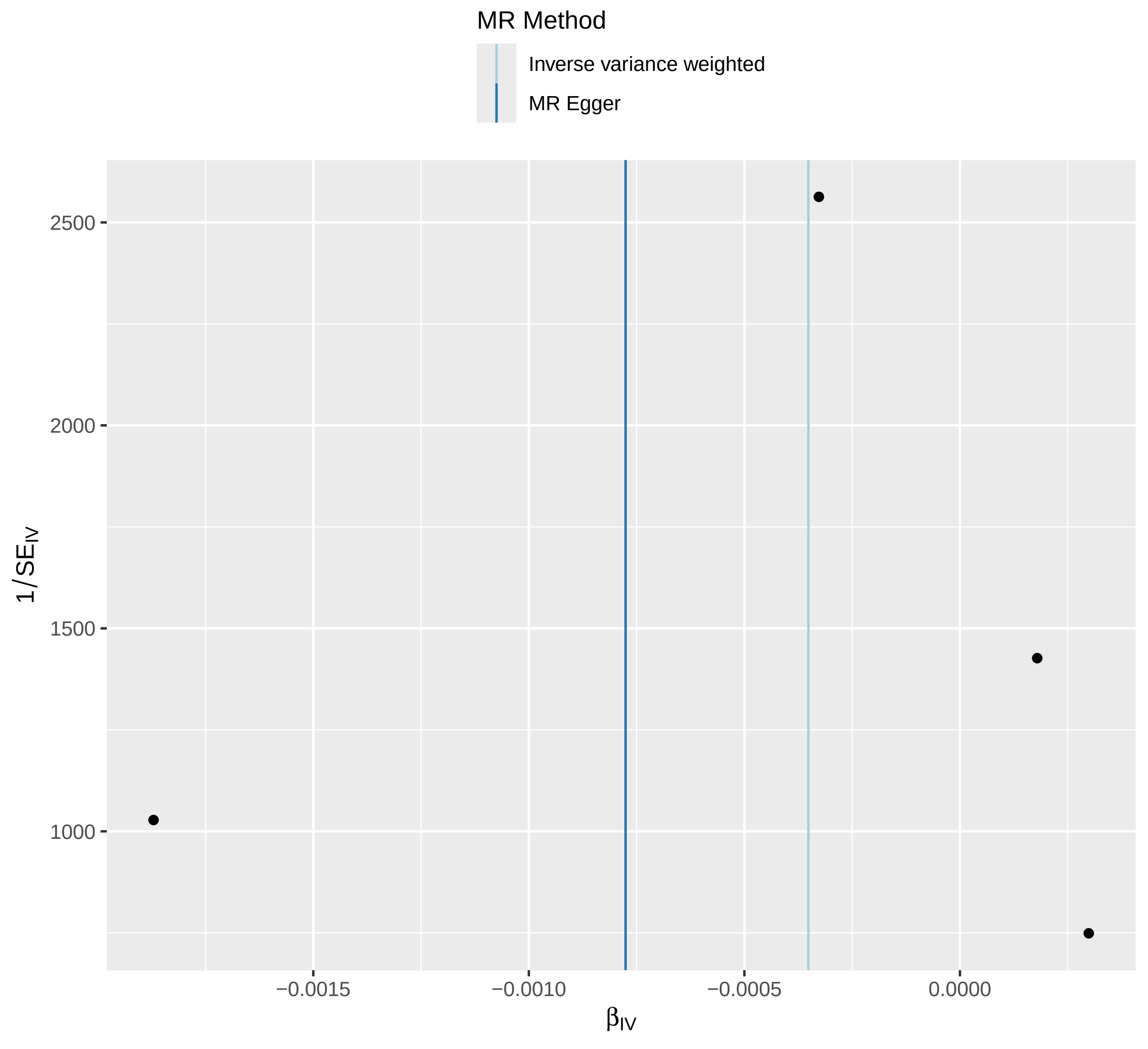

MR Method
Inverse variance weighted
MR Egger
2500
2000
1500
1000
1/SE_IV
−0.0015
−0.0010
−0.0005
0.0000
β_IV

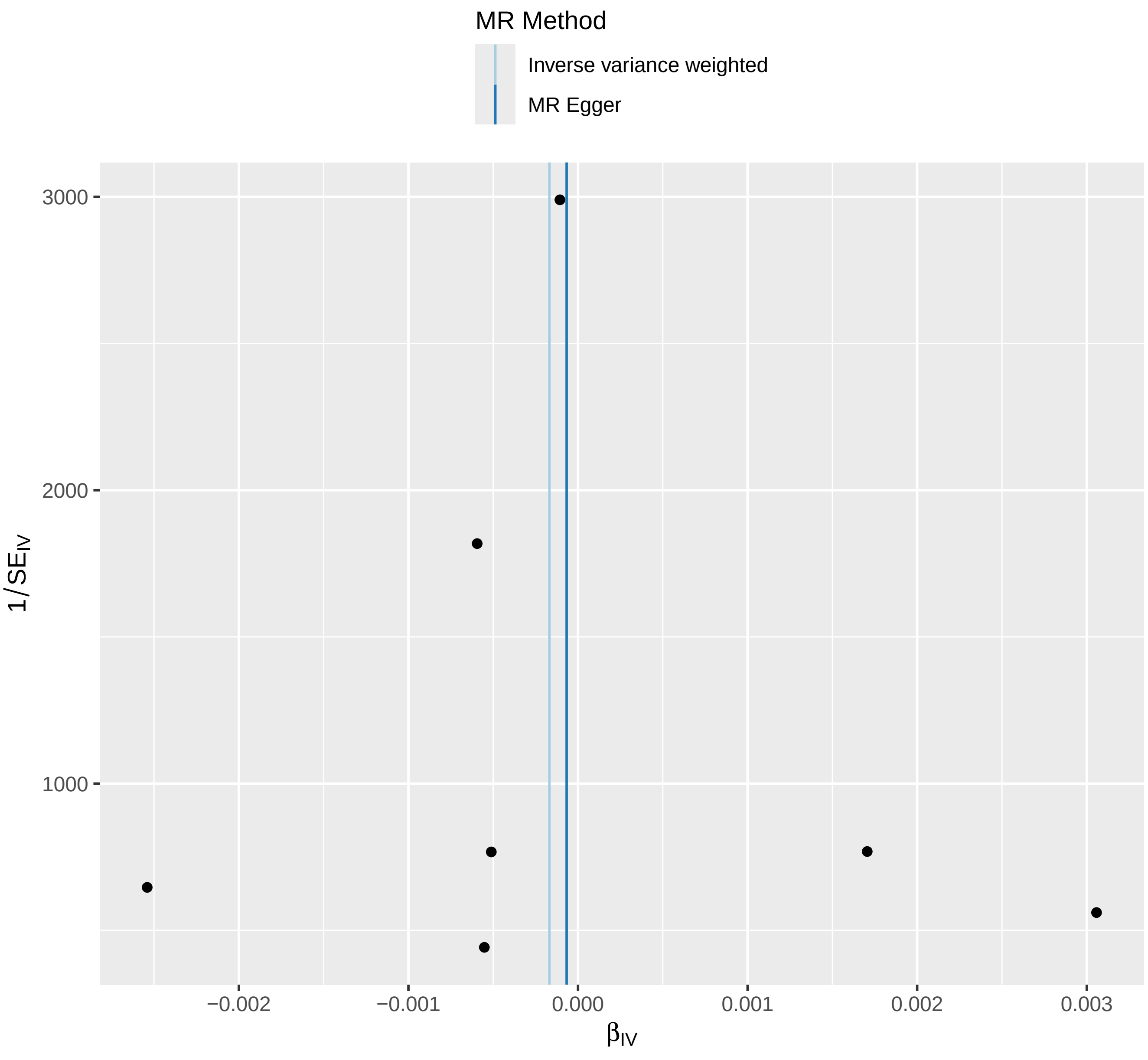
MR Method
Inverse variance weighted
MR Egger
3000
2000
1000
1/SE_{IV}
−0.002
−0.001
0.000
0.001
0.002
0.003
β_{IV}

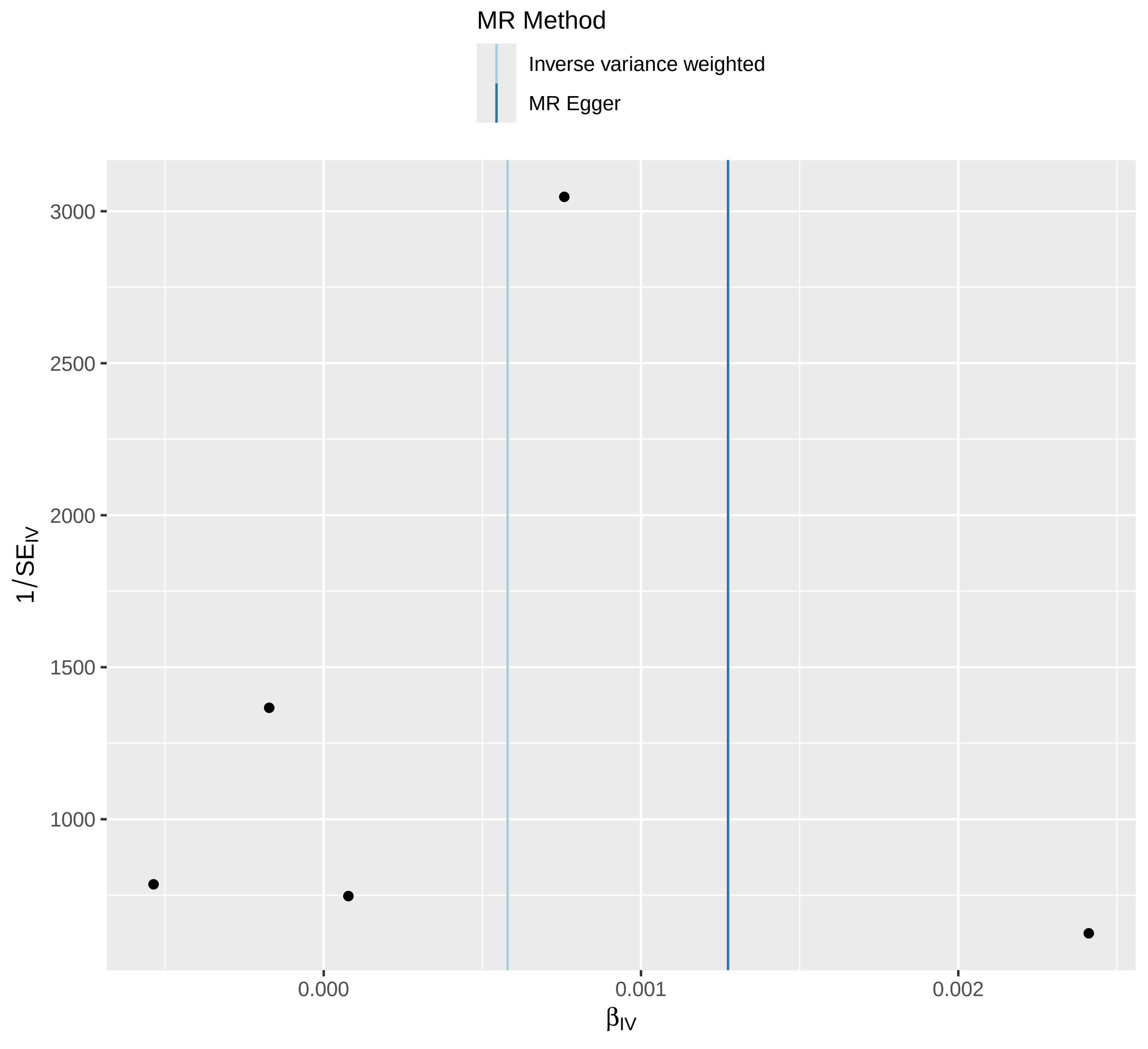

MR Method
Inverse variance weighted
MR Egger
3000
2500
2000
1500
1000
0.000
0.001
0.002
1/SE_IV
β_IV

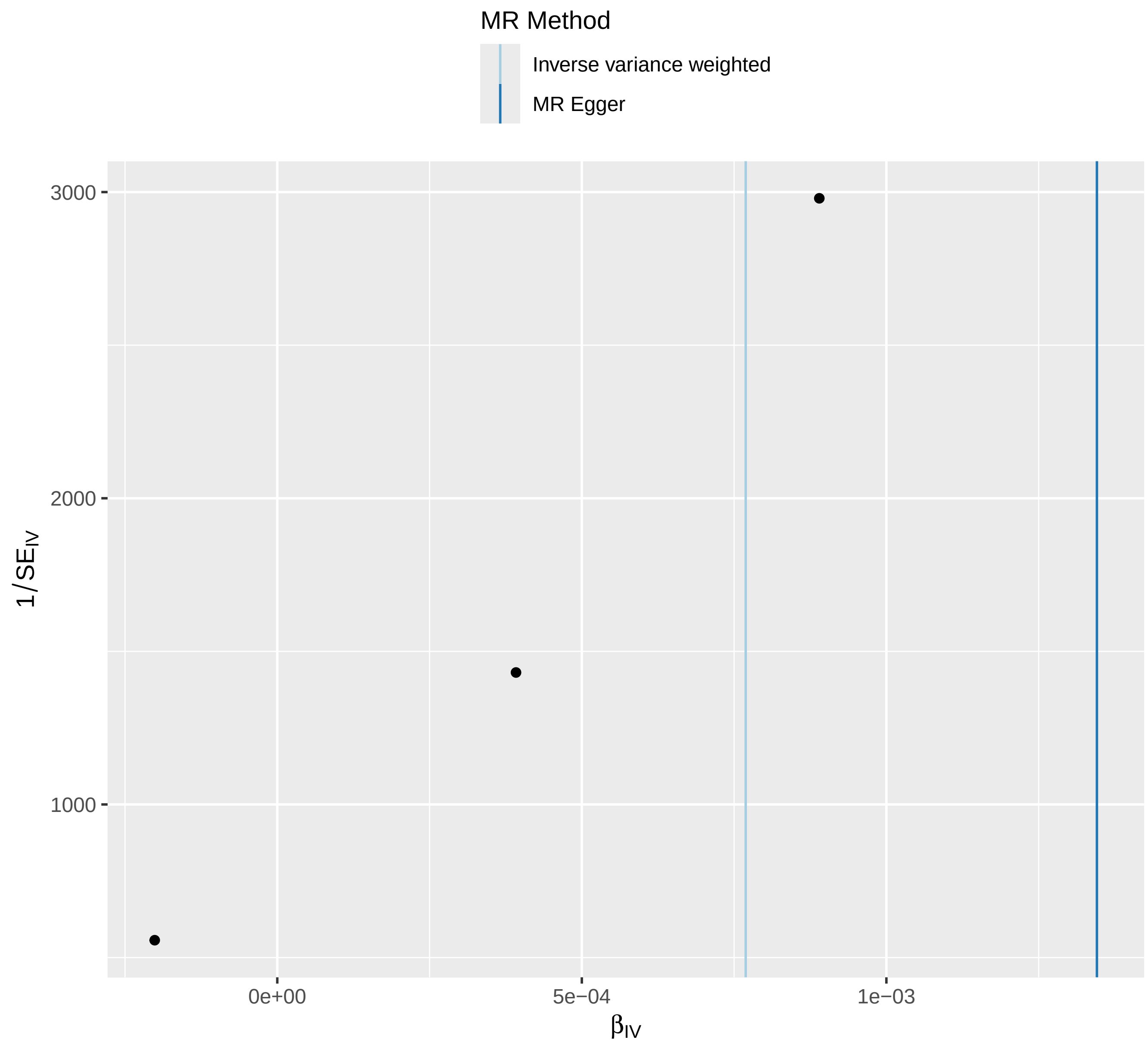
MR Method
Inverse variance weighted
MR Egger
3000
2000
1000
$1/SE_{IV}$
0e+00
5e−04
1e−03
β_{IV}

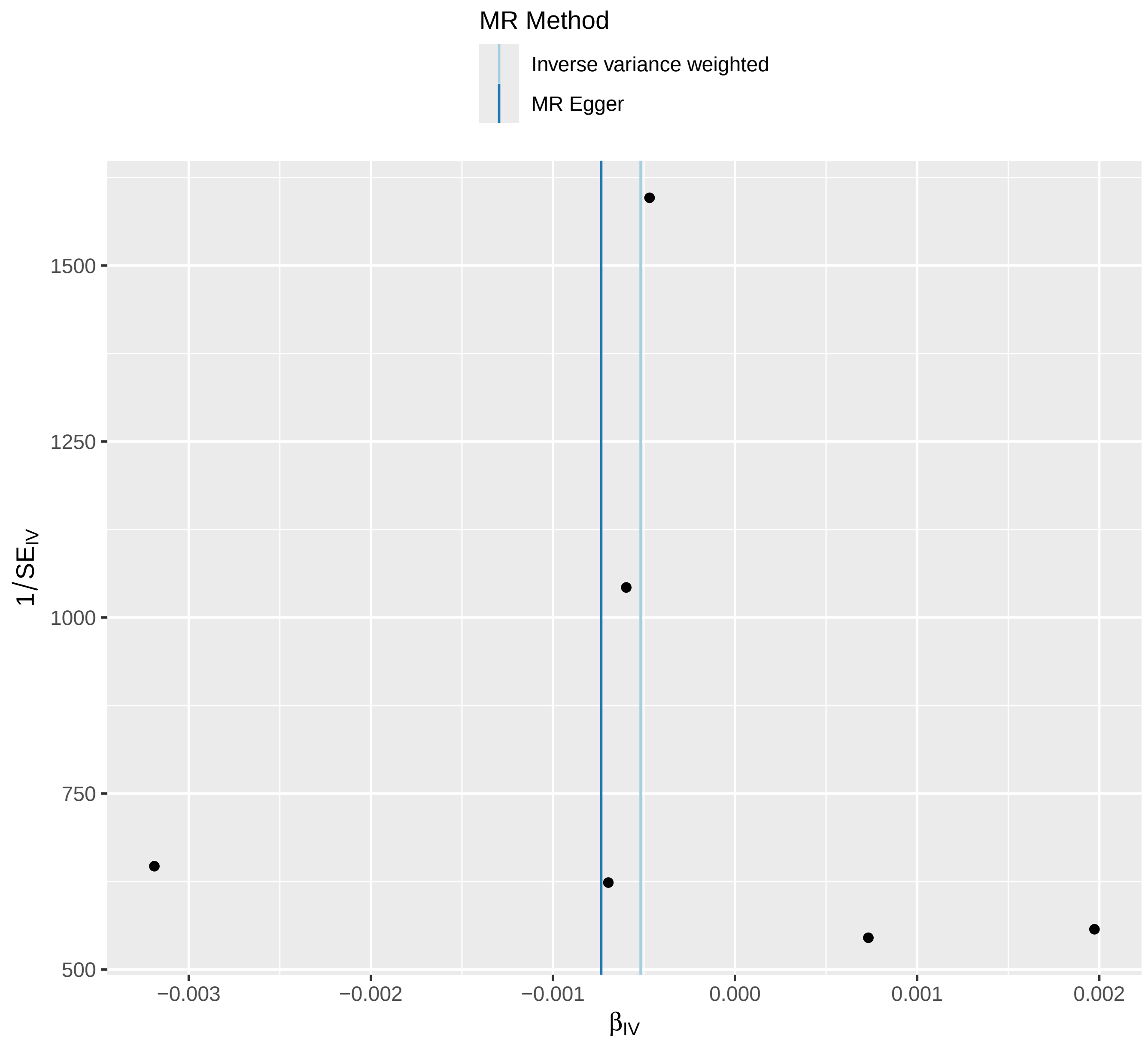

MR Method
Inverse variance weighted
MR Egger
1500
1250
1000
750
500
−0.003
−0.002
−0.001
0.000
0.001
0.002
$1/SE_{IV}$
β_{IV}

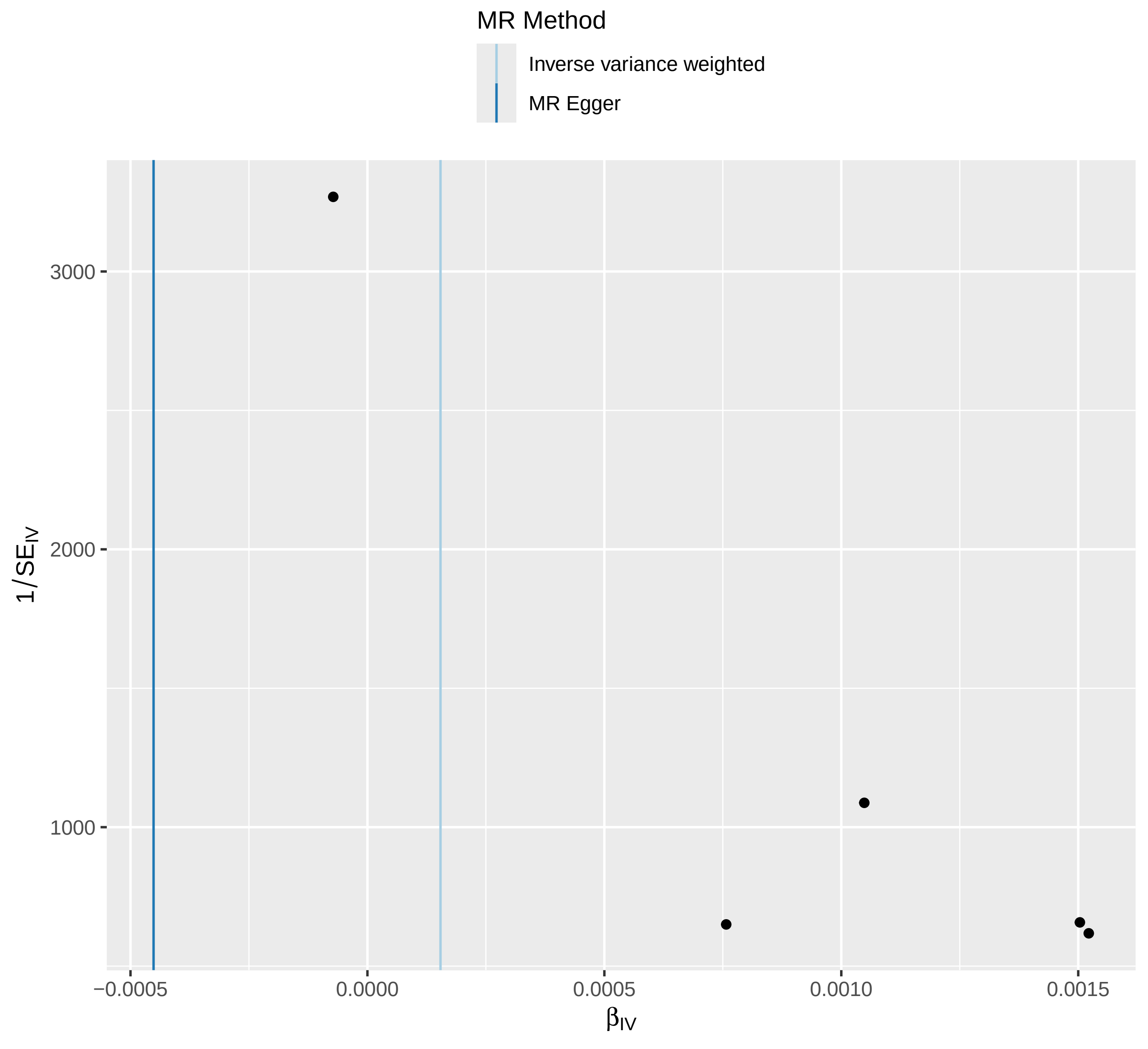
MR Method
Inverse variance weighted
MR Egger
3000
2000
1000
1/SE_IV
−0.0005
0.0000
0.0005
0.0010
0.0015
β_IV

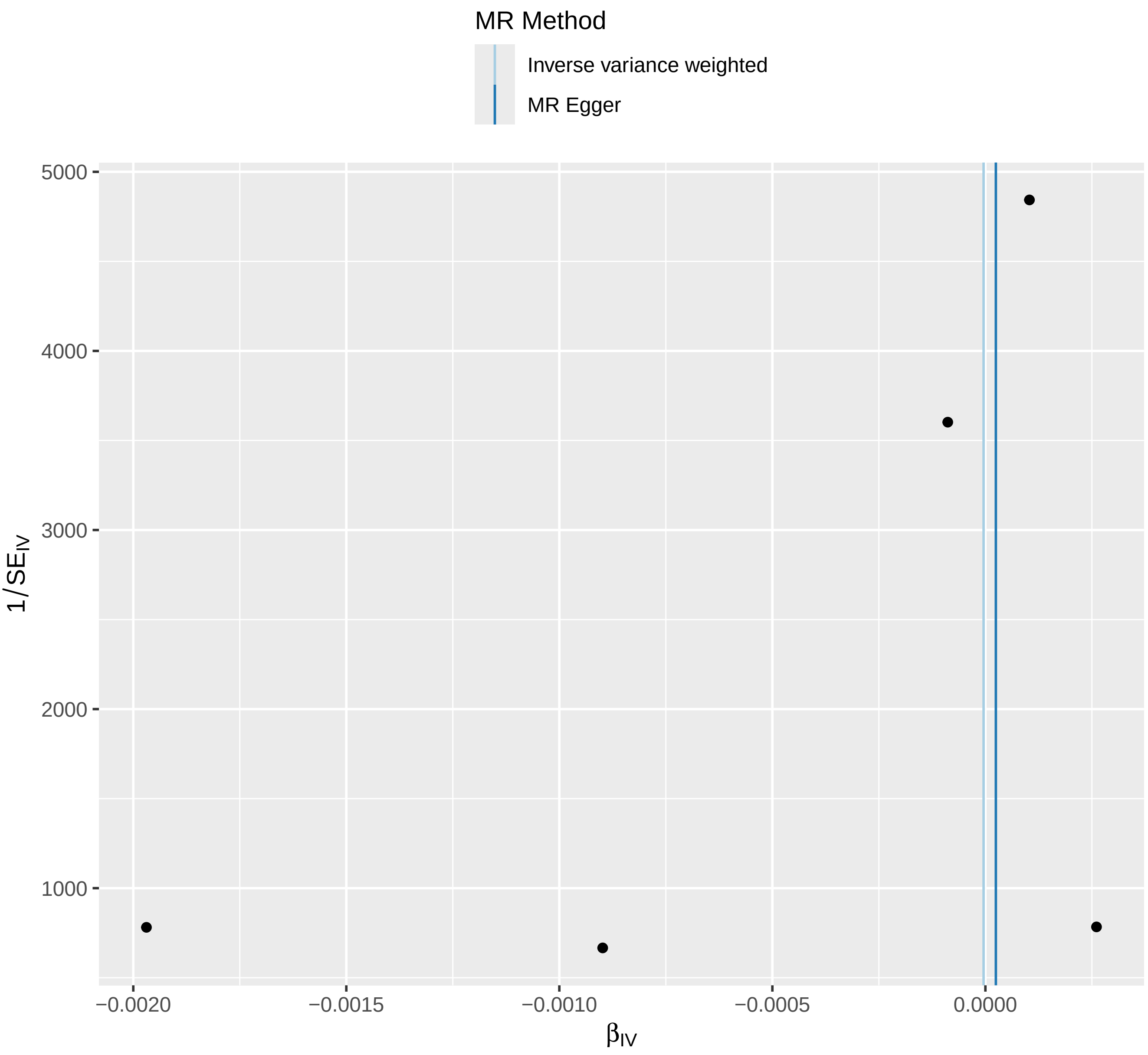
MR Method
Inverse variance weighted
MR Egger
5000
4000
3000
2000
1000
1/SE$_{IV}$
−0.0020
−0.0015
−0.0010
−0.0005
0.0000
β$_{IV}$

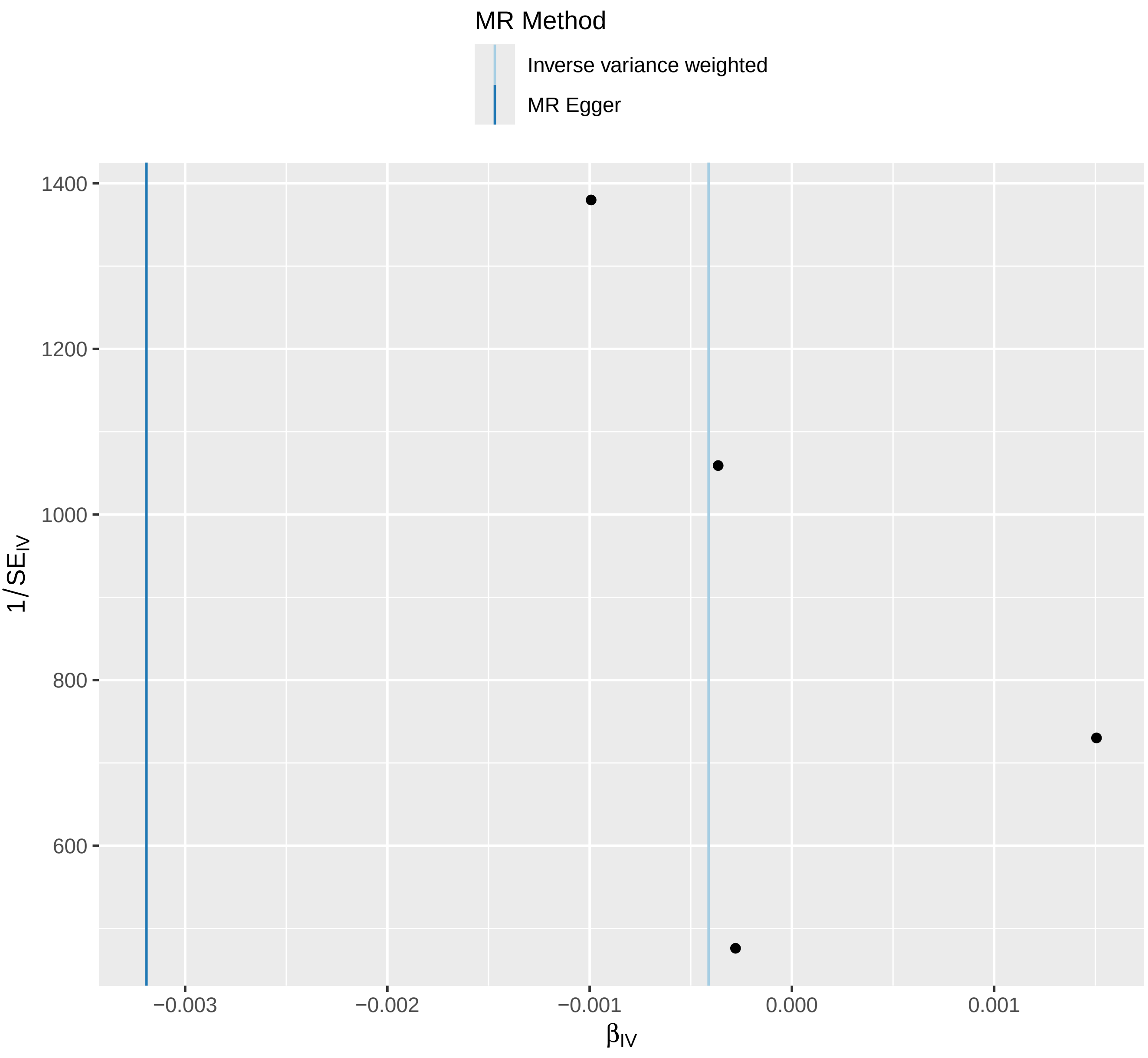

MR Method
Inverse variance weighted
MR Egger
1400
1200
1000
800
600
1/SE_IV
−0.003
−0.002
−0.001
0.000
0.001
β_IV

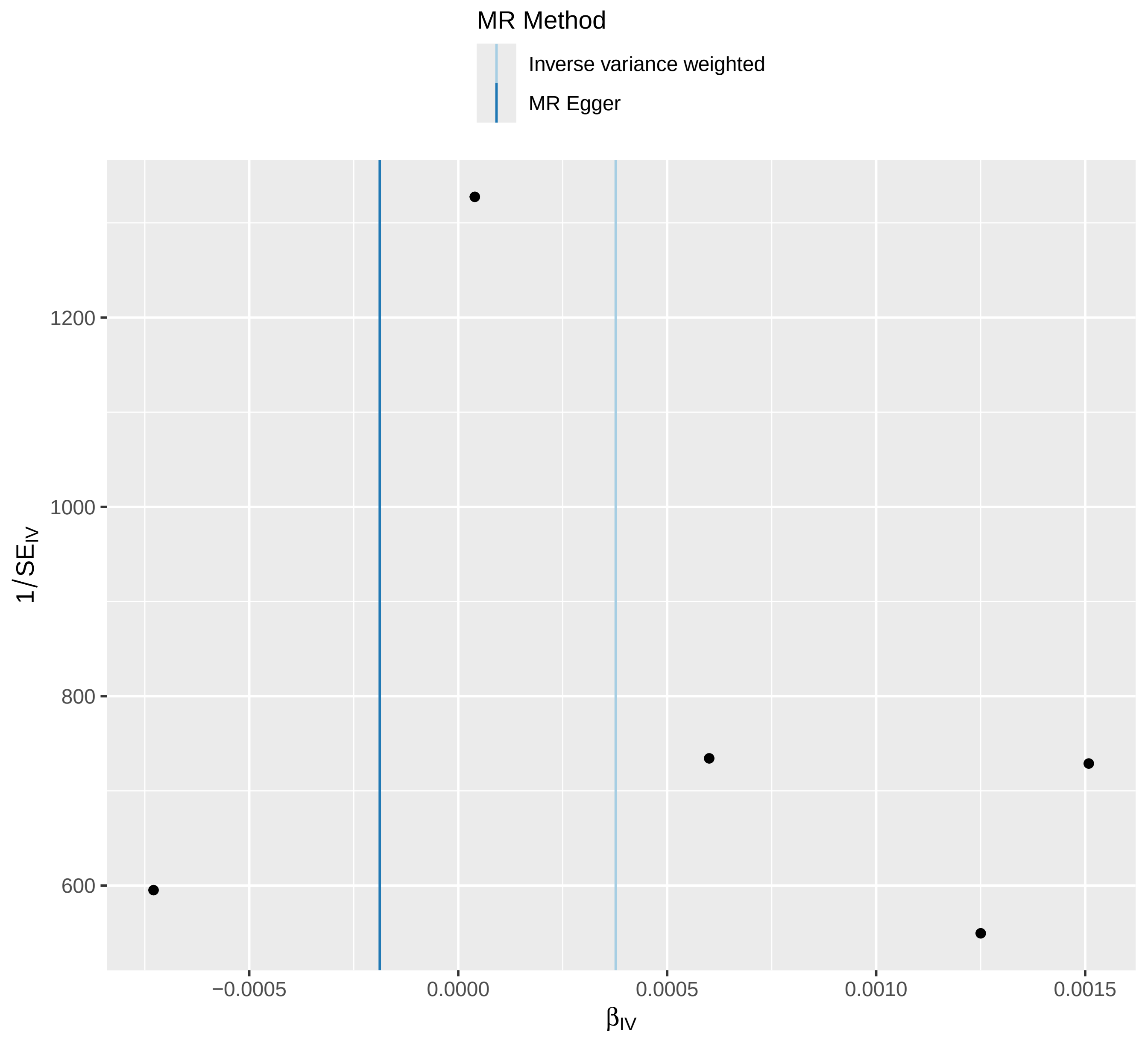

MR Method
Inverse variance weighted
MR Egger
1200
1000
800
600
$1/SE_{IV}$
−0.0005
0.0000
0.0005
0.0010
0.0015
β_{IV}

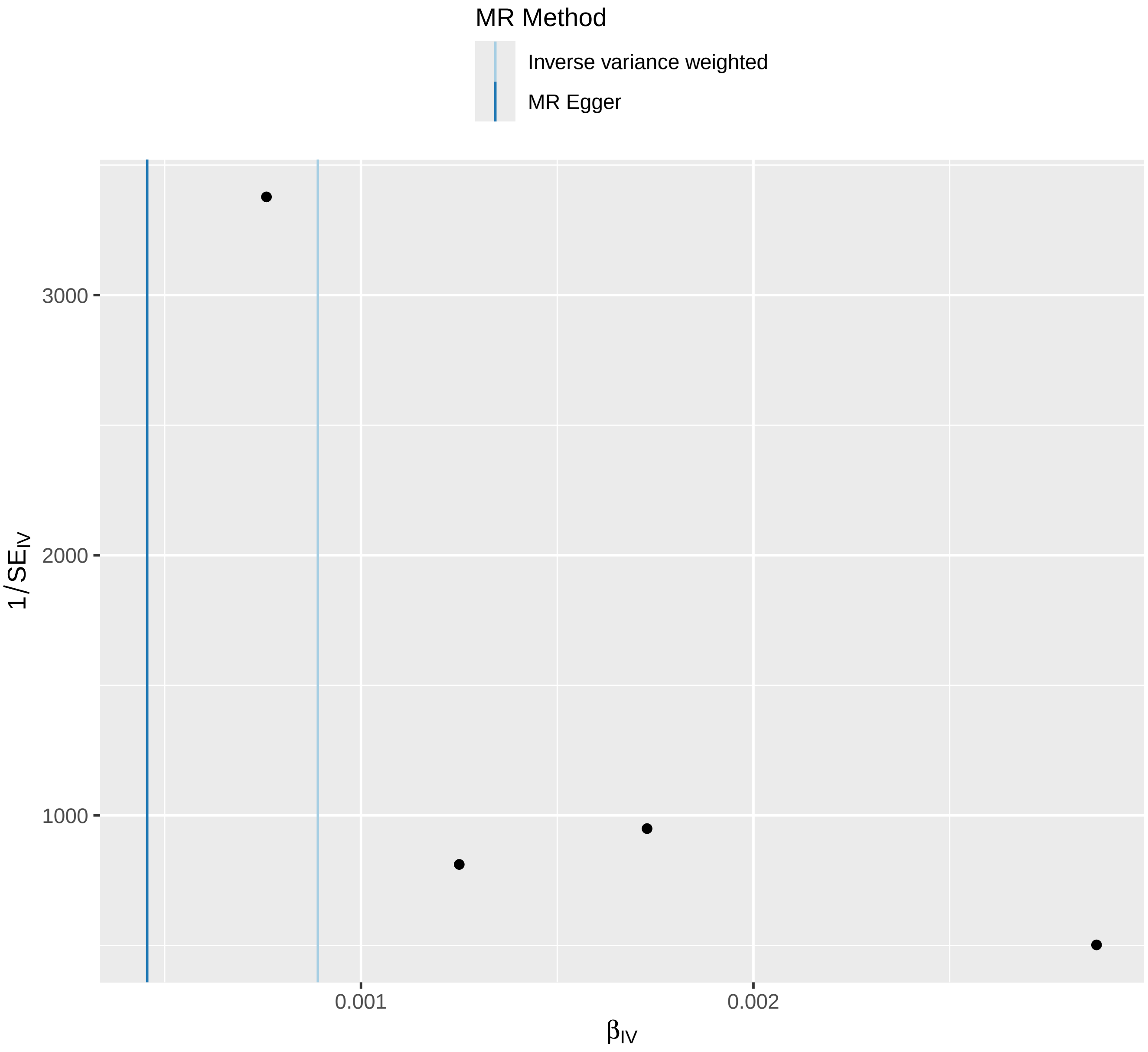

MR Method
Inverse variance weighted
MR Egger
3000
2000
1000
$1/SE_{IV}$
0.001
0.002
β_{IV}

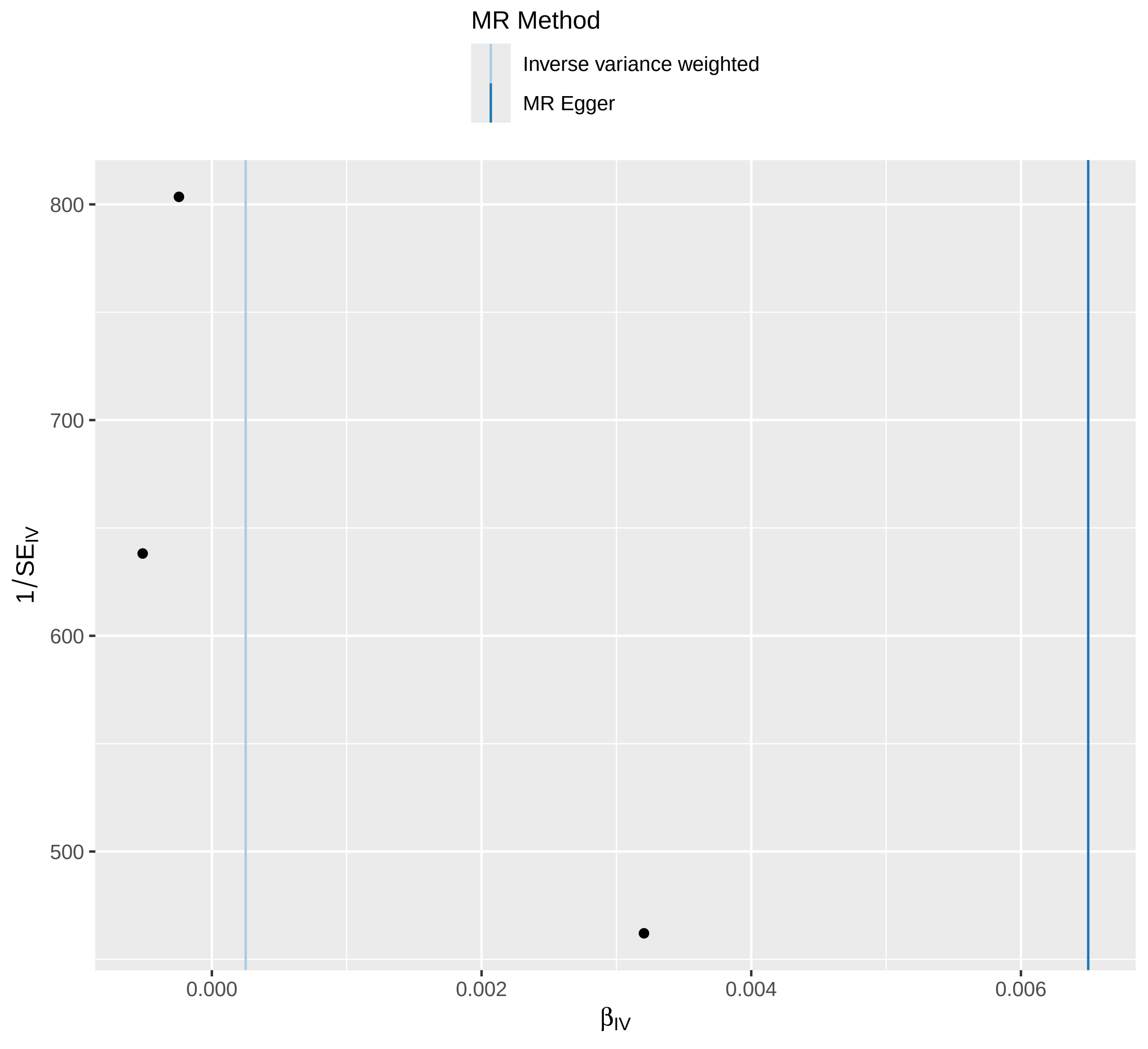

MR Method
Inverse variance weighted
MR Egger
800
700
600
500
$1/SE_{IV}$
0.000
0.002
0.004
0.006
β_{IV}

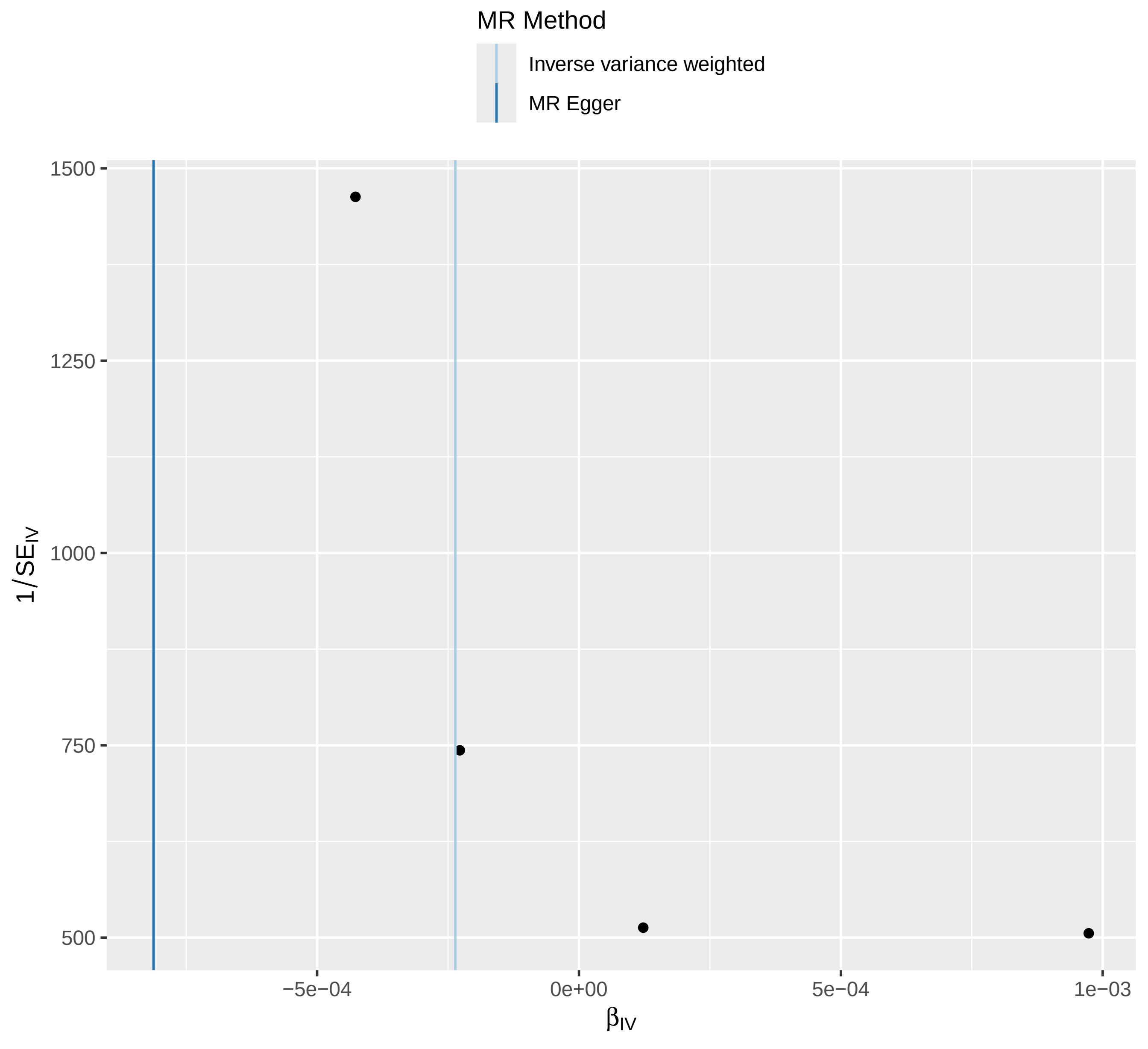
MR Method
Inverse variance weighted
MR Egger
1500
1250
1000
750
500
1/SE$_{IV}$
−5e−04
0e+00
5e−04
1e−03
β$_{IV}$

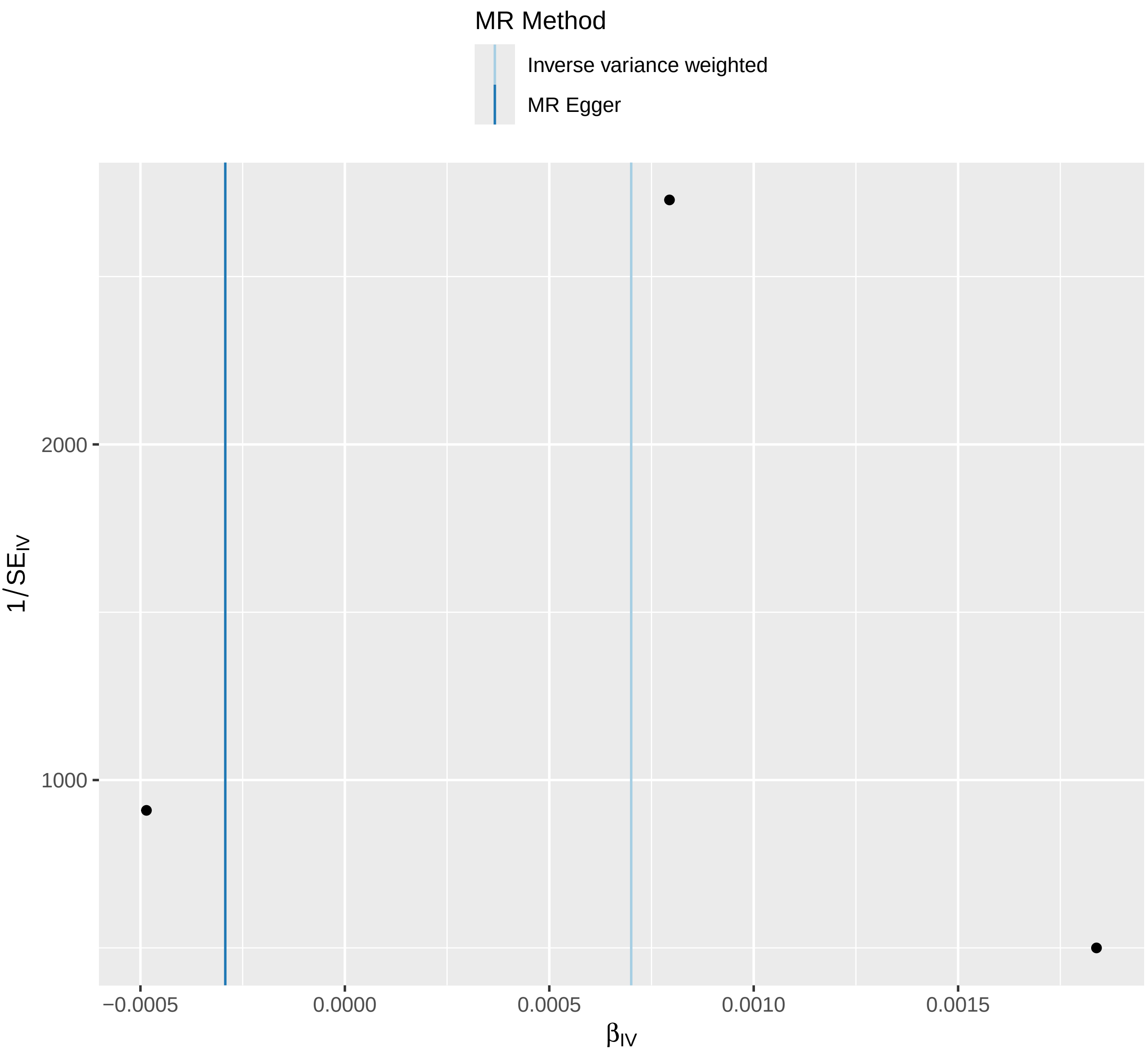
MR Method
Inverse variance weighted
MR Egger
2000
1000
1/SE_IV
−0.0005
0.0000
0.0005
0.0010
0.0015
β_IV

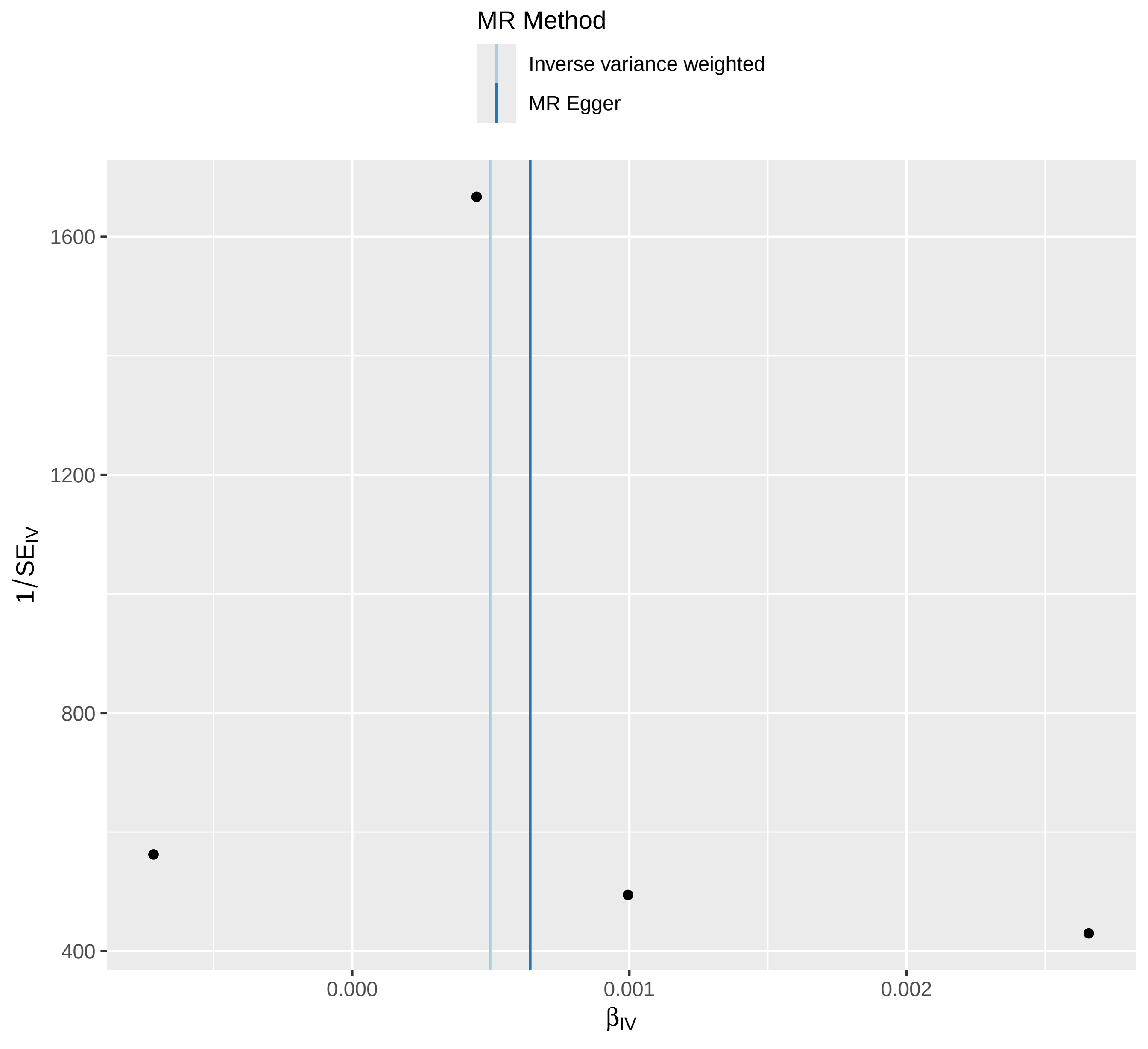
MR Method
Inverse variance weighted
MR Egger
1600
1200
800
400
1/SE_IV
0.000
0.001
0.002
β_IV

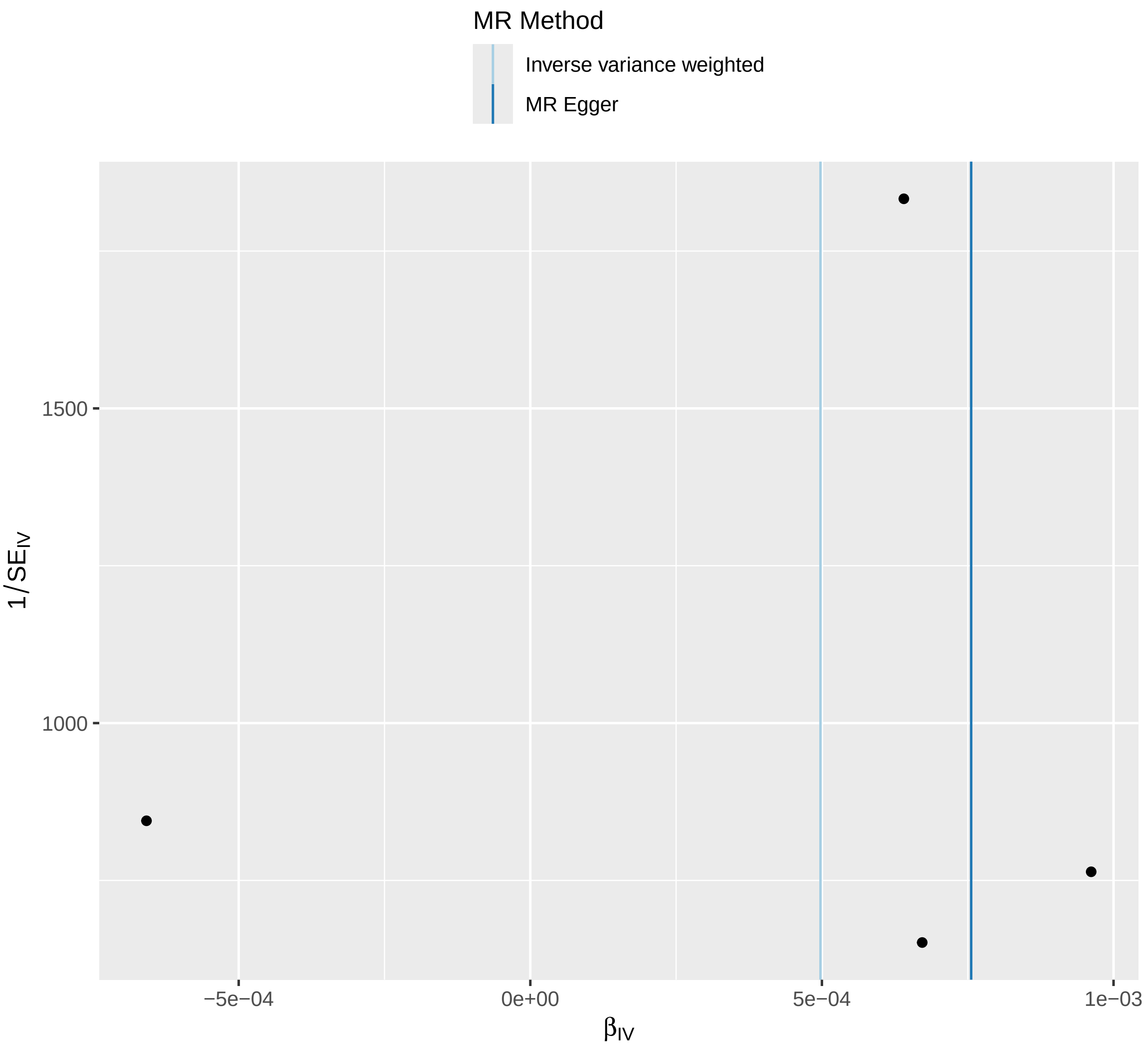
MR Method
Inverse variance weighted
MR Egger
1500
1000
1/SE$_{IV}$
−5e−04
0e+00
5e−04
1e−03
β$_{IV}$

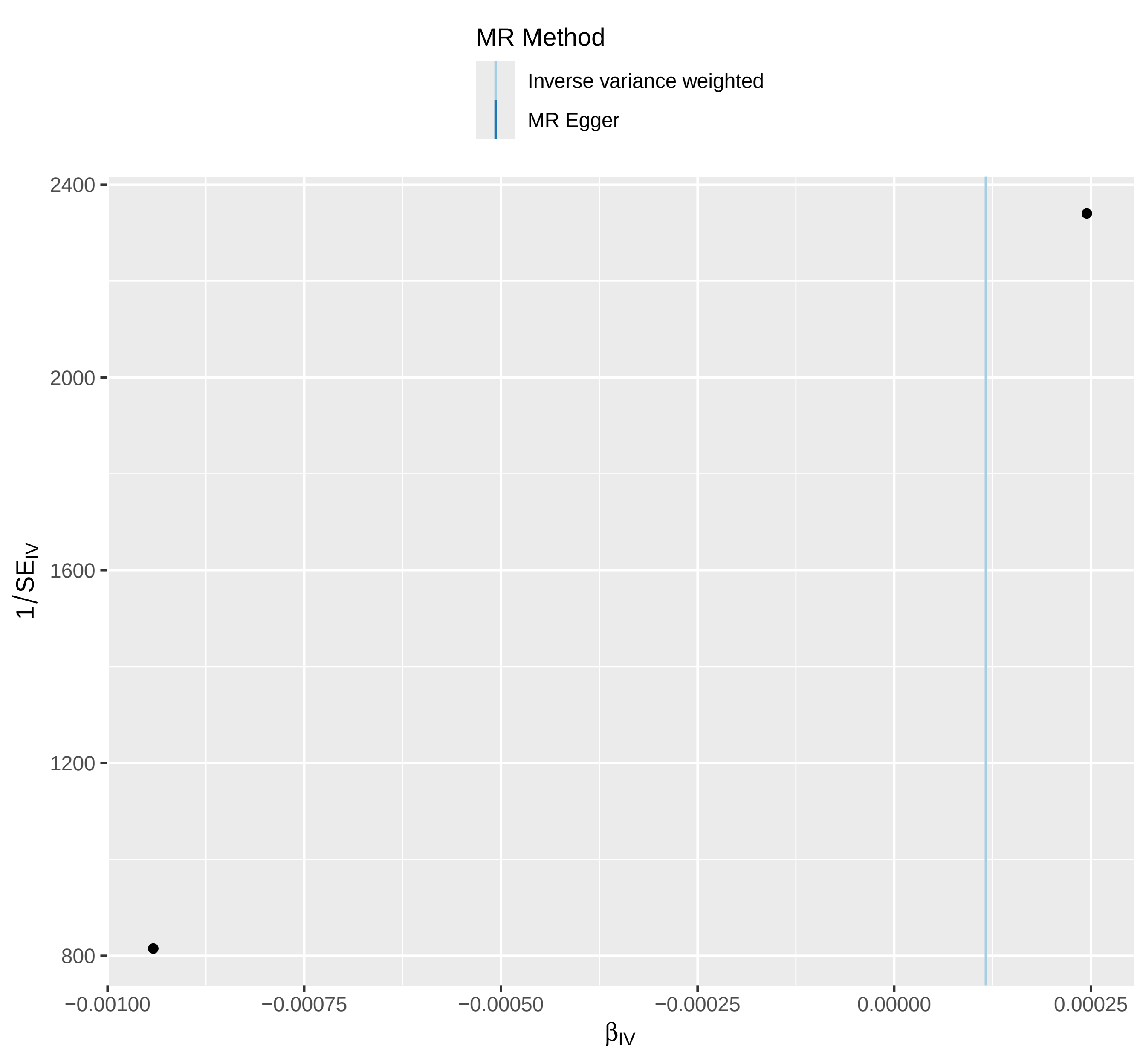
MR Method
Inverse variance weighted
MR Egger
2400
2000
1600
1200
800
1/SE_IV
−0.00100
−0.00075
−0.00050
−0.00025
0.00000
0.00025
β_IV

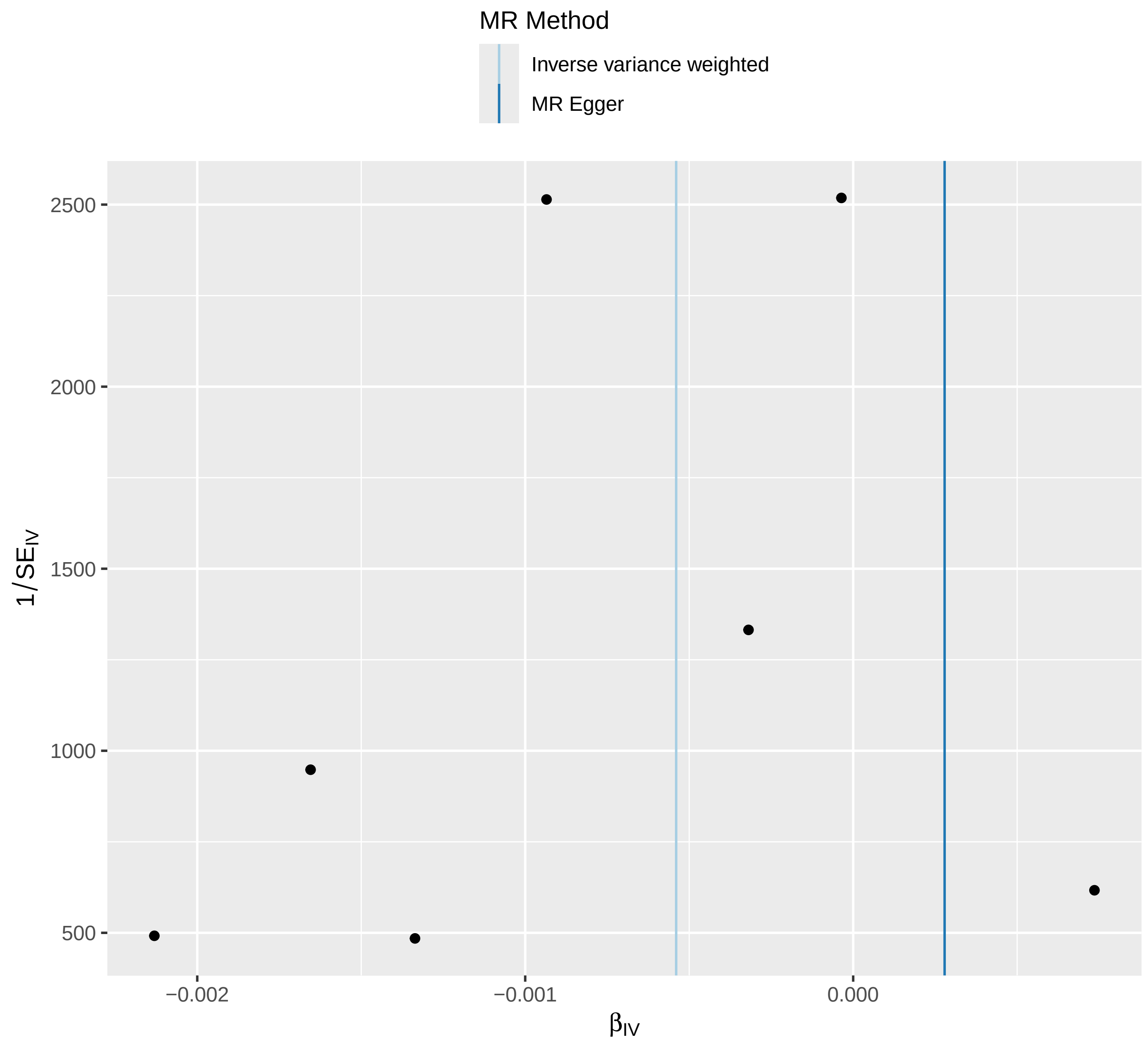

MR Method
Inverse variance weighted
MR Egger
2500
2000
1500
1000
500
1/SE$_{IV}$
−0.002
−0.001
0.000
β$_{IV}$

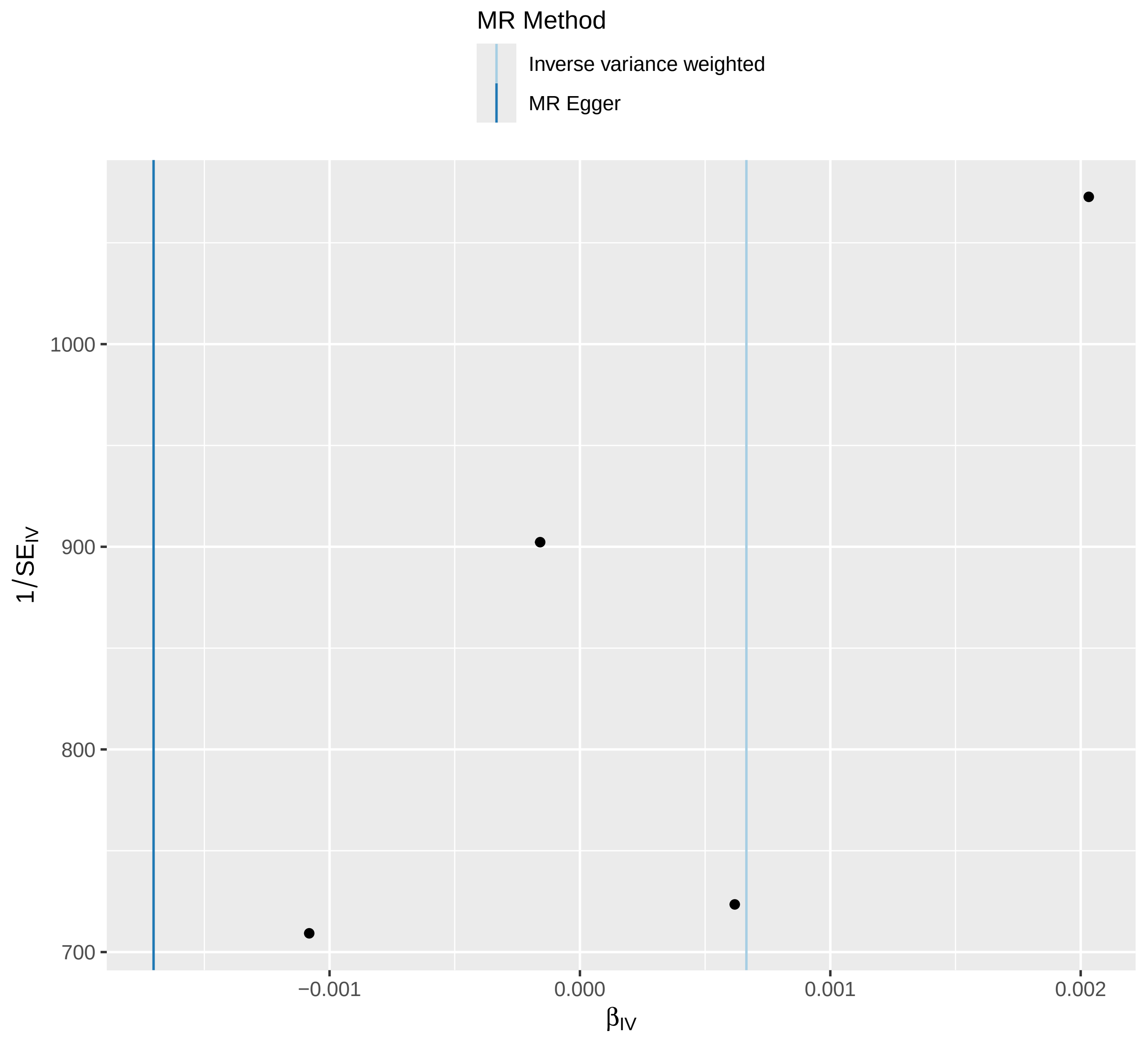
MR Method
Inverse variance weighted
MR Egger
1000
900
800
700
1/SE$_{IV}$
−0.001
0.000
0.001
0.002
β$_{IV}$

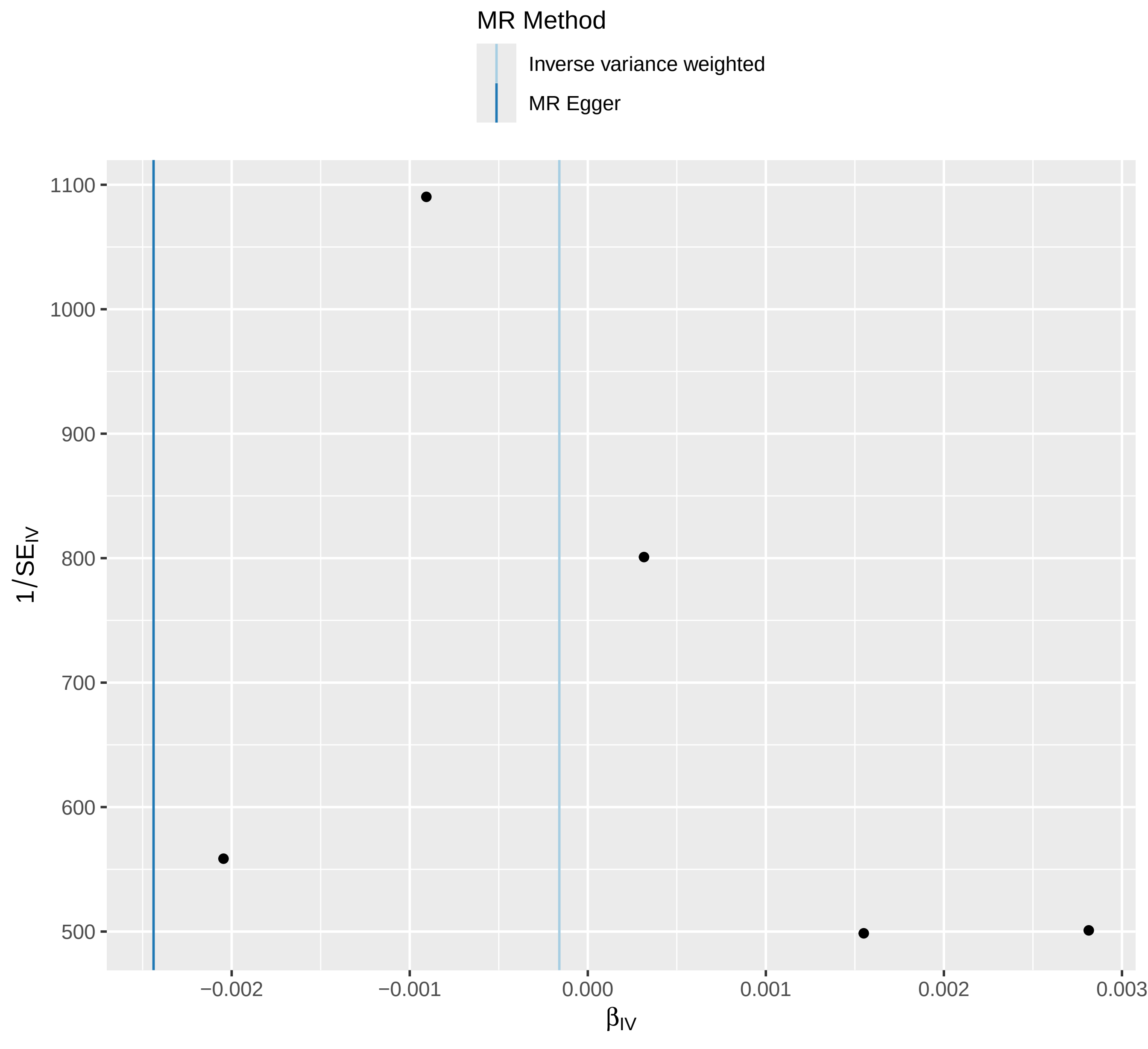

MR Method
Inverse variance weighted
MR Egger
1100
1000
900
800
700
600
500
1/SE$_{IV}$
−0.002
−0.001
0.000
0.001
0.002
0.003
β$_{IV}$

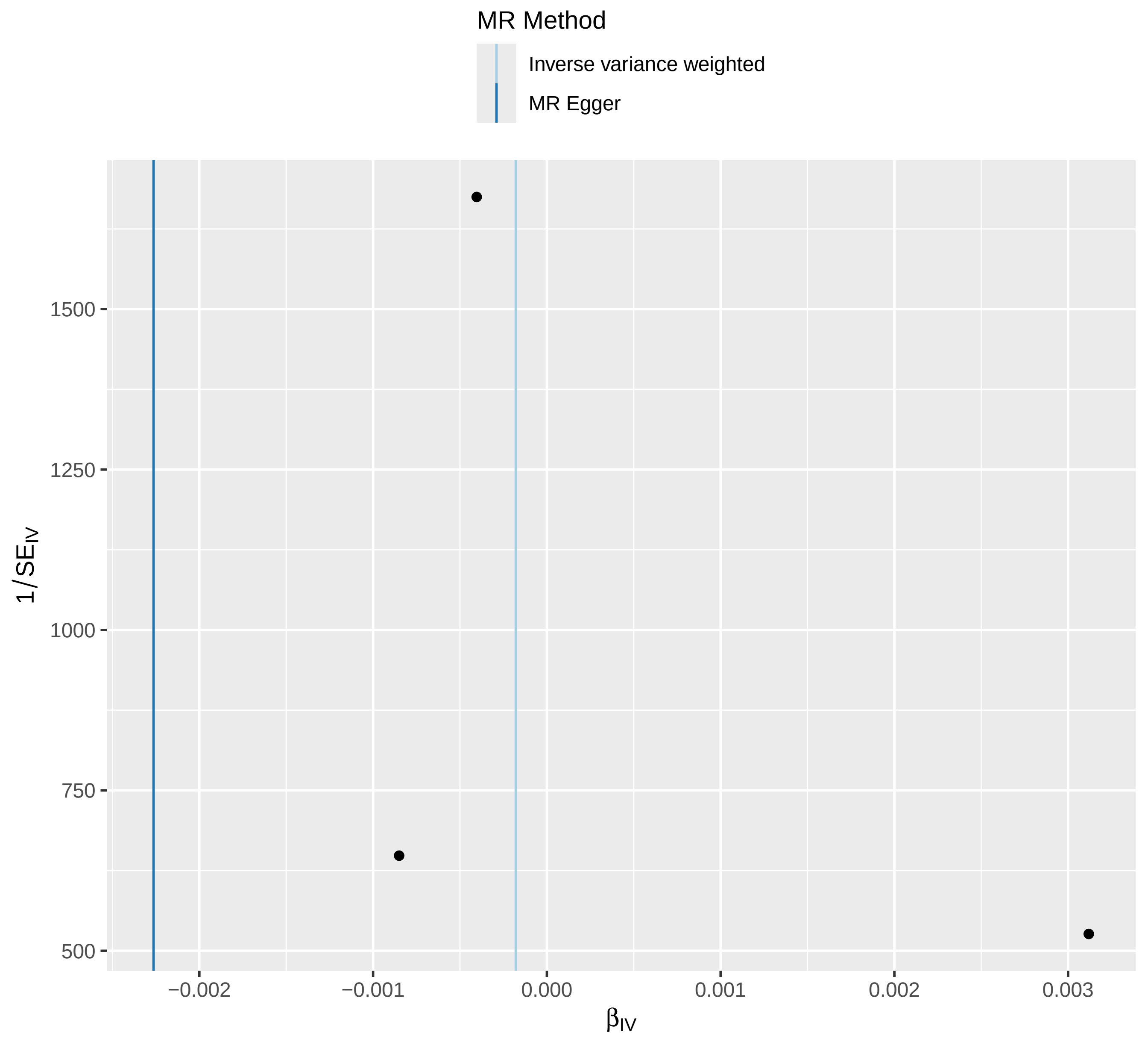
MR Method
Inverse variance weighted
MR Egger
1500
1250
1000
750
500
−0.002
−0.001
0.000
0.001
0.002
0.003
β_{IV}
$1/SE_{IV}$

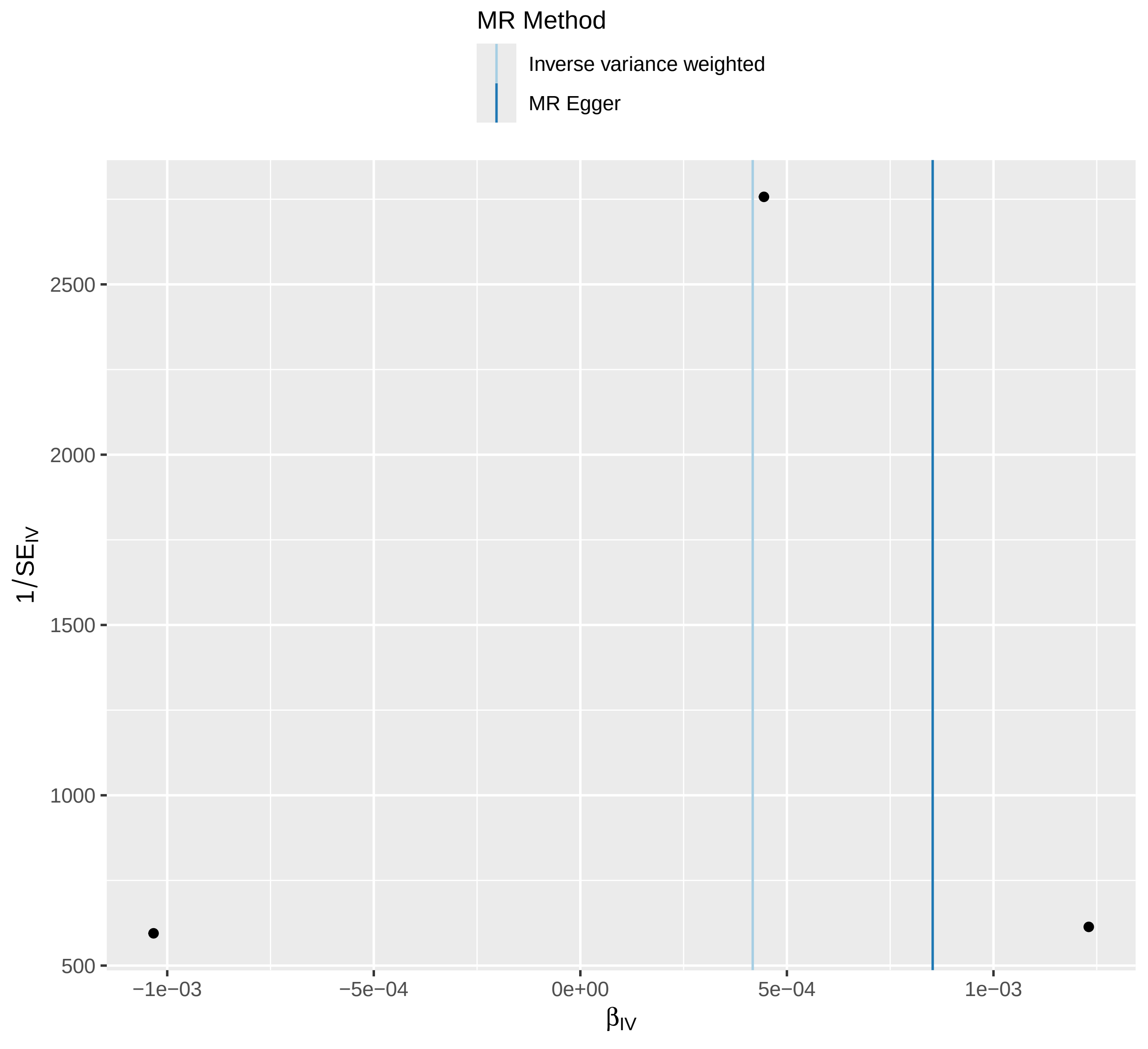
MR Method
Inverse variance weighted
MR Egger
2500
2000
1500
1000
500
1/SE$_{IV}$
−1e−03
−5e−04
0e+00
5e−04
1e−03
β$_{IV}$

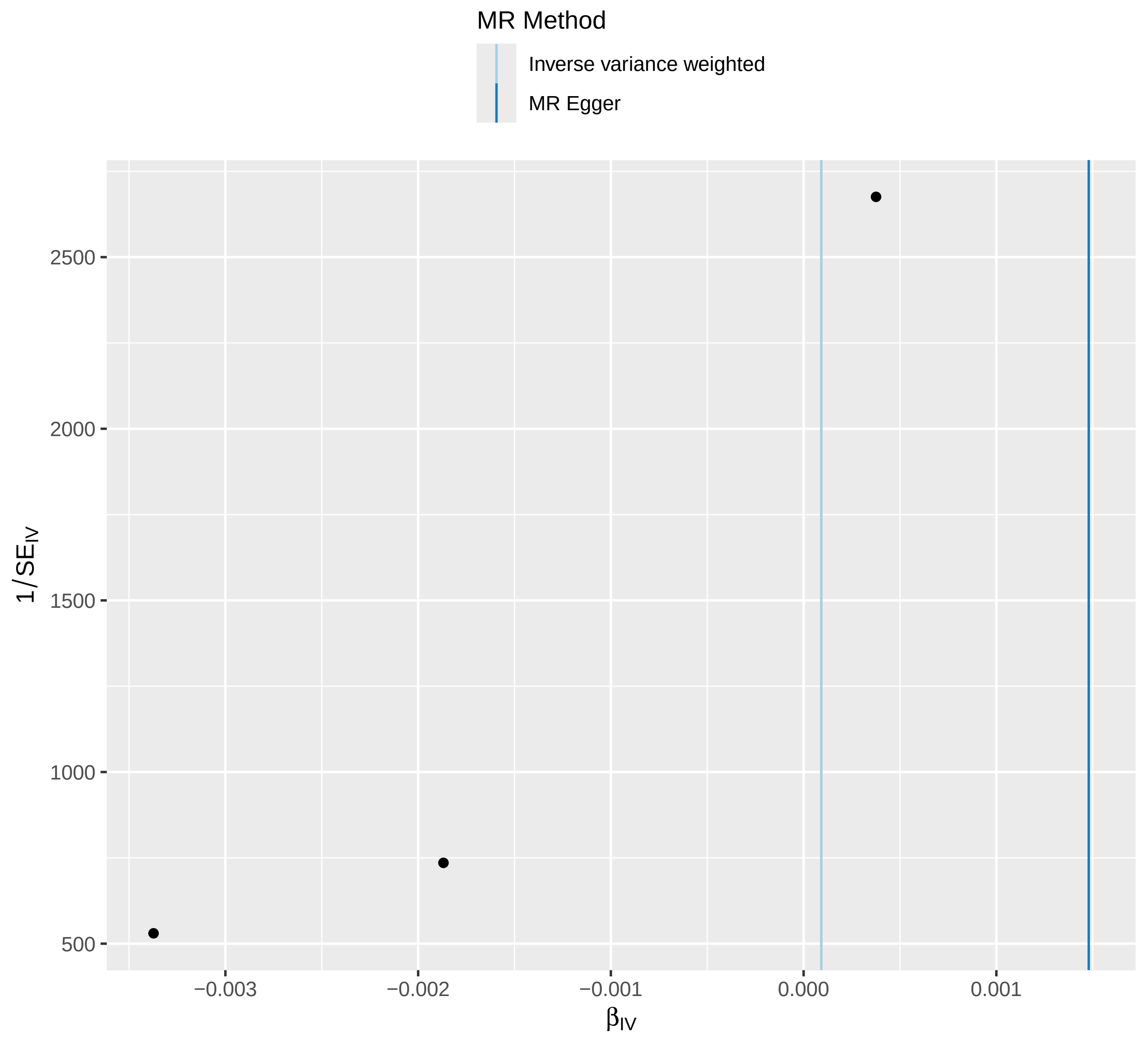

MR Method
Inverse variance weighted
MR Egger
2500
2000
1500
1000
500
1/SE_IV
−0.003
−0.002
−0.001
0.000
0.001
β_IV

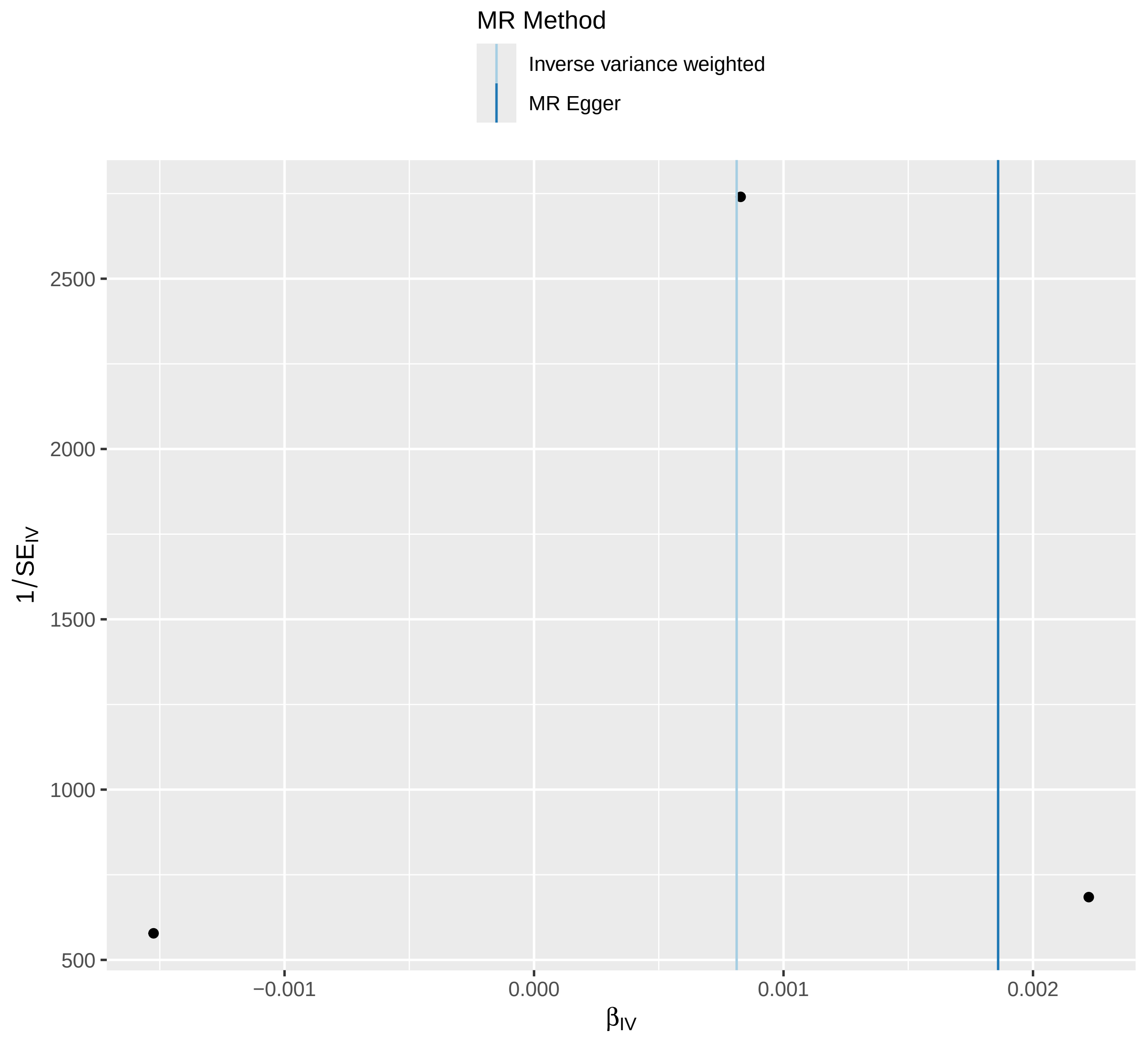
MR Method
Inverse variance weighted
MR Egger
2500
2000
1500
1000
500
1/SE_IV
−0.001
0.000
0.001
0.002
β_IV

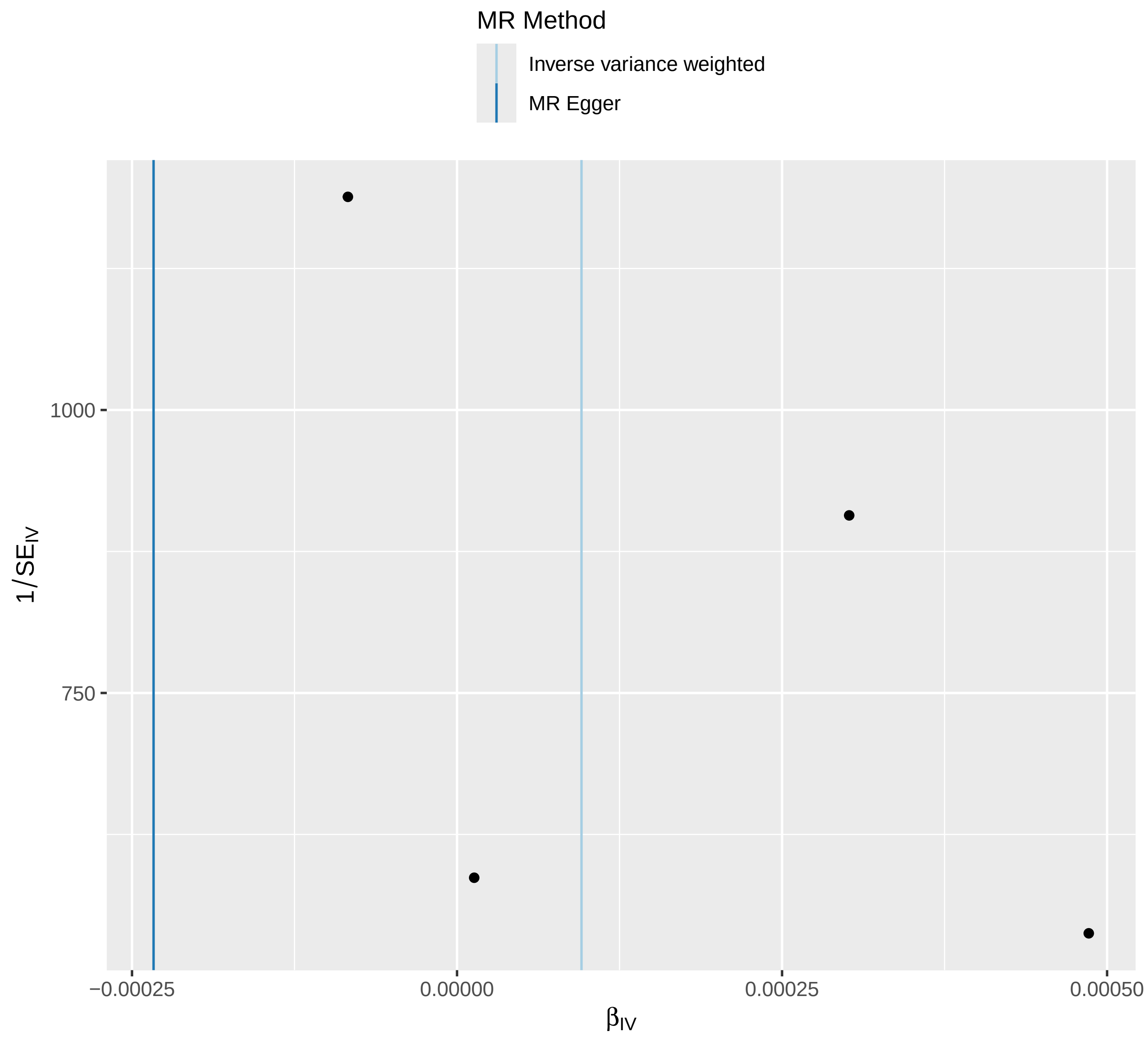

MR Method
Inverse variance weighted
MR Egger
1000
750
1/SE_IV
−0.00025
0.00000
0.00025
0.00050
β_IV

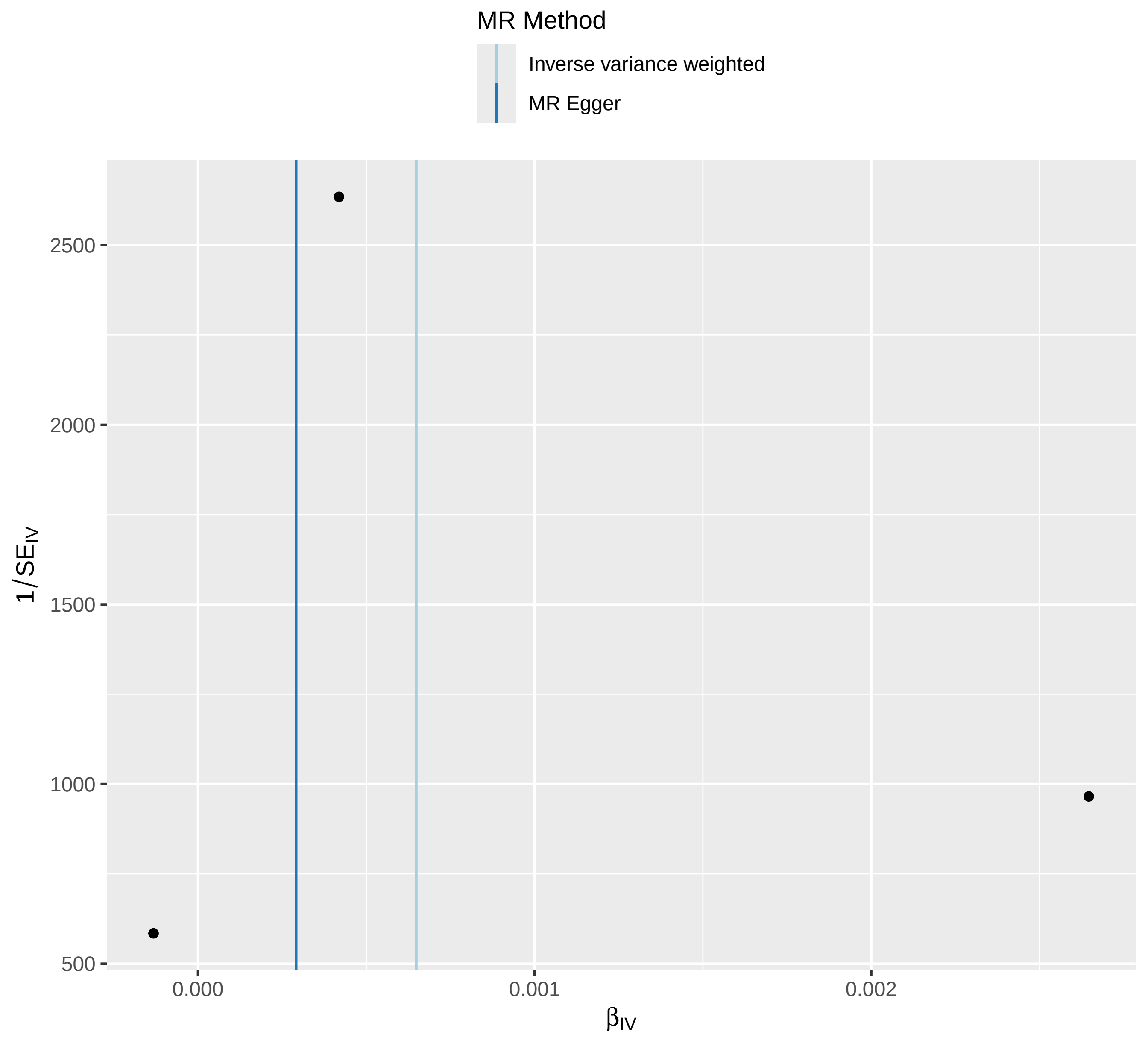
MR Method
Inverse variance weighted
MR Egger
2500
2000
1500
1000
500
1/SE_IV
0.000
0.001
0.002
β_IV

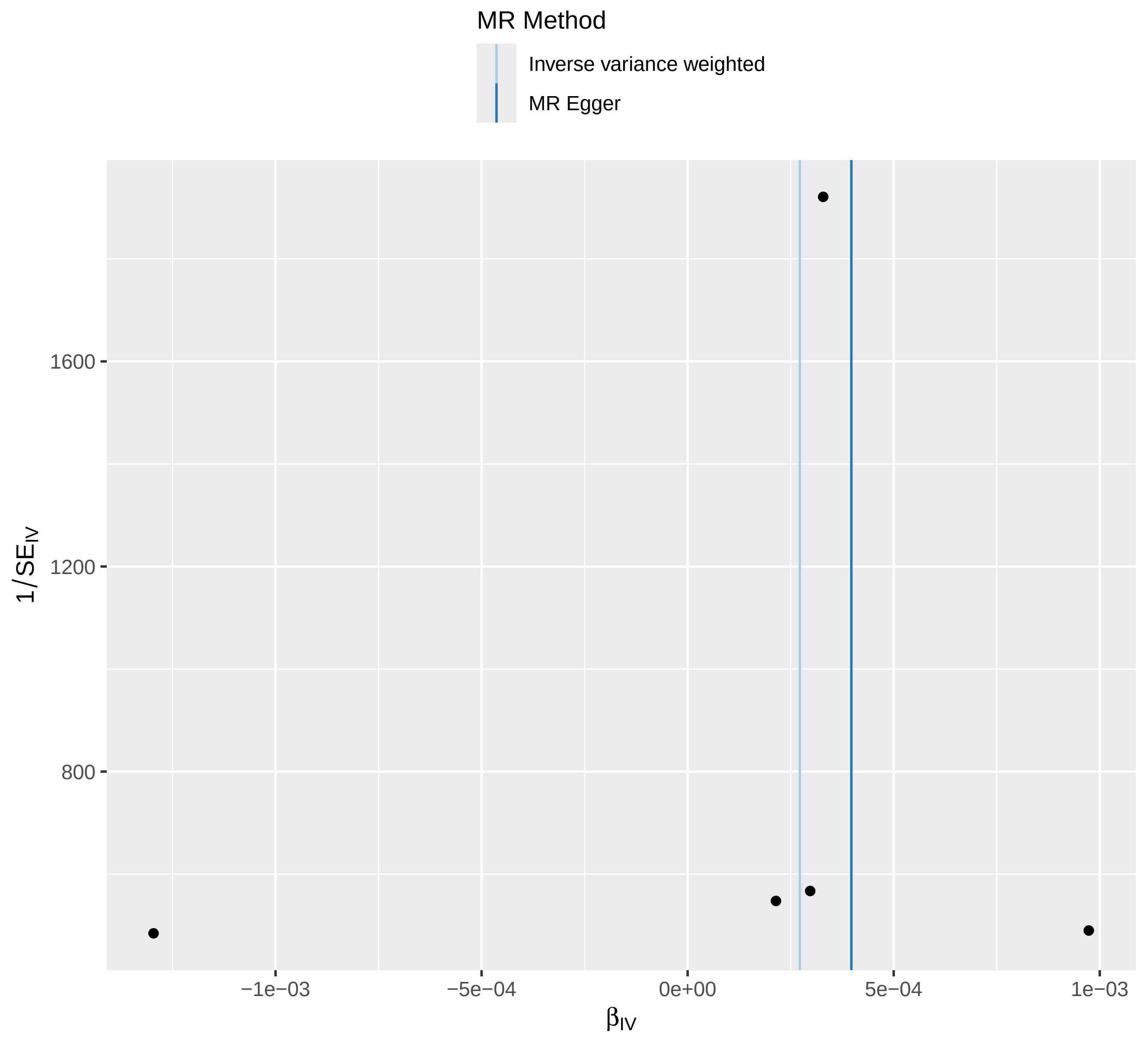
MR Method
Inverse variance weighted
MR Egger
1600
1200
800
1/SE_IV
−1e−03
−5e−04
0e+00
5e−04
1e−03
β_IV

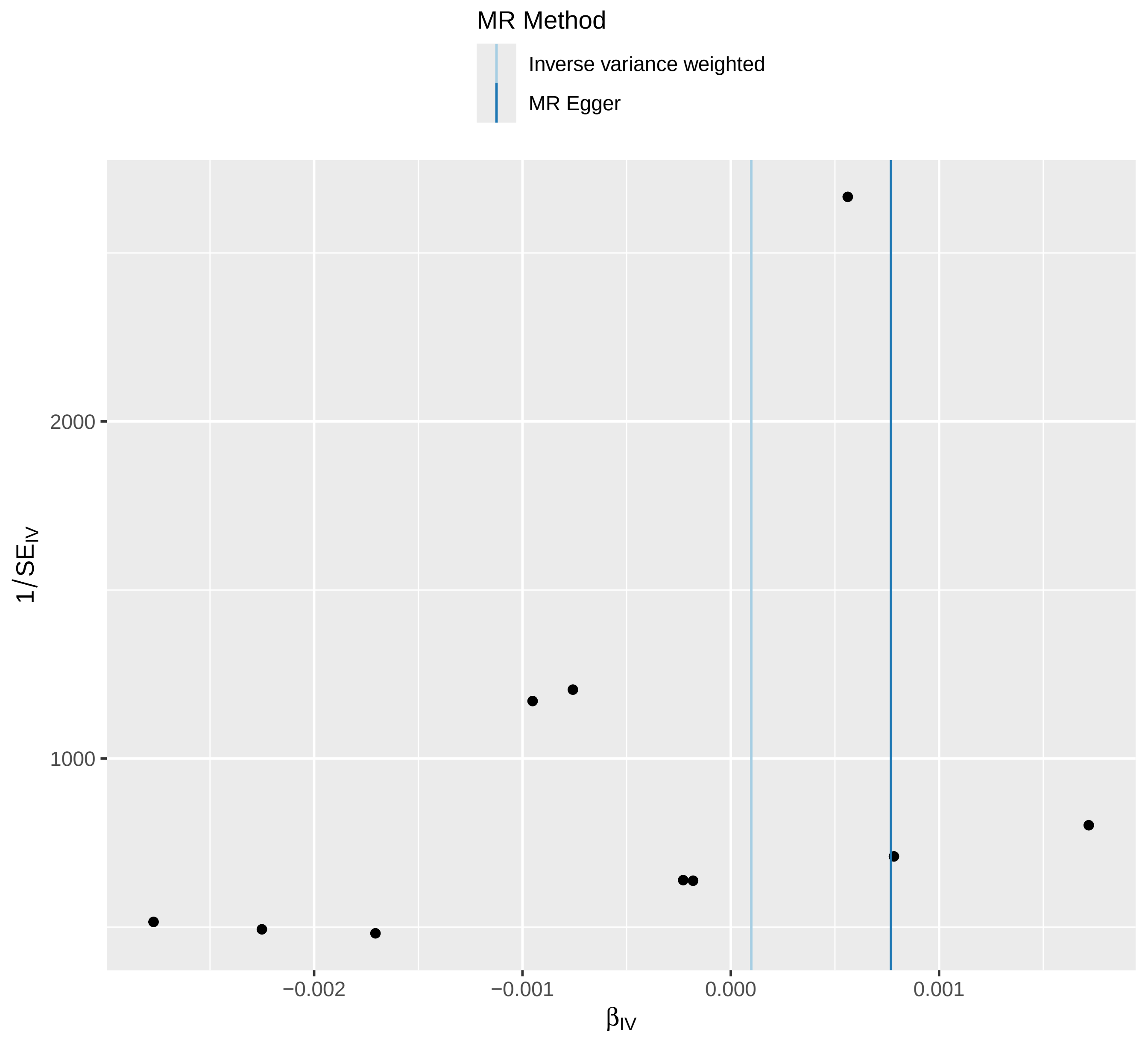
MR Method
Inverse variance weighted
MR Egger
2000
1000
$1/SE_{IV}$
−0.002
−0.001
0.000
0.001
β_{IV}

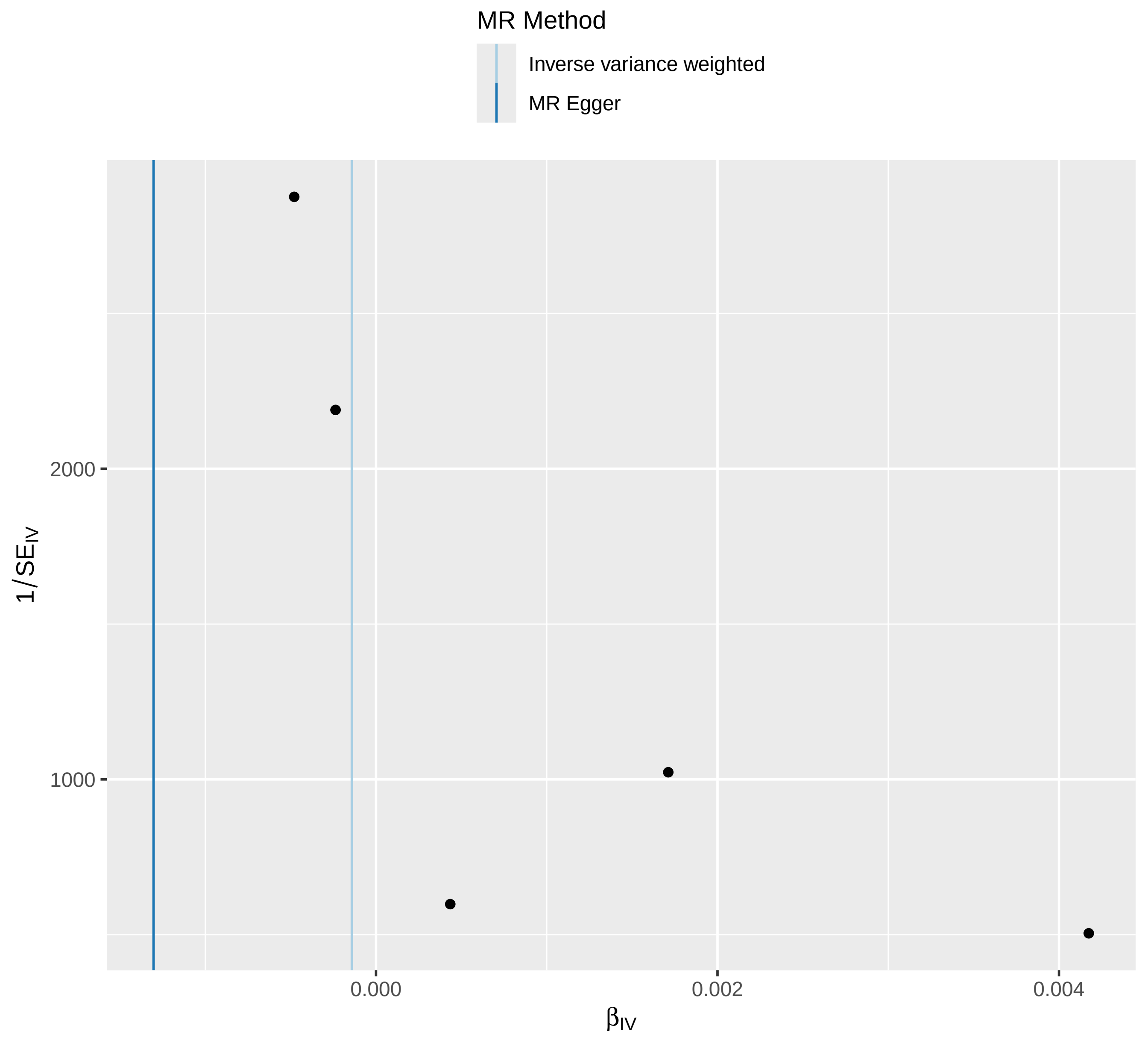
MR Method
Inverse variance weighted
MR Egger
2000
1000
0.000
0.002
0.004
1/SE_IV
β_IV

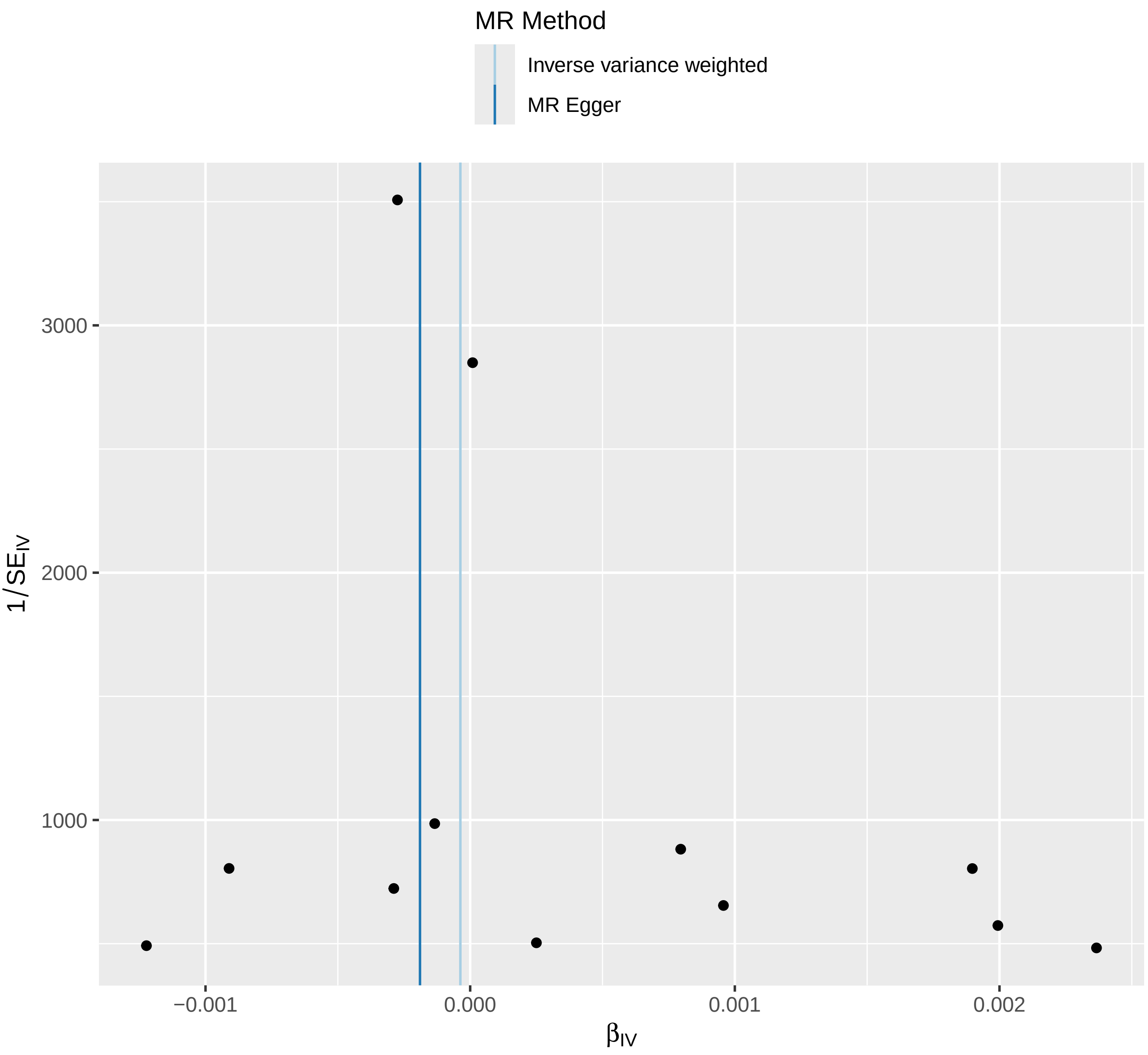

MR Method
Inverse variance weighted
MR Egger
3000
2000
1000
1/SE$_{IV}$
−0.001
0.000
0.001
0.002
β$_{IV}$

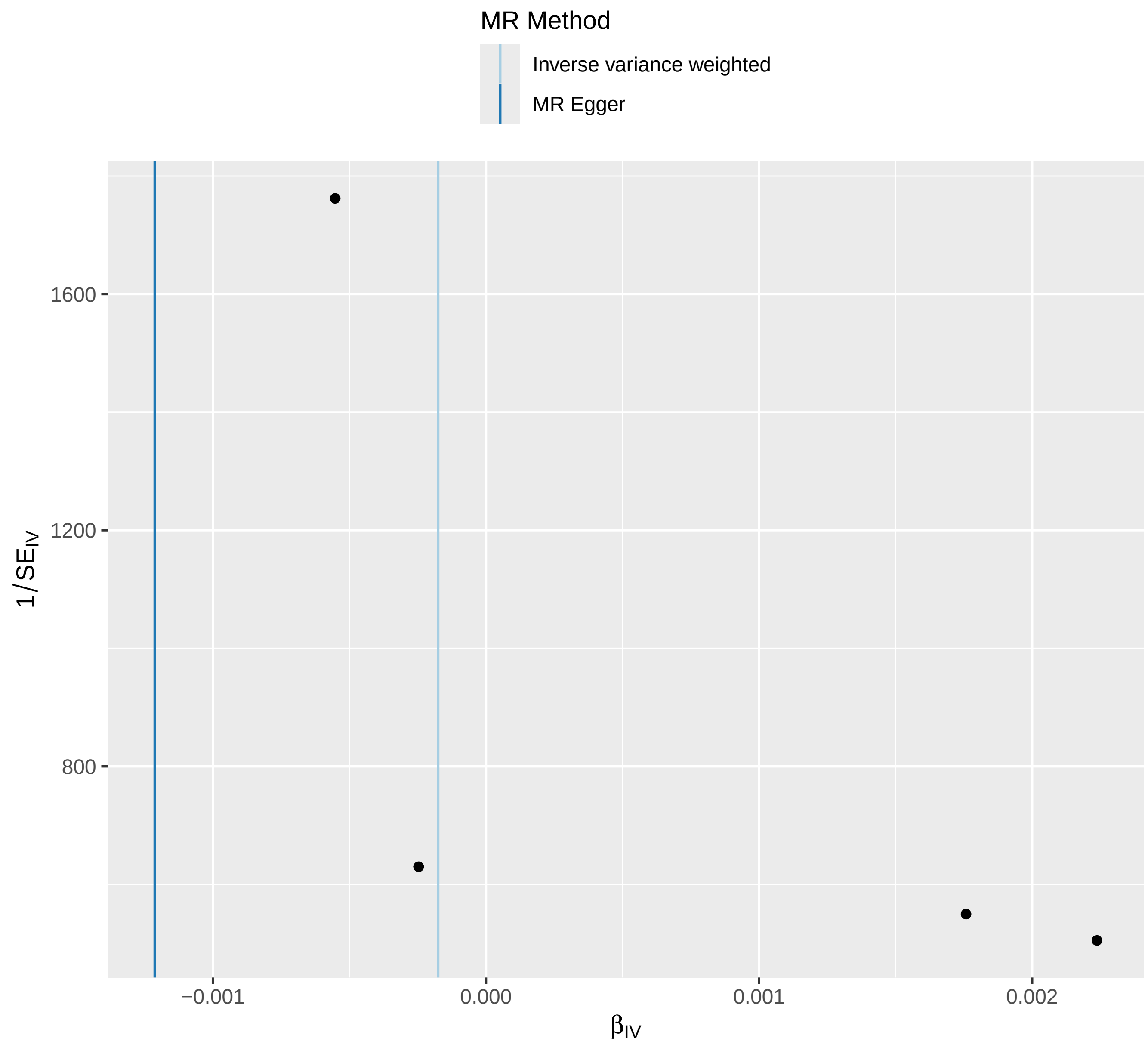
MR Method
Inverse variance weighted
MR Egger
1600
1200
800
1/SE$_{IV}$
−0.001
0.000
0.001
0.002
β_{IV}

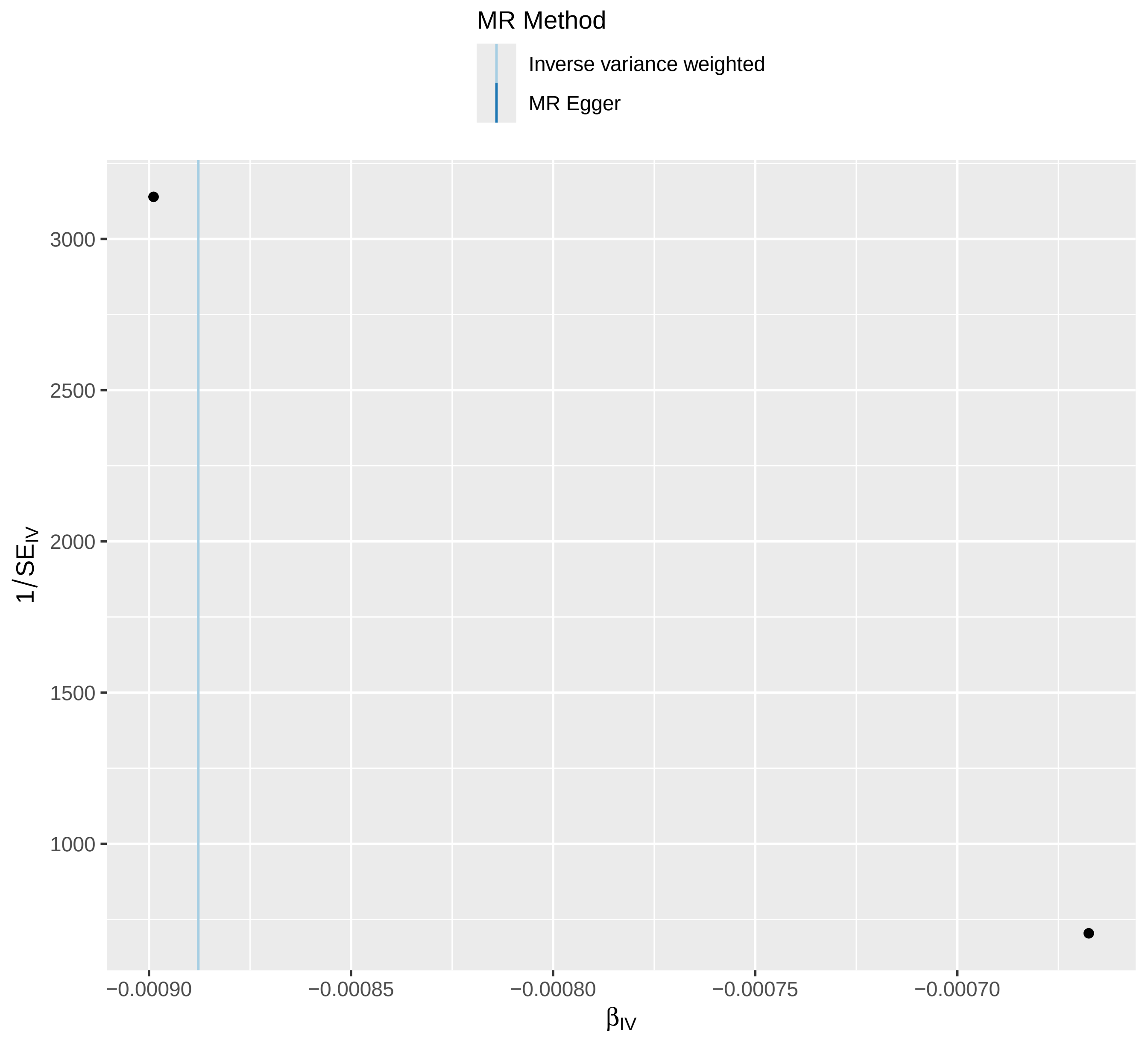
MR Method
Inverse variance weighted
MR Egger
3000
2500
2000
1500
1000
$1/SE_{IV}$
−0.00090
−0.00085
−0.00080
−0.00075
−0.00070
β_{IV}

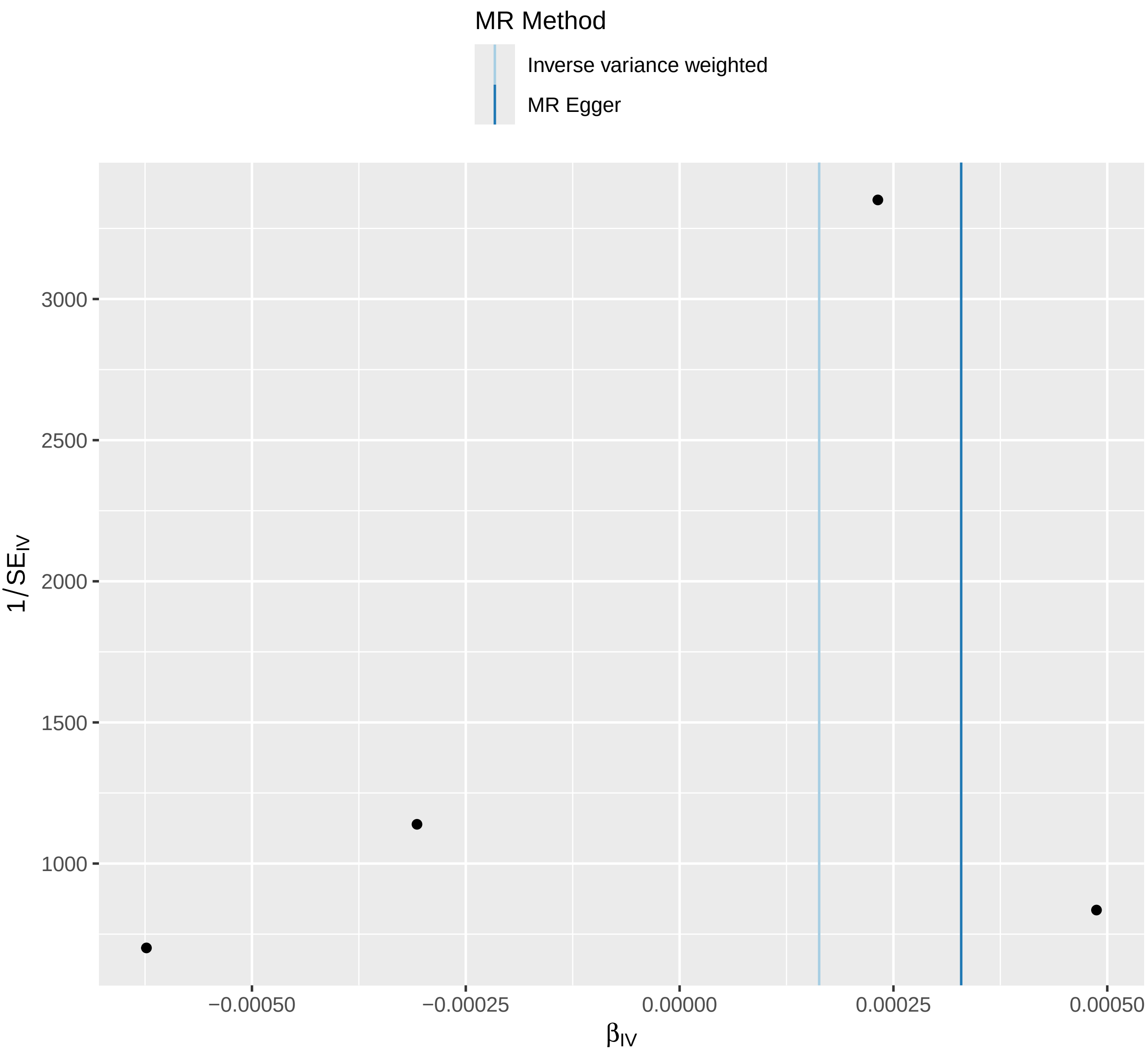

MR Method
Inverse variance weighted
MR Egger
3000
2500
2000
1500
1000
$1/SE_{IV}$
−0.00050
−0.00025
0.00000
0.00025
0.00050
β_{IV}

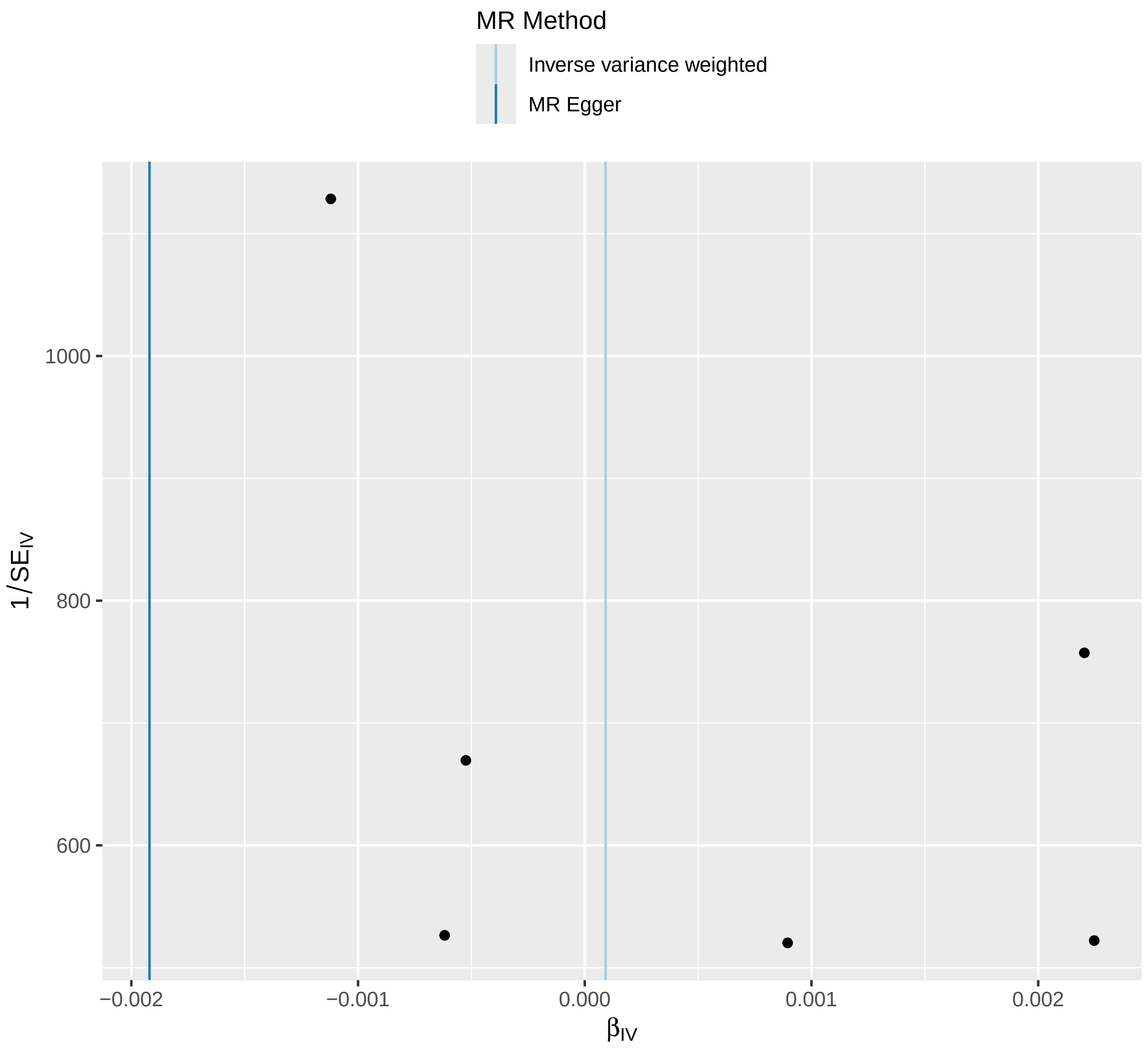

MR Method
Inverse variance weighted
MR Egger
1000
800
600
1/SE$_{IV}$
−0.002
−0.001
0.000
0.001
0.002
β_{IV}

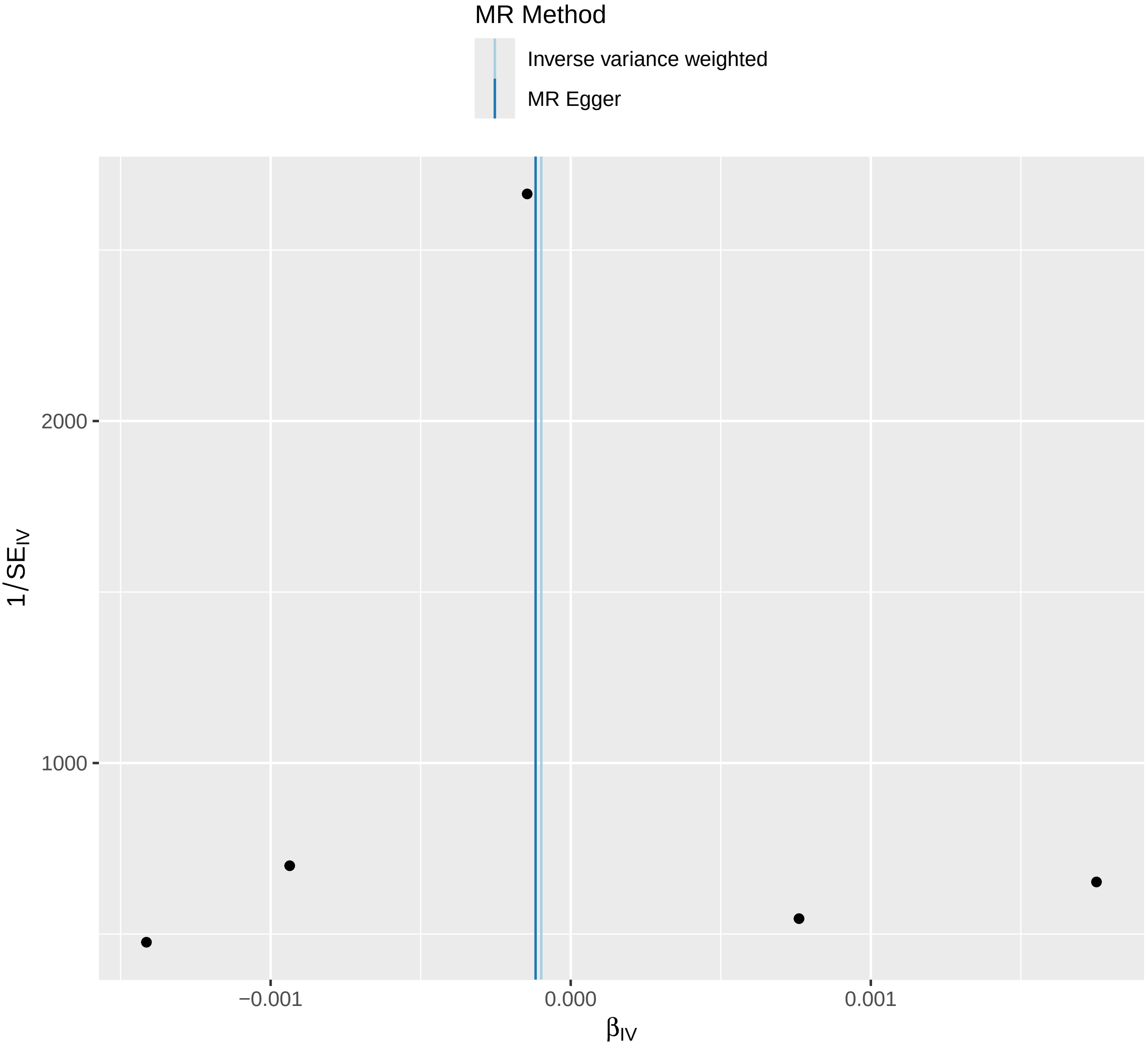
MR Method
Inverse variance weighted
MR Egger
2000
1000
1/SE_IV
−0.001
0.000
0.001
β_IV

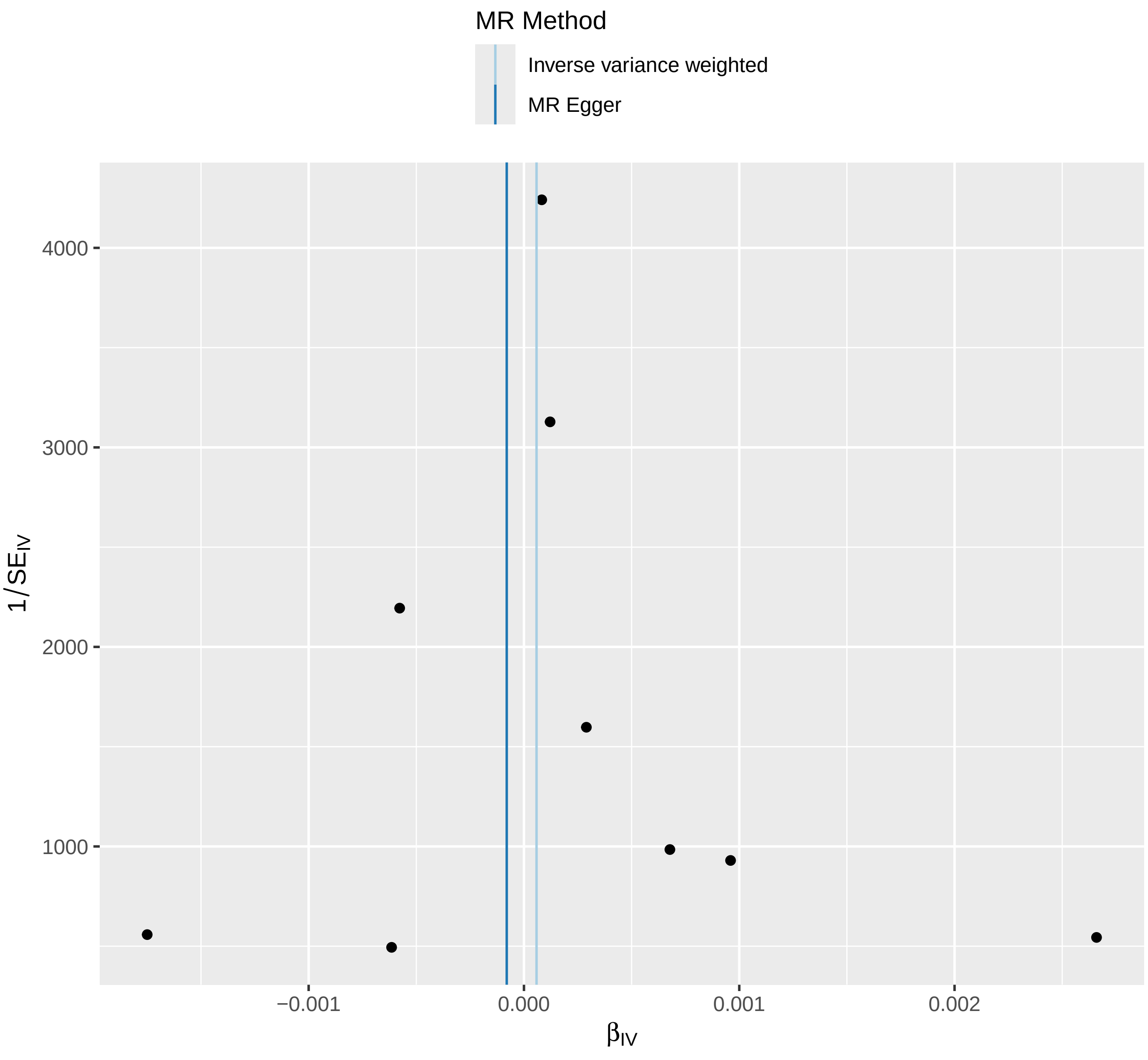

MR Method
Inverse variance weighted
MR Egger
4000
3000
2000
1000
−0.001
0.000
0.001
0.002
$1/SE_{IV}$
β_{IV}

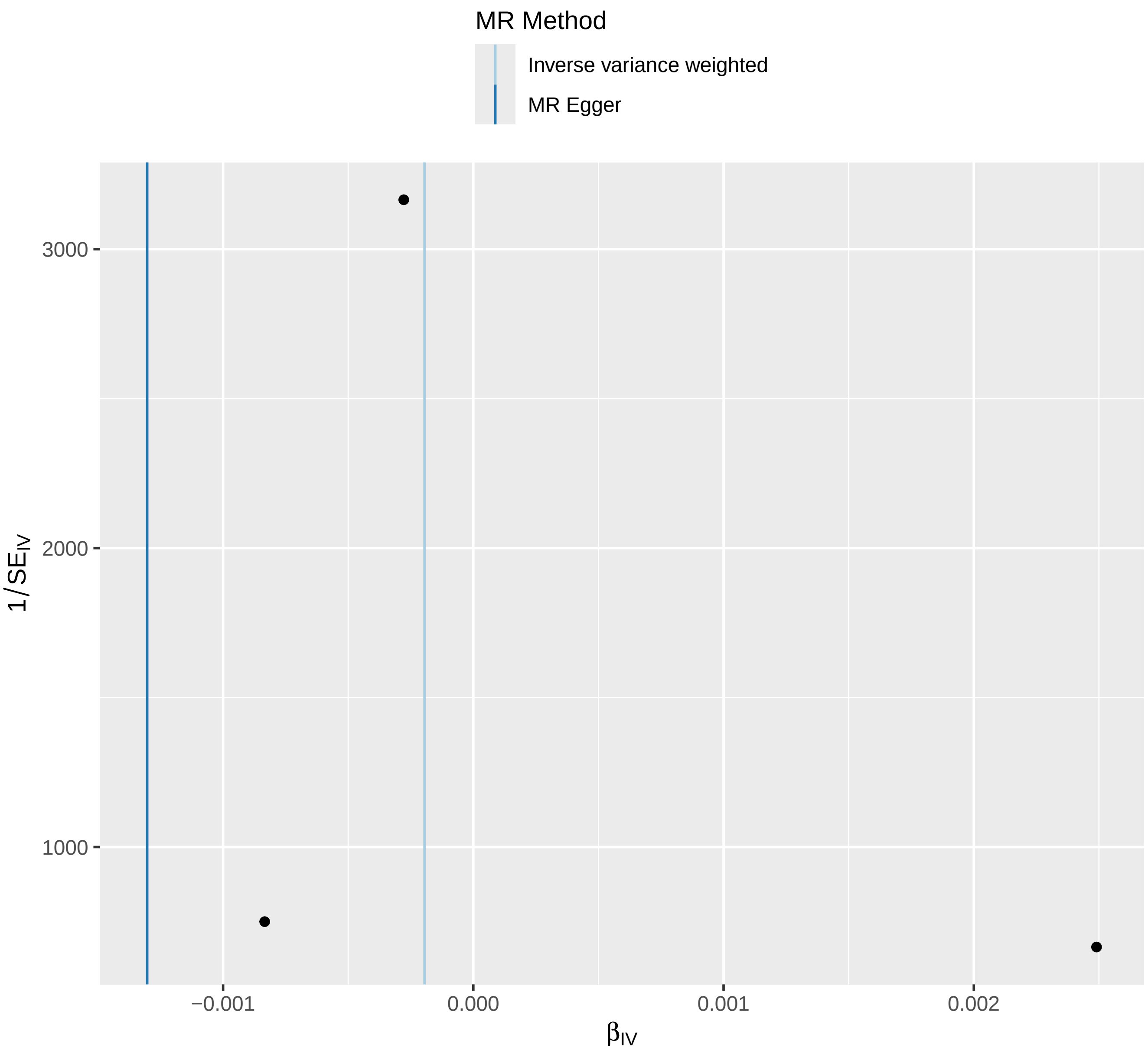

MR Method
Inverse variance weighted
MR Egger
3000
2000
1000
1/SE$_{IV}$
−0.001
0.000
0.001
0.002
β_{IV}

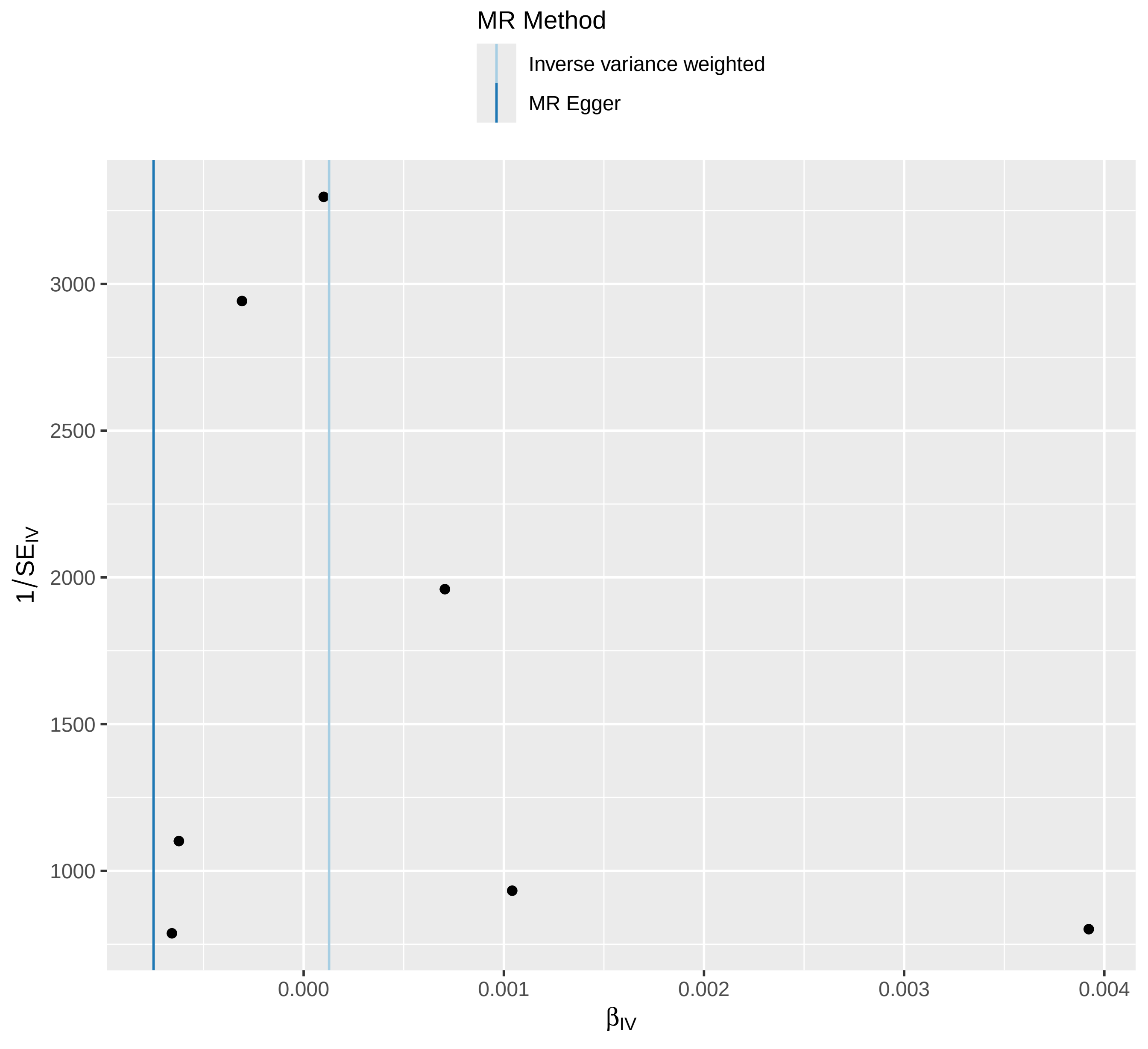
MR Method
Inverse variance weighted
MR Egger
3000
2500
2000
1500
1000
$1/SE_{IV}$
0.000
0.001
0.002
0.003
0.004
β_{IV}

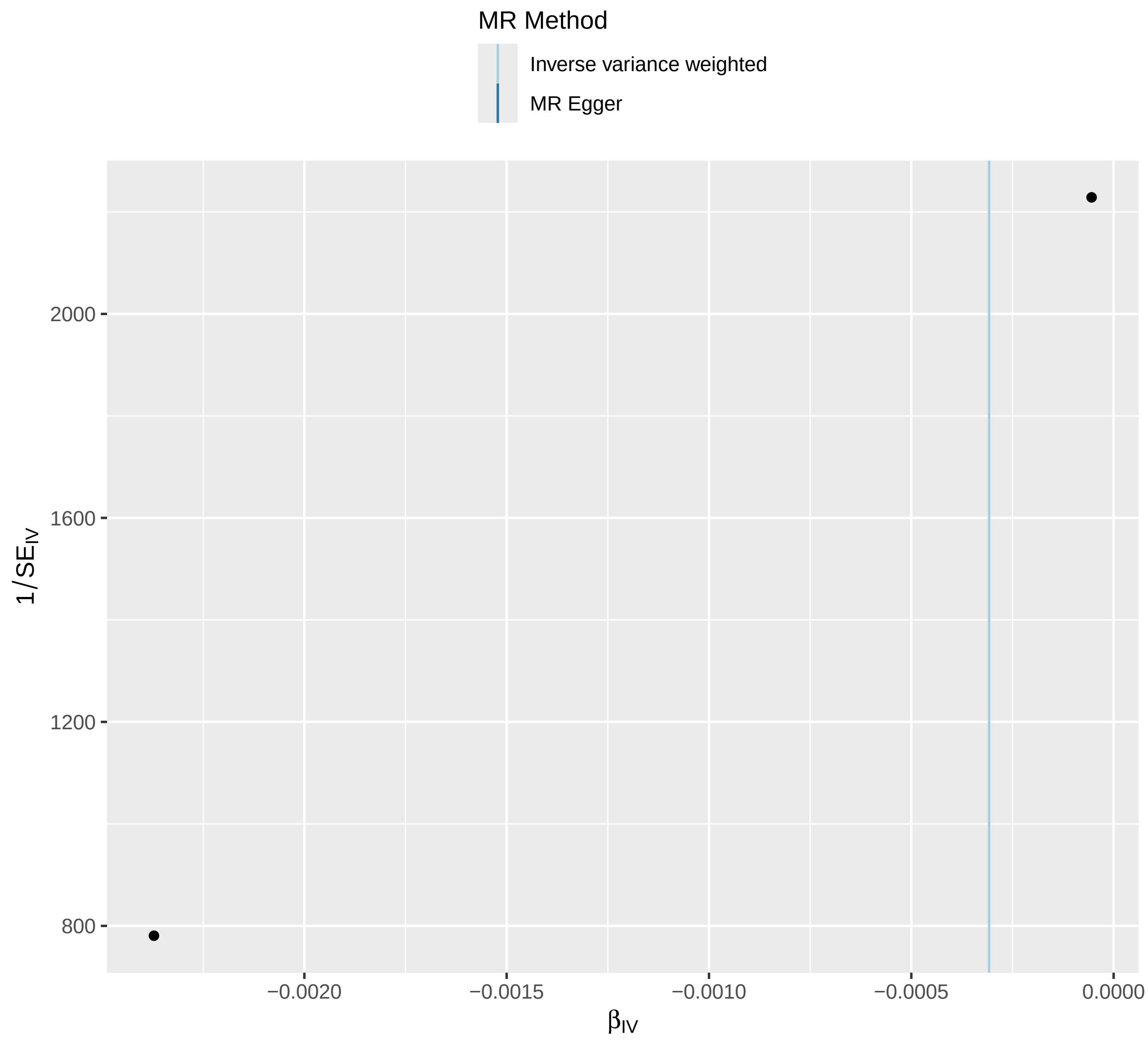
MR Method
Inverse variance weighted
MR Egger
2000
1600
1200
800
1/SE_IV
−0.0020
−0.0015
−0.0010
−0.0005
0.0000
β_IV

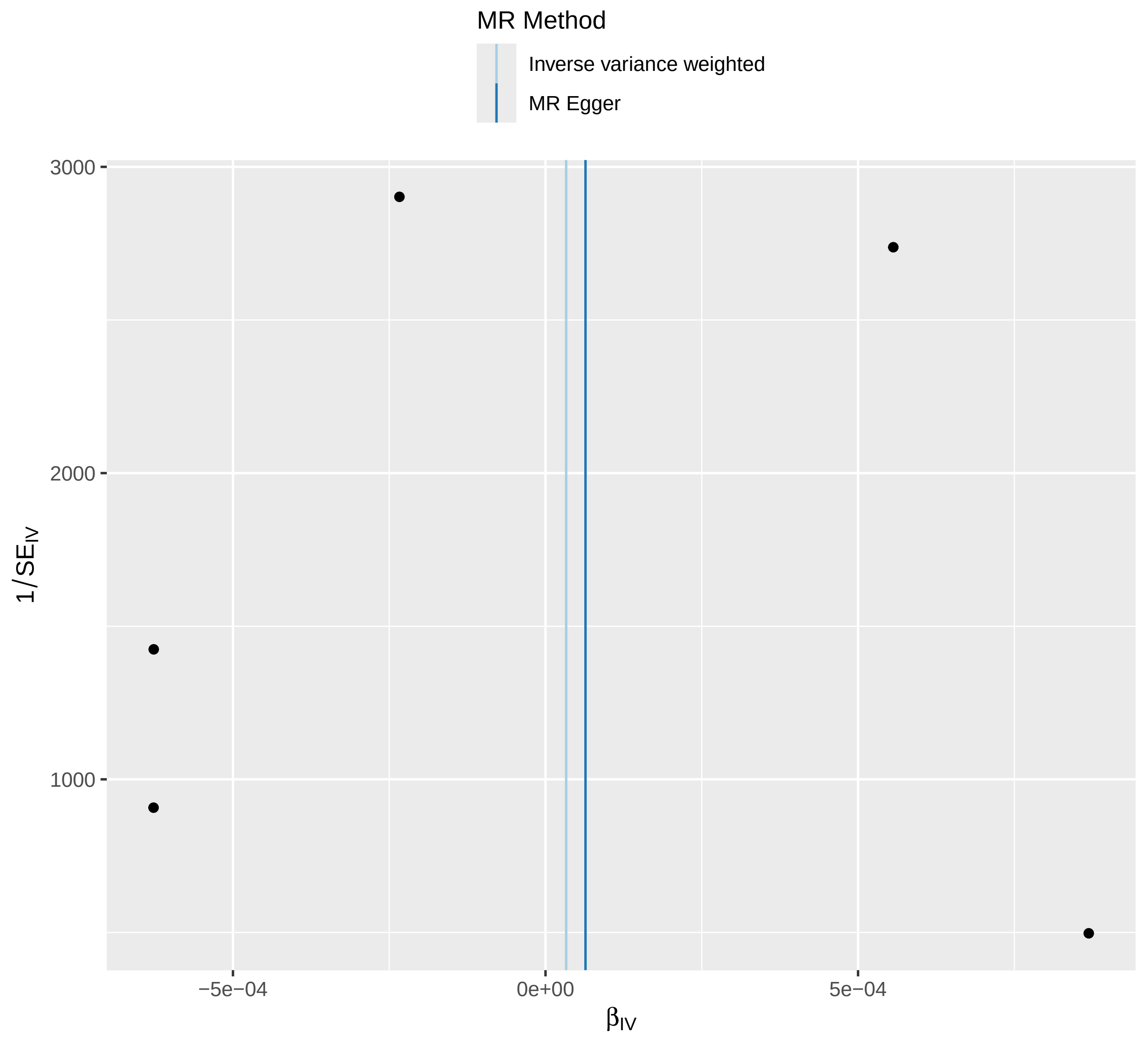

MR Method
Inverse variance weighted
MR Egger
3000
2000
1000
1/SE$_{IV}$
−5e−04
0e+00
5e−04
β$_{IV}$

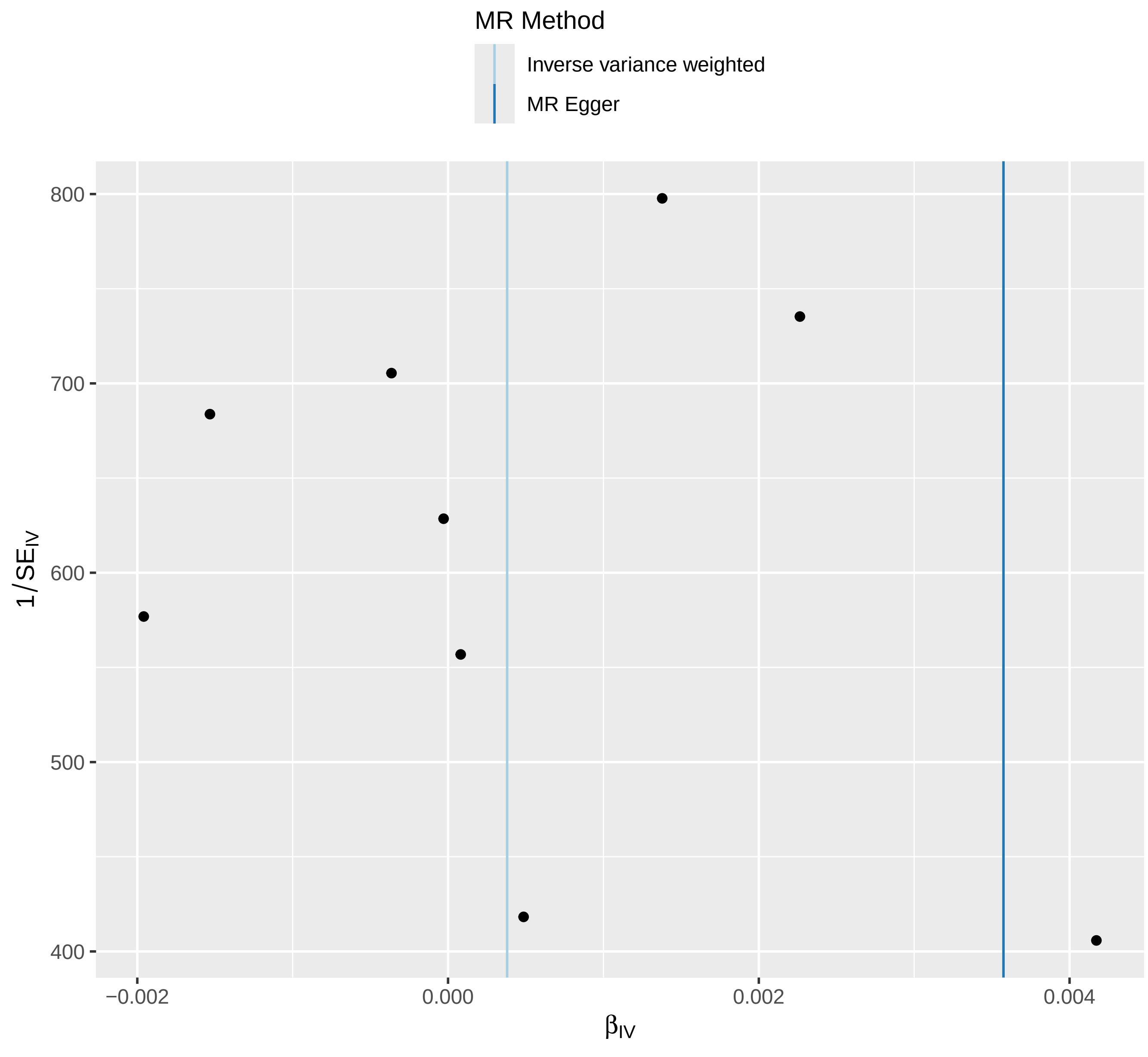
MR Method
Inverse variance weighted
MR Egger
800
700
600
500
400
$1/SE_{IV}$
−0.002
0.000
0.002
0.004
β_{IV}

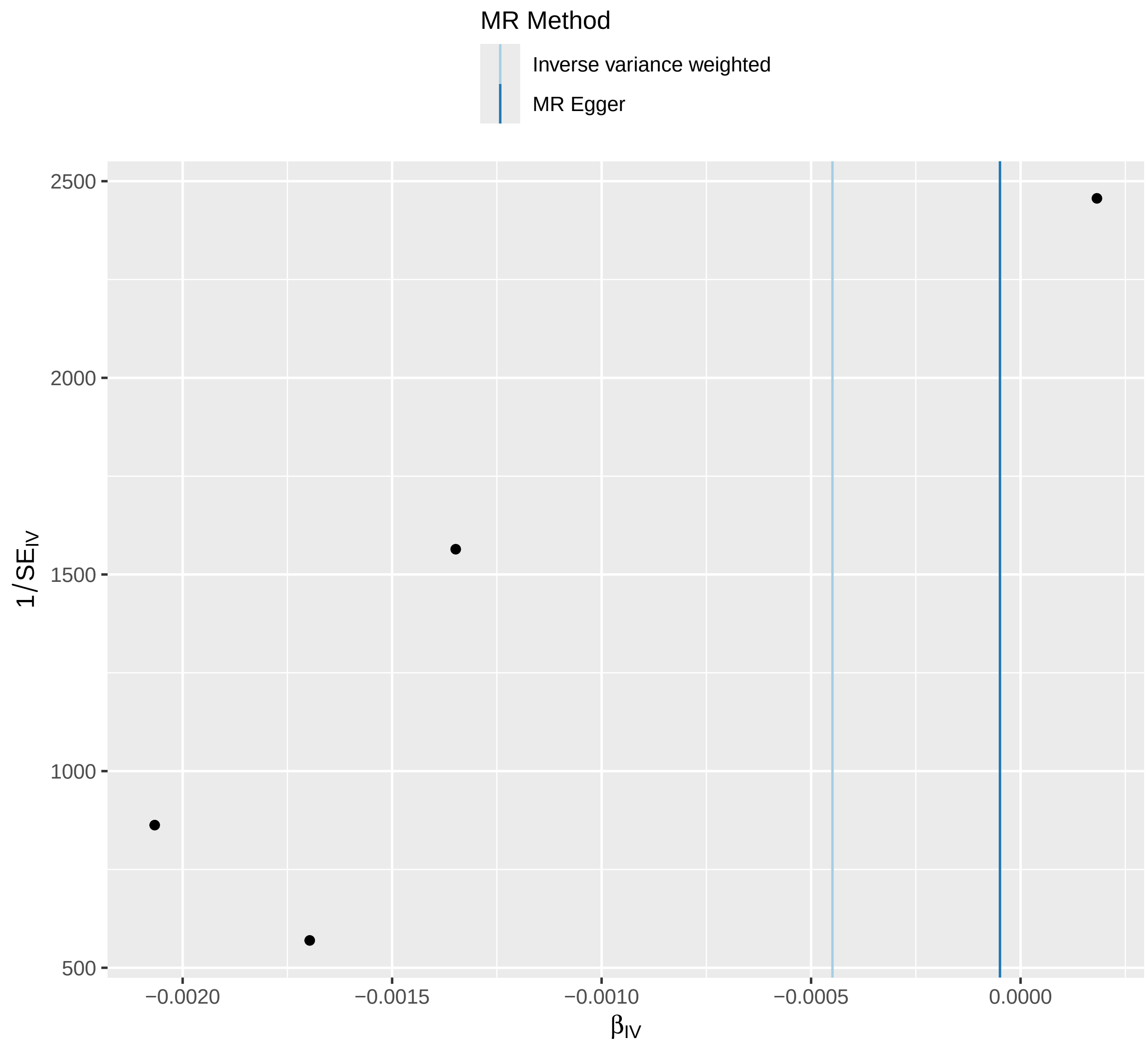

MR Method
Inverse variance weighted
MR Egger
2500
2000
1500
1000
500
1/SE_IV
−0.0020
−0.0015
−0.0010
−0.0005
0.0000
β_IV

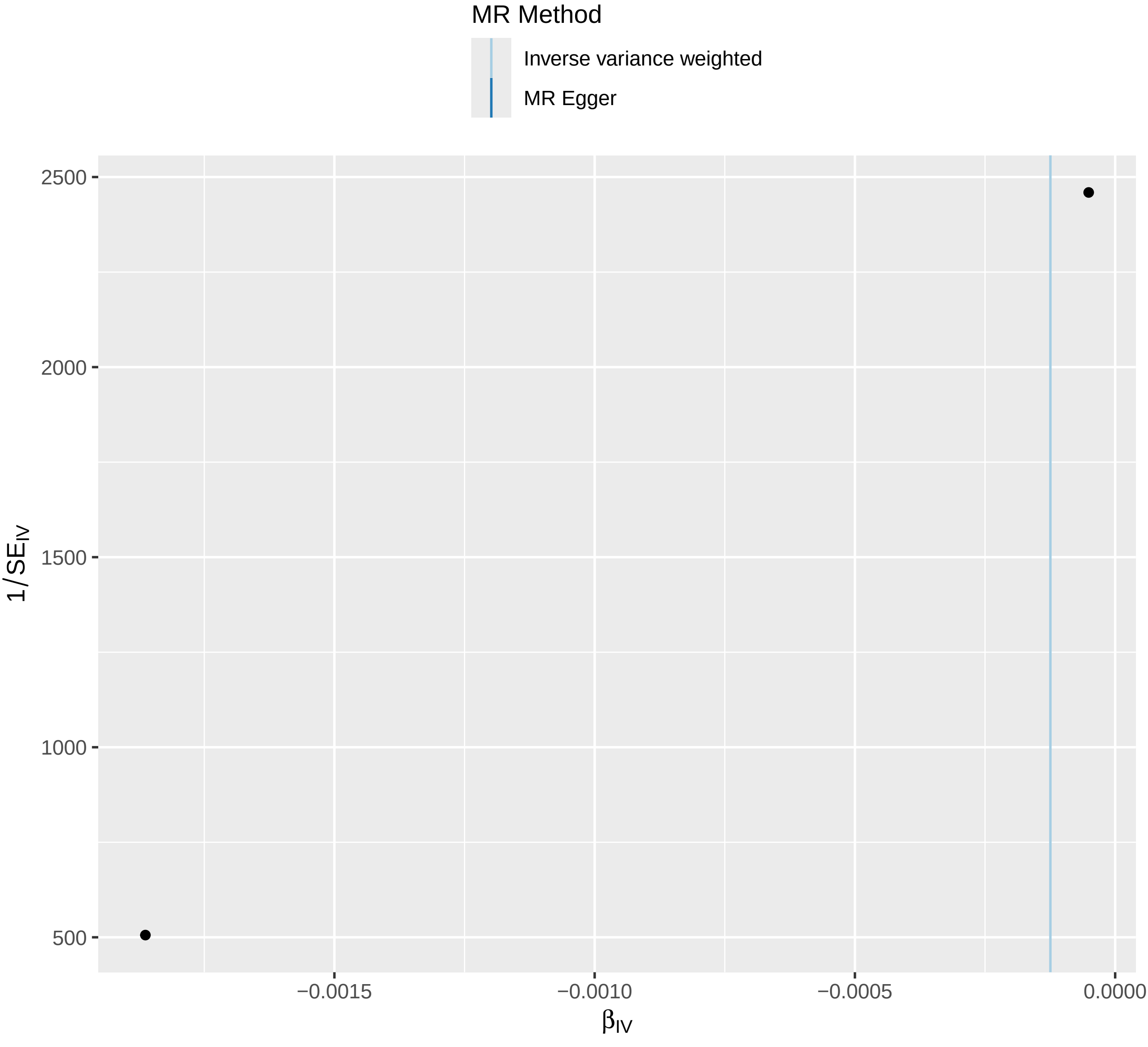

MR Method
Inverse variance weighted
MR Egger
2500
2000
1500
1000
500
$1/SE_{IV}$
−0.0015
−0.0010
−0.0005
0.0000
β_{IV}

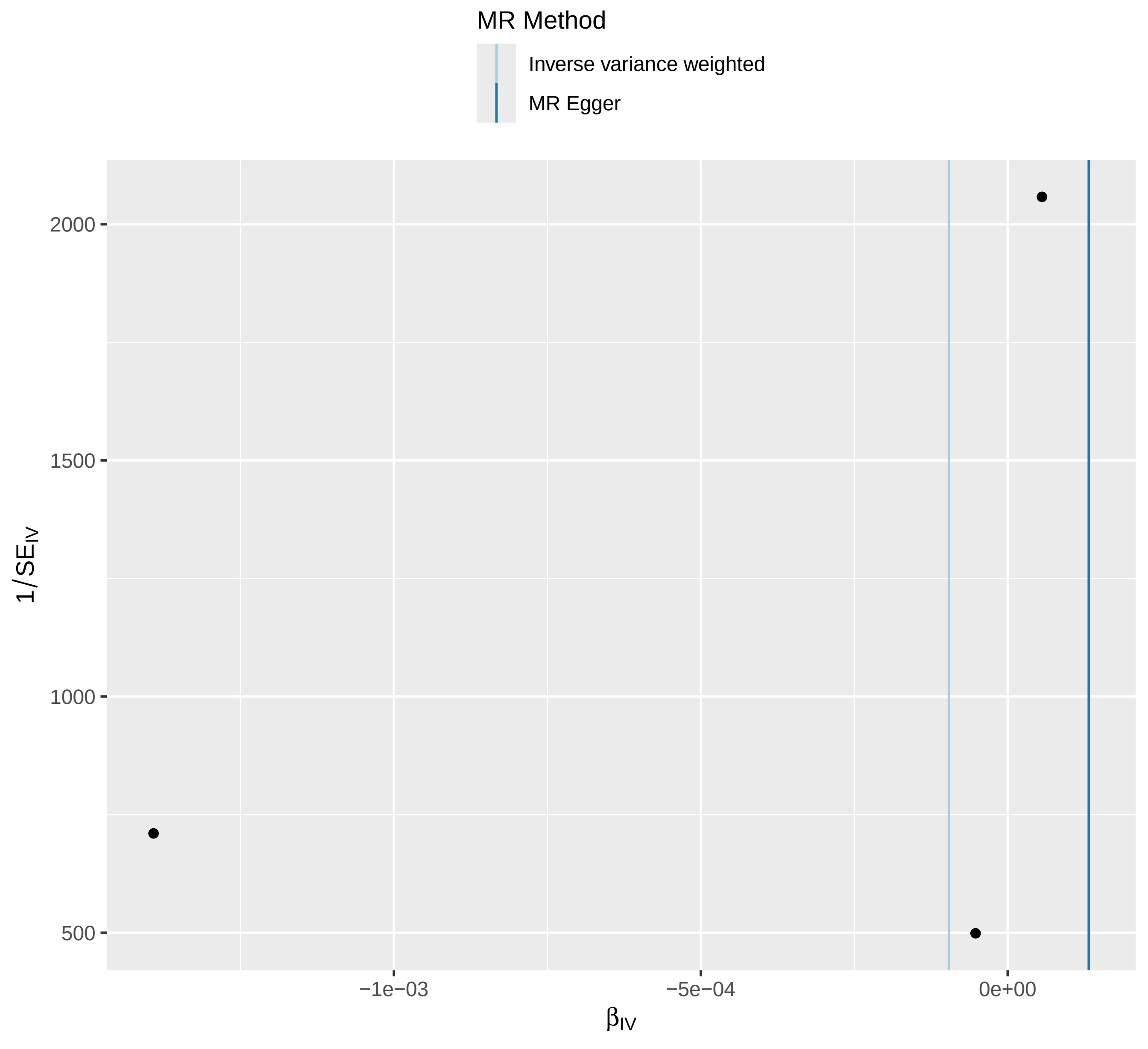

MR Method
Inverse variance weighted
MR Egger
2000
1500
1000
500
1/SE_IV
−1e−03
−5e−04
0e+00
β_IV

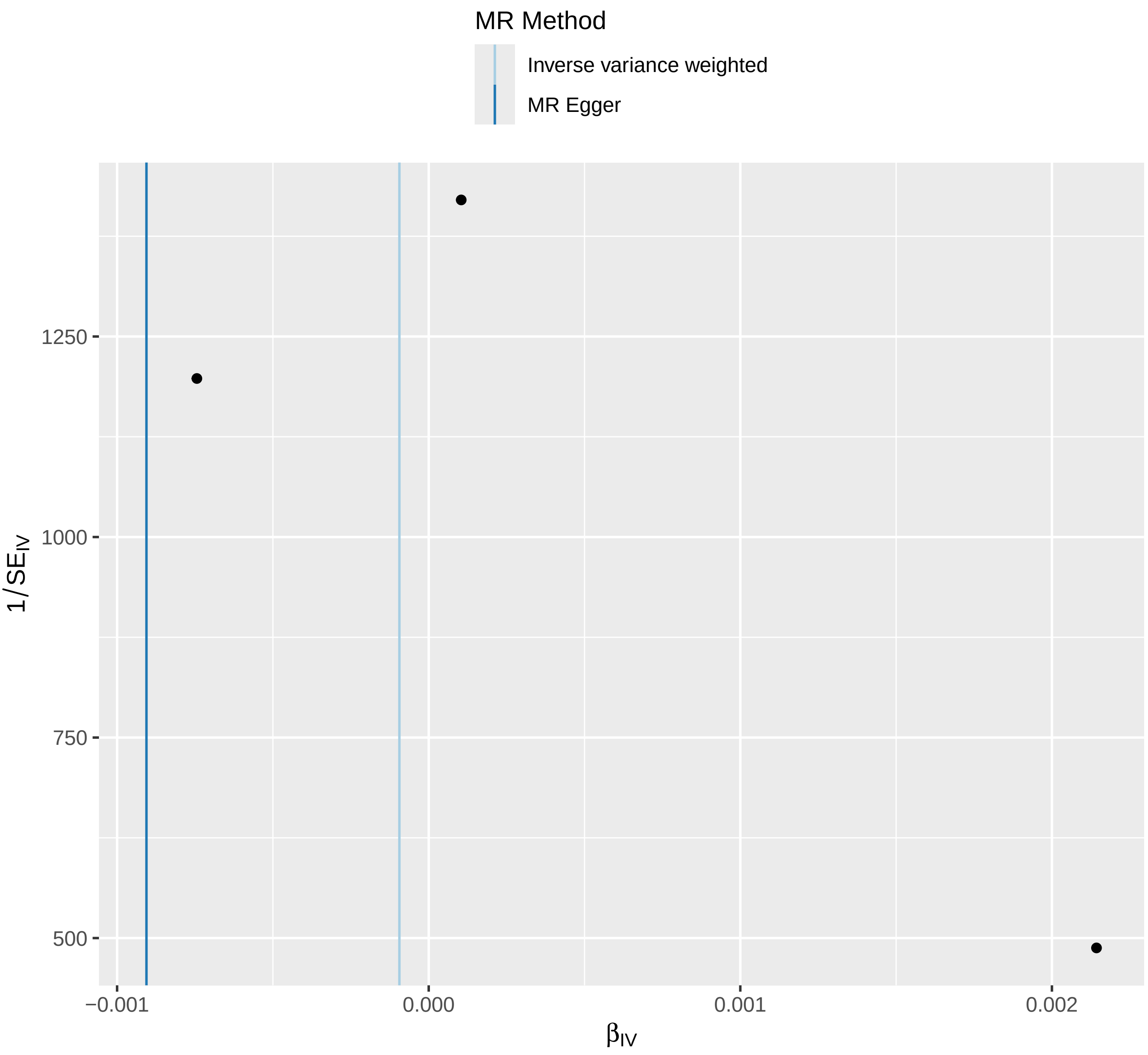

MR Method
Inverse variance weighted
MR Egger
1250
1000
750
500
$1/SE_{IV}$
−0.001
0.000
0.001
0.002
β_{IV}

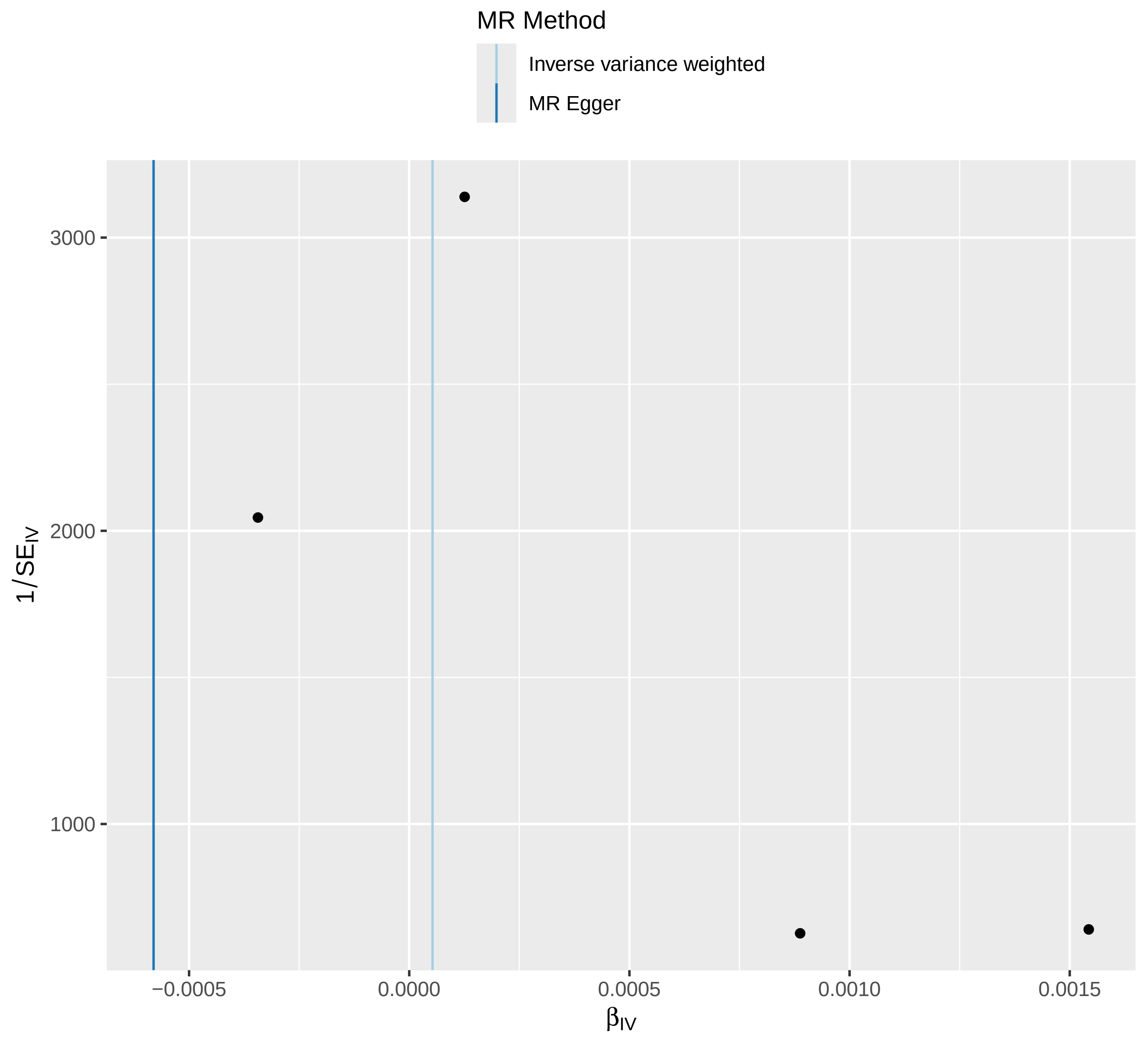

MR Method
Inverse variance weighted
MR Egger
3000
2000
1000
1/SE$_{IV}$
−0.0005
0.0000
0.0005
0.0010
0.0015
β$_{IV}$

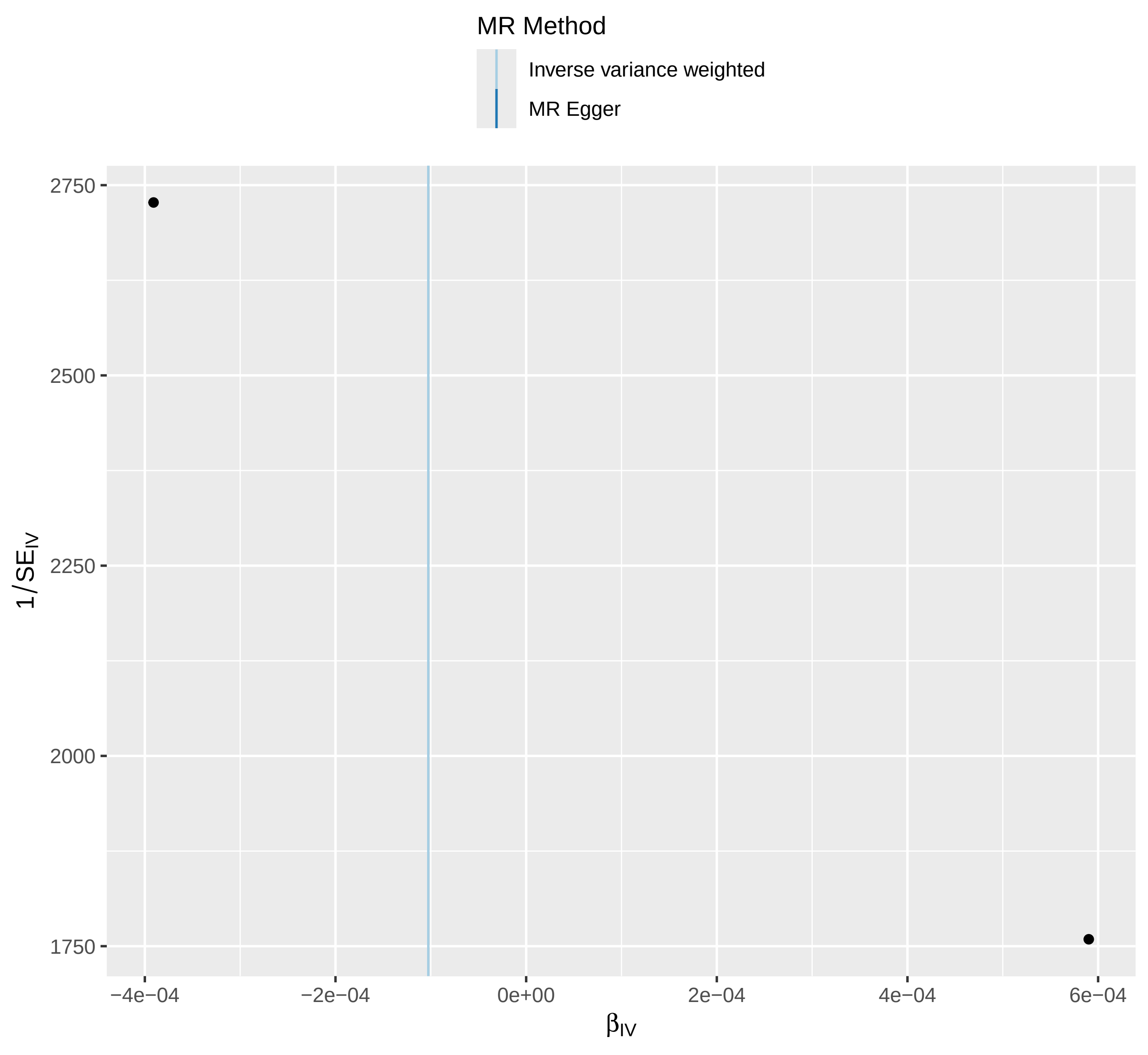
MR Method
Inverse variance weighted
MR Egger
2750
2500
2250
2000
1750
1/SE_IV
−4e−04
−2e−04
0e+00
2e−04
4e−04
6e−04
β_IV

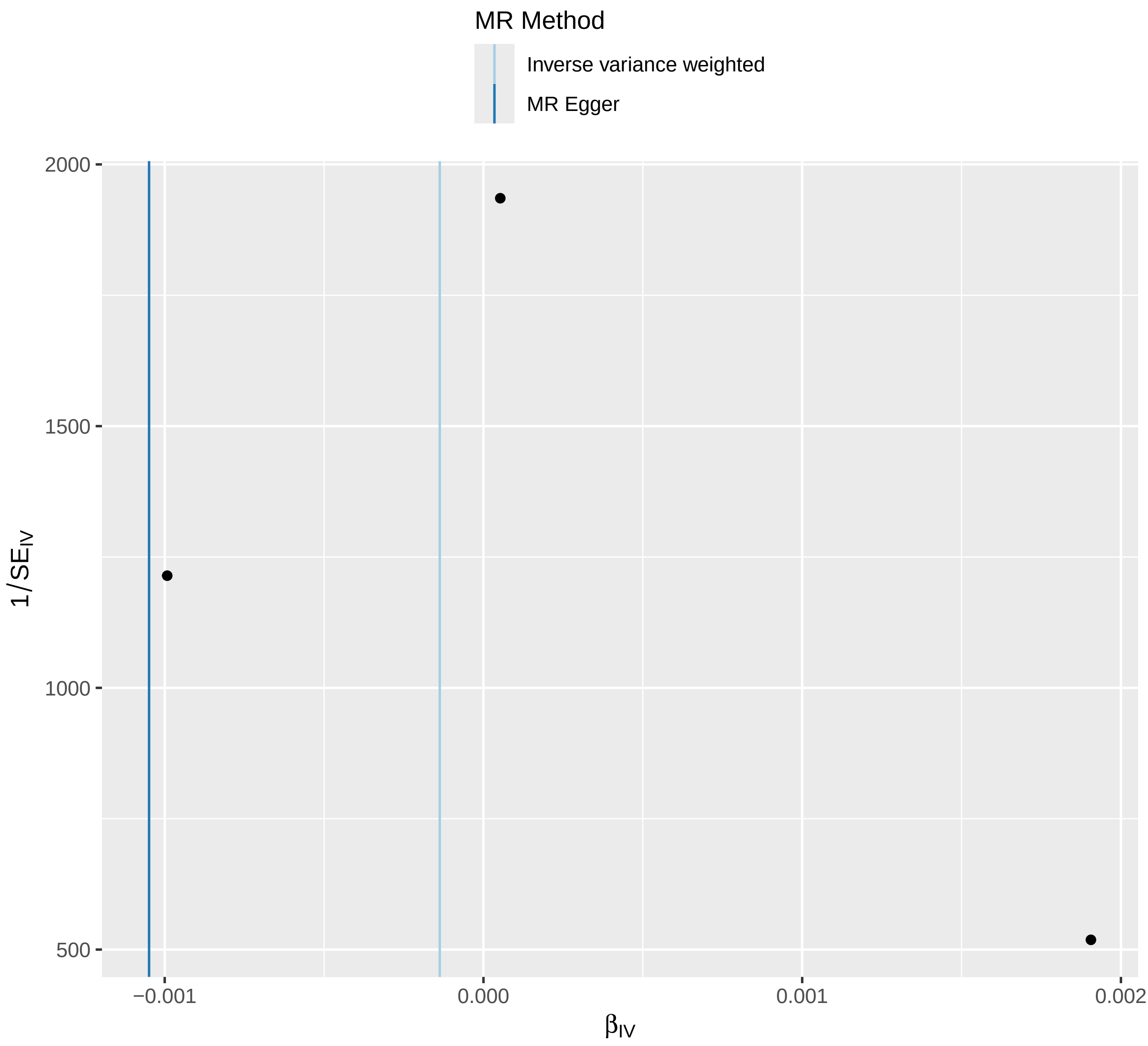

MR Method
Inverse variance weighted
MR Egger
2000
1500
1000
500
1/SE_IV
−0.001
0.000
0.001
0.002
β_IV

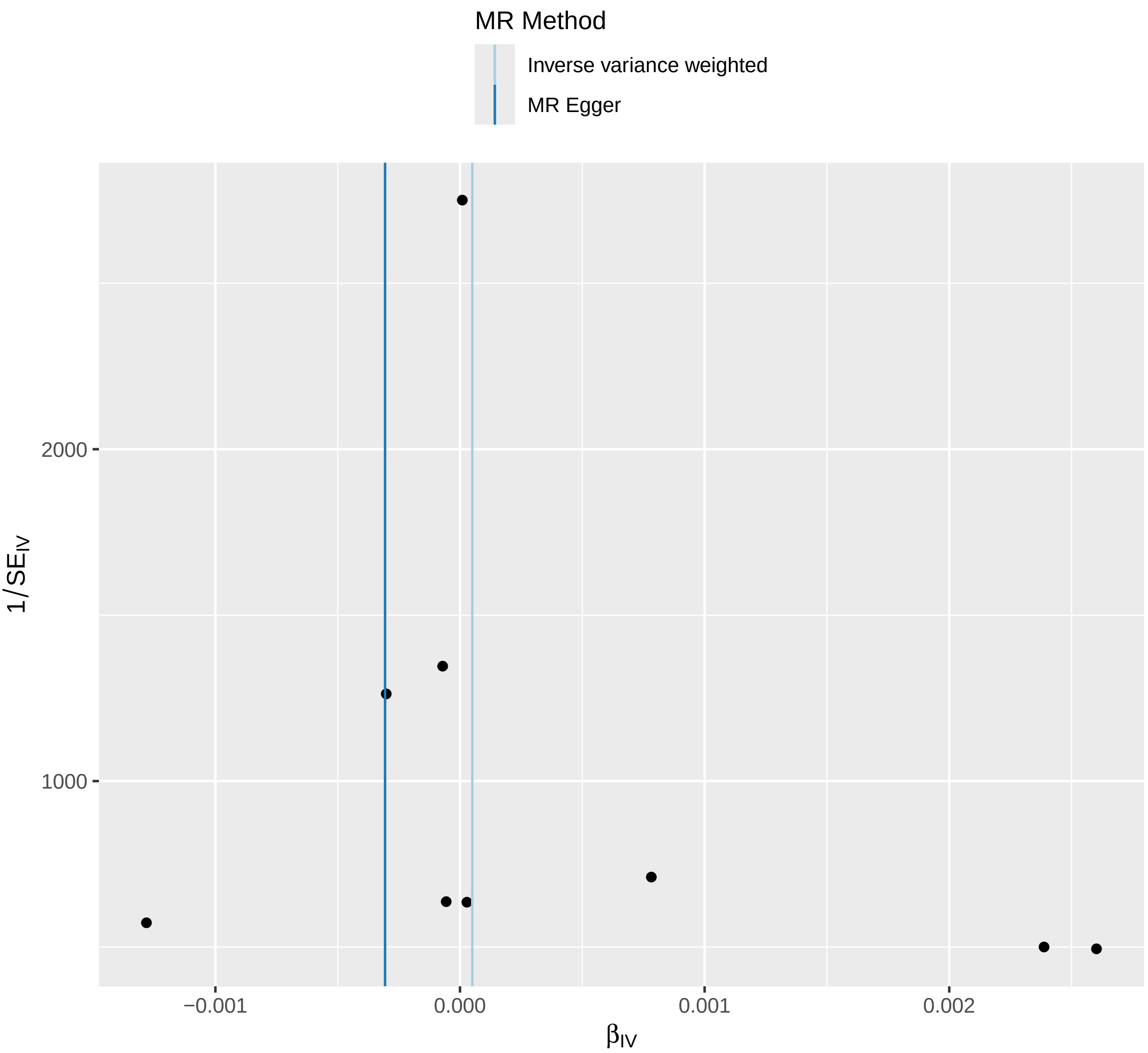

MR Method
Inverse variance weighted
MR Egger
2000
1000
1/SE_IV
−0.001
0.000
0.001
0.002
β_IV

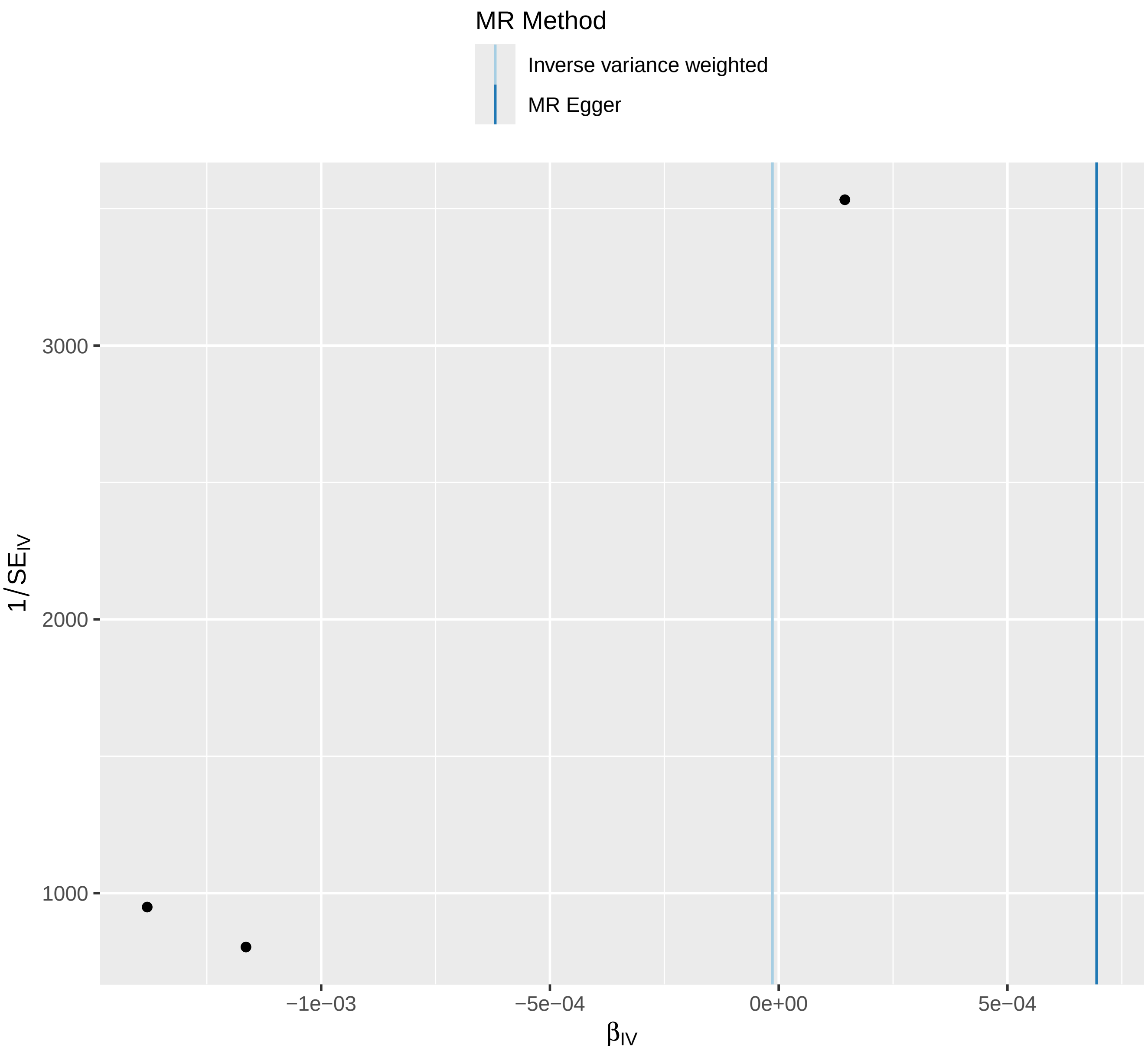

MR Method
Inverse variance weighted
MR Egger
1/SE_IV
3000
2000
1000
−1e−03
−5e−04
0e+00
5e−04
β_IV

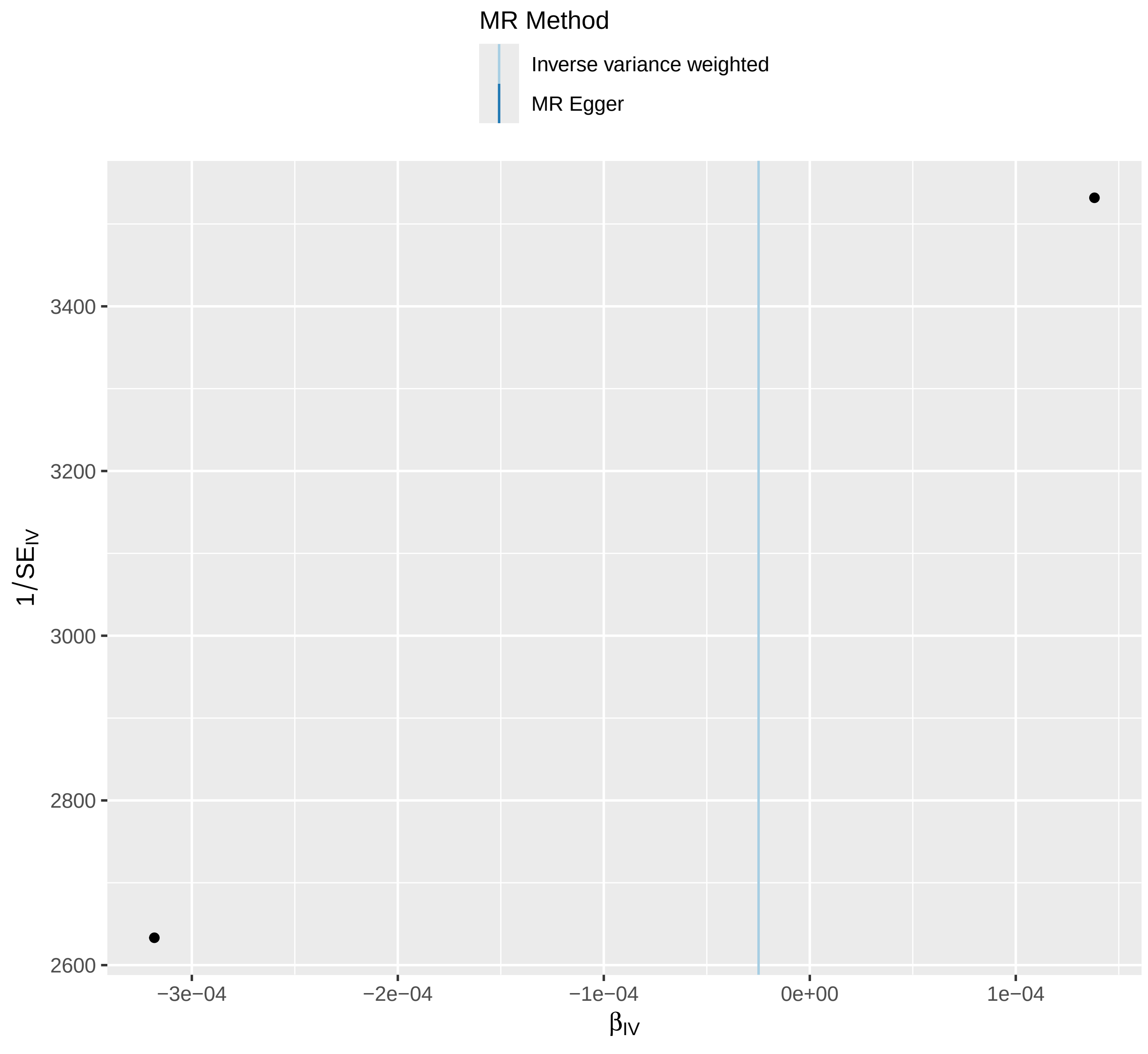

MR Method
Inverse variance weighted
MR Egger
3400
3200
3000
2800
2600
1/SE$_{IV}$
−3e−04
−2e−04
−1e−04
0e+00
1e−04
β$_{IV}$

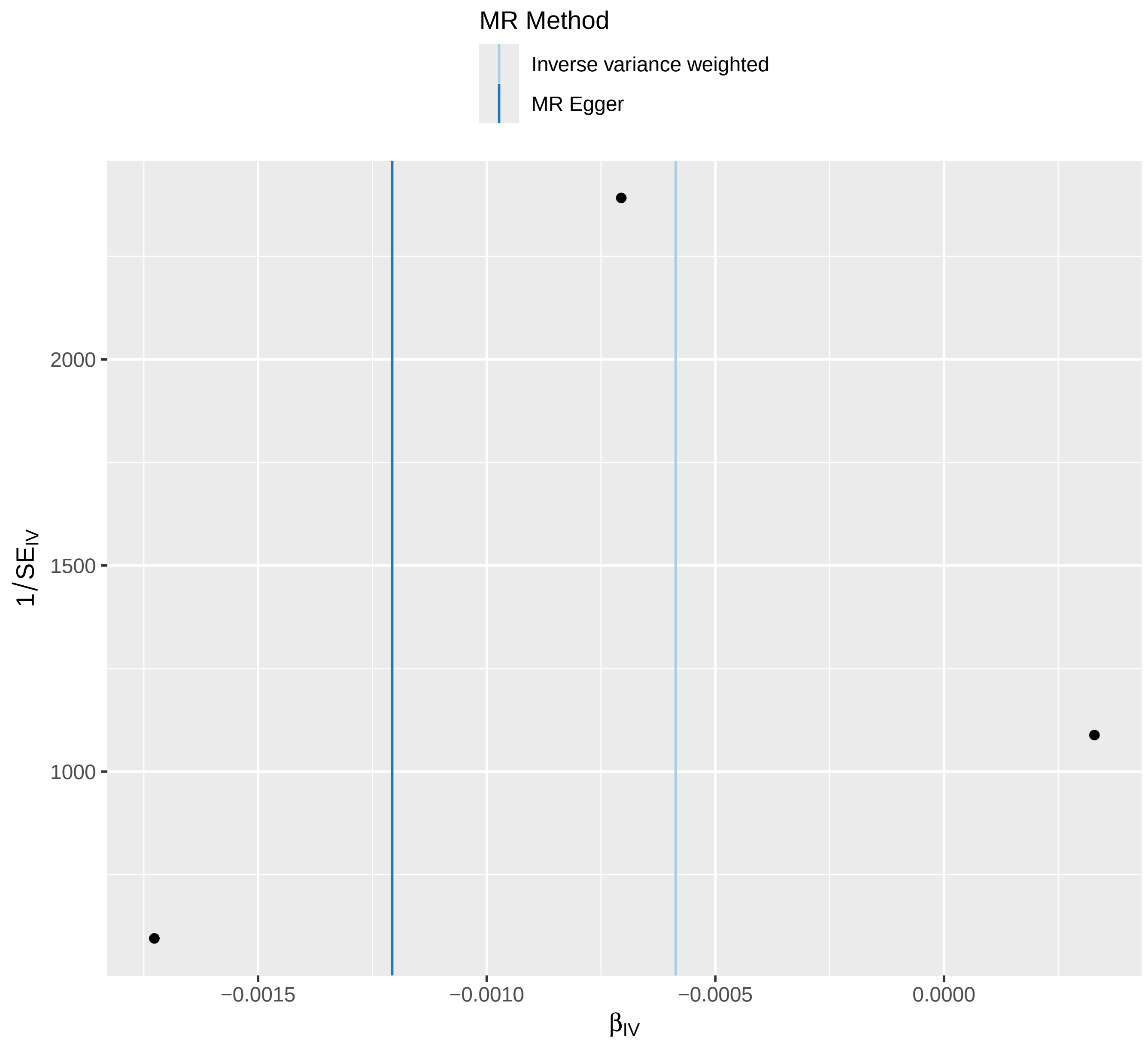
MR Method
Inverse variance weighted
MR Egger
2000
1500
1000
$1/SE_{IV}$
−0.0015
−0.0010
−0.0005
0.0000
β_{IV}

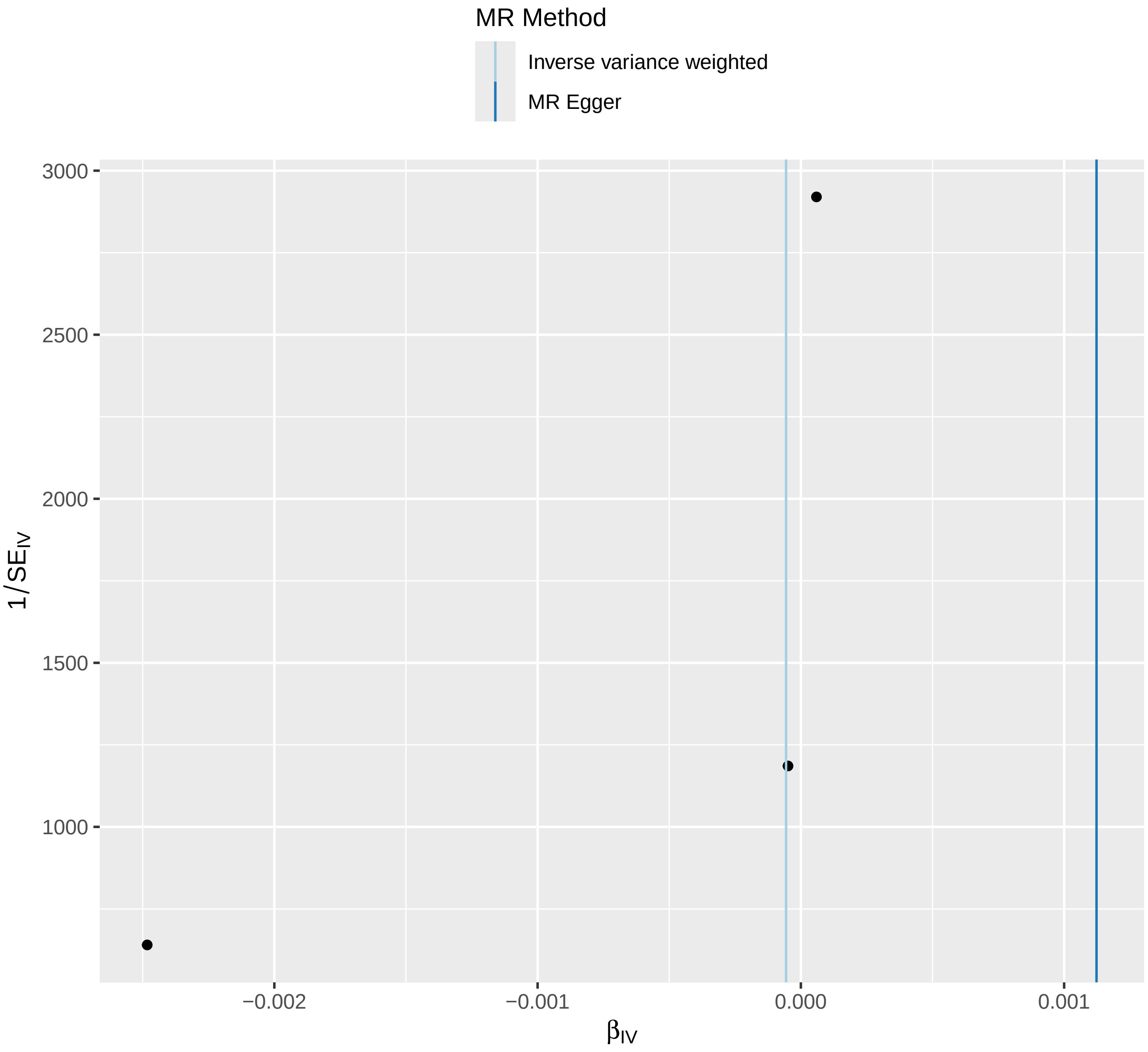
MR Method
Inverse variance weighted
MR Egger
3000
2500
2000
1500
1000
$1/SE_{IV}$
−0.002
−0.001
0.000
0.001
β_{IV}

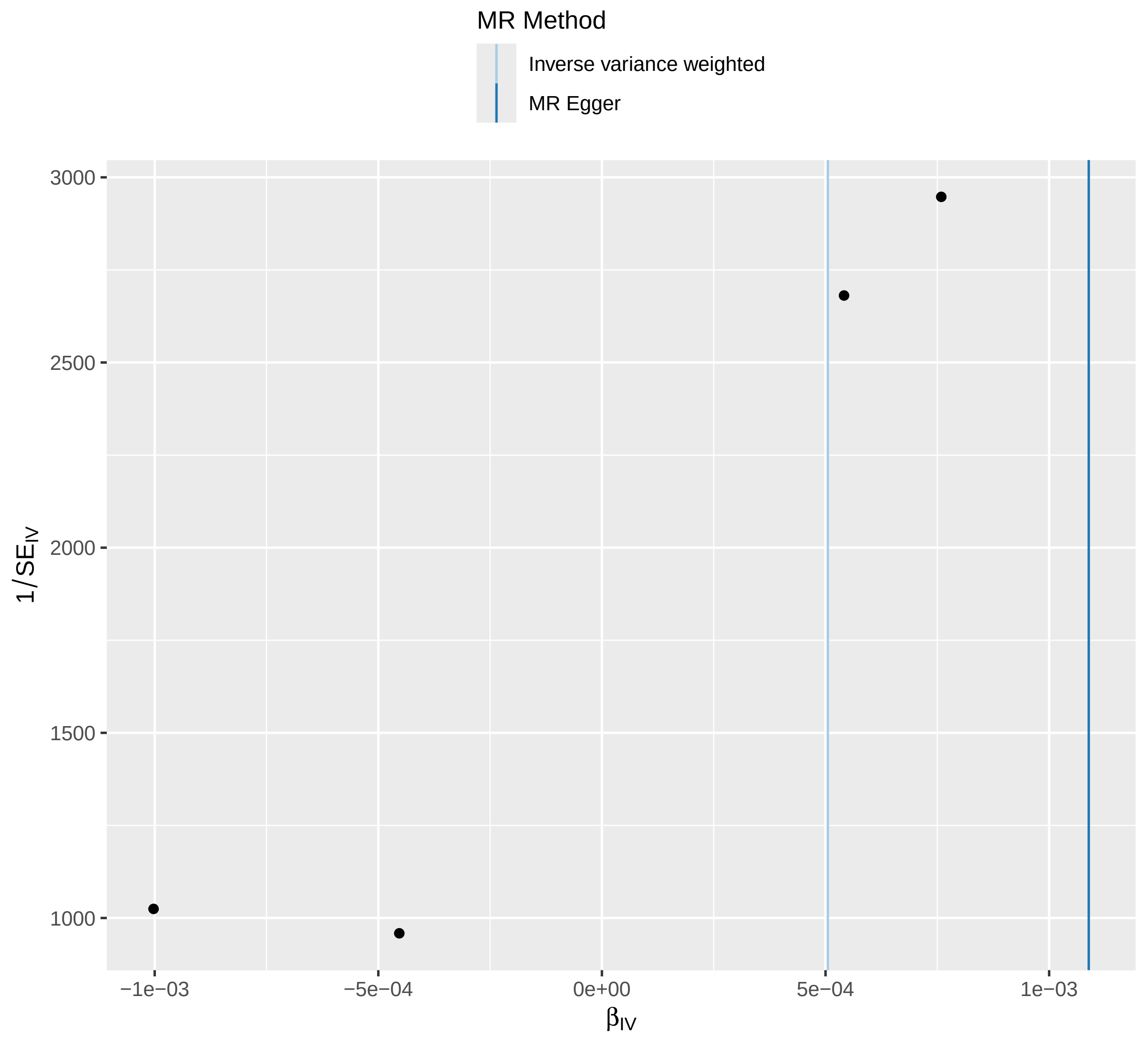
MR Method
Inverse variance weighted
MR Egger
3000
2500
2000
1500
1000
1/SE_IV
−1e−03
−5e−04
0e+00
5e−04
1e−03
β_IV

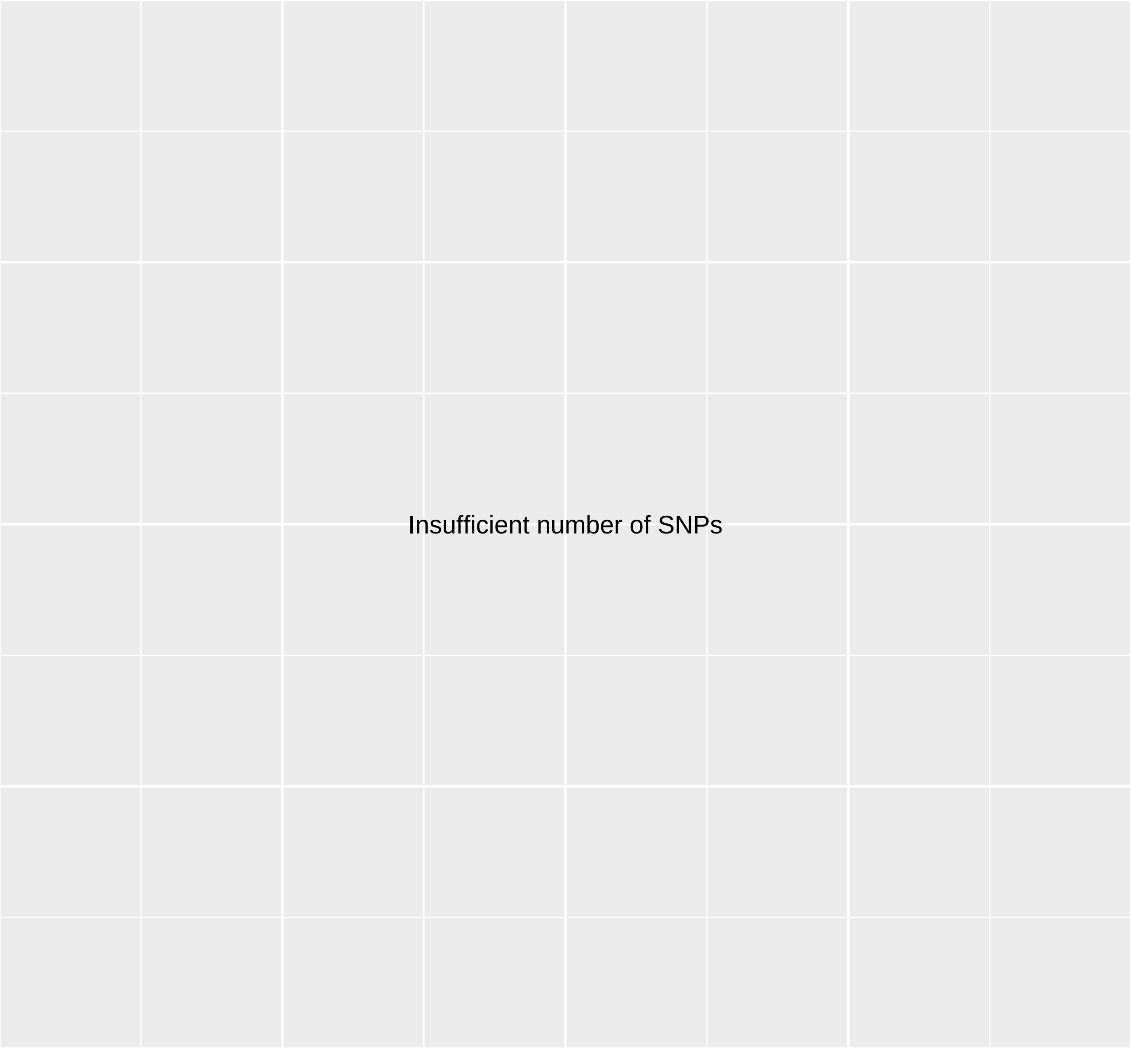
Insufficient number of SNPs

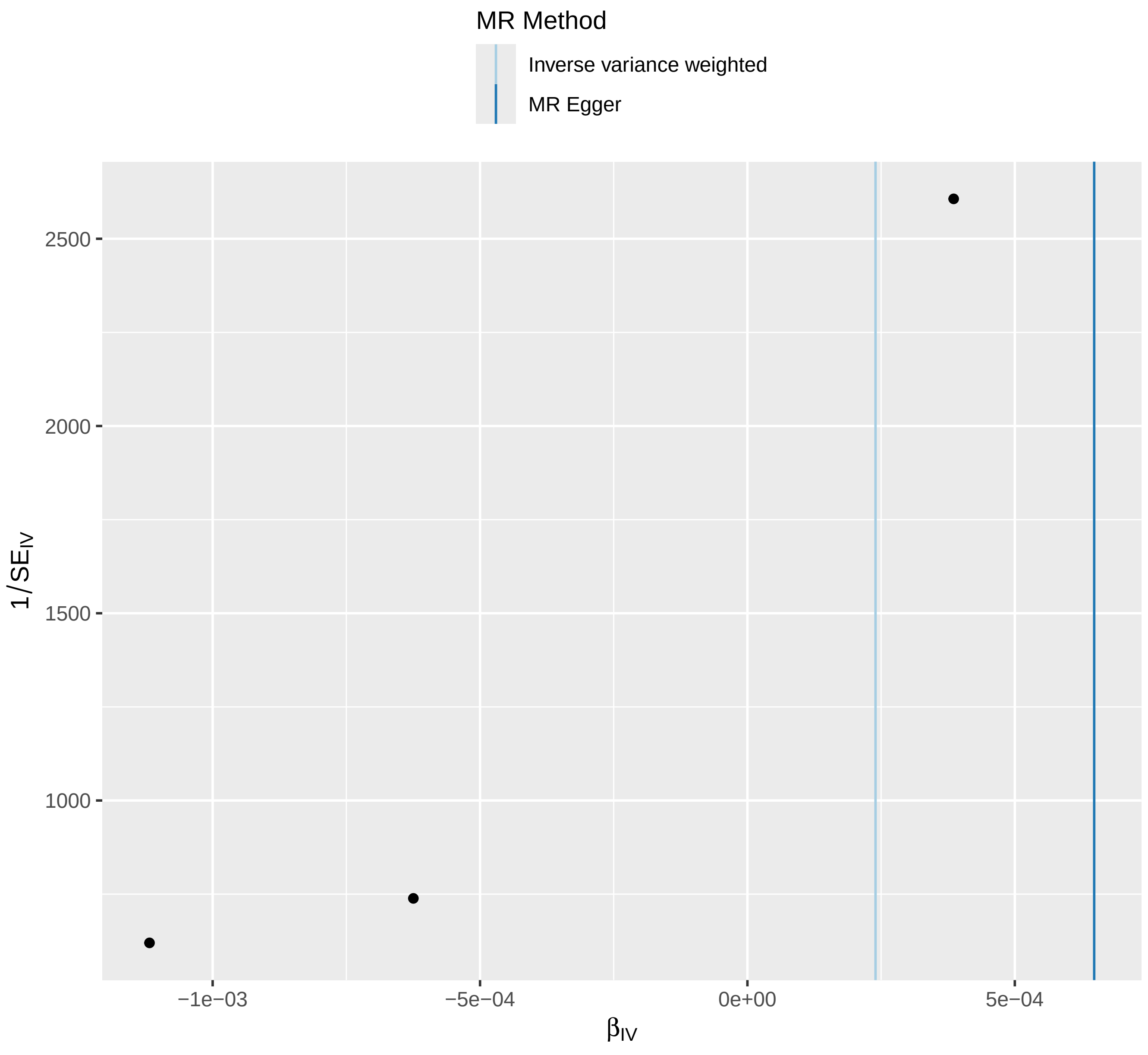
MR Method
Inverse variance weighted
MR Egger
2500
2000
1500
1000
1/SE_IV
−1e−03
−5e−04
0e+00
5e−04
β_IV

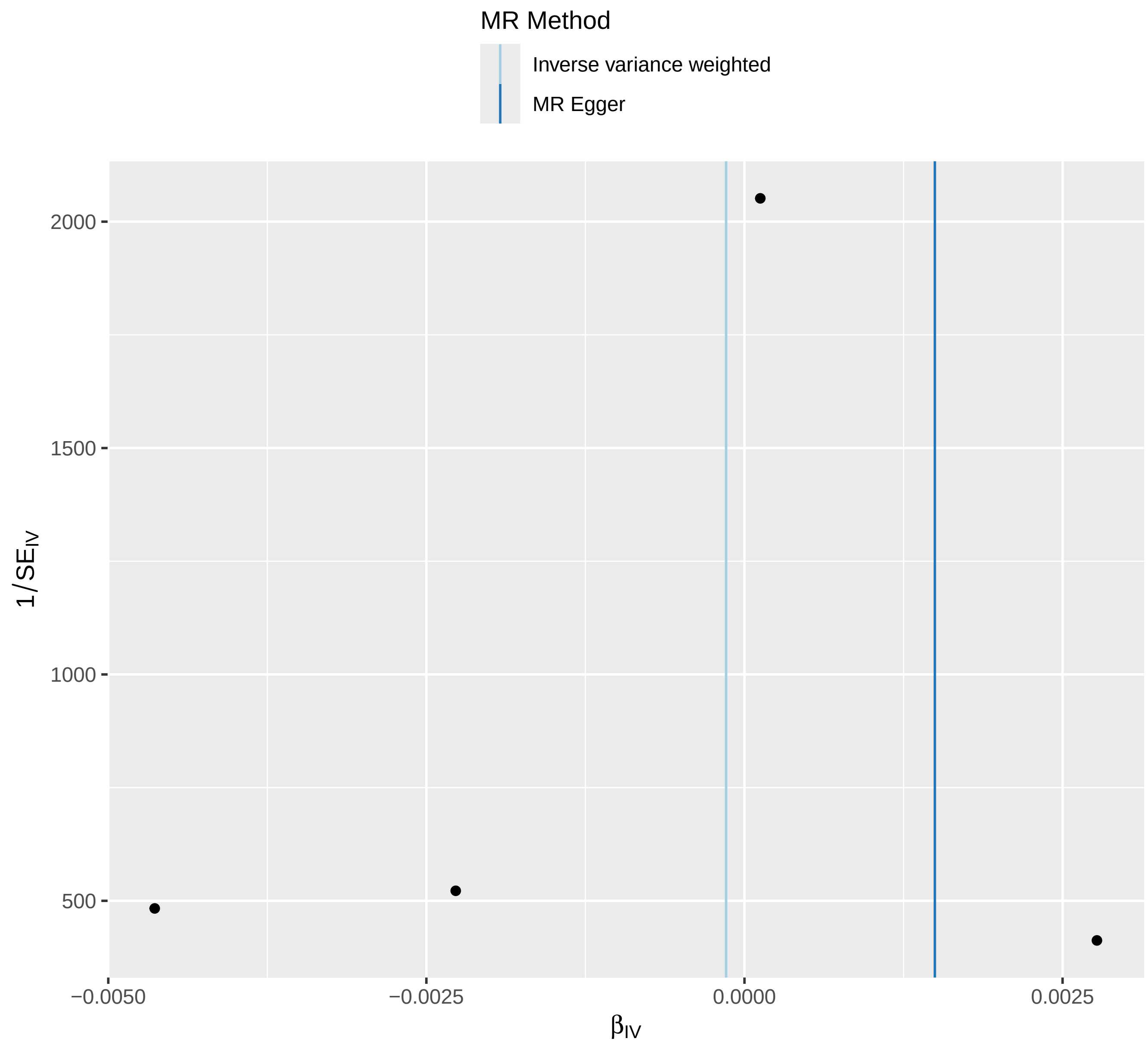

MR Method
Inverse variance weighted
MR Egger
2000
1500
1000
500
1/SE_IV
−0.0050
−0.0025
0.0000
0.0025
β_IV

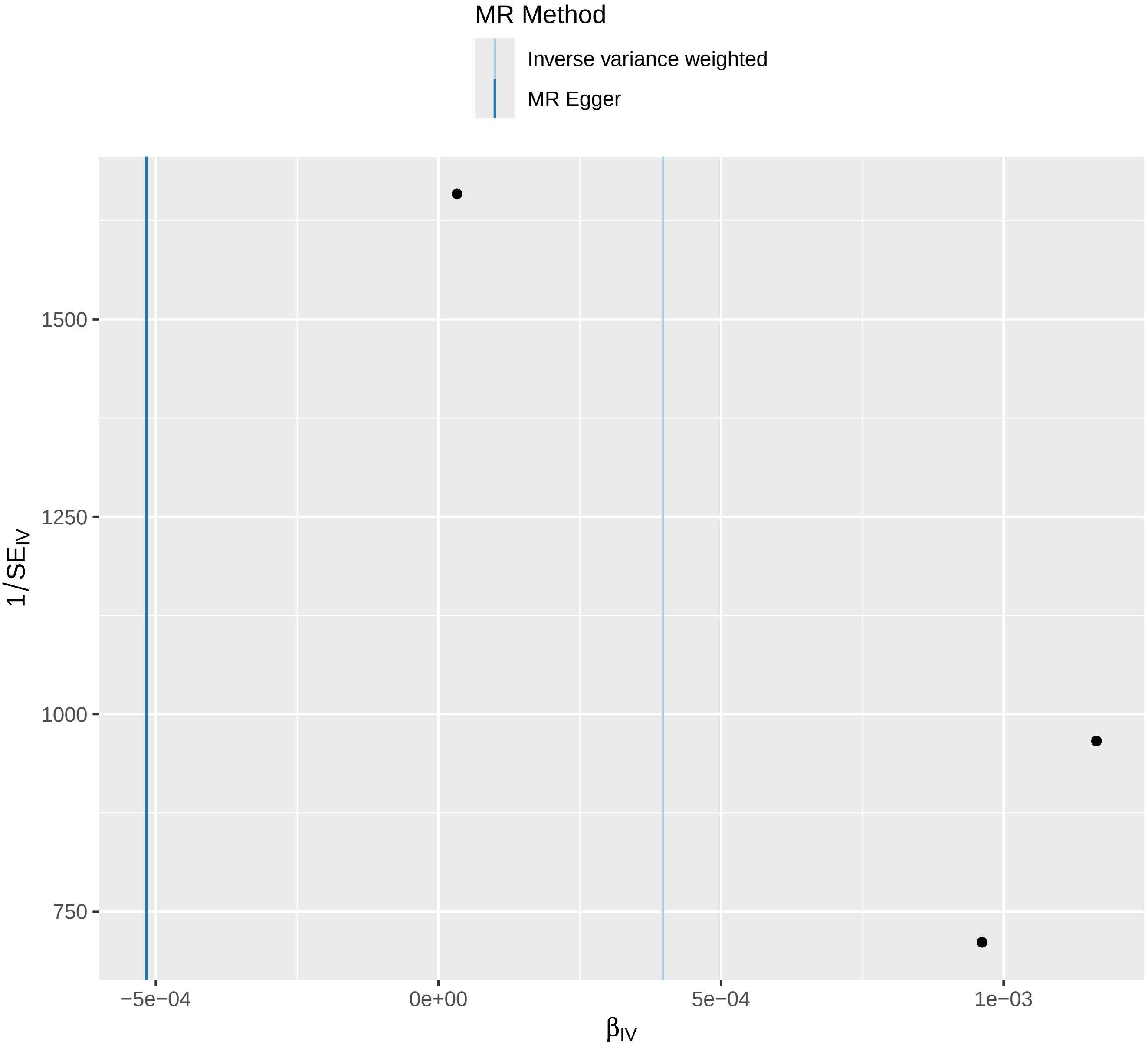

MR Method
Inverse variance weighted
MR Egger
1500
1250
1000
750
1/SE$_{IV}$
−5e−04
0e+00
5e−04
1e−03
β$_{IV}$

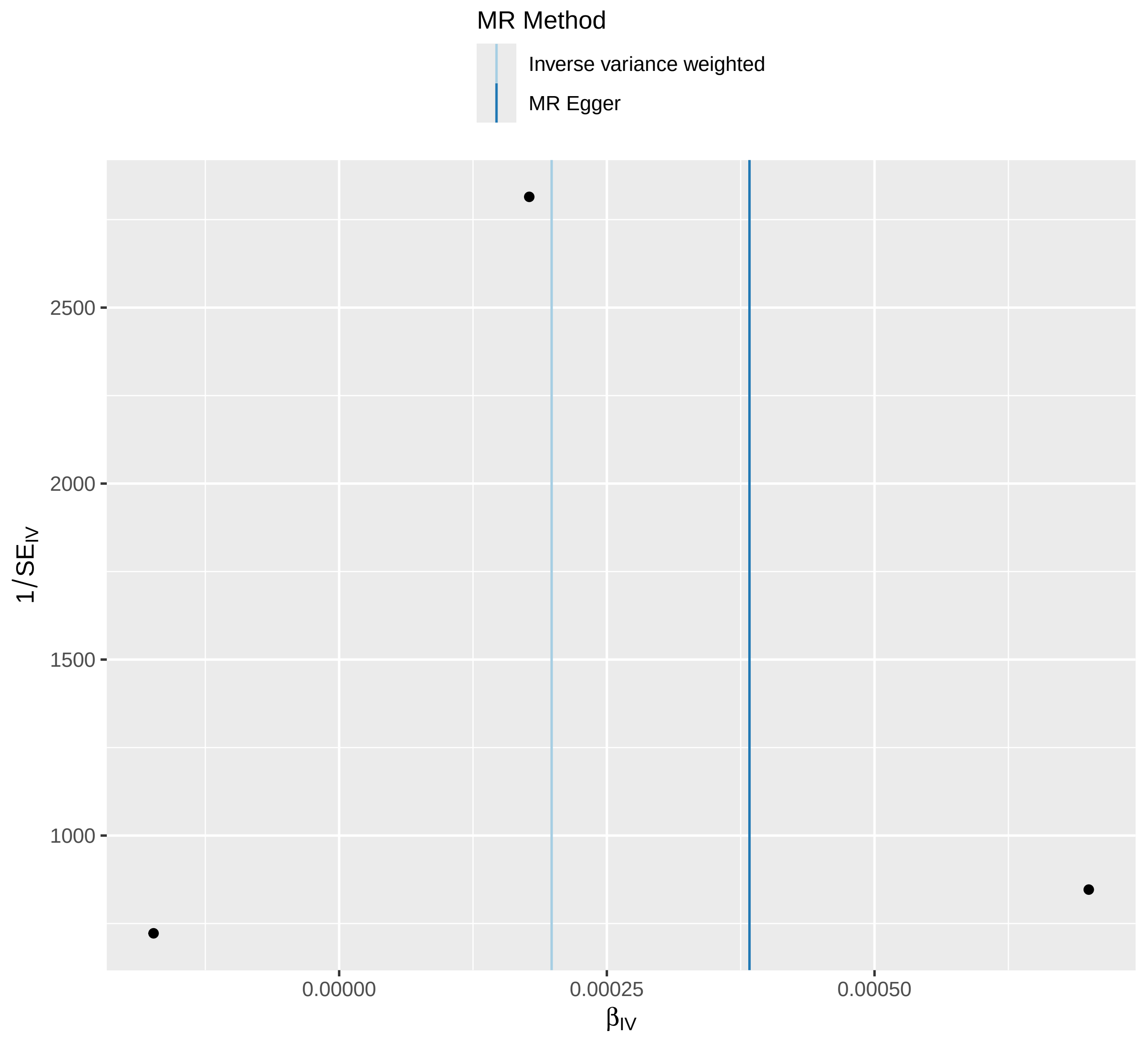

MR Method
Inverse variance weighted
MR Egger
2500
2000
1500
1000
1/SE$_{IV}$
0.00000
0.00025
0.00050
β$_{IV}$

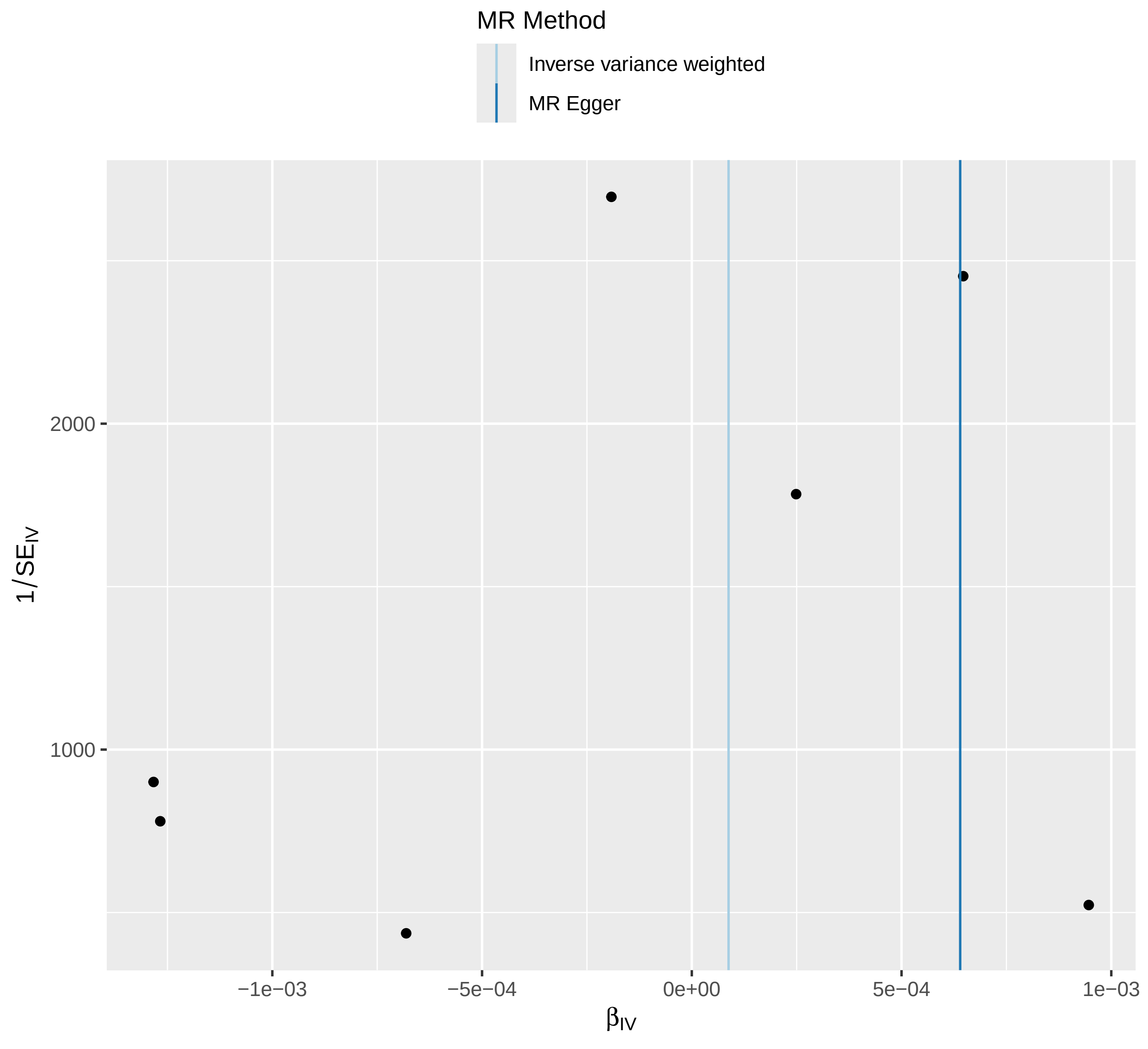
MR Method
Inverse variance weighted
MR Egger
2000
1000
1/SE_IV
−1e−03
−5e−04
0e+00
5e−04
1e−03
β_IV

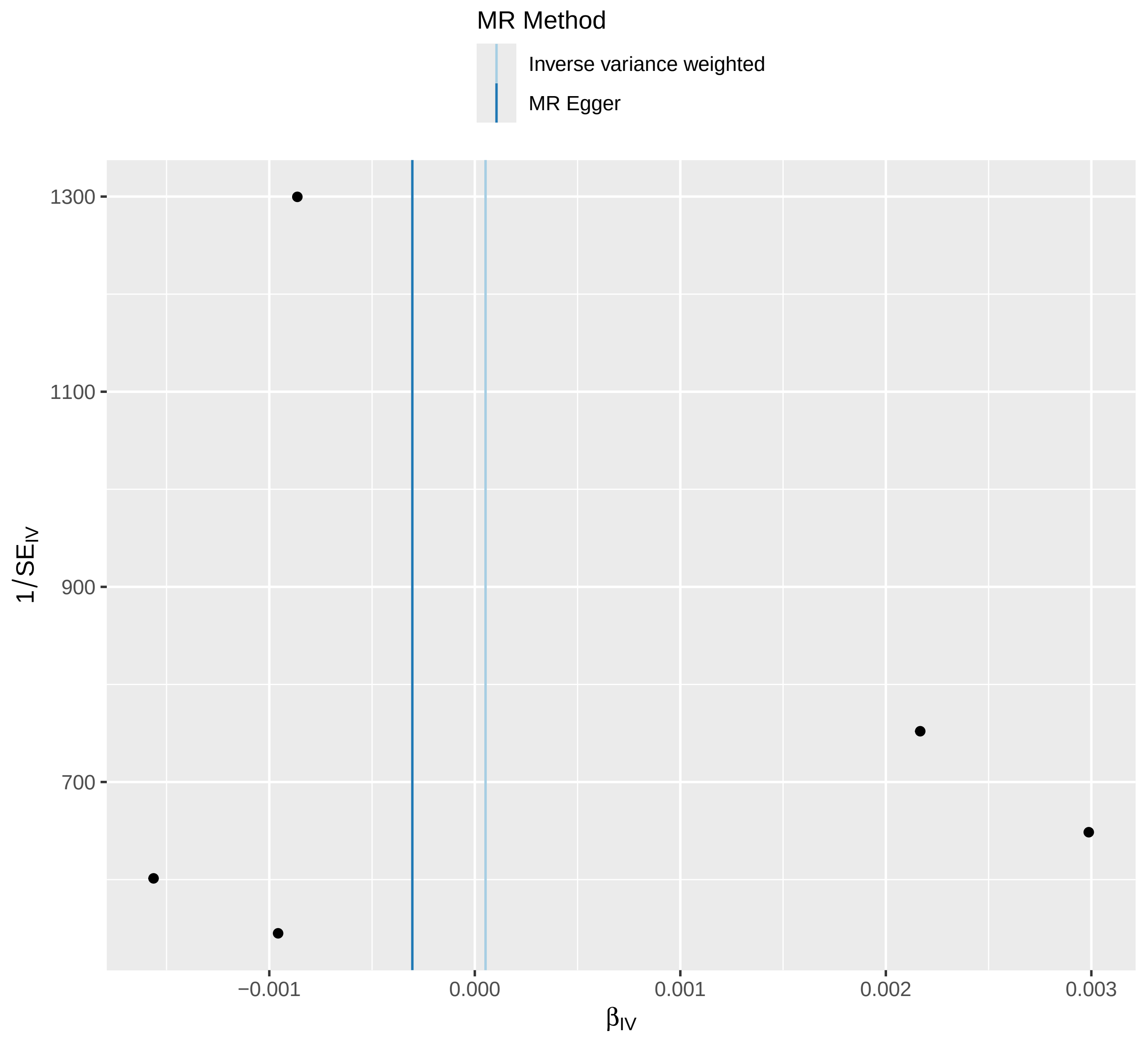

MR Method
Inverse variance weighted
MR Egger
1300
1100
900
700
1/SE_IV
−0.001
0.000
0.001
0.002
0.003
β_IV

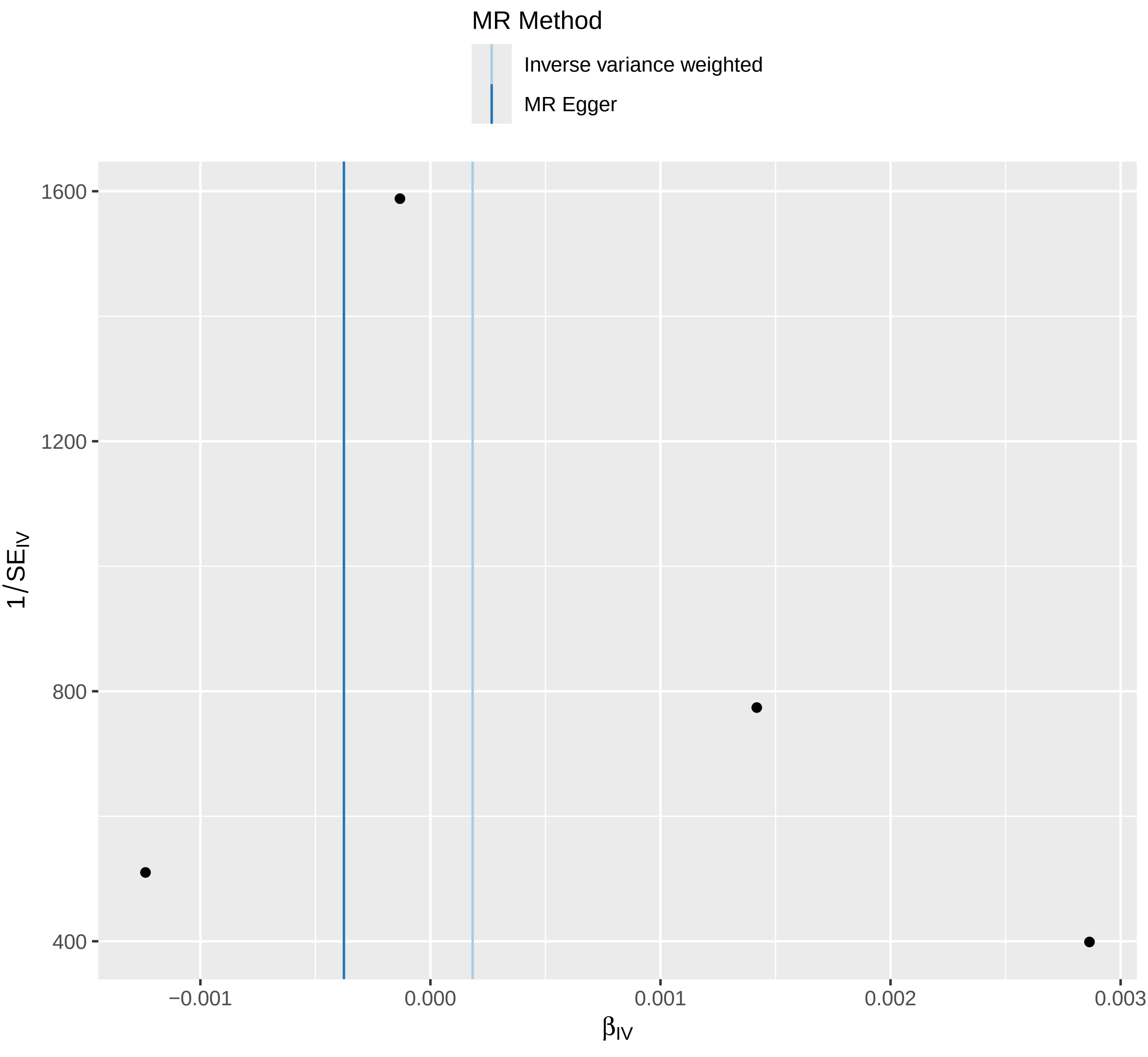

MR Method
Inverse variance weighted
MR Egger
1600
1200
800
400
−0.001
0.000
0.001
0.002
0.003
$1/SE_{IV}$
β_{IV}

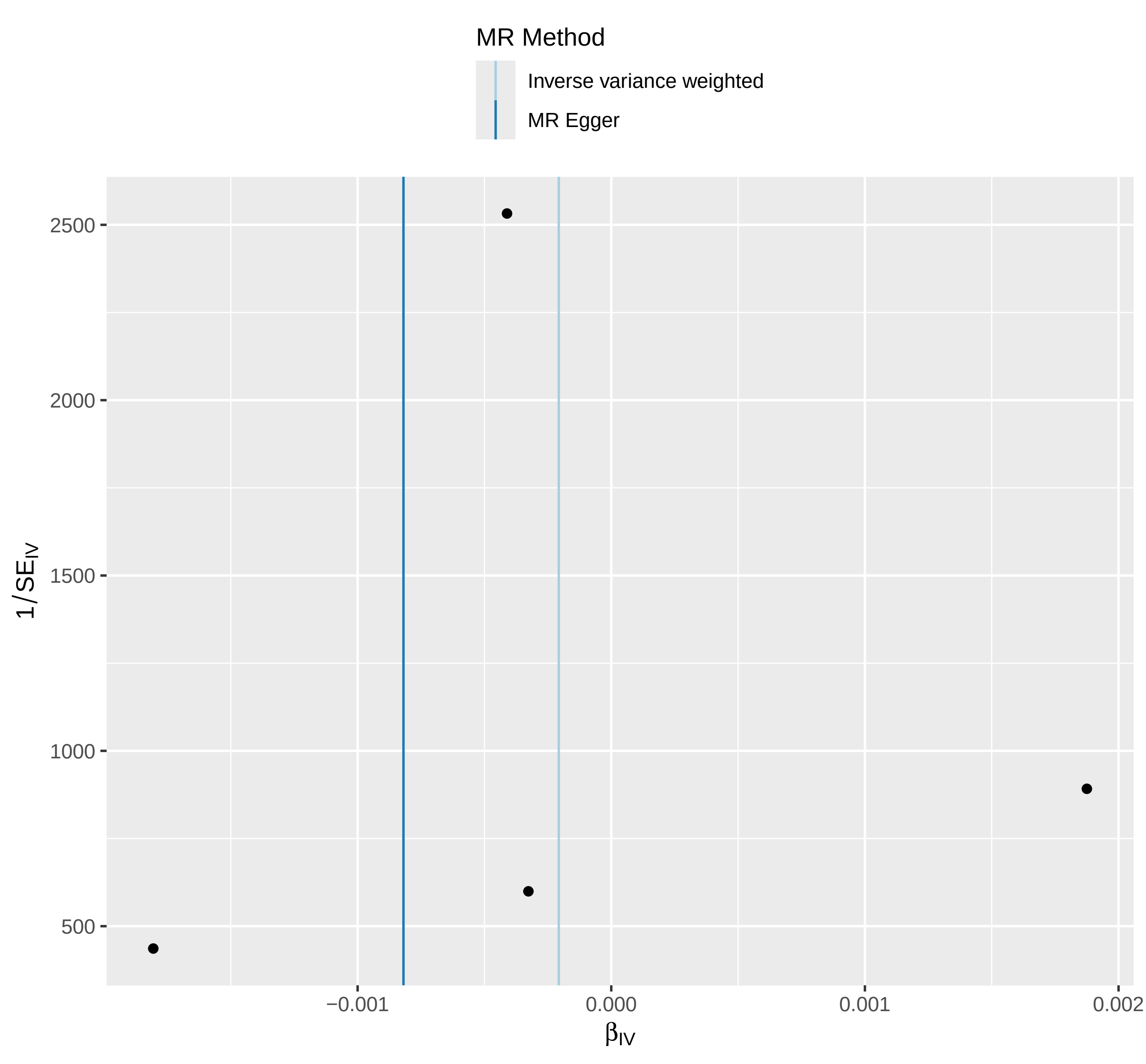
MR Method
Inverse variance weighted
MR Egger
2500
2000
1500
1000
500
1/SE_IV
−0.001
0.000
0.001
0.002
β_IV

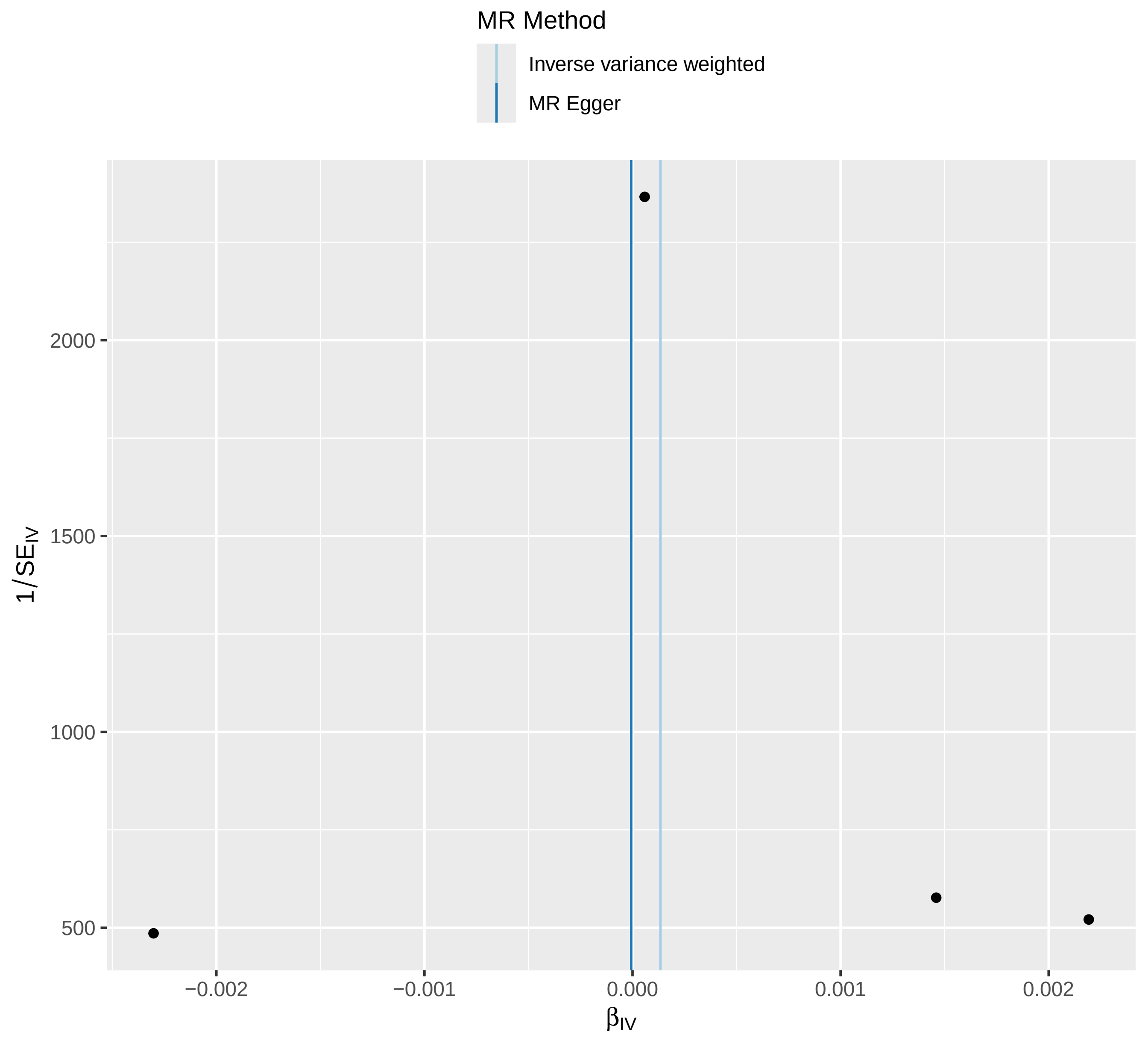

MR Method
Inverse variance weighted
MR Egger
2000
1500
1000
500
1/SE_IV
−0.002
−0.001
0.000
0.001
0.002
β_IV

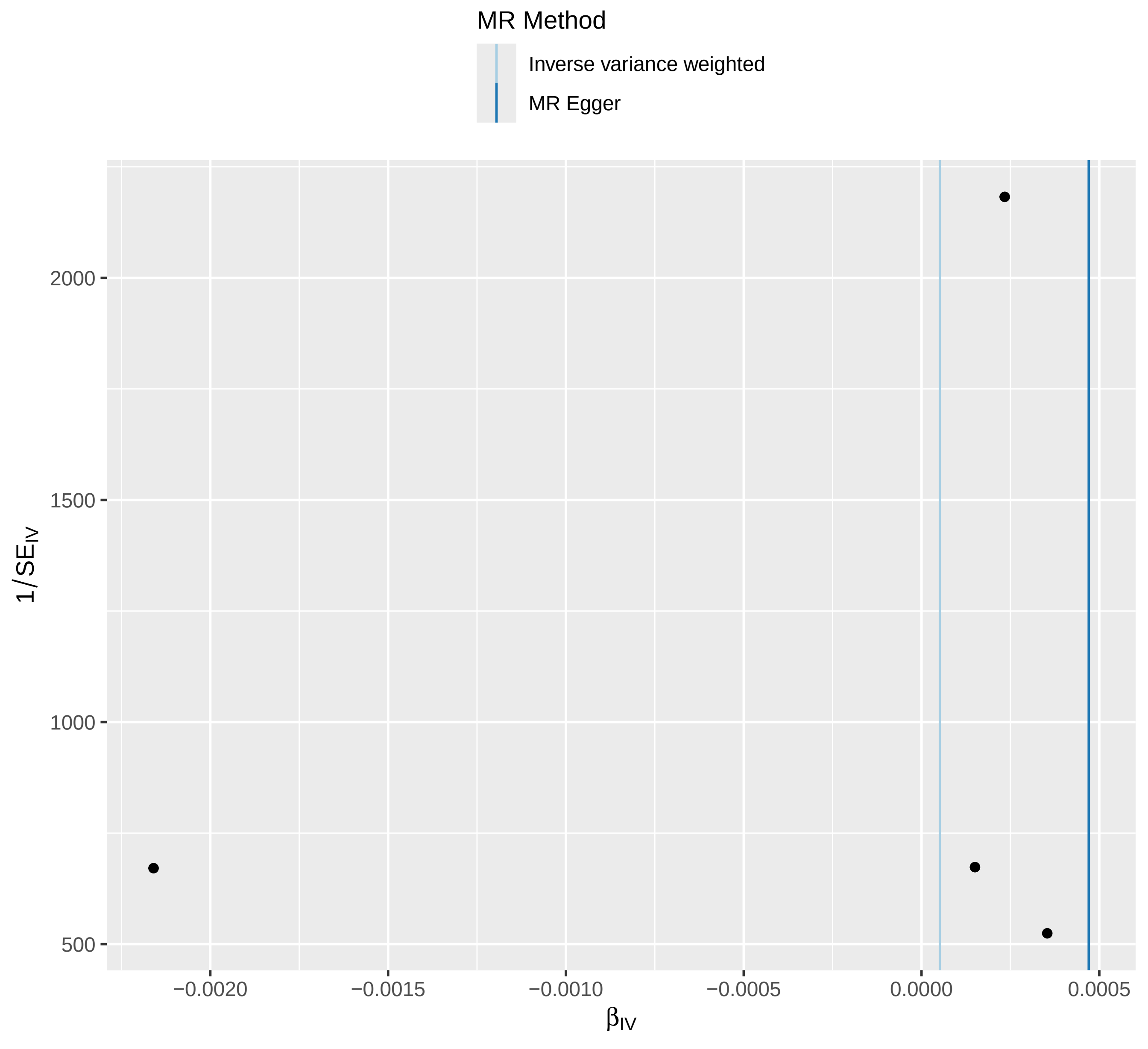
MR Method
Inverse variance weighted
MR Egger
2000
1500
1000
500
1/SE_IV
−0.0020
−0.0015
−0.0010
−0.0005
0.0000
0.0005
β_IV

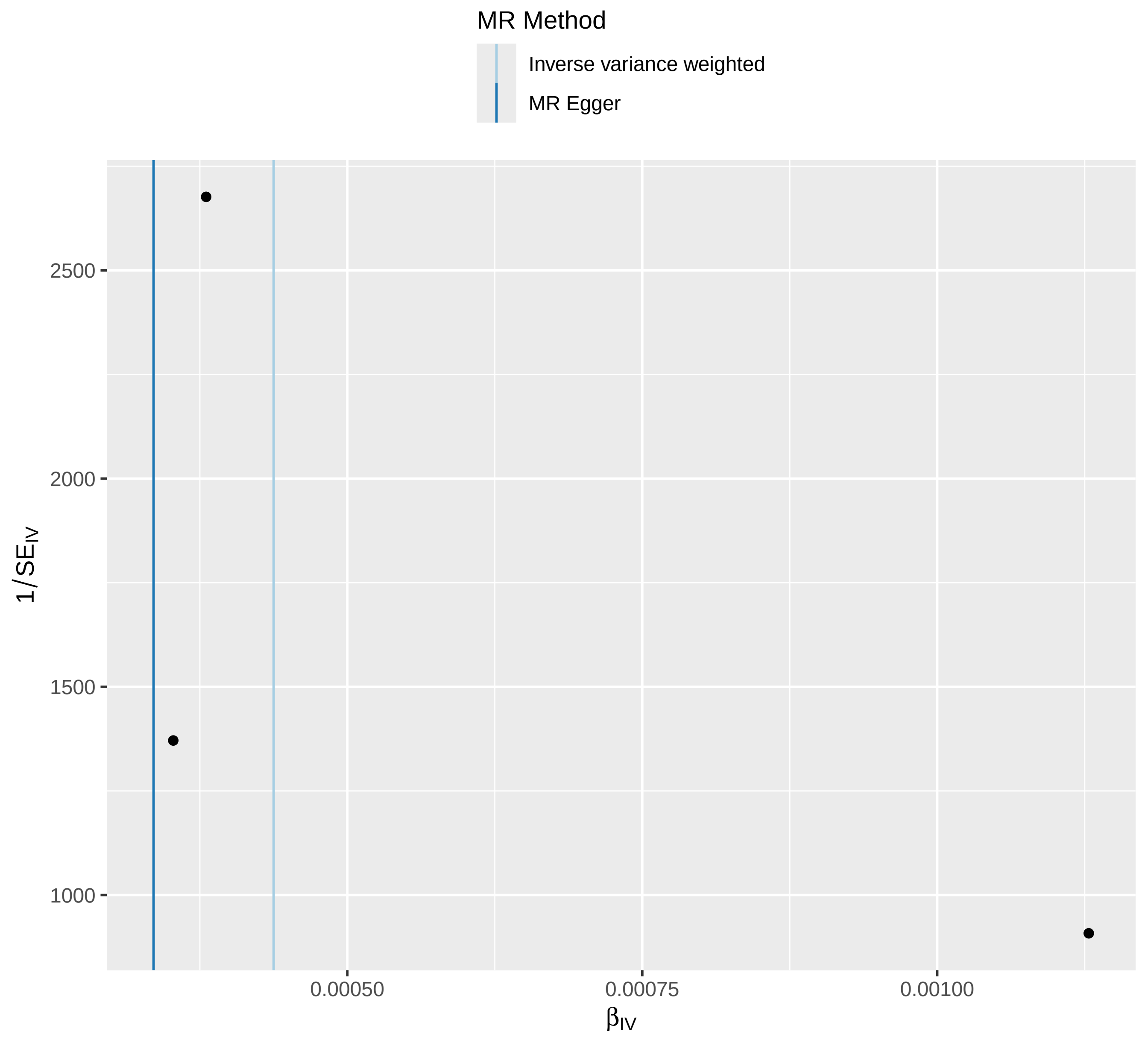
MR Method
Inverse variance weighted
MR Egger
2500
2000
1500
1000
1/SE_IV
0.00050
0.00075
0.00100
β_IV

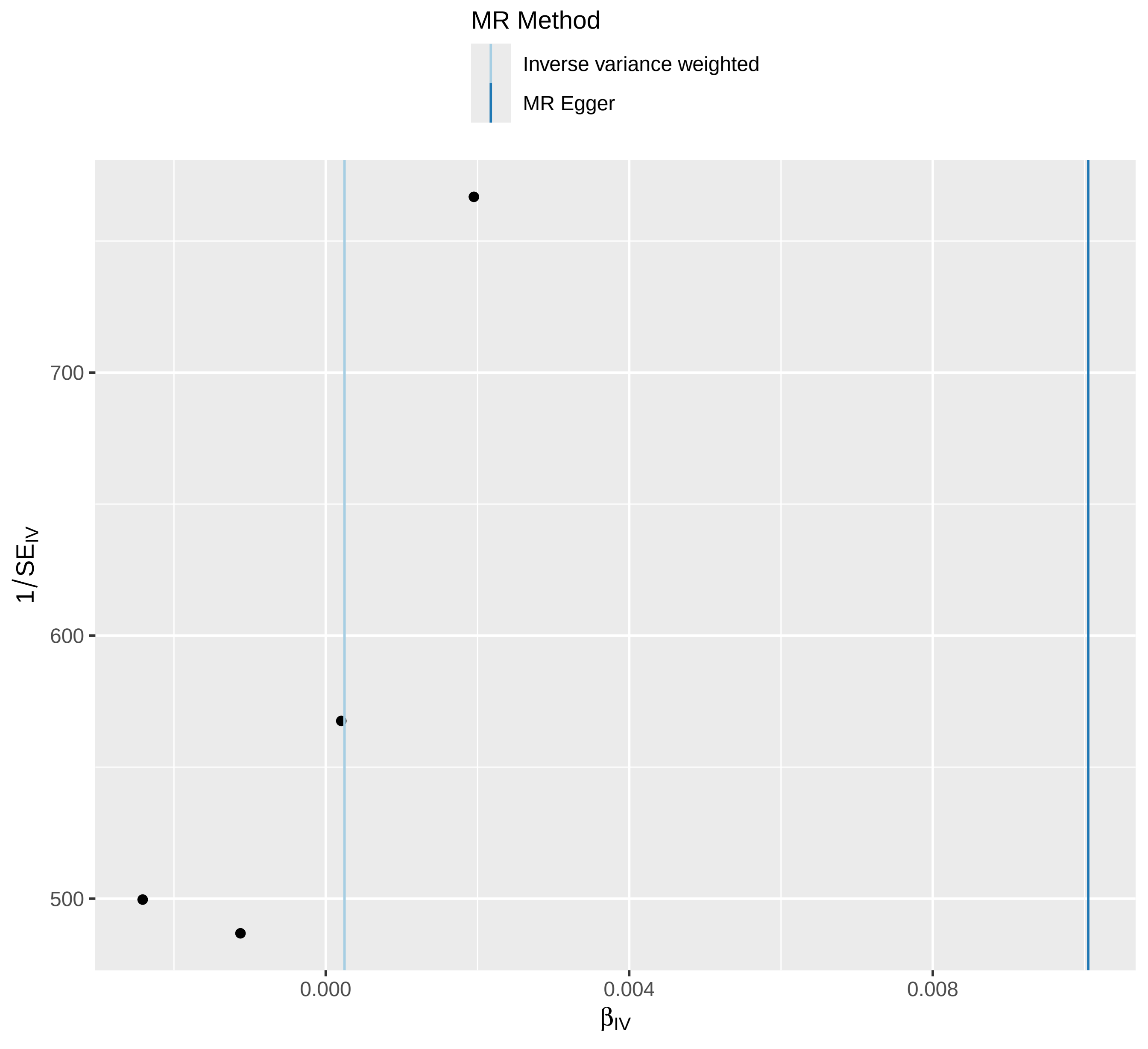
MR Method
Inverse variance weighted
MR Egger
700
600
500
0.000
0.004
0.008
1/SE_IV
β_IV

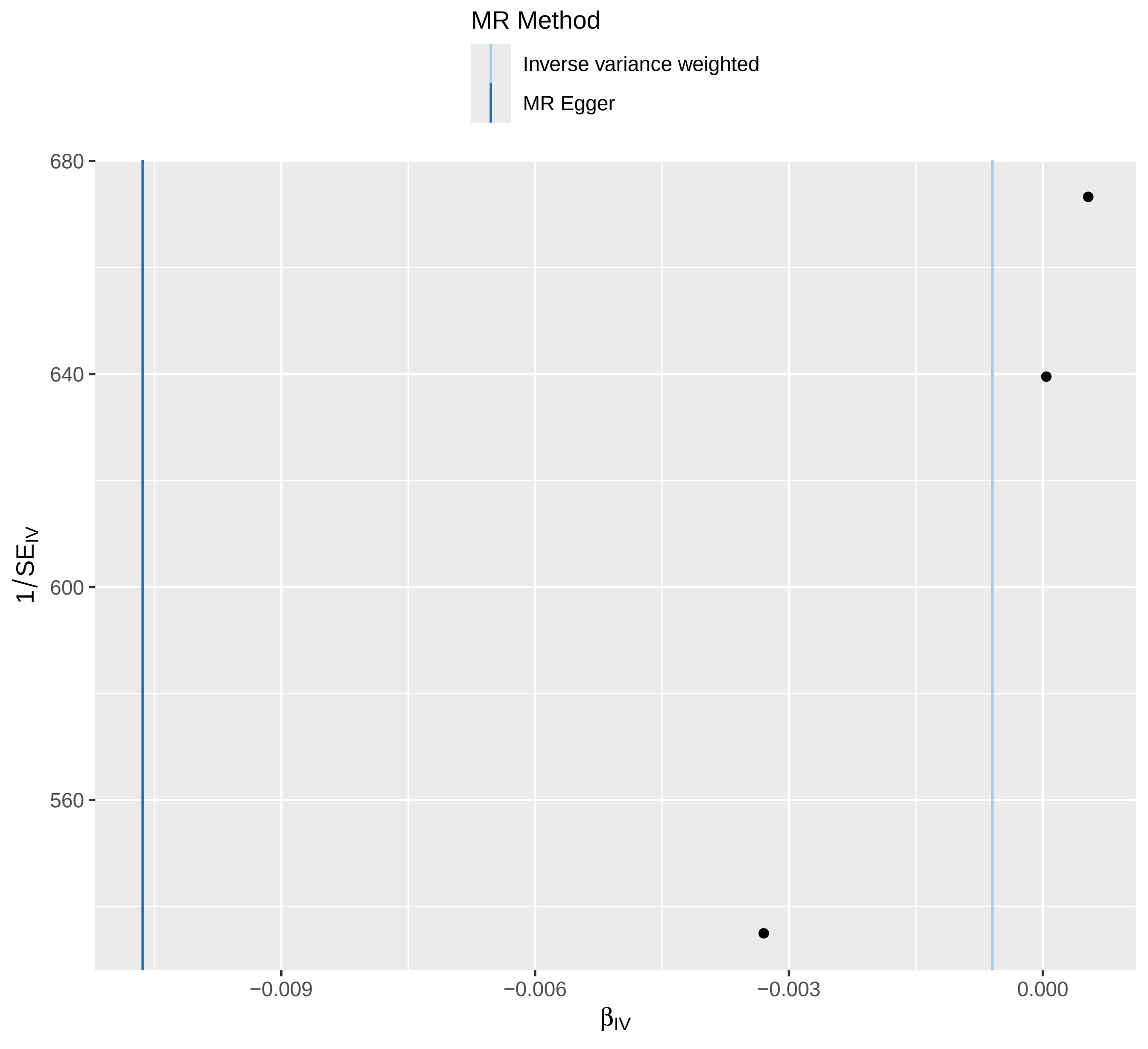

MR Method
Inverse variance weighted
MR Egger
680
640
600
560
1/SE_IV
−0.009
−0.006
−0.003
0.000
β_IV

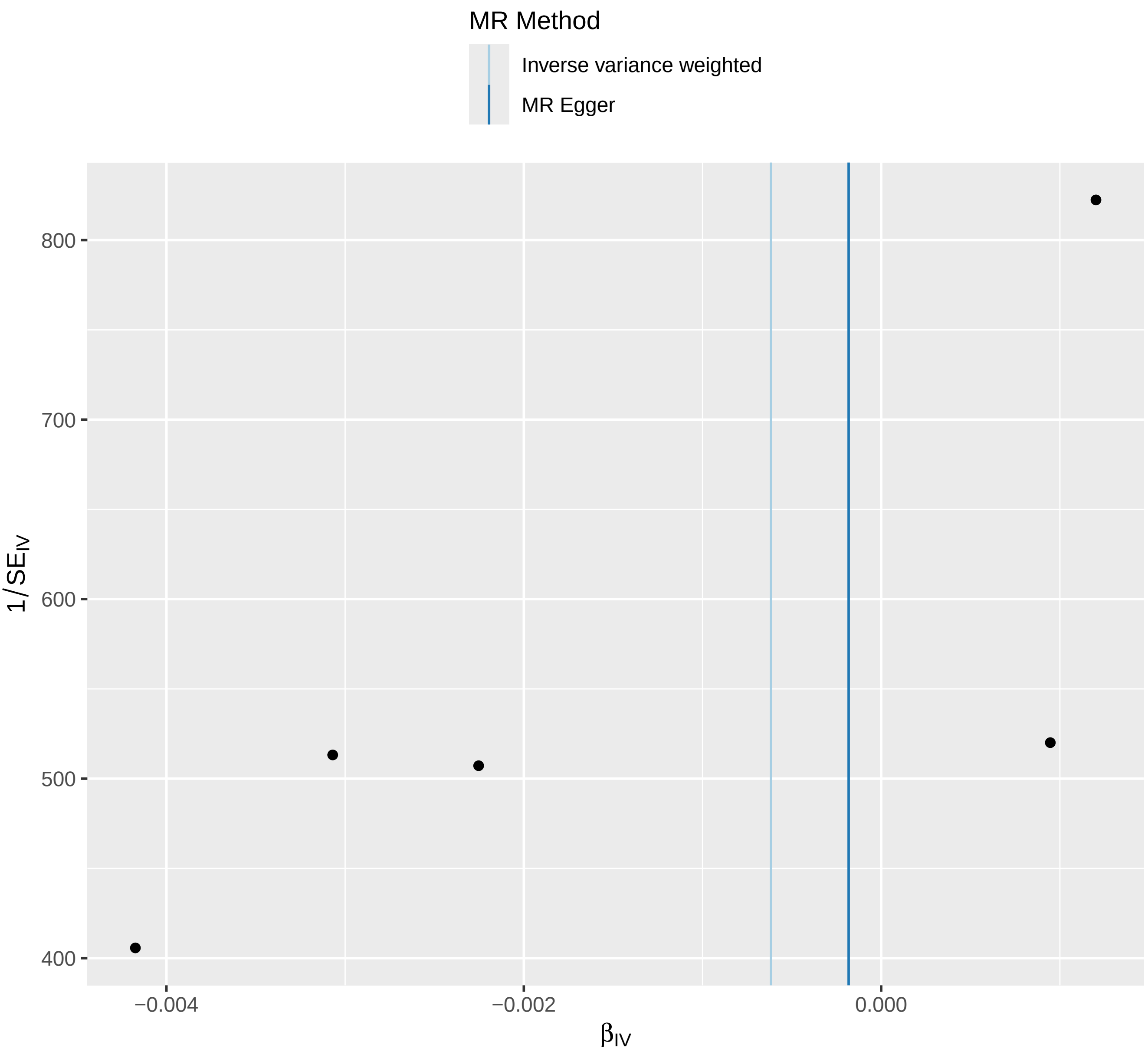
MR Method
Inverse variance weighted
MR Egger
800
700
600
500
400
1/SE$_{IV}$
−0.004
−0.002
0.000
β$_{IV}$

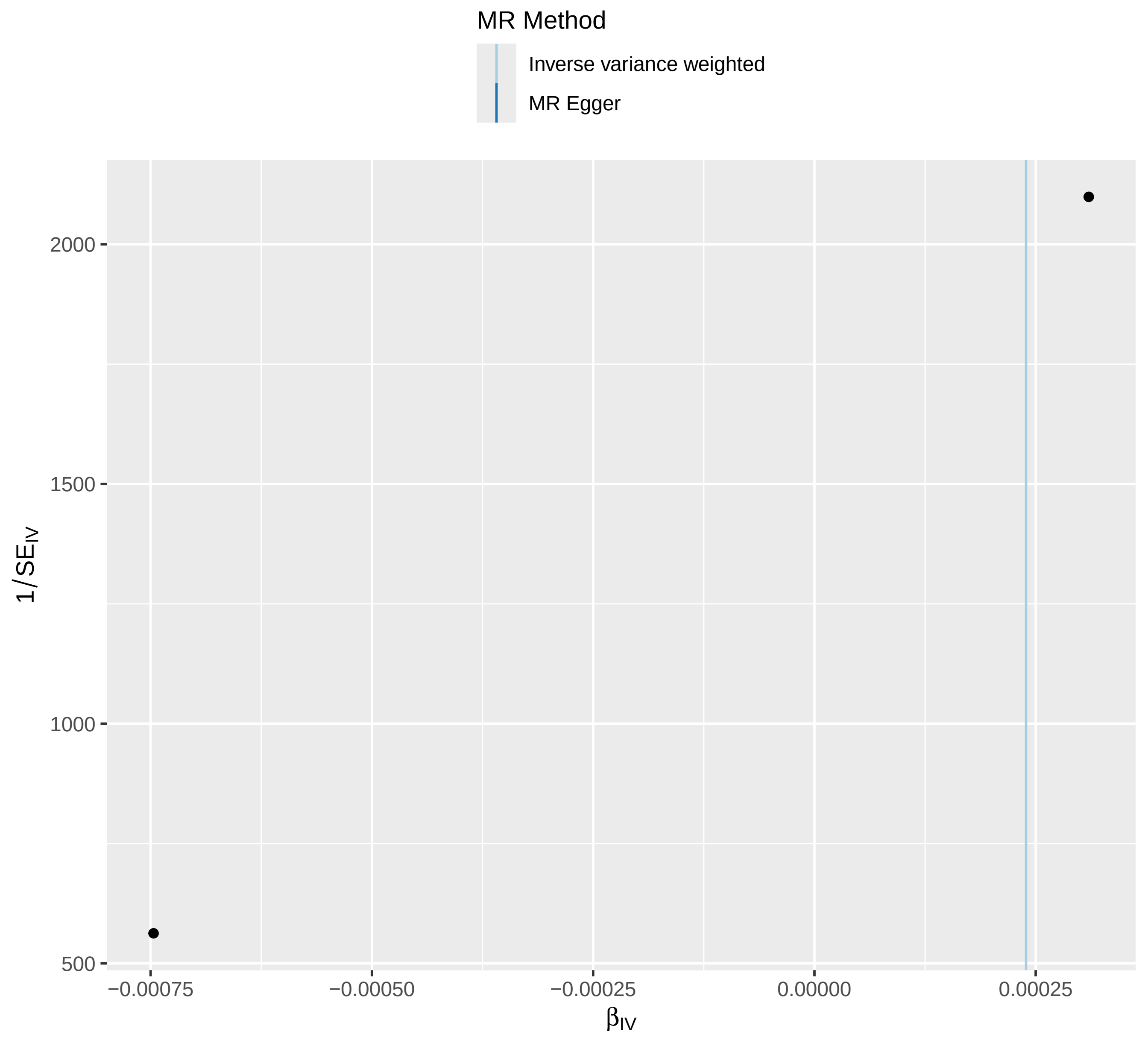

MR Method
Inverse variance weighted
MR Egger
2000
1500
1000
500
1/SE_IV
−0.00075
−0.00050
−0.00025
0.00000
0.00025
β_IV

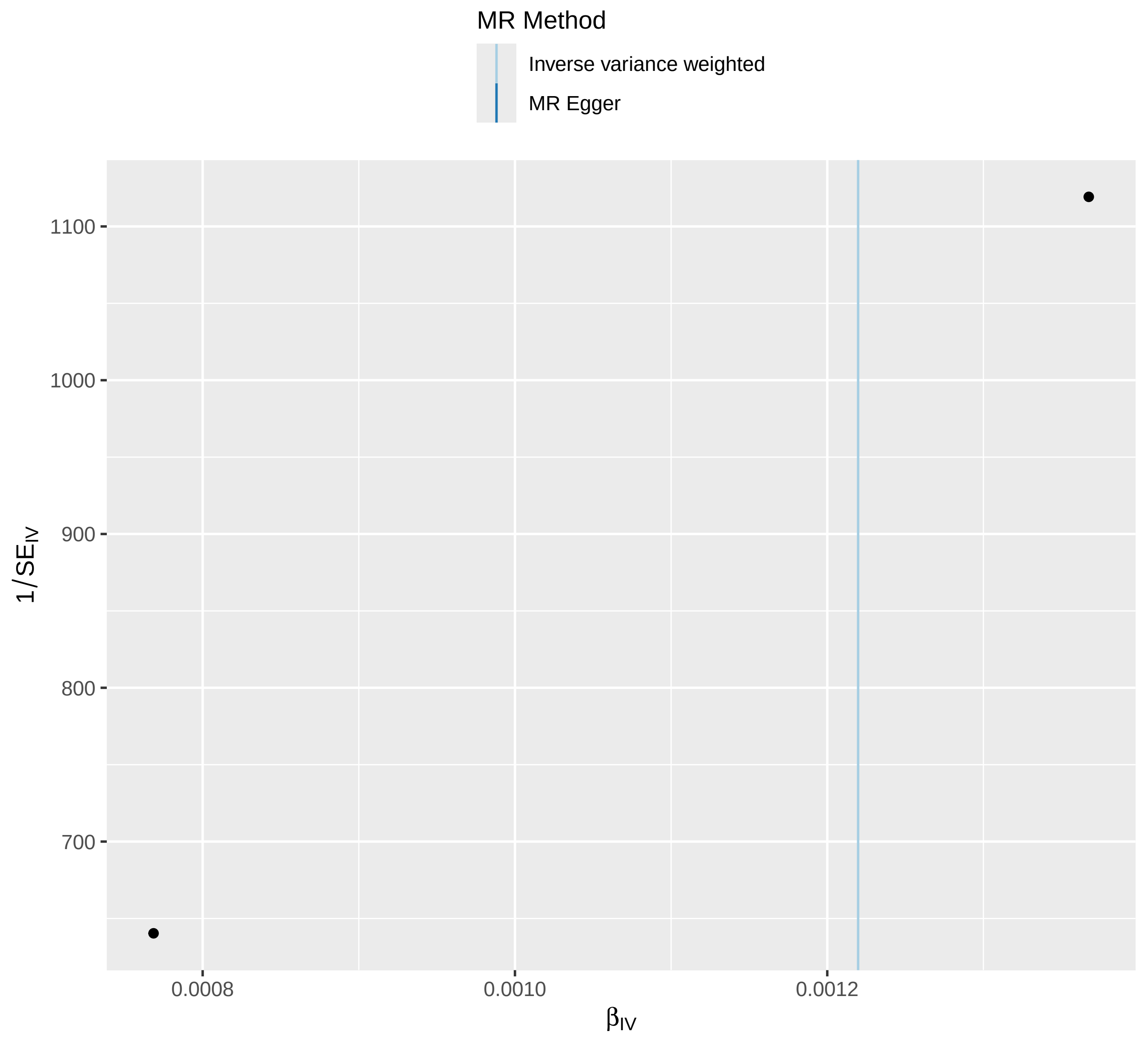
MR Method
Inverse variance weighted
MR Egger
1100
1000
900
800
700
0.0008
0.0010
0.0012
β_{IV}
$1/SE_{IV}$

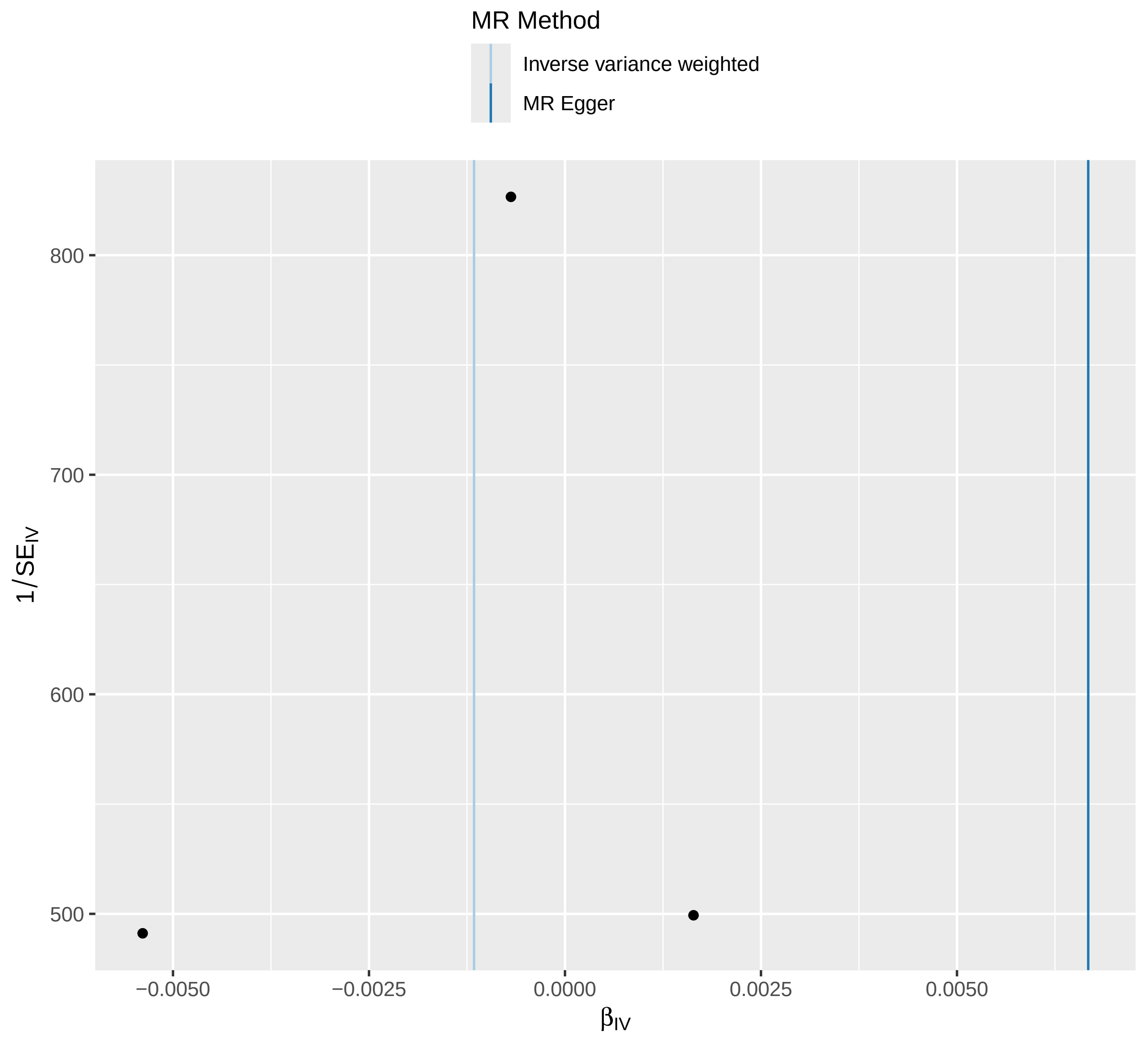
MR Method
Inverse variance weighted
MR Egger
800
700
600
500
1/SE_IV
−0.0050
−0.0025
0.0000
0.0025
0.0050
β_IV

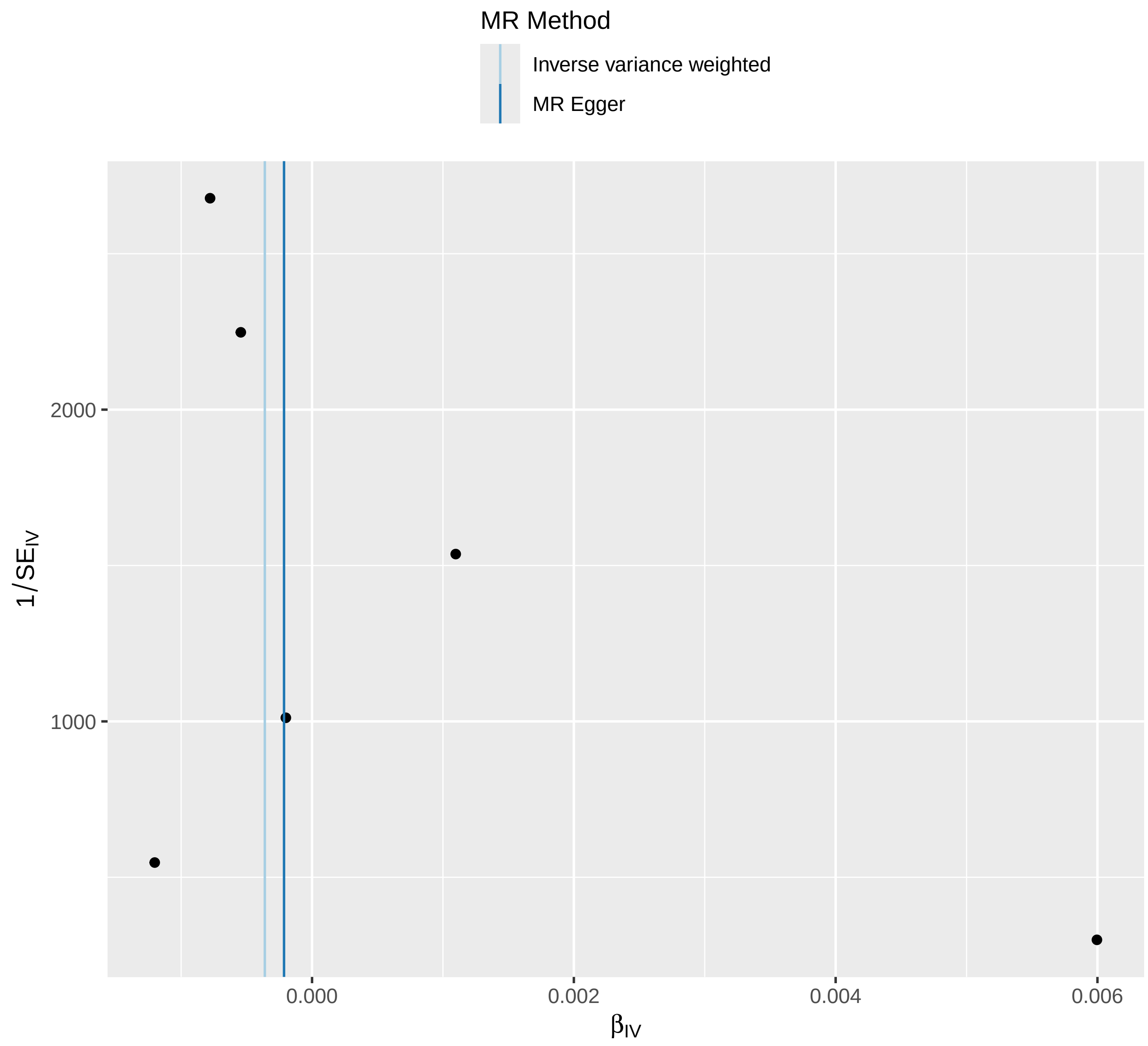

MR Method
Inverse variance weighted
MR Egger
2000
1000
1/SE_IV
0.000
0.002
0.004
0.006
β_IV

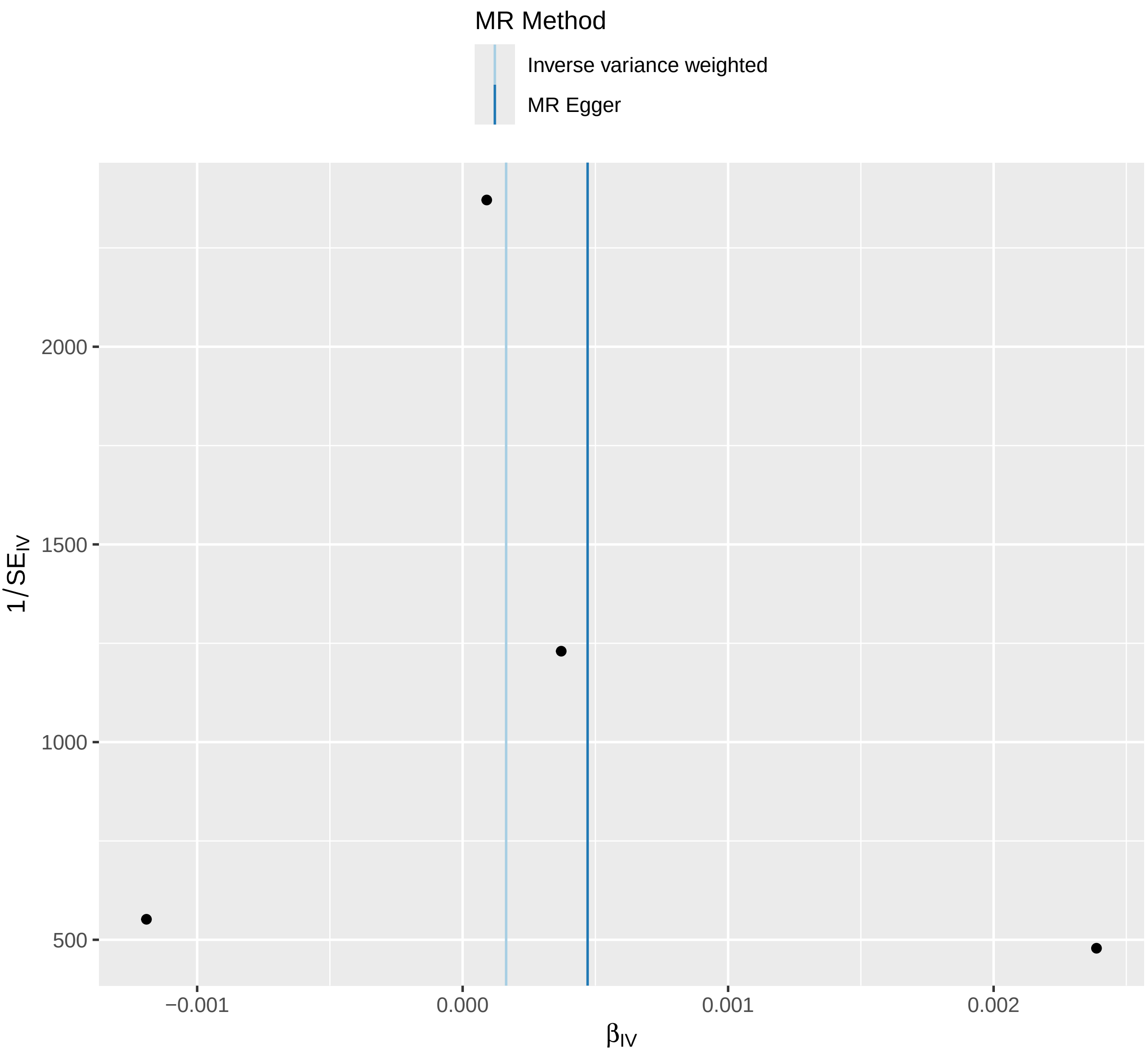
MR Method
Inverse variance weighted
MR Egger
2000
1500
1000
500
1/SE$_{IV}$
−0.001
0.000
0.001
0.002
β$_{IV}$

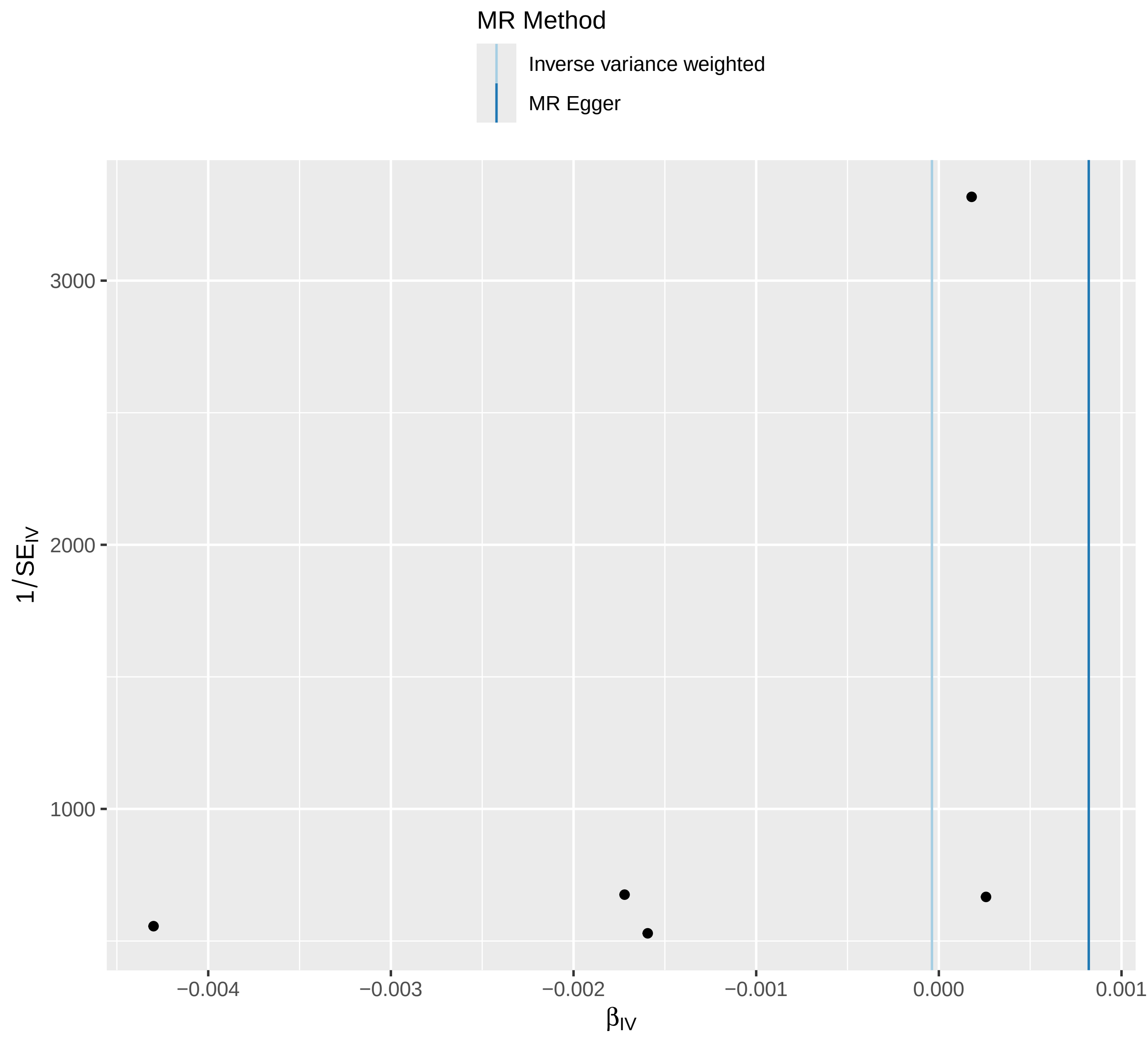

MR Method
Inverse variance weighted
MR Egger
3000
2000
1000
1/SE_IV
−0.004
−0.003
−0.002
−0.001
0.000
0.001
β_IV

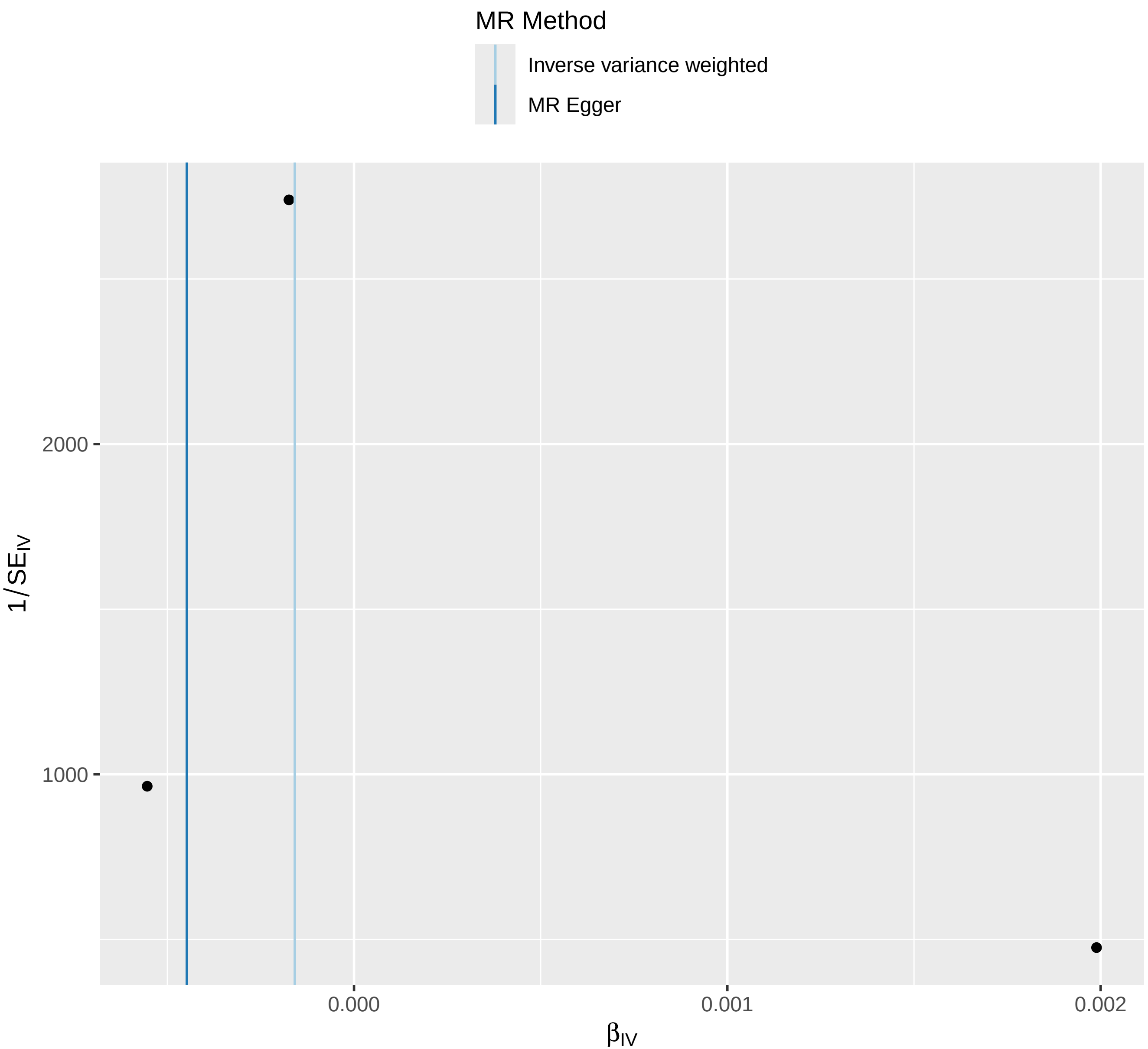

MR Method
Inverse variance weighted
MR Egger
2000
1000
1/SE_IV
0.000
0.001
0.002
β_IV

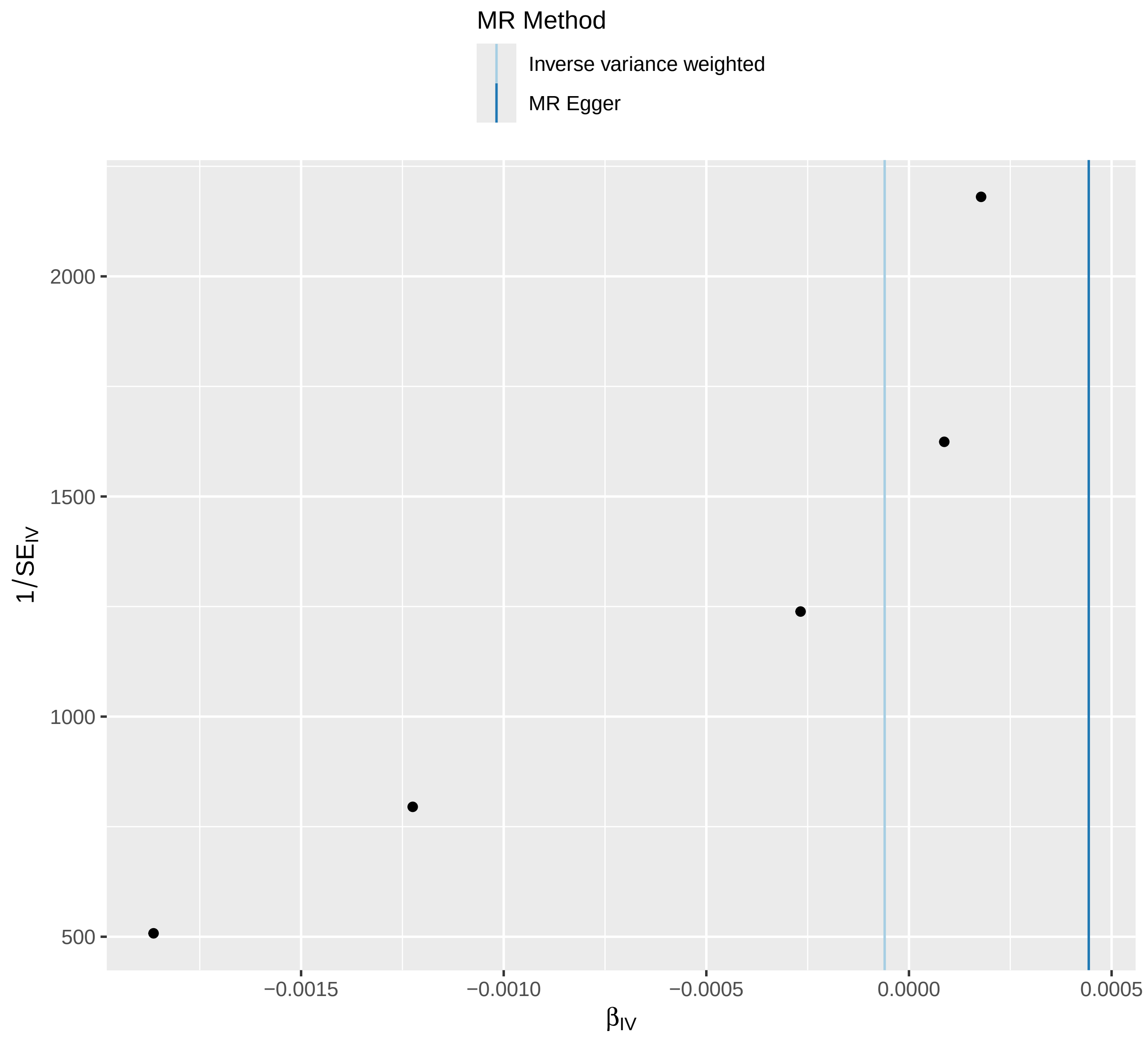

MR Method
Inverse variance weighted
MR Egger
2000
1500
1000
500
1/SE_IV
−0.0015
−0.0010
−0.0005
0.0000
0.0005
β_IV

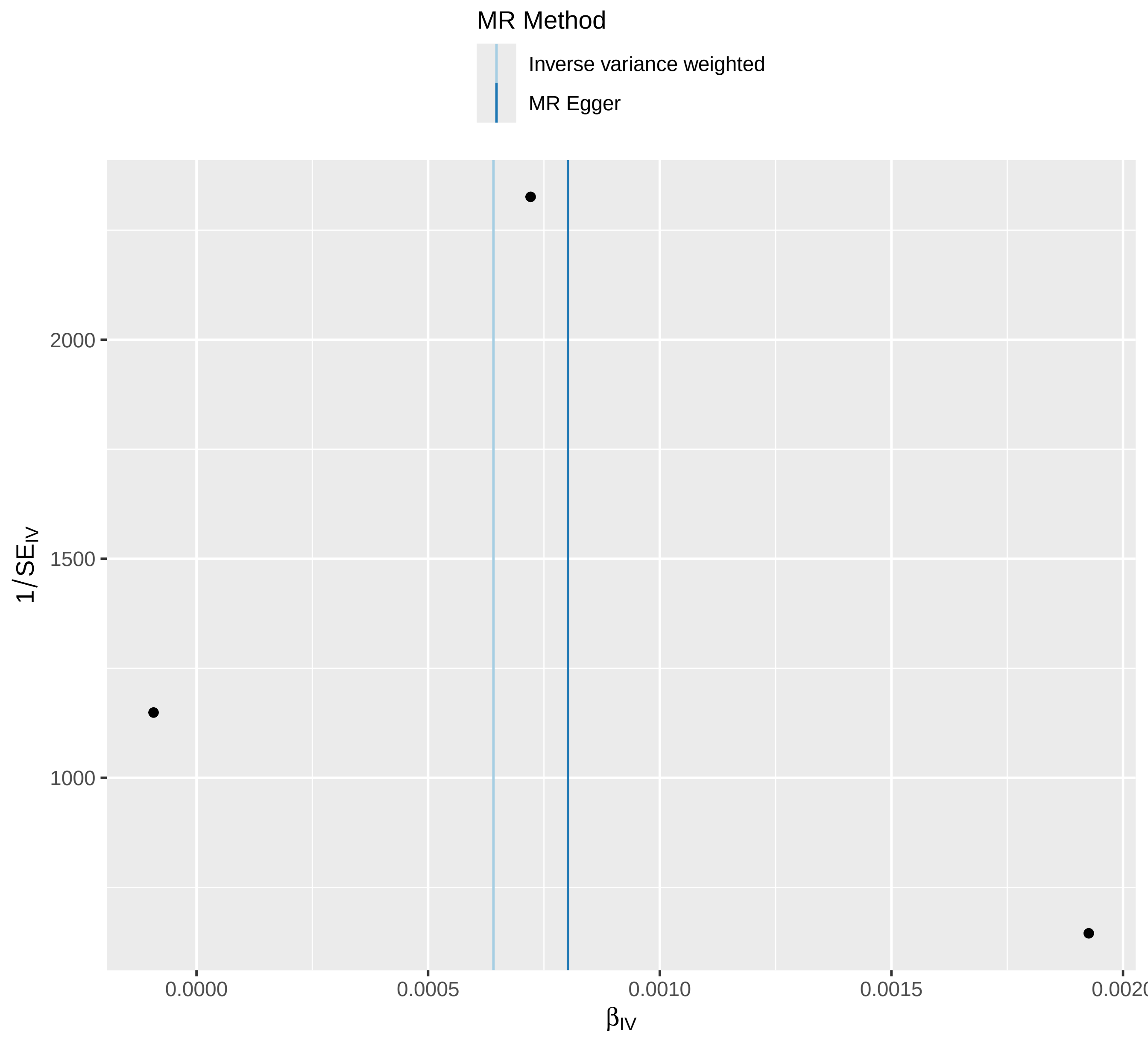
MR Method
Inverse variance weighted
MR Egger
2000
1500
1000
1/SE_IV
0.0000
0.0005
0.0010
0.0015
0.0020
β_IV

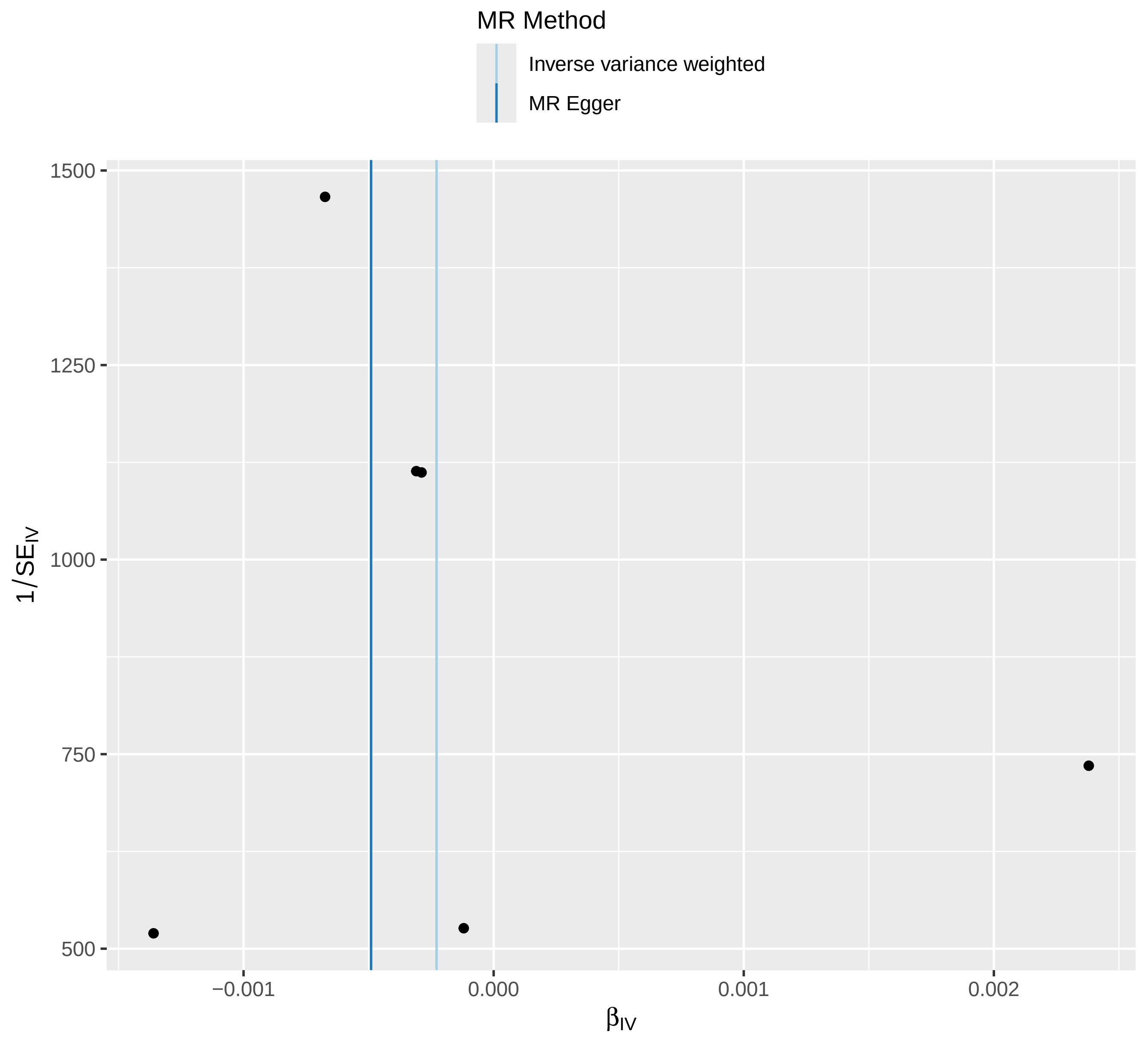
MR Method
Inverse variance weighted
MR Egger
1500
1250
1000
750
500
1/SE_IV
−0.001
0.000
0.001
0.002
β_IV

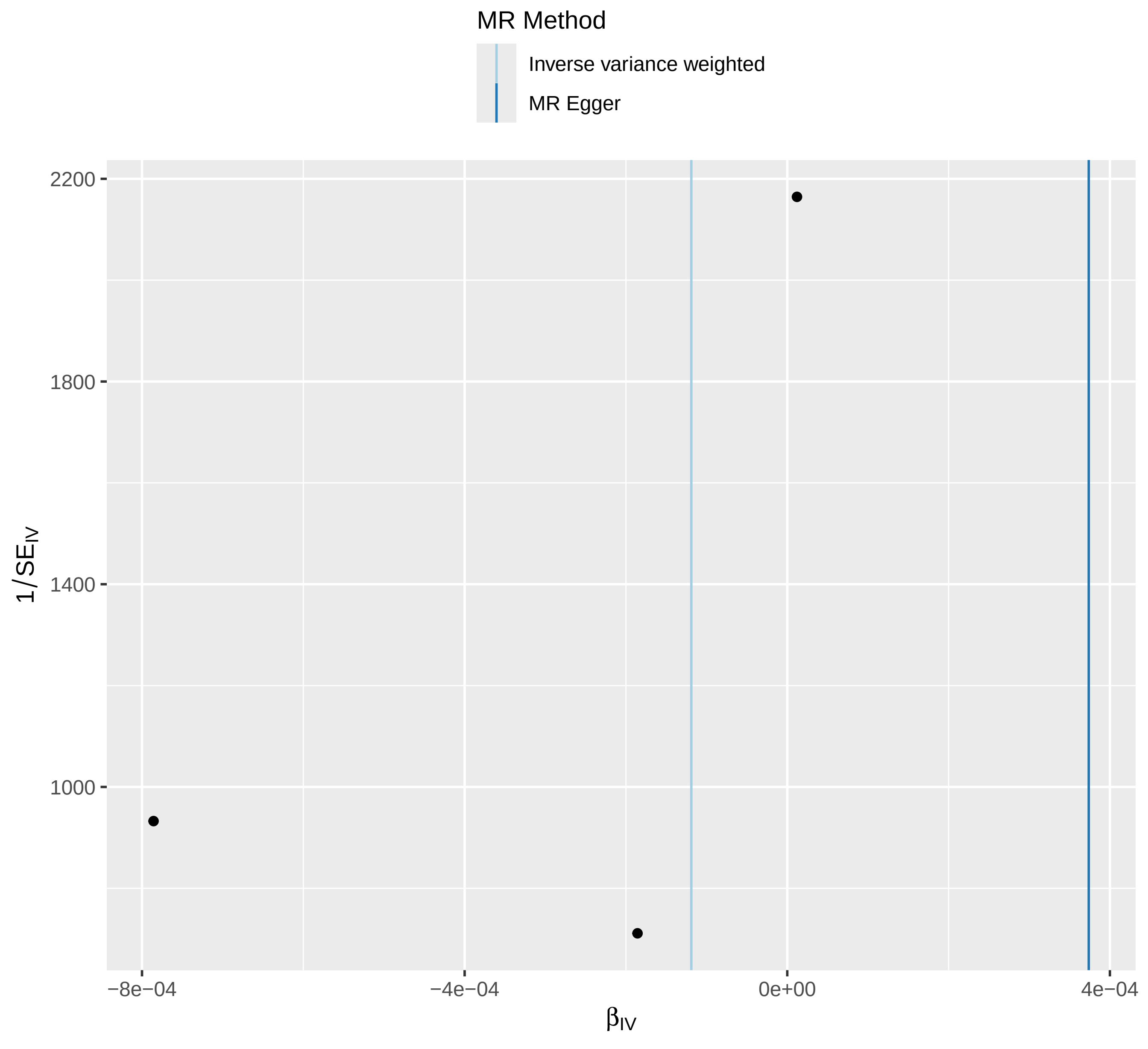
MR Method
Inverse variance weighted
MR Egger
2200
1800
1400
1000
1/SE_IV
−8e−04
−4e−04
0e+00
4e−04
β_IV

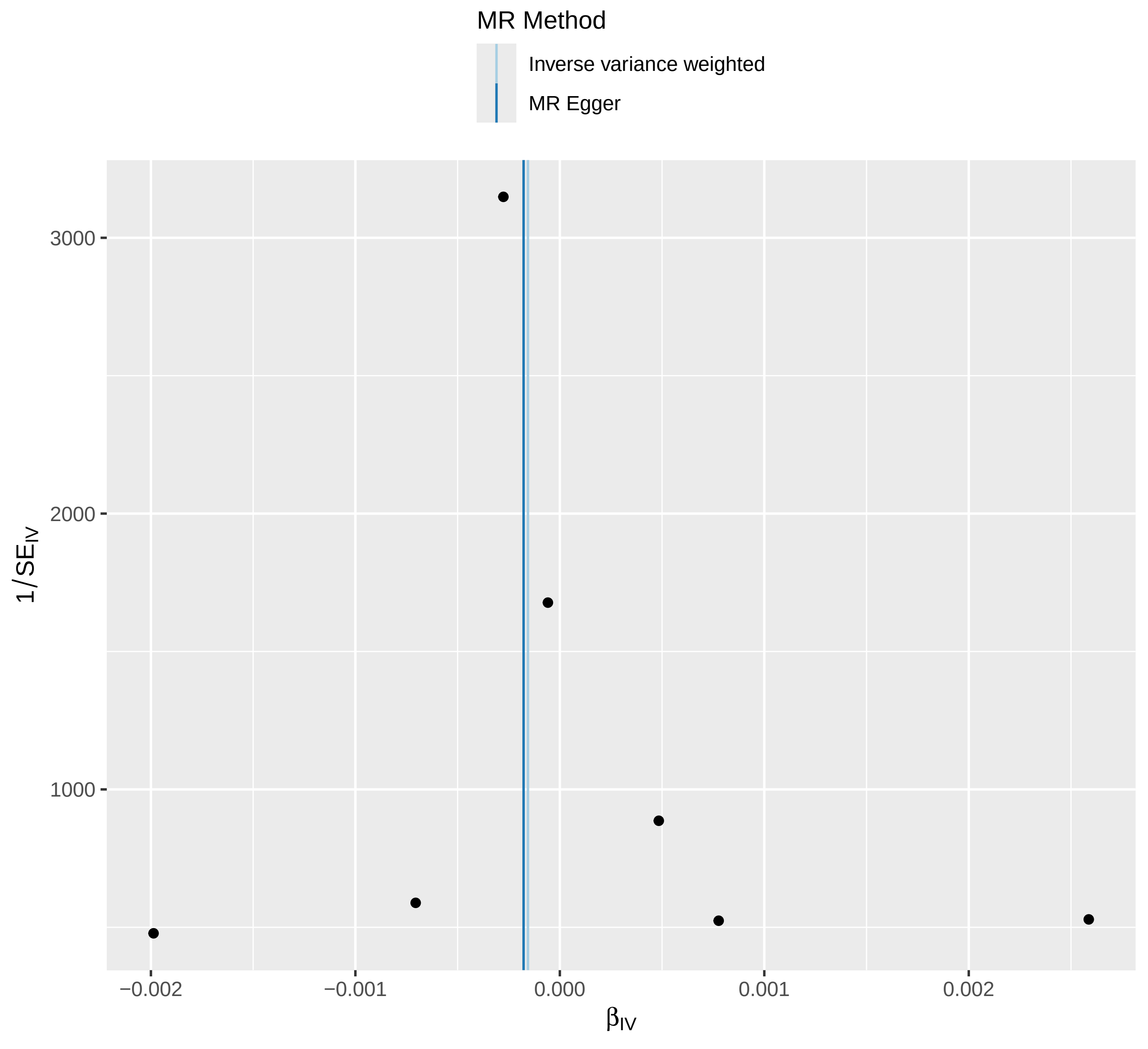

MR Method
Inverse variance weighted
MR Egger
3000
2000
1000
1/SE_IV
−0.002
−0.001
0.000
0.001
0.002
β_IV

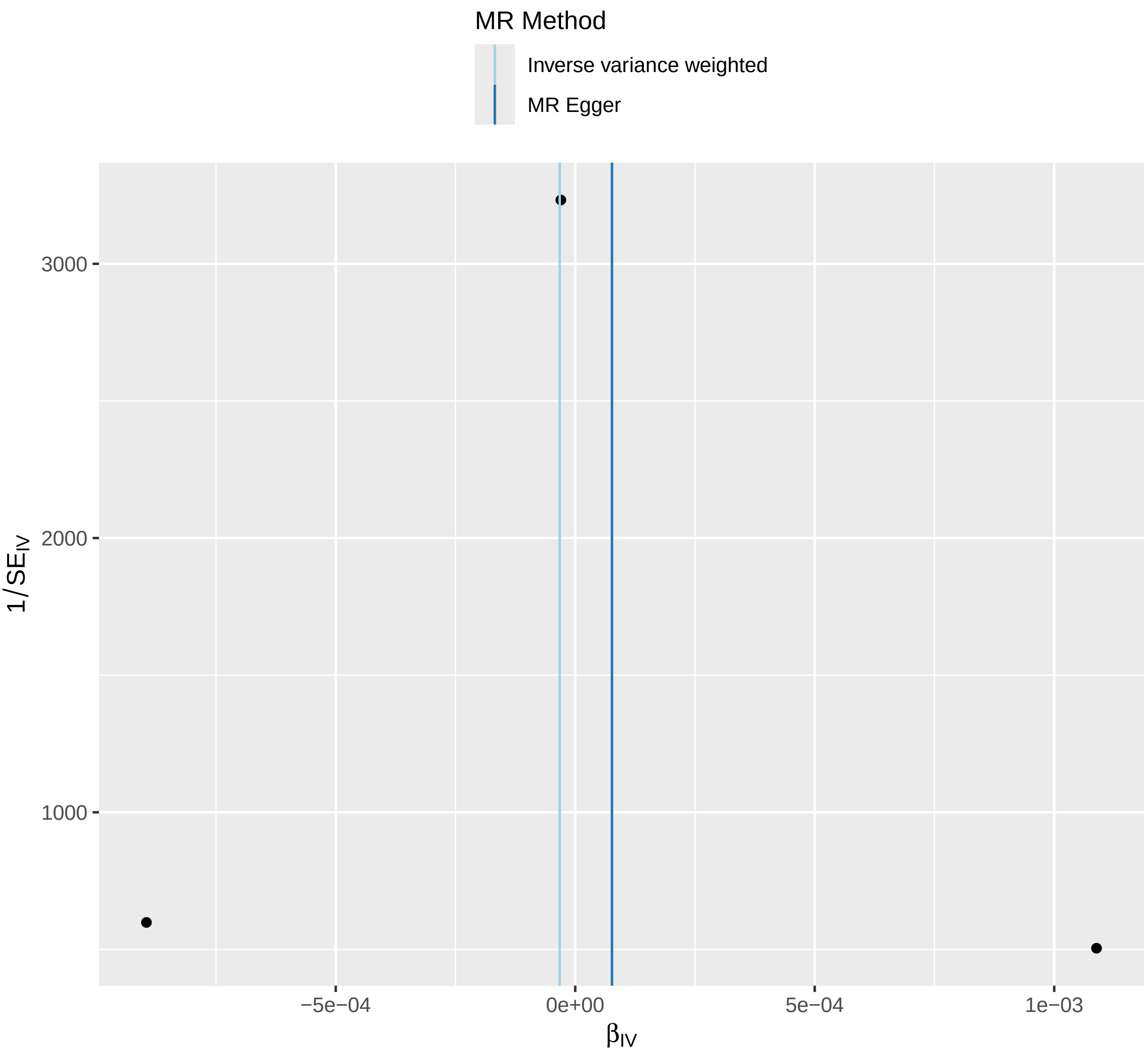
MR Method
Inverse variance weighted
MR Egger
3000
2000
1000
1/SE_IV
−5e−04
0e+00
5e−04
1e−03
β_IV

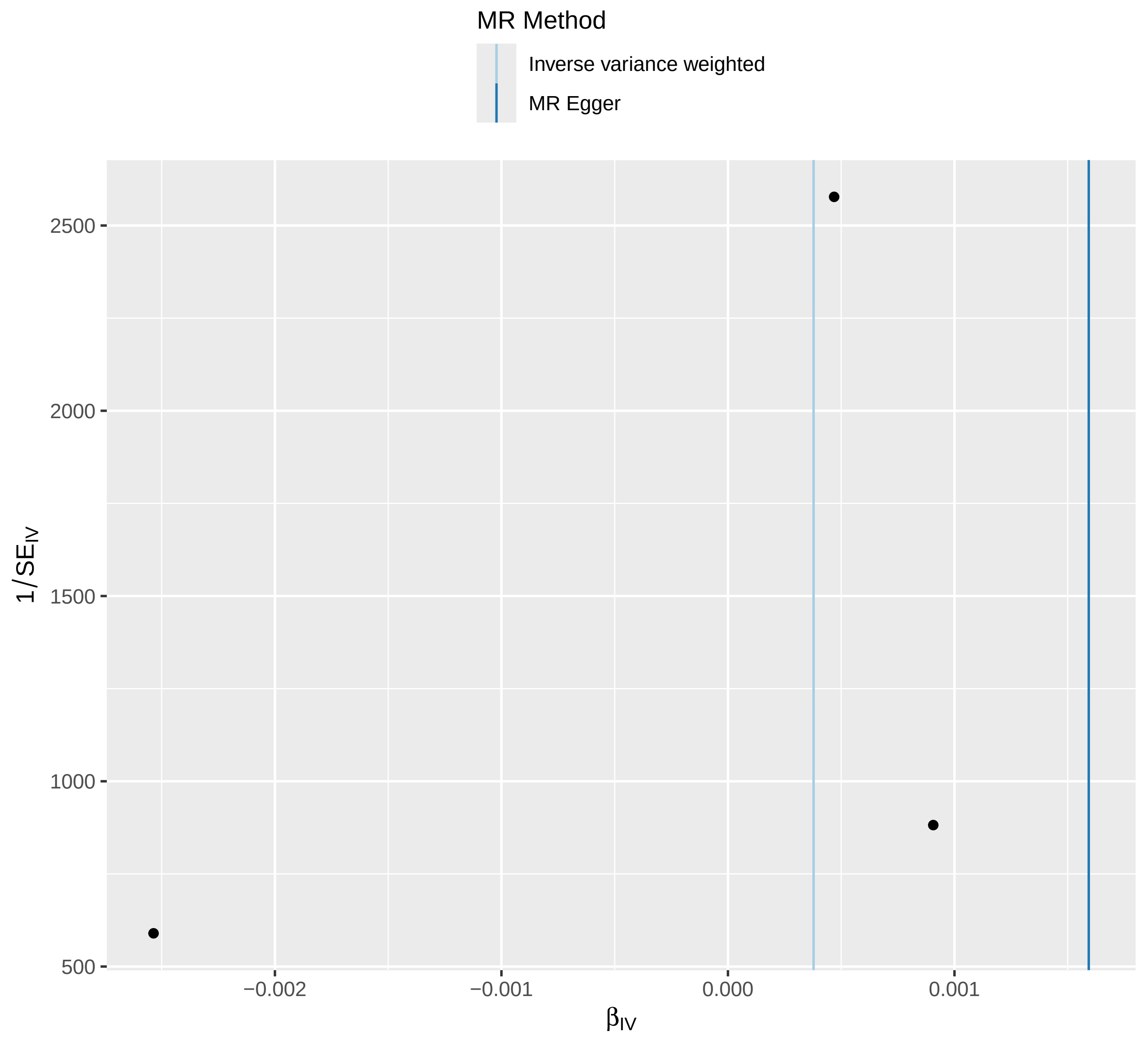

MR Method
Inverse variance weighted
MR Egger
2500
2000
1500
1000
500
1/SE_IV
−0.002
−0.001
0.000
0.001
β_IV

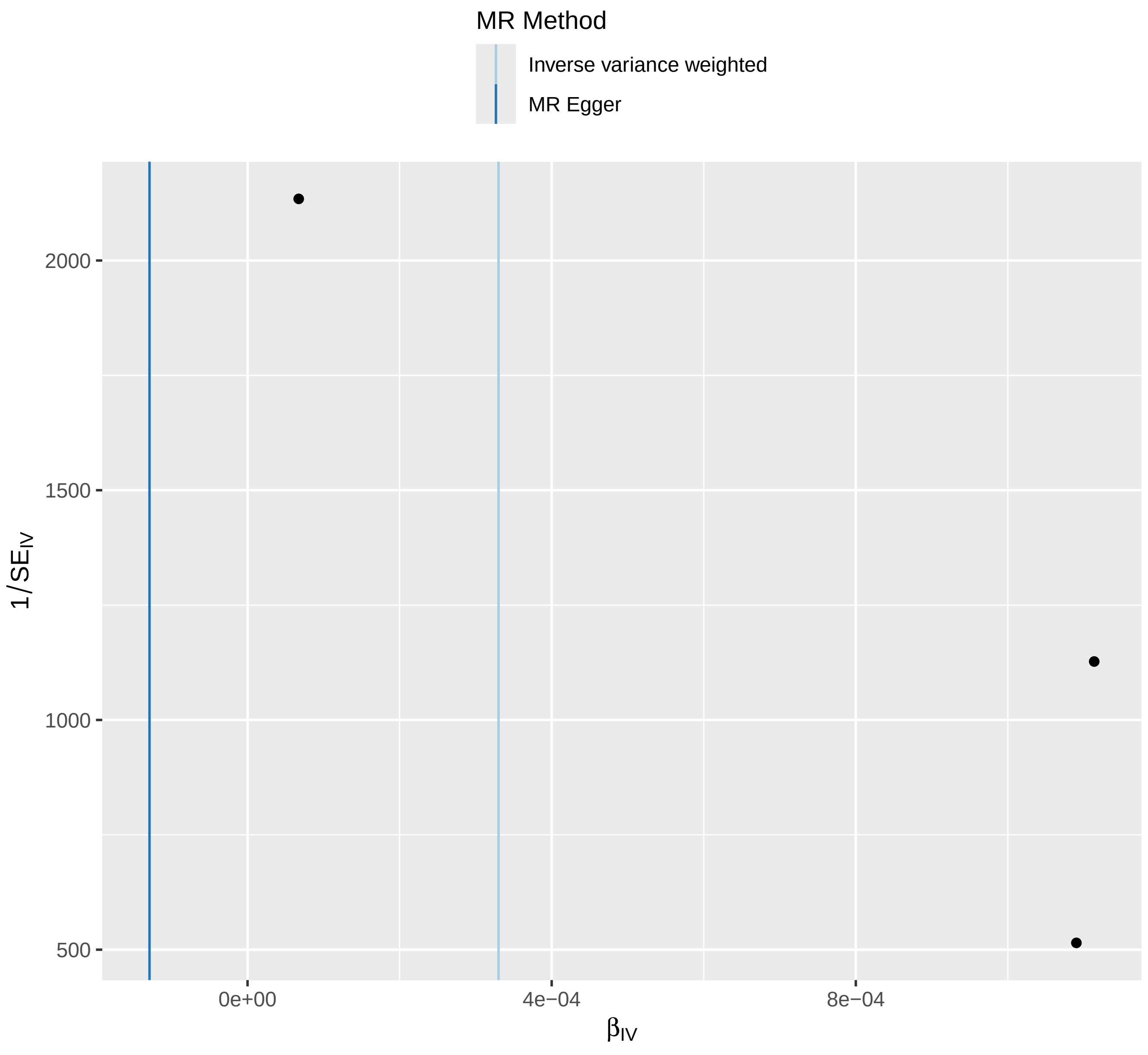
MR Method
Inverse variance weighted
MR Egger
2000
1500
1000
500
1/SE_IV
0e+00
4e−04
8e−04
β_IV

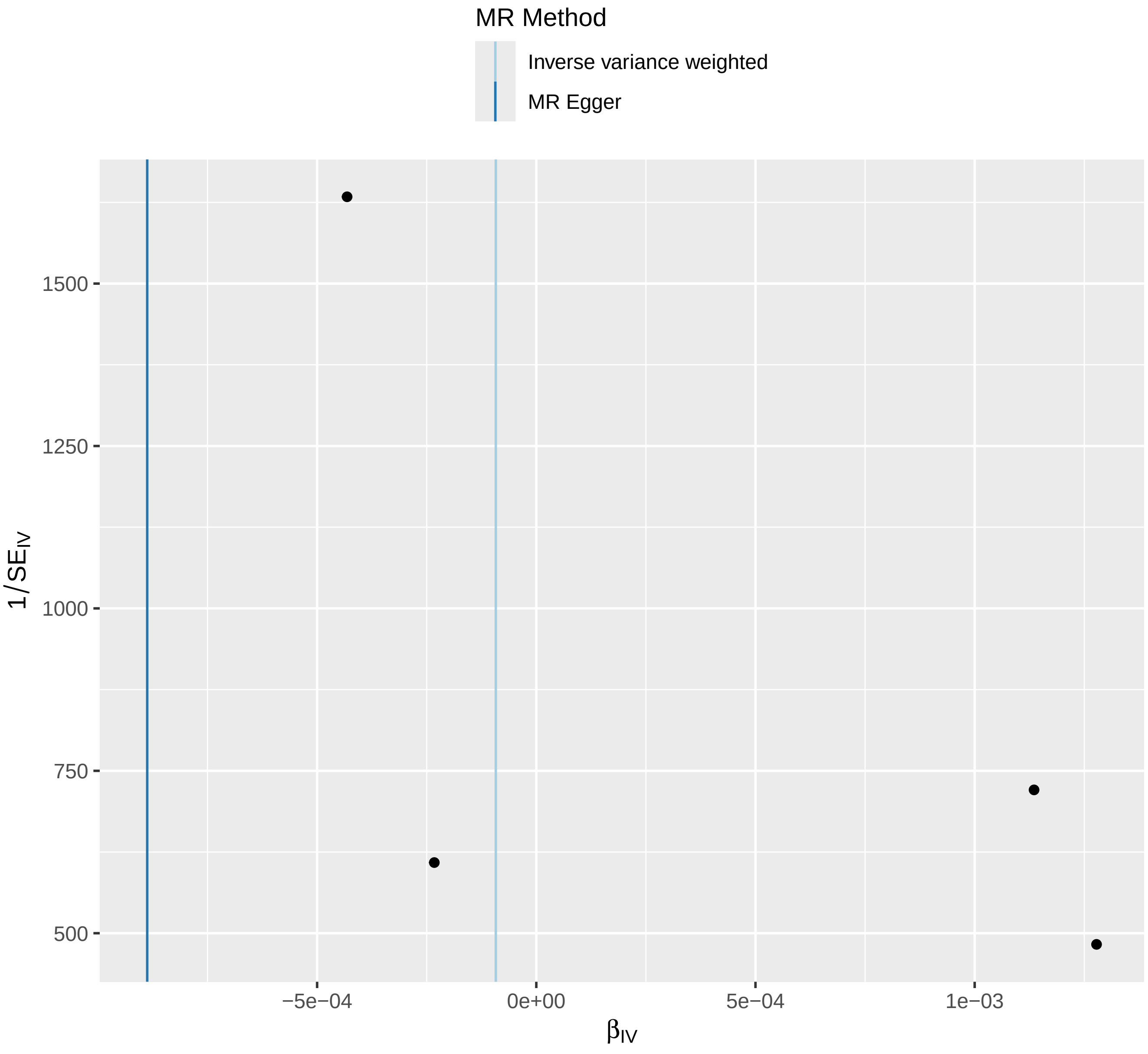

MR Method
Inverse variance weighted
MR Egger
1500
1250
1000
750
500
1/SE_IV
−5e−04
0e+00
5e−04
1e−03
β_IV

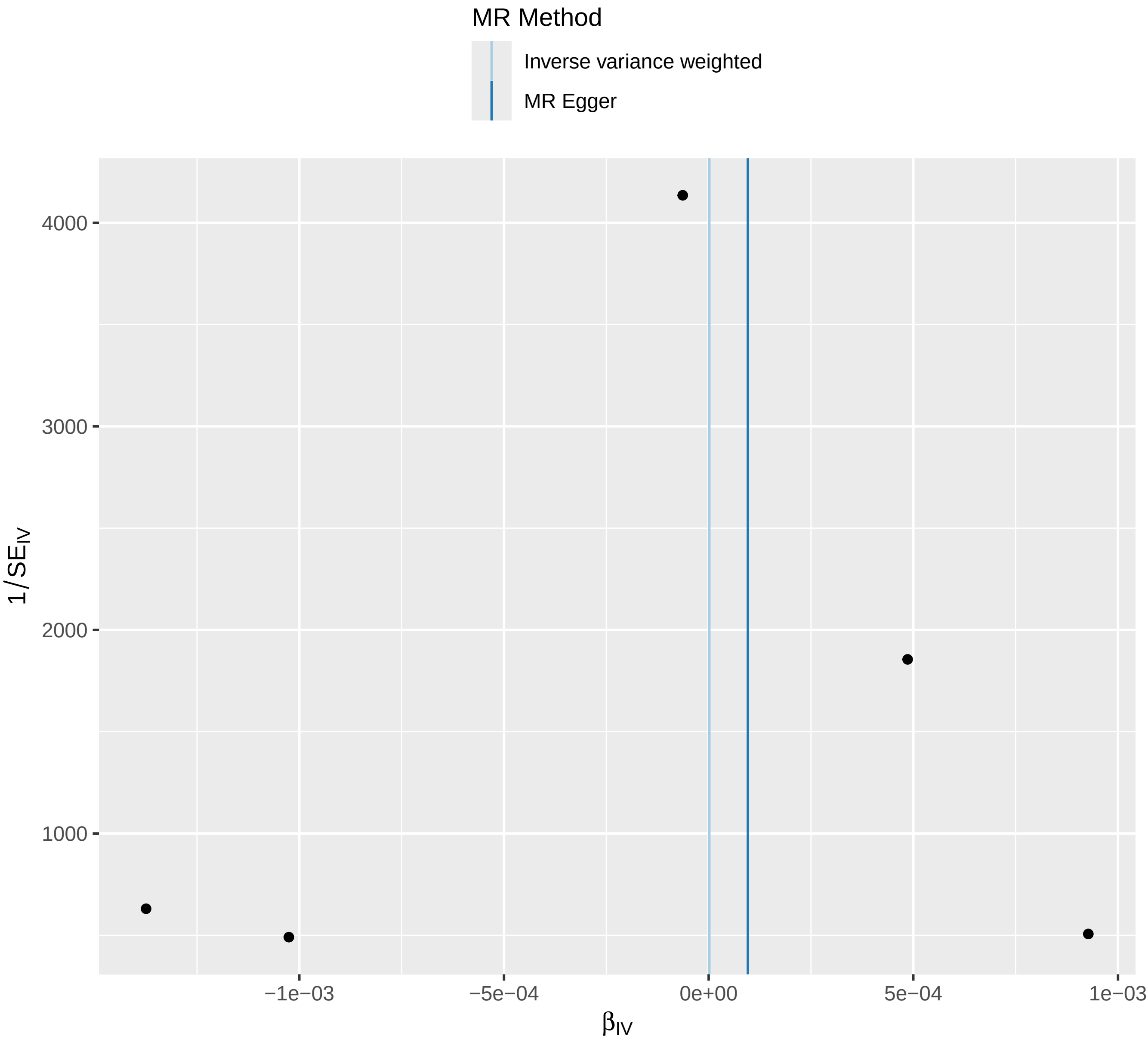

MR Method
Inverse variance weighted
MR Egger
4000
3000
2000
1000
1/SE_IV
−1e−03
−5e−04
0e+00
5e−04
1e−03
β_IV

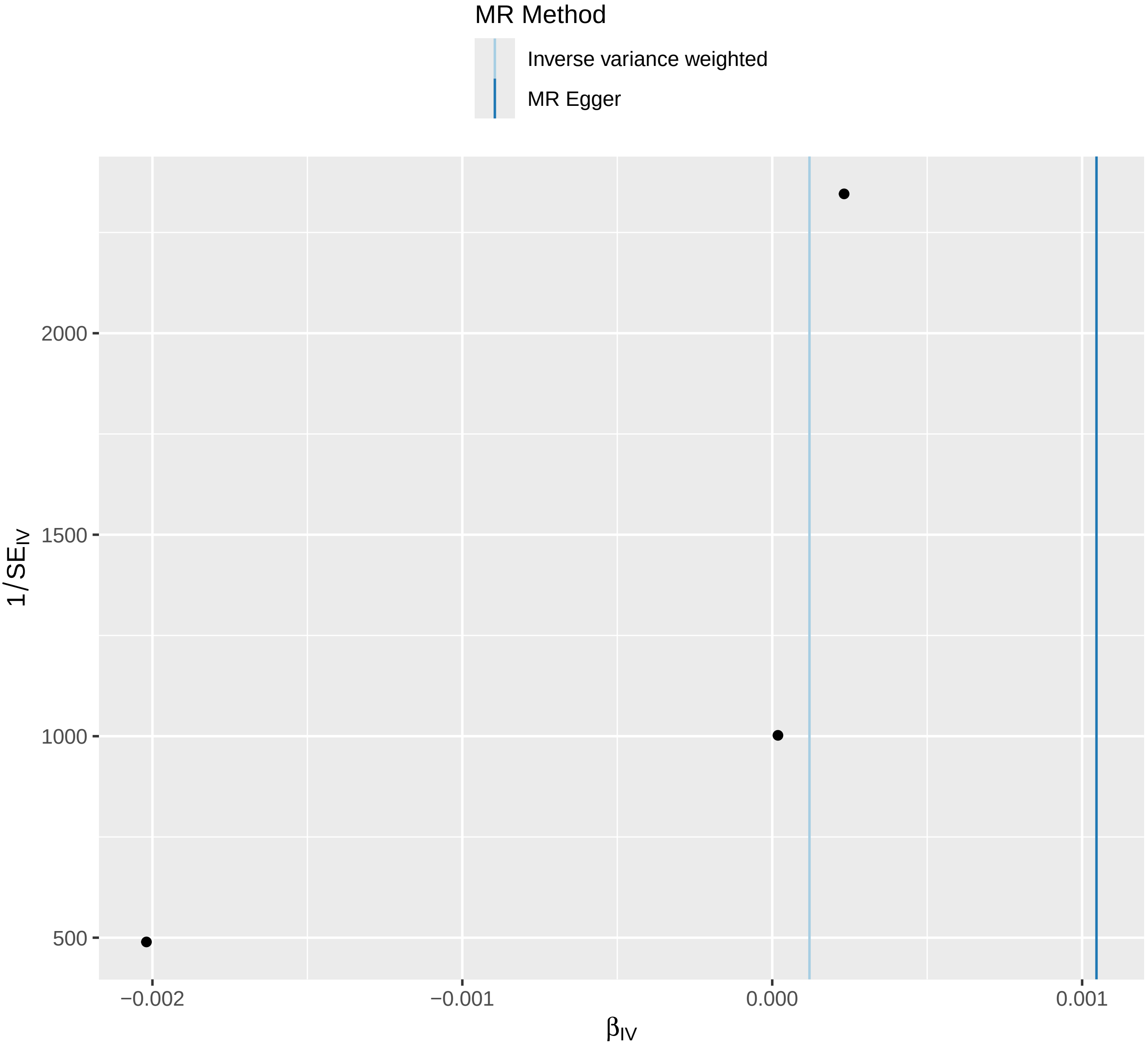
MR Method
Inverse variance weighted
MR Egger
2000
1500
1000
500
$1/SE_{IV}$
−0.002
−0.001
0.000
0.001
β_{IV}

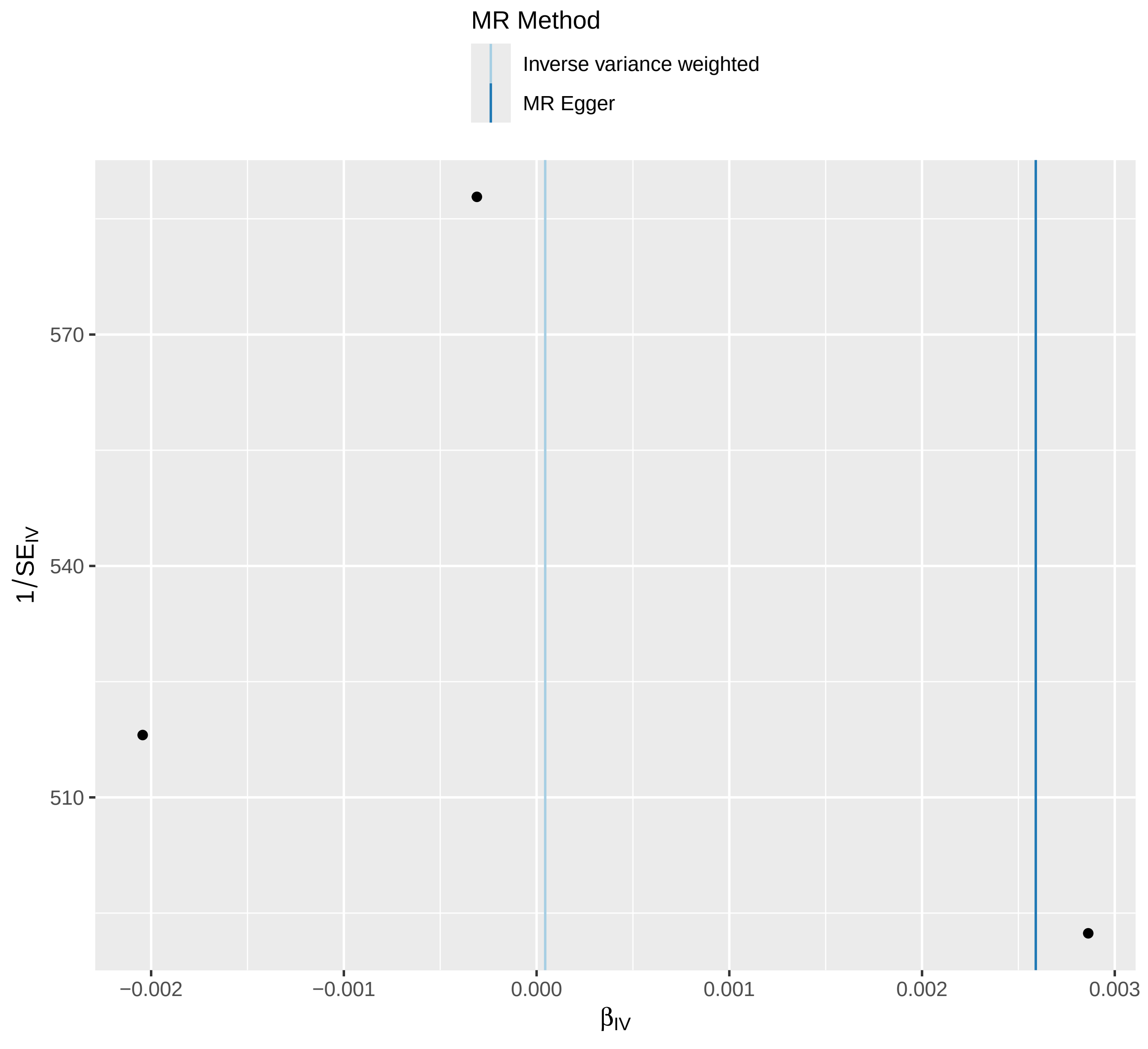

MR Method
Inverse variance weighted
MR Egger
570
540
510
1/SE_IV
−0.002
−0.001
0.000
0.001
0.002
0.003
β_IV

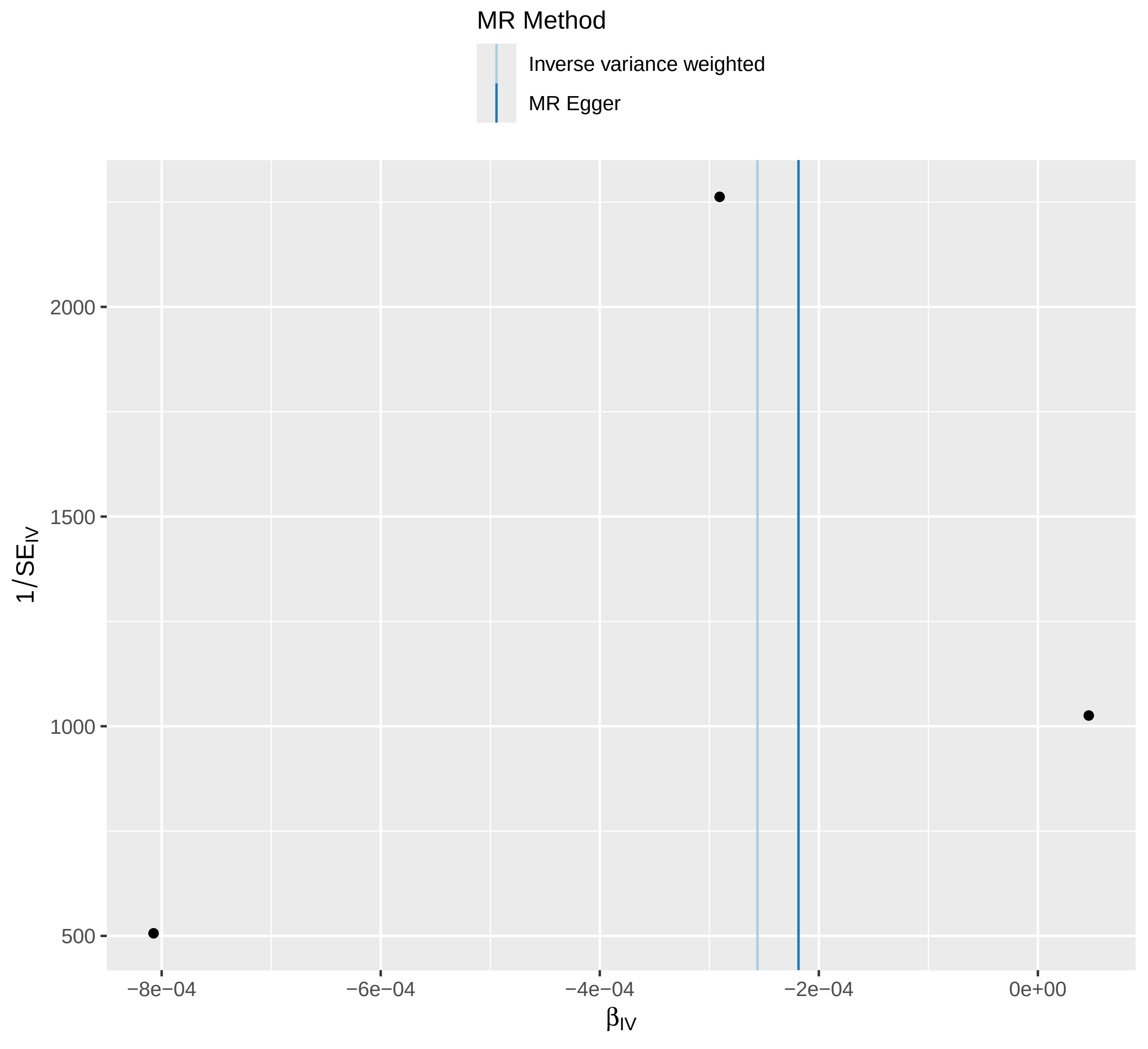
MR Method
Inverse variance weighted
MR Egger
2000
1500
1000
500
1/SE_IV
−8e−04
−6e−04
−4e−04
−2e−04
0e+00
β_IV

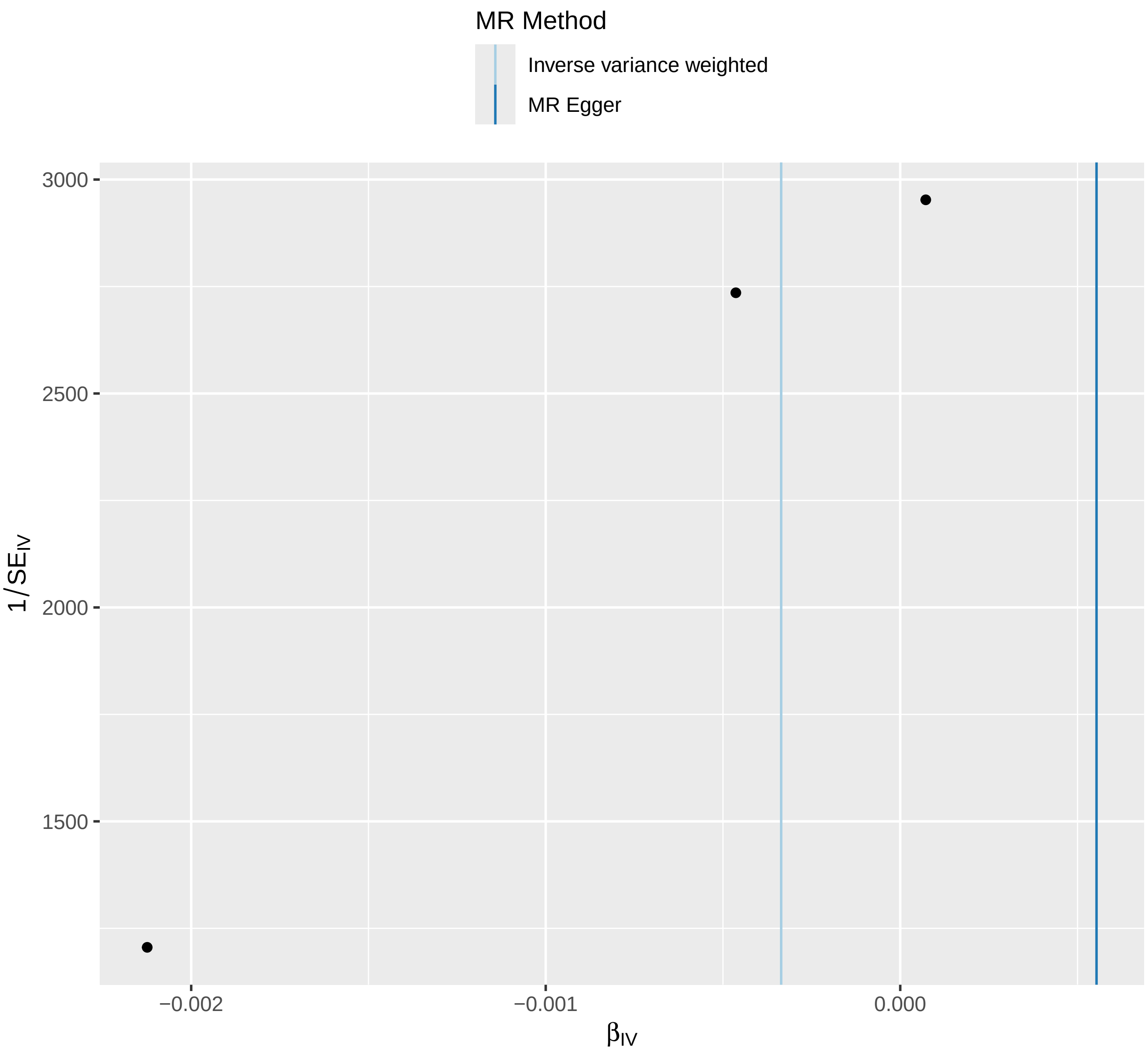
MR Method
Inverse variance weighted
MR Egger
3000
2500
2000
1500
1/SE$_{IV}$
−0.002
−0.001
0.000
β$_{IV}$

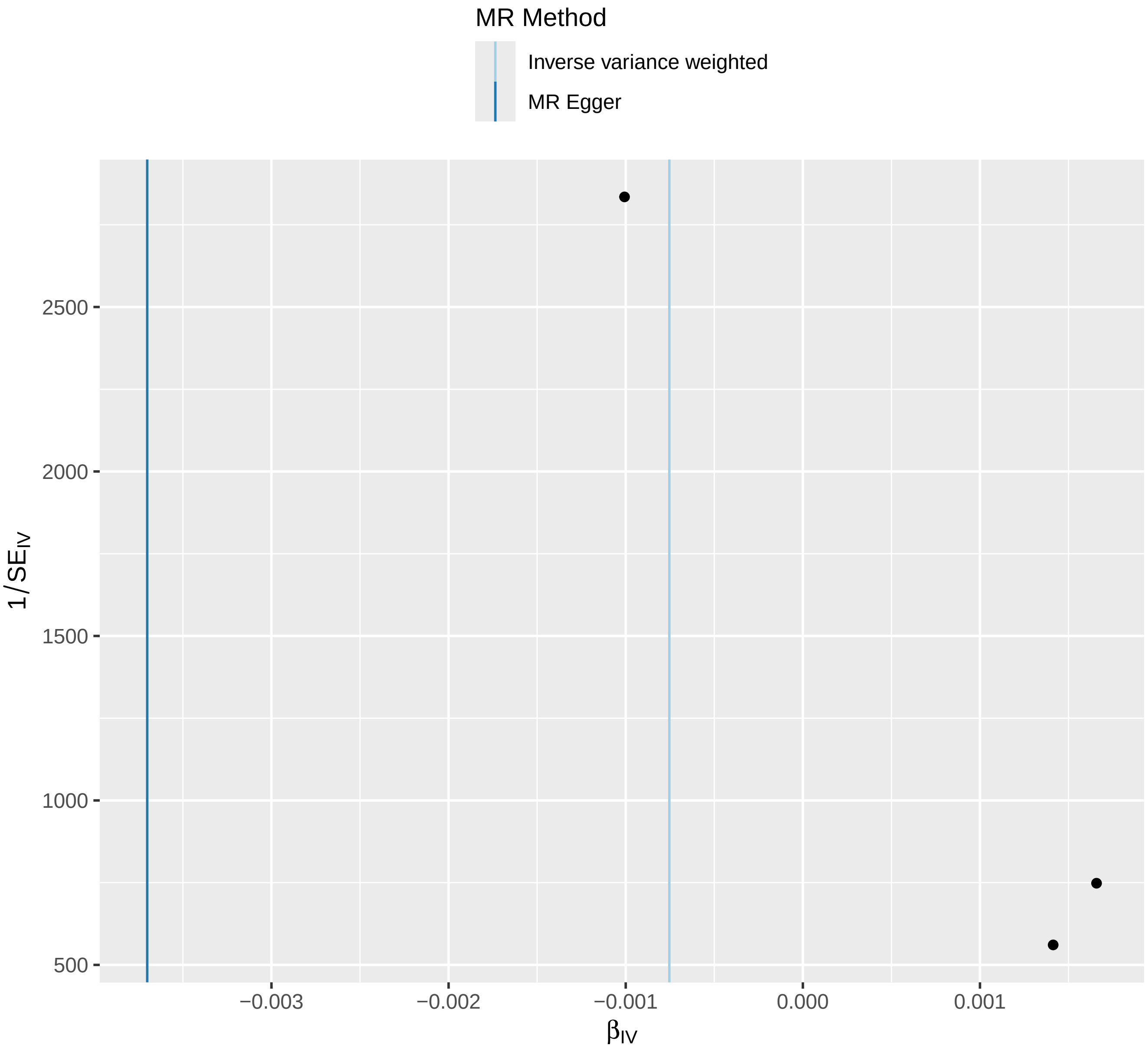
MR Method
Inverse variance weighted
MR Egger
2500
2000
1500
1000
500
1/SE_IV
−0.003
−0.002
−0.001
0.000
0.001
β_IV

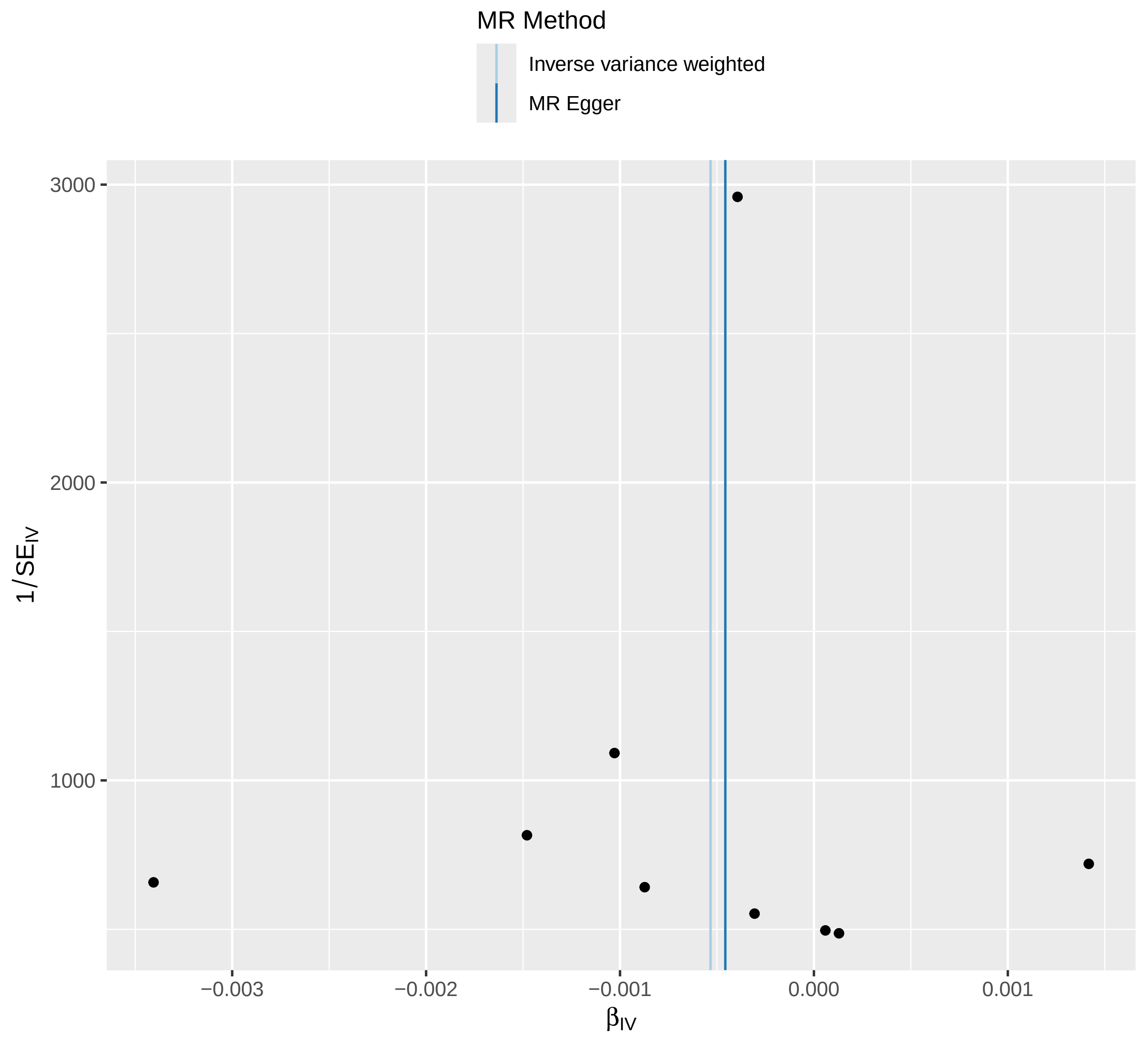

MR Method
Inverse variance weighted
MR Egger
3000
2000
1000
1/SE$_{IV}$
−0.003
−0.002
−0.001
0.000
0.001
β$_{IV}$

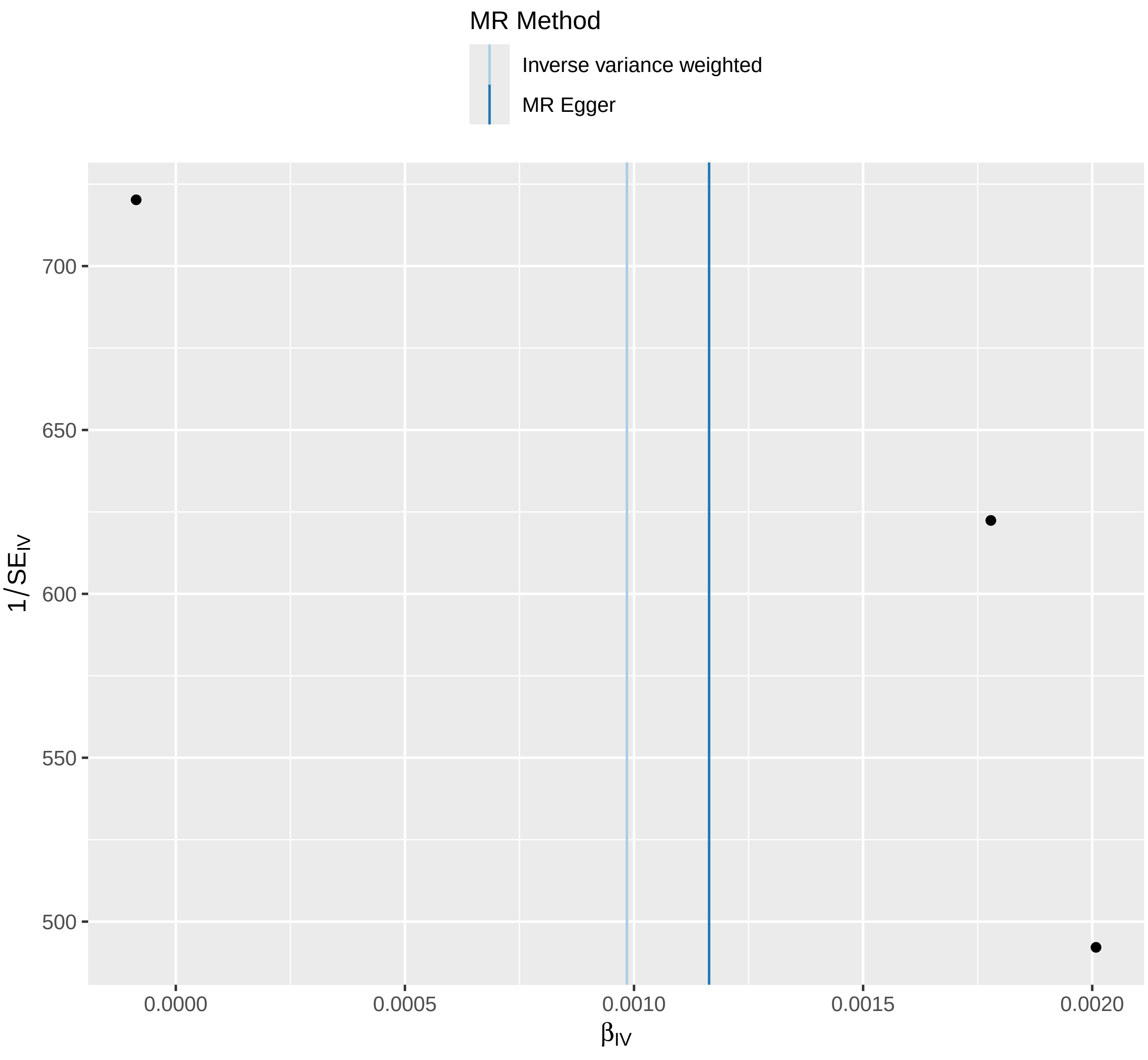

MR Method
Inverse variance weighted
MR Egger
700
650
600
550
500
1/SE_IV
0.0000
0.0005
0.0010
0.0015
0.0020
β_IV

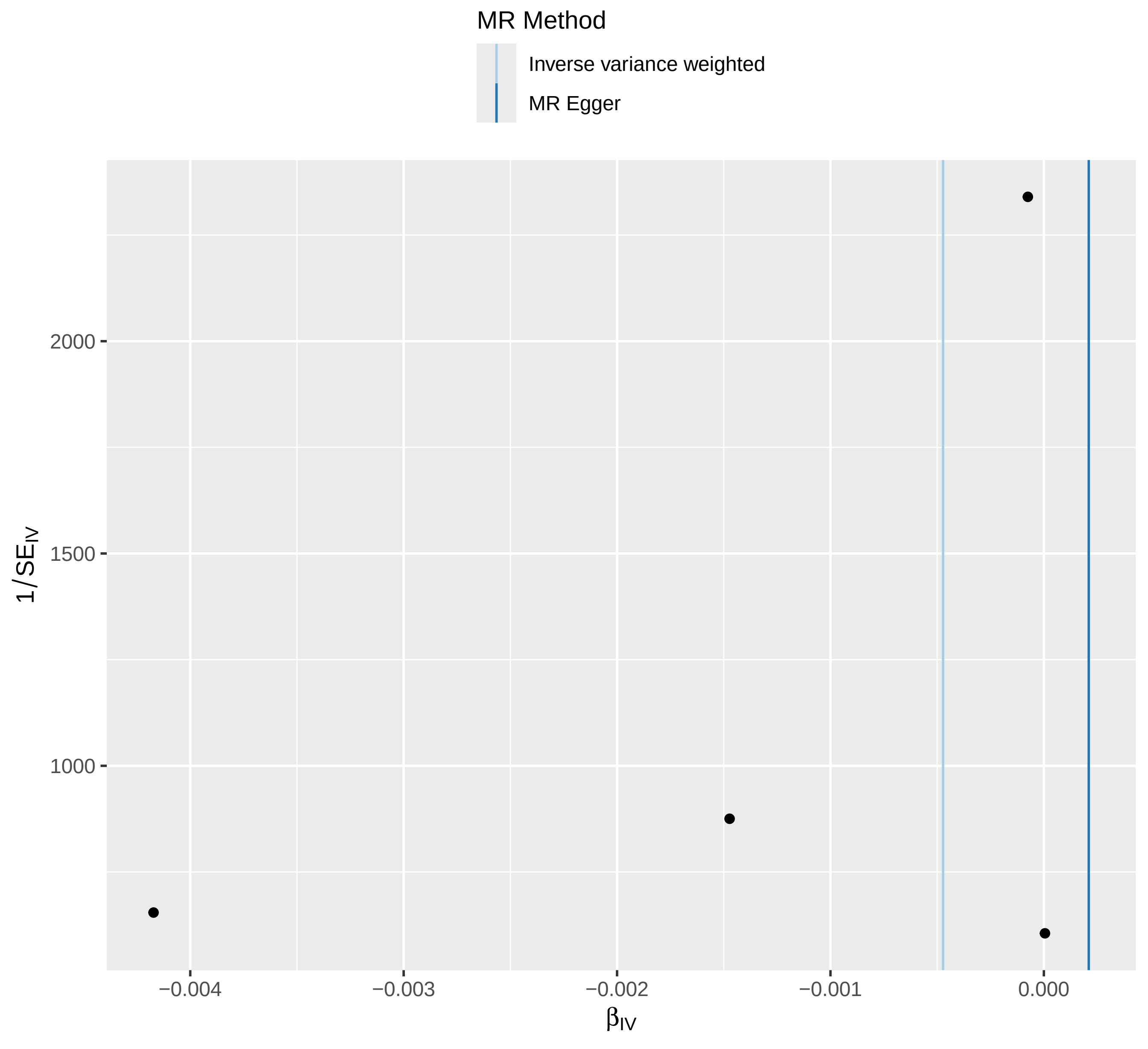
MR Method
Inverse variance weighted
MR Egger
2000
1500
1000
1/SE_IV
−0.004
−0.003
−0.002
−0.001
0.000
β_IV

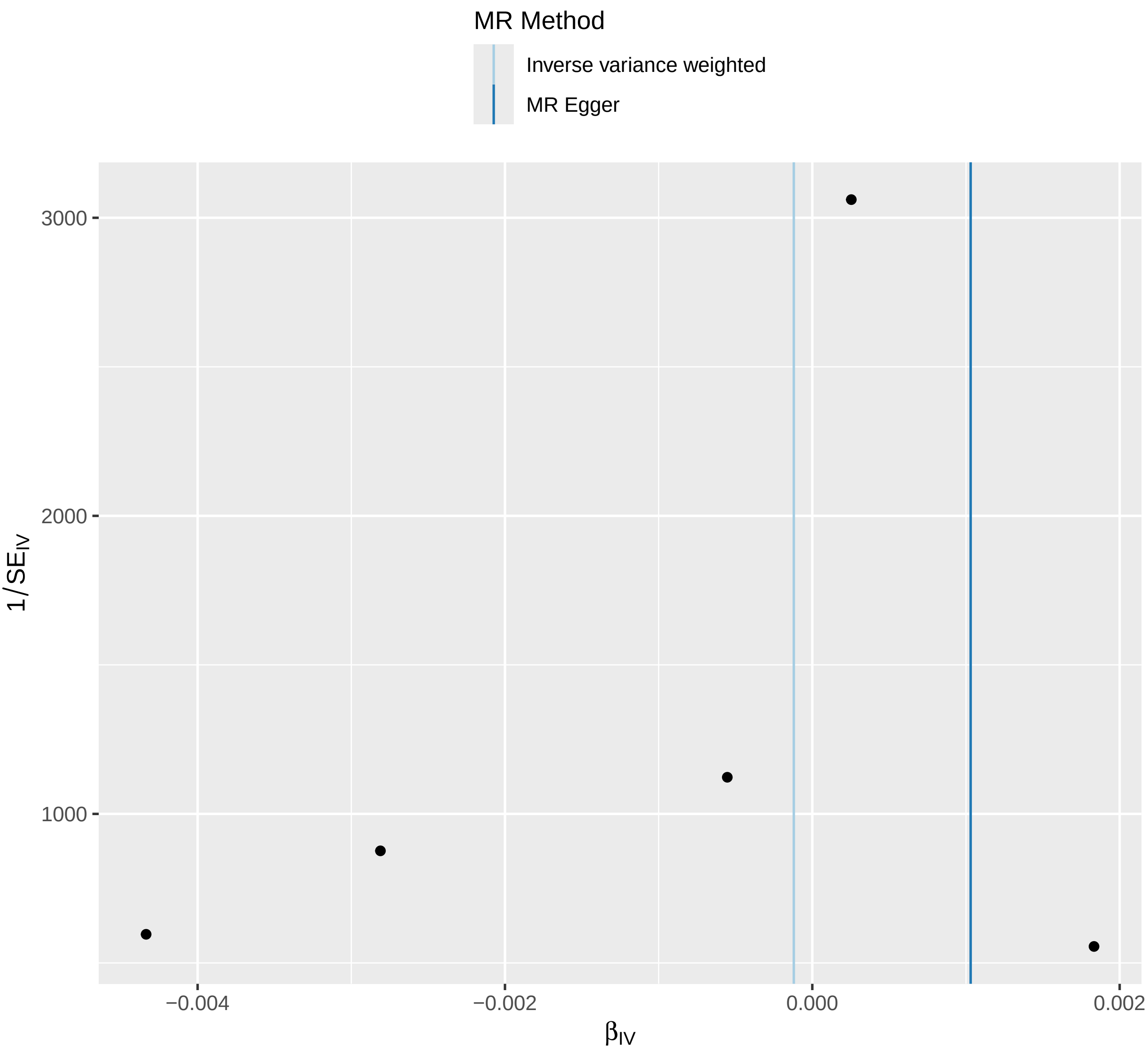

MR Method
Inverse variance weighted
MR Egger
3000
2000
1000
1/SE$_{IV}$
−0.004
−0.002
0.000
0.002
β$_{IV}$

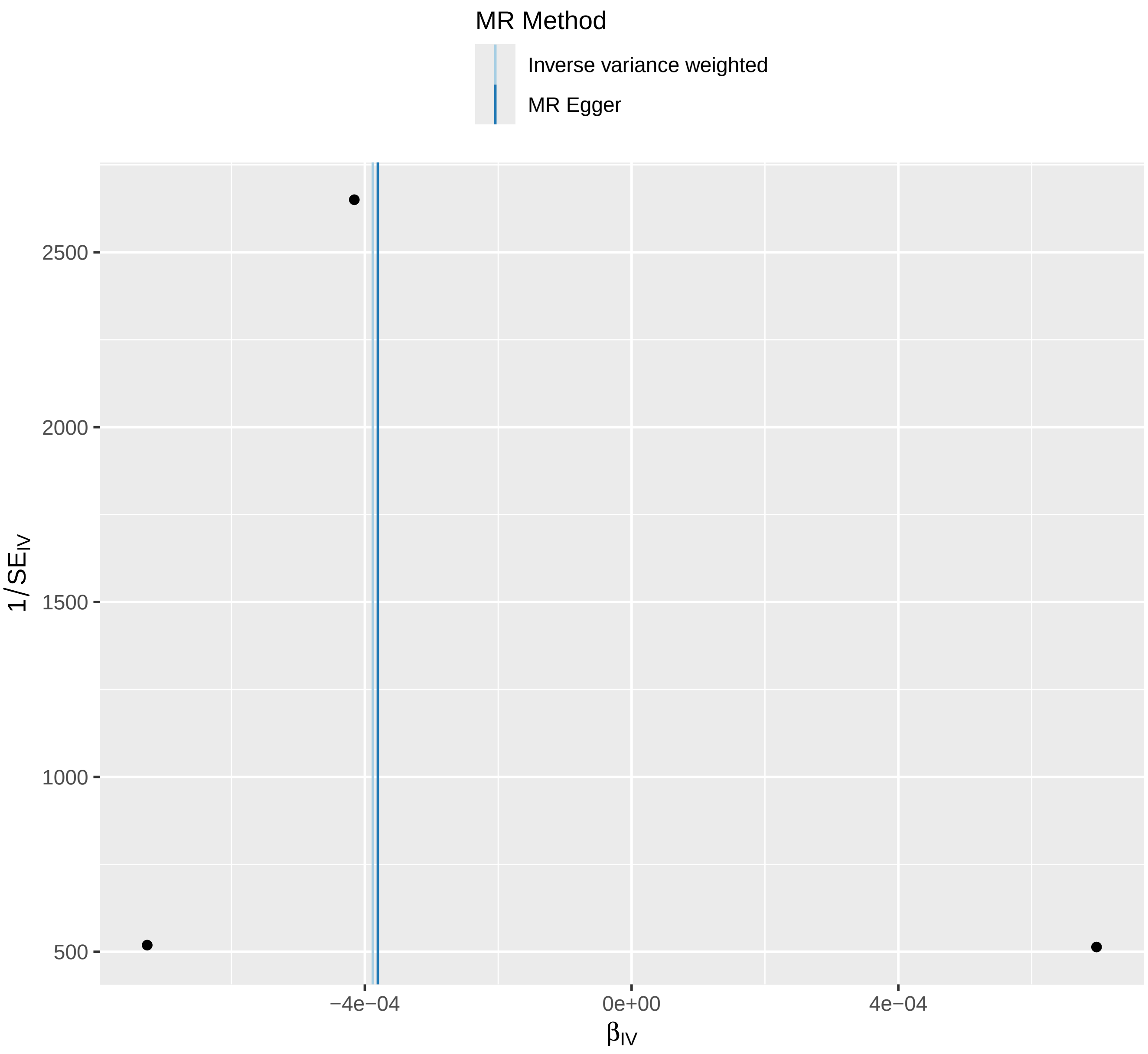

MR Method
Inverse variance weighted
MR Egger
2500
2000
1500
1000
500
−4e−04
0e+00
4e−04
β_{IV}
$1/SE_{IV}$

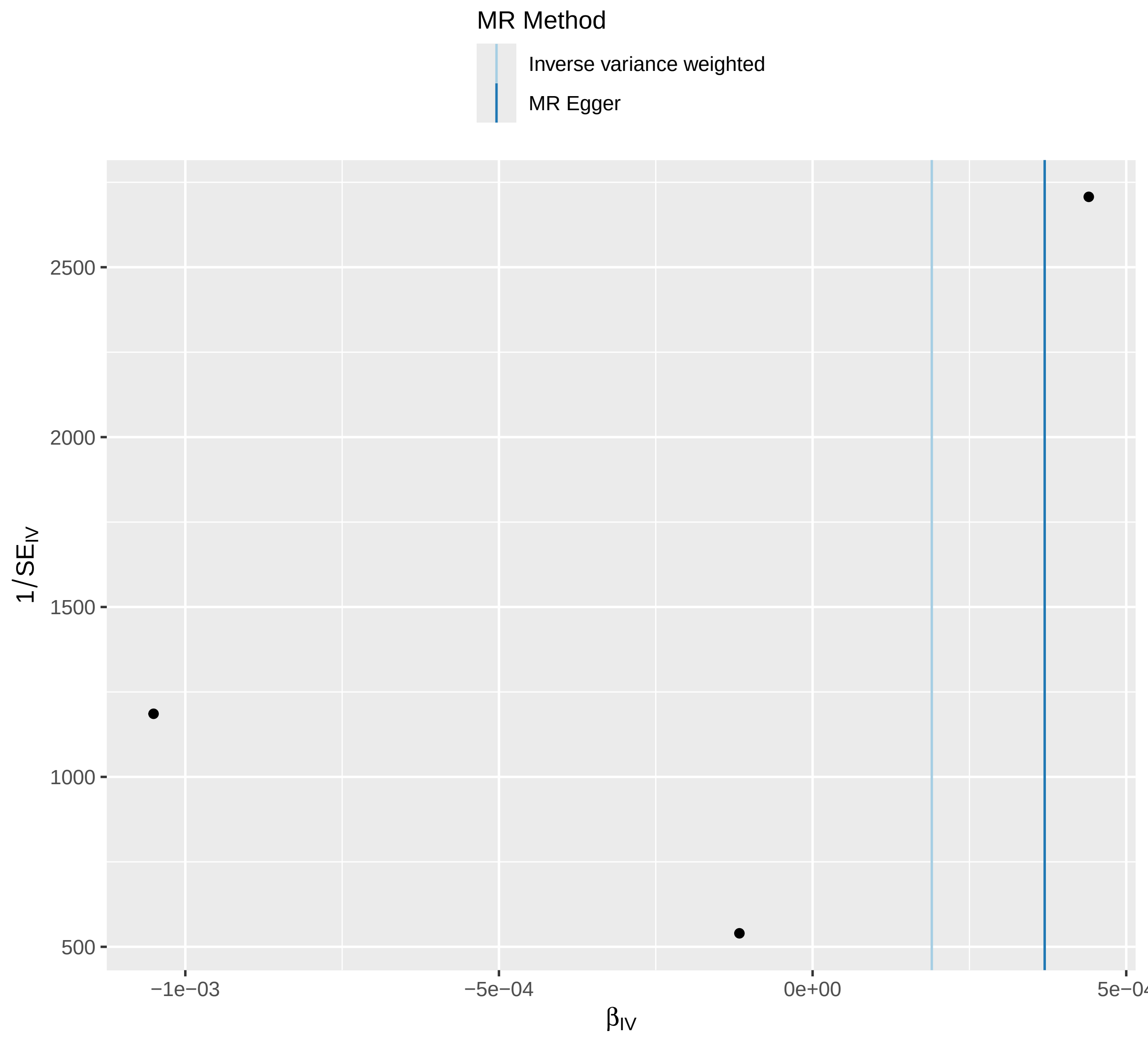
MR Method
Inverse variance weighted
MR Egger
2500
2000
1500
1000
500
$1/SE_{IV}$
−1e−03
−5e−04
0e+00
5e−04
β_{IV}

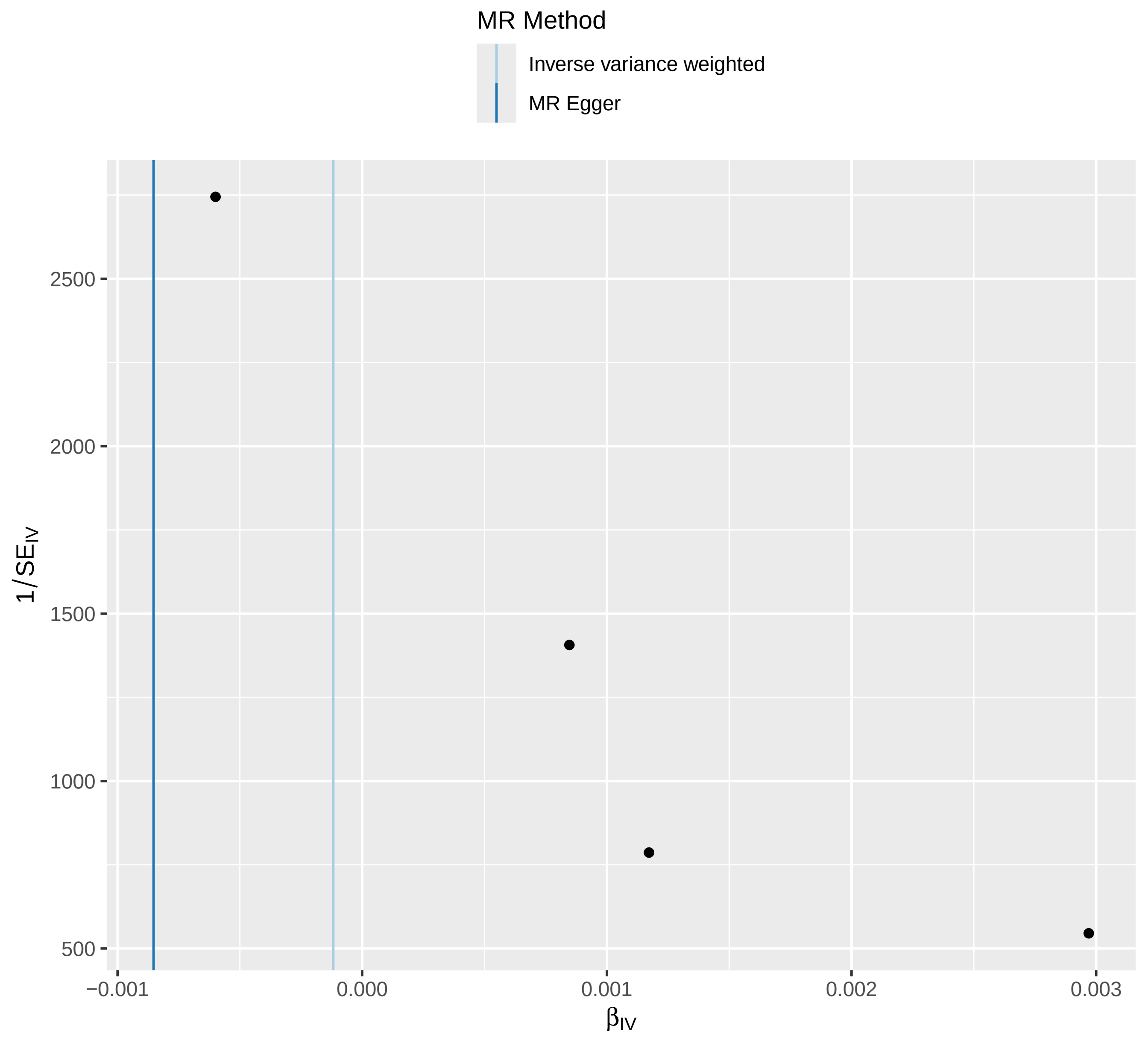

MR Method
Inverse variance weighted
MR Egger
2500
2000
1500
1000
500
1/SE_IV
−0.001
0.000
0.001
0.002
0.003
β_IV

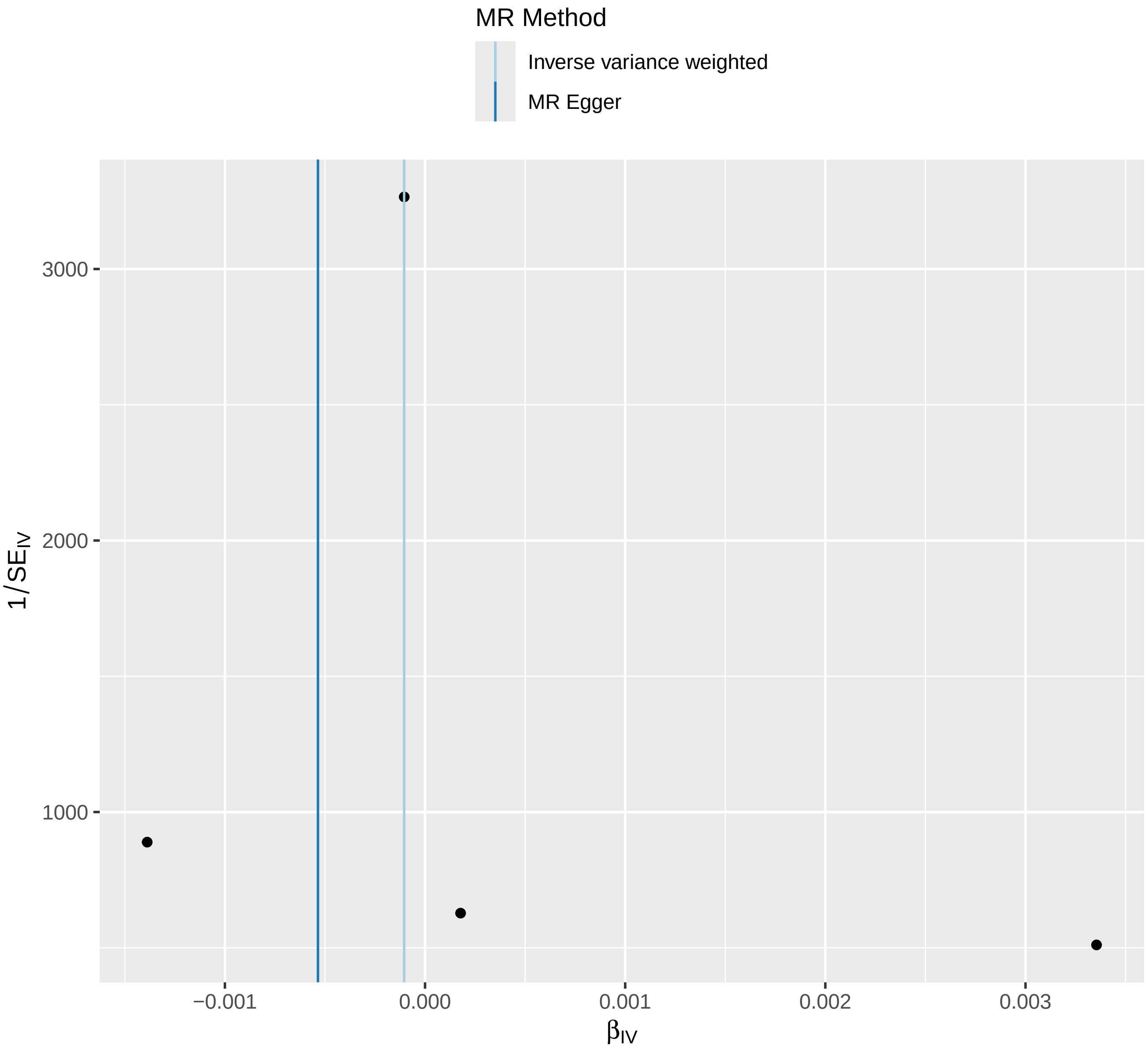

MR Method
Inverse variance weighted
MR Egger
3000
2000
1000
1/SE_IV
−0.001
0.000
0.001
0.002
0.003
β_IV

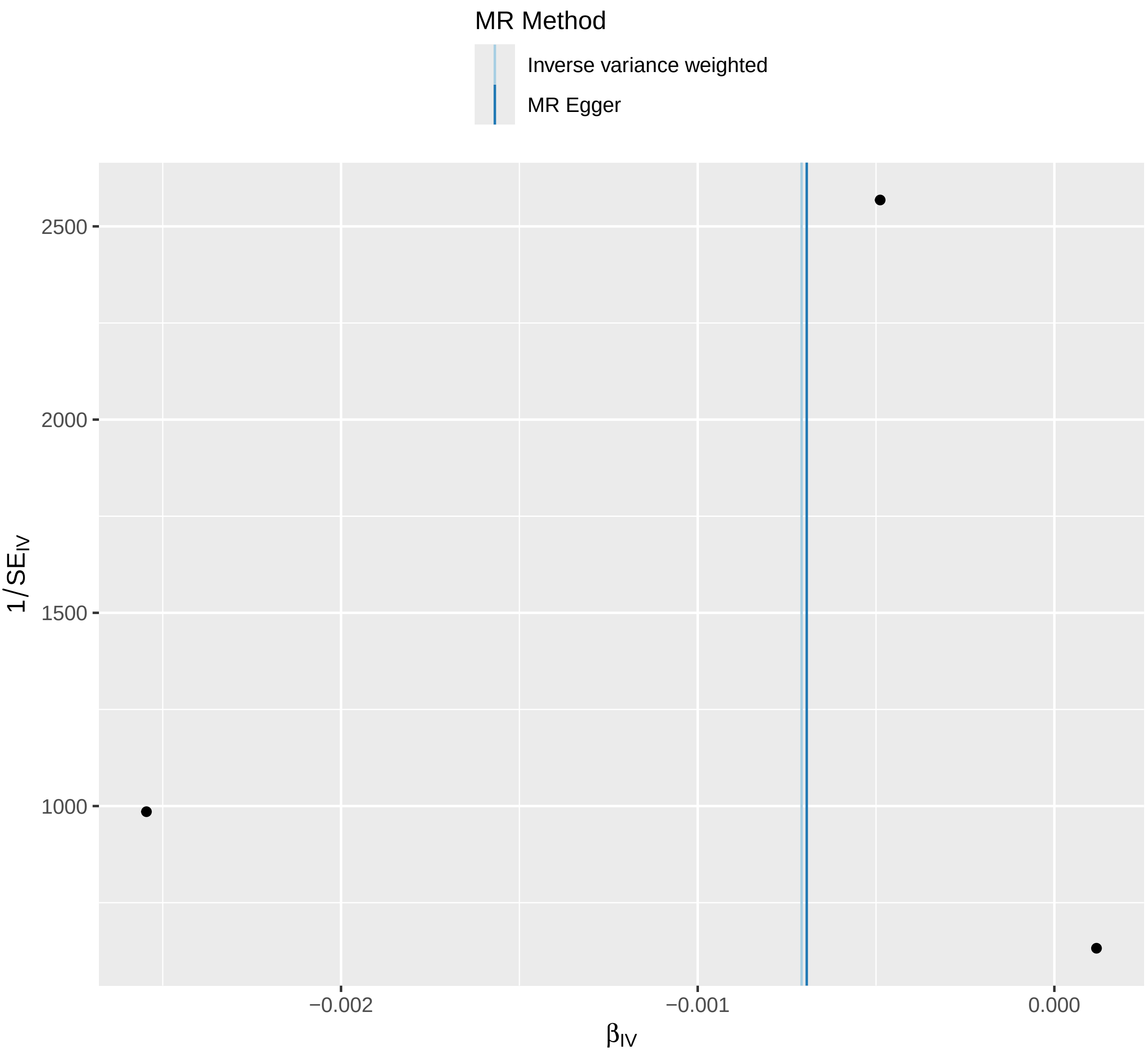

MR Method
Inverse variance weighted
MR Egger
2500
2000
1500
1000
1/SE_{IV}
−0.002
−0.001
0.000
β_{IV}

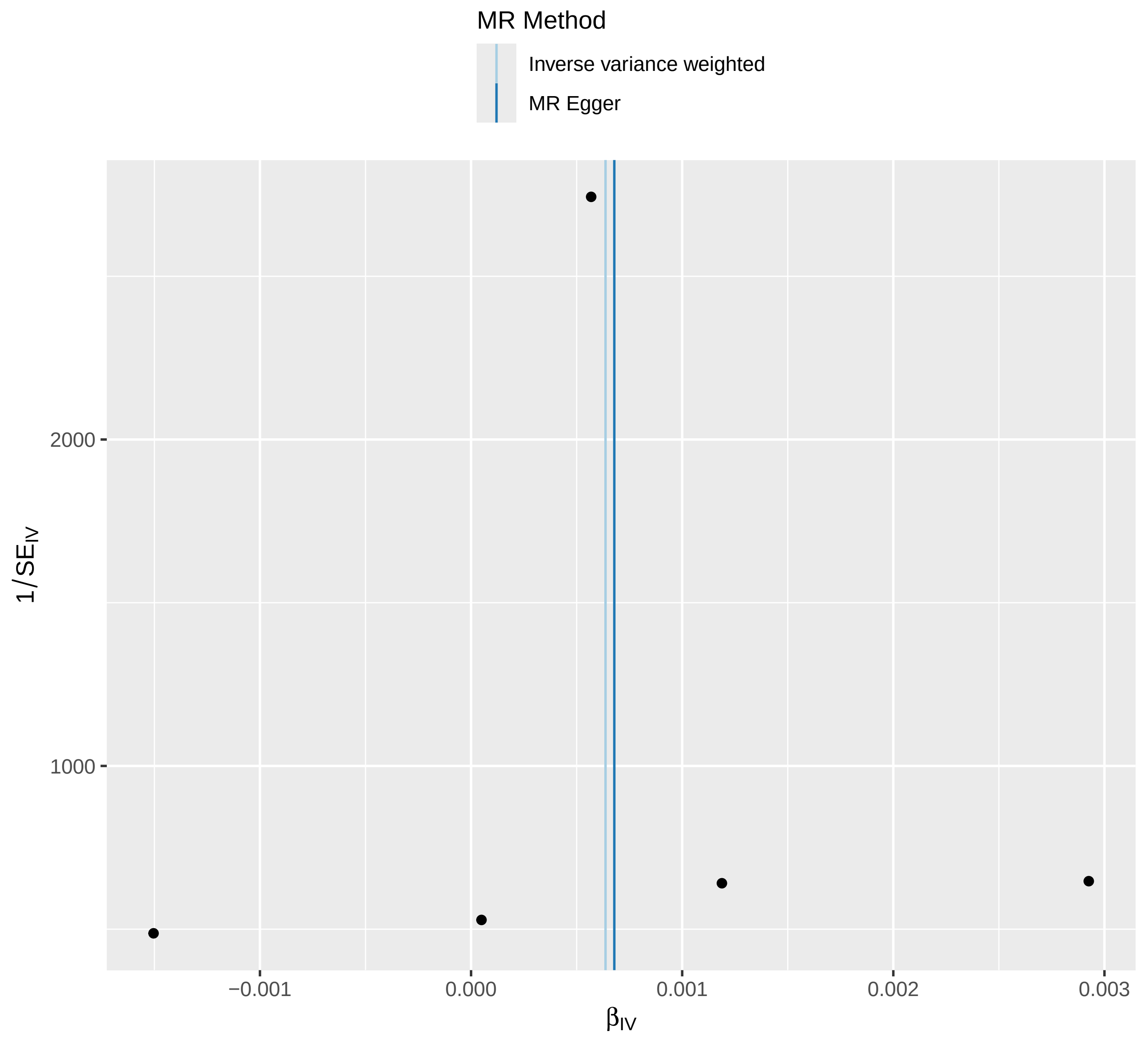

MR Method
Inverse variance weighted
MR Egger
2000
1000
1/SE_IV
−0.001
0.000
0.001
0.002
0.003
β_IV

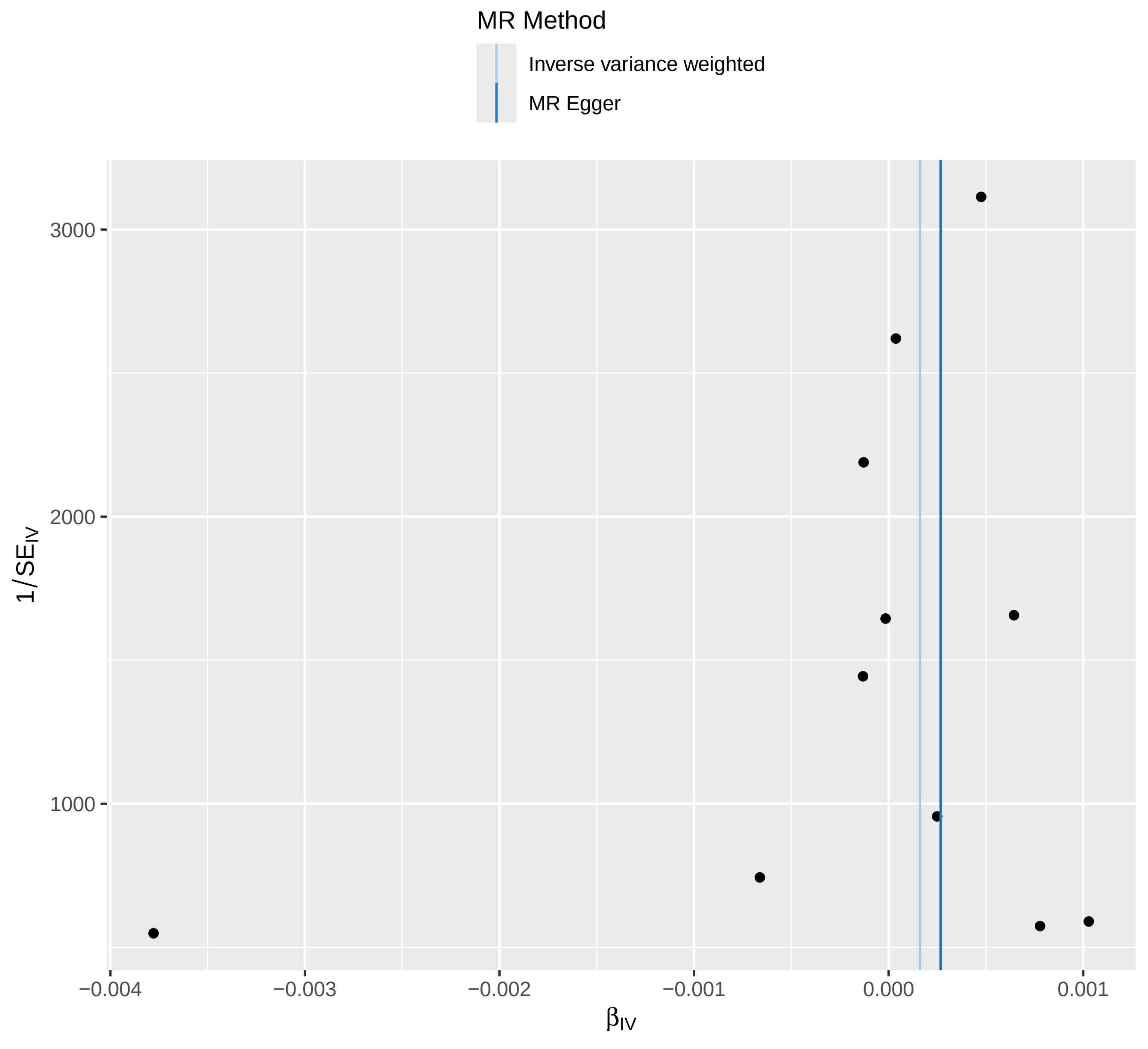
MR Method
Inverse variance weighted
MR Egger
3000
2000
1000
1/SE_IV
−0.004
−0.003
−0.002
−0.001
0.000
0.001
β_IV

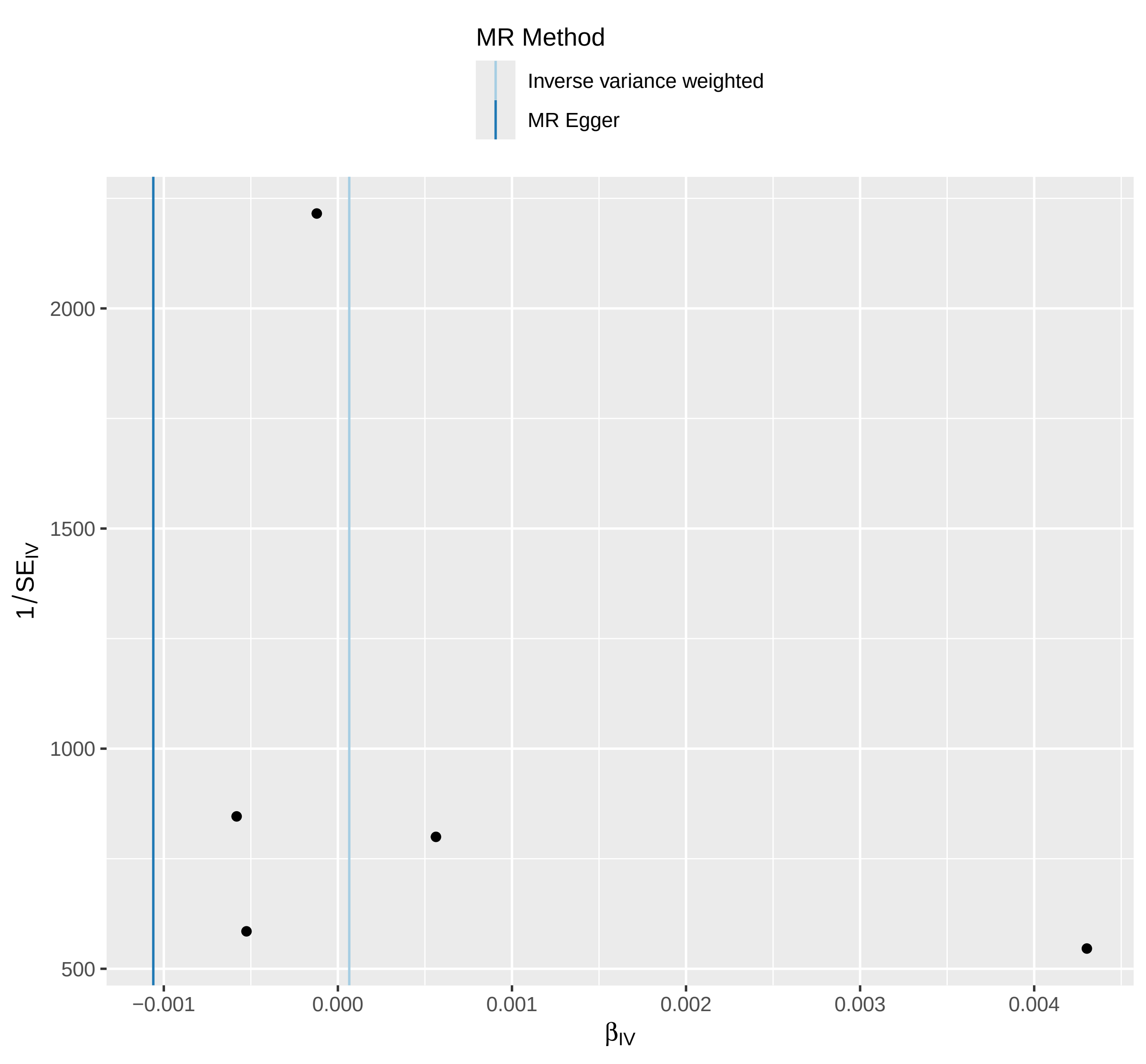

MR Method
Inverse variance weighted
MR Egger
2000
1500
1000
500
1/SE_IV
−0.001
0.000
0.001
0.002
0.003
0.004
β_IV

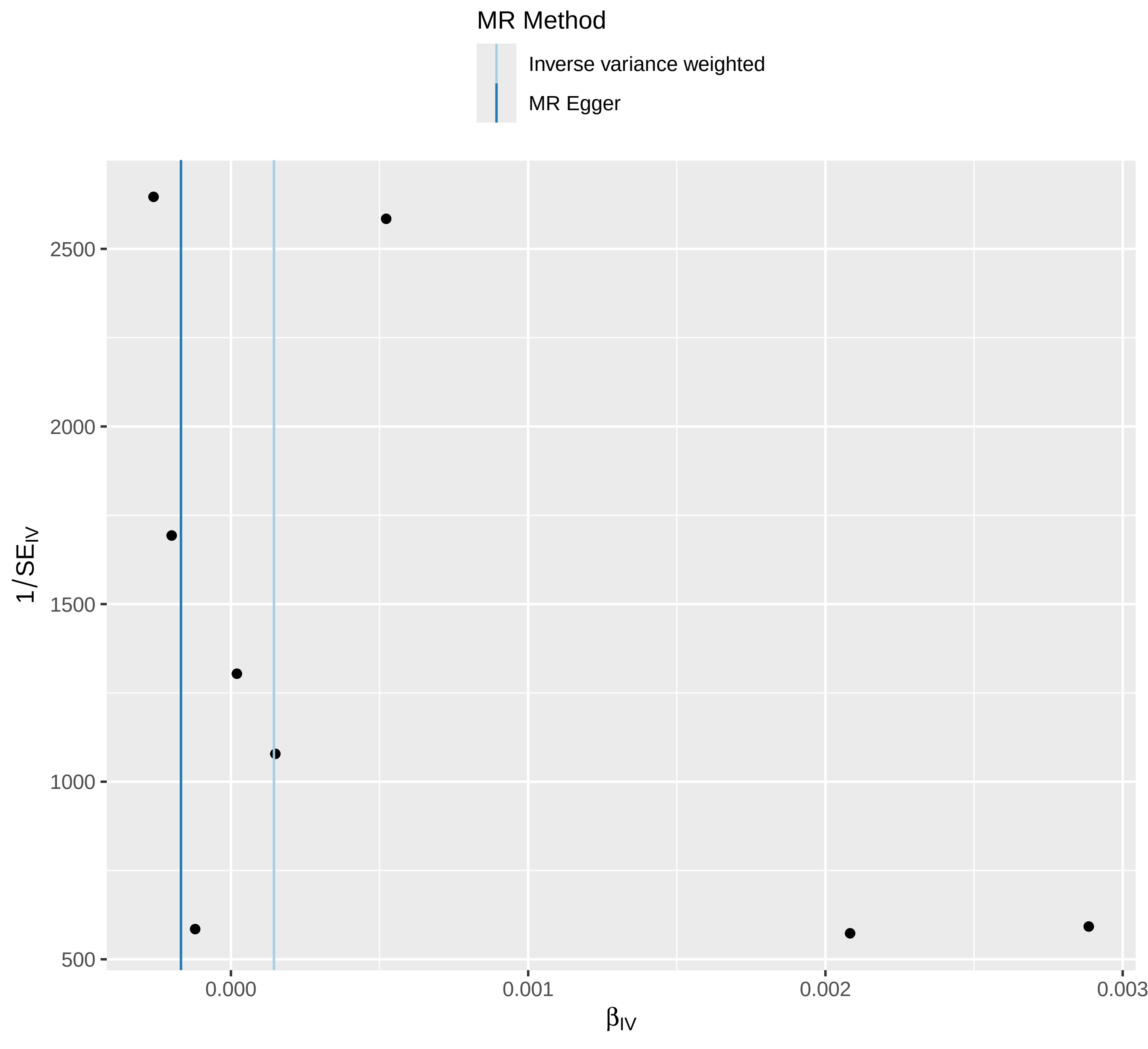

MR Method
Inverse variance weighted
MR Egger
2500
2000
1500
1000
500
1/SE_IV
0.000
0.001
0.002
0.003
β_IV

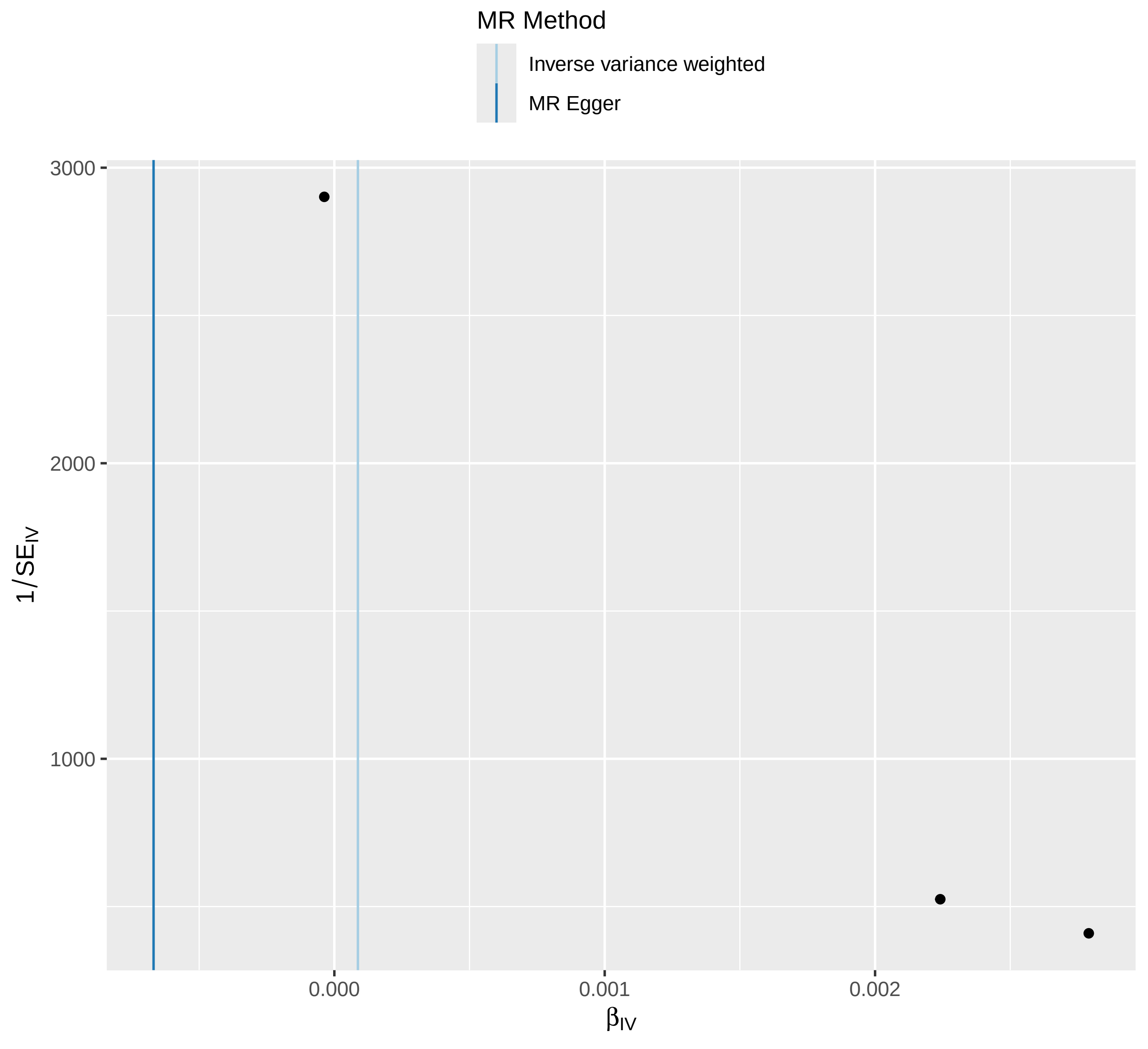

MR Method
Inverse variance weighted
MR Egger
3000
2000
1000
1/SE_IV
0.000
0.001
0.002
β_IV

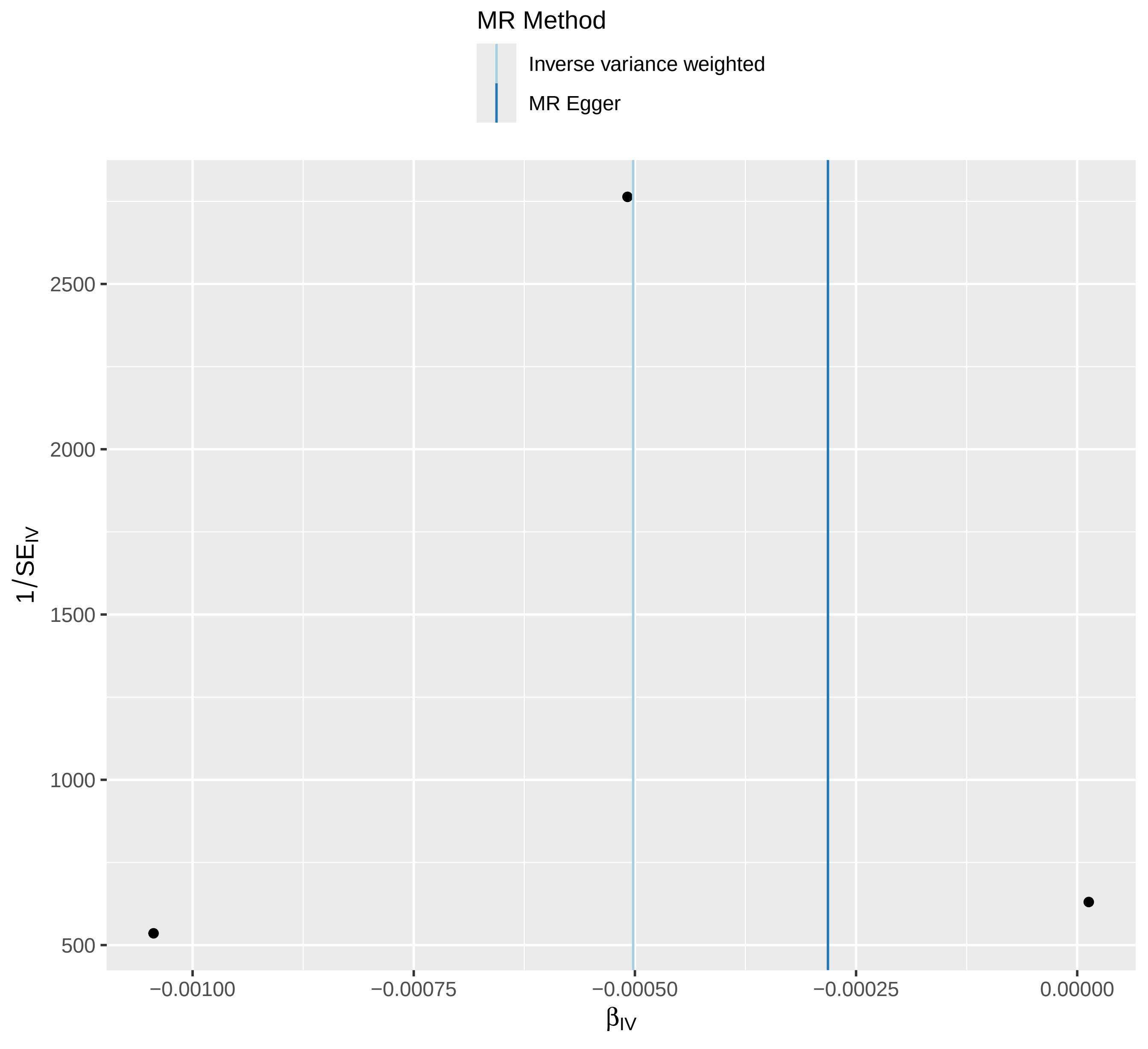
MR Method
Inverse variance weighted
MR Egger
2500
2000
1500
1000
500
1/SE_IV
−0.00100
−0.00075
−0.00050
−0.00025
0.00000
β_IV

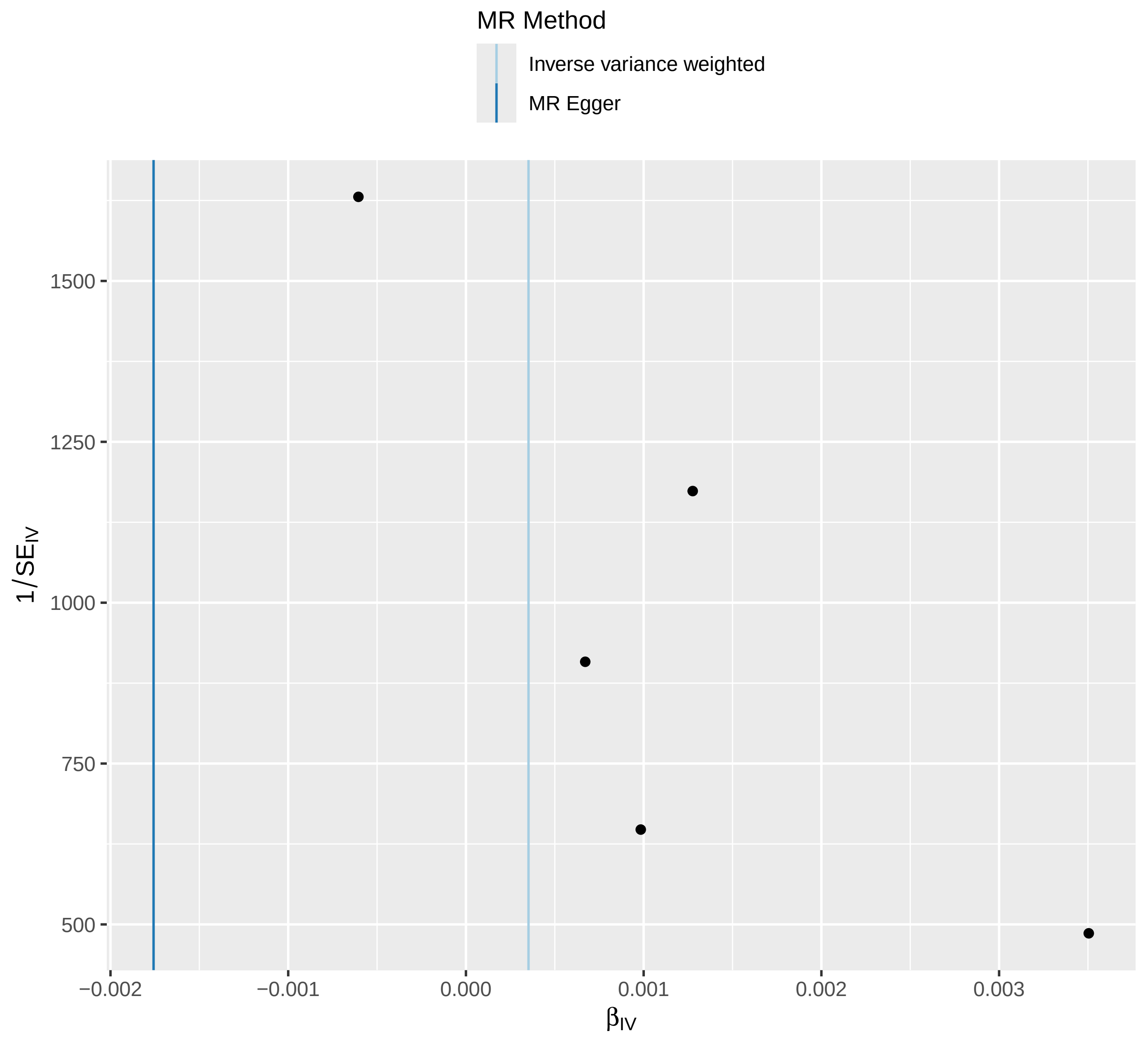

MR Method
Inverse variance weighted
MR Egger
1/SE_IV
1500
1250
1000
750
500
−0.002
−0.001
0.000
0.001
0.002
0.003
β_IV

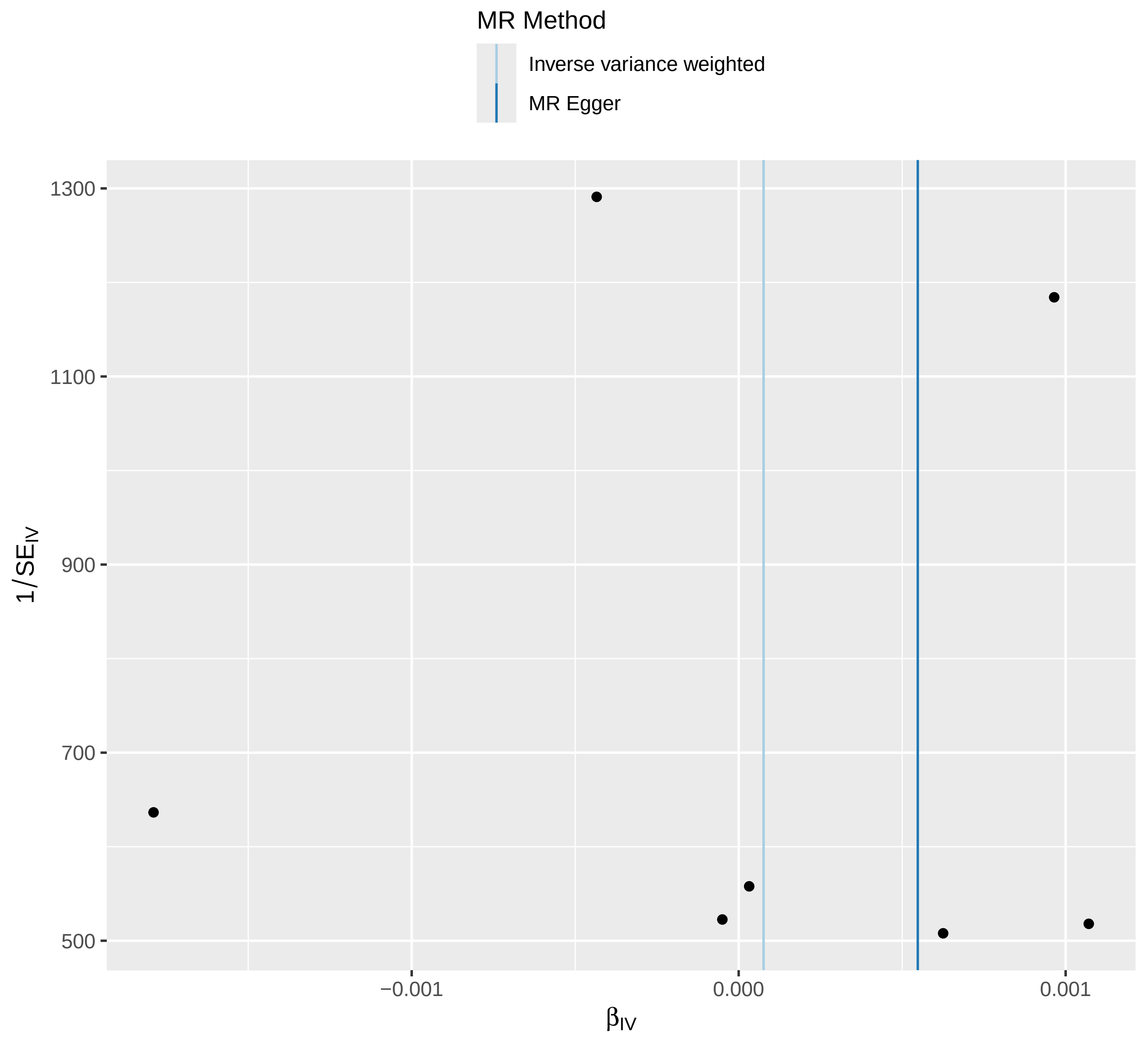

MR Method
Inverse variance weighted
MR Egger
1300
1100
900
700
500
1/SE_IV
−0.001
0.000
0.001
β_IV

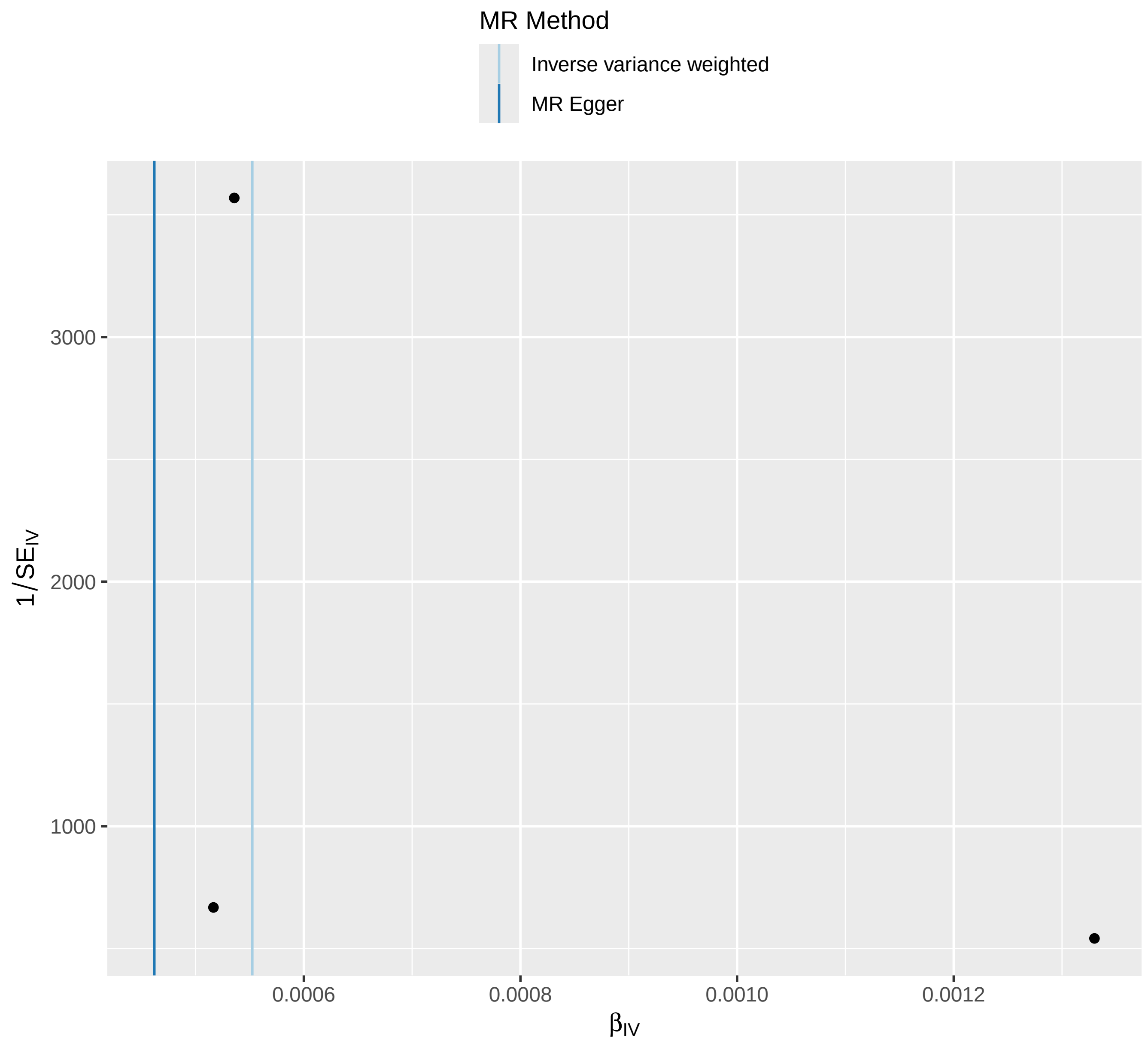
MR Method
Inverse variance weighted
MR Egger
3000
2000
1000
1/SE_IV
0.0006
0.0008
0.0010
0.0012
β_IV

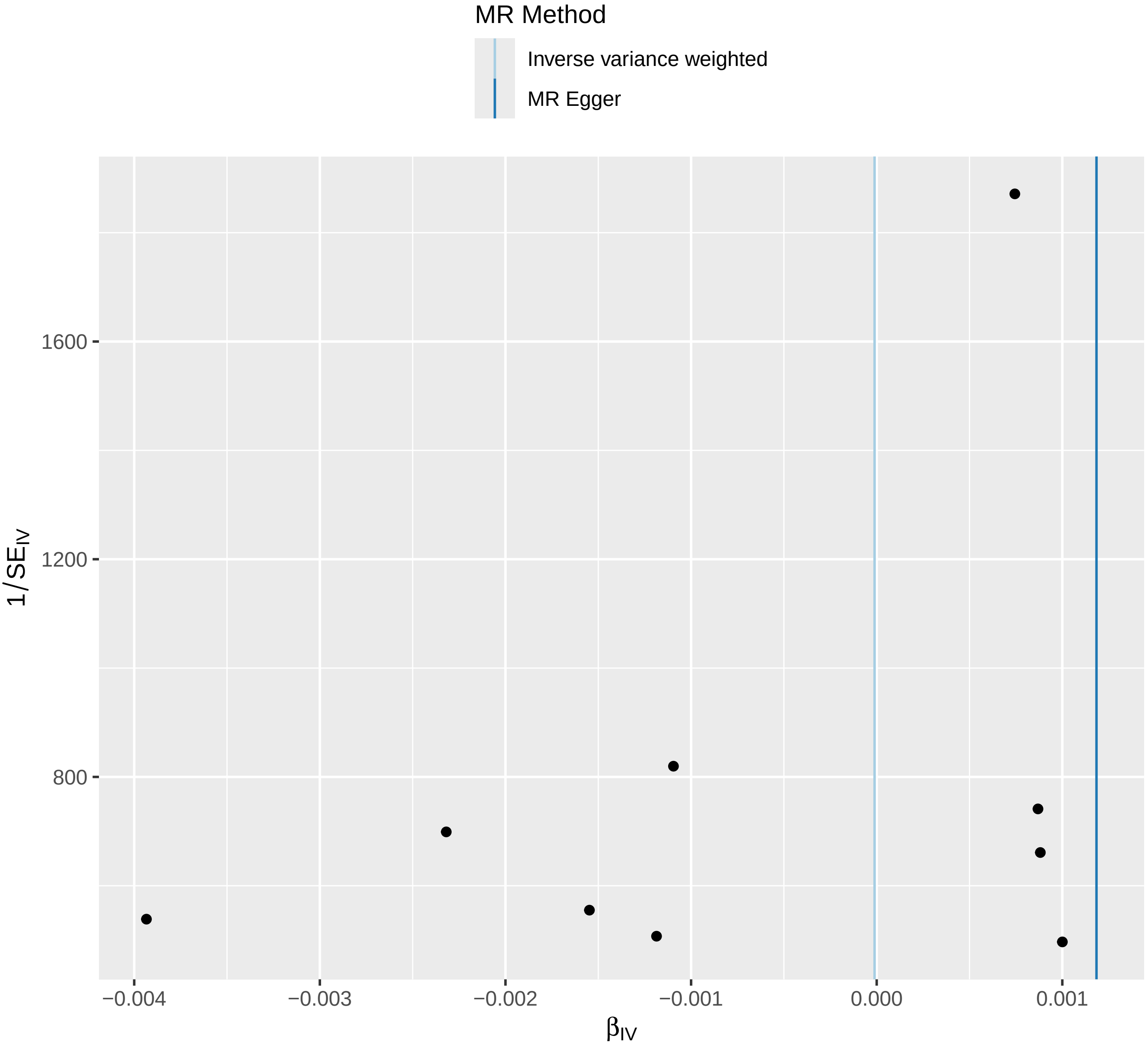

MR Method
Inverse variance weighted
MR Egger
1600
1200
800
1/SE_IV
−0.004
−0.003
−0.002
−0.001
0.000
0.001
β_IV

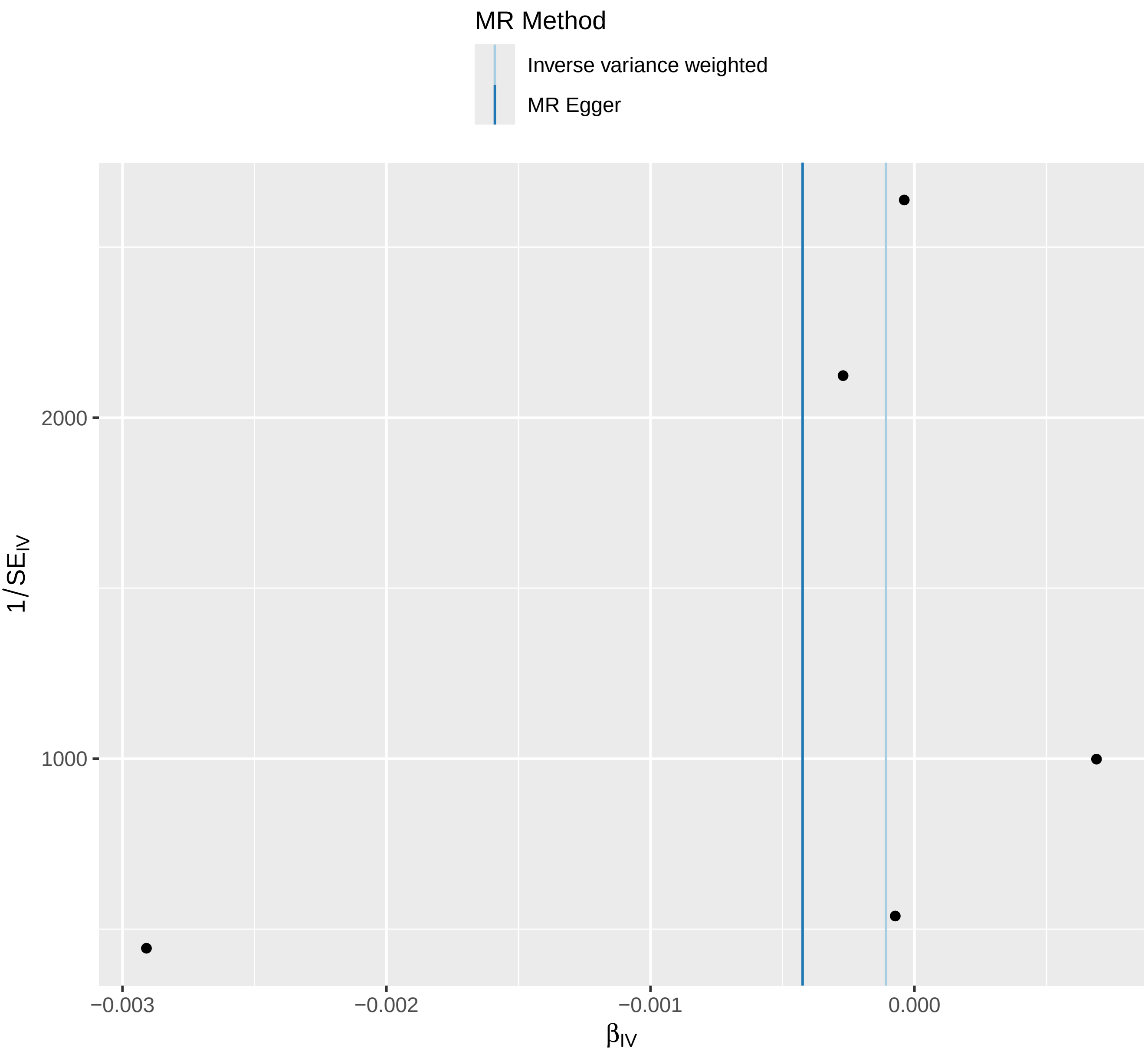

MR Method
Inverse variance weighted
MR Egger
2000
1000
1/SE$_{IV}$
−0.003
−0.002
−0.001
0.000
β$_{IV}$

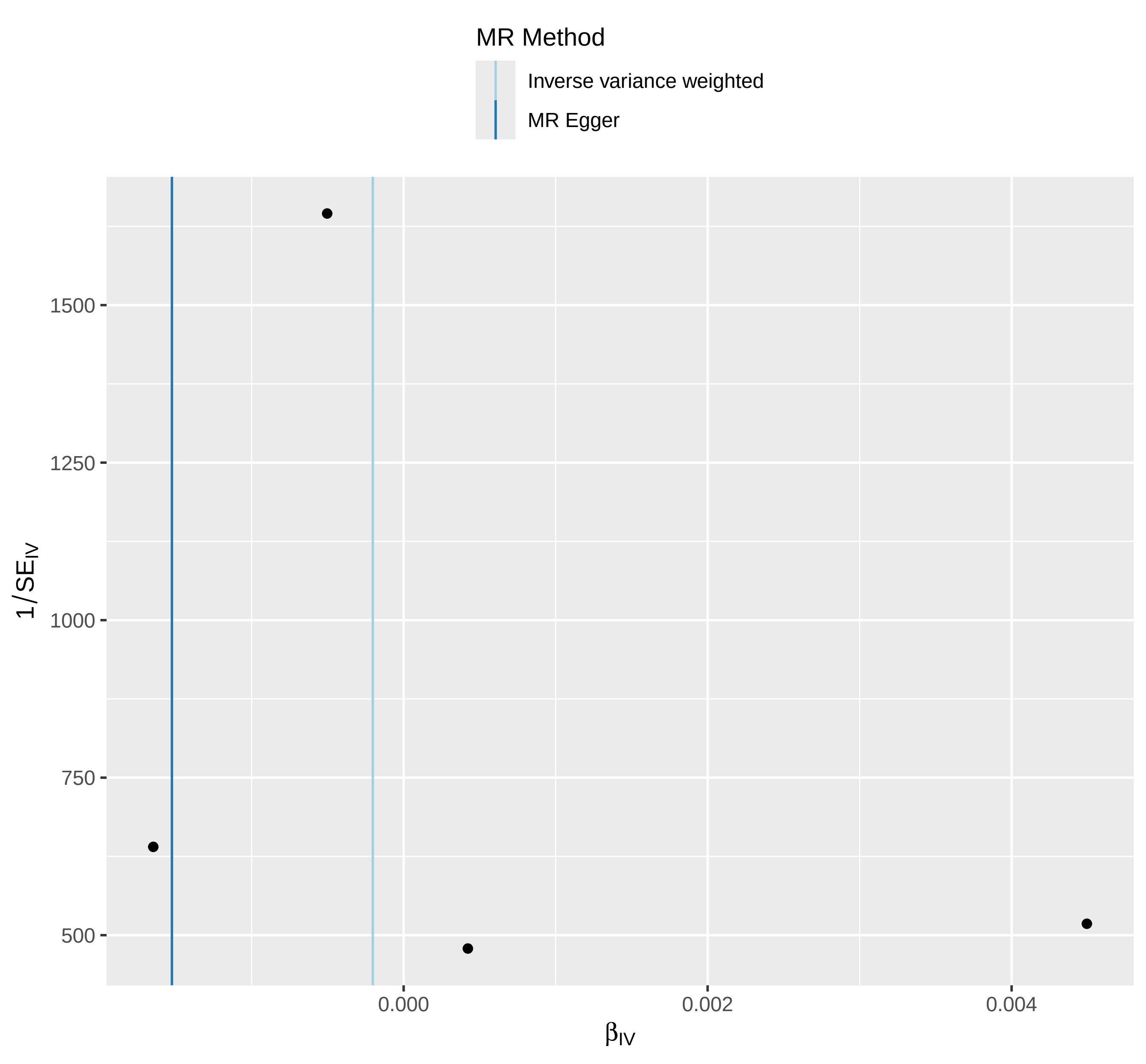
MR Method
Inverse variance weighted
MR Egger
1500
1250
1000
750
500
0.000
0.002
0.004
1/SE_IV
β_IV

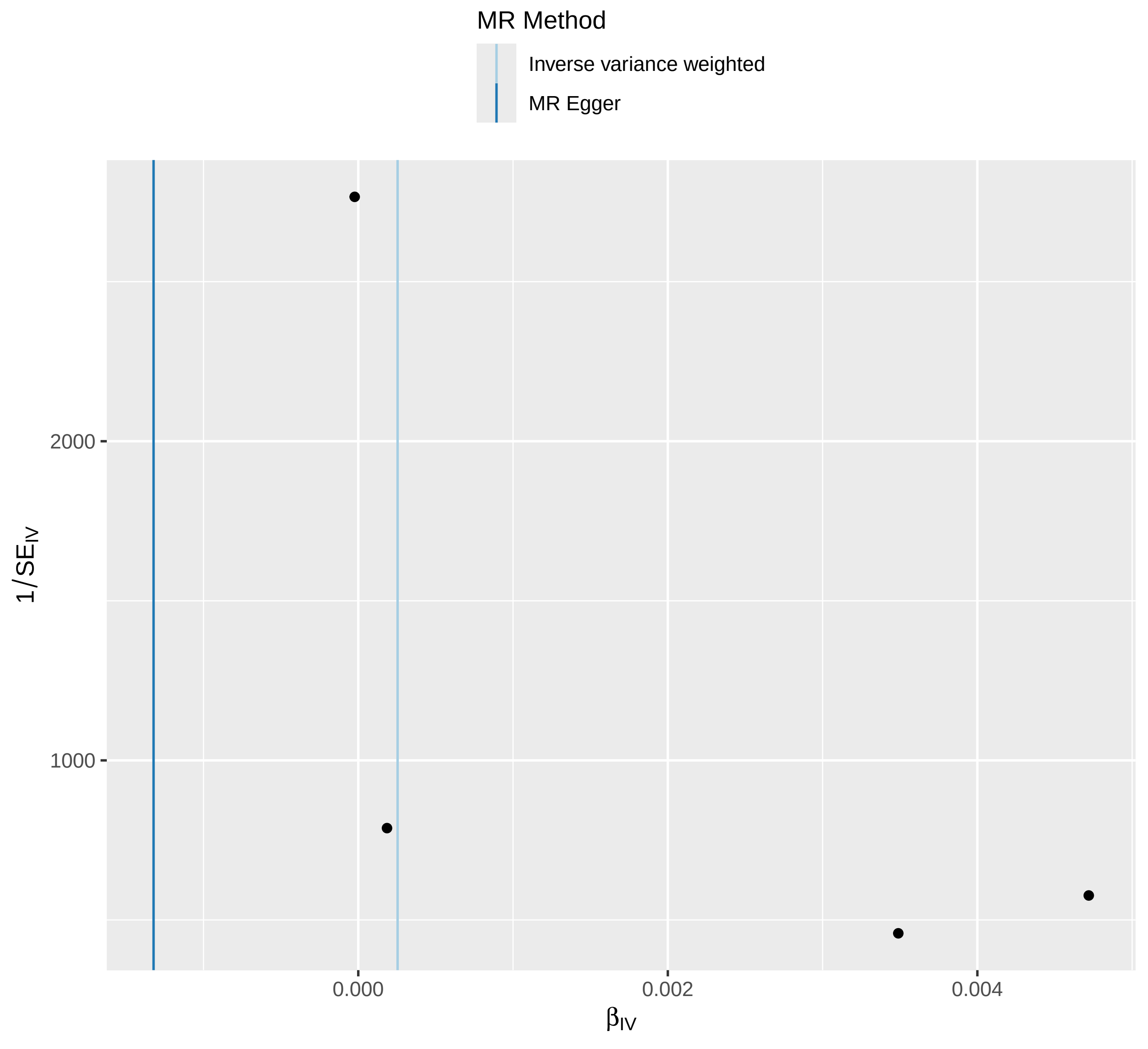

MR Method
Inverse variance weighted
MR Egger
2000
1000
1/SE_IV
0.000
0.002
0.004
β_IV

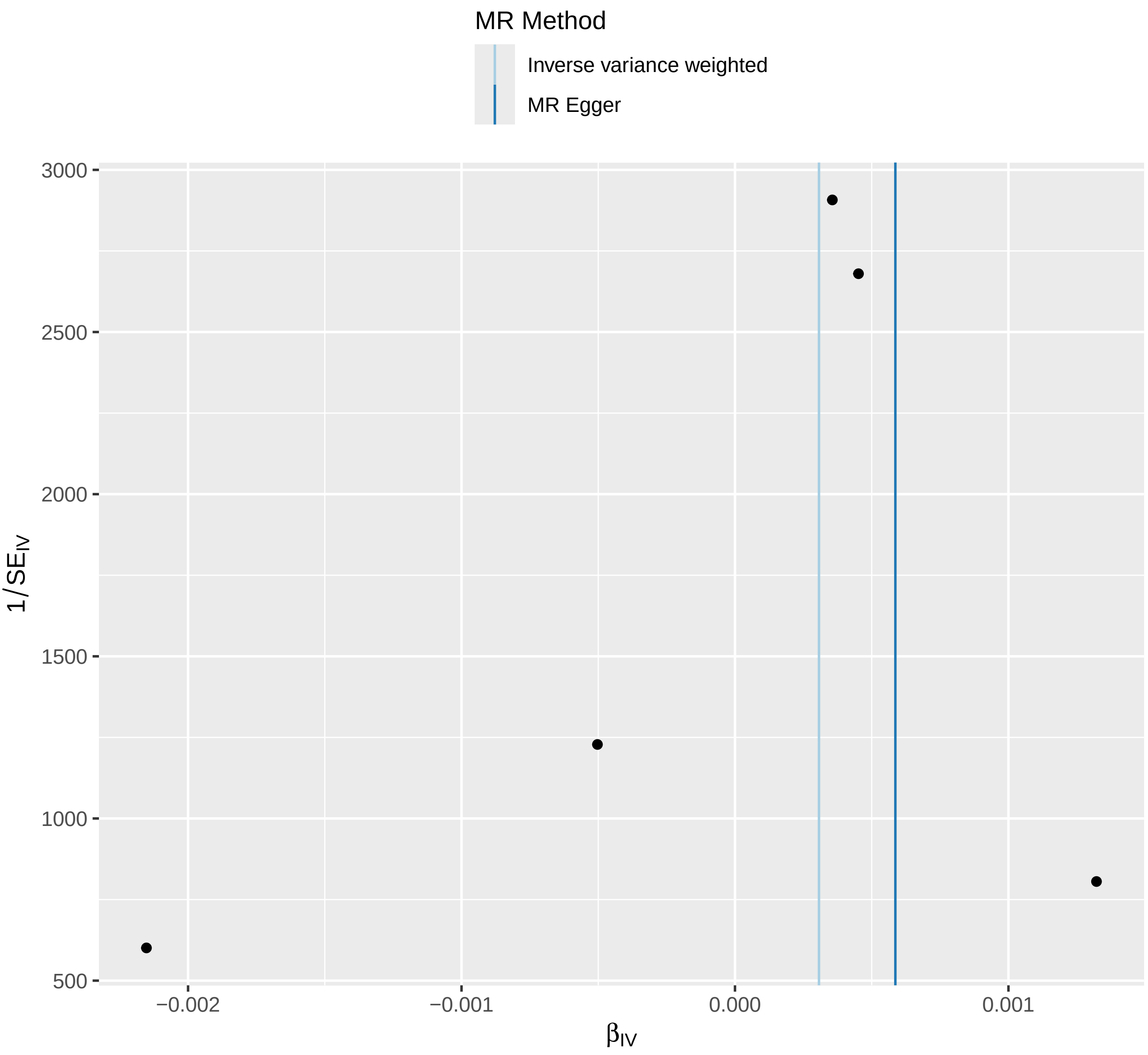

MR Method
Inverse variance weighted
MR Egger
3000
2500
2000
1500
1000
500
$1/SE_{IV}$
−0.002
−0.001
0.000
0.001
β_{IV}

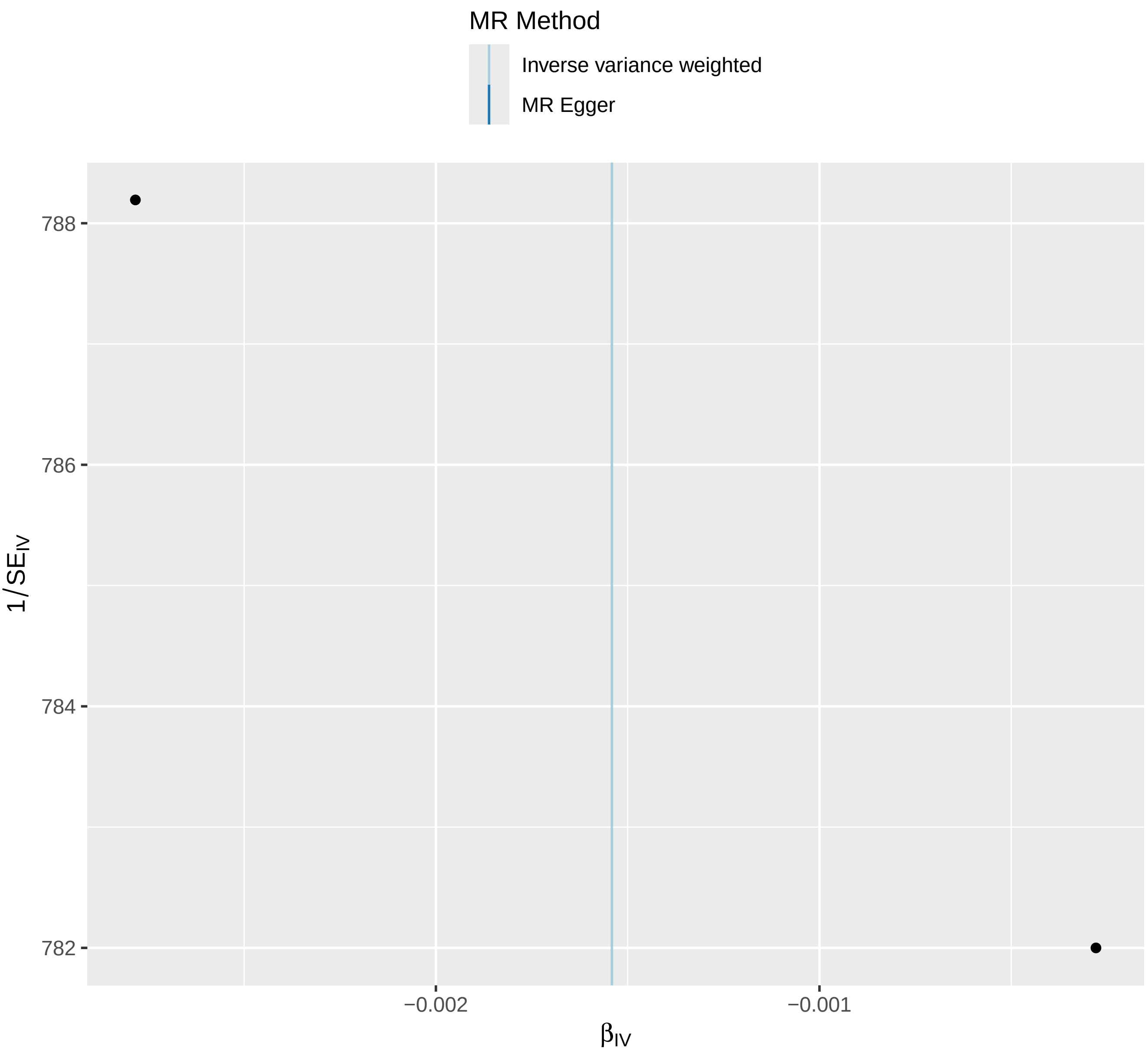

MR Method
Inverse variance weighted
MR Egger
788
786
784
782
1/SE$_{IV}$
−0.002
−0.001
β$_{IV}$

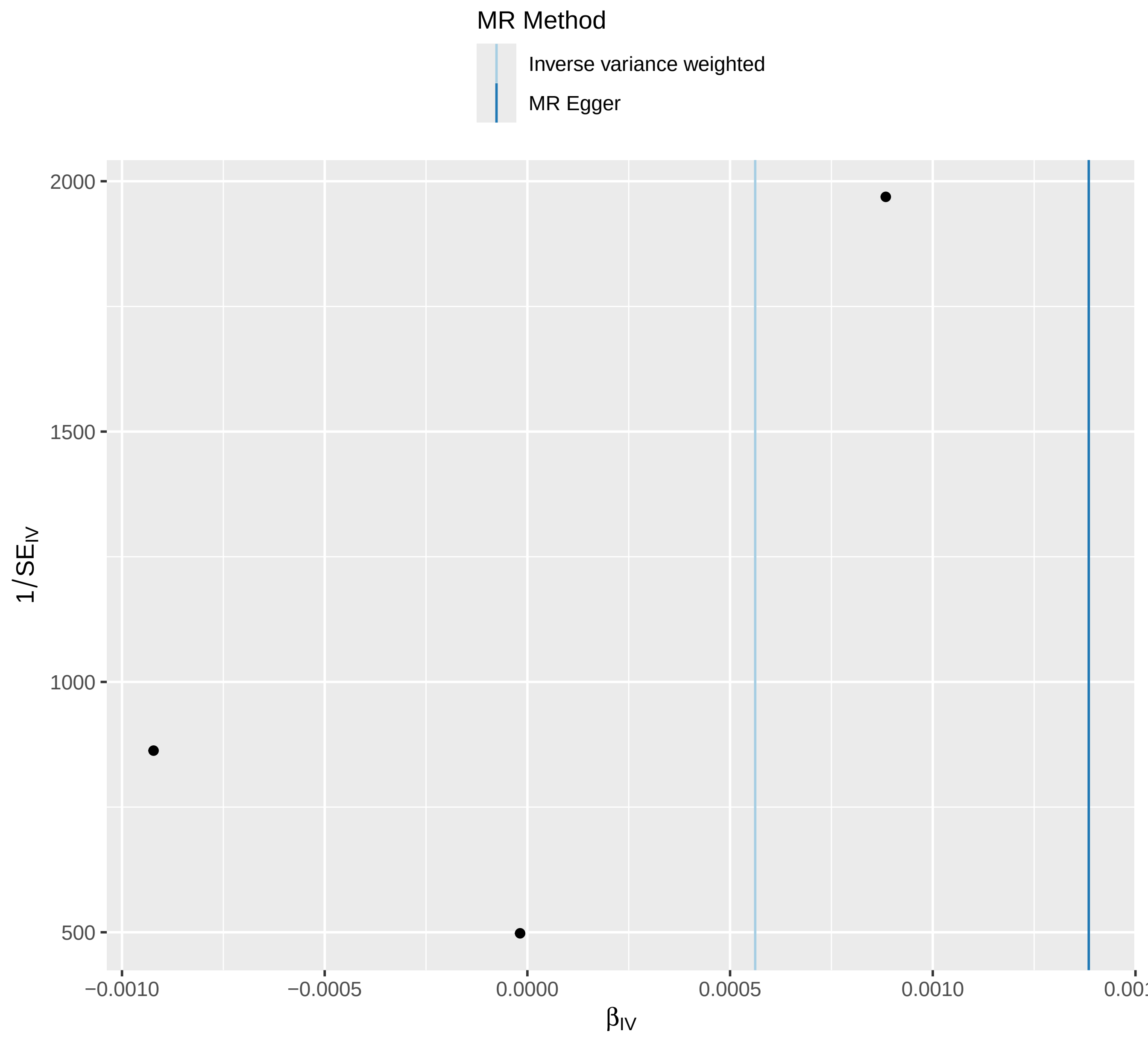

MR Method
Inverse variance weighted
MR Egger
2000
1500
1000
500
1/SE$_{IV}$
−0.0010
−0.0005
0.0000
0.0005
0.0010
0.001
β$_{IV}$

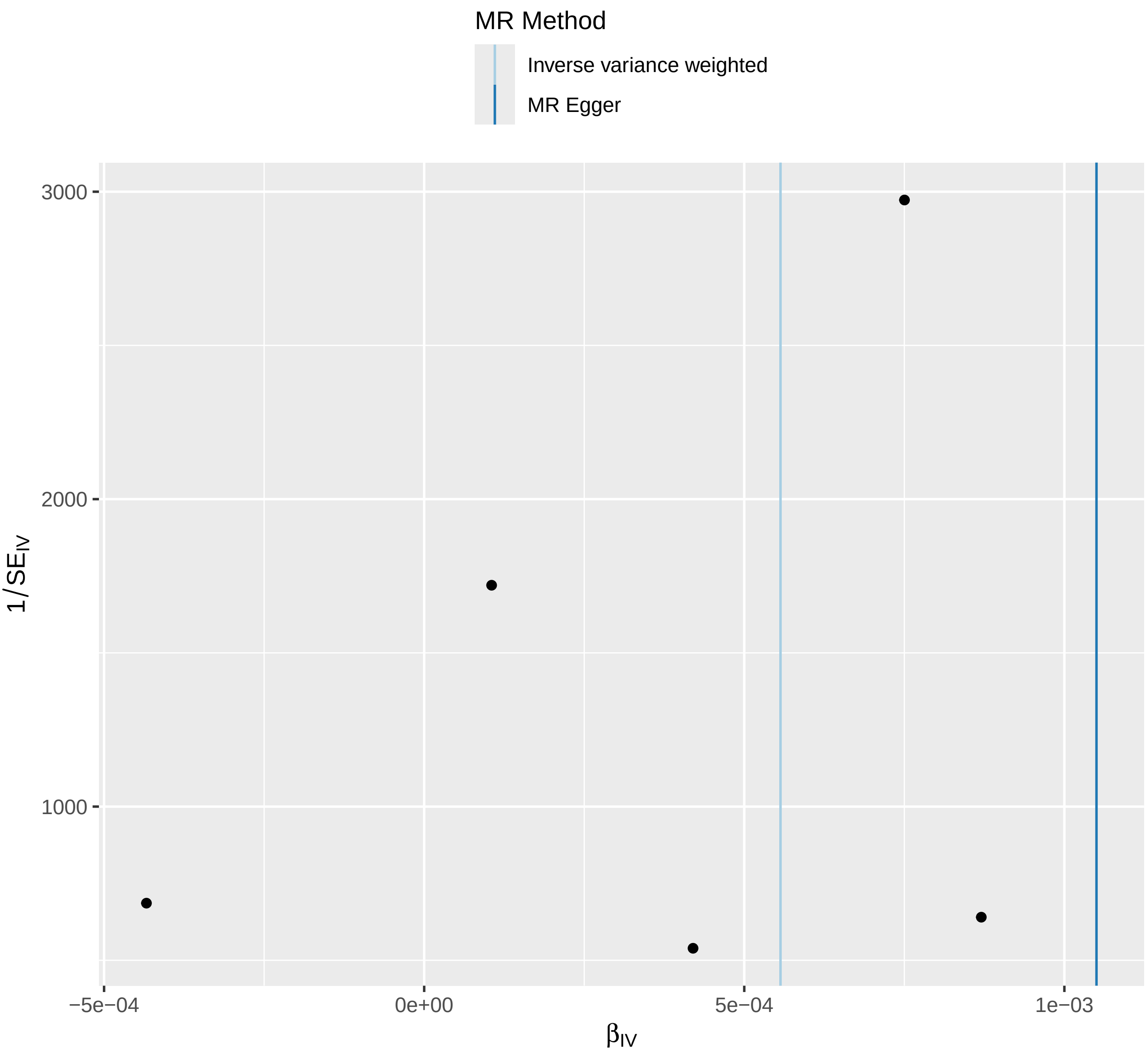

MR Method
Inverse variance weighted
MR Egger
3000
2000
1000
1/SE_IV
−5e−04
0e+00
5e−04
1e−03
β_IV

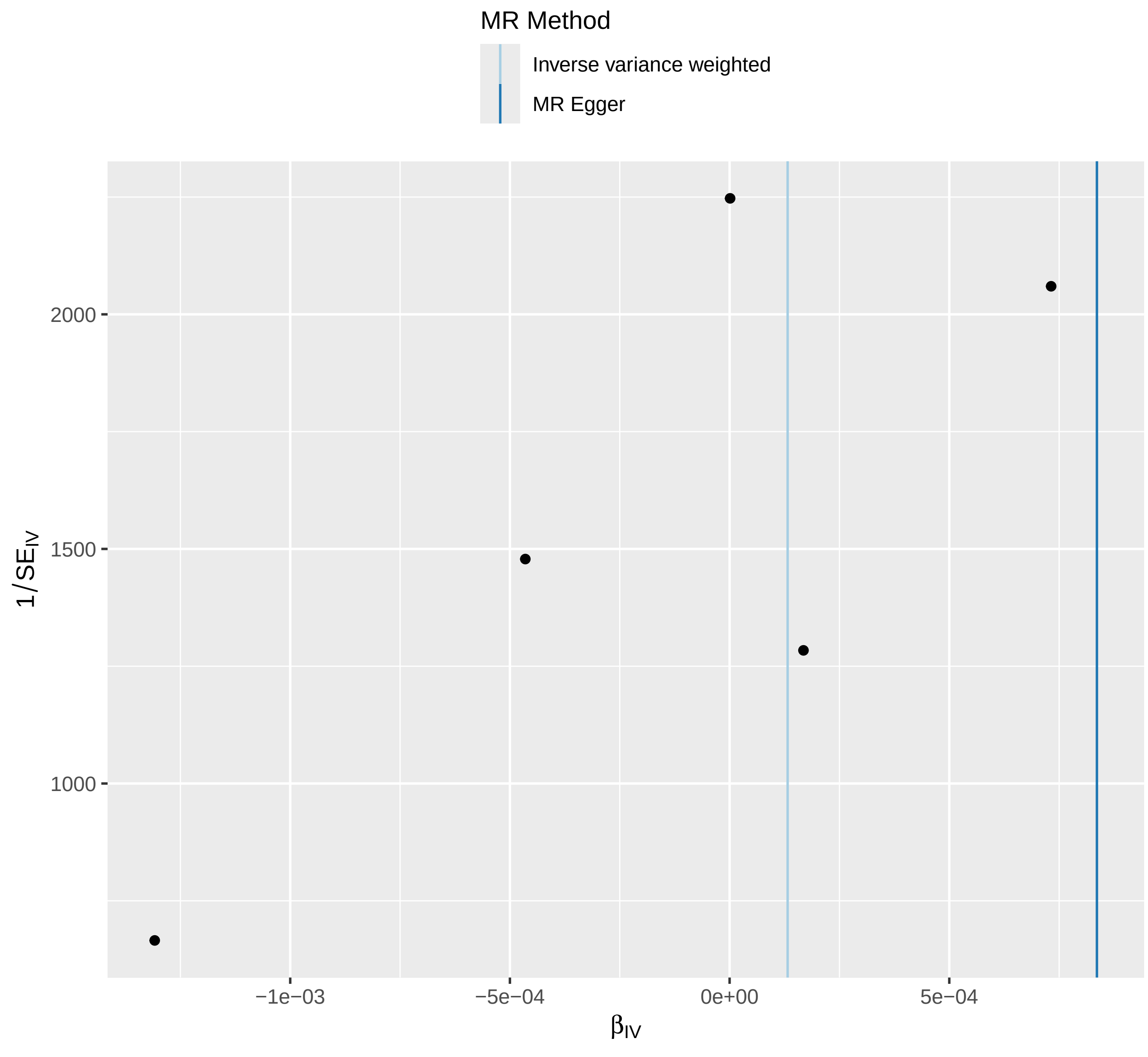

MR Method
Inverse variance weighted
MR Egger
2000
1500
1000
1/SE$_{IV}$
−1e−03
−5e−04
0e+00
5e−04
β$_{IV}$

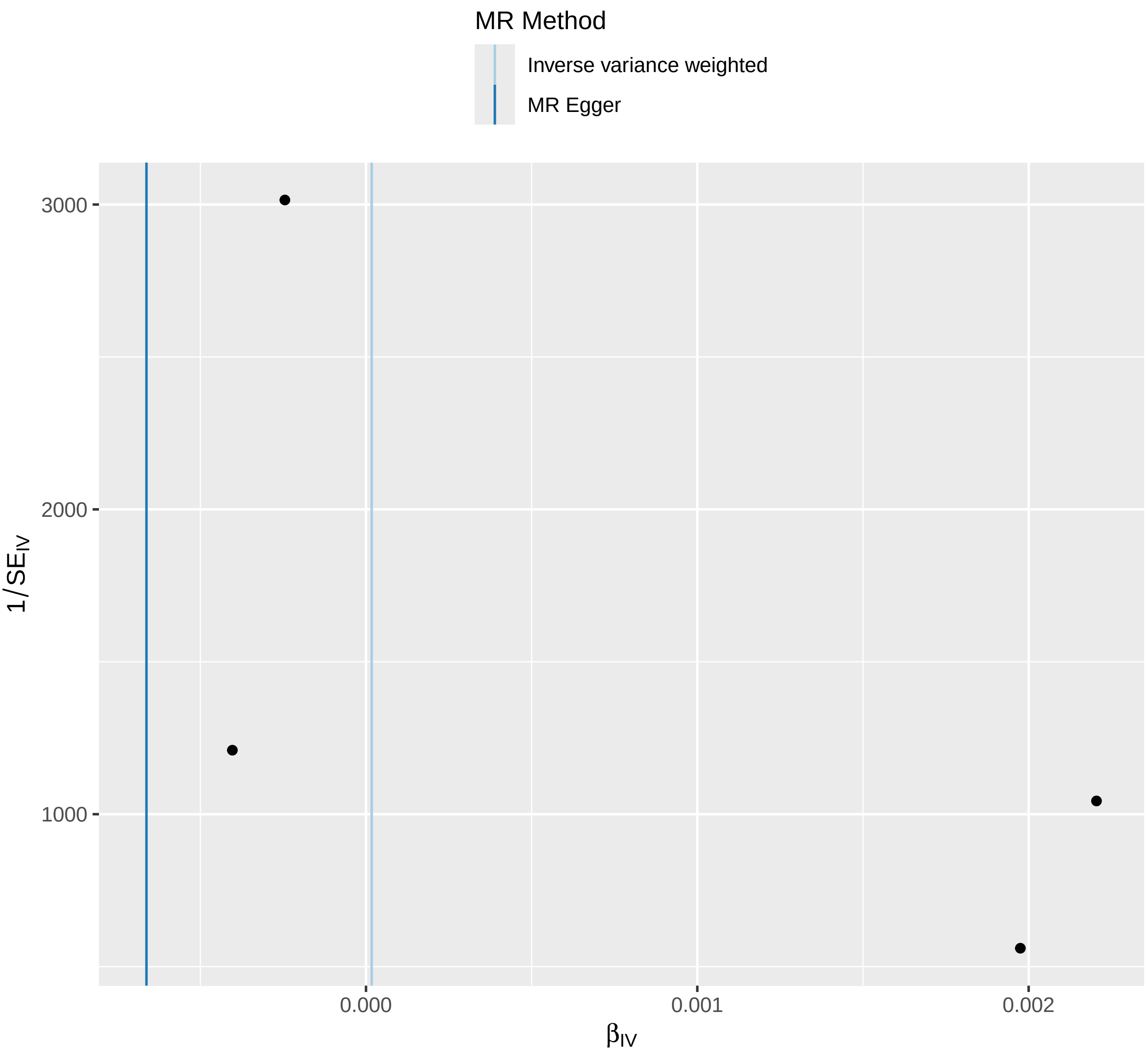
MR Method
Inverse variance weighted
MR Egger
3000
2000
1000
0.000
0.001
0.002
β_{IV}
$1/SE_{IV}$

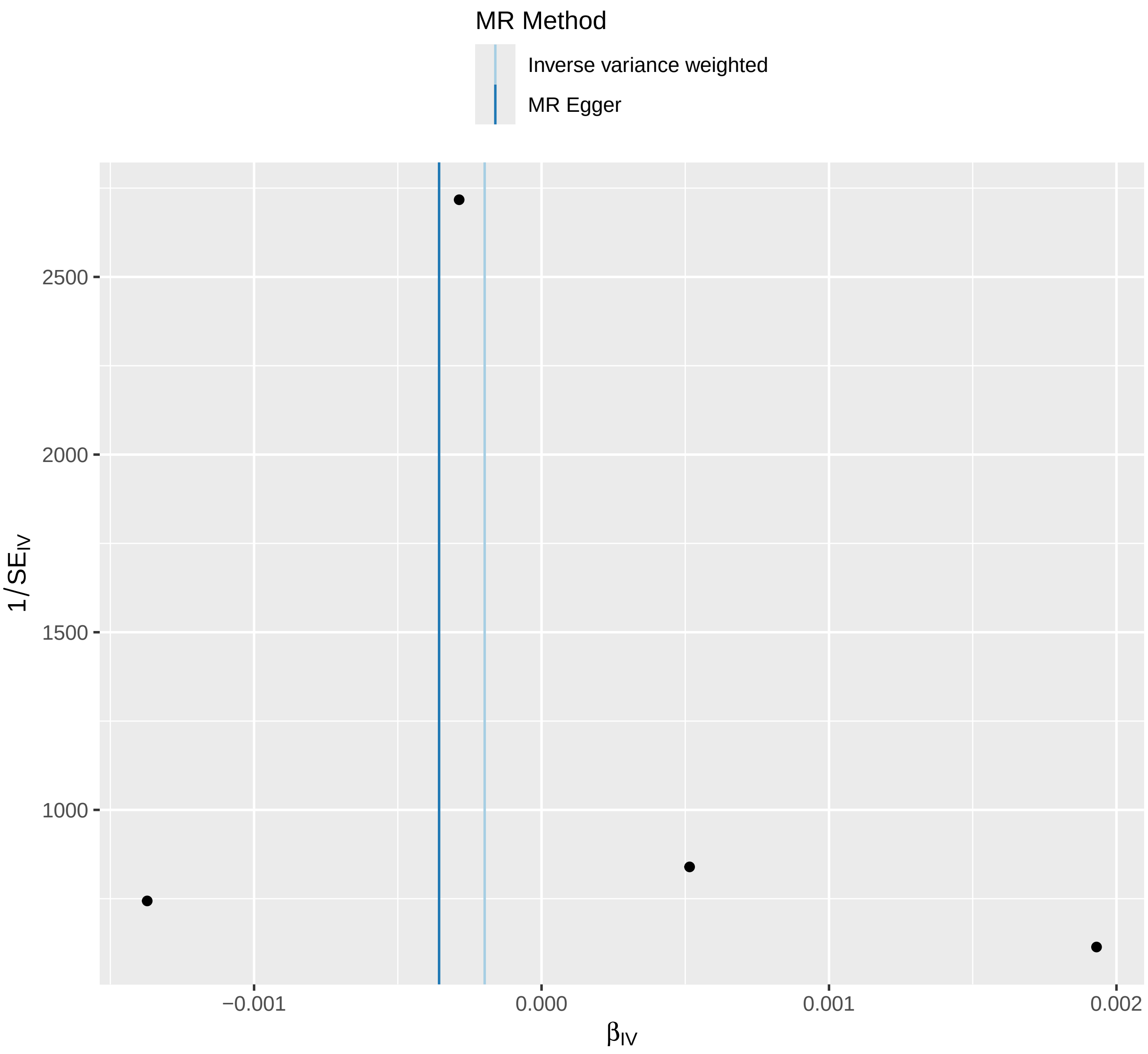
MR Method
Inverse variance weighted
MR Egger
2500
2000
1500
1000
1/SE$_{IV}$
−0.001
0.000
0.001
0.002
β$_{IV}$

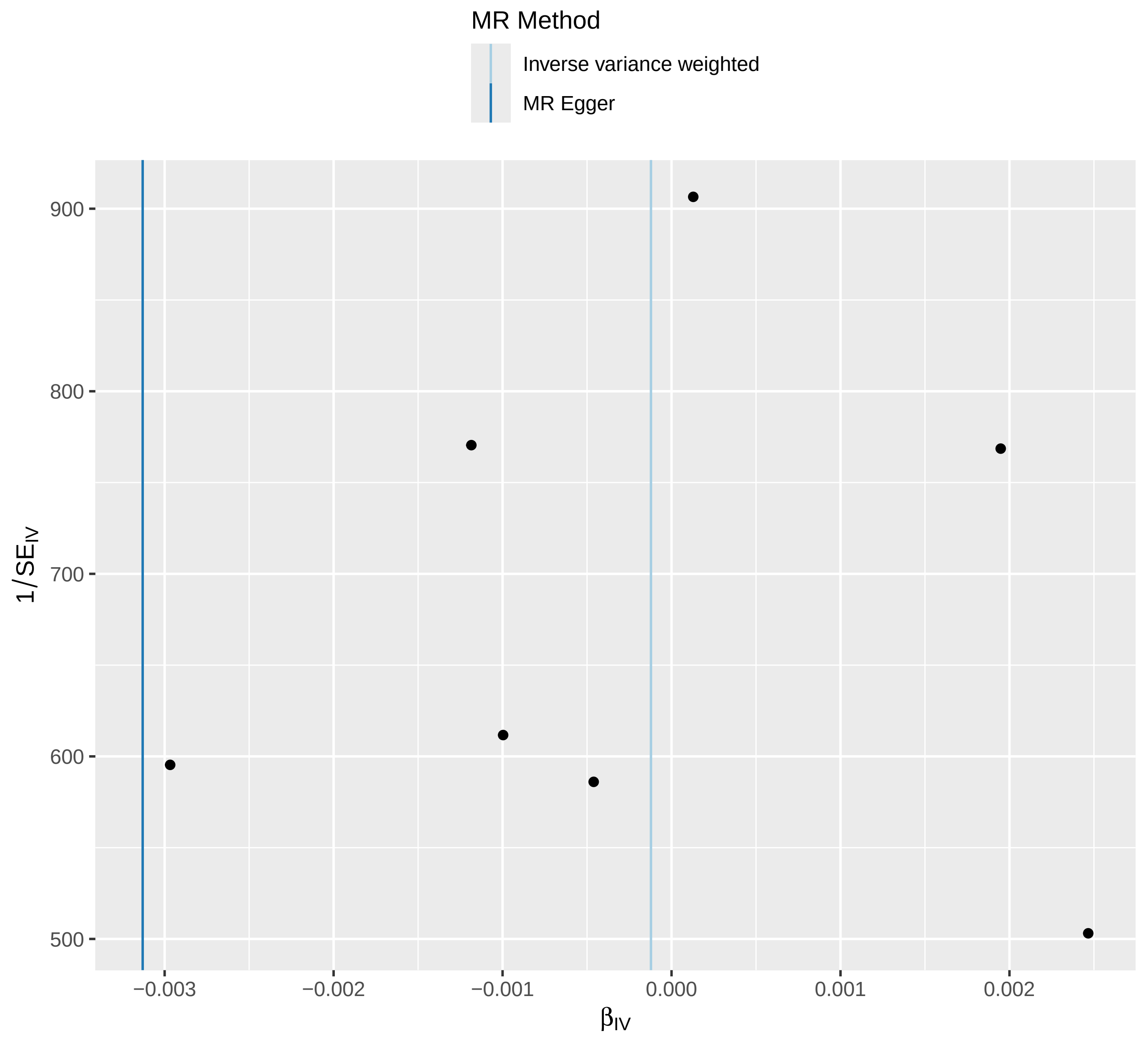
MR Method
Inverse variance weighted
MR Egger
900
800
700
600
500
1/SE_IV
−0.003
−0.002
−0.001
0.000
0.001
0.002
β_IV

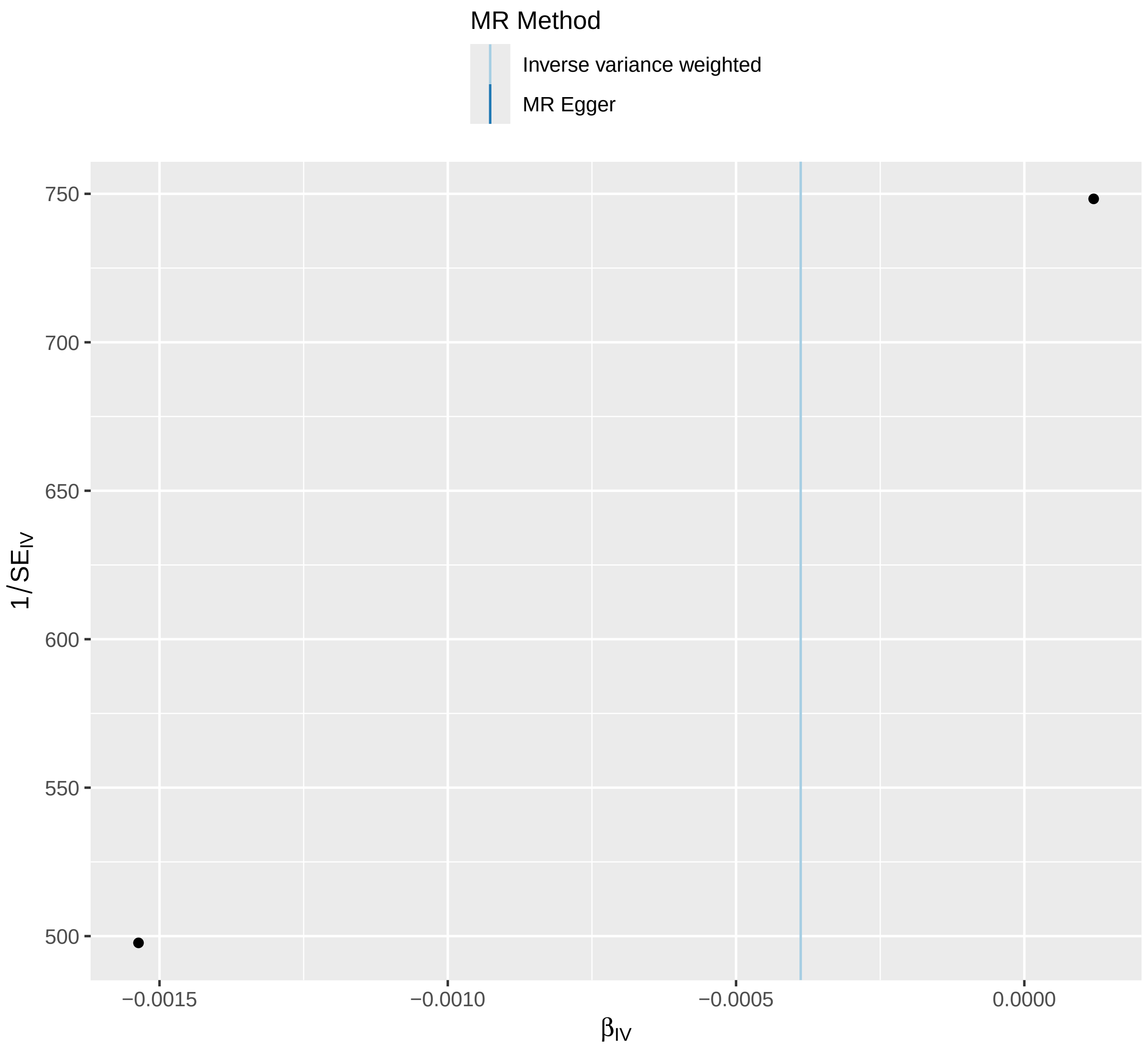

MR Method
Inverse variance weighted
MR Egger
750
700
650
600
550
500
1/SE_IV
−0.0015
−0.0010
−0.0005
0.0000
β_IV

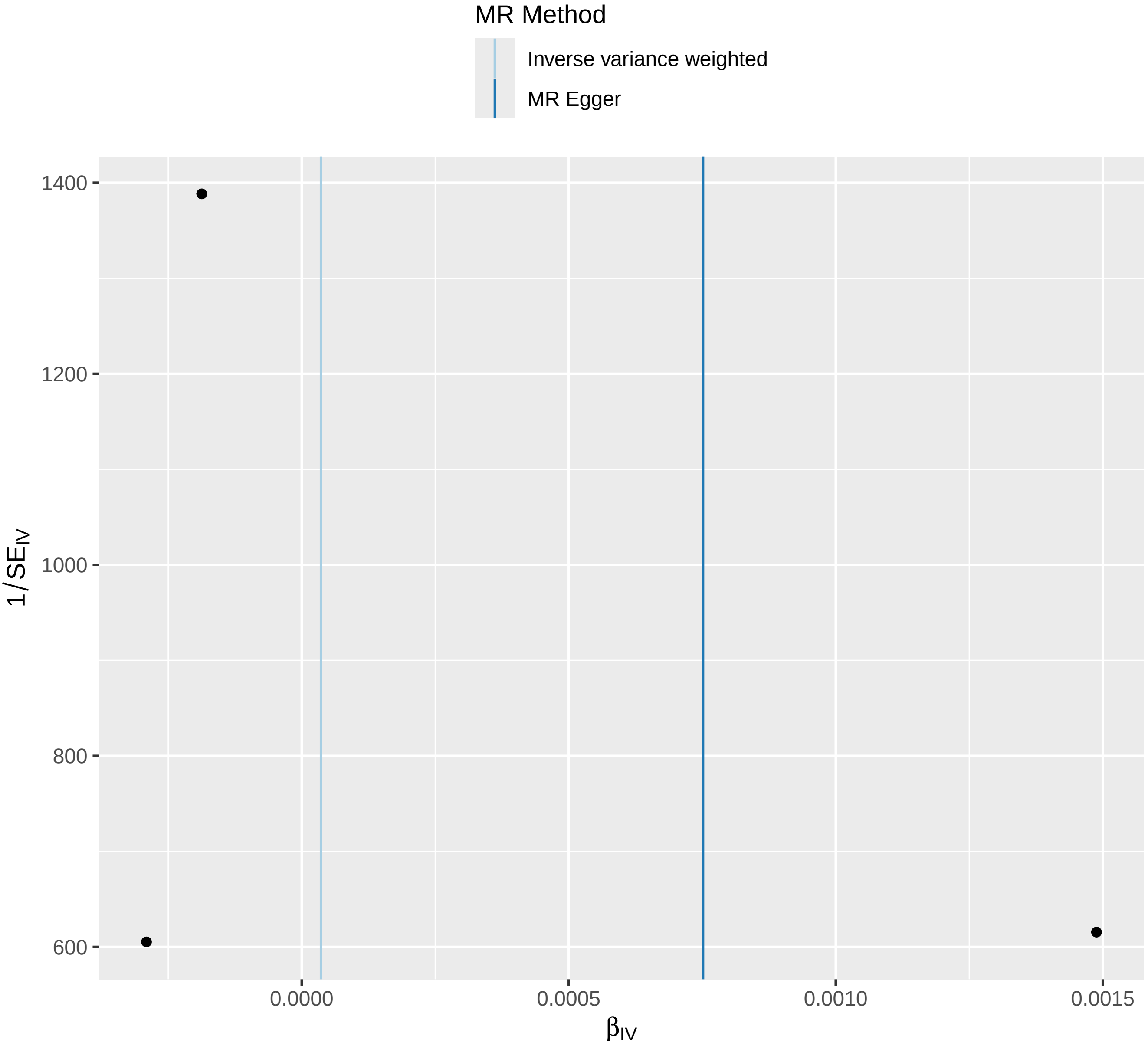

MR Method
Inverse variance weighted
MR Egger
1400
1200
1000
800
600
1/SE_IV
0.0000
0.0005
0.0010
0.0015
β_IV

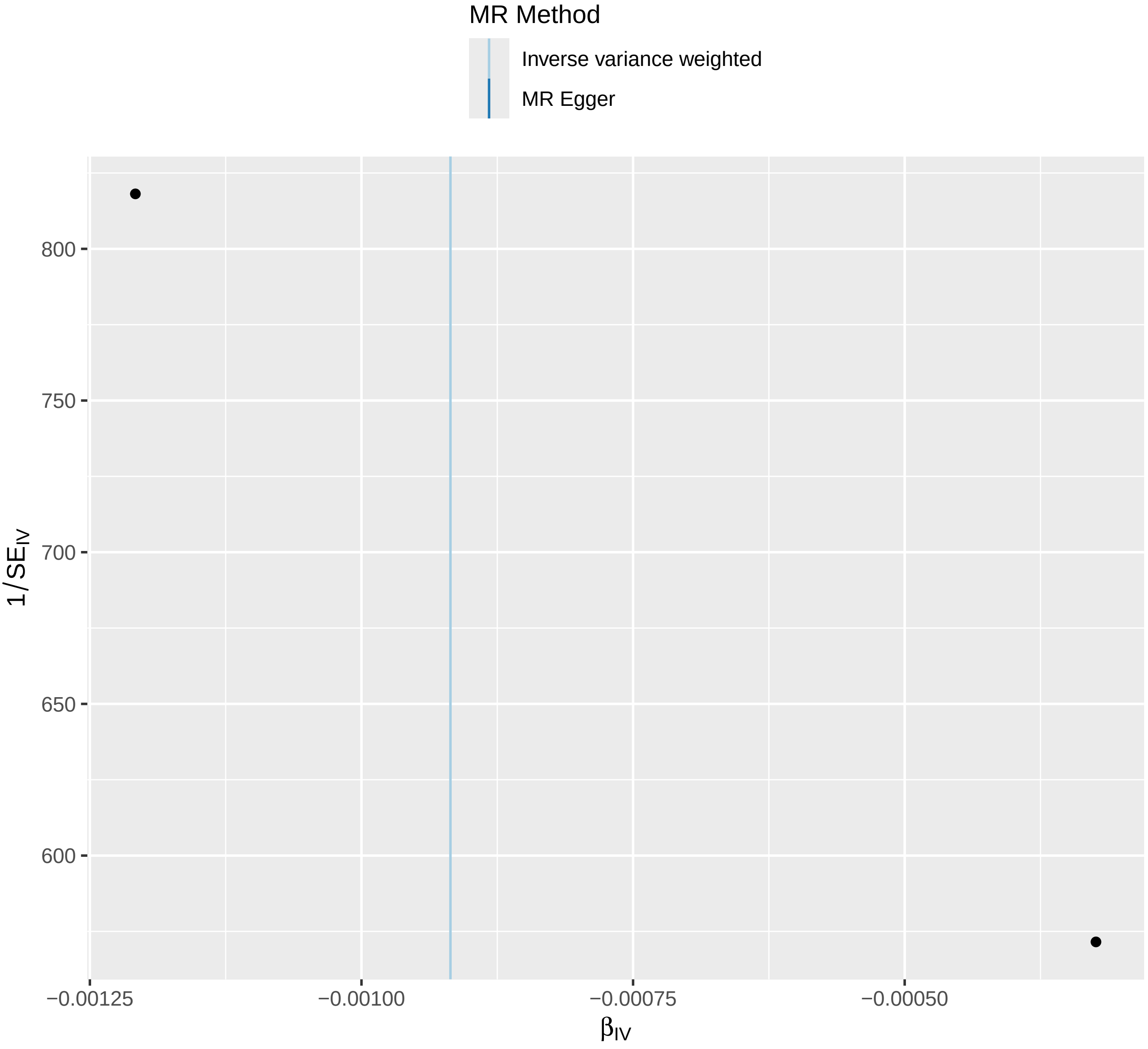

MR Method
Inverse variance weighted
MR Egger
800
750
700
650
600
1/SE_IV
−0.00125
−0.00100
−0.00075
−0.00050
β_IV

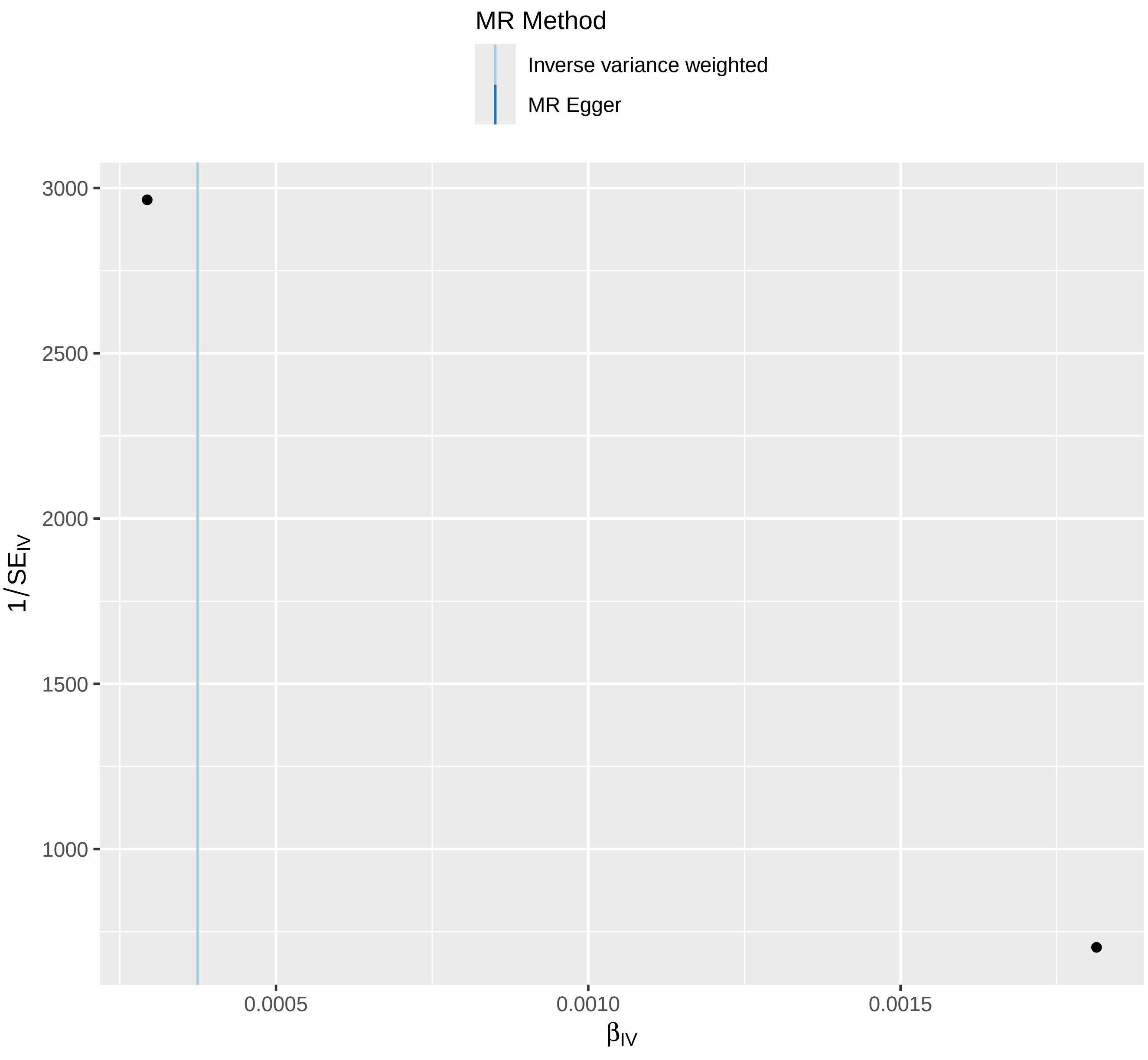
MR Method
Inverse variance weighted
MR Egger
3000
2500
2000
1500
1000
1/SE_IV
0.0005
0.0010
0.0015
β_IV

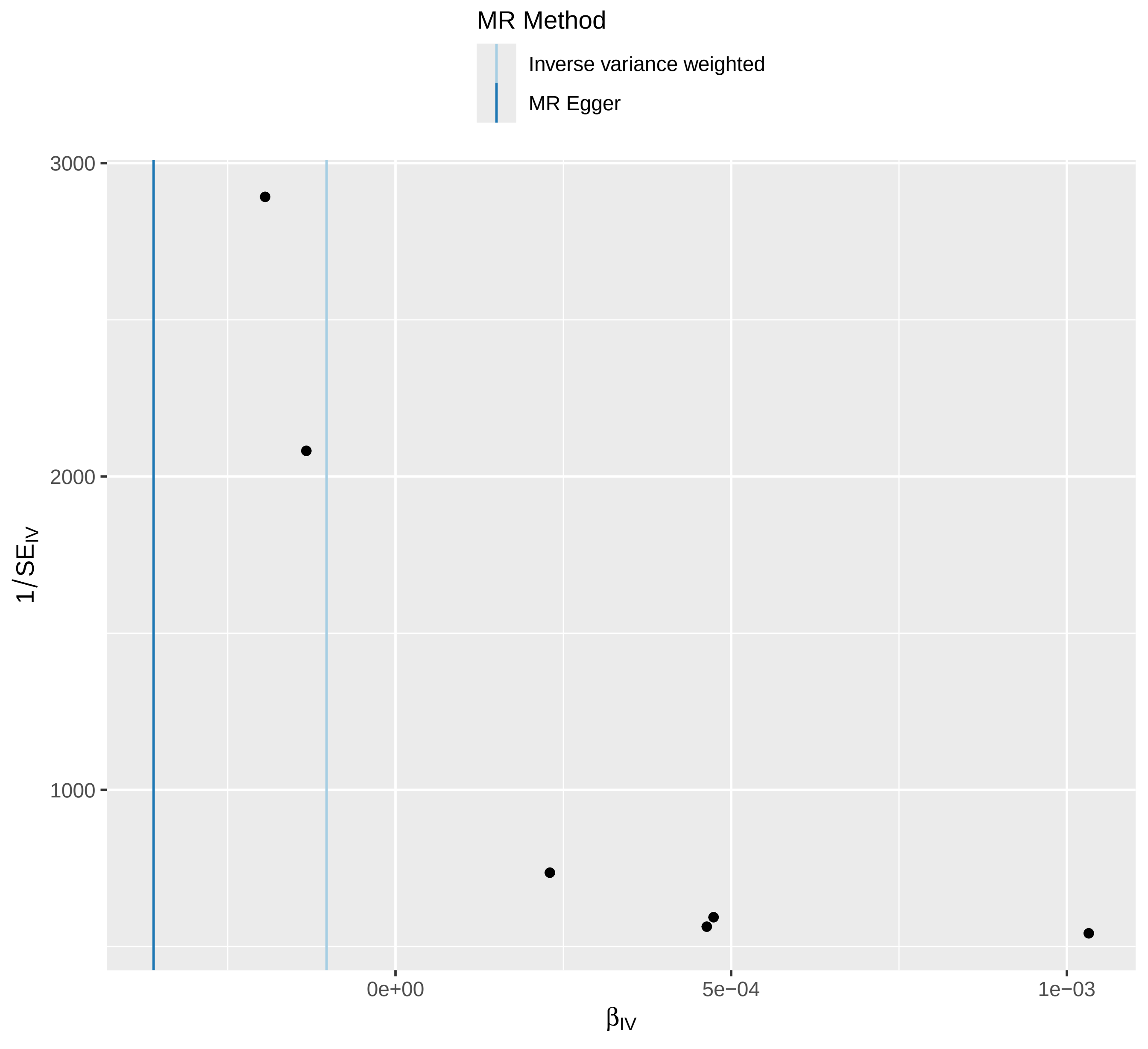
MR Method
Inverse variance weighted
MR Egger
3000
2000
1000
1/SE_IV
0e+00
5e−04
1e−03
β_IV

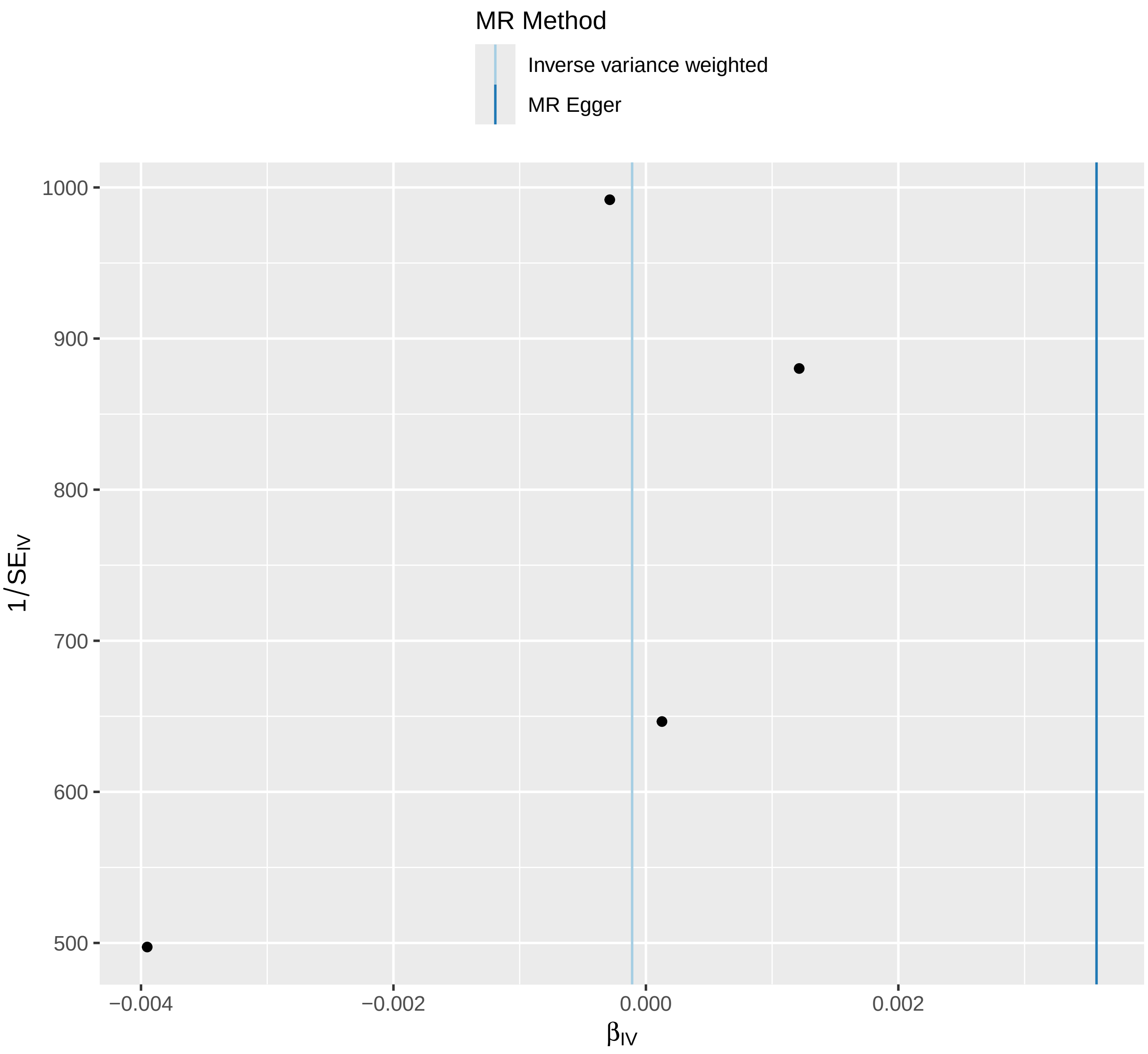

MR Method
Inverse variance weighted
MR Egger
1000
900
800
700
600
500
1/SE_IV
−0.004
−0.002
0.000
0.002
β_IV

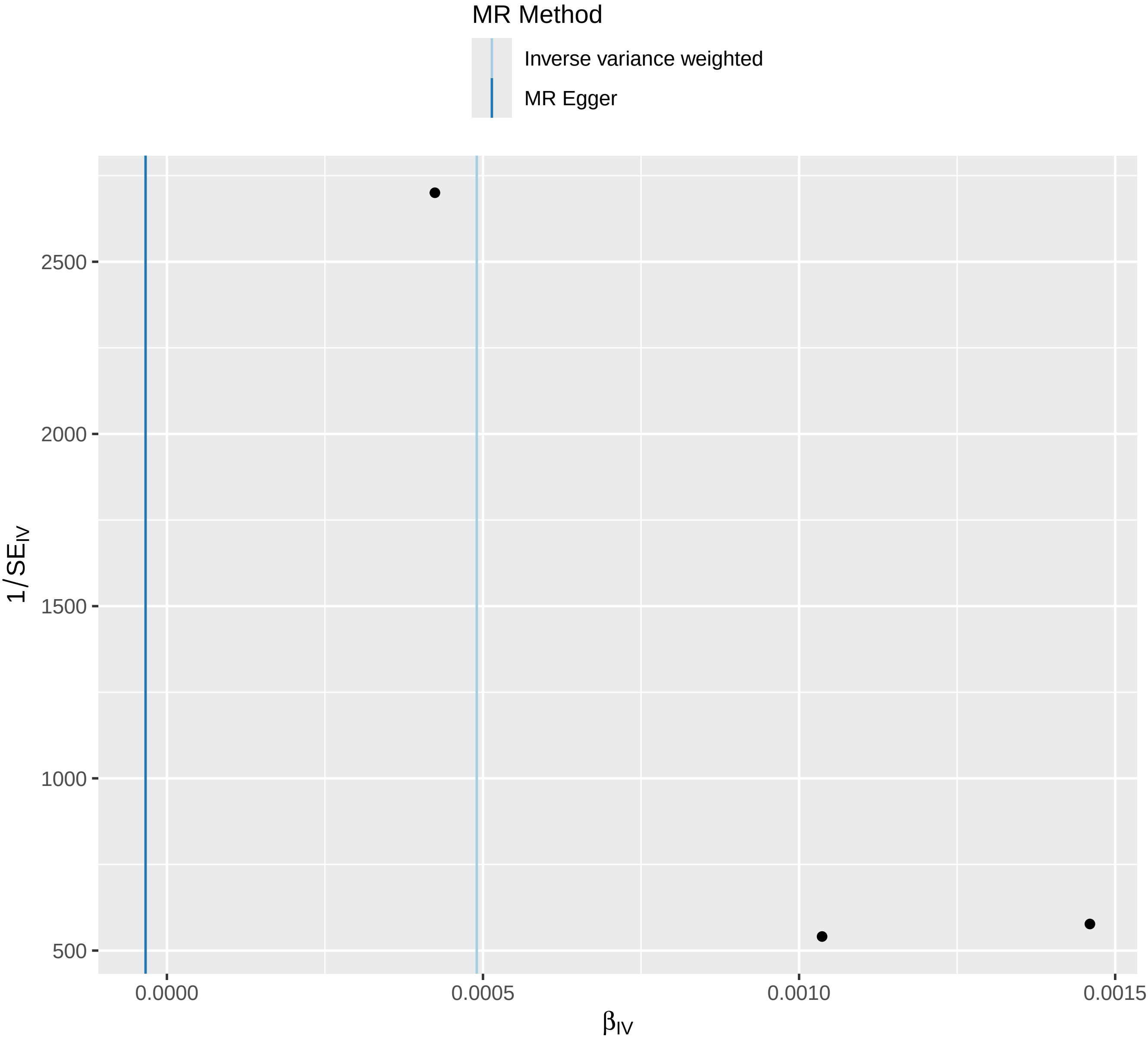
MR Method
Inverse variance weighted
MR Egger
2500
2000
1500
1000
500
$1/SE_{IV}$
0.0000
0.0005
0.0010
0.0015
β_{IV}

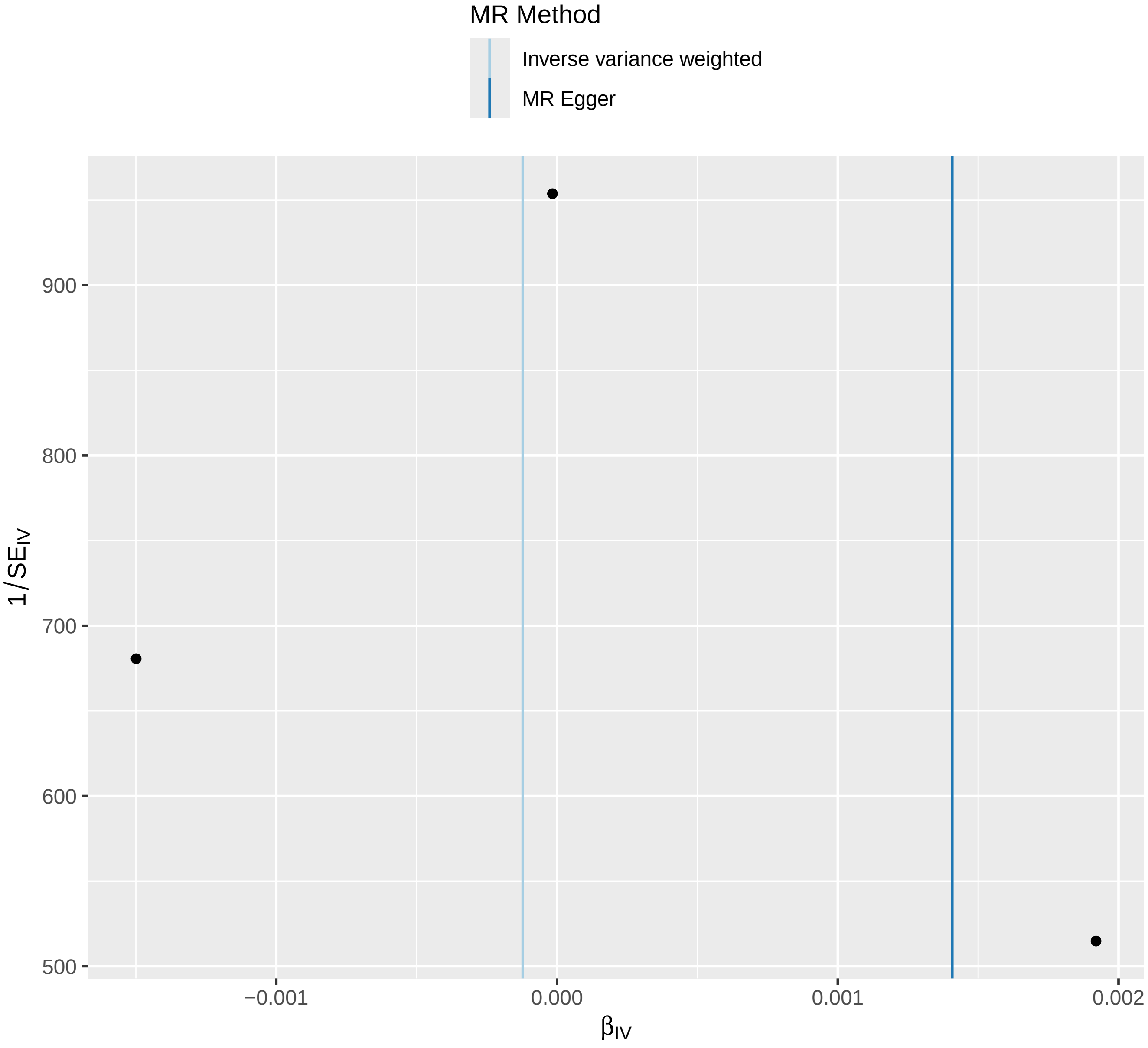
MR Method
Inverse variance weighted
MR Egger
900
800
700
600
500
1/SE_IV
−0.001
0.000
0.001
0.002
β_IV

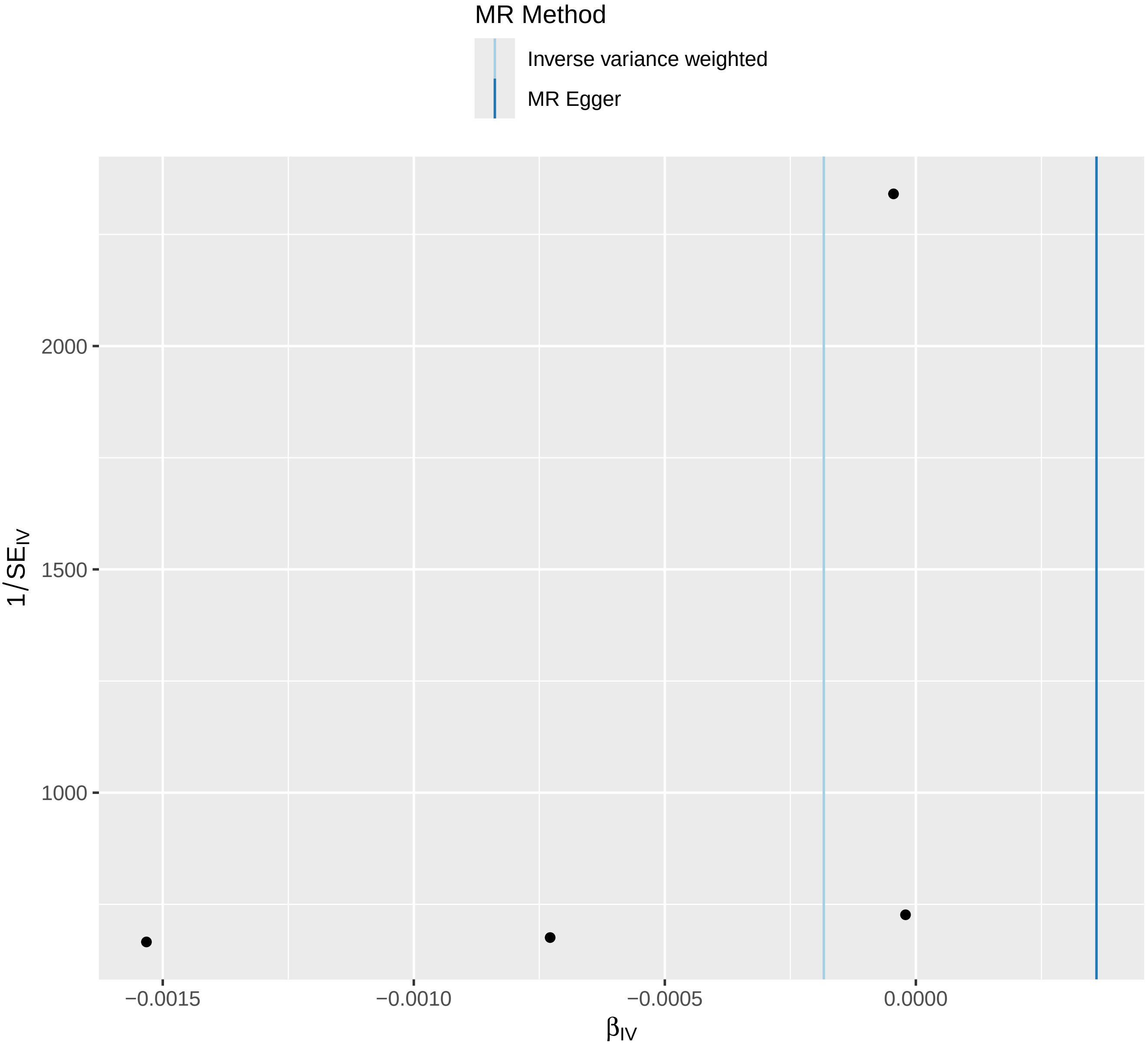
MR Method
Inverse variance weighted
MR Egger
1/SE_IV
2000
1500
1000
−0.0015
−0.0010
−0.0005
0.0000
β_IV

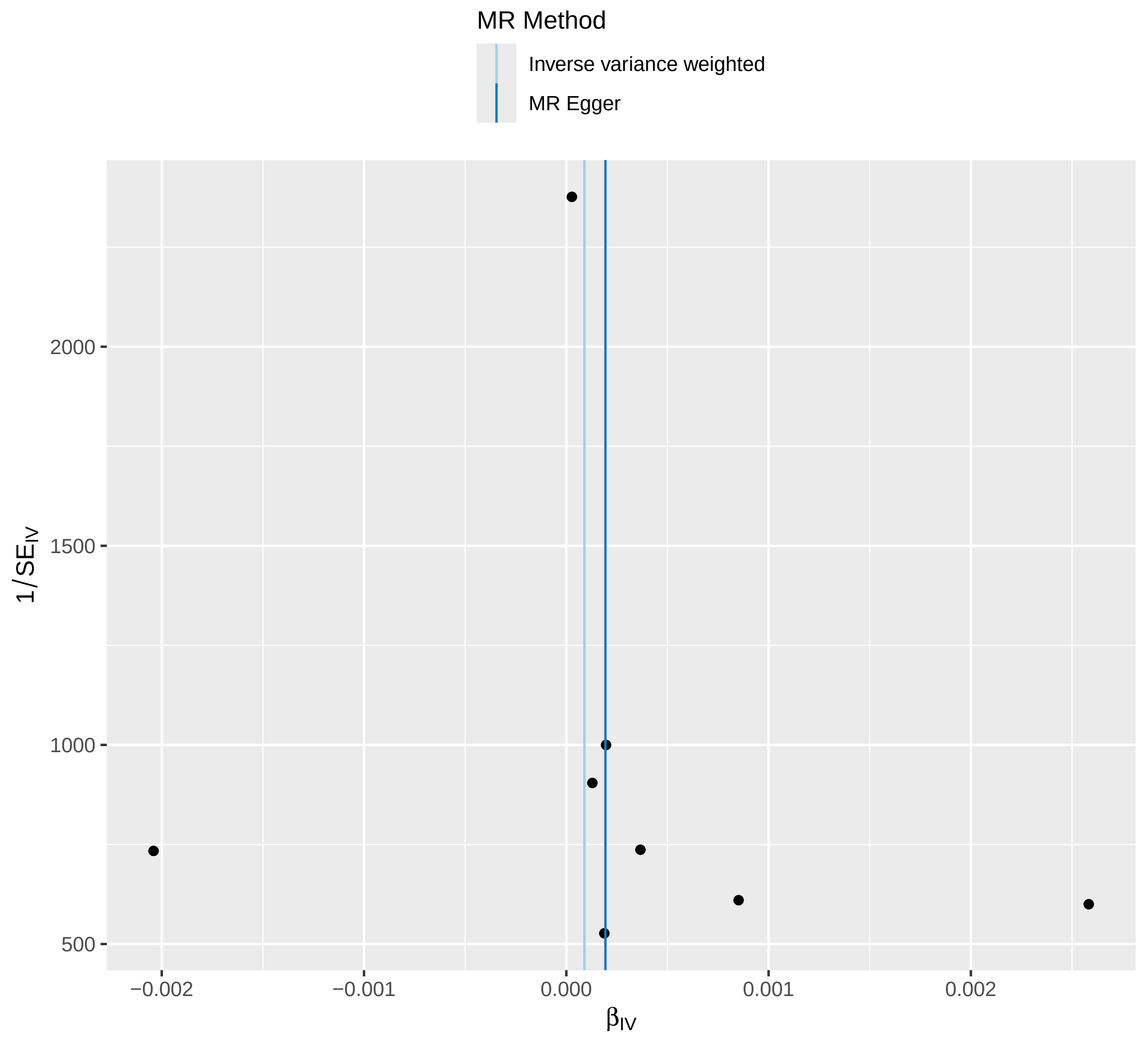
MR Method
Inverse variance weighted
MR Egger
2000
1500
1000
500
1/SE_IV
−0.002
−0.001
0.000
0.001
0.002
β_IV

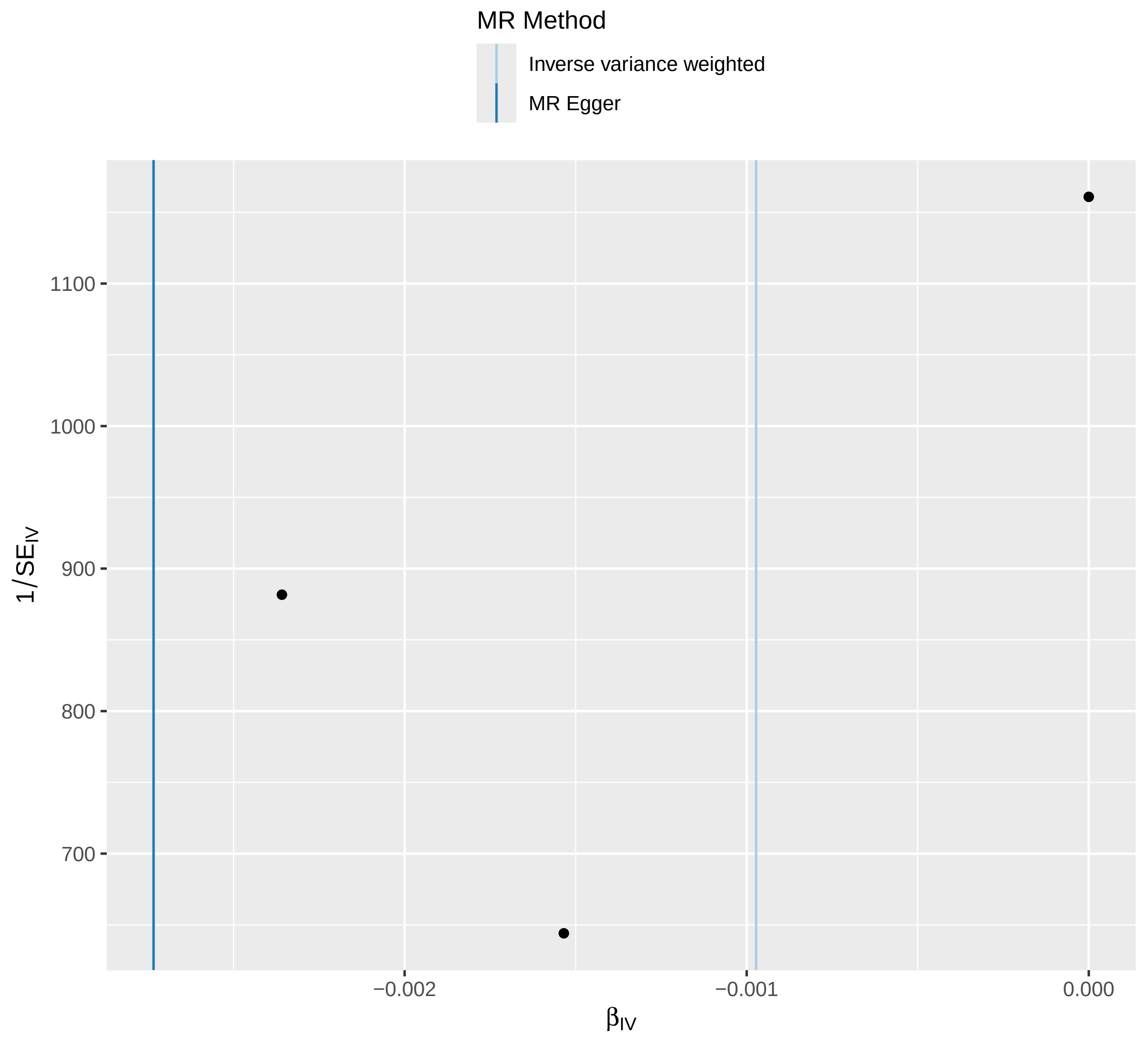

MR Method
Inverse variance weighted
MR Egger
1100
1000
900
800
700
−0.002
−0.001
0.000
1/SE_IV
β_IV

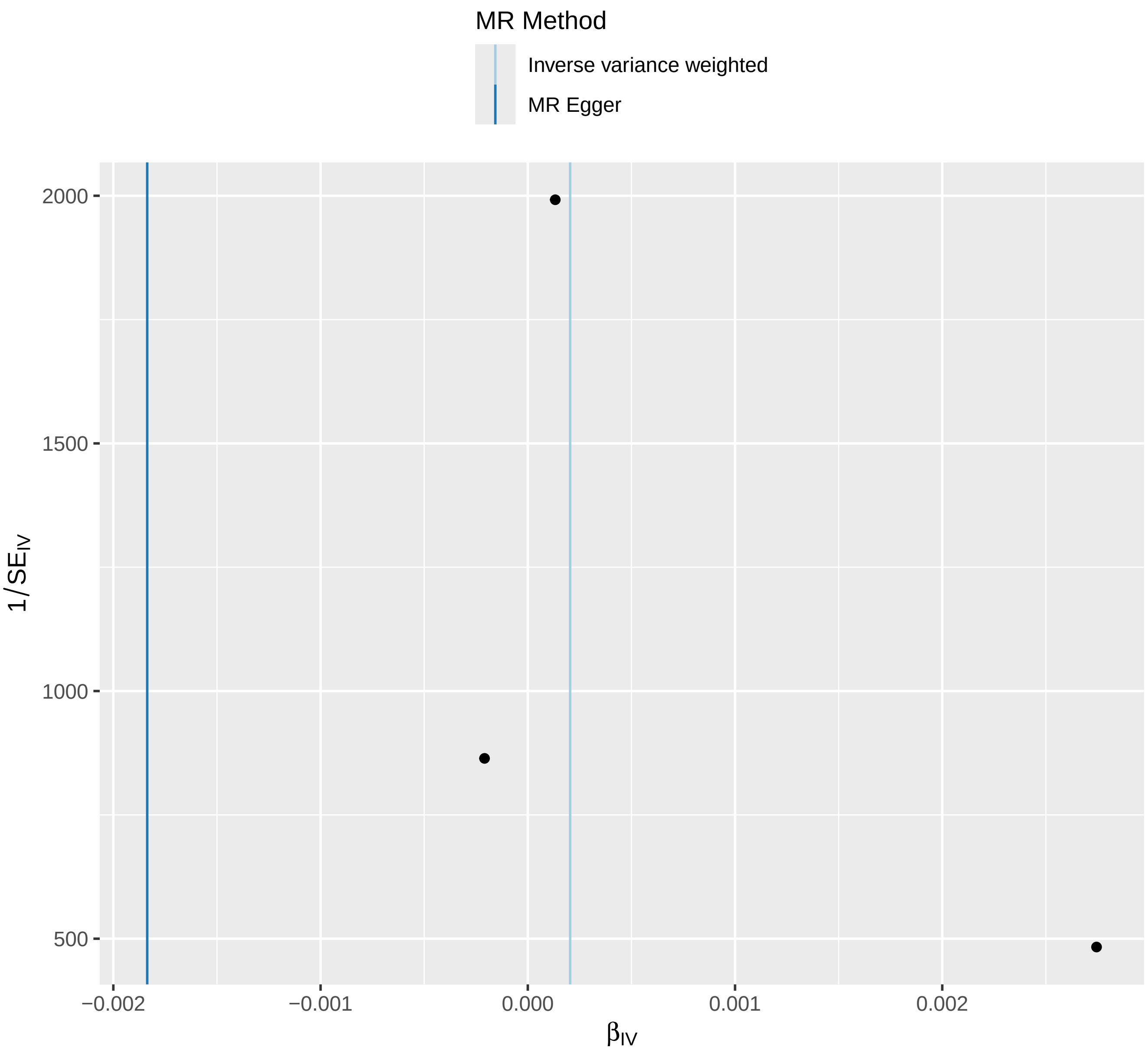
MR Method
Inverse variance weighted
MR Egger
2000
1500
1000
500
1/SE_IV
−0.002
−0.001
0.000
0.001
0.002
β_IV

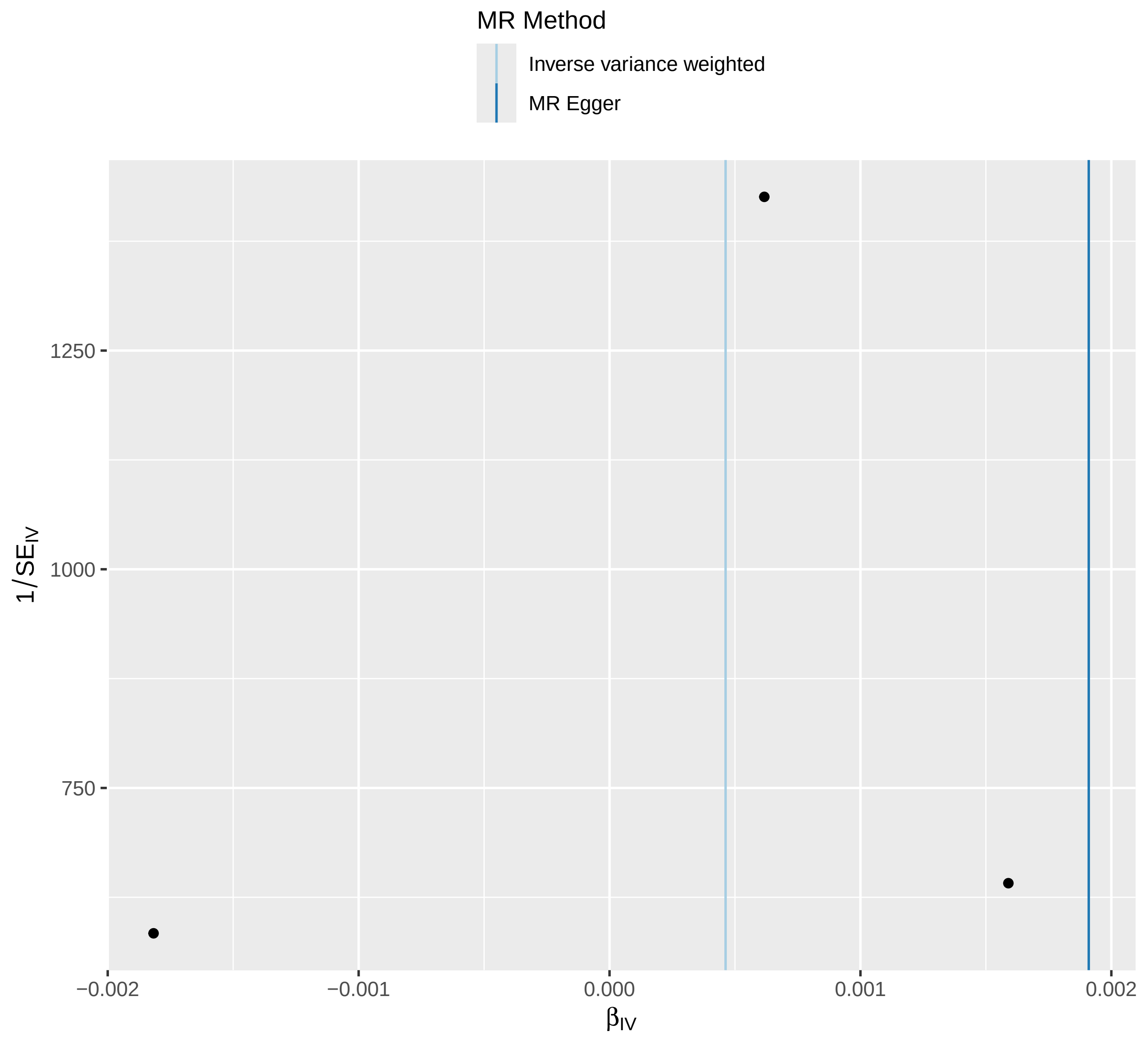

MR Method
Inverse variance weighted
MR Egger
1250
1000
750
1/SE_IV
−0.002
−0.001
0.000
0.001
0.002
β_IV

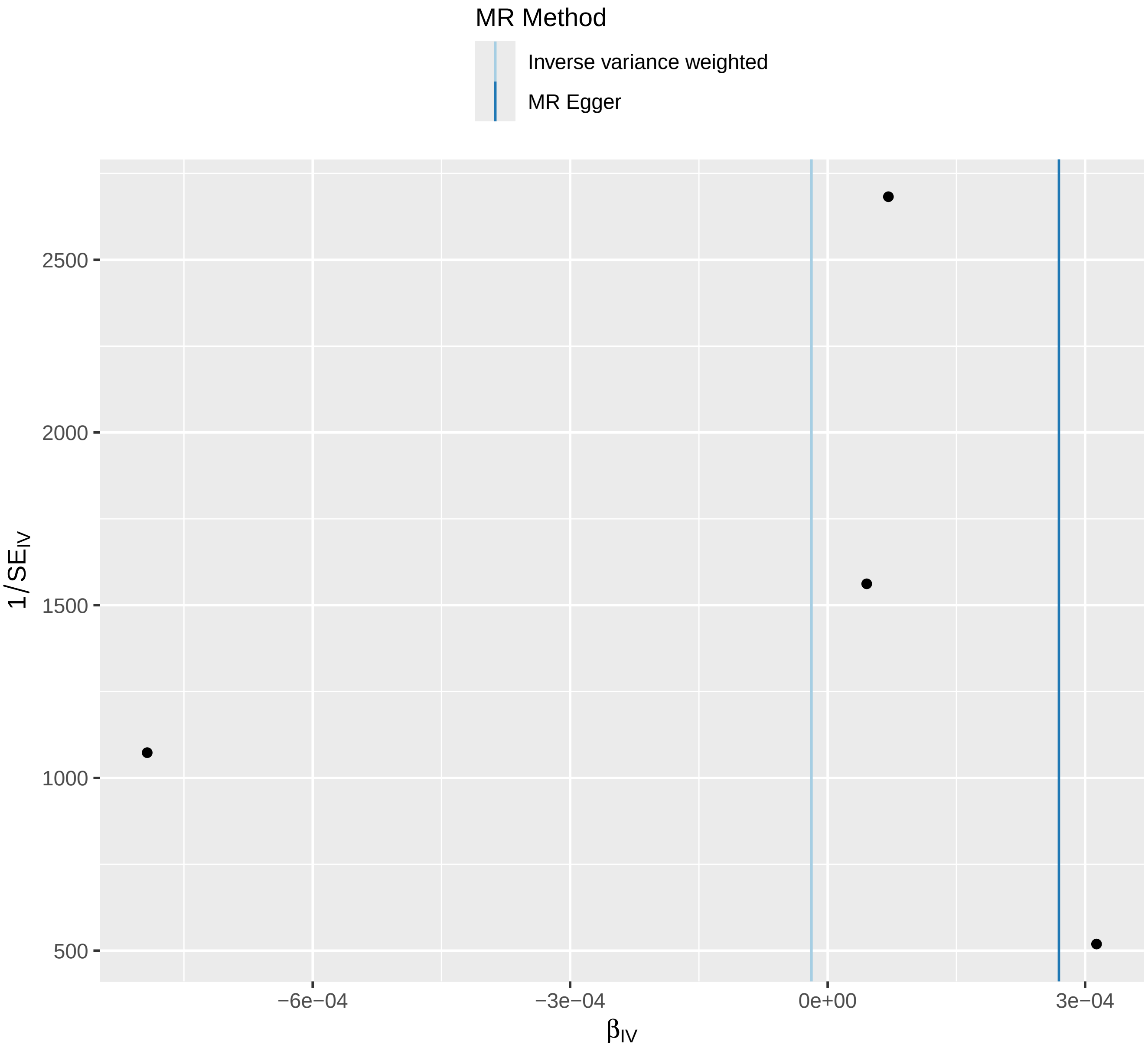

MR Method
Inverse variance weighted
MR Egger
2500
2000
1500
1000
500
$1/SE_{IV}$
−6e−04
−3e−04
0e+00
3e−04
β_{IV}

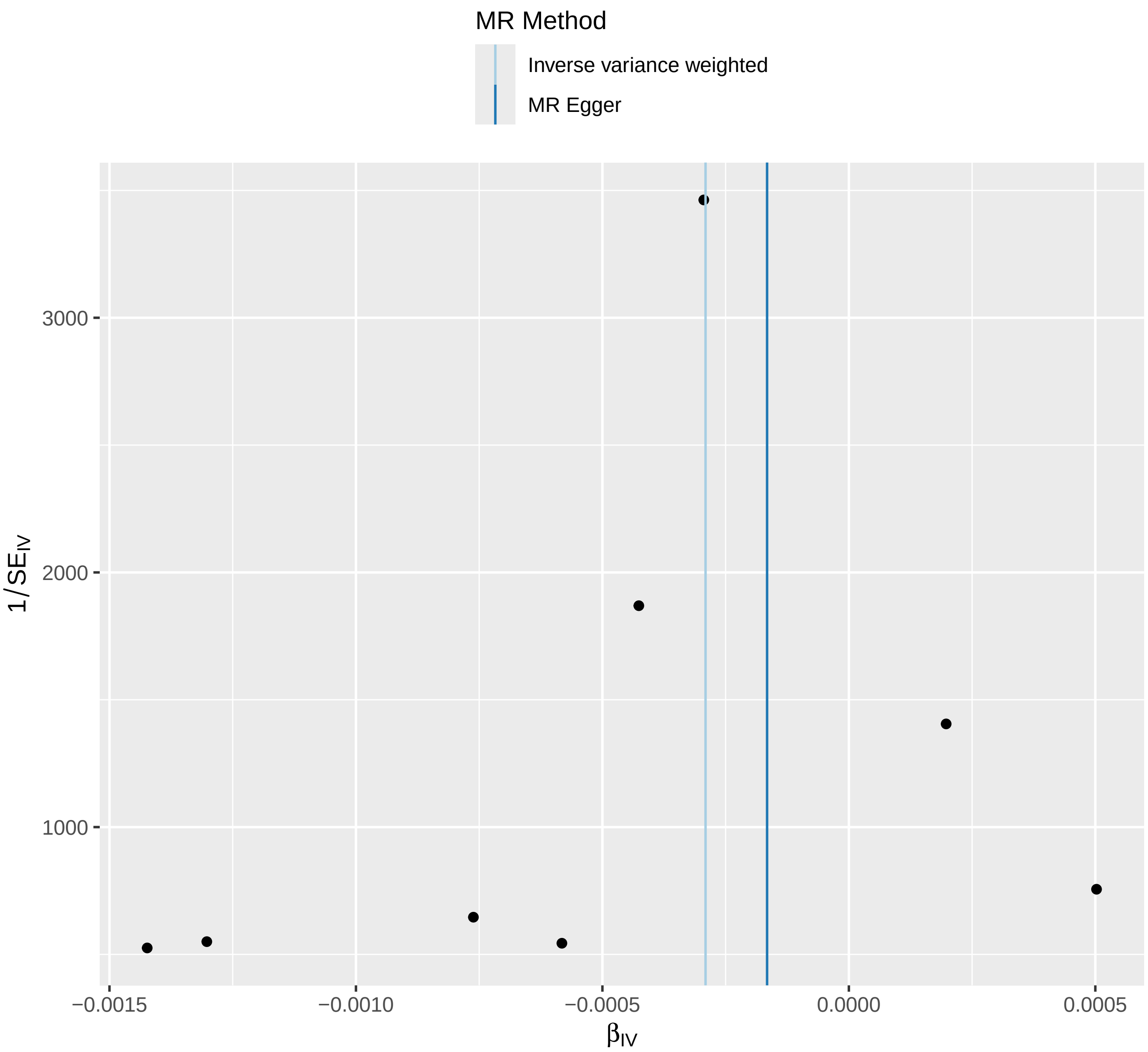
MR Method
Inverse variance weighted
MR Egger
3000
2000
1000
1/SE_IV
−0.0015
−0.0010
−0.0005
0.0000
0.0005
β_IV

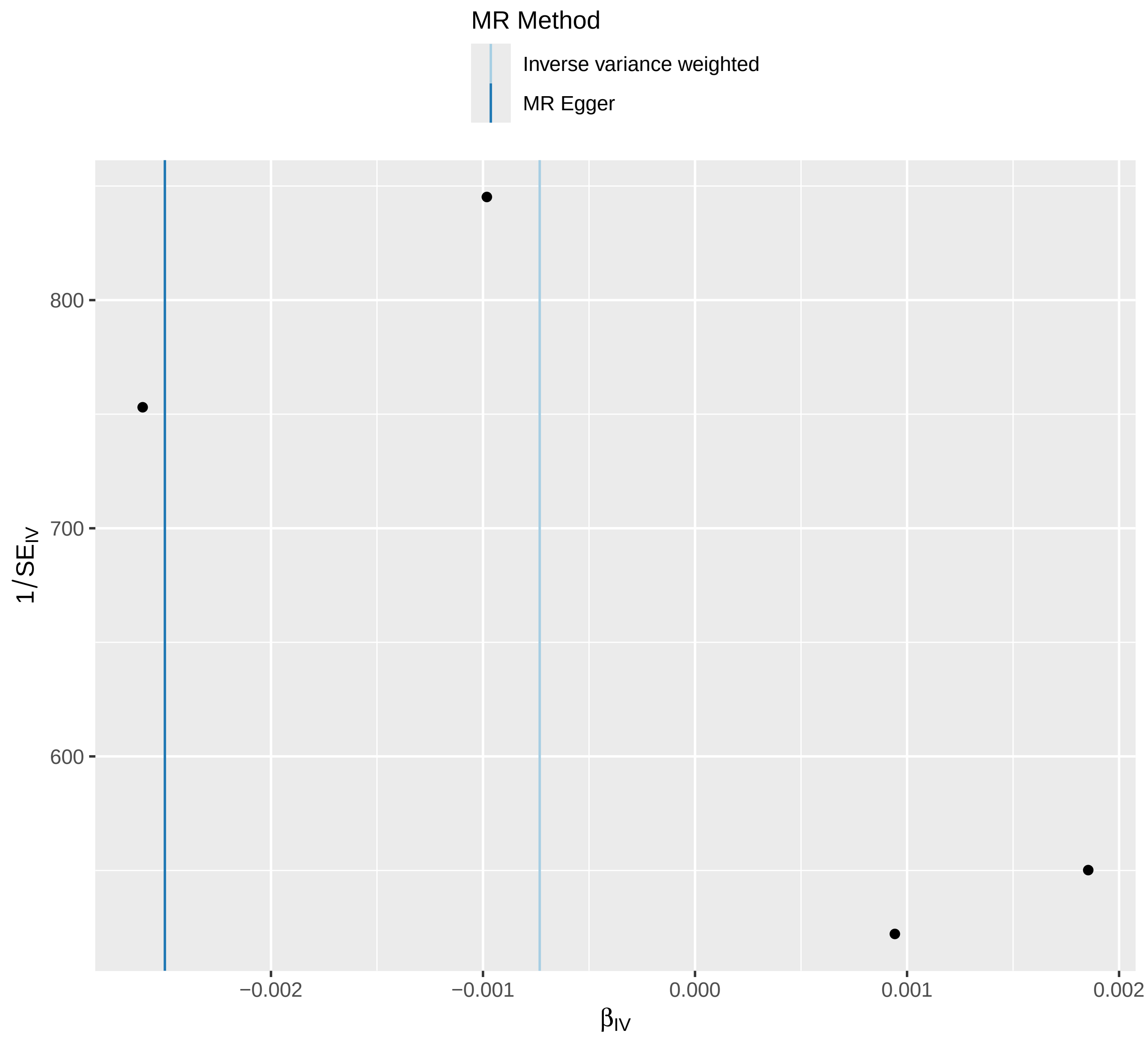
MR Method
Inverse variance weighted
MR Egger
800
700
600
1/SE_IV
−0.002
−0.001
0.000
0.001
0.002
β_IV

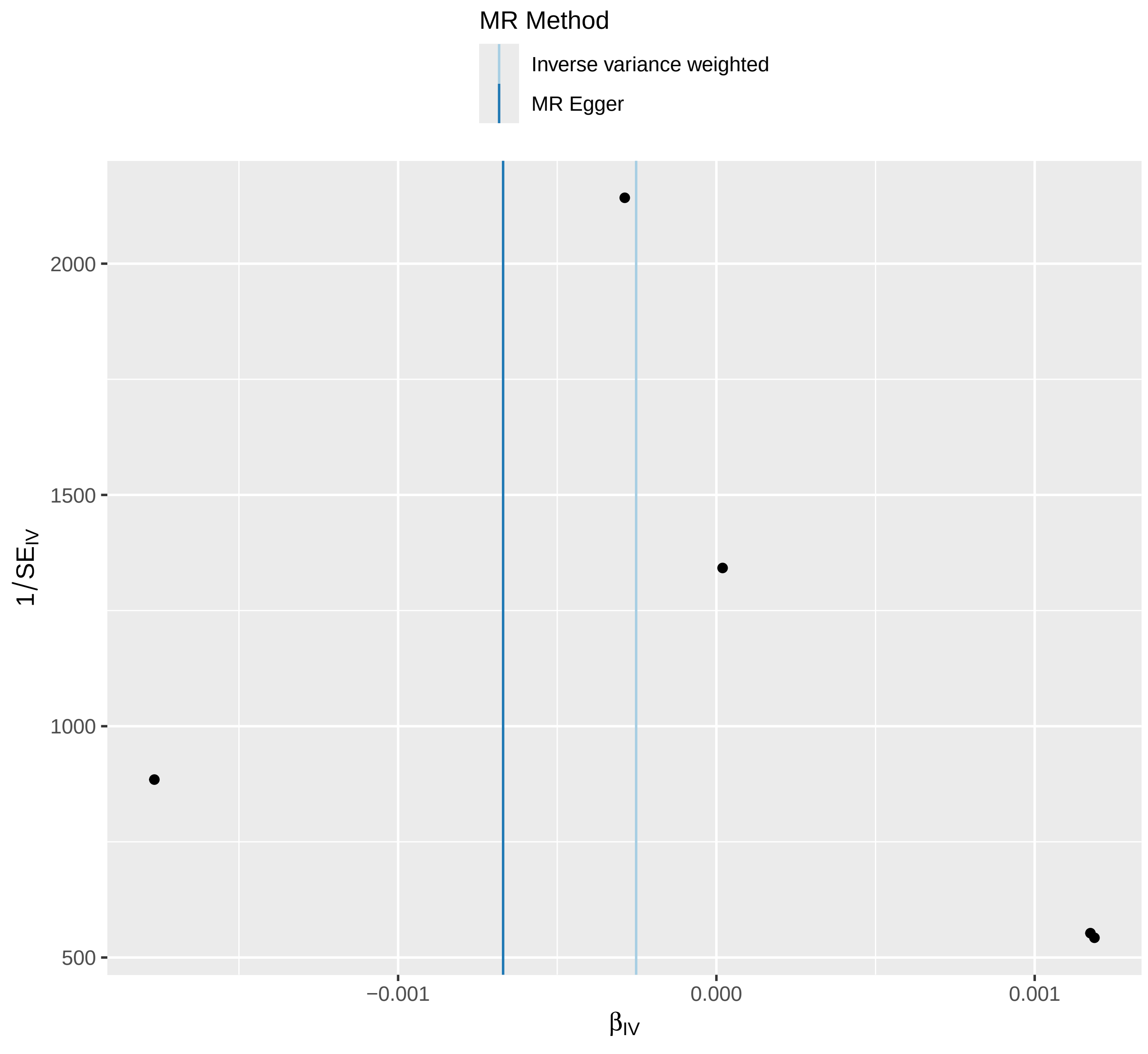

MR Method
Inverse variance weighted
MR Egger
2000
1500
1000
500
1/SE_IV
−0.001
0.000
0.001
β_IV

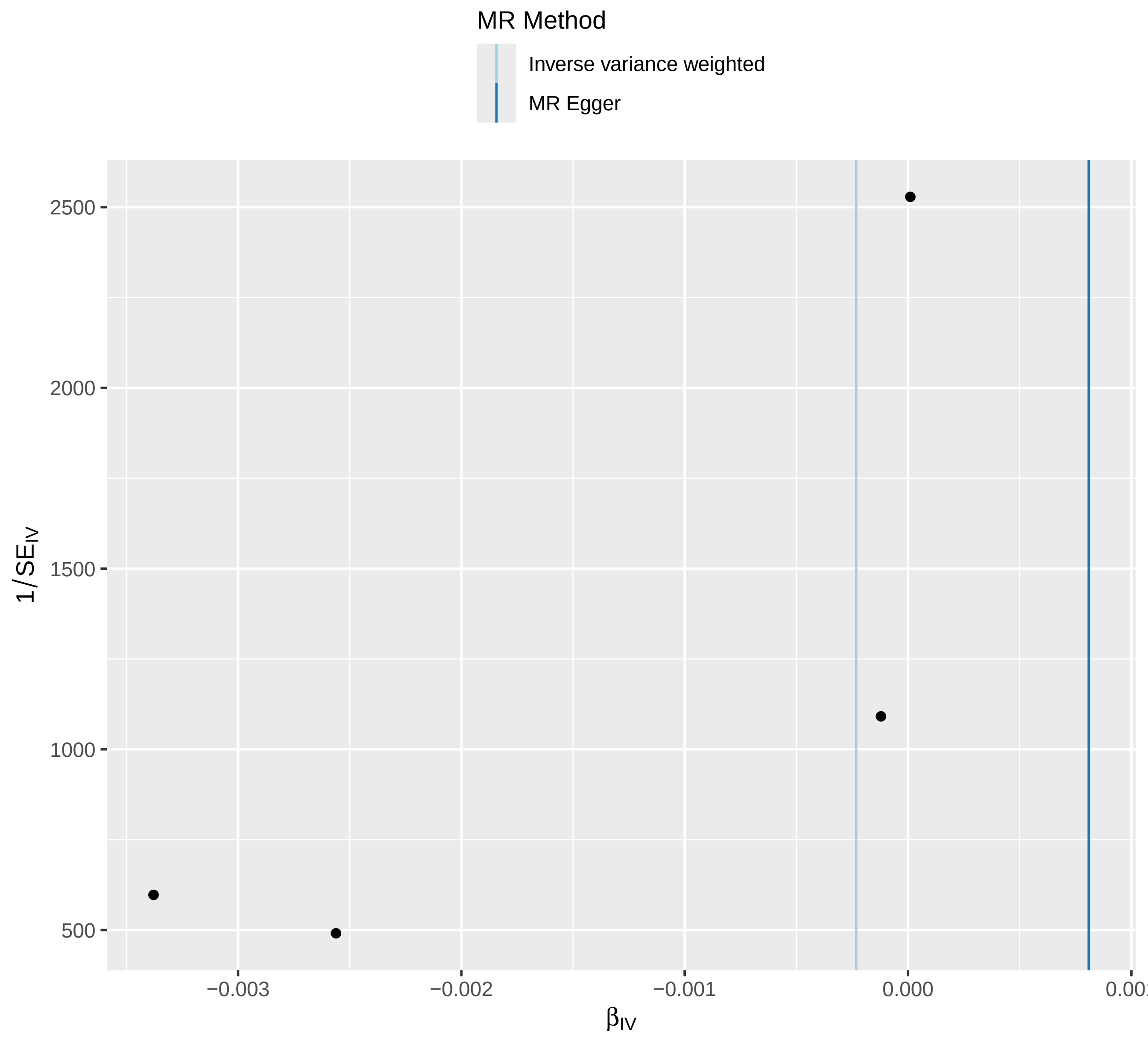
MR Method
Inverse variance weighted
MR Egger
2500
2000
1500
1000
500
$1/SE_{IV}$
−0.003
−0.002
−0.001
0.000
β_{IV}

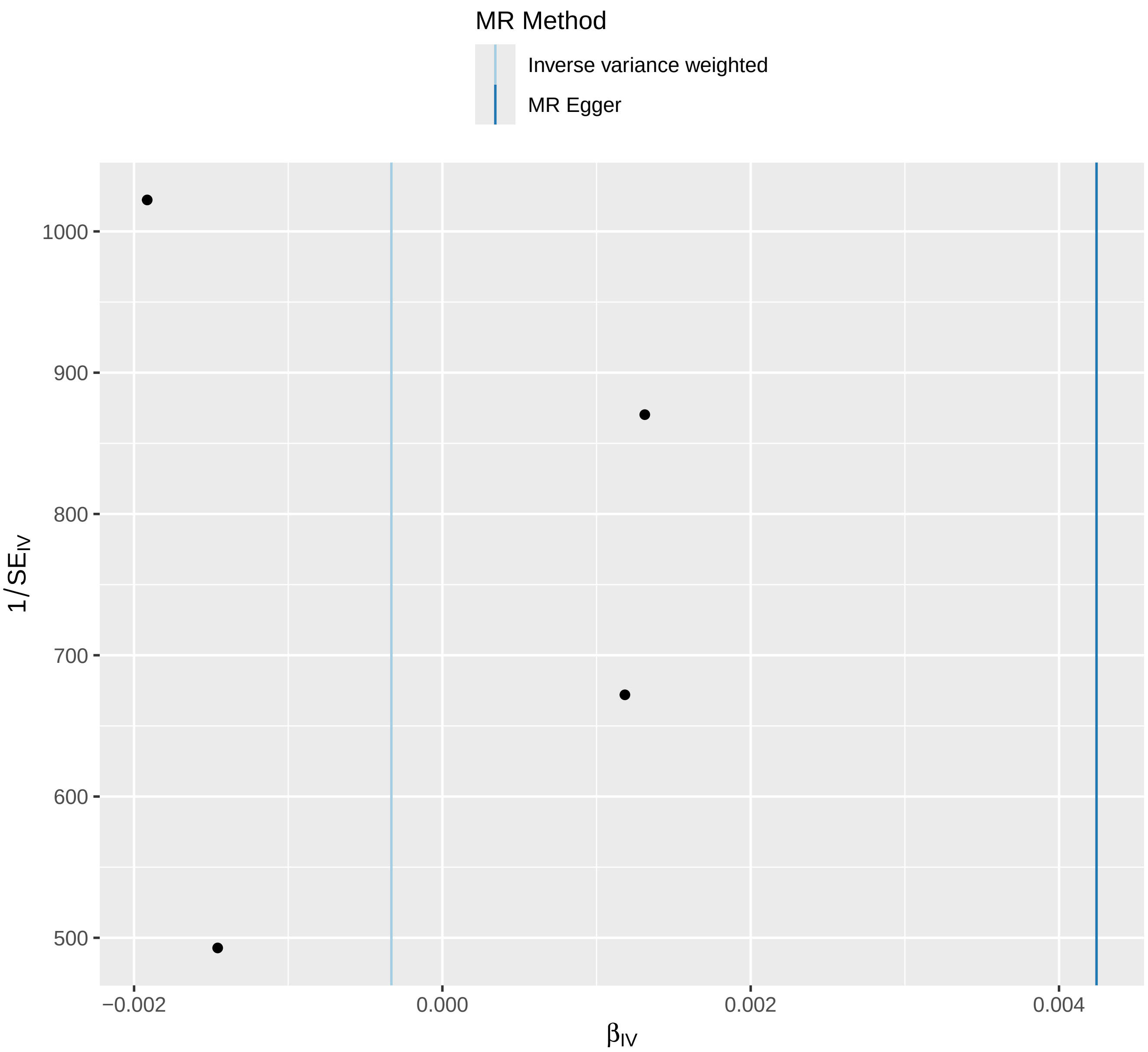
MR Method
Inverse variance weighted
MR Egger
1000
900
800
700
600
500
1/SE_IV
−0.002
0.000
0.002
0.004
β_IV

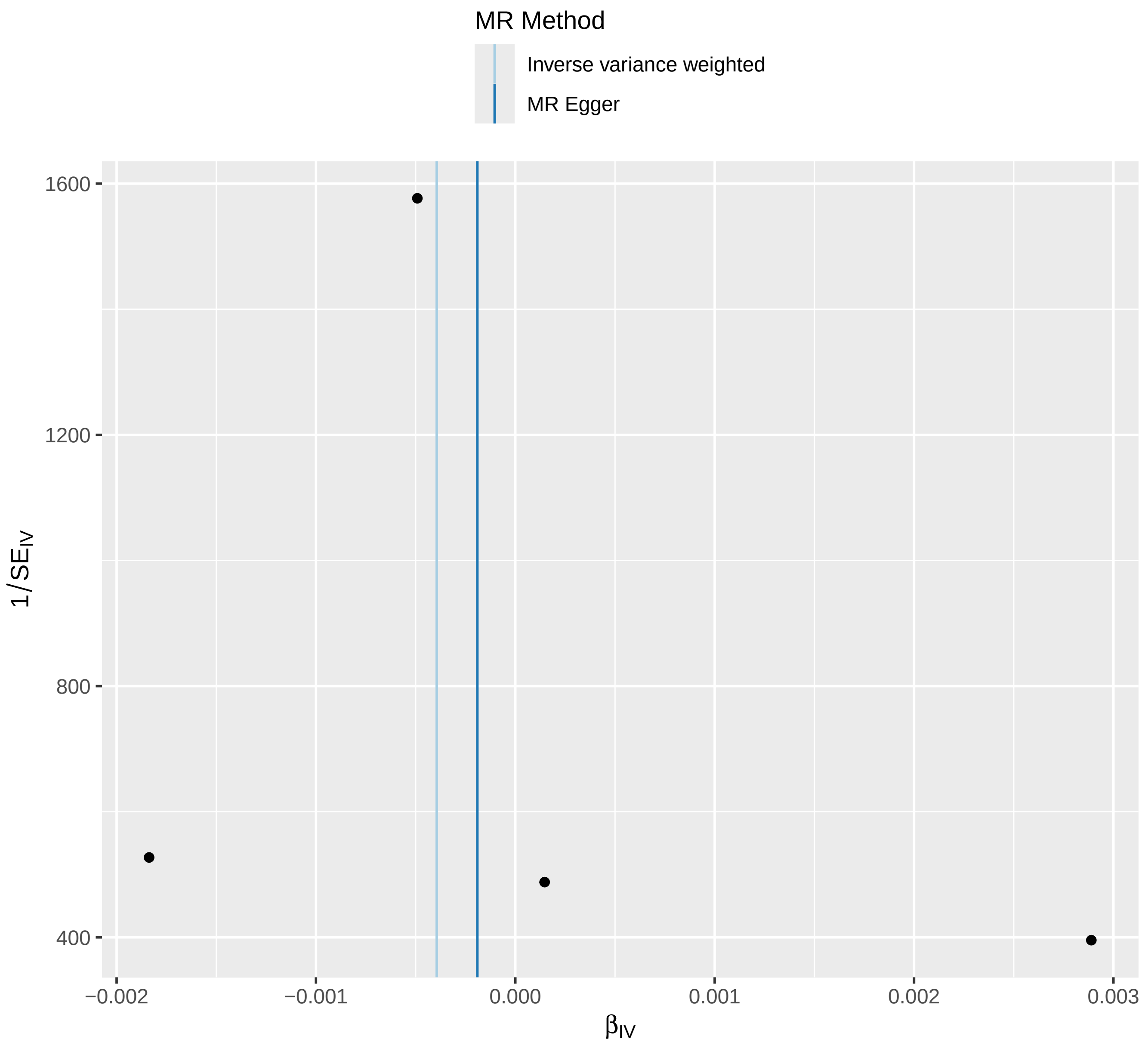
MR Method
Inverse variance weighted
MR Egger
1600
1200
800
400
1/SE_IV
−0.002
−0.001
0.000
0.001
0.002
0.003
β_IV

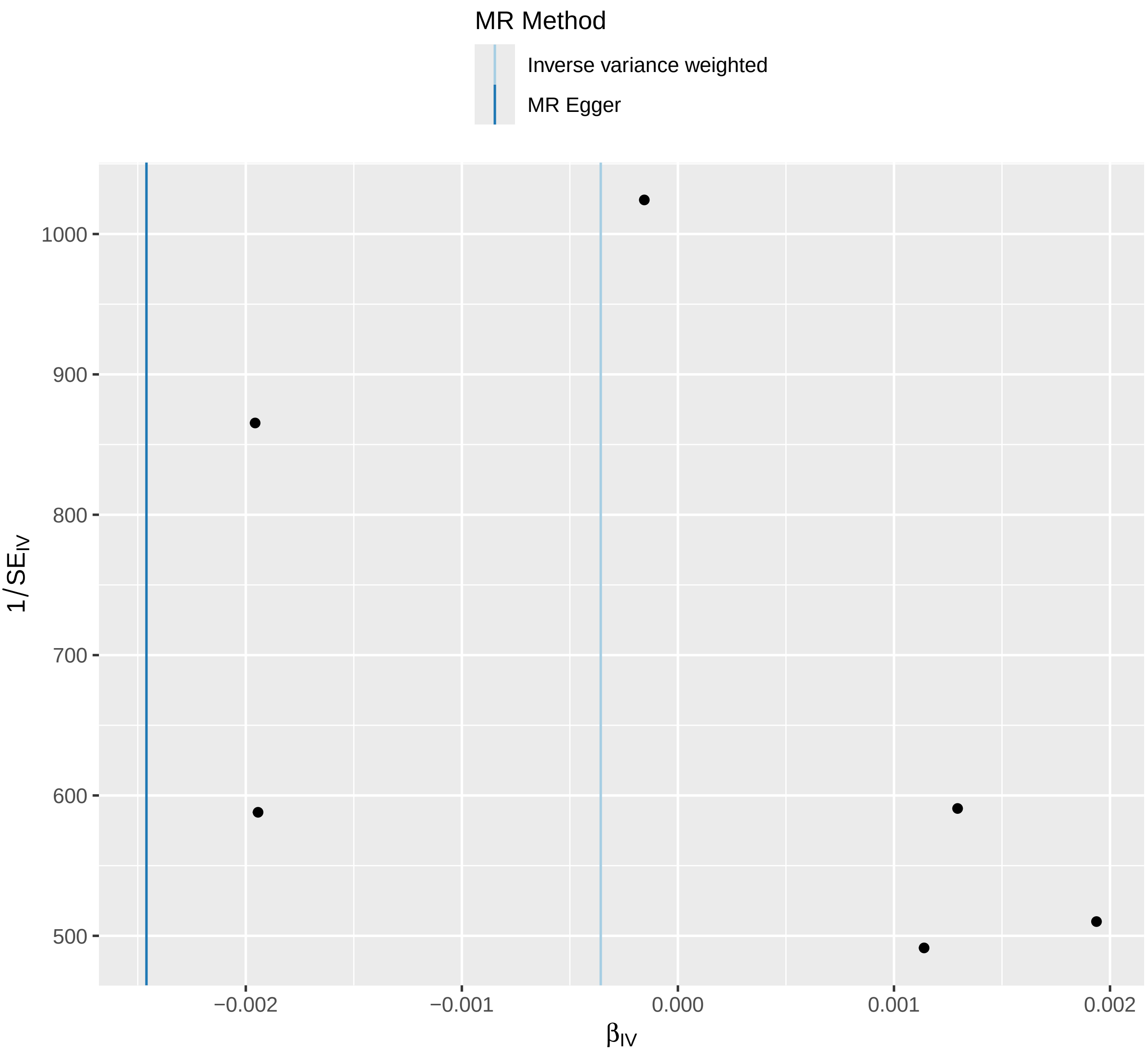
MR Method
Inverse variance weighted
MR Egger
1000
900
800
700
600
500
1/SE_IV
−0.002
−0.001
0.000
0.001
0.002
β_IV

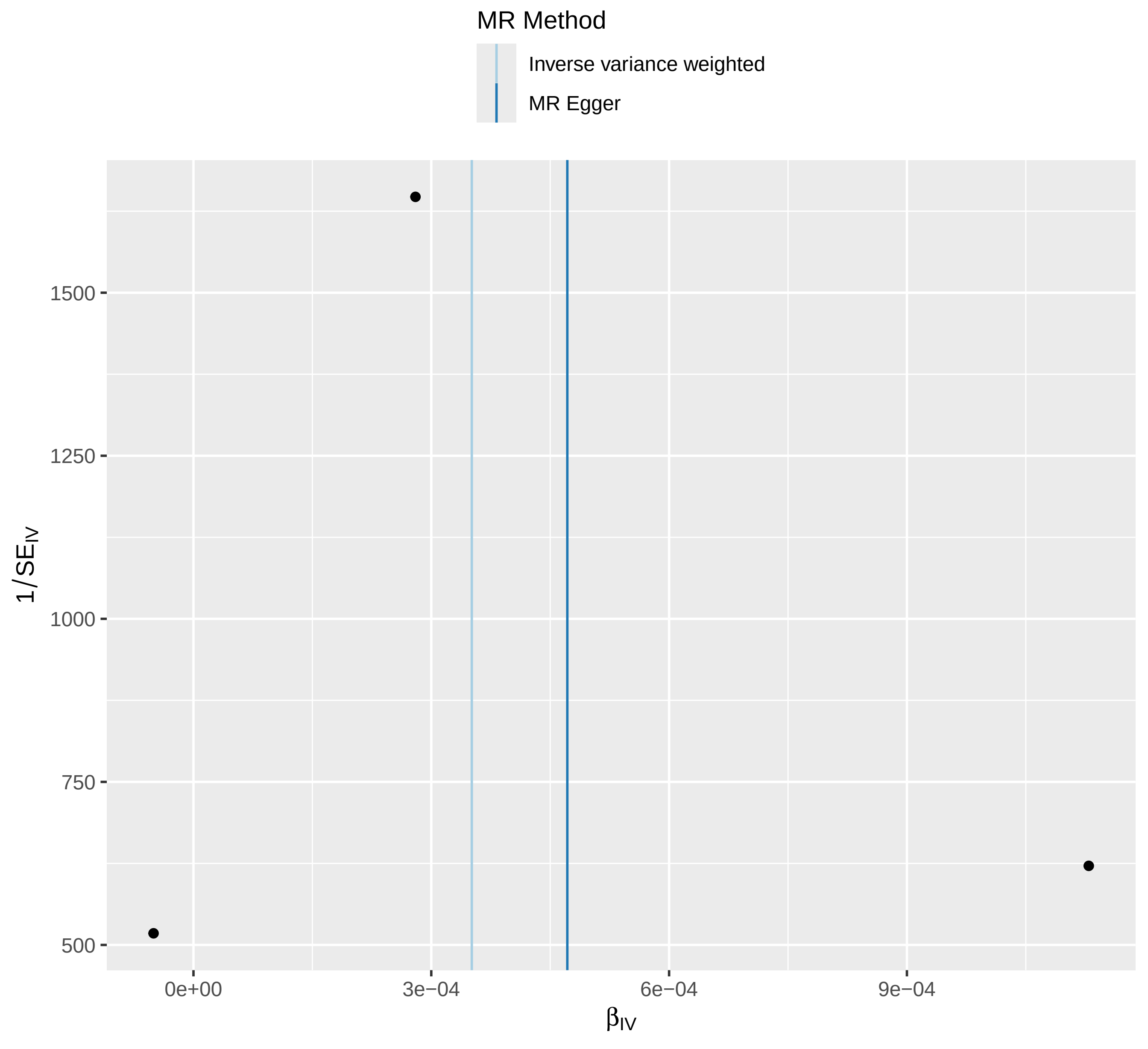

MR Method
Inverse variance weighted
MR Egger
1500
1250
1000
750
500
1/SE_IV
0e+00
3e−04
6e−04
9e−04
β_IV

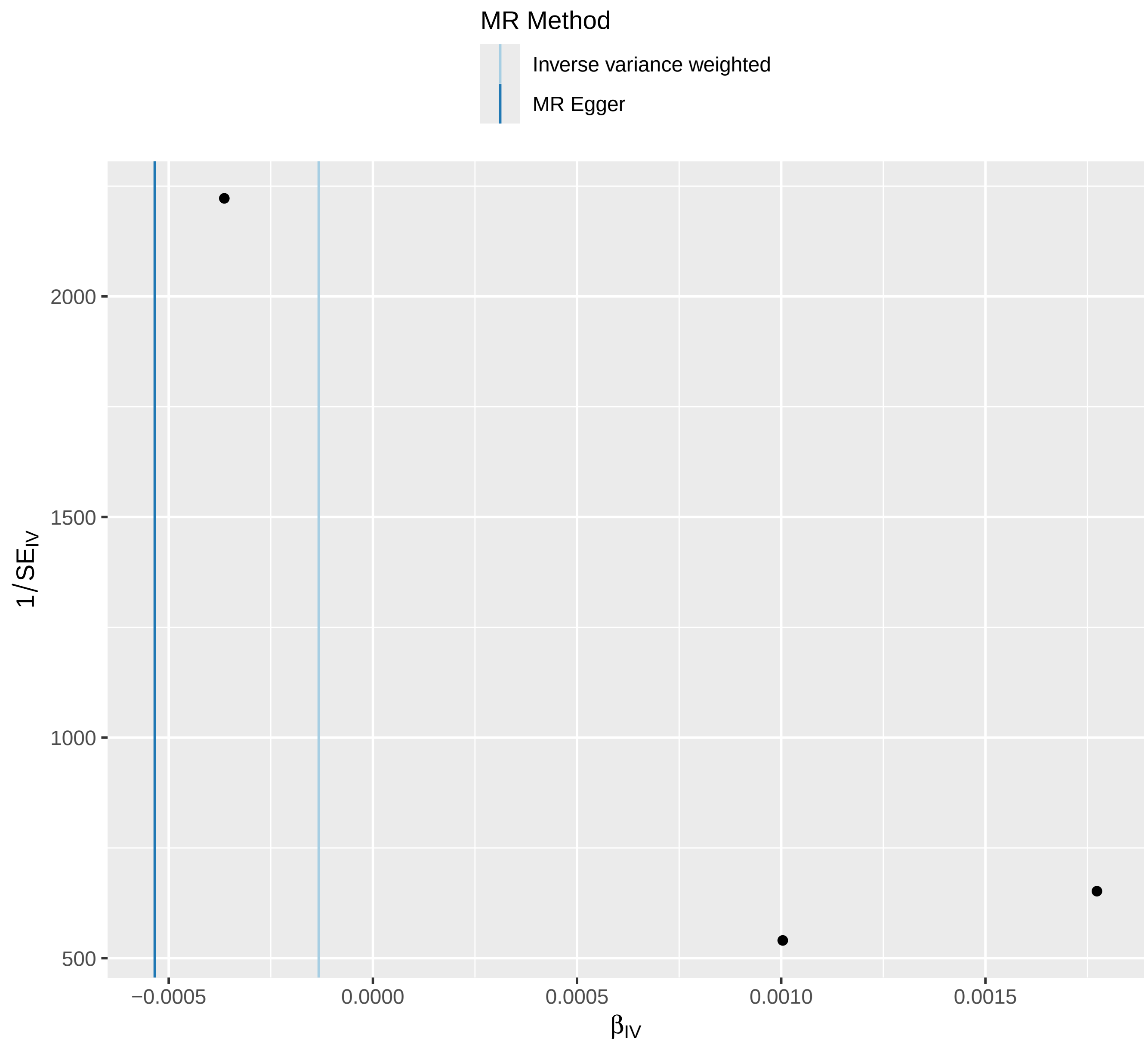

MR Method
Inverse variance weighted
MR Egger
2000
1500
1000
500
$1/SE_{IV}$
−0.0005
0.0000
0.0005
0.0010
0.0015
β_{IV}

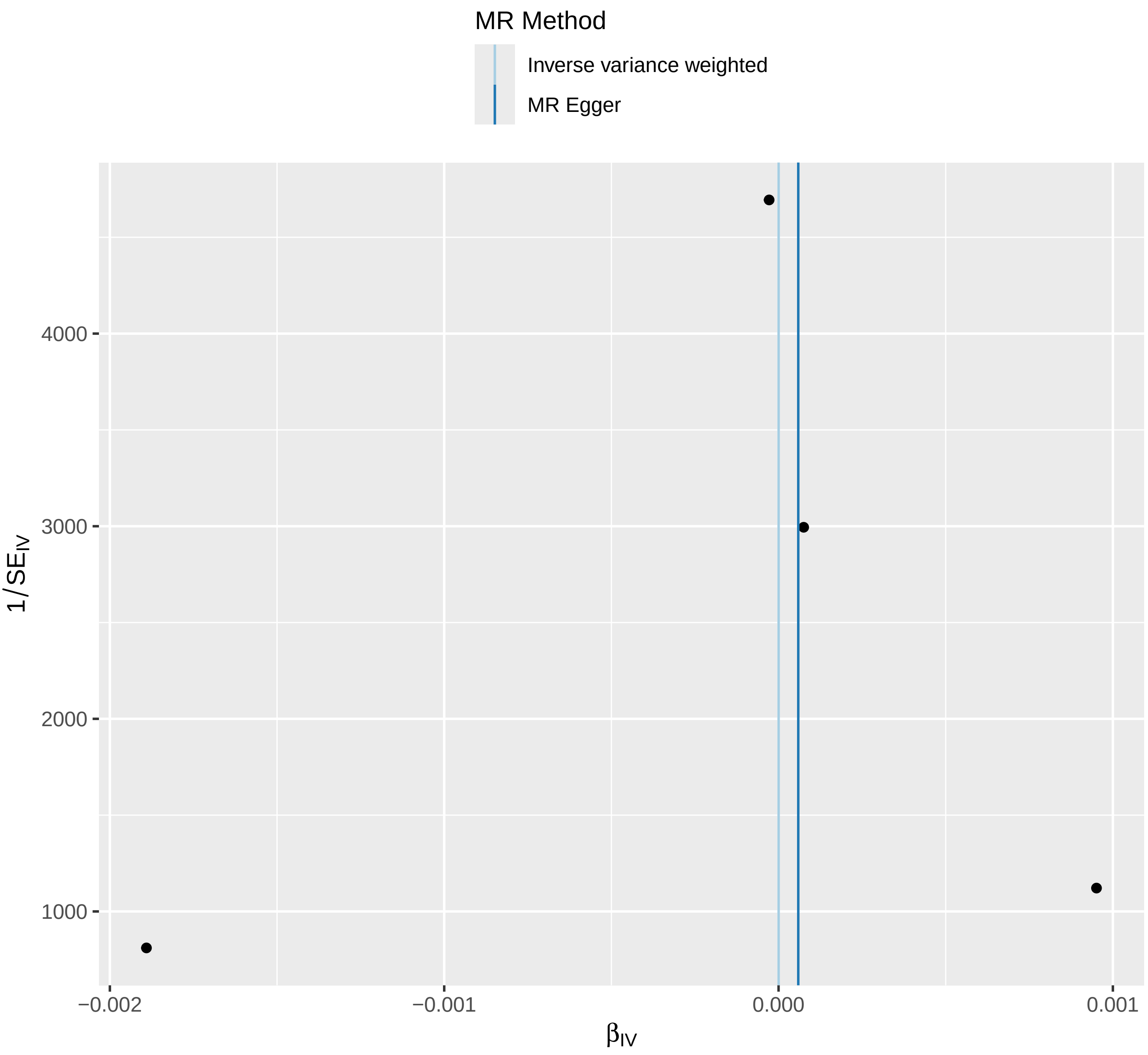

MR Method
Inverse variance weighted
MR Egger
4000
3000
2000
1000
1/SE_IV
−0.002
−0.001
0.000
0.001
β_IV

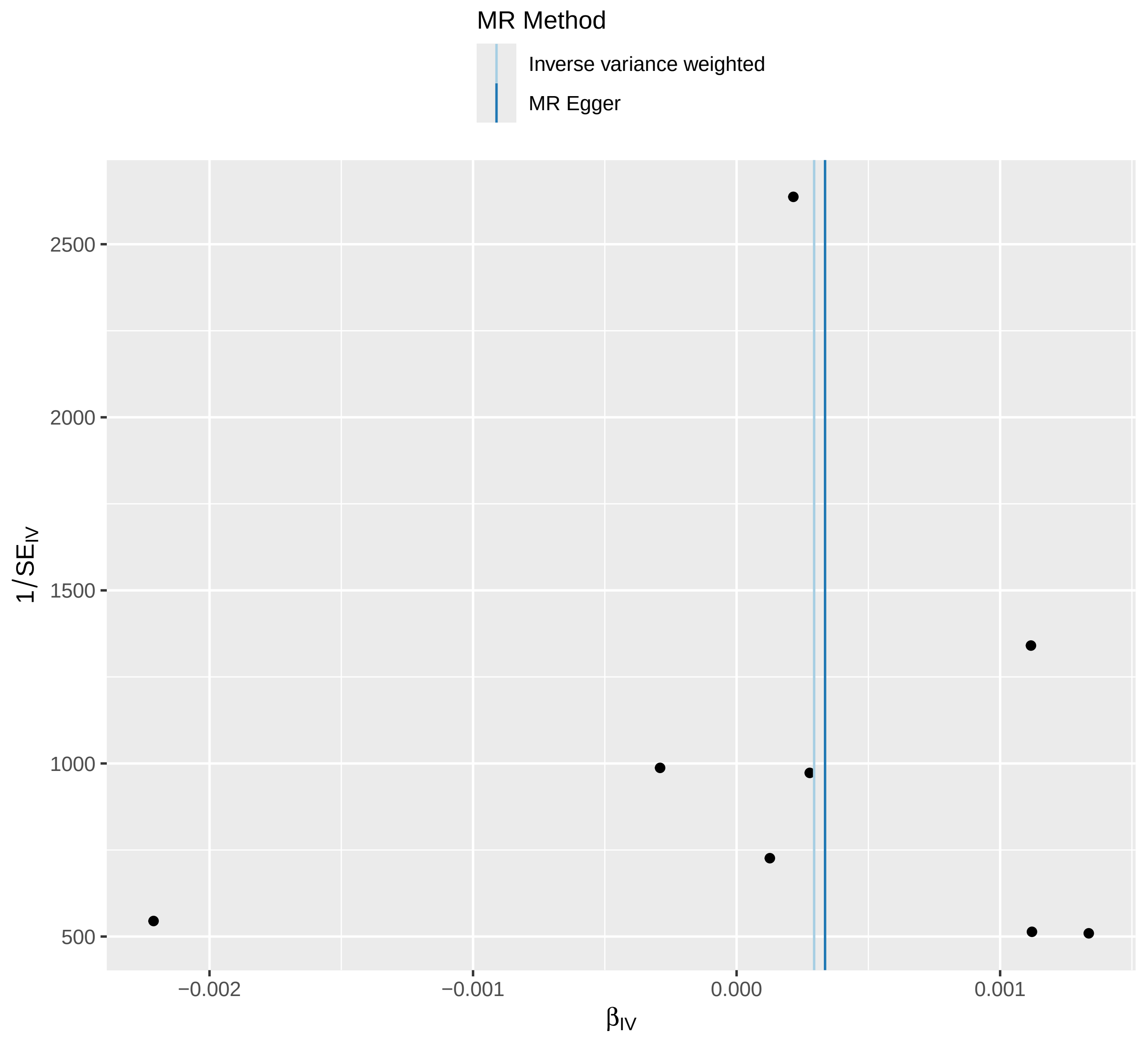
MR Method
Inverse variance weighted
MR Egger
2500
2000
1500
1000
500
1/SE_IV
−0.002
−0.001
0.000
0.001
β_IV

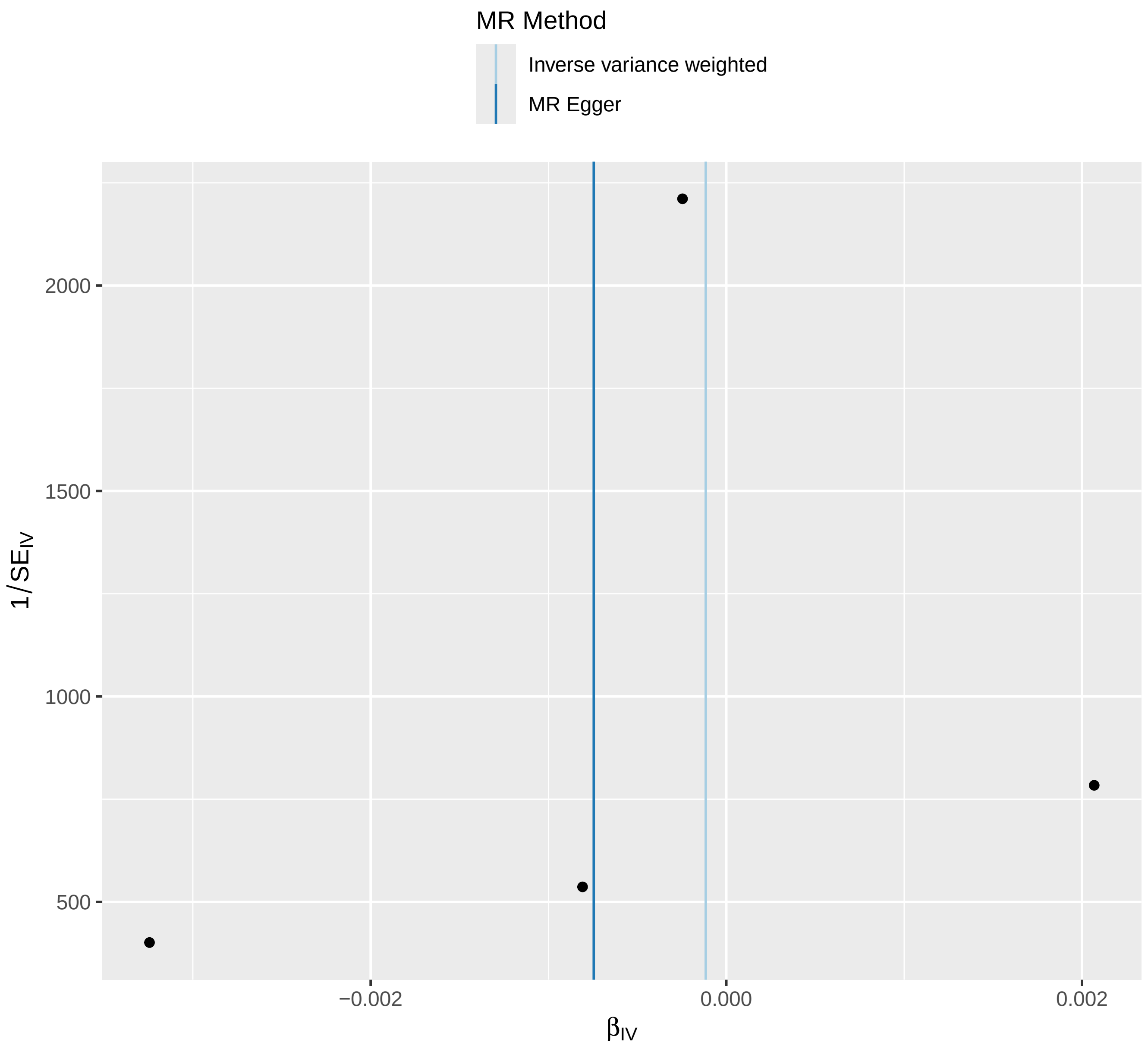
MR Method
Inverse variance weighted
MR Egger
2000
1500
1000
500
−0.002
0.000
0.002
$1/SE_{IV}$
β_{IV}

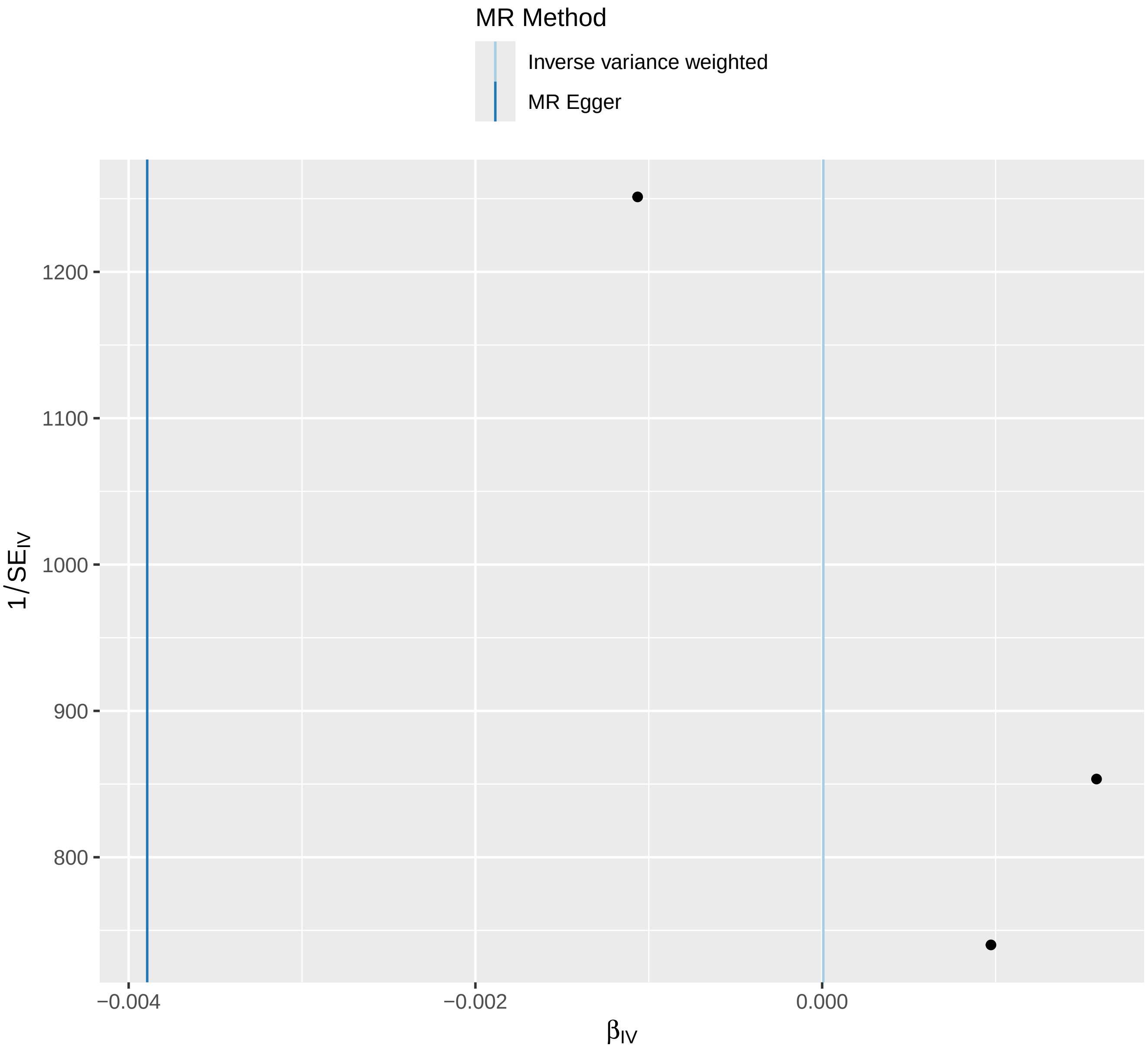
MR Method
Inverse variance weighted
MR Egger
1200
1100
1000
900
800
1/SE_IV
−0.004
−0.002
0.000
β_IV

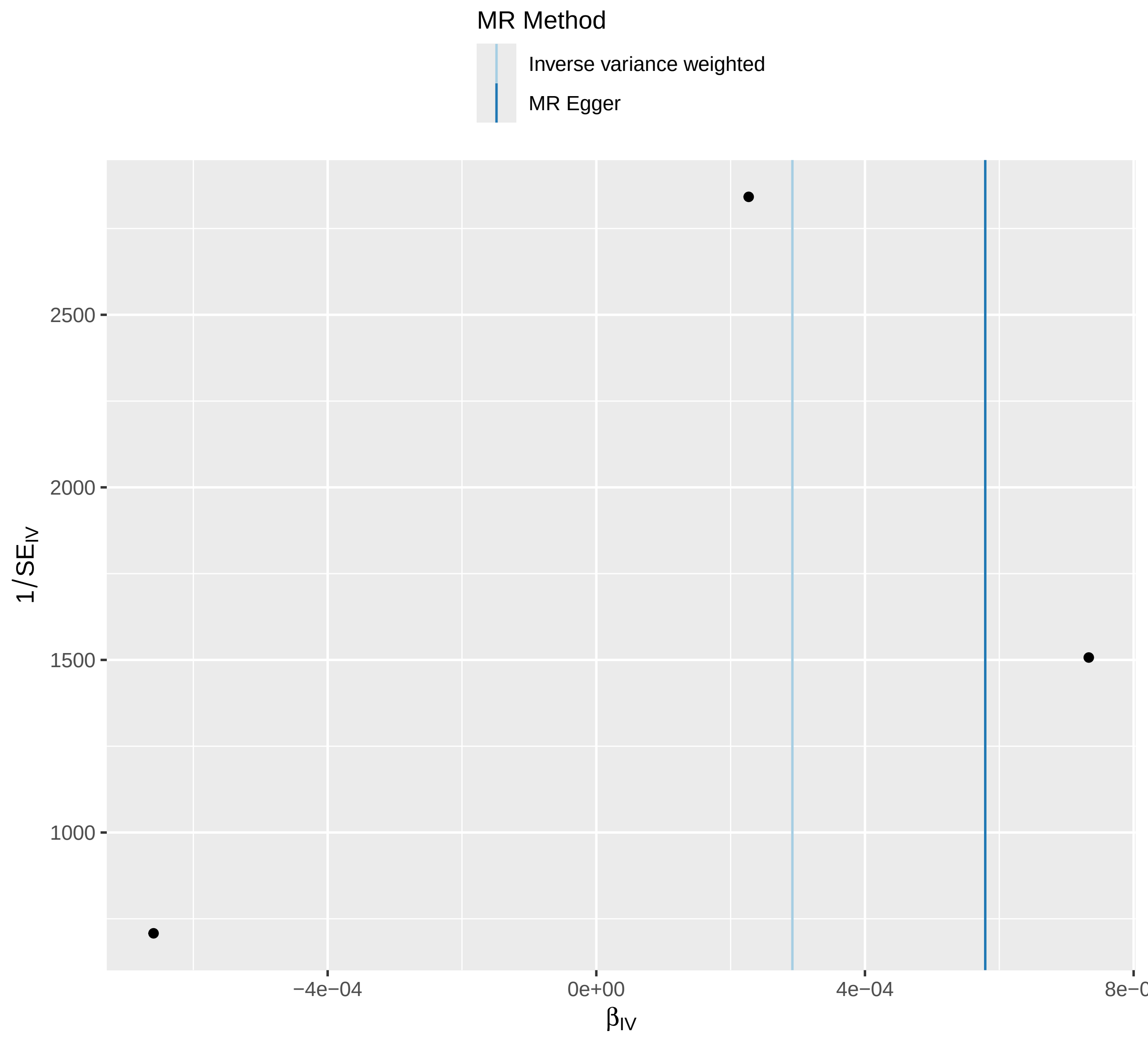
MR Method
Inverse variance weighted
MR Egger
2500
2000
1500
1000
−4e−04
0e+00
4e−04
8e−0
1/SE_IV
β_IV

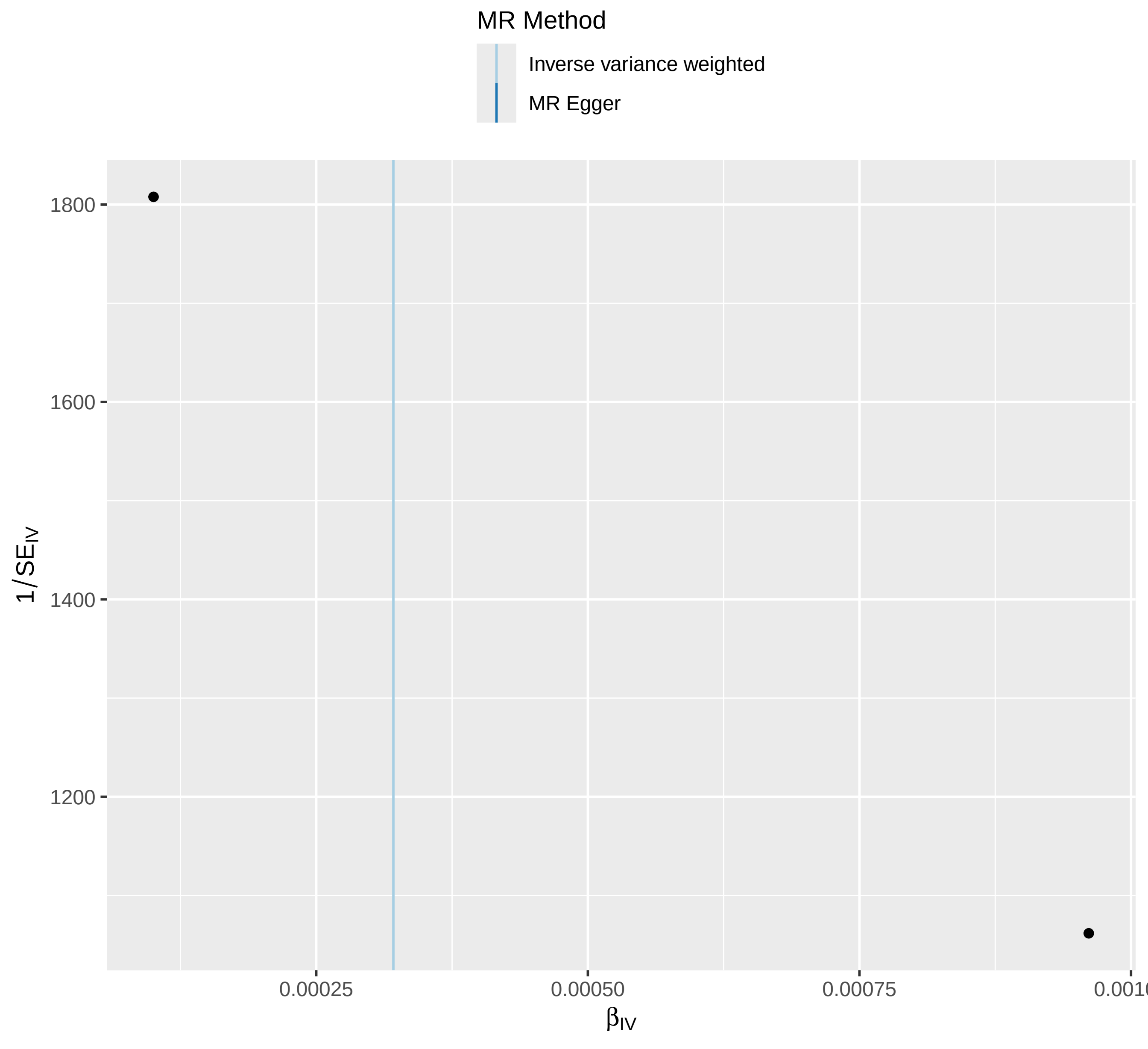

MR Method
Inverse variance weighted
MR Egger
1800
1600
1400
1200
$1/SE_{IV}$
0.00025
0.00050
0.00075
0.0010
β_{IV}

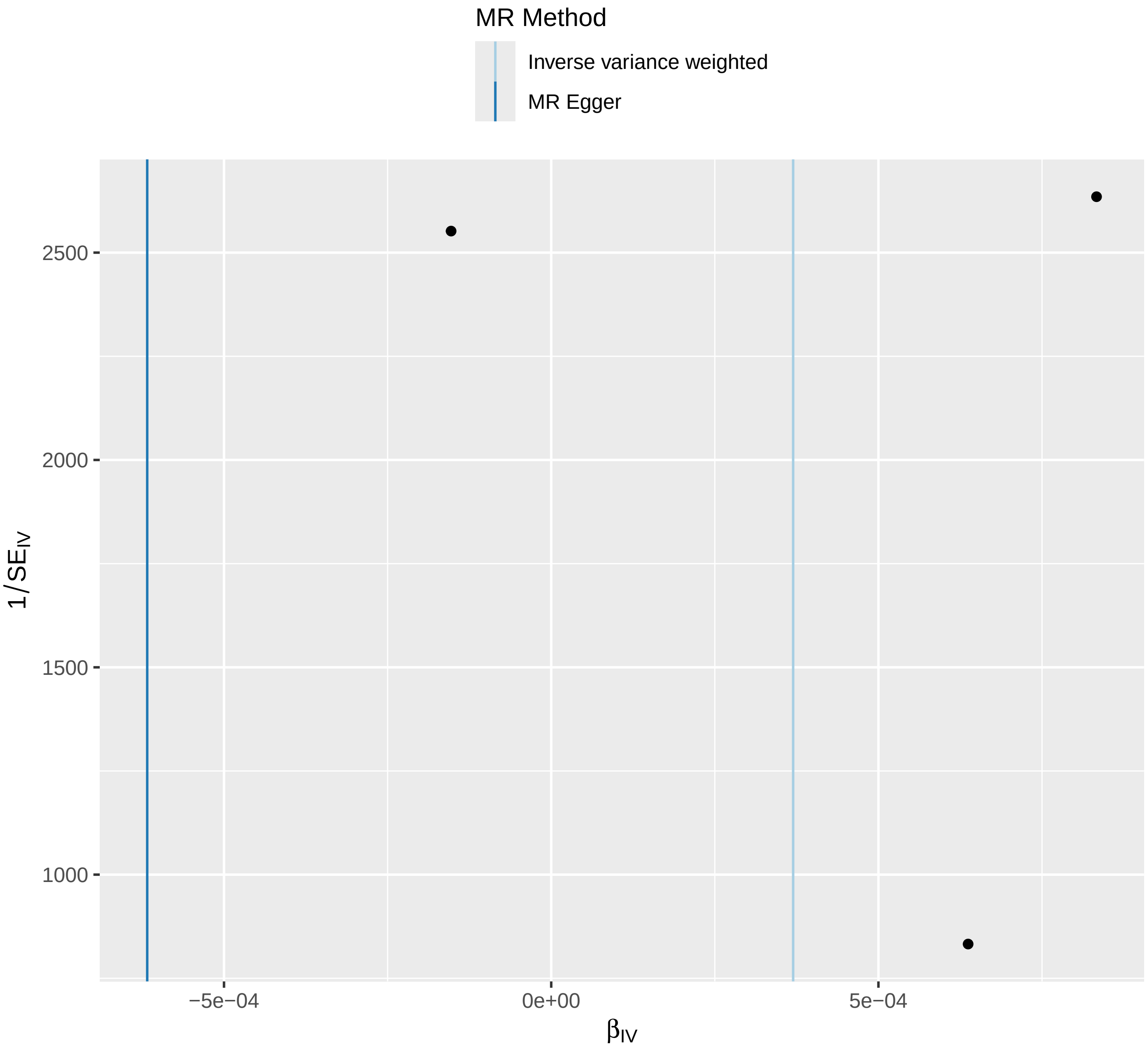
MR Method
Inverse variance weighted
MR Egger
2500
2000
1500
1000
1/SE_IV
−5e−04
0e+00
5e−04
β_IV

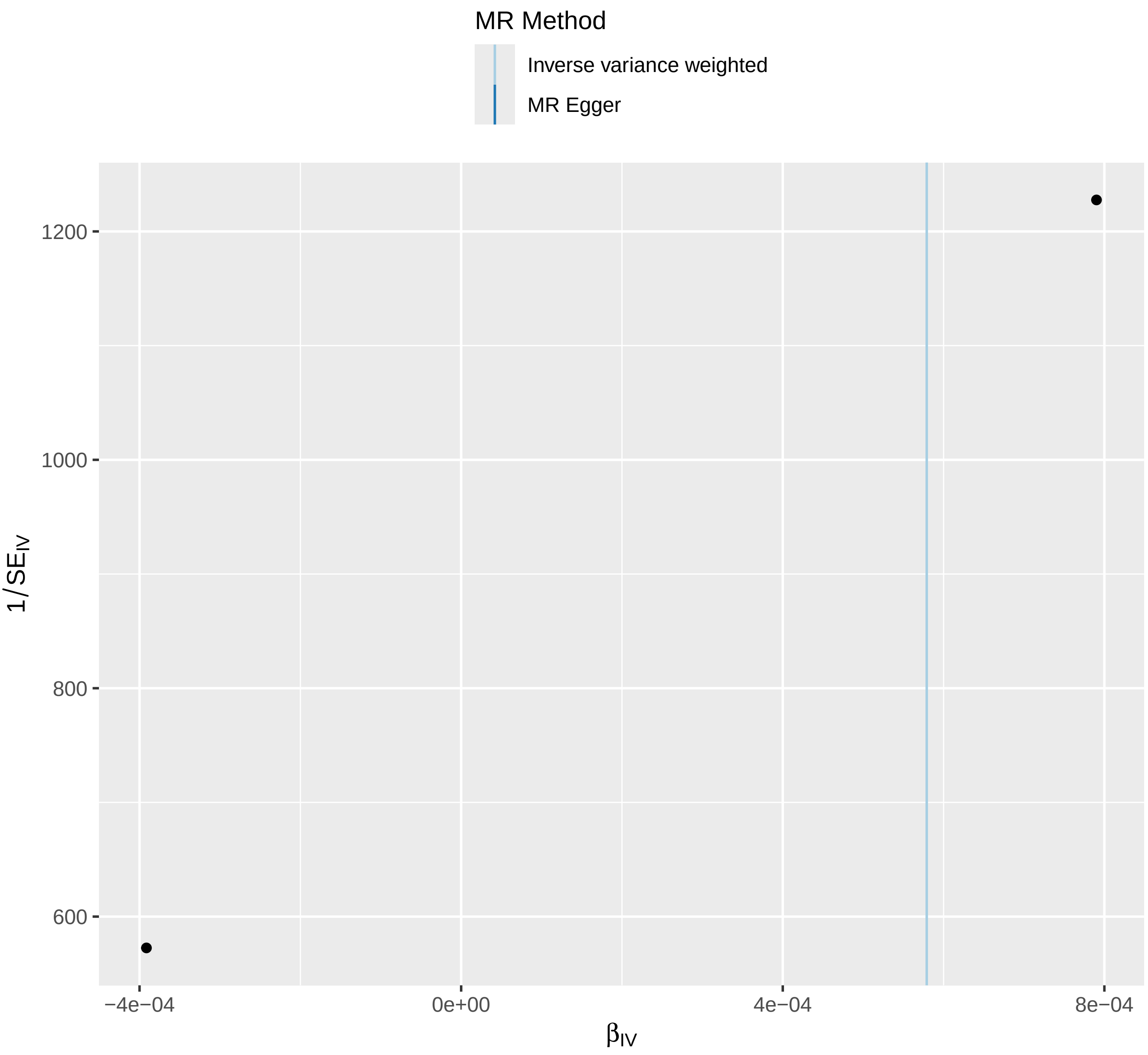
MR Method
Inverse variance weighted
MR Egger
1200
1000
800
600
1/SE_IV
−4e−04
0e+00
4e−04
8e−04
β_IV

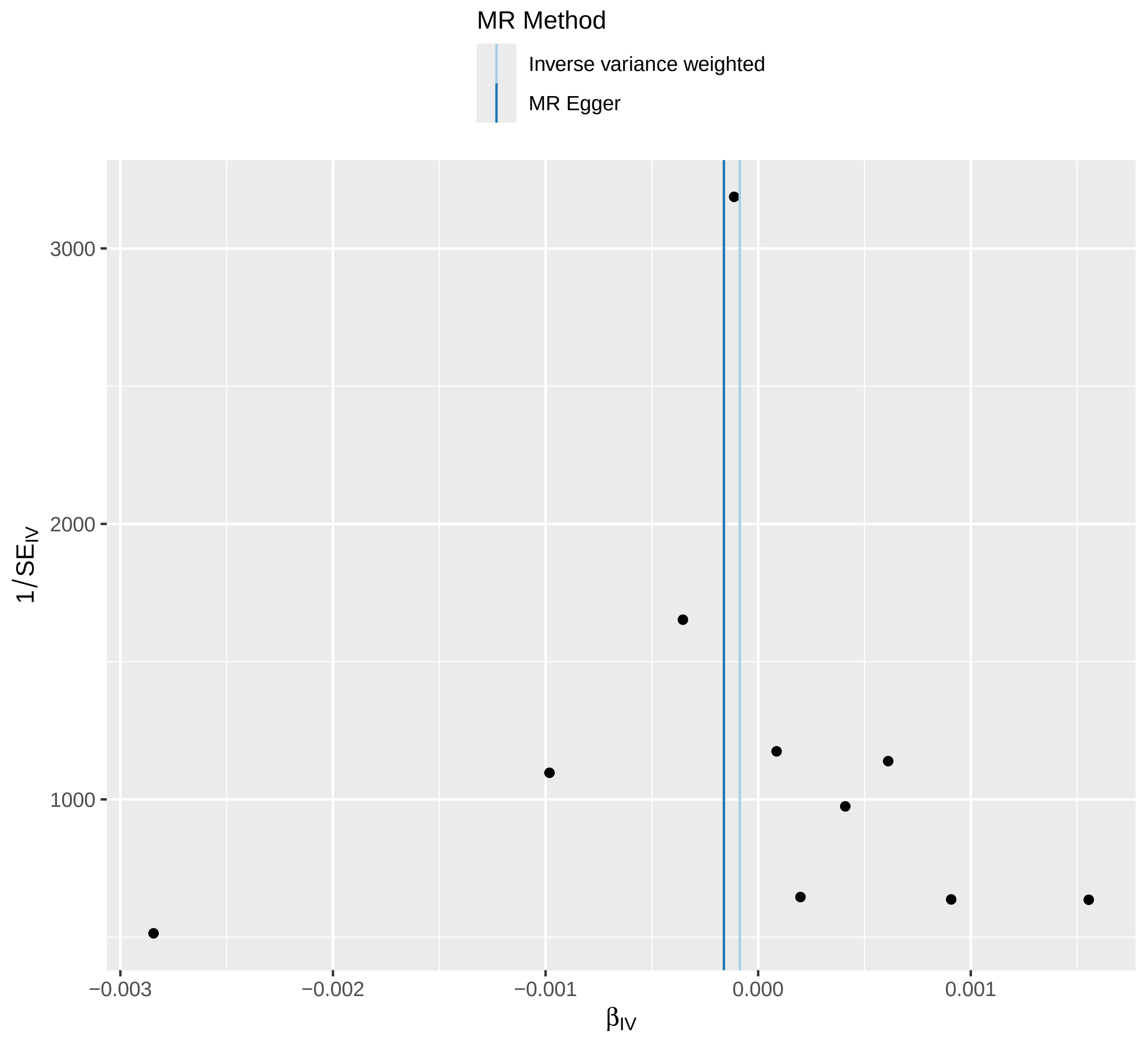

MR Method
Inverse variance weighted
MR Egger
3000
2000
1000
1/SE_IV
−0.003
−0.002
−0.001
0.000
0.001
β_IV

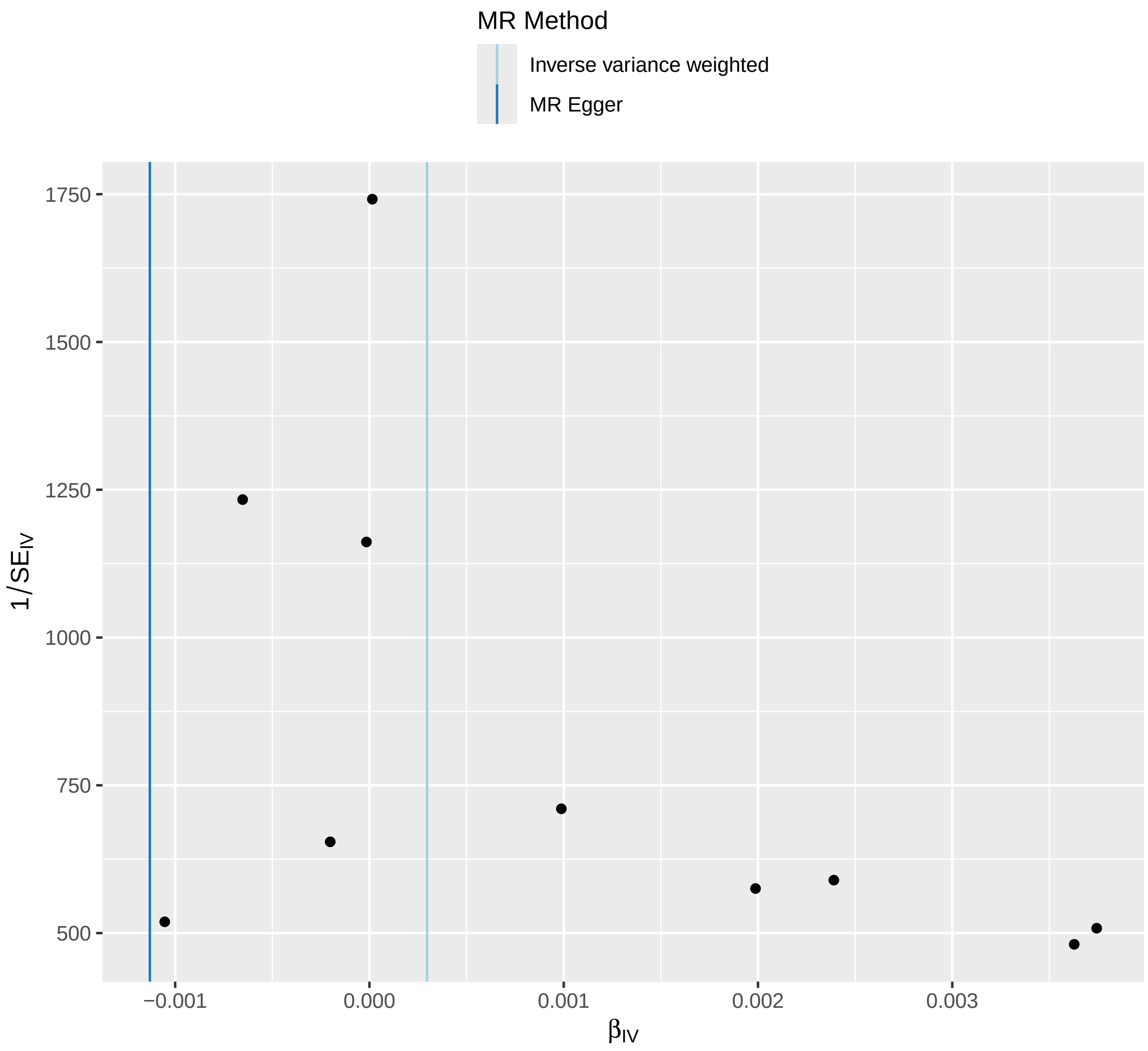

MR Method
Inverse variance weighted
MR Egger
1750
1500
1250
1000
750
500
1/SE$_{IV}$
−0.001
0.000
0.001
0.002
0.003
β$_{IV}$

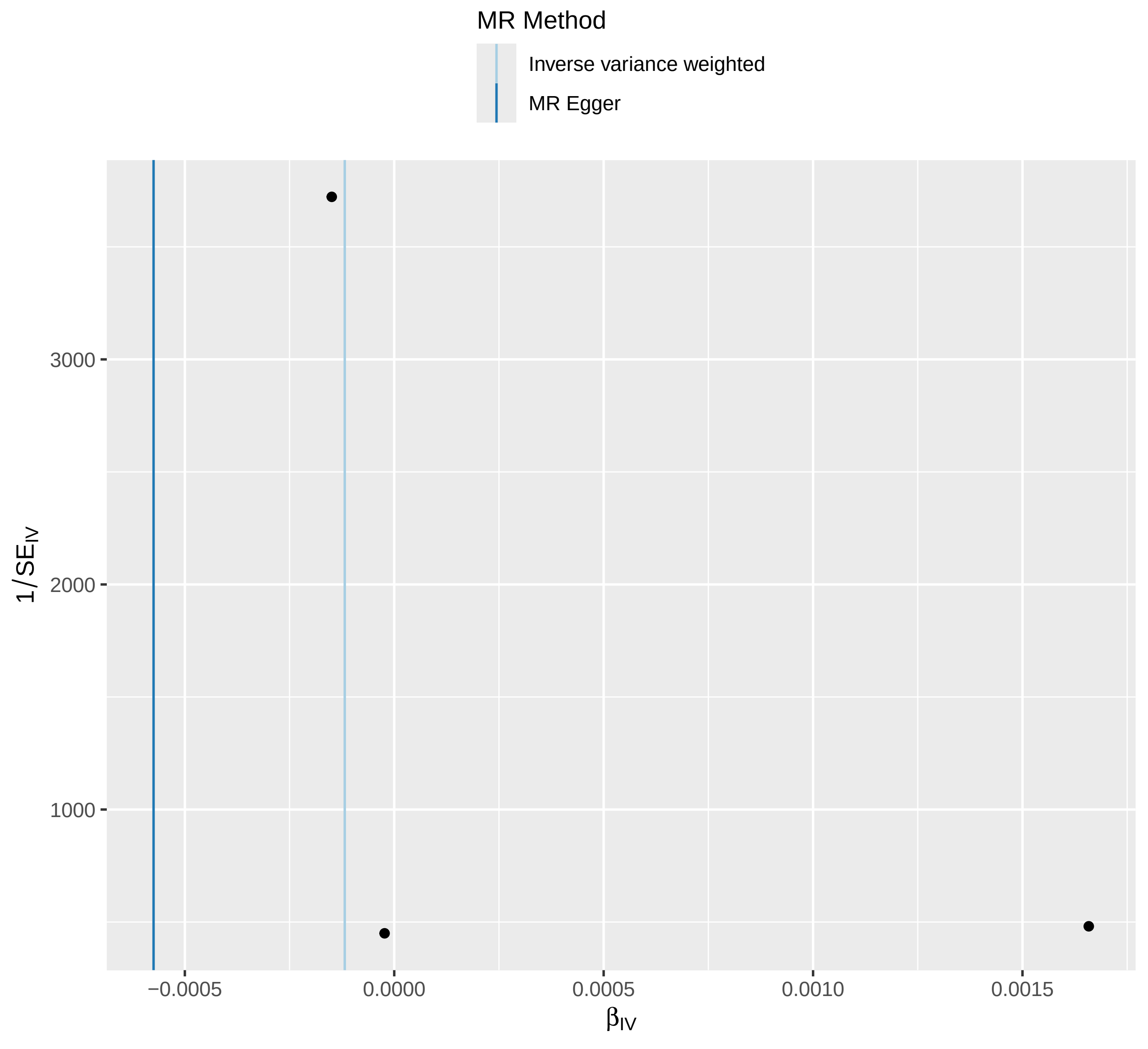
MR Method
Inverse variance weighted
MR Egger
3000
2000
1000
1/SE_IV
−0.0005
0.0000
0.0005
0.0010
0.0015
β_IV

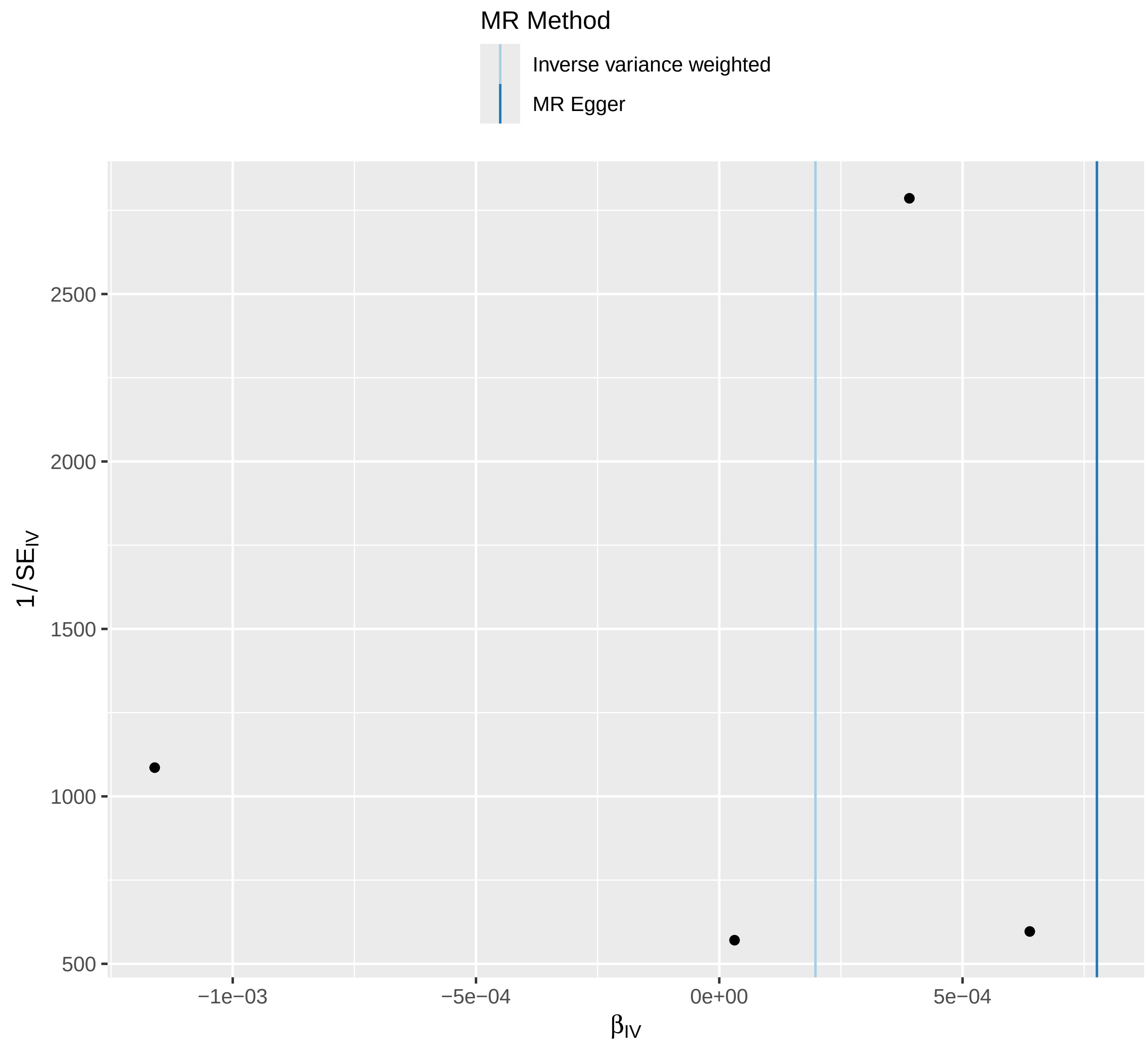

MR Method
Inverse variance weighted
MR Egger
2500
2000
1500
1000
500
−1e−03
−5e−04
0e+00
5e−04
$1/SE_{IV}$
β_{IV}

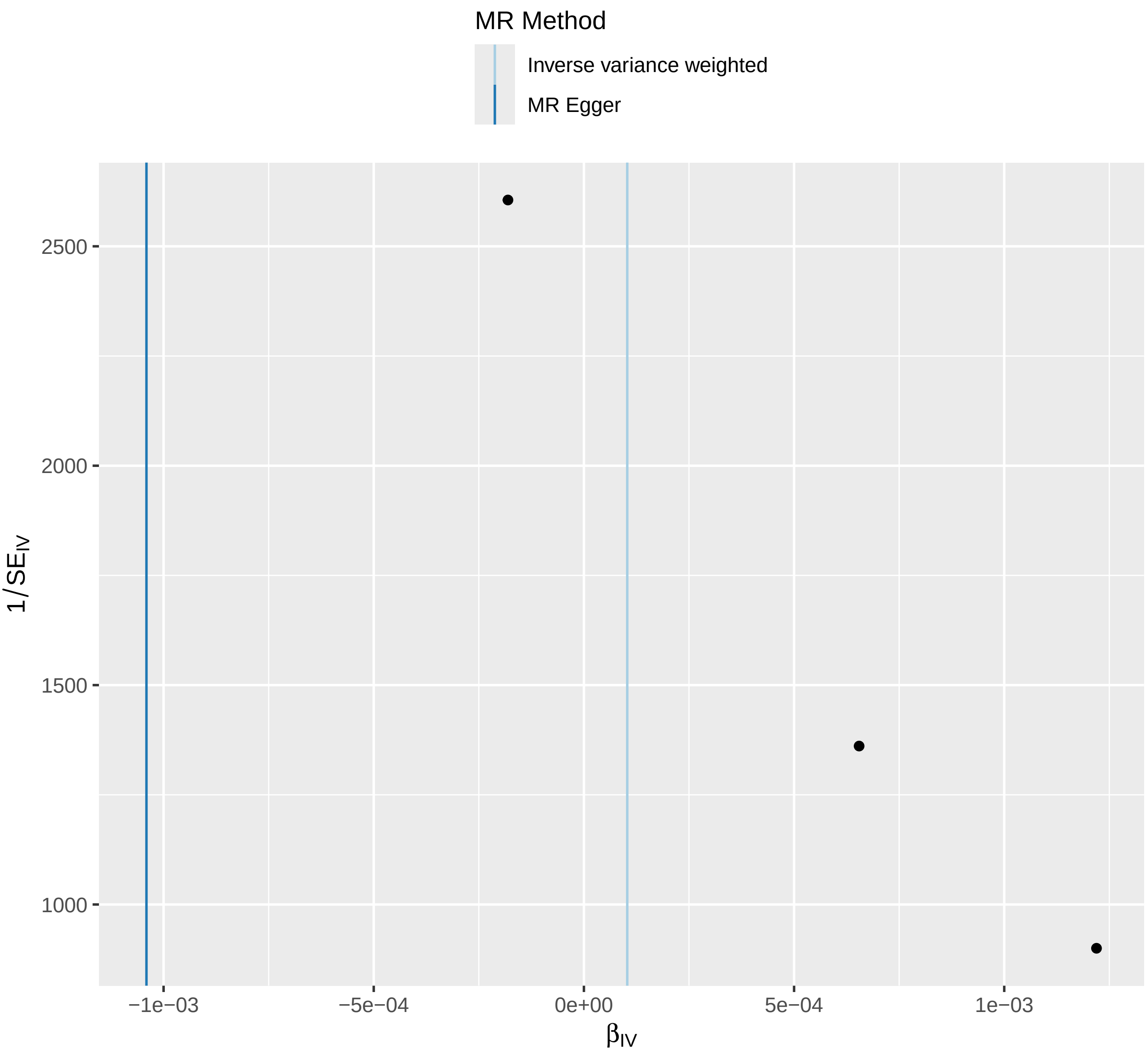
MR Method
Inverse variance weighted
MR Egger
2500
2000
1500
1000
1/SE_IV
−1e−03
−5e−04
0e+00
5e−04
1e−03
β_IV

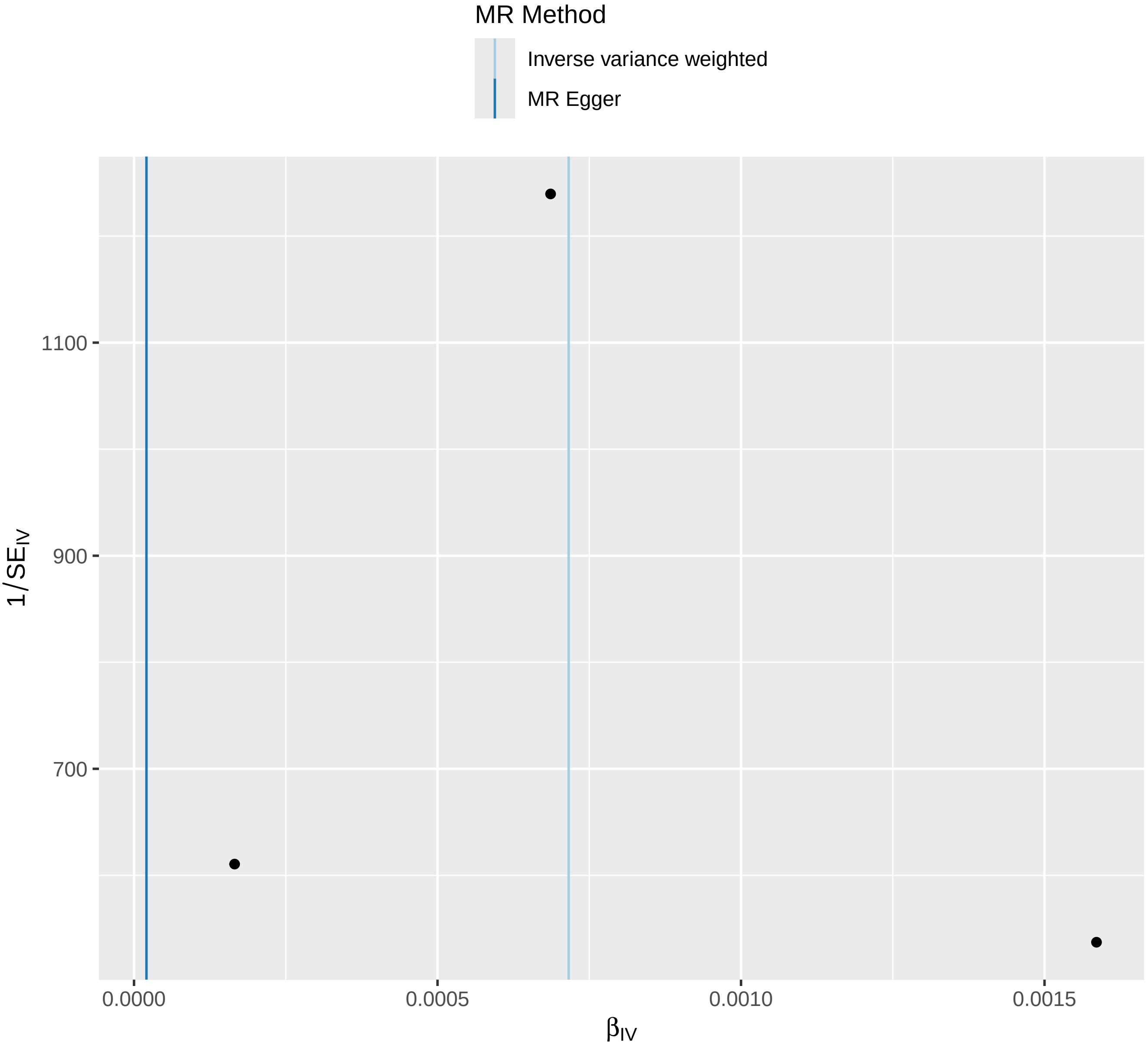
MR Method
Inverse variance weighted
MR Egger
1100
900
700
0.0000
0.0005
0.0010
0.0015
β_{IV}
$1/SE_{IV}$

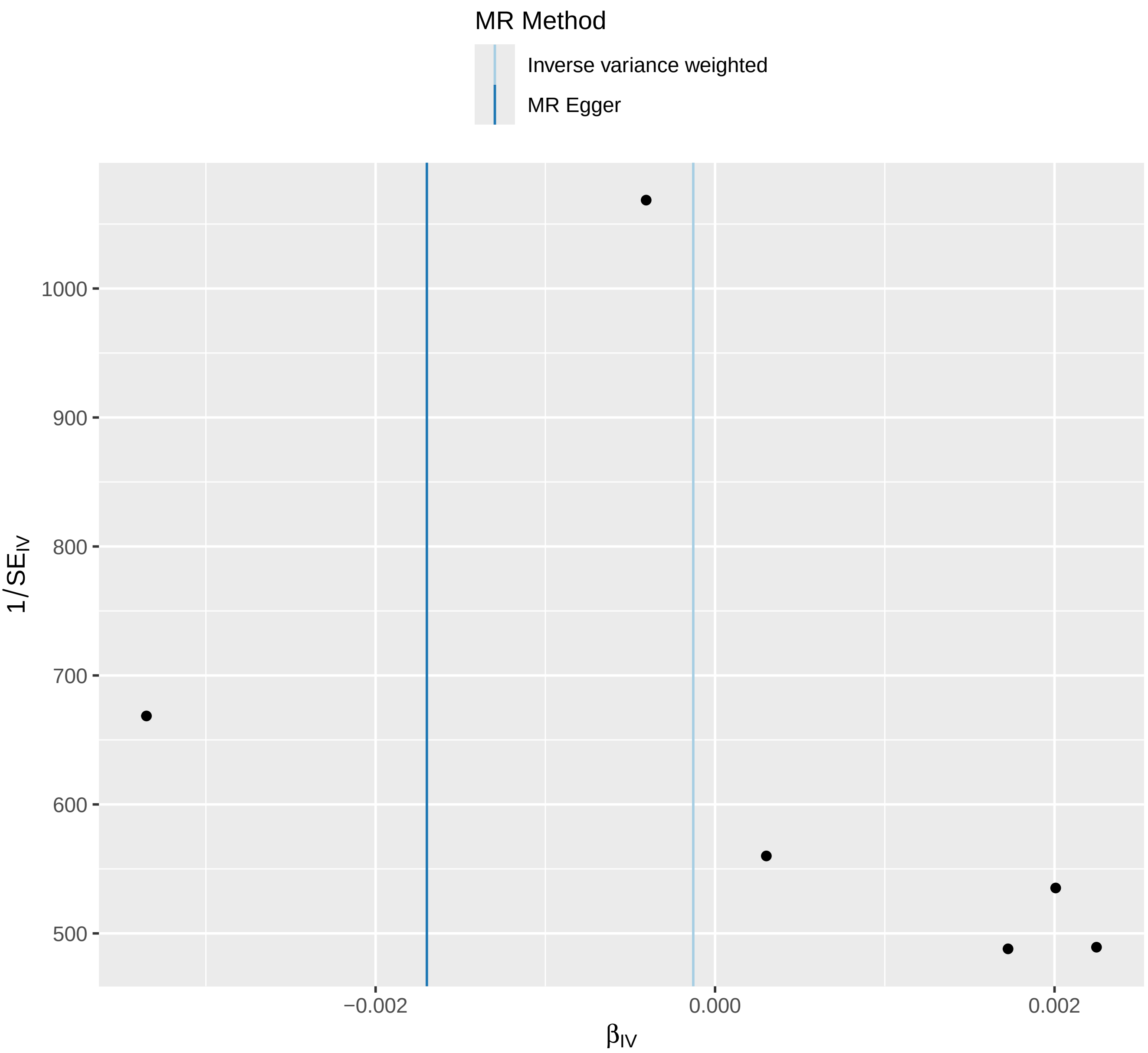
MR Method
Inverse variance weighted
MR Egger
1000
900
800
700
600
500
−0.002
0.000
0.002
$1/SE_{IV}$
β_{IV}

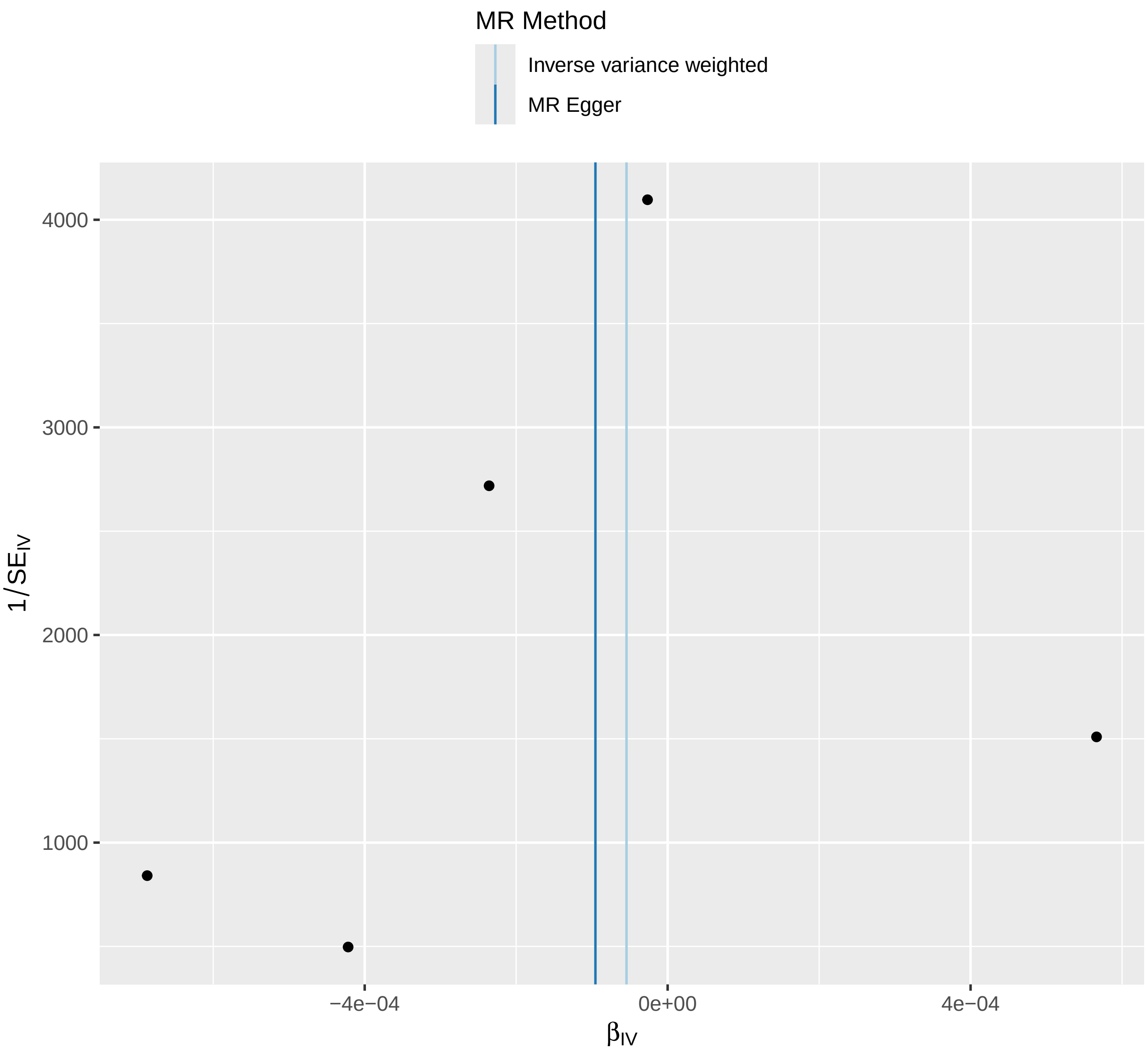
MR Method
Inverse variance weighted
MR Egger
4000
3000
2000
1000
$1/SE_{IV}$
−4e−04
0e+00
4e−04
β_{IV}

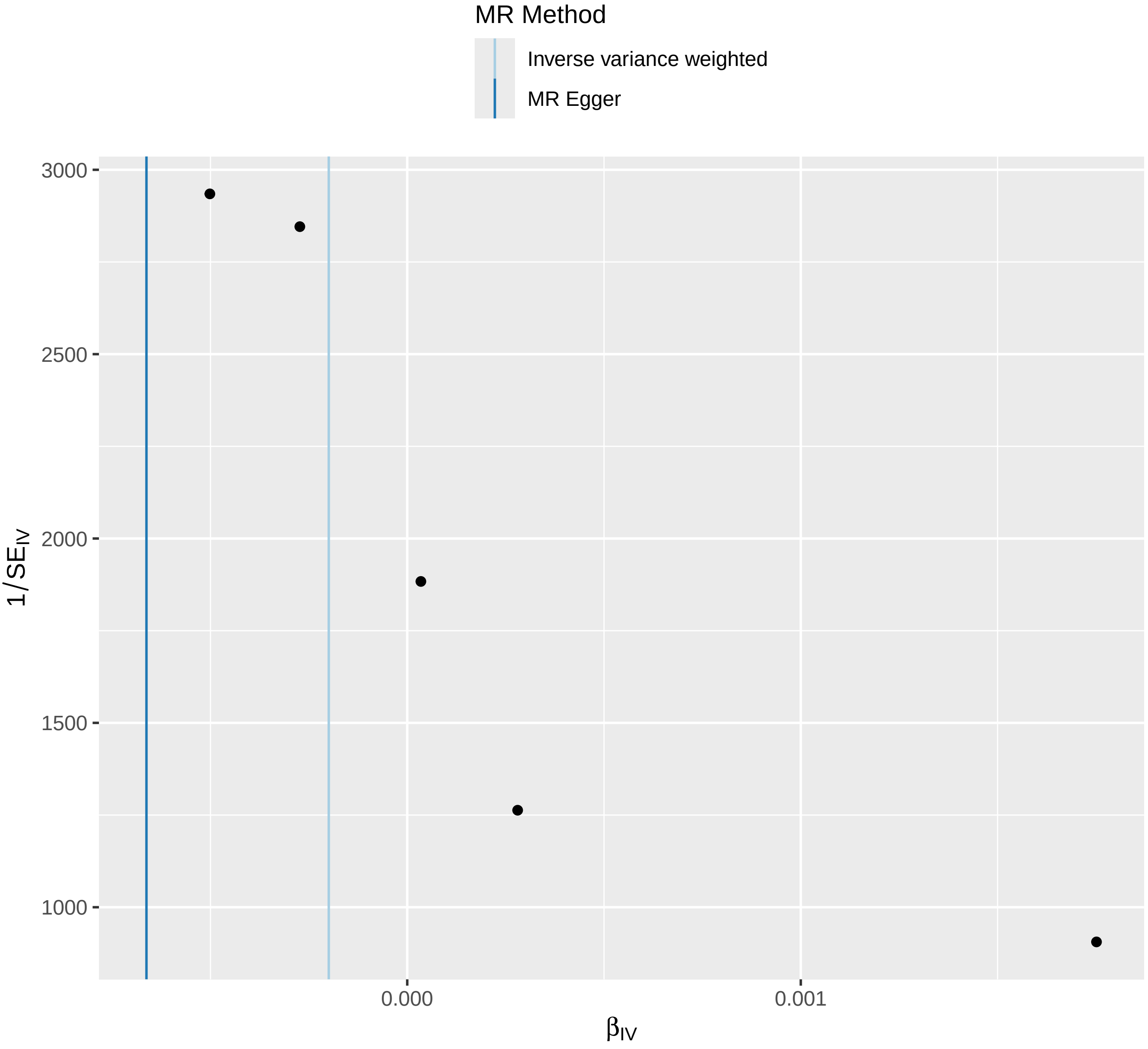

MR Method
Inverse variance weighted
MR Egger
3000
2500
2000
1500
1000
$1/SE_{IV}$
0.000
0.001
β_{IV}

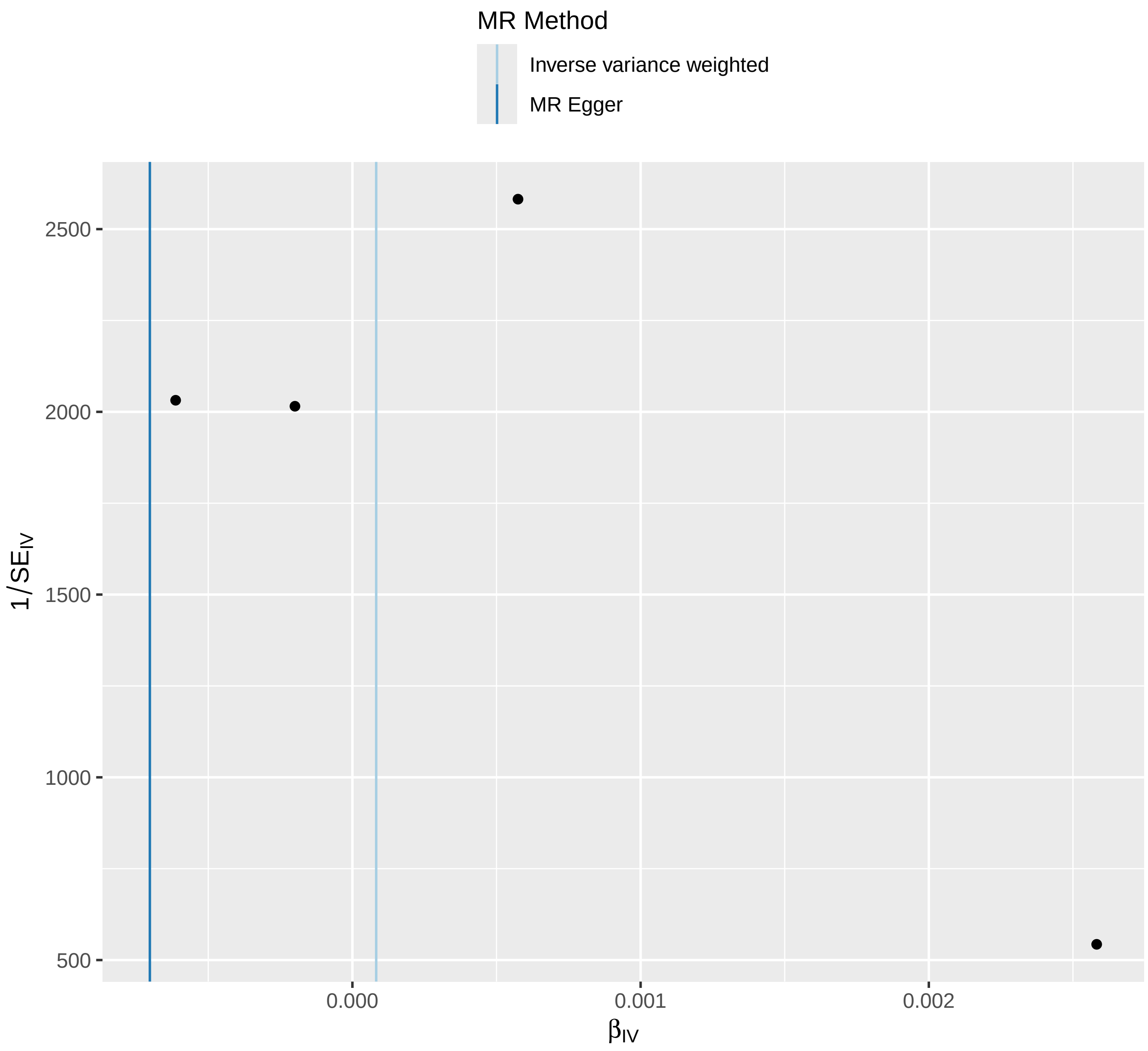
MR Method
Inverse variance weighted
MR Egger
2500
2000
1500
1000
500
1/SE_IV
0.000
0.001
0.002
β_IV

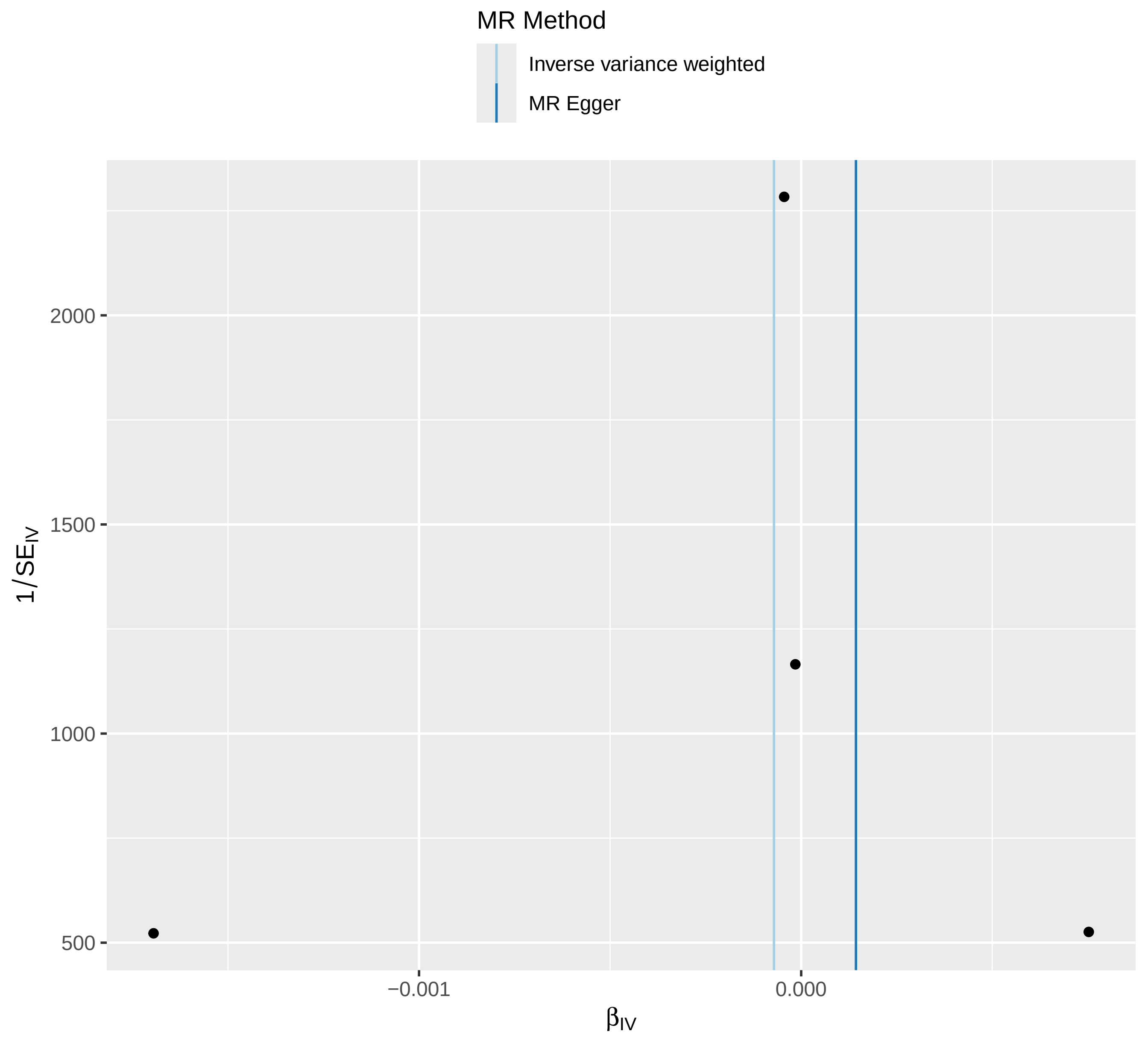

MR Method
Inverse variance weighted
MR Egger
2000
1500
1000
500
1/SE_IV
−0.001
0.000
β_IV

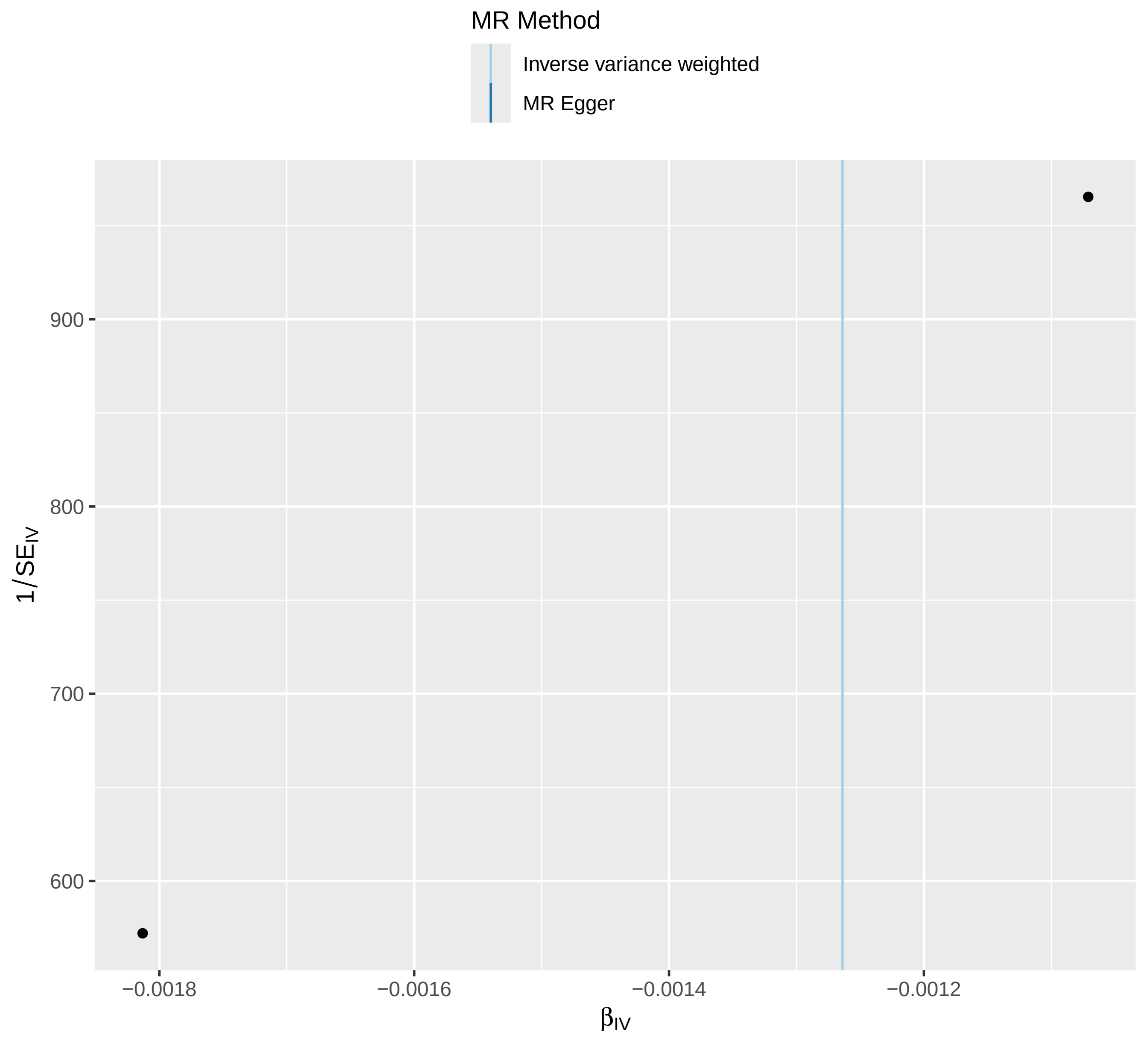
MR Method
Inverse variance weighted
MR Egger
900
800
700
600
1/SE_IV
−0.0018
−0.0016
−0.0014
−0.0012
β_IV

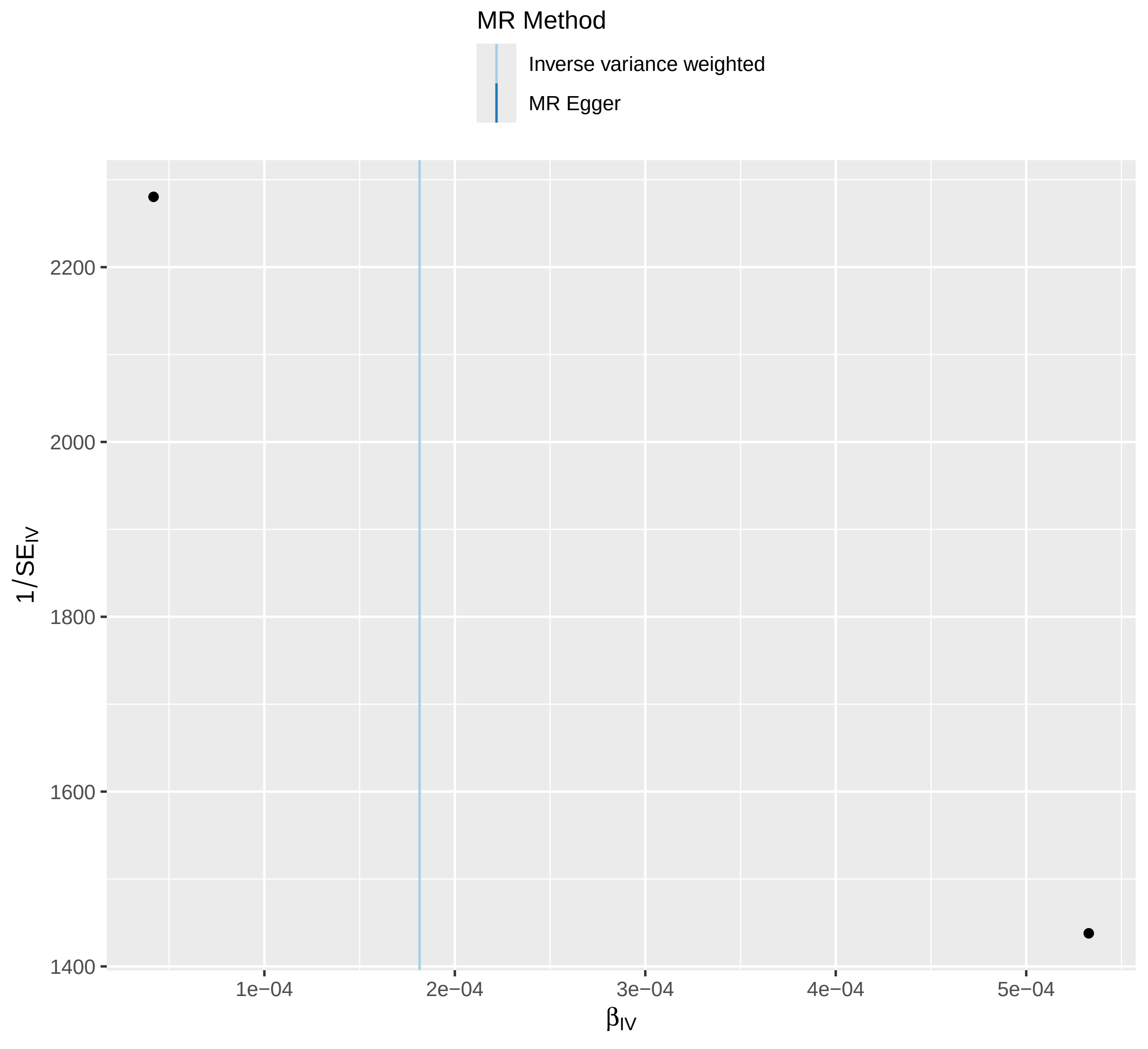
MR Method
Inverse variance weighted
MR Egger
2200
2000
1800
1600
1400
1/SE_IV
1e−04
2e−04
3e−04
4e−04
5e−04
β_IV

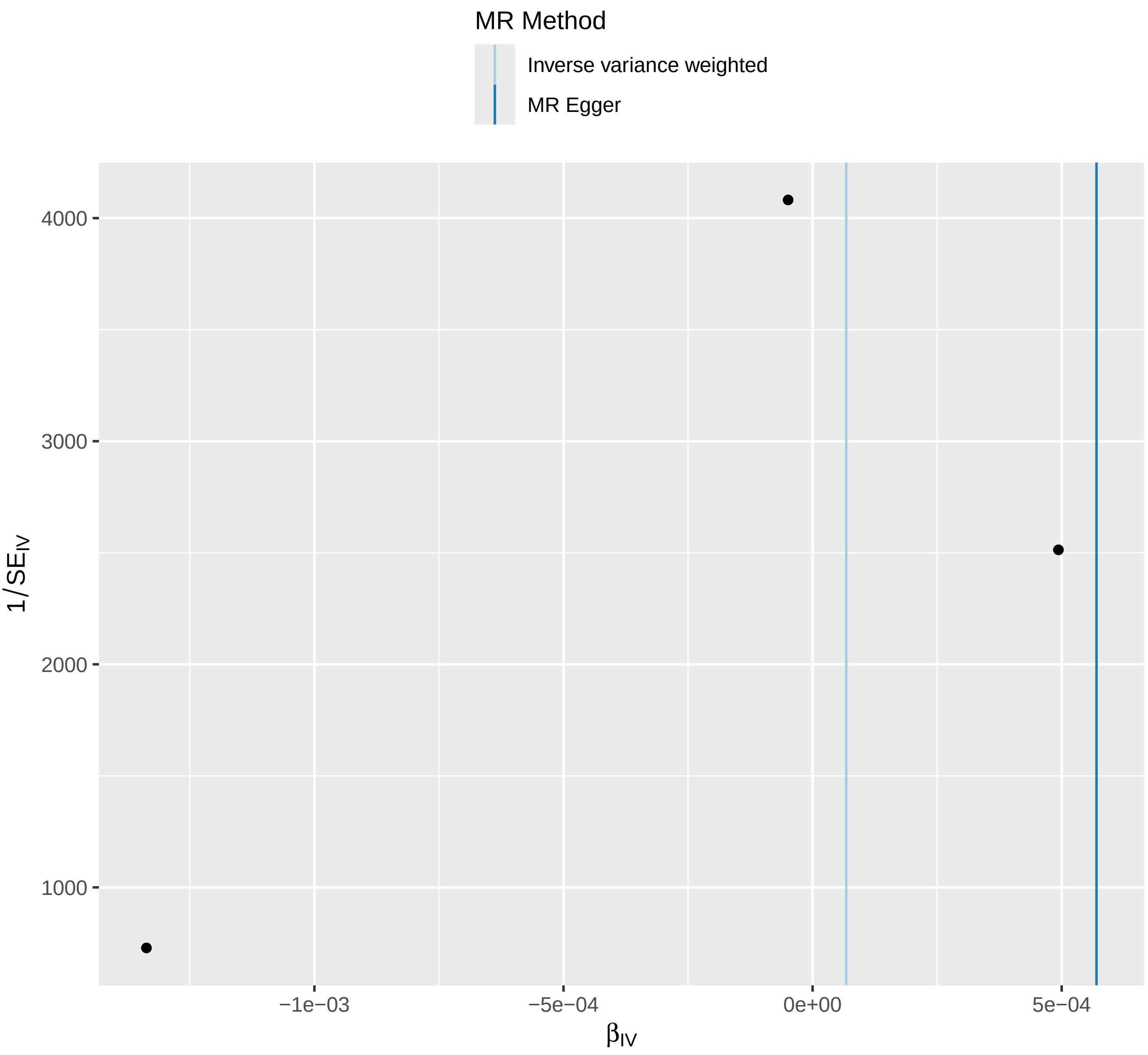

MR Method
Inverse variance weighted
MR Egger
4000
3000
2000
1000
1/SE_IV
−1e−03
−5e−04
0e+00
5e−04
β_IV

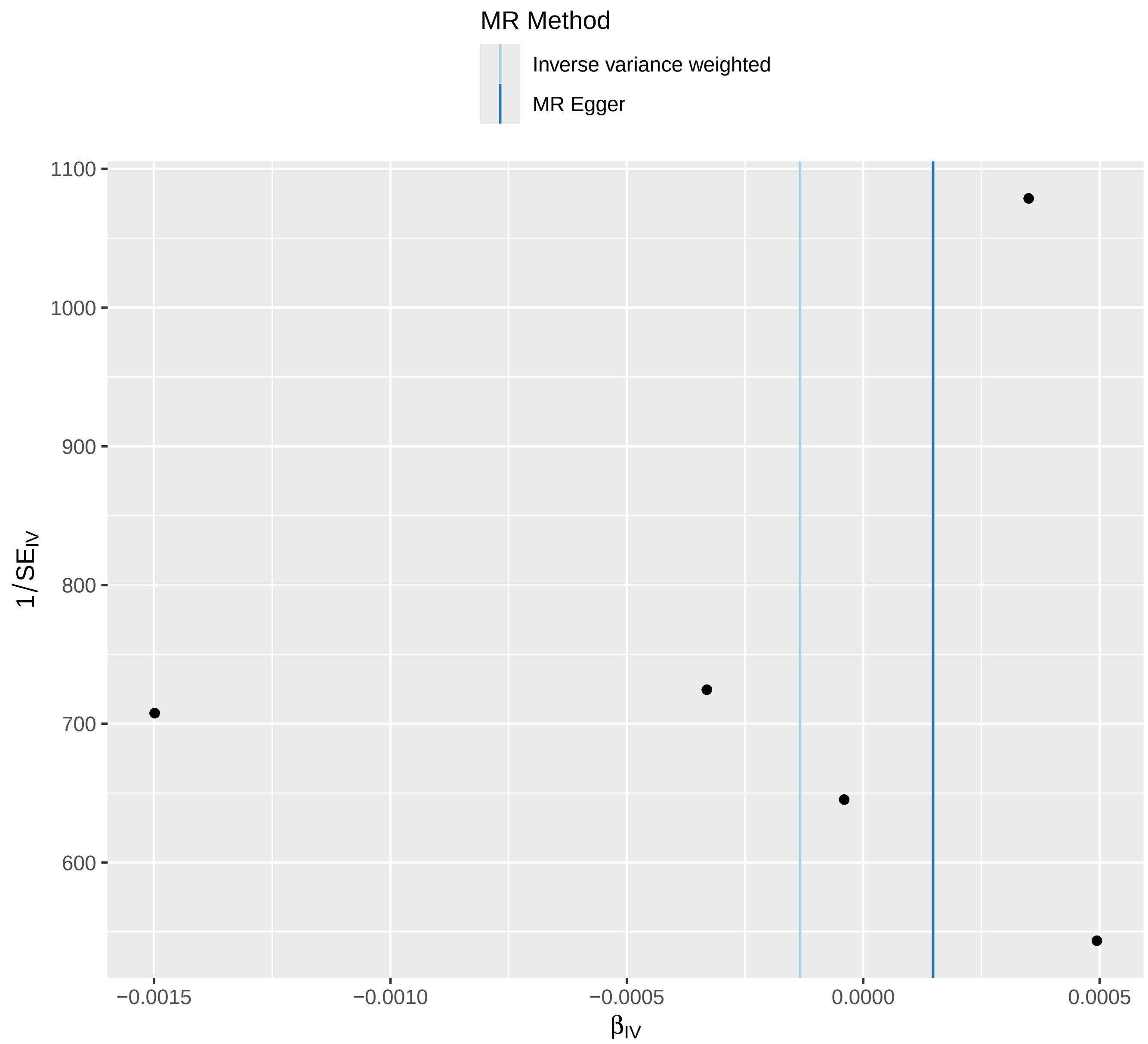

MR Method
Inverse variance weighted
MR Egger
1100
1000
900
800
700
600
1/SE_IV
−0.0015
−0.0010
−0.0005
0.0000
0.0005
β_IV

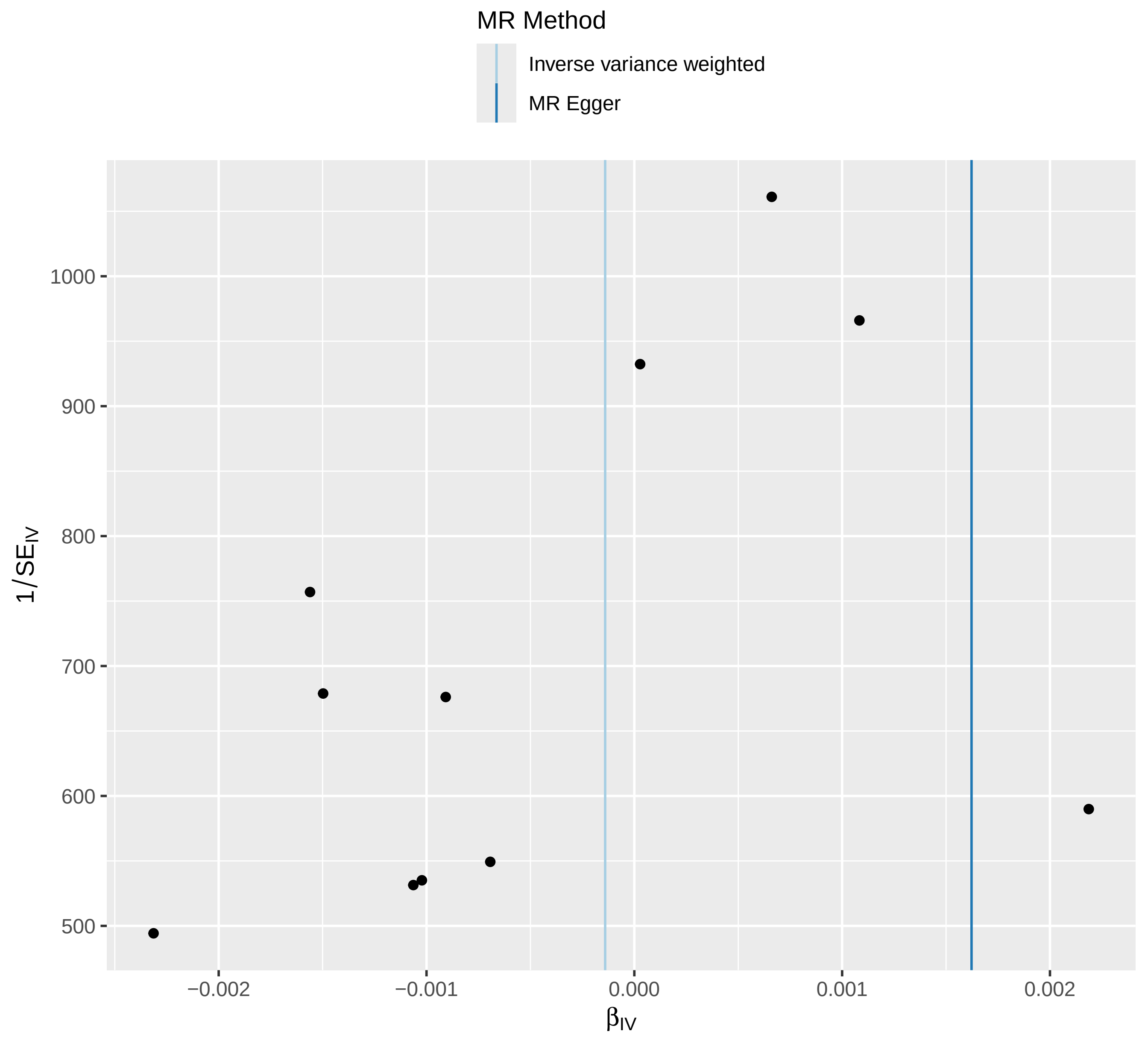

MR Method
Inverse variance weighted
MR Egger
1/SE$_{IV}$
1000
900
800
700
600
500
−0.002
−0.001
0.000
0.001
0.002
β$_{IV}$

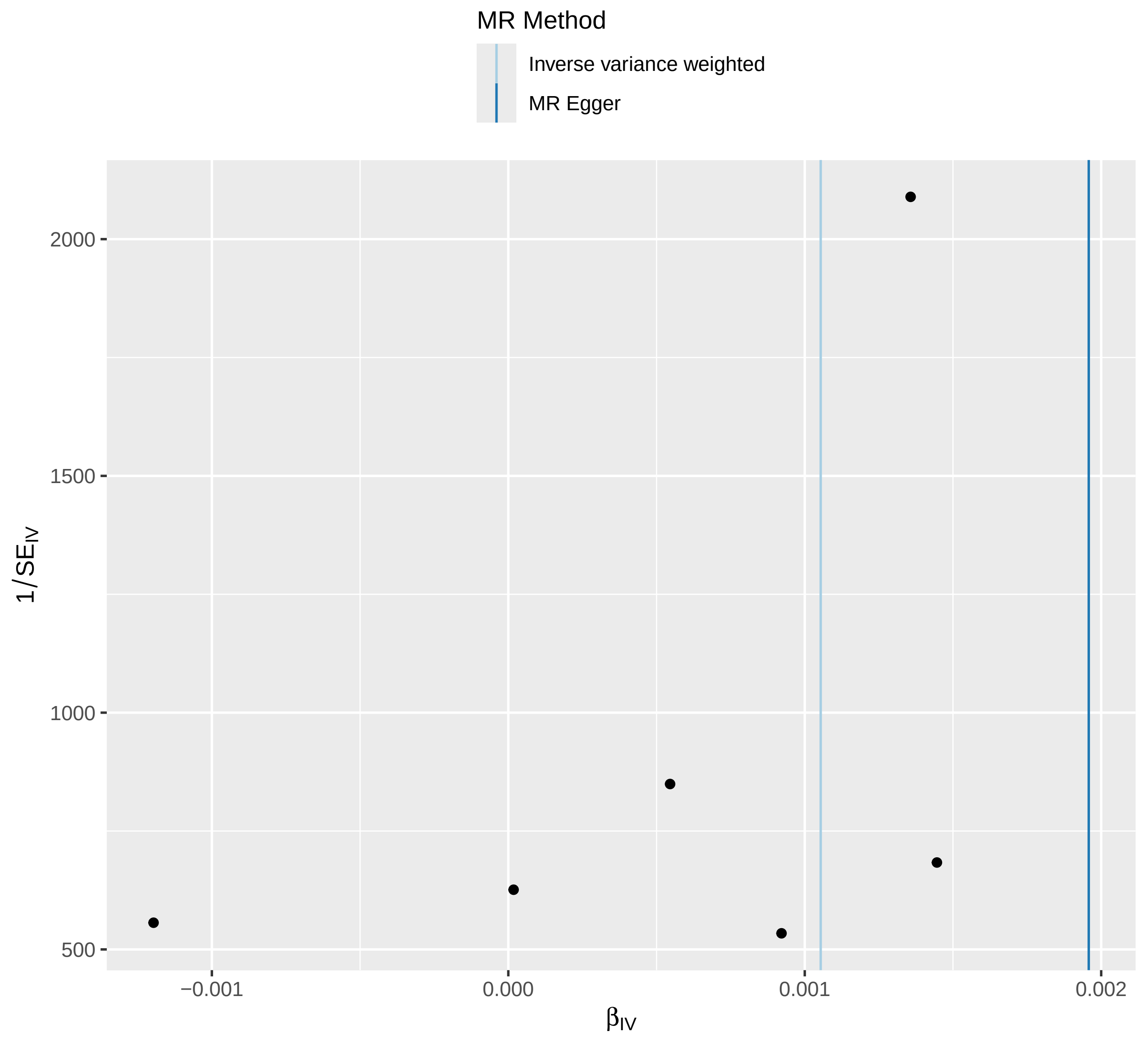

MR Method
Inverse variance weighted
MR Egger
2000
1500
1000
500
1/SE_IV
−0.001
0.000
0.001
0.002
β_IV

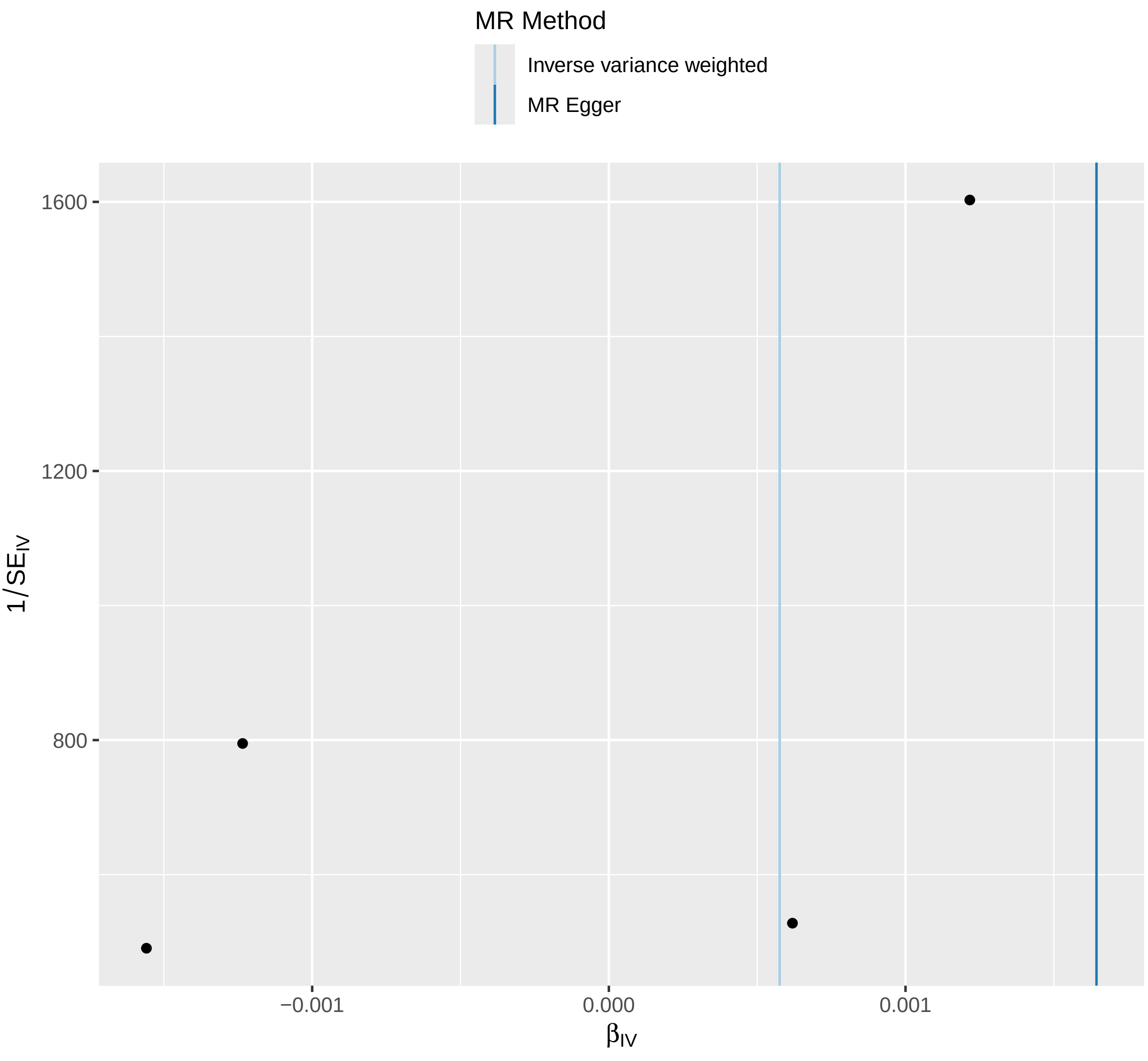

MR Method
Inverse variance weighted
MR Egger
1600
1200
800
1/SE_IV
−0.001
0.000
0.001
β_IV

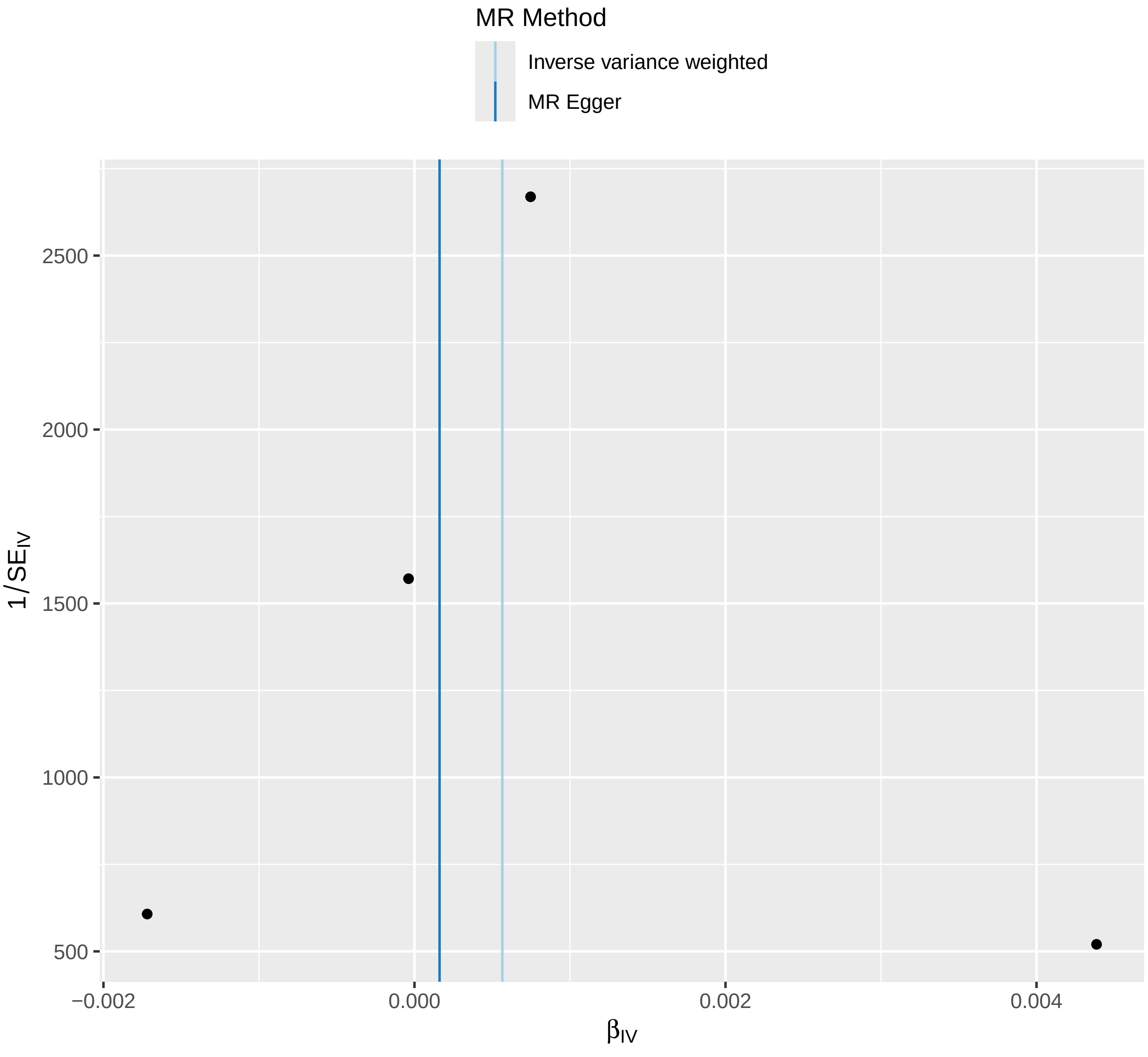

MR Method
Inverse variance weighted
MR Egger
2500
2000
1500
1000
500
1/SE$_{IV}$
−0.002
0.000
0.002
0.004
β$_{IV}$

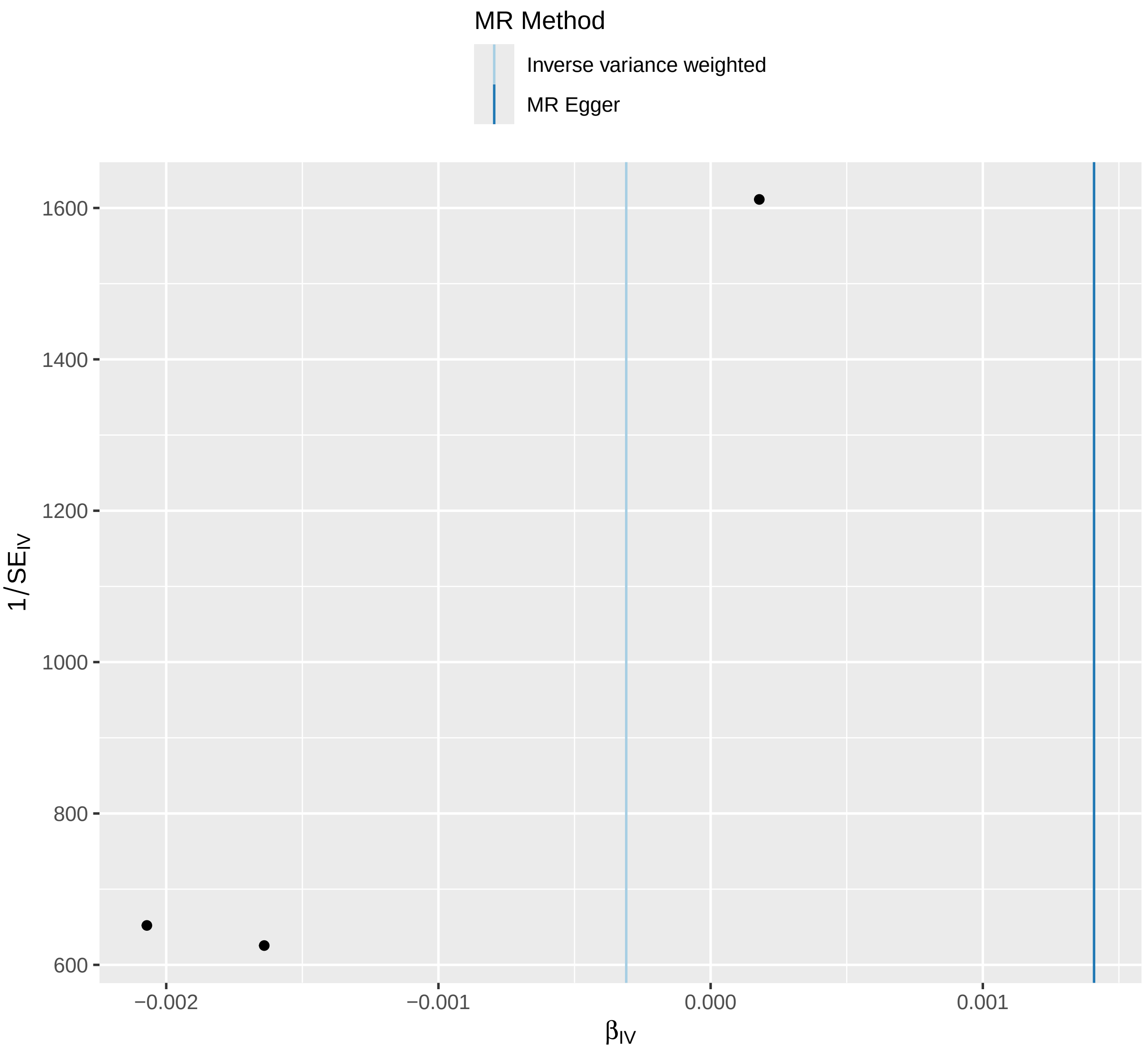
MR Method
Inverse variance weighted
MR Egger
1600
1400
1200
1000
800
600
1/SE$_{IV}$
−0.002
−0.001
0.000
0.001
β$_{IV}$

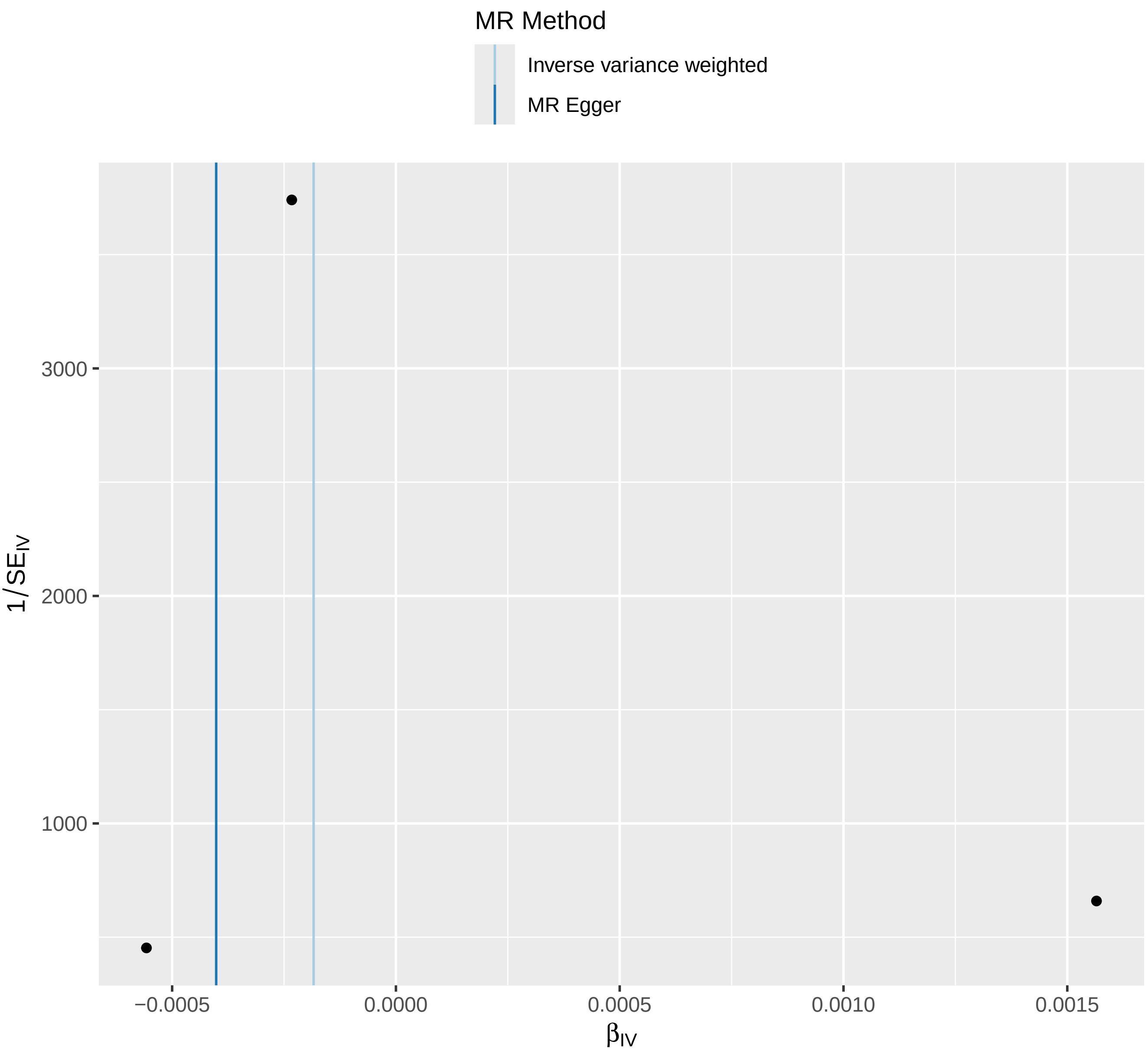
MR Method
Inverse variance weighted
MR Egger
1/SE$_{IV}$
3000
2000
1000
−0.0005
0.0000
0.0005
0.0010
0.0015
β$_{IV}$

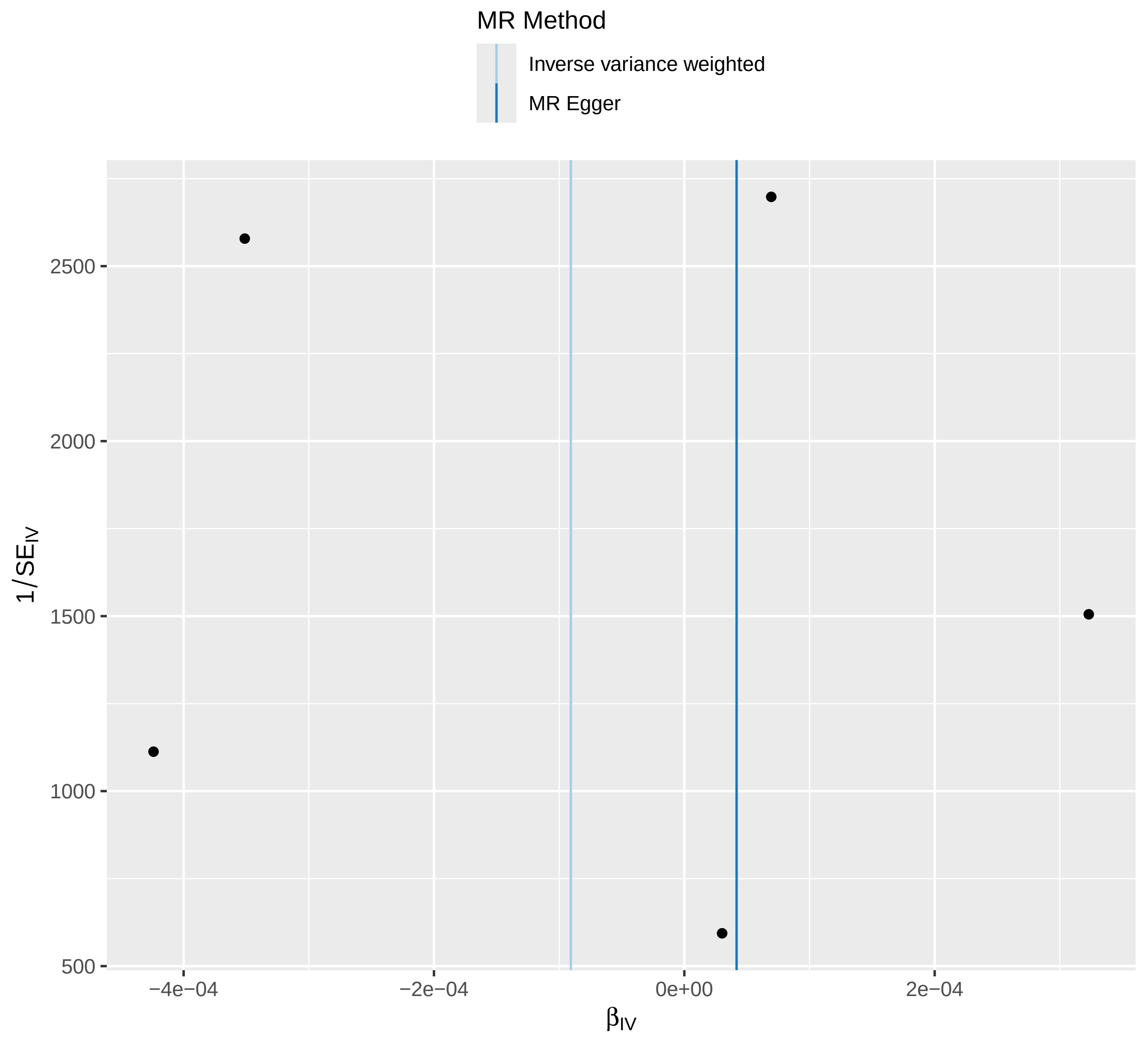
MR Method
Inverse variance weighted
MR Egger
2500
2000
1500
1000
500
−4e−04
−2e−04
0e+00
2e−04
1/SE_IV
β_IV

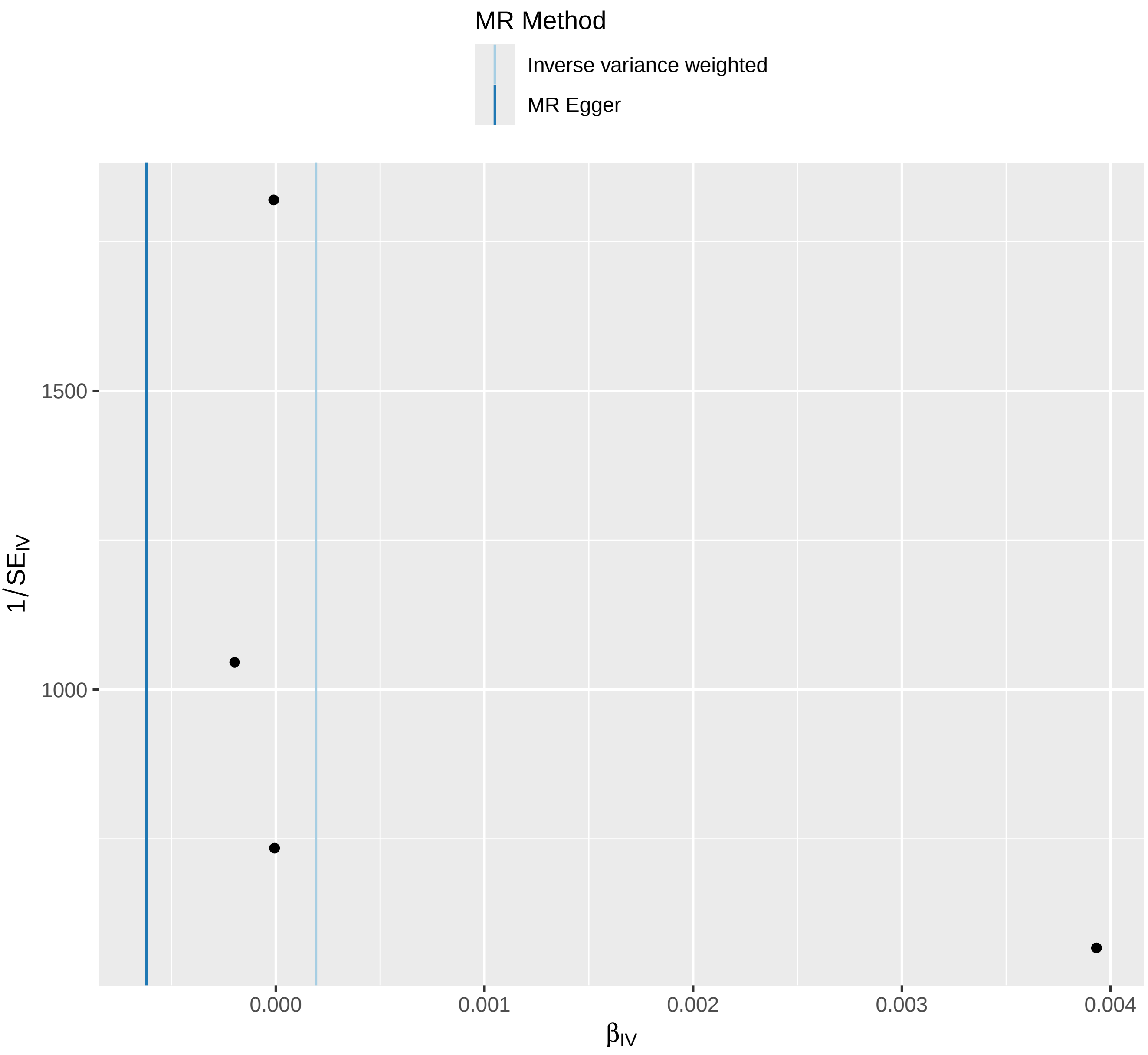

MR Method
Inverse variance weighted
MR Egger
1500
1000
1/SE$_{IV}$
0.000
0.001
0.002
0.003
0.004
β$_{IV}$

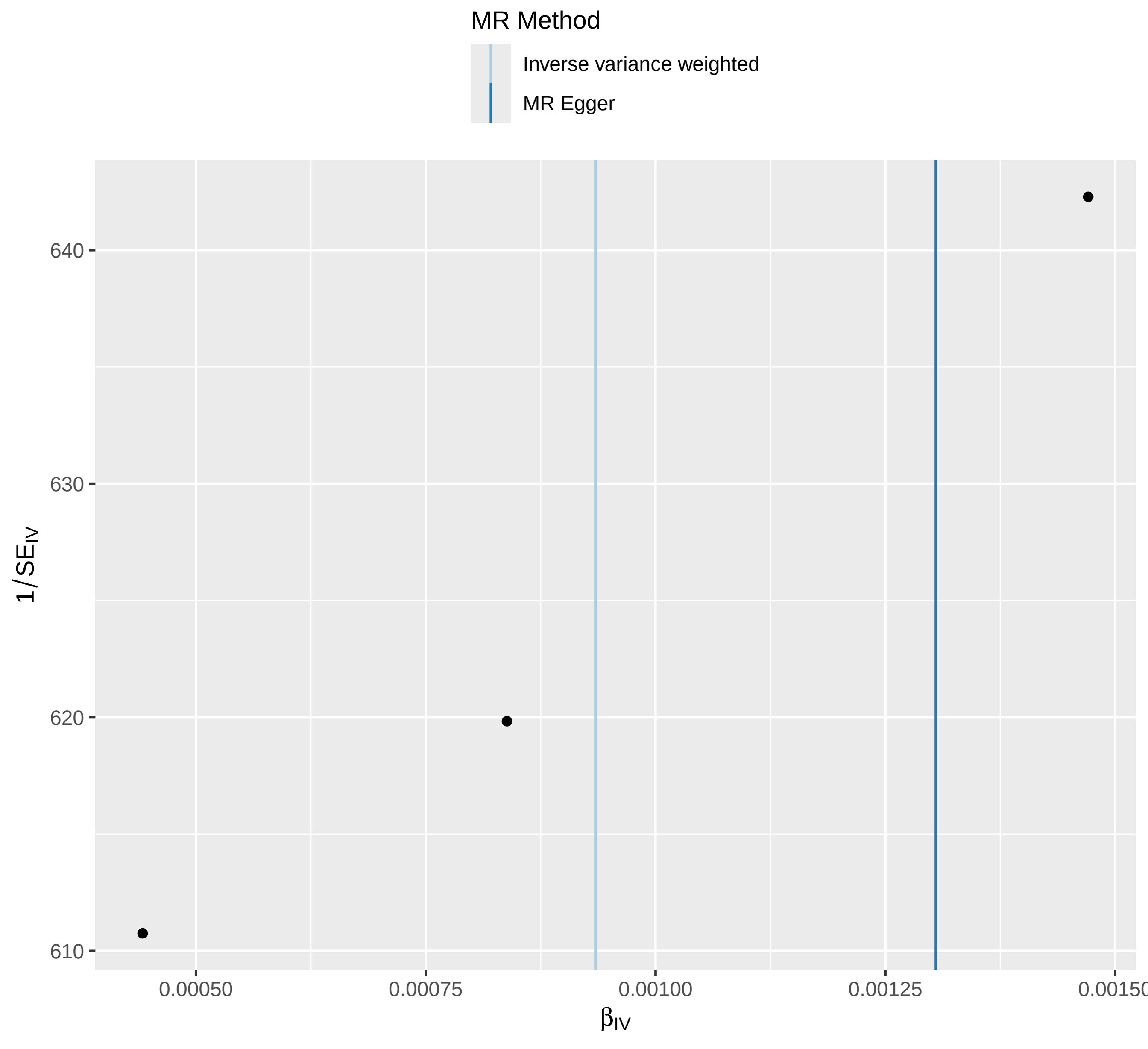
MR Method
Inverse variance weighted
MR Egger
640
630
620
610
1/SE_IV
0.00050
0.00075
0.00100
0.00125
0.00150
β_IV

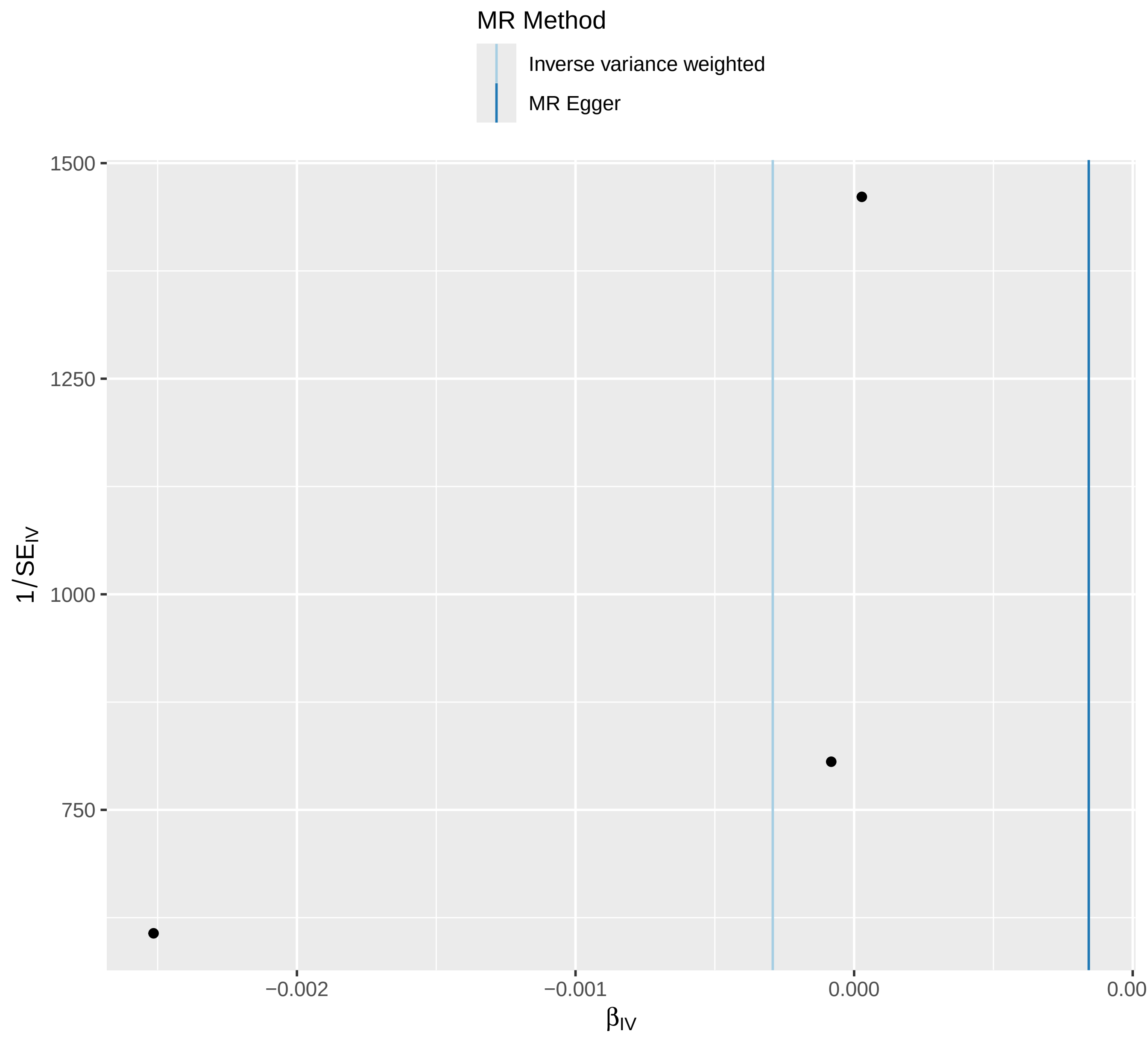

MR Method
Inverse variance weighted
MR Egger
1500
1250
1000
750
1/SE_IV
−0.002
−0.001
0.000
0.00
β_IV

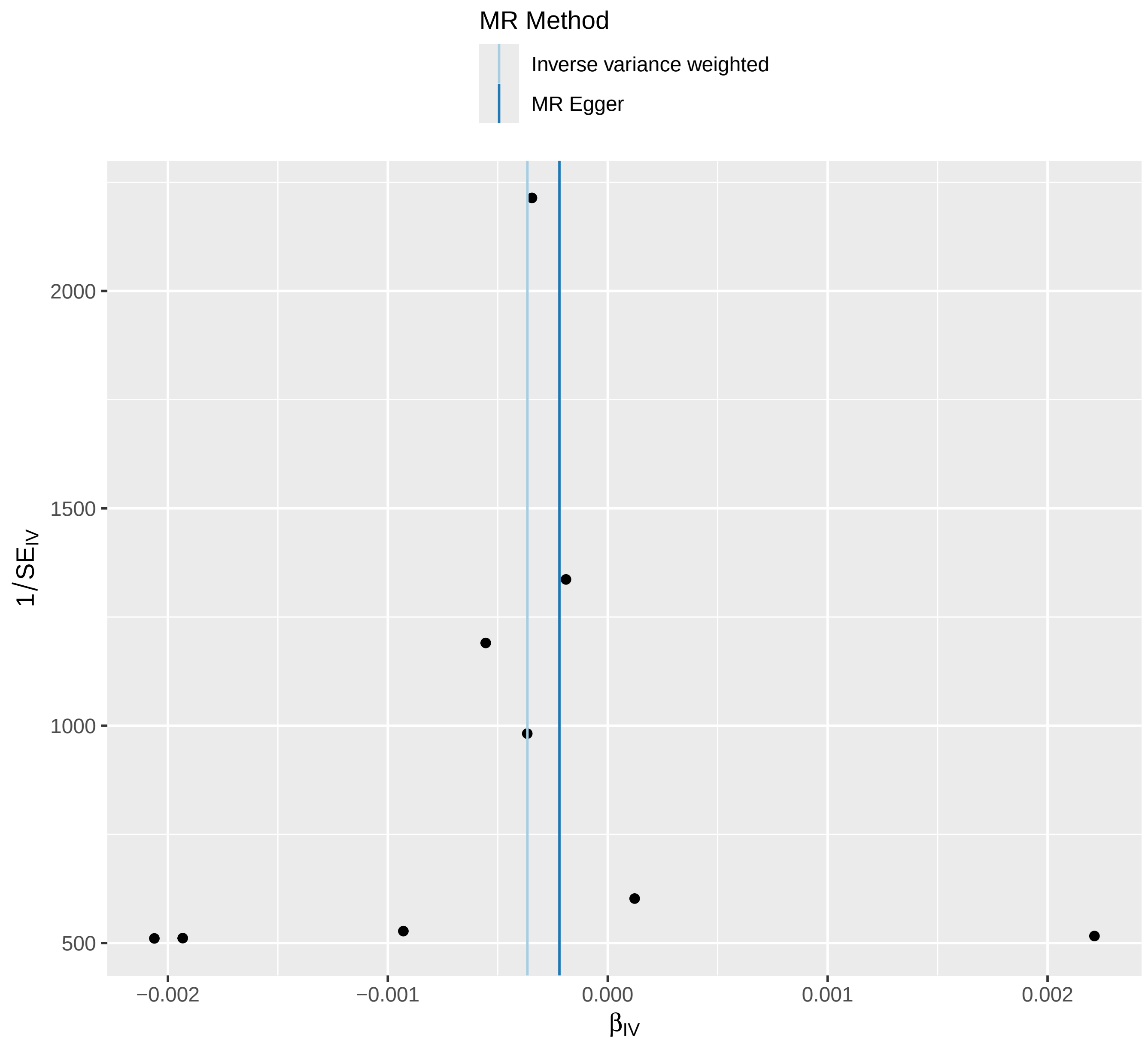
MR Method
Inverse variance weighted
MR Egger
2000
1500
1000
500
$1/SE_{IV}$
−0.002
−0.001
0.000
0.001
0.002
β_{IV}

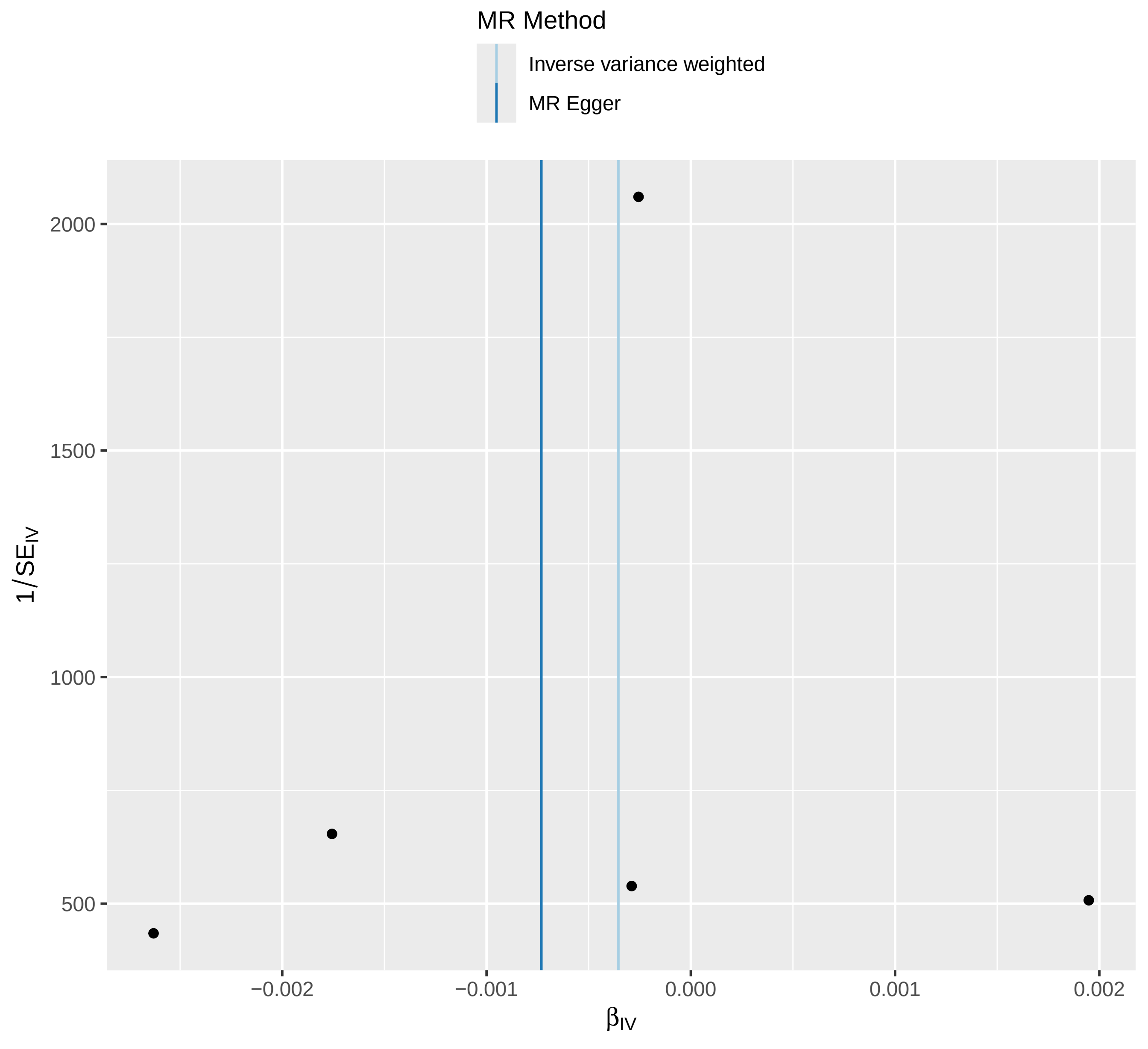

MR Method
Inverse variance weighted
MR Egger
2000
1500
1000
500
1/SE$_{IV}$
−0.002
−0.001
0.000
0.001
0.002
β_{IV}

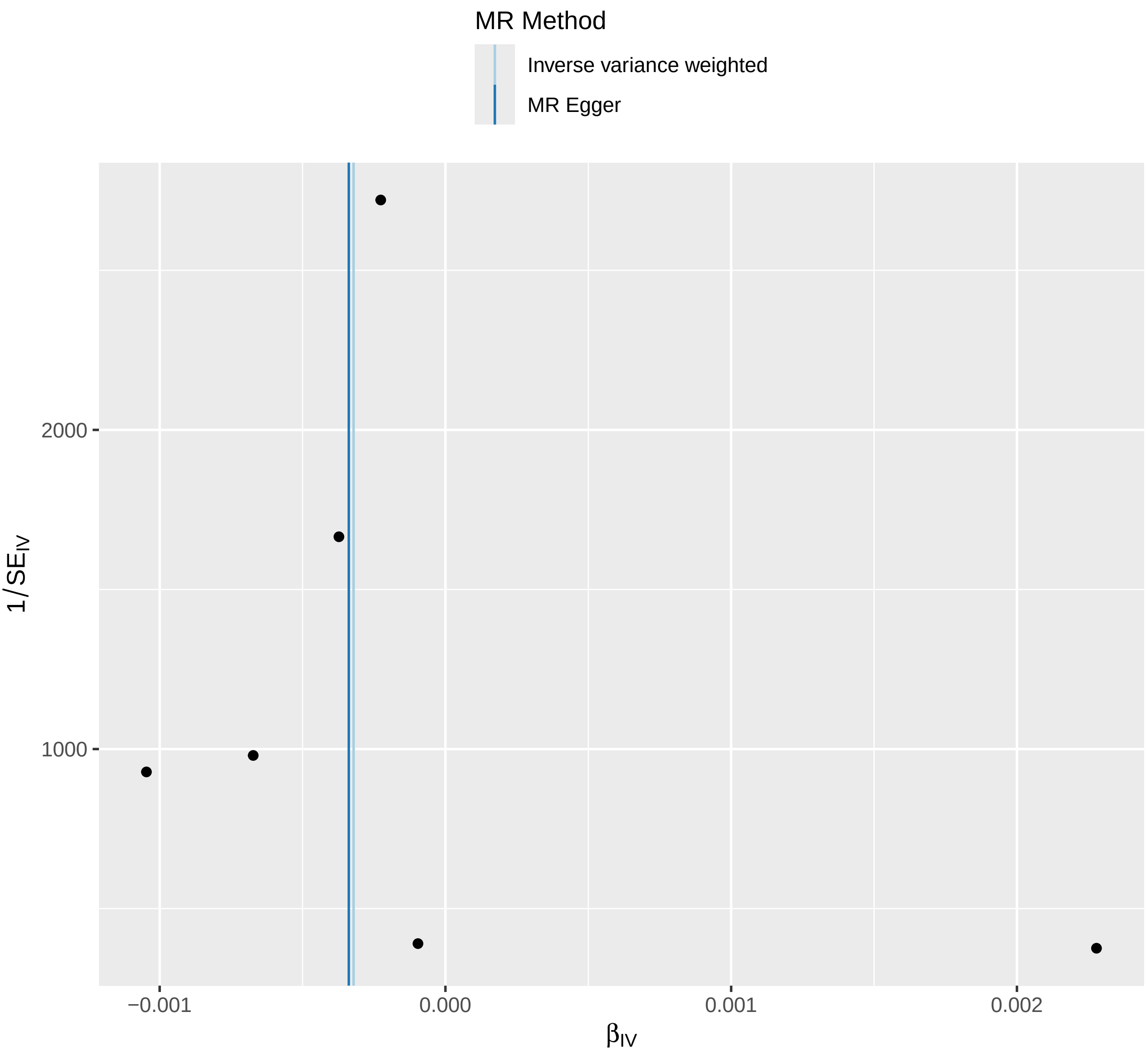
MR Method
Inverse variance weighted
MR Egger
2000
1000
1/SE_IV
−0.001
0.000
0.001
0.002
β_IV

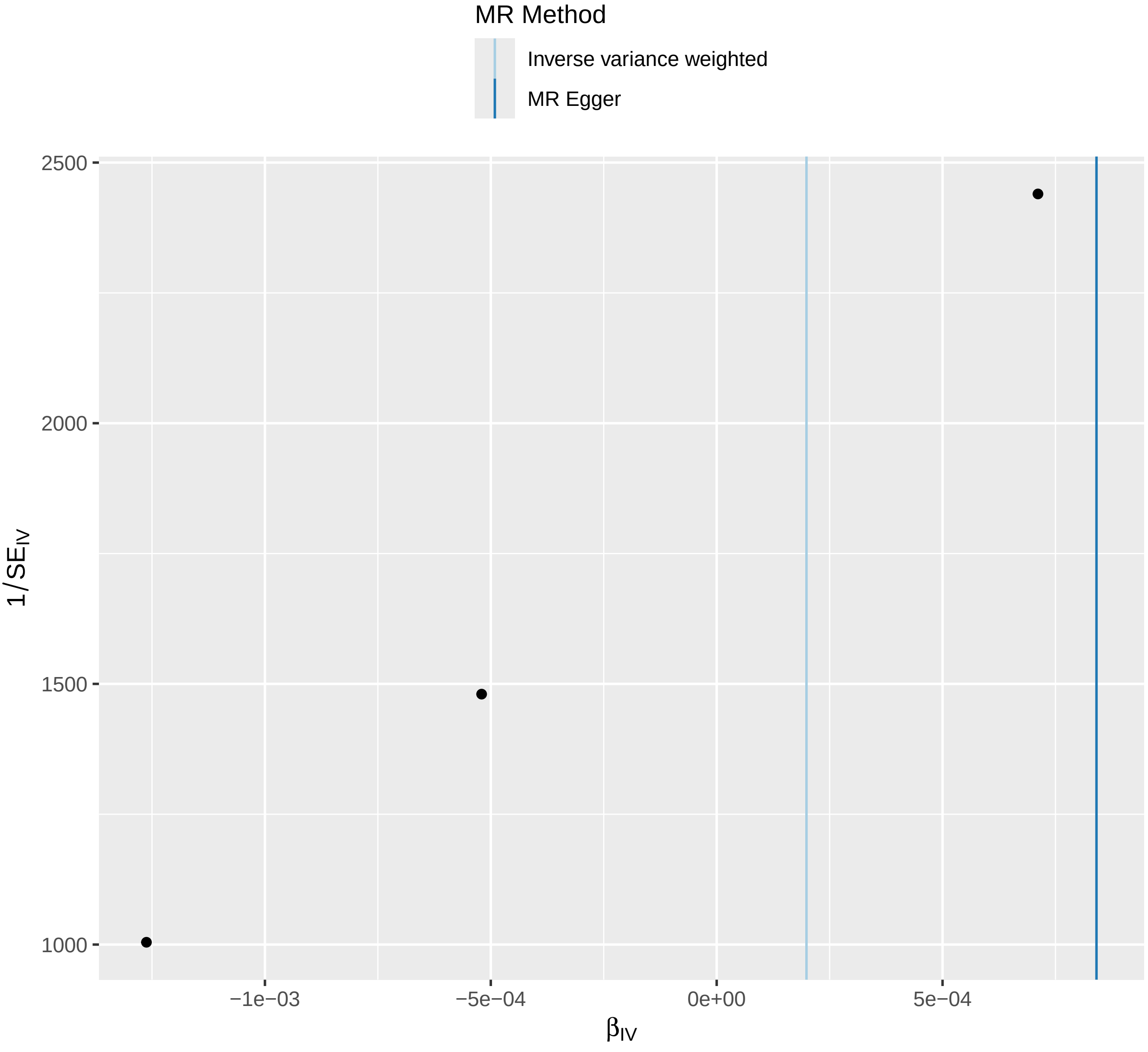

MR Method
Inverse variance weighted
MR Egger
2500
2000
1500
1000
1/SE_IV
−1e−03
−5e−04
0e+00
5e−04
β_IV

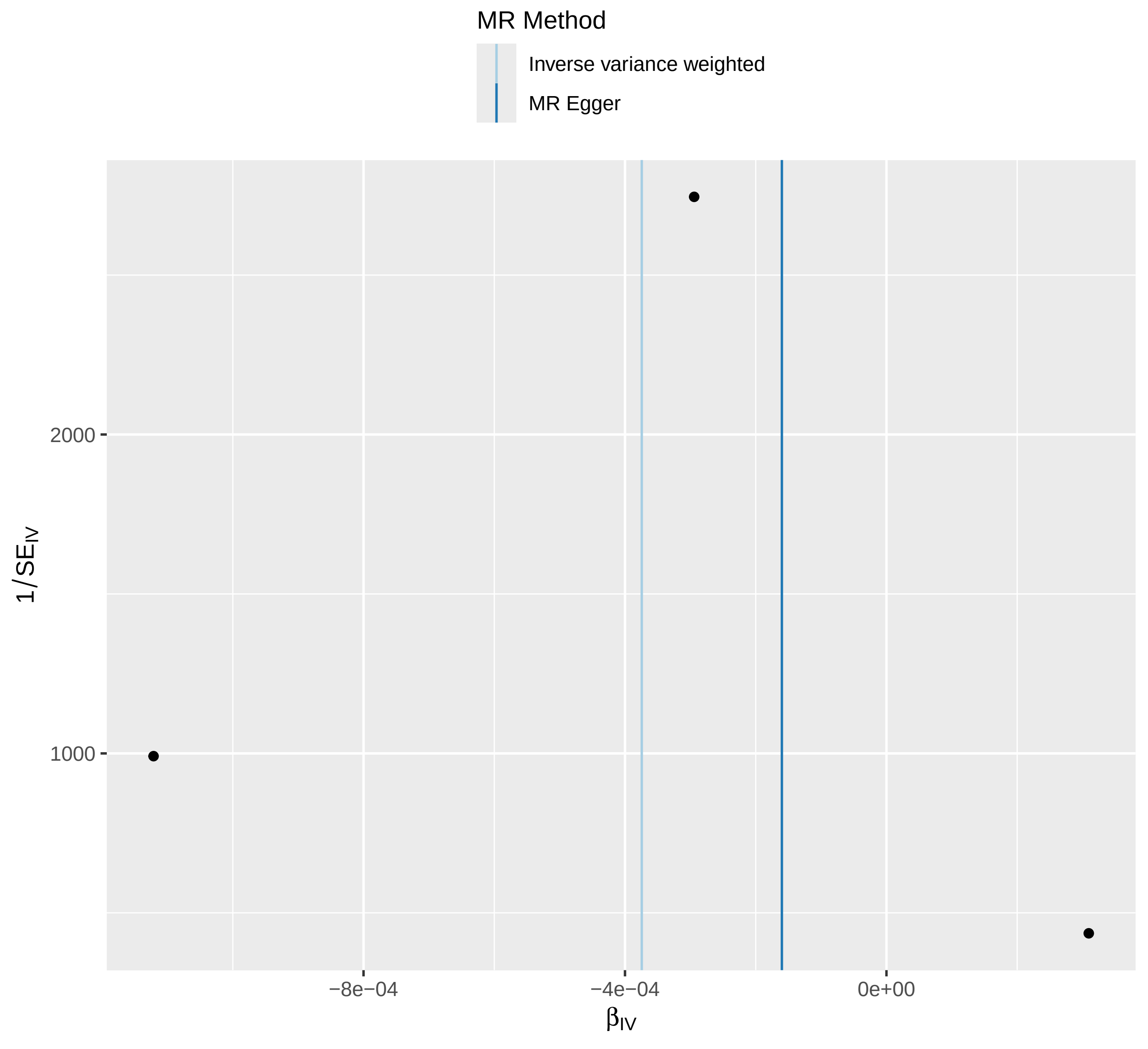

MR Method
Inverse variance weighted
MR Egger
2000
1000
$1/SE_{IV}$
−8e−04
−4e−04
0e+00
β_{IV}

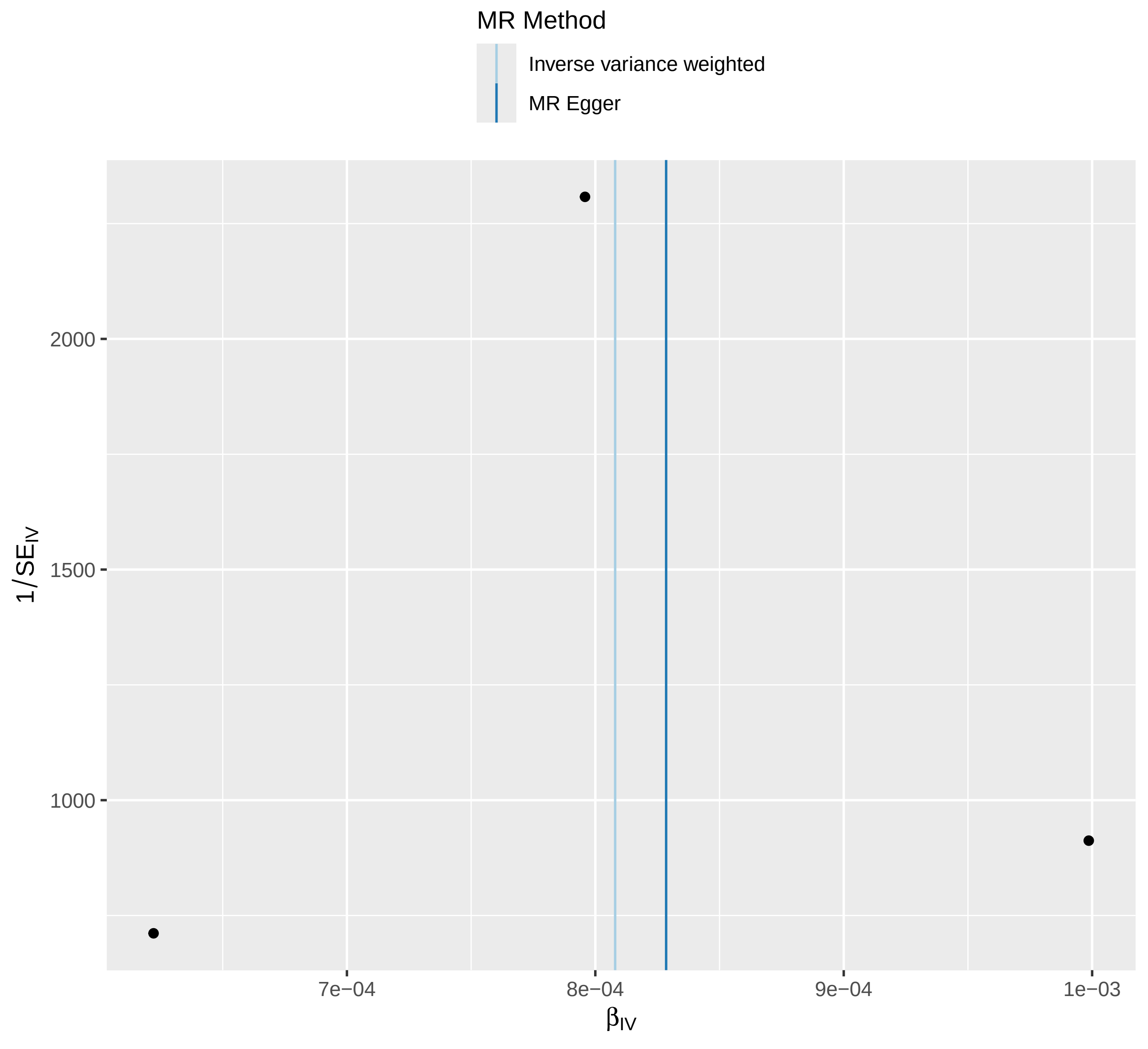

MR Method
Inverse variance weighted
MR Egger
2000
1500
1000
1/SE_IV
7e−04
8e−04
9e−04
1e−03
β_IV

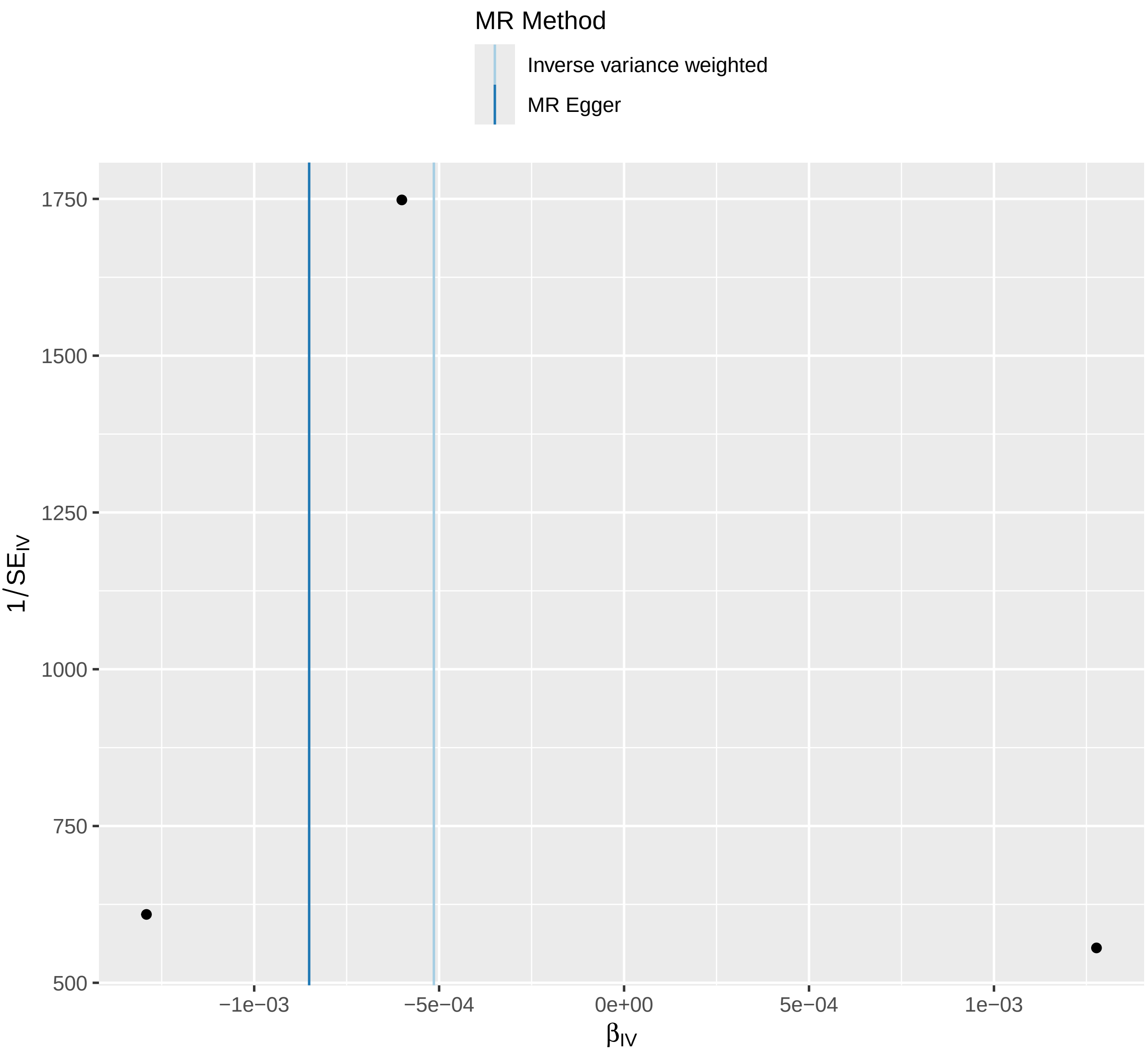

MR Method
Inverse variance weighted
MR Egger
1750
1500
1250
1000
750
500
1/SE_IV
−1e−03
−5e−04
0e+00
5e−04
1e−03
β_IV

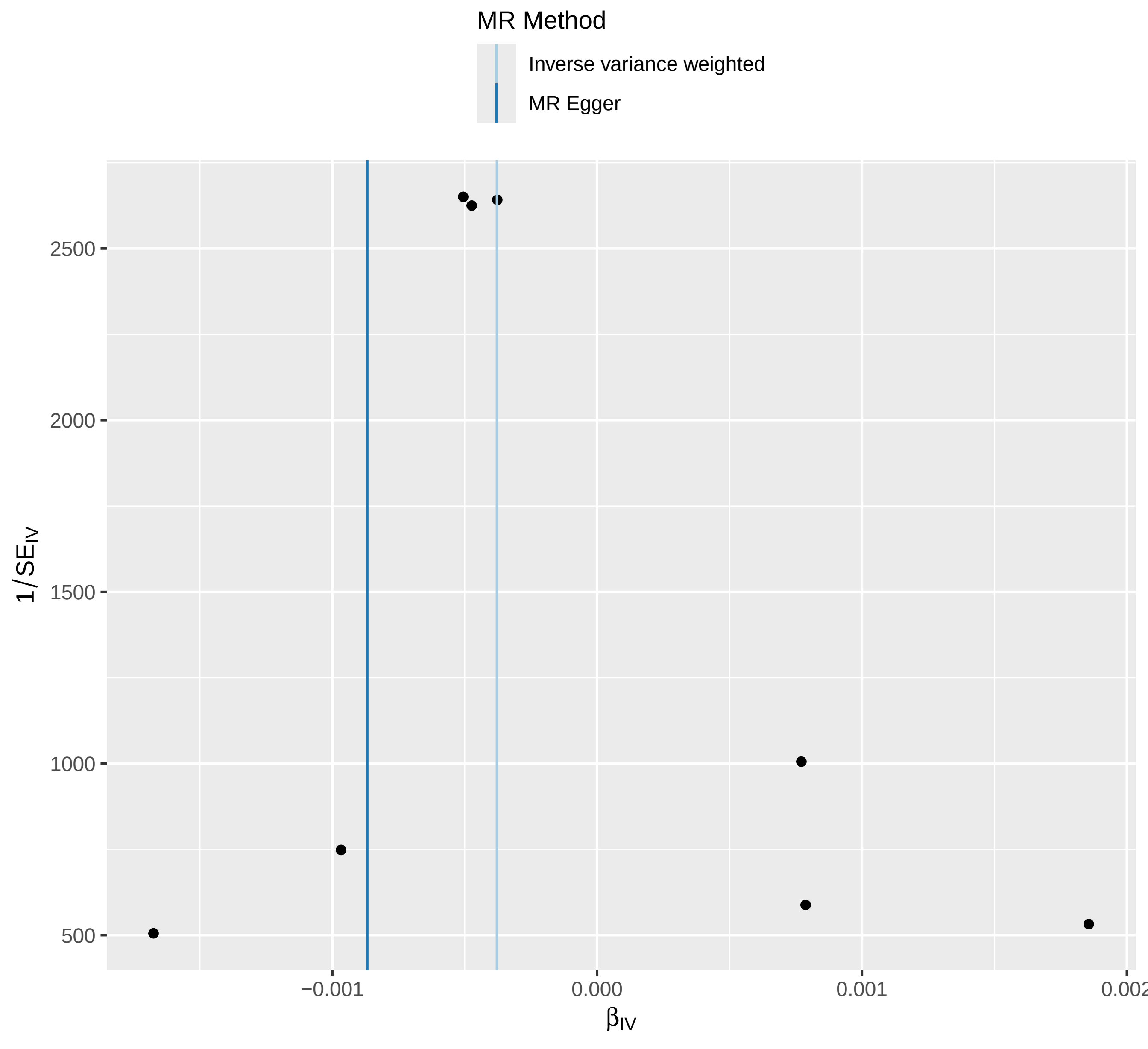
MR Method
Inverse variance weighted
MR Egger
2500
2000
1500
1000
500
1/SE$_{IV}$
−0.001
0.000
0.001
0.002
β$_{IV}$

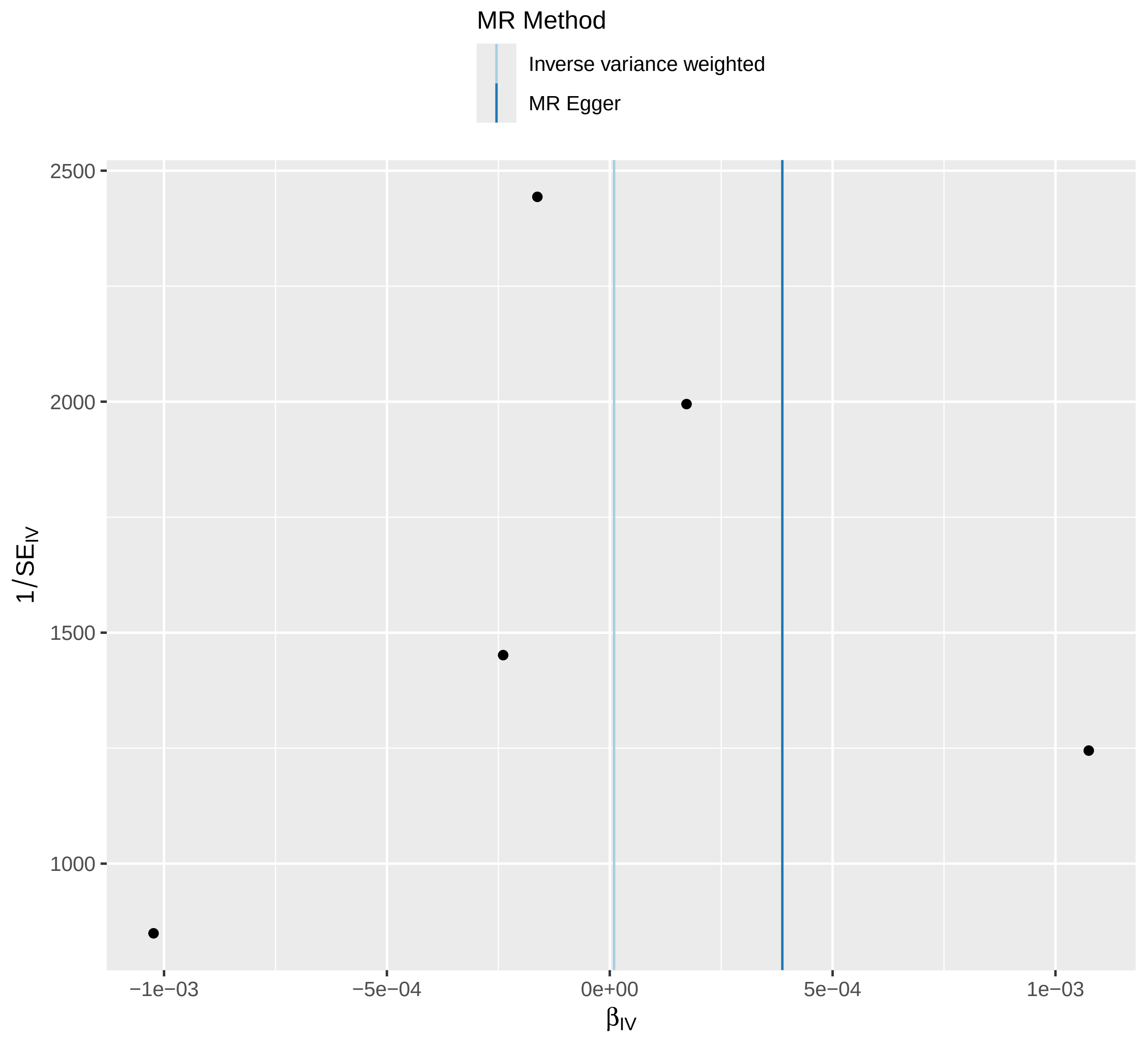
MR Method
Inverse variance weighted
MR Egger
2500
2000
1500
1000
1/SE_IV
−1e−03
−5e−04
0e+00
5e−04
1e−03
β_IV

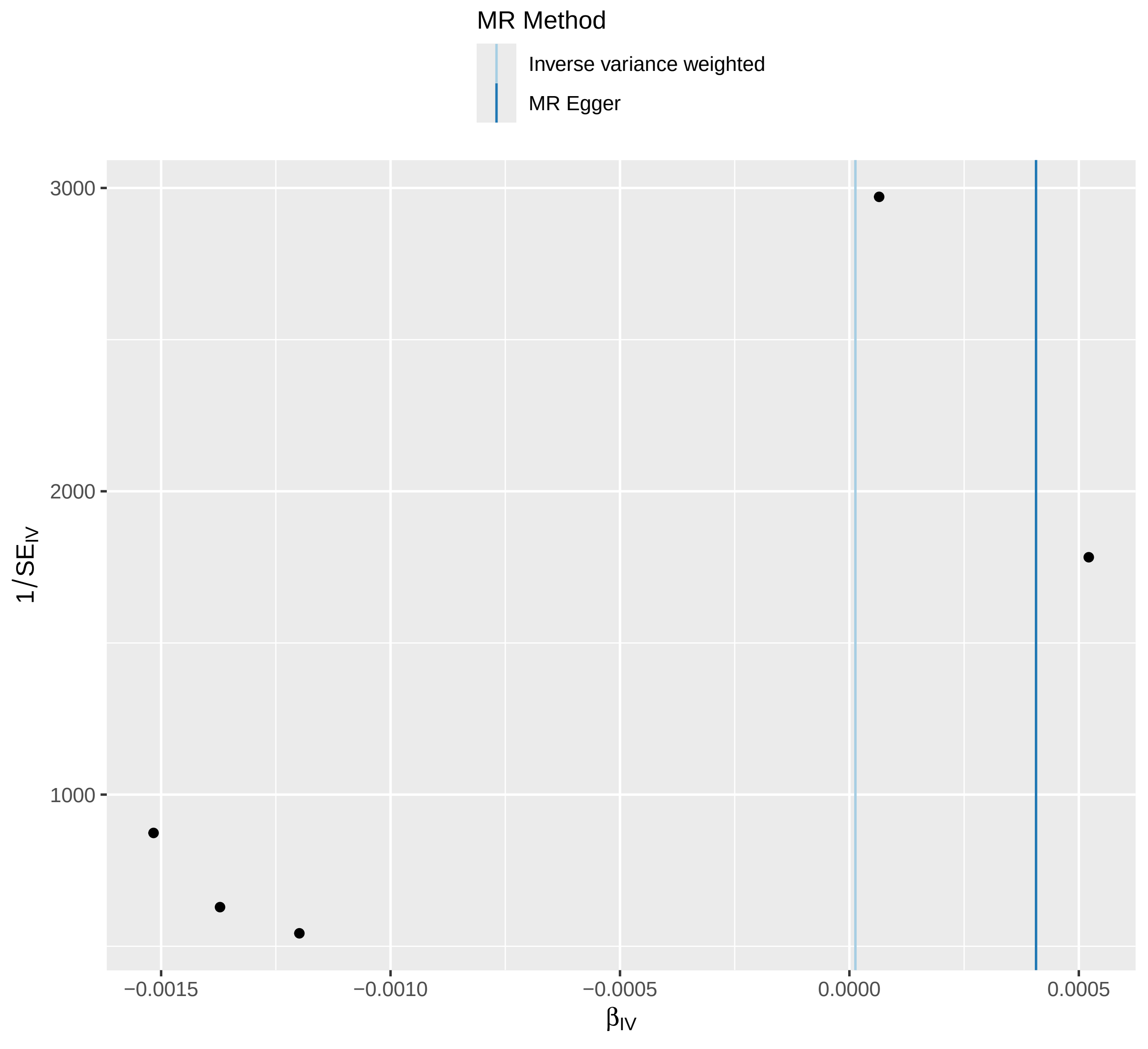
MR Method
Inverse variance weighted
MR Egger
3000
2000
1000
1/SE_IV
−0.0015
−0.0010
−0.0005
0.0000
0.0005
β_IV

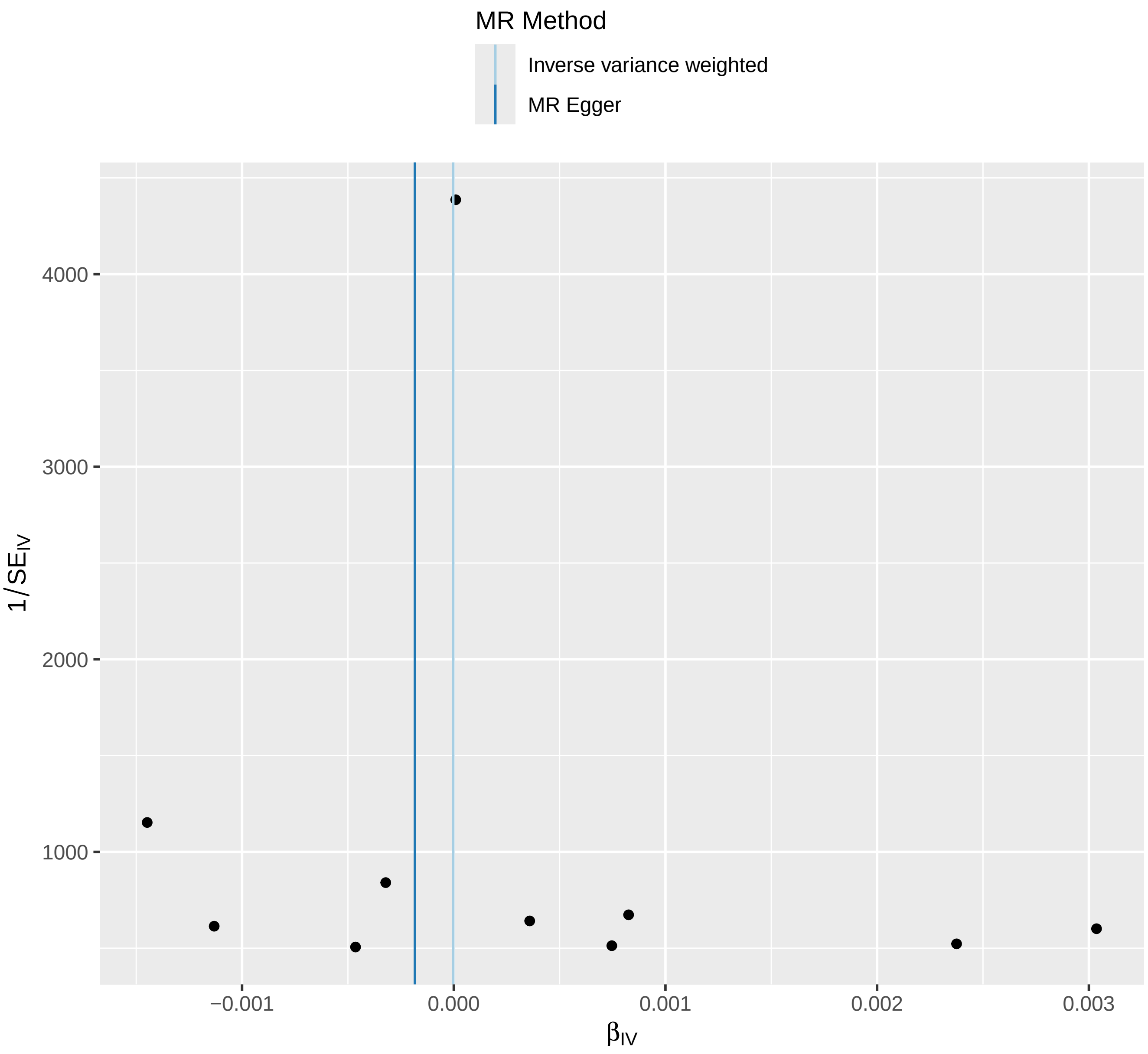

MR Method
Inverse variance weighted
MR Egger
4000
3000
2000
1000
1/SE_IV
−0.001
0.000
0.001
0.002
0.003
β_IV

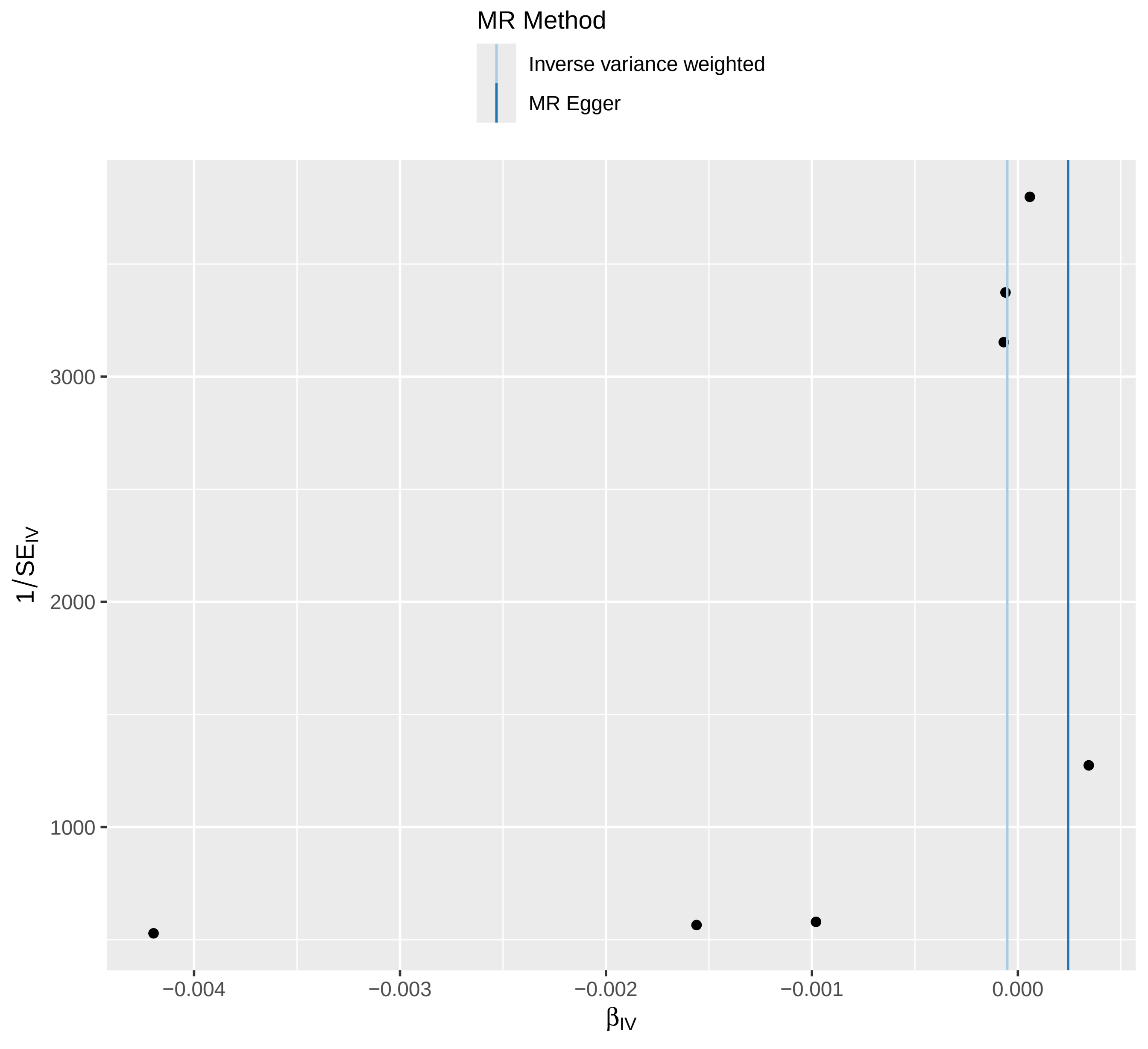

MR Method
Inverse variance weighted
MR Egger
3000
2000
1000
1/SE_IV
−0.004
−0.003
−0.002
−0.001
0.000
β_IV

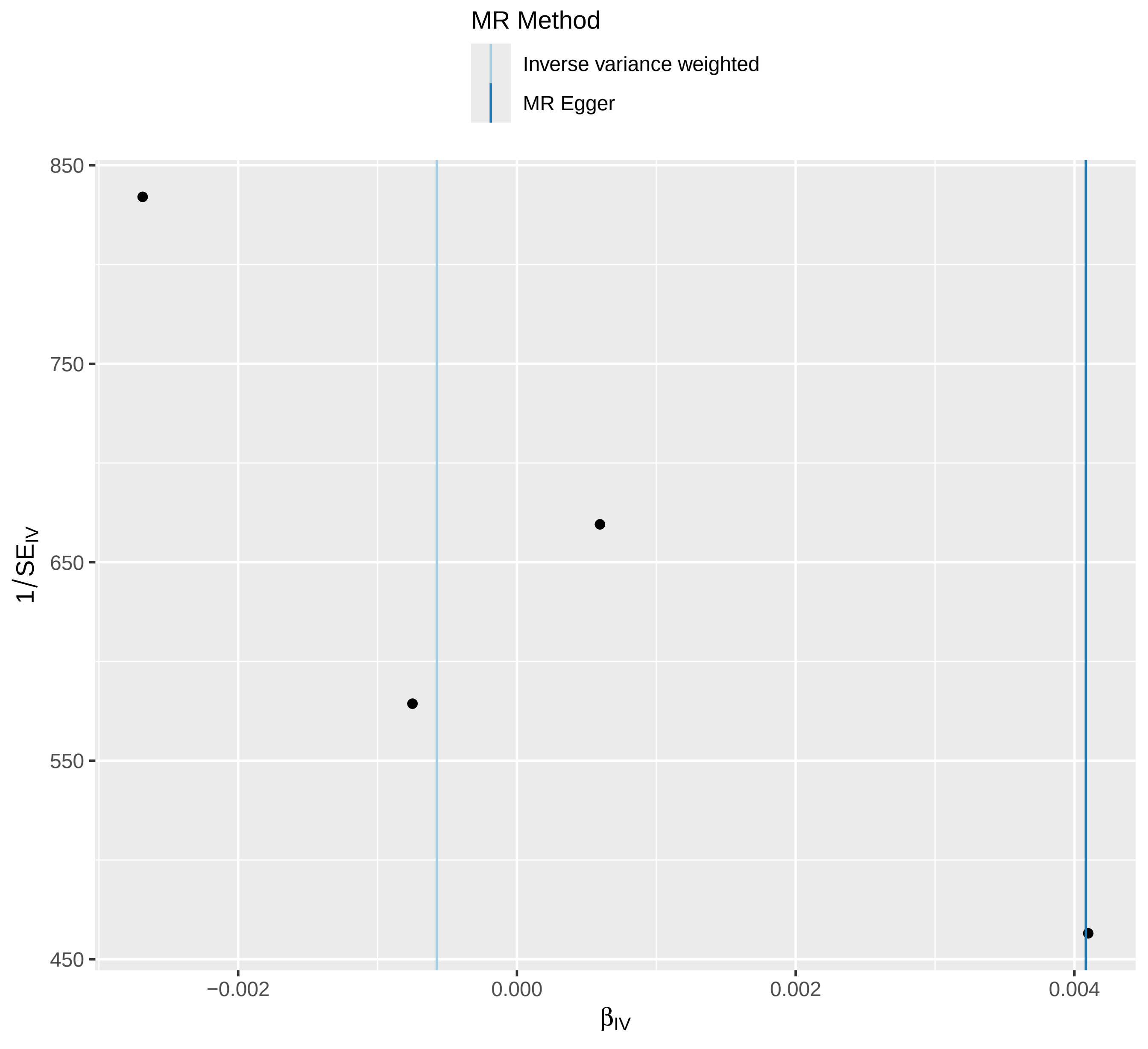

MR Method
Inverse variance weighted
MR Egger
850
750
650
550
450
1/SE_IV
−0.002
0.000
0.002
0.004
β_IV

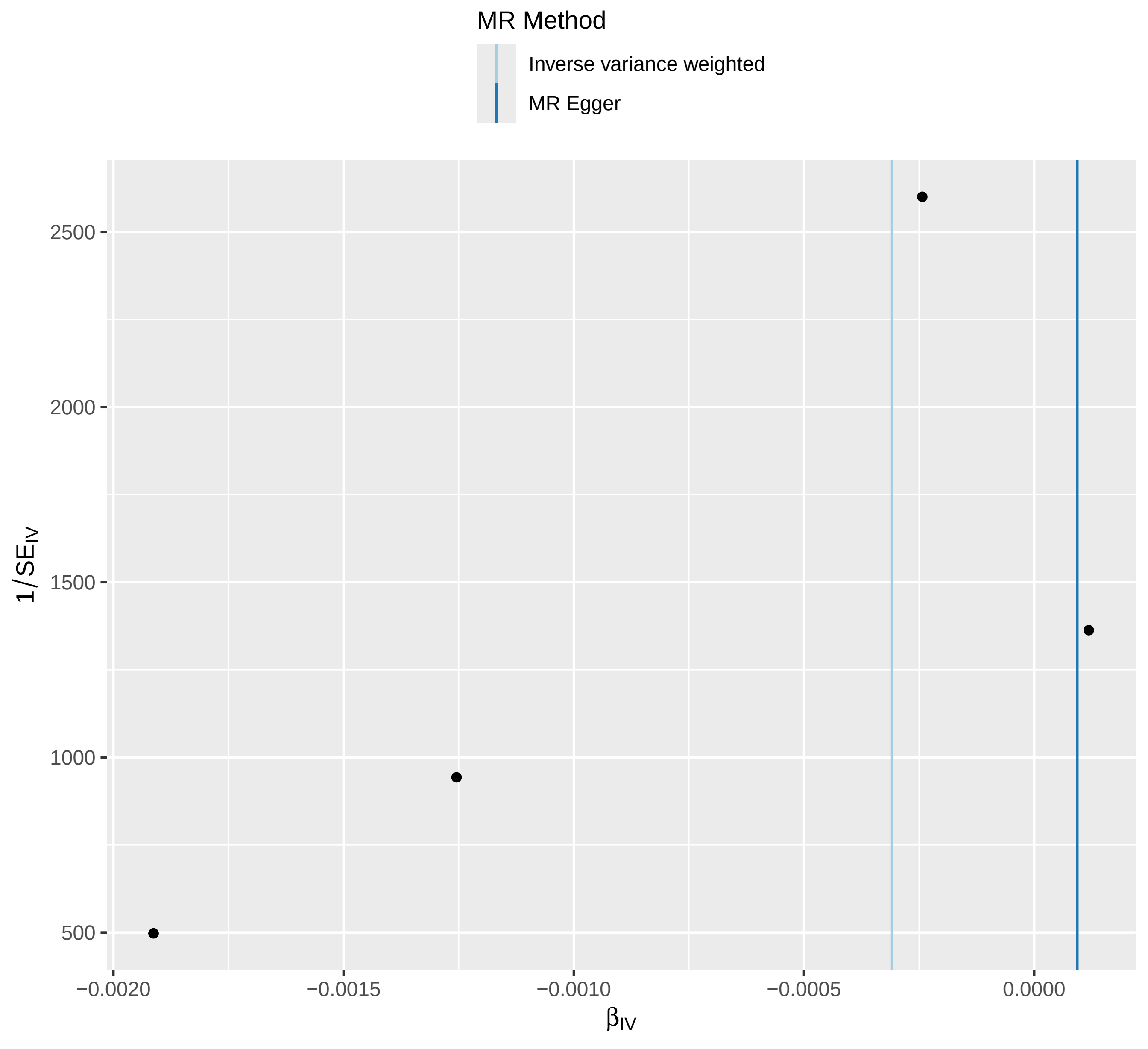
MR Method
Inverse variance weighted
MR Egger
2500
2000
1500
1000
500
1/SE_IV
−0.0020
−0.0015
−0.0010
−0.0005
0.0000
β_IV

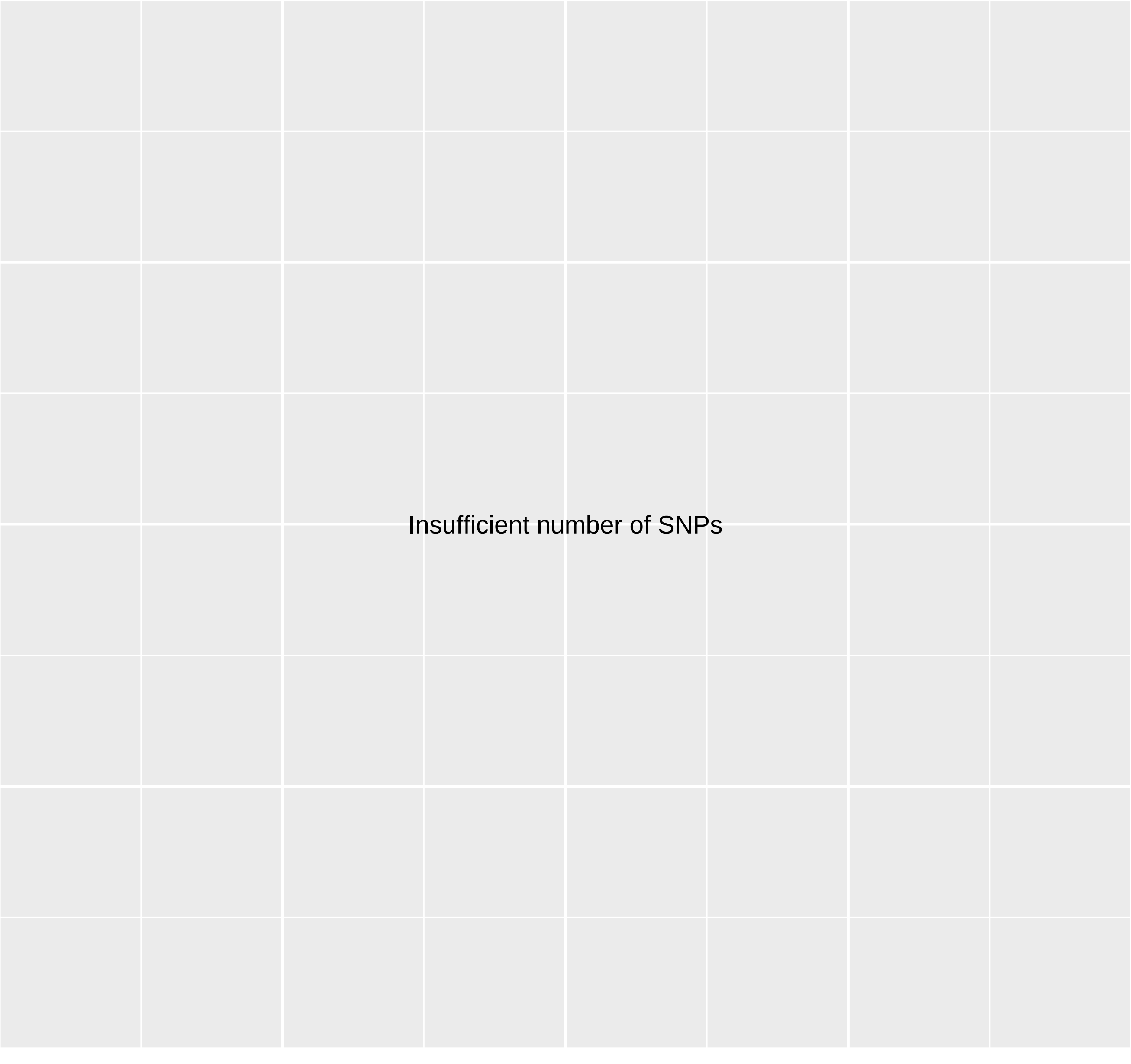
Insufficient number of SNPs

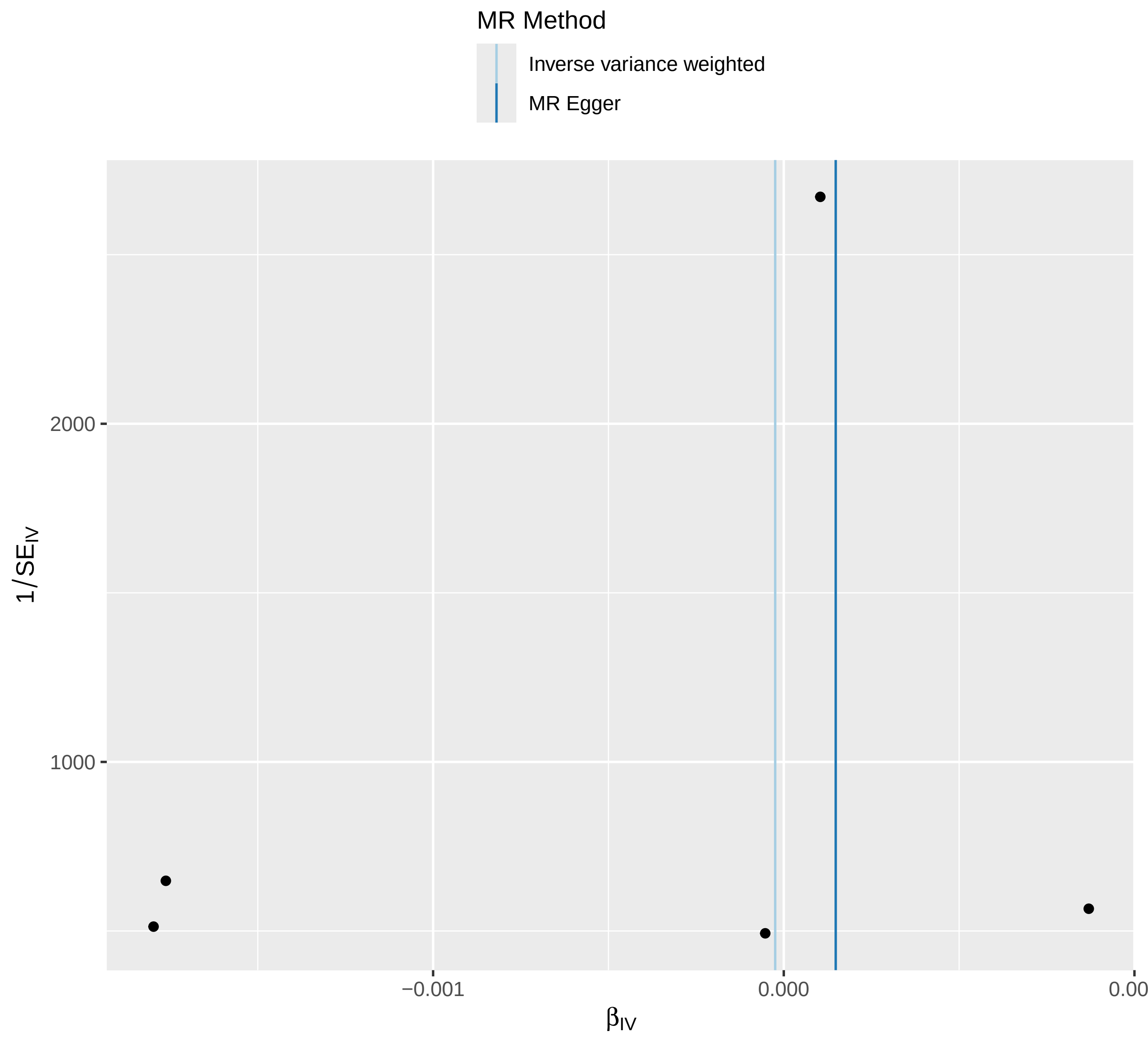
MR Method
Inverse variance weighted
MR Egger
2000
1000
1/SE_IV
−0.001
0.000
0.00
β_IV

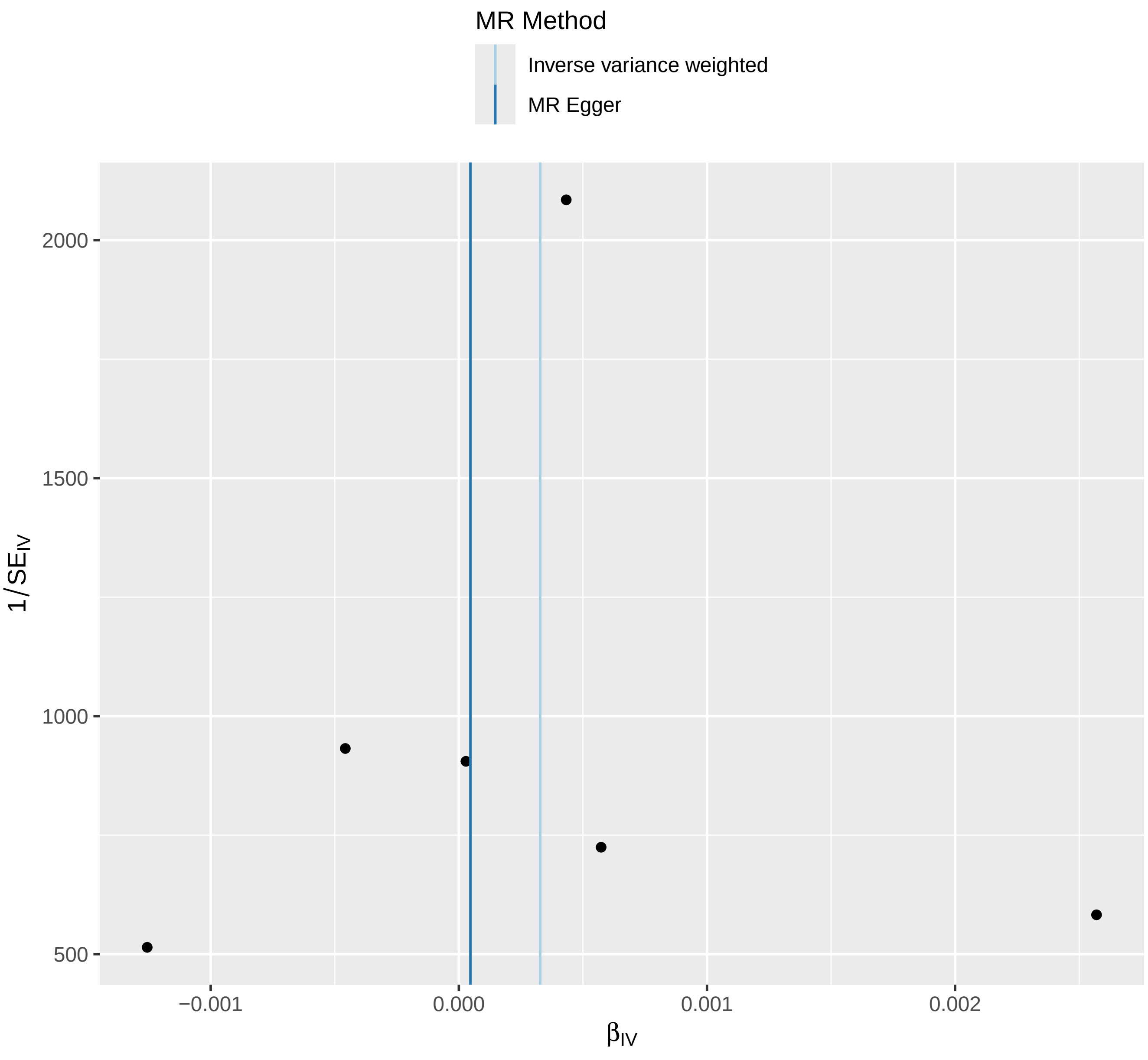

MR Method
Inverse variance weighted
MR Egger
2000
1500
1000
500
1/SE_{IV}
−0.001
0.000
0.001
0.002
β_{IV}

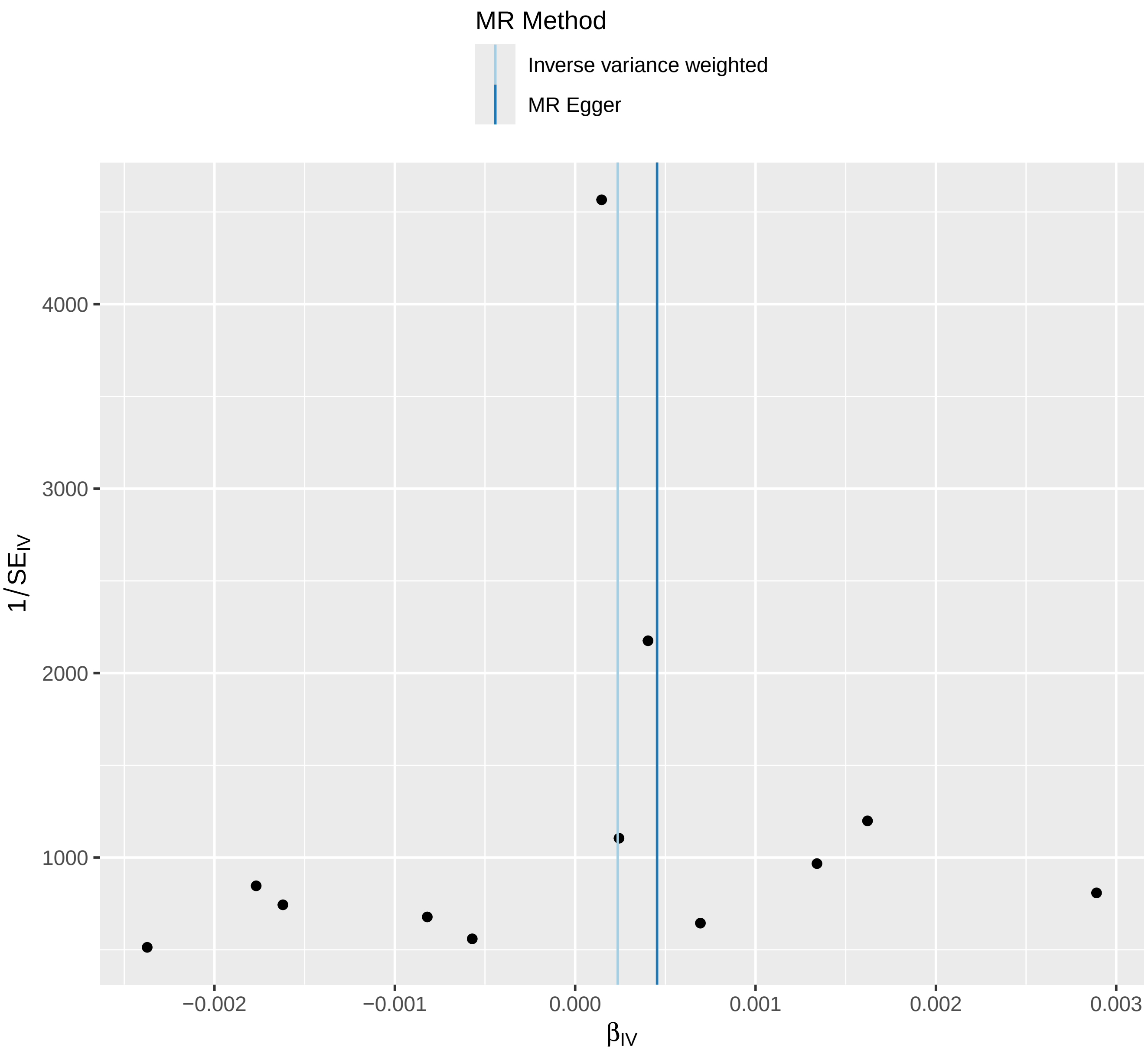

MR Method
Inverse variance weighted
MR Egger
4000
3000
2000
1000
1/SE_IV
−0.002
−0.001
0.000
0.001
0.002
0.003
β_IV

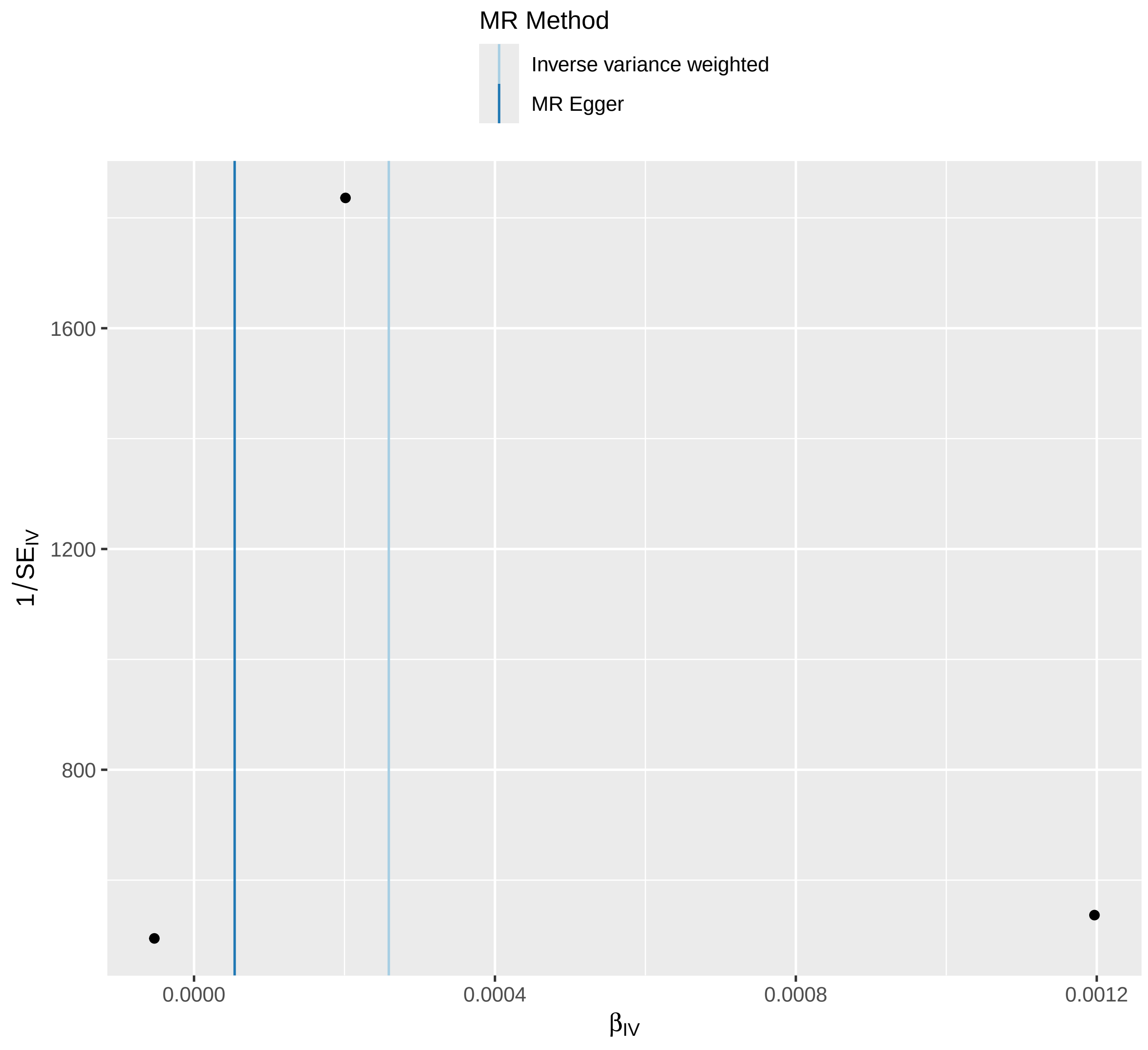

MR Method
Inverse variance weighted
MR Egger
1600
1200
800
$1/SE_{IV}$
0.0000
0.0004
0.0008
0.0012
β_{IV}

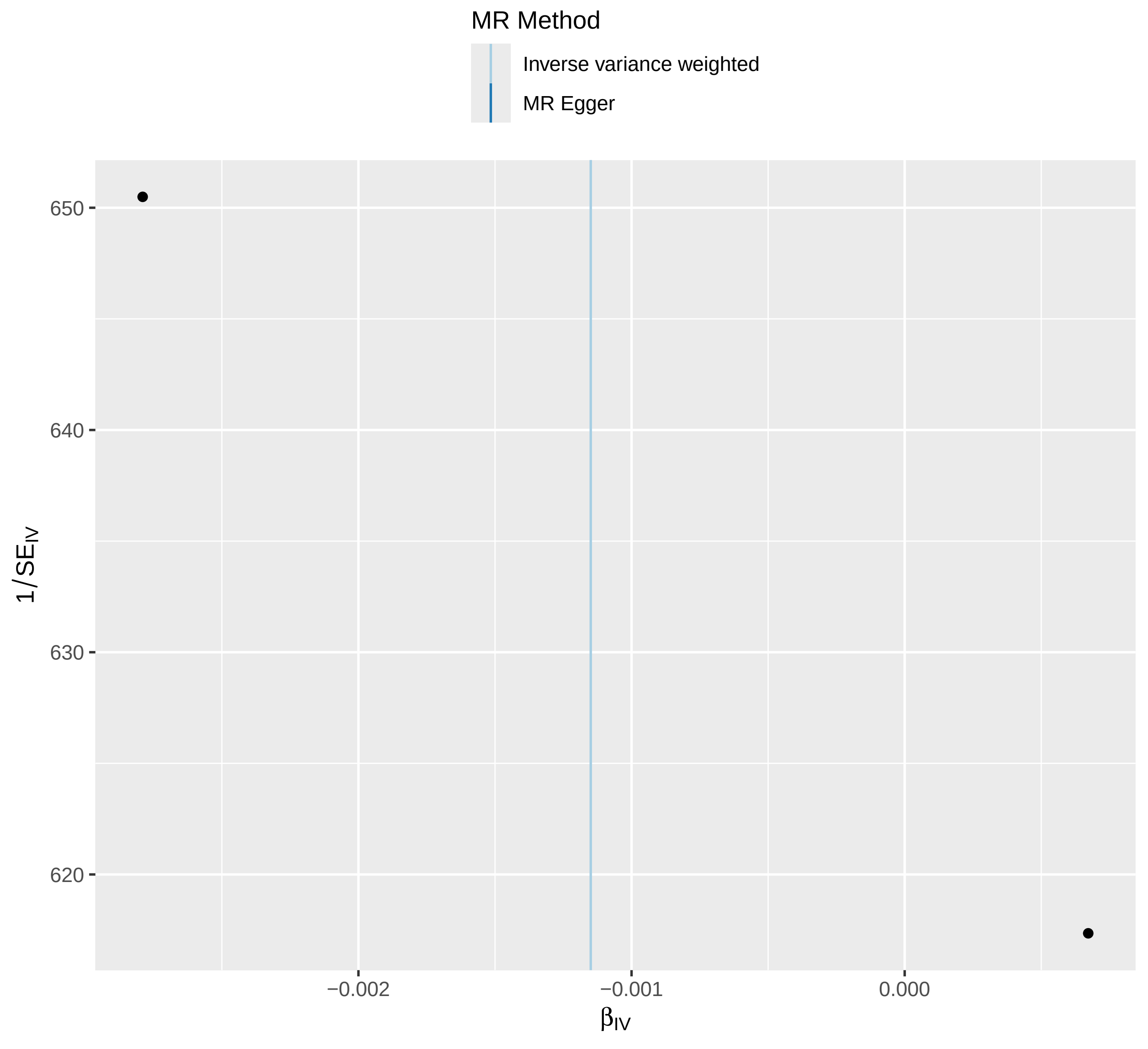

MR Method
Inverse variance weighted
MR Egger
650
640
630
620
1/SE$_{IV}$
−0.002
−0.001
0.000
β$_{IV}$

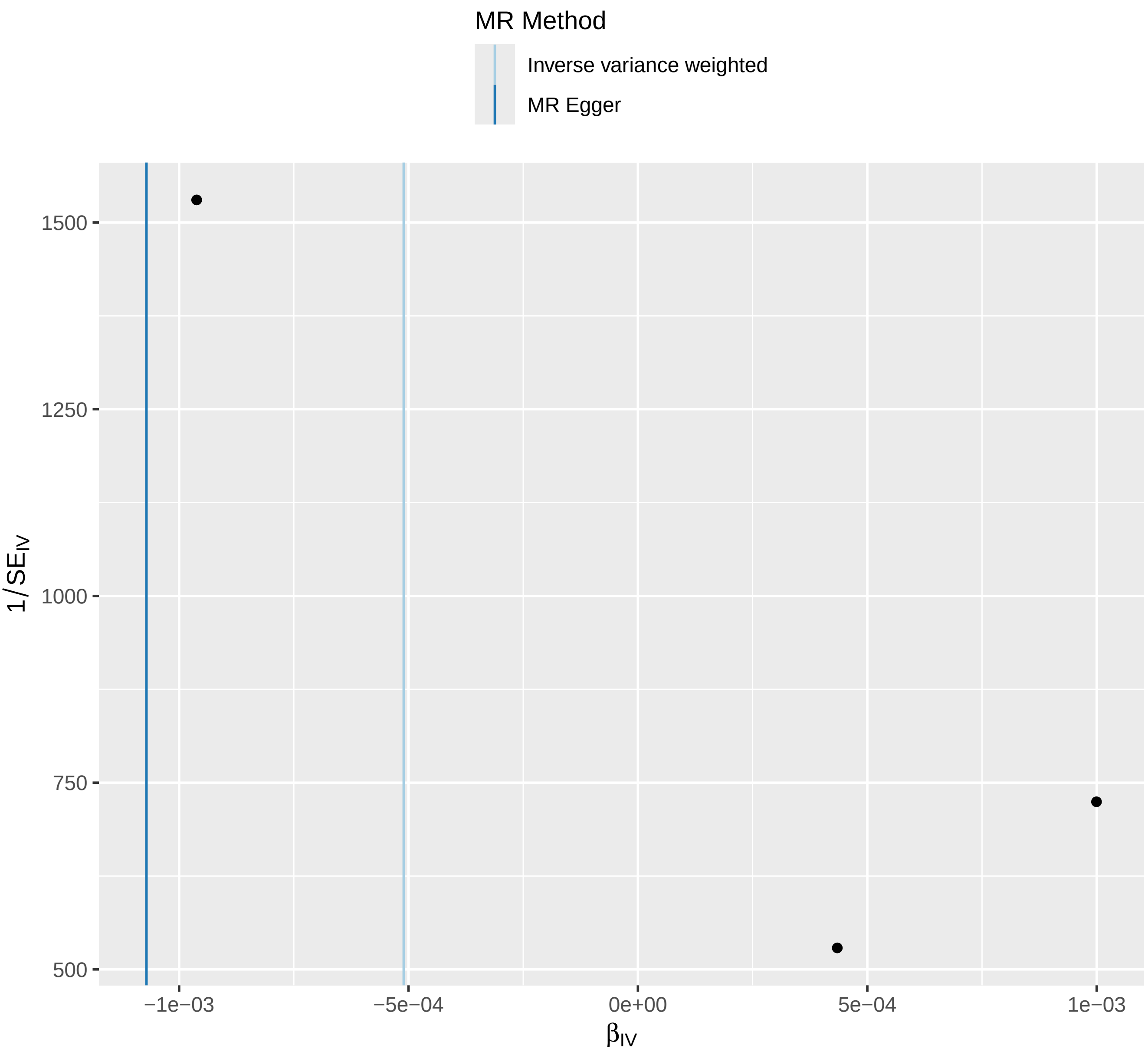
MR Method
Inverse variance weighted
MR Egger
1500
1250
1000
750
500
1/SE_IV
−1e−03
−5e−04
0e+00
5e−04
1e−03
β_IV

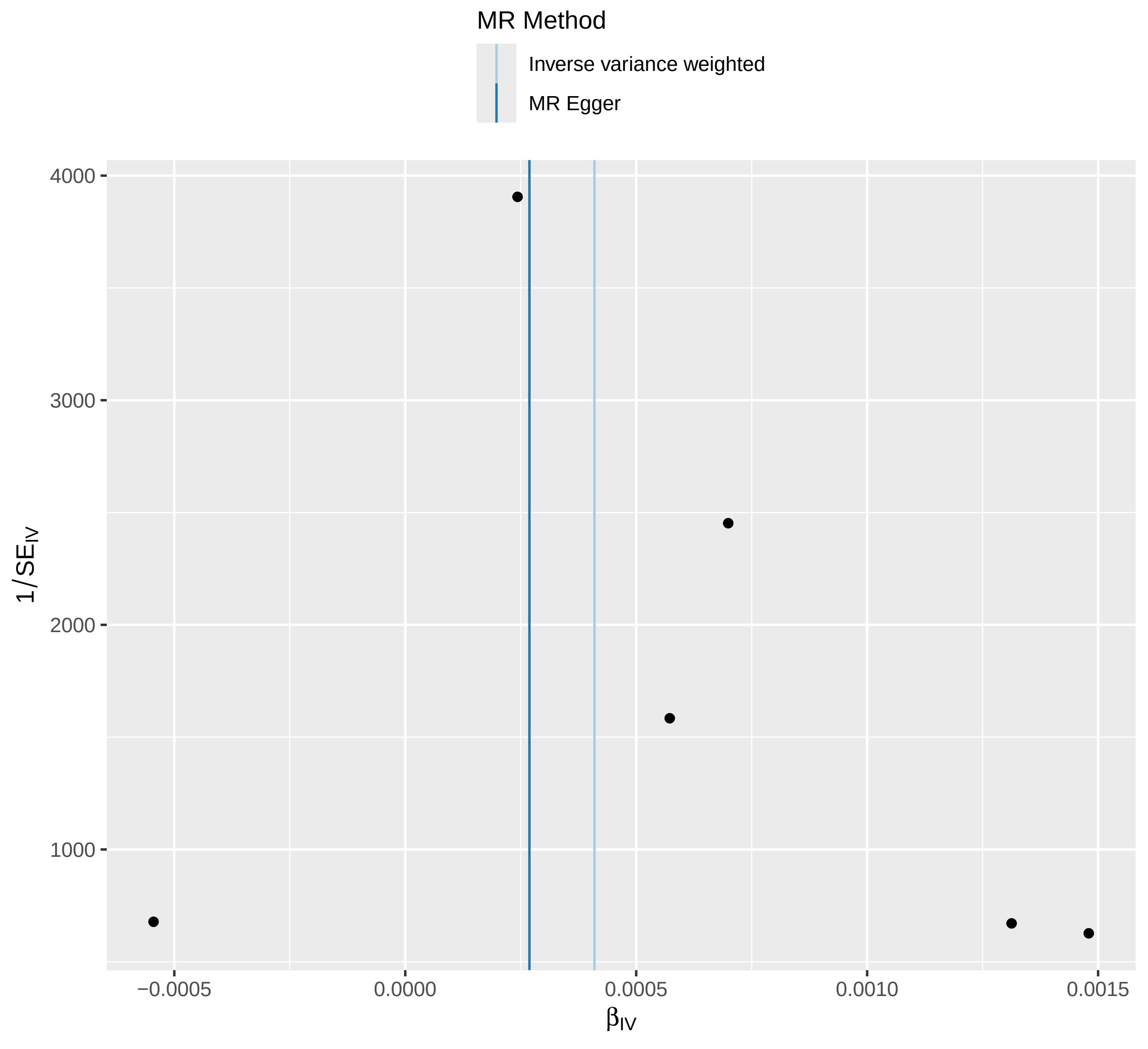
MR Method
Inverse variance weighted
MR Egger
4000
3000
2000
1000
1/SE_IV
−0.0005
0.0000
0.0005
0.0010
0.0015
β_IV

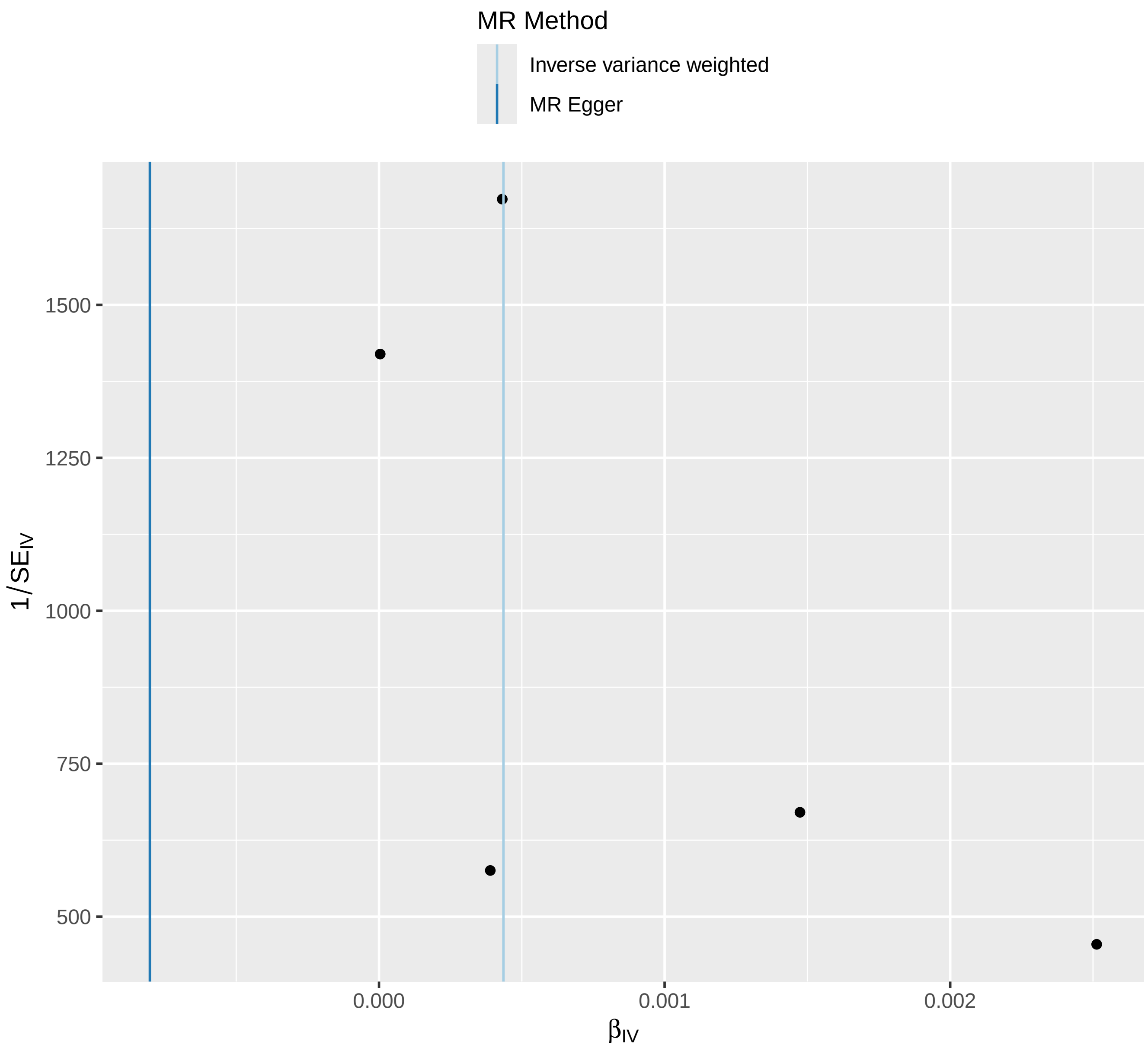

MR Method
Inverse variance weighted
MR Egger
1500
1250
1000
750
500
1/SE_IV
0.000
0.001
0.002
β_IV

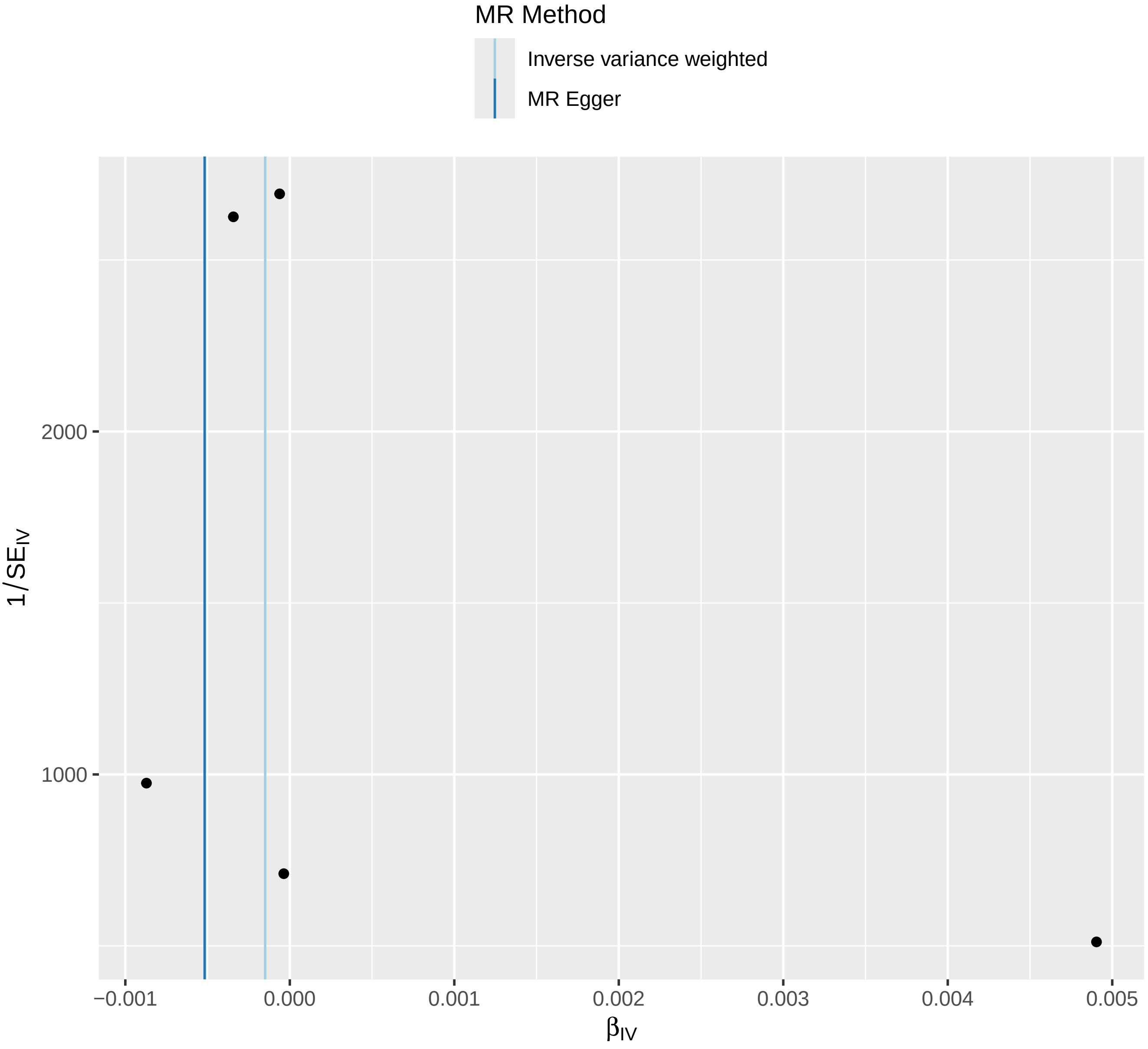
MR Method
Inverse variance weighted
MR Egger
2000
1000
1/SE_IV
−0.001
0.000
0.001
0.002
0.003
0.004
0.005
β_IV

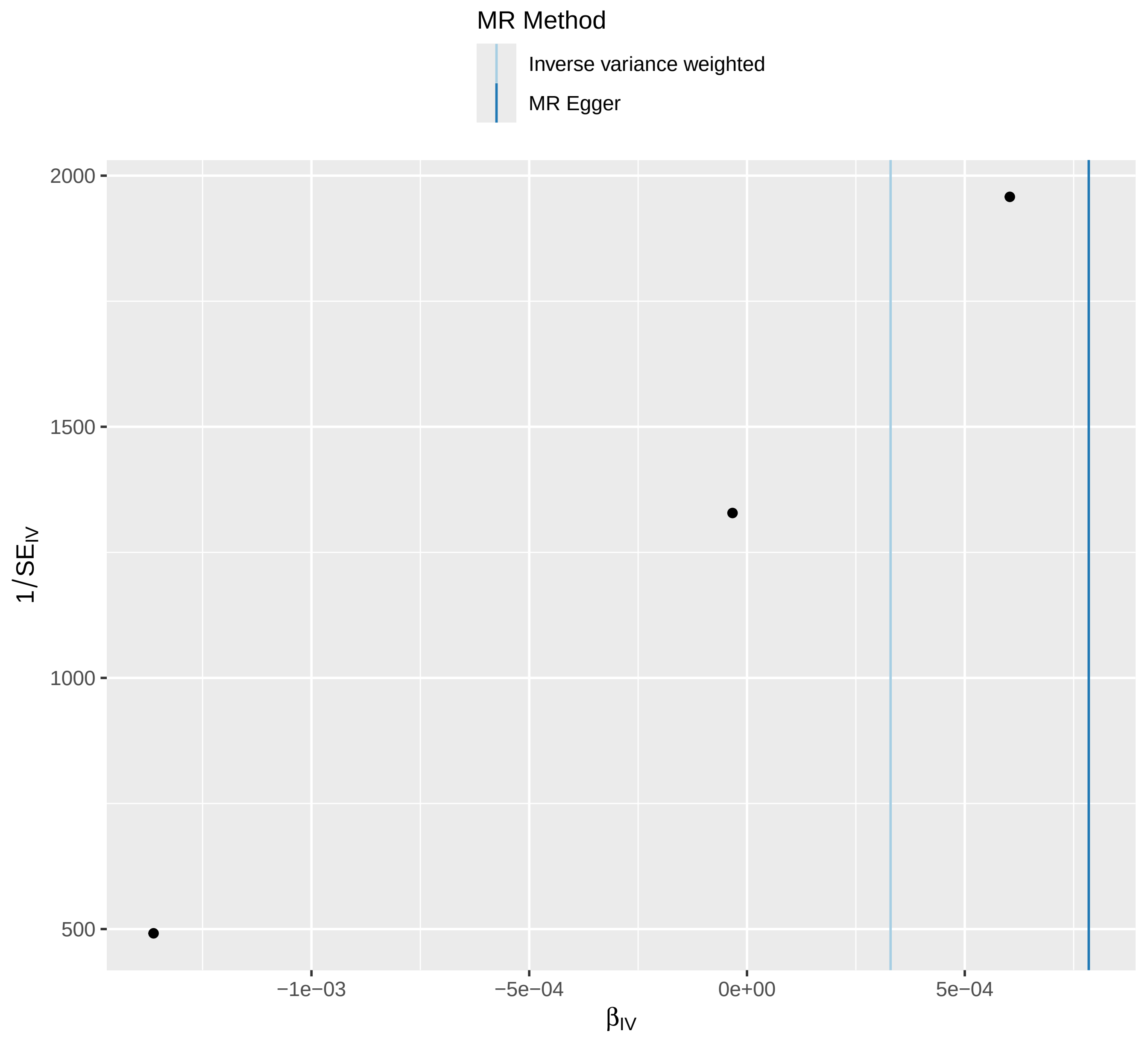

MR Method
Inverse variance weighted
MR Egger
2000
1500
1000
500
1/SE_IV
−1e−03
−5e−04
0e+00
5e−04
β_IV

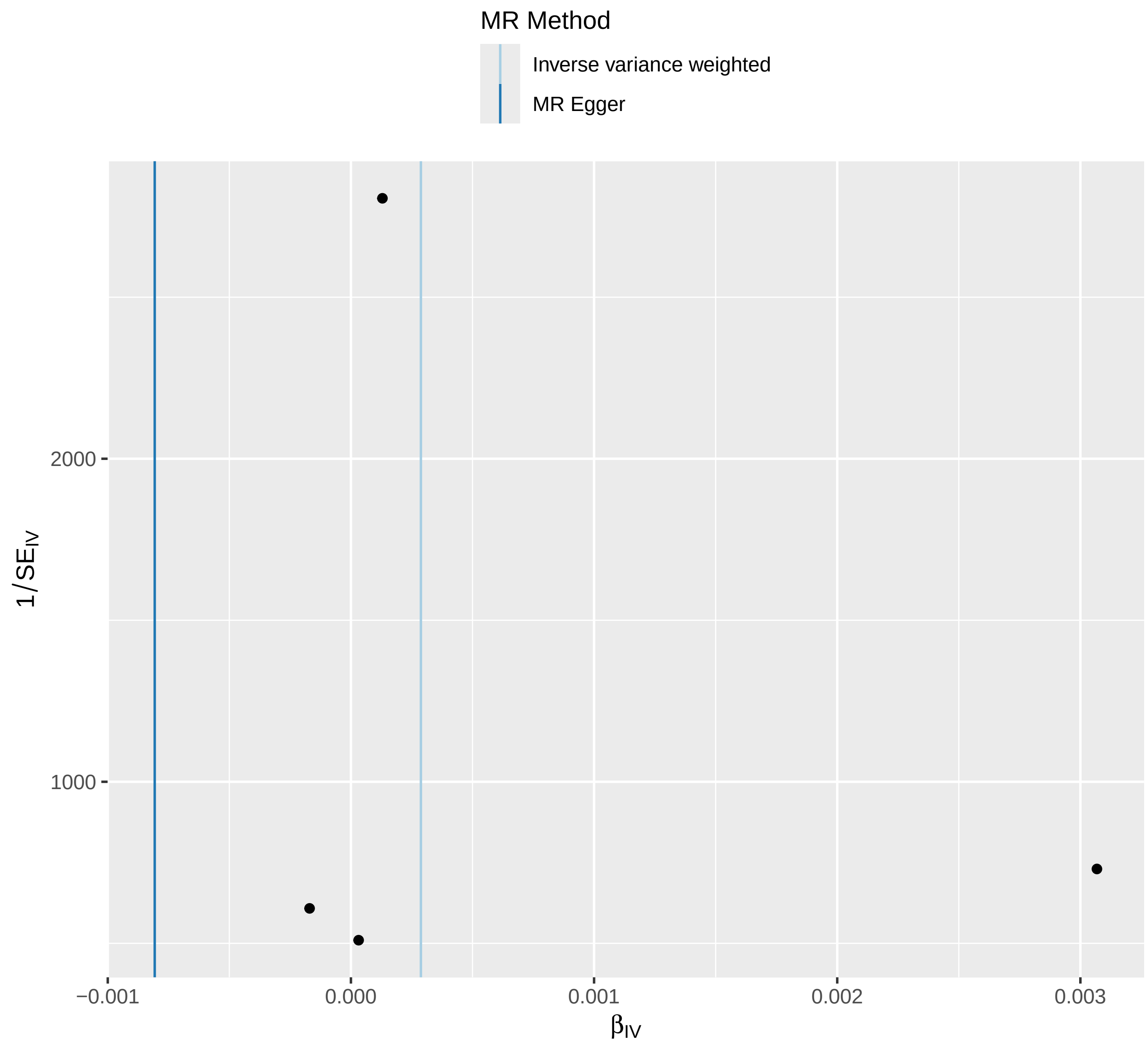
MR Method
Inverse variance weighted
MR Egger
2000
1000
1/SE$_{IV}$
−0.001
0.000
0.001
0.002
0.003
β$_{IV}$

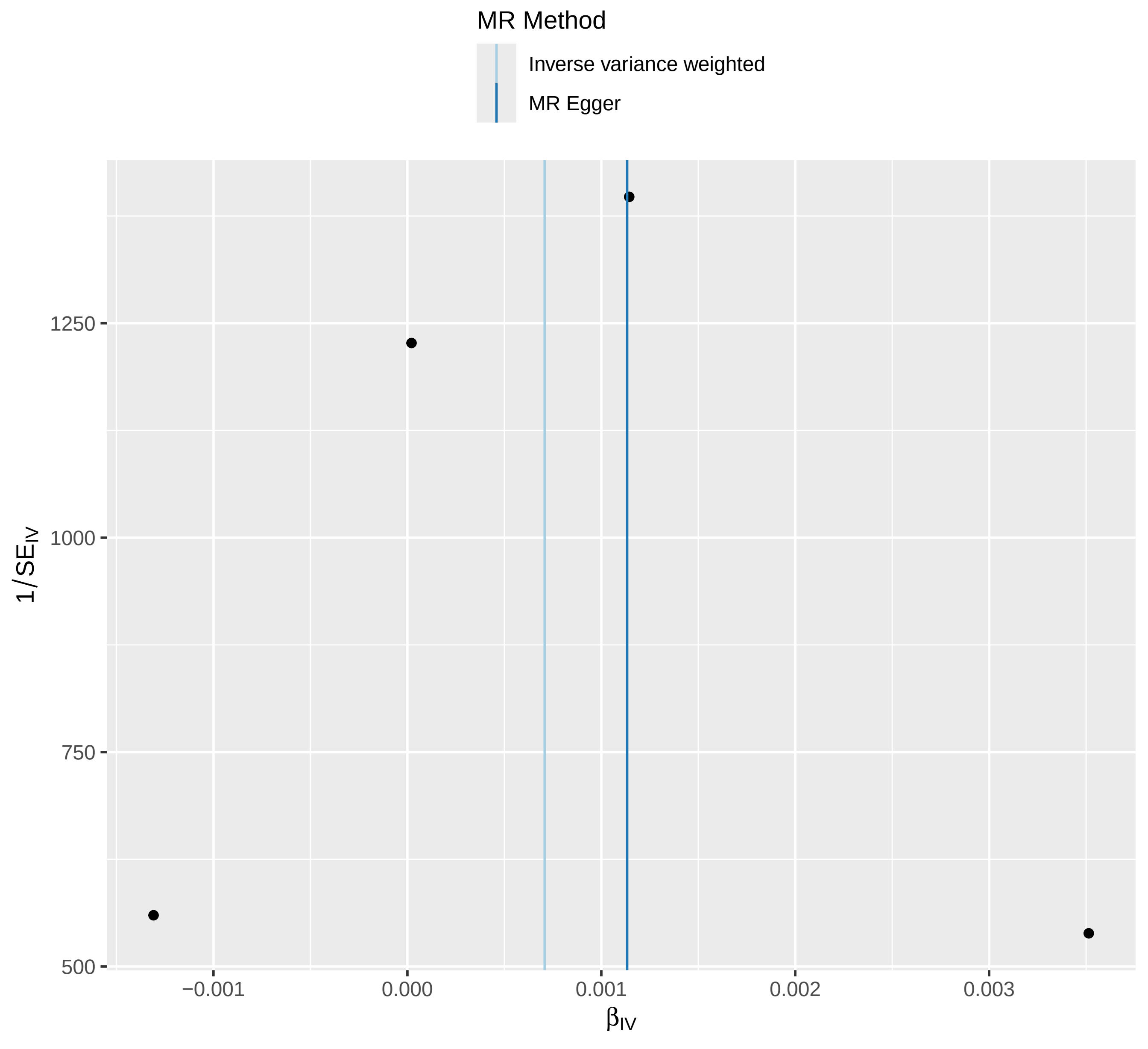

MR Method
Inverse variance weighted
MR Egger
1250
1000
750
500
1/SE$_{IV}$
−0.001
0.000
0.001
0.002
0.003
β_{IV}

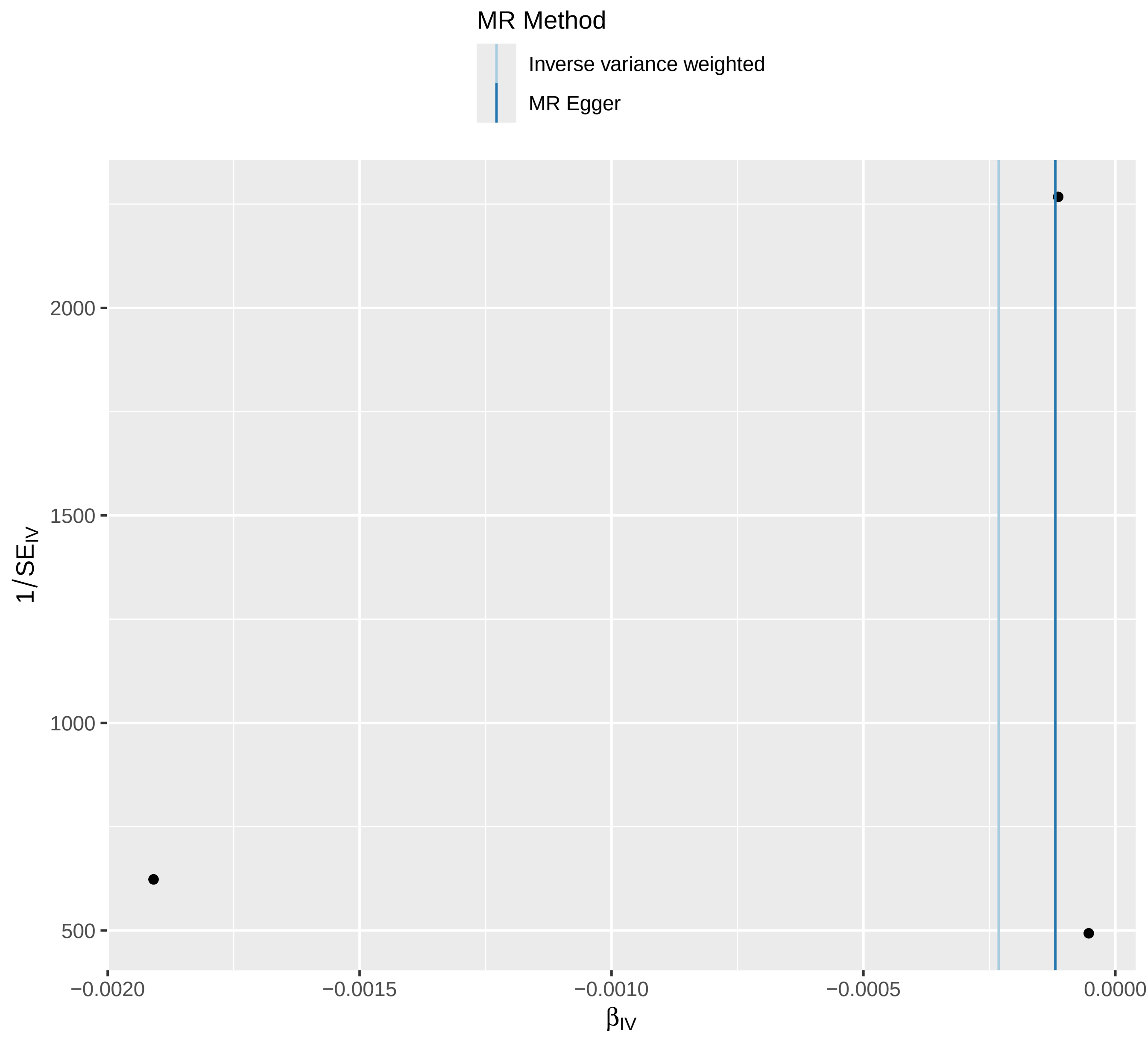

MR Method
Inverse variance weighted
MR Egger
2000
1500
1000
500
1/SE_IV
−0.0020
−0.0015
−0.0010
−0.0005
0.0000
β_IV

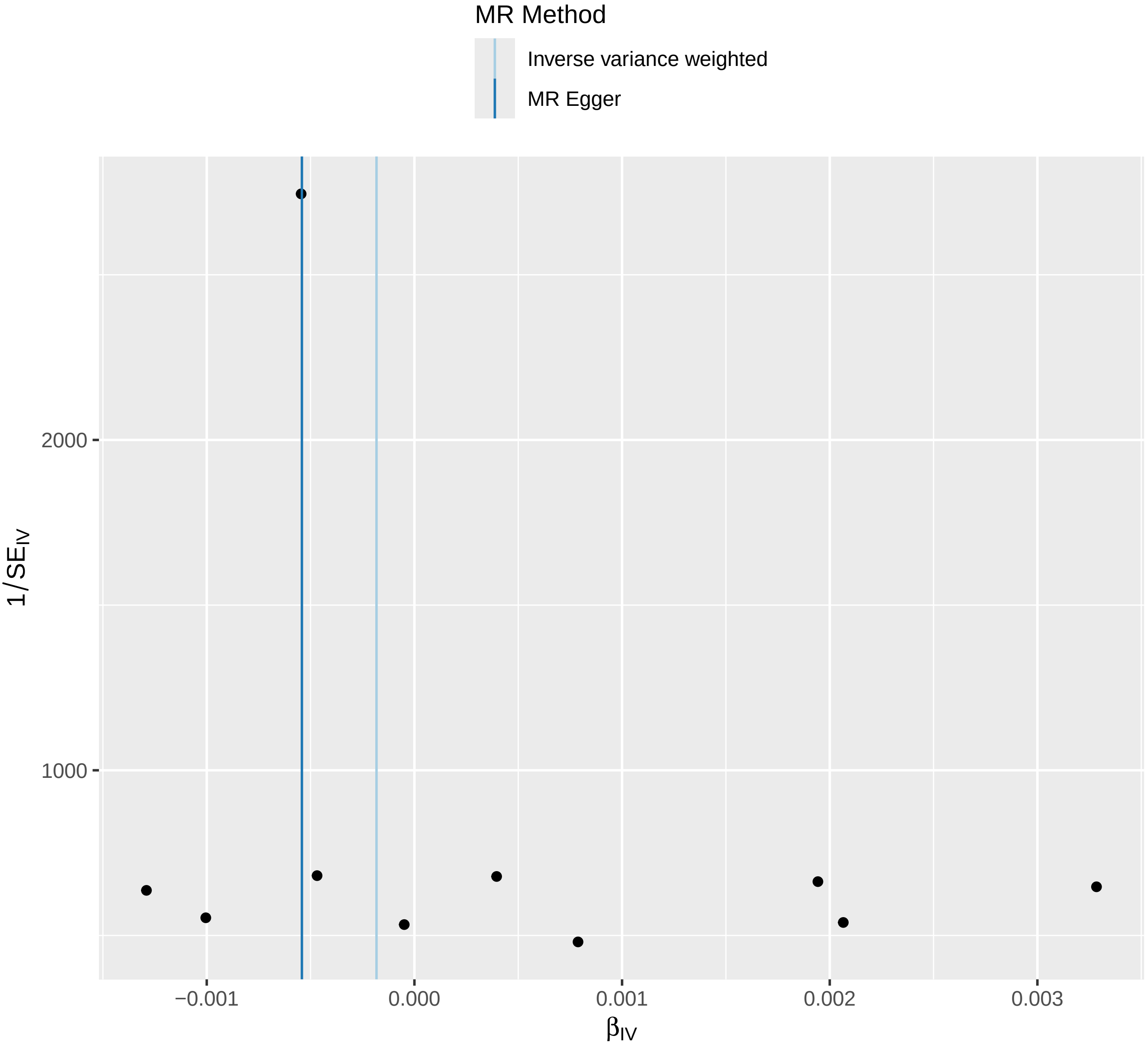
MR Method
Inverse variance weighted
MR Egger
2000
1000
1/SE$_{IV}$
−0.001
0.000
0.001
0.002
0.003
β$_{IV}$

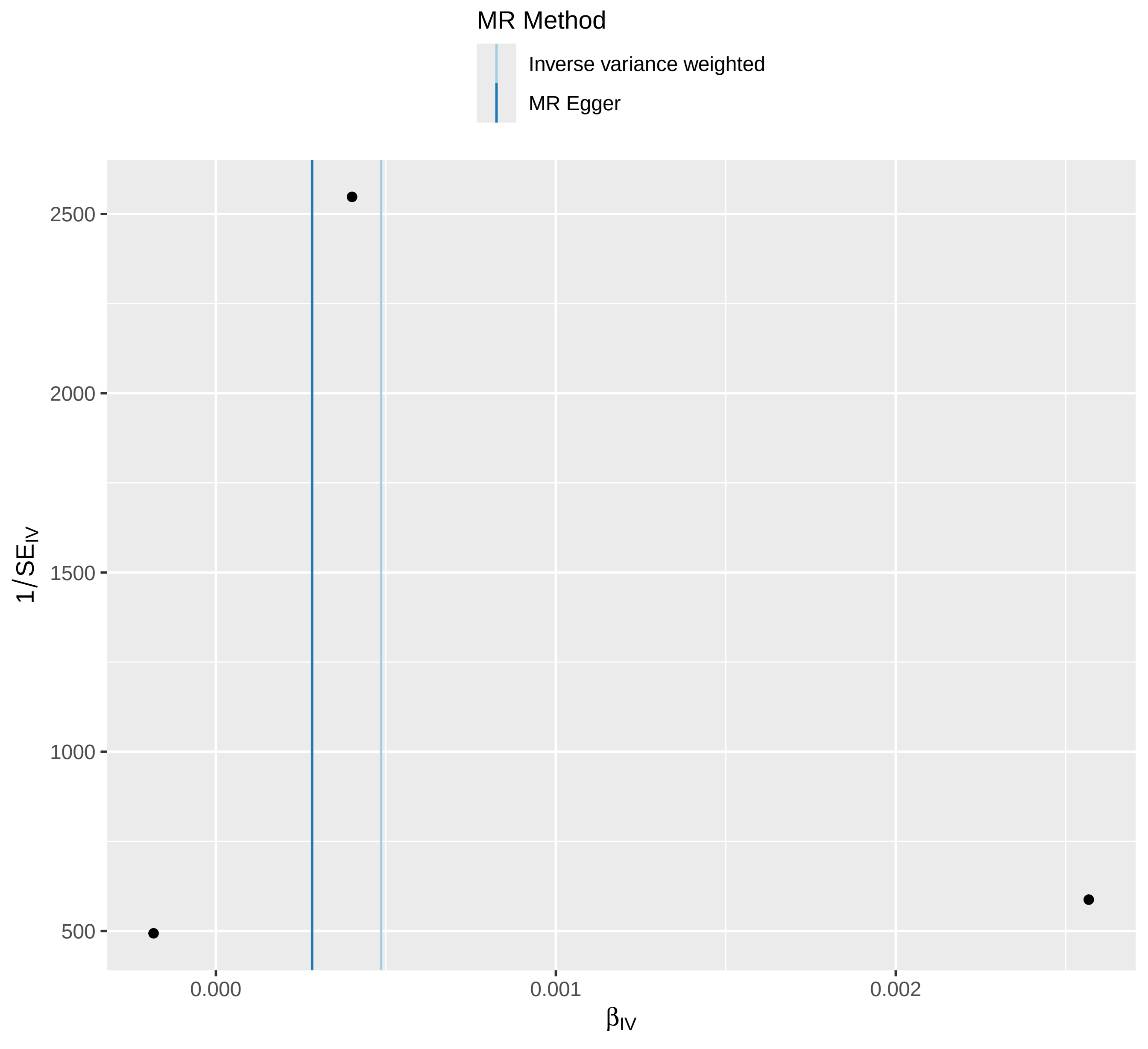

MR Method
Inverse variance weighted
MR Egger
2500
2000
1500
1000
500
0.000
0.001
0.002
1/SE_IV
β_IV

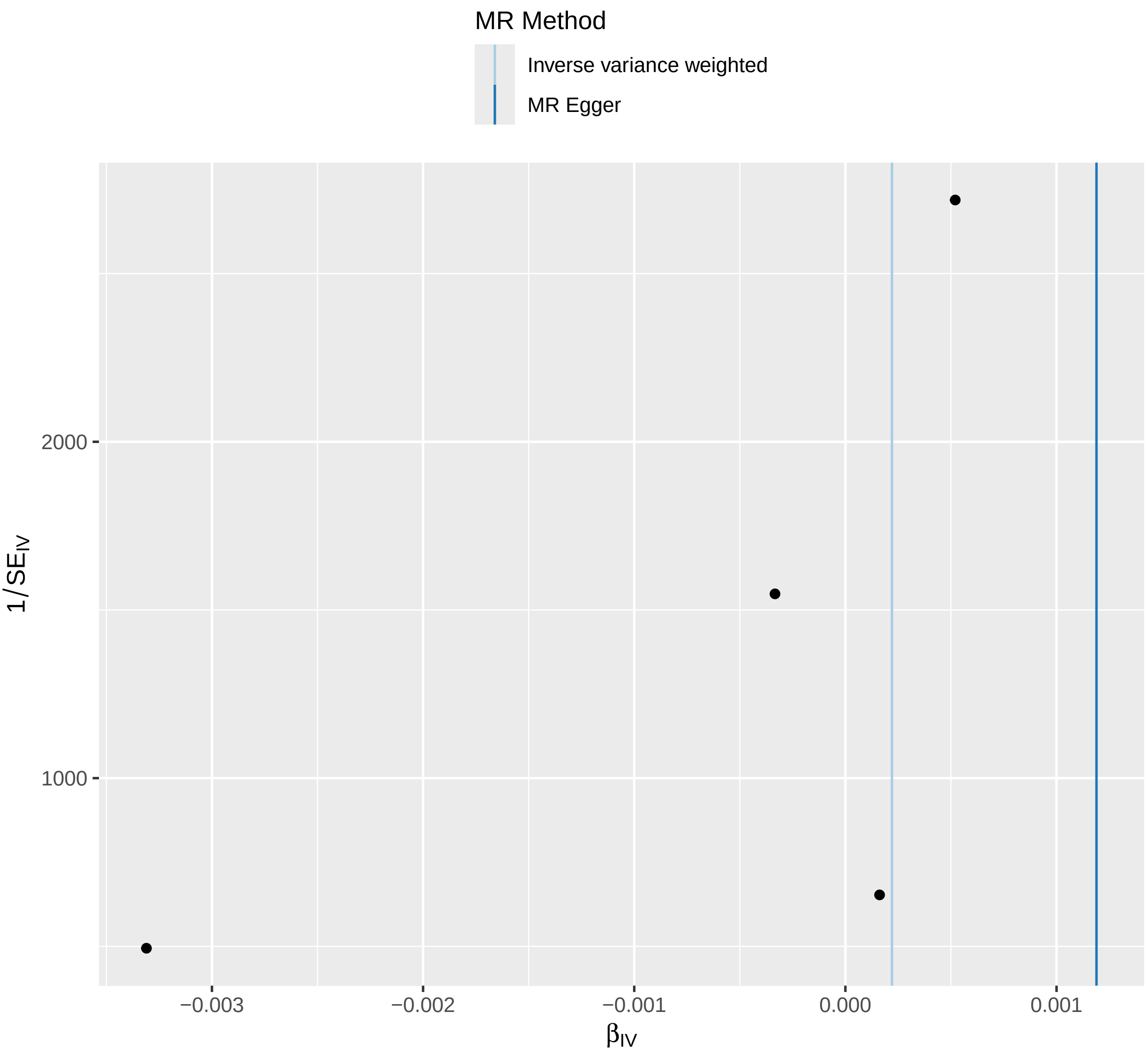

MR Method
Inverse variance weighted
MR Egger
2000
1000
1/SE$_{IV}$
−0.003
−0.002
−0.001
0.000
0.001
β$_{IV}$

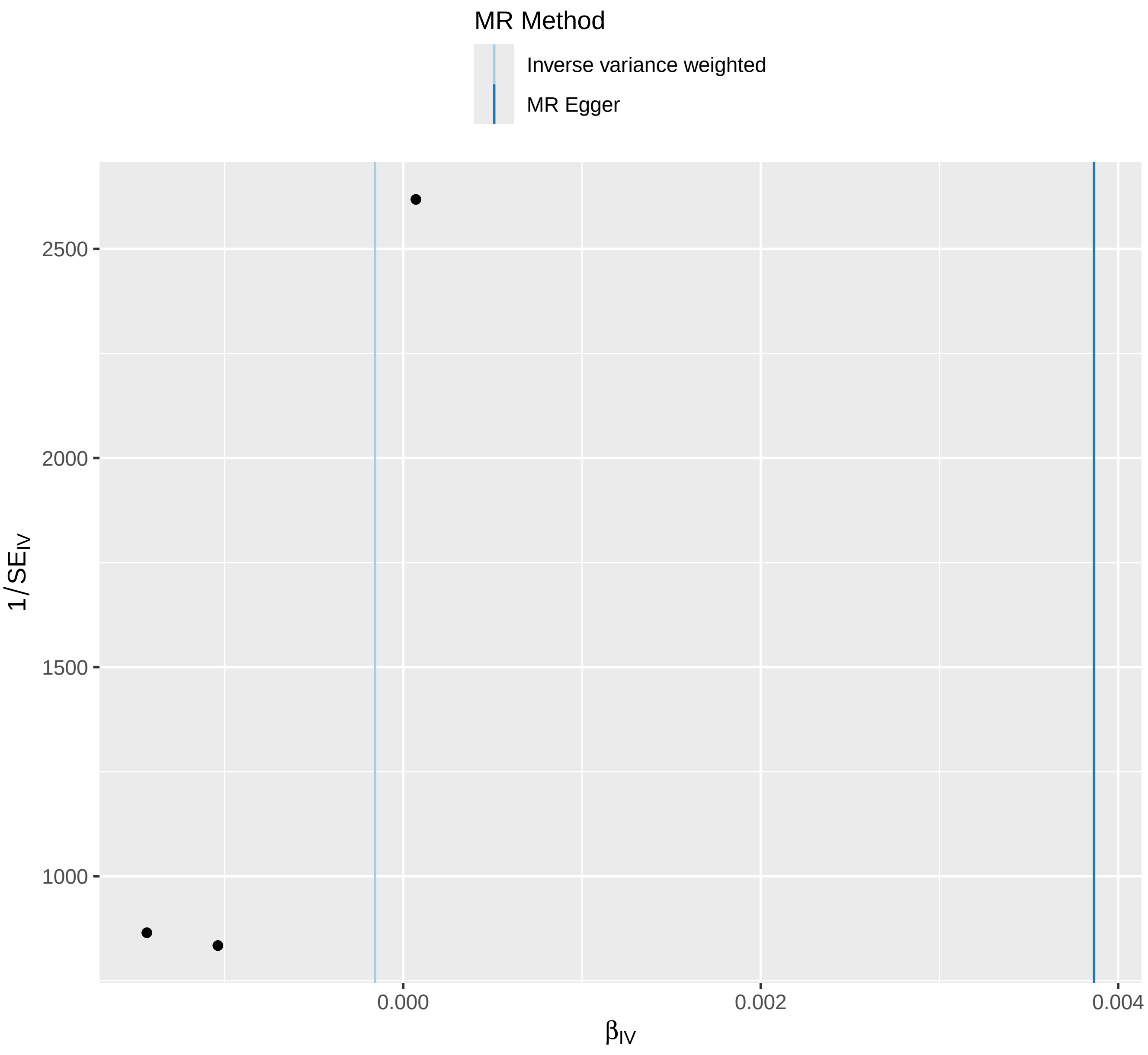

MR Method
Inverse variance weighted
MR Egger
2500
2000
1500
1000
1/SE_IV
0.000
0.002
0.004
β_IV

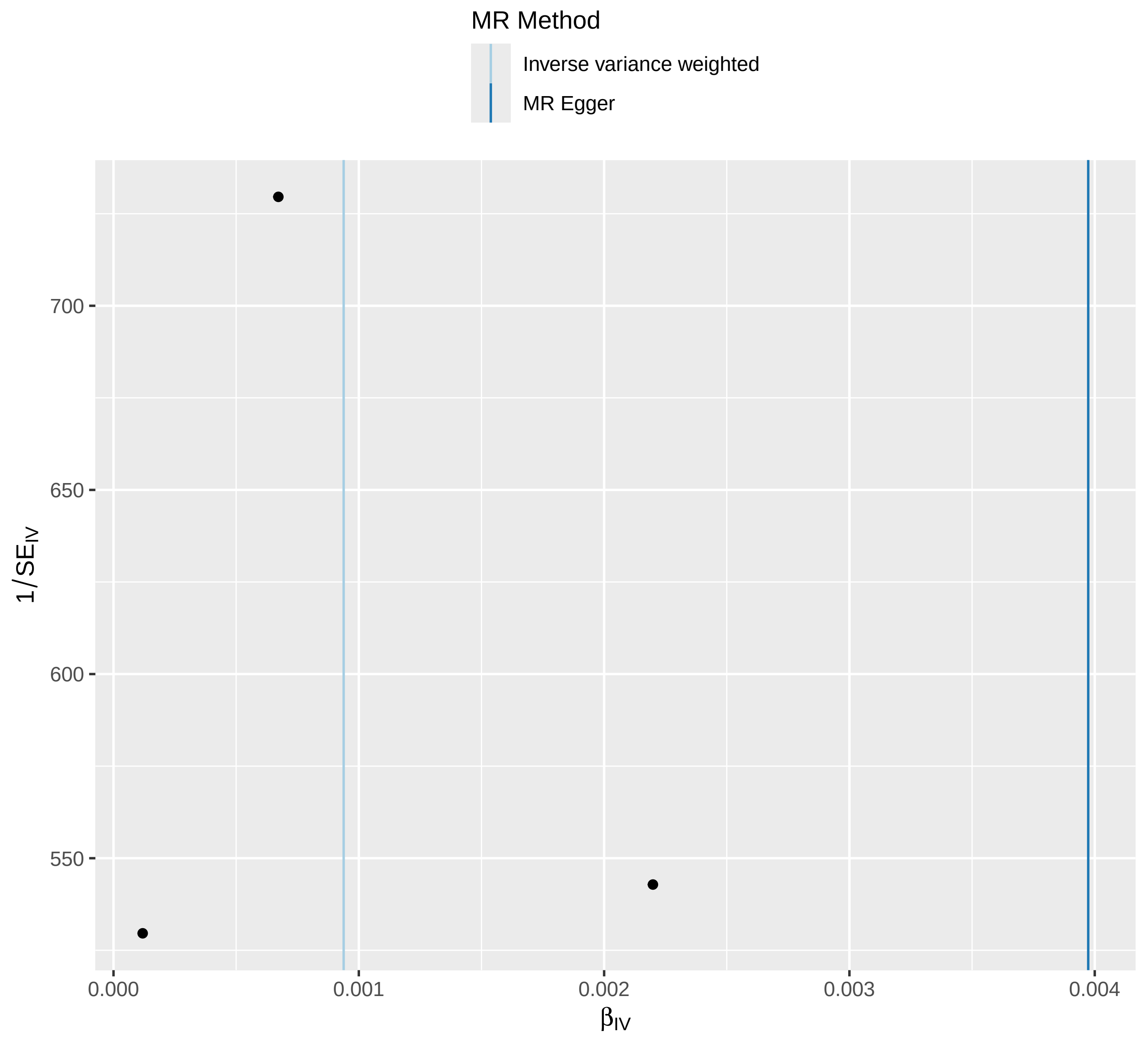
MR Method
Inverse variance weighted
MR Egger
700
650
600
550
1/SE_IV
0.000
0.001
0.002
0.003
0.004
β_IV

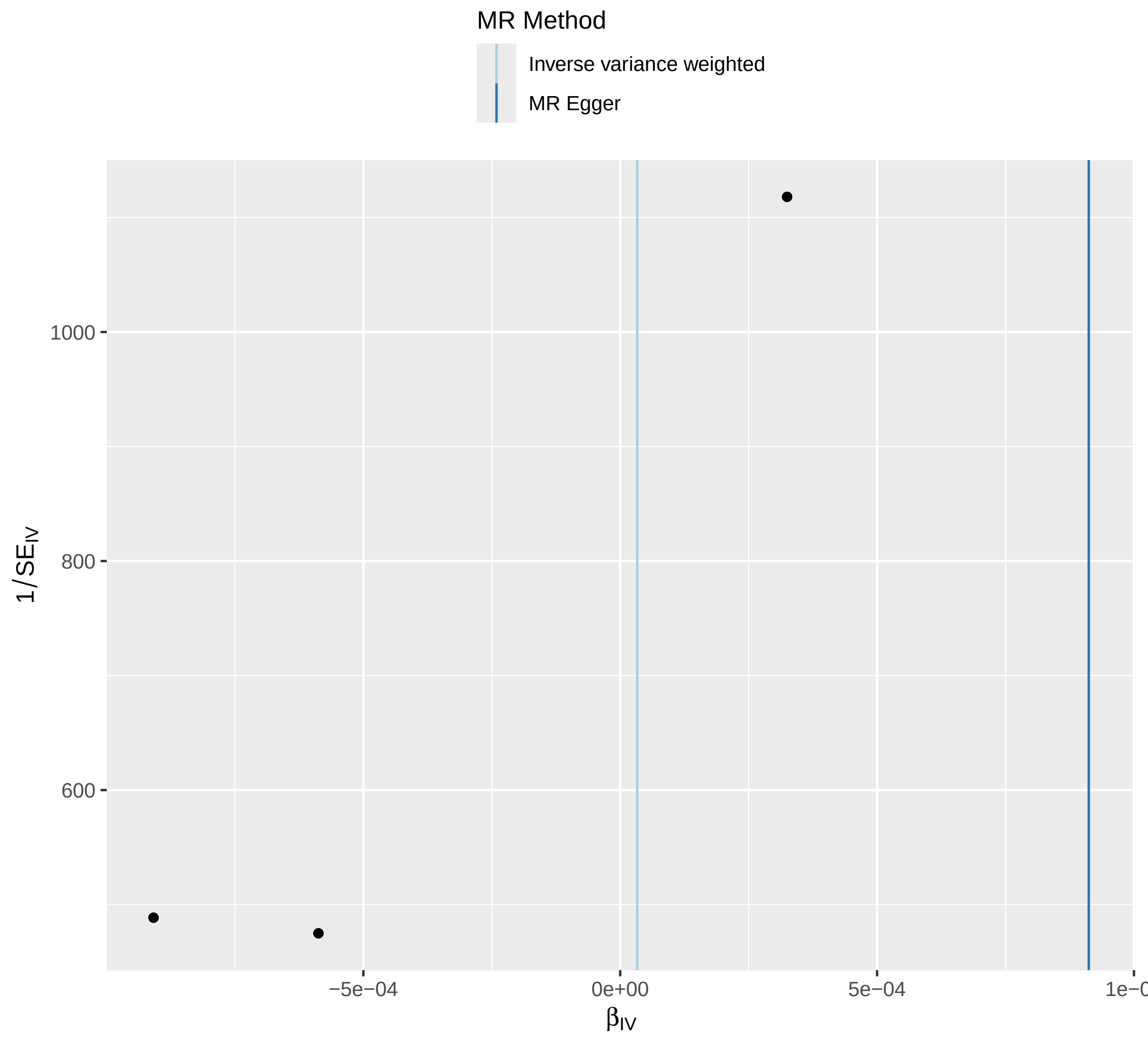
MR Method
Inverse variance weighted
MR Egger
1000
800
600
1/SE$_{IV}$
−5e−04
0e+00
5e−04
1e−0
β$_{IV}$

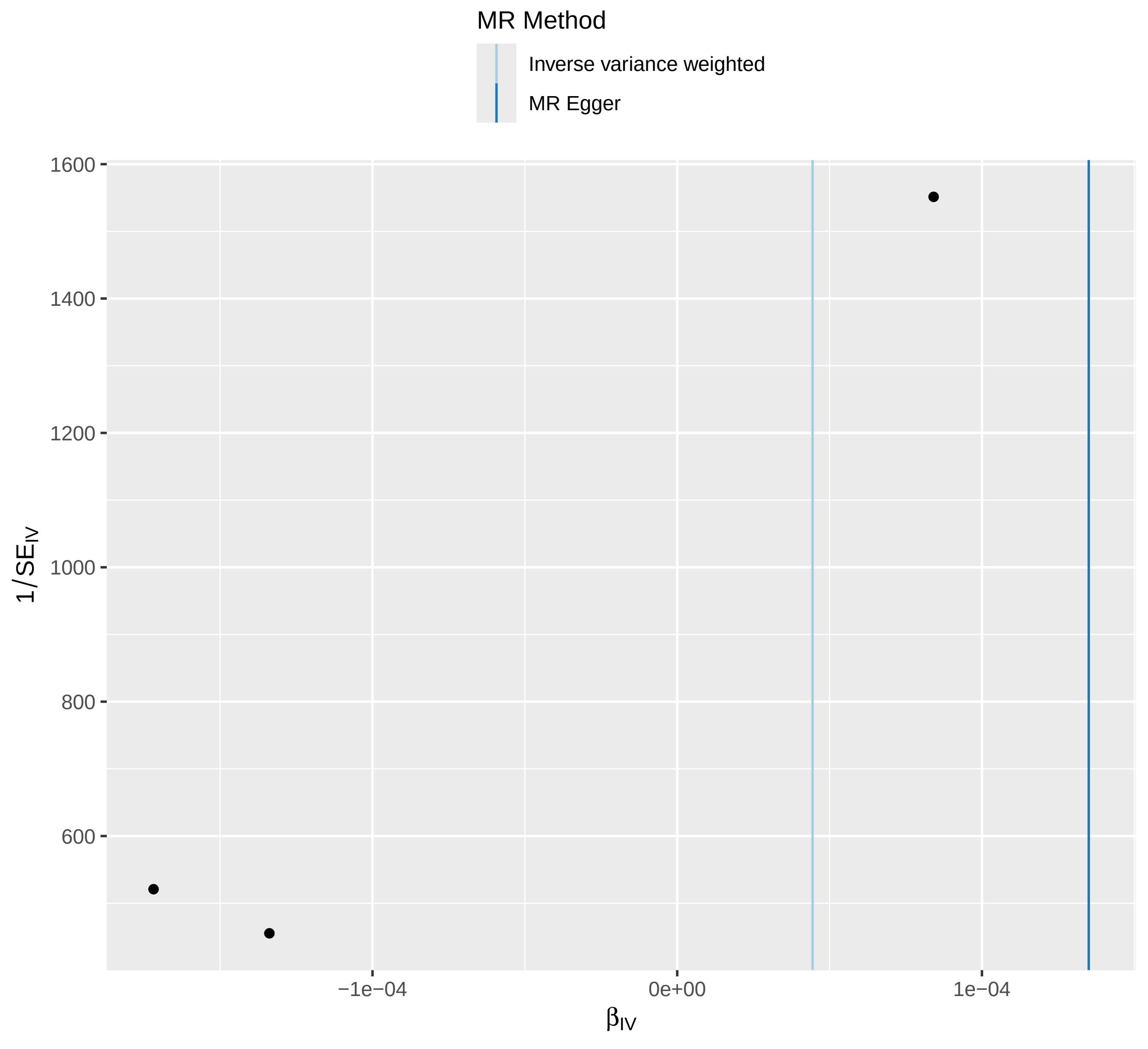
MR Method
Inverse variance weighted
MR Egger
1600
1400
1200
1000
800
600
1/SE_IV
−1e−04
0e+00
1e−04
β_IV

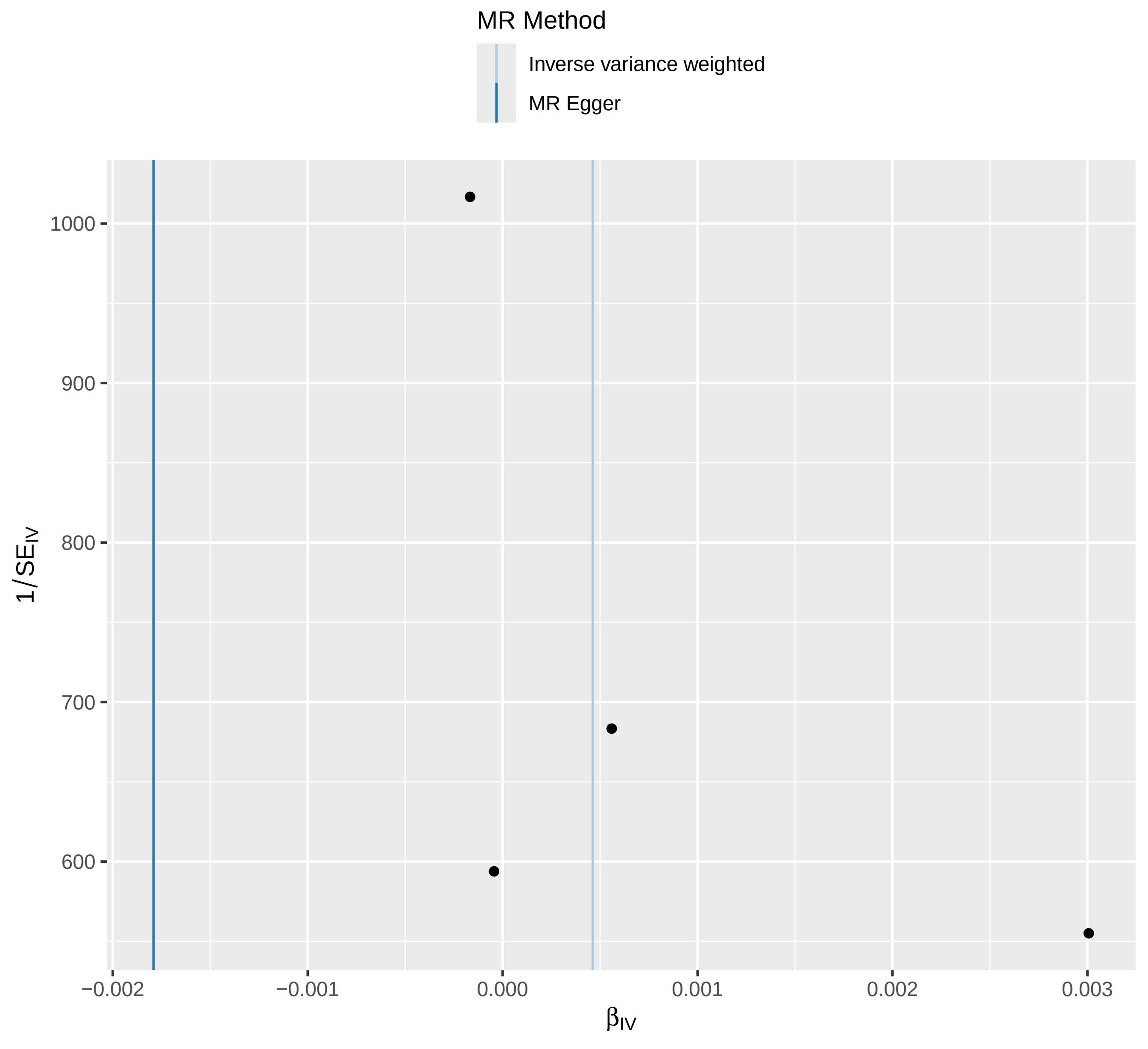
MR Method
Inverse variance weighted
MR Egger
1000
900
800
700
600
1/SE_IV
−0.002
−0.001
0.000
0.001
0.002
0.003
β_IV

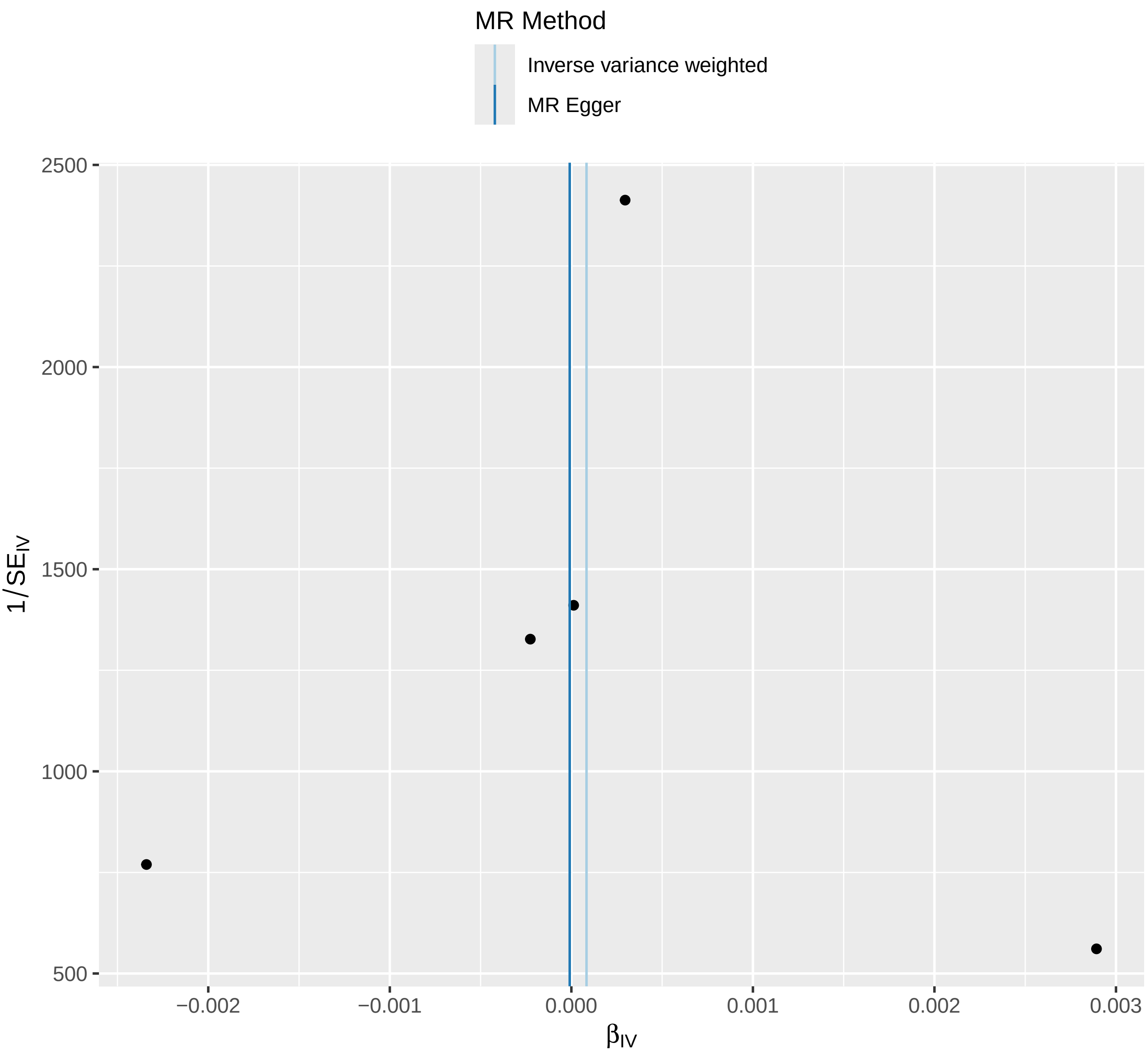
MR Method
Inverse variance weighted
MR Egger
2500
2000
1500
1000
500
1/SE_IV
−0.002
−0.001
0.000
0.001
0.002
0.003
β_IV

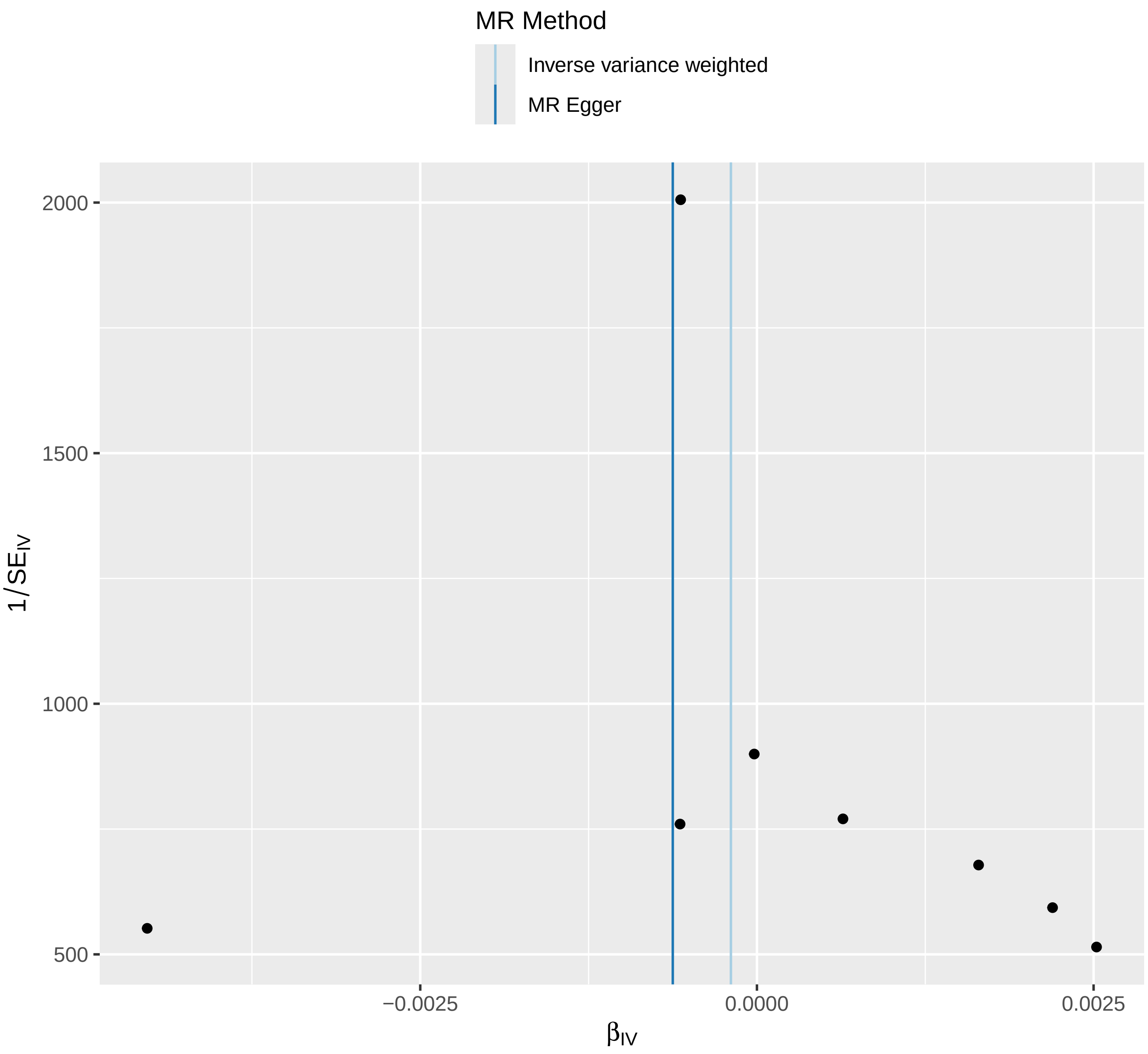
MR Method
Inverse variance weighted
MR Egger
2000
1500
1000
500
1/SE$_{IV}$
−0.0025
0.0000
0.0025
β_{IV}

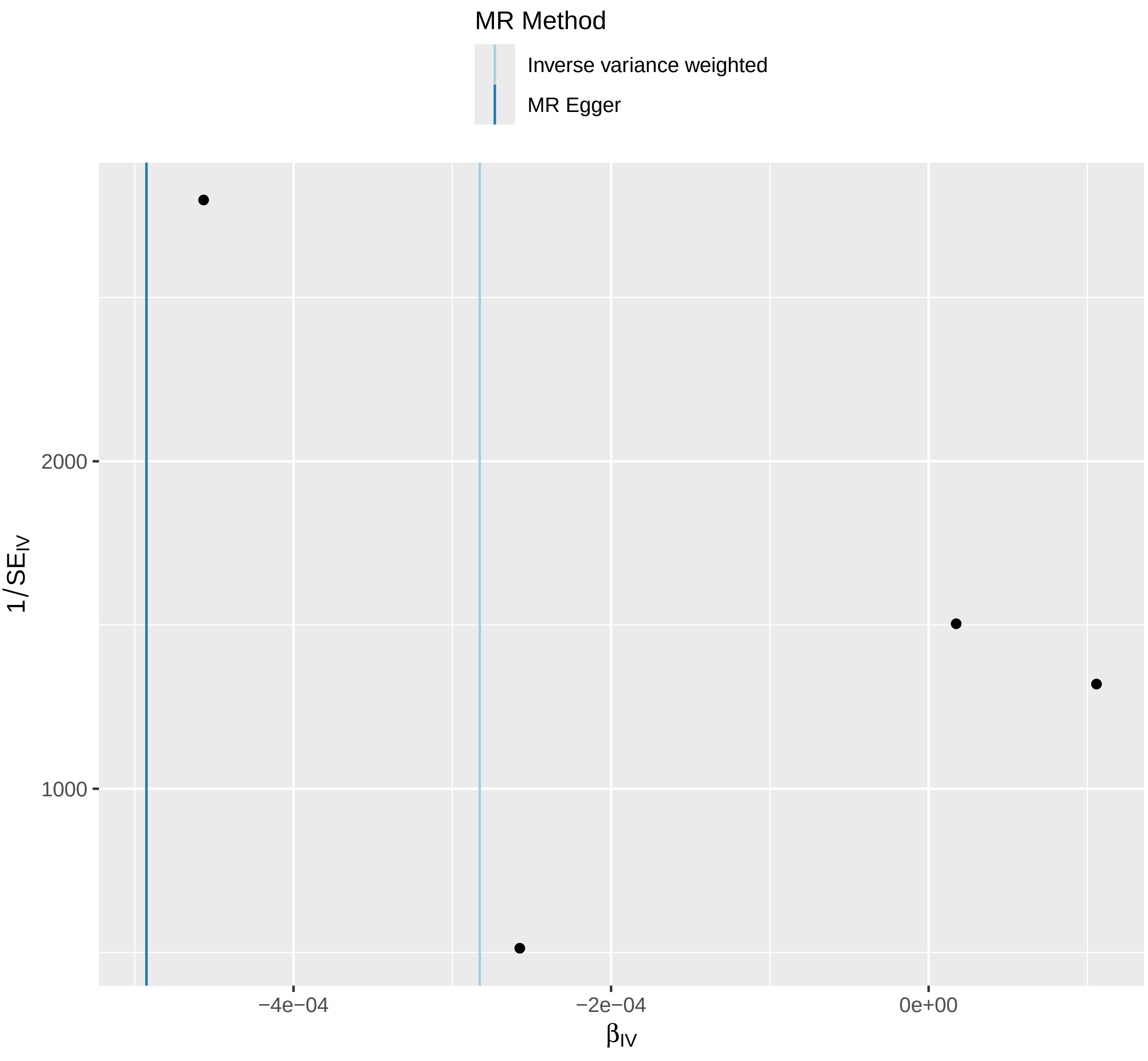
MR Method
Inverse variance weighted
MR Egger
2000
1000
1/SE_IV
−4e−04
−2e−04
0e+00
β_IV

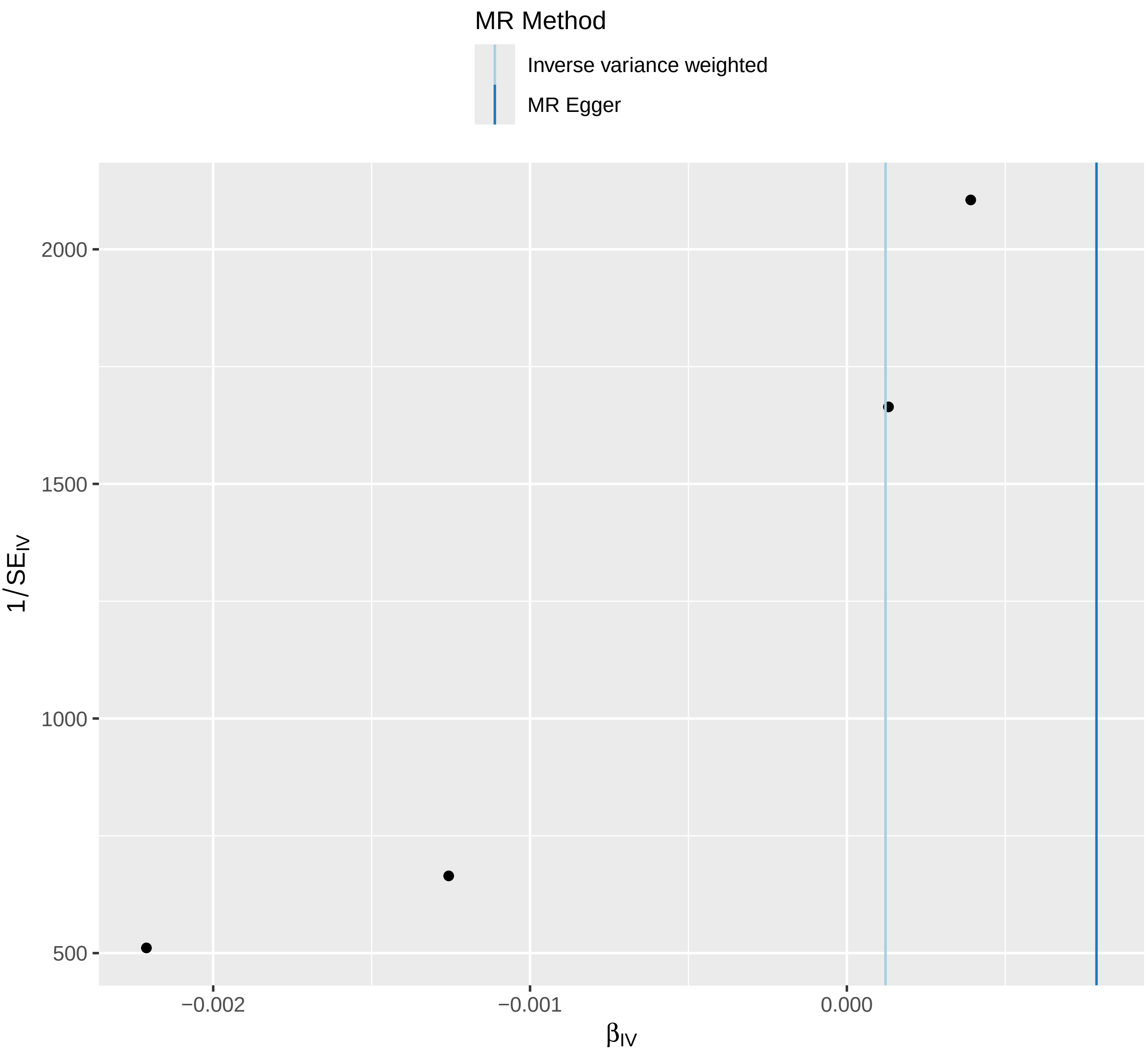
MR Method
Inverse variance weighted
MR Egger
2000
1500
1000
500
1/SE$_{IV}$
−0.002
−0.001
0.000
β$_{IV}$

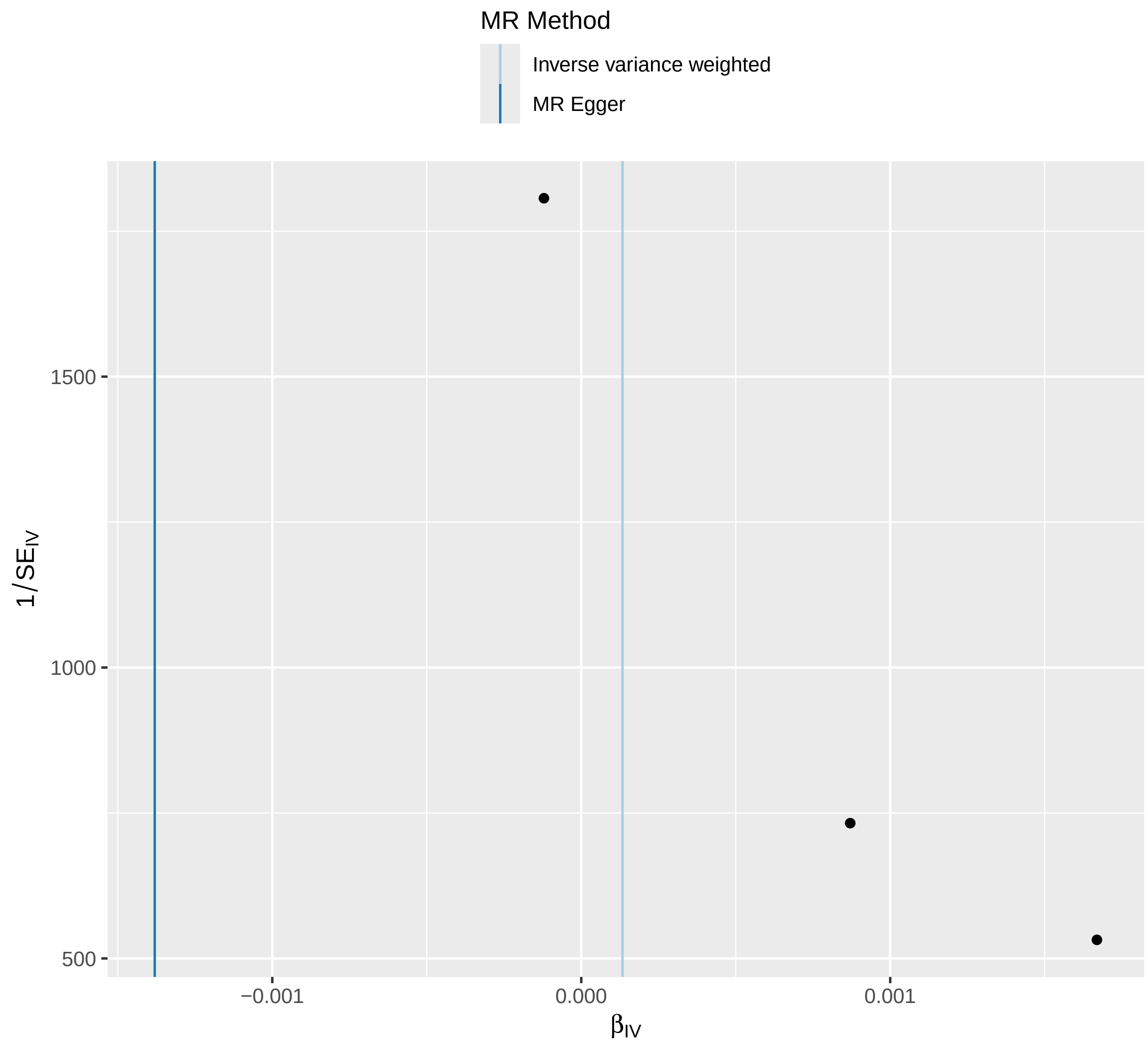

MR Method
Inverse variance weighted
MR Egger
1500
1000
500
1/SE_IV
−0.001
0.000
0.001
β_IV

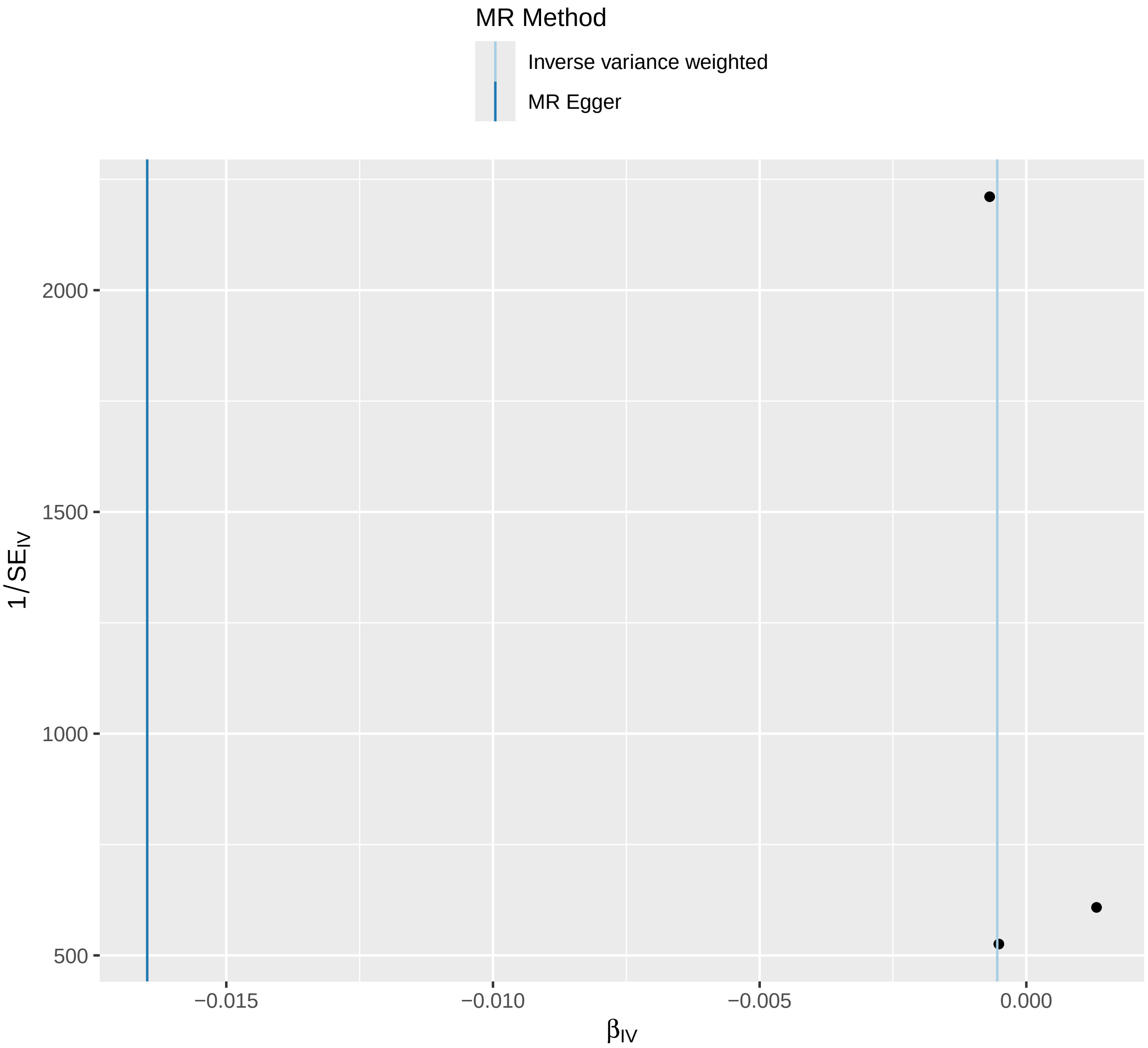

MR Method
Inverse variance weighted
MR Egger
2000
1500
1000
500
1/SE_IV
−0.015
−0.010
−0.005
0.000
β_IV

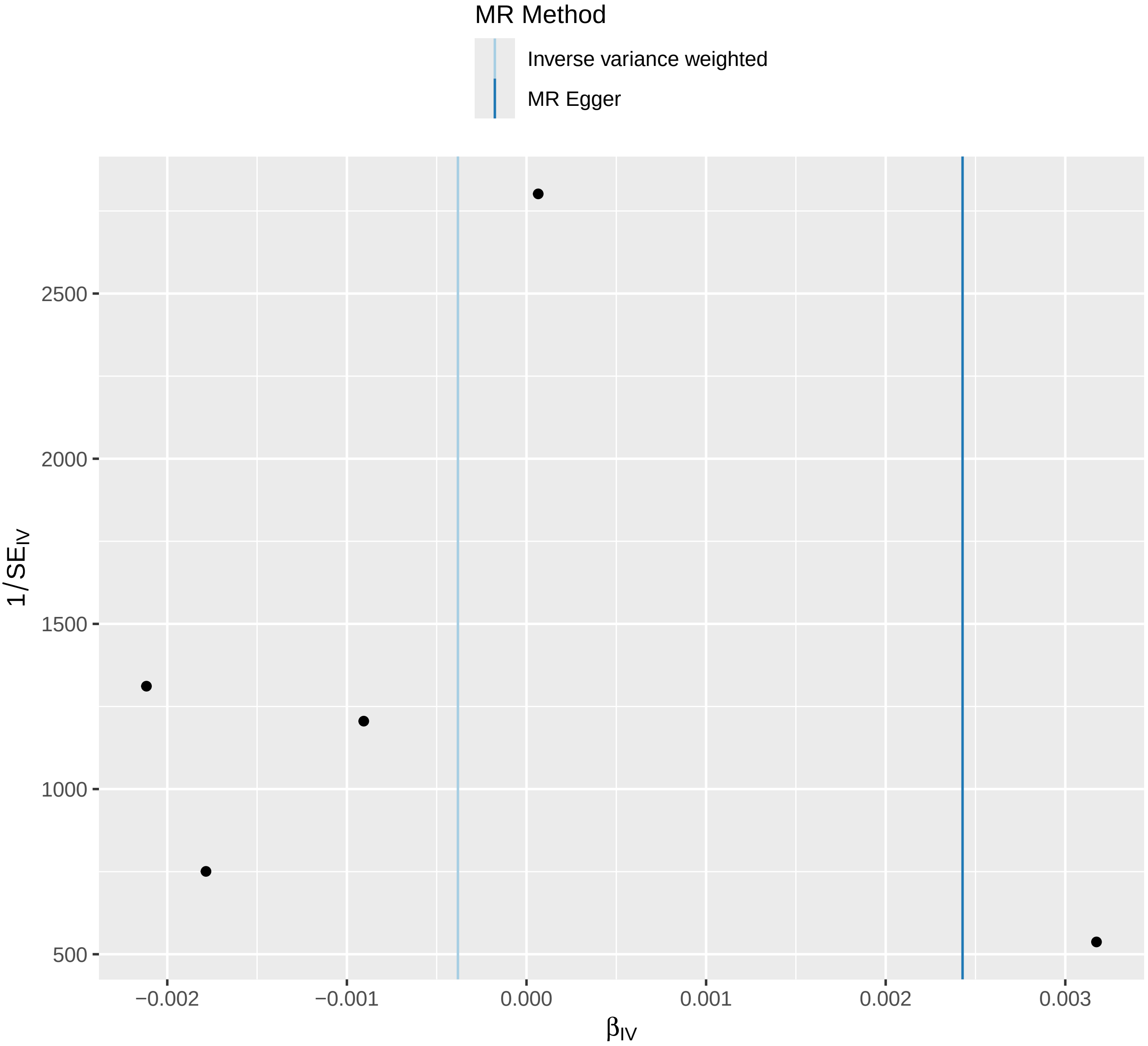
MR Method
Inverse variance weighted
MR Egger
2500
2000
1500
1000
500
1/SE_IV
−0.002
−0.001
0.000
0.001
0.002
0.003
β_IV

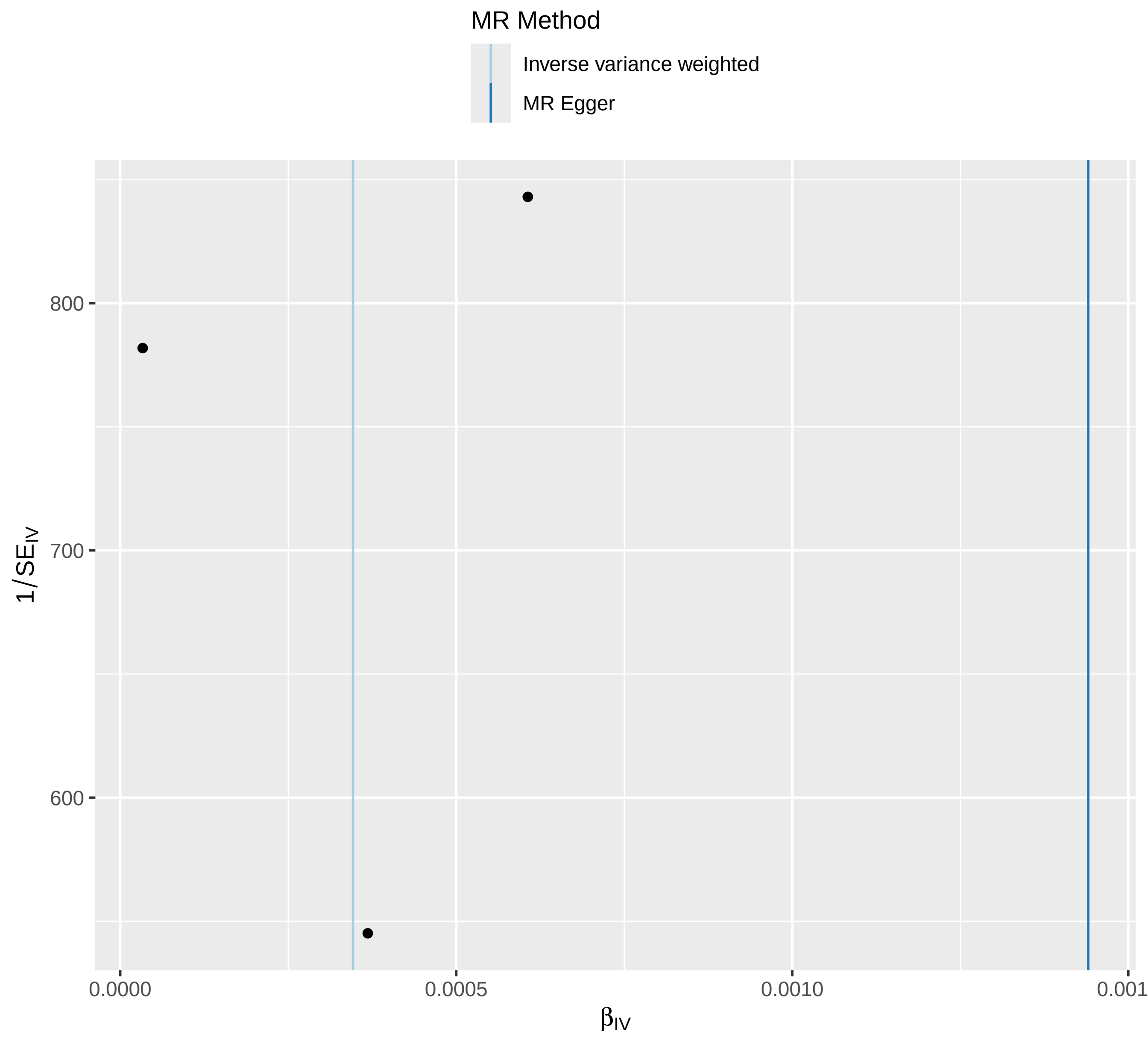
MR Method
Inverse variance weighted
MR Egger
800
700
600
1/SE$_{IV}$
0.0000
0.0005
0.0010
0.001
β_{IV}

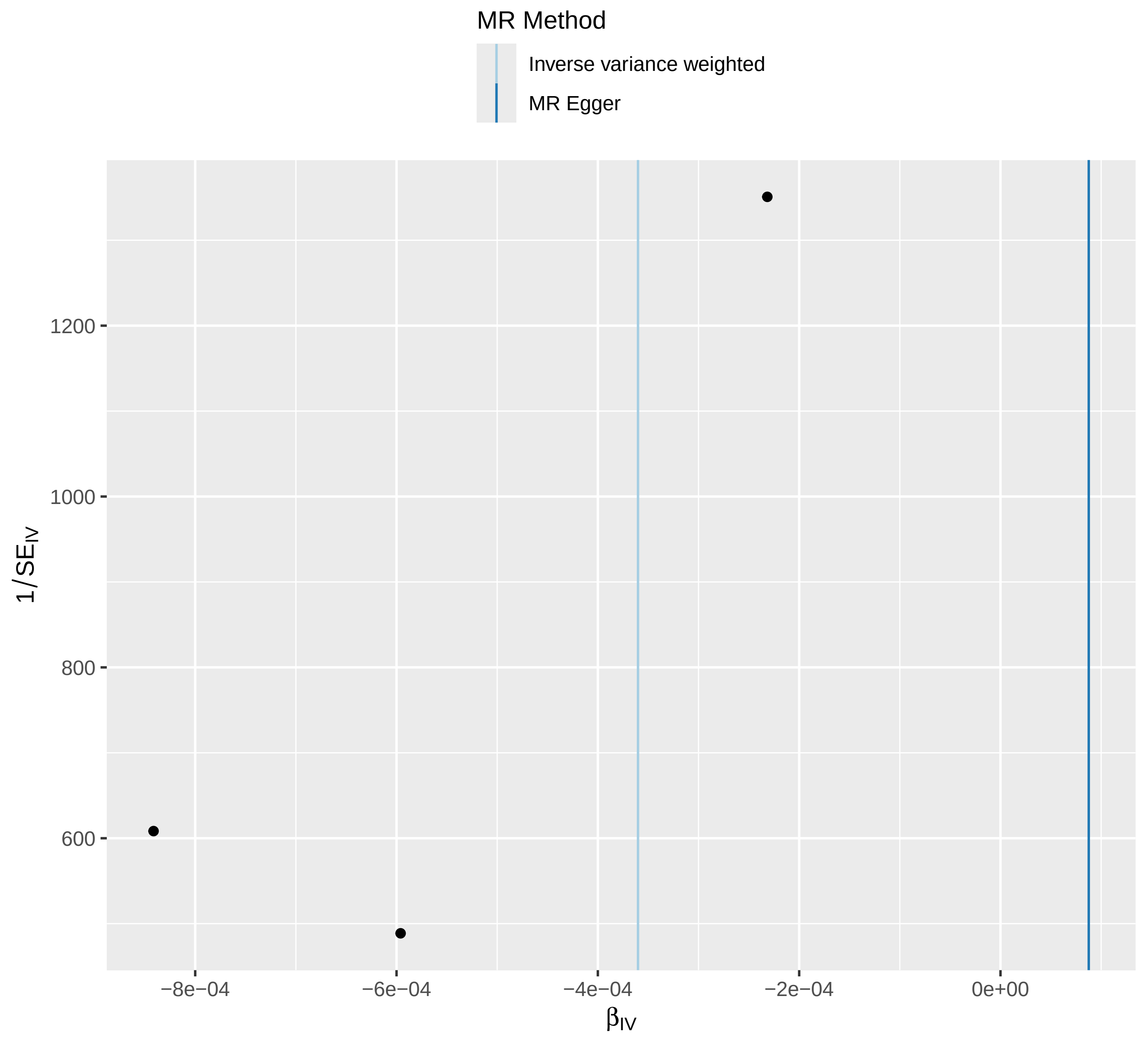
MR Method
Inverse variance weighted
MR Egger
1200
1000
800
600
1/SE$_{IV}$
−8e−04
−6e−04
−4e−04
−2e−04
0e+00
β$_{IV}$

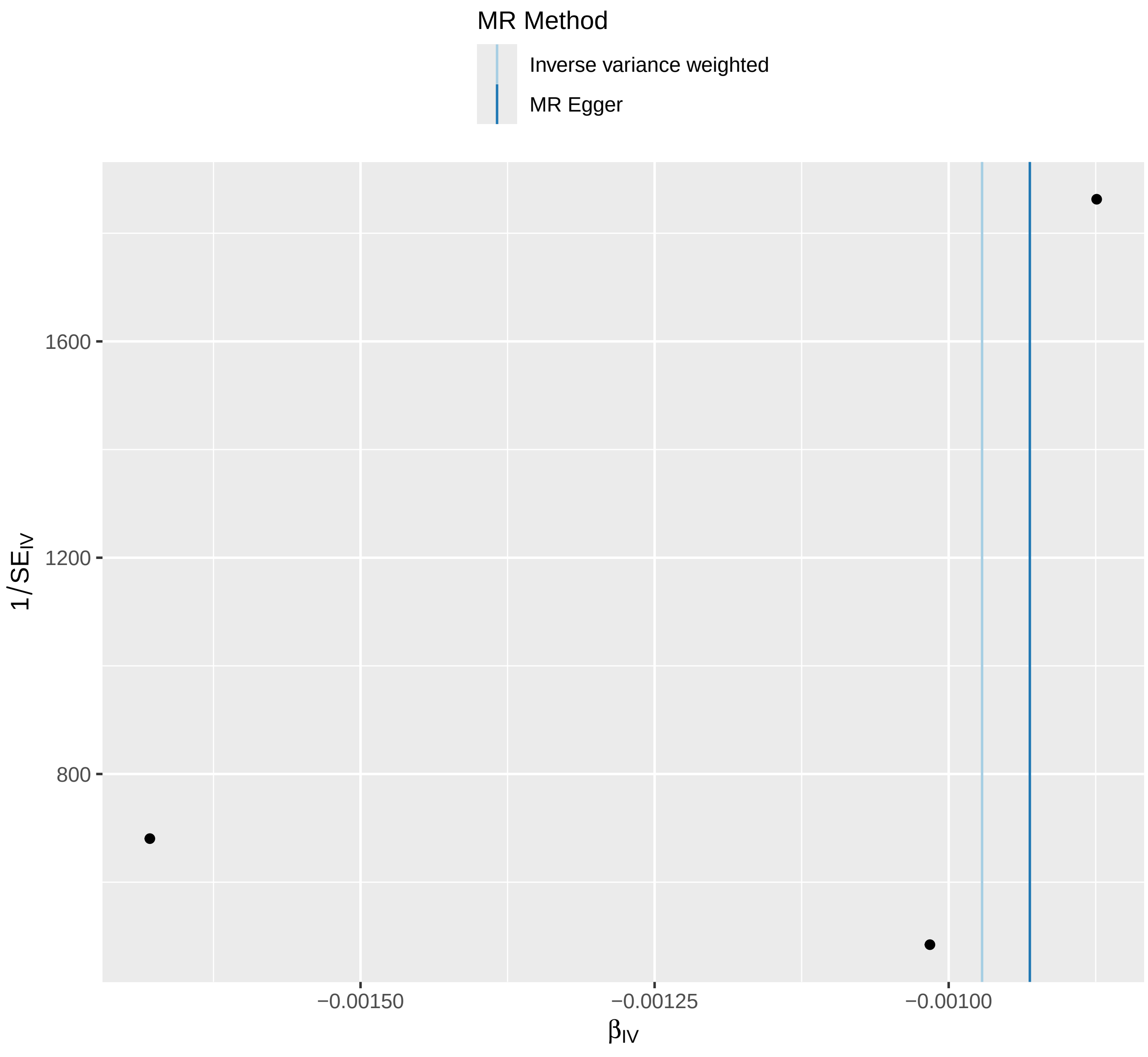
MR Method
Inverse variance weighted
MR Egger
1600
1200
800
1/SE_IV
−0.00150
−0.00125
−0.00100
β_IV

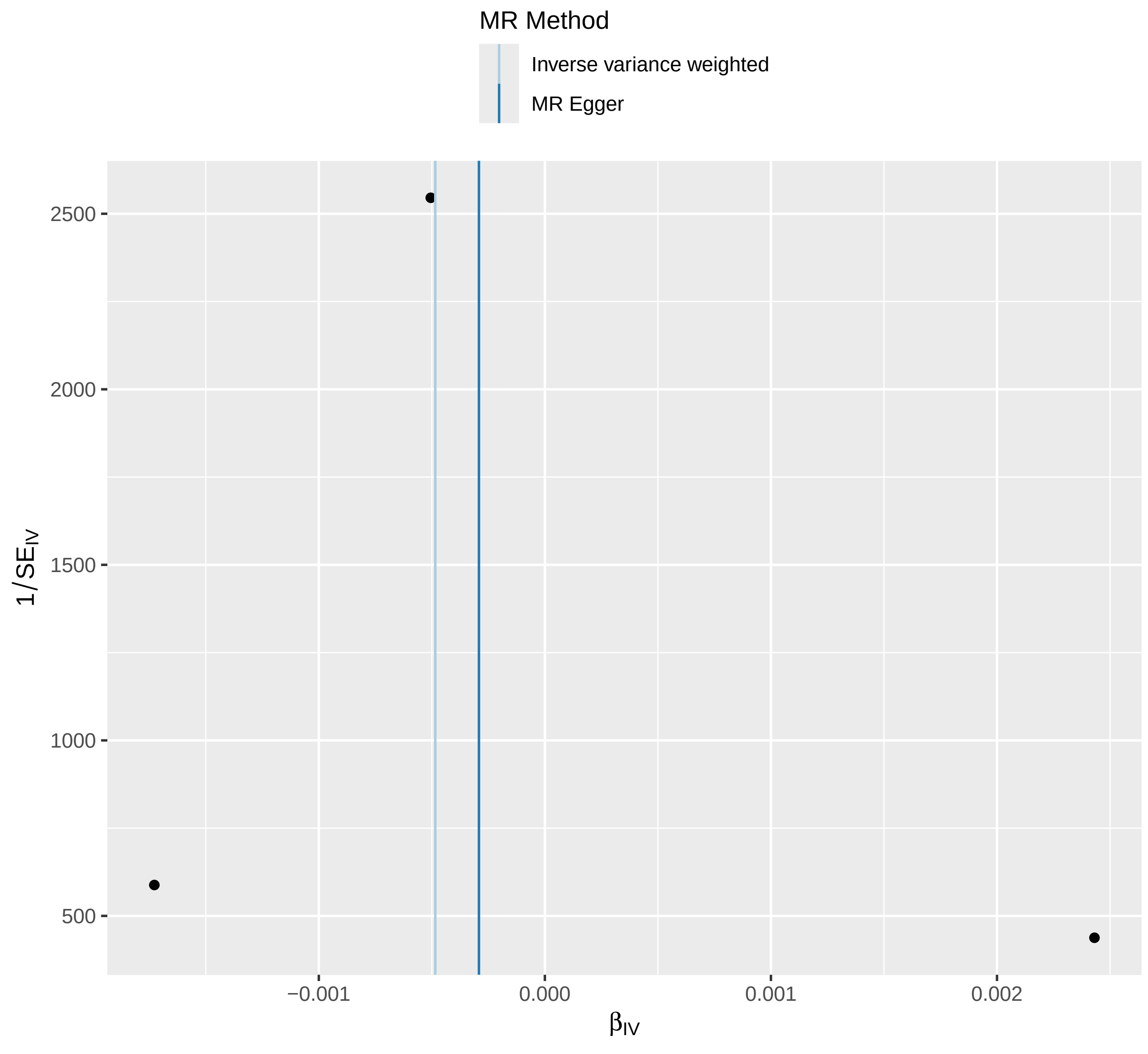

MR Method
Inverse variance weighted
MR Egger
2500
2000
1500
1000
500
1/SE_IV
−0.001
0.000
0.001
0.002
β_IV

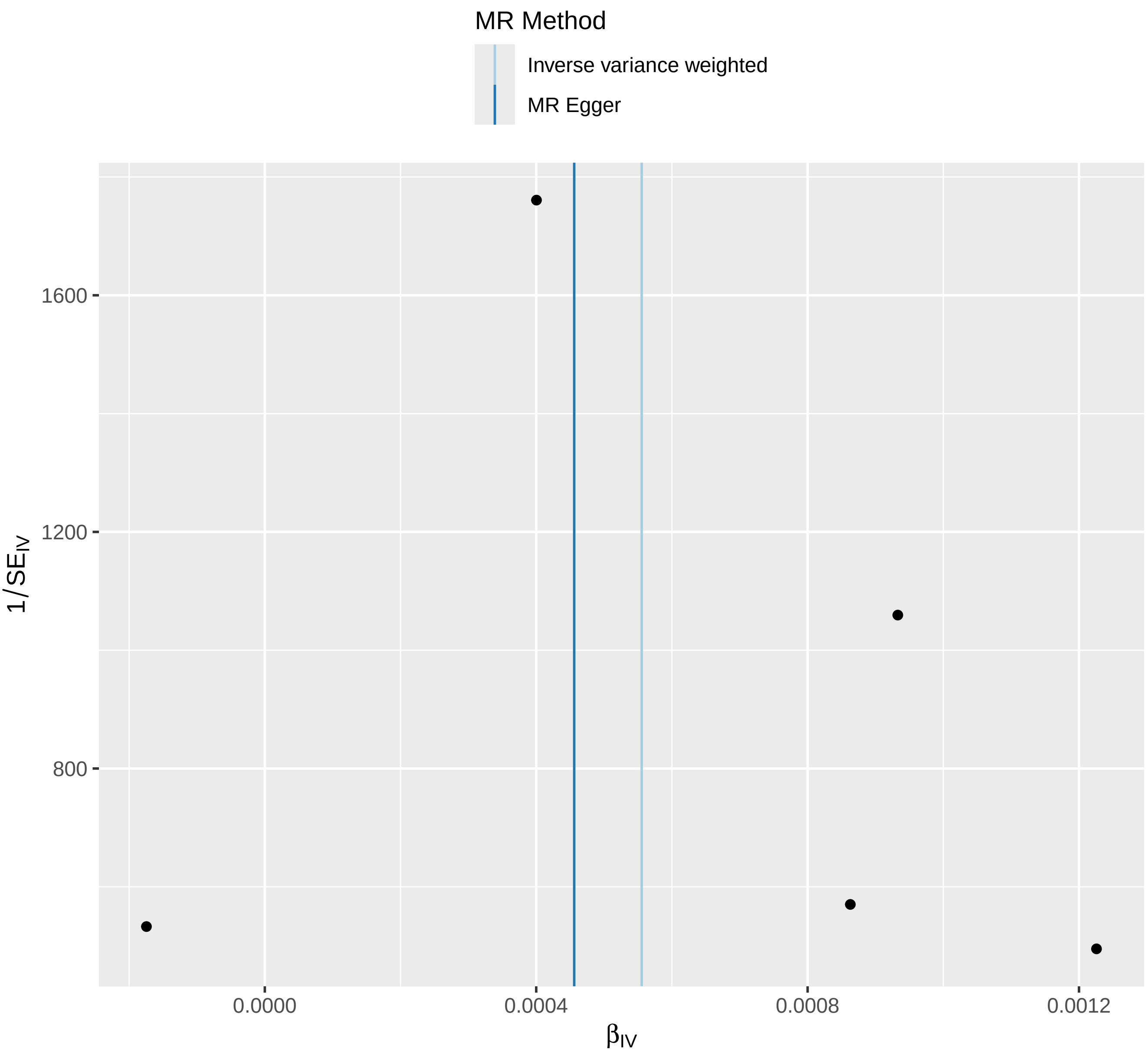

MR Method
Inverse variance weighted
MR Egger
1600
1200
800
1/SE_IV
0.0000
0.0004
0.0008
0.0012
β_IV

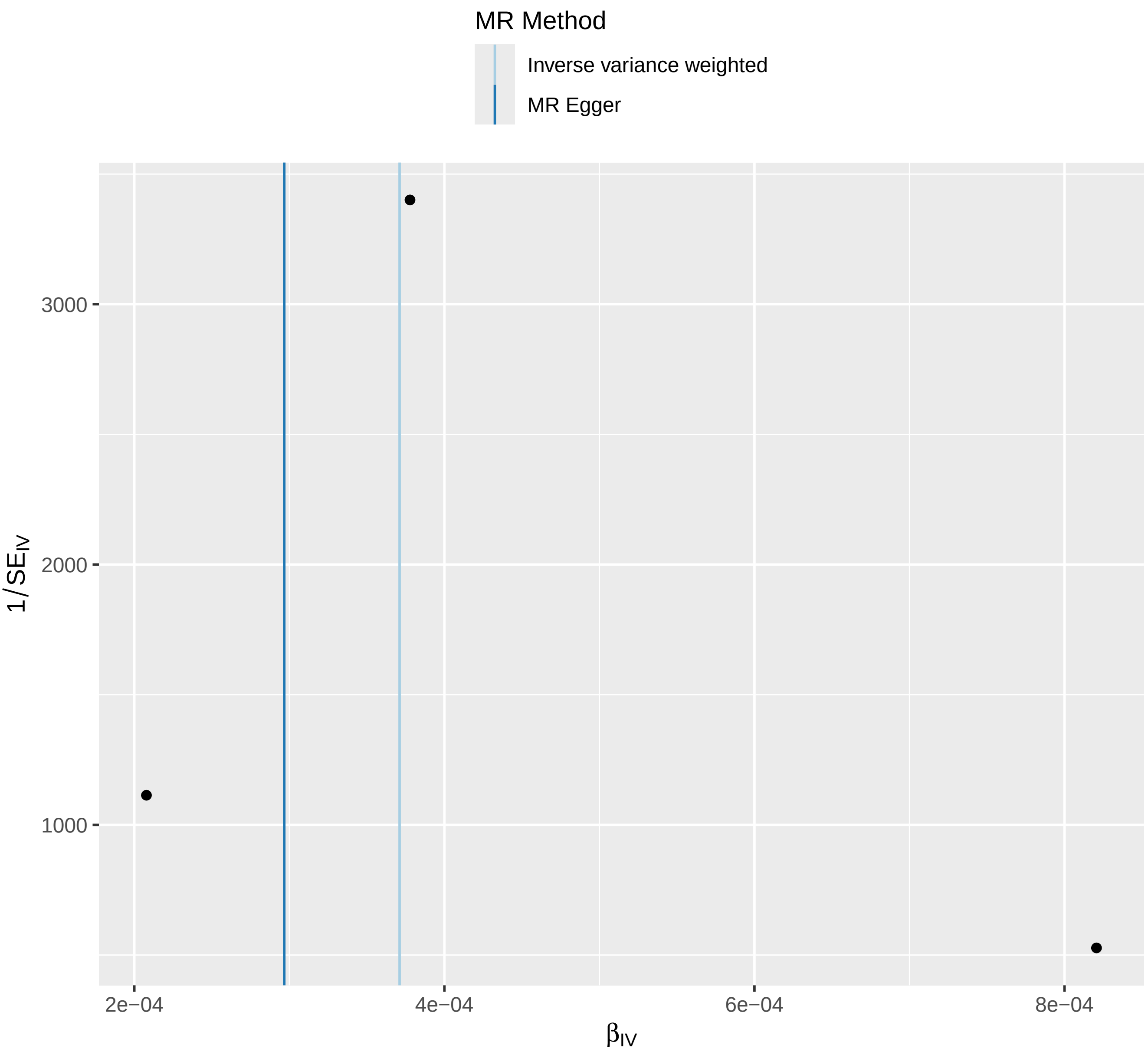
MR Method
Inverse variance weighted
MR Egger
3000
2000
1000
1/SE_IV
2e−04
4e−04
6e−04
8e−04
β_IV

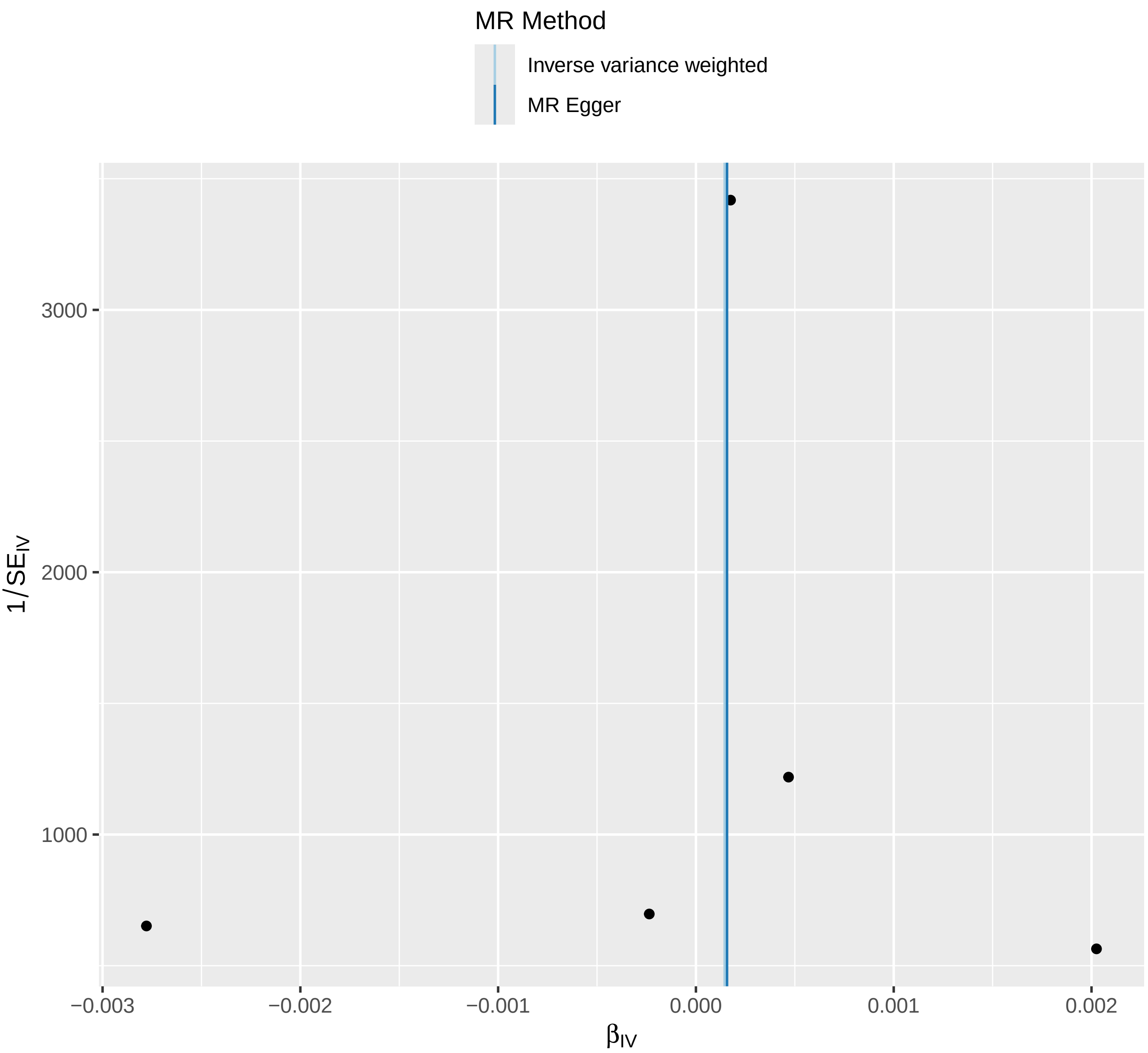
MR Method
Inverse variance weighted
MR Egger
3000
2000
1000
1/SE$_{IV}$
−0.003
−0.002
−0.001
0.000
0.001
0.002
β$_{IV}$

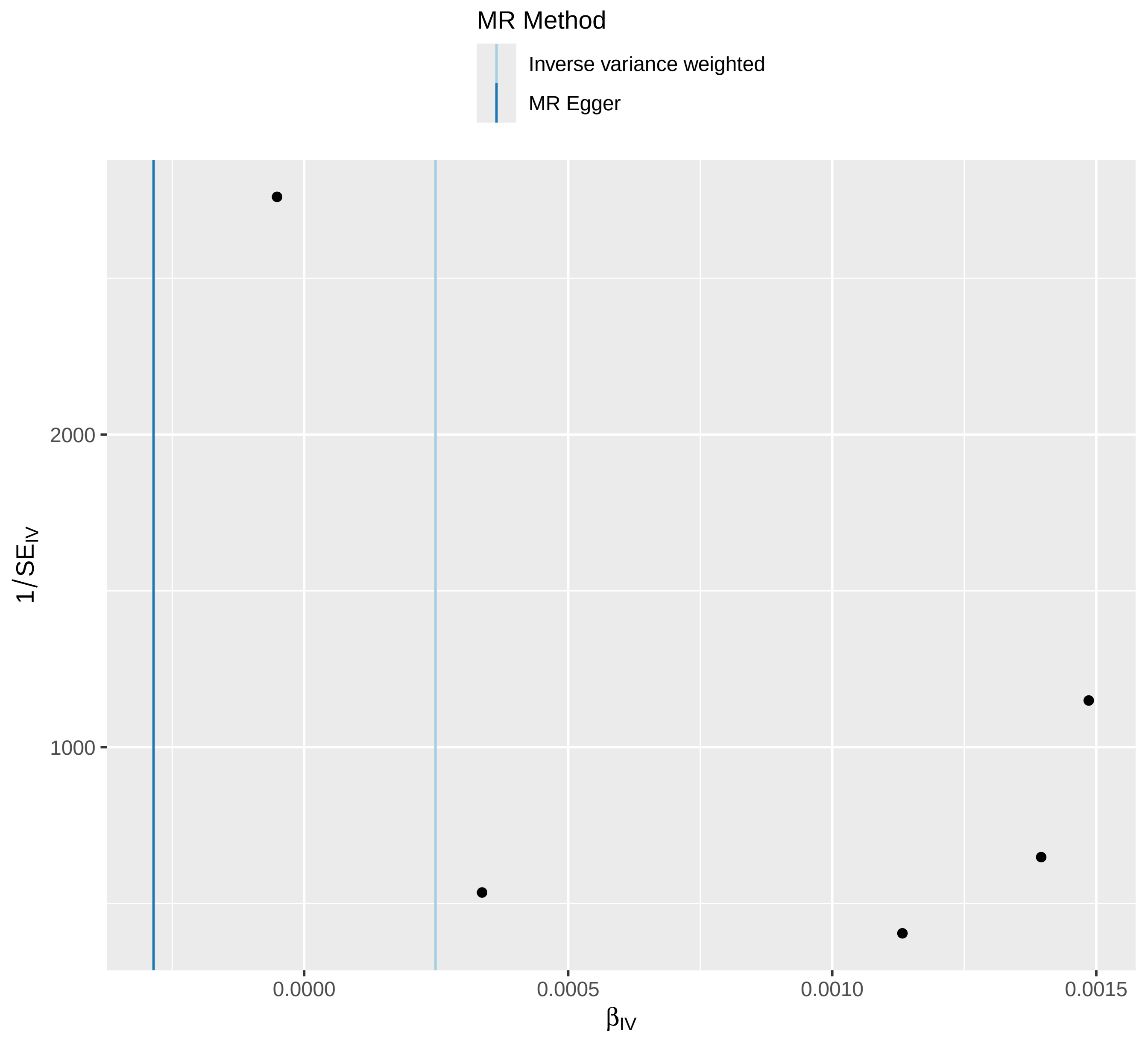

MR Method
Inverse variance weighted
MR Egger
2000
1000
1/SE_IV
0.0000
0.0005
0.0010
0.0015
β_IV

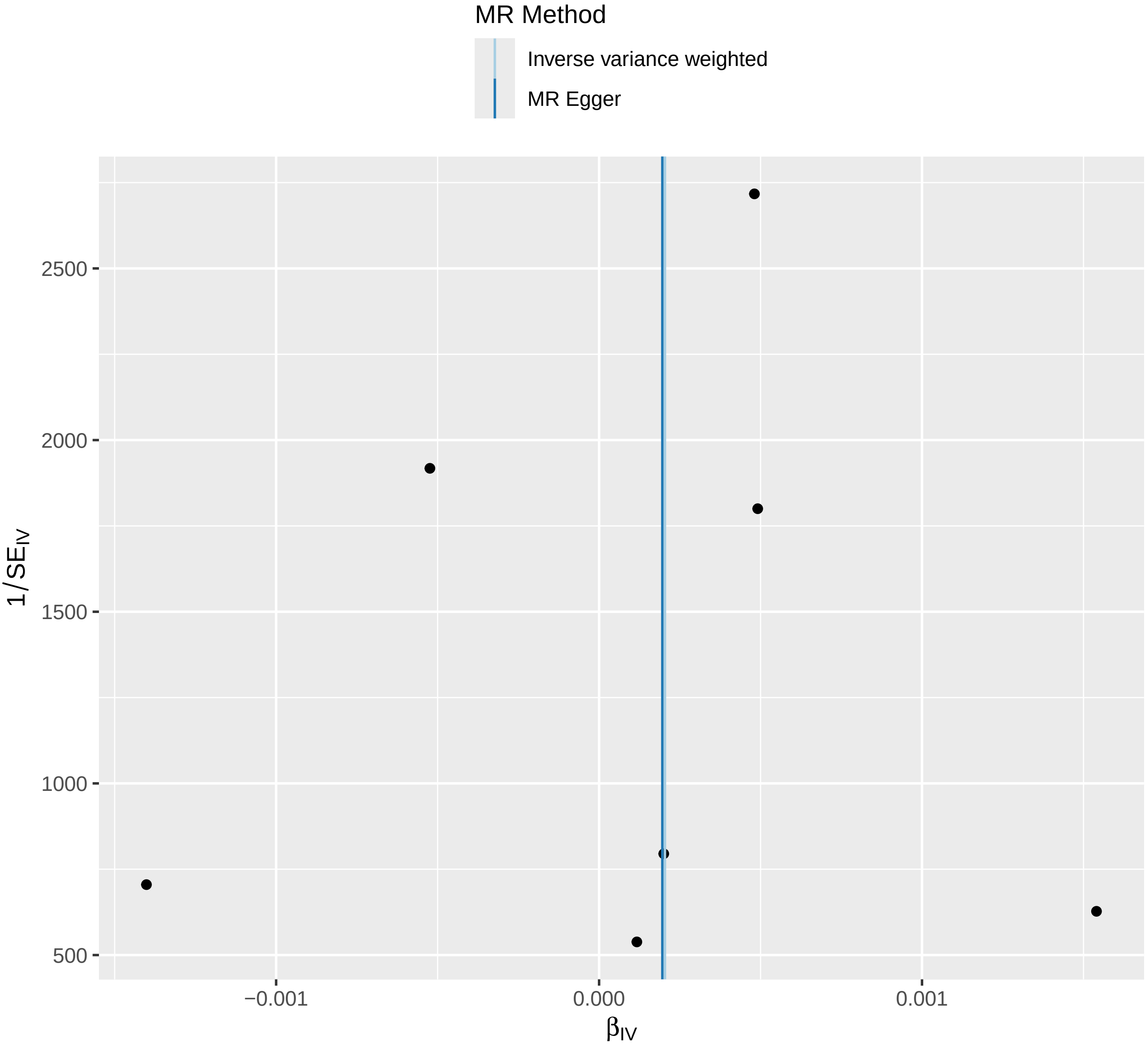
MR Method
Inverse variance weighted
MR Egger
2500
2000
1500
1000
500
1/SE_IV
−0.001
0.000
0.001
β_IV

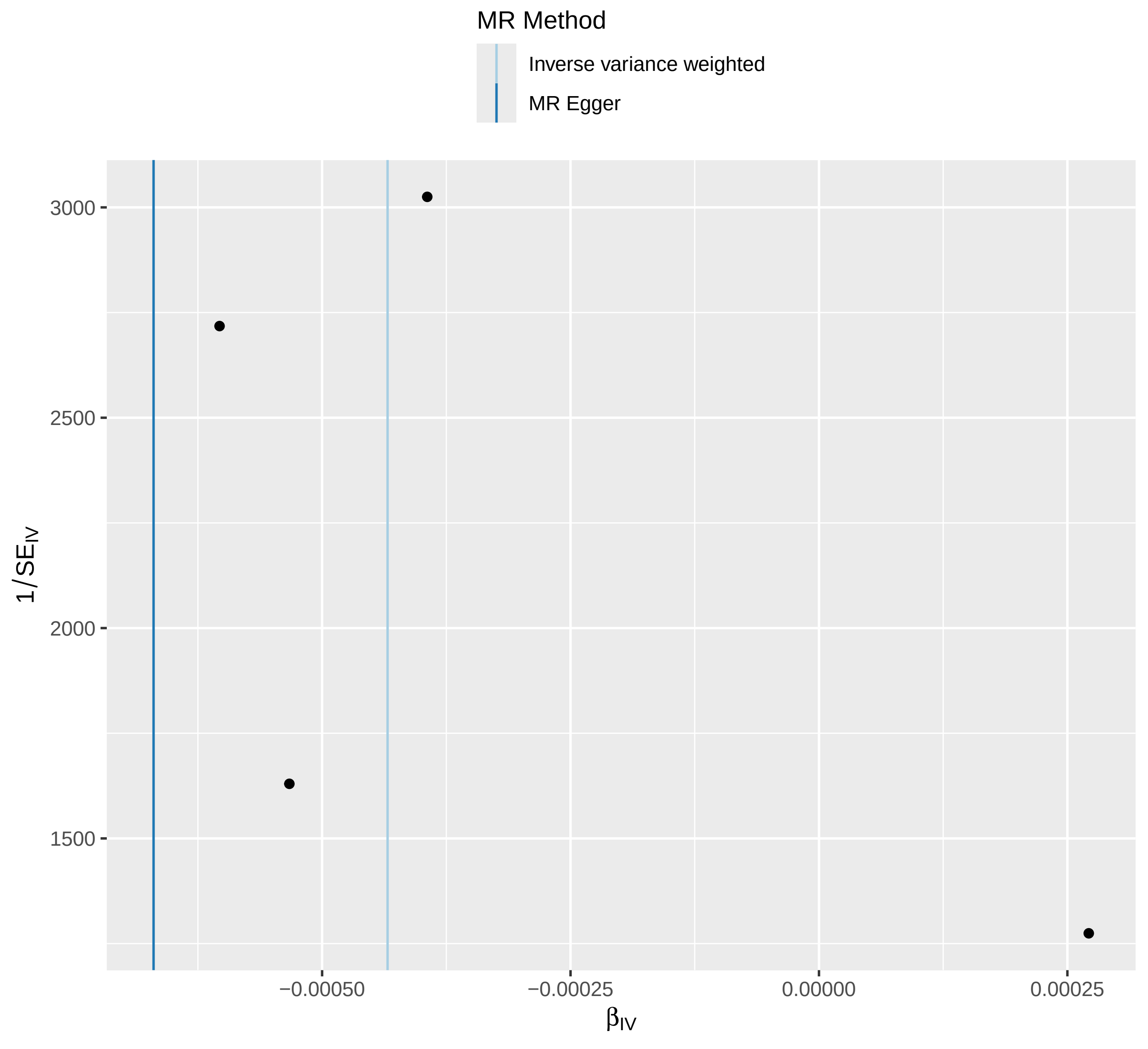

MR Method
Inverse variance weighted
MR Egger
3000
2500
2000
1500
1/SE_IV
−0.00050
−0.00025
0.00000
0.00025
β_IV

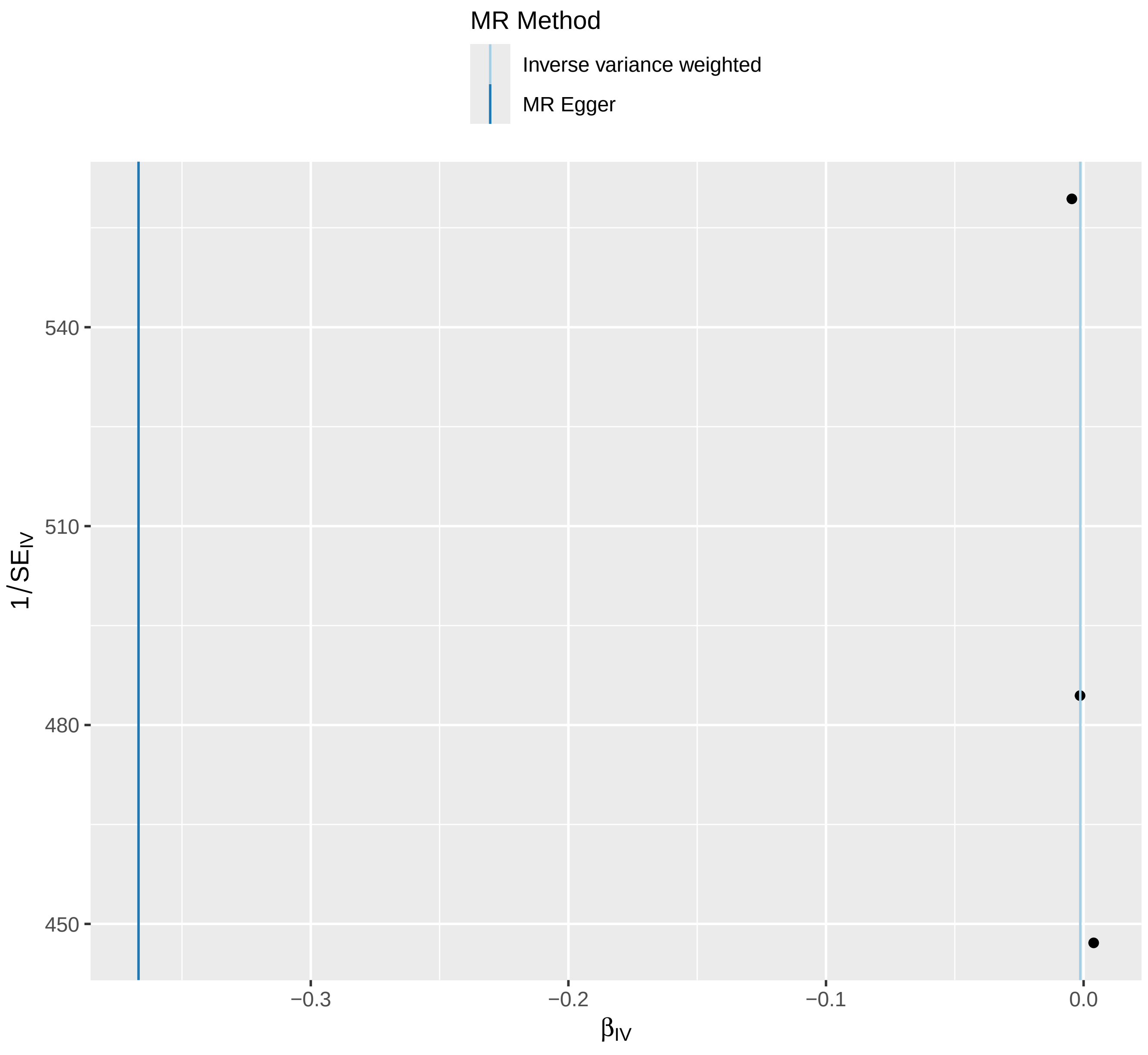

MR Method
Inverse variance weighted
MR Egger
540
510
480
450
$1/SE_{IV}$
−0.3
−0.2
−0.1
0.0
β_{IV}

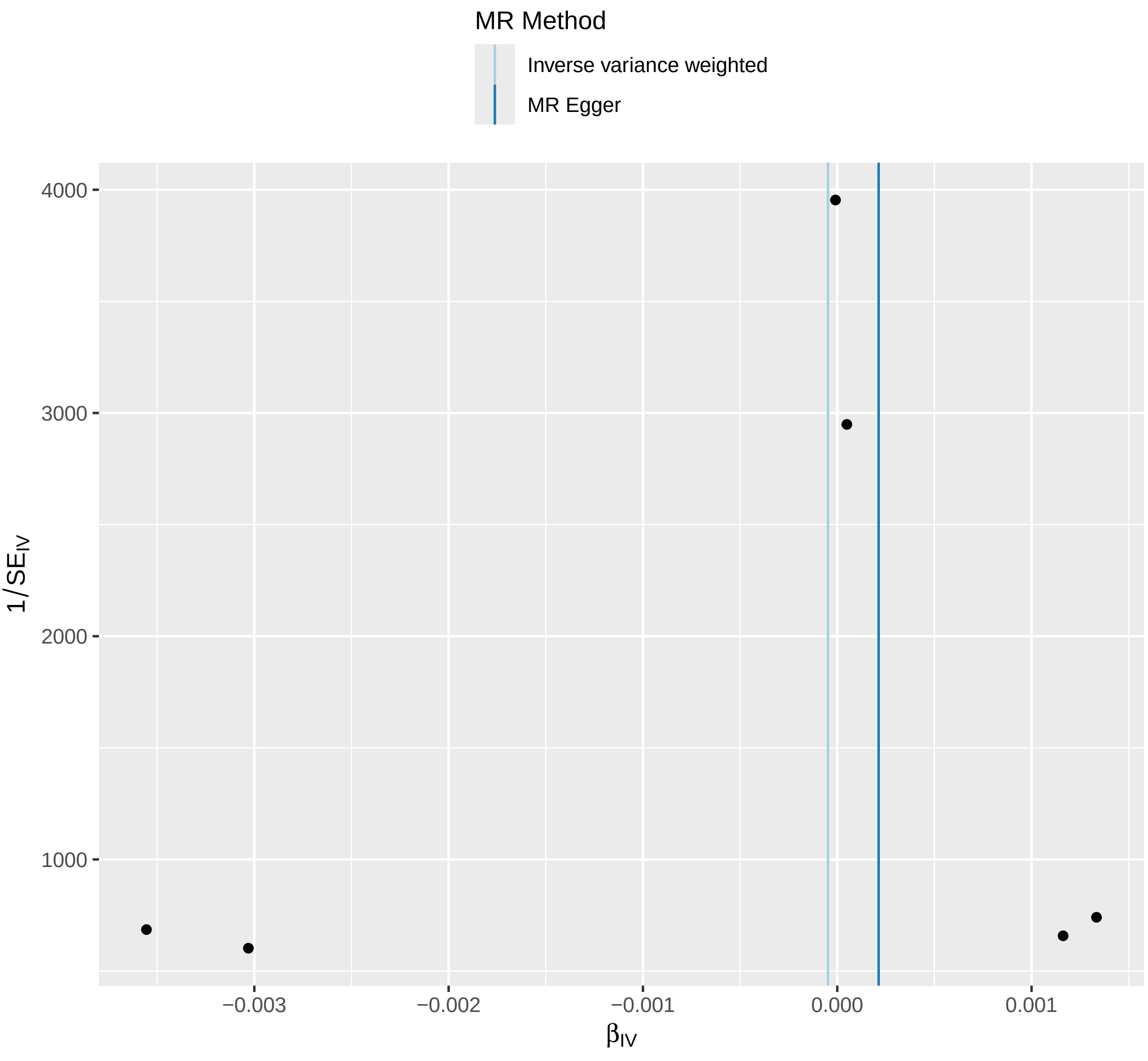
MR Method
Inverse variance weighted
MR Egger
4000
3000
2000
1000
1/SE_IV
−0.003
−0.002
−0.001
0.000
0.001
β_IV

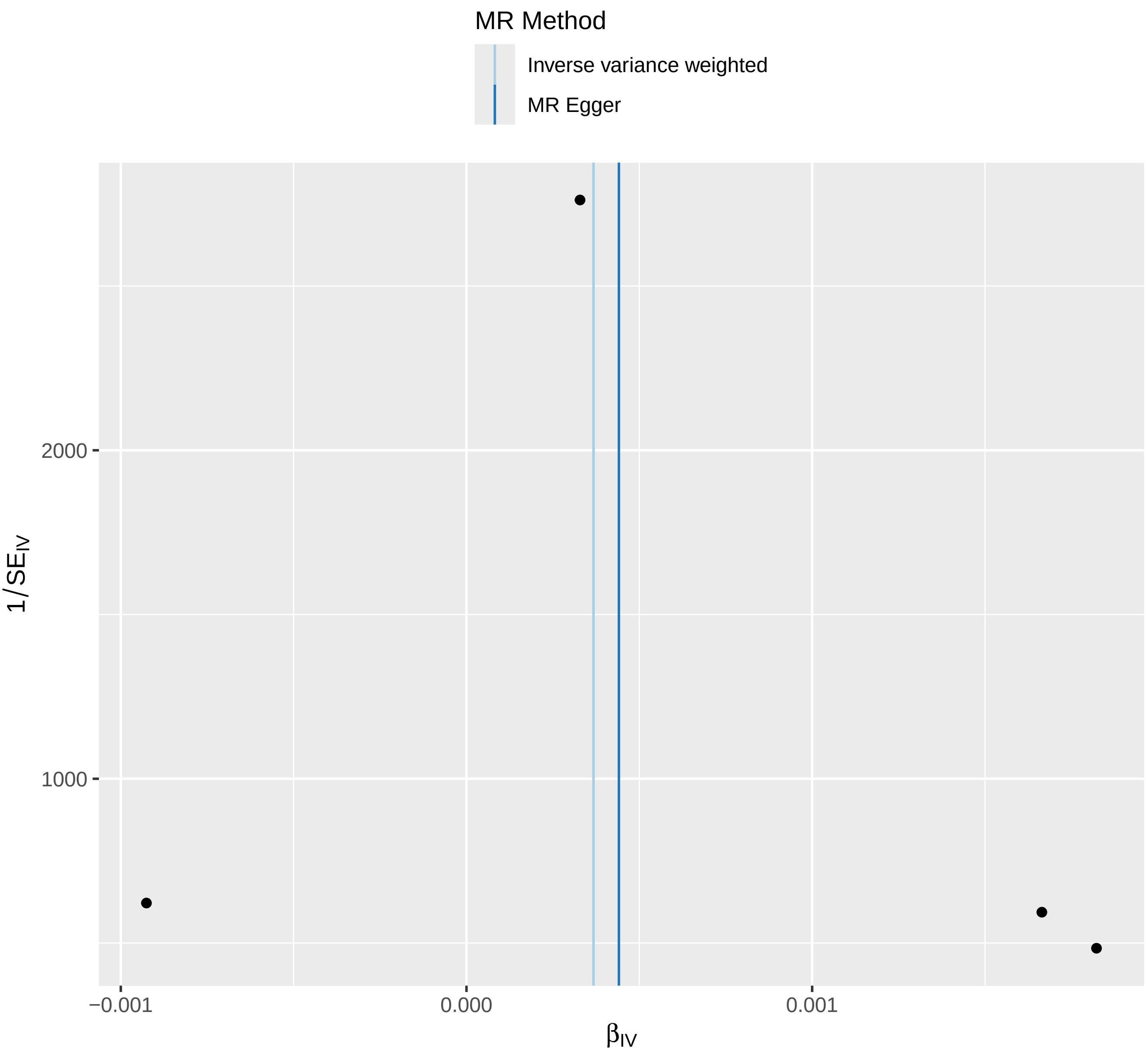
MR Method
Inverse variance weighted
MR Egger
2000
1000
1/SE_IV
−0.001
0.000
0.001
β_IV

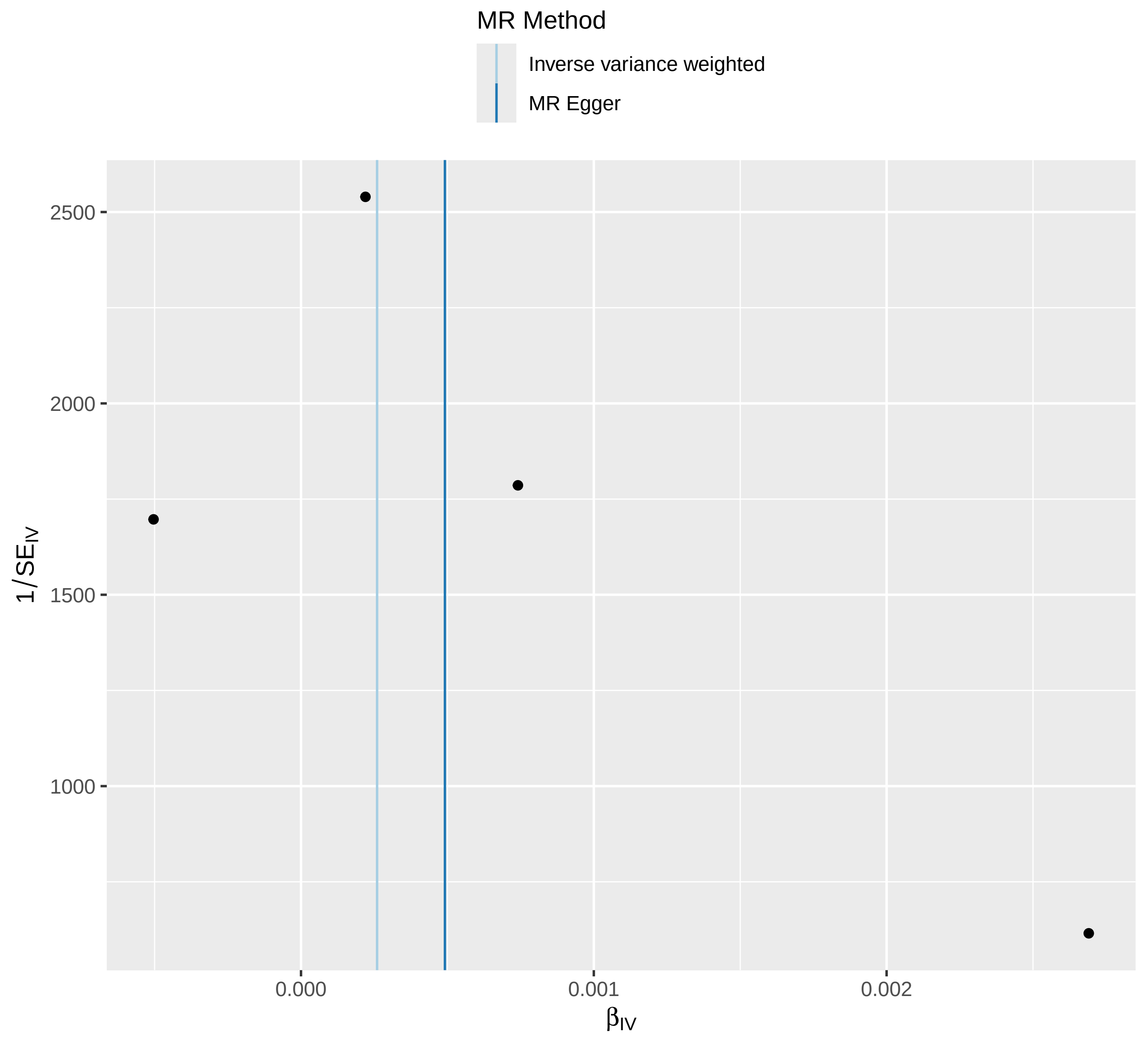
MR Method
Inverse variance weighted
MR Egger
2500
2000
1500
1000
1/SE_IV
0.000
0.001
0.002
β_IV

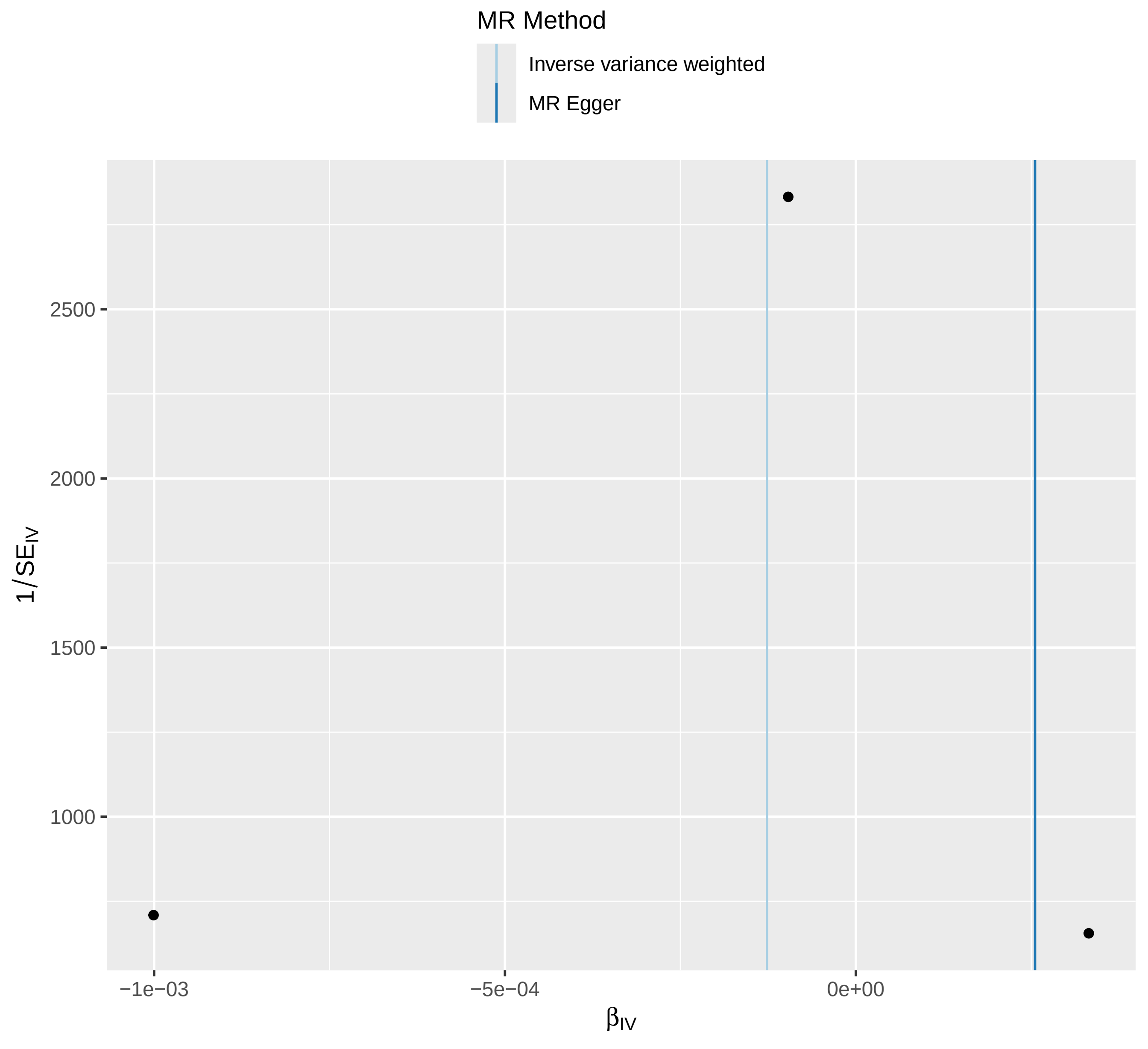

MR Method
Inverse variance weighted
MR Egger
2500
2000
1500
1000
1/SE$_{IV}$
−1e−03
−5e−04
0e+00
β$_{IV}$

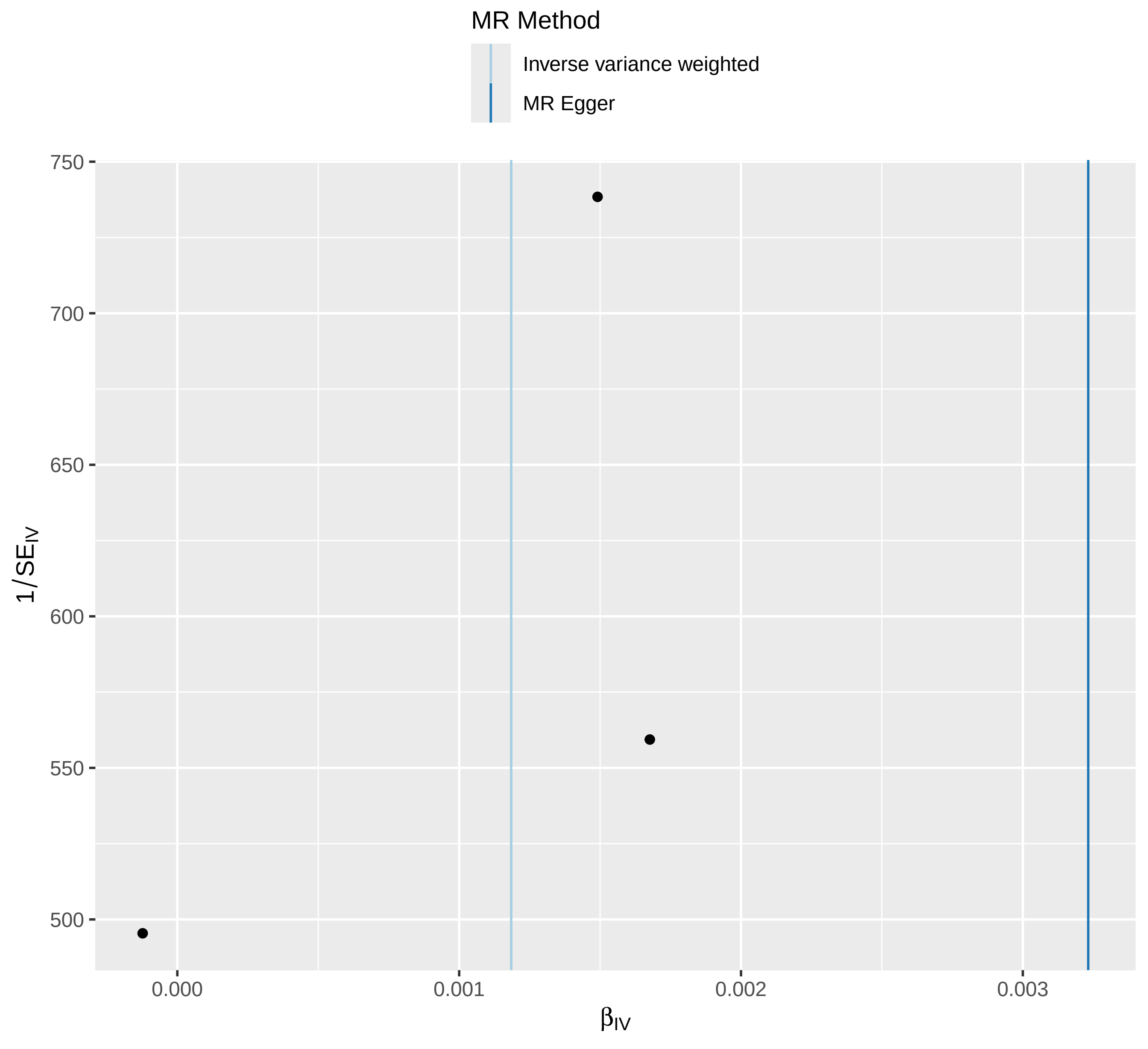

MR Method
Inverse variance weighted
MR Egger
750
700
650
600
550
500
0.000
0.001
0.002
0.003
$1/SE_{IV}$
β_{IV}

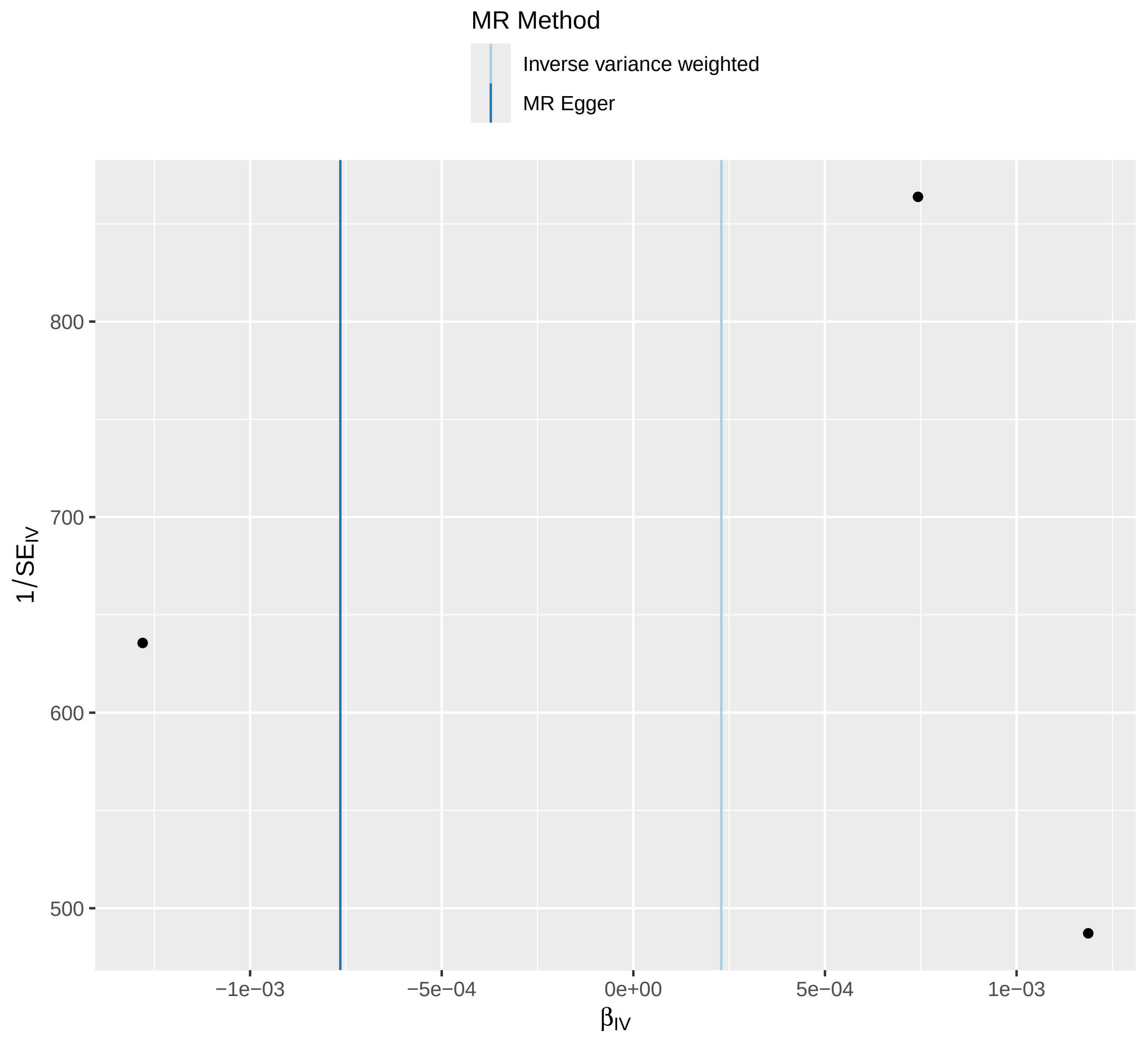
MR Method
Inverse variance weighted
MR Egger
800
700
600
500
$1/SE_{IV}$
−1e−03
−5e−04
0e+00
5e−04
1e−03
β_{IV}

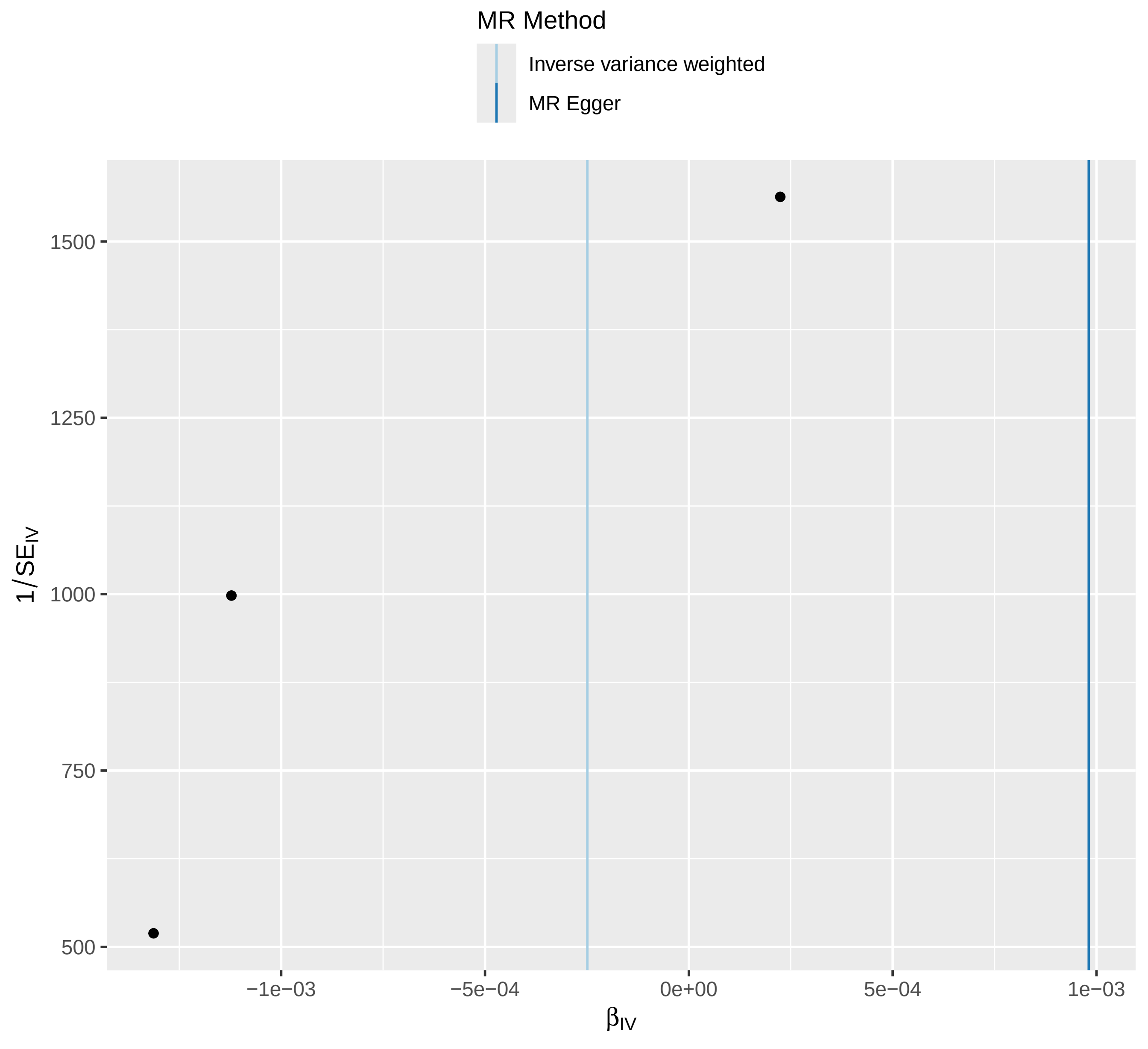
MR Method
Inverse variance weighted
MR Egger
1500
1250
1000
750
500
1/SE_IV
−1e−03
−5e−04
0e+00
5e−04
1e−03
β_IV

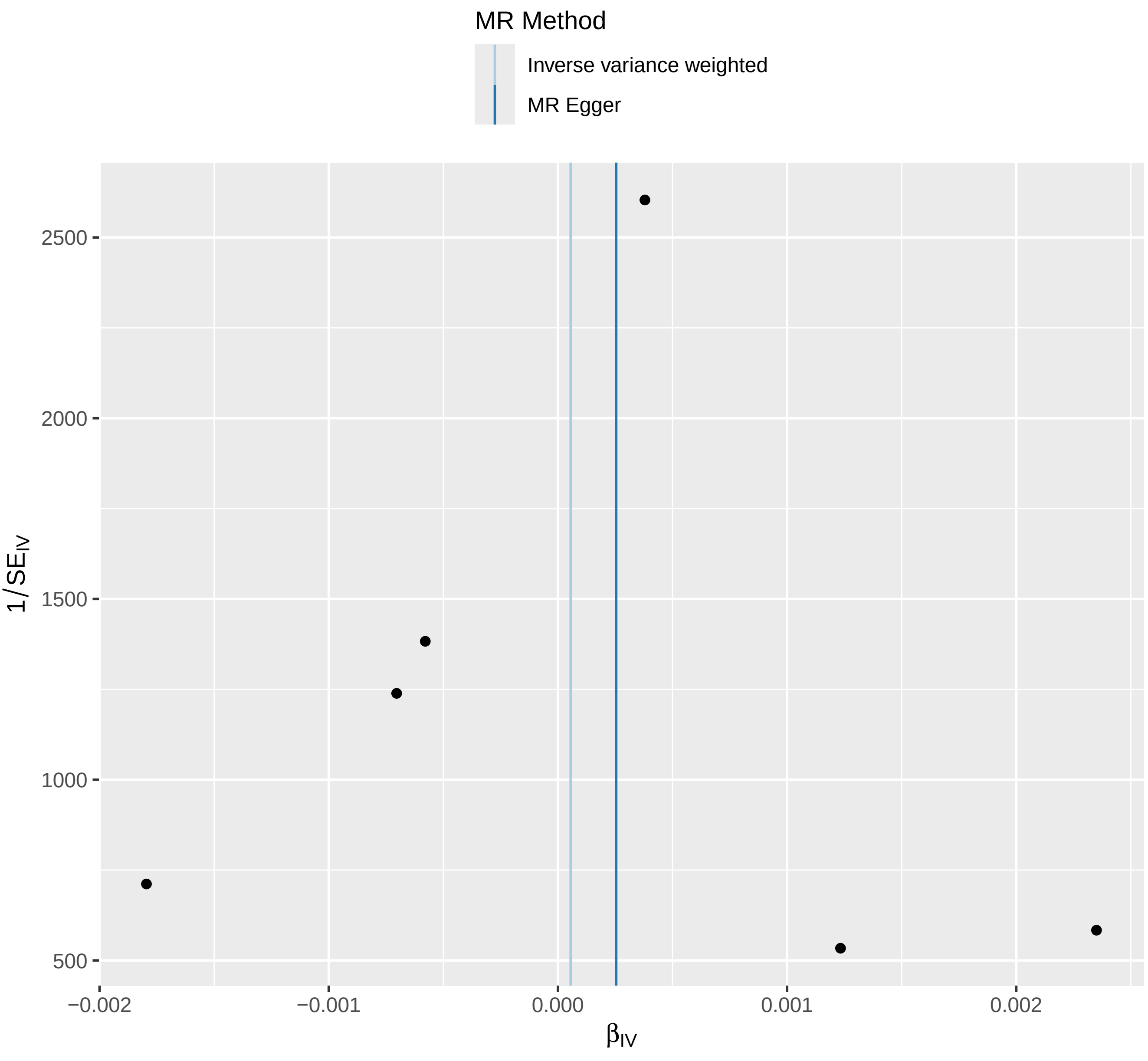
MR Method
Inverse variance weighted
MR Egger
2500
2000
1500
1000
500
$1/SE_{IV}$
−0.002
−0.001
0.000
0.001
0.002
β_{IV}

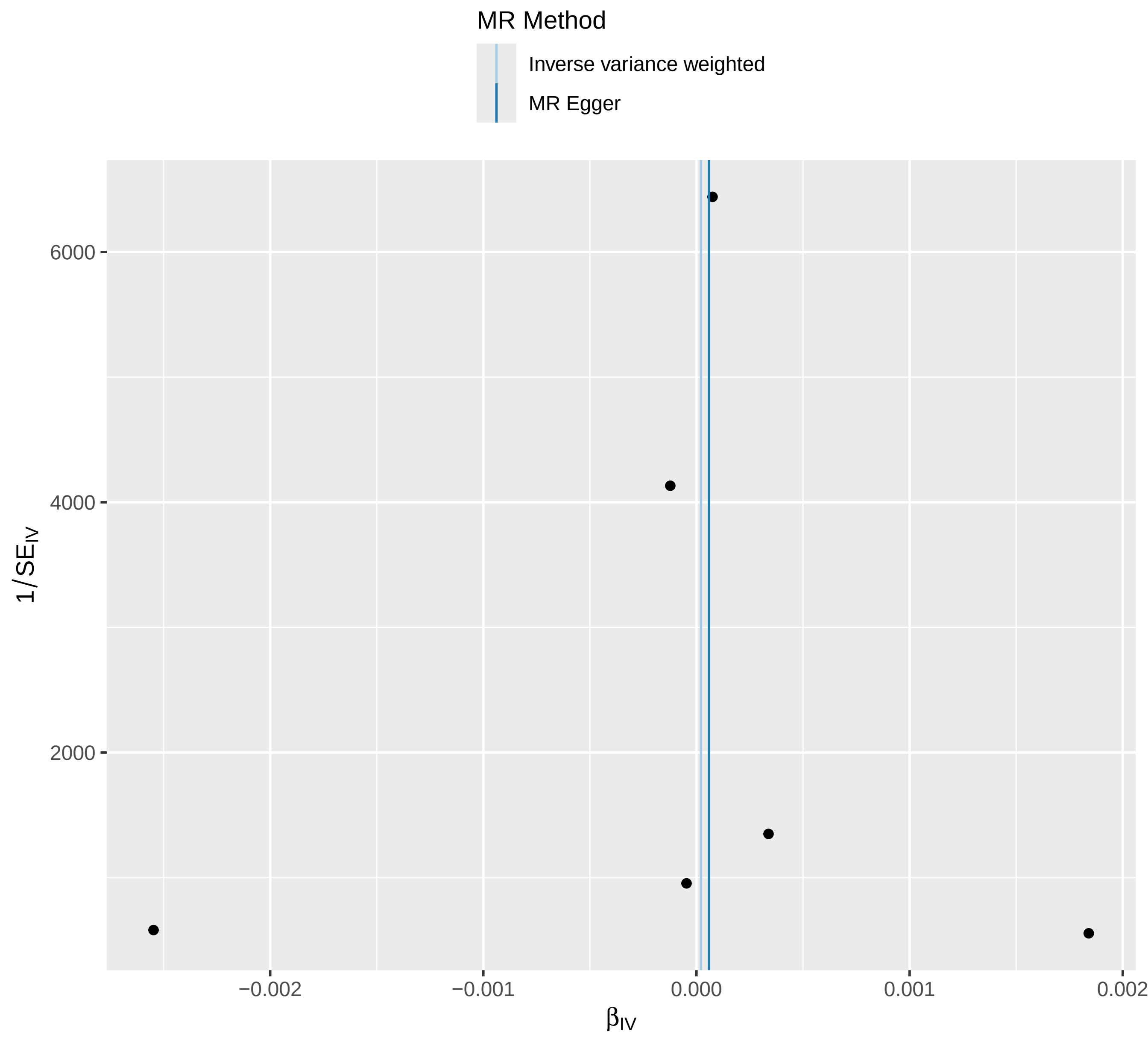

MR Method
Inverse variance weighted
MR Egger
6000
4000
2000
1/SE$_{IV}$
−0.002
−0.001
0.000
0.001
0.002
β_{IV}

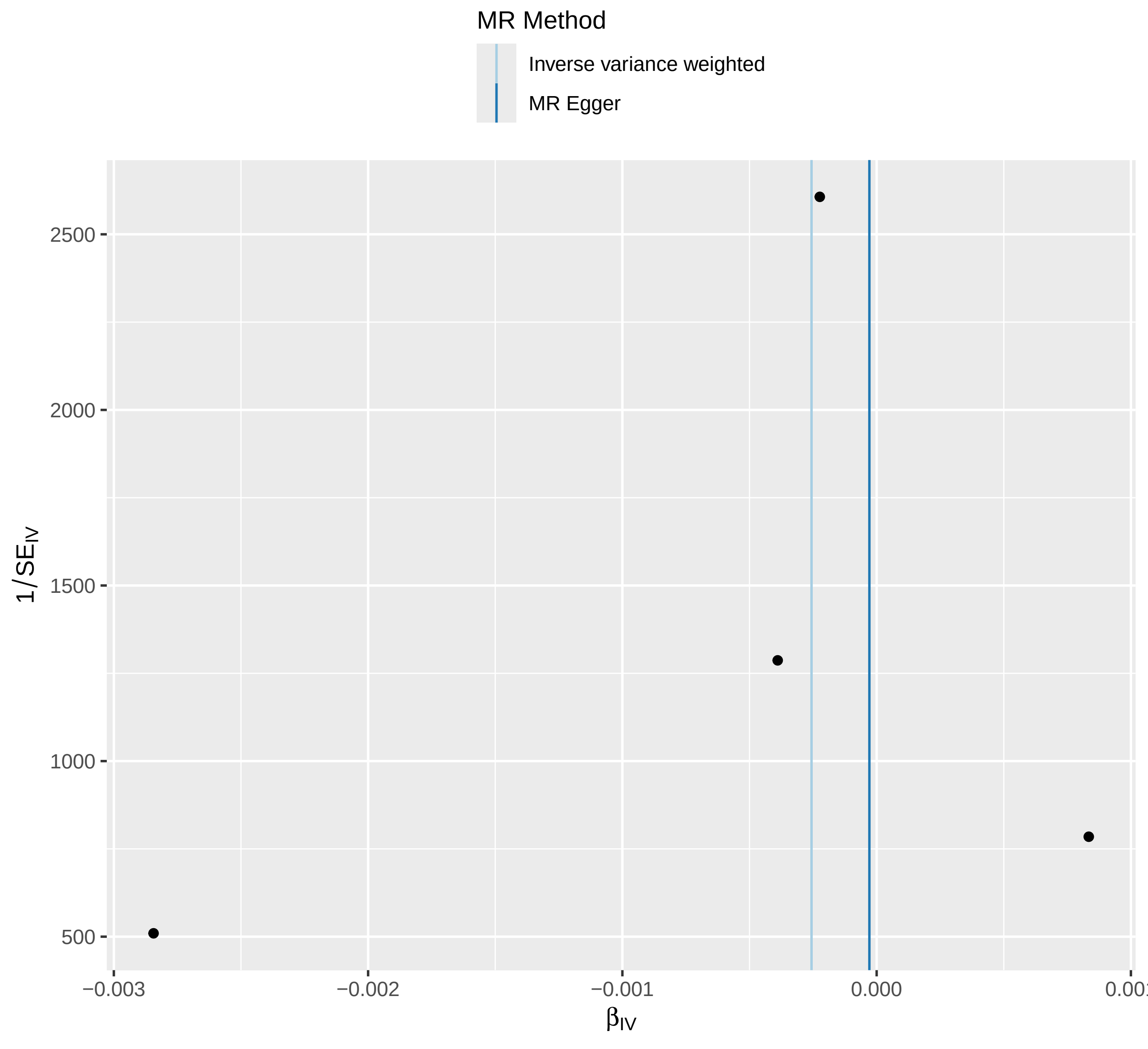
MR Method
Inverse variance weighted
MR Egger
2500
2000
1500
1000
500
1/SE_IV
−0.003
−0.002
−0.001
0.000
β_IV

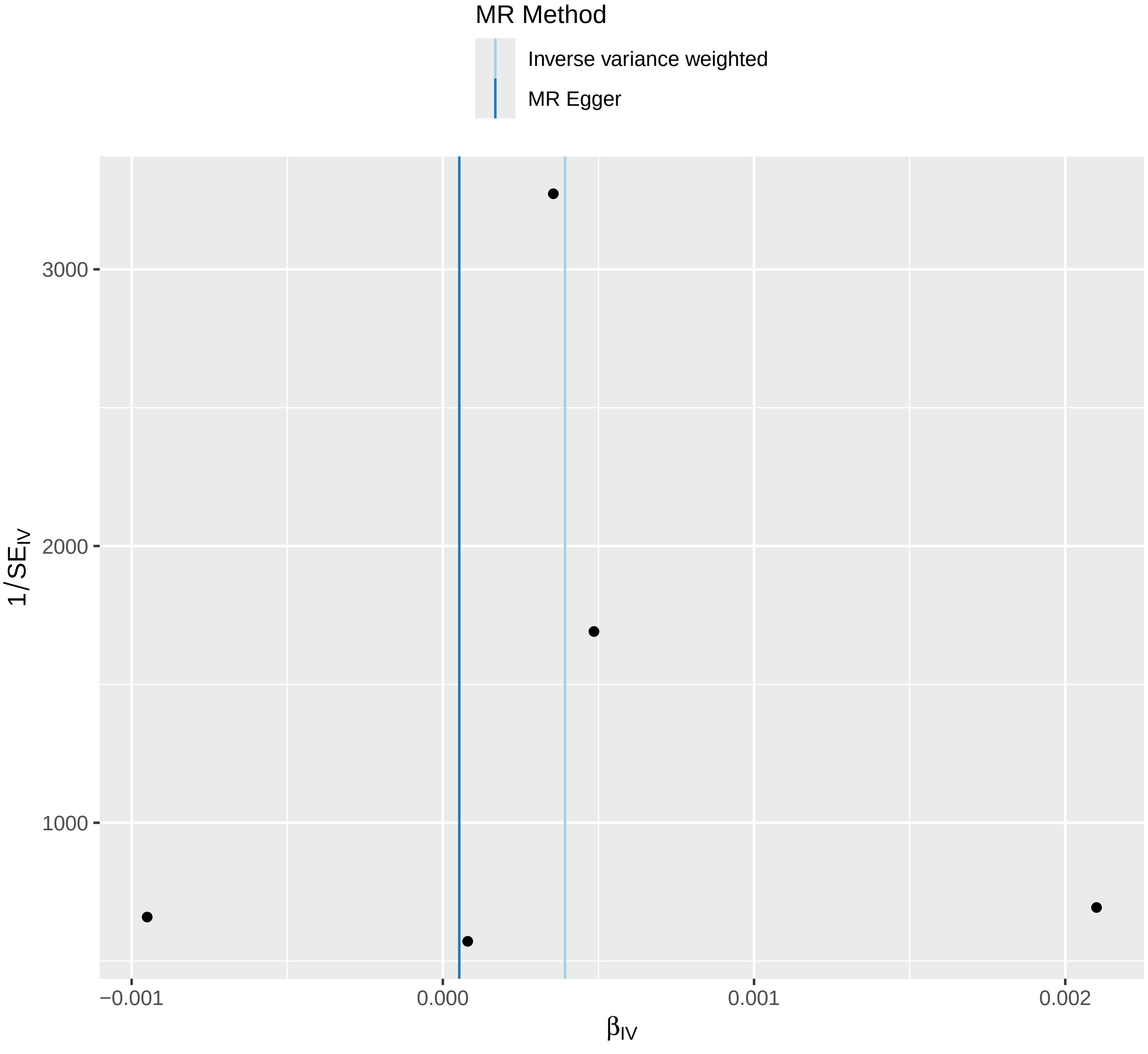

MR Method
Inverse variance weighted
MR Egger
3000
2000
1000
1/SE$_{IV}$
−0.001
0.000
0.001
0.002
β$_{IV}$

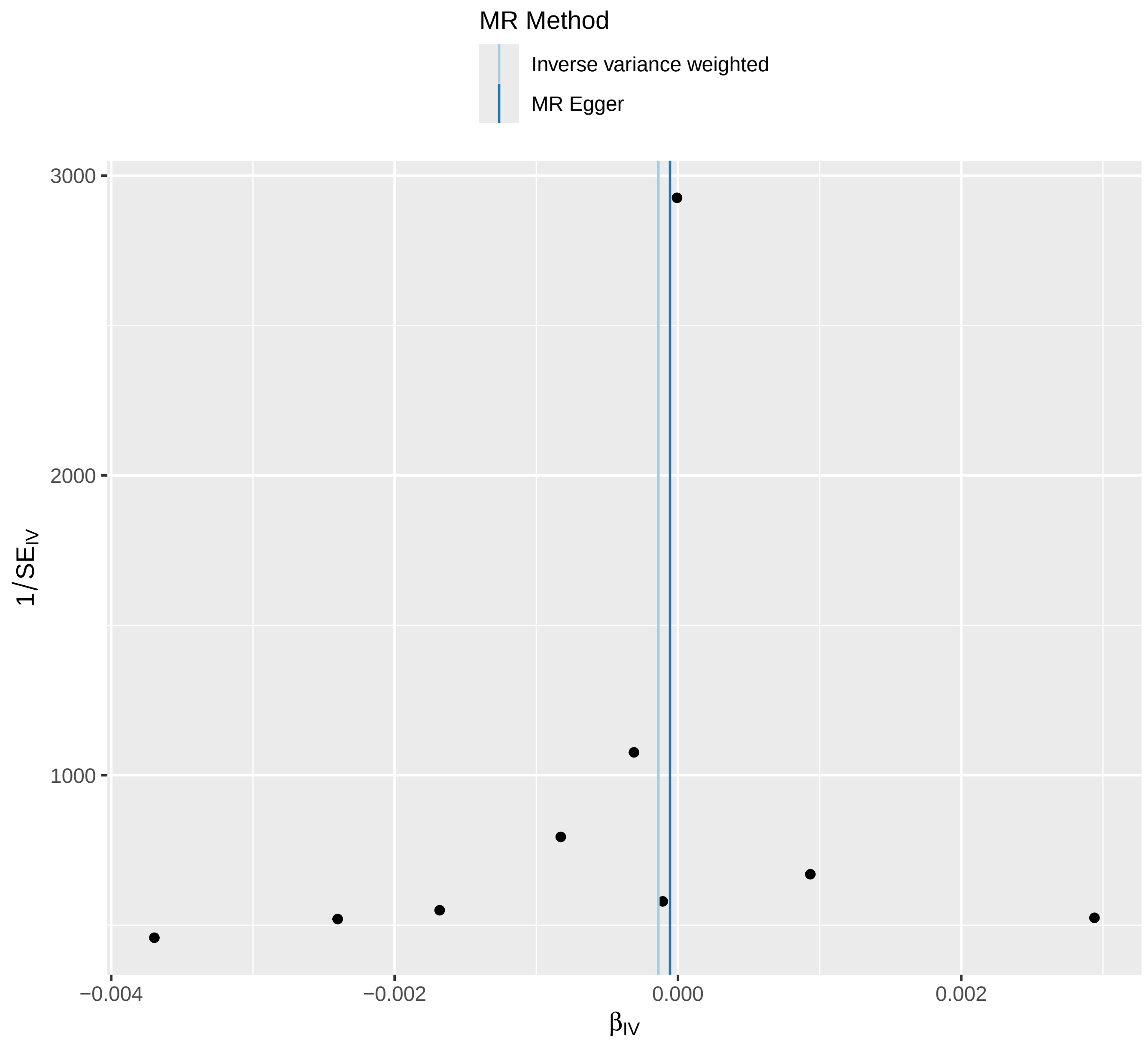

MR Method
Inverse variance weighted
MR Egger
3000
2000
1000
1/SE_IV
−0.004
−0.002
0.000
0.002
β_IV

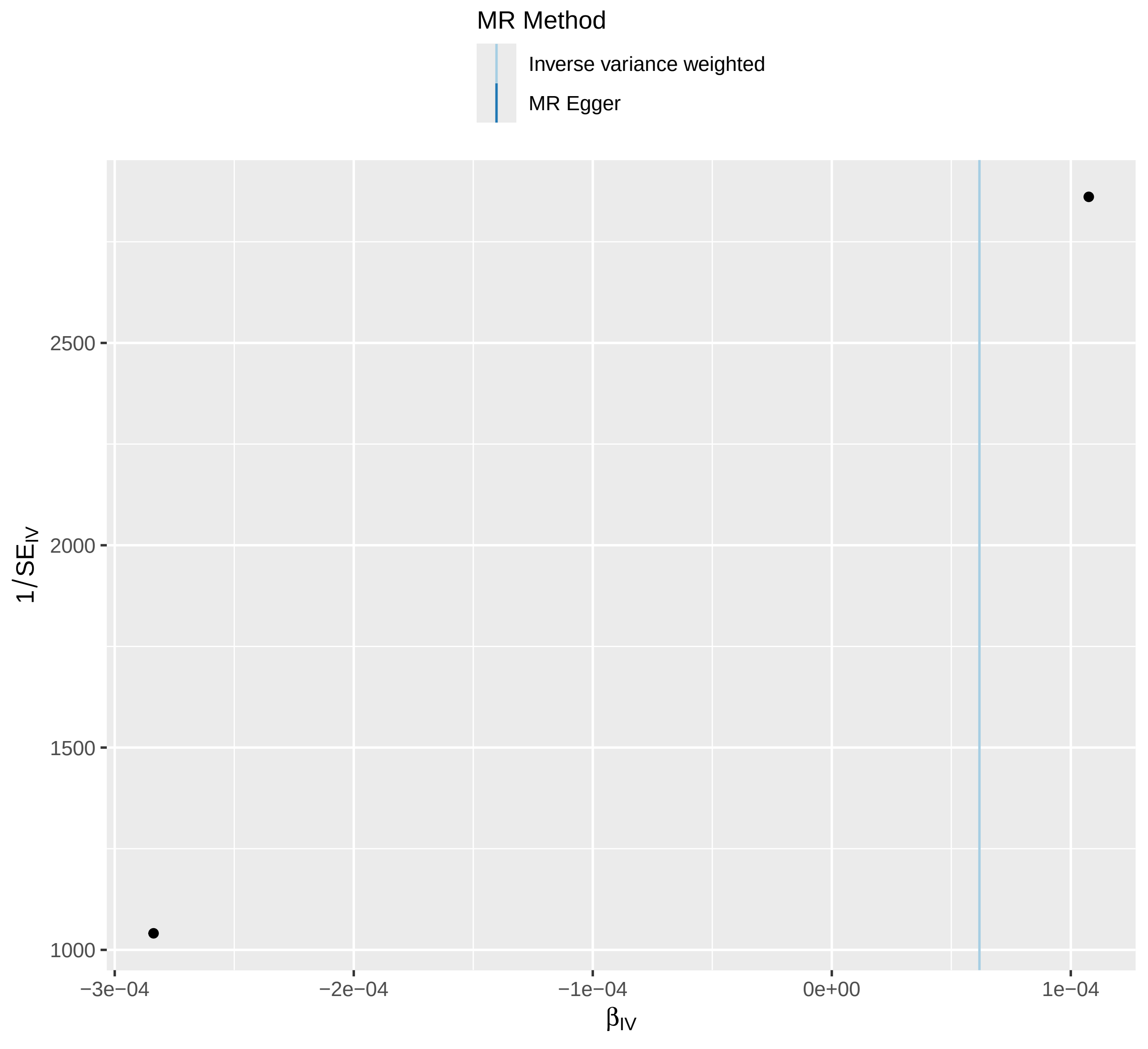
MR Method
Inverse variance weighted
MR Egger
2500
2000
1500
1000
1/SE$_{IV}$
−3e−04
−2e−04
−1e−04
0e+00
1e−04
β_{IV}

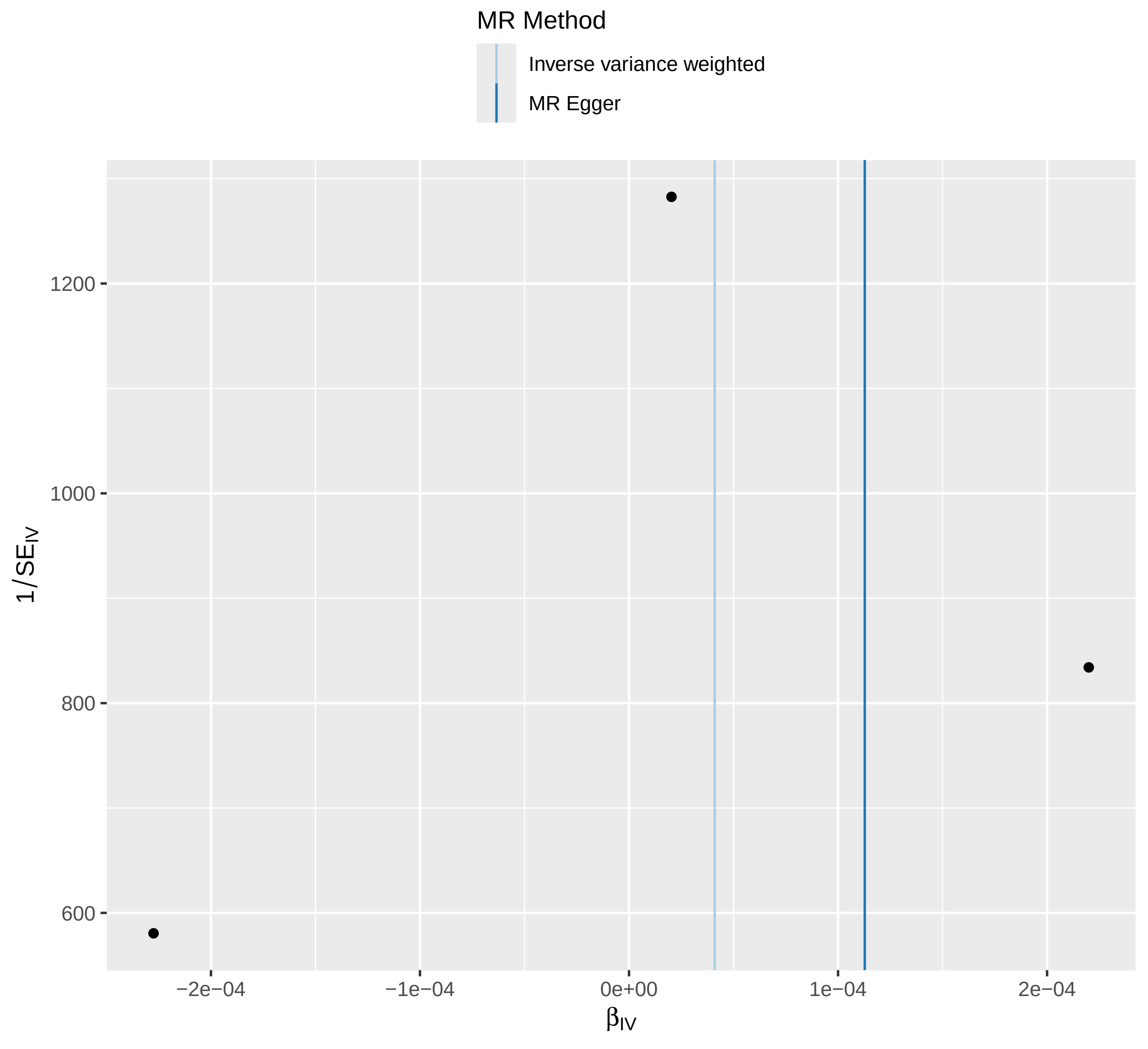

MR Method
Inverse variance weighted
MR Egger
1200
1000
800
600
1/SE_IV
−2e−04
−1e−04
0e+00
1e−04
2e−04
β_IV

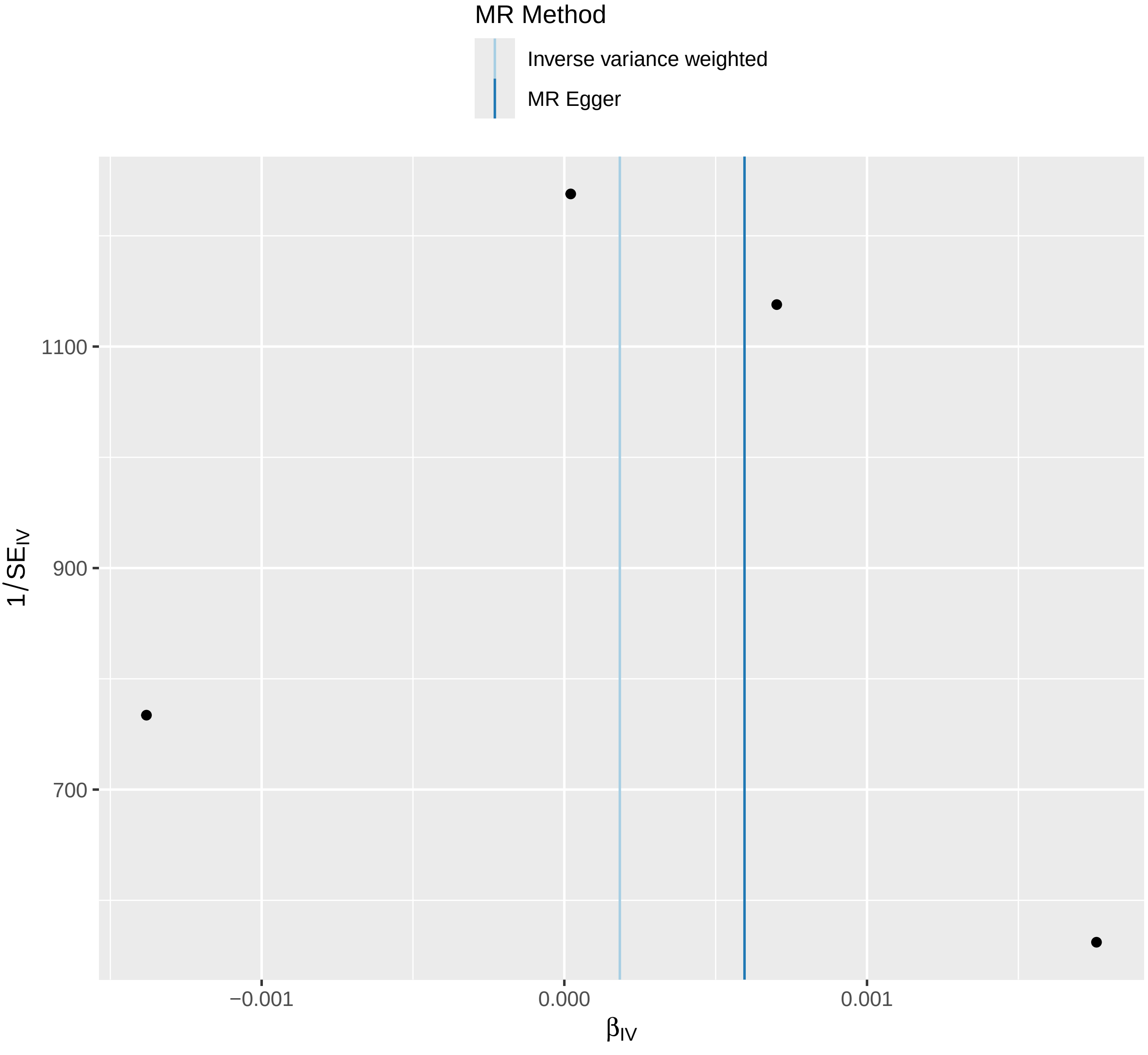
MR Method
Inverse variance weighted
MR Egger
1100
900
700
1/SE$_{IV}$
−0.001
0.000
0.001
β$_{IV}$

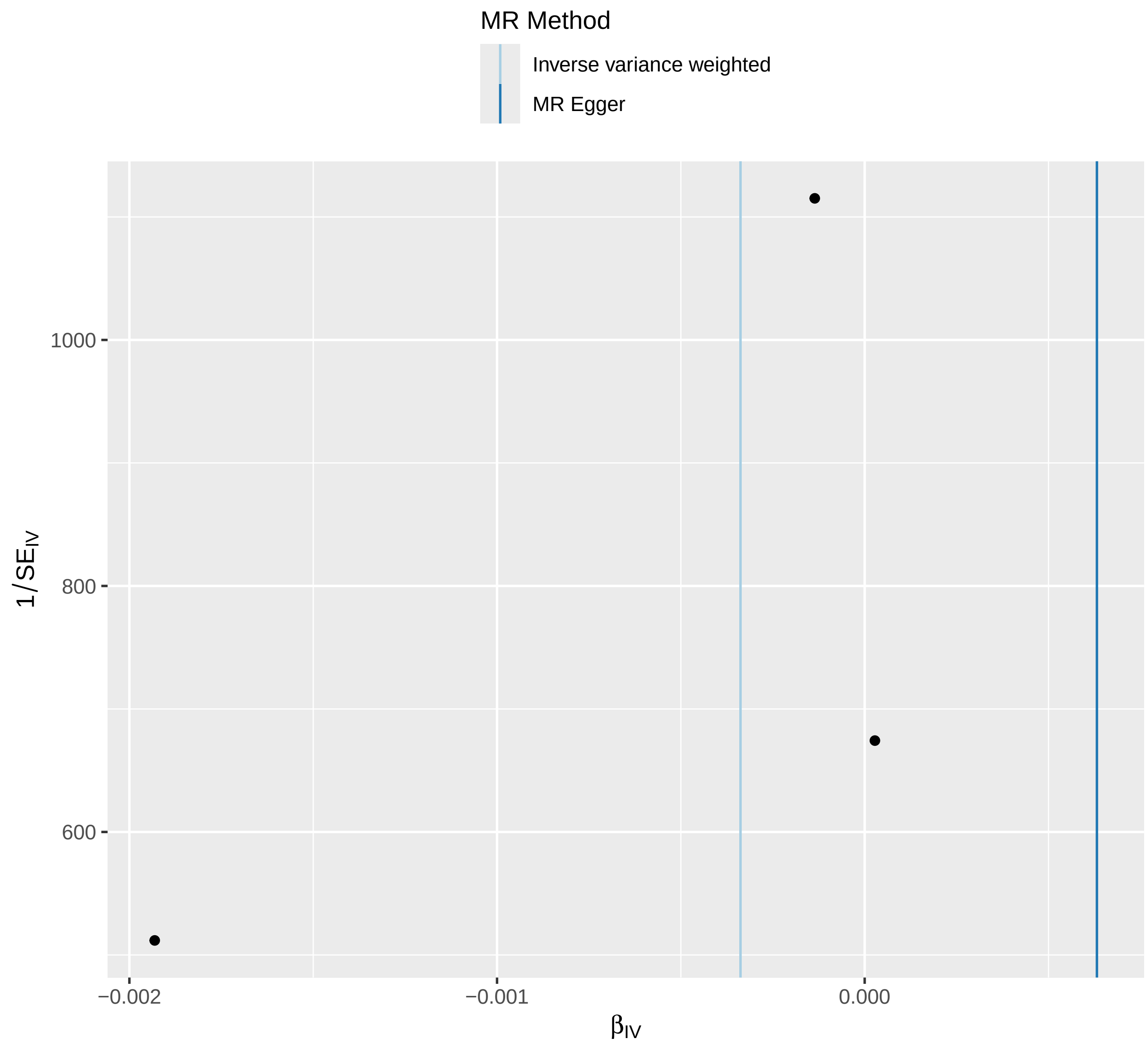

MR Method
Inverse variance weighted
MR Egger
1000
800
600
1/SE_IV
−0.002
−0.001
0.000
β_IV

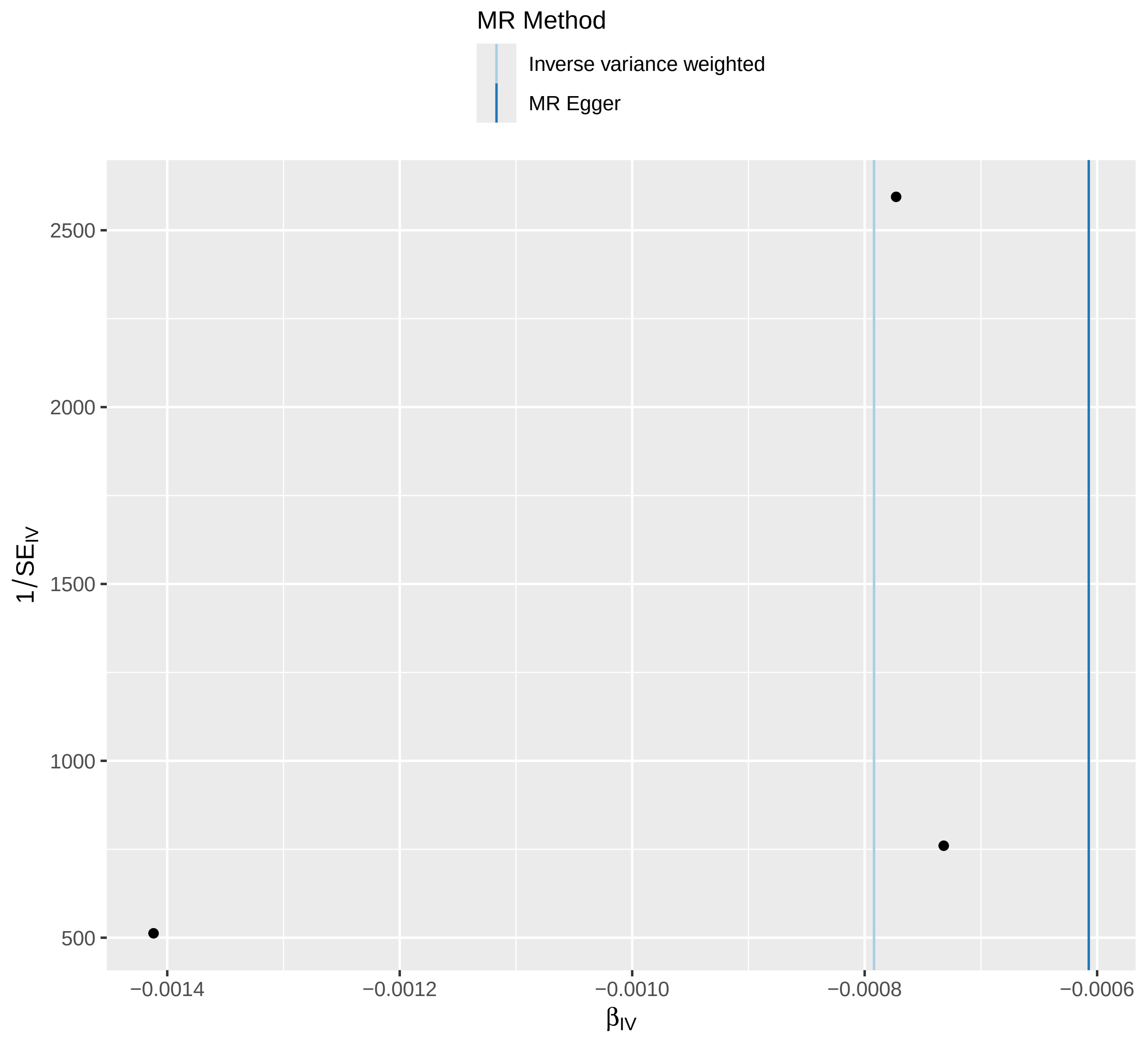

MR Method
Inverse variance weighted
MR Egger
2500
2000
1500
1000
500
1/SE_IV
−0.0014
−0.0012
−0.0010
−0.0008
−0.0006
β_IV

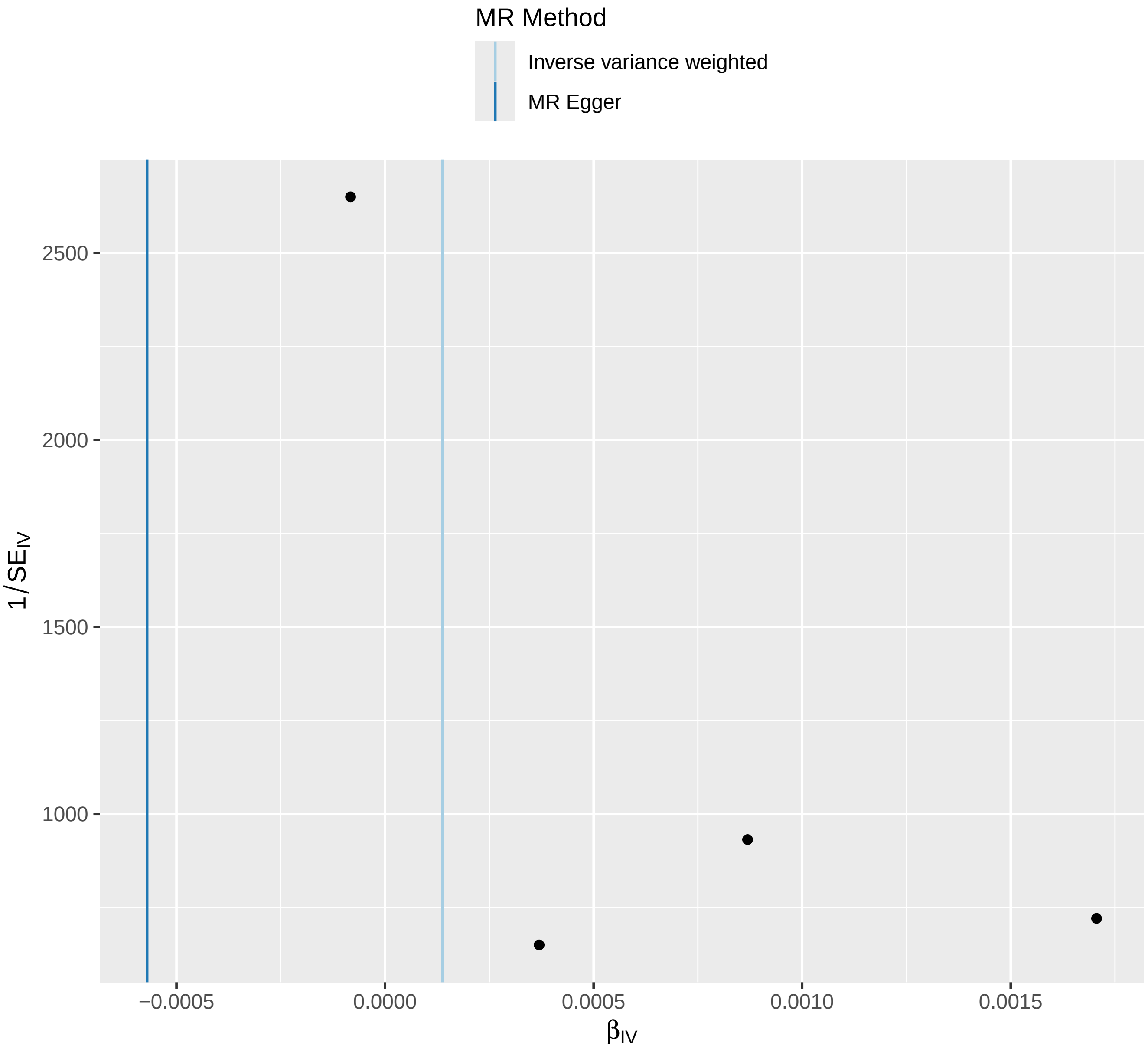
MR Method
Inverse variance weighted
MR Egger
2500
2000
1500
1000
1/SE_IV
−0.0005
0.0000
0.0005
0.0010
0.0015
β_IV

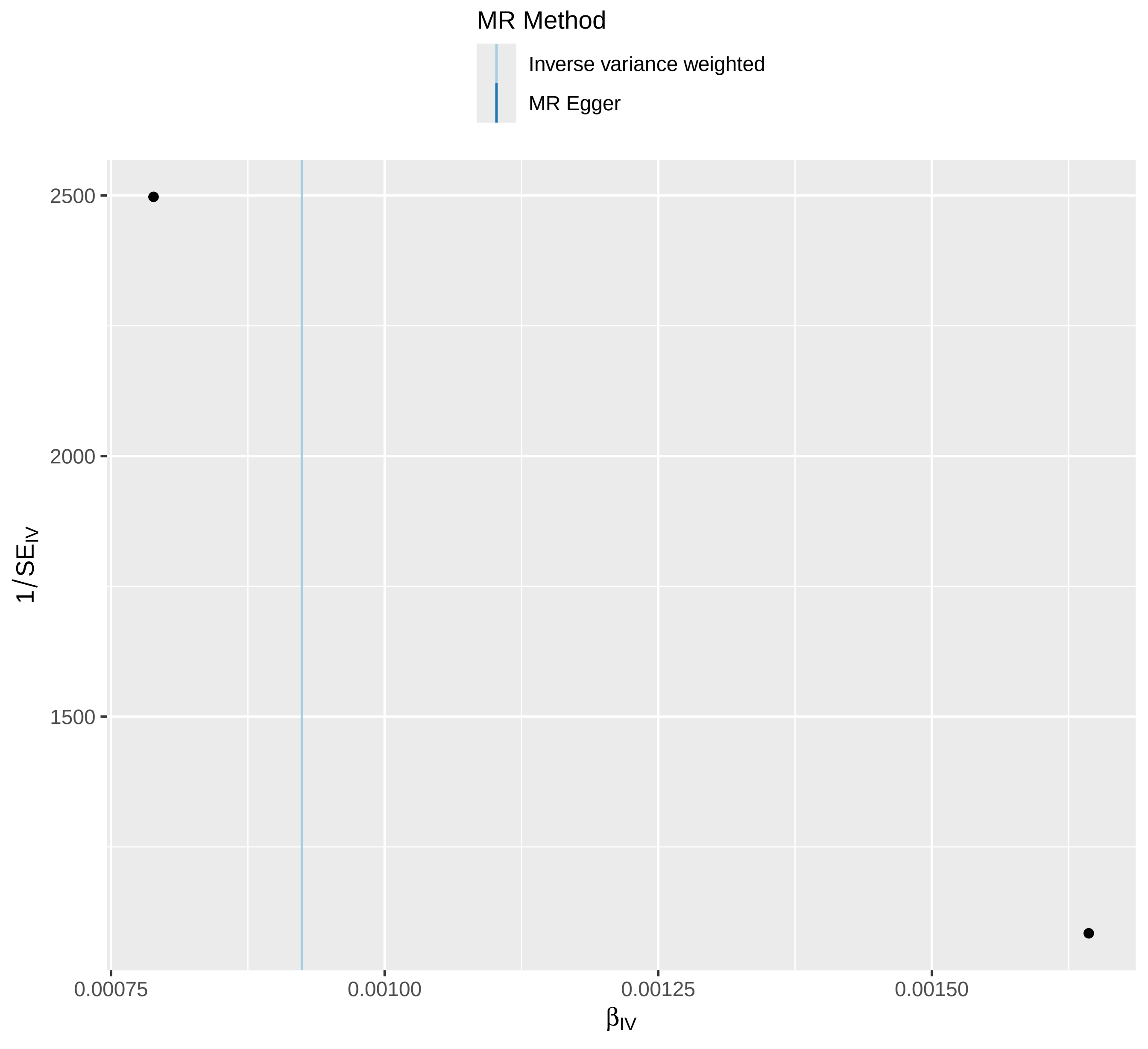

MR Method
Inverse variance weighted
MR Egger
2500
2000
1500
1/SE_IV
0.00075
0.00100
0.00125
0.00150
β_IV

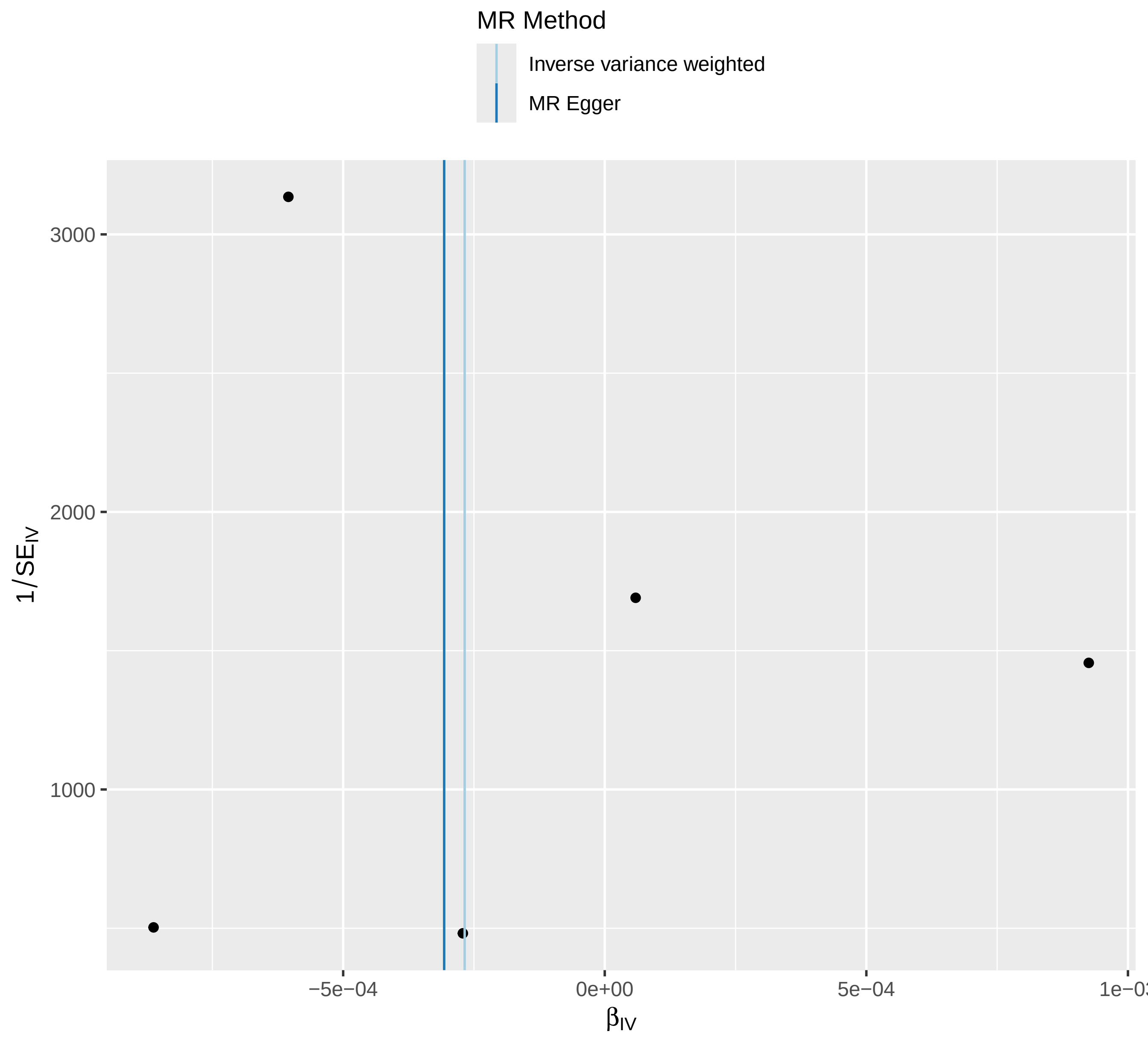
MR Method
Inverse variance weighted
MR Egger
3000
2000
1000
$1/SE_{IV}$
−5e−04
0e+00
5e−04
1e−03
β_{IV}

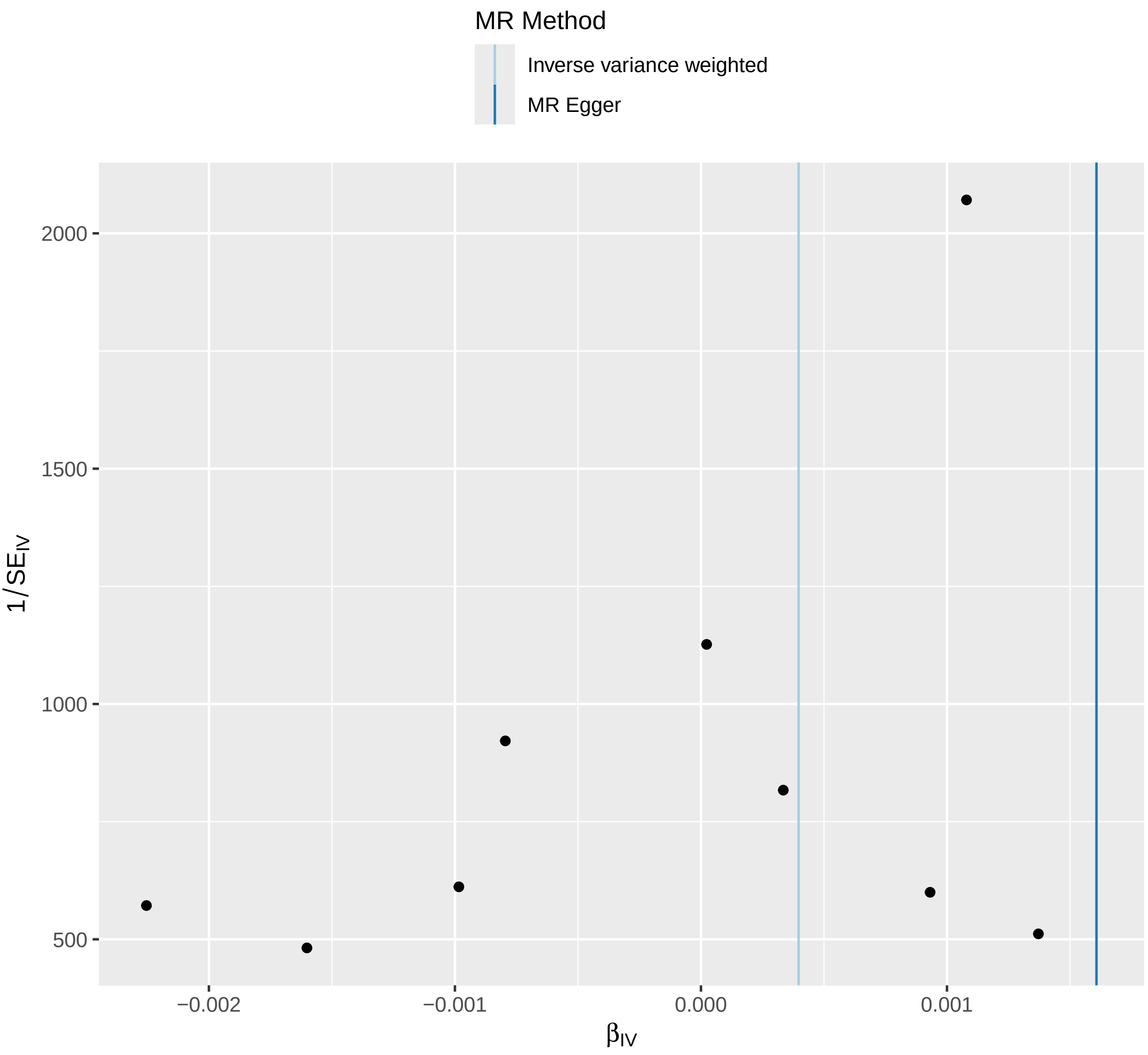
MR Method
Inverse variance weighted
MR Egger
2000
1500
1000
500
1/SE$_{IV}$
−0.002
−0.001
0.000
0.001
β$_{IV}$

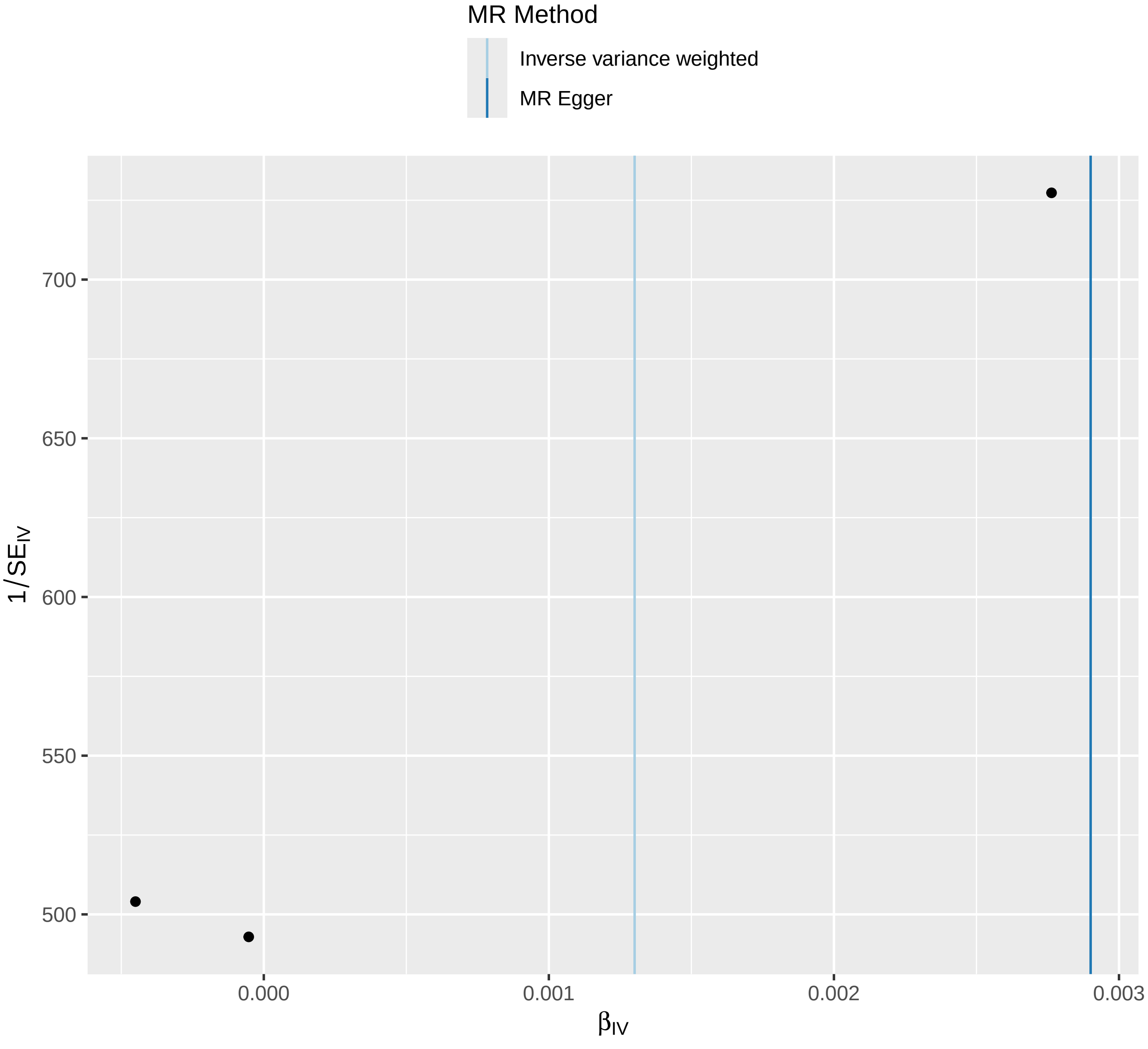
MR Method
Inverse variance weighted
MR Egger
700
650
600
550
500
$1/SE_{IV}$
0.000
0.001
0.002
0.003
β_{IV}

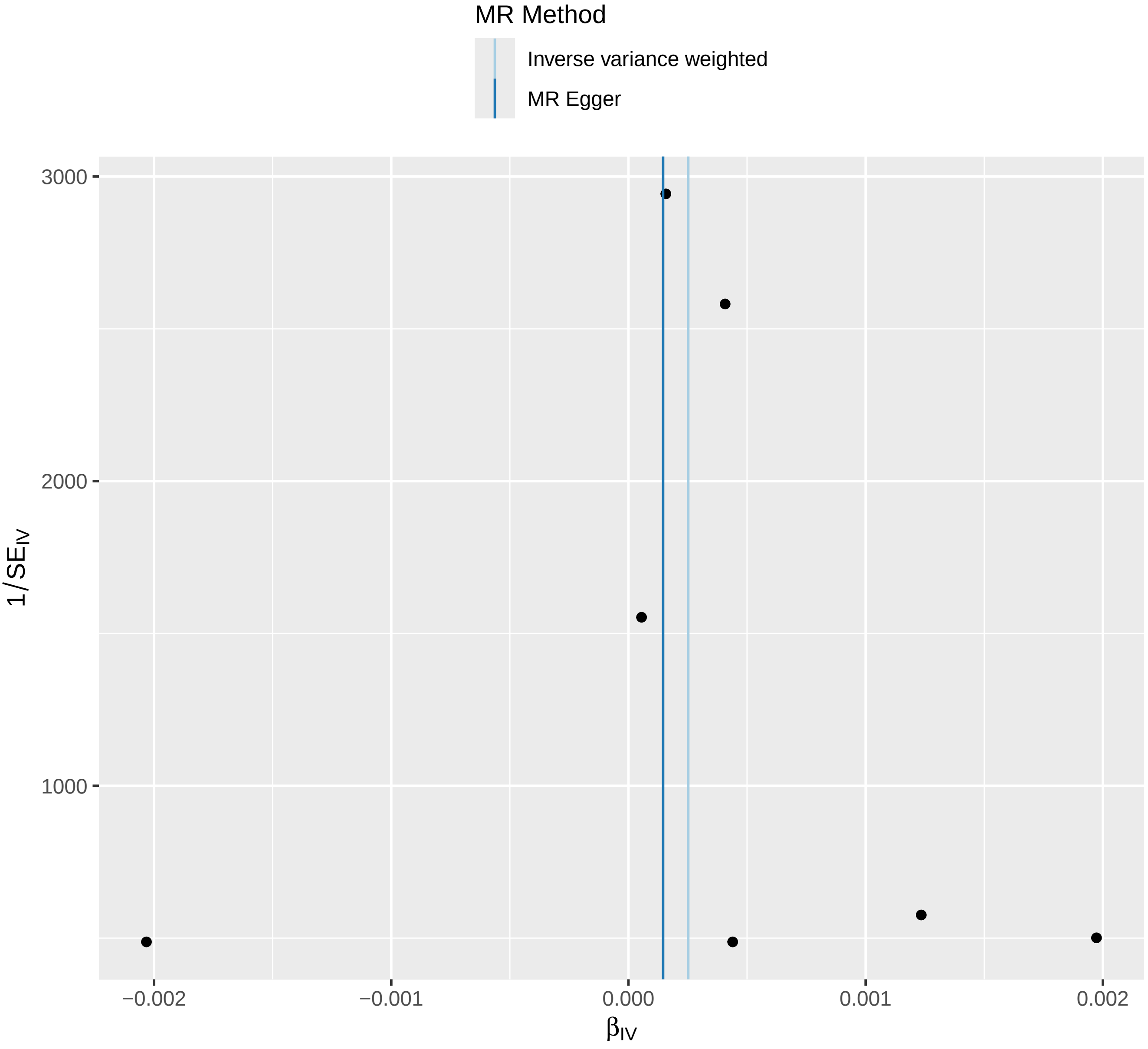
MR Method
Inverse variance weighted
MR Egger
3000
2000
1000
$1/SE_{IV}$
−0.002
−0.001
0.000
0.001
0.002
β_{IV}

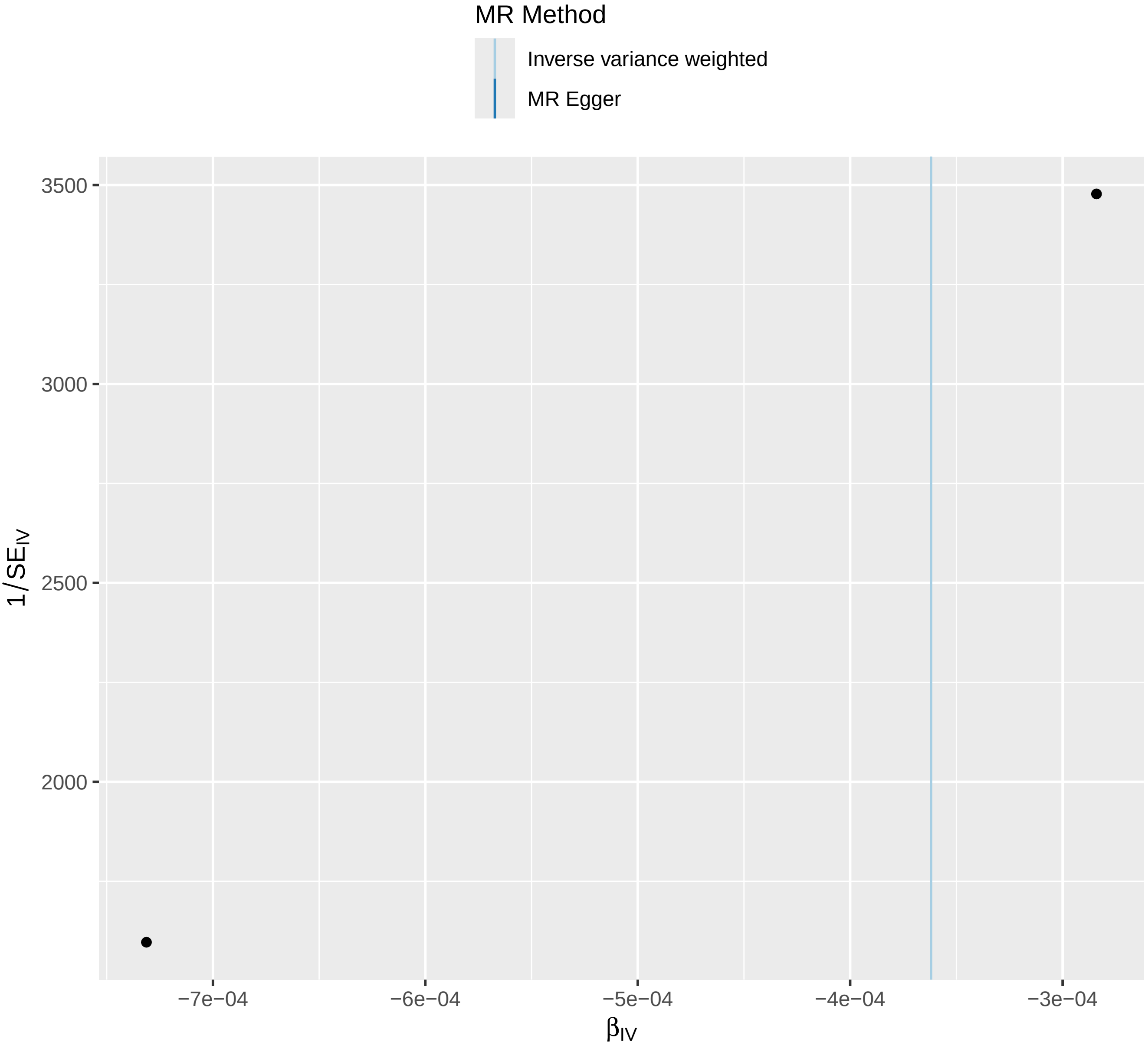
MR Method
Inverse variance weighted
MR Egger
3500
3000
2500
2000
1/SE$_{IV}$
−7e−04
−6e−04
−5e−04
−4e−04
−3e−04
β$_{IV}$

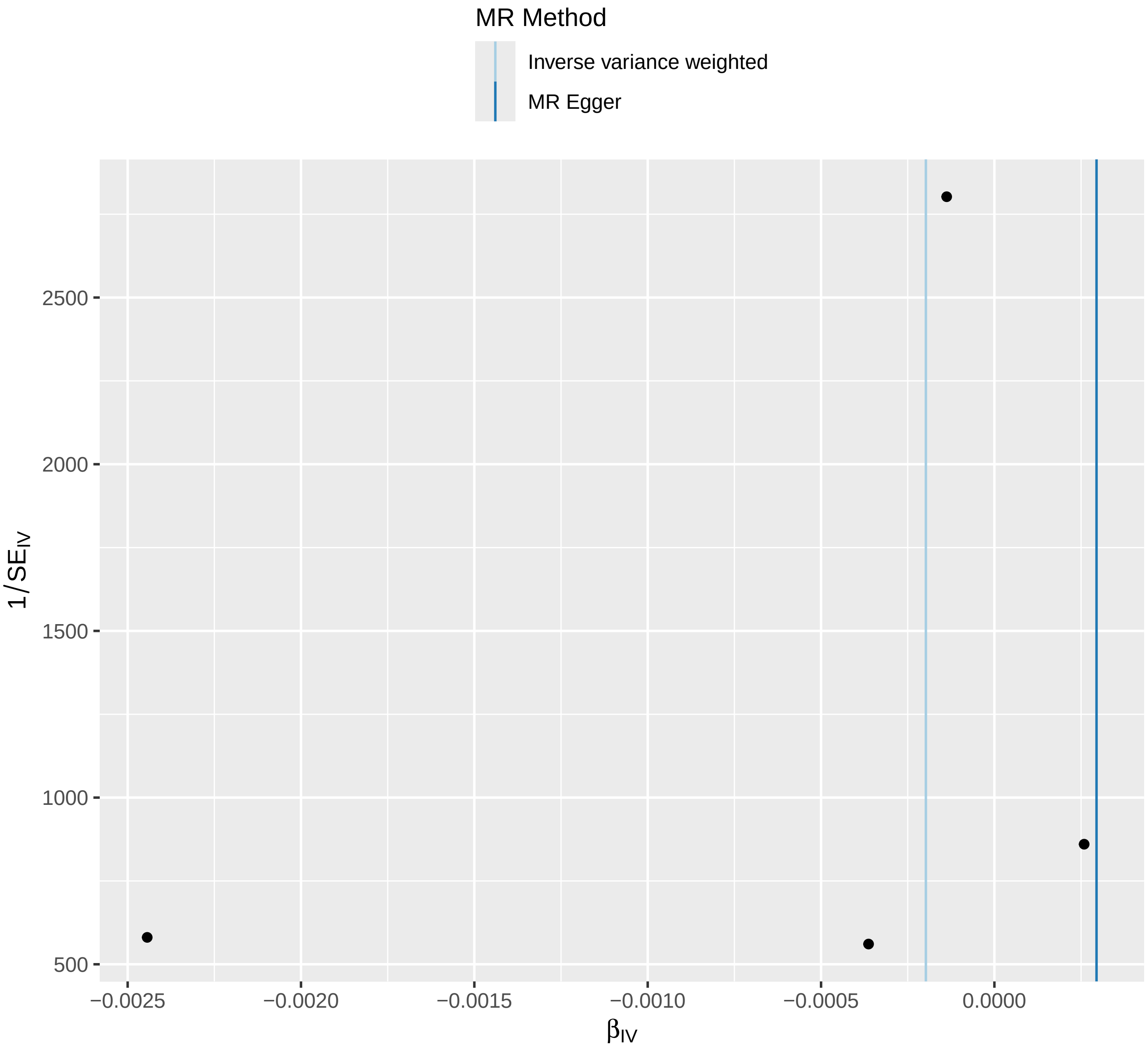

MR Method
Inverse variance weighted
MR Egger
2500
2000
1500
1000
500
1/SE$_{IV}$
−0.0025
−0.0020
−0.0015
−0.0010
−0.0005
0.0000
β_{IV}

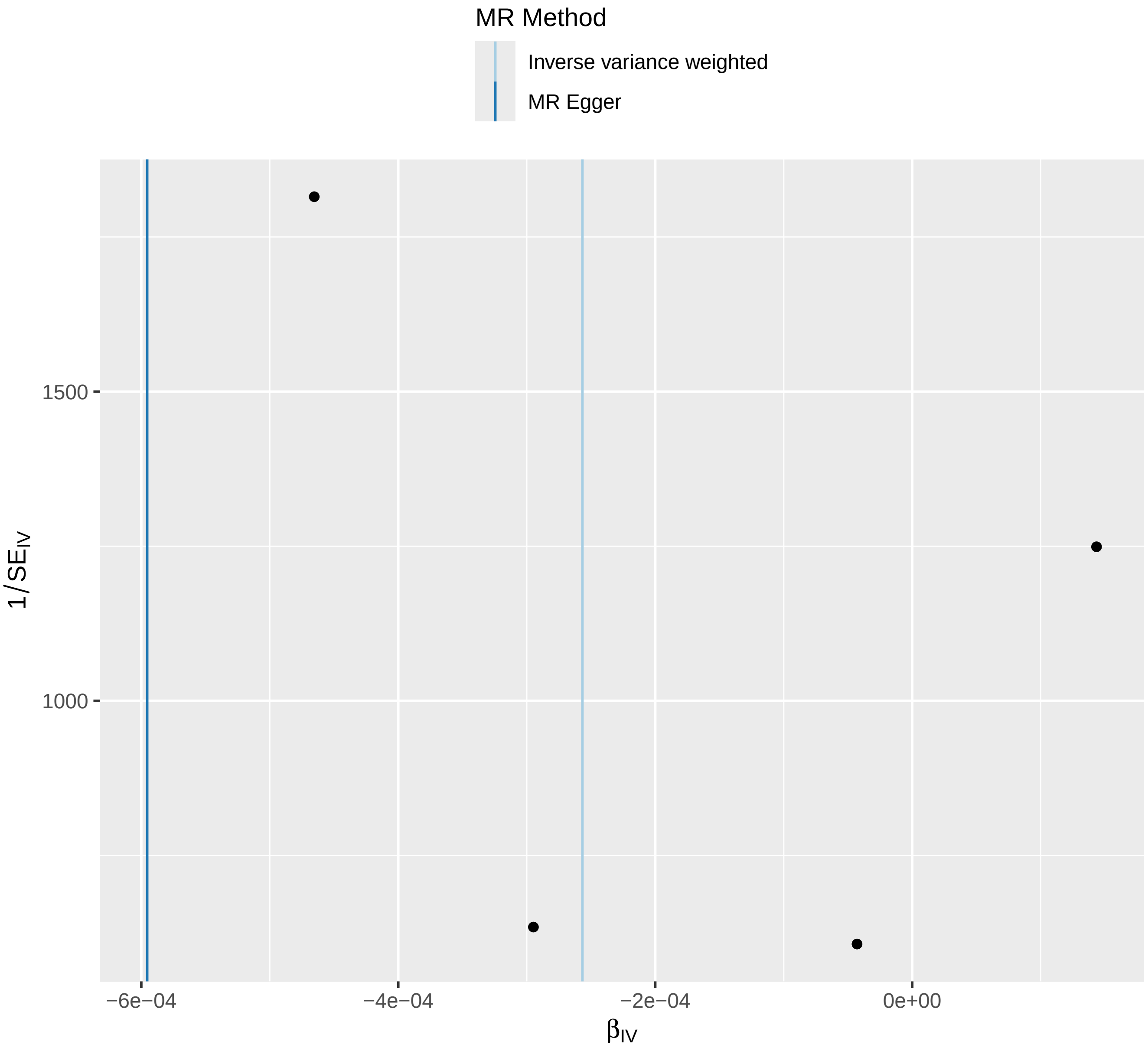
MR Method
Inverse variance weighted
MR Egger
1500
1000
−6e−04
−4e−04
−2e−04
0e+00
β_{IV}
$1/SE_{IV}$

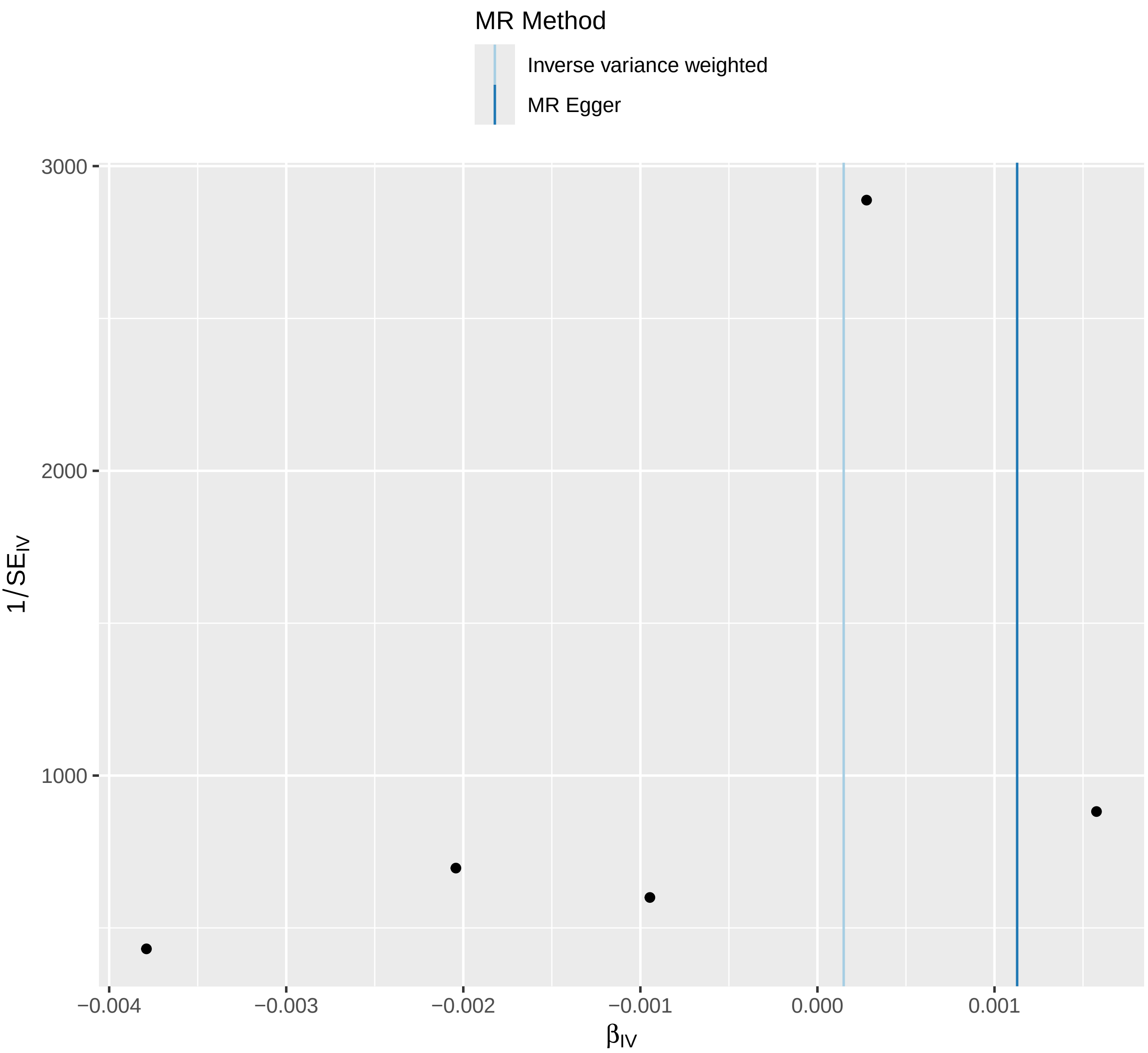

MR Method
Inverse variance weighted
MR Egger
3000
2000
1000
1/SE_IV
−0.004
−0.003
−0.002
−0.001
0.000
0.001
β_IV

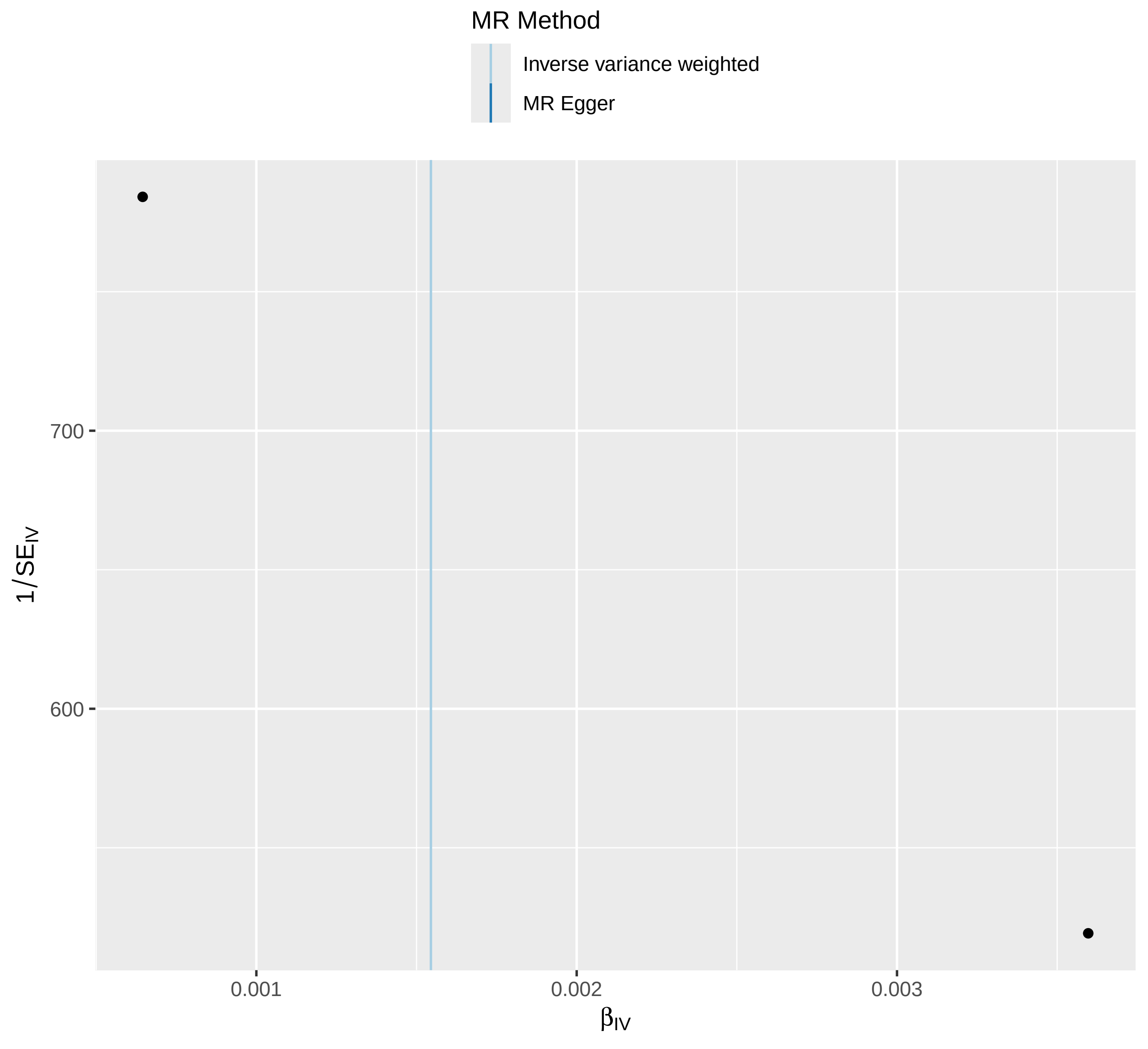
MR Method
Inverse variance weighted
MR Egger
700
600
1/SE$_{IV}$
0.001
0.002
0.003
β$_{IV}$

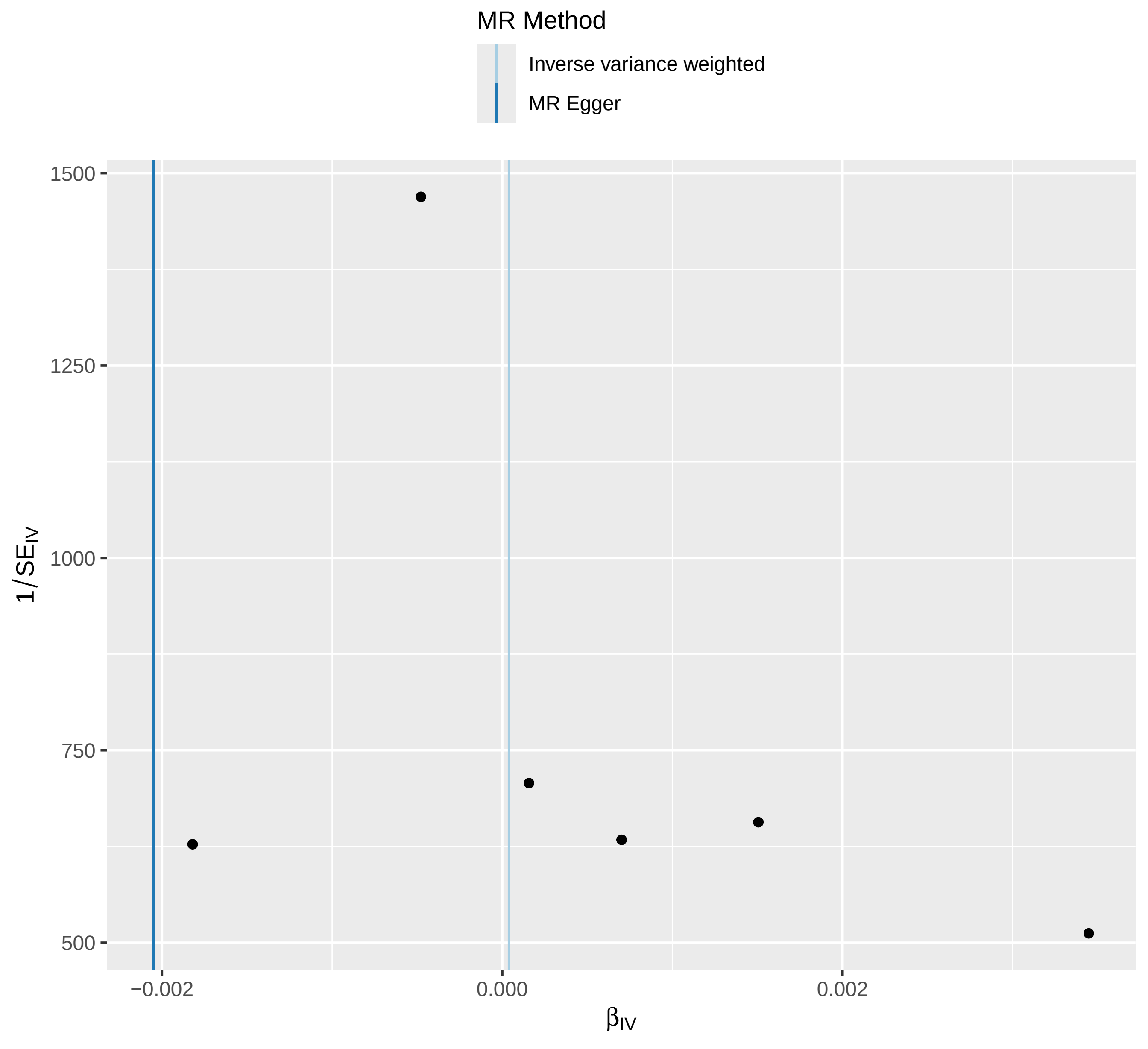

MR Method
Inverse variance weighted
MR Egger
1500
1250
1000
750
500
1/SE_IV
−0.002
0.000
0.002
β_IV

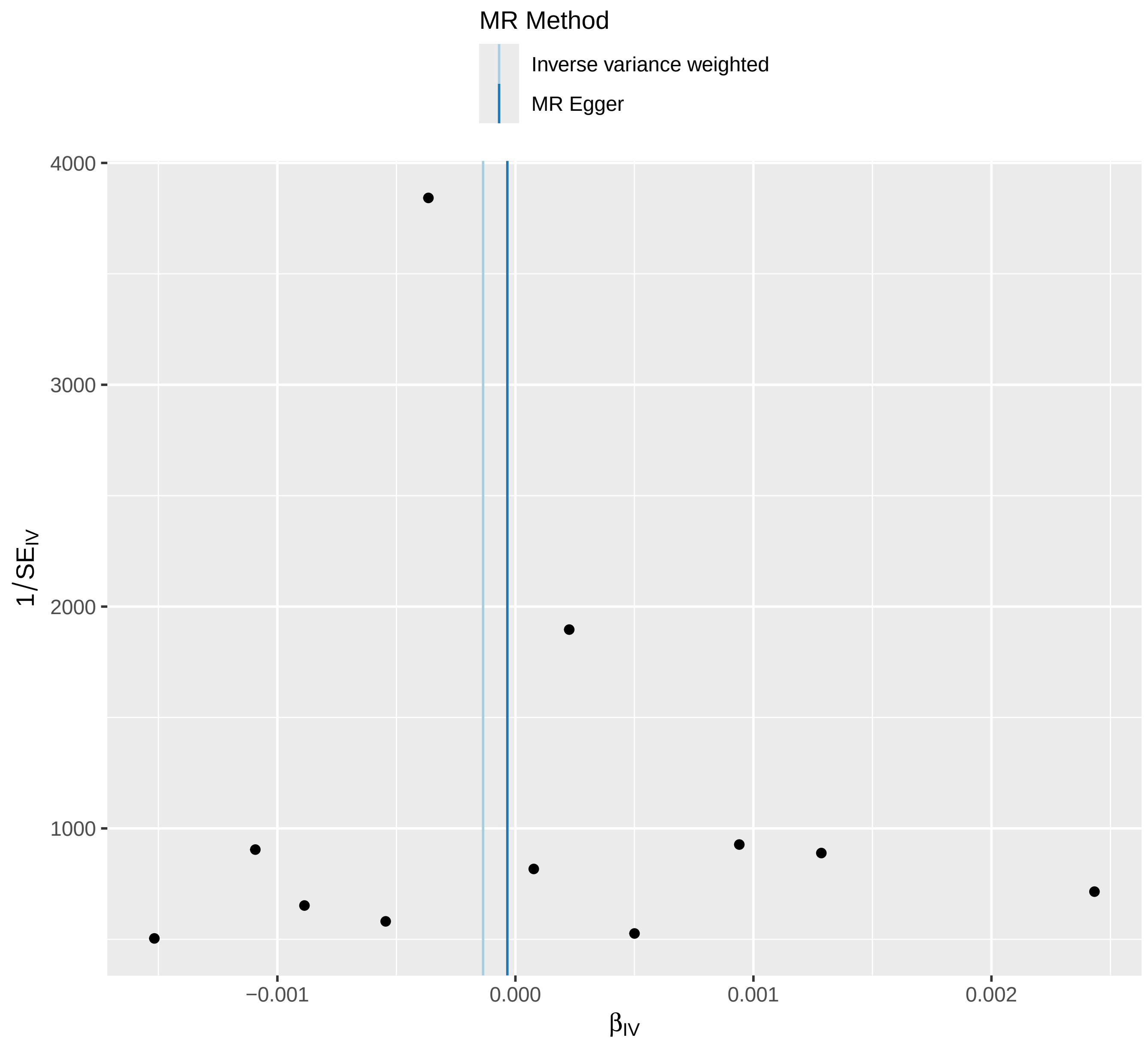

MR Method
Inverse variance weighted
MR Egger
4000
3000
2000
1000
1/SE_IV
−0.001
0.000
0.001
0.002
β_IV

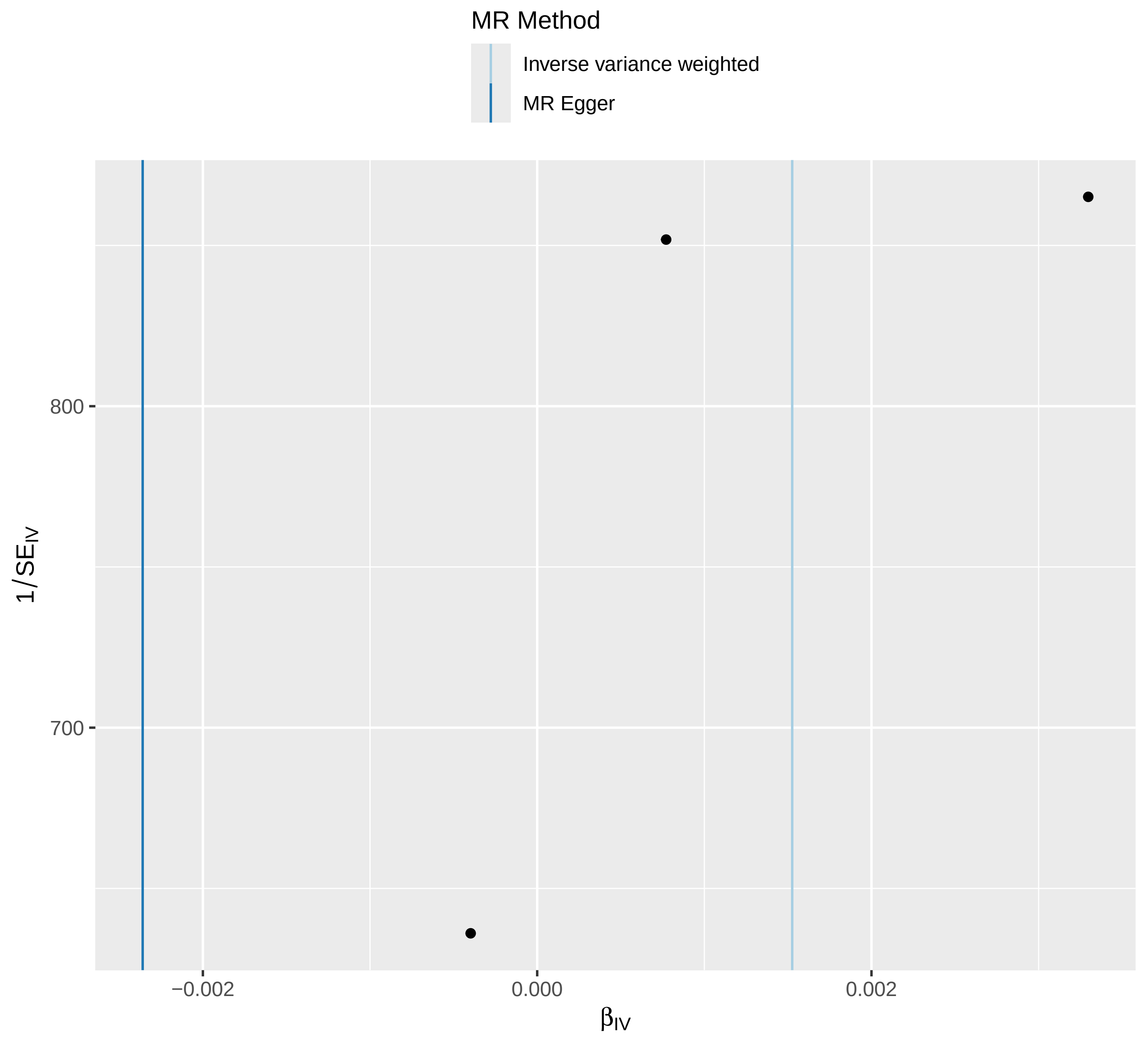
MR Method
Inverse variance weighted
MR Egger
800
700
1/SE$_{IV}$
−0.002
0.000
0.002
β$_{IV}$

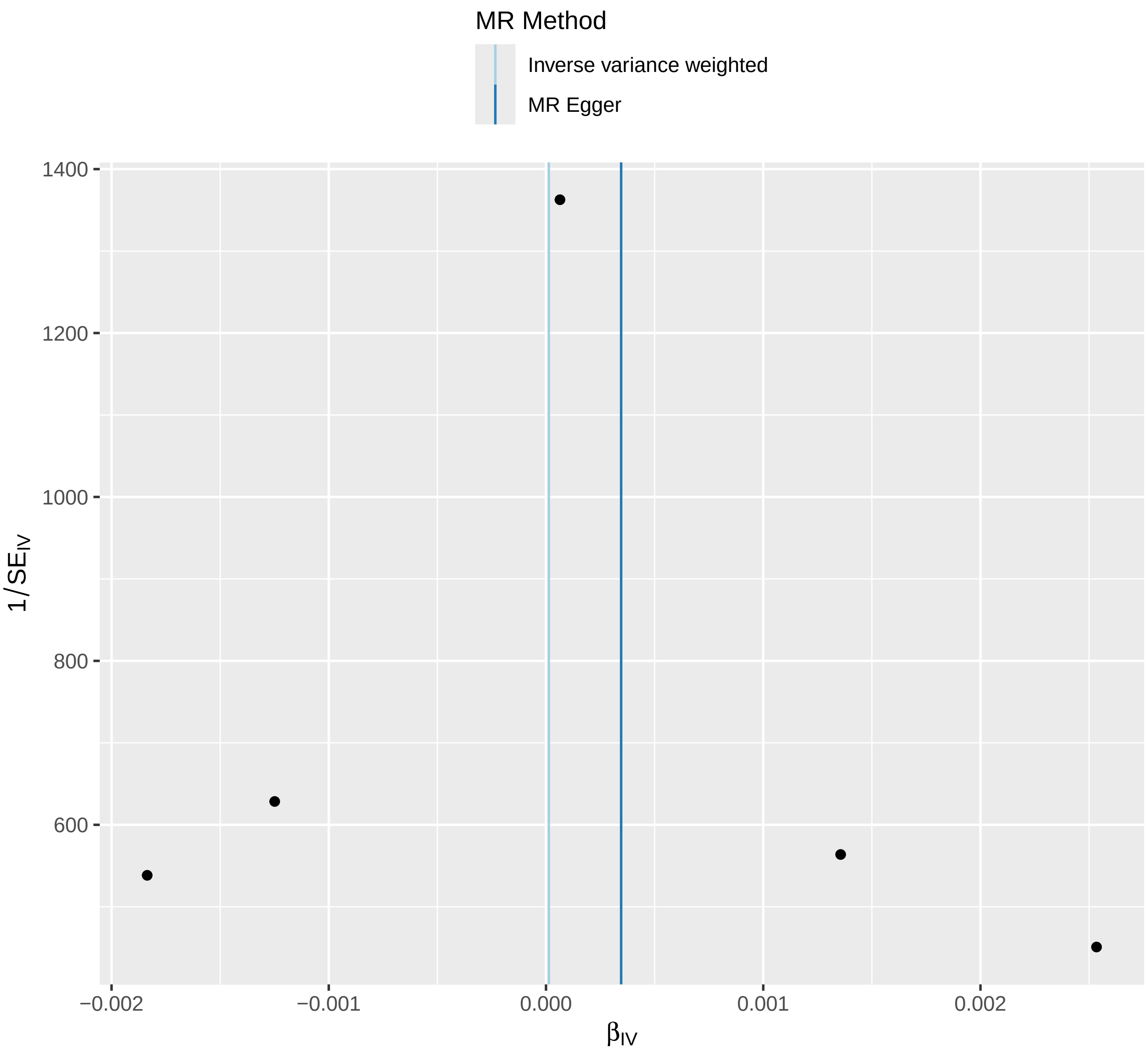

MR Method
Inverse variance weighted
MR Egger
1400
1200
1000
800
600
1/SE_IV
−0.002
−0.001
0.000
0.001
0.002
β_IV

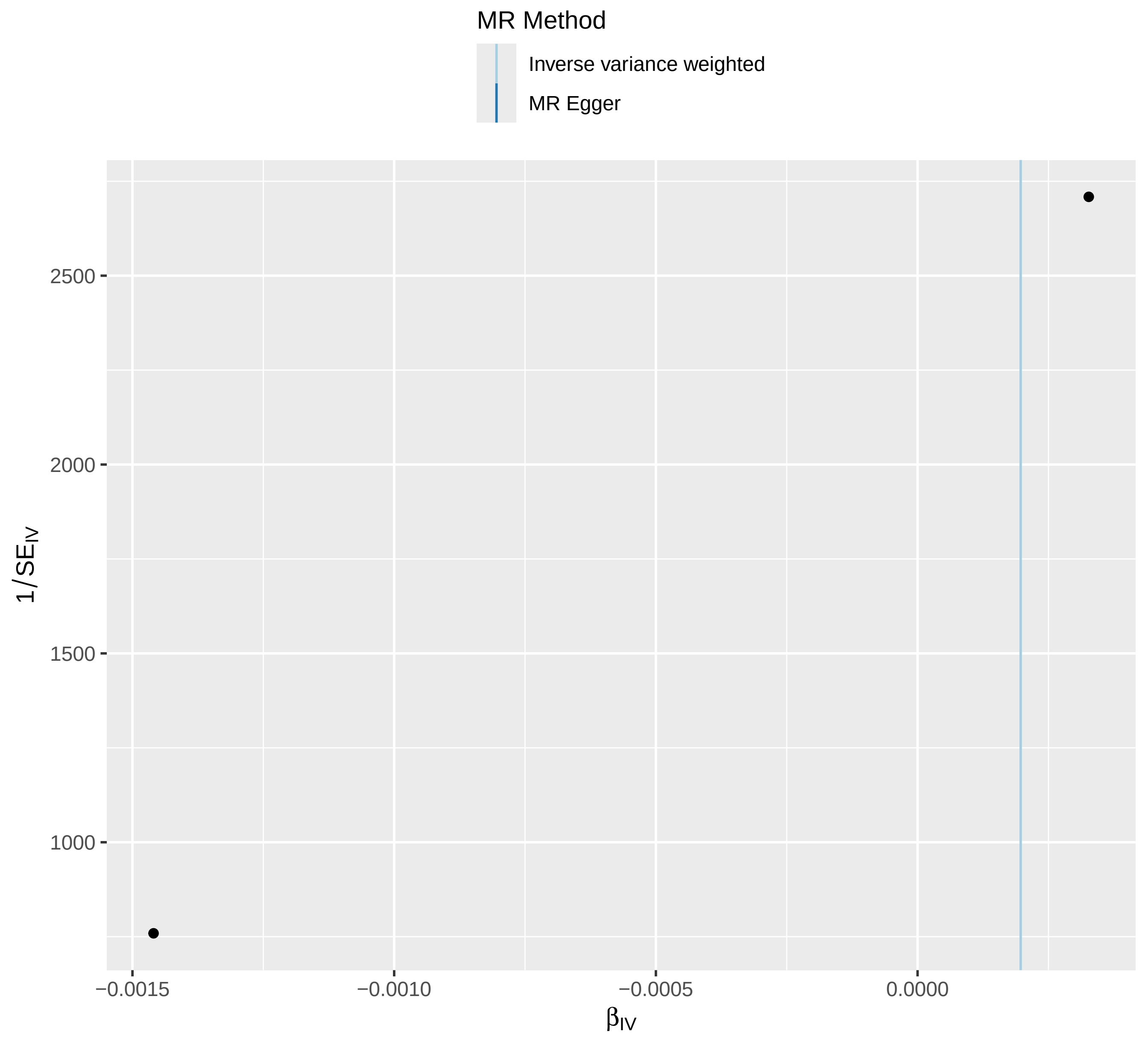
MR Method
Inverse variance weighted
MR Egger
2500
2000
1500
1000
1/SE_IV
−0.0015
−0.0010
−0.0005
0.0000
β_IV

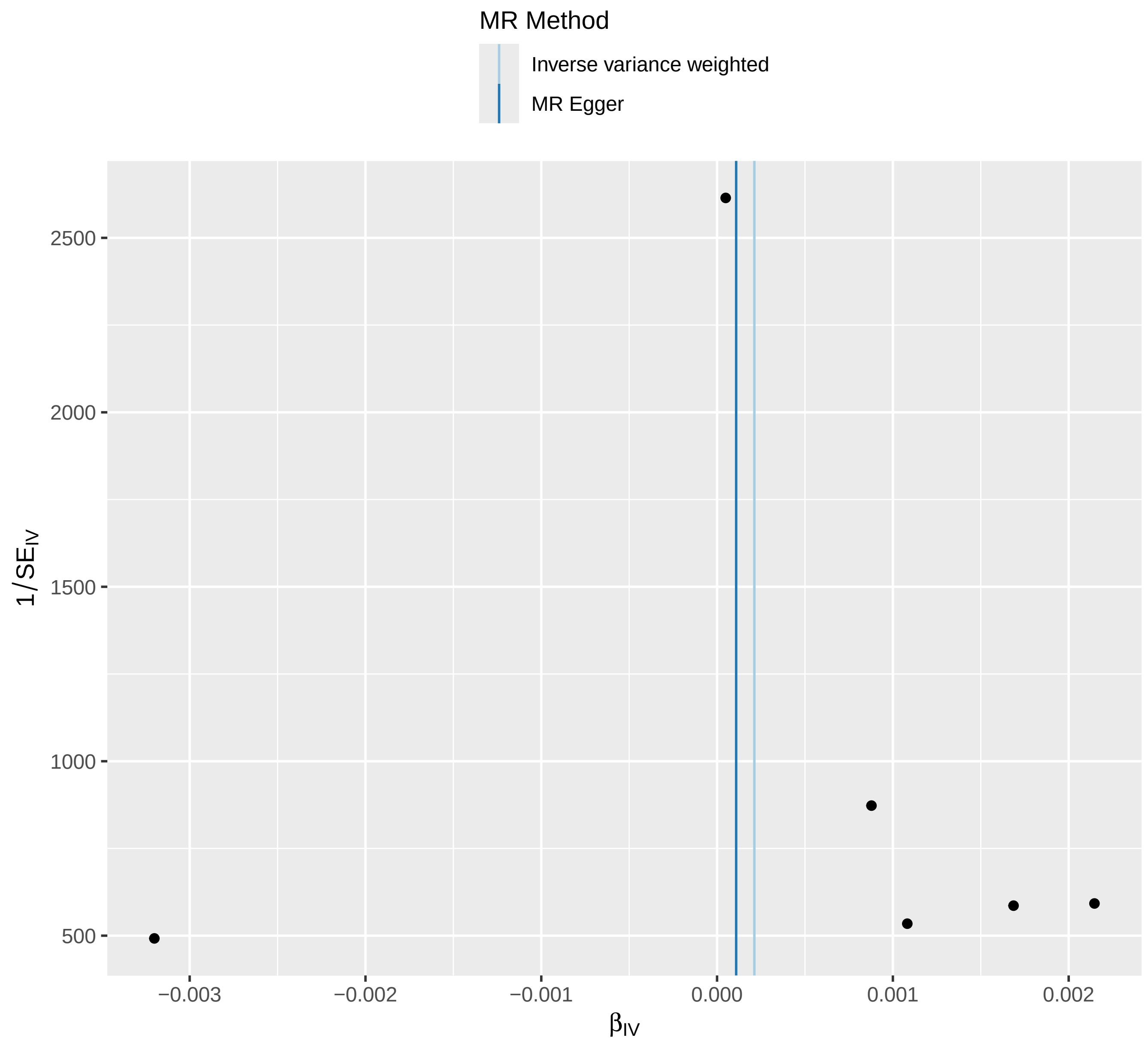

MR Method
Inverse variance weighted
MR Egger
2500
2000
1500
1000
500
1/SE_IV
−0.003
−0.002
−0.001
0.000
0.001
0.002
β_IV

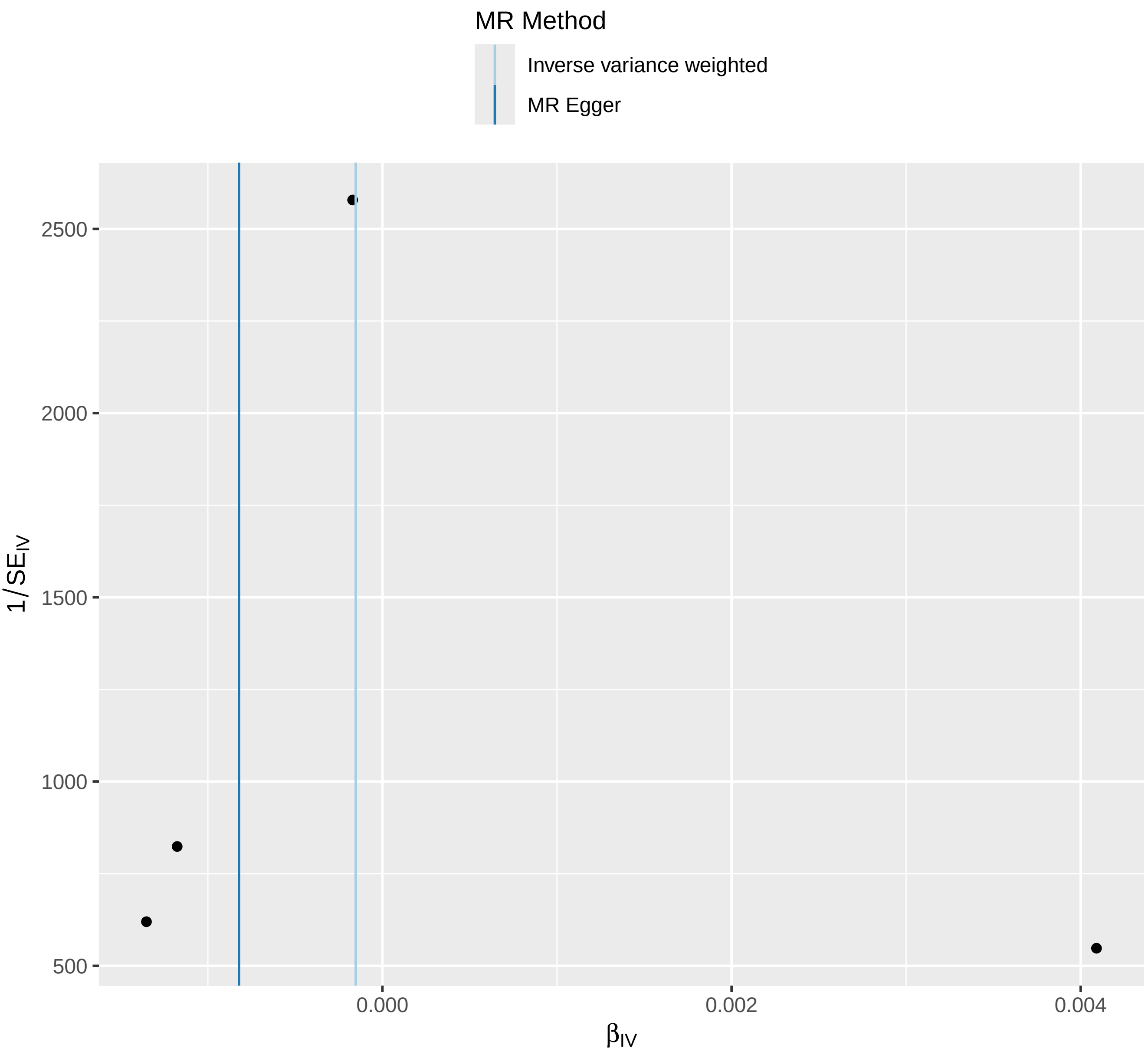
MR Method
Inverse variance weighted
MR Egger
2500
2000
1500
1000
500
$1/SE_{IV}$
0.000
0.002
0.004
β_{IV}

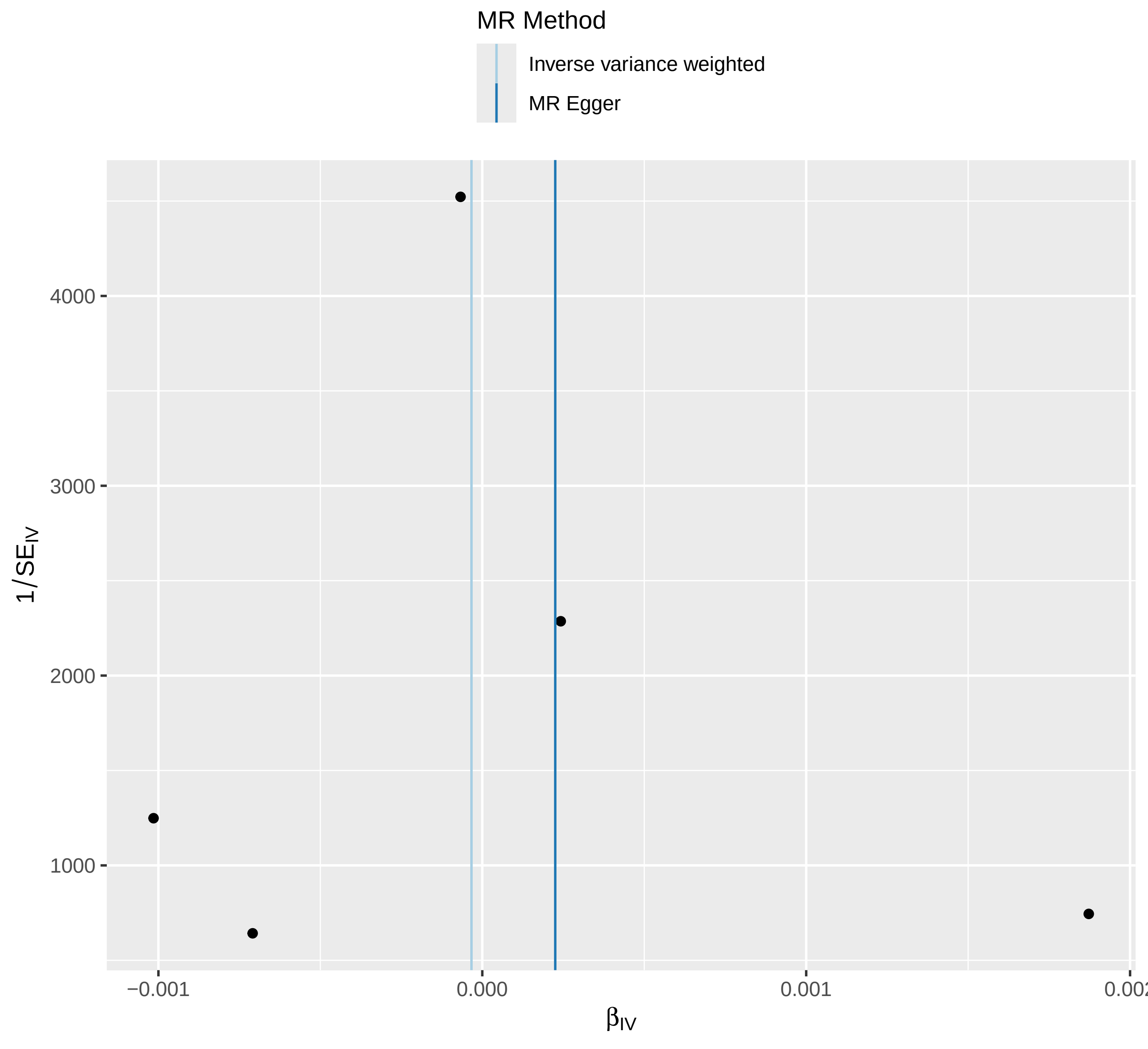
MR Method
Inverse variance weighted
MR Egger
4000
3000
2000
1000
1/SE_IV
−0.001
0.000
0.001
0.002
β_IV

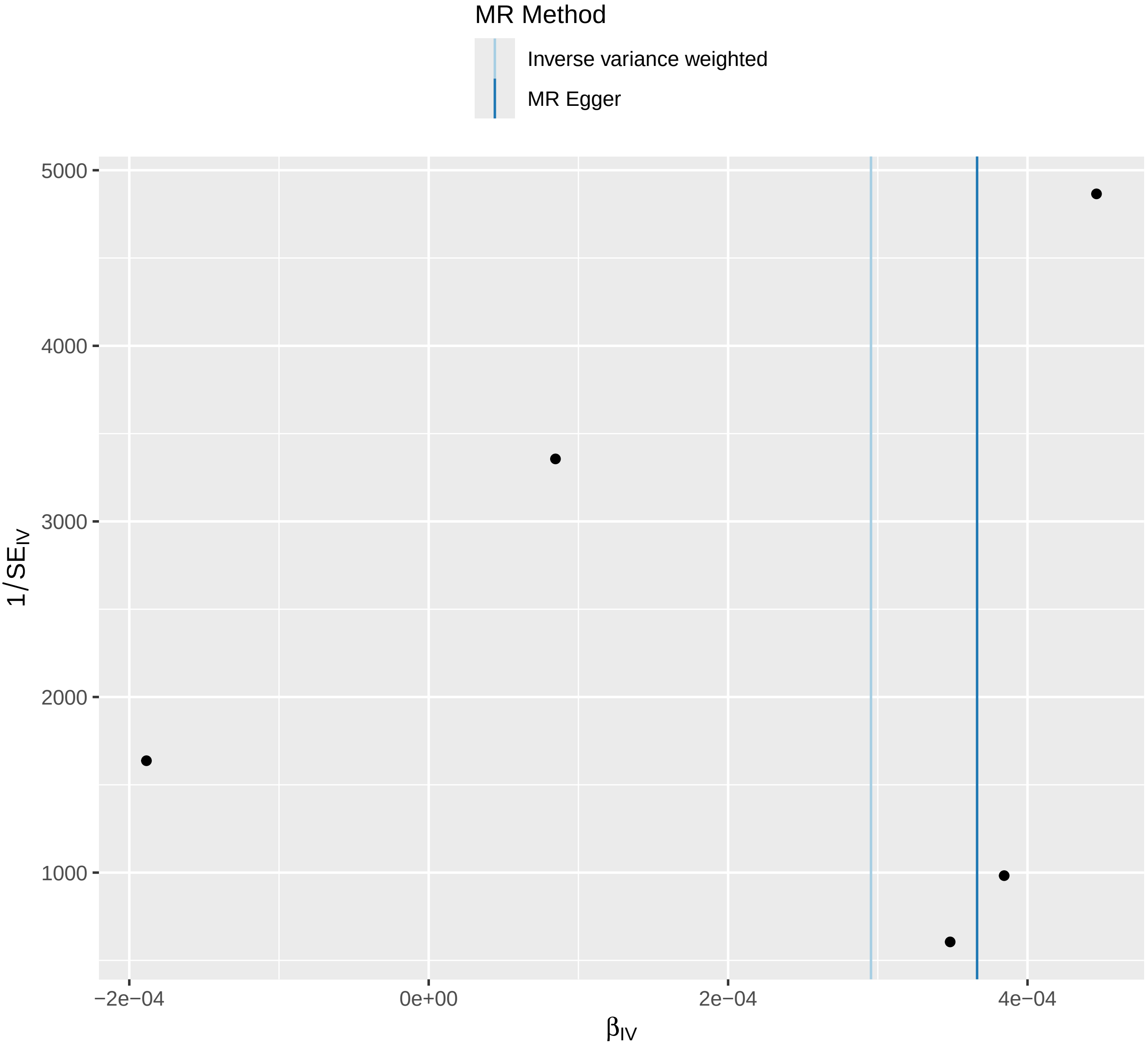

MR Method
Inverse variance weighted
MR Egger
5000
4000
3000
2000
1000
1/SE$_{IV}$
−2e−04
0e+00
2e−04
4e−04
β$_{IV}$

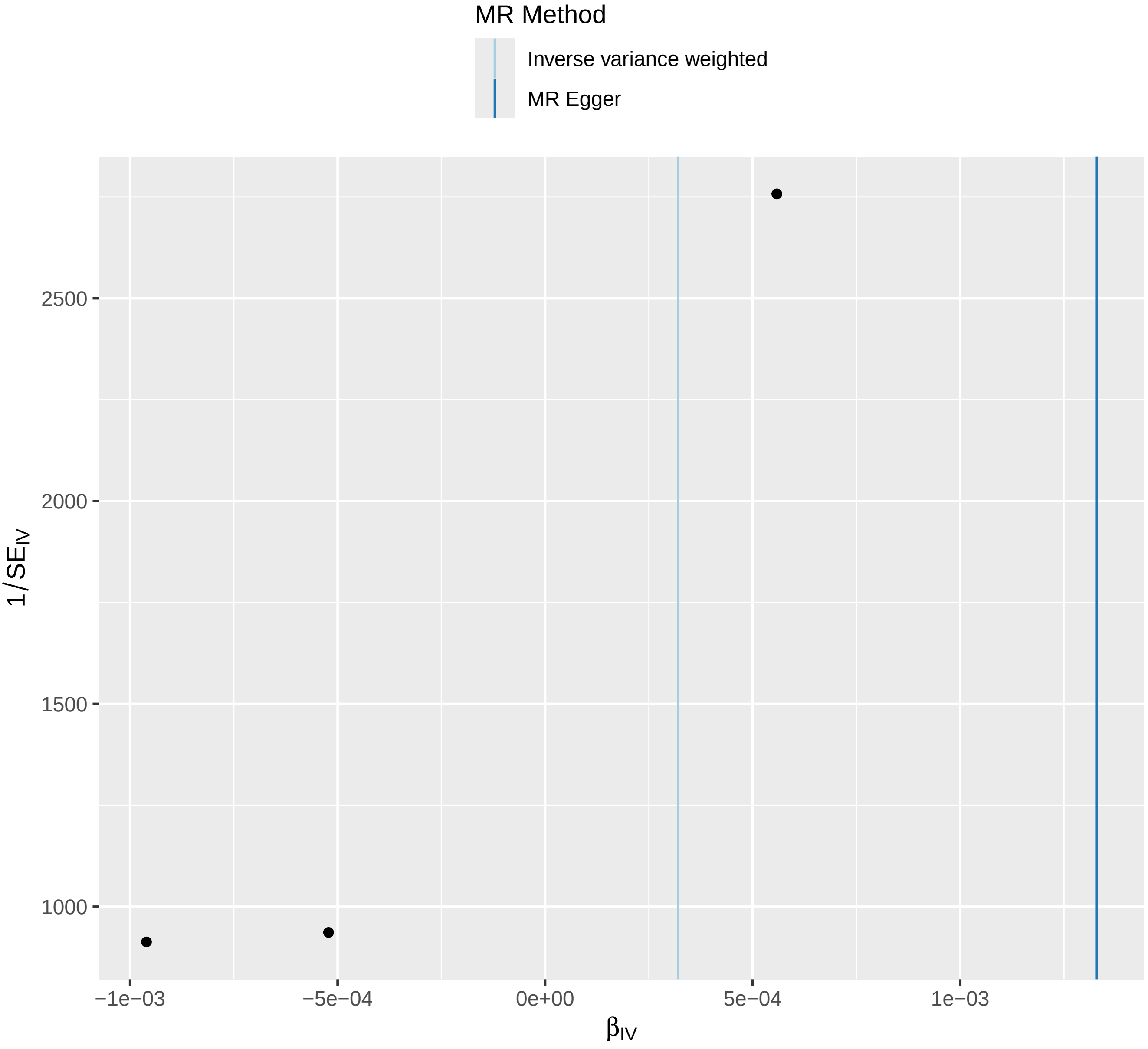

MR Method
Inverse variance weighted
MR Egger
2500
2000
1500
1000
1/SE$_{IV}$
−1e−03
−5e−04
0e+00
5e−04
1e−03
β$_{IV}$

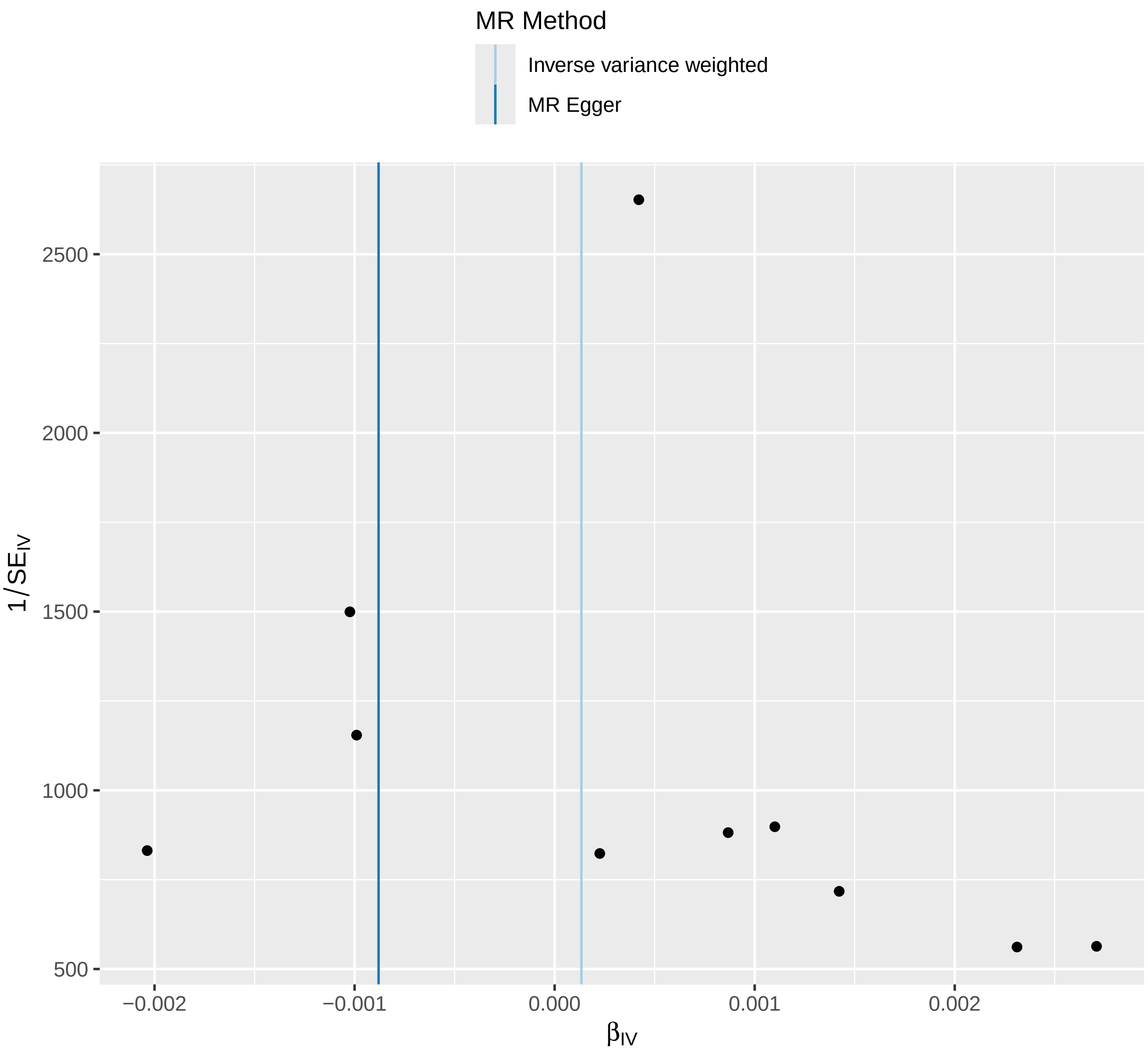
MR Method
Inverse variance weighted
MR Egger
2500
2000
1500
1000
500
1/SE_IV
−0.002
−0.001
0.000
0.001
0.002
β_IV

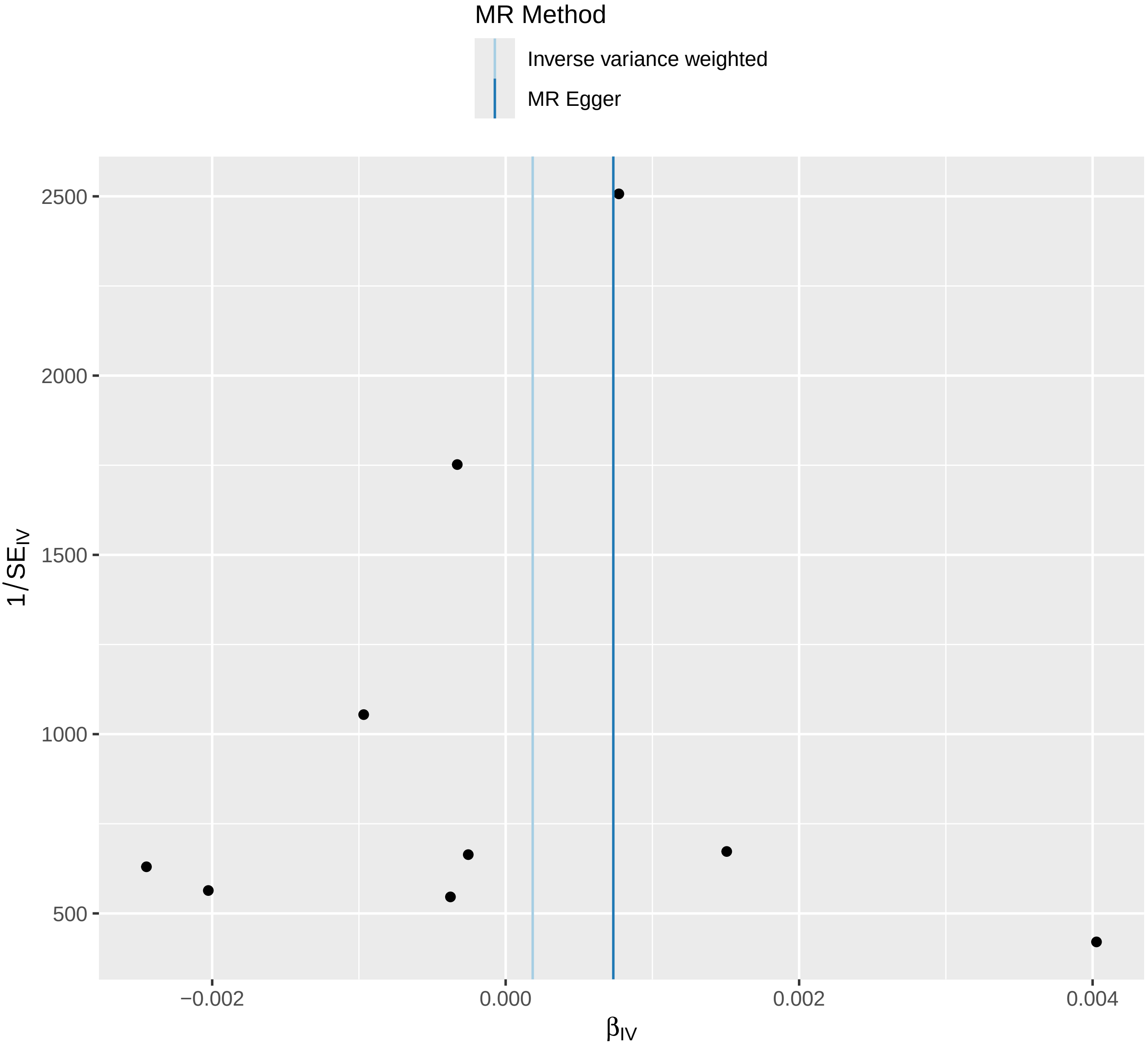

MR Method
Inverse variance weighted
MR Egger
2500
2000
1500
1000
500
1/SE_IV
−0.002
0.000
0.002
0.004
β_IV

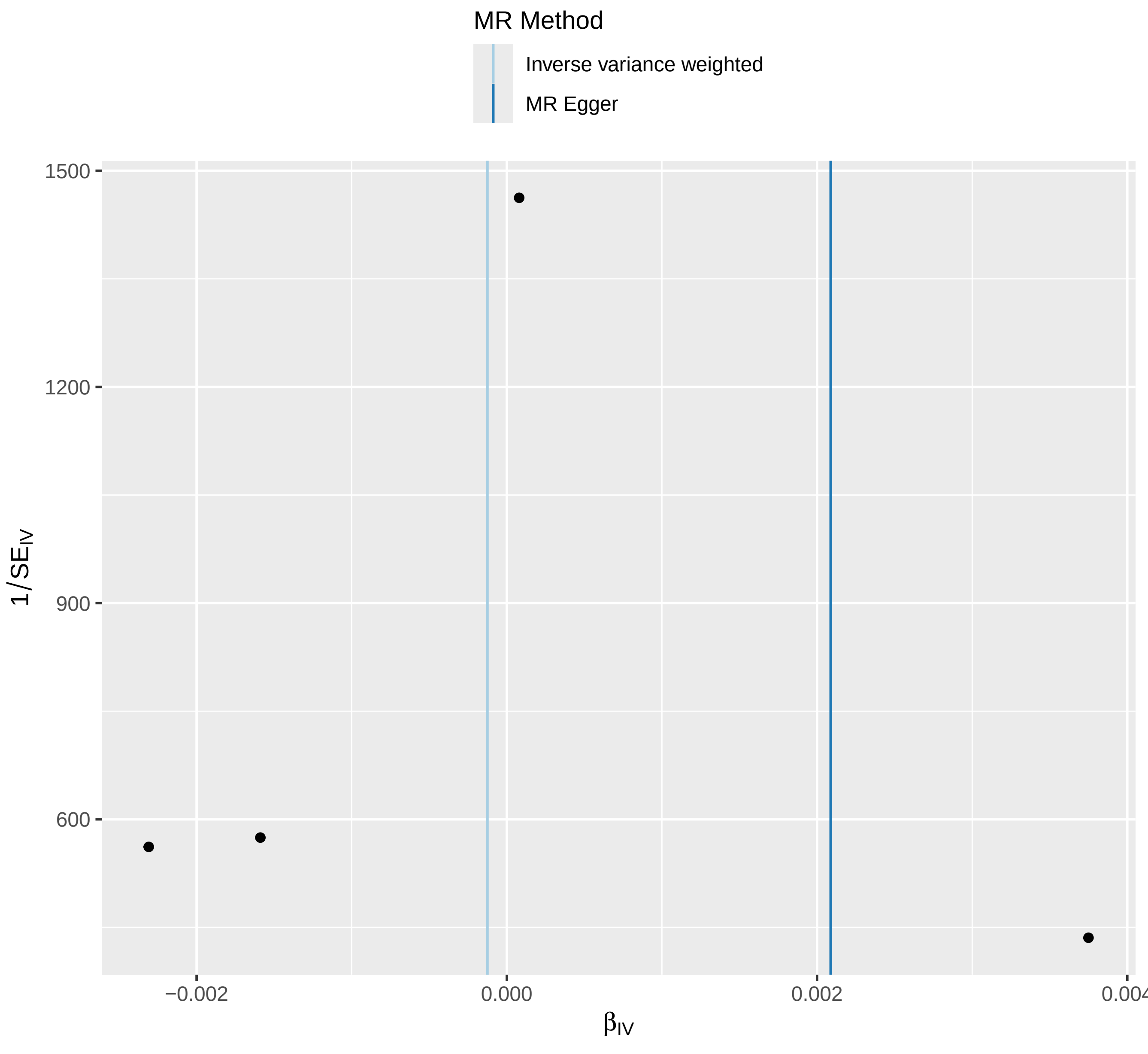

MR Method
Inverse variance weighted
MR Egger
1500
1200
900
600
−0.002
0.000
0.002
0.004
$1/SE_{IV}$
β_{IV}

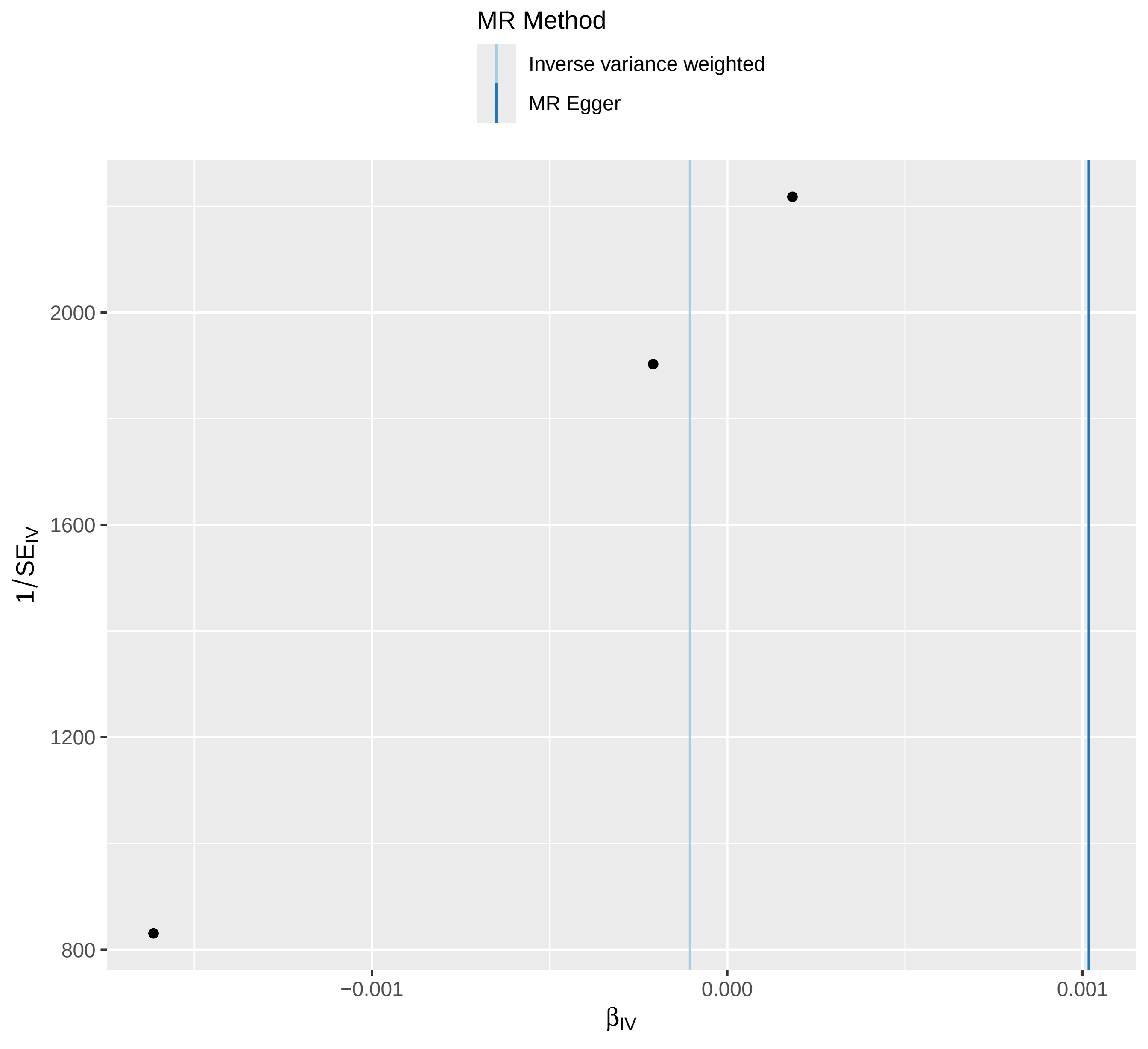

MR Method
Inverse variance weighted
MR Egger
2000
1600
1200
800
1/SE_IV
−0.001
0.000
0.001
β_IV

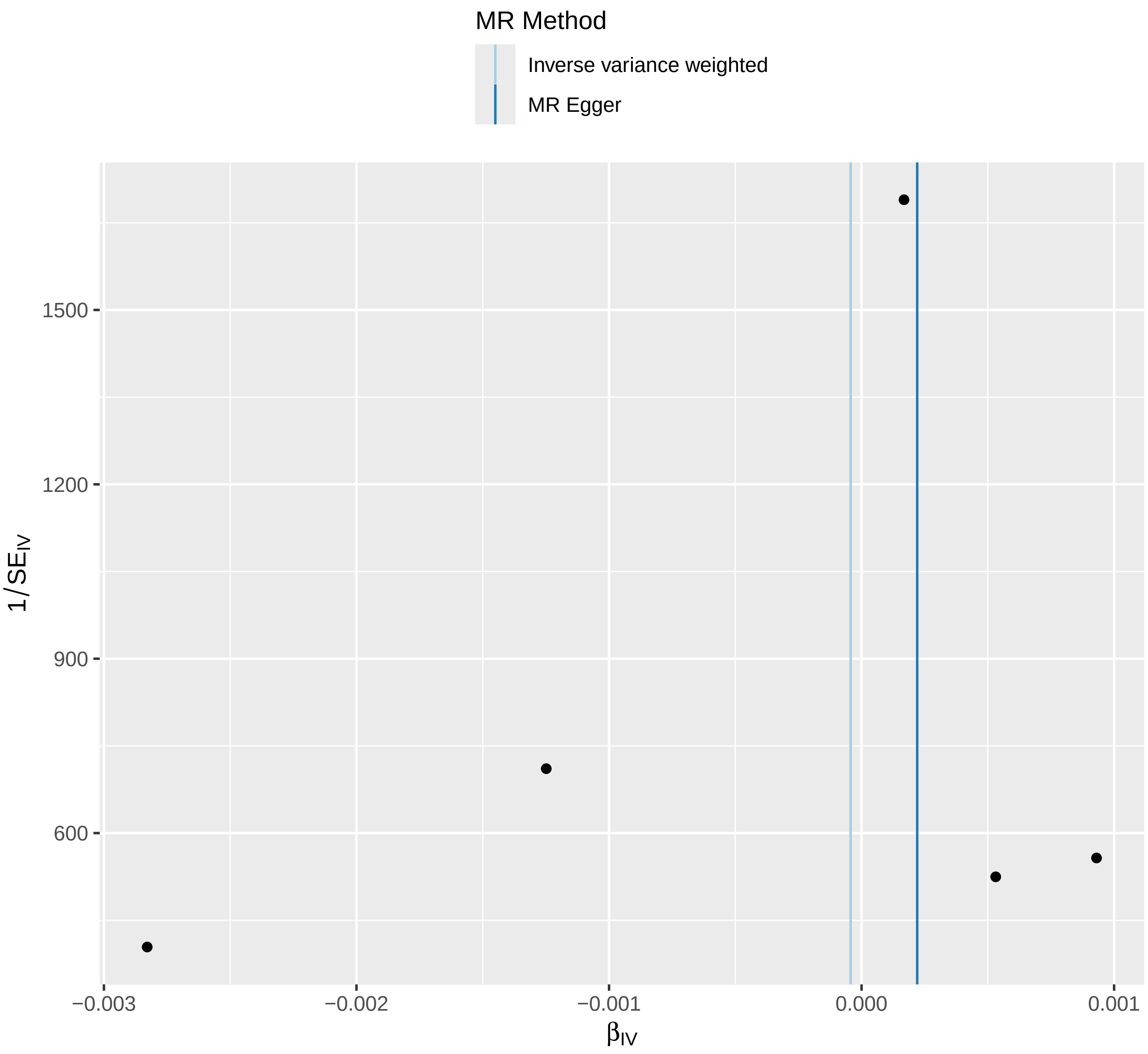
MR Method
Inverse variance weighted
MR Egger
1500
1200
900
600
1/SE$_{IV}$
−0.003
−0.002
−0.001
0.000
0.001
β$_{IV}$

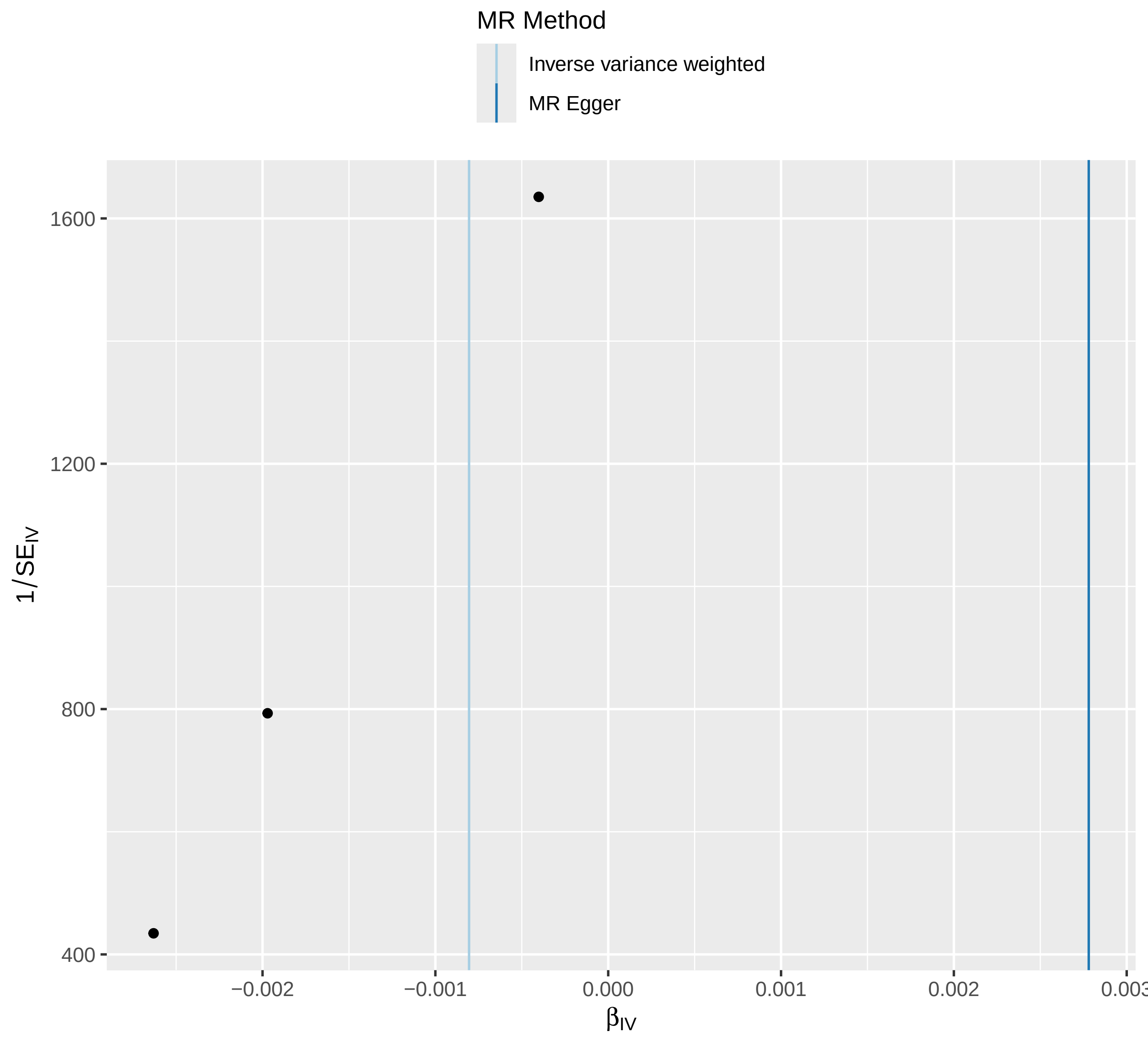
MR Method
Inverse variance weighted
MR Egger
1600
1200
800
400
1/SE_IV
−0.002
−0.001
0.000
0.001
0.002
0.003
β_IV

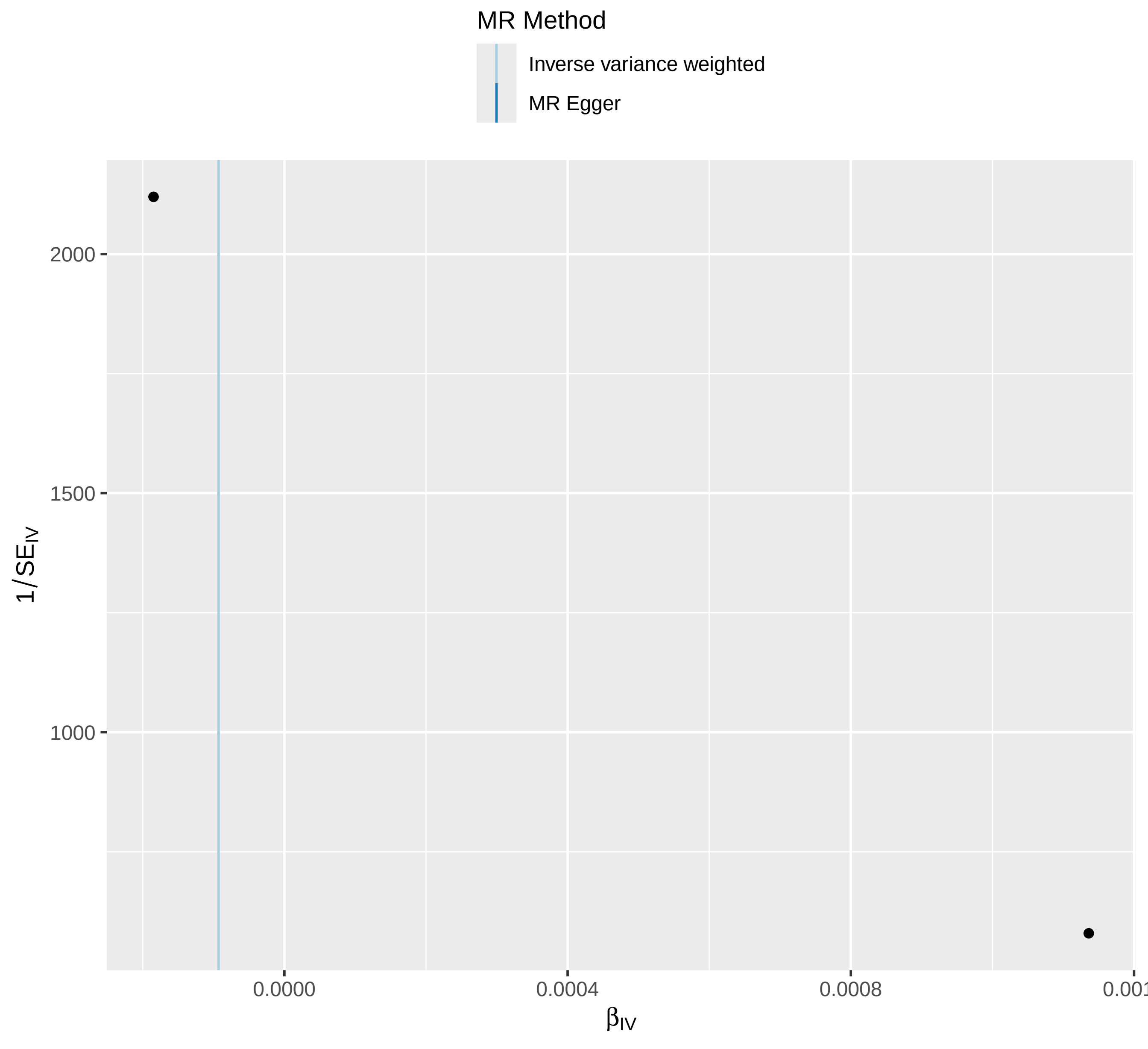

MR Method
Inverse variance weighted
MR Egger
2000
1500
1000
1/SE_IV
0.0000
0.0004
0.0008
0.001
β_IV

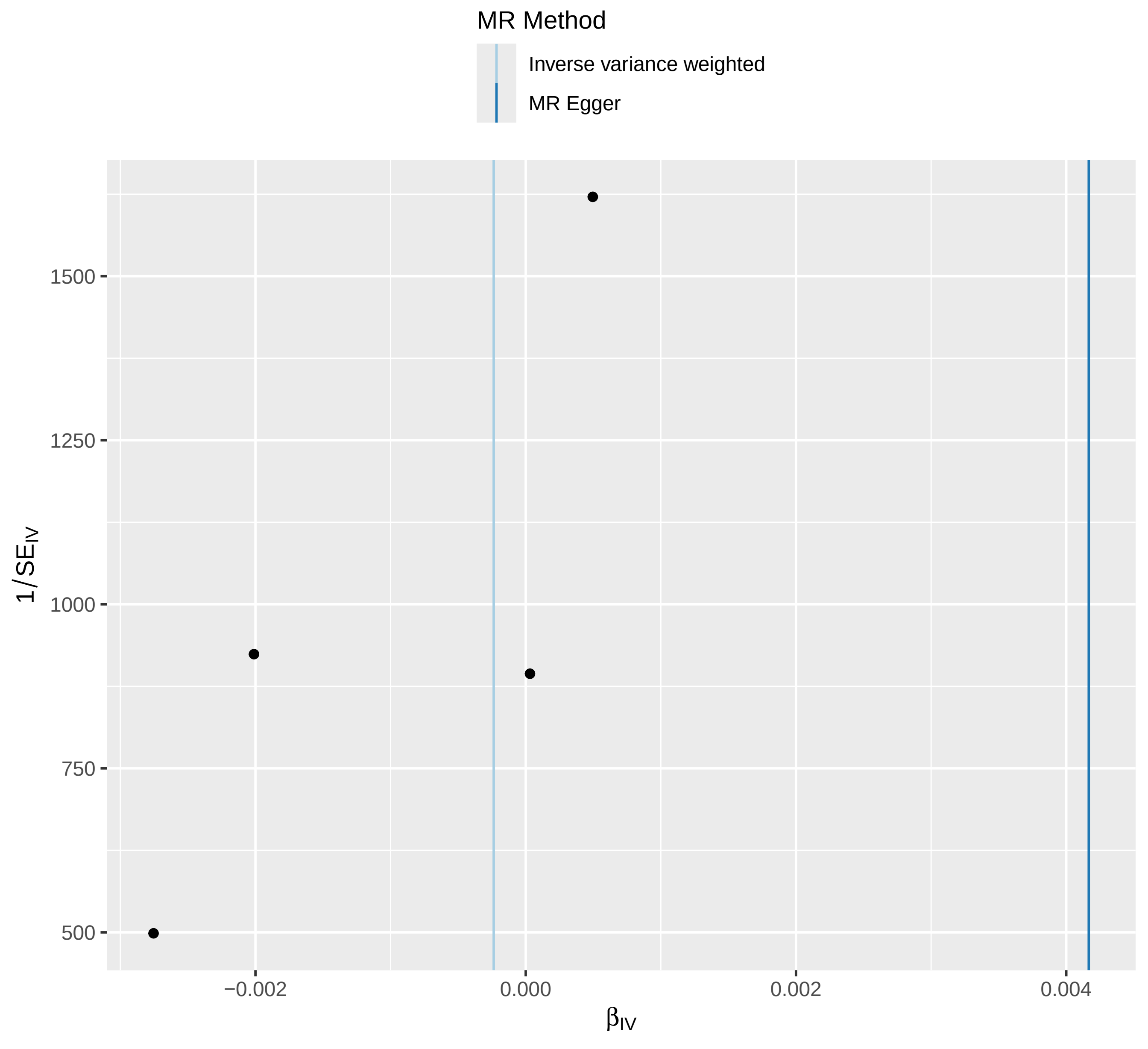

MR Method
Inverse variance weighted
MR Egger
1500
1250
1000
750
500
−0.002
0.000
0.002
0.004
$1/SE_{IV}$
β_{IV}

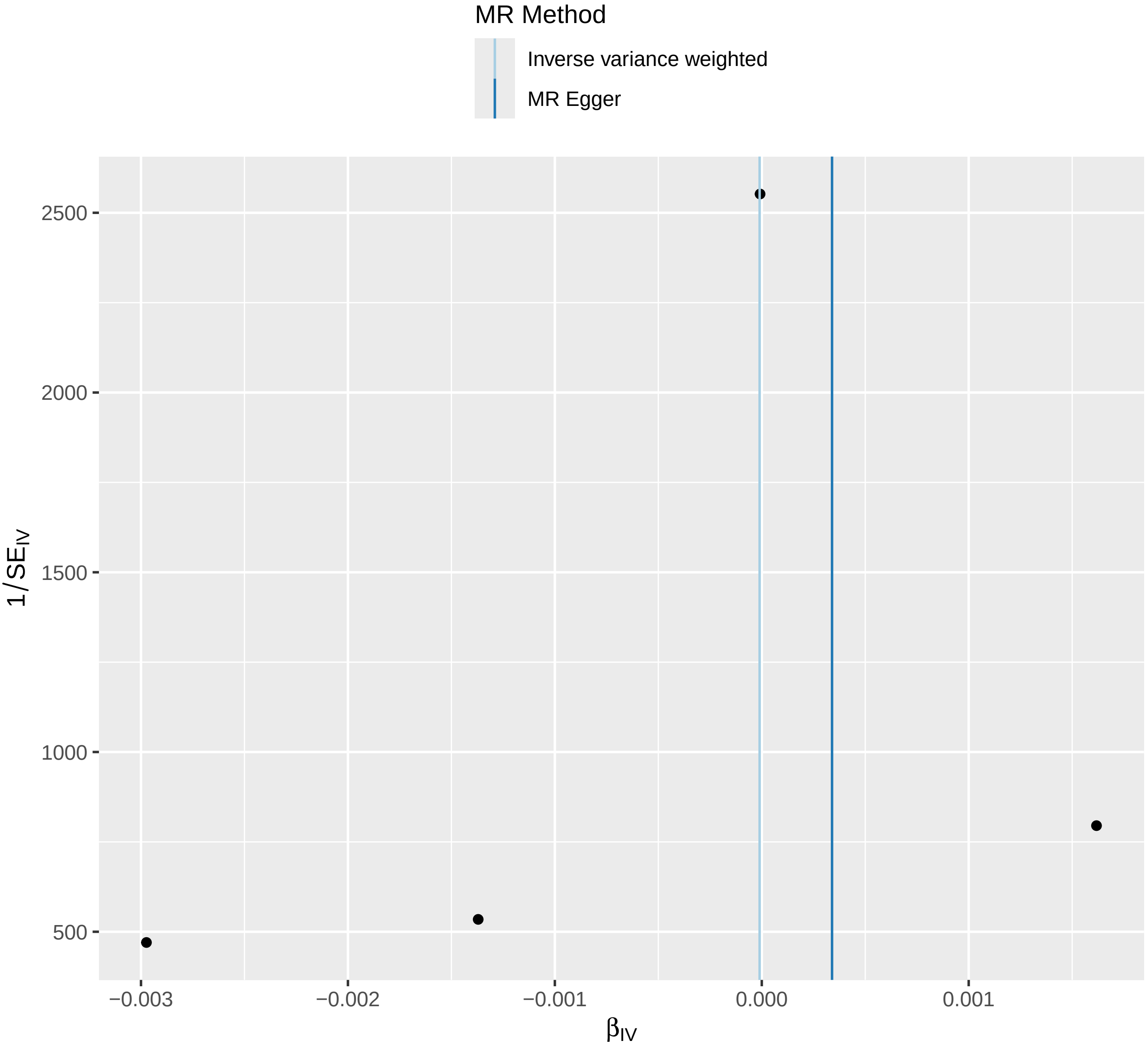
MR Method
Inverse variance weighted
MR Egger
2500
2000
1500
1000
500
$1/SE_{IV}$
−0.003
−0.002
−0.001
0.000
0.001
β_{IV}

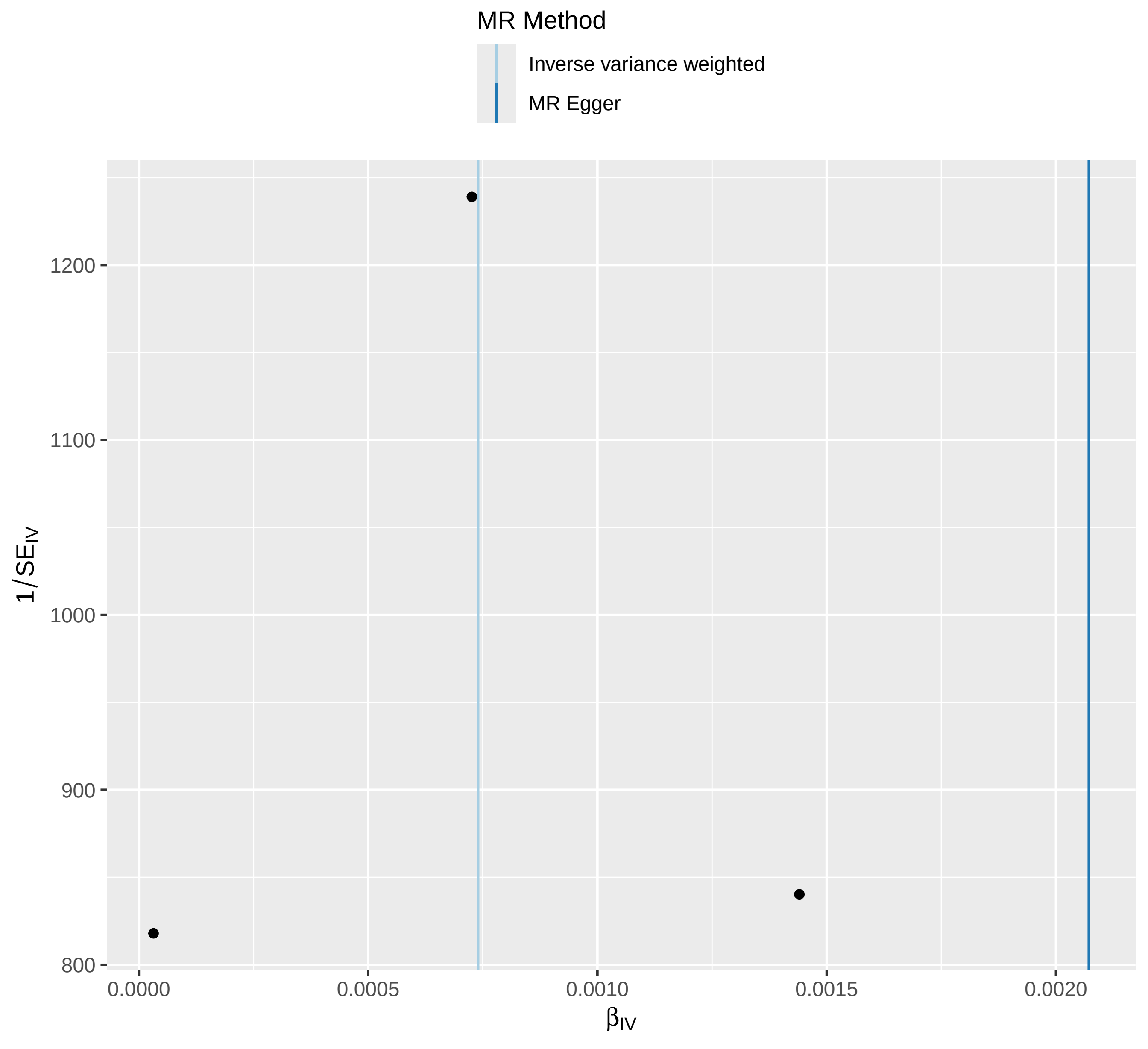

MR Method
Inverse variance weighted
MR Egger
1200
1100
1000
900
800
1/SE_IV
0.0000
0.0005
0.0010
0.0015
0.0020
β_IV

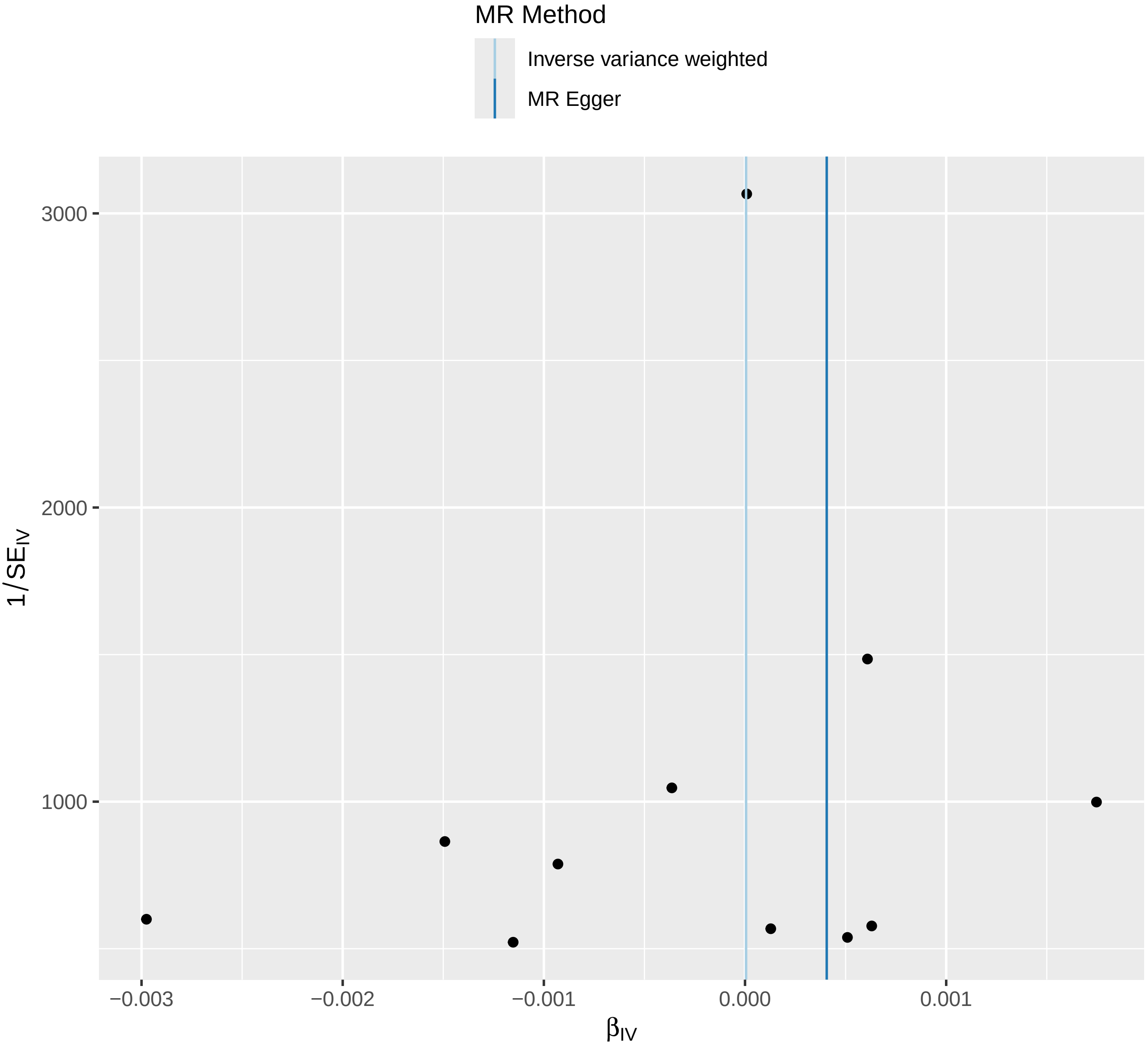

MR Method
Inverse variance weighted
MR Egger
3000
2000
1000
$1/SE_{IV}$
−0.003
−0.002
−0.001
0.000
0.001
β_{IV}

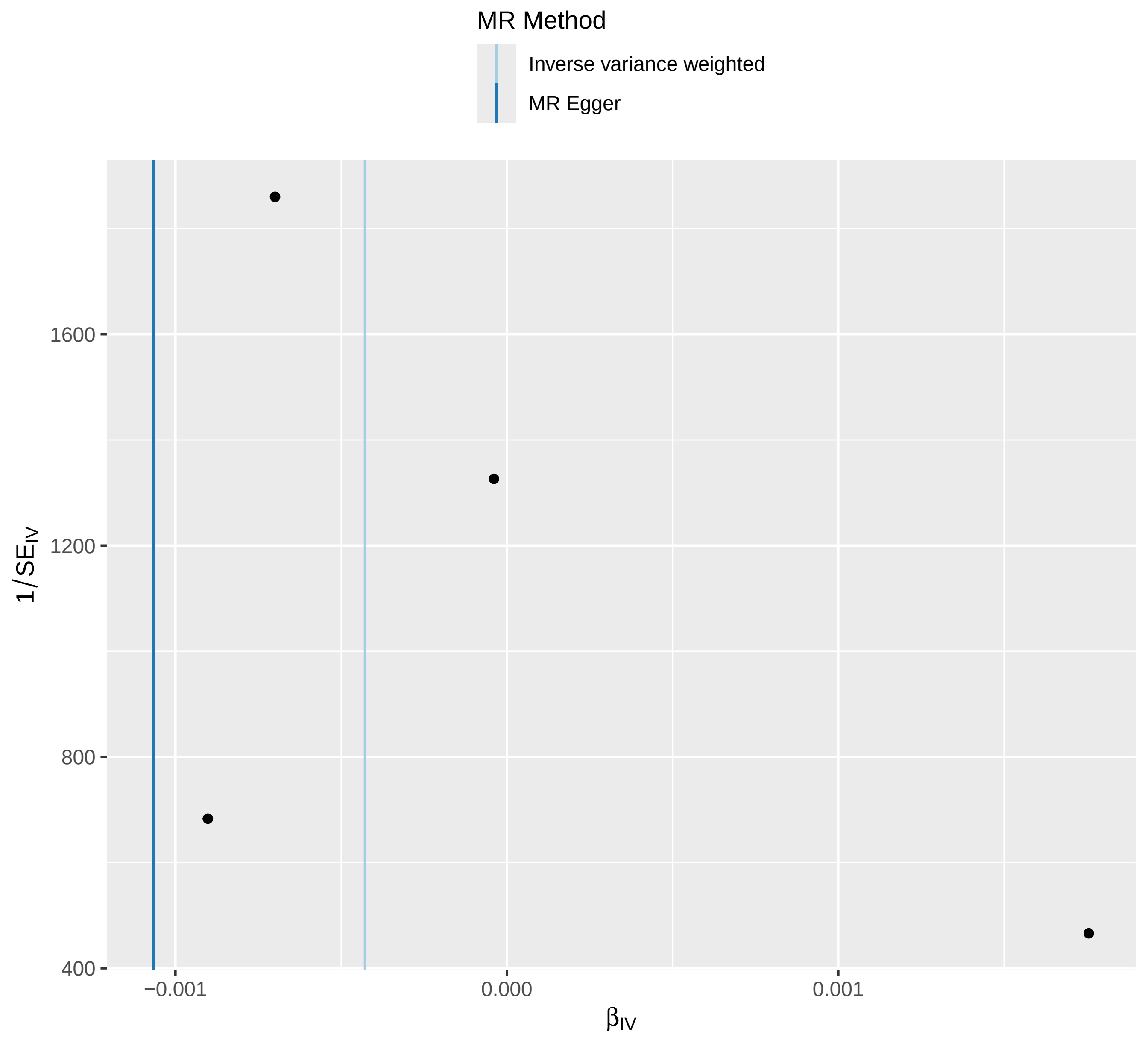
MR Method
Inverse variance weighted
MR Egger
1600
1200
800
400
1/SE_IV
−0.001
0.000
0.001
β_IV

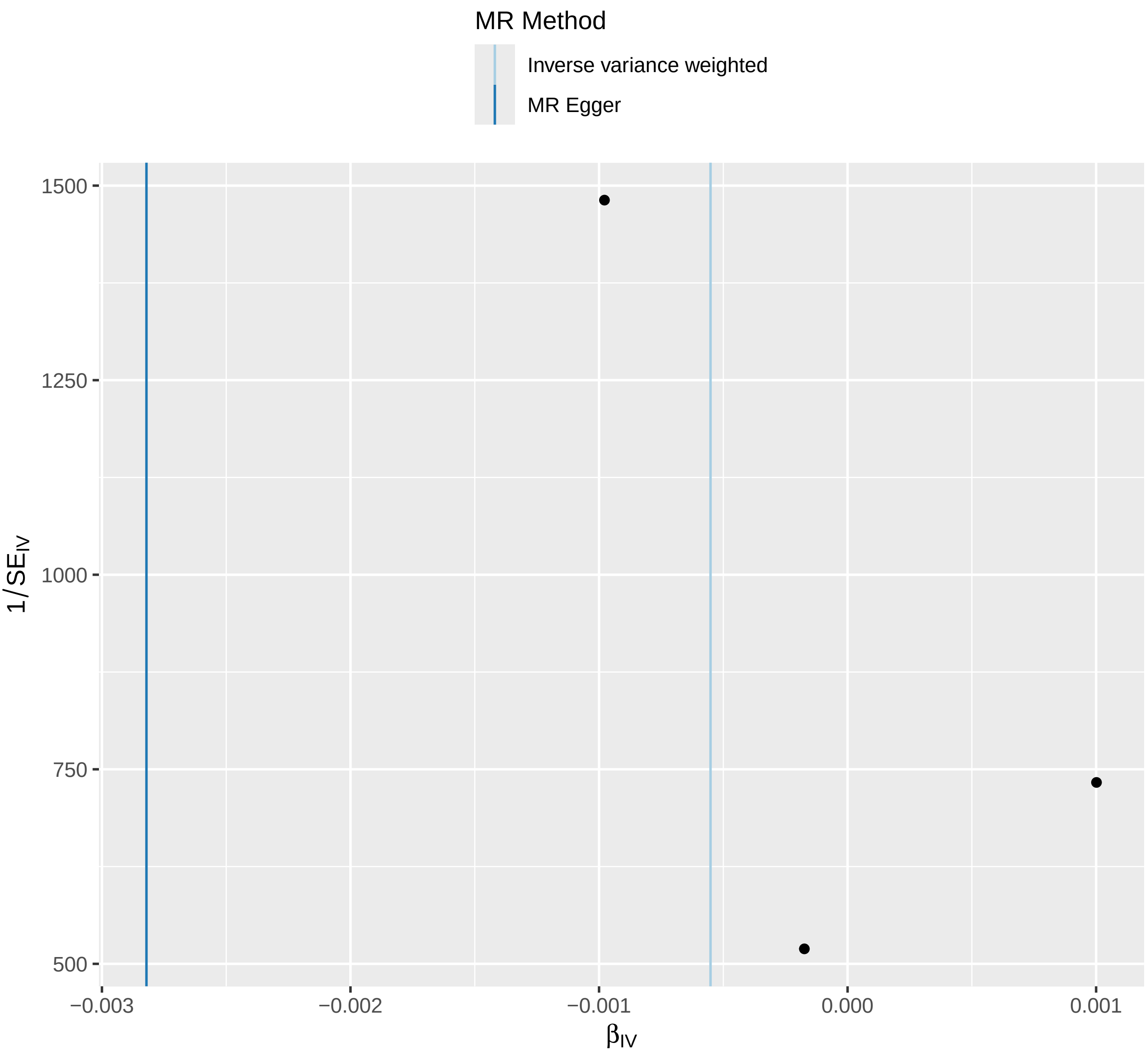

MR Method
Inverse variance weighted
MR Egger
1500
1250
1000
750
500
1/SE_IV
−0.003
−0.002
−0.001
0.000
0.001
β_IV

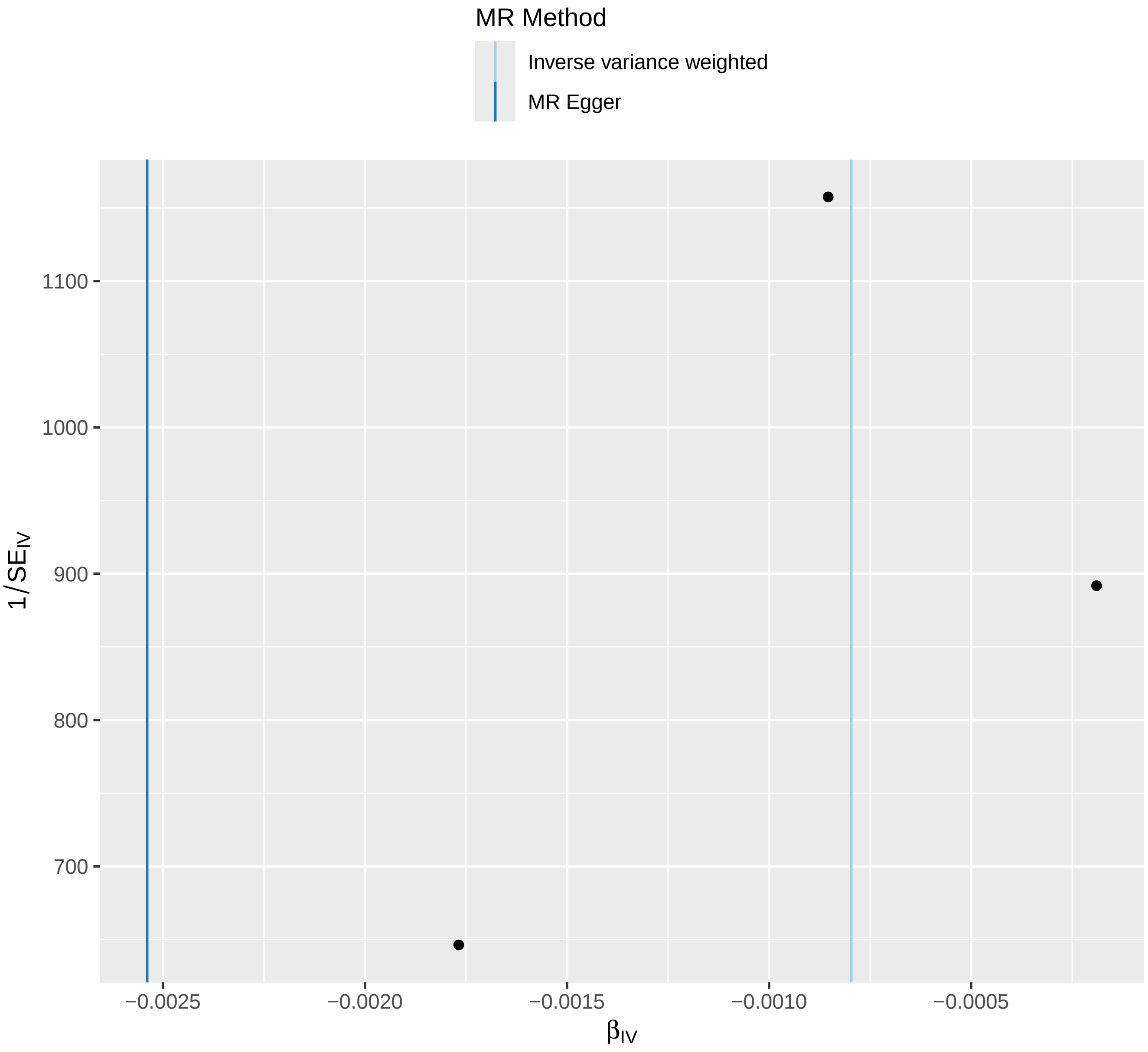

MR Method
Inverse variance weighted
MR Egger
1100
1000
900
800
700
1/SE_{IV}
−0.0025
−0.0020
−0.0015
−0.0010
−0.0005
β_{IV}

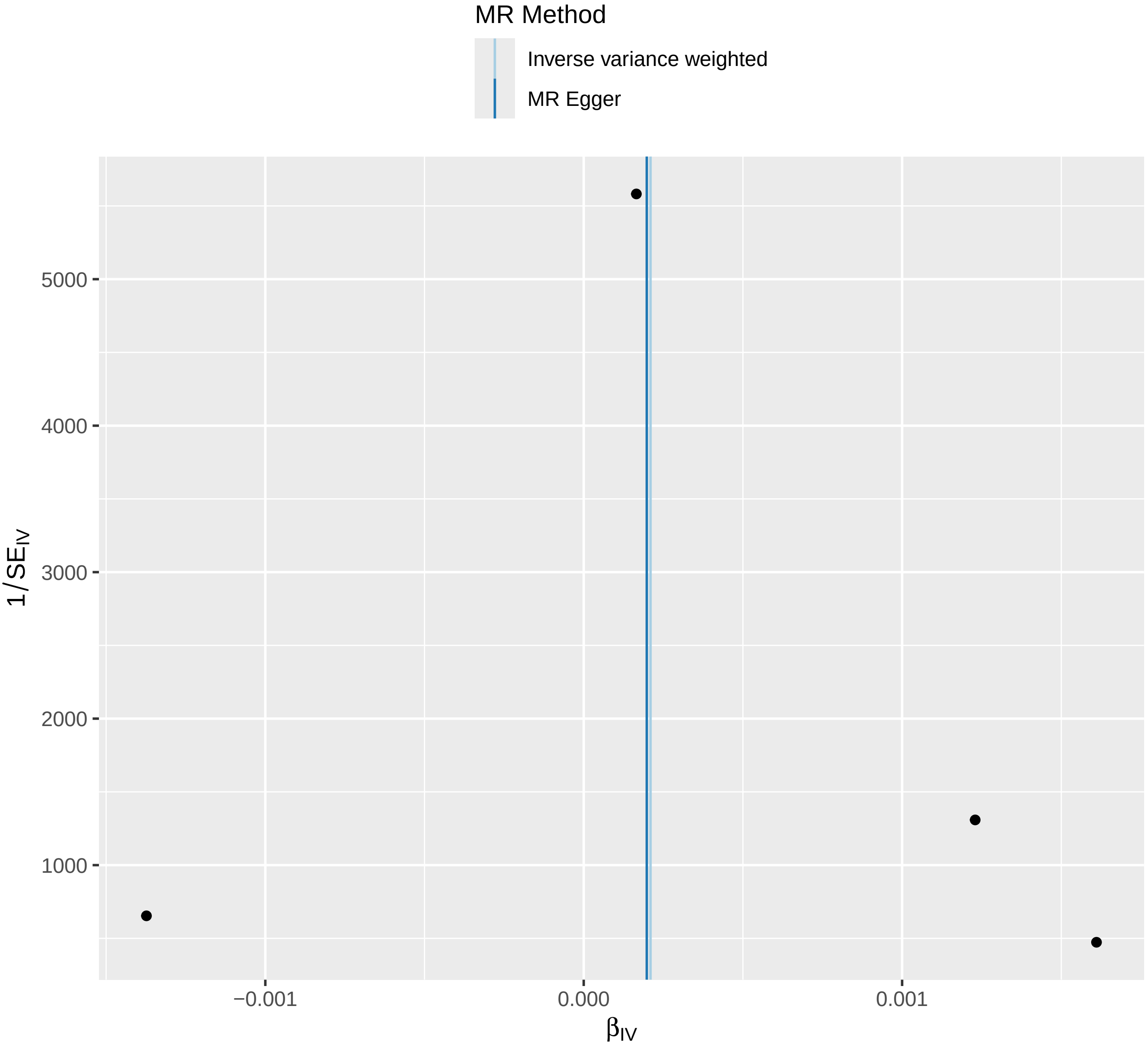

MR Method
Inverse variance weighted
MR Egger
5000
4000
3000
2000
1000
1/SE_IV
−0.001
0.000
0.001
β_IV

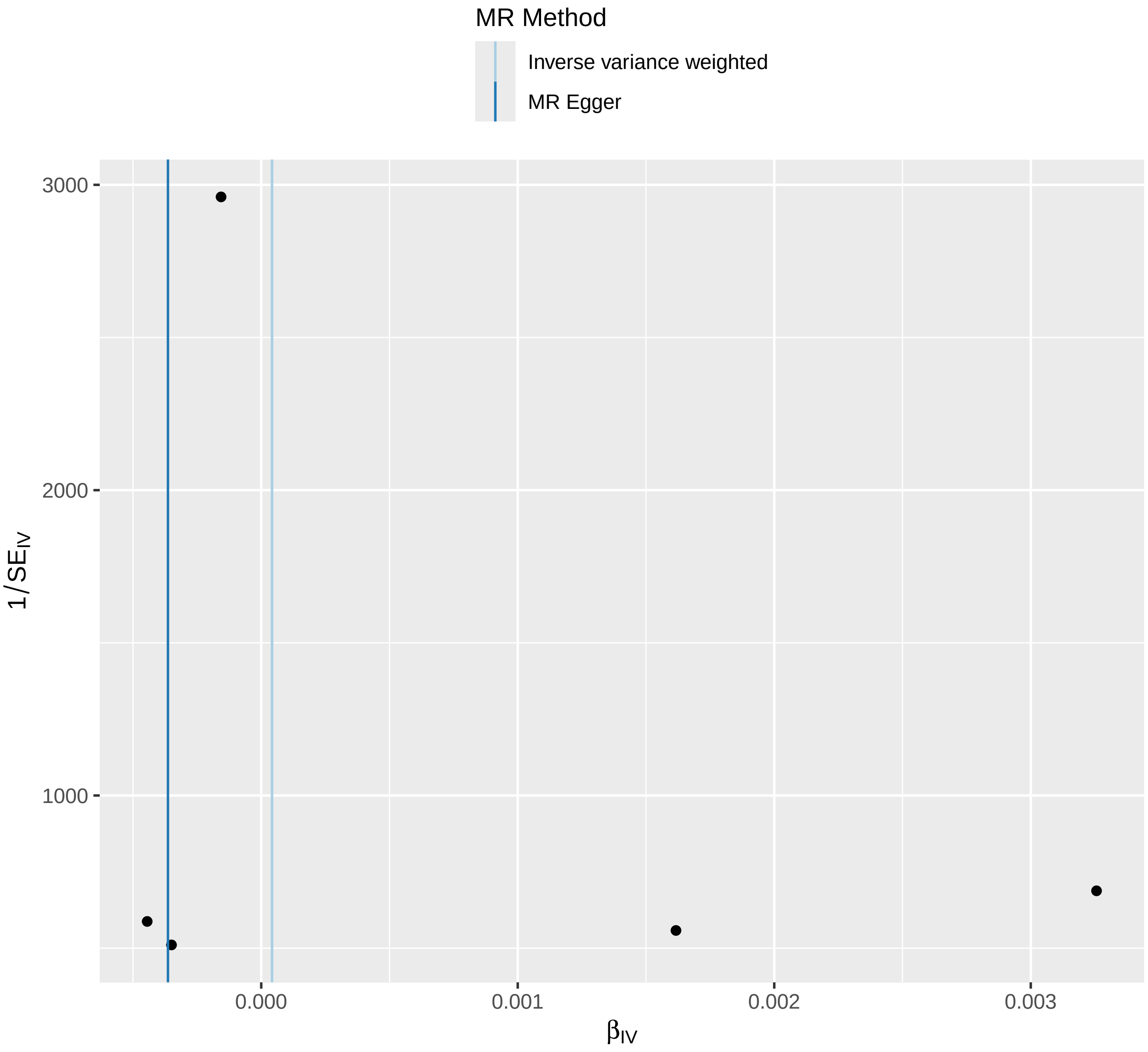
MR Method
Inverse variance weighted
MR Egger
3000
2000
1000
0.000
0.001
0.002
0.003
1/SE_IV
β_IV

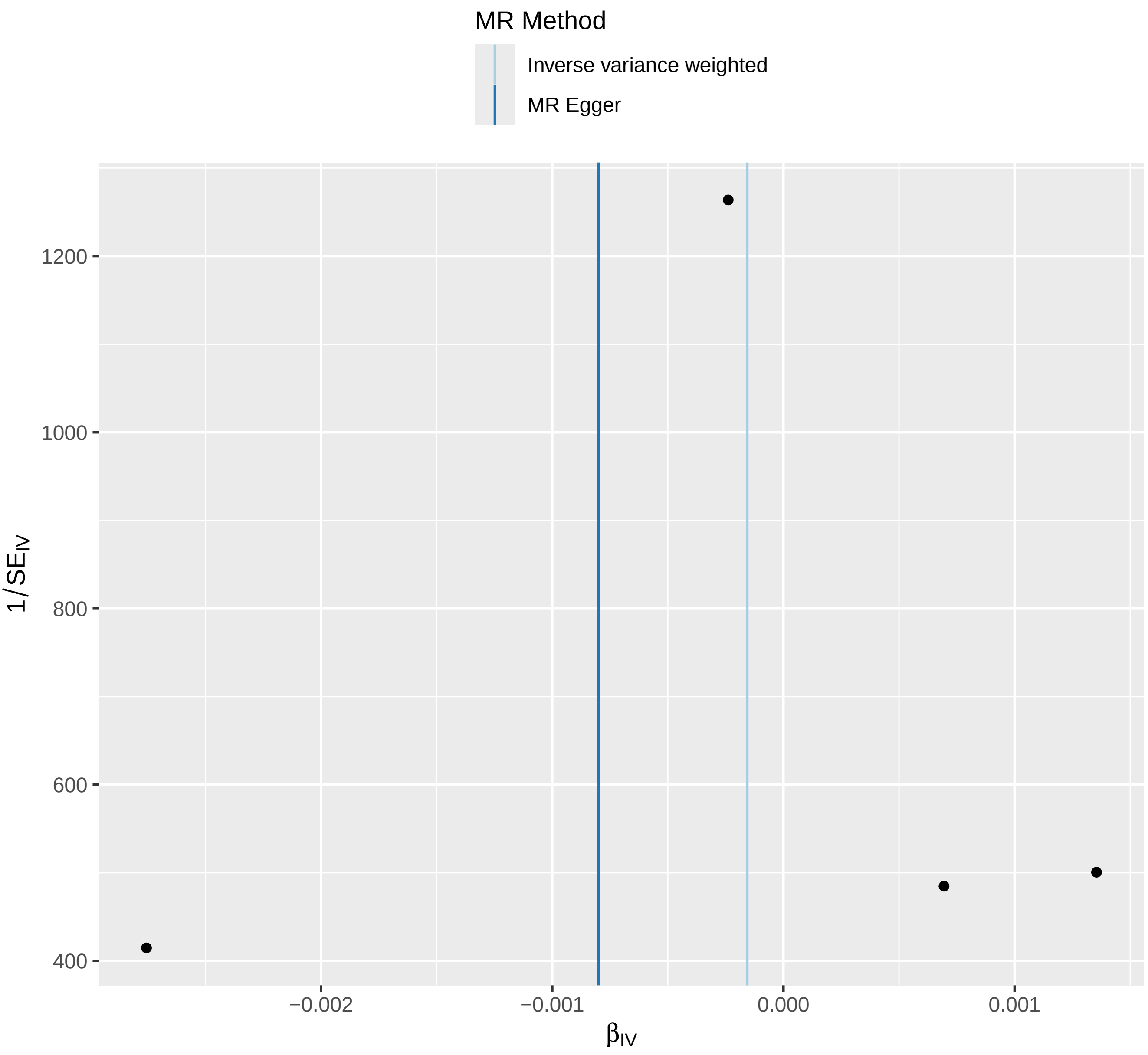

MR Method
Inverse variance weighted
MR Egger
1200
1000
800
600
400
1/SE_IV
−0.002
−0.001
0.000
0.001
β_IV

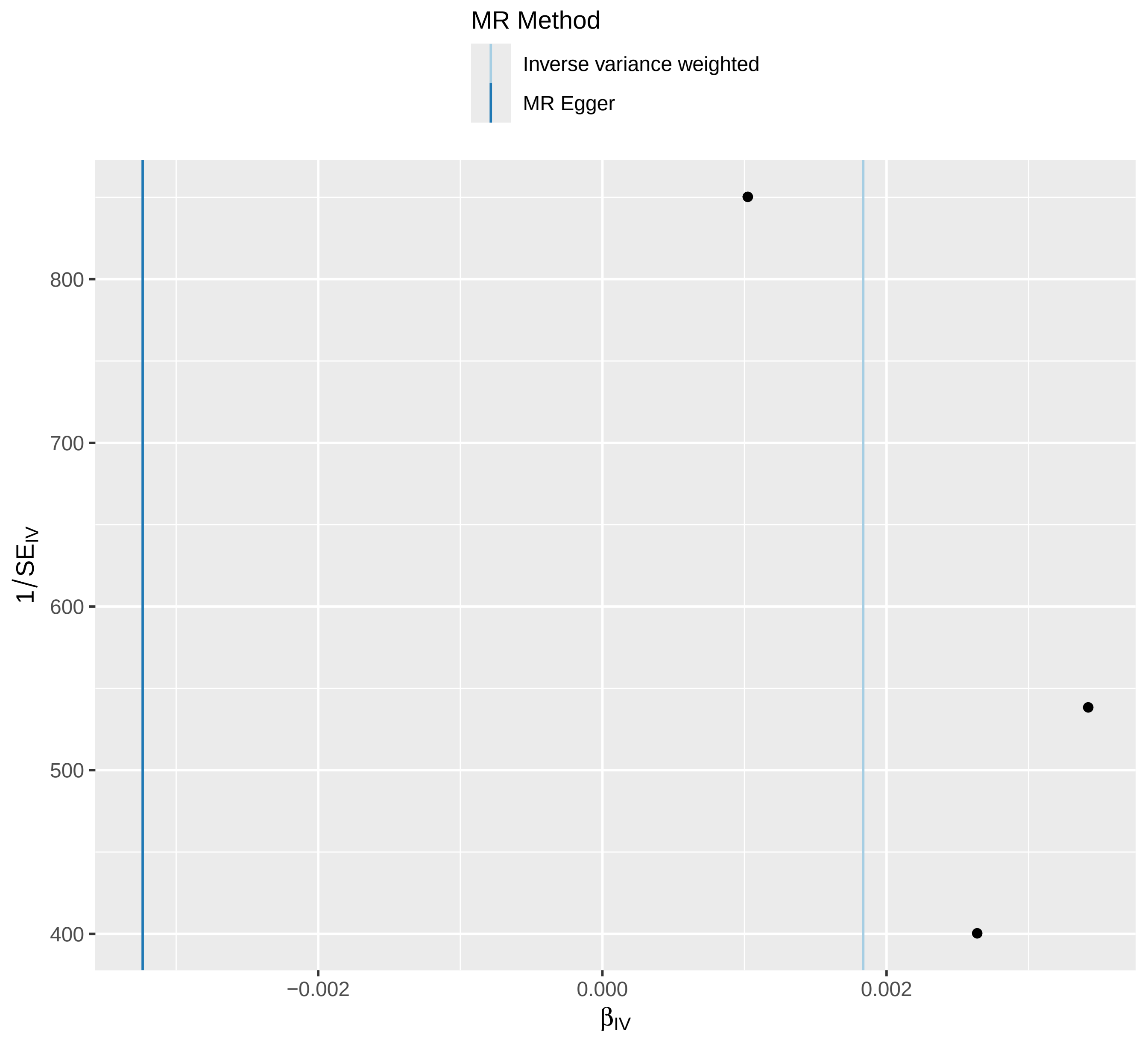
MR Method
Inverse variance weighted
MR Egger
800
700
600
500
400
1/SE_IV
−0.002
0.000
0.002
β_IV

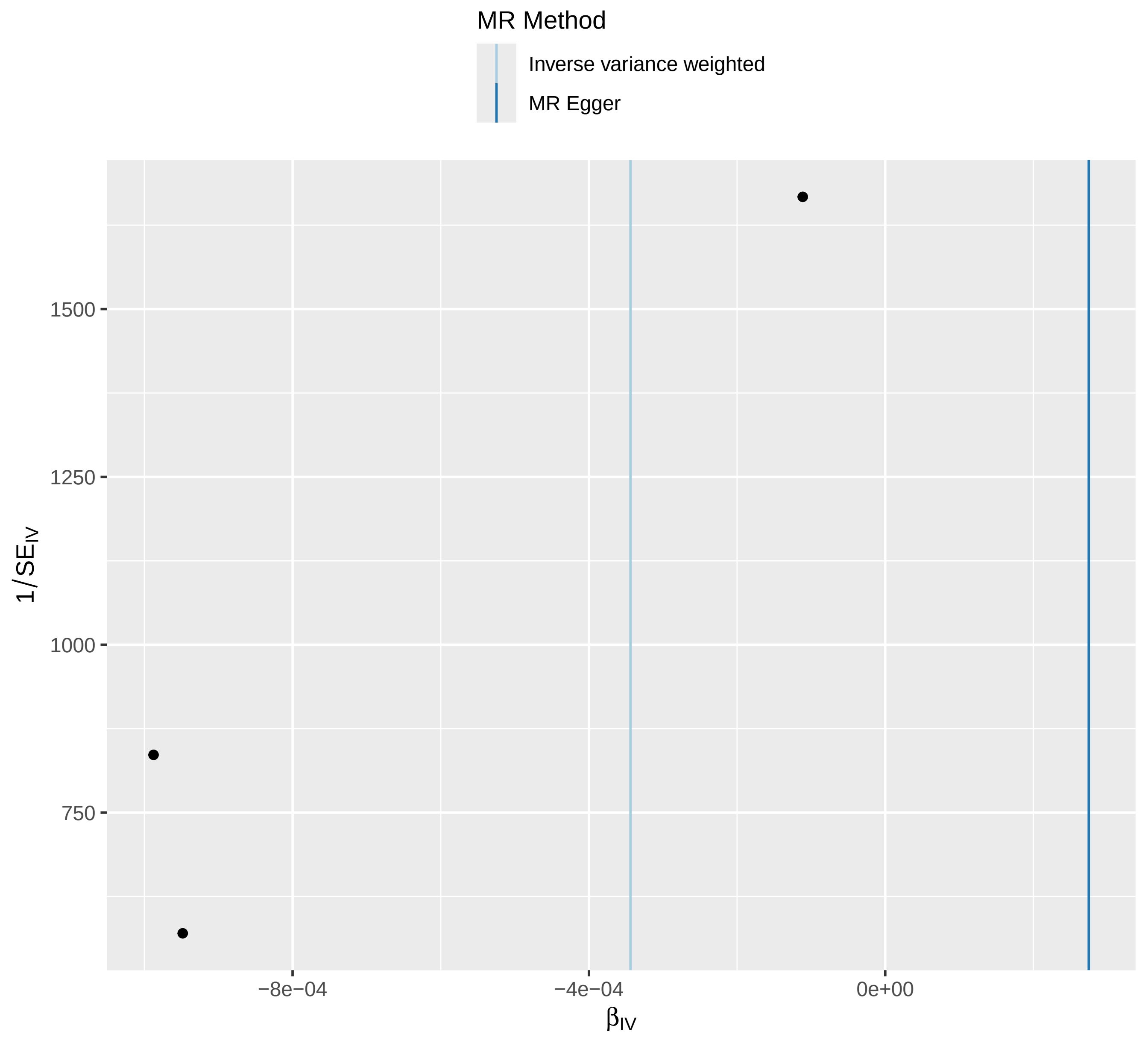

MR Method
Inverse variance weighted
MR Egger
1500
1250
1000
750
1/SE$_{IV}$
−8e−04
−4e−04
0e+00
β_{IV}

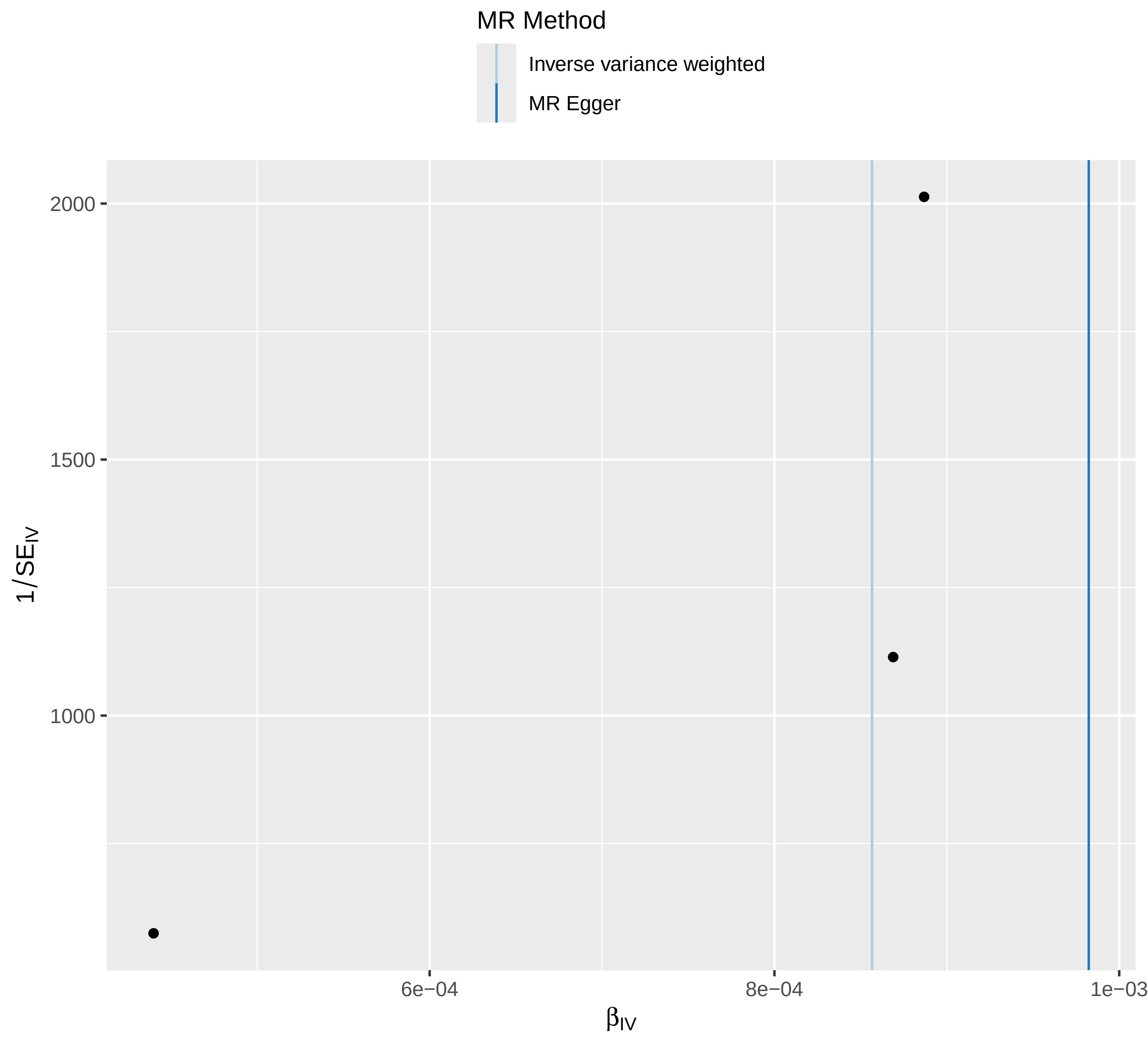
MR Method
Inverse variance weighted
MR Egger
2000
1500
1000
1/SE_IV
6e−04
8e−04
1e−03
β_IV

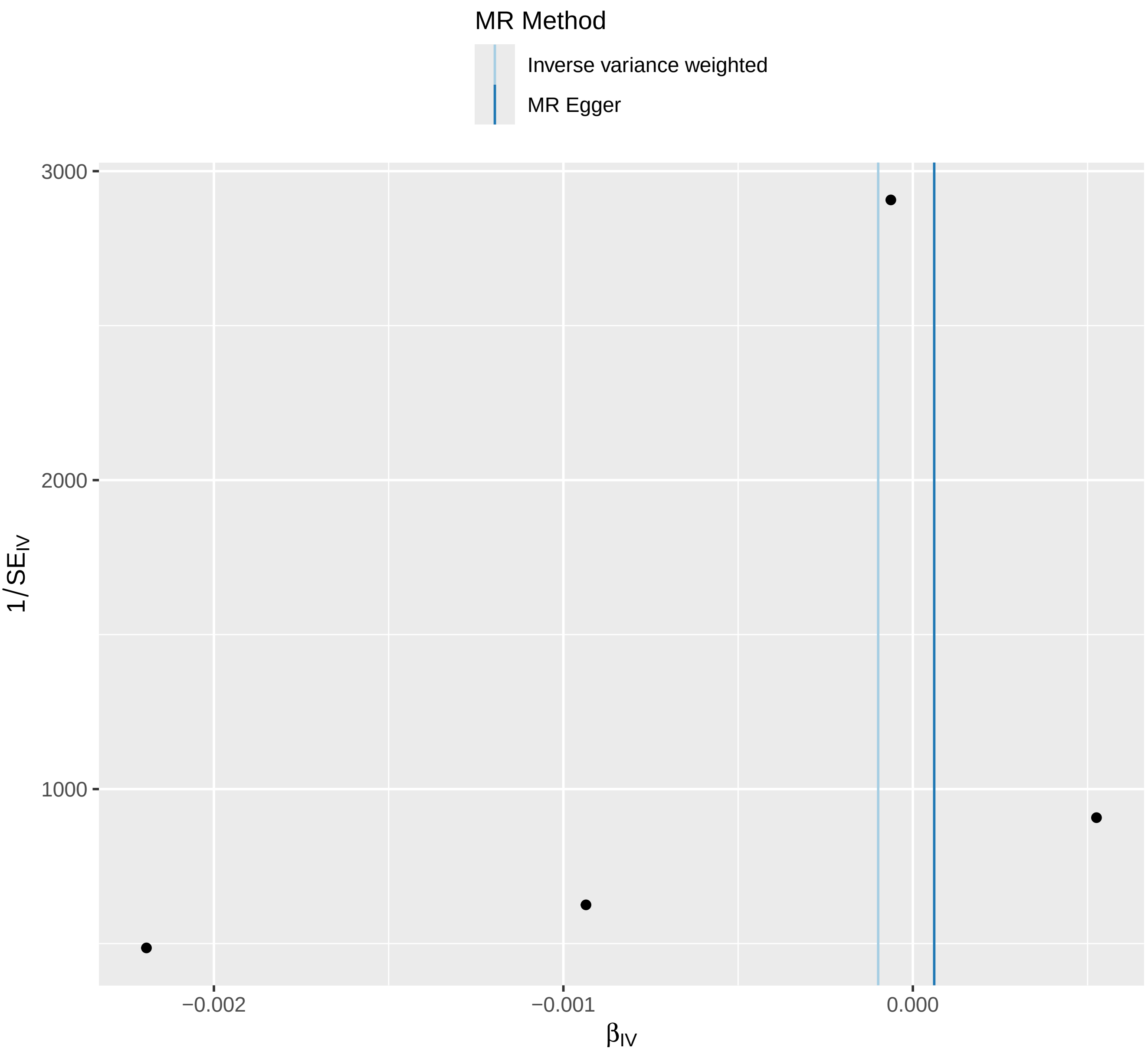

MR Method
Inverse variance weighted
MR Egger
3000
2000
1000
$1/SE_{IV}$
−0.002
−0.001
0.000
β_{IV}

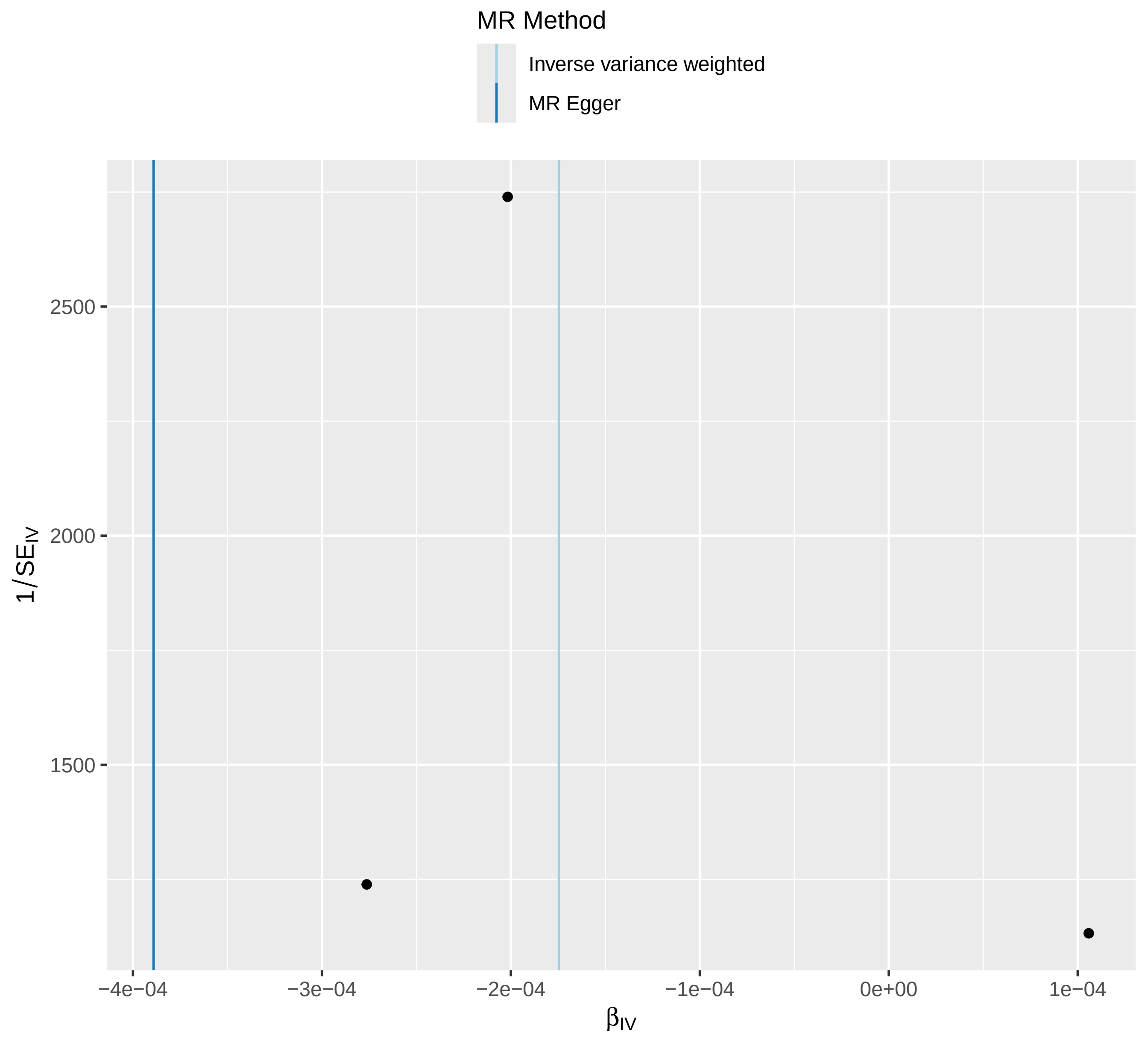

MR Method
Inverse variance weighted
MR Egger
2500
2000
1500
1/SE_IV
−4e−04
−3e−04
−2e−04
−1e−04
0e+00
1e−04
β_IV

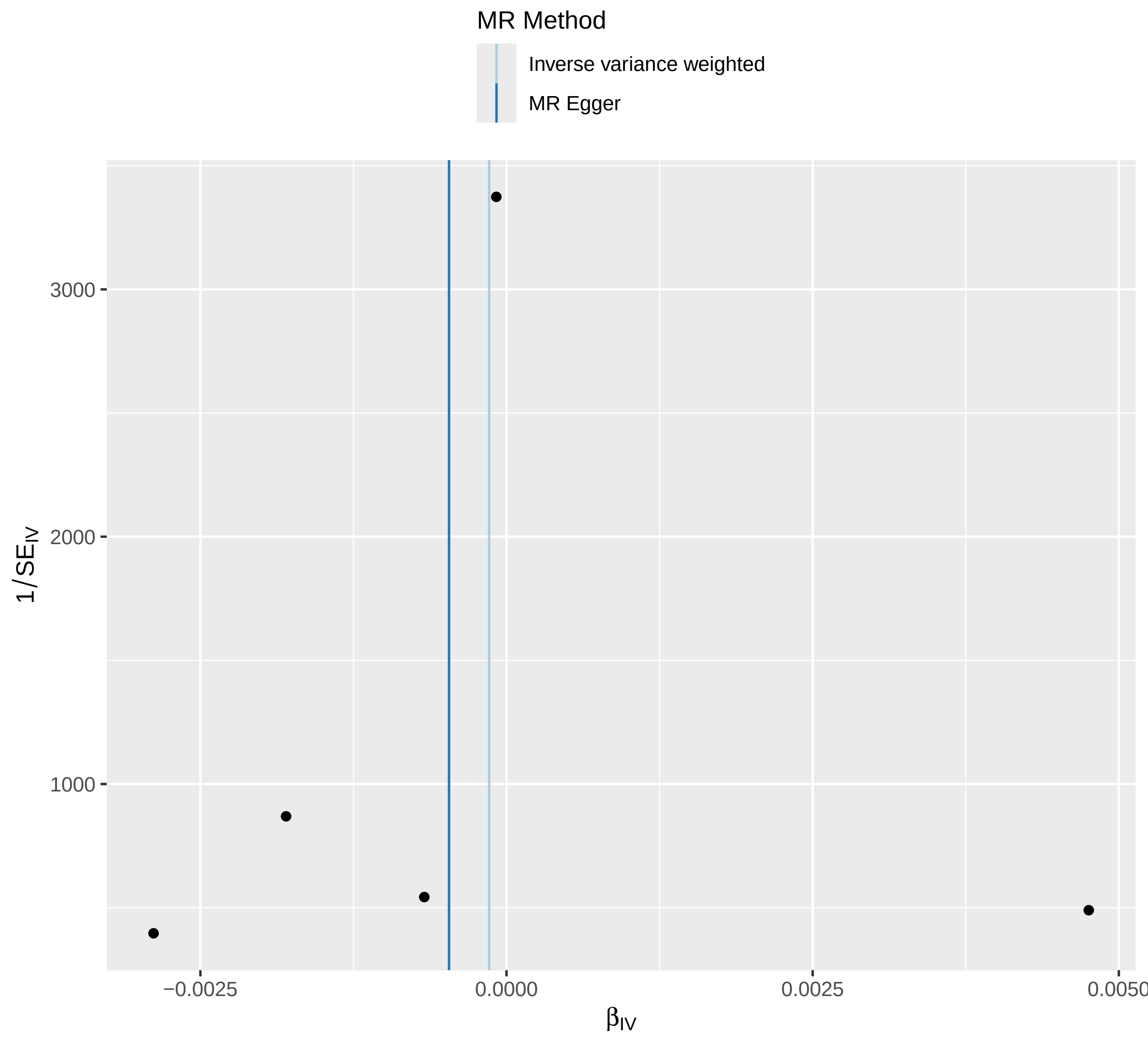

MR Method
Inverse variance weighted
MR Egger
3000
2000
1000
$1/SE_{IV}$
−0.0025
0.0000
0.0025
0.0050
β_{IV}

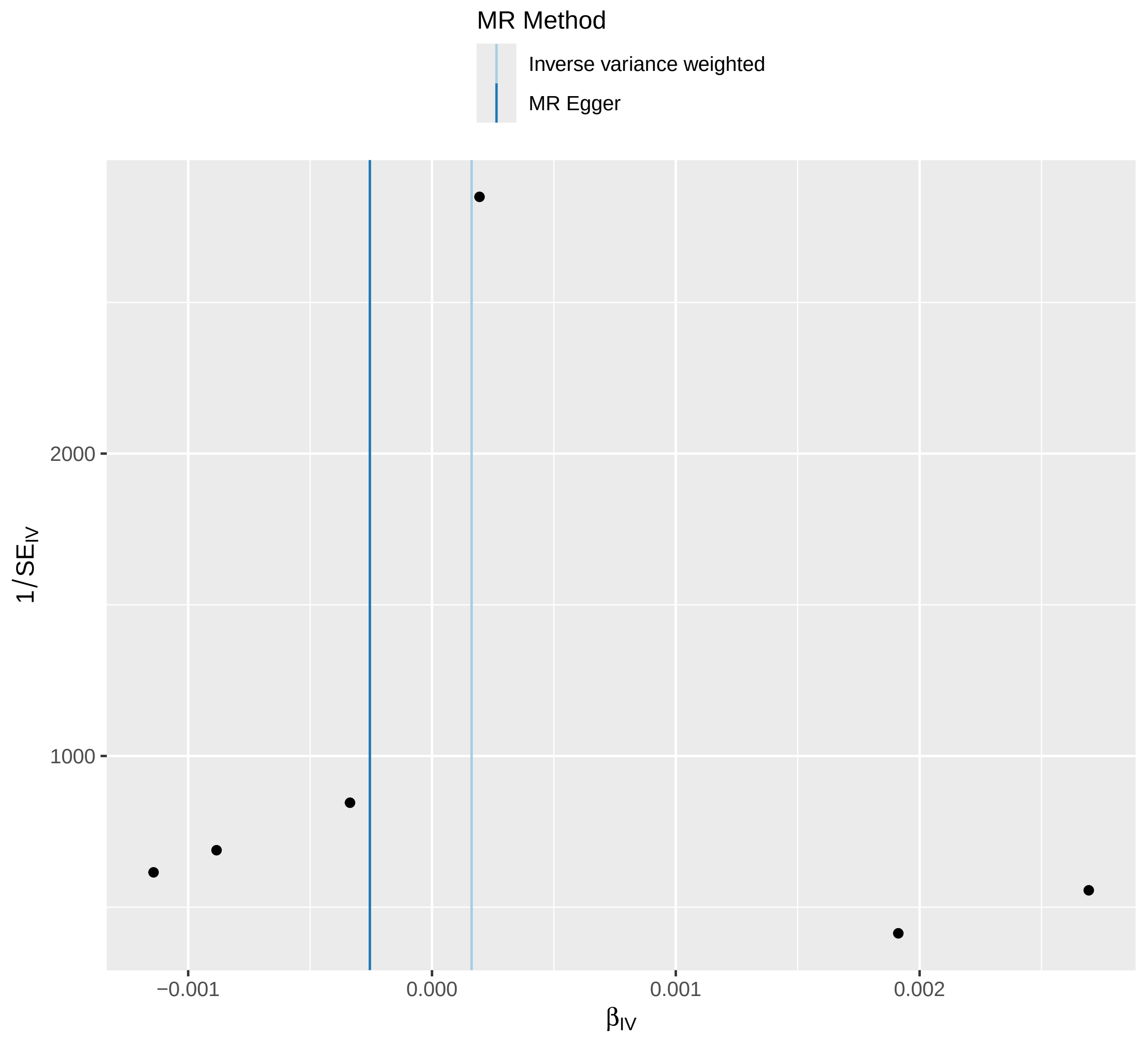
MR Method
Inverse variance weighted
MR Egger
2000
1000
1/SE_IV
−0.001
0.000
0.001
0.002
β_IV

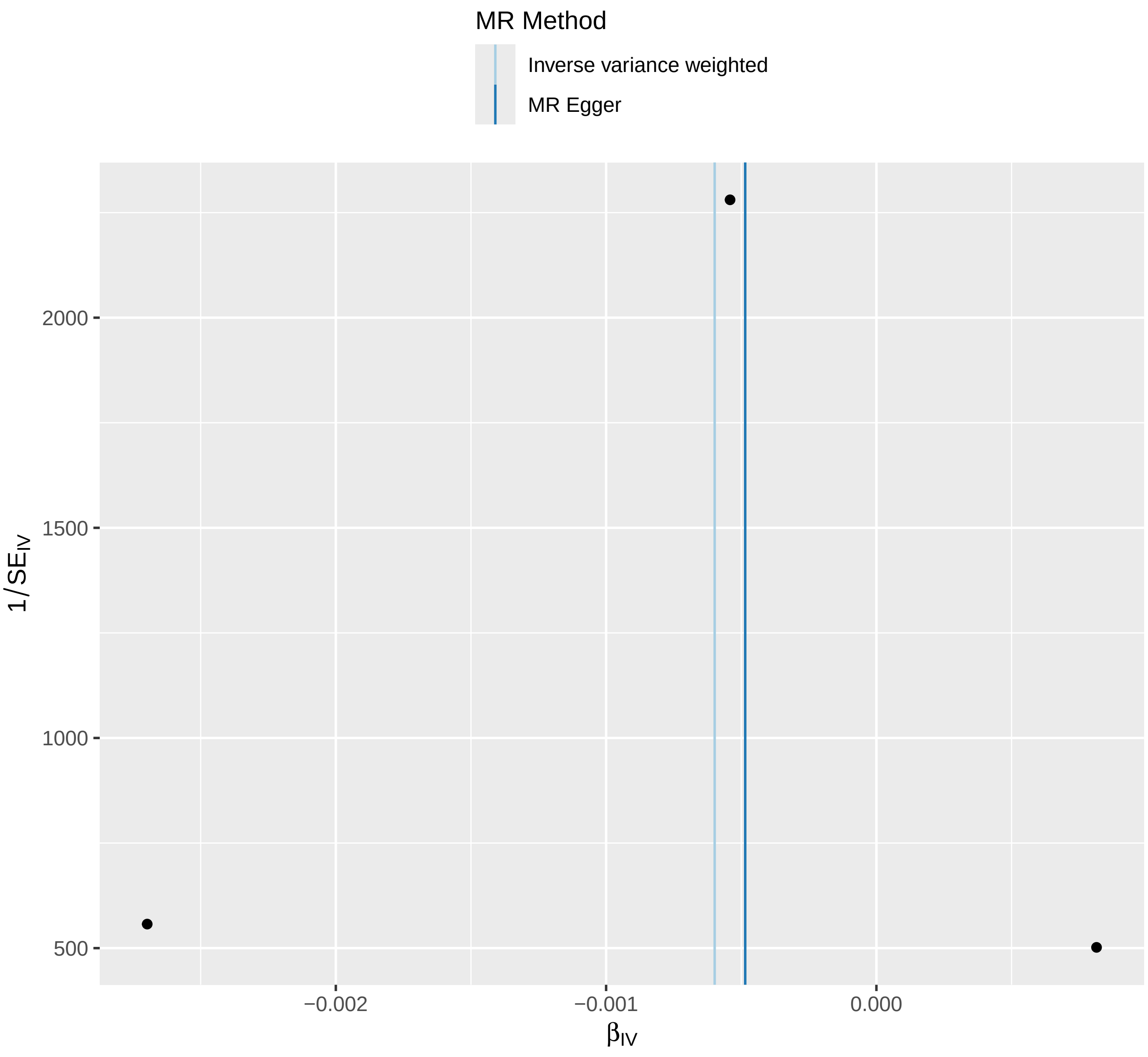
MR Method
Inverse variance weighted
MR Egger
2000
1500
1000
500
1/SE_IV
−0.002
−0.001
0.000
β_IV

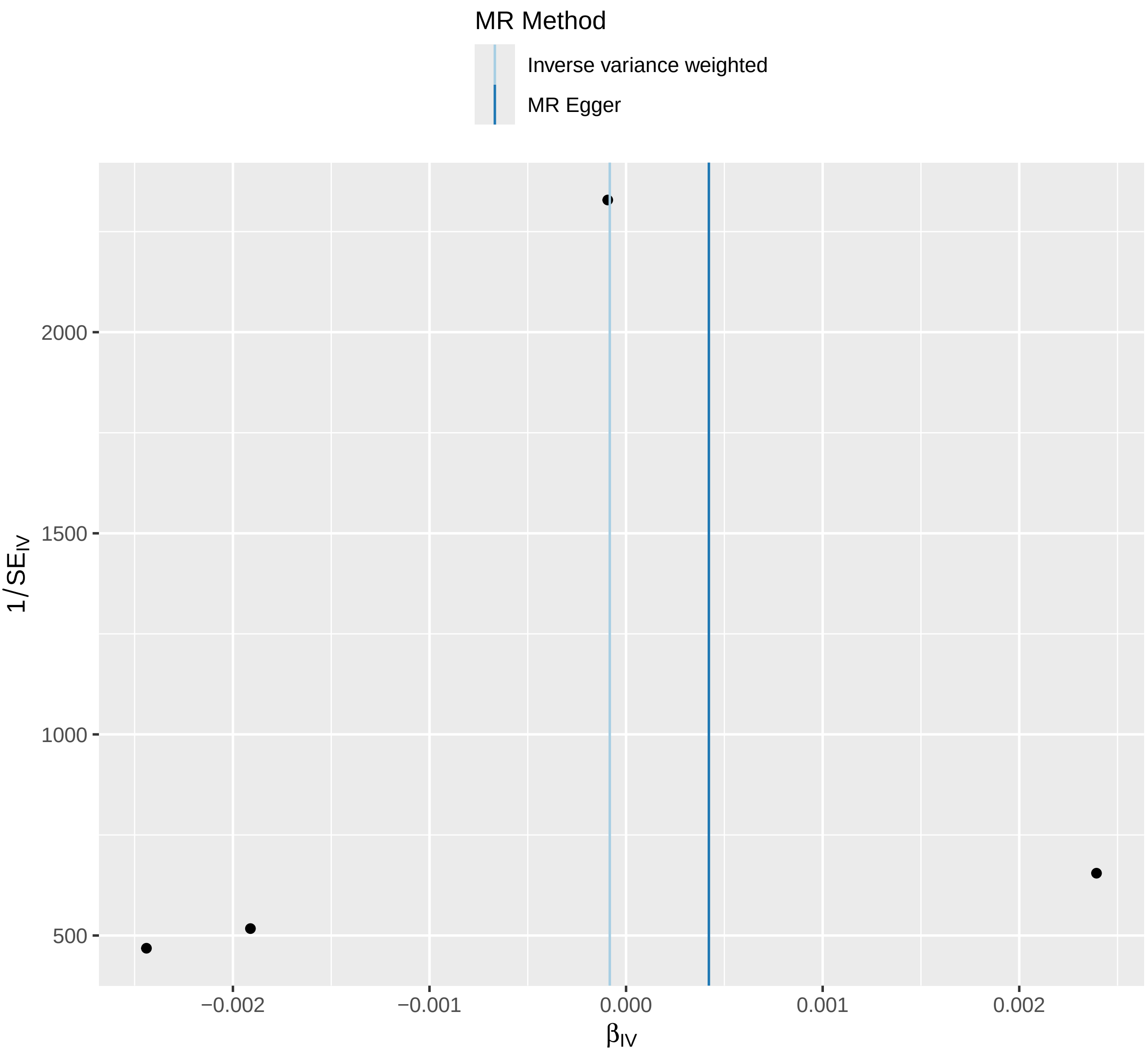

MR Method
Inverse variance weighted
MR Egger
2000
1500
1000
500
1/SE_IV
−0.002
−0.001
0.000
0.001
0.002
β_IV

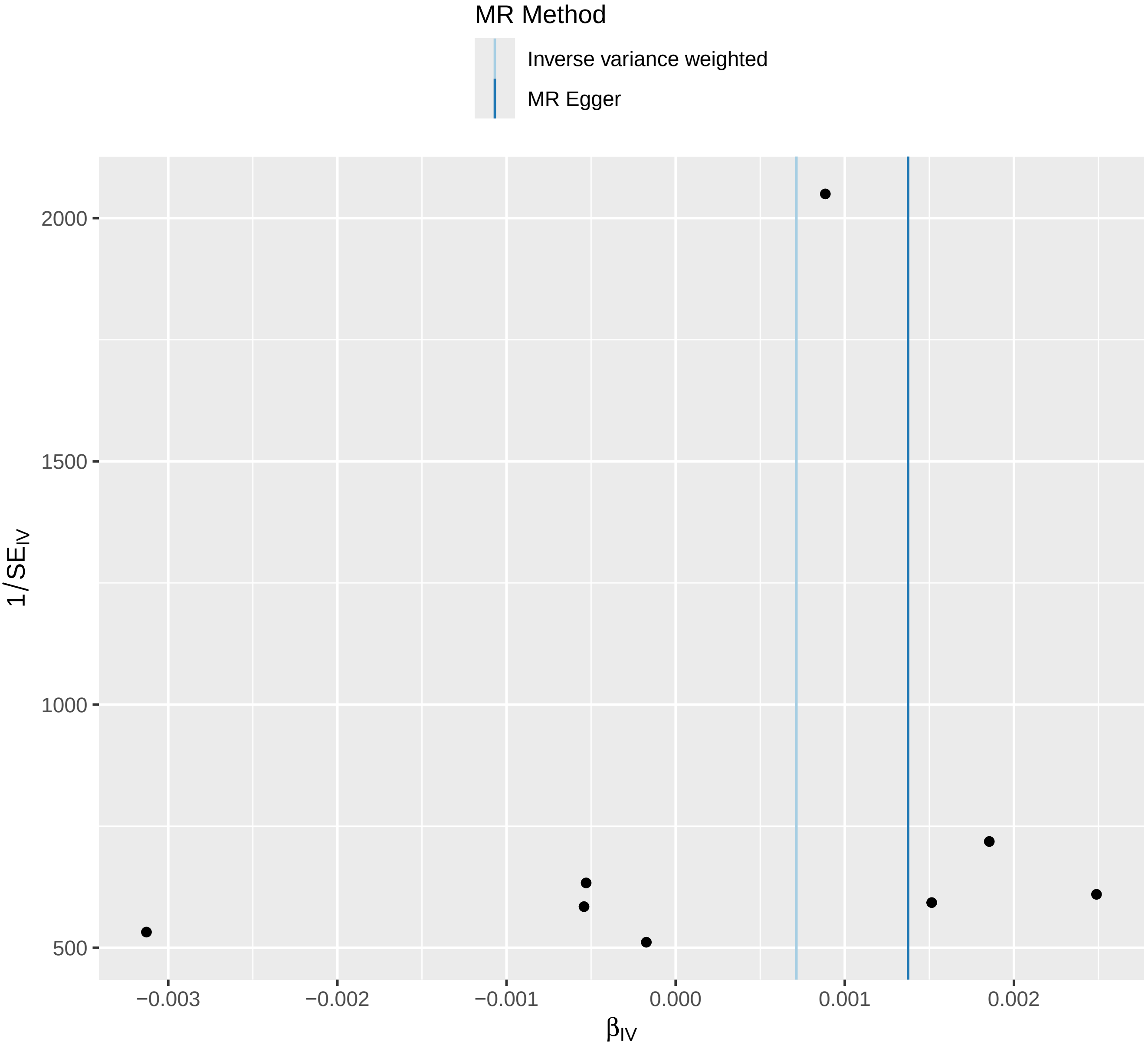
MR Method
Inverse variance weighted
MR Egger
2000
1500
1000
500
$1/SE_{IV}$
−0.003
−0.002
−0.001
0.000
0.001
0.002
β_{IV}

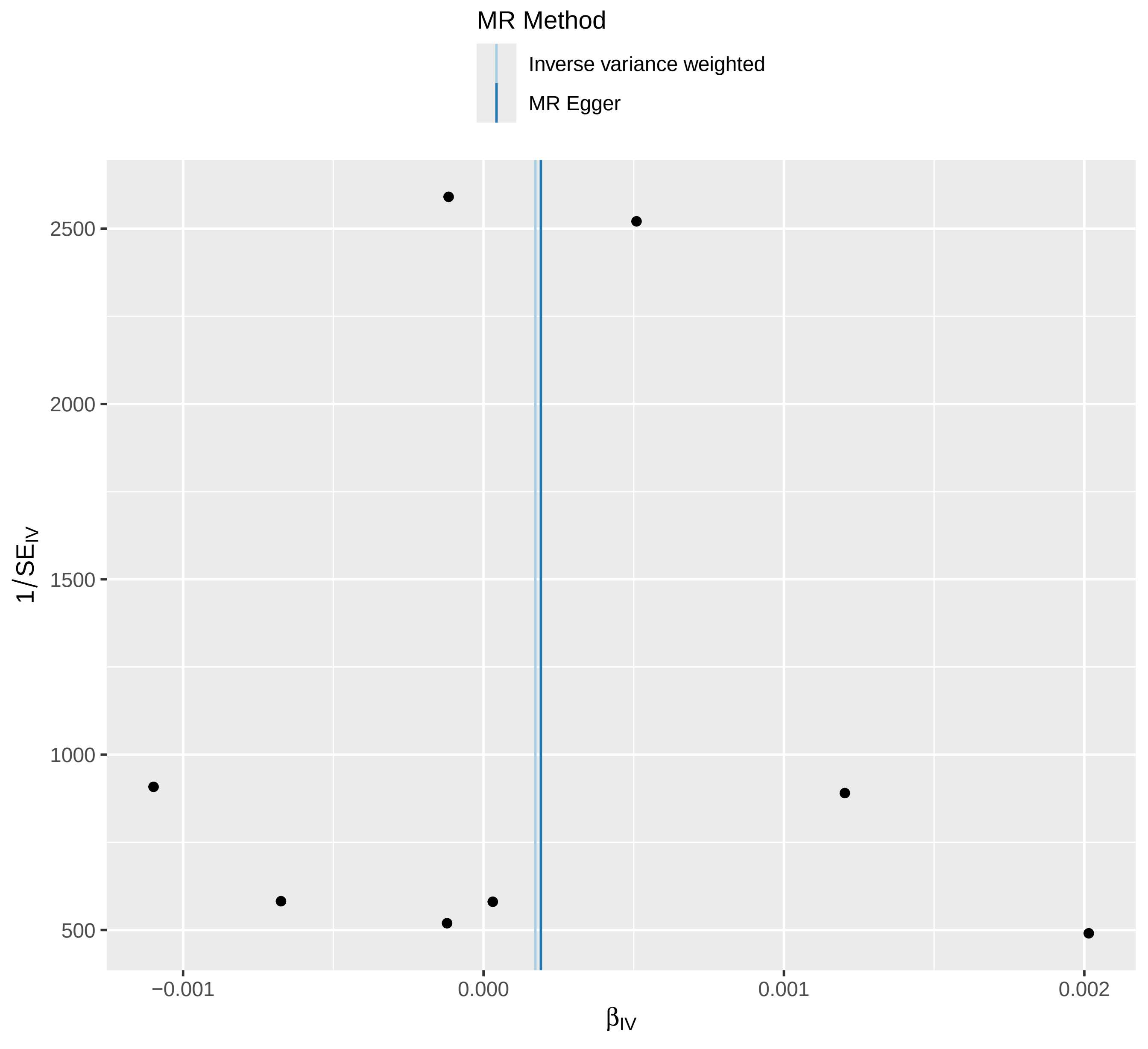

MR Method
Inverse variance weighted
MR Egger
2500
2000
1500
1000
500
$1/SE_{IV}$
−0.001
0.000
0.001
0.002
β_{IV}

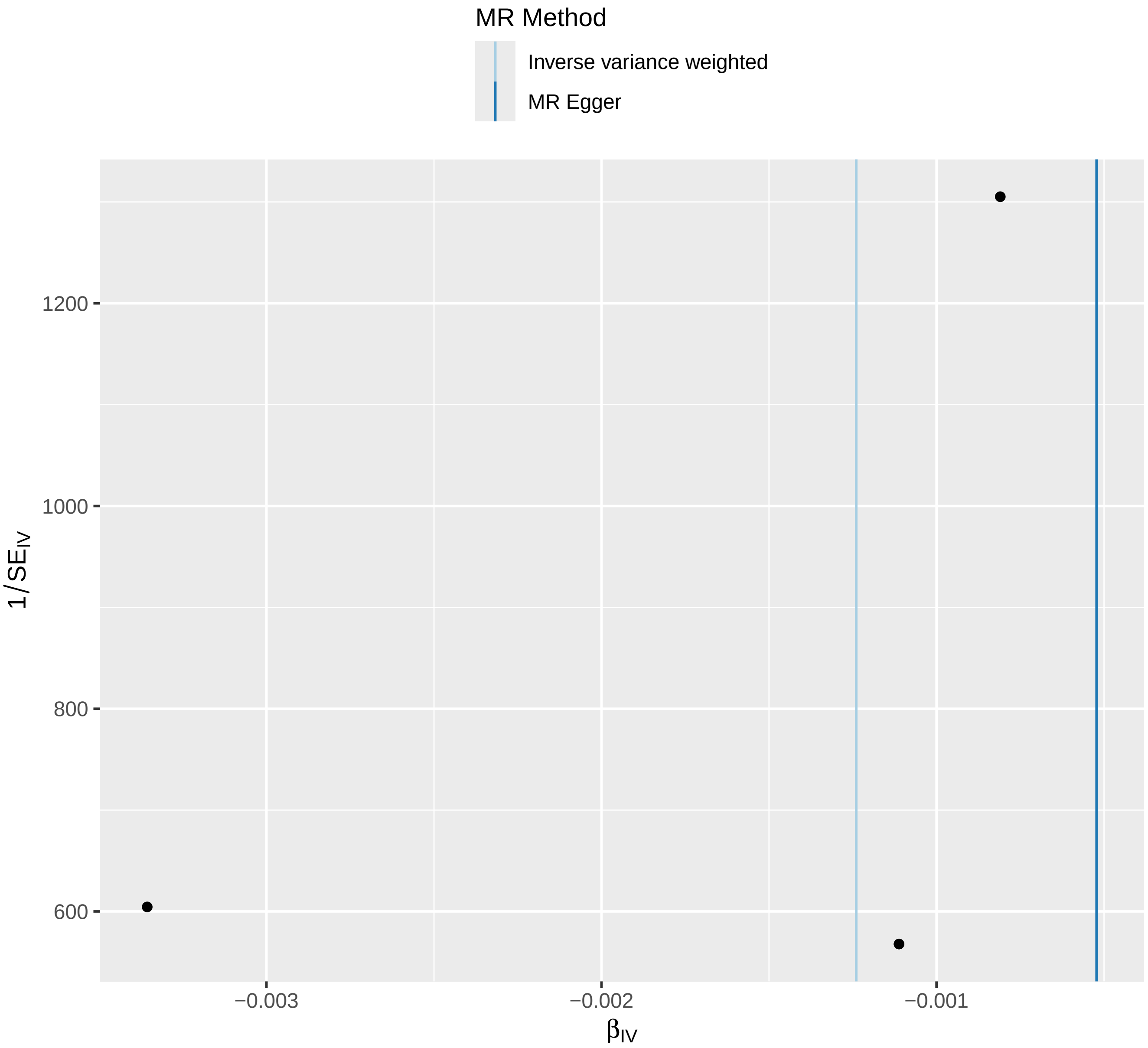

MR Method
Inverse variance weighted
MR Egger
1200
1000
800
600
1/SE_IV
−0.003
−0.002
−0.001
β_IV

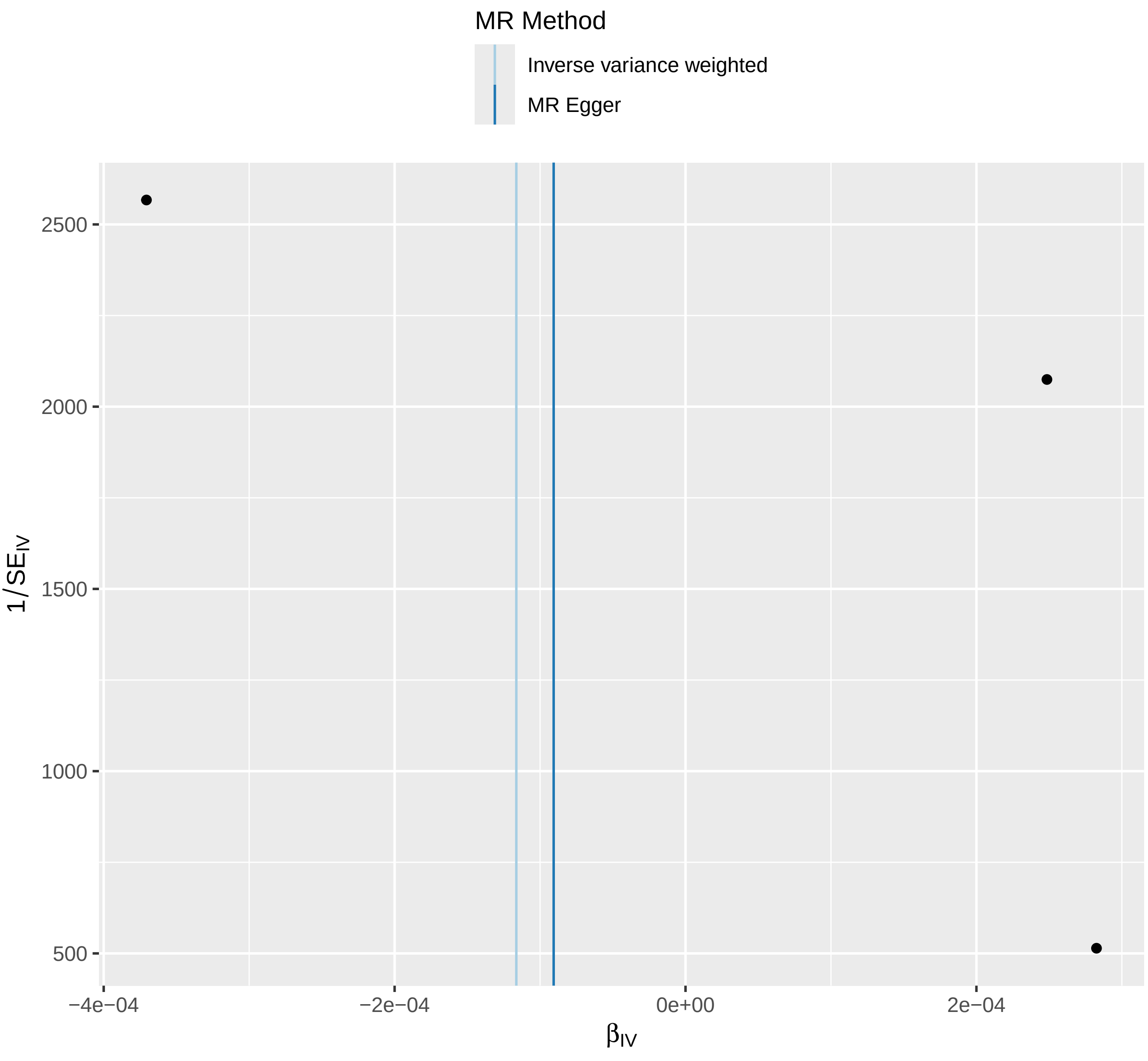

MR Method
Inverse variance weighted
MR Egger
2500
2000
1500
1000
500
1/SE_IV
−4e−04
−2e−04
0e+00
2e−04
β_IV

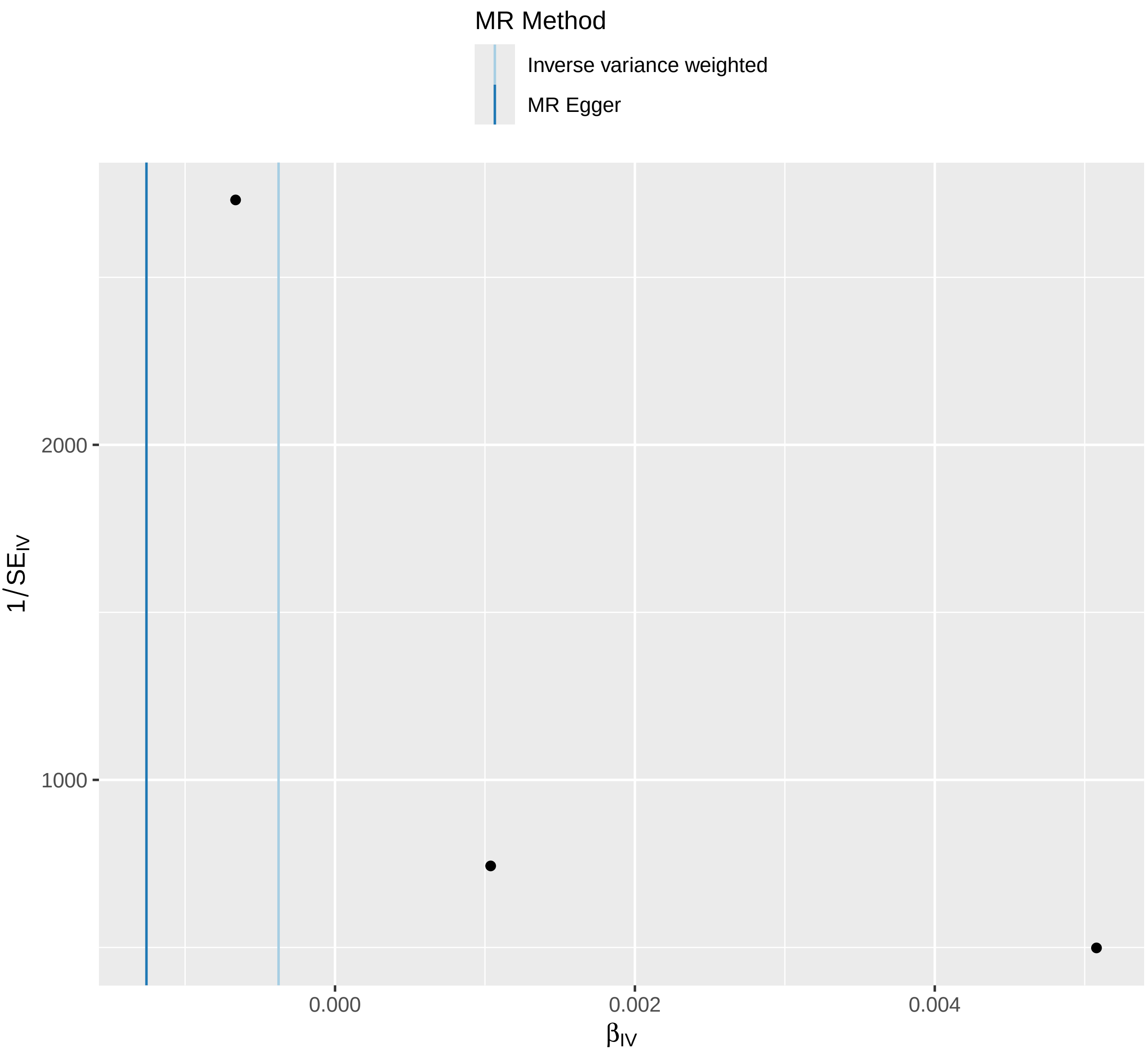

MR Method
Inverse variance weighted
MR Egger
2000
1000
1/SE_IV
0.000
0.002
0.004
β_IV

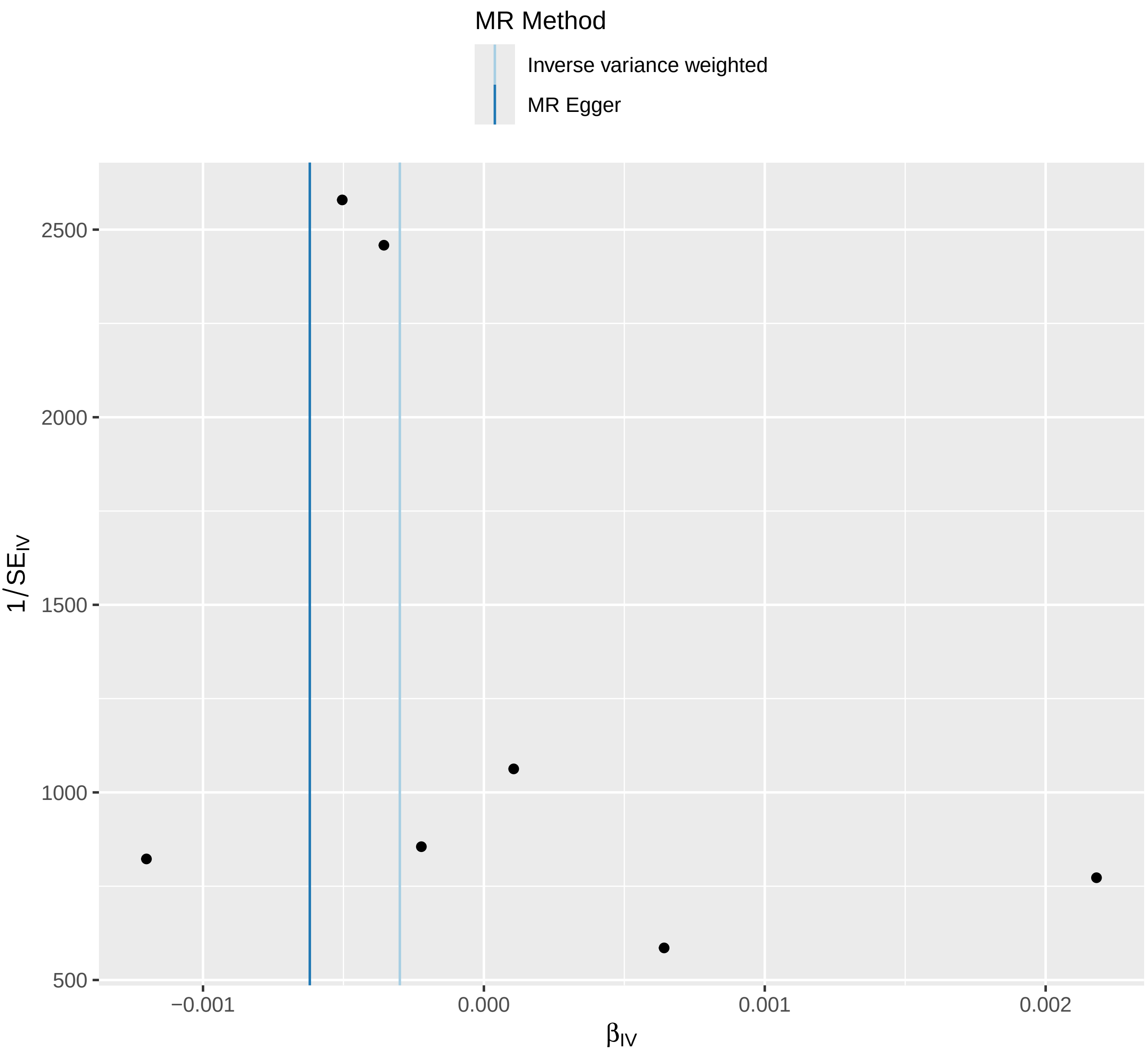

MR Method
Inverse variance weighted
MR Egger
2500
2000
1500
1000
500
1/SE$_{IV}$
−0.001
0.000
0.001
0.002
β$_{IV}$

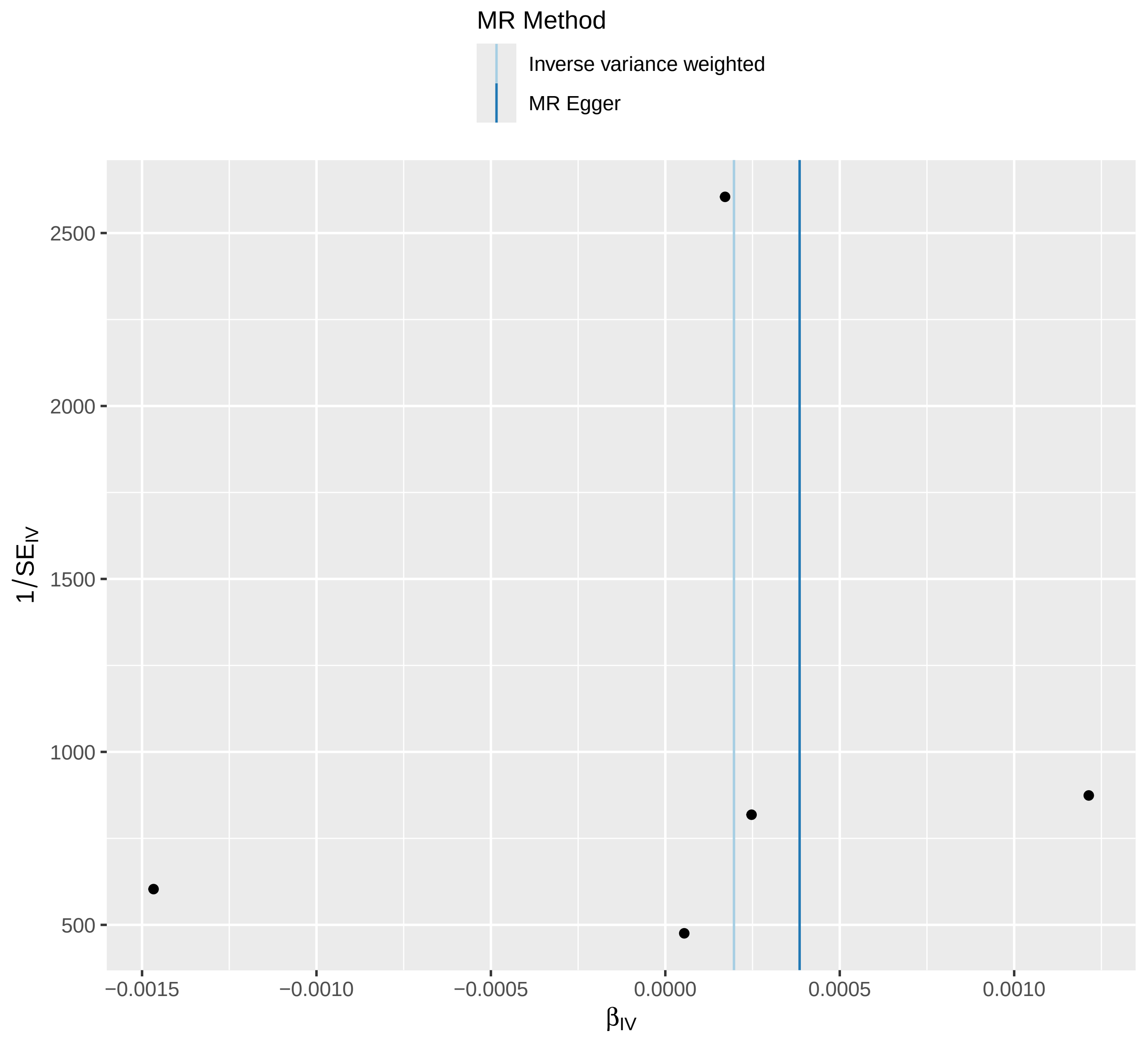
MR Method
Inverse variance weighted
MR Egger
2500
2000
1500
1000
500
1/SE_IV
−0.0015
−0.0010
−0.0005
0.0000
0.0005
0.0010
β_IV

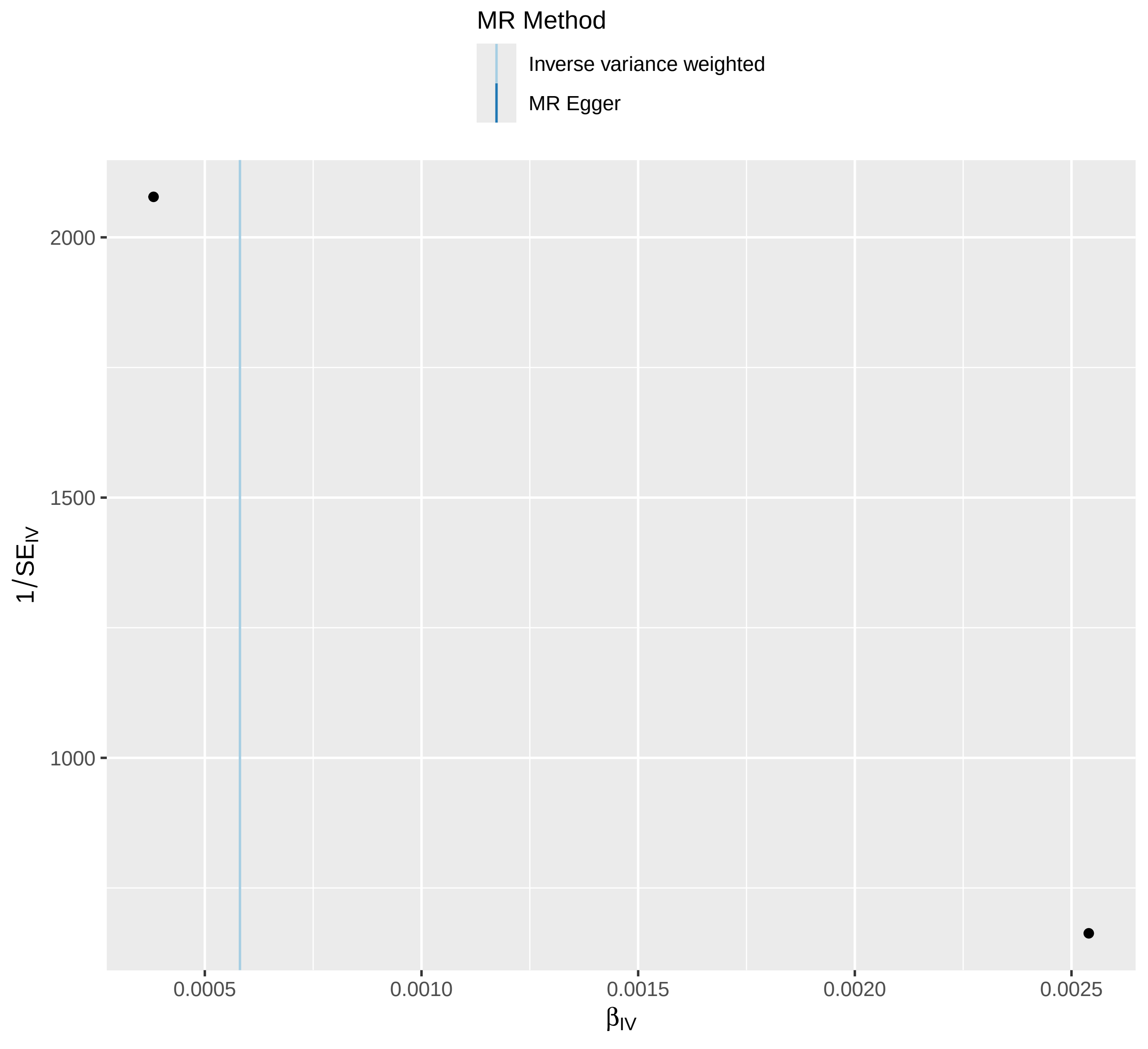
MR Method
Inverse variance weighted
MR Egger
2000
1500
1000
1/SE_IV
0.0005
0.0010
0.0015
0.0020
0.0025
β_IV

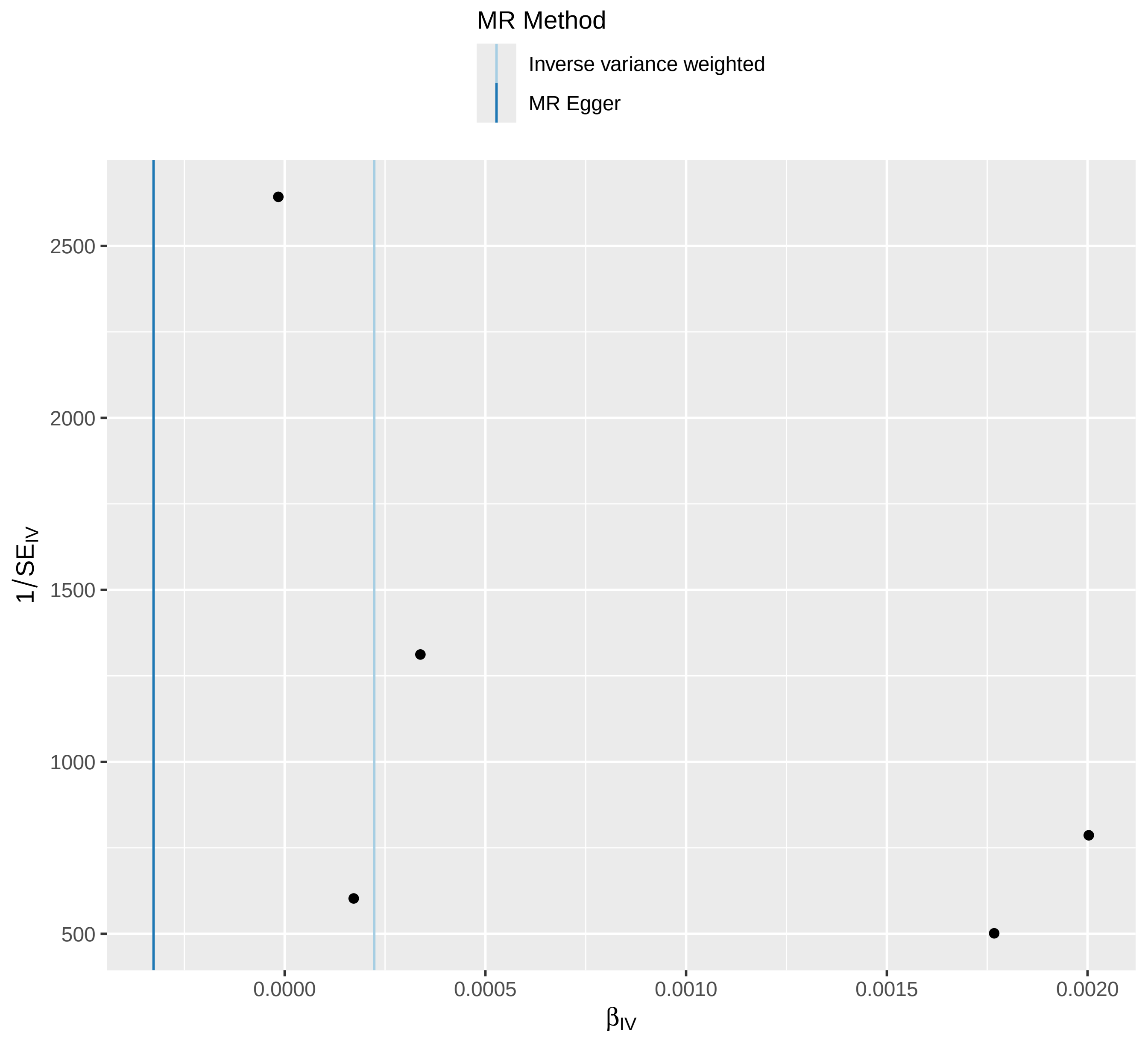

MR Method
Inverse variance weighted
MR Egger
2500
2000
1500
1000
500
1/SE_IV
0.0000
0.0005
0.0010
0.0015
0.0020
β_IV

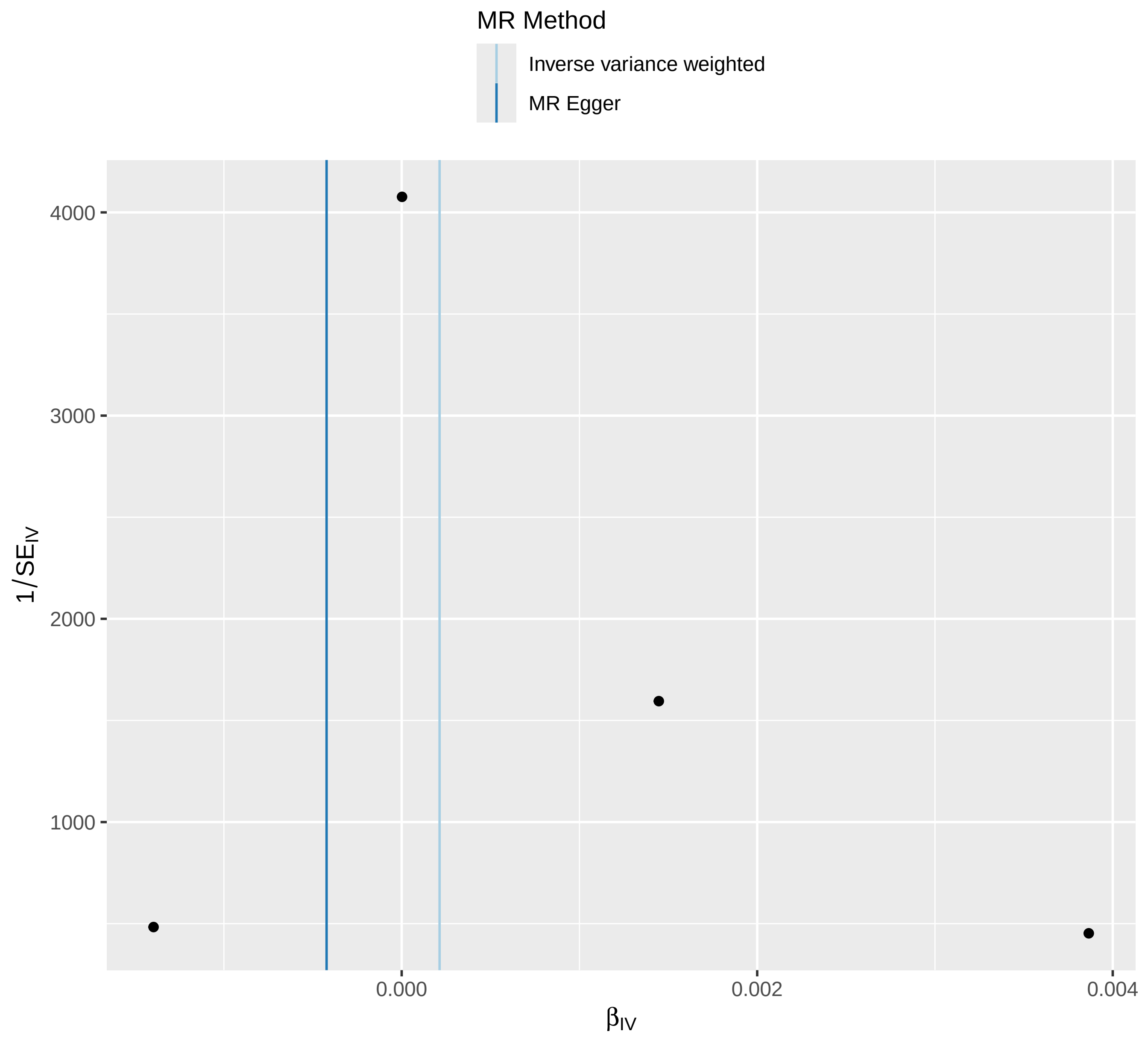
MR Method
Inverse variance weighted
MR Egger
4000
3000
2000
1000
1/SE_IV
0.000
0.002
0.004
β_IV

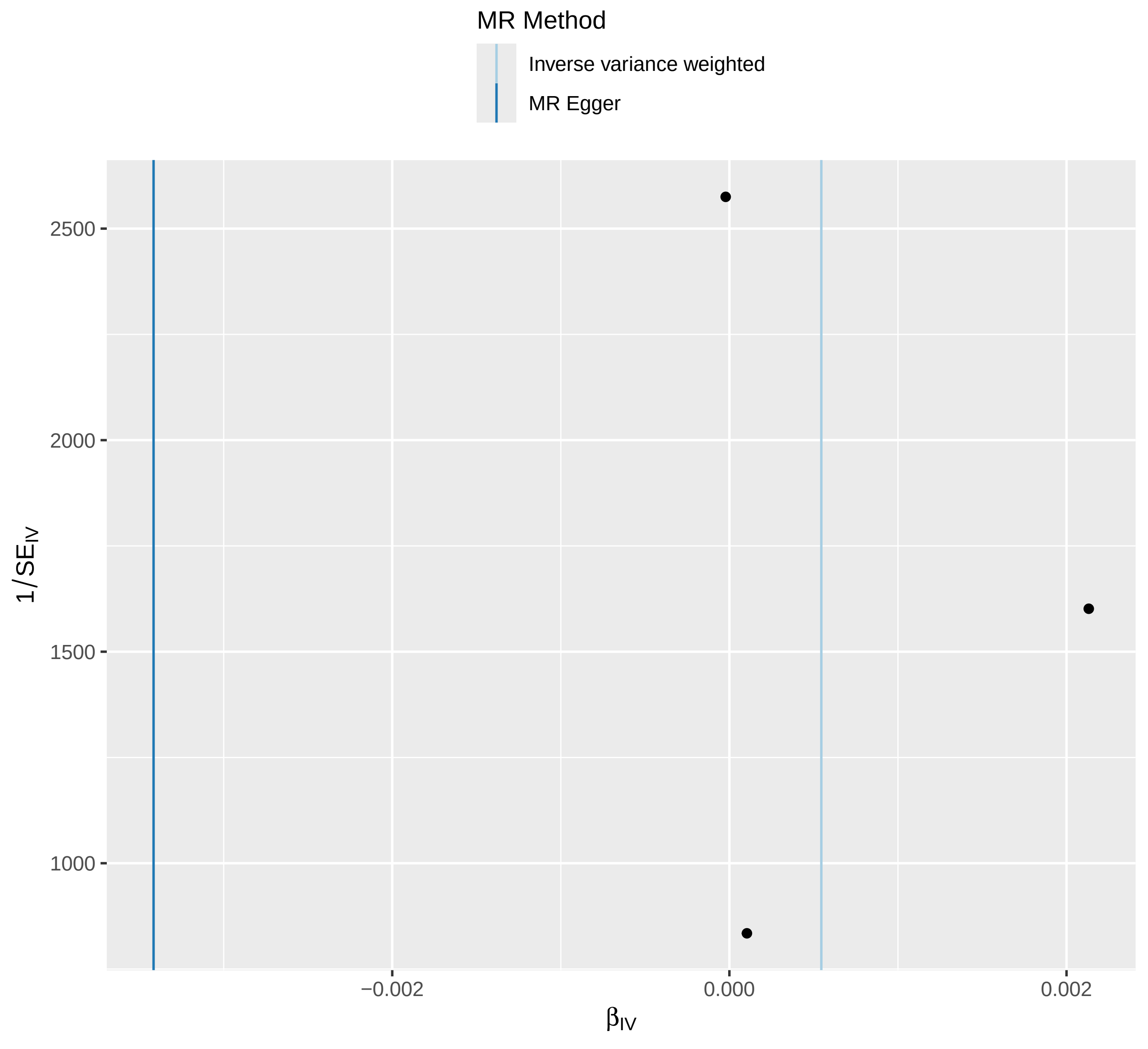

MR Method
Inverse variance weighted
MR Egger
2500
2000
1500
1000
$1/SE_{IV}$
−0.002
0.000
0.002
β_{IV}

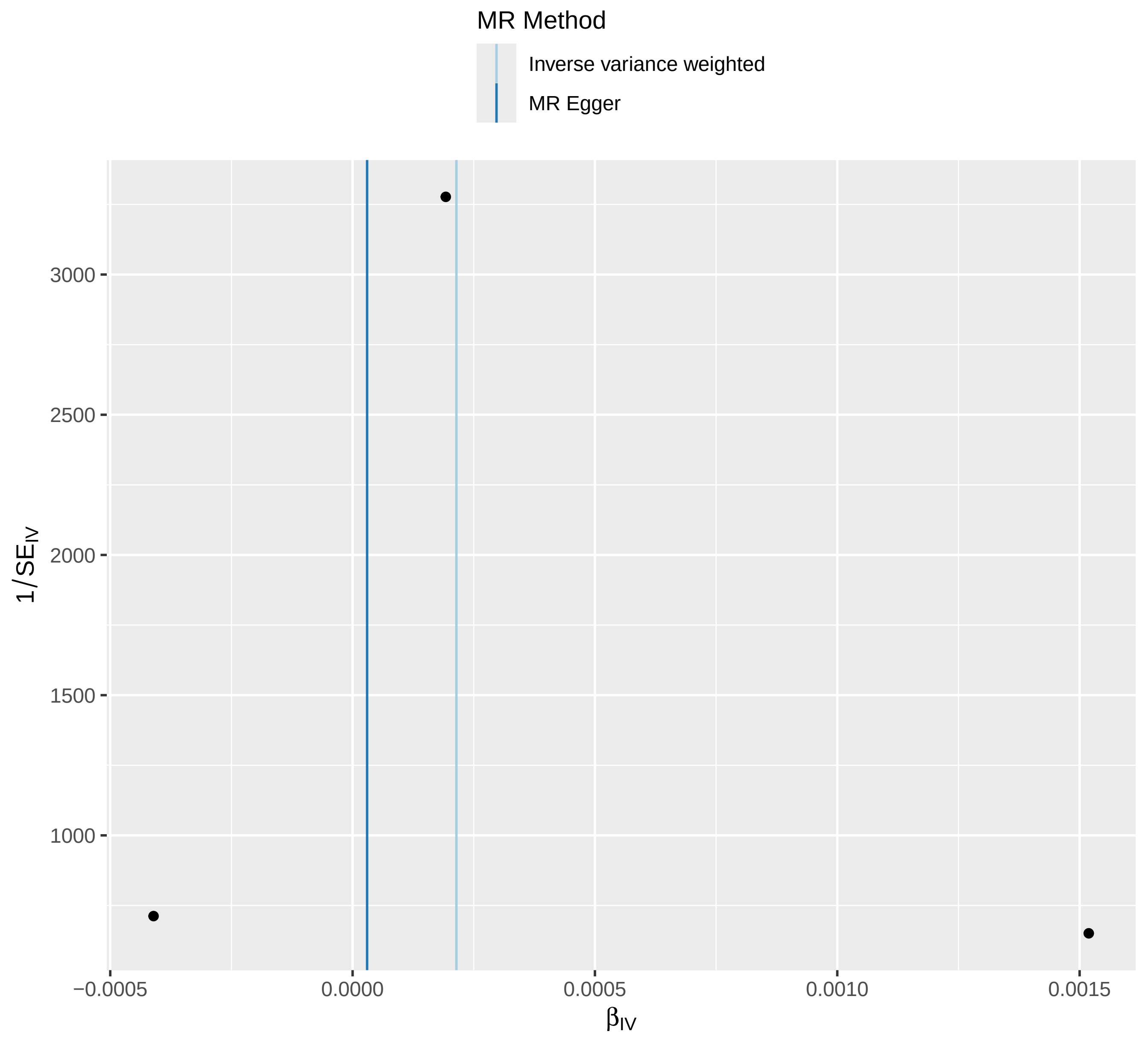
MR Method
Inverse variance weighted
MR Egger
3000
2500
2000
1500
1000
1/SE_IV
−0.0005
0.0000
0.0005
0.0010
0.0015
β_IV

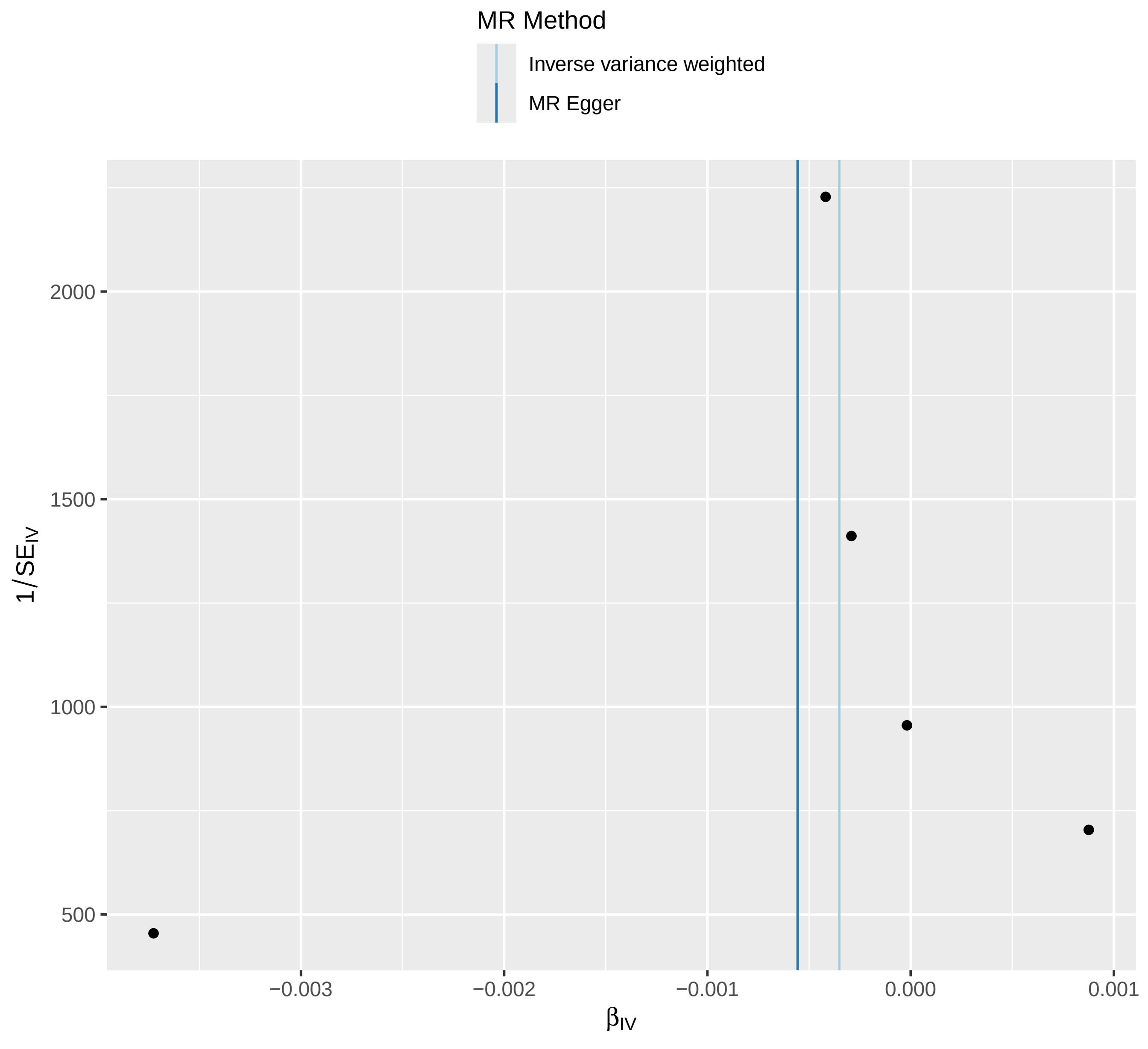

MR Method
Inverse variance weighted
MR Egger
2000
1500
1000
500
1/SE_IV
−0.003
−0.002
−0.001
0.000
0.001
β_IV

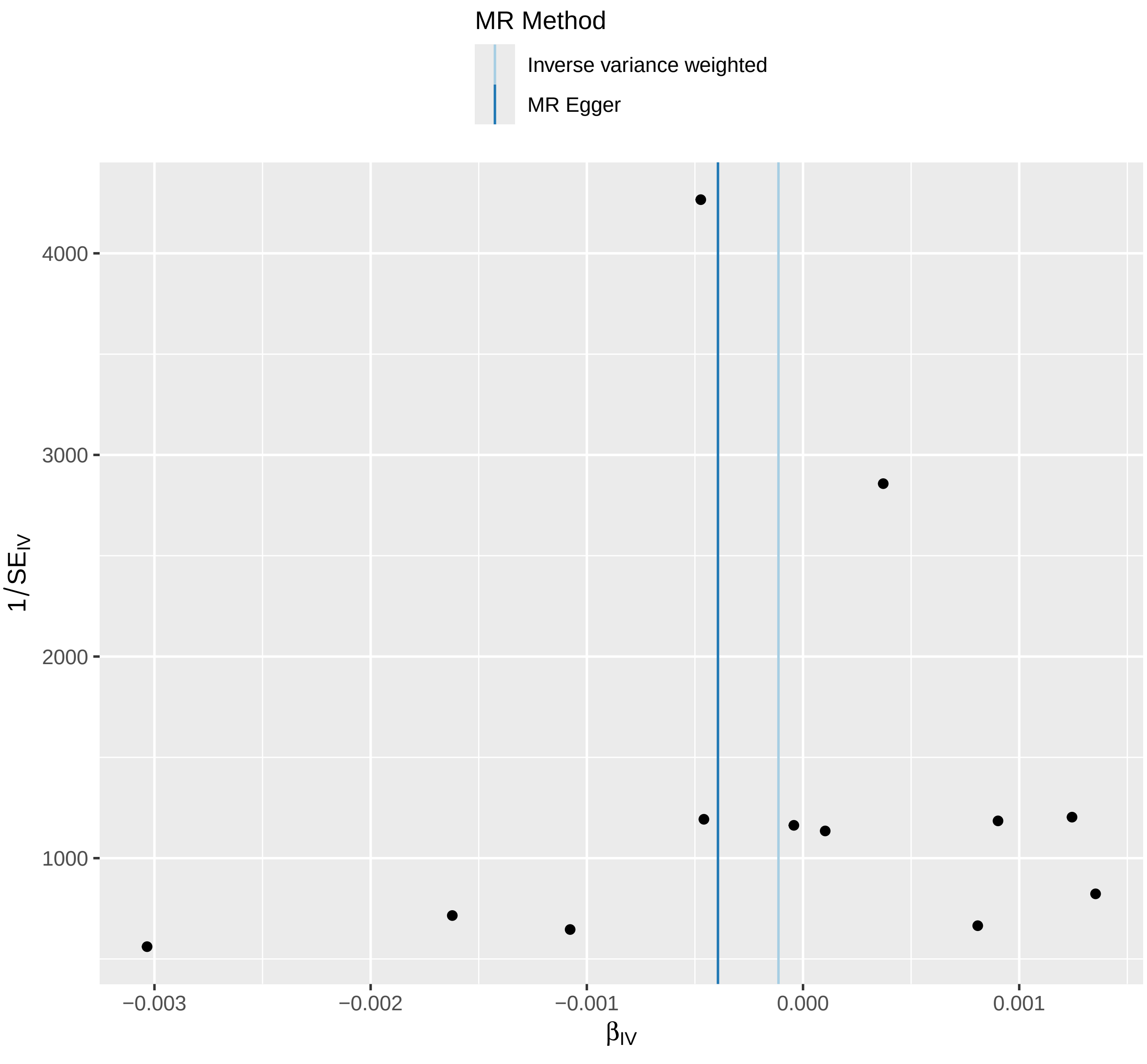

MR Method
Inverse variance weighted
MR Egger
4000
3000
2000
1000
1/SE_IV
−0.003
−0.002
−0.001
0.000
0.001
β_IV

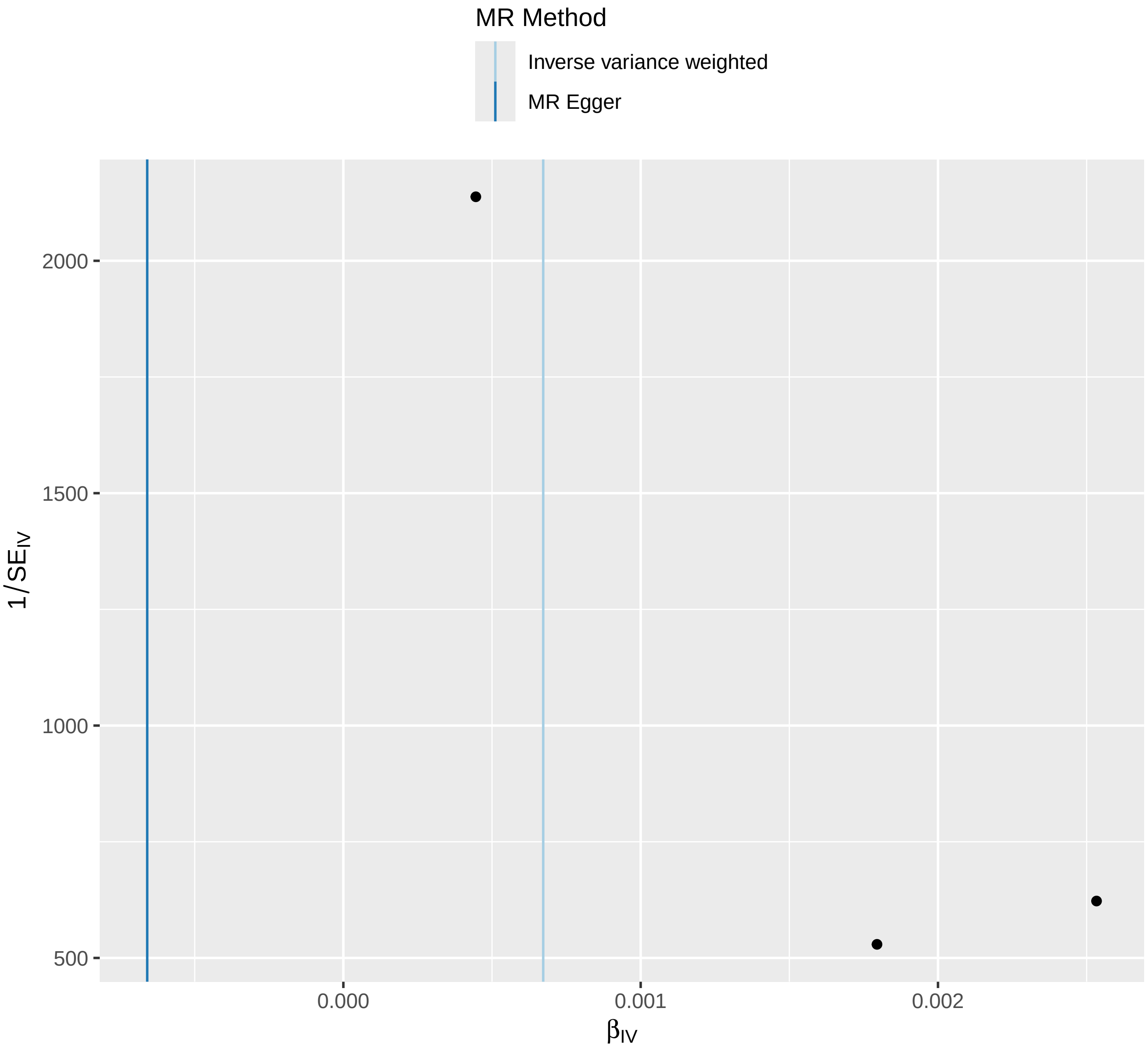

MR Method
Inverse variance weighted
MR Egger
2000
1500
1000
500
0.000
0.001
0.002
1/SE_IV
β_IV

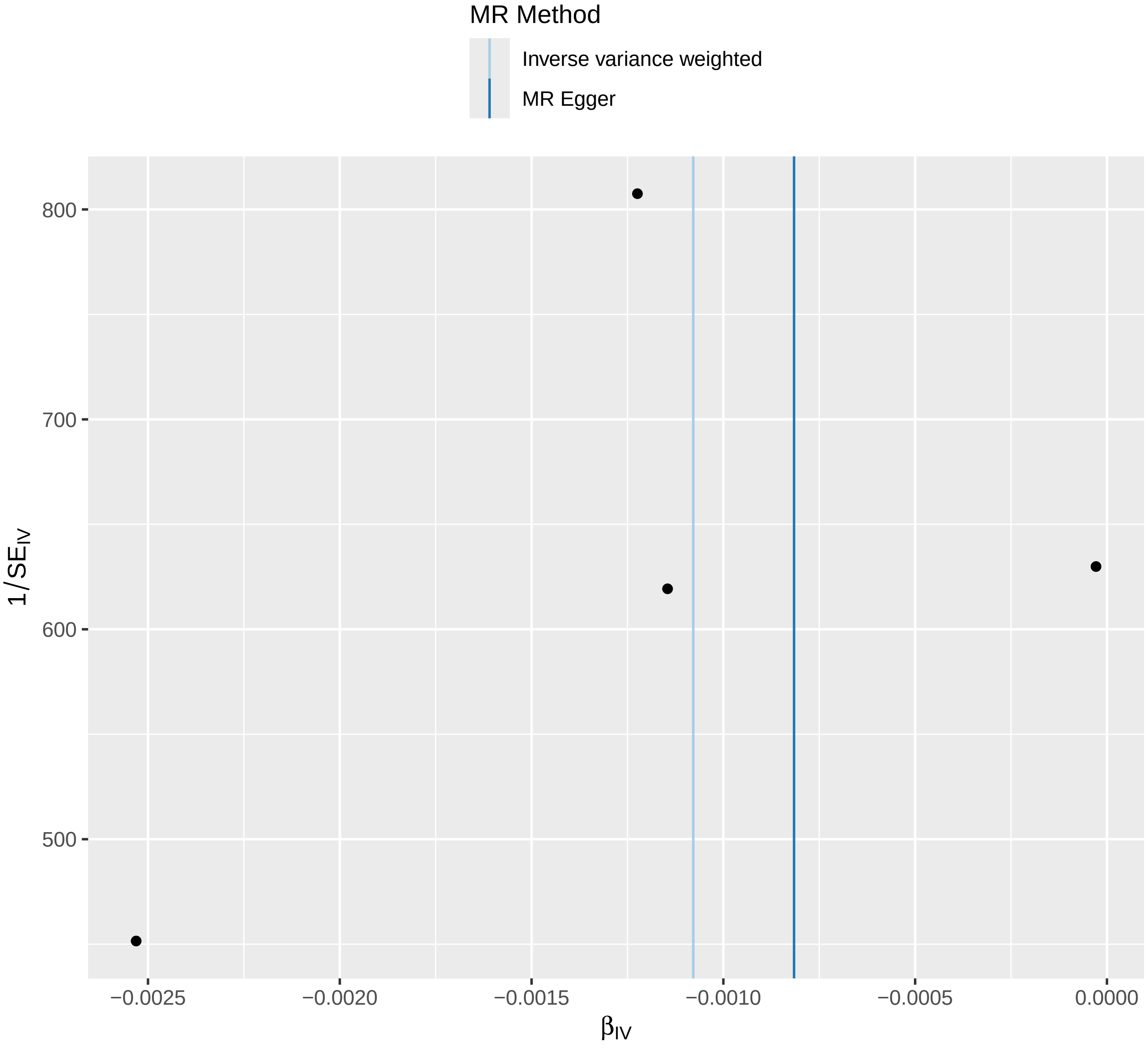
MR Method
Inverse variance weighted
MR Egger
800
700
600
500
1/SE_IV
−0.0025
−0.0020
−0.0015
−0.0010
−0.0005
0.0000
β_IV

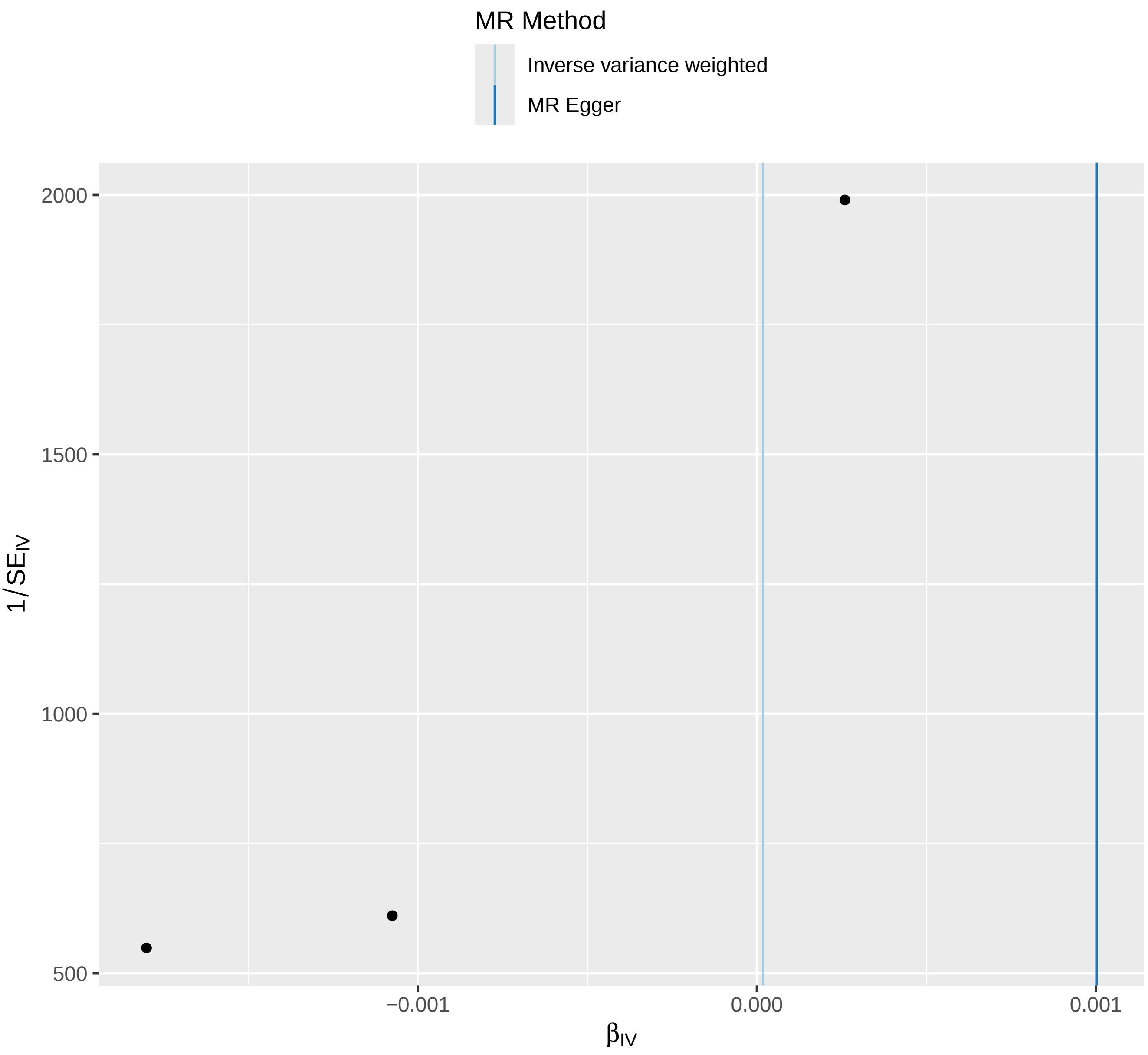
MR Method
Inverse variance weighted
MR Egger
2000
1500
1000
500
1/SE_IV
−0.001
0.000
0.001
β_IV

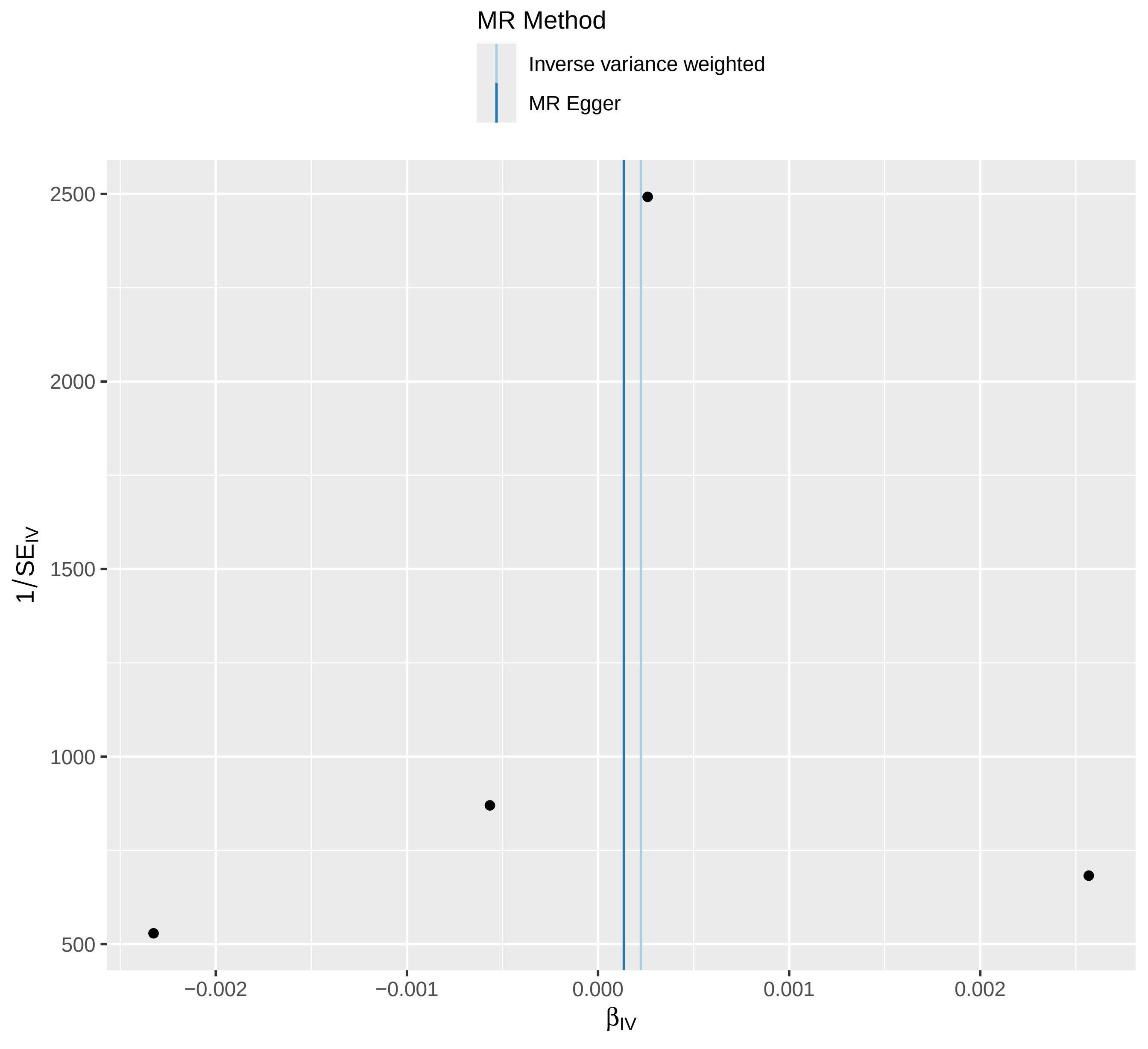
MR Method
Inverse variance weighted
MR Egger
2500
2000
1500
1000
500
1/SE_IV
−0.002
−0.001
0.000
0.001
0.002
β_IV

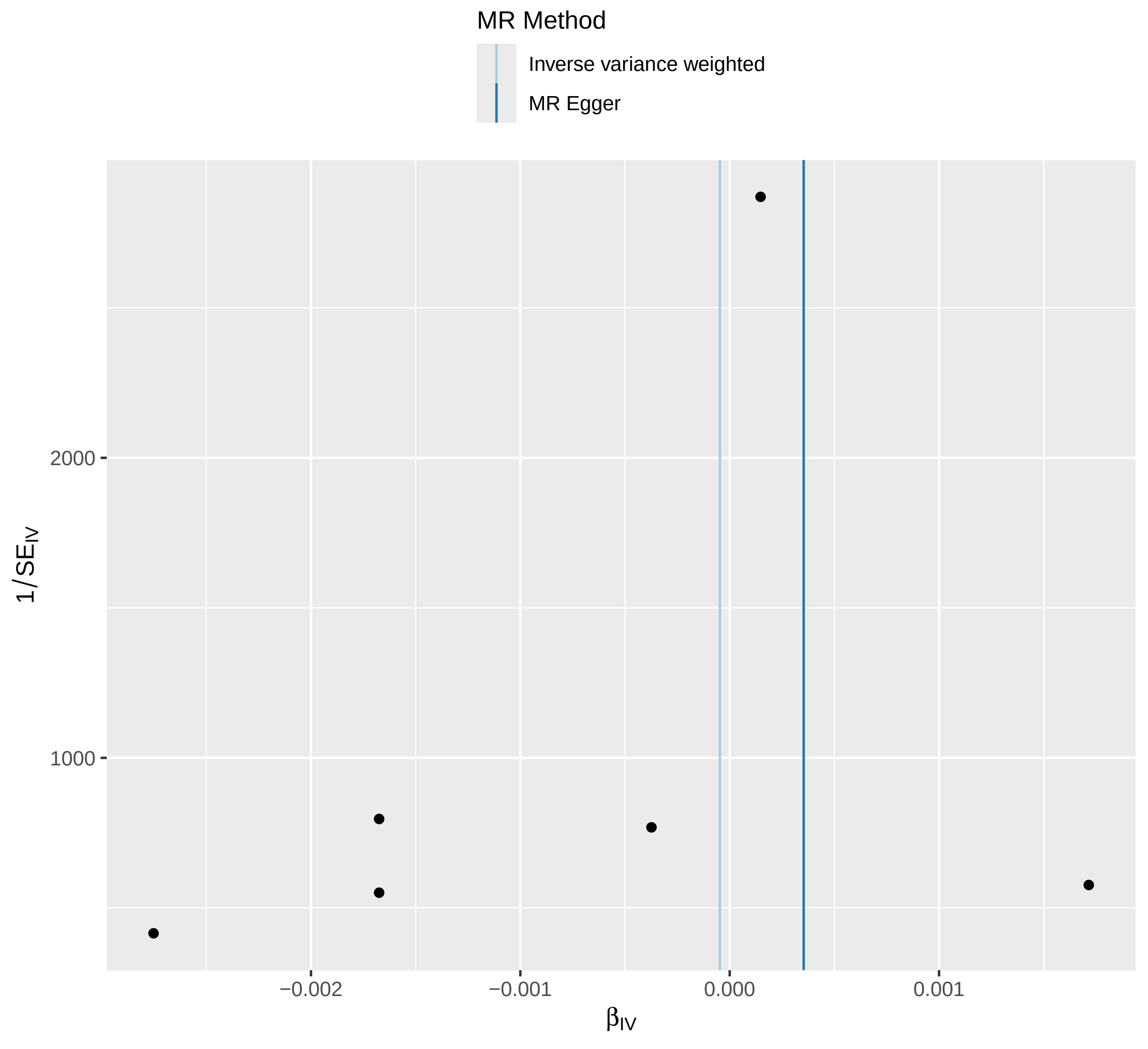
MR Method
Inverse variance weighted
MR Egger
2000
1000
1/SE_IV
−0.002
−0.001
0.000
0.001
β_IV

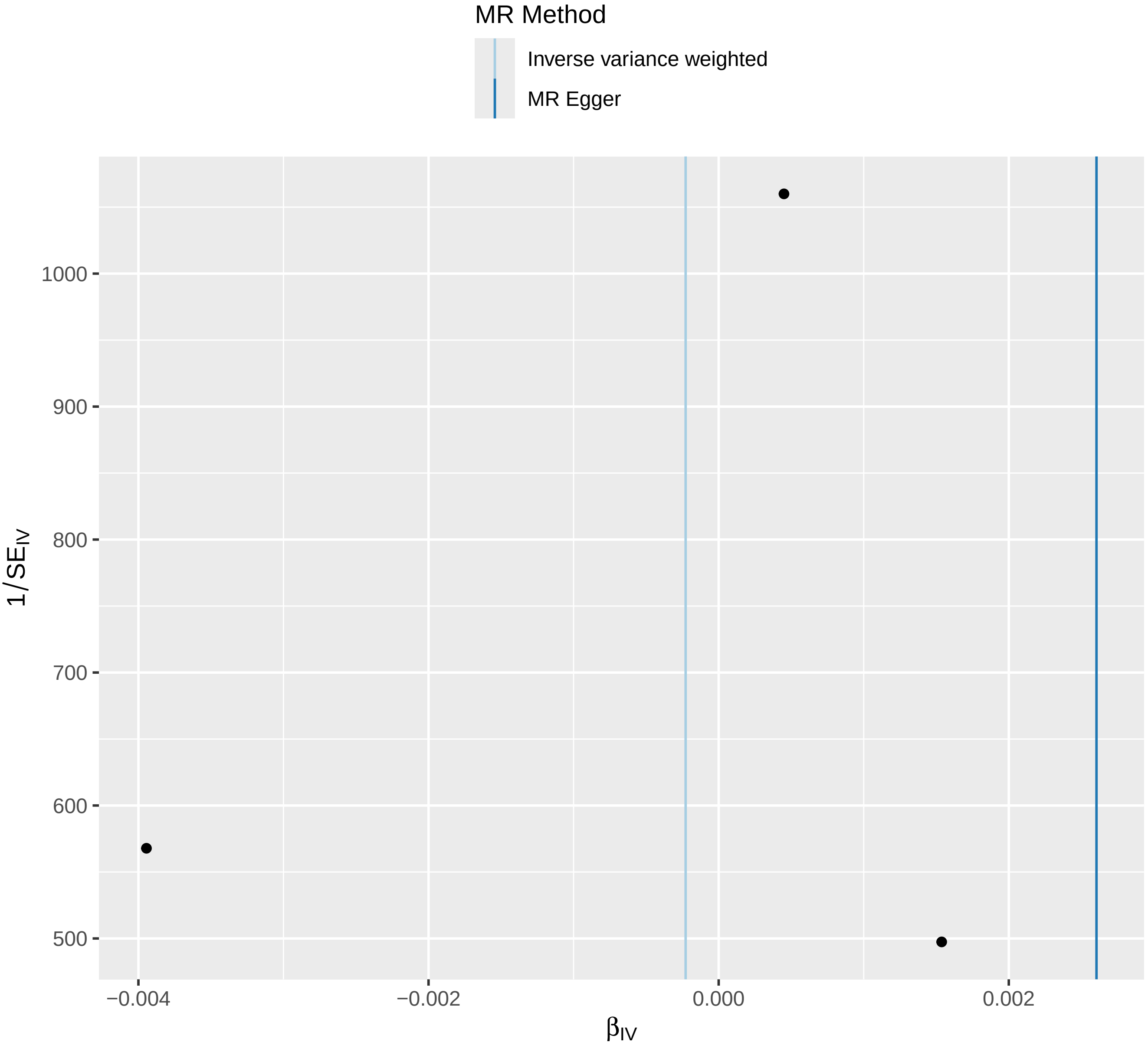
MR Method
Inverse variance weighted
MR Egger
1000
900
800
700
600
500
1/SE$_{IV}$
−0.004
−0.002
0.000
0.002
β$_{IV}$

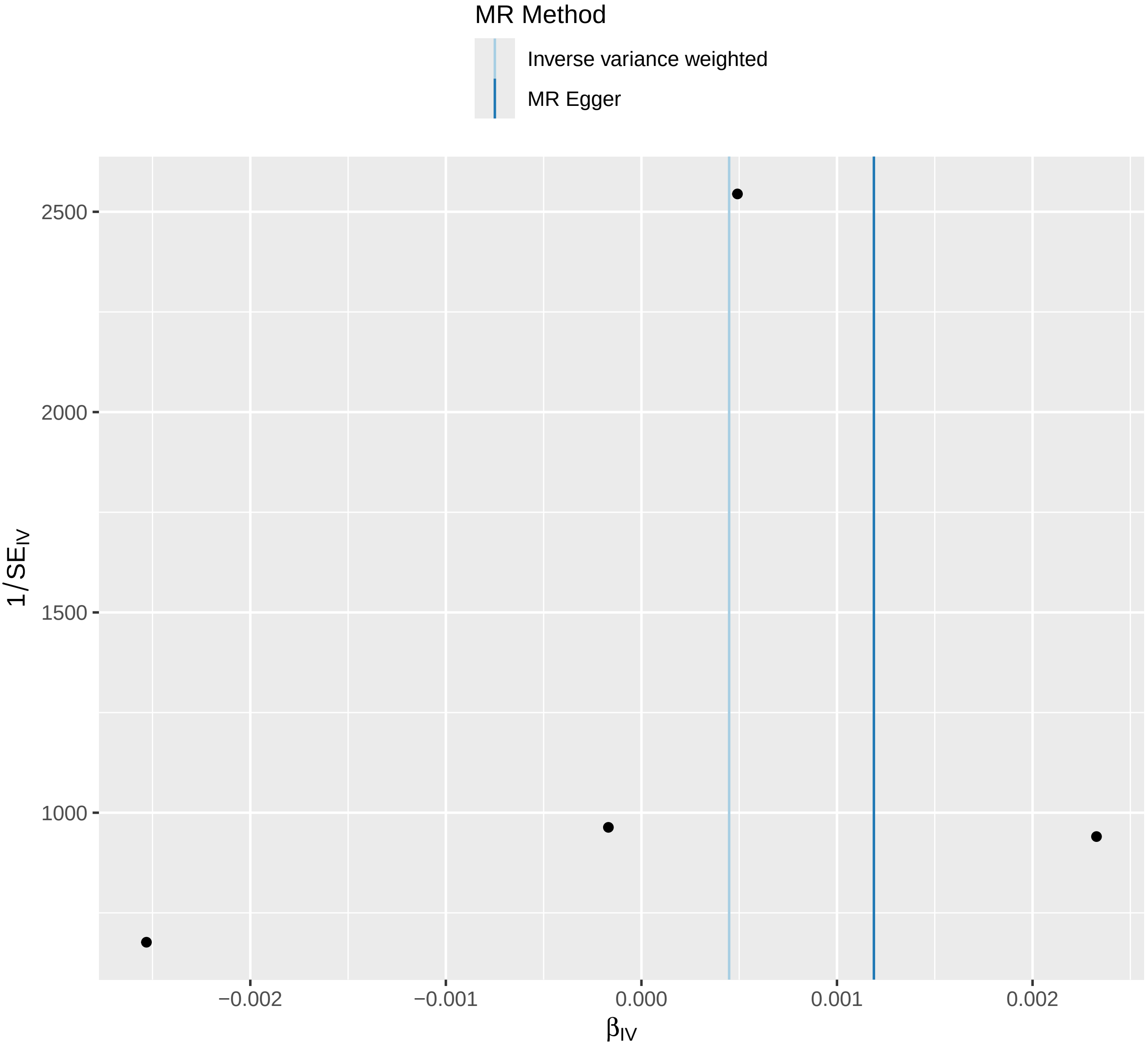
MR Method
Inverse variance weighted
MR Egger
2500
2000
1500
1000
−0.002
−0.001
0.000
0.001
0.002
$1/SE_{IV}$
β_{IV}

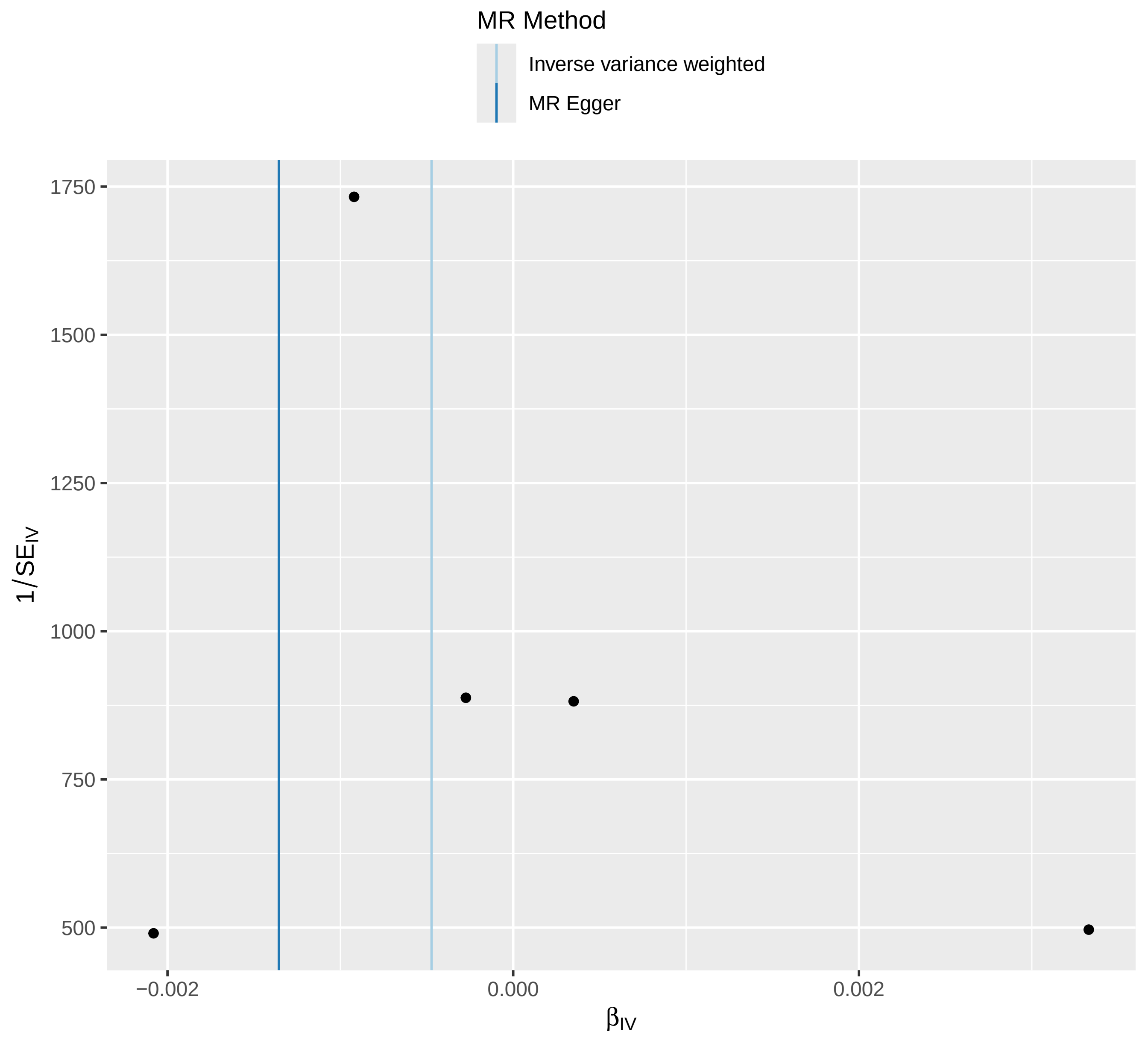

MR Method
Inverse variance weighted
MR Egger
1750
1500
1250
1000
750
500
1/SE$_{IV}$
−0.002
0.000
0.002
β$_{IV}$

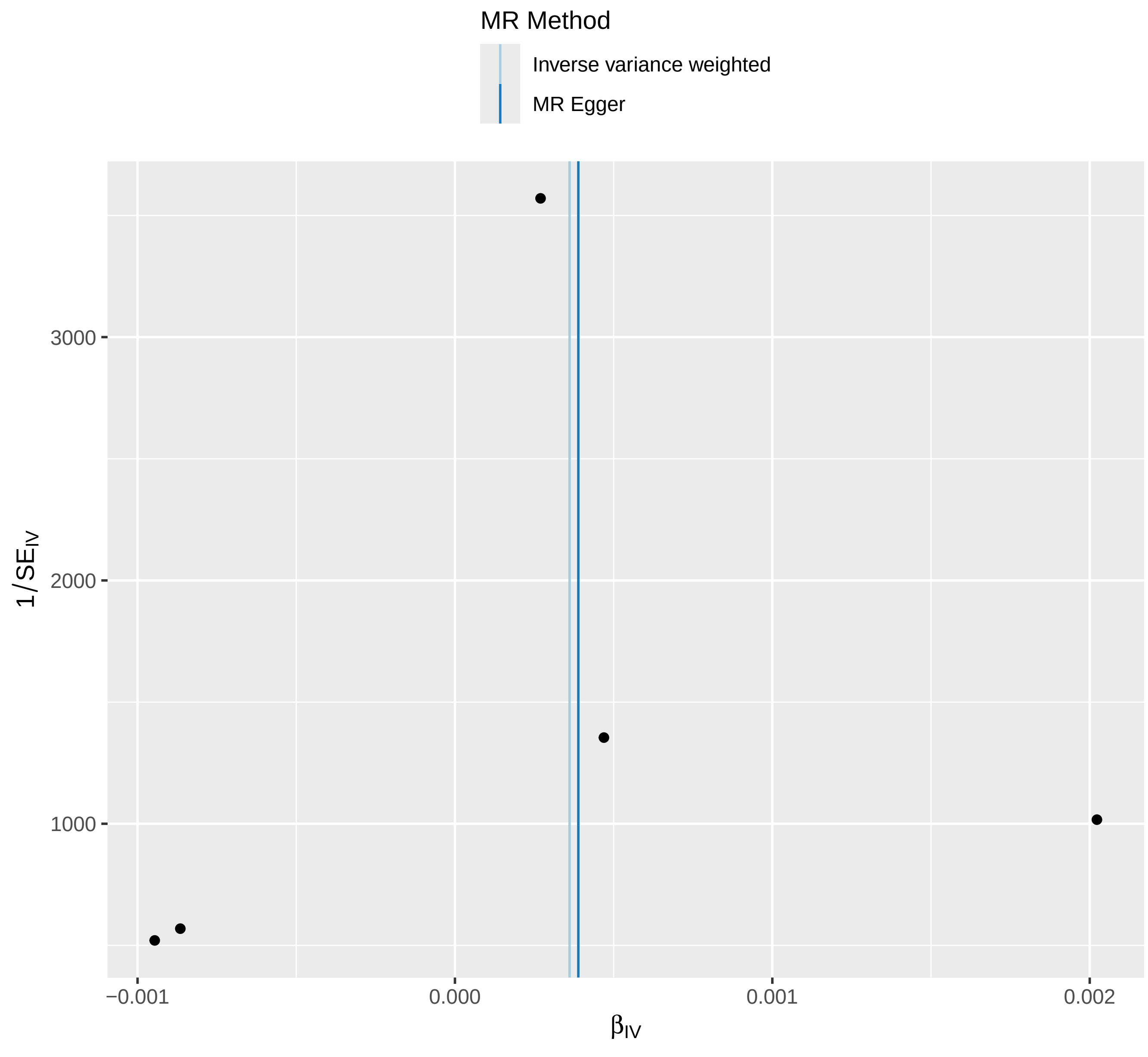
MR Method
Inverse variance weighted
MR Egger
3000
2000
1000
1/SE_IV
−0.001
0.000
0.001
0.002
β_IV

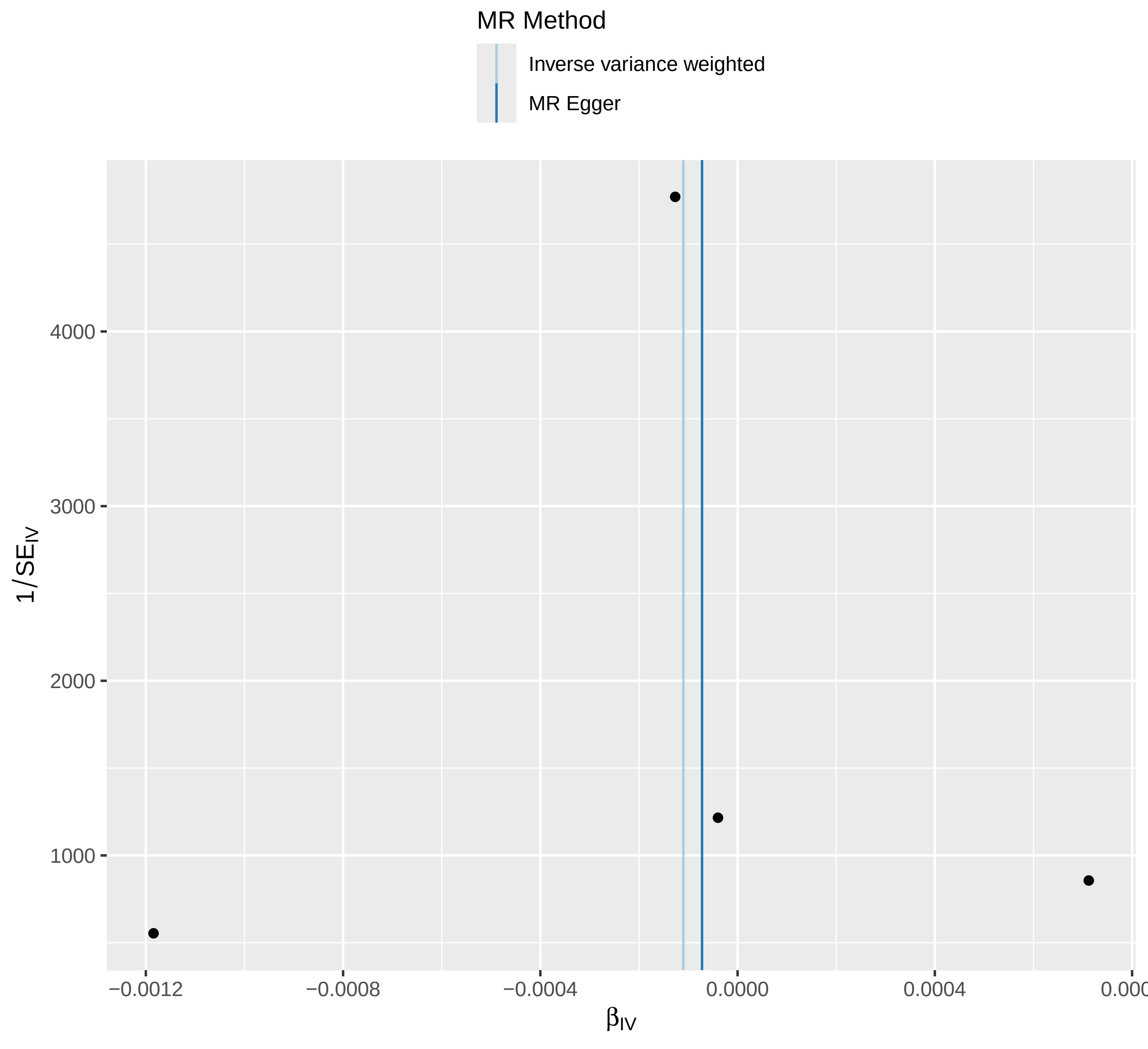
MR Method
Inverse variance weighted
MR Egger
4000
3000
2000
1000
1/SE$_{IV}$
−0.0012
−0.0008
−0.0004
0.0000
0.0004
β$_{IV}$

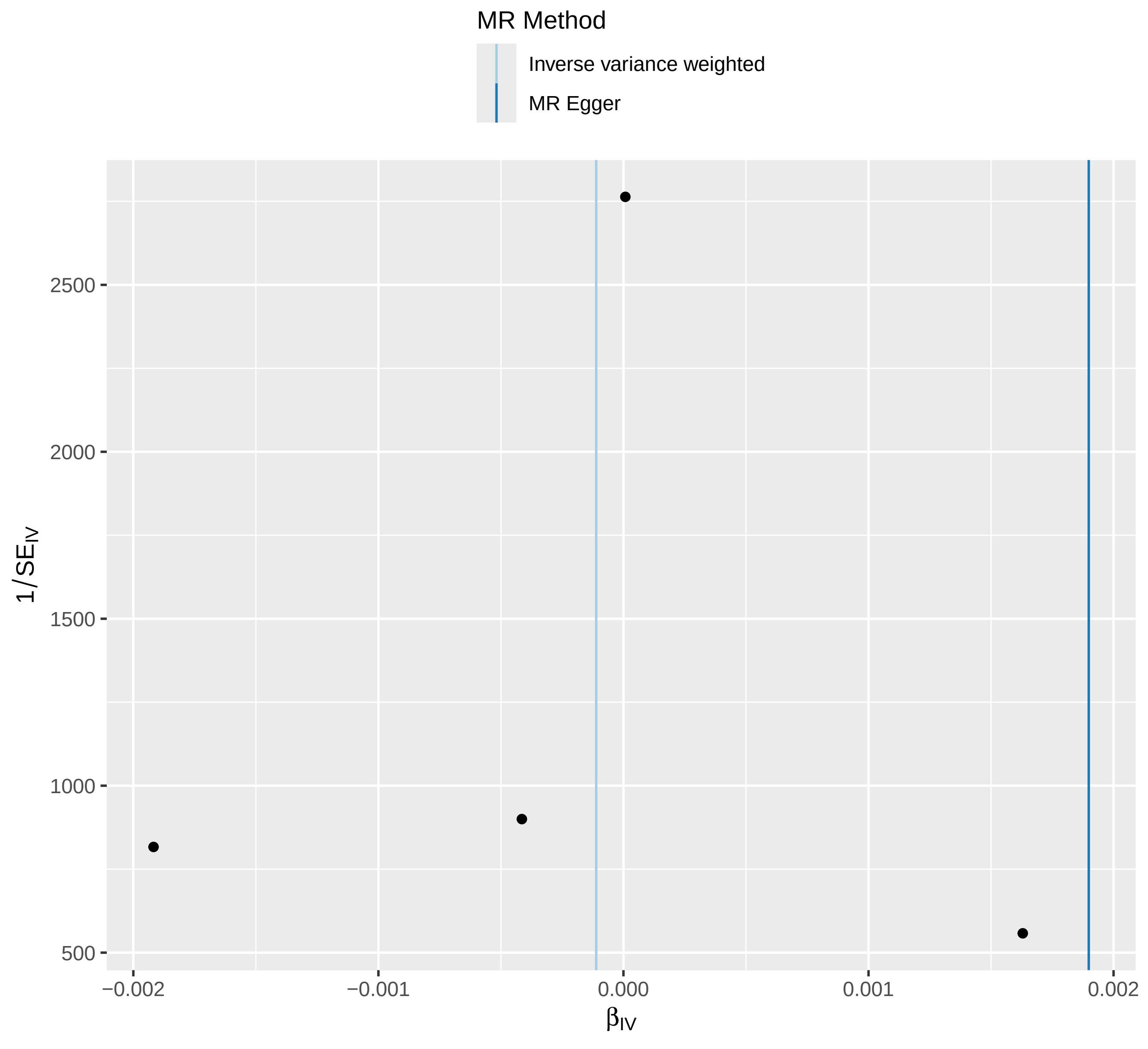
MR Method
Inverse variance weighted
MR Egger
2500
2000
1500
1000
500
1/SE_IV
−0.002
−0.001
0.000
0.001
0.002
β_IV

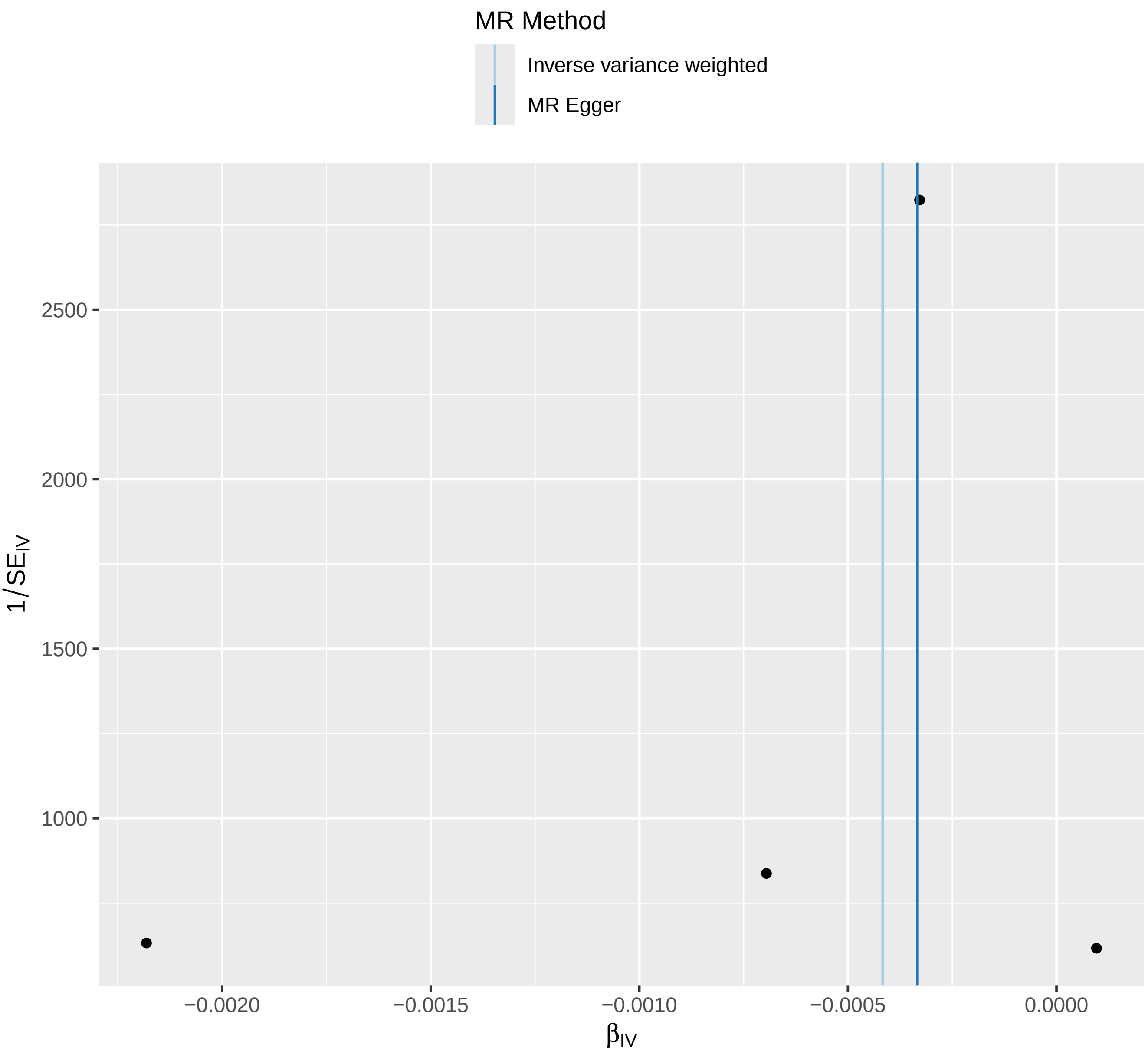

MR Method
Inverse variance weighted
MR Egger
2500
2000
1500
1000
1/SE$_{IV}$
−0.0020
−0.0015
−0.0010
−0.0005
0.0000
β$_{IV}$

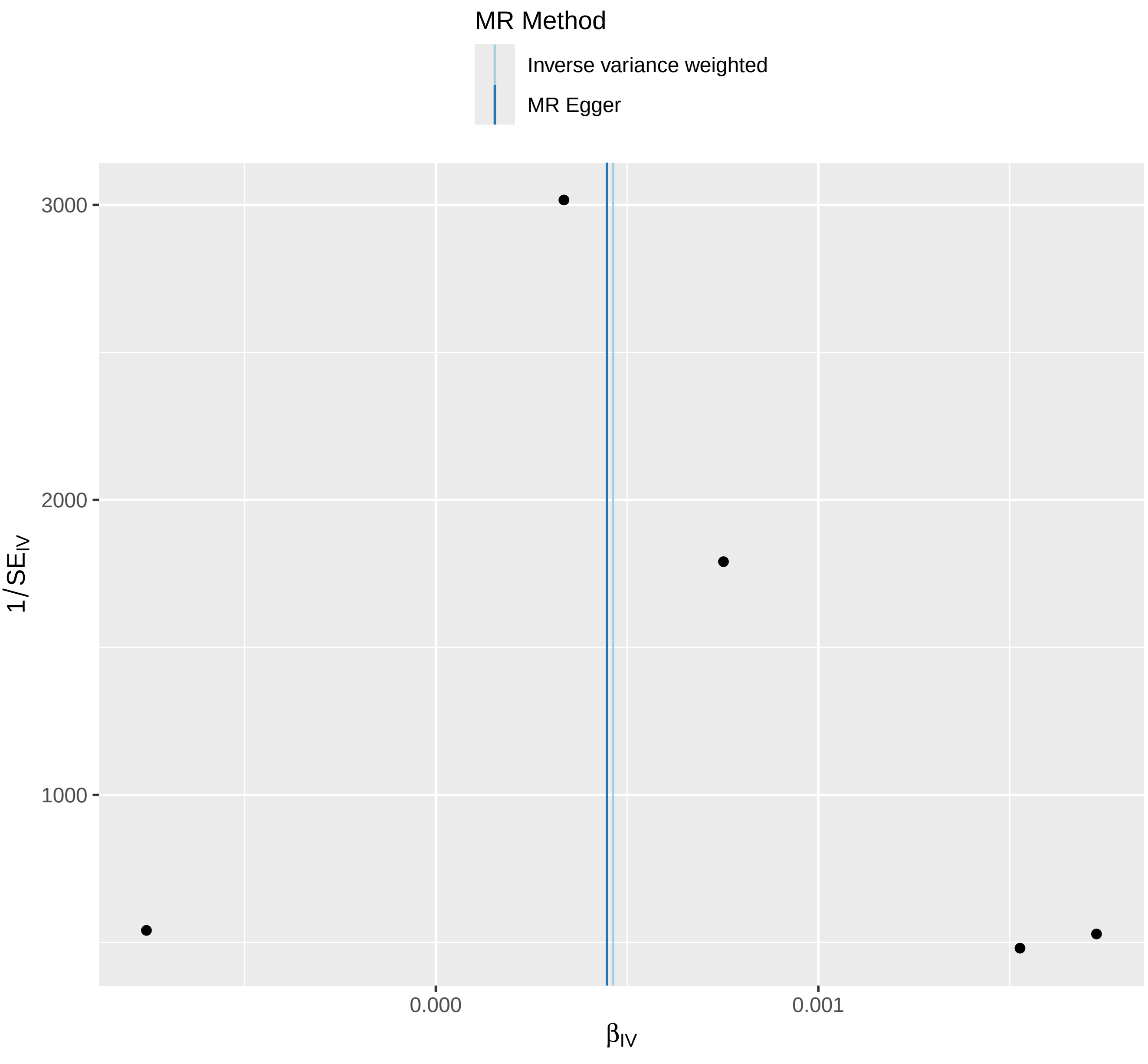
MR Method
Inverse variance weighted
MR Egger
3000
2000
1000
0.000
0.001
$1/SE_{IV}$
β_{IV}

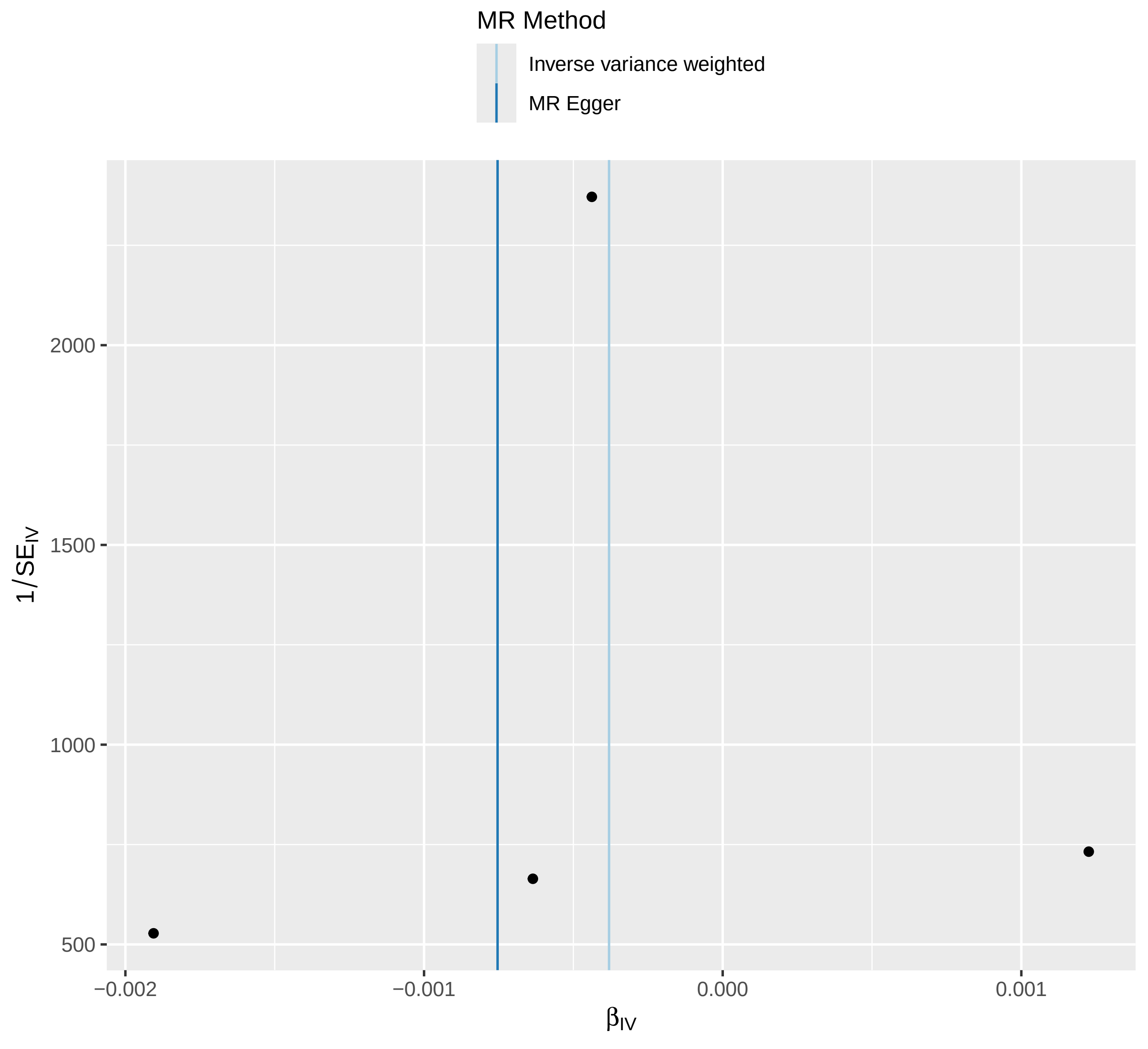
MR Method
Inverse variance weighted
MR Egger
2000
1500
1000
500
1/SE_IV
−0.002
−0.001
0.000
0.001
β_IV

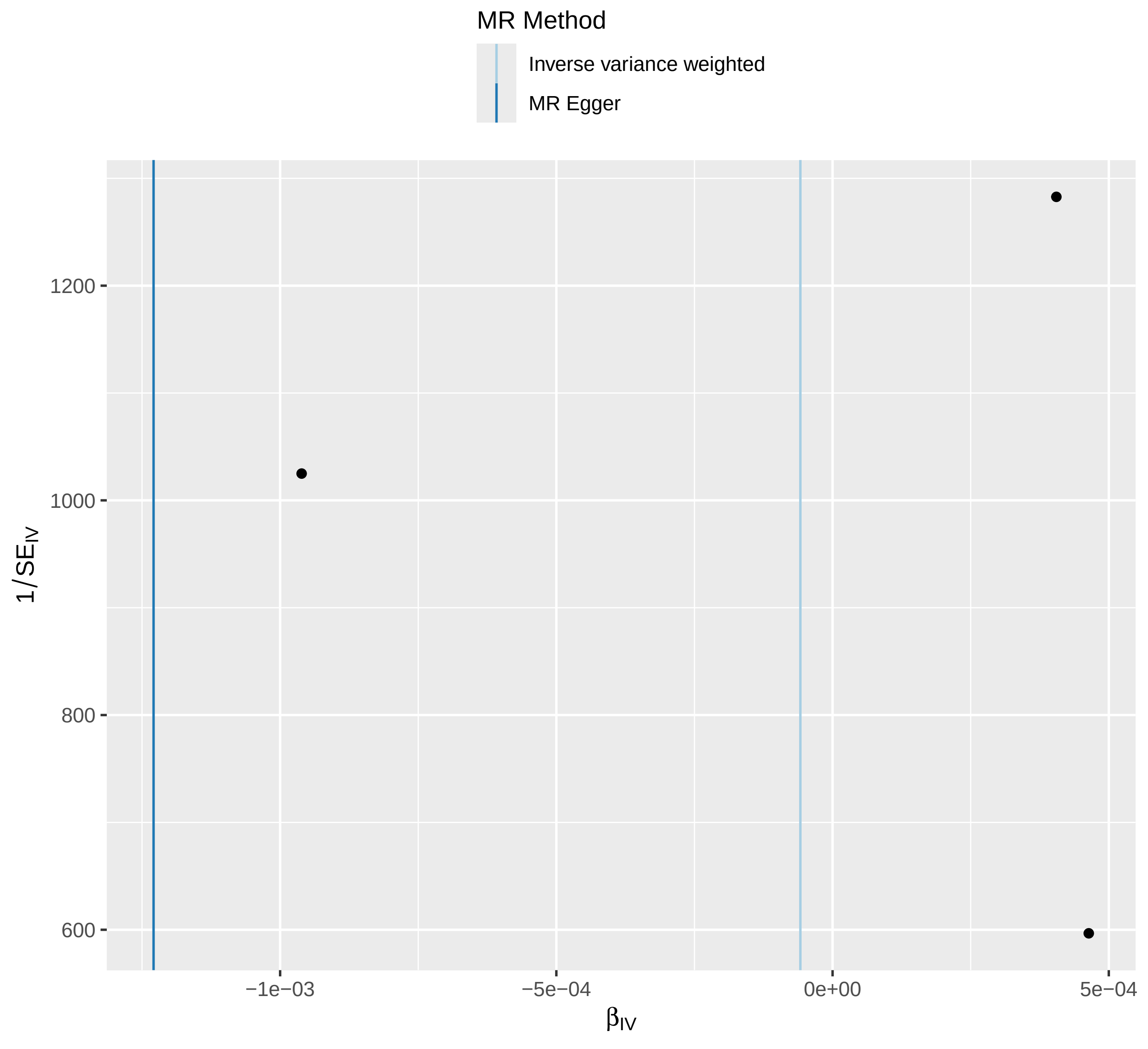
MR Method
Inverse variance weighted
MR Egger
1200
1000
800
600
1/SE_IV
−1e−03
−5e−04
0e+00
5e−04
β_IV

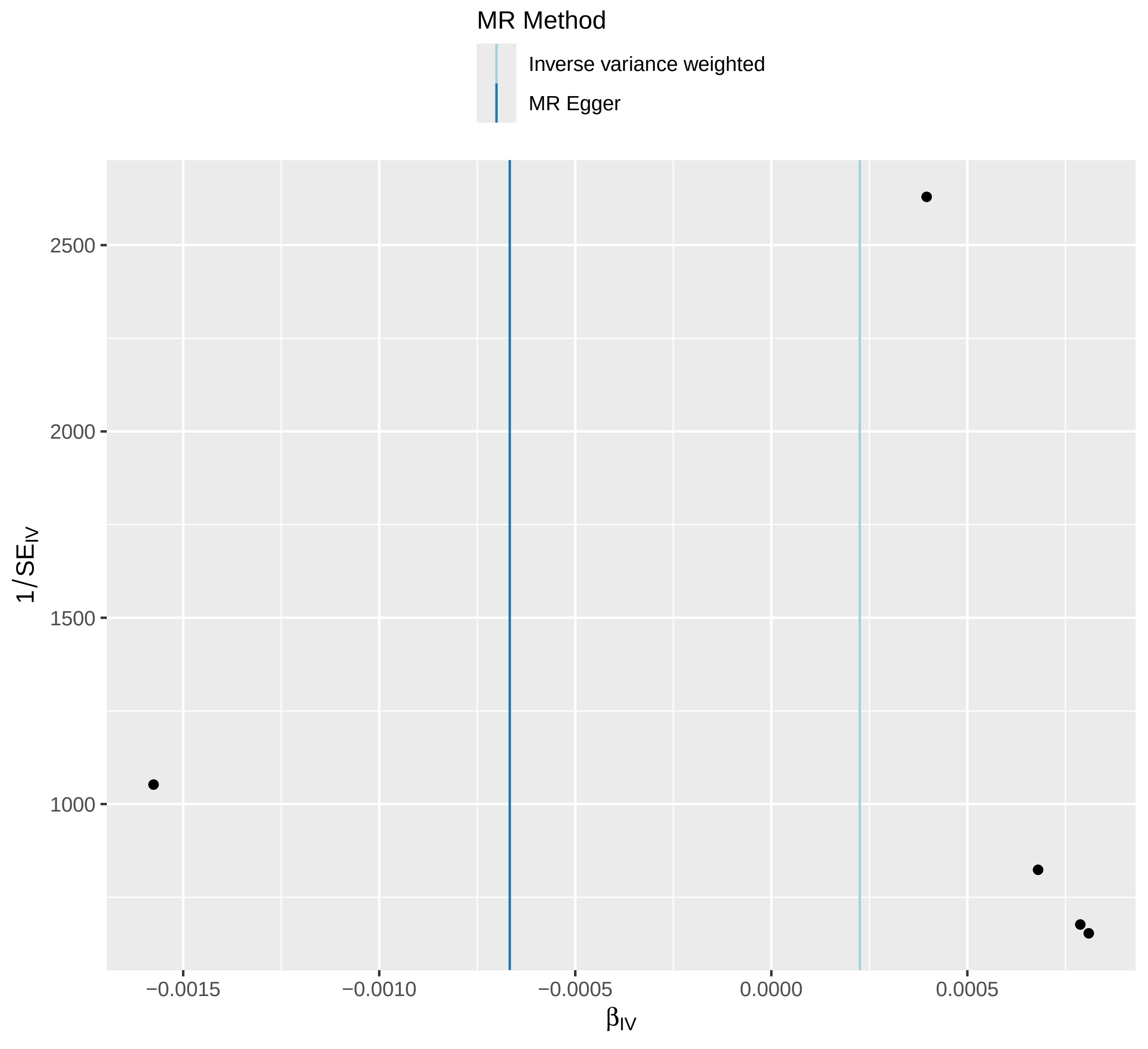
MR Method
Inverse variance weighted
MR Egger
1/SE_IV
2500
2000
1500
1000
−0.0015
−0.0010
−0.0005
0.0000
0.0005
β_IV

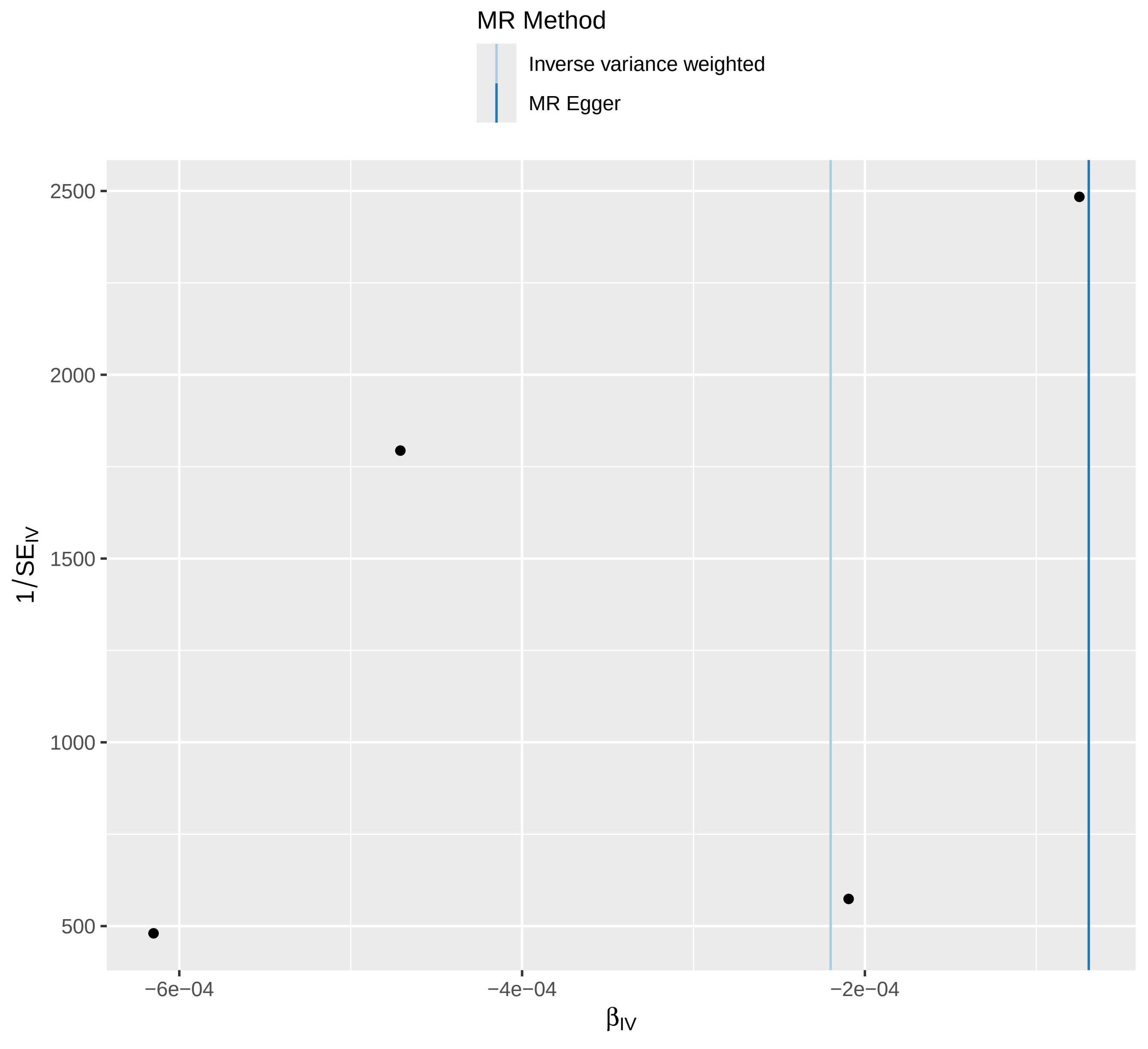
MR Method
Inverse variance weighted
MR Egger
2500
2000
1500
1000
500
1/SE_IV
−6e−04
−4e−04
−2e−04
β_IV

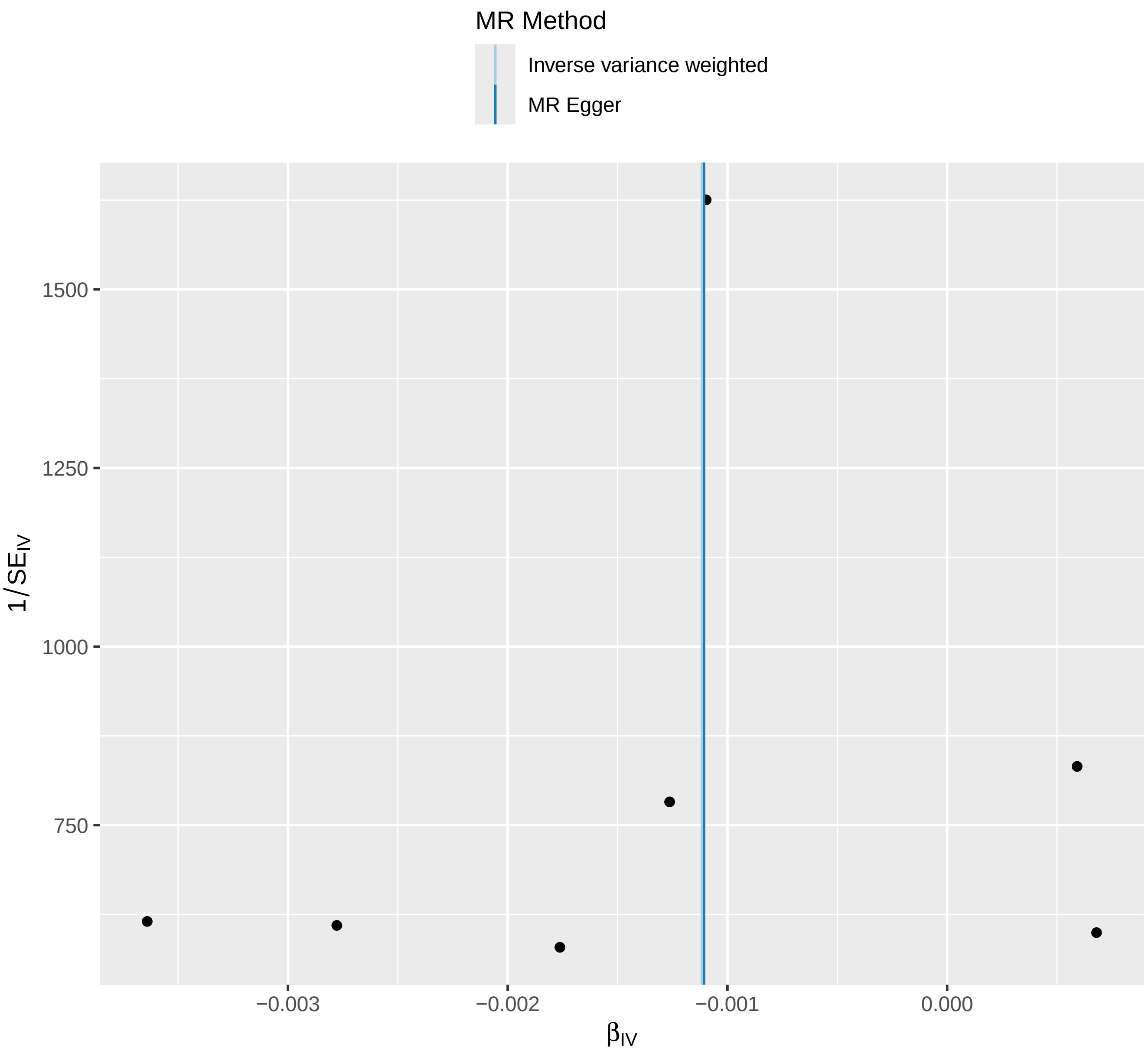
MR Method
Inverse variance weighted
MR Egger
1500
1250
1000
750
1/SE$_{IV}$
−0.003
−0.002
−0.001
0.000
β$_{IV}$

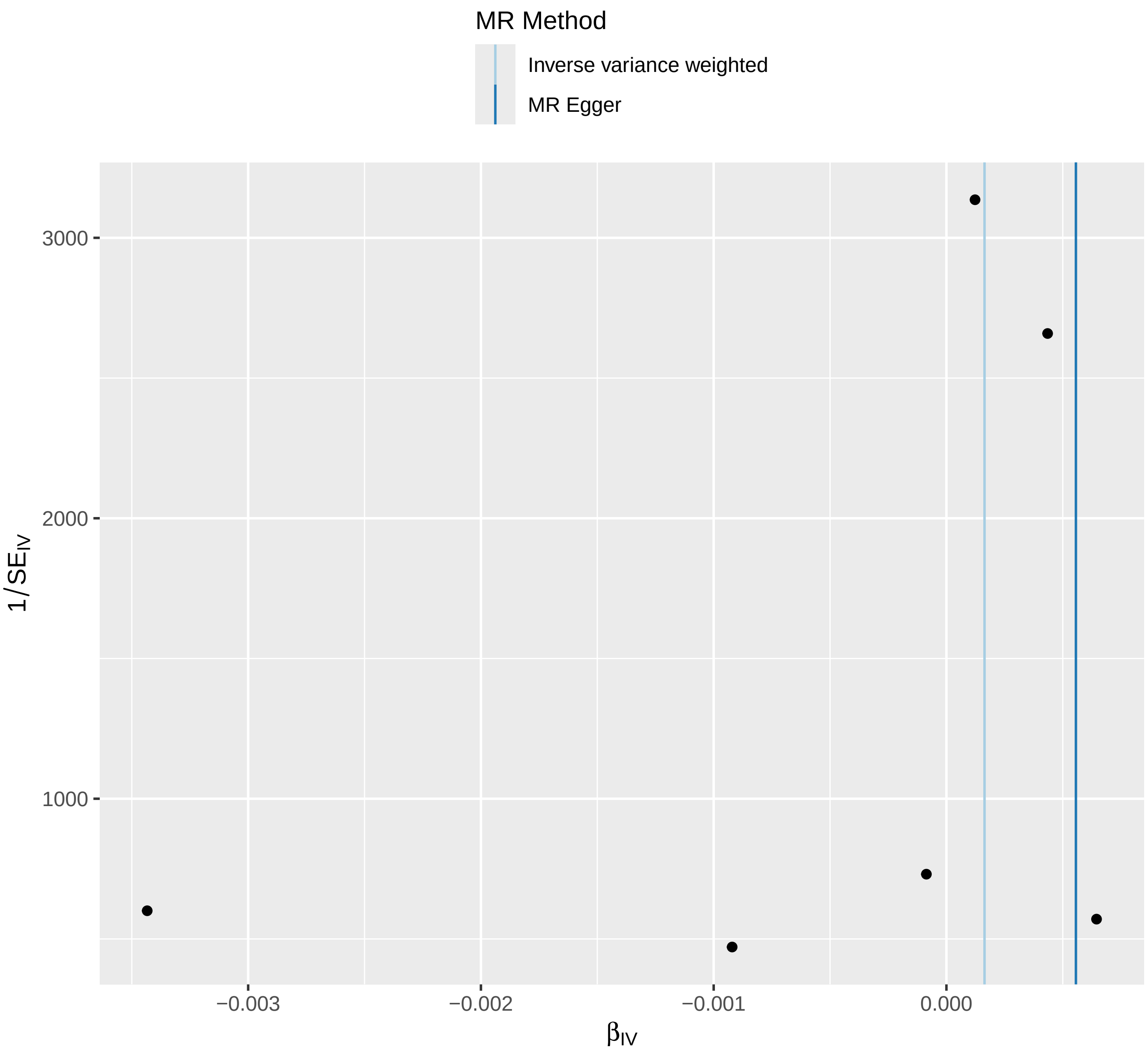

MR Method
Inverse variance weighted
MR Egger
3000
2000
1000
1/SE_IV
−0.003
−0.002
−0.001
0.000
β_IV

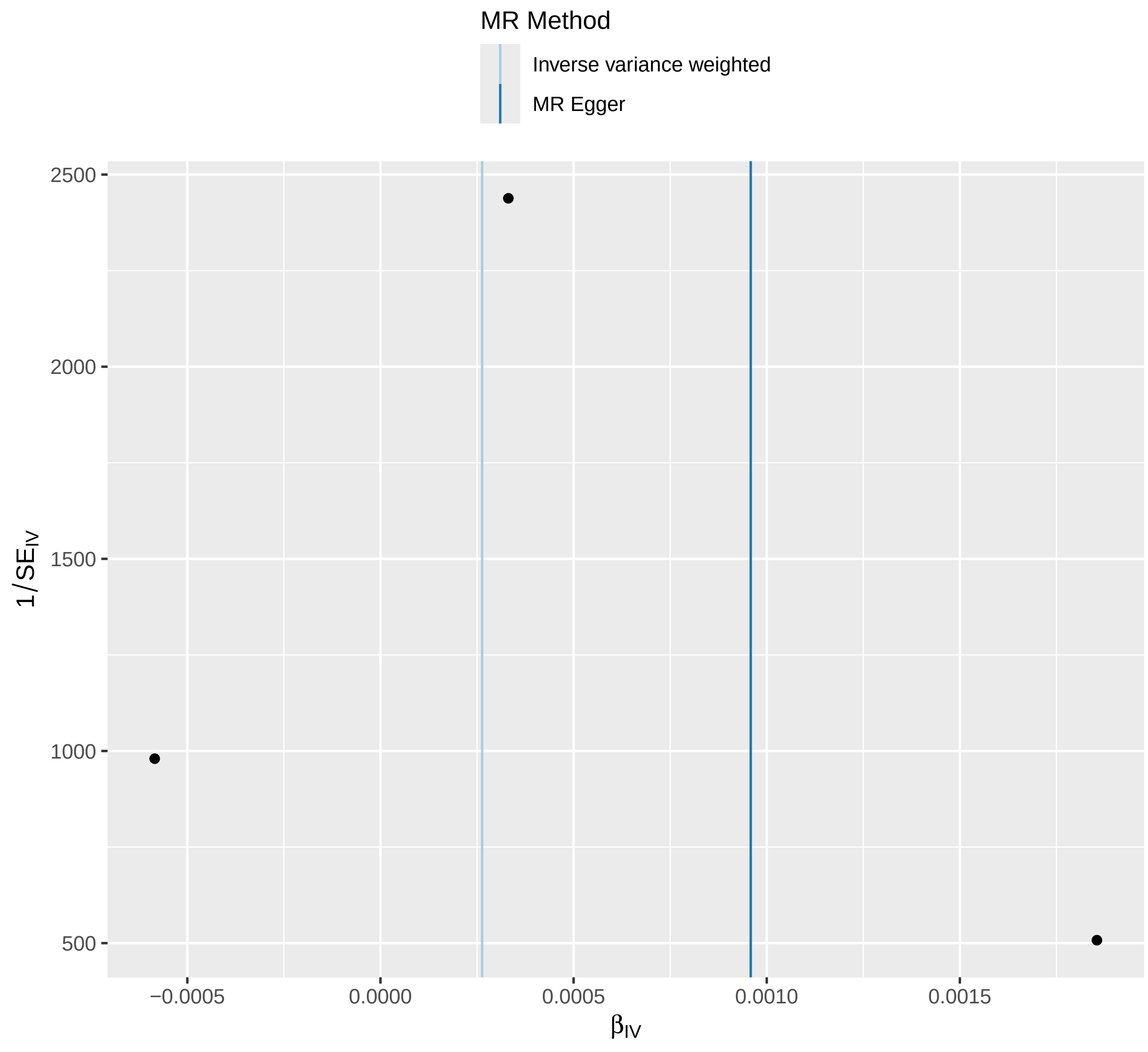
MR Method
Inverse variance weighted
MR Egger
2500
2000
1500
1000
500
1/SE_IV
−0.0005
0.0000
0.0005
0.0010
0.0015
β_IV

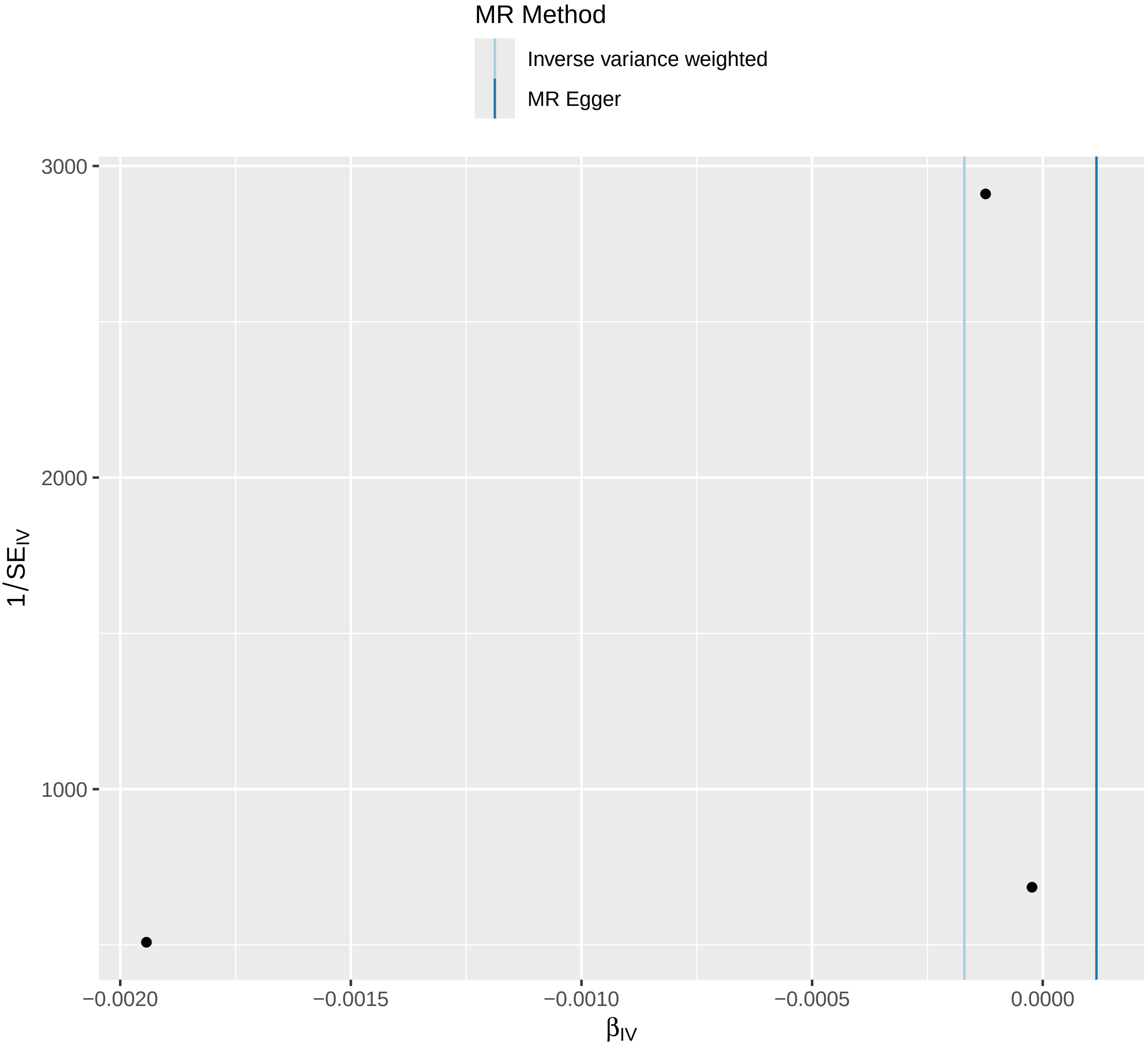

MR Method
Inverse variance weighted
MR Egger
3000
2000
1000
1/SE_IV
−0.0020
−0.0015
−0.0010
−0.0005
0.0000
β_IV

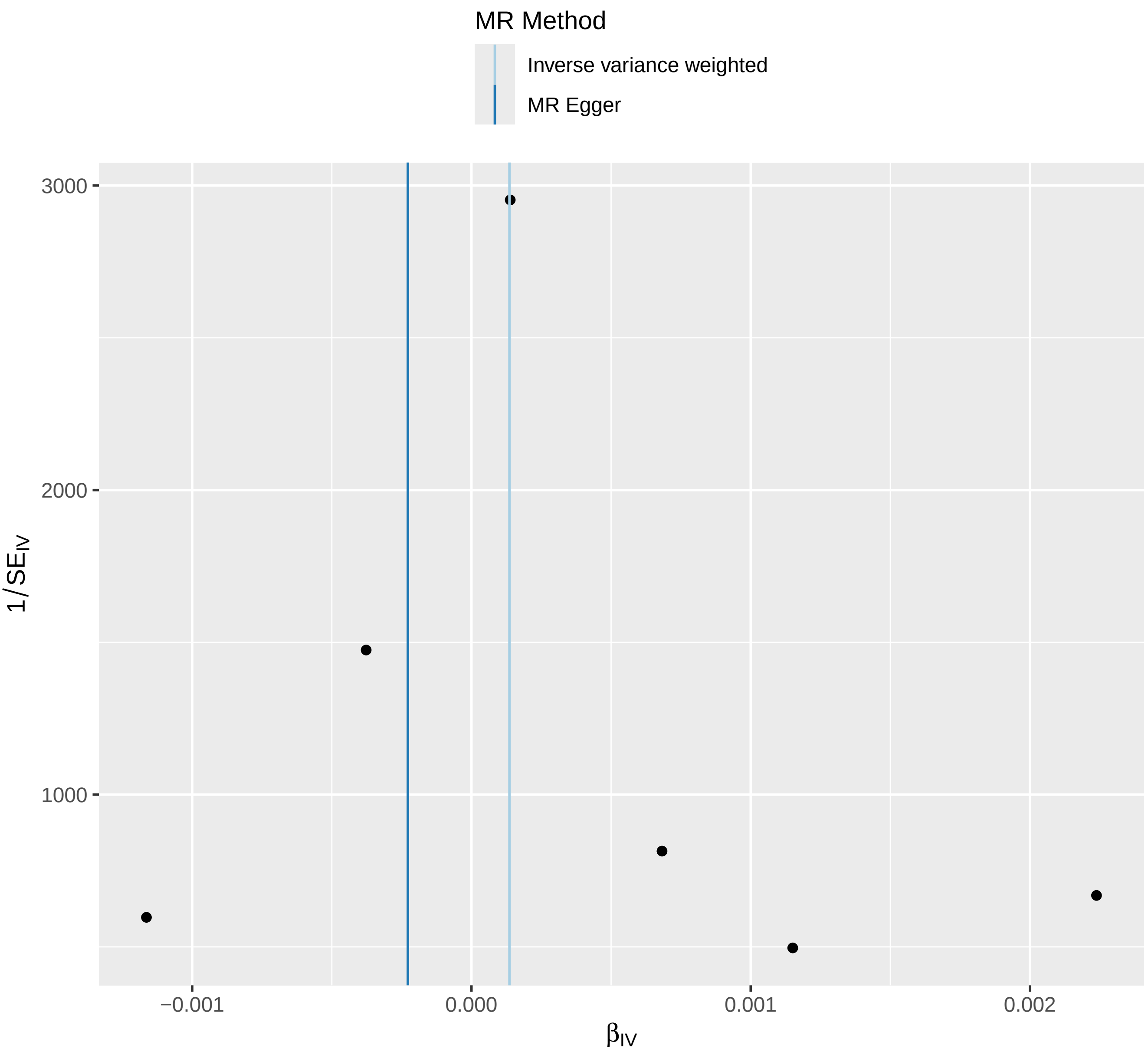
MR Method
Inverse variance weighted
MR Egger
3000
2000
1000
1/SE_IV
−0.001
0.000
0.001
0.002
β_IV

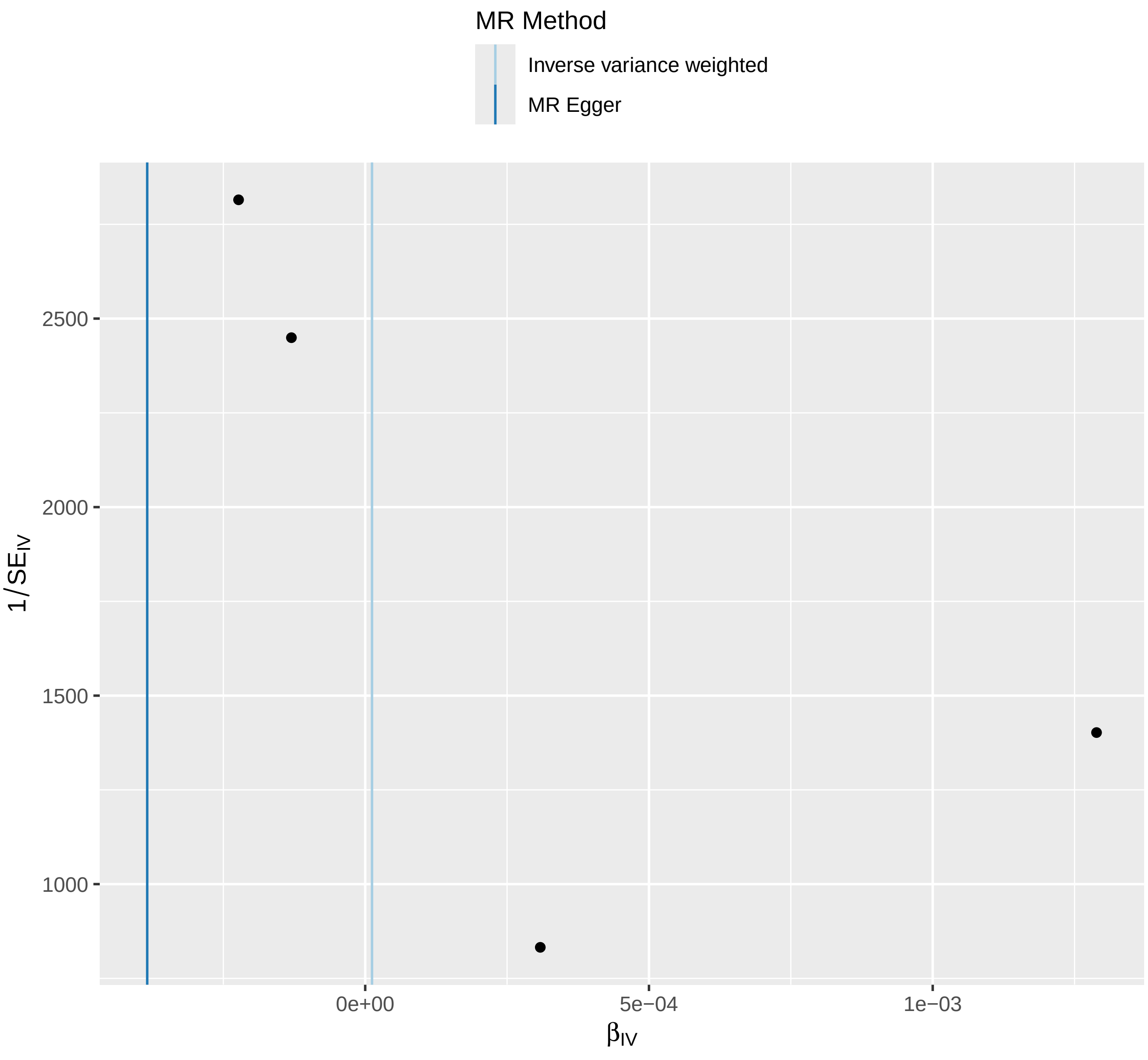
MR Method
Inverse variance weighted
MR Egger
2500
2000
1500
1000
1/SE_IV
0e+00
5e−04
1e−03
β_IV

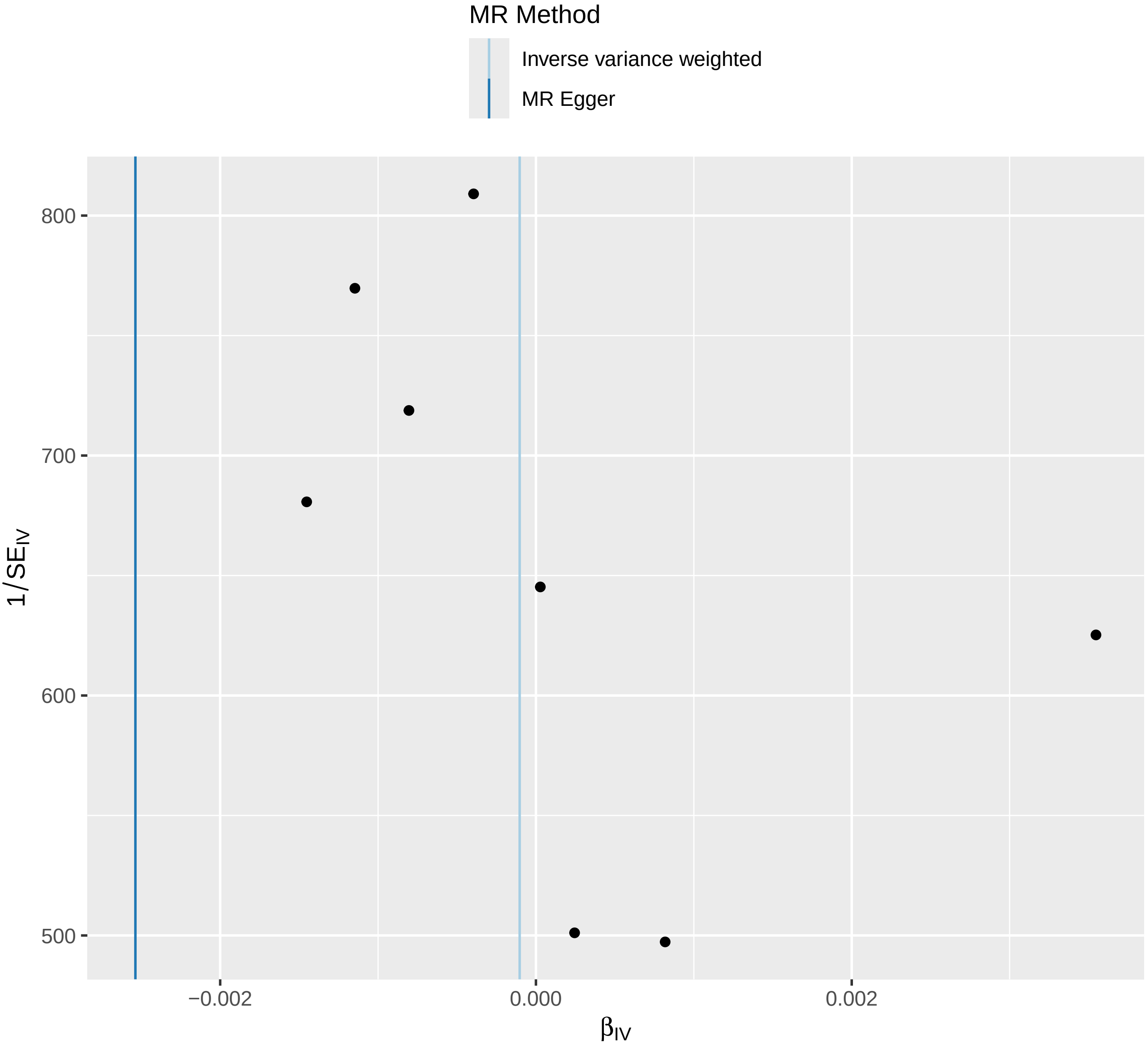

MR Method
Inverse variance weighted
MR Egger
800
700
600
500
1/SE_IV
−0.002
0.000
0.002
β_IV

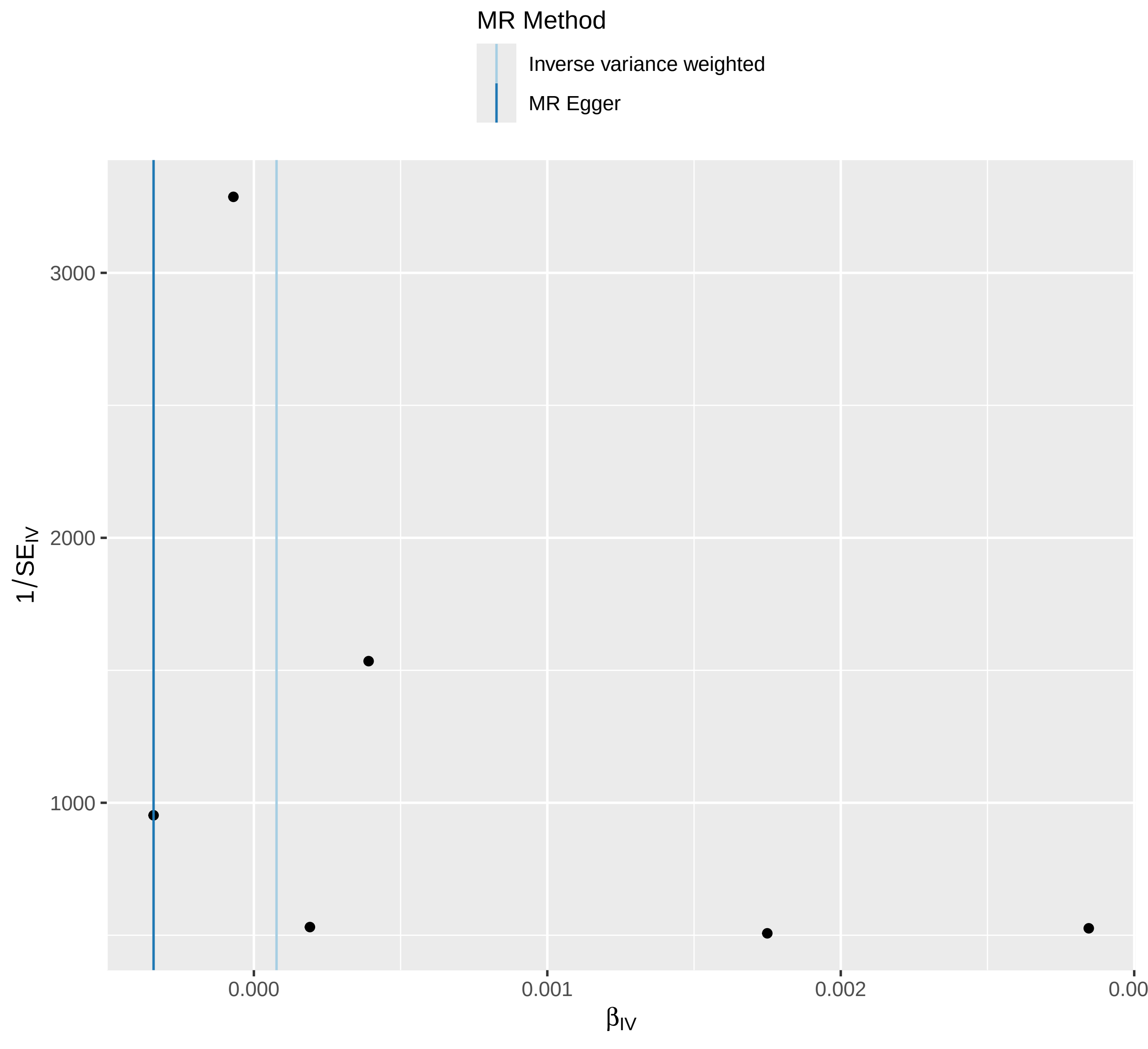

MR Method
Inverse variance weighted
MR Egger
3000
2000
1000
1/SE$_{IV}$
0.000
0.001
0.002
0.00
β_{IV}

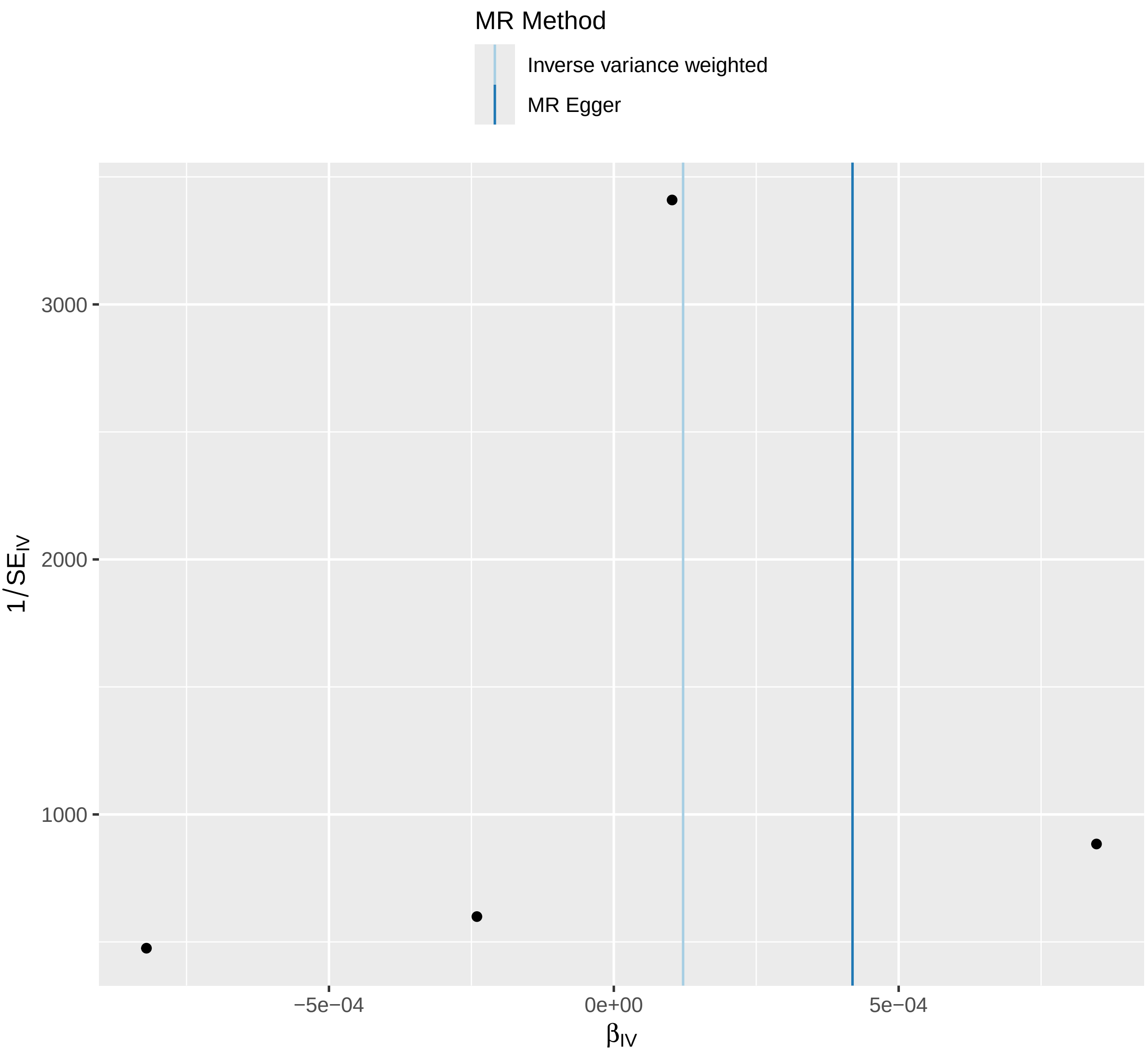

MR Method
Inverse variance weighted
MR Egger
3000
2000
1000
1/SE_IV
−5e−04
0e+00
5e−04
β_IV

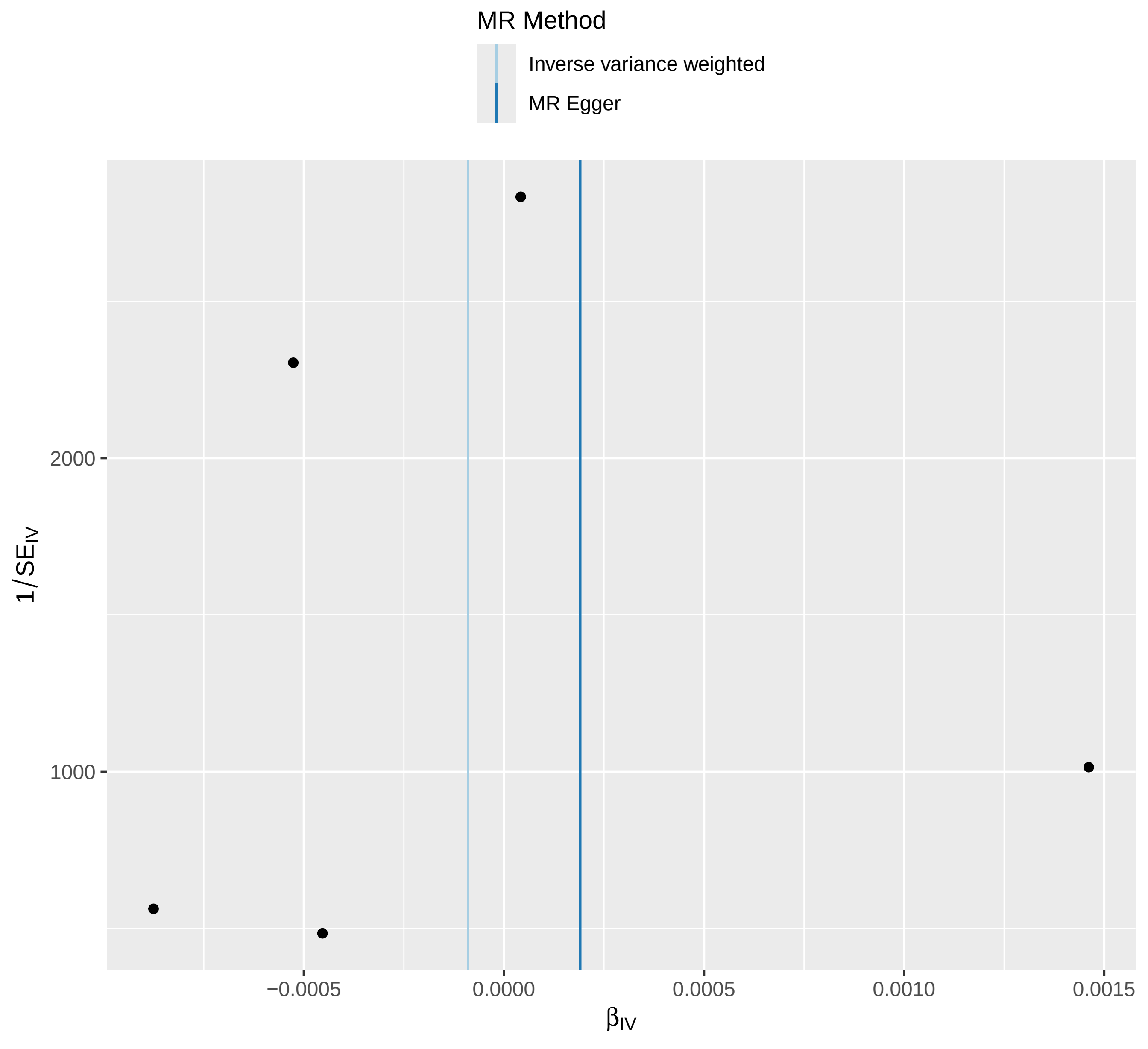

MR Method
Inverse variance weighted
MR Egger
2000
1000
1/SE_IV
−0.0005
0.0000
0.0005
0.0010
0.0015
β_IV

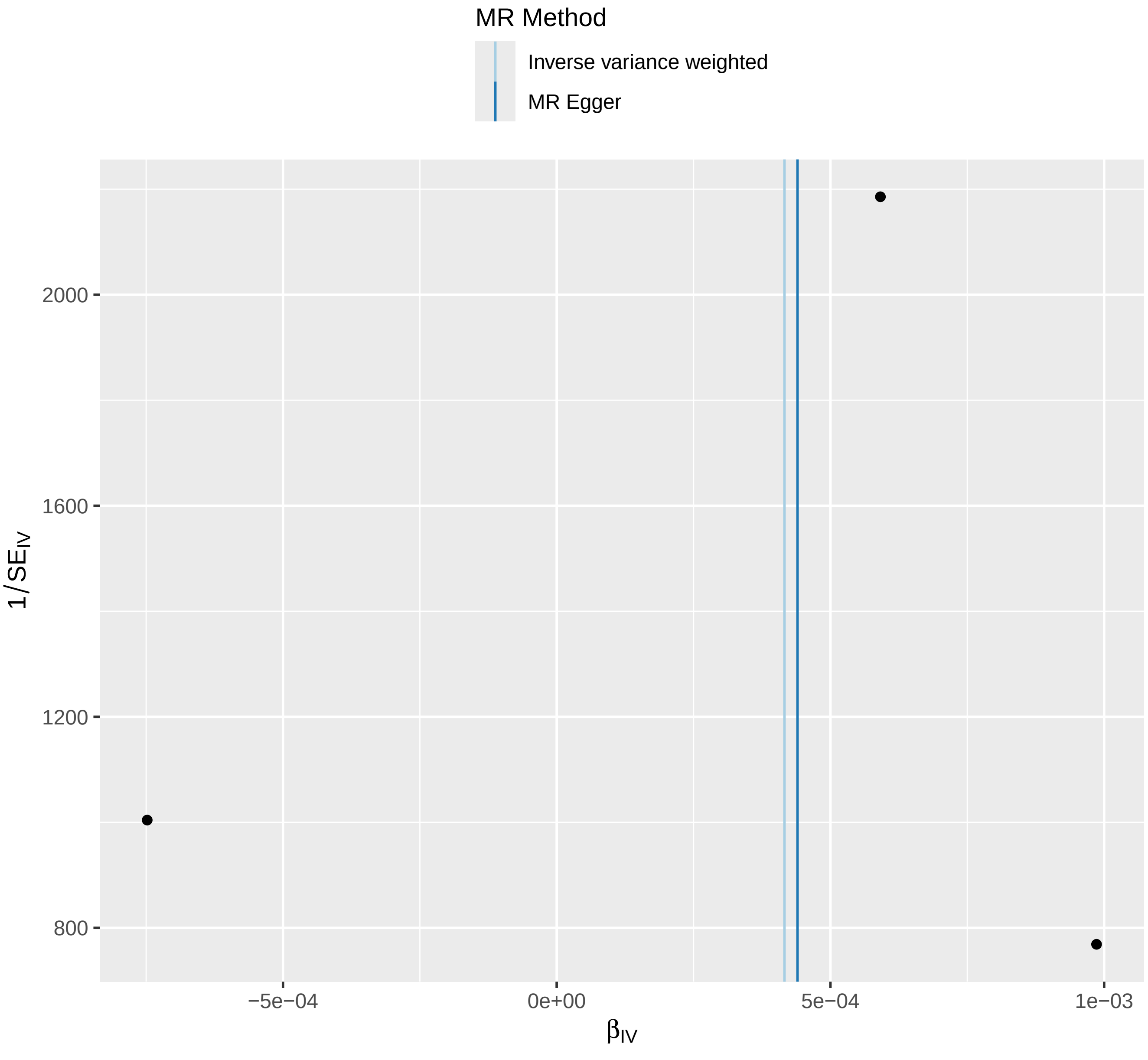
MR Method
Inverse variance weighted
MR Egger
2000
1600
1200
800
1/SE$_{IV}$
−5e−04
0e+00
5e−04
1e−03
β$_{IV}$

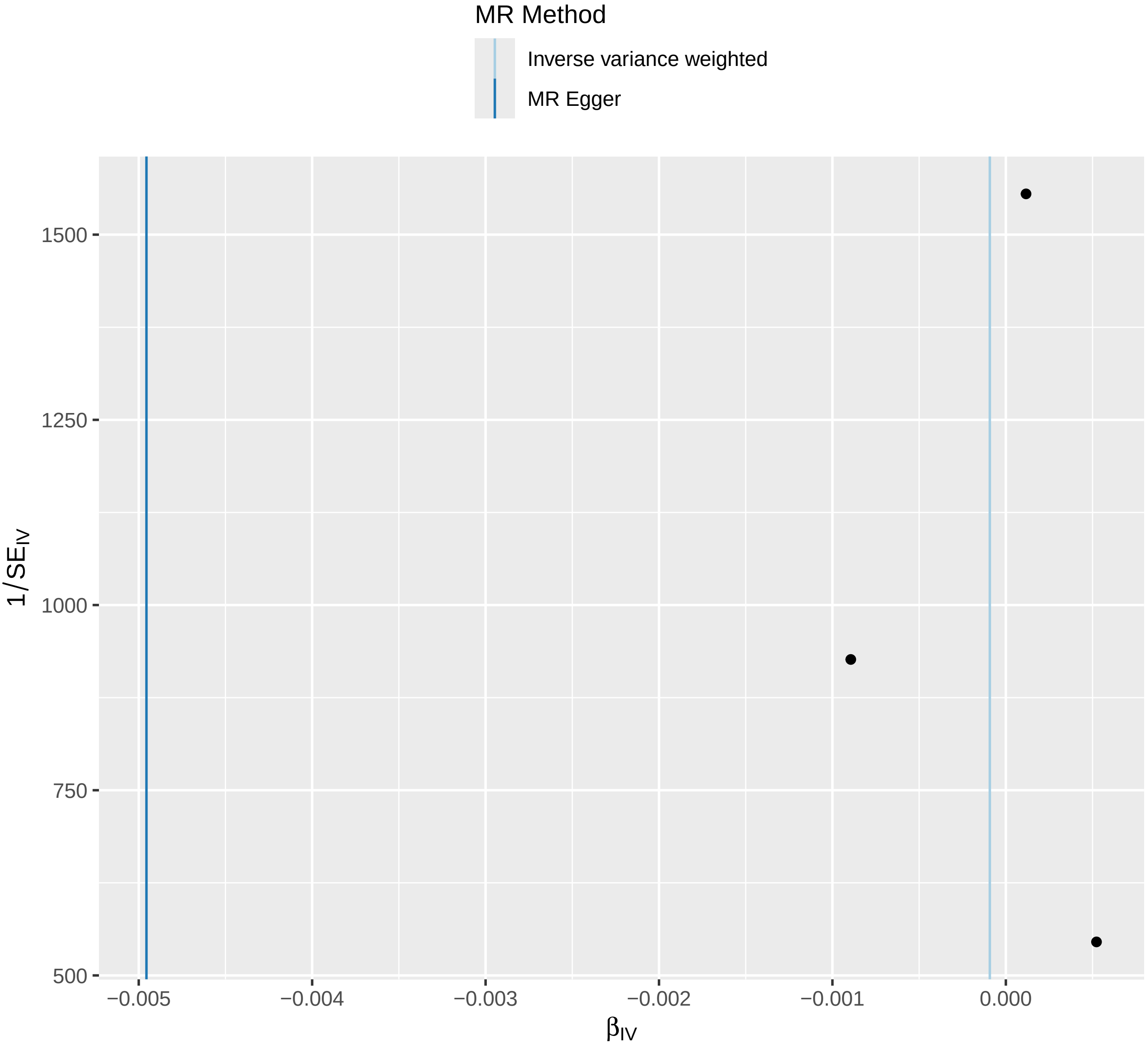
MR Method
Inverse variance weighted
MR Egger
1500
1250
1000
750
500
−0.005
−0.004
−0.003
−0.002
−0.001
0.000
1/SE_IV
β_IV

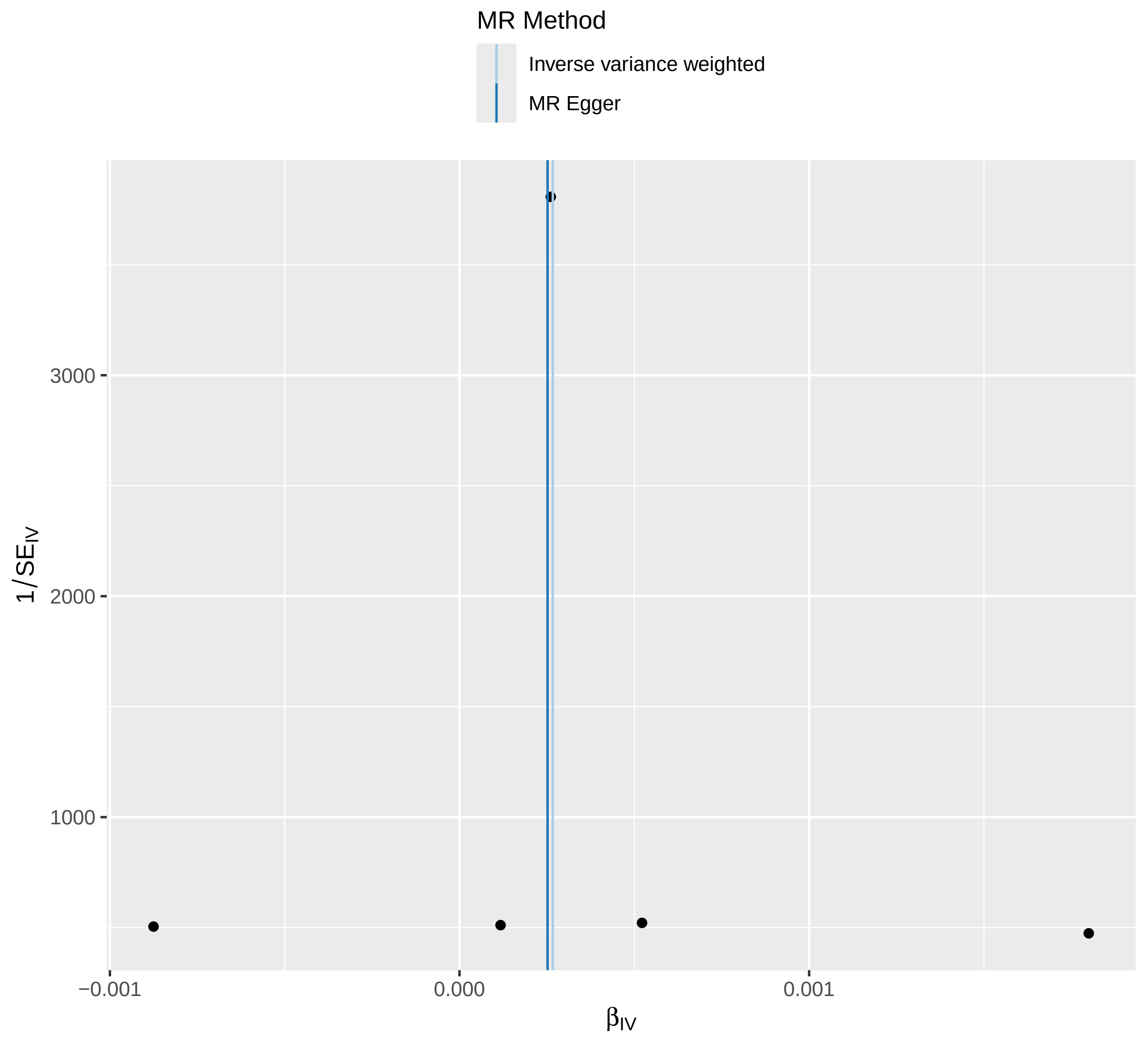

MR Method
Inverse variance weighted
MR Egger
3000
2000
1000
1/SE_IV
−0.001
0.000
0.001
β_IV

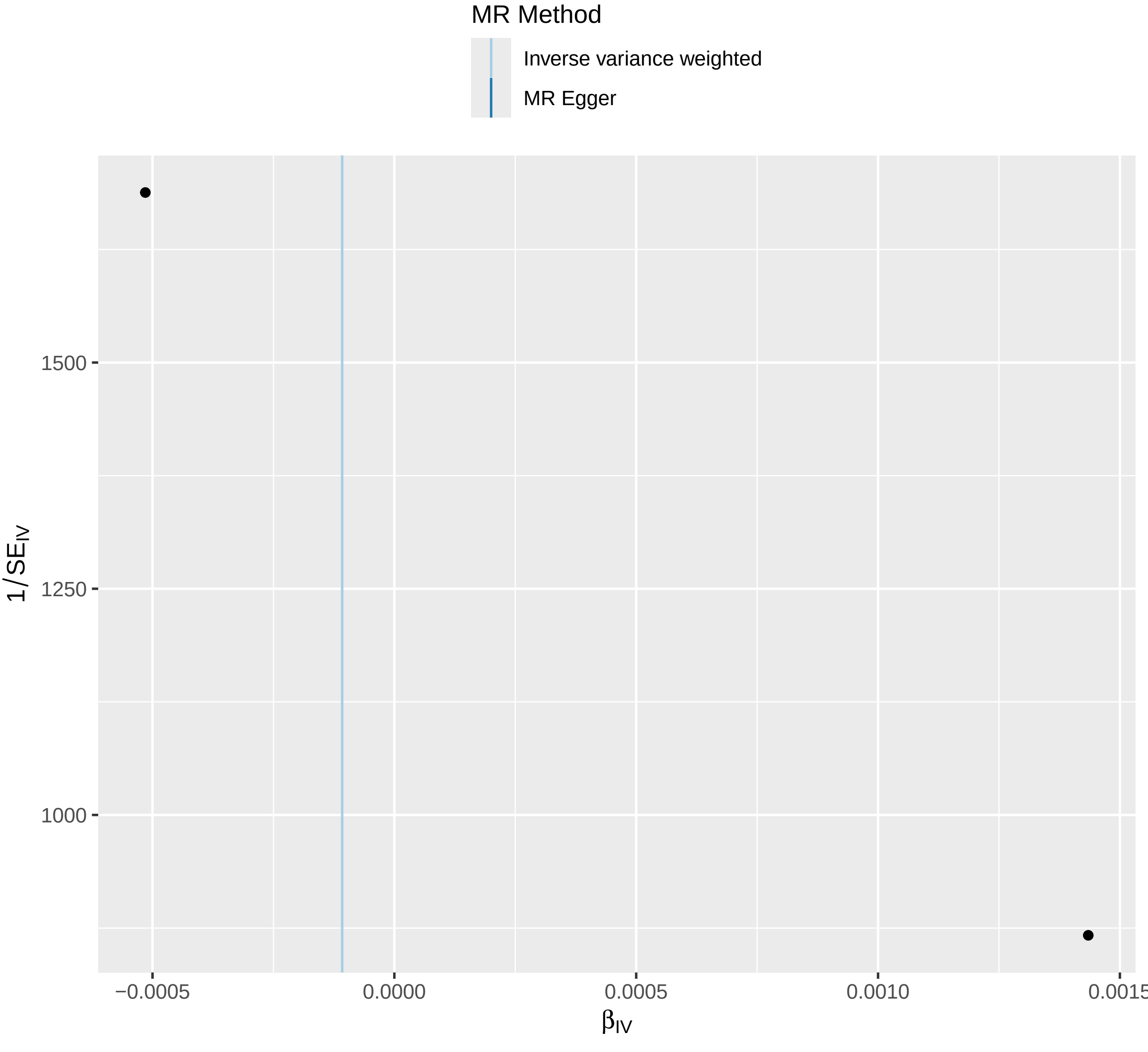
MR Method
Inverse variance weighted
MR Egger
1500
1250
1000
1/SE_{IV}
−0.0005
0.0000
0.0005
0.0010
0.0015
β_{IV}

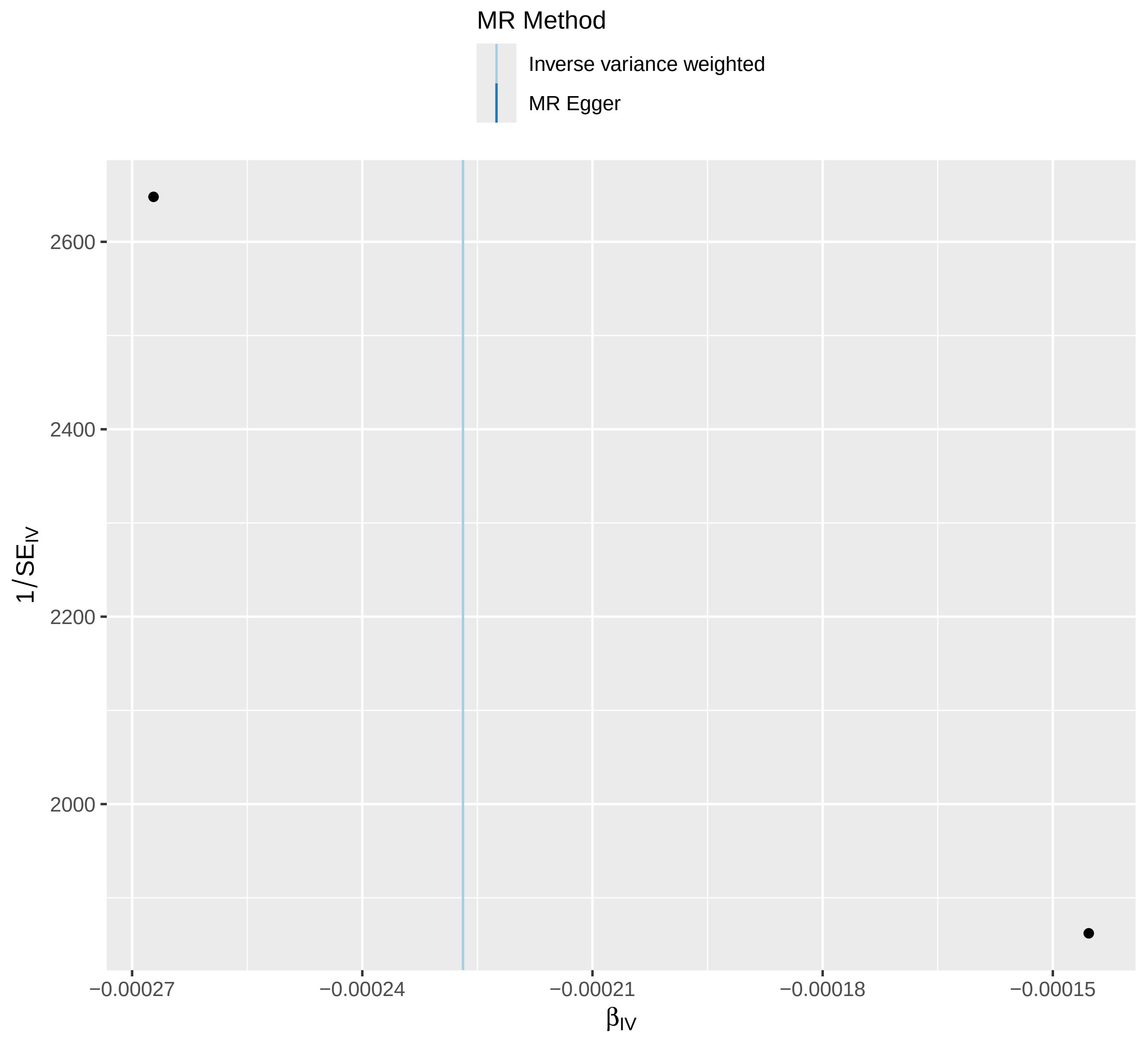

MR Method
Inverse variance weighted
MR Egger
2600
2400
2200
2000
1/SE_IV
−0.00027
−0.00024
−0.00021
−0.00018
−0.00015
β_IV

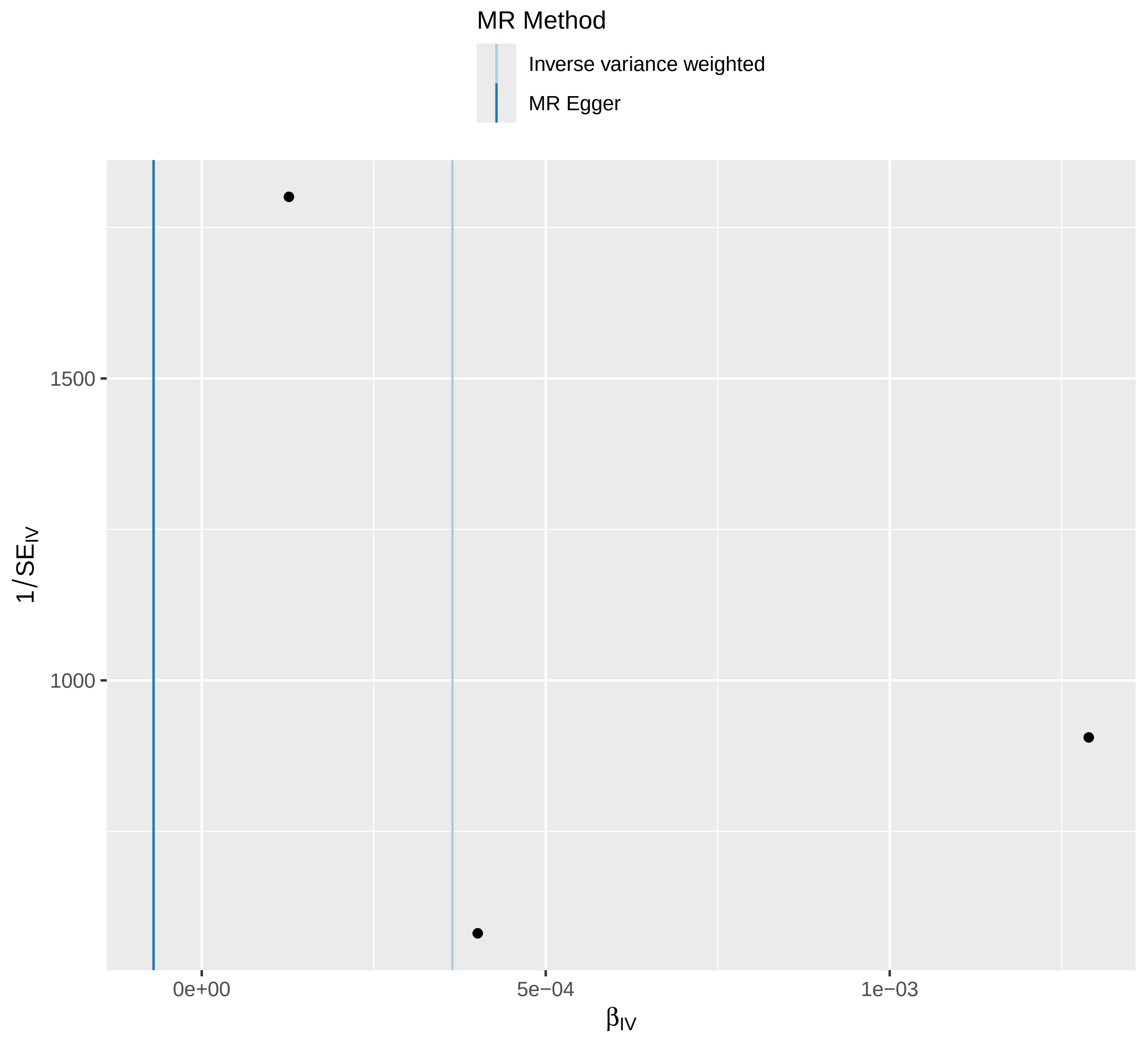
MR Method
Inverse variance weighted
MR Egger
1500
1000
0e+00
5e−04
1e−03
$1/SE_{IV}$
β_{IV}

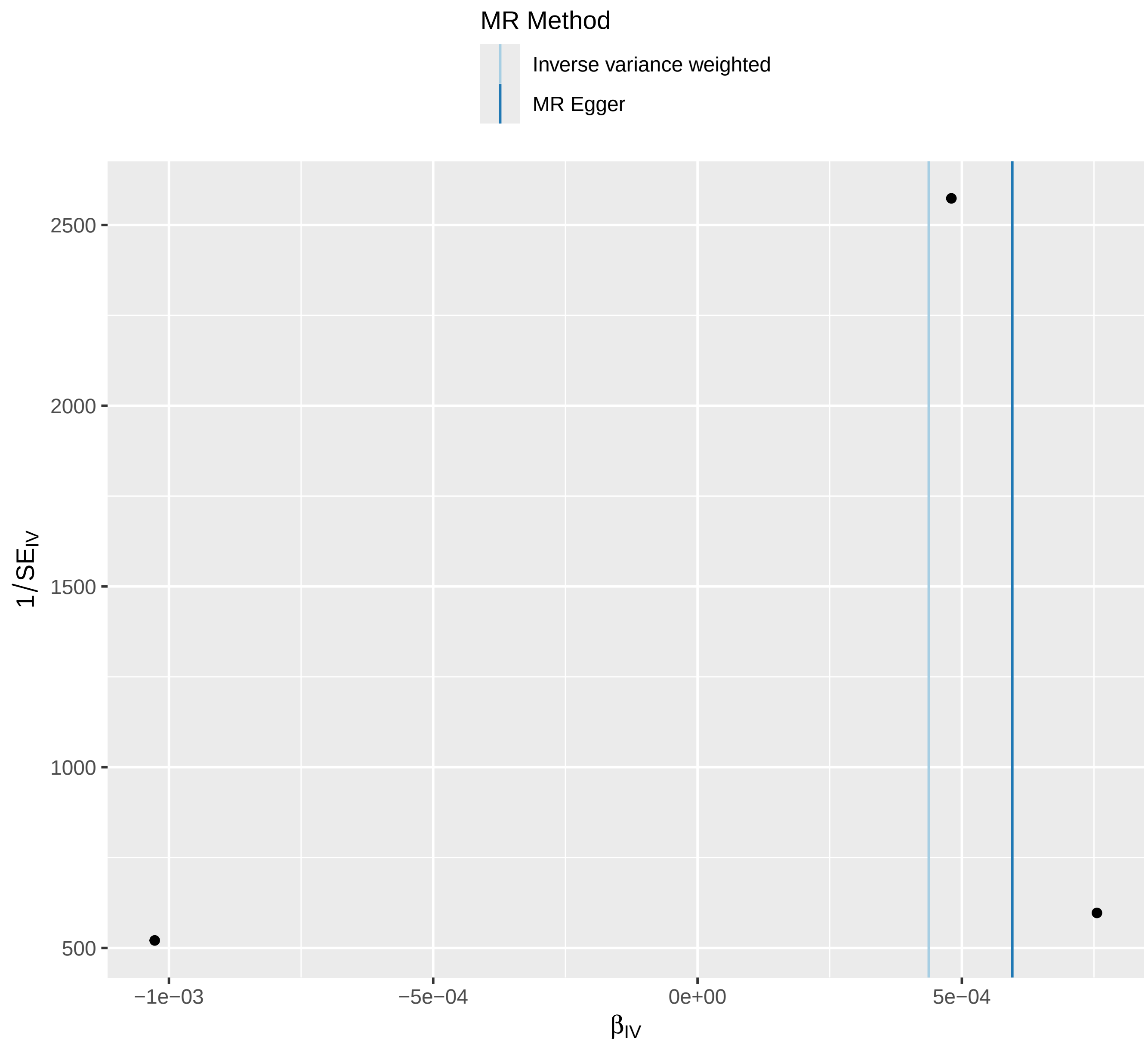
MR Method
Inverse variance weighted
MR Egger
2500
2000
1500
1000
500
1/SE_IV
−1e−03
−5e−04
0e+00
5e−04
β_IV

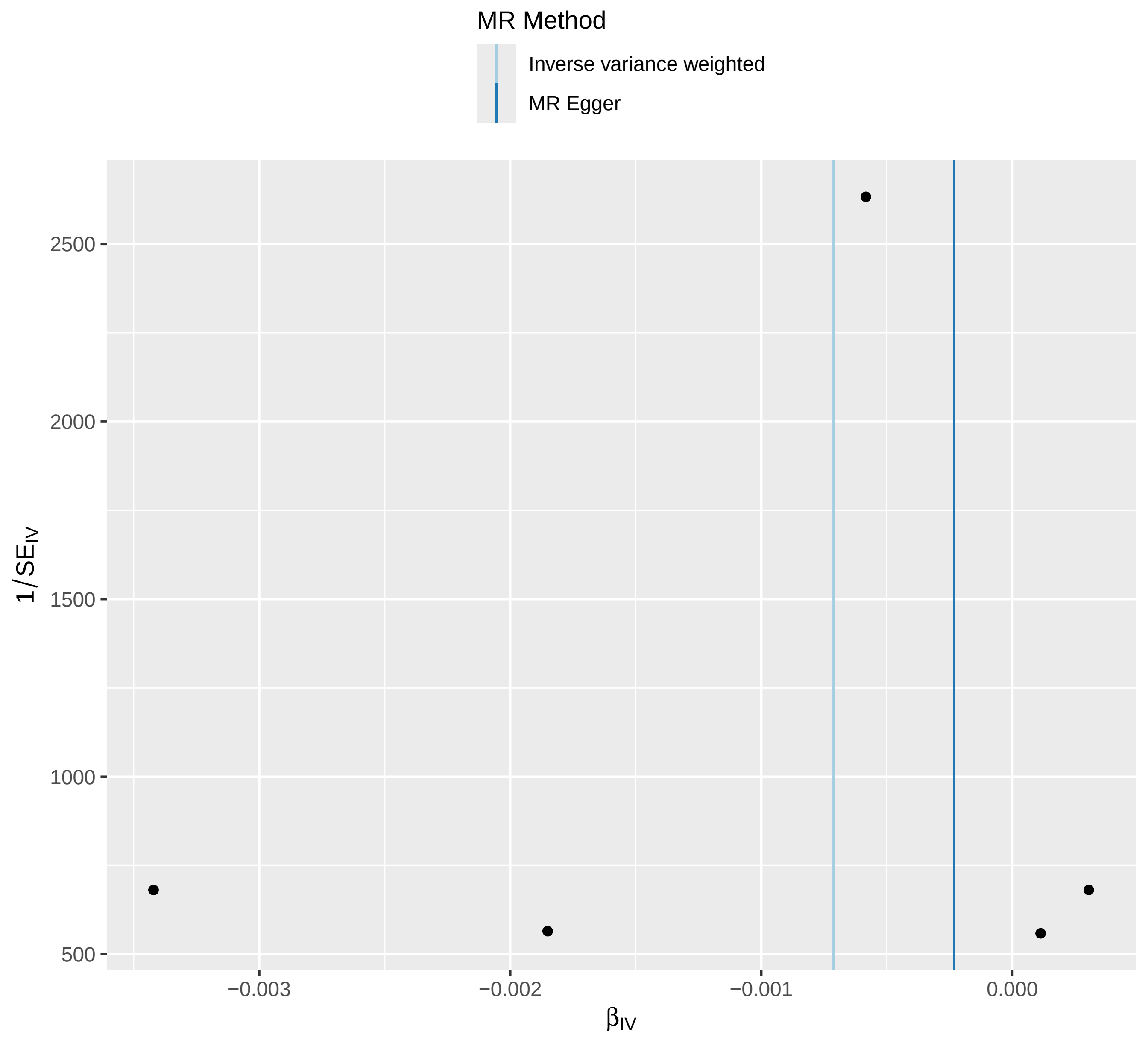

MR Method
Inverse variance weighted
MR Egger
2500
2000
1500
1000
500
1/SE_IV
−0.003
−0.002
−0.001
0.000
β_IV

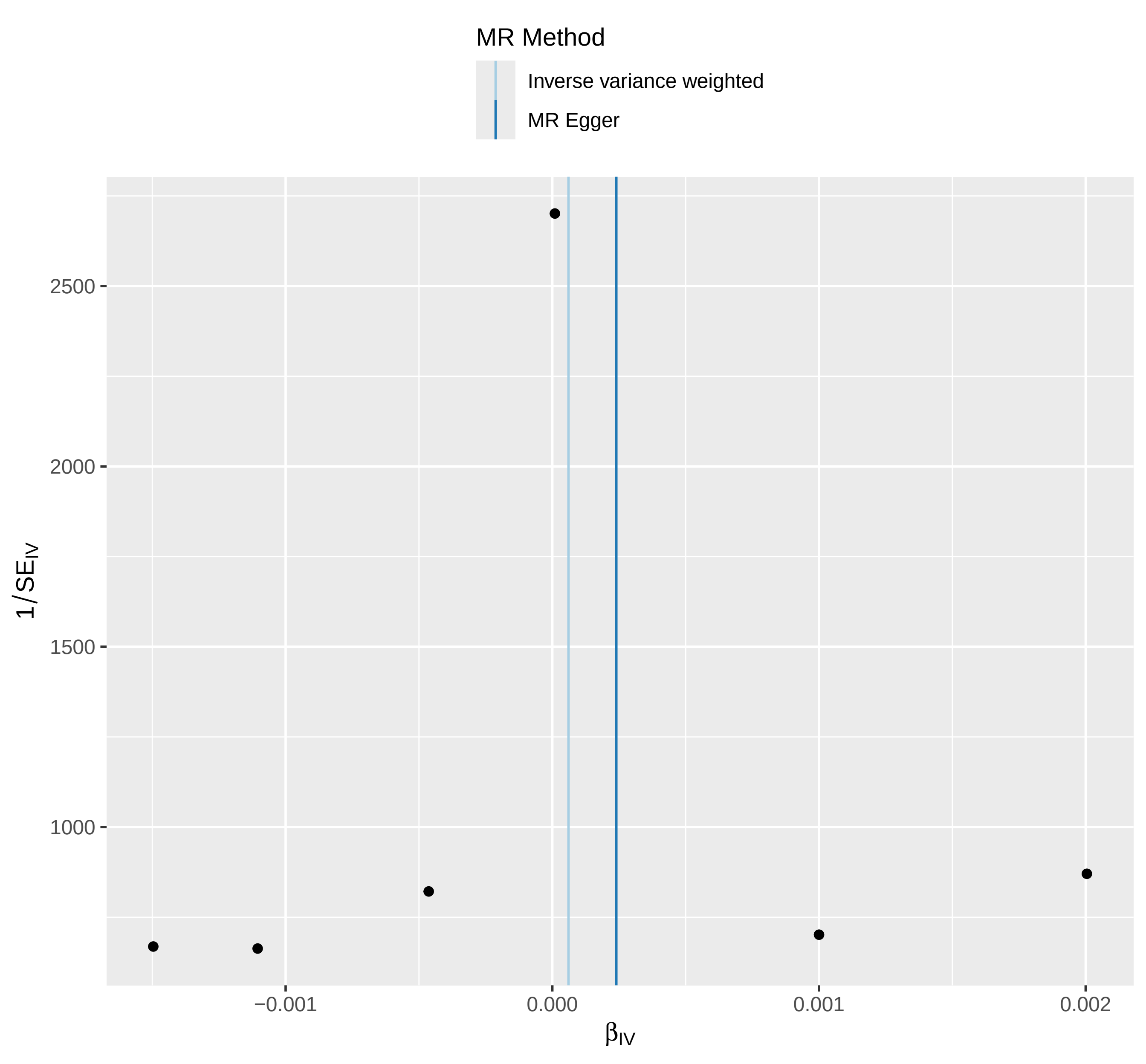
MR Method
Inverse variance weighted
MR Egger
2500
2000
1500
1000
1/SE_IV
−0.001
0.000
0.001
0.002
β_IV

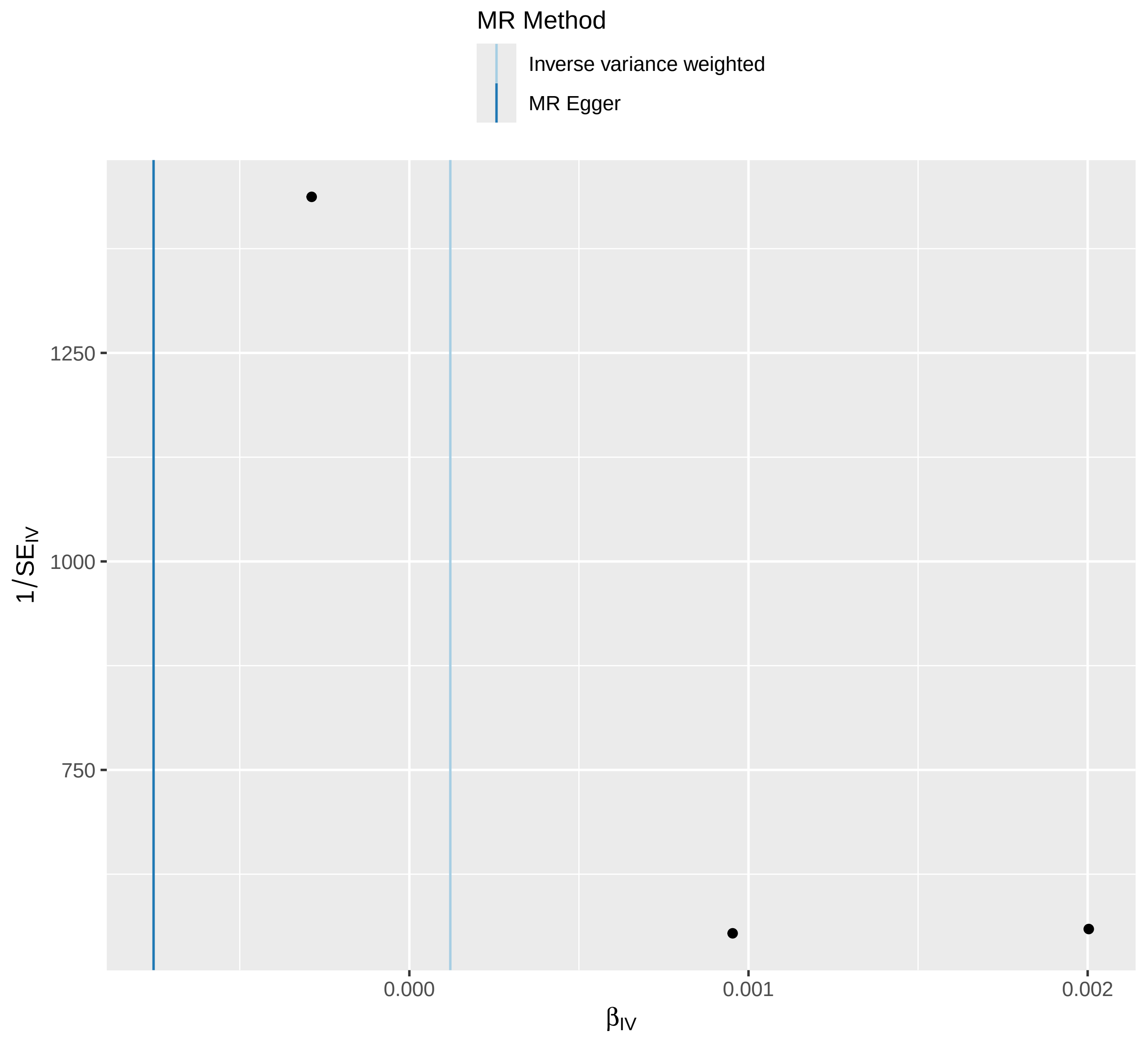
MR Method
Inverse variance weighted
MR Egger
1250
1000
750
1/SE$_{IV}$
0.000
0.001
0.002
β_{IV}

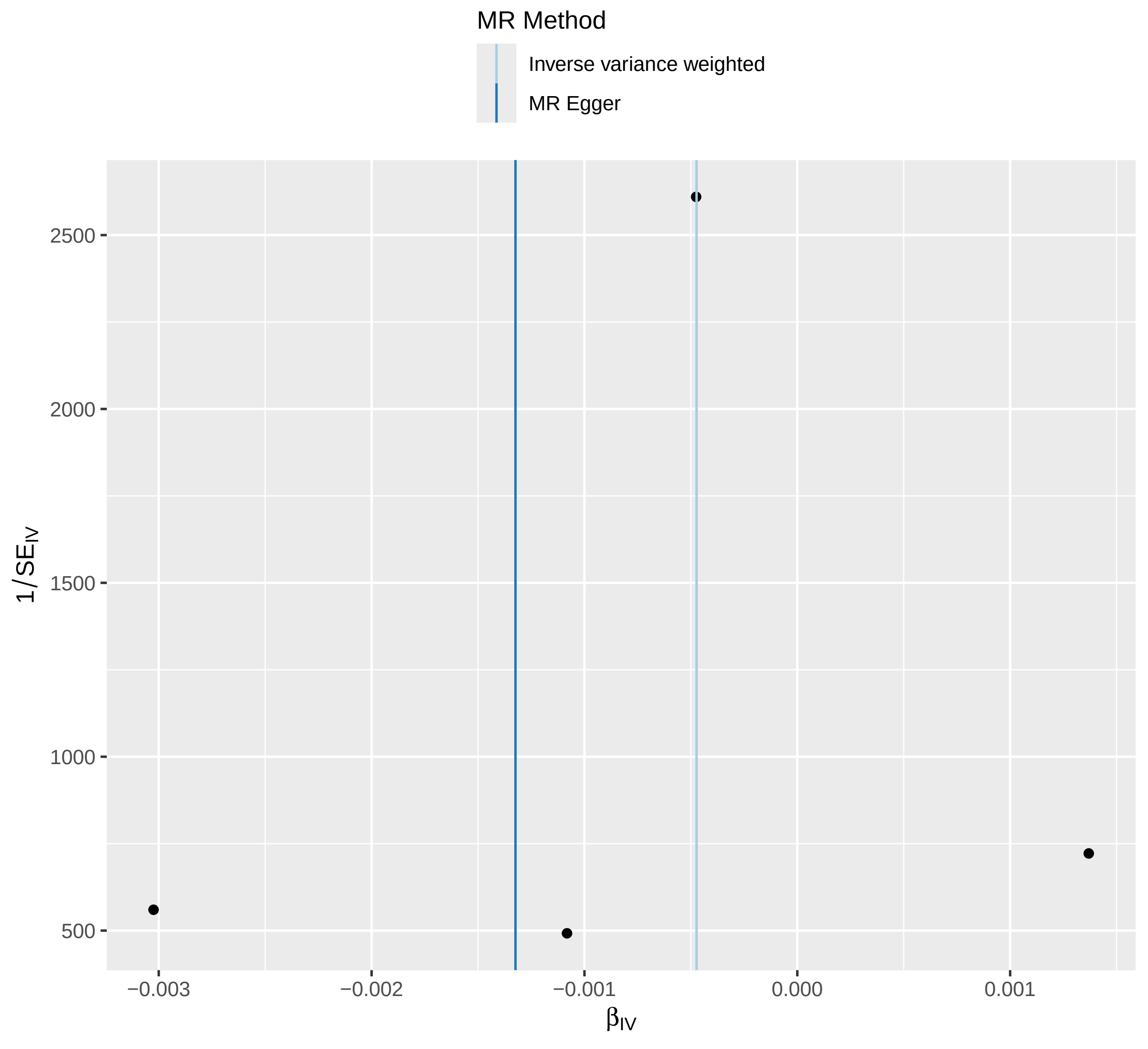
MR Method
Inverse variance weighted
MR Egger
2500
2000
1500
1000
500
1/SE$_{IV}$
−0.003
−0.002
−0.001
0.000
0.001
β$_{IV}$

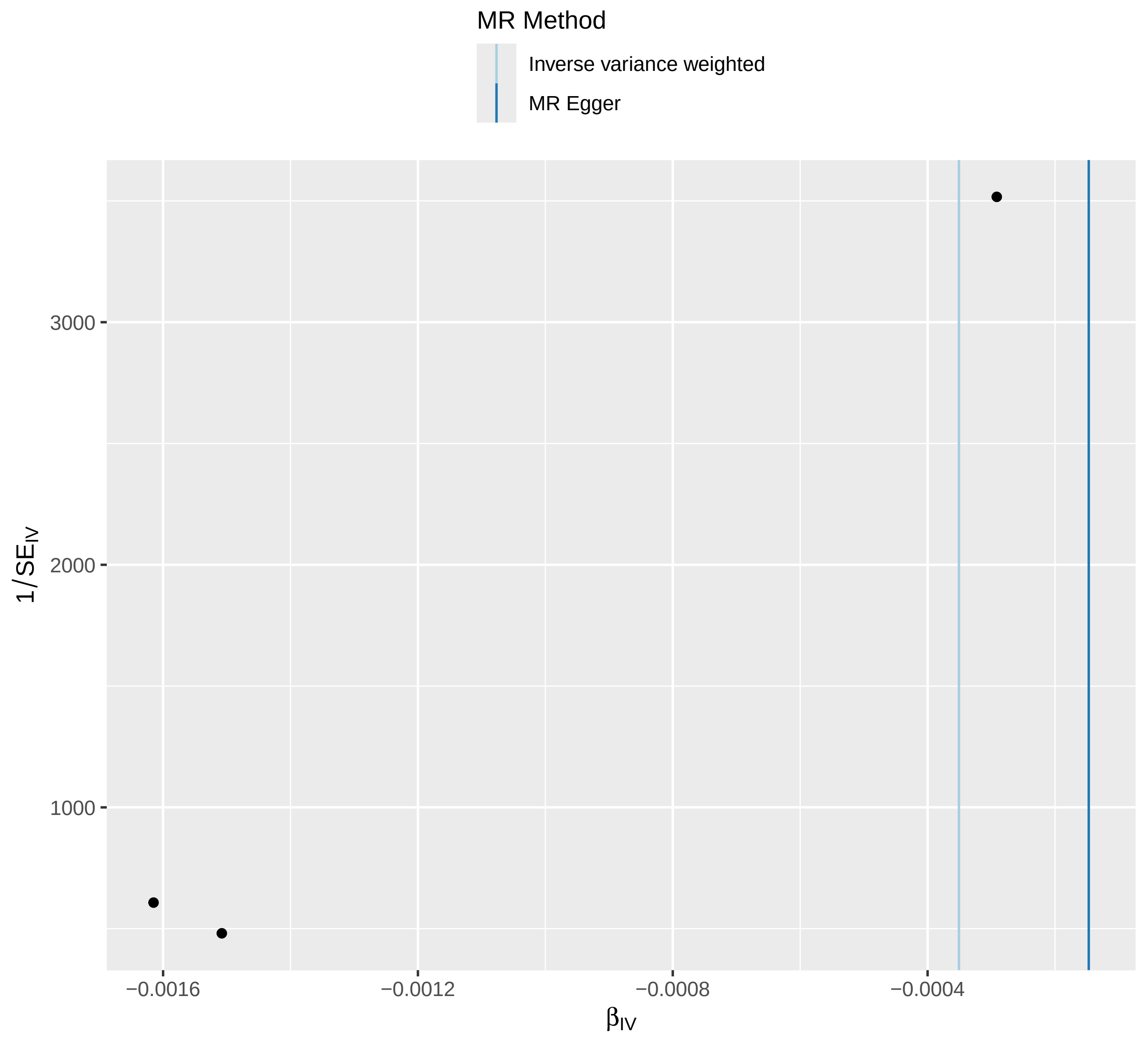
MR Method
Inverse variance weighted
MR Egger
3000
2000
1000
1/SE_IV
−0.0016
−0.0012
−0.0008
−0.0004
β_IV

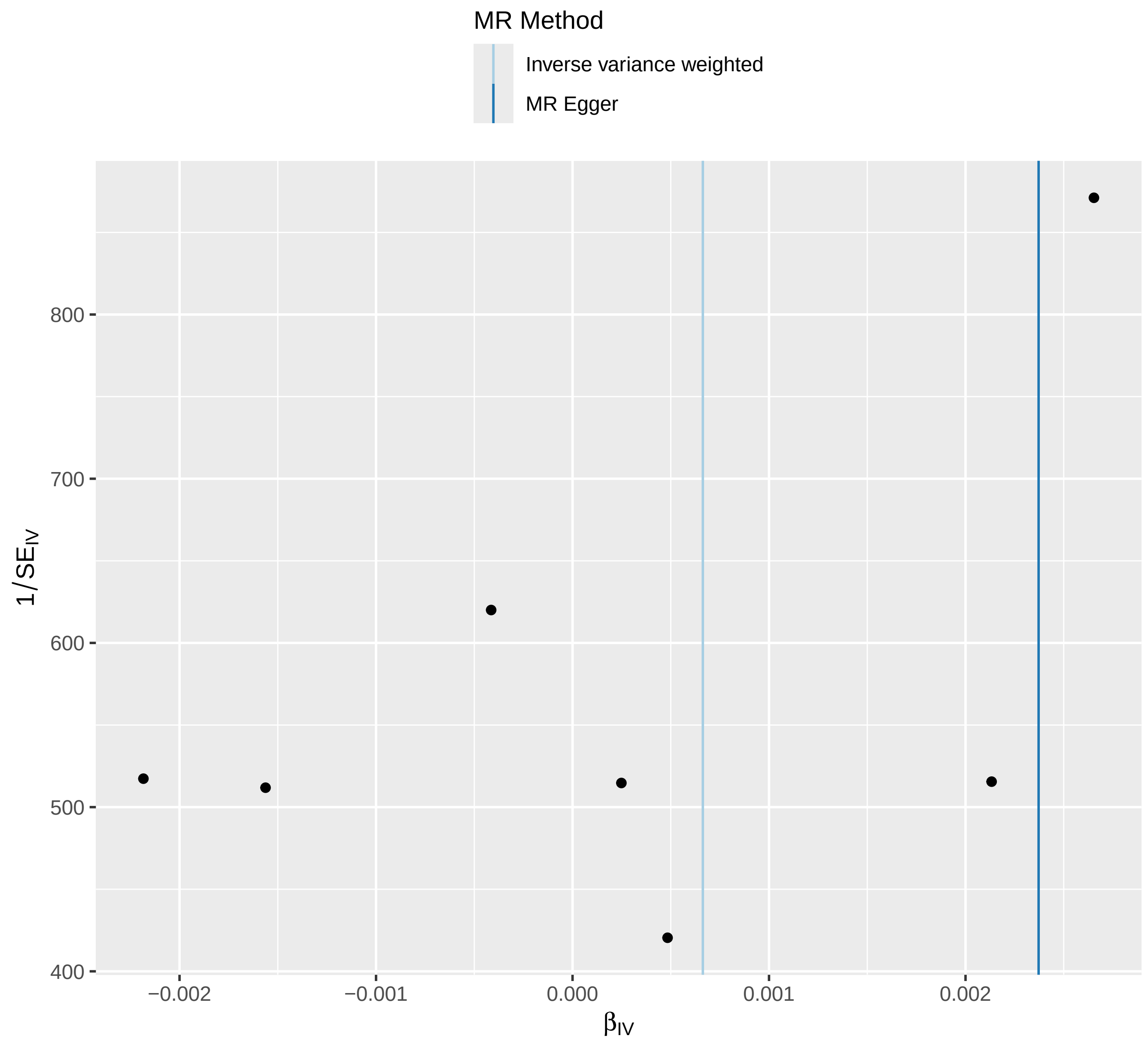
MR Method
Inverse variance weighted
MR Egger
800
700
600
500
400
1/SE_IV
−0.002
−0.001
0.000
0.001
0.002
β_IV

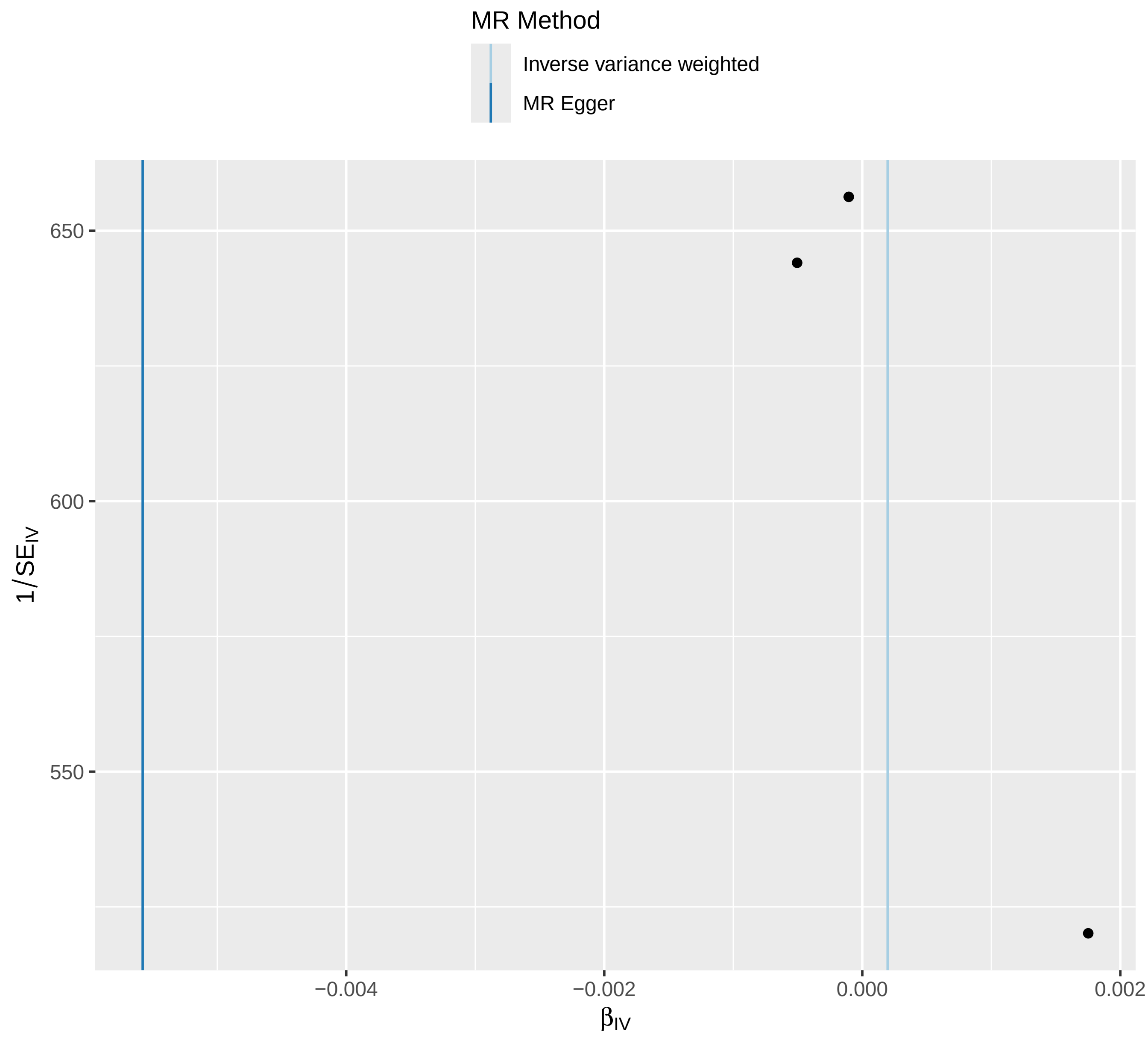
MR Method
Inverse variance weighted
MR Egger
650
600
550
1/SE$_{IV}$
−0.004
−0.002
0.000
0.002
β$_{IV}$

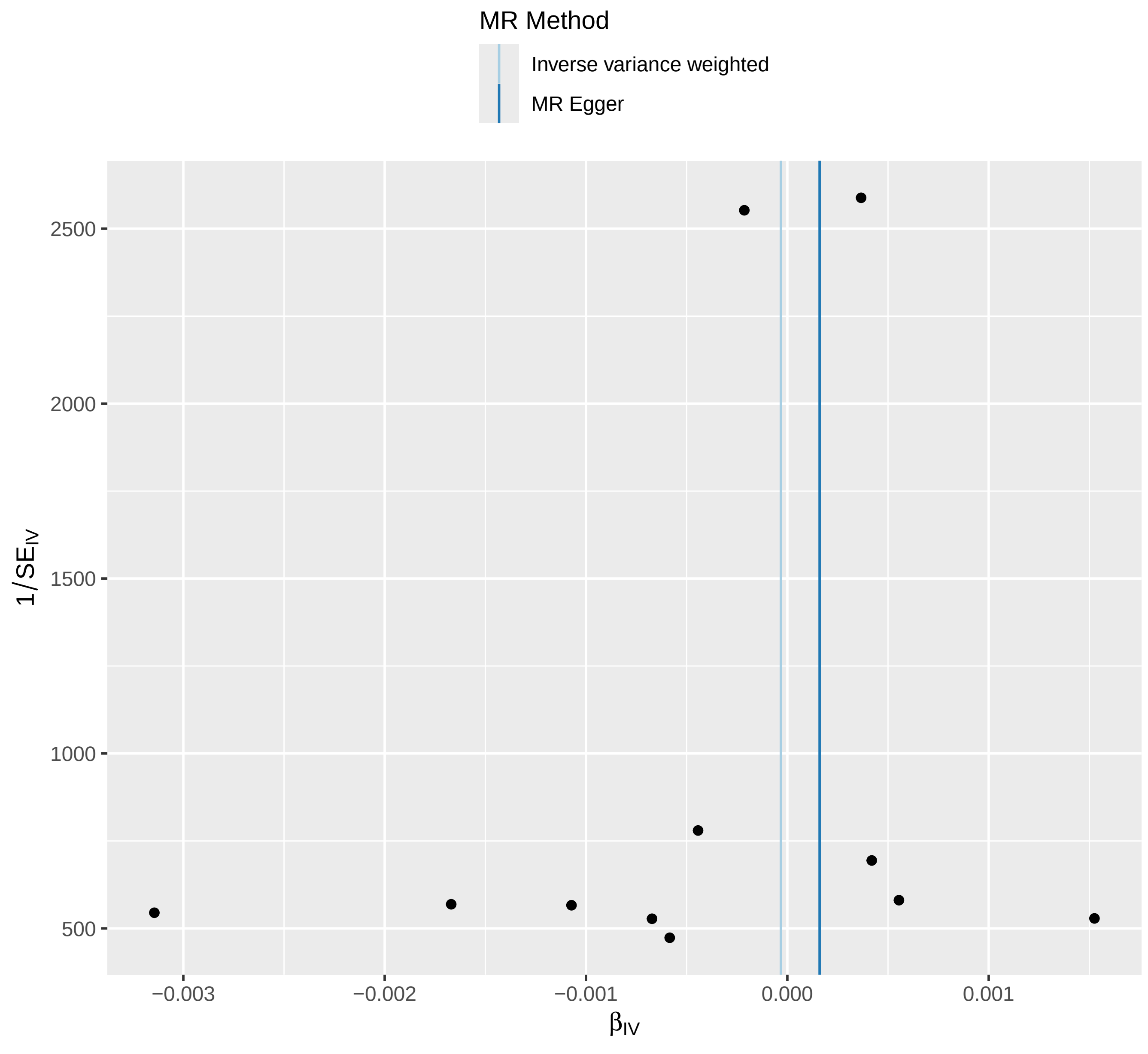
MR Method
Inverse variance weighted
MR Egger
2500
2000
1500
1000
500
1/SE$_{IV}$
−0.003
−0.002
−0.001
0.000
0.001
β$_{IV}$

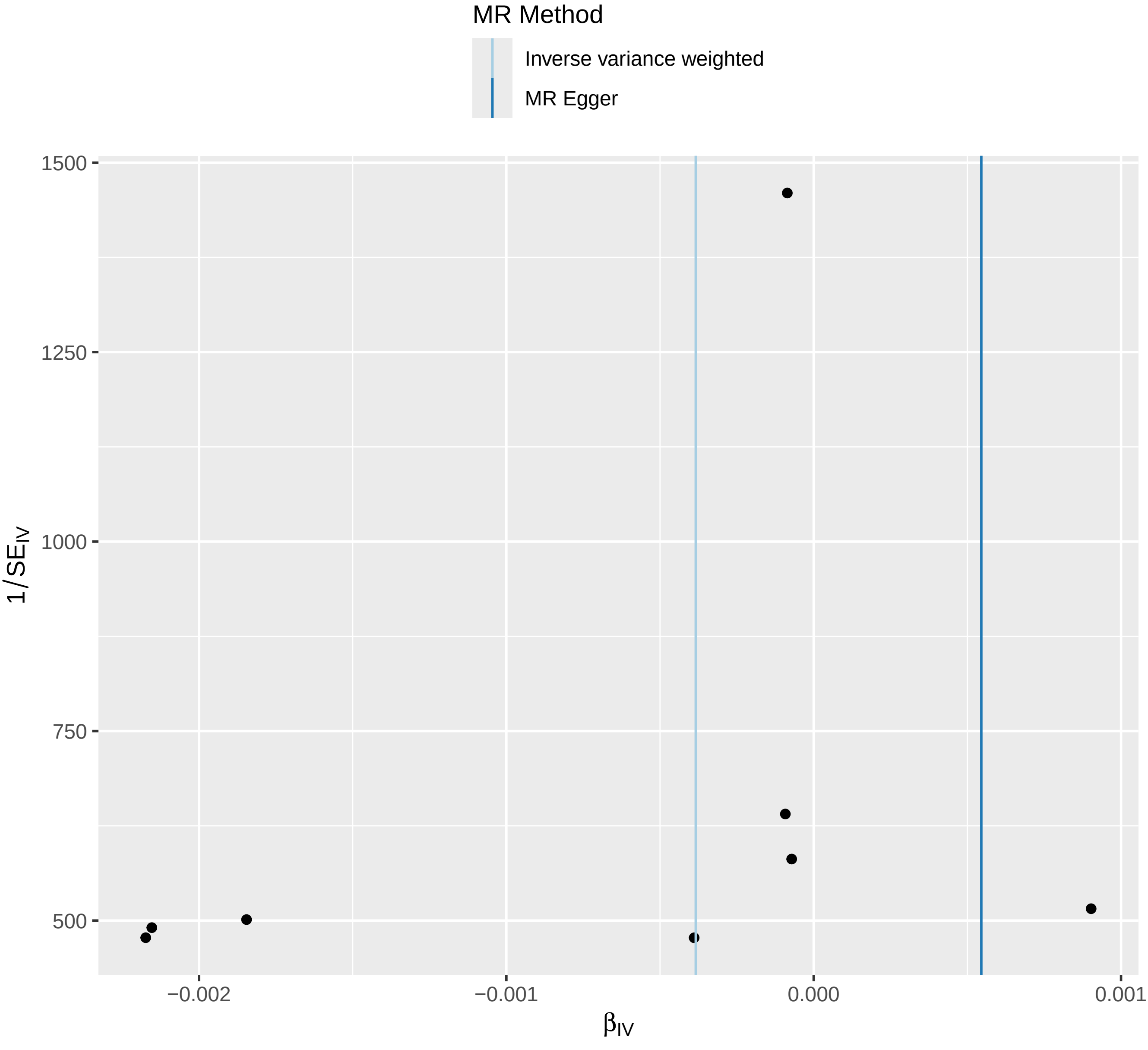

MR Method
Inverse variance weighted
MR Egger
1500
1250
1000
750
500
1/SE$_{IV}$
−0.002
−0.001
0.000
0.001
β$_{IV}$

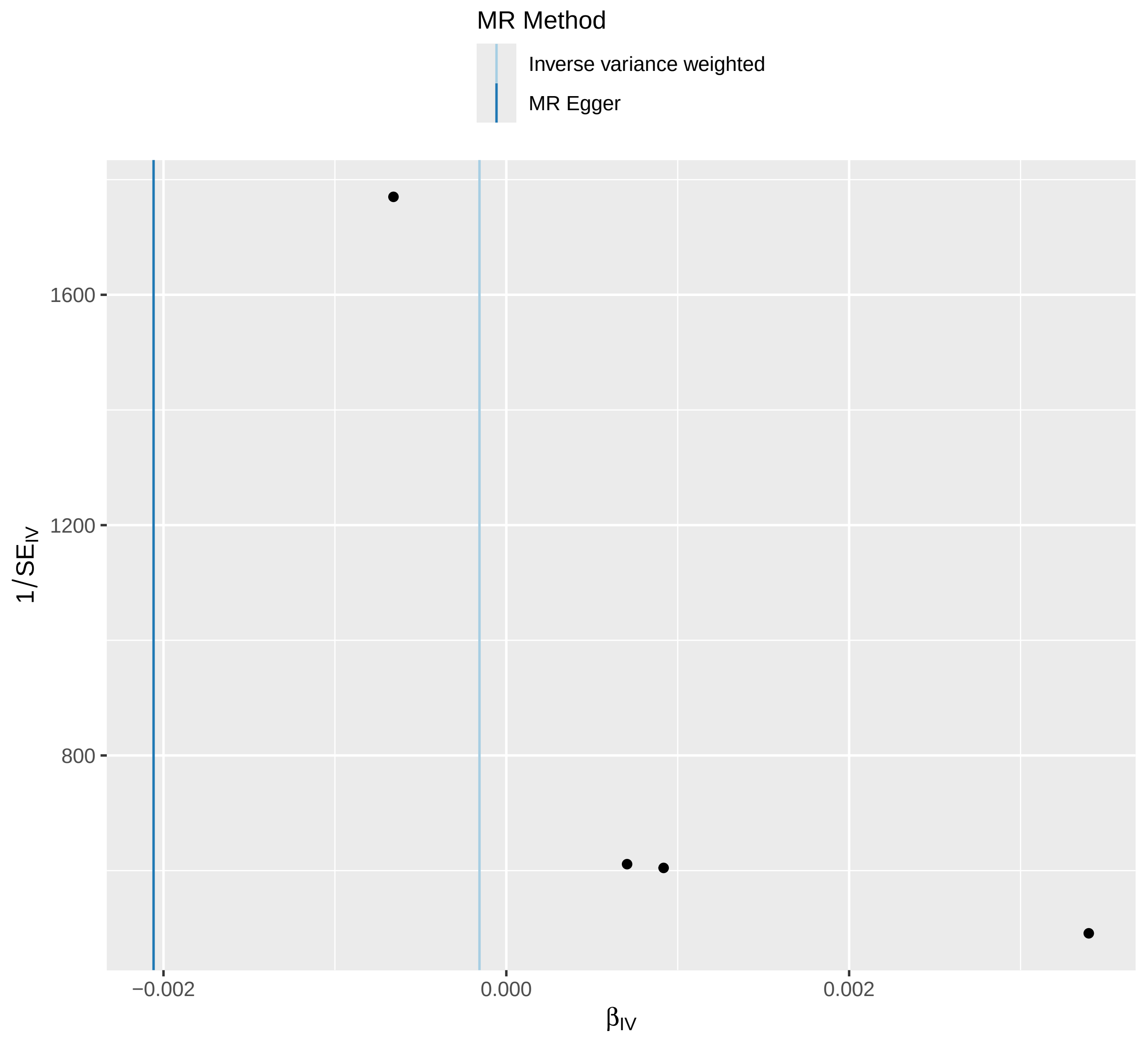

MR Method
Inverse variance weighted
MR Egger
1600
1200
800
1/SE$_{IV}$
−0.002
0.000
0.002
β$_{IV}$

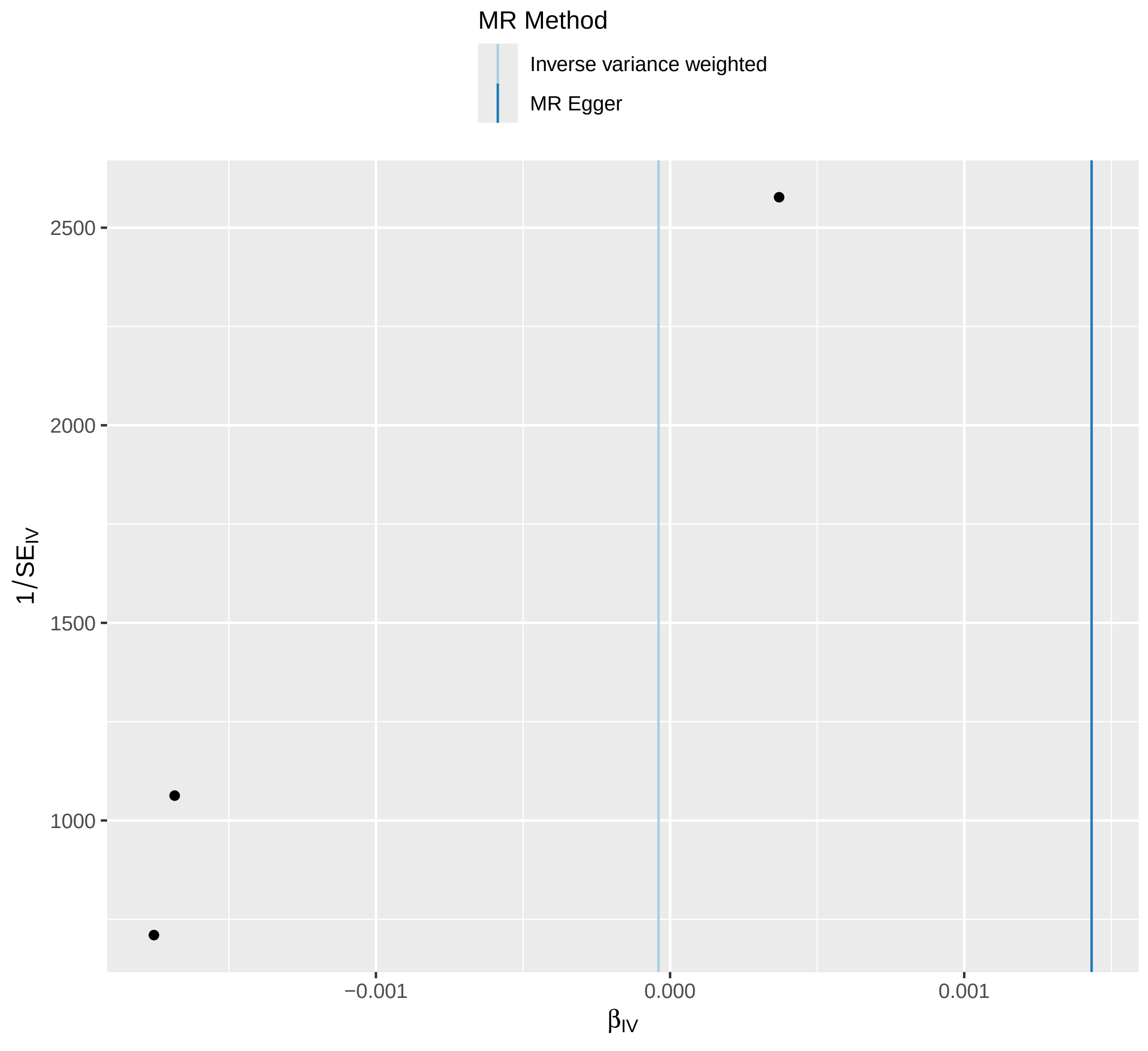

MR Method
Inverse variance weighted
MR Egger
2500
2000
1500
1000
−0.001
0.000
0.001
$1/SE_{IV}$
β_{IV}

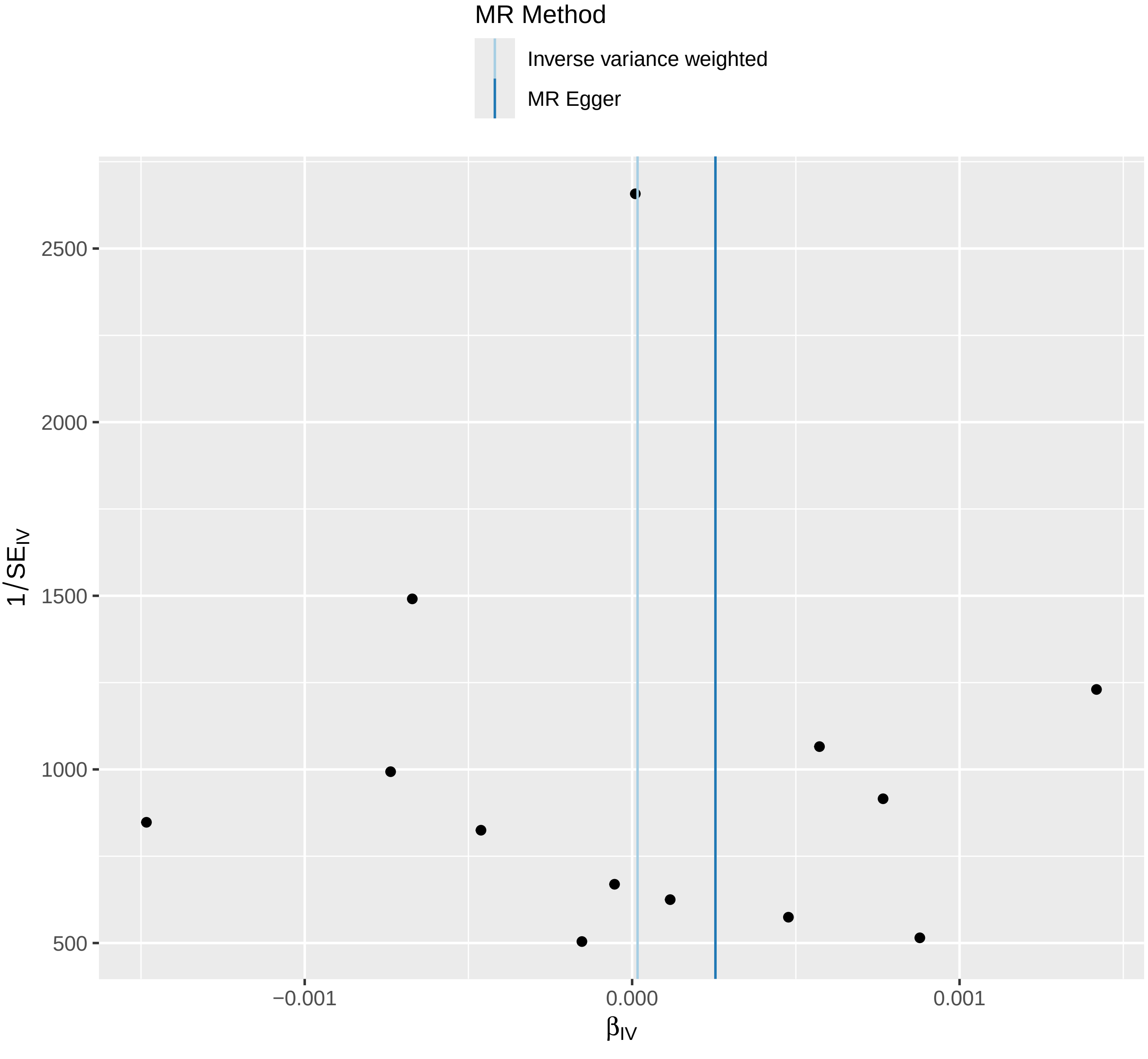
MR Method
Inverse variance weighted
MR Egger
2500
2000
1500
1000
500
1/SE_IV
−0.001
0.000
0.001
β_IV

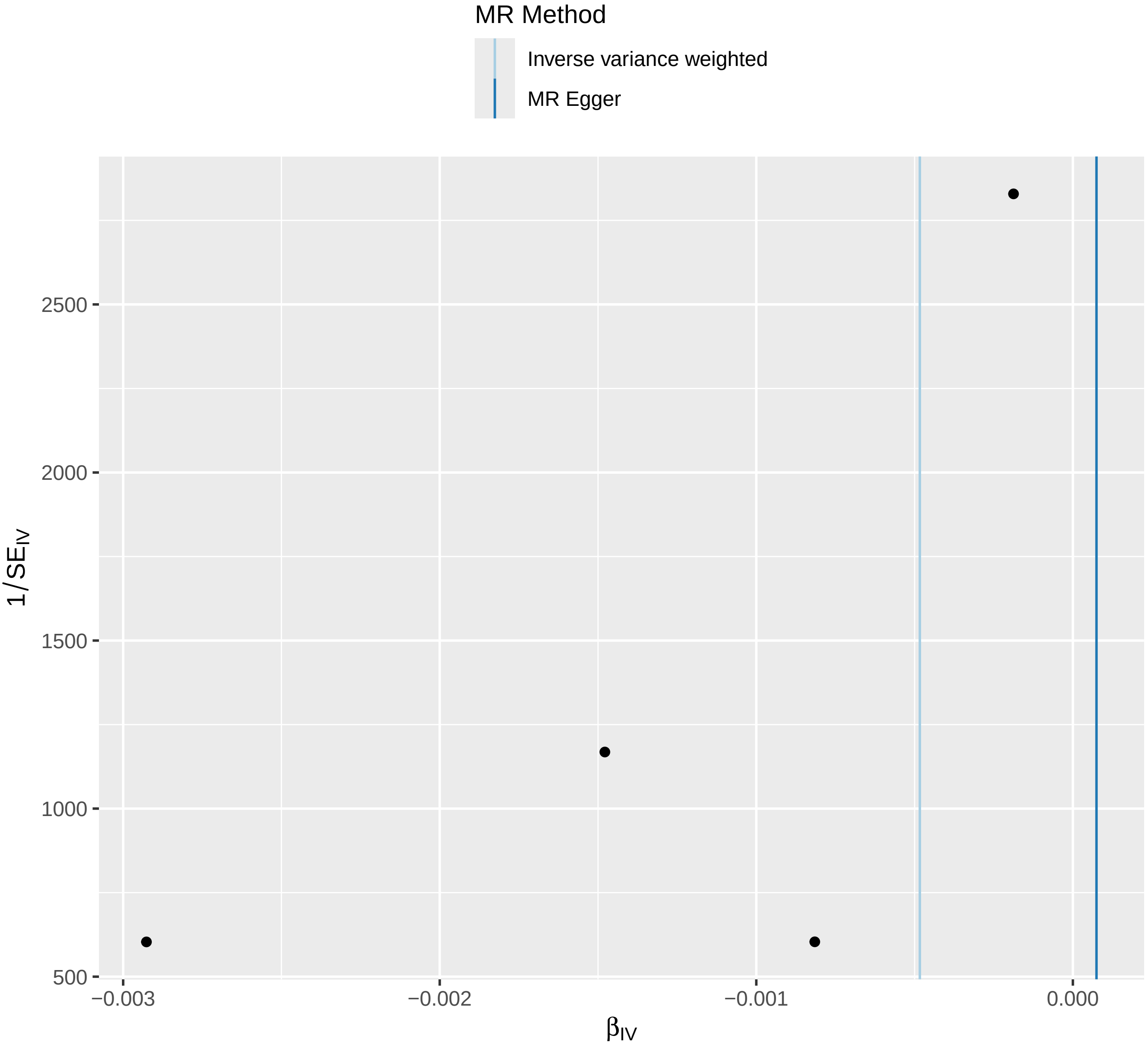
MR Method
Inverse variance weighted
MR Egger
2500
2000
1500
1000
500
1/SE$_{IV}$
−0.003
−0.002
−0.001
0.000
β$_{IV}$

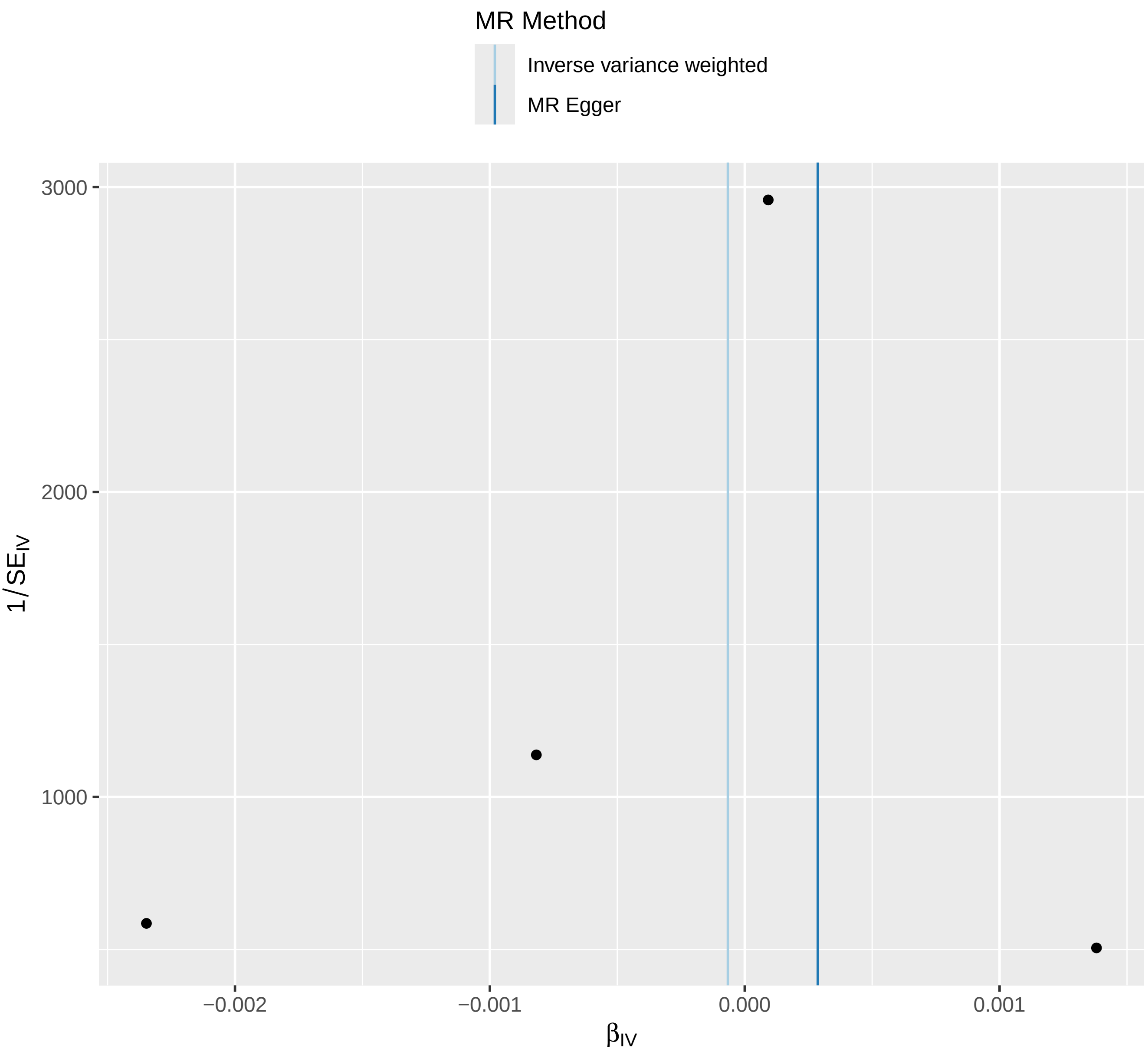

MR Method
Inverse variance weighted
MR Egger
3000
2000
1000
1/SE_IV
−0.002
−0.001
0.000
0.001
β_IV

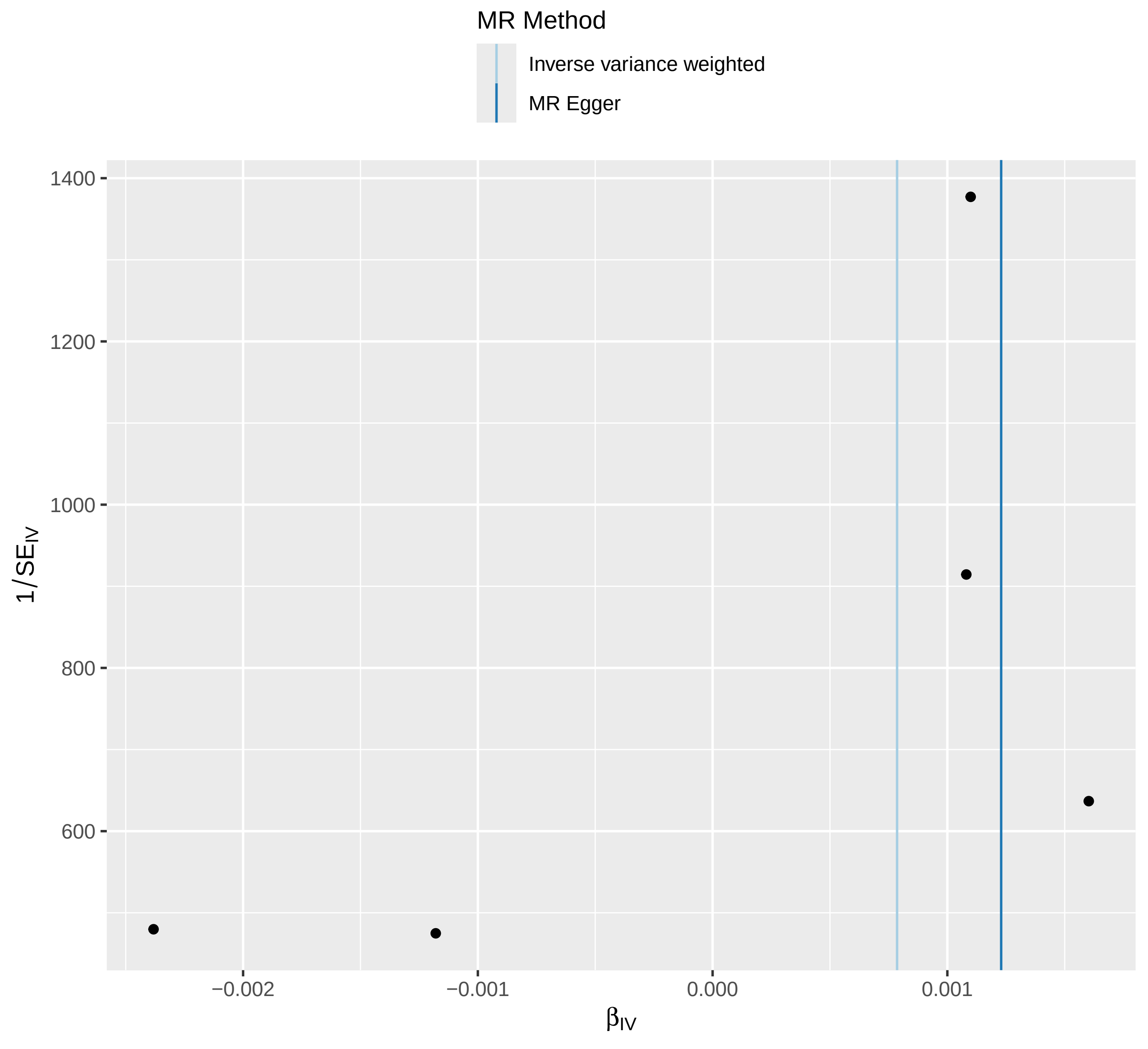

MR Method
Inverse variance weighted
MR Egger
1400
1200
1000
800
600
1/SE_IV
−0.002
−0.001
0.000
0.001
β_IV

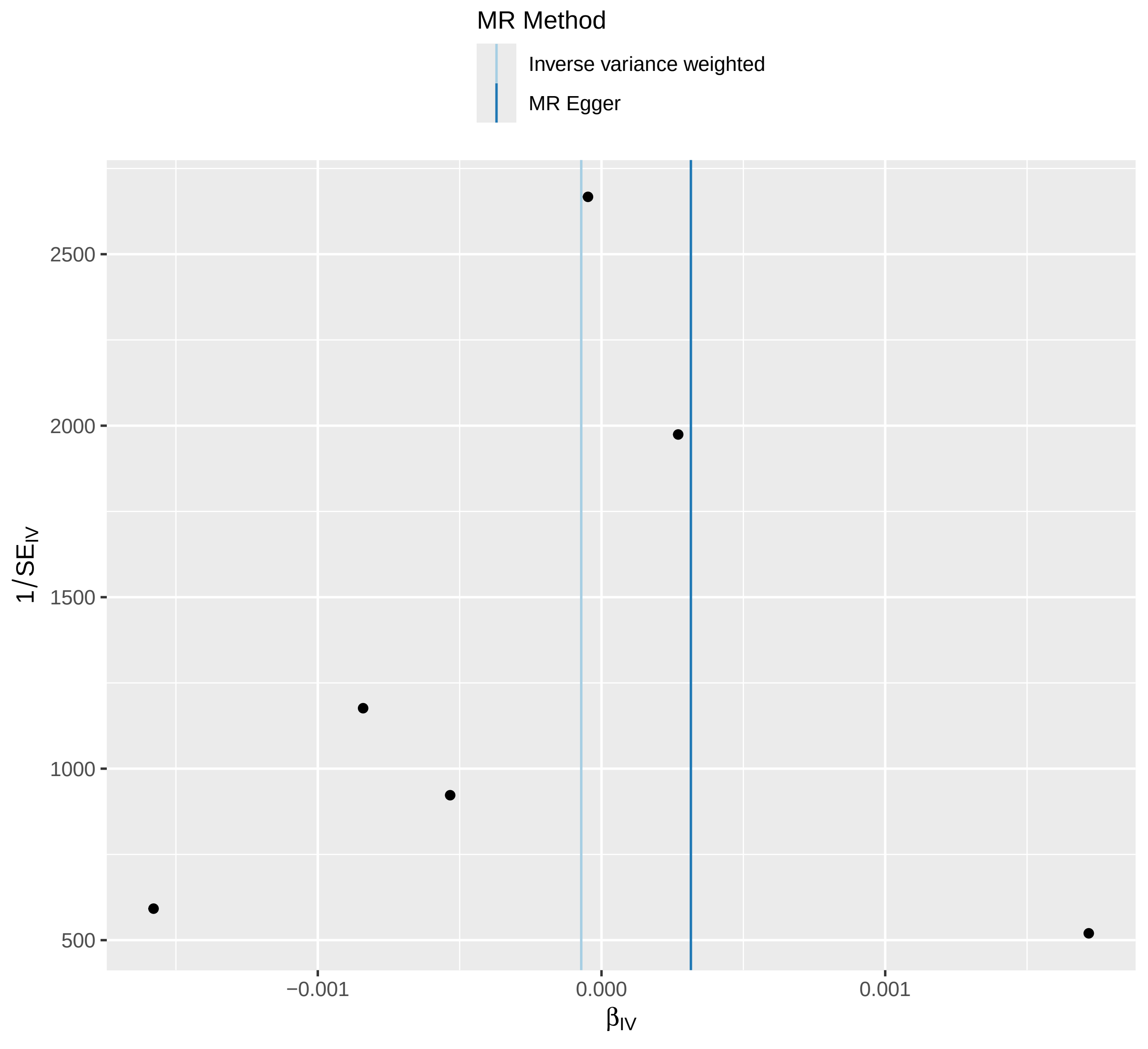
MR Method
Inverse variance weighted
MR Egger
2500
2000
1500
1000
500
1/SE$_{IV}$
−0.001
0.000
0.001
β$_{IV}$

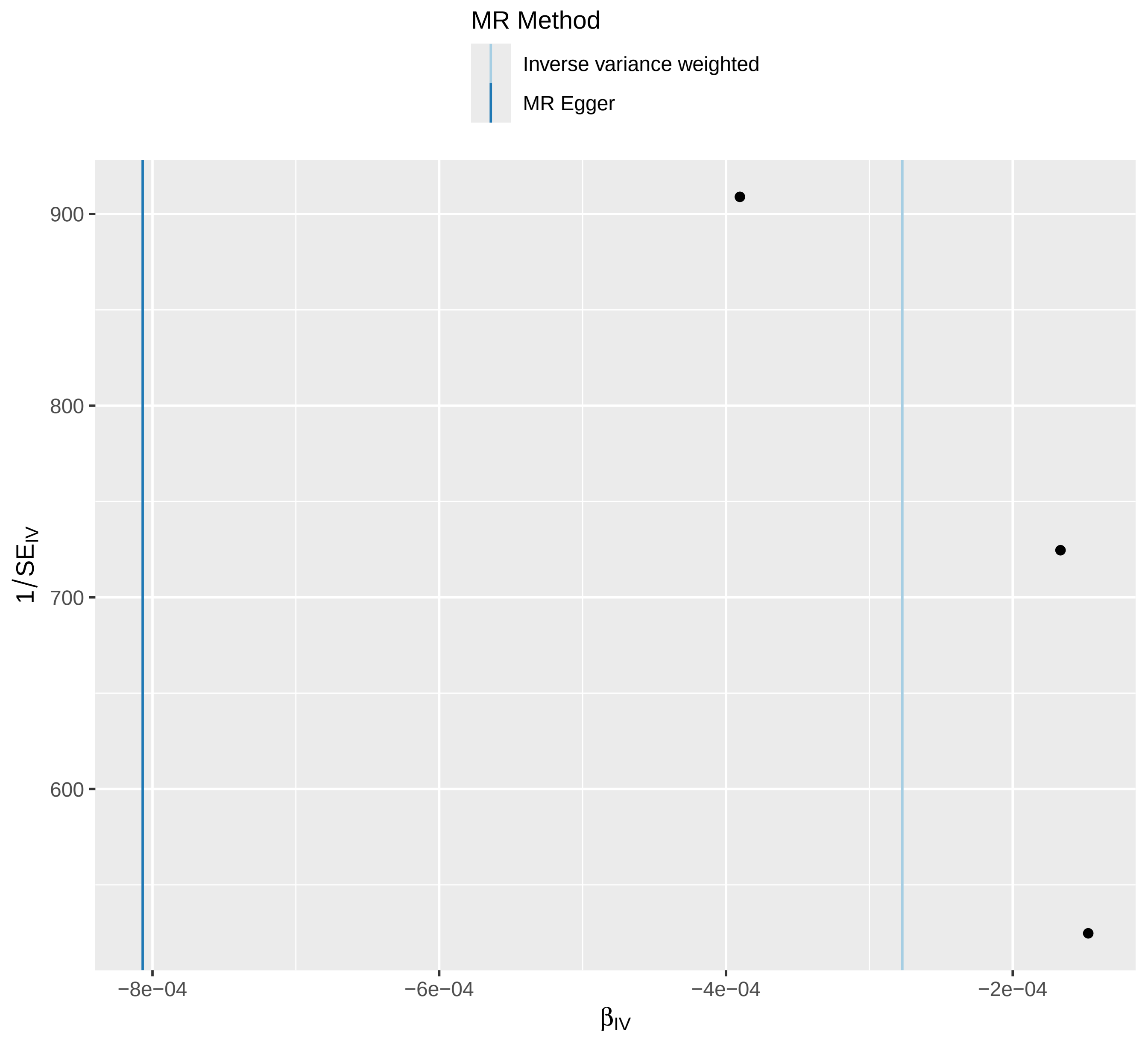
MR Method
Inverse variance weighted
MR Egger
900
800
700
600
1/SE_IV
−8e−04
−6e−04
−4e−04
−2e−04
β_IV

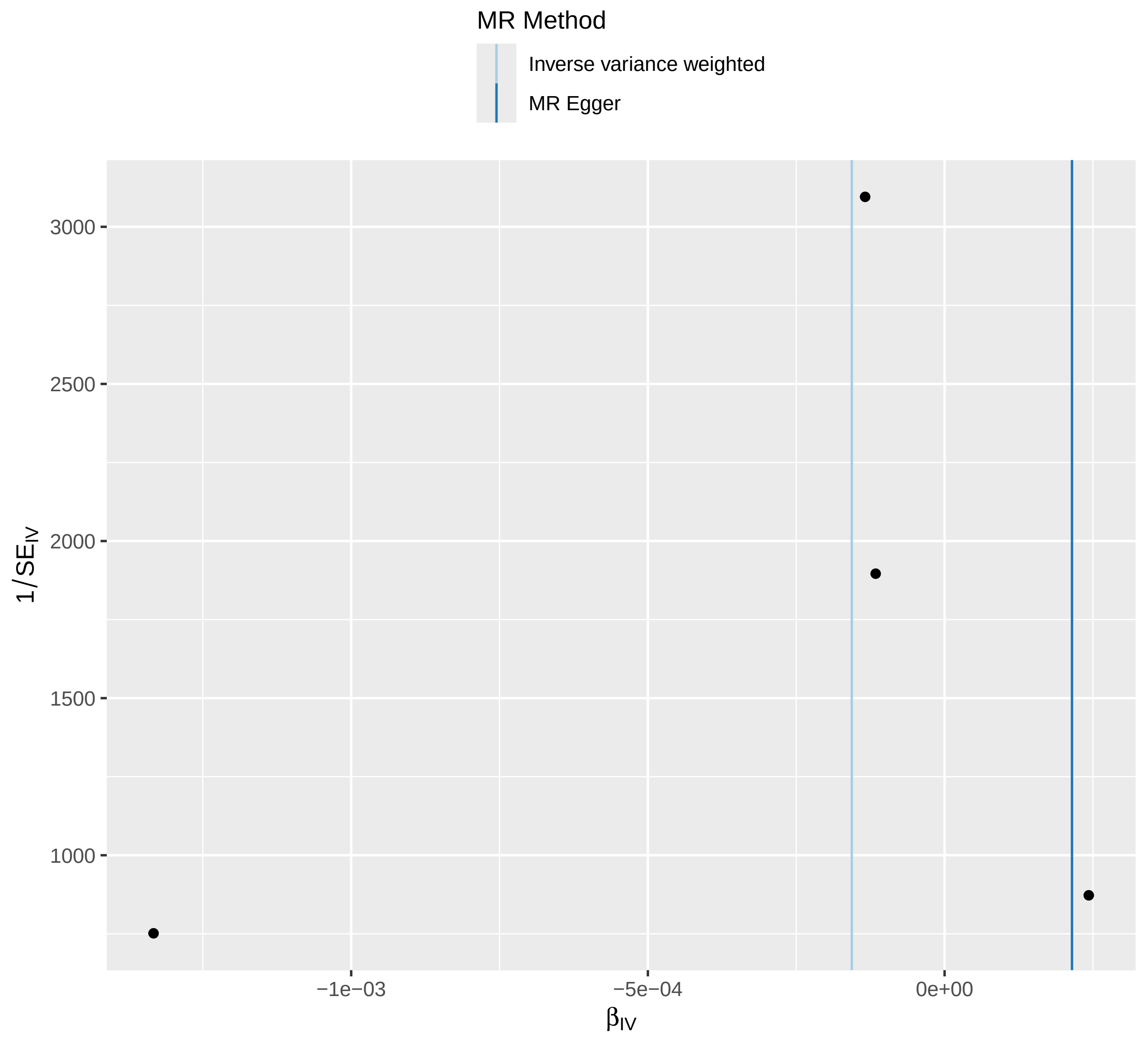

MR Method
Inverse variance weighted
MR Egger
3000
2500
2000
1500
1000
1/SE_IV
−1e−03
−5e−04
0e+00
β_IV

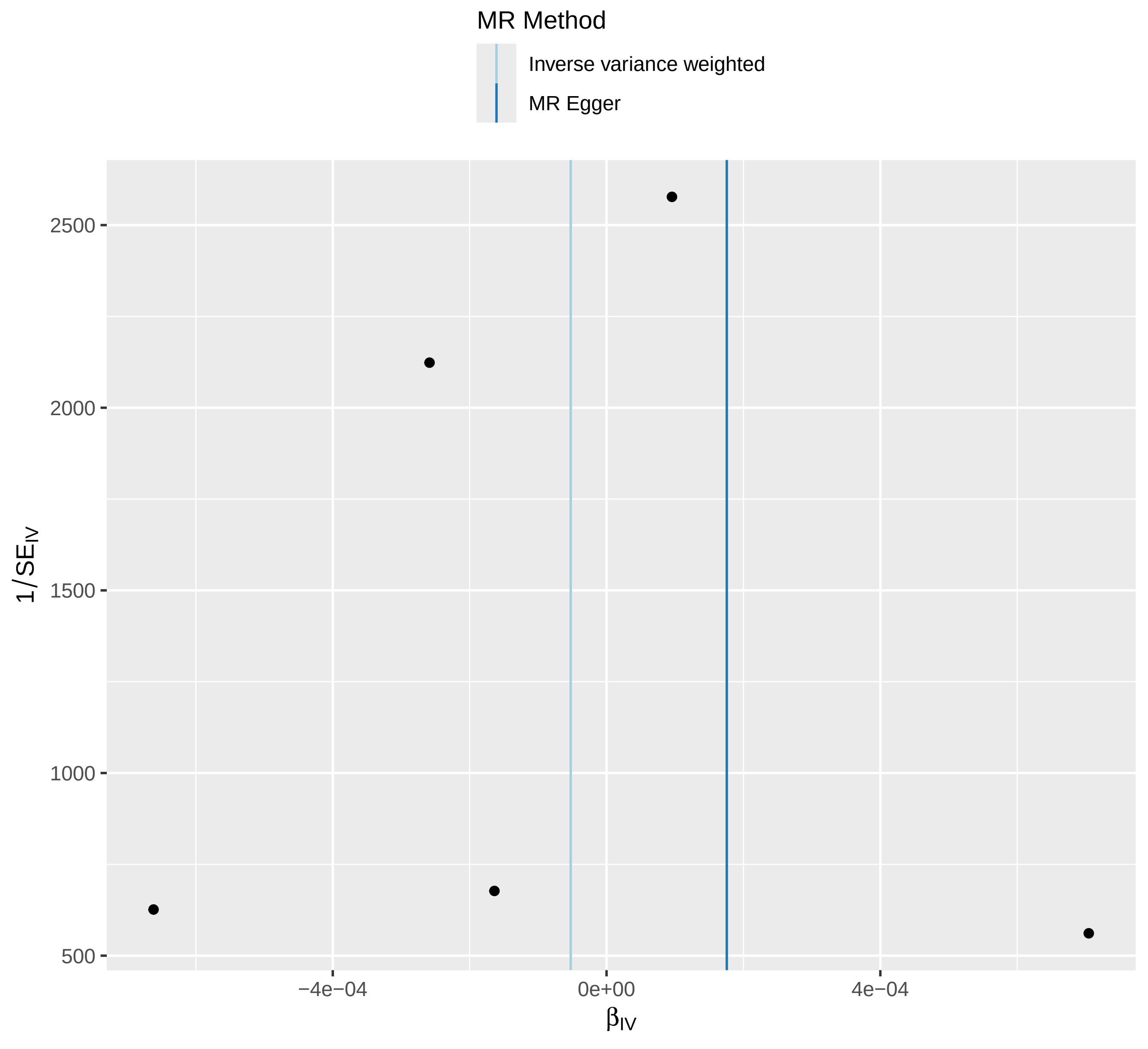
MR Method
Inverse variance weighted
MR Egger
2500
2000
1500
1000
500
1/SE_IV
−4e−04
0e+00
4e−04
β_IV

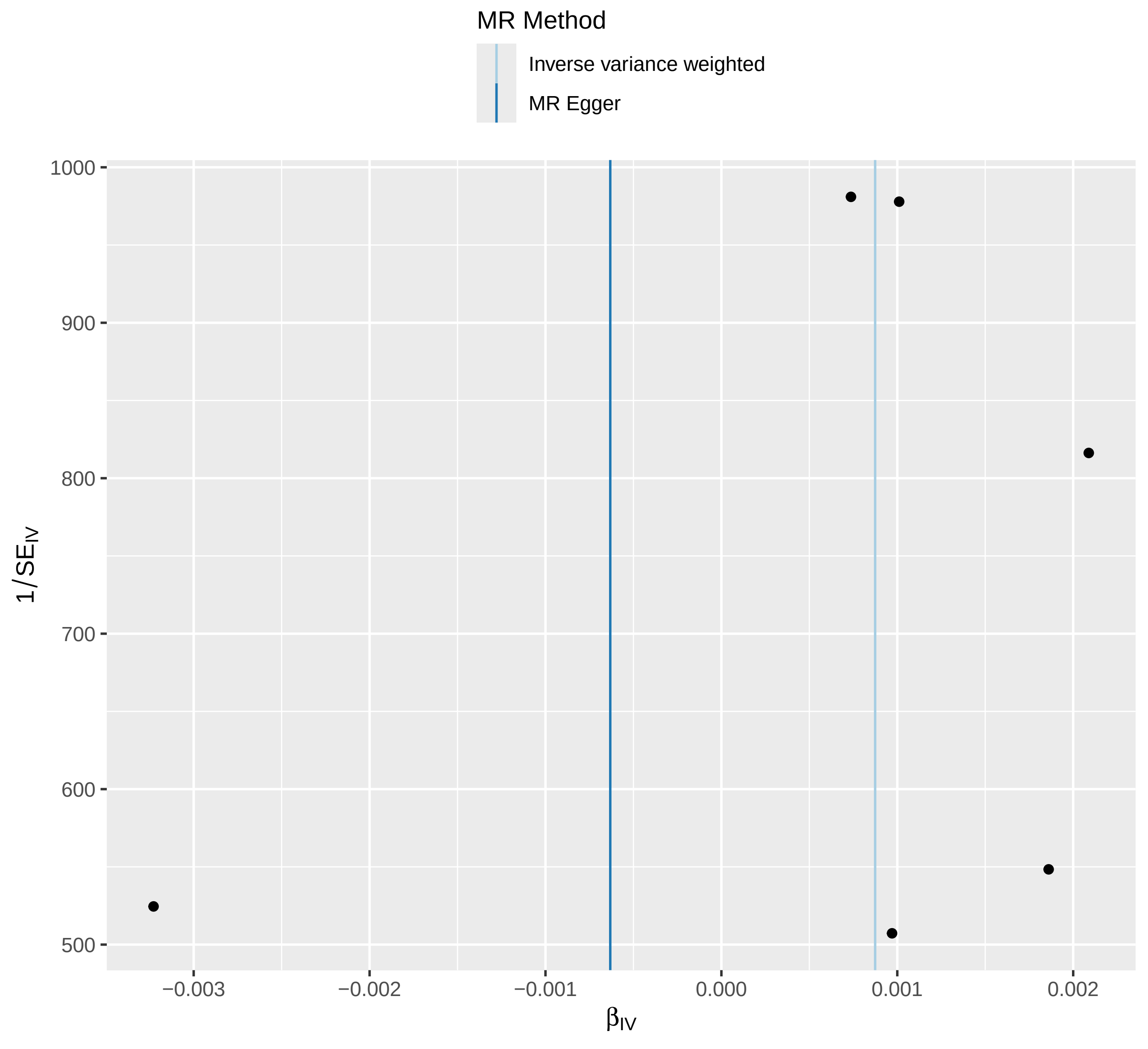

MR Method
Inverse variance weighted
MR Egger
1000
900
800
700
600
500
1/SE_IV
−0.003
−0.002
−0.001
0.000
0.001
0.002
β_IV

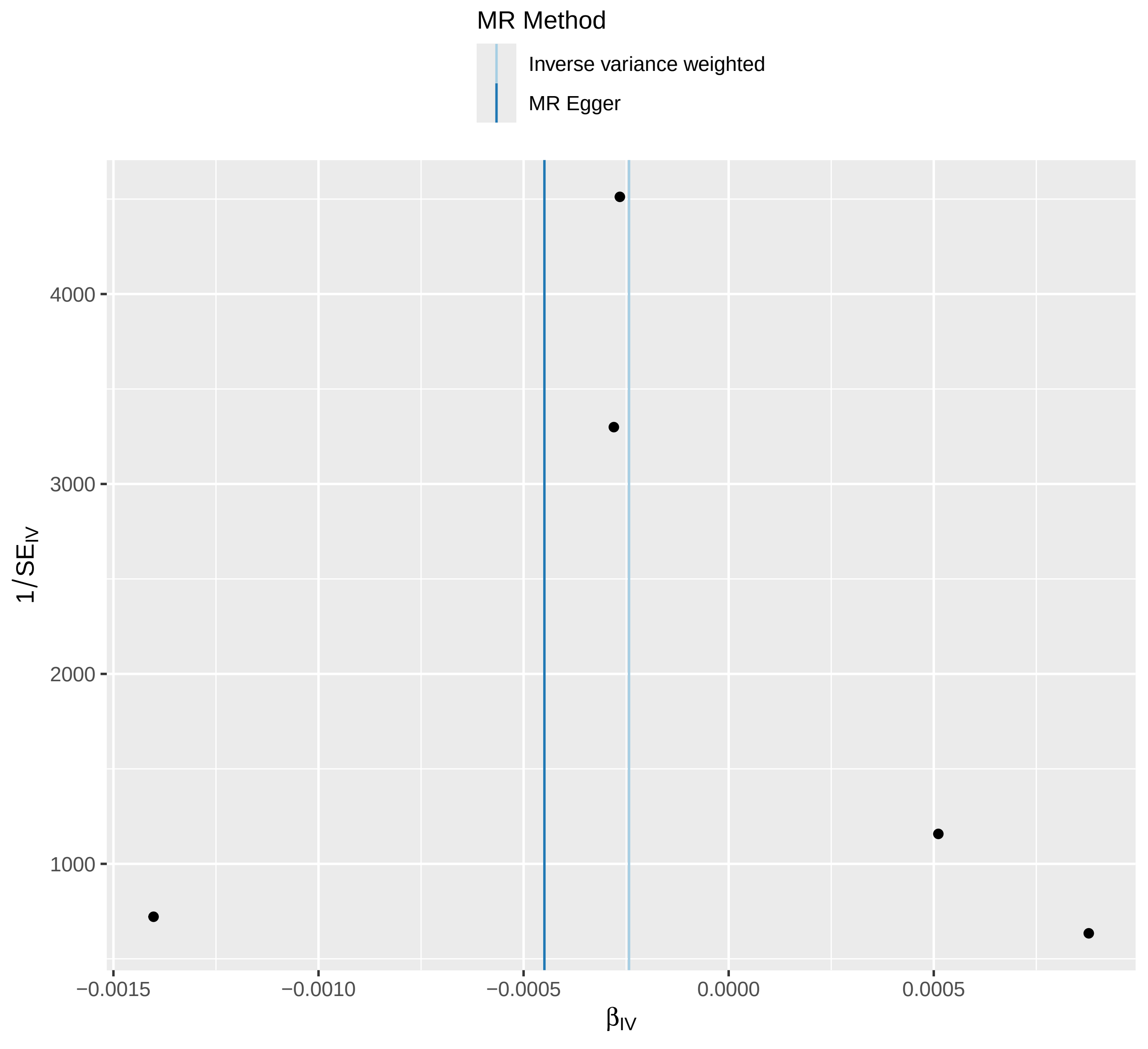

MR Method
Inverse variance weighted
MR Egger
4000
3000
2000
1000
1/SE_IV
−0.0015
−0.0010
−0.0005
0.0000
0.0005
β_IV

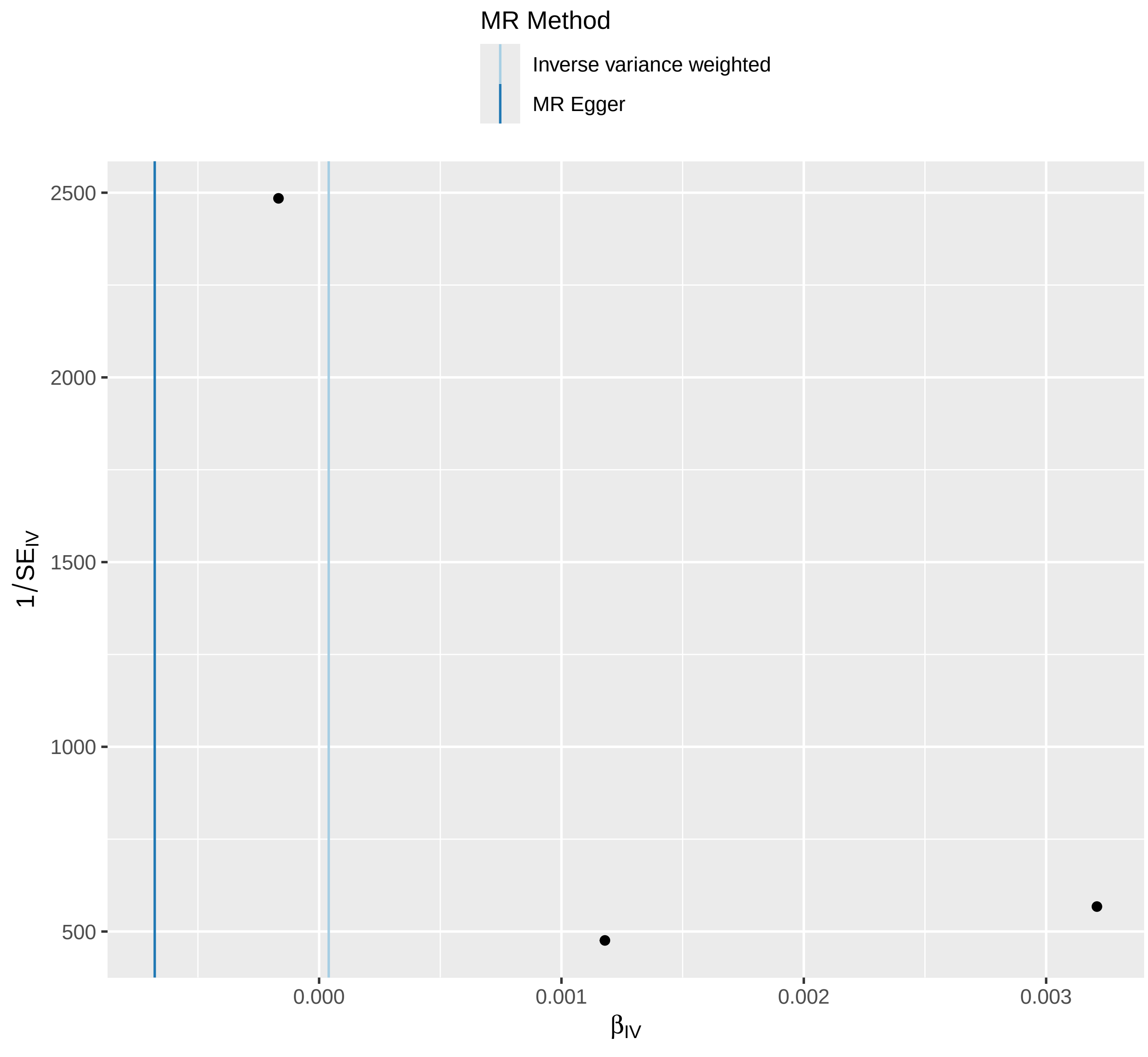

MR Method
Inverse variance weighted
MR Egger
2500
2000
1500
1000
500
1/SE$_{IV}$
0.000
0.001
0.002
0.003
β$_{IV}$

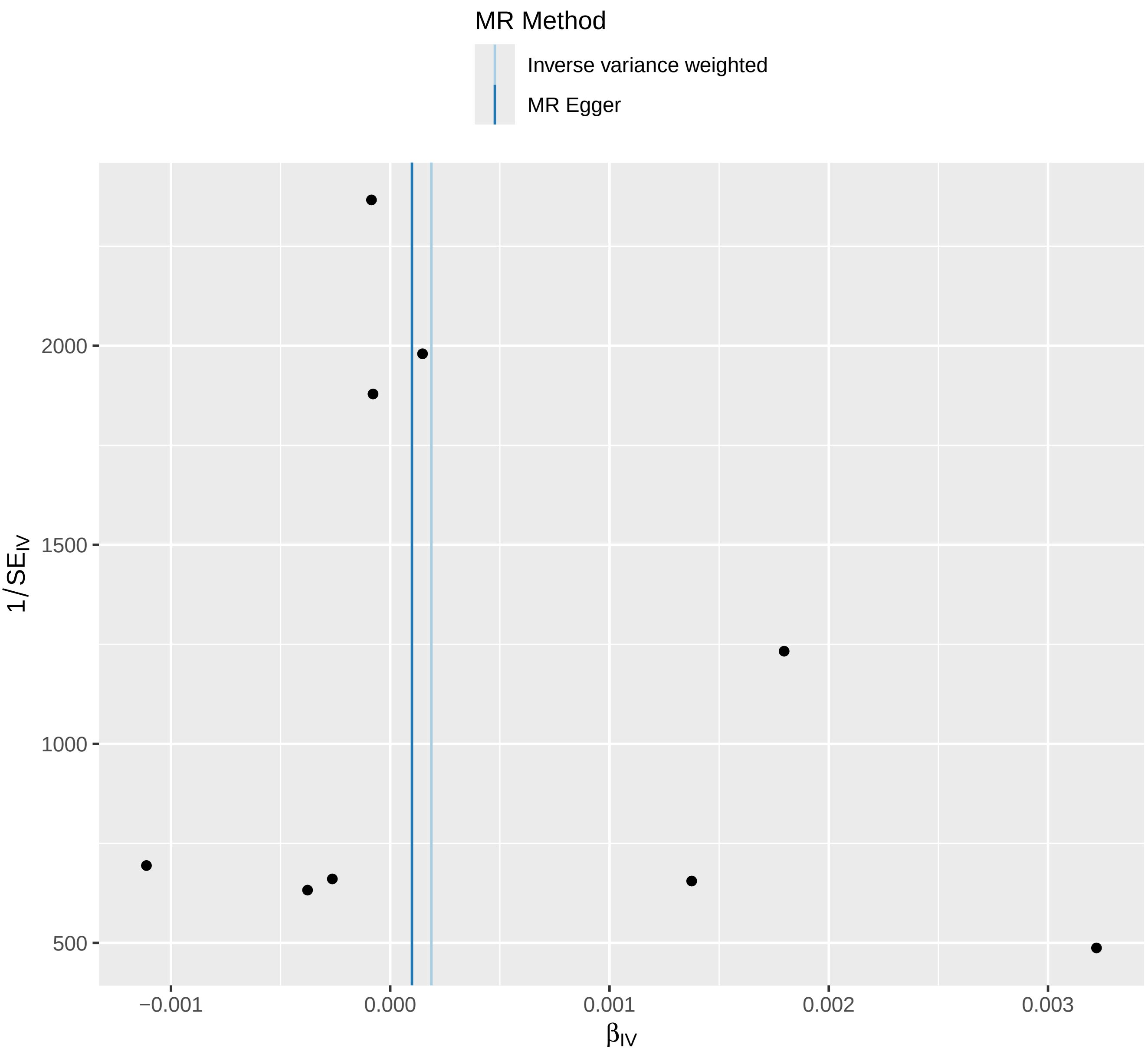
MR Method
Inverse variance weighted
MR Egger
2000
1500
1000
500
1/SE_IV
−0.001
0.000
0.001
0.002
0.003
β_IV

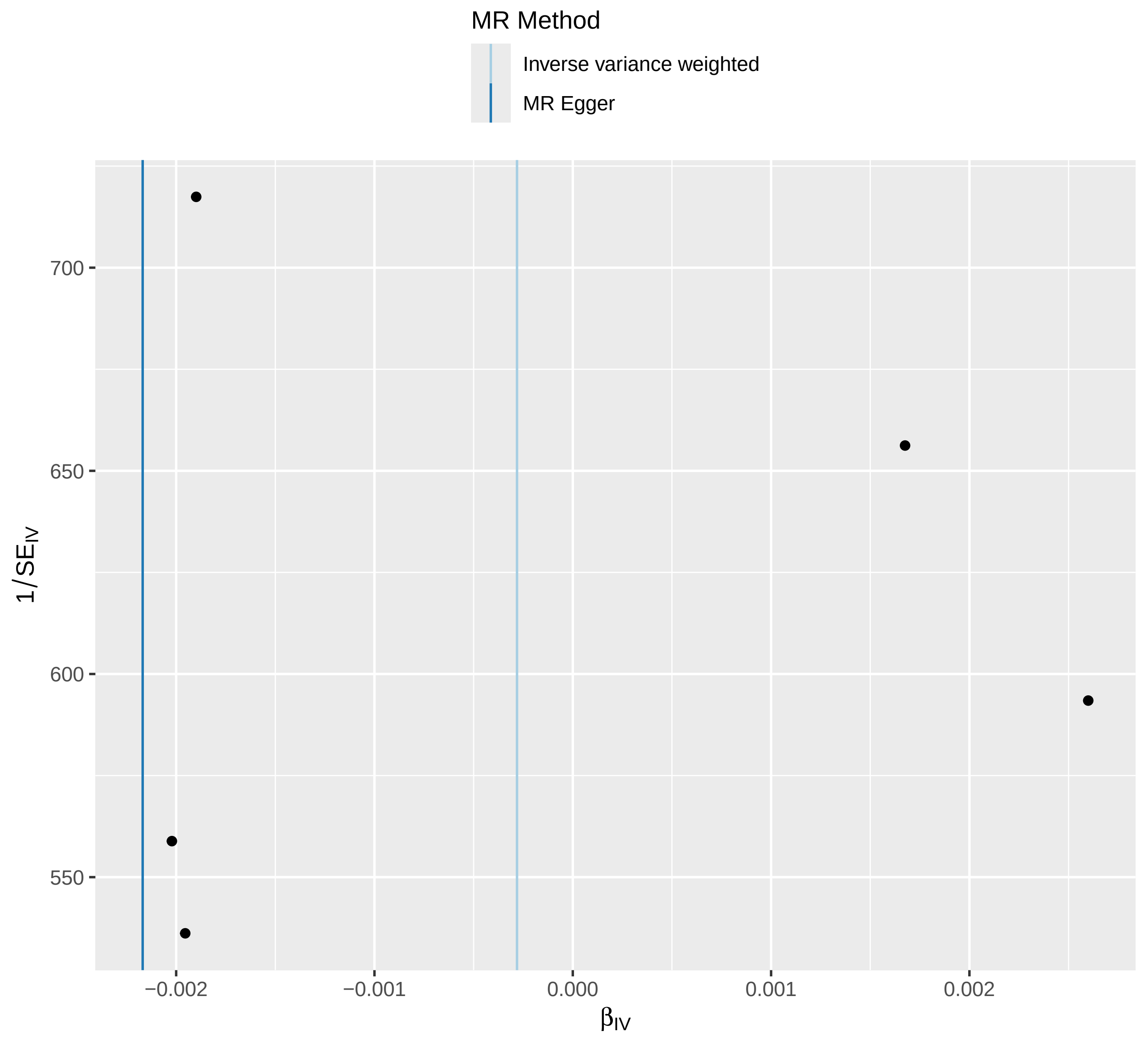

MR Method
Inverse variance weighted
MR Egger
700
650
600
550
1/SE$_{IV}$
−0.002
−0.001
0.000
0.001
0.002
β_{IV}

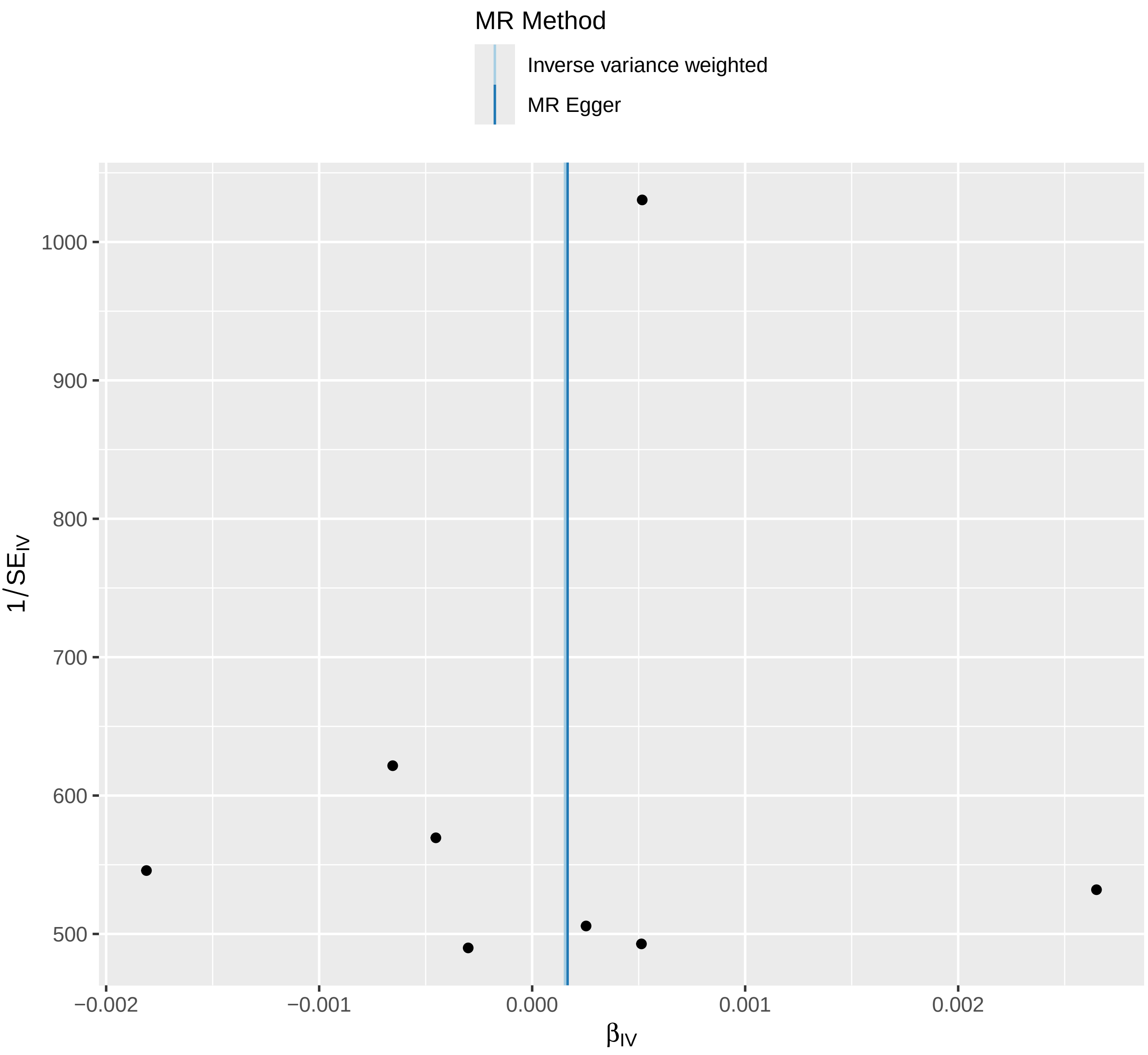

MR Method
Inverse variance weighted
MR Egger
1000
900
800
700
600
500
1/SE$_{IV}$
−0.002
−0.001
0.000
0.001
0.002
β$_{IV}$

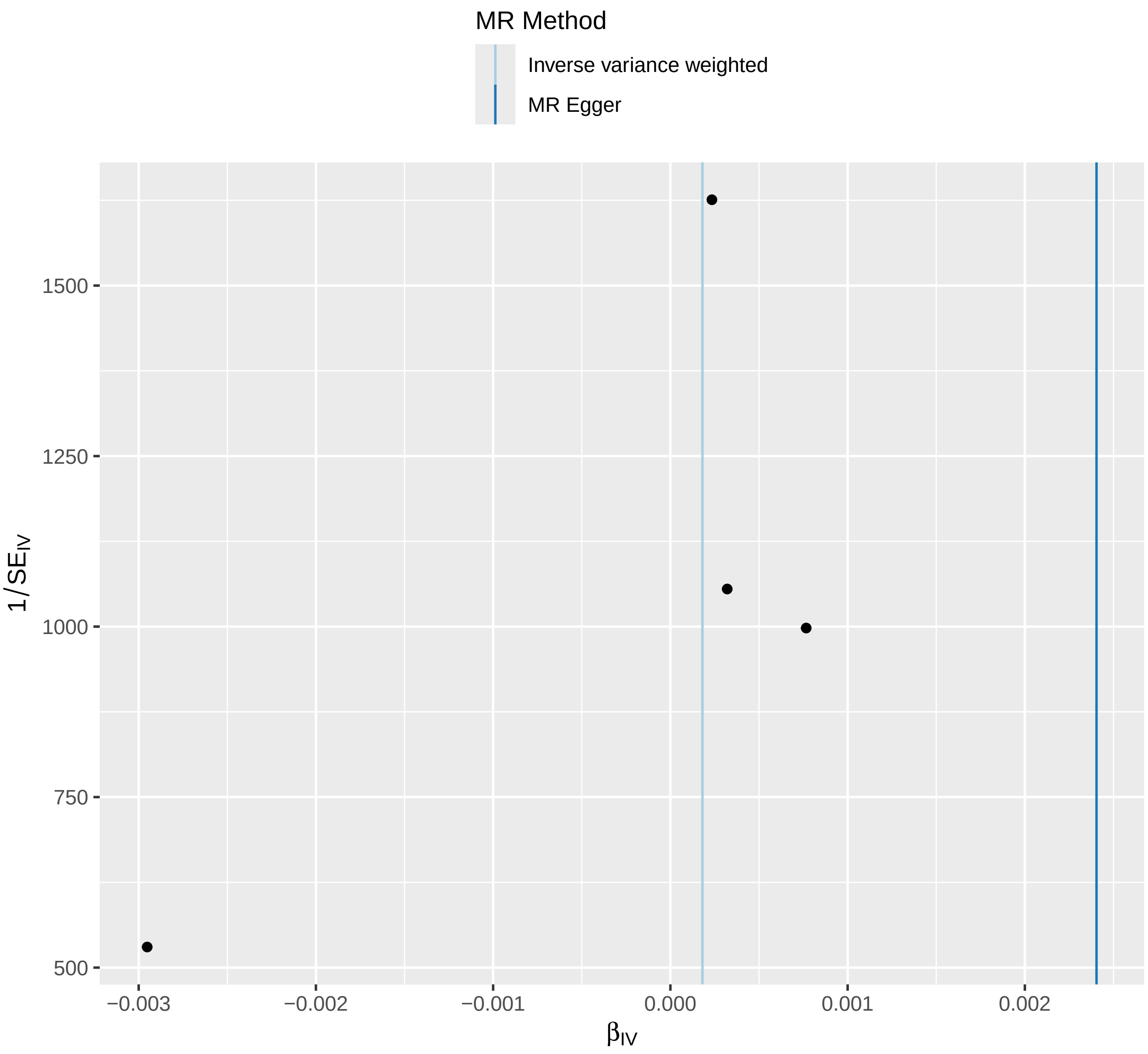

MR Method
Inverse variance weighted
MR Egger
1500
1250
1000
750
500
$1/SE_{IV}$
−0.003
−0.002
−0.001
0.000
0.001
0.002
β_{IV}

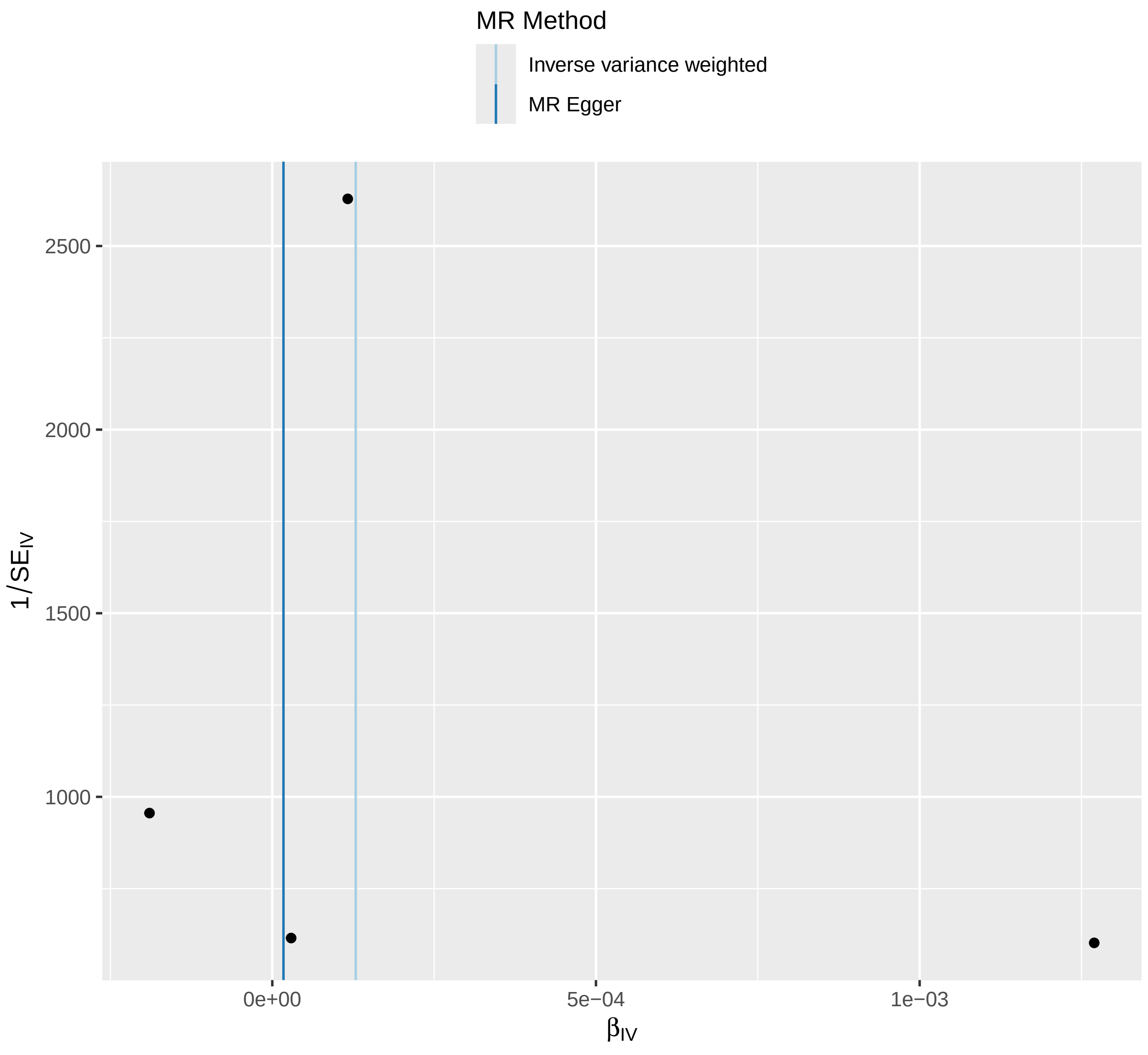
MR Method
Inverse variance weighted
MR Egger
2500
2000
1500
1000
1/SE$_{IV}$
0e+00
5e−04
1e−03
β_{IV}

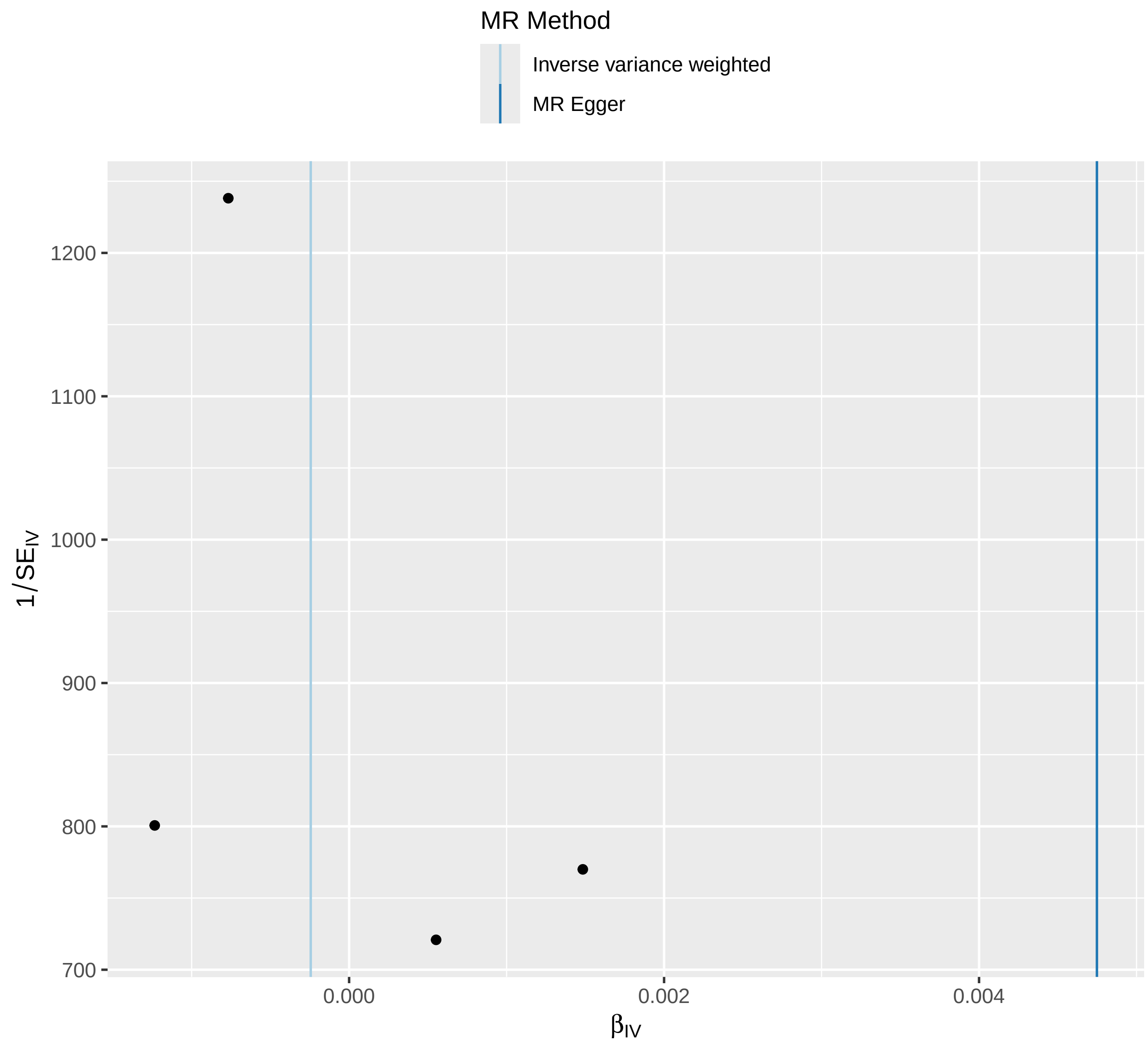
MR Method
Inverse variance weighted
MR Egger
1200
1100
1000
900
800
700
0.000
0.002
0.004
$1/SE_{IV}$
β_{IV}

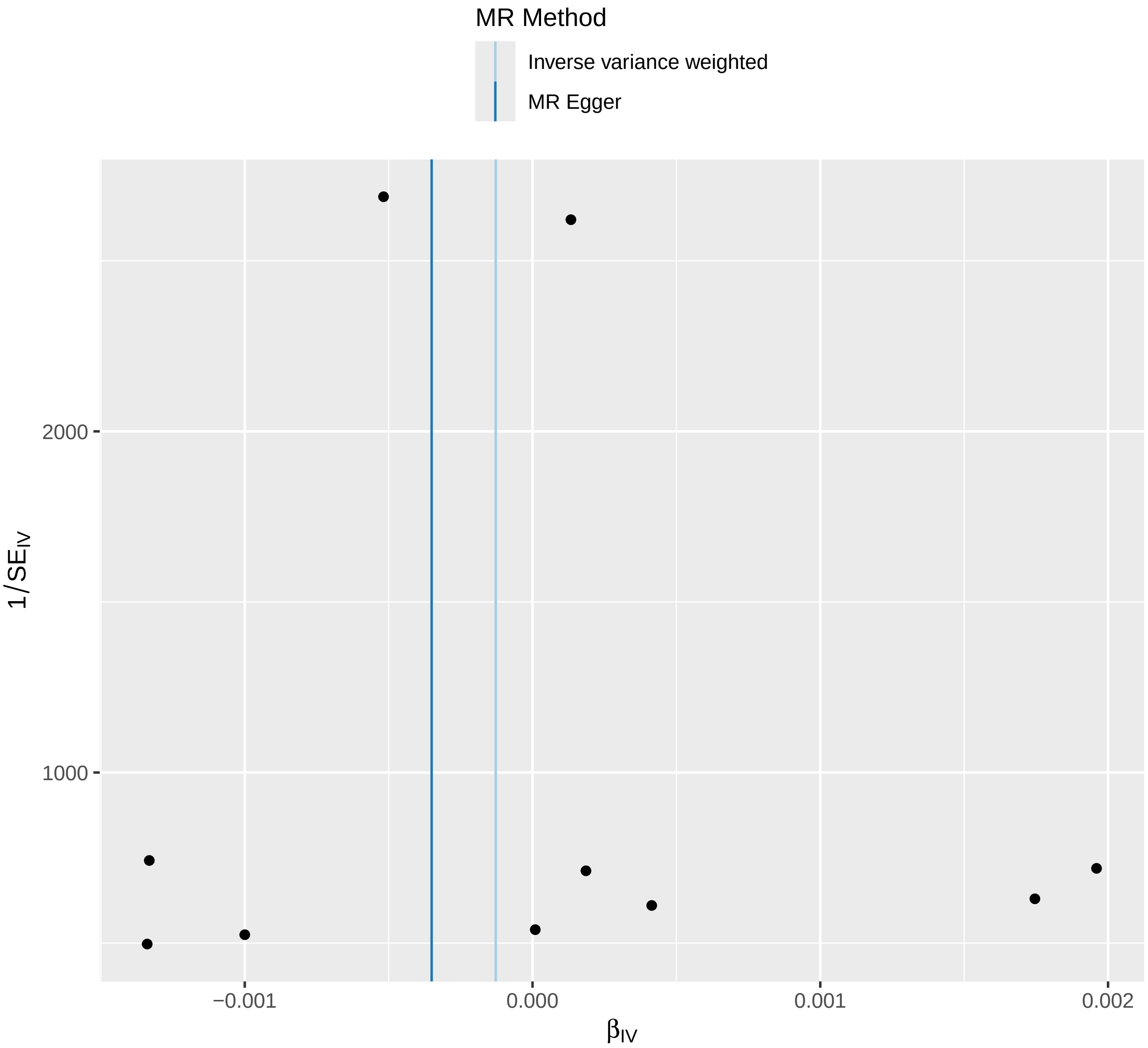

MR Method
Inverse variance weighted
MR Egger
2000
1000
1/SE$_{IV}$
−0.001
0.000
0.001
0.002
β$_{IV}$

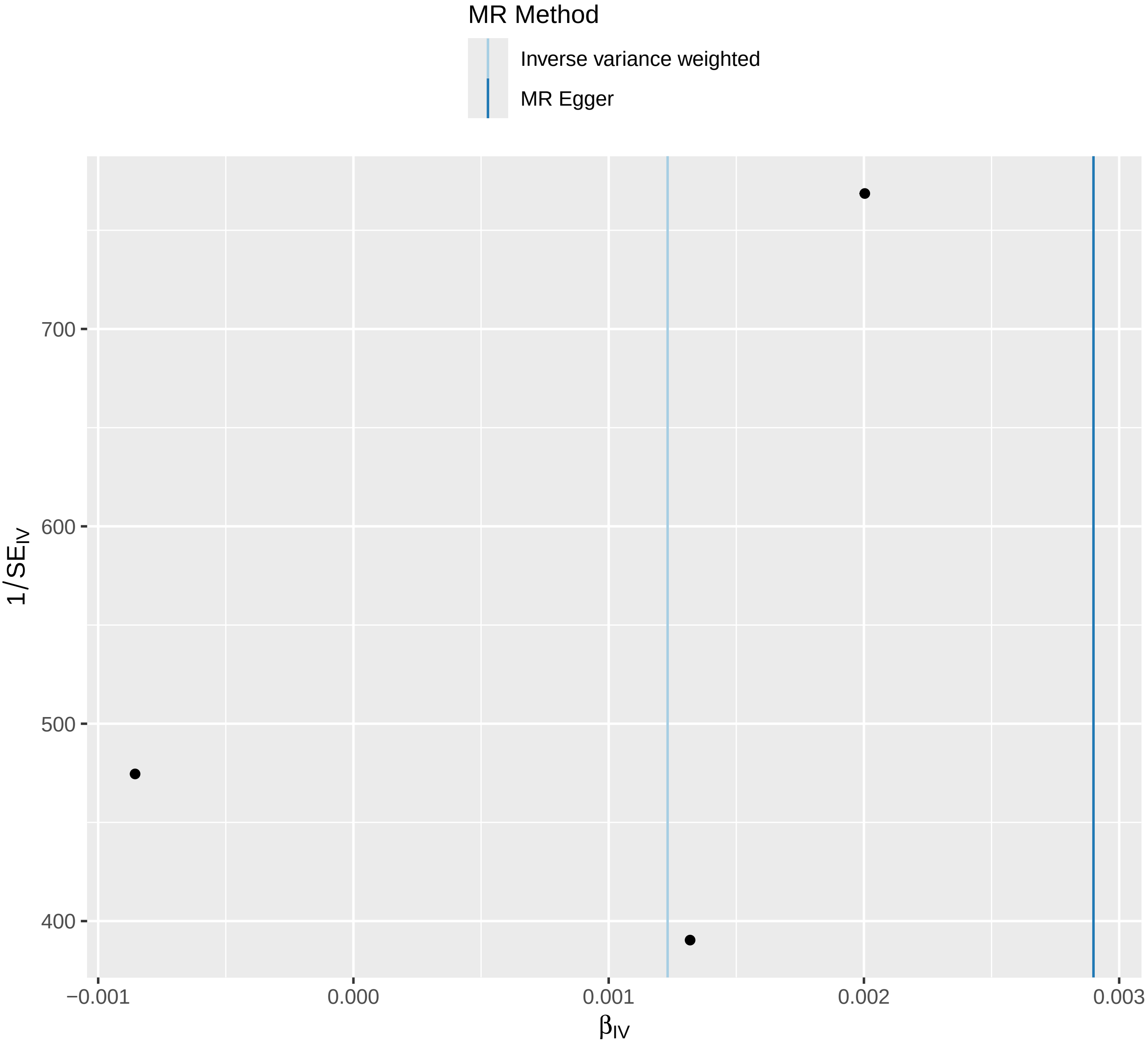

MR Method
Inverse variance weighted
MR Egger
700
600
500
400
1/SE$_{IV}$
−0.001
0.000
0.001
0.002
0.003
β$_{IV}$

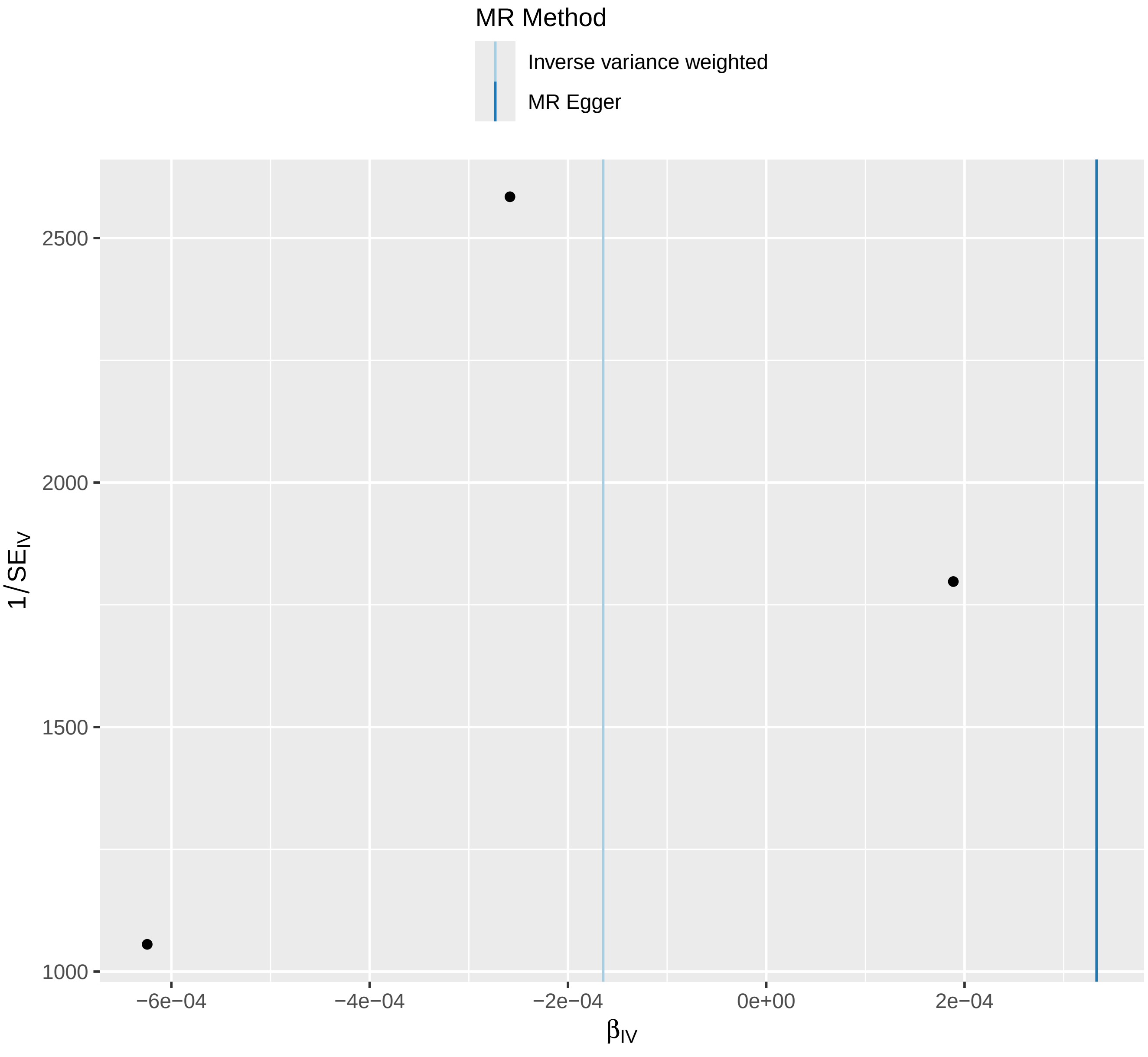
MR Method
Inverse variance weighted
MR Egger
2500
2000
1500
1000
−6e−04
−4e−04
−2e−04
0e+00
2e−04
$1/SE_{IV}$
β_{IV}

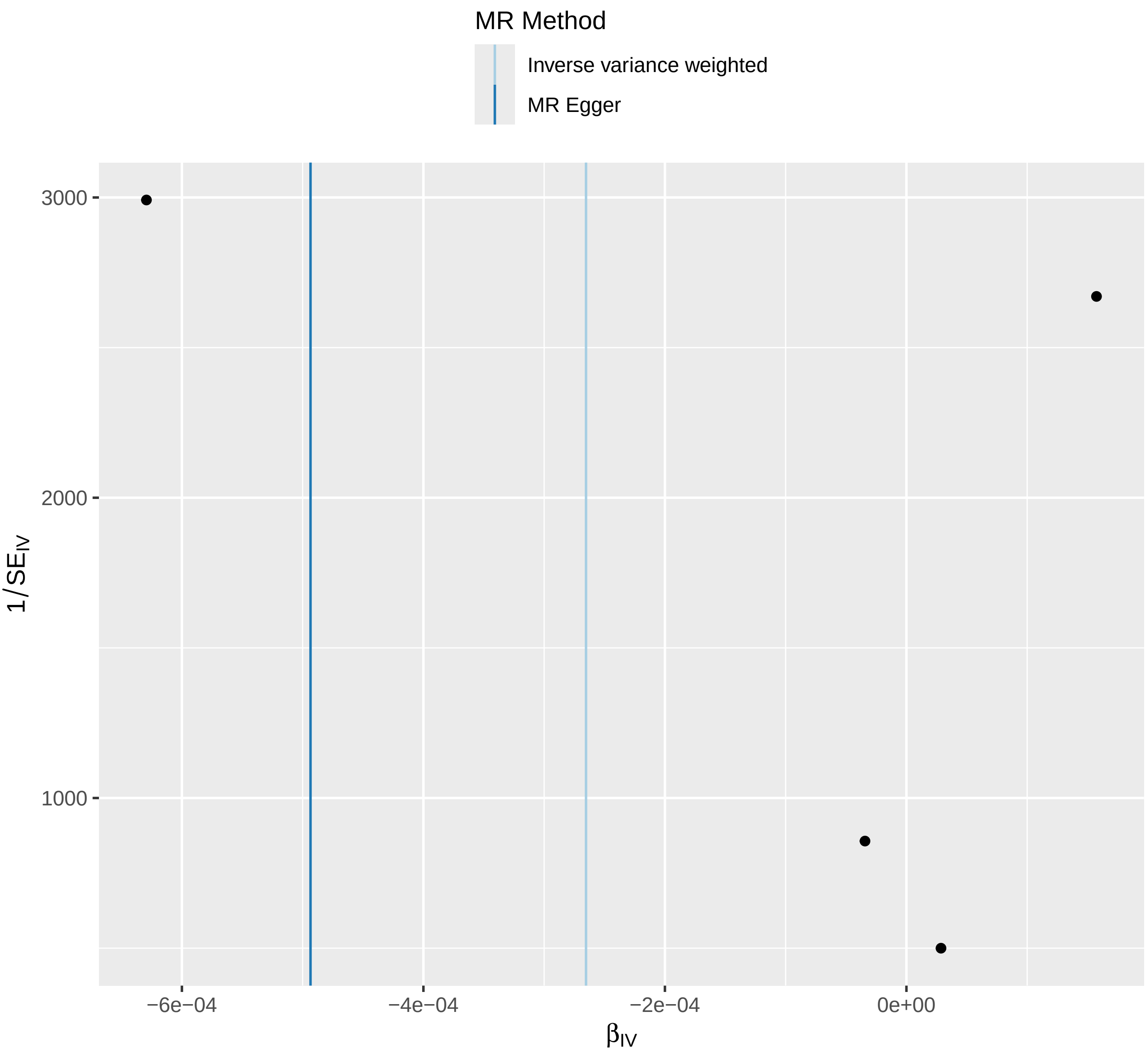
MR Method
Inverse variance weighted
MR Egger
3000
2000
1000
1/SE_IV
−6e−04
−4e−04
−2e−04
0e+00
β_IV

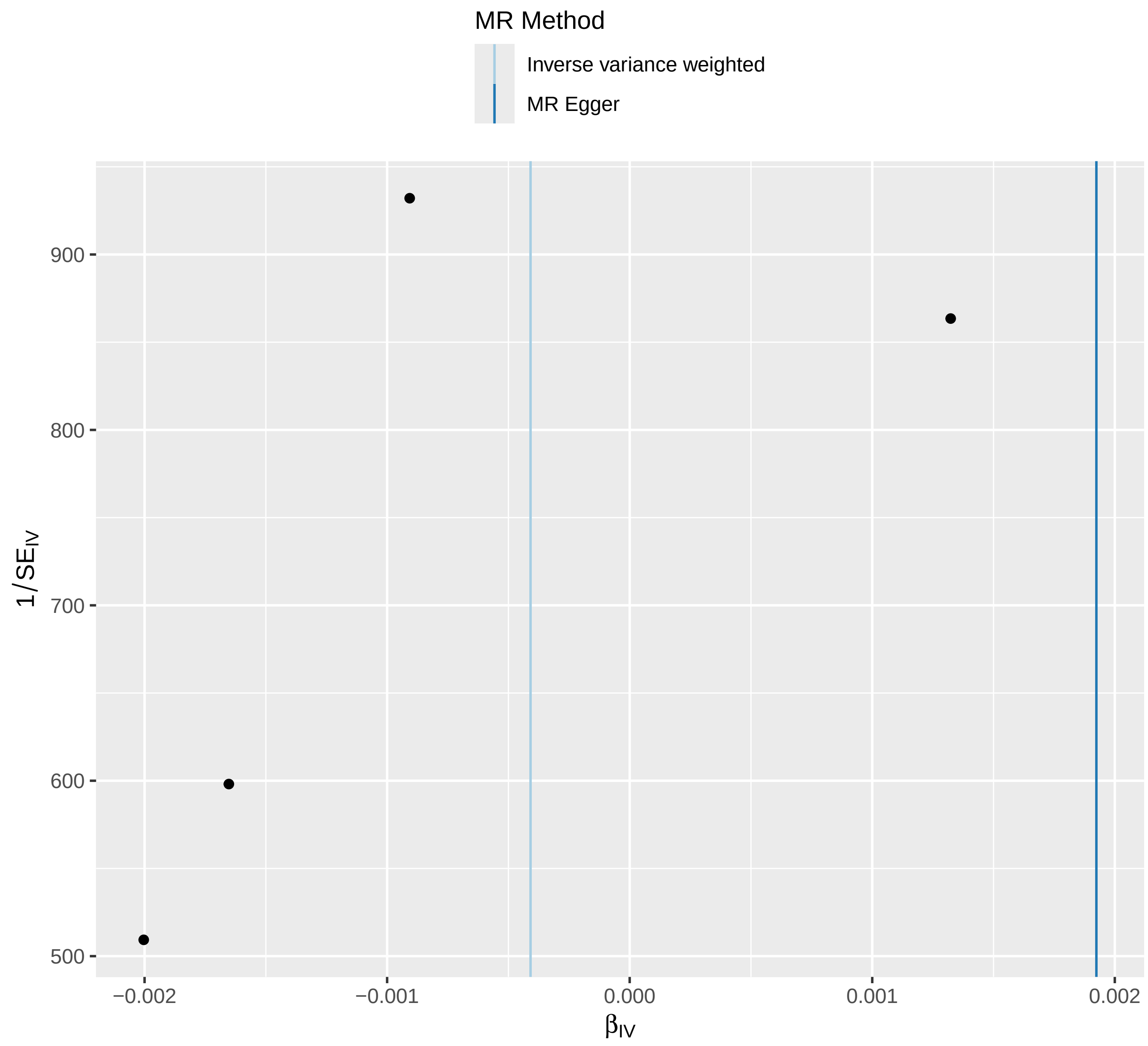
MR Method
Inverse variance weighted
MR Egger
900
800
700
600
500
1/SE$_{IV}$
−0.002
−0.001
0.000
0.001
0.002
β_{IV}

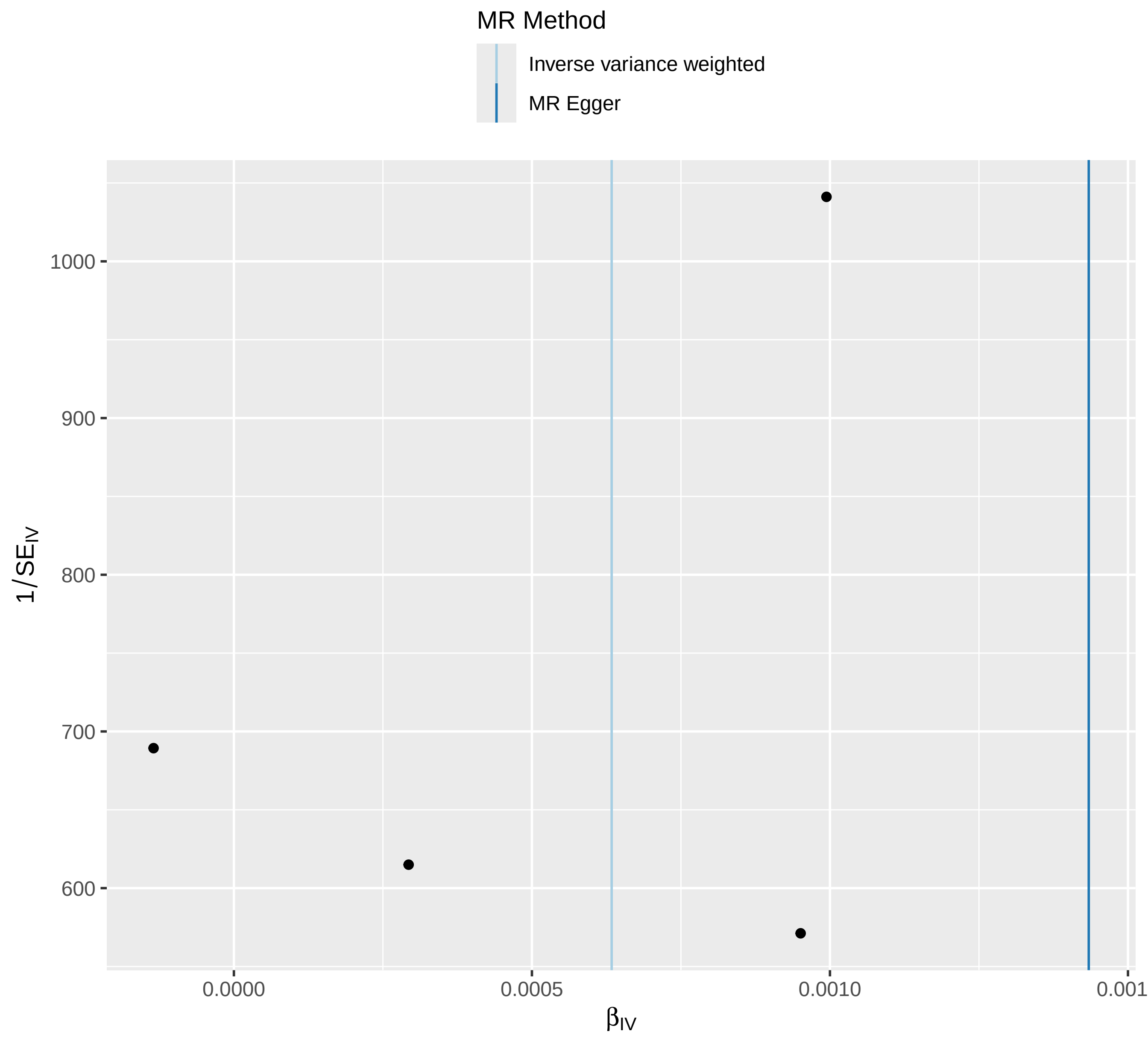
MR Method
Inverse variance weighted
MR Egger
1000
900
800
700
600
1/SE_IV
0.0000
0.0005
0.0010
0.001
β_IV

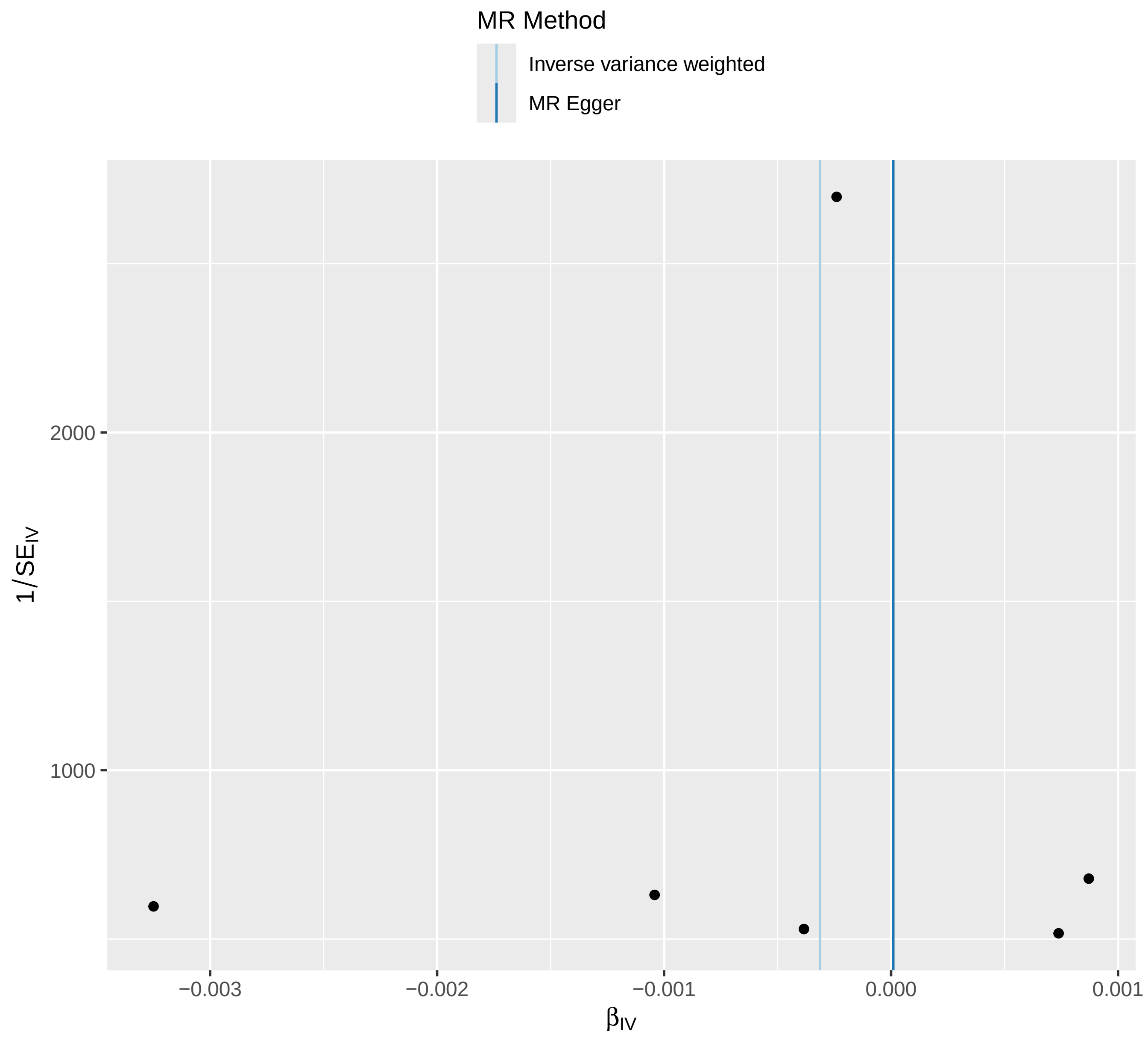

MR Method
Inverse variance weighted
MR Egger
2000
1000
1/SE_IV
−0.003
−0.002
−0.001
0.000
0.001
β_IV

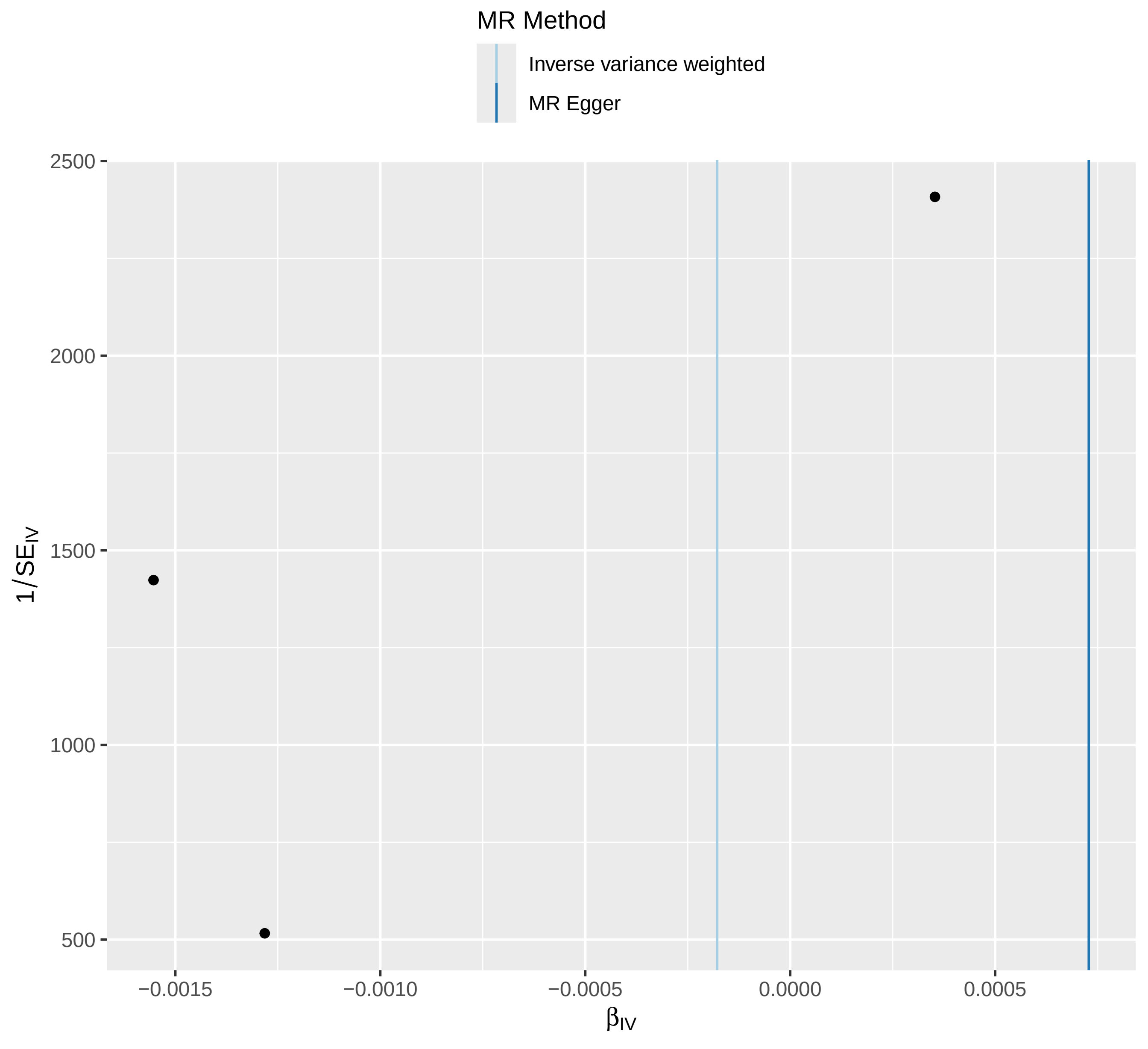
MR Method
Inverse variance weighted
MR Egger
2500
2000
1500
1000
500
$1/SE_{IV}$
−0.0015
−0.0010
−0.0005
0.0000
0.0005
β_{IV}

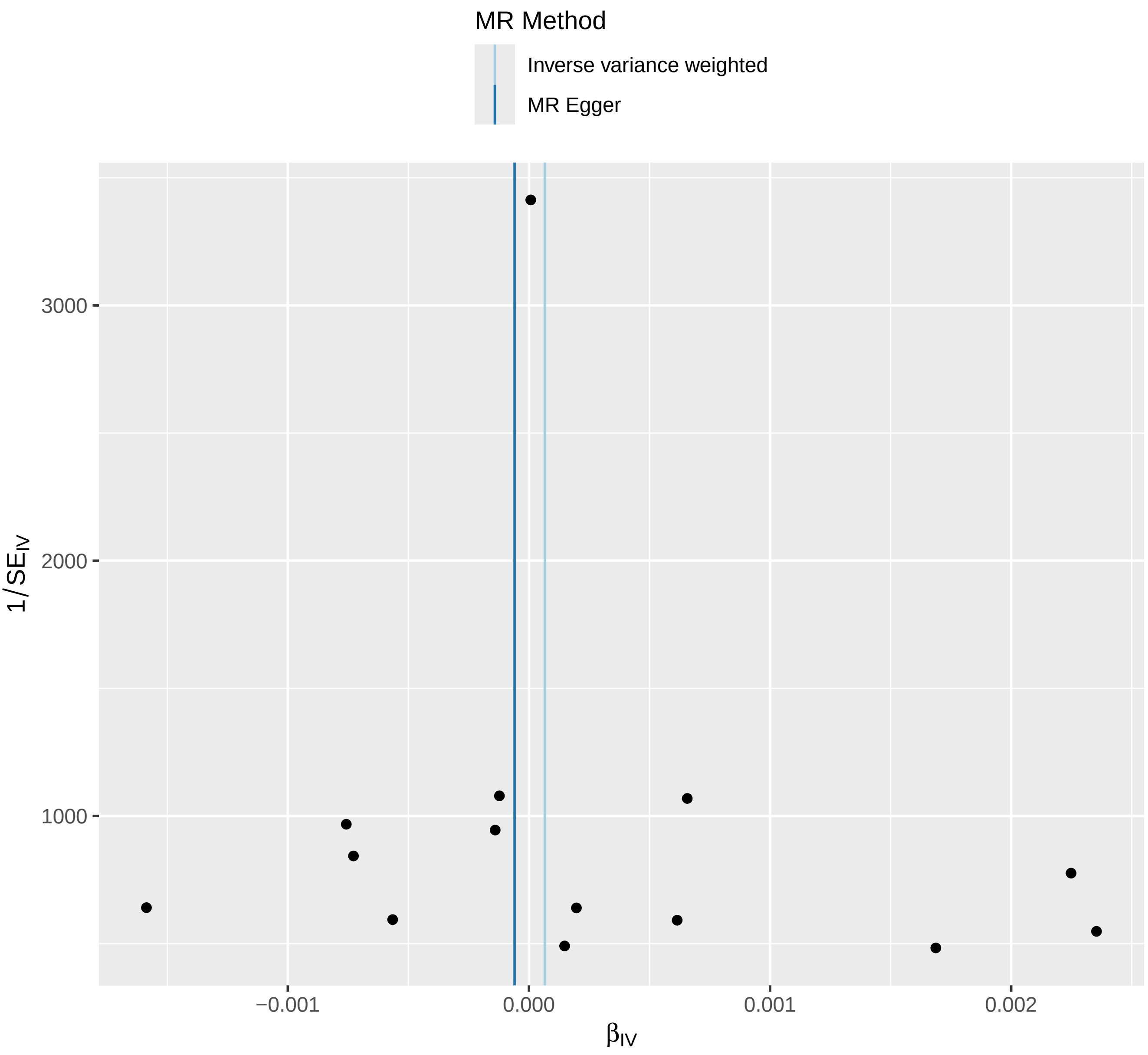
MR Method
Inverse variance weighted
MR Egger
3000
2000
1000
1/SE_IV
−0.001
0.000
0.001
0.002
β_IV

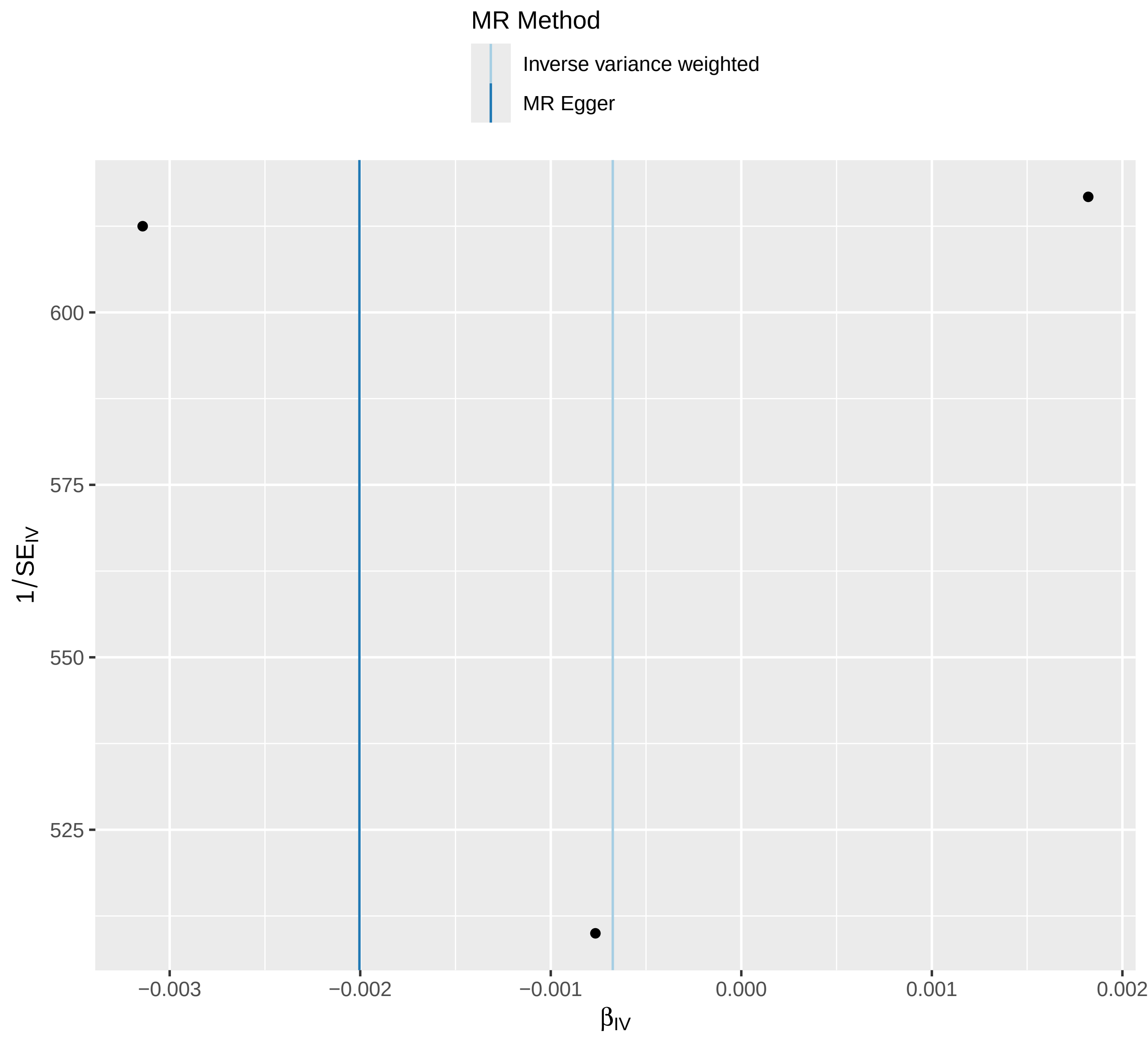

MR Method
Inverse variance weighted
MR Egger
600
575
550
525
$1/SE_{IV}$
−0.003
−0.002
−0.001
0.000
0.001
0.002
β_{IV}

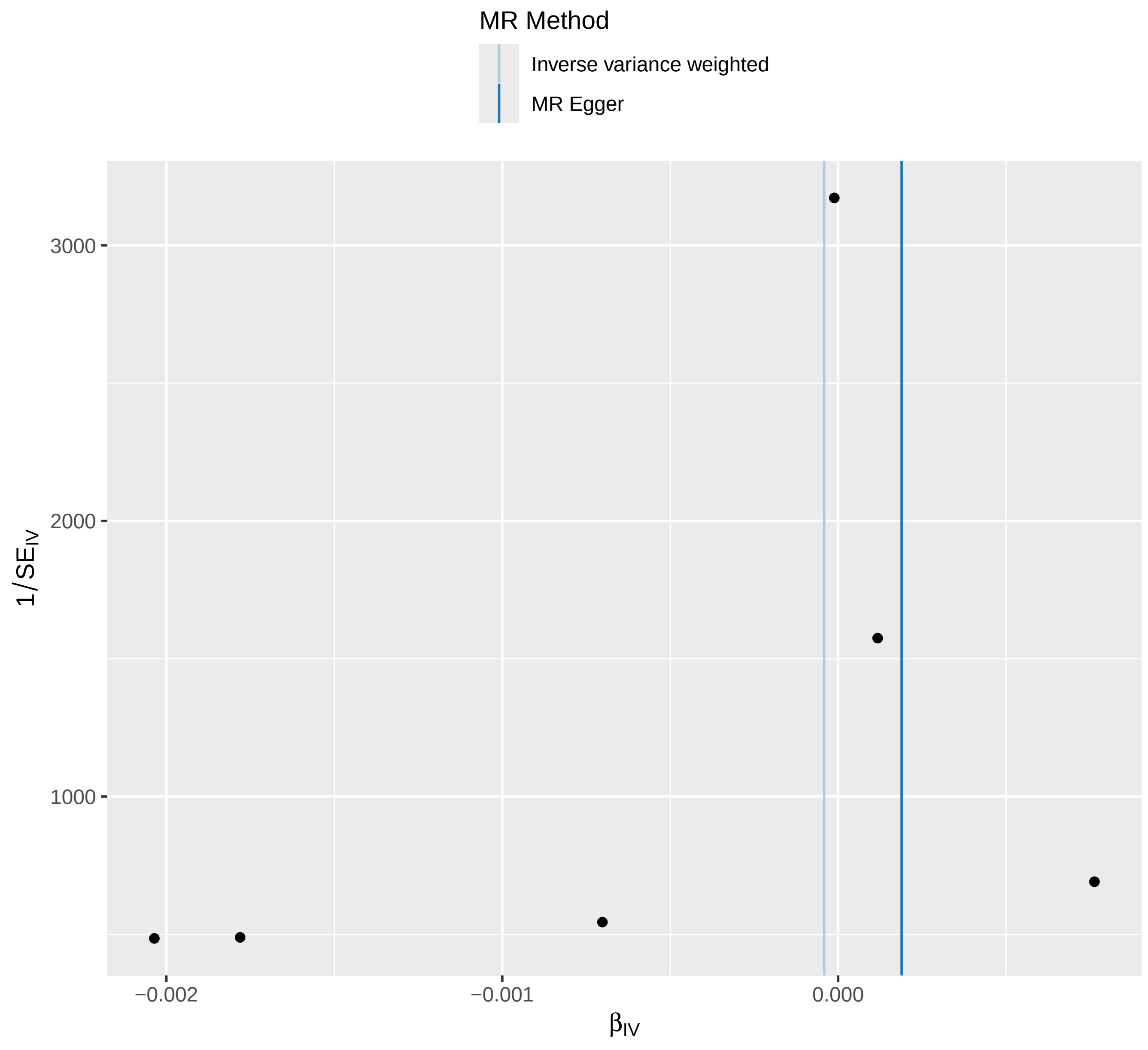
MR Method
Inverse variance weighted
MR Egger
3000
2000
1000
1/SE_IV
−0.002
−0.001
0.000
β_IV

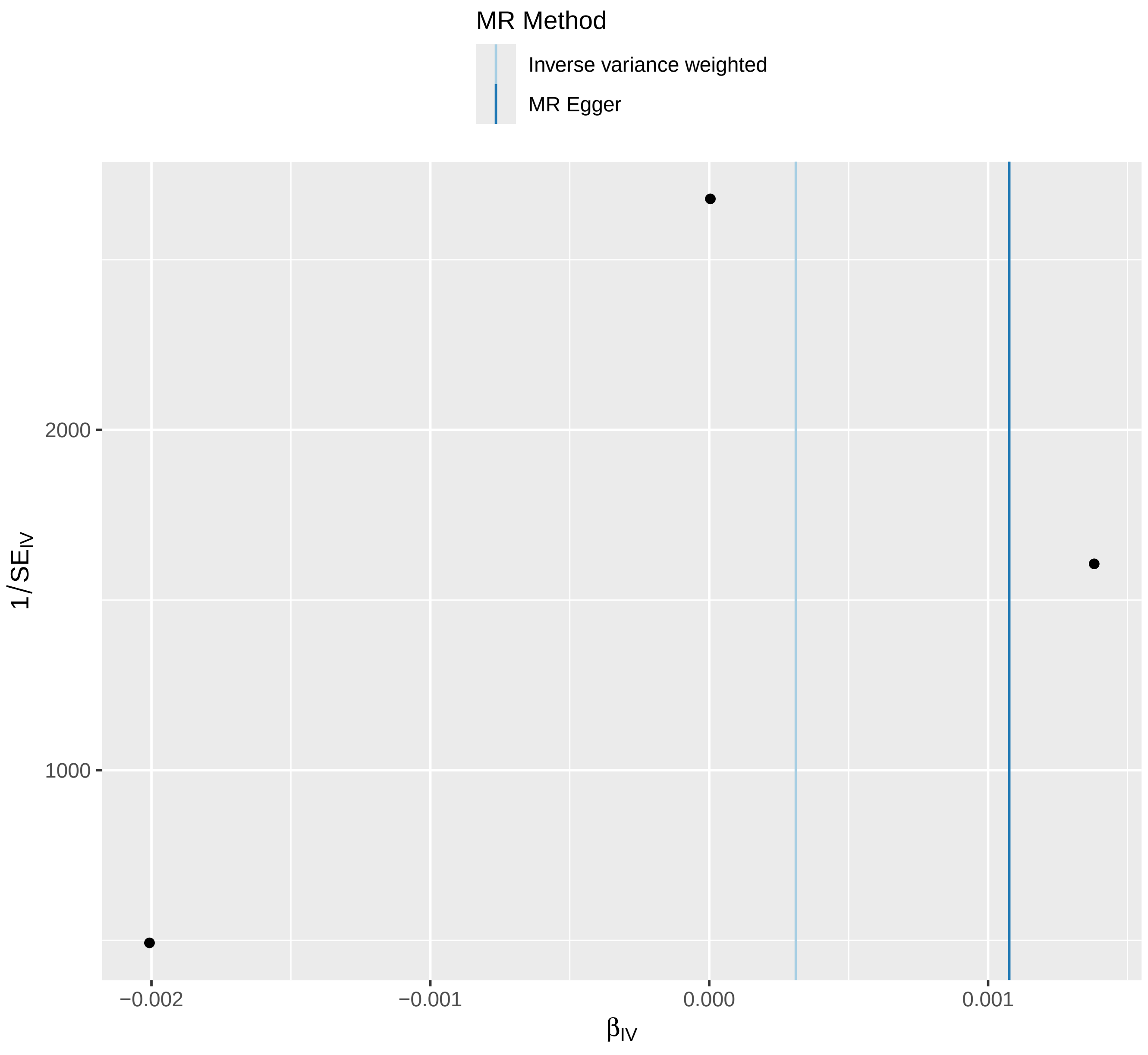
MR Method
Inverse variance weighted
MR Egger
2000
1000
1/SE$_{IV}$
−0.002
−0.001
0.000
0.001
β$_{IV}$

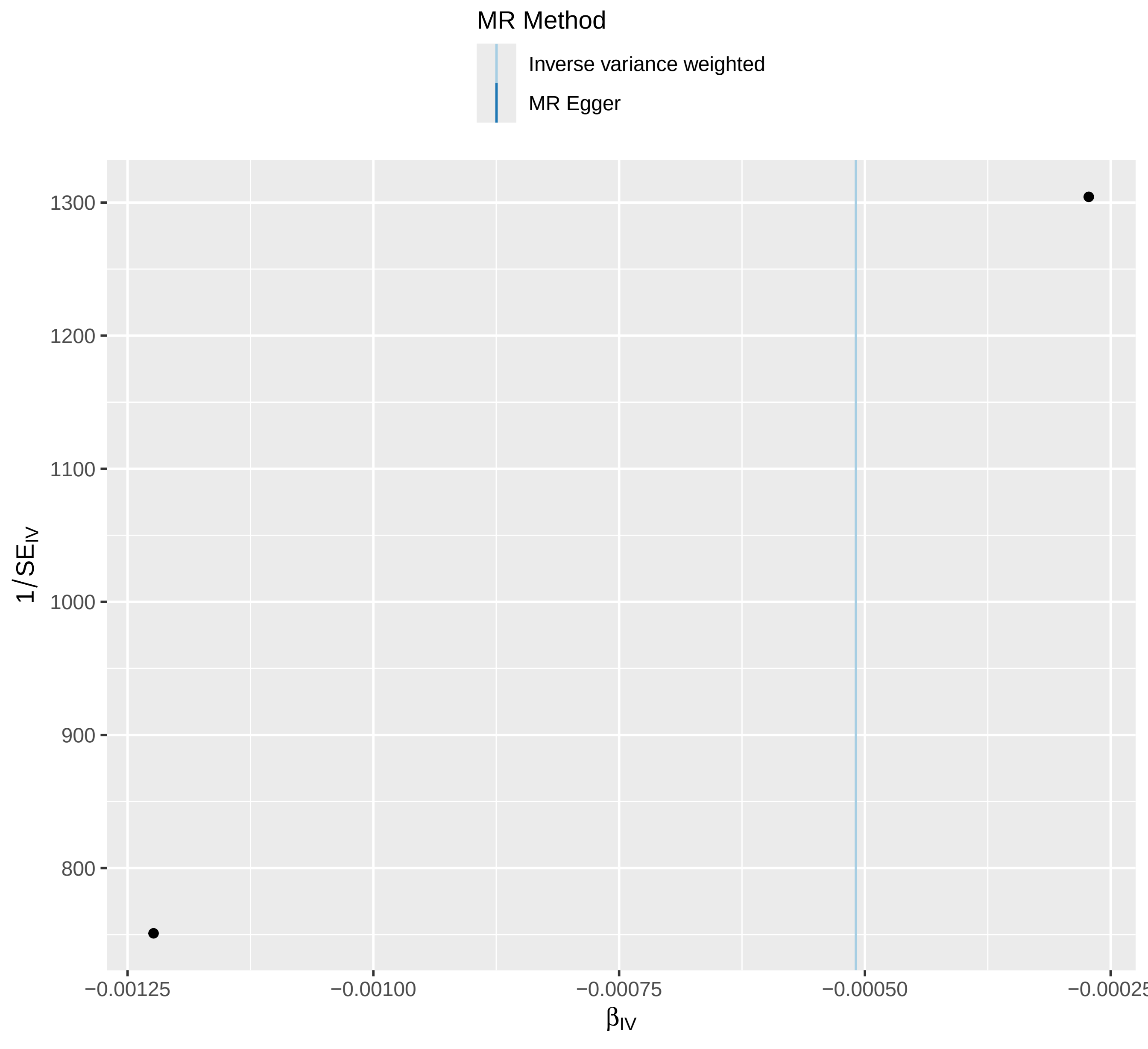

MR Method
Inverse variance weighted
MR Egger
1300
1200
1100
1000
900
800
$1/SE_{IV}$
−0.00125
−0.00100
−0.00075
−0.00050
−0.00025
β_{IV}

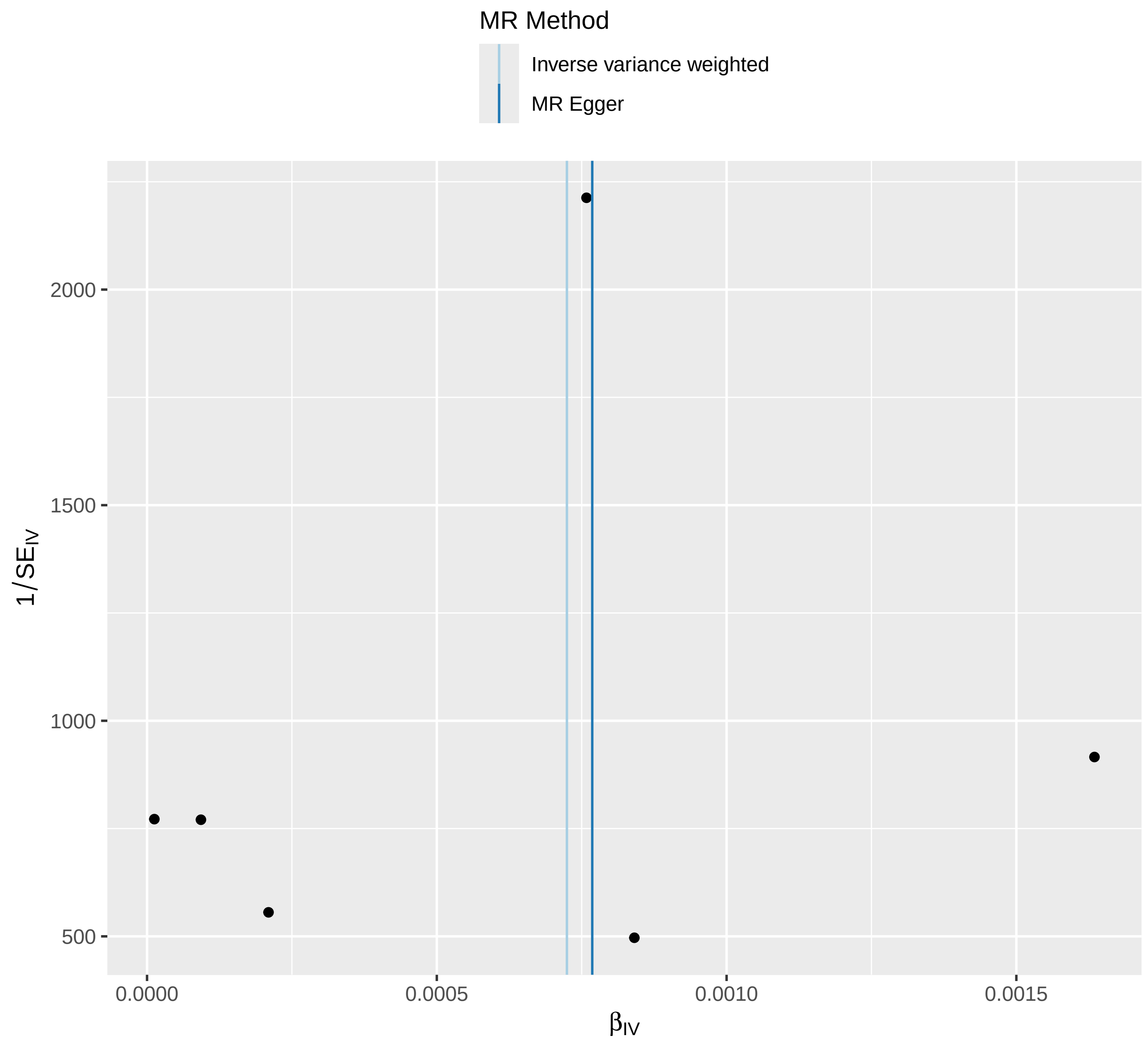
MR Method
Inverse variance weighted
MR Egger
2000
1500
1000
500
$1/SE_{IV}$
0.0000
0.0005
0.0010
0.0015
β_{IV}

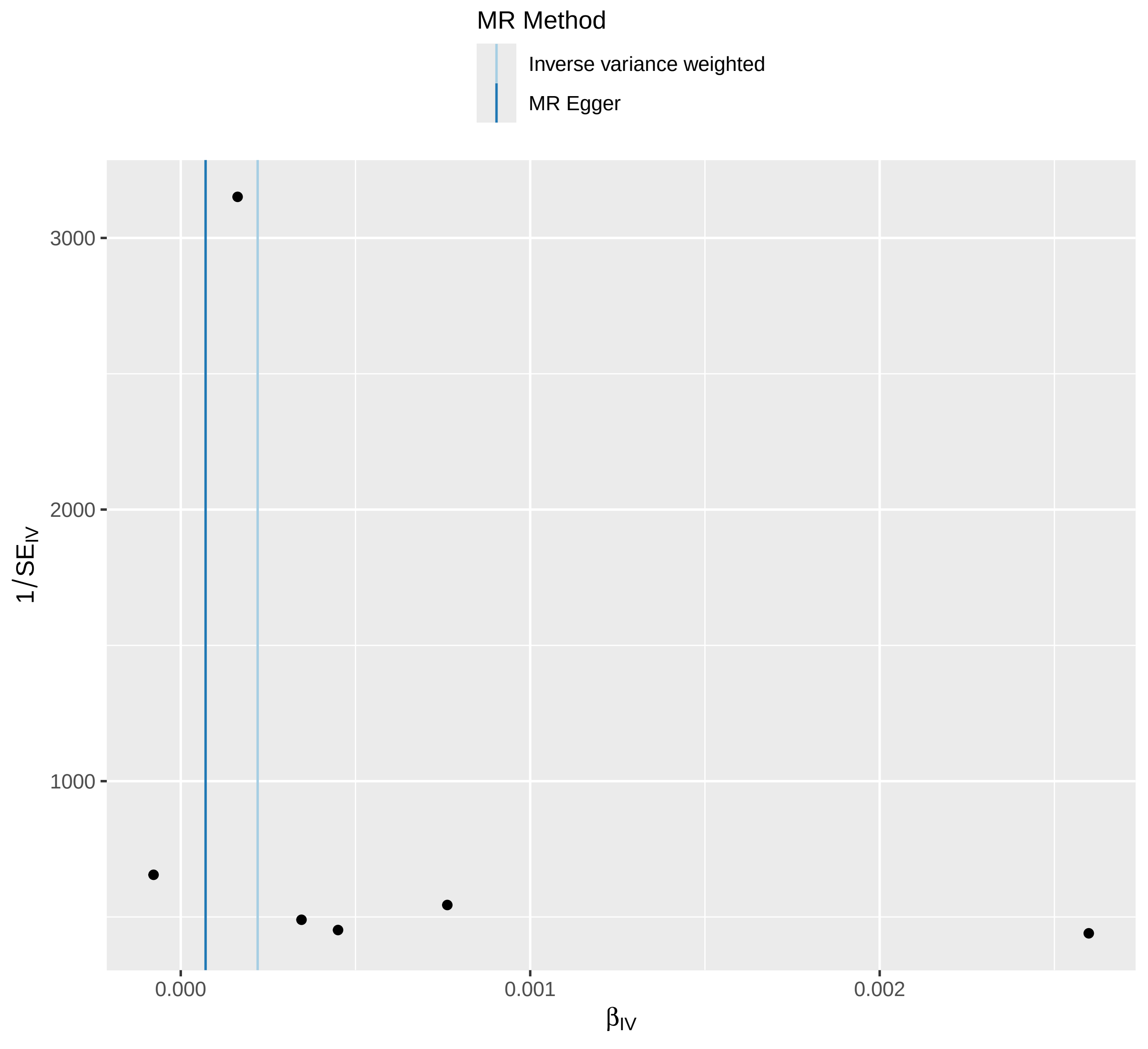
MR Method
Inverse variance weighted
MR Egger
3000
2000
1000
0.000
0.001
0.002
1/SE_IV
β_IV

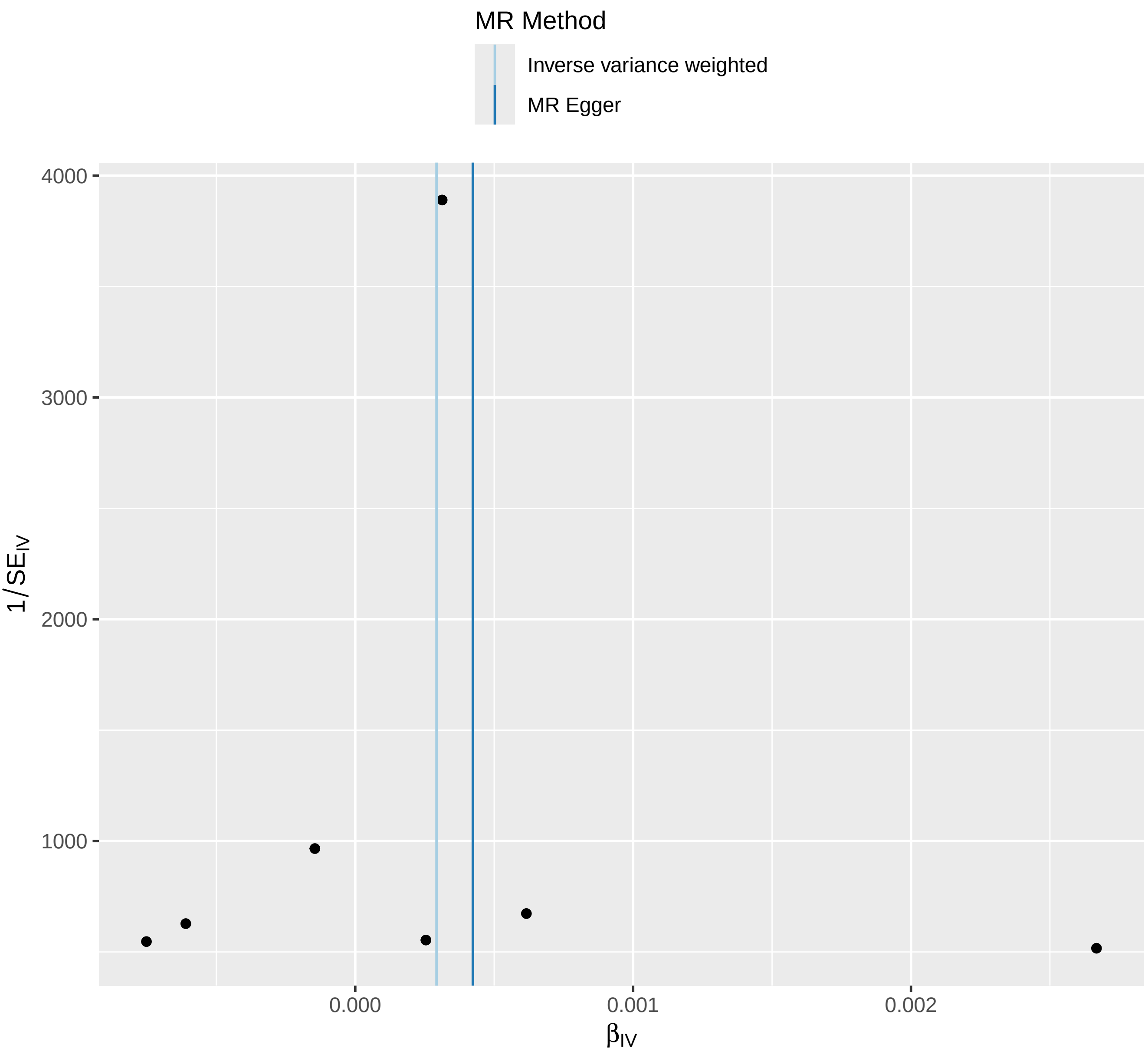
MR Method
Inverse variance weighted
MR Egger
4000
3000
2000
1000
0.000
0.001
0.002
1/SE_IV
β_IV

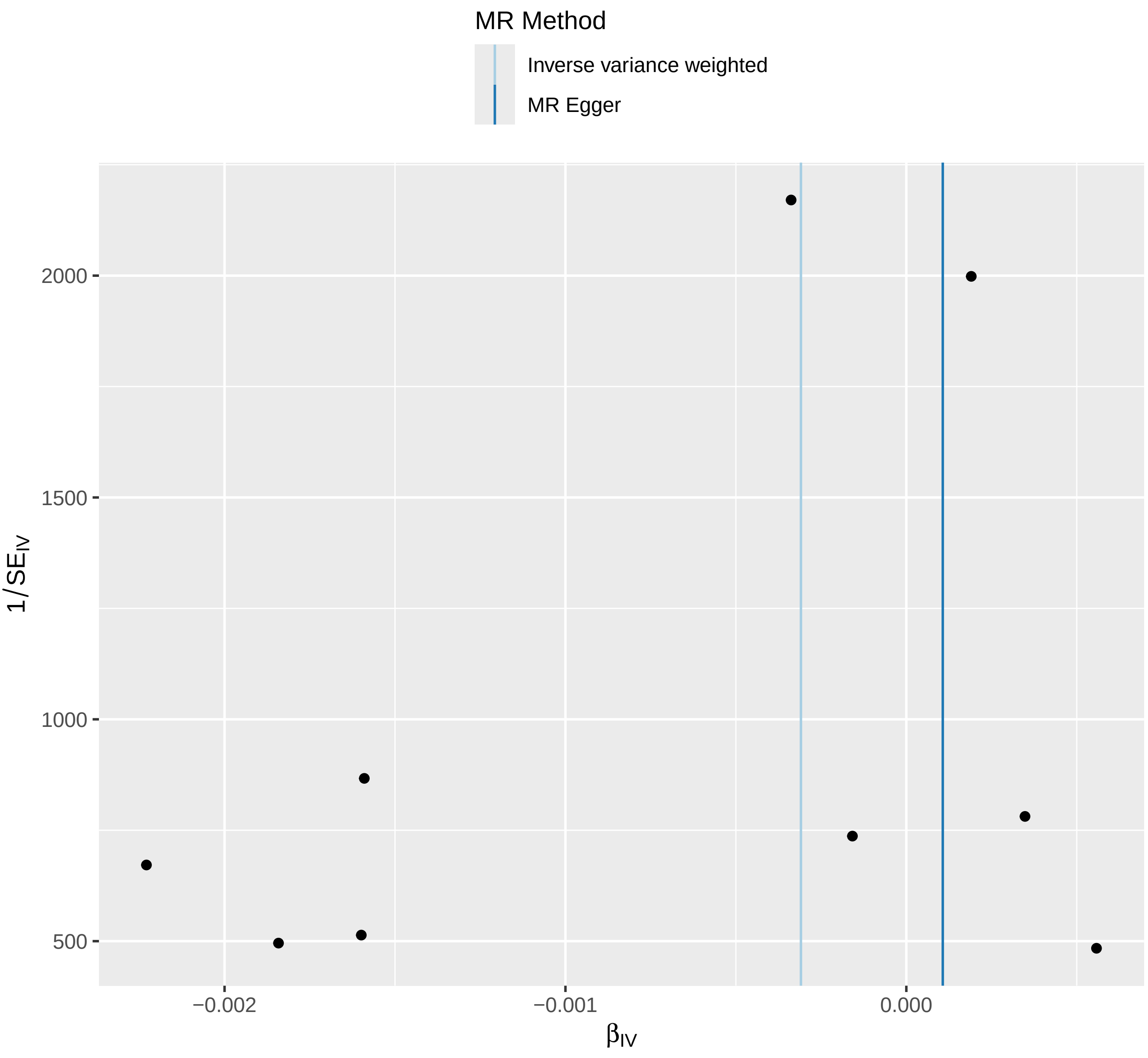
MR Method
Inverse variance weighted
MR Egger
2000
1500
1000
500
1/SE_IV
−0.002
−0.001
0.000
β_IV

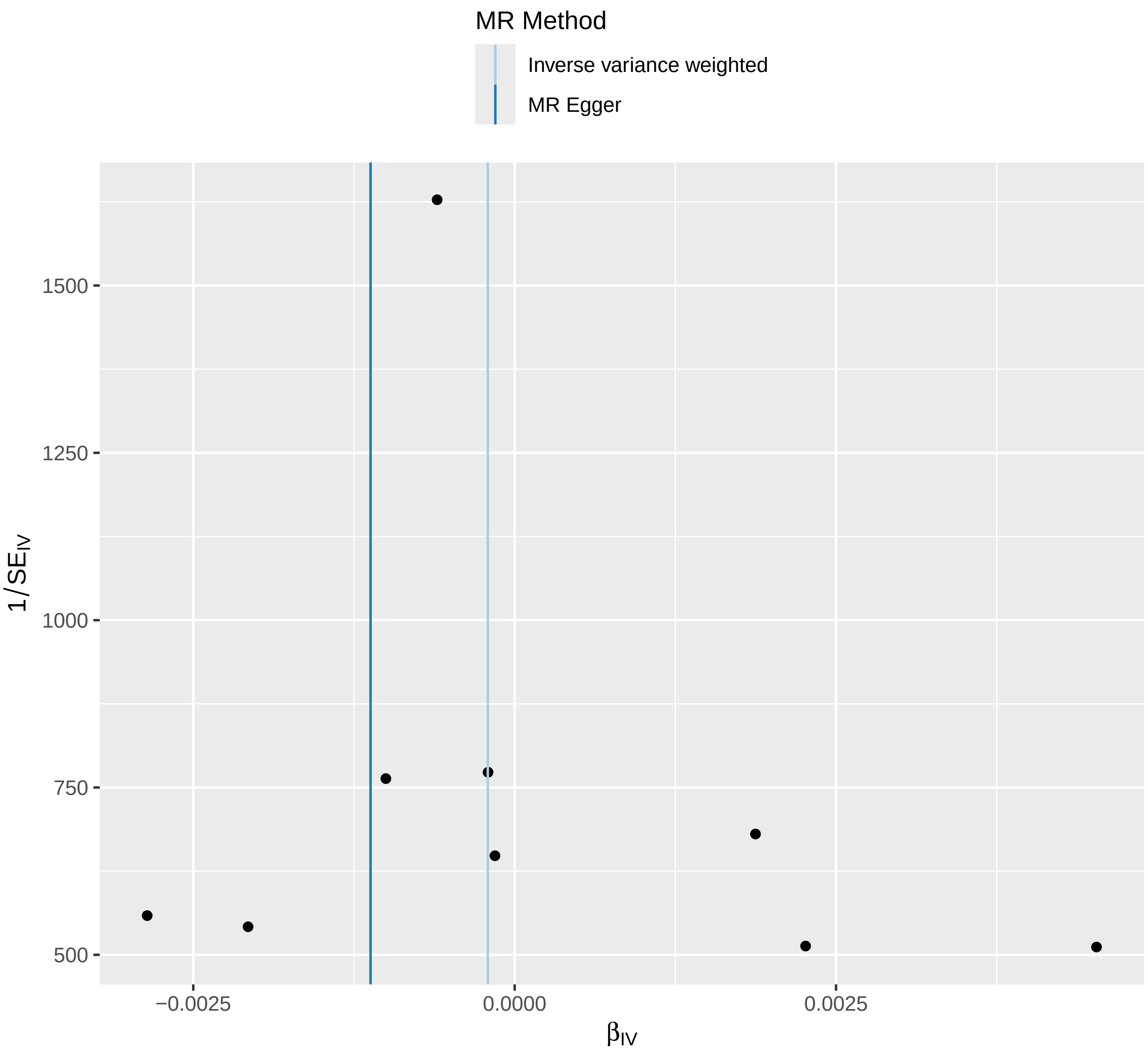

MR Method
Inverse variance weighted
MR Egger
1500
1250
1000
750
500
1/SE_IV
−0.0025
0.0000
0.0025
β_IV

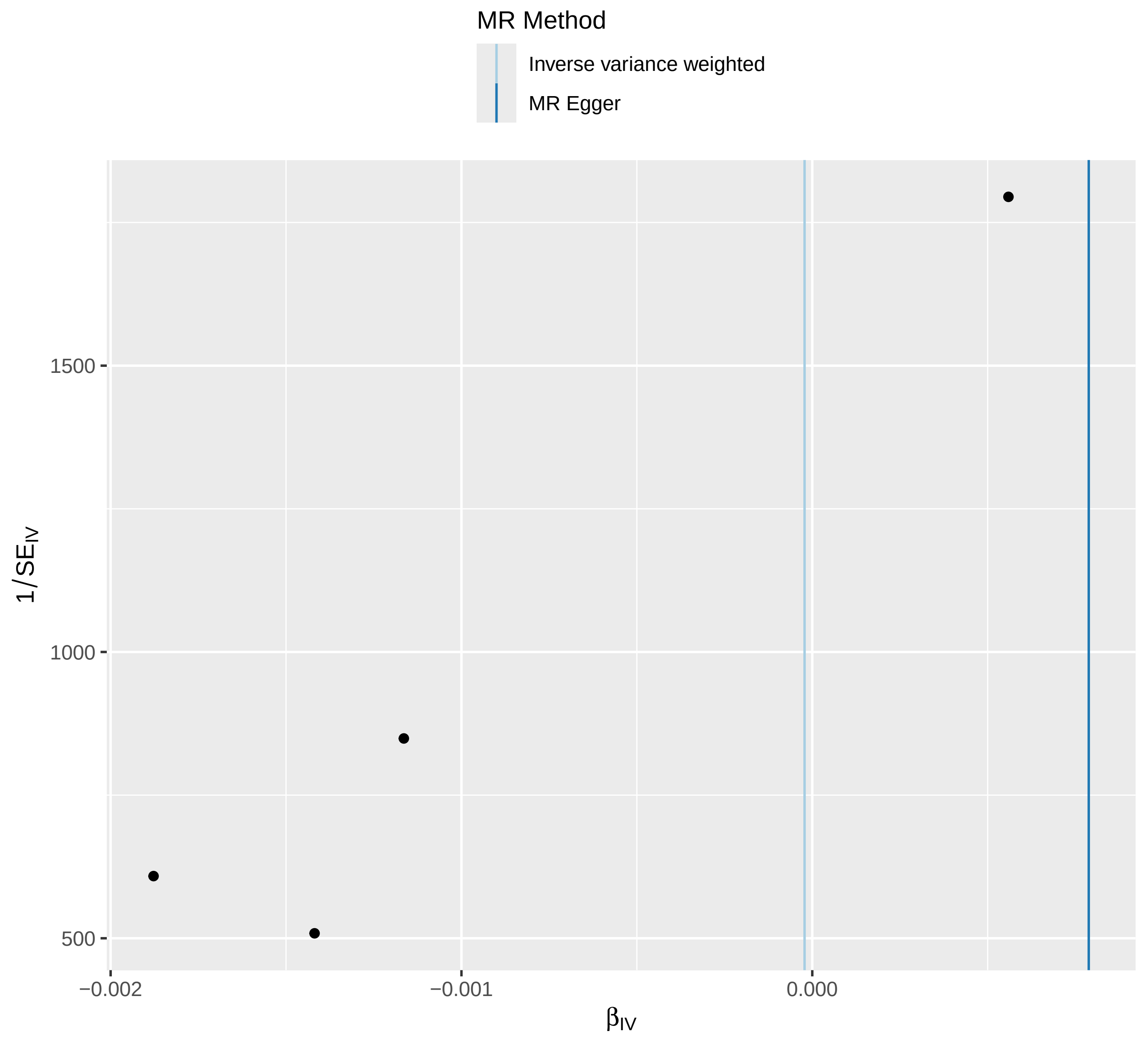

MR Method
Inverse variance weighted
MR Egger
1500
1000
500
1/SE_IV
−0.002
−0.001
0.000
β_IV

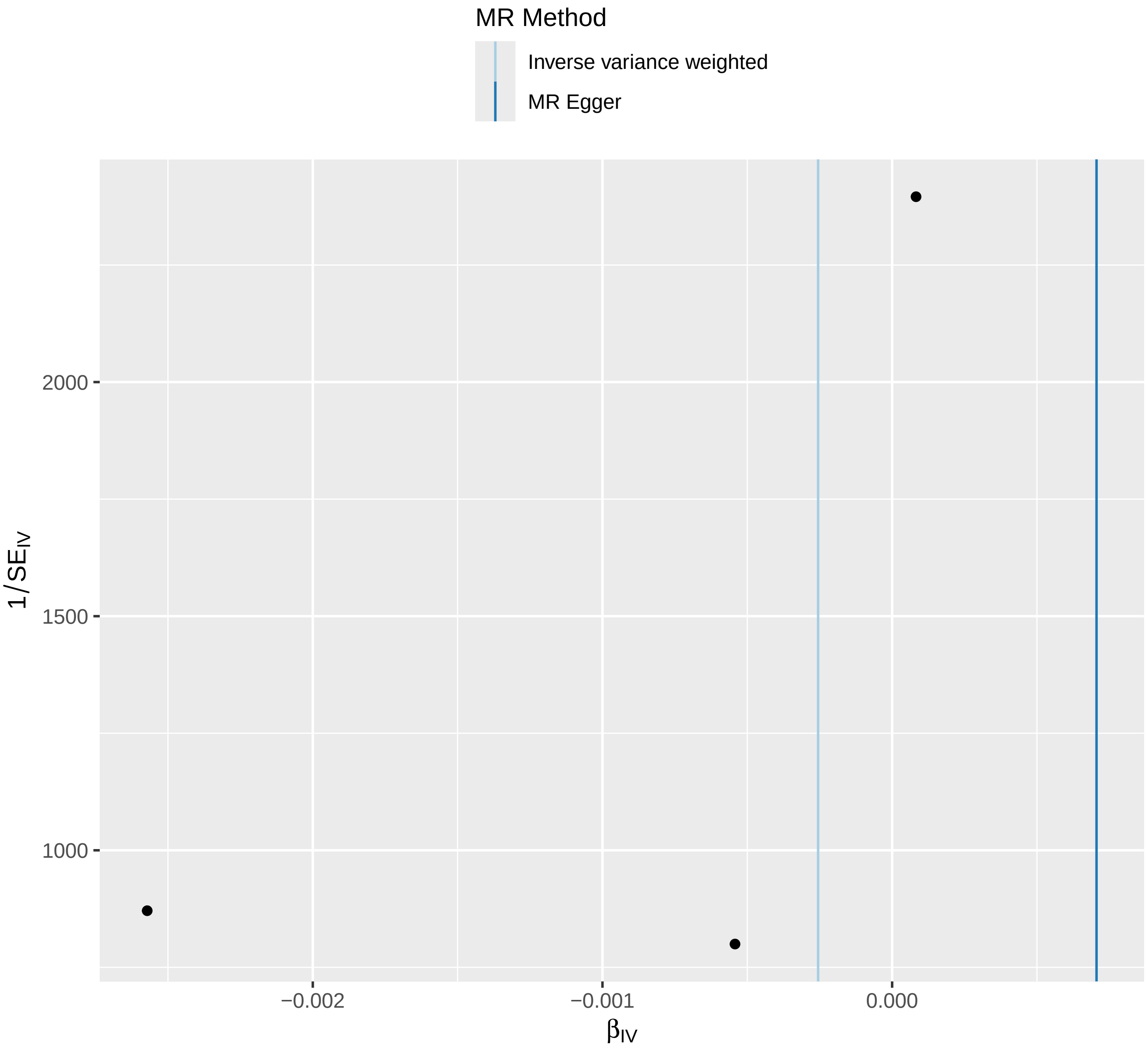

MR Method
Inverse variance weighted
MR Egger
2000
1500
1000
1/SE$_{IV}$
−0.002
−0.001
0.000
β$_{IV}$

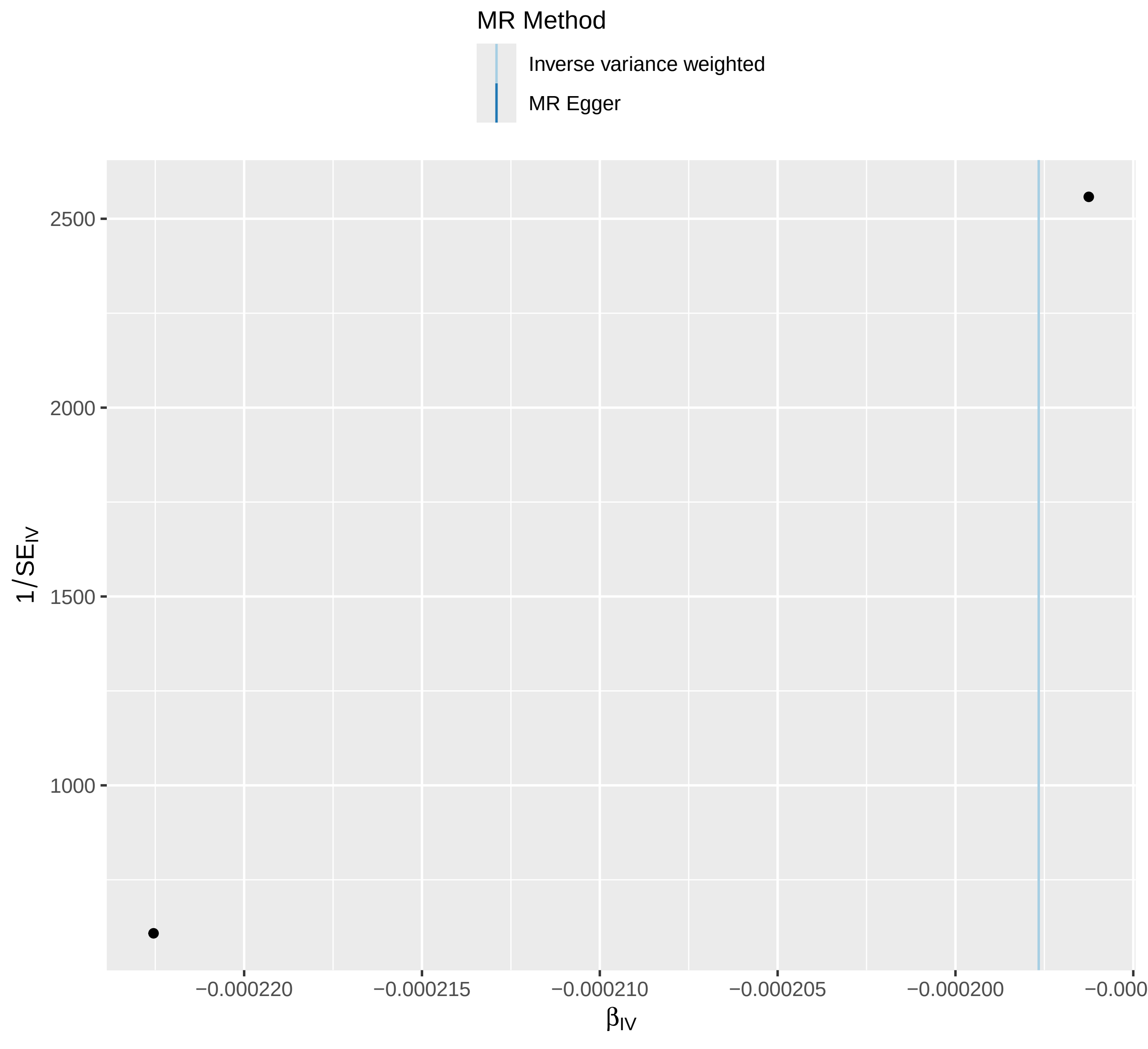

MR Method
Inverse variance weighted
MR Egger
2500
2000
1500
1000
1/SE_IV
−0.000220
−0.000215
−0.000210
−0.000205
−0.000200
−0.000
β_IV

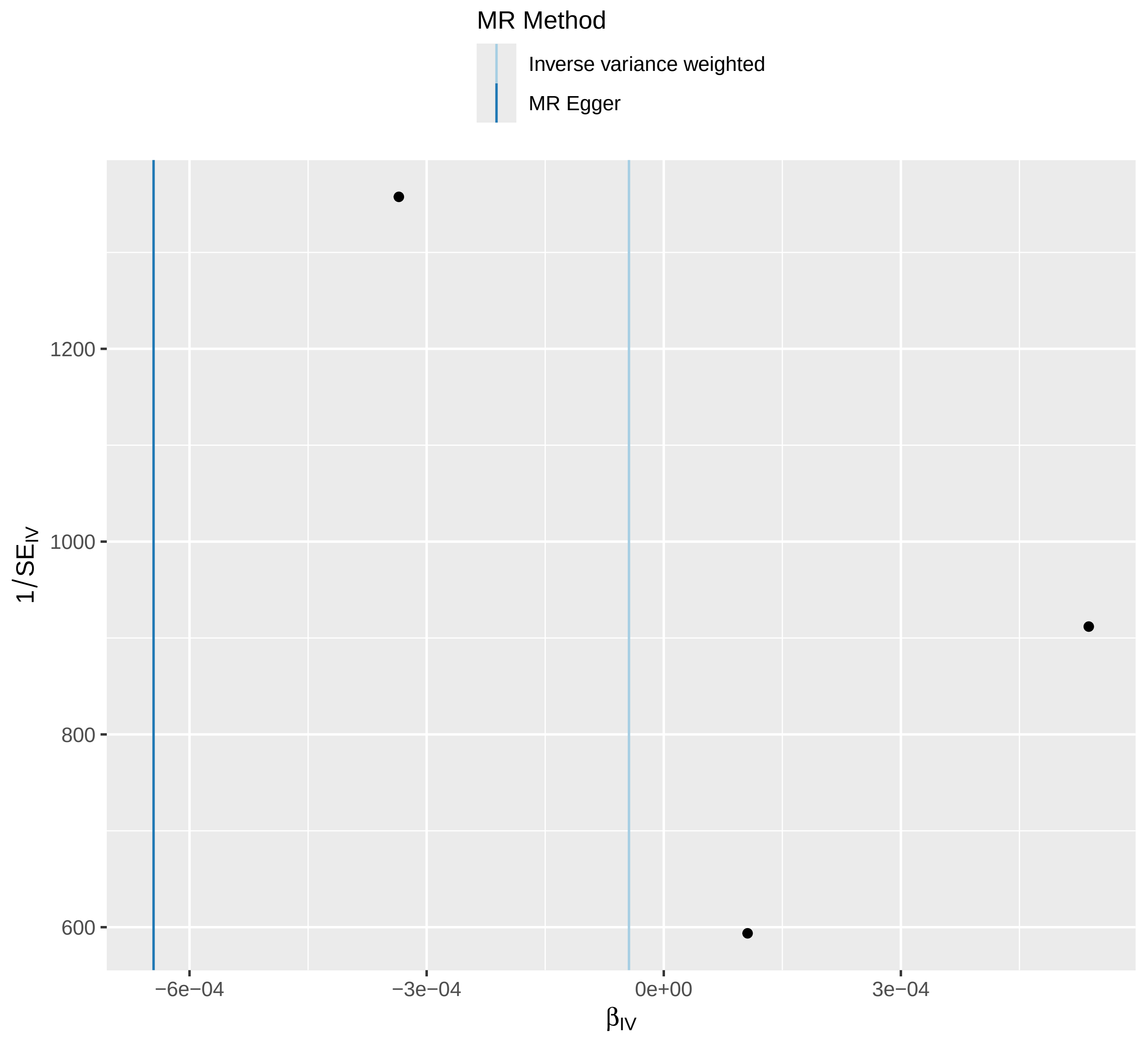

MR Method
Inverse variance weighted
MR Egger
1200
1000
800
600
1/SE_IV
−6e−04
−3e−04
0e+00
3e−04
β_IV

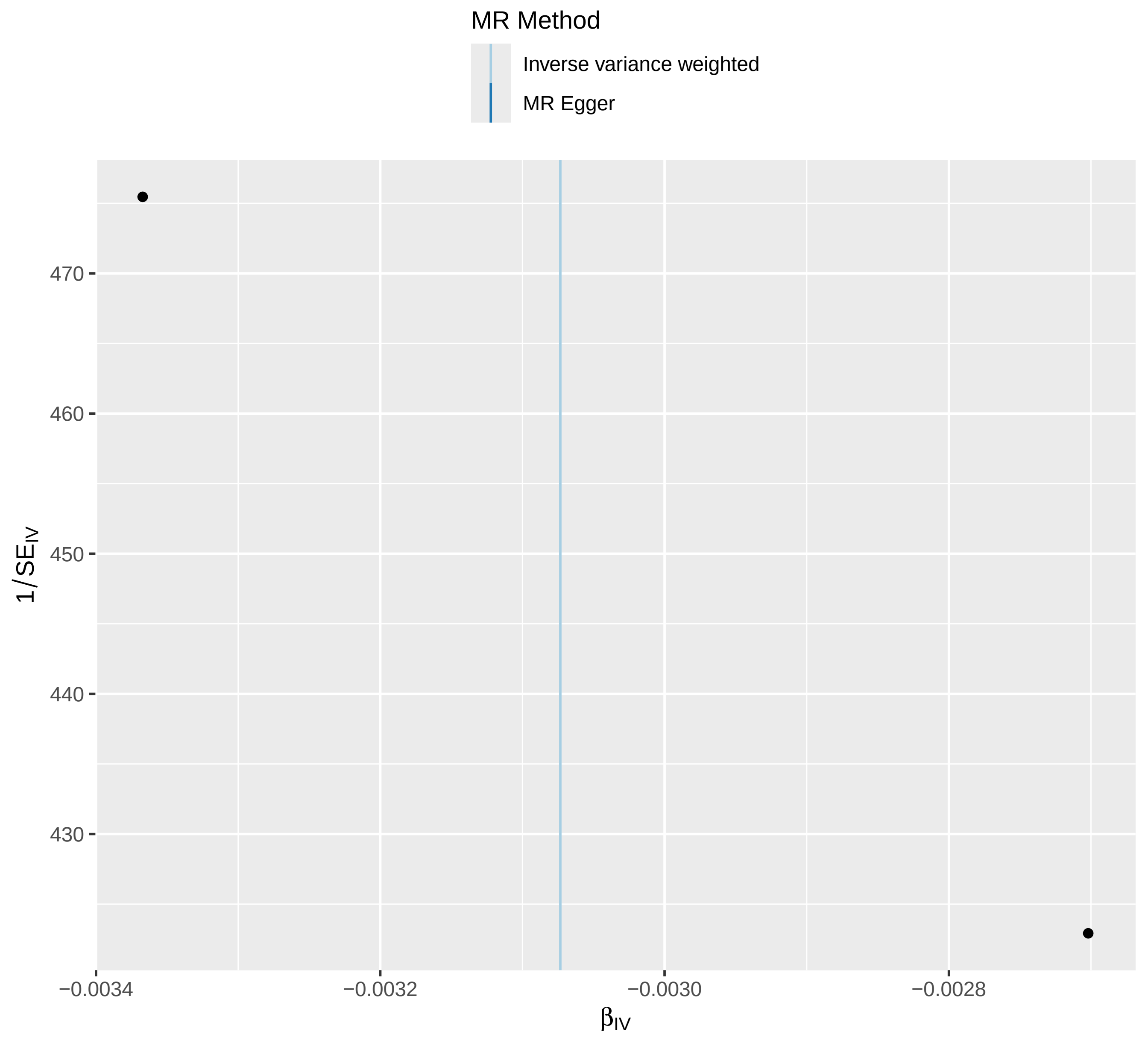
MR Method
Inverse variance weighted
MR Egger
470
460
450
440
430
1/SE_IV
−0.0034
−0.0032
−0.0030
−0.0028
β_IV

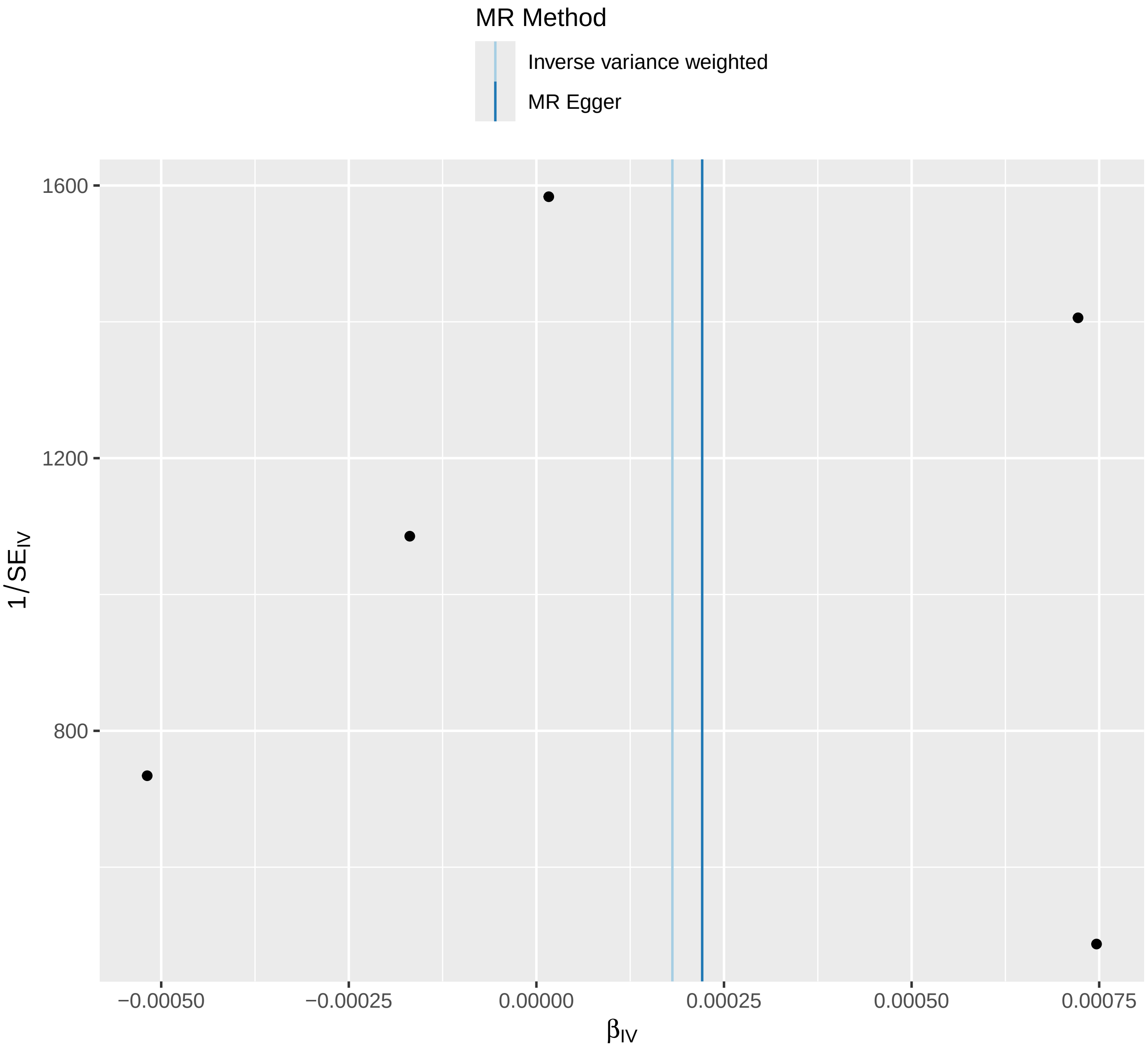

MR Method
Inverse variance weighted
MR Egger
1600
1200
800
−0.00050
−0.00025
0.00000
0.00025
0.00050
0.00075
$1/SE_{IV}$
β_{IV}

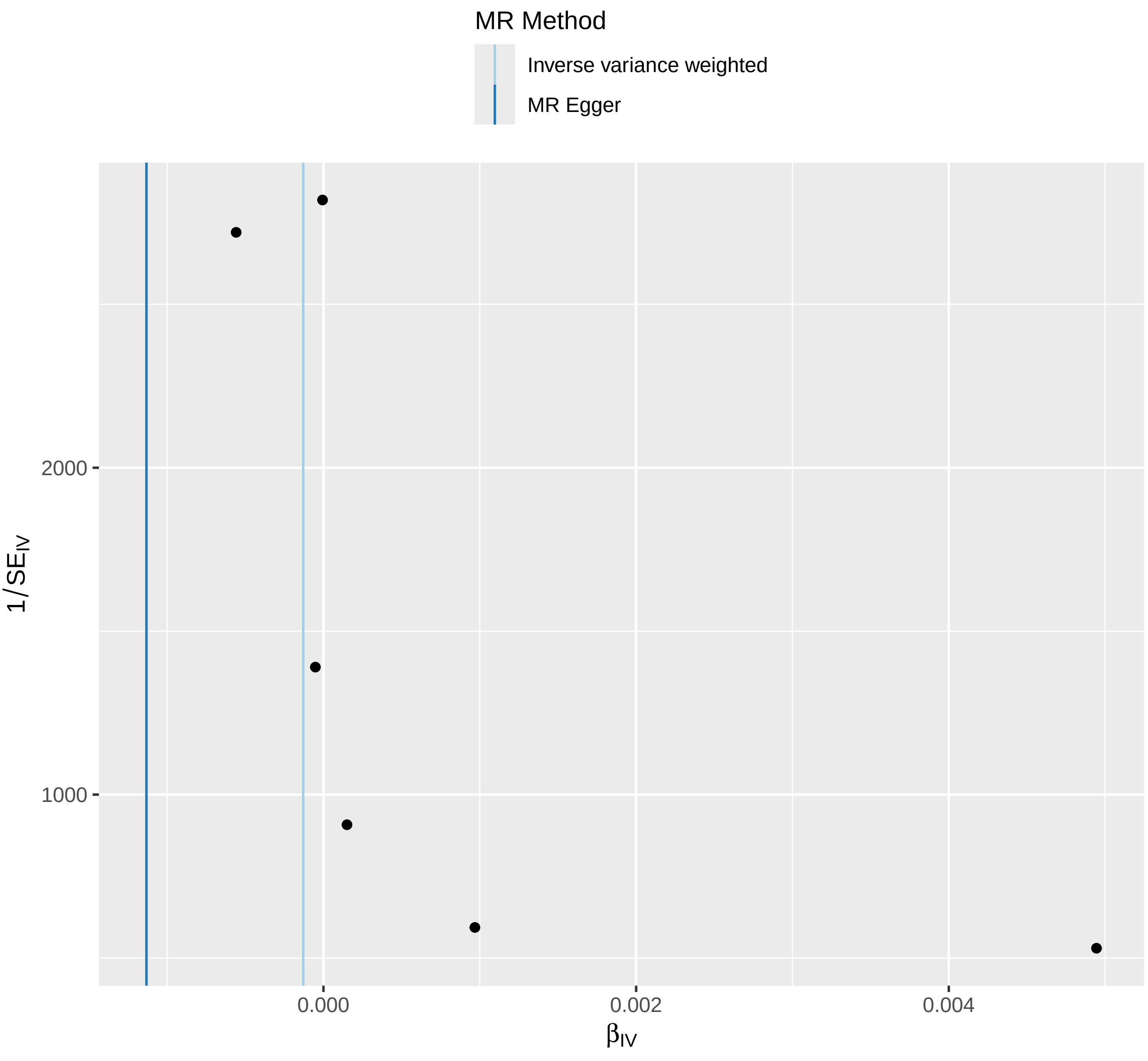

MR Method
Inverse variance weighted
MR Egger
2000
1000
1/SE$_{IV}$
0.000
0.002
0.004
β$_{IV}$

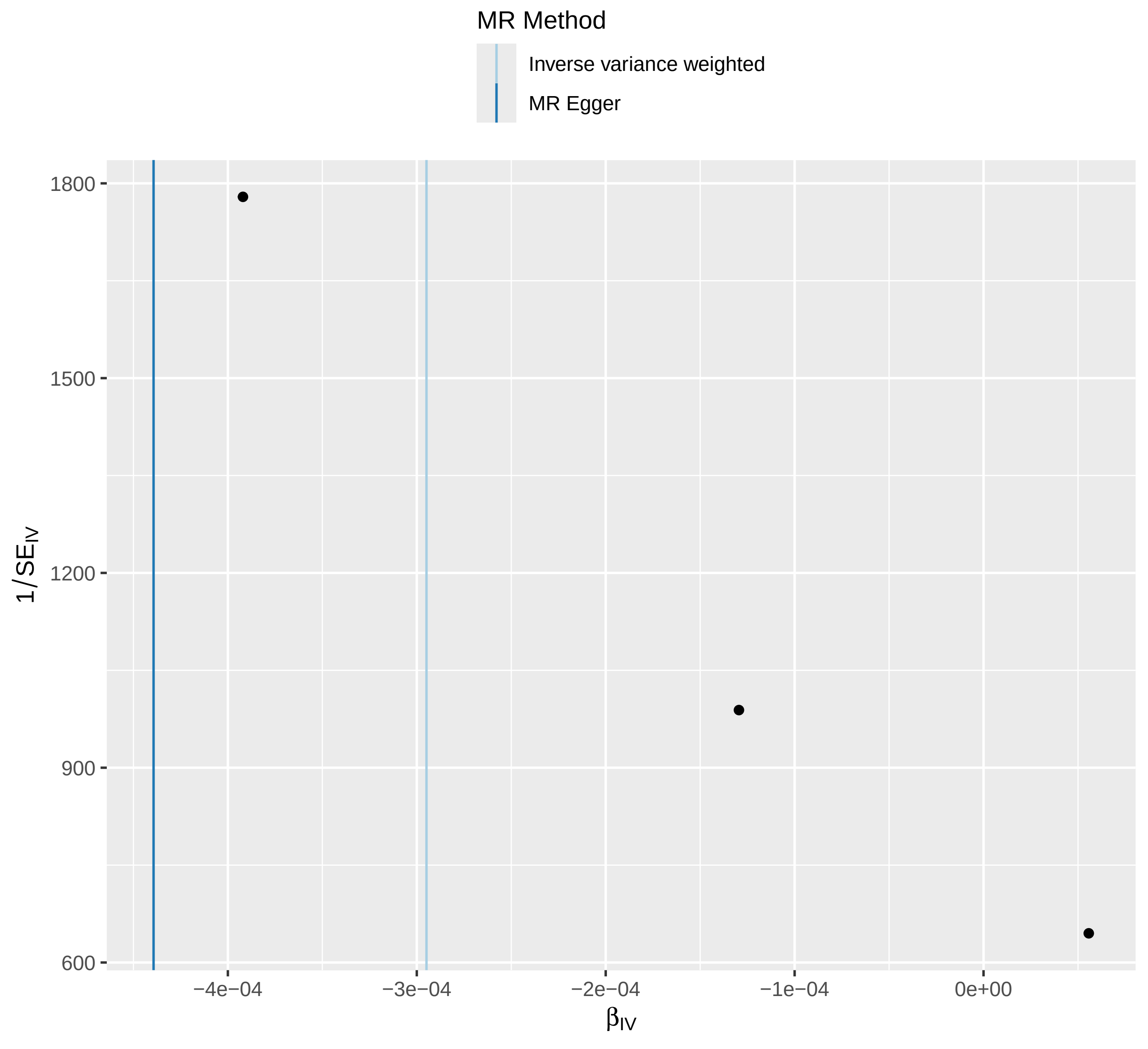
MR Method
Inverse variance weighted
MR Egger
1800
1500
1200
900
600
$1/SE_{IV}$
−4e−04
−3e−04
−2e−04
−1e−04
0e+00
β_{IV}

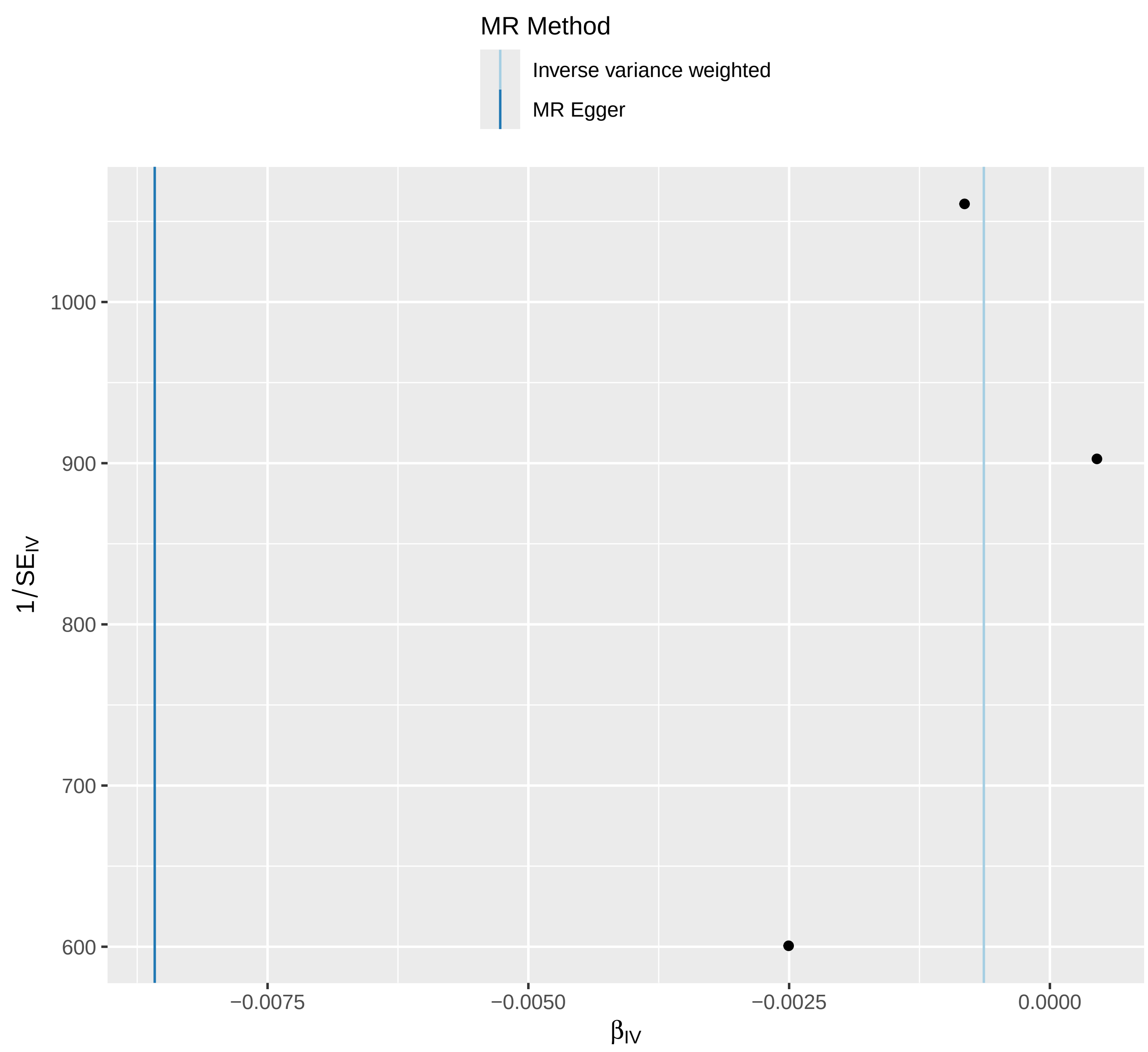
MR Method
Inverse variance weighted
MR Egger
1000
900
800
700
600
$1/SE_{IV}$
−0.0075
−0.0050
−0.0025
0.0000
β_{IV}

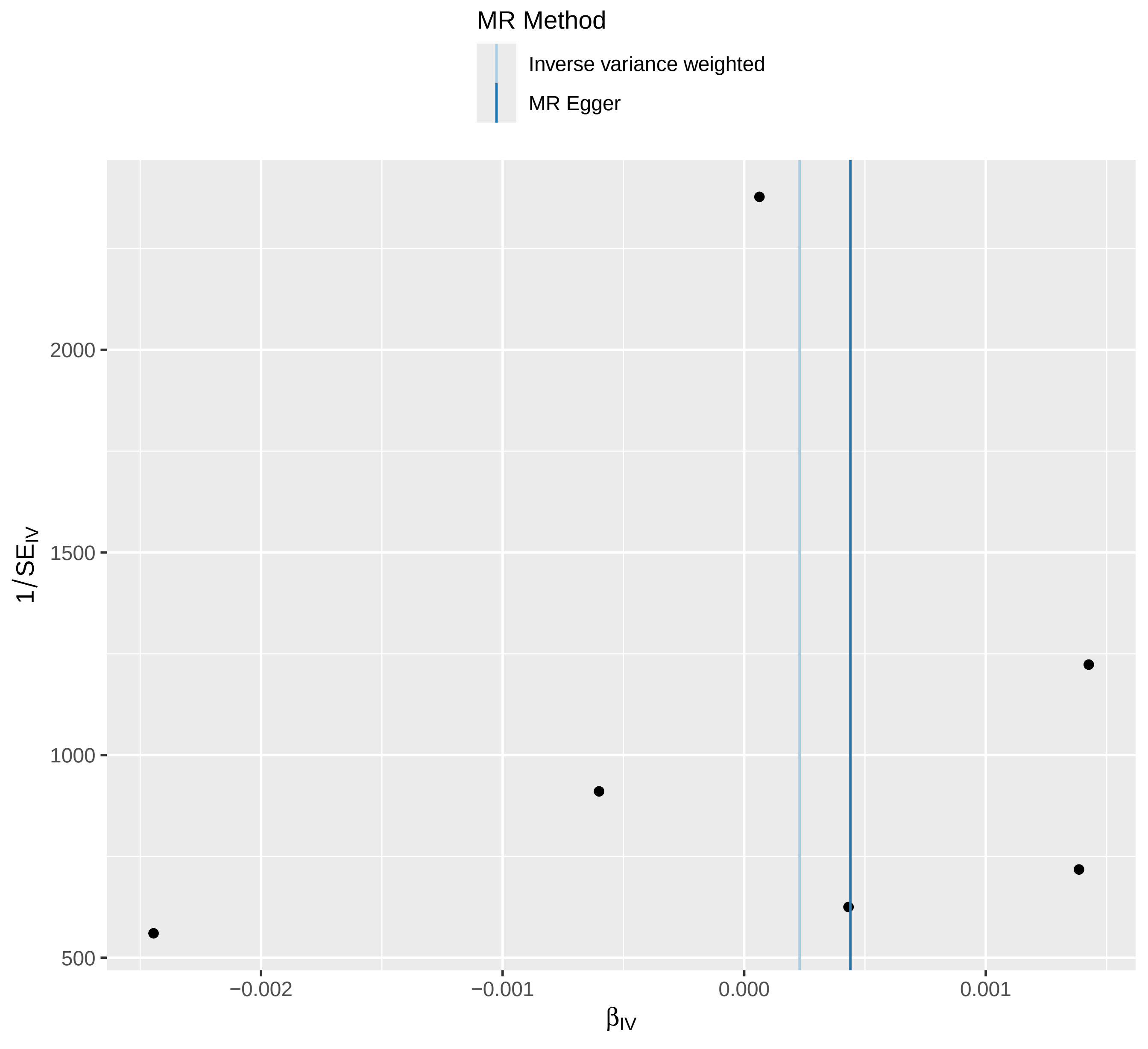
MR Method
Inverse variance weighted
MR Egger
1/SE_IV
2000
1500
1000
500
−0.002
−0.001
0.000
0.001
β_IV

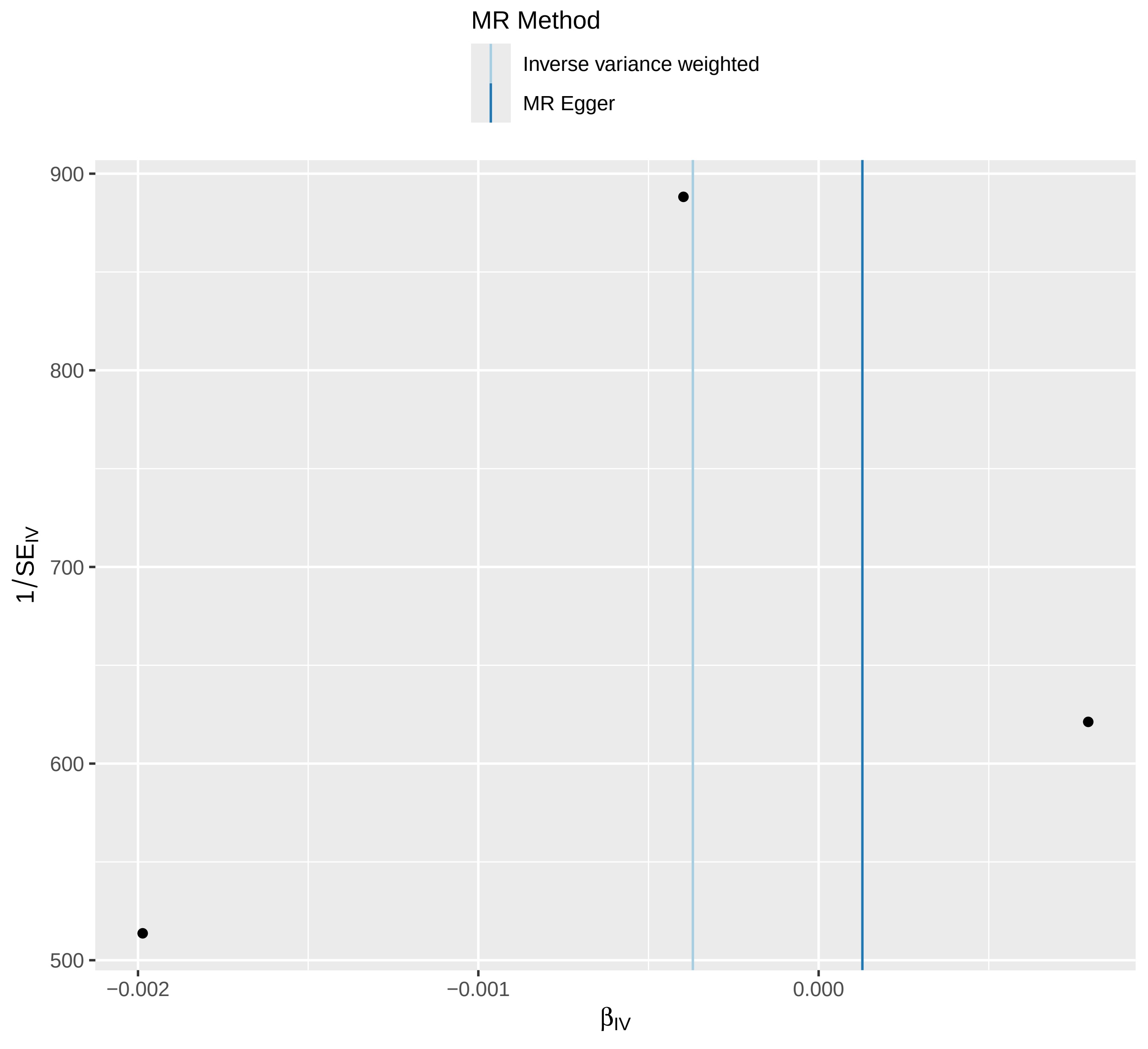
MR Method
Inverse variance weighted
MR Egger
900
800
700
600
500
1/SE_IV
−0.002
−0.001
0.000
β_IV

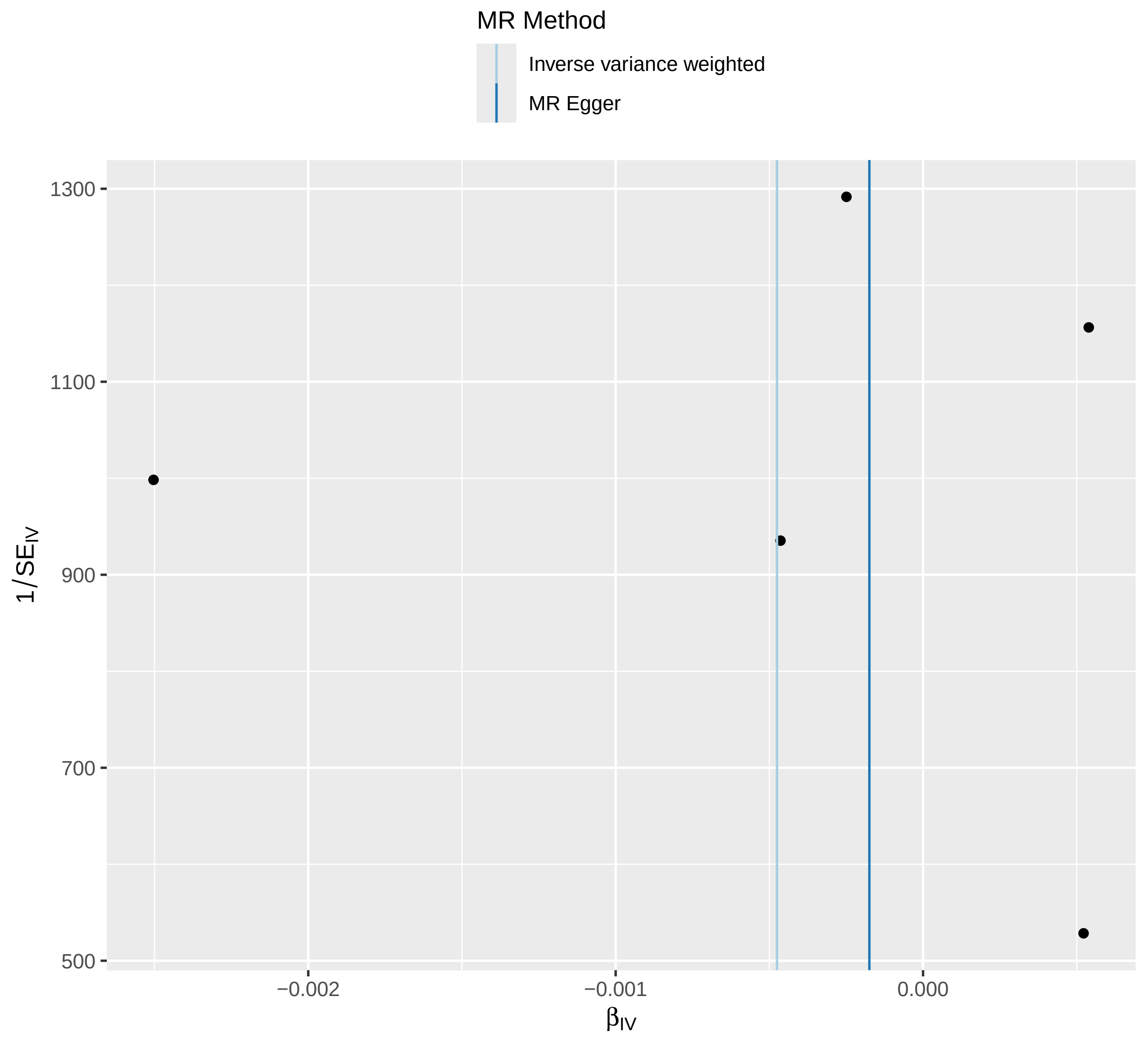

MR Method
Inverse variance weighted
MR Egger
1300
1100
900
700
500
−0.002
−0.001
0.000
$1/SE_{IV}$
β_{IV}

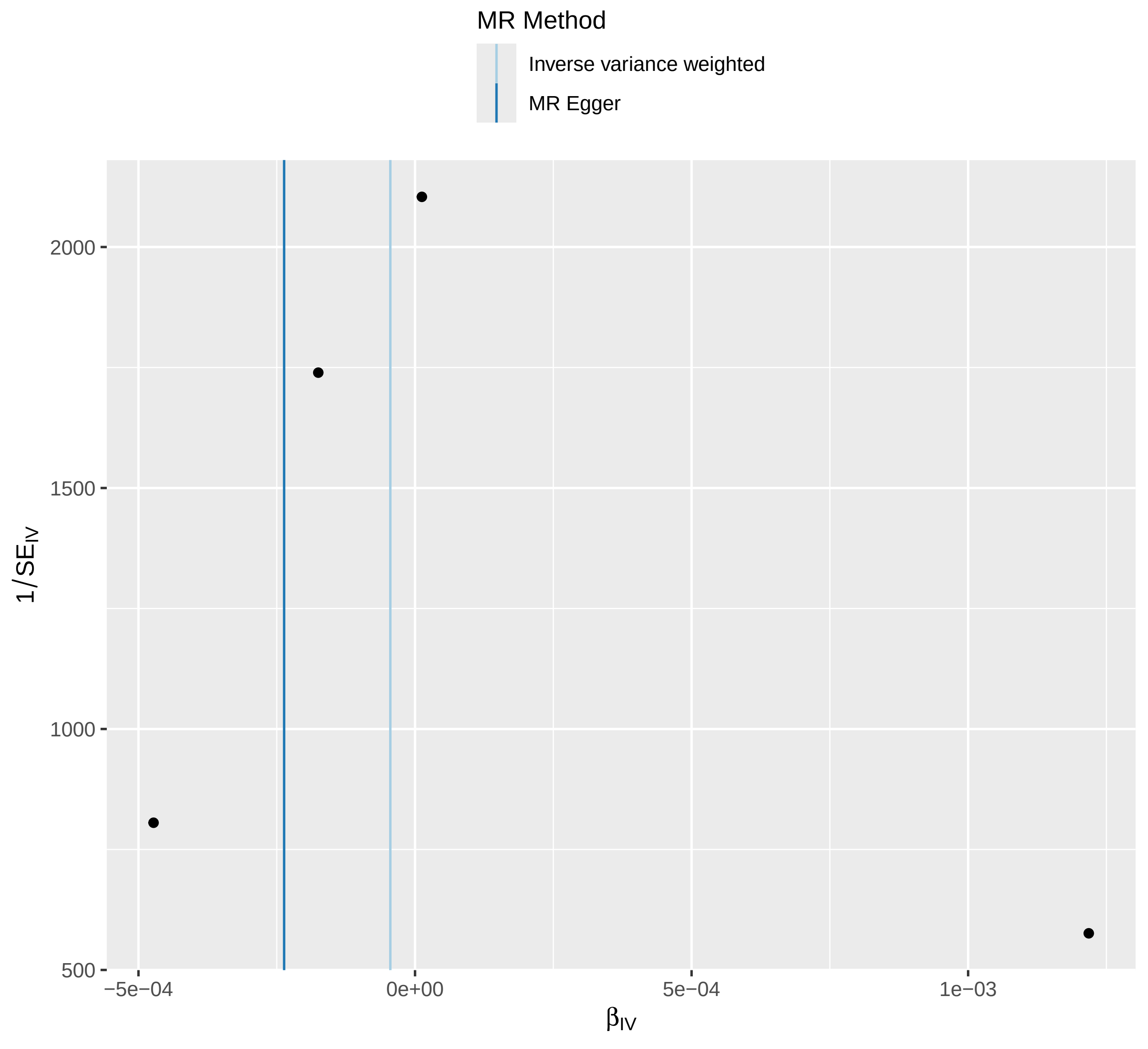
MR Method
Inverse variance weighted
MR Egger
2000
1500
1000
500
1/SE$_{IV}$
−5e−04
0e+00
5e−04
1e−03
β$_{IV}$

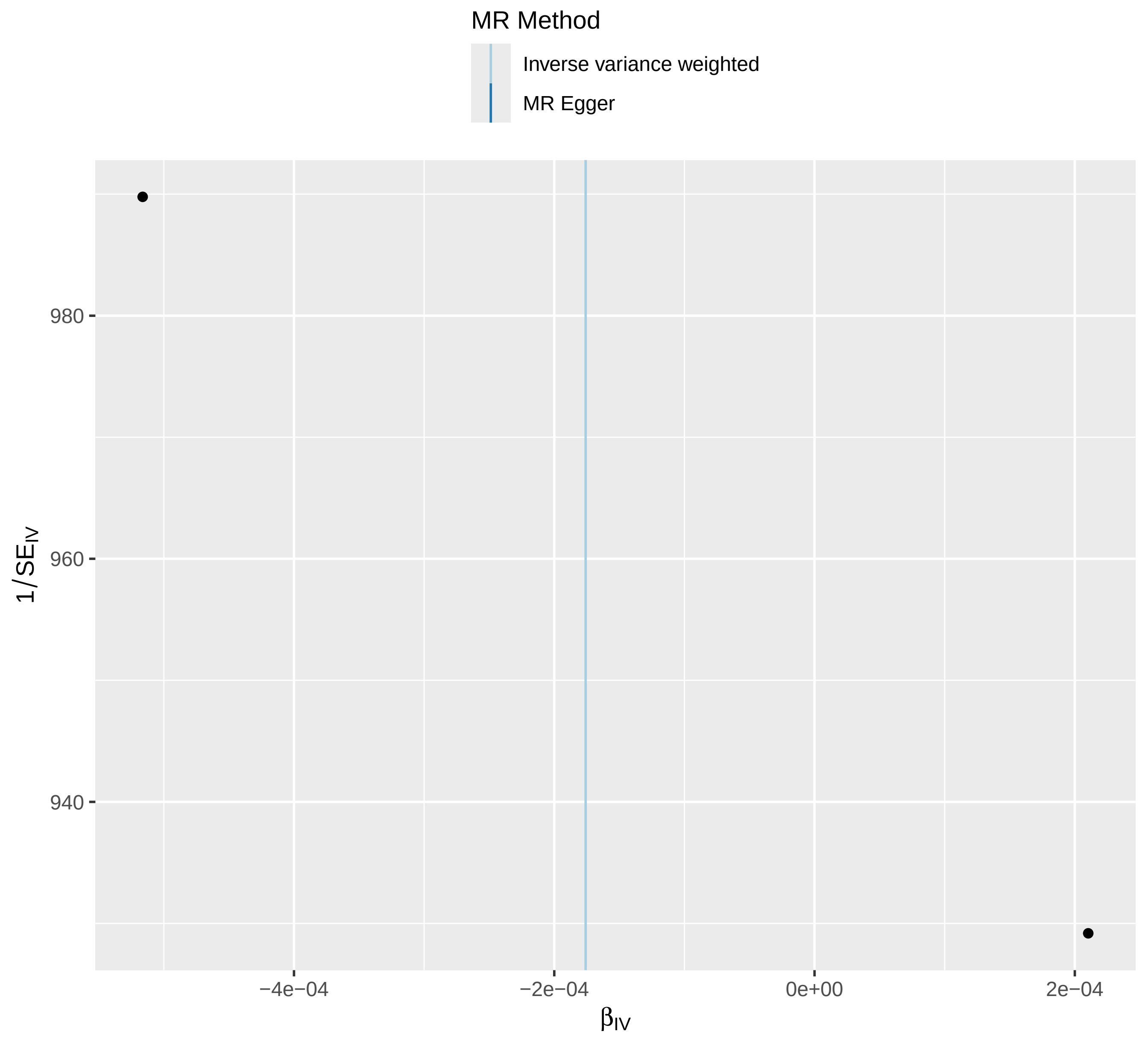

MR Method
Inverse variance weighted
MR Egger
980
960
940
1/SE_IV
−4e−04
−2e−04
0e+00
2e−04
β_IV

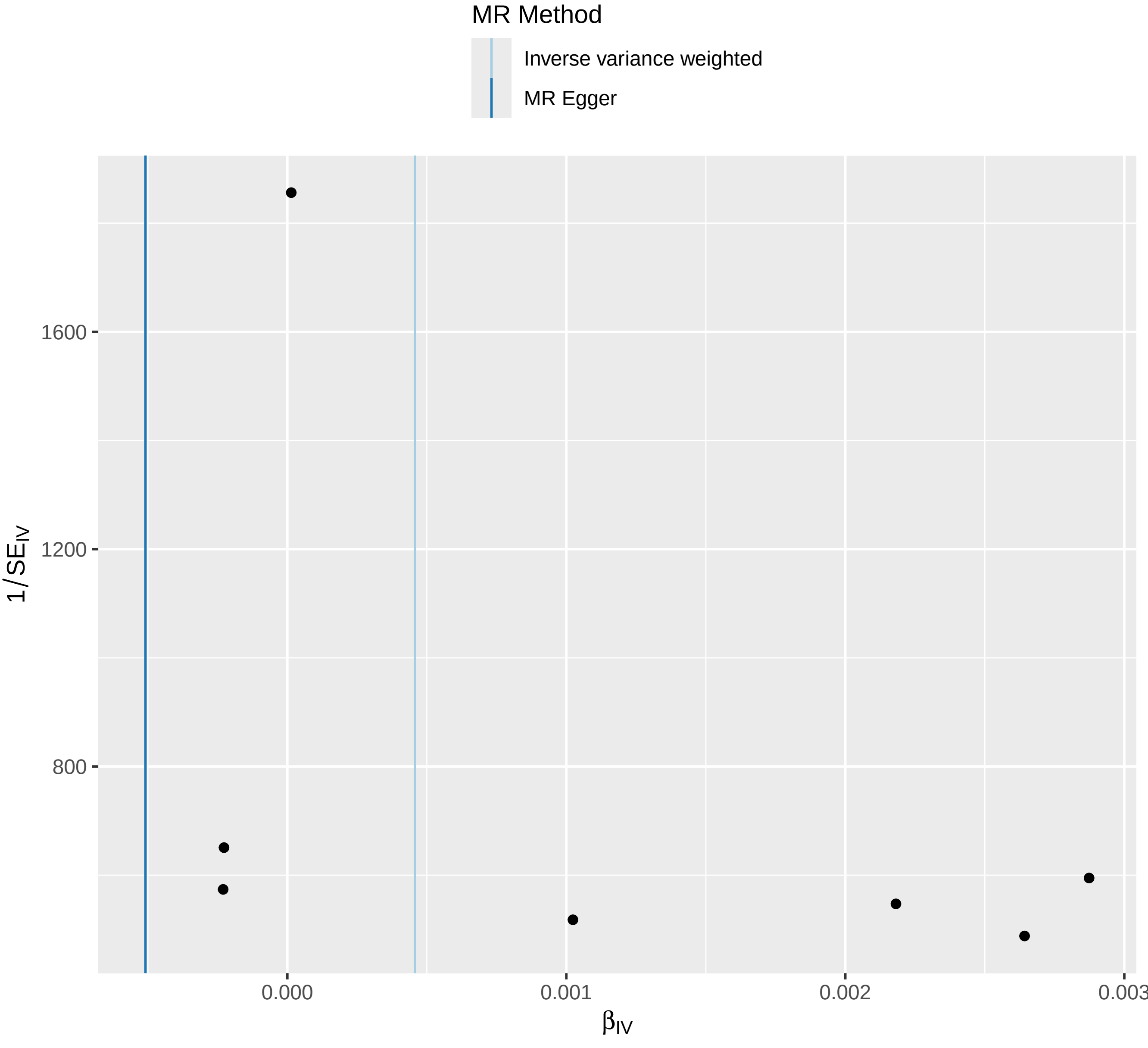
MR Method
Inverse variance weighted
MR Egger
1600
1200
800
1/SE$_{IV}$
0.000
0.001
0.002
0.003
β$_{IV}$

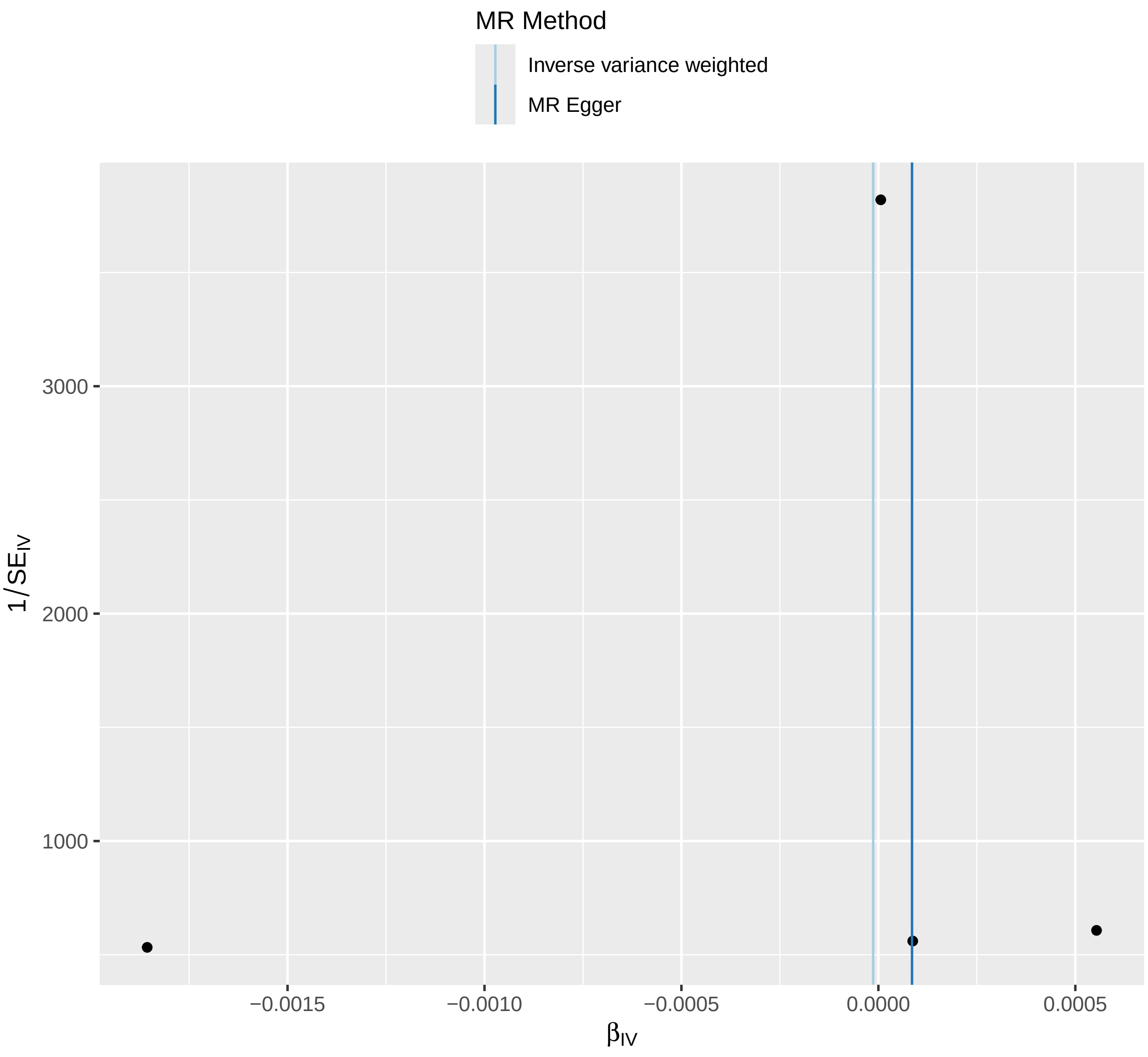

MR Method
Inverse variance weighted
MR Egger
3000
2000
1000
1/SE_IV
−0.0015
−0.0010
−0.0005
0.0000
0.0005
β_IV

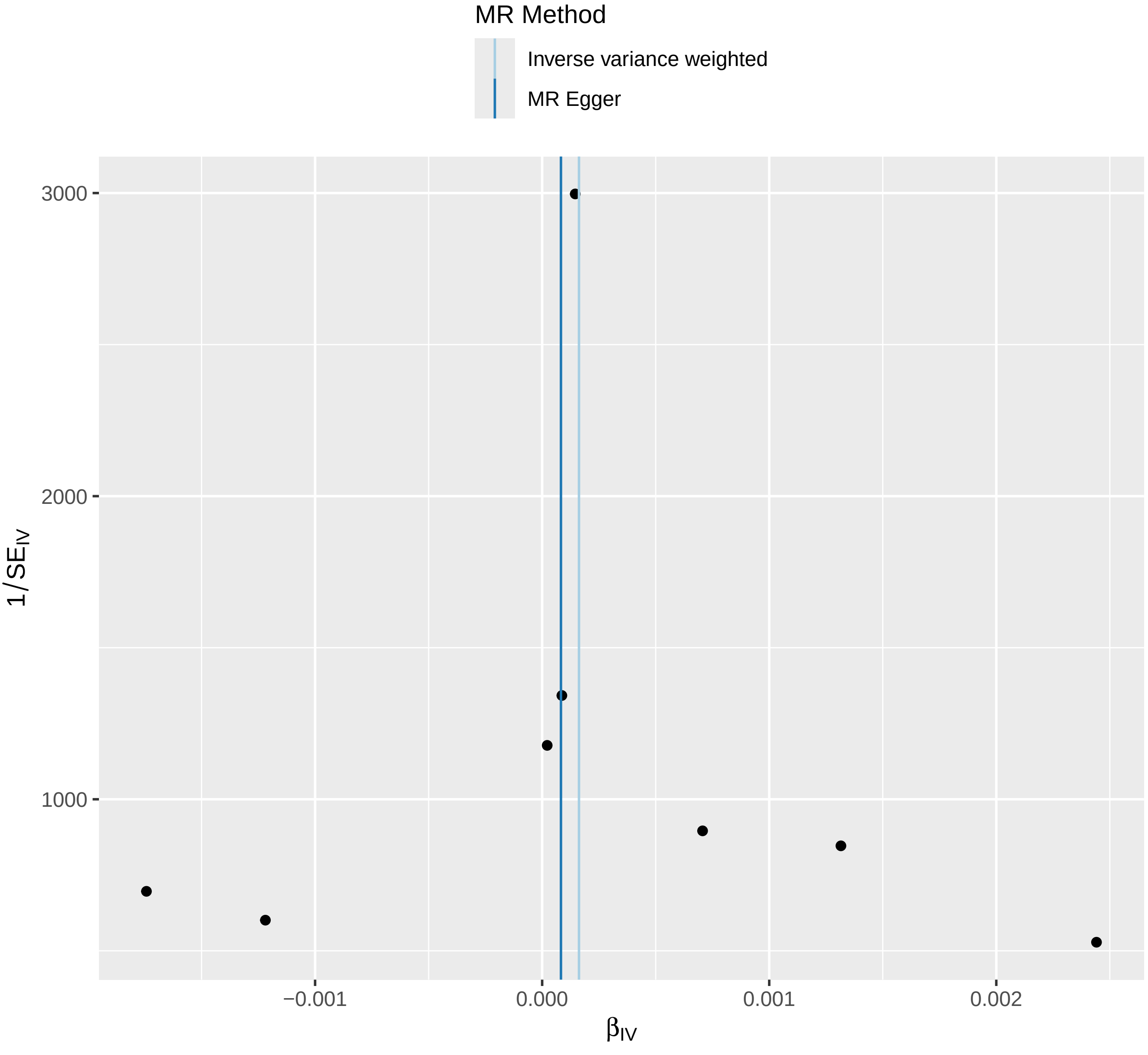

MR Method
Inverse variance weighted
MR Egger
3000
2000
1000
1/SE$_{IV}$
−0.001
0.000
0.001
0.002
β$_{IV}$

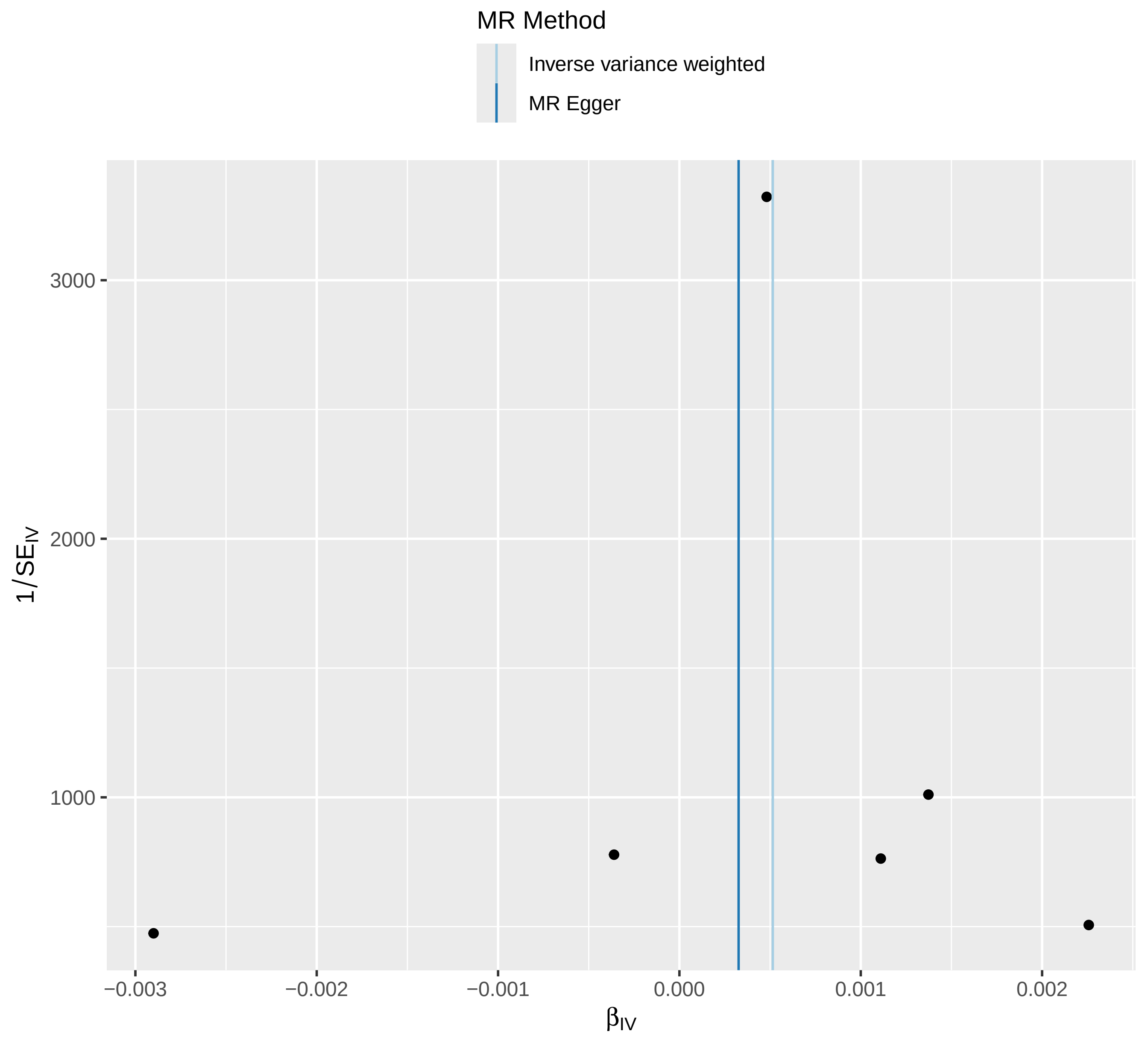
MR Method
Inverse variance weighted
MR Egger
3000
2000
1000
1/SE$_{IV}$
−0.003
−0.002
−0.001
0.000
0.001
0.002
β_{IV}

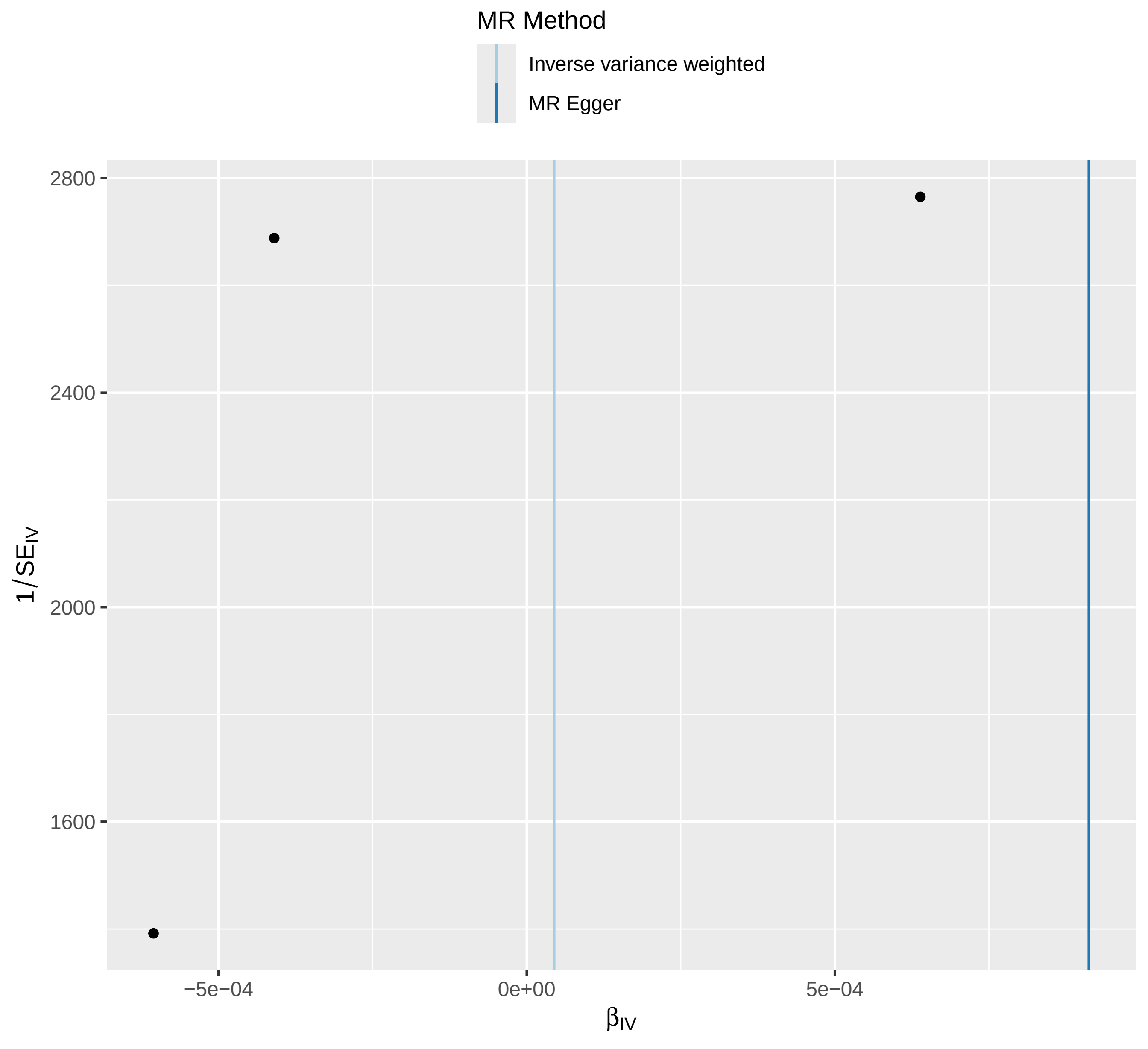
MR Method
Inverse variance weighted
MR Egger
2800
2400
2000
1600
1/SE_IV
−5e−04
0e+00
5e−04
β_IV

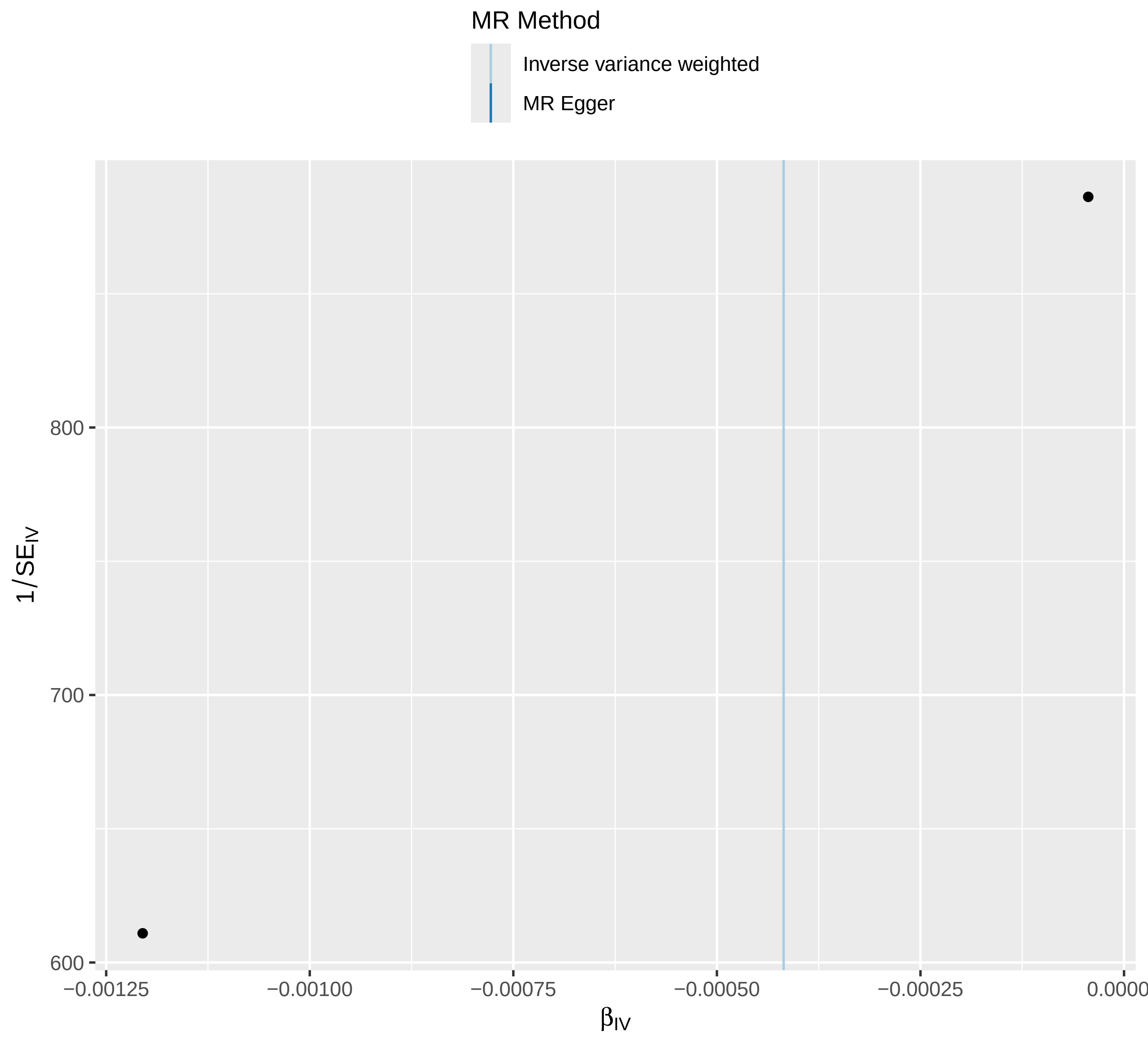

MR Method
Inverse variance weighted
MR Egger
800
700
600
1/SE_IV
−0.00125
−0.00100
−0.00075
−0.00050
−0.00025
0.0000
β_IV

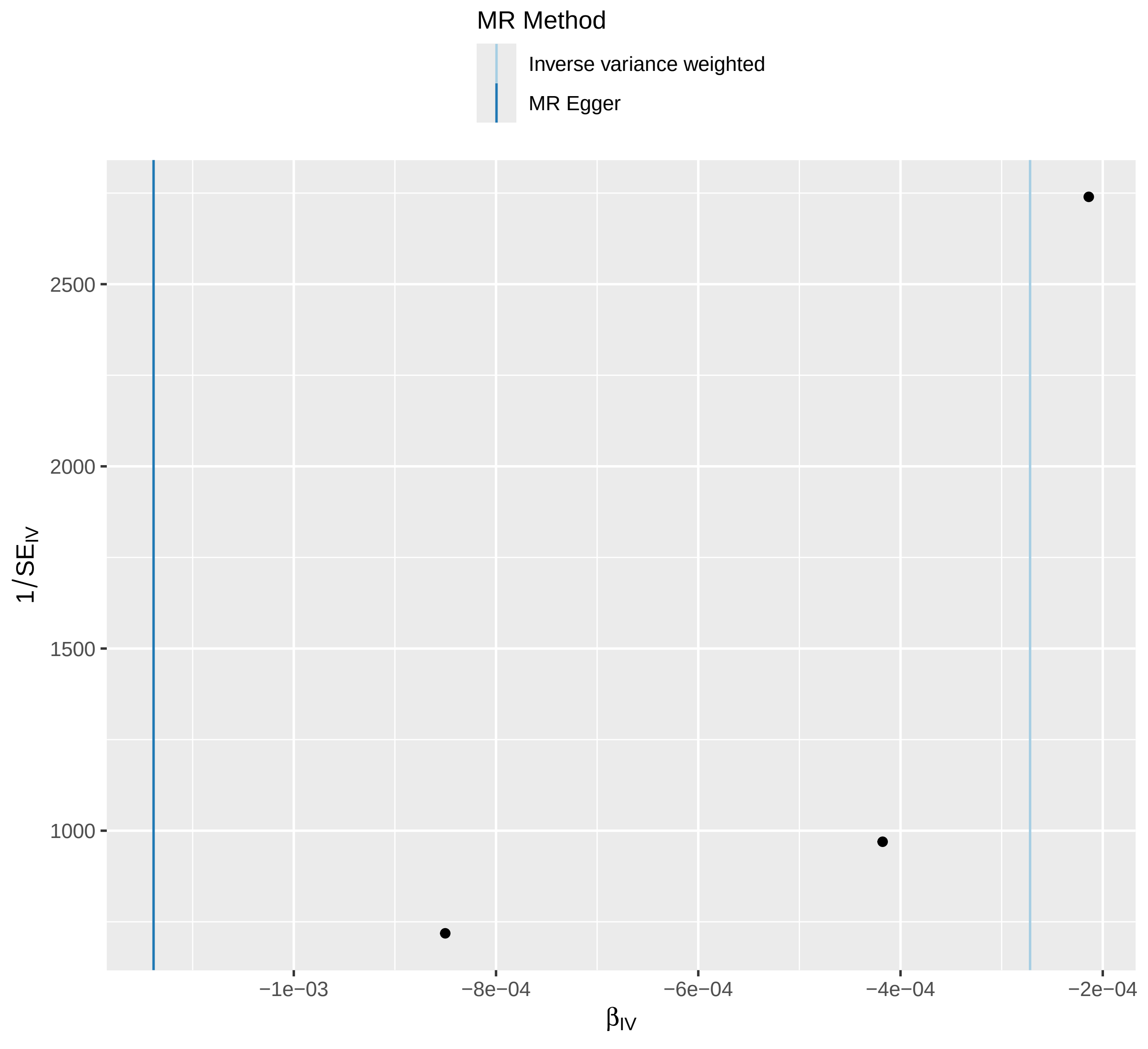
MR Method
Inverse variance weighted
MR Egger
2500
2000
1500
1000
−1e−03
−8e−04
−6e−04
−4e−04
−2e−04
1/SE$_{IV}$
β$_{IV}$

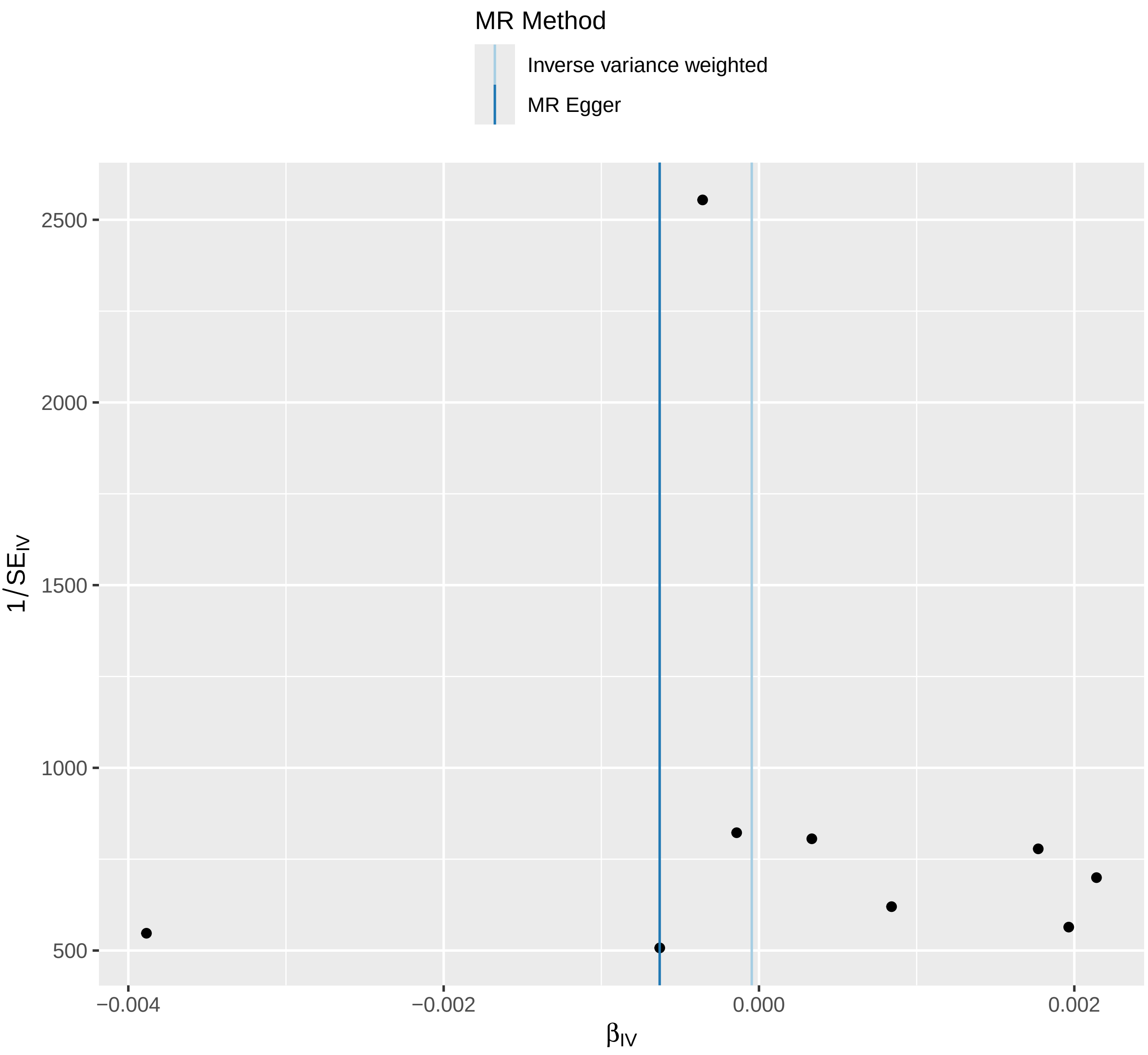
MR Method
Inverse variance weighted
MR Egger
2500
2000
1500
1000
500
$1/SE_{IV}$
−0.004
−0.002
0.000
0.002
β_{IV}

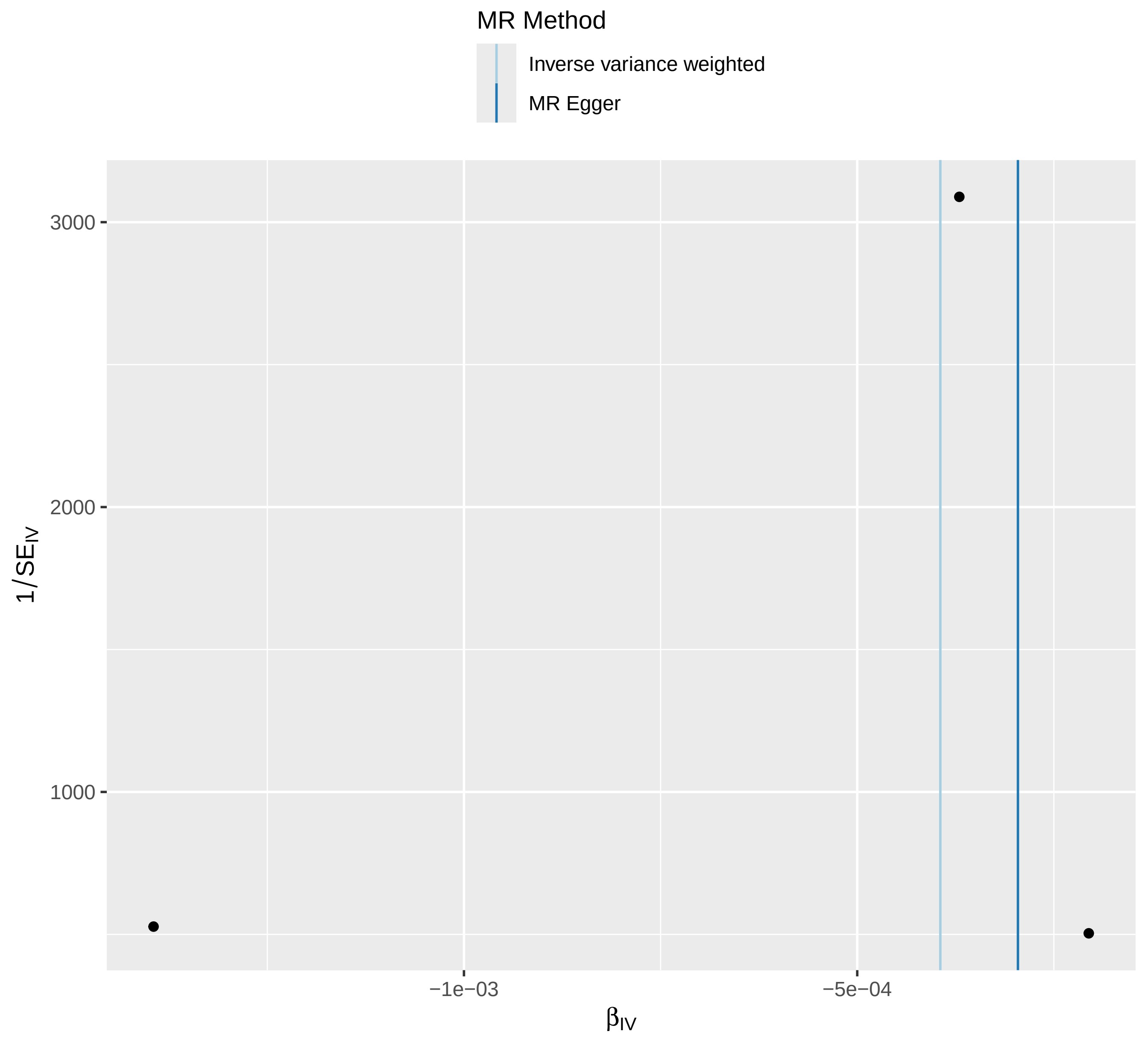

MR Method
Inverse variance weighted
MR Egger
3000
2000
1000
1/SE_IV
−1e−03
−5e−04
β_IV

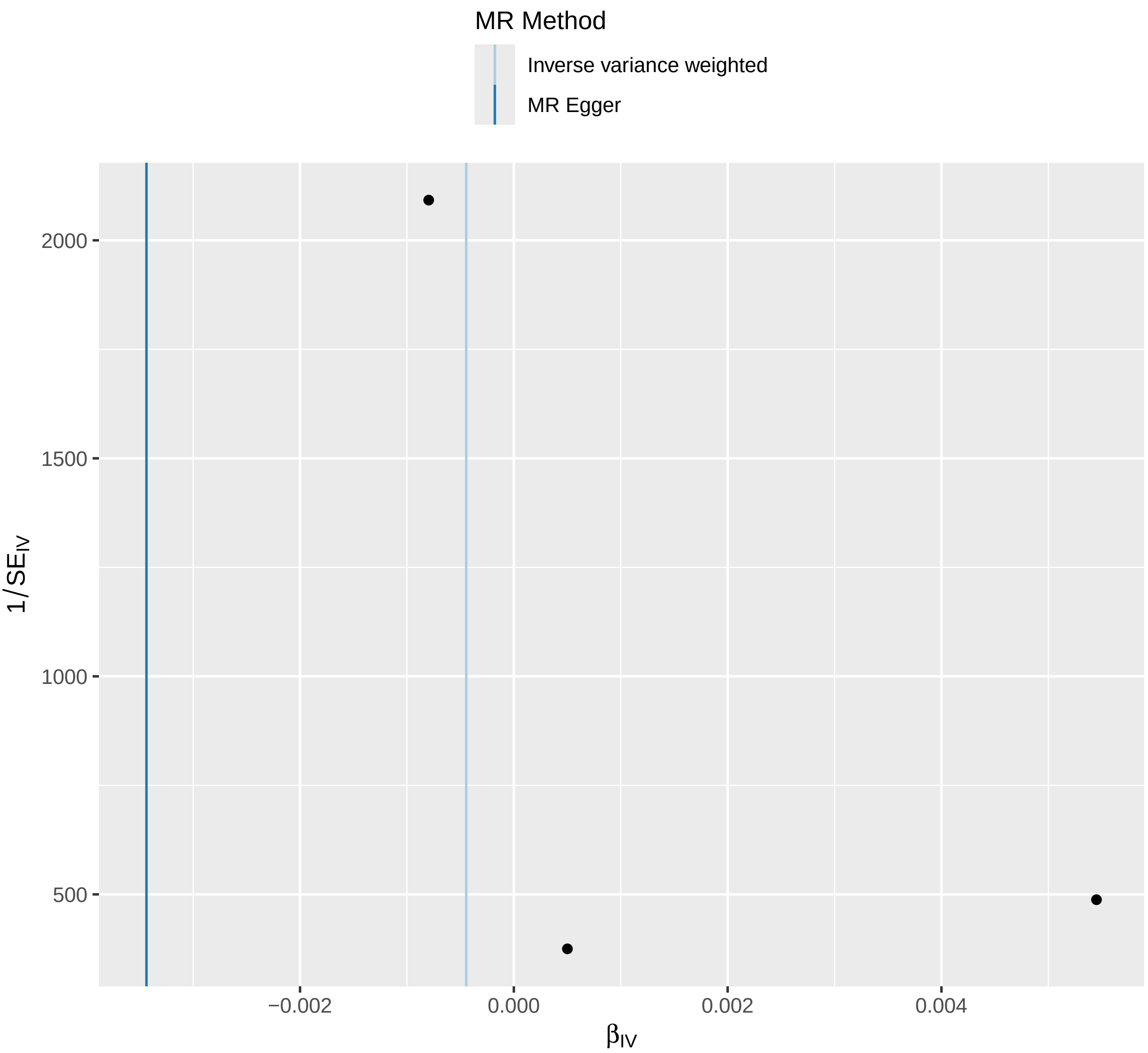
MR Method
Inverse variance weighted
MR Egger
2000
1500
1000
500
1/SE$_{IV}$
−0.002
0.000
0.002
0.004
β$_{IV}$

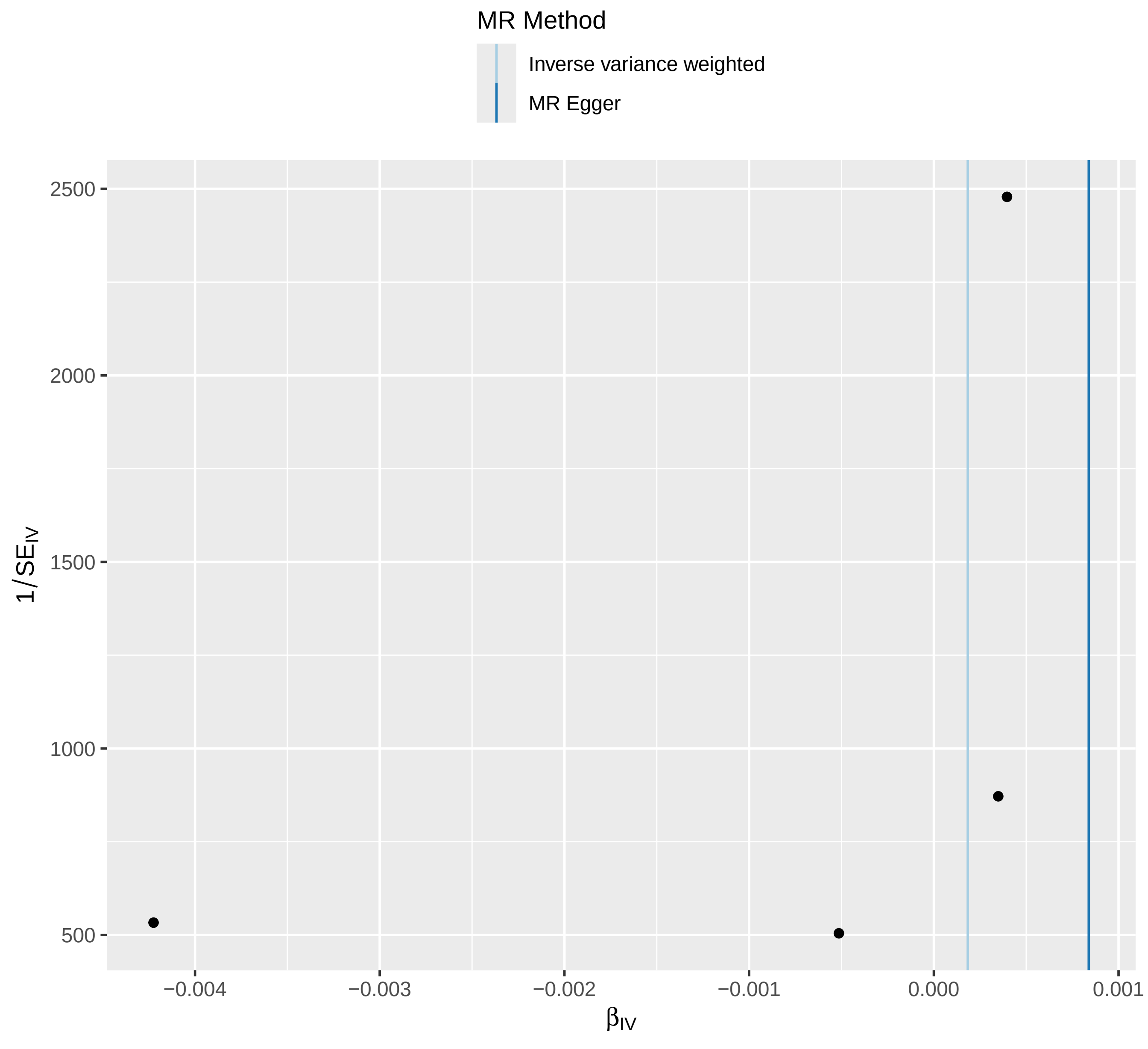
MR Method
Inverse variance weighted
MR Egger
2500
2000
1500
1000
500
1/SE_IV
−0.004
−0.003
−0.002
−0.001
0.000
0.001
β_IV

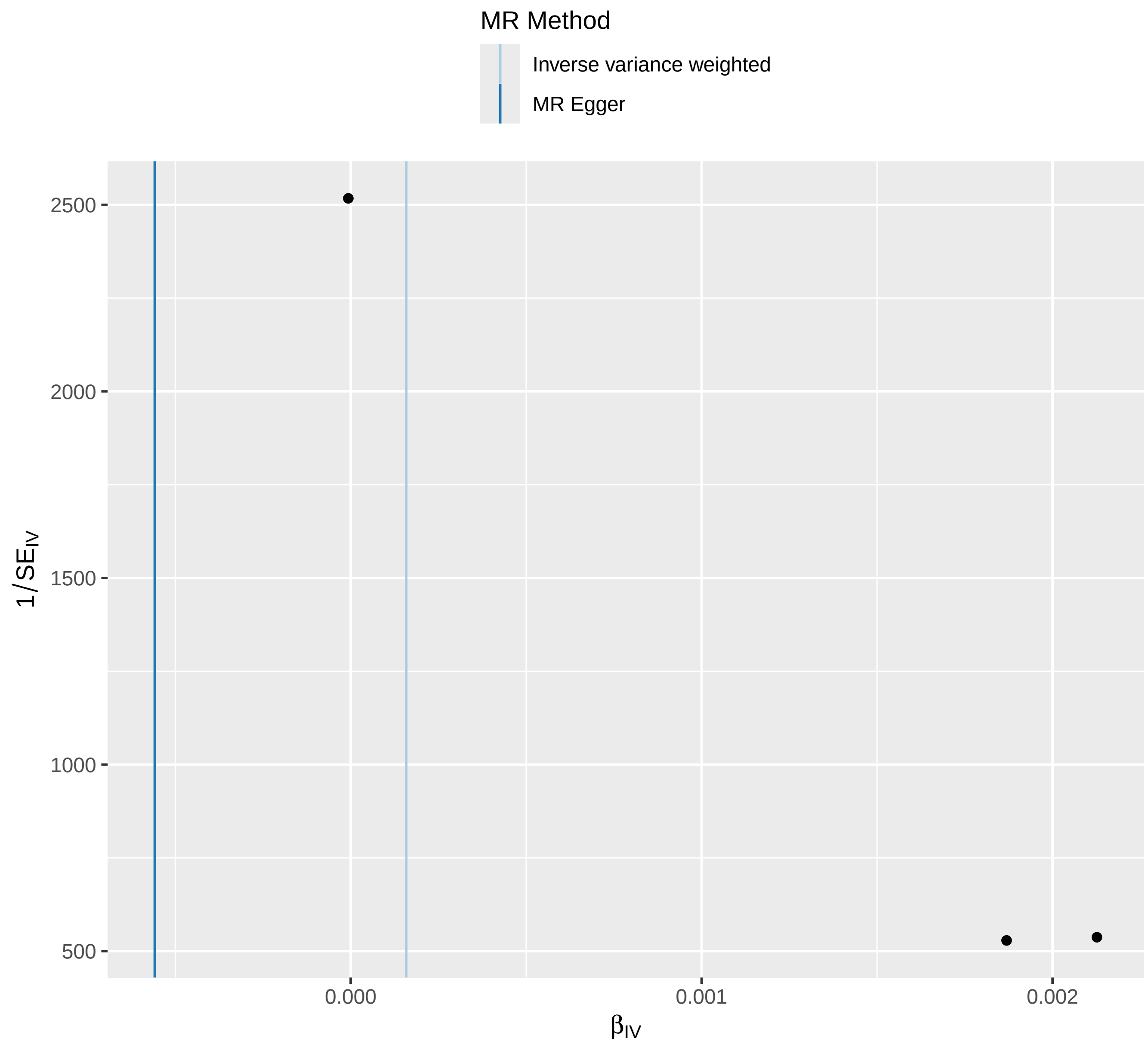
MR Method
Inverse variance weighted
MR Egger
2500
2000
1500
1000
500
1/SE_IV
0.000
0.001
0.002
β_IV

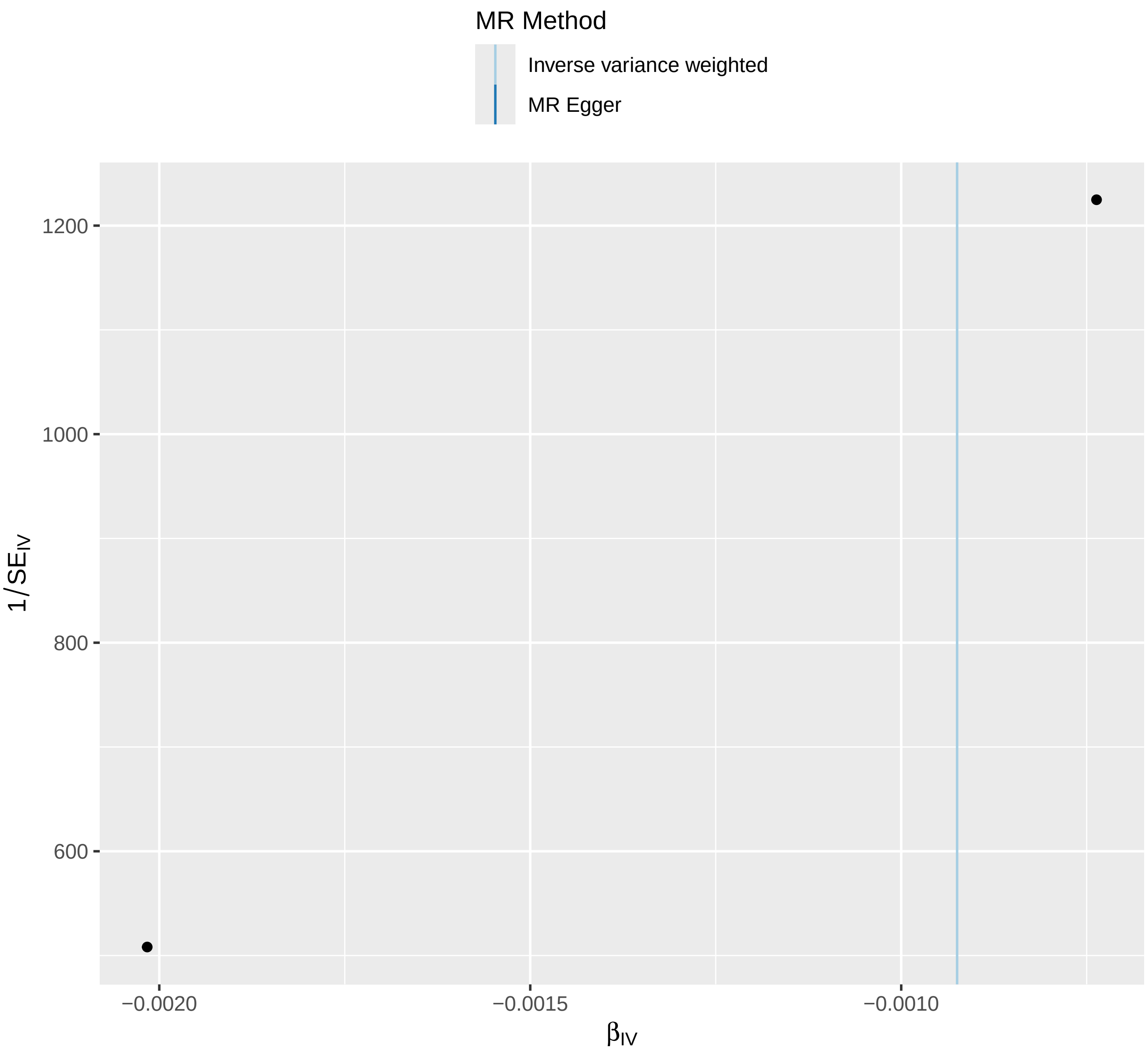

MR Method
Inverse variance weighted
MR Egger
1200
1000
800
600
−0.0020
−0.0015
−0.0010
β_{IV}
$1/SE_{IV}$

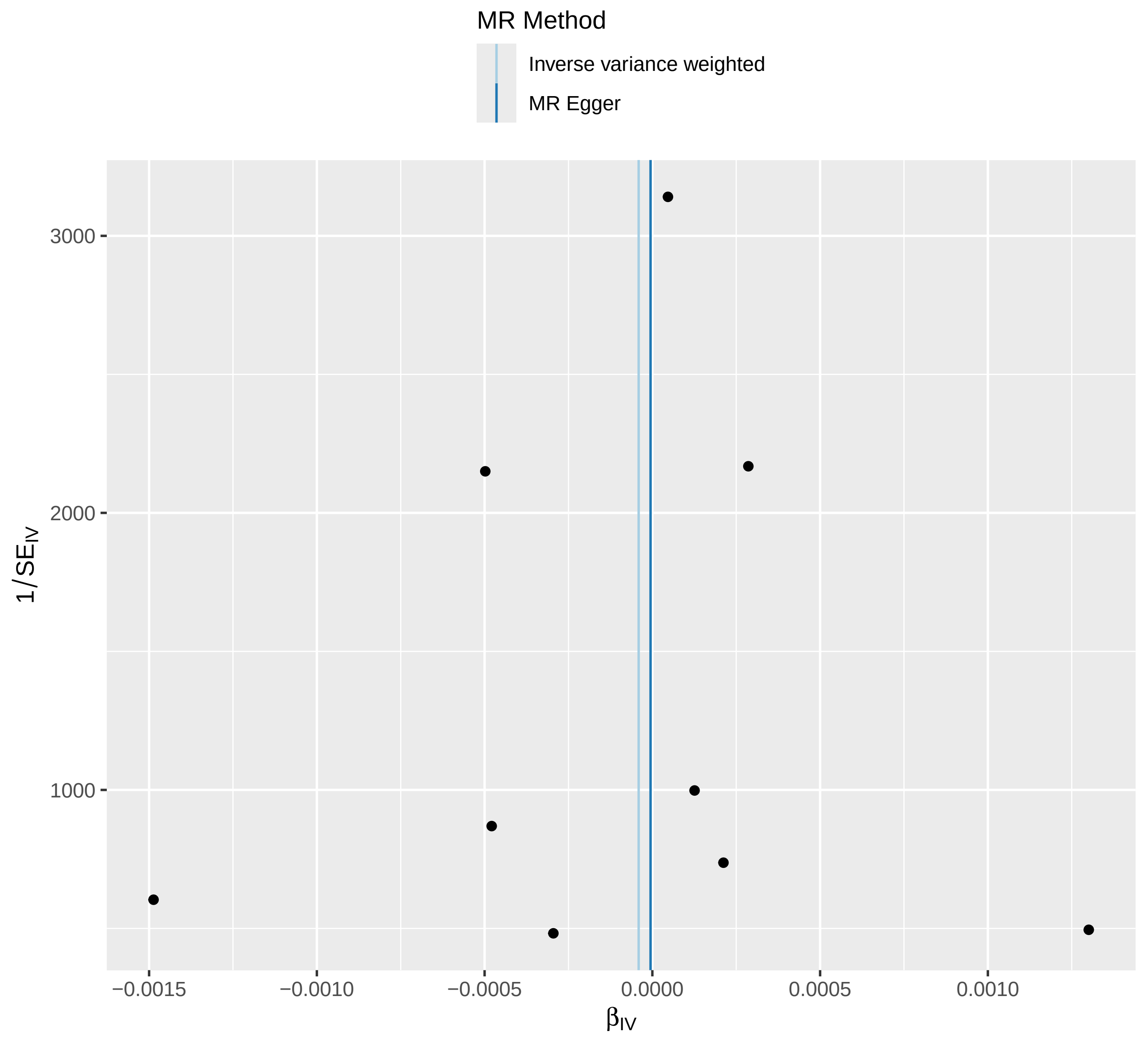
MR Method
Inverse variance weighted
MR Egger
3000
2000
1000
1/SE_IV
−0.0015
−0.0010
−0.0005
0.0000
0.0005
0.0010
β_IV

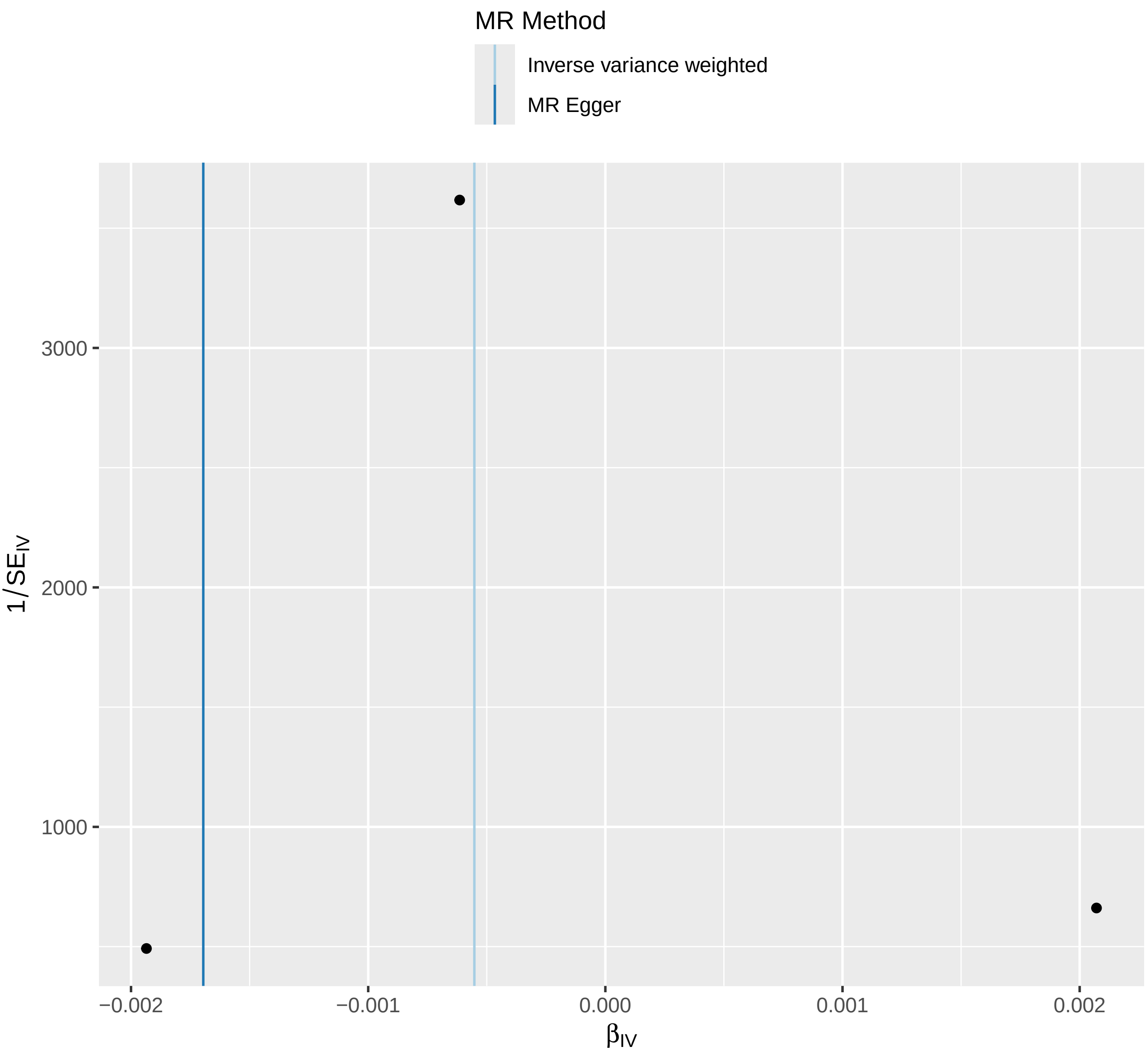

MR Method
Inverse variance weighted
MR Egger
3000
2000
1000
1/SE_IV
−0.002
−0.001
0.000
0.001
0.002
β_IV

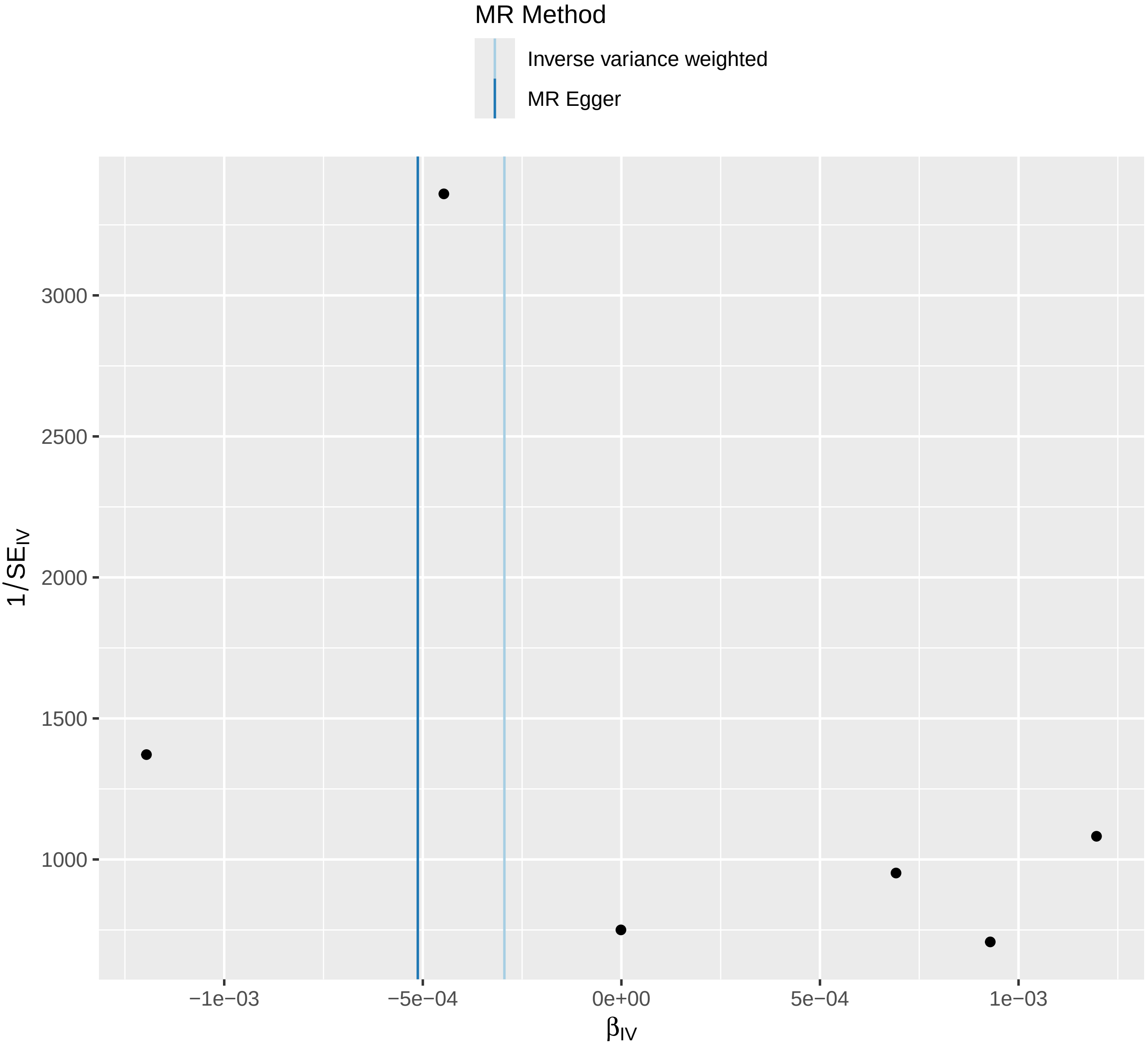

MR Method
Inverse variance weighted
MR Egger
3000
2500
2000
1500
1000
1/SE_IV
−1e−03
−5e−04
0e+00
5e−04
1e−03
β_IV

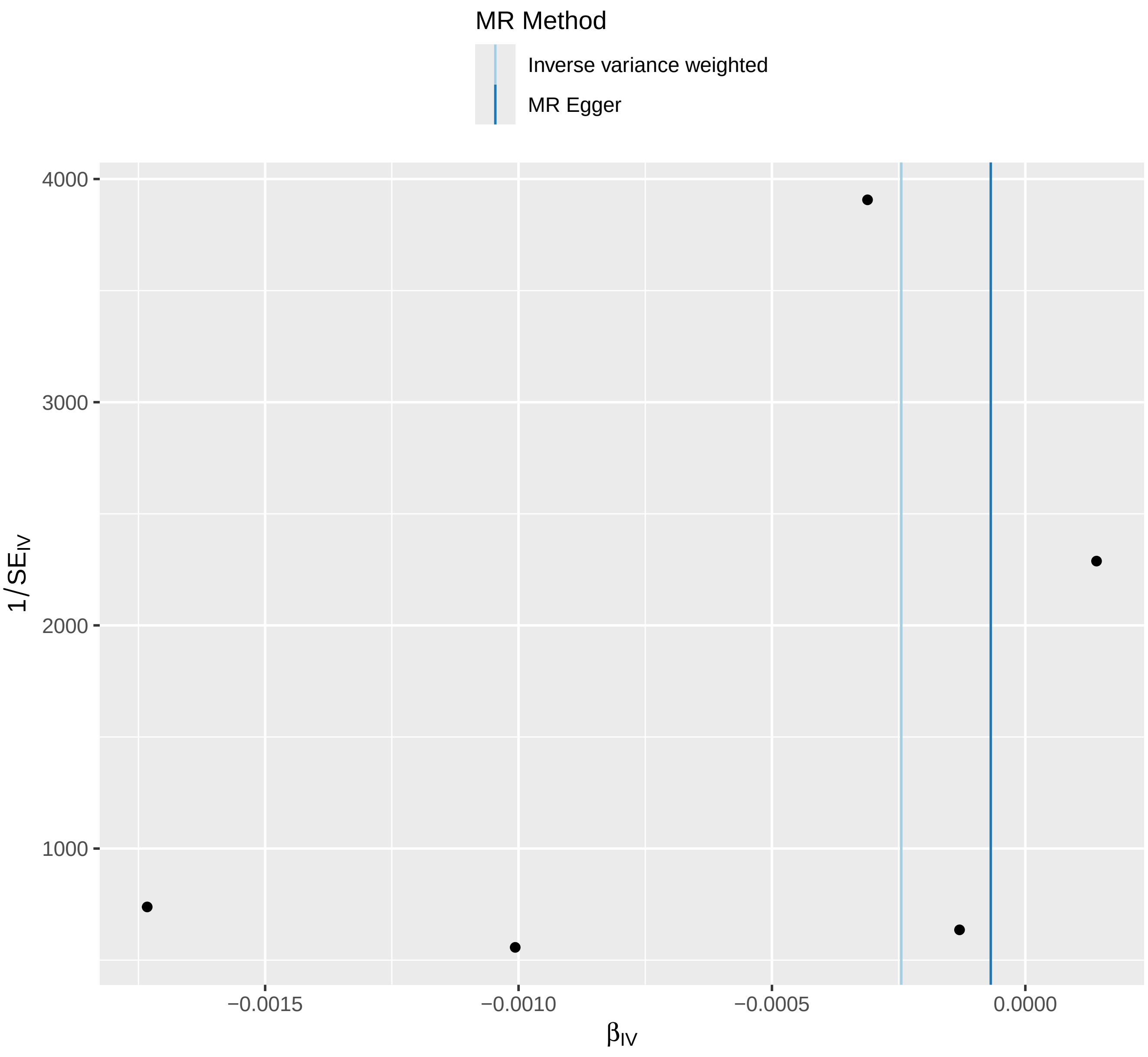
MR Method
Inverse variance weighted
MR Egger
4000
3000
2000
1000
1/SE_IV
−0.0015
−0.0010
−0.0005
0.0000
β_IV

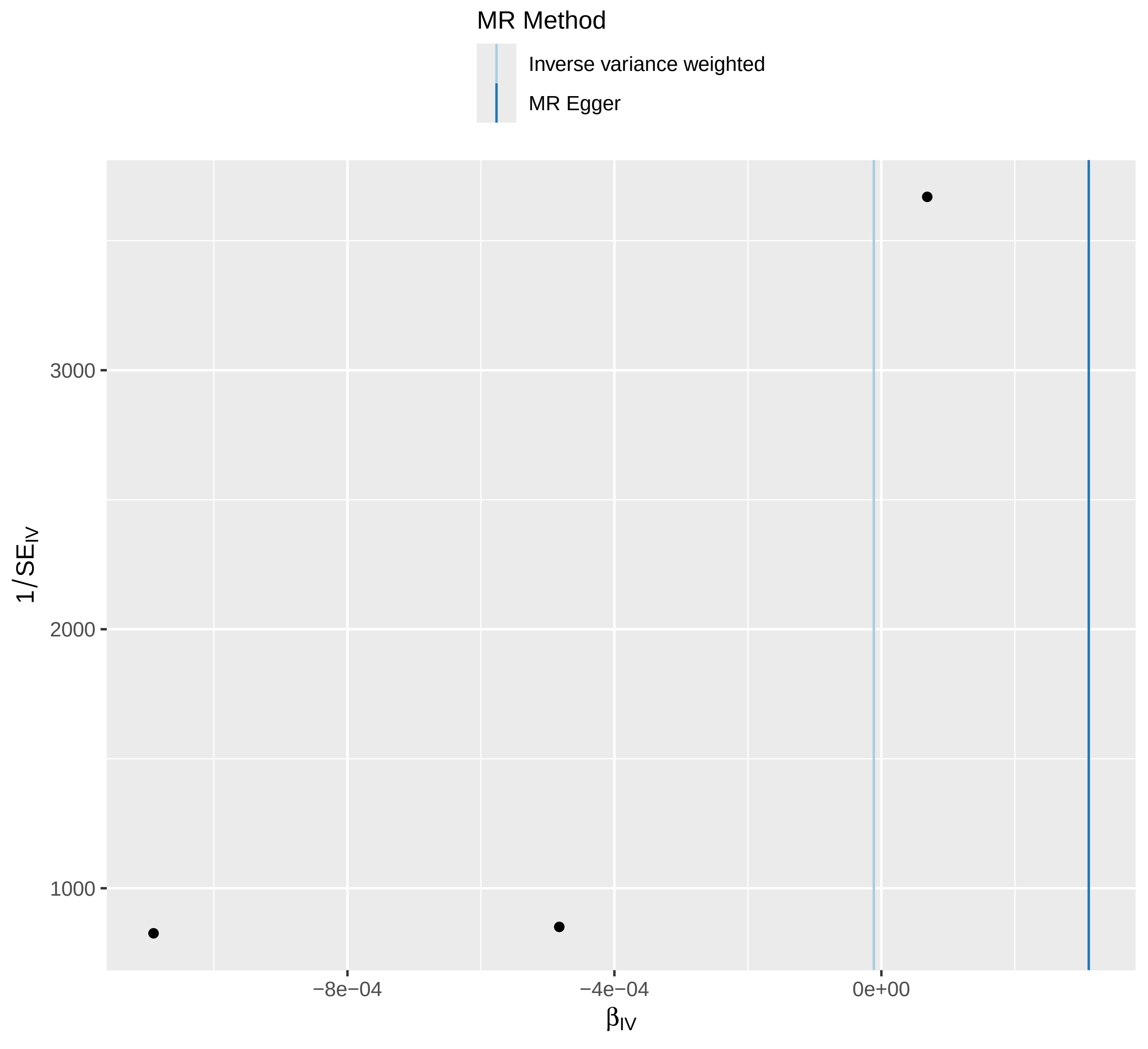

MR Method
Inverse variance weighted
MR Egger
3000
2000
1000
1/SE_IV
−8e−04
−4e−04
0e+00
β_IV

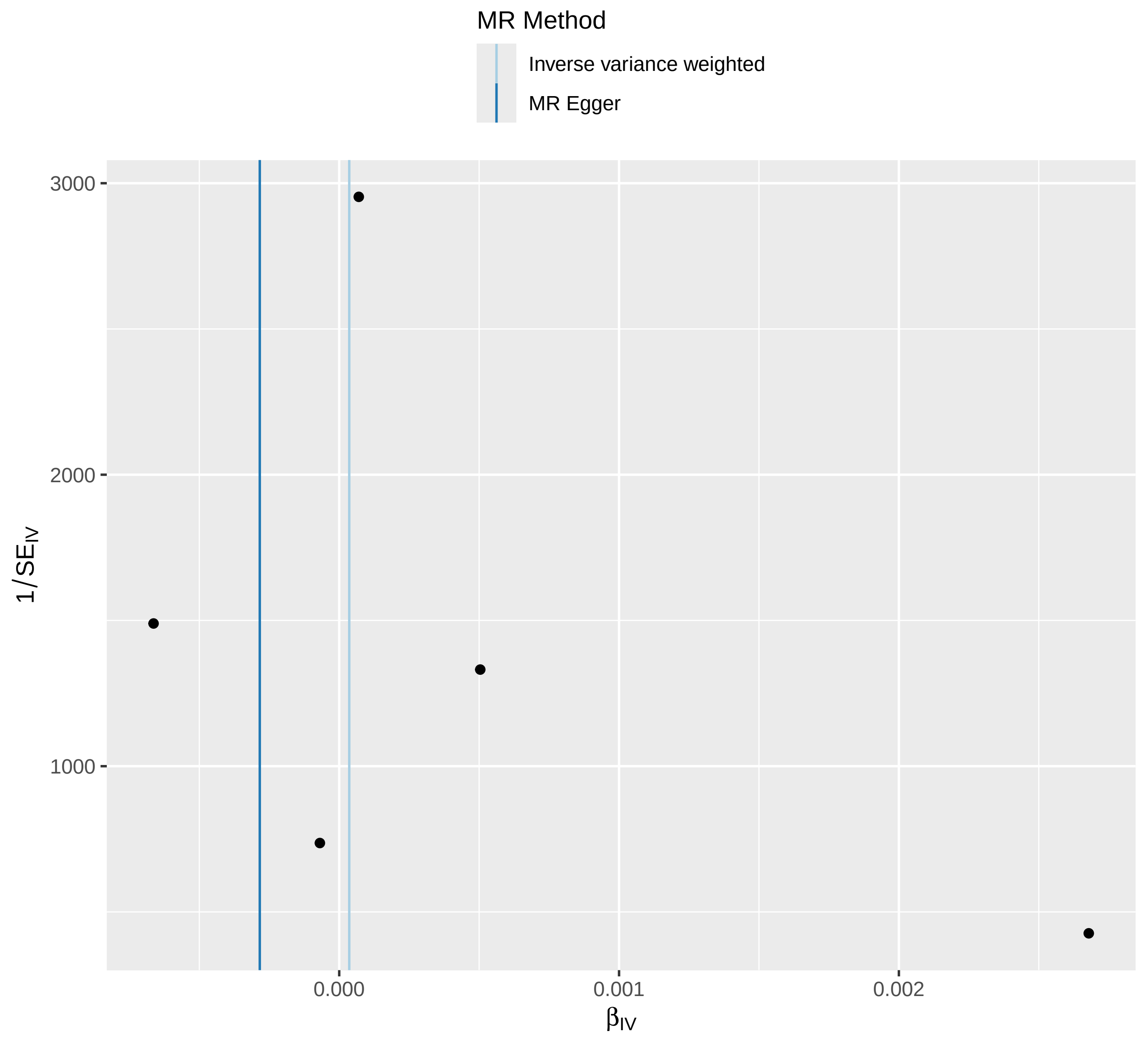
MR Method
Inverse variance weighted
MR Egger
3000
2000
1000
$1/SE_{IV}$
0.000
0.001
0.002
β_{IV}

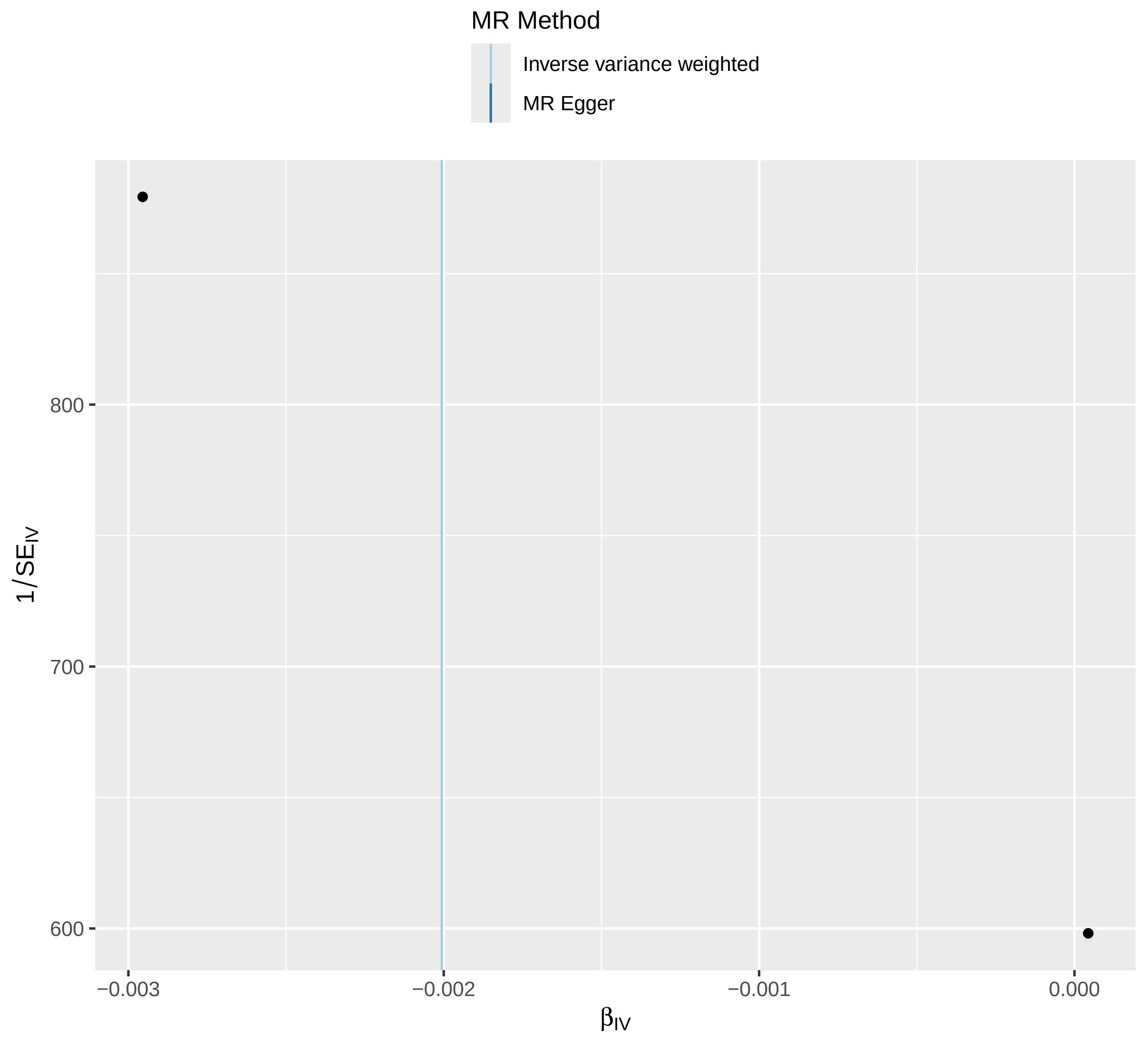
MR Method
Inverse variance weighted
MR Egger
800
700
600
$1/SE_{IV}$
−0.003
−0.002
−0.001
0.000
β_{IV}

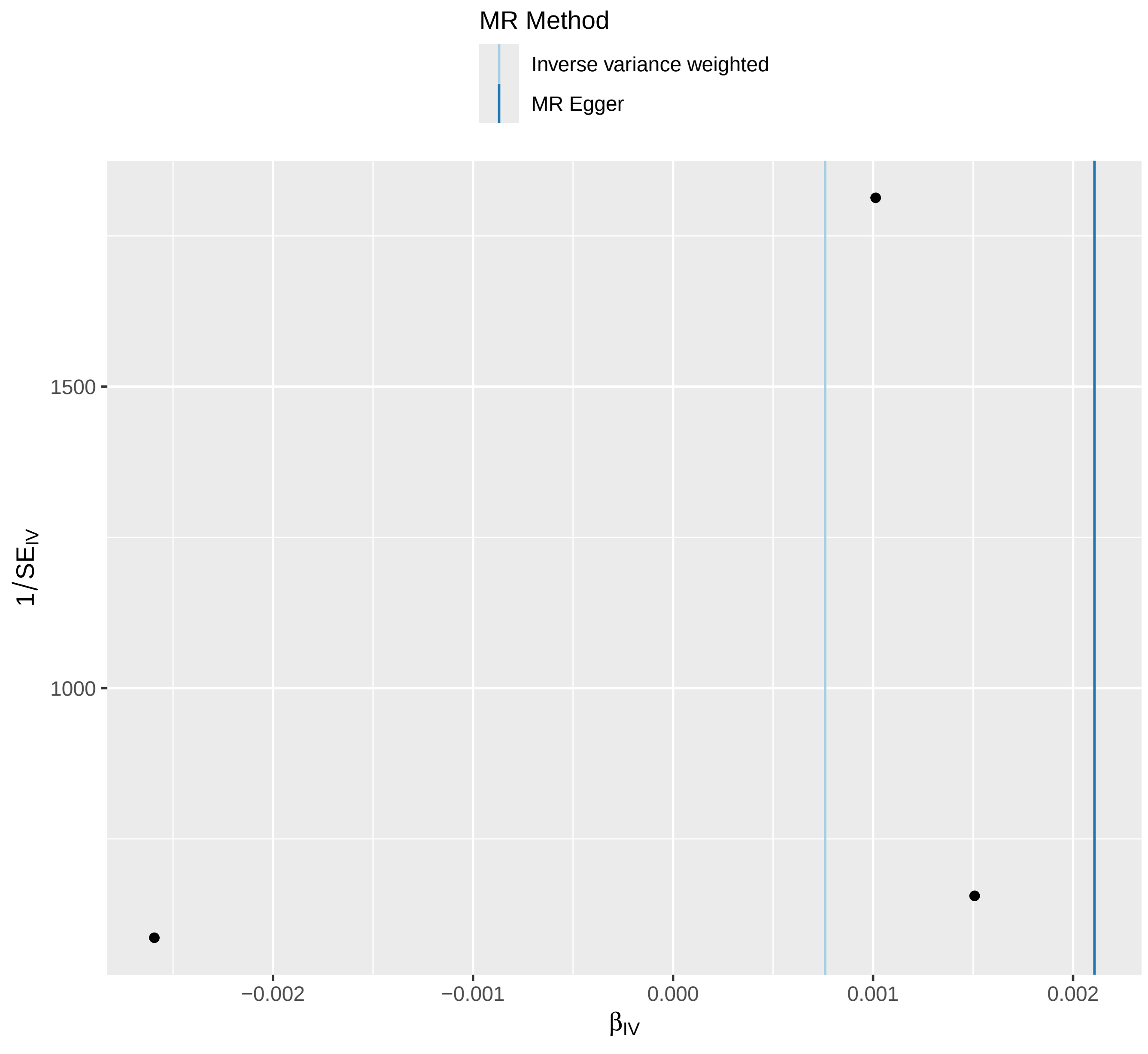

MR Method
Inverse variance weighted
MR Egger
1500
1000
1/SE_IV
−0.002
−0.001
0.000
0.001
0.002
β_IV

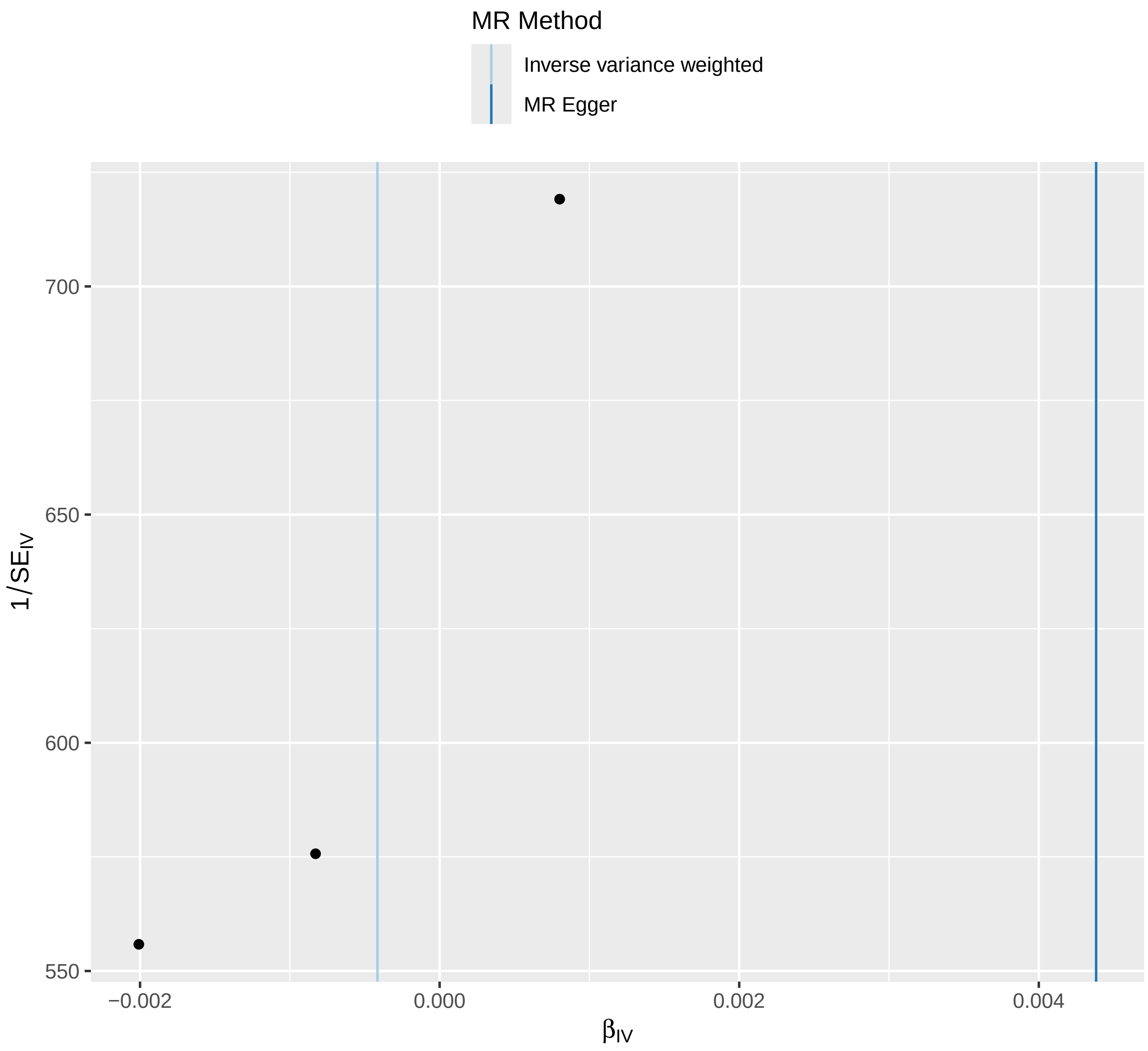

MR Method
Inverse variance weighted
MR Egger
700
650
600
550
1/SE_IV
−0.002
0.000
0.002
0.004
β_IV

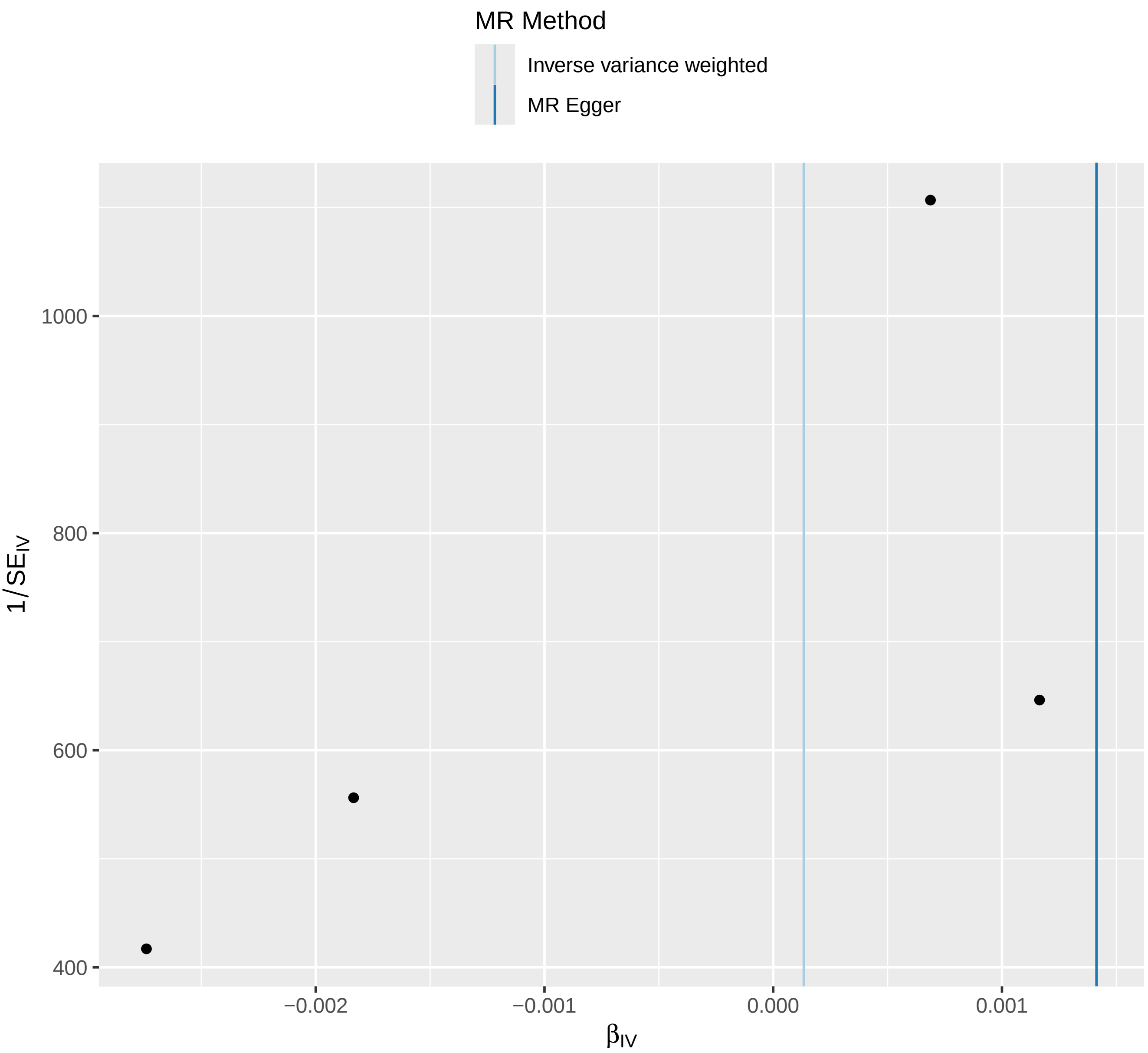
MR Method
Inverse variance weighted
MR Egger
1000
800
600
400
1/SE$_{IV}$
−0.002
−0.001
0.000
0.001
β$_{IV}$

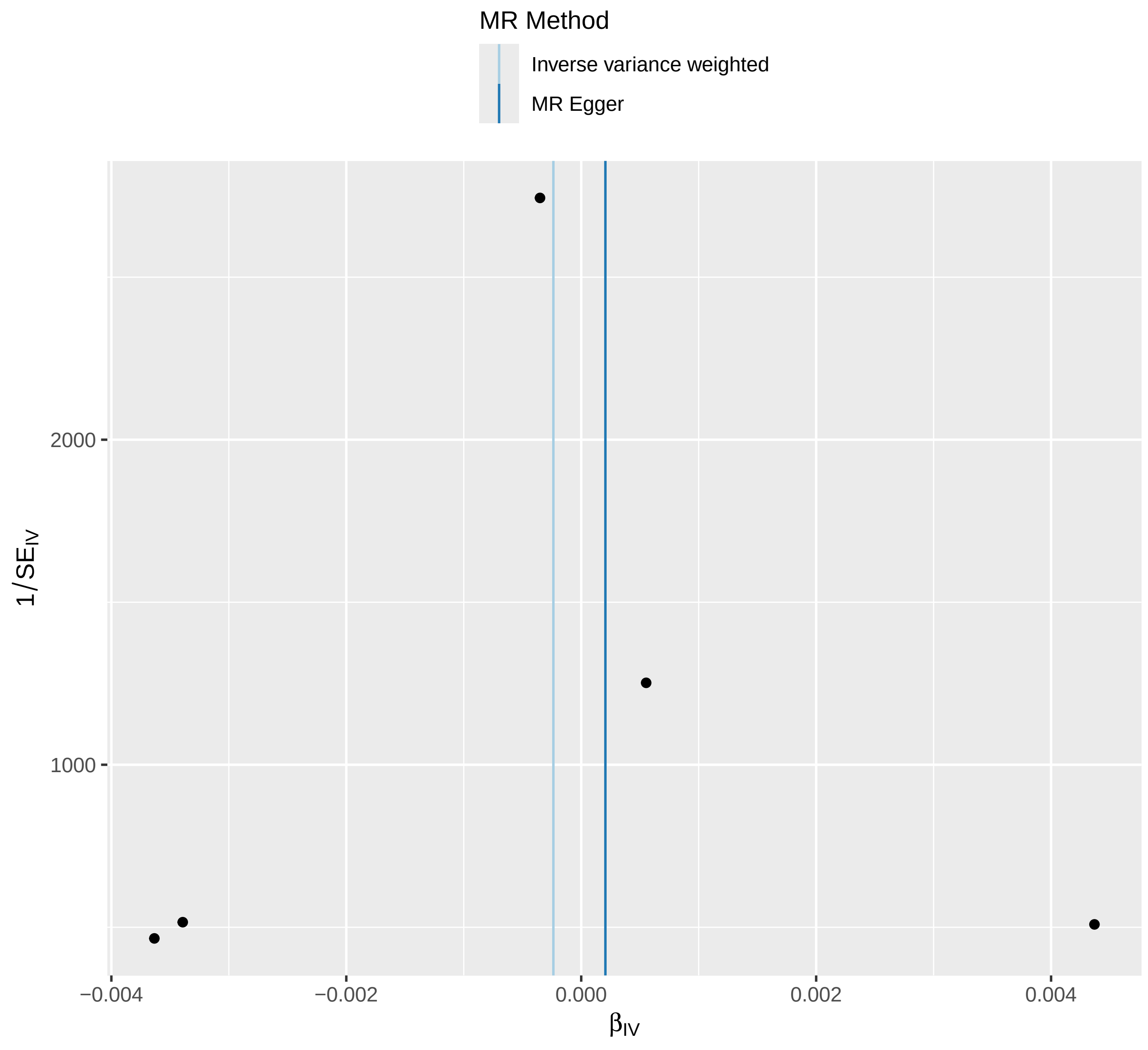
MR Method
Inverse variance weighted
MR Egger
2000
1000
1/SE_IV
−0.004
−0.002
0.000
0.002
0.004
β_IV

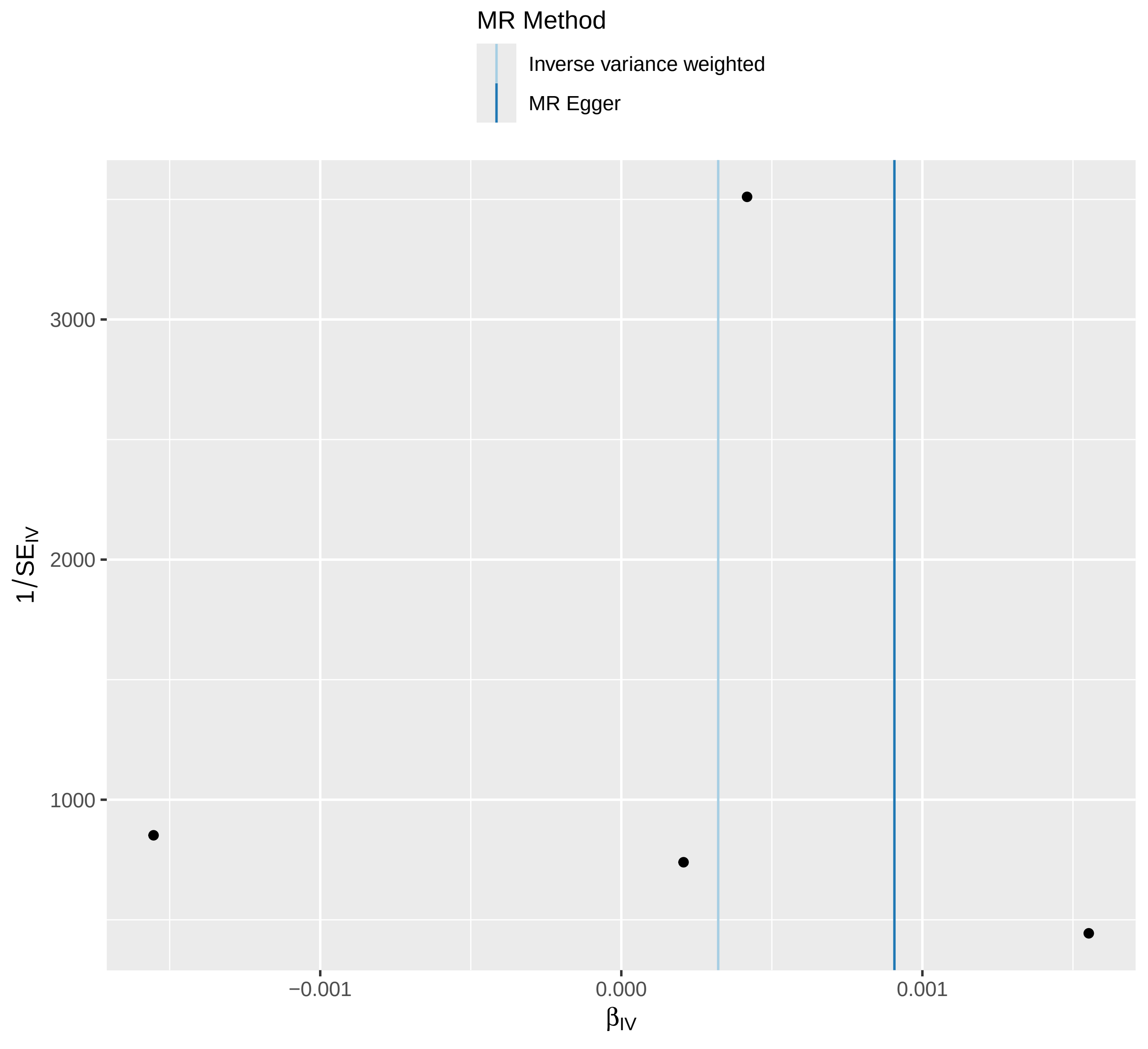
MR Method
Inverse variance weighted
MR Egger
3000
2000
1000
−0.001
0.000
0.001
1/SE_IV
β_IV

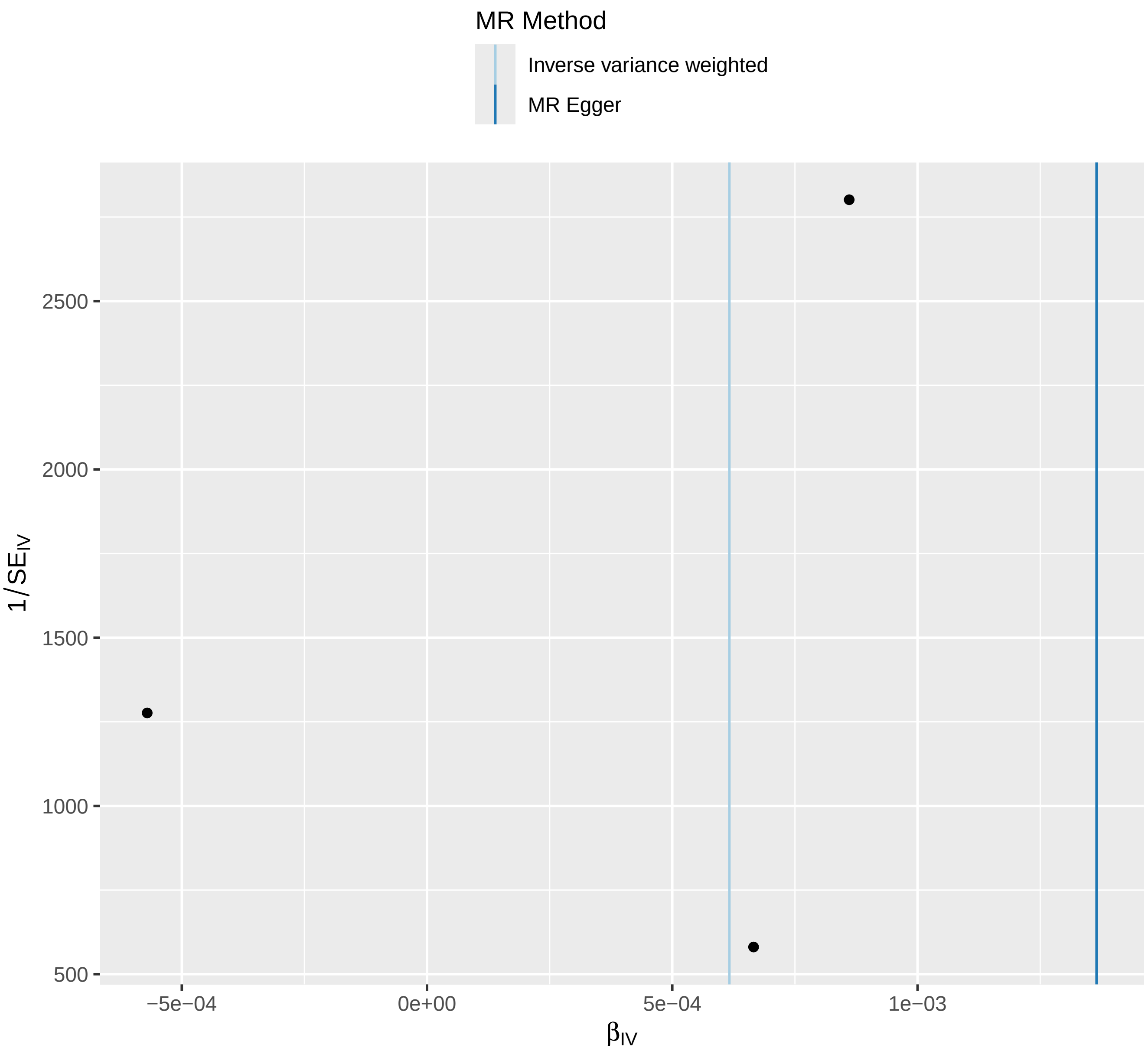
MR Method
Inverse variance weighted
MR Egger
2500
2000
1500
1000
500
1/SE_IV
−5e−04
0e+00
5e−04
1e−03
β_IV

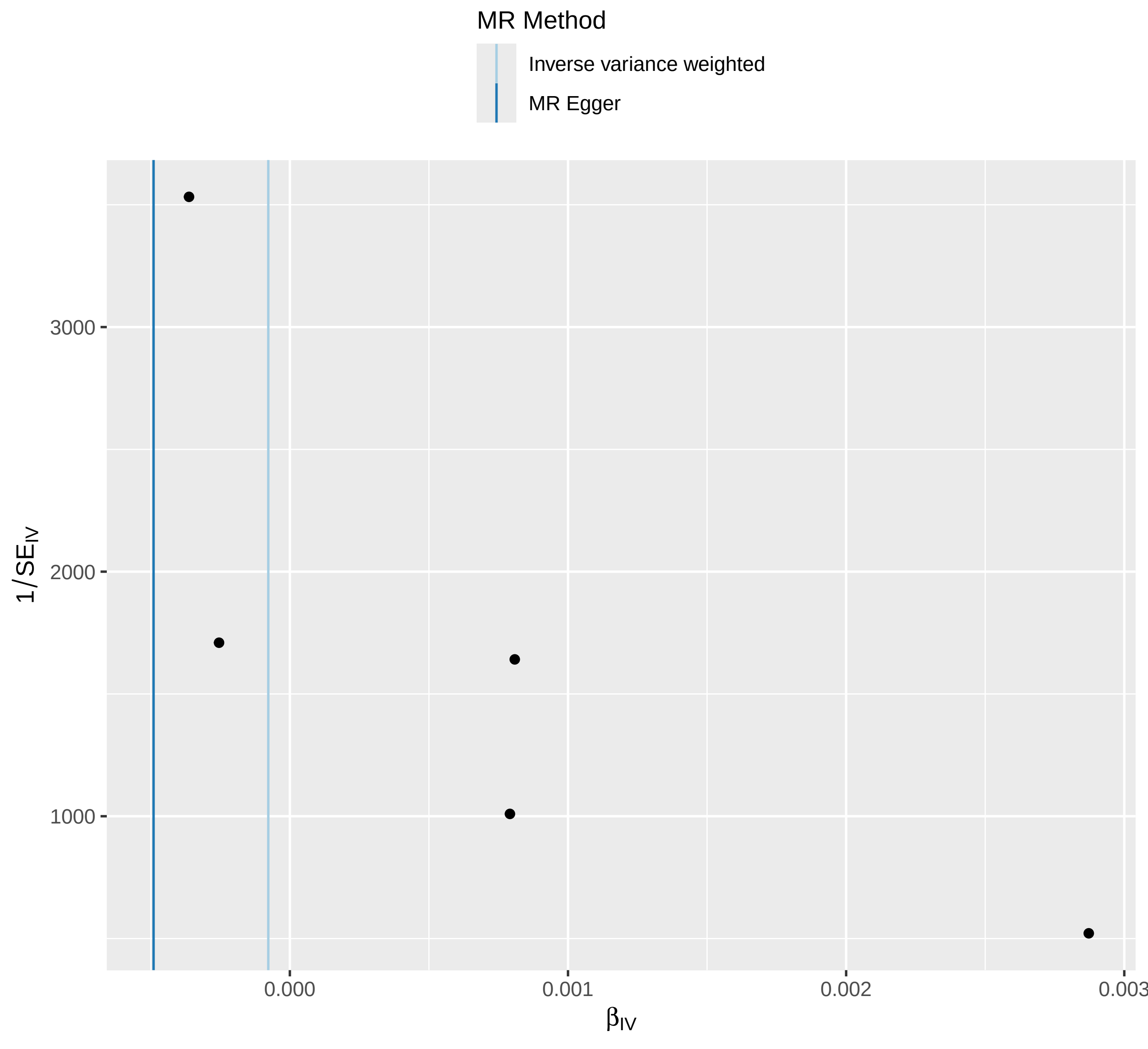

MR Method
Inverse variance weighted
MR Egger
3000
2000
1000
1/SE_IV
0.000
0.001
0.002
0.003
β_IV

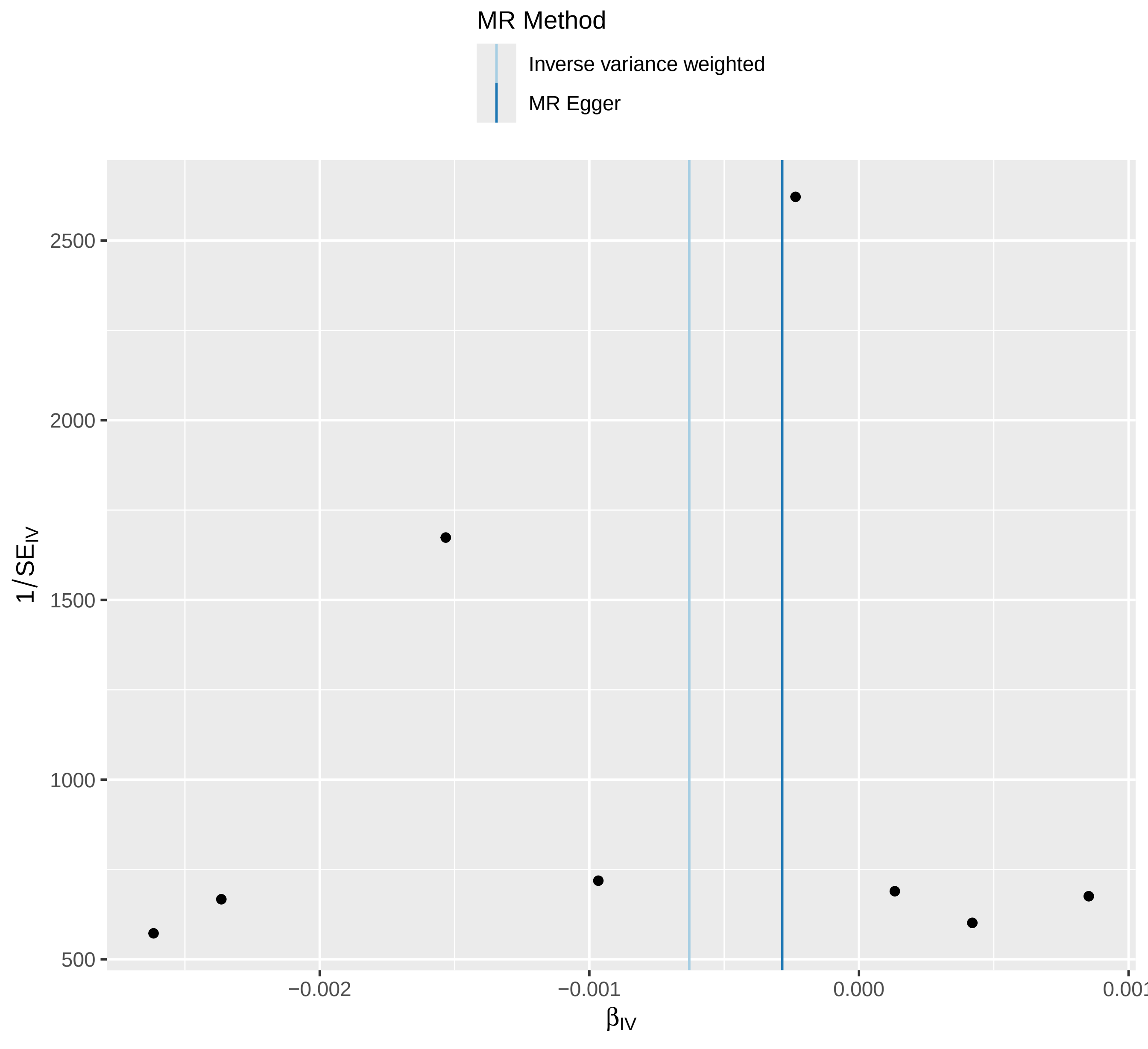
MR Method
Inverse variance weighted
MR Egger
2500
2000
1500
1000
500
1/SE$_{IV}$
−0.002
−0.001
0.000
0.001
β$_{IV}$

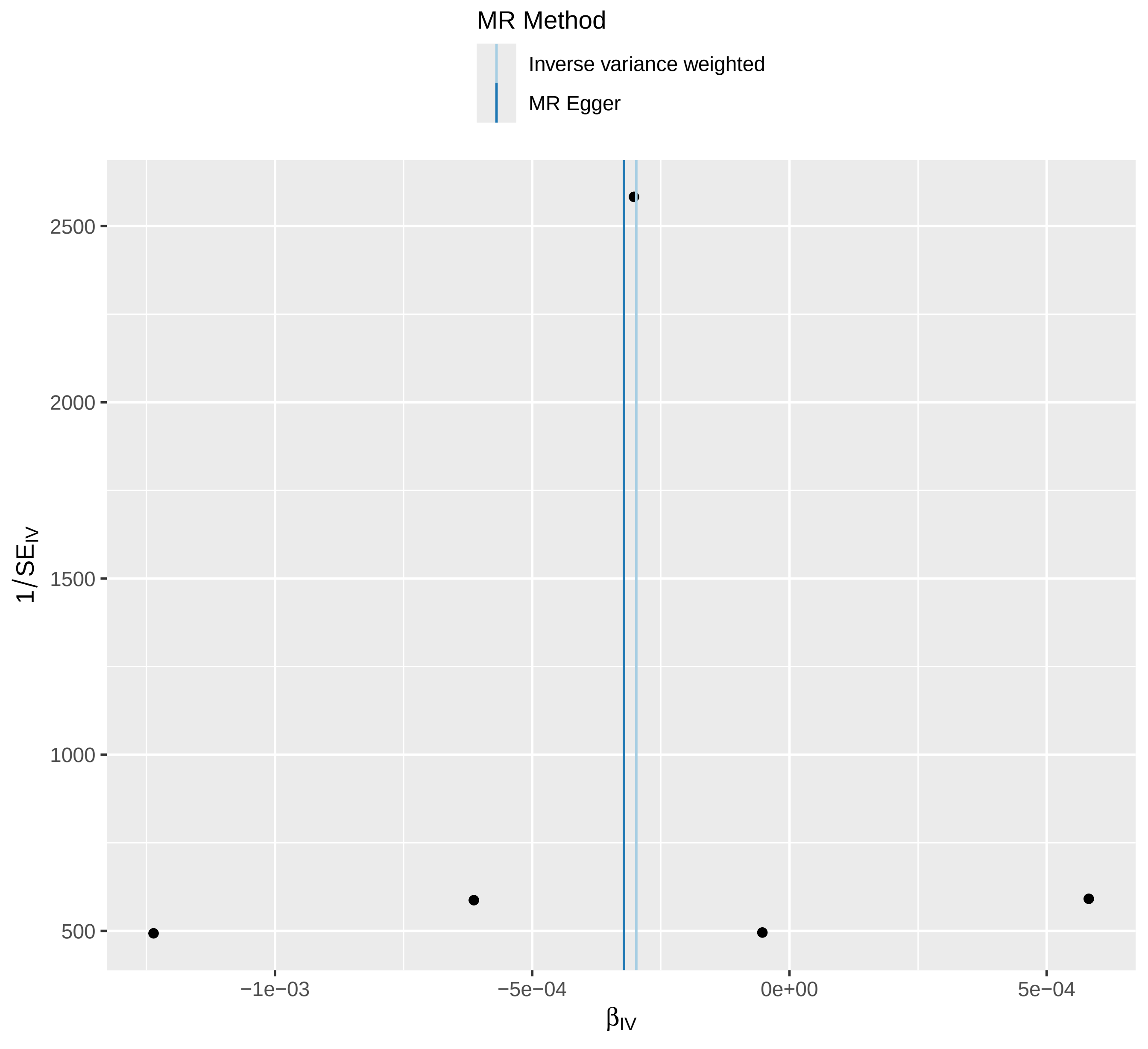
MR Method
Inverse variance weighted
MR Egger
2500
2000
1500
1000
500
1/SE_IV
−1e−03
−5e−04
0e+00
5e−04
β_IV

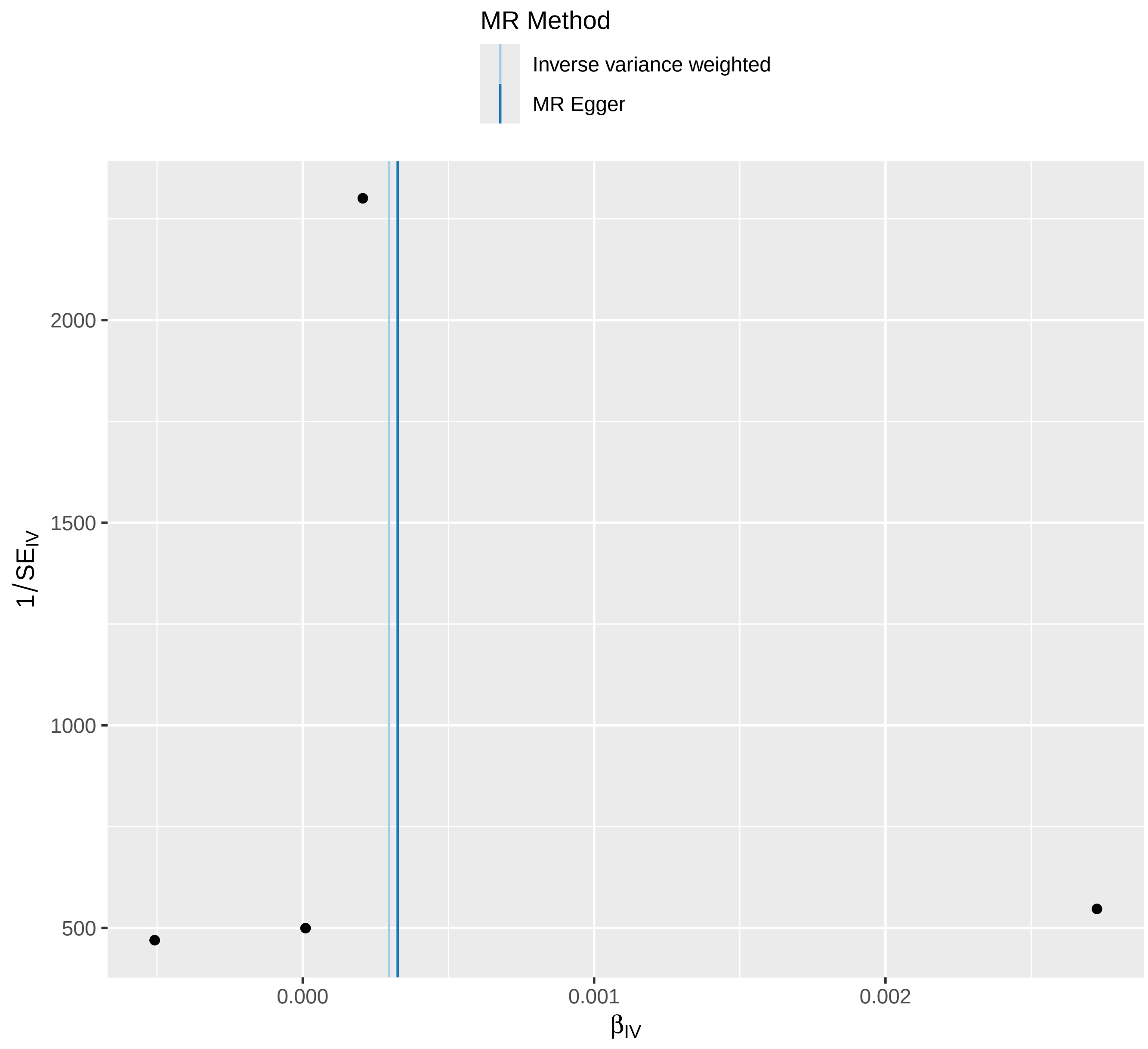
MR Method
Inverse variance weighted
MR Egger
2000
1500
1000
500
1/SE_IV
0.000
0.001
0.002
β_IV

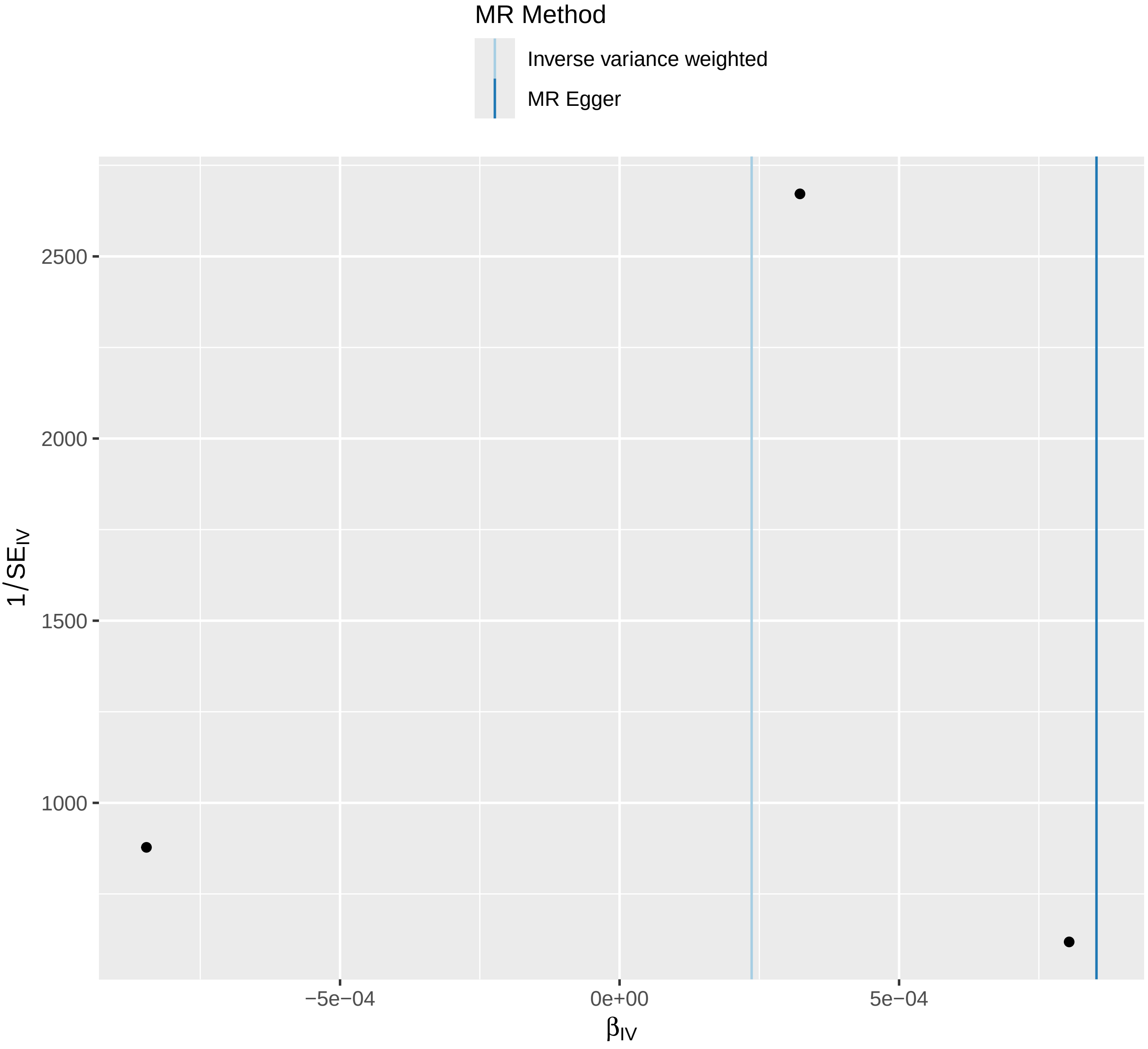
MR Method
Inverse variance weighted
MR Egger
2500
2000
1500
1000
1/SE$_{IV}$
−5e−04
0e+00
5e−04
β$_{IV}$

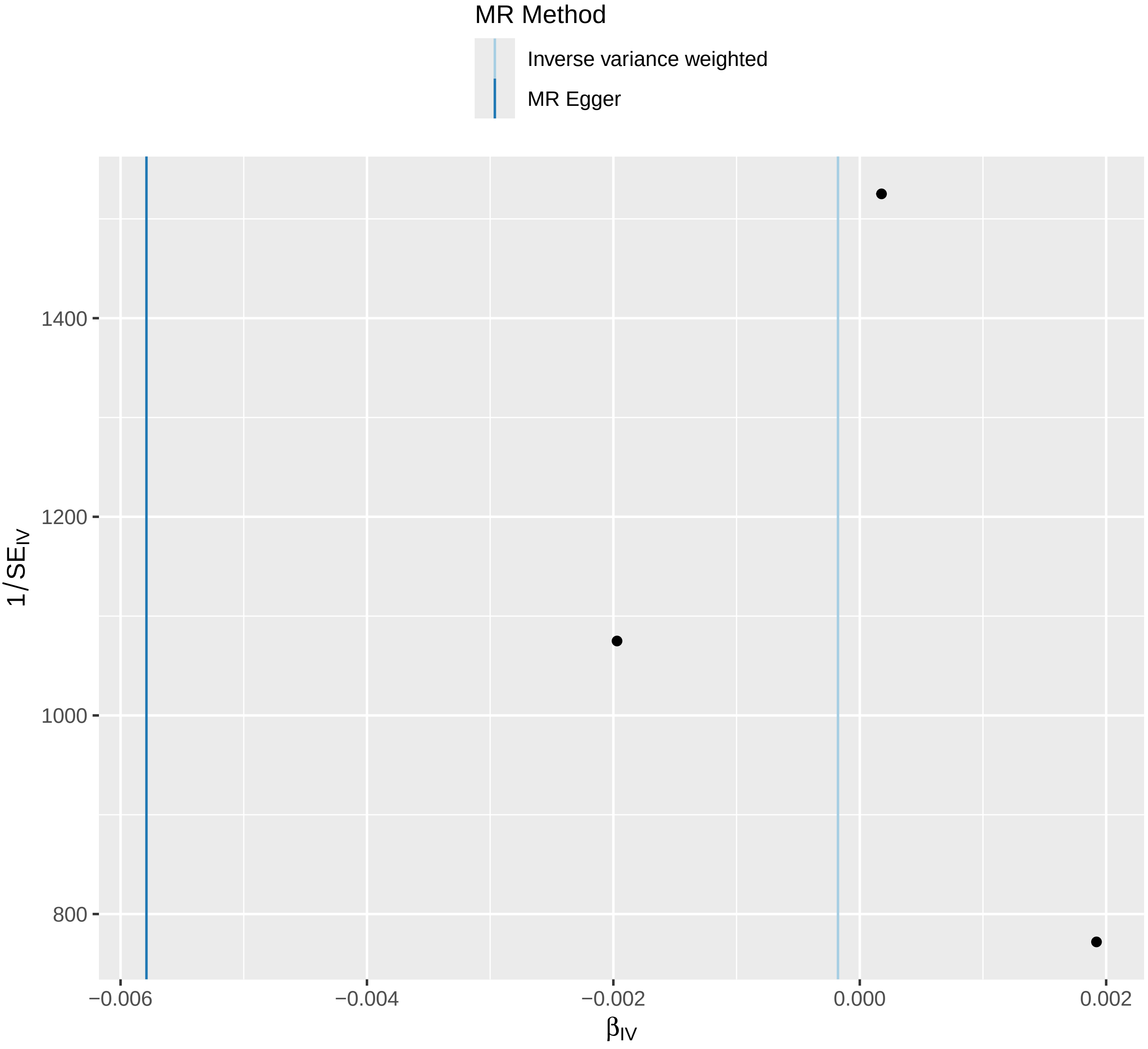

MR Method
Inverse variance weighted
MR Egger
1400
1200
1000
800
1/SE_IV
−0.006
−0.004
−0.002
0.000
0.002
β_IV

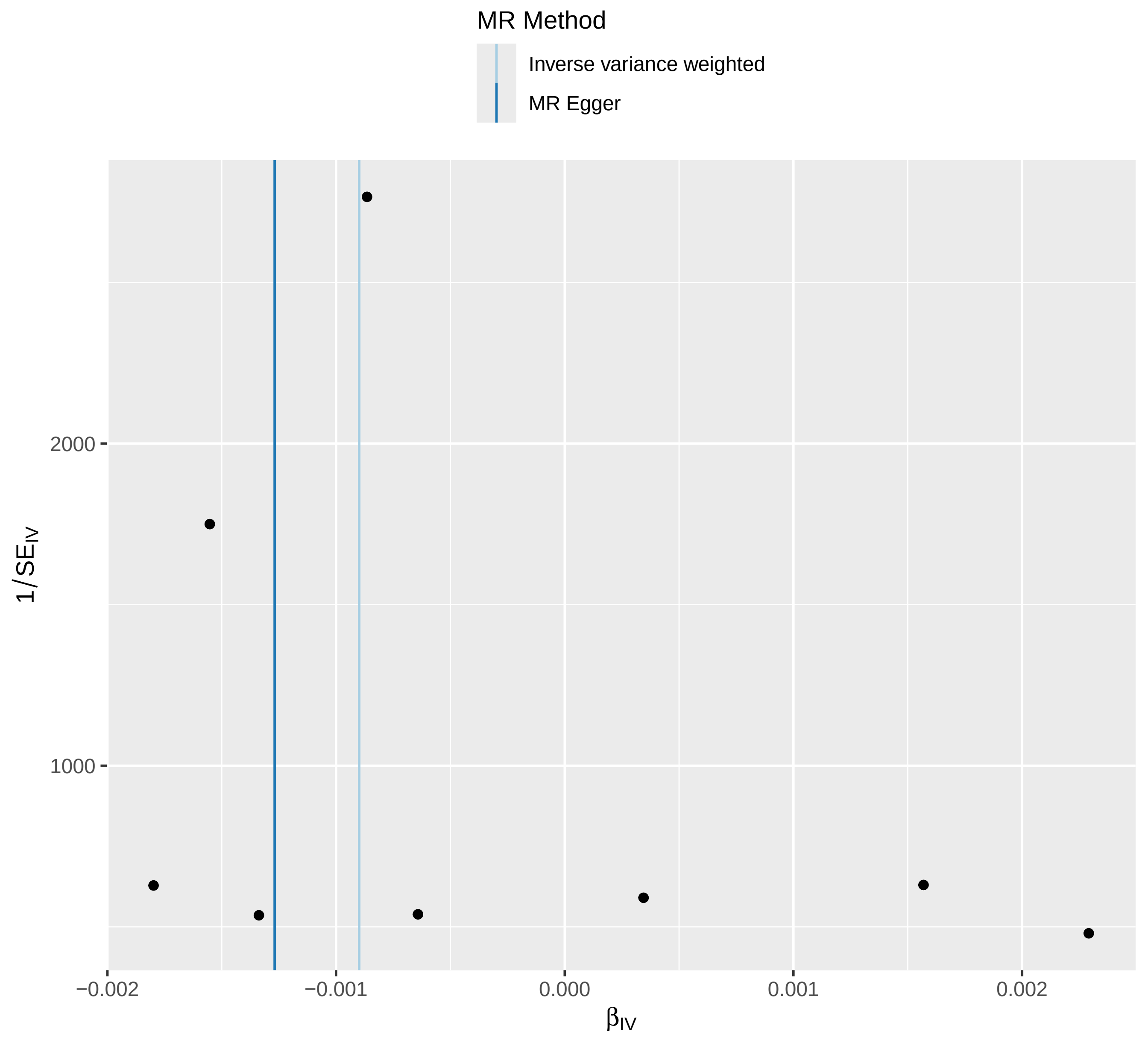

MR Method
Inverse variance weighted
MR Egger
2000
1000
1/SE$_{IV}$
−0.002
−0.001
0.000
0.001
0.002
β$_{IV}$

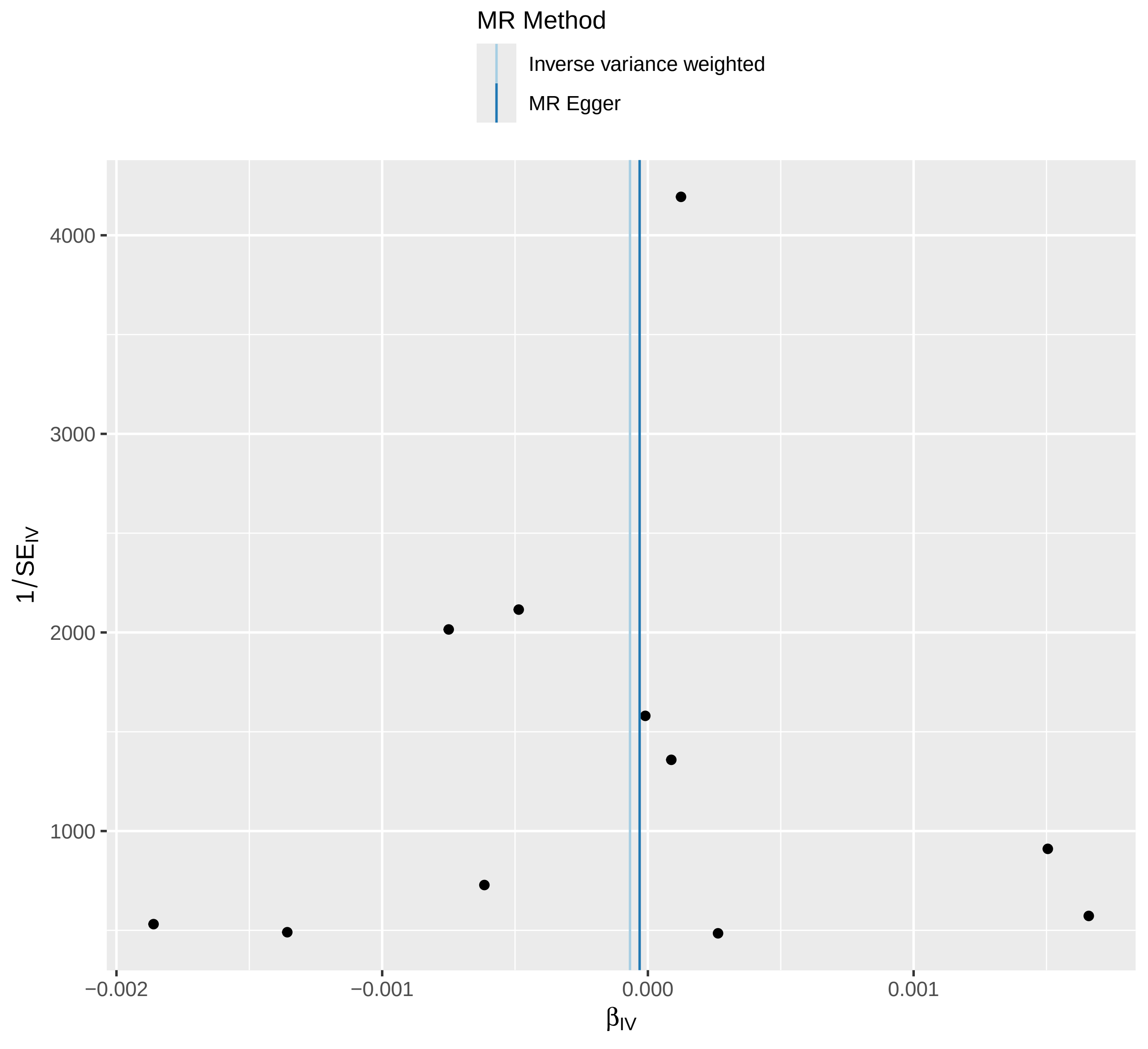
MR Method
Inverse variance weighted
MR Egger
4000
3000
2000
1000
1/SE_IV
−0.002
−0.001
0.000
0.001
β_IV

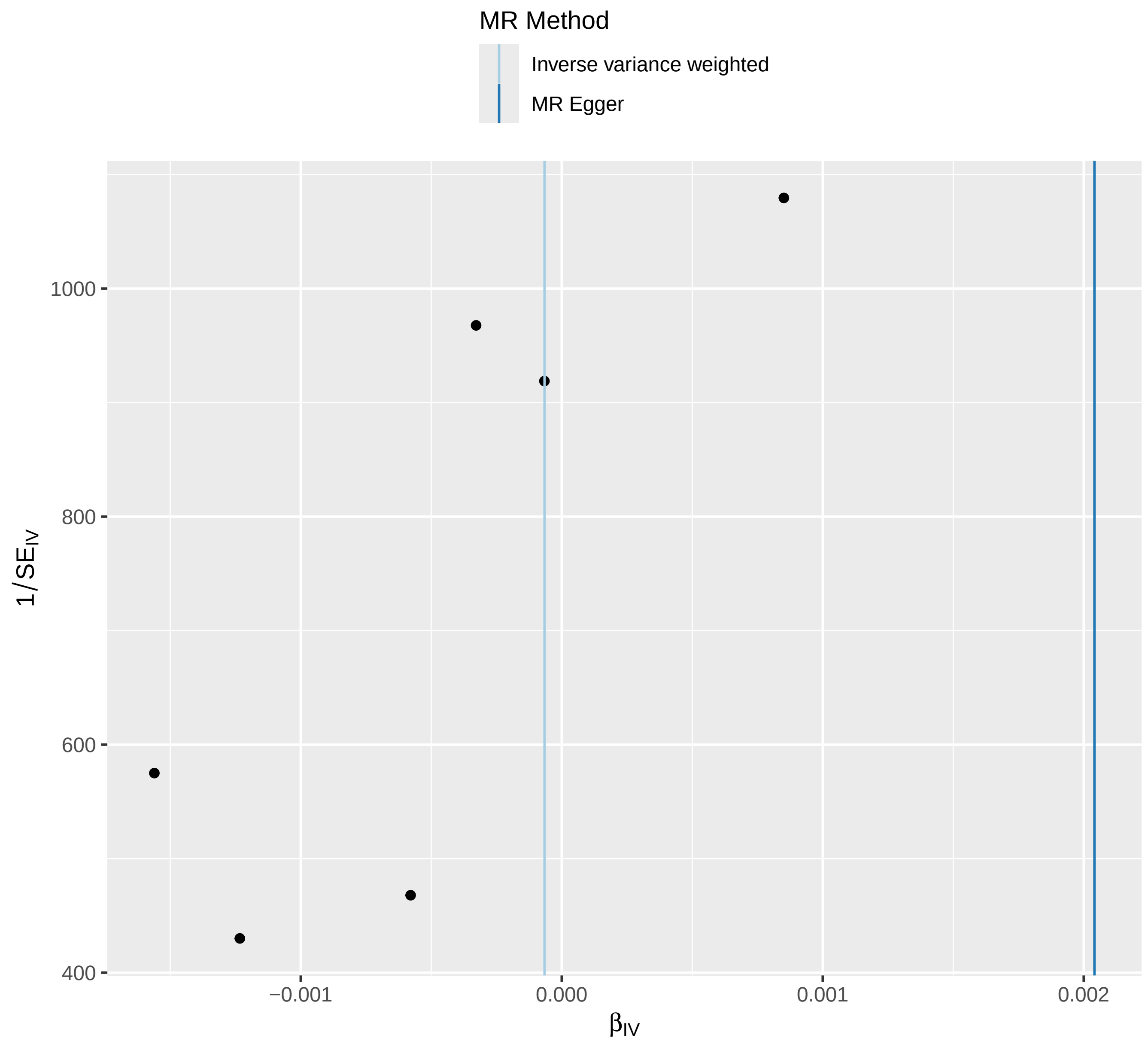

MR Method
Inverse variance weighted
MR Egger
1000
800
600
400
1/SE_IV
−0.001
0.000
0.001
0.002
β_IV

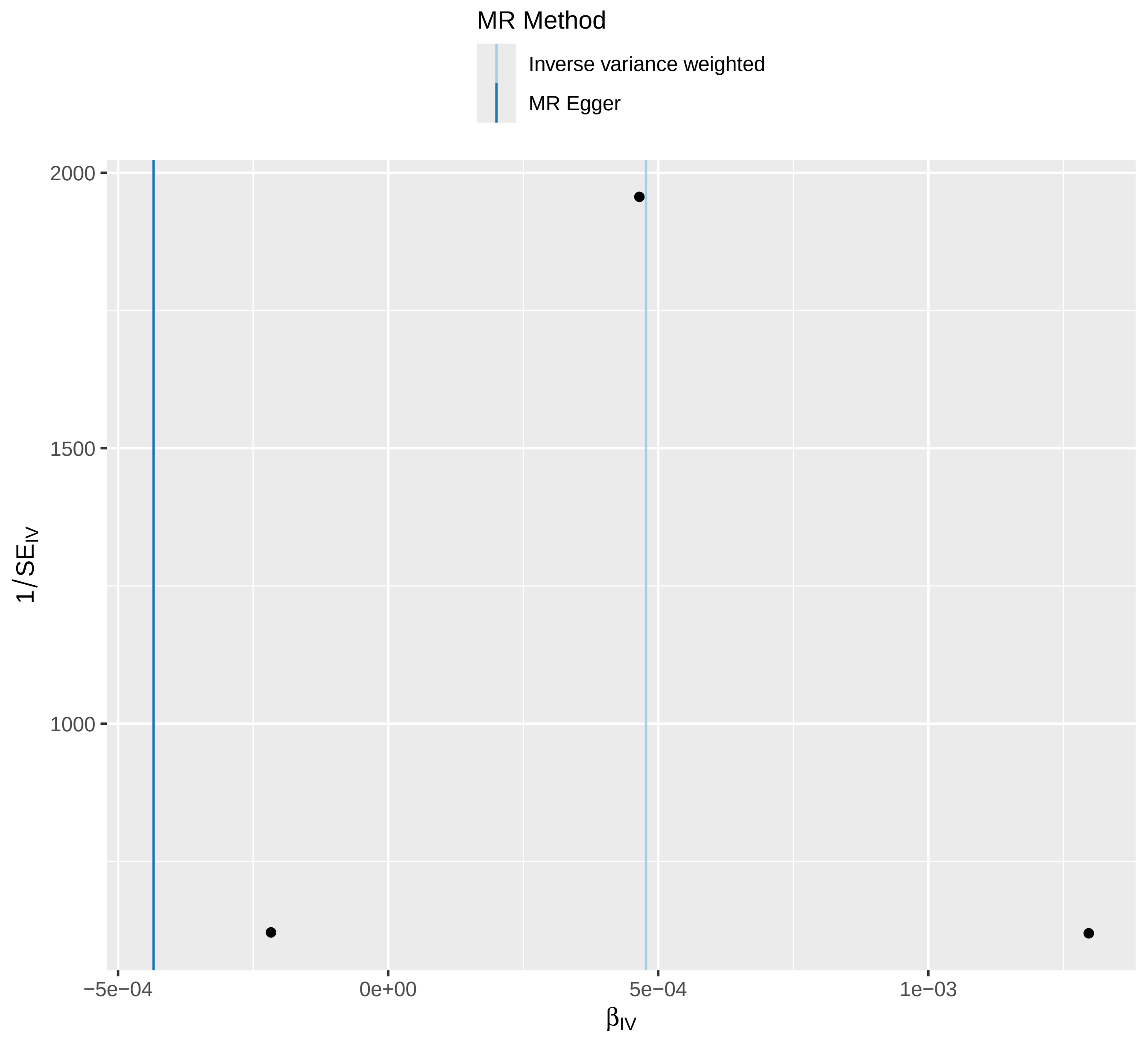
MR Method
Inverse variance weighted
MR Egger
2000
1500
1000
1/SE$_{IV}$
−5e−04
0e+00
5e−04
1e−03
β$_{IV}$

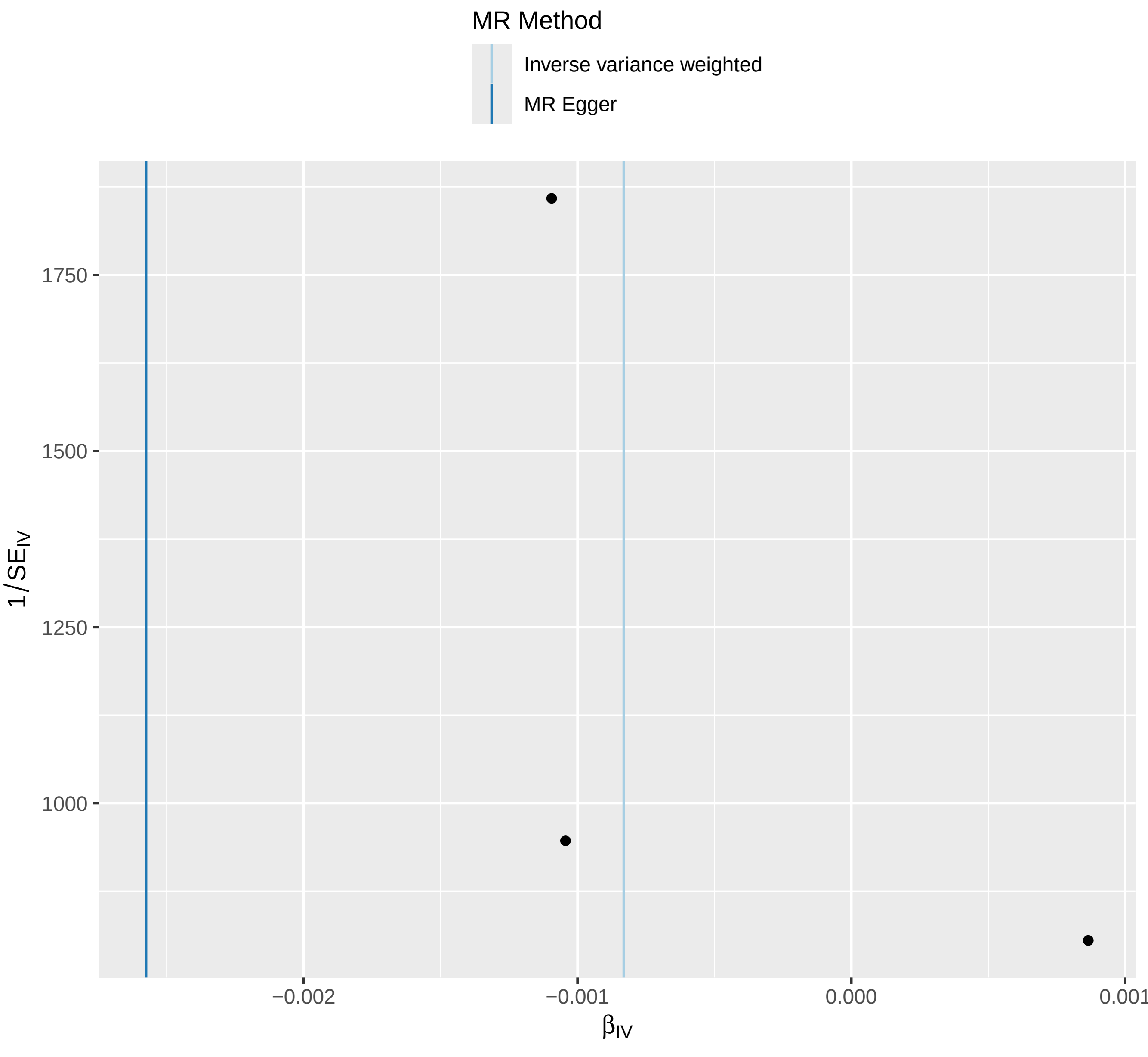
MR Method
Inverse variance weighted
MR Egger
1750
1500
1250
1000
1/SE_IV
−0.002
−0.001
0.000
0.001
β_IV

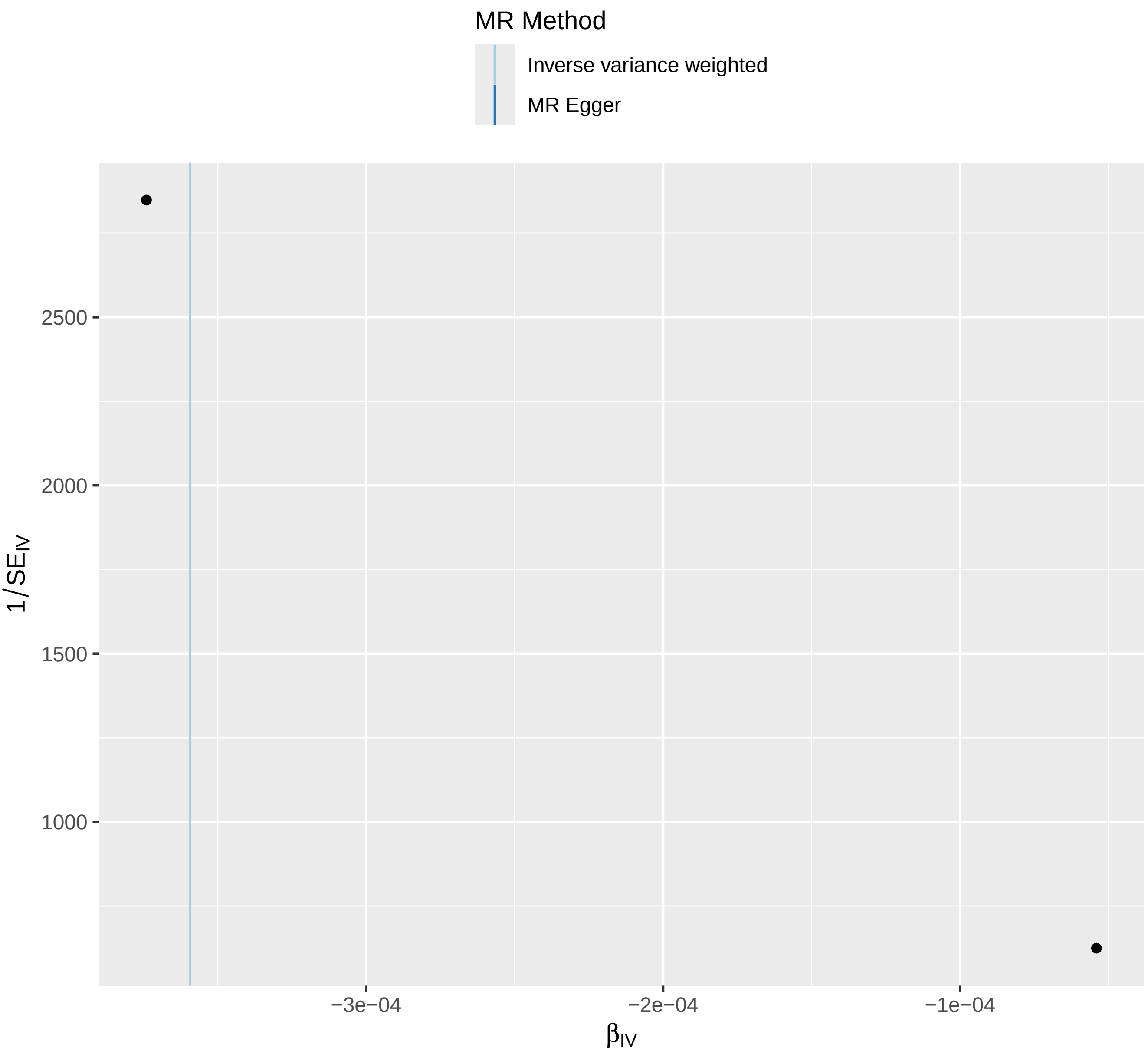
MR Method
Inverse variance weighted
MR Egger
2500
2000
1500
1000
1/SE_IV
−3e−04
−2e−04
−1e−04
β_IV

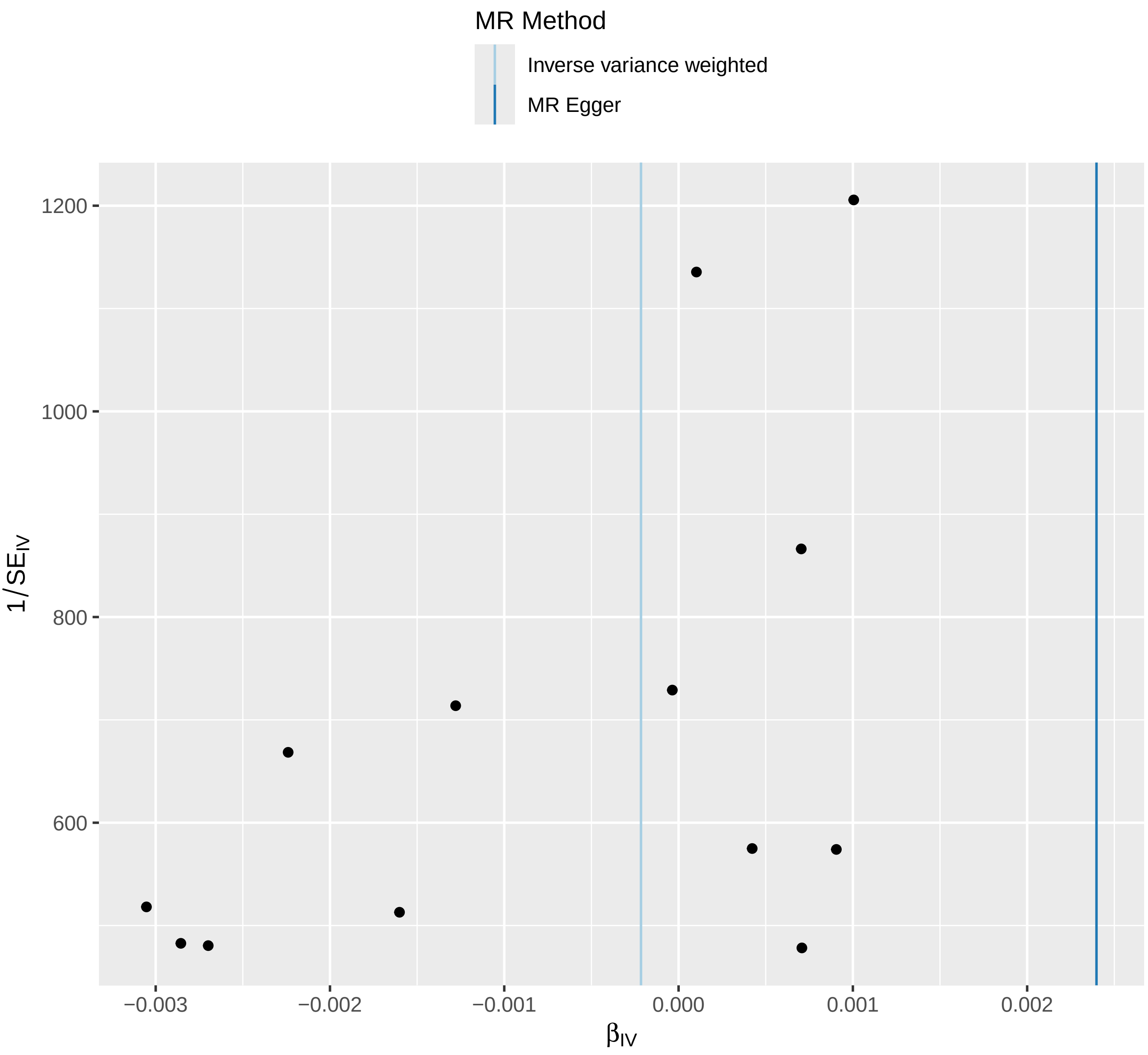
MR Method
Inverse variance weighted
MR Egger
1200
1000
800
600
1/SE_IV
−0.003
−0.002
−0.001
0.000
0.001
0.002
β_IV

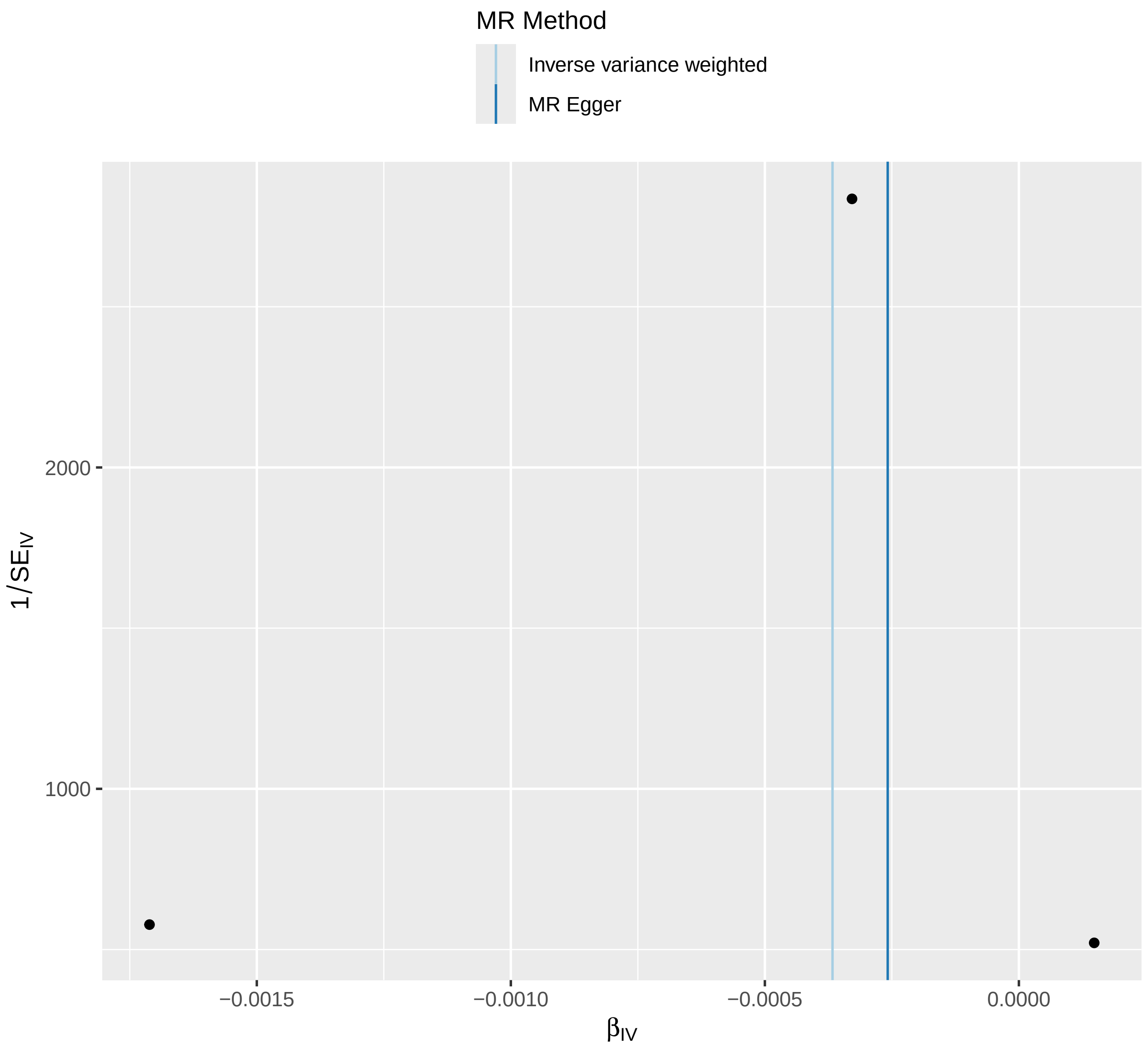
MR Method
Inverse variance weighted
MR Egger
2000
1000
1/SE_IV
−0.0015
−0.0010
−0.0005
0.0000
β_IV

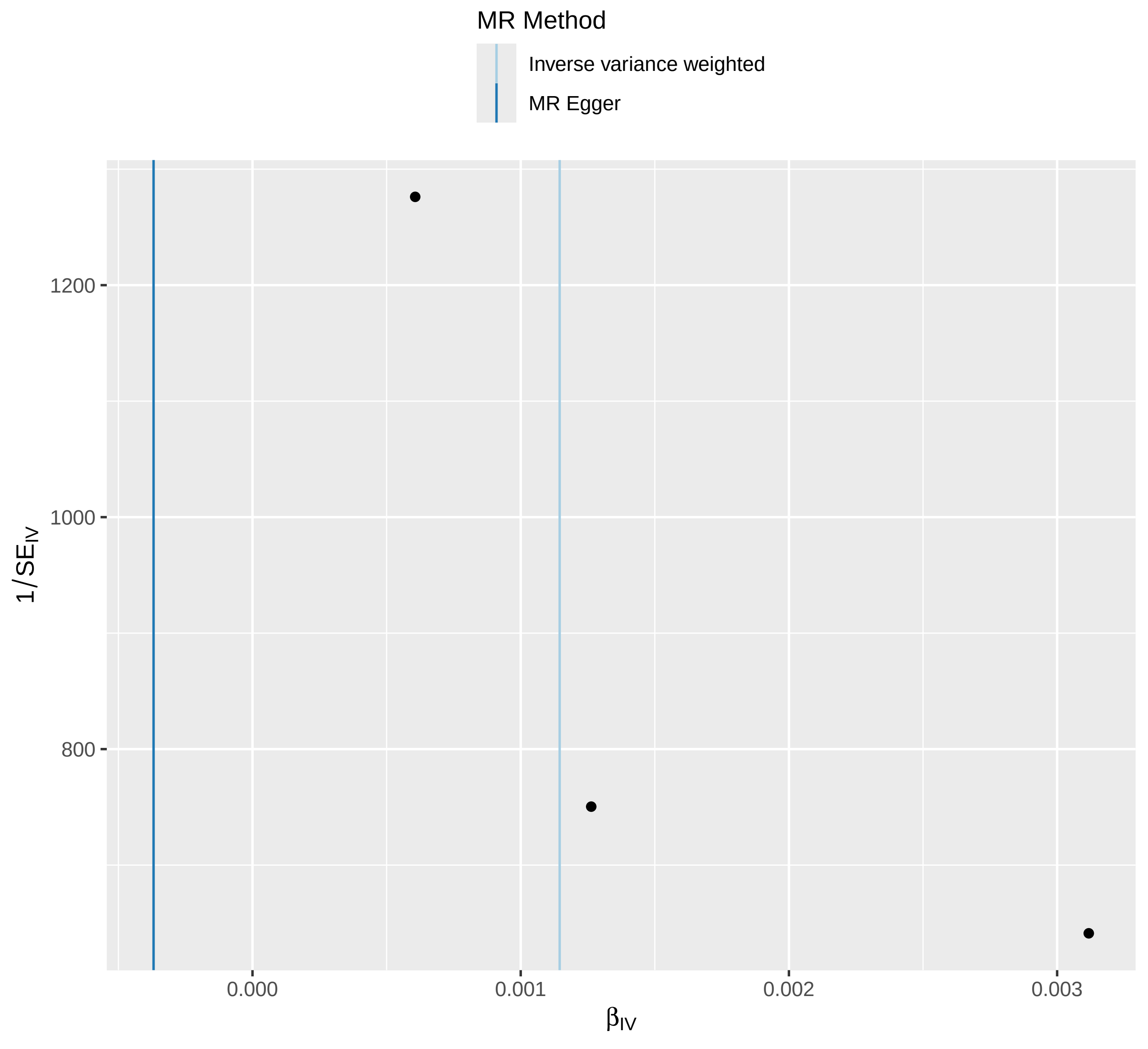
MR Method
Inverse variance weighted
MR Egger
1200
1000
800
1/SE$_{IV}$
0.000
0.001
0.002
0.003
β$_{IV}$

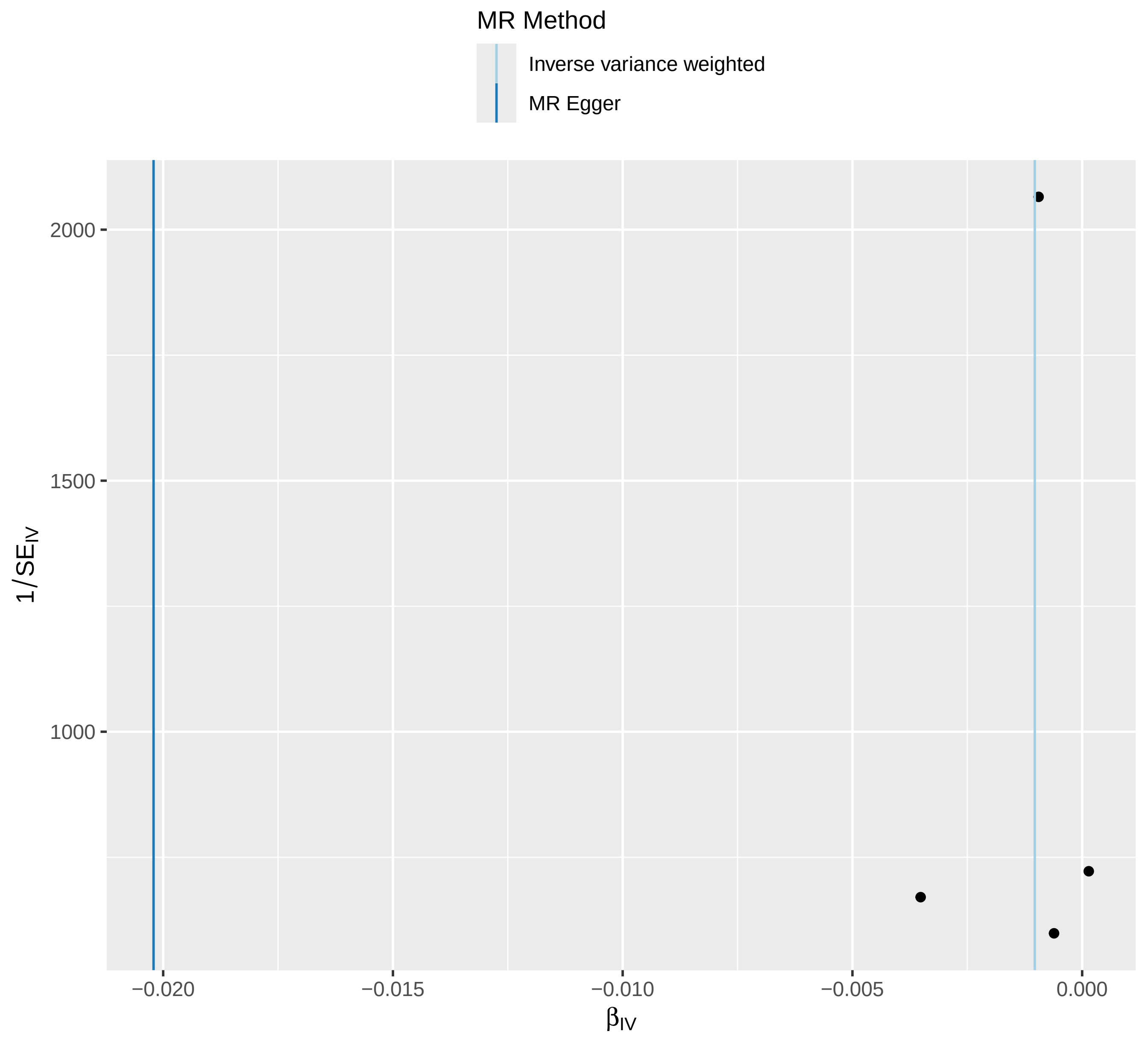

MR Method
Inverse variance weighted
MR Egger
2000
1500
1000
1/SE_IV
−0.020
−0.015
−0.010
−0.005
0.000
β_IV

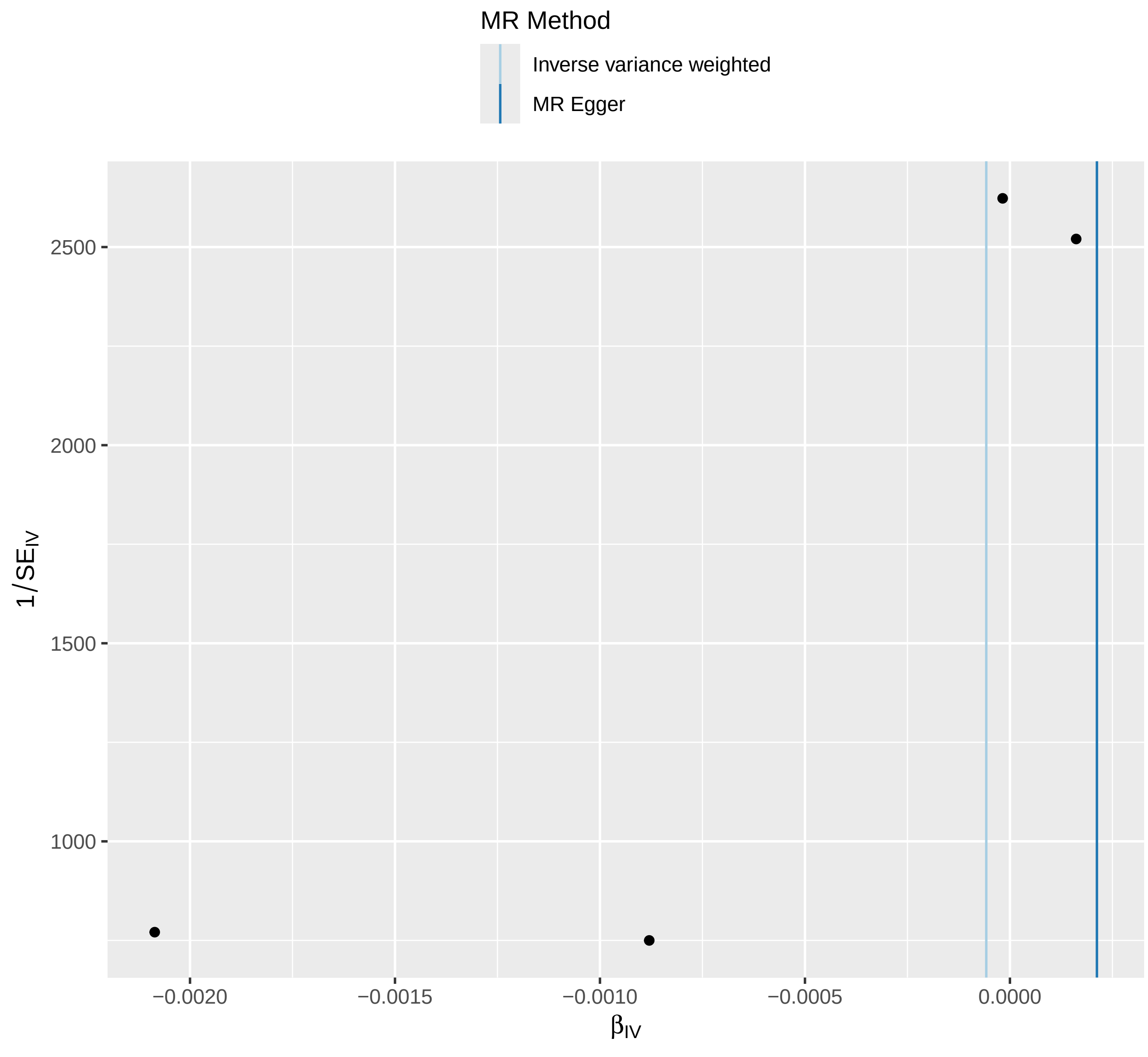

MR Method
Inverse variance weighted
MR Egger
2500
2000
1500
1000
1/SE_IV
−0.0020
−0.0015
−0.0010
−0.0005
0.0000
β_IV

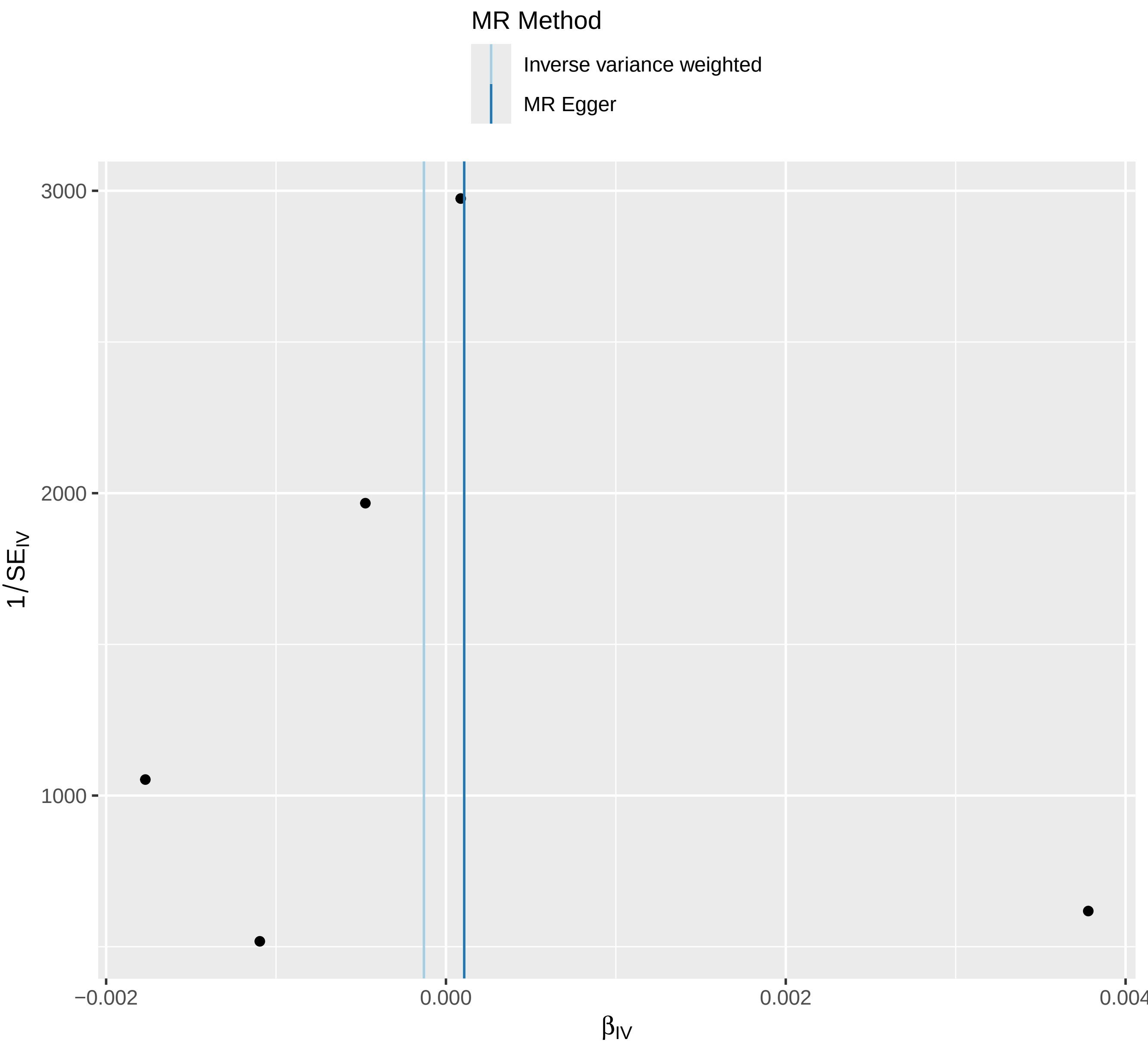
MR Method
Inverse variance weighted
MR Egger
3000
2000
1000
$1/SE_{IV}$
−0.002
0.000
0.002
0.004
β_{IV}

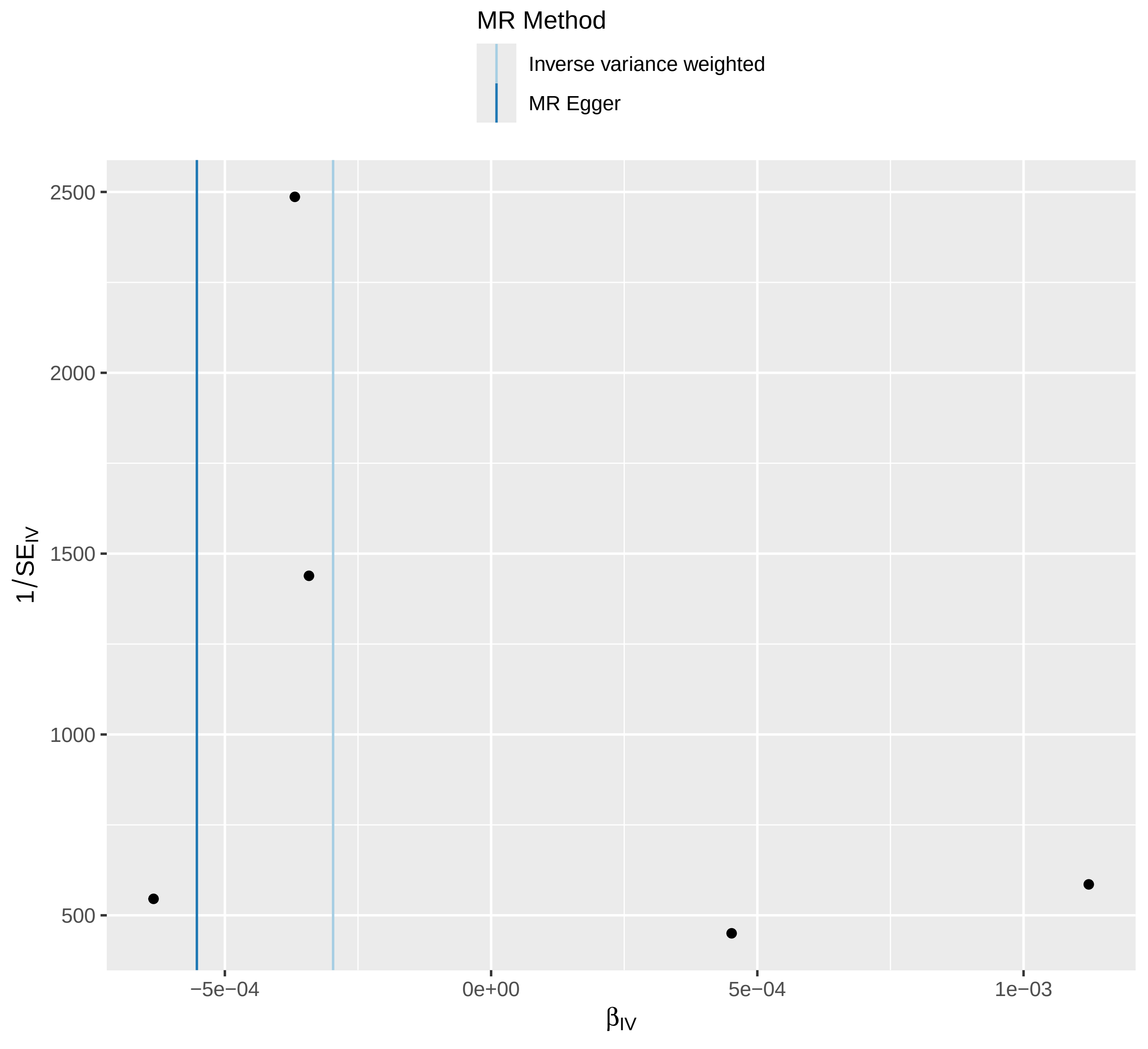

MR Method
Inverse variance weighted
MR Egger
2500
2000
1500
1000
500
1/SE_IV
−5e−04
0e+00
5e−04
1e−03
β_IV

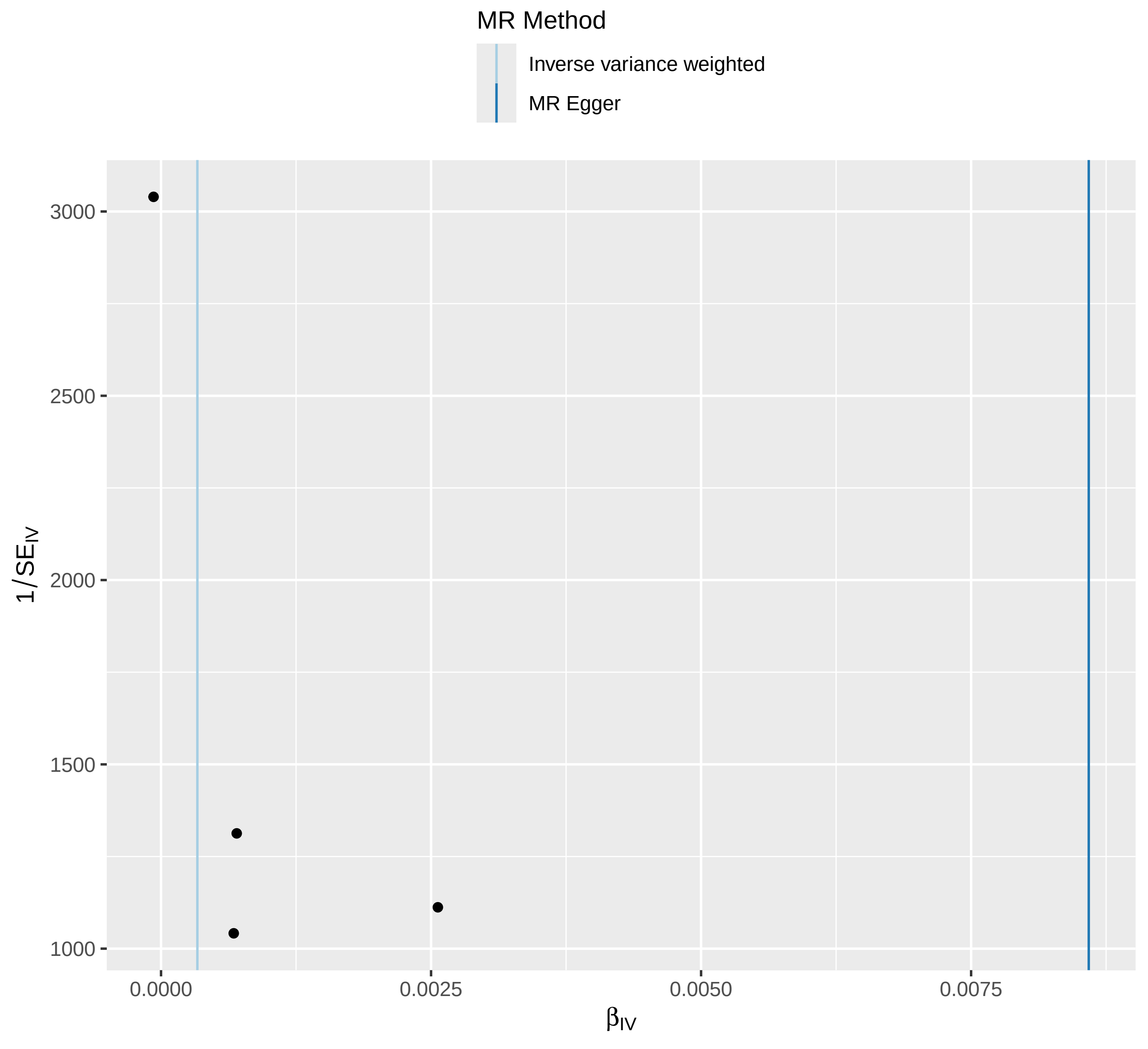

MR Method
Inverse variance weighted
MR Egger
3000
2500
2000
1500
1000
$1/SE_{IV}$
0.0000
0.0025
0.0050
0.0075
β_{IV}

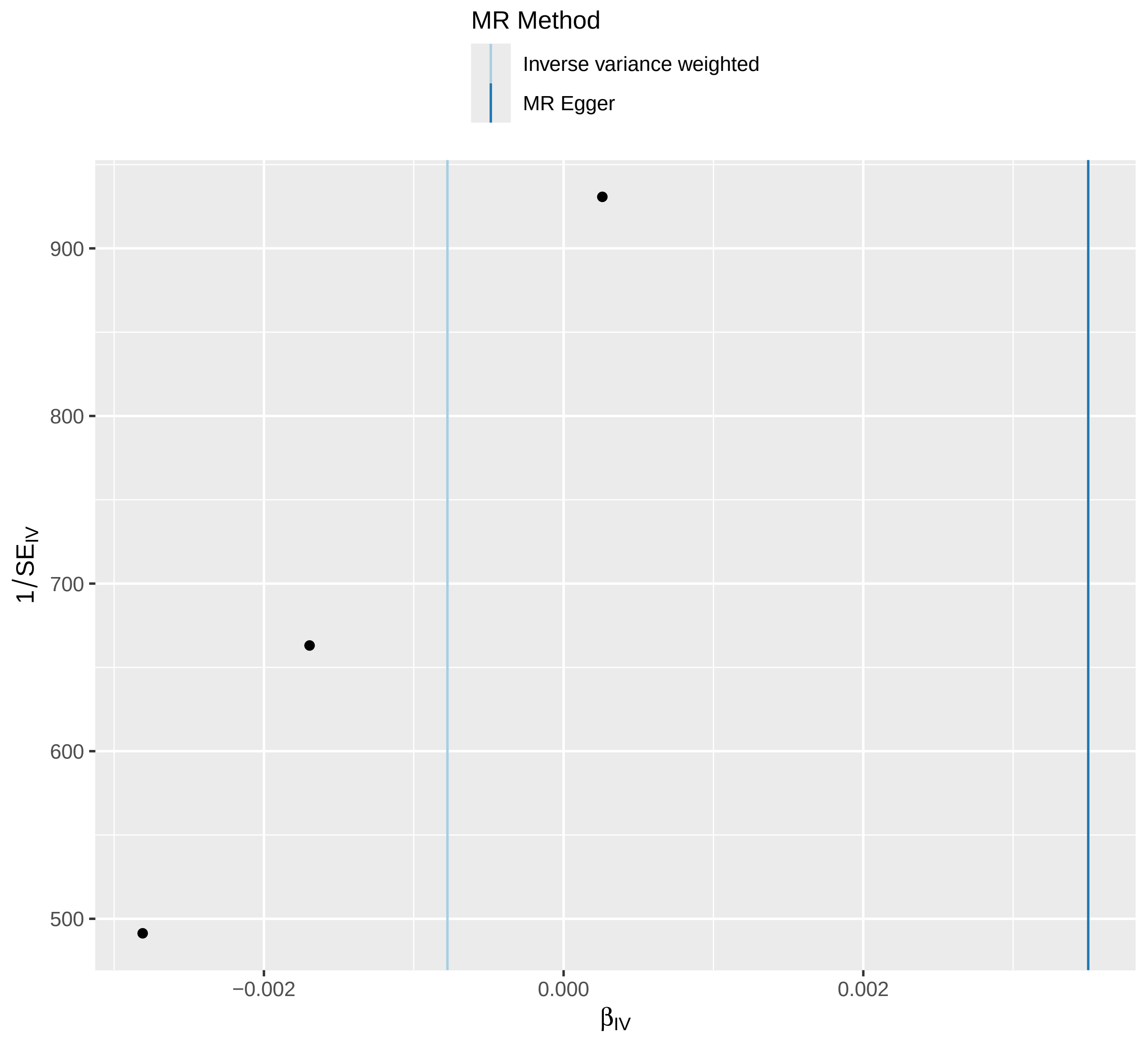
MR Method
Inverse variance weighted
MR Egger
900
800
700
600
500
1/SE_IV
−0.002
0.000
0.002
β_IV

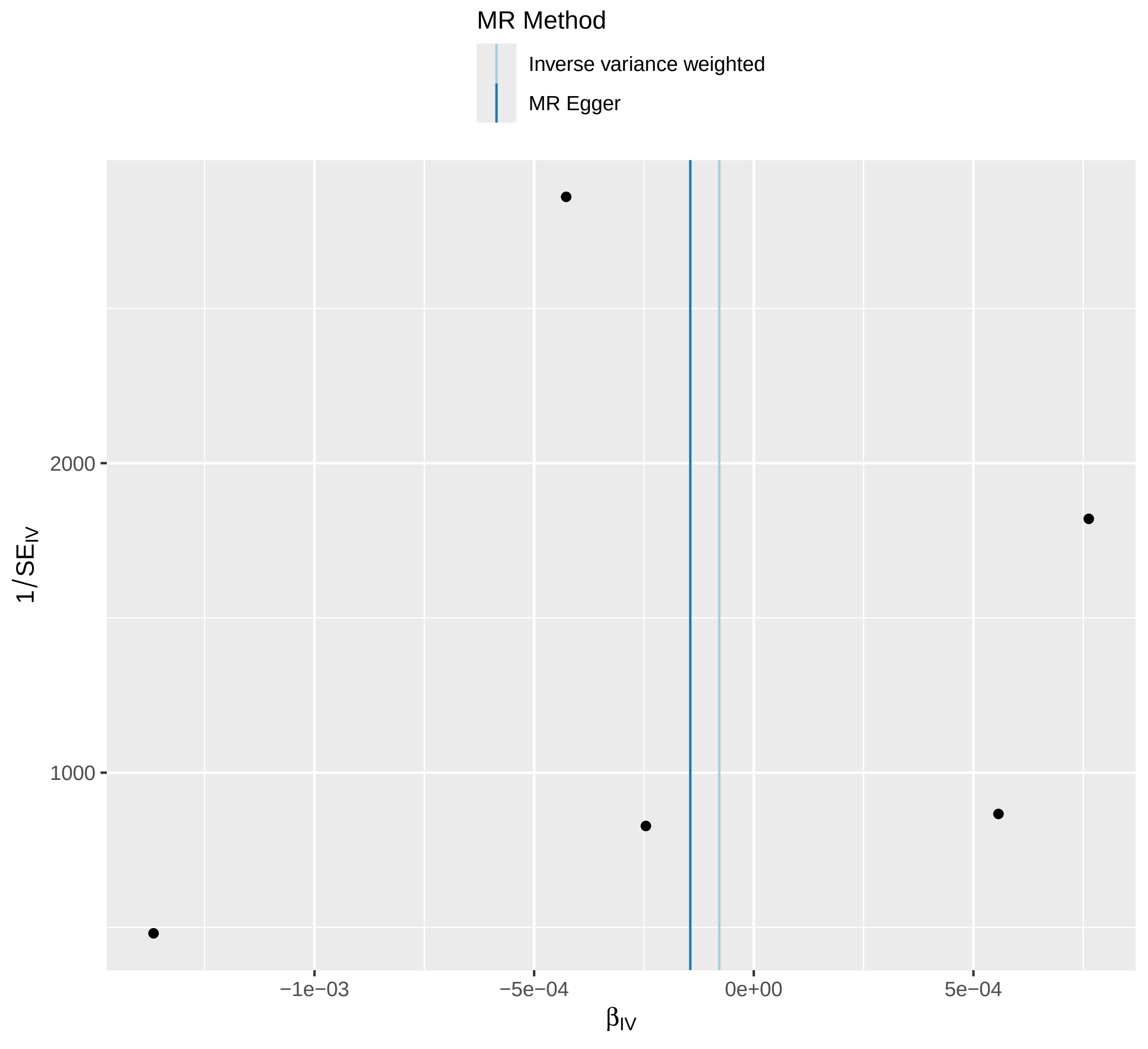
MR Method
Inverse variance weighted
MR Egger
2000
1000
$1/SE_{IV}$
−1e−03
−5e−04
0e+00
5e−04
β_{IV}

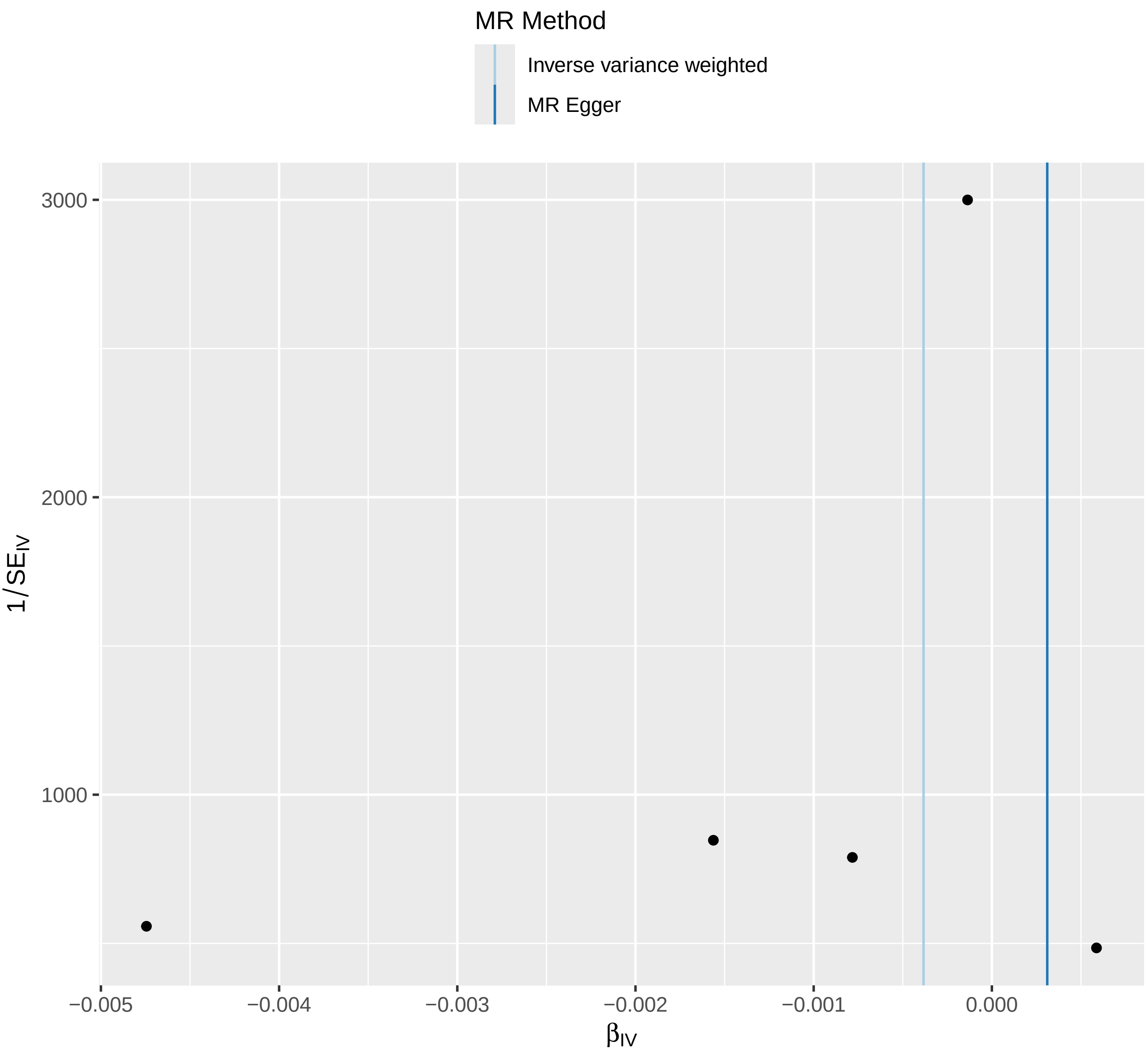
MR Method
Inverse variance weighted
MR Egger
3000
2000
1000
1/SE_IV
−0.005
−0.004
−0.003
−0.002
−0.001
0.000
β_IV

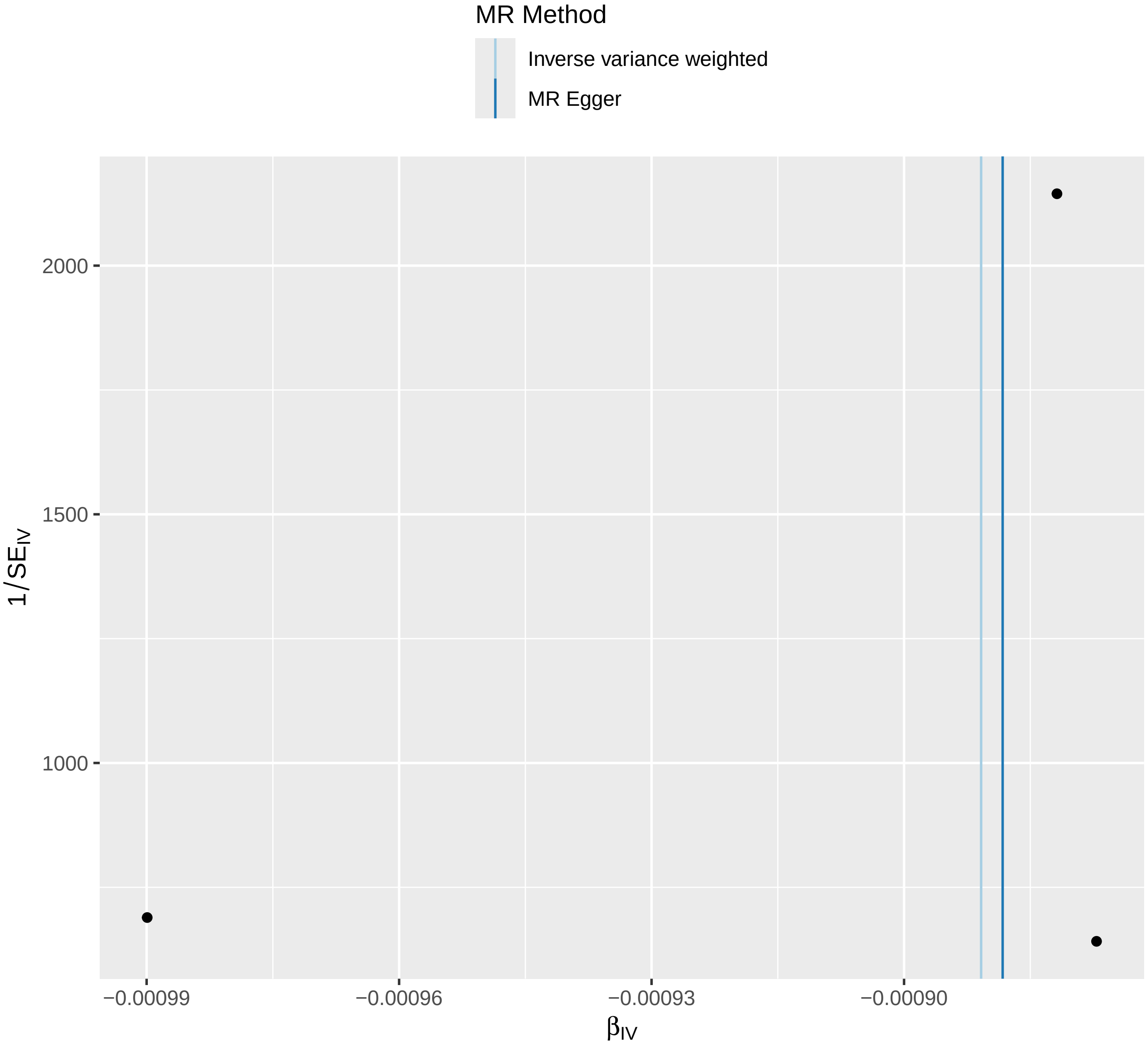

MR Method
Inverse variance weighted
MR Egger
2000
1500
1000
1/SE_IV
−0.00099
−0.00096
−0.00093
−0.00090
β_IV

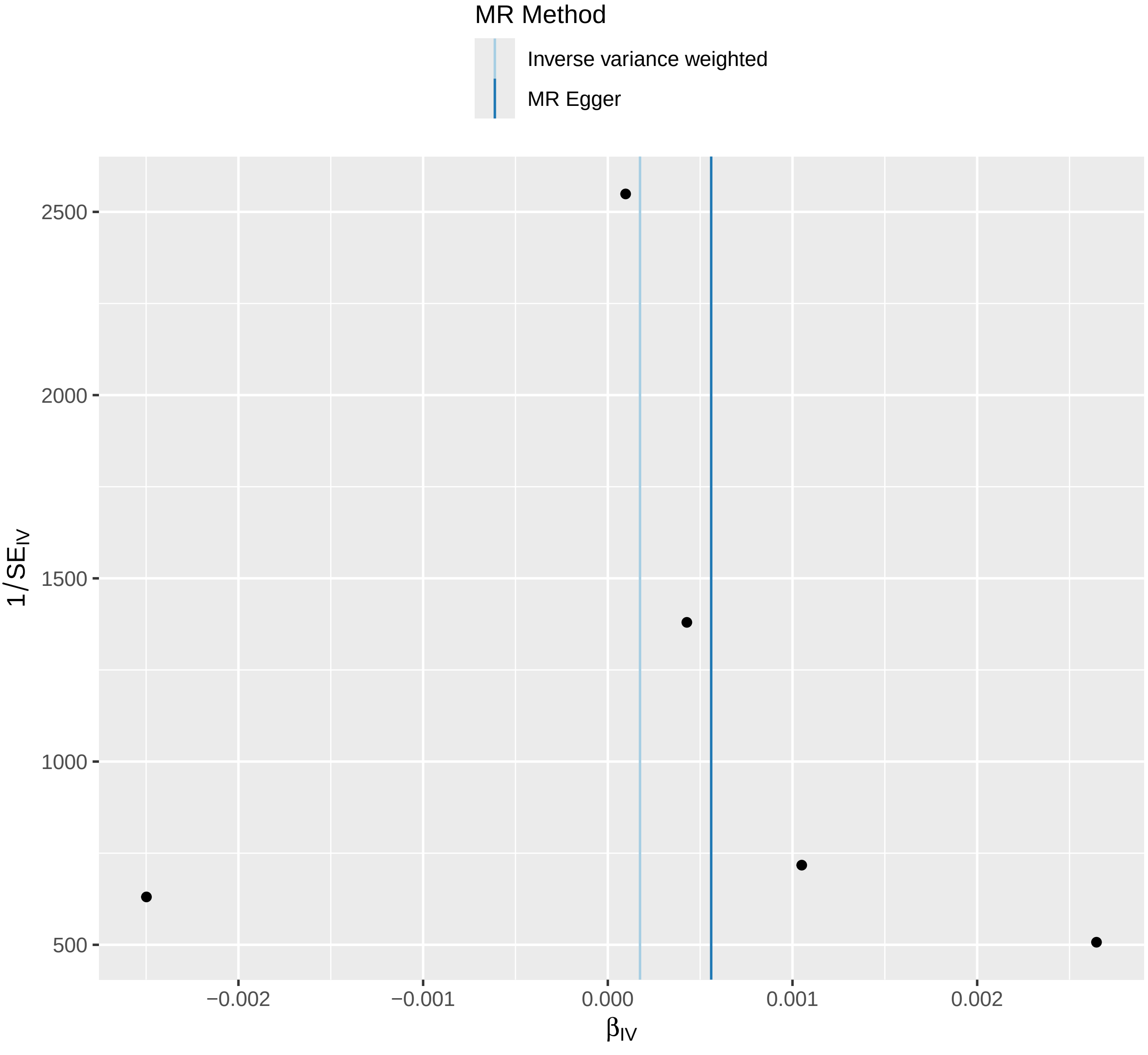

MR Method
Inverse variance weighted
MR Egger
2500
2000
1500
1000
500
1/SE_IV
−0.002
−0.001
0.000
0.001
0.002
β_IV

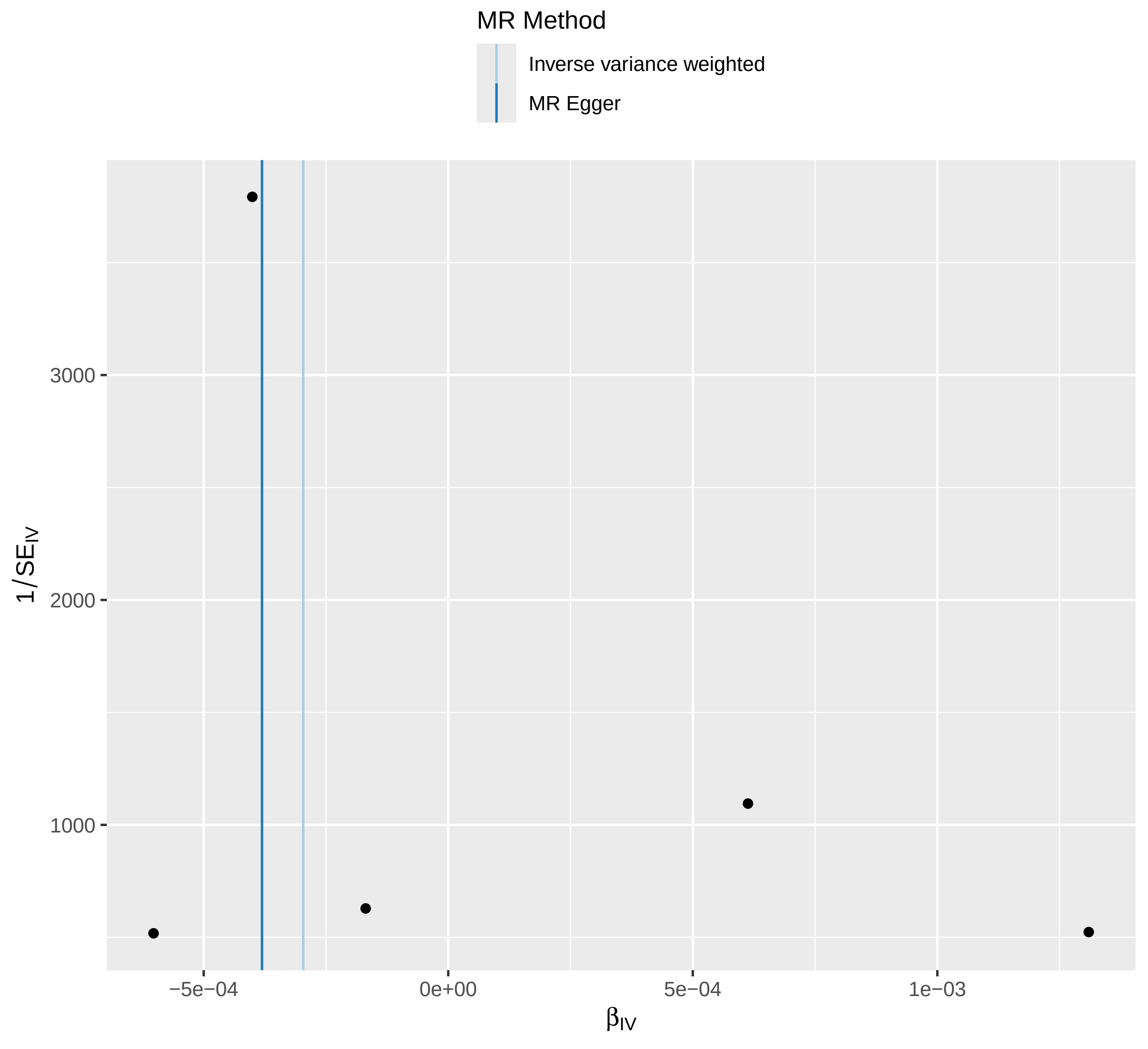
MR Method
Inverse variance weighted
MR Egger
3000
2000
1000
−5e−04
0e+00
5e−04
1e−03
$1/SE_{IV}$
β_{IV}

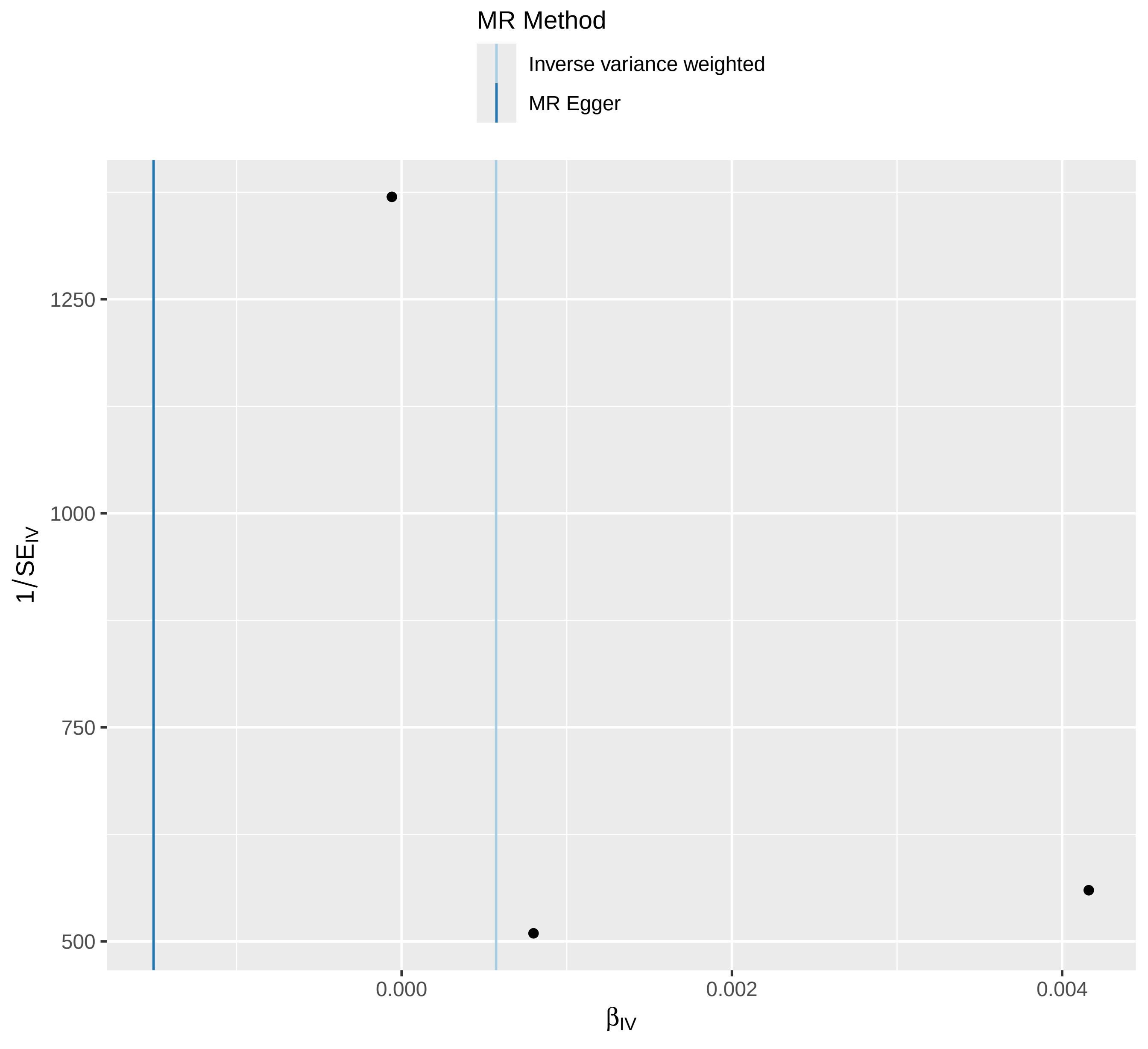

MR Method
Inverse variance weighted
MR Egger
1250
1000
750
500
0.000
0.002
0.004
1/SE_IV
β_IV

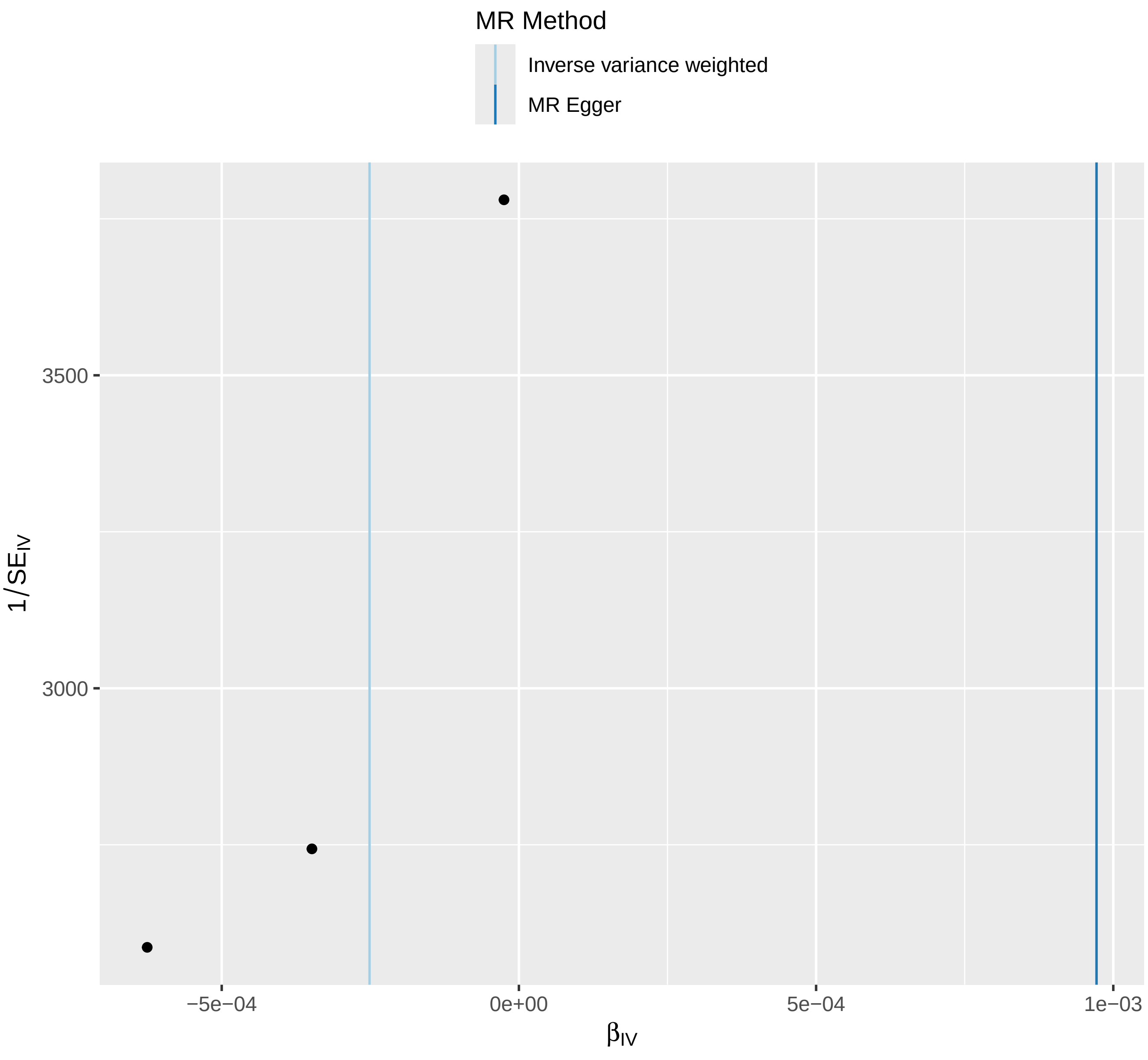
MR Method
Inverse variance weighted
MR Egger
3500
3000
1/SE$_{IV}$
−5e−04
0e+00
5e−04
1e−03
β$_{IV}$

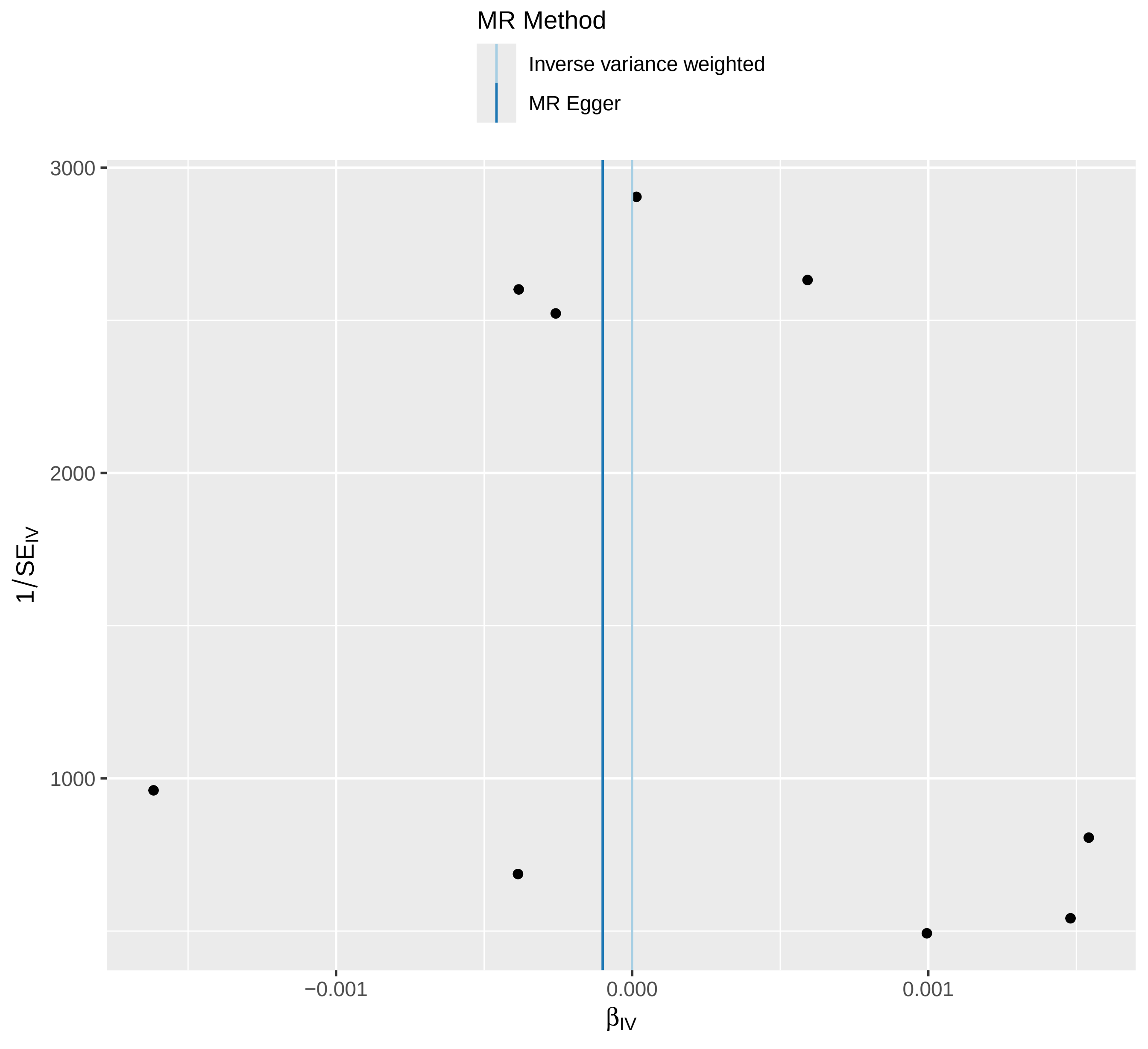

MR Method
Inverse variance weighted
MR Egger
3000
2000
1000
1/SE_IV
−0.001
0.000
0.001
β_IV

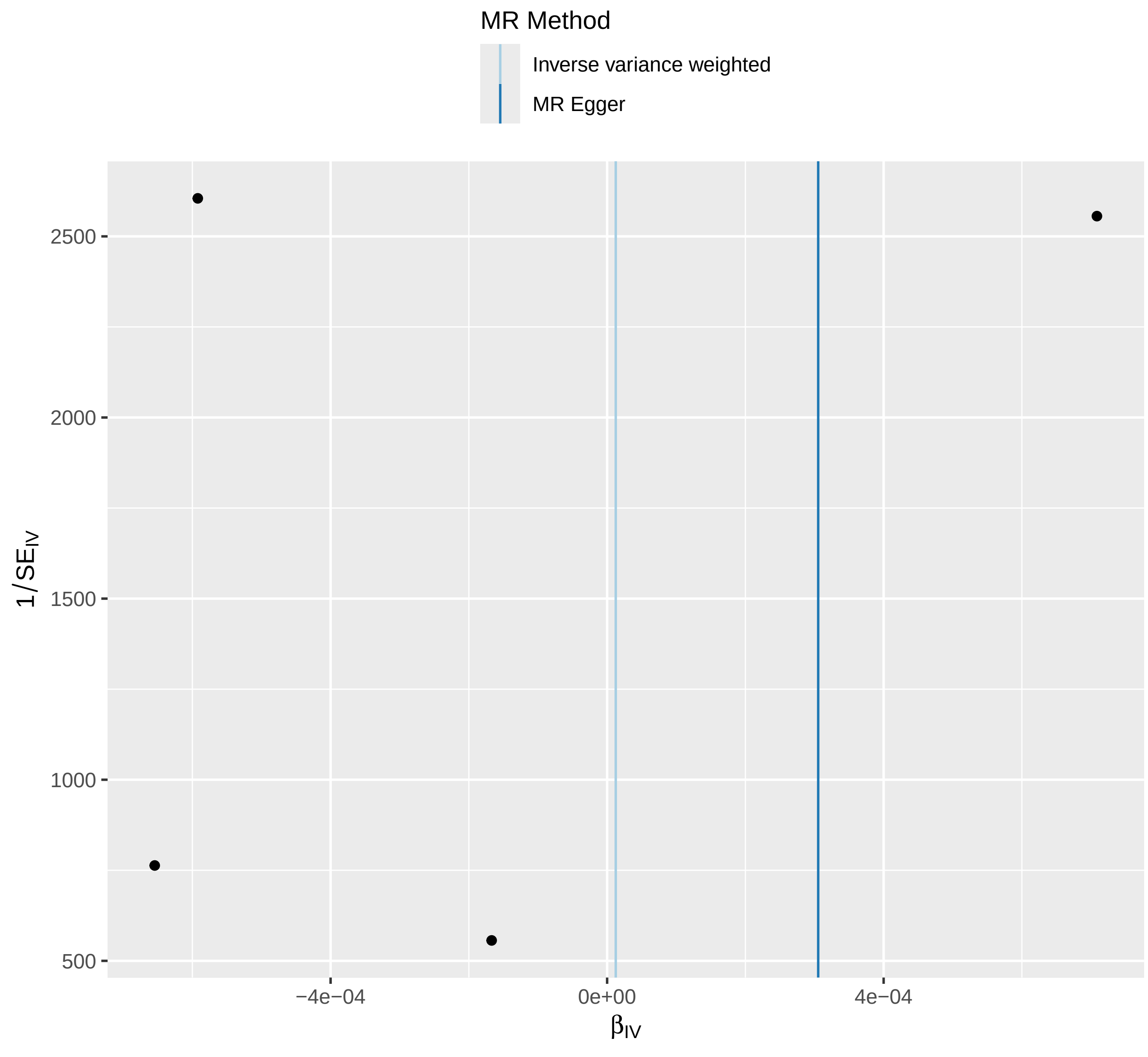

MR Method
Inverse variance weighted
MR Egger
2500
2000
1500
1000
500
1/SE_IV
−4e−04
0e+00
4e−04
β_IV

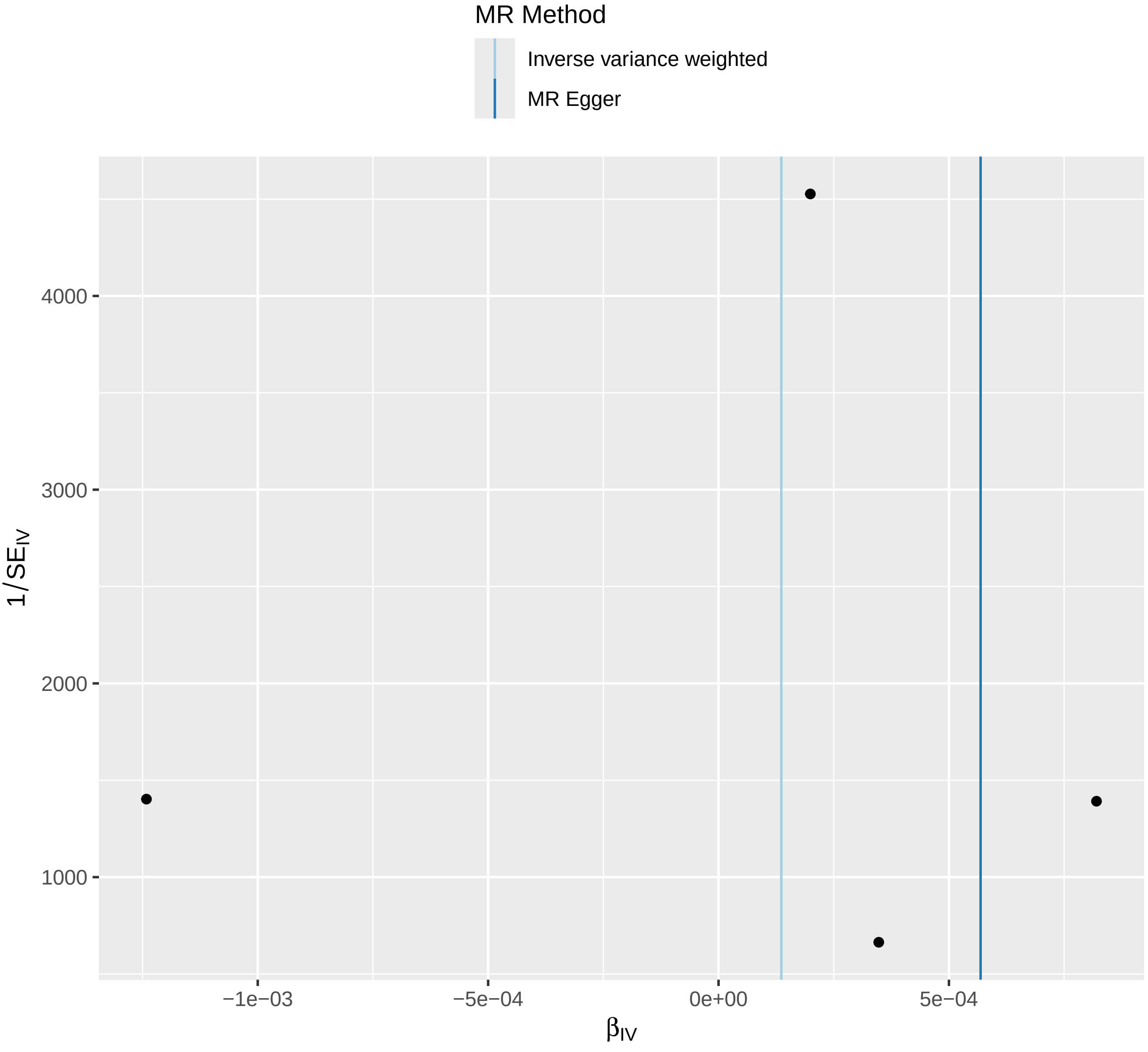

MR Method
Inverse variance weighted
MR Egger
4000
3000
2000
1000
1/SE$_{IV}$
−1e−03
−5e−04
0e+00
5e−04
β$_{IV}$

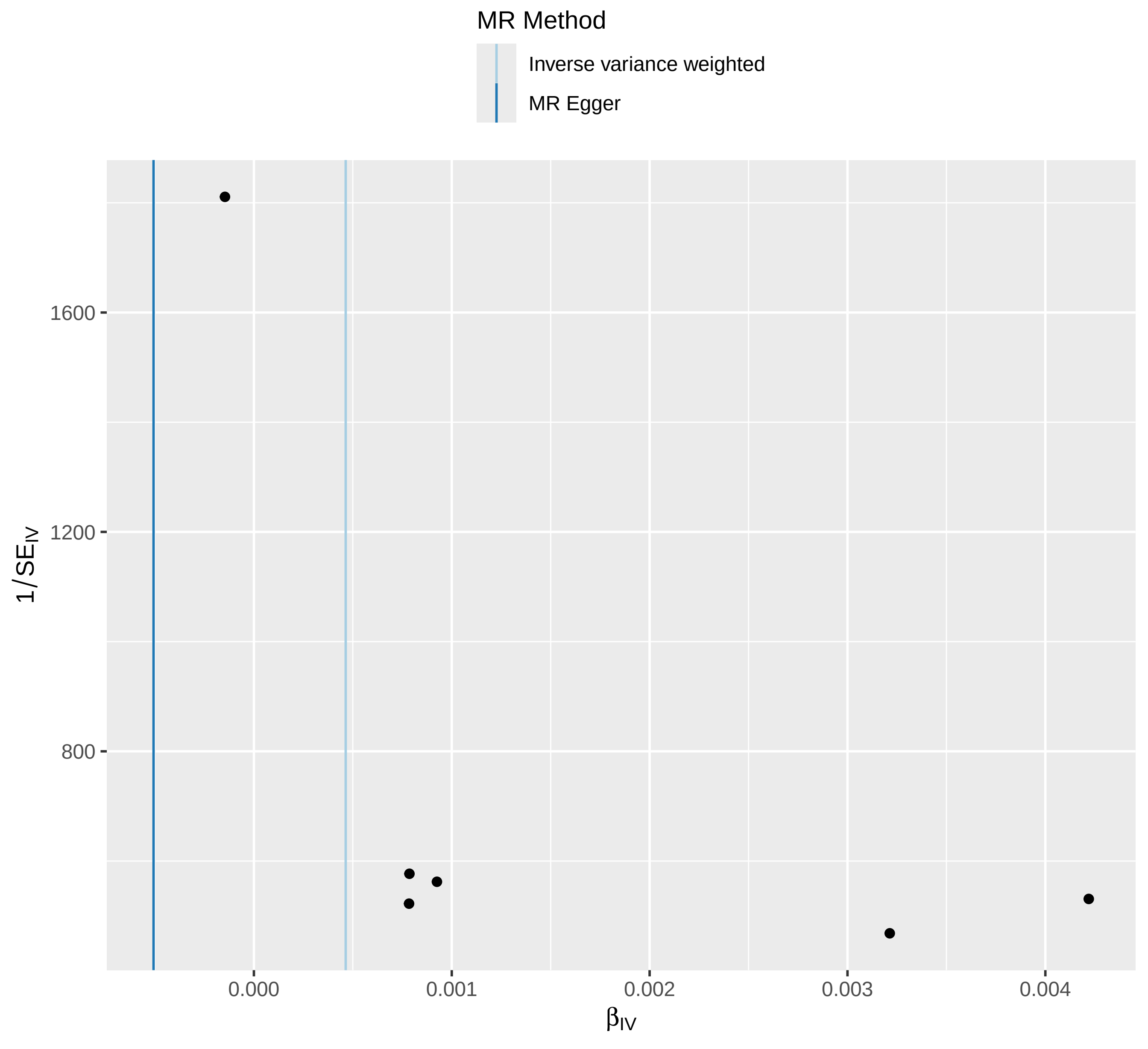
MR Method
Inverse variance weighted
MR Egger
1600
1200
800
1/SE_IV
0.000
0.001
0.002
0.003
0.004
β_IV

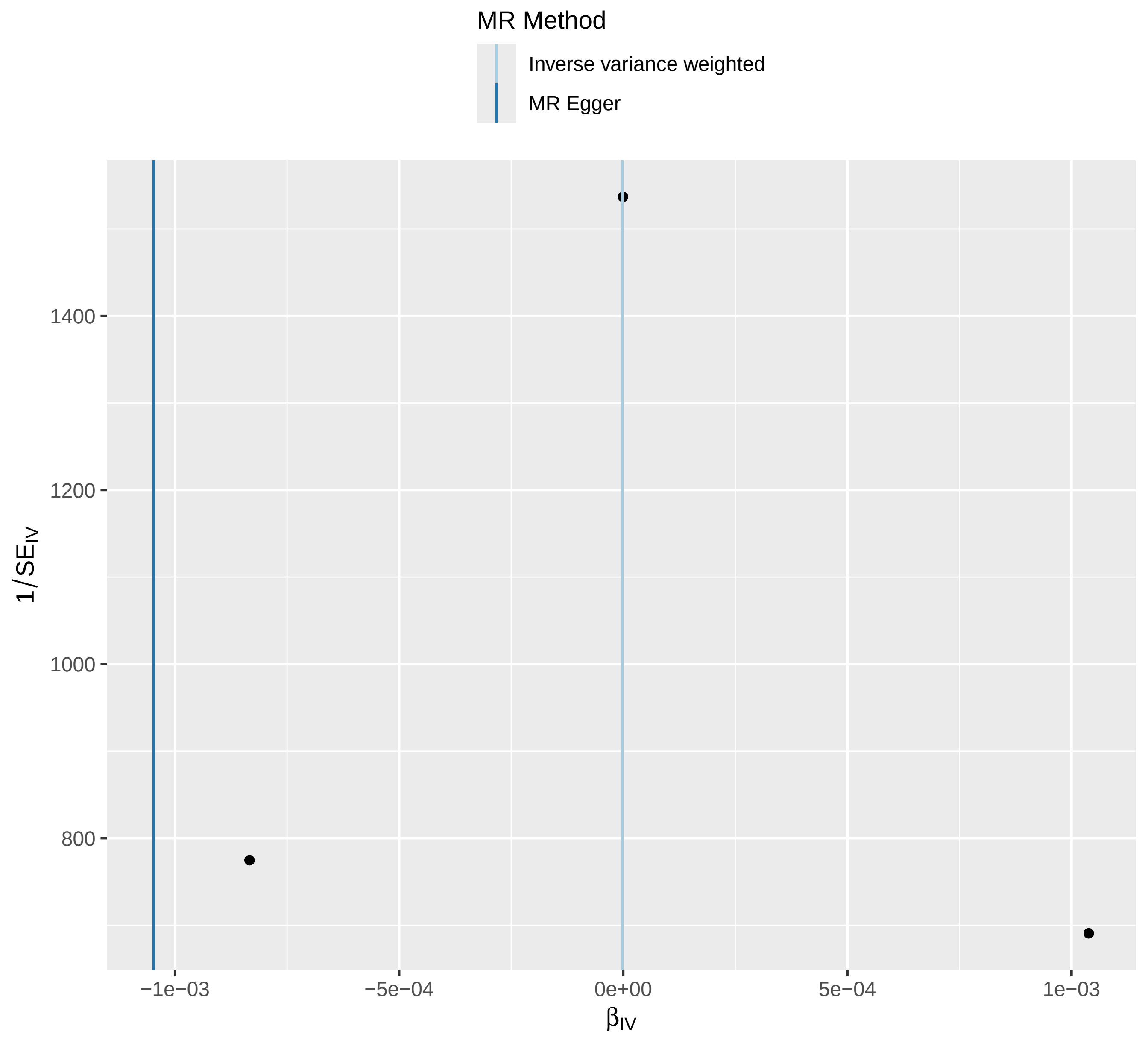

MR Method
Inverse variance weighted
MR Egger
1400
1200
1000
800
1/SE$_{IV}$
−1e−03
−5e−04
0e+00
5e−04
1e−03
β_{IV}

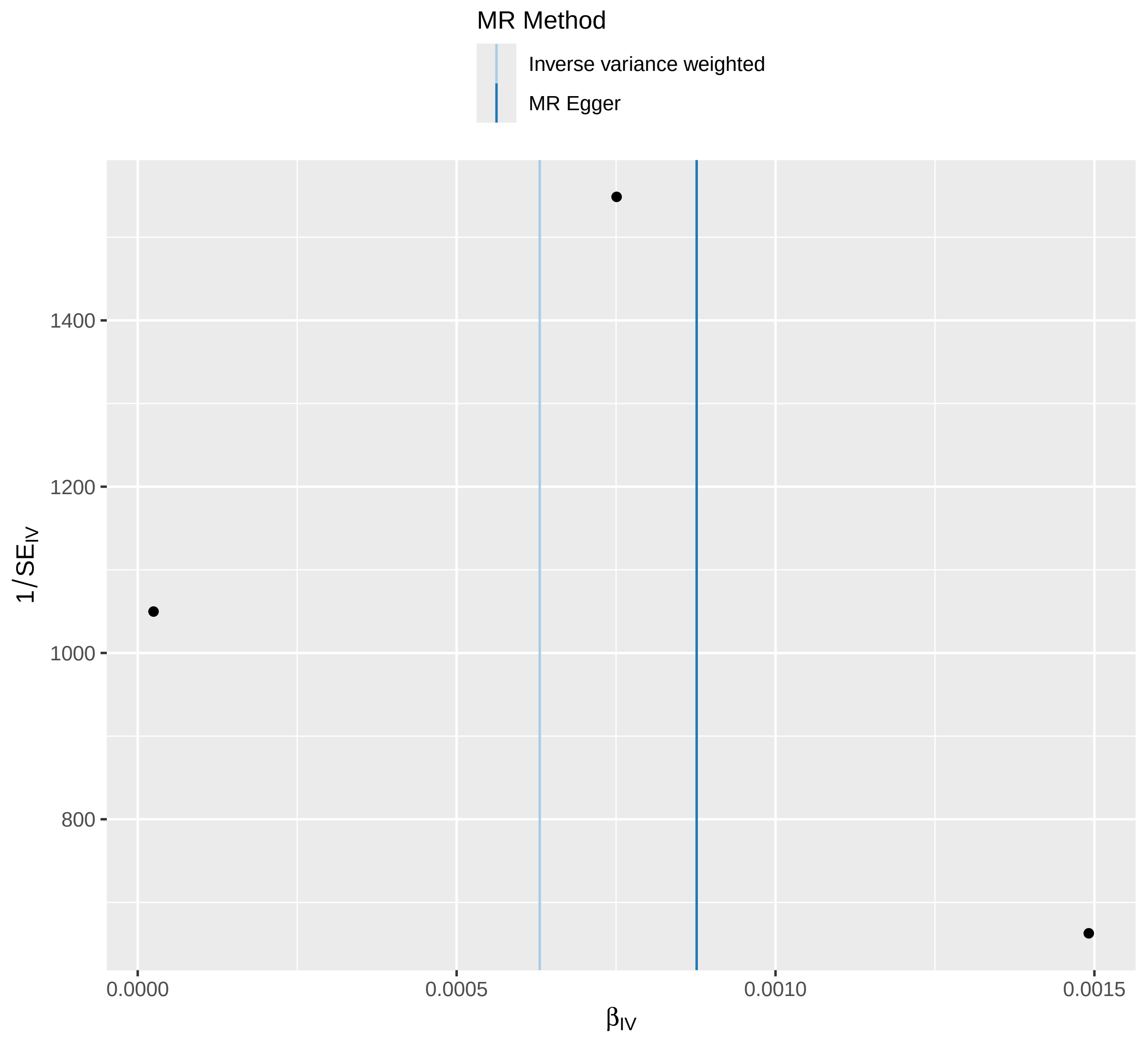

MR Method
Inverse variance weighted
MR Egger
1400
1200
1000
800
1/SE_IV
0.0000
0.0005
0.0010
0.0015
β_IV

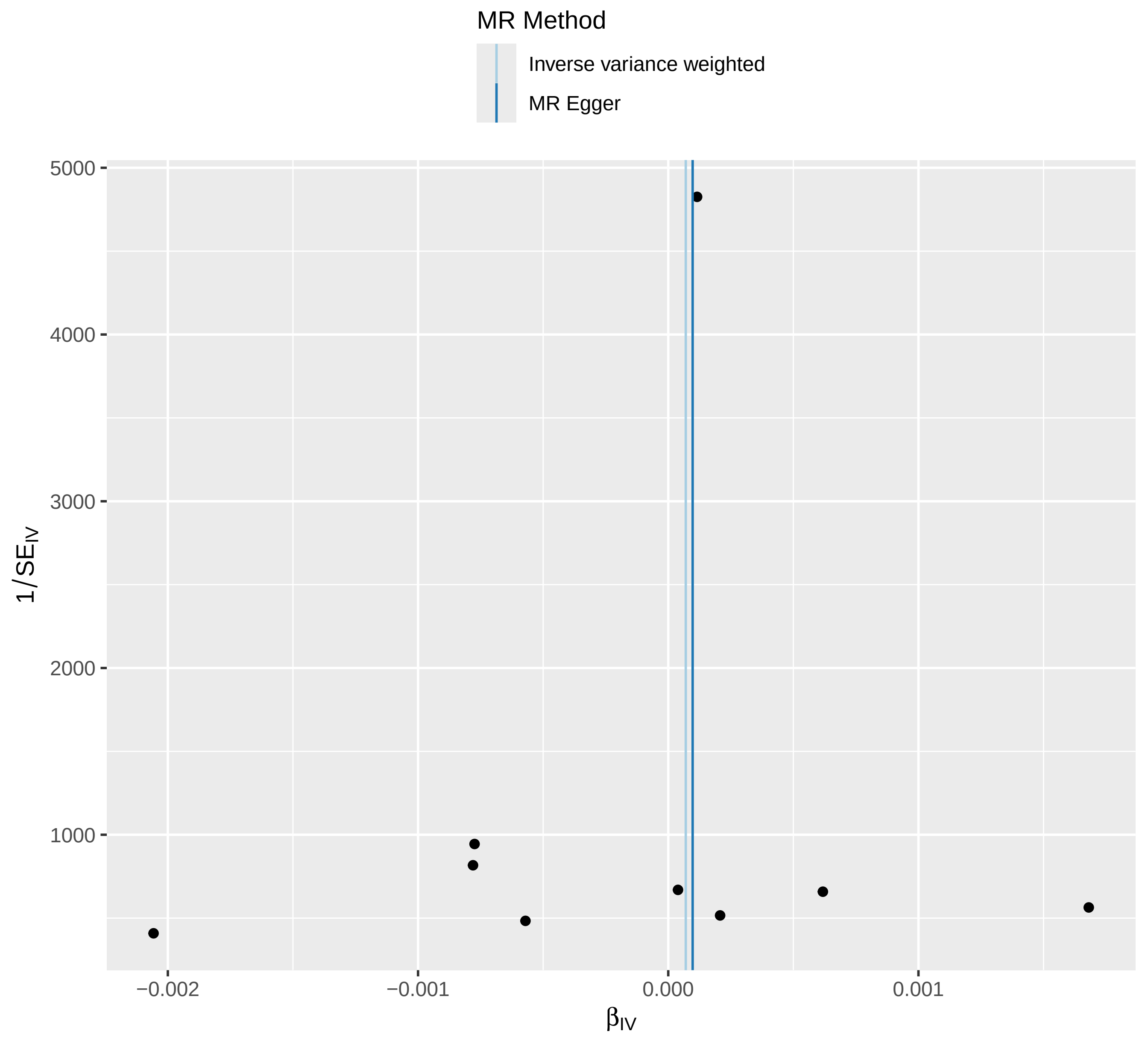
MR Method
Inverse variance weighted
MR Egger
5000
4000
3000
2000
1000
1/SE_IV
−0.002
−0.001
0.000
0.001
β_IV

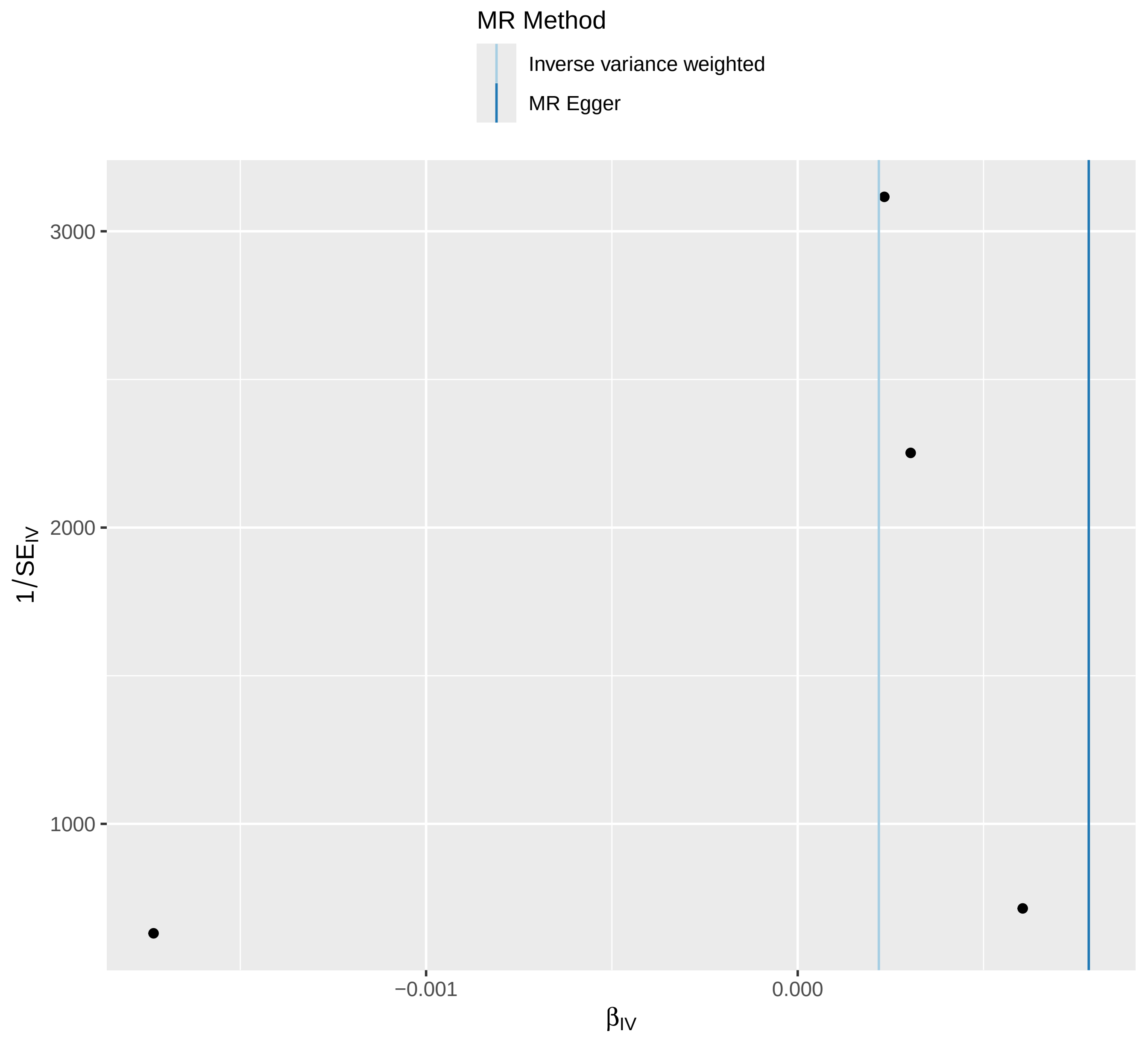

MR Method
Inverse variance weighted
MR Egger
3000
2000
1000
1/SE_IV
−0.001
0.000
β_IV

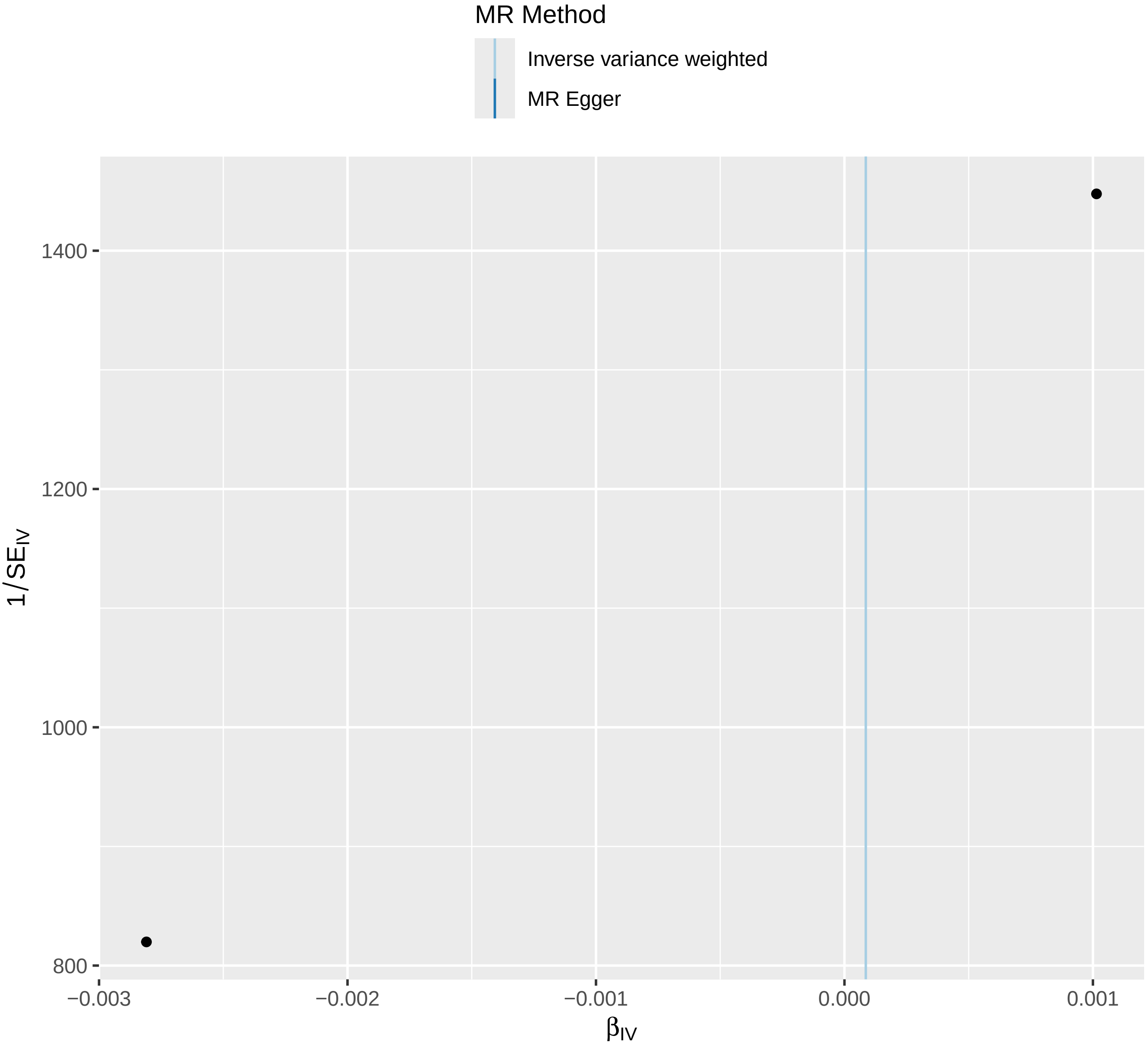

MR Method
Inverse variance weighted
MR Egger
1400
1200
1000
800
1/SE_IV
−0.003
−0.002
−0.001
0.000
0.001
β_IV

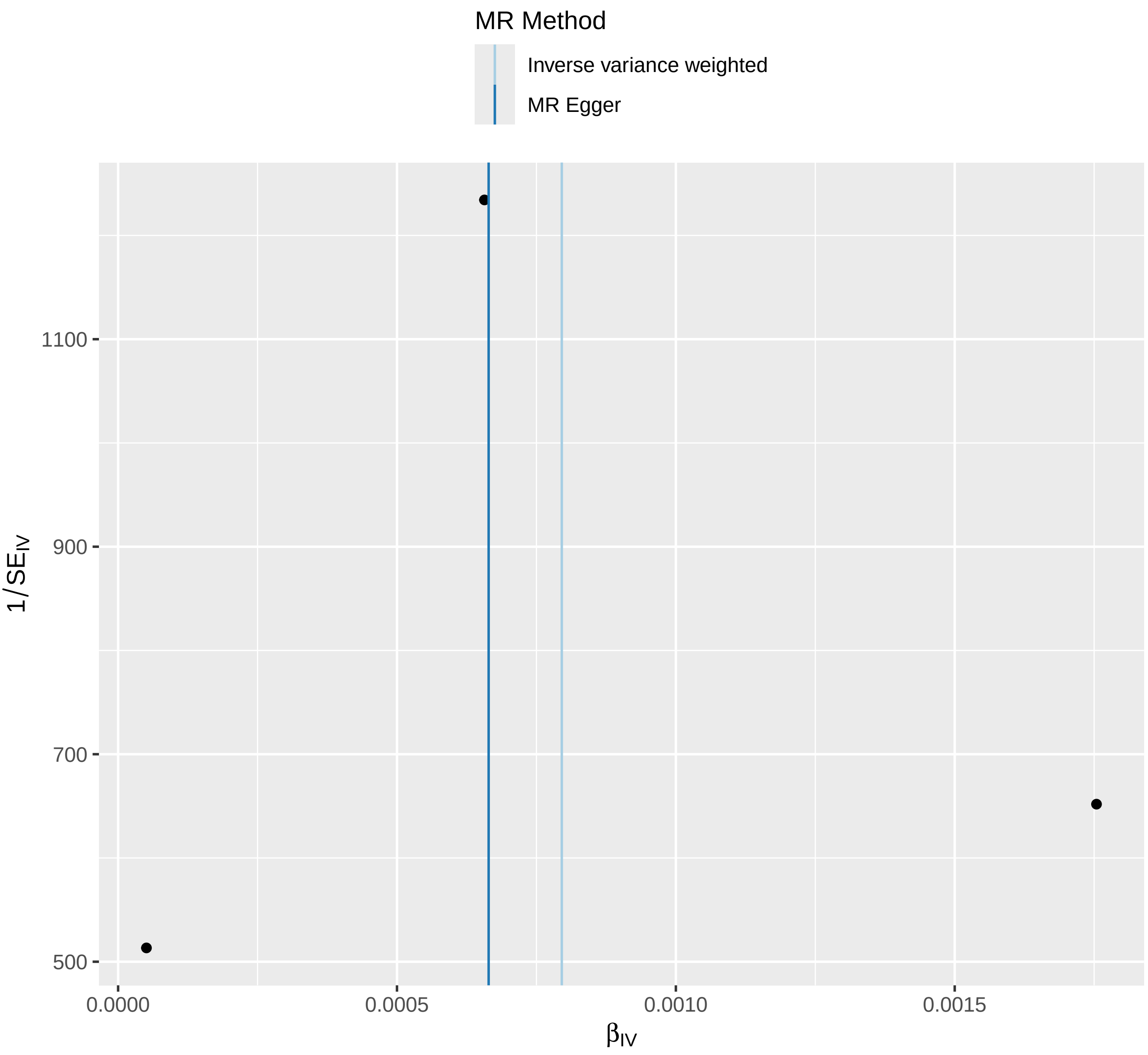
MR Method
Inverse variance weighted
MR Egger
1100
900
700
500
$1/SE_{IV}$
0.0000
0.0005
0.0010
0.0015
β_{IV}

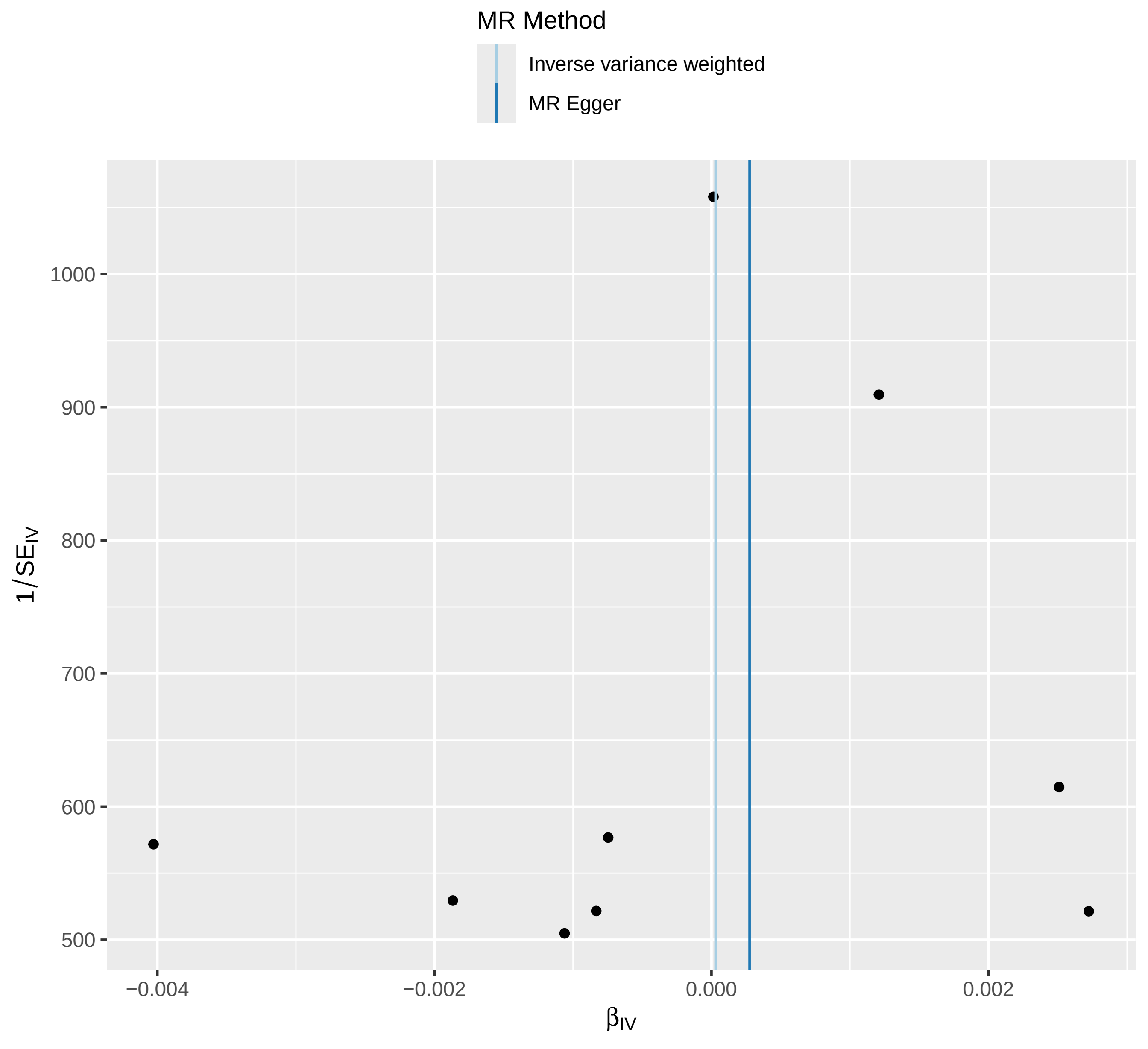

MR Method
Inverse variance weighted
MR Egger
1000
900
800
700
600
500
1/SE_IV
−0.004
−0.002
0.000
0.002
β_IV

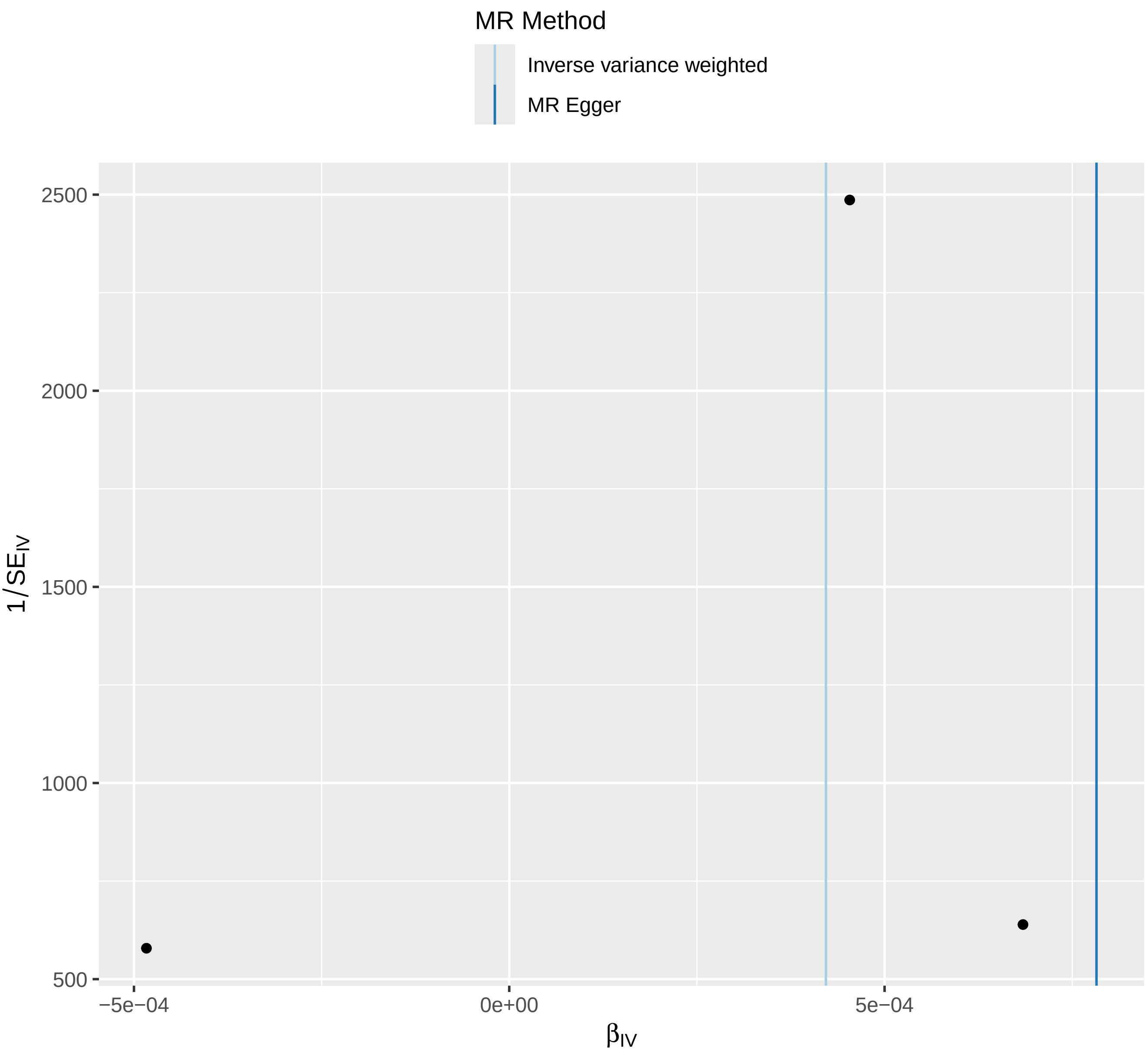

MR Method
Inverse variance weighted
MR Egger
2500
2000
1500
1000
500
1/SE_IV
−5e−04
0e+00
5e−04
β_IV

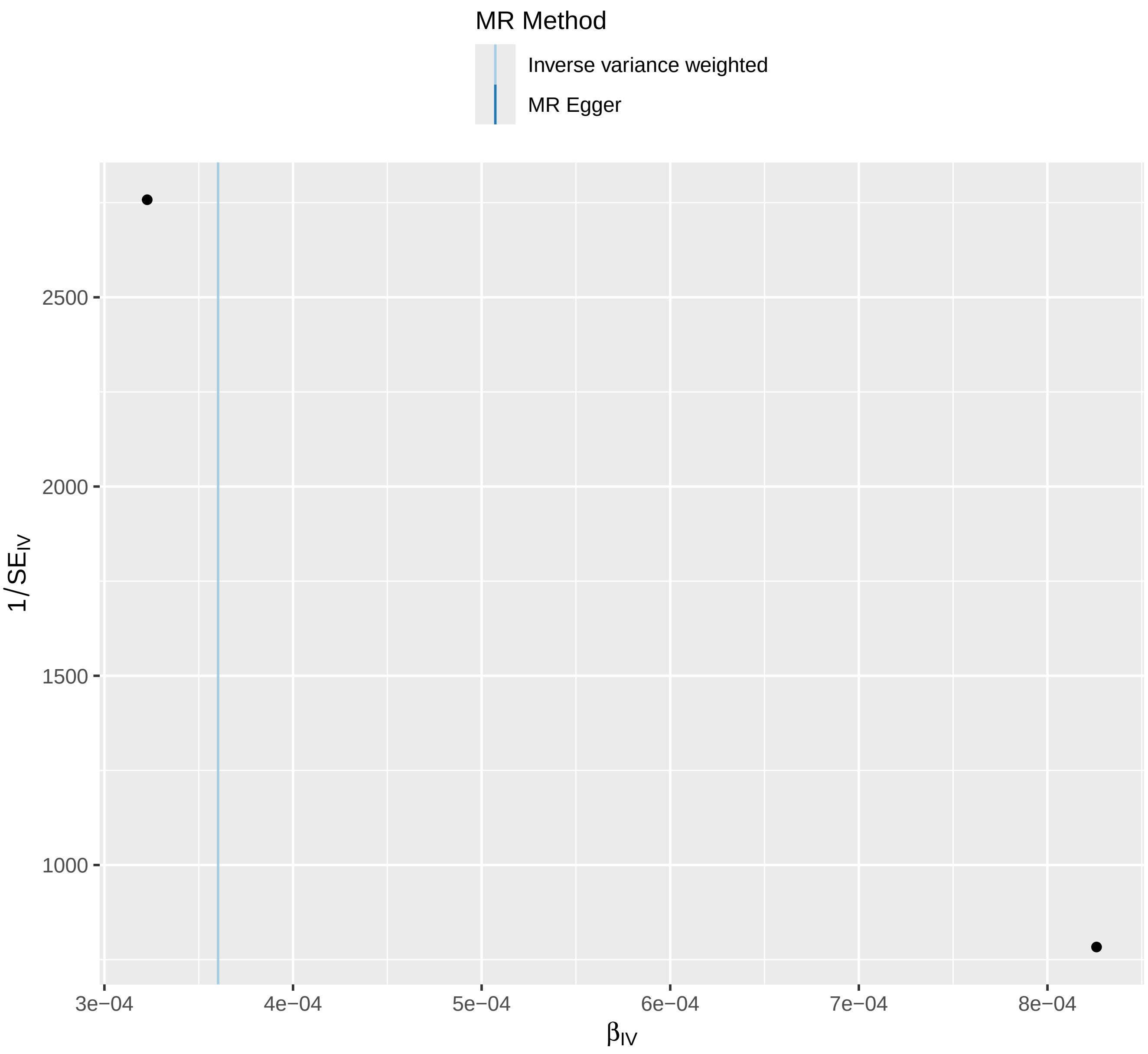

MR Method
Inverse variance weighted
MR Egger
2500
2000
1500
1000
1/SE_IV
3e−04
4e−04
5e−04
6e−04
7e−04
8e−04
β_IV

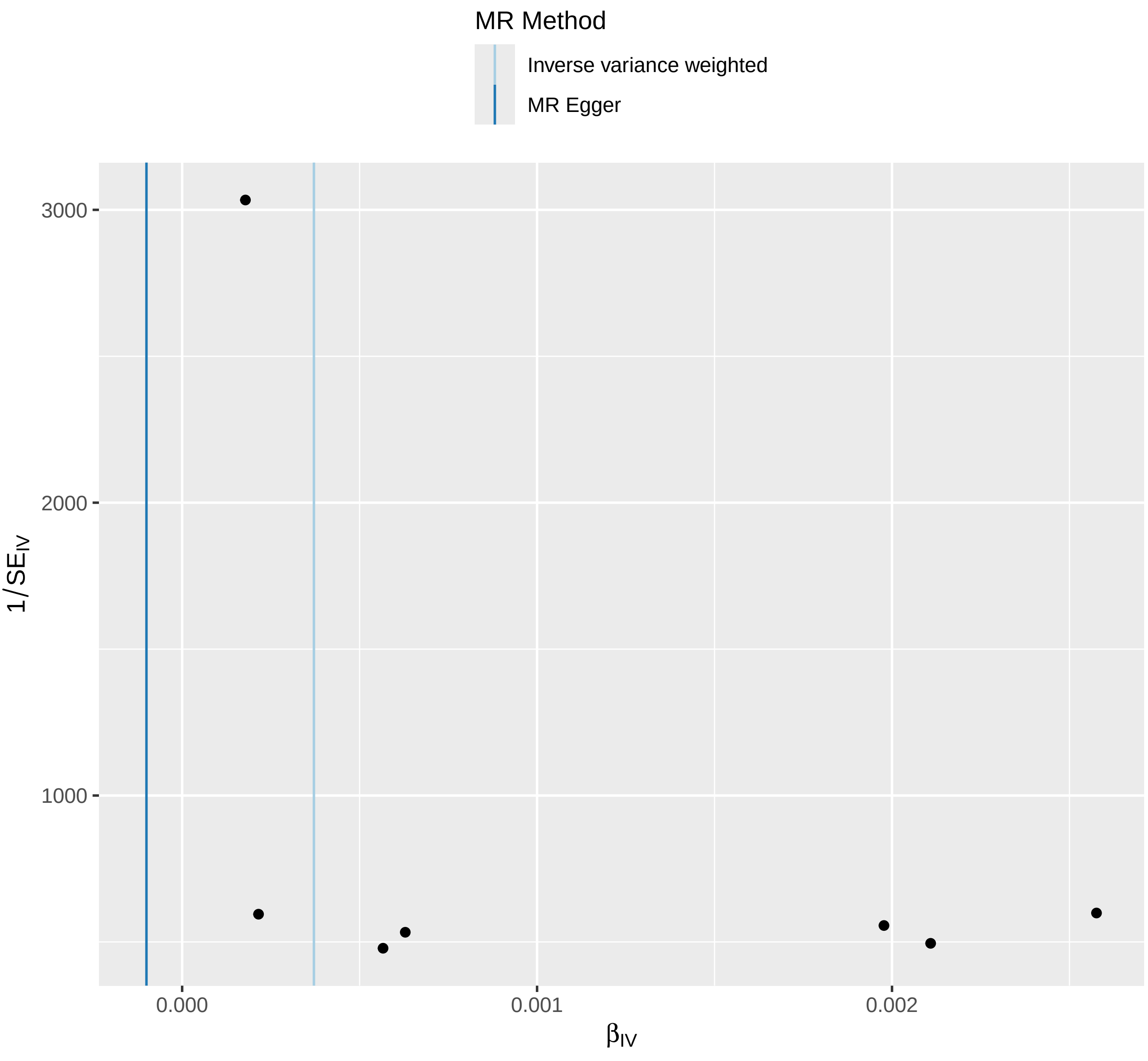

MR Method
Inverse variance weighted
MR Egger
3000
2000
1000
$1/SE_{IV}$
0.000
0.001
0.002
β_{IV}

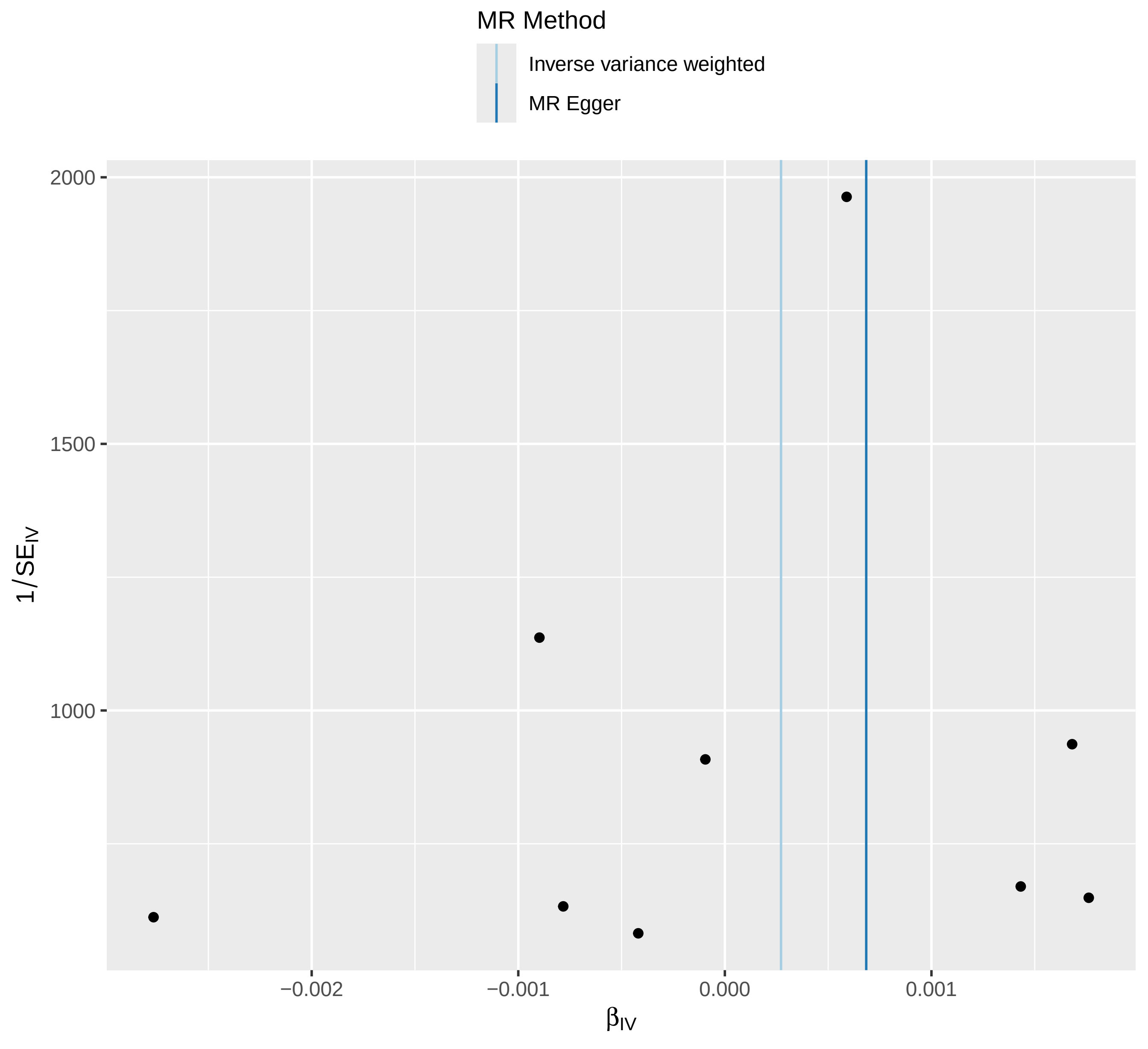

MR Method
Inverse variance weighted
MR Egger
2000
1500
1000
1/SE_IV
−0.002
−0.001
0.000
0.001
β_IV

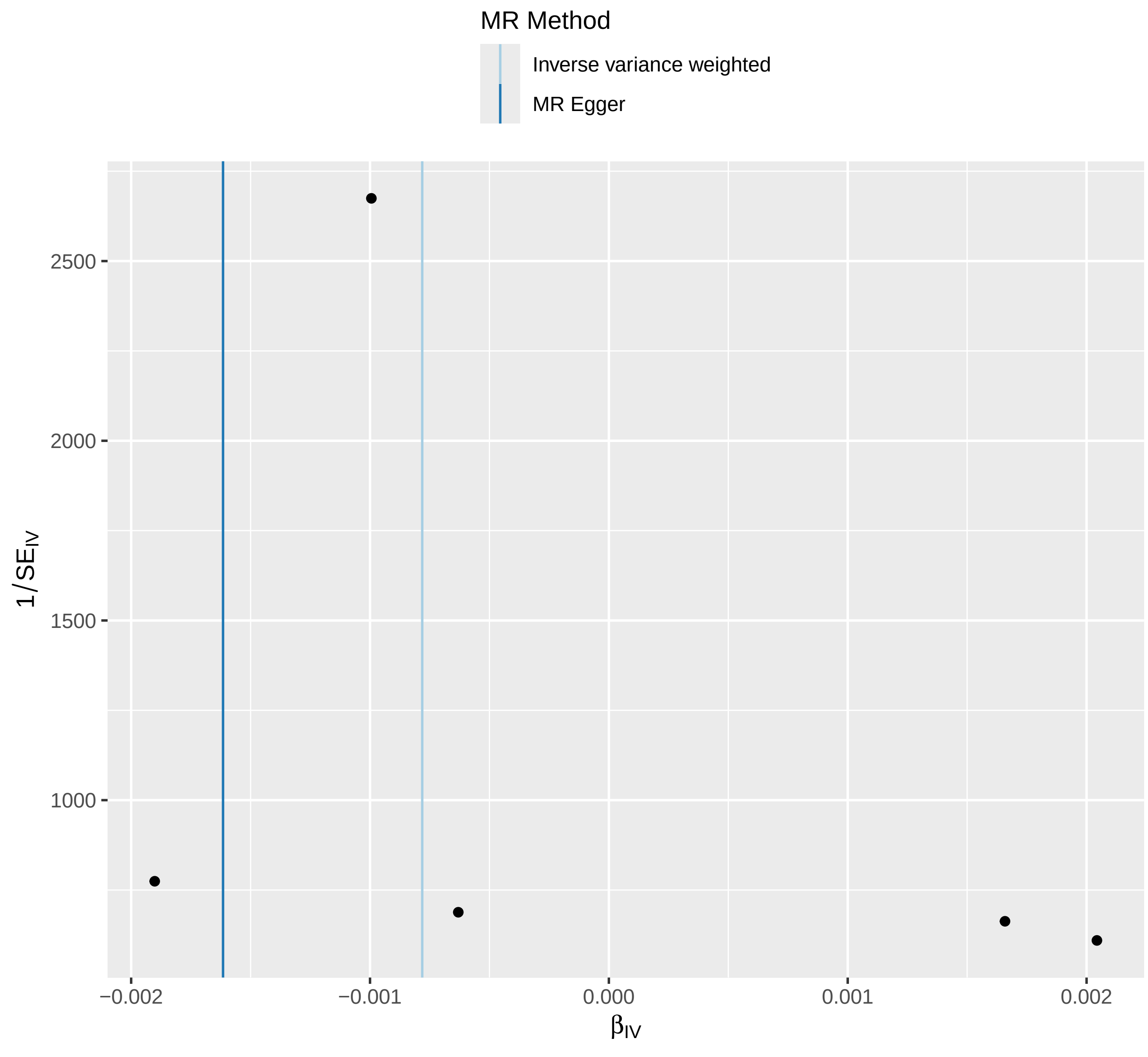

MR Method
Inverse variance weighted
MR Egger
2500
2000
1500
1000
1/SE$_{IV}$
−0.002
−0.001
0.000
0.001
0.002
β$_{IV}$

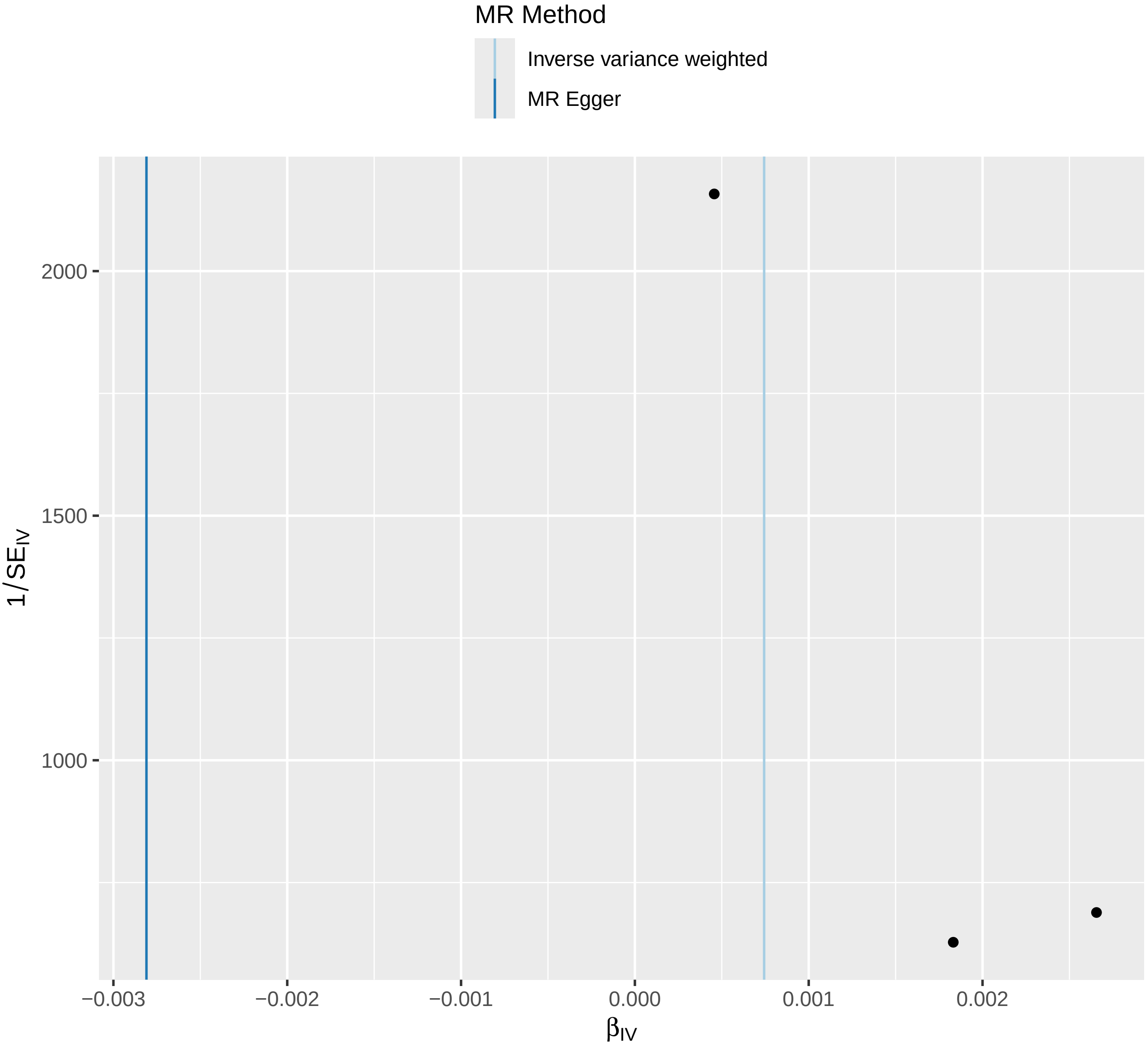
MR Method
Inverse variance weighted
MR Egger
2000
1500
1000
1/SE_IV
−0.003
−0.002
−0.001
0.000
0.001
0.002
β_IV

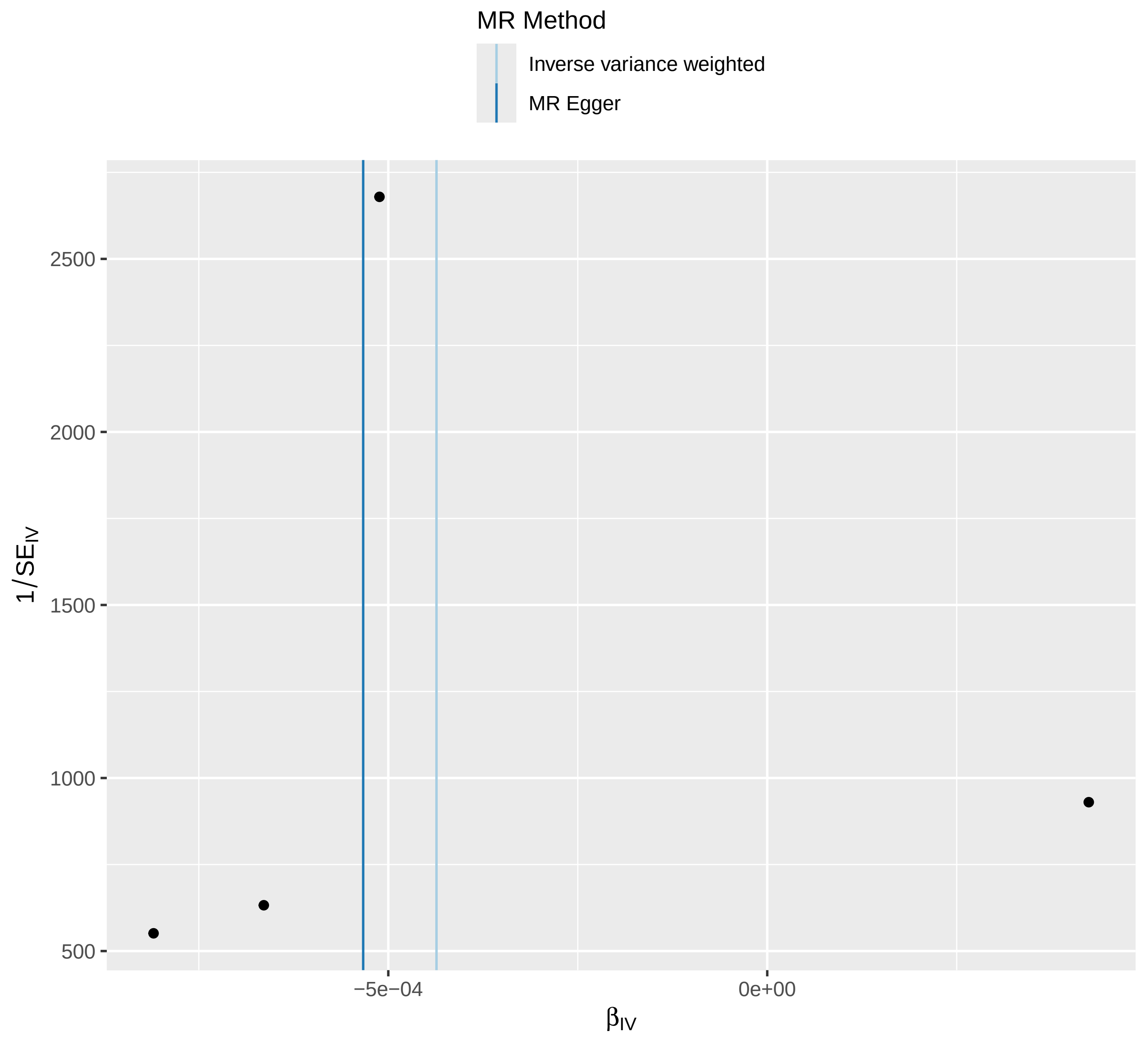

MR Method
Inverse variance weighted
MR Egger
2500
2000
1500
1000
500
1/SE_IV
−5e−04
0e+00
β_IV

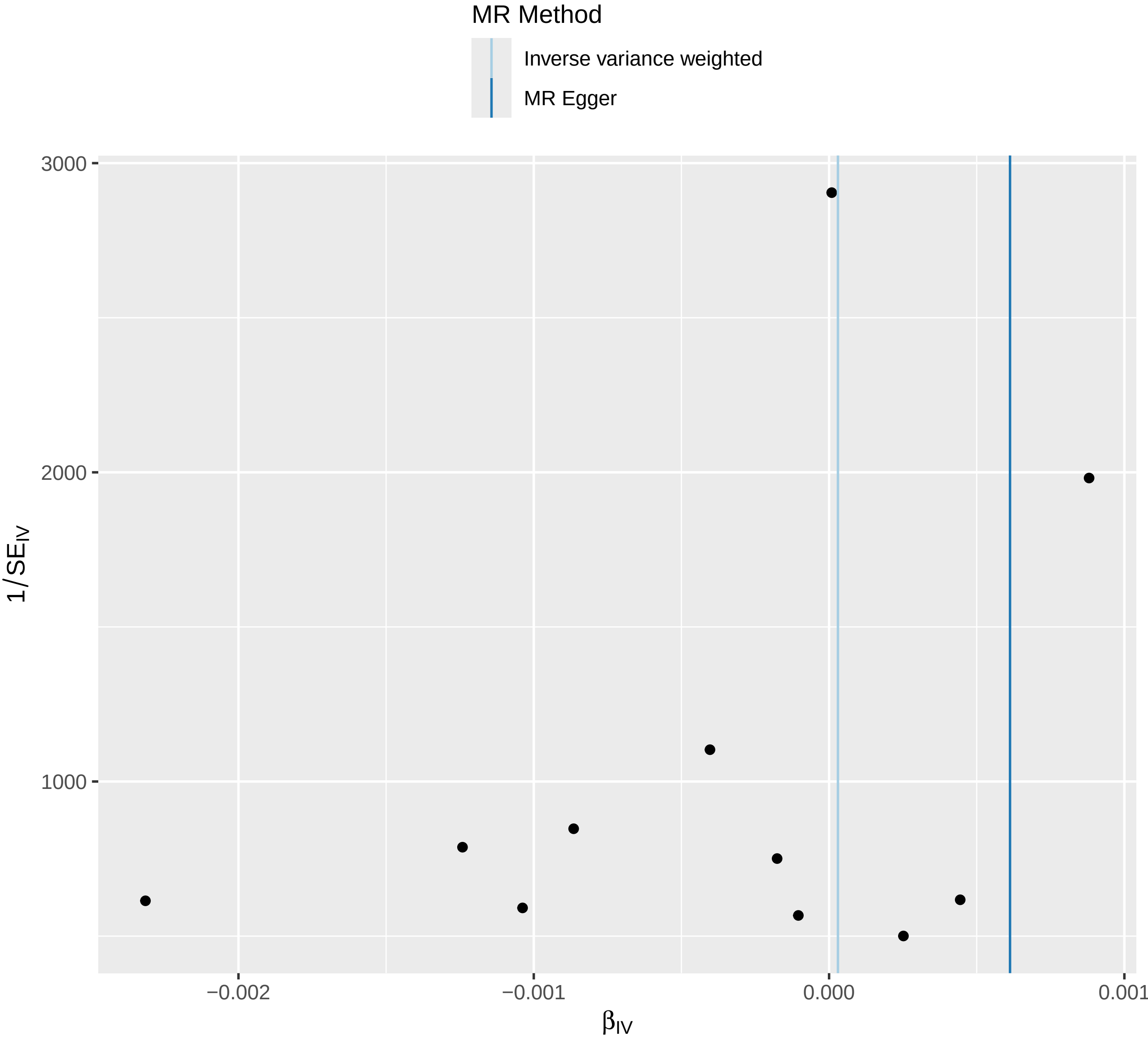

MR Method
Inverse variance weighted
MR Egger
3000
2000
1000
1/SE$_{IV}$
−0.002
−0.001
0.000
0.001
β$_{IV}$

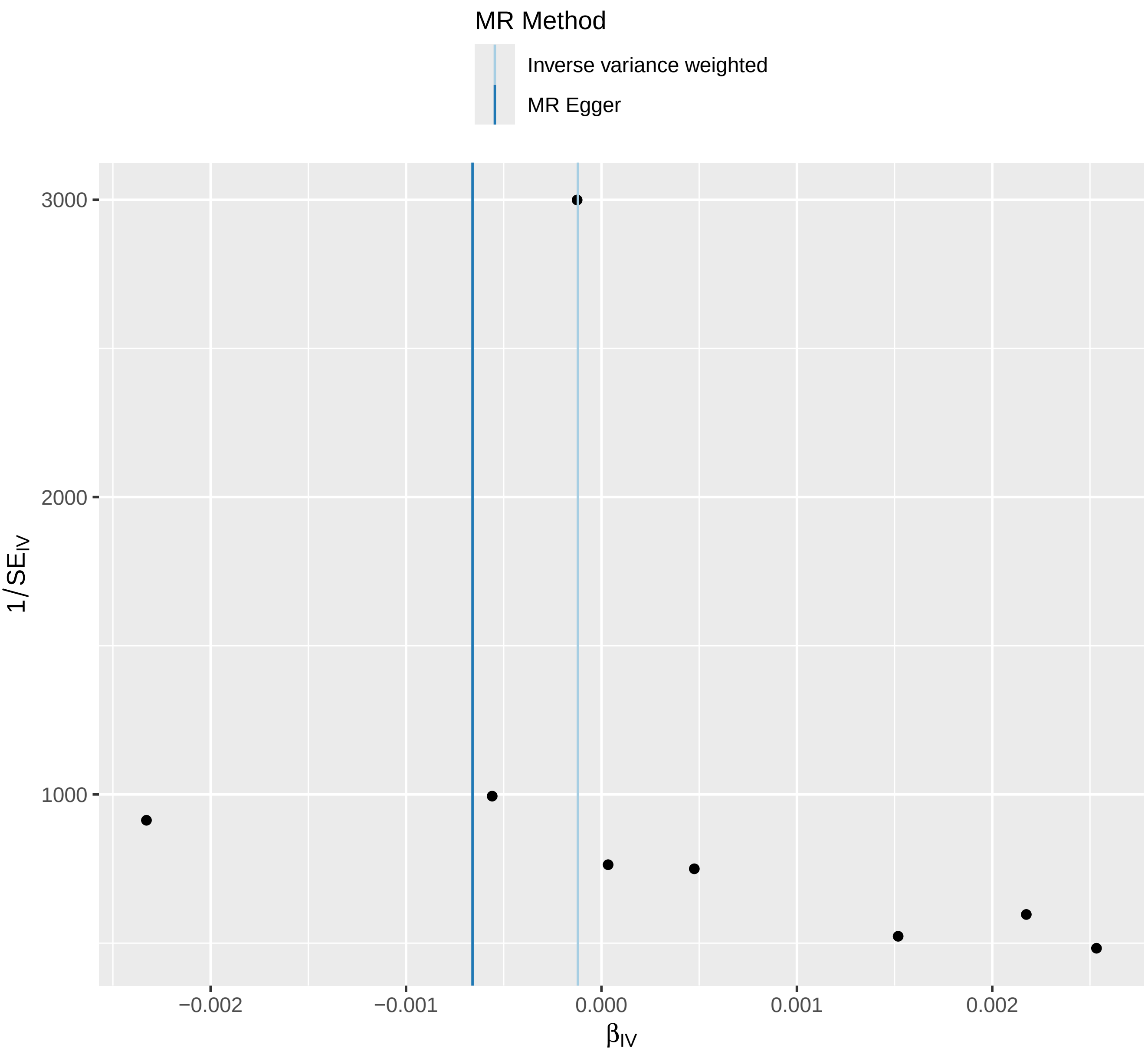
MR Method
Inverse variance weighted
MR Egger
3000
2000
1000
1/SE_IV
−0.002
−0.001
0.000
0.001
0.002
β_IV

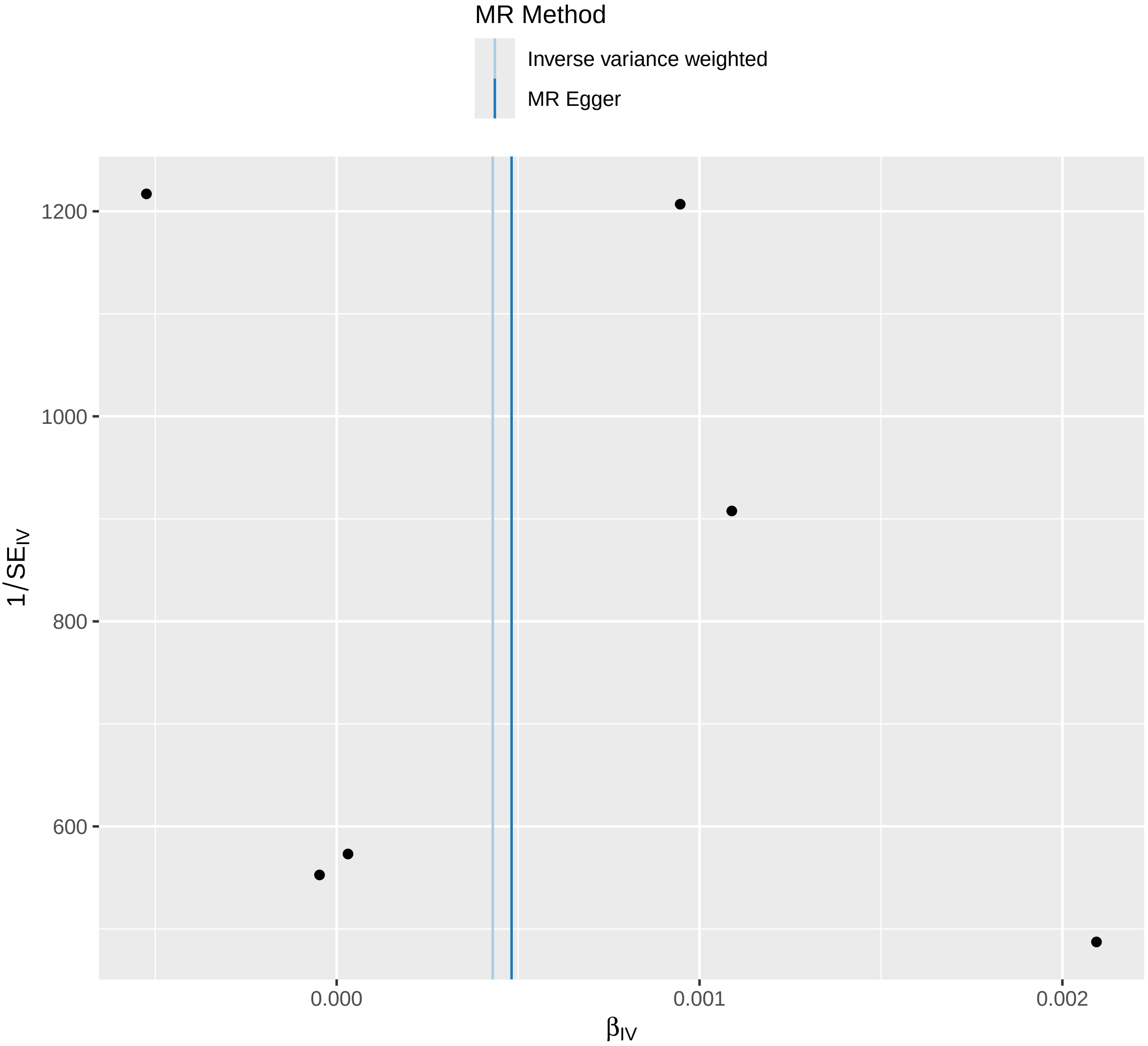

MR Method
Inverse variance weighted
MR Egger
1200
1000
800
600
0.000
0.001
0.002
β_{IV}
$1/SE_{IV}$

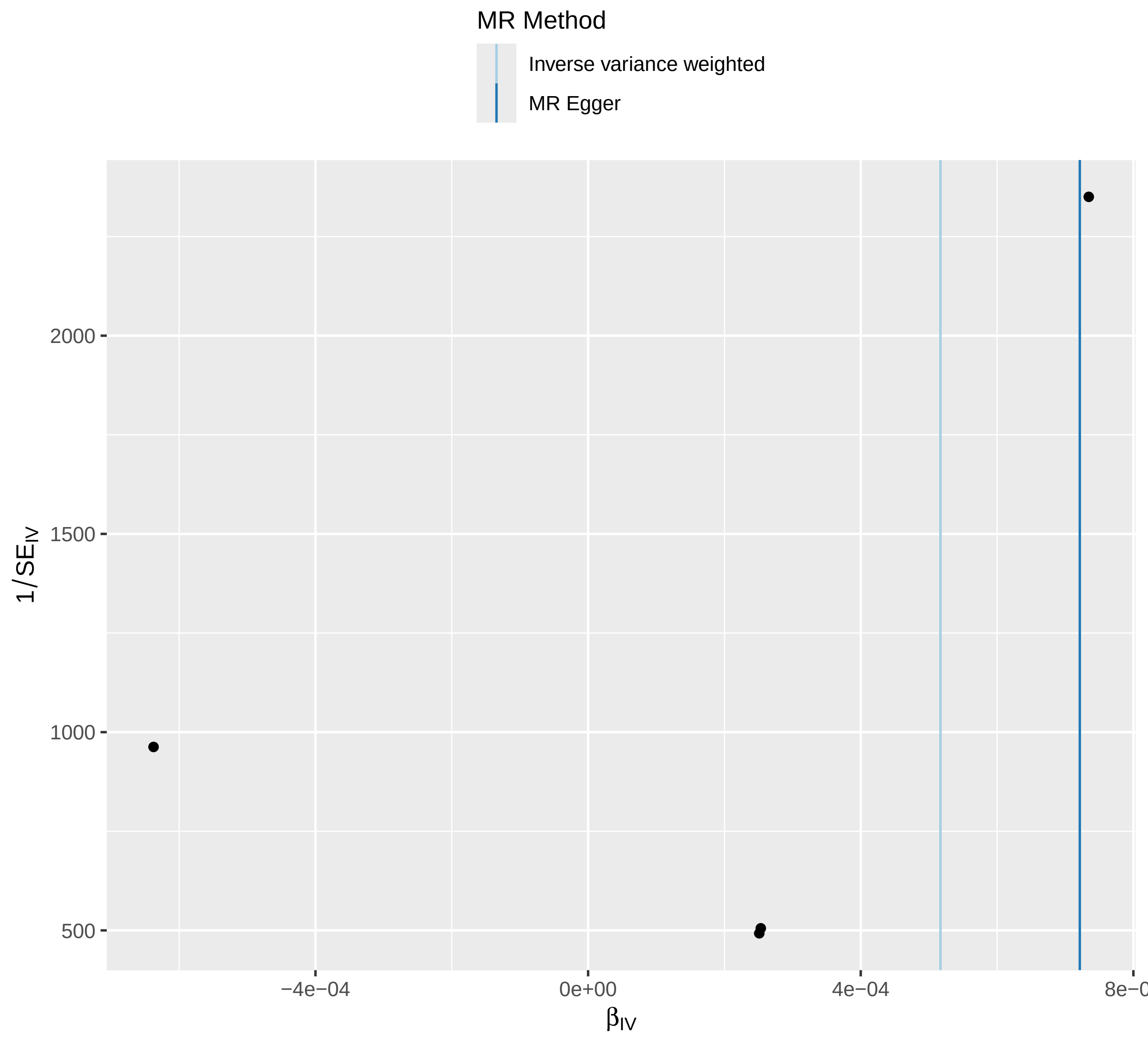

MR Method
Inverse variance weighted
MR Egger
2000
1500
1000
500
1/SE$_{IV}$
−4e−04
0e+00
4e−04
8e−0
β$_{IV}$

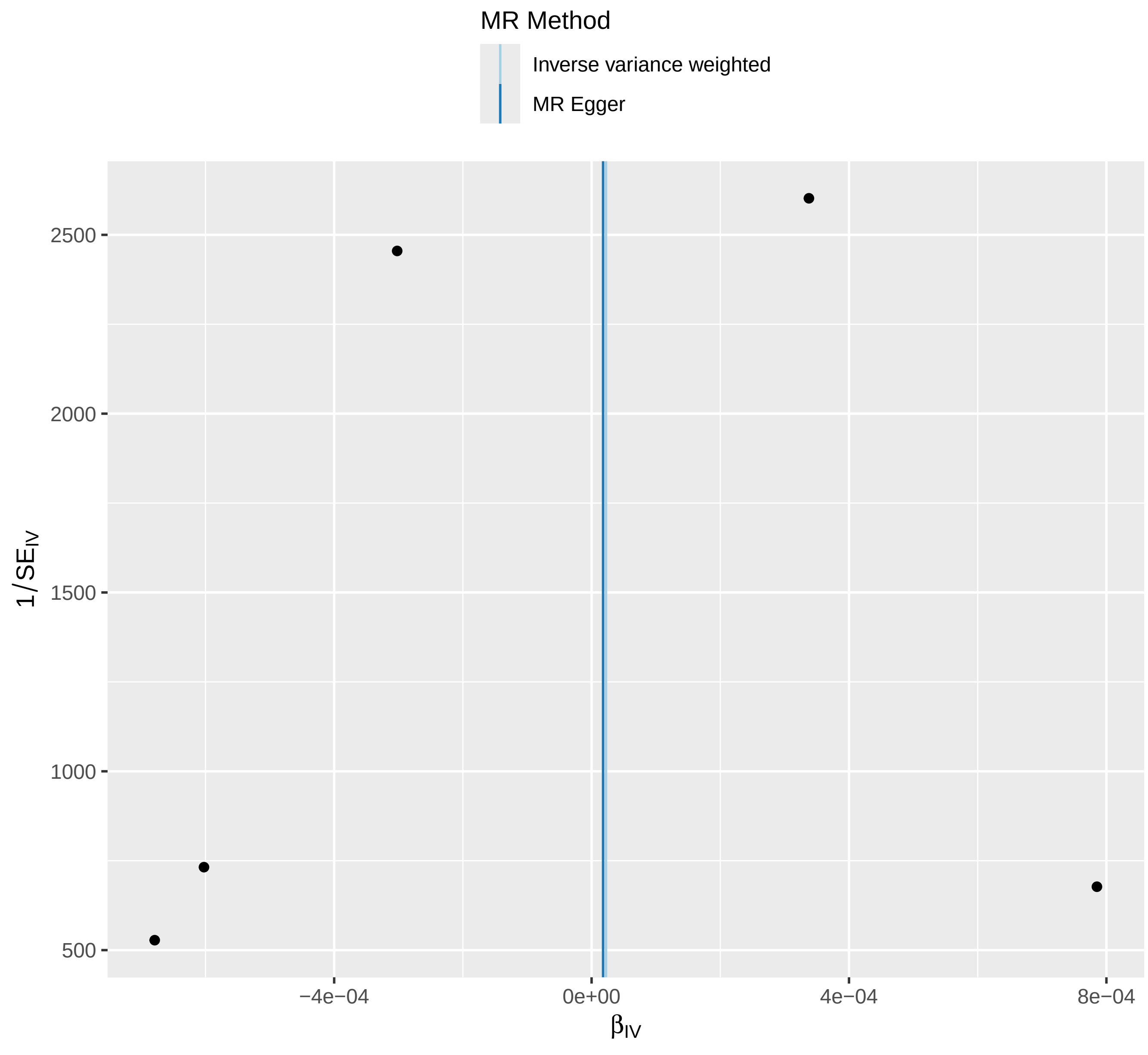

MR Method
Inverse variance weighted
MR Egger
2500
2000
1500
1000
500
1/SE$_{IV}$
−4e−04
0e+00
4e−04
8e−04
β$_{IV}$

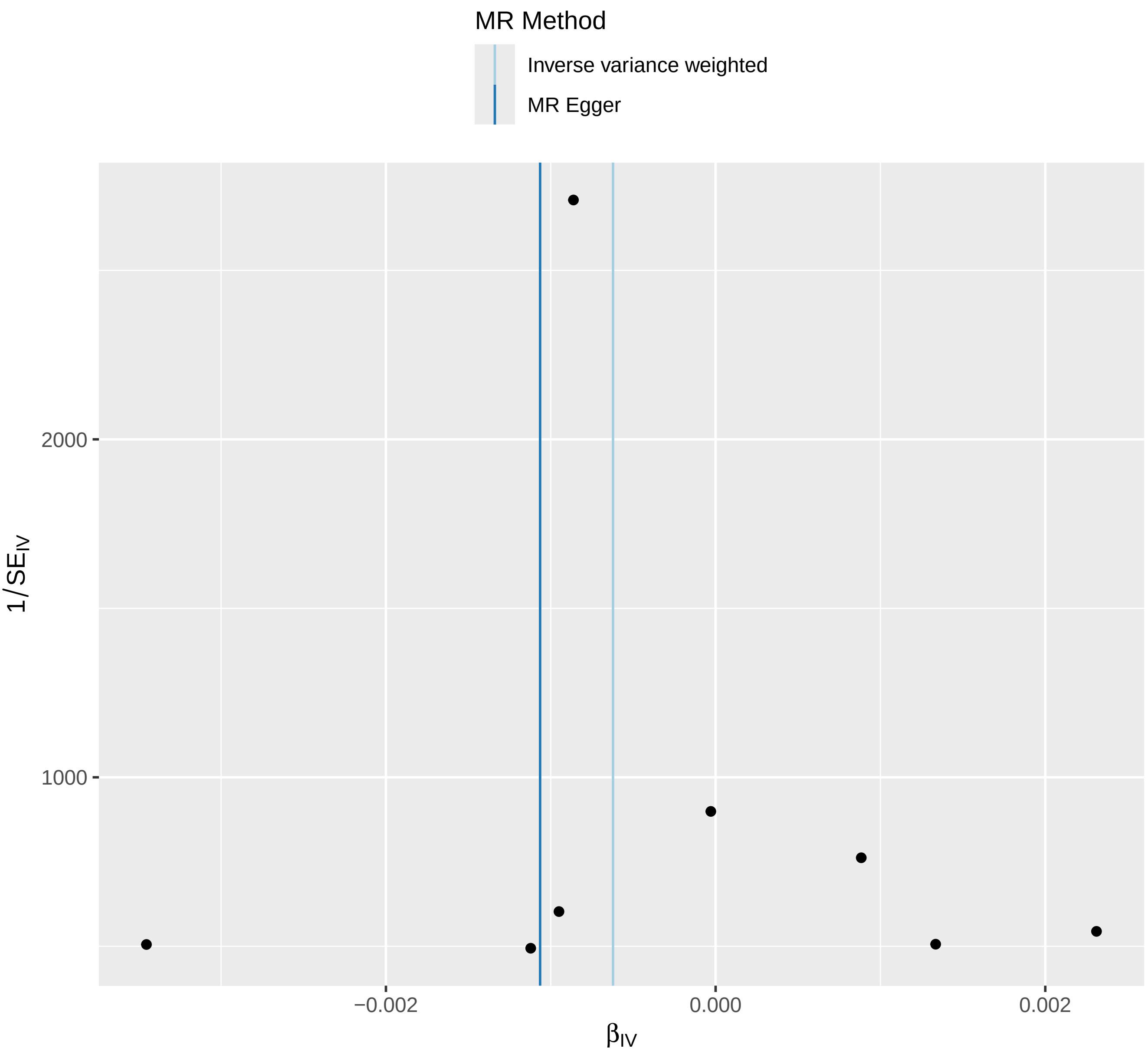
MR Method
Inverse variance weighted
MR Egger
2000
1000
1/SE_IV
−0.002
0.000
0.002
β_IV

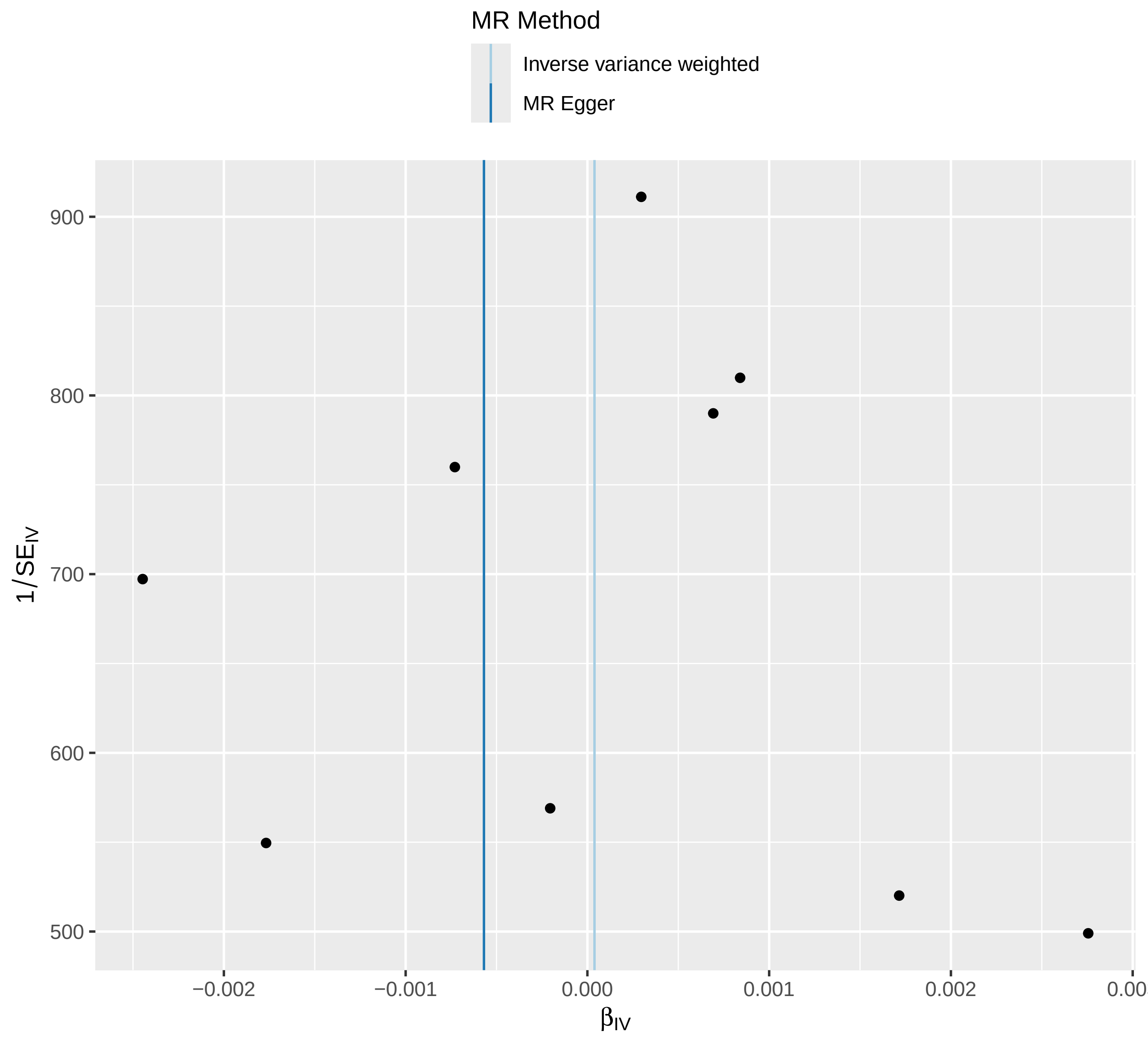
MR Method
Inverse variance weighted
MR Egger
900
800
700
600
500
1/SE_IV
−0.002
−0.001
0.000
0.001
0.002
β_IV

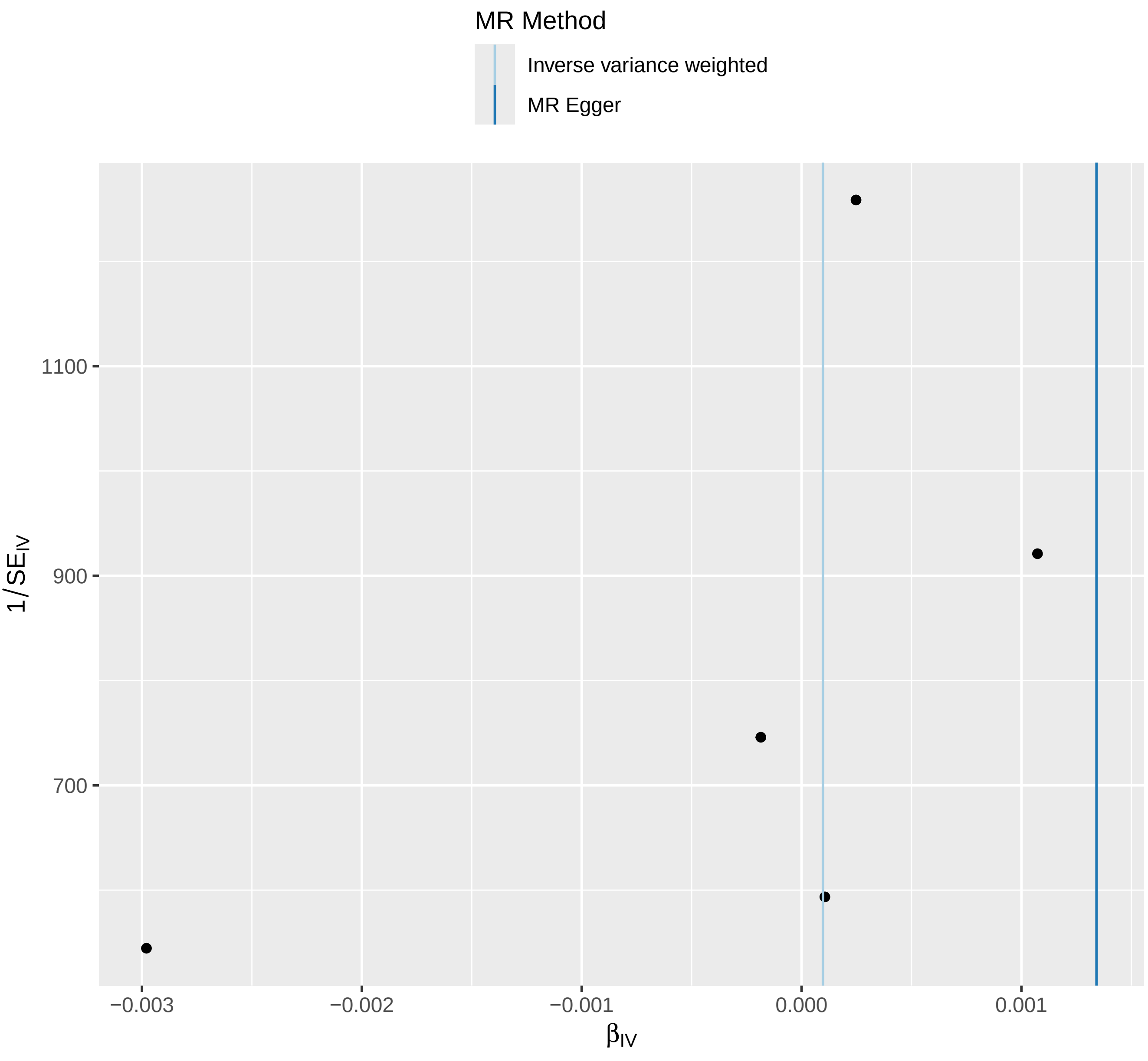

MR Method
Inverse variance weighted
MR Egger
1100
900
700
1/SE_IV
−0.003
−0.002
−0.001
0.000
0.001
β_IV

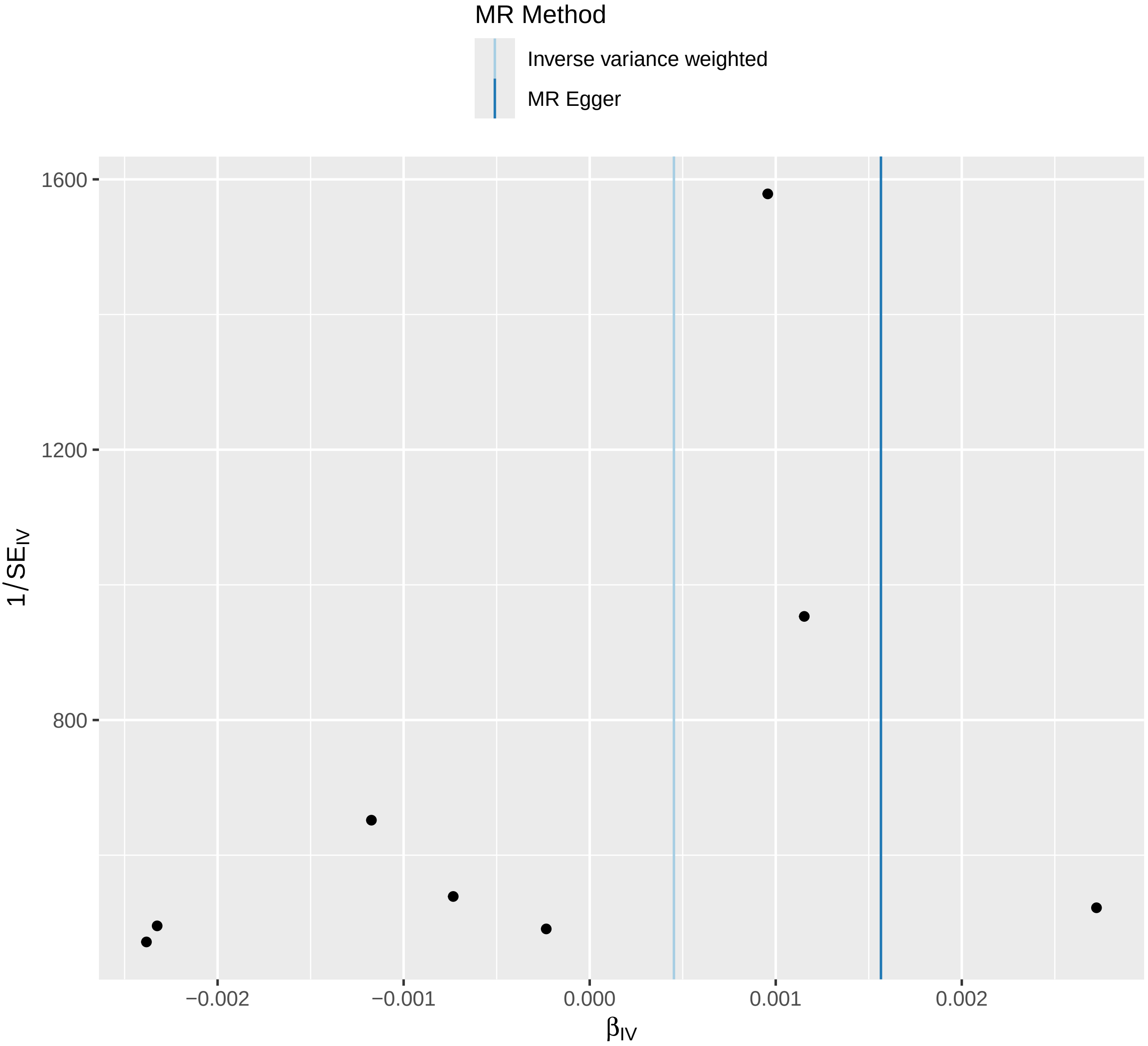
MR Method
Inverse variance weighted
MR Egger
1600
1200
800
1/SE_IV
−0.002
−0.001
0.000
0.001
0.002
β_IV

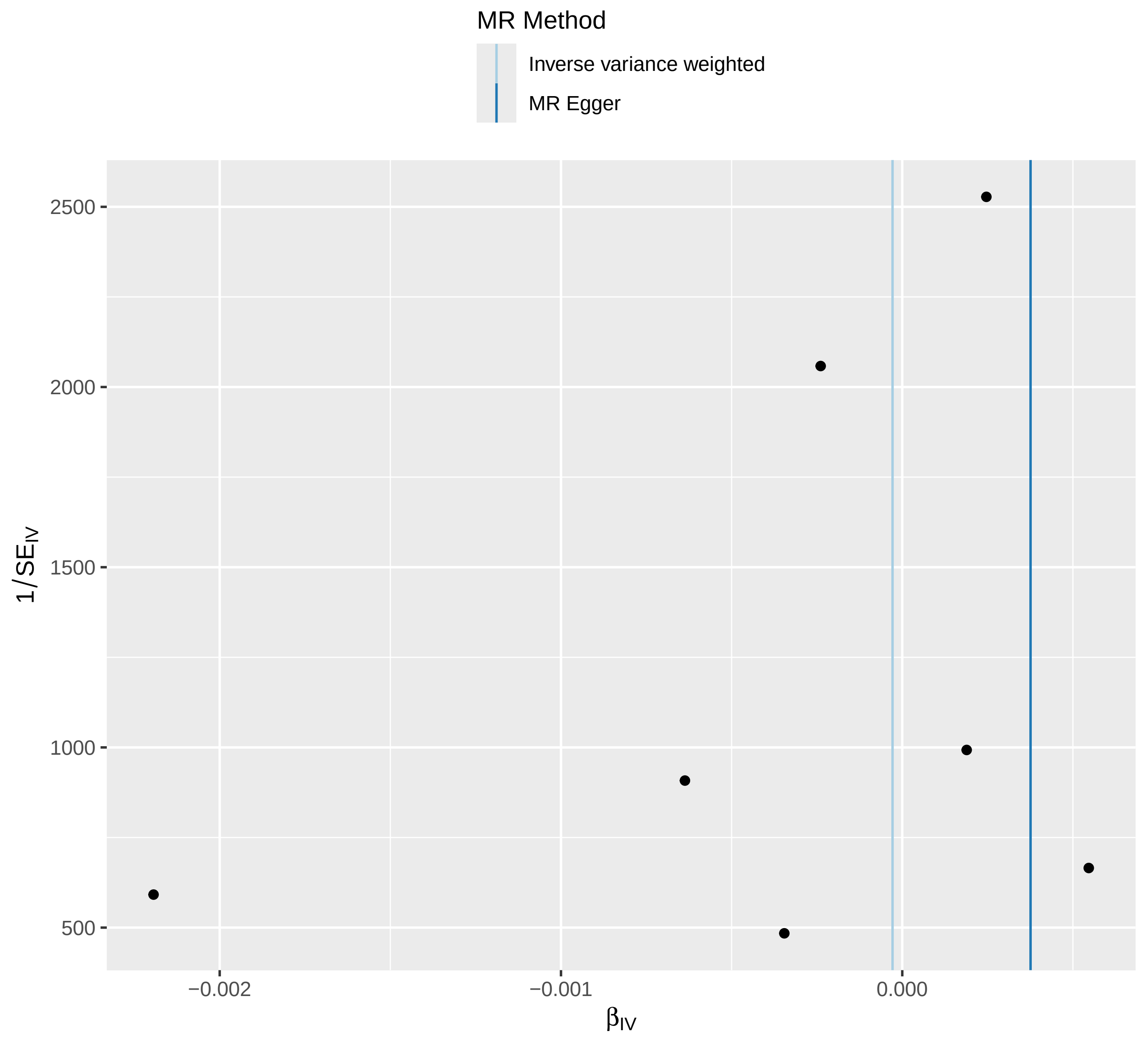
MR Method
Inverse variance weighted
MR Egger
2500
2000
1500
1000
500
1/SE_IV
−0.002
−0.001
0.000
β_IV

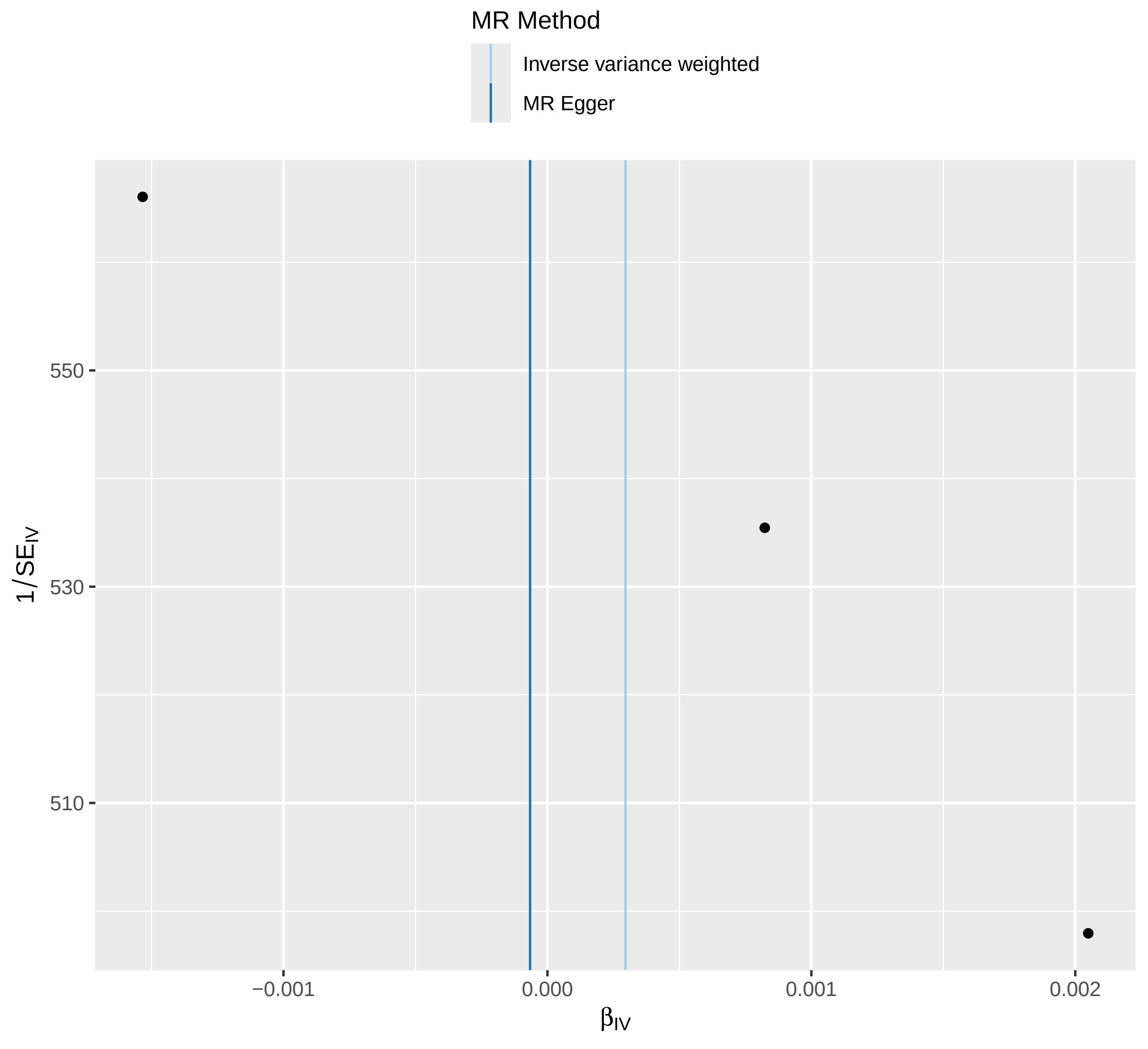

MR Method
Inverse variance weighted
MR Egger
550
530
510
1/SE_IV
−0.001
0.000
0.001
0.002
β_IV

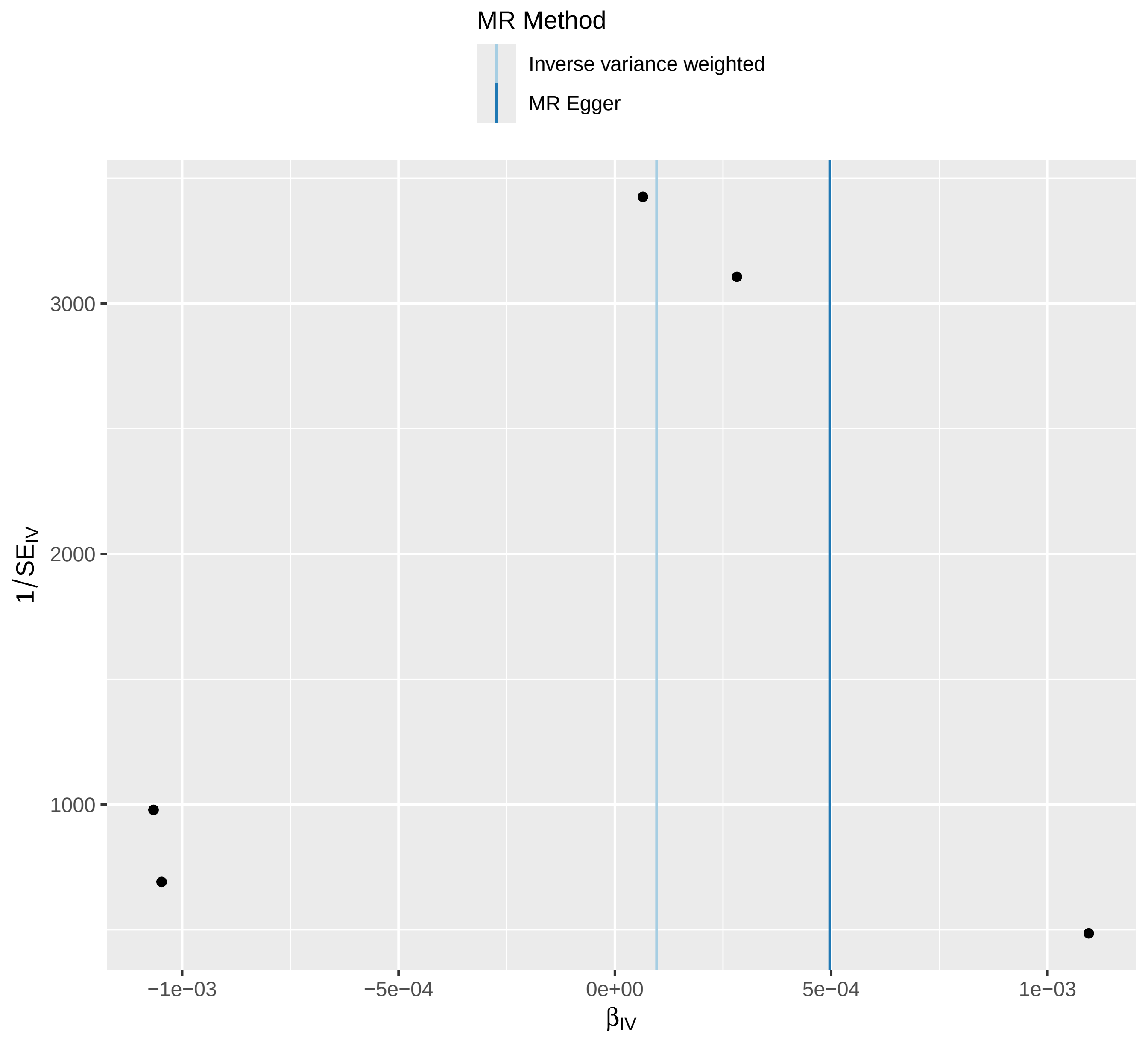

MR Method
Inverse variance weighted
MR Egger
3000
2000
1000
1/SE$_{IV}$
−1e−03
−5e−04
0e+00
5e−04
1e−03
β$_{IV}$

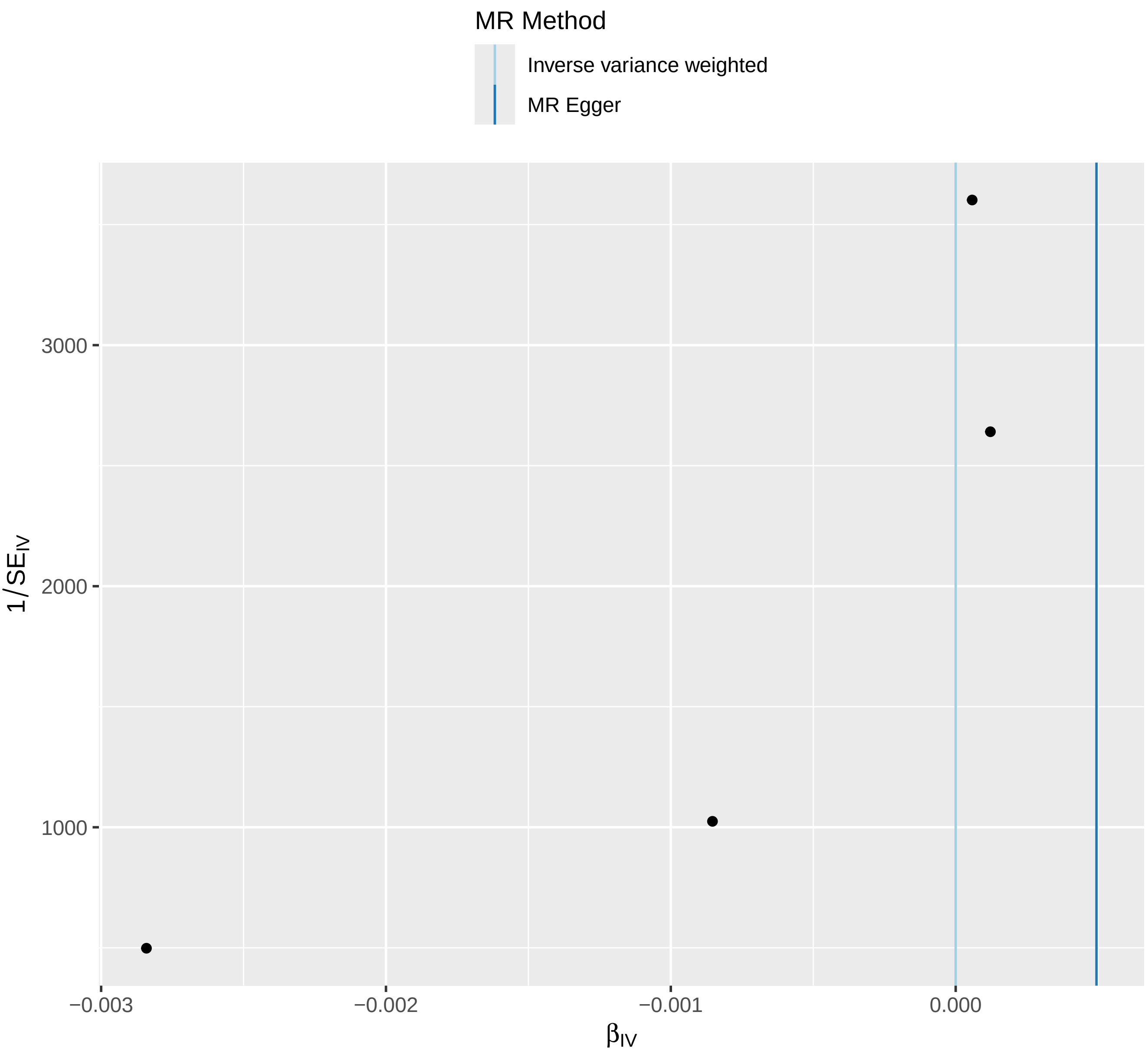
MR Method
Inverse variance weighted
MR Egger
3000
2000
1000
1/SE_IV
−0.003
−0.002
−0.001
0.000
β_IV

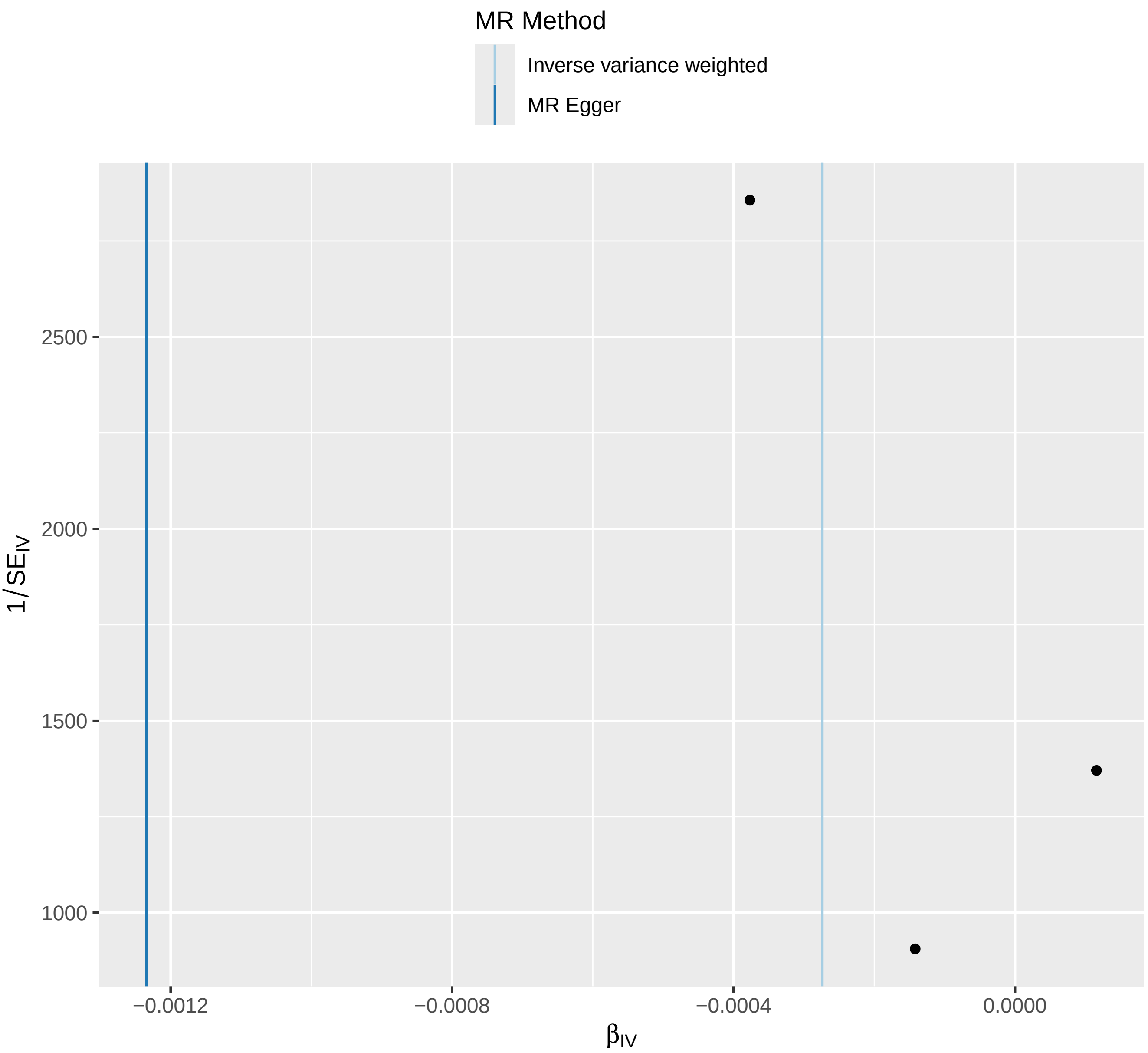
MR Method
Inverse variance weighted
MR Egger
2500
2000
1500
1000
1/SE$_{IV}$
−0.0012
−0.0008
−0.0004
0.0000
β$_{IV}$

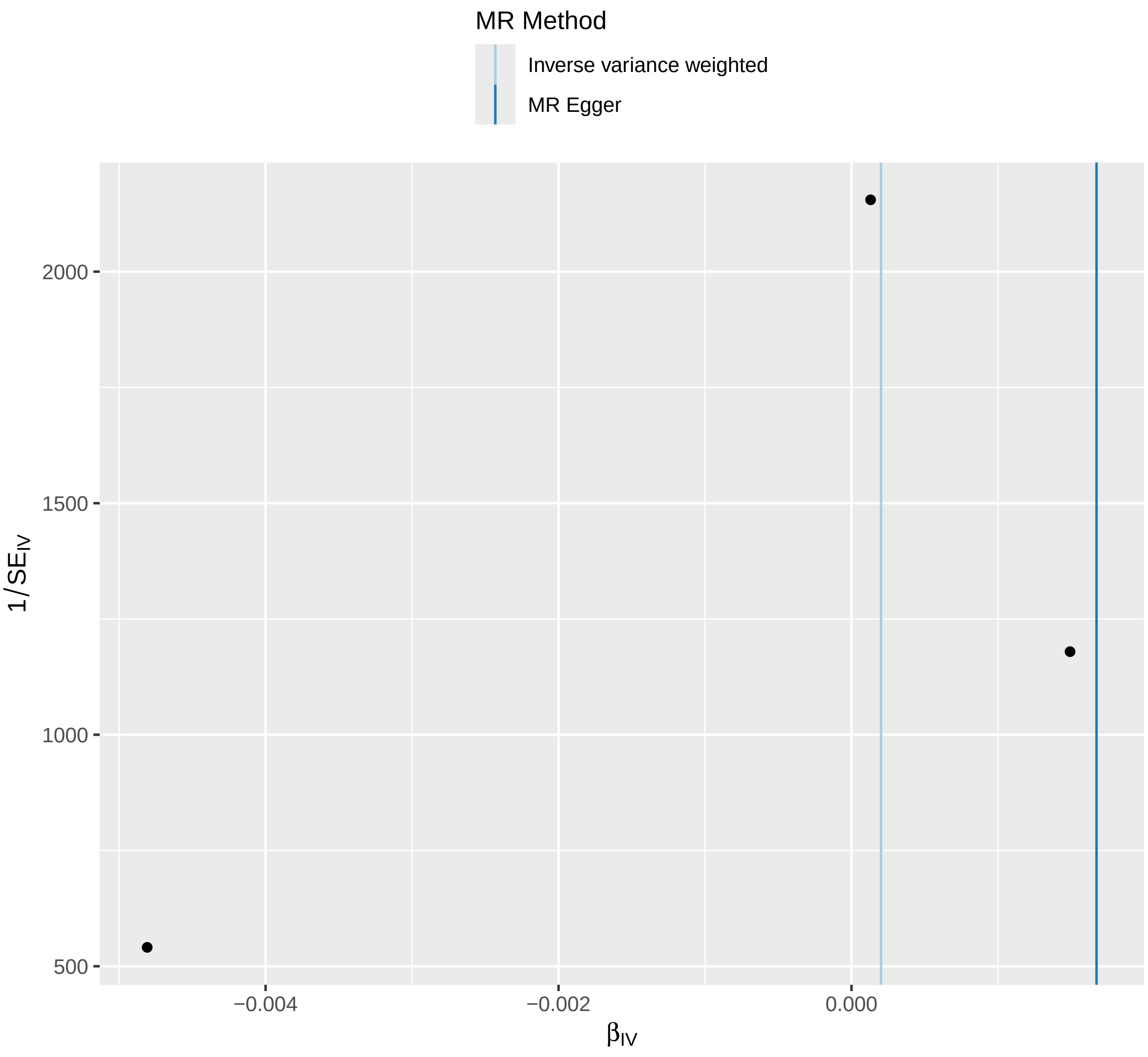

MR Method
Inverse variance weighted
MR Egger
2000
1500
1000
500
1/SE$_{IV}$
−0.004
−0.002
0.000
β$_{IV}$

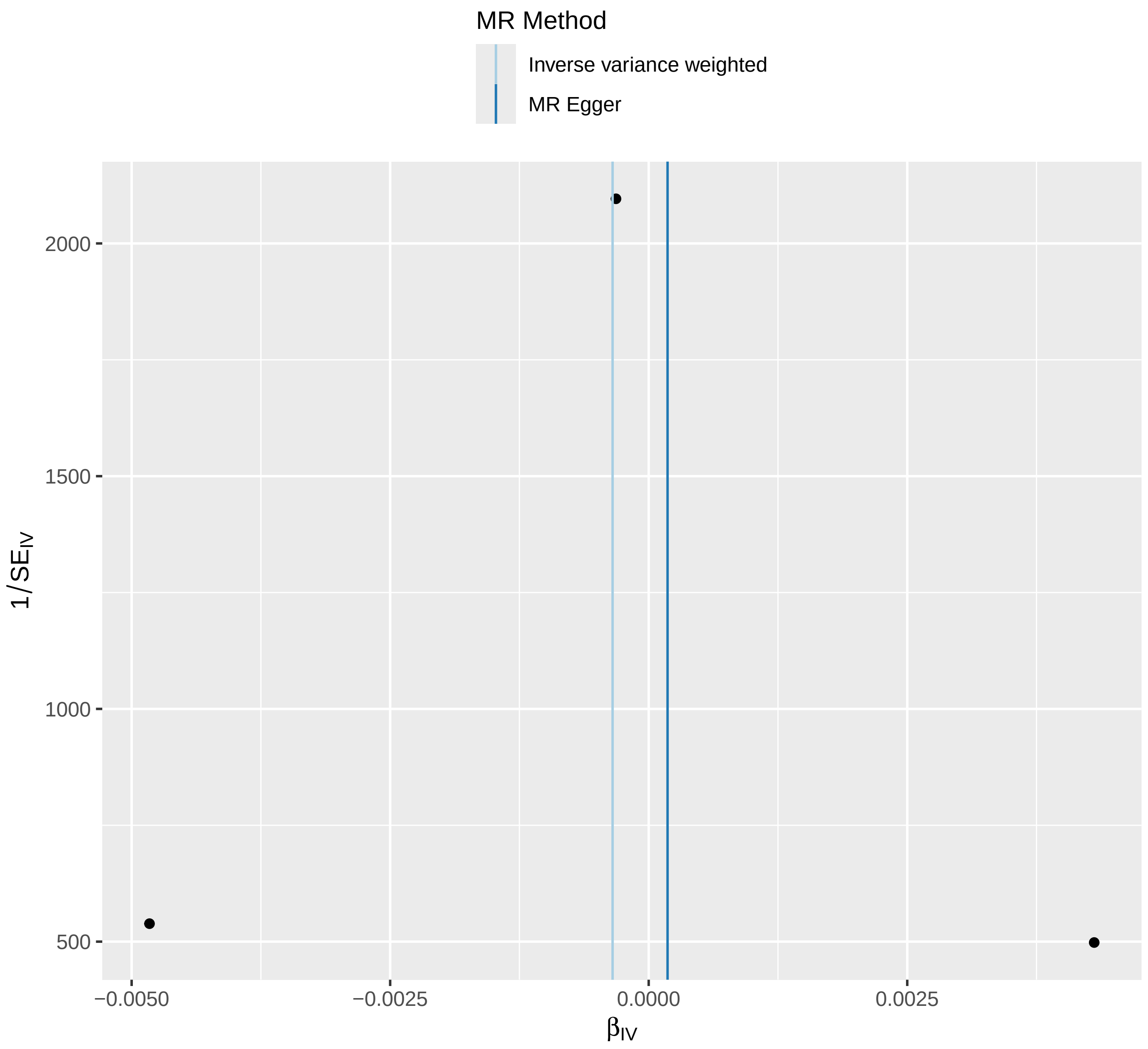
MR Method
Inverse variance weighted
MR Egger
2000
1500
1000
500
$1/SE_{IV}$
−0.0050
−0.0025
0.0000
0.0025
β_{IV}

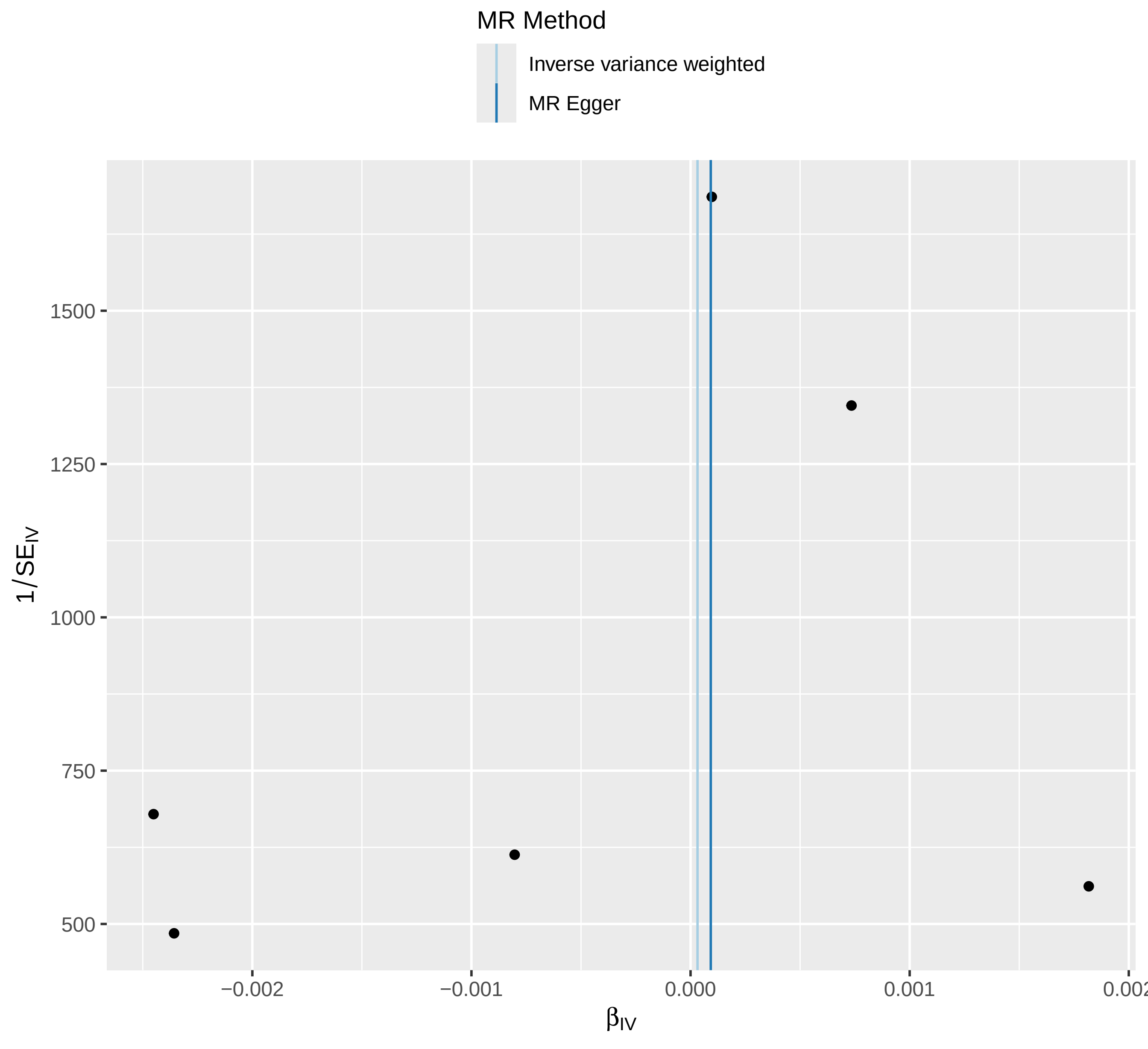
MR Method
Inverse variance weighted
MR Egger
1500
1250
1000
750
500
−0.002
−0.001
0.000
0.001
0.002
$1/SE_{IV}$
β_{IV}

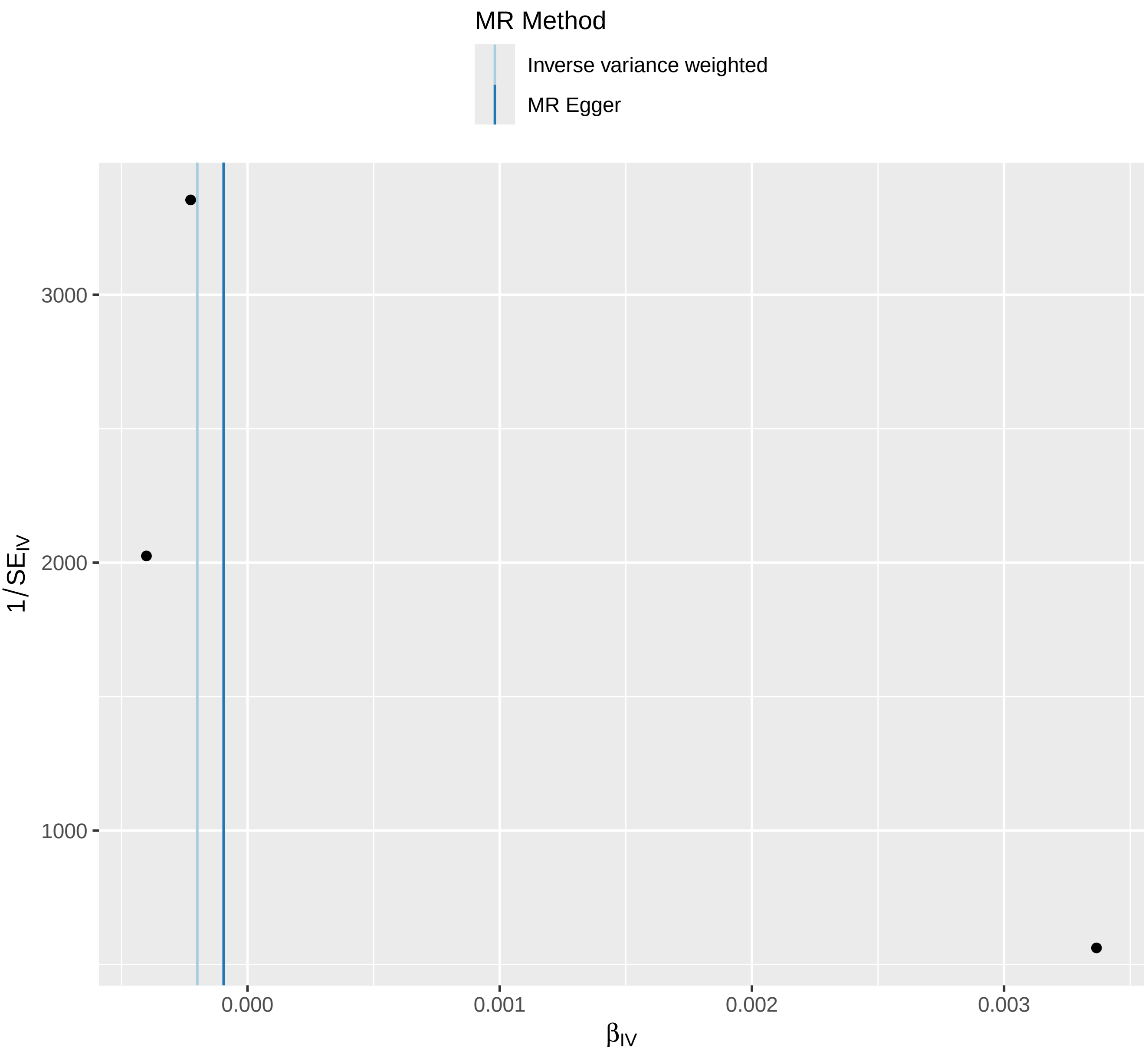
MR Method
Inverse variance weighted
MR Egger
3000
2000
1000
1/SE_IV
0.000
0.001
0.002
0.003
β_IV

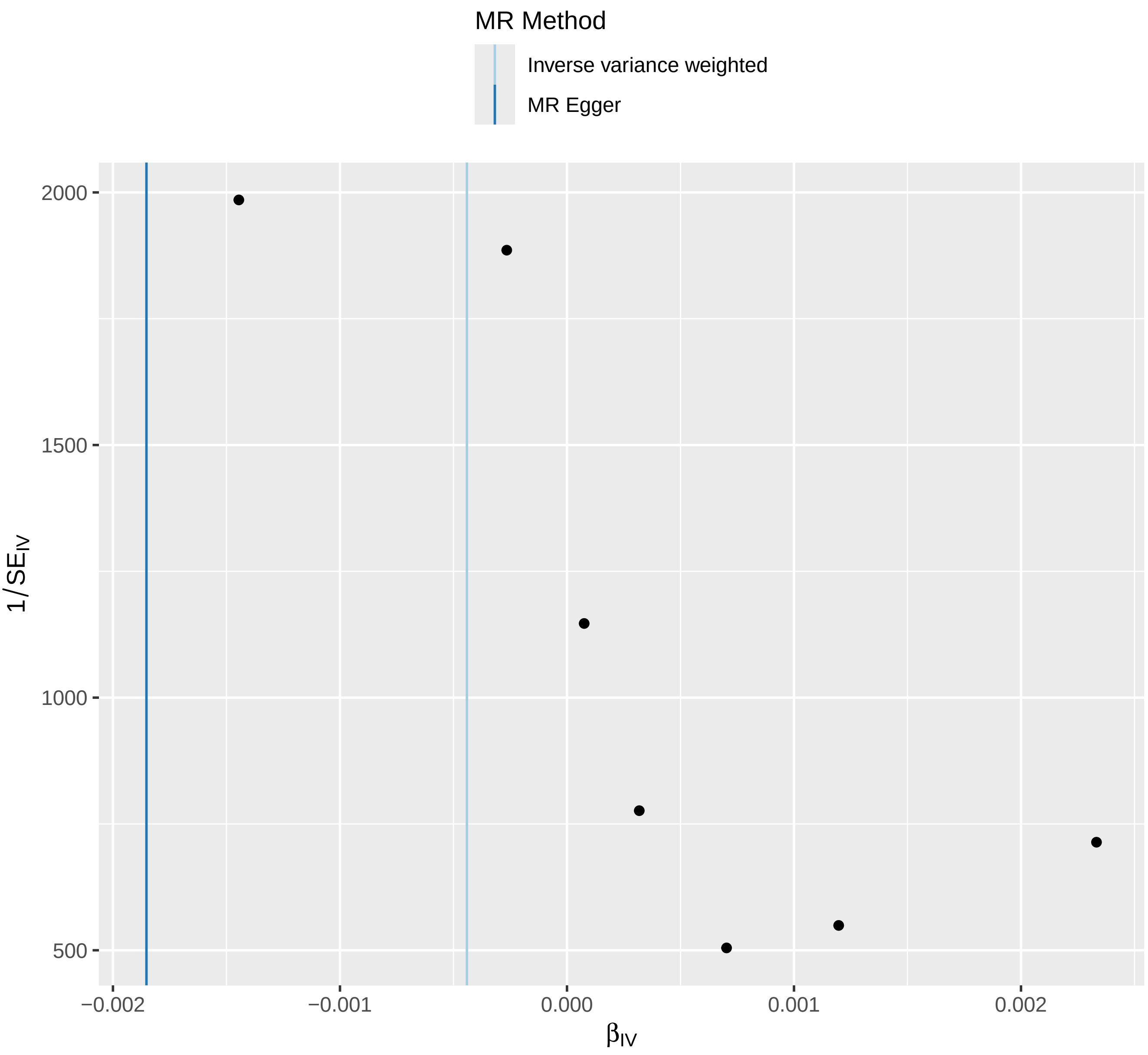

MR Method
Inverse variance weighted
MR Egger
2000
1500
1000
500
1/SE_IV
−0.002
−0.001
0.000
0.001
0.002
β_IV

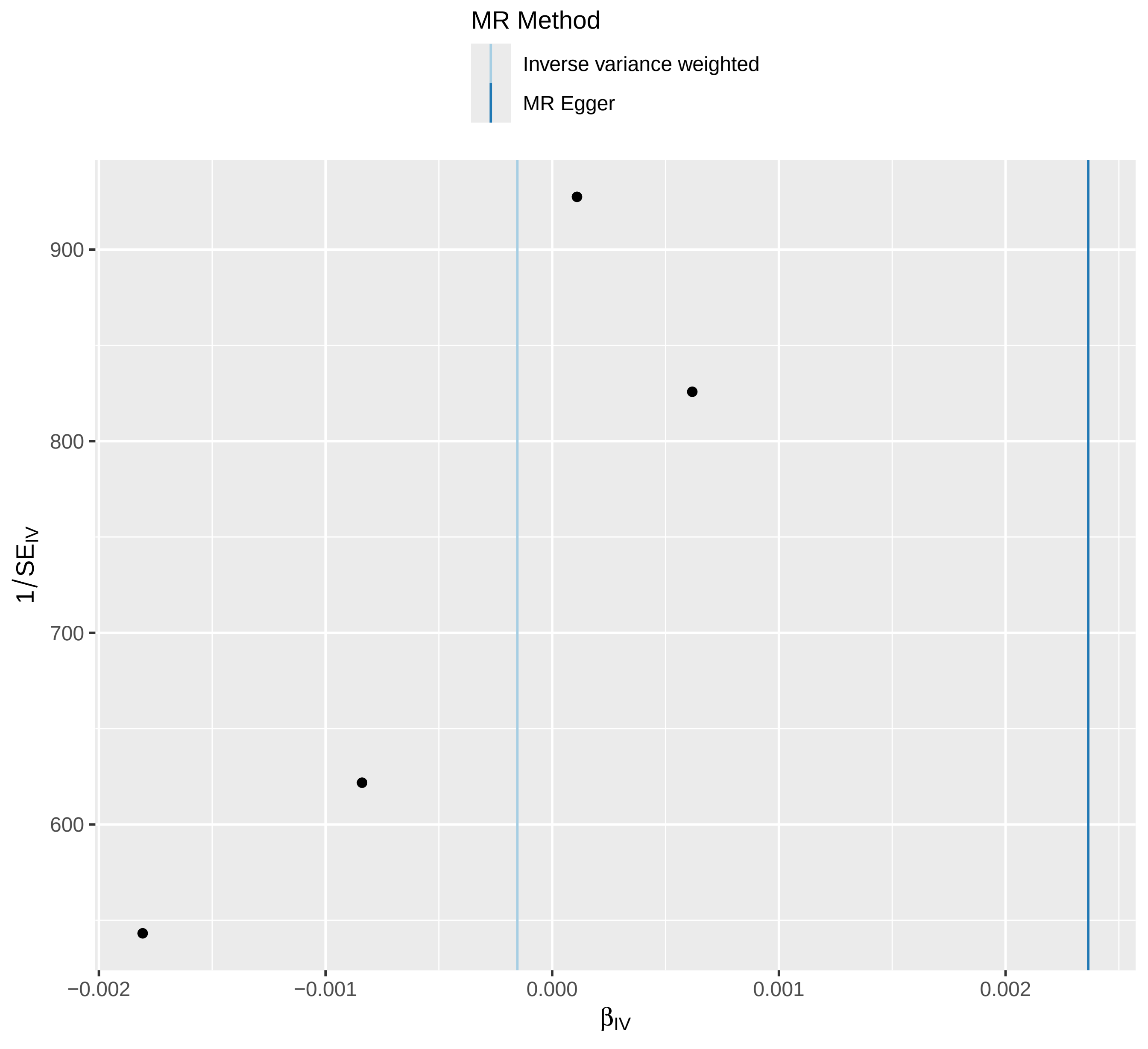

MR Method
Inverse variance weighted
MR Egger
900
800
700
600
−0.002
−0.001
0.000
0.001
0.002
$1/SE_{IV}$
β_{IV}

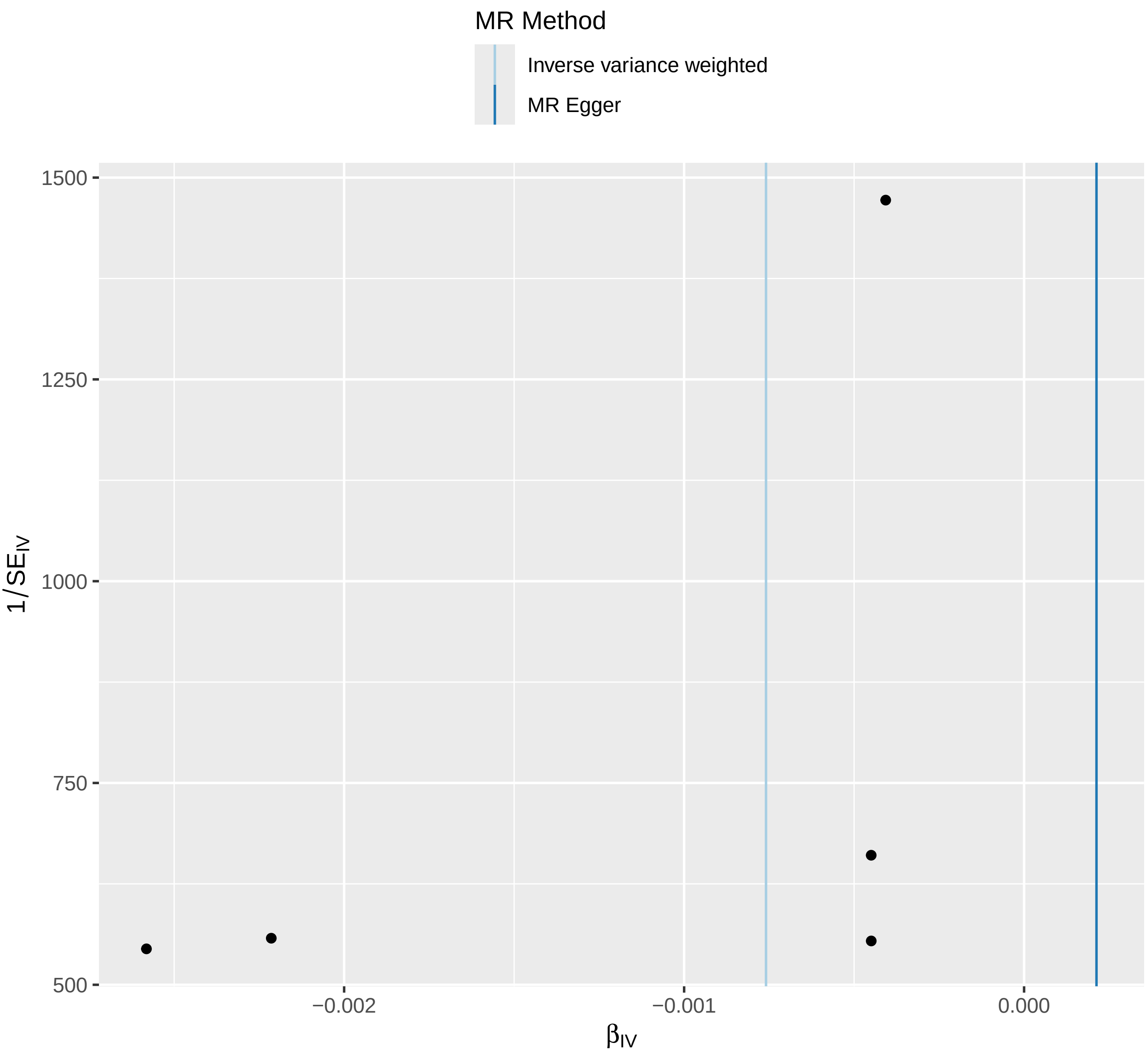
MR Method
Inverse variance weighted
MR Egger
1500
1250
1000
750
500
1/SE$_{IV}$
−0.002
−0.001
0.000
β$_{IV}$

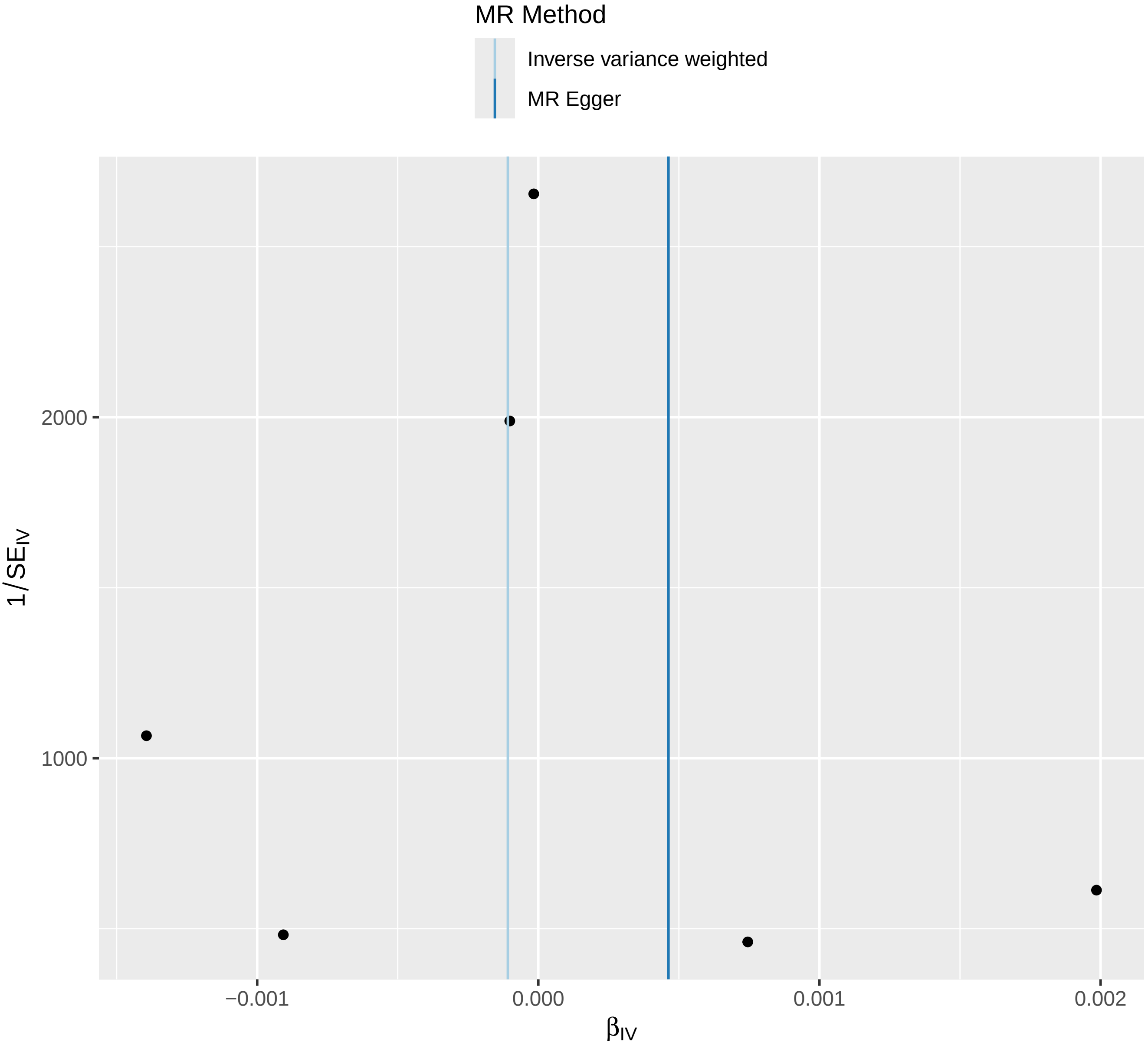

MR Method
Inverse variance weighted
MR Egger
2000
1000
1/SE_IV
−0.001
0.000
0.001
0.002
β_IV

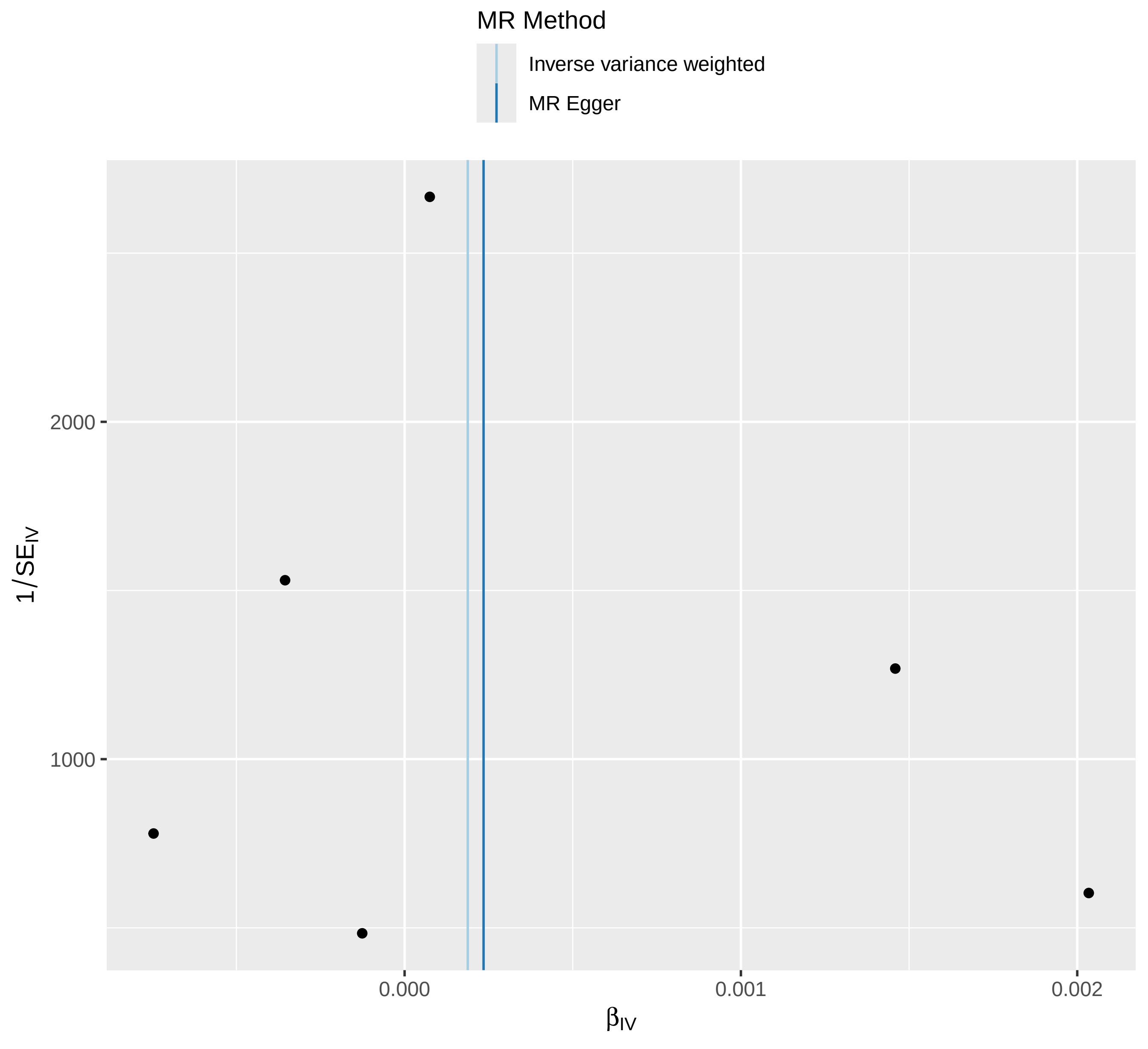
MR Method
Inverse variance weighted
MR Egger
2000
1000
1/SE$_{IV}$
0.000
0.001
0.002
β$_{IV}$

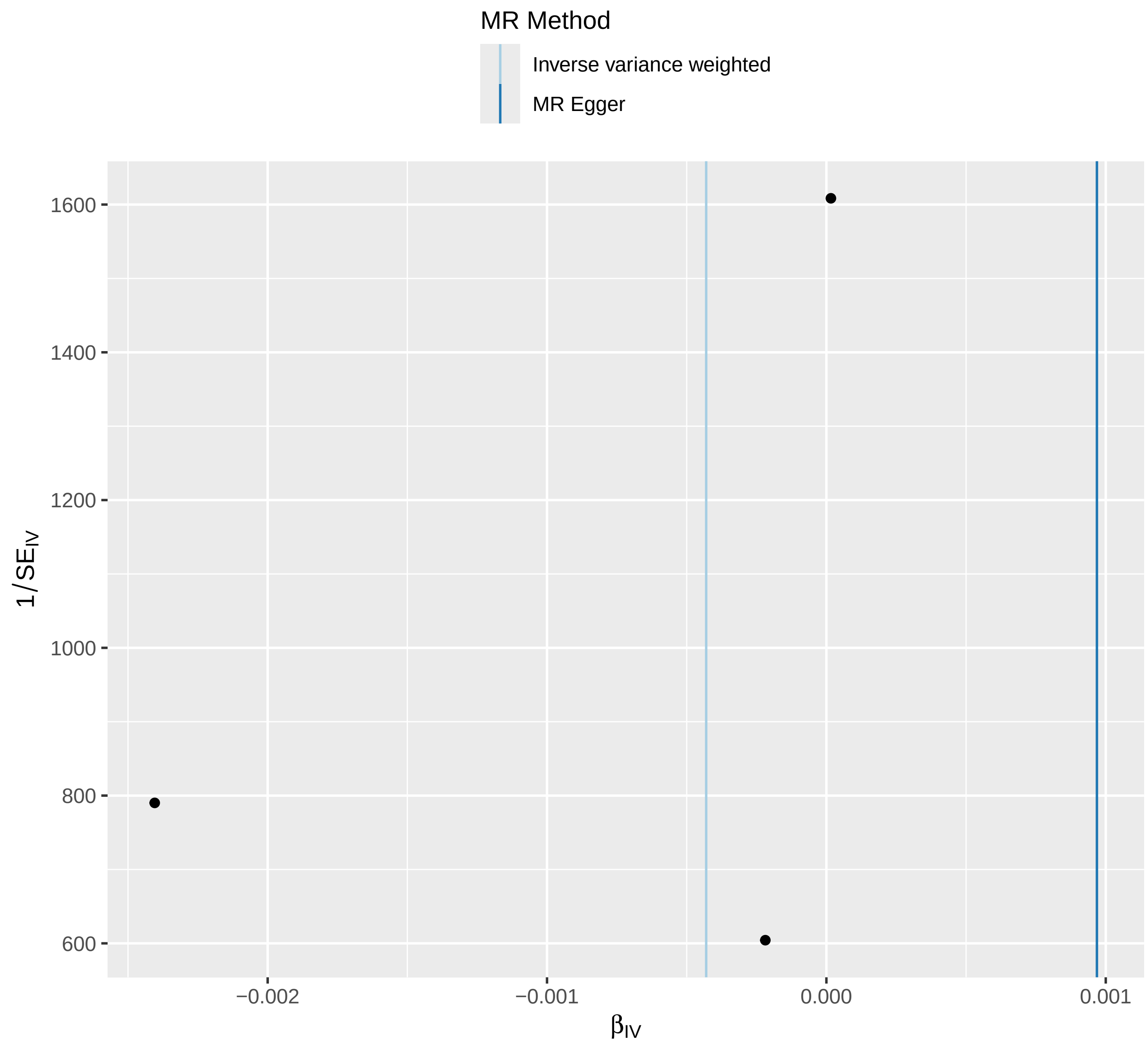
MR Method
Inverse variance weighted
MR Egger
1600
1400
1200
1000
800
600
1/SE$_{IV}$
−0.002
−0.001
0.000
0.001
β$_{IV}$

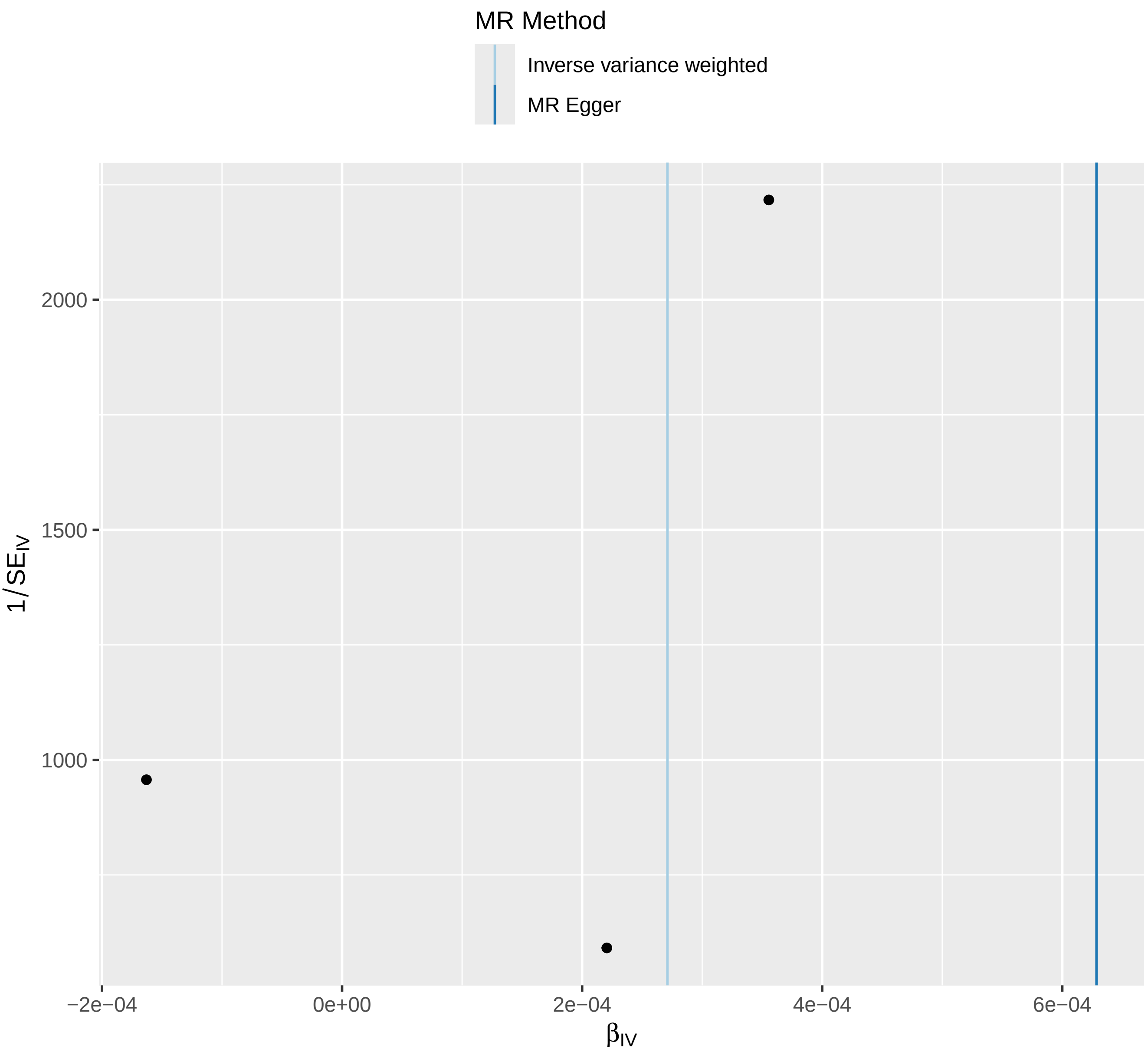

MR Method
Inverse variance weighted
MR Egger
2000
1500
1000
1/SE_IV
−2e−04
0e+00
2e−04
4e−04
6e−04
β_IV

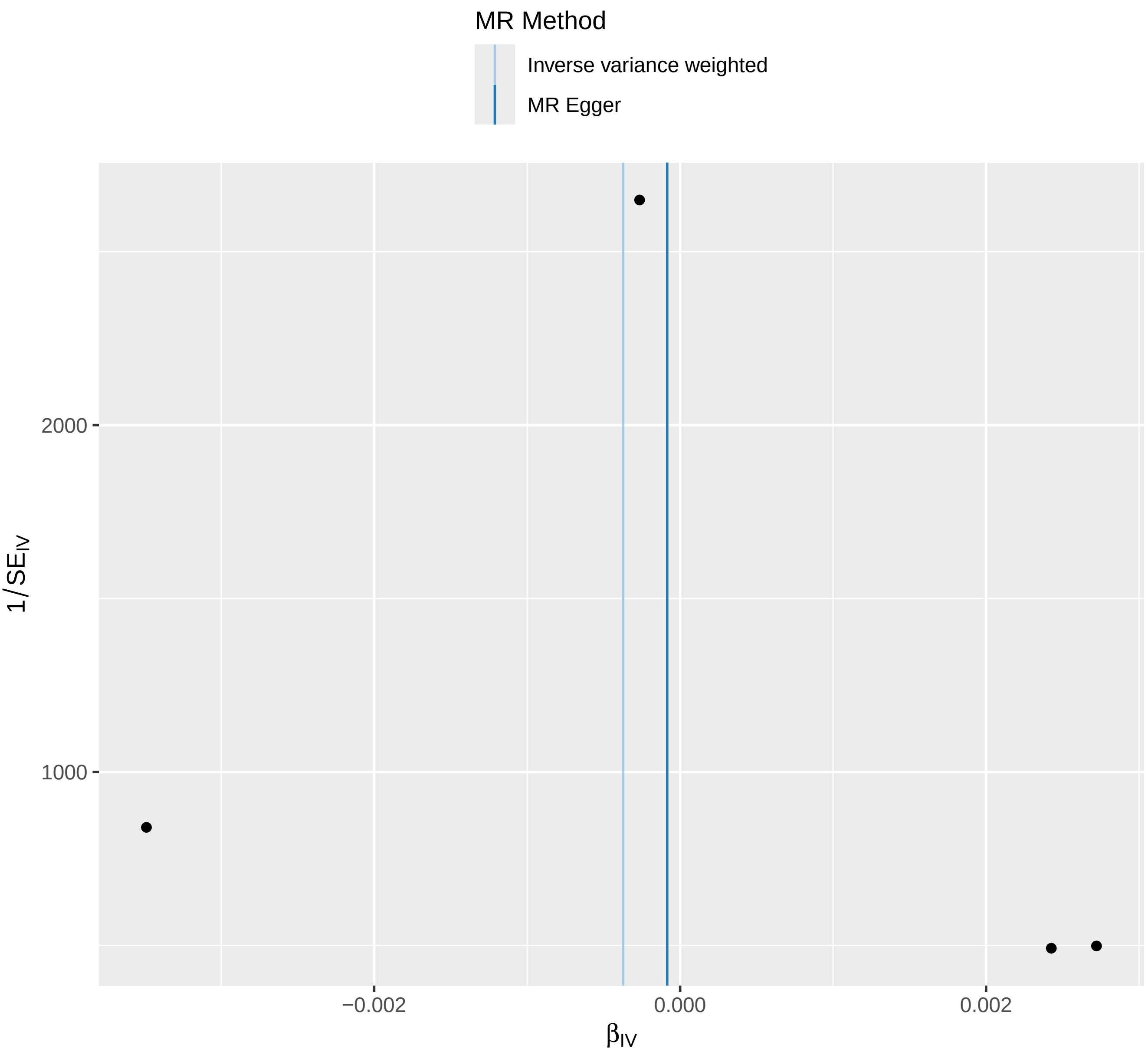

MR Method
Inverse variance weighted
MR Egger
2000
1000
1/SE_IV
−0.002
0.000
0.002
β_IV

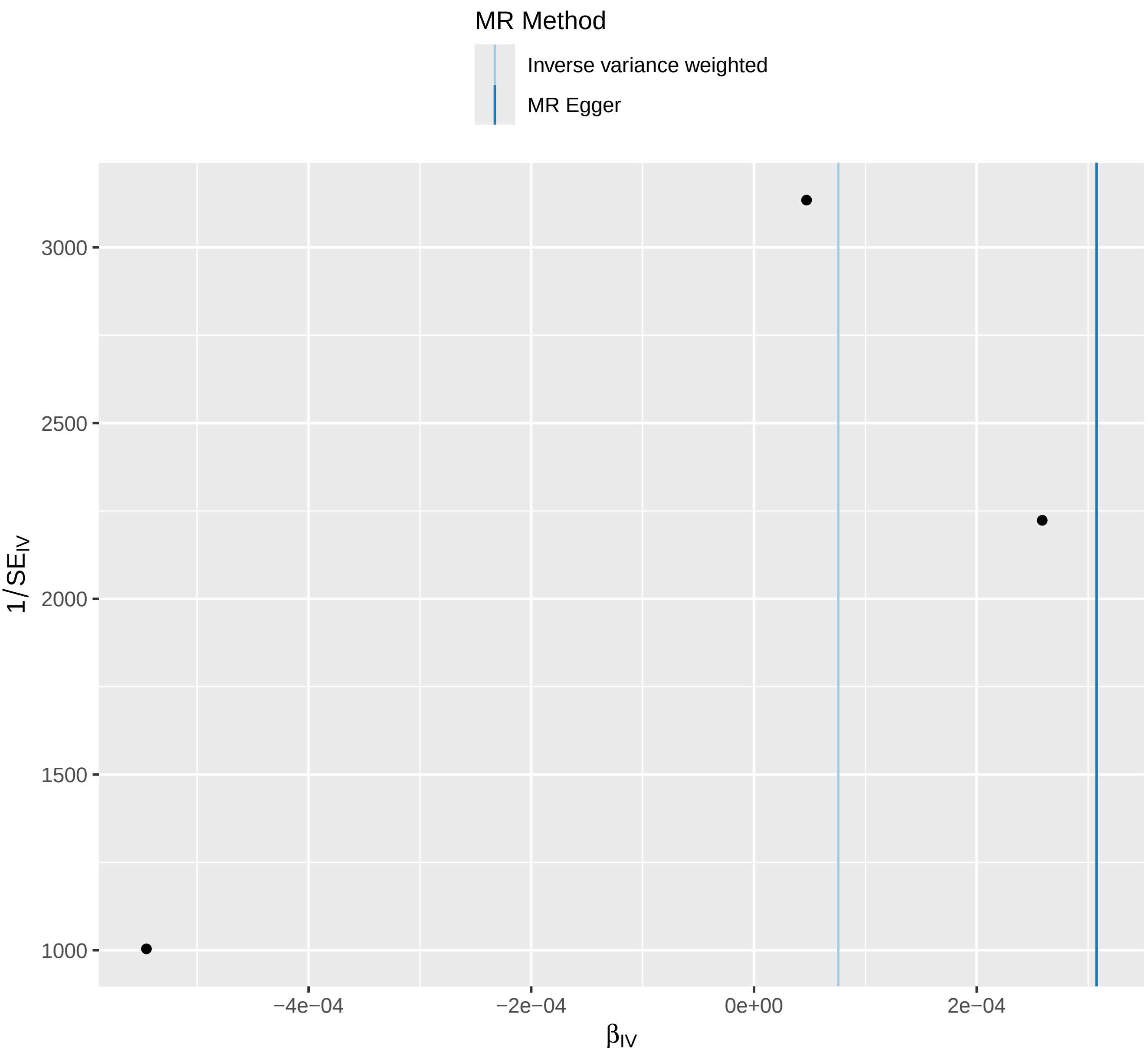
MR Method
Inverse variance weighted
MR Egger
3000
2500
2000
1500
1000
1/SE_IV
−4e−04
−2e−04
0e+00
2e−04
β_IV

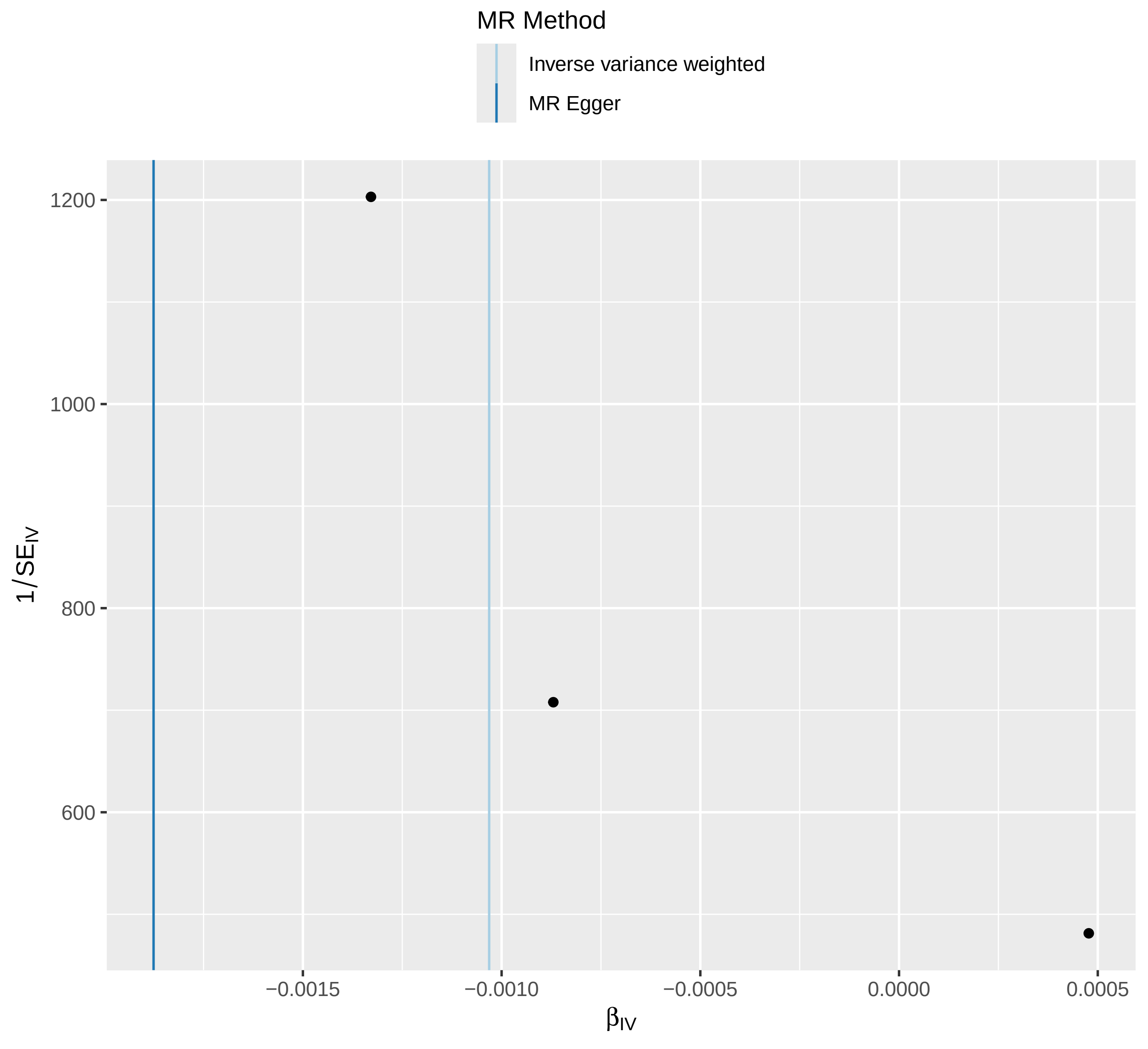

MR Method
Inverse variance weighted
MR Egger
1200
1000
800
600
1/SE_IV
−0.0015
−0.0010
−0.0005
0.0000
0.0005
β_IV

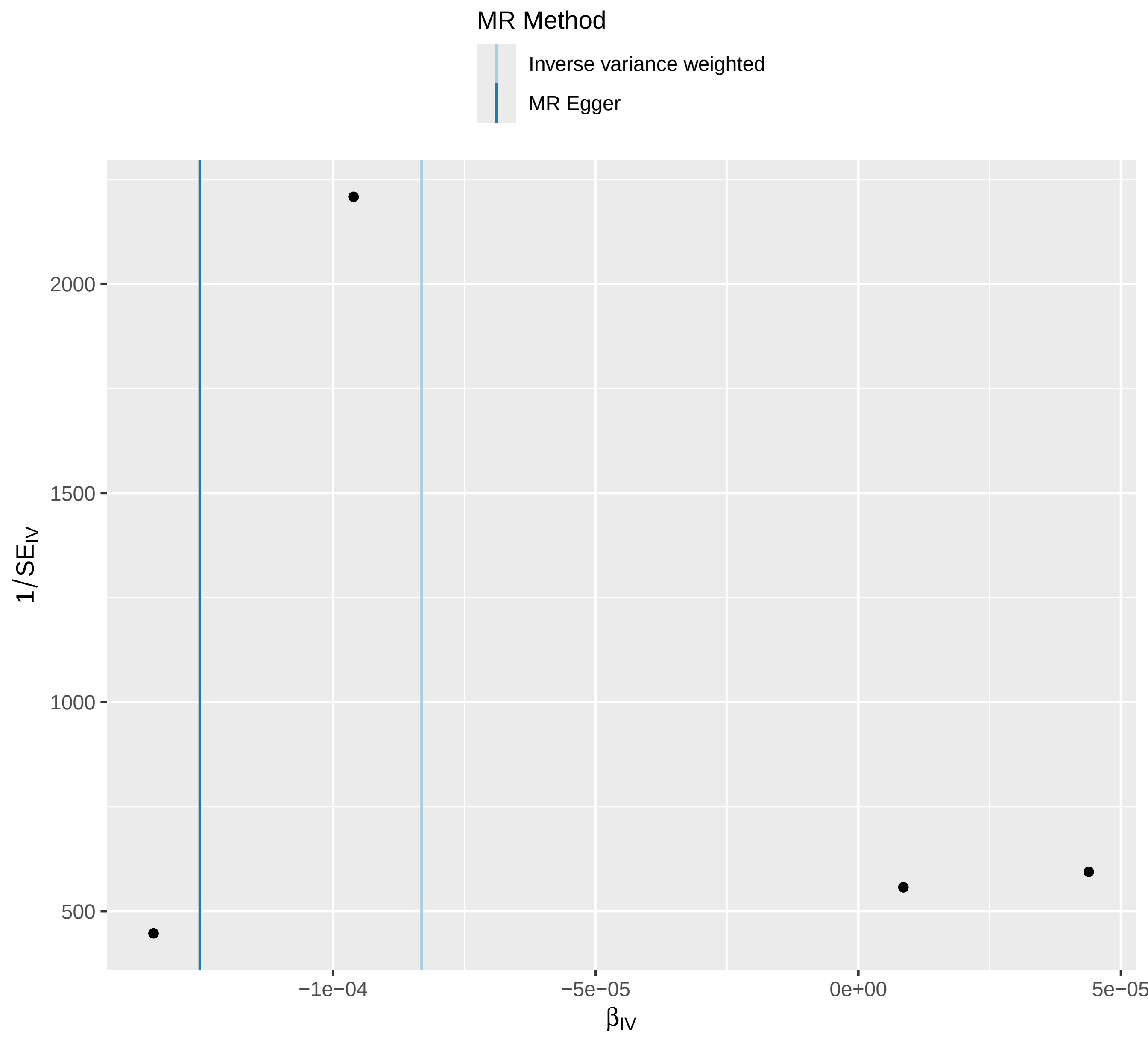
MR Method
Inverse variance weighted
MR Egger
2000
1500
1000
500
1/SE_IV
−1e−04
−5e−05
0e+00
5e−05
β_IV

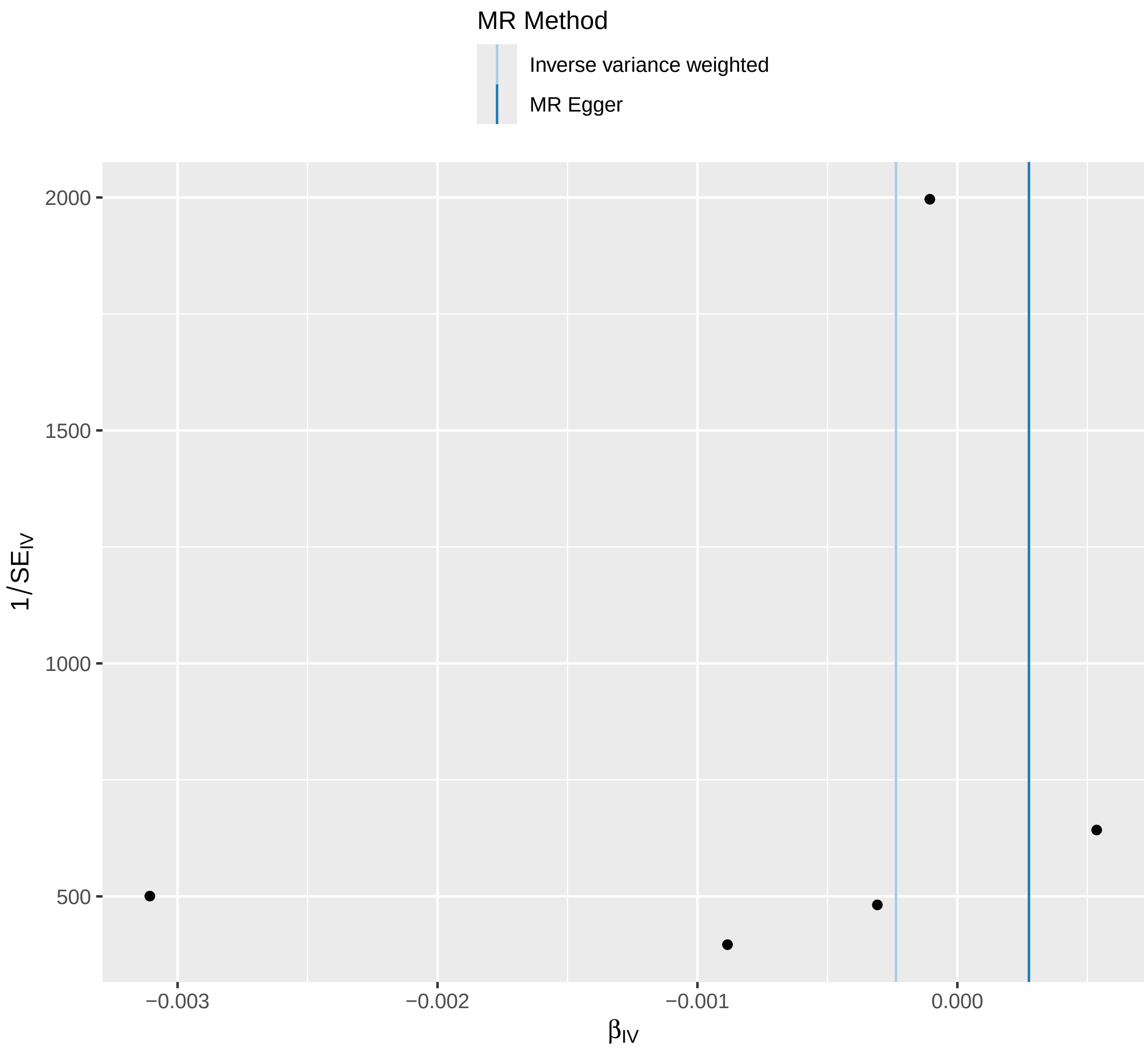
MR Method
Inverse variance weighted
MR Egger
2000
1500
1000
500
1/SE_IV
−0.003
−0.002
−0.001
0.000
β_IV

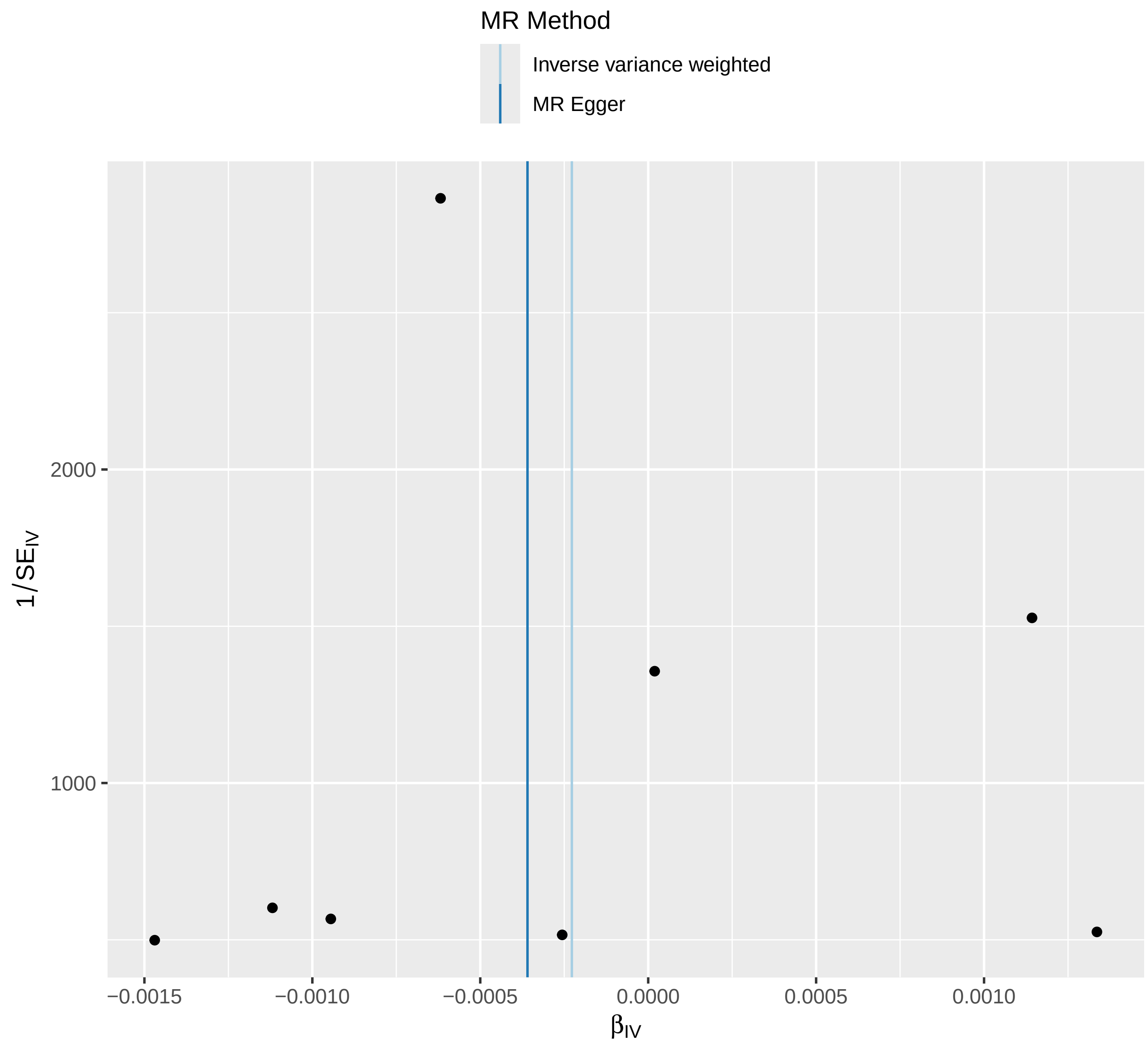

MR Method
Inverse variance weighted
MR Egger
2000
1000
1/SE_IV
−0.0015
−0.0010
−0.0005
0.0000
0.0005
0.0010
β_IV

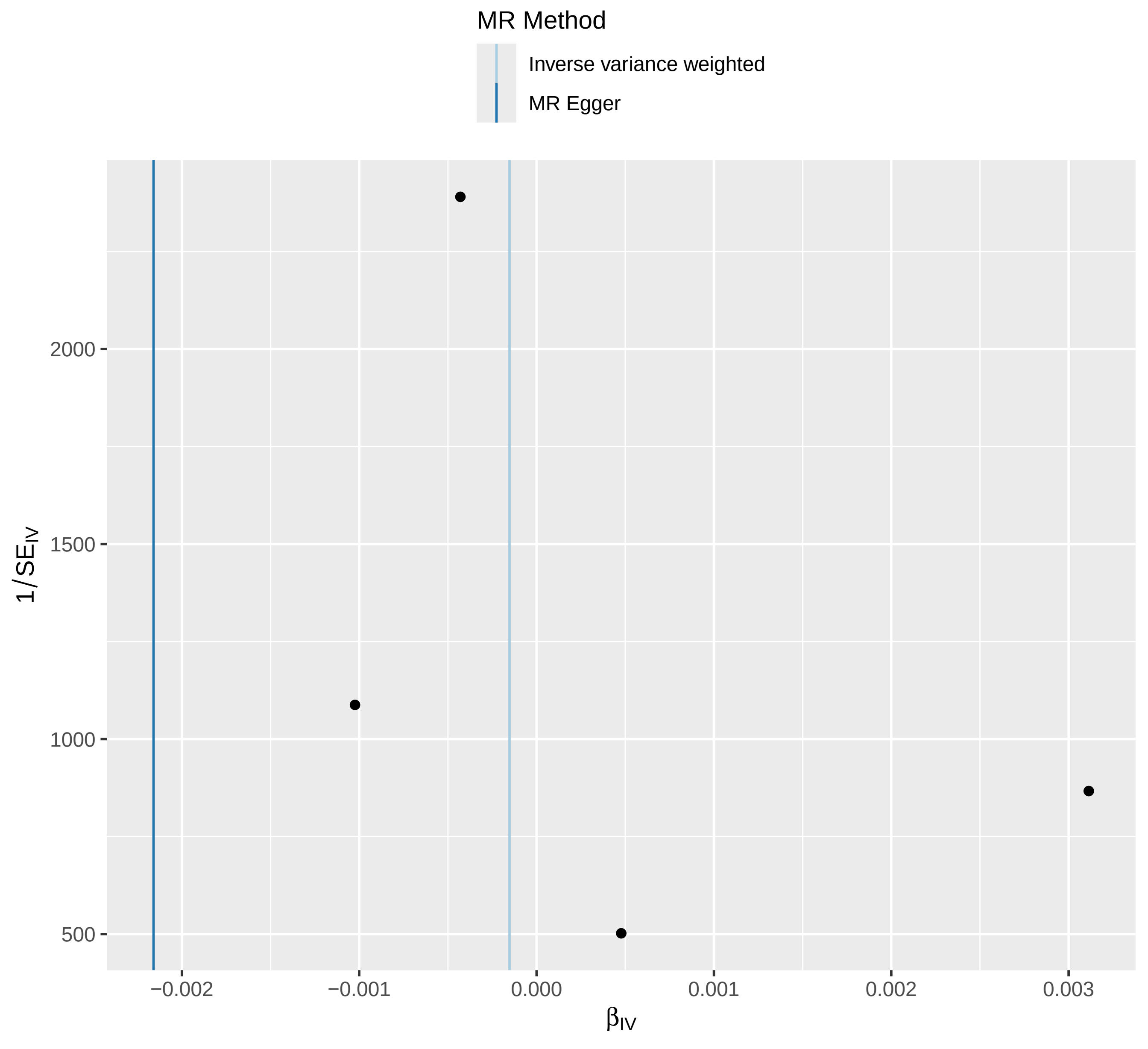
MR Method
Inverse variance weighted
MR Egger
2000
1500
1000
500
$1/SE_{IV}$
−0.002
−0.001
0.000
0.001
0.002
0.003
β_{IV}

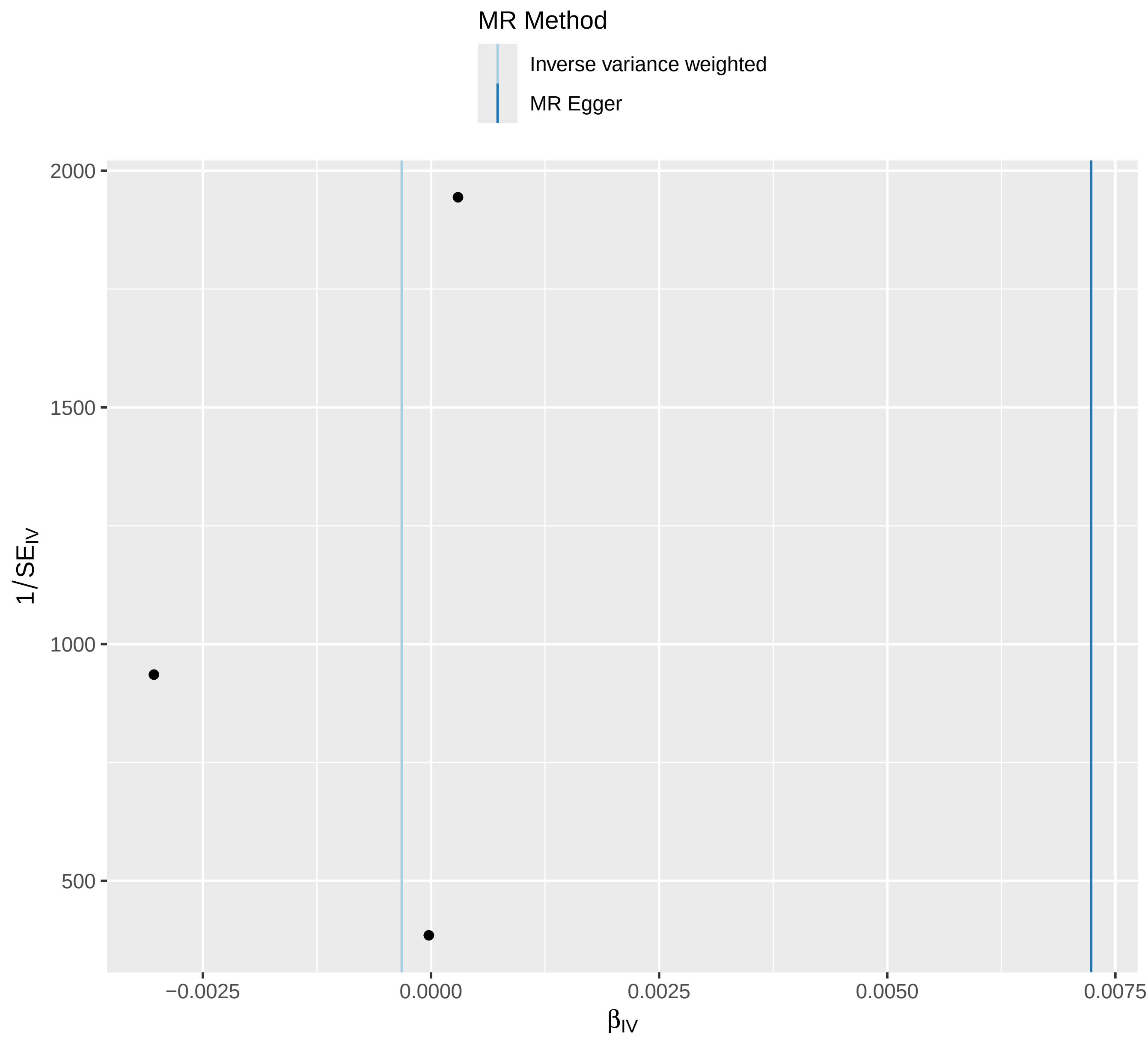

MR Method
Inverse variance weighted
MR Egger
2000
1500
1000
500
1/SE_IV
−0.0025
0.0000
0.0025
0.0050
0.0075
β_IV

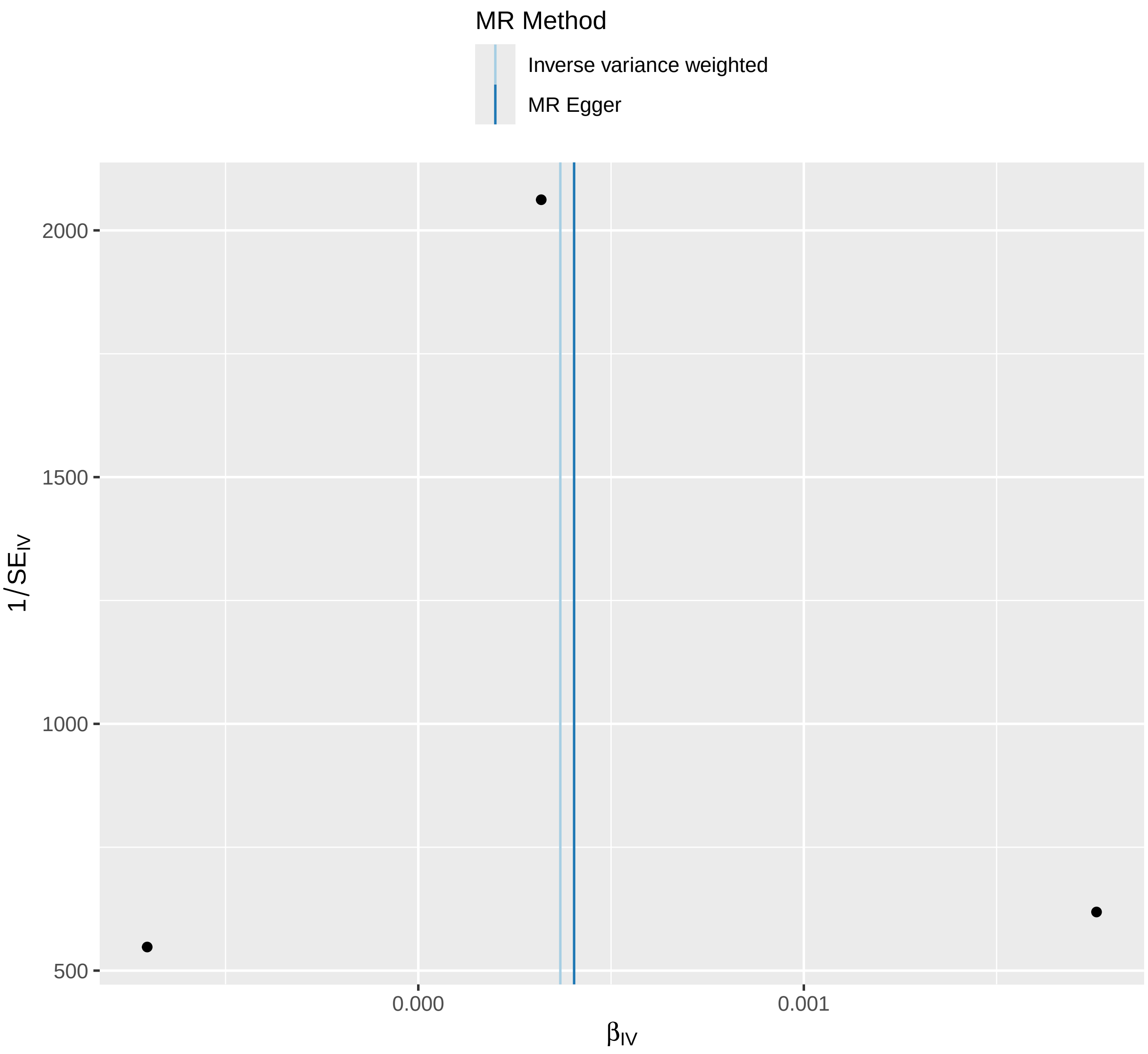

MR Method
Inverse variance weighted
MR Egger
2000
1500
1000
500
1/SE$_{IV}$
0.000
0.001
β$_{IV}$

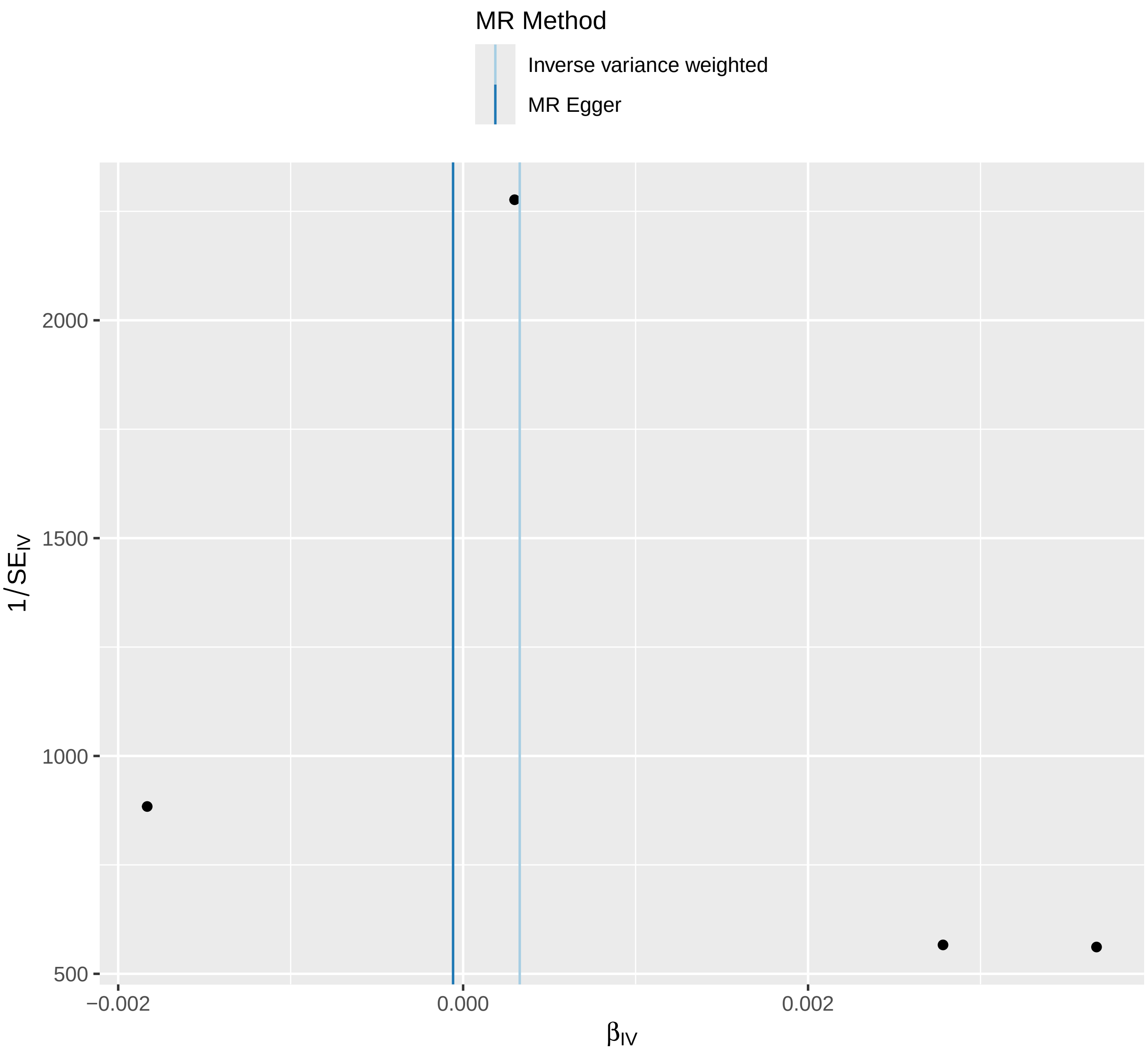

MR Method
Inverse variance weighted
MR Egger
2000
1500
1000
500
1/SE$_{IV}$
−0.002
0.000
0.002
β$_{IV}$

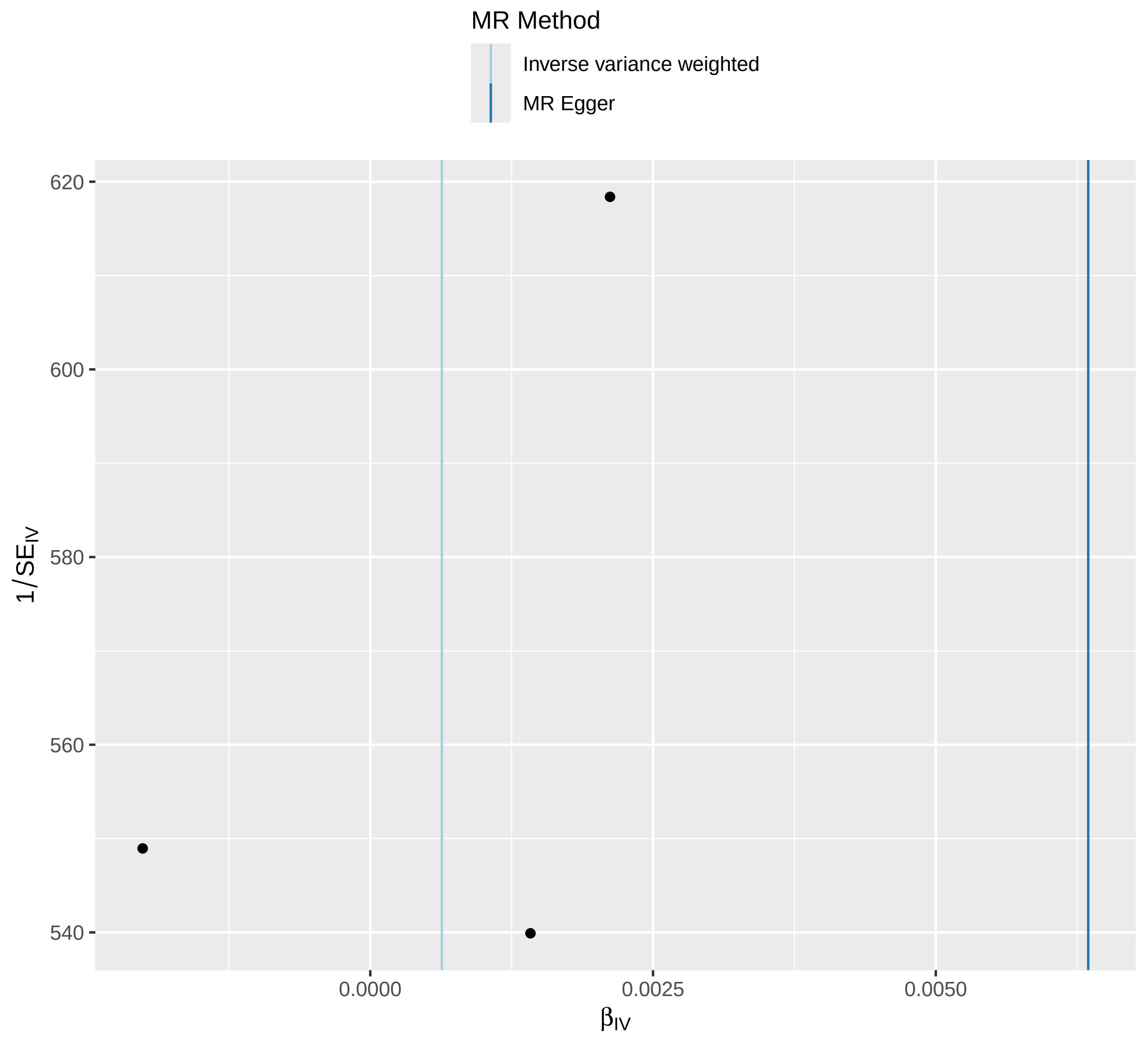
MR Method
Inverse variance weighted
MR Egger
620
600
580
560
540
1/SE_IV
0.0000
0.0025
0.0050
β_IV

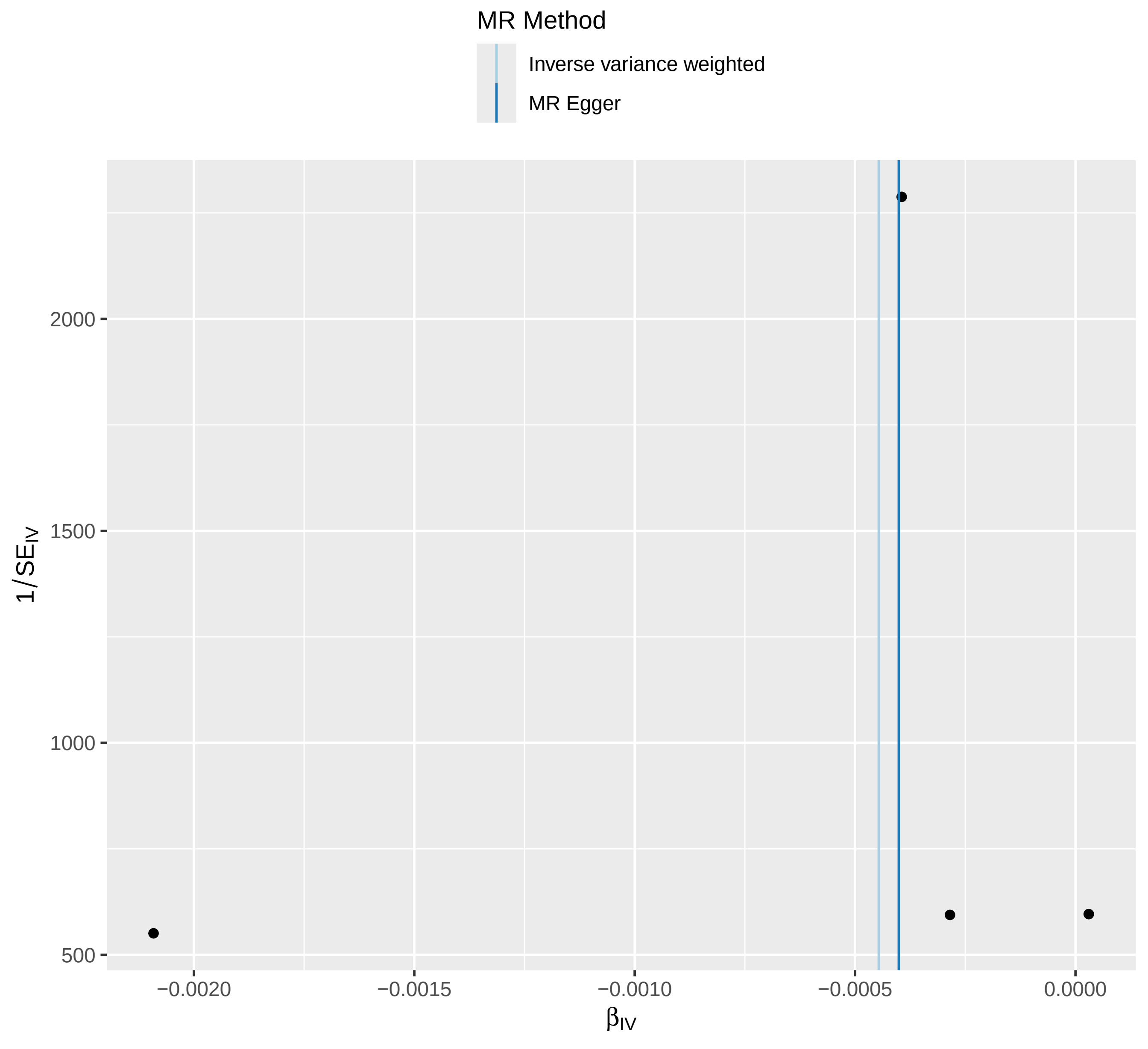
MR Method
Inverse variance weighted
MR Egger
2000
1500
1000
500
1/SE_IV
−0.0020
−0.0015
−0.0010
−0.0005
0.0000
β_IV

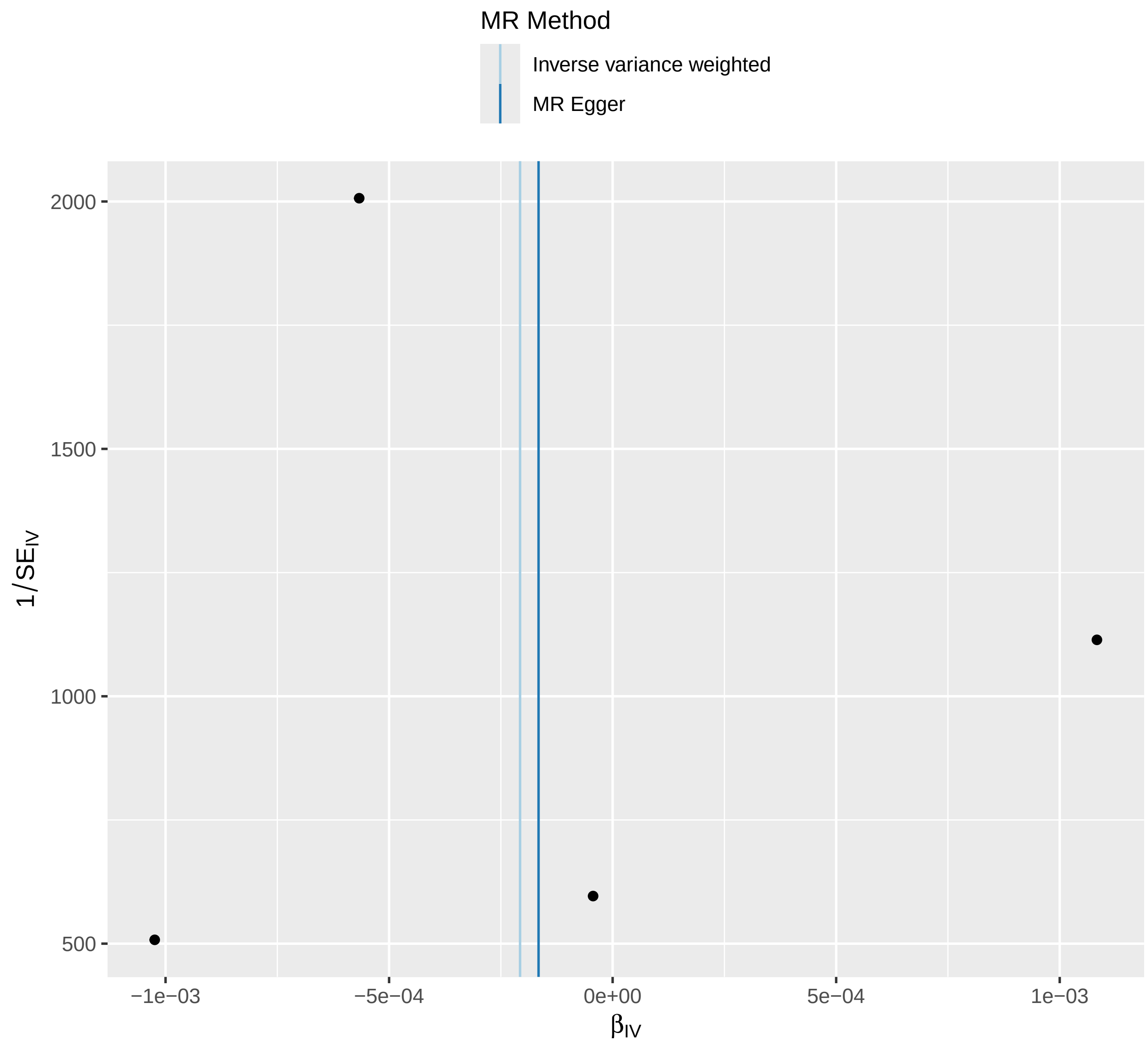
MR Method
Inverse variance weighted
MR Egger
2000
1500
1000
500
$1/SE_{IV}$
−1e−03
−5e−04
0e+00
5e−04
1e−03
β_{IV}

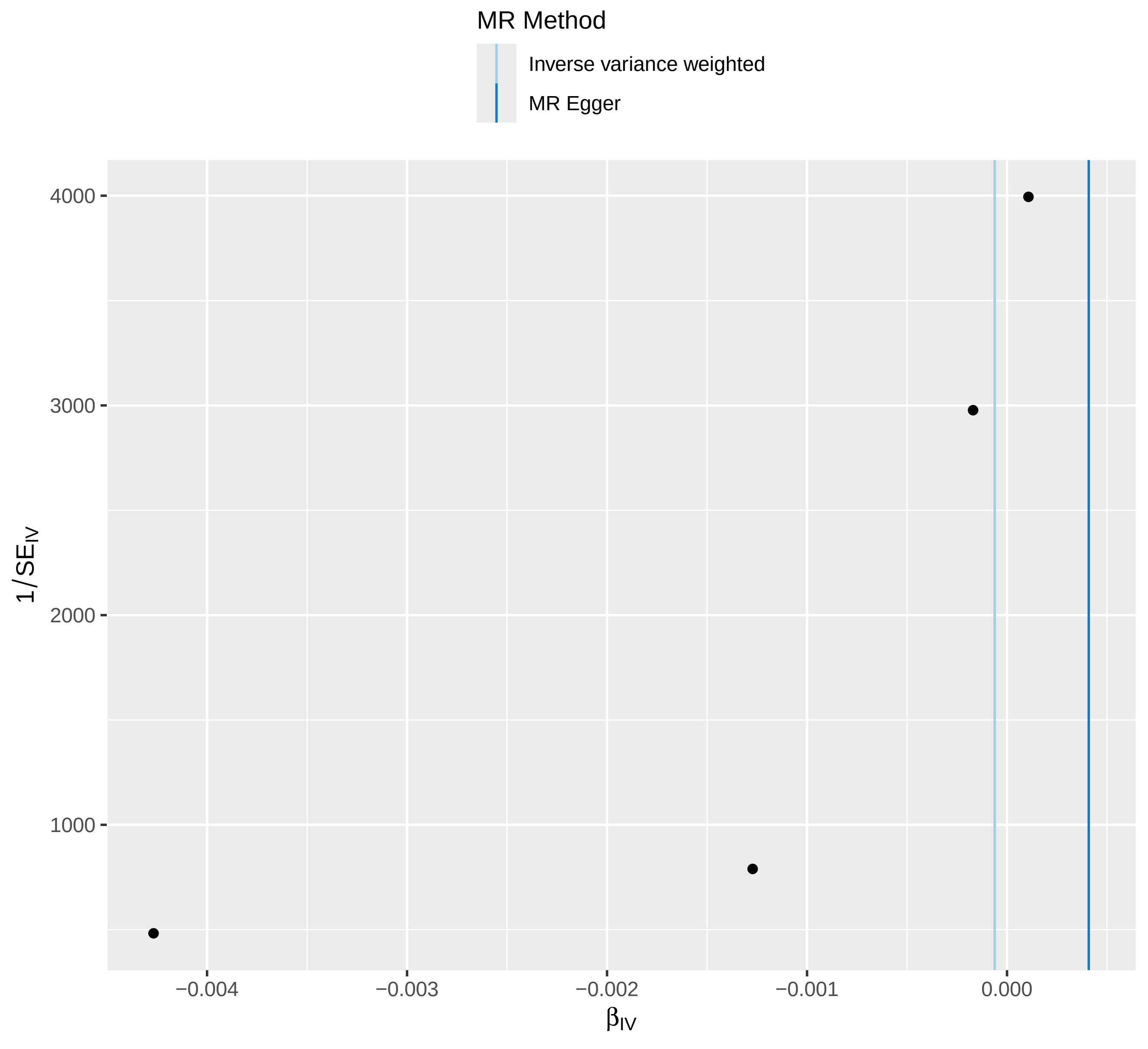
MR Method
Inverse variance weighted
MR Egger
4000
3000
2000
1000
1/SE$_{IV}$
−0.004
−0.003
−0.002
−0.001
0.000
β$_{IV}$

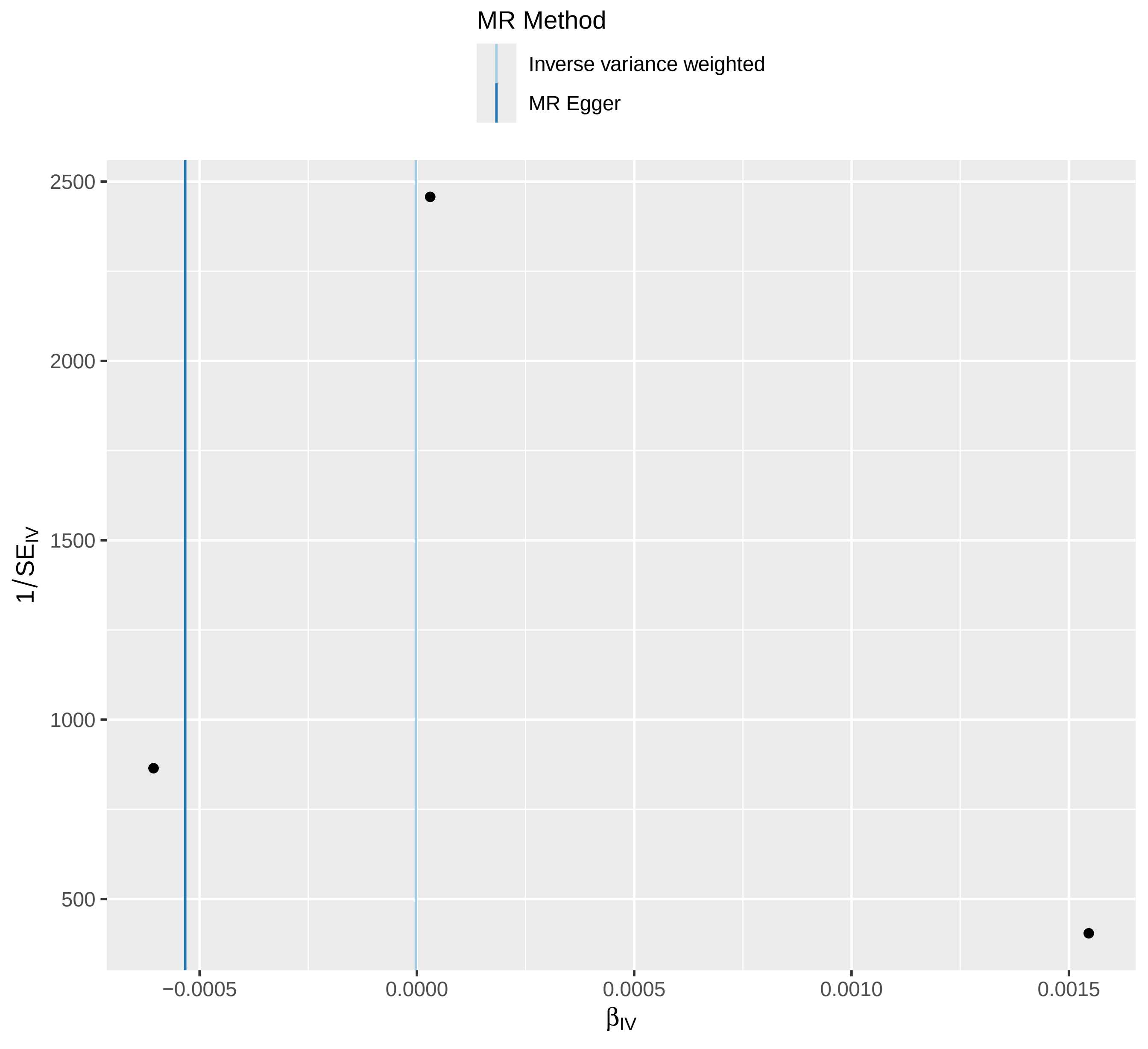
MR Method
Inverse variance weighted
MR Egger
2500
2000
1500
1000
500
1/SE_IV
−0.0005
0.0000
0.0005
0.0010
0.0015
β_IV

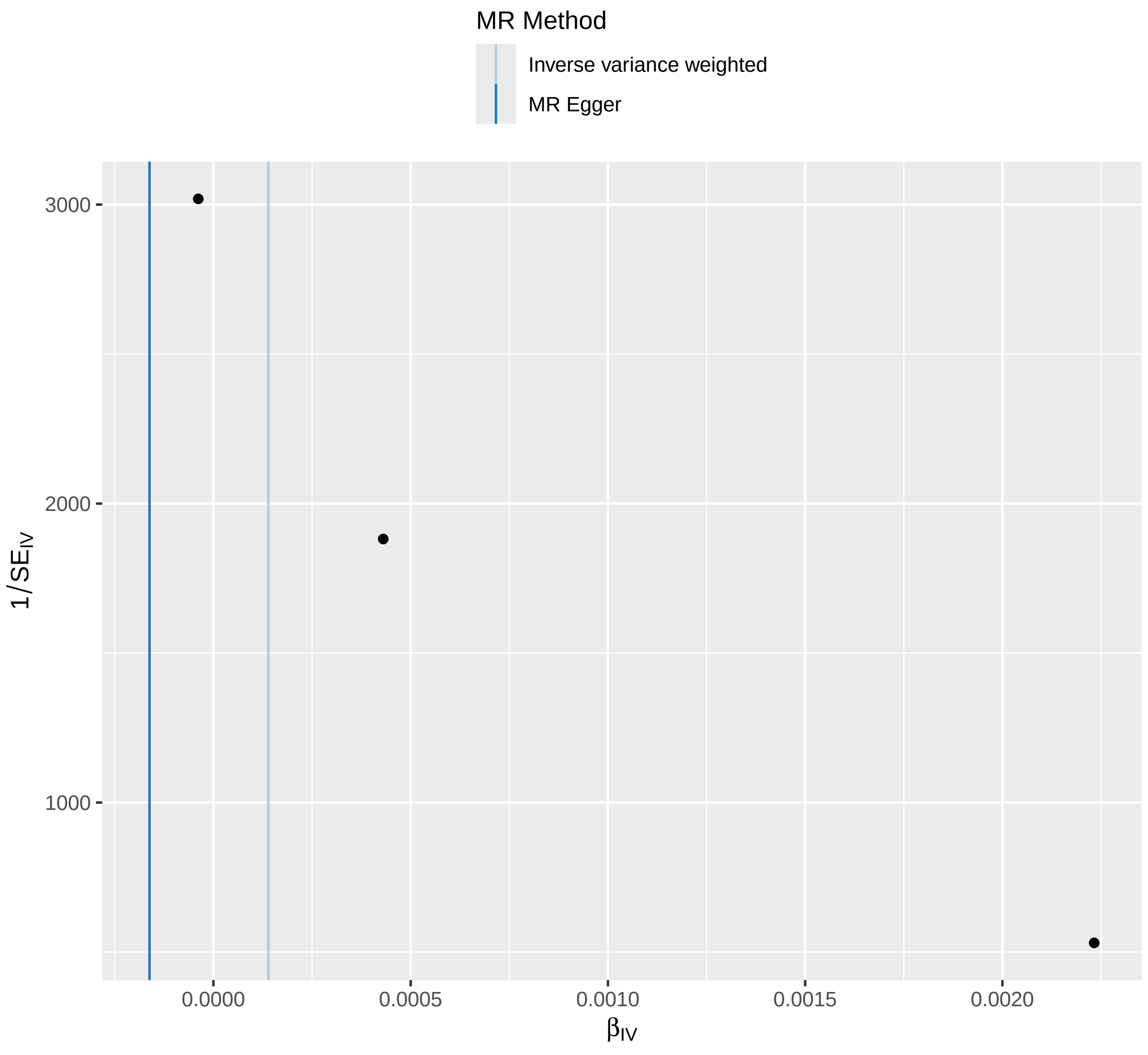
MR Method
Inverse variance weighted
MR Egger
3000
2000
1000
1/SE_IV
0.0000
0.0005
0.0010
0.0015
0.0020
β_IV

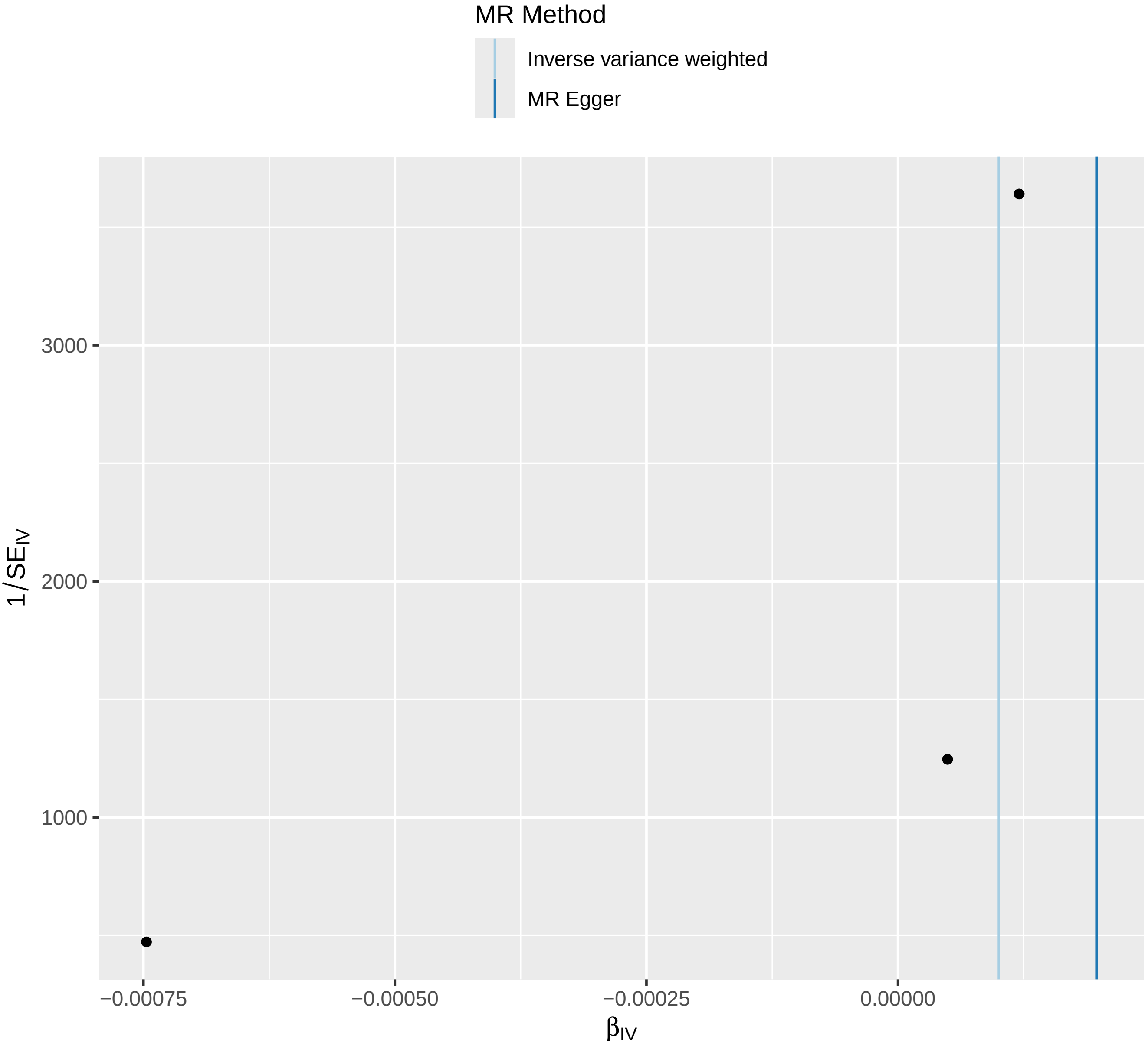
MR Method
Inverse variance weighted
MR Egger
3000
2000
1000
1/SE$_{IV}$
−0.00075
−0.00050
−0.00025
0.00000
β$_{IV}$

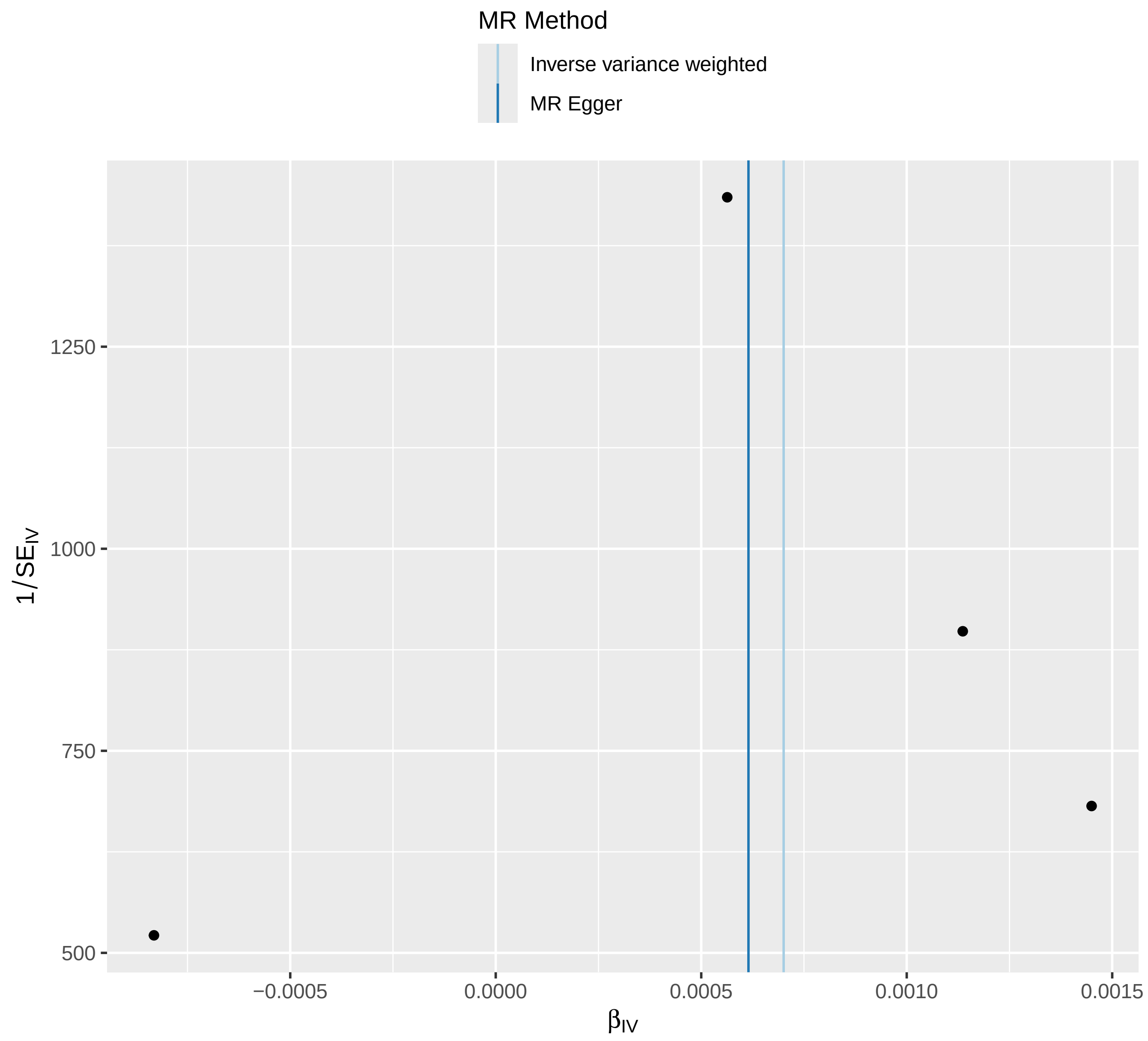
MR Method
Inverse variance weighted
MR Egger
1250
1000
750
500
1/SE_IV
−0.0005
0.0000
0.0005
0.0010
0.0015
β_IV

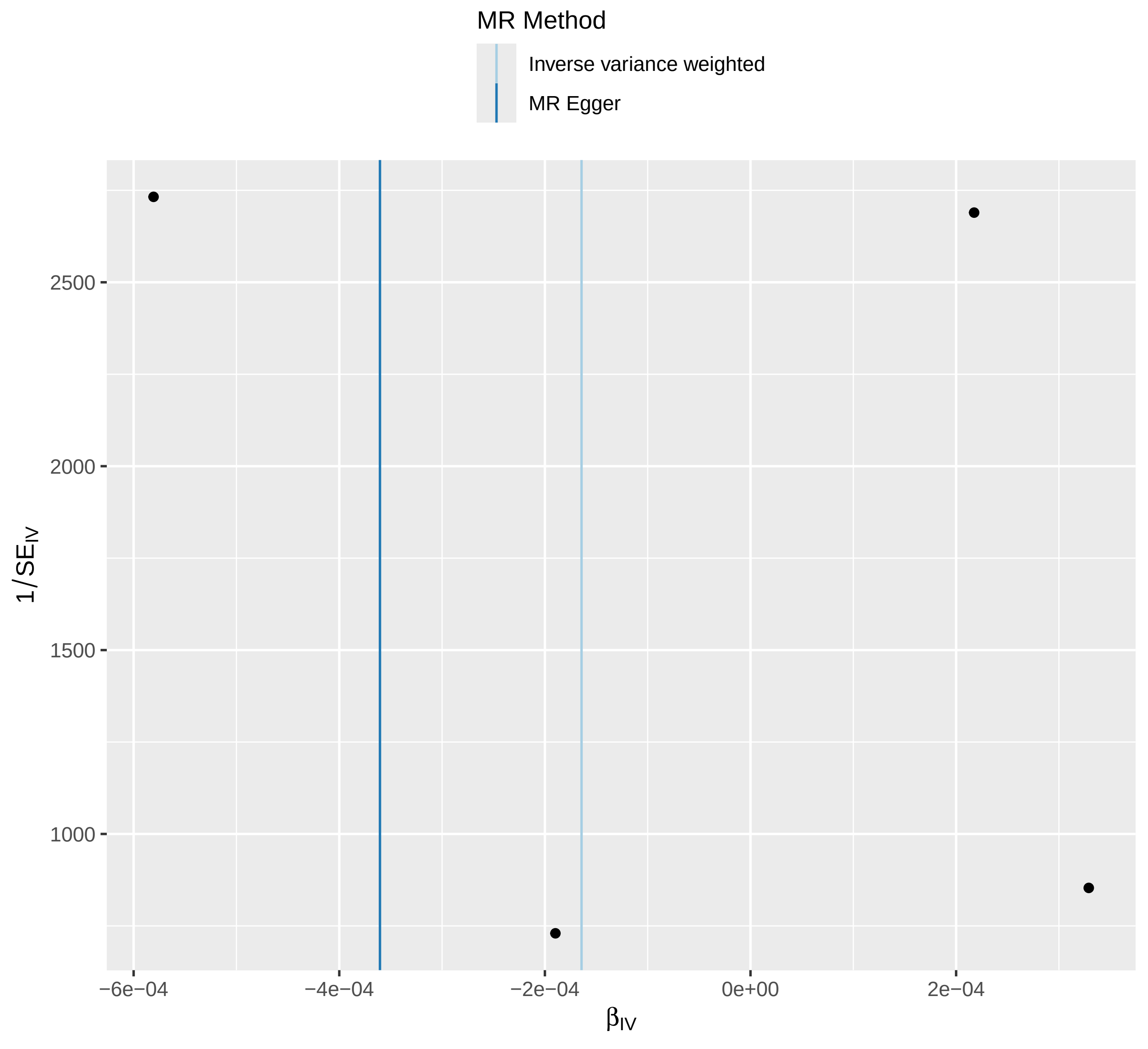
MR Method
Inverse variance weighted
MR Egger
2500
2000
1500
1000
1/SE$_{IV}$
−6e−04
−4e−04
−2e−04
0e+00
2e−04
β$_{IV}$

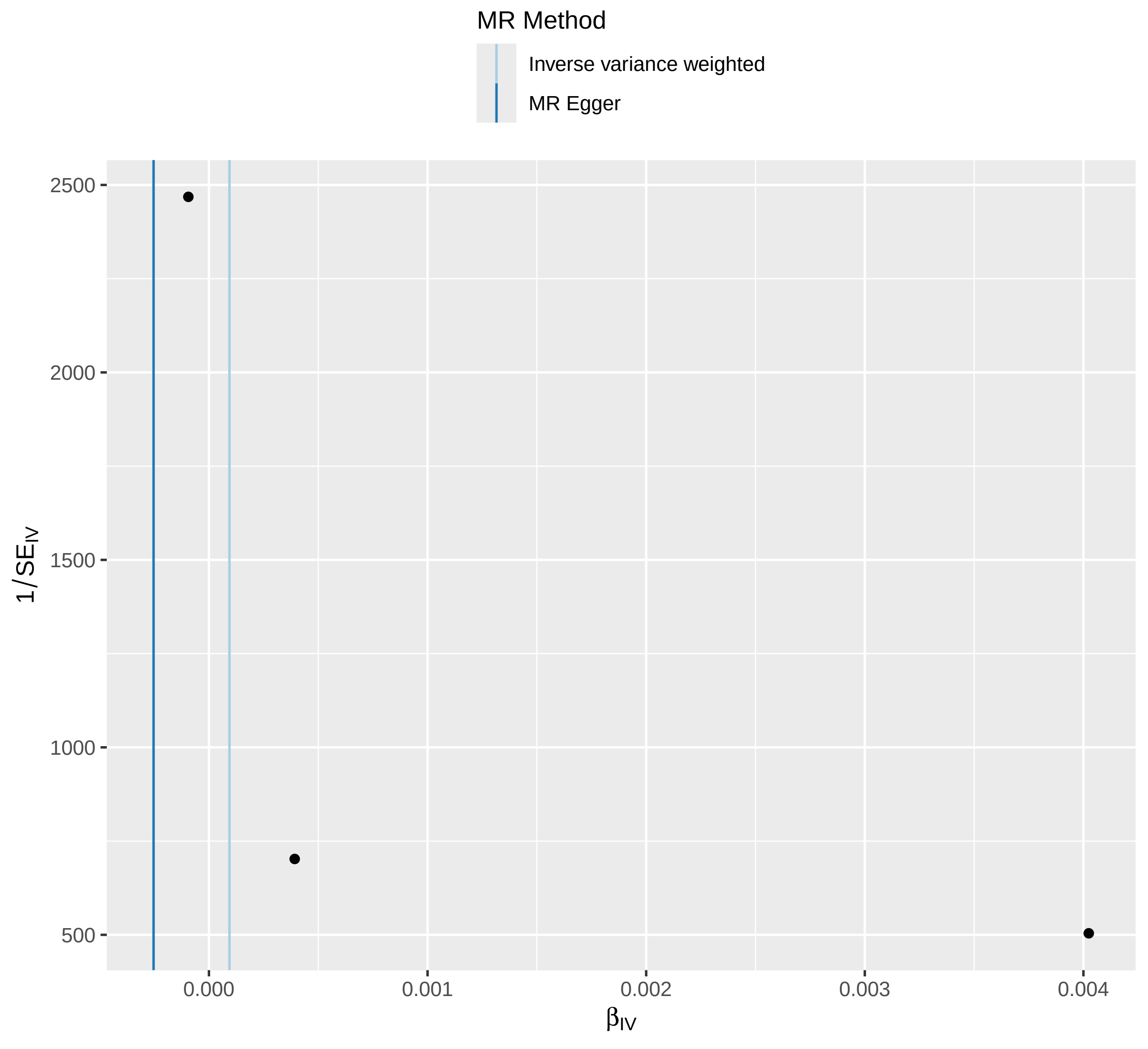
MR Method
Inverse variance weighted
MR Egger
2500
2000
1500
1000
500
$1/SE_{IV}$
0.000
0.001
0.002
0.003
0.004
β_{IV}

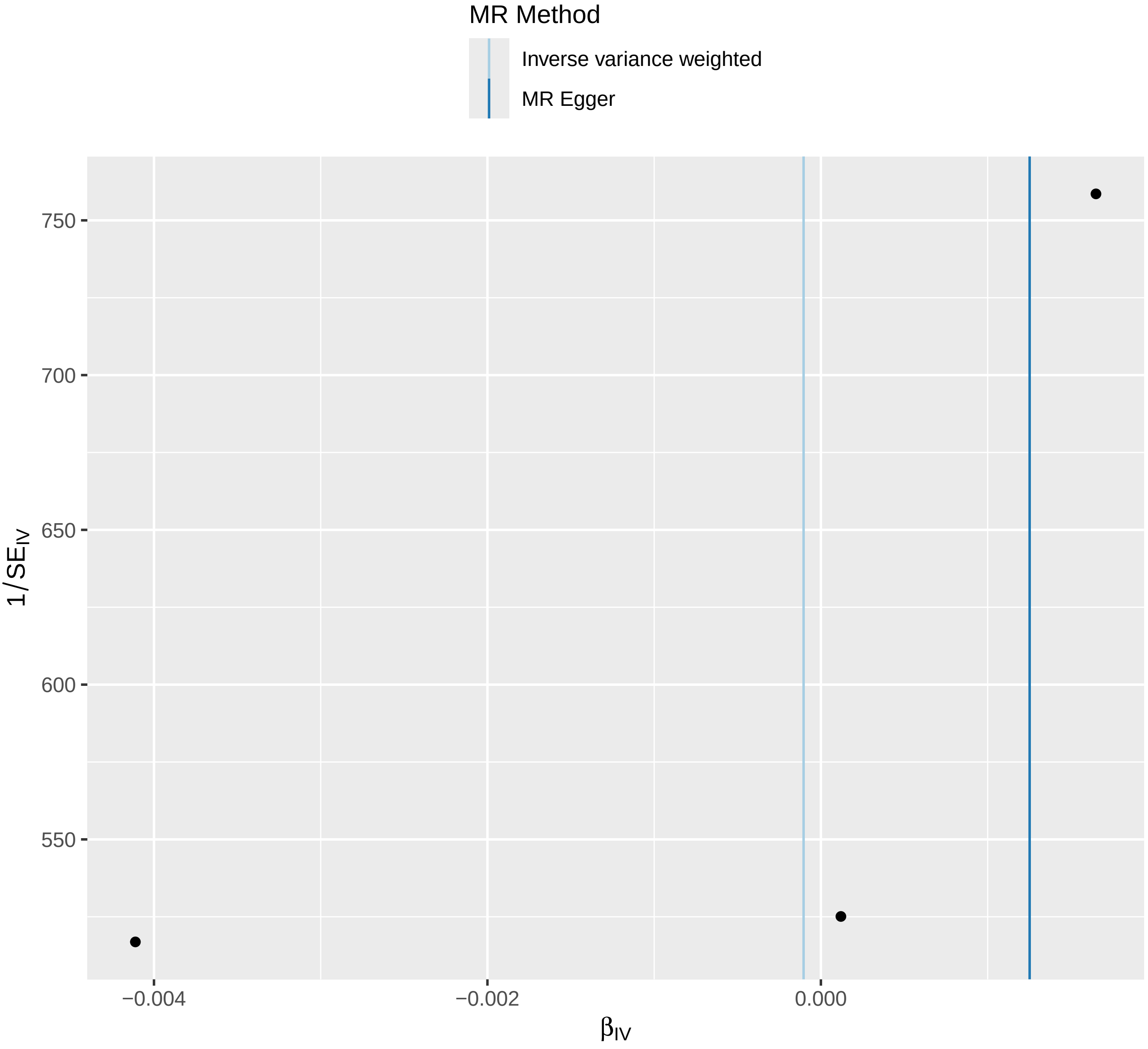

MR Method
Inverse variance weighted
MR Egger
750
700
650
600
550
1/SE_IV
−0.004
−0.002
0.000
β_IV

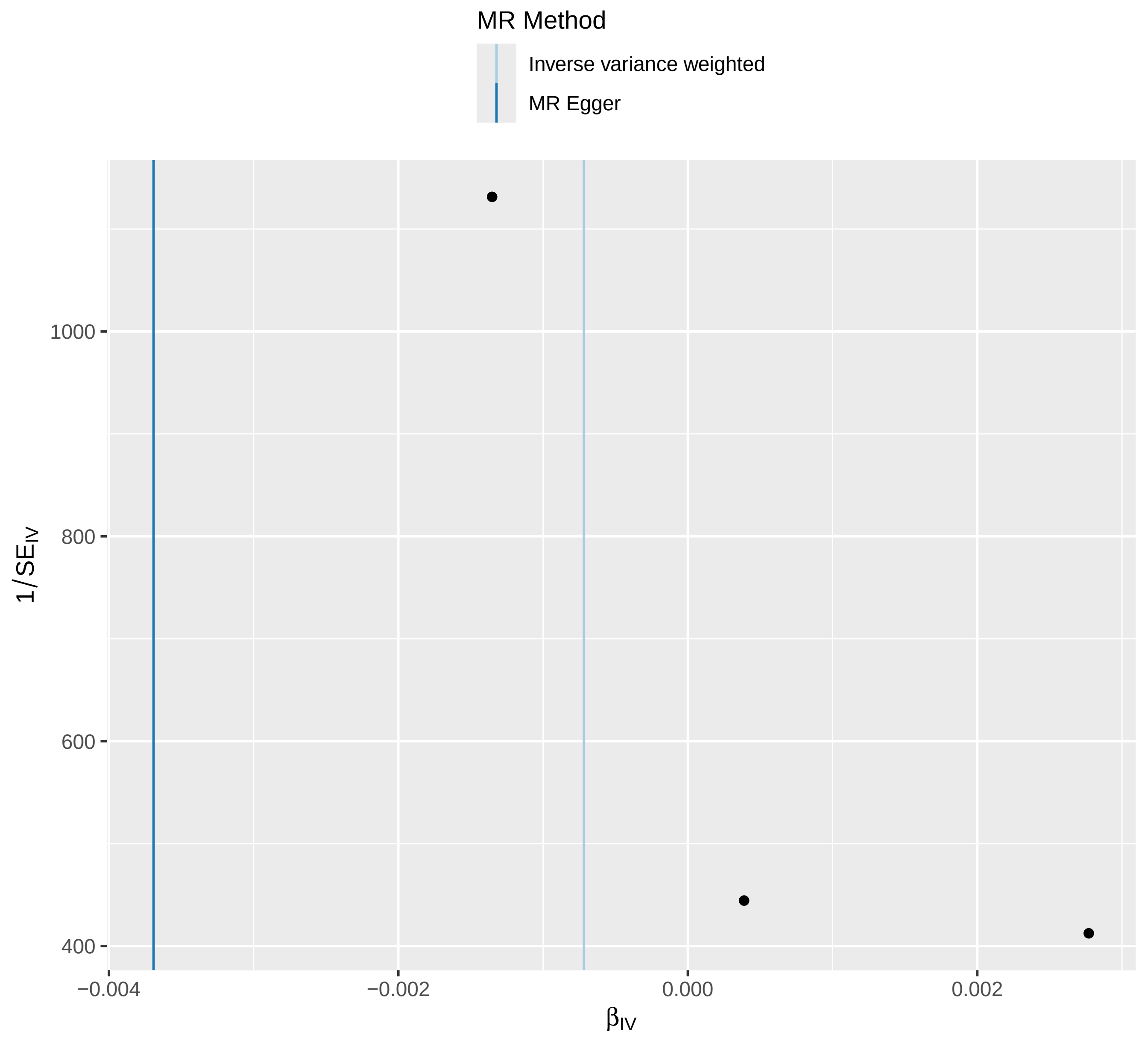

MR Method
Inverse variance weighted
MR Egger
1000
800
600
400
1/SE$_{IV}$
−0.004
−0.002
0.000
0.002
β$_{IV}$

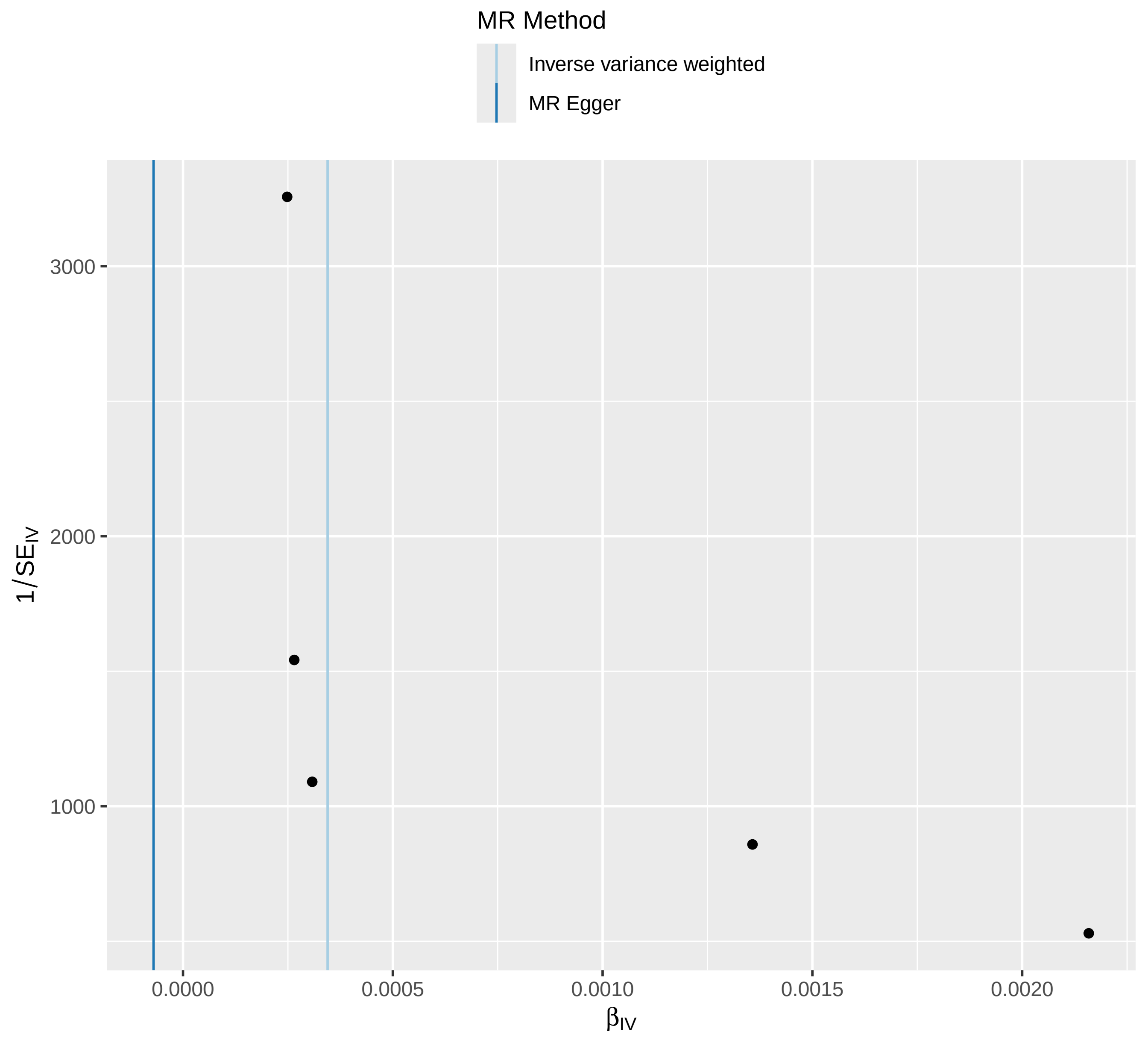
MR Method
Inverse variance weighted
MR Egger
3000
2000
1000
1/SE$_{IV}$
0.0000
0.0005
0.0010
0.0015
0.0020
β$_{IV}$

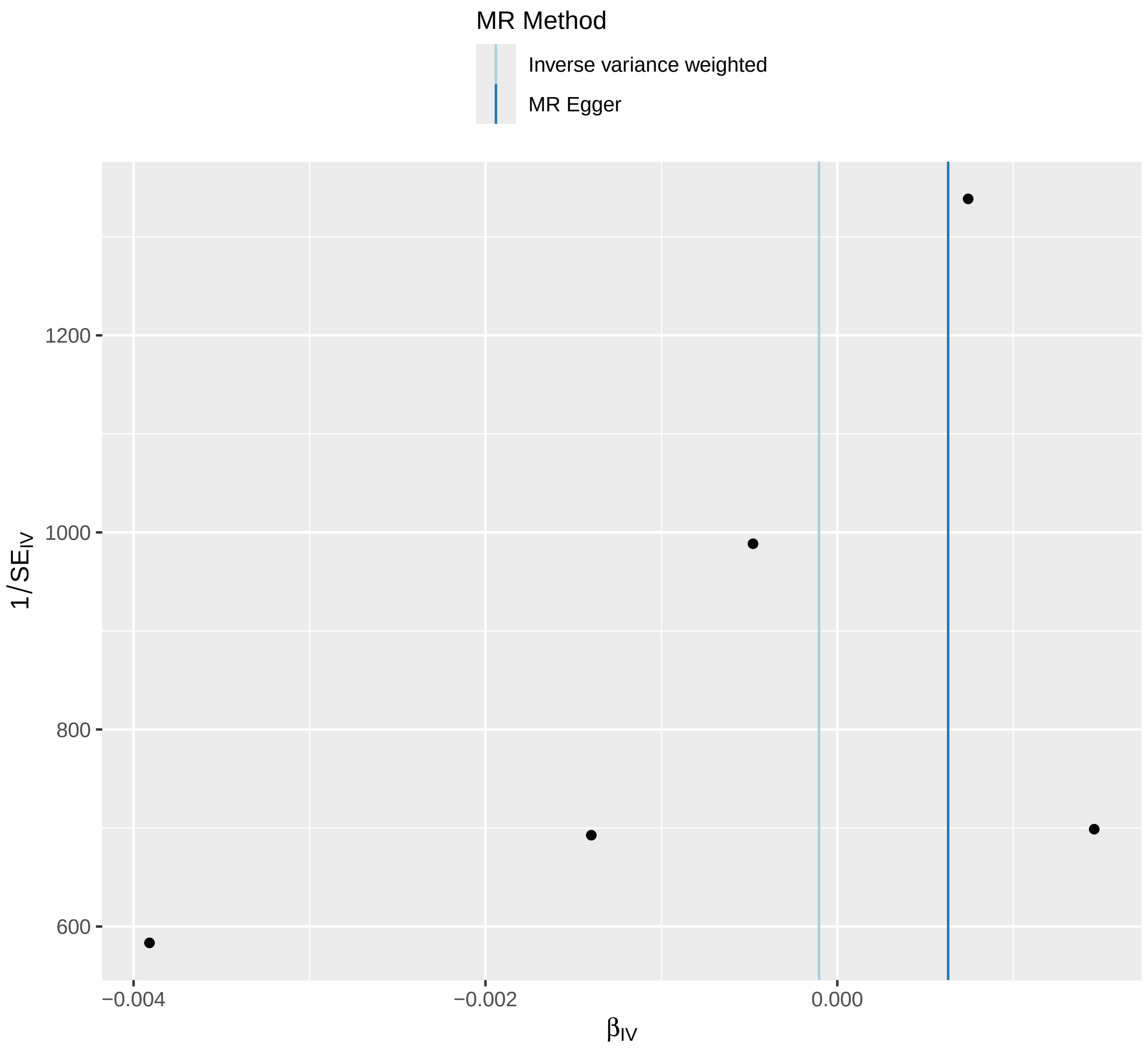
MR Method
Inverse variance weighted
MR Egger
1200
1000
800
600
$1/SE_{IV}$
−0.004
−0.002
0.000
β_{IV}

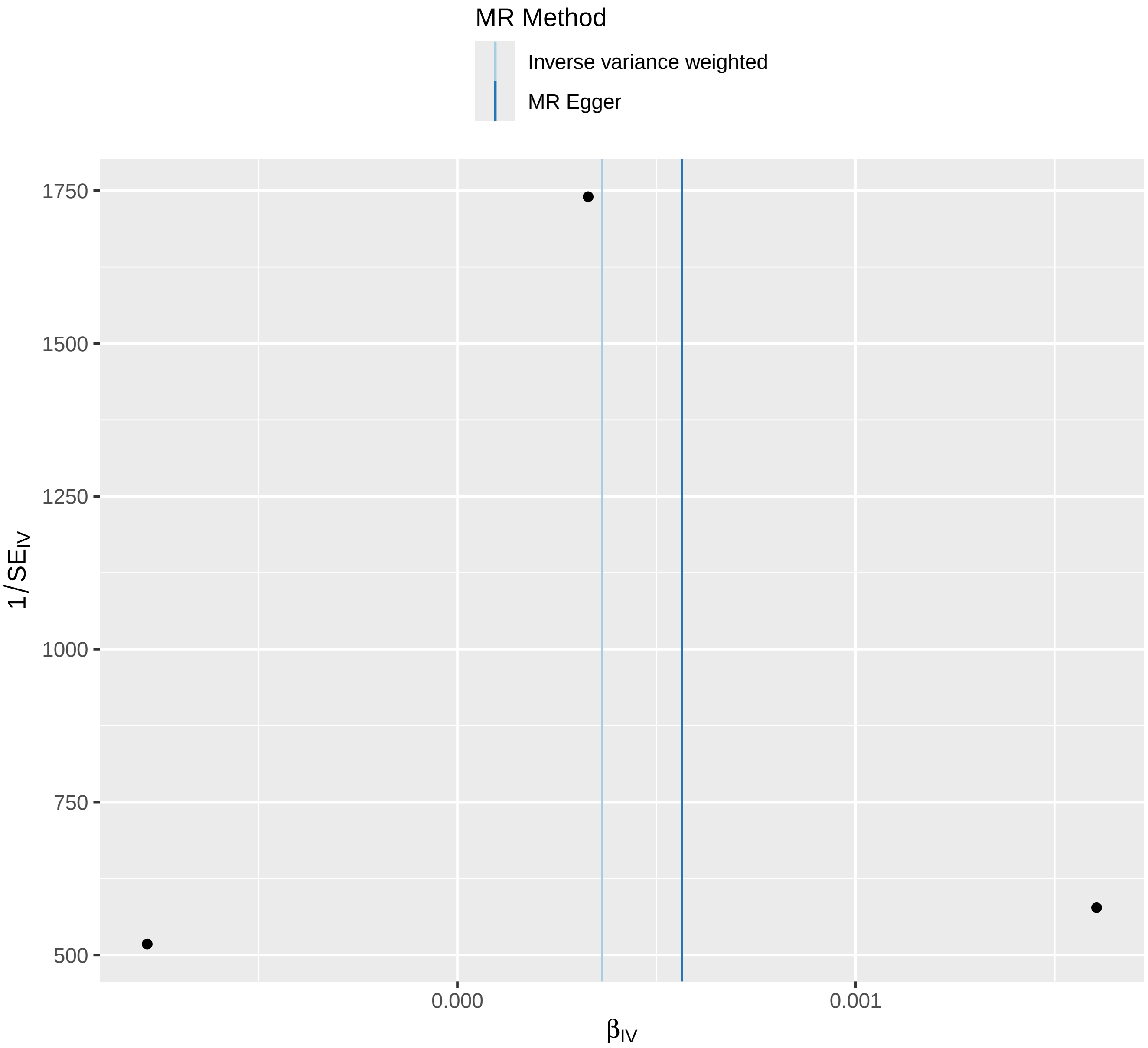

MR Method
Inverse variance weighted
MR Egger
1750
1500
1250
1000
750
500
1/SE$_{IV}$
0.000
0.001
β$_{IV}$

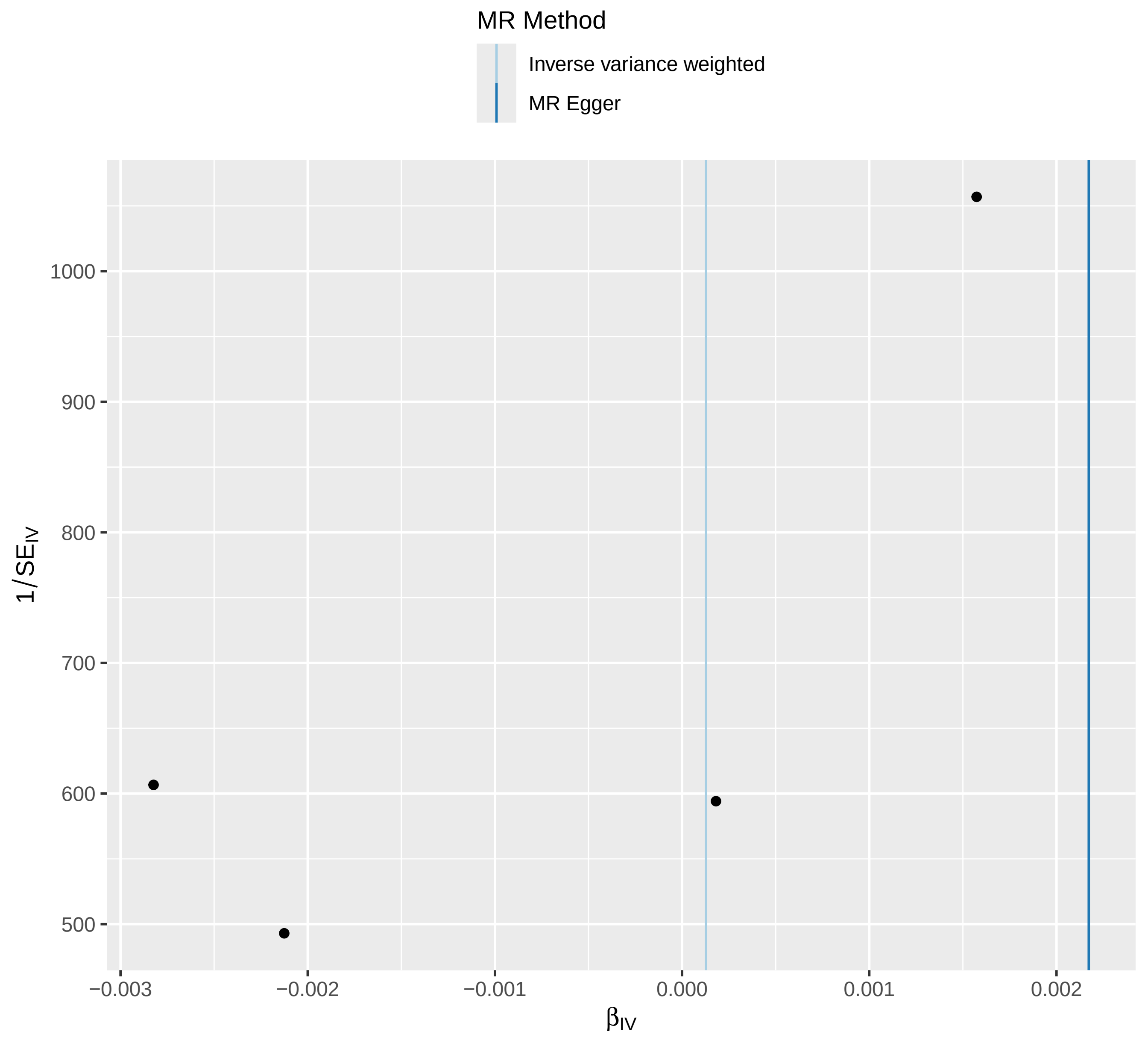

MR Method
Inverse variance weighted
MR Egger
1000
900
800
700
600
500
1/SE$_{IV}$
−0.003
−0.002
−0.001
0.000
0.001
0.002
β$_{IV}$

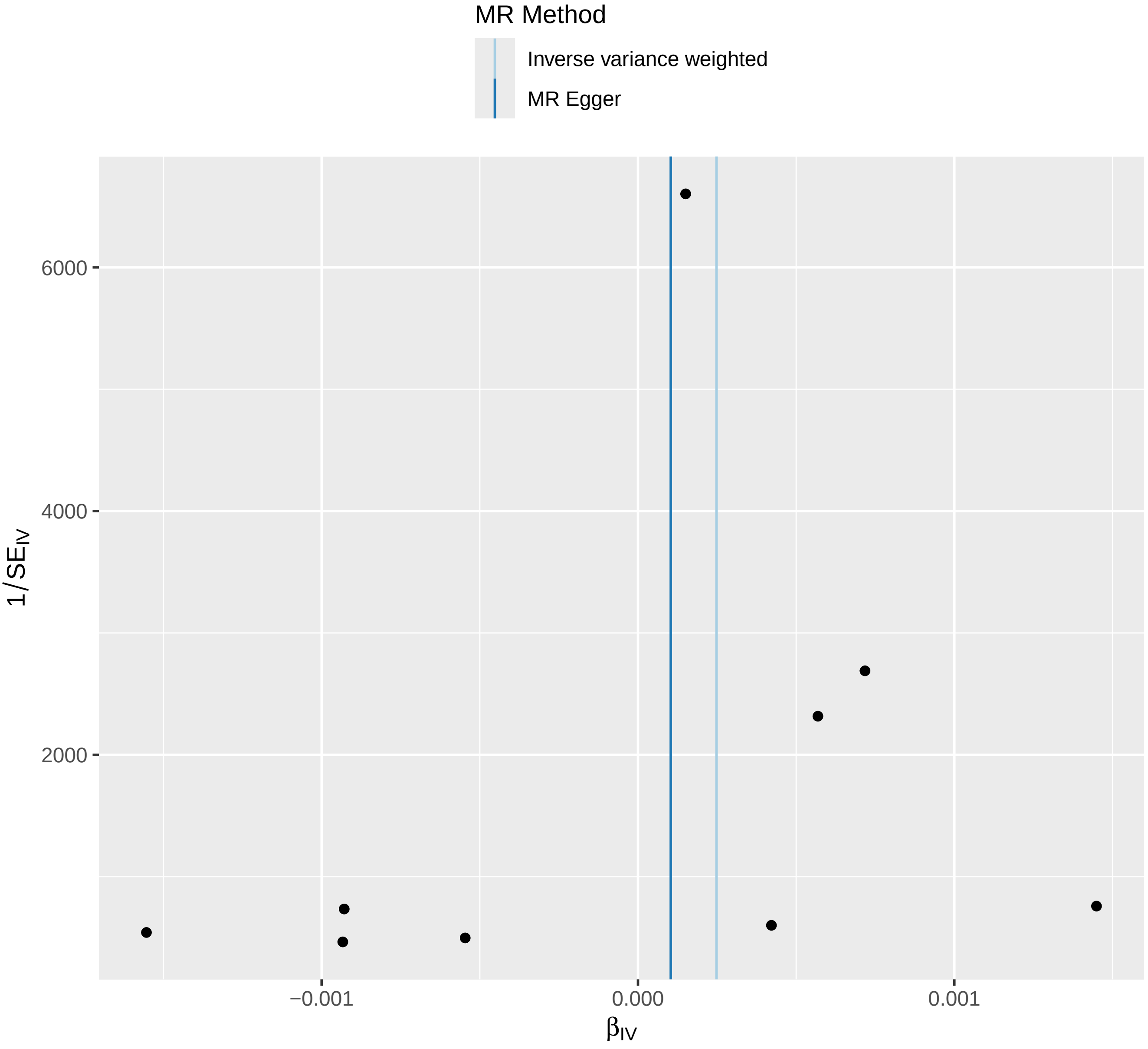

MR Method
Inverse variance weighted
MR Egger
6000
4000
2000
1/SE_IV
−0.001
0.000
0.001
β_IV

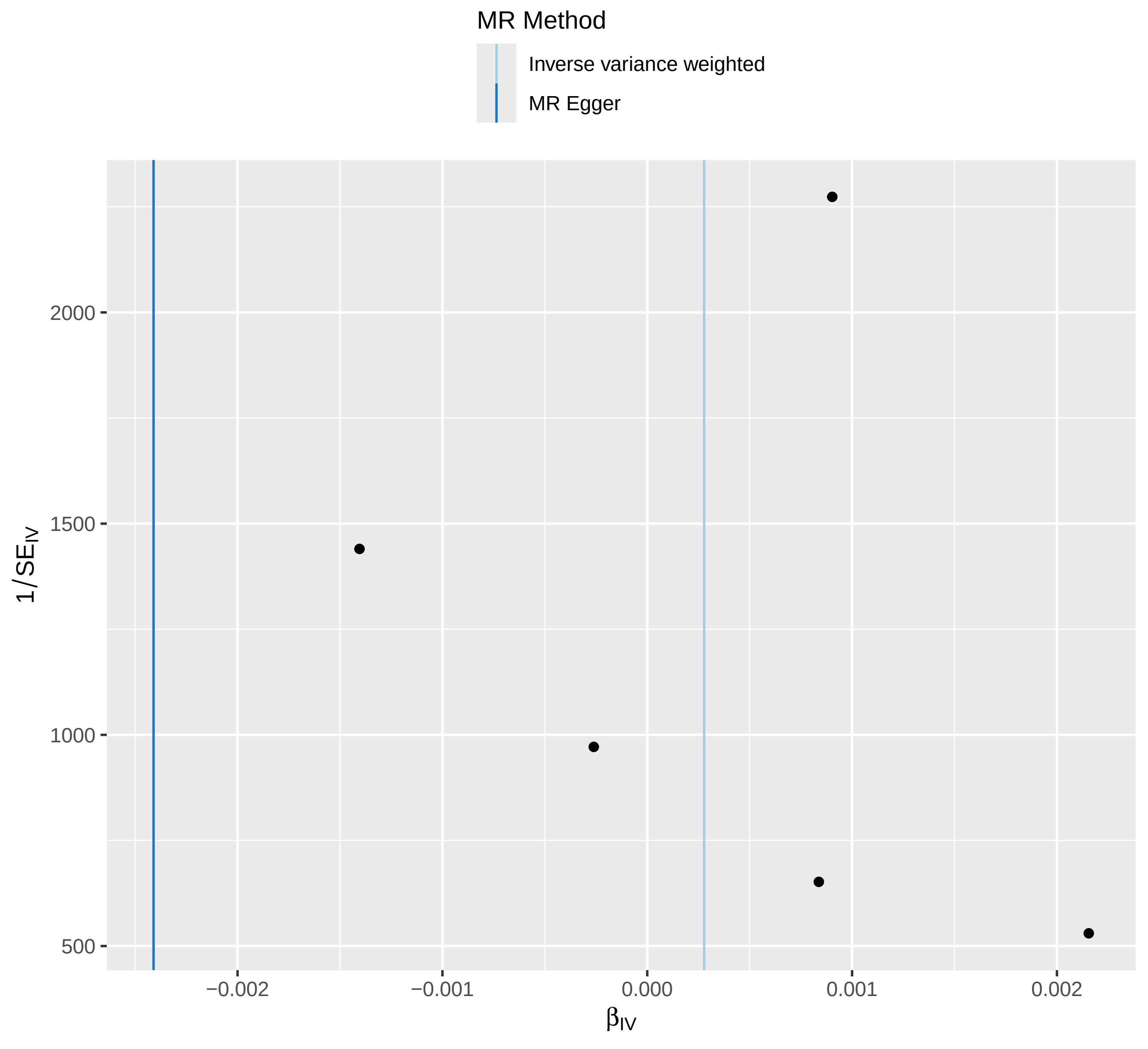

MR Method
Inverse variance weighted
MR Egger
2000
1500
1000
500
1/SE_IV
−0.002
−0.001
0.000
0.001
0.002
β_IV

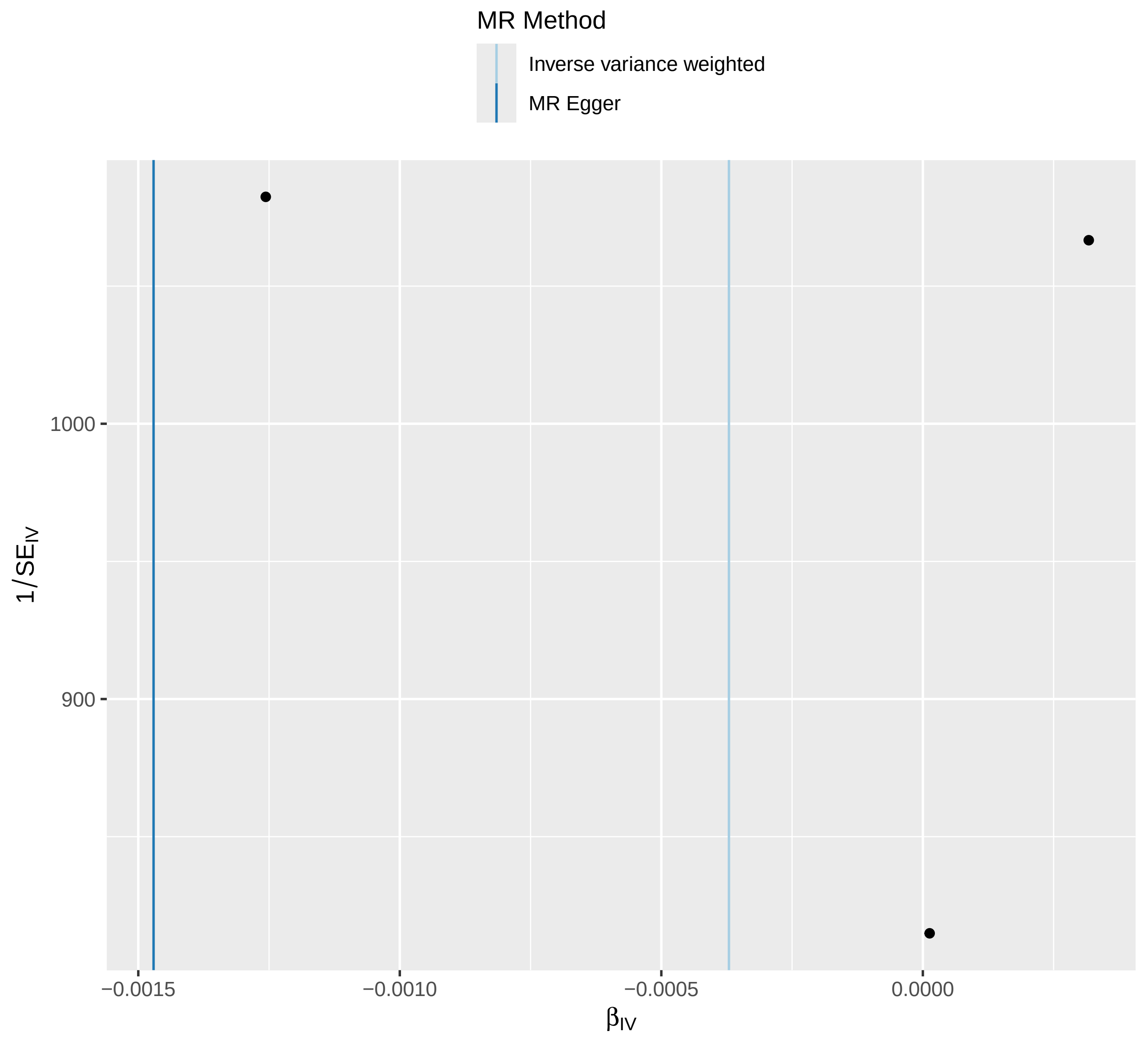
MR Method
Inverse variance weighted
MR Egger
1000
900
1/SE_IV
−0.0015
−0.0010
−0.0005
0.0000
β_IV

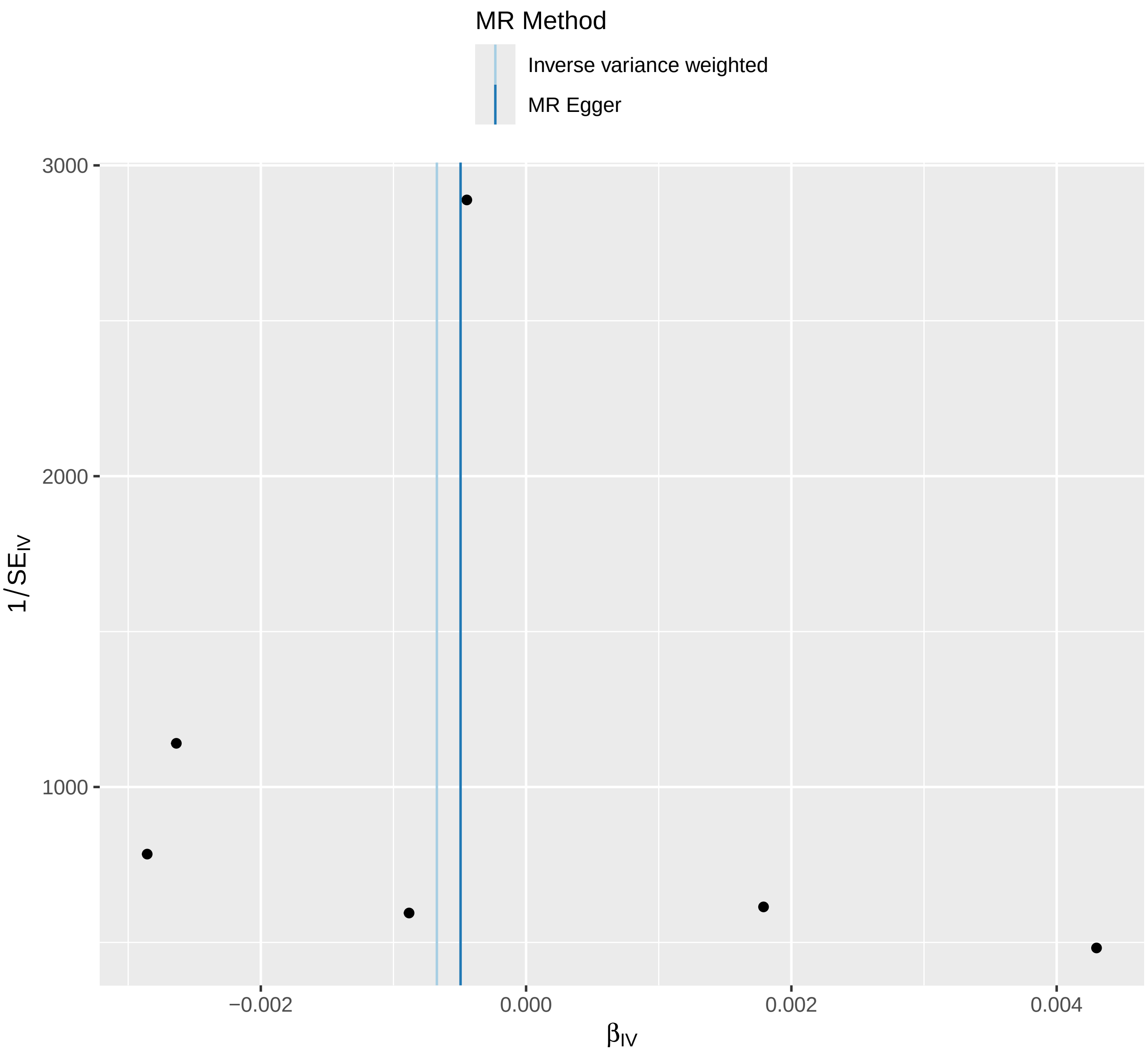

MR Method
Inverse variance weighted
MR Egger
3000
2000
1000
1/SE_IV
−0.002
0.000
0.002
0.004
β_IV

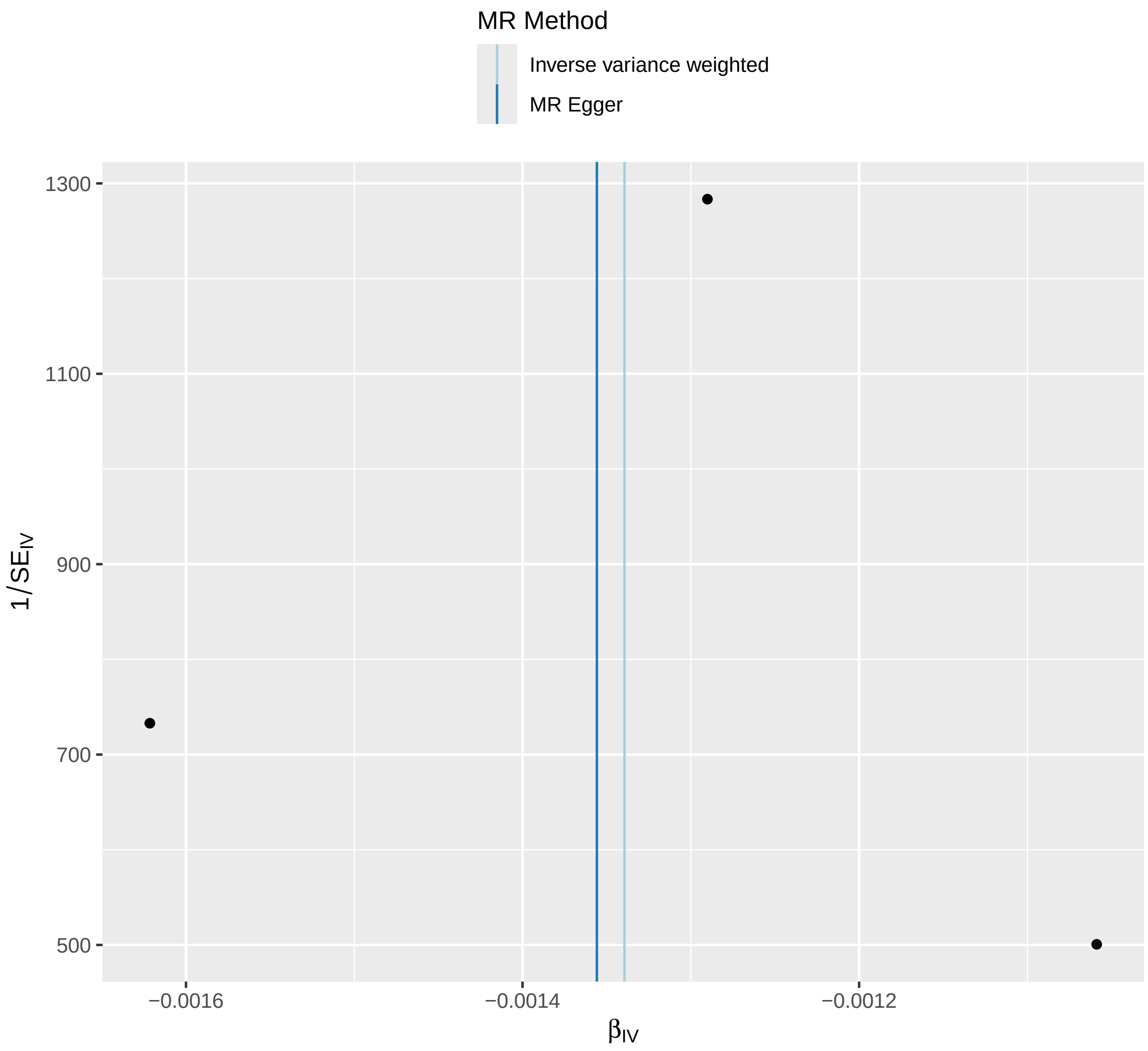
MR Method
Inverse variance weighted
MR Egger
1300
1100
900
700
500
1/SE$_{IV}$
−0.0016
−0.0014
−0.0012
β_{IV}

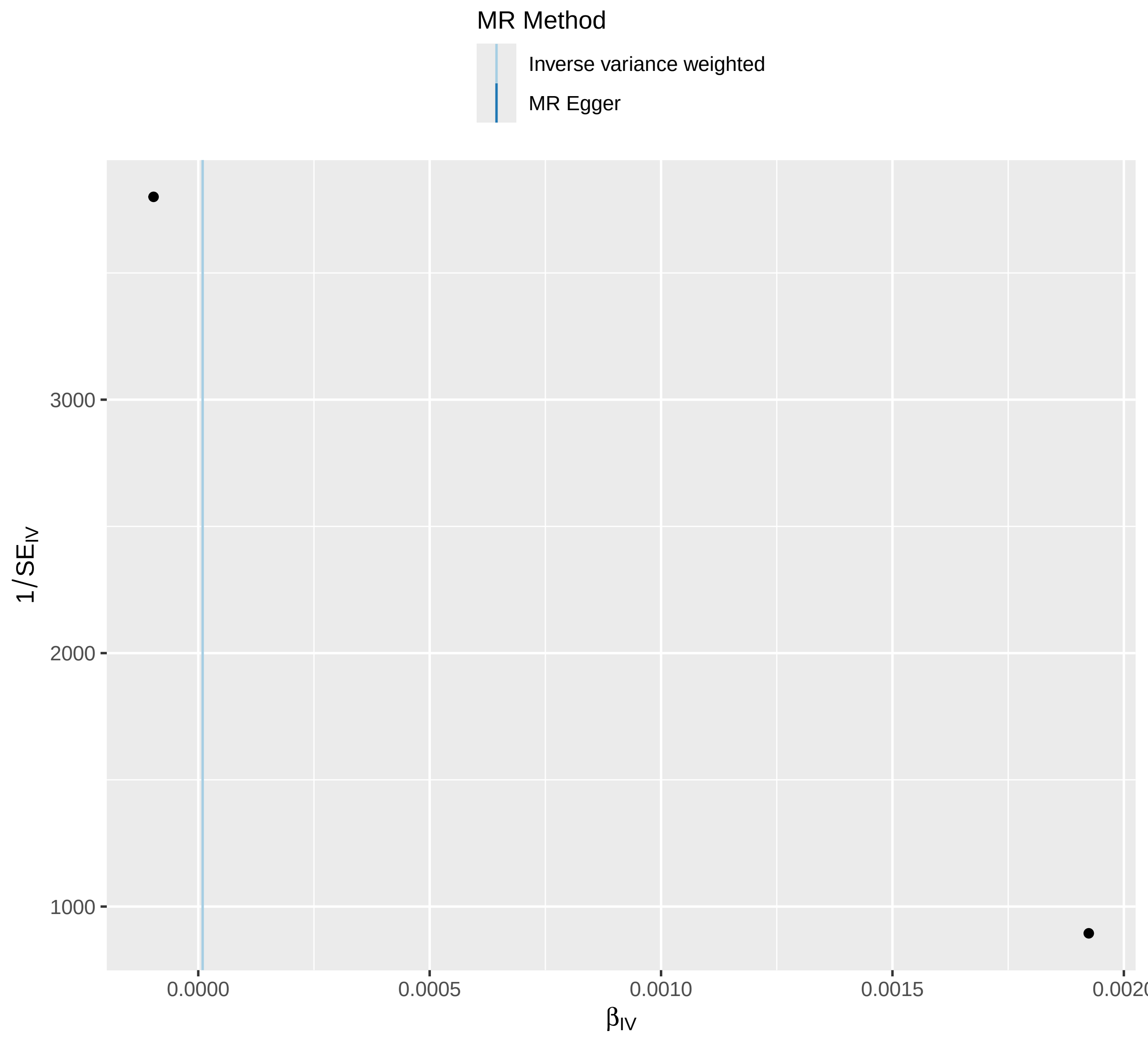
MR Method
Inverse variance weighted
MR Egger
3000
2000
1000
1/SE_IV
0.0000
0.0005
0.0010
0.0015
0.0020
β_IV

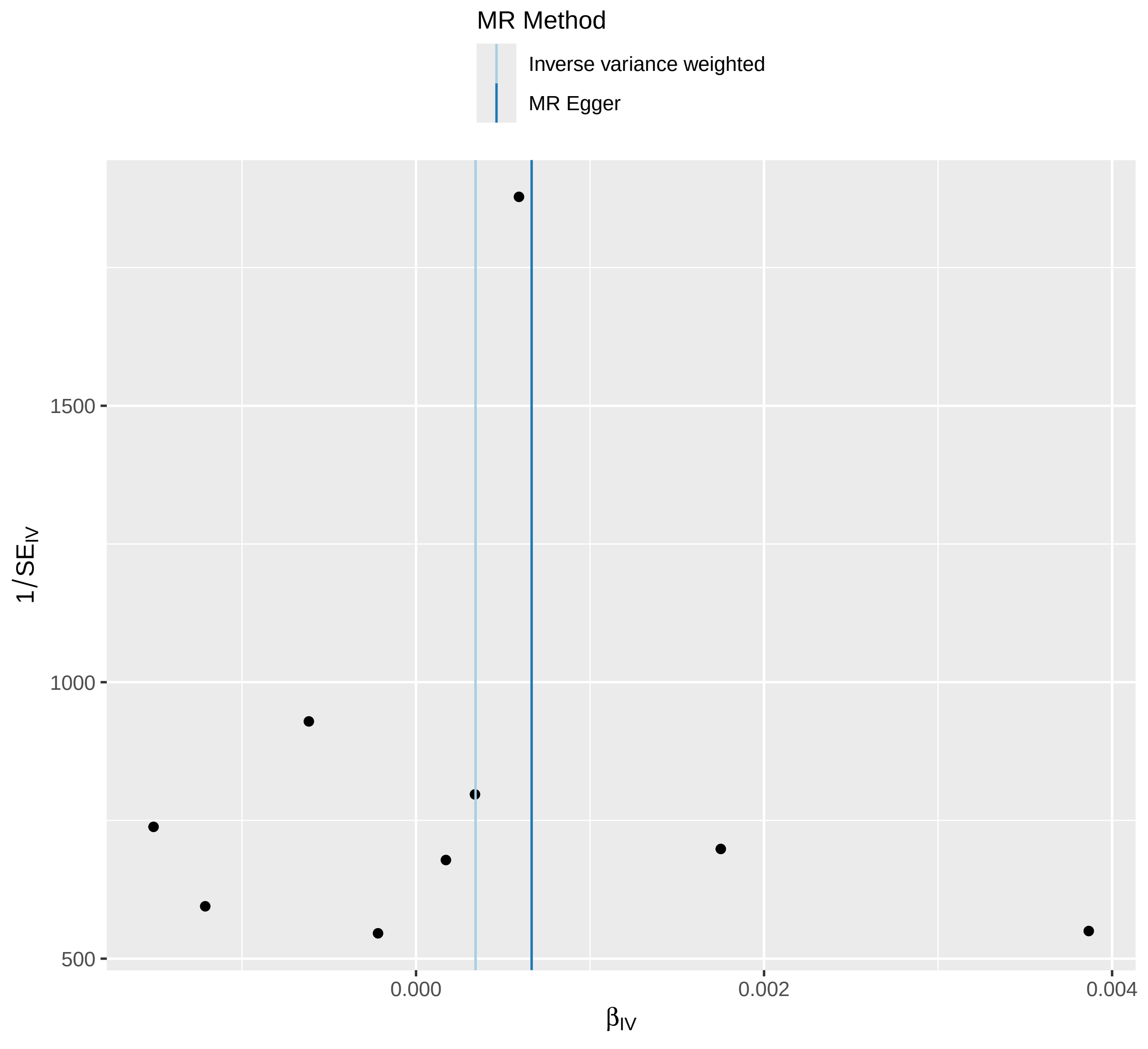

MR Method
Inverse variance weighted
MR Egger
1500
1000
500
0.000
0.002
0.004
$1/SE_{IV}$
β_{IV}

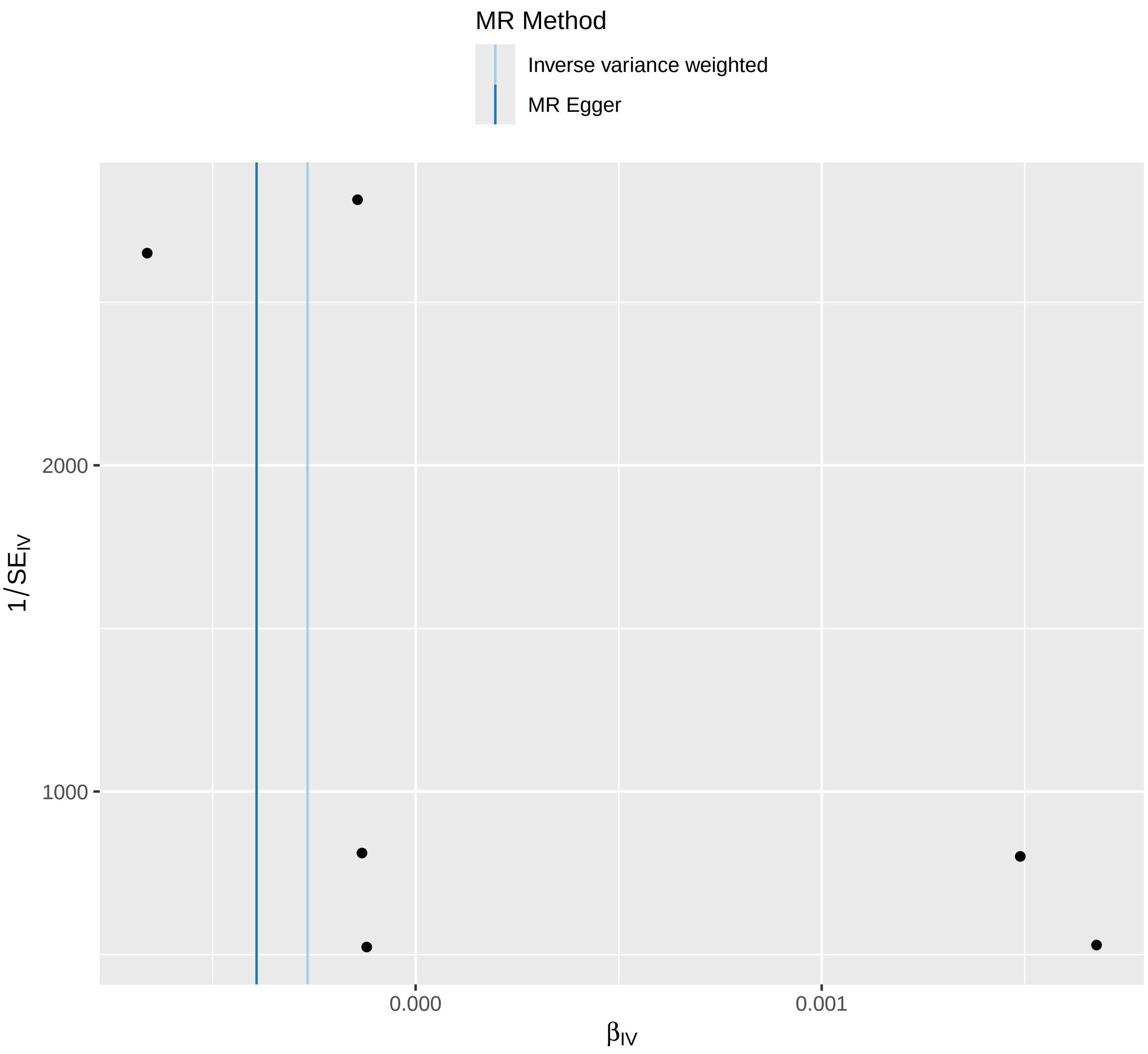

MR Method
Inverse variance weighted
MR Egger
2000
1000
0.000
0.001
$1/SE_{IV}$
β_{IV}

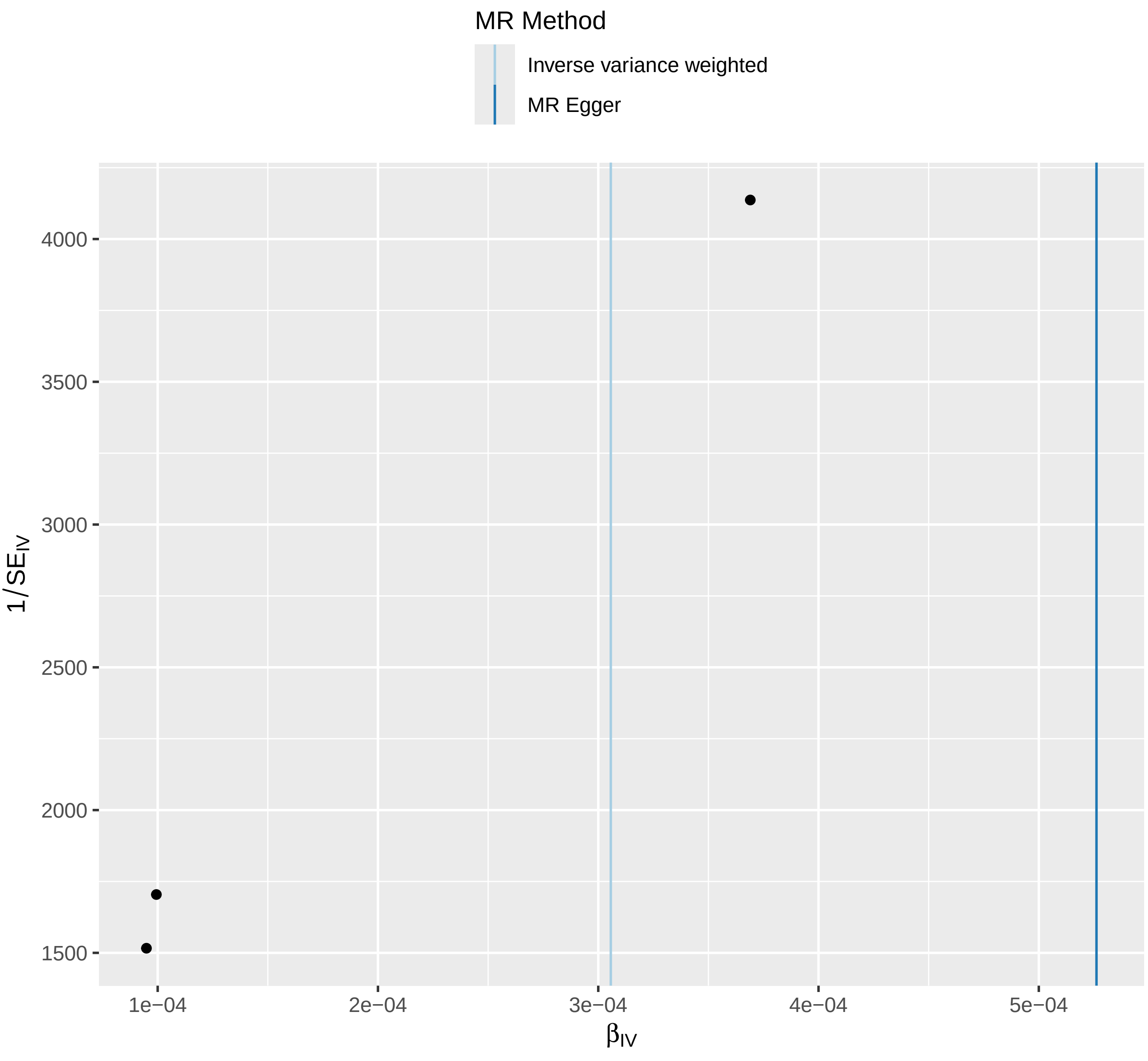

MR Method
Inverse variance weighted
MR Egger
4000
3500
3000
2500
2000
1500
1/SE_IV
1e−04
2e−04
3e−04
4e−04
5e−04
β_IV

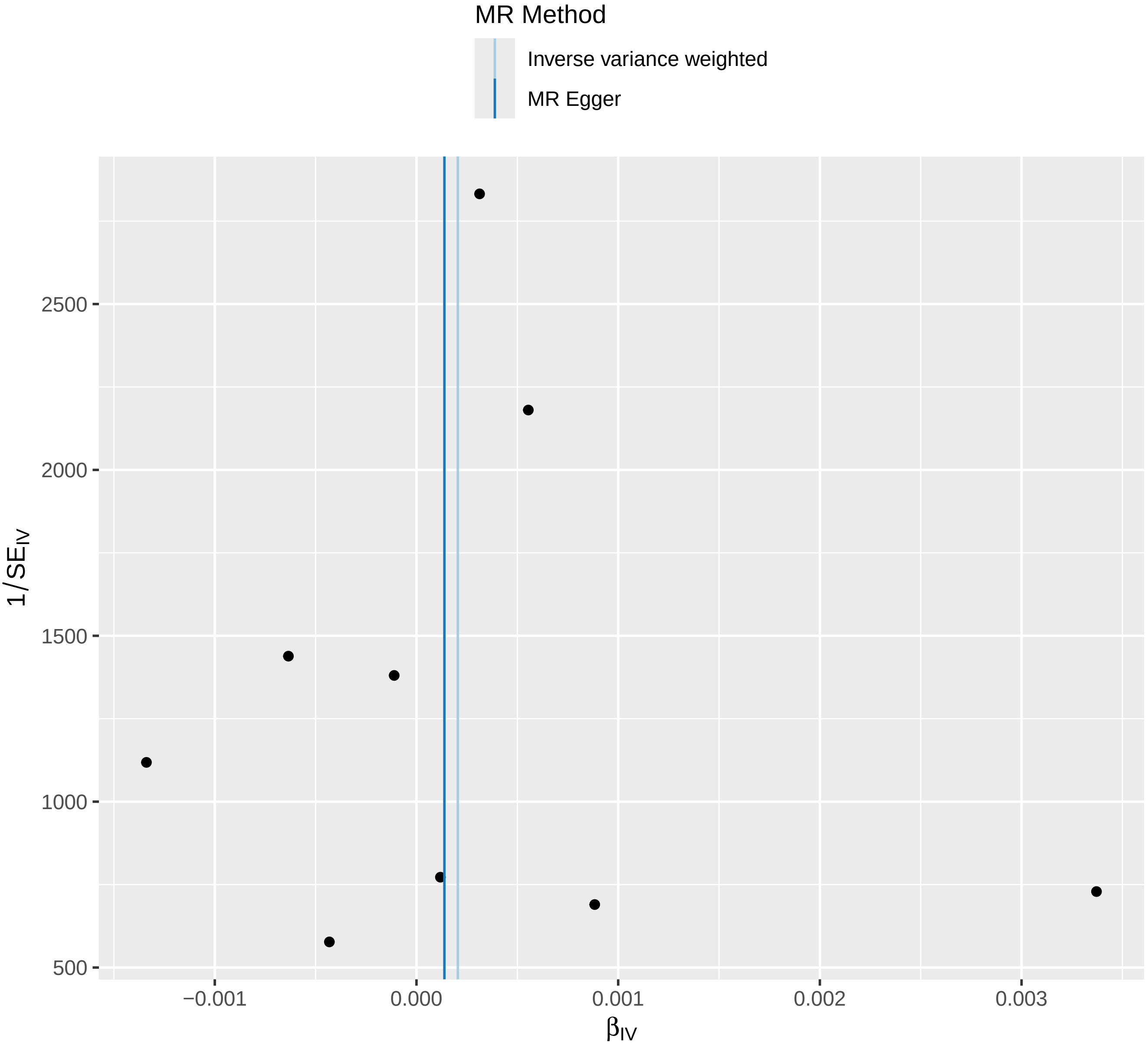
MR Method
Inverse variance weighted
MR Egger
2500
2000
1500
1000
500
1/SE_IV
−0.001
0.000
0.001
0.002
0.003
β_IV

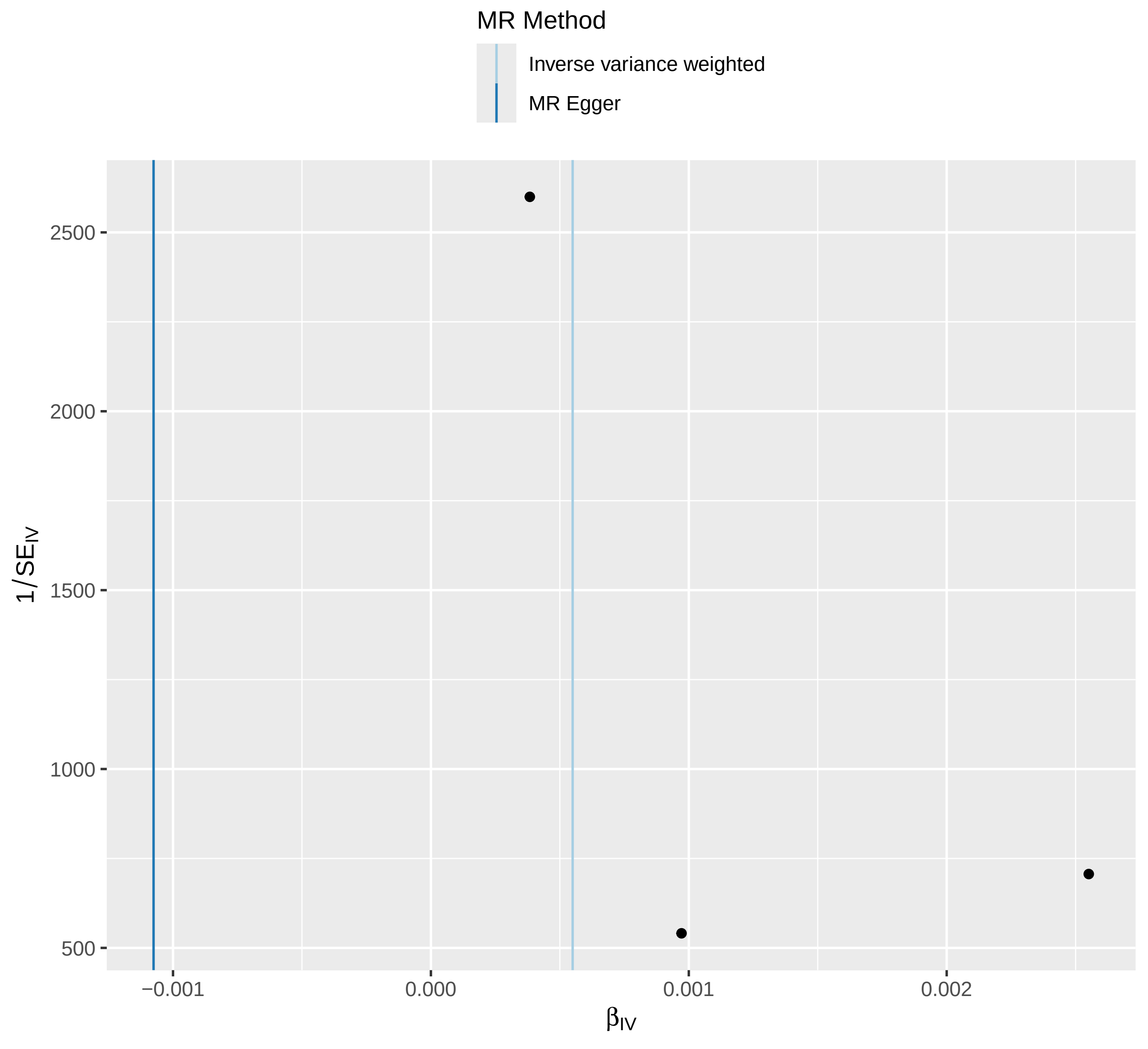

MR Method
Inverse variance weighted
MR Egger
2500
2000
1500
1000
500
1/SE_IV
−0.001
0.000
0.001
0.002
β_IV

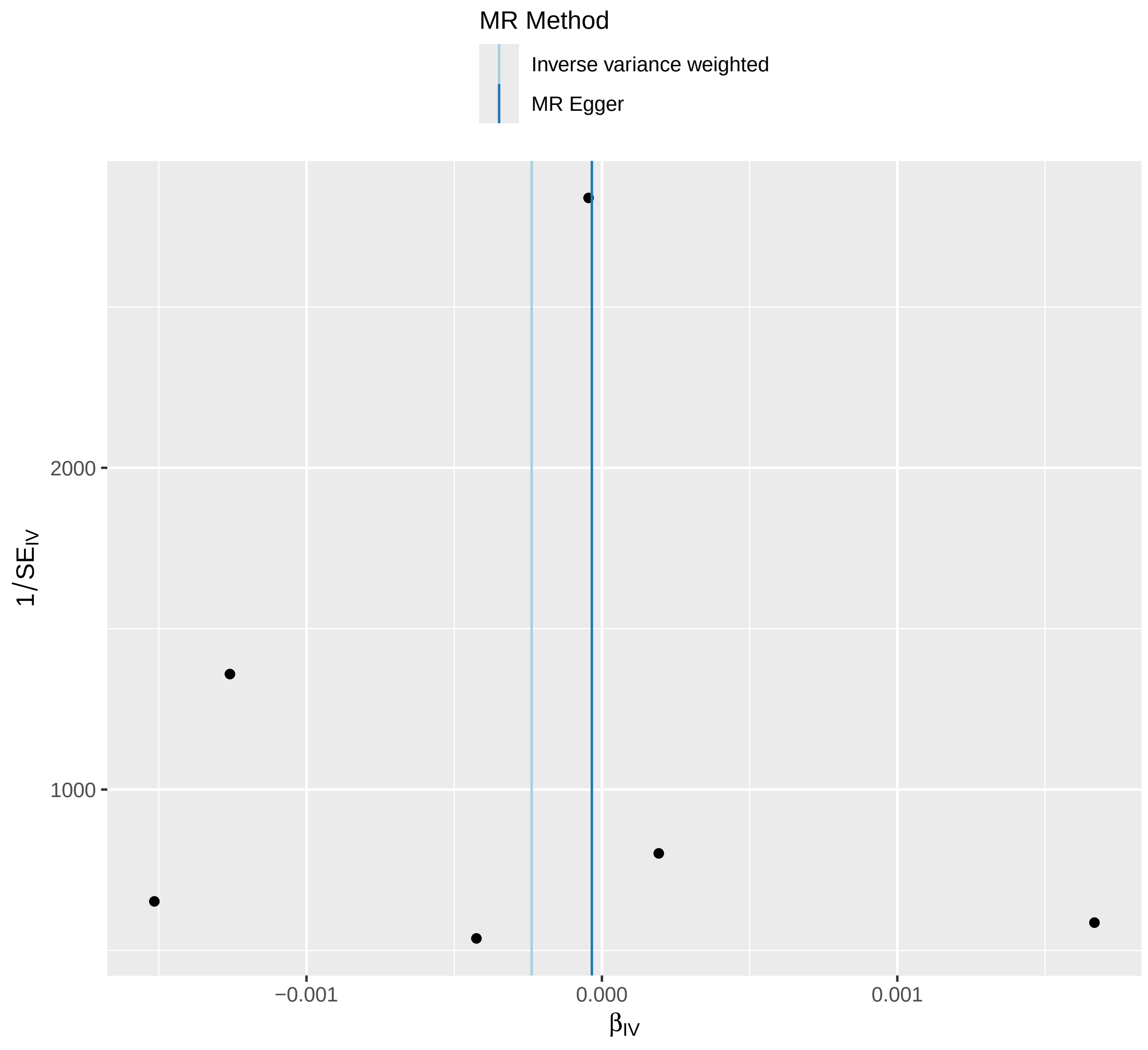

MR Method
Inverse variance weighted
MR Egger
2000
1000
1/SE_IV
−0.001
0.000
0.001
β_IV

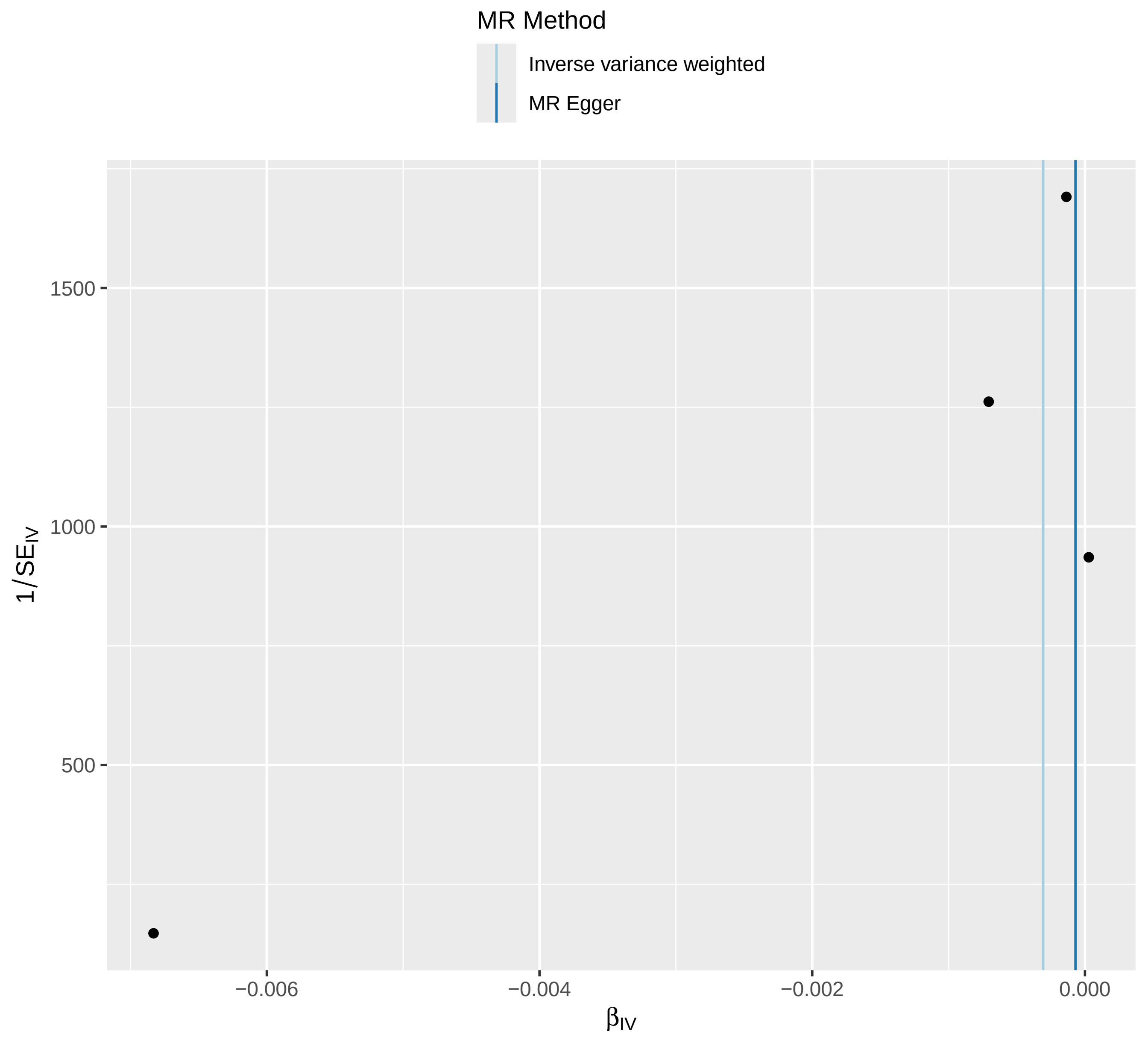
MR Method
Inverse variance weighted
MR Egger
1500
1000
500
1/SE$_{IV}$
−0.006
−0.004
−0.002
0.000
β$_{IV}$

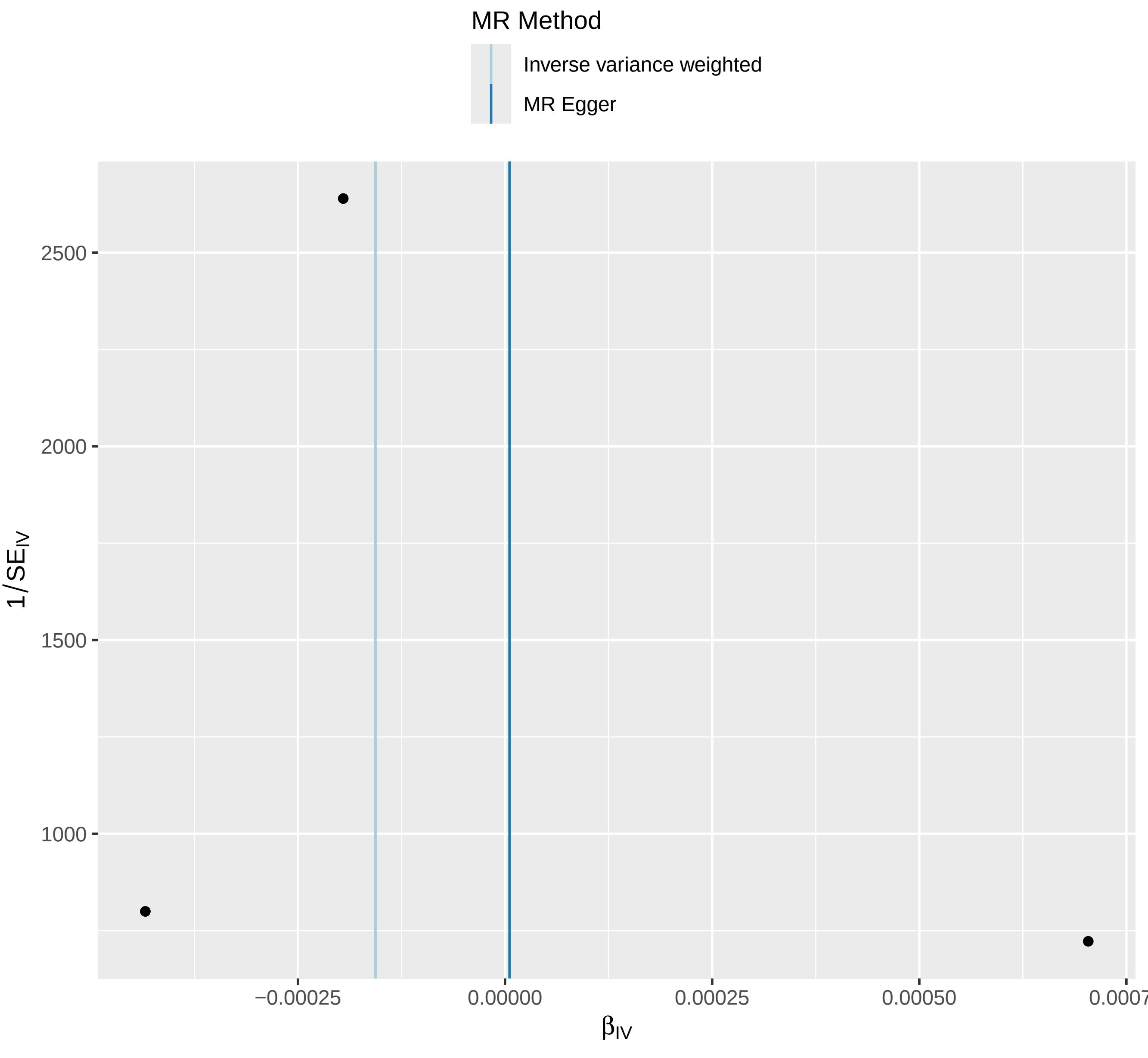
MR Method
Inverse variance weighted
MR Egger
2500
2000
1500
1000
$1/SE_{IV}$
−0.00025
0.00000
0.00025
0.00050
0.0007
β_{IV}

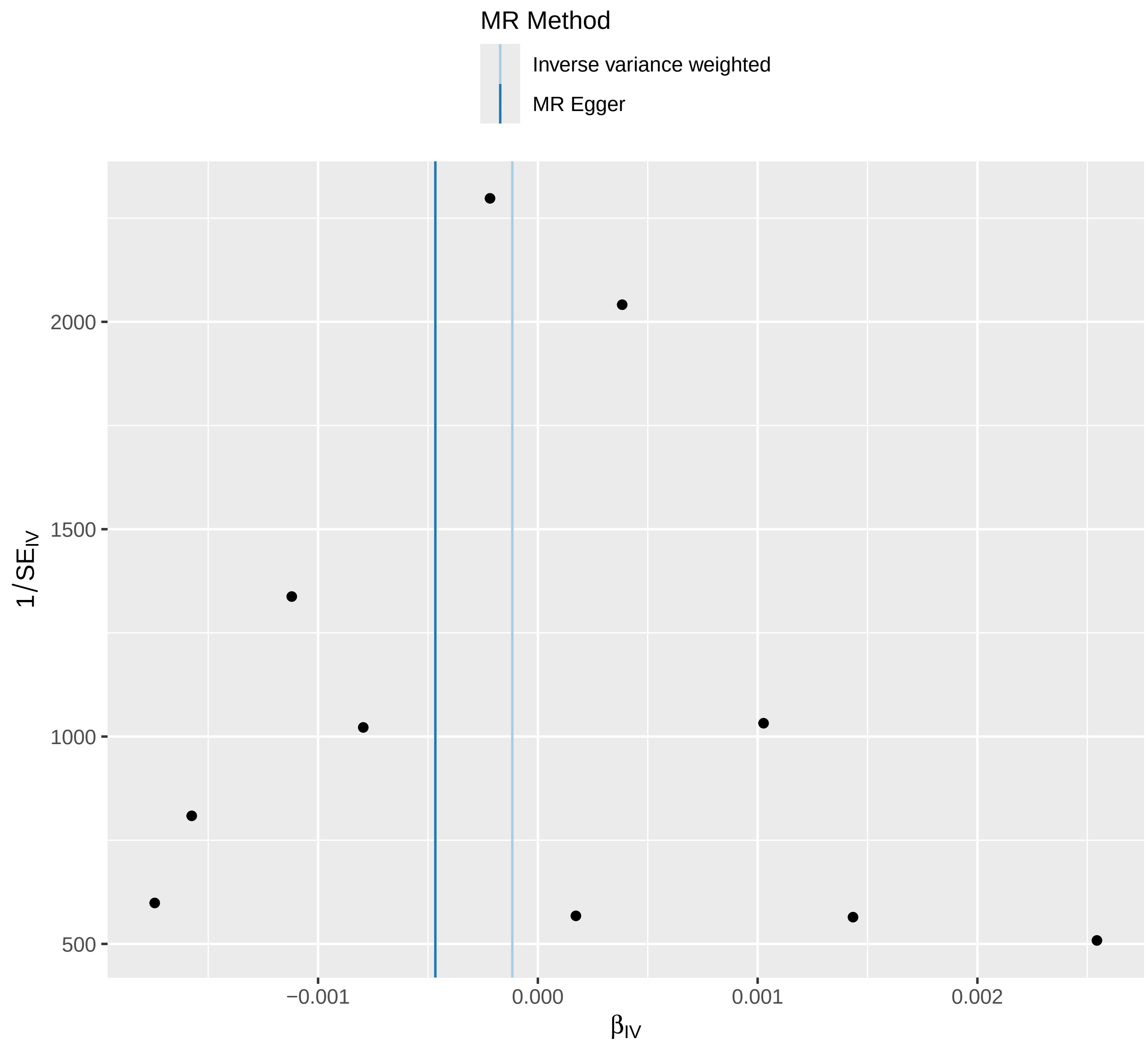
MR Method
Inverse variance weighted
MR Egger
1/SE_IV
2000
1500
1000
500
−0.001
0.000
0.001
0.002
β_IV

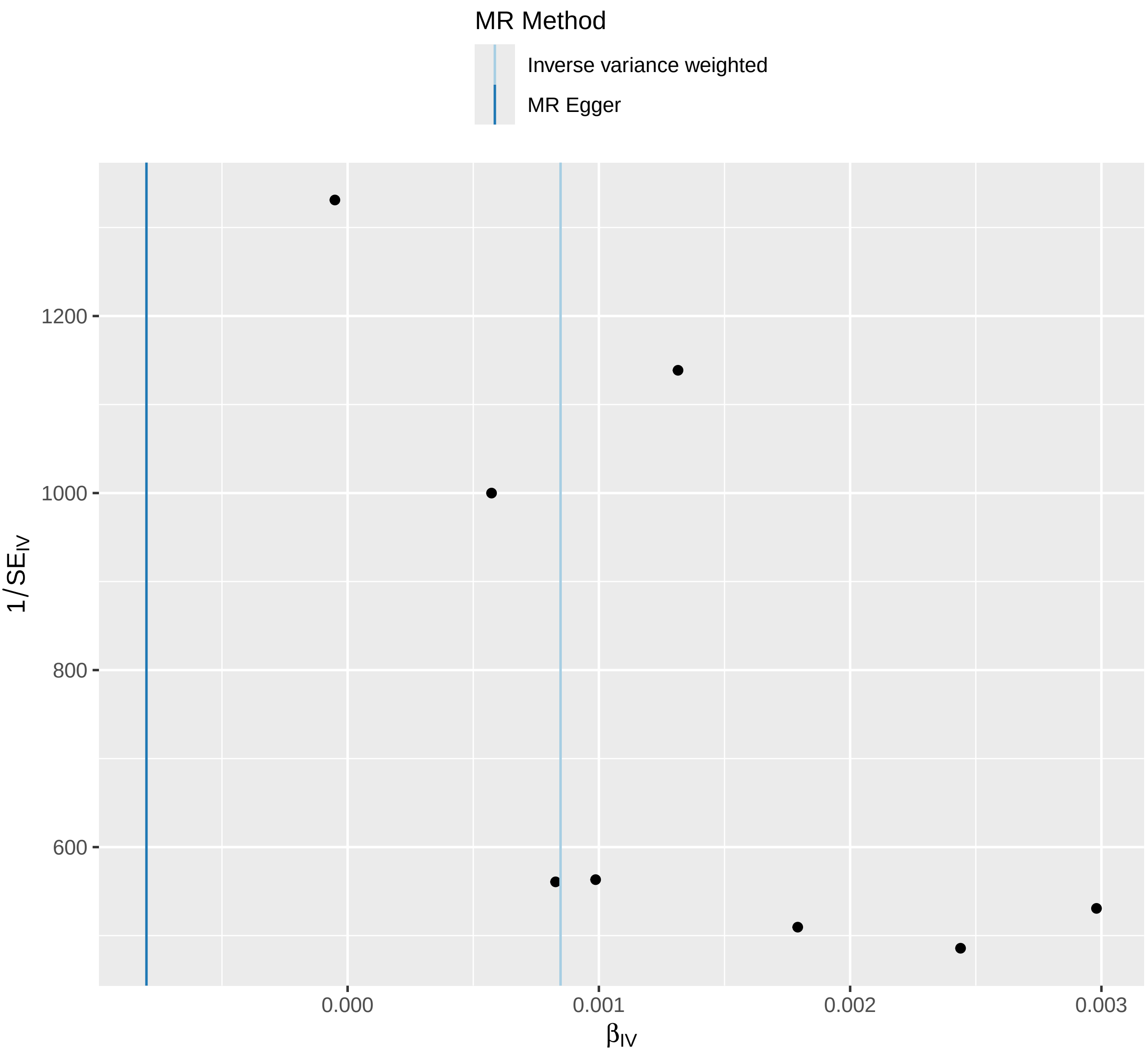
MR Method
Inverse variance weighted
MR Egger
1200
1000
800
600
1/SE_IV
0.000
0.001
0.002
0.003
β_IV

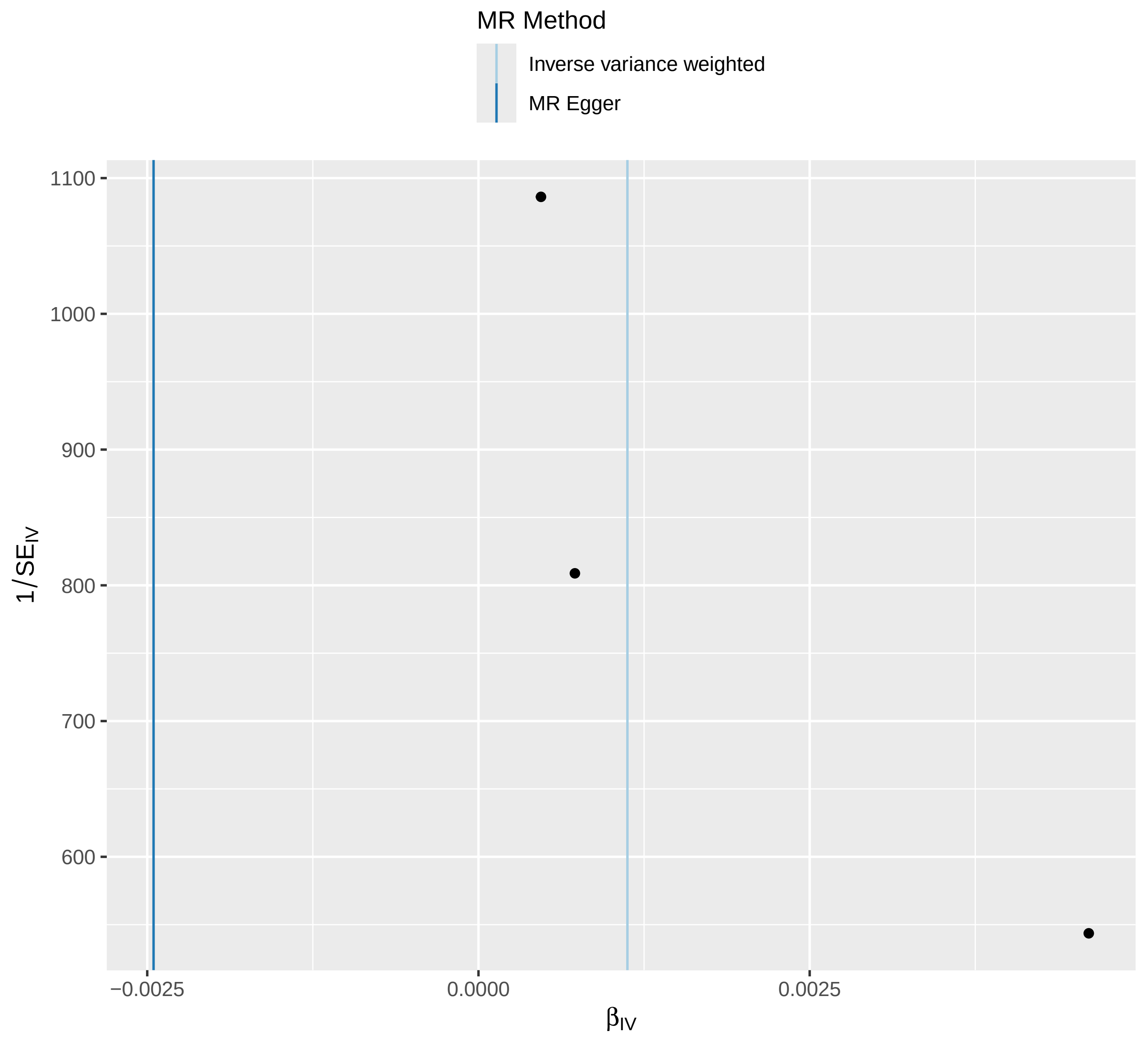

MR Method
Inverse variance weighted
MR Egger
1100
1000
900
800
700
600
−0.0025
0.0000
0.0025
$1/SE_{IV}$
β_{IV}

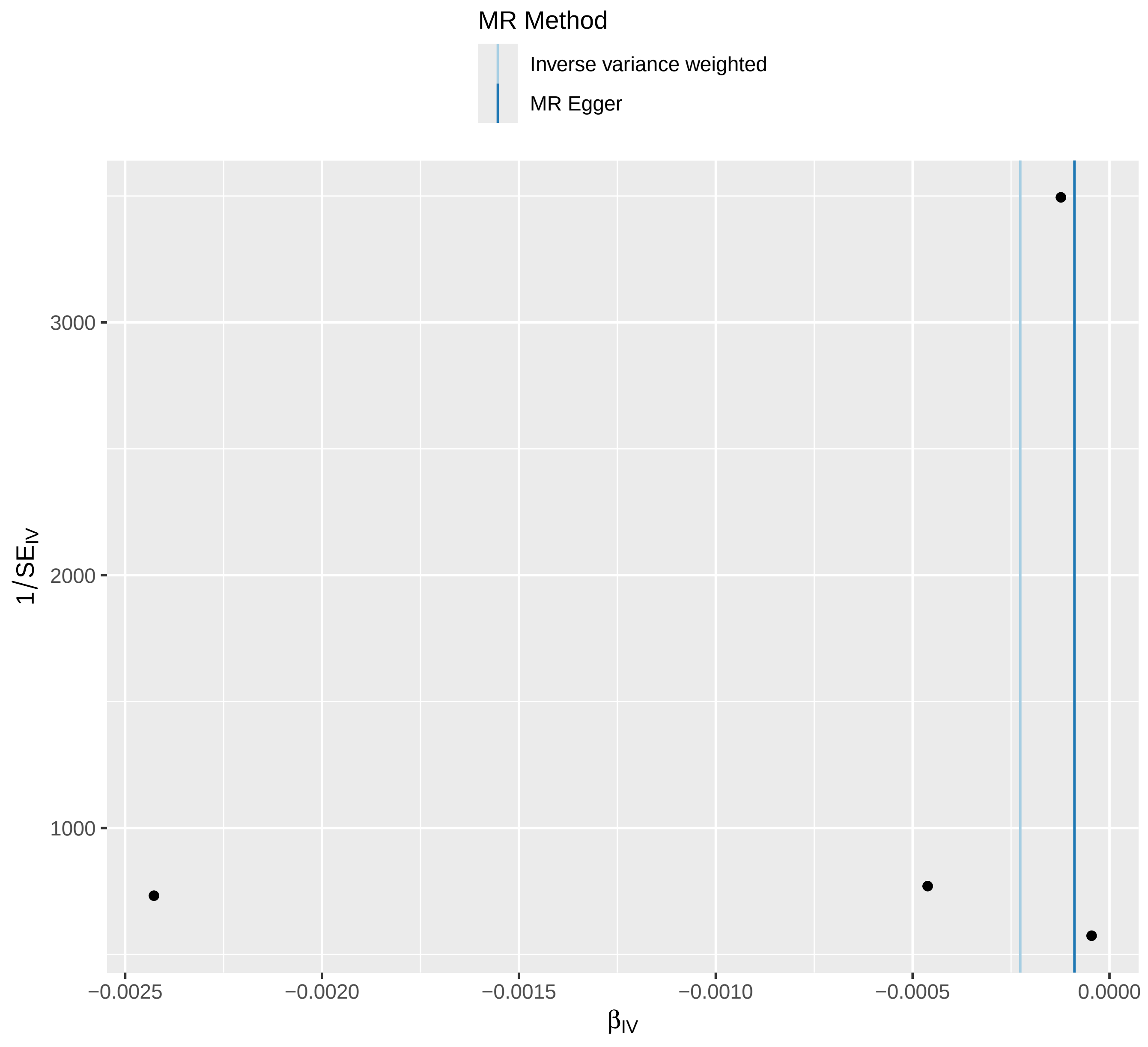
MR Method
Inverse variance weighted
MR Egger
3000
2000
1000
1/SE_IV
−0.0025
−0.0020
−0.0015
−0.0010
−0.0005
0.0000
β_IV

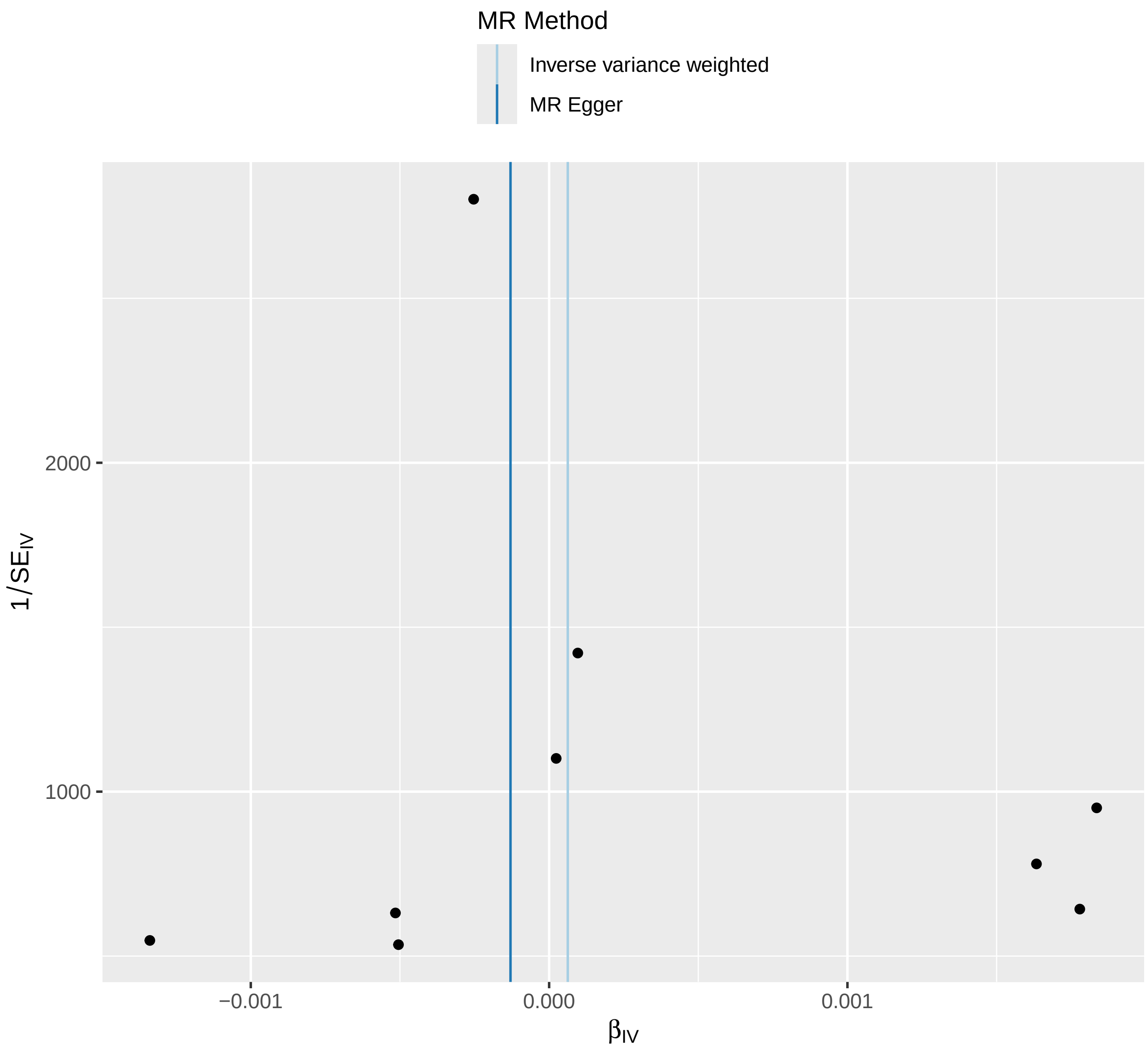

MR Method
Inverse variance weighted
MR Egger
2000
1000
$1/SE_{IV}$
−0.001
0.000
0.001
β_{IV}

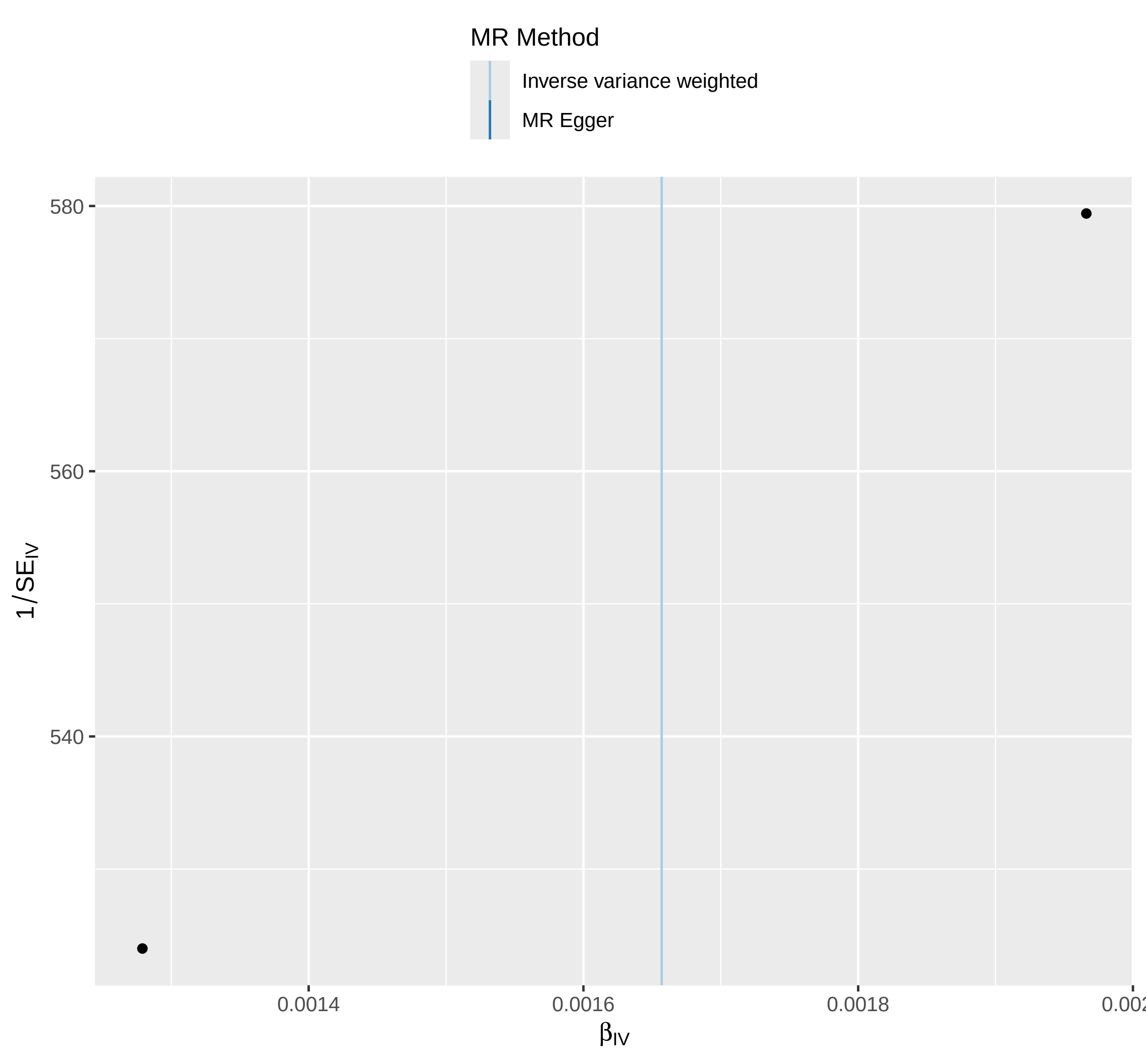

MR Method
Inverse variance weighted
MR Egger
580
560
540
1/SE_IV
0.0014
0.0016
0.0018
0.002
β_IV

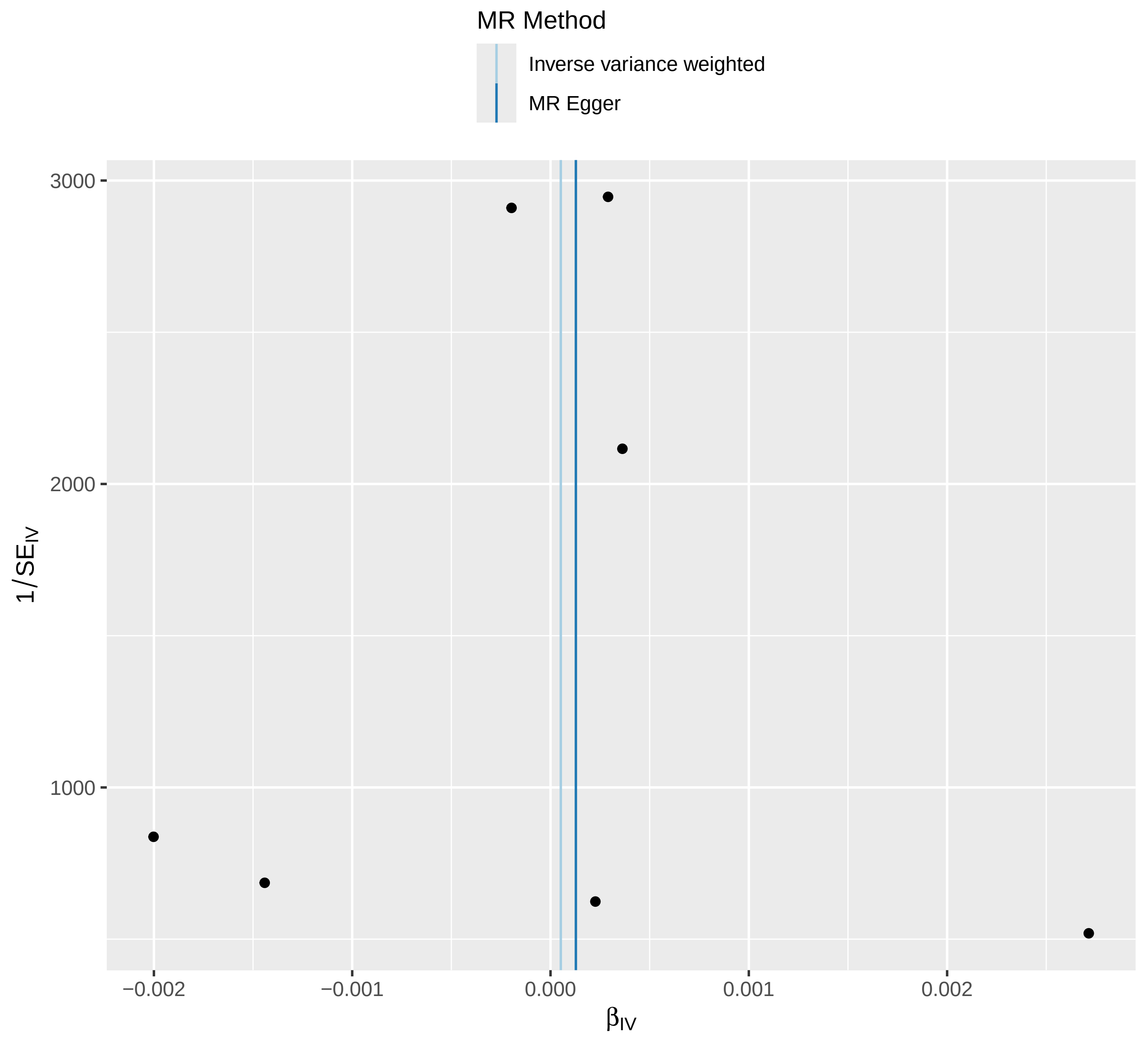

MR Method
Inverse variance weighted
MR Egger
3000
2000
1000
1/SE_IV
−0.002
−0.001
0.000
0.001
0.002
β_IV

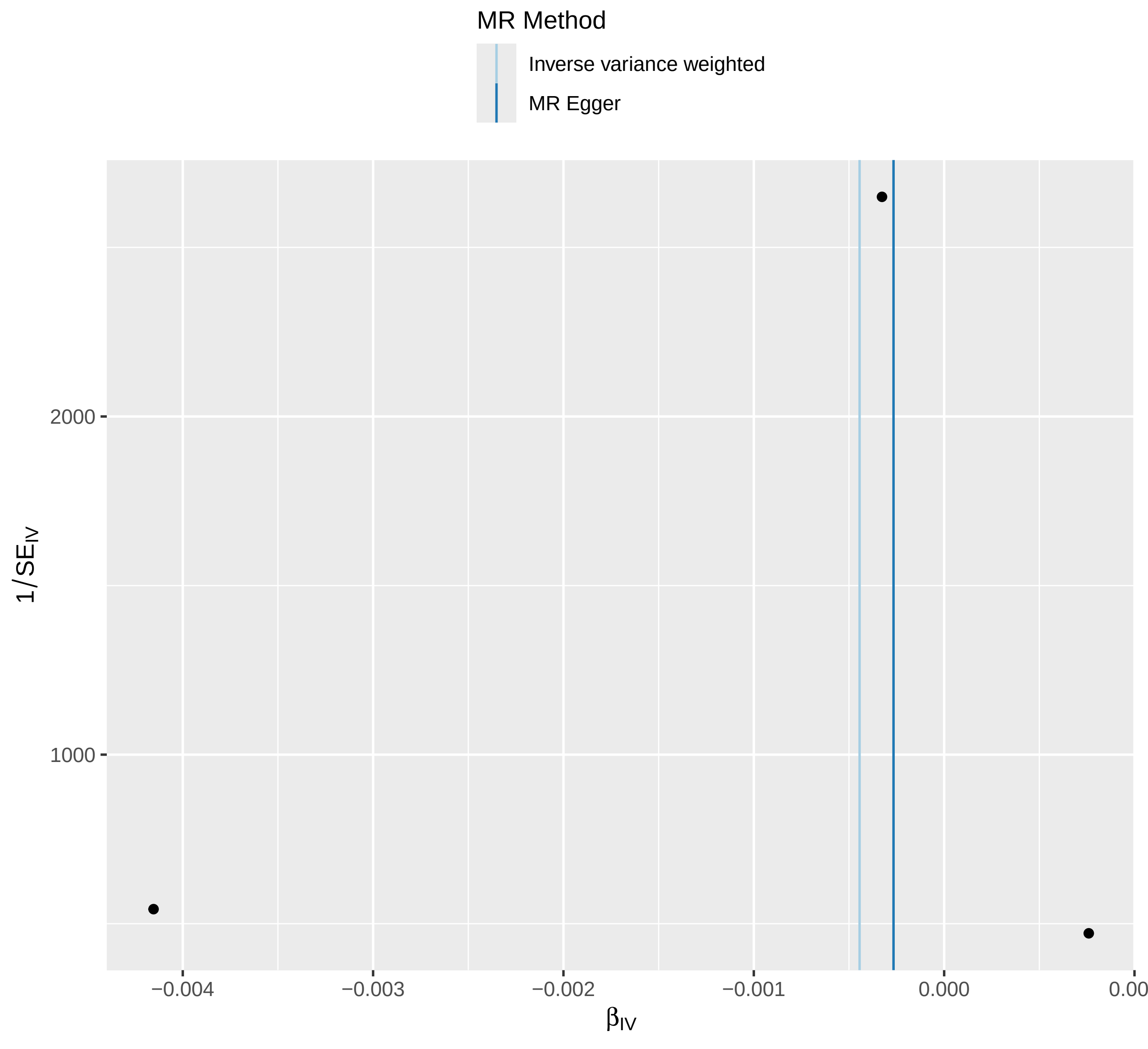

MR Method
Inverse variance weighted
MR Egger
2000
1000
1/SE_IV
−0.004
−0.003
−0.002
−0.001
0.000
0.00
β_IV

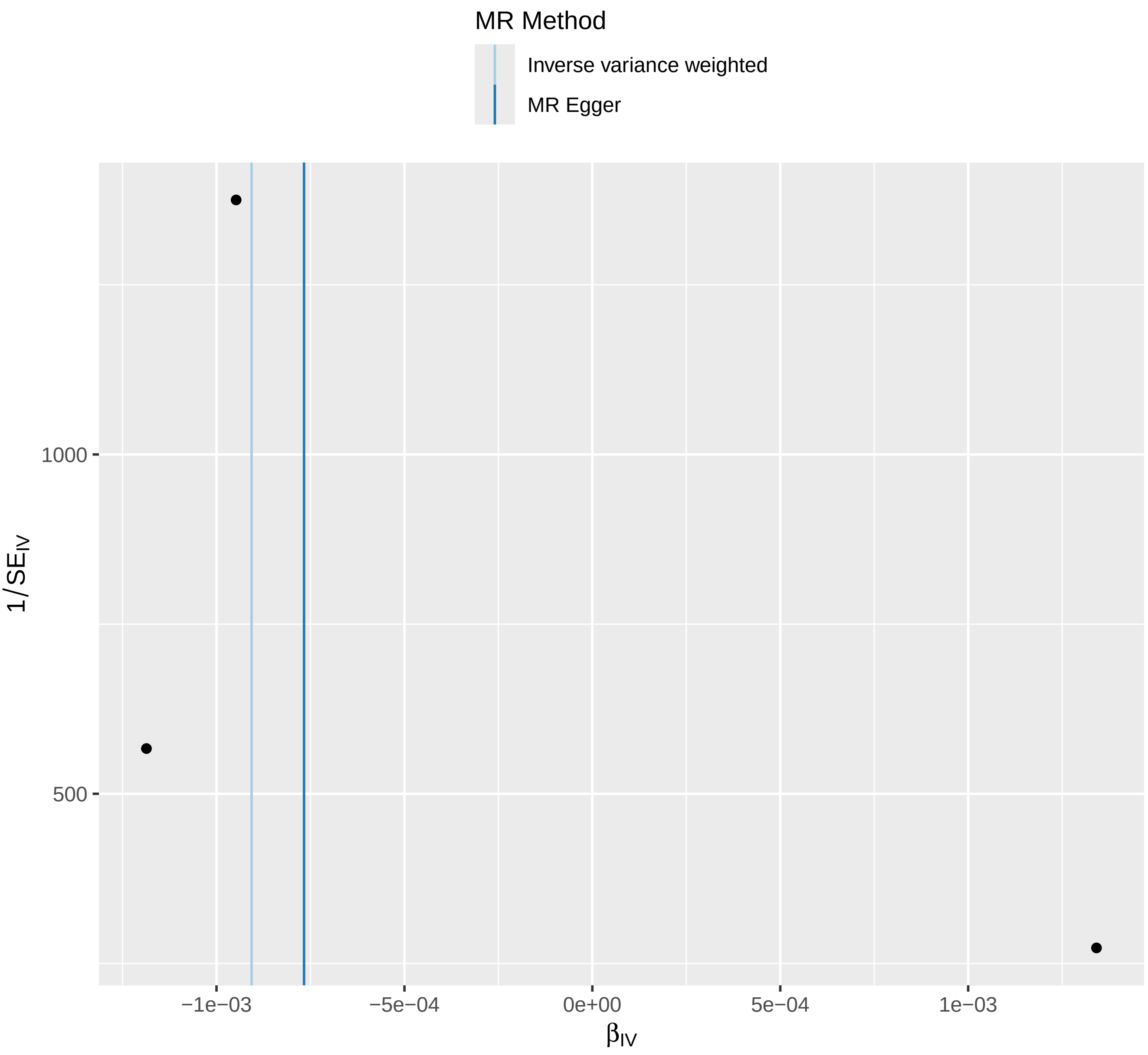

MR Method
Inverse variance weighted
MR Egger
1000
500
1/SE$_{IV}$
−1e−03
−5e−04
0e+00
5e−04
1e−03
β$_{IV}$

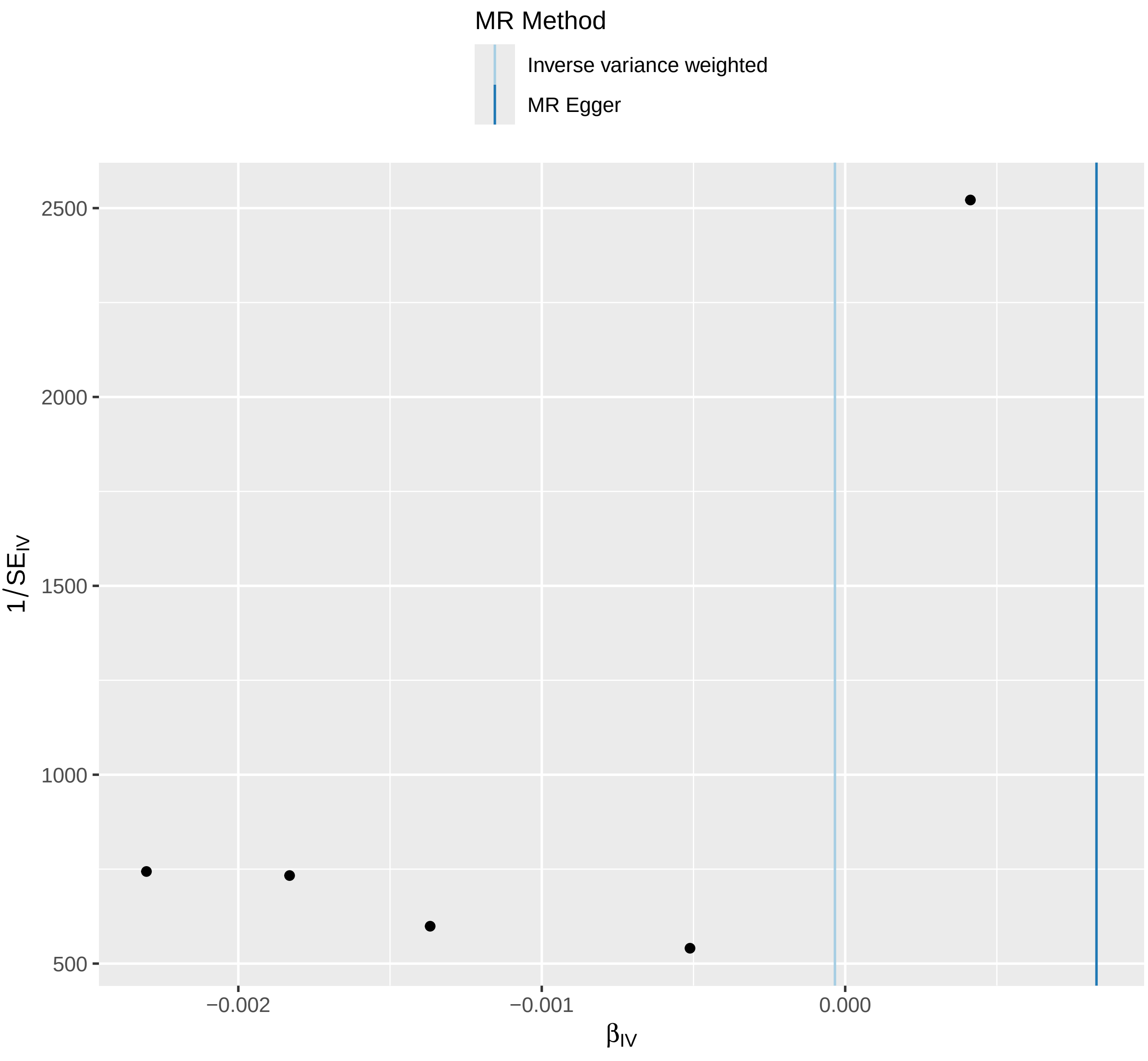

MR Method
Inverse variance weighted
MR Egger
2500
2000
1500
1000
500
1/SE_IV
−0.002
−0.001
0.000
β_IV

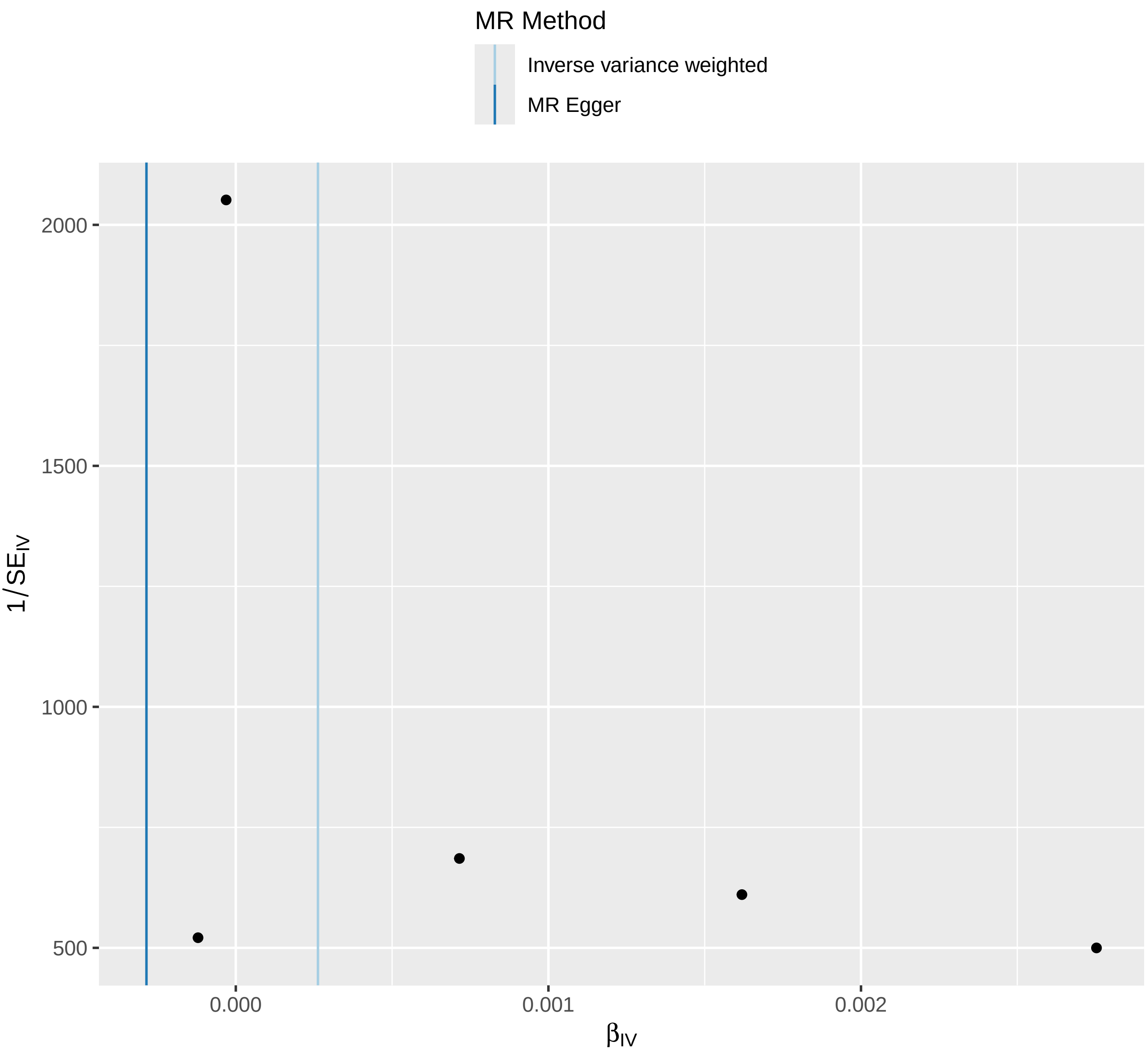
MR Method
Inverse variance weighted
MR Egger
2000
1500
1000
500
1/SE_IV
0.000
0.001
0.002
β_IV

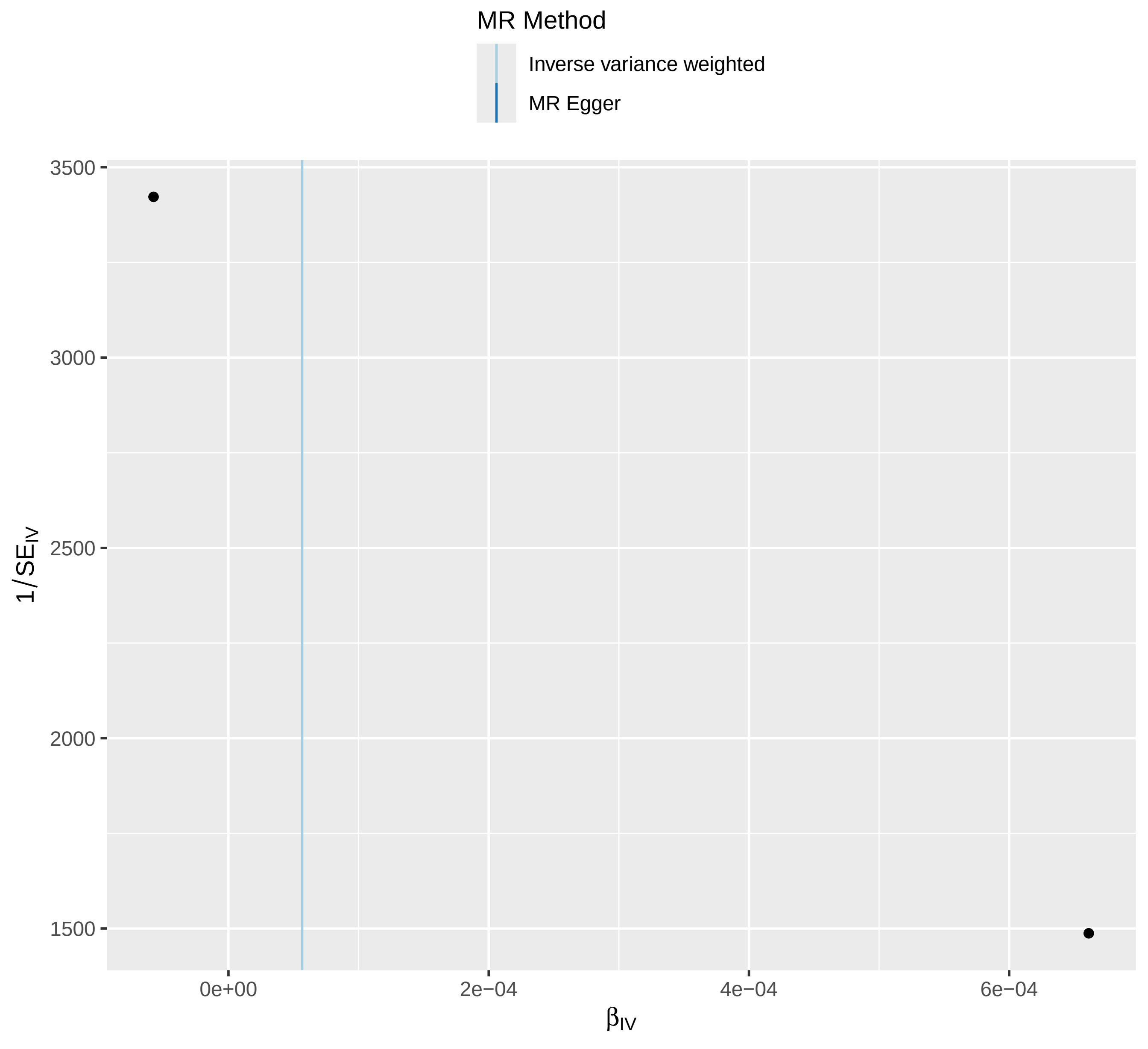

MR Method
Inverse variance weighted
MR Egger
3500
3000
2500
2000
1500
1/SE_IV
0e+00
2e−04
4e−04
6e−04
β_IV

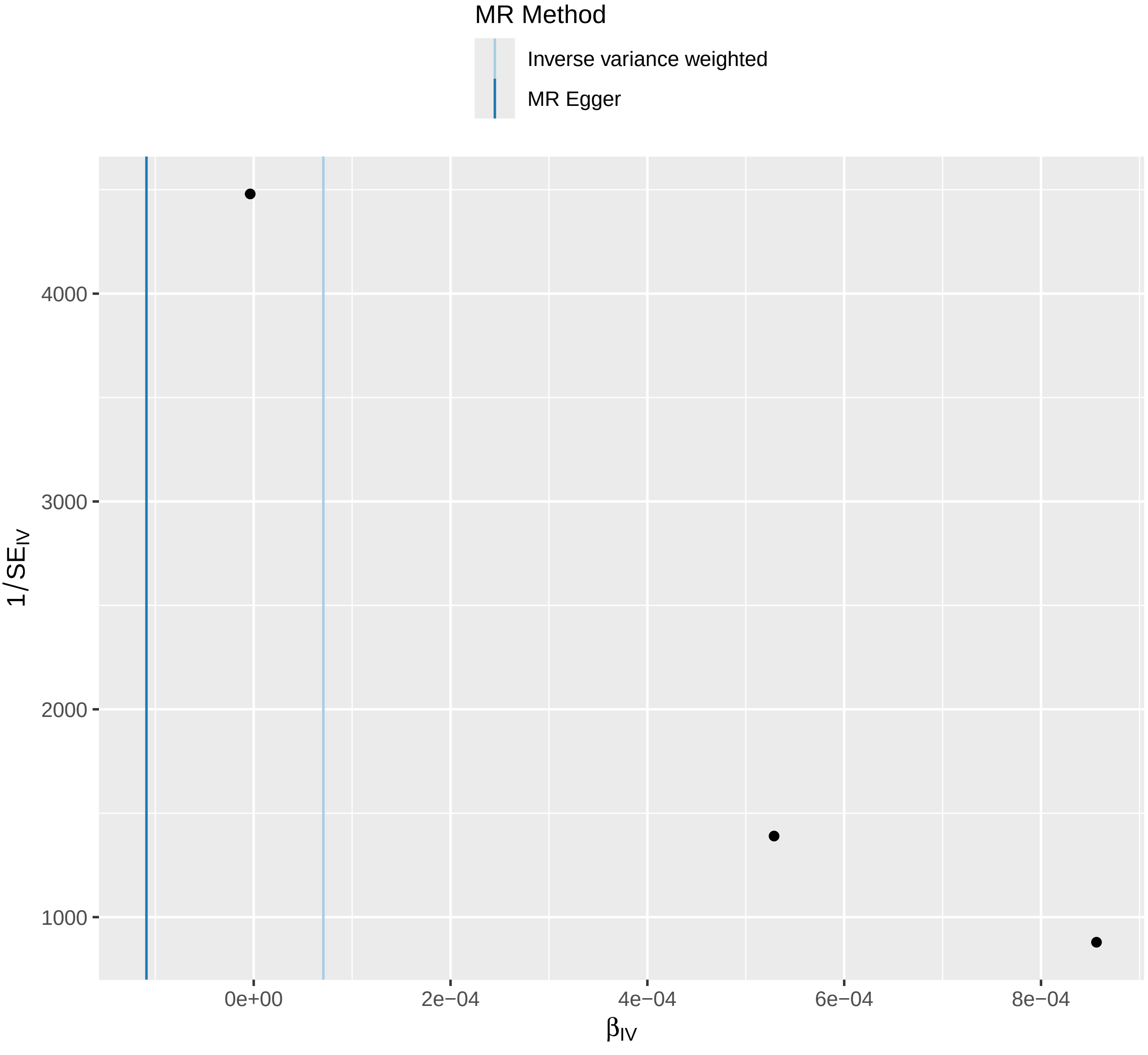

MR Method
Inverse variance weighted
MR Egger
4000
3000
2000
1000
1/SE$_{IV}$
0e+00
2e−04
4e−04
6e−04
8e−04
β_{IV}

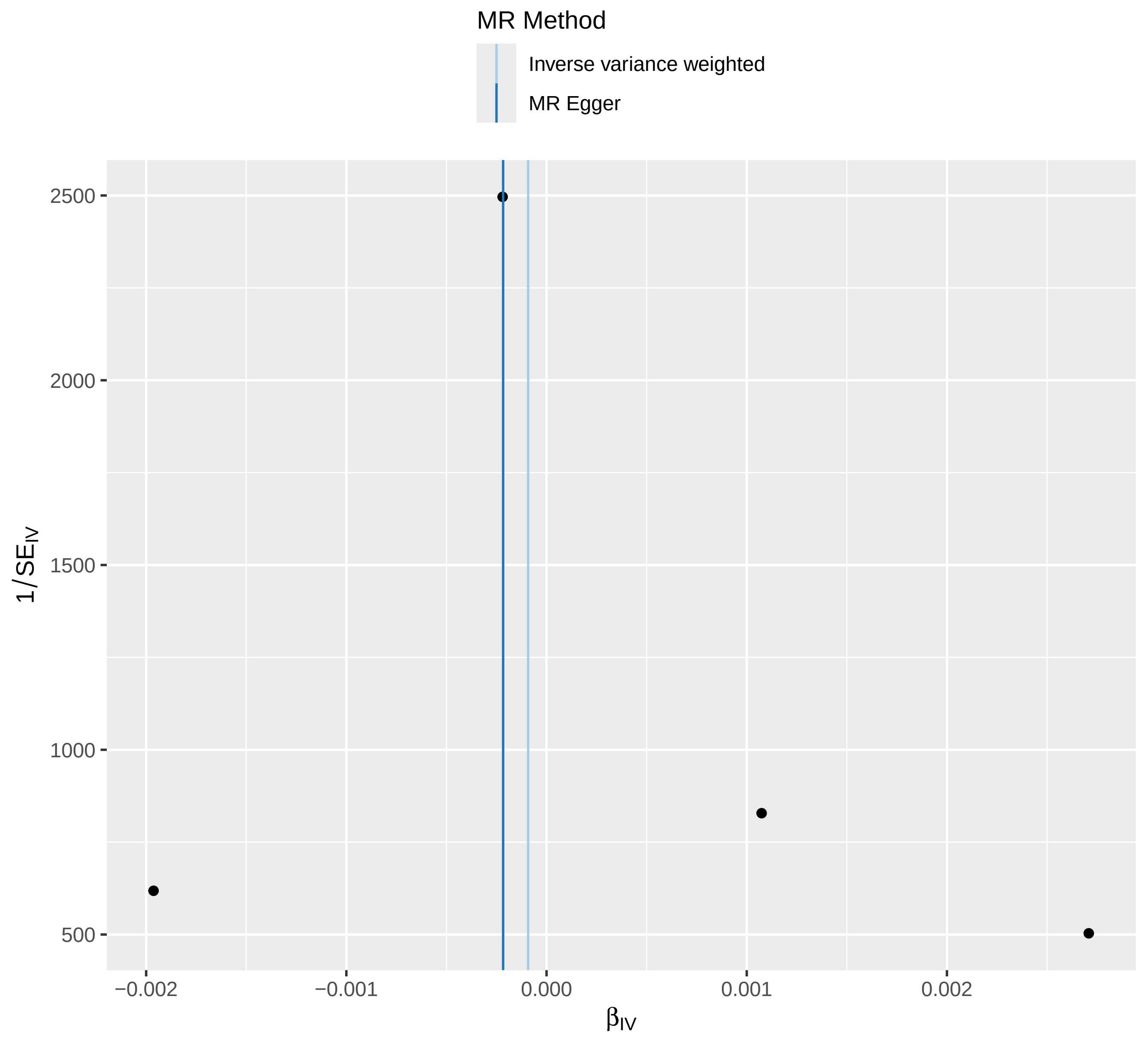
MR Method
Inverse variance weighted
MR Egger
2500
2000
1500
1000
500
1/SE_IV
−0.002
−0.001
0.000
0.001
0.002
β_IV

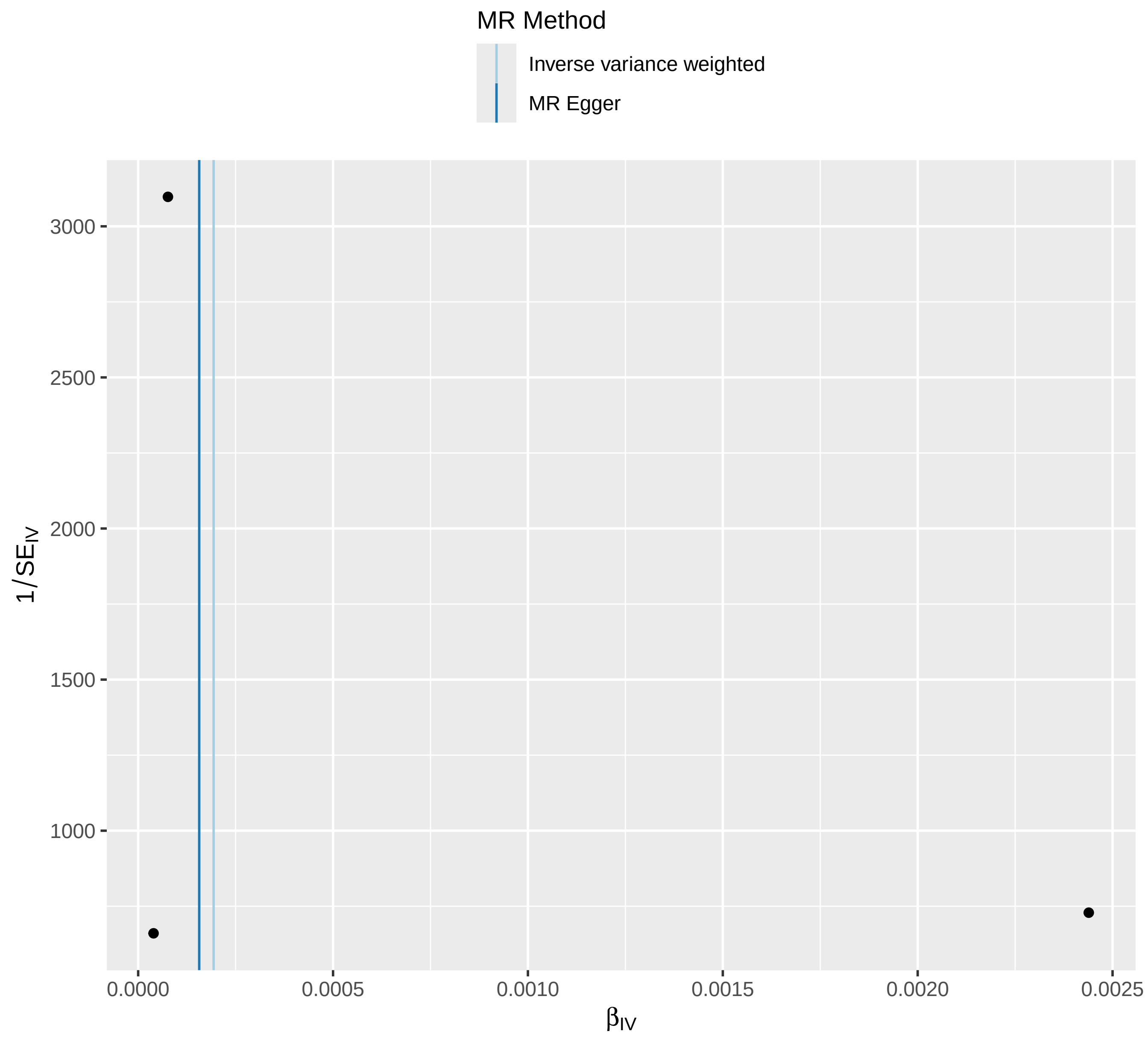
MR Method
Inverse variance weighted
MR Egger
3000
2500
2000
1500
1000
$1/SE_{IV}$
0.0000
0.0005
0.0010
0.0015
0.0020
0.0025
β_{IV}

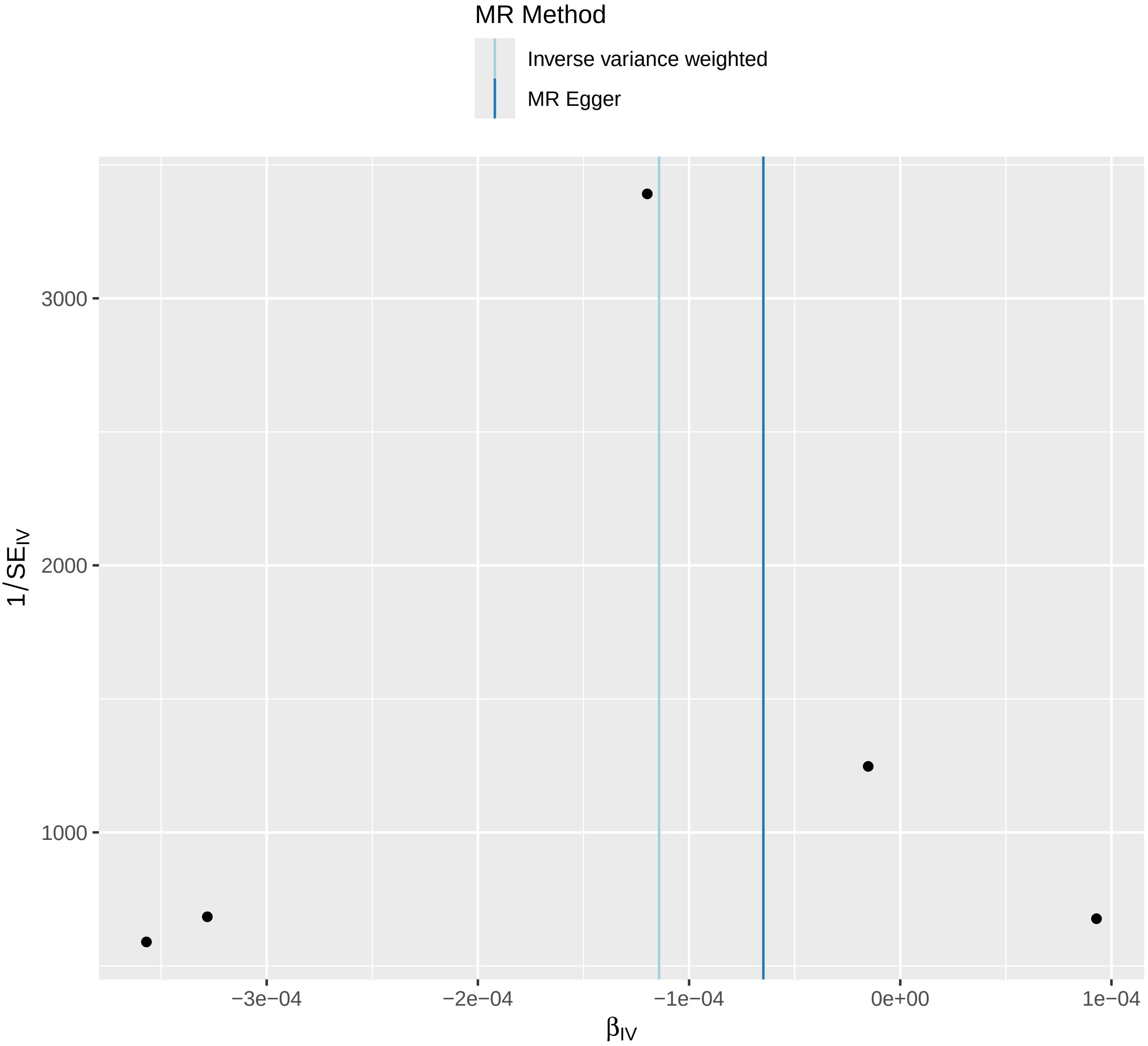

MR Method
Inverse variance weighted
MR Egger
3000
2000
1000
1/SE_IV
−3e−04
−2e−04
−1e−04
0e+00
1e−04
β_IV

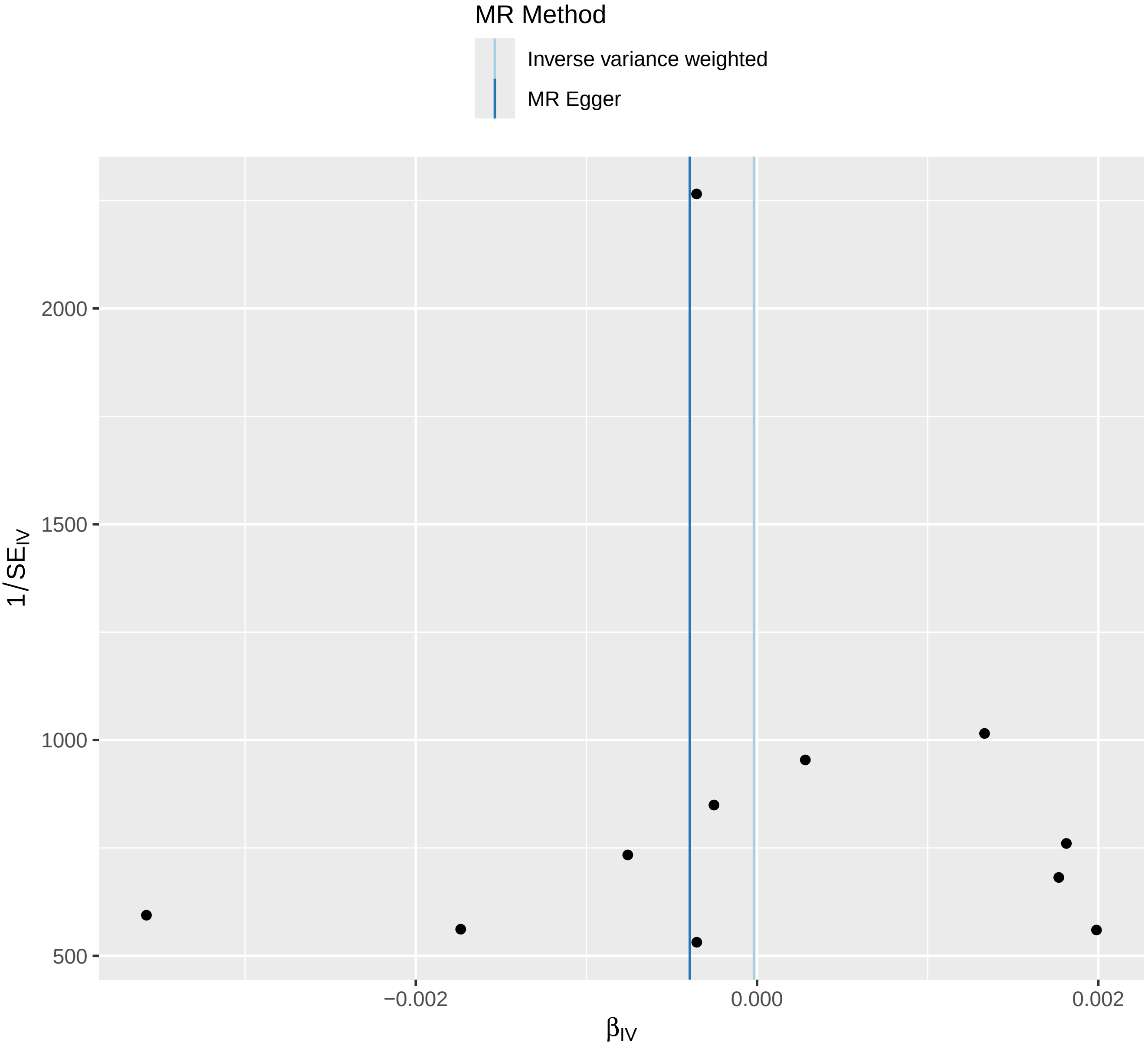
MR Method
Inverse variance weighted
MR Egger
2000
1500
1000
500
1/SE_IV
−0.002
0.000
0.002
β_IV

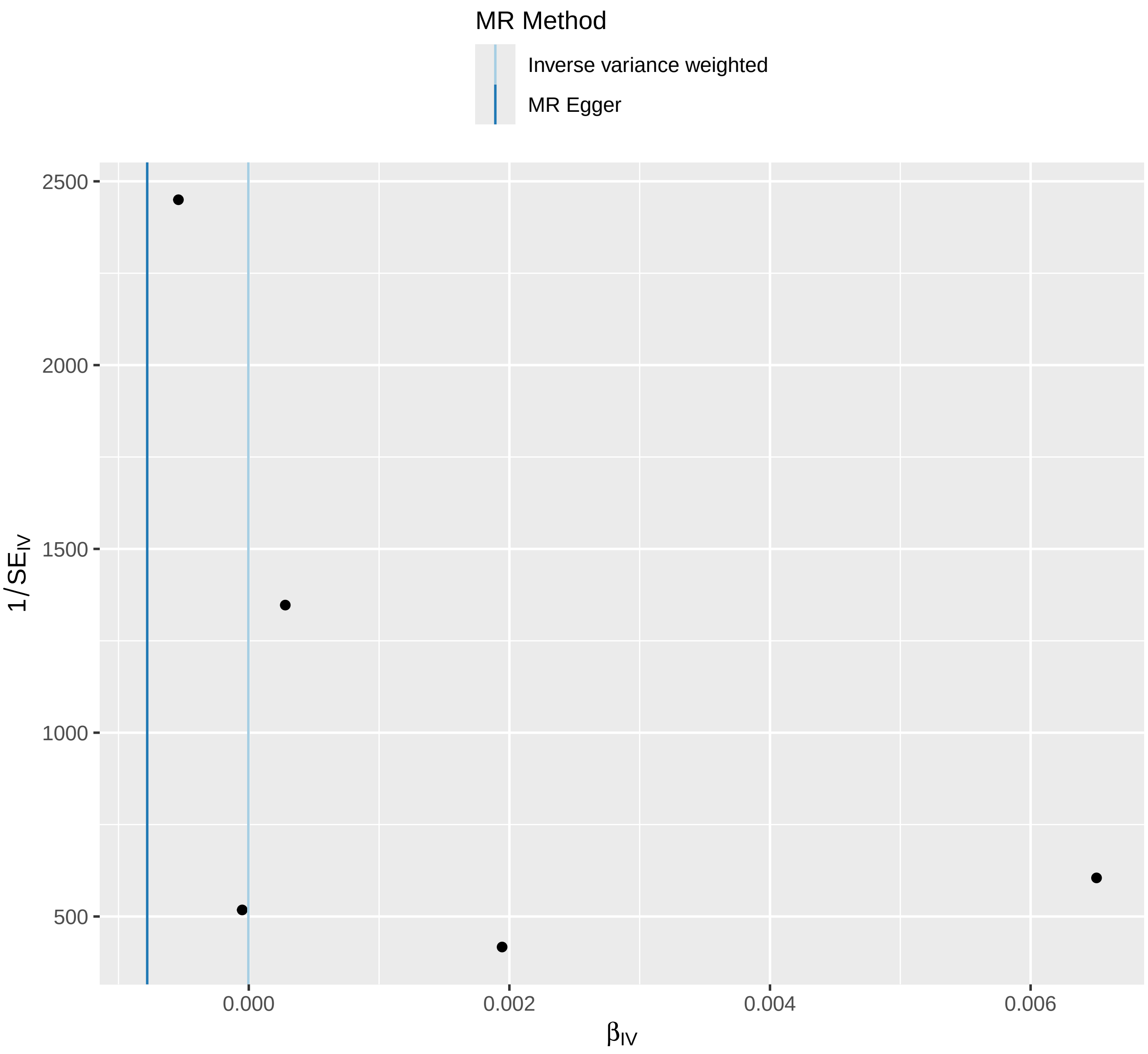
MR Method
Inverse variance weighted
MR Egger
2500
2000
1500
1000
500
$1/SE_{IV}$
0.000
0.002
0.004
0.006
β_{IV}

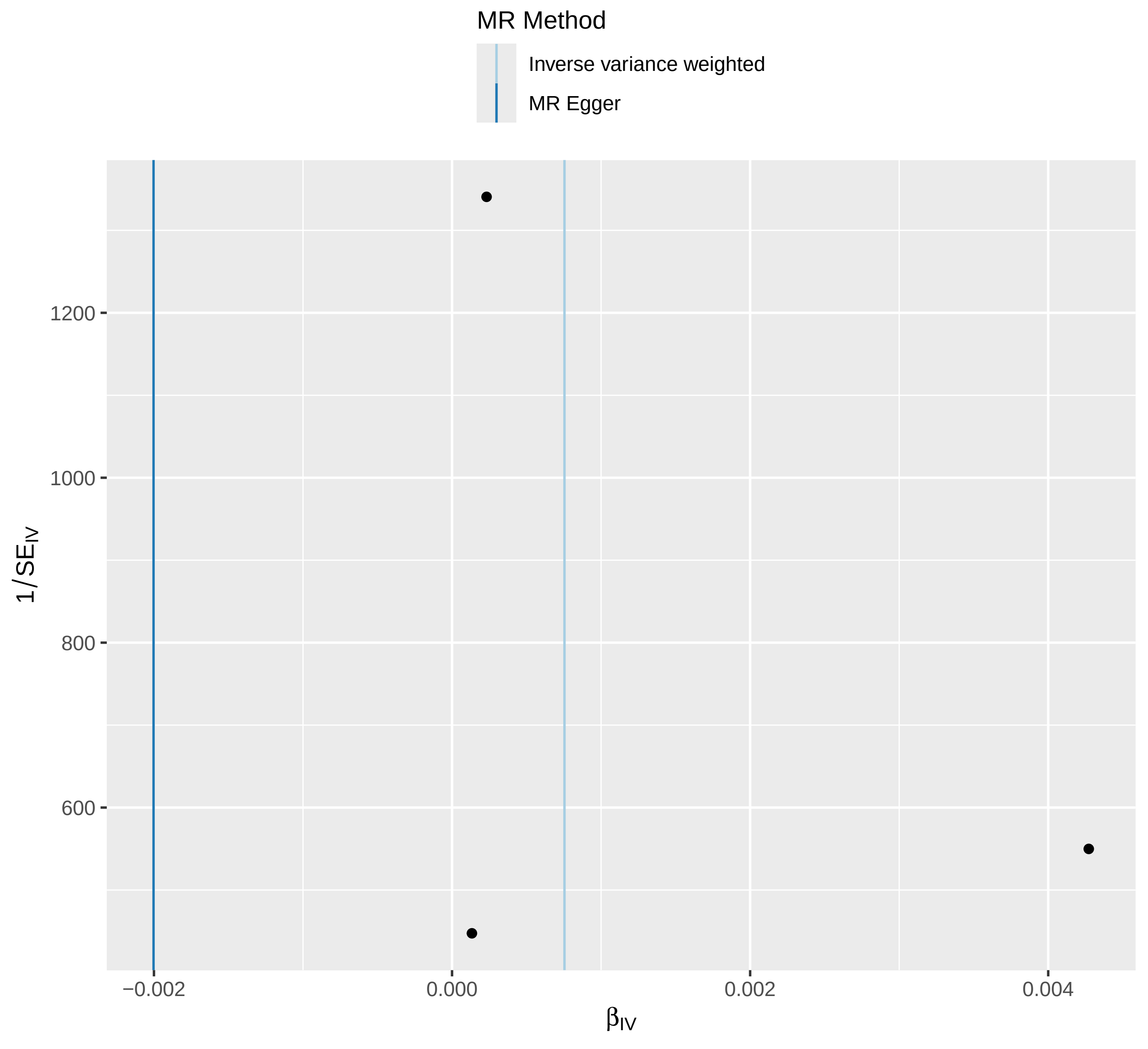

MR Method
Inverse variance weighted
MR Egger
1200
1000
800
600
1/SE_IV
−0.002
0.000
0.002
0.004
β_IV

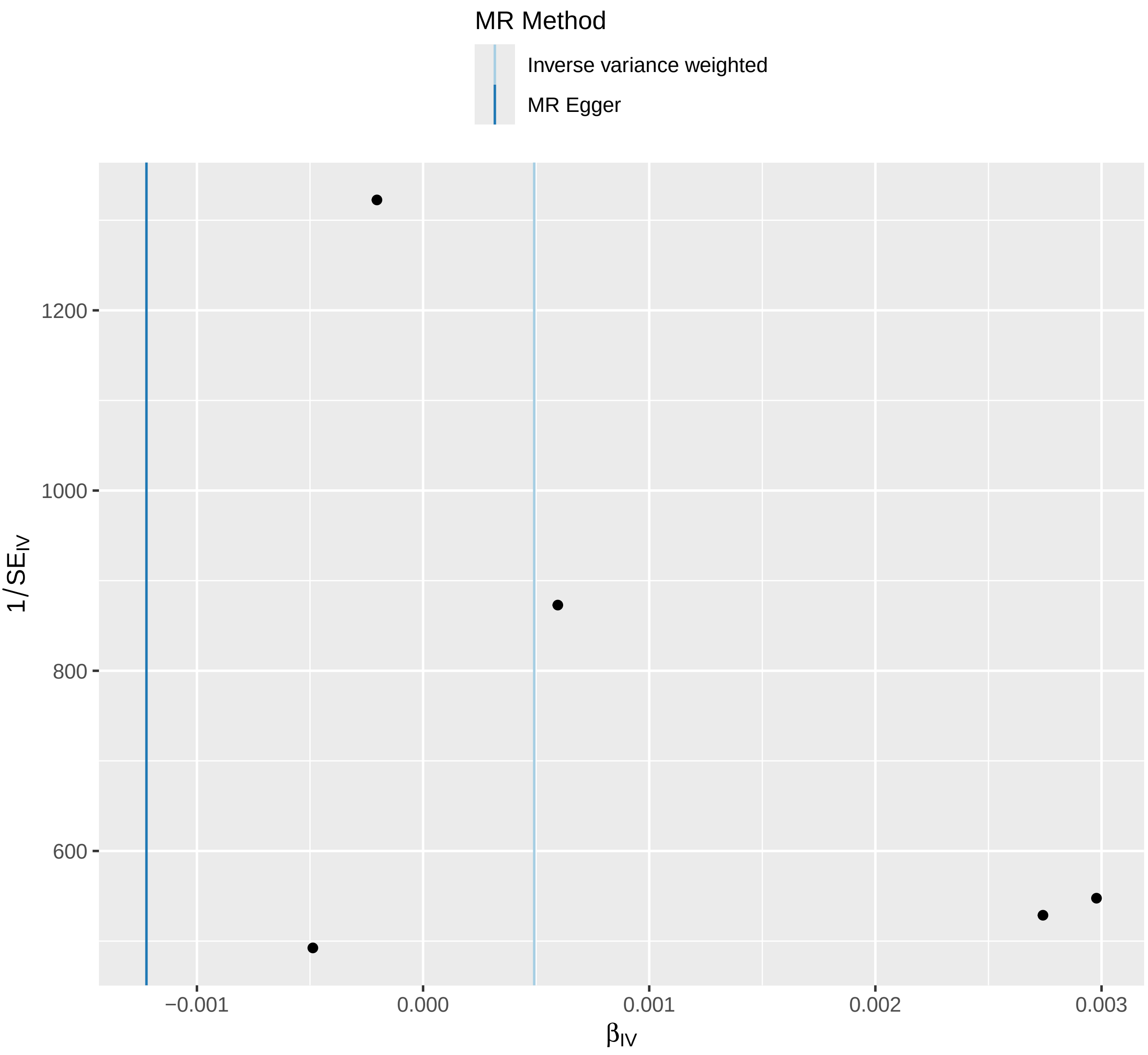
MR Method
Inverse variance weighted
MR Egger
1200
1000
800
600
1/SE_IV
−0.001
0.000
0.001
0.002
0.003
β_IV

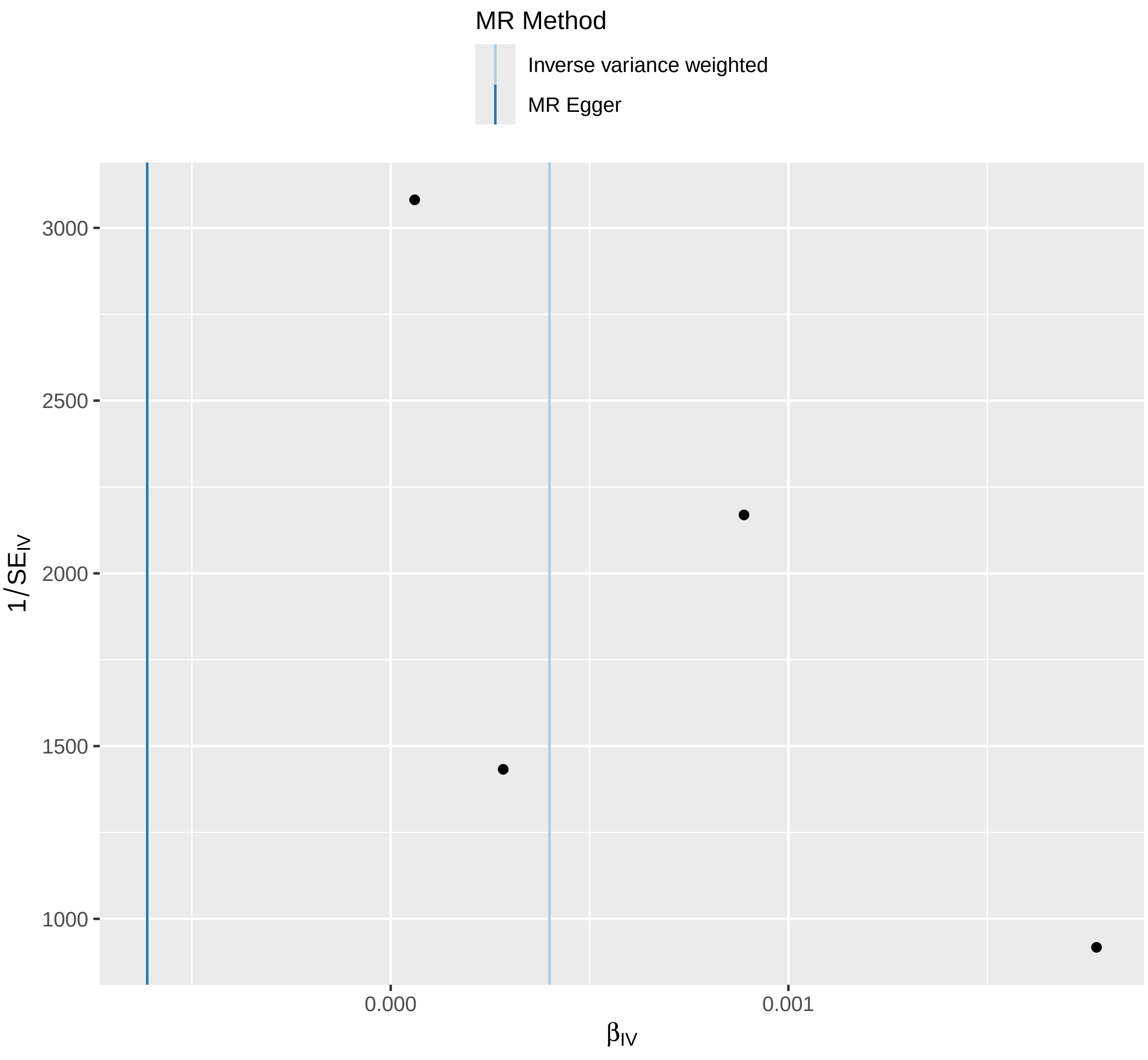
MR Method
Inverse variance weighted
MR Egger
3000
2500
2000
1500
1000
1/SE_IV
0.000
0.001
β_IV

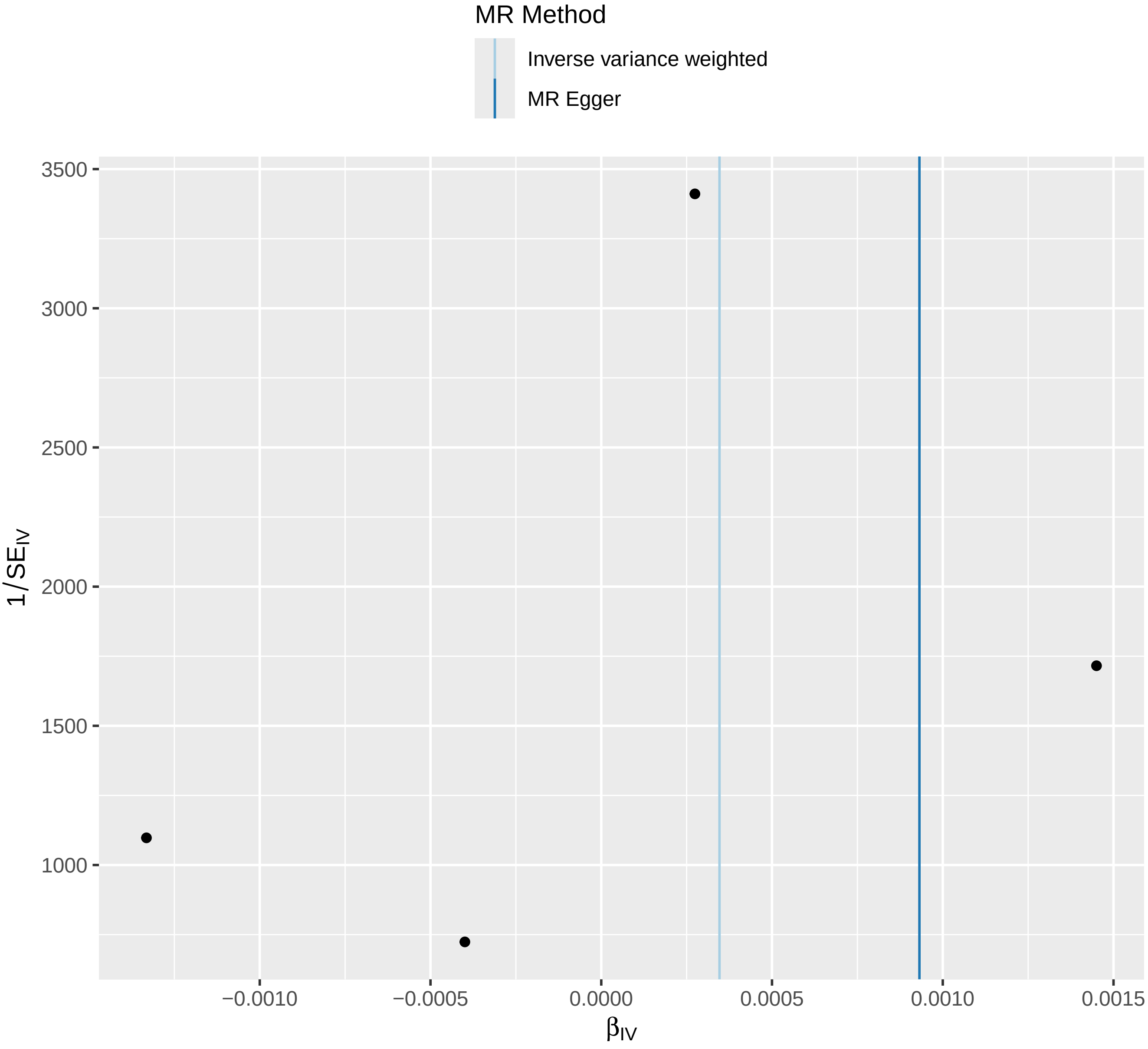
MR Method
Inverse variance weighted
MR Egger
3500
3000
2500
2000
1500
1000
1/SE_IV
−0.0010
−0.0005
0.0000
0.0005
0.0010
0.0015
β_IV

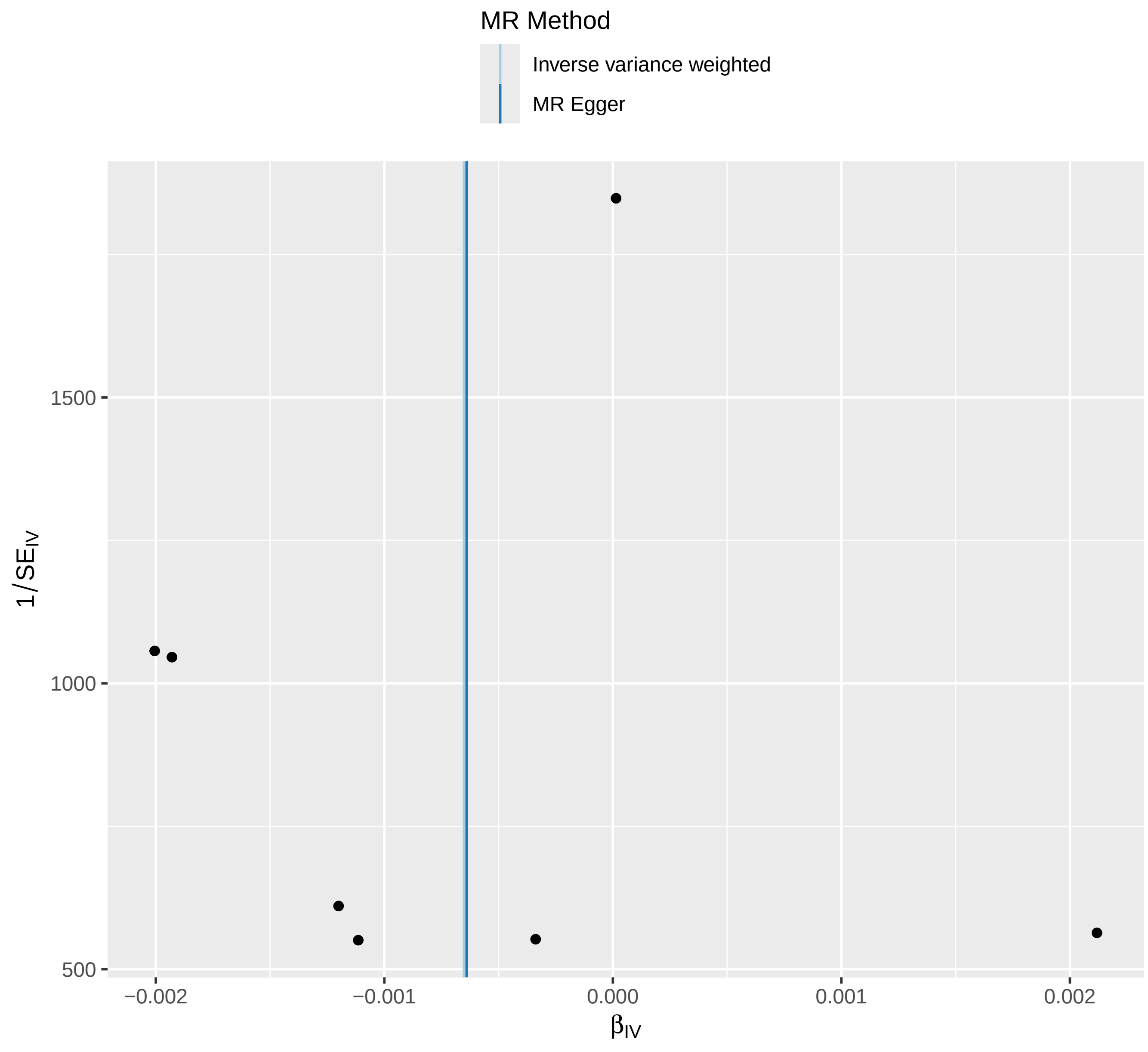
MR Method
Inverse variance weighted
MR Egger
1500
1000
500
−0.002
−0.001
0.000
0.001
0.002
$1/SE_{IV}$
β_{IV}

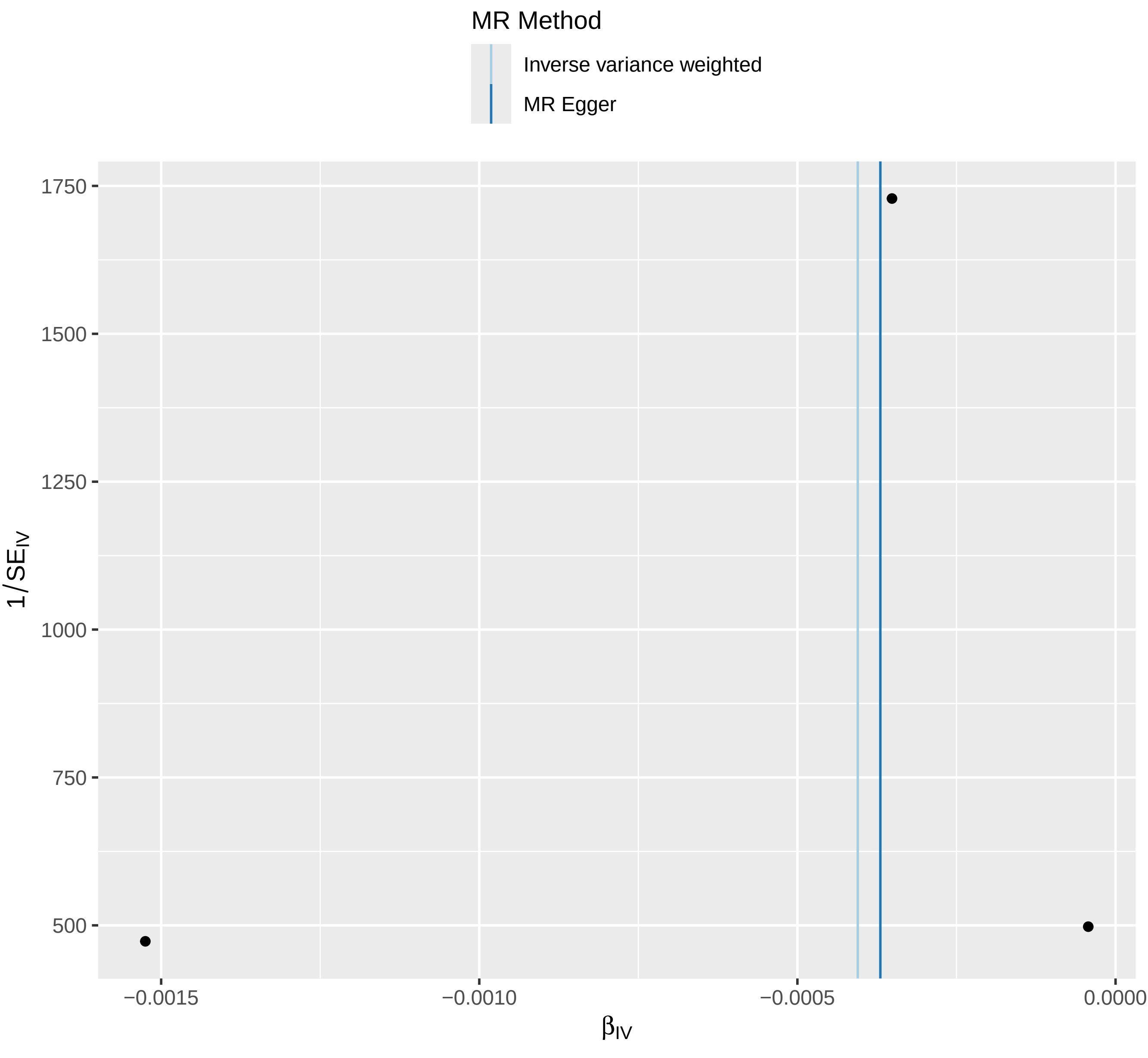

MR Method
Inverse variance weighted
MR Egger
1750
1500
1250
1000
750
500
1/SE$_{IV}$
−0.0015
−0.0010
−0.0005
0.0000
β_{IV}

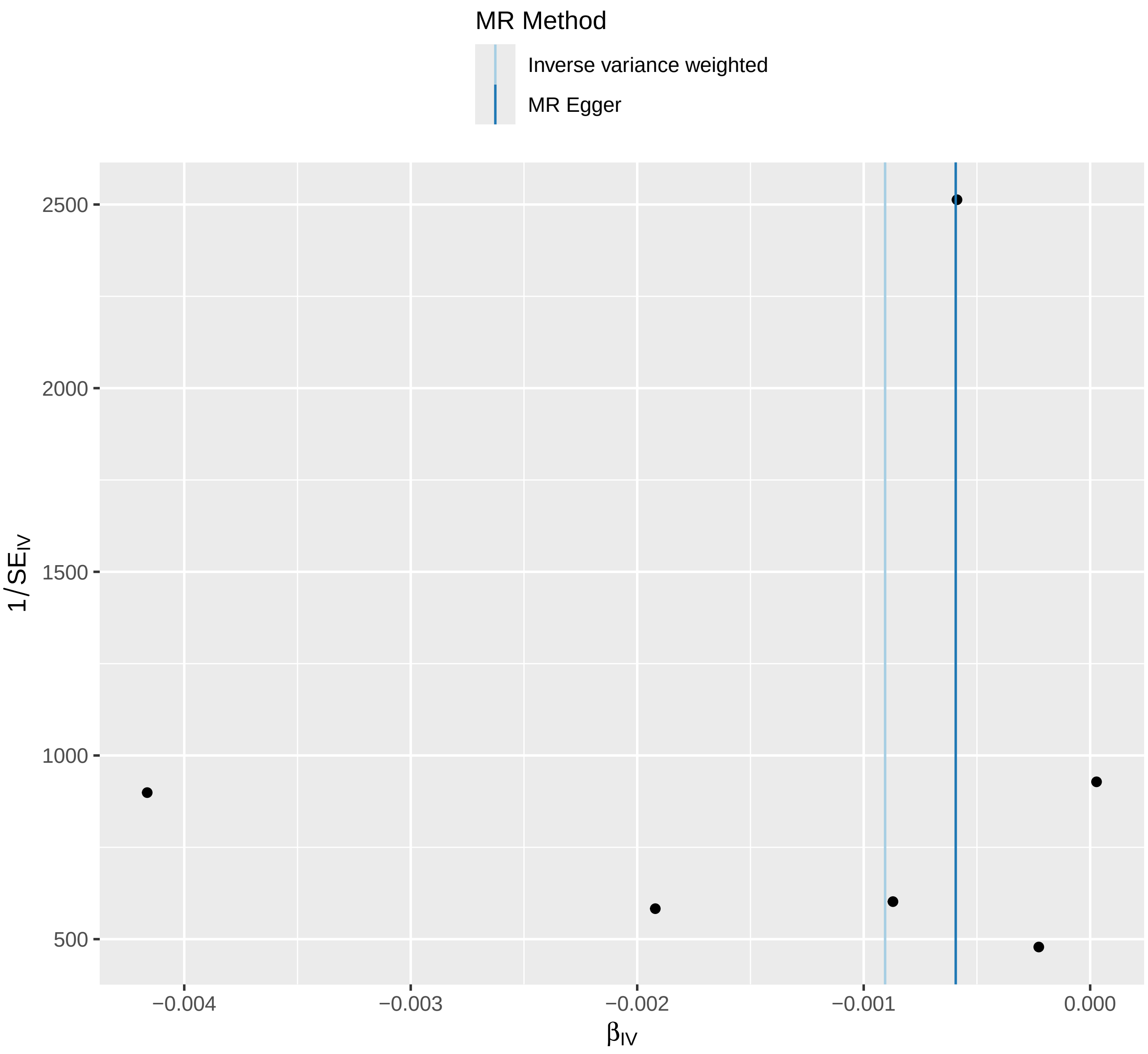

MR Method
Inverse variance weighted
MR Egger
2500
2000
1500
1000
500
1/SE_IV
−0.004
−0.003
−0.002
−0.001
0.000
β_IV

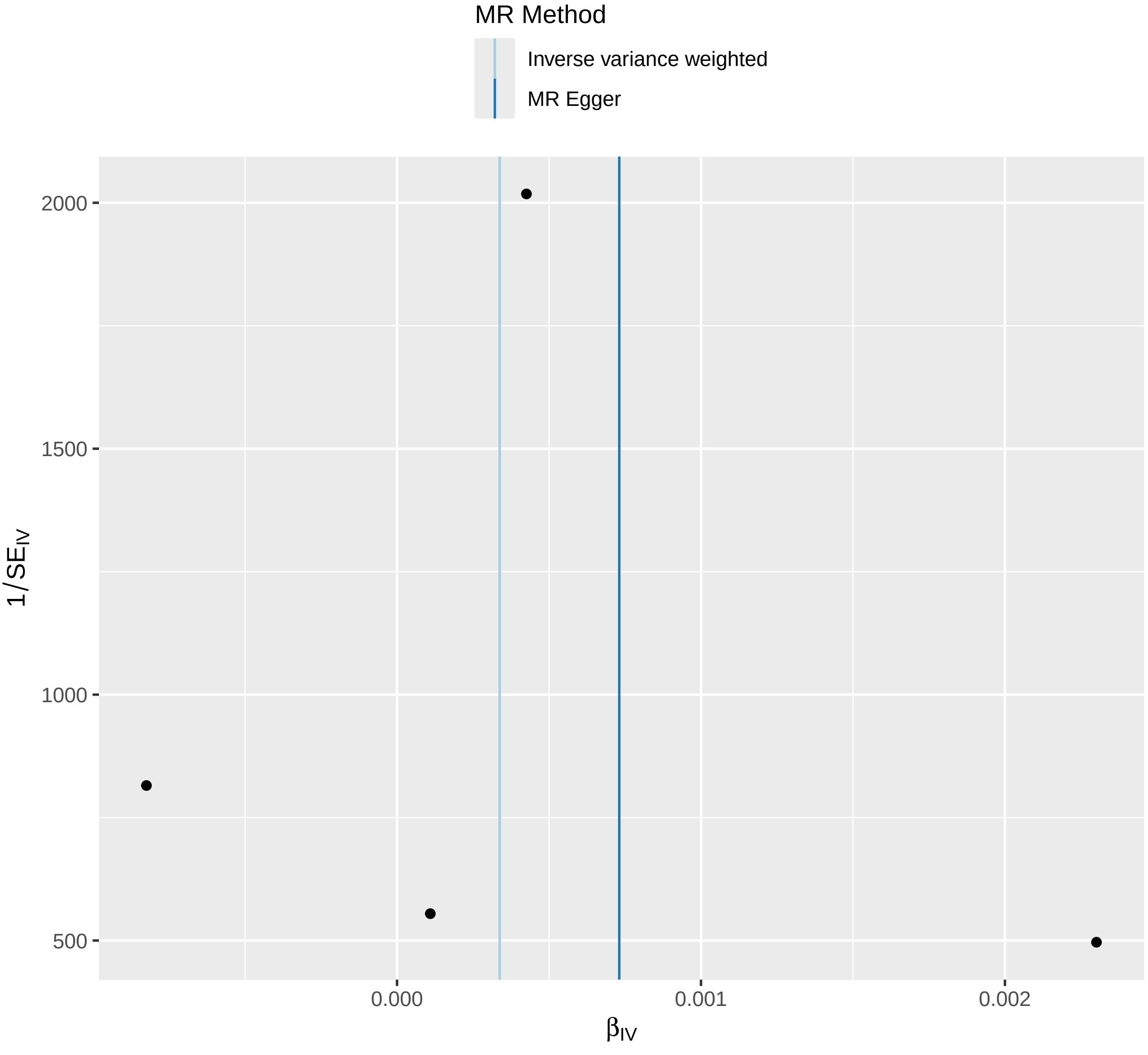
MR Method
Inverse variance weighted
MR Egger
2000
1500
1000
500
1/SE_IV
0.000
0.001
0.002
β_IV

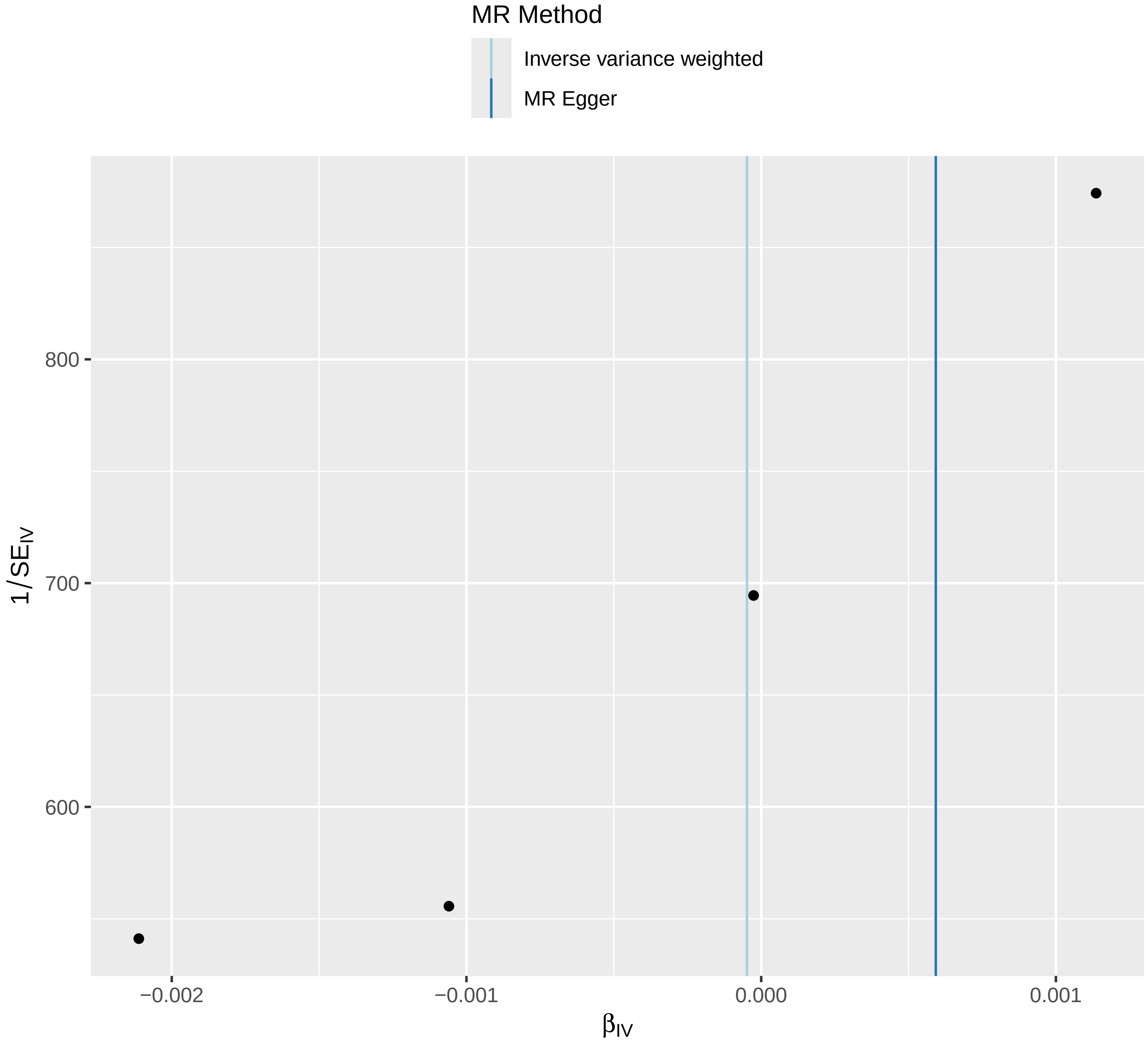

MR Method
Inverse variance weighted
MR Egger
800
700
600
$1/SE_{IV}$
−0.002
−0.001
0.000
0.001
β_{IV}

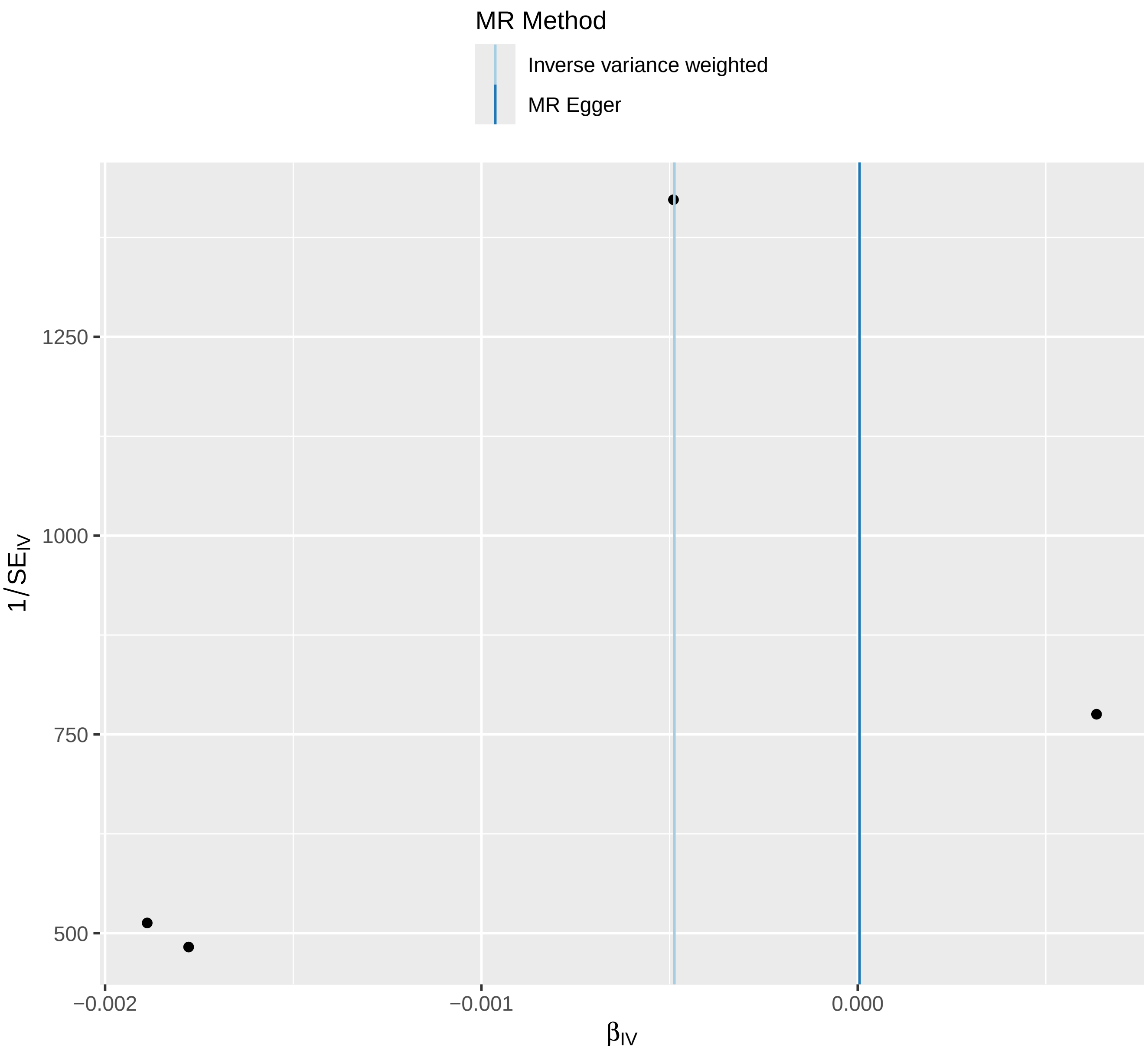

MR Method
Inverse variance weighted
MR Egger
1250
1000
750
500
1/SE$_{IV}$
−0.002
−0.001
0.000
β$_{IV}$

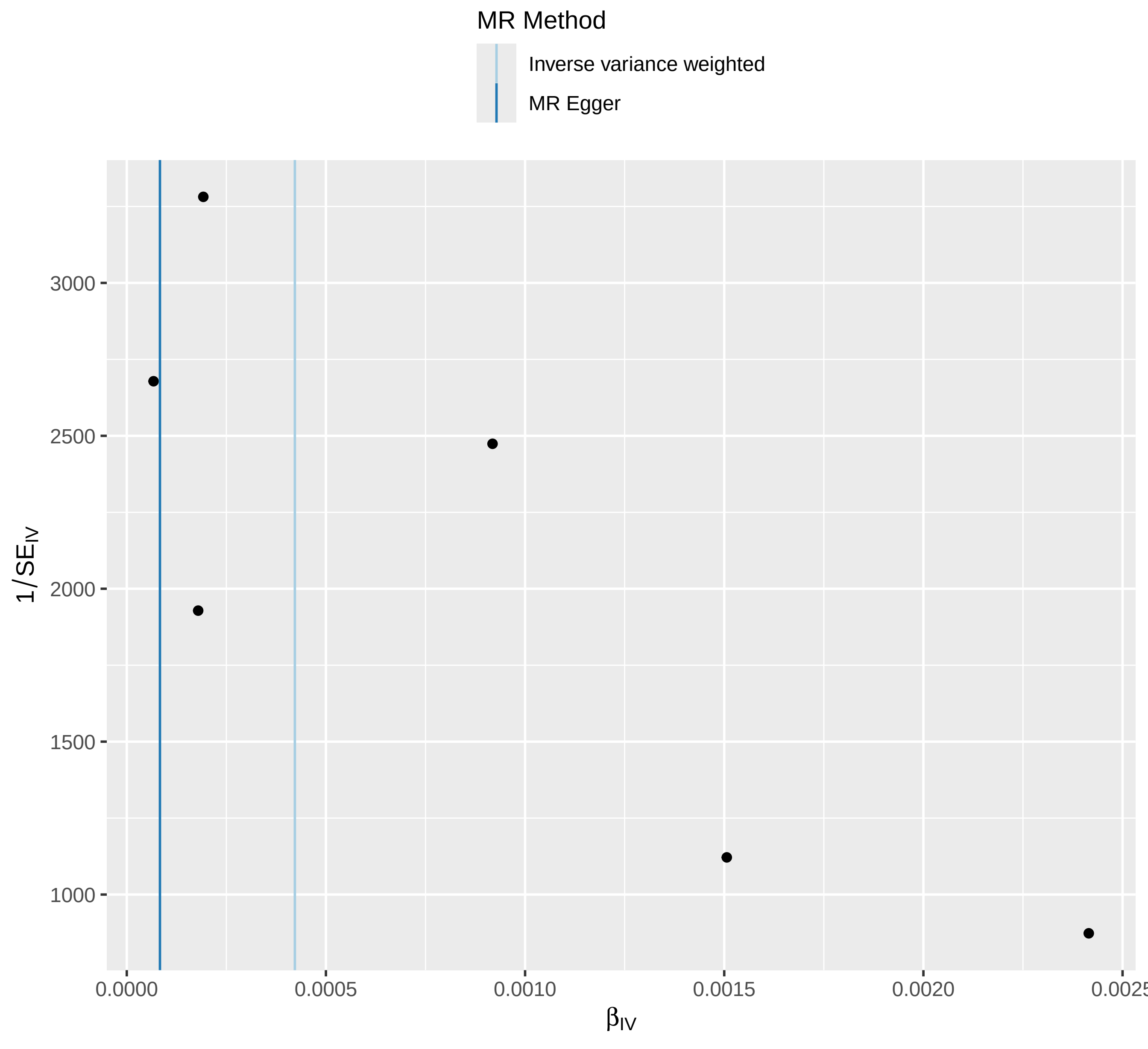
MR Method
Inverse variance weighted
MR Egger
3000
2500
2000
1500
1000
$1/SE_{IV}$
0.0000
0.0005
0.0010
0.0015
0.0020
0.0025
β_{IV}

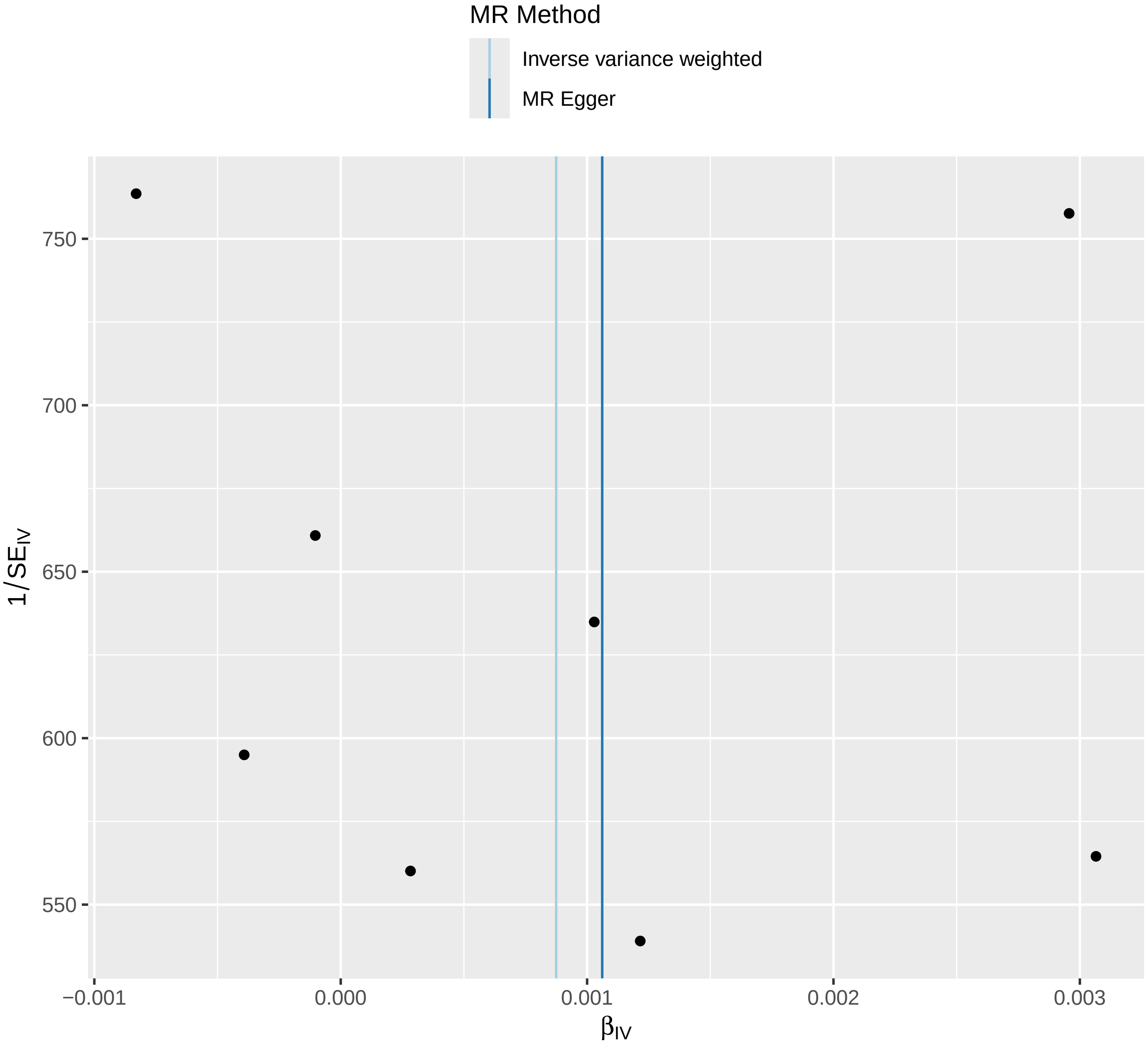
MR Method
Inverse variance weighted
MR Egger
750
700
650
600
550
1/SE$_{IV}$
−0.001
0.000
0.001
0.002
0.003
β$_{IV}$

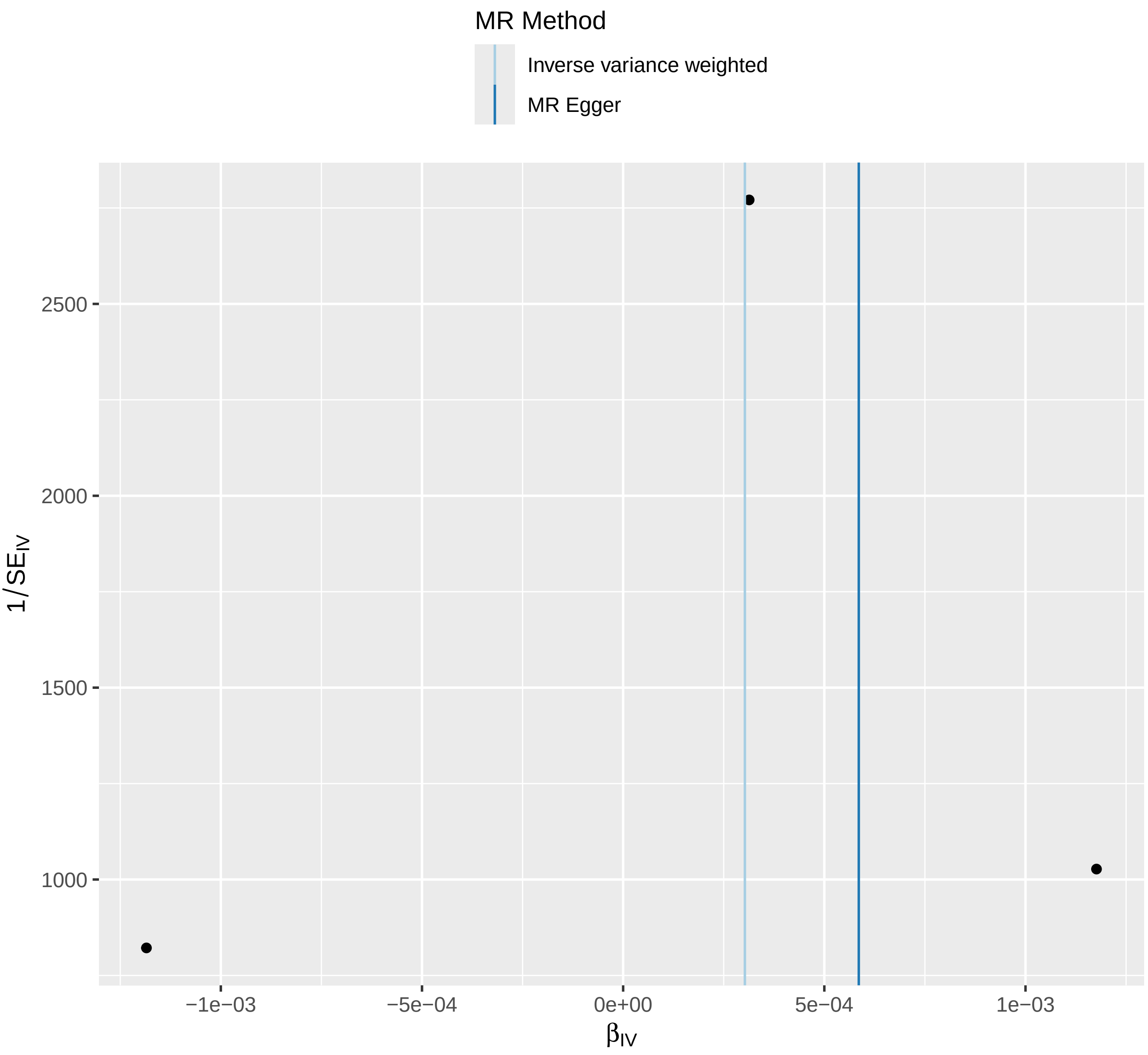

MR Method
Inverse variance weighted
MR Egger
2500
2000
1500
1000
1/SE_IV
−1e−03
−5e−04
0e+00
5e−04
1e−03
β_IV

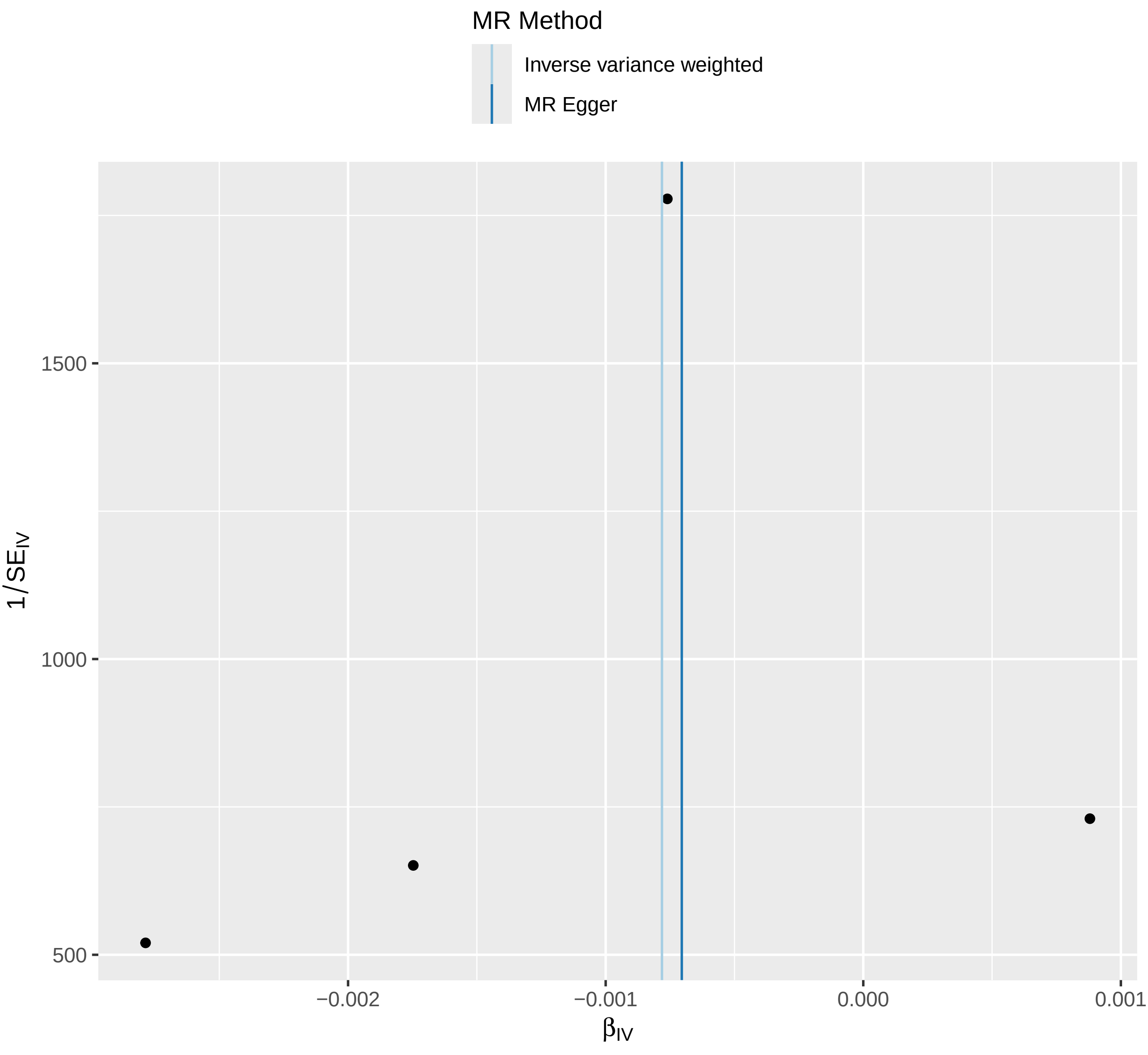
MR Method
Inverse variance weighted
MR Egger
1500
1000
500
1/SE_IV
−0.002
−0.001
0.000
0.001
β_IV

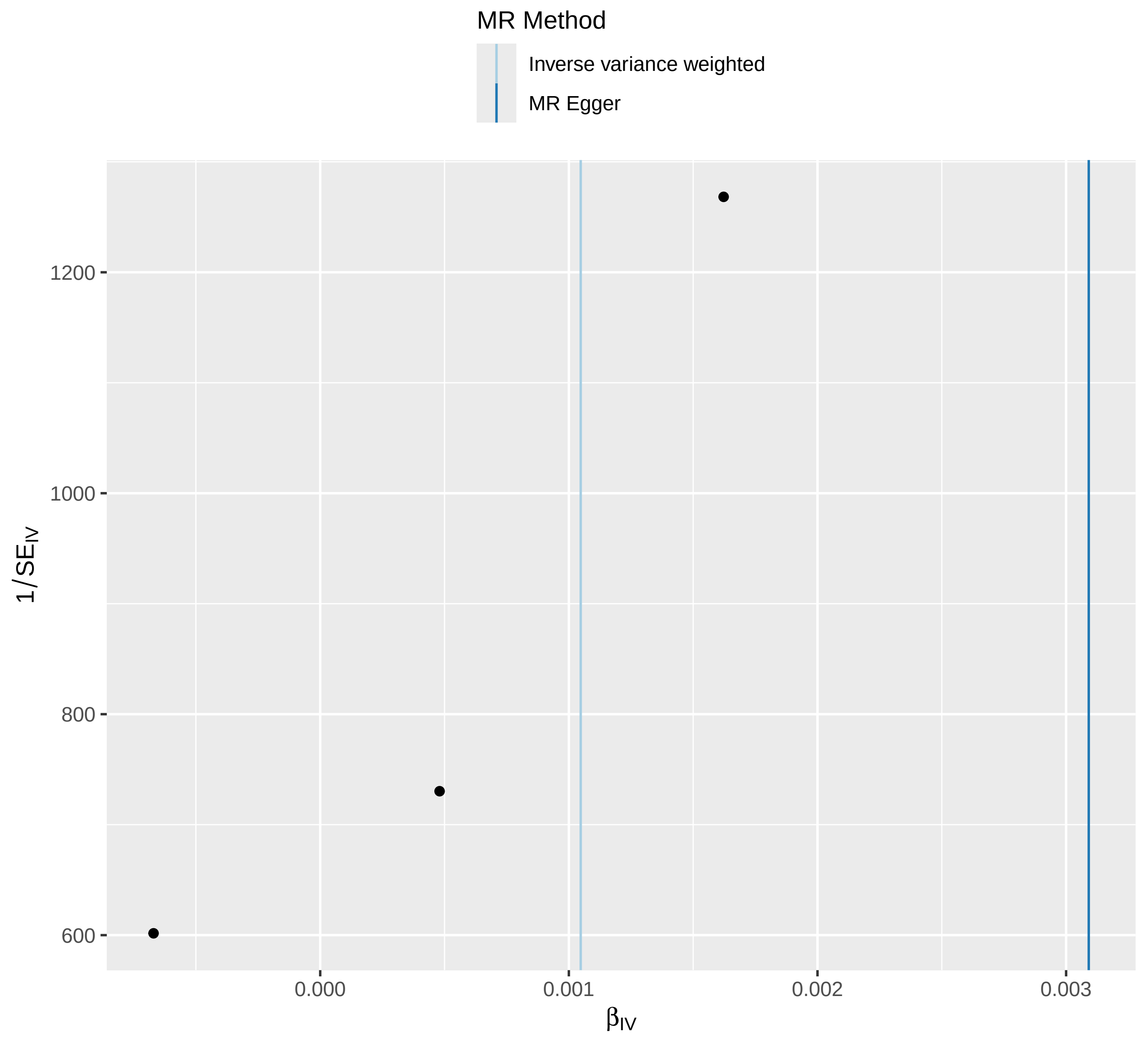

MR Method
Inverse variance weighted
MR Egger
1200
1000
800
600
0.000
0.001
0.002
0.003
β_{IV}
$1/SE_{IV}$

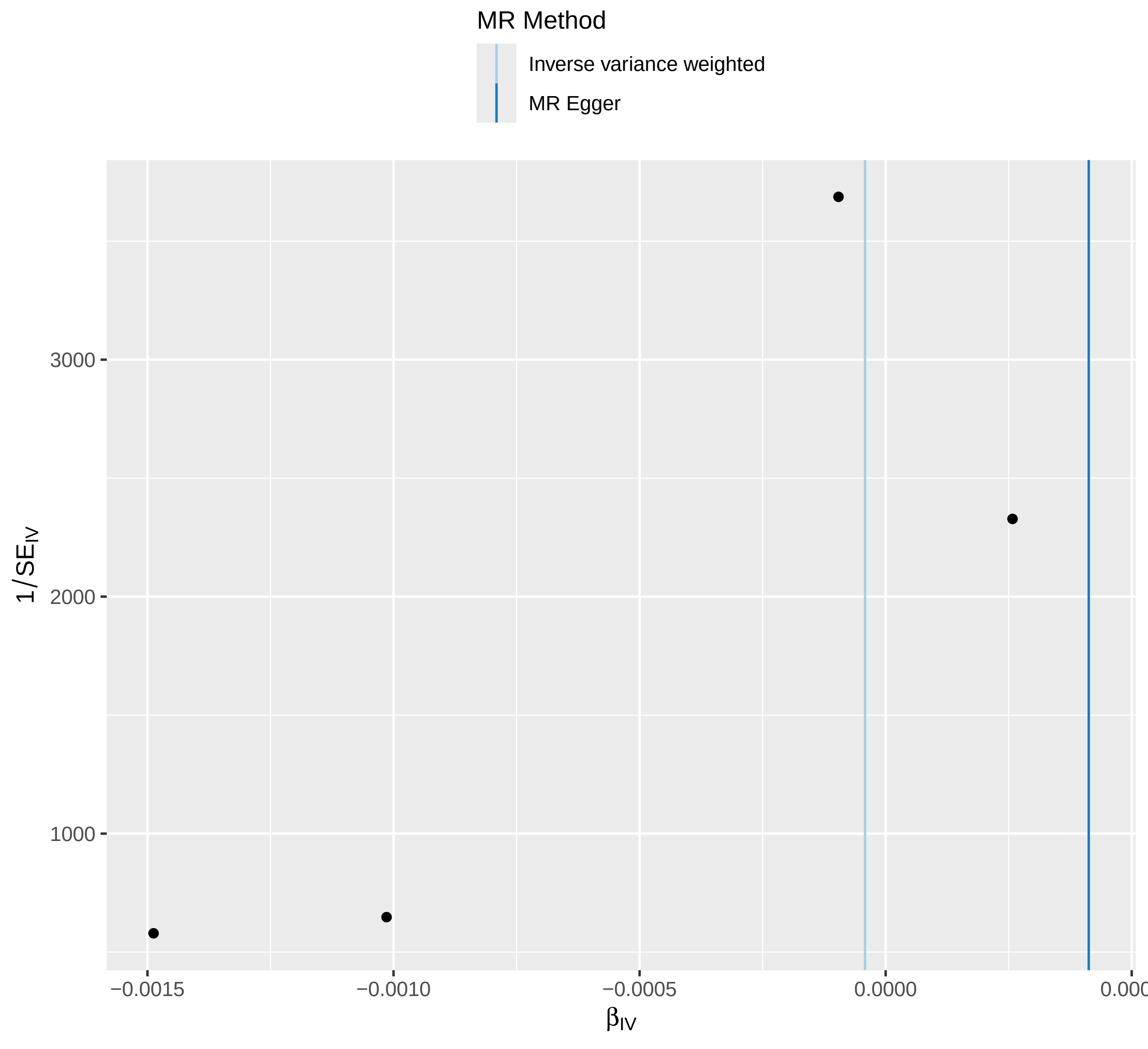
MR Method
Inverse variance weighted
MR Egger
3000
2000
1000
1/SE_IV
−0.0015
−0.0010
−0.0005
0.0000
β_IV

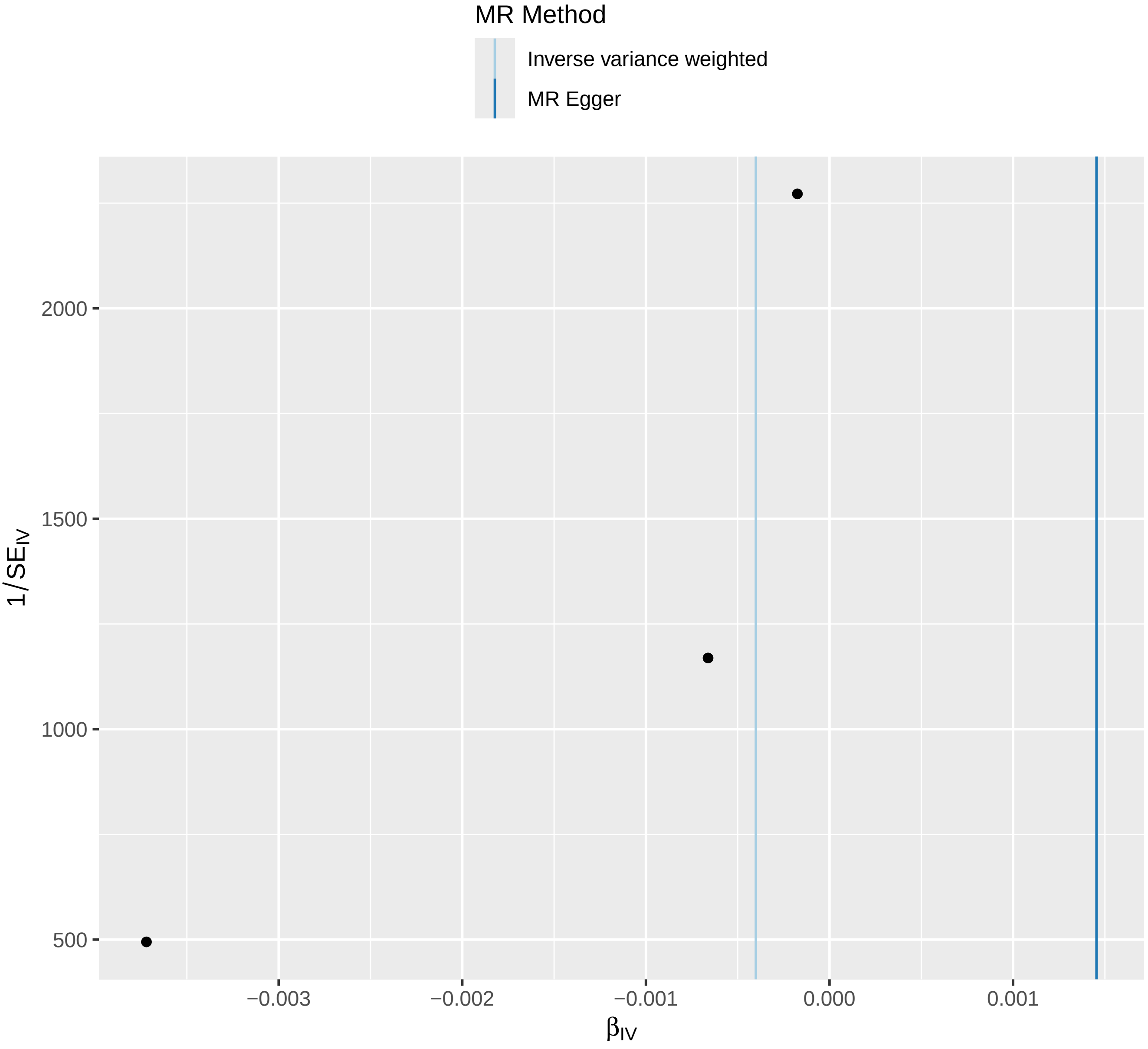

MR Method
Inverse variance weighted
MR Egger
2000
1500
1000
500
1/SE_IV
−0.003
−0.002
−0.001
0.000
0.001
β_IV

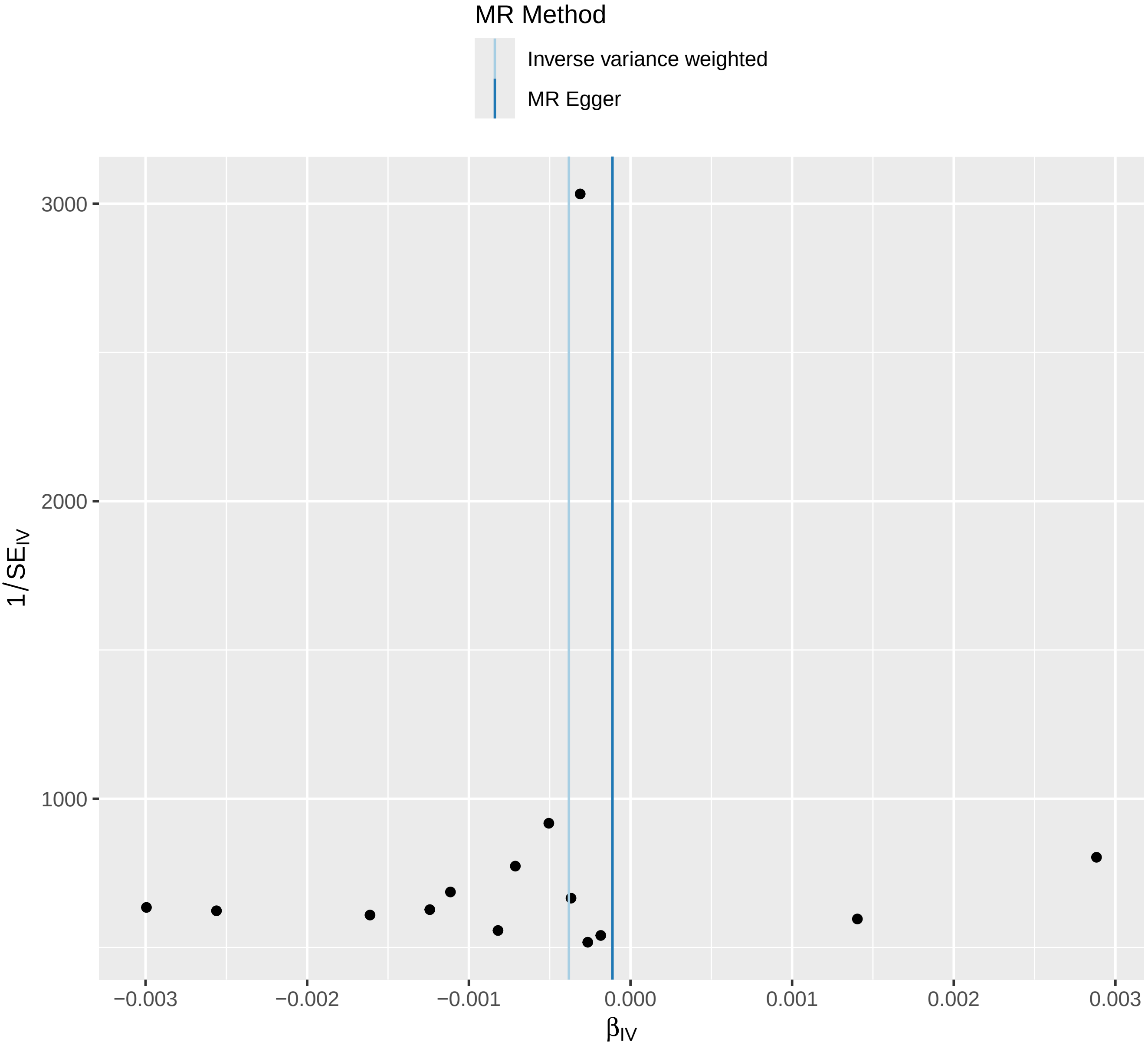

MR Method
Inverse variance weighted
MR Egger
3000
2000
1000
1/SE$_{IV}$
−0.003
−0.002
−0.001
0.000
0.001
0.002
0.003
β$_{IV}$

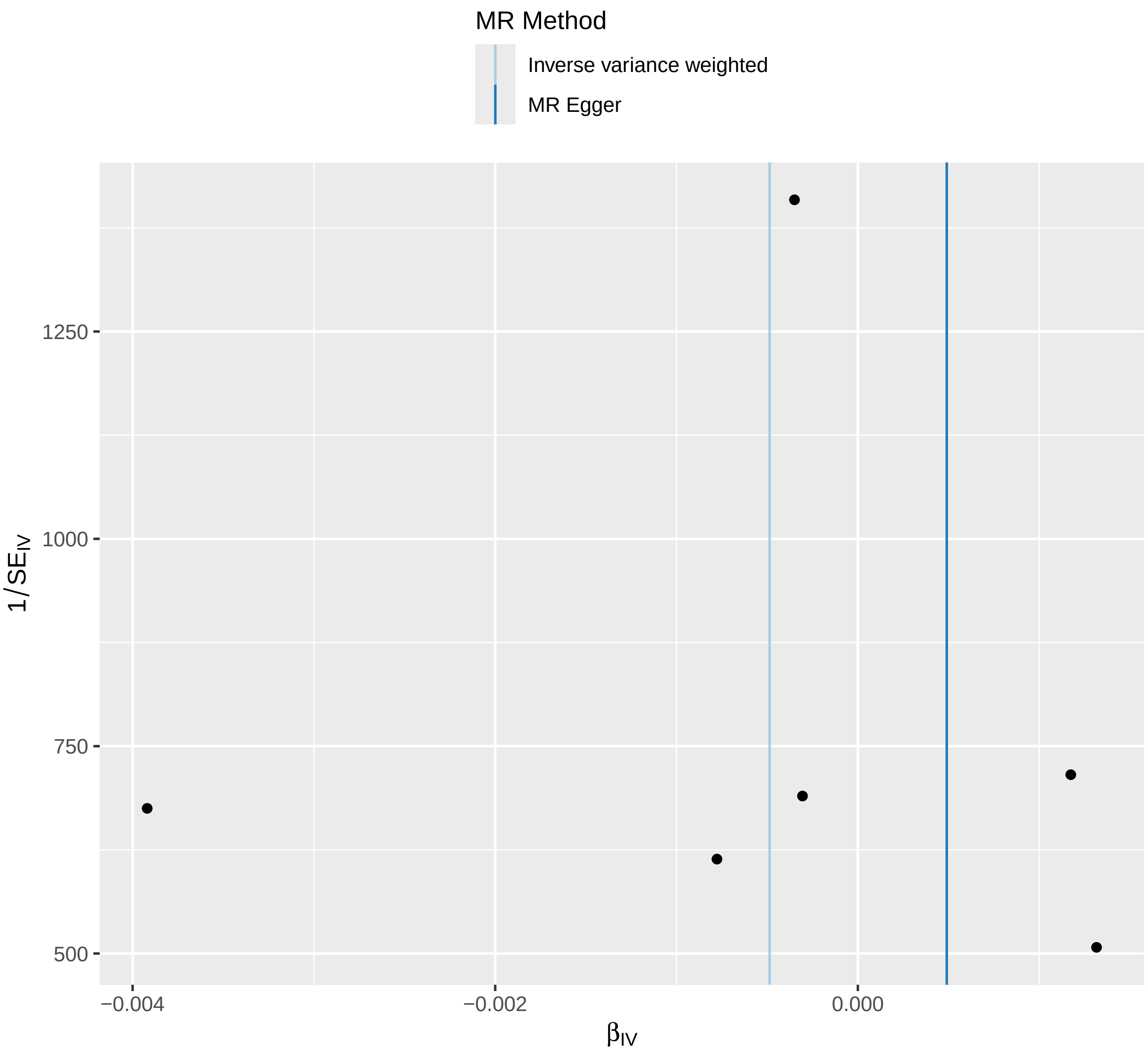
MR Method
Inverse variance weighted
MR Egger
1250
1000
750
500
1/SE_IV
−0.004
−0.002
0.000
β_IV

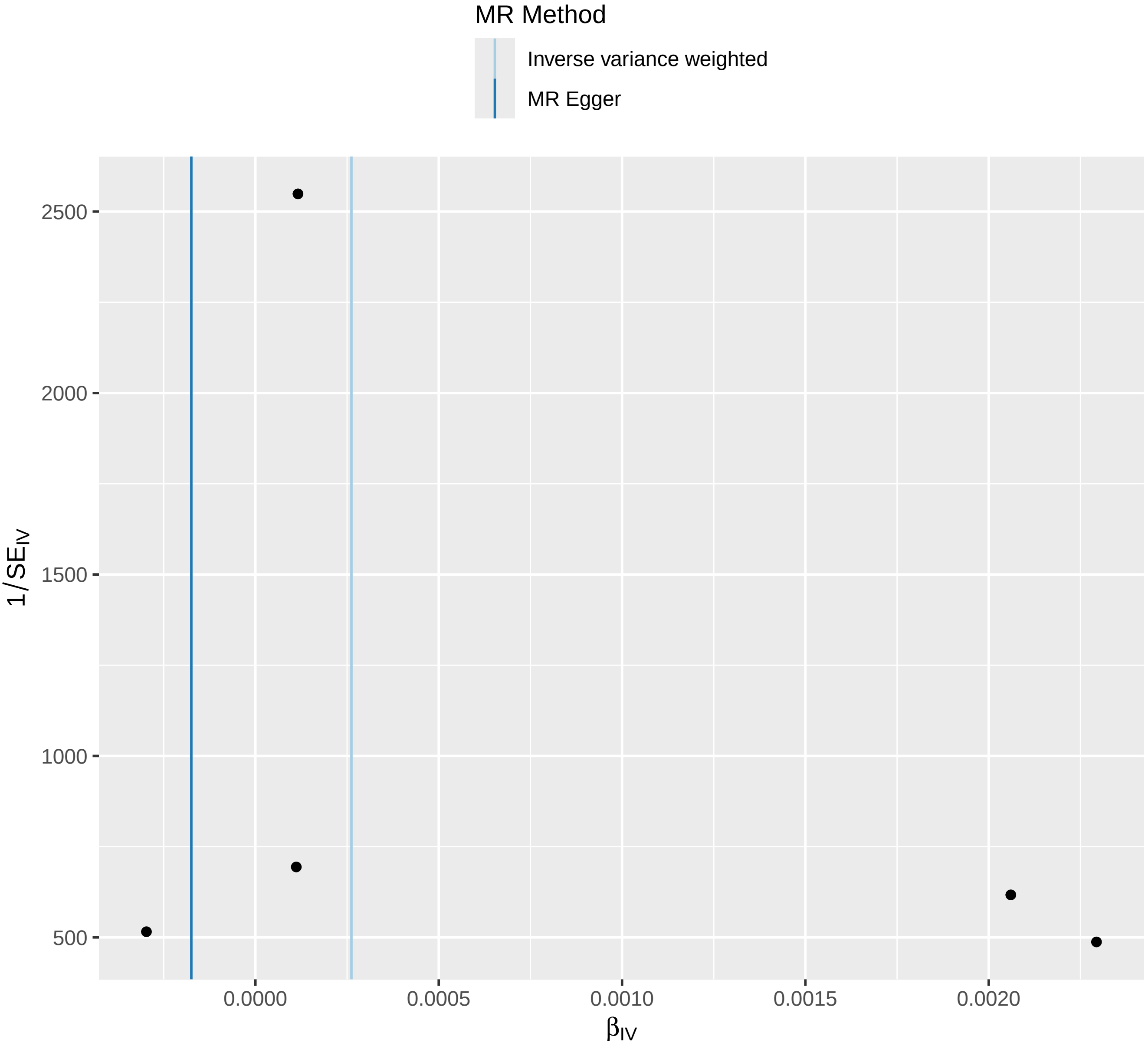

MR Method
Inverse variance weighted
MR Egger
2500
2000
1500
1000
500
1/SE$_{IV}$
0.0000
0.0005
0.0010
0.0015
0.0020
β$_{IV}$

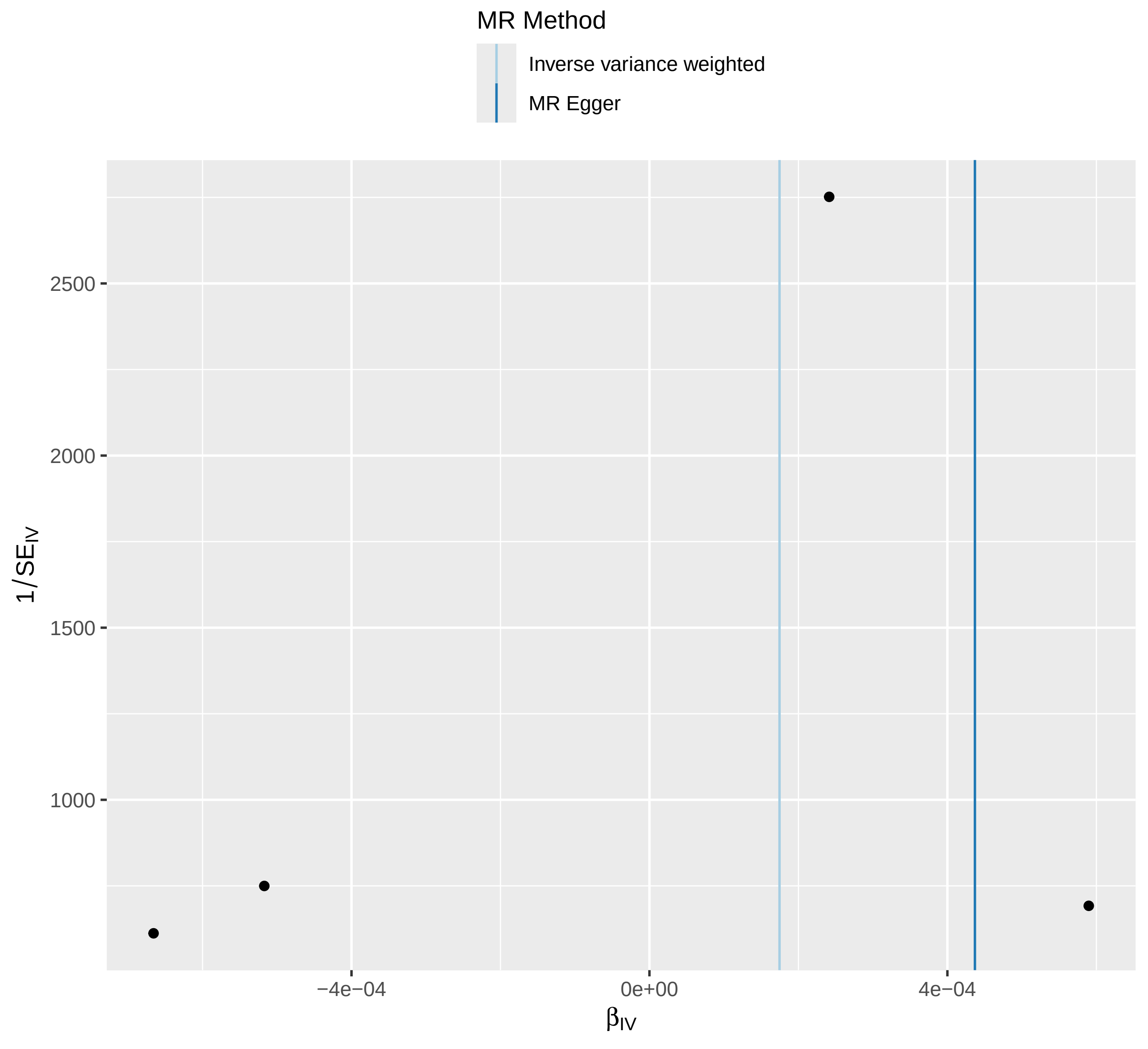
MR Method
Inverse variance weighted
MR Egger
2500
2000
1500
1000
1/SE_IV
−4e−04
0e+00
4e−04
β_IV

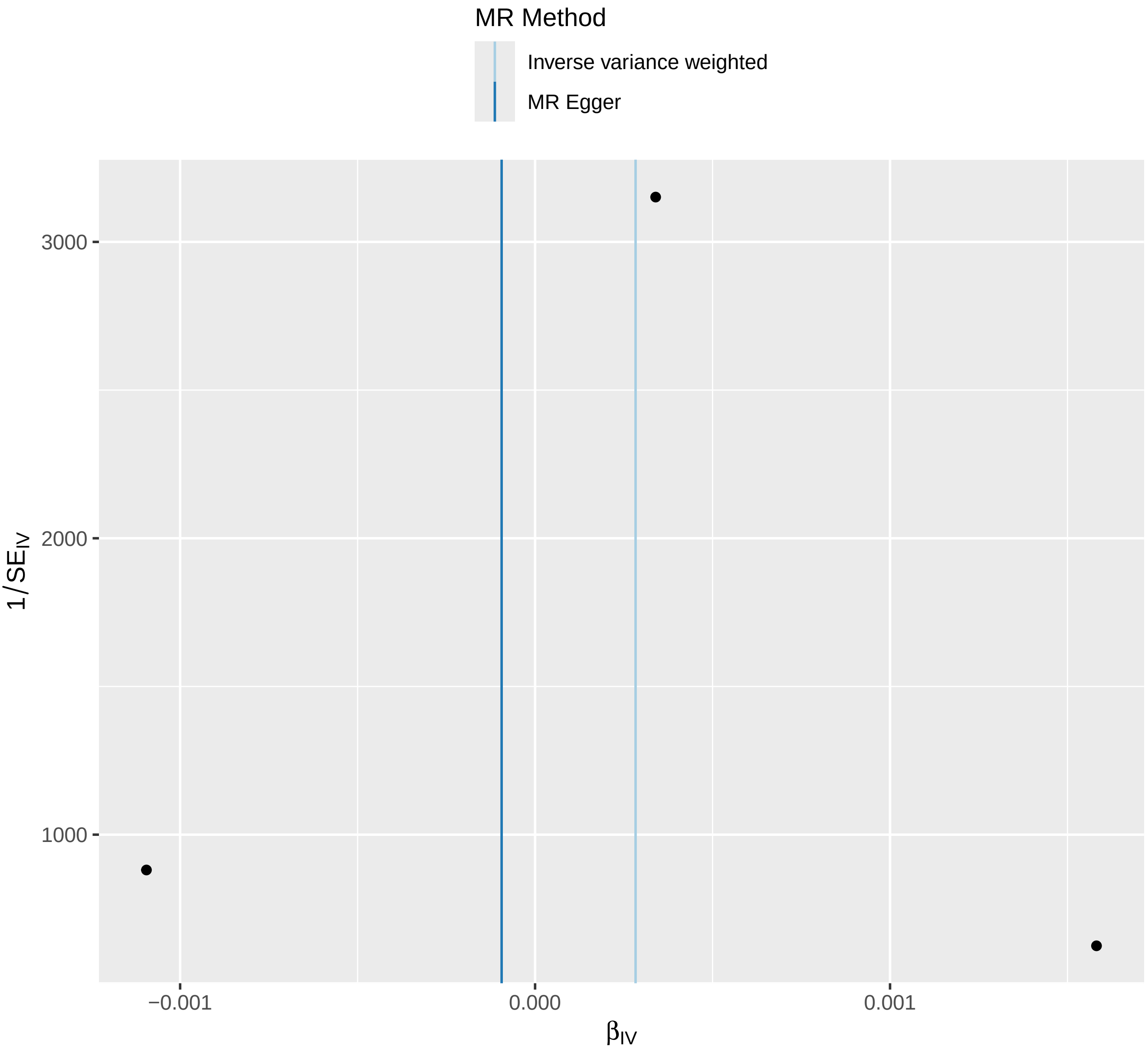
MR Method
Inverse variance weighted
MR Egger
3000
2000
1000
1/SE$_{IV}$
−0.001
0.000
0.001
β$_{IV}$

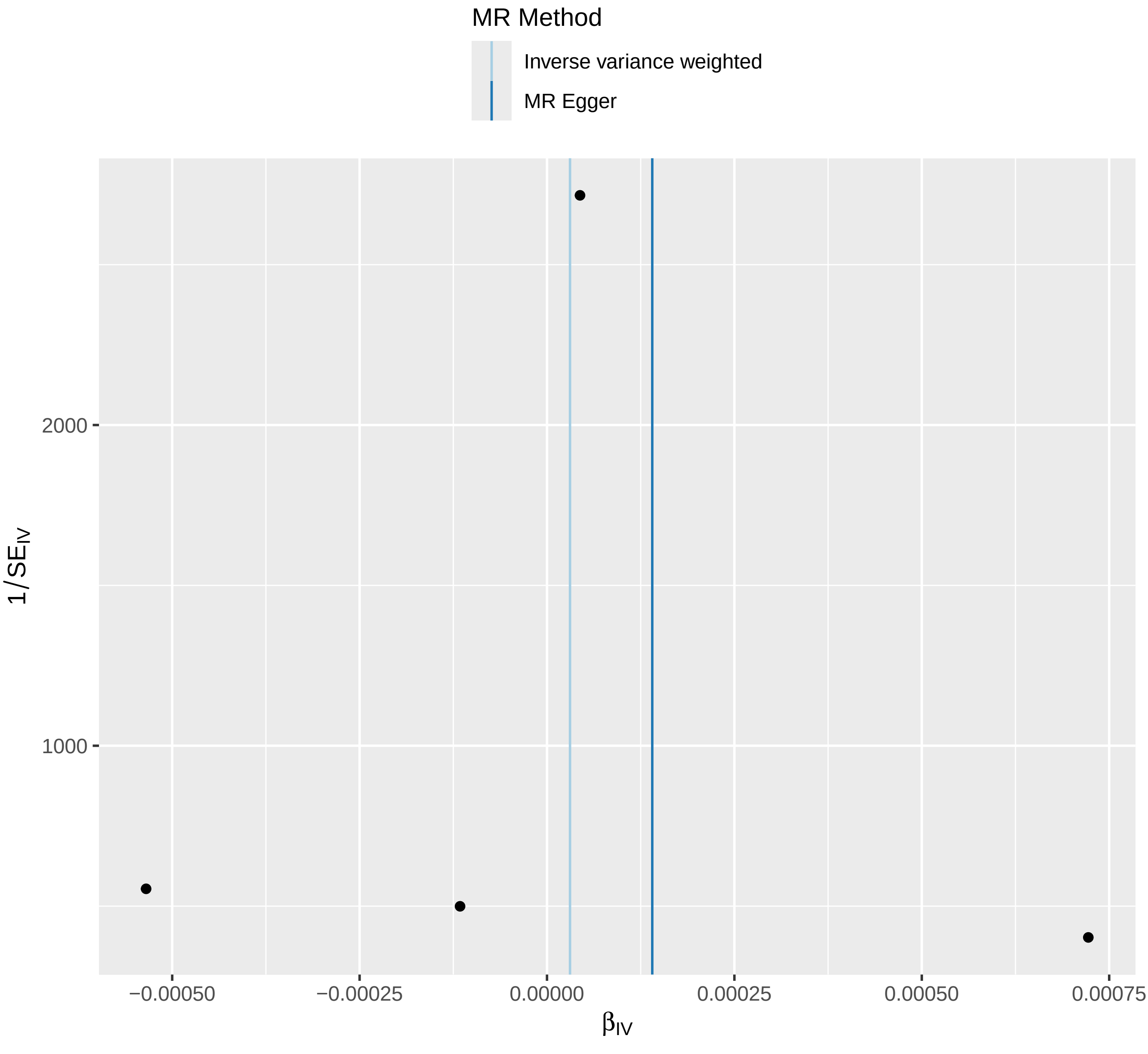

MR Method
Inverse variance weighted
MR Egger
2000
1000
1/SE_IV
−0.00050
−0.00025
0.00000
0.00025
0.00050
0.00075
β_IV

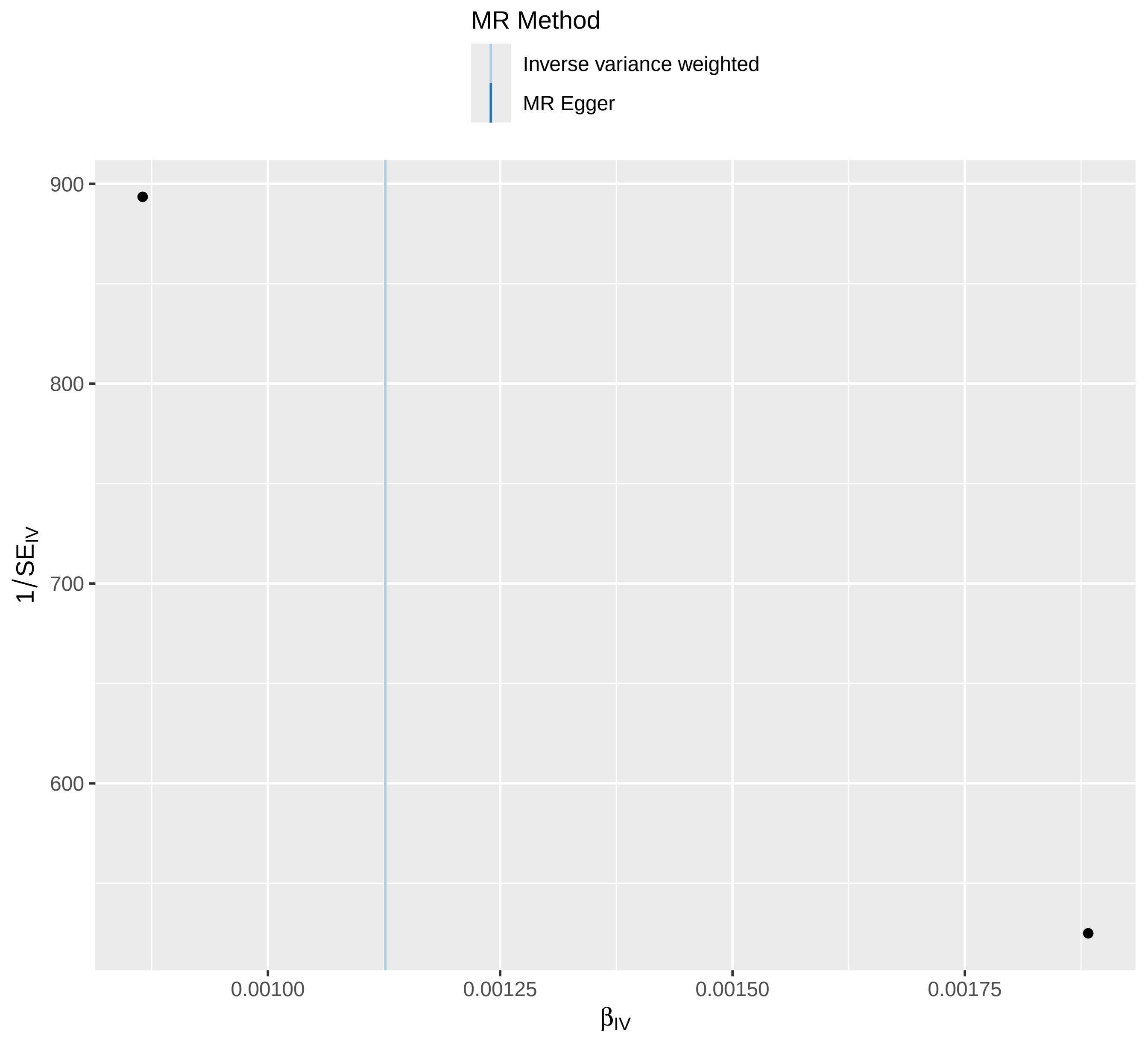
MR Method
Inverse variance weighted
MR Egger
900
800
700
600
1/SE_IV
0.00100
0.00125
0.00150
0.00175
β_IV

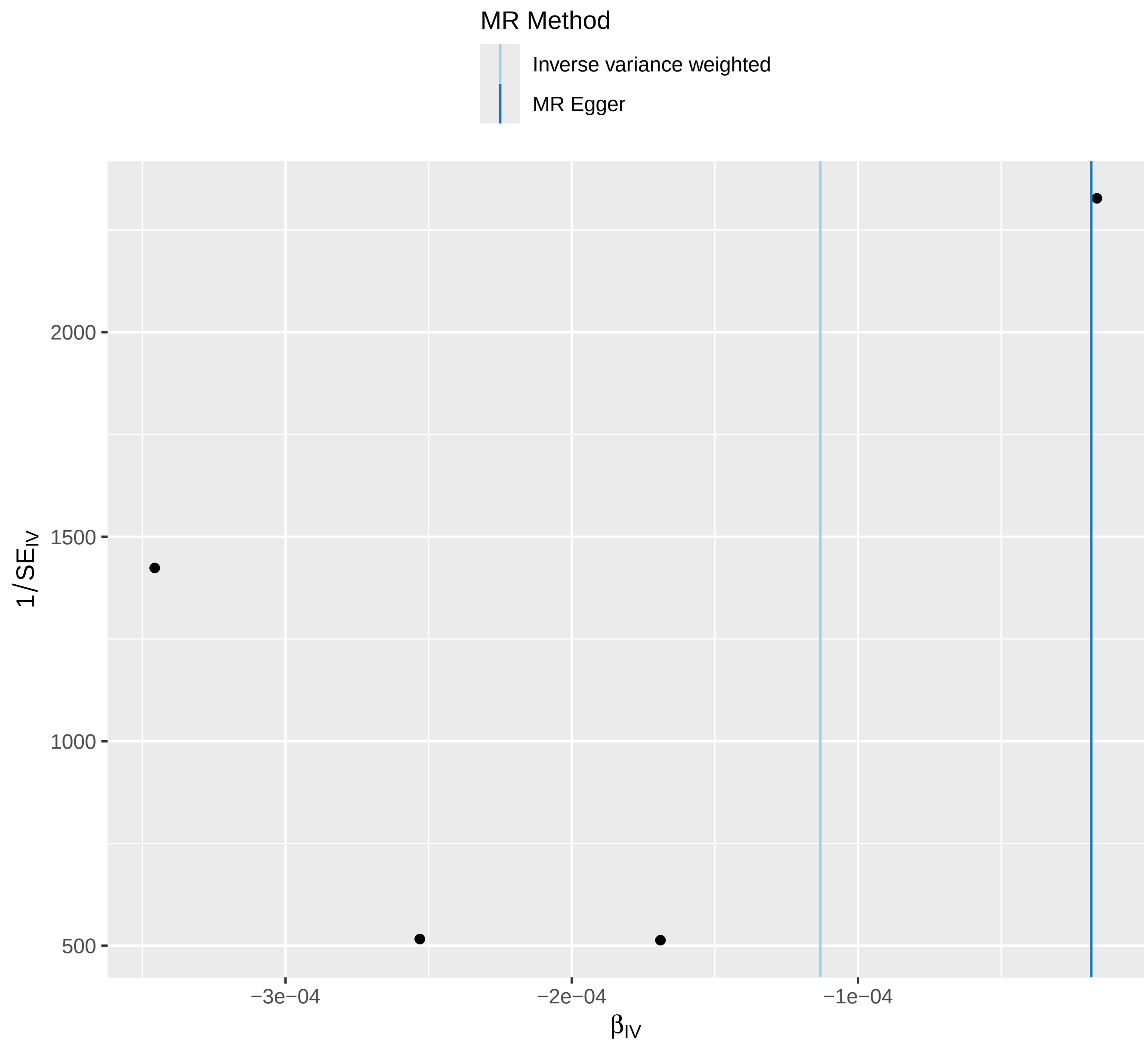
MR Method
Inverse variance weighted
MR Egger
2000
1500
1000
500
1/SE_IV
−3e−04
−2e−04
−1e−04
β_IV

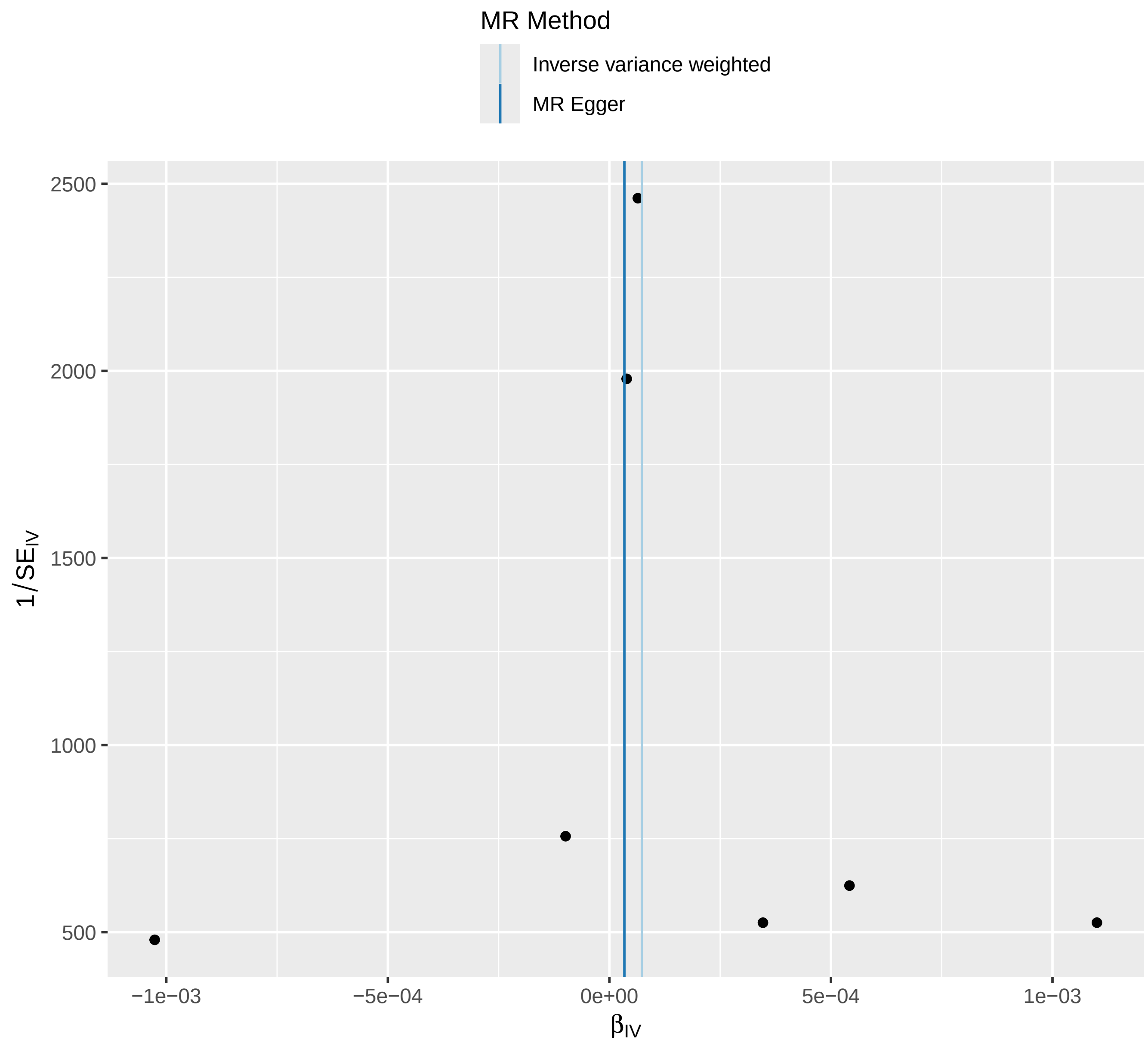
MR Method
Inverse variance weighted
MR Egger
2500
2000
1500
1000
500
1/SE_IV
−1e−03
−5e−04
0e+00
5e−04
1e−03
β_IV

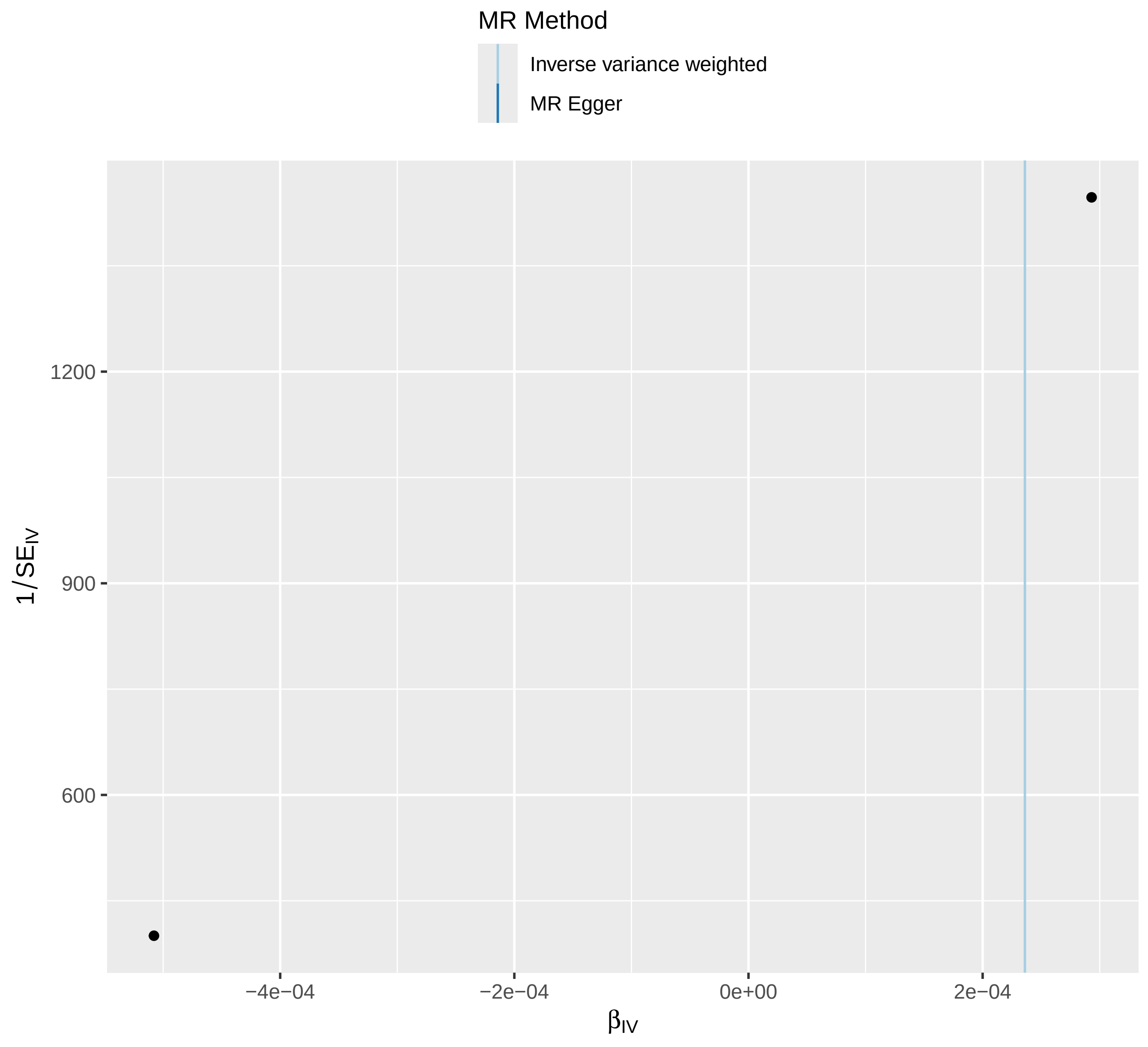

MR Method
Inverse variance weighted
MR Egger
1200
900
600
1/SE_IV
−4e−04
−2e−04
0e+00
2e−04
β_IV

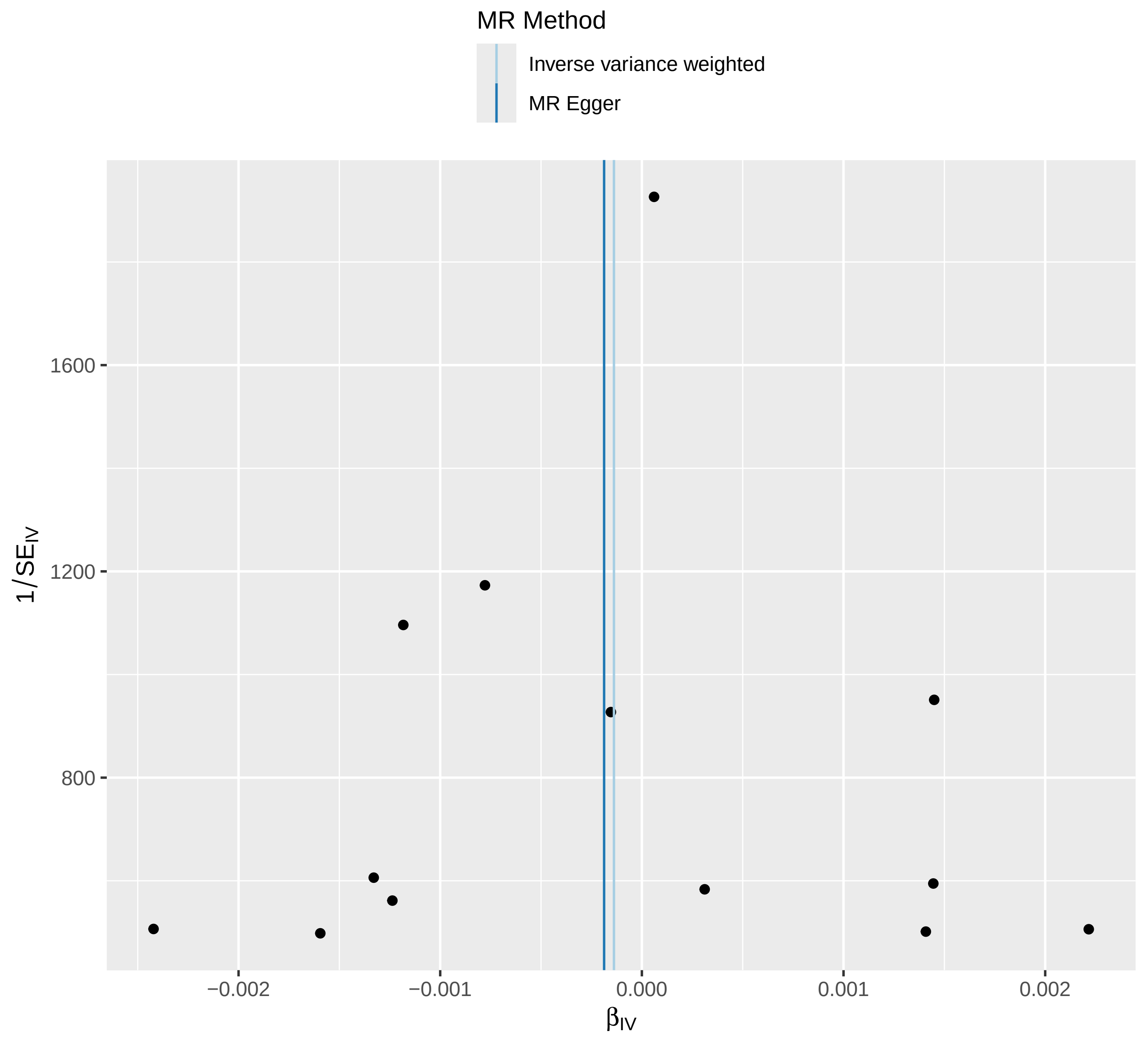

MR Method
Inverse variance weighted
MR Egger
1600
1200
800
1/SE_IV
−0.002
−0.001
0.000
0.001
0.002
β_IV

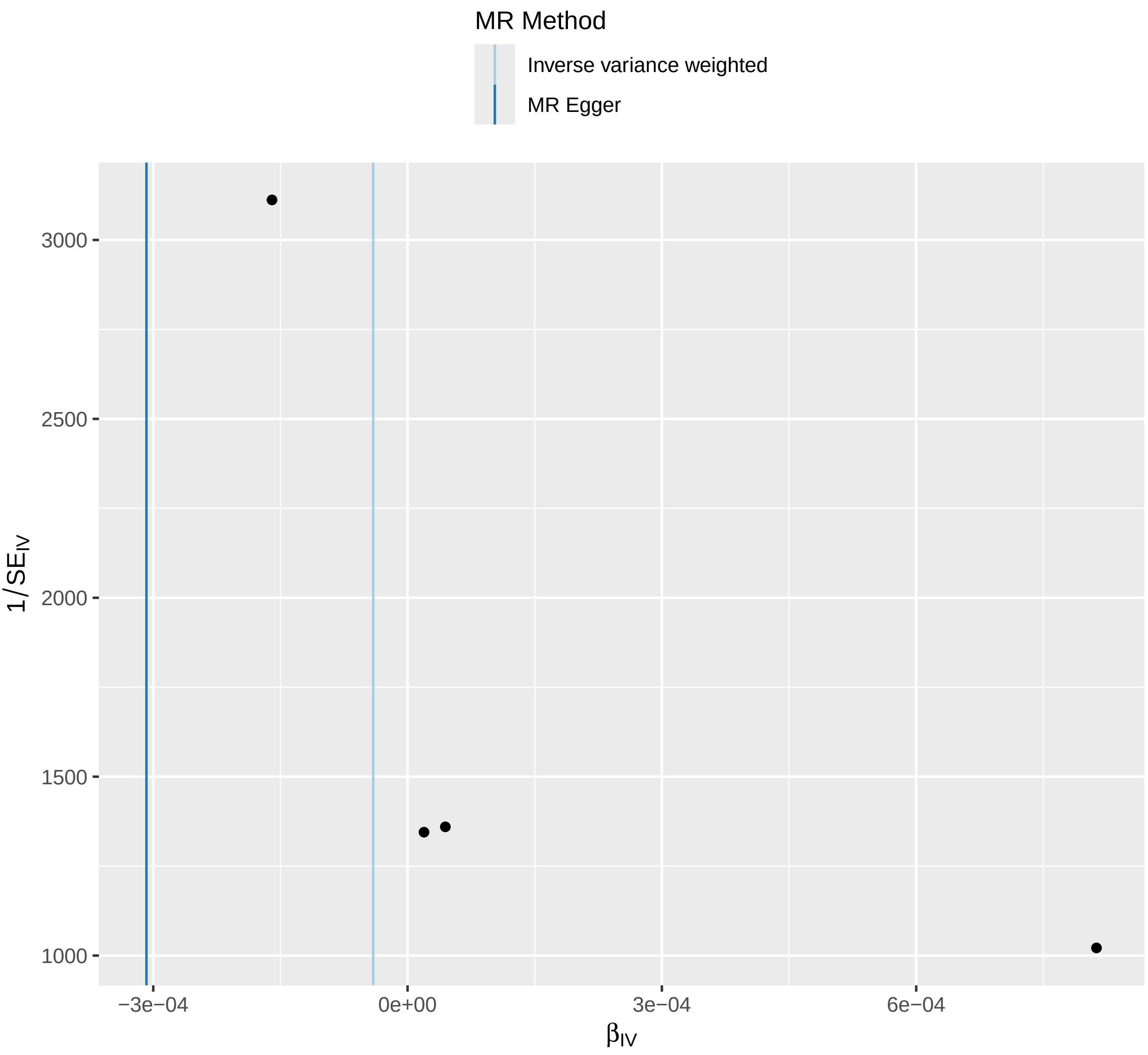
MR Method
Inverse variance weighted
MR Egger
3000
2500
2000
1500
1000
$1/SE_{IV}$
−3e−04
0e+00
3e−04
6e−04
β_{IV}

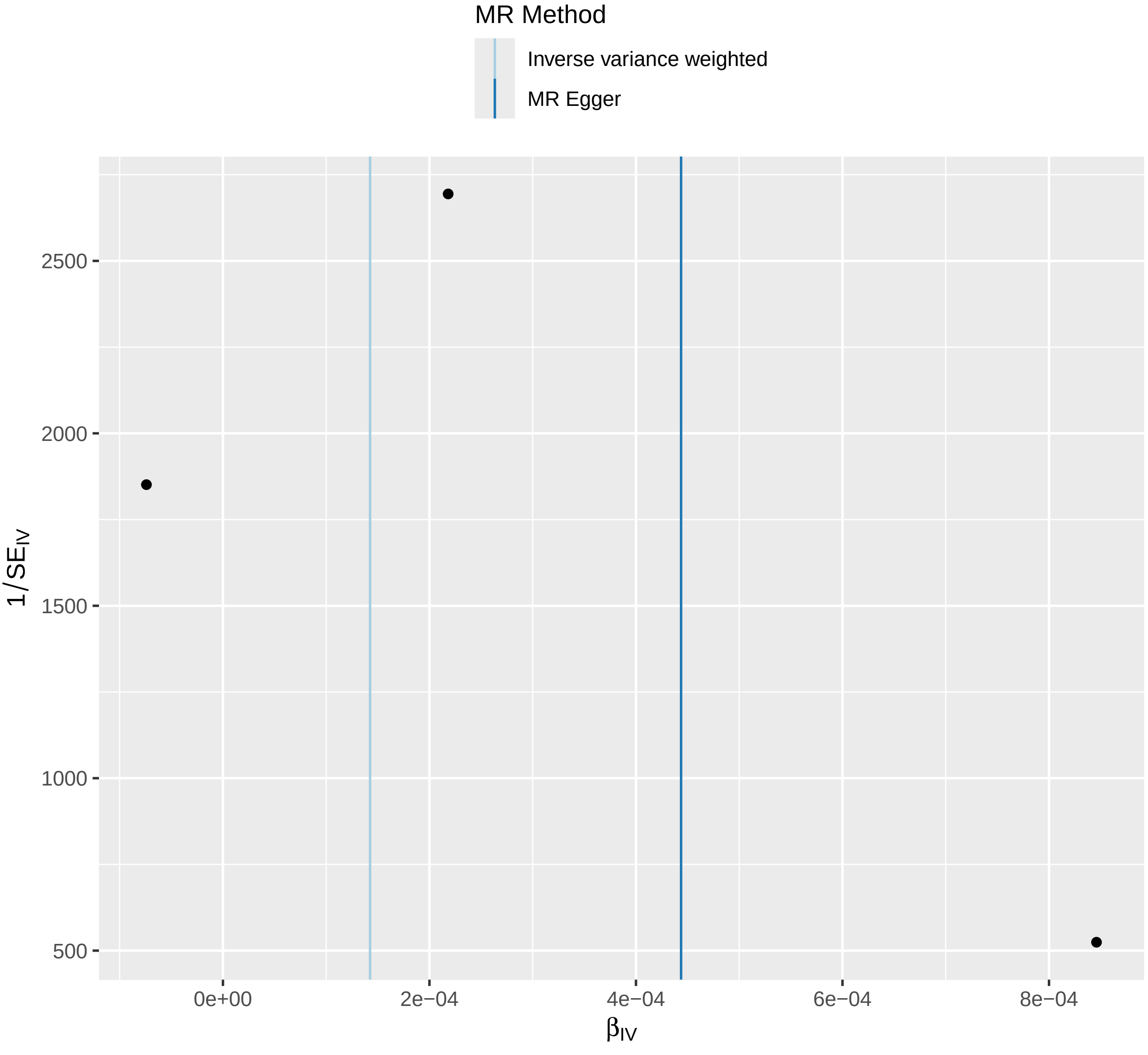
MR Method
Inverse variance weighted
MR Egger
2500
2000
1500
1000
500
1/SE$_{IV}$
0e+00
2e−04
4e−04
6e−04
8e−04
β$_{IV}$

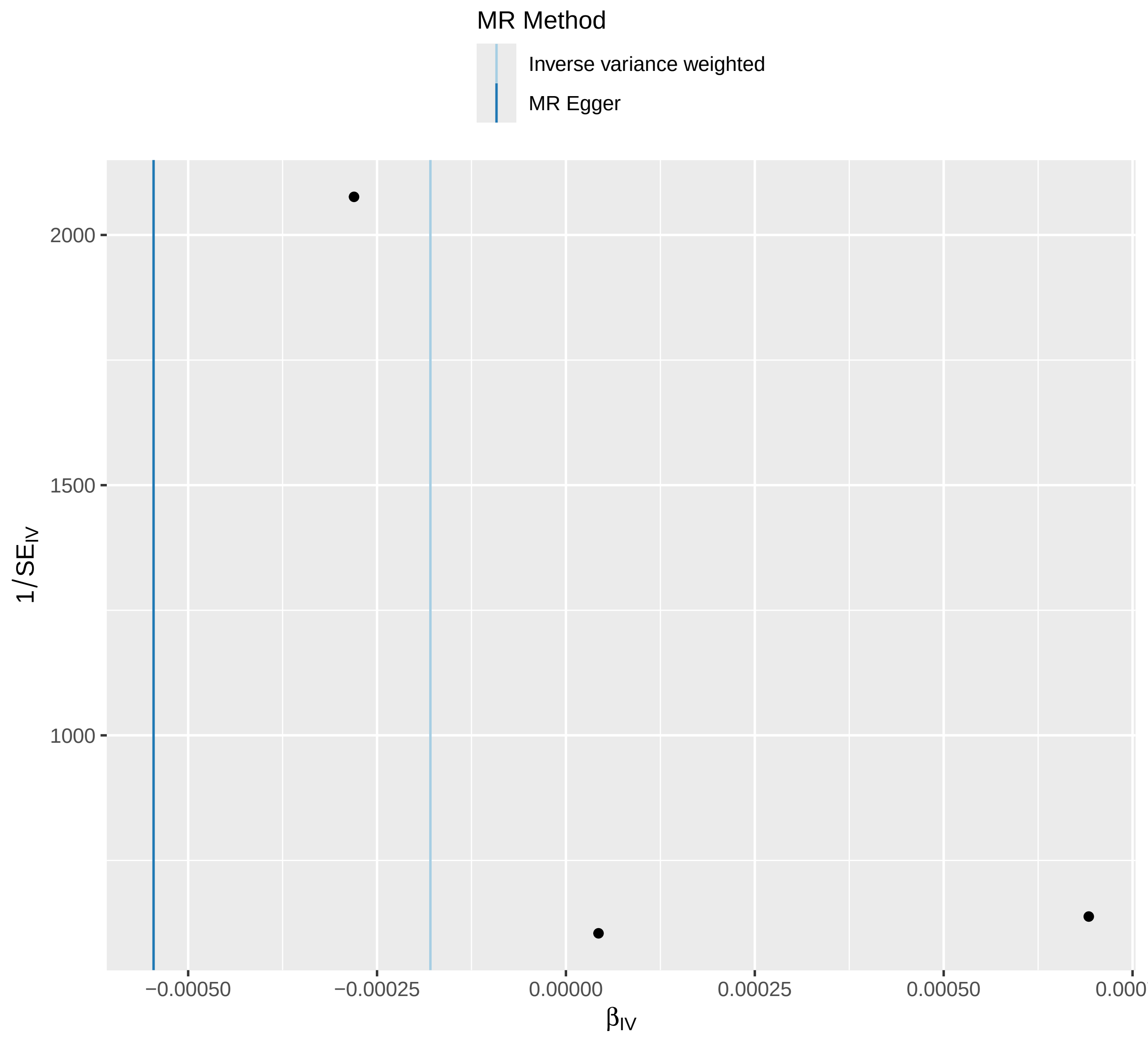

MR Method
Inverse variance weighted
MR Egger
2000
1500
1000
1/SE_IV
−0.00050
−0.00025
0.00000
0.00025
0.00050
0.0007
β_IV

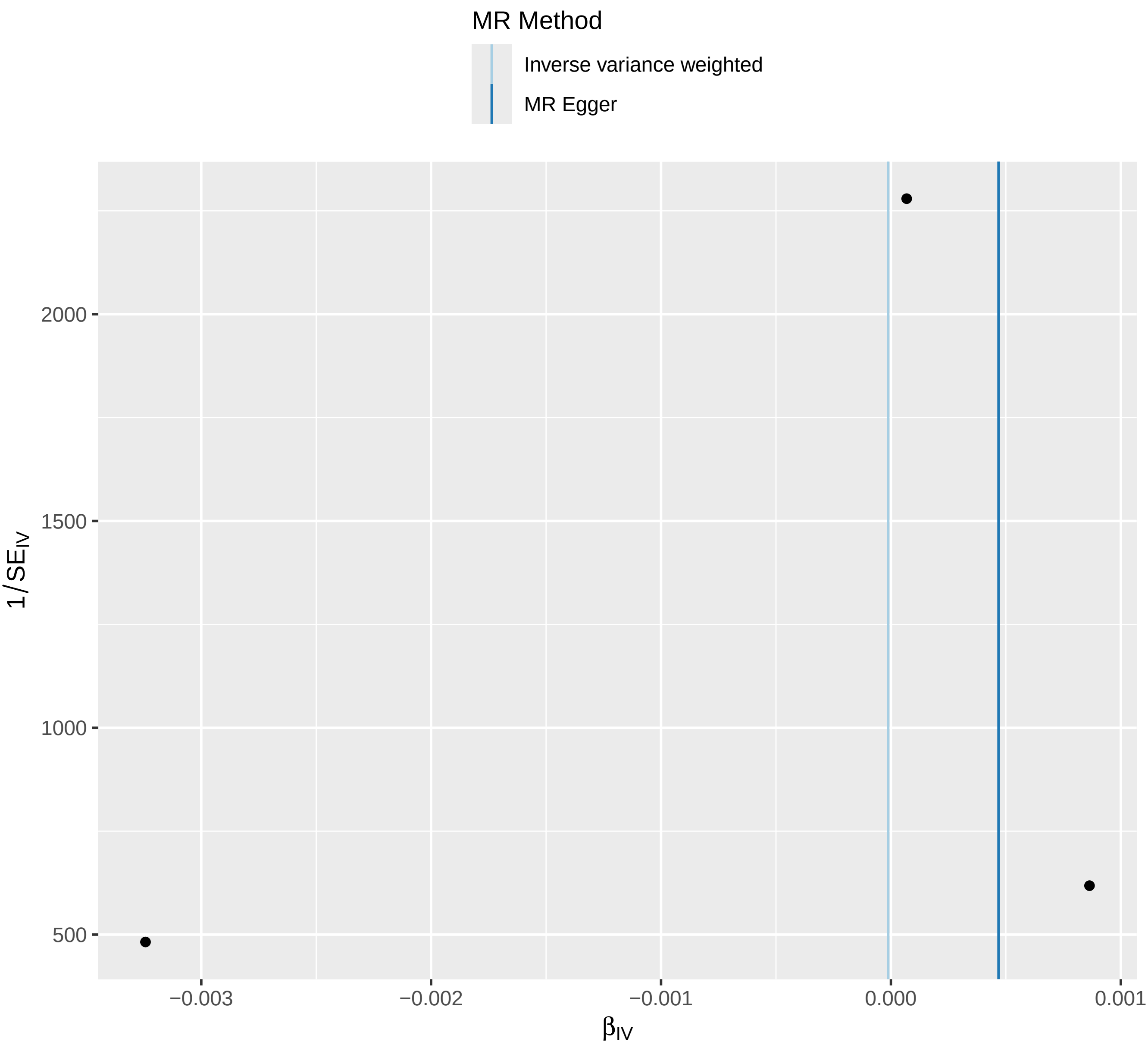

MR Method
Inverse variance weighted
MR Egger
2000
1500
1000
500
1/SE_IV
−0.003
−0.002
−0.001
0.000
0.001
β_IV

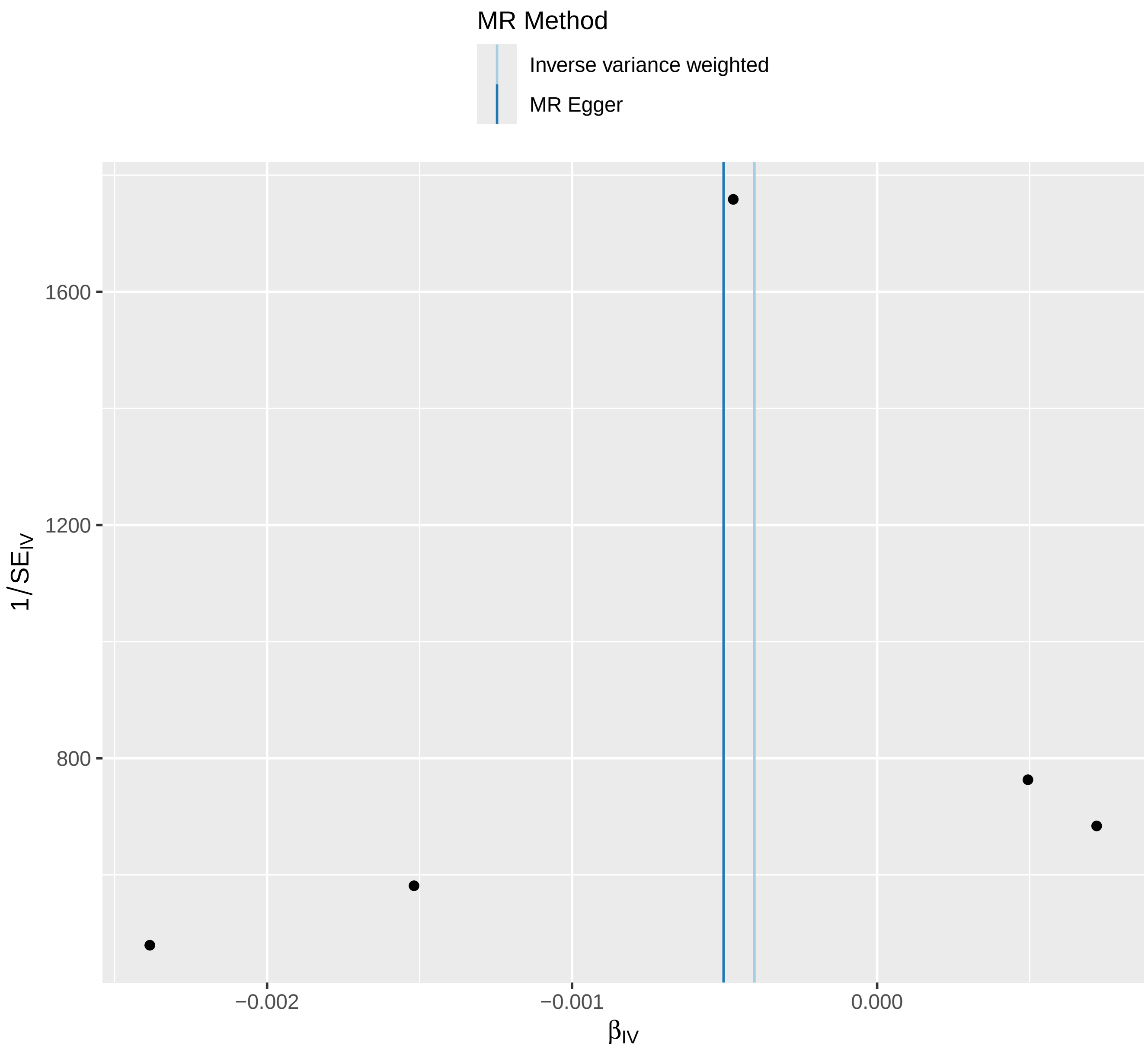

MR Method
Inverse variance weighted
MR Egger
1600
1200
800
1/SE_IV
−0.002
−0.001
0.000
β_IV

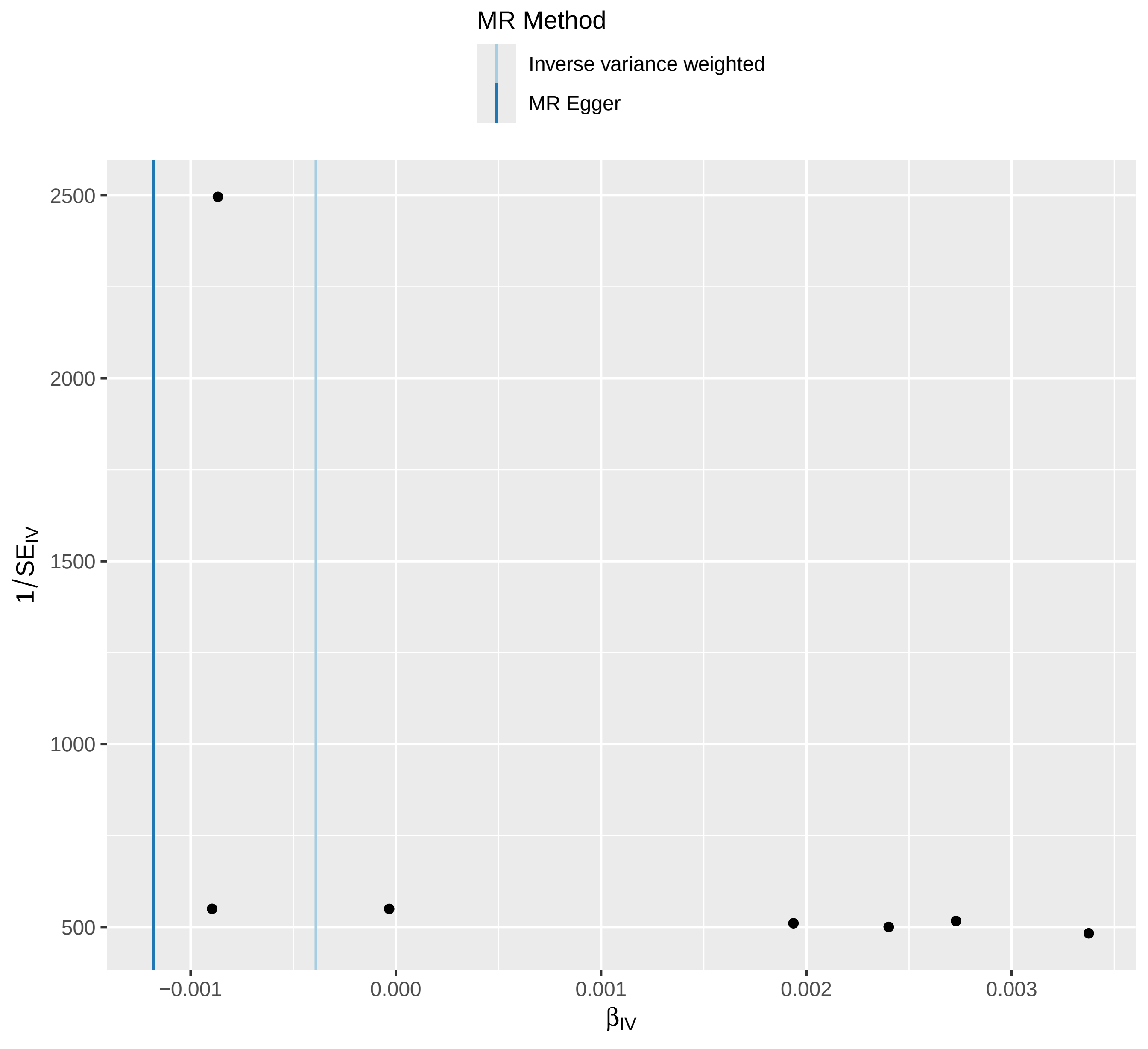
MR Method
Inverse variance weighted
MR Egger
2500
2000
1500
1000
500
−0.001
0.000
0.001
0.002
0.003
$1/SE_{IV}$
β_{IV}

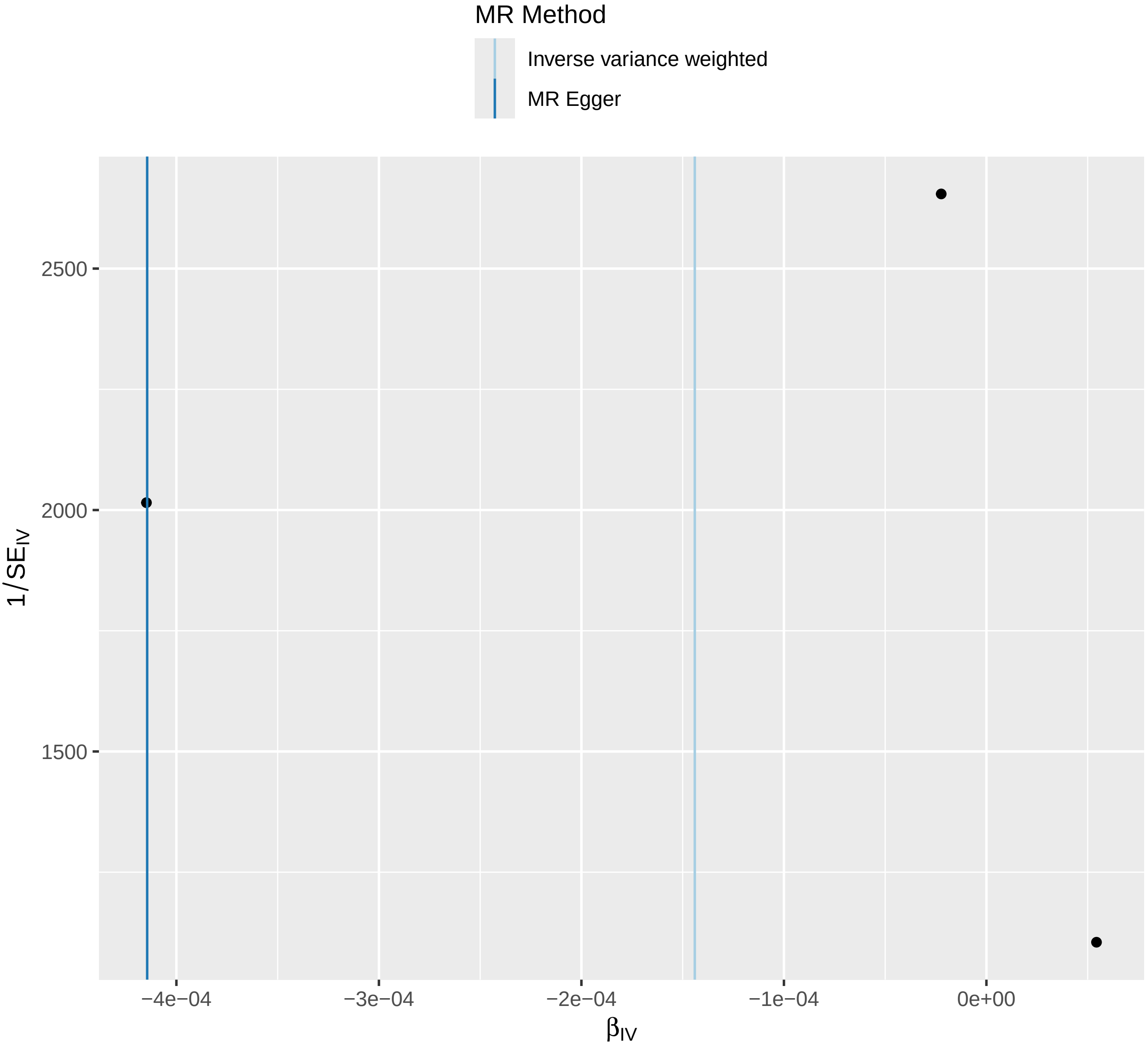

MR Method
Inverse variance weighted
MR Egger
2500
2000
1500
1/SE$_{IV}$
−4e−04
−3e−04
−2e−04
−1e−04
0e+00
β$_{IV}$

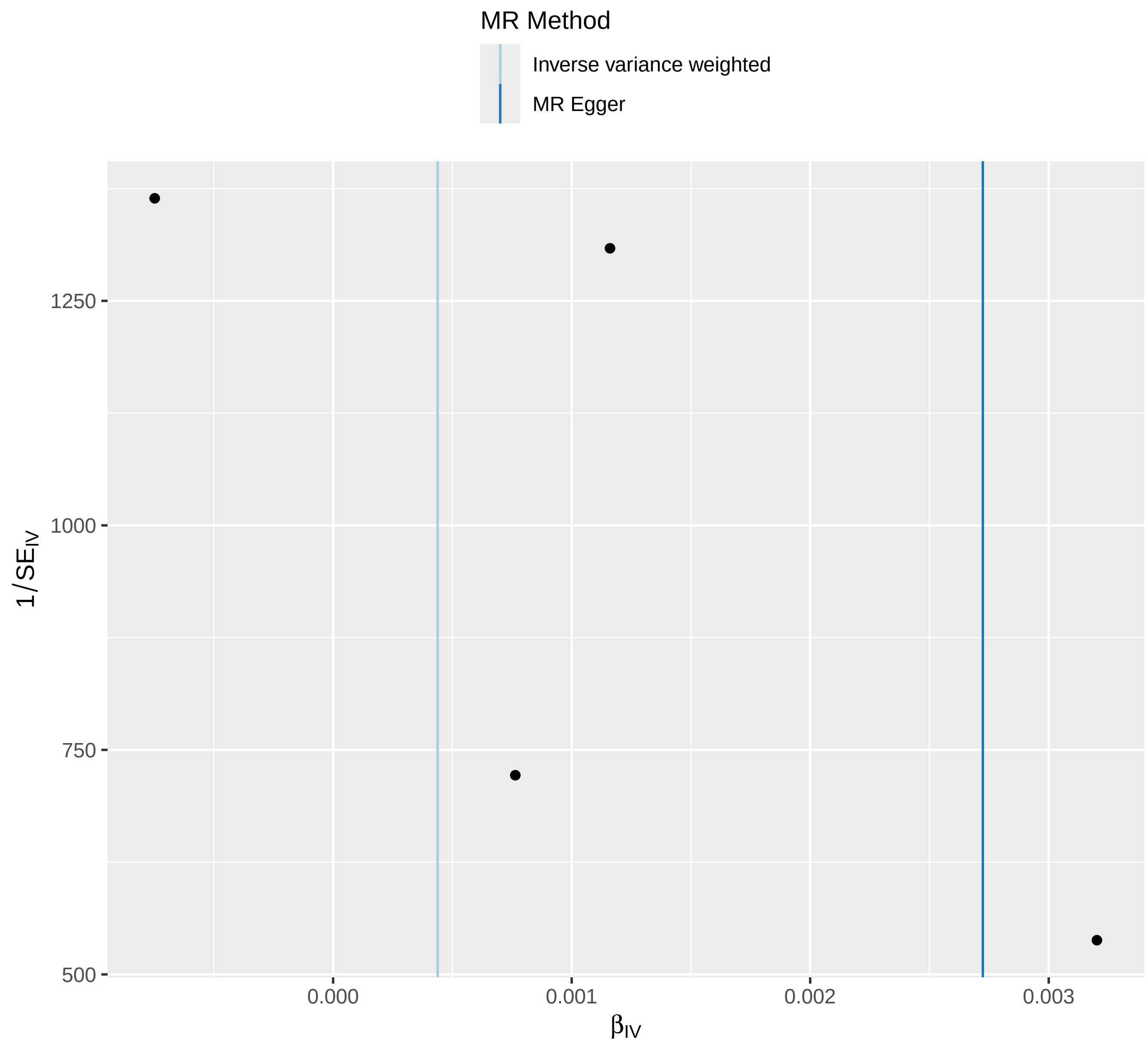

MR Method
Inverse variance weighted
MR Egger
1250
1000
750
500
1/SE$_{IV}$
0.000
0.001
0.002
0.003
β_{IV}

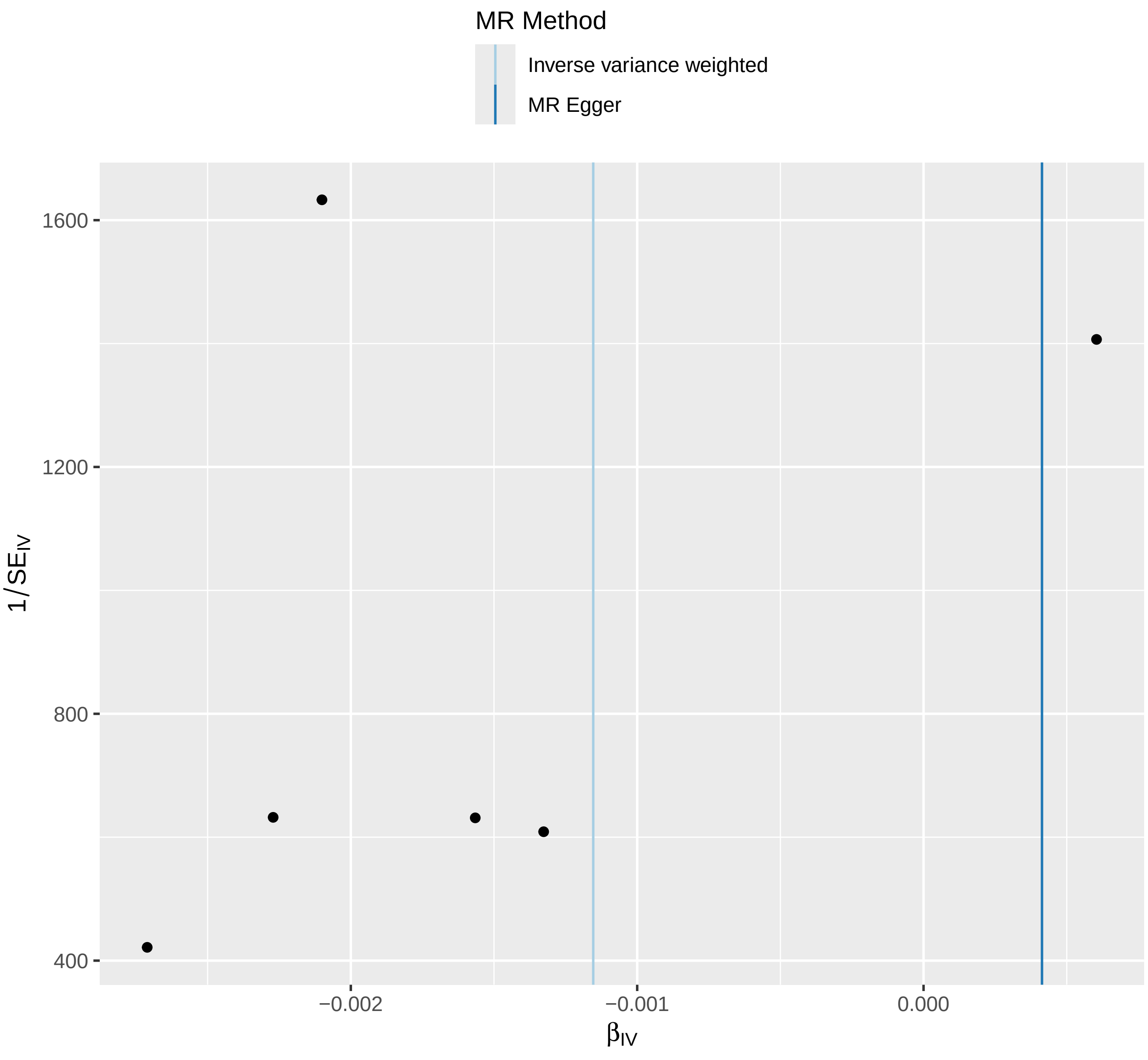
MR Method
Inverse variance weighted
MR Egger
1600
1200
800
400
1/SE$_{IV}$
−0.002
−0.001
0.000
β$_{IV}$

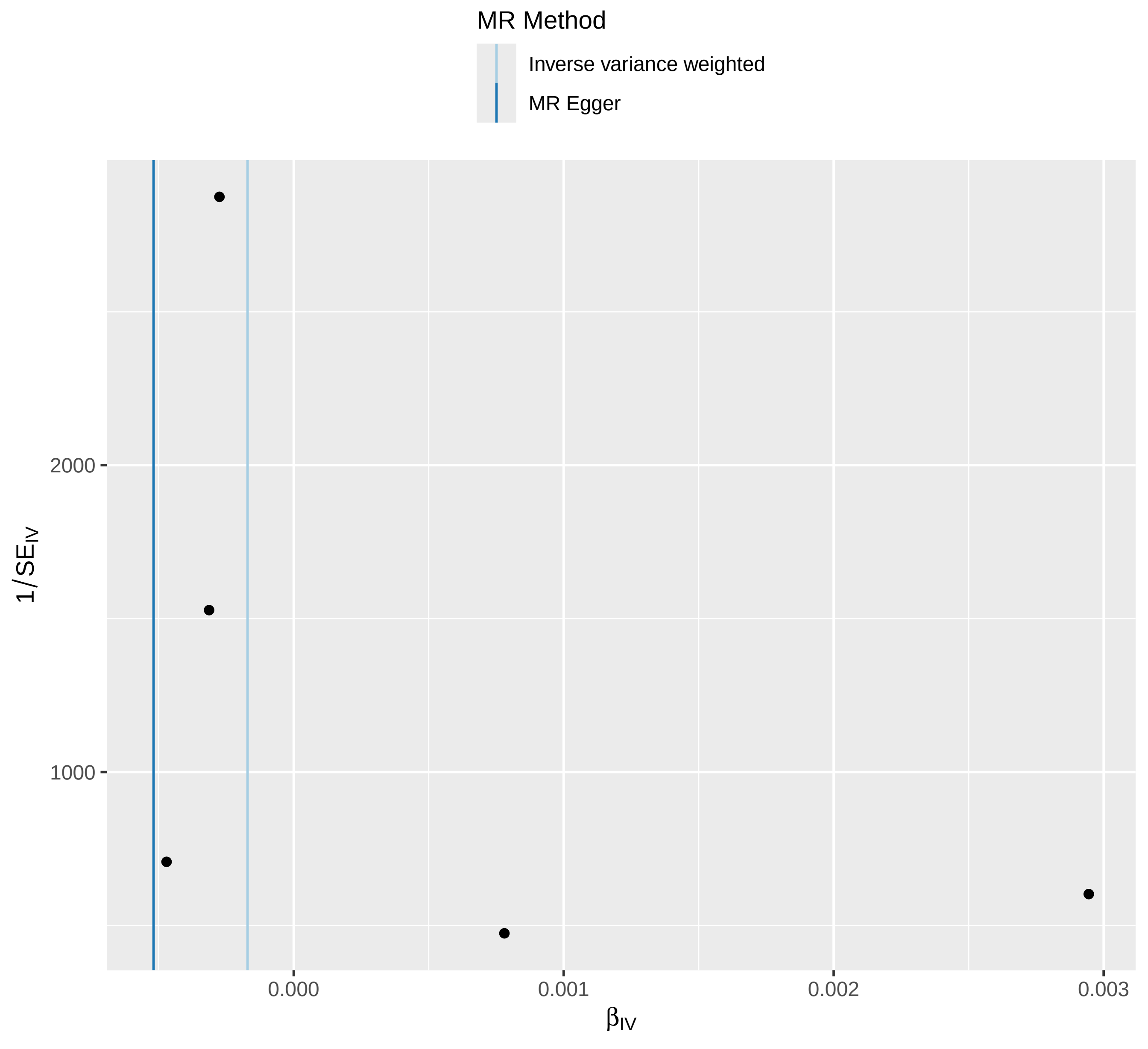
MR Method
Inverse variance weighted
MR Egger
2000
1000
1/SE_IV
0.000
0.001
0.002
0.003
β_IV

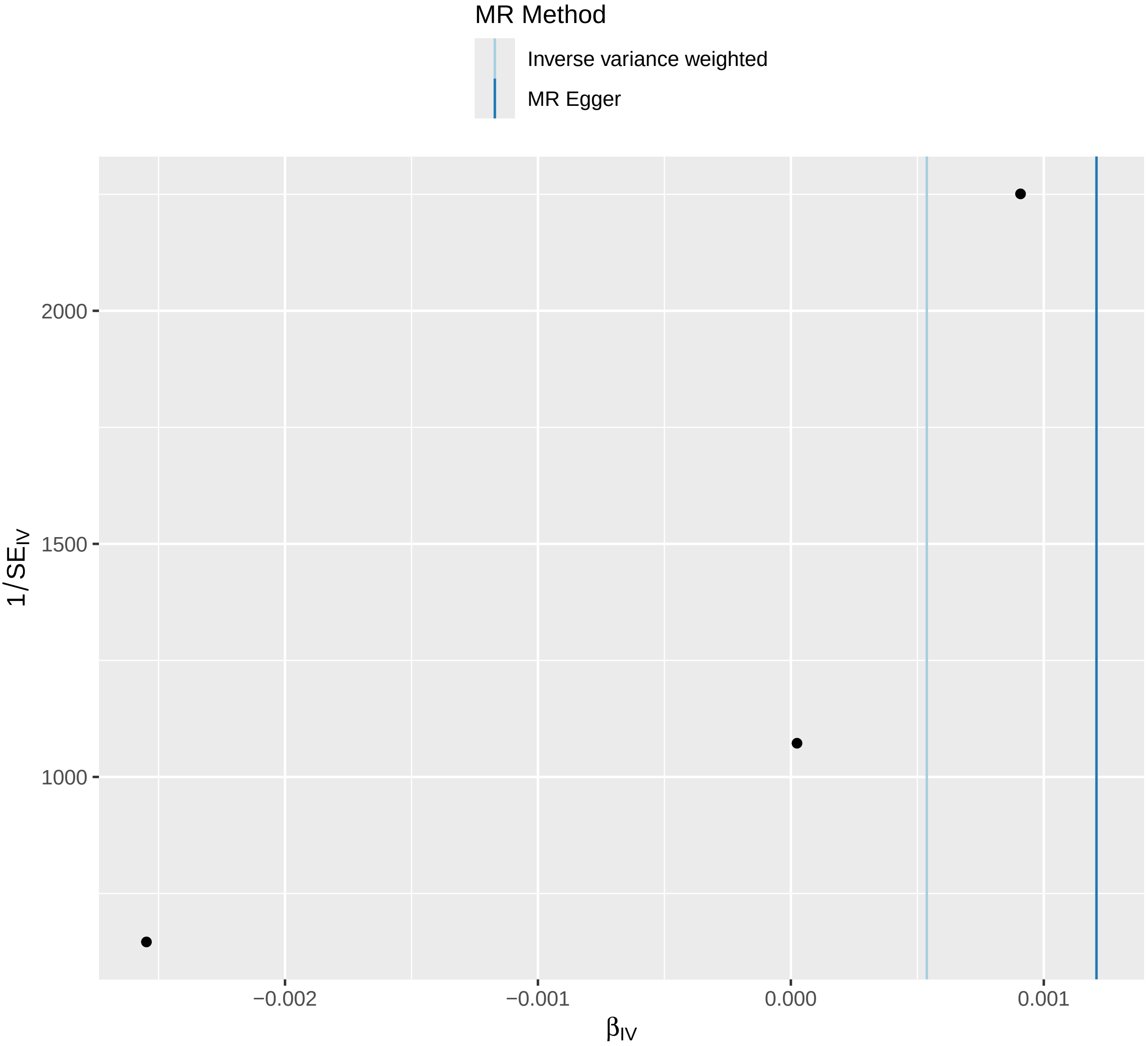

MR Method
Inverse variance weighted
MR Egger
2000
1500
1000
1/SE$_{IV}$
−0.002
−0.001
0.000
0.001
β$_{IV}$

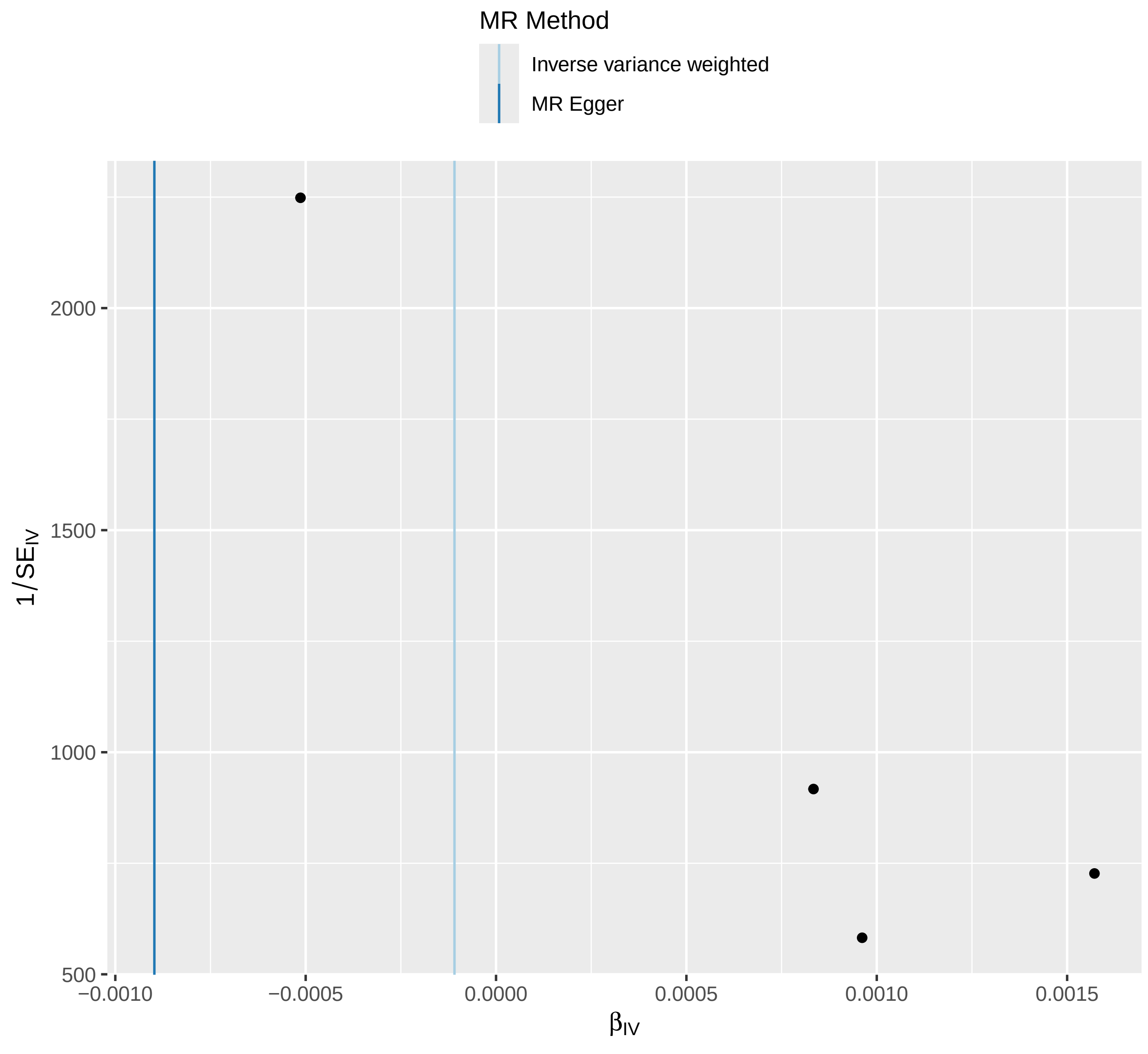

MR Method
Inverse variance weighted
MR Egger
2000
1500
1000
500
1/SE$_{IV}$
−0.0010
−0.0005
0.0000
0.0005
0.0010
0.0015
β$_{IV}$

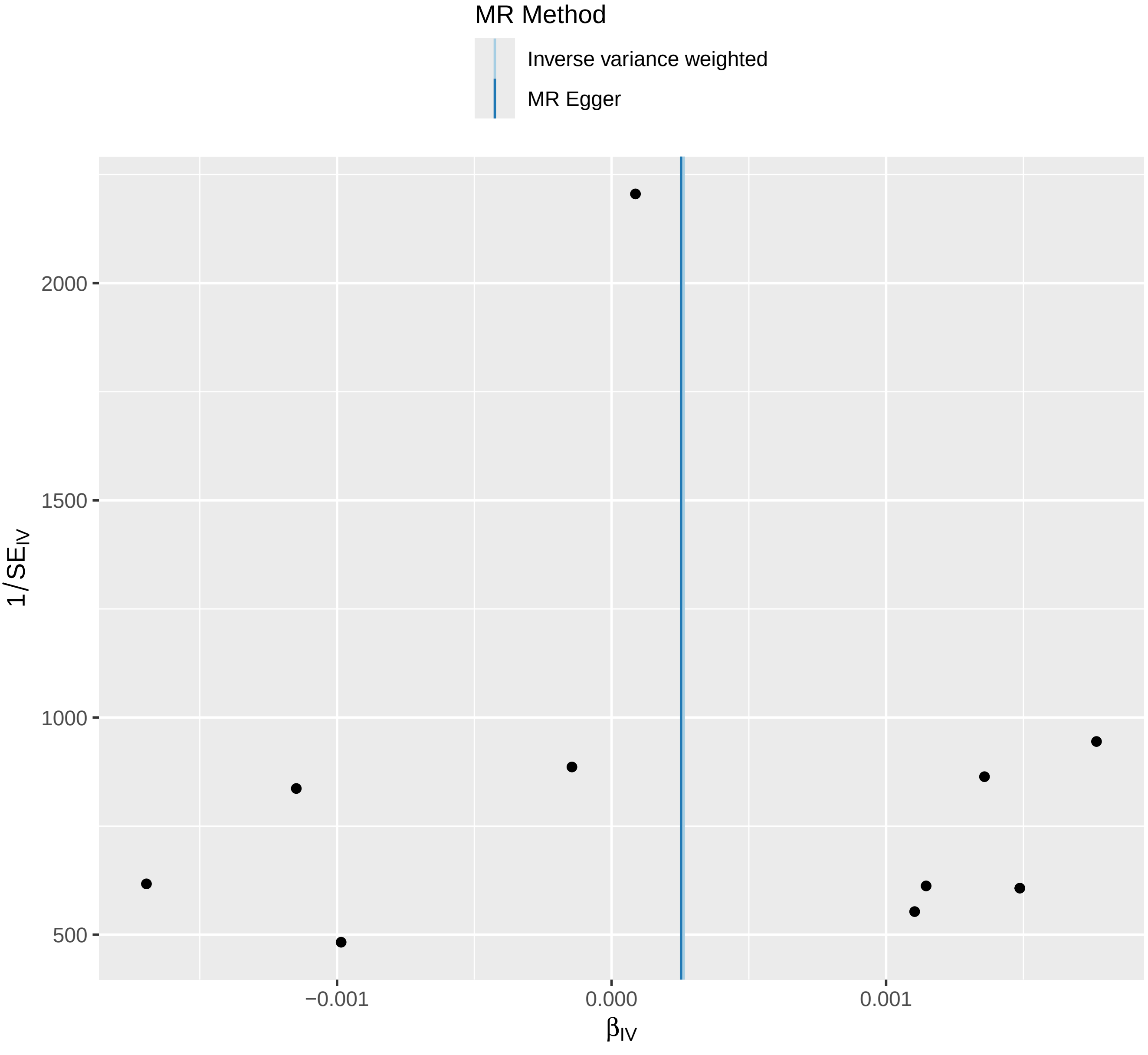

MR Method
Inverse variance weighted
MR Egger
2000
1500
1000
500
1/SE_IV
−0.001
0.000
0.001
β_IV

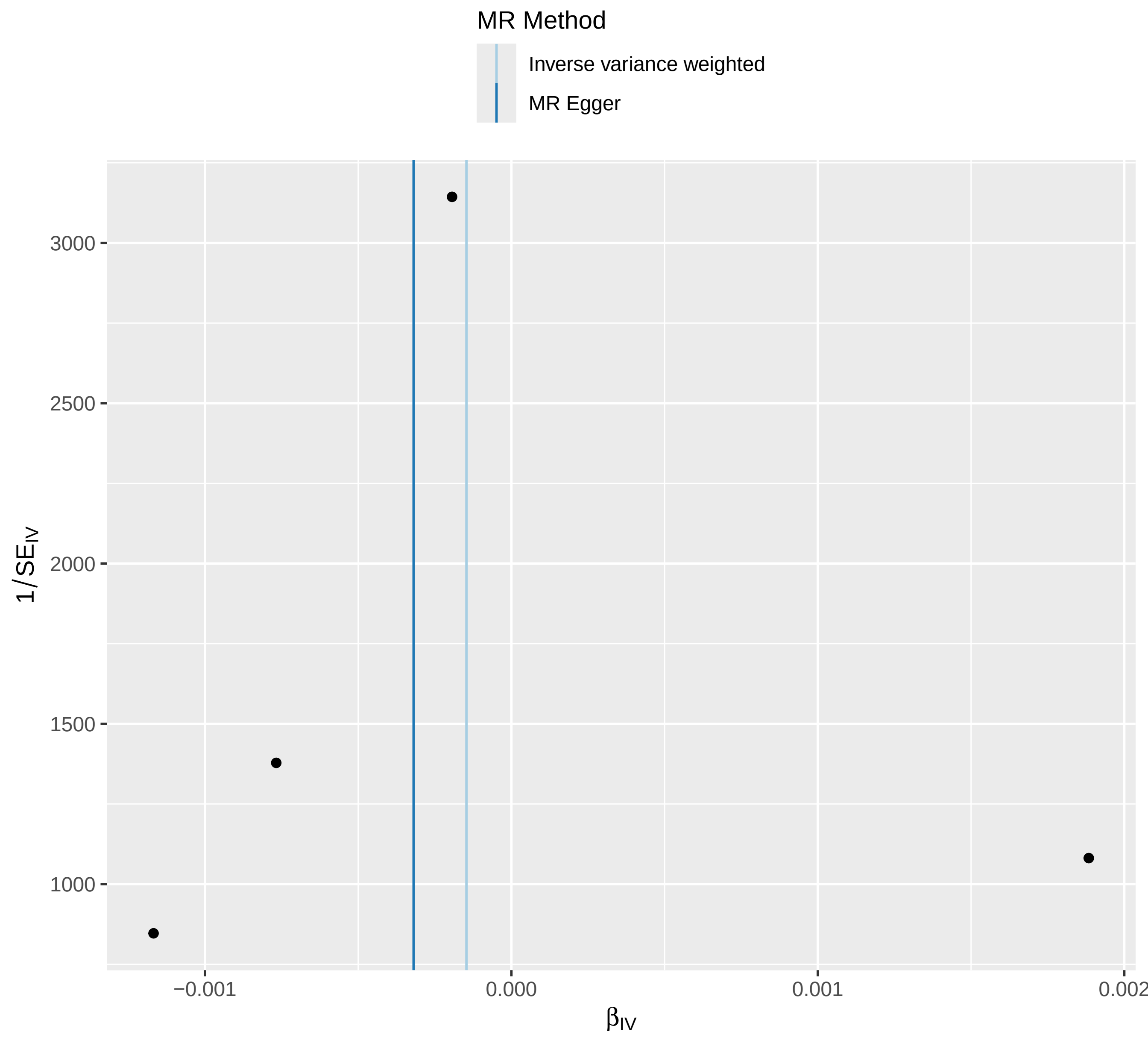
MR Method
Inverse variance weighted
MR Egger
3000
2500
2000
1500
1000
1/SE_IV
−0.001
0.000
0.001
0.002
β_IV

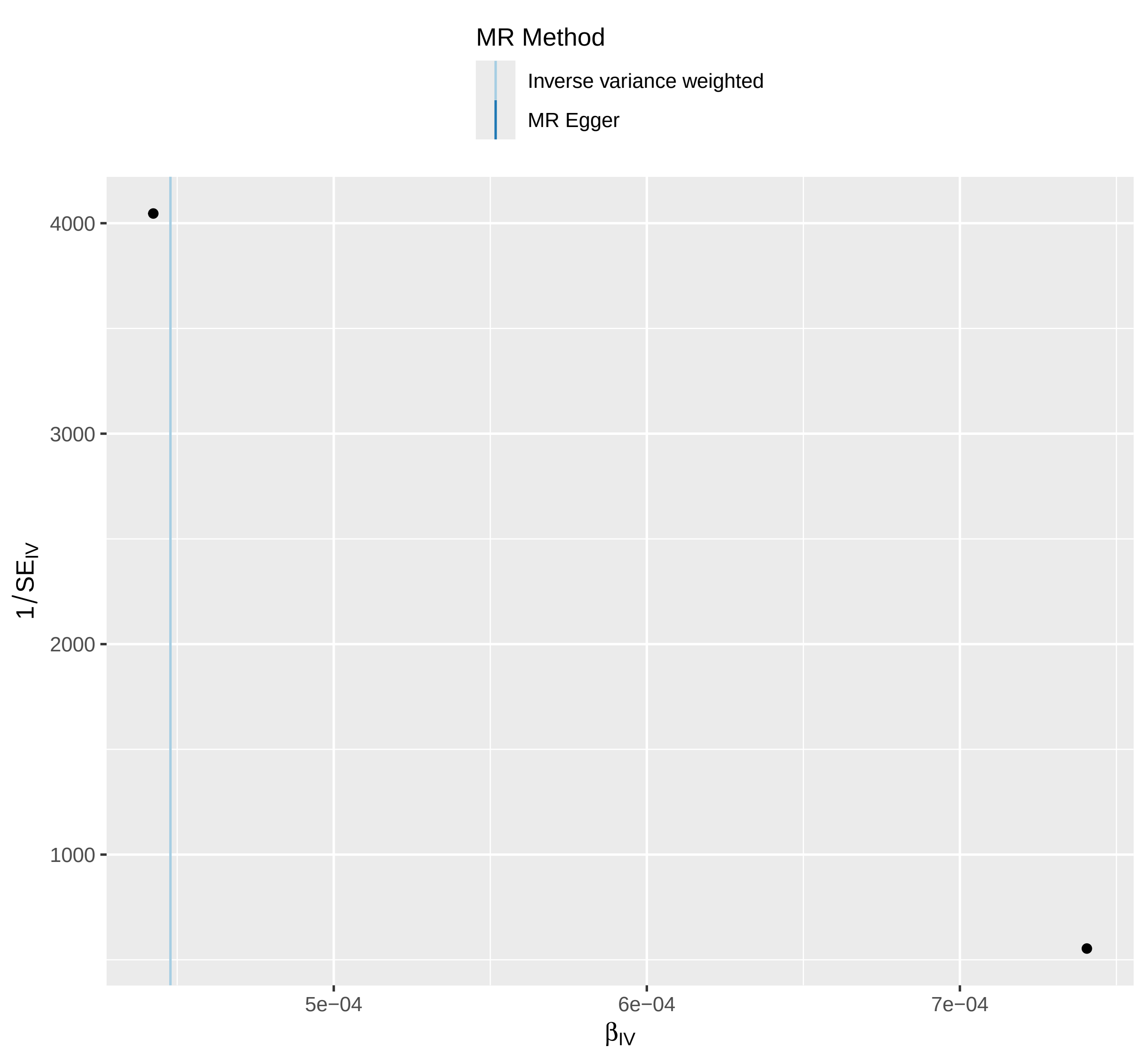
MR Method
Inverse variance weighted
MR Egger
4000
3000
2000
1000
1/SE_IV
5e−04
6e−04
7e−04
β_IV

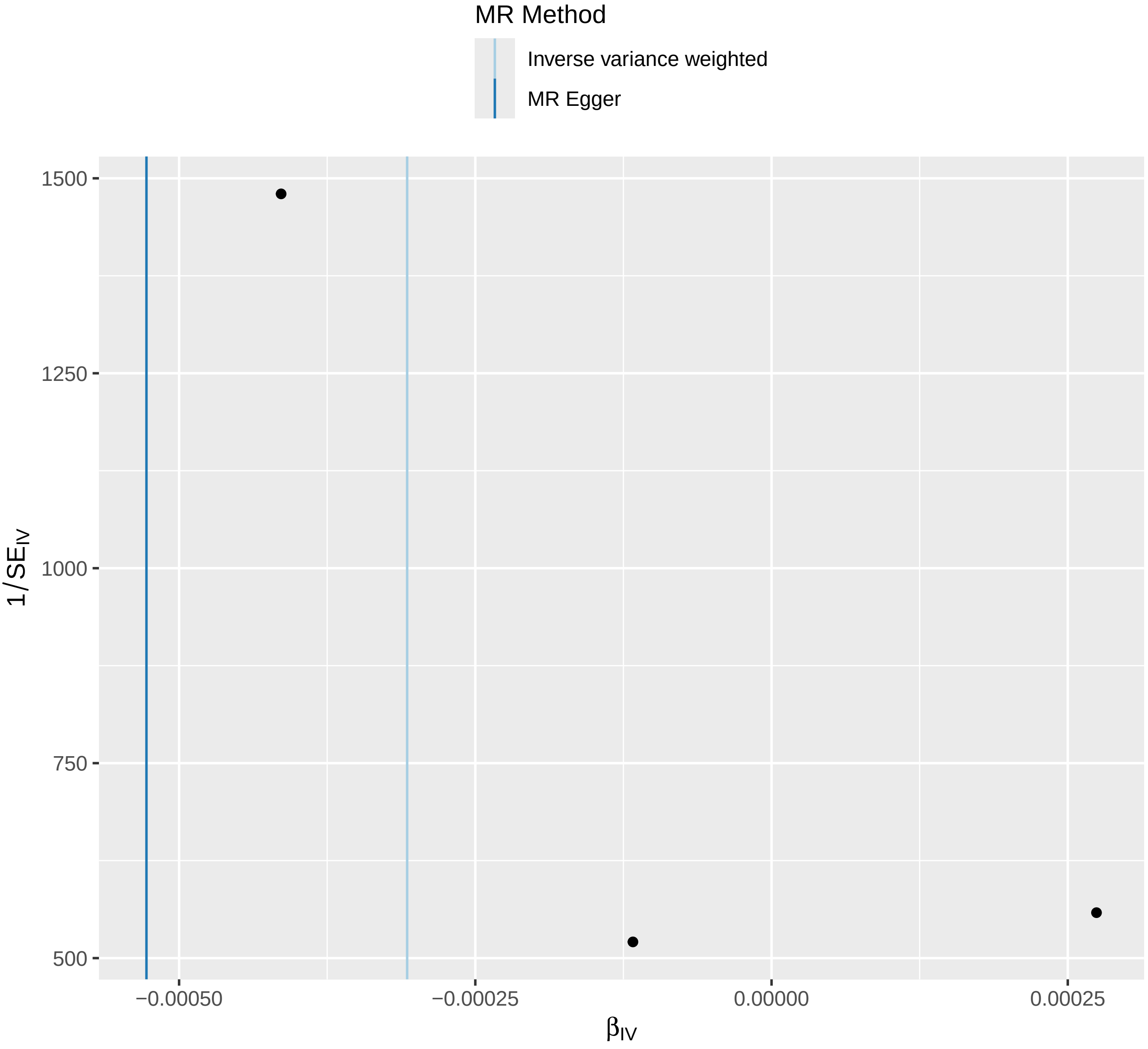
MR Method
Inverse variance weighted
MR Egger
1500
1250
1000
750
500
−0.00050
−0.00025
0.00000
0.00025
$1/SE_{IV}$
β_{IV}

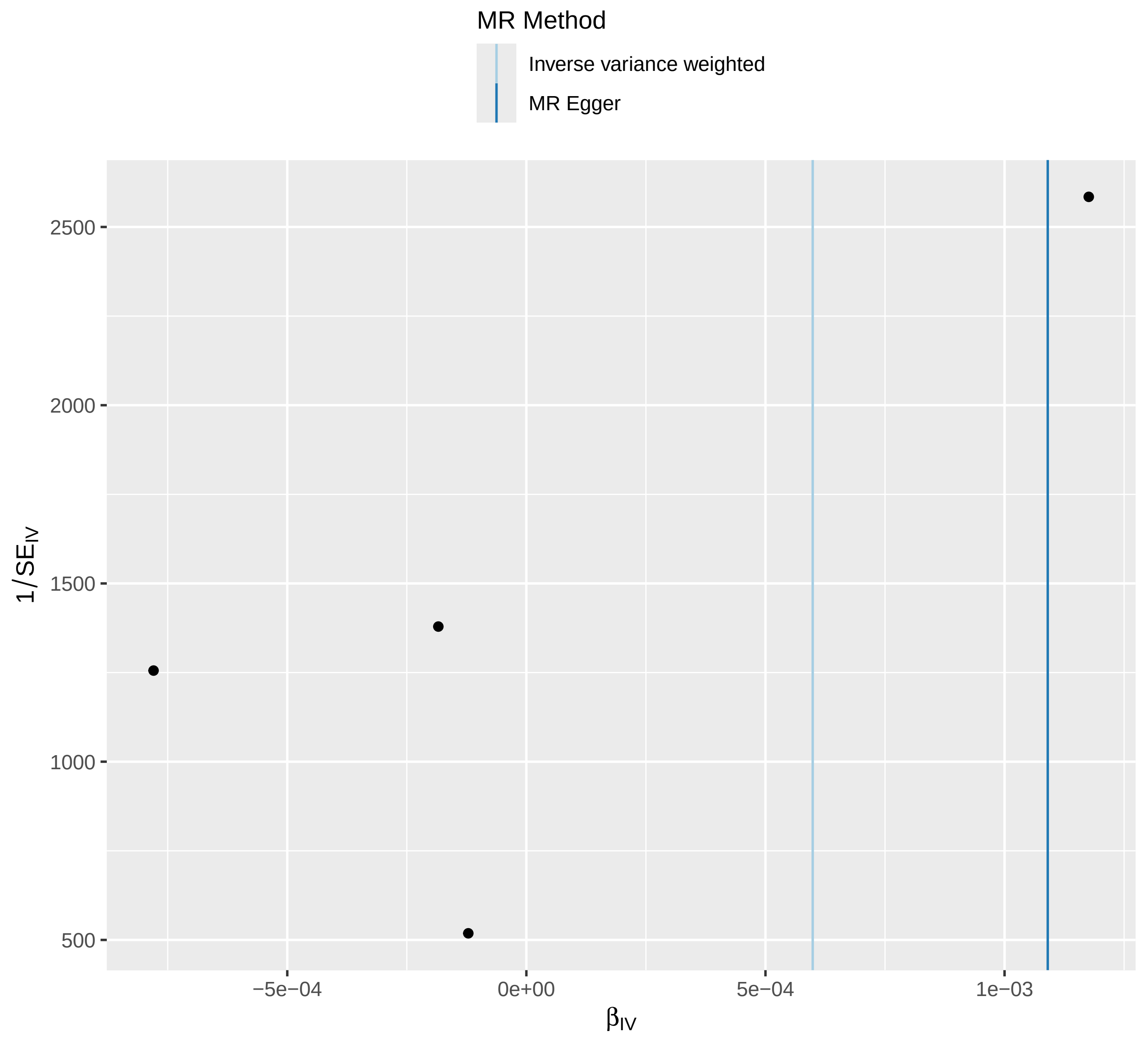
MR Method
Inverse variance weighted
MR Egger
2500
2000
1500
1000
500
1/SE_IV
−5e−04
0e+00
5e−04
1e−03
β_IV

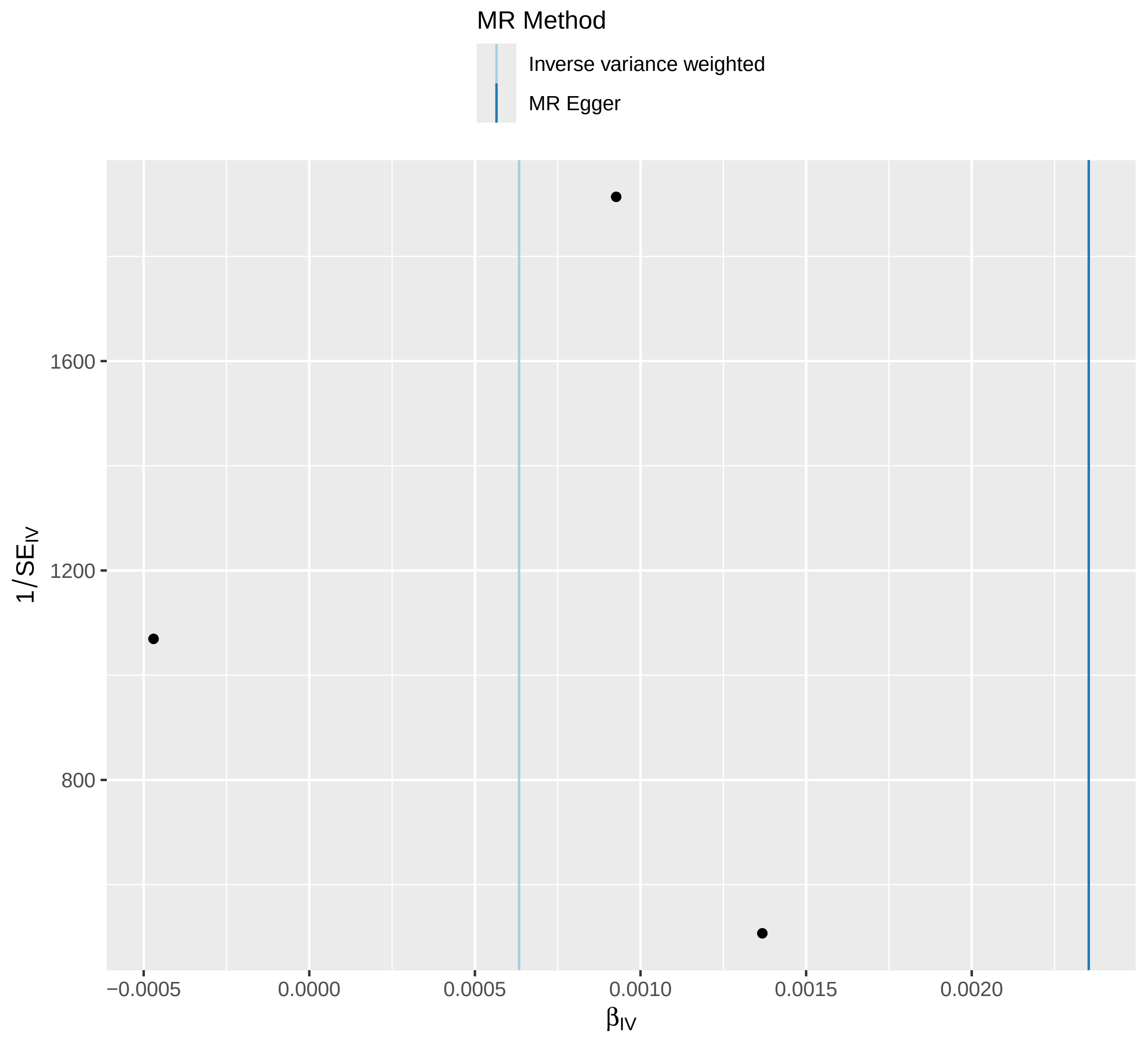
MR Method
Inverse variance weighted
MR Egger
1600
1200
800
1/SE_IV
−0.0005
0.0000
0.0005
0.0010
0.0015
0.0020
β_IV

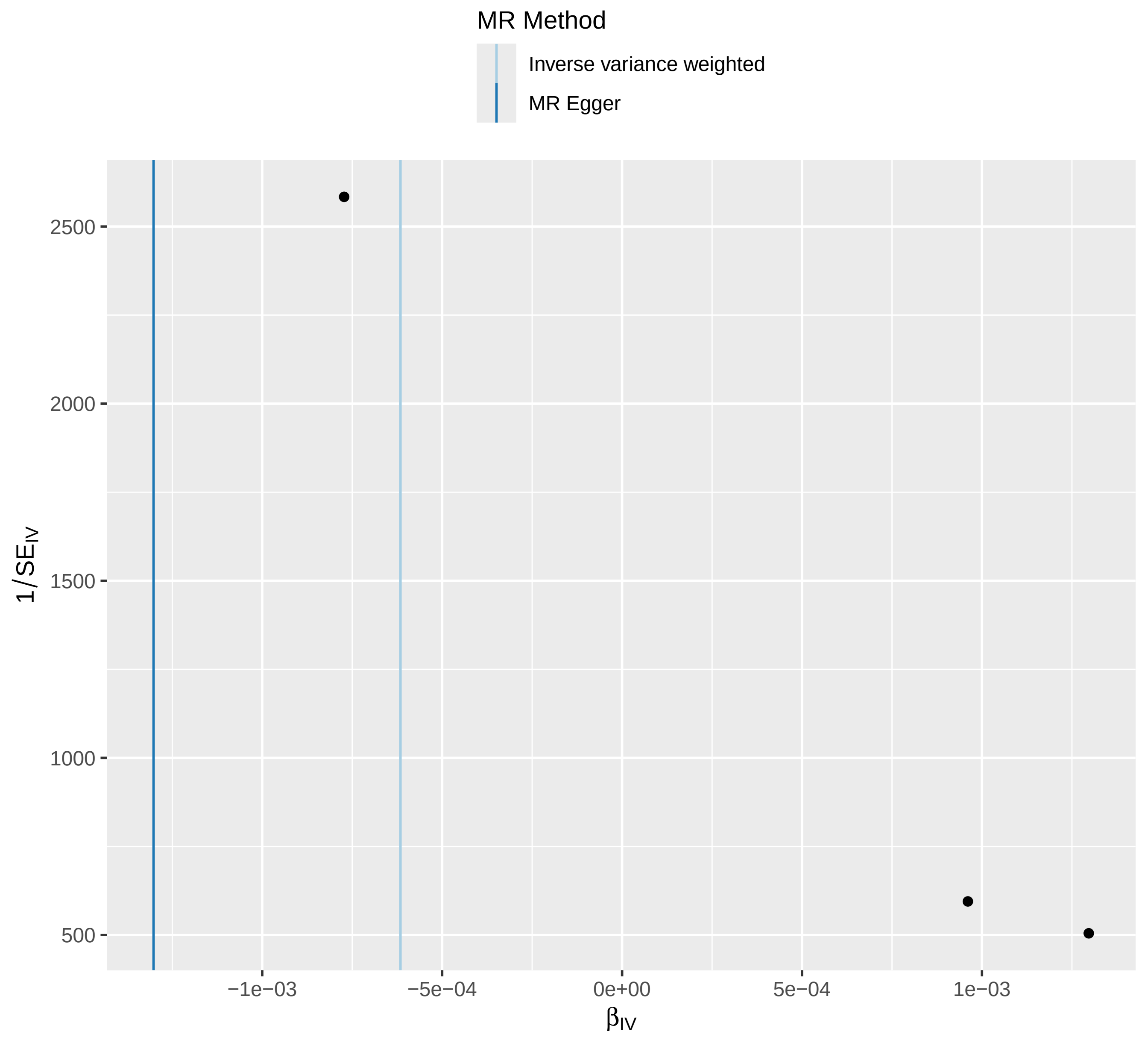

MR Method
Inverse variance weighted
MR Egger
2500
2000
1500
1000
500
1/SE$_{IV}$
−1e−03
−5e−04
0e+00
5e−04
1e−03
β$_{IV}$

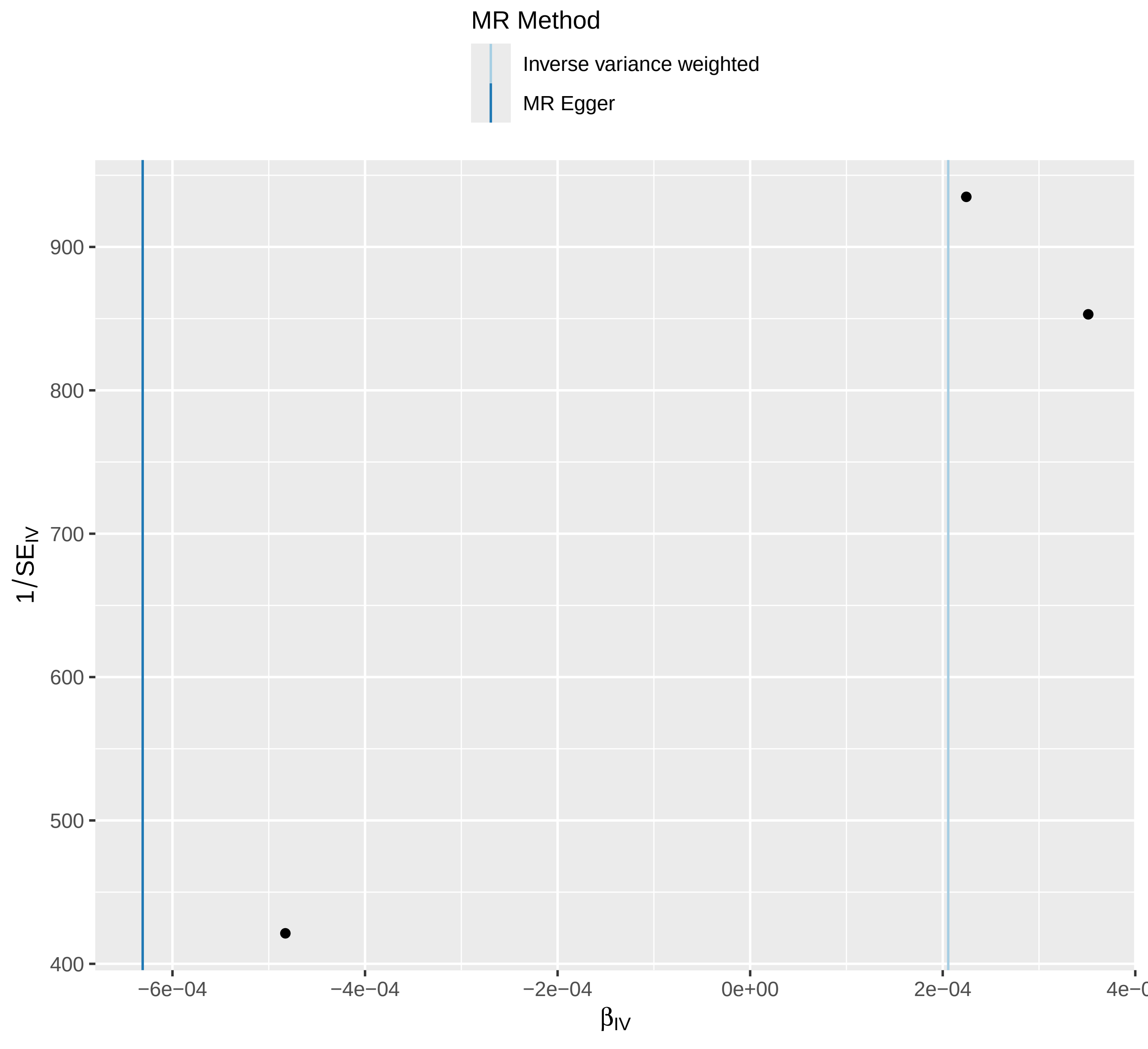
MR Method
Inverse variance weighted
MR Egger
900
800
700
600
500
400
1/SE_IV
−6e−04
−4e−04
−2e−04
0e+00
2e−04
4e−0
β_IV

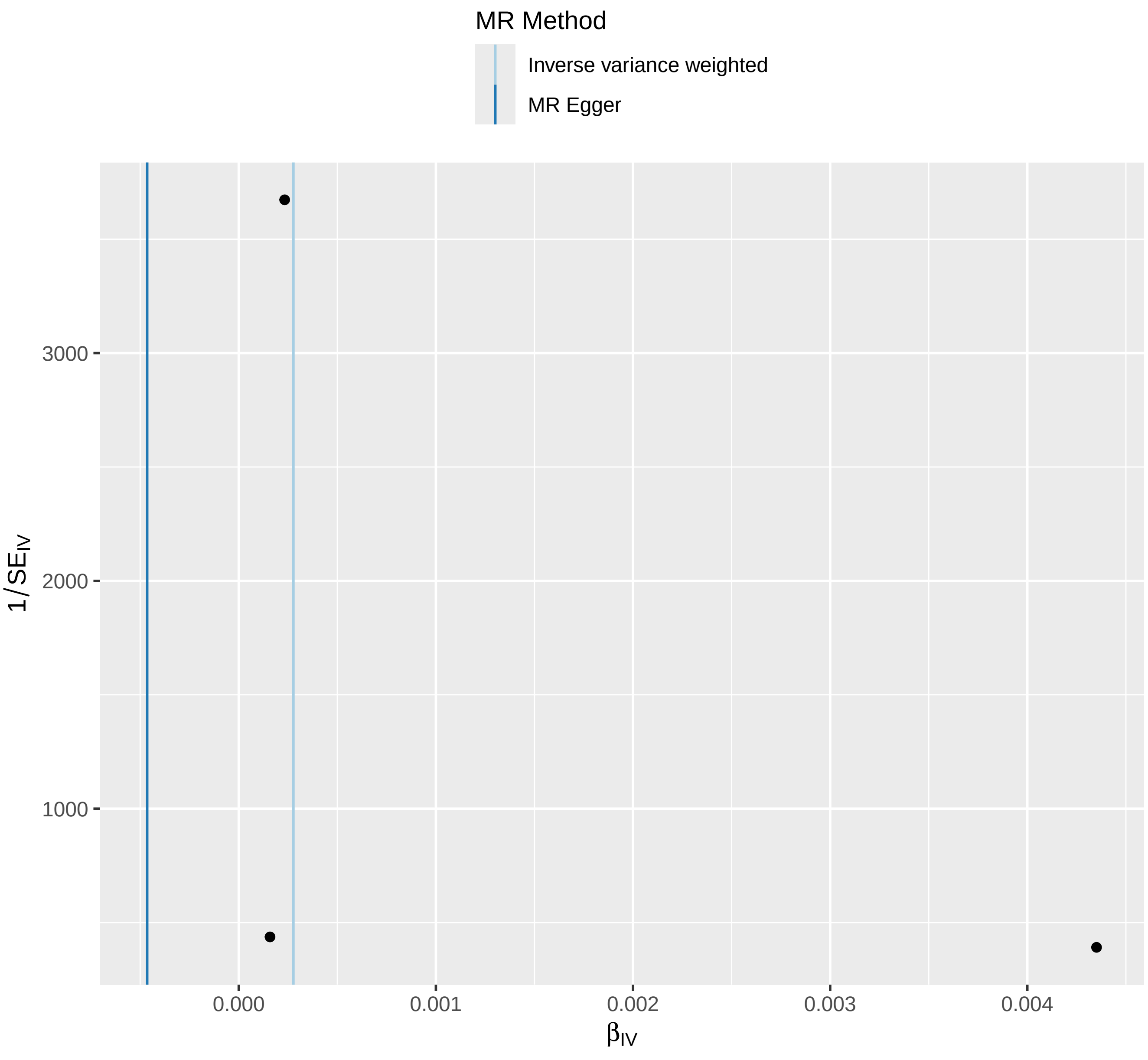
MR Method
Inverse variance weighted
MR Egger
3000
2000
1000
1/SE_IV
0.000
0.001
0.002
0.003
0.004
β_IV

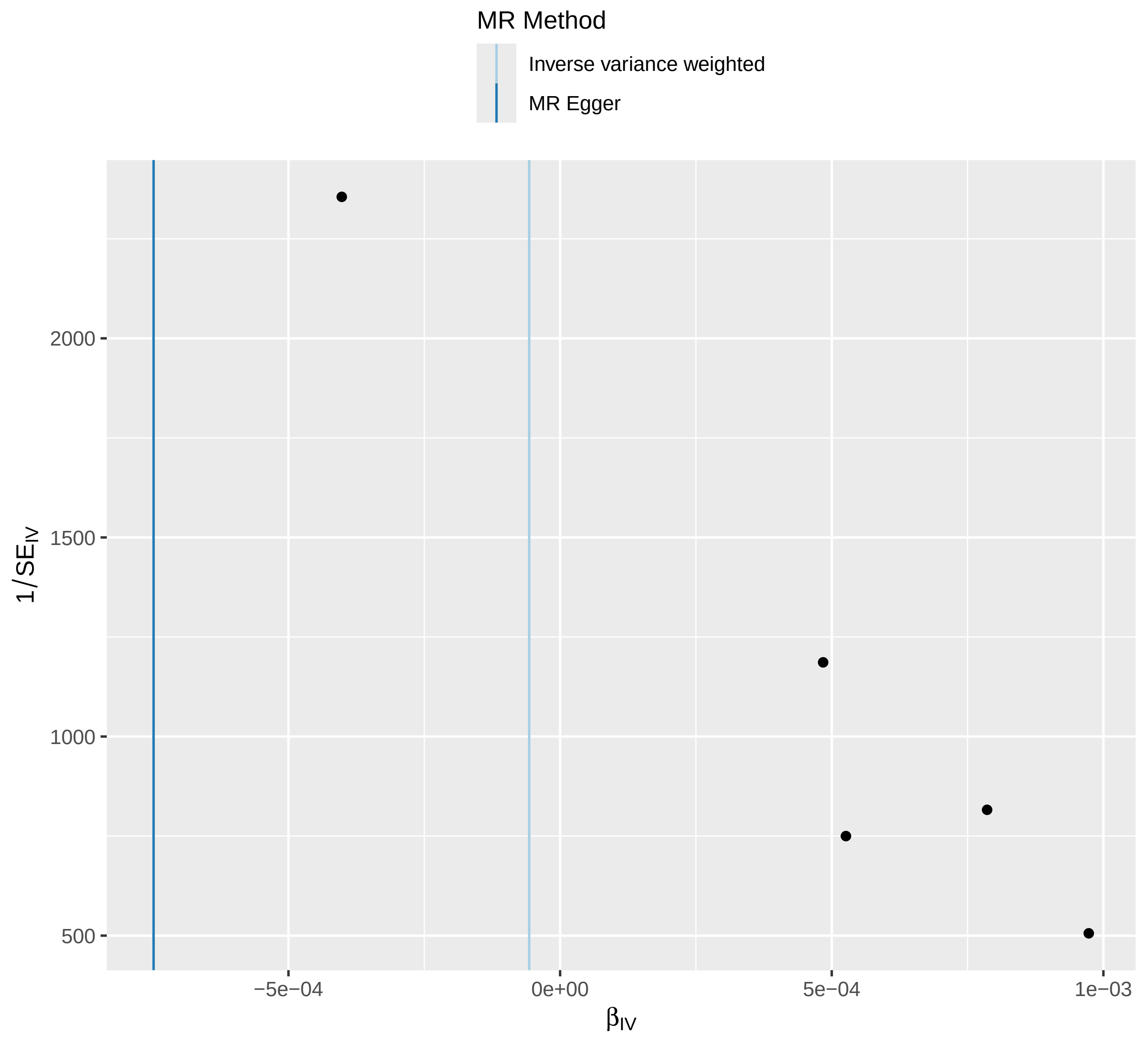
MR Method
Inverse variance weighted
MR Egger
2000
1500
1000
500
1/SE_IV
−5e−04
0e+00
5e−04
1e−03
β_IV

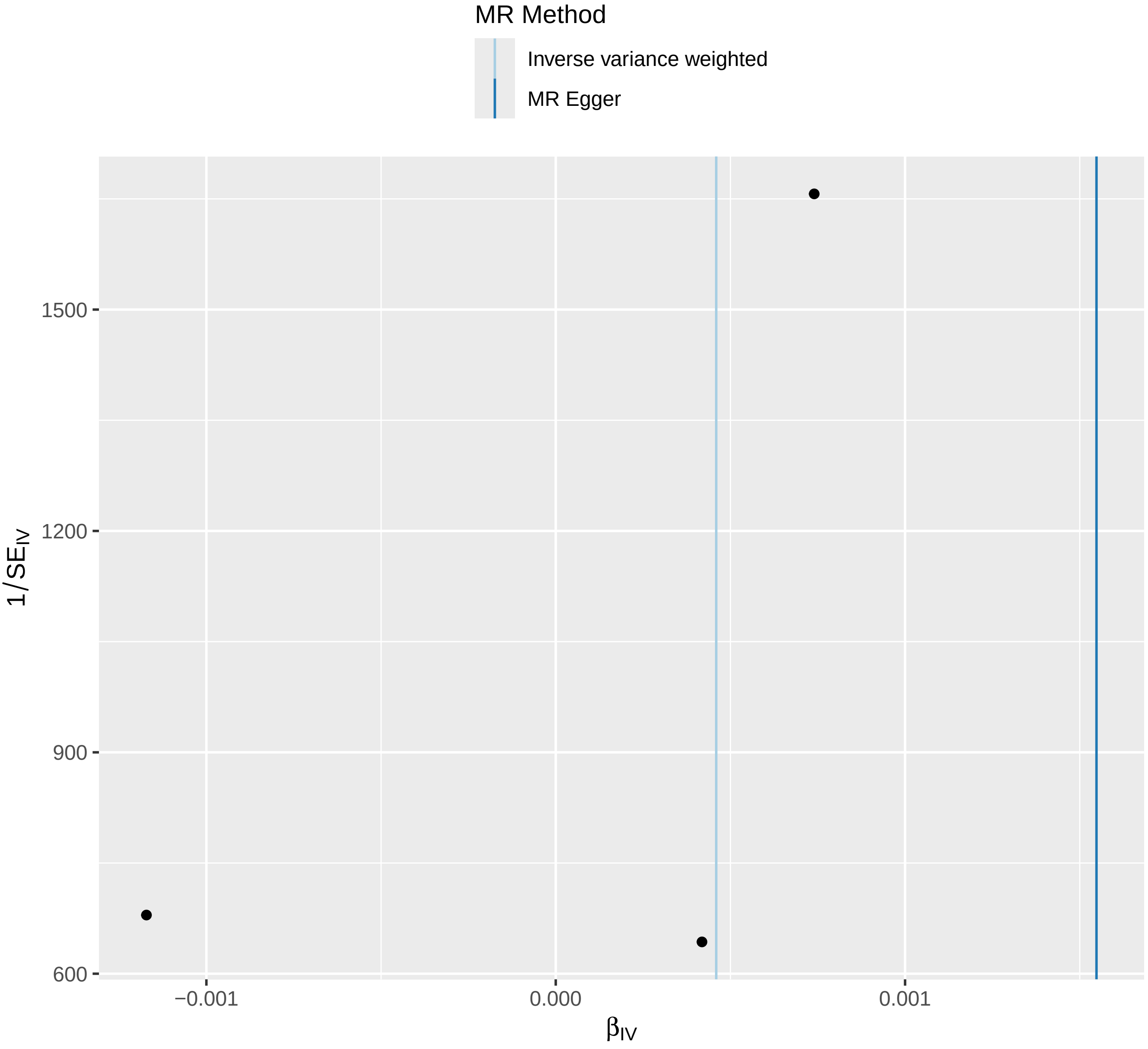

MR Method
Inverse variance weighted
MR Egger
1500
1200
900
600
−0.001
0.000
0.001
1/SE_IV
β_IV

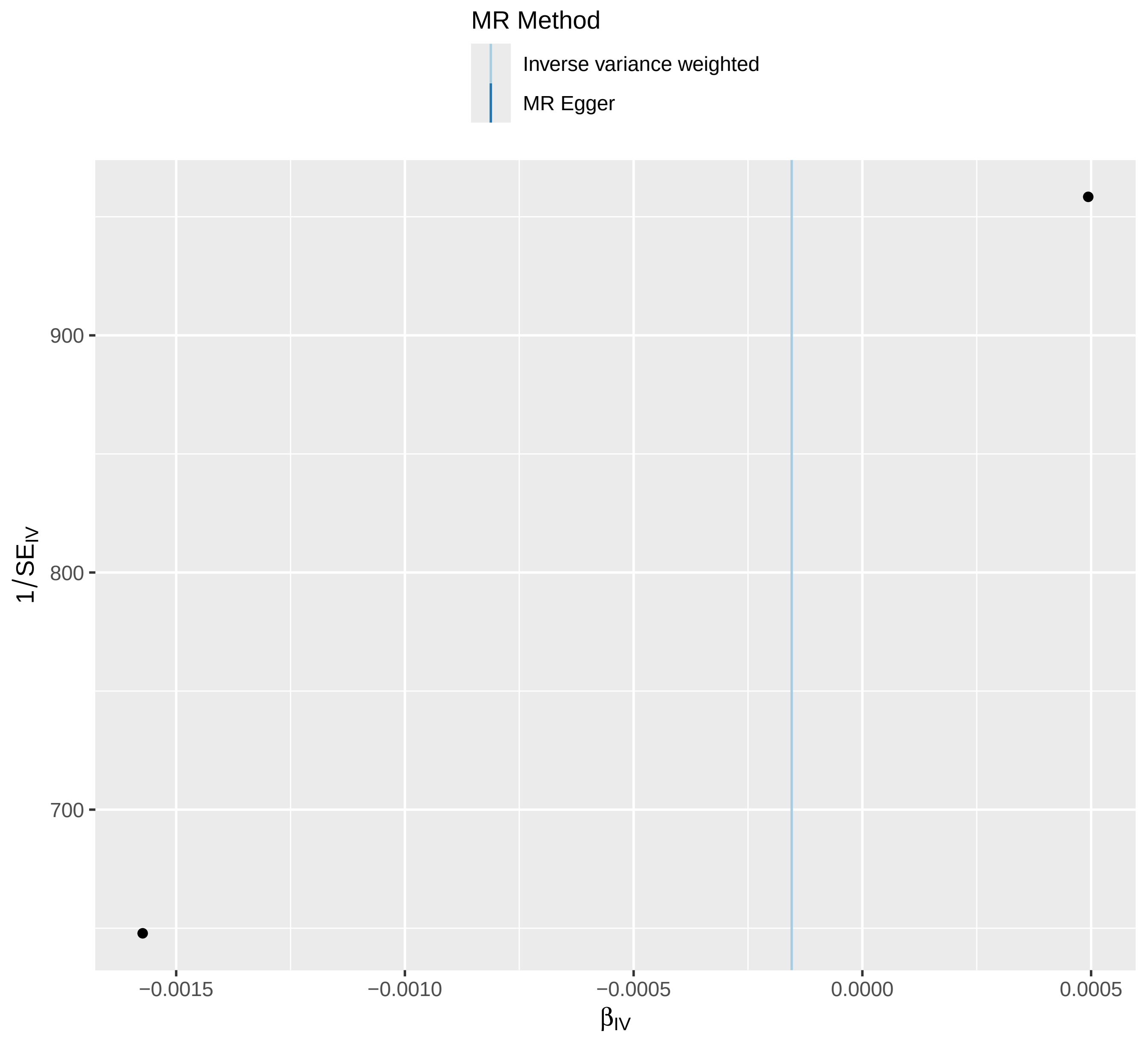
MR Method
Inverse variance weighted
MR Egger
900
800
700
1/SE_IV
−0.0015
−0.0010
−0.0005
0.0000
0.0005
β_IV

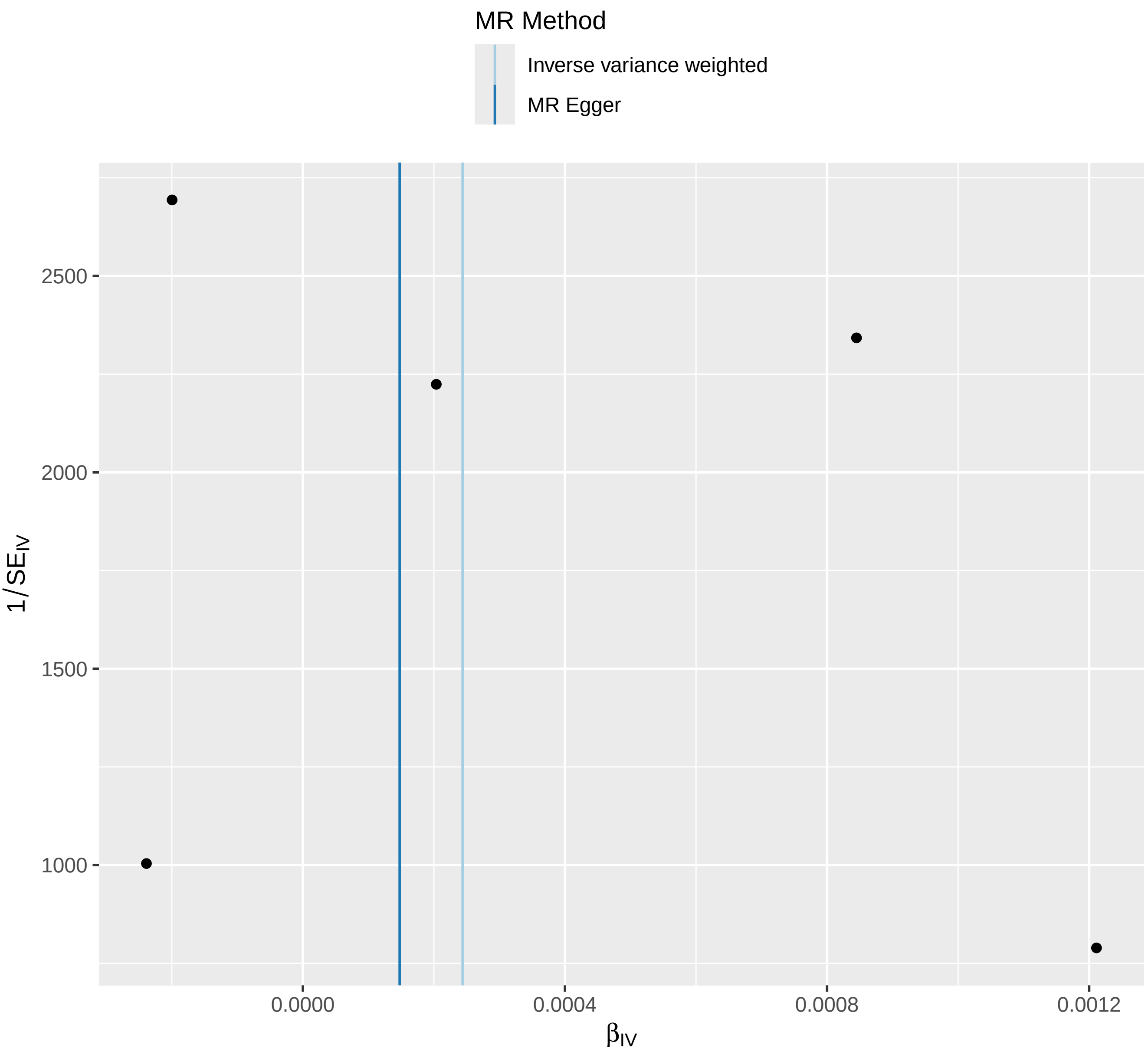
MR Method
Inverse variance weighted
MR Egger
2500
2000
1500
1000
1/SE$_{IV}$
0.0000
0.0004
0.0008
0.0012
β$_{IV}$

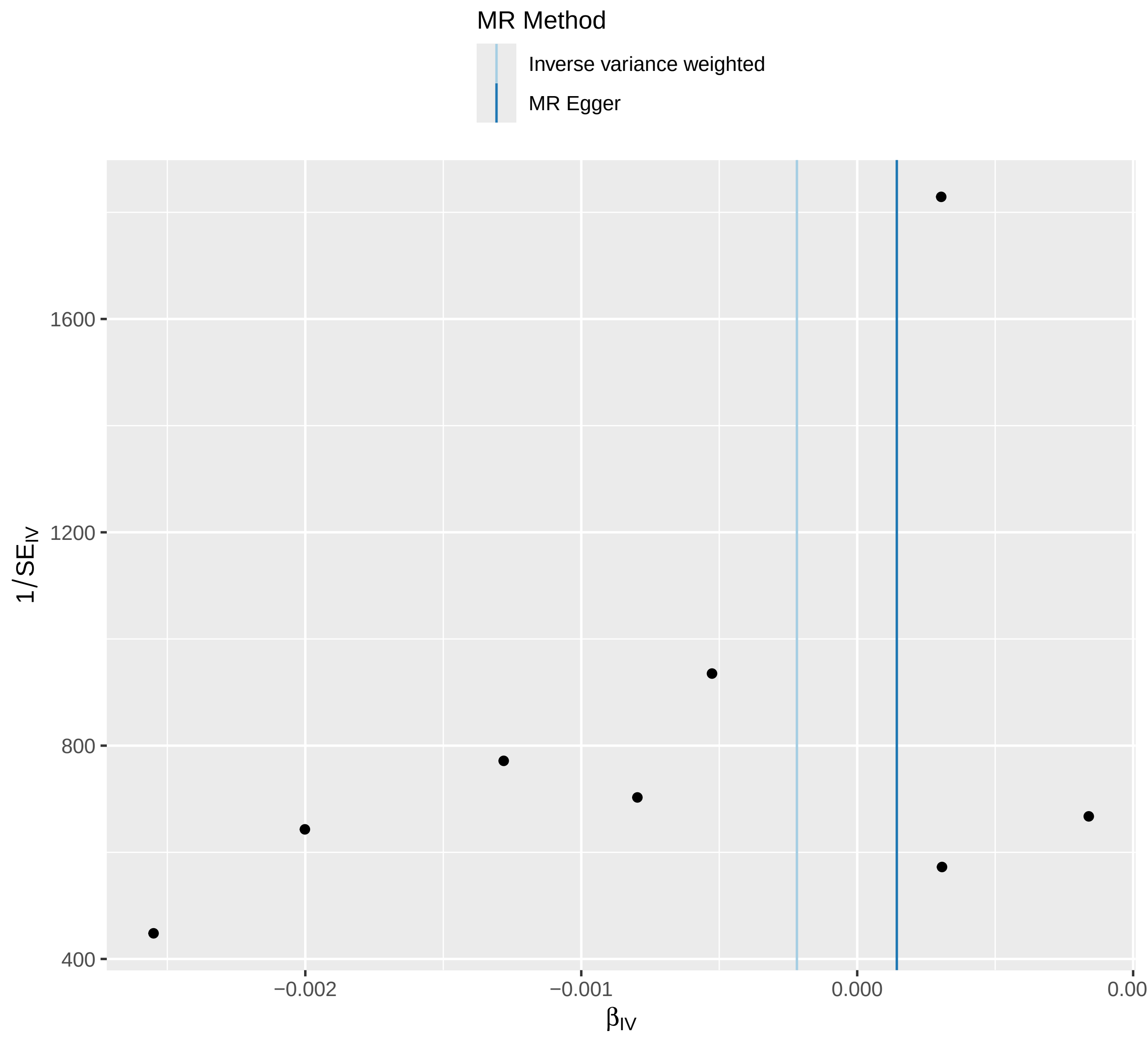

MR Method
Inverse variance weighted
MR Egger
1600
1200
800
400
1/SE_IV
−0.002
−0.001
0.000
0.00
β_IV

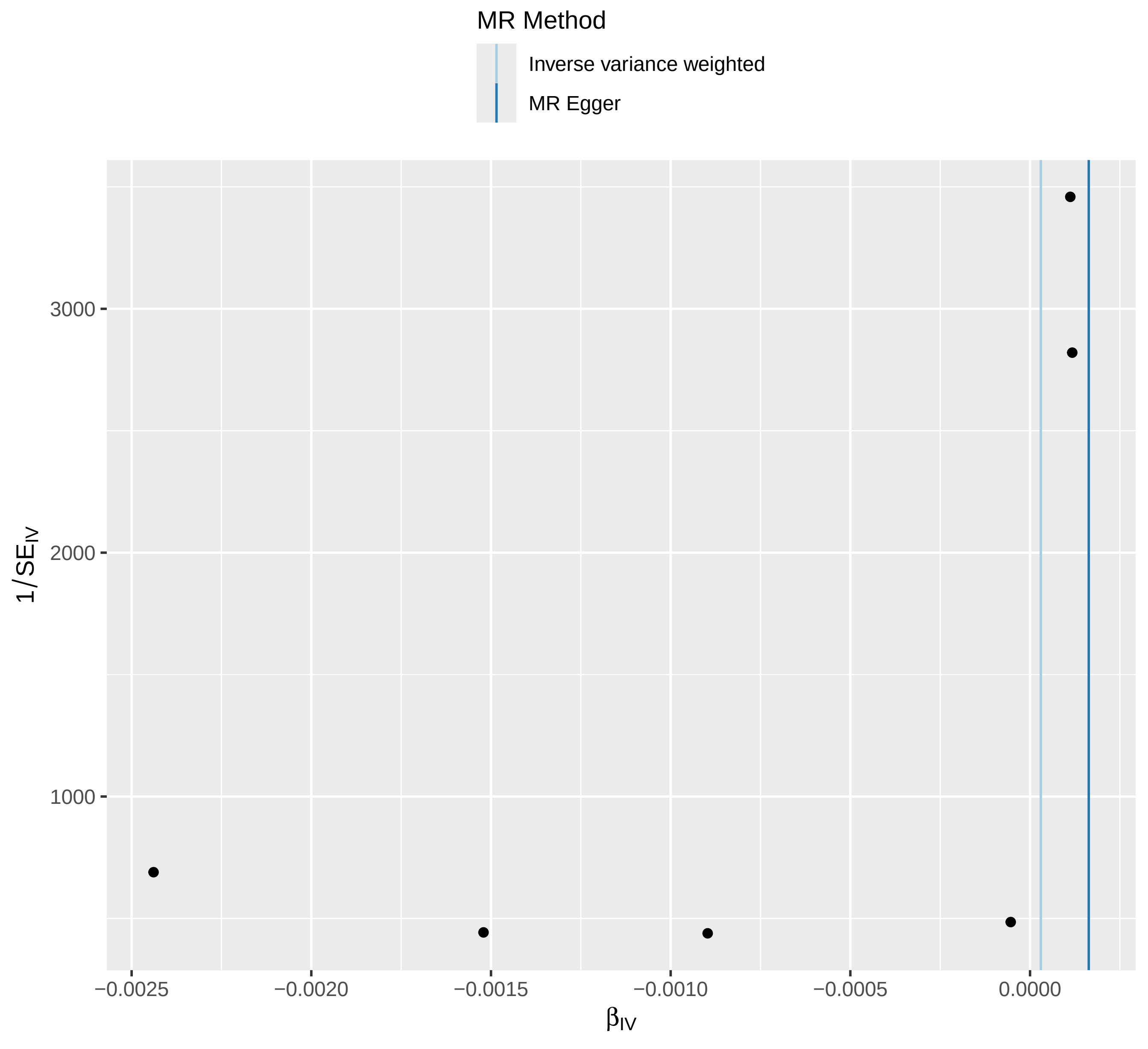
MR Method
Inverse variance weighted
MR Egger
3000
2000
1000
1/SE_IV
−0.0025
−0.0020
−0.0015
−0.0010
−0.0005
0.0000
β_IV

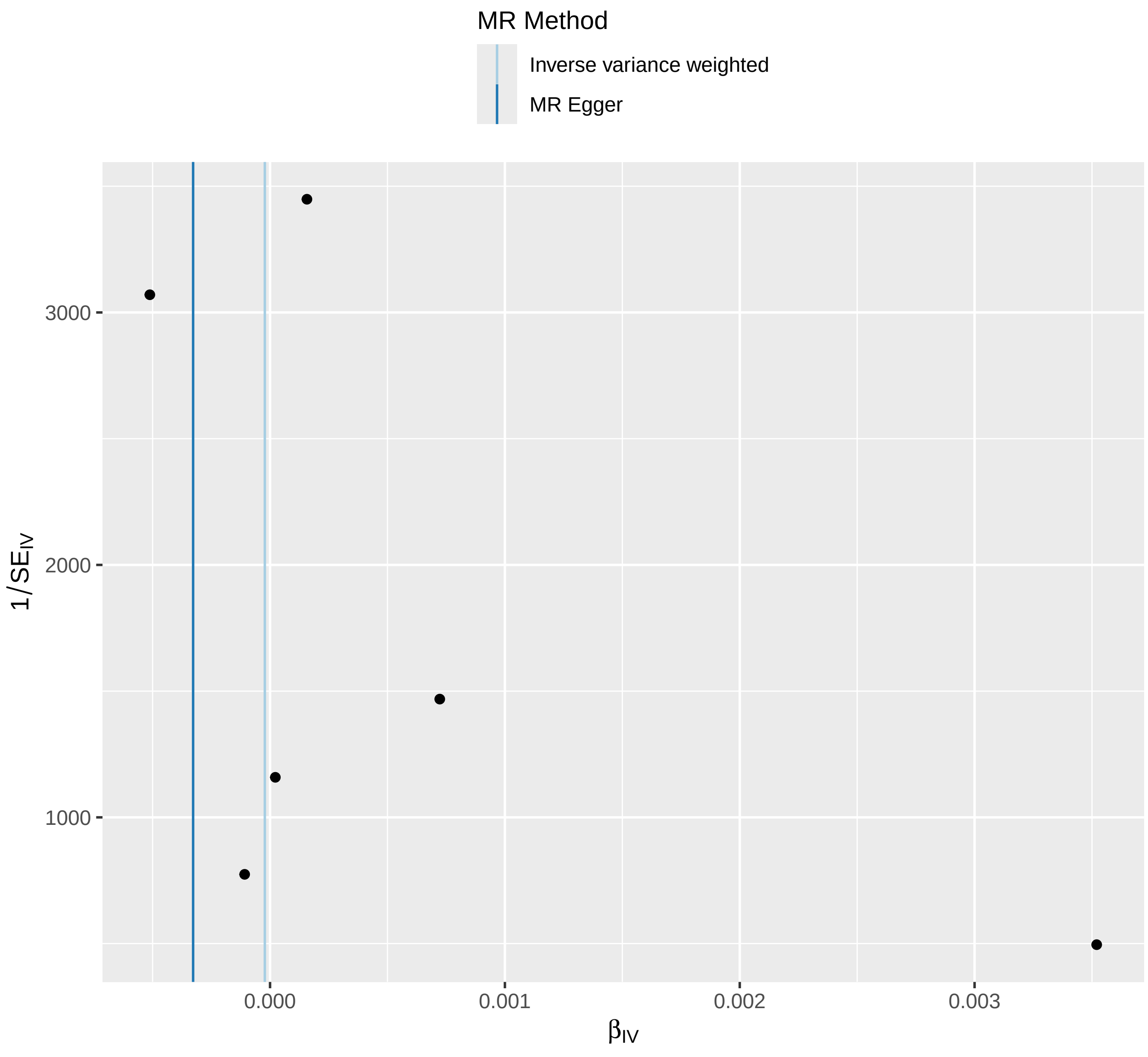
MR Method
Inverse variance weighted
MR Egger
3000
2000
1000
1/SE_IV
0.000
0.001
0.002
0.003
β_IV

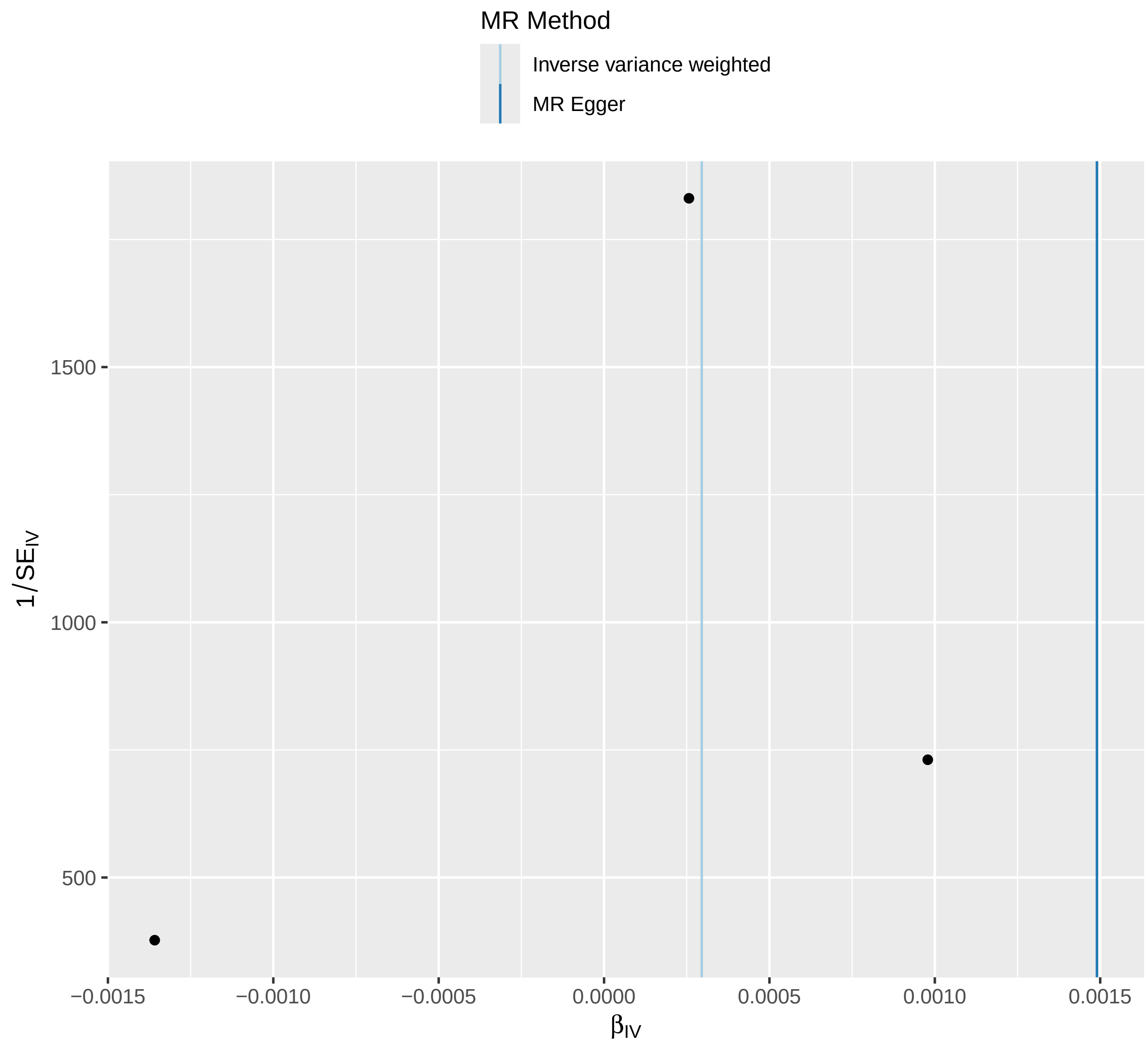
MR Method
Inverse variance weighted
MR Egger
1/SE$_{IV}$
1500
1000
500
−0.0015
−0.0010
−0.0005
0.0000
0.0005
0.0010
0.0015
β$_{IV}$

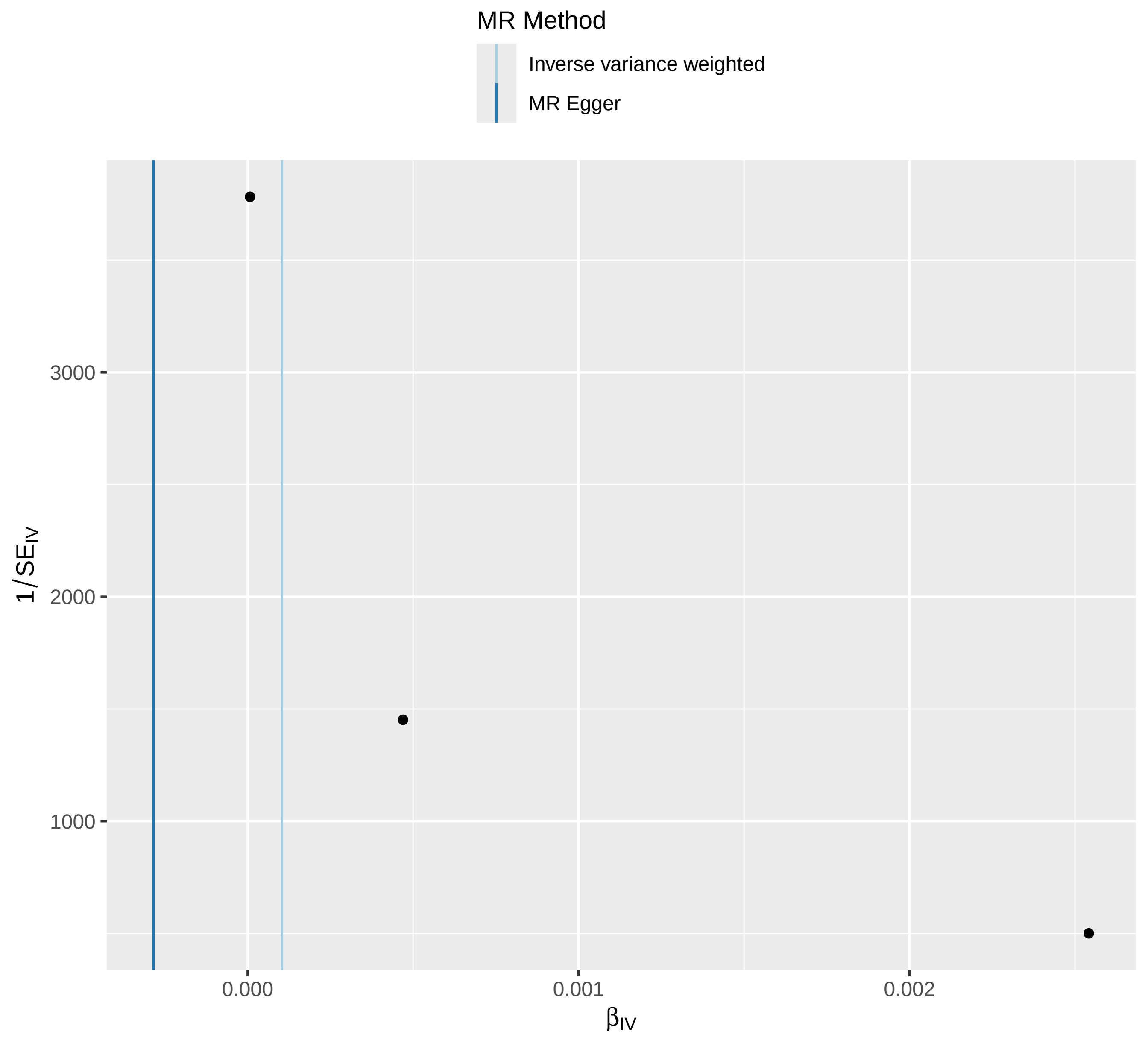

MR Method
Inverse variance weighted
MR Egger
3000
2000
1000
1/SE_IV
0.000
0.001
0.002
β_IV

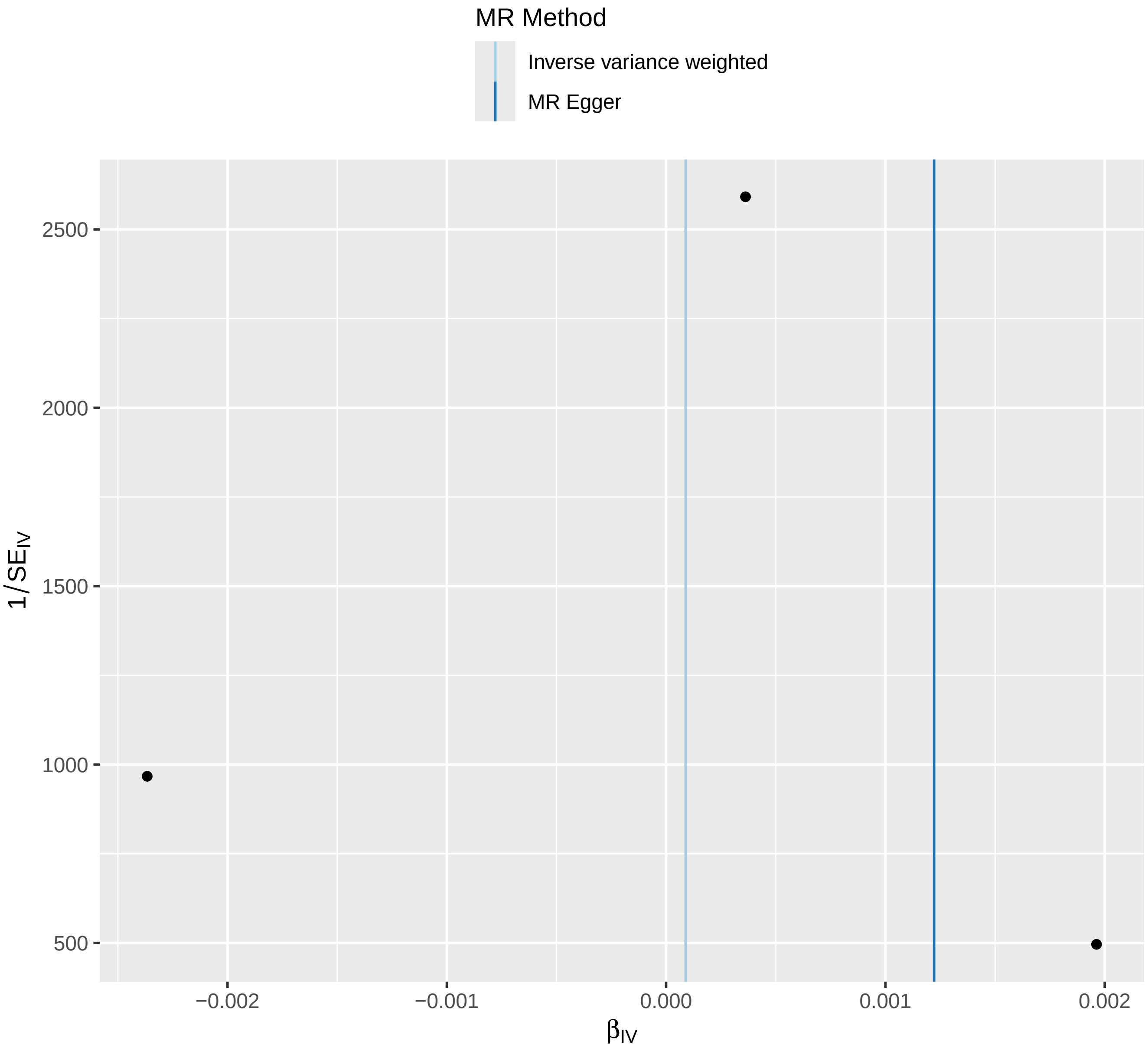
MR Method
Inverse variance weighted
MR Egger
1/SE_IV
β_IV
2500
2000
1500
1000
500
−0.002
−0.001
0.000
0.001
0.002

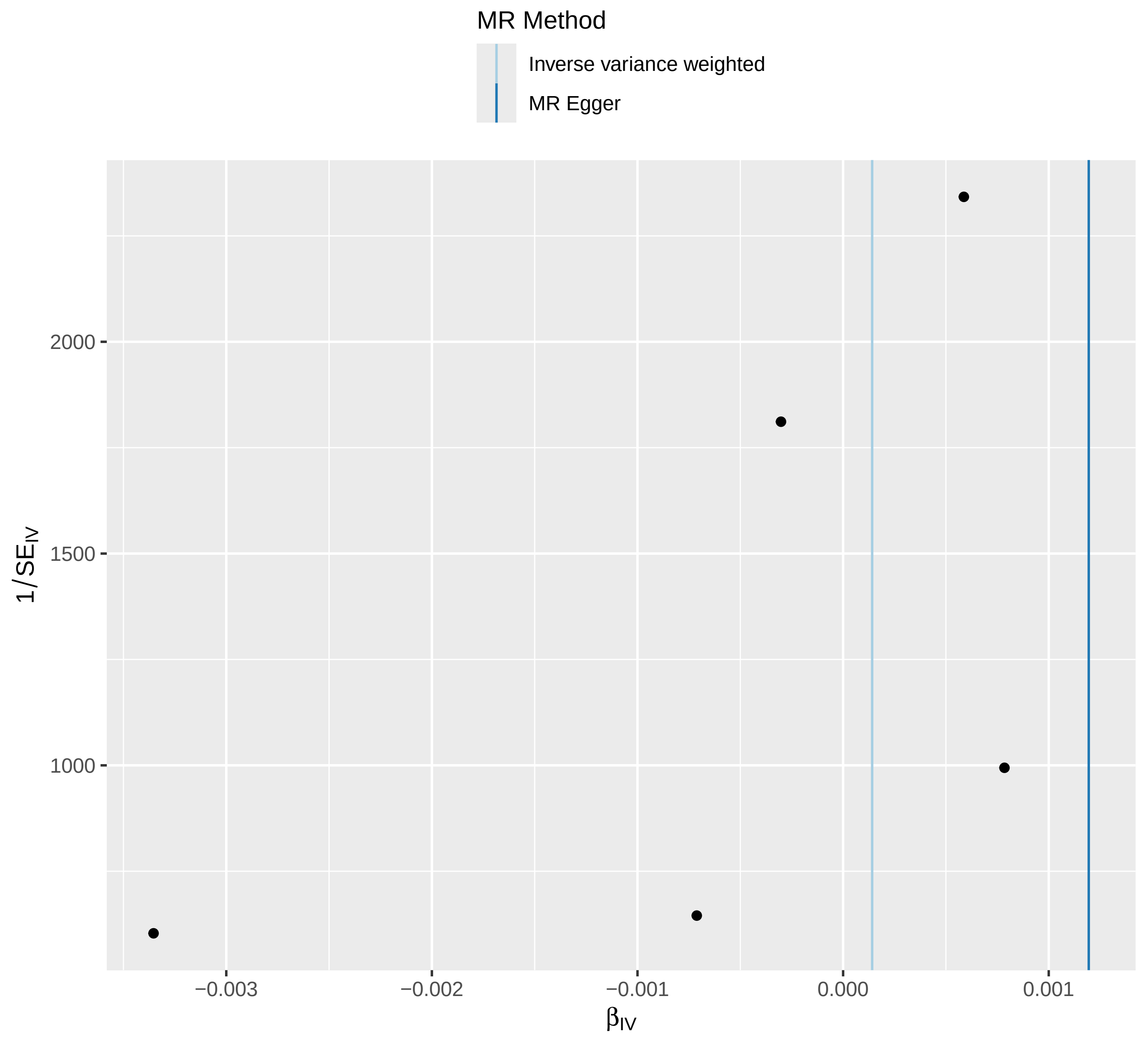
MR Method
Inverse variance weighted
MR Egger
2000
1500
1000
1/SE_IV
−0.003
−0.002
−0.001
0.000
0.001
β_IV

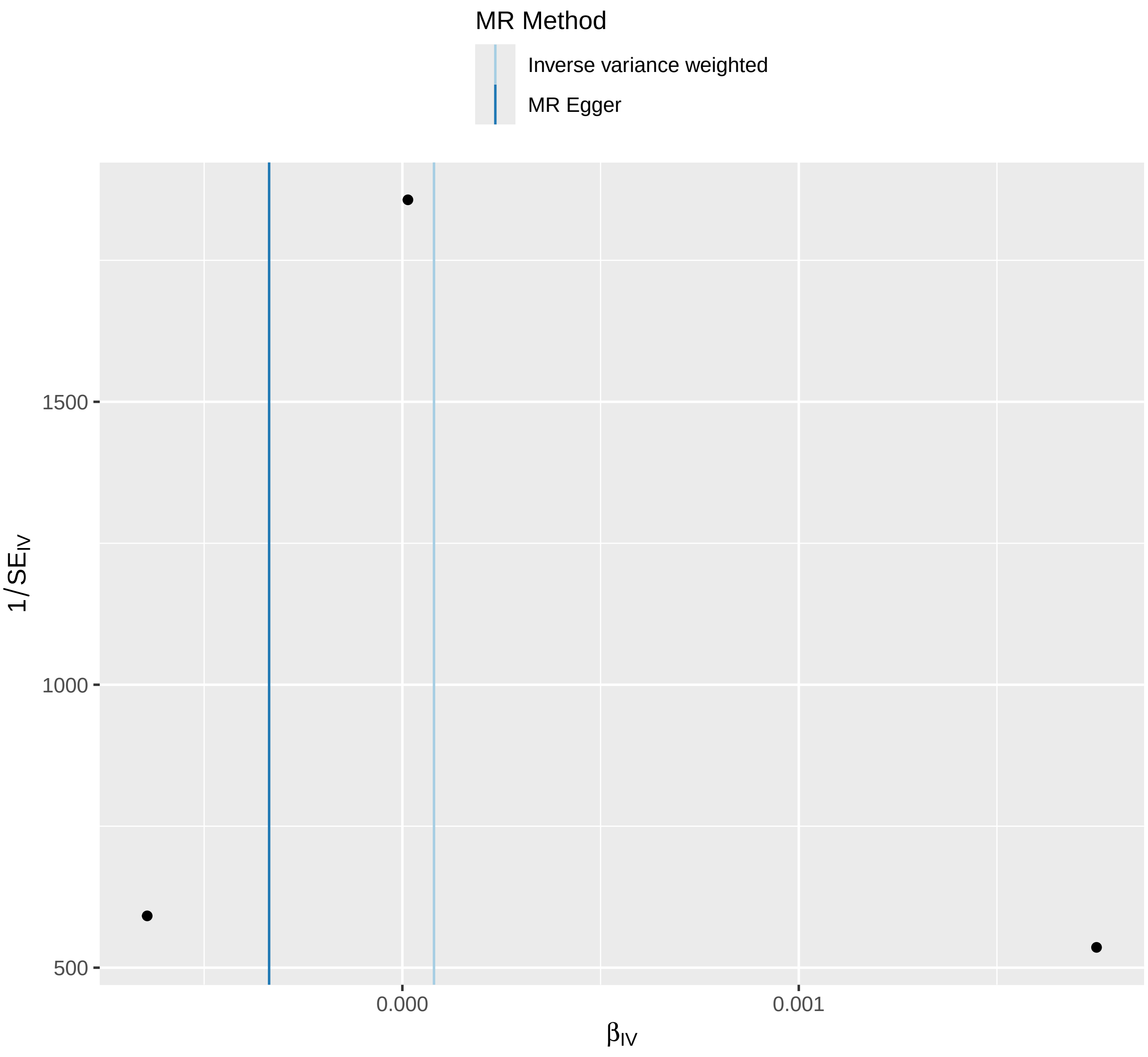
MR Method
Inverse variance weighted
MR Egger
1500
1000
500
1/SE_IV
0.000
0.001
β_IV

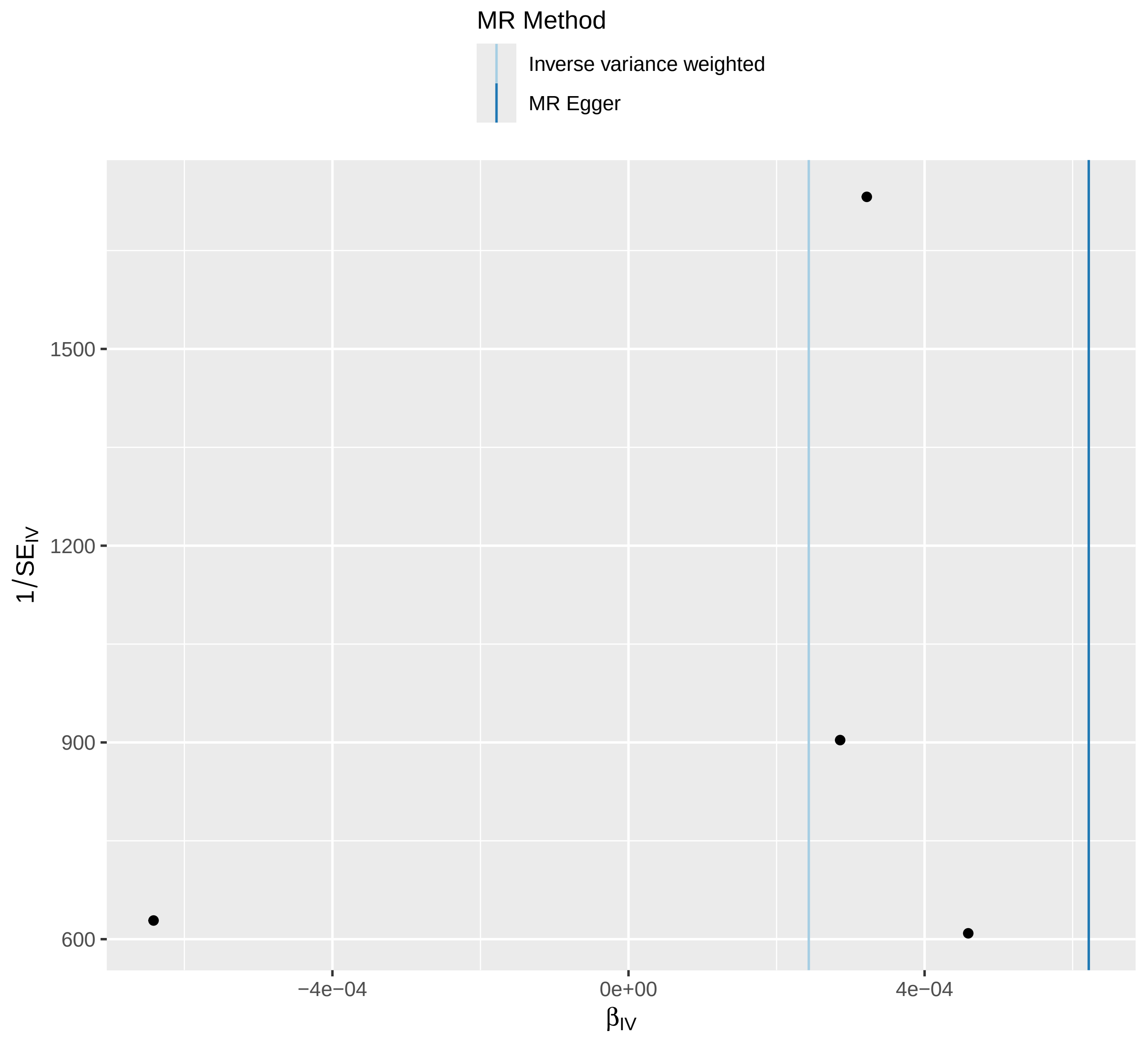
MR Method
Inverse variance weighted
MR Egger
1500
1200
900
600
1/SE_IV
−4e−04
0e+00
4e−04
β_IV

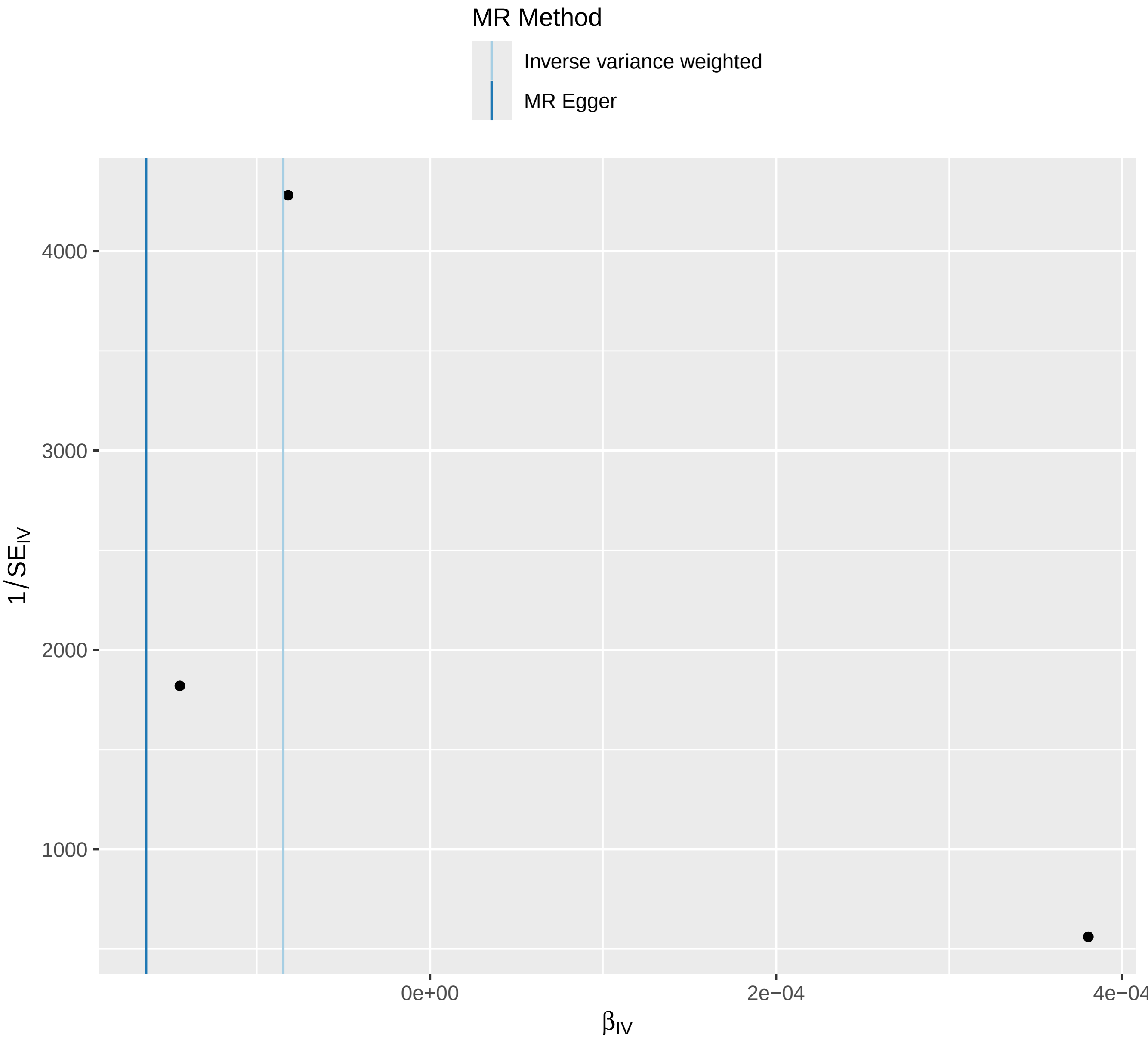
MR Method
Inverse variance weighted
MR Egger
4000
3000
2000
1000
1/SE_IV
0e+00
2e−04
4e−04
β_IV

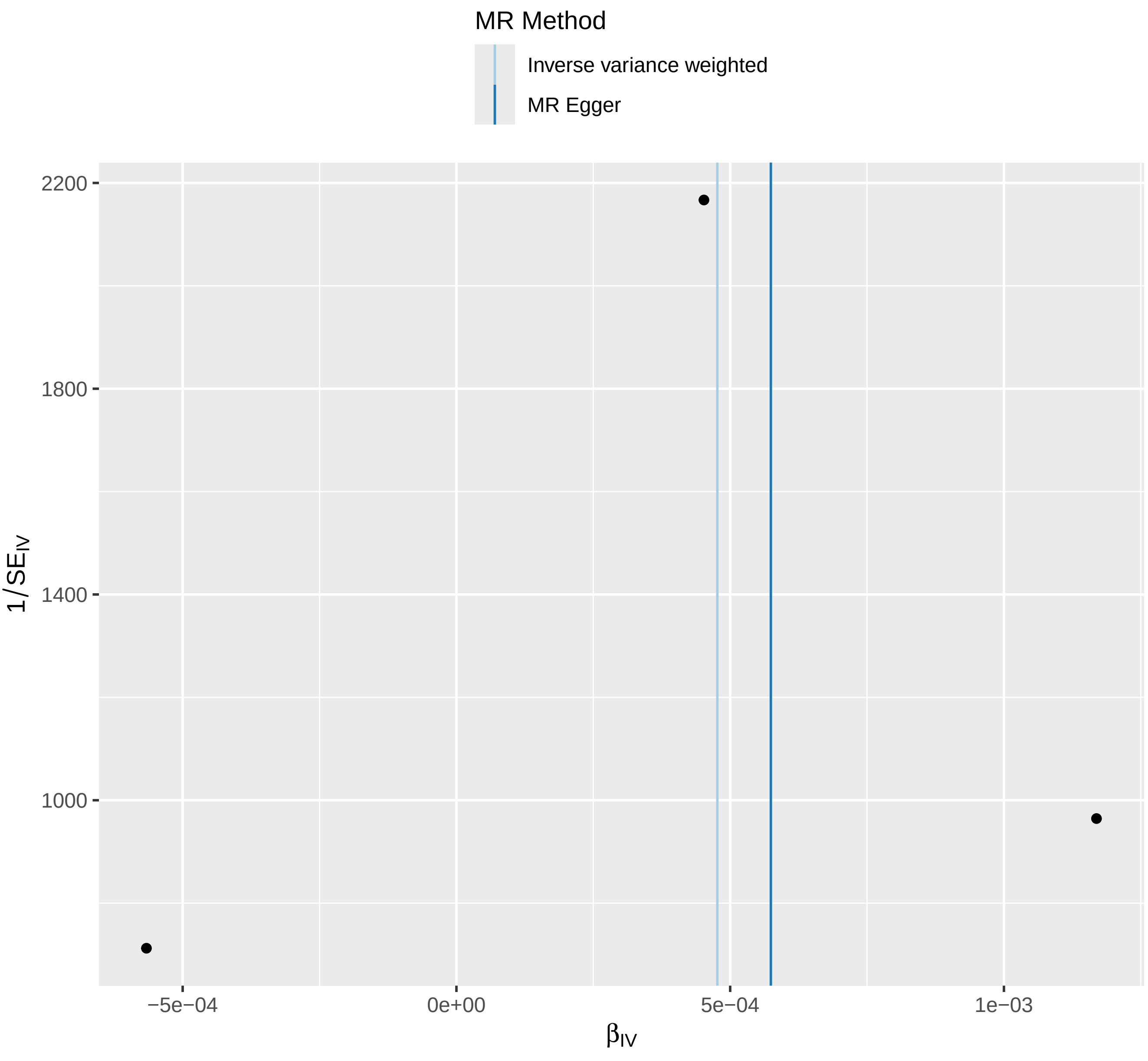
MR Method
Inverse variance weighted
MR Egger
2200
1800
1400
1000
1/SE$_{IV}$
−5e−04
0e+00
5e−04
1e−03
β$_{IV}$

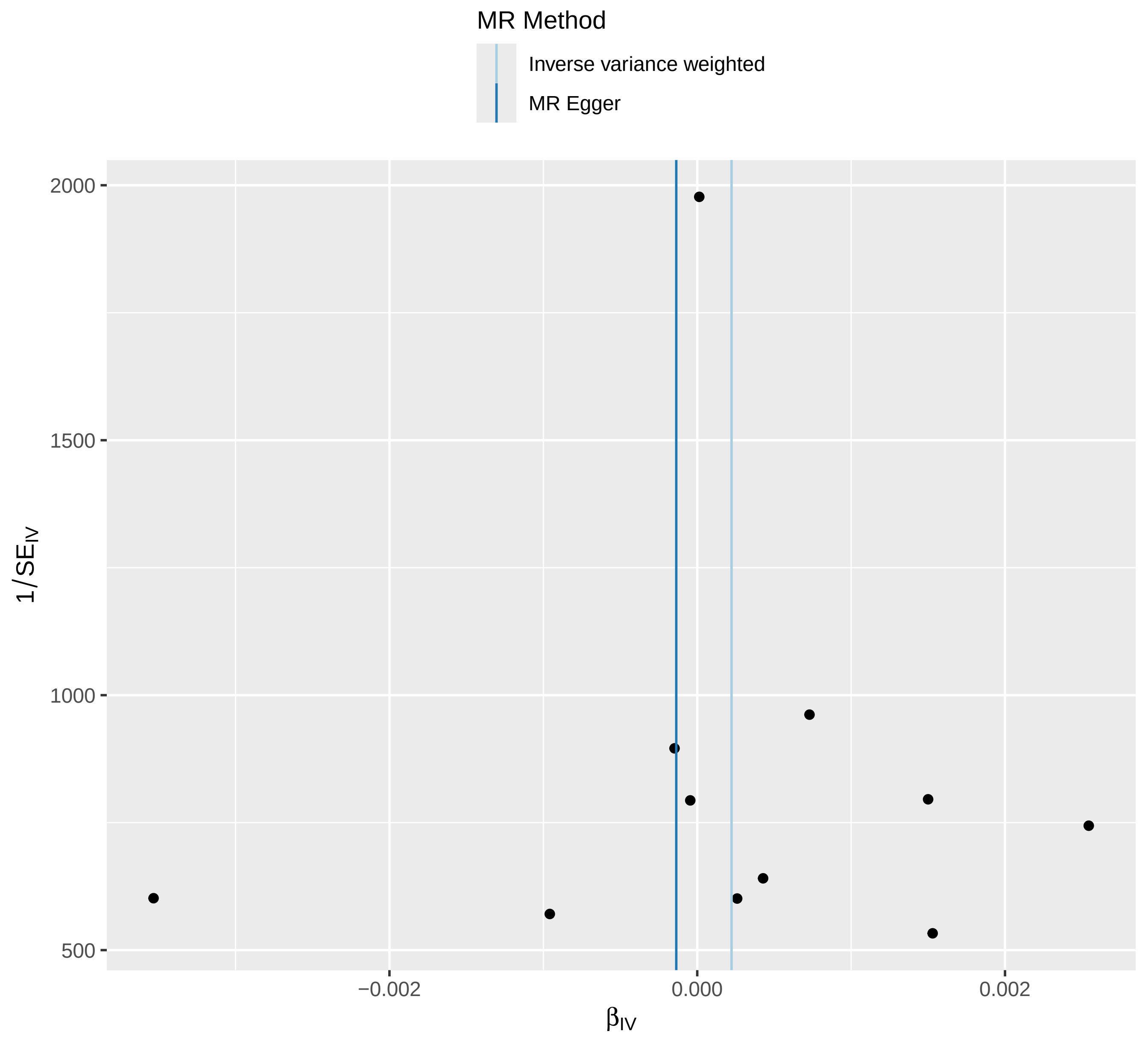

MR Method
Inverse variance weighted
MR Egger
2000
1500
1000
500
1/SE_IV
−0.002
0.000
0.002
β_IV

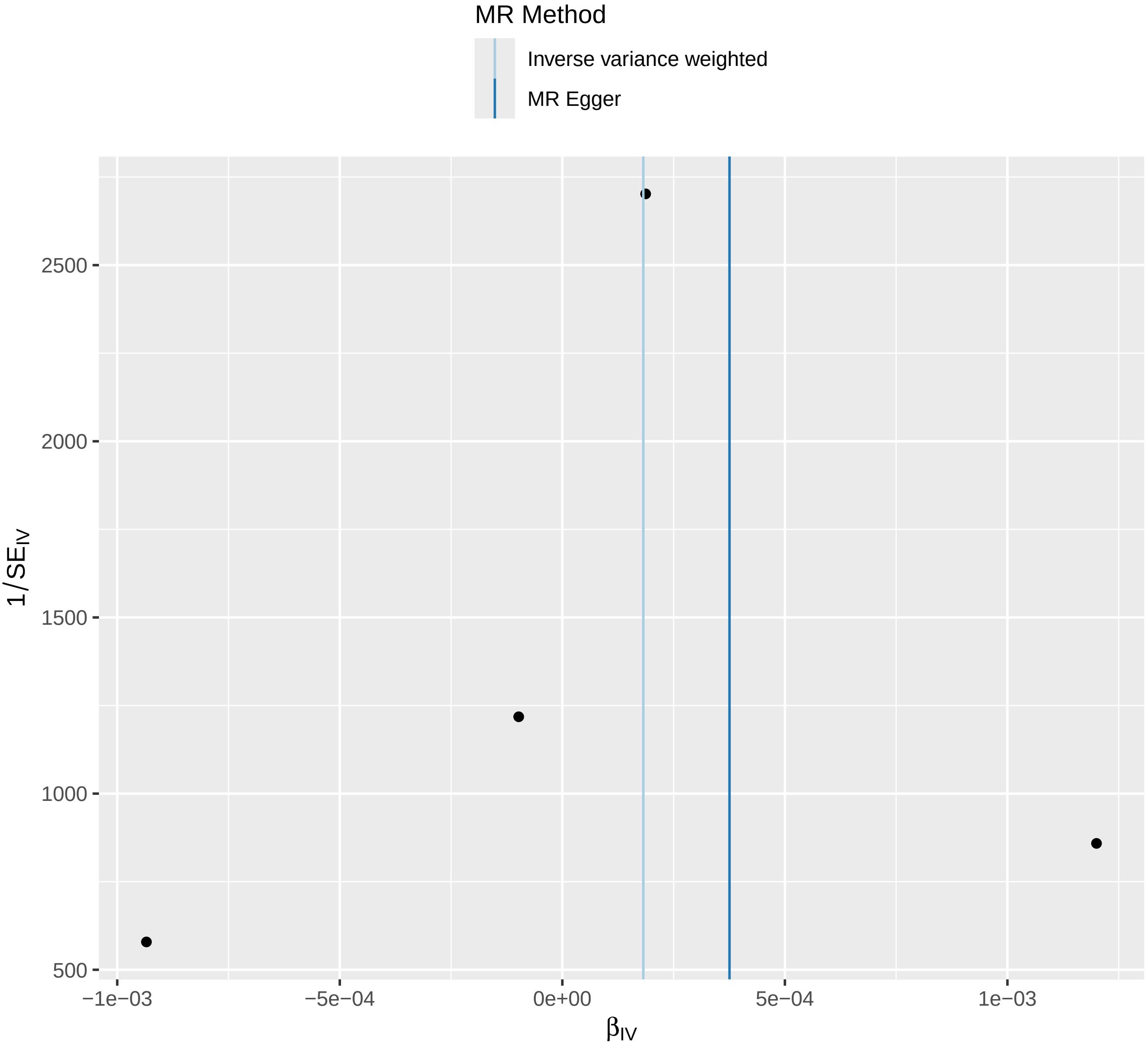
MR Method
Inverse variance weighted
MR Egger
2500
2000
1500
1000
500
1/SE$_{IV}$
−1e−03
−5e−04
0e+00
5e−04
1e−03
β_{IV}

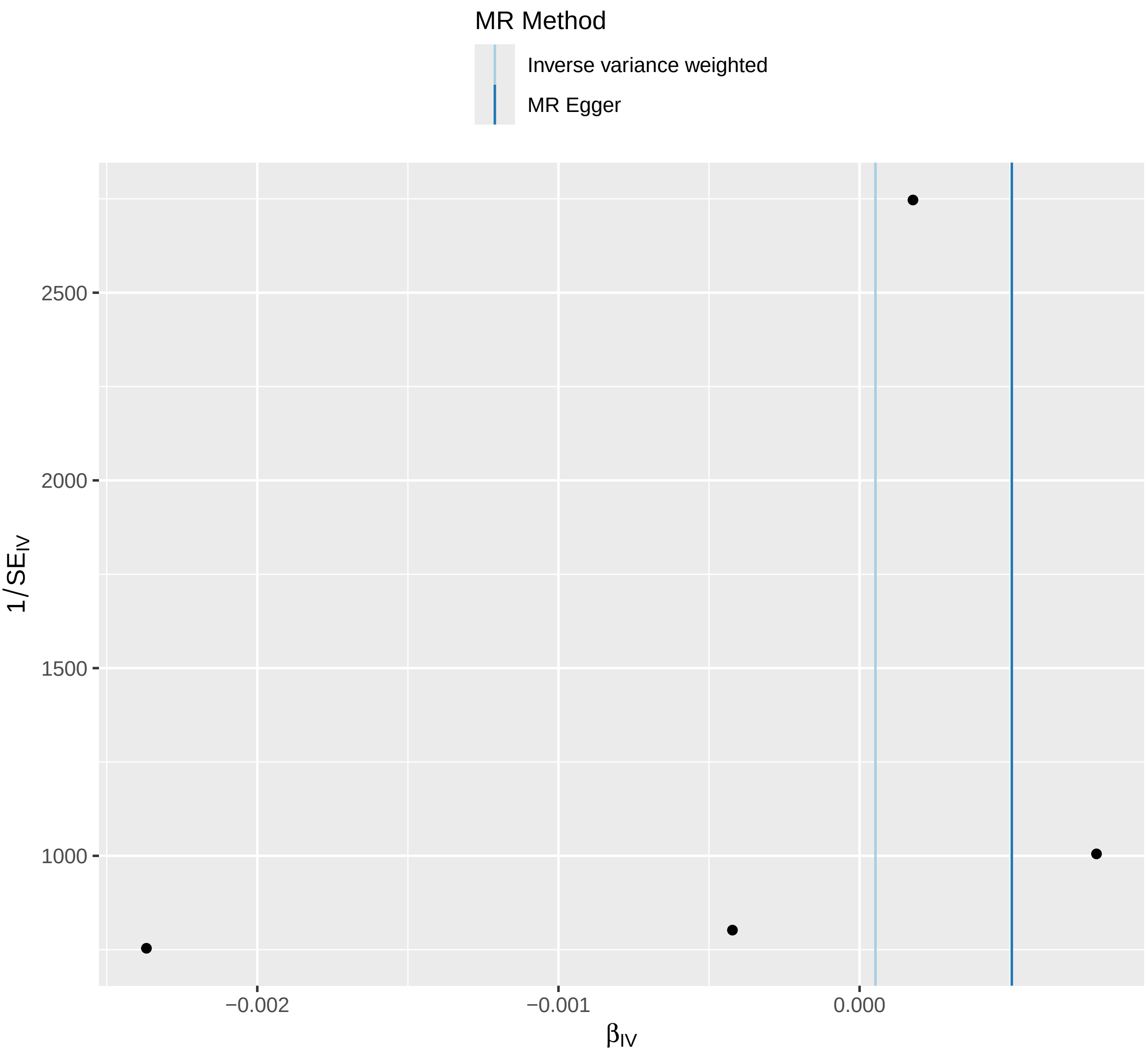
MR Method
Inverse variance weighted
MR Egger
2500
2000
1500
1000
1/SE_IV
−0.002
−0.001
0.000
β_IV

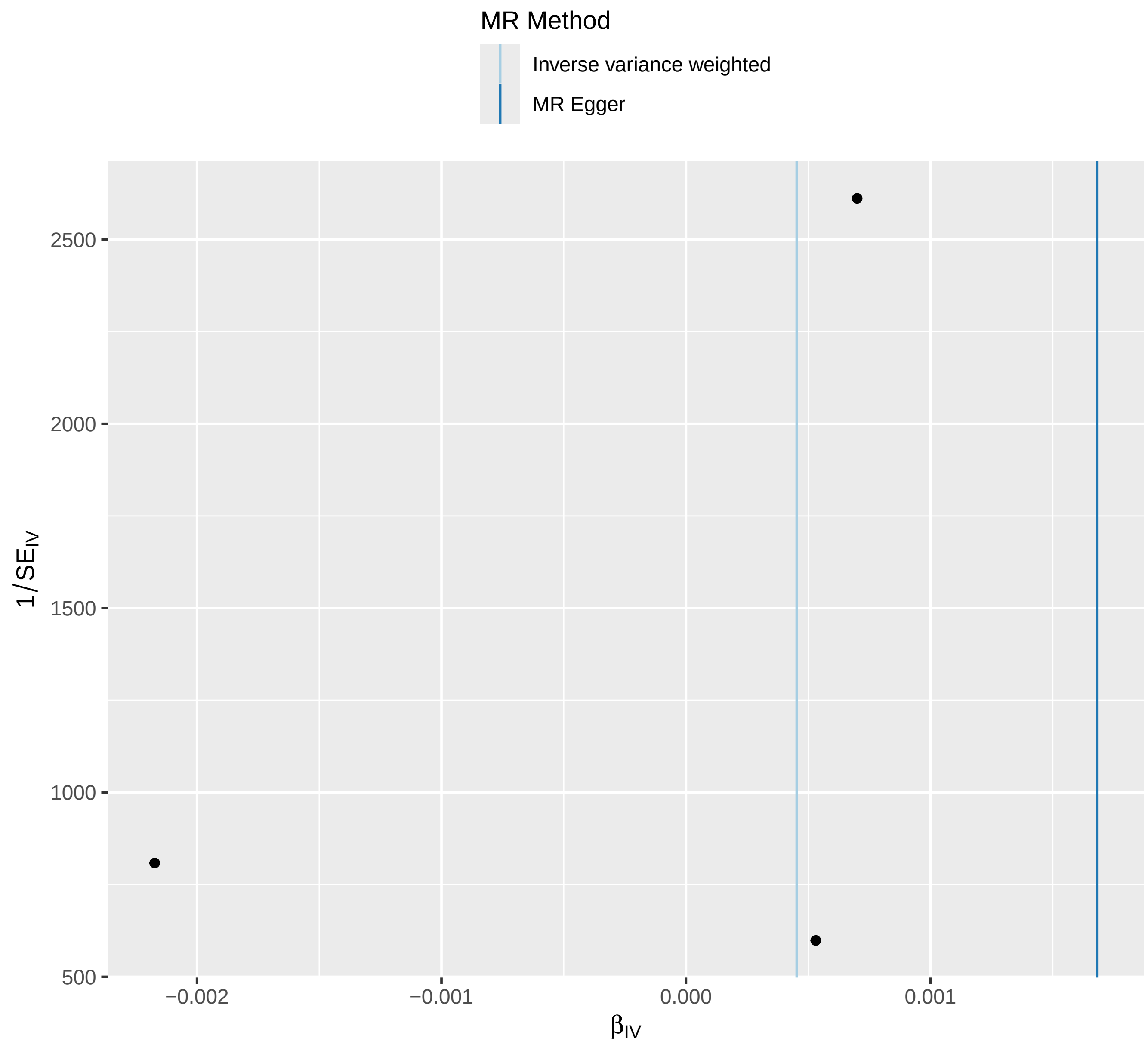
MR Method
Inverse variance weighted
MR Egger
2500
2000
1500
1000
500
1/SE_IV
−0.002
−0.001
0.000
0.001
β_IV

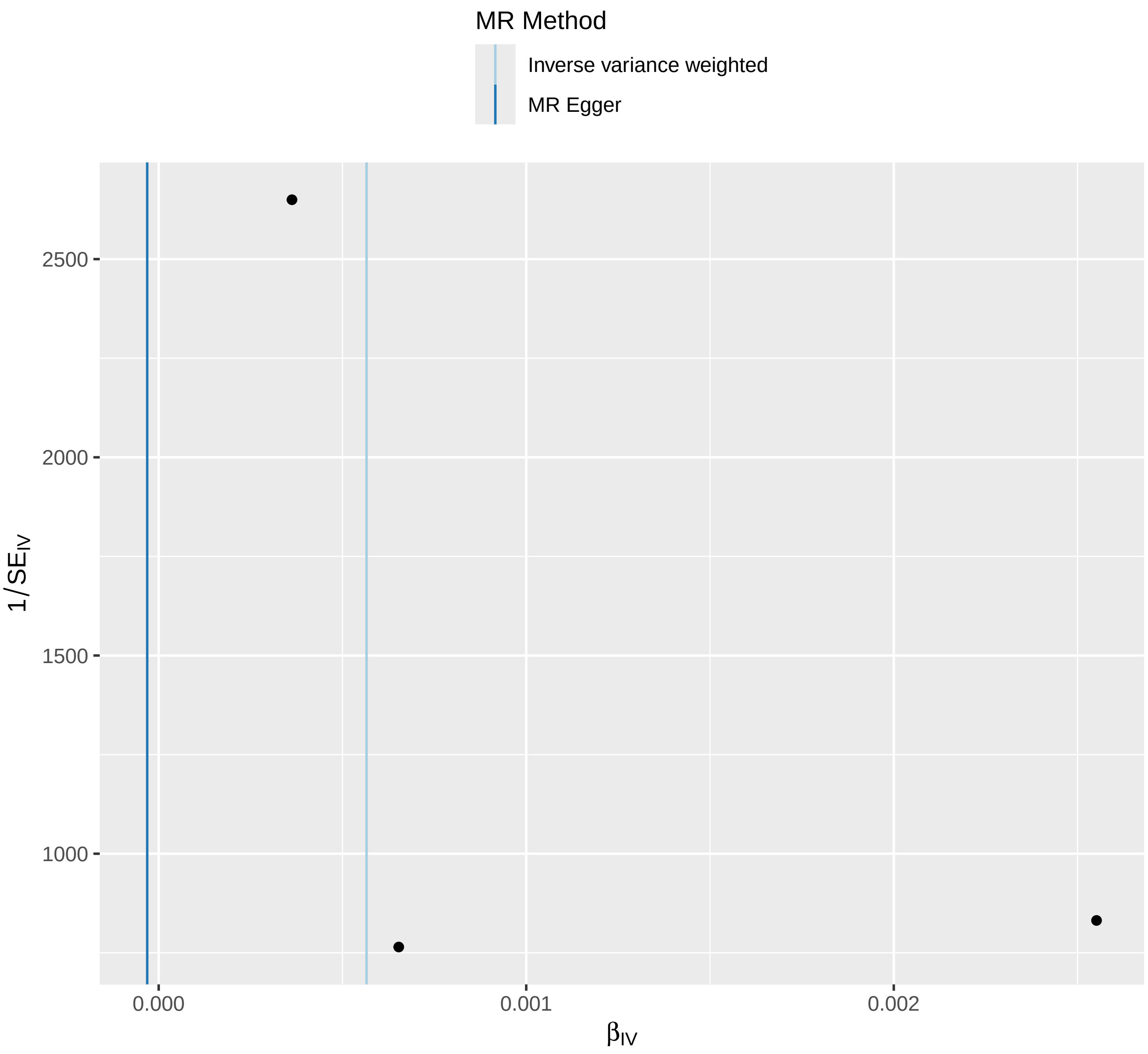
MR Method
Inverse variance weighted
MR Egger
2500
2000
1500
1000
1/SE_IV
0.000
0.001
0.002
β_IV

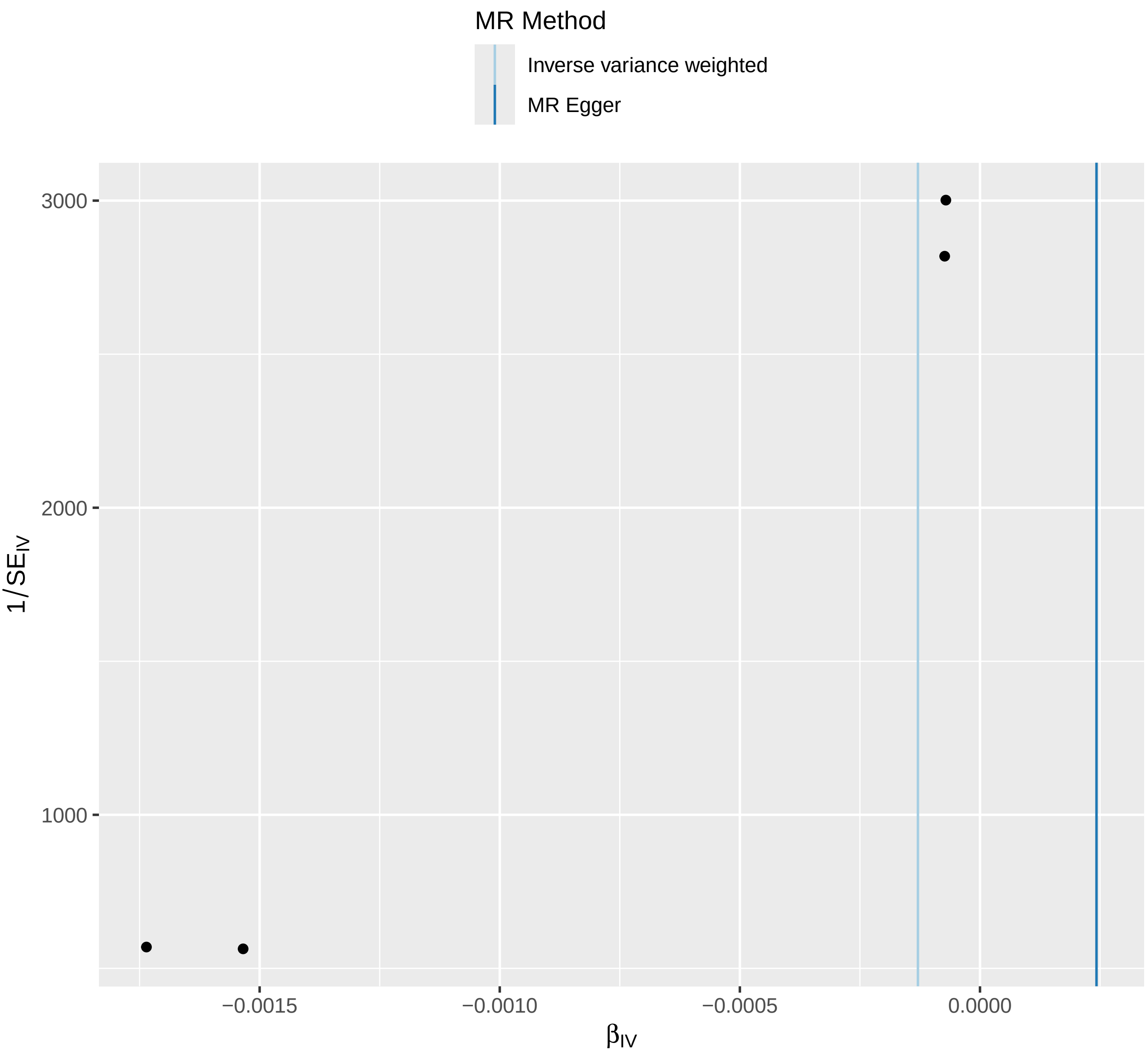
MR Method
Inverse variance weighted
MR Egger
3000
2000
1000
1/SE_IV
−0.0015
−0.0010
−0.0005
0.0000
β_IV

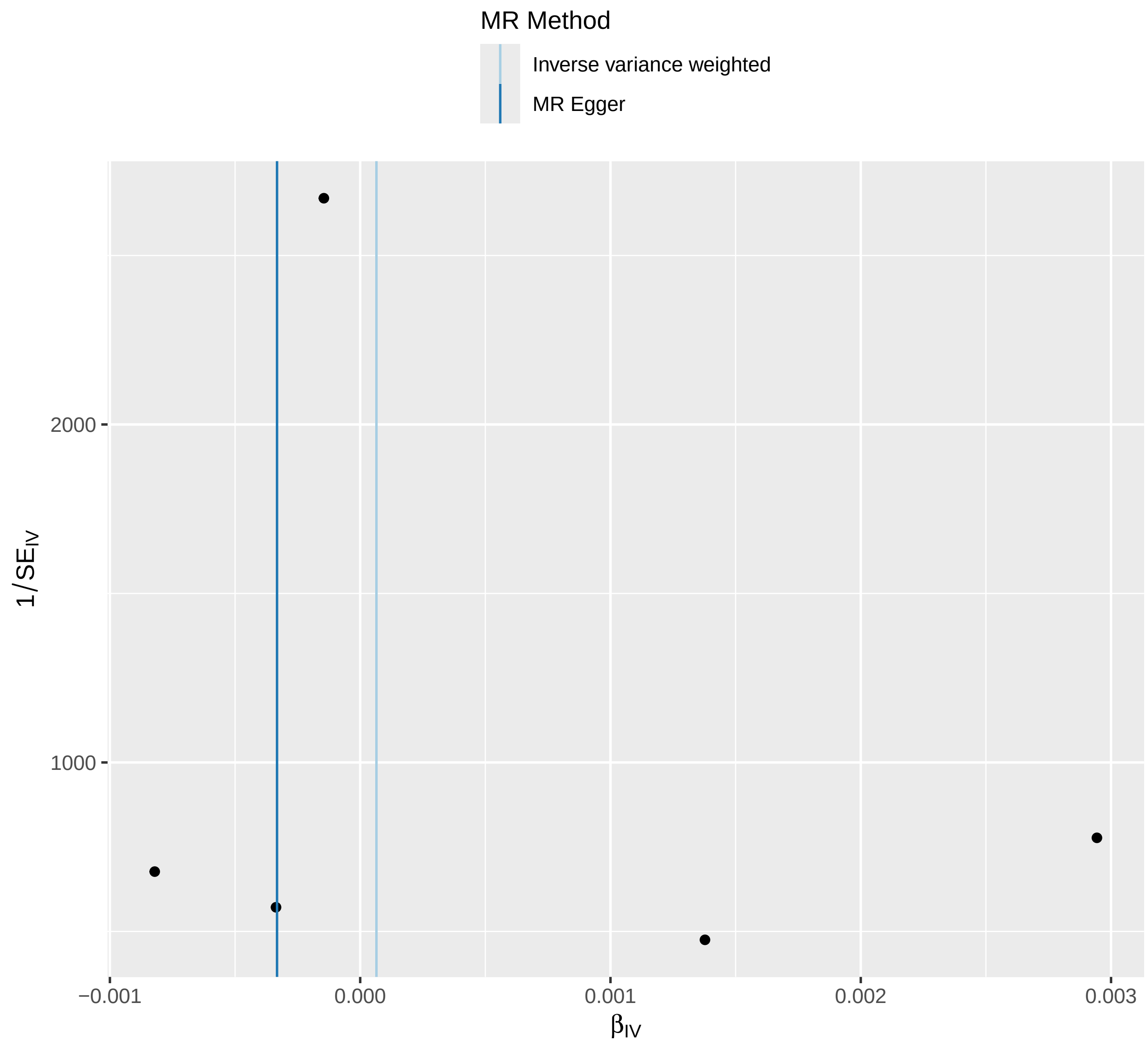
MR Method
Inverse variance weighted
MR Egger
2000
1000
1/SE_IV
−0.001
0.000
0.001
0.002
0.003
β_IV

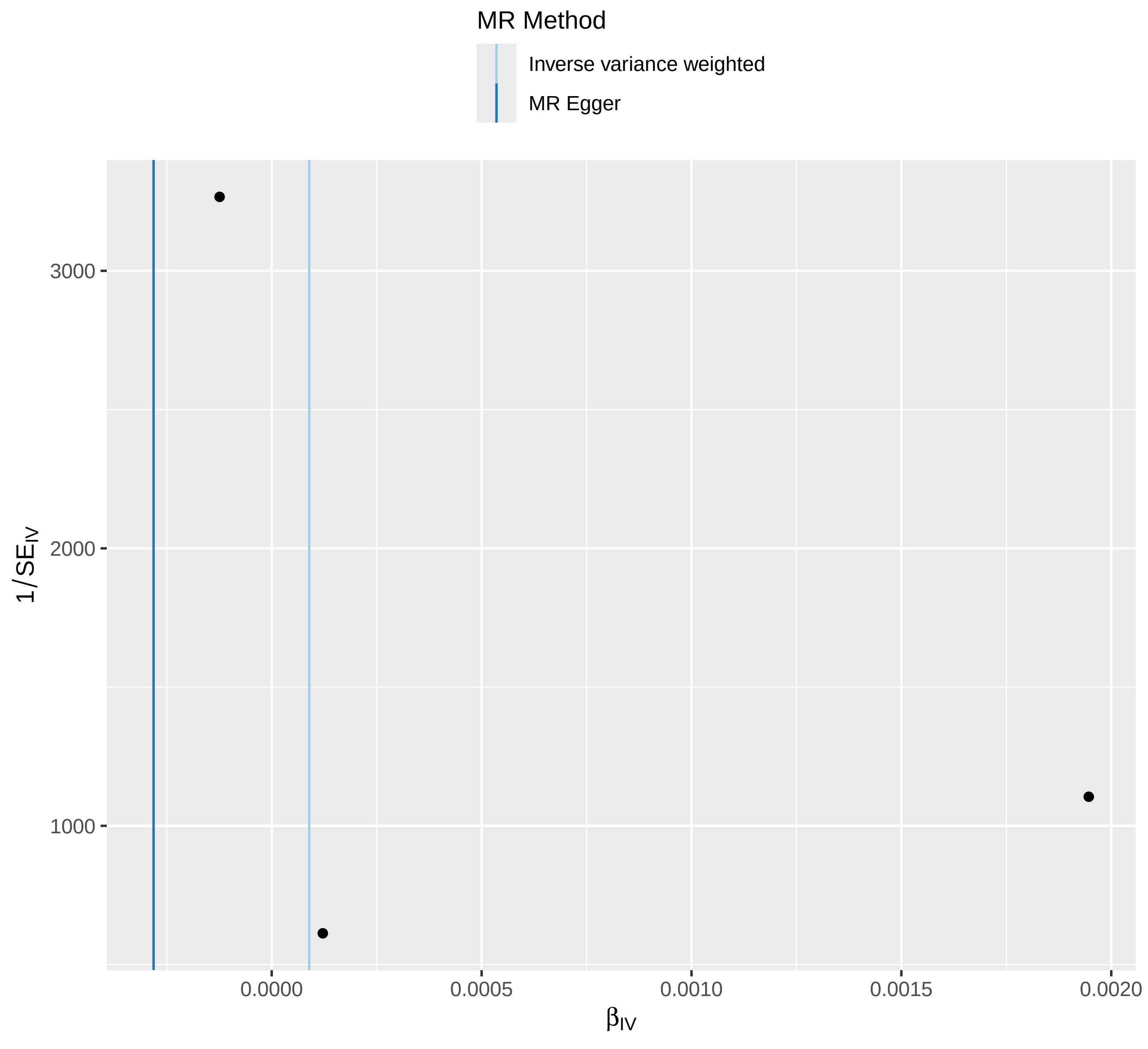

MR Method
Inverse variance weighted
MR Egger
3000
2000
1000
1/SE$_{IV}$
0.0000
0.0005
0.0010
0.0015
0.0020
β$_{IV}$

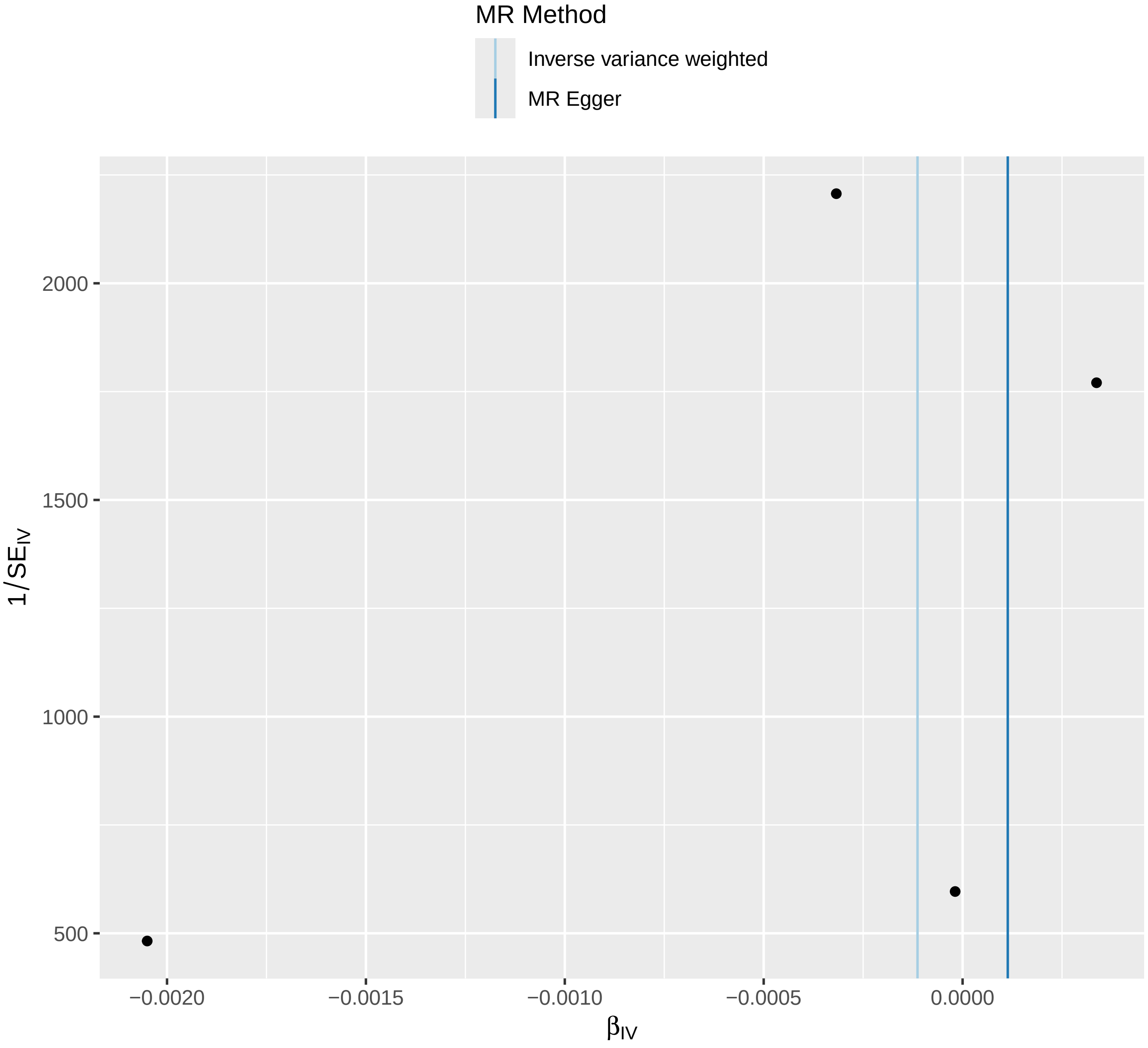
MR Method
Inverse variance weighted
MR Egger
2000
1500
1000
500
1/SE_IV
−0.0020
−0.0015
−0.0010
−0.0005
0.0000
β_IV

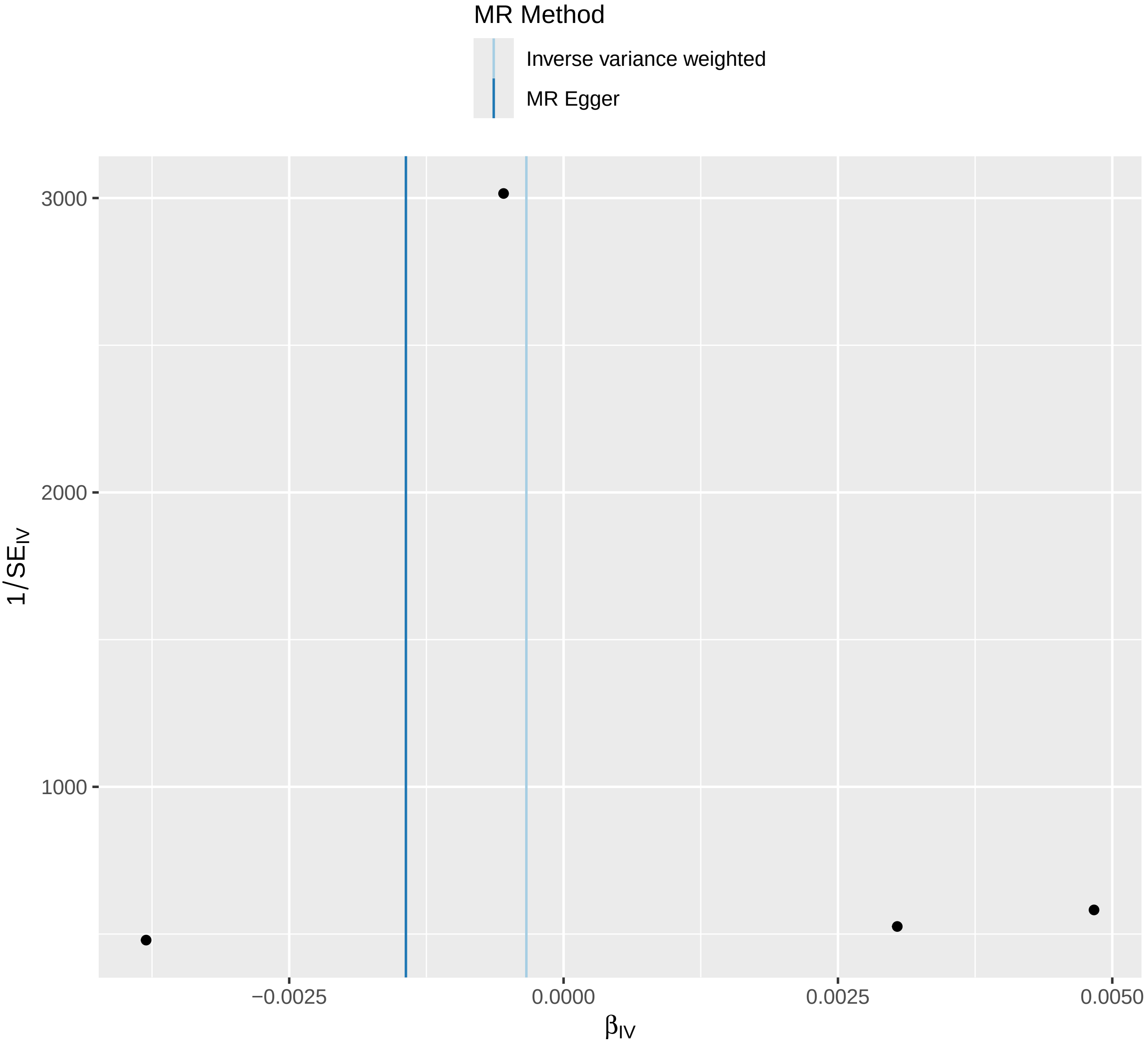
MR Method
Inverse variance weighted
MR Egger
3000
2000
1000
1/SE_IV
−0.0025
0.0000
0.0025
0.0050
β_IV

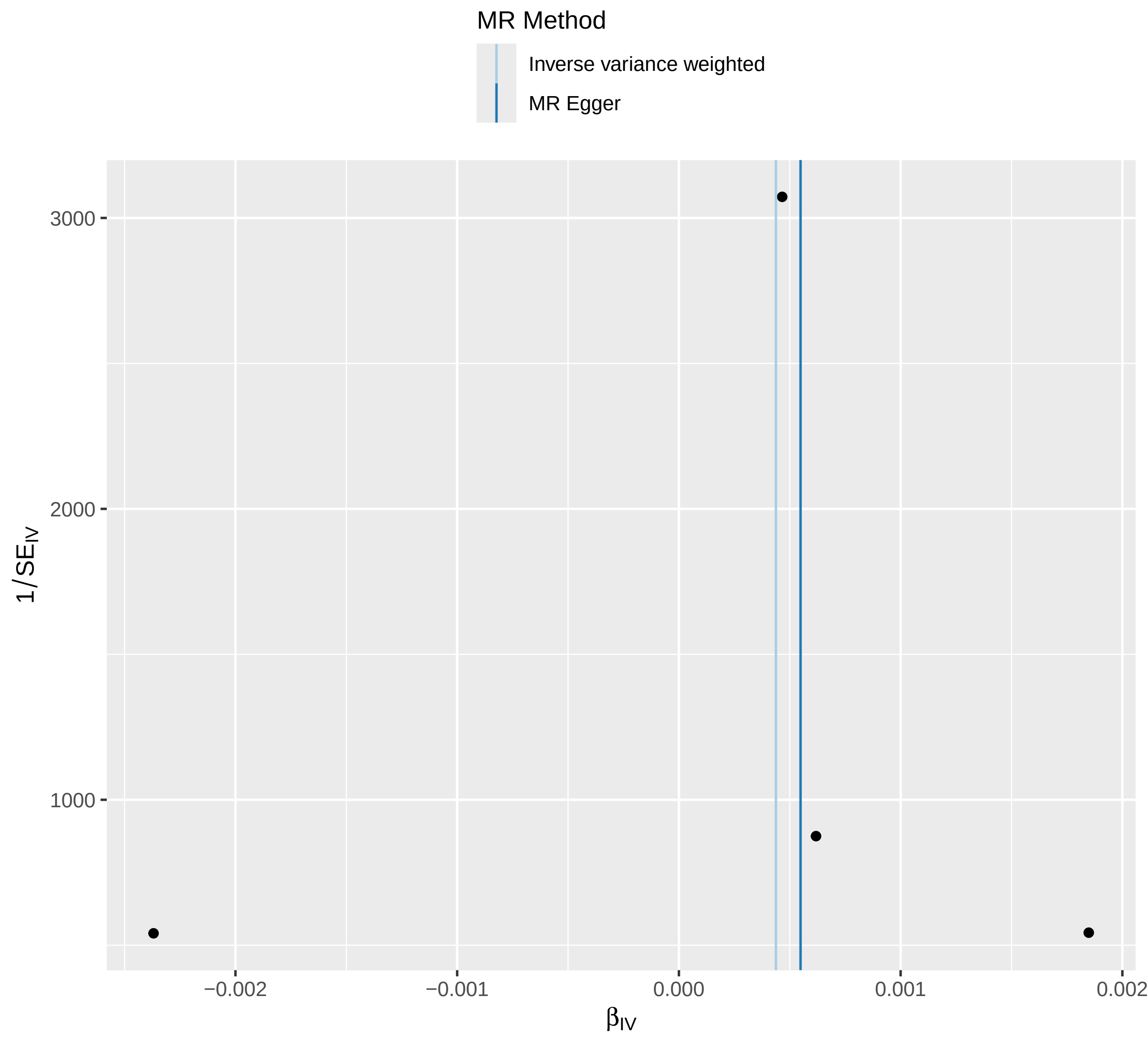

MR Method
Inverse variance weighted
MR Egger
3000
2000
1000
1/SE$_{IV}$
−0.002
−0.001
0.000
0.001
0.002
β$_{IV}$

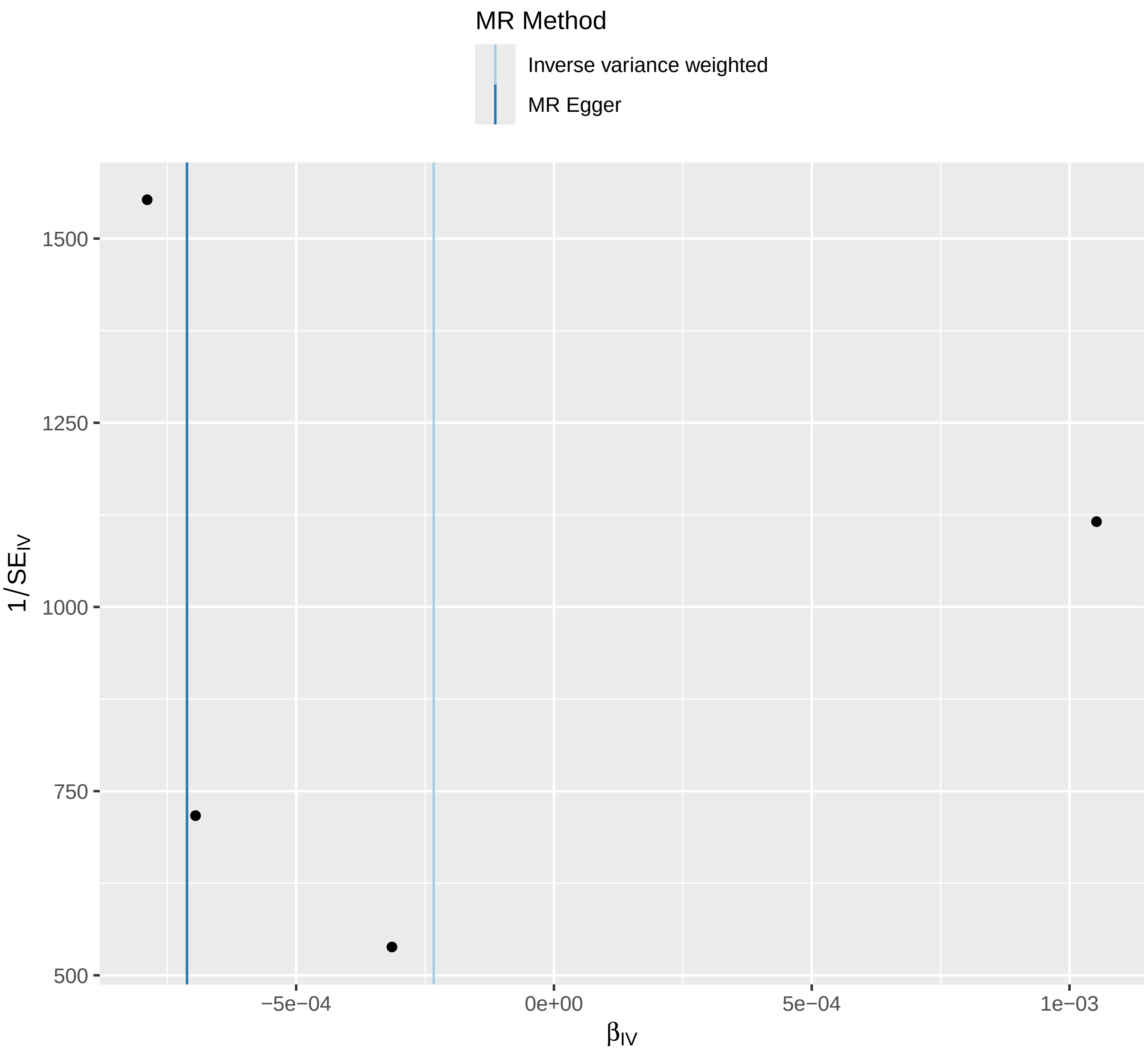

MR Method
Inverse variance weighted
MR Egger
1500
1250
1000
750
500
1/SE_IV
−5e−04
0e+00
5e−04
1e−03
β_IV

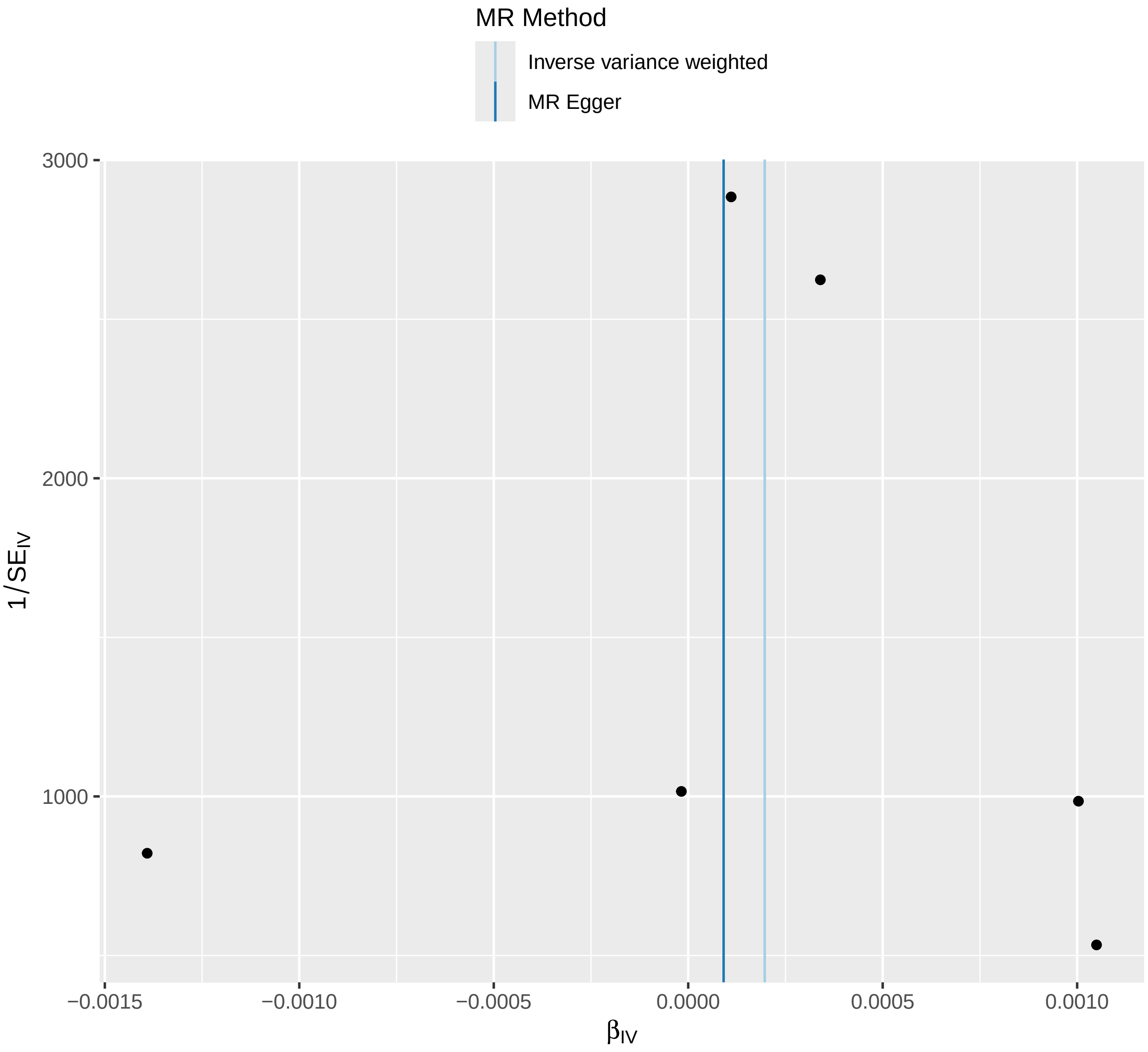

MR Method
Inverse variance weighted
MR Egger
3000
2000
1000
1/SE_IV
−0.0015
−0.0010
−0.0005
0.0000
0.0005
0.0010
β_IV

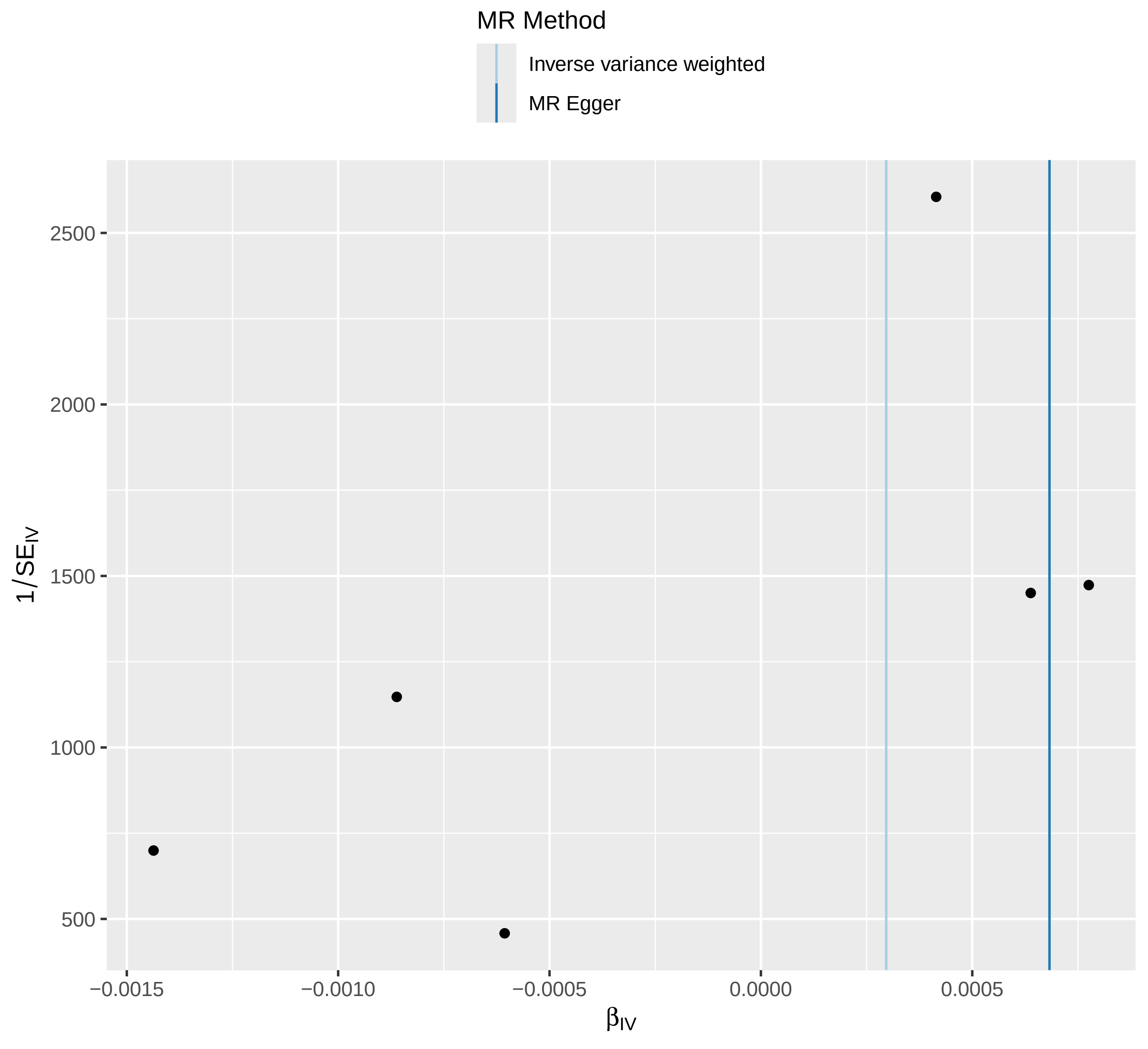

MR Method
Inverse variance weighted
MR Egger
2500
2000
1500
1000
500
$1/SE_{IV}$
−0.0015
−0.0010
−0.0005
0.0000
0.0005
β_{IV}

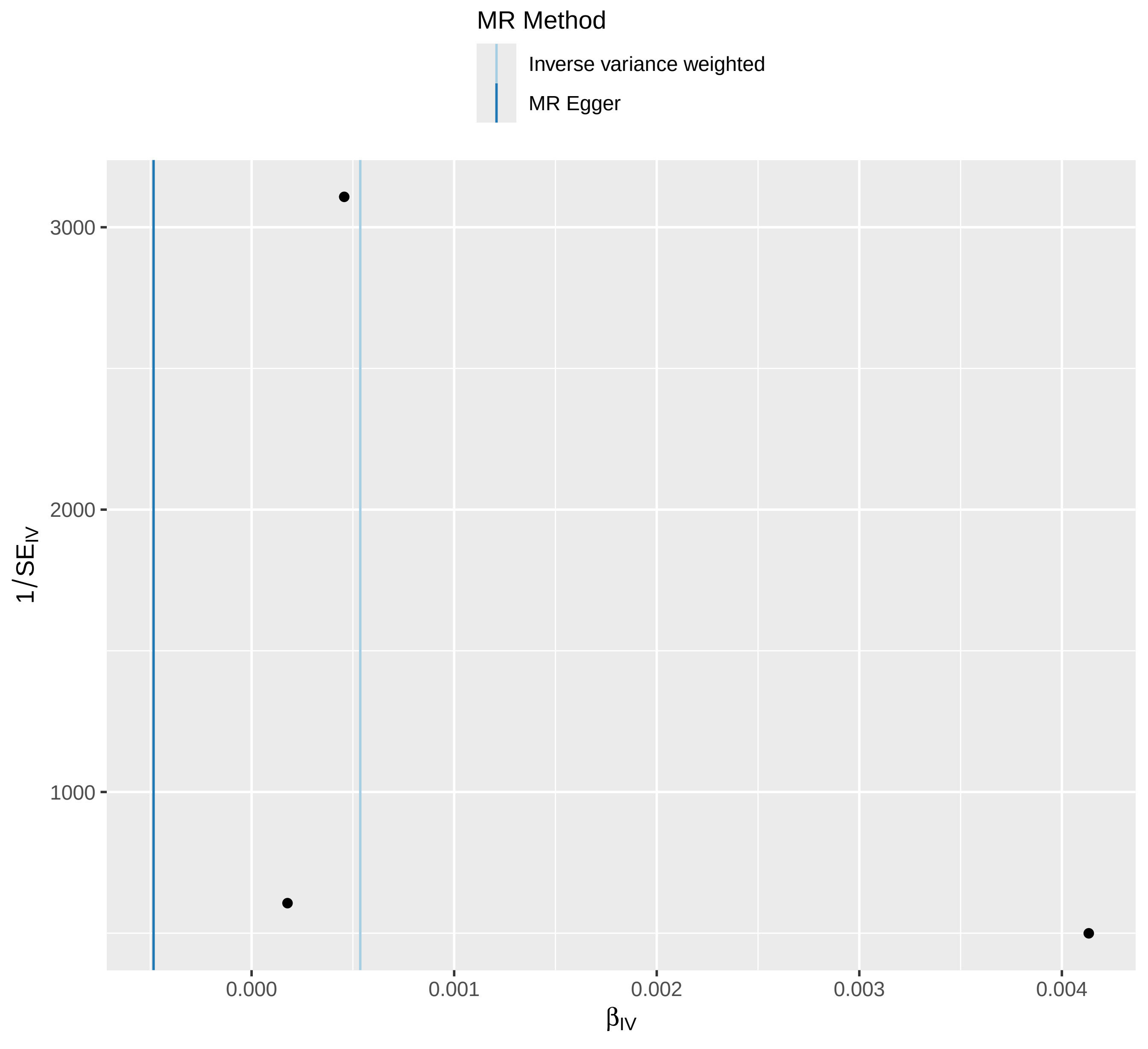

MR Method
Inverse variance weighted
MR Egger
3000
2000
1000
1/SE$_{IV}$
0.000
0.001
0.002
0.003
0.004
β$_{IV}$

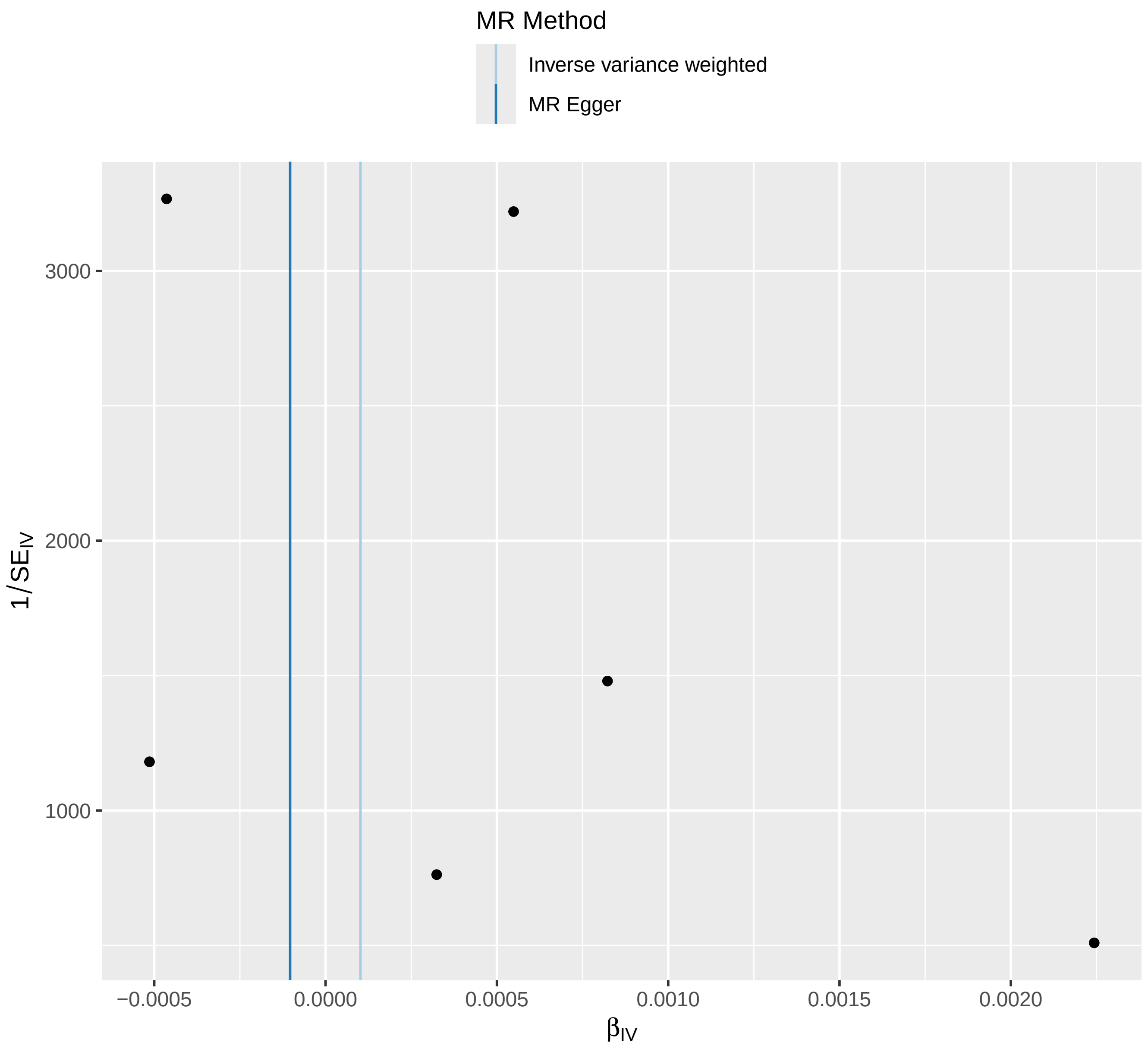

MR Method
Inverse variance weighted
MR Egger
3000
2000
1000
1/SE_IV
−0.0005
0.0000
0.0005
0.0010
0.0015
0.0020
β_IV

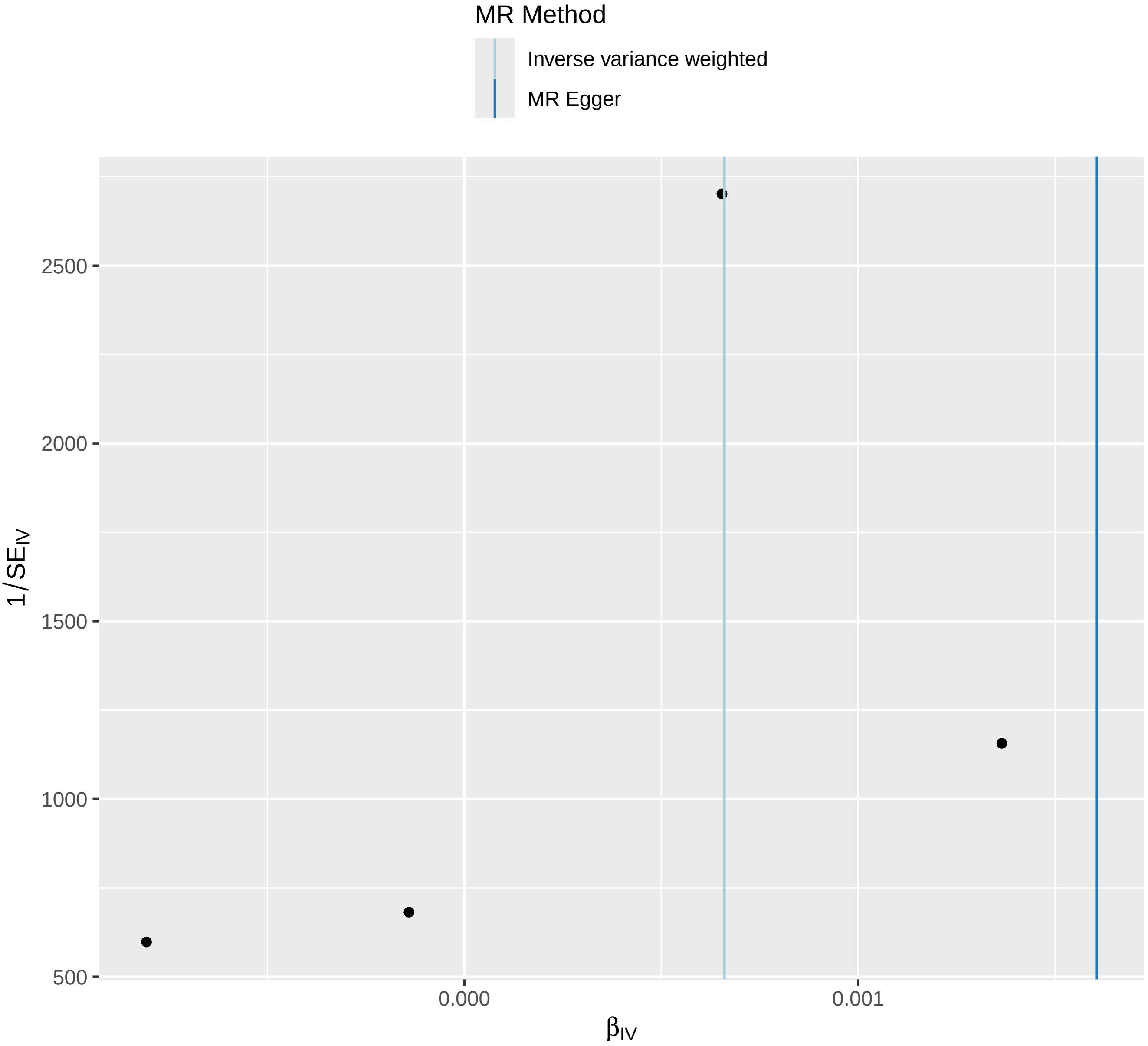

MR Method
Inverse variance weighted
MR Egger
2500
2000
1500
1000
500
$1/SE_{IV}$
0.000
0.001
β_{IV}

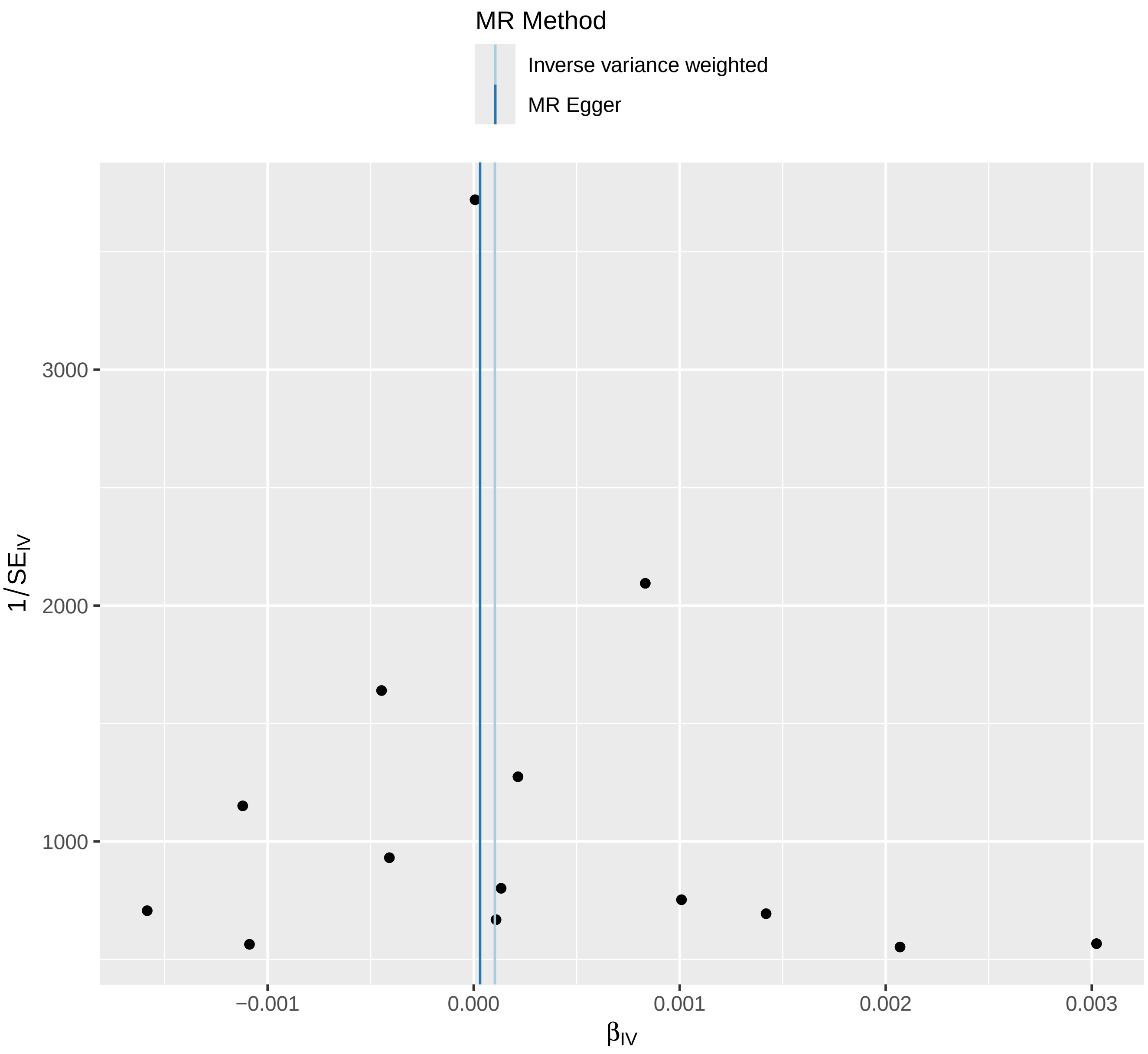

MR Method
Inverse variance weighted
MR Egger
3000
2000
1000
1/SE$_{IV}$
−0.001
0.000
0.001
0.002
0.003
β$_{IV}$

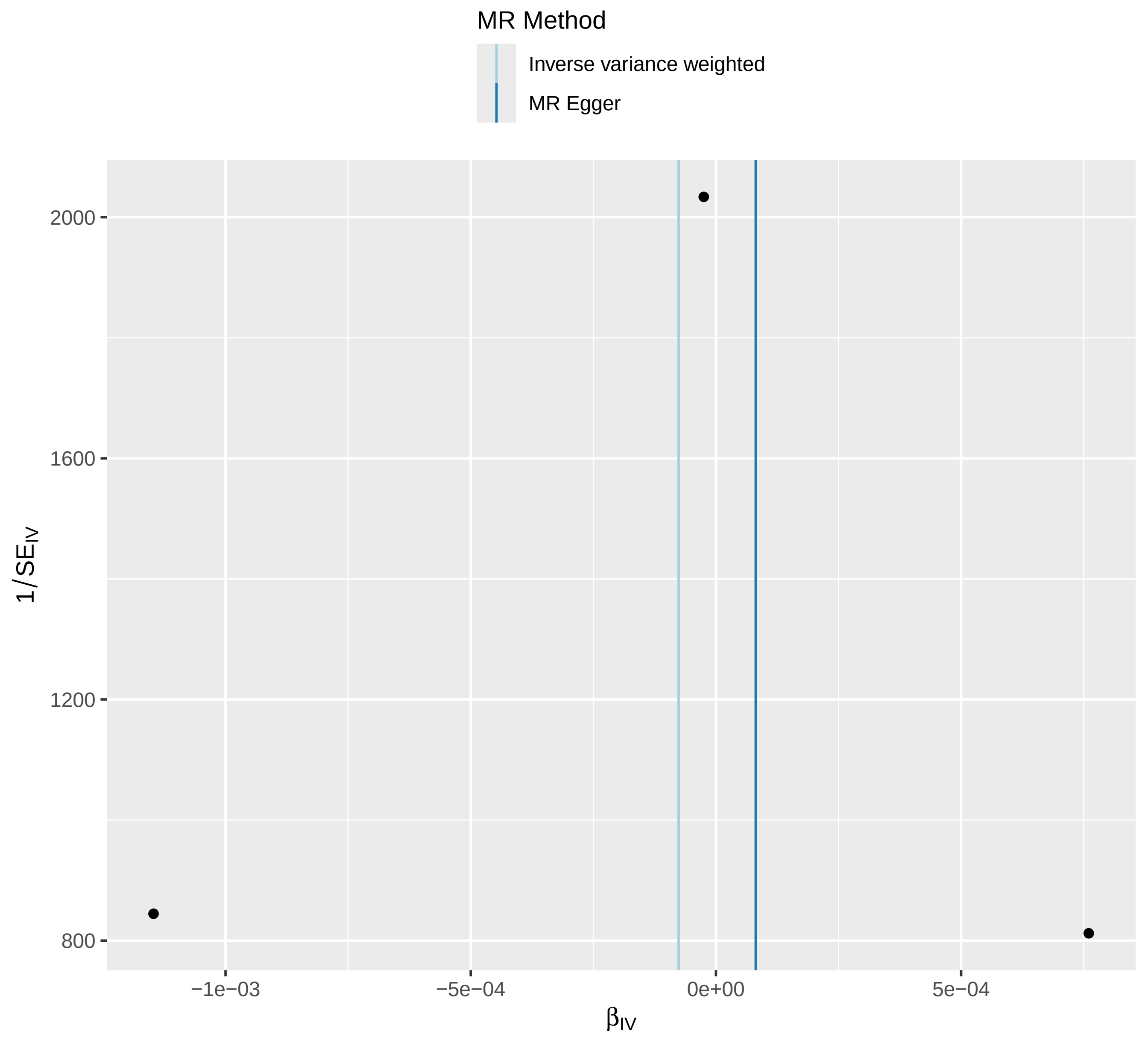
MR Method
Inverse variance weighted
MR Egger
2000
1600
1200
800
$1/SE_{IV}$
−1e−03
−5e−04
0e+00
5e−04
β_{IV}

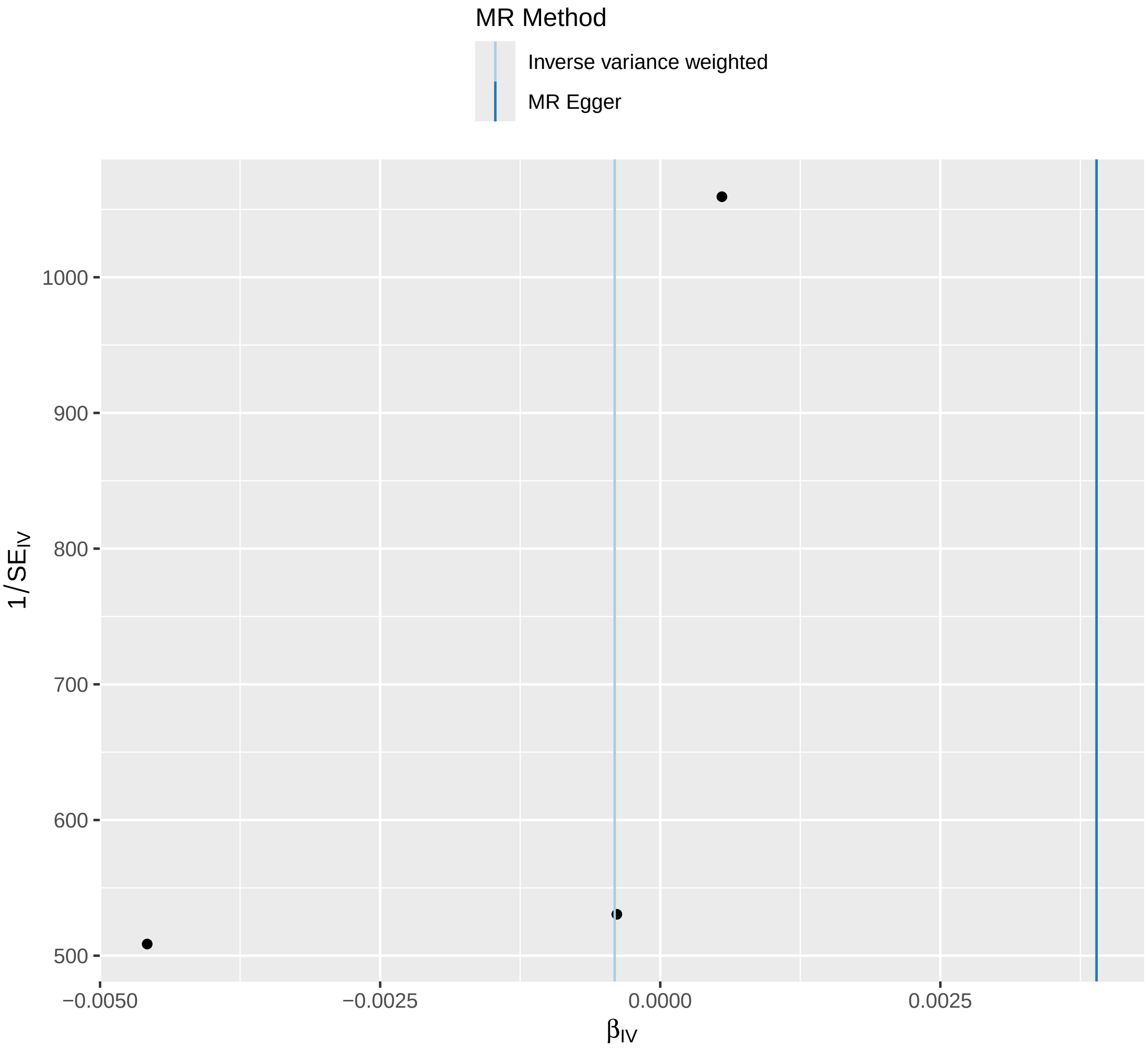
MR Method
Inverse variance weighted
MR Egger
1000
900
800
700
600
500
1/SE$_{IV}$
−0.0050
−0.0025
0.0000
0.0025
β$_{IV}$

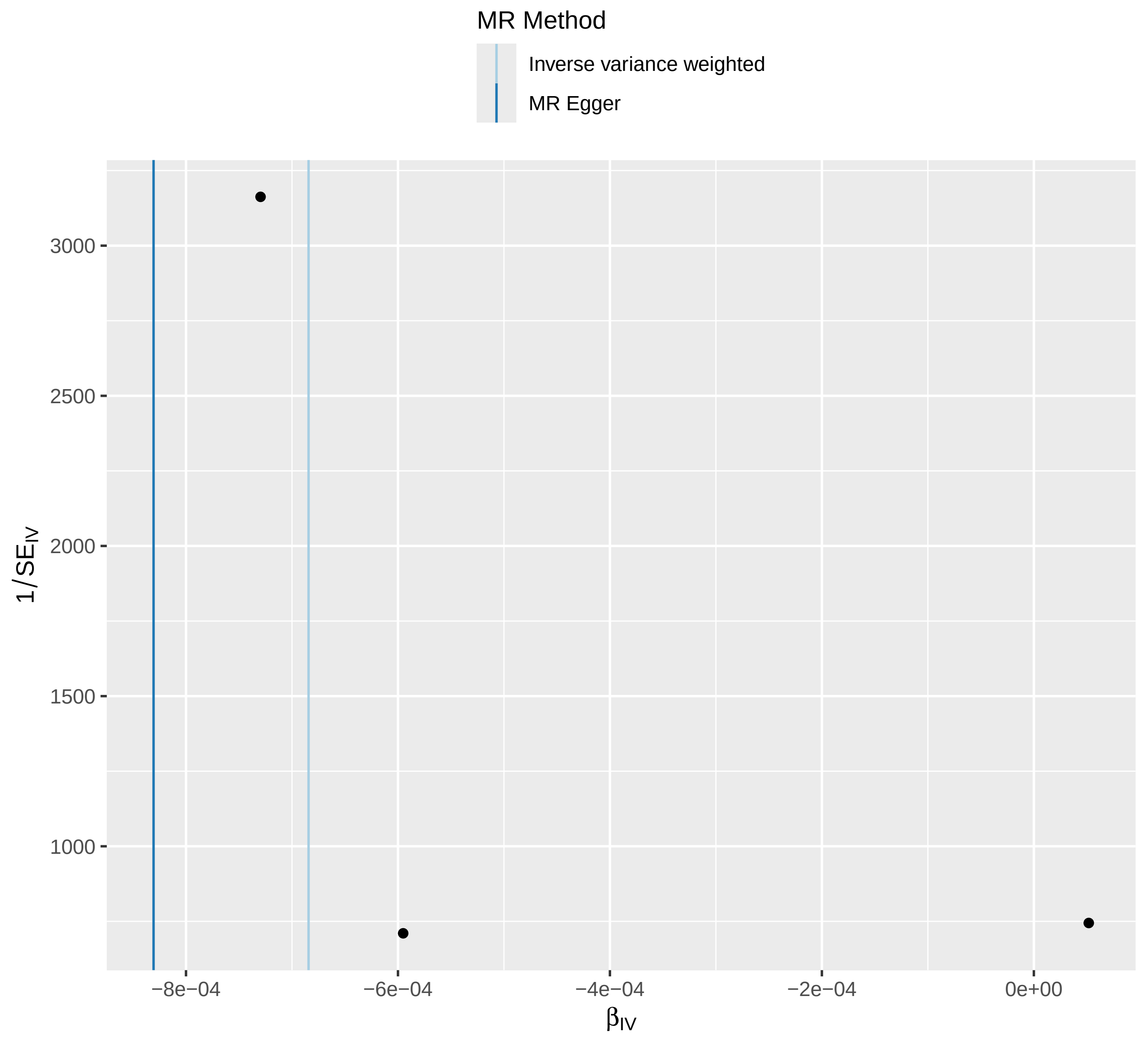

MR Method
Inverse variance weighted
MR Egger
3000
2500
2000
1500
1000
1/SE_IV
−8e−04
−6e−04
−4e−04
−2e−04
0e+00
β_IV

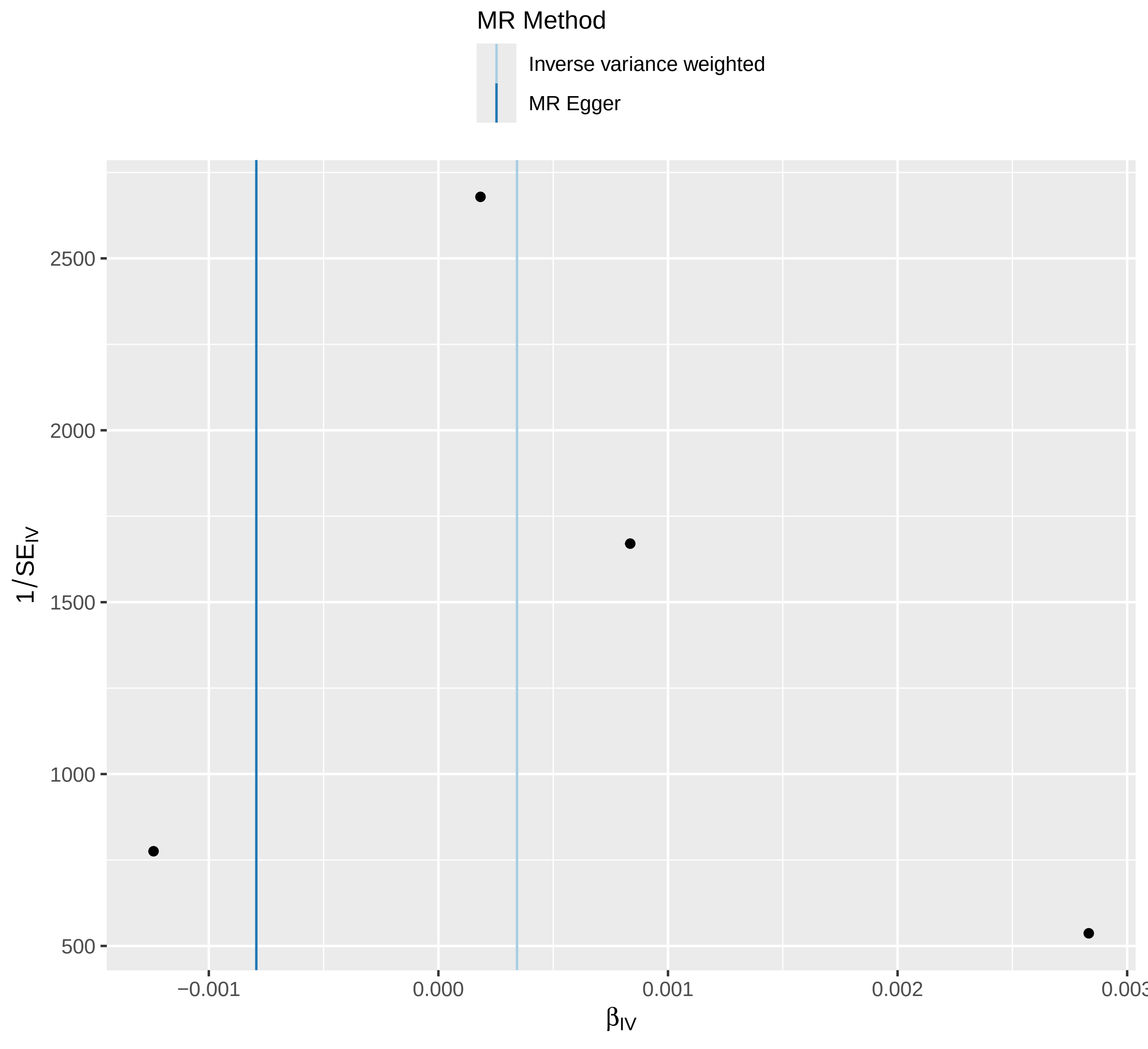

MR Method
Inverse variance weighted
MR Egger
2500
2000
1500
1000
500
1/SE_IV
−0.001
0.000
0.001
0.002
0.003
β_IV

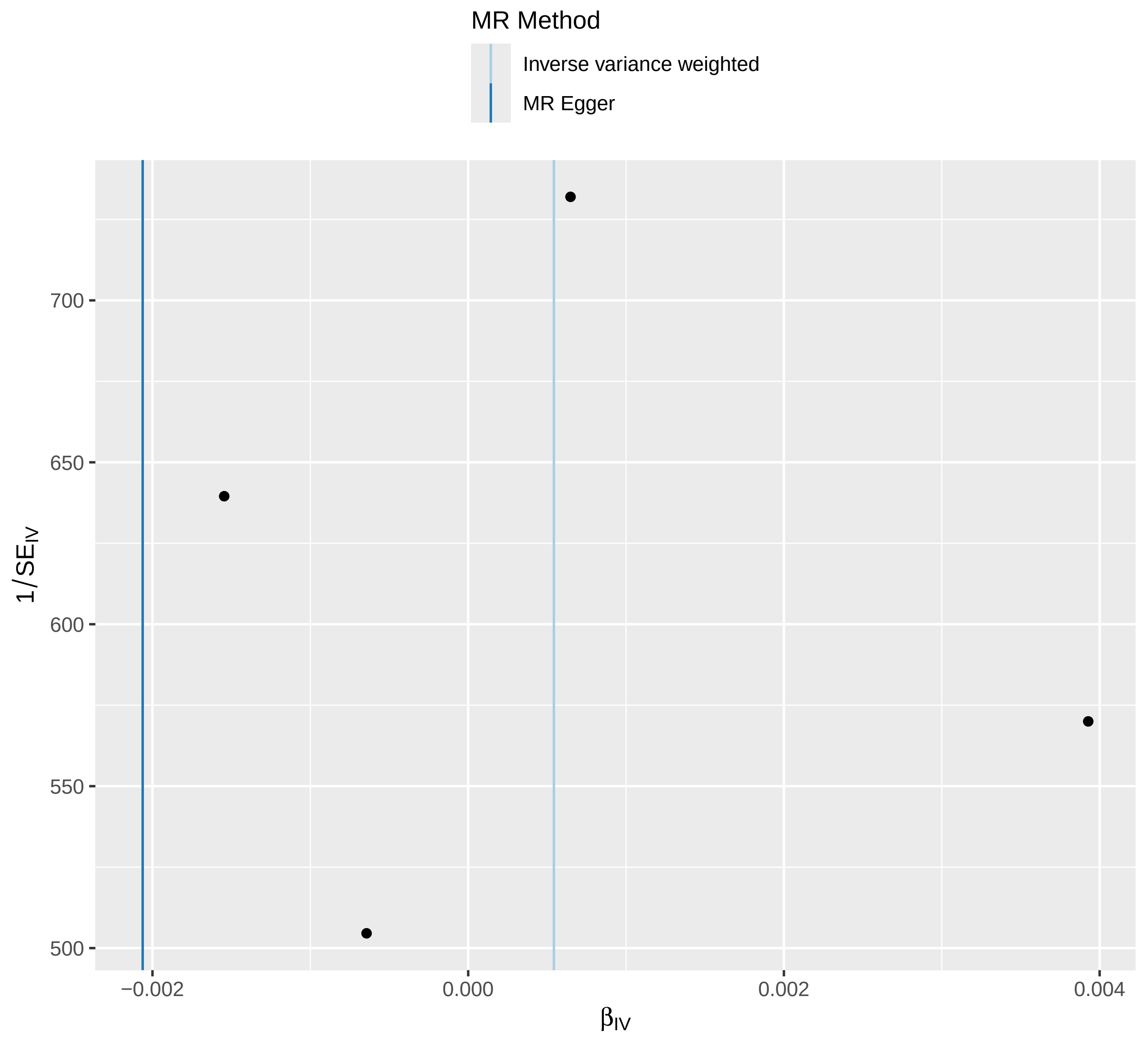

MR Method
Inverse variance weighted
MR Egger
700
650
600
550
500
1/SE_IV
−0.002
0.000
0.002
0.004
β_IV

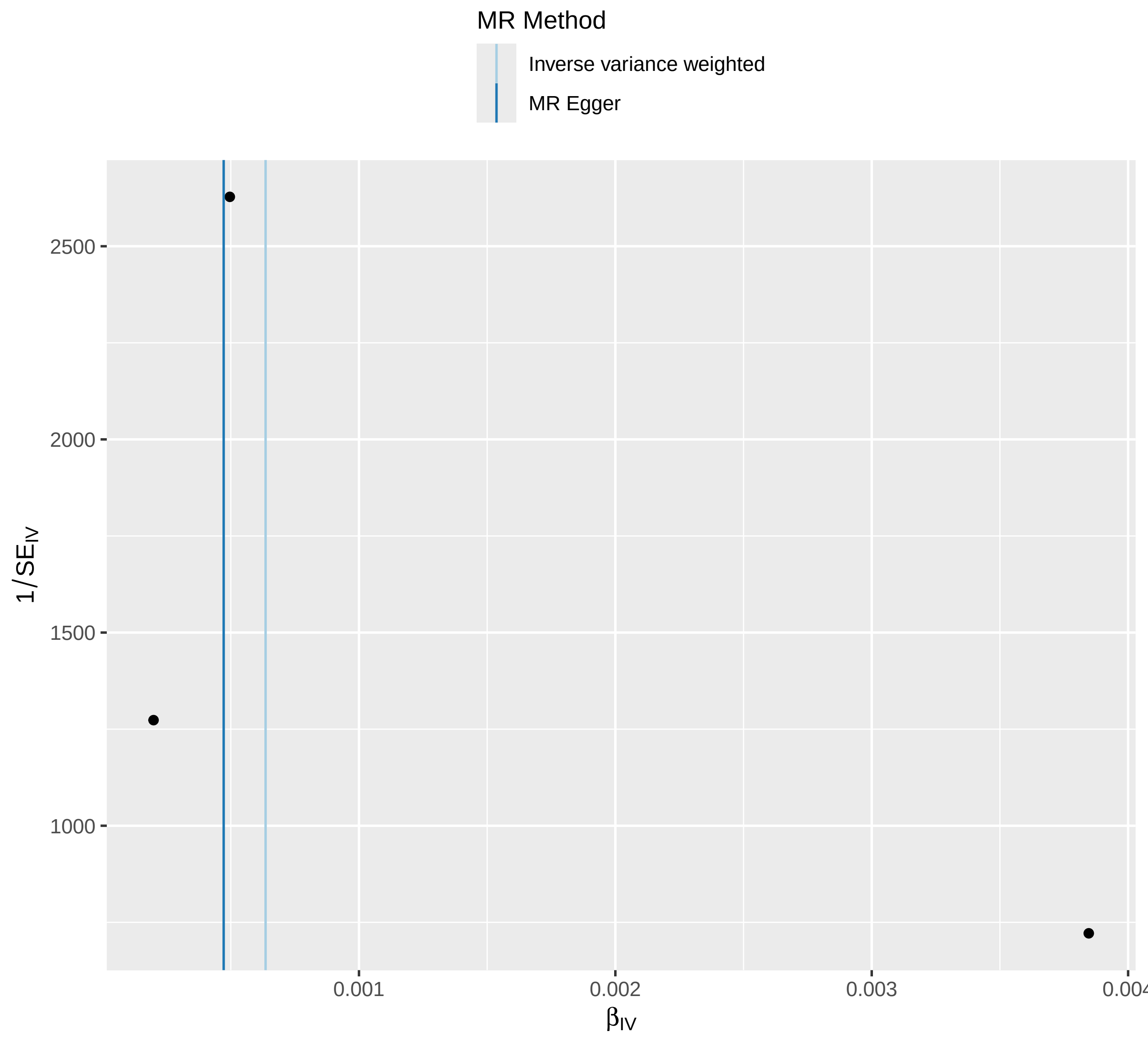
MR Method
Inverse variance weighted
MR Egger
2500
2000
1500
1000
1/SE_IV
0.001
0.002
0.003
0.004
β_IV

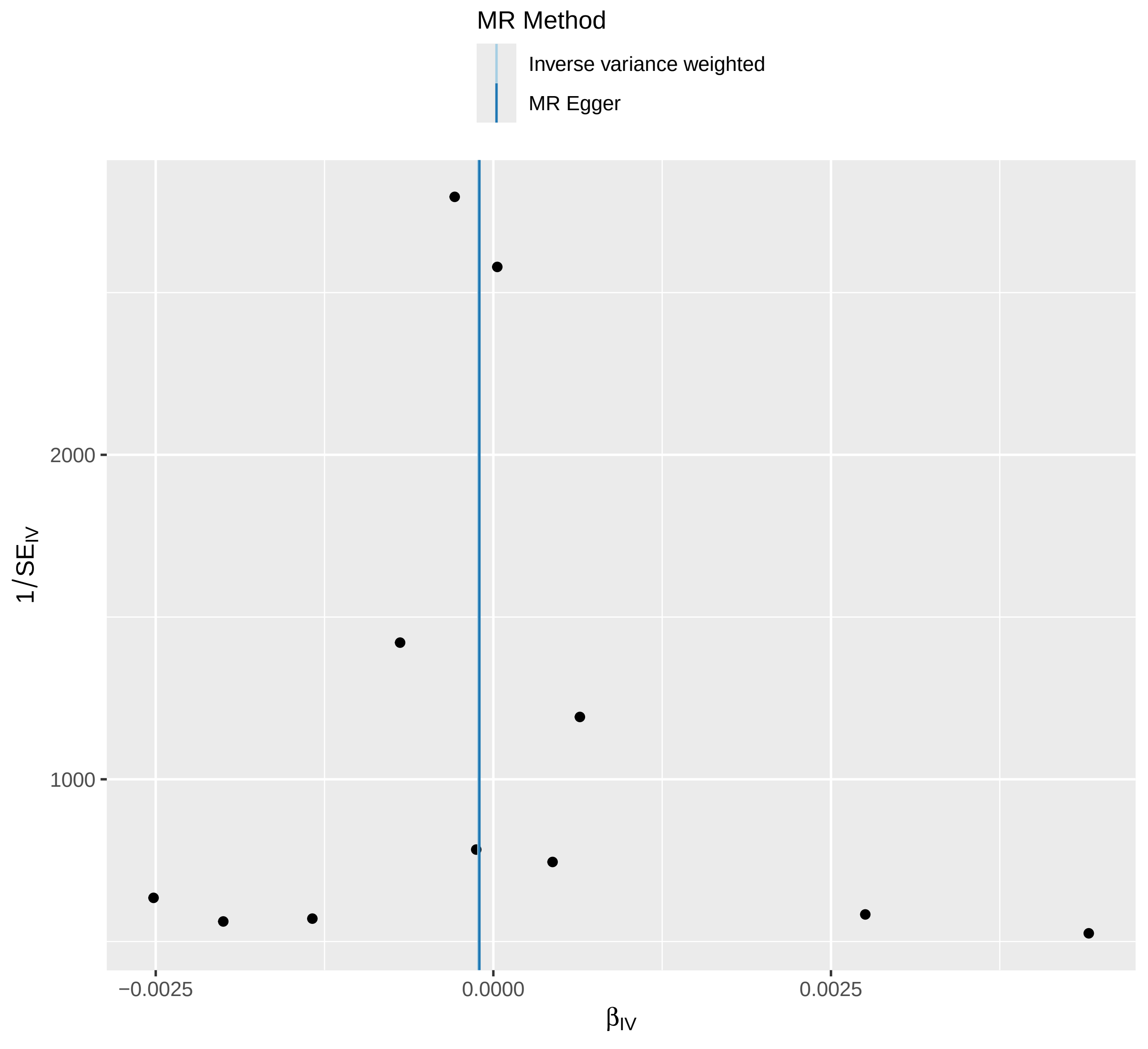

MR Method
Inverse variance weighted
MR Egger
2000
1000
$1/SE_{IV}$
−0.0025
0.0000
0.0025
β_{IV}

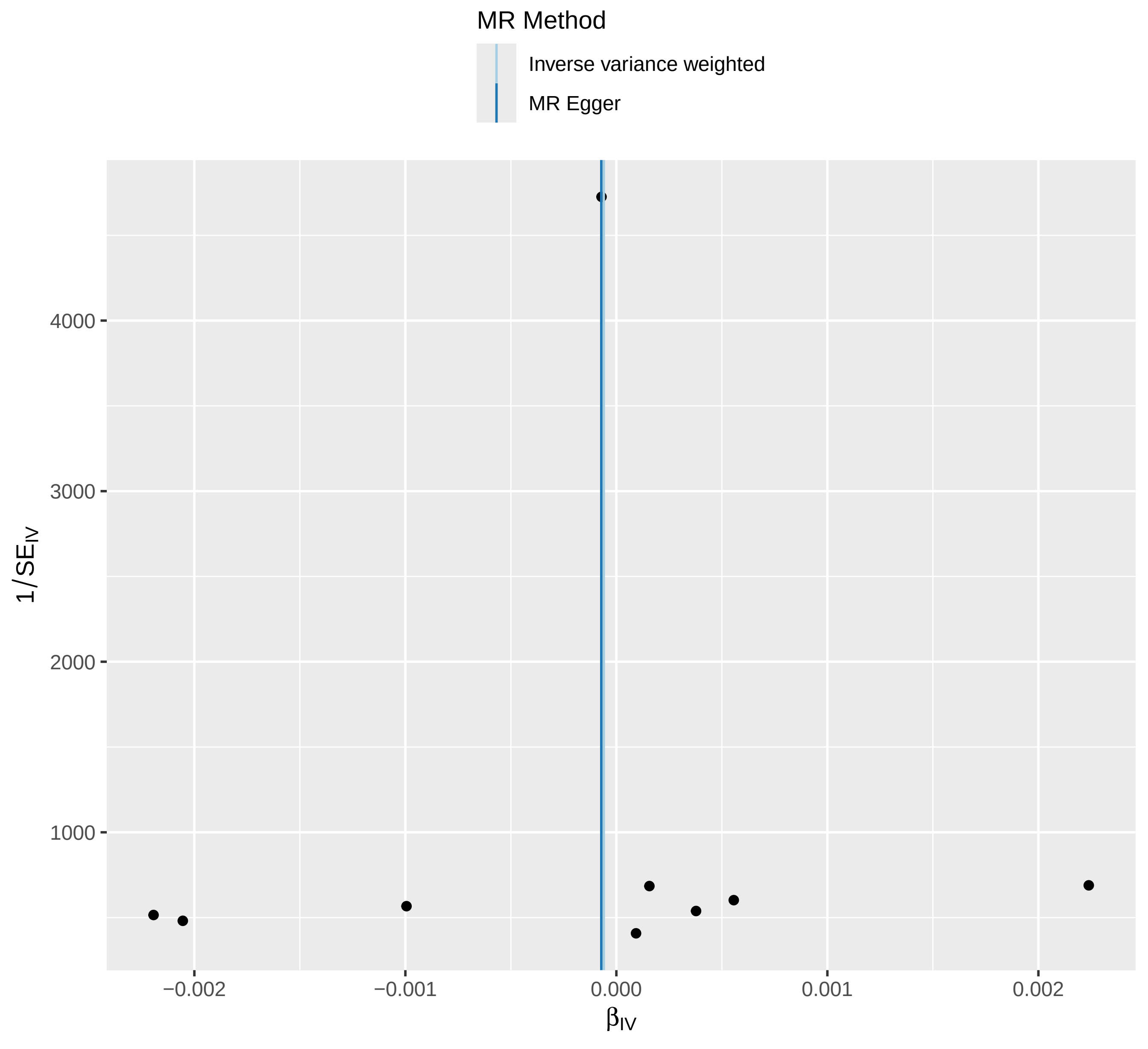
MR Method
Inverse variance weighted
MR Egger
4000
3000
2000
1000
1/SE_IV
−0.002
−0.001
0.000
0.001
0.002
β_IV

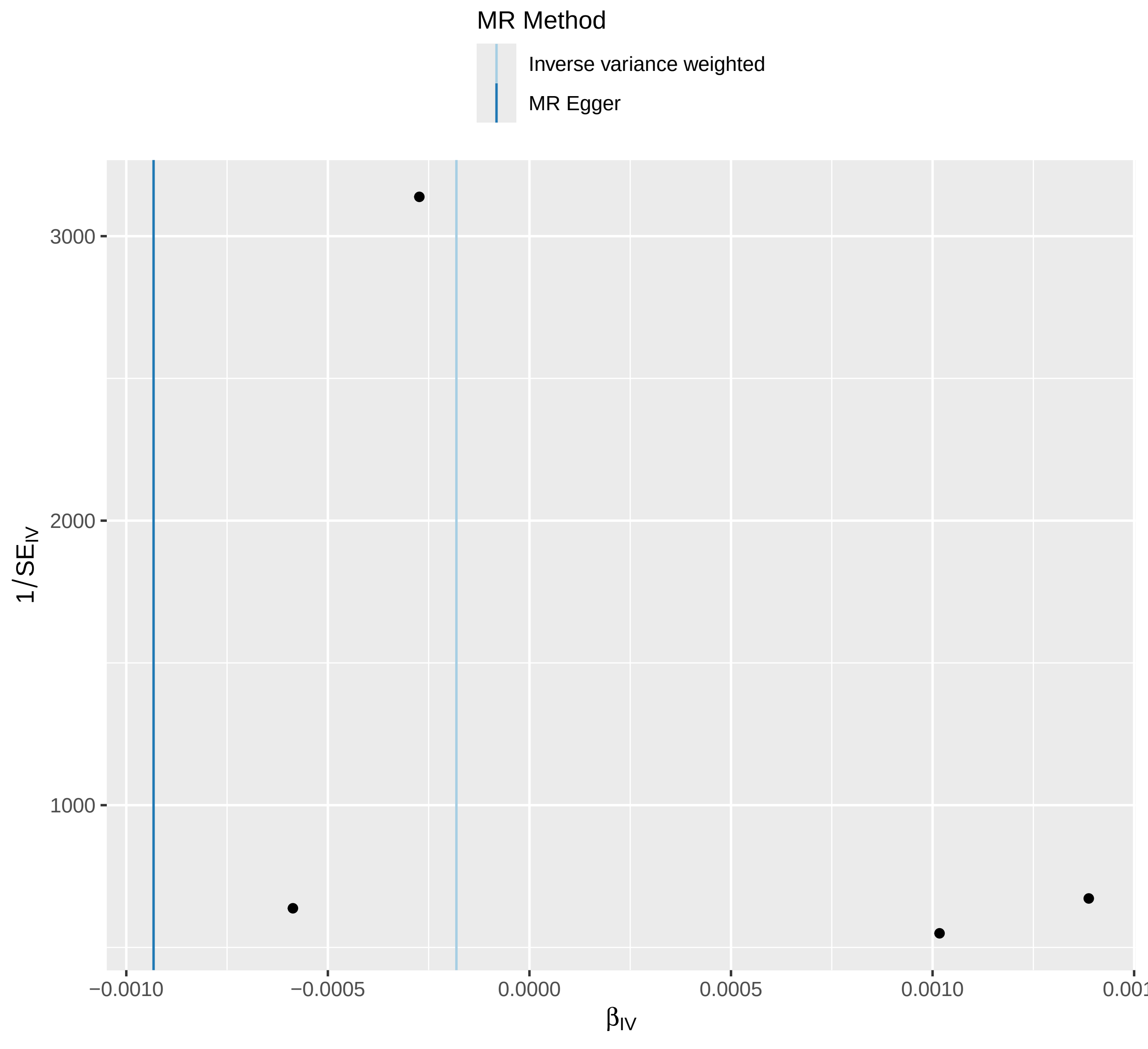

MR Method
Inverse variance weighted
MR Egger
3000
2000
1000
1/SE$_{IV}$
−0.0010
−0.0005
0.0000
0.0005
0.0010
0.001
β$_{IV}$

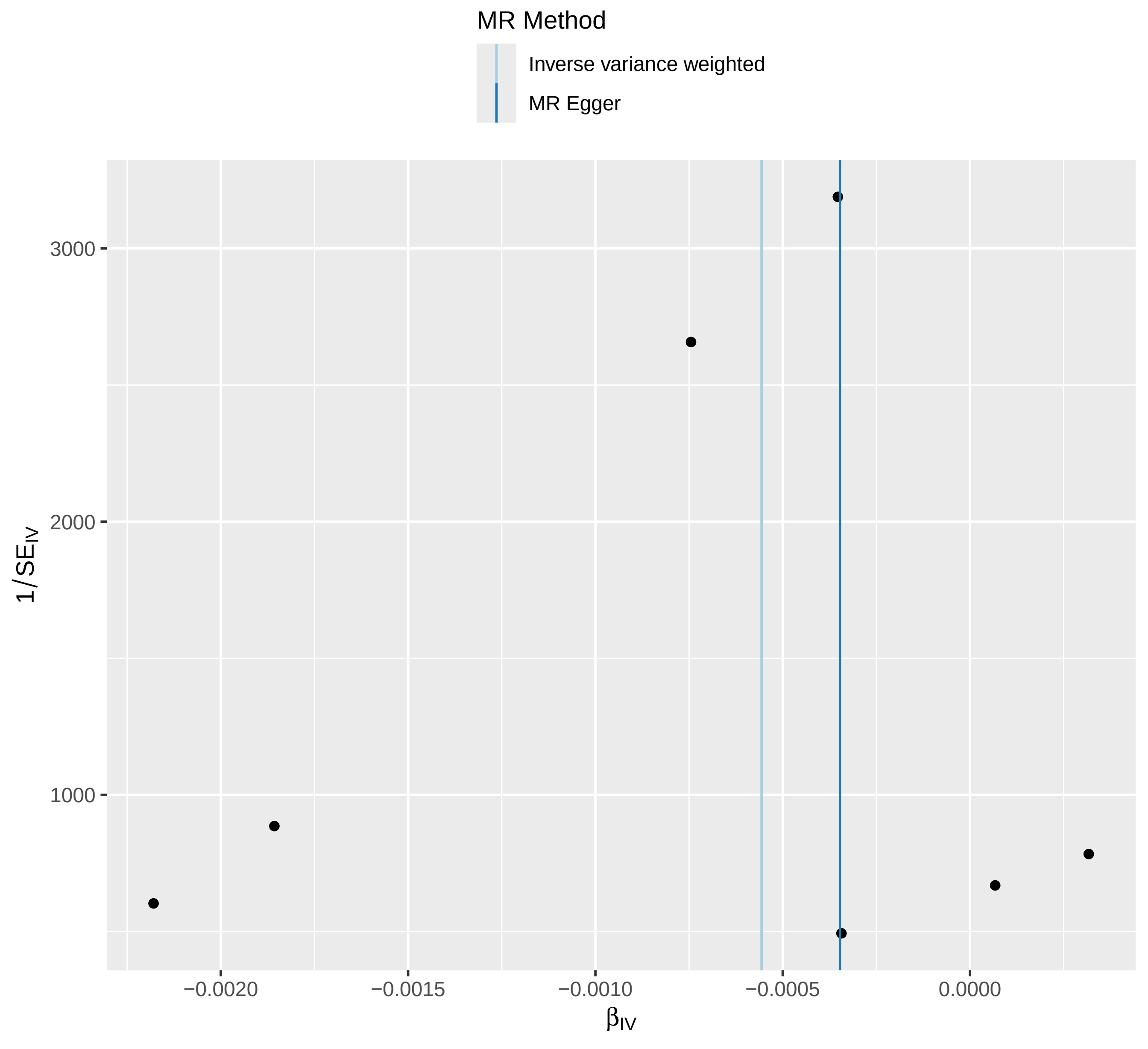

MR Method
Inverse variance weighted
MR Egger
3000
2000
1000
1/SE_IV
−0.0020
−0.0015
−0.0010
−0.0005
0.0000
β_IV

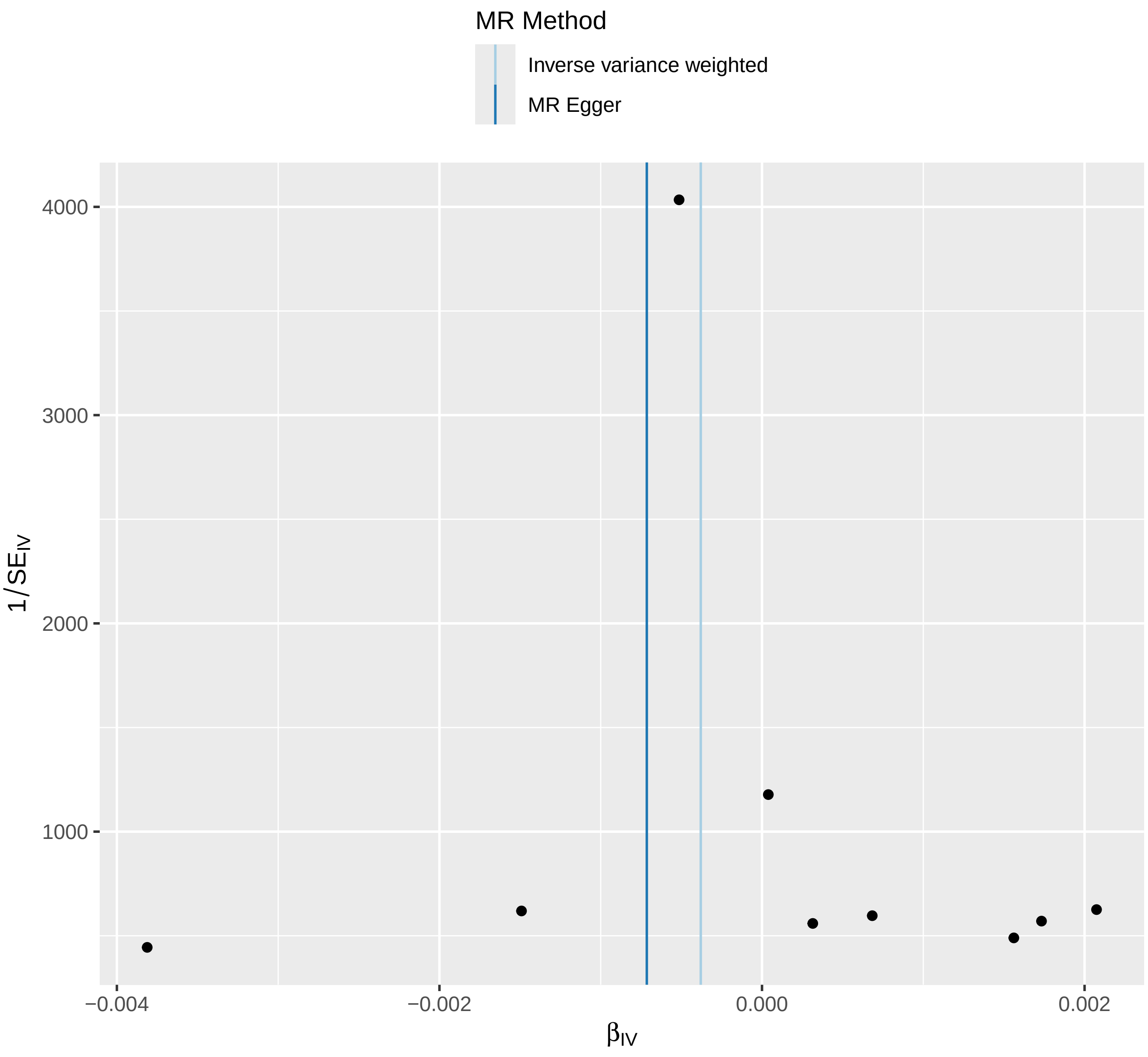

MR Method
Inverse variance weighted
MR Egger
4000
3000
2000
1000
1/SE_IV
−0.004
−0.002
0.000
0.002
β_IV

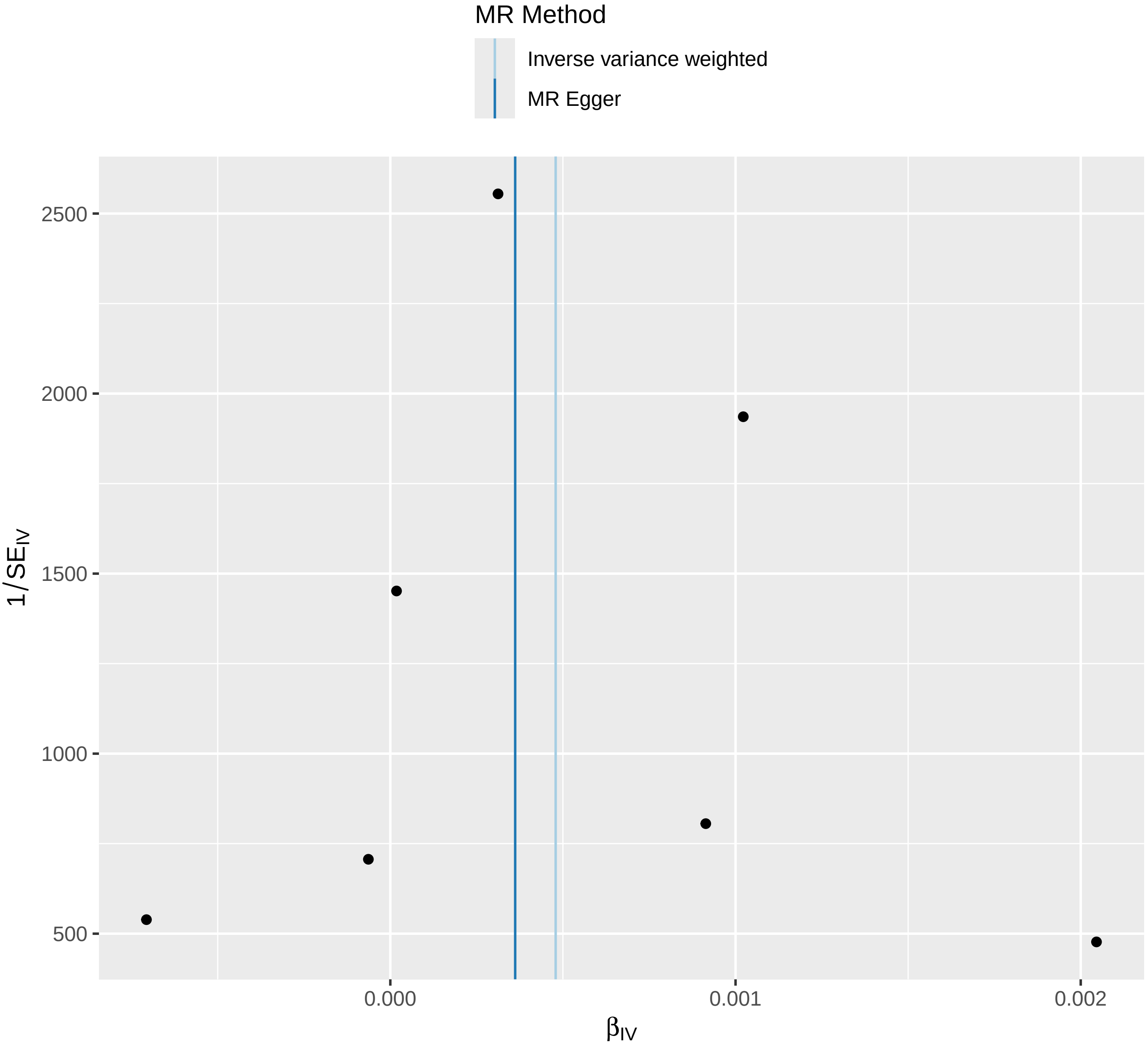
MR Method
Inverse variance weighted
MR Egger
2500
2000
1500
1000
500
1/SE_IV
0.000
0.001
0.002
β_IV

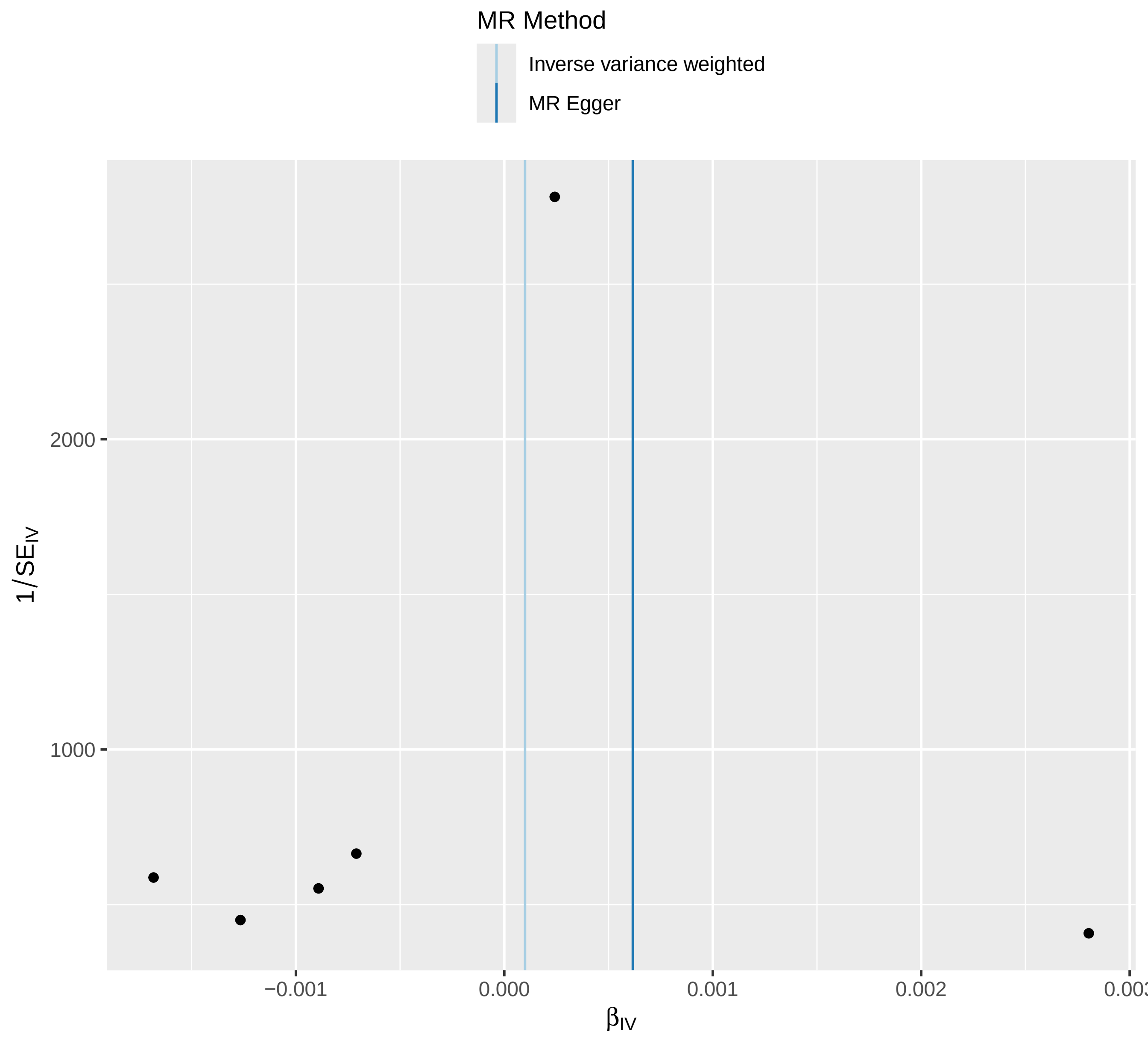

MR Method
Inverse variance weighted
MR Egger
2000
1000
1/SE_IV
−0.001
0.000
0.001
0.002
0.003
β_IV

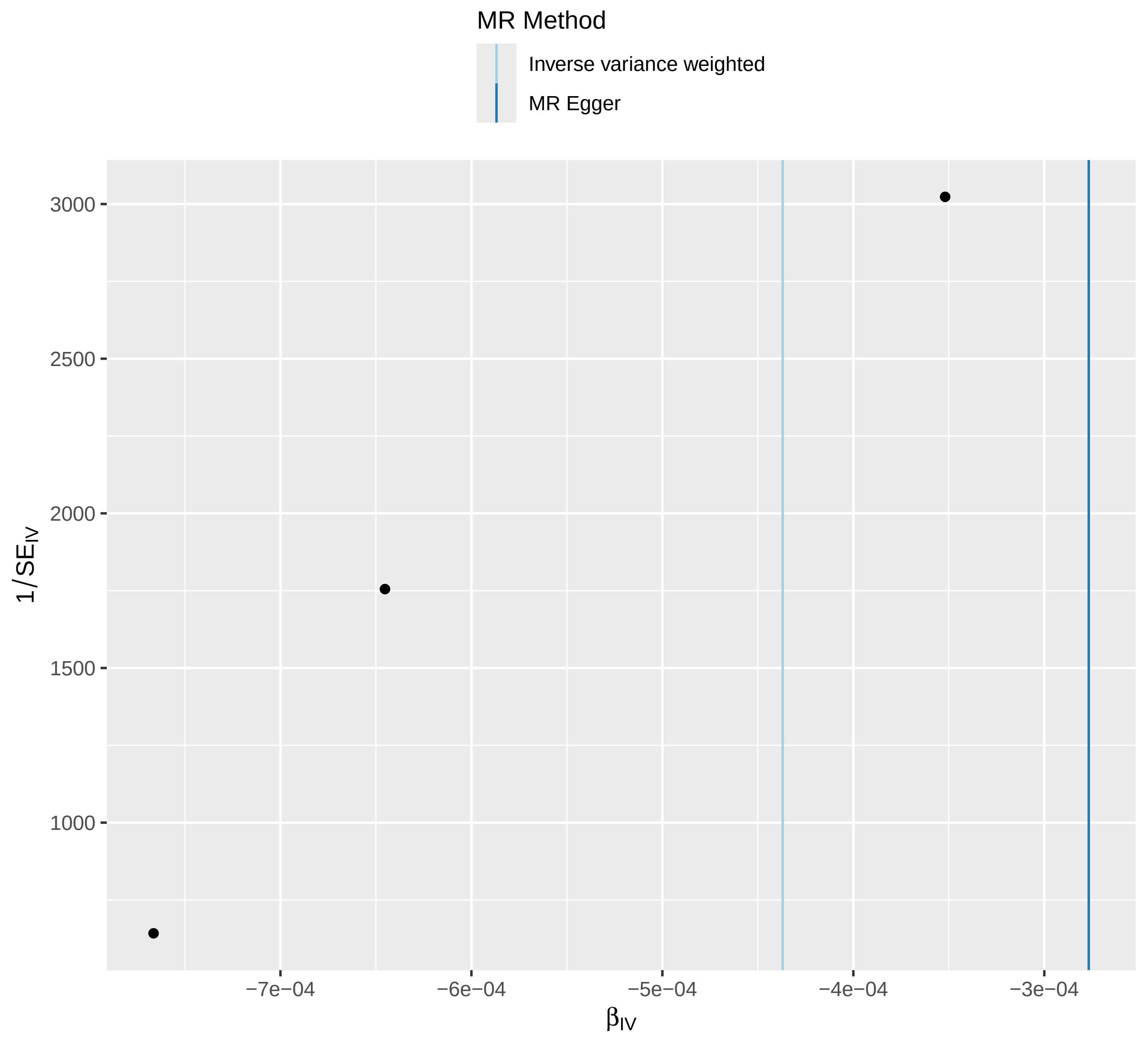
MR Method
Inverse variance weighted
MR Egger
3000
2500
2000
1500
1000
1/SE$_{IV}$
−7e−04
−6e−04
−5e−04
−4e−04
−3e−04
β$_{IV}$

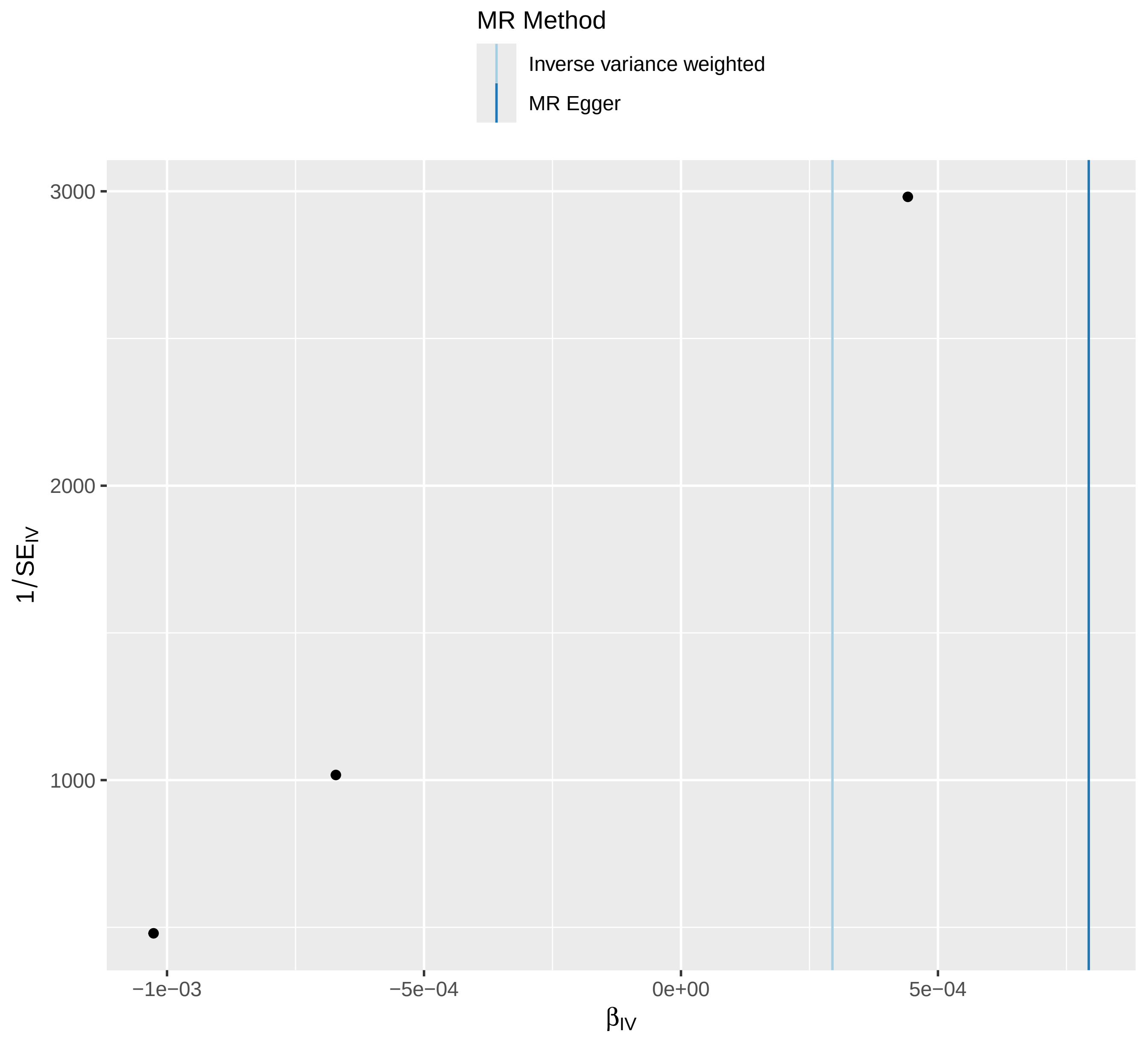
MR Method
Inverse variance weighted
MR Egger
3000
2000
1000
1/SE_IV
−1e−03
−5e−04
0e+00
5e−04
β_IV

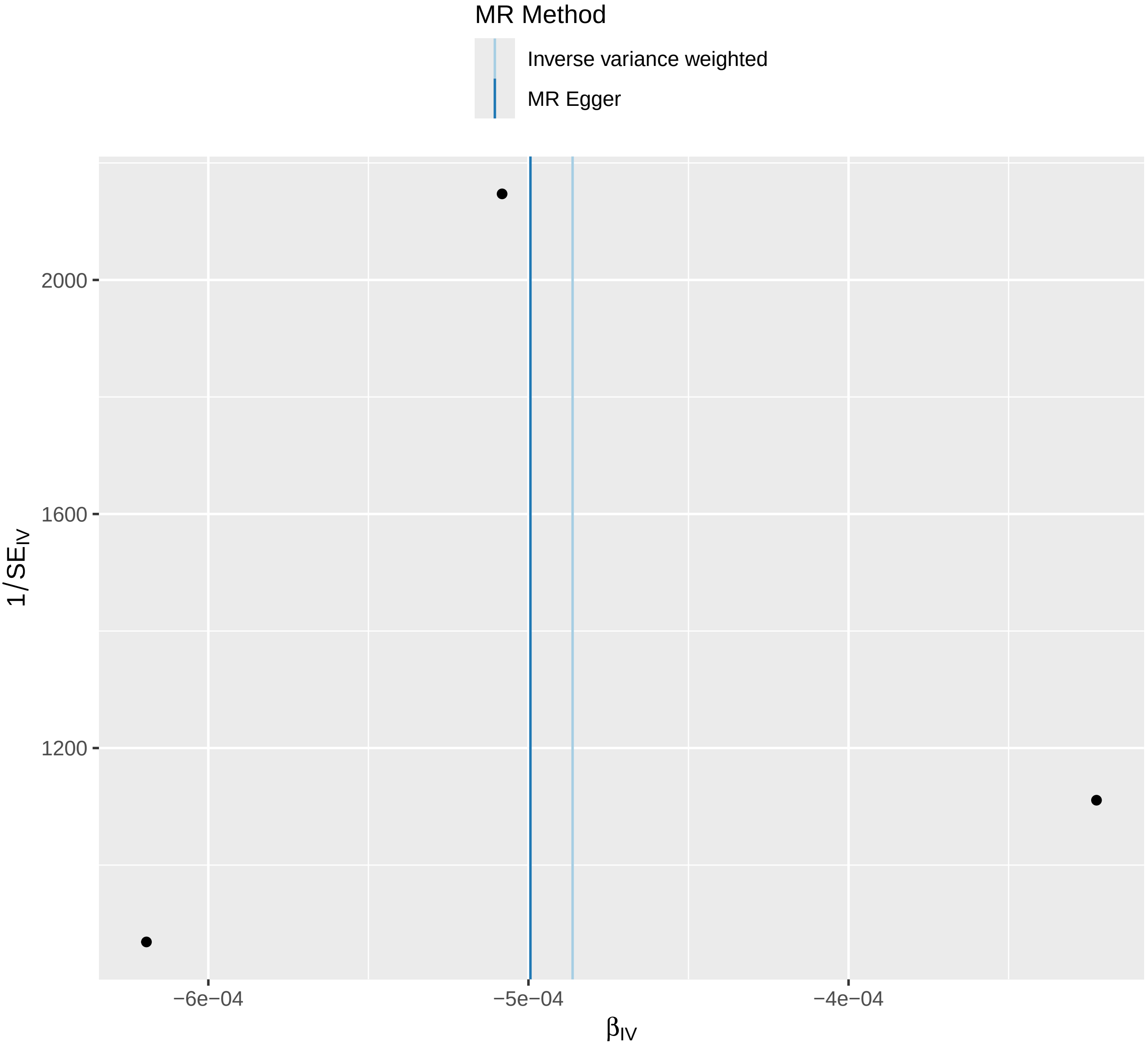

MR Method
Inverse variance weighted
MR Egger
2000
1600
1200
1/SE_IV
−6e−04
−5e−04
−4e−04
β_IV

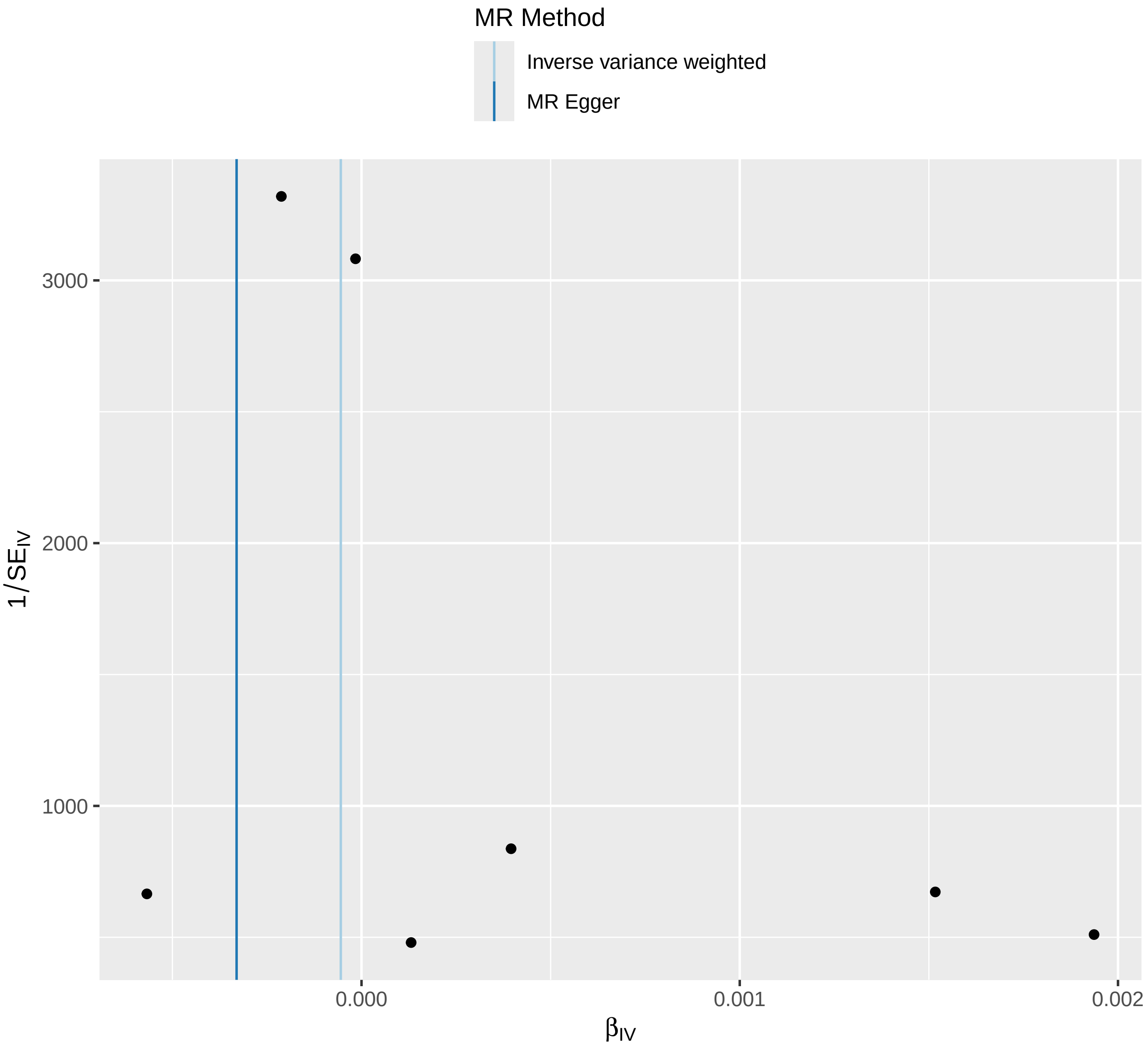

MR Method
Inverse variance weighted
MR Egger
3000
2000
1000
1/SE_IV
0.000
0.001
0.002
β_IV

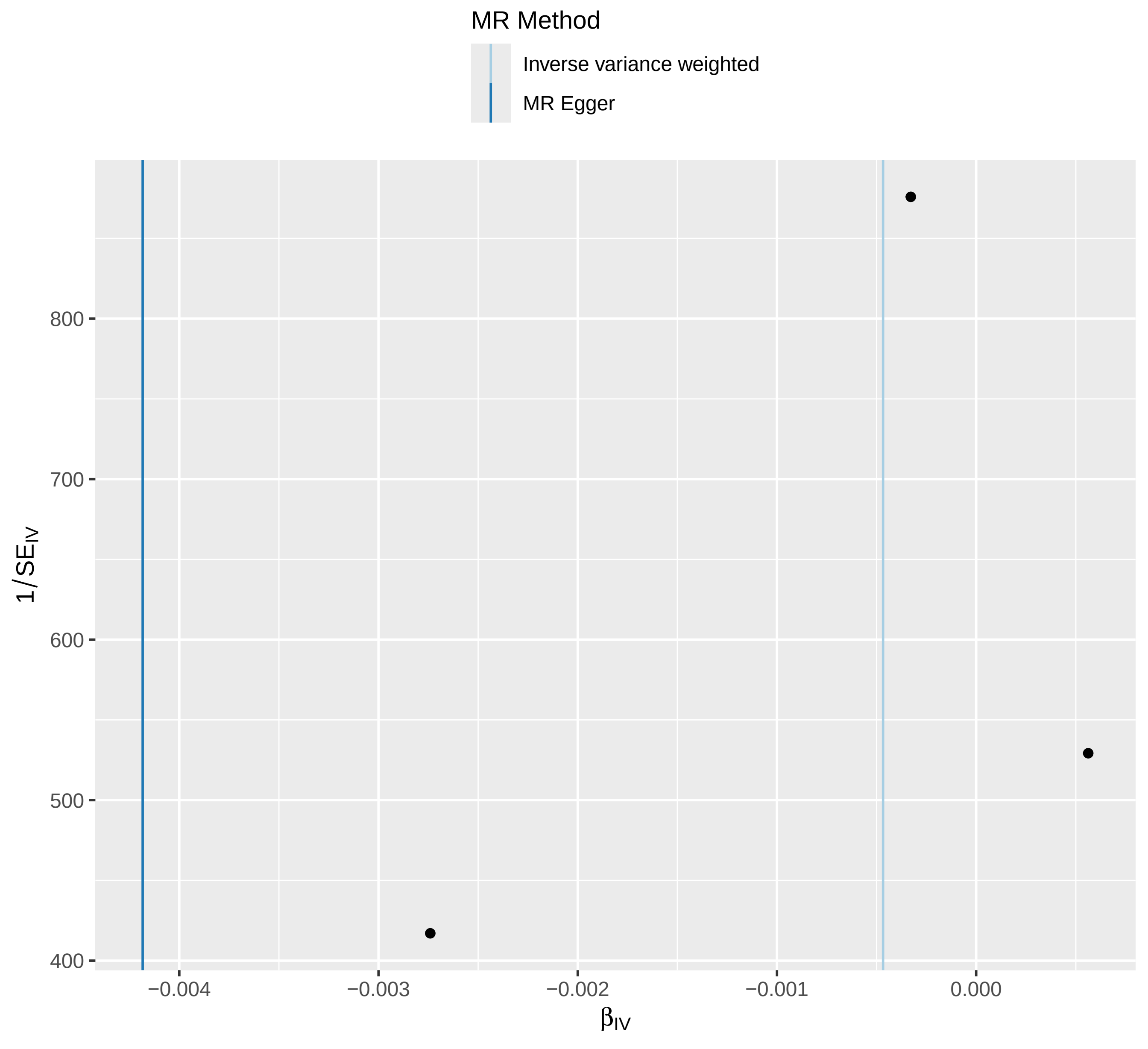
MR Method
Inverse variance weighted
MR Egger
800
700
600
500
400
1/SE_IV
−0.004
−0.003
−0.002
−0.001
0.000
β_IV

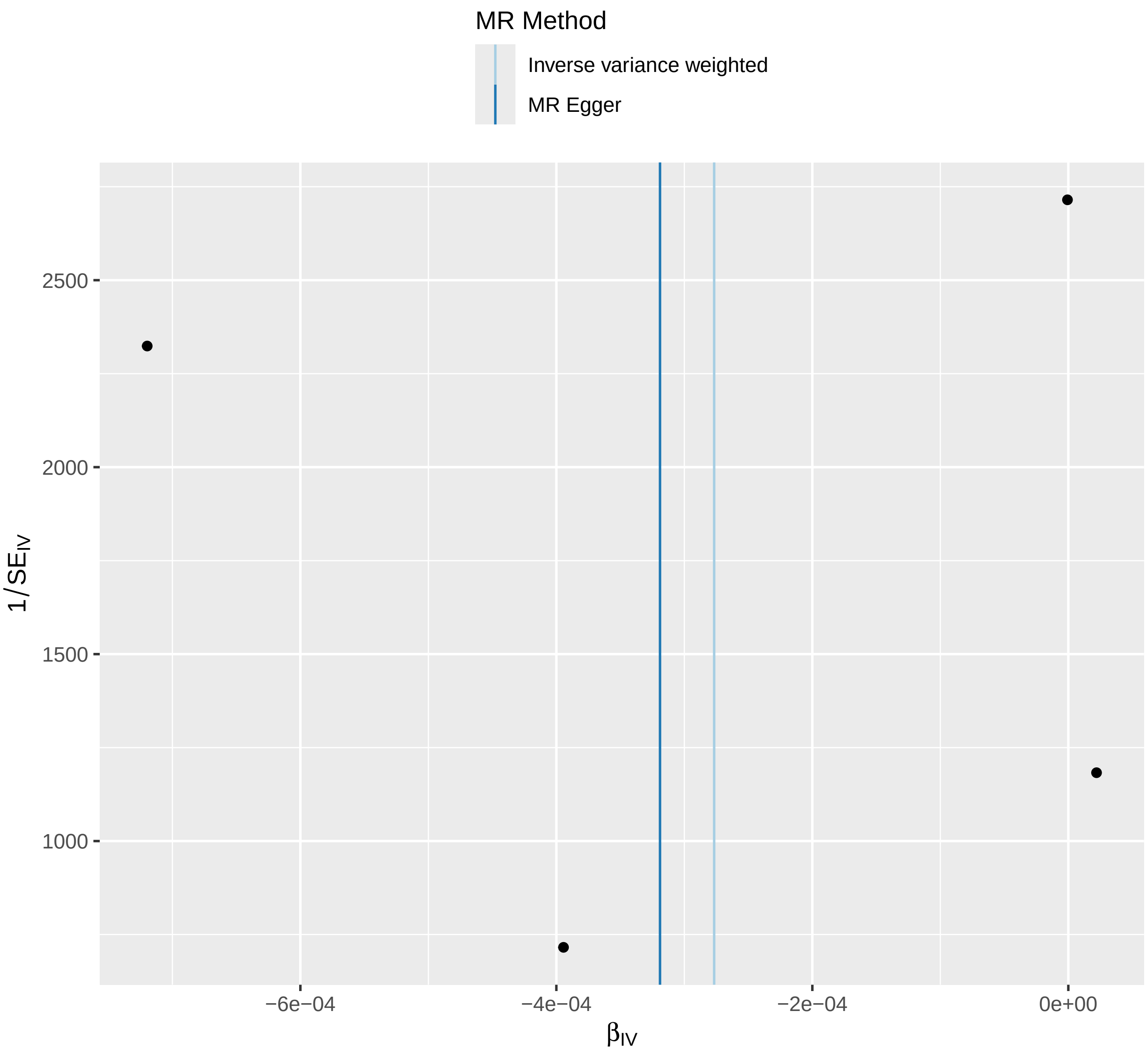
MR Method
Inverse variance weighted
MR Egger
2500
2000
1500
1000
1/SE_IV
−6e−04
−4e−04
−2e−04
0e+00
β_IV

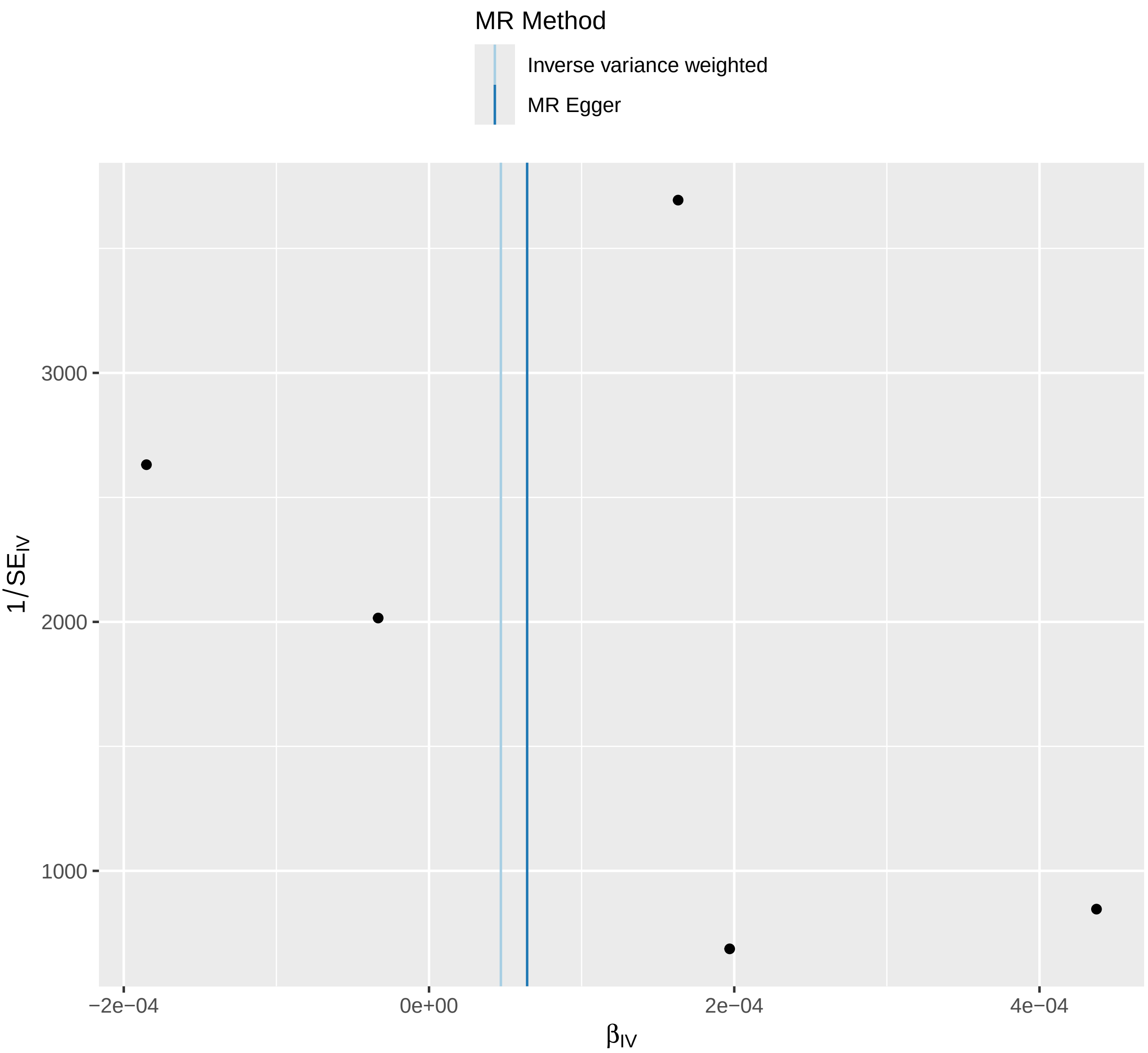
MR Method
Inverse variance weighted
MR Egger
3000
2000
1000
1/SE_IV
−2e−04
0e+00
2e−04
4e−04
β_IV

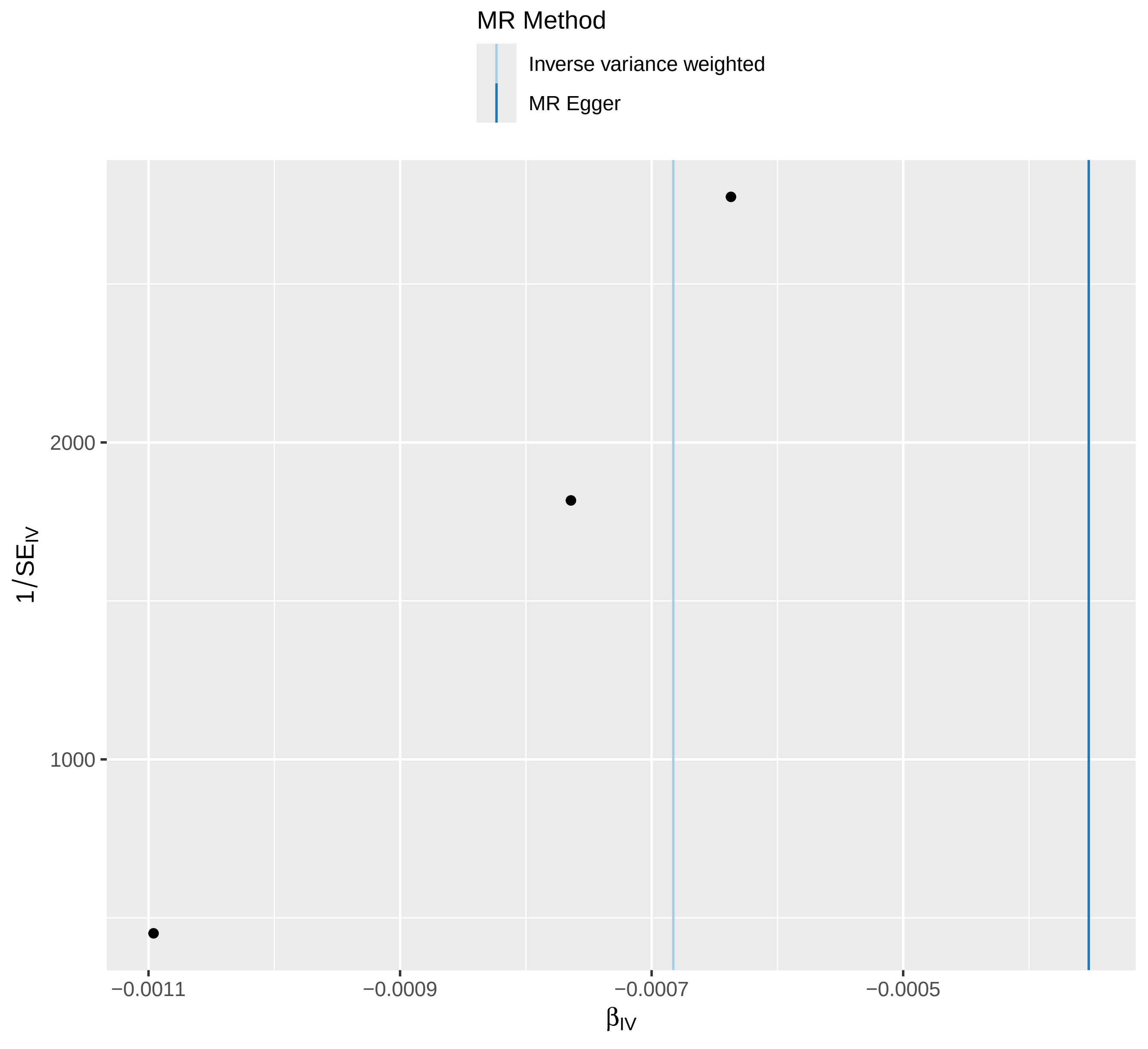

MR Method
Inverse variance weighted
MR Egger
2000
1000
1/SE$_{IV}$
−0.0011
−0.0009
−0.0007
−0.0005
β$_{IV}$

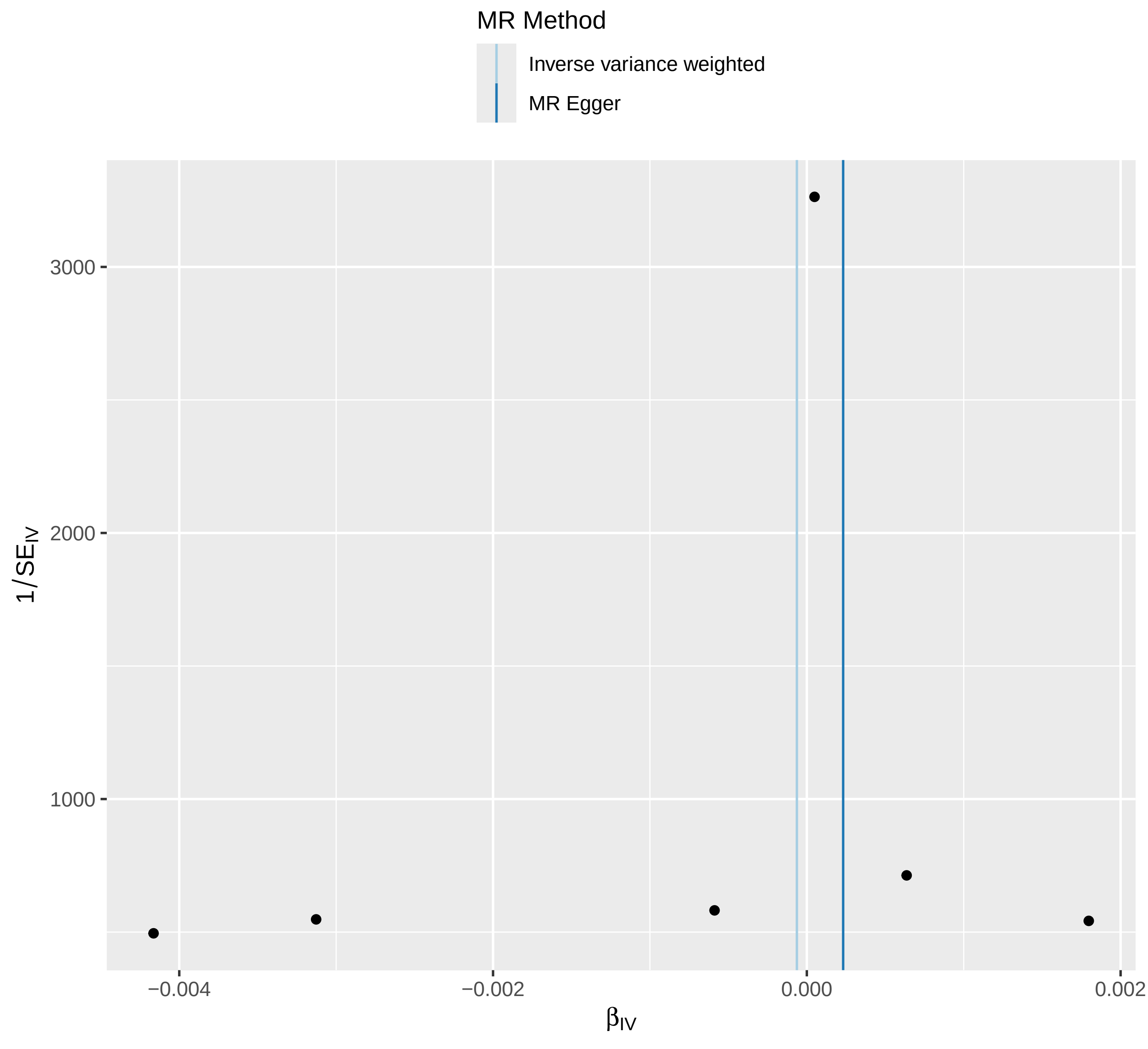

MR Method
Inverse variance weighted
MR Egger
3000
2000
1000
1/SE_IV
−0.004
−0.002
0.000
0.002
β_IV

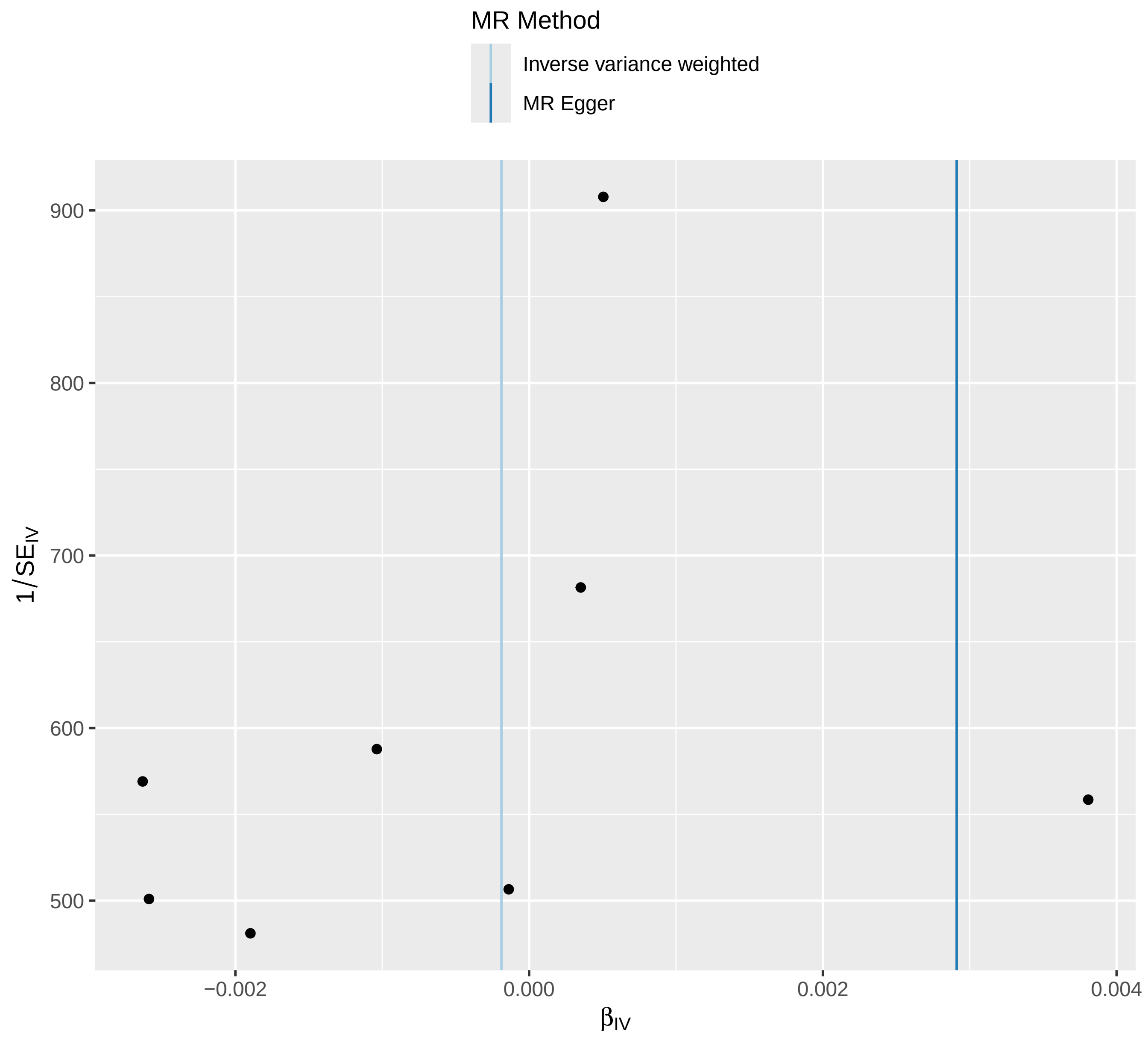

MR Method
Inverse variance weighted
MR Egger
900
800
700
600
500
1/SE_IV
−0.002
0.000
0.002
0.004
β_IV

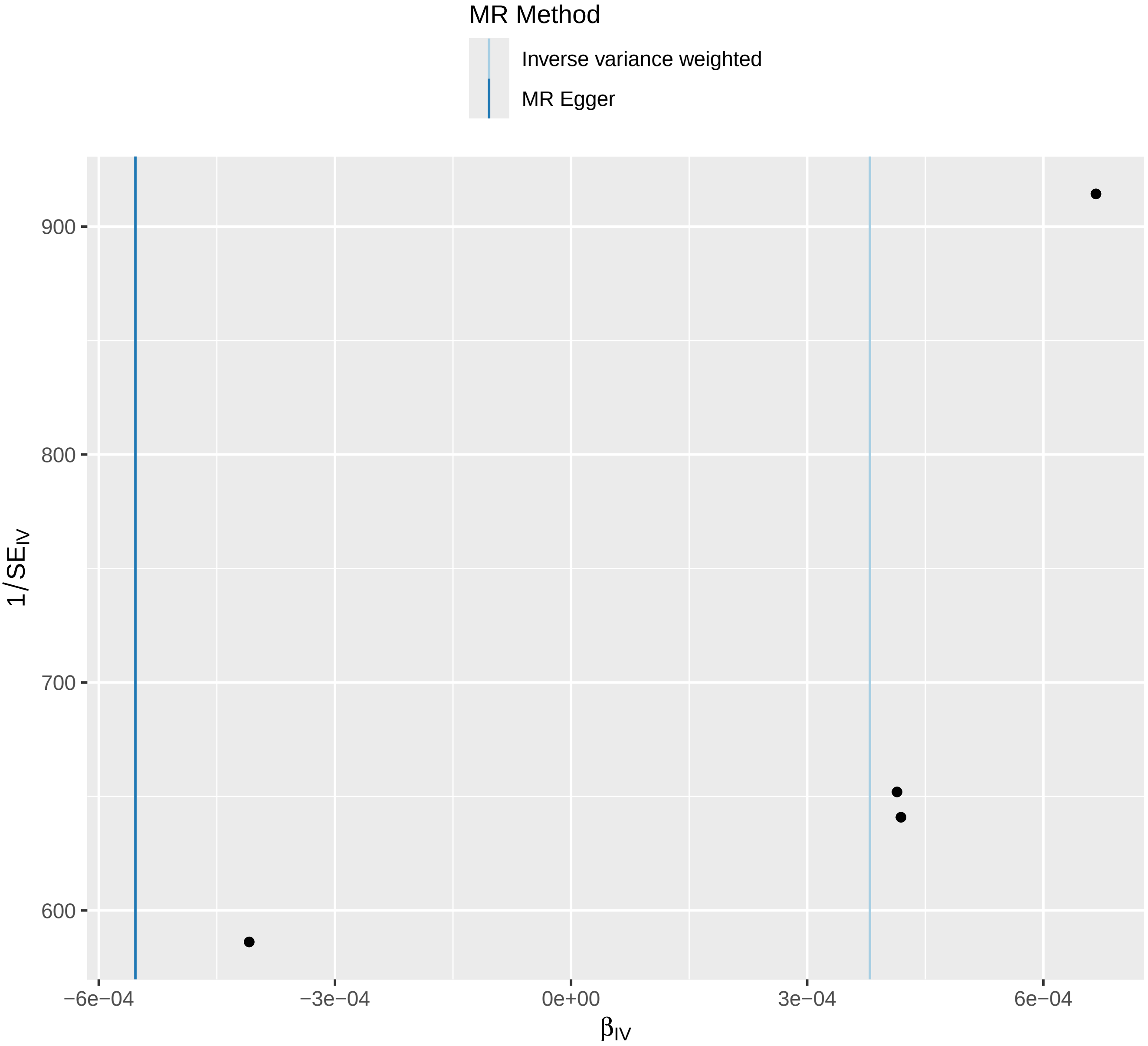
MR Method
Inverse variance weighted
MR Egger
900
800
700
600
1/SE$_{IV}$
−6e−04
−3e−04
0e+00
3e−04
6e−04
β$_{IV}$

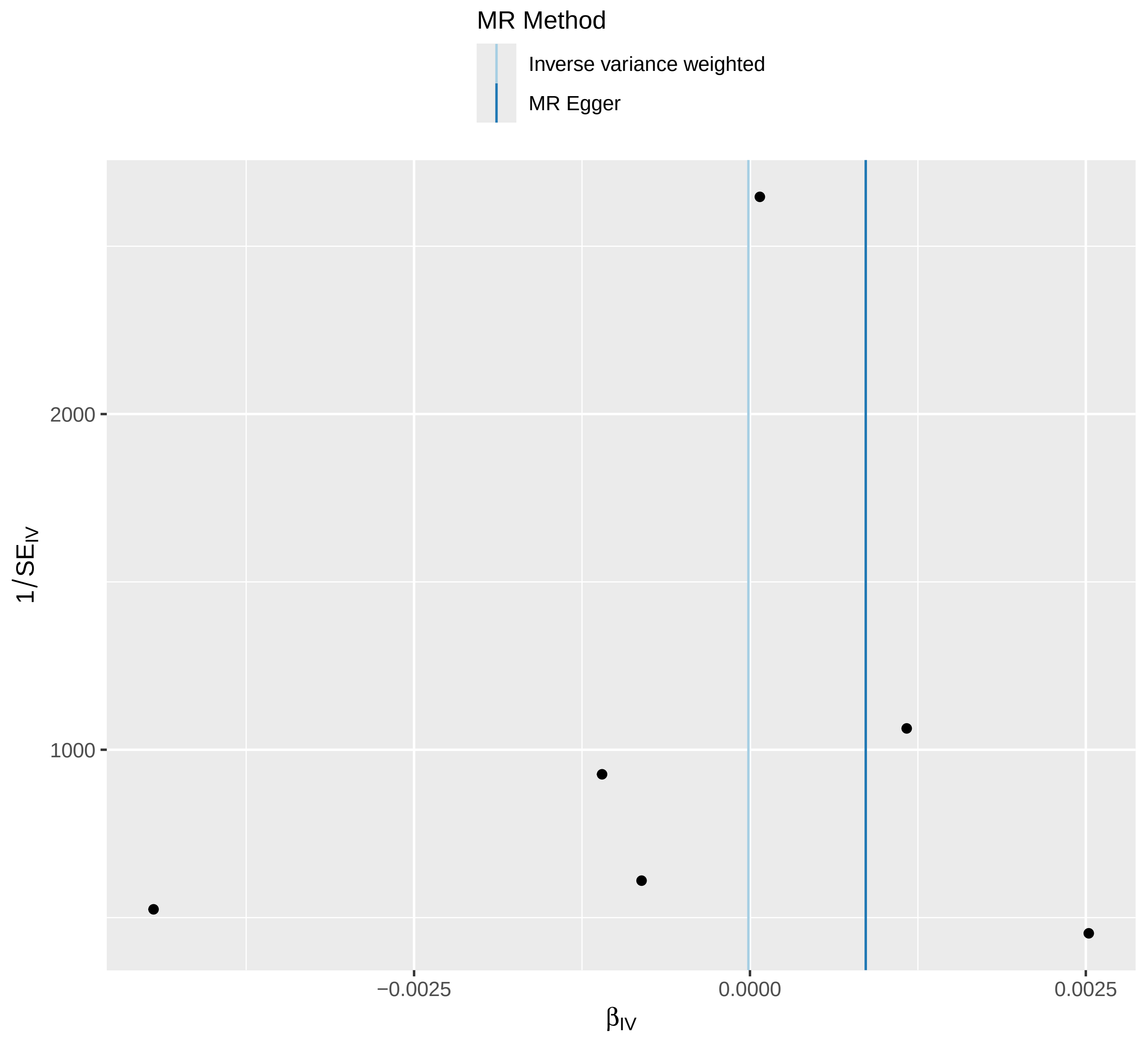
MR Method
Inverse variance weighted
MR Egger
2000
1000
1/SE_IV
−0.0025
0.0000
0.0025
β_IV

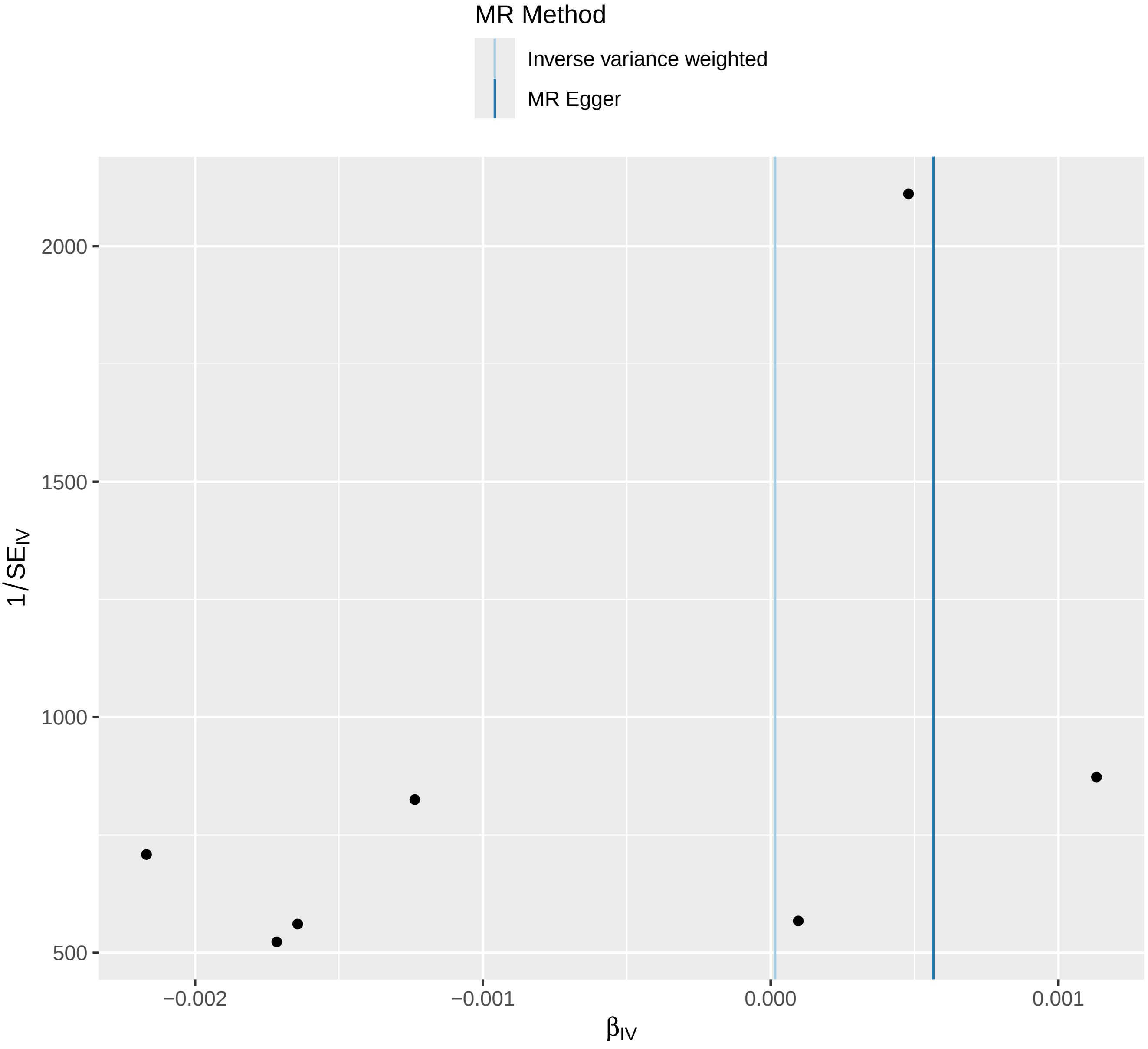

MR Method
Inverse variance weighted
MR Egger
2000
1500
1000
500
1/SE$_{IV}$
−0.002
−0.001
0.000
0.001
β$_{IV}$

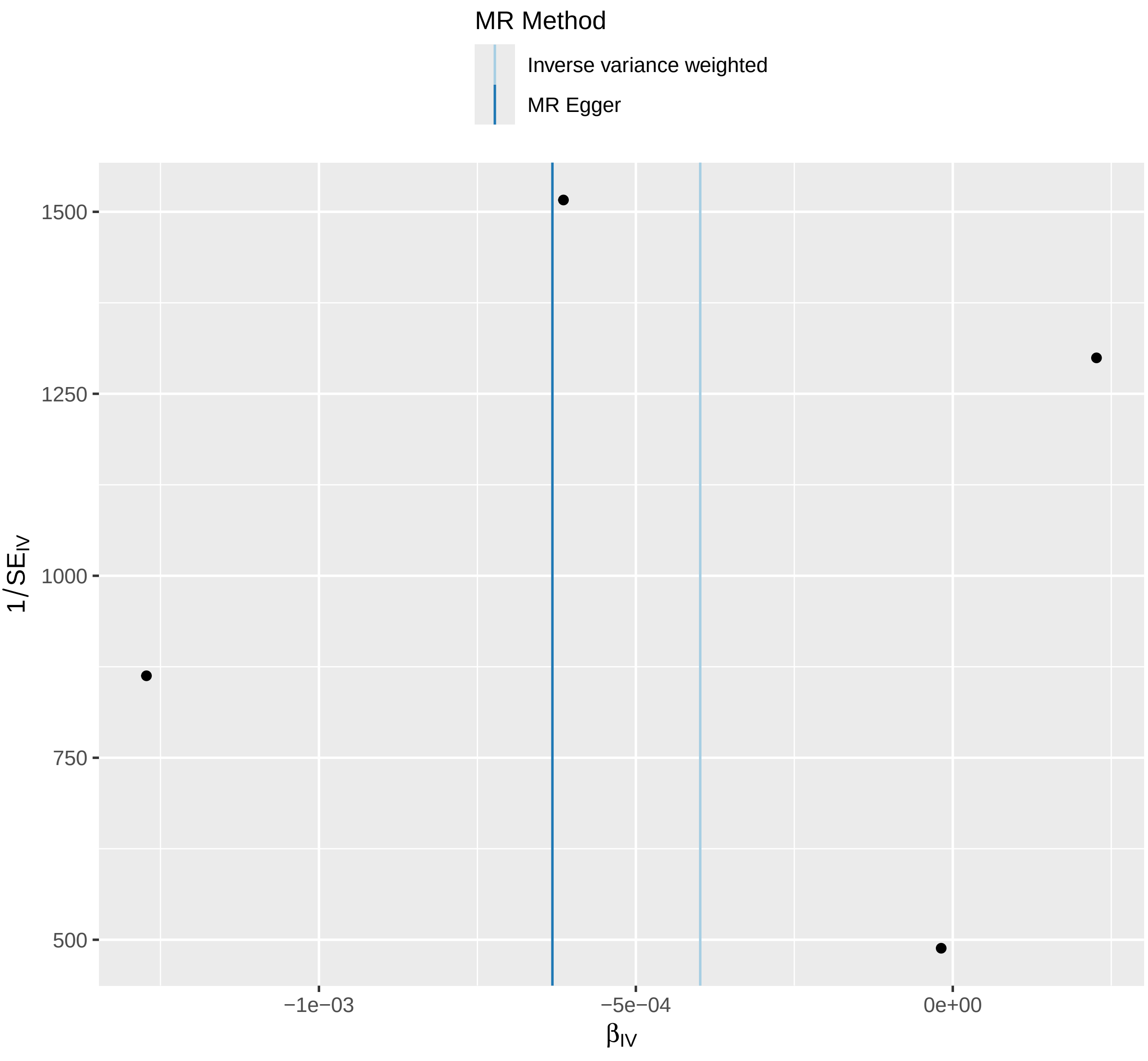
MR Method
Inverse variance weighted
MR Egger
1500
1250
1000
750
500
1/SE$_{IV}$
−1e−03
−5e−04
0e+00
β$_{IV}$

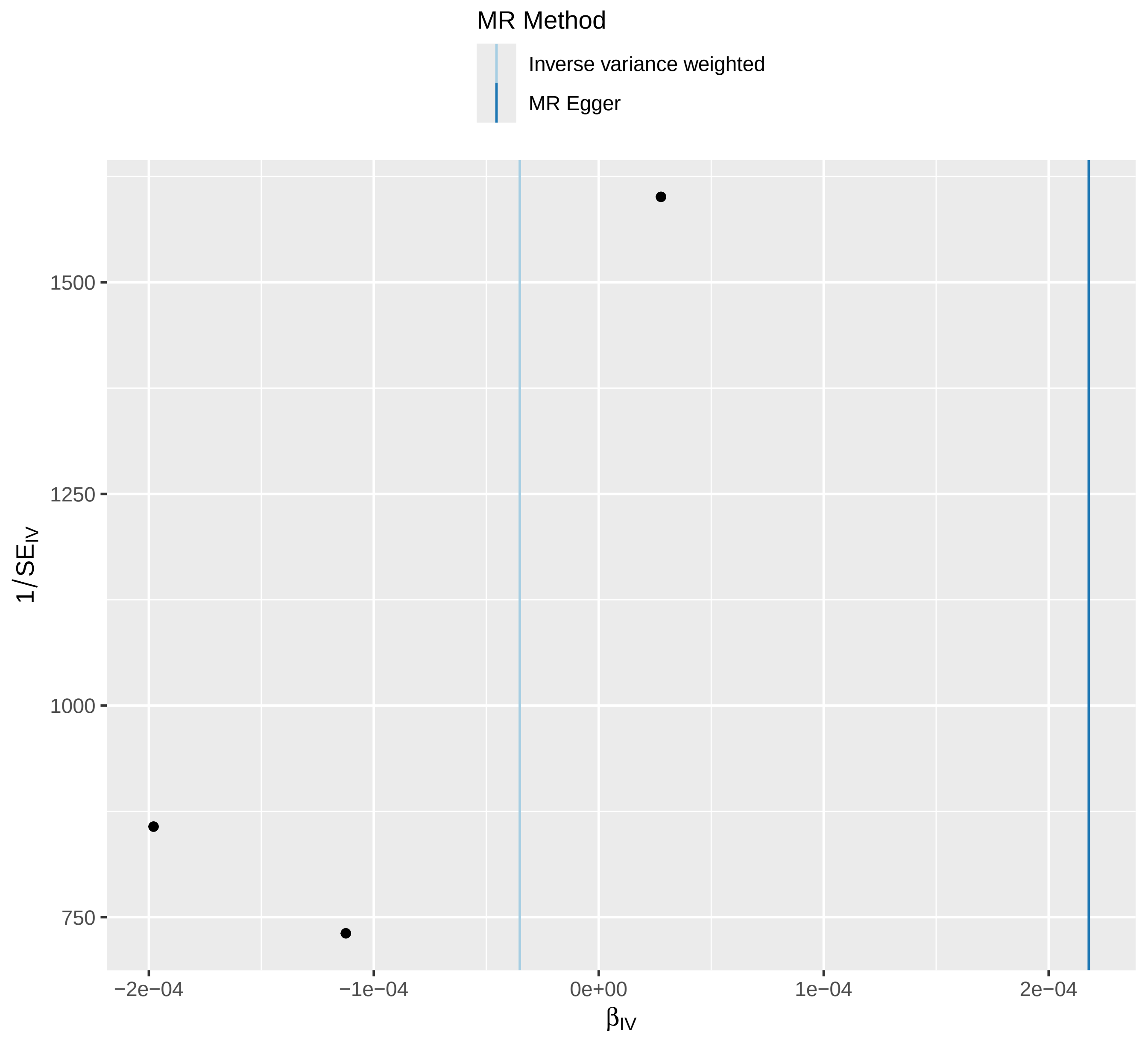

MR Method
Inverse variance weighted
MR Egger
1500
1250
1000
750
1/SE_IV
−2e−04
−1e−04
0e+00
1e−04
2e−04
β_IV

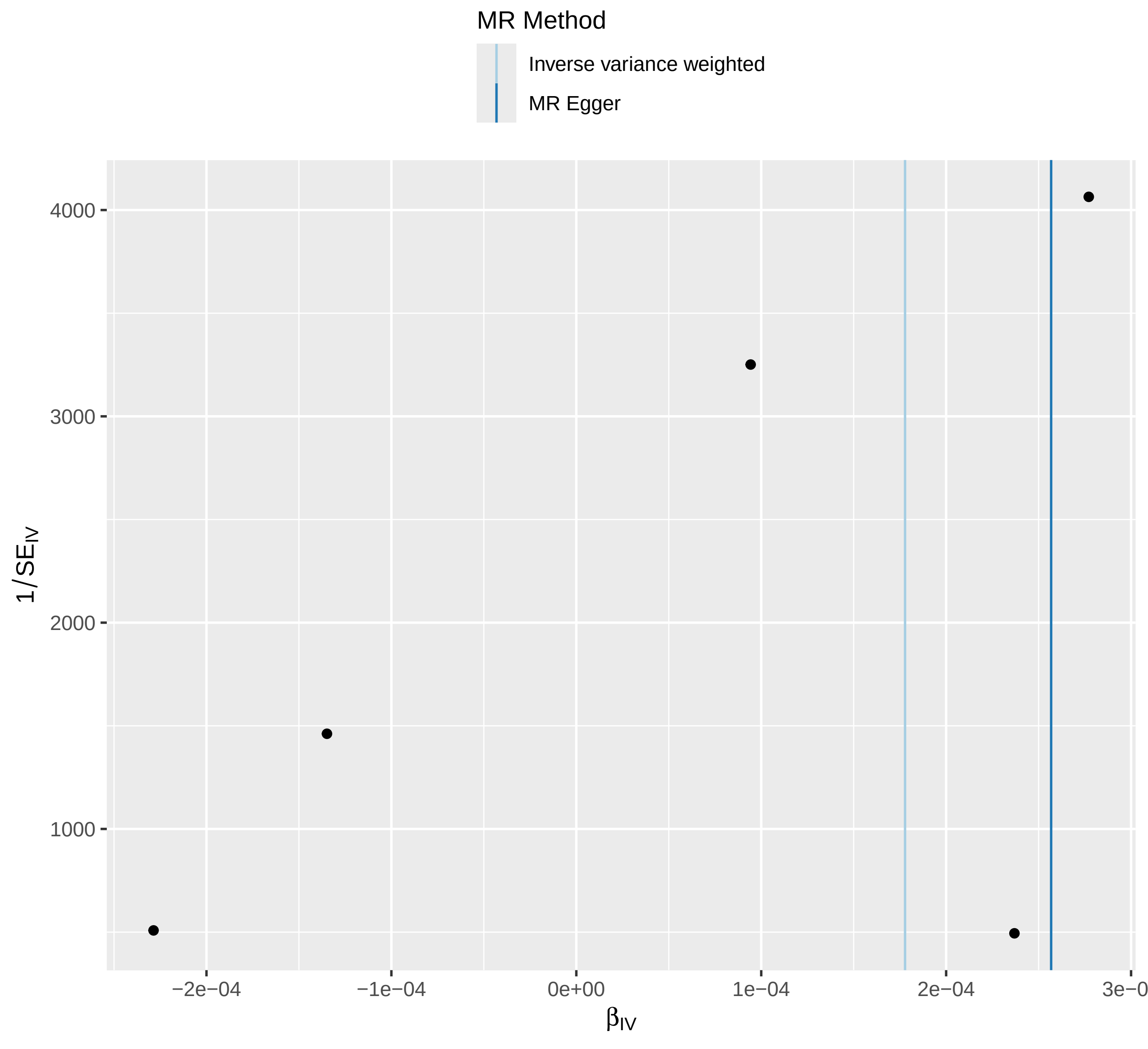

MR Method
Inverse variance weighted
MR Egger
4000
3000
2000
1000
$1/SE_{IV}$
−2e−04
−1e−04
0e+00
1e−04
2e−04
3e−0
β_{IV}

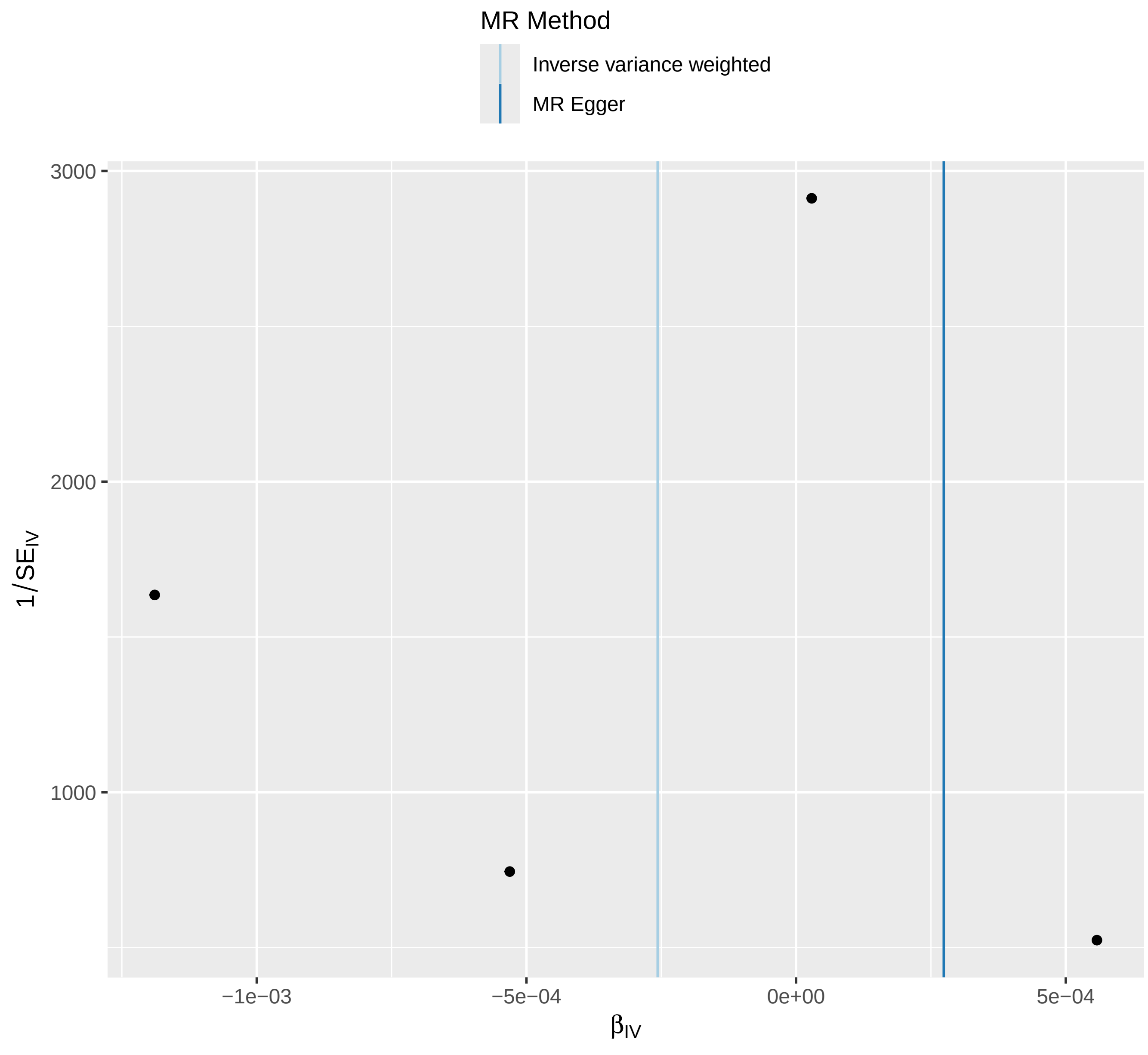

MR Method
Inverse variance weighted
MR Egger
3000
2000
1000
1/SE_IV
−1e−03
−5e−04
0e+00
5e−04
β_IV

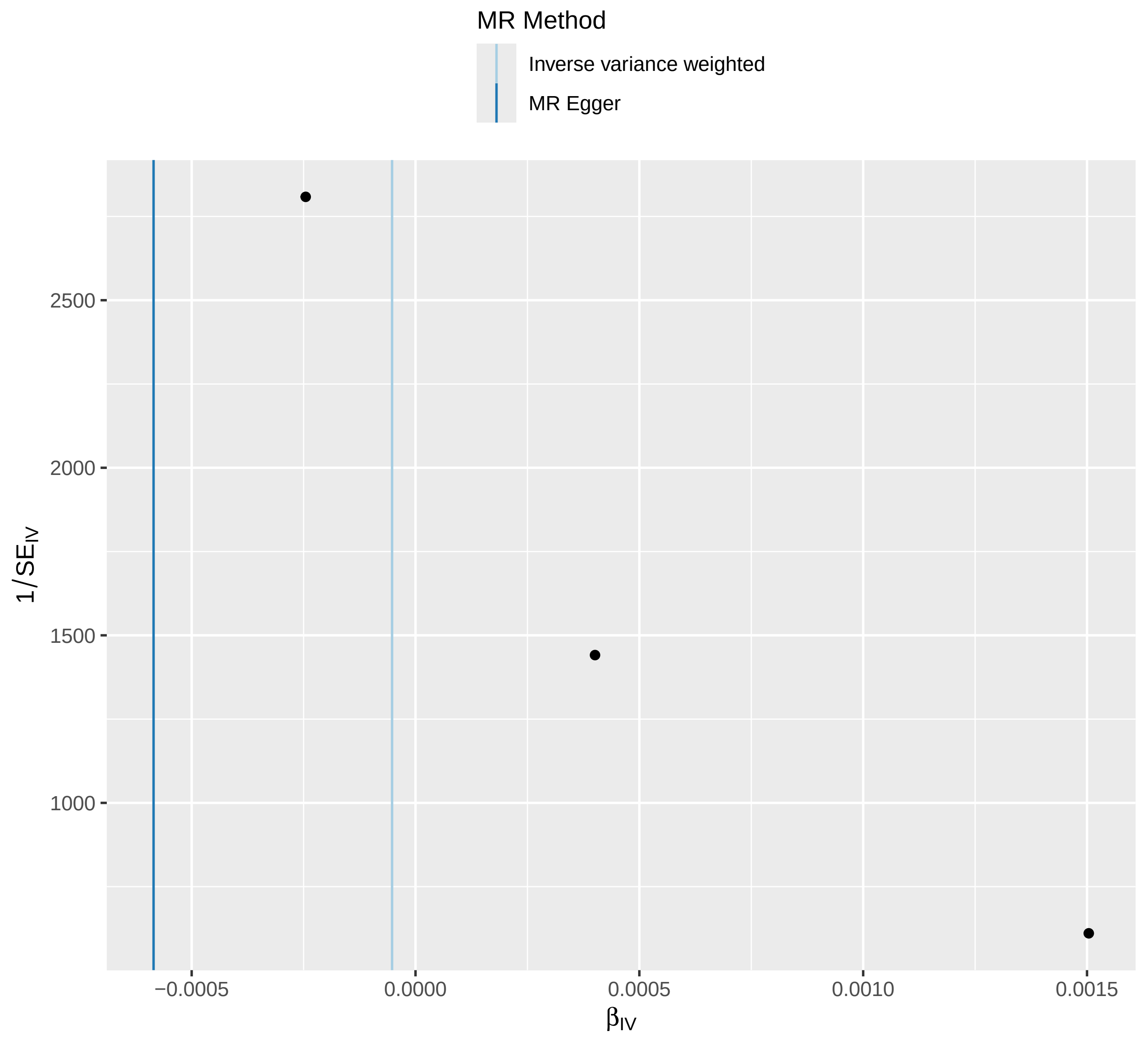
MR Method
Inverse variance weighted
MR Egger
2500
2000
1500
1000
1/SE_IV
−0.0005
0.0000
0.0005
0.0010
0.0015
β_IV

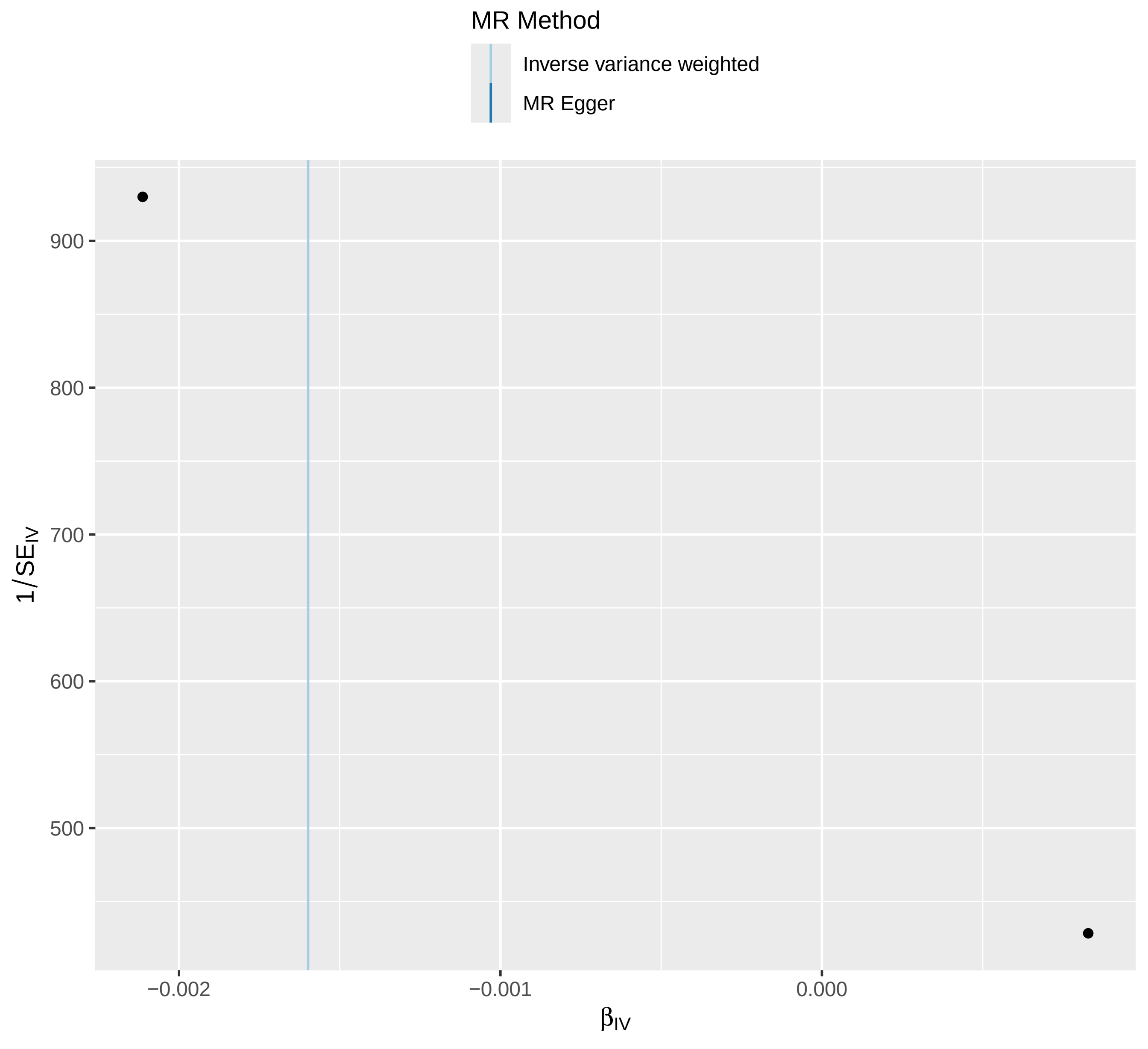
MR Method
Inverse variance weighted
MR Egger
900
800
700
600
500
1/SE$_{IV}$
−0.002
−0.001
0.000
β$_{IV}$

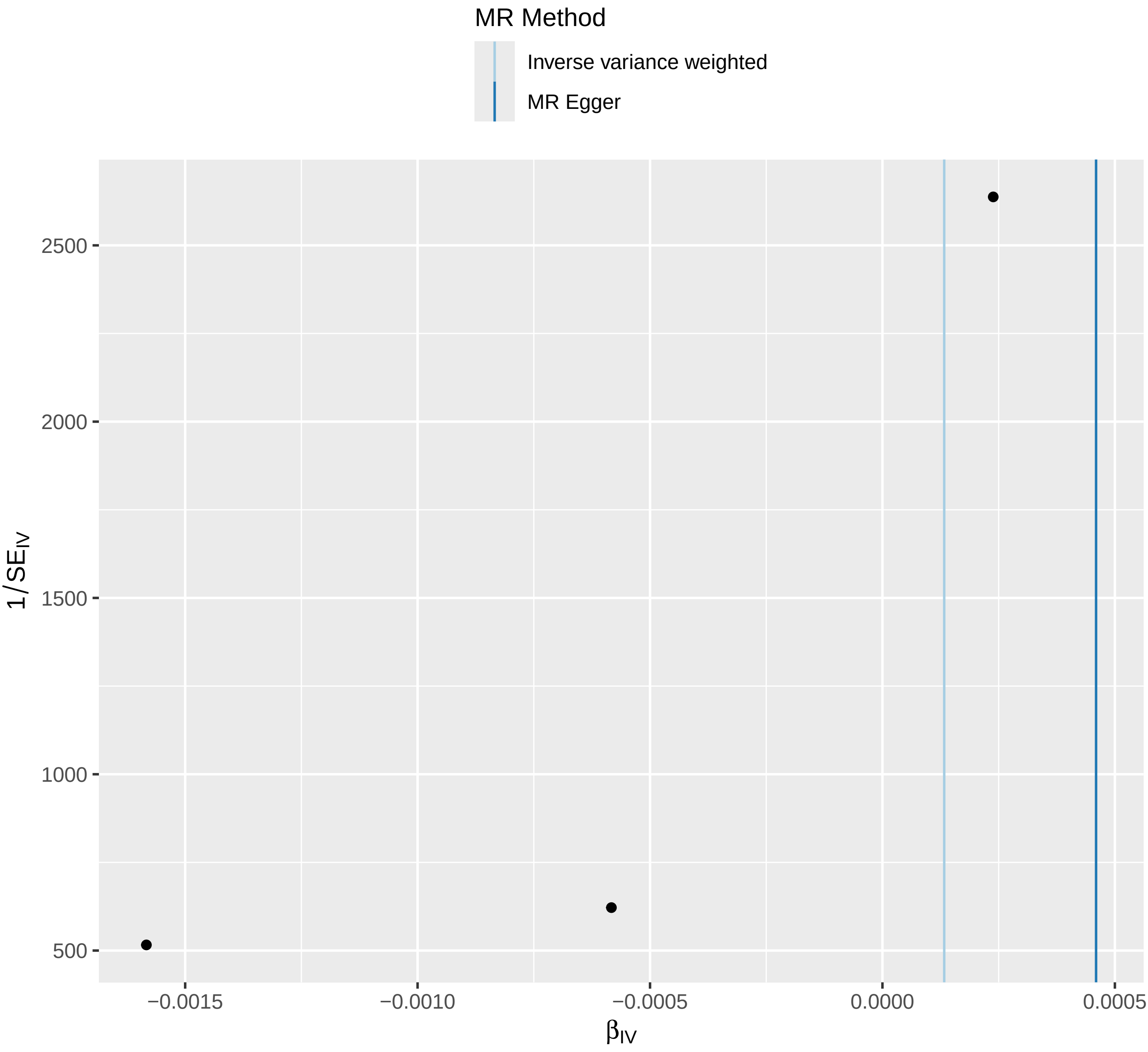

MR Method
Inverse variance weighted
MR Egger
2500
2000
1500
1000
500
1/SE$_{IV}$
−0.0015
−0.0010
−0.0005
0.0000
0.0005
β$_{IV}$

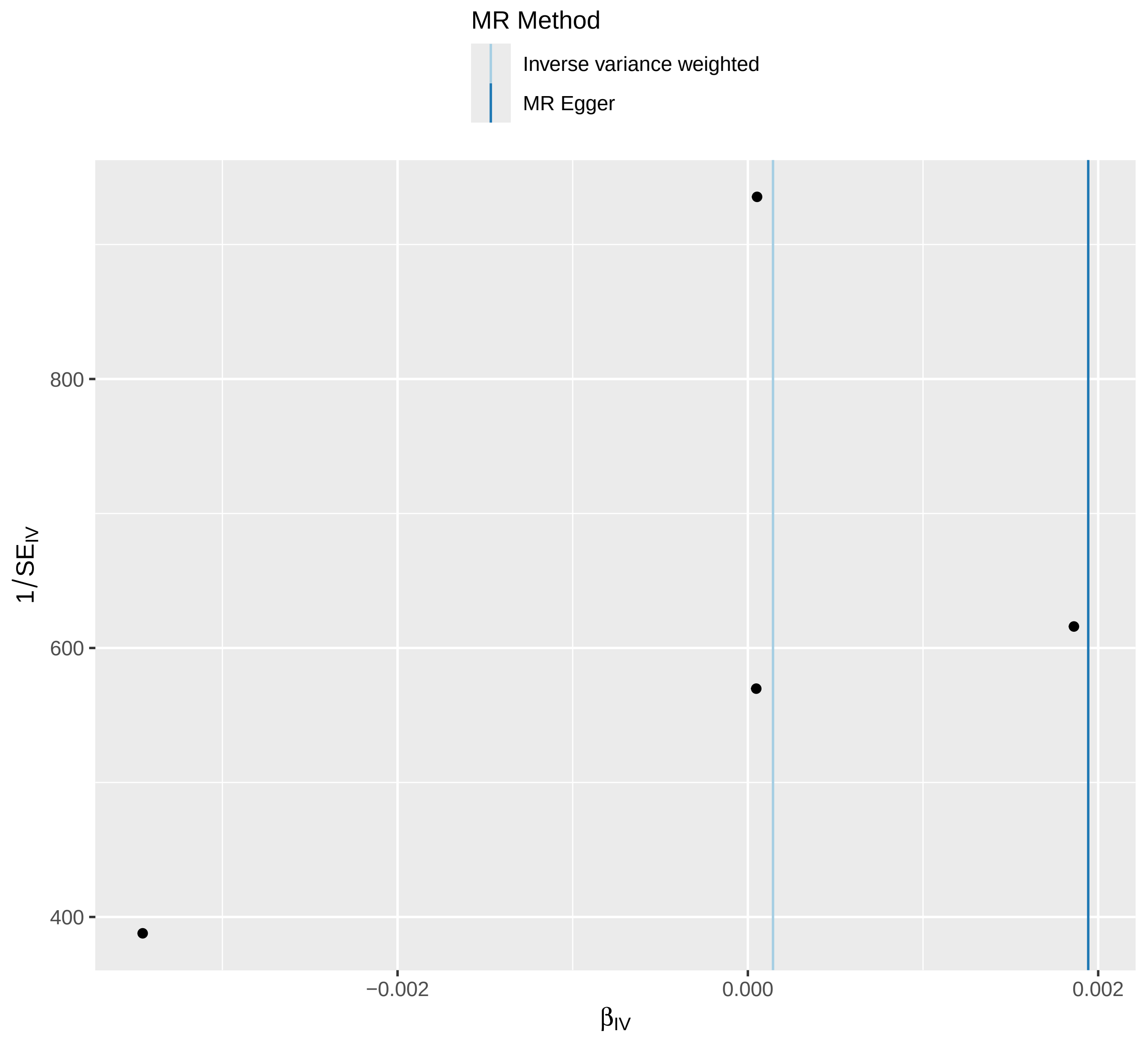

MR Method
Inverse variance weighted
MR Egger
800
600
400
1/SE_IV
−0.002
0.000
0.002
β_IV

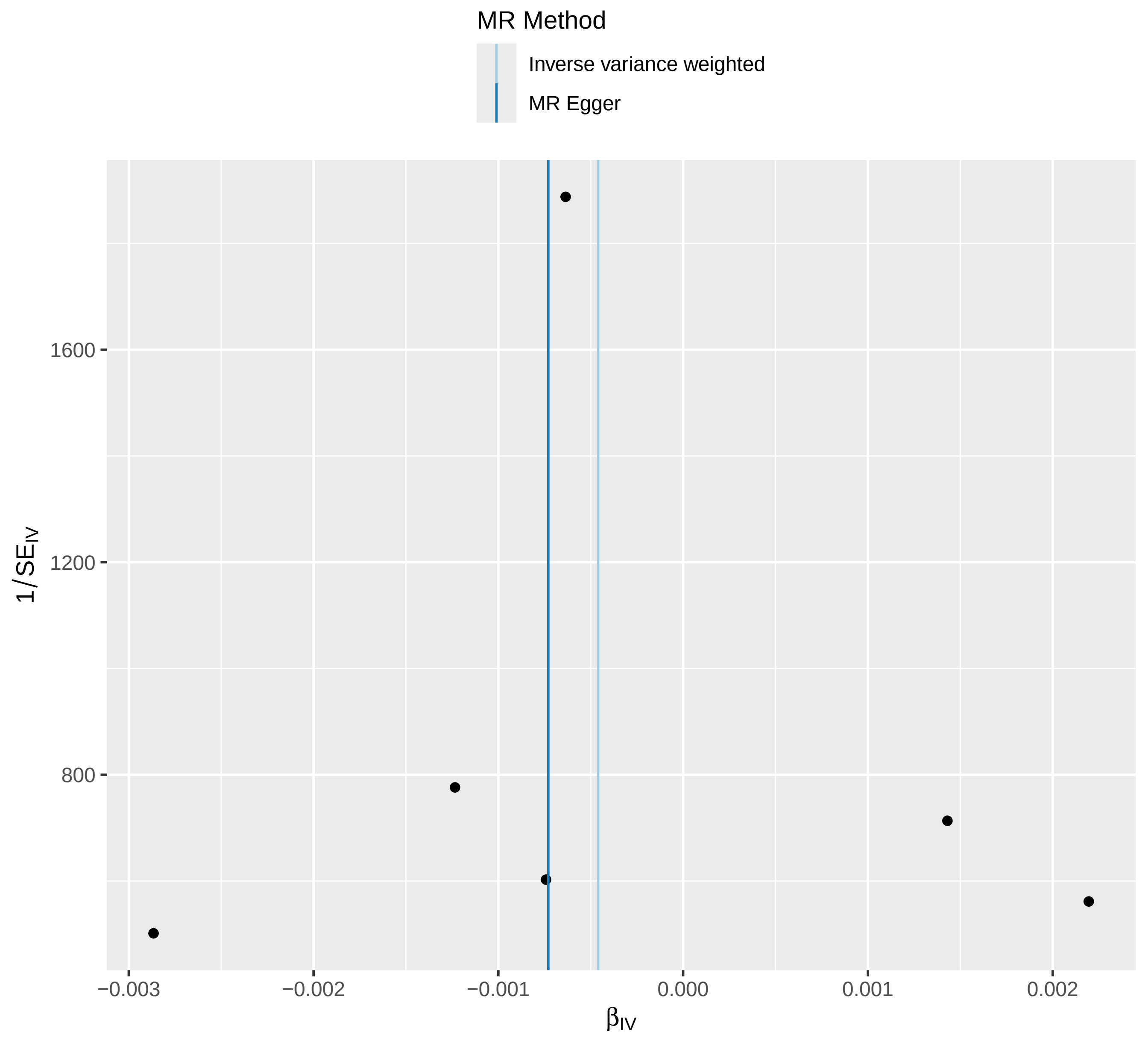
MR Method
Inverse variance weighted
MR Egger
1600
1200
800
1/SE_IV
−0.003
−0.002
−0.001
0.000
0.001
0.002
β_IV

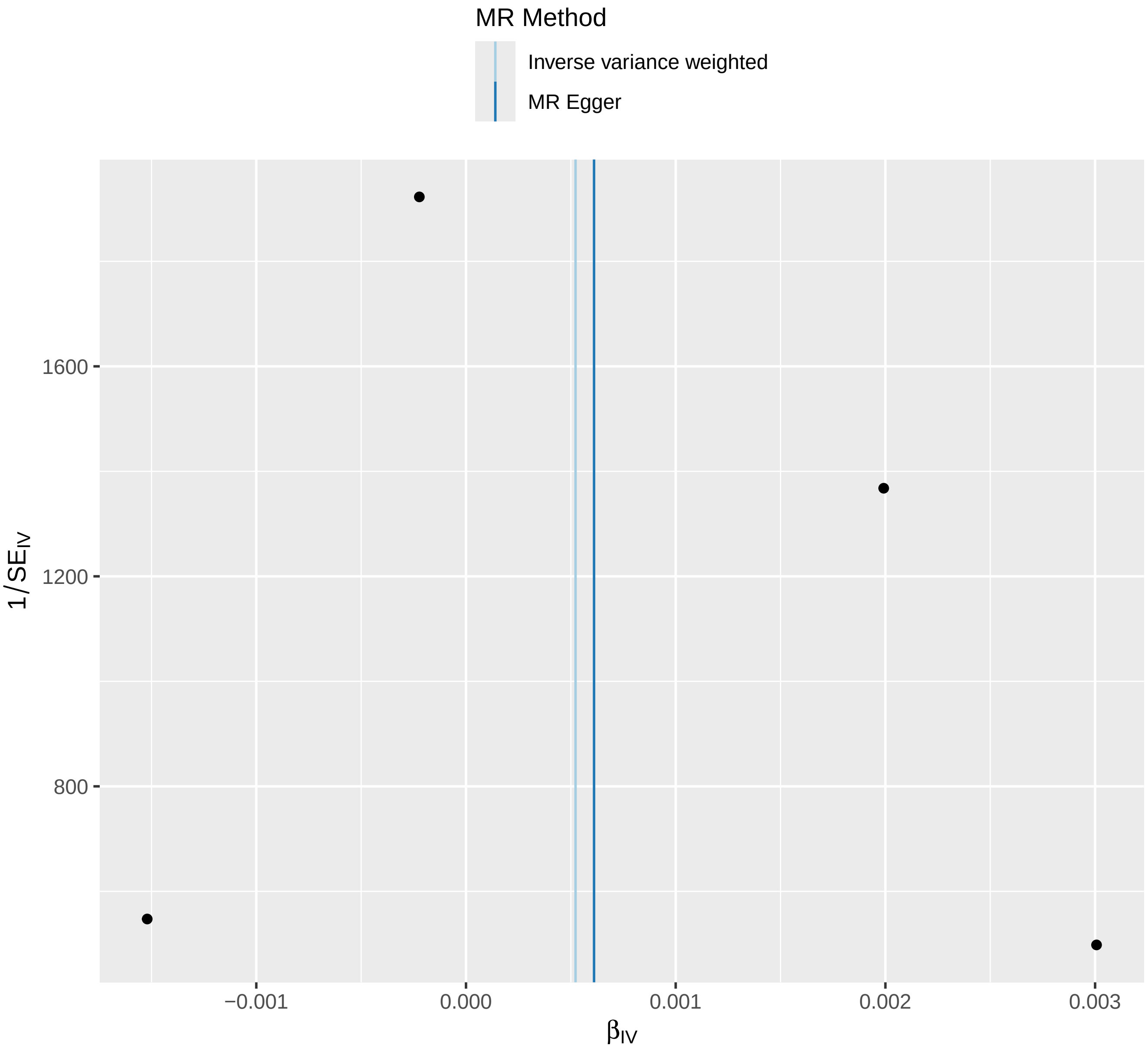
MR Method
Inverse variance weighted
MR Egger
1600
1200
800
1/SE$_{IV}$
−0.001
0.000
0.001
0.002
0.003
β$_{IV}$

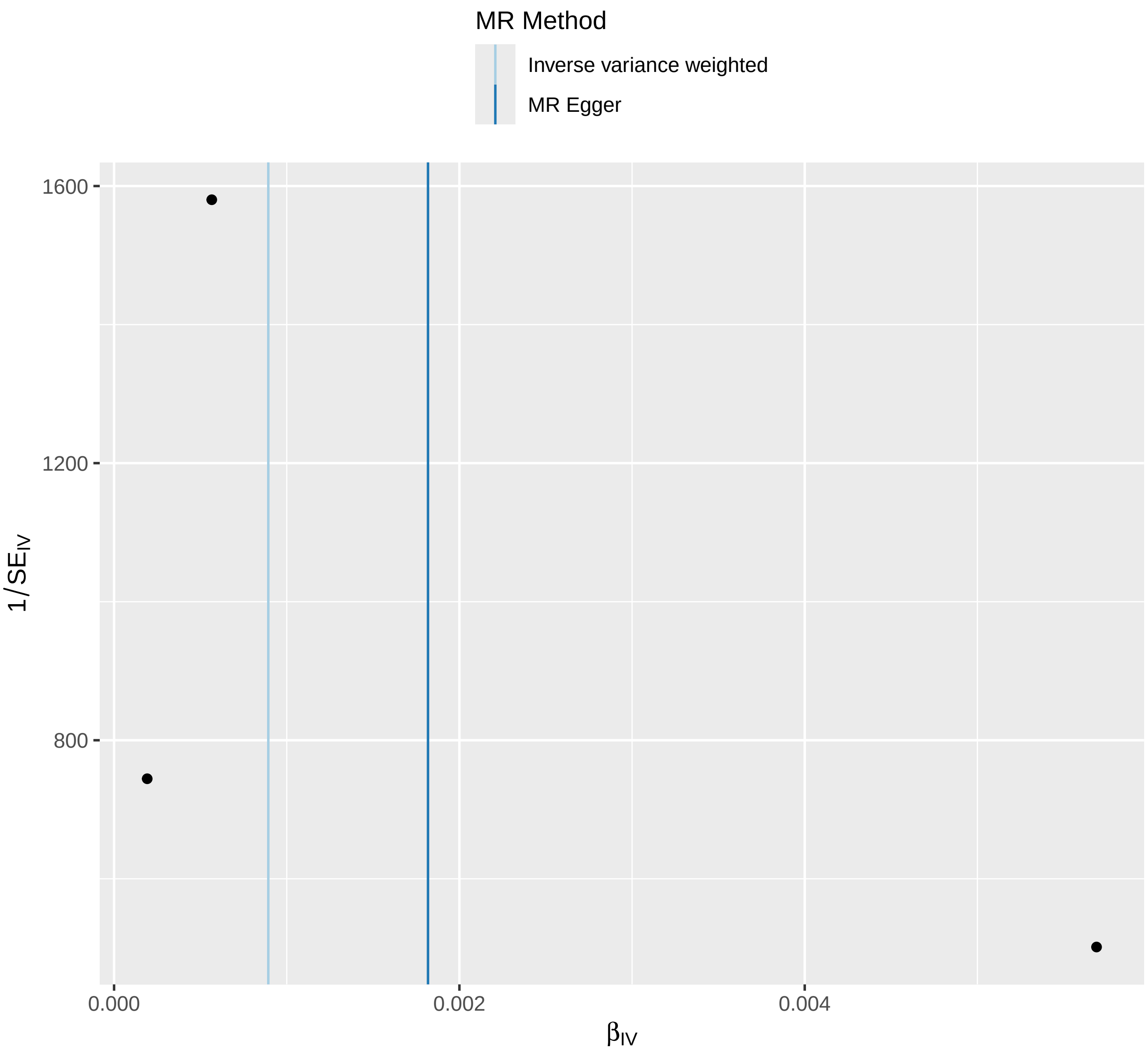

MR Method
Inverse variance weighted
MR Egger
1600
1200
800
1/SE_IV
0.000
0.002
0.004
β_IV

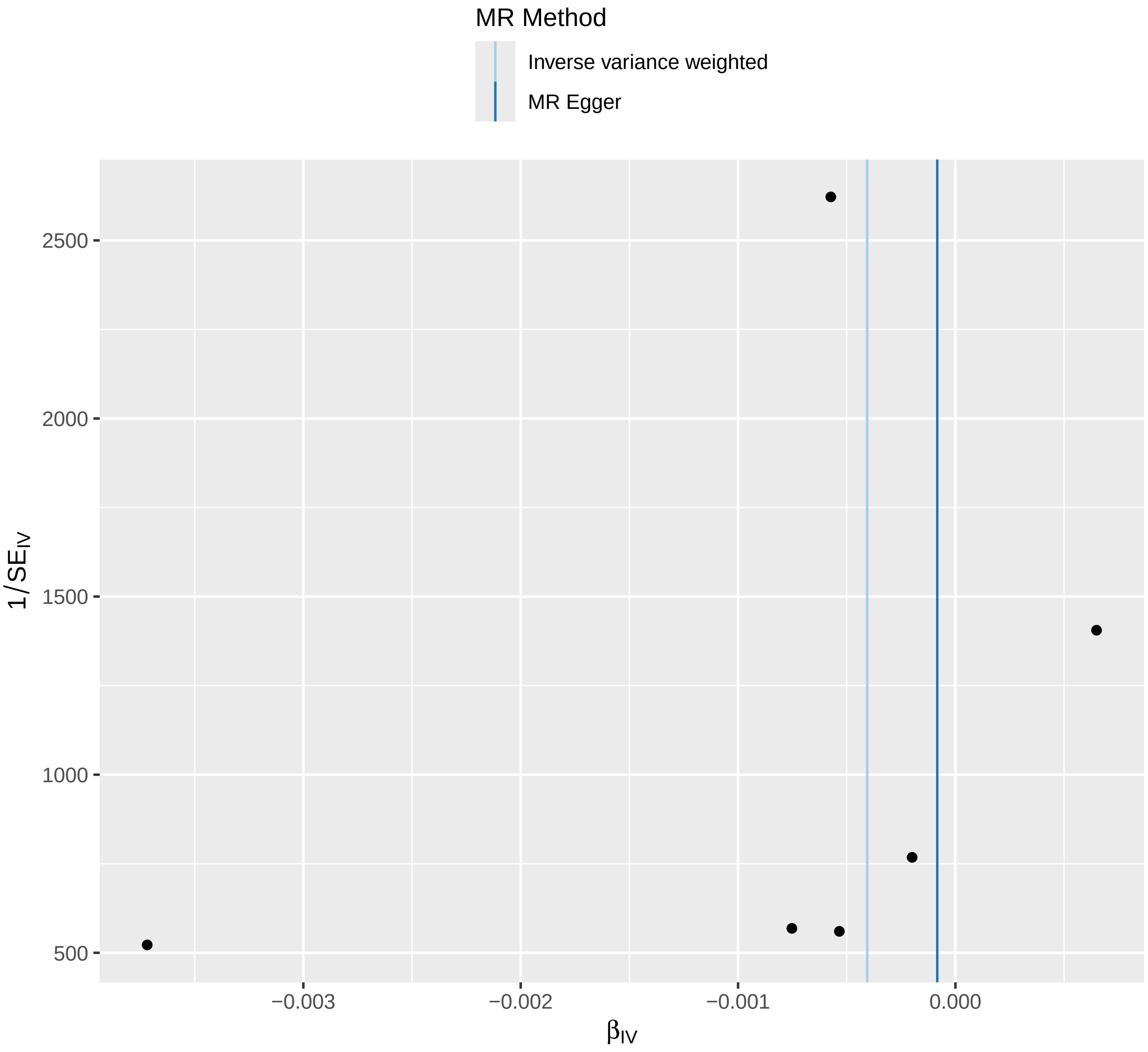
MR Method
Inverse variance weighted
MR Egger
2500
2000
1500
1000
500
1/SE$_{IV}$
−0.003
−0.002
−0.001
0.000
β$_{IV}$

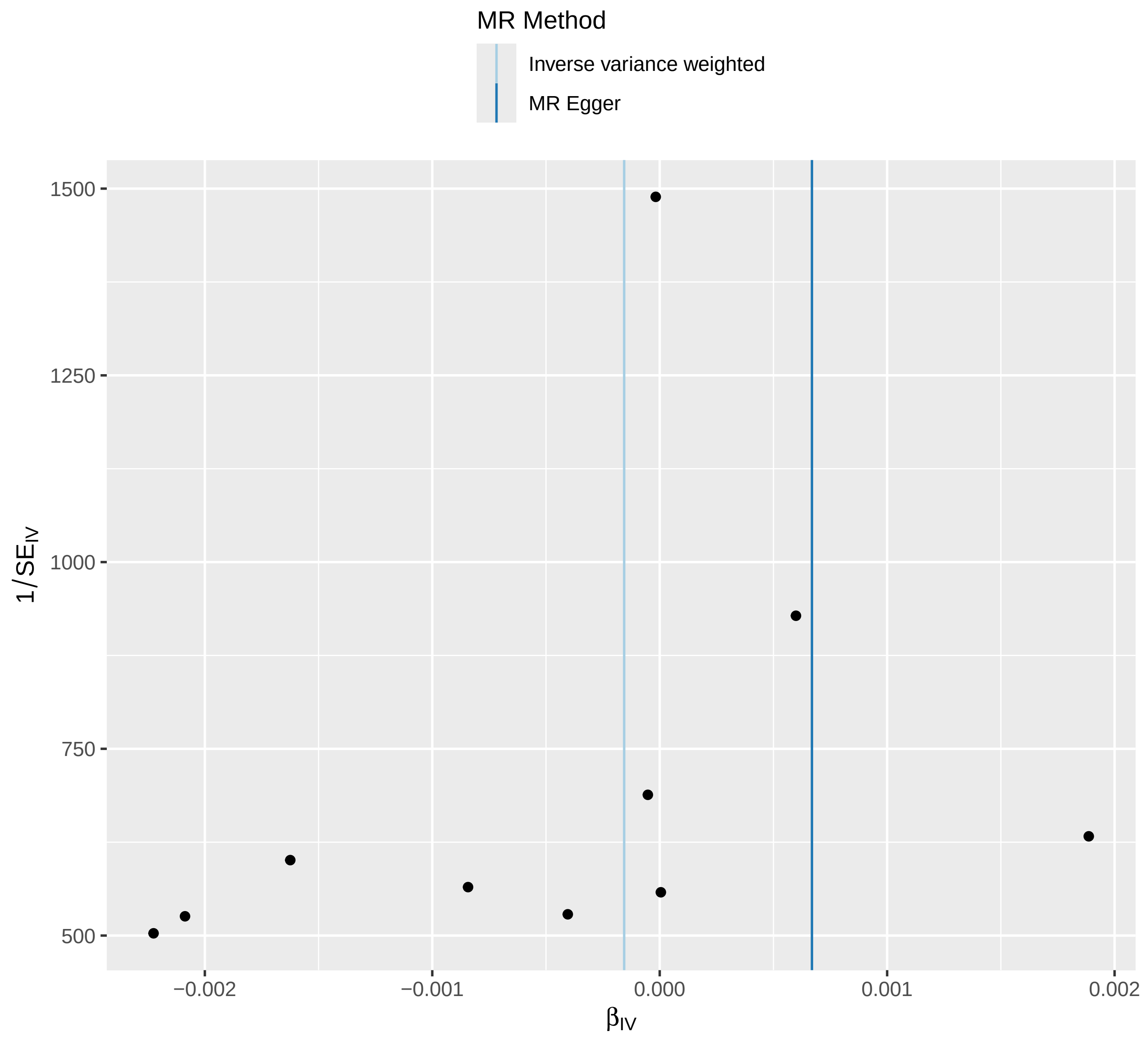
MR Method
Inverse variance weighted
MR Egger
1500
1250
1000
750
500
$1/SE_{IV}$
−0.002
−0.001
0.000
0.001
0.002
β_{IV}

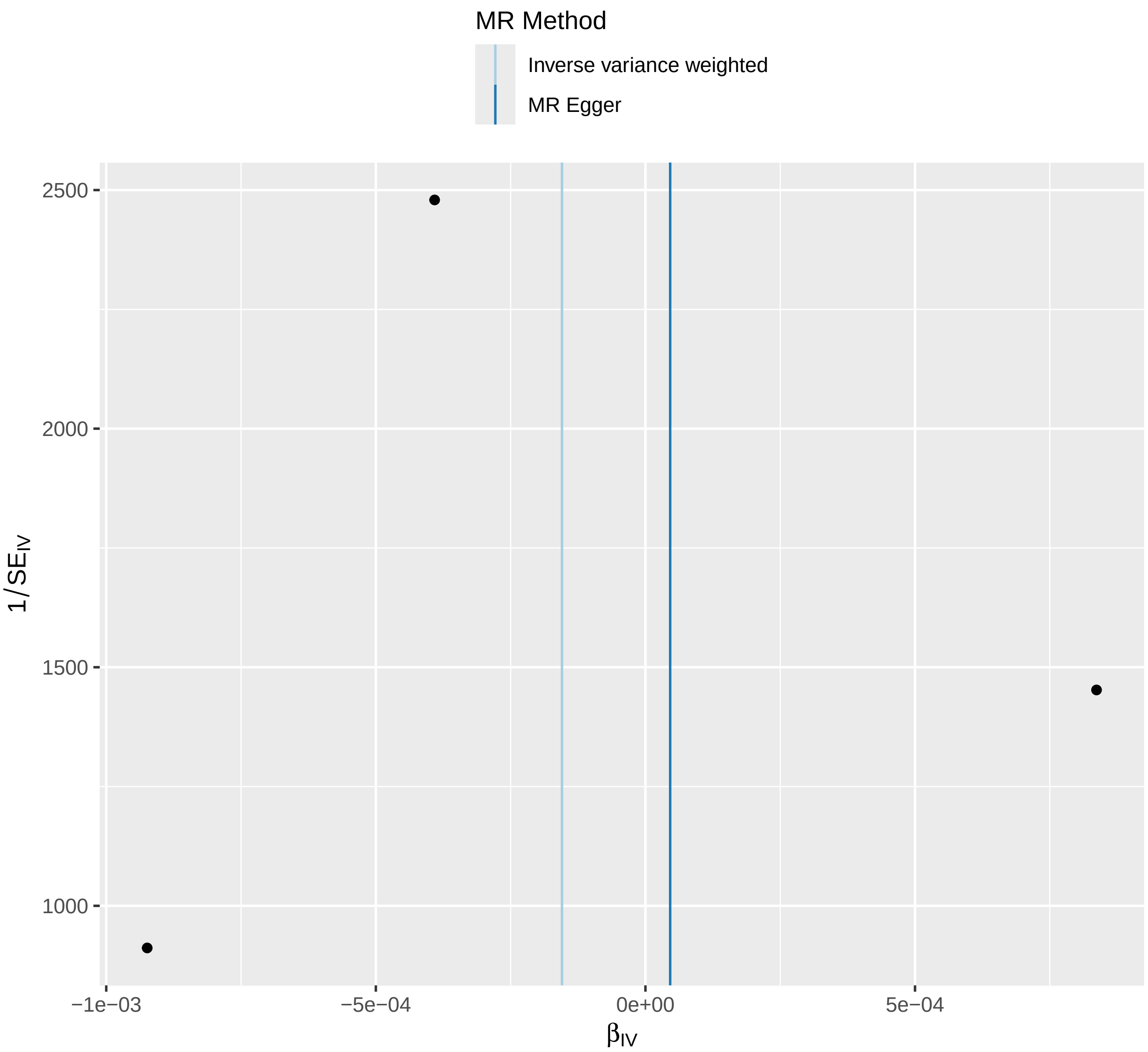
MR Method
Inverse variance weighted
MR Egger
2500
2000
1500
1000
$1/SE_{IV}$
−1e−03
−5e−04
0e+00
5e−04
β_{IV}

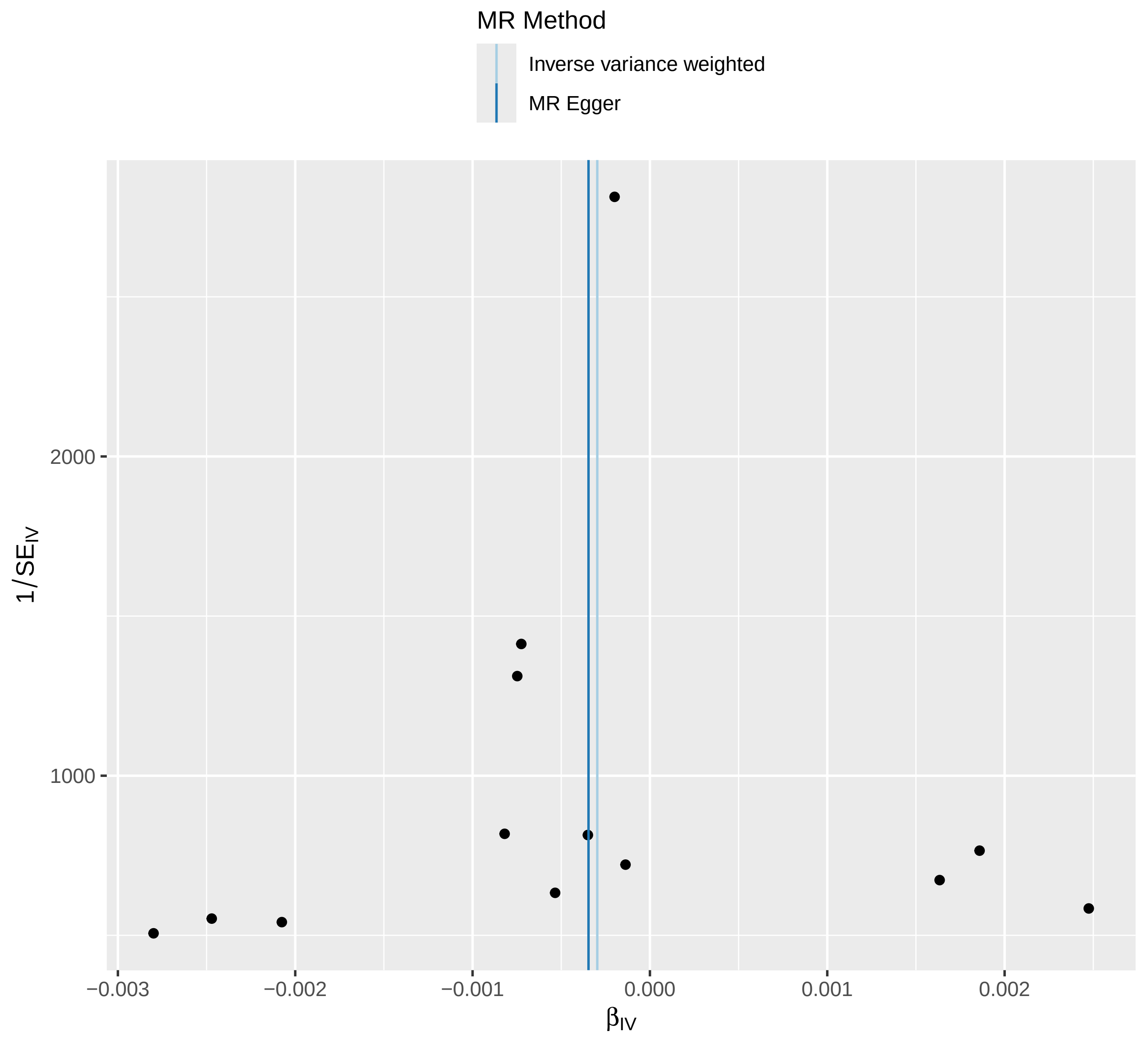
MR Method
Inverse variance weighted
MR Egger
2000
1000
$1/SE_{IV}$
−0.003
−0.002
−0.001
0.000
0.001
0.002
β_{IV}

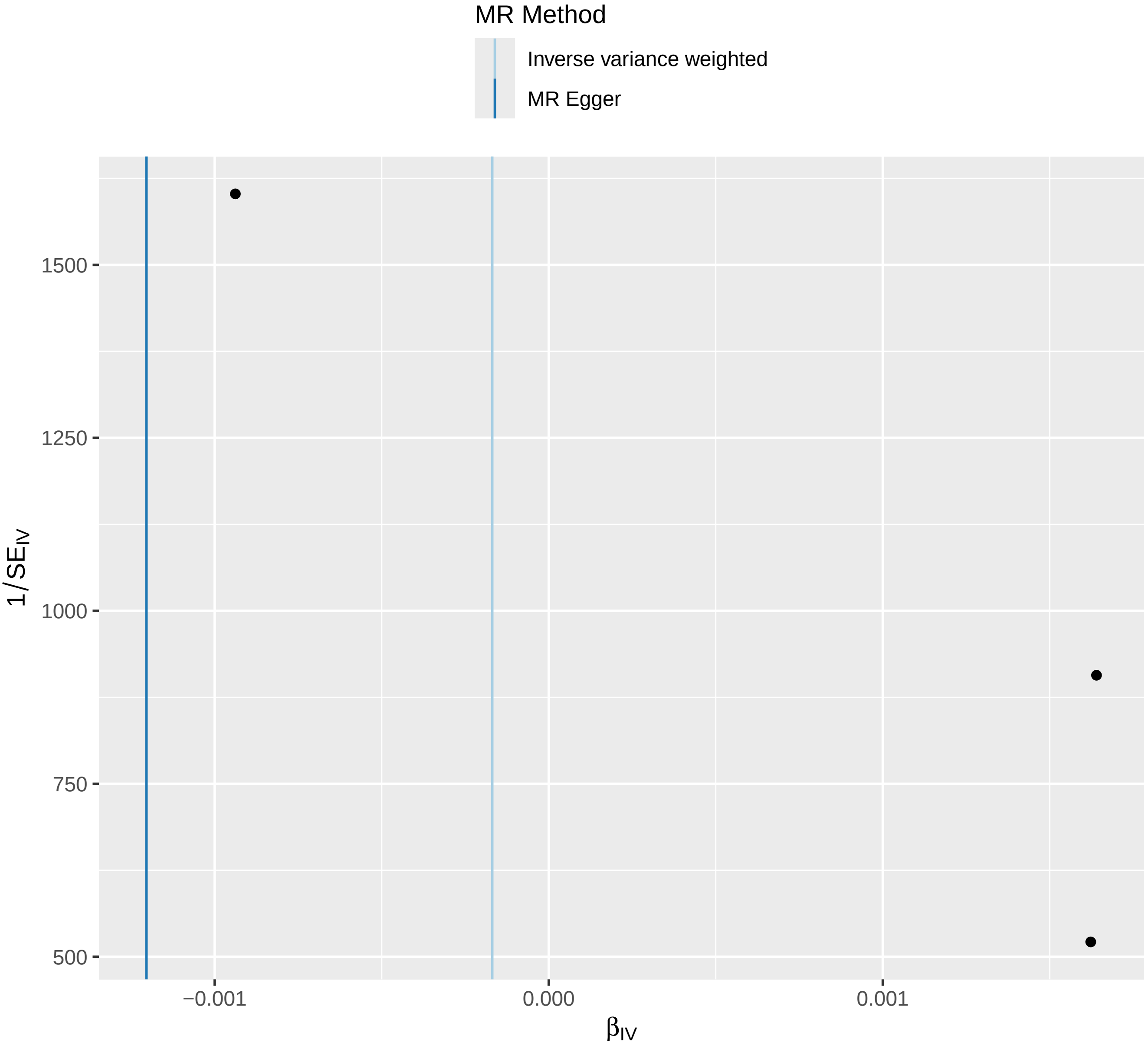

MR Method
Inverse variance weighted
MR Egger
1500
1250
1000
750
500
$1/SE_{IV}$
−0.001
0.000
0.001
β_{IV}

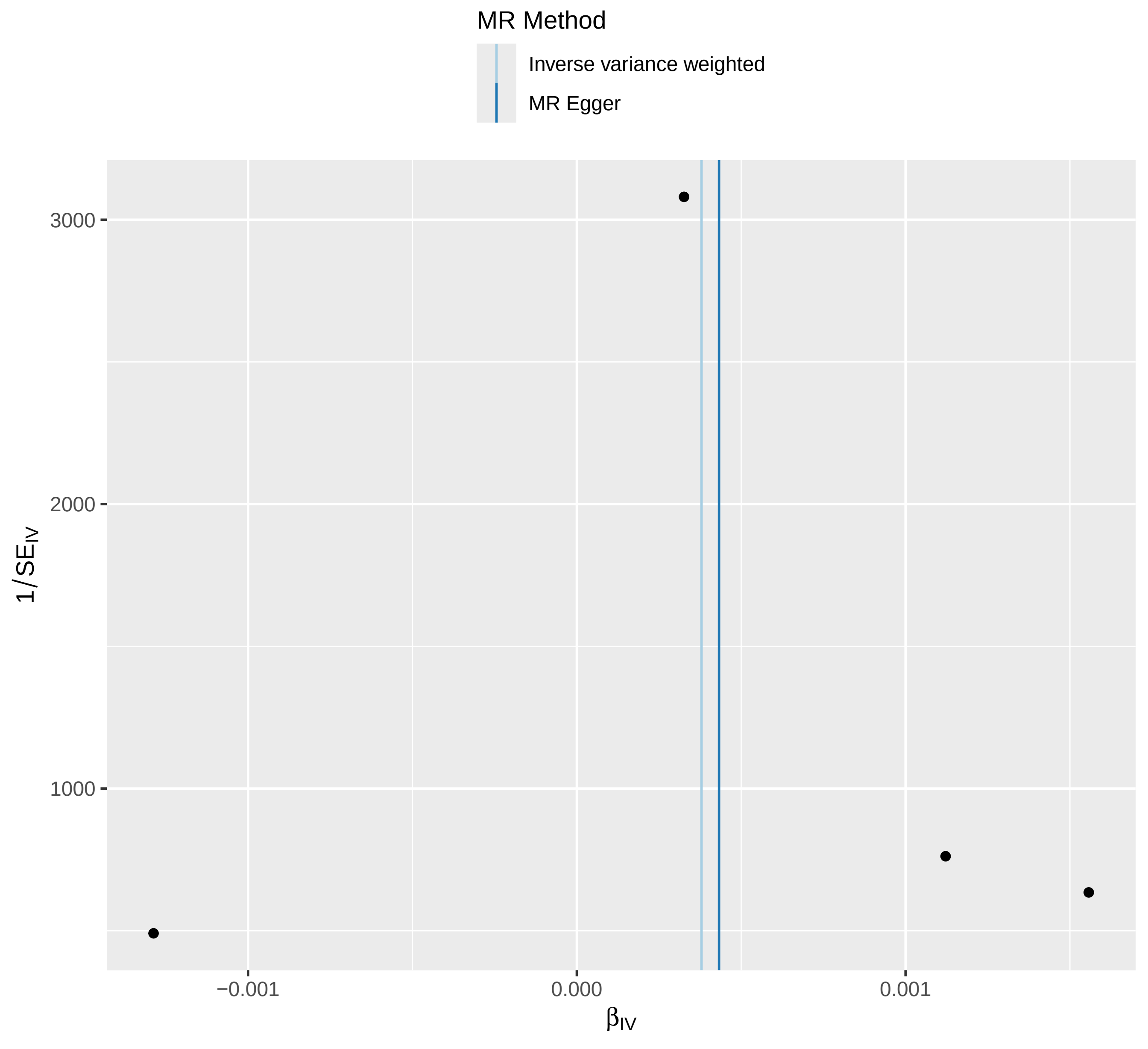

MR Method
Inverse variance weighted
MR Egger
3000
2000
1000
1/SE_IV
−0.001
0.000
0.001
β_IV

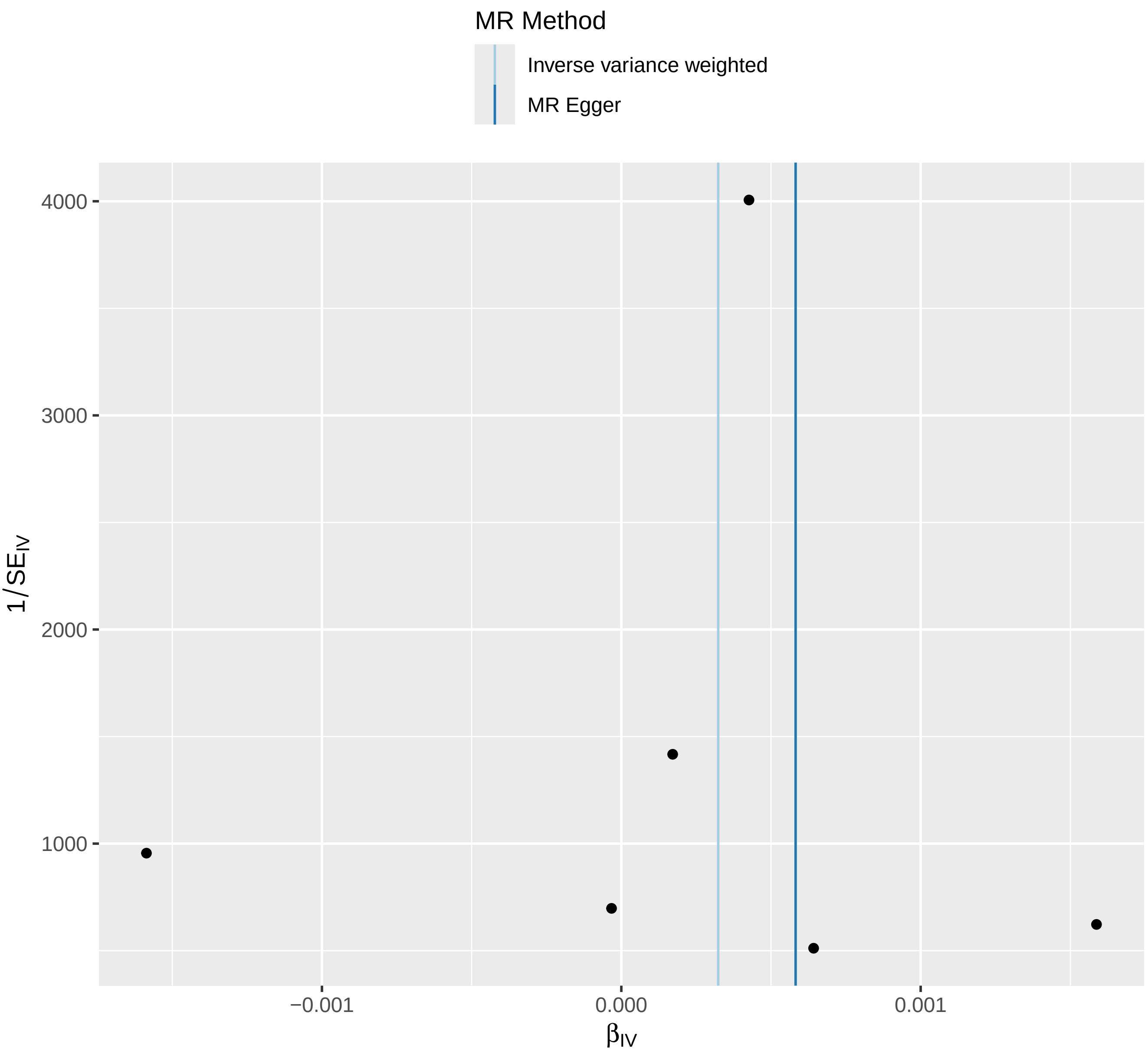
MR Method
Inverse variance weighted
MR Egger
4000
3000
2000
1000
1/SE_IV
−0.001
0.000
0.001
β_IV

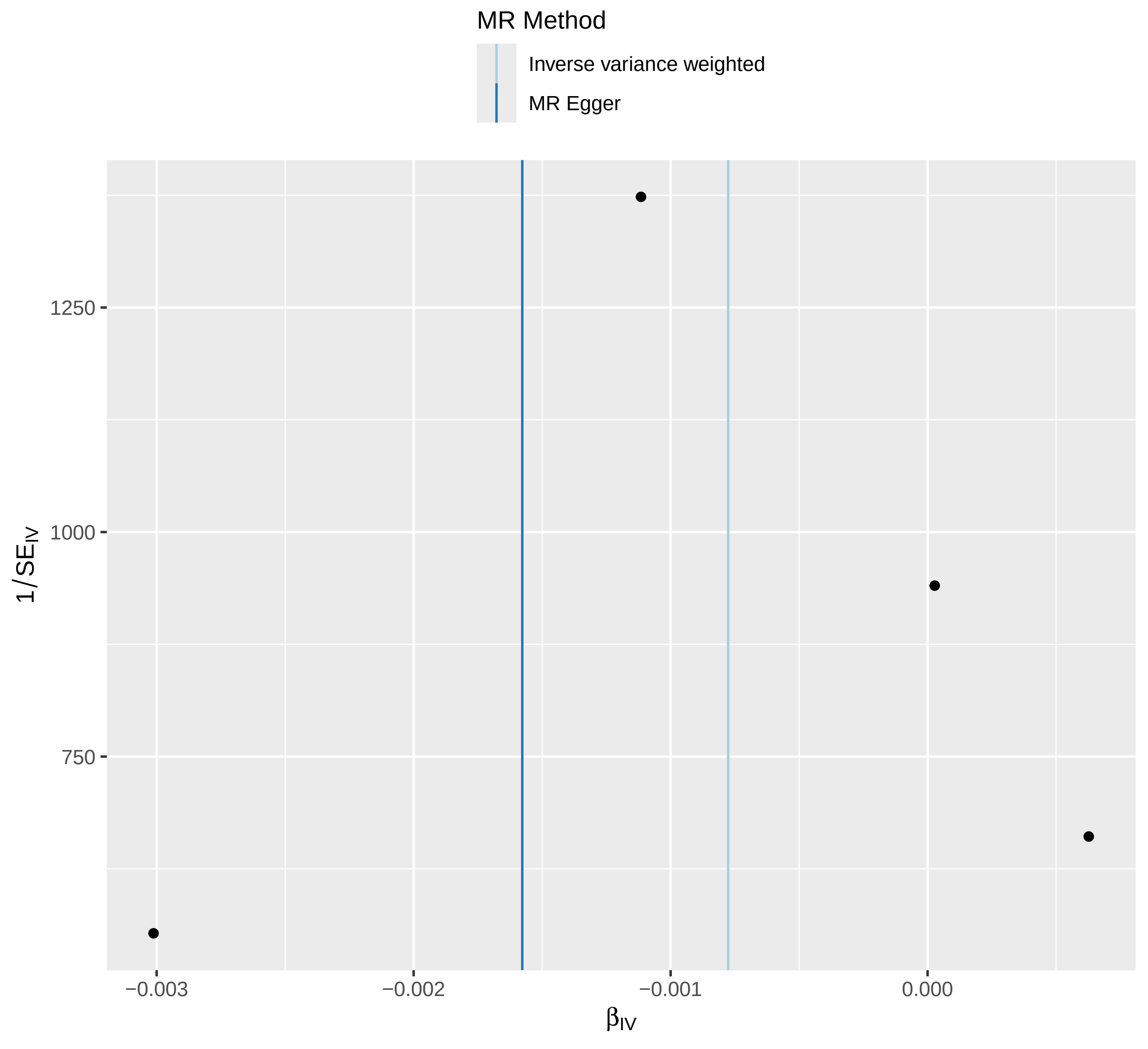
MR Method
Inverse variance weighted
MR Egger
1250
1000
750
1/SE_IV
−0.003
−0.002
−0.001
0.000
β_IV

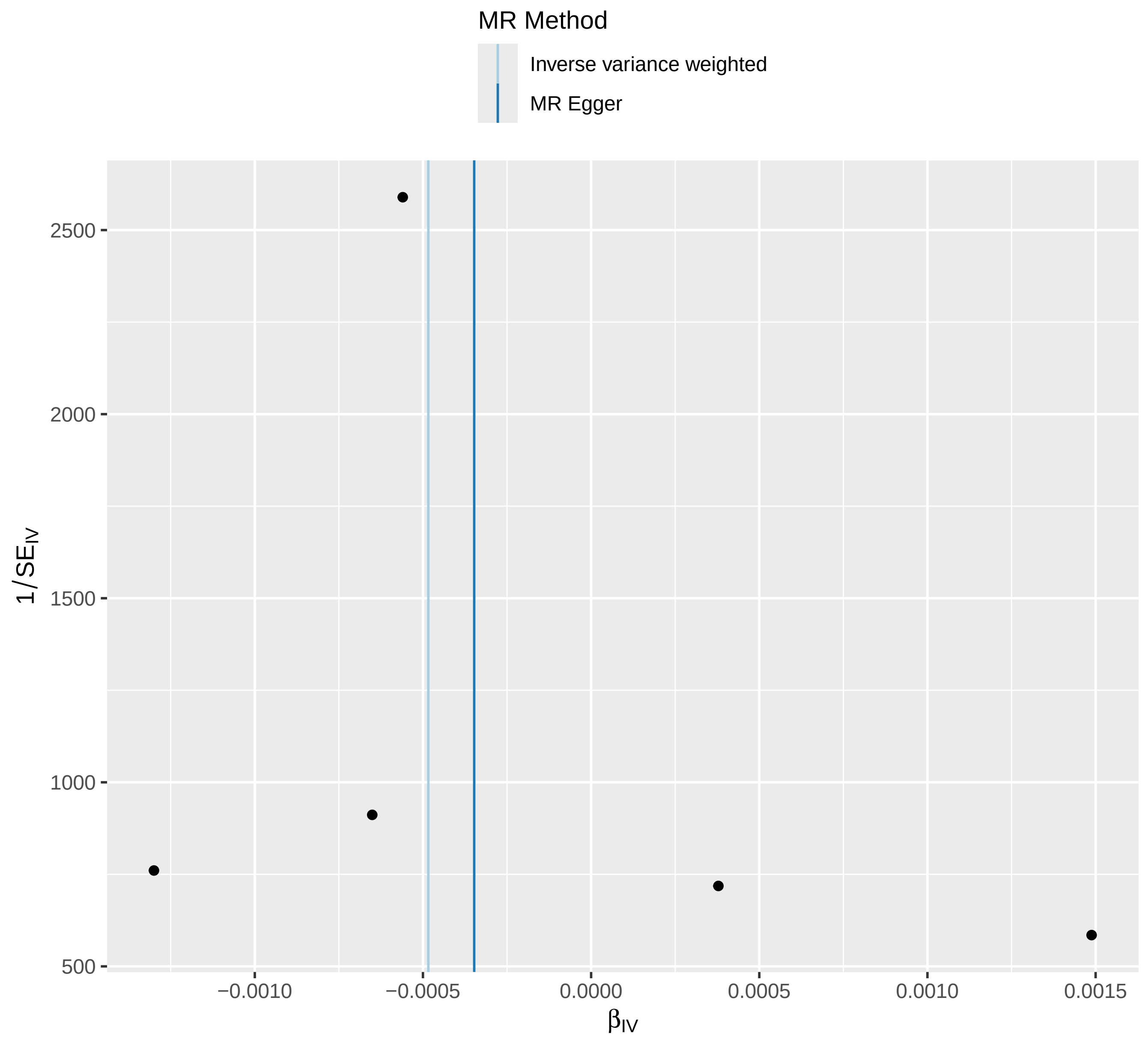

MR Method
Inverse variance weighted
MR Egger
2500
2000
1500
1000
500
1/SE_IV
−0.0010
−0.0005
0.0000
0.0005
0.0010
0.0015
β_IV

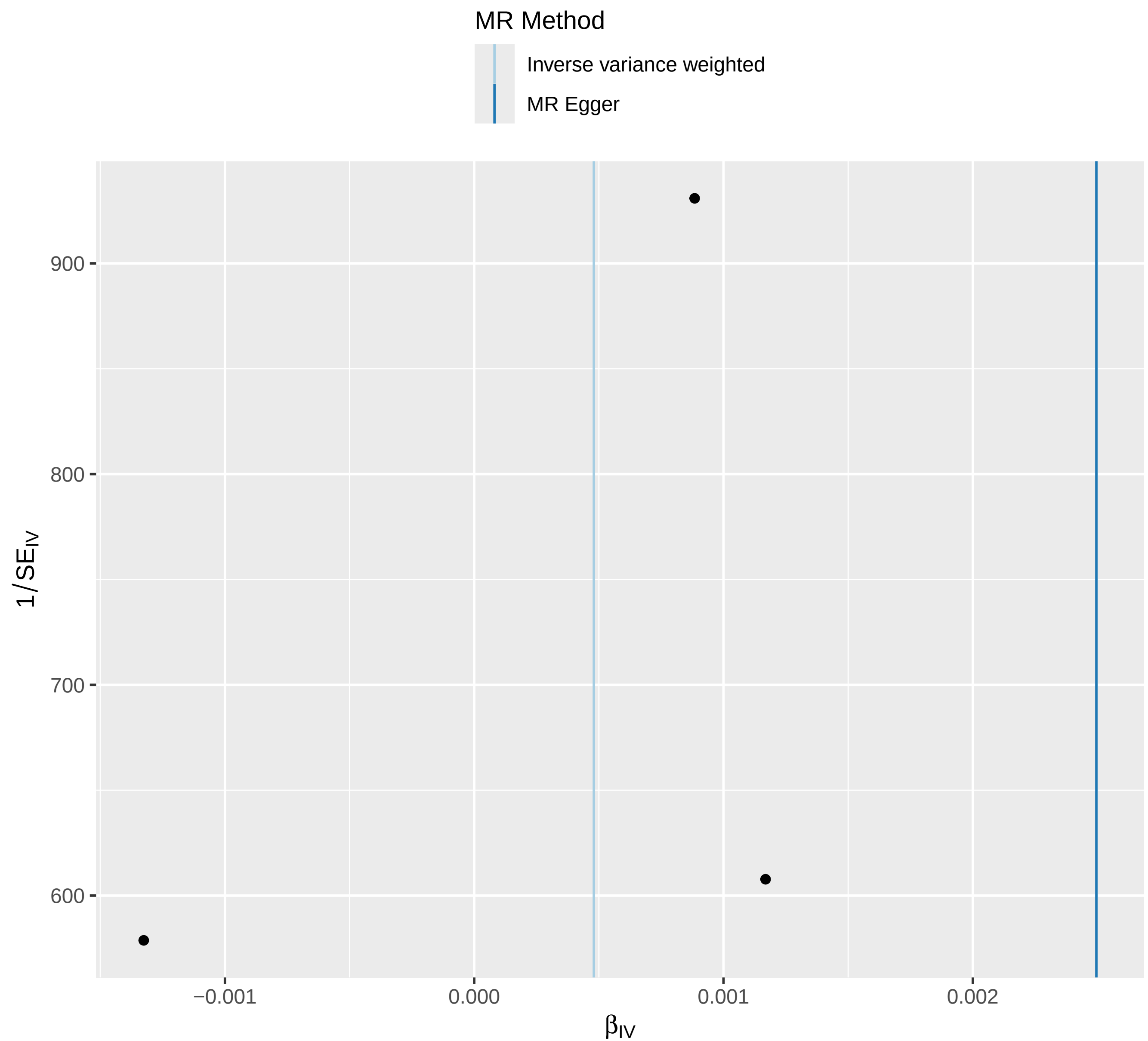

MR Method
Inverse variance weighted
MR Egger
900
800
700
600
1/SE_IV
−0.001
0.000
0.001
0.002
β_IV

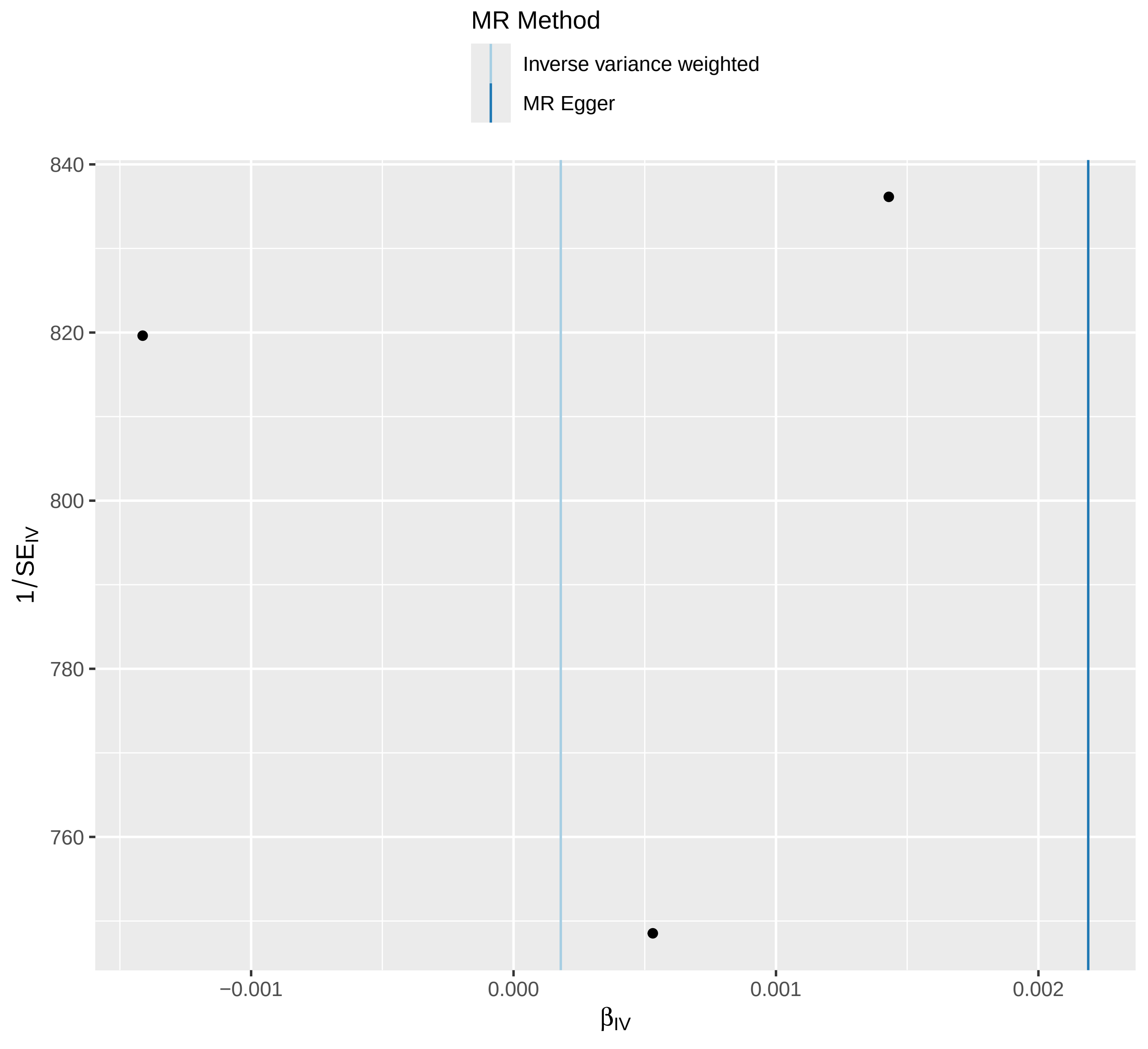
MR Method
Inverse variance weighted
MR Egger
840
820
800
780
760
1/SE_IV
−0.001
0.000
0.001
0.002
β_IV

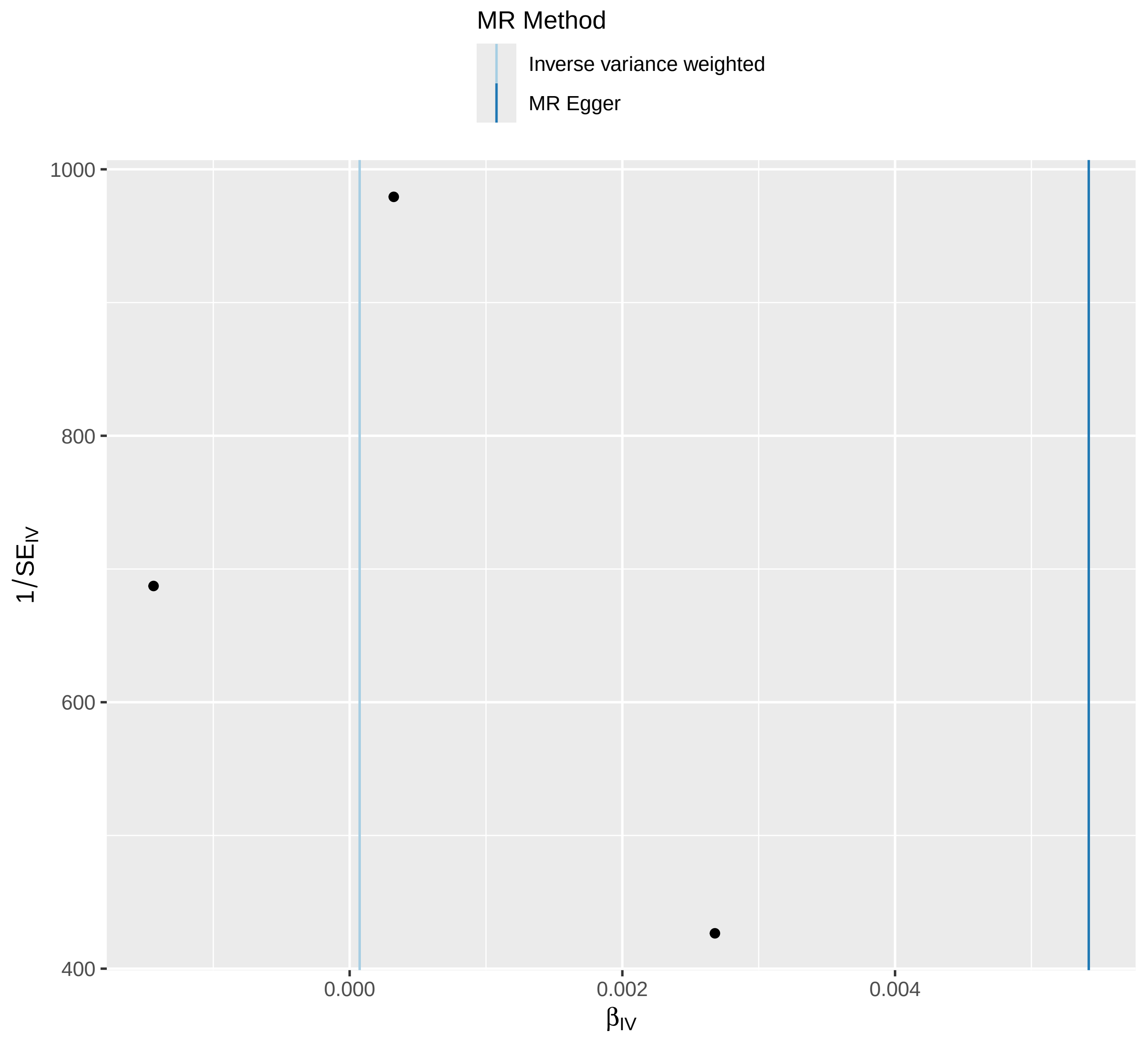
MR Method
Inverse variance weighted
MR Egger
1000
800
600
400
1/SE_IV
0.000
0.002
0.004
β_IV

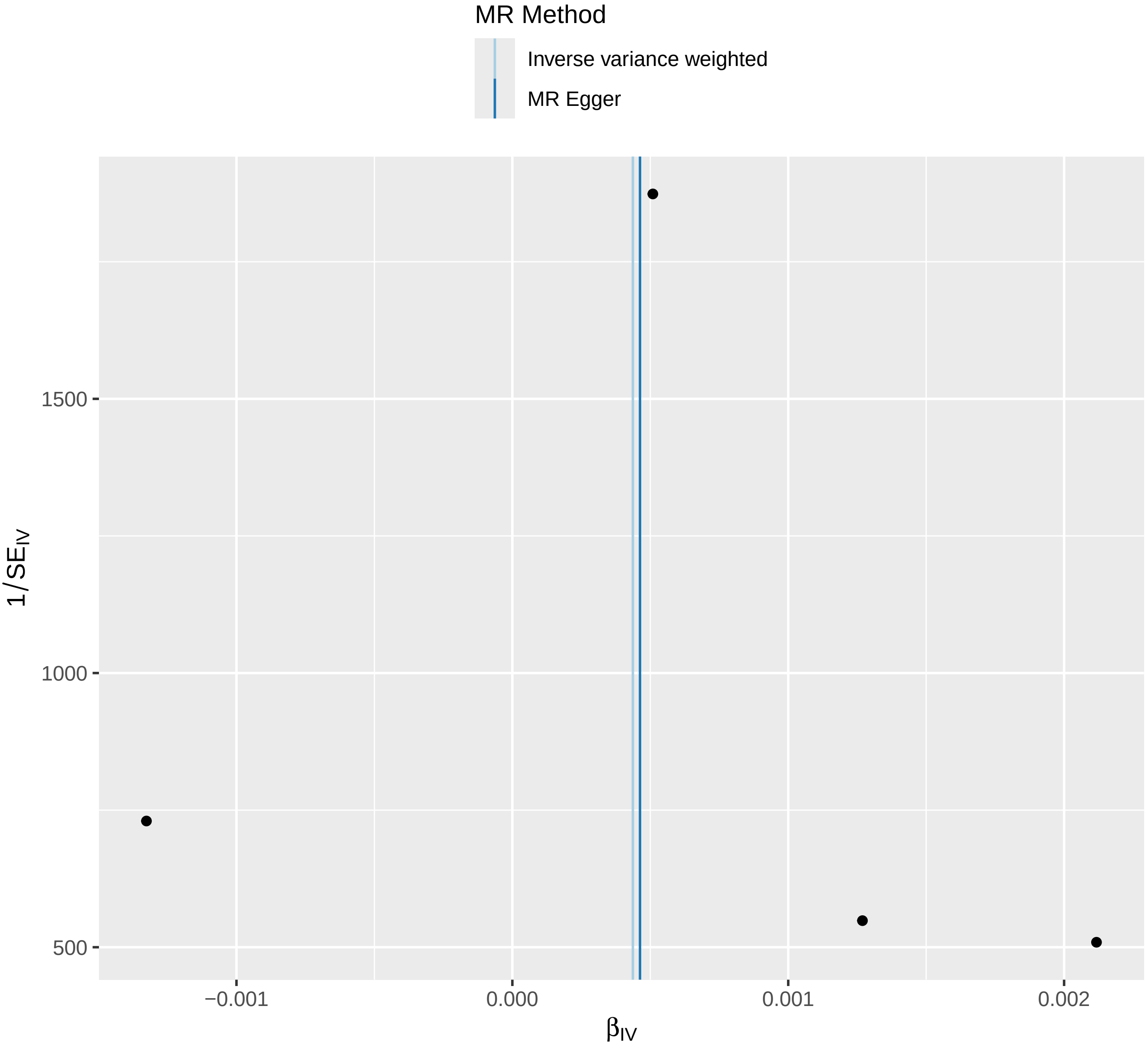
MR Method
Inverse variance weighted
MR Egger
1500
1000
500
1/SE_IV
−0.001
0.000
0.001
0.002
β_IV

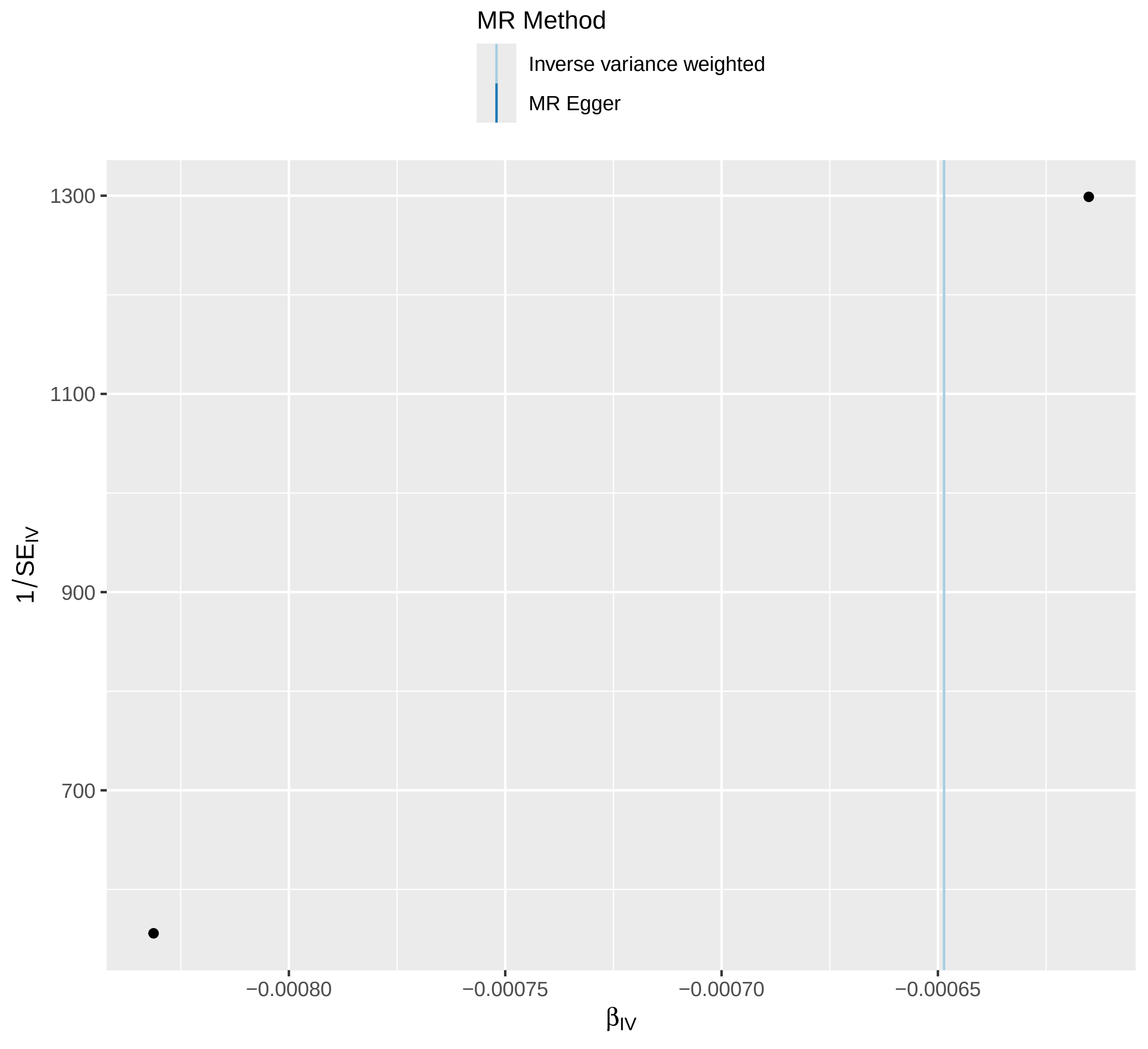
MR Method
Inverse variance weighted
MR Egger
1300
1100
900
700
1/SE_IV
−0.00080
−0.00075
−0.00070
−0.00065
β_IV

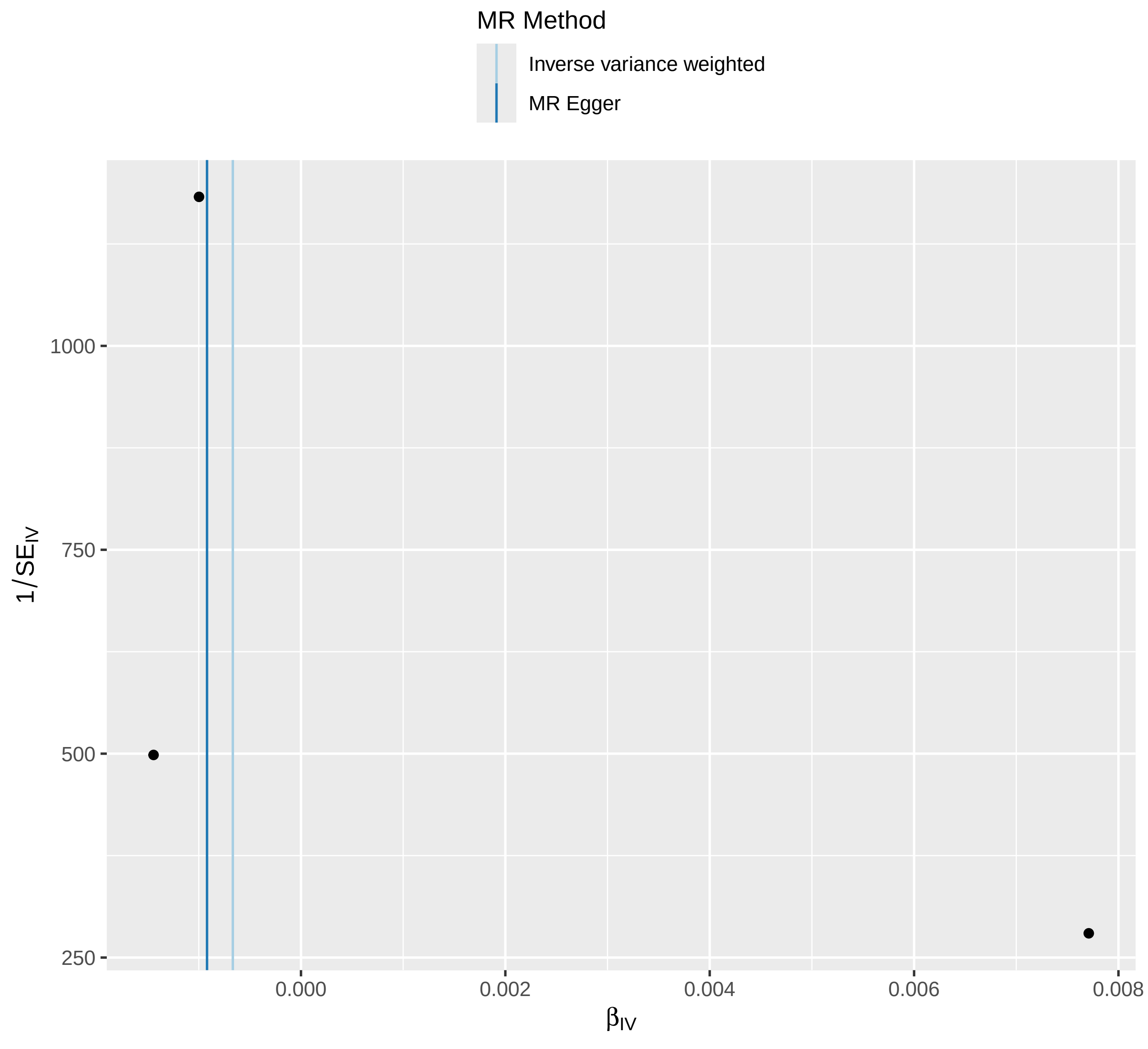

MR Method
Inverse variance weighted
MR Egger
1000
750
500
250
1/SE_IV
0.000
0.002
0.004
0.006
0.008
β_IV

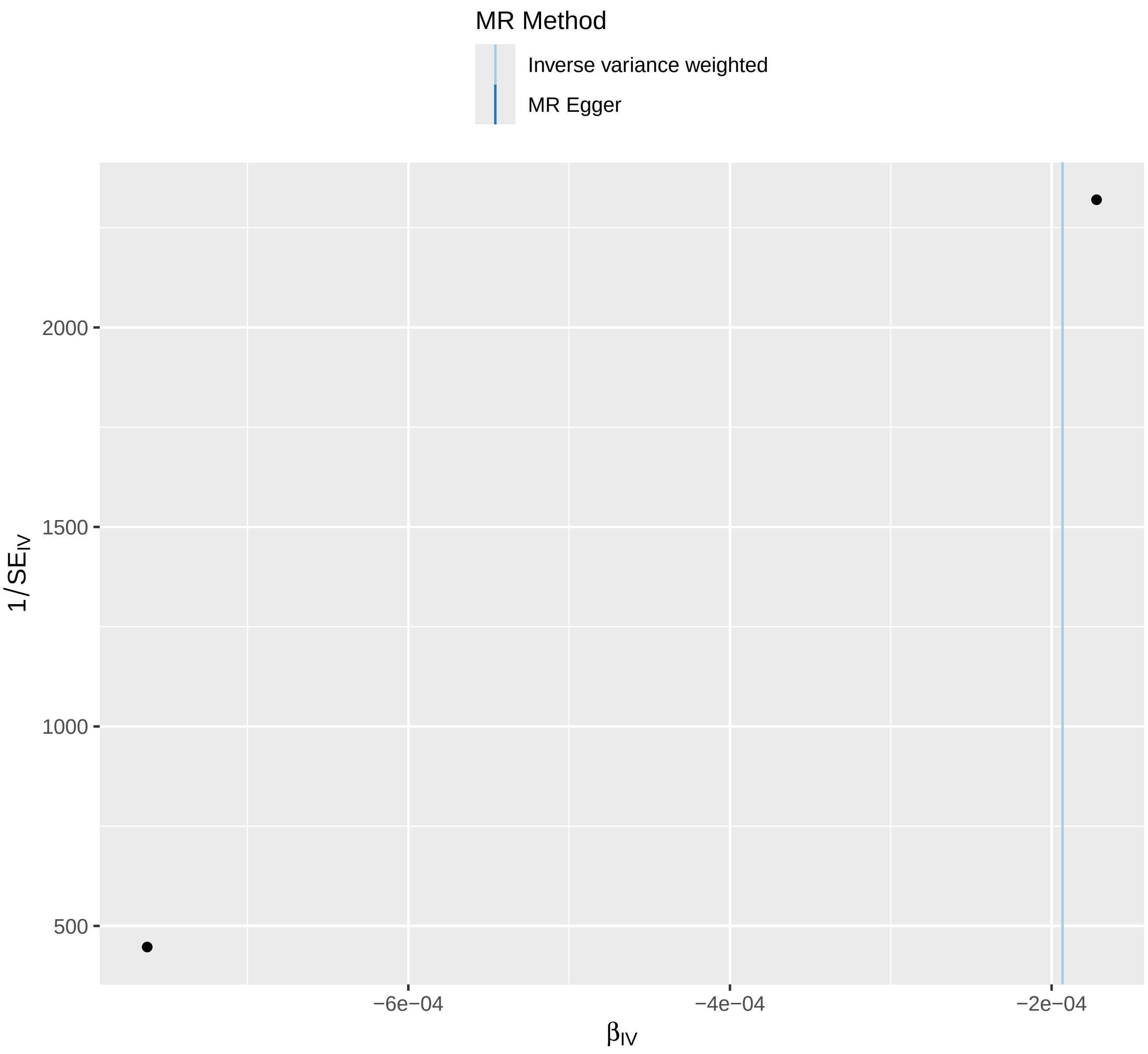
MR Method
Inverse variance weighted
MR Egger
2000
1500
1000
500
1/SE_IV
−6e−04
−4e−04
−2e−04
β_IV

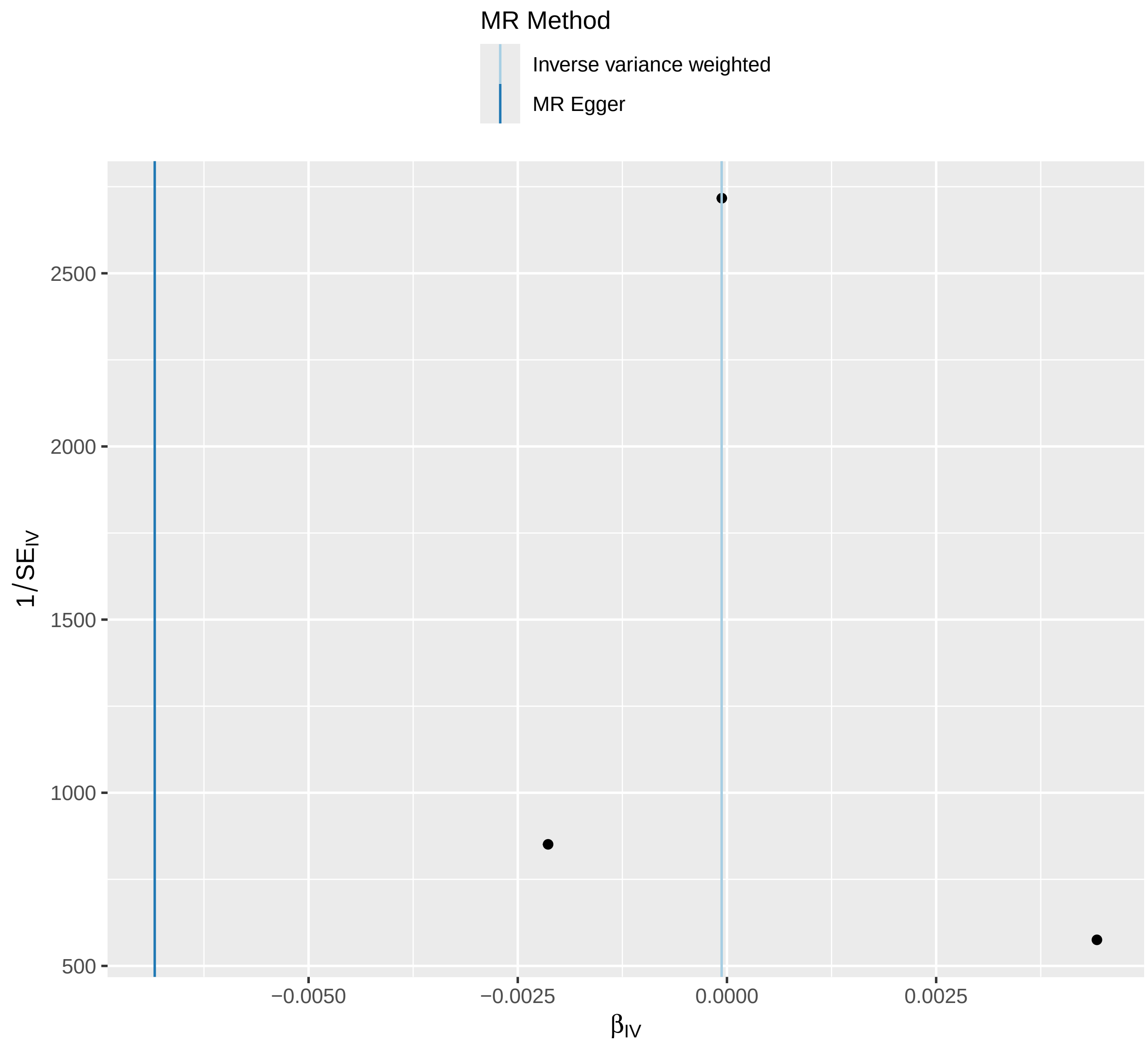
MR Method
Inverse variance weighted
MR Egger
2500
2000
1500
1000
500
1/SE_IV
−0.0050
−0.0025
0.0000
0.0025
β_IV

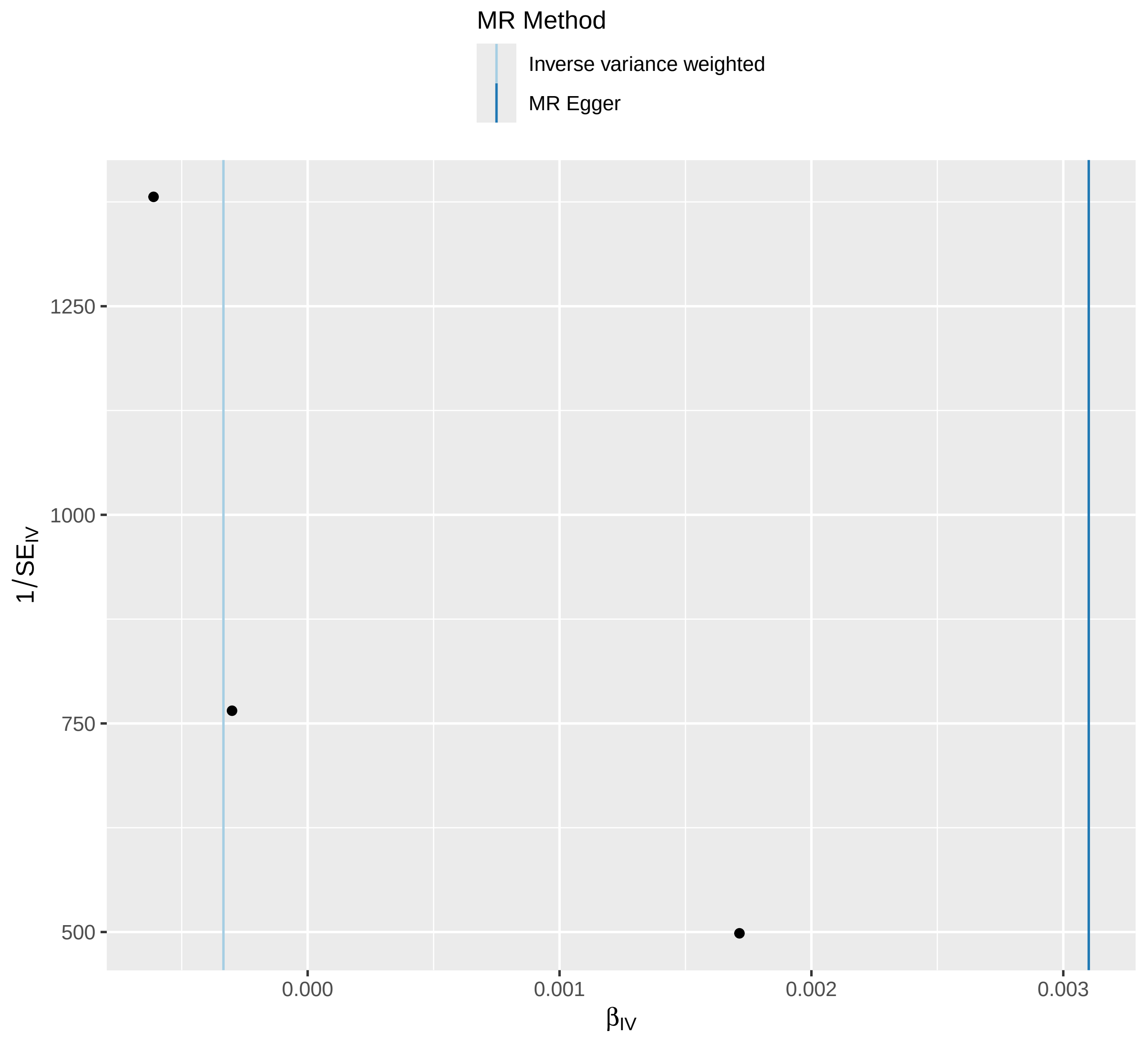
MR Method
Inverse variance weighted
MR Egger
1250
1000
750
500
1/SE_IV
0.000
0.001
0.002
0.003
β_IV

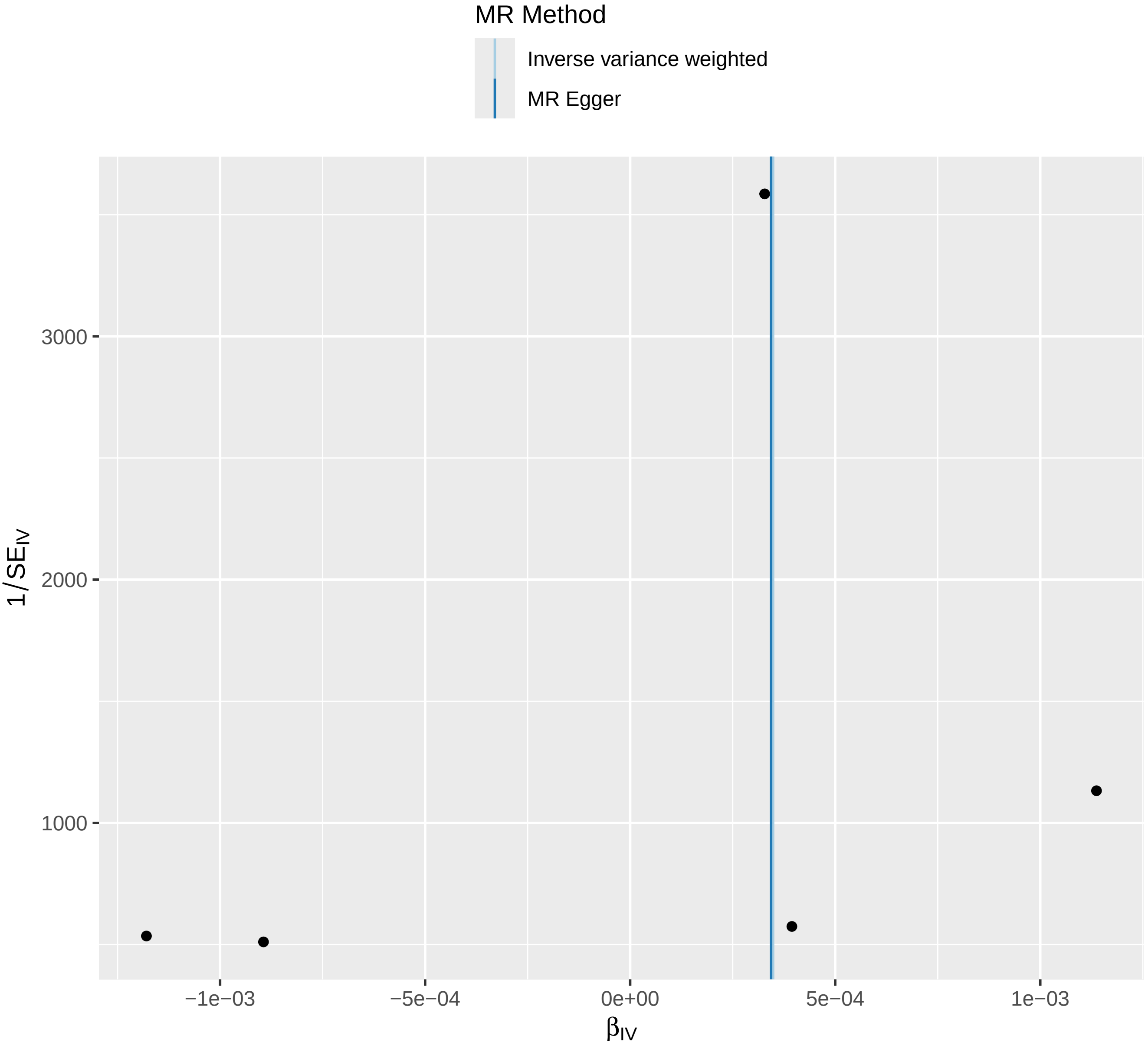

MR Method
Inverse variance weighted
MR Egger
3000
2000
1000
−1e−03
−5e−04
0e+00
5e−04
1e−03
$1/SE_{IV}$
β_{IV}

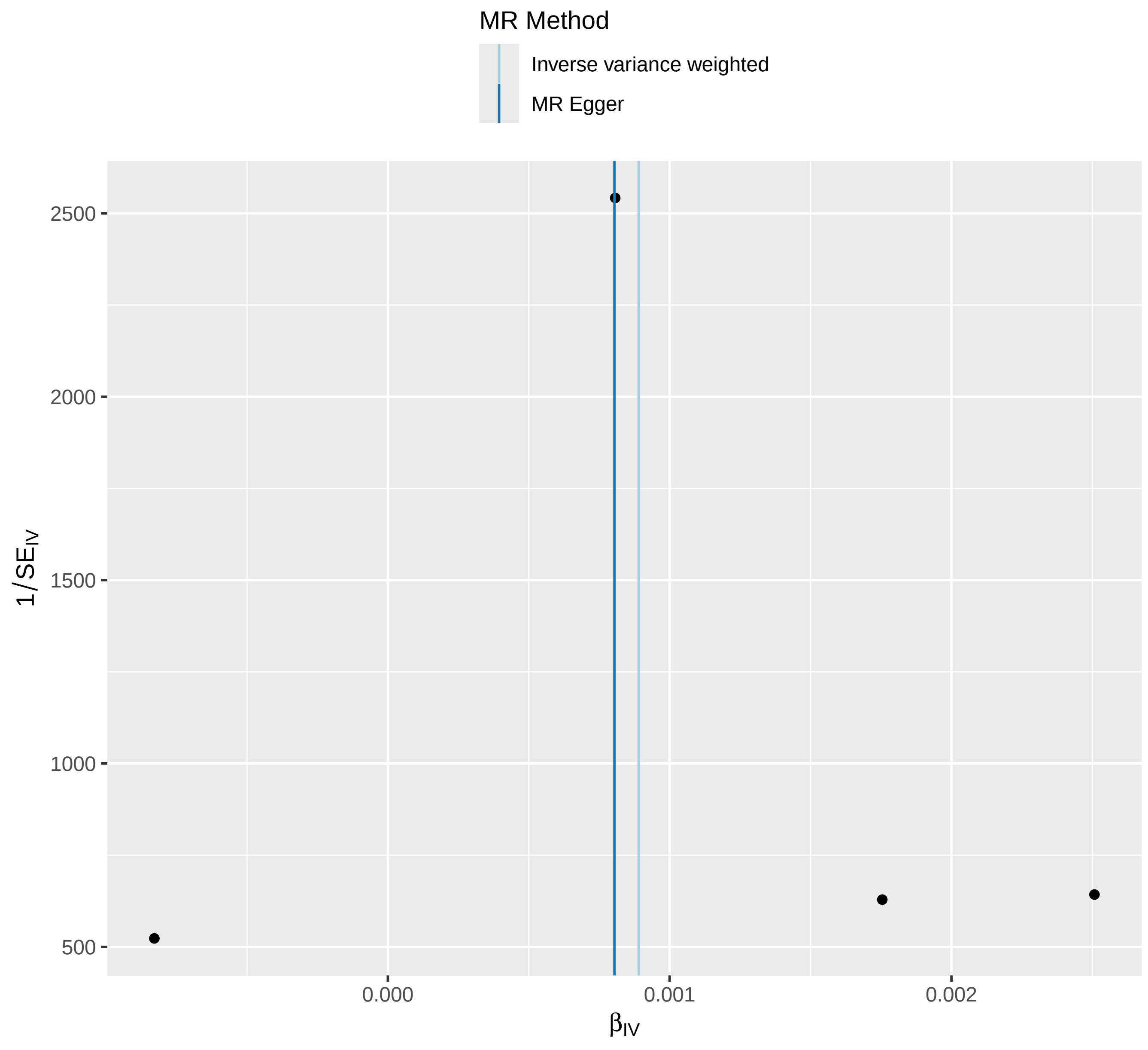
MR Method
Inverse variance weighted
MR Egger
2500
2000
1500
1000
500
1/SE_IV
0.000
0.001
0.002
β_IV

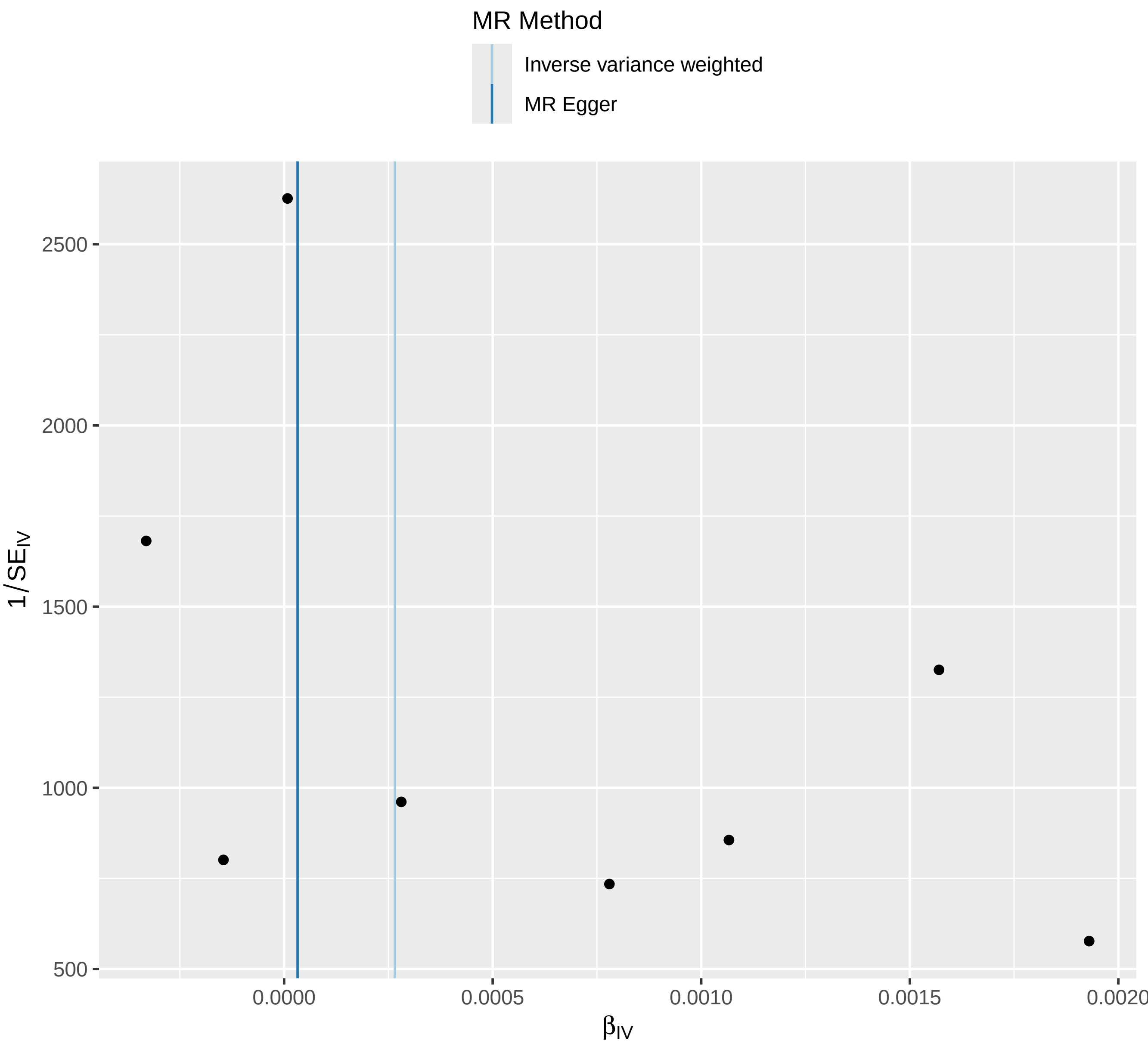
MR Method
Inverse variance weighted
MR Egger
2500
2000
1500
1000
500
1/SE_IV
0.0000
0.0005
0.0010
0.0015
0.0020
β_IV

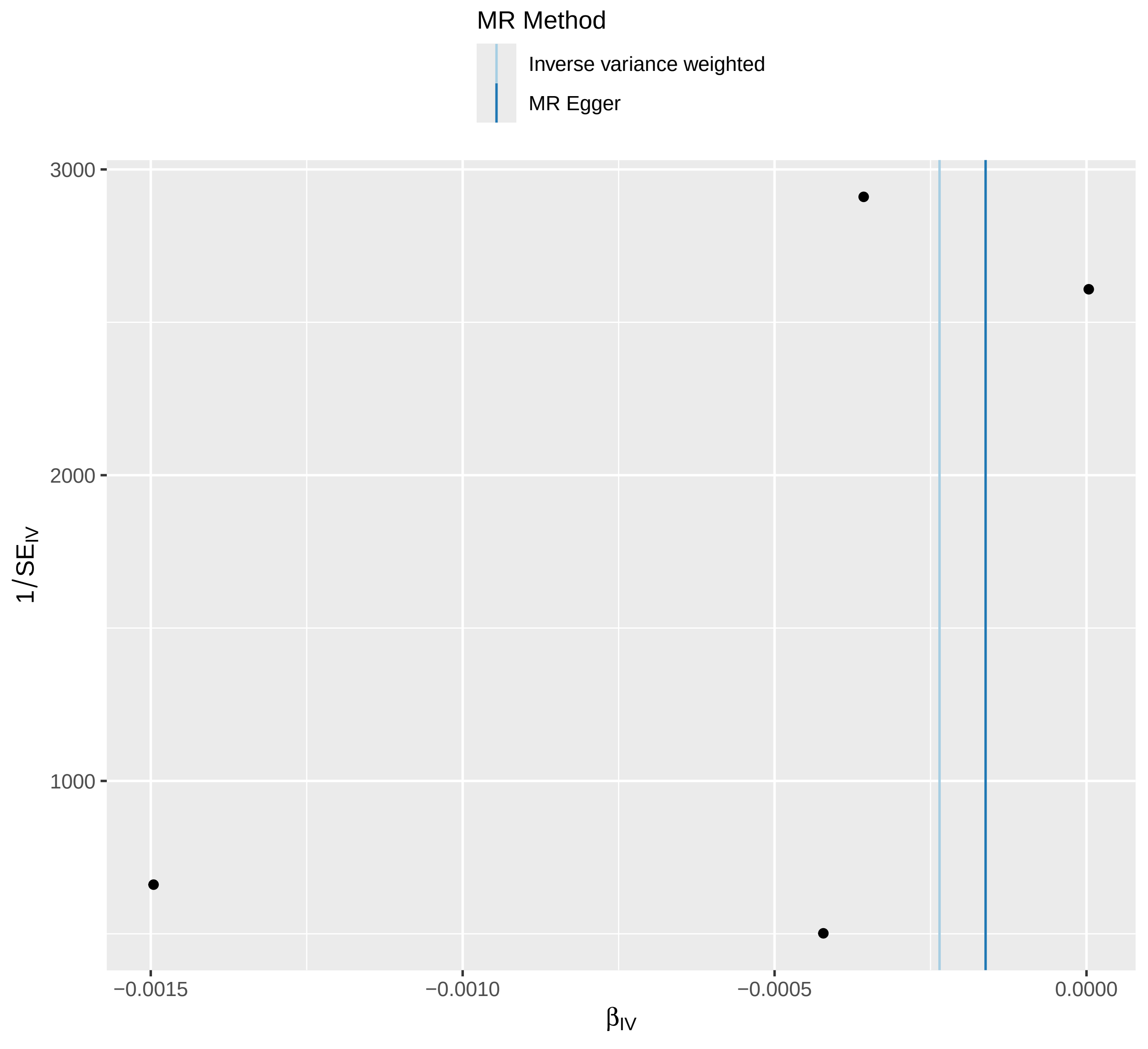

MR Method
Inverse variance weighted
MR Egger
3000
2000
1000
1/SE_IV
−0.0015
−0.0010
−0.0005
0.0000
β_IV

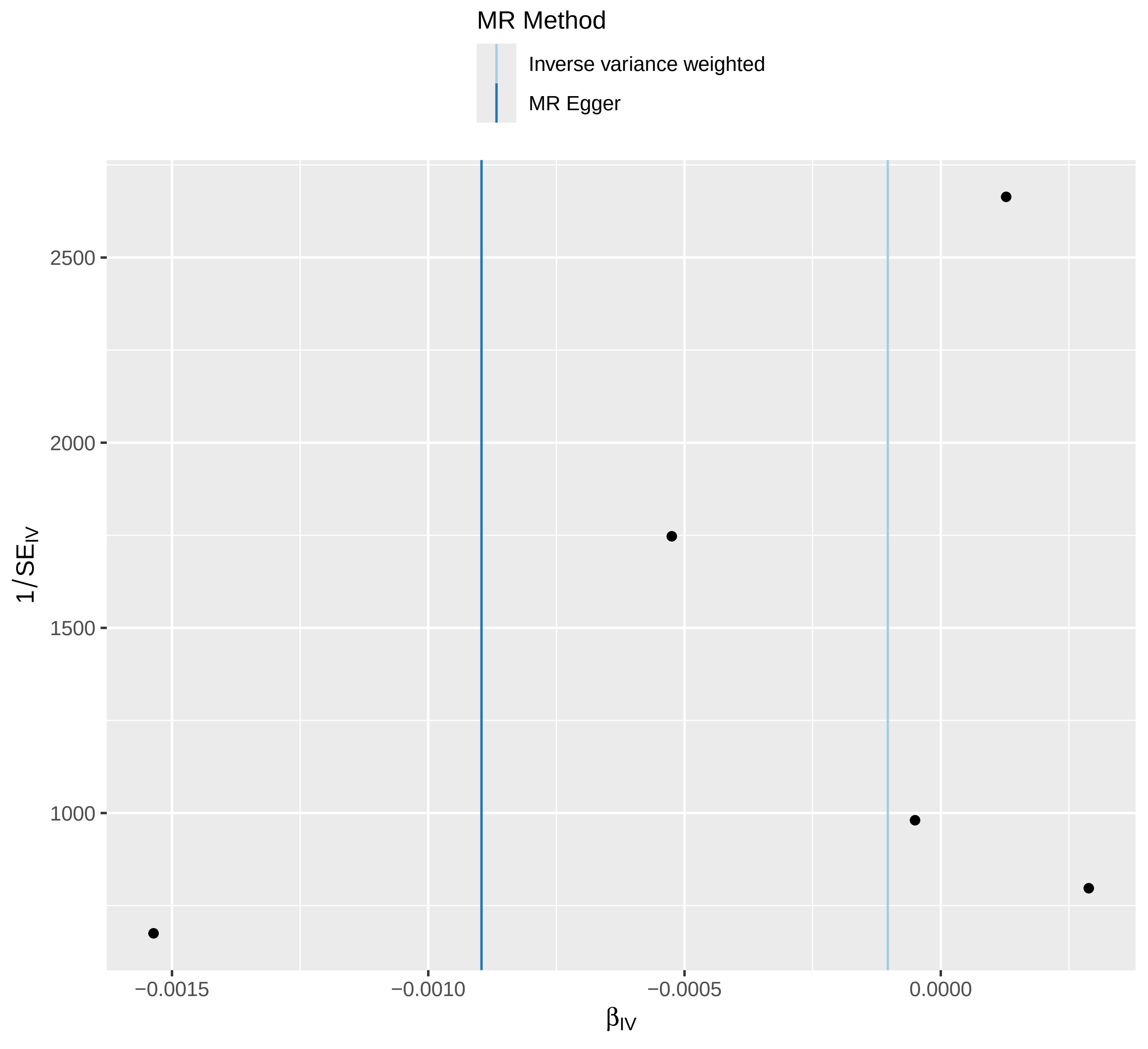
MR Method
Inverse variance weighted
MR Egger
2500
2000
1500
1000
$1/SE_{IV}$
−0.0015
−0.0010
−0.0005
0.0000
β_{IV}

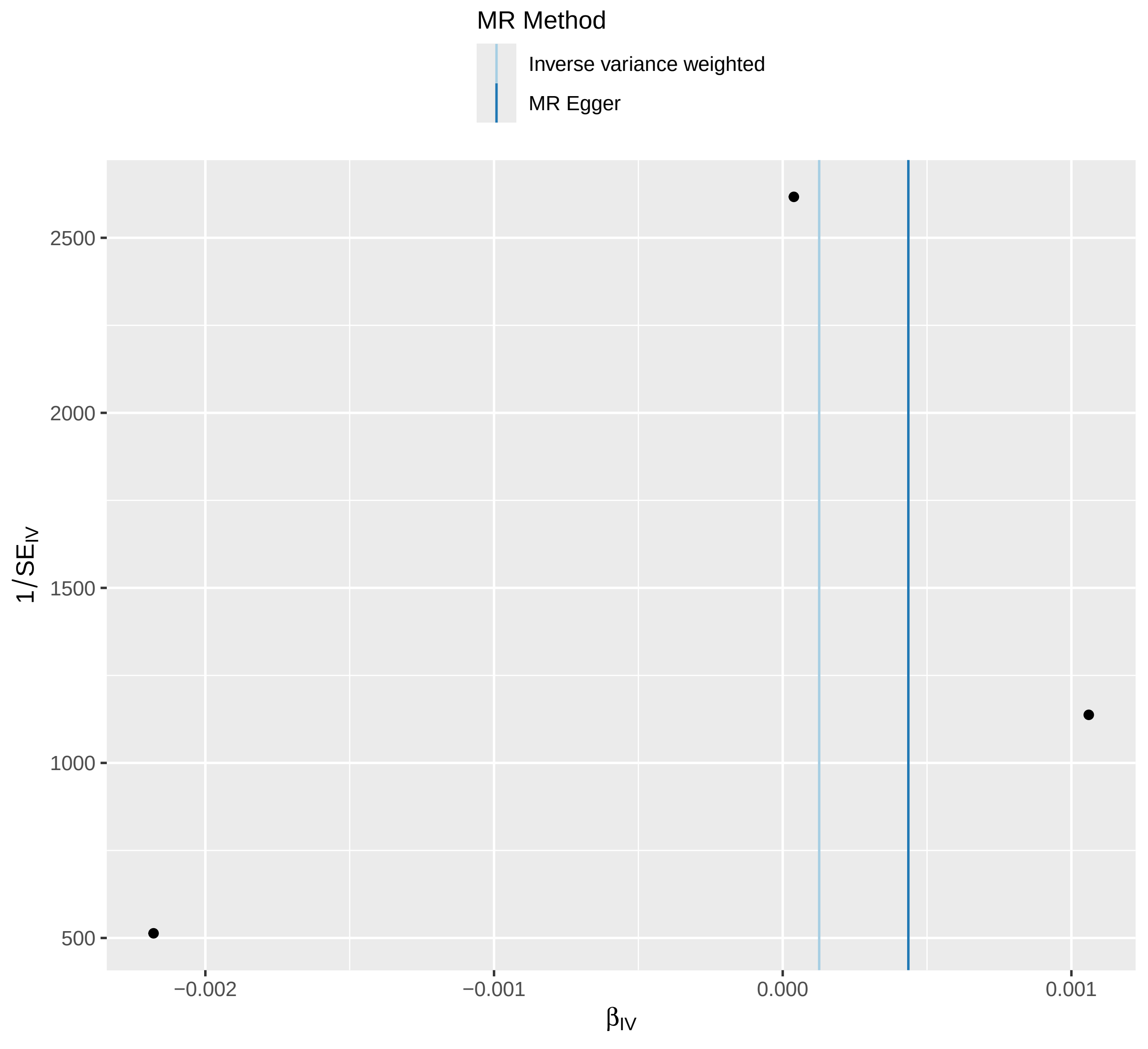

MR Method
Inverse variance weighted
MR Egger
2500
2000
1500
1000
500
1/SE_IV
−0.002
−0.001
0.000
0.001
β_IV

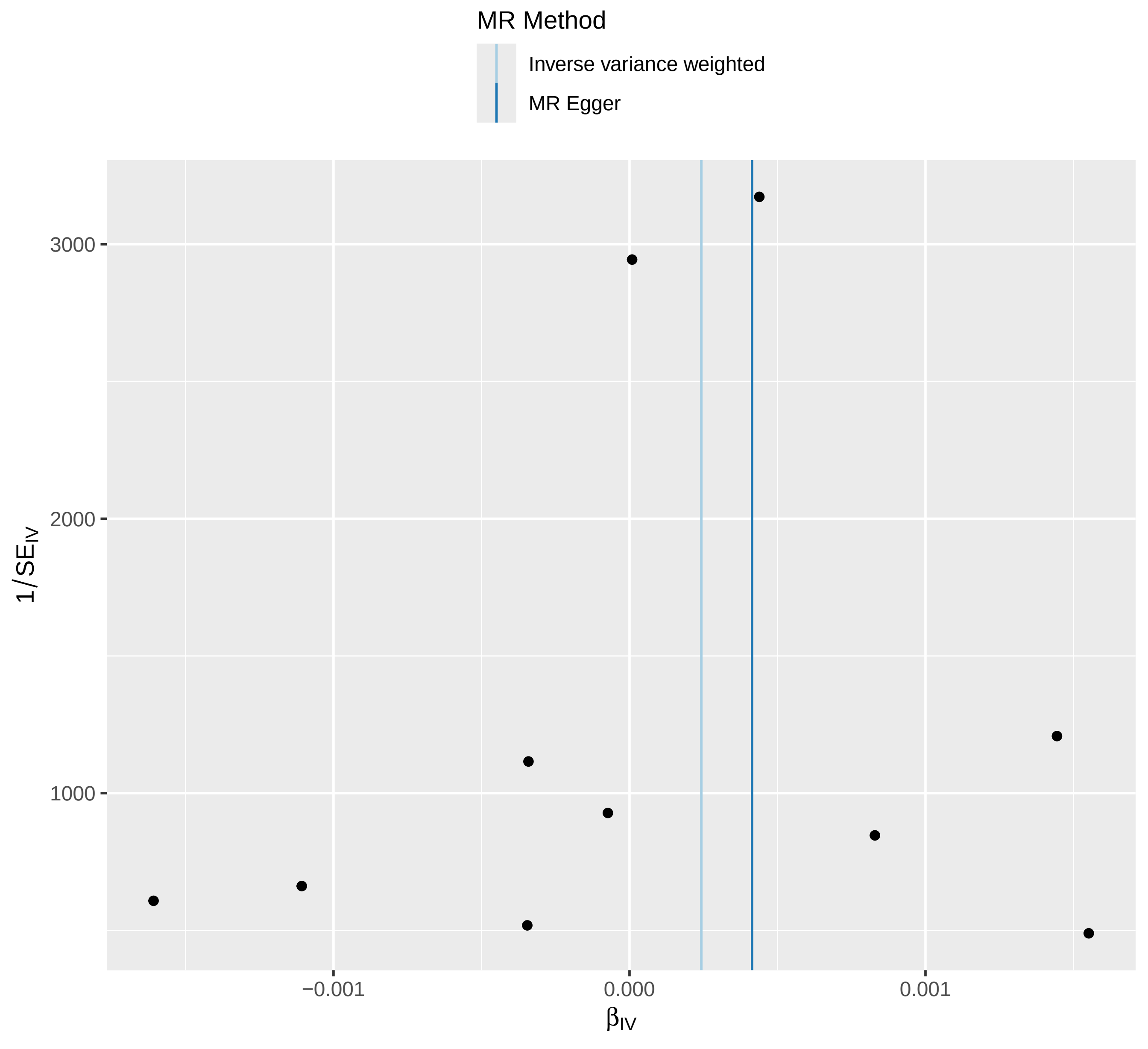
MR Method
Inverse variance weighted
MR Egger
3000
2000
1000
1/SE_IV
−0.001
0.000
0.001
β_IV

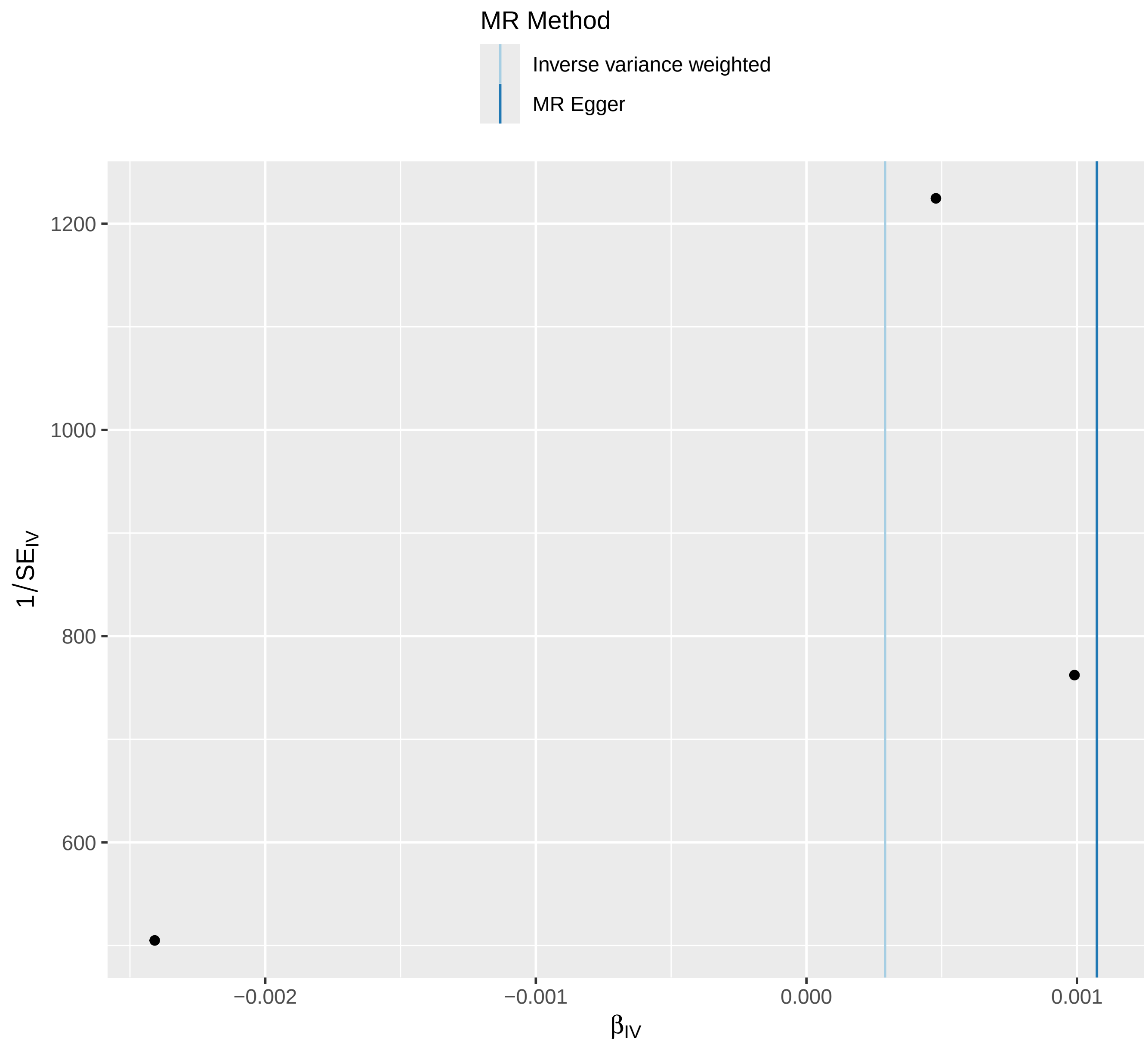
MR Method
Inverse variance weighted
MR Egger
1200
1000
800
600
1/SE_IV
−0.002
−0.001
0.000
0.001
β_IV

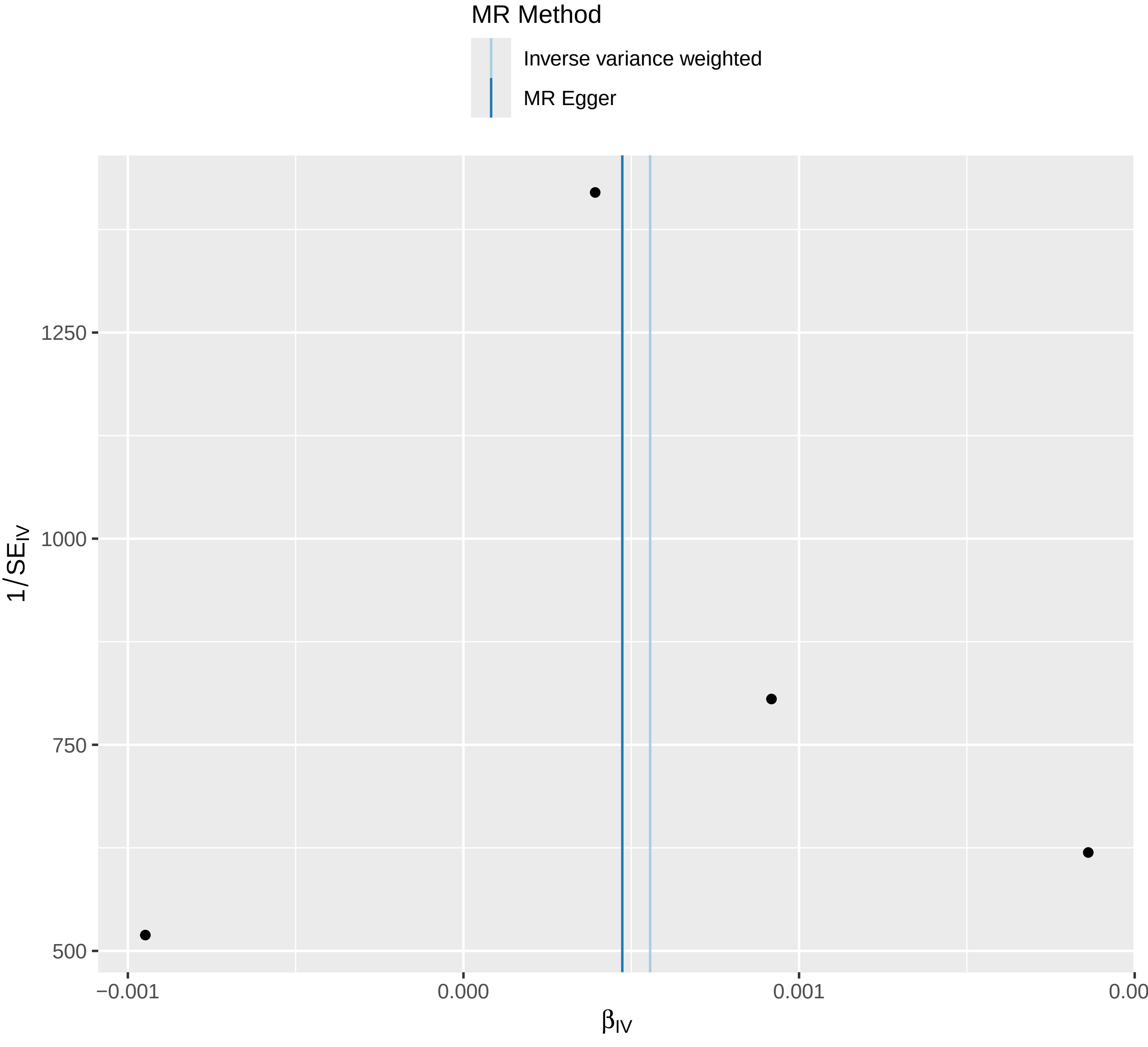
MR Method
Inverse variance weighted
MR Egger
1250
1000
750
500
$1/SE_{IV}$
−0.001
0.000
0.001
0.00
β_{IV}

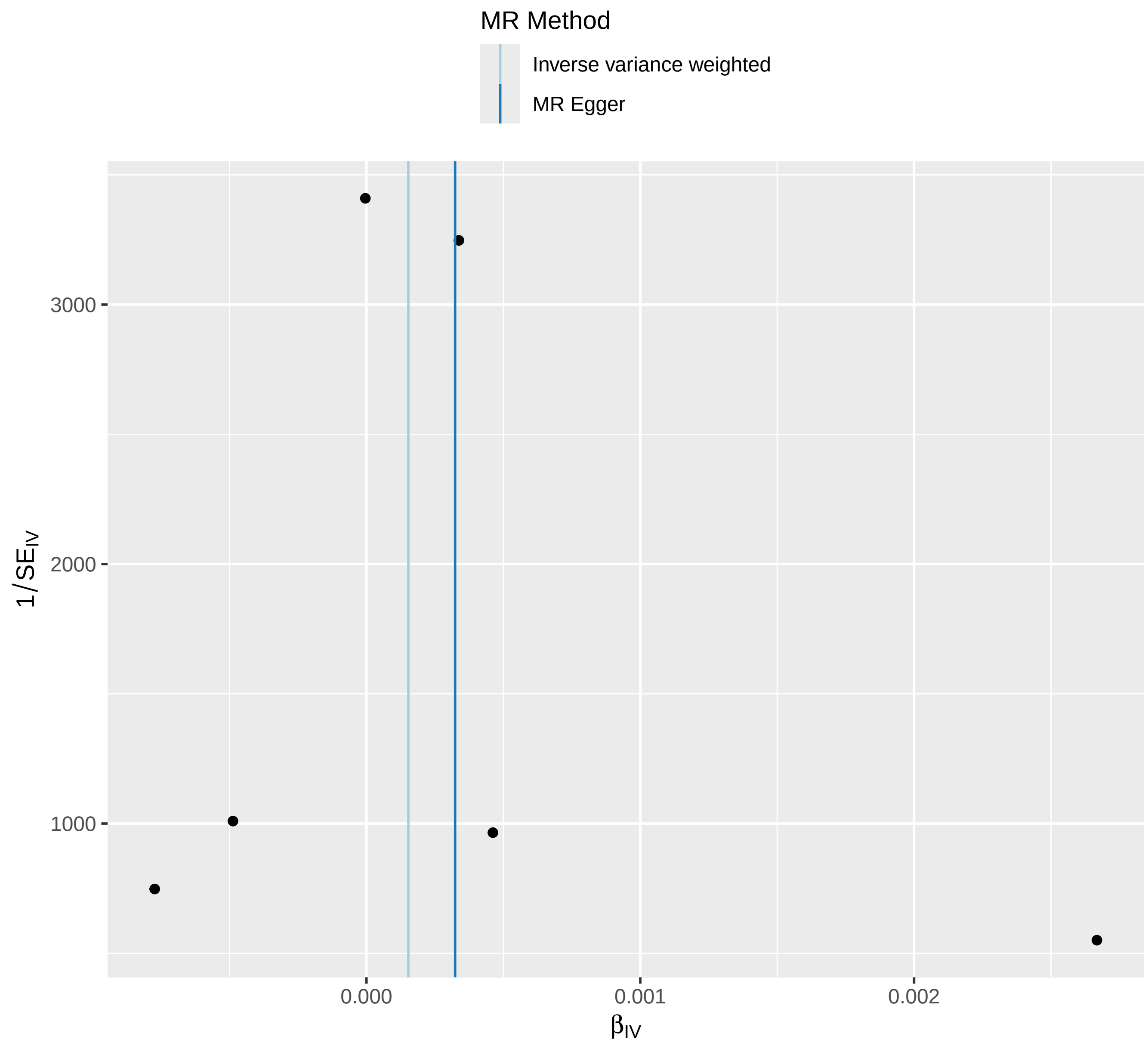
MR Method
Inverse variance weighted
MR Egger
3000
2000
1000
0.000
0.001
0.002
$1/SE_{IV}$
β_{IV}

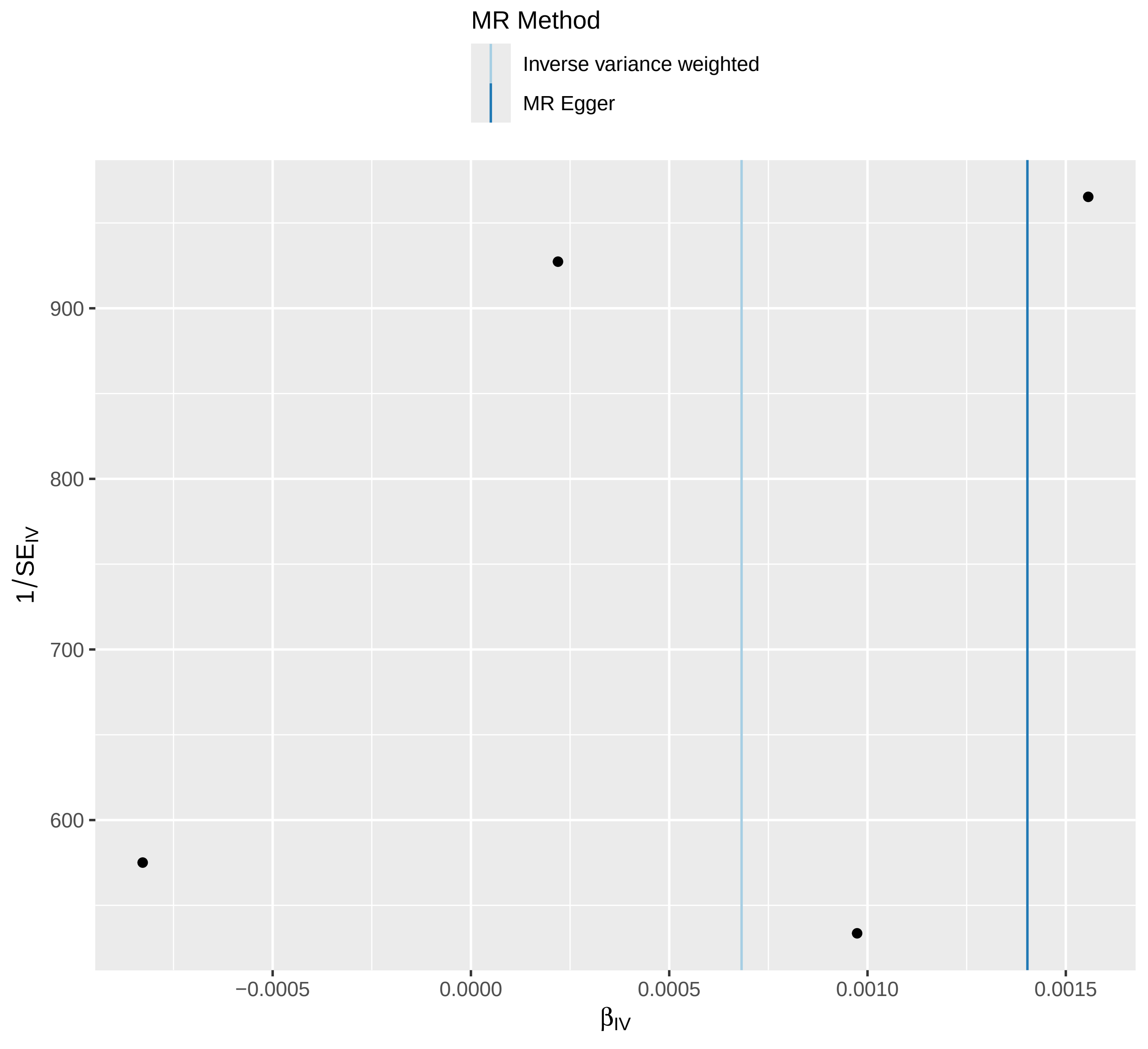
MR Method
Inverse variance weighted
MR Egger
900
800
700
600
1/SE_IV
−0.0005
0.0000
0.0005
0.0010
0.0015
β_IV

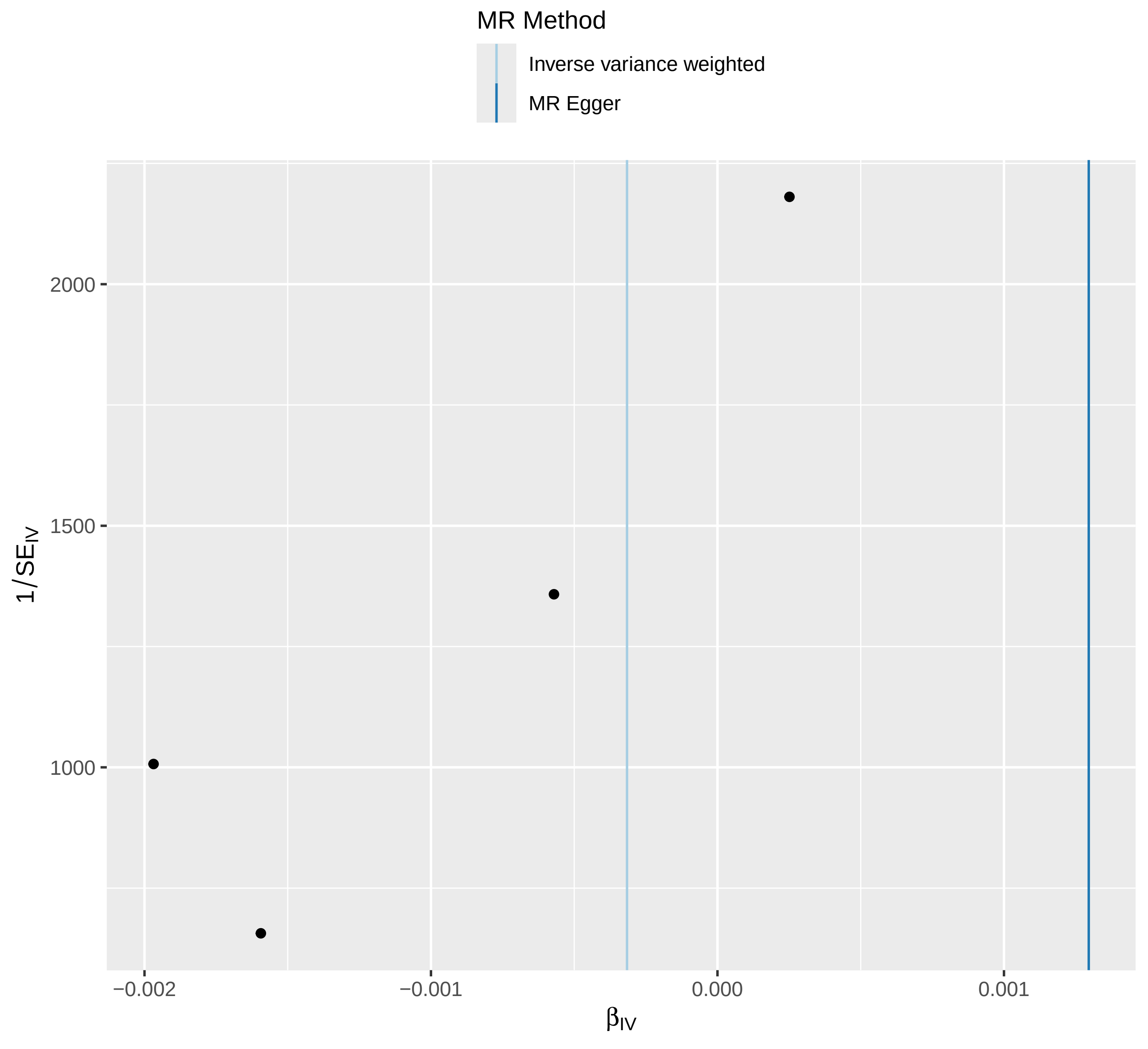
MR Method
Inverse variance weighted
MR Egger
2000
1500
1000
1/SE_IV
−0.002
−0.001
0.000
0.001
β_IV

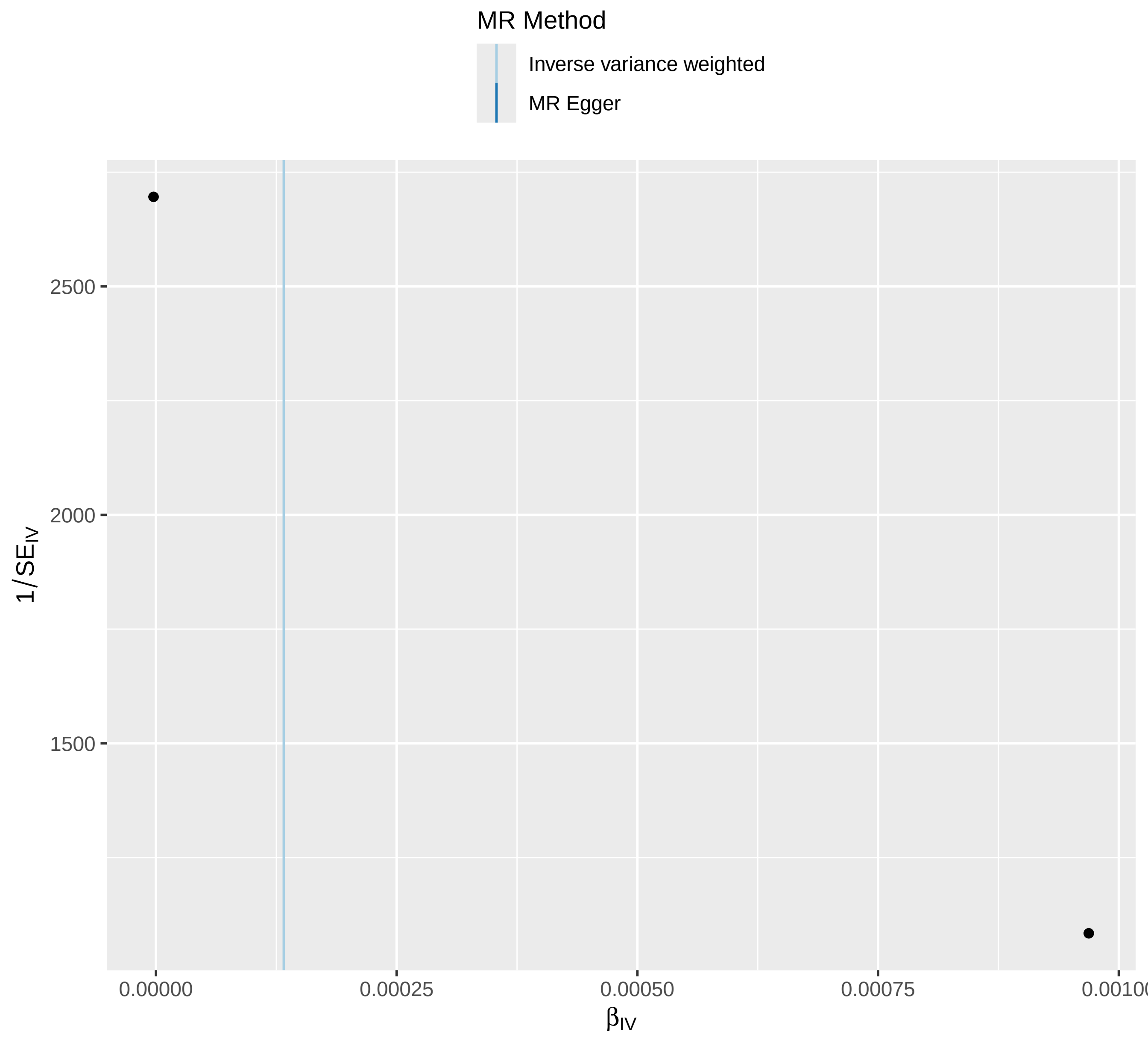

MR Method
Inverse variance weighted
MR Egger
2500
2000
1500
1/SE_IV
0.00000
0.00025
0.00050
0.00075
0.00100
β_IV

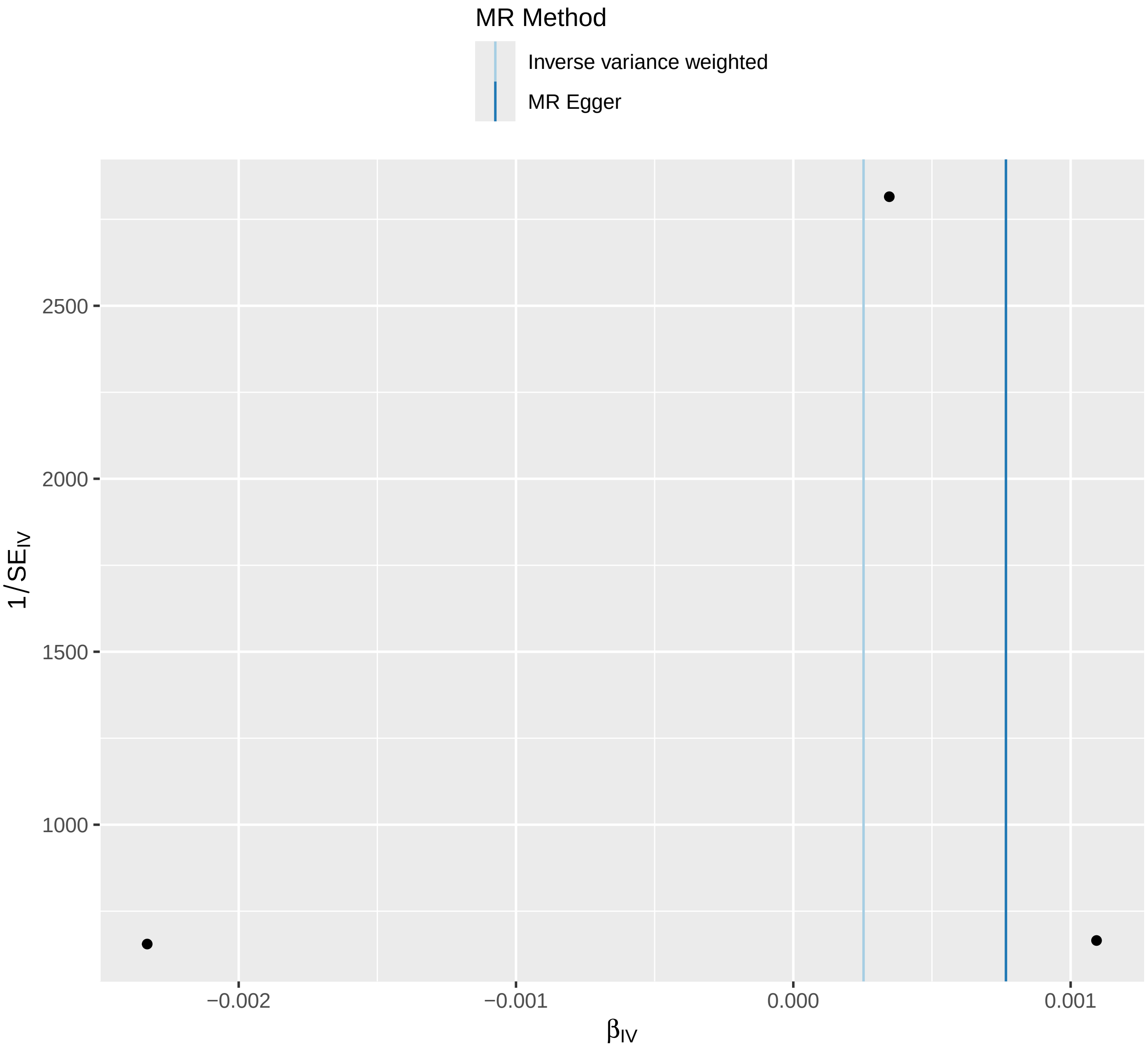
MR Method
Inverse variance weighted
MR Egger
2500
2000
1500
1000
1/SE_IV
−0.002
−0.001
0.000
0.001
β_IV

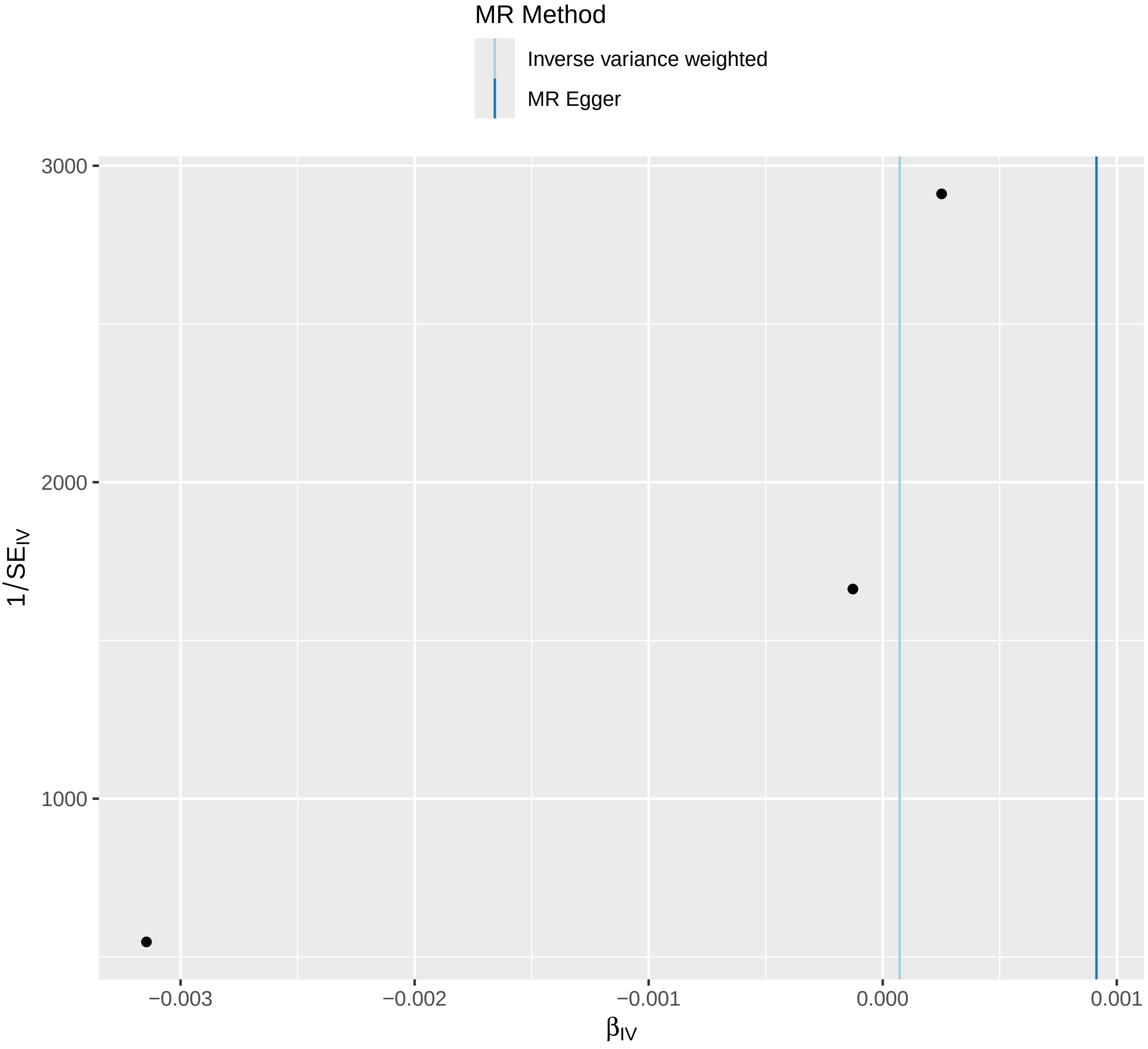

MR Method
Inverse variance weighted
MR Egger
3000
2000
1000
1/SE_IV
−0.003
−0.002
−0.001
0.000
0.001
β_IV

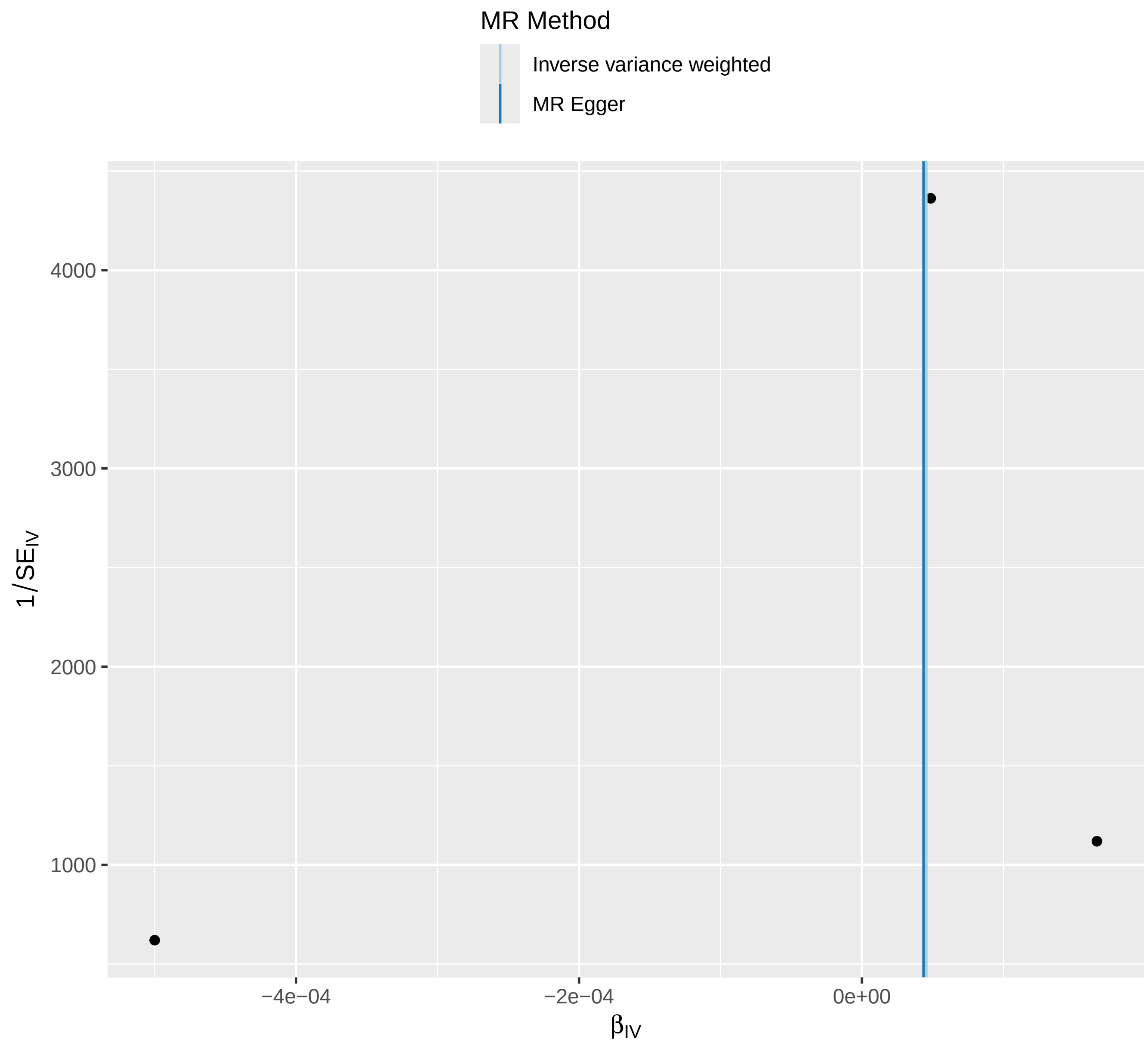

MR Method
Inverse variance weighted
MR Egger
4000
3000
2000
1000
1/SE_IV
−4e−04
−2e−04
0e+00
β_IV

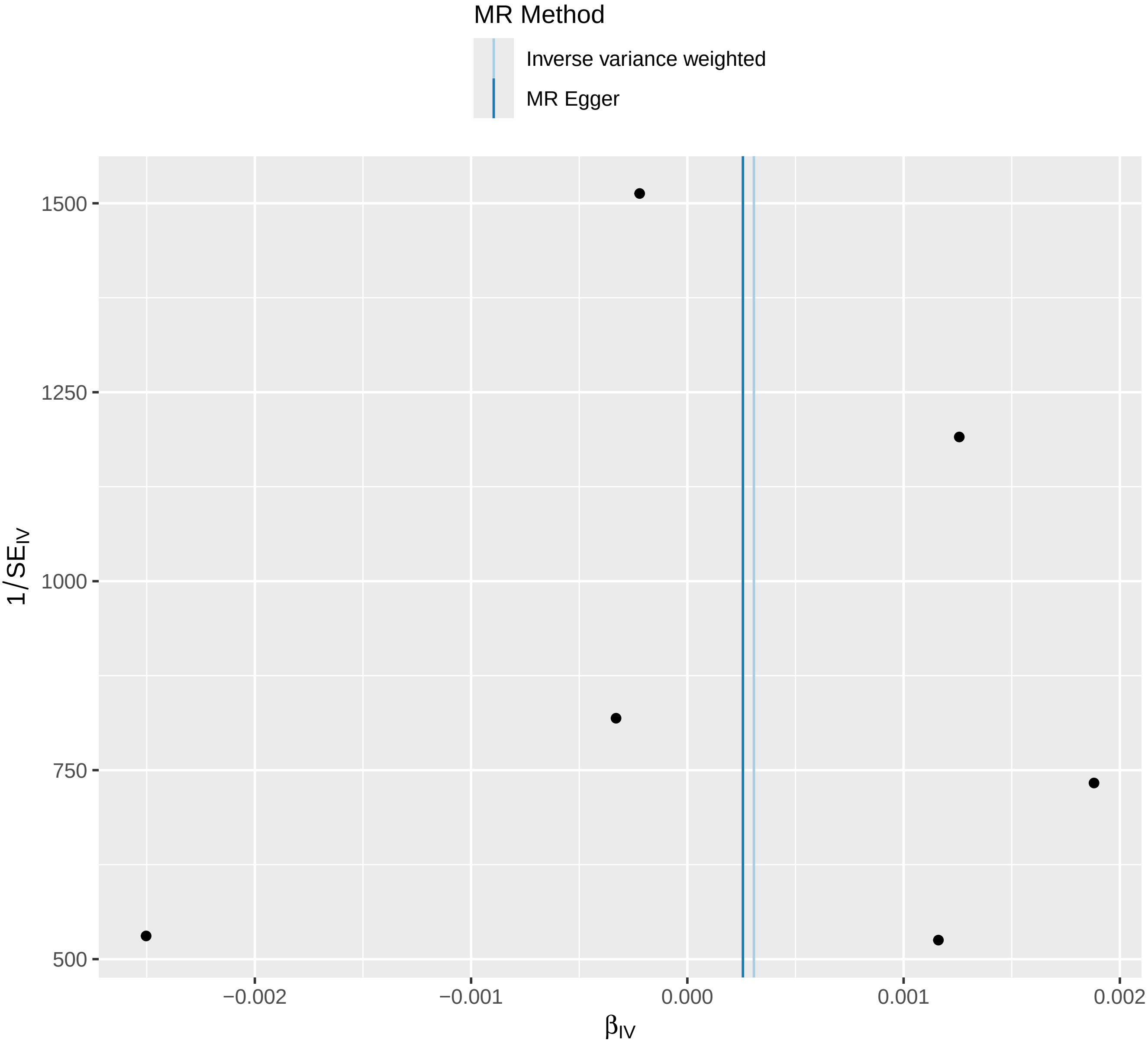
MR Method
Inverse variance weighted
MR Egger
1500
1250
1000
750
500
1/SE_IV
−0.002
−0.001
0.000
0.001
0.002
β_IV

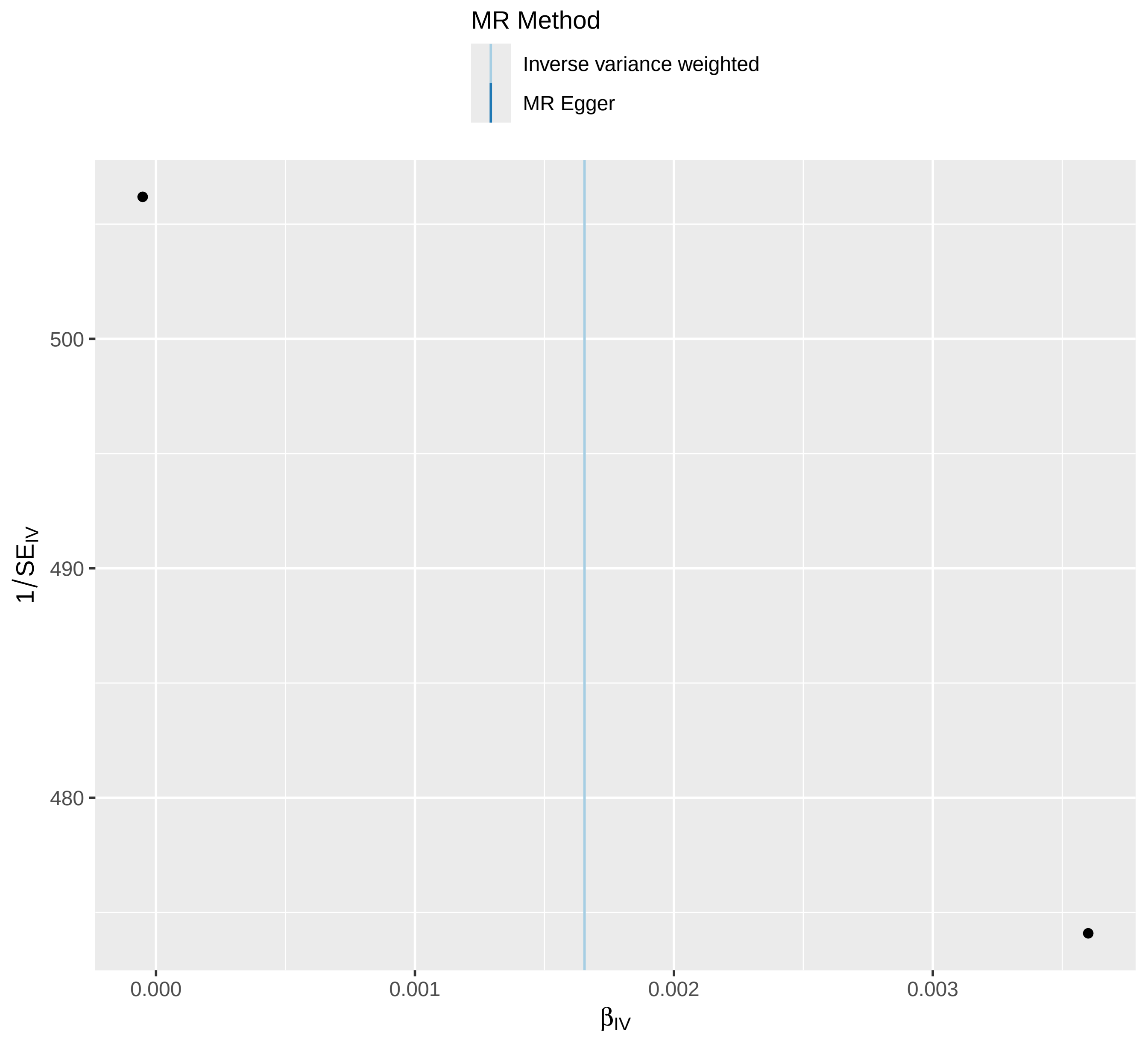
MR Method
Inverse variance weighted
MR Egger
500
490
480
$1/SE_{IV}$
0.000
0.001
0.002
0.003
β_{IV}

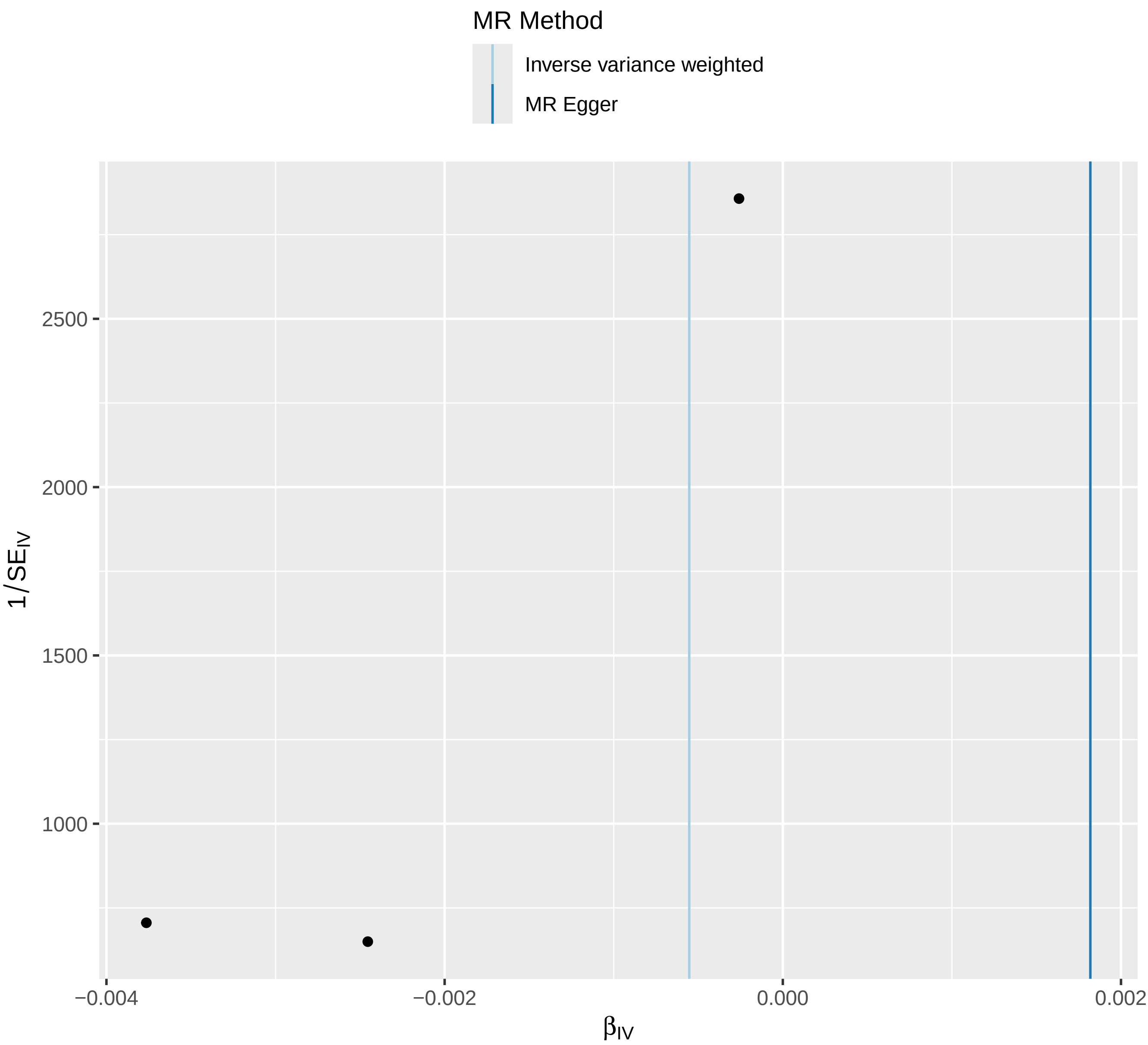
MR Method
Inverse variance weighted
MR Egger
2500
2000
1500
1000
1/SE_IV
−0.004
−0.002
0.000
0.002
β_IV

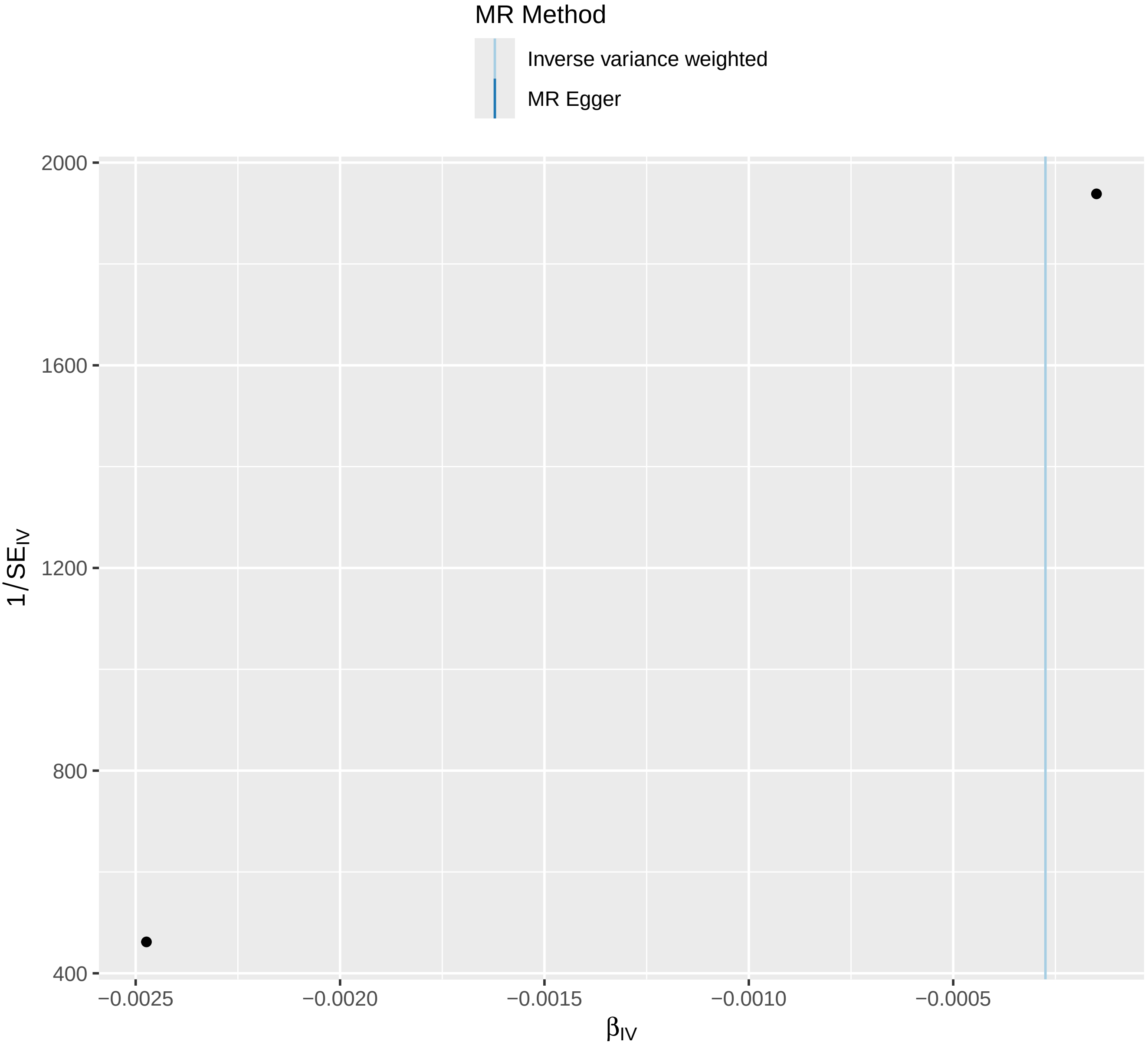

MR Method
Inverse variance weighted
MR Egger
2000
1600
1200
800
400
1/SE_IV
−0.0025
−0.0020
−0.0015
−0.0010
−0.0005
β_IV

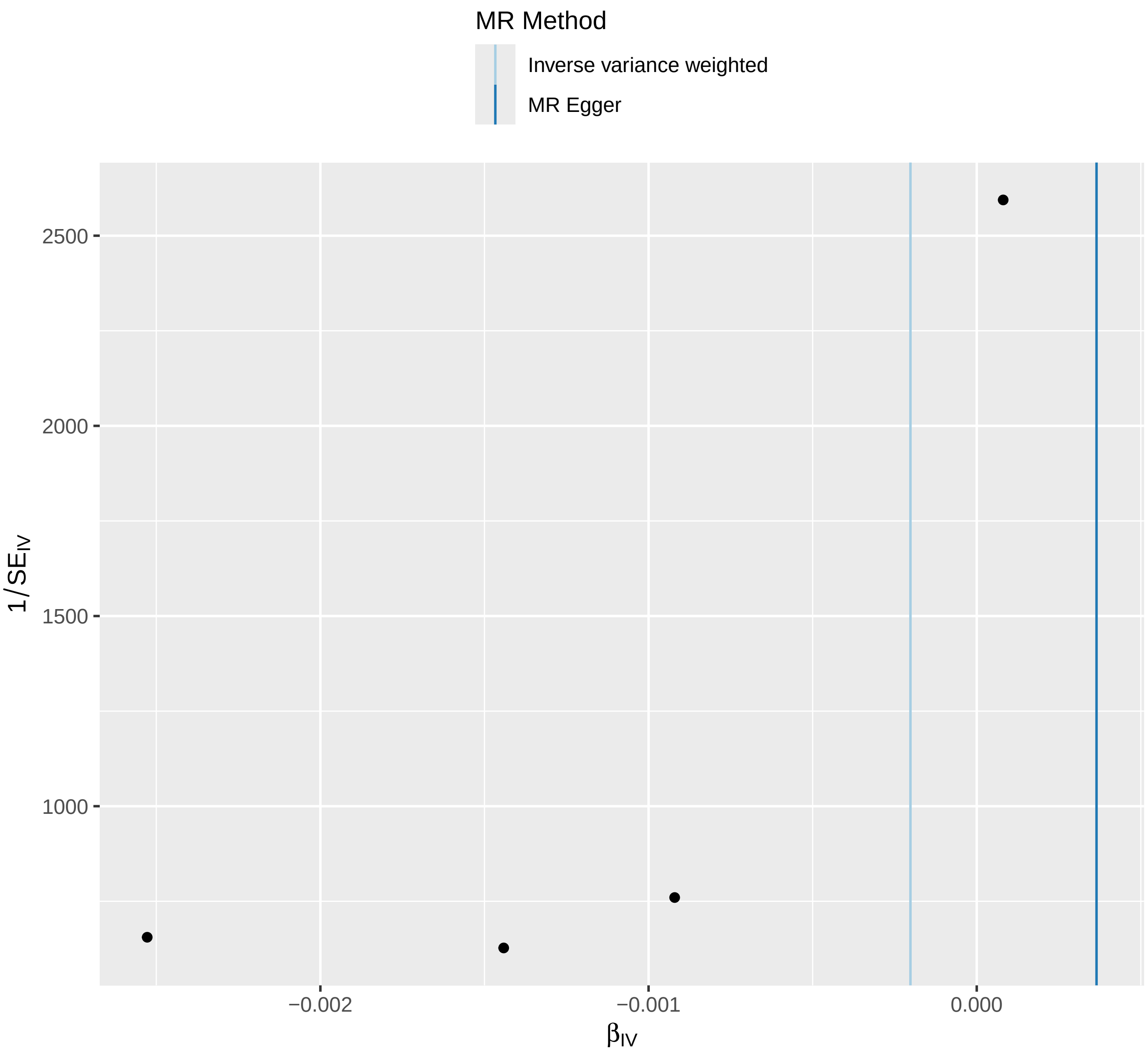
MR Method
Inverse variance weighted
MR Egger
2500
2000
1500
1000
$1/SE_{IV}$
−0.002
−0.001
0.000
β_{IV}

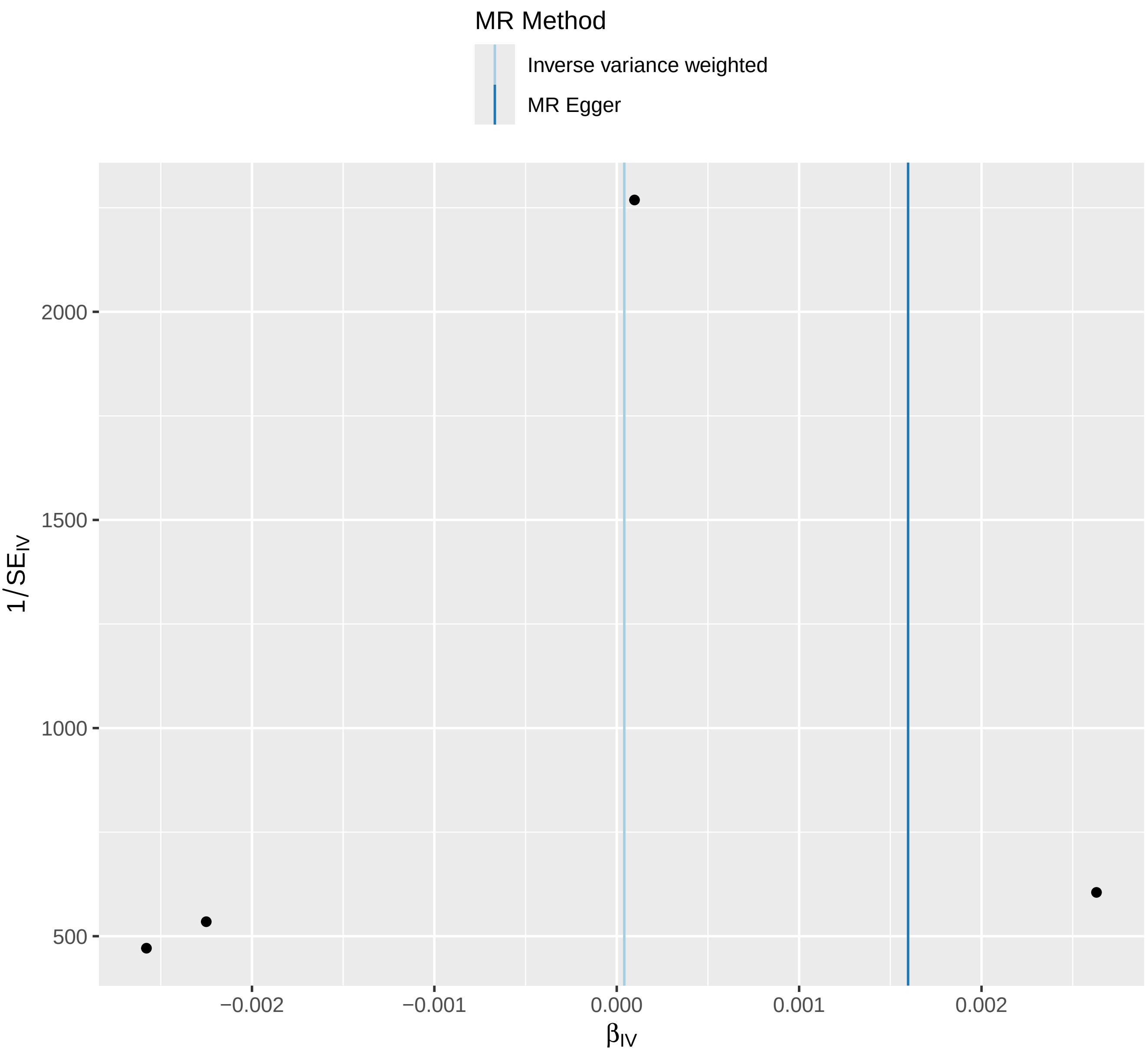

MR Method
Inverse variance weighted
MR Egger
2000
1500
1000
500
1/SE_IV
−0.002
−0.001
0.000
0.001
0.002
β_IV

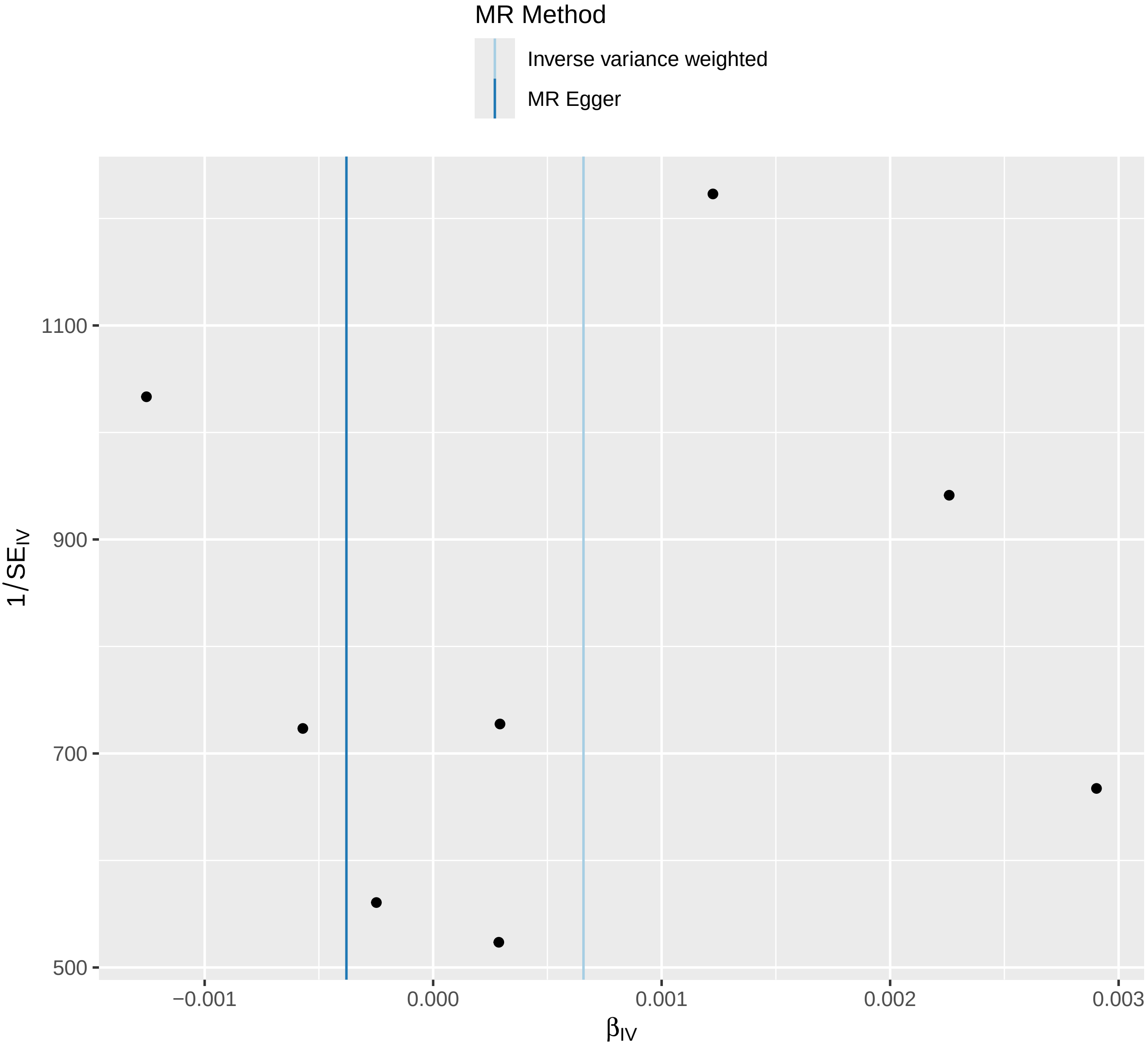
MR Method
Inverse variance weighted
MR Egger
1100
900
700
500
−0.001
0.000
0.001
0.002
0.003
β_{IV}
$1/SE_{IV}$

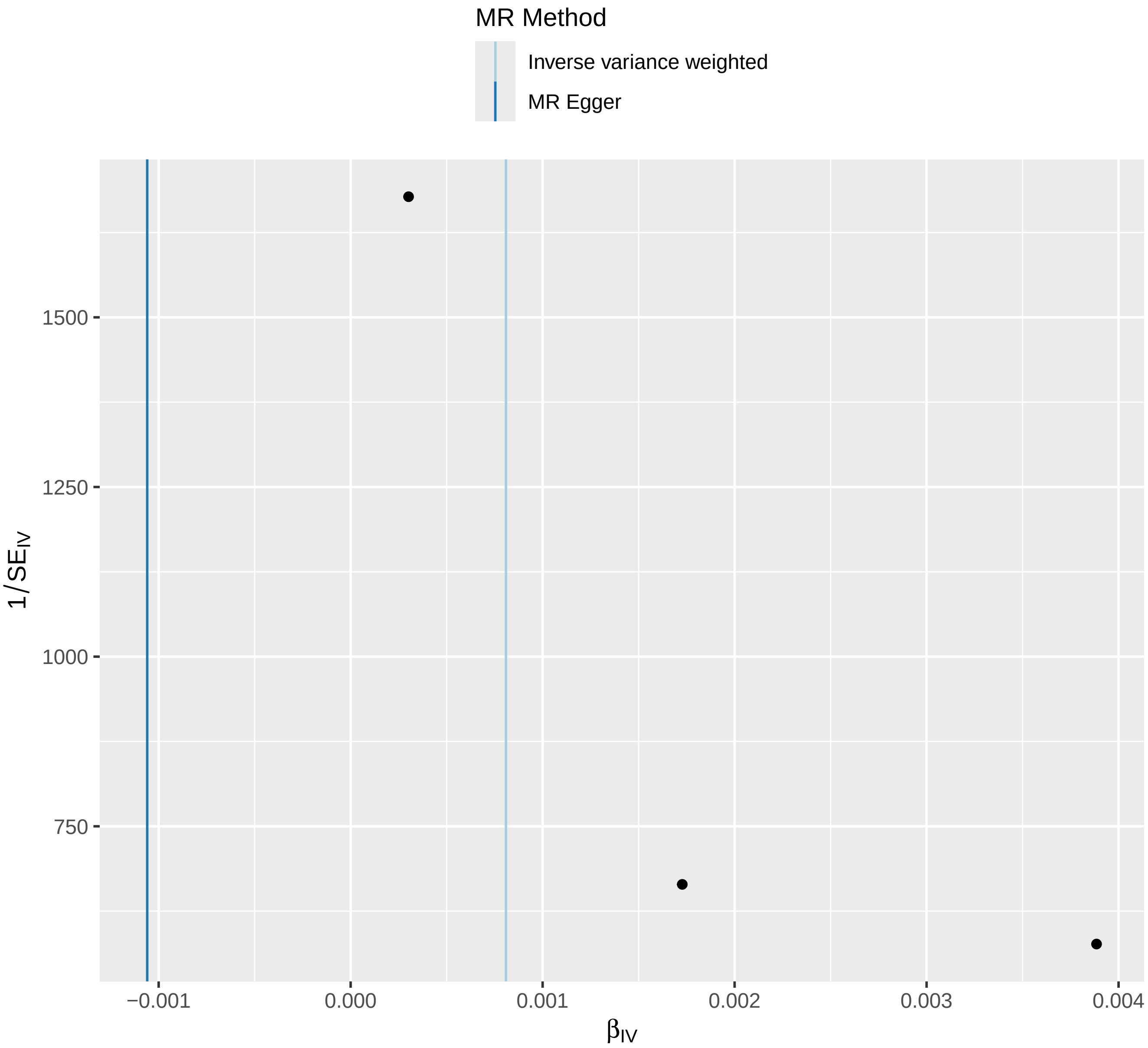
MR Method
Inverse variance weighted
MR Egger
1500
1250
1000
750
1/SE_IV
−0.001
0.000
0.001
0.002
0.003
0.004
β_IV

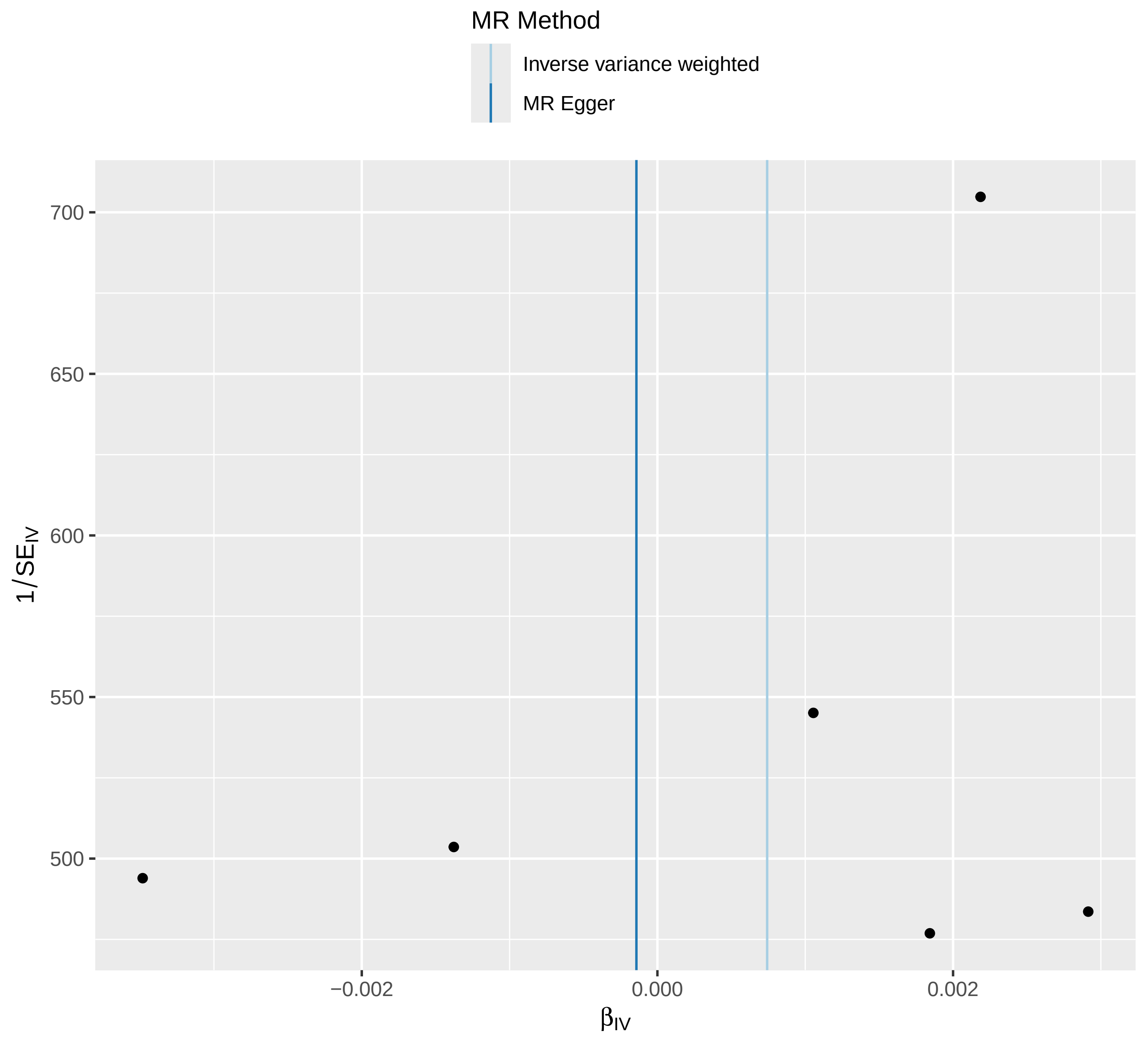
MR Method
Inverse variance weighted
MR Egger
1/SE_IV
700
650
600
550
500
−0.002
0.000
0.002
β_IV

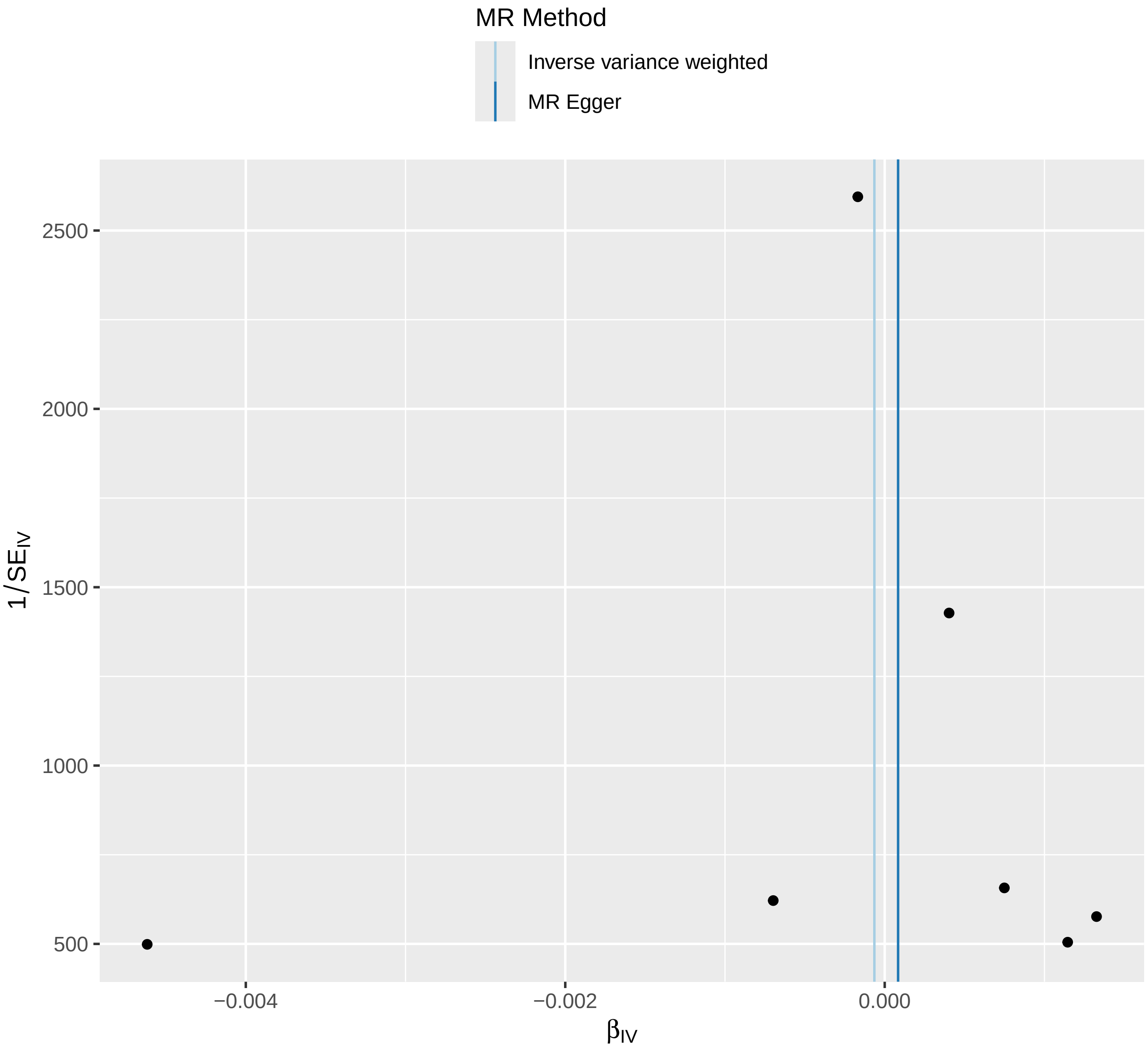
MR Method
Inverse variance weighted
MR Egger
2500
2000
1500
1000
500
1/SE_IV
−0.004
−0.002
0.000
β_IV

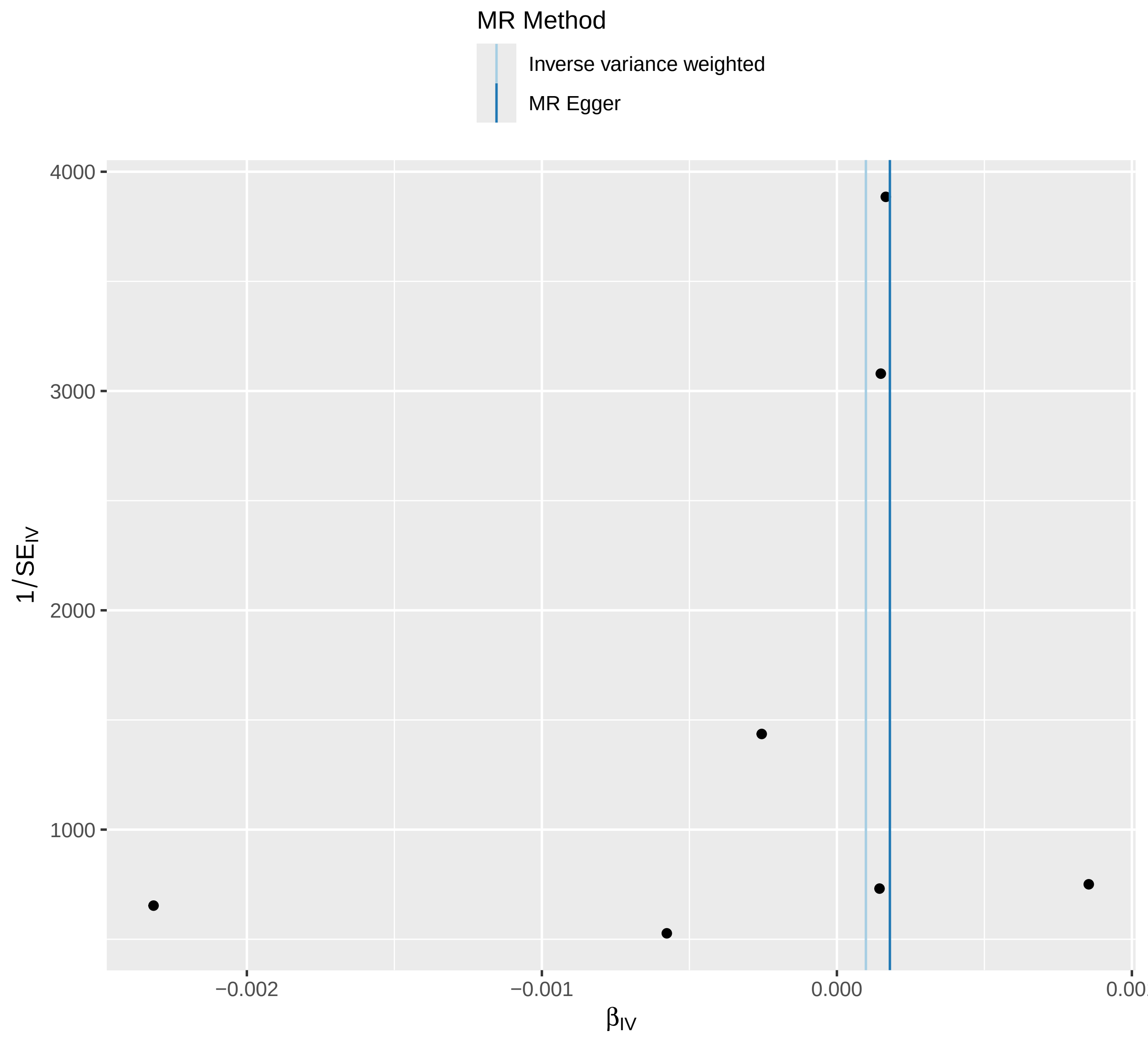

MR Method
Inverse variance weighted
MR Egger
4000
3000
2000
1000
1/SE_IV
−0.002
−0.001
0.000
0.00
β_IV

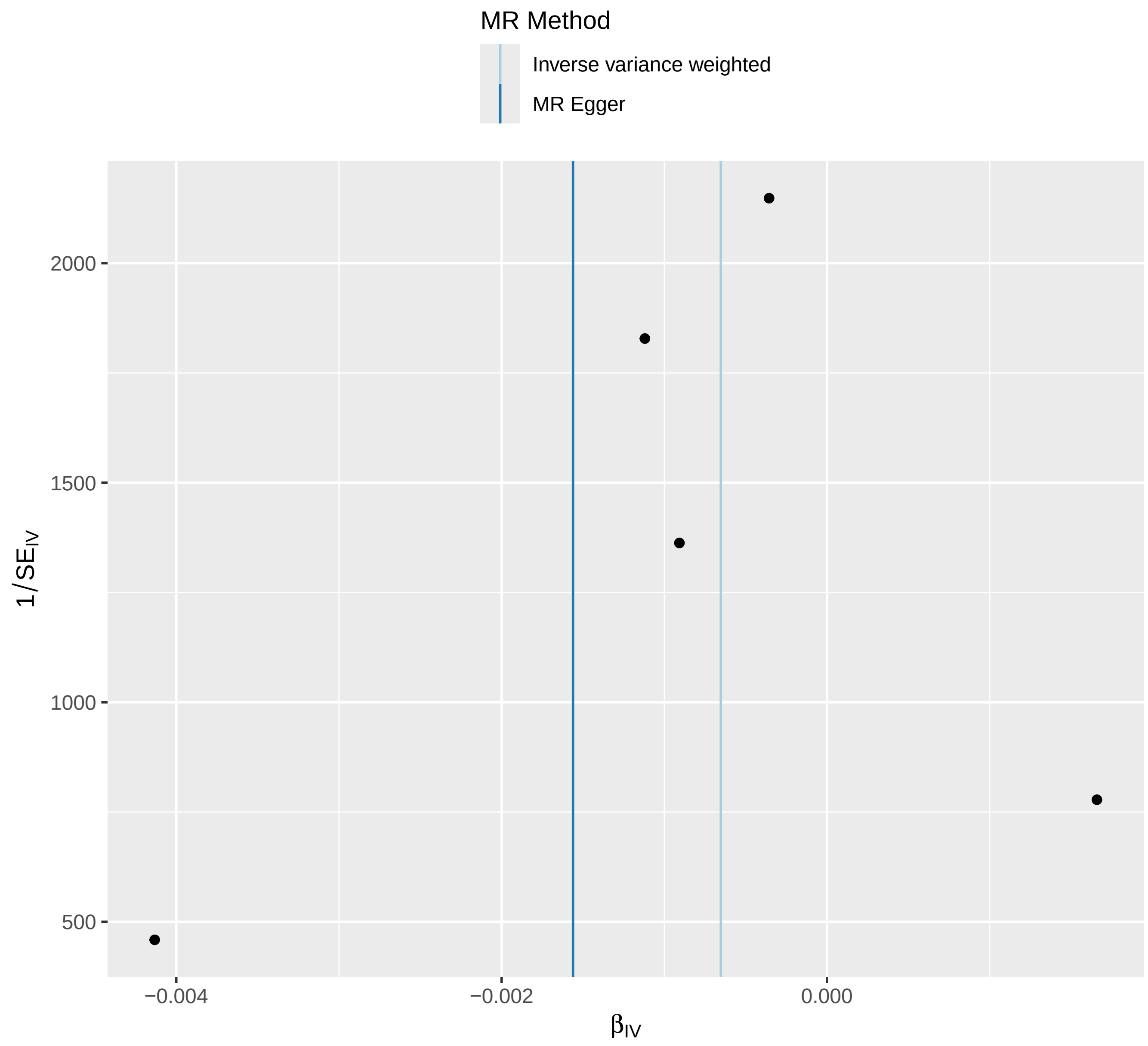
MR Method
Inverse variance weighted
MR Egger
2000
1500
1000
500
1/SE_IV
−0.004
−0.002
0.000
β_IV

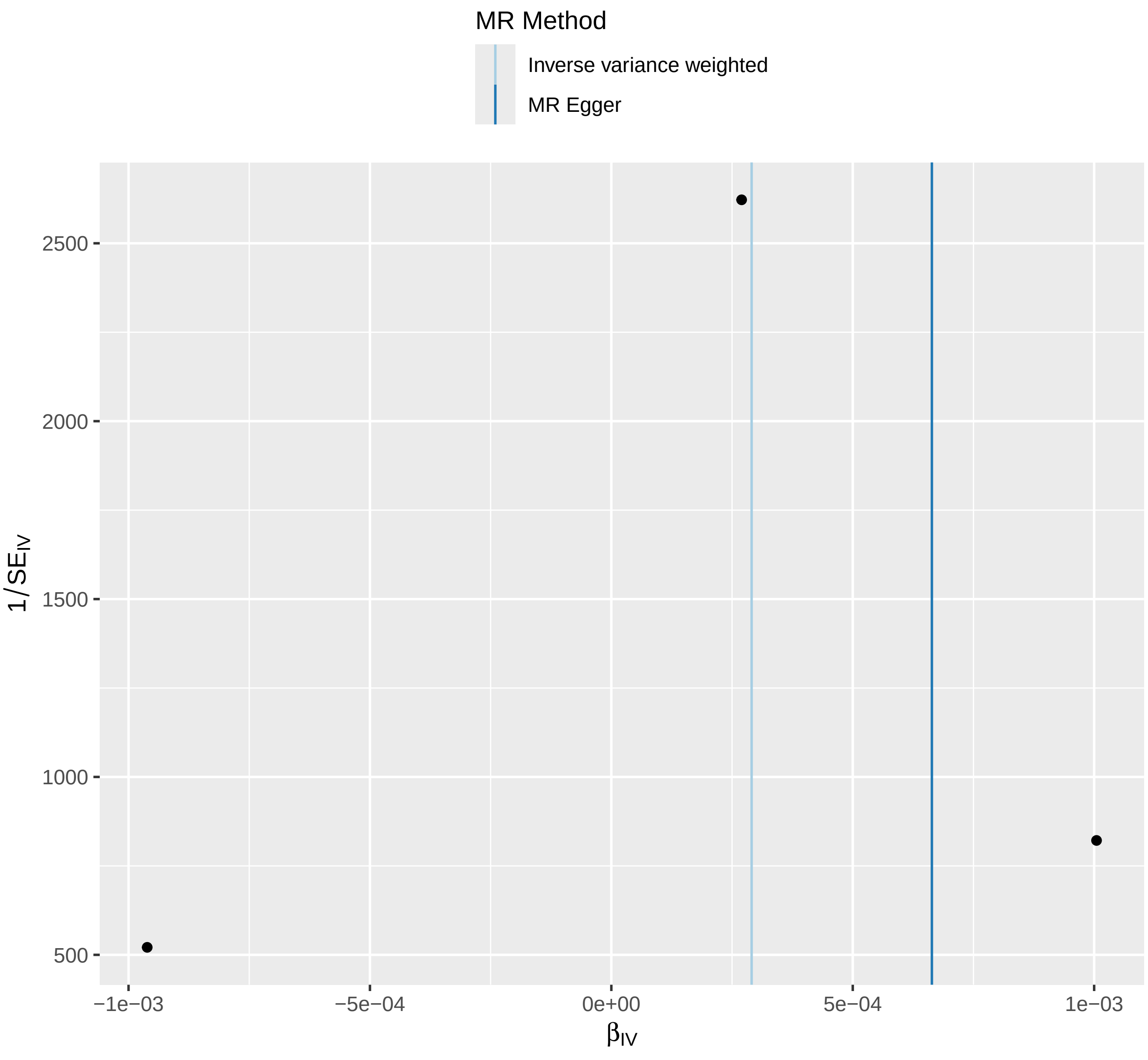

MR Method
Inverse variance weighted
MR Egger
2500
2000
1500
1000
500
1/SE_IV
−1e−03
−5e−04
0e+00
5e−04
1e−03
β_IV

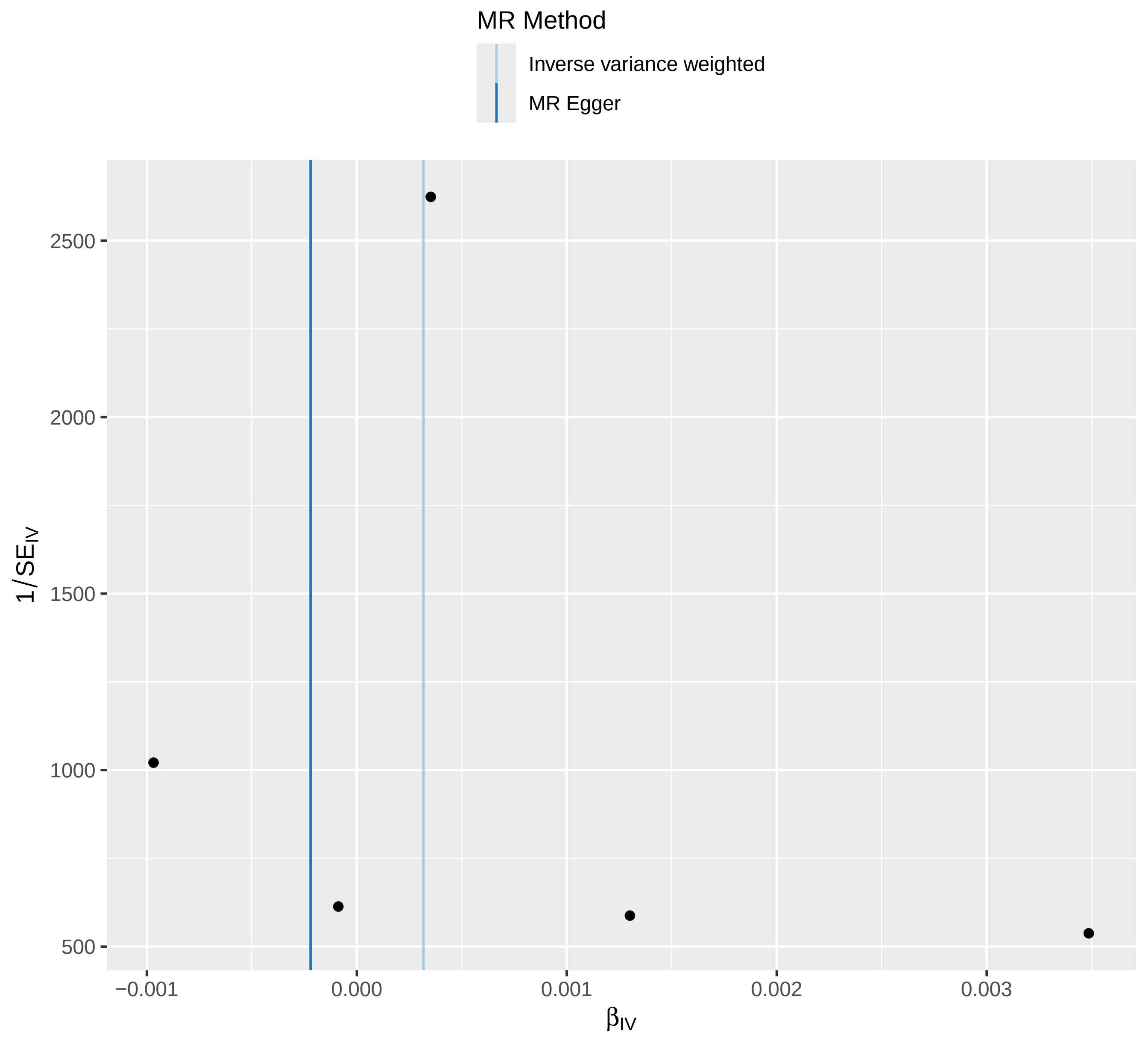

MR Method
Inverse variance weighted
MR Egger
2500
2000
1500
1000
500
$1/SE_{IV}$
−0.001
0.000
0.001
0.002
0.003
β_{IV}

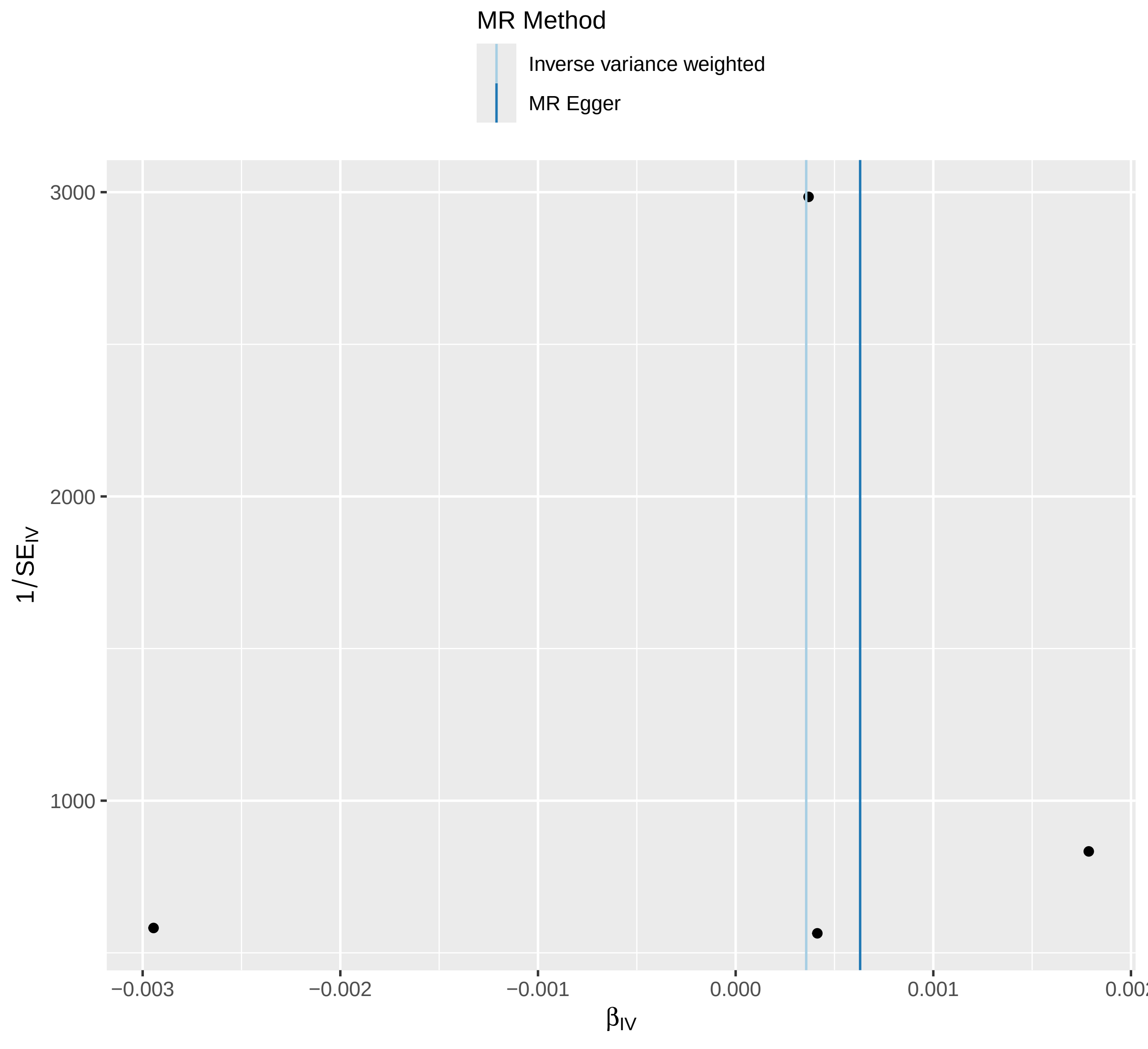
MR Method
Inverse variance weighted
MR Egger
3000
2000
1000
$1/SE_{IV}$
−0.003
−0.002
−0.001
0.000
0.001
0.002
β_{IV}

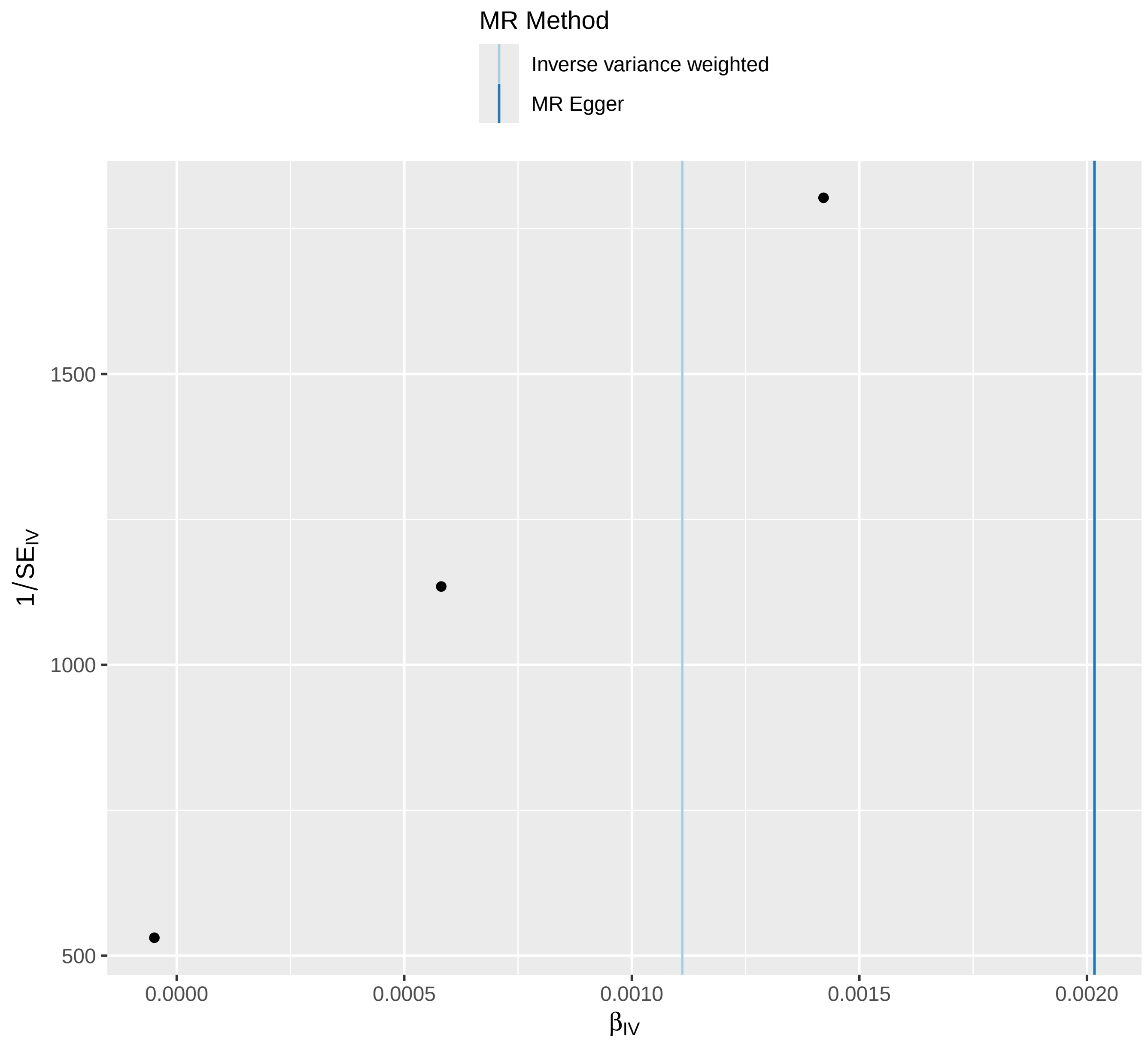

MR Method
Inverse variance weighted
MR Egger
1500
1000
500
1/SE_IV
0.0000
0.0005
0.0010
0.0015
0.0020
β_IV

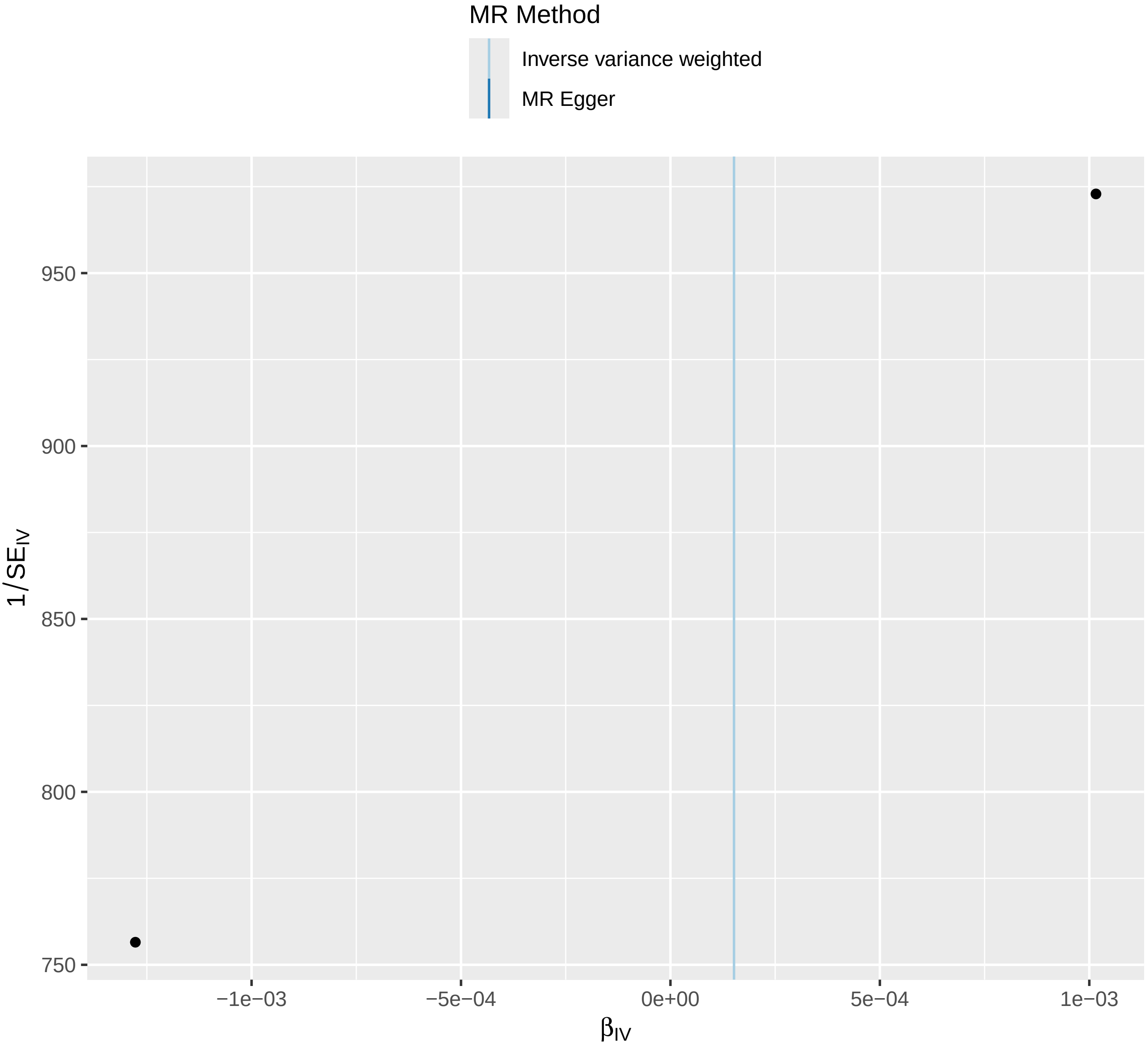
MR Method
Inverse variance weighted
MR Egger
950
900
850
800
750
1/SE_IV
−1e−03
−5e−04
0e+00
5e−04
1e−03
β_IV

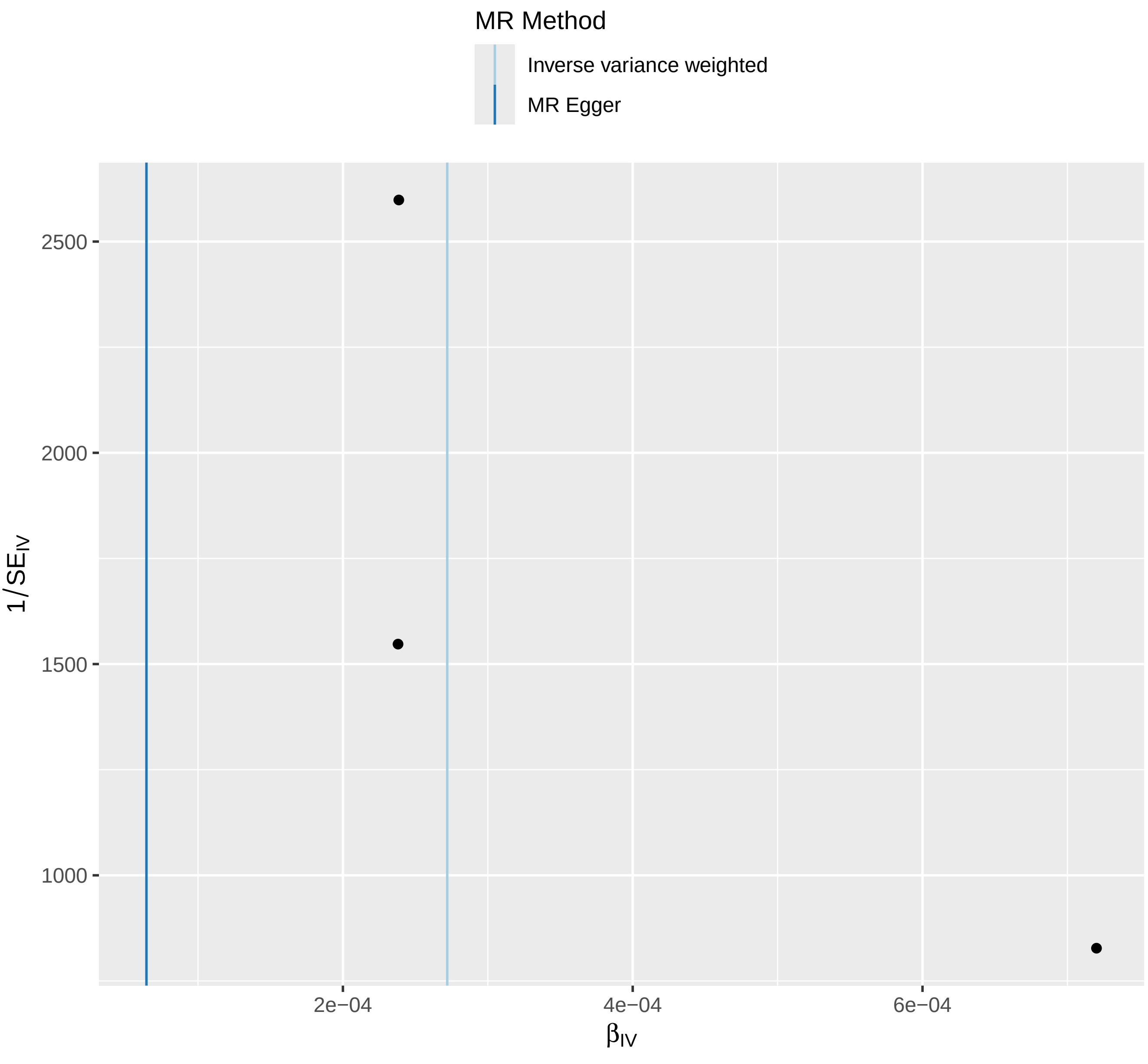

MR Method
Inverse variance weighted
MR Egger
2500
2000
1500
1000
1/SE_IV
2e−04
4e−04
6e−04
β_IV

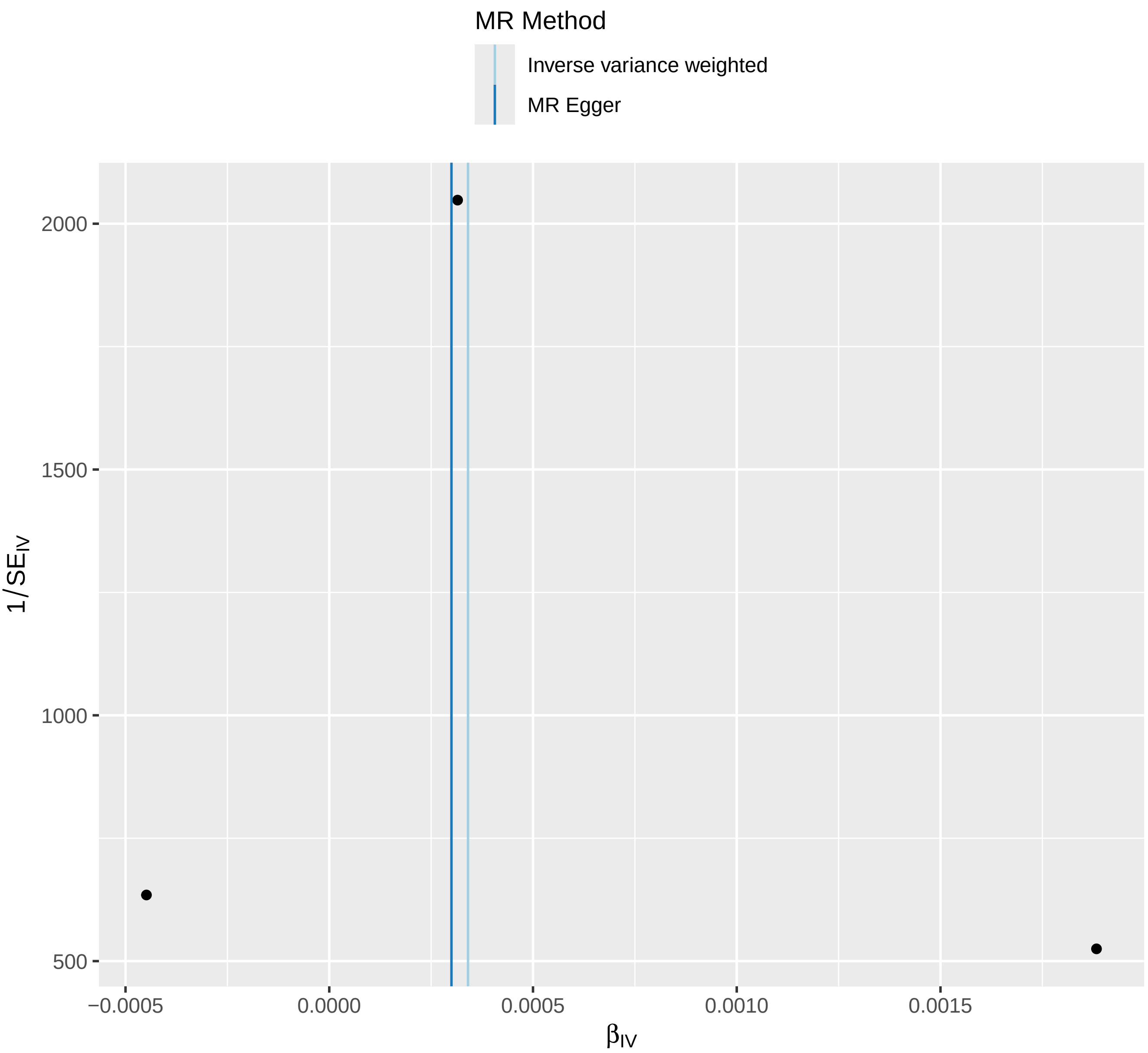
MR Method
Inverse variance weighted
MR Egger
2000
1500
1000
500
1/SE_IV
−0.0005
0.0000
0.0005
0.0010
0.0015
β_IV

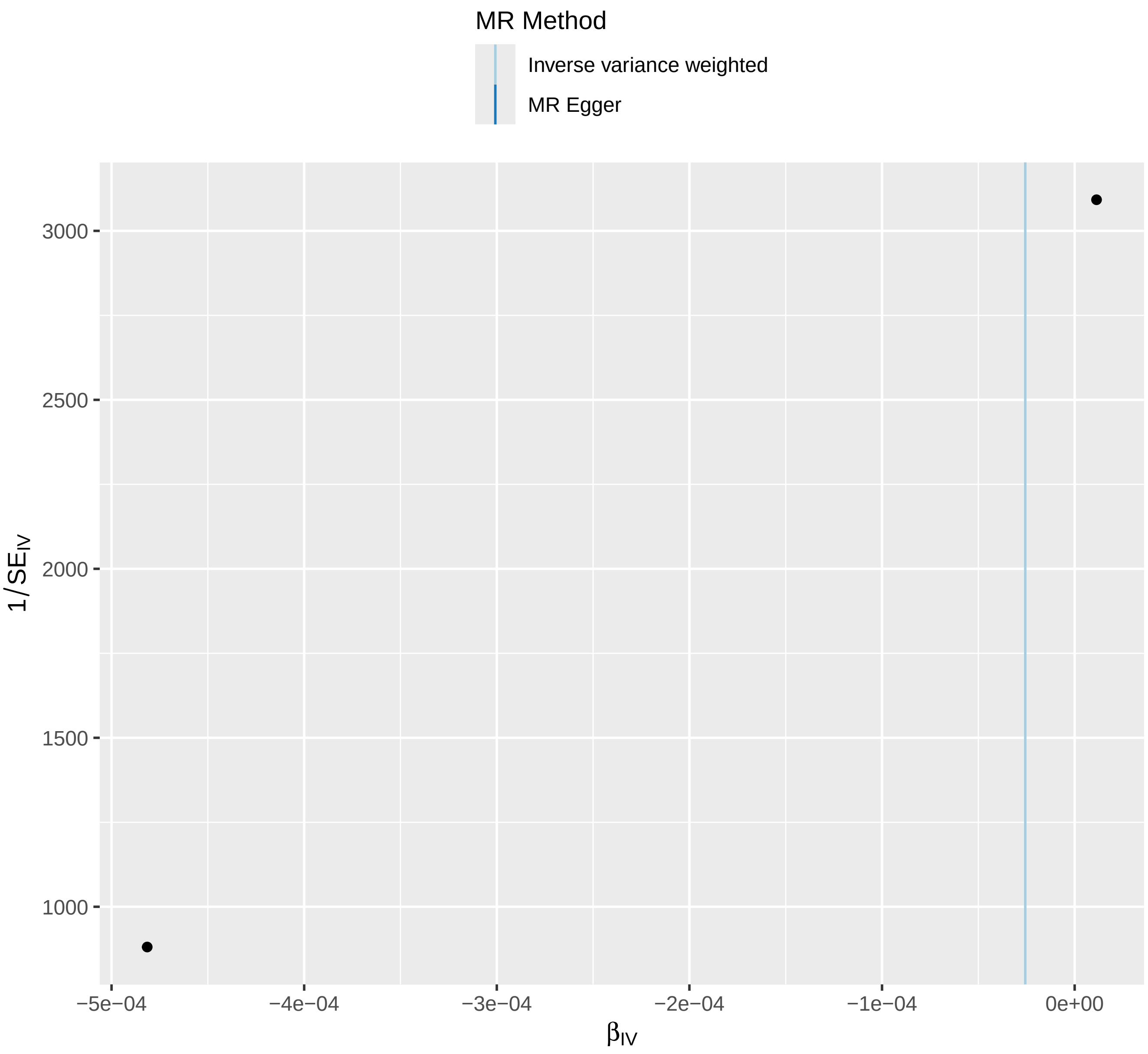

MR Method
Inverse variance weighted
MR Egger
3000
2500
2000
1500
1000
1/SE_IV
−5e−04
−4e−04
−3e−04
−2e−04
−1e−04
0e+00
β_IV

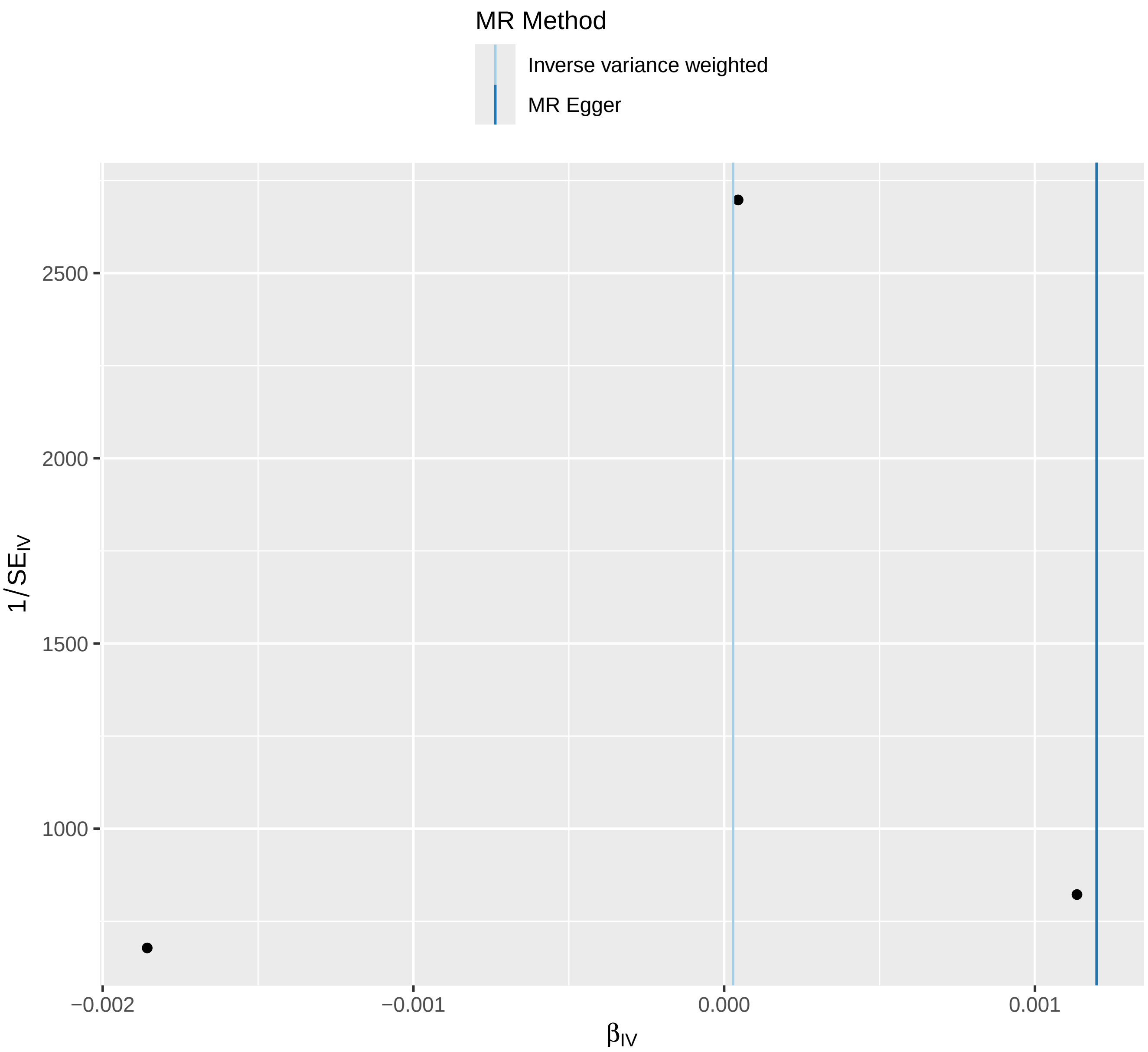
MR Method
Inverse variance weighted
MR Egger
2500
2000
1500
1000
1/SE_IV
−0.002
−0.001
0.000
0.001
β_IV

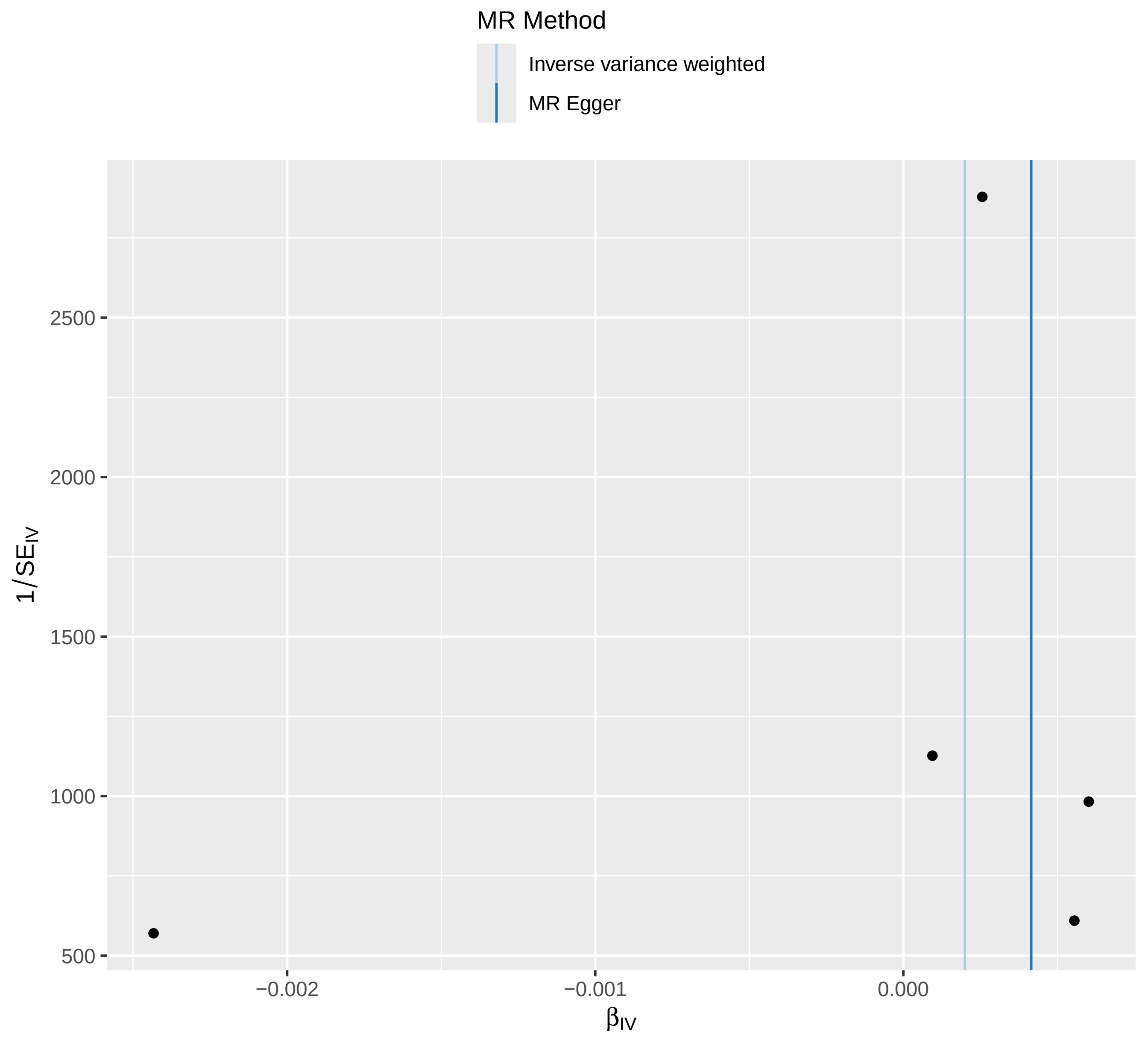
MR Method
Inverse variance weighted
MR Egger
2500
2000
1500
1000
500
1/SE_IV
−0.002
−0.001
0.000
β_IV

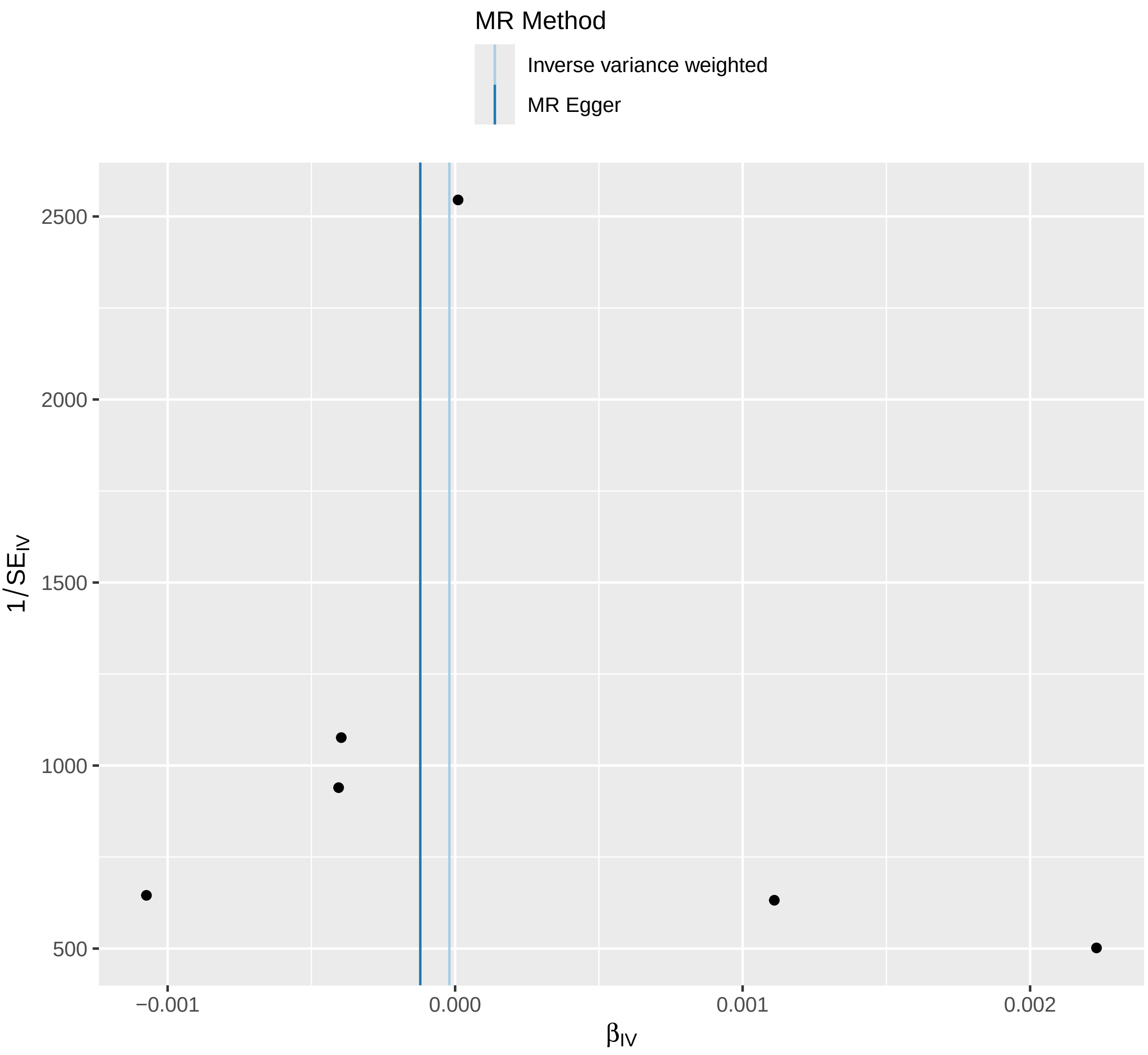
MR Method
Inverse variance weighted
MR Egger
2500
2000
1500
1000
500
1/SE_IV
−0.001
0.000
0.001
0.002
β_IV

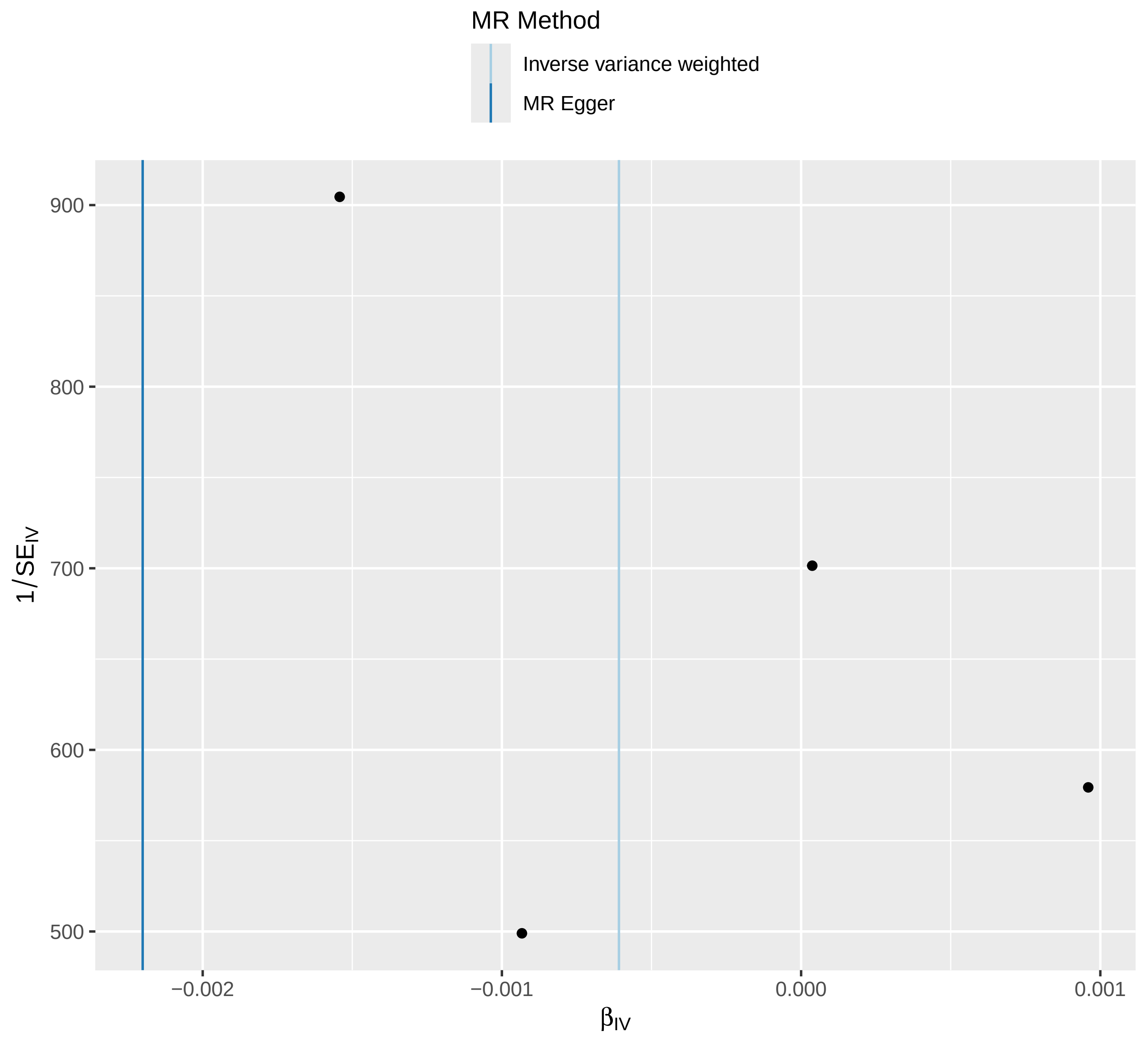
MR Method
Inverse variance weighted
MR Egger
900
800
700
600
500
1/SE_IV
−0.002
−0.001
0.000
0.001
β_IV

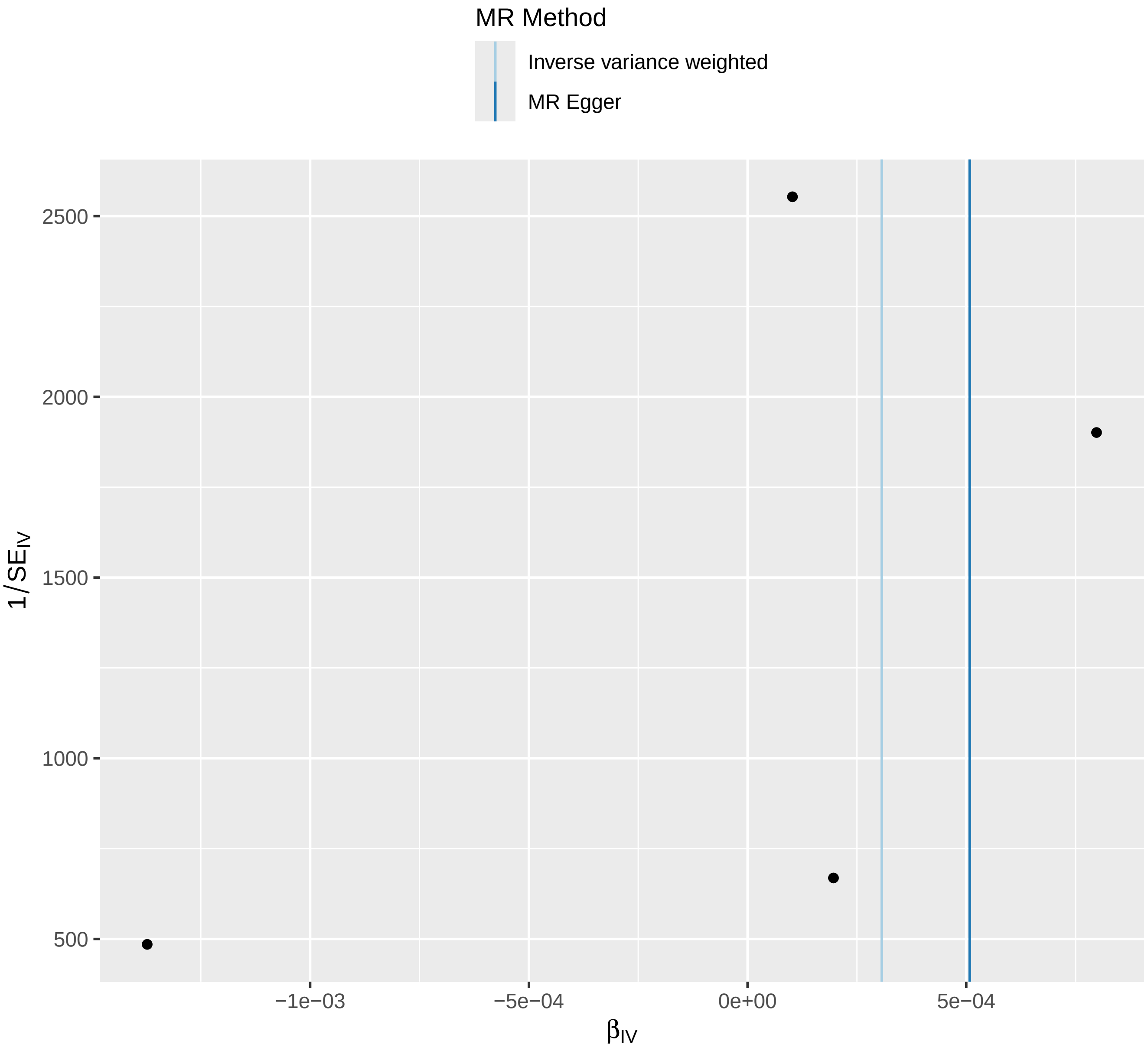
MR Method
Inverse variance weighted
MR Egger
2500
2000
1500
1000
500
1/SE_IV
−1e−03
−5e−04
0e+00
5e−04
β_IV

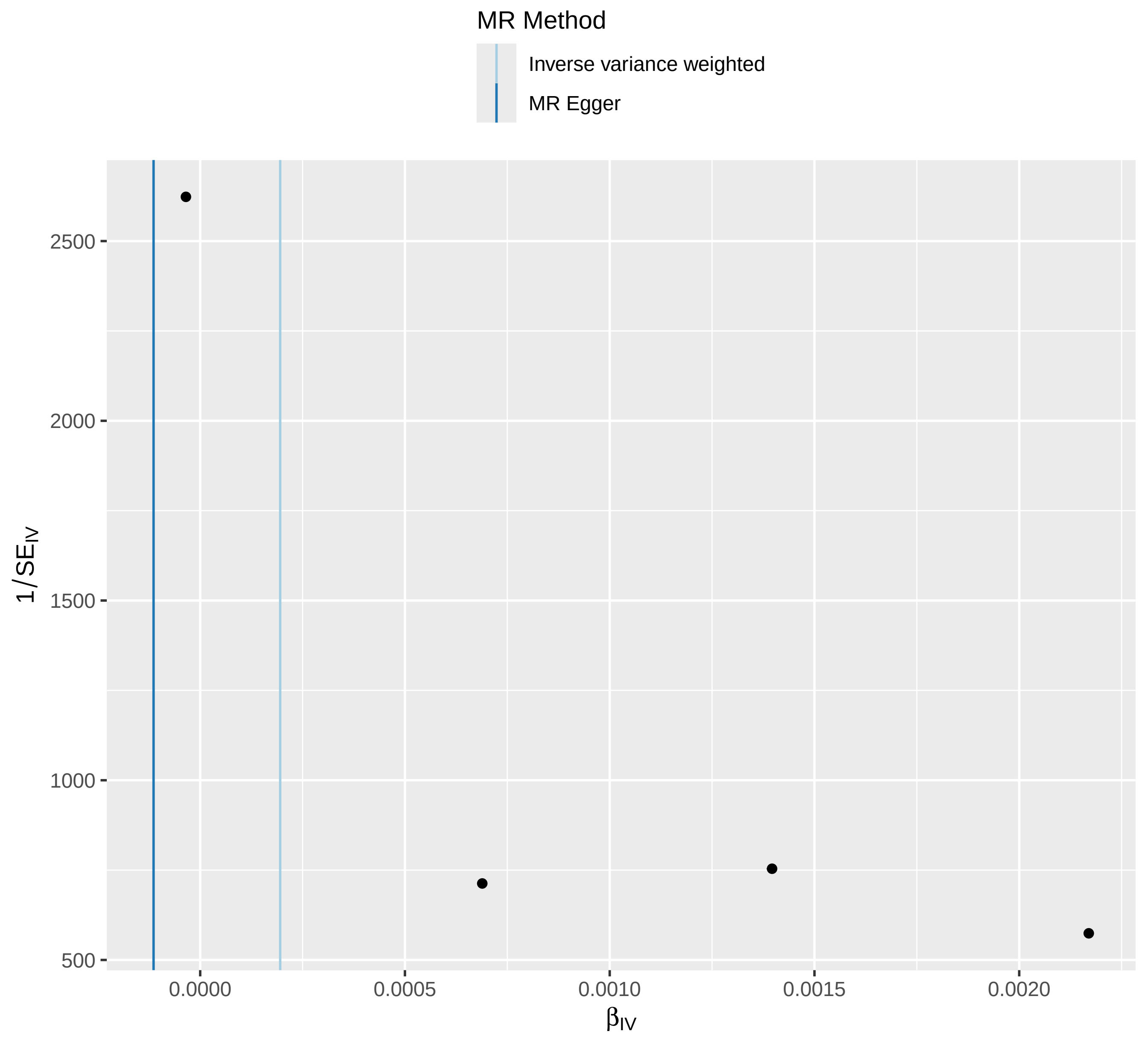
MR Method
Inverse variance weighted
MR Egger
2500
2000
1500
1000
500
1/SE$_{IV}$
0.0000
0.0005
0.0010
0.0015
0.0020
β$_{IV}$

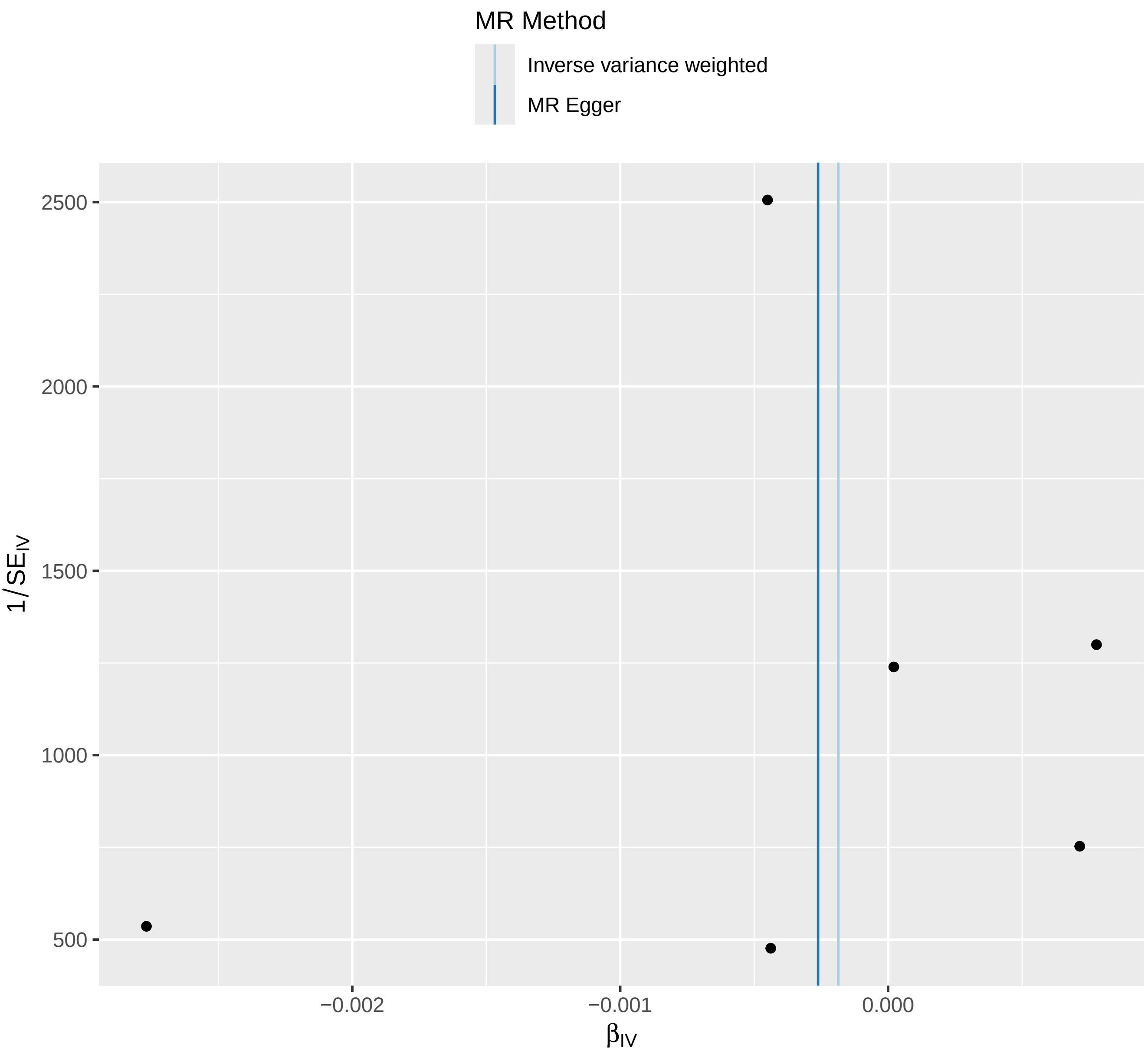
MR Method
Inverse variance weighted
MR Egger
2500
2000
1500
1000
500
1/SE_IV
−0.002
−0.001
0.000
β_IV

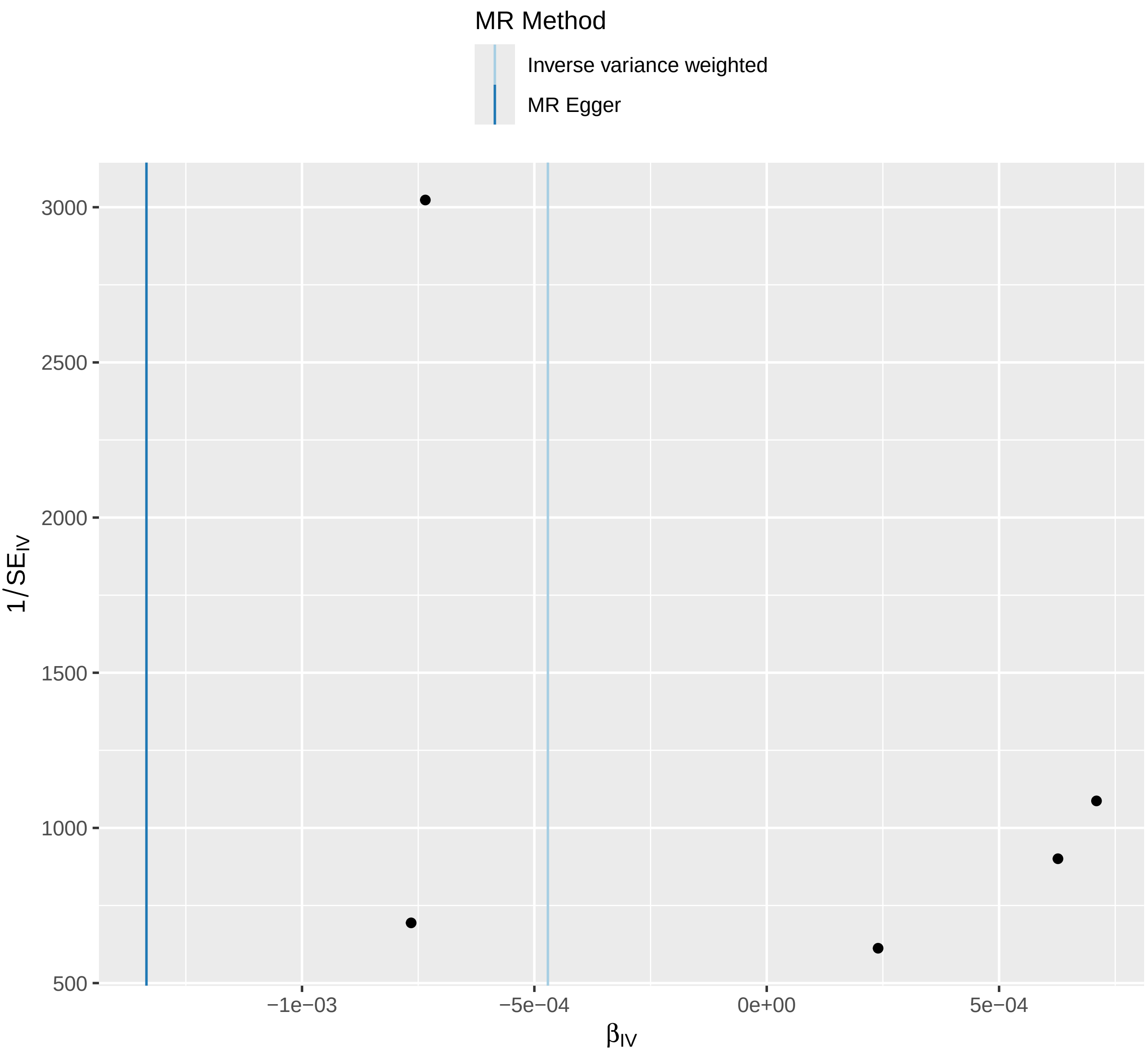

MR Method
Inverse variance weighted
MR Egger
3000
2500
2000
1500
1000
500
1/SE$_{IV}$
−1e−03
−5e−04
0e+00
5e−04
β$_{IV}$

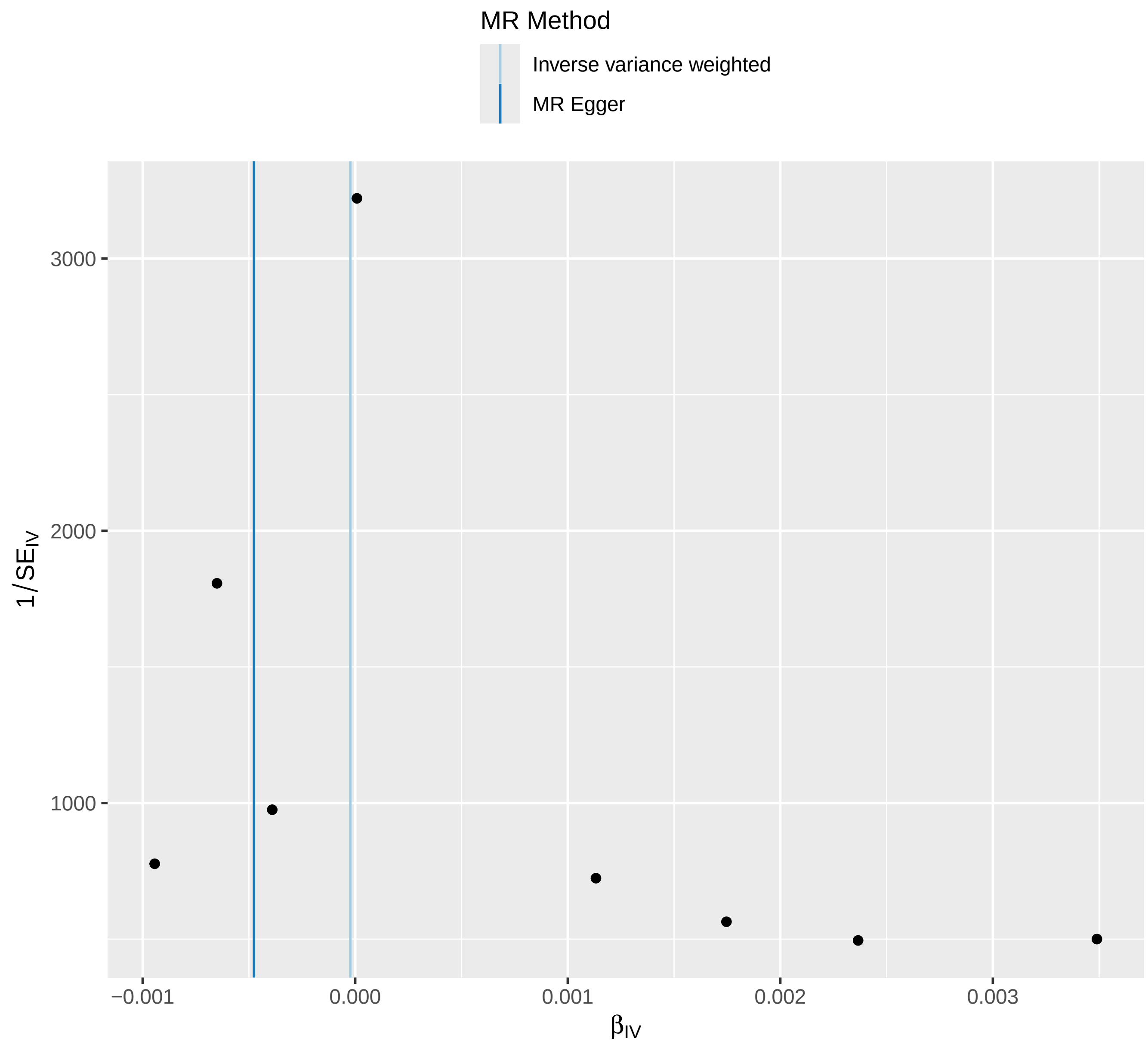

MR Method
Inverse variance weighted
MR Egger
3000
2000
1000
1/SE$_{IV}$
−0.001
0.000
0.001
0.002
0.003
β$_{IV}$

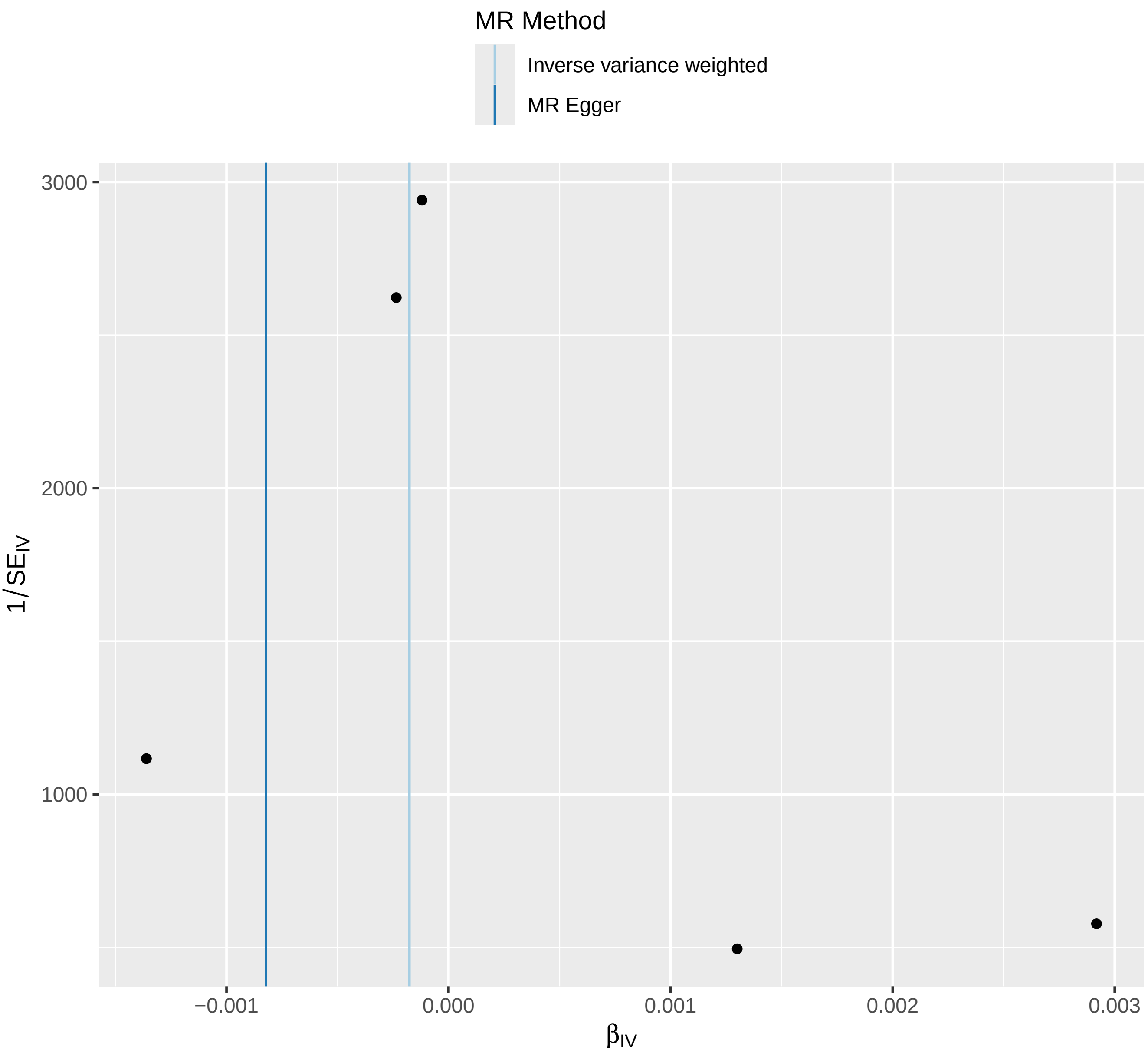

MR Method
Inverse variance weighted
MR Egger
3000
2000
1000
1/SE$_{IV}$
−0.001
0.000
0.001
0.002
0.003
β$_{IV}$

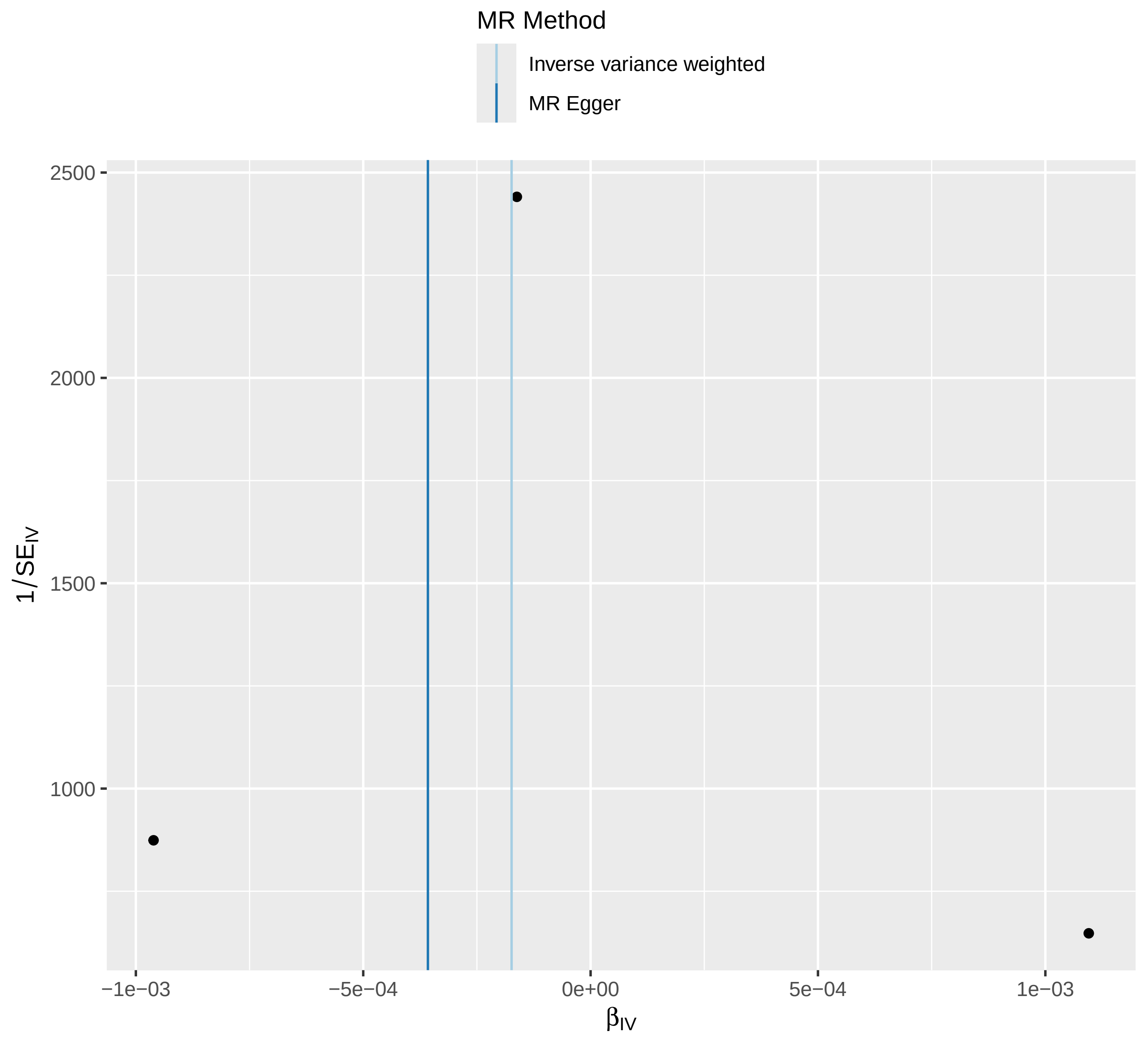
MR Method
Inverse variance weighted
MR Egger
2500
2000
1500
1000
1/SE_IV
−1e−03
−5e−04
0e+00
5e−04
1e−03
β_IV

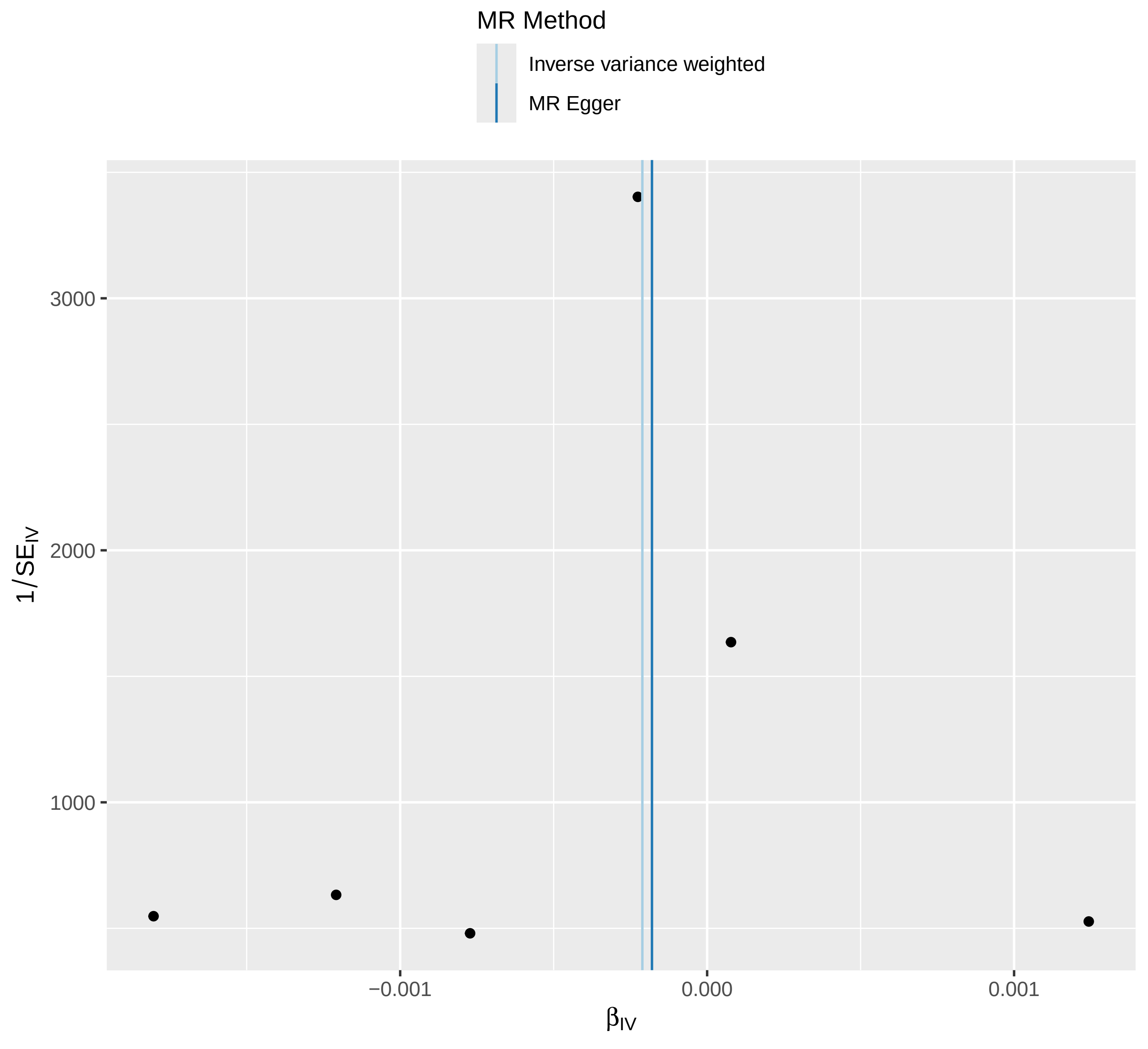
MR Method
Inverse variance weighted
MR Egger
3000
2000
1000
1/SE_IV
−0.001
0.000
0.001
β_IV

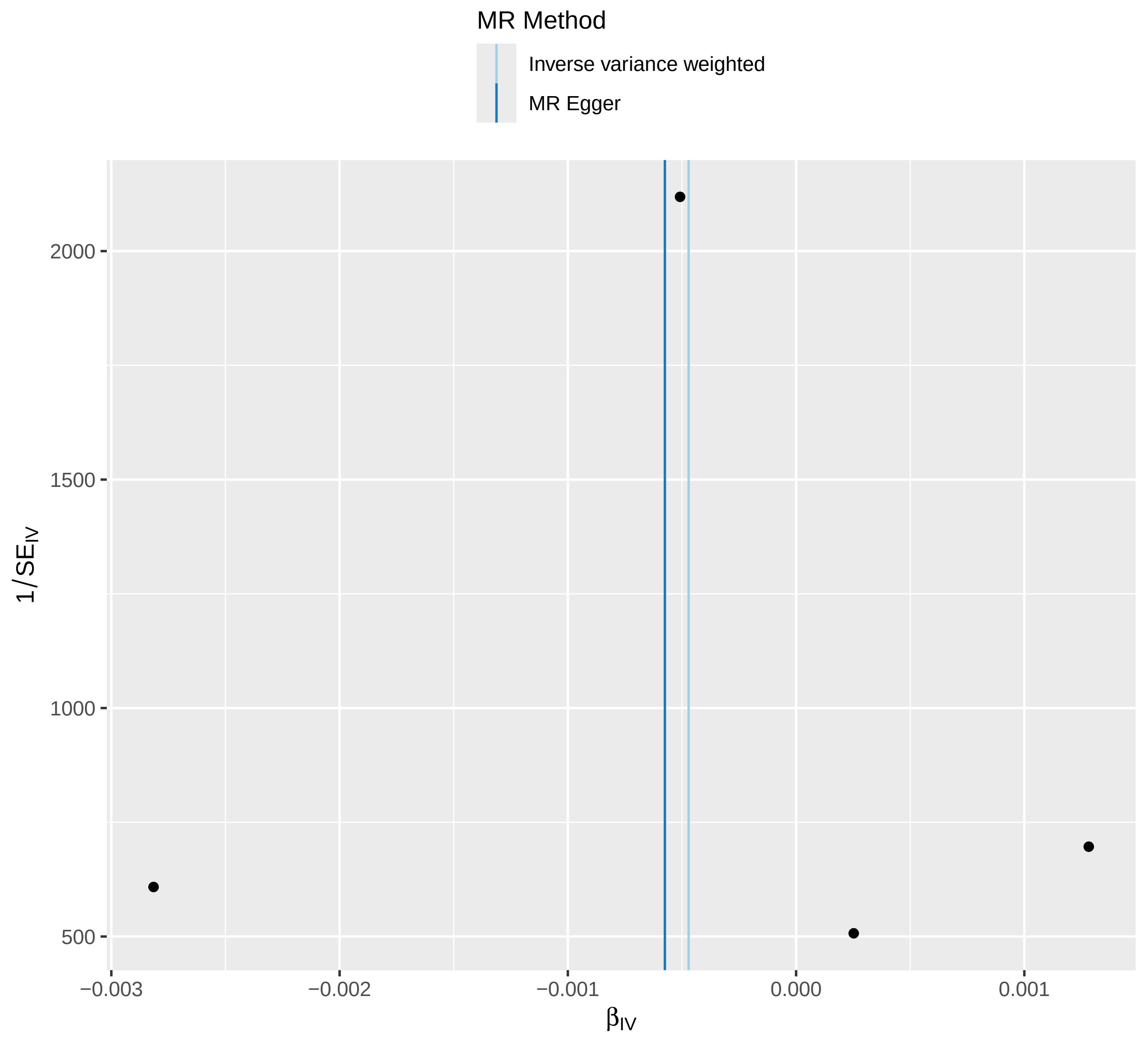
MR Method
Inverse variance weighted
MR Egger
2000
1500
1000
500
1/SE_IV
−0.003
−0.002
−0.001
0.000
0.001
β_IV

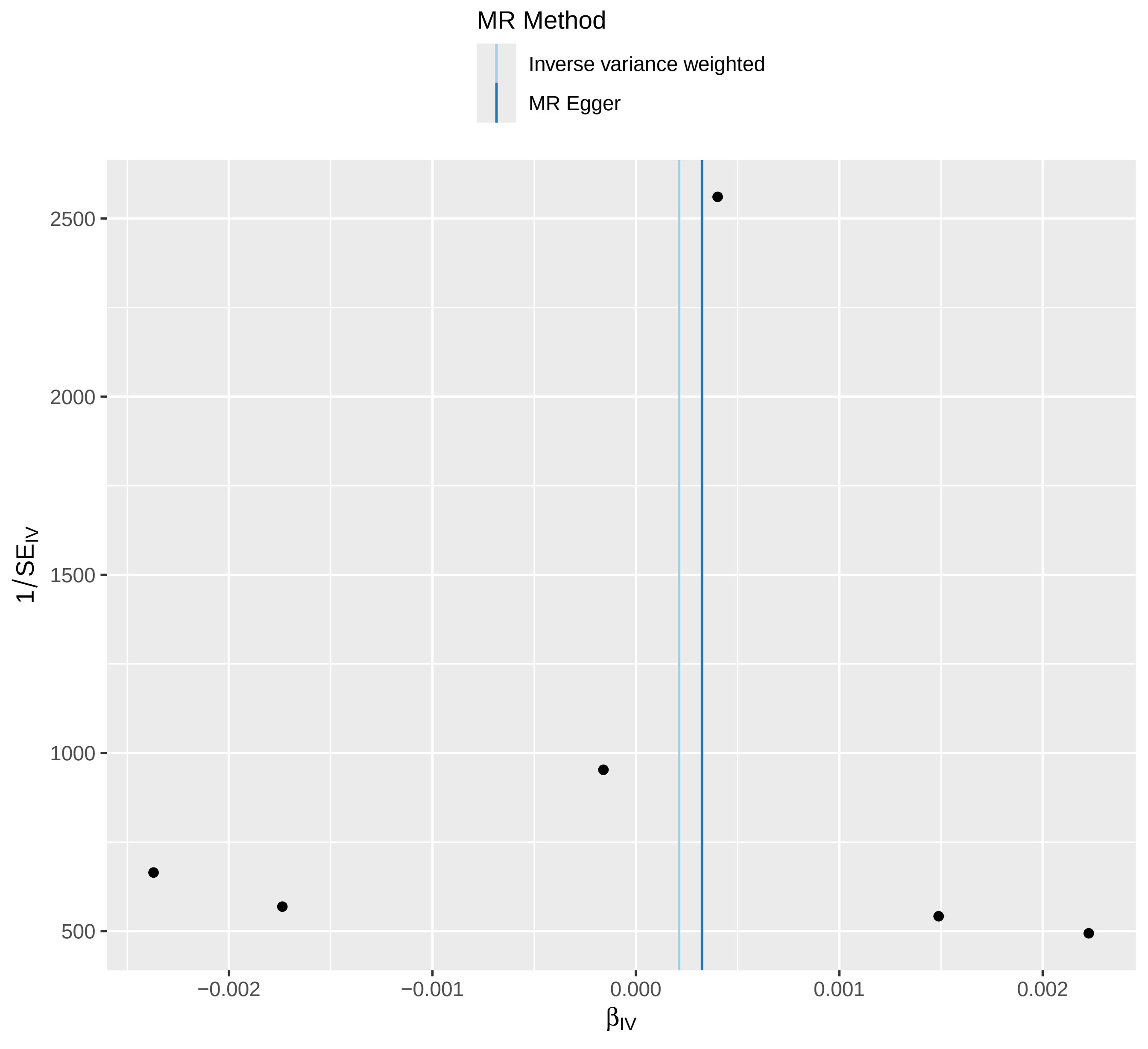

MR Method
Inverse variance weighted
MR Egger
2500
2000
1500
1000
500
1/SE_IV
−0.002
−0.001
0.000
0.001
0.002
β_IV

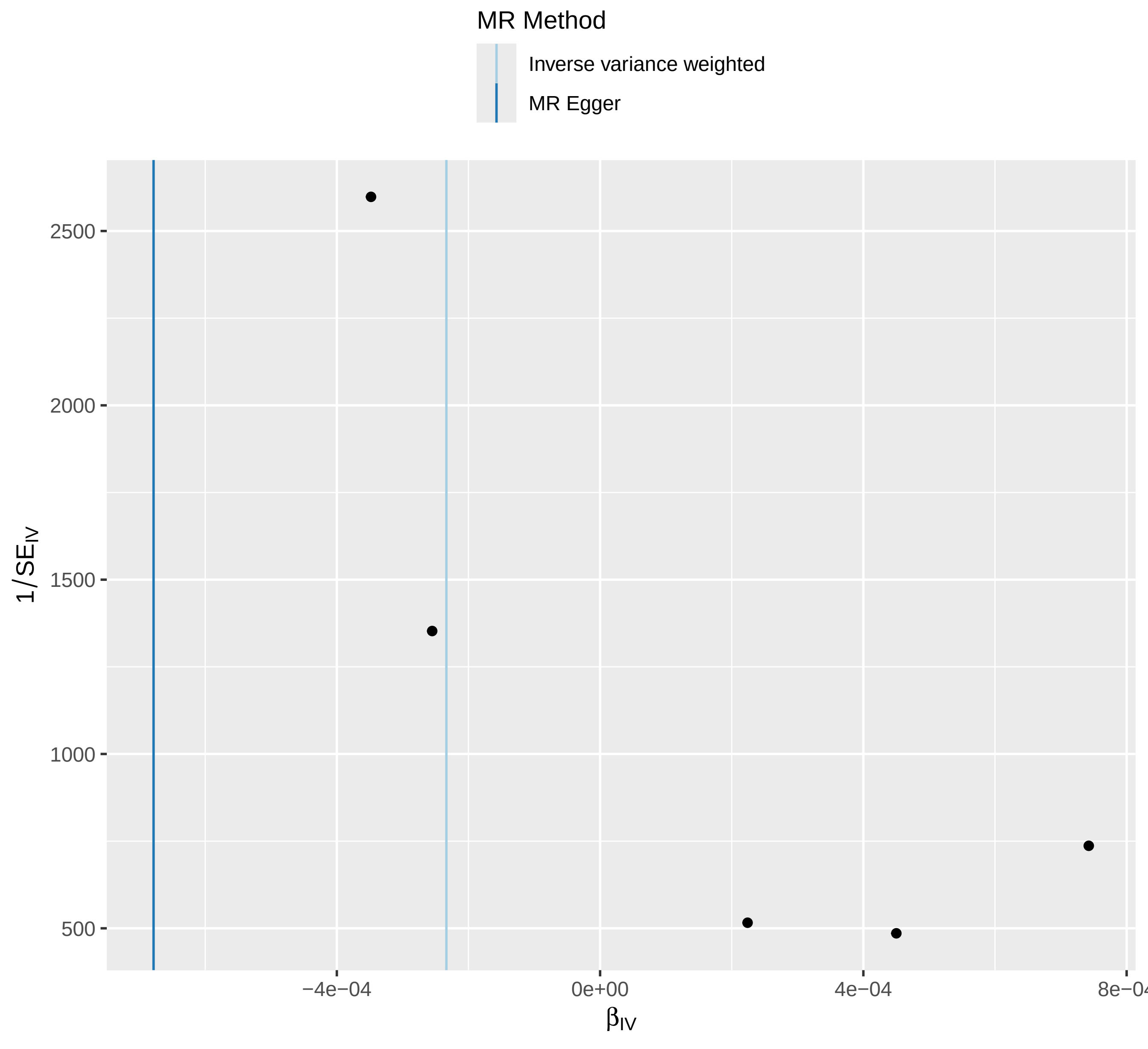
MR Method
Inverse variance weighted
MR Egger
2500
2000
1500
1000
500
1/SE_IV
−4e−04
0e+00
4e−04
8e−04
β_IV

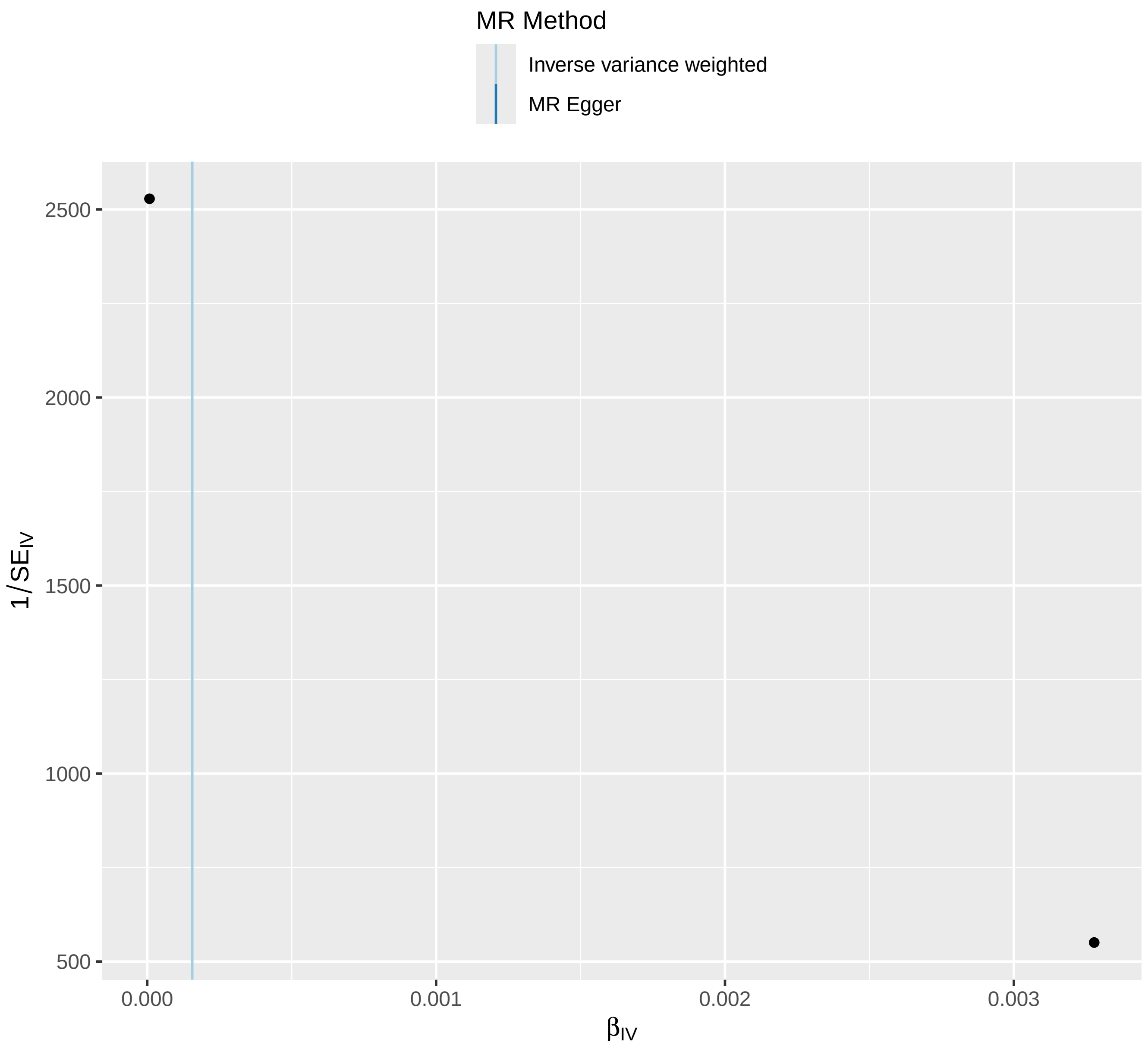
MR Method
Inverse variance weighted
MR Egger
2500
2000
1500
1000
500
1/SE_IV
0.000
0.001
0.002
0.003
β_IV

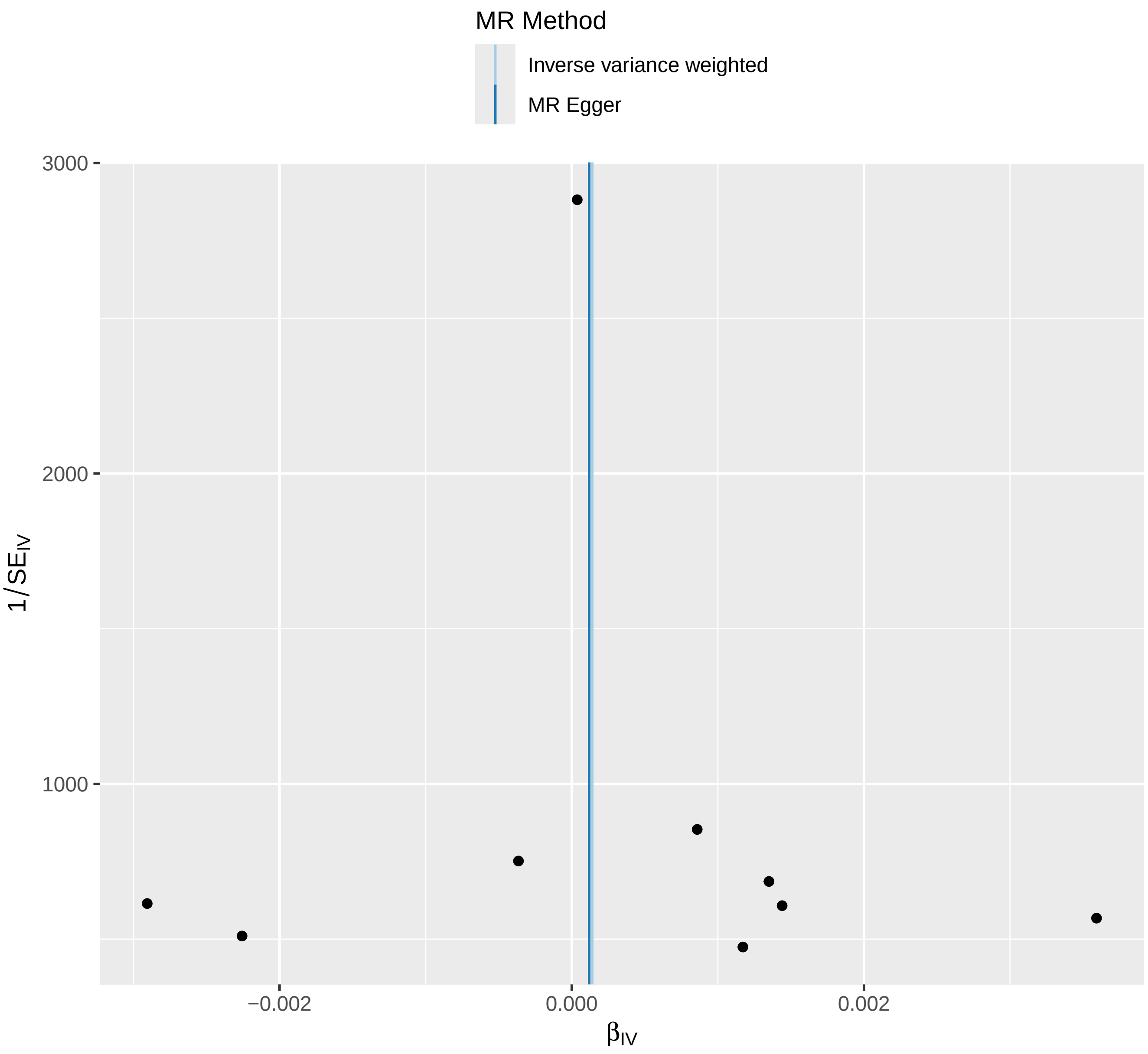

MR Method
Inverse variance weighted
MR Egger
3000
2000
1000
1/SE_IV
−0.002
0.000
0.002
β_IV

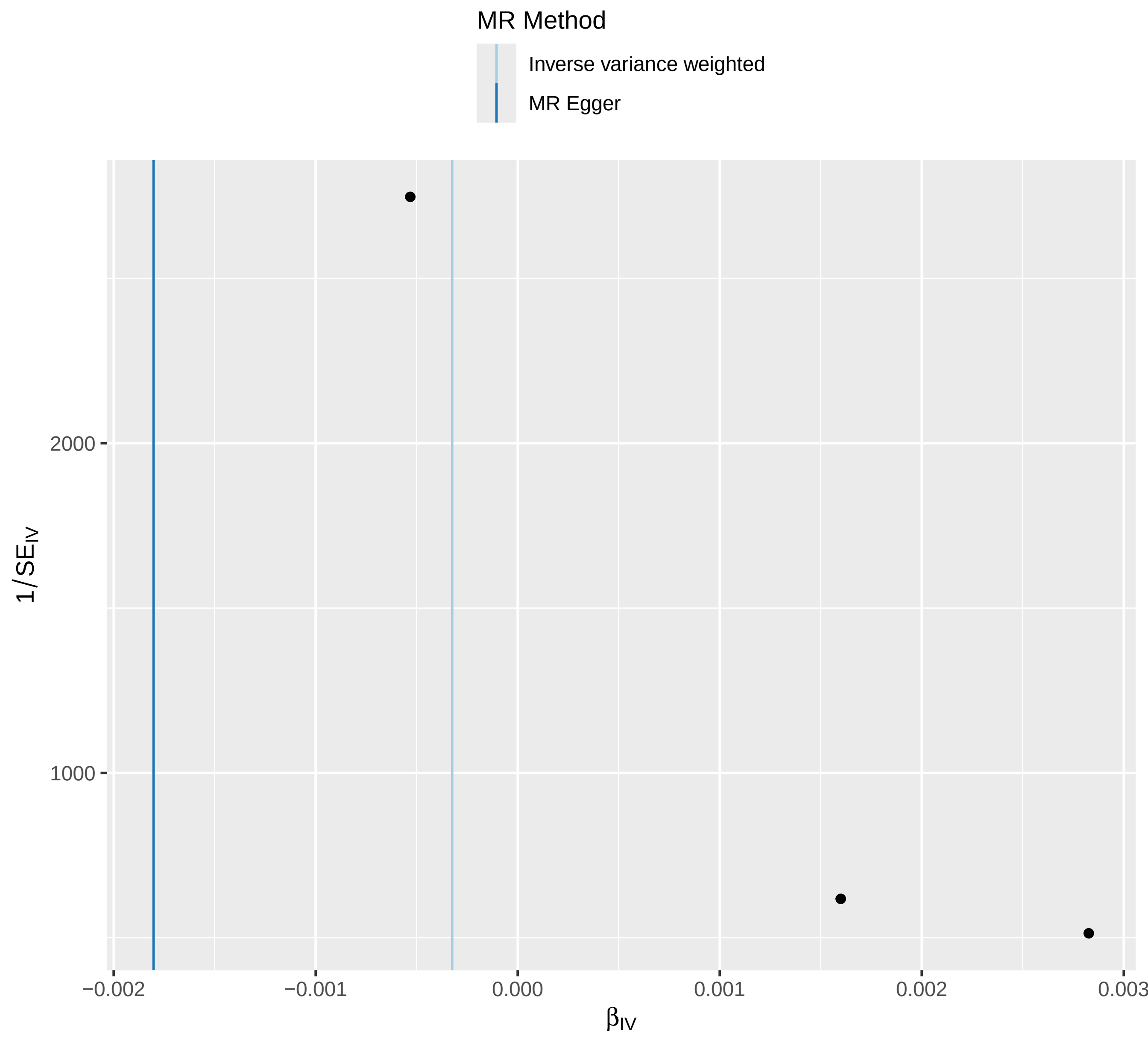

MR Method
Inverse variance weighted
MR Egger
2000
1000
$1/SE_{IV}$
−0.002
−0.001
0.000
0.001
0.002
0.003
β_{IV}

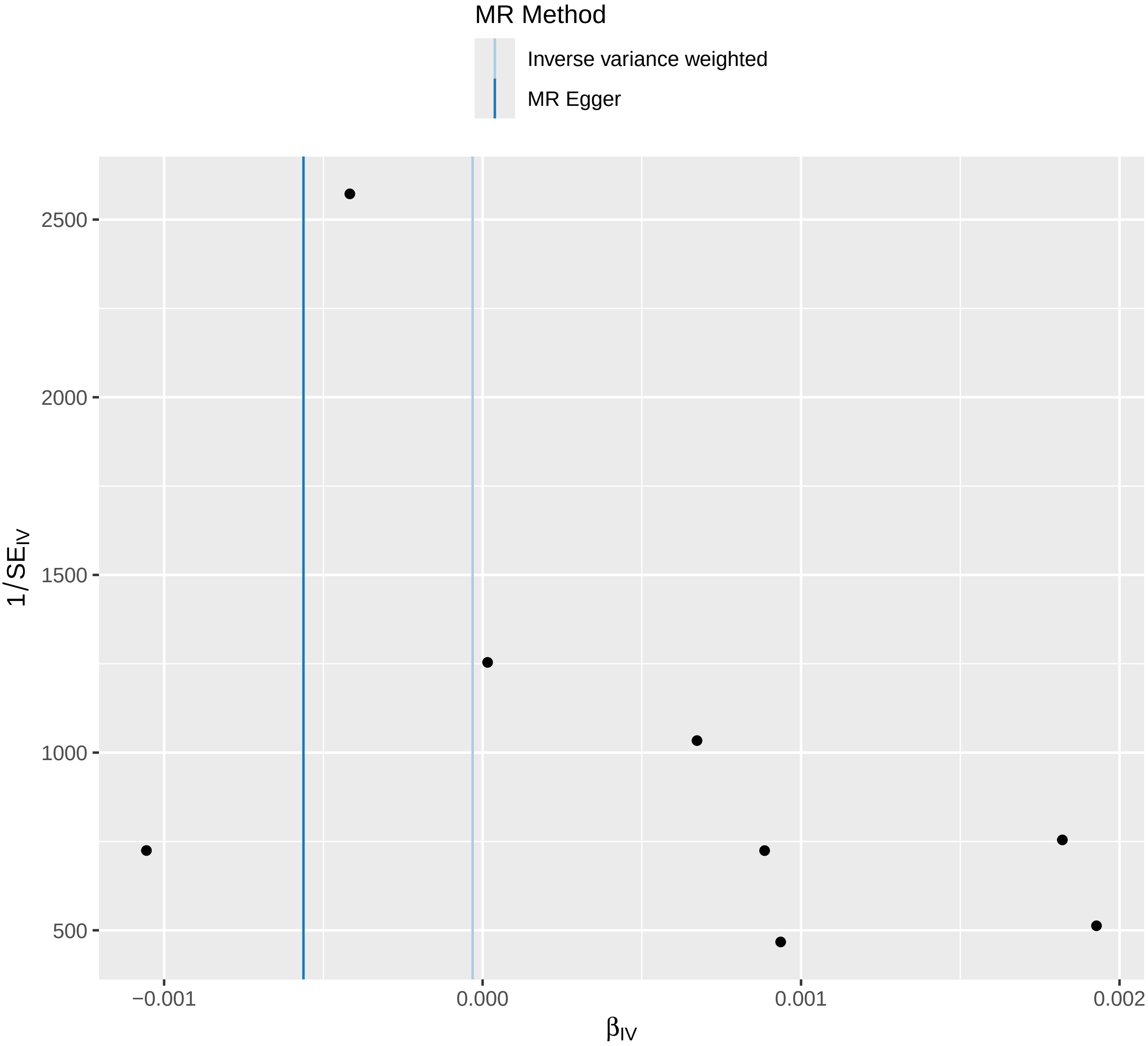
MR Method
Inverse variance weighted
MR Egger
2500
2000
1500
1000
500
1/SE_IV
−0.001
0.000
0.001
0.002
β_IV

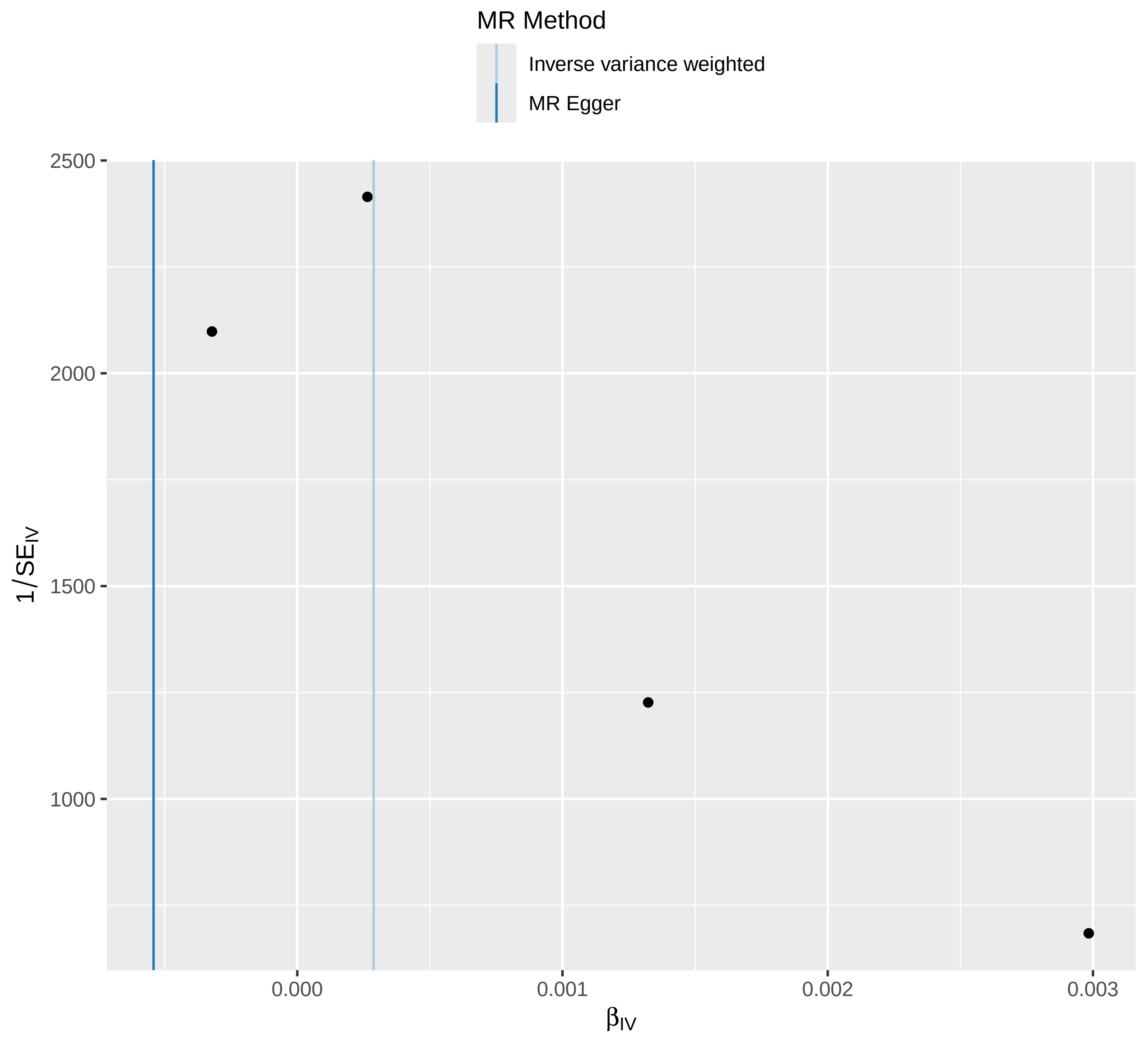
MR Method
Inverse variance weighted
MR Egger
2500
2000
1500
1000
1/SE_IV
0.000
0.001
0.002
0.003
β_IV

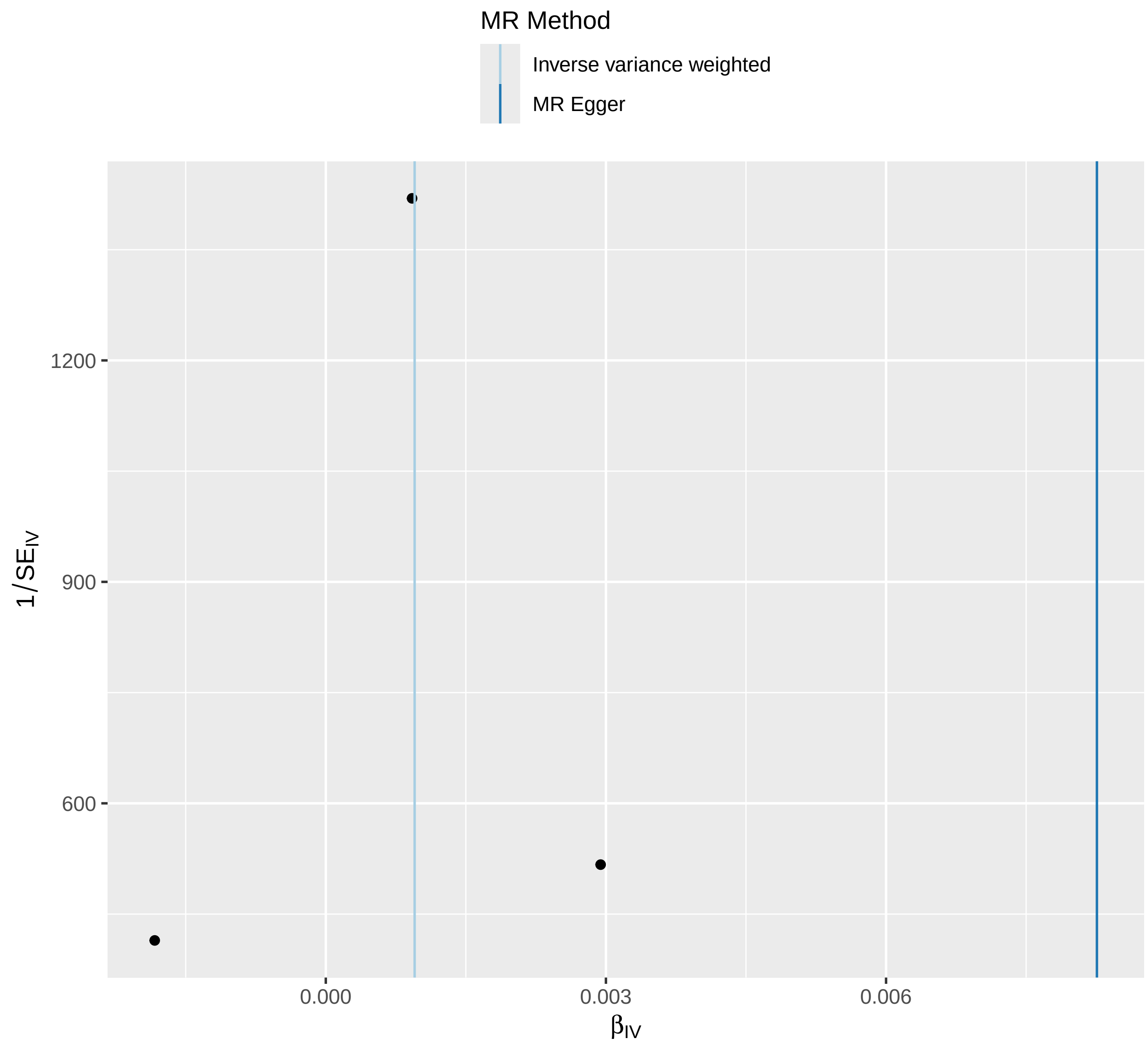

MR Method
Inverse variance weighted
MR Egger
1200
900
600
0.000
0.003
0.006
$1/SE_{IV}$
β_{IV}

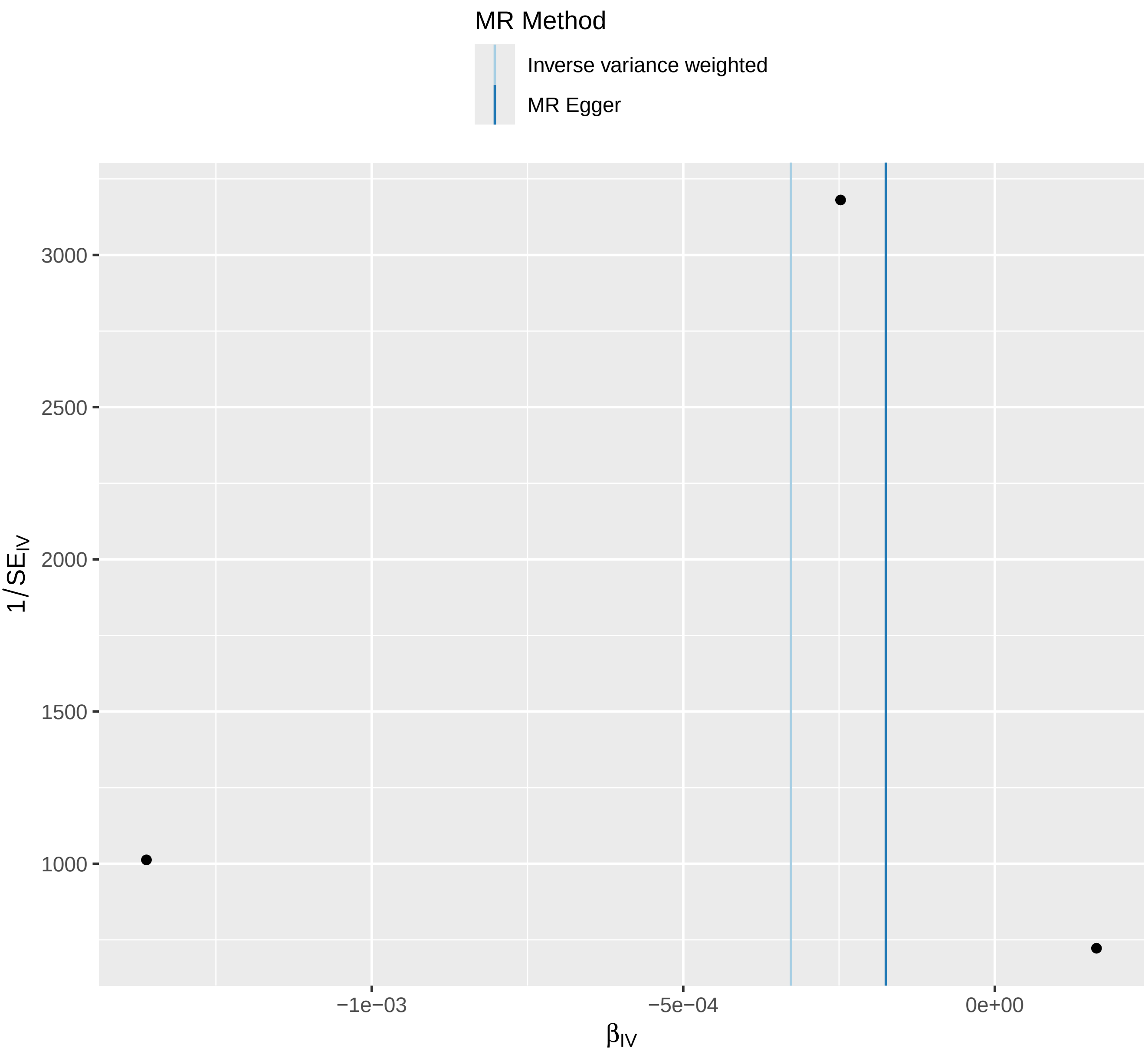
MR Method
Inverse variance weighted
MR Egger
3000
2500
2000
1500
1000
$1/SE_{IV}$
−1e−03
−5e−04
0e+00
β_{IV}

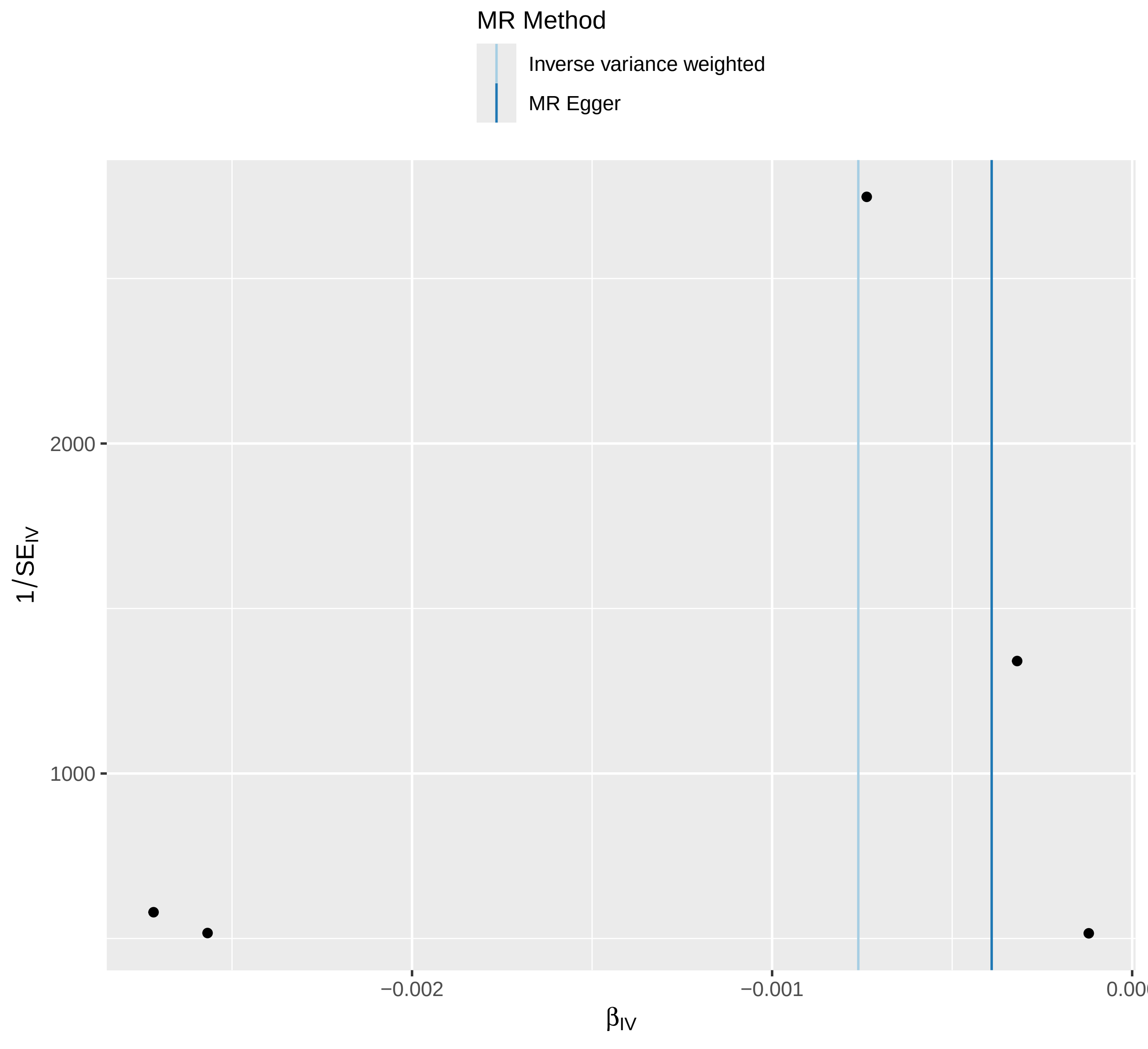

MR Method
Inverse variance weighted
MR Egger
2000
1000
$1/SE_{IV}$
−0.002
−0.001
0.000
β_{IV}

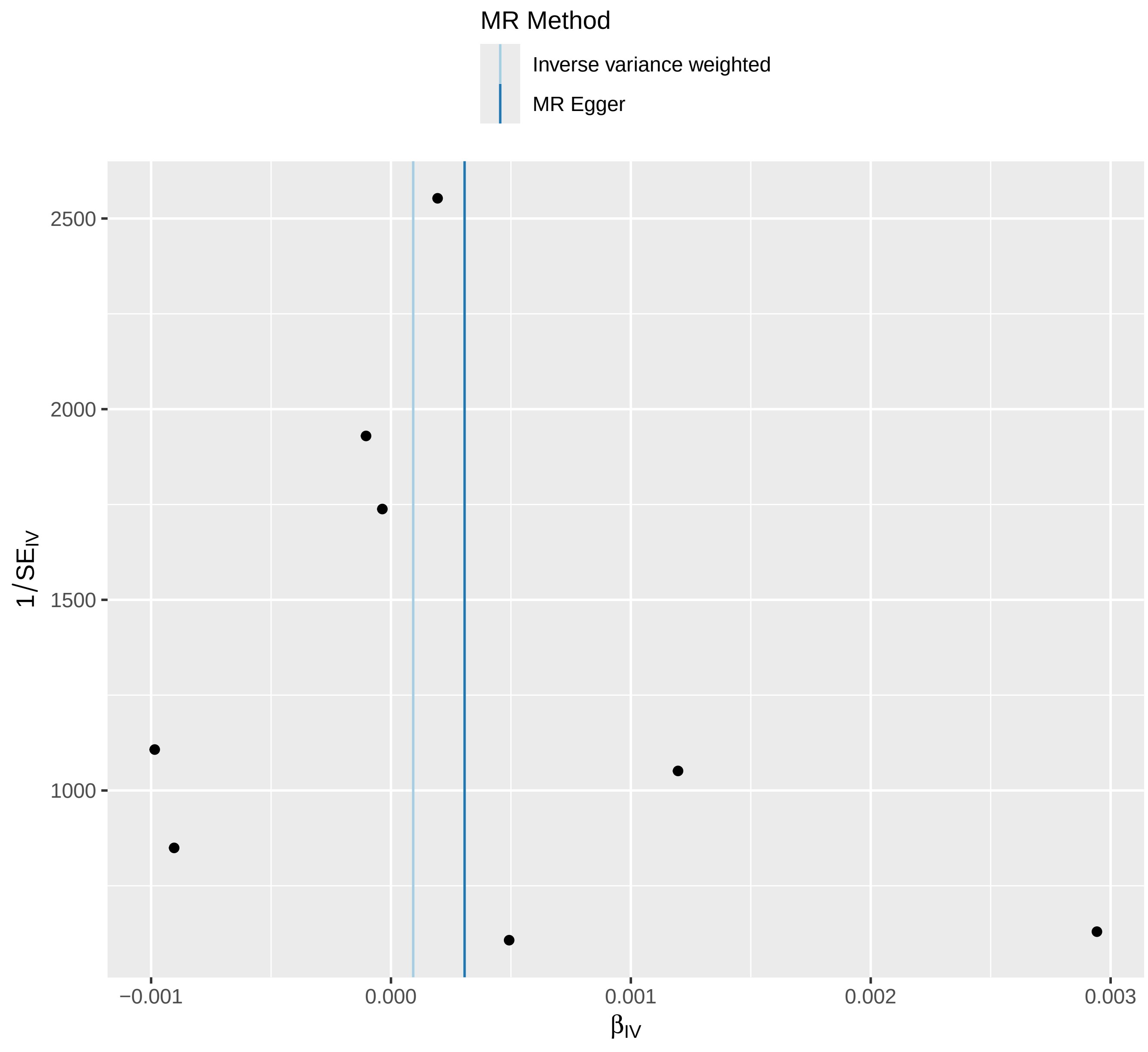
MR Method
Inverse variance weighted
MR Egger
2500
2000
1500
1000
$1/SE_{IV}$
−0.001
0.000
0.001
0.002
0.003
β_{IV}

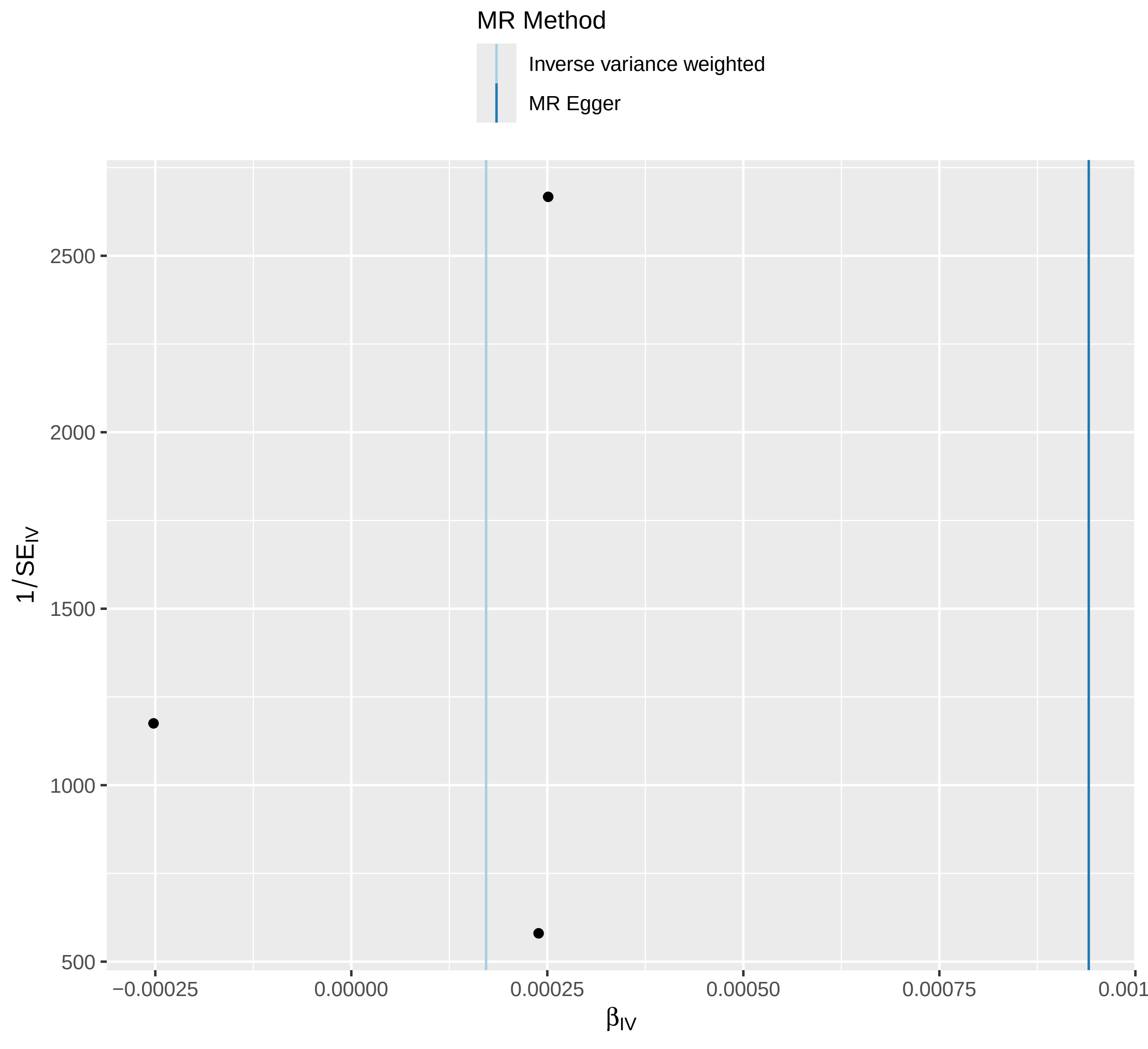

MR Method
Inverse variance weighted
MR Egger
2500
2000
1500
1000
500
1/SE_IV
−0.00025
0.00000
0.00025
0.00050
0.00075
0.001
β_IV

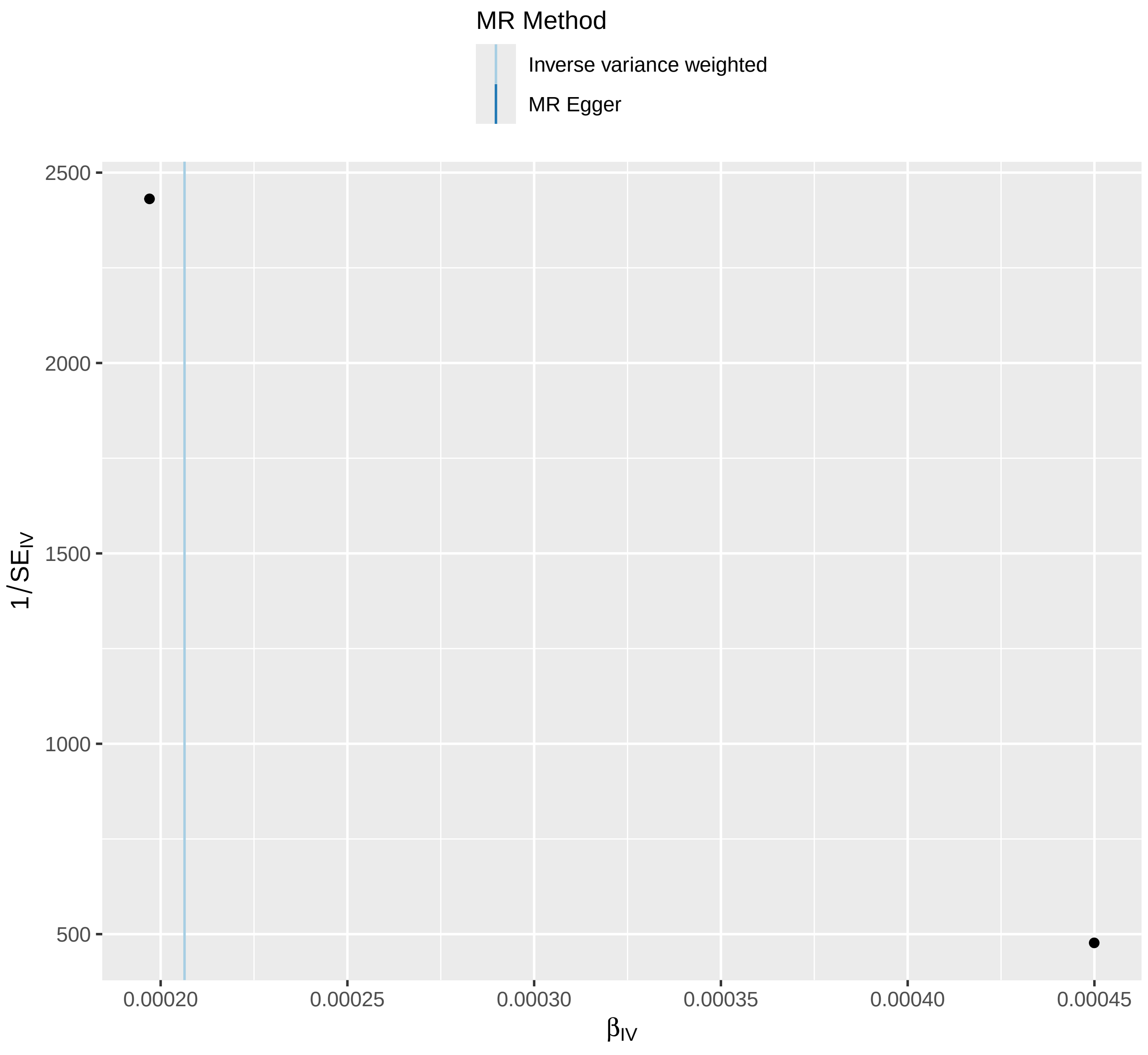

MR Method
Inverse variance weighted
MR Egger
2500
2000
1500
1000
500
1/SE_IV
0.00020
0.00025
0.00030
0.00035
0.00040
0.00045
β_IV

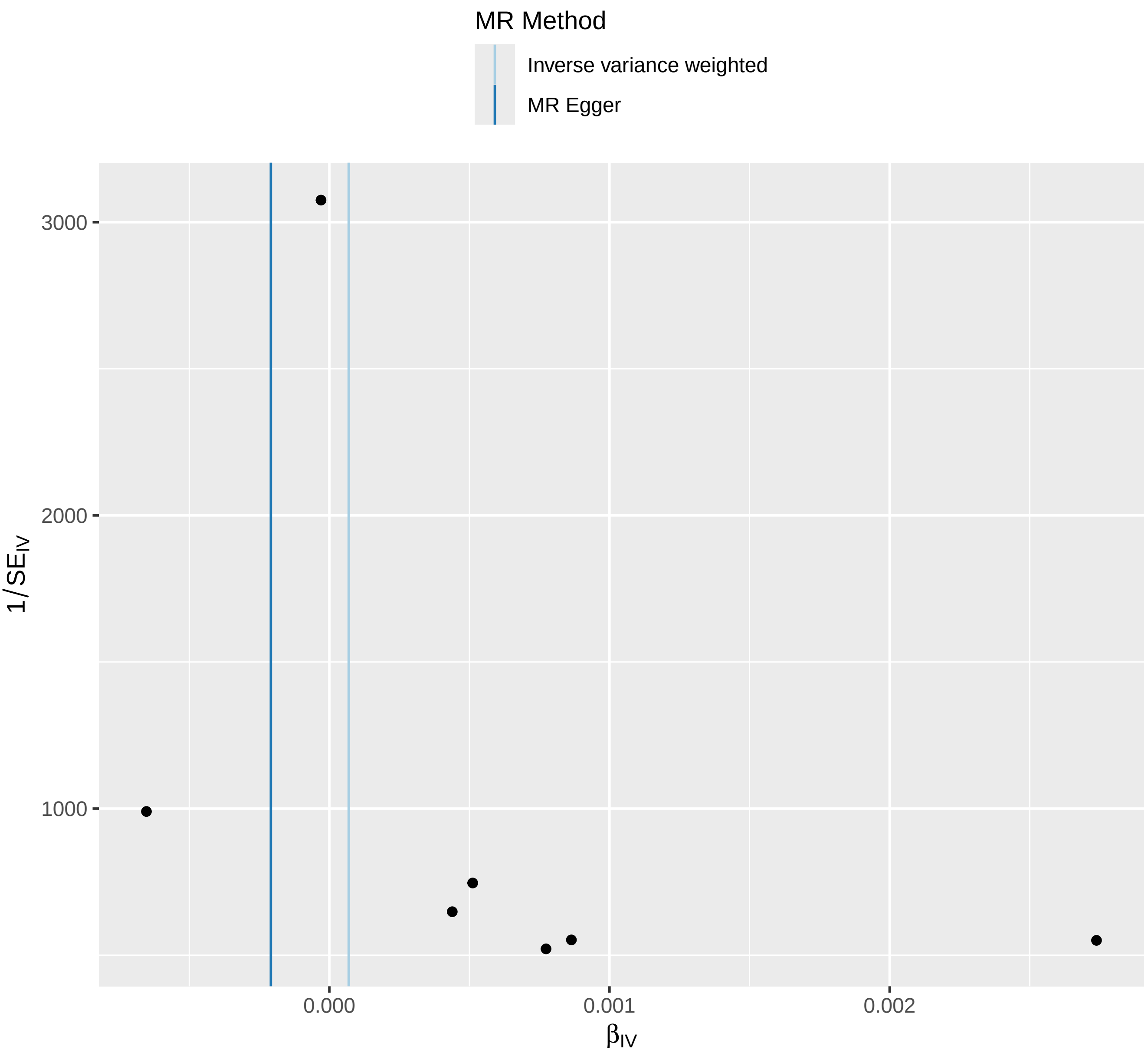
MR Method
Inverse variance weighted
MR Egger
3000
2000
1000
1/SE_IV
0.000
0.001
0.002
β_IV

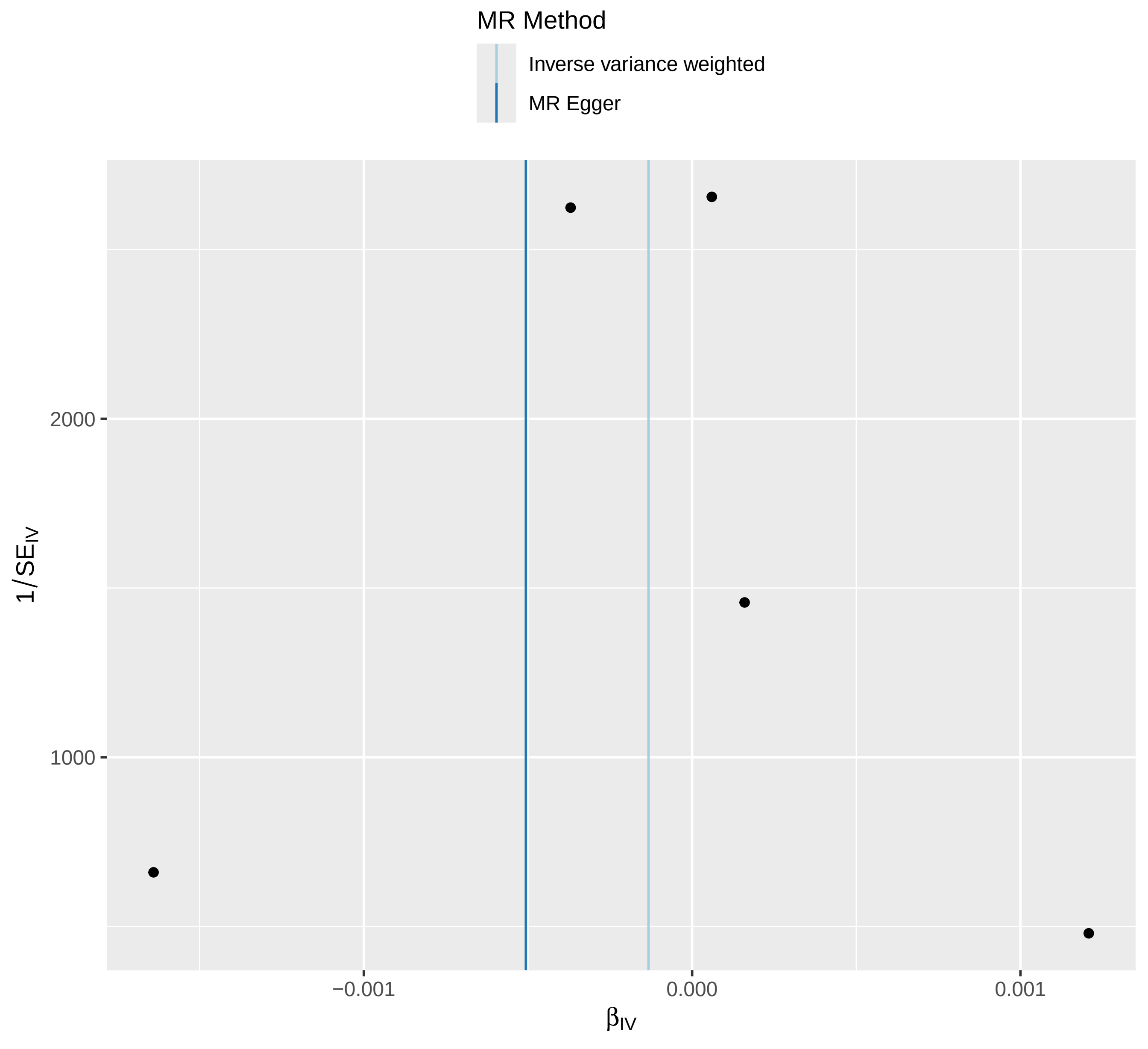
MR Method
Inverse variance weighted
MR Egger
2000
1000
1/SE_IV
−0.001
0.000
0.001
β_IV

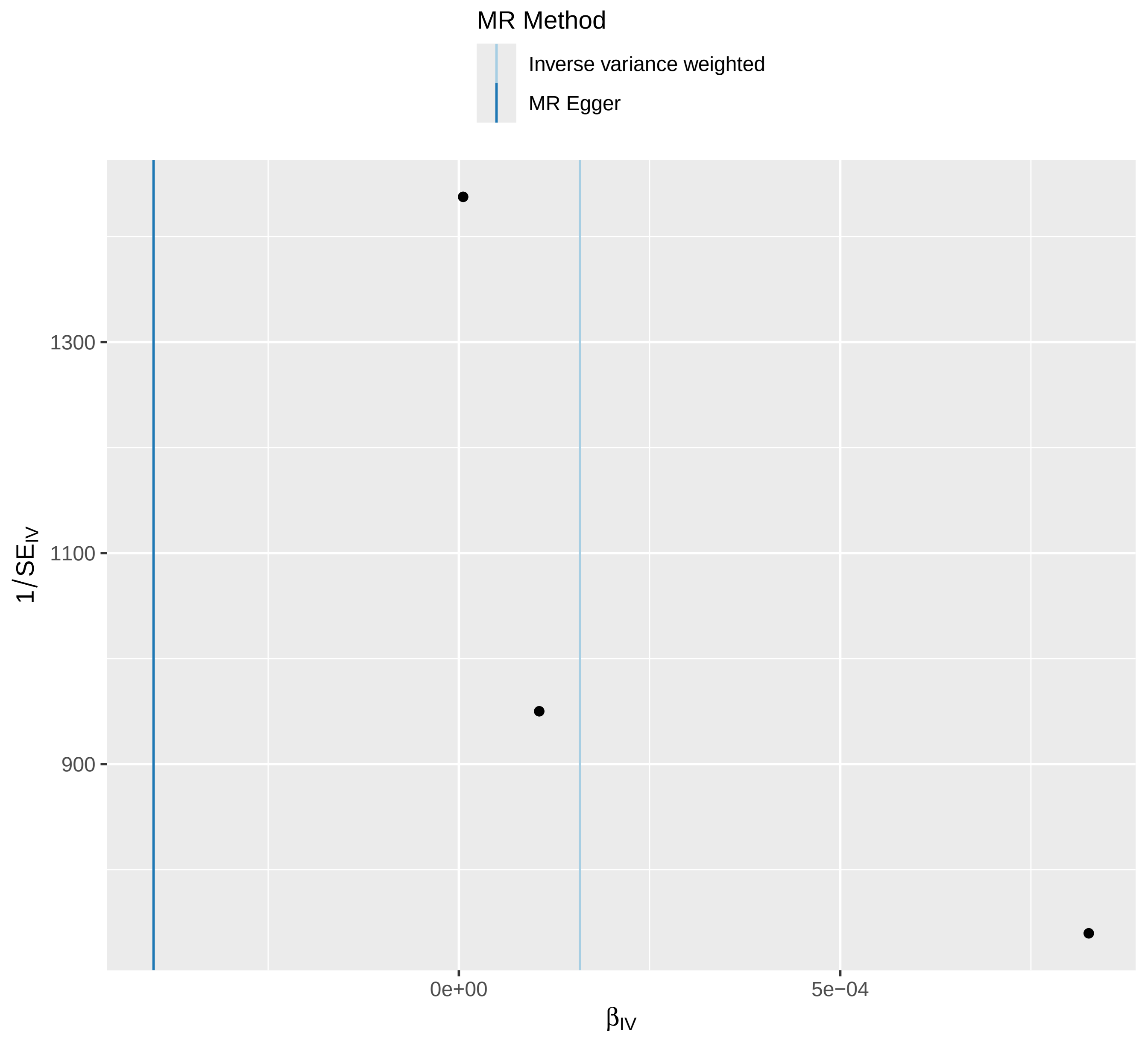

MR Method
Inverse variance weighted
MR Egger
1300
1100
900
1/SE$_{IV}$
0e+00
5e−04
β$_{IV}$

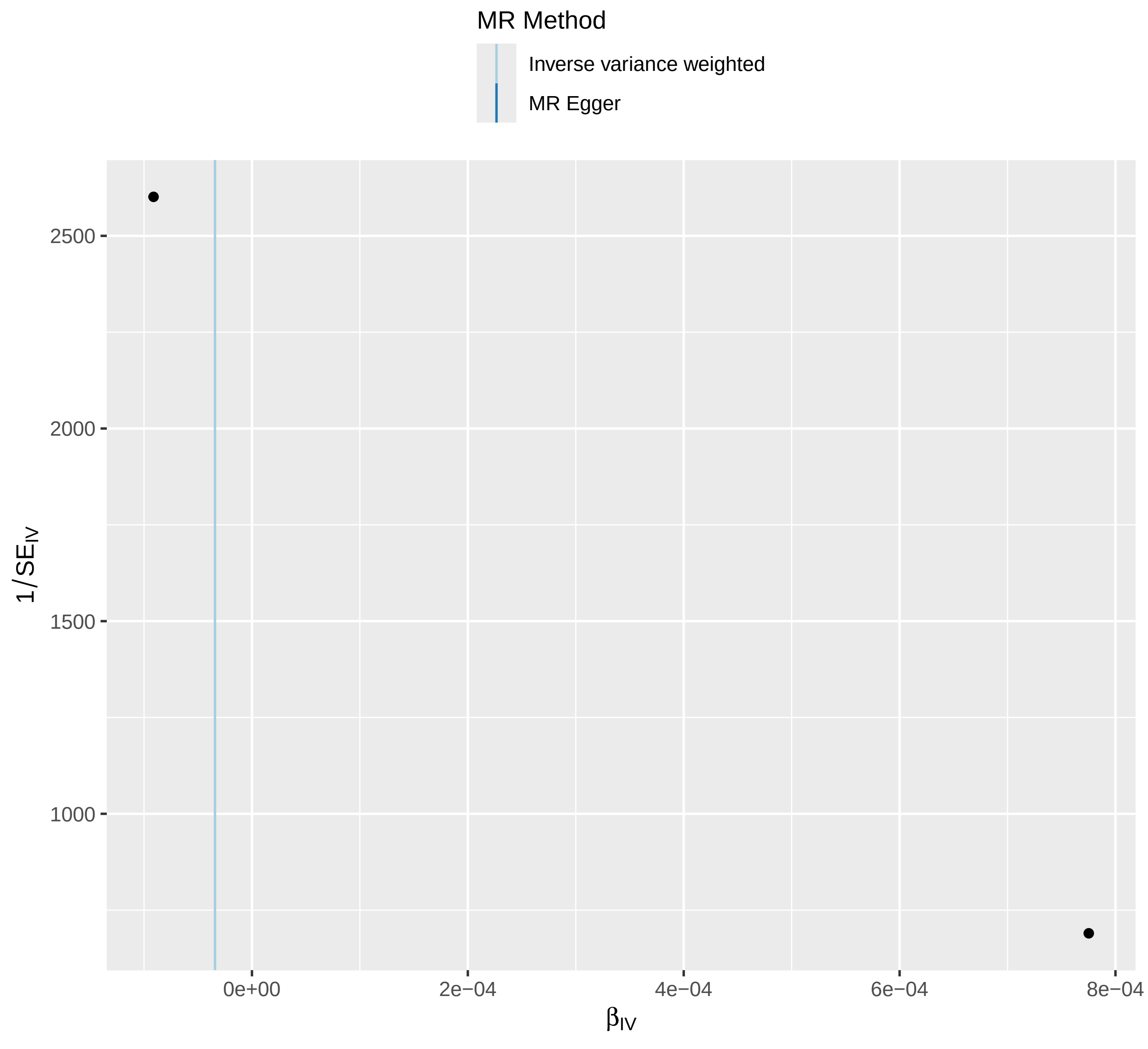

MR Method
Inverse variance weighted
MR Egger
2500
2000
1500
1000
1/SE_IV
0e+00
2e−04
4e−04
6e−04
8e−04
β_IV

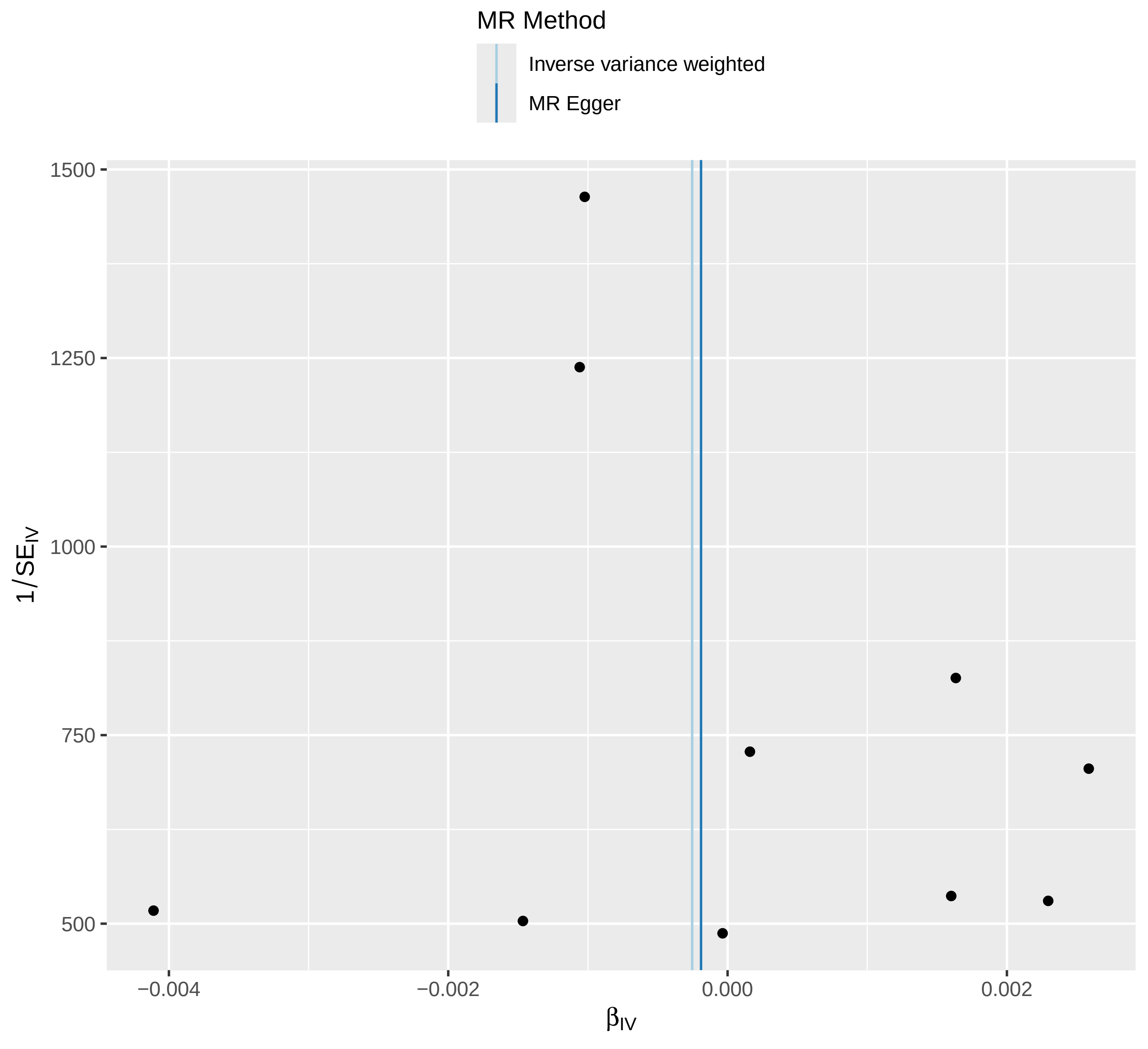
MR Method
Inverse variance weighted
MR Egger
1500
1250
1000
750
500
1/SE_IV
−0.004
−0.002
0.000
0.002
β_IV

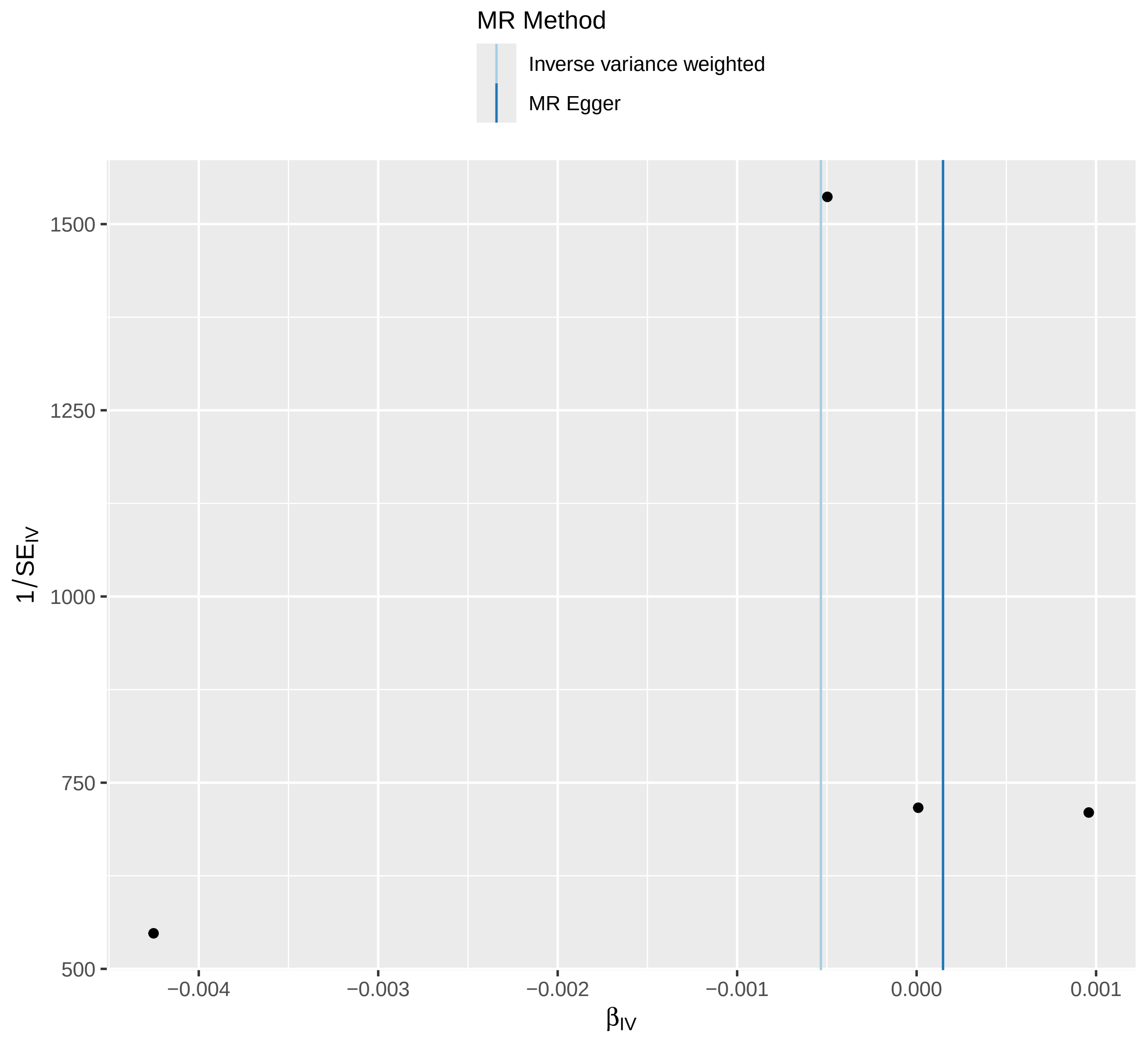
MR Method
Inverse variance weighted
MR Egger
1500
1250
1000
750
500
1/SE_IV
−0.004
−0.003
−0.002
−0.001
0.000
0.001
β_IV

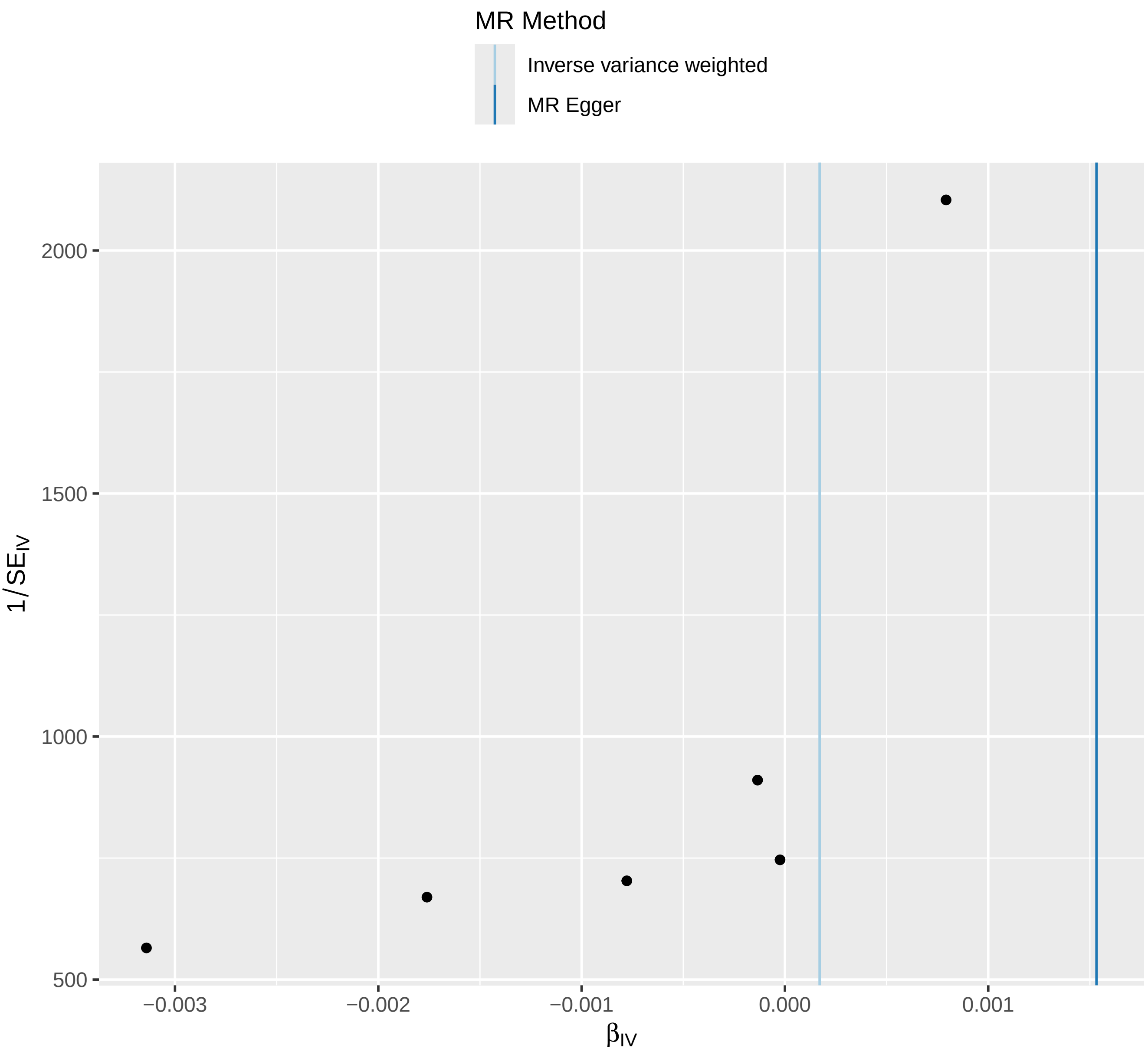

MR Method
Inverse variance weighted
MR Egger
2000
1500
1000
500
1/SE_IV
−0.003
−0.002
−0.001
0.000
0.001
β_IV

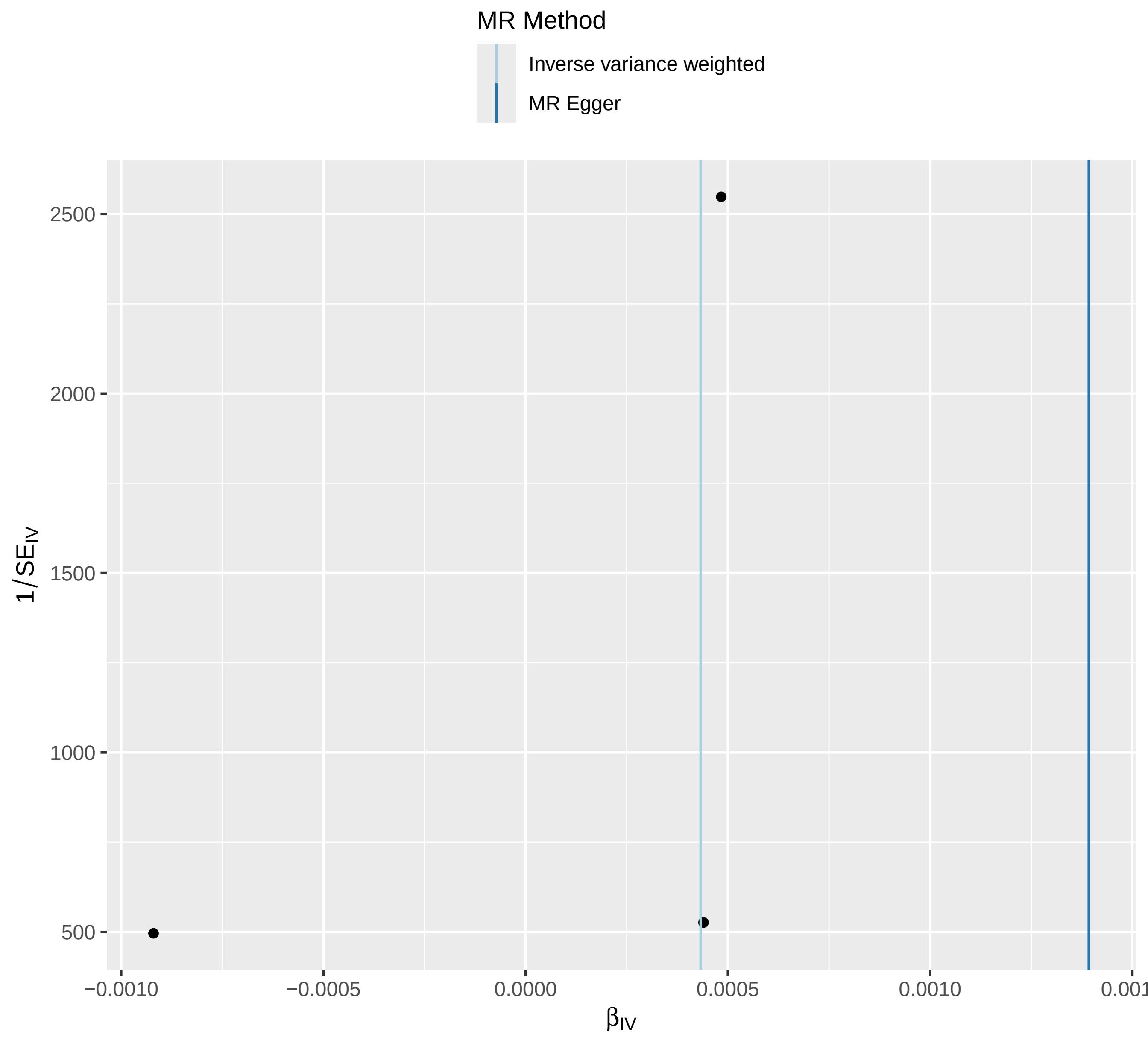

MR Method
Inverse variance weighted
MR Egger
2500
2000
1500
1000
500
1/SE_IV
−0.0010
−0.0005
0.0000
0.0005
0.0010
0.001
β_IV

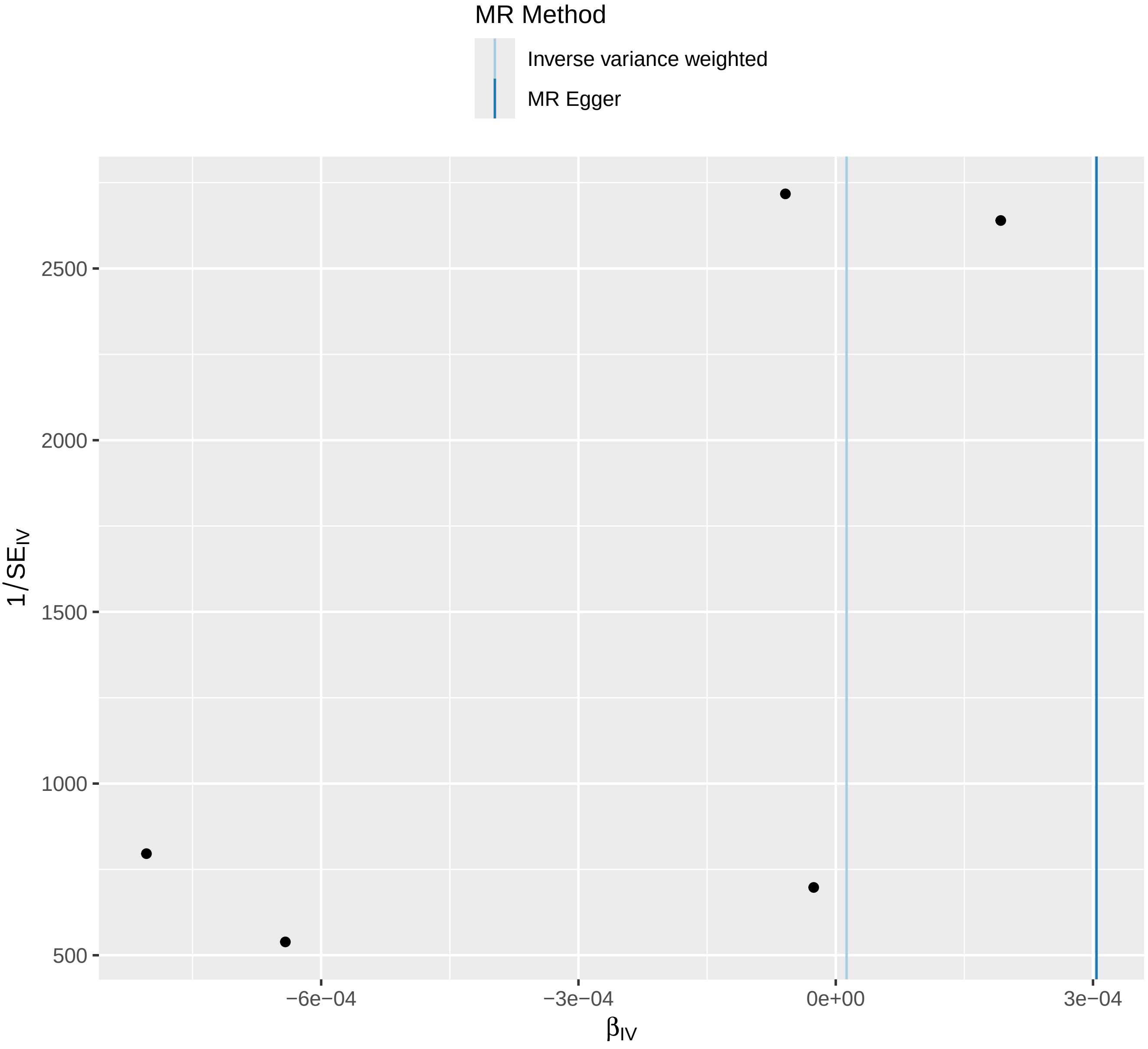
MR Method
Inverse variance weighted
MR Egger
2500
2000
1500
1000
500
1/SE_IV
−6e−04
−3e−04
0e+00
3e−04
β_IV

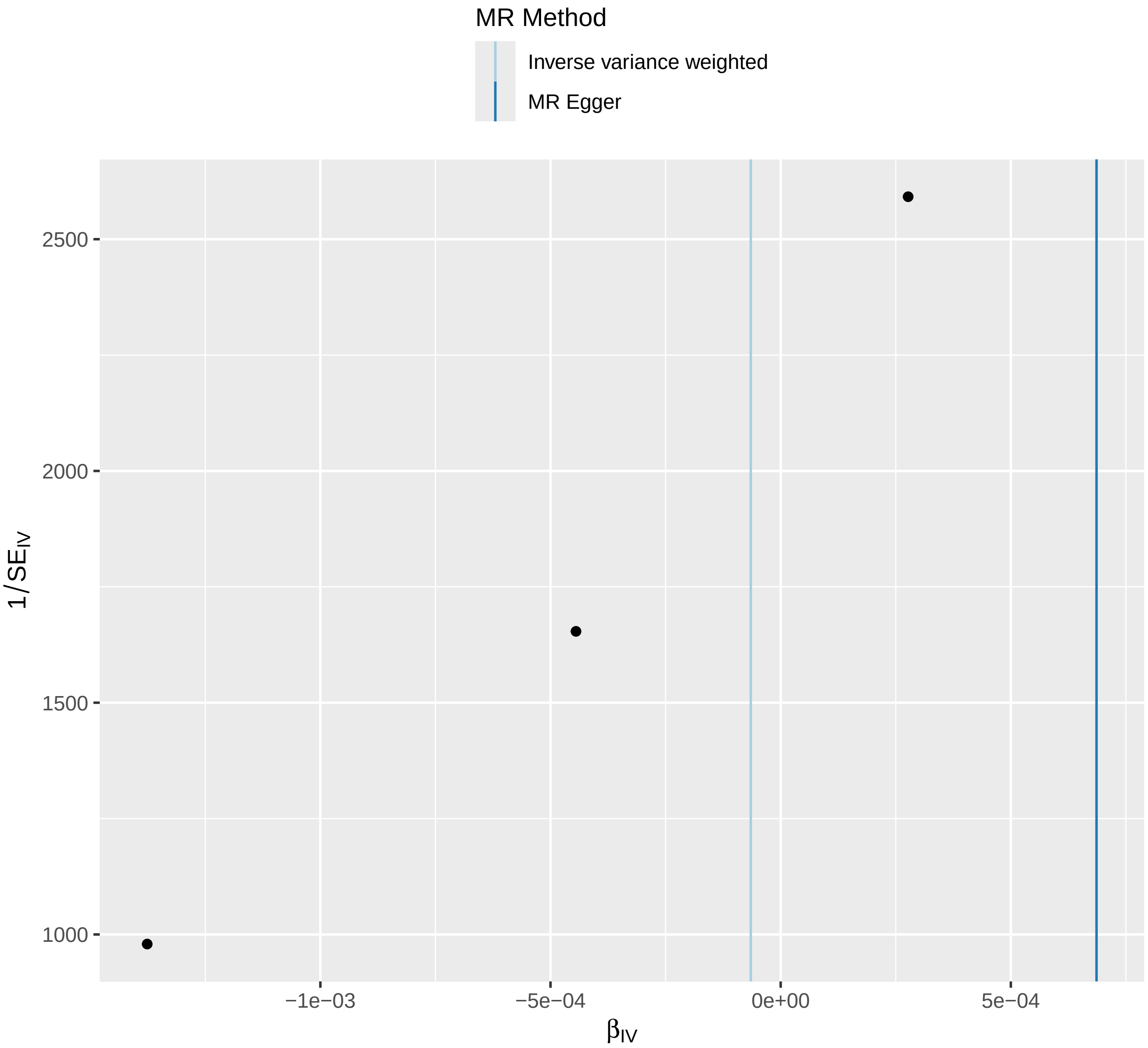

MR Method
Inverse variance weighted
MR Egger
2500
2000
1500
1000
1/SE_IV
−1e−03
−5e−04
0e+00
5e−04
β_IV

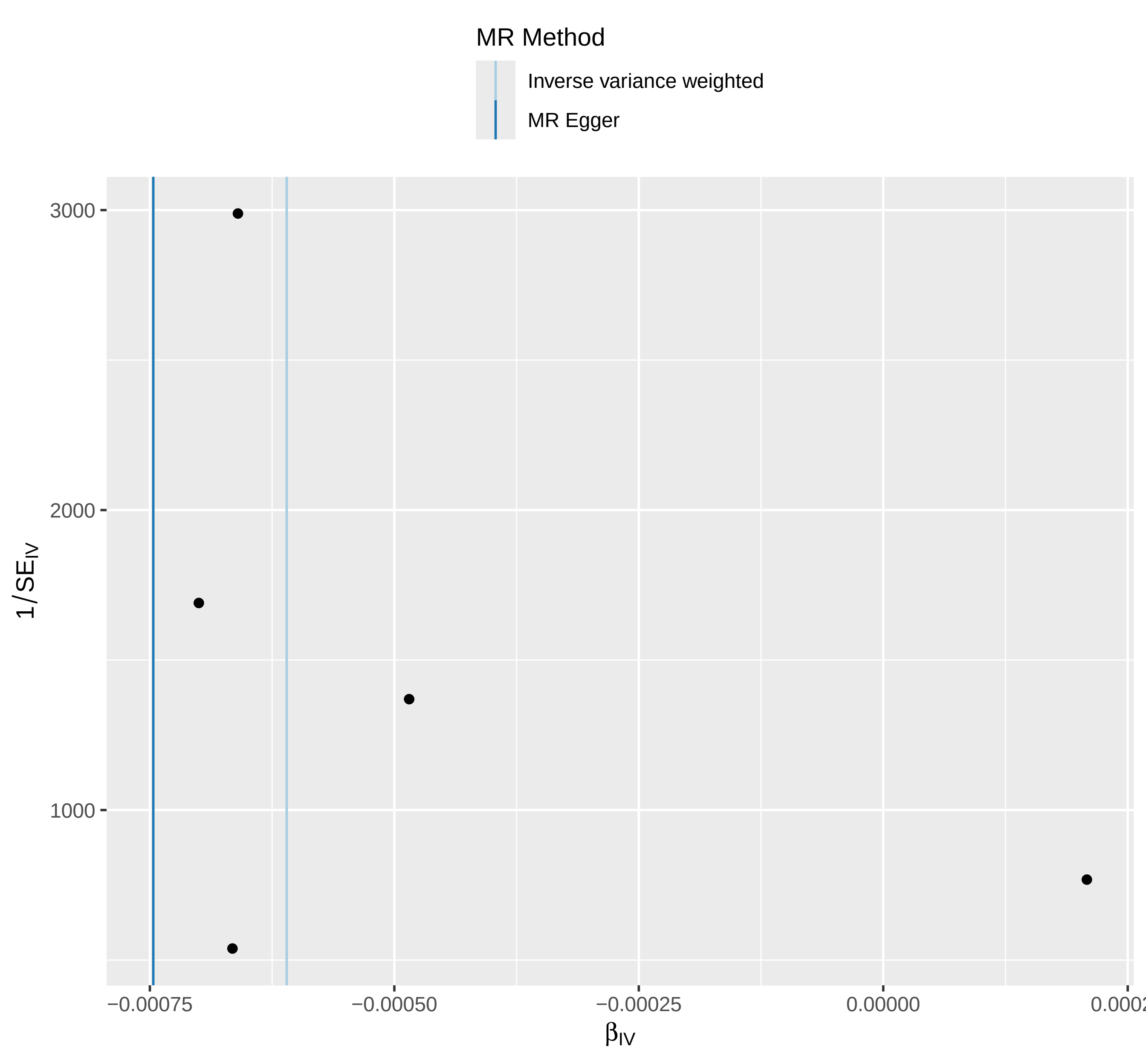
MR Method
Inverse variance weighted
MR Egger
3000
2000
1000
1/SE$_{IV}$
−0.00075
−0.00050
−0.00025
0.00000
0.0002
β$_{IV}$

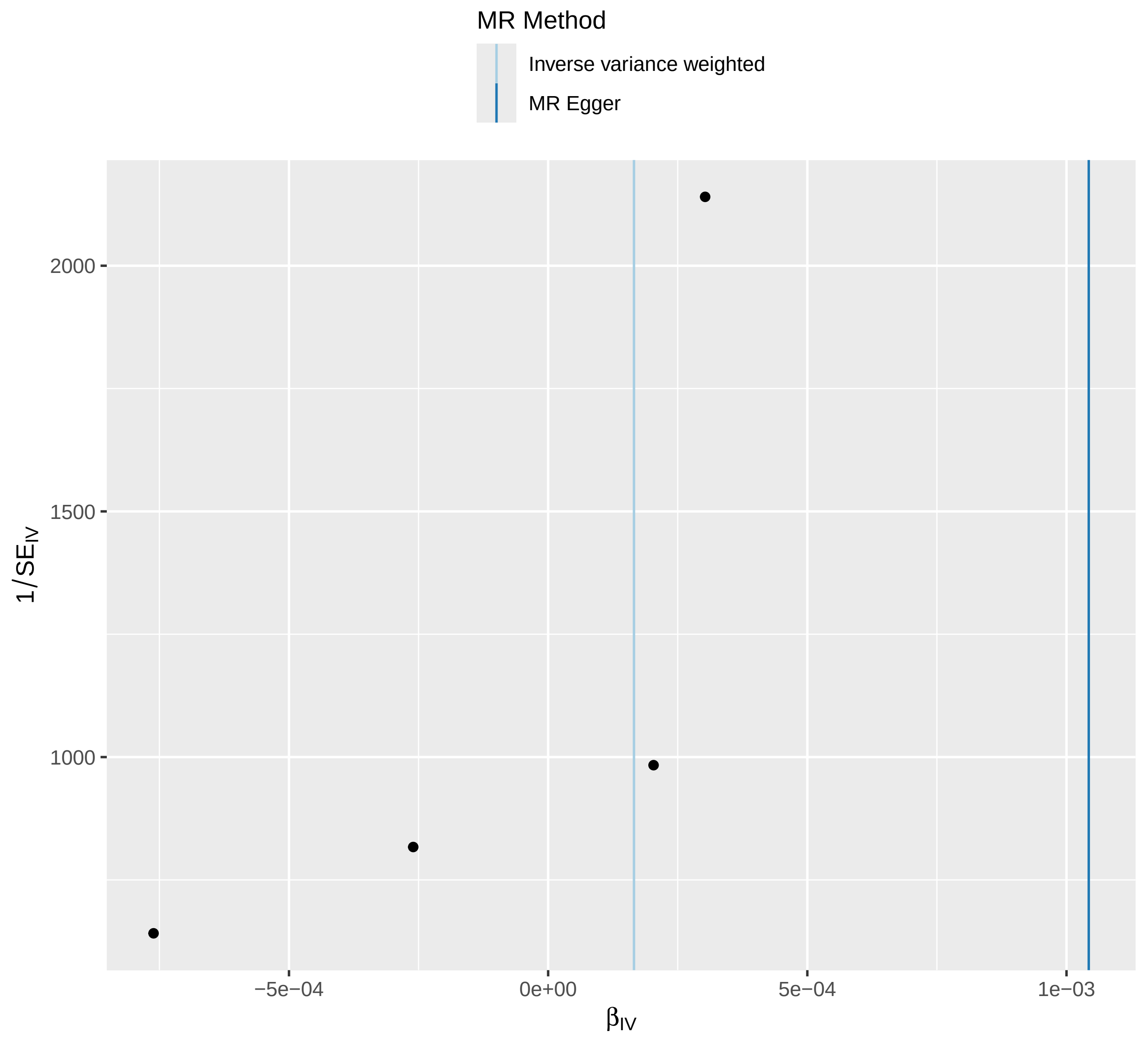
MR Method
Inverse variance weighted
MR Egger
2000
1500
1000
1/SE_IV
−5e−04
0e+00
5e−04
1e−03
β_IV

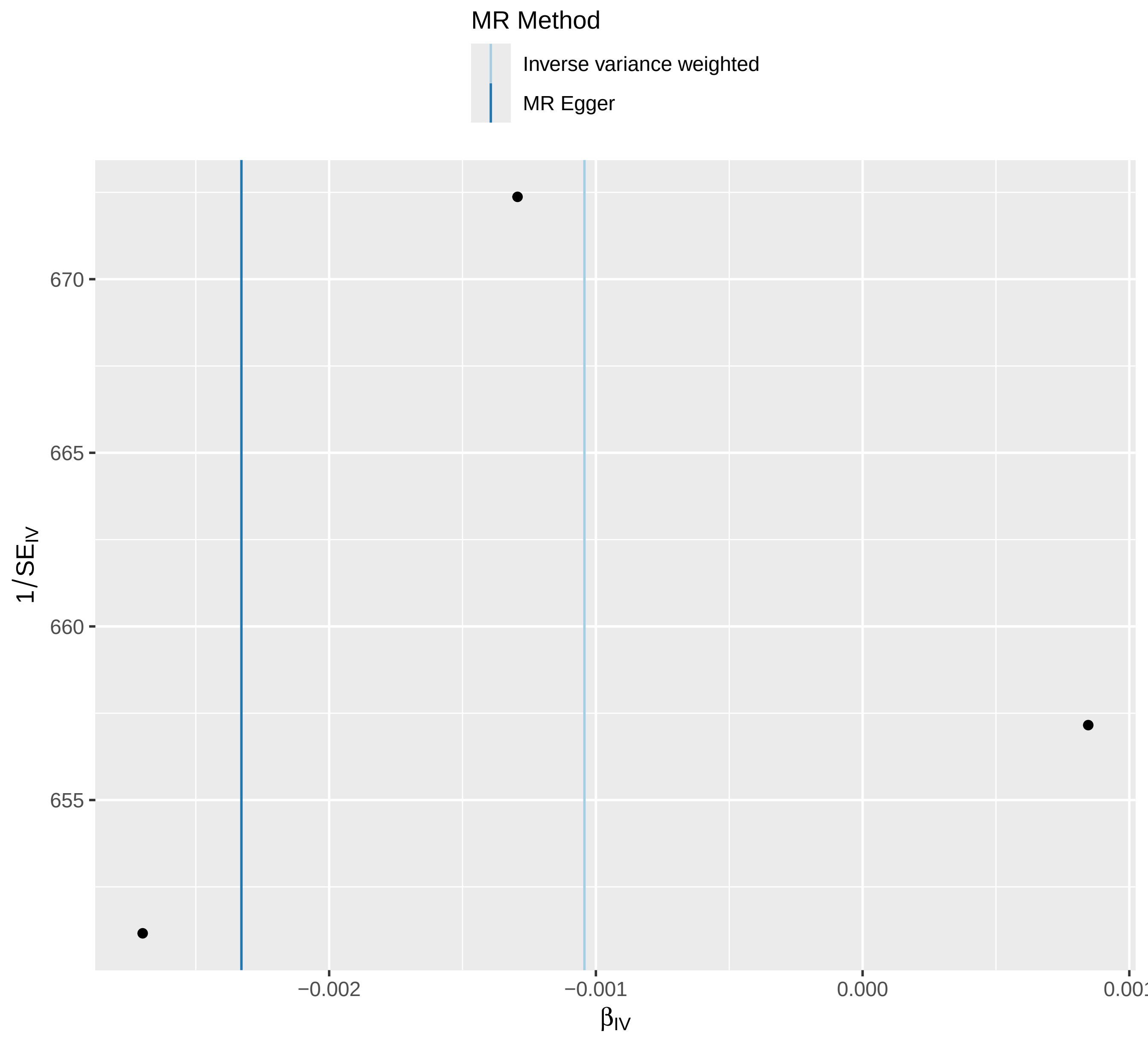

MR Method
Inverse variance weighted
MR Egger
670
665
660
655
$1/SE_{IV}$
−0.002
−0.001
0.000
β_{IV}

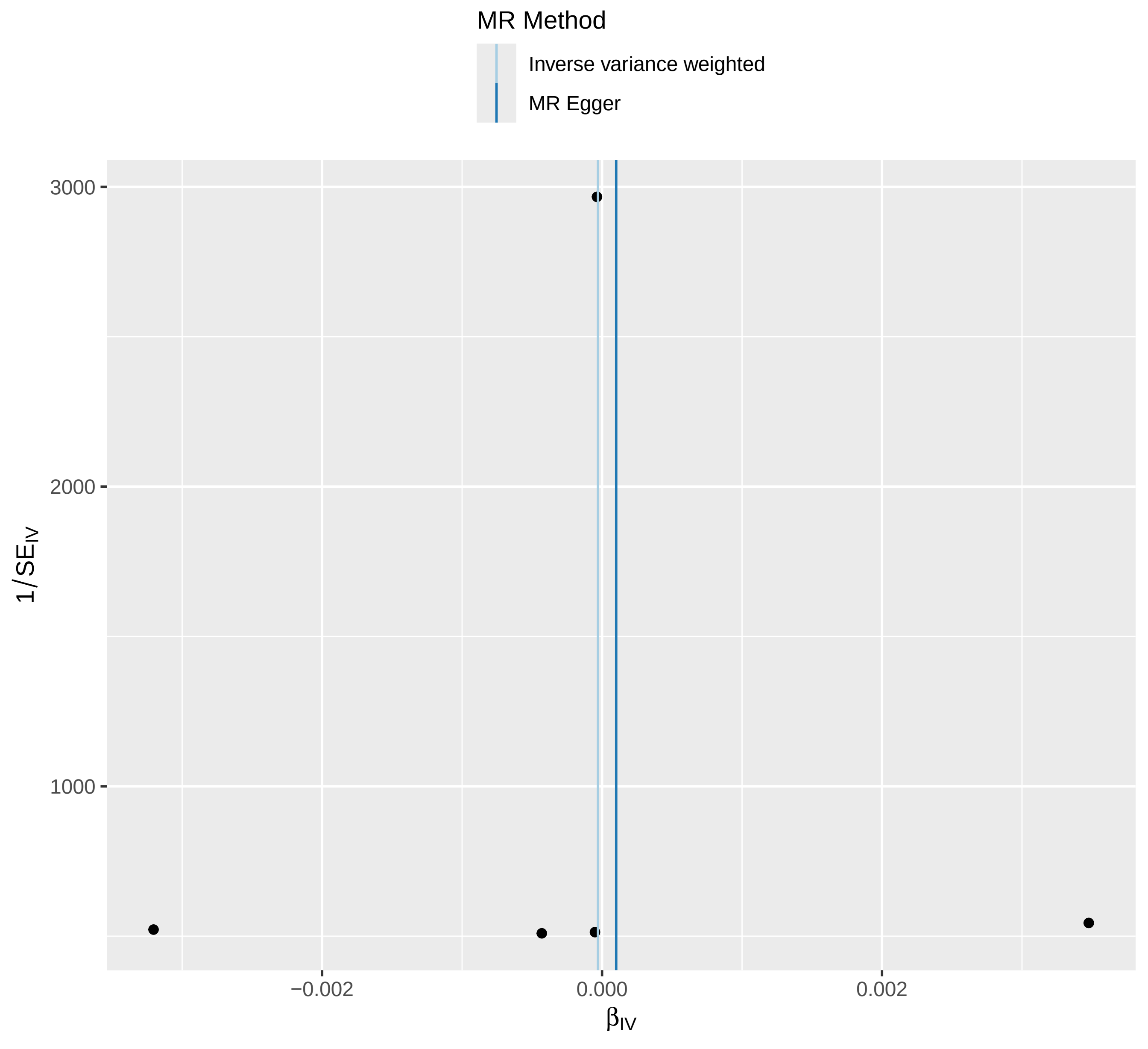

MR Method
Inverse variance weighted
MR Egger
3000
2000
1000
1/SE$_{IV}$
−0.002
0.000
0.002
β$_{IV}$

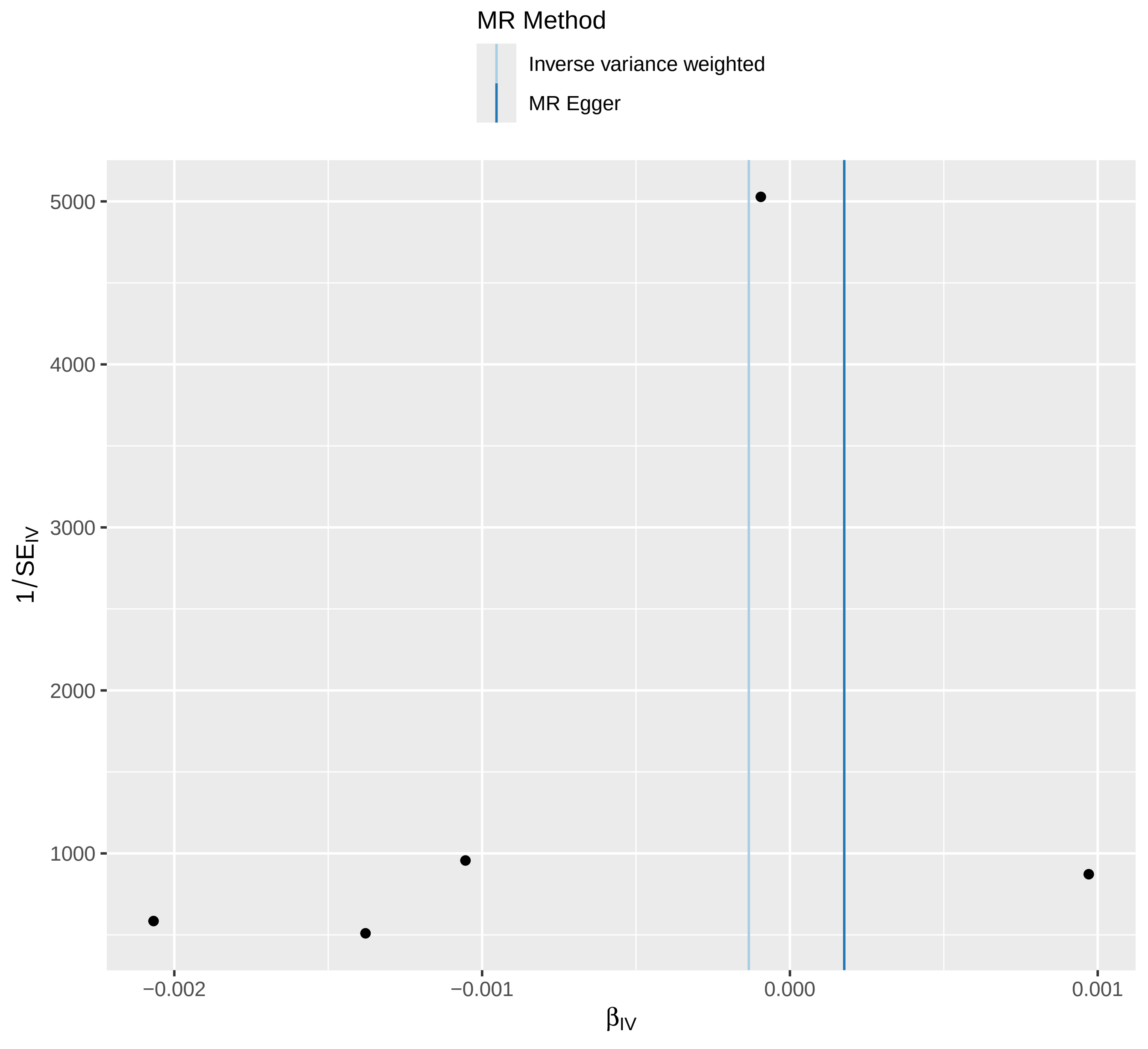

MR Method
Inverse variance weighted
MR Egger
5000
4000
3000
2000
1000
1/SE$_{IV}$
−0.002
−0.001
0.000
0.001
β_{IV}

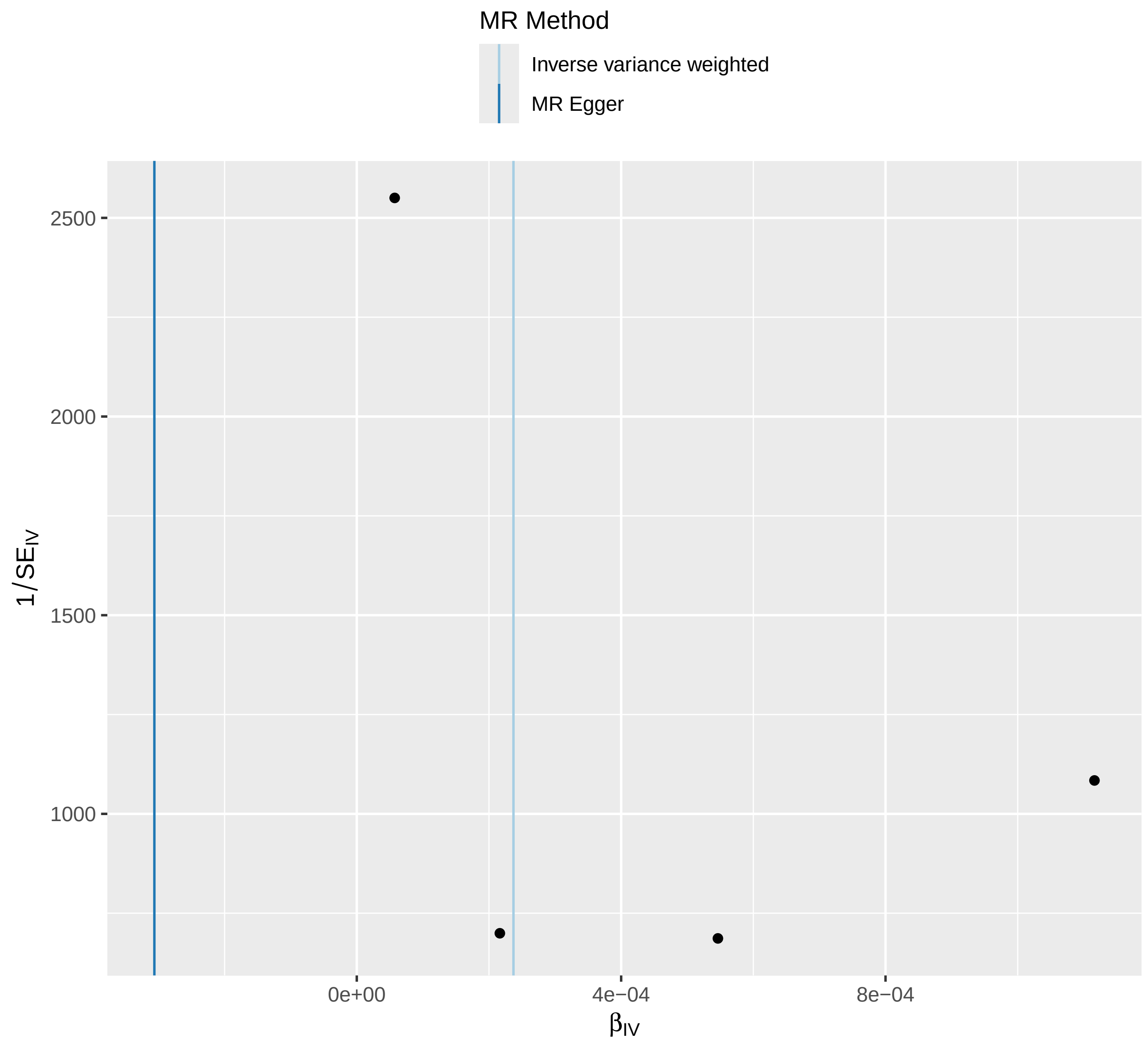
MR Method
Inverse variance weighted
MR Egger
2500
2000
1500
1000
1/SE$_{IV}$
0e+00
4e−04
8e−04
β$_{IV}$

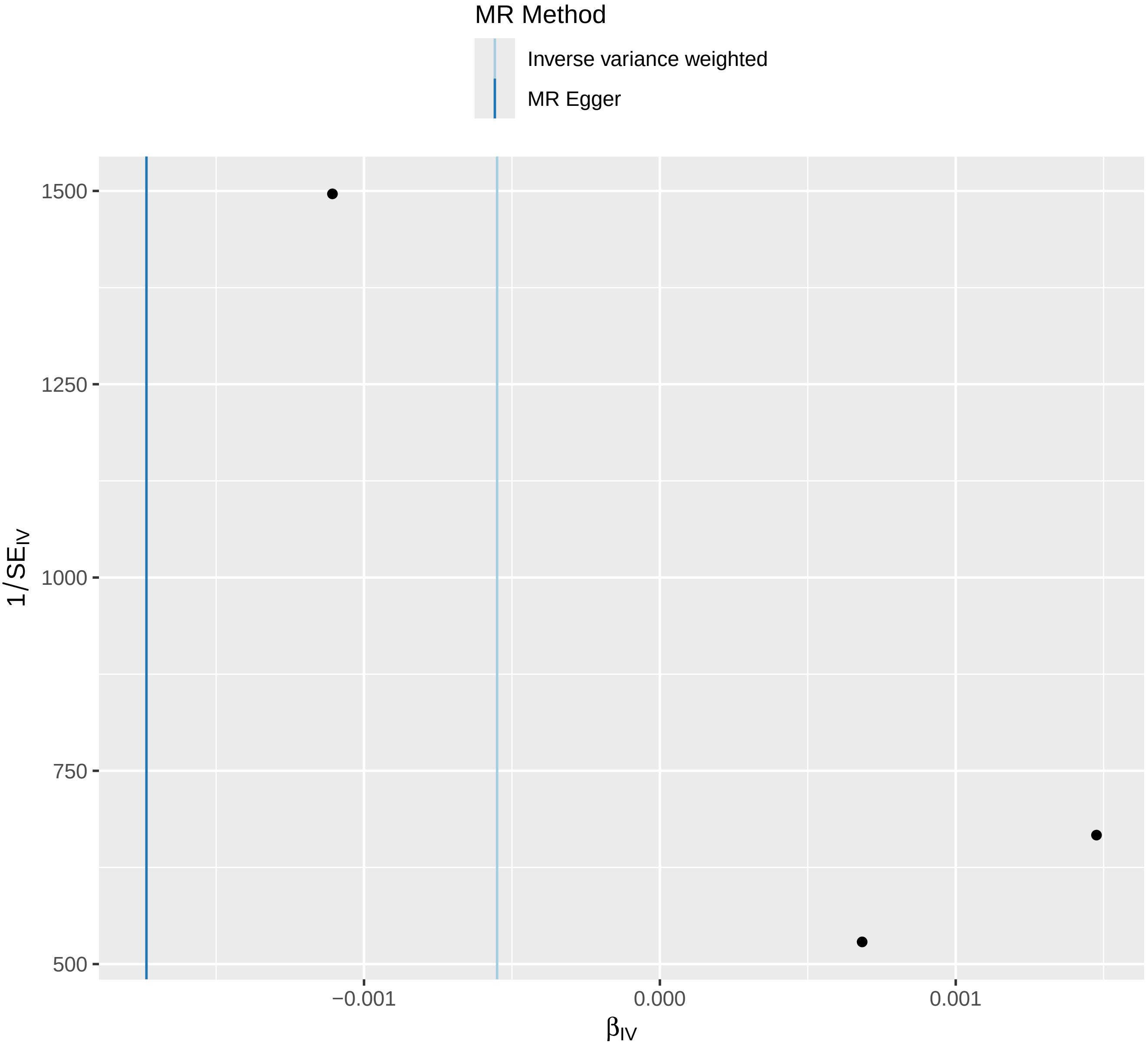
MR Method
Inverse variance weighted
MR Egger
1500
1250
1000
750
500
1/SE_IV
−0.001
0.000
0.001
β_IV

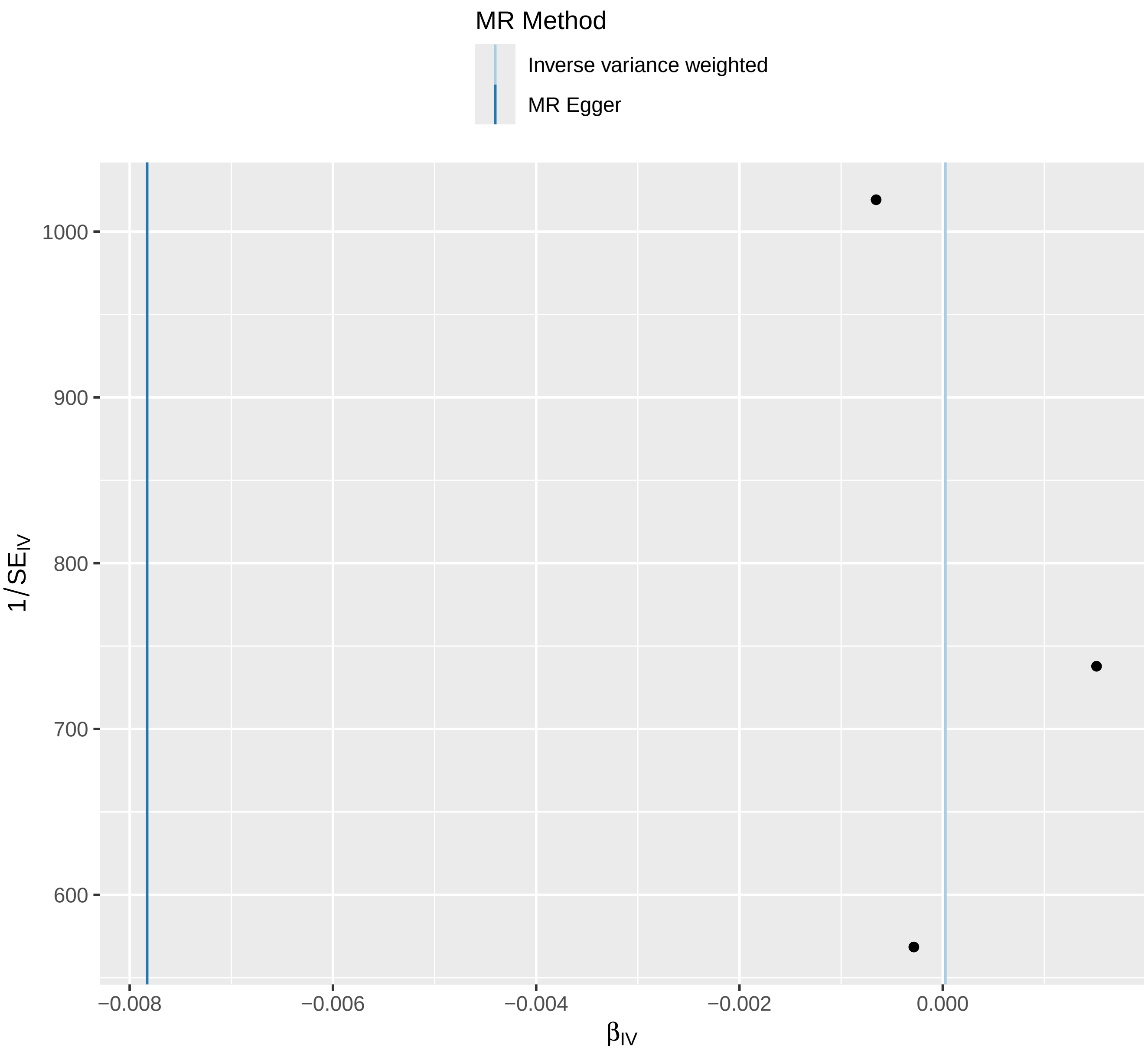

MR Method
Inverse variance weighted
MR Egger
1000
900
800
700
600
1/SE$_{IV}$
−0.008
−0.006
−0.004
−0.002
0.000
β$_{IV}$

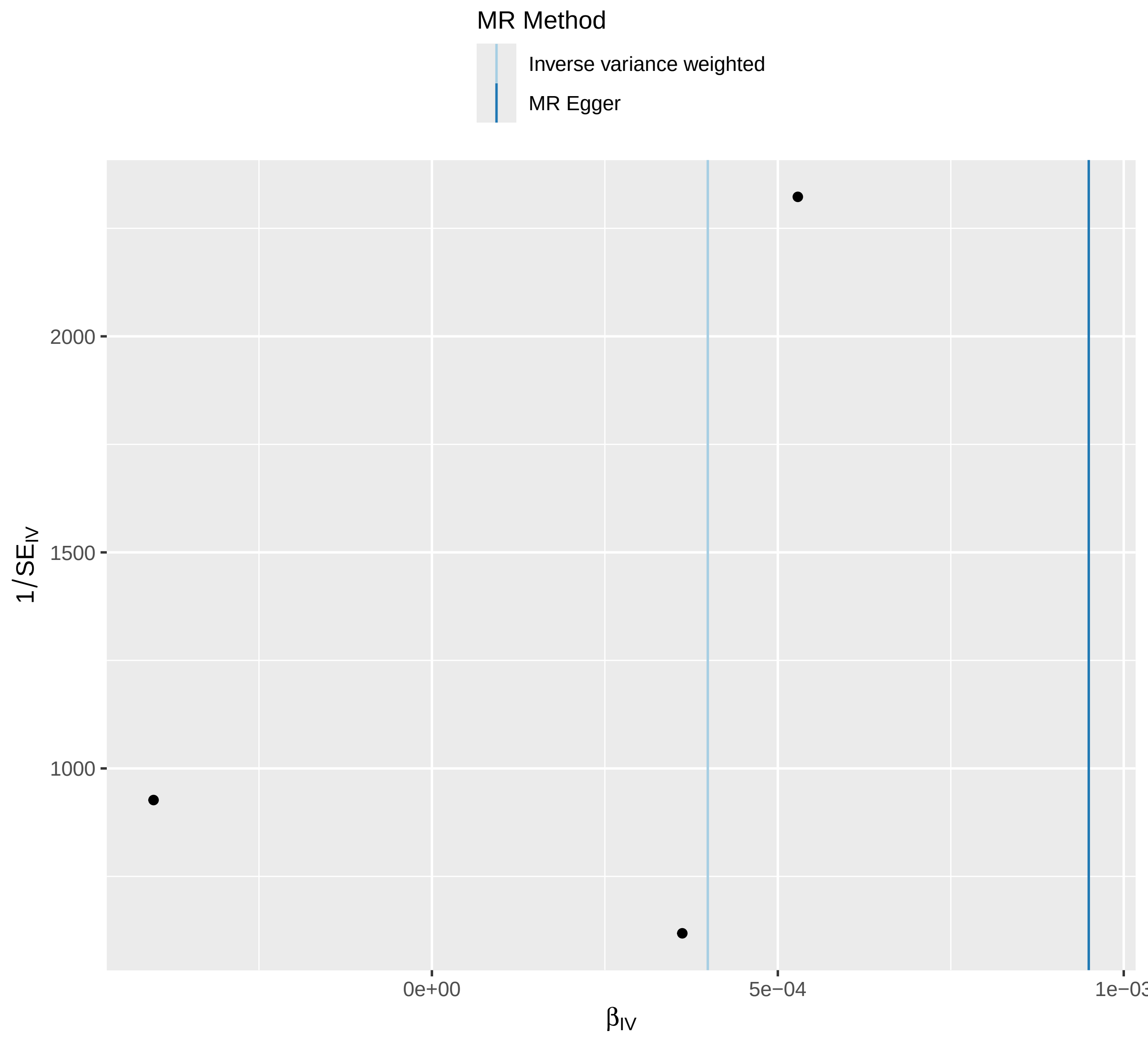

MR Method
Inverse variance weighted
MR Egger
2000
1500
1000
1/SE$_{IV}$
0e+00
5e−04
1e−03
β_{IV}

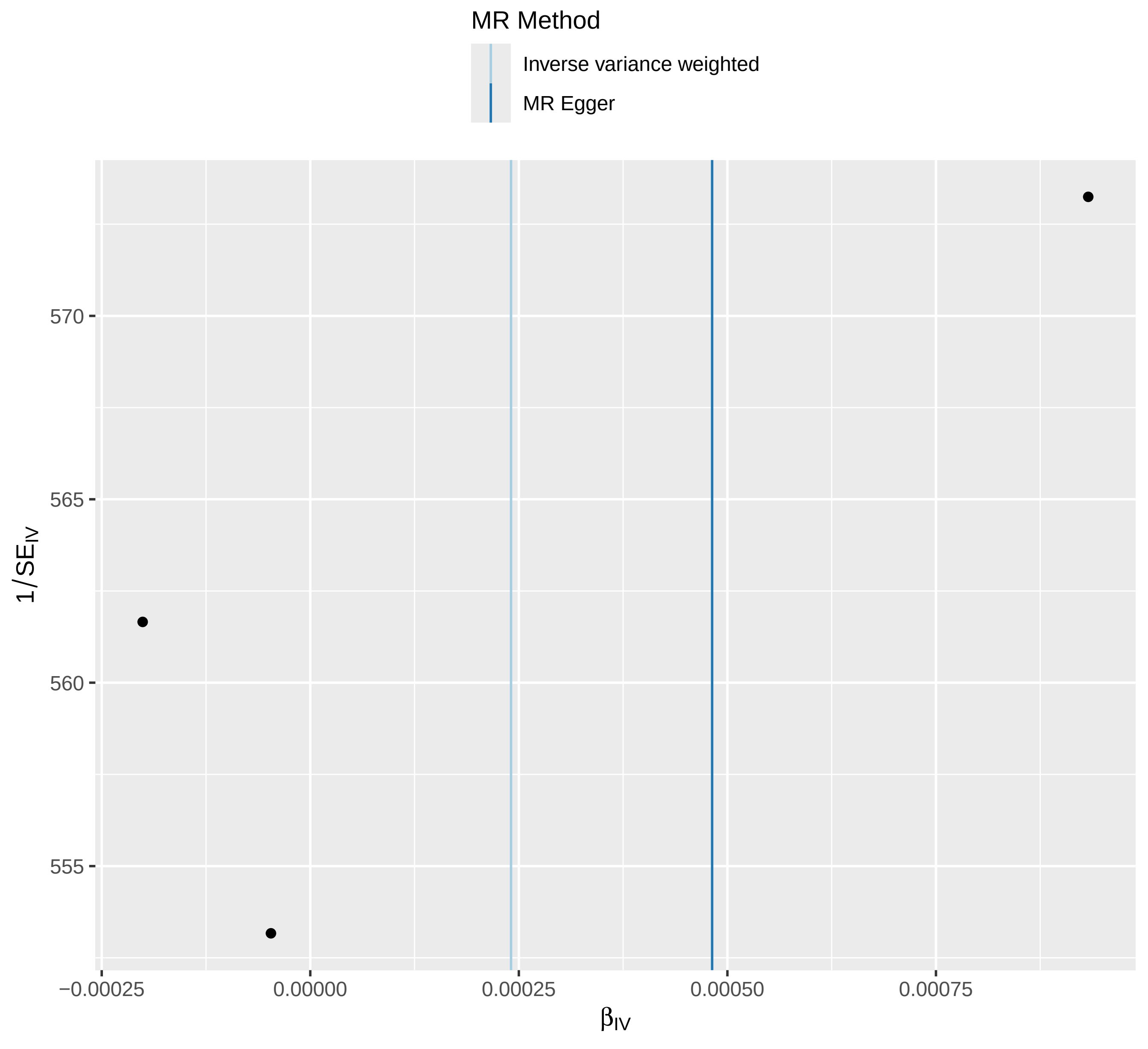
MR Method
Inverse variance weighted
MR Egger
570
565
560
555
1/SE_IV
−0.00025
0.00000
0.00025
0.00050
0.00075
β_IV

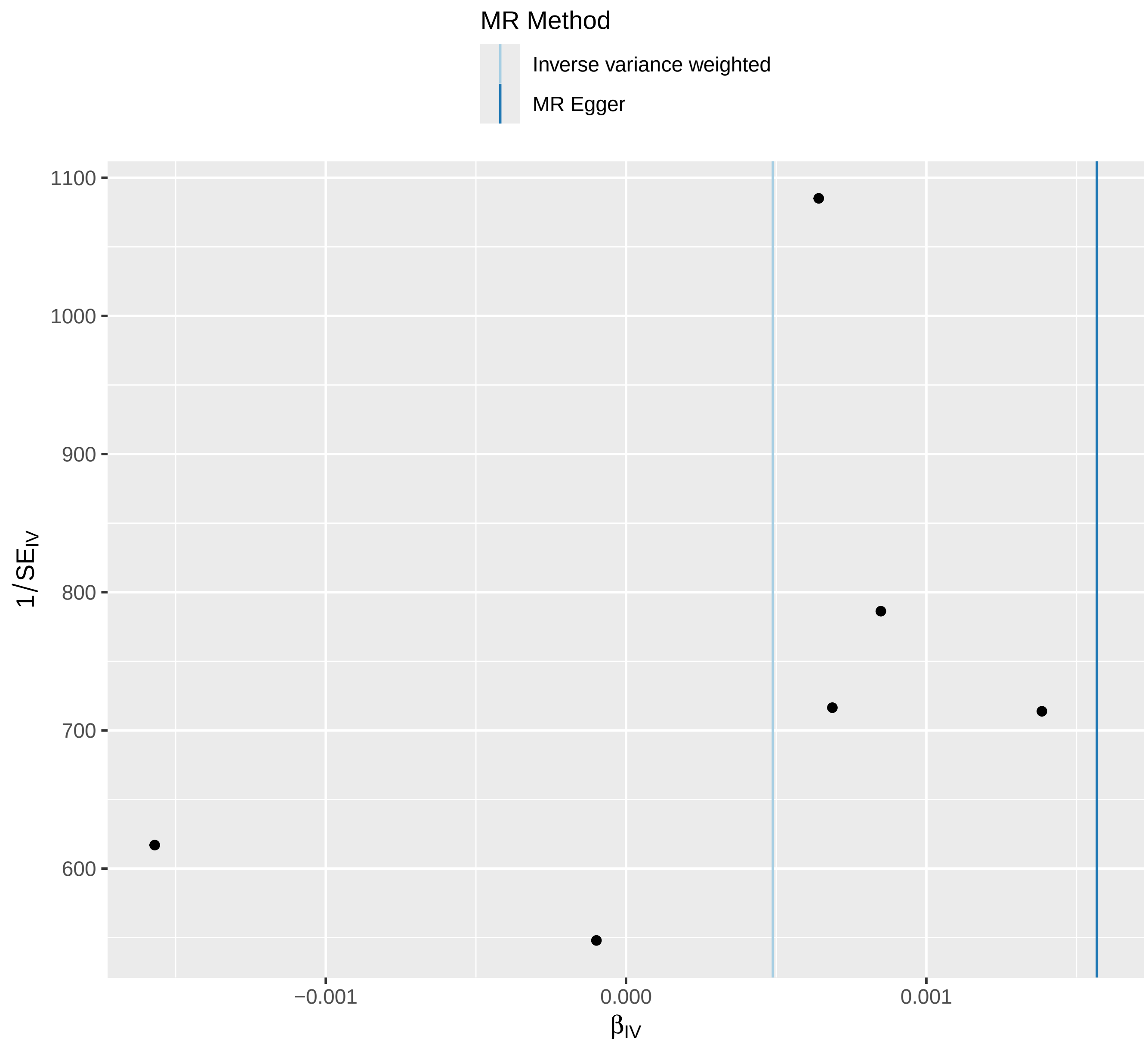
MR Method
Inverse variance weighted
MR Egger
1100
1000
900
800
700
600
1/SE_IV
−0.001
0.000
0.001
β_IV

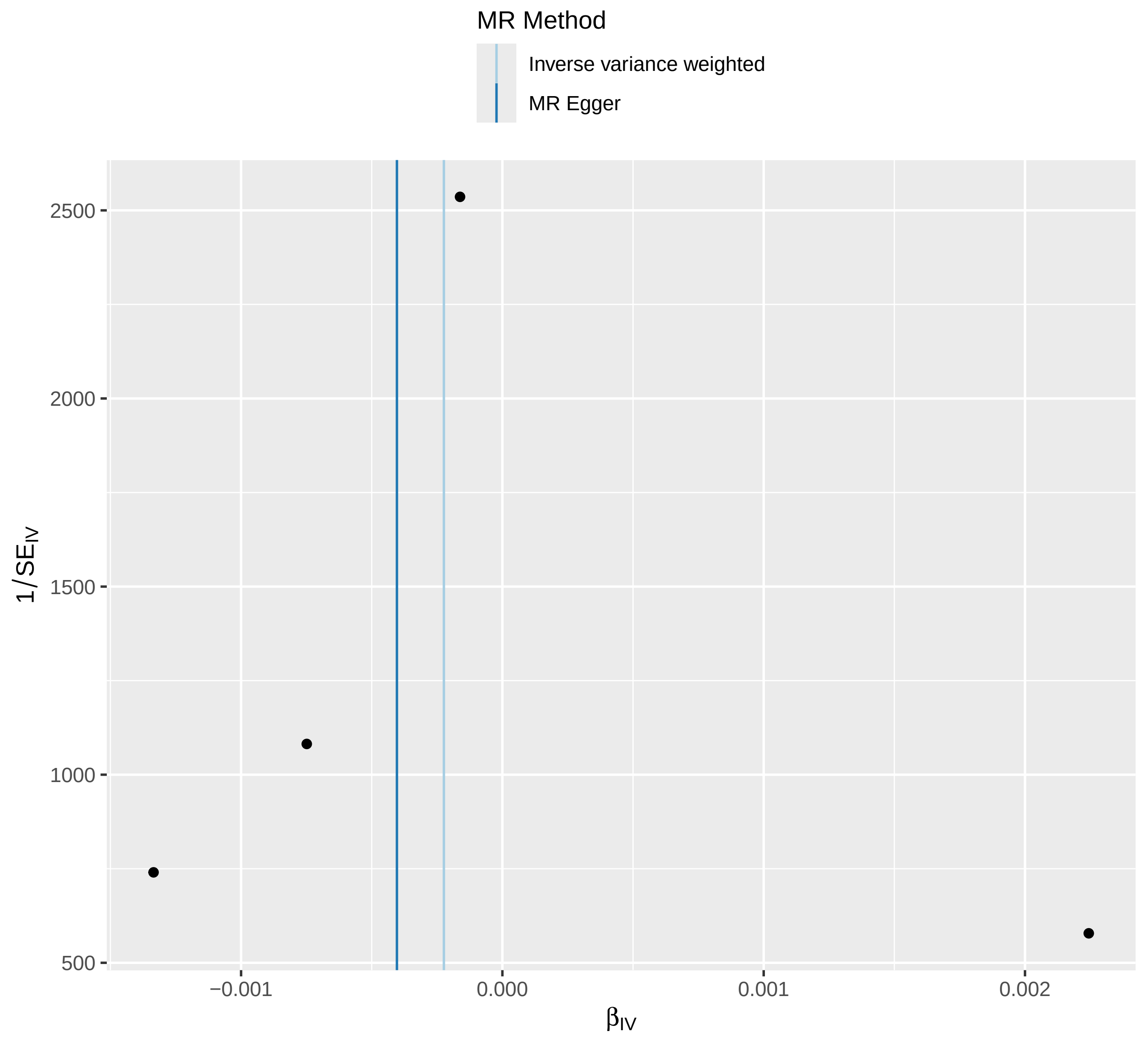

MR Method
Inverse variance weighted
MR Egger
2500
2000
1500
1000
500
1/SE_IV
−0.001
0.000
0.001
0.002
β_IV

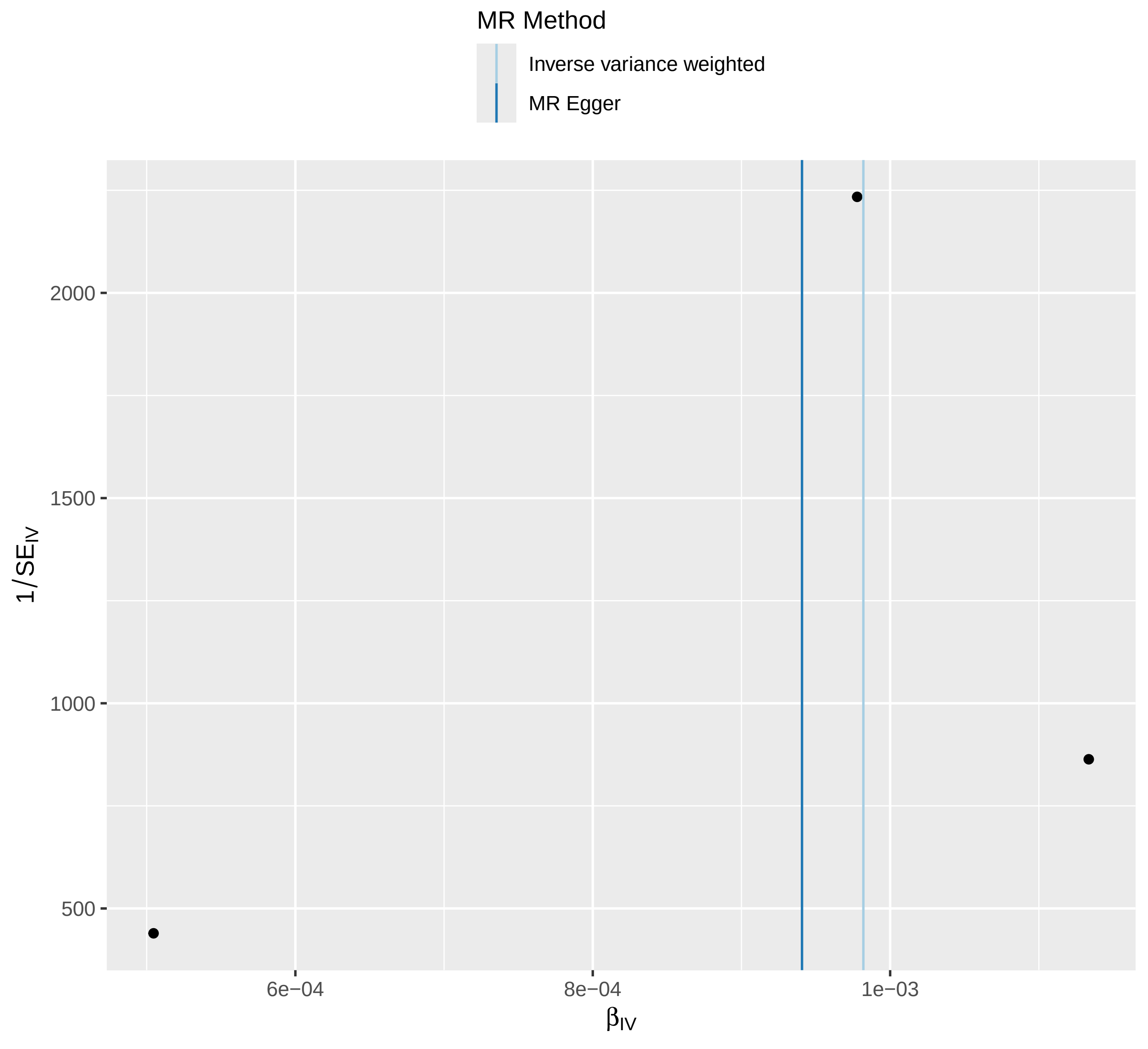
MR Method
Inverse variance weighted
MR Egger
2000
1500
1000
500
1/SE_IV
6e−04
8e−04
1e−03
β_IV

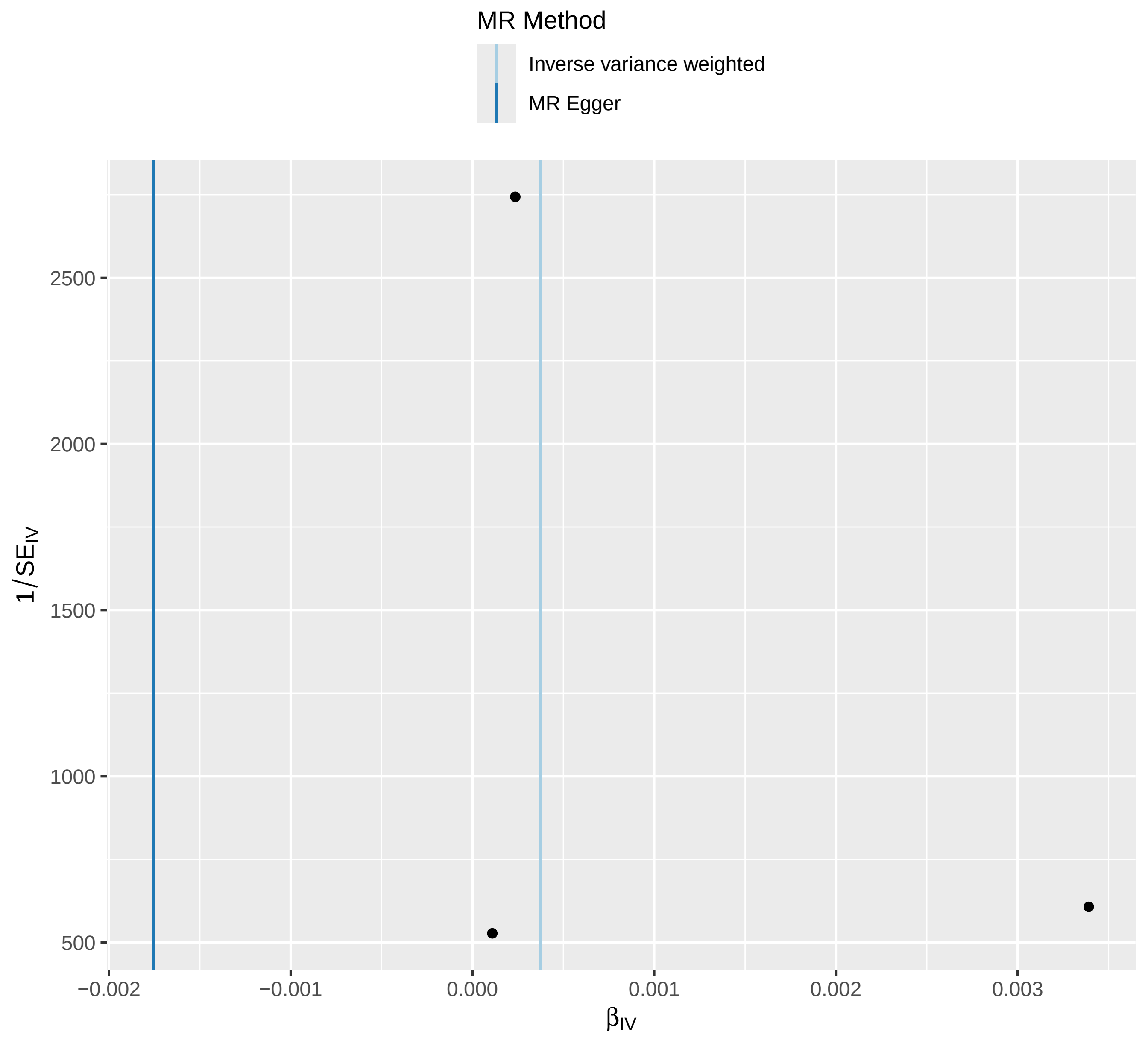
MR Method
Inverse variance weighted
MR Egger
1/SE$_{IV}$
2500
2000
1500
1000
500
−0.002
−0.001
0.000
0.001
0.002
0.003
β_{IV}

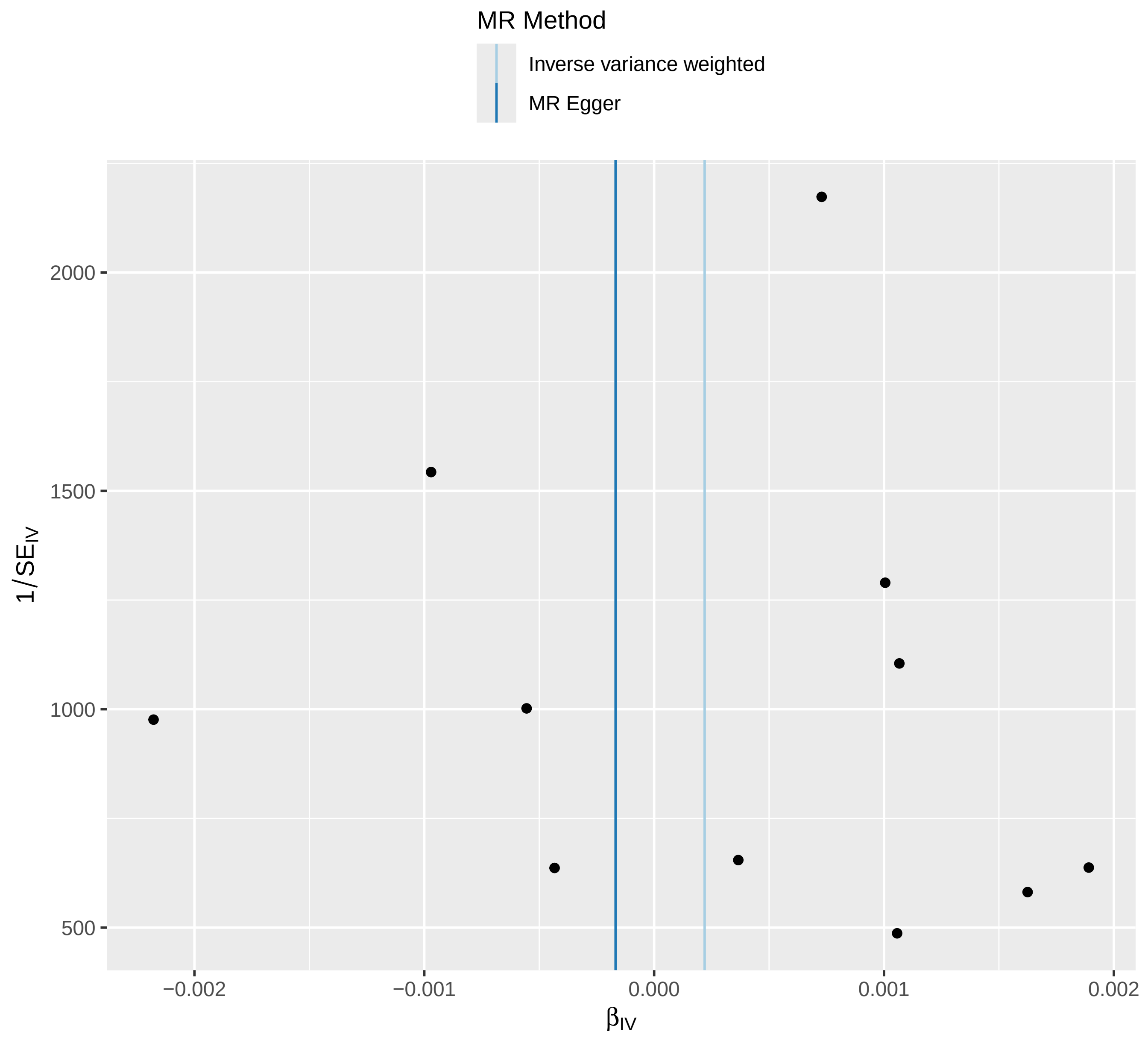

MR Method
Inverse variance weighted
MR Egger
2000
1500
1000
500
1/SE_IV
−0.002
−0.001
0.000
0.001
0.002
β_IV

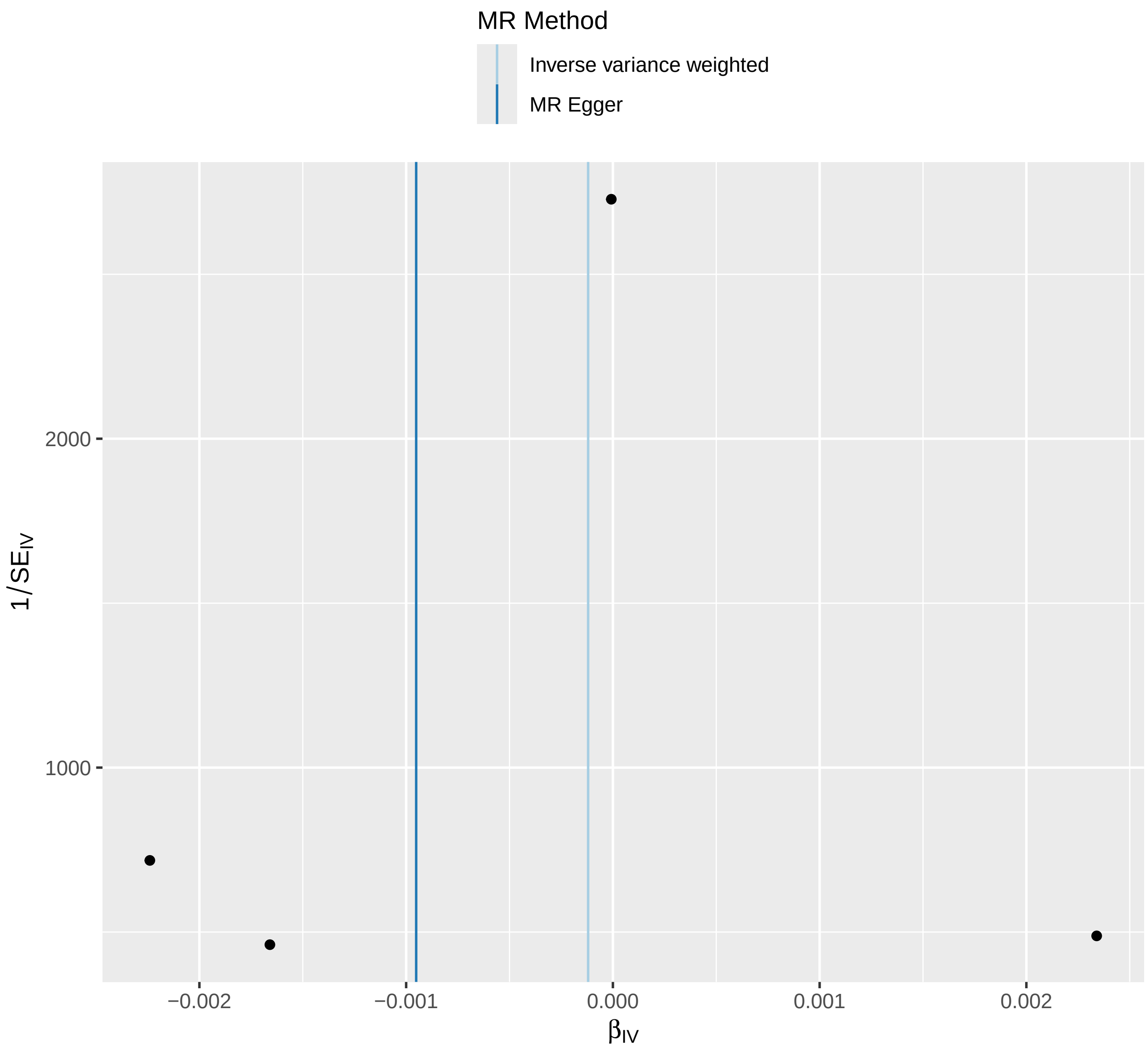
MR Method
Inverse variance weighted
MR Egger
2000
1000
1/SE_IV
−0.002
−0.001
0.000
0.001
0.002
β_IV

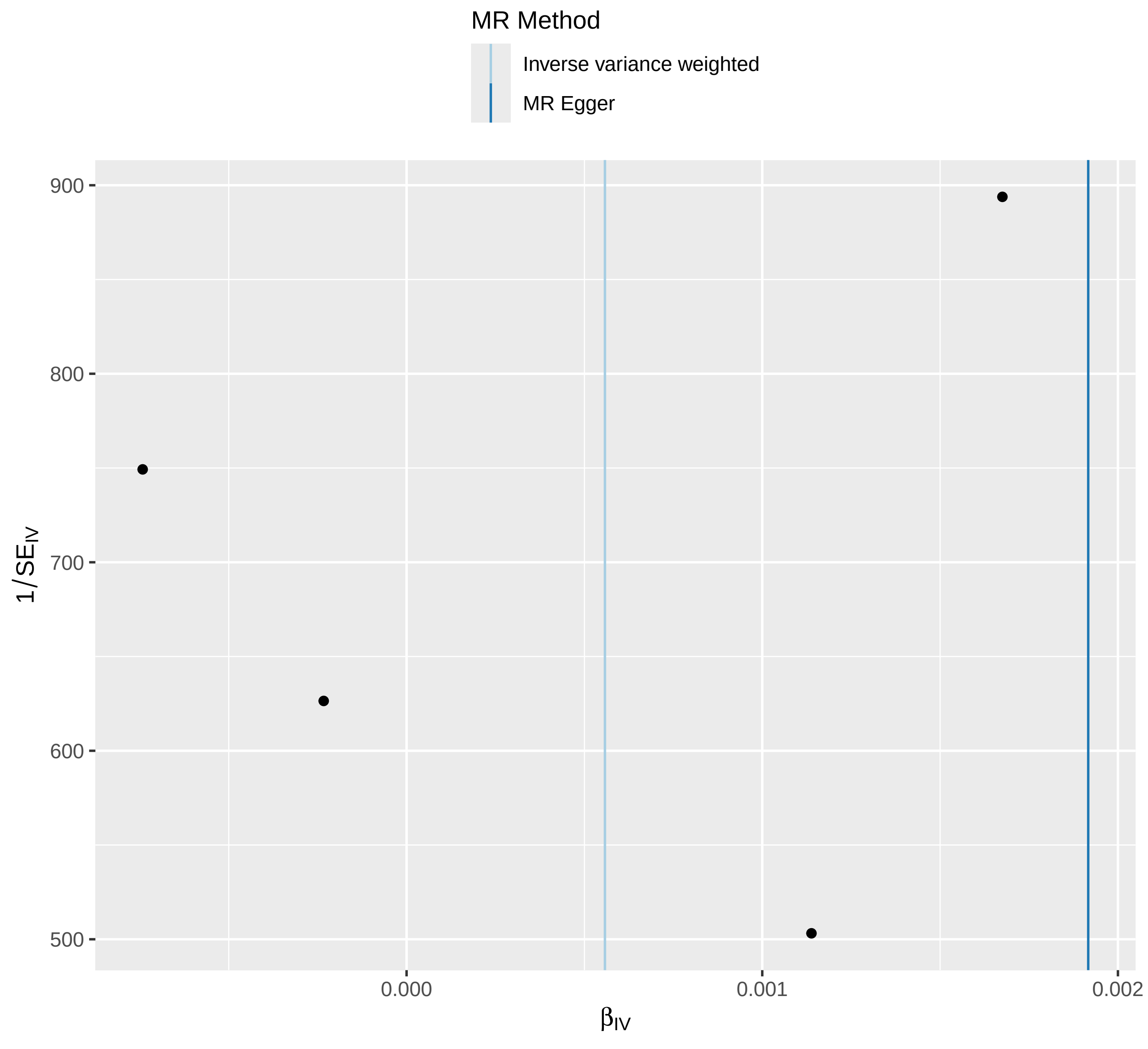

MR Method
Inverse variance weighted
MR Egger
900
800
700
600
500
1/SE_IV
0.000
0.001
0.002
β_IV

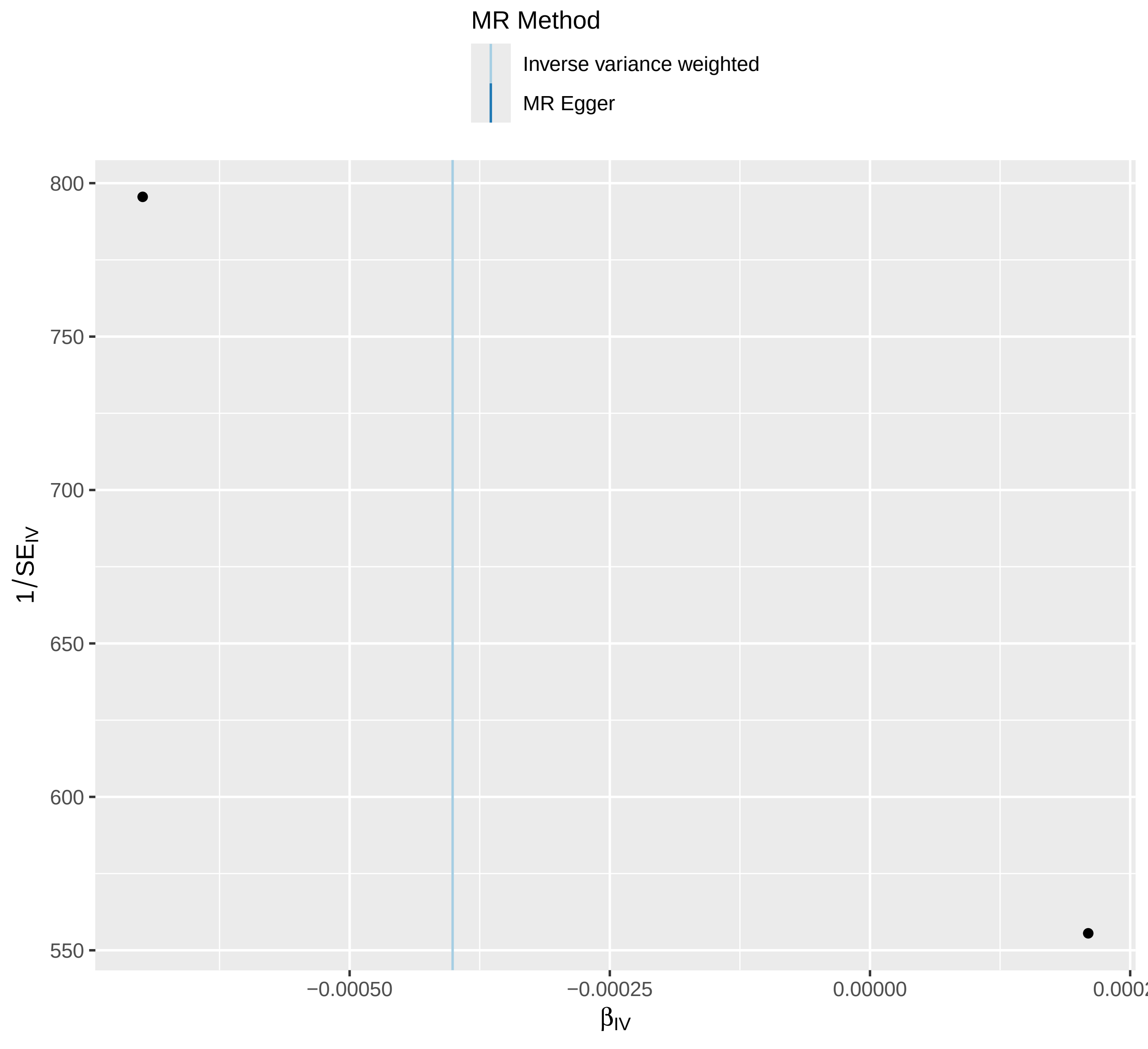

MR Method
Inverse variance weighted
MR Egger
800
750
700
650
600
550
1/SE_IV
−0.00050
−0.00025
0.00000
β_IV

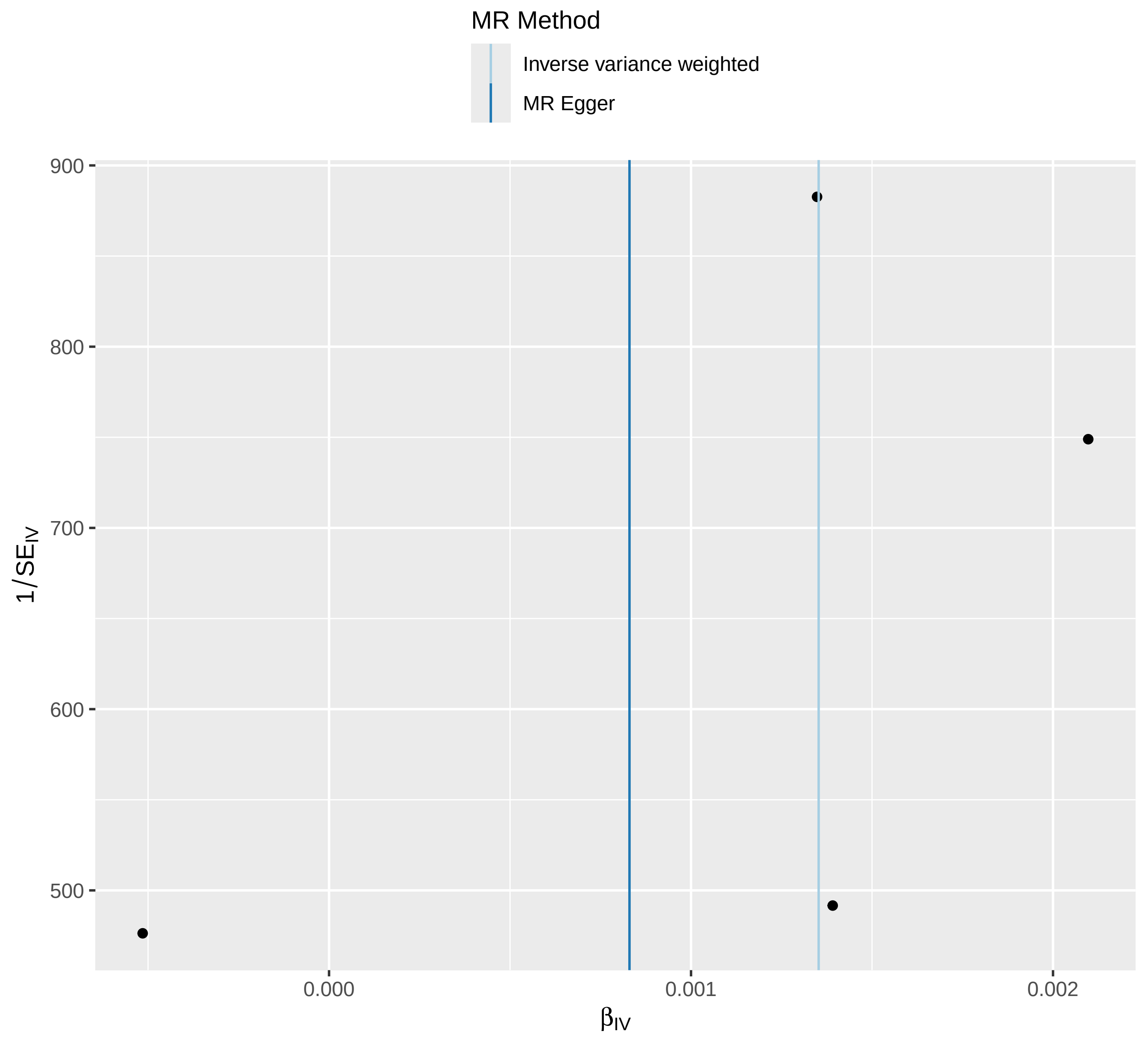

MR Method
Inverse variance weighted
MR Egger
900
800
700
600
500
1/SE_IV
0.000
0.001
0.002
β_IV

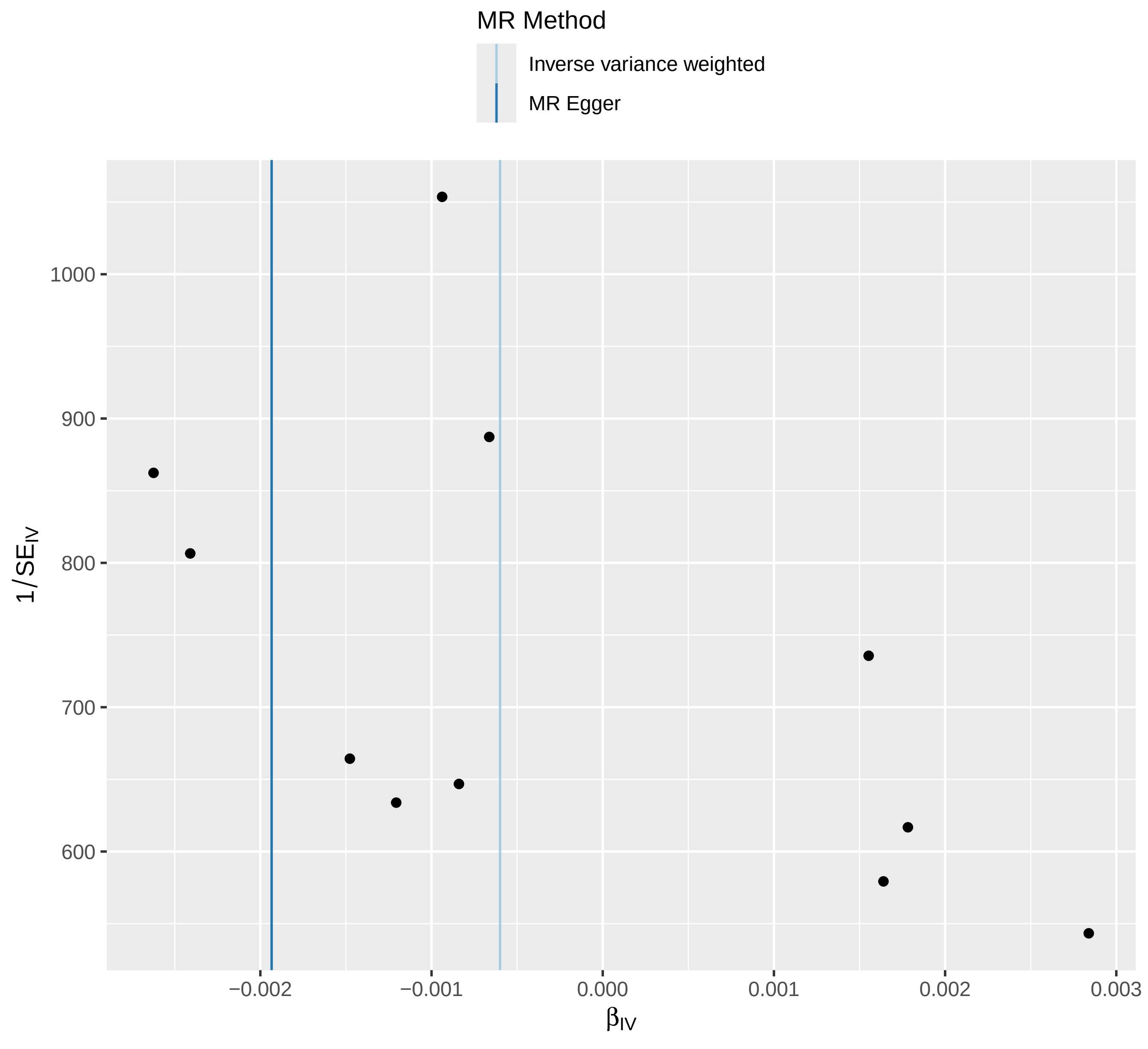
MR Method
Inverse variance weighted
MR Egger
1000
900
800
700
600
1/SE$_{IV}$
−0.002
−0.001
0.000
0.001
0.002
0.003
β$_{IV}$

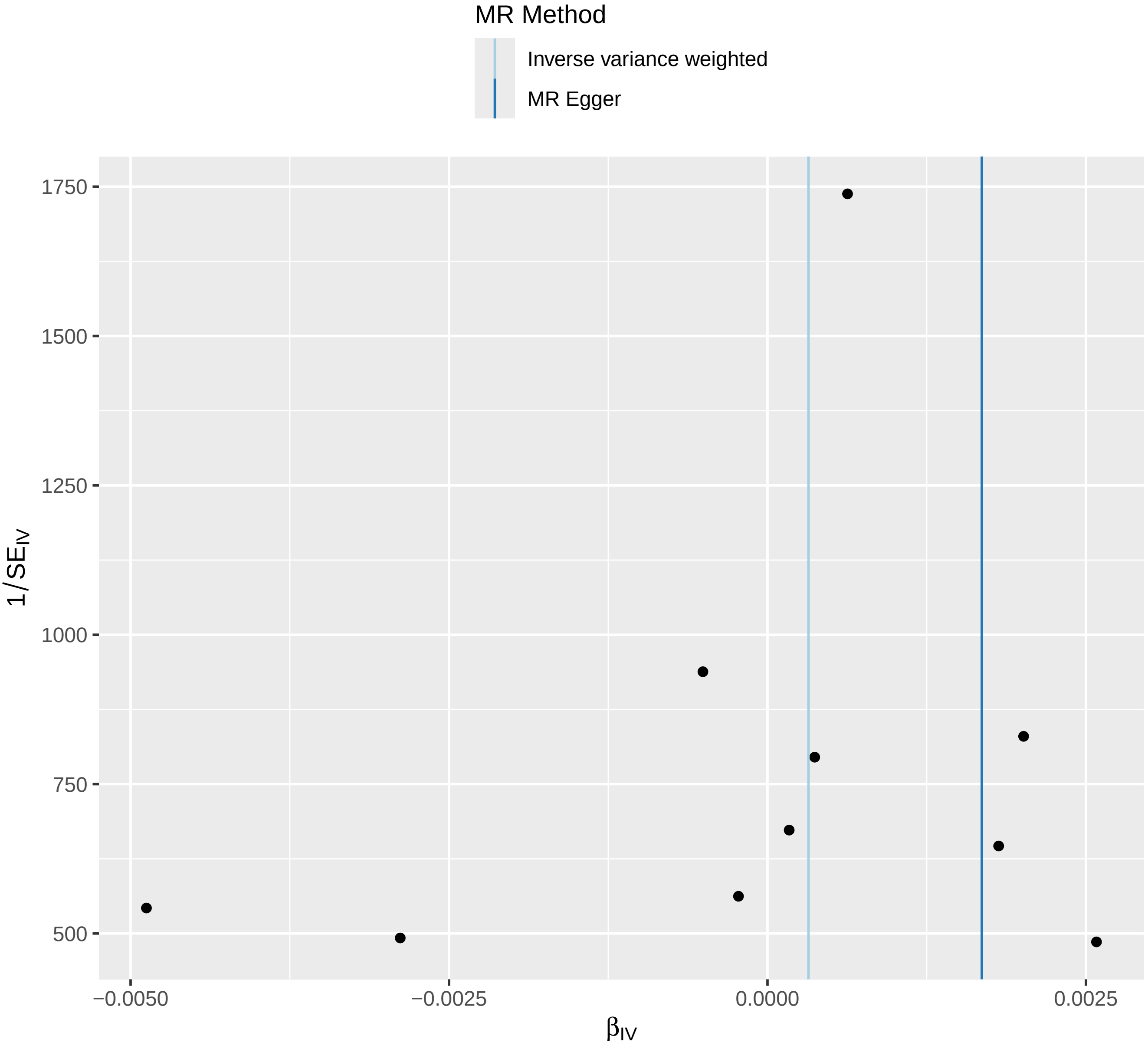

MR Method
Inverse variance weighted
MR Egger
1750
1500
1250
1000
750
500
1/SE$_{IV}$
−0.0050
−0.0025
0.0000
0.0025
β$_{IV}$

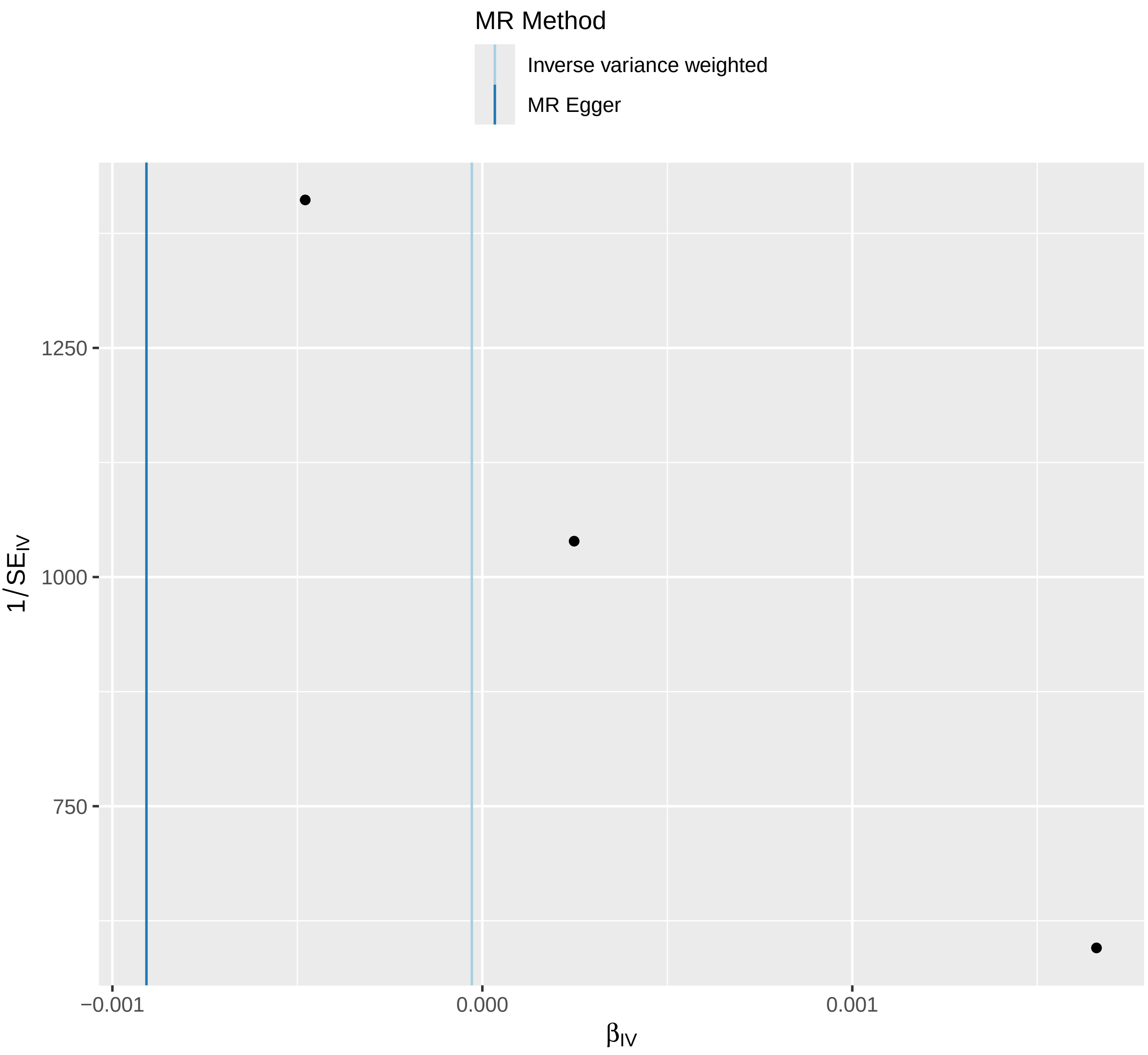

MR Method
Inverse variance weighted
MR Egger
1250
1000
750
$1/SE_{IV}$
−0.001
0.000
0.001
β_{IV}

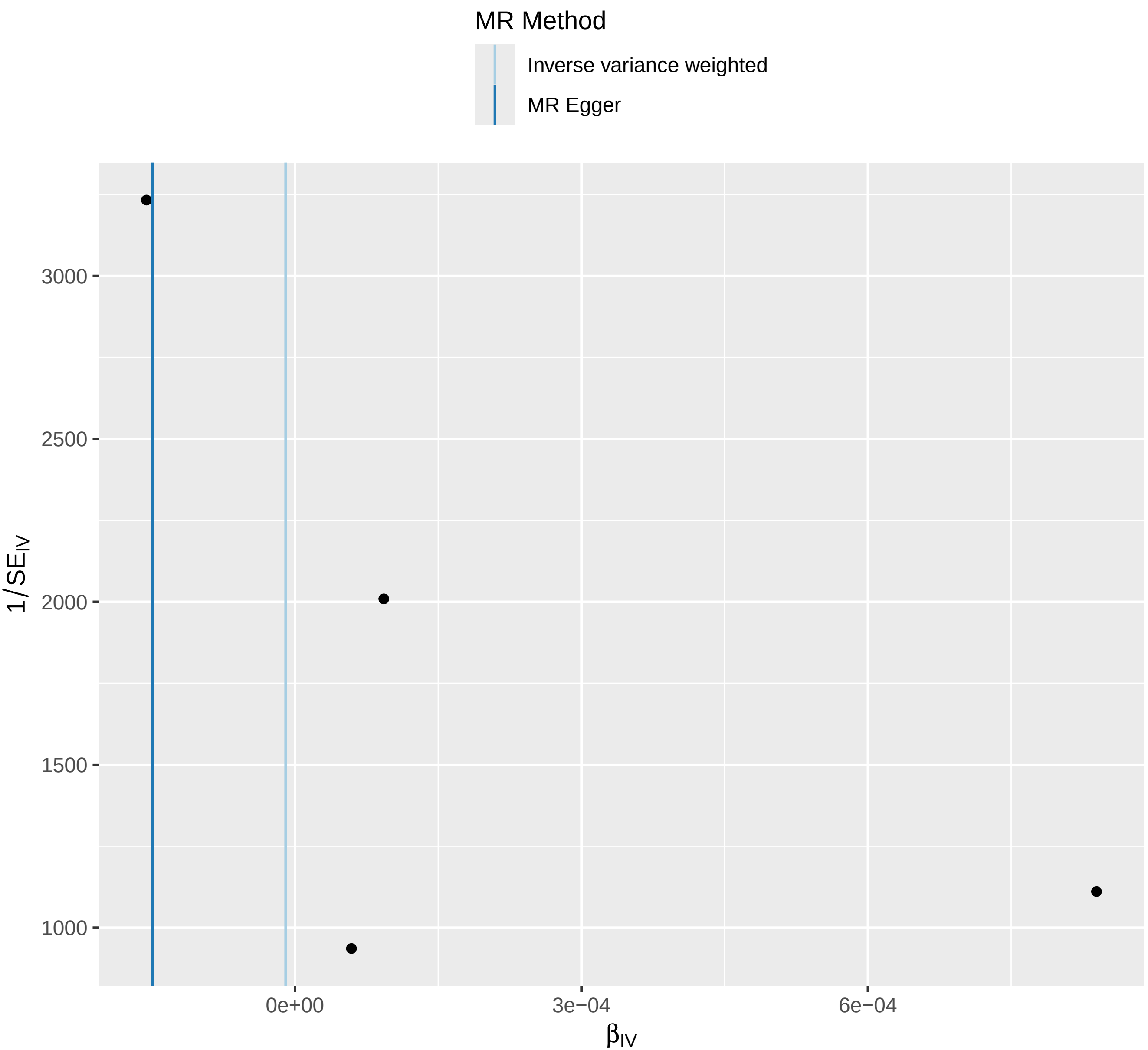
MR Method
Inverse variance weighted
MR Egger
3000
2500
2000
1500
1000
1/SE_IV
0e+00
3e−04
6e−04
β_IV

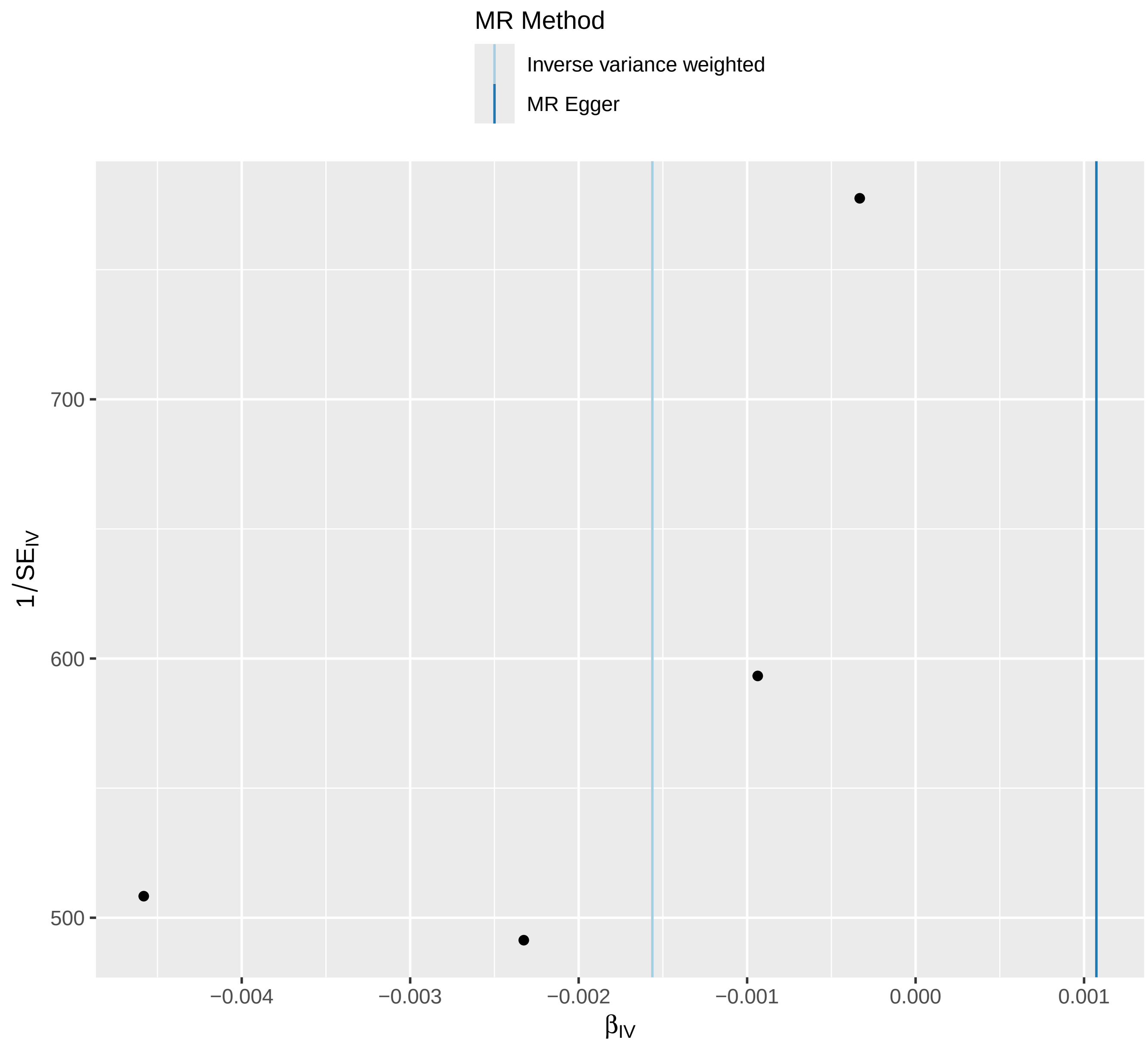

MR Method
Inverse variance weighted
MR Egger
700
600
500
1/SE_IV
−0.004
−0.003
−0.002
−0.001
0.000
0.001
β_IV

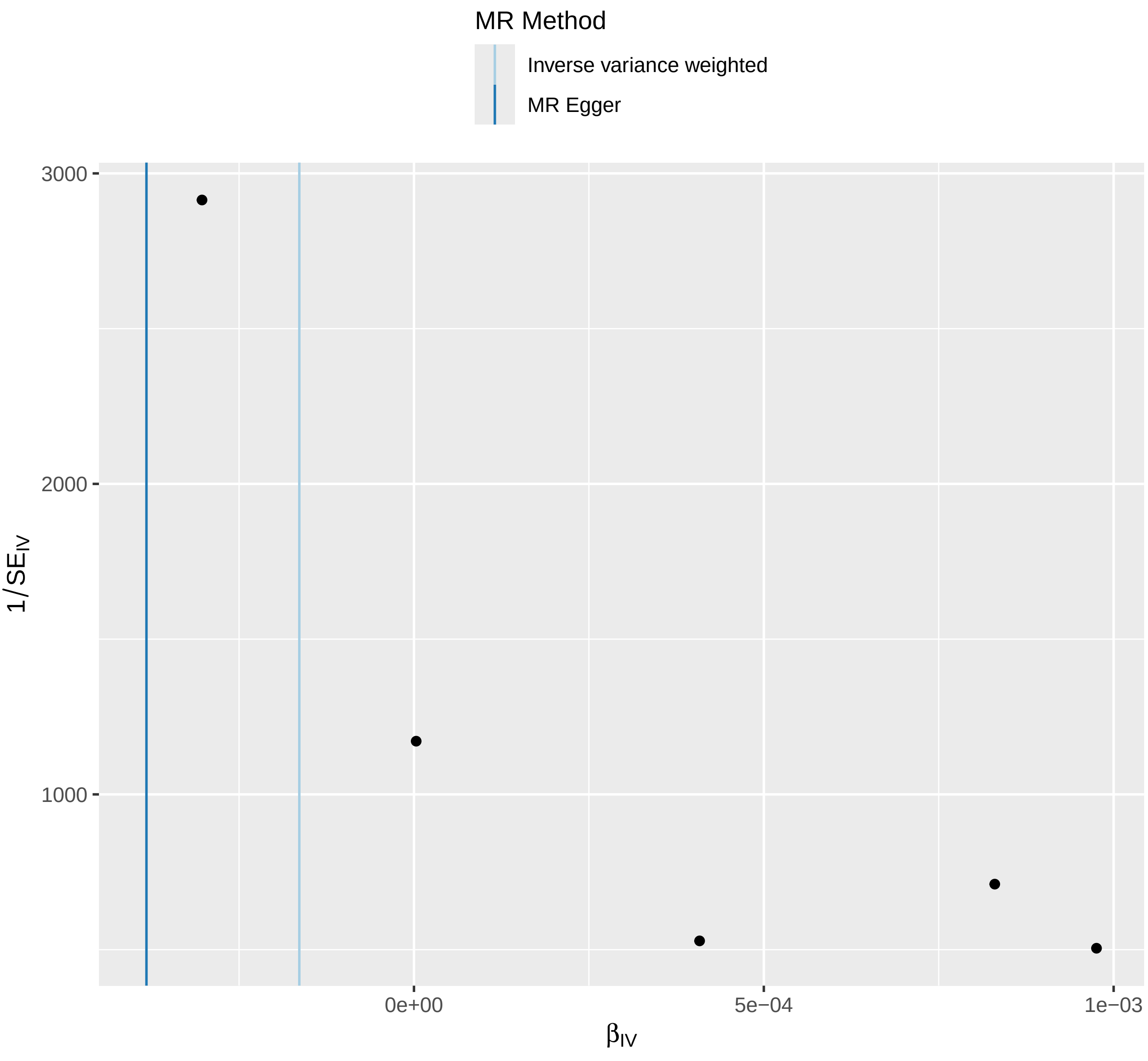
MR Method
Inverse variance weighted
MR Egger
3000
2000
1000
1/SE_IV
0e+00
5e−04
1e−03
β_IV

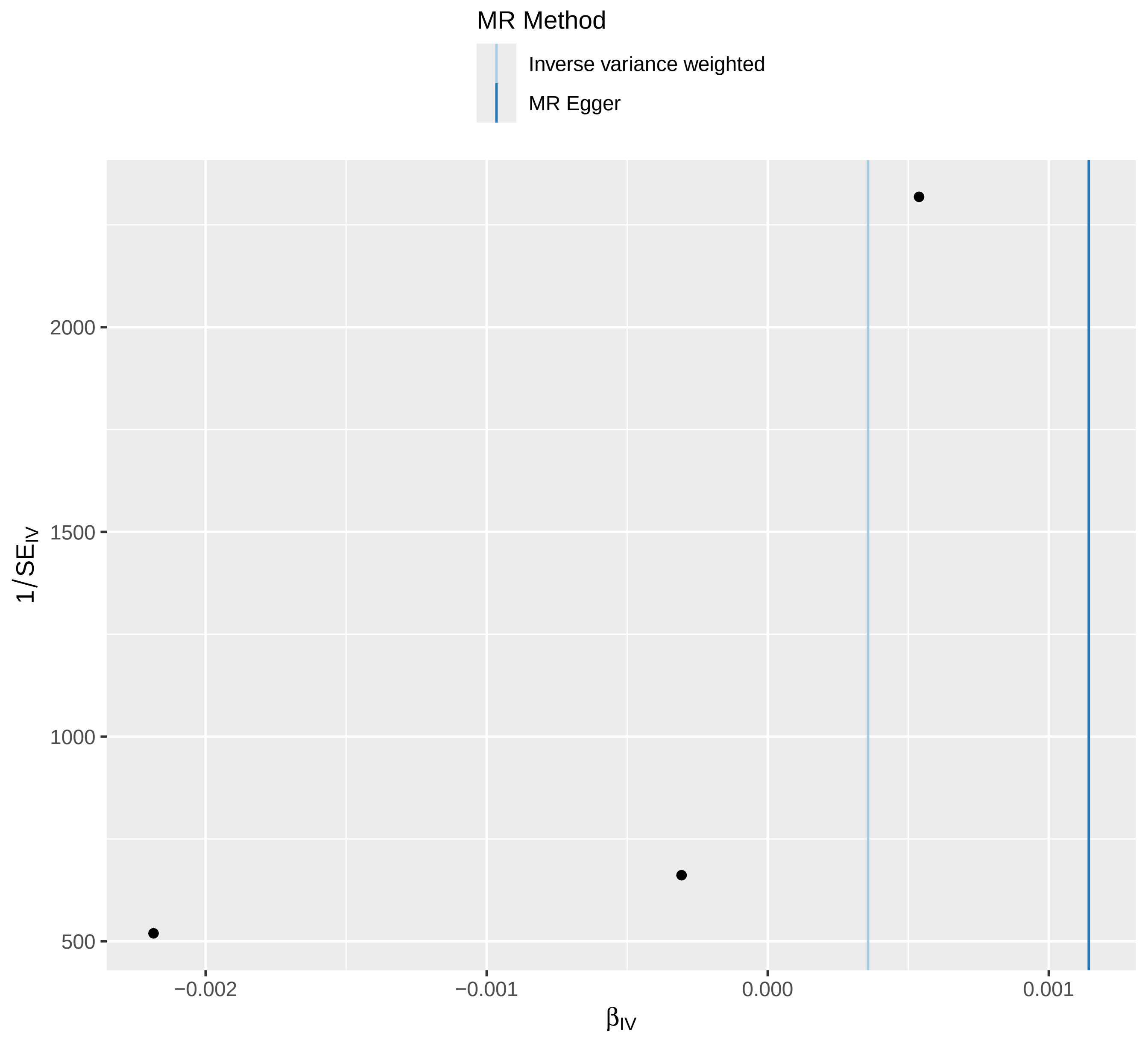

MR Method
Inverse variance weighted
MR Egger
2000
1500
1000
500
1/SE$_{IV}$
−0.002
−0.001
0.000
0.001
β$_{IV}$

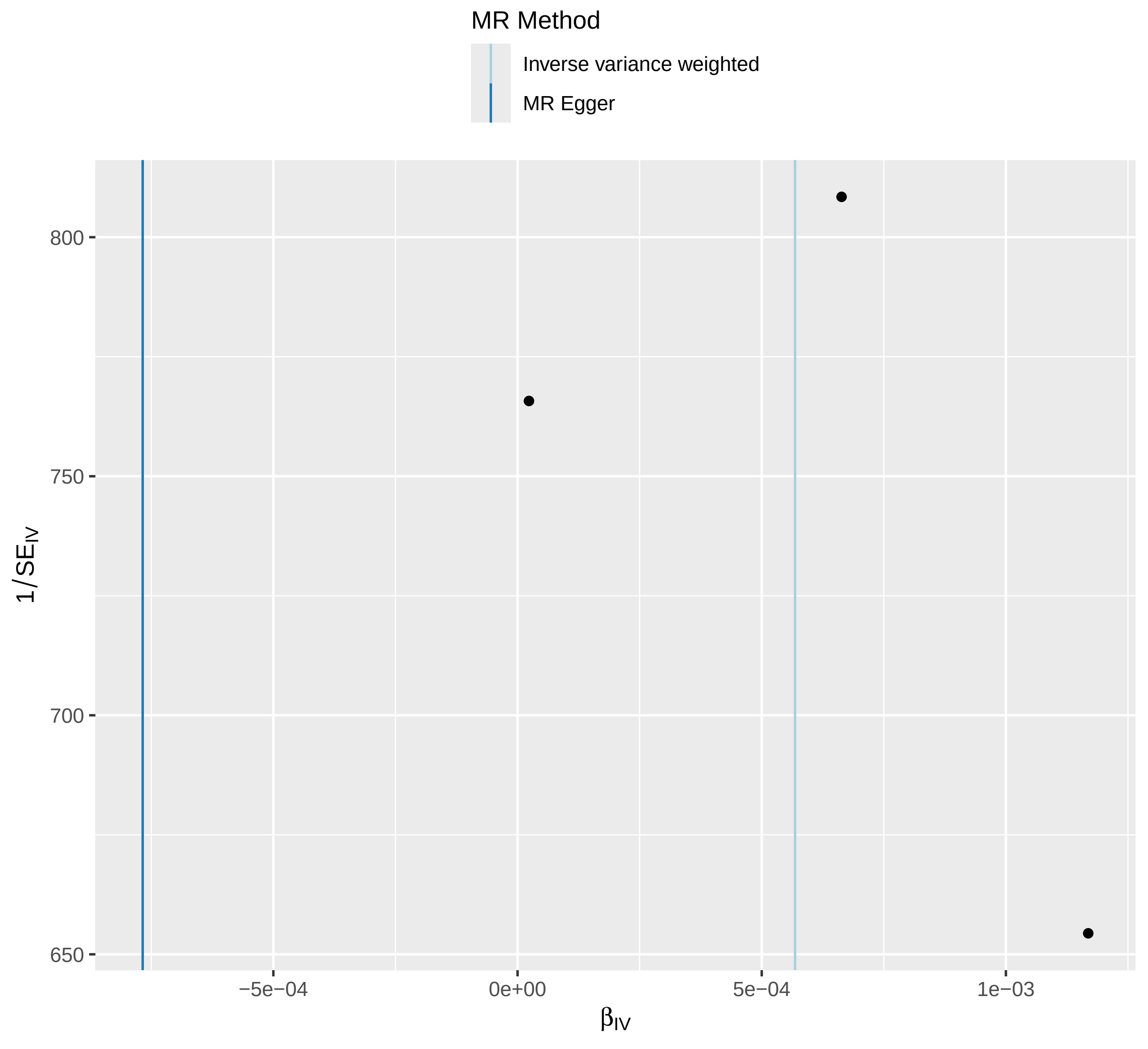
MR Method
Inverse variance weighted
MR Egger
800
750
700
650
1/SE$_{IV}$
−5e−04
0e+00
5e−04
1e−03
β_{IV}

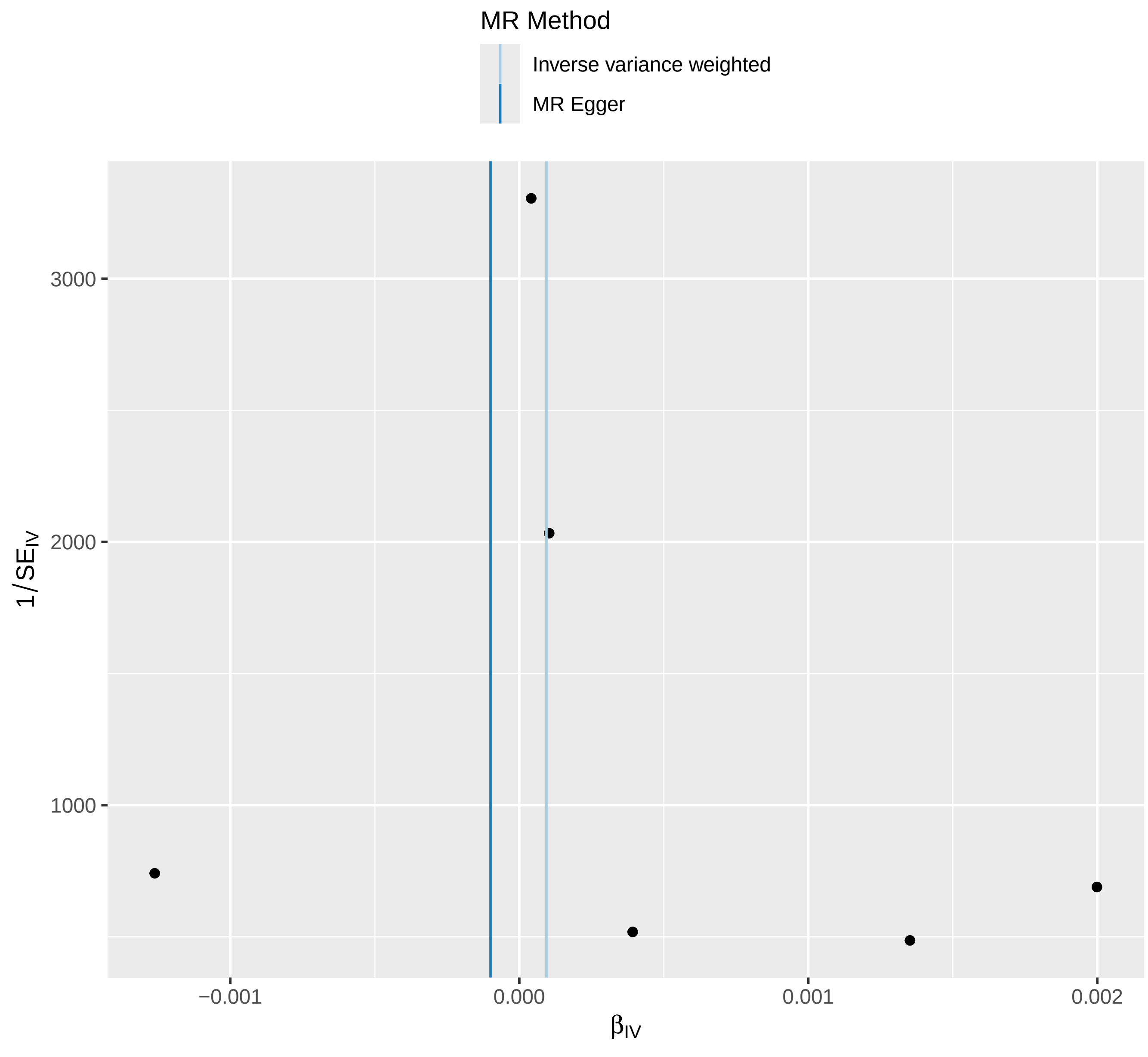

MR Method
Inverse variance weighted
MR Egger
3000
2000
1000
1/SE$_{IV}$
−0.001
0.000
0.001
0.002
β$_{IV}$

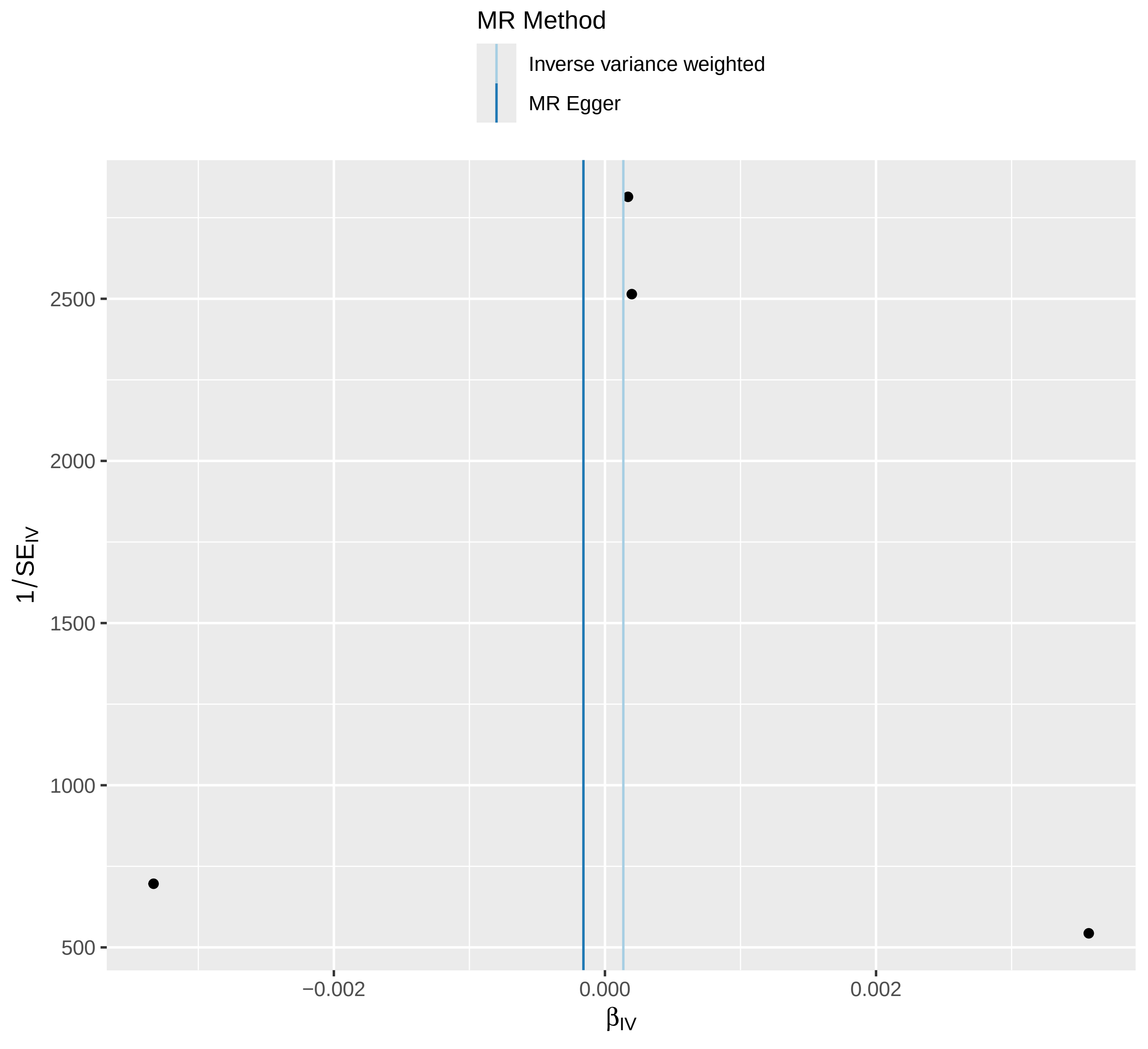

MR Method
Inverse variance weighted
MR Egger
1/SE$_{IV}$
2500
2000
1500
1000
500
−0.002
0.000
0.002
β$_{IV}$

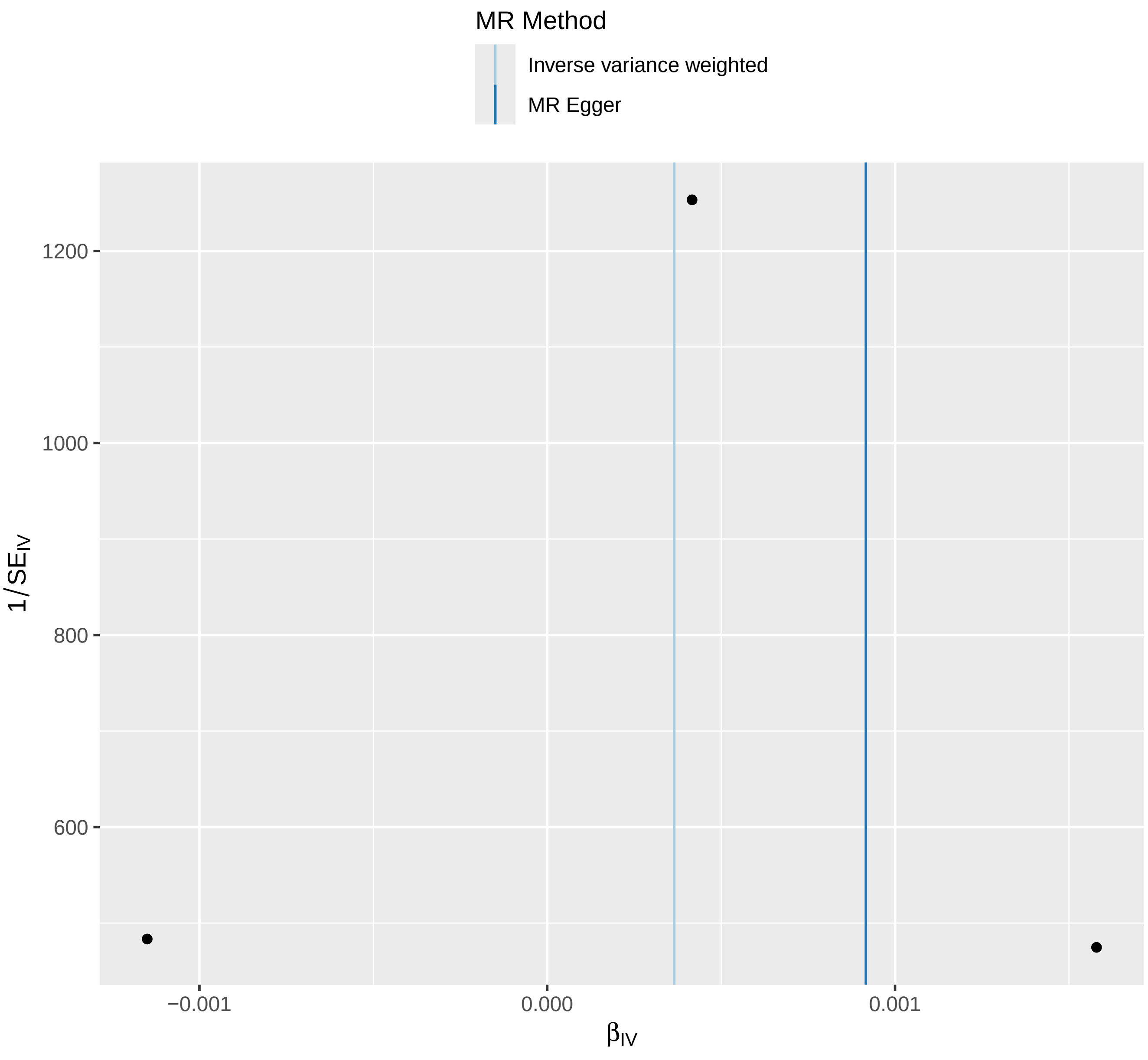

MR Method
Inverse variance weighted
MR Egger
1200
1000
800
600
1/SE_IV
−0.001
0.000
0.001
β_IV

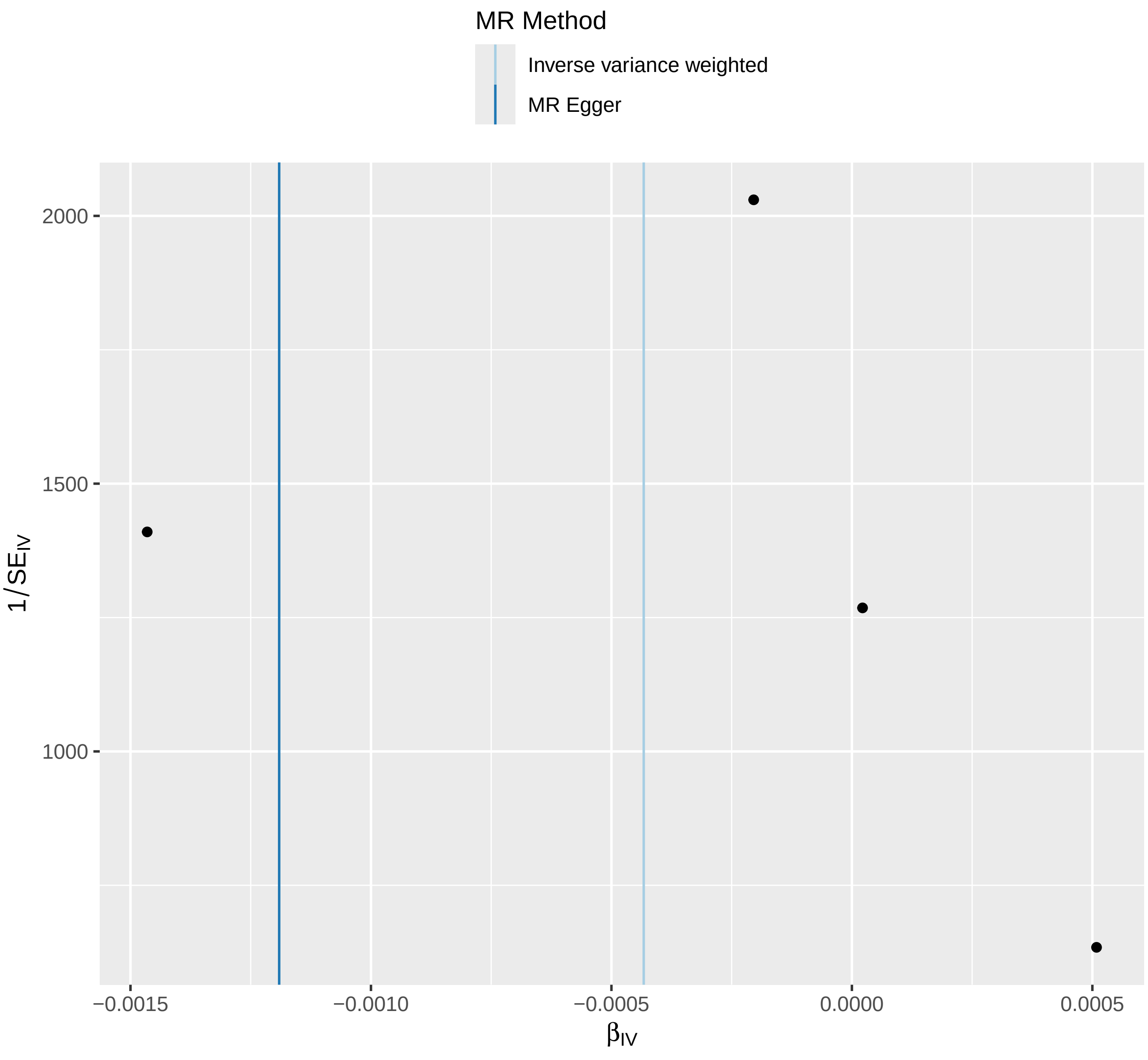

MR Method
Inverse variance weighted
MR Egger
2000
1500
1000
1/SE$_{IV}$
−0.0015
−0.0010
−0.0005
0.0000
0.0005
β$_{IV}$

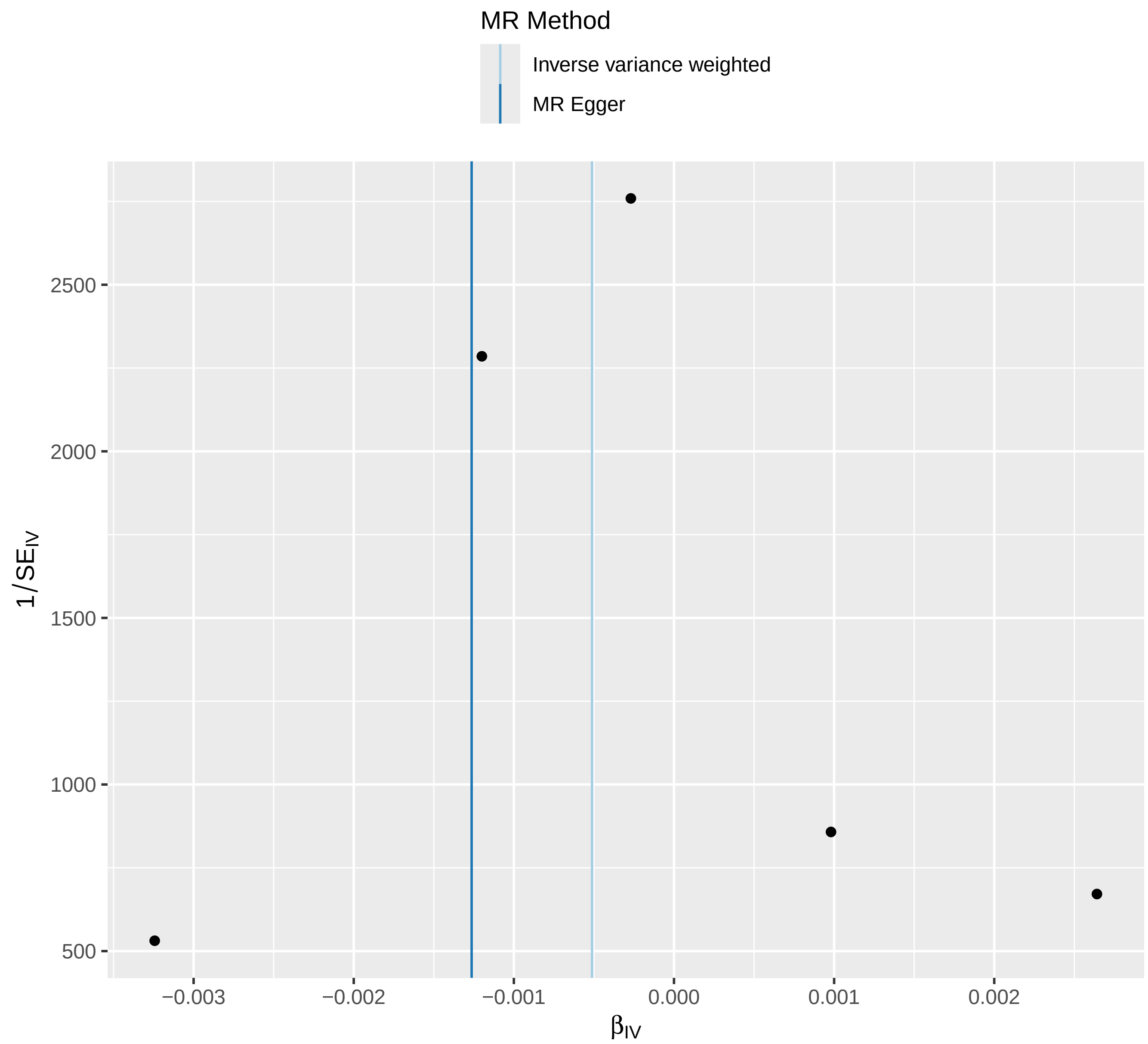
MR Method
Inverse variance weighted
MR Egger
2500
2000
1500
1000
500
1/SE$_{IV}$
−0.003
−0.002
−0.001
0.000
0.001
0.002
β$_{IV}$

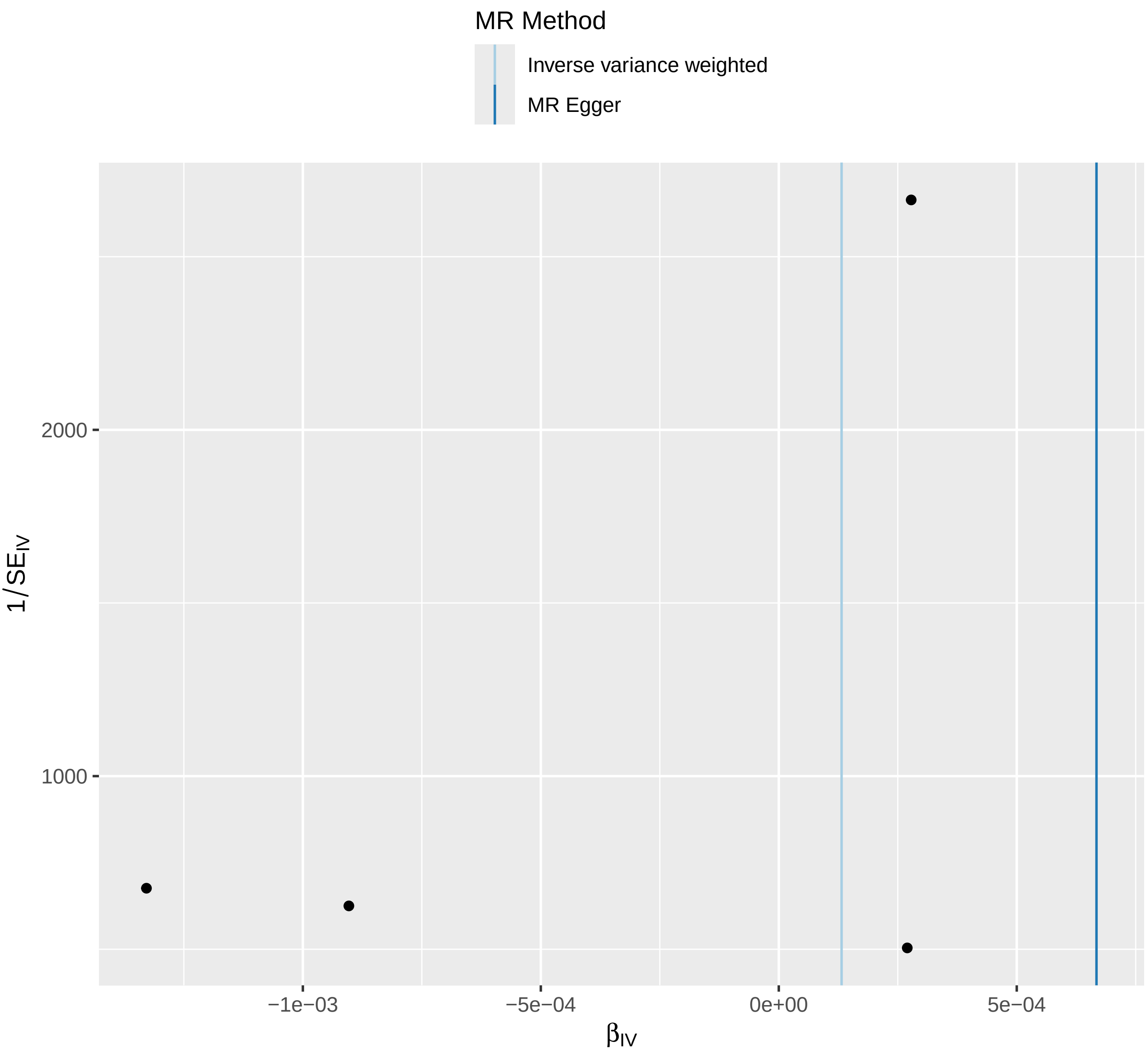
MR Method
Inverse variance weighted
MR Egger
2000
1000
1/SE$_{IV}$
−1e−03
−5e−04
0e+00
5e−04
β_{IV}

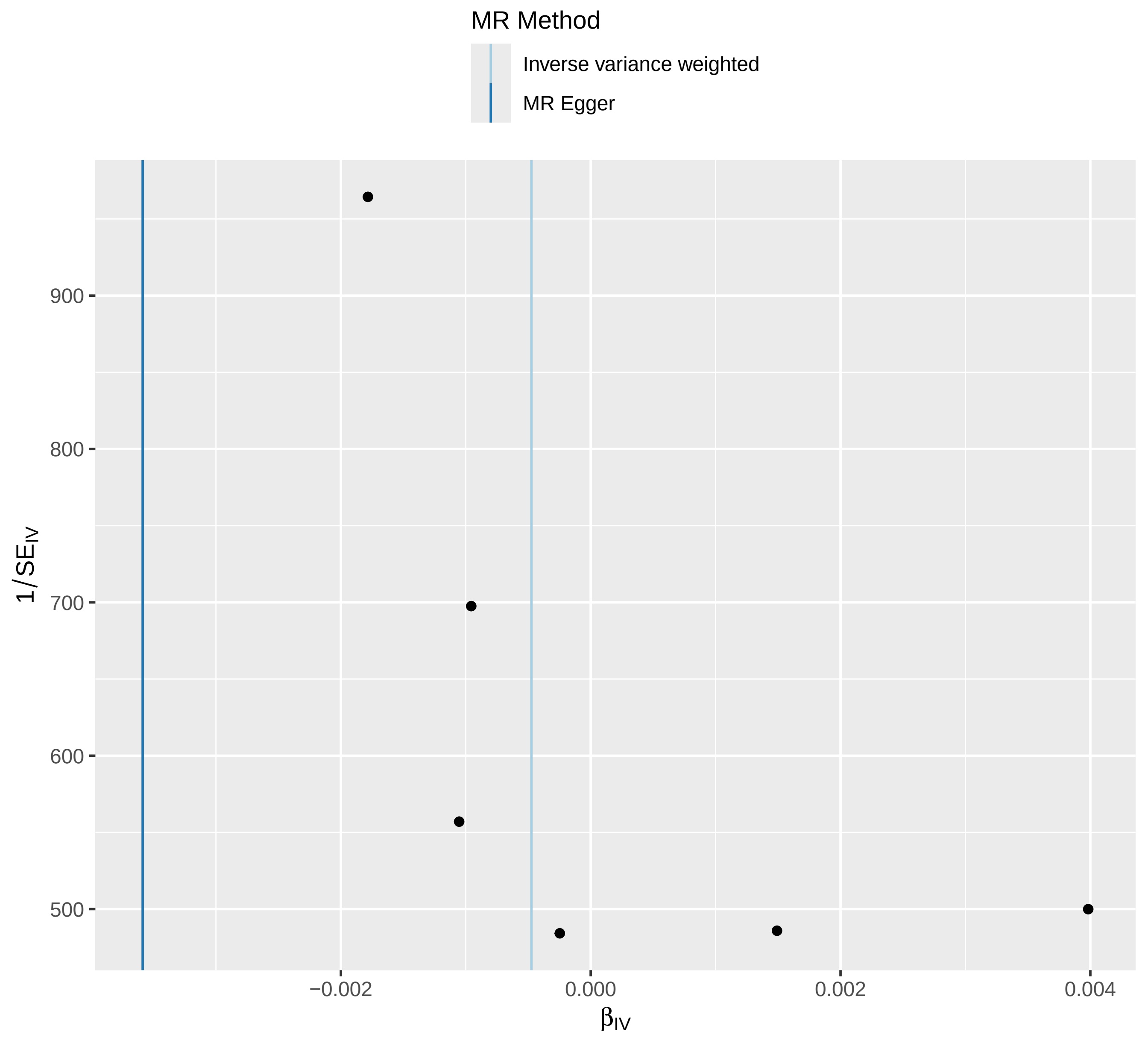
MR Method
Inverse variance weighted
MR Egger
900
800
700
600
500
−0.002
0.000
0.002
0.004
$1/SE_{IV}$
β_{IV}

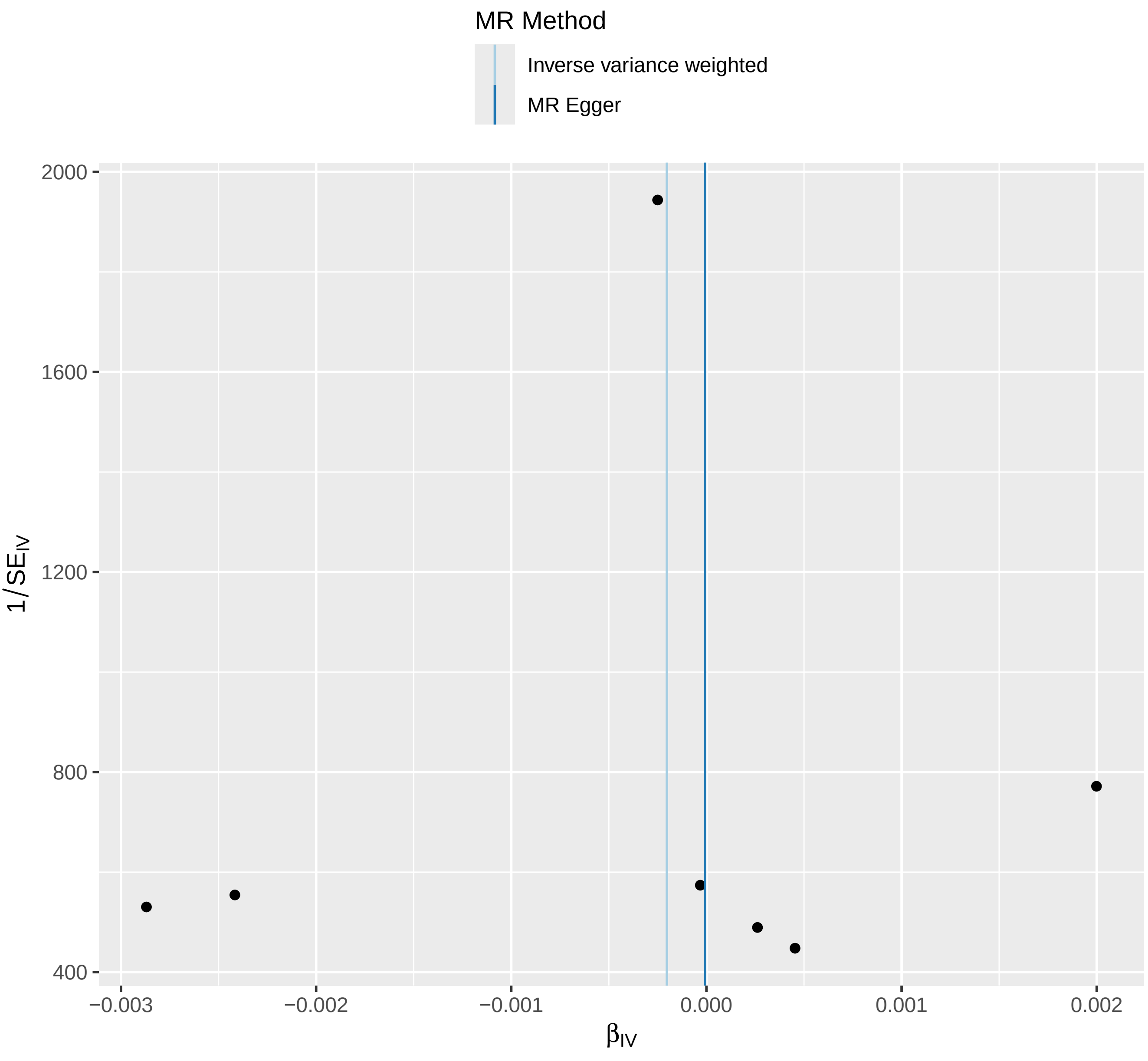
MR Method
Inverse variance weighted
MR Egger
2000
1600
1200
800
400
1/SE_IV
−0.003
−0.002
−0.001
0.000
0.001
0.002
β_IV

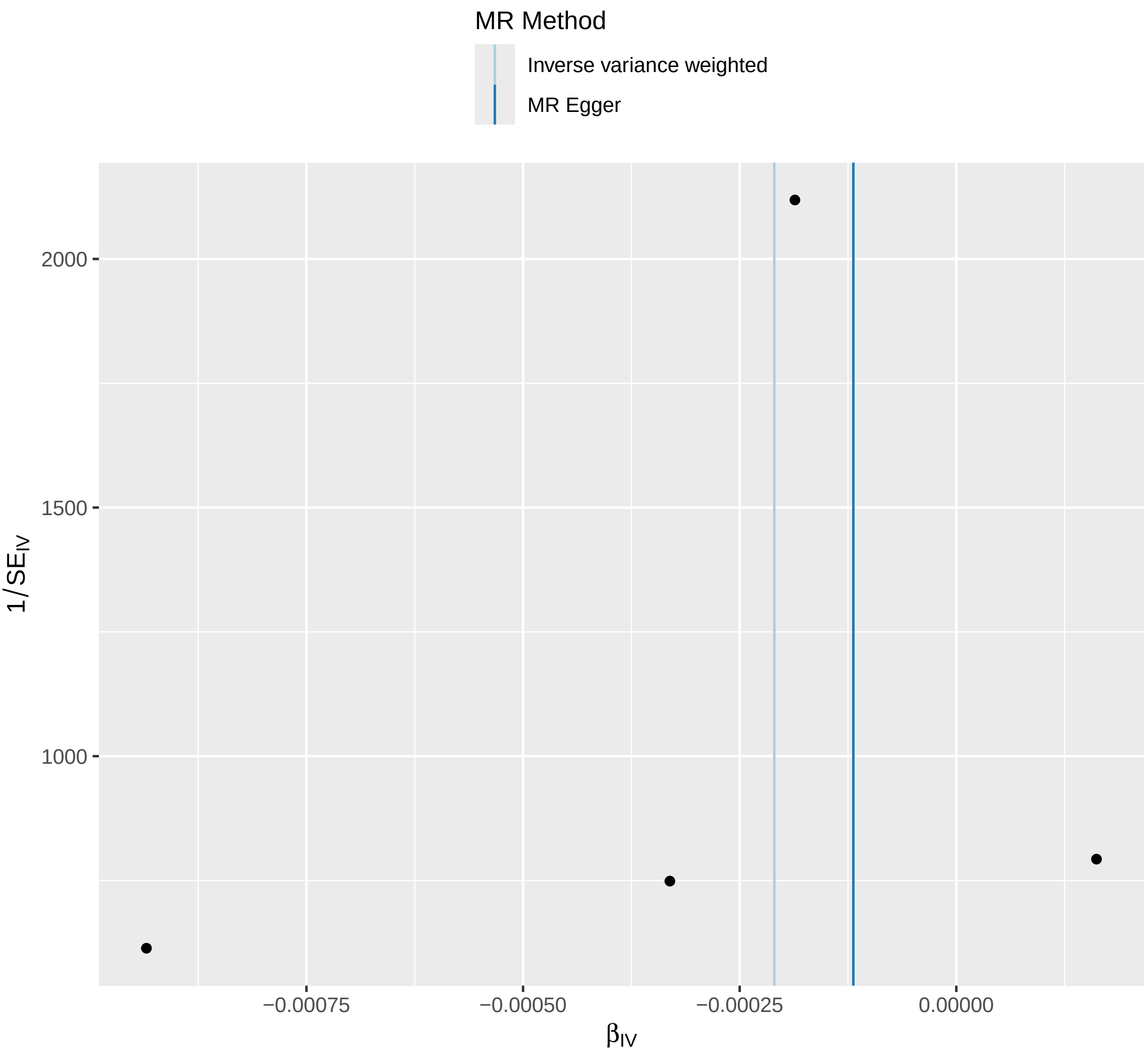

MR Method
Inverse variance weighted
MR Egger
2000
1500
1000
1/SE$_{IV}$
−0.00075
−0.00050
−0.00025
0.00000
β$_{IV}$

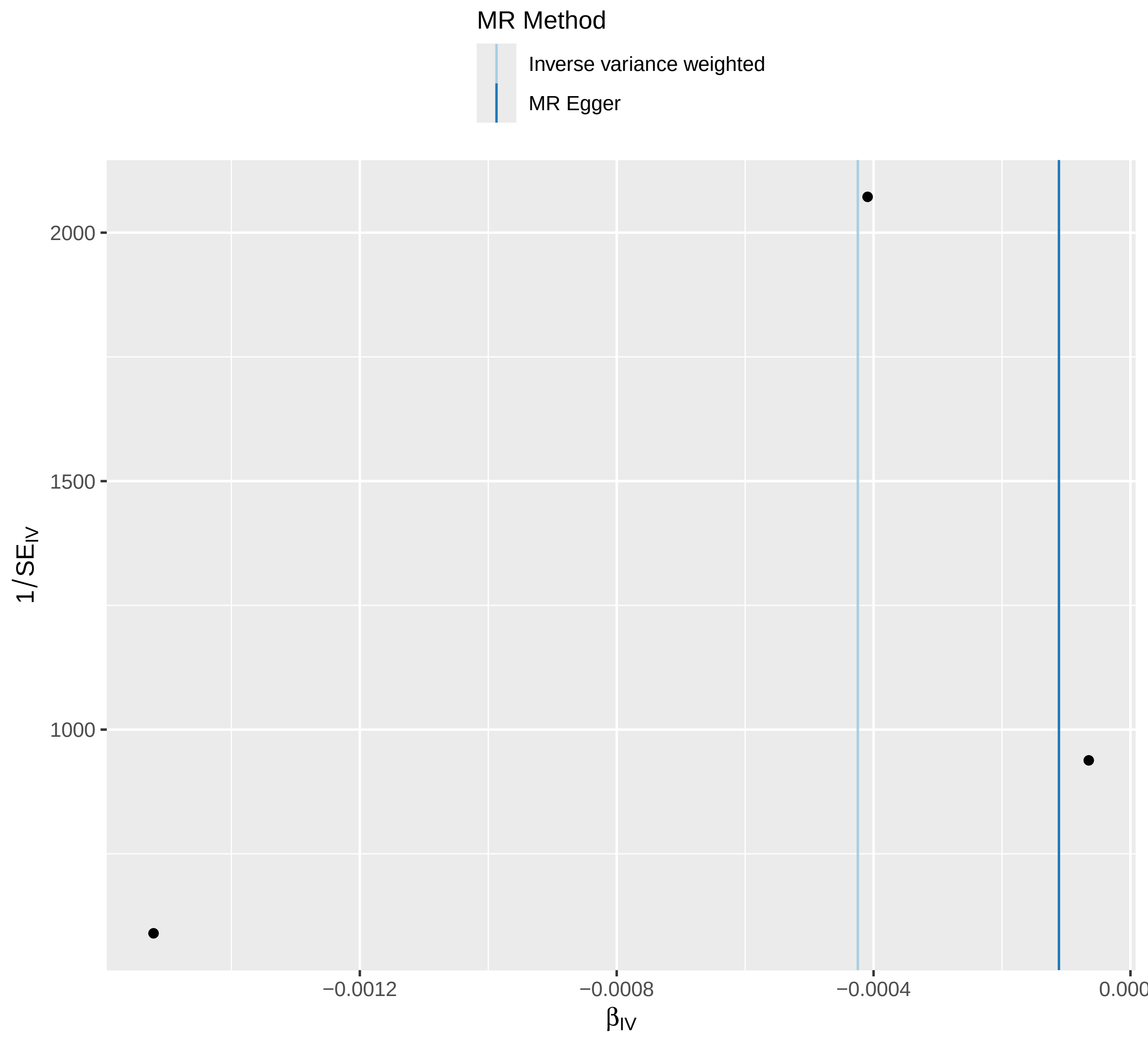

MR Method
Inverse variance weighted
MR Egger
2000
1500
1000
1/SE_IV
−0.0012
−0.0008
−0.0004
0.000
β_IV

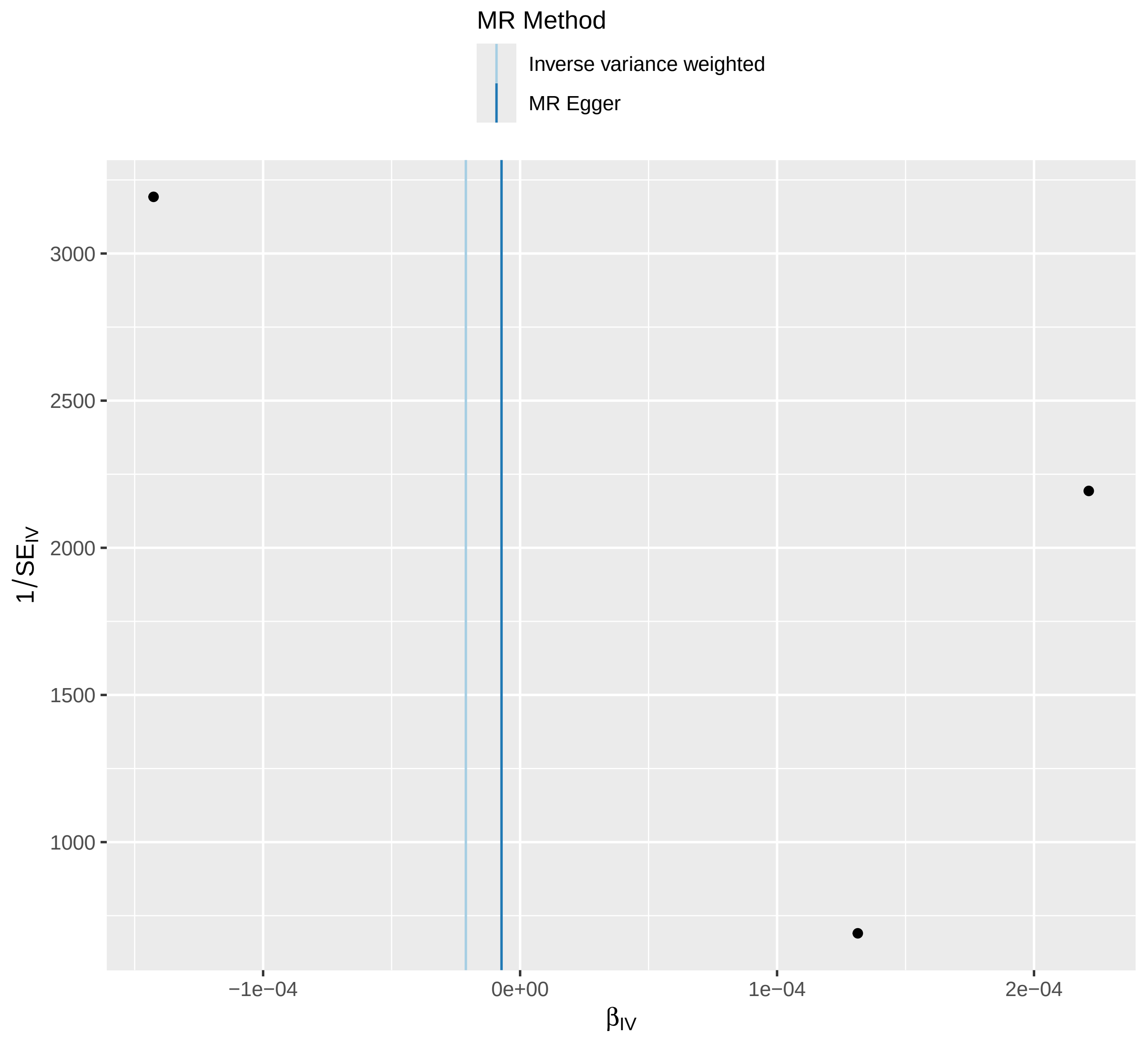

MR Method
Inverse variance weighted
MR Egger
3000
2500
2000
1500
1000
1/SE_IV
−1e−04
0e+00
1e−04
2e−04
β_IV

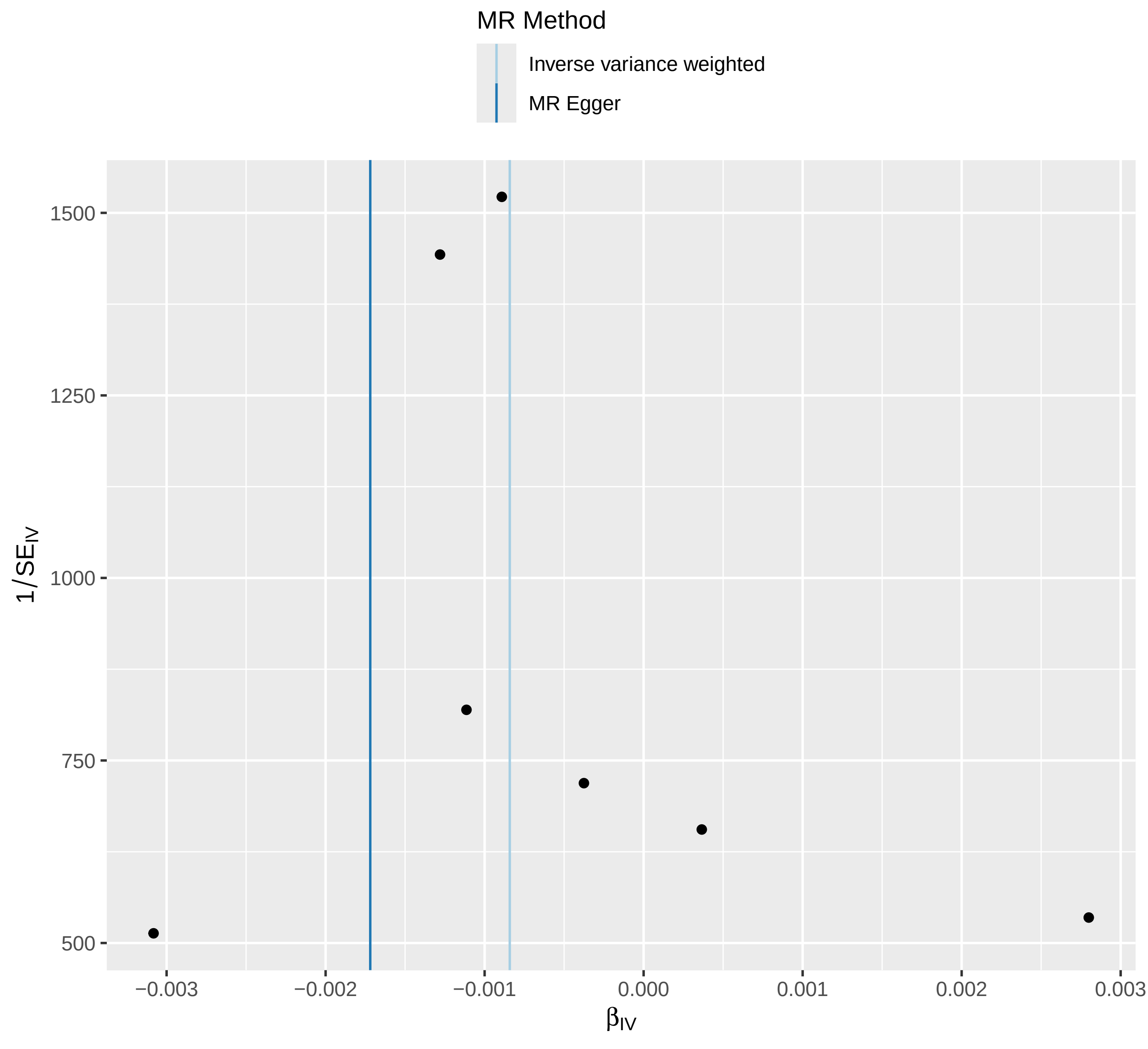

MR Method
Inverse variance weighted
MR Egger
1500
1250
1000
750
500
1/SE_IV
−0.003
−0.002
−0.001
0.000
0.001
0.002
0.003
β_IV

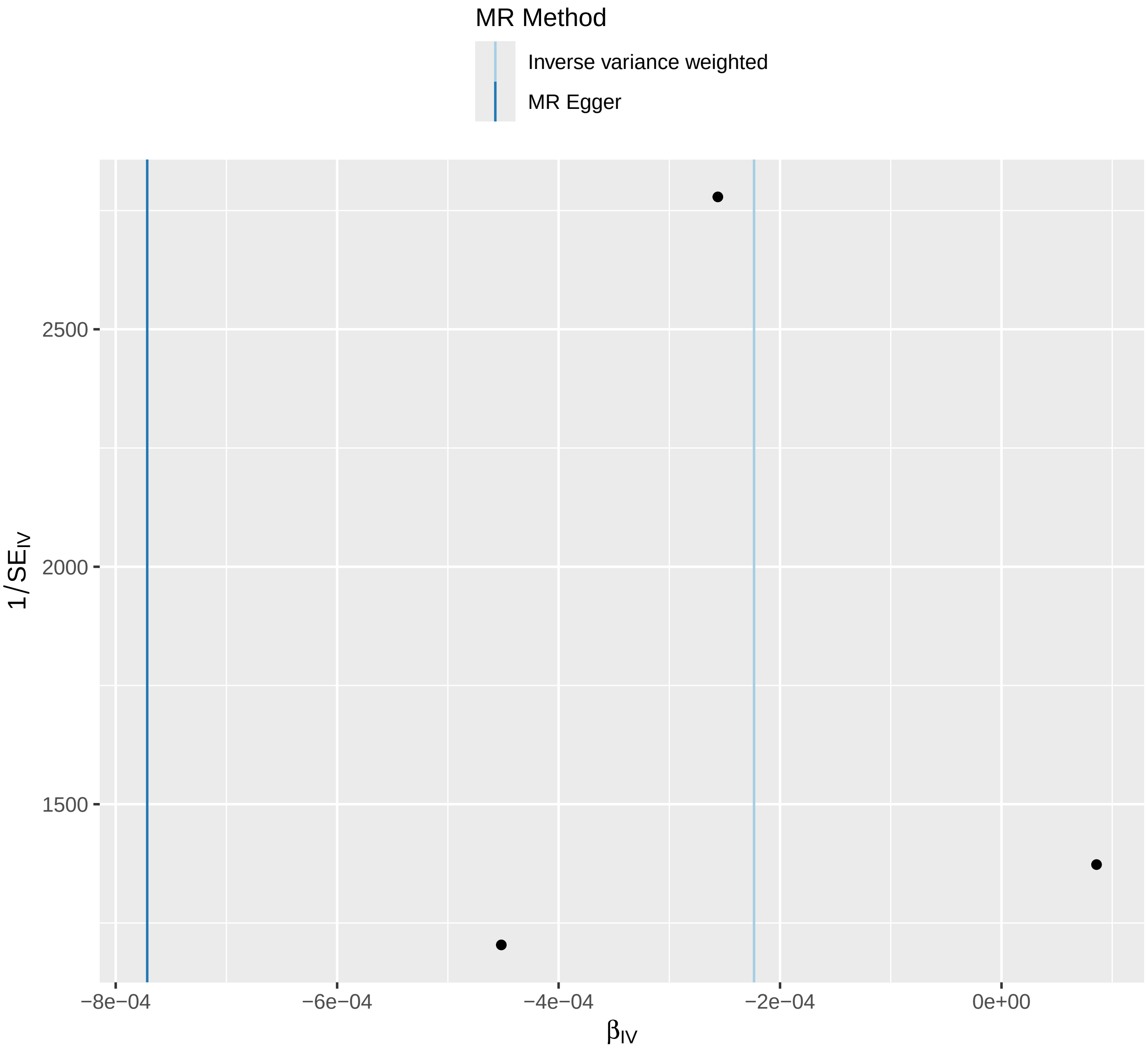
MR Method
Inverse variance weighted
MR Egger
2500
2000
1500
1/SE$_{IV}$
−8e−04
−6e−04
−4e−04
−2e−04
0e+00
β$_{IV}$

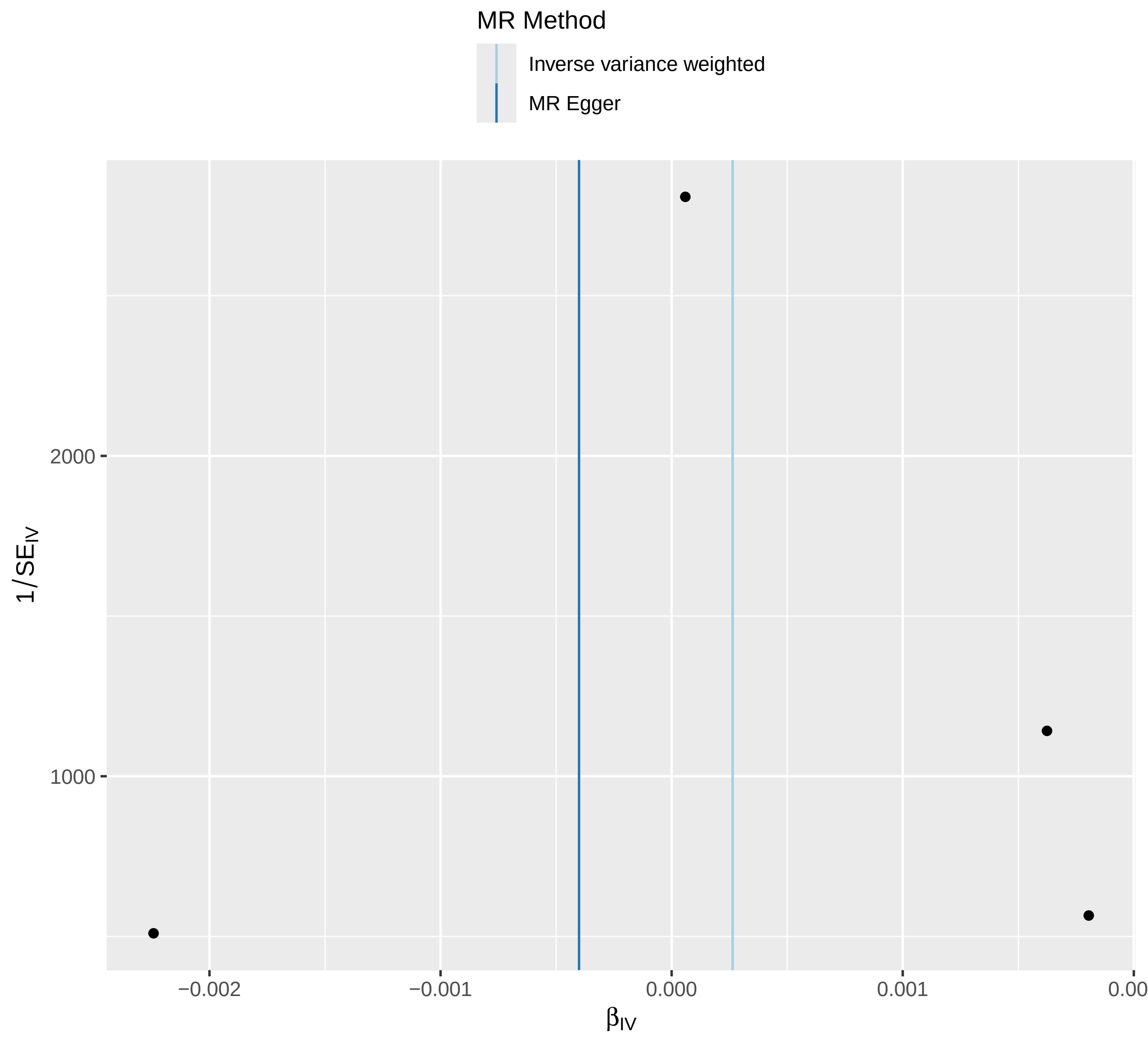

MR Method
Inverse variance weighted
MR Egger
2000
1000
1/SE$_{IV}$
−0.002
−0.001
0.000
0.001
0.00
β$_{IV}$

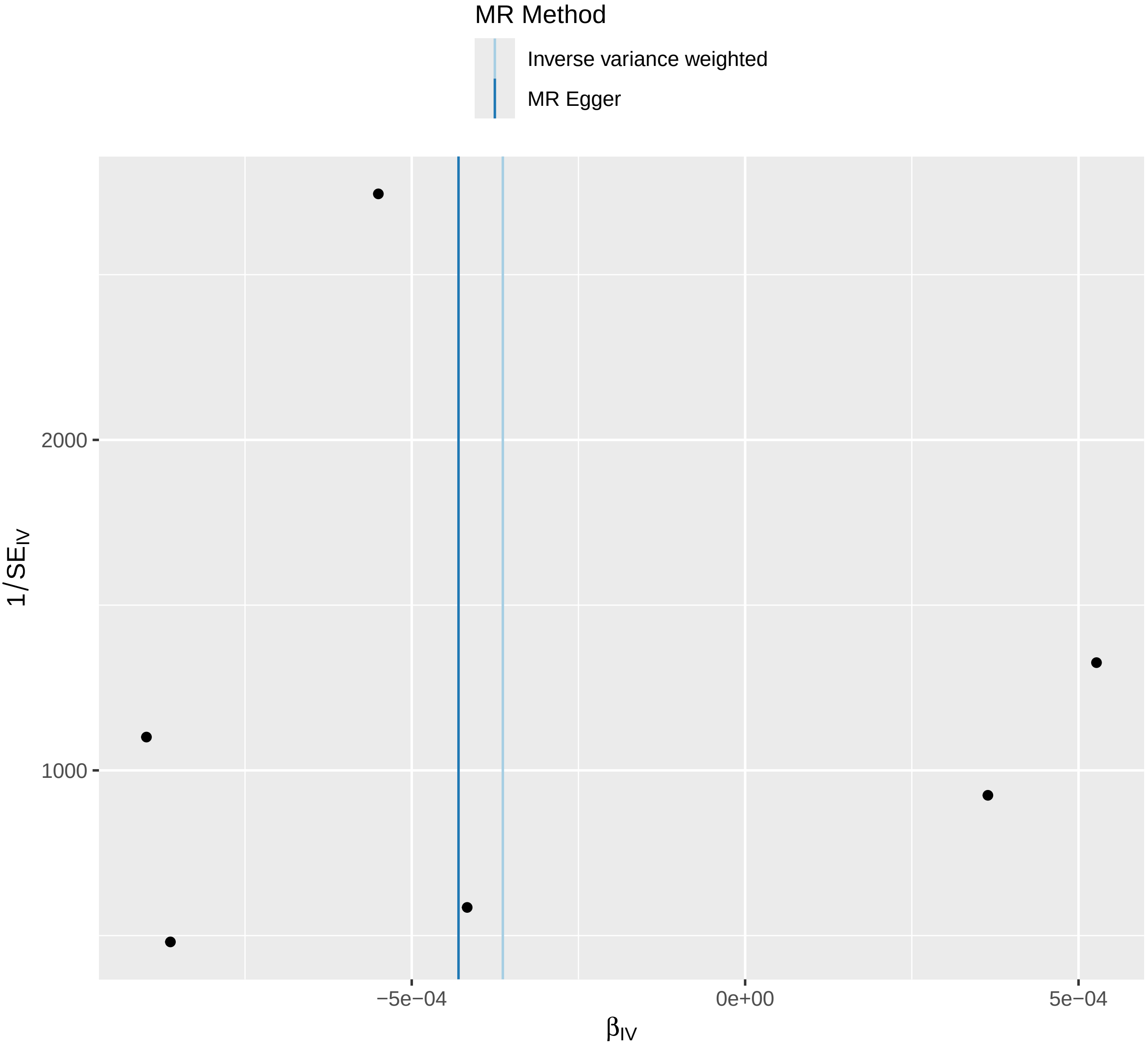

MR Method
Inverse variance weighted
MR Egger
2000
1000
$1/SE_{IV}$
−5e−04
0e+00
5e−04
β_{IV}

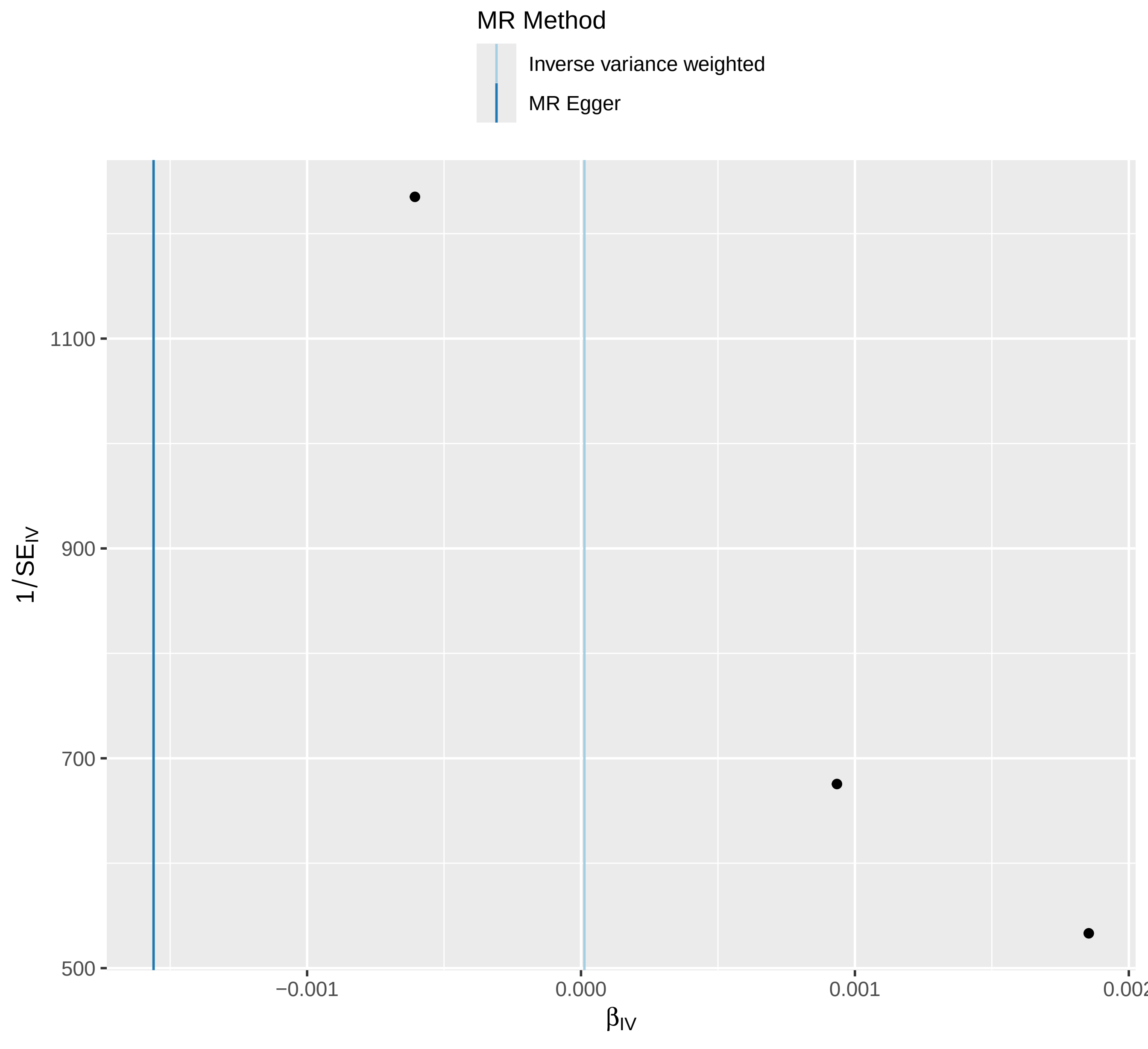

MR Method
Inverse variance weighted
MR Egger
1100
900
700
500
−0.001
0.000
0.001
0.002
β_{IV}
$1/SE_{IV}$

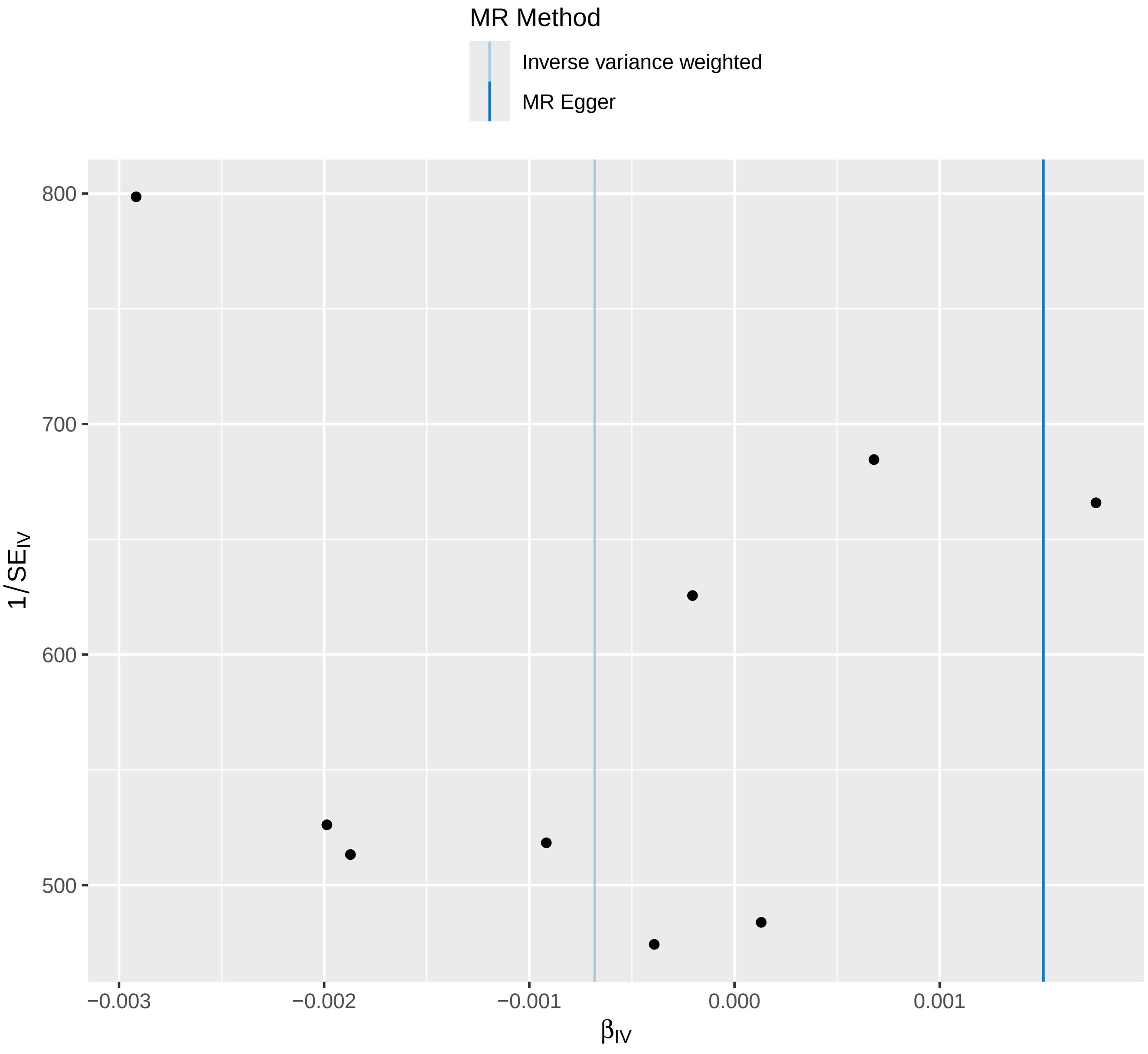
MR Method
Inverse variance weighted
MR Egger
800
700
600
500
1/SE_IV
−0.003
−0.002
−0.001
0.000
0.001
β_IV

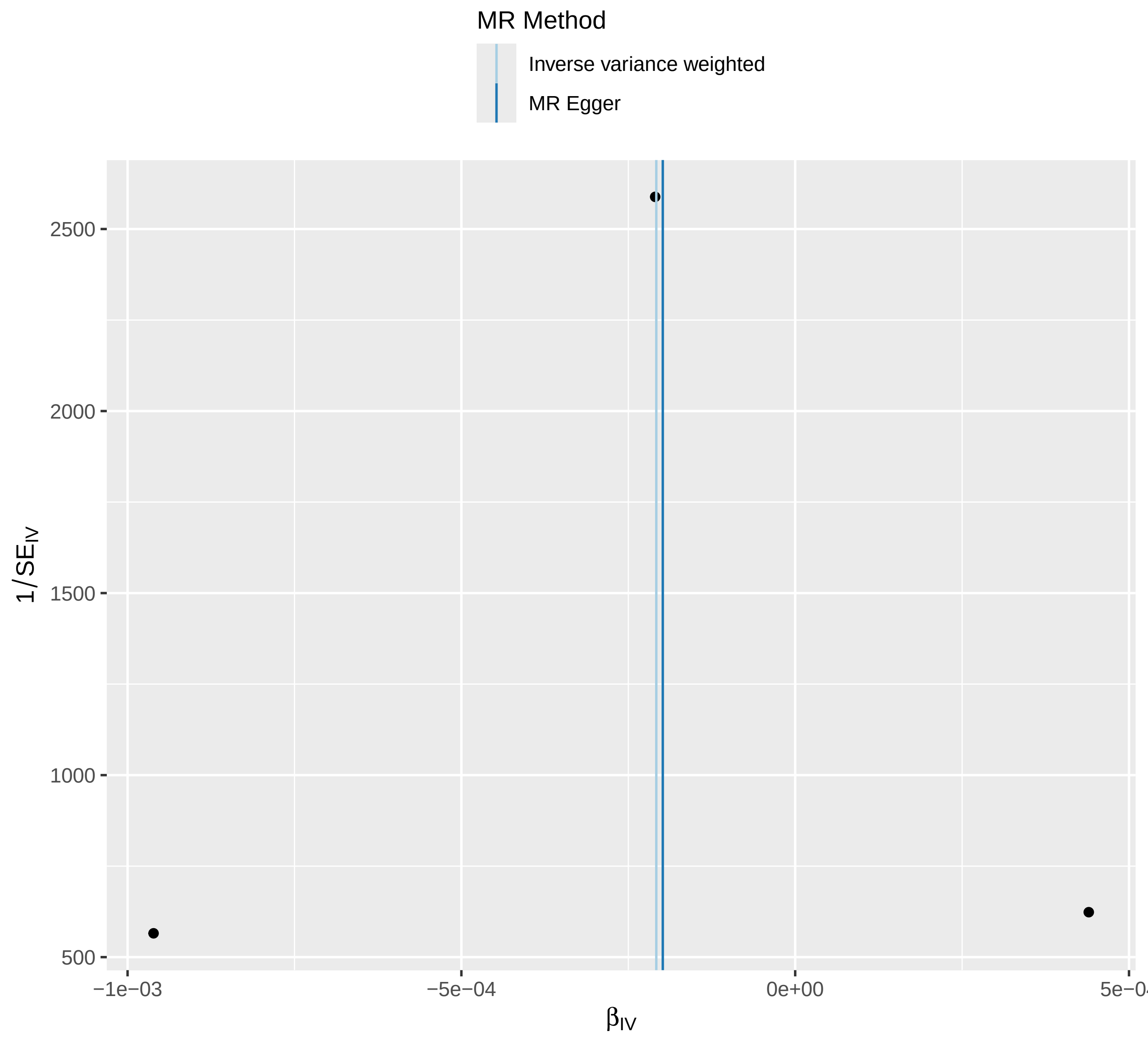

MR Method
Inverse variance weighted
MR Egger
2500
2000
1500
1000
500
$1/SE_{IV}$
−1e−03
−5e−04
0e+00
5e−04
β_{IV}

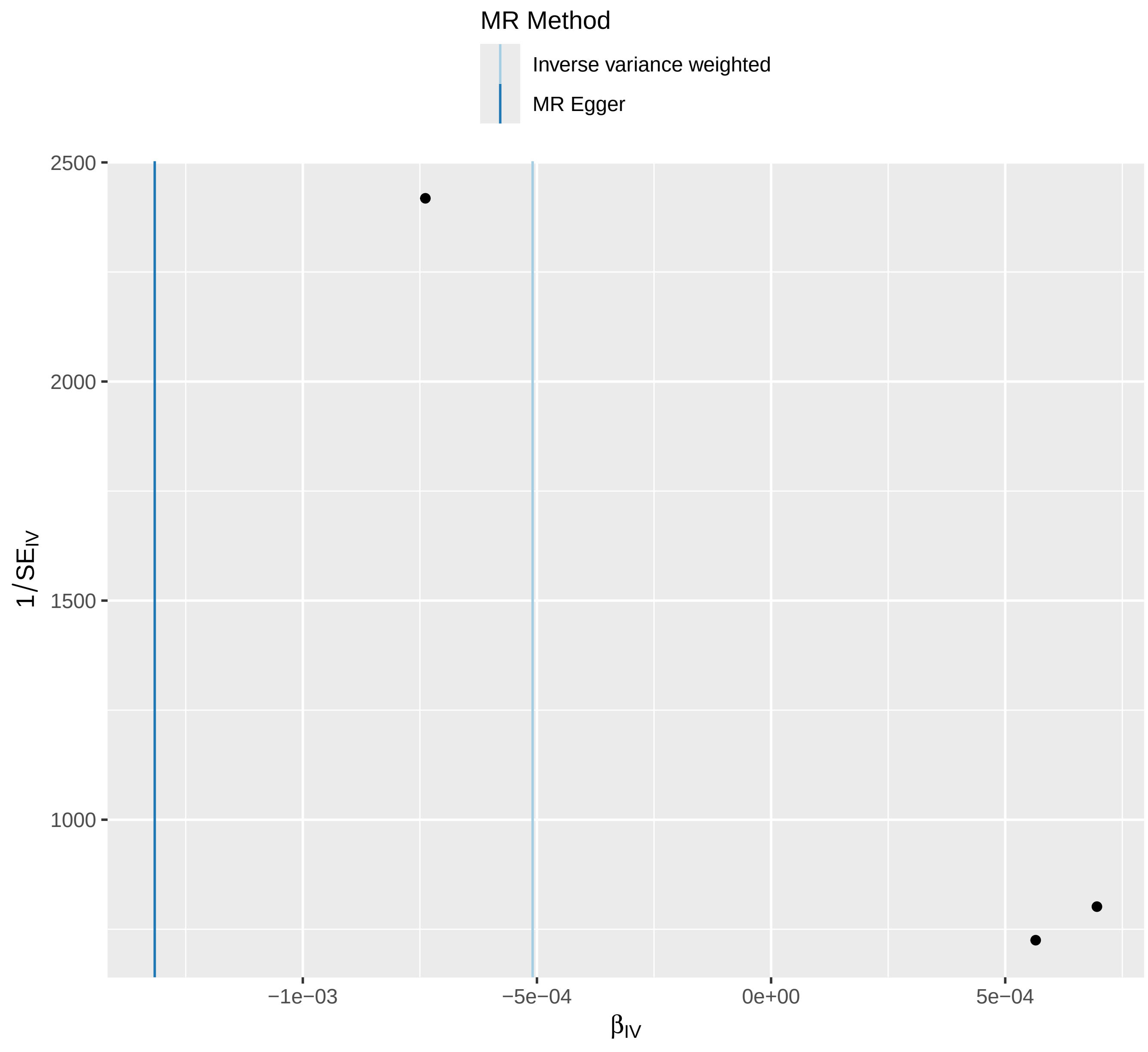
MR Method
Inverse variance weighted
MR Egger
2500
2000
1500
1000
1/SE_IV
−1e−03
−5e−04
0e+00
5e−04
β_IV

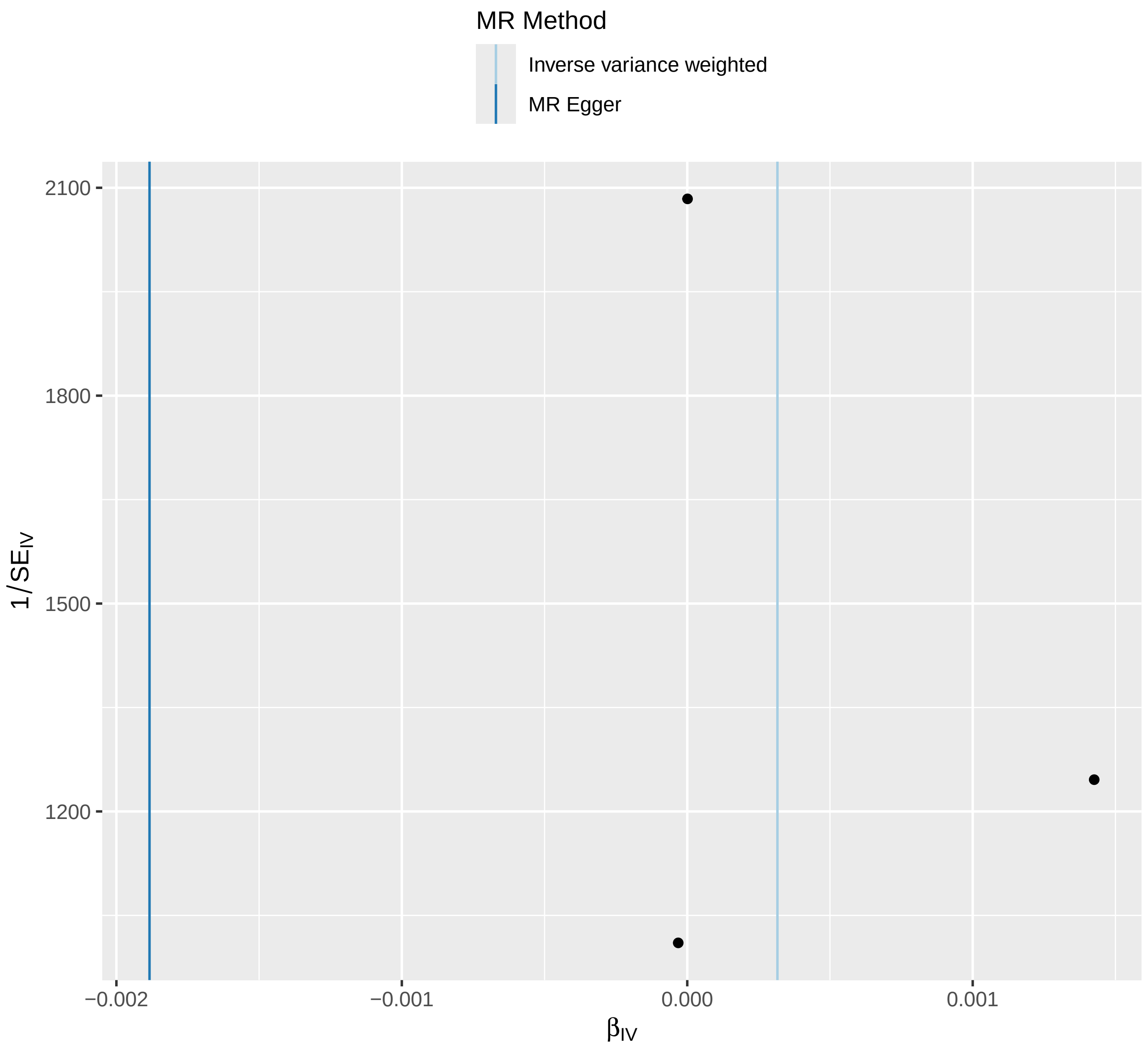

MR Method
Inverse variance weighted
MR Egger
2100
1800
1500
1200
1/SE$_{IV}$
−0.002
−0.001
0.000
0.001
β$_{IV}$

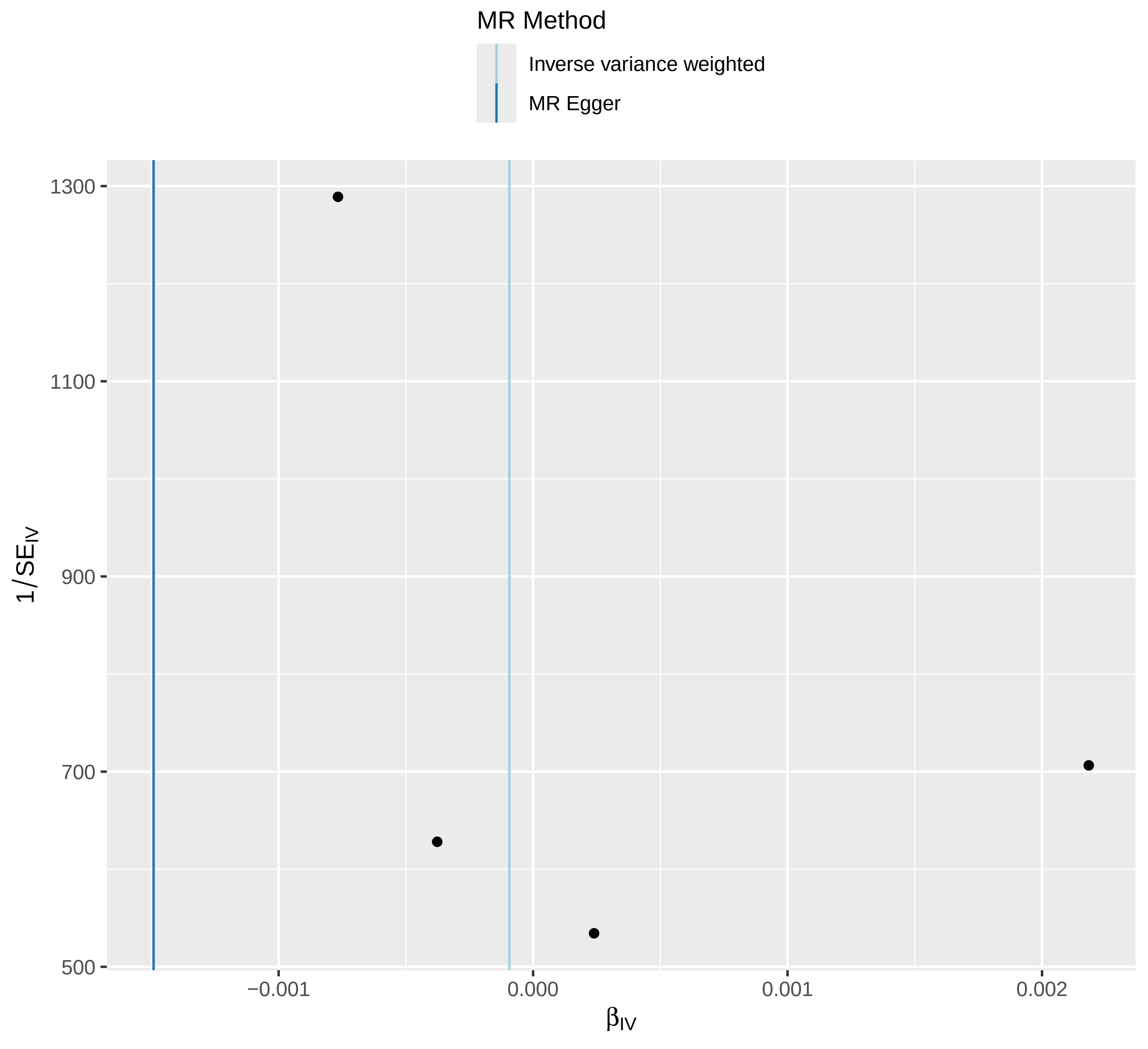

MR Method
Inverse variance weighted
MR Egger
1300
1100
900
700
500
−0.001
0.000
0.001
0.002
1/SE_IV
β_IV

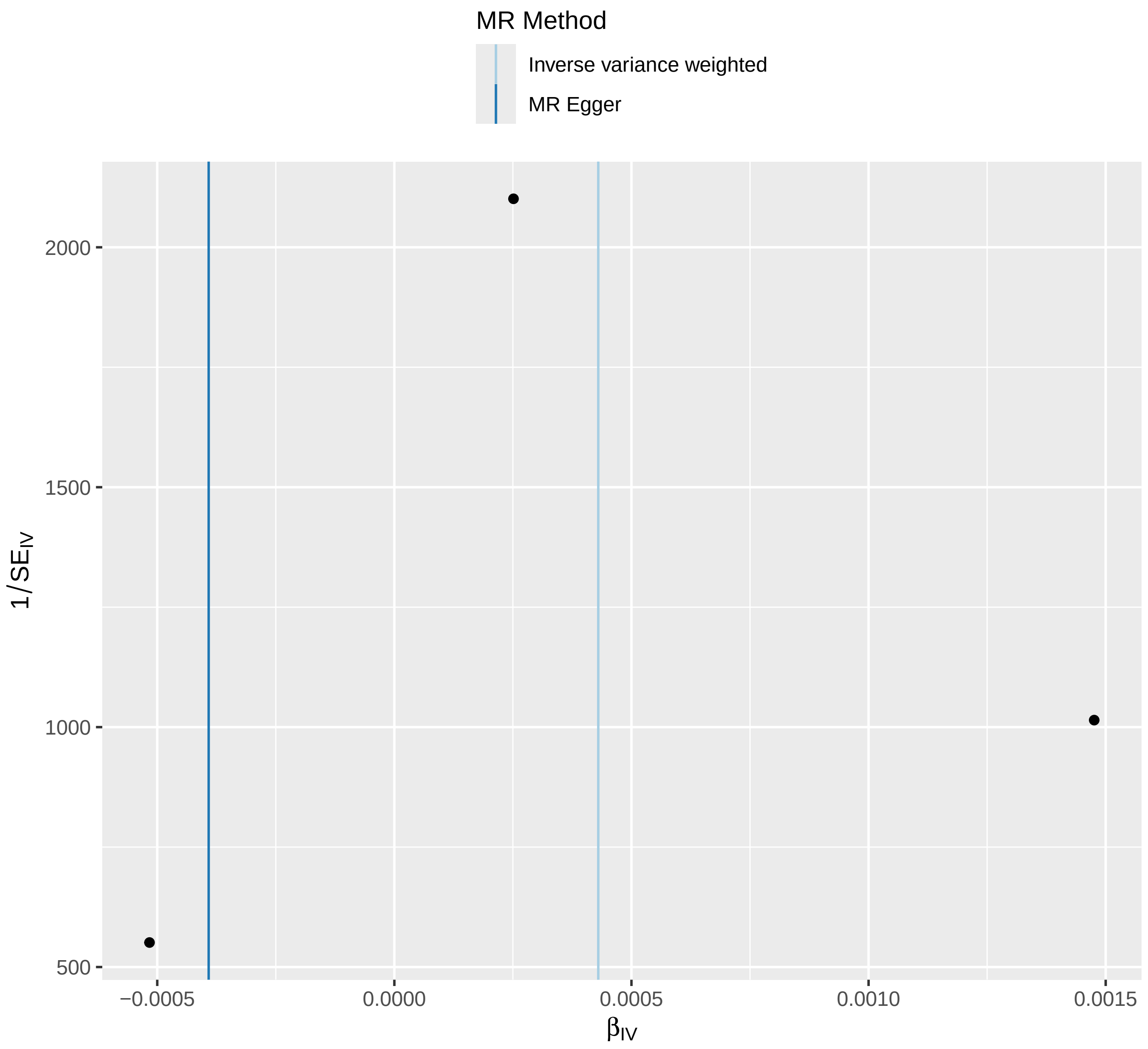
MR Method
Inverse variance weighted
MR Egger
2000
1500
1000
500
1/SE_IV
−0.0005
0.0000
0.0005
0.0010
0.0015
β_IV

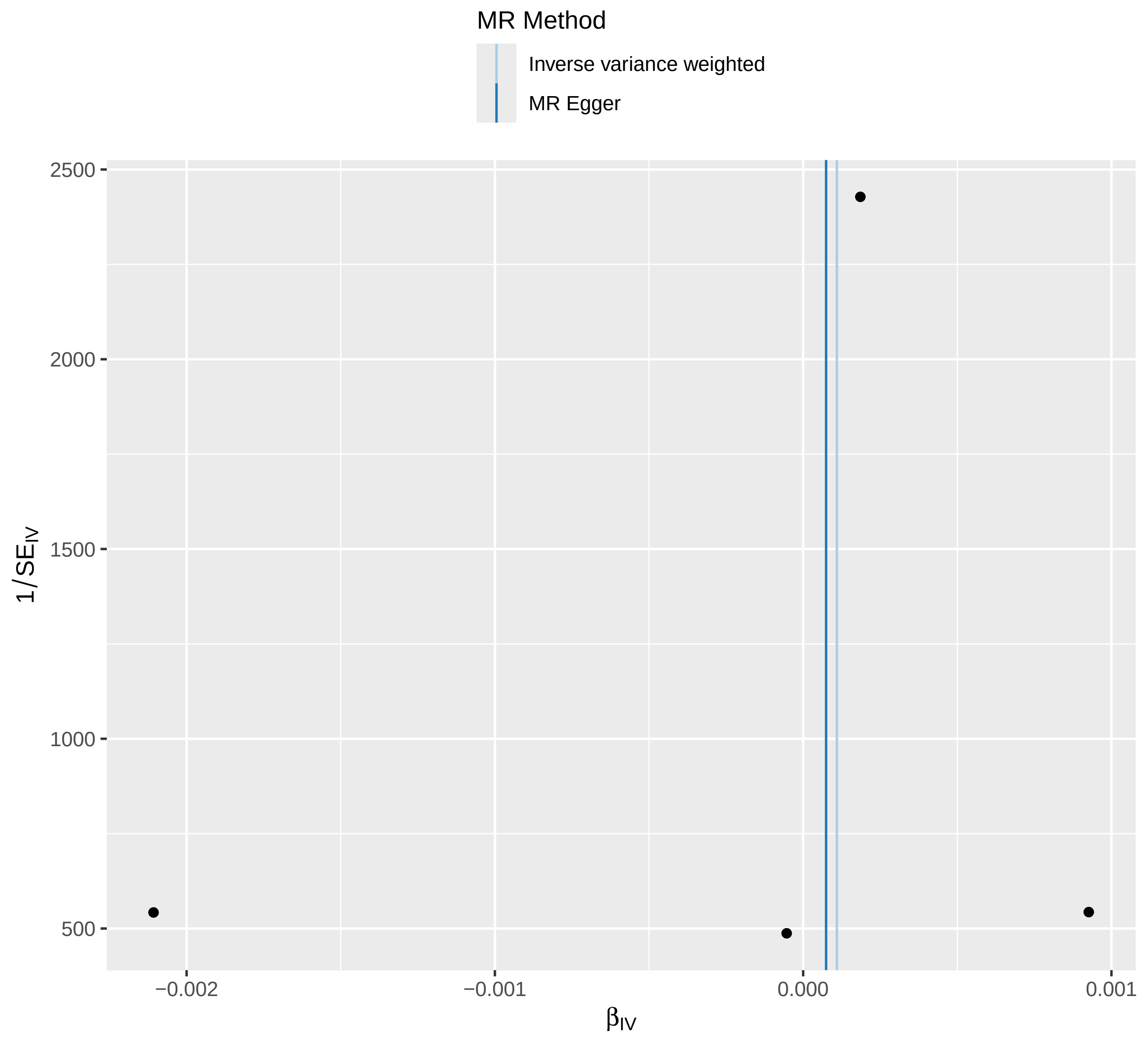

MR Method
Inverse variance weighted
MR Egger
2500
2000
1500
1000
500
1/SE_IV
−0.002
−0.001
0.000
0.001
β_IV

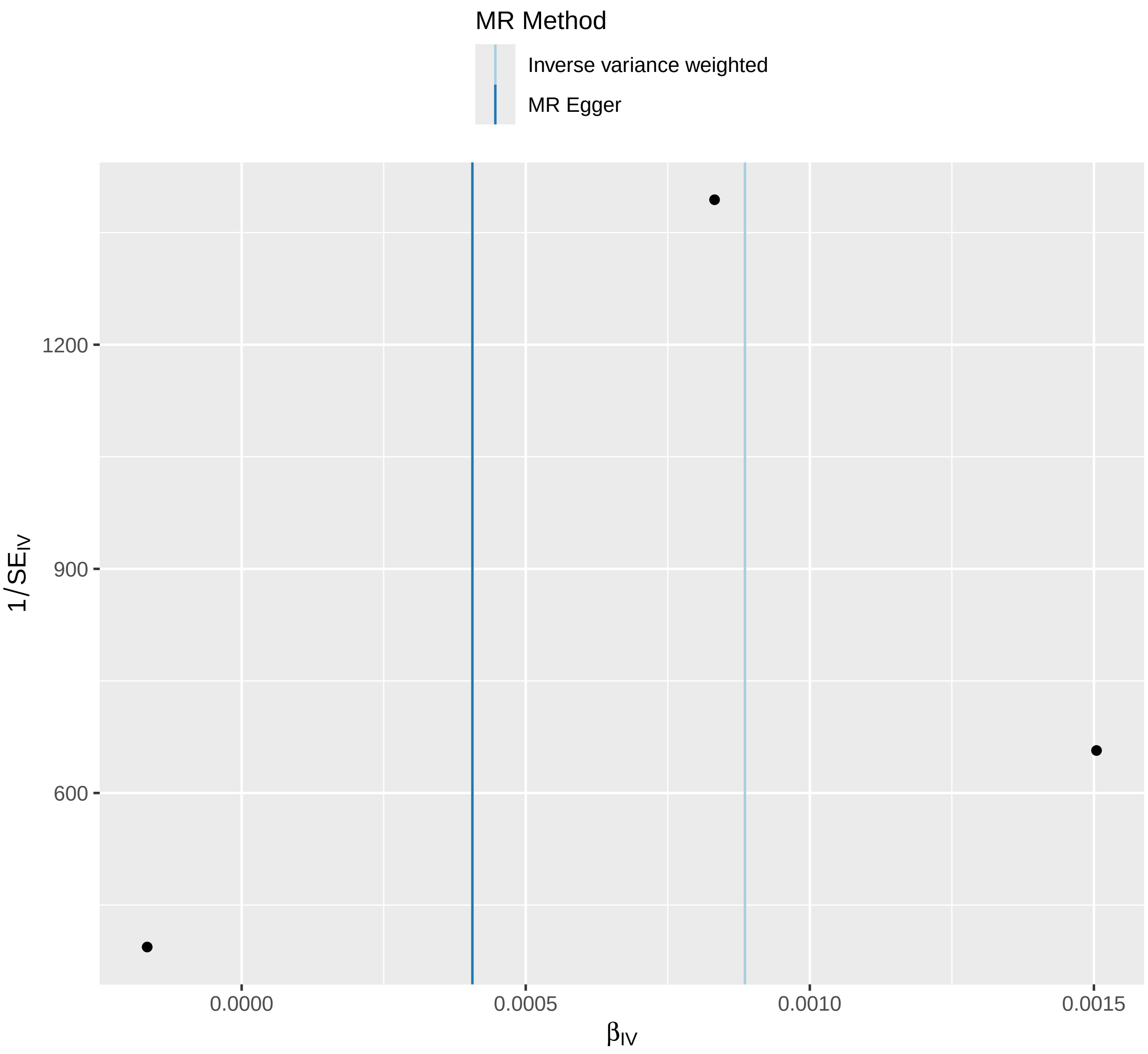
MR Method
Inverse variance weighted
MR Egger
1200
900
600
0.0000
0.0005
0.0010
0.0015
β_{IV}
$1/SE_{IV}$

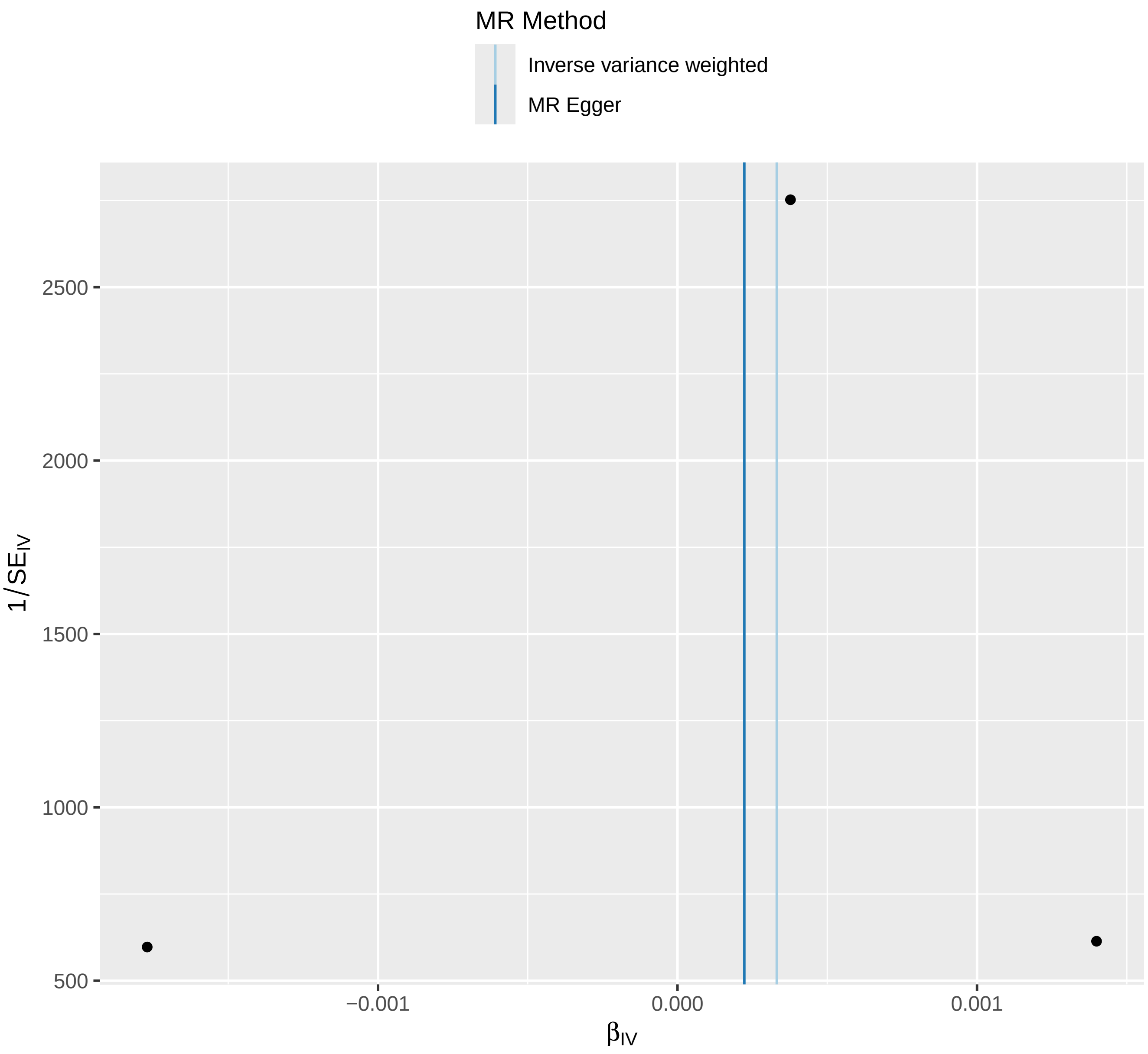
MR Method
Inverse variance weighted
MR Egger
2500
2000
1500
1000
500
$1/SE_{IV}$
−0.001
0.000
0.001
β_{IV}

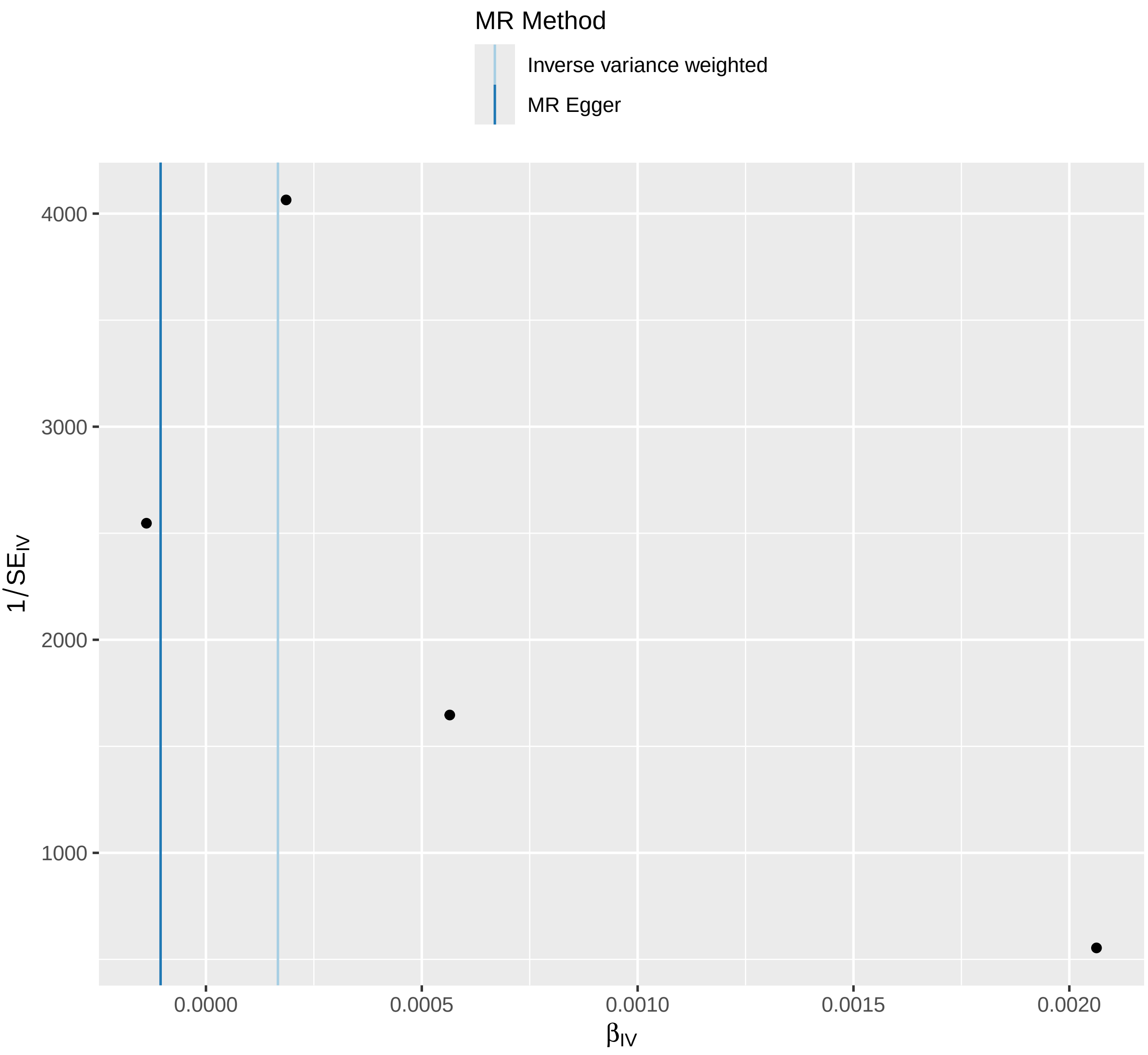
MR Method
Inverse variance weighted
MR Egger
4000
3000
2000
1000
0.0000
0.0005
0.0010
0.0015
0.0020
β_{IV}
$1/SE_{IV}$

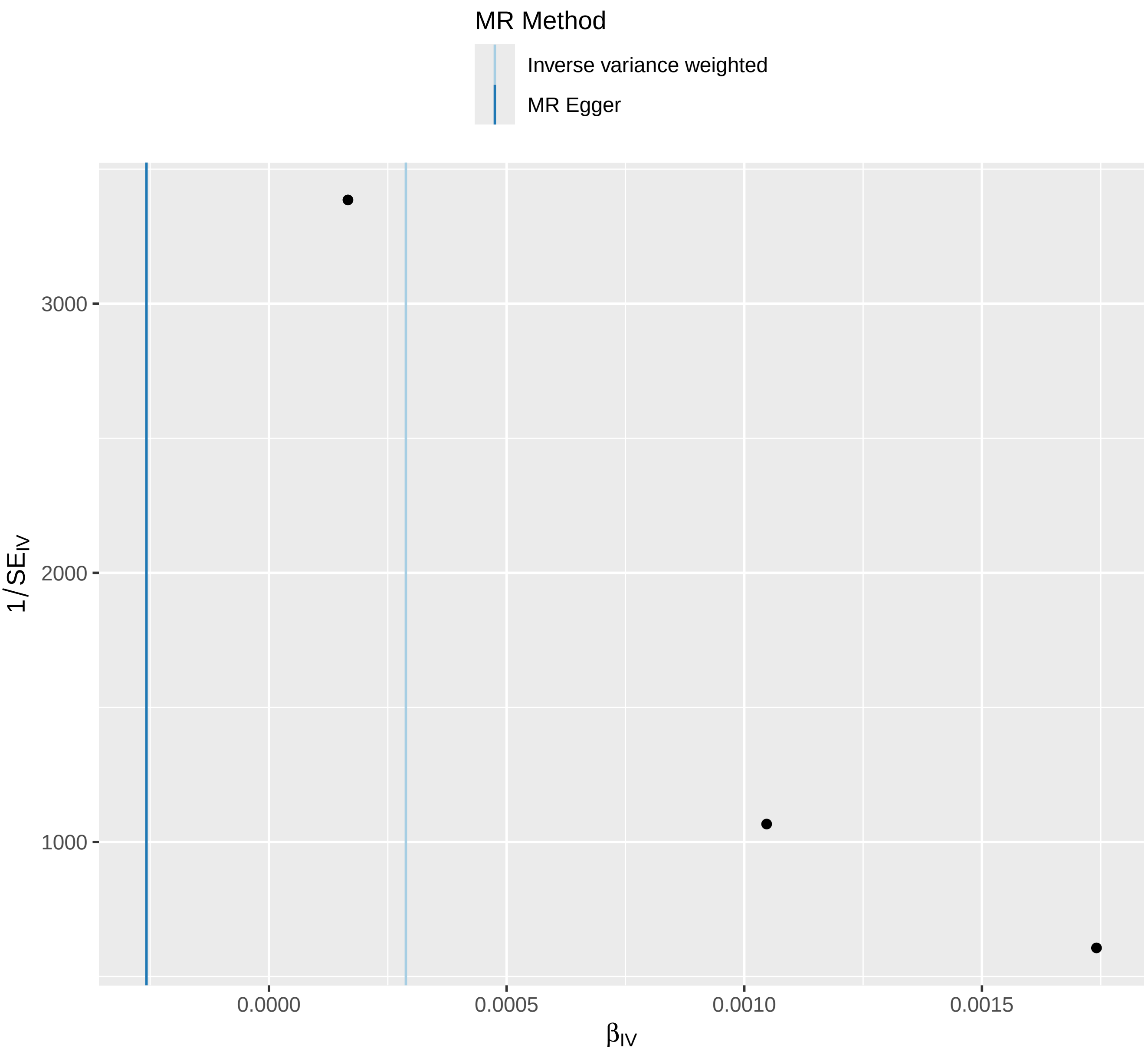
MR Method
Inverse variance weighted
MR Egger
3000
2000
1000
1/SE_IV
0.0000
0.0005
0.0010
0.0015
β_IV

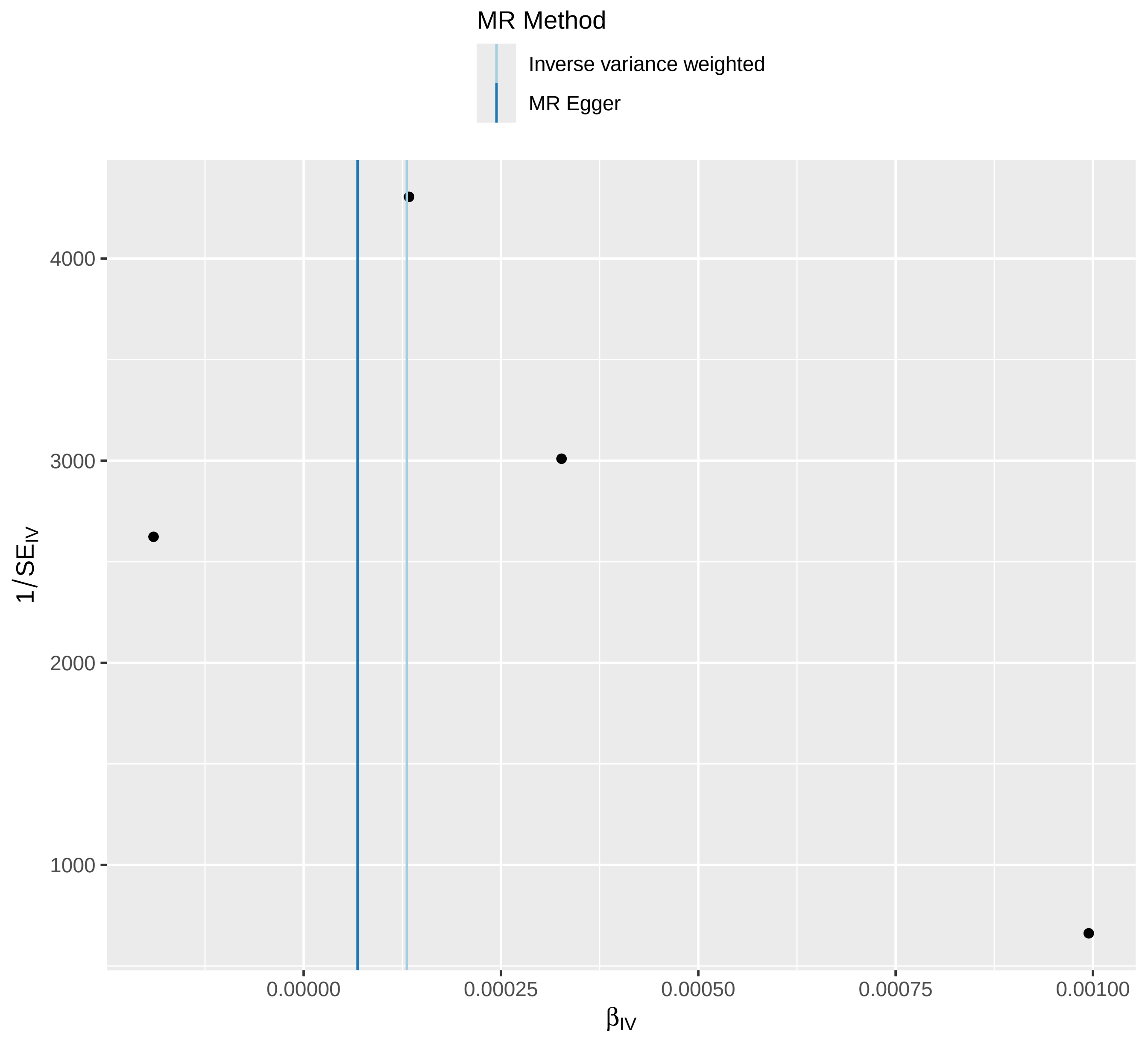

MR Method
Inverse variance weighted
MR Egger
4000
3000
2000
1000
$1/SE_{IV}$
0.00000
0.00025
0.00050
0.00075
0.00100
β_{IV}

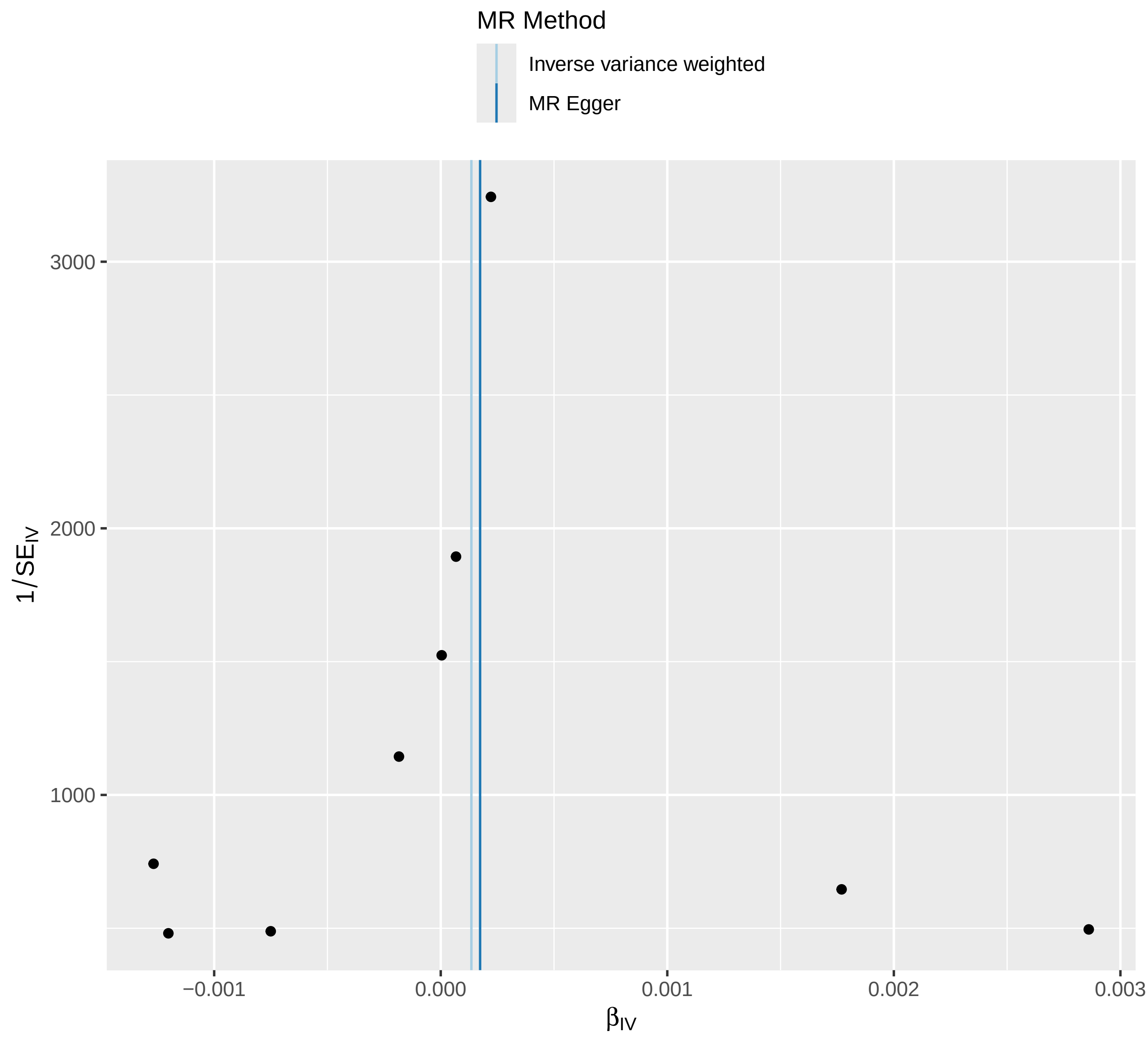

MR Method
Inverse variance weighted
MR Egger
3000
2000
1000
1/SE_IV
−0.001
0.000
0.001
0.002
0.003
β_IV

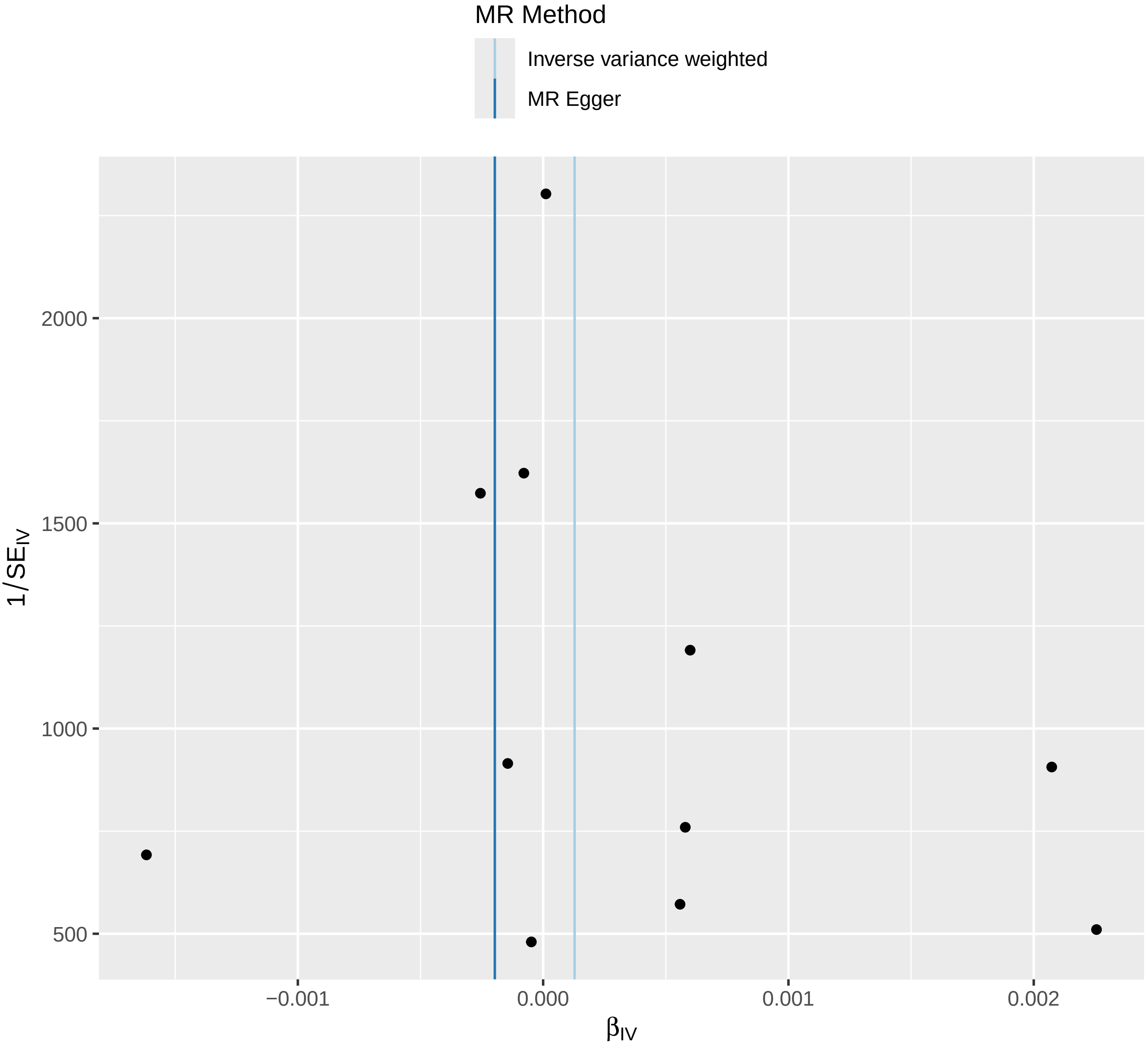
MR Method
Inverse variance weighted
MR Egger
2000
1500
1000
500
1/SE$_{IV}$
−0.001
0.000
0.001
0.002
β$_{IV}$

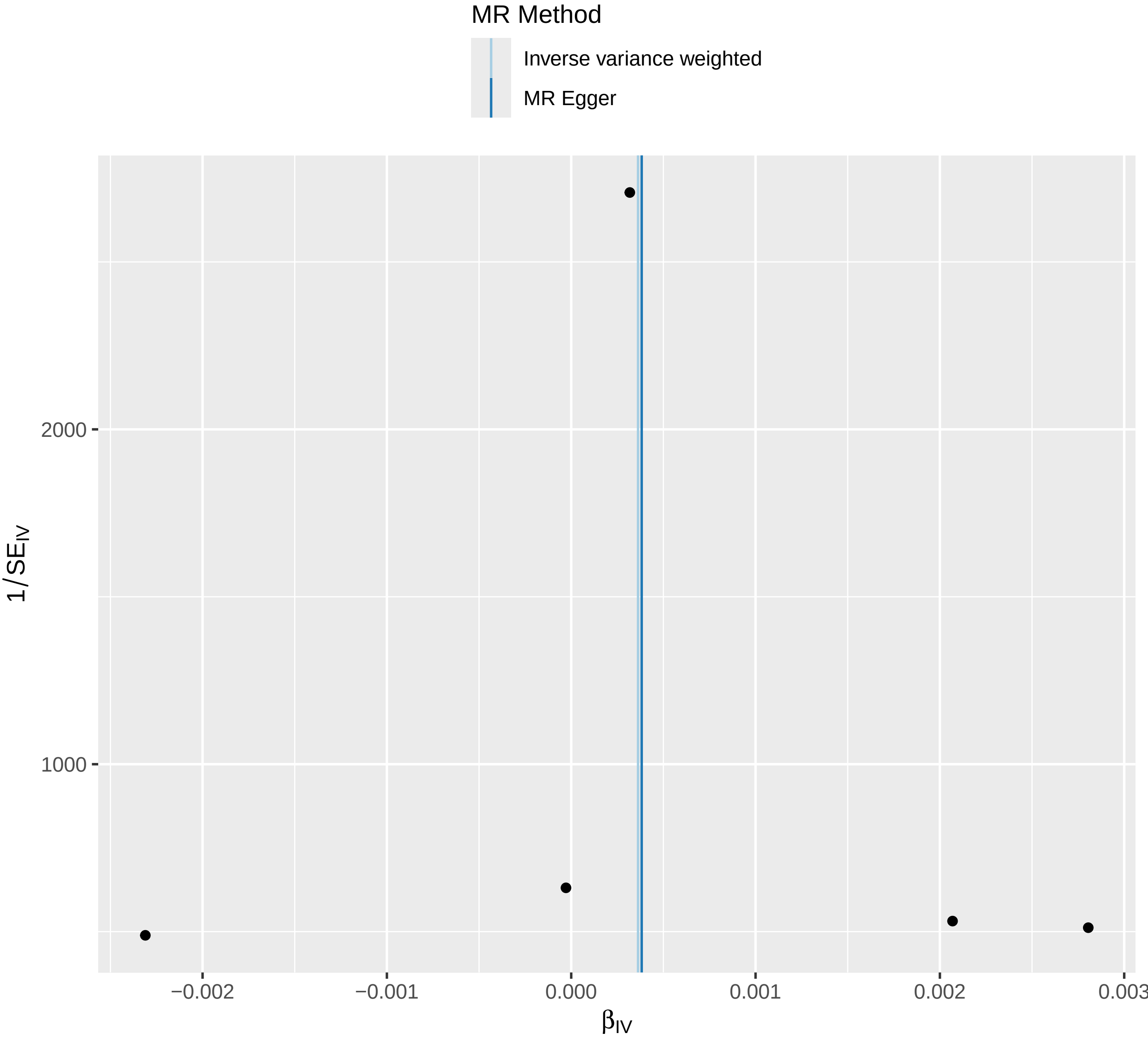
MR Method
Inverse variance weighted
MR Egger
2000
1000
1/SE$_{IV}$
−0.002
−0.001
0.000
0.001
0.002
0.003
β$_{IV}$

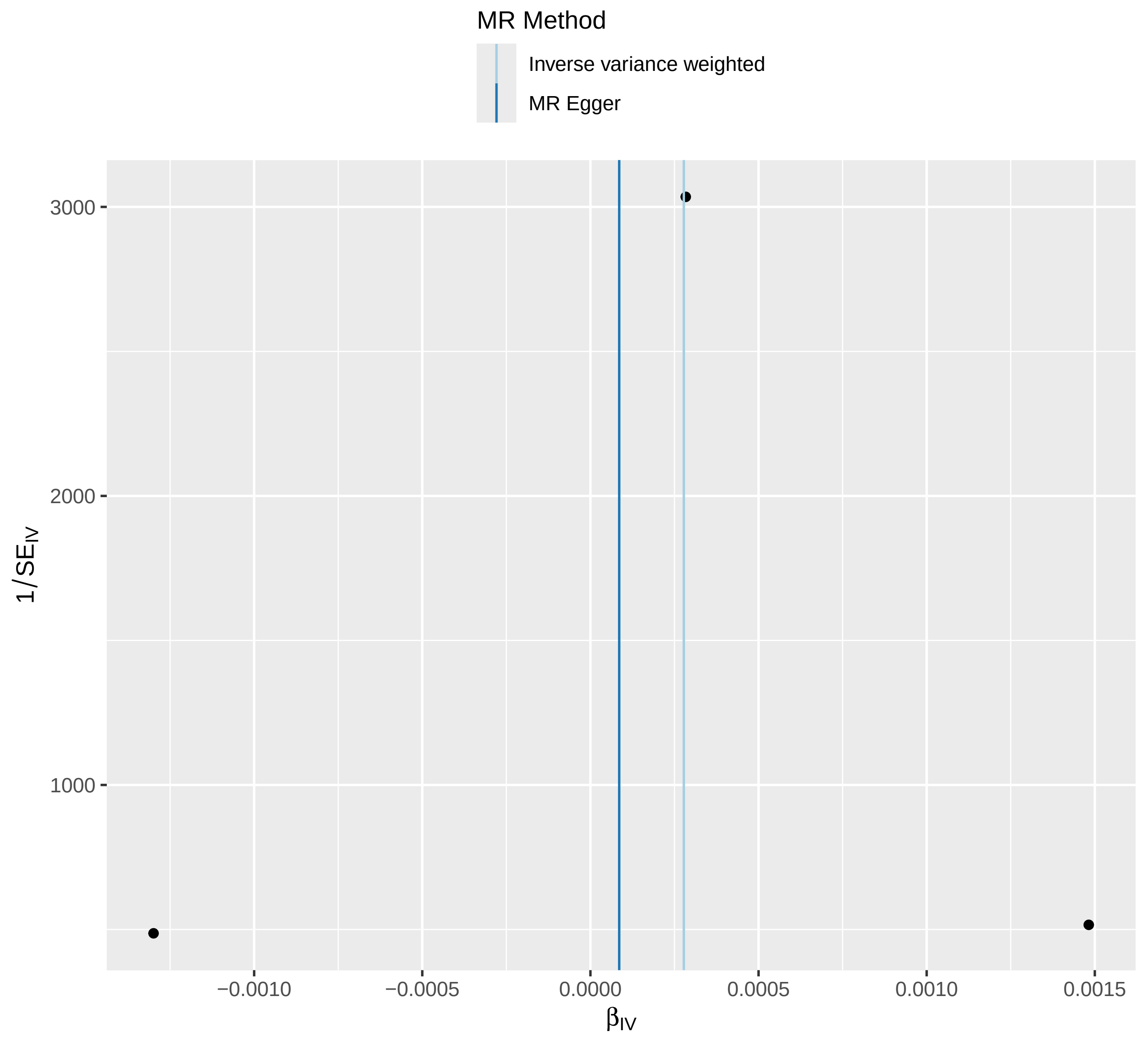

MR Method
Inverse variance weighted
MR Egger
3000
2000
1000
1/SE_IV
−0.0010
−0.0005
0.0000
0.0005
0.0010
0.0015
β_IV

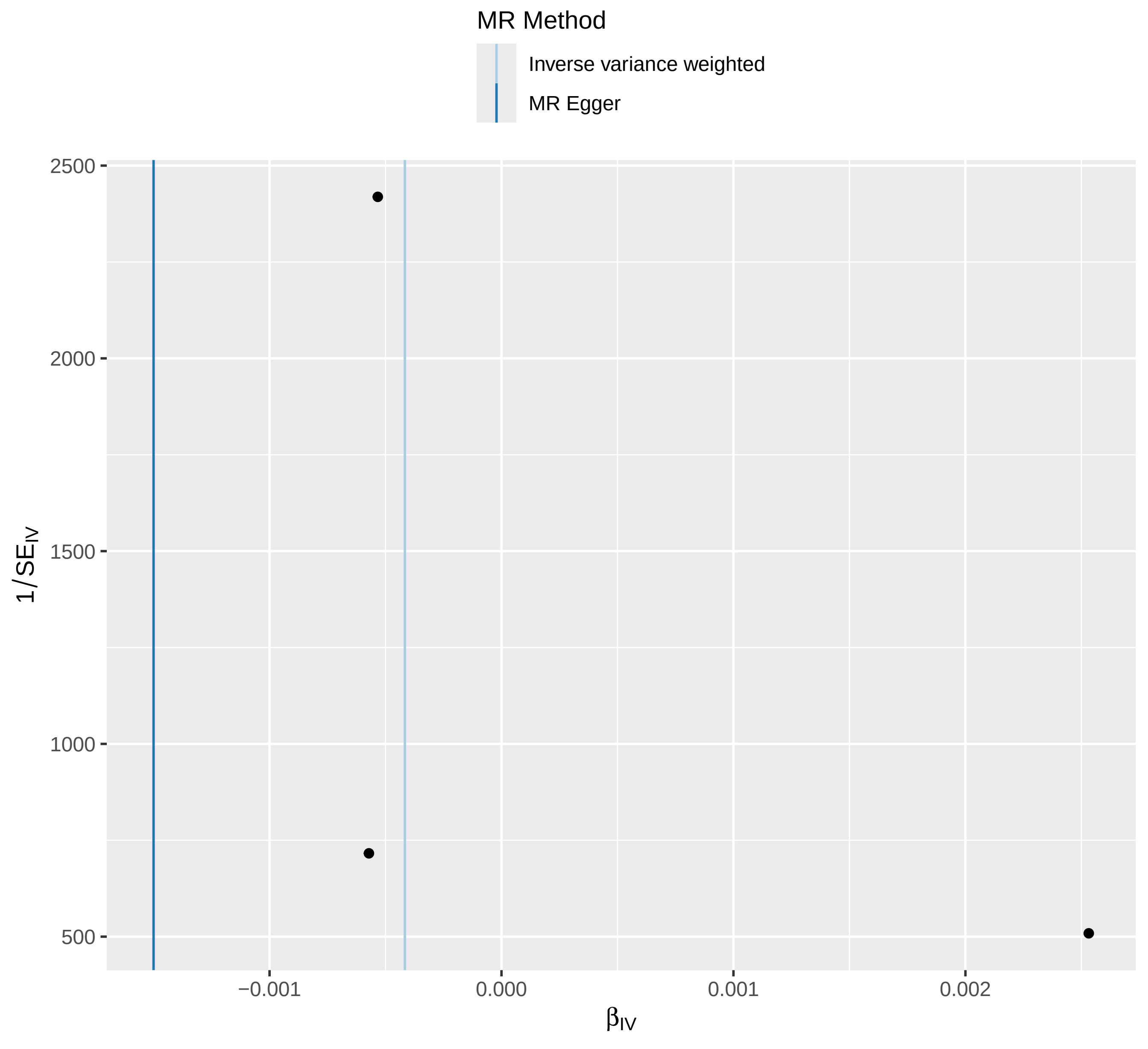

MR Method
Inverse variance weighted
MR Egger
2500
2000
1500
1000
500
1/SE$_{IV}$
−0.001
0.000
0.001
0.002
β$_{IV}$

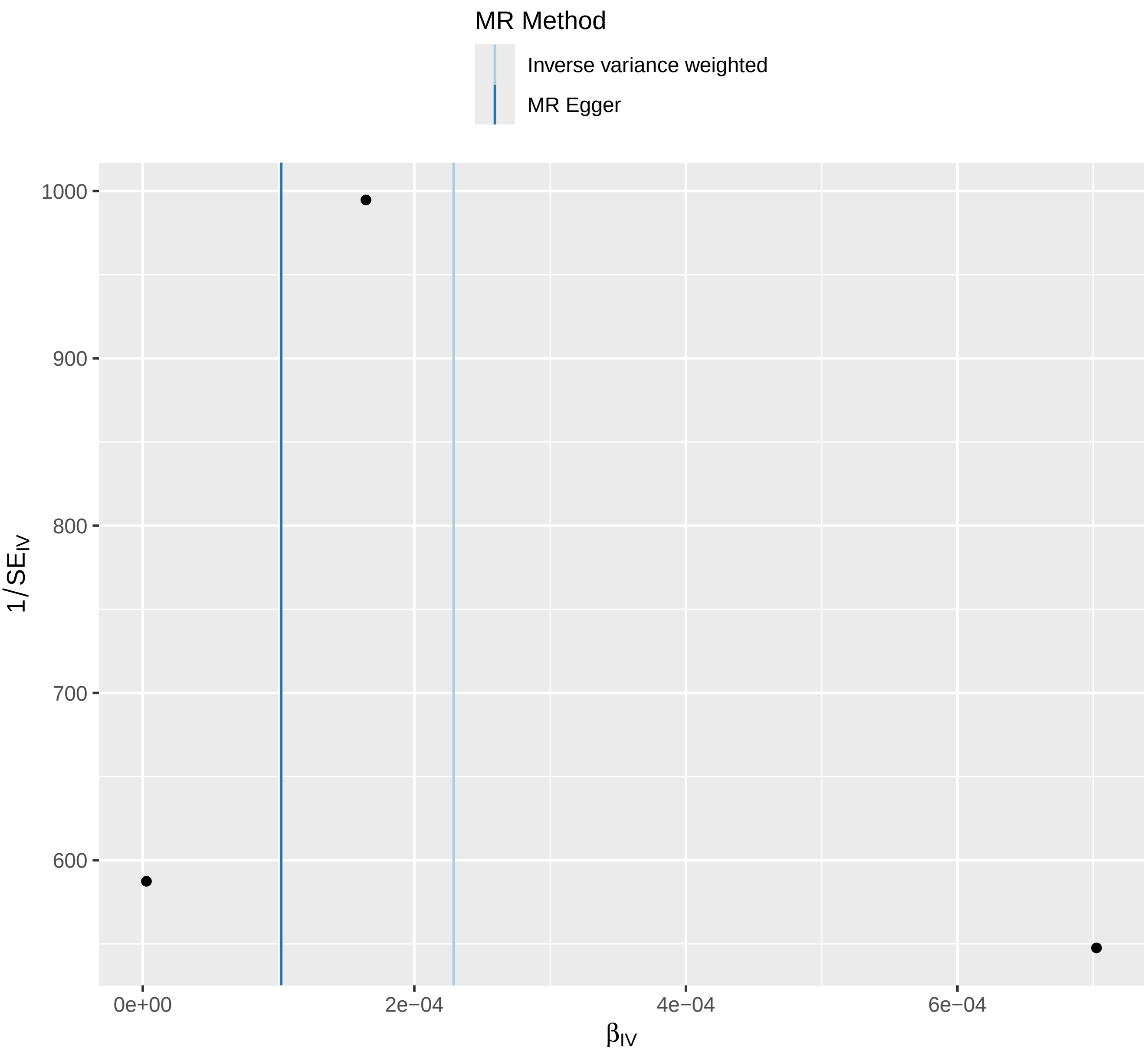

MR Method
Inverse variance weighted
MR Egger
1000
900
800
700
600
1/SE$_{IV}$
0e+00
2e−04
4e−04
6e−04
β$_{IV}$

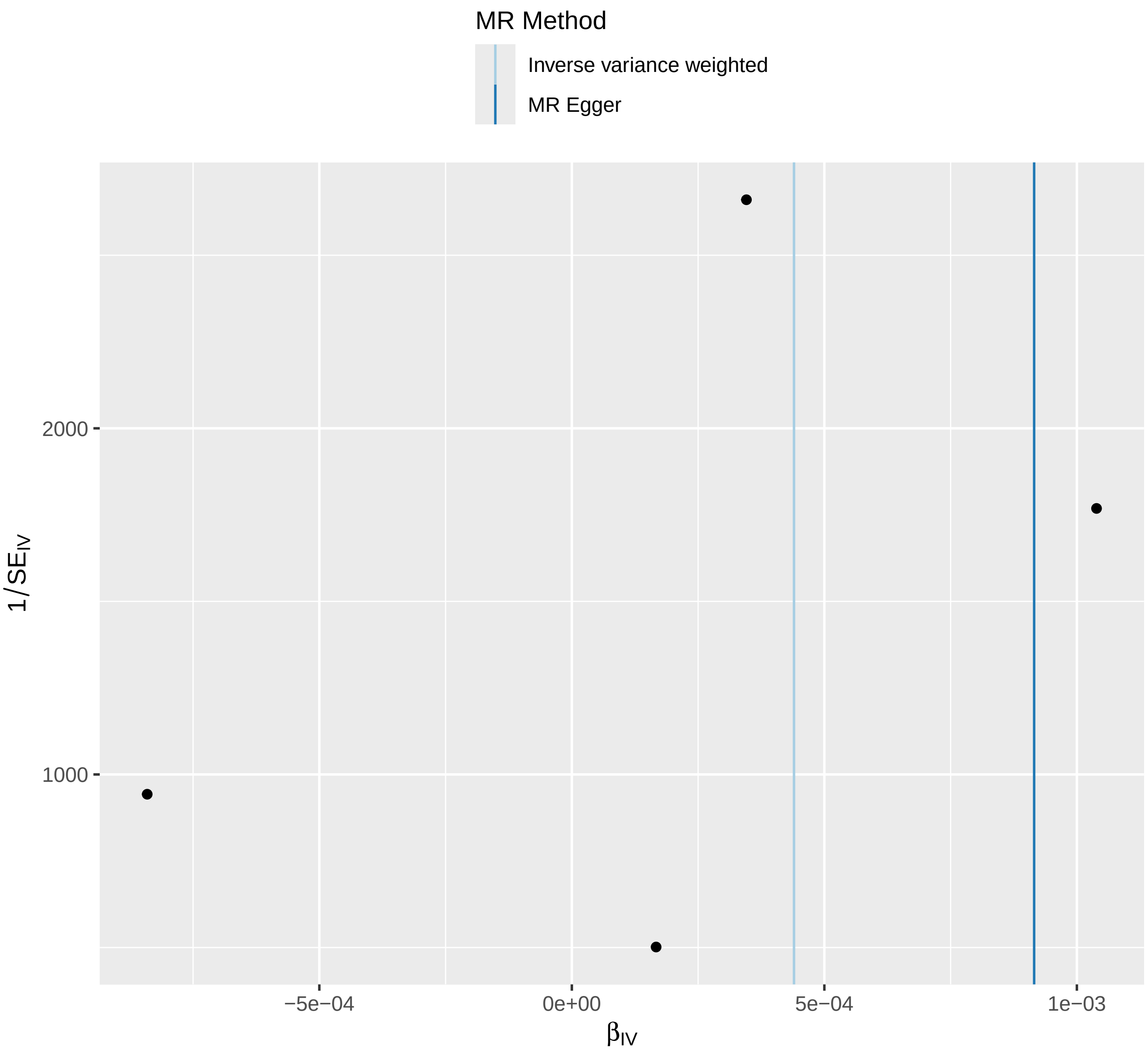

MR Method
Inverse variance weighted
MR Egger
1/SE_IV
2000
1000
−5e−04
0e+00
5e−04
1e−03
β_IV

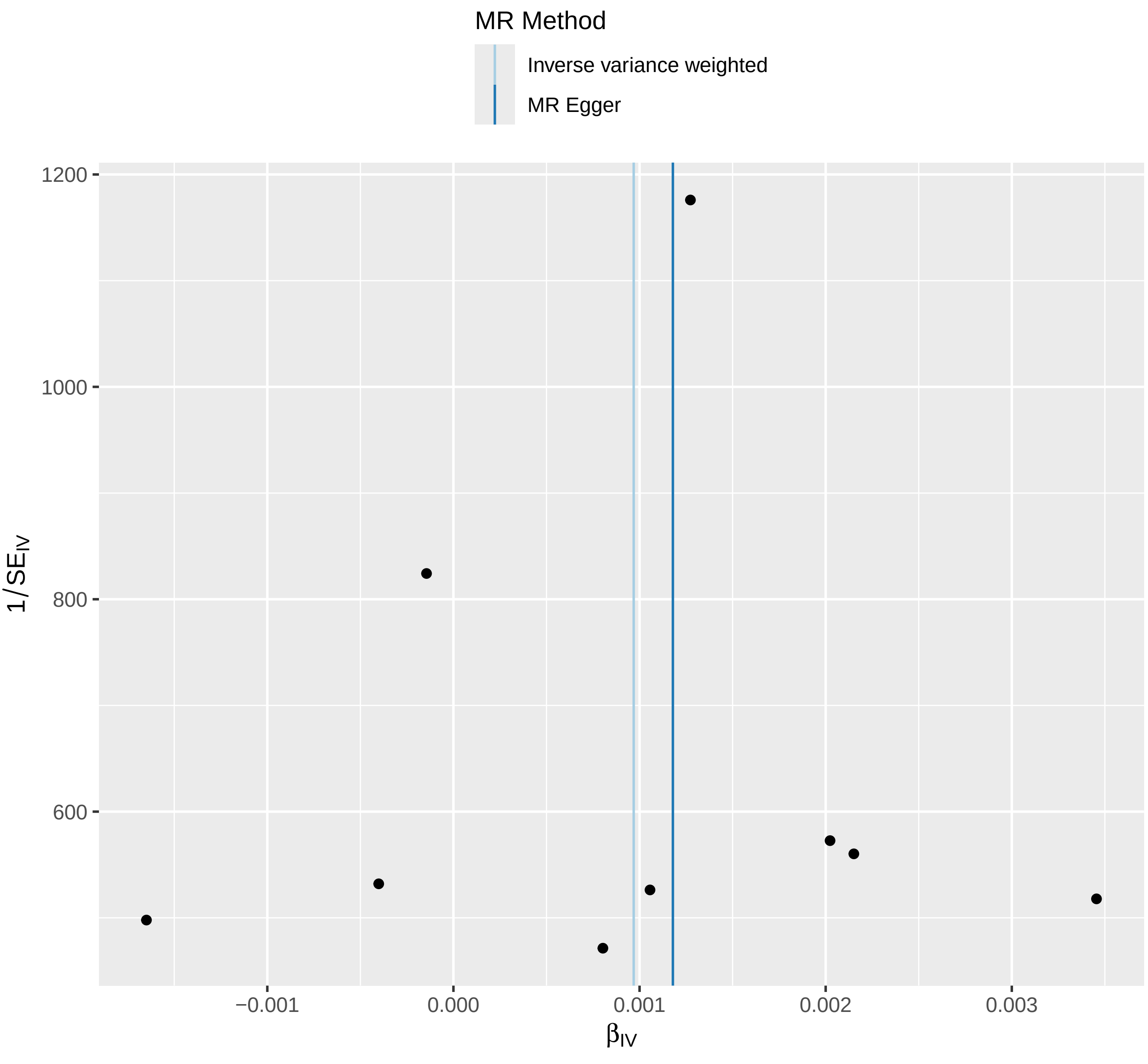
MR Method
Inverse variance weighted
MR Egger
1200
1000
800
600
−0.001
0.000
0.001
0.002
0.003
$1/SE_{IV}$
β_{IV}

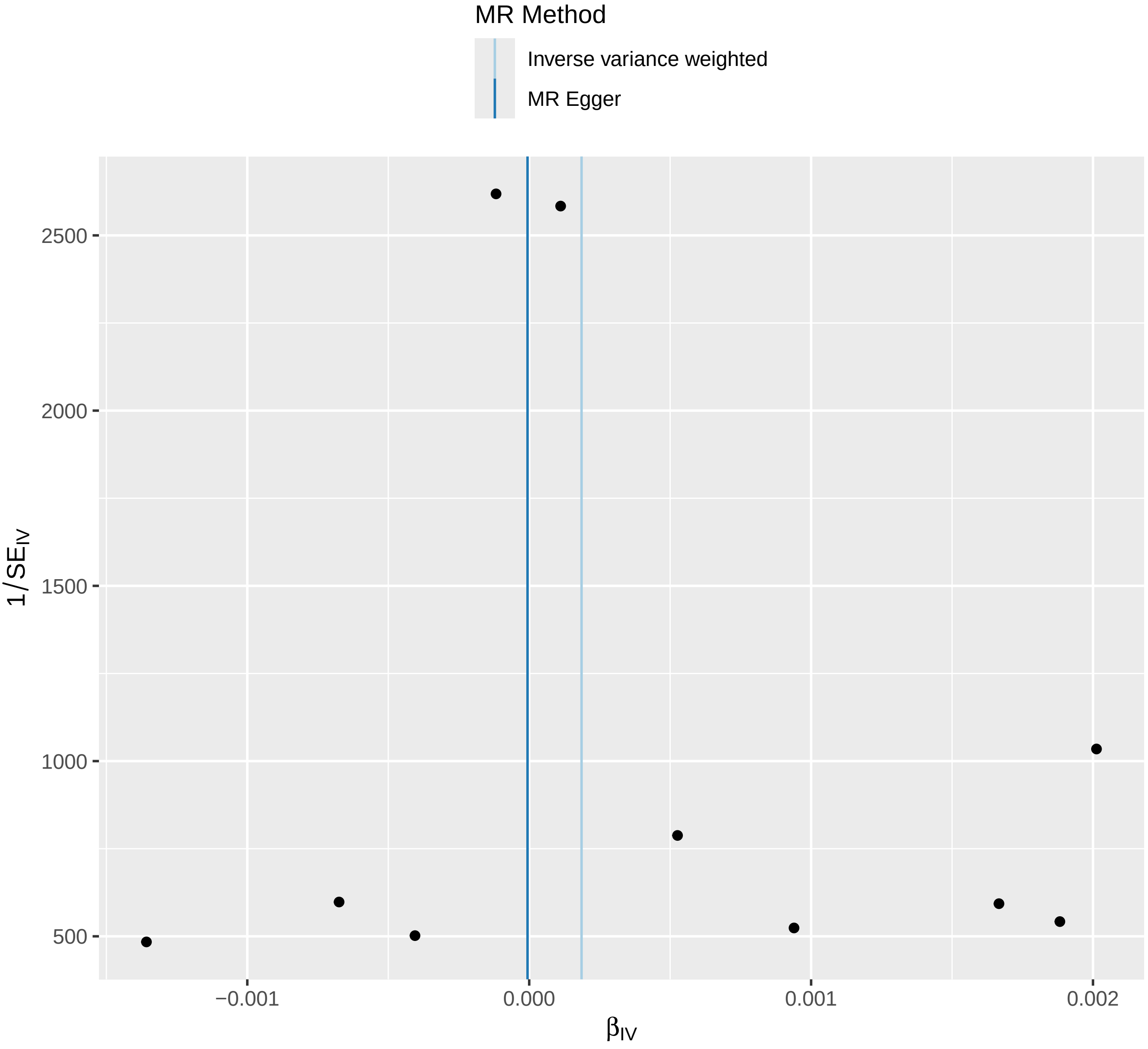
MR Method
Inverse variance weighted
MR Egger
2500
2000
1500
1000
500
1/SE$_{IV}$
−0.001
0.000
0.001
0.002
β$_{IV}$

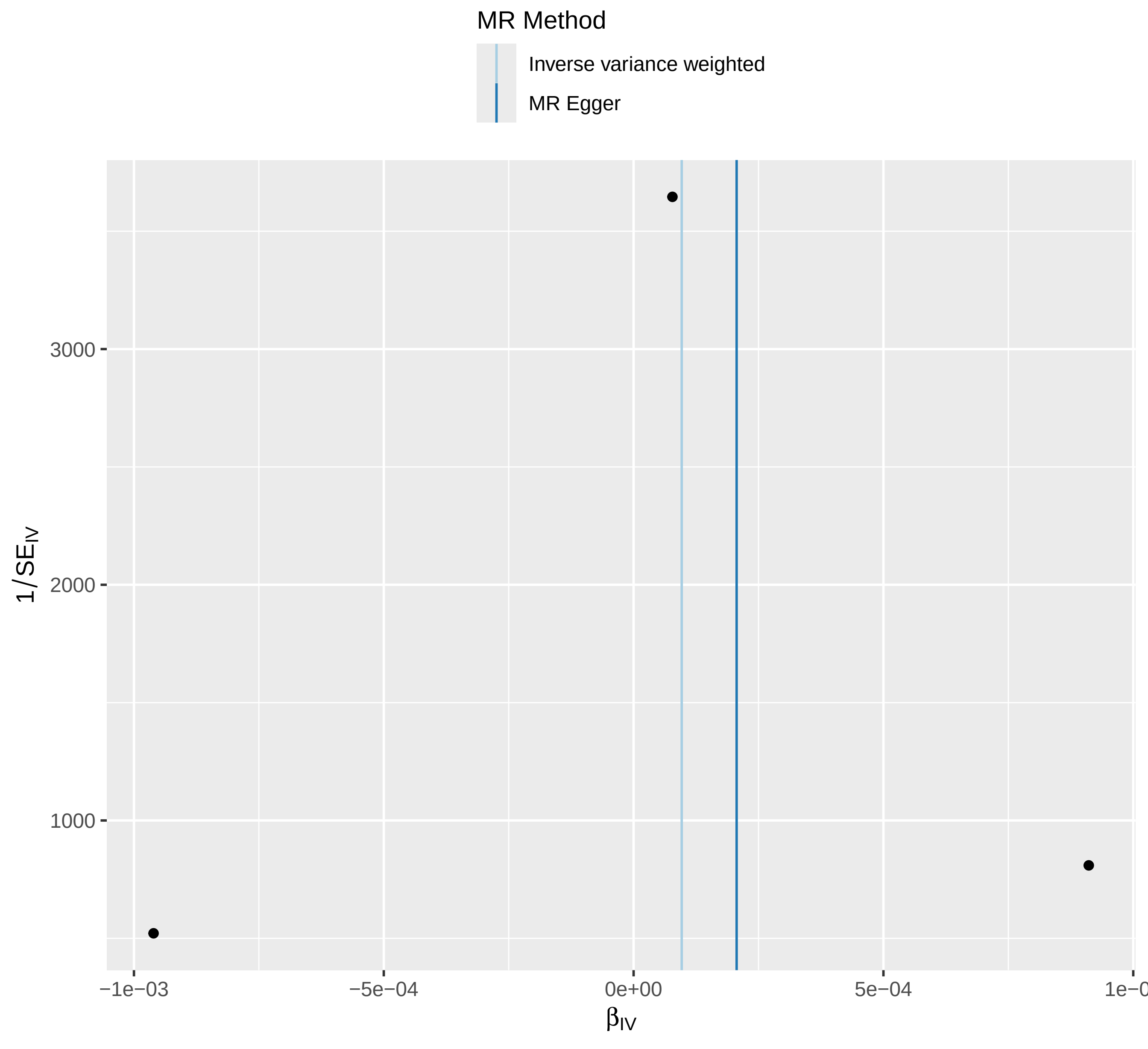
MR Method
Inverse variance weighted
MR Egger
3000
2000
1000
1/SE$_{IV}$
−1e−03
−5e−04
0e+00
5e−04
1e−0
β$_{IV}$

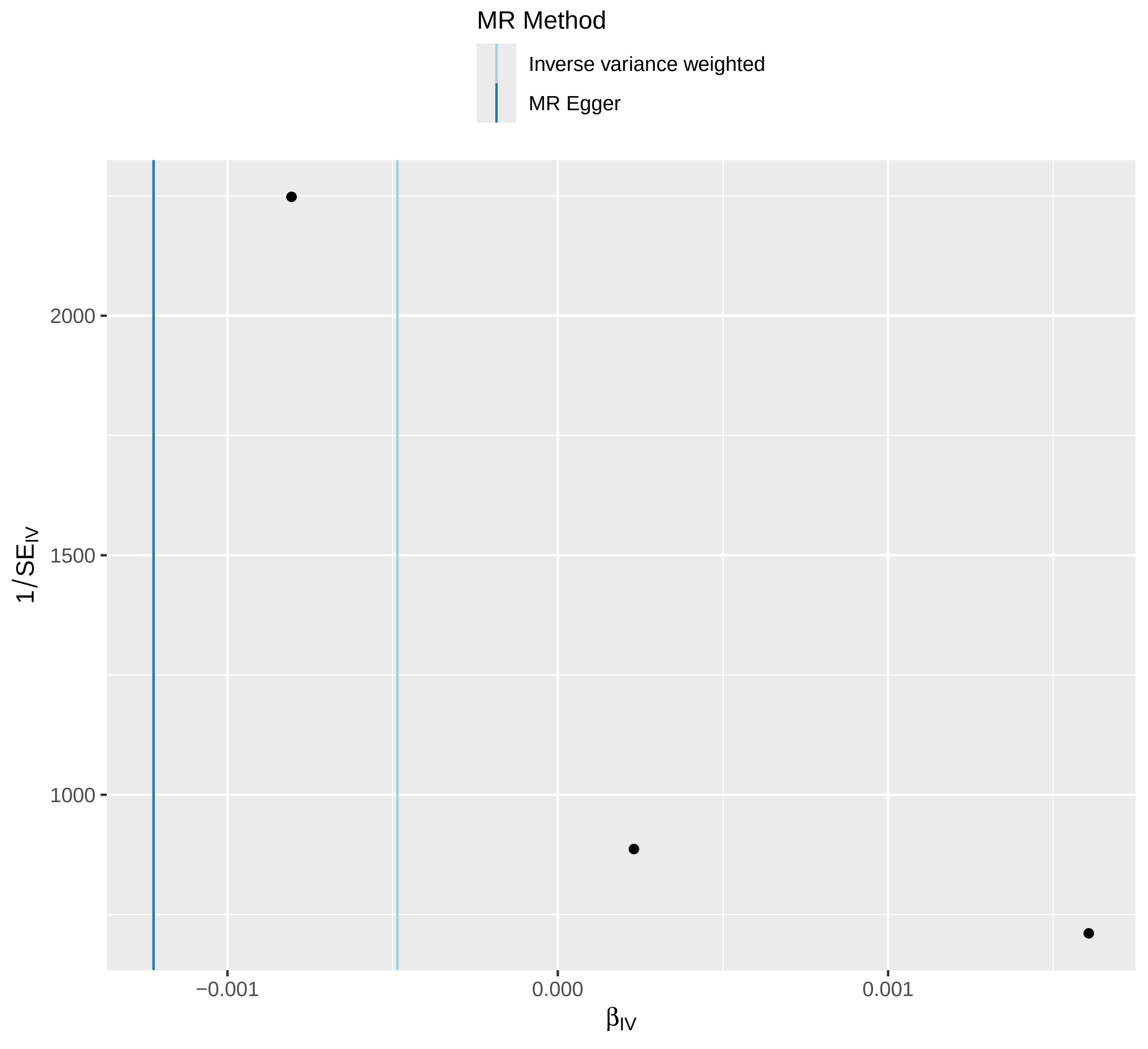

MR Method
Inverse variance weighted
MR Egger
2000
1500
1000
1/SE_IV
−0.001
0.000
0.001
β_IV

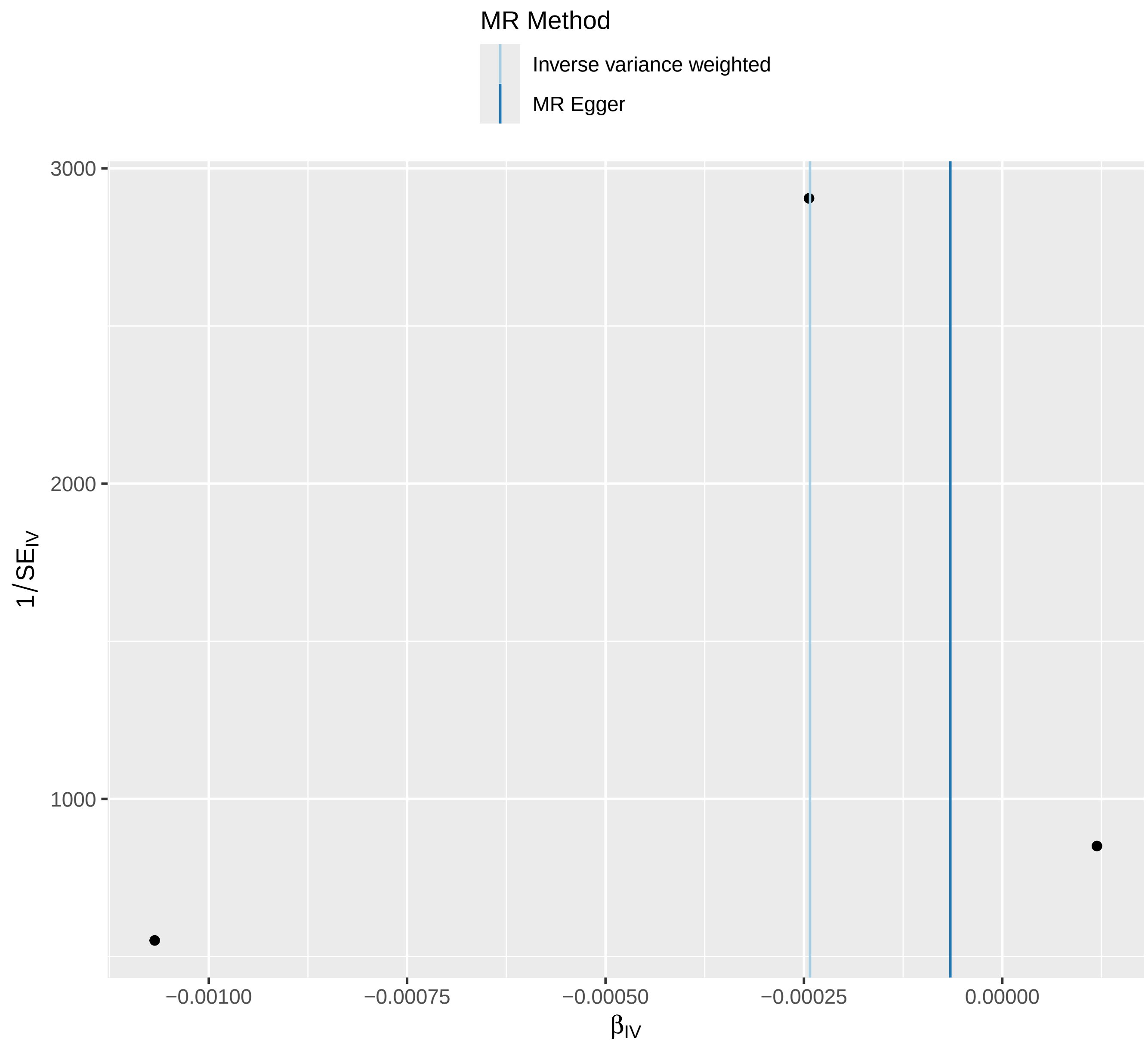

MR Method
Inverse variance weighted
MR Egger
3000
2000
1000
1/SE_IV
−0.00100
−0.00075
−0.00050
−0.00025
0.00000
β_IV

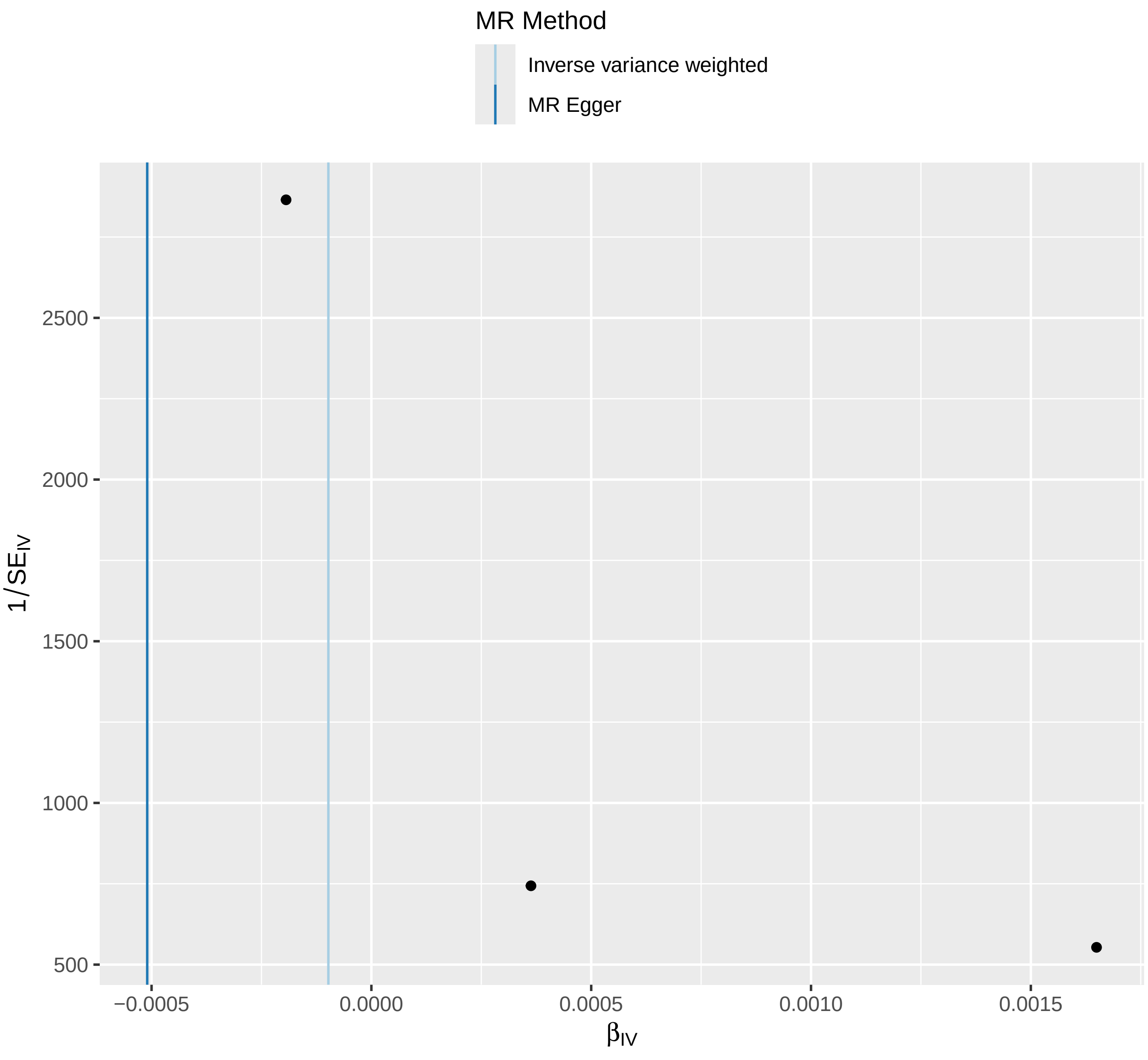

MR Method
Inverse variance weighted
MR Egger
2500
2000
1500
1000
500
$1/SE_{IV}$
−0.0005
0.0000
0.0005
0.0010
0.0015
β_{IV}

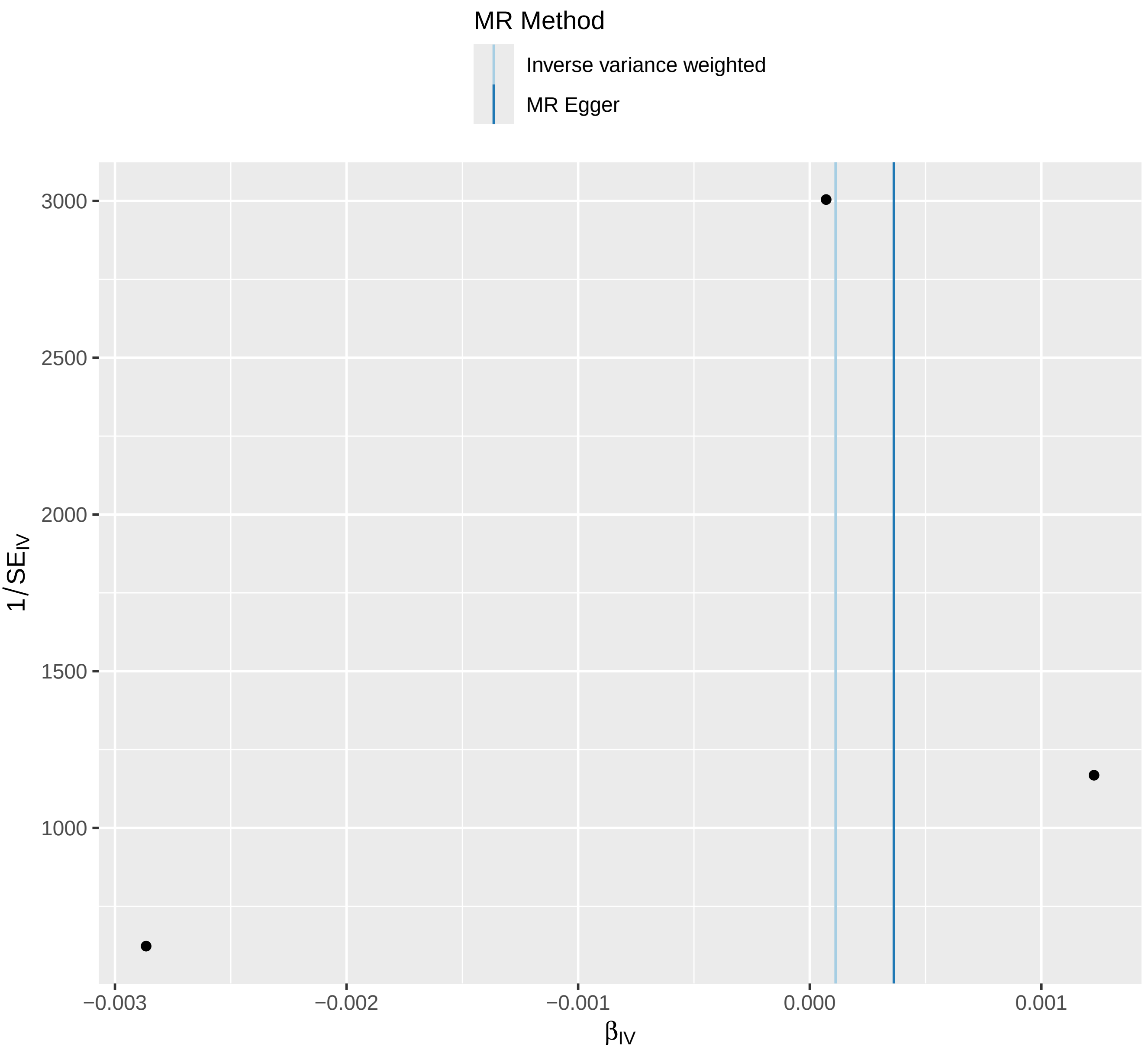
MR Method
Inverse variance weighted
MR Egger
3000
2500
2000
1500
1000
1/SE$_{IV}$
−0.003
−0.002
−0.001
0.000
0.001
β_{IV}

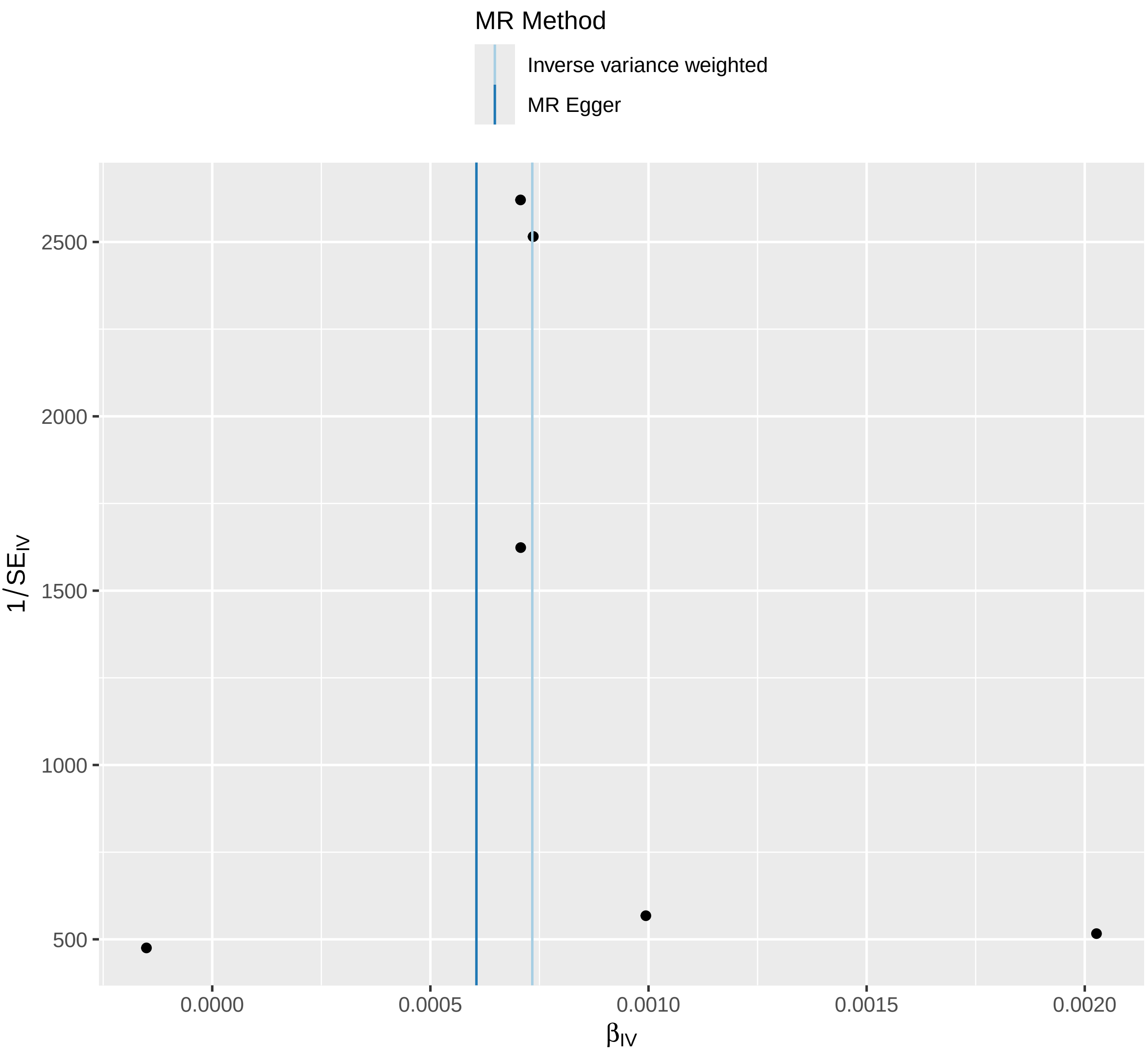
MR Method
Inverse variance weighted
MR Egger
2500
2000
1500
1000
500
1/SE_IV
0.0000
0.0005
0.0010
0.0015
0.0020
β_IV

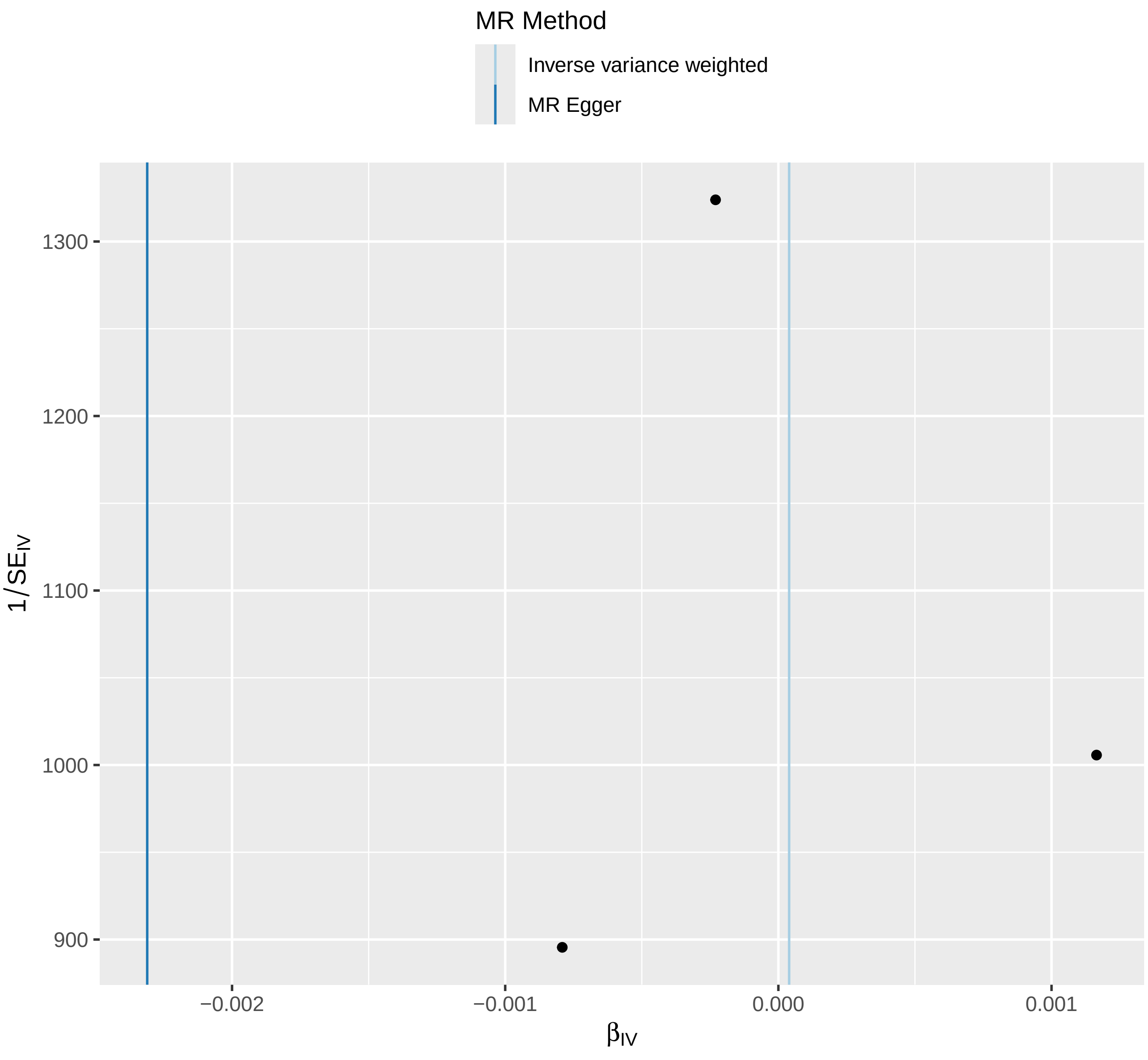

MR Method
Inverse variance weighted
MR Egger
1300
1200
1100
1000
900
1/SE_IV
−0.002
−0.001
0.000
0.001
β_IV

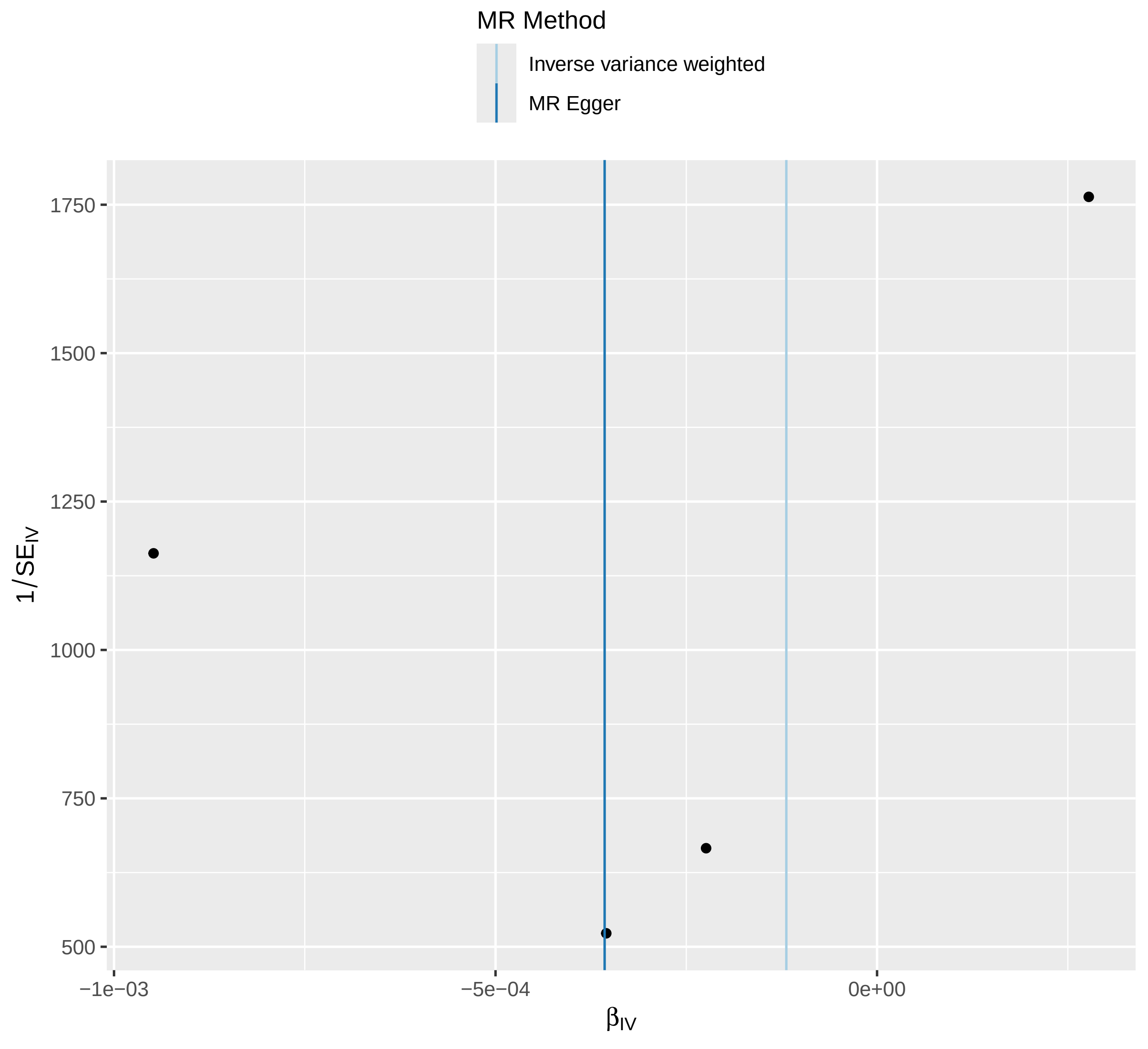
MR Method
Inverse variance weighted
MR Egger
1750
1500
1250
1000
750
500
1/SE_IV
−1e−03
−5e−04
0e+00
β_IV

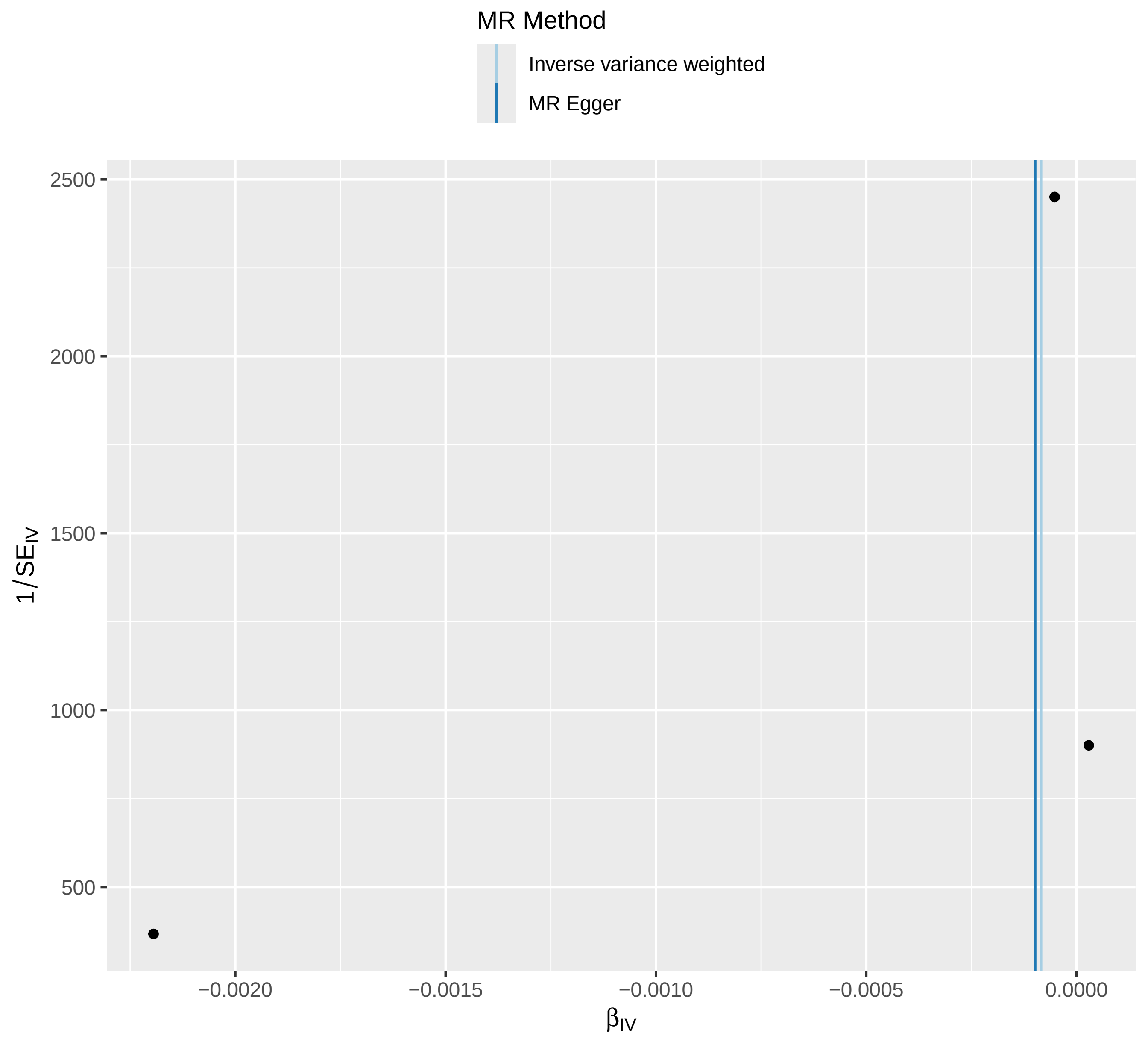

MR Method
Inverse variance weighted
MR Egger
2500
2000
1500
1000
500
$1/SE_{IV}$
−0.0020
−0.0015
−0.0010
−0.0005
0.0000
β_{IV}

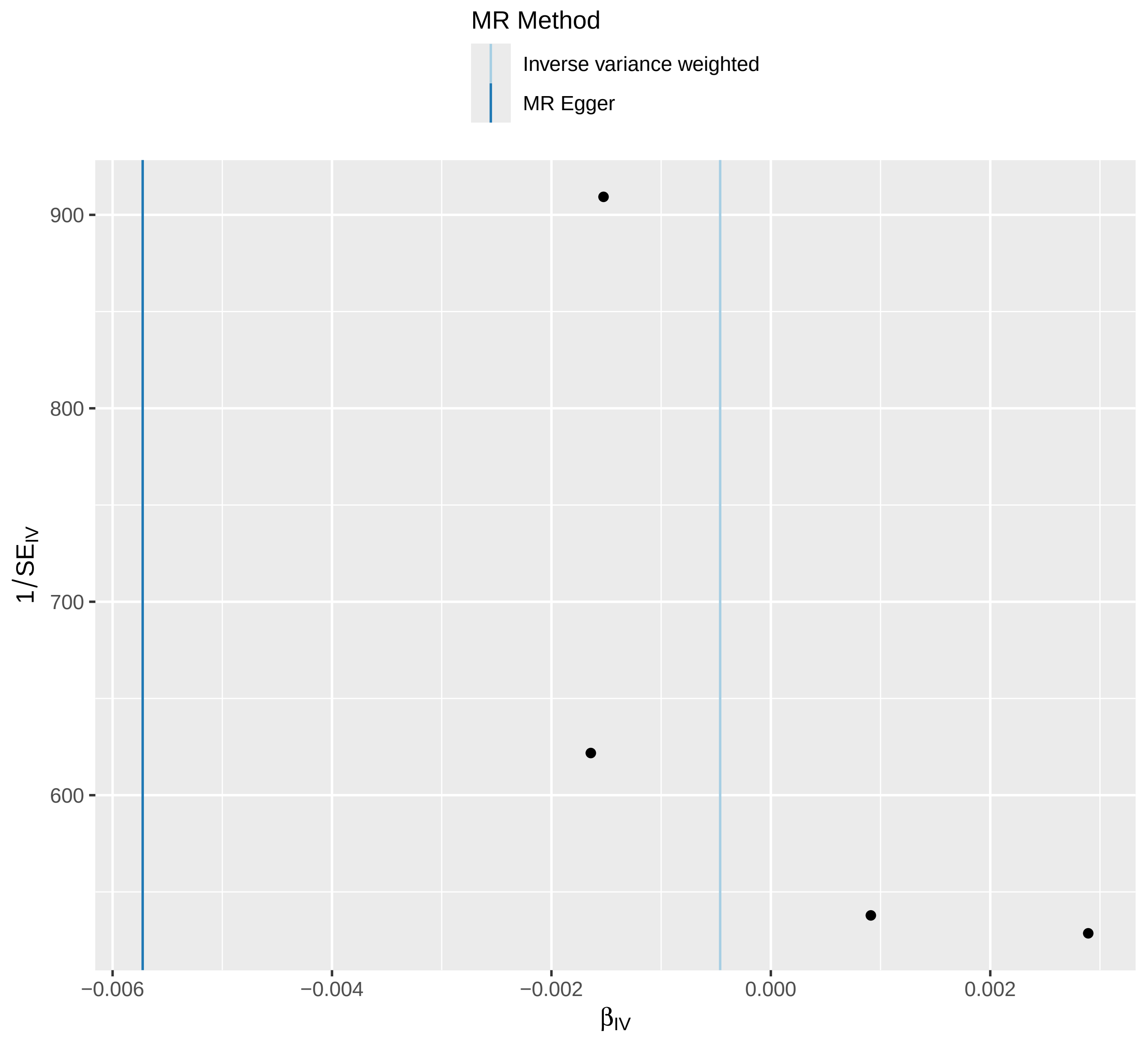

MR Method
Inverse variance weighted
MR Egger
900
800
700
600
1/SE_IV
−0.006
−0.004
−0.002
0.000
0.002
β_IV

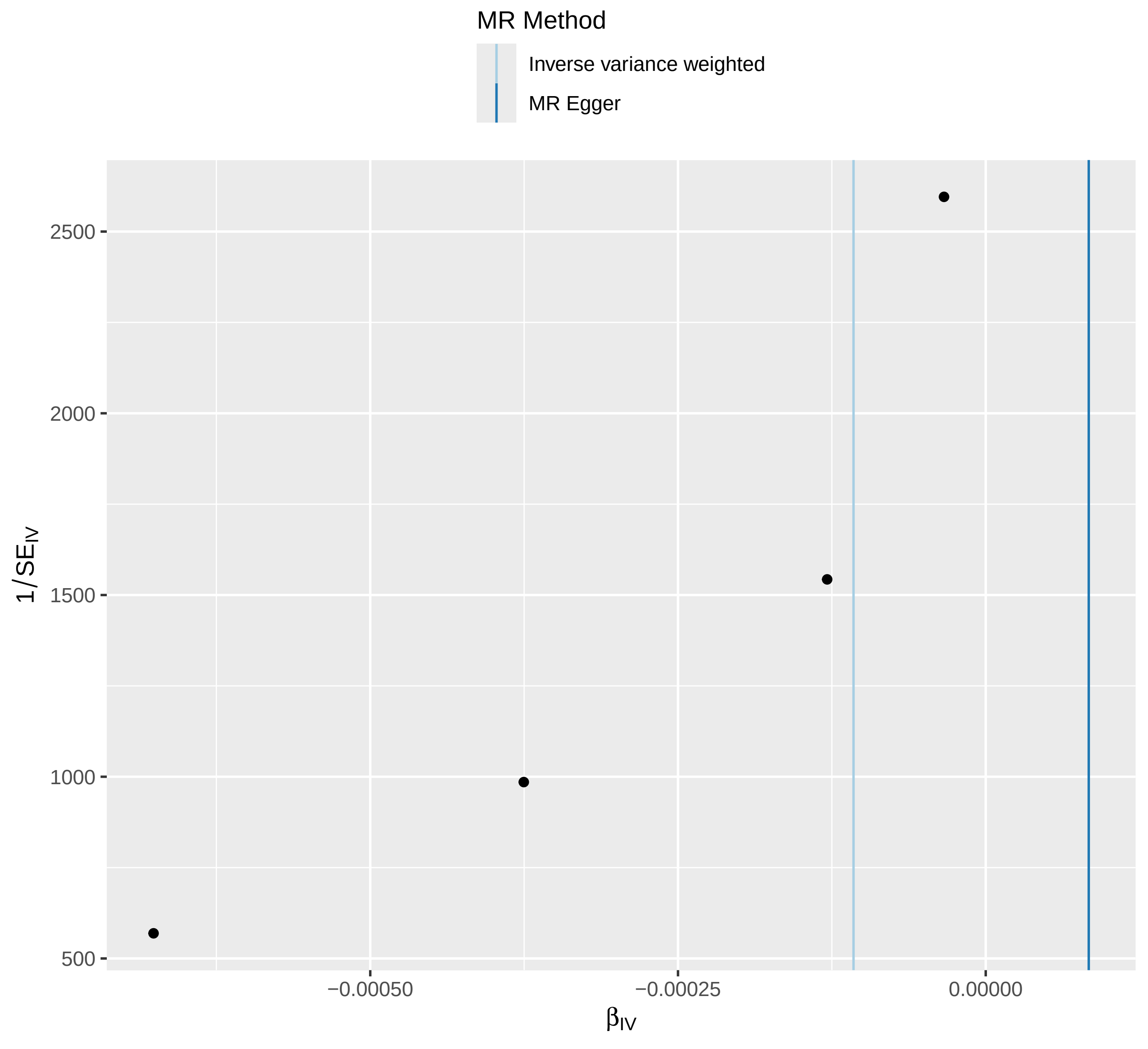
MR Method
Inverse variance weighted
MR Egger
2500
2000
1500
1000
500
1/SE$_{IV}$
−0.00050
−0.00025
0.00000
β$_{IV}$

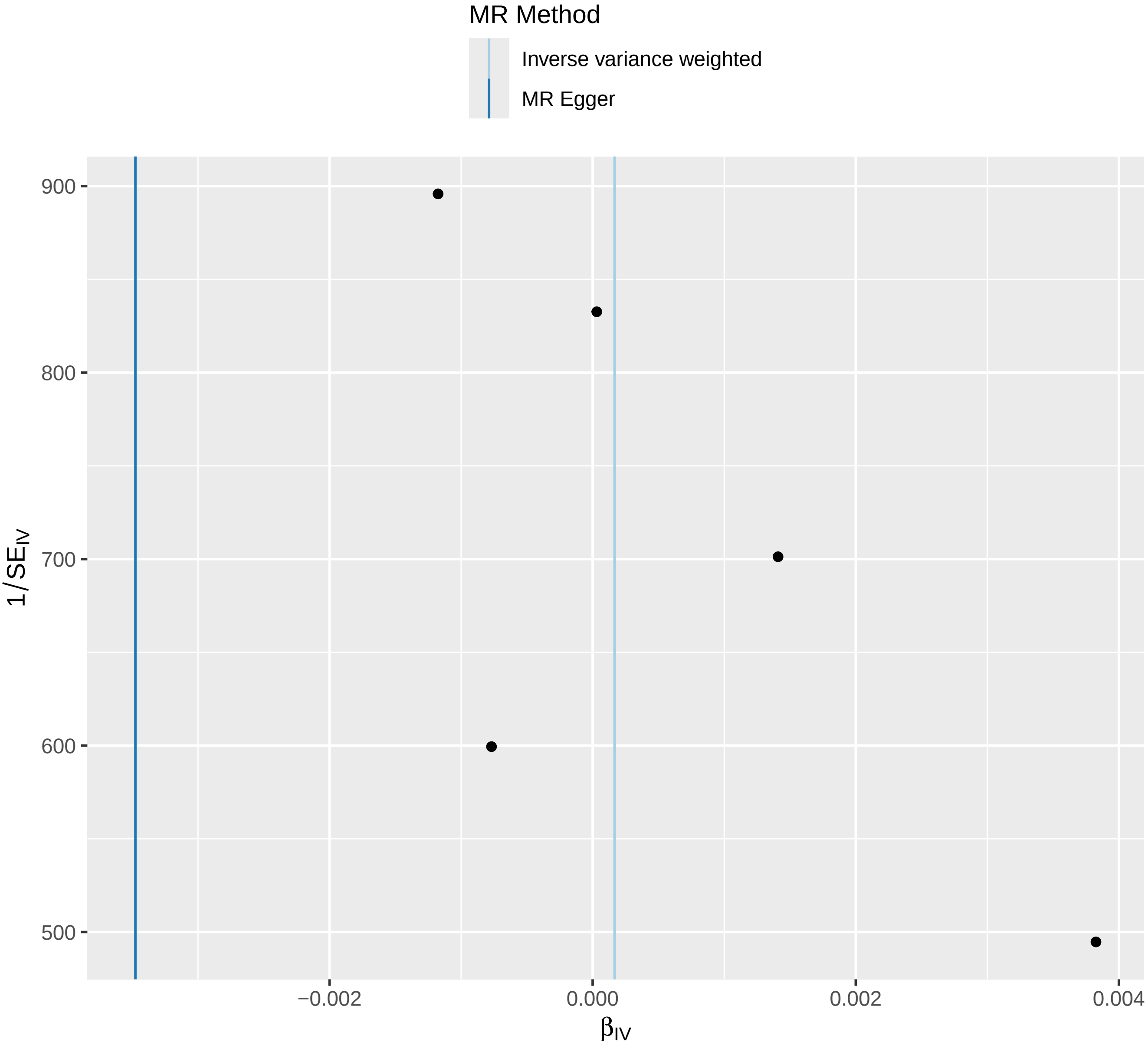
MR Method
Inverse variance weighted
MR Egger
900
800
700
600
500
1/SE_IV
−0.002
0.000
0.002
0.004
β_IV

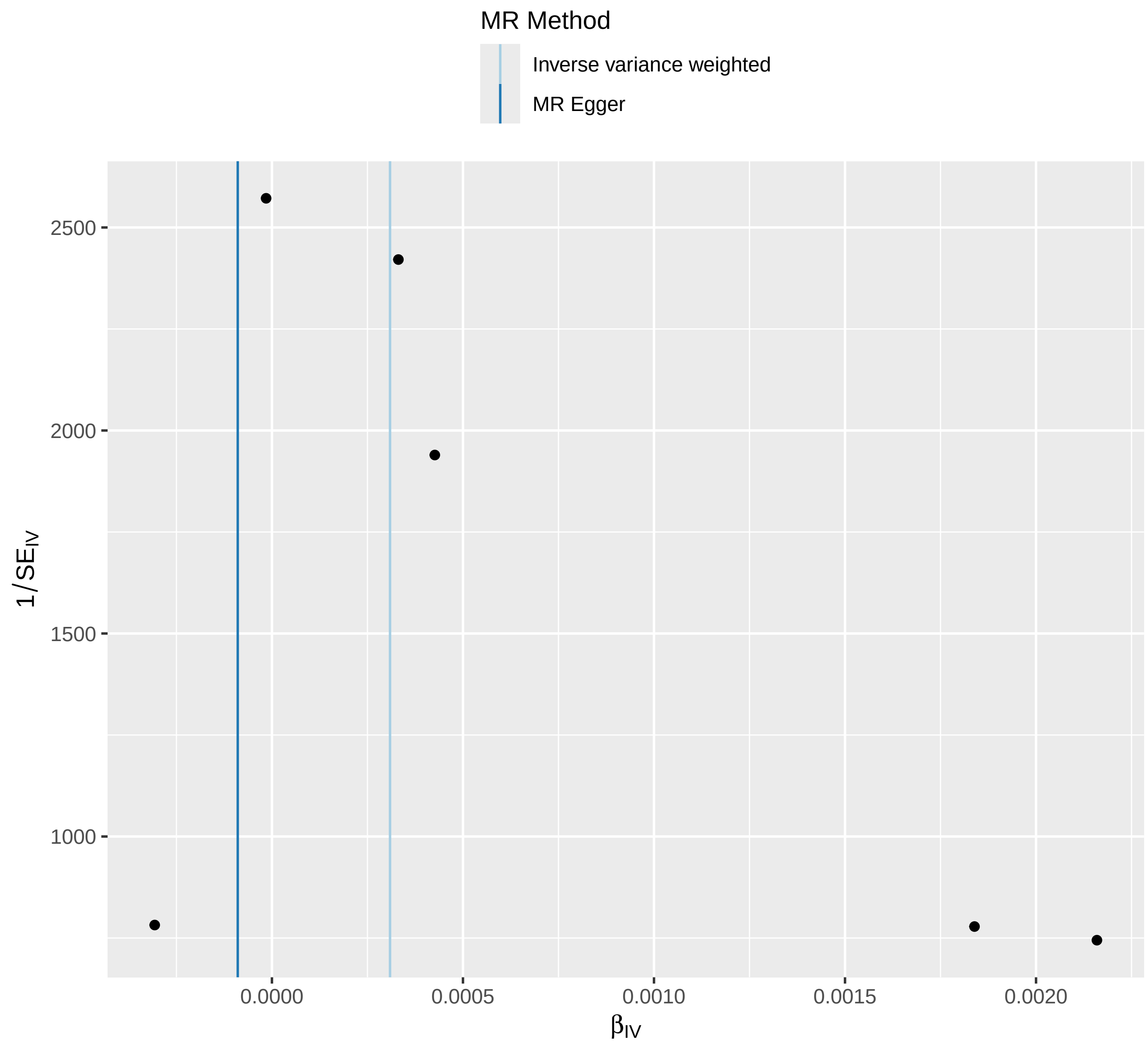
MR Method
Inverse variance weighted
MR Egger
2500
2000
1500
1000
1/SE$_{IV}$
0.0000
0.0005
0.0010
0.0015
0.0020
β$_{IV}$

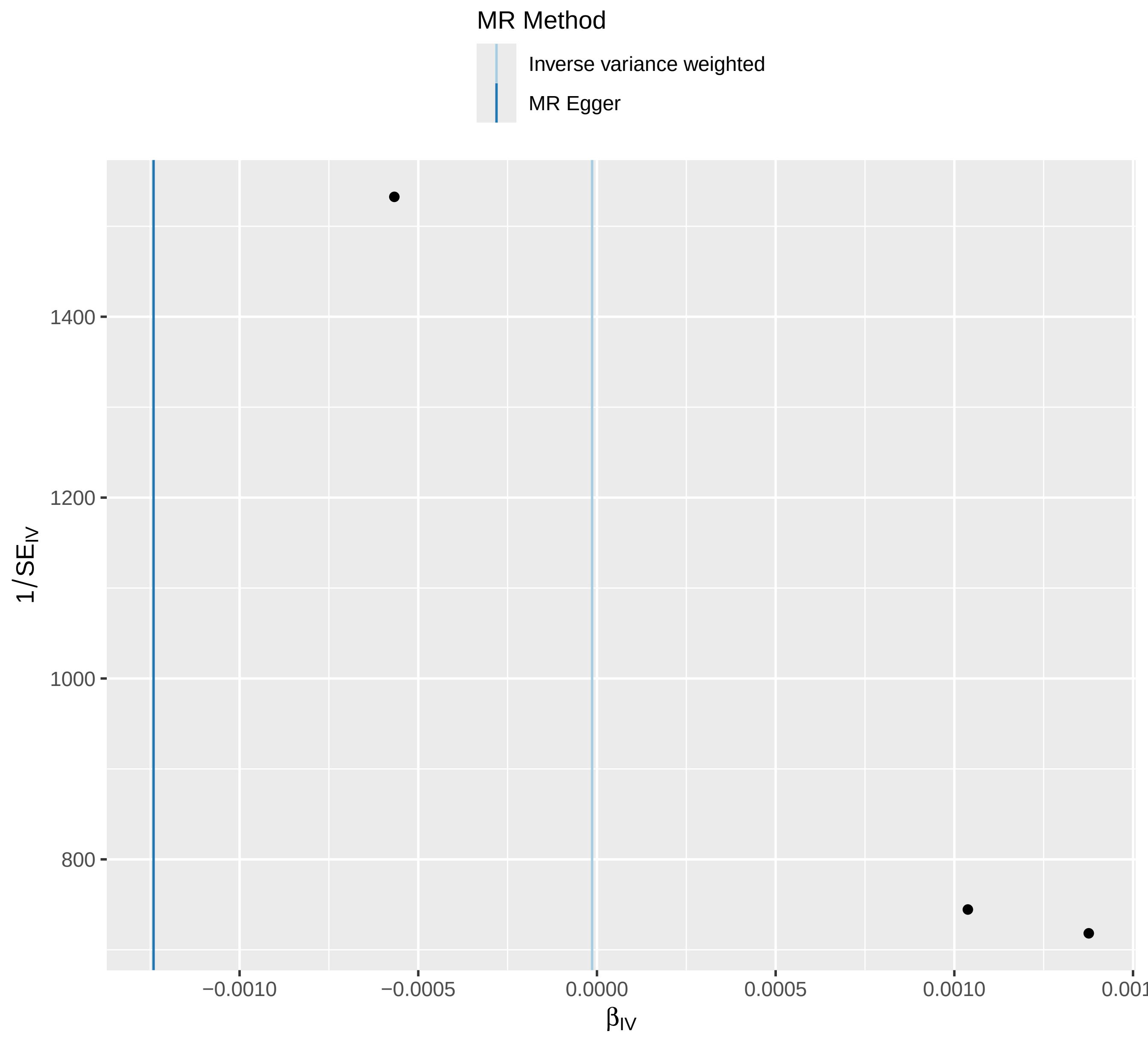
MR Method
Inverse variance weighted
MR Egger
1400
1200
1000
800
1/SE_IV
−0.0010
−0.0005
0.0000
0.0005
0.0010
0.001
β_IV

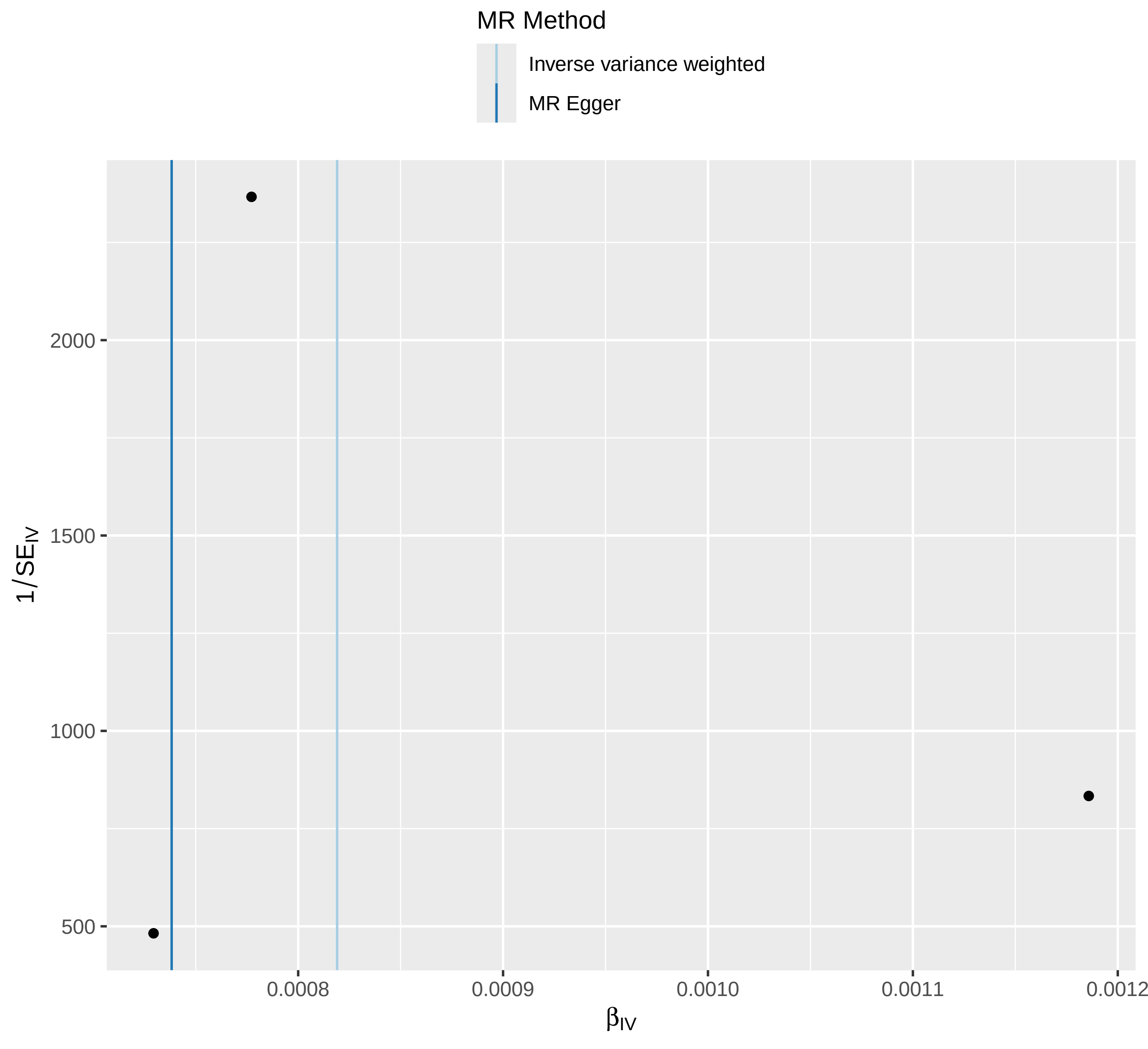

MR Method
Inverse variance weighted
MR Egger
2000
1500
1000
500
1/SE$_{IV}$
0.0008
0.0009
0.0010
0.0011
0.0012
β$_{IV}$

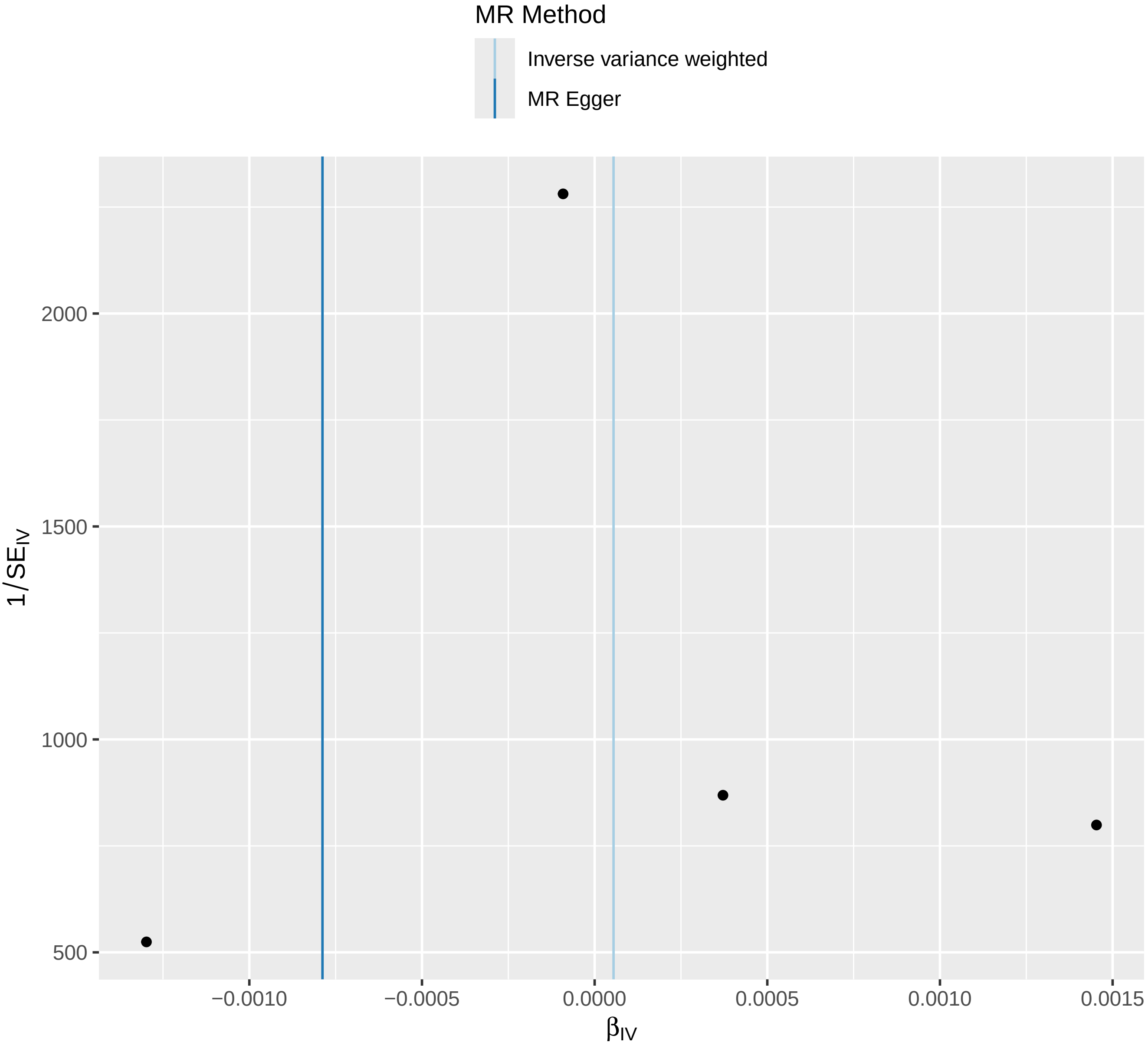
MR Method
Inverse variance weighted
MR Egger
2000
1500
1000
500
1/SE$_{IV}$
−0.0010
−0.0005
0.0000
0.0005
0.0010
0.0015
β$_{IV}$

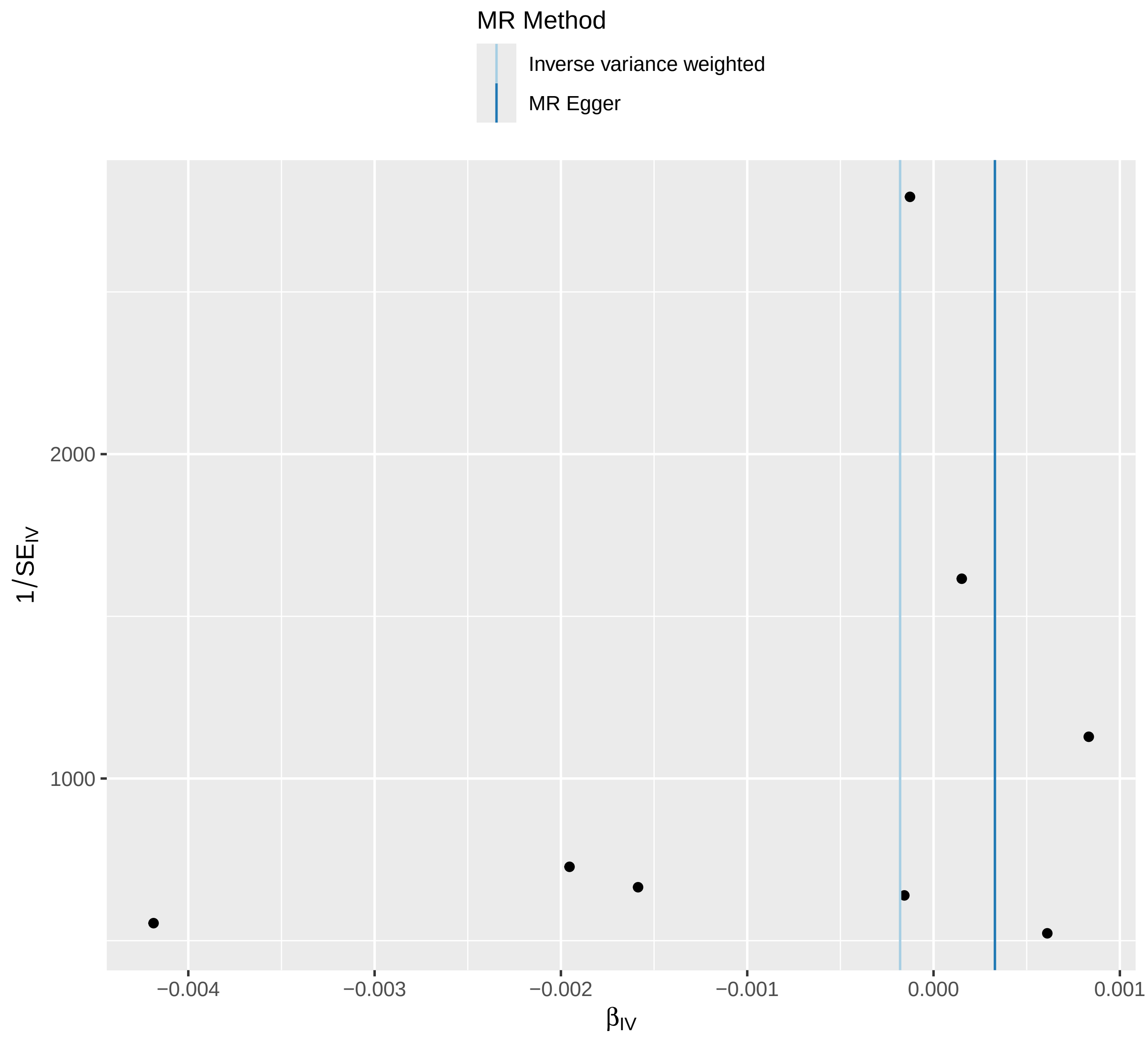
MR Method
Inverse variance weighted
MR Egger
2000
1000
1/SE_IV
−0.004
−0.003
−0.002
−0.001
0.000
0.001
β_IV

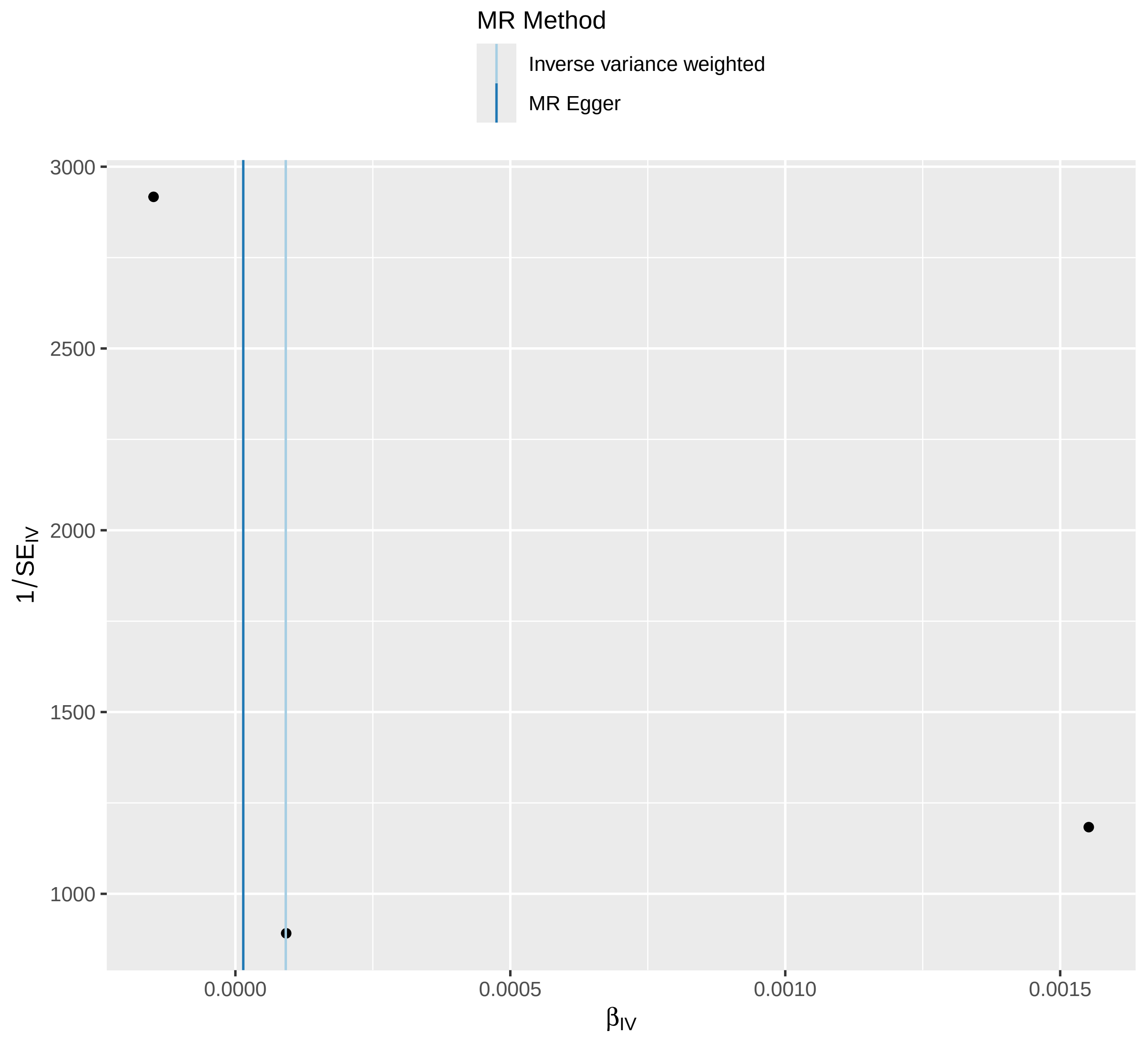

MR Method
Inverse variance weighted
MR Egger
3000
2500
2000
1500
1000
$1/SE_{IV}$
0.0000
0.0005
0.0010
0.0015
β_{IV}

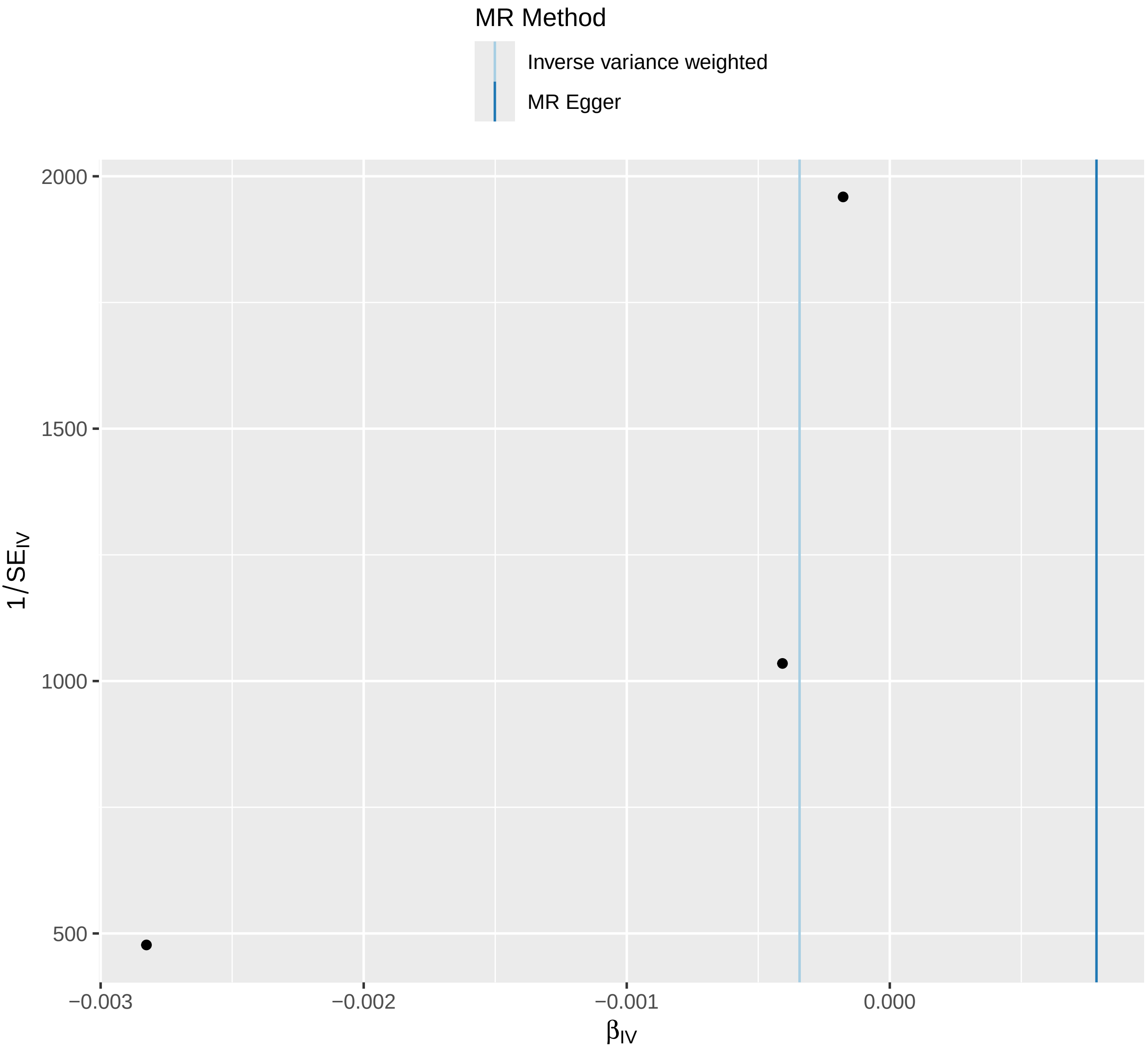
MR Method
Inverse variance weighted
MR Egger
2000
1500
1000
500
1/SE_IV
−0.003
−0.002
−0.001
0.000
β_IV

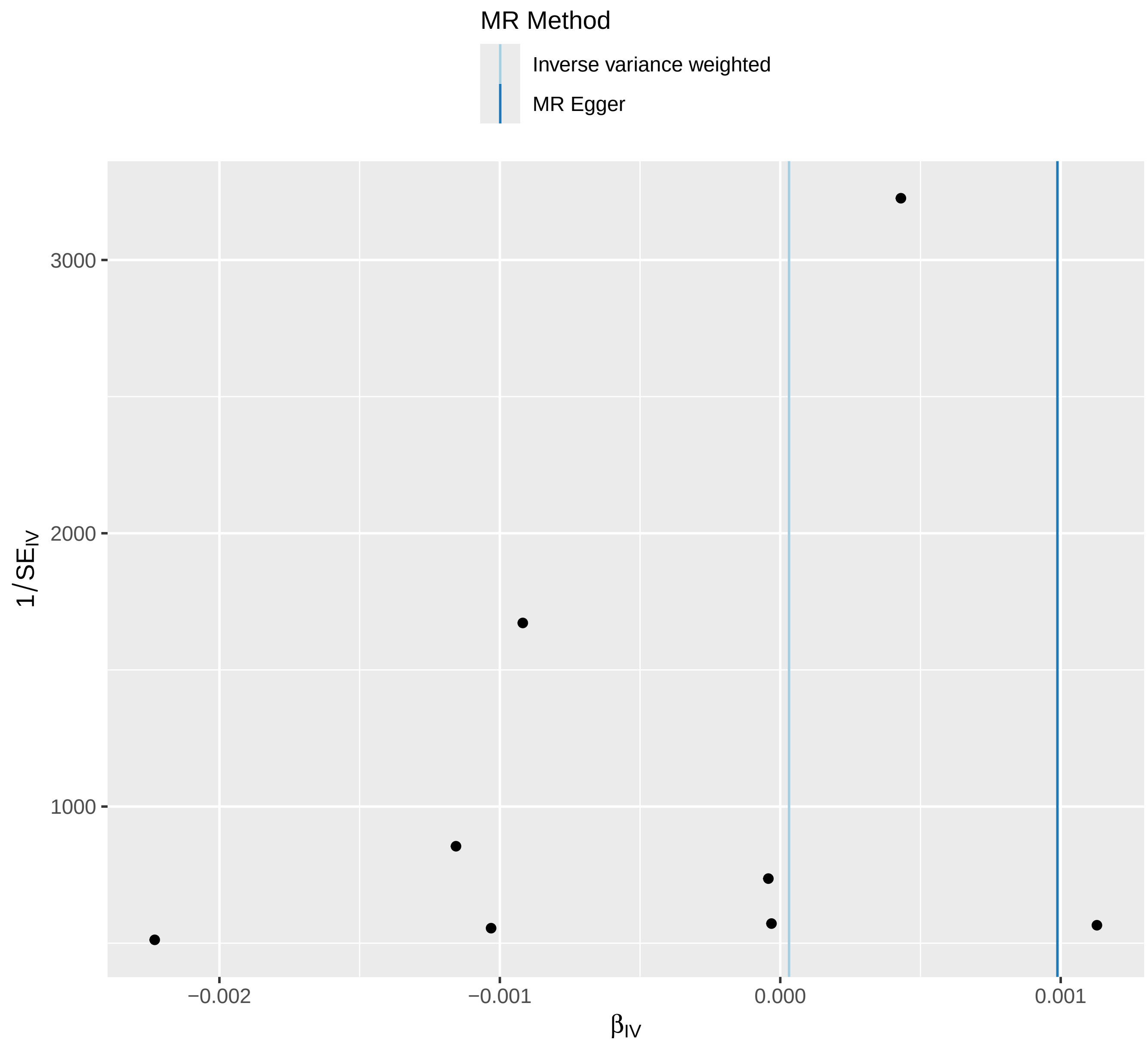

MR Method
Inverse variance weighted
MR Egger
3000
2000
1000
1/SE_IV
−0.002
−0.001
0.000
0.001
β_IV

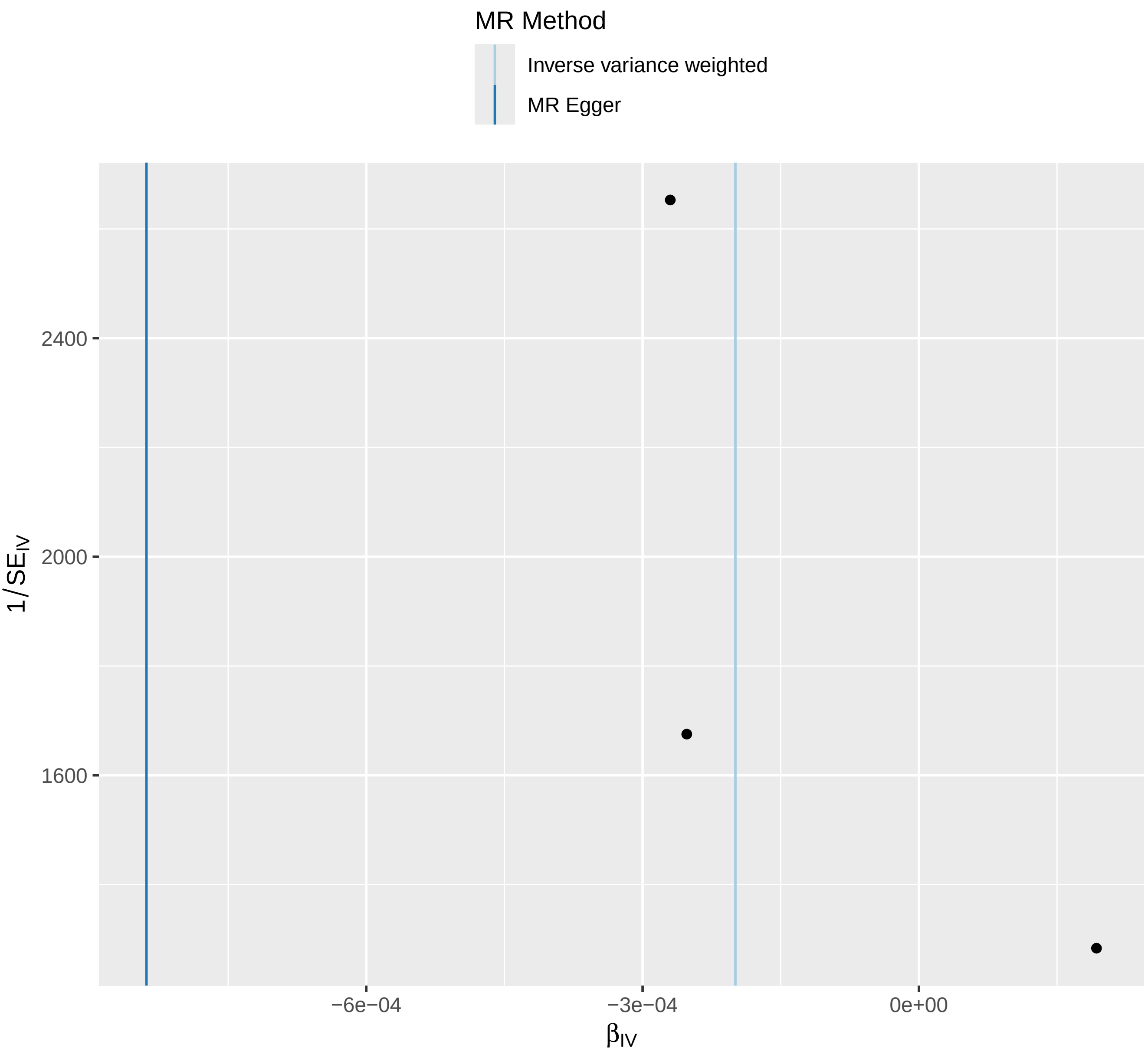
MR Method
Inverse variance weighted
MR Egger
2400
2000
1600
1/SE_{IV}
−6e−04
−3e−04
0e+00
β_{IV}

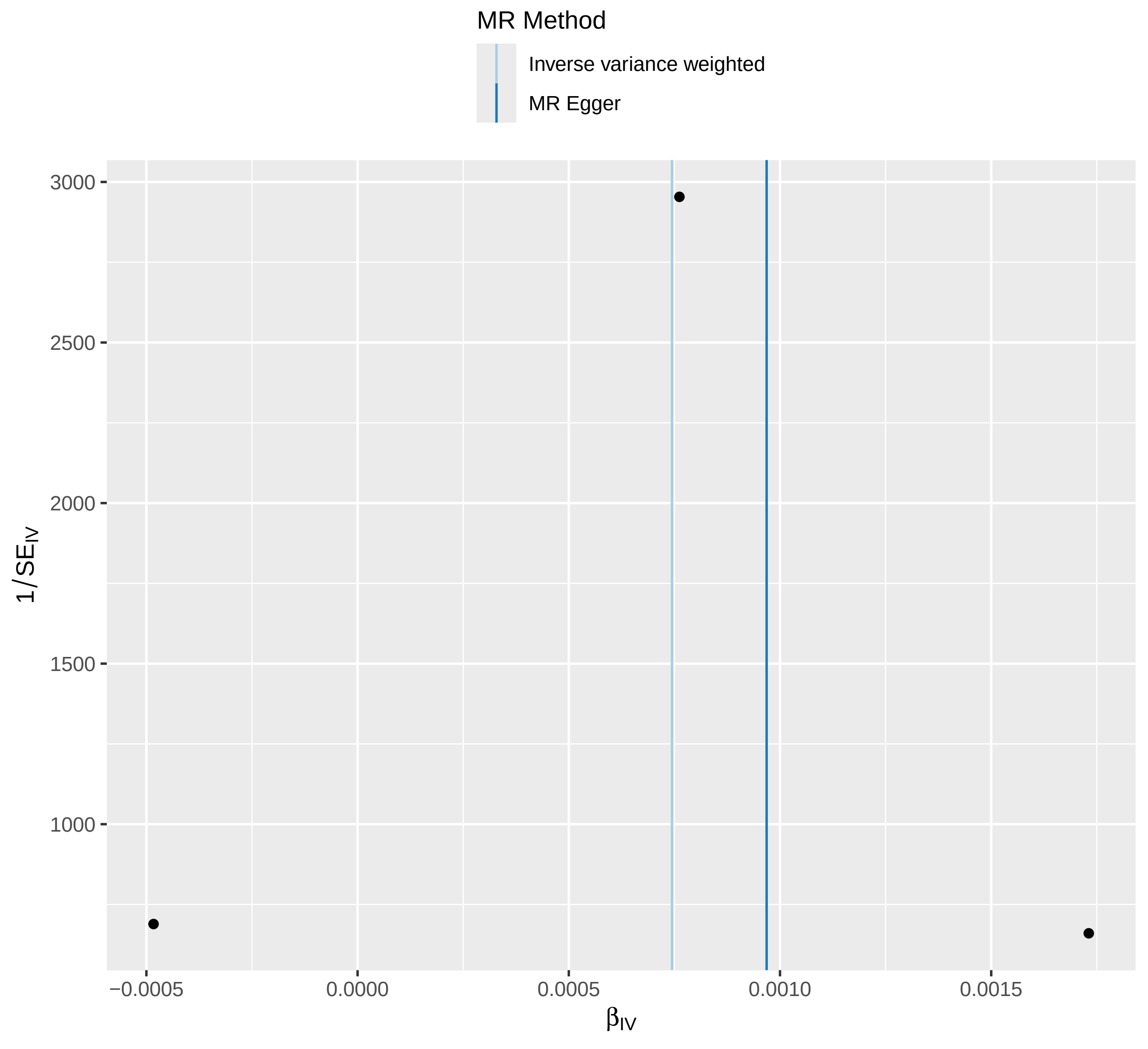
MR Method
Inverse variance weighted
MR Egger
3000
2500
2000
1500
1000
1/SE_IV
−0.0005
0.0000
0.0005
0.0010
0.0015
β_IV

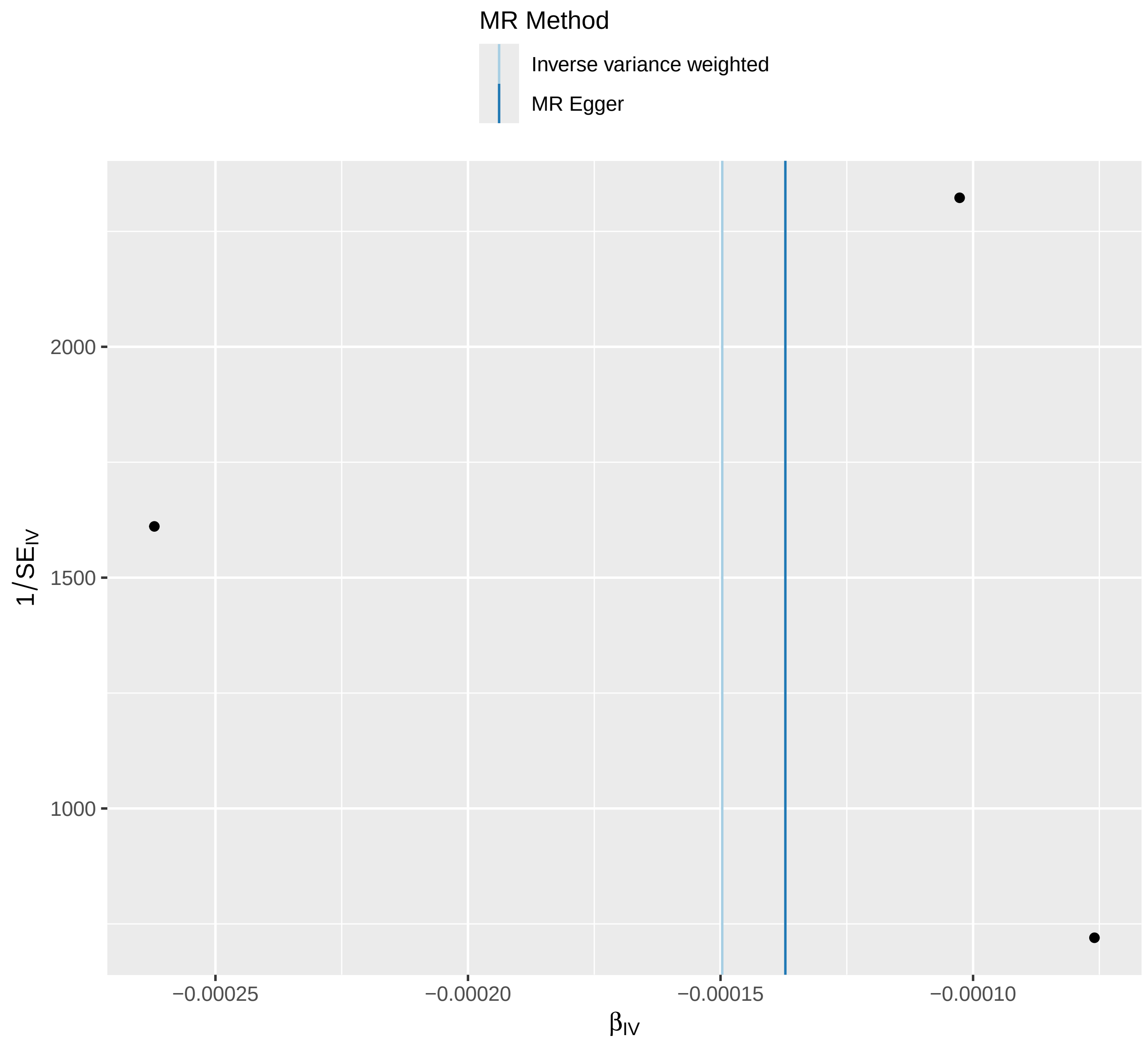

MR Method
Inverse variance weighted
MR Egger
2000
1500
1000
1/SE_IV
−0.00025
−0.00020
−0.00015
−0.00010
β_IV

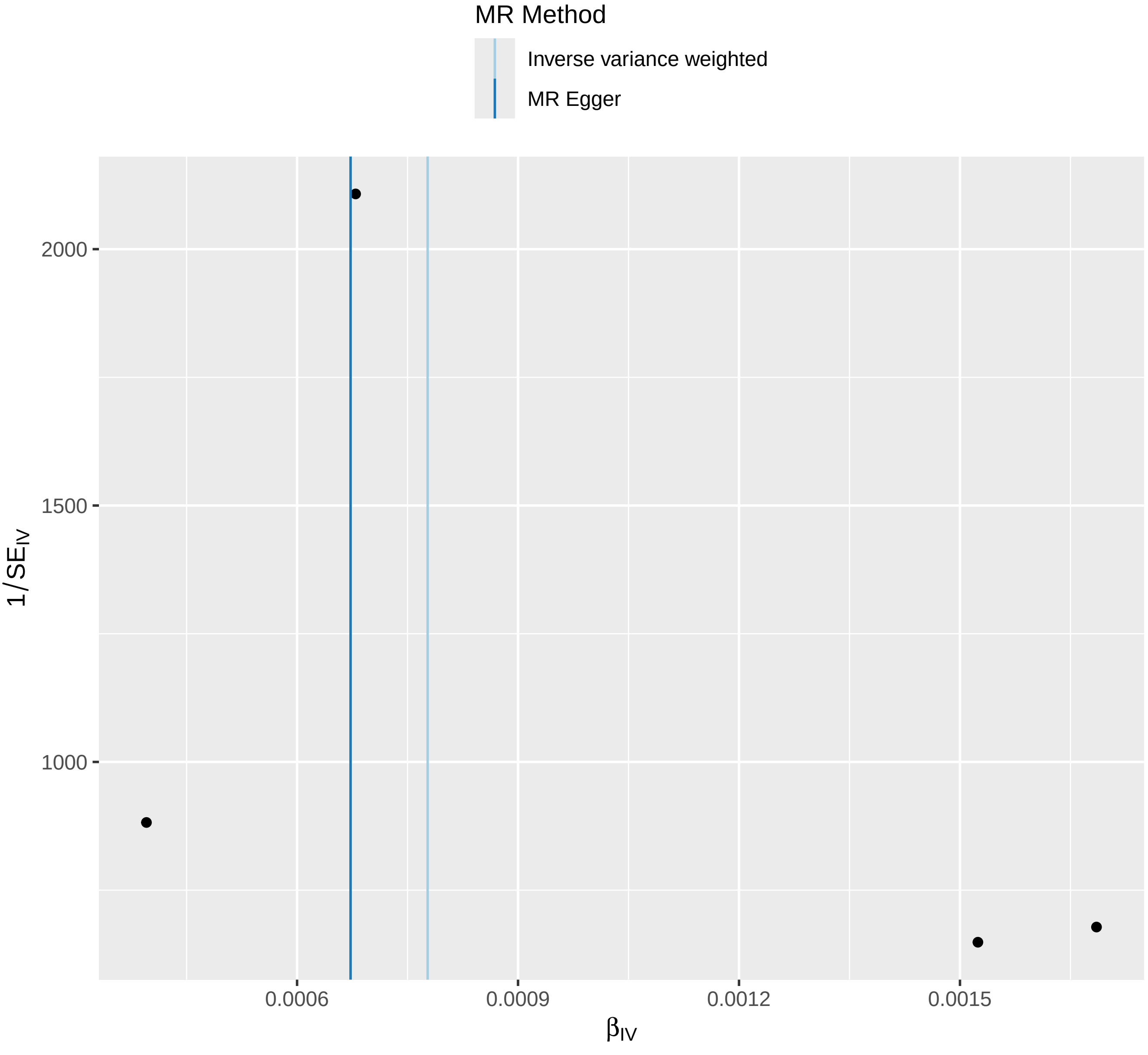
MR Method
Inverse variance weighted
MR Egger
2000
1500
1000
1/SE_IV
0.0006
0.0009
0.0012
0.0015
β_IV

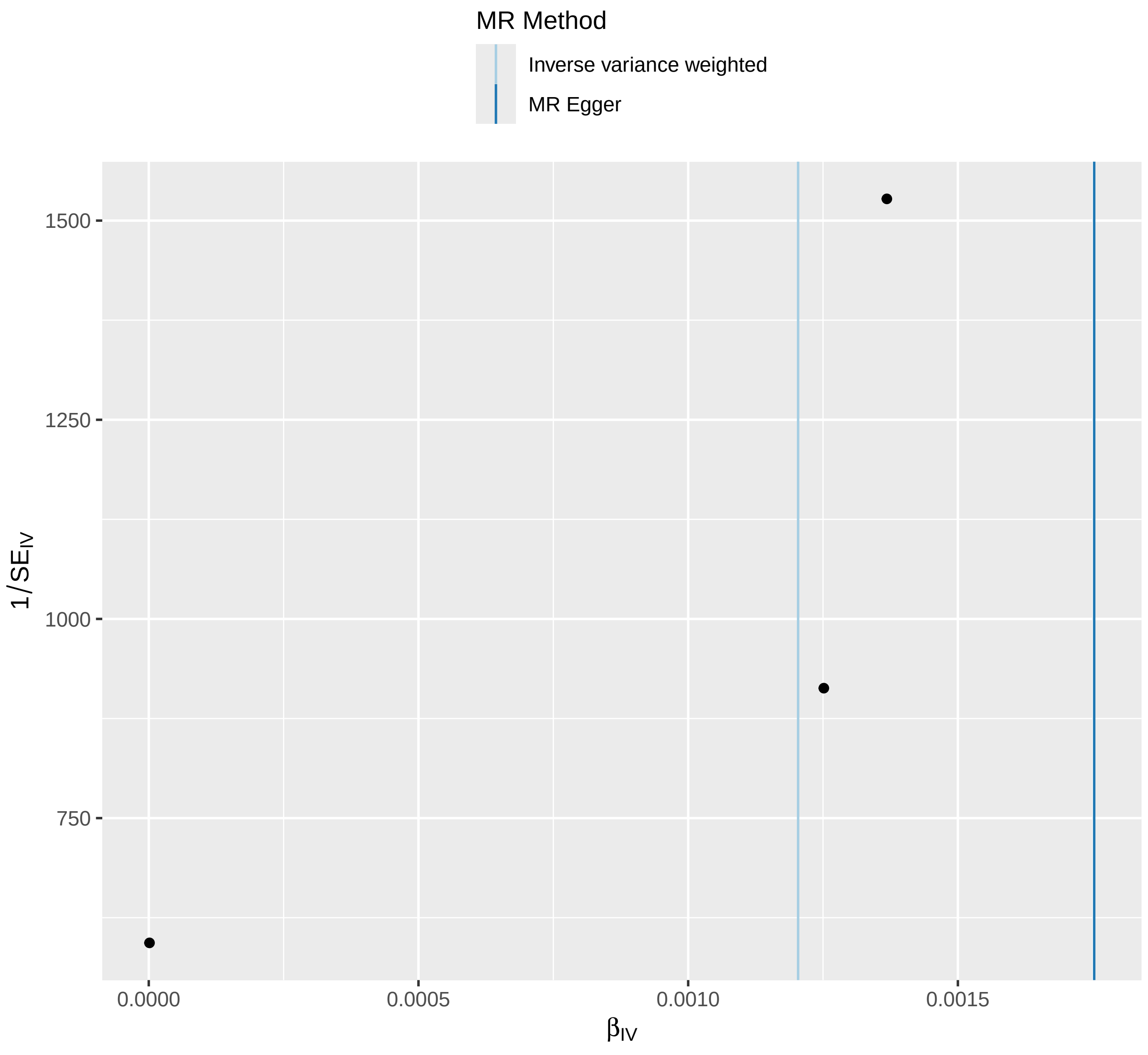

MR Method
Inverse variance weighted
MR Egger
1500
1250
1000
750
1/SE$_{IV}$
0.0000
0.0005
0.0010
0.0015
β_{IV}

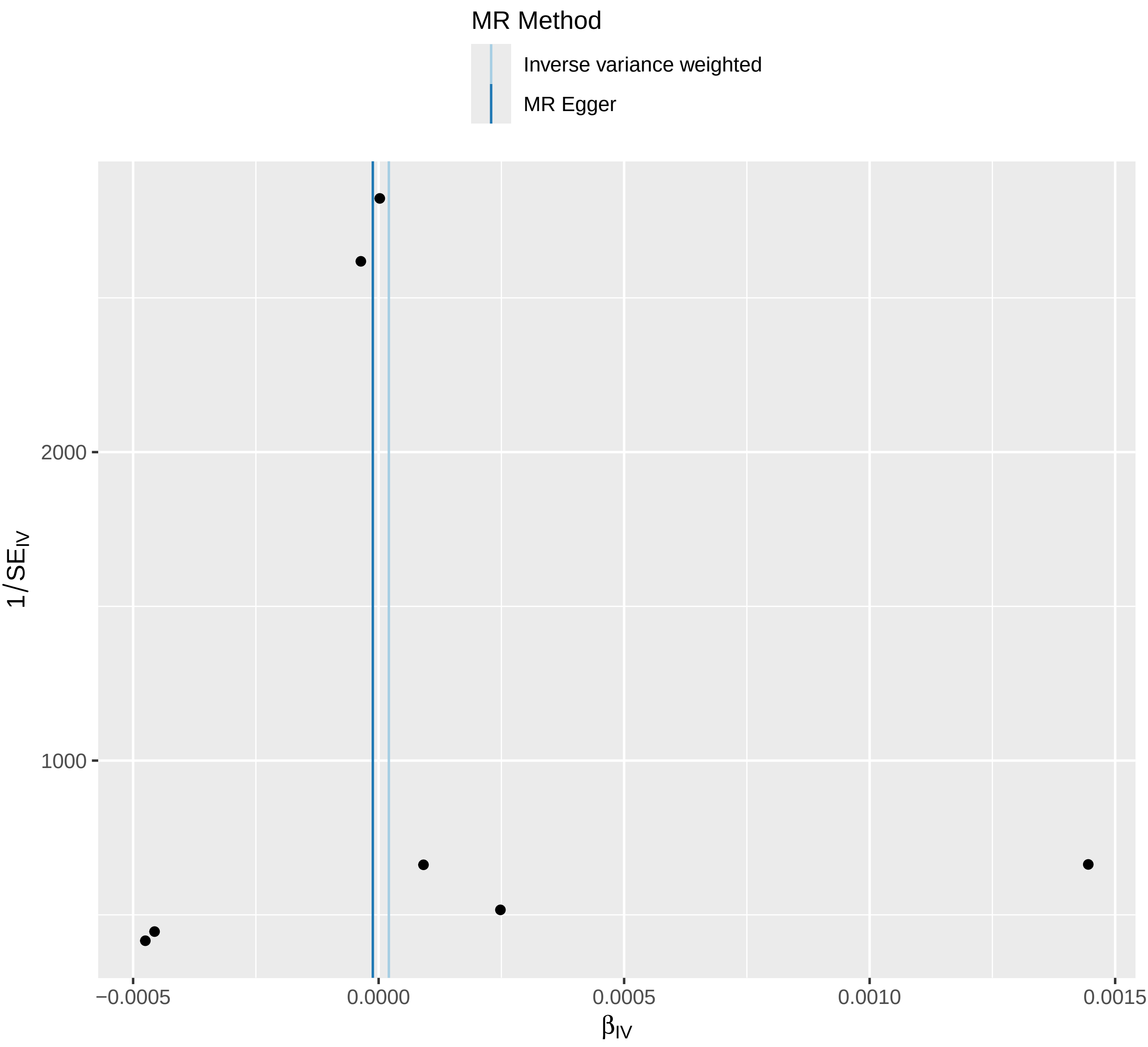
MR Method
Inverse variance weighted
MR Egger
1/SE_IV
2000
1000
β_IV
−0.0005
0.0000
0.0005
0.0010
0.0015

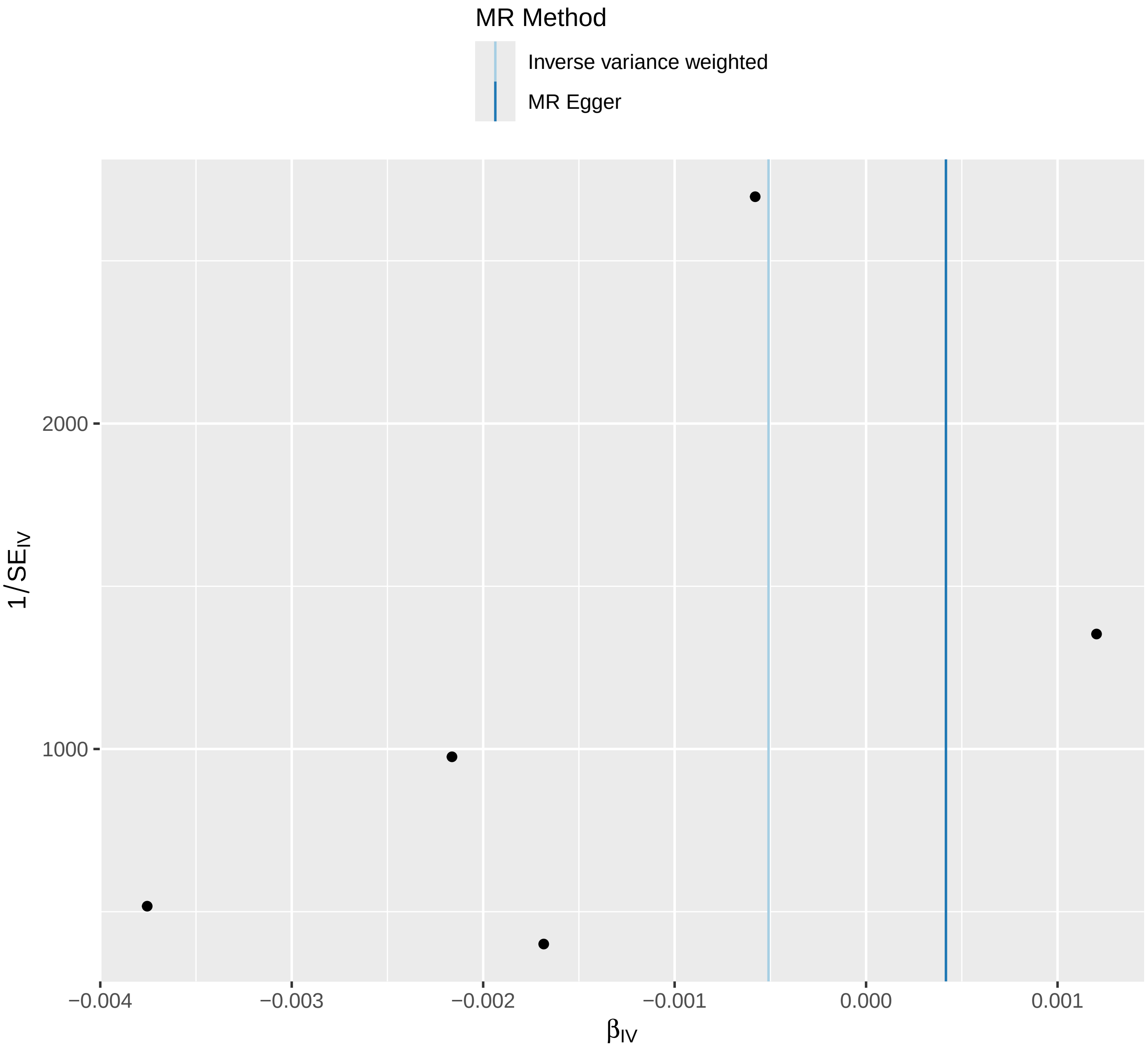

MR Method
Inverse variance weighted
MR Egger
2000
1000
1/SE$_{IV}$
−0.004
−0.003
−0.002
−0.001
0.000
0.001
β$_{IV}$

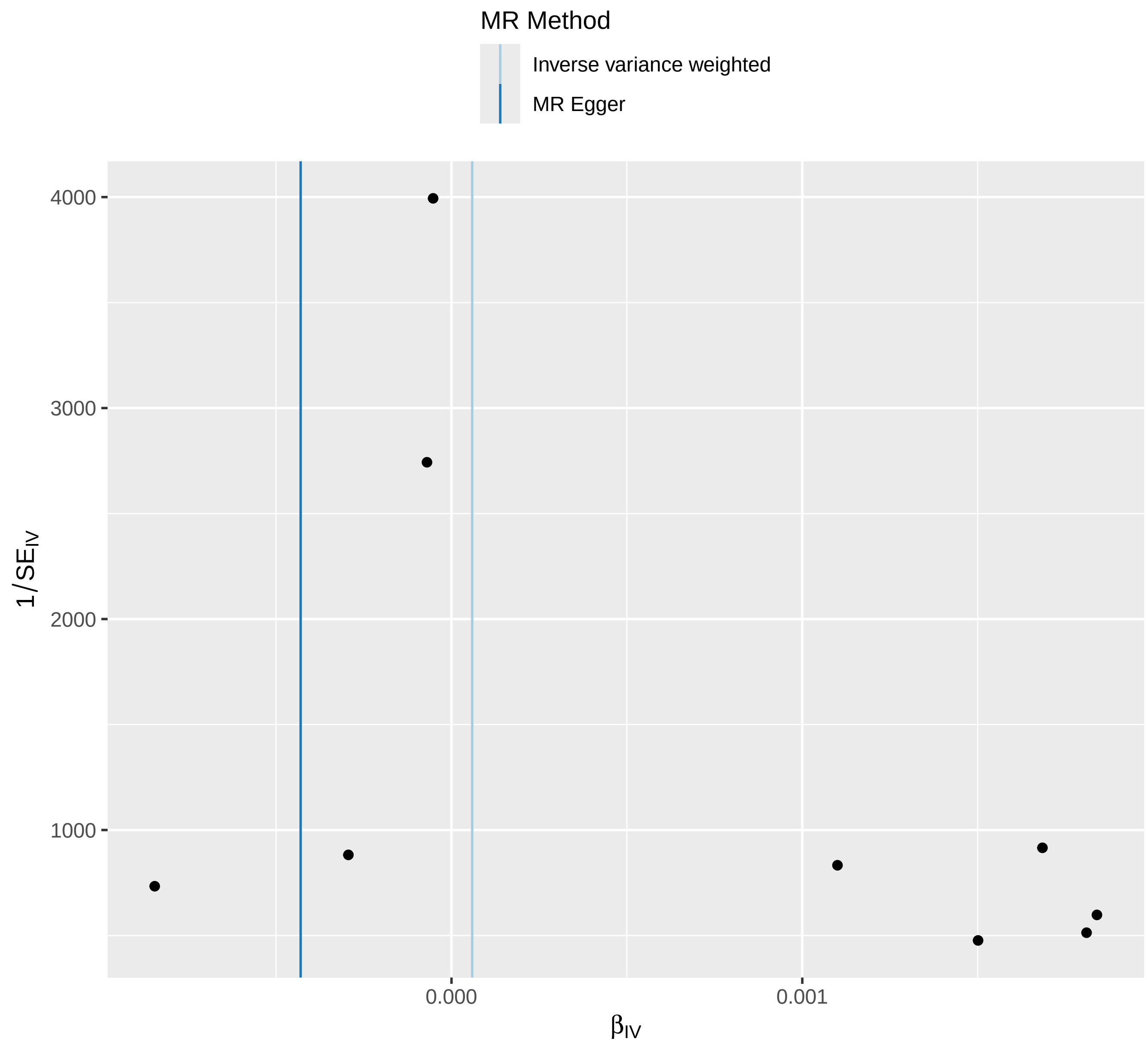
MR Method
Inverse variance weighted
MR Egger
4000
3000
2000
1000
1/SE_IV
0.000
0.001
β_IV

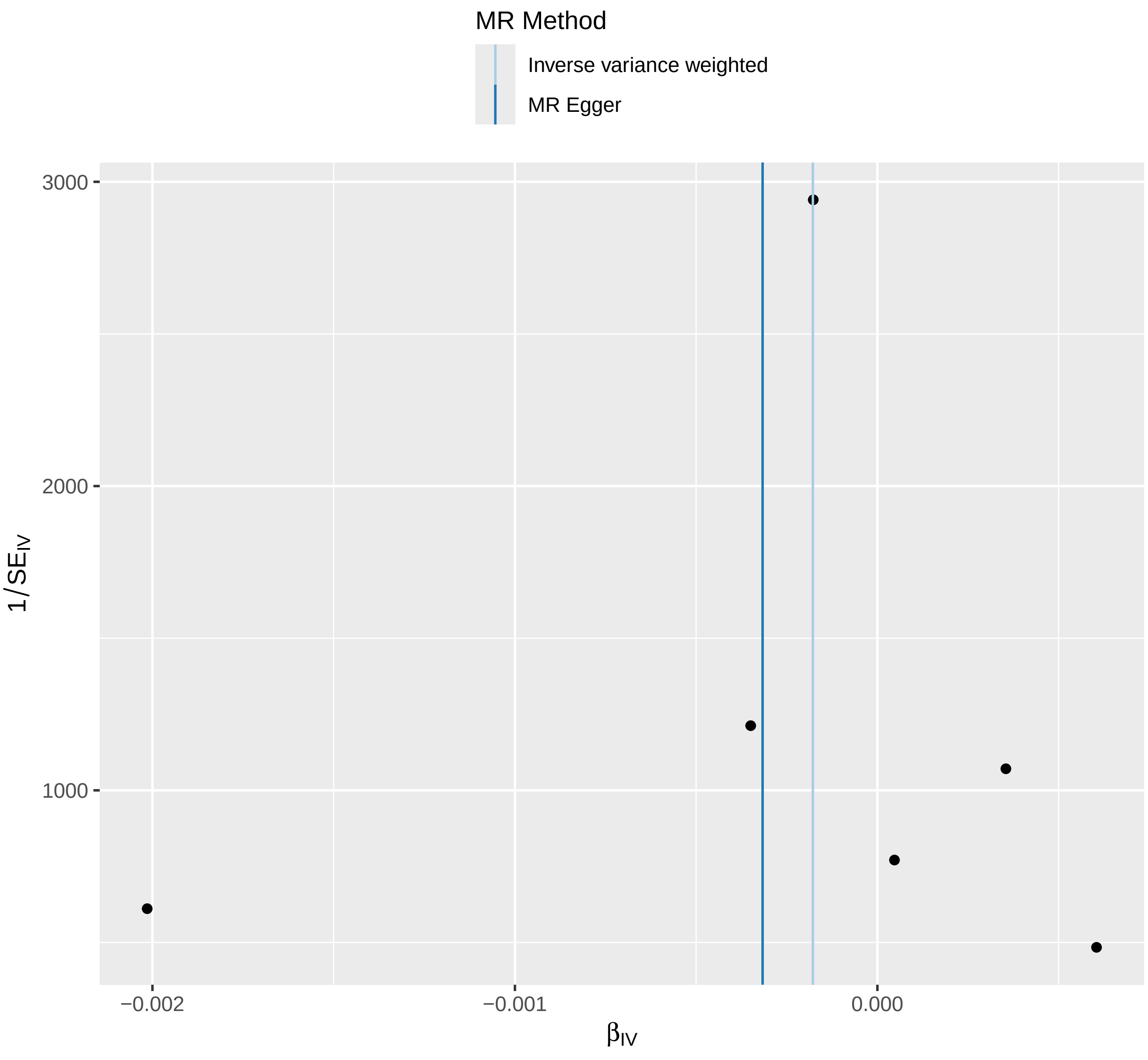
MR Method
Inverse variance weighted
MR Egger
3000
2000
1000
$1/SE_{IV}$
−0.002
−0.001
0.000
β_{IV}
