# Supplementary material for: Integrative multi-omics and single-cell transcriptomics reveal ARHGEF12 driving chemoresistance in bladder cancer
Source: Hereditas. 2025 Nov 27;162:234. doi: 10.1186/s41065-025-00606-1 (PMC12661753; doi:10.1186/s41065-025-00606-1)
Supplement: Supplementary file 1 — Supplementary Material 1. [file 41065_2025_606_MOESM1_ESM.zip › Supplementary1/Supplementary - MR/eQTL-MR/MR/pic.leaveoneout.pdf]

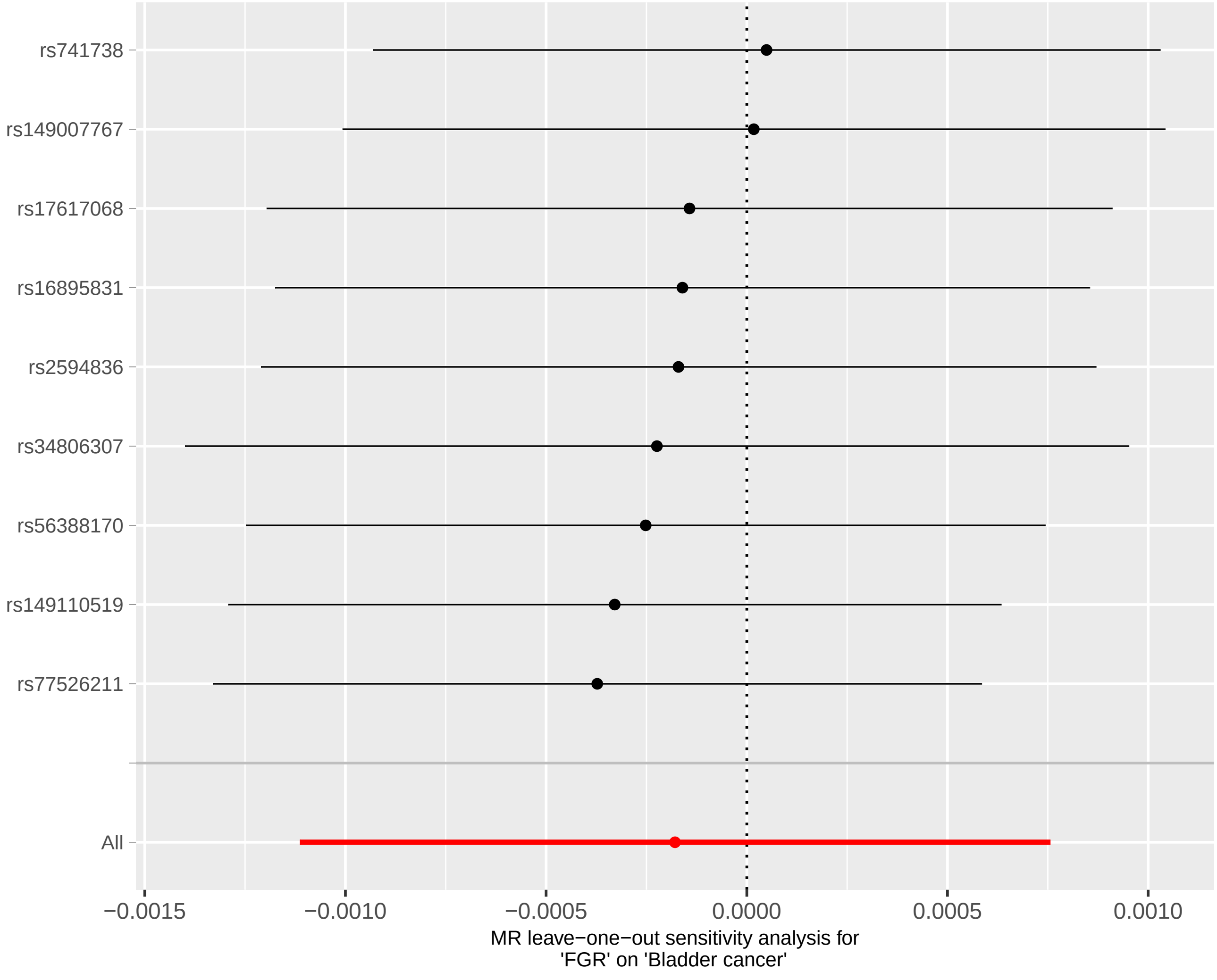
rs741738
rs149007767
rs17617068
rs16895831
rs2594836
rs34806307
rs56388170
rs149110519
rs77526211
All
−0.0015
−0.0010
−0.0005
0.0000
0.0005
0.0010
MR leave−one−out sensitivity analysis for
'FGR' on 'Bladder cancer'

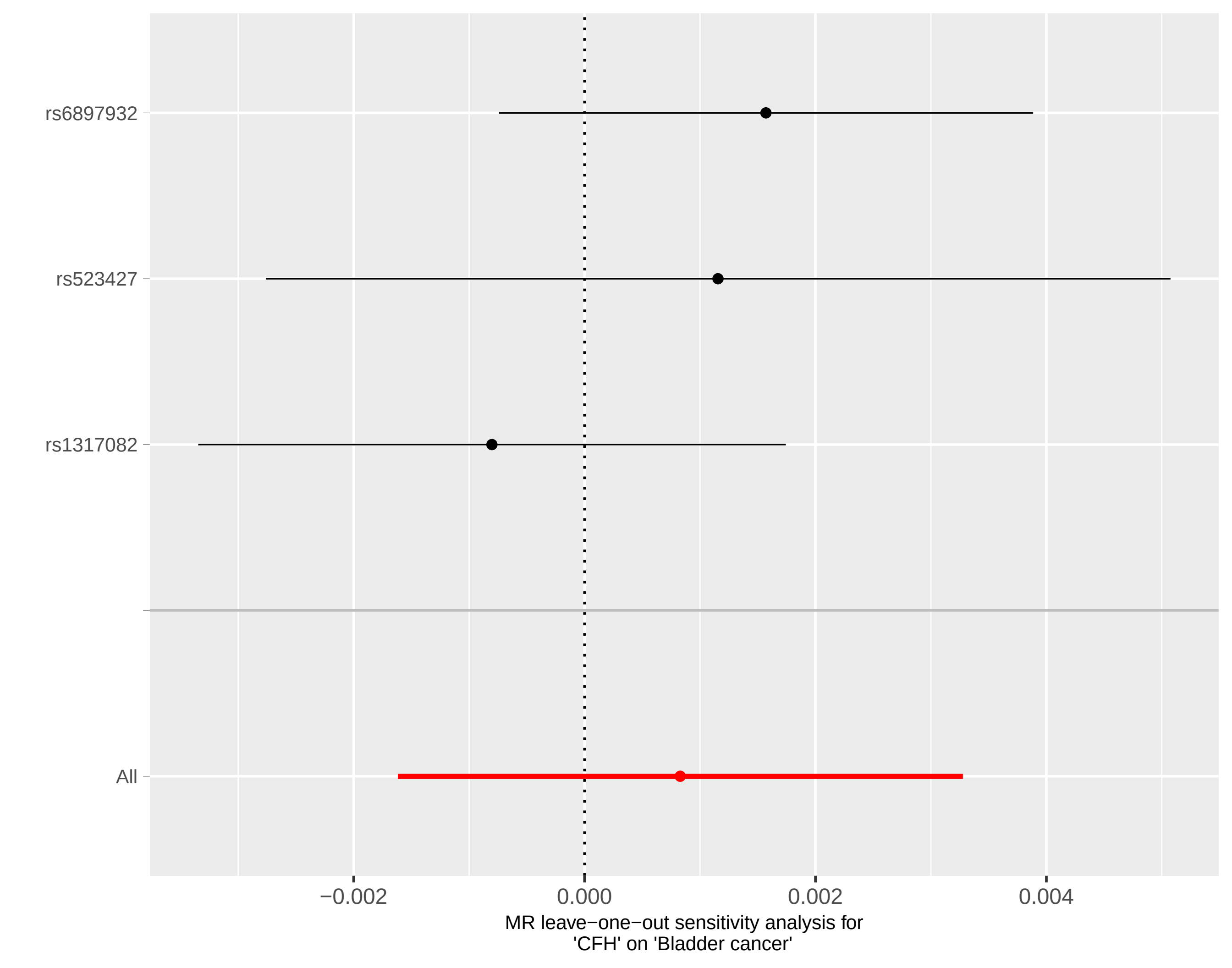
rs6897932
rs523427
rs1317082
All
−0.002
0.000
0.002
0.004
MR leave−one−out sensitivity analysis for
'CFH' on 'Bladder cancer'

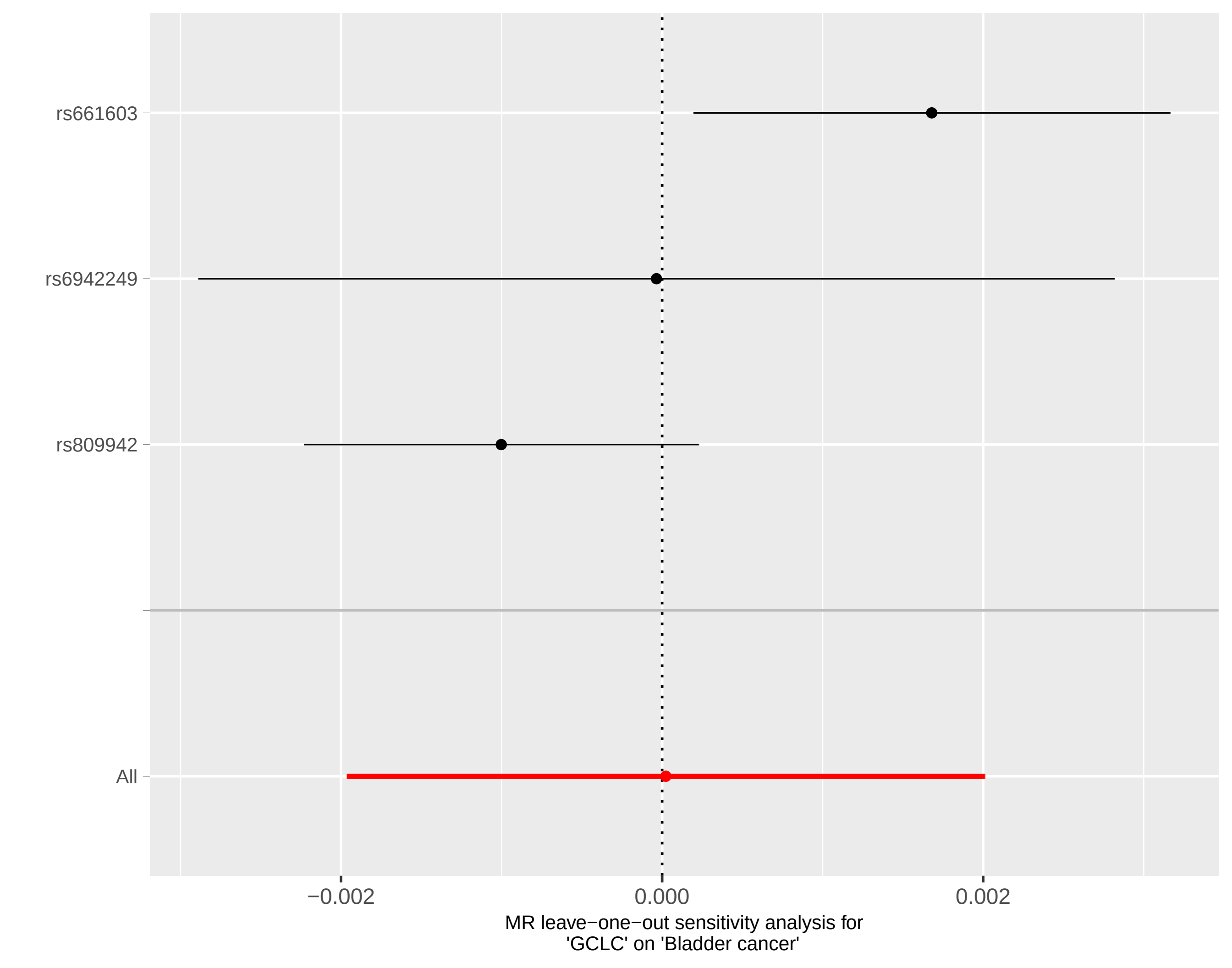
rs661603
rs6942249
rs809942
All
−0.002
0.000
0.002
MR leave−one−out sensitivity analysis for
'GCLC' on 'Bladder cancer'

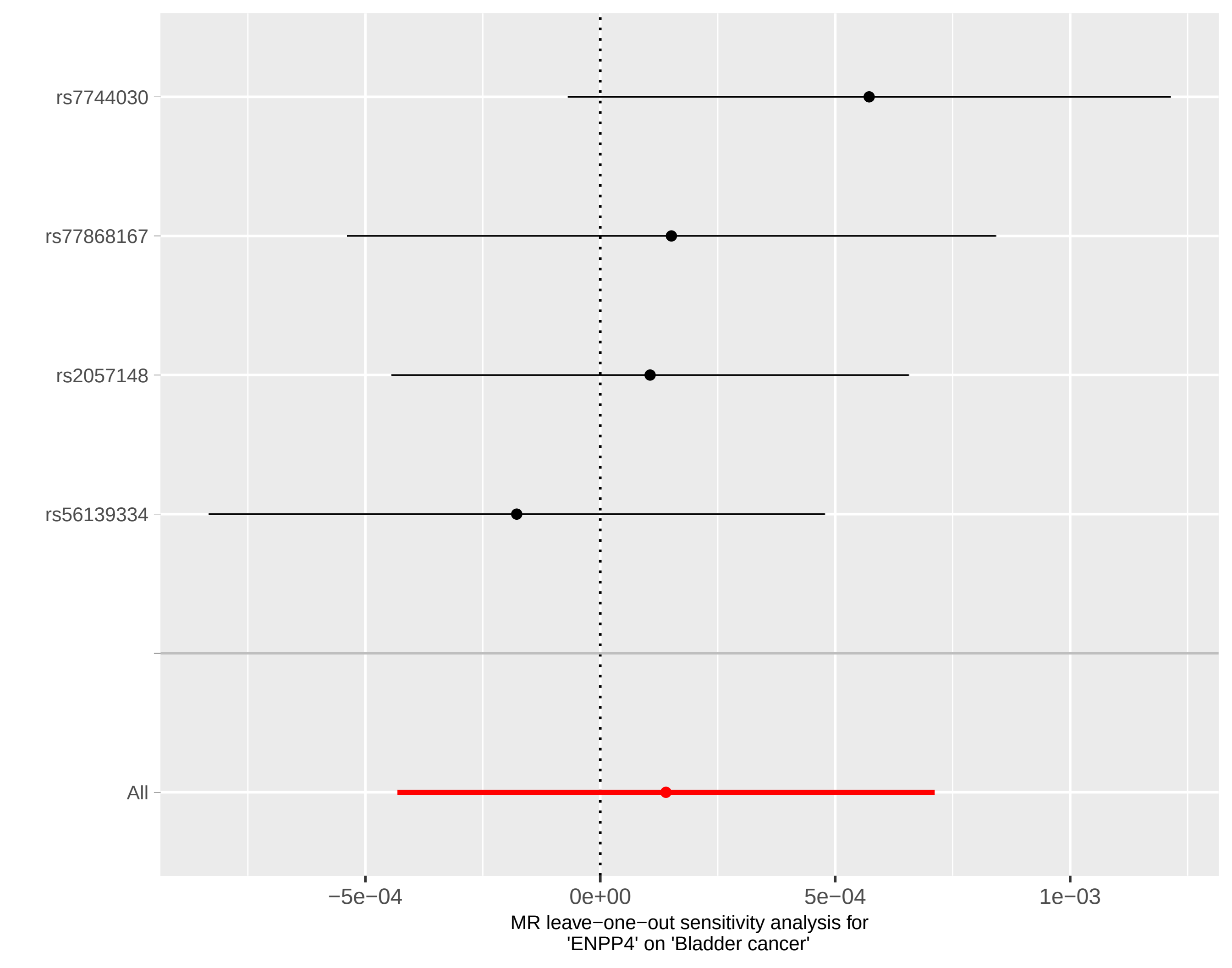

rs7744030
rs77868167
rs2057148
rs56139334
All
−5e−04
0e+00
5e−04
1e−03
MR leave−one−out sensitivity analysis for
'ENPP4' on 'Bladder cancer'

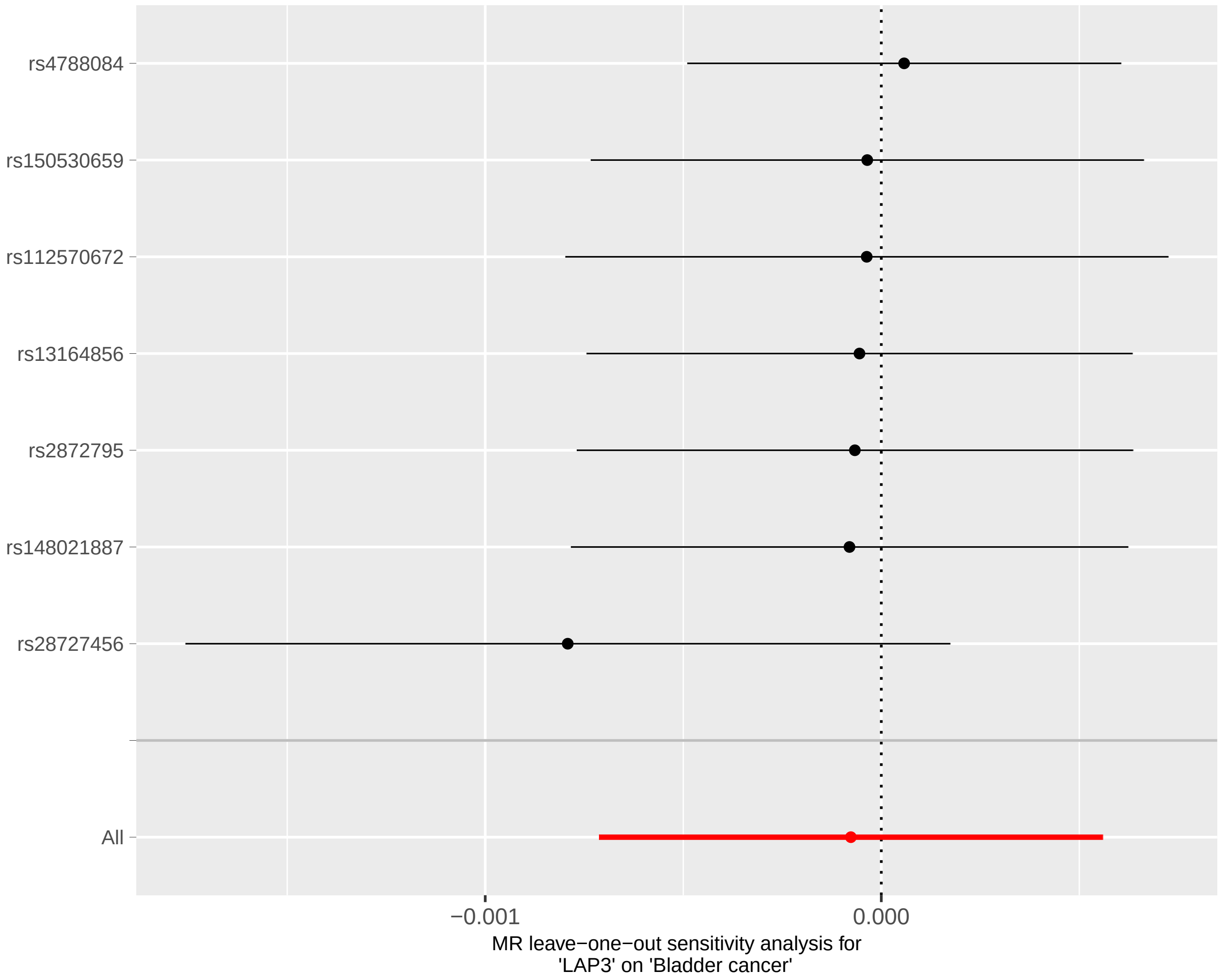

rs4788084
rs150530659
rs112570672
rs13164856
rs2872795
rs148021887
rs28727456
All
−0.001
0.000
MR leave−one−out sensitivity analysis for
'LAP3' on 'Bladder cancer'

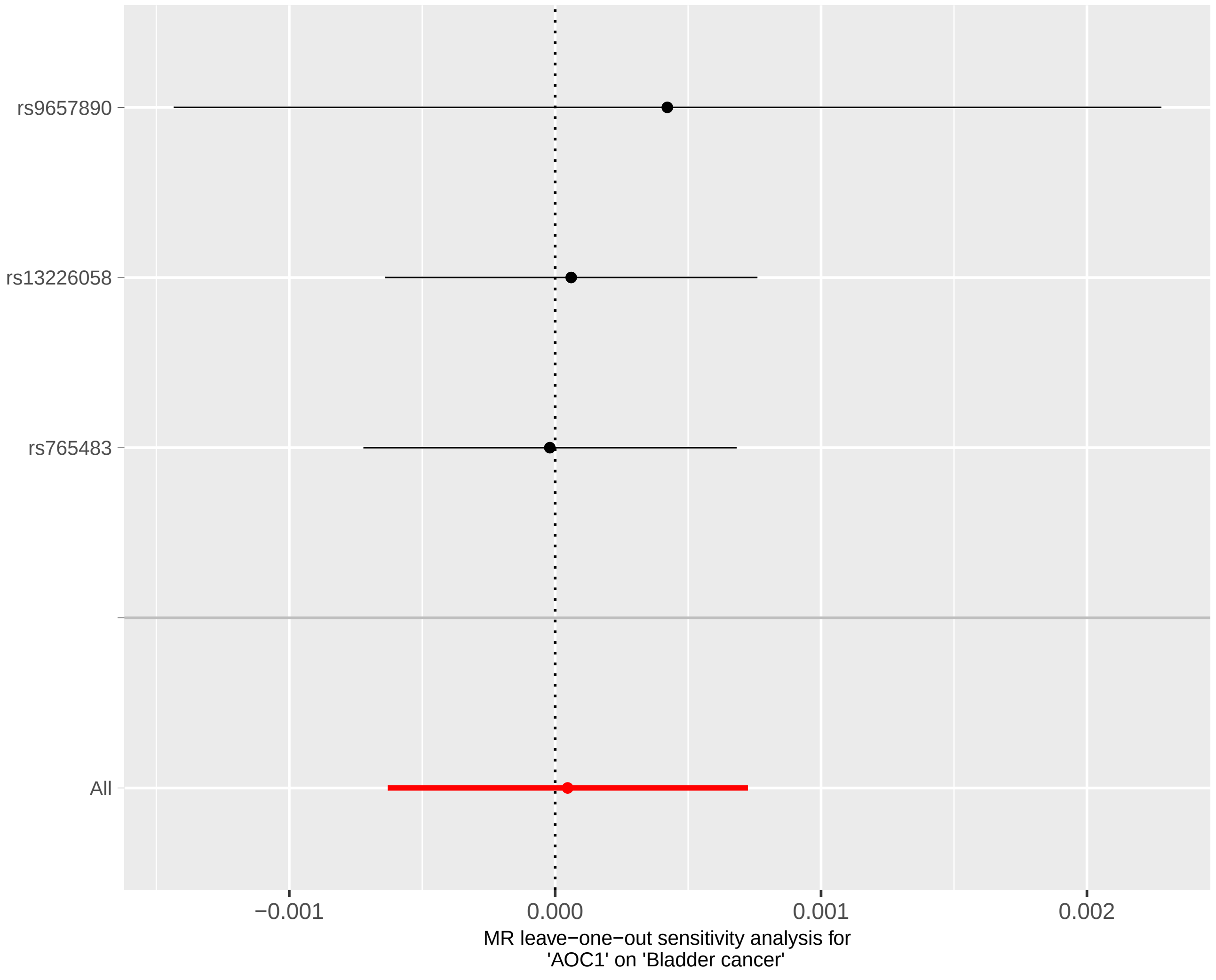

rs9657890
rs13226058
rs765483
All
−0.001
0.000
0.001
0.002
MR leave−one−out sensitivity analysis for
'AOC1' on 'Bladder cancer'

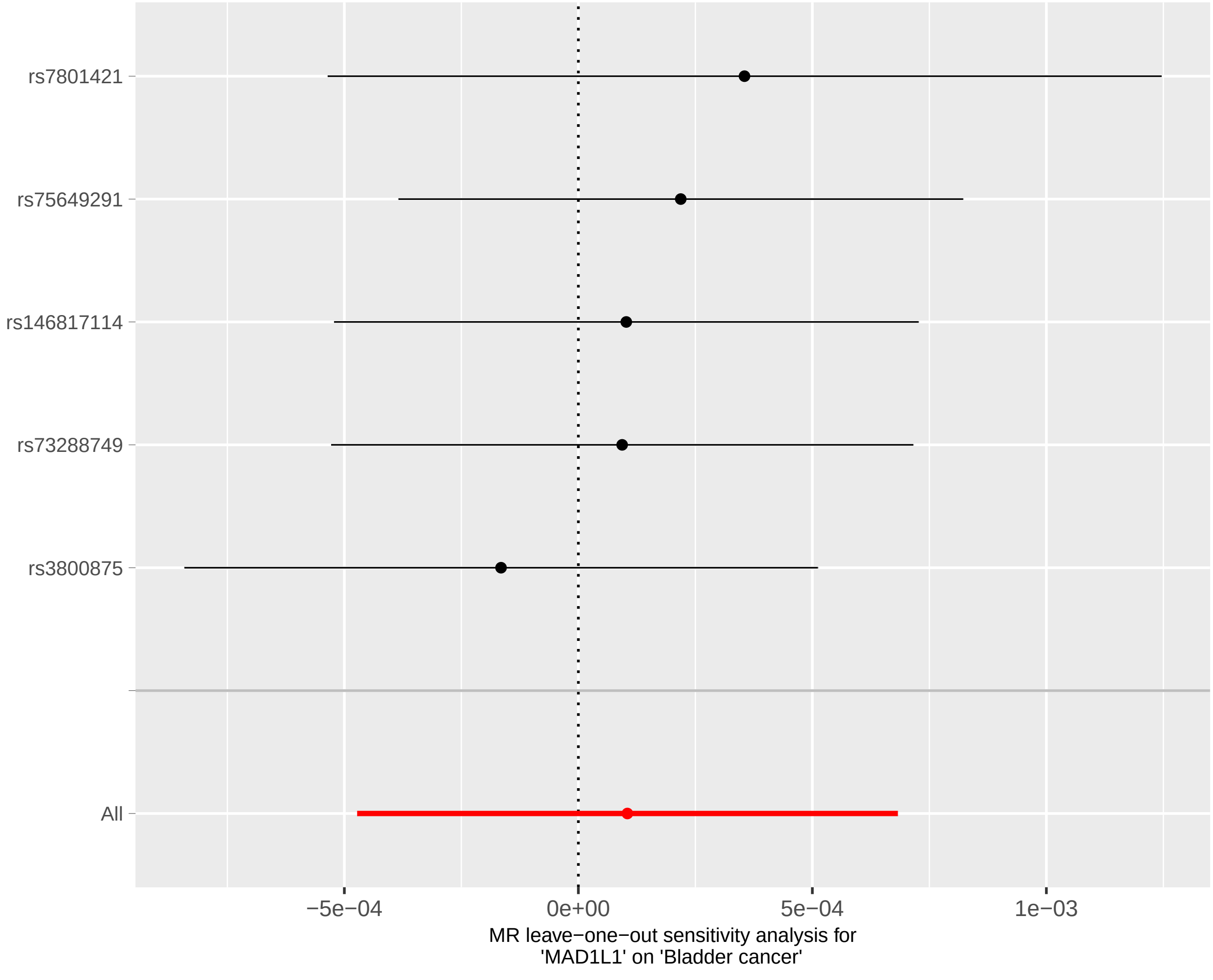
rs7801421
rs75649291
rs146817114
rs73288749
rs3800875
All
−5e−04
0e+00
5e−04
1e−03
MR leave−one−out sensitivity analysis for
'MAD1L1' on 'Bladder cancer'

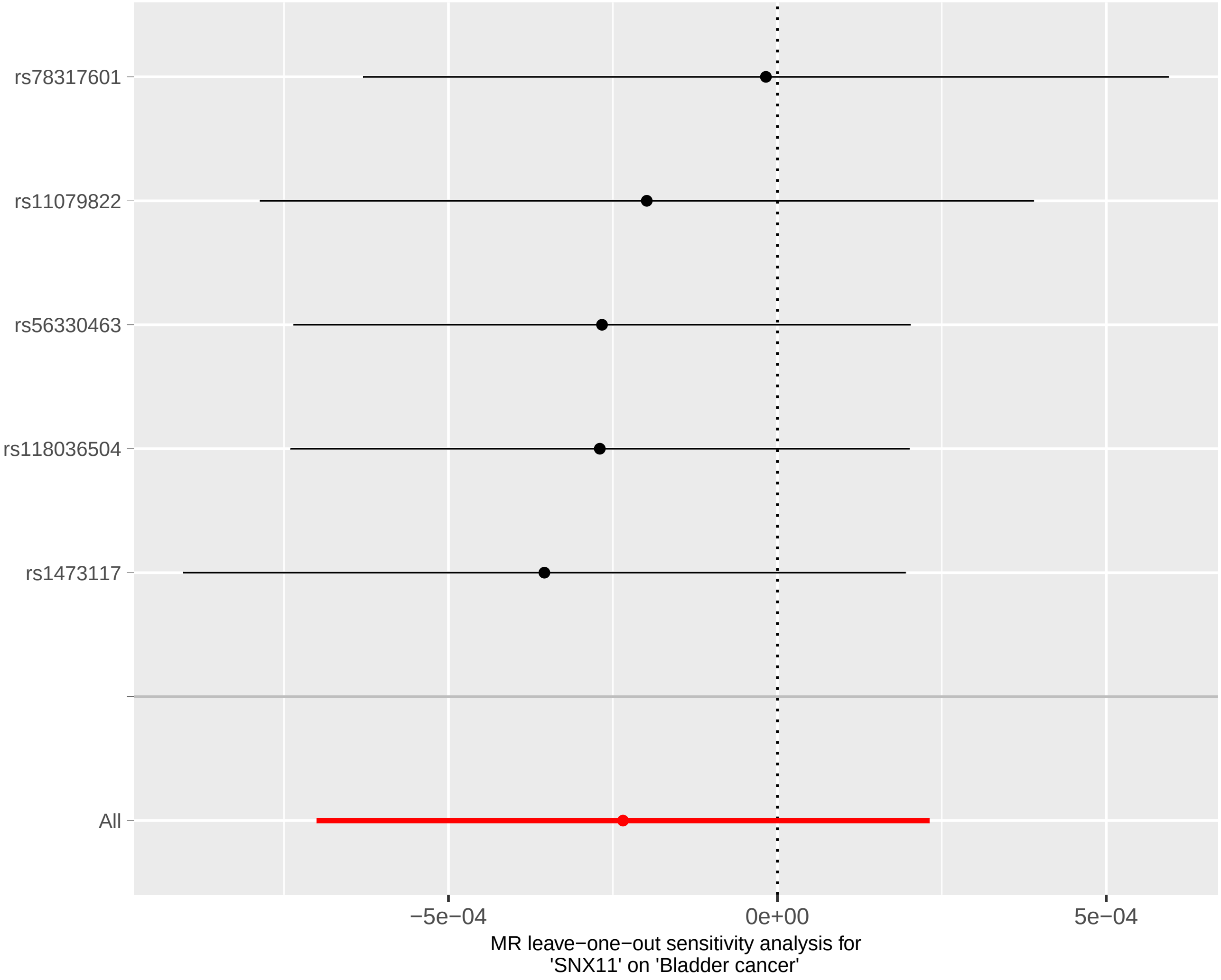
rs78317601
rs11079822
rs56330463
rs118036504
rs1473117
All
−5e−04
0e+00
5e−04
MR leave−one−out sensitivity analysis for
'SNX11' on 'Bladder cancer'

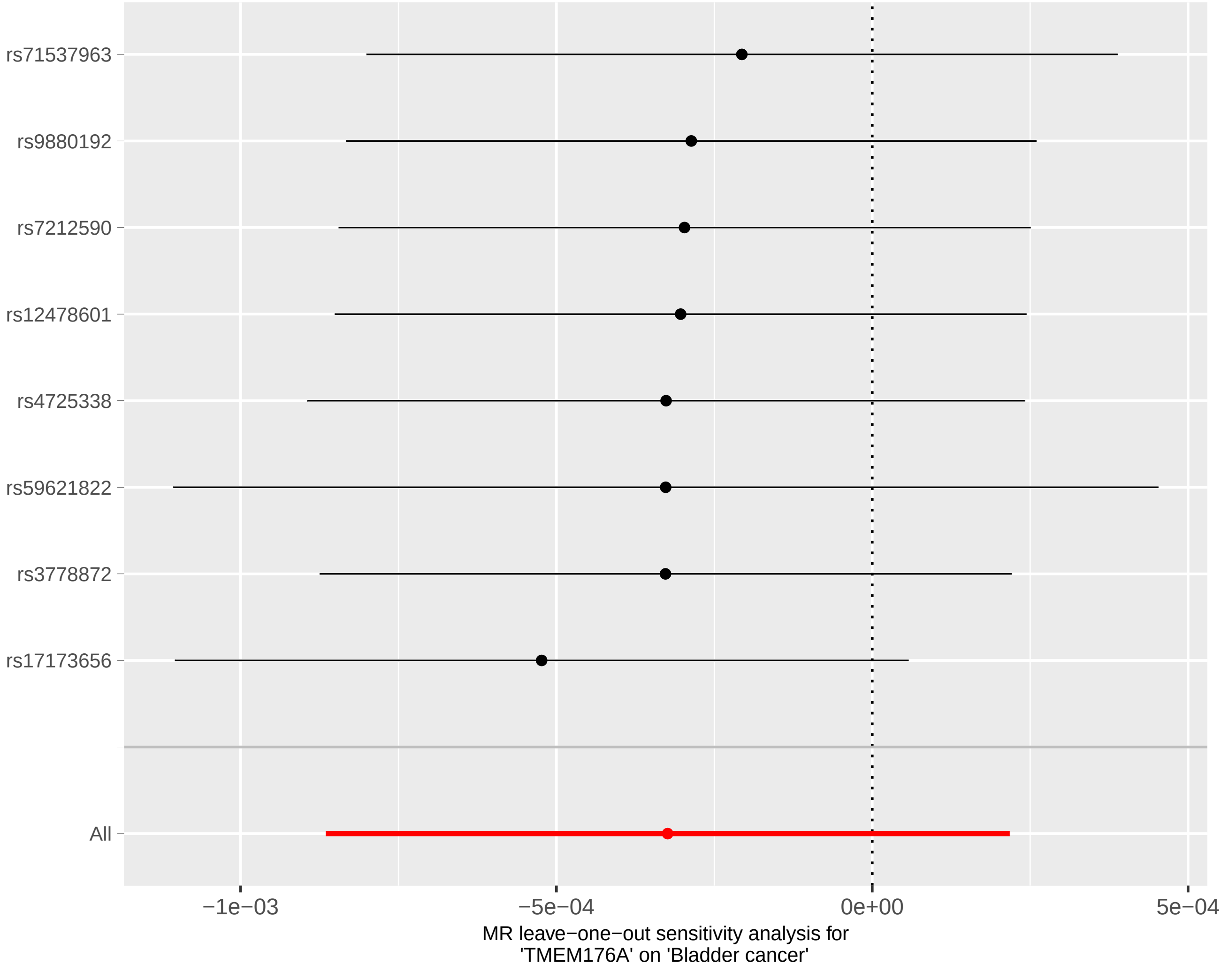
rs71537963
rs9880192
rs7212590
rs12478601
rs4725338
rs59621822
rs3778872
rs17173656
All
−1e−03
−5e−04
0e+00
5e−04
MR leave−one−out sensitivity analysis for
'TMEM176A' on 'Bladder cancer'

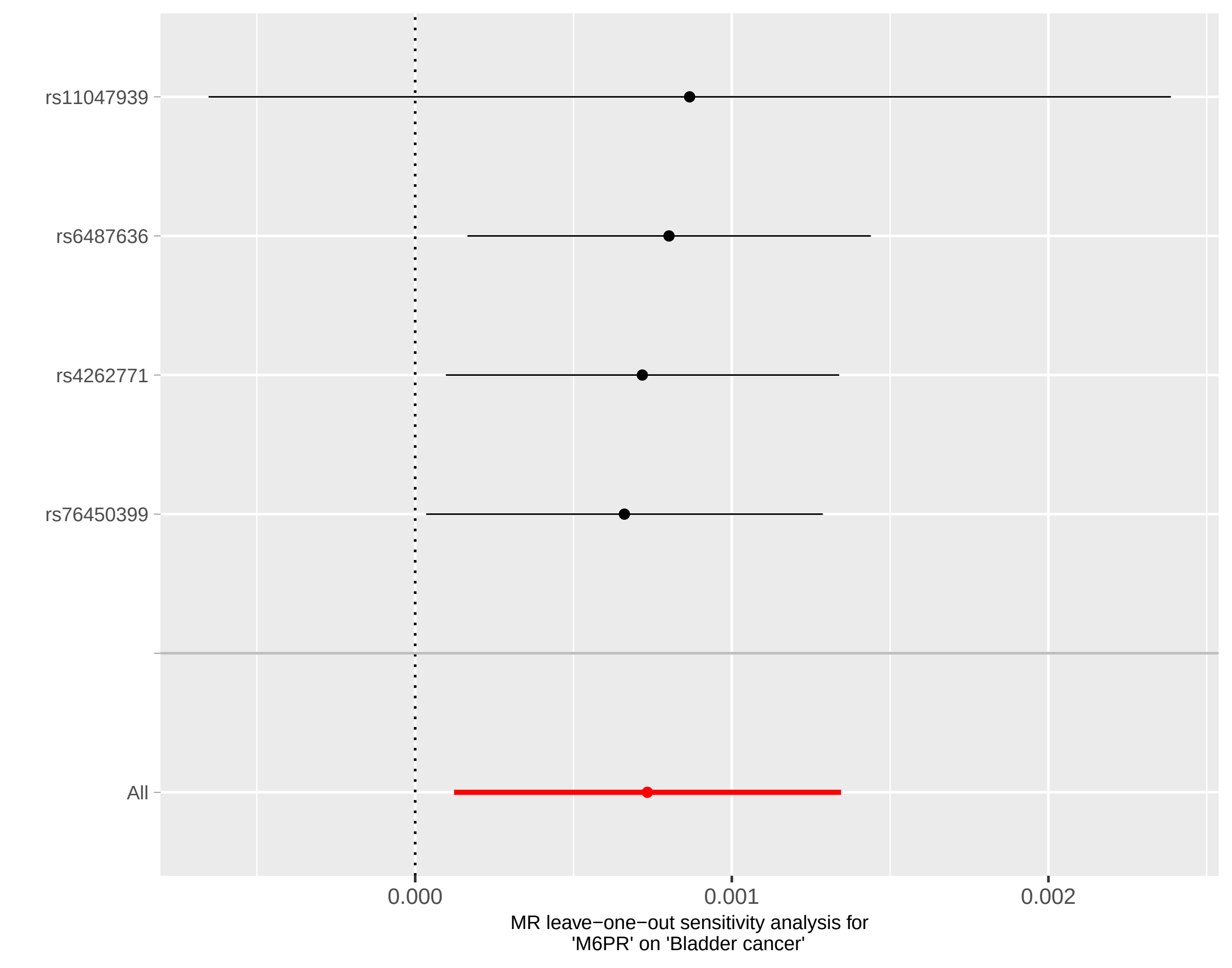
rs11047939
rs6487636
rs4262771
rs76450399
All
0.000
0.001
0.002
MR leave−one−out sensitivity analysis for
'M6PR' on 'Bladder cancer'

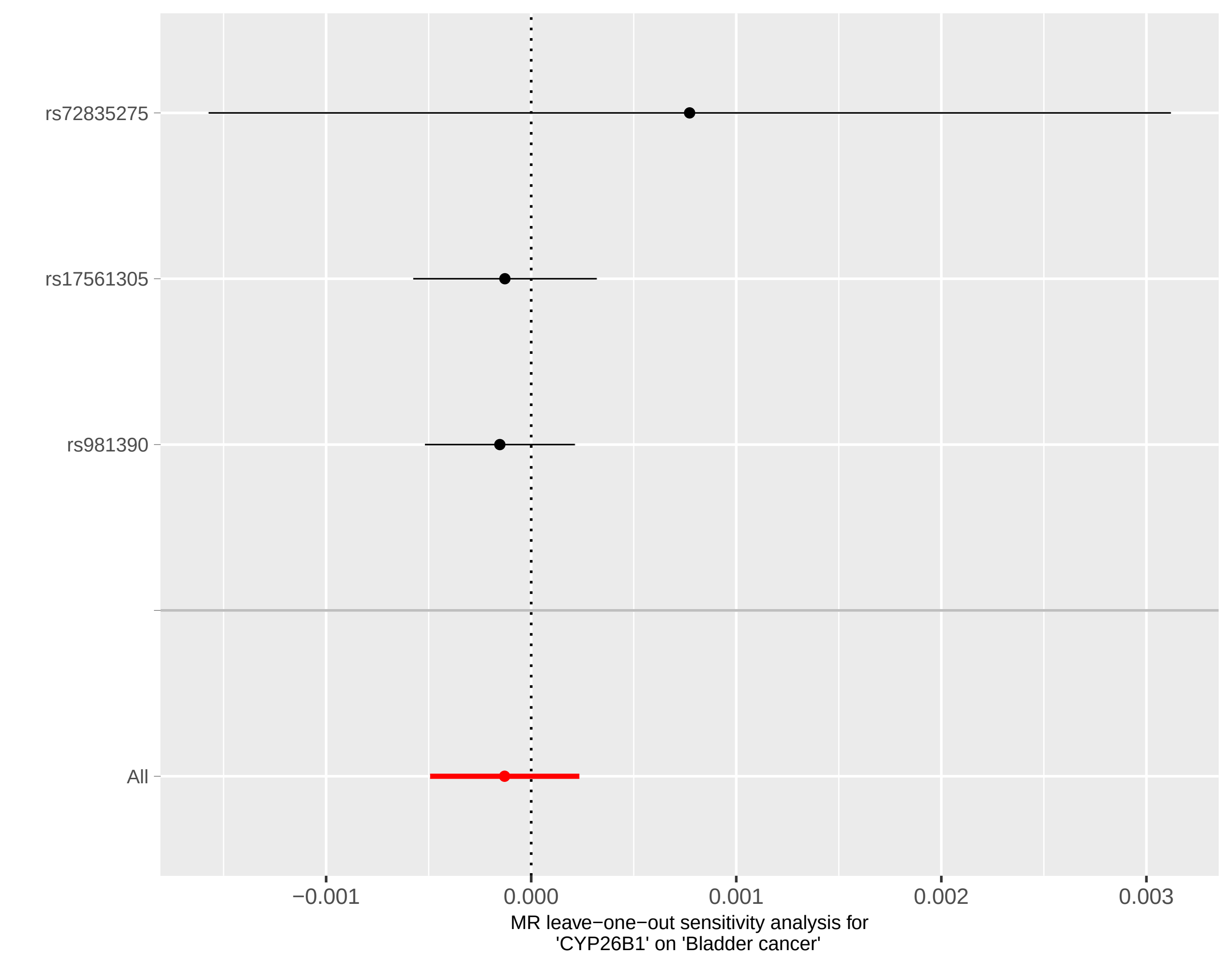
rs72835275
rs17561305
rs981390
All
−0.001
0.000
0.001
0.002
0.003
MR leave−one−out sensitivity analysis for
'CYP26B1' on 'Bladder cancer'

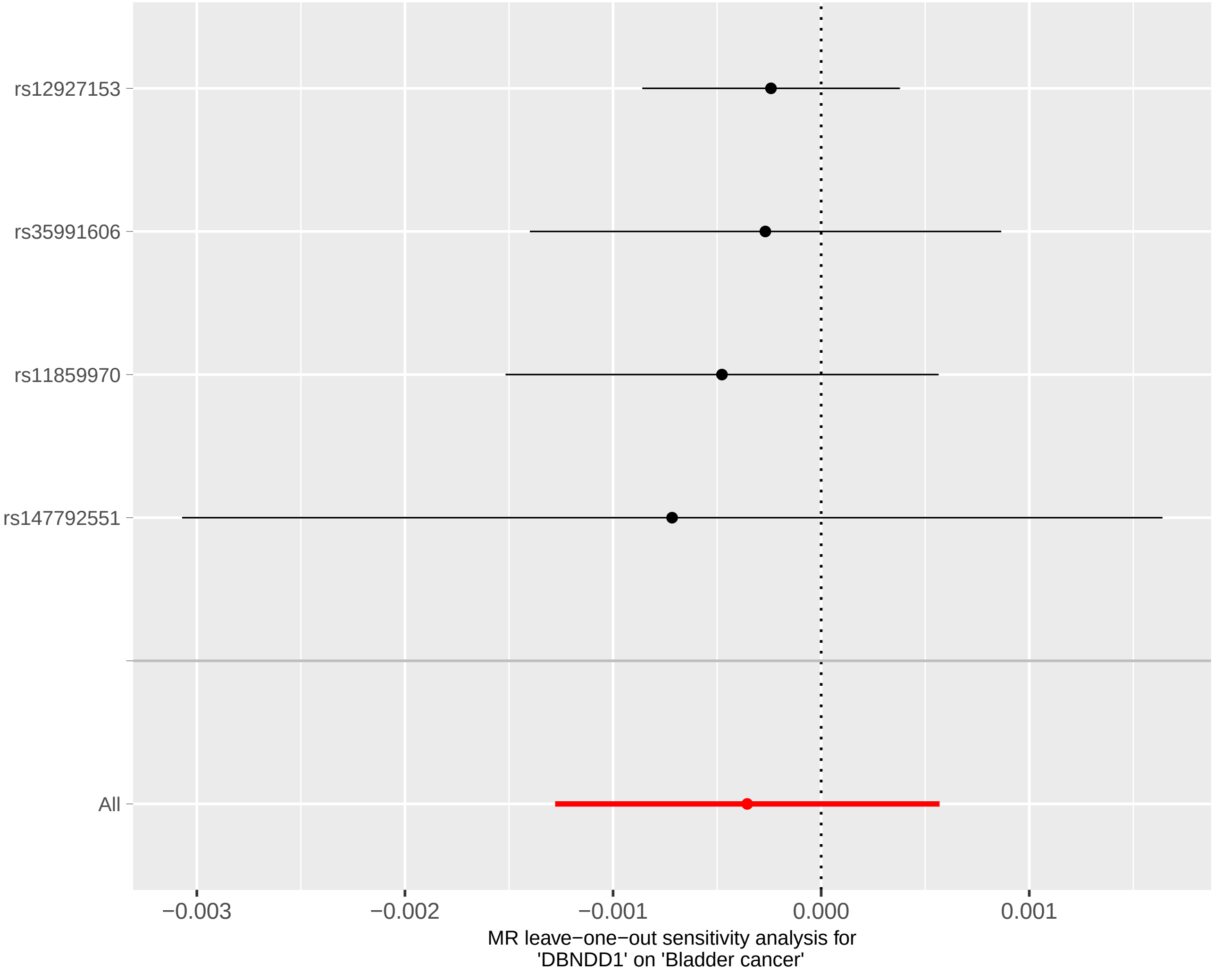
rs12927153
rs35991606
rs11859970
rs147792551
All
−0.003
−0.002
−0.001
0.000
0.001
MR leave−one−out sensitivity analysis for
'DBNDD1' on 'Bladder cancer'

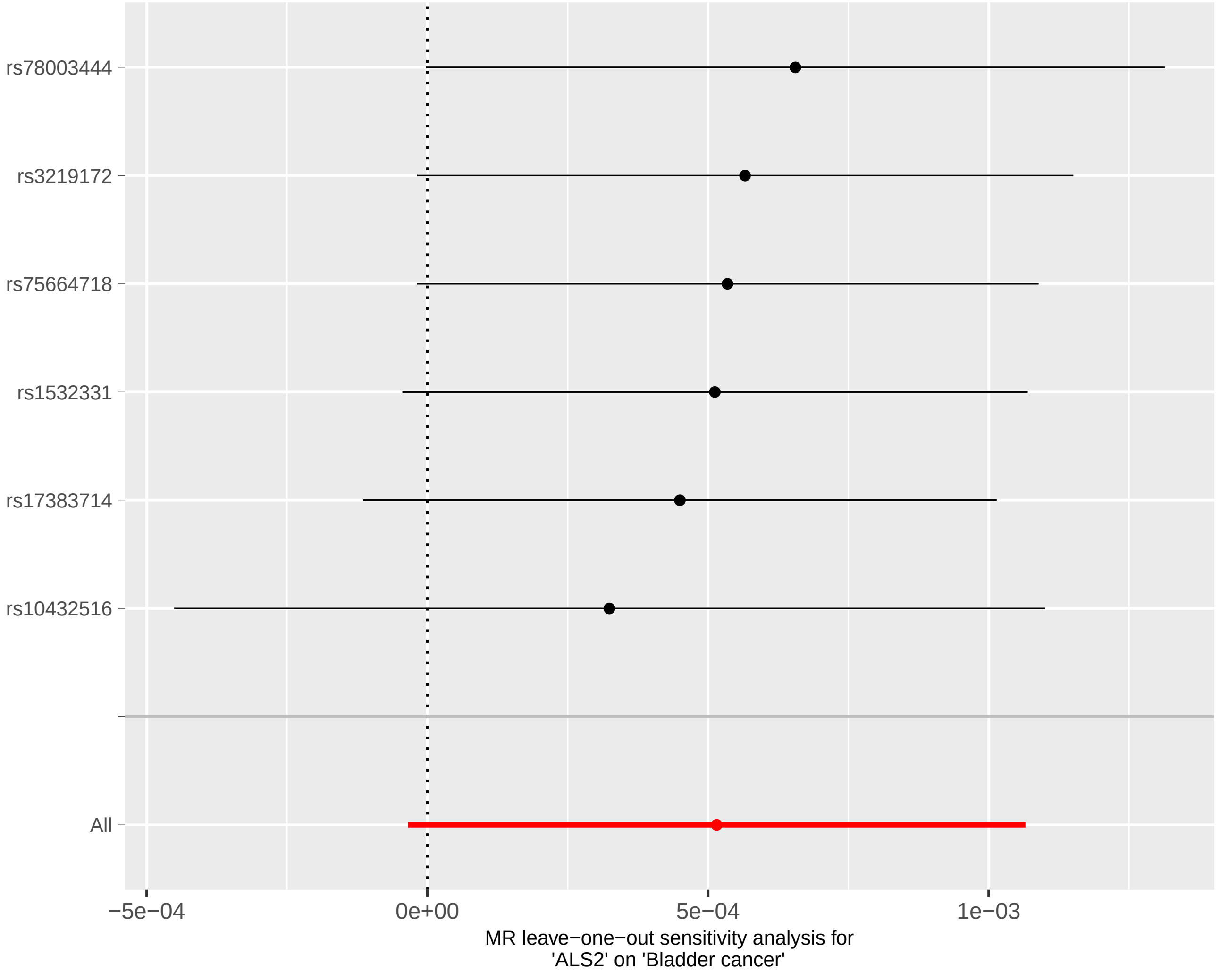
rs78003444
rs3219172
rs75664718
rs1532331
rs17383714
rs10432516
All
−5e−04
0e+00
5e−04
1e−03
MR leave−one−out sensitivity analysis for
'ALS2' on 'Bladder cancer'

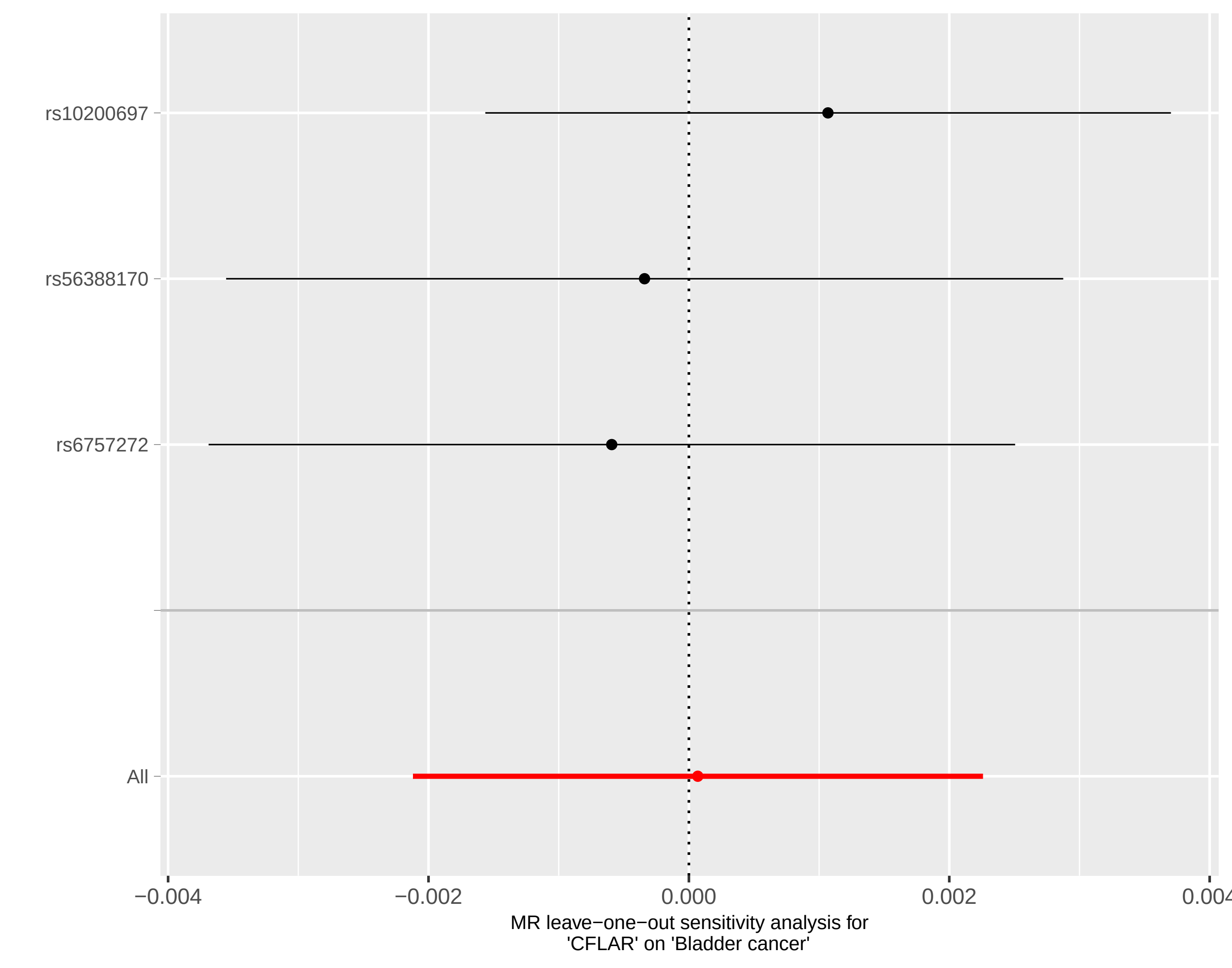
rs10200697
rs56388170
rs6757272
All
−0.004
−0.002
0.000
0.002
0.004
MR leave−one−out sensitivity analysis for
'CFLAR' on 'Bladder cancer'

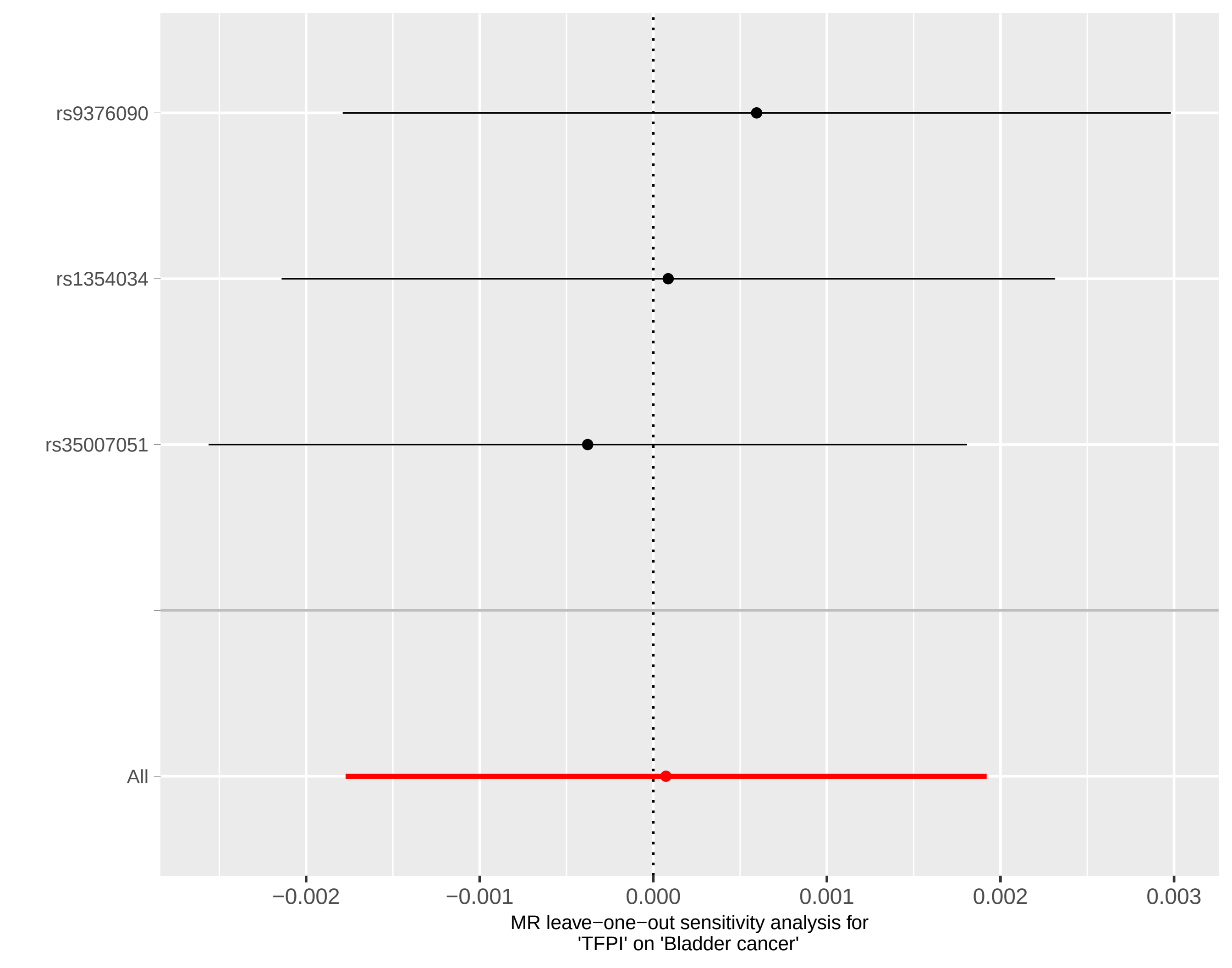
rs9376090
rs1354034
rs35007051
All
−0.002
−0.001
0.000
0.001
0.002
0.003
MR leave−one−out sensitivity analysis for
'TFPI' on 'Bladder cancer'

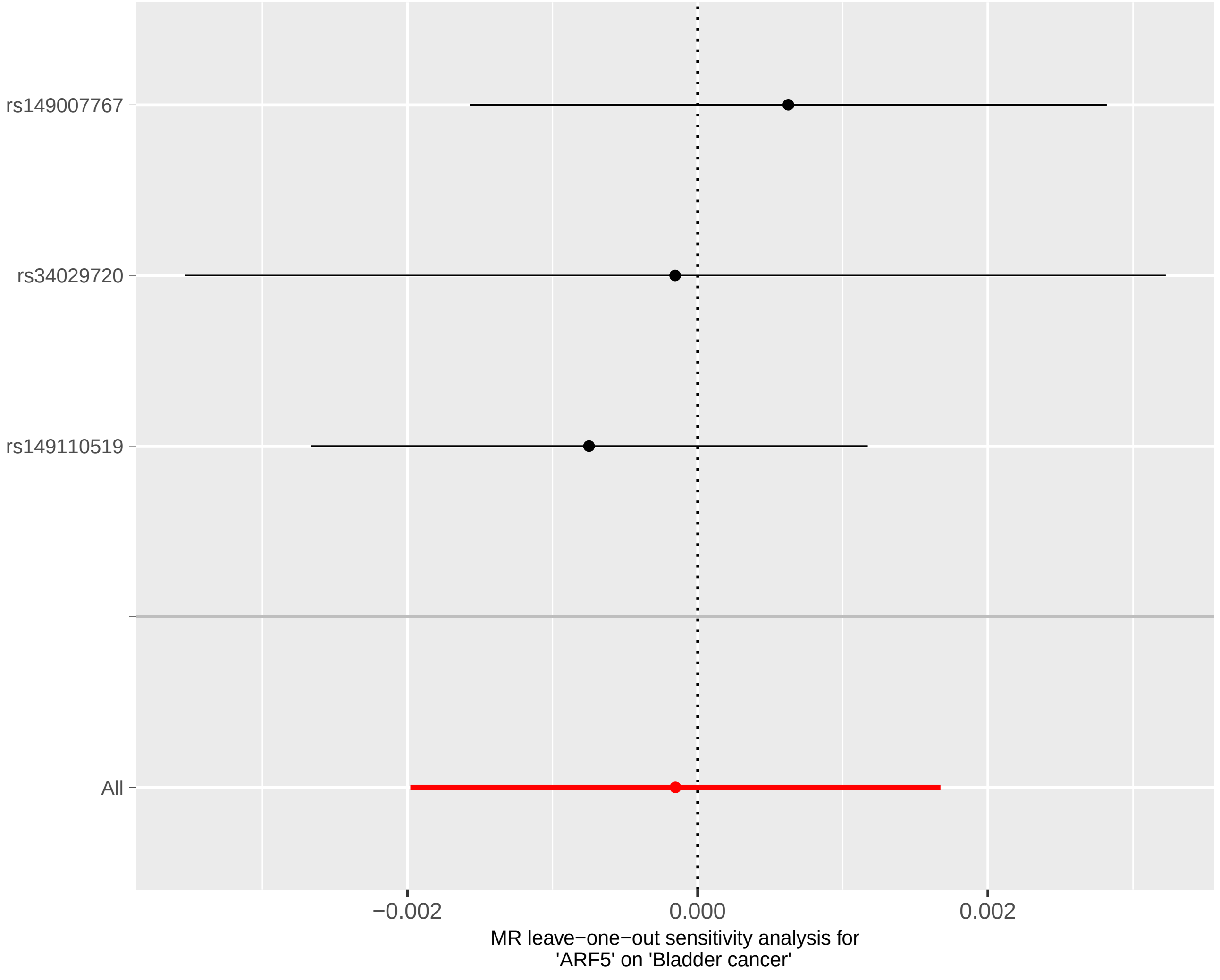
rs149007767
rs34029720
rs149110519
All
−0.002
0.000
0.002
MR leave−one−out sensitivity analysis for
'ARF5' on 'Bladder cancer'

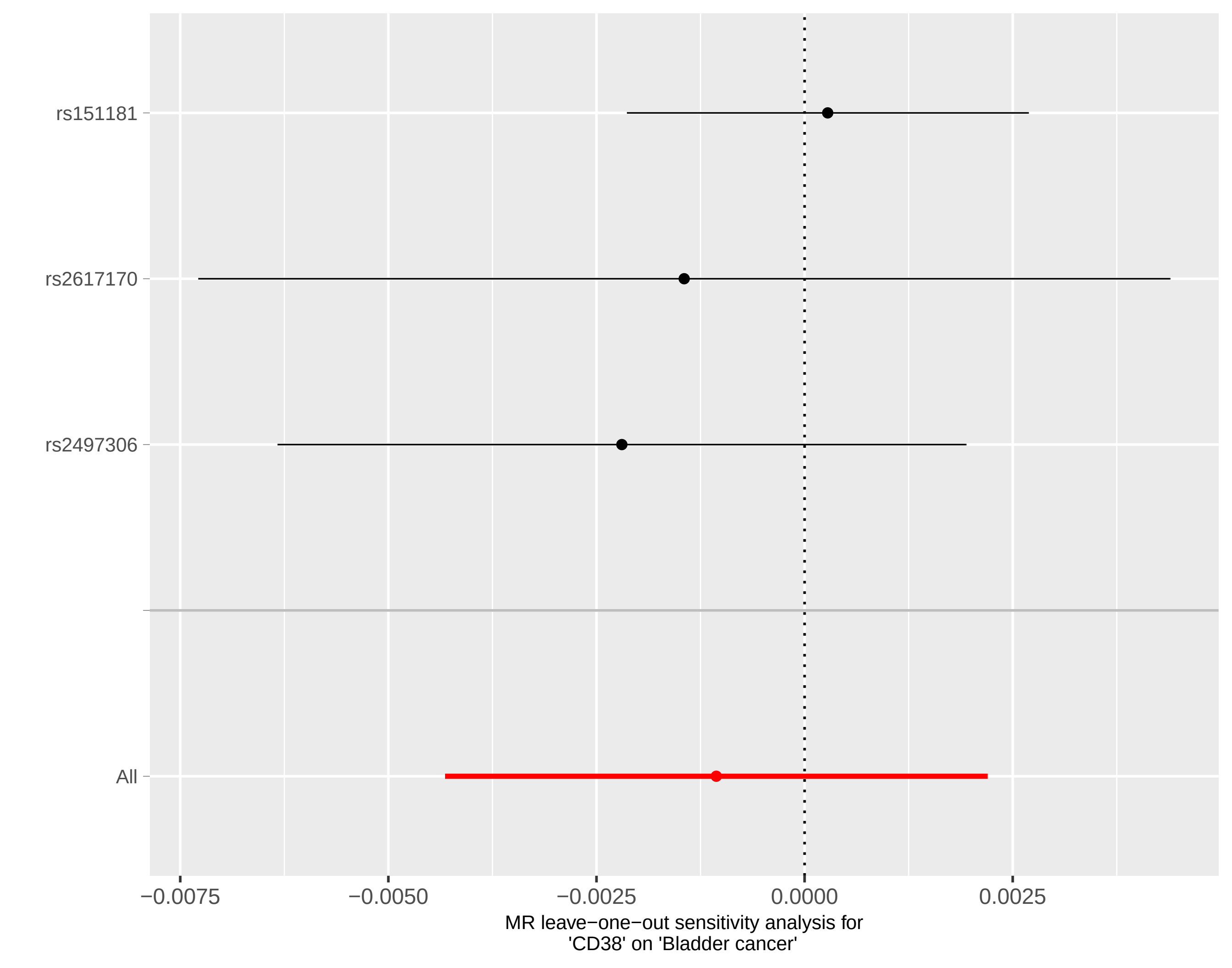
rs151181
rs2617170
rs2497306
All
−0.0075
−0.0050
−0.0025
0.0000
0.0025
MR leave−one−out sensitivity analysis for
'CD38' on 'Bladder cancer'

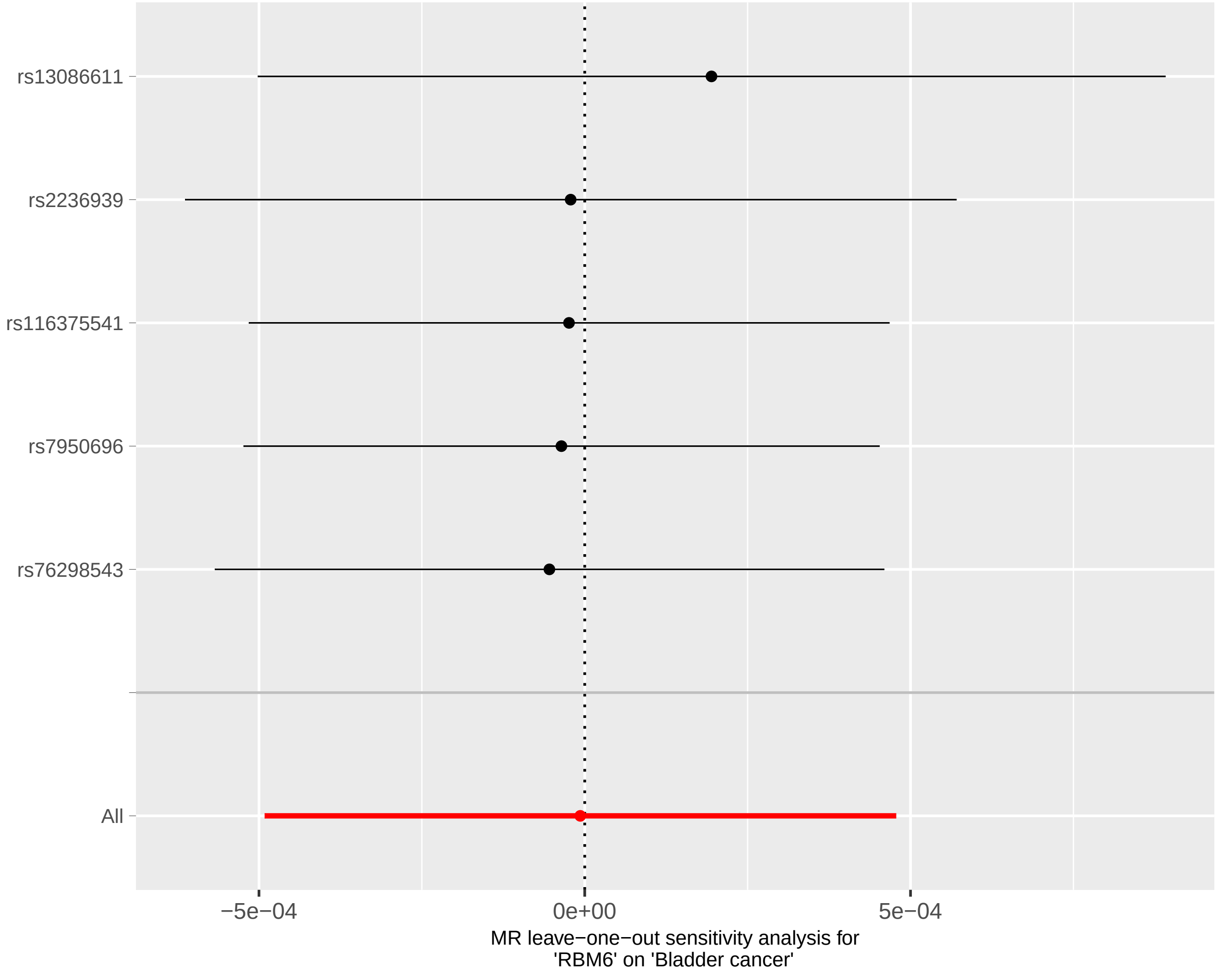
rs13086611
rs2236939
rs116375541
rs7950696
rs76298543
All
−5e−04
0e+00
5e−04
MR leave−one−out sensitivity analysis for
'RBM6' on 'Bladder cancer'

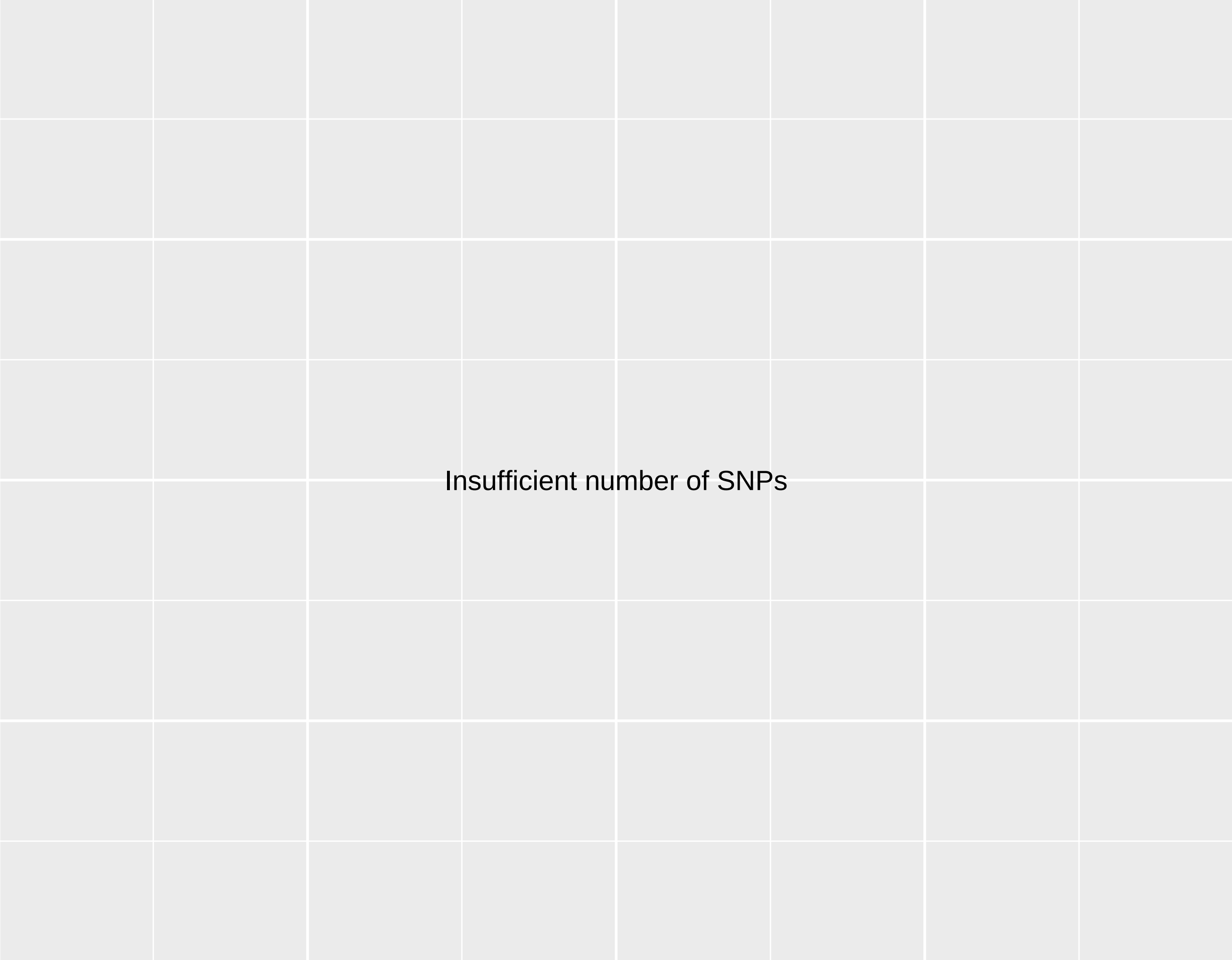
Insufficient number of SNPs

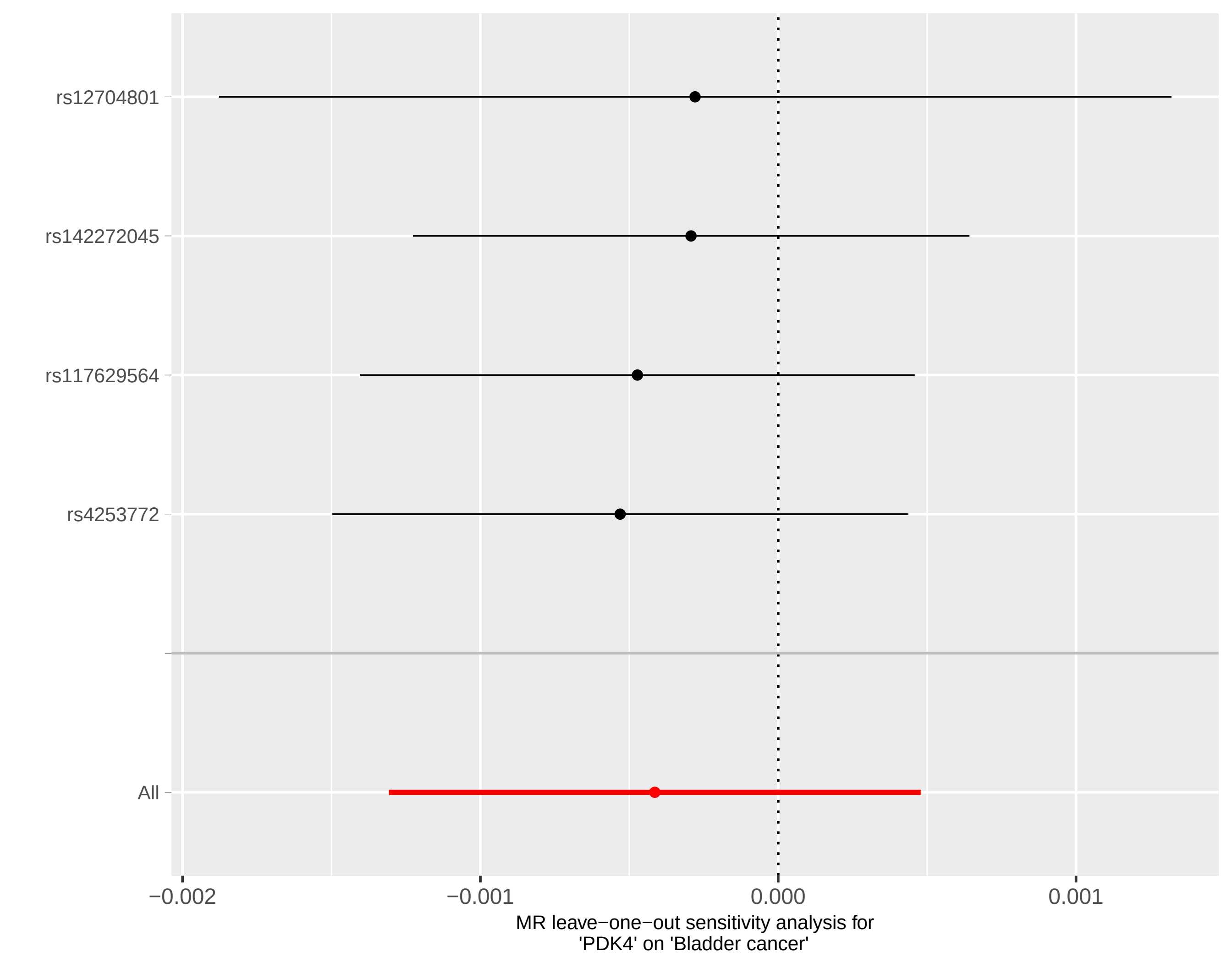
rs12704801
rs142272045
rs117629564
rs4253772
All
−0.002
−0.001
0.000
0.001
MR leave−one−out sensitivity analysis for
'PDK4' on 'Bladder cancer'

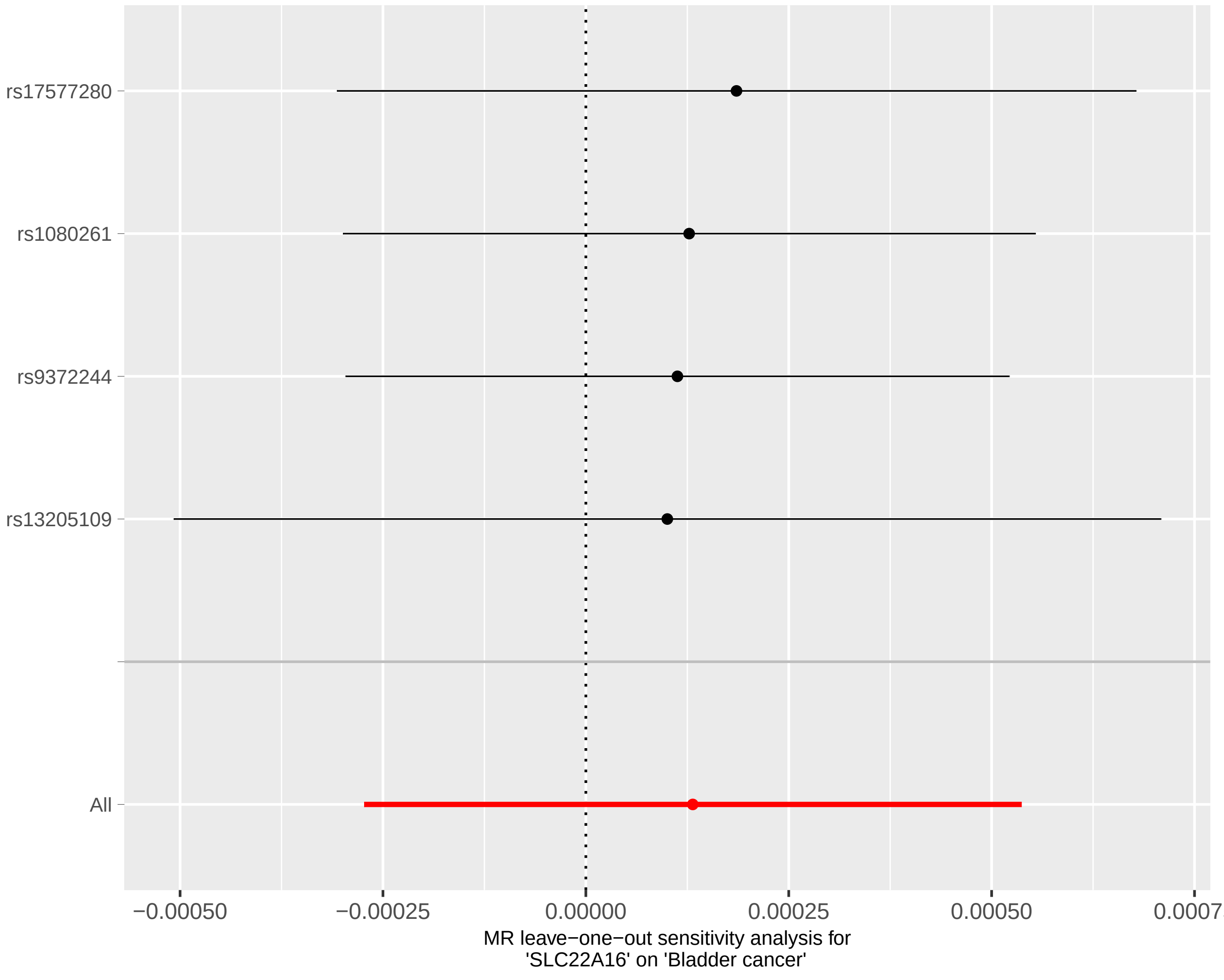
rs17577280
rs1080261
rs9372244
rs13205109
All
−0.00050
−0.00025
0.00000
0.00025
0.00050
MR leave−one−out sensitivity analysis for
'SLC22A16' on 'Bladder cancer'

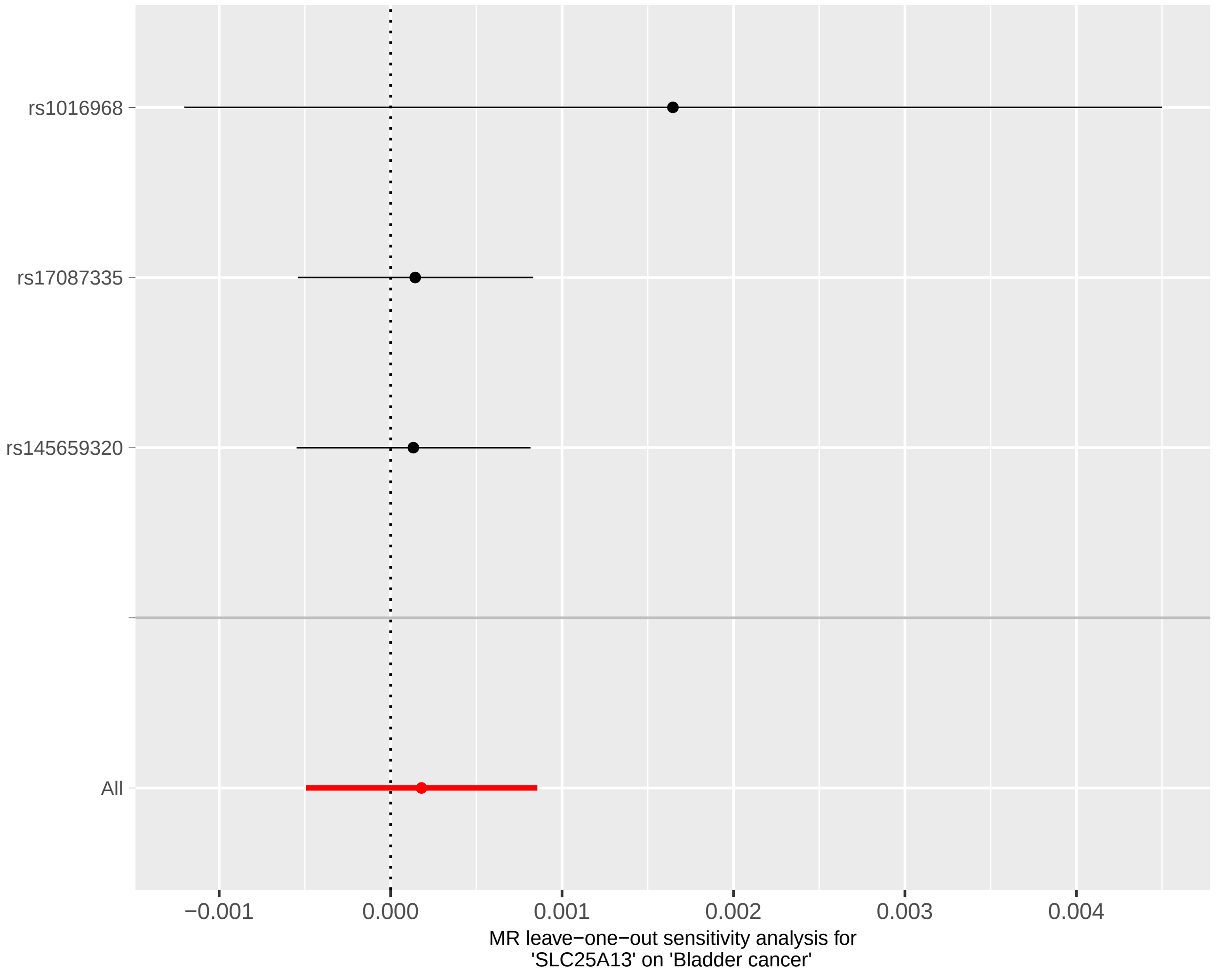
rs1016968
rs17087335
rs145659320
All
−0.001
0.000
0.001
0.002
0.003
0.004
MR leave−one−out sensitivity analysis for
'SLC25A13' on 'Bladder cancer'

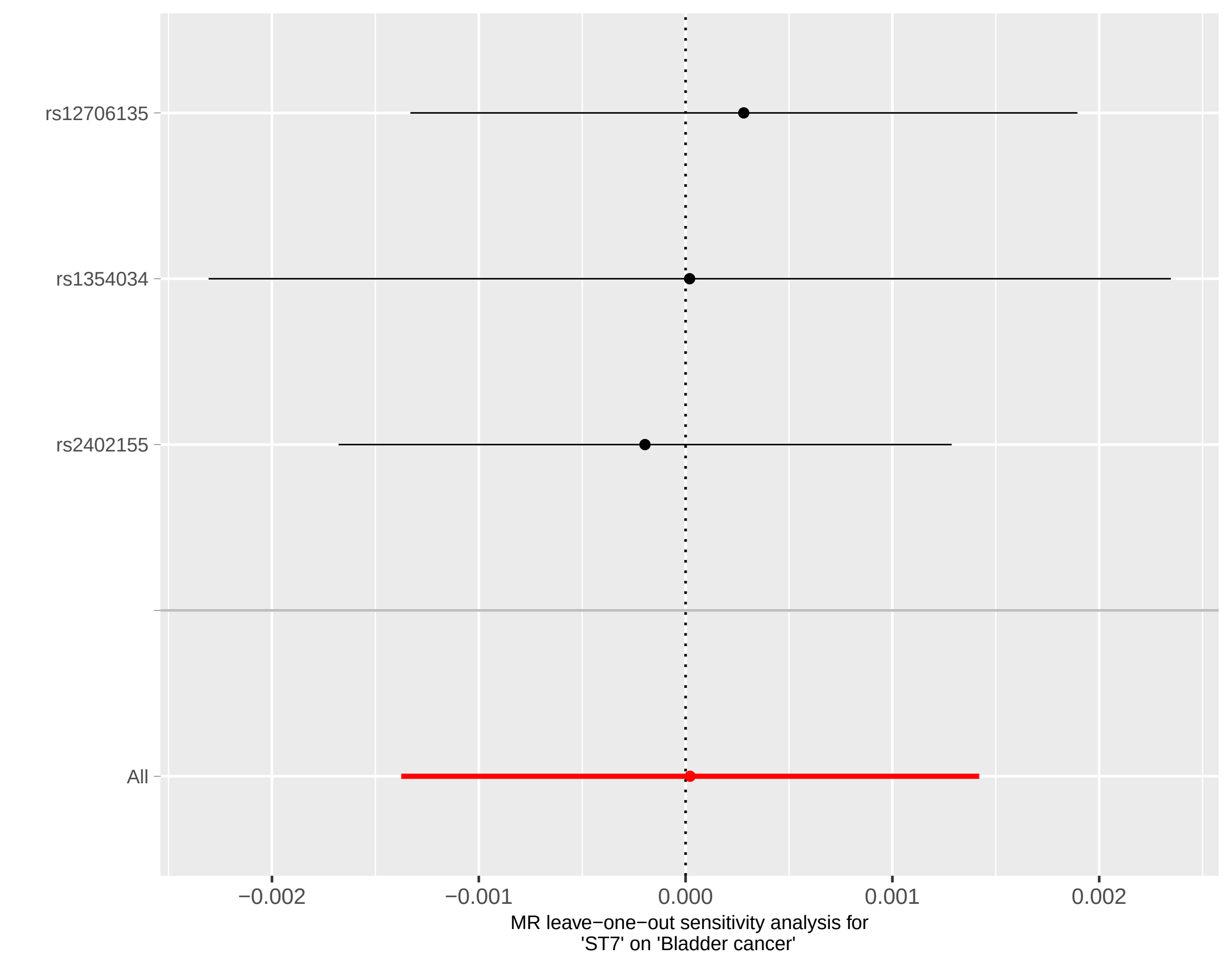
rs12706135
rs1354034
rs2402155
All
−0.002
−0.001
0.000
0.001
0.002
MR leave−one−out sensitivity analysis for
'ST7' on 'Bladder cancer'

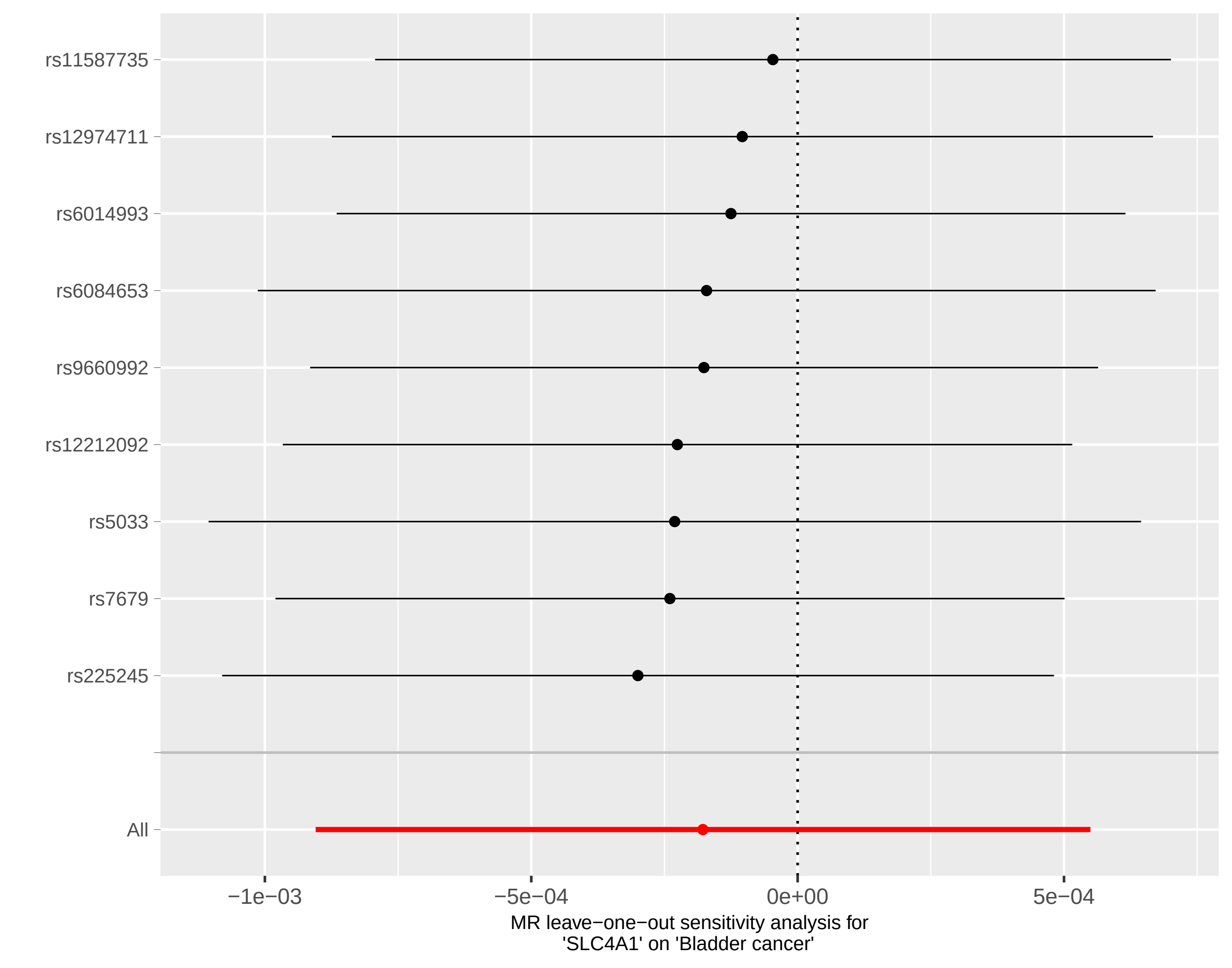
rs11587735
rs12974711
rs6014993
rs6084653
rs9660992
rs12212092
rs5033
rs7679
rs225245
All
−1e−03
−5e−04
0e+00
5e−04
MR leave−one−out sensitivity analysis for
'SLC4A1' on 'Bladder cancer'

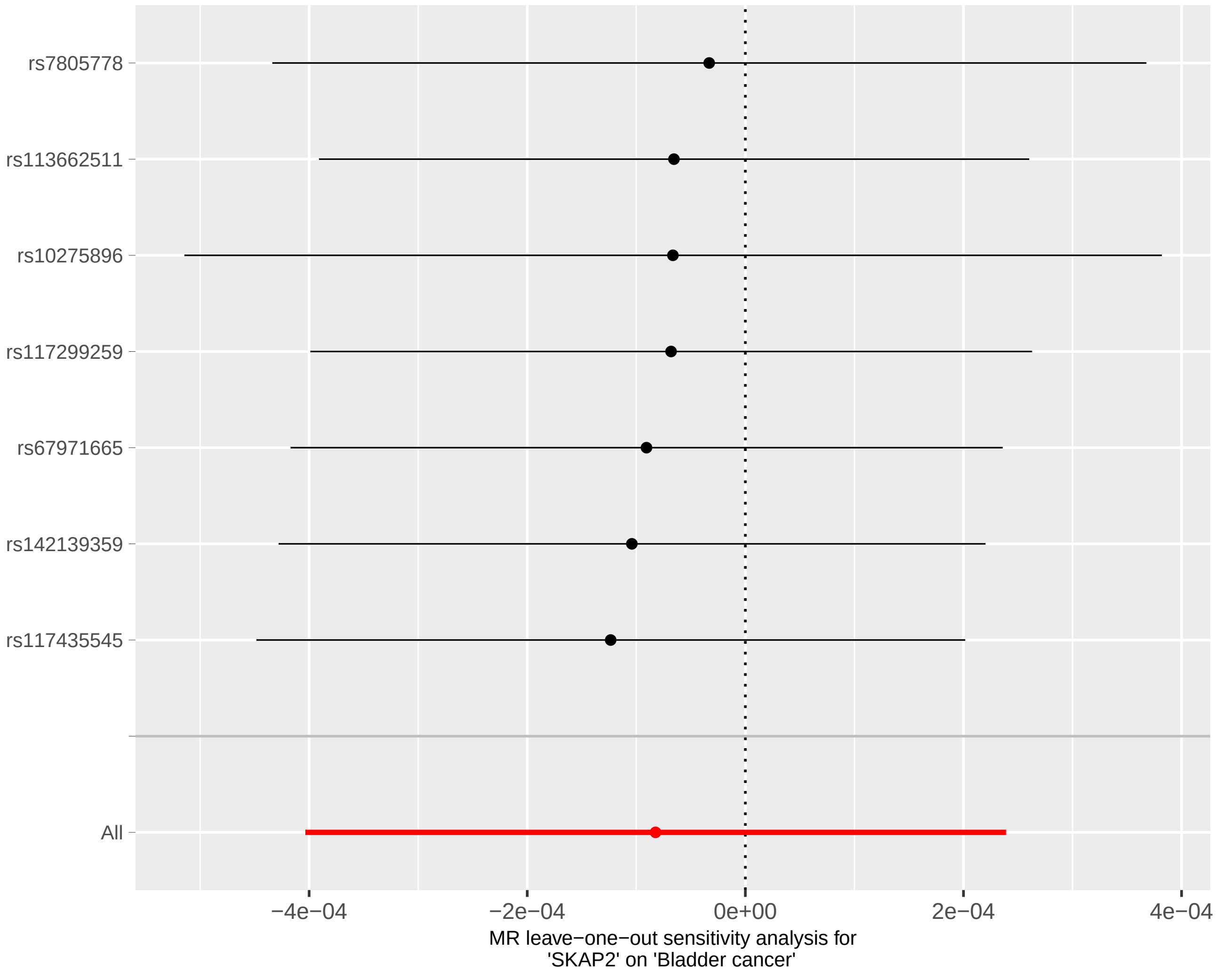
rs7805778
rs113662511
rs10275896
rs117299259
rs67971665
rs142139359
rs117435545
All
−4e−04
−2e−04
0e+00
2e−04
4e−04
MR leave−one−out sensitivity analysis for
'SKAP2' on 'Bladder cancer'

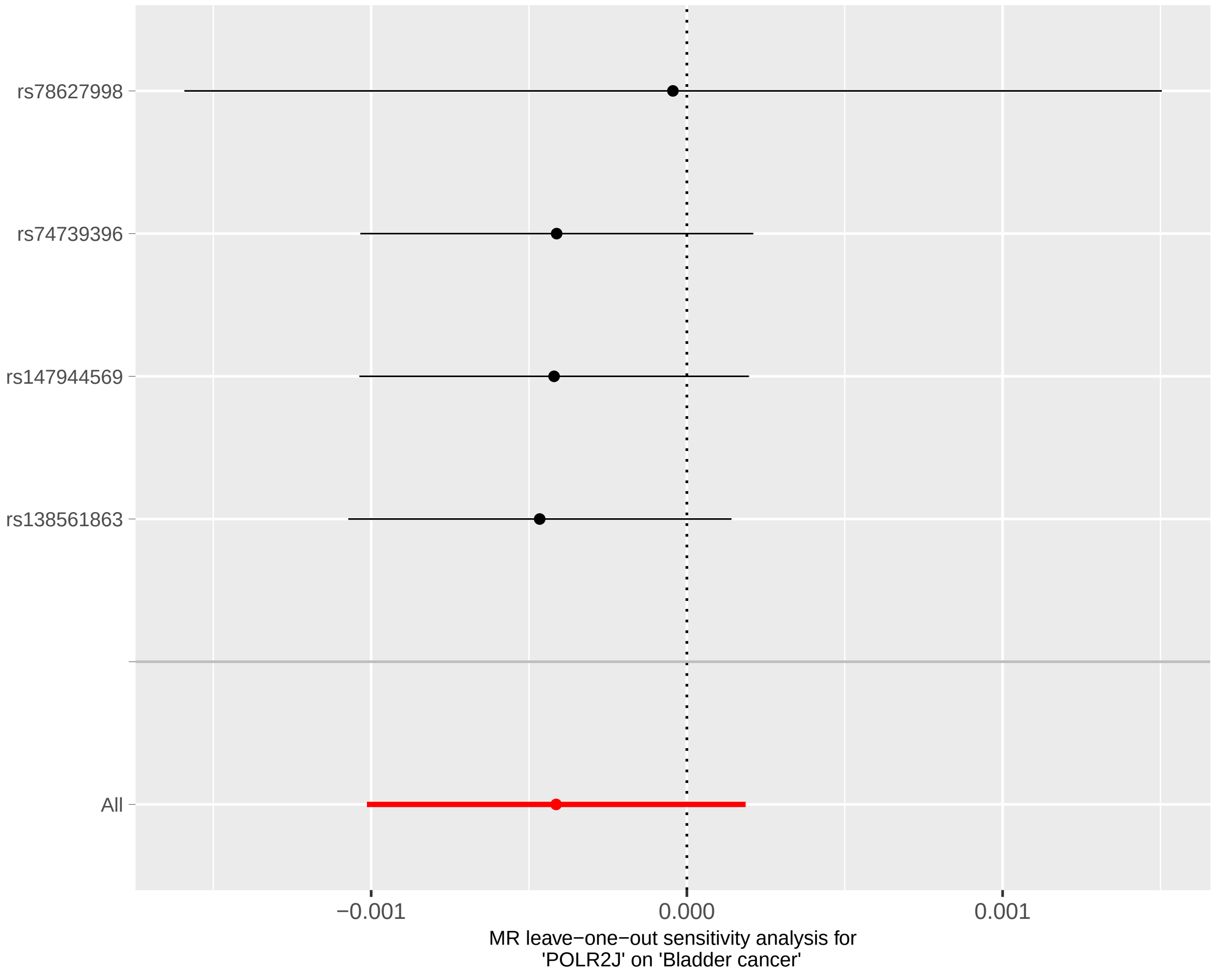
rs78627998
rs74739396
rs147944569
rs138561863
All
−0.001
0.000
0.001
MR leave−one−out sensitivity analysis for
'POLR2J' on 'Bladder cancer'

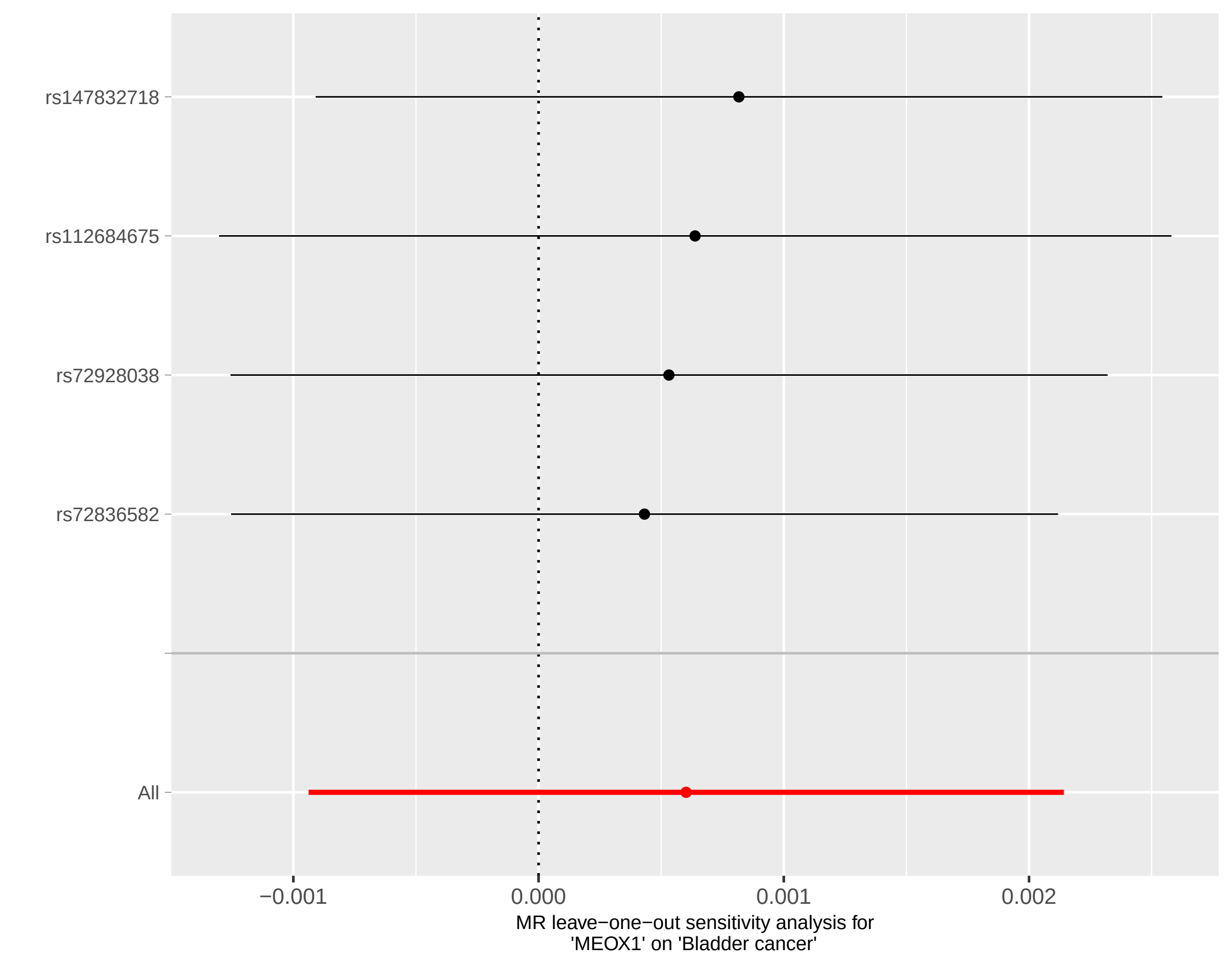
rs147832718
rs112684675
rs72928038
rs72836582
All
−0.001
0.000
0.001
0.002
MR leave−one−out sensitivity analysis for
'MEOX1' on 'Bladder cancer'

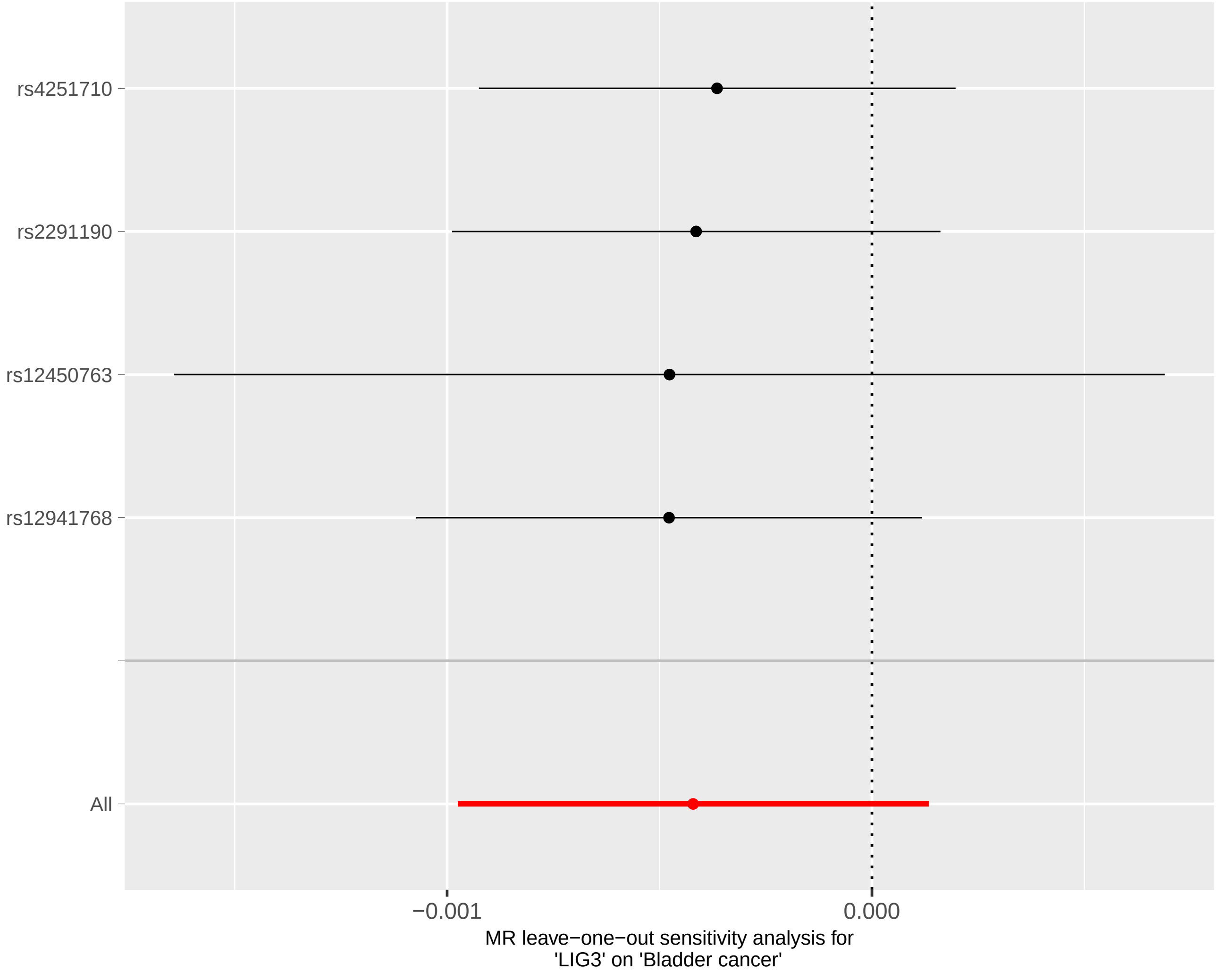
rs4251710
rs2291190
rs12450763
rs12941768
All
−0.001
0.000
MR leave−one−out sensitivity analysis for
'LIG3' on 'Bladder cancer'

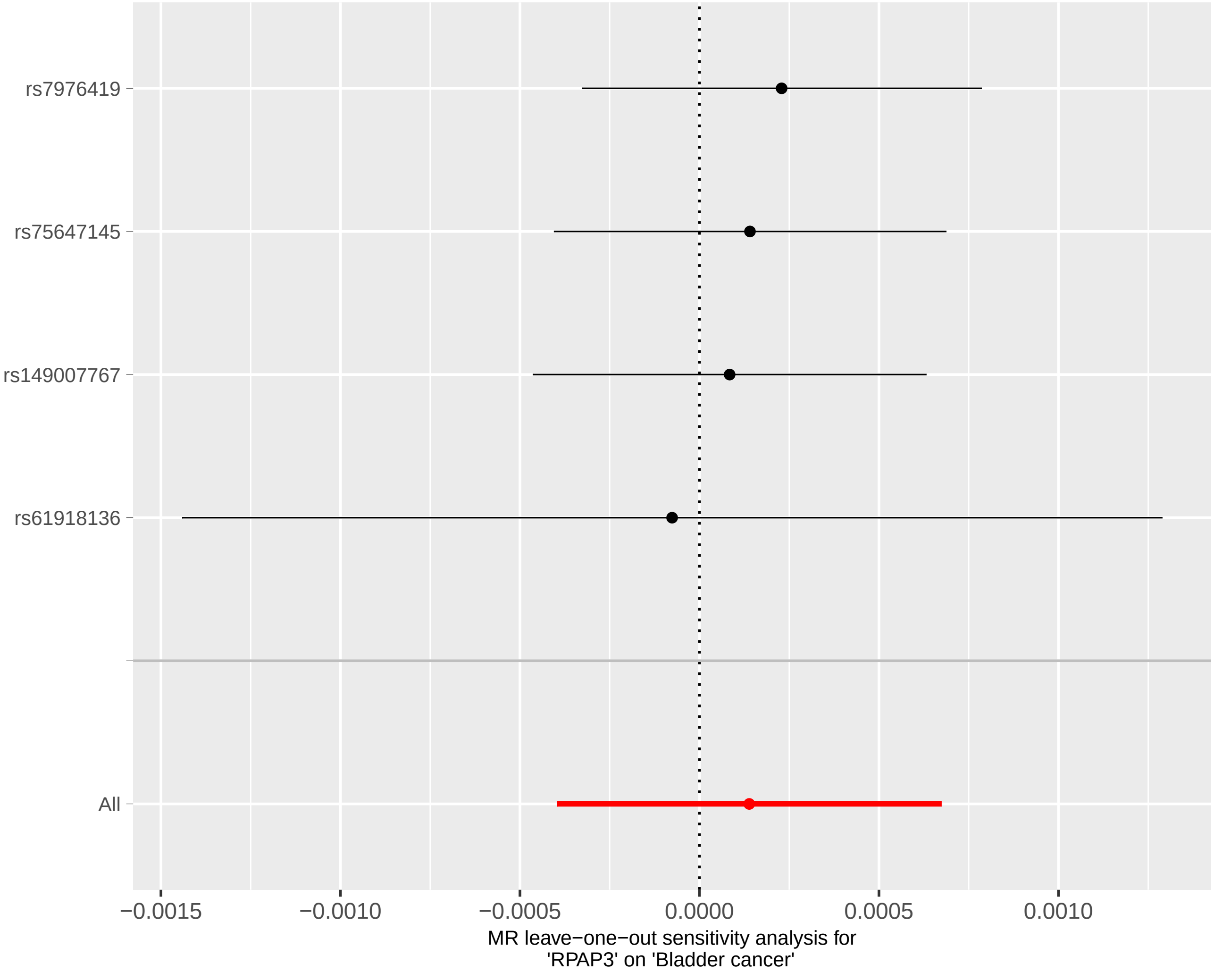
rs7976419
rs75647145
rs149007767
rs61918136
All
−0.0015
−0.0010
−0.0005
0.0000
0.0005
0.0010
MR leave−one−out sensitivity analysis for
'RPAP3' on 'Bladder cancer'

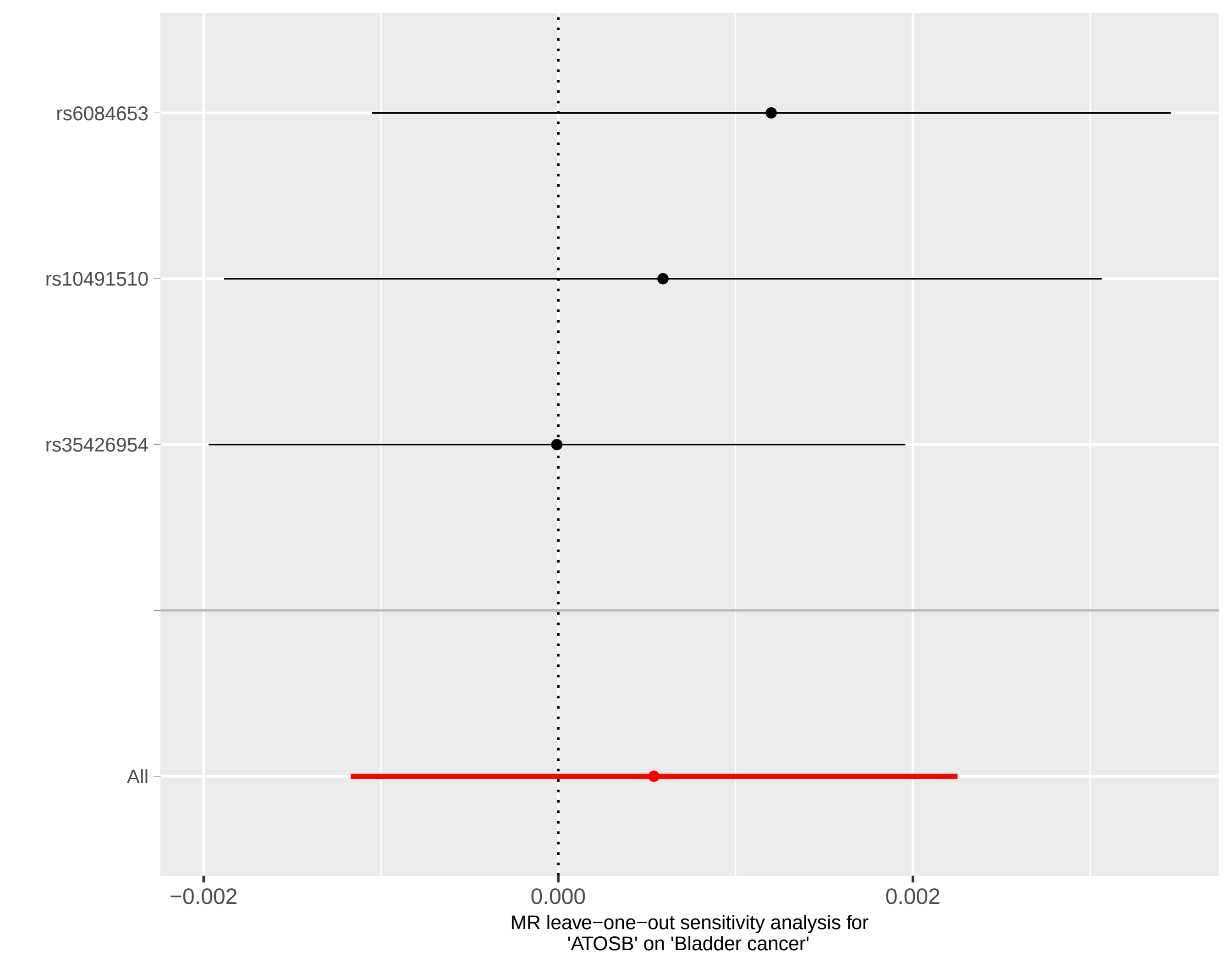
rs6084653
rs10491510
rs35426954
All
−0.002
0.000
0.002
MR leave−one−out sensitivity analysis for
'ATOSB' on 'Bladder cancer'

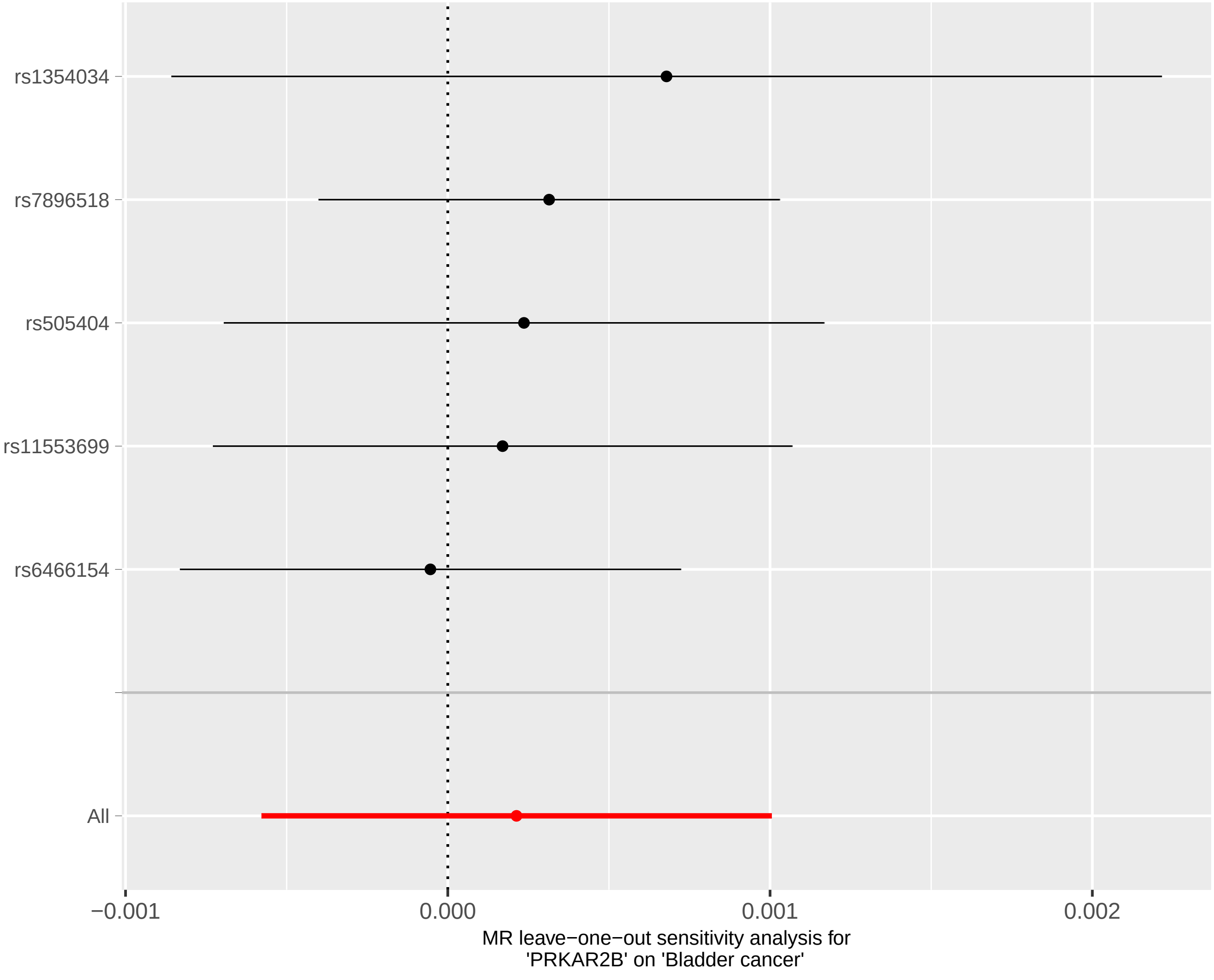
rs1354034
rs7896518
rs505404
rs11553699
rs6466154
All
−0.001
0.000
0.001
0.002
MR leave−one−out sensitivity analysis for
'PRKAR2B' on 'Bladder cancer'

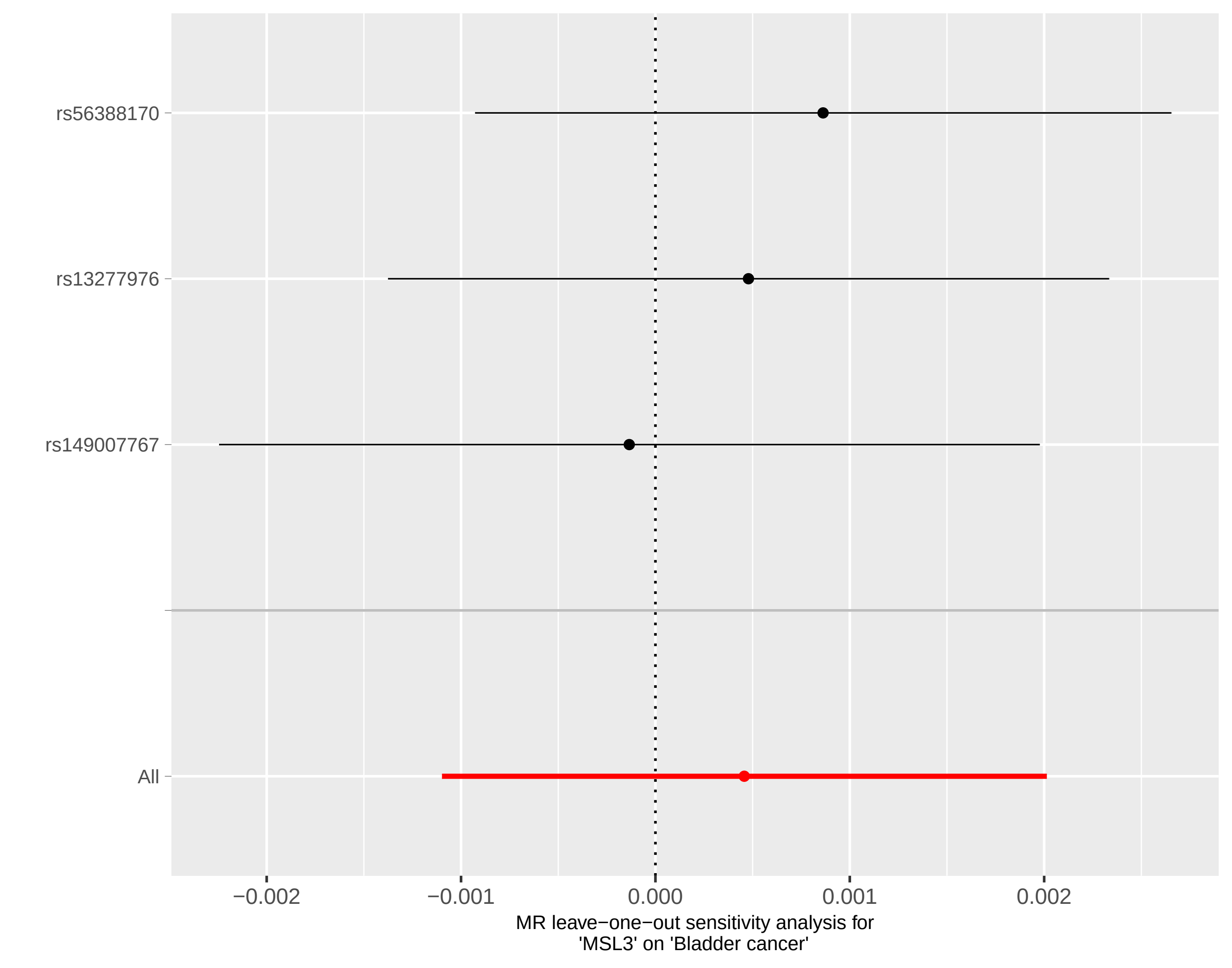
rs56388170
rs13277976
rs149007767
All
−0.002
−0.001
0.000
0.001
0.002
MR leave−one−out sensitivity analysis for
'MSL3' on 'Bladder cancer'

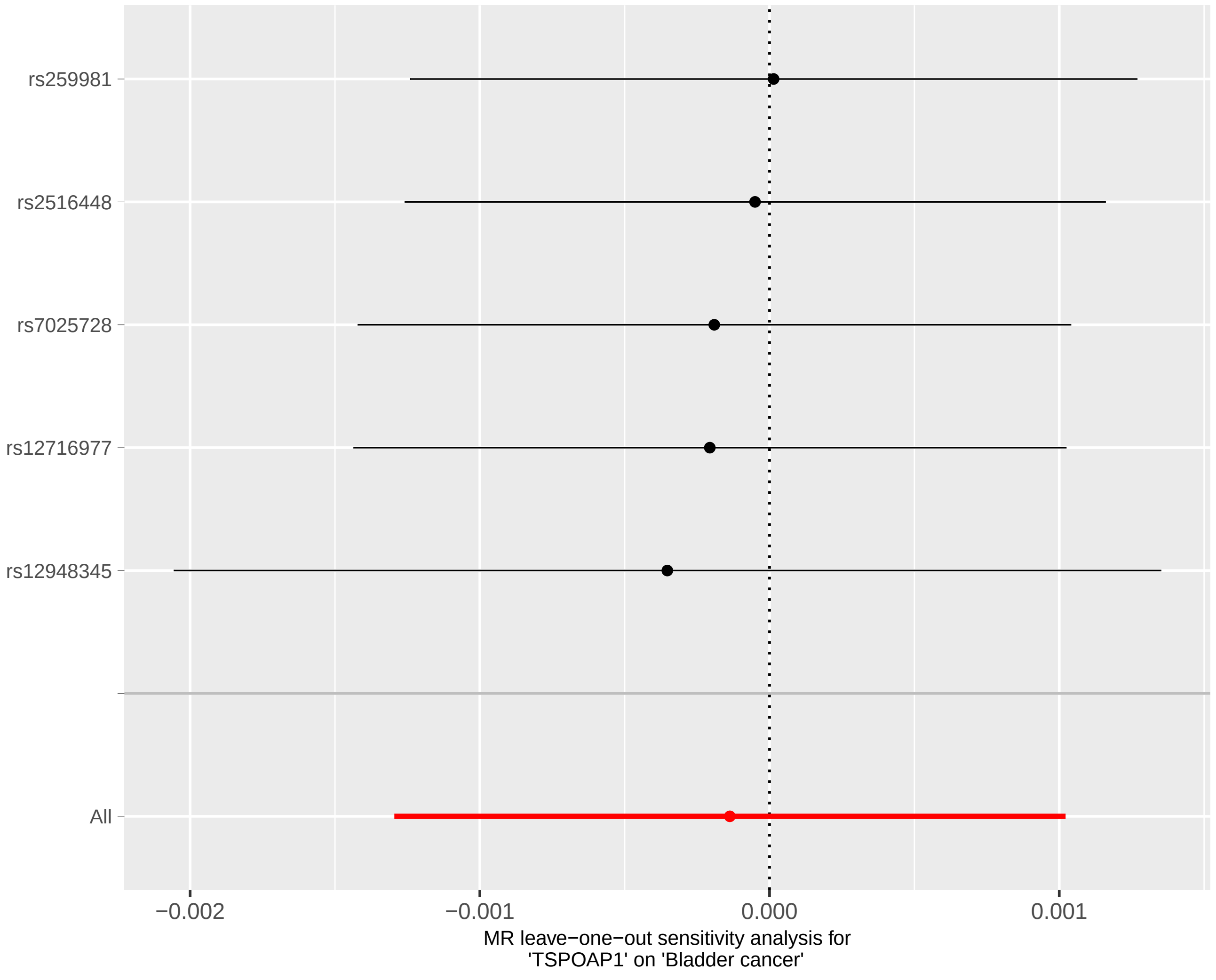

rs259981
rs2516448
rs7025728
rs12716977
rs12948345
All
−0.002
−0.001
0.000
0.001
MR leave−one−out sensitivity analysis for
'TSPOAP1' on 'Bladder cancer'

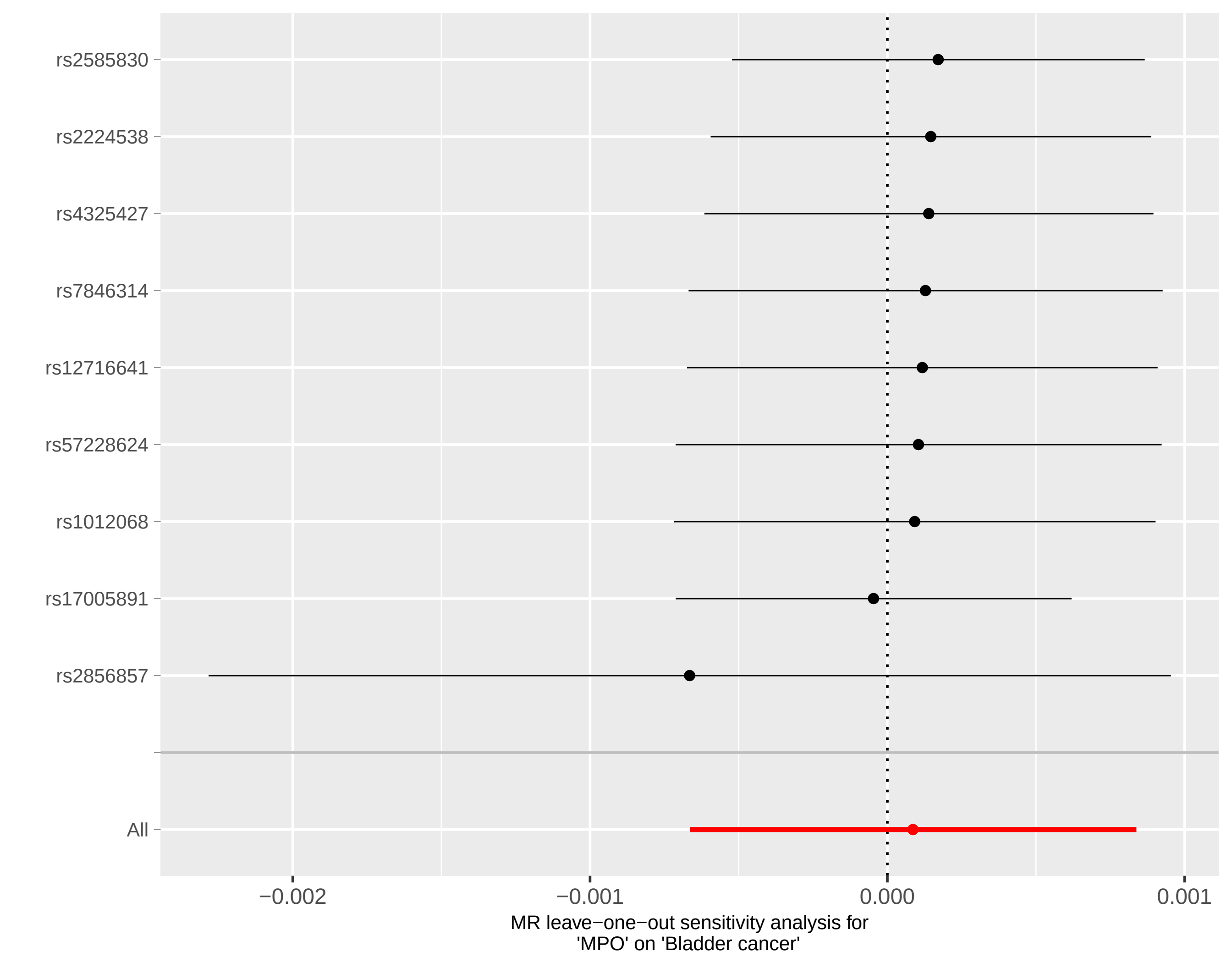
rs2585830
rs2224538
rs4325427
rs7846314
rs12716641
rs57228624
rs1012068
rs17005891
rs2856857
All
−0.002
−0.001
0.000
0.001
MR leave−one−out sensitivity analysis for
'MPO' on 'Bladder cancer'

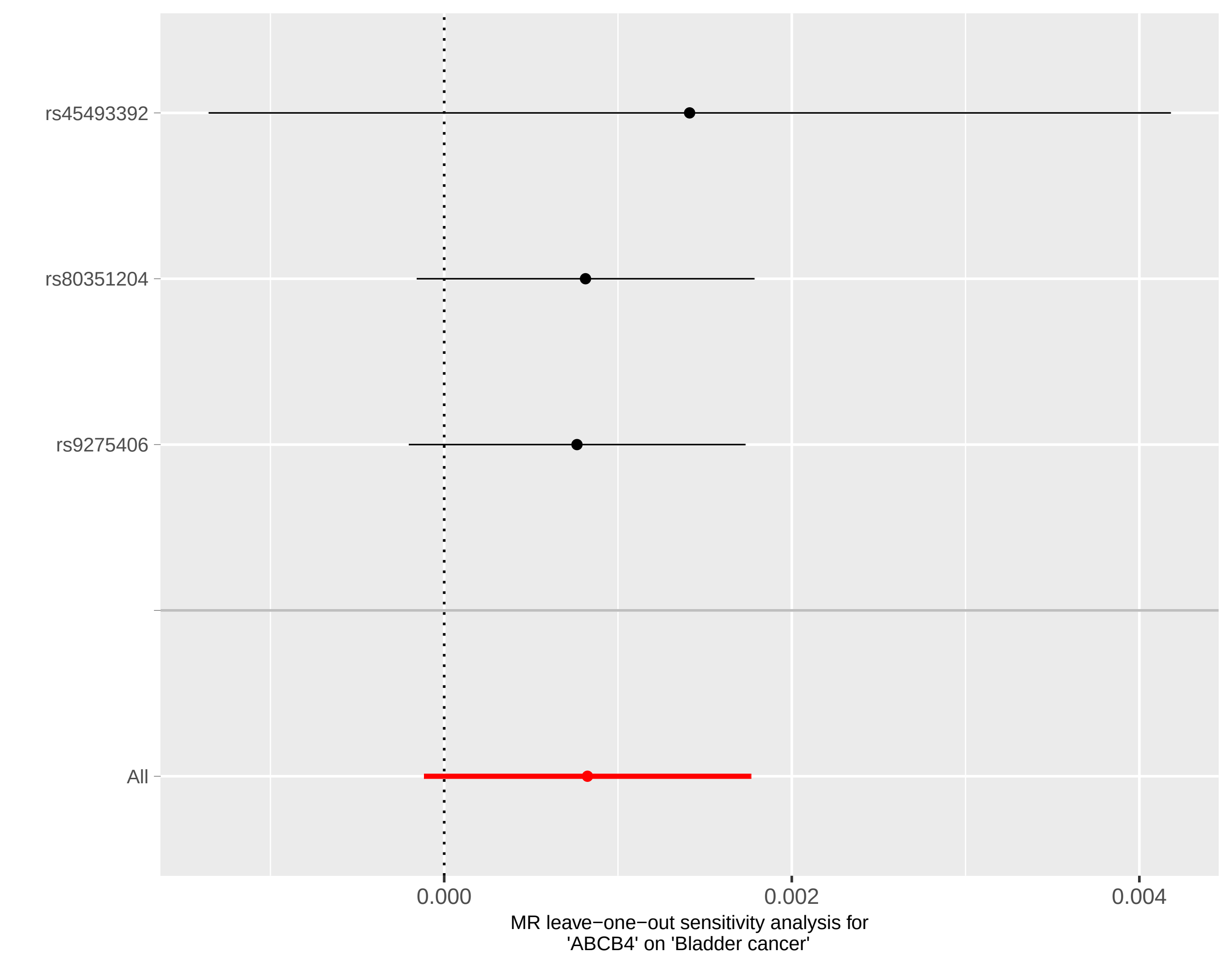
rs45493392
rs80351204
rs9275406
All
0.000
0.002
0.004
MR leave−one−out sensitivity analysis for
'ABCB4' on 'Bladder cancer'

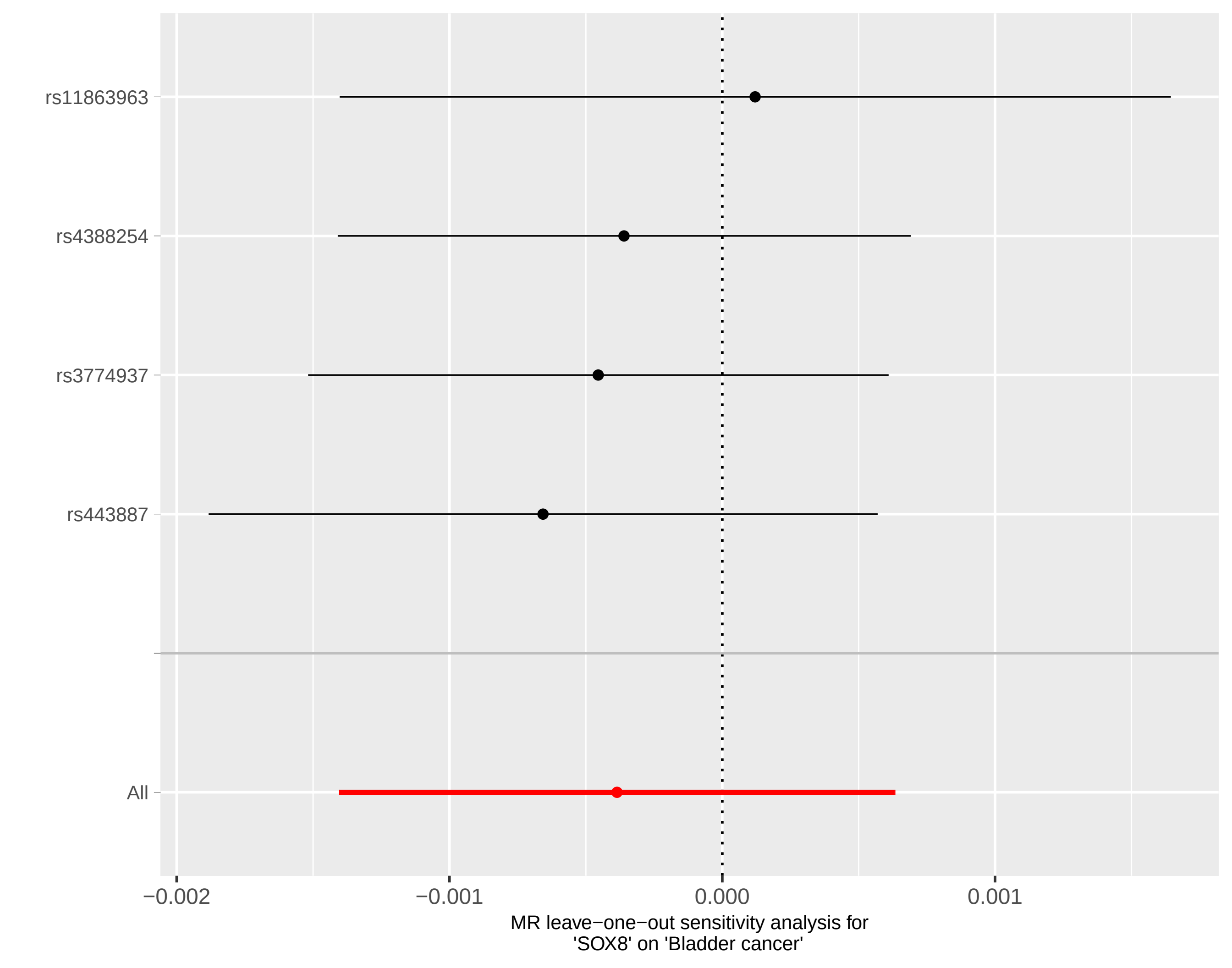
rs11863963
rs4388254
rs3774937
rs443887
All
−0.002
−0.001
0.000
0.001
MR leave−one−out sensitivity analysis for
'SOX8' on 'Bladder cancer'

Insufficient number of SNPs

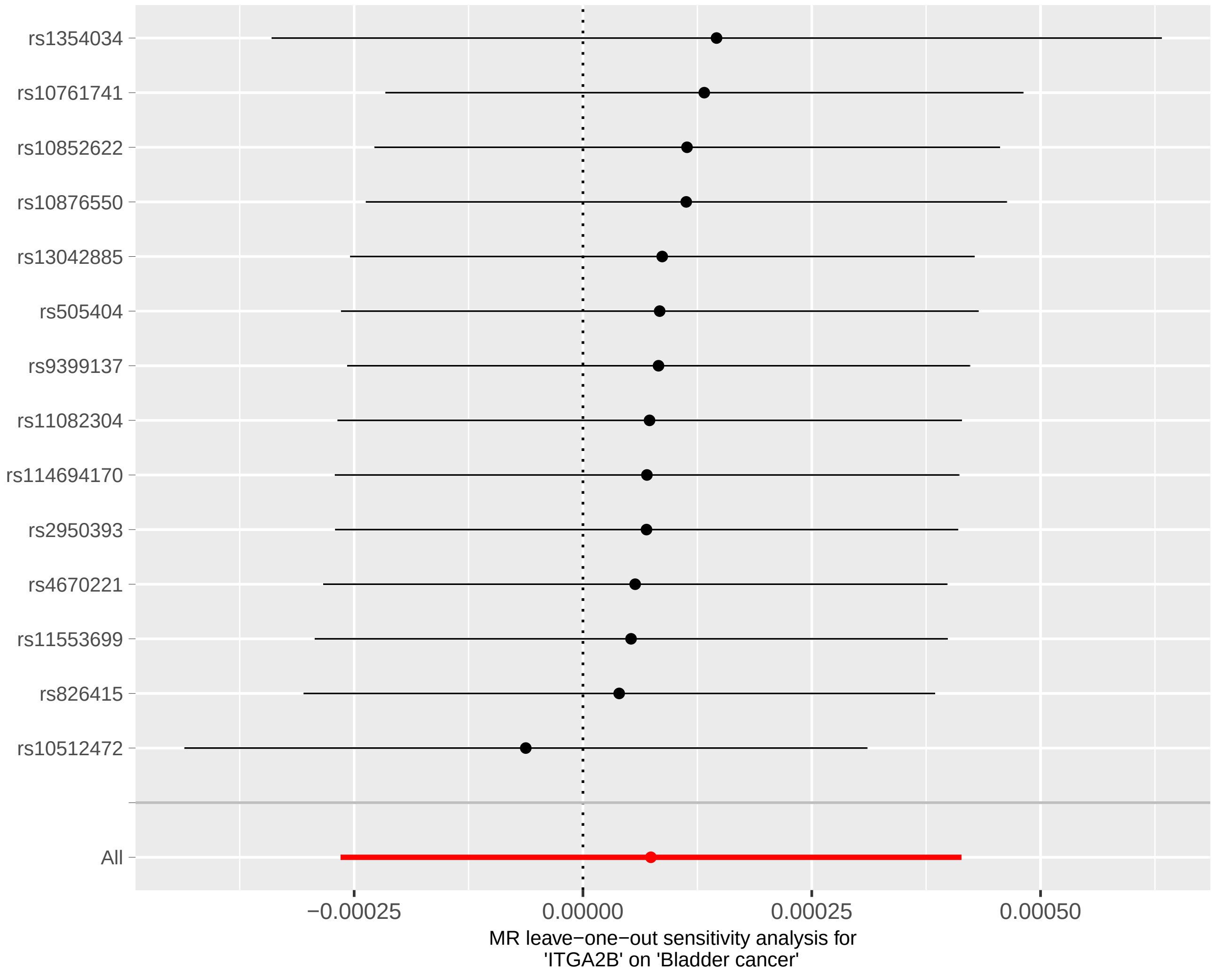
rs1354034
rs10761741
rs10852622
rs10876550
rs13042885
rs505404
rs9399137
rs11082304
rs114694170
rs2950393
rs4670221
rs11553699
rs826415
rs10512472
All
−0.00025
0.00000
0.00025
0.00050
MR leave−one−out sensitivity analysis for
'ITGA2B' on 'Bladder cancer'

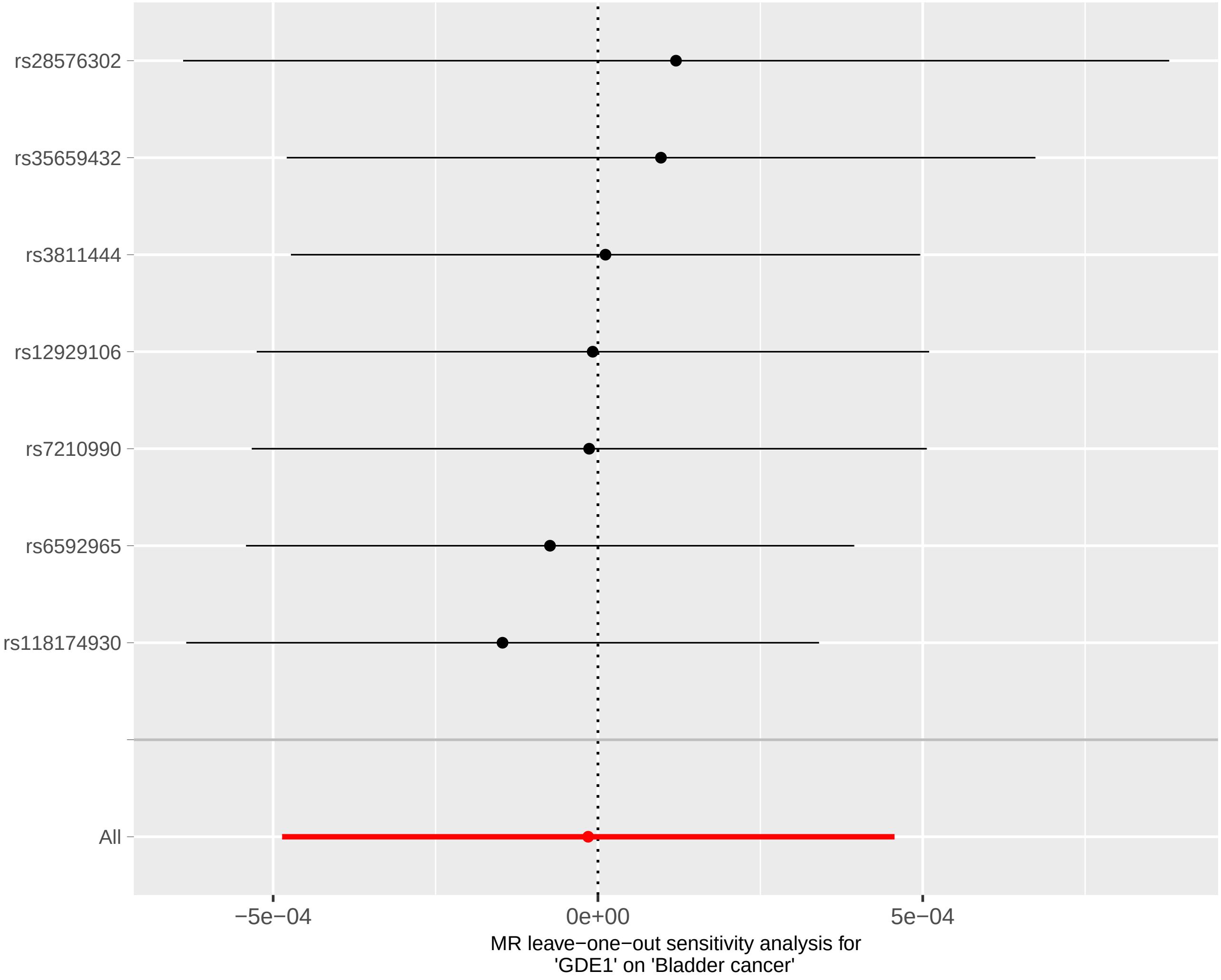
rs28576302
rs35659432
rs3811444
rs12929106
rs7210990
rs6592965
rs118174930
All
−5e−04
0e+00
5e−04
MR leave−one−out sensitivity analysis for
'GDE1' on 'Bladder cancer'

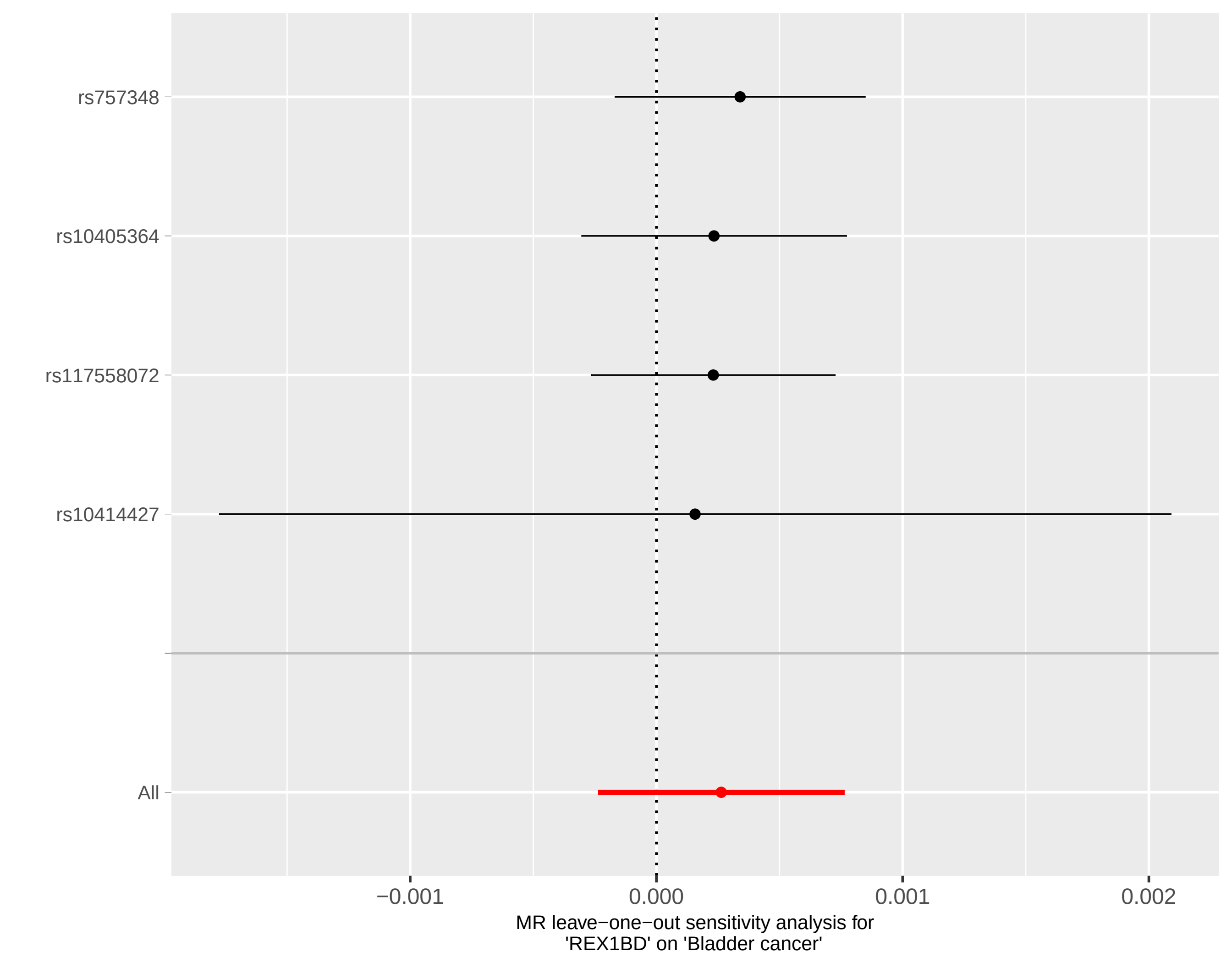
rs757348
rs10405364
rs117558072
rs10414427
All
−0.001
0.000
0.001
0.002
MR leave−one−out sensitivity analysis for
'REX1BD' on 'Bladder cancer'

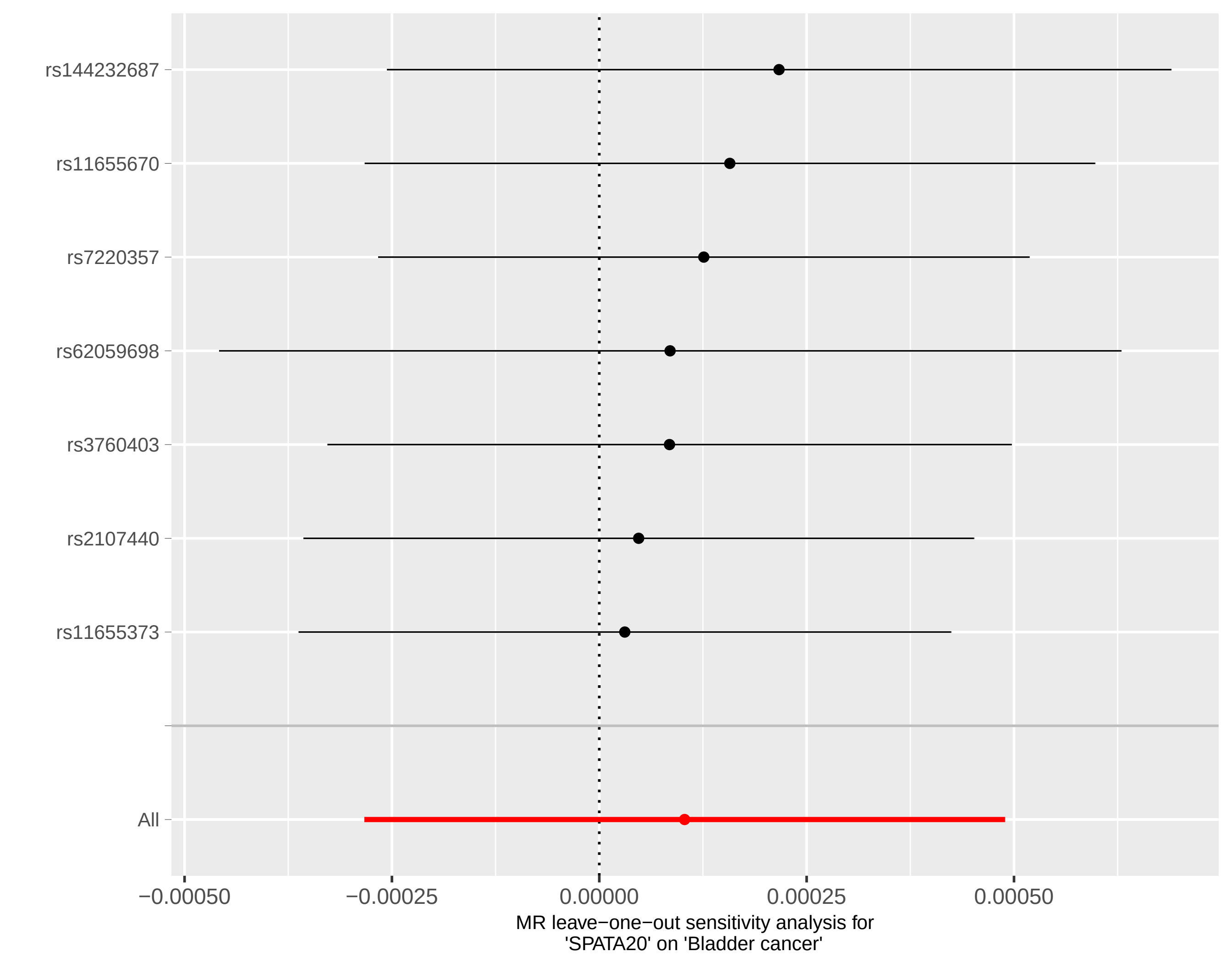
rs144232687
rs11655670
rs7220357
rs62059698
rs3760403
rs2107440
rs11655373
All
−0.00050
−0.00025
0.00000
0.00025
0.00050
MR leave−one−out sensitivity analysis for
'SPATA20' on 'Bladder cancer'

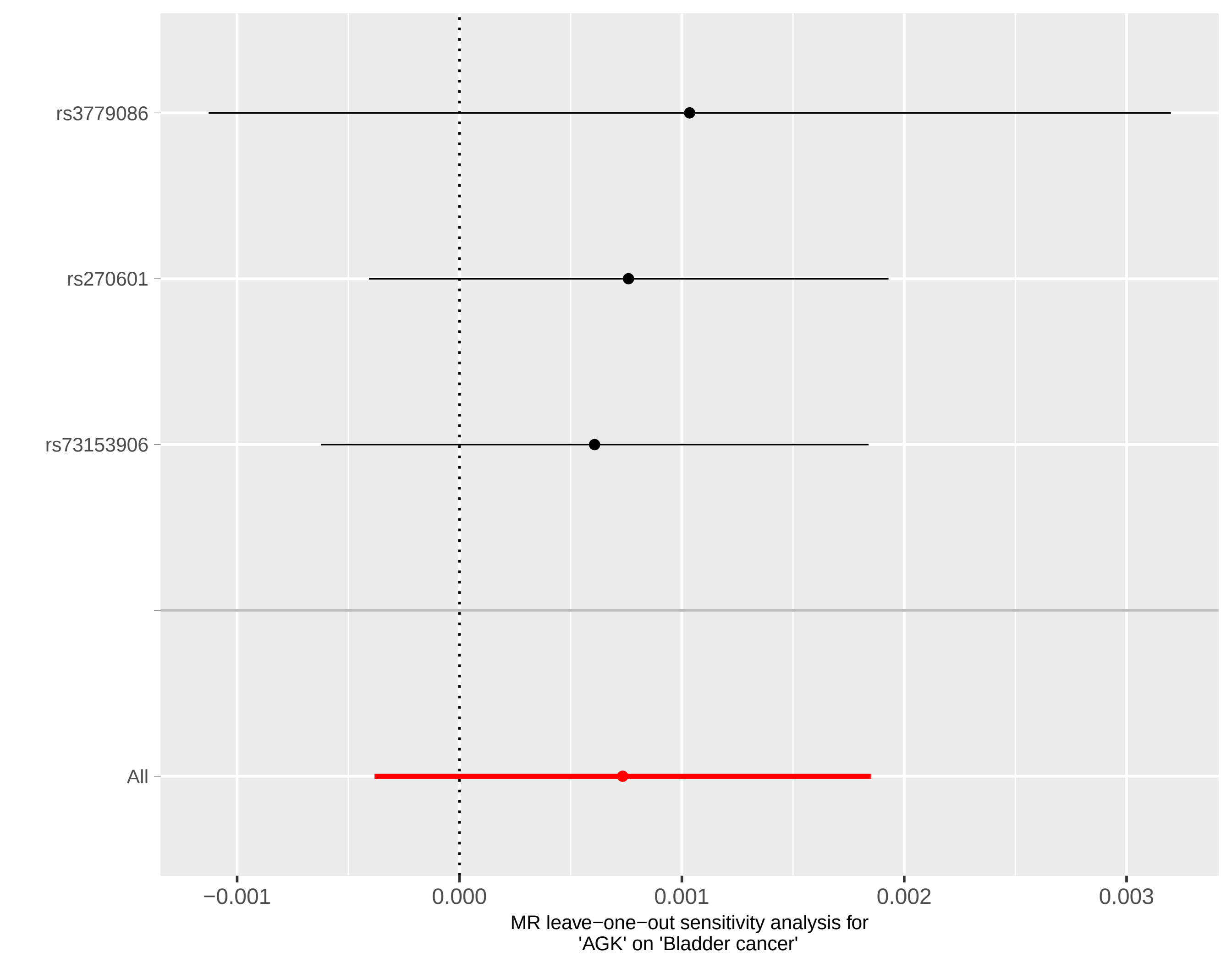
rs3779086
rs270601
rs73153906
All
−0.001
0.000
0.001
0.002
0.003
MR leave−one−out sensitivity analysis for
'AGK' on 'Bladder cancer'

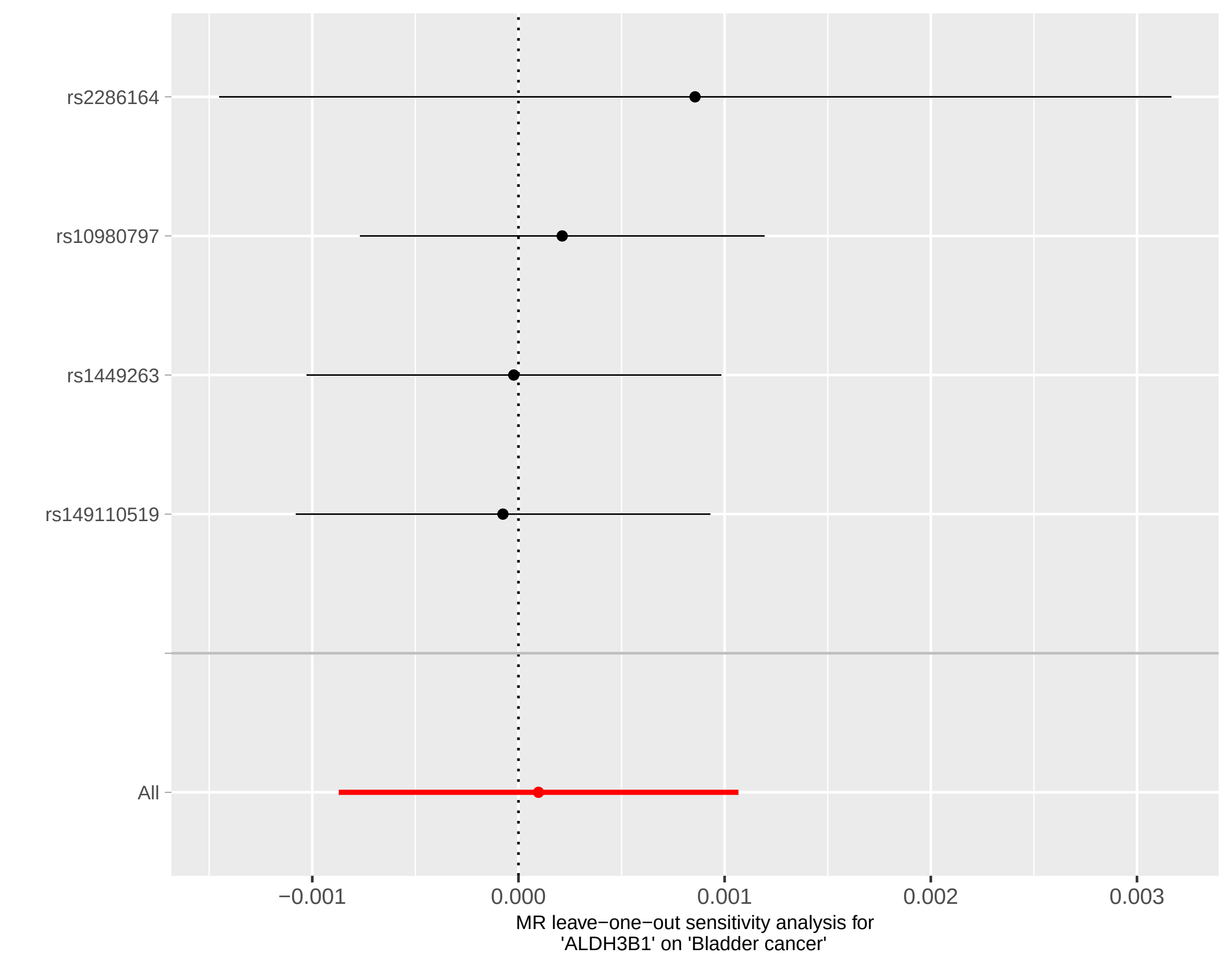
rs2286164
rs10980797
rs1449263
rs149110519
All
−0.001
0.000
0.001
0.002
0.003
MR leave−one−out sensitivity analysis for
'ALDH3B1' on 'Bladder cancer'

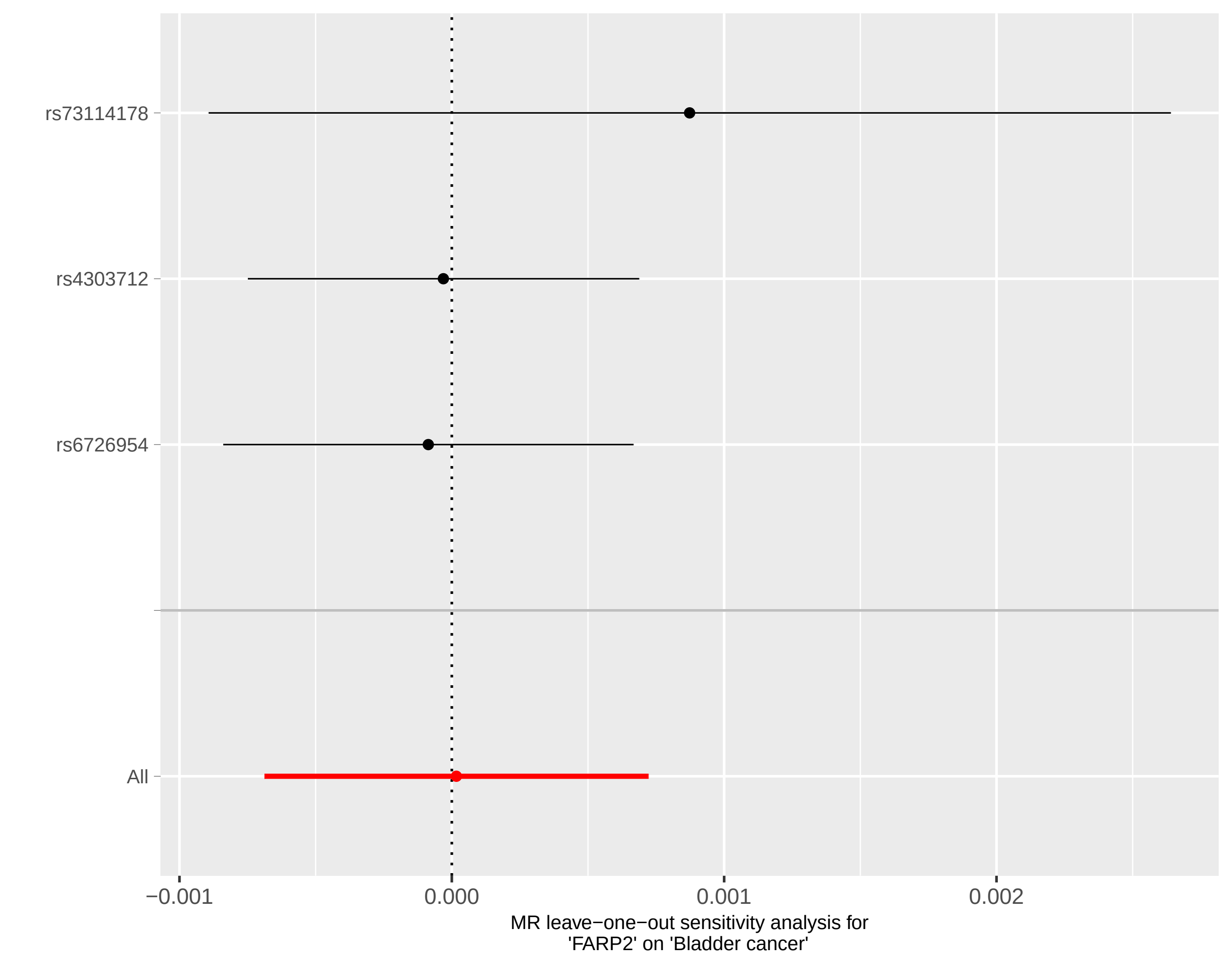
rs73114178
rs4303712
rs6726954
All
−0.001
0.000
0.001
0.002
MR leave−one−out sensitivity analysis for
'FARP2' on 'Bladder cancer'

Insufficient number of SNPs

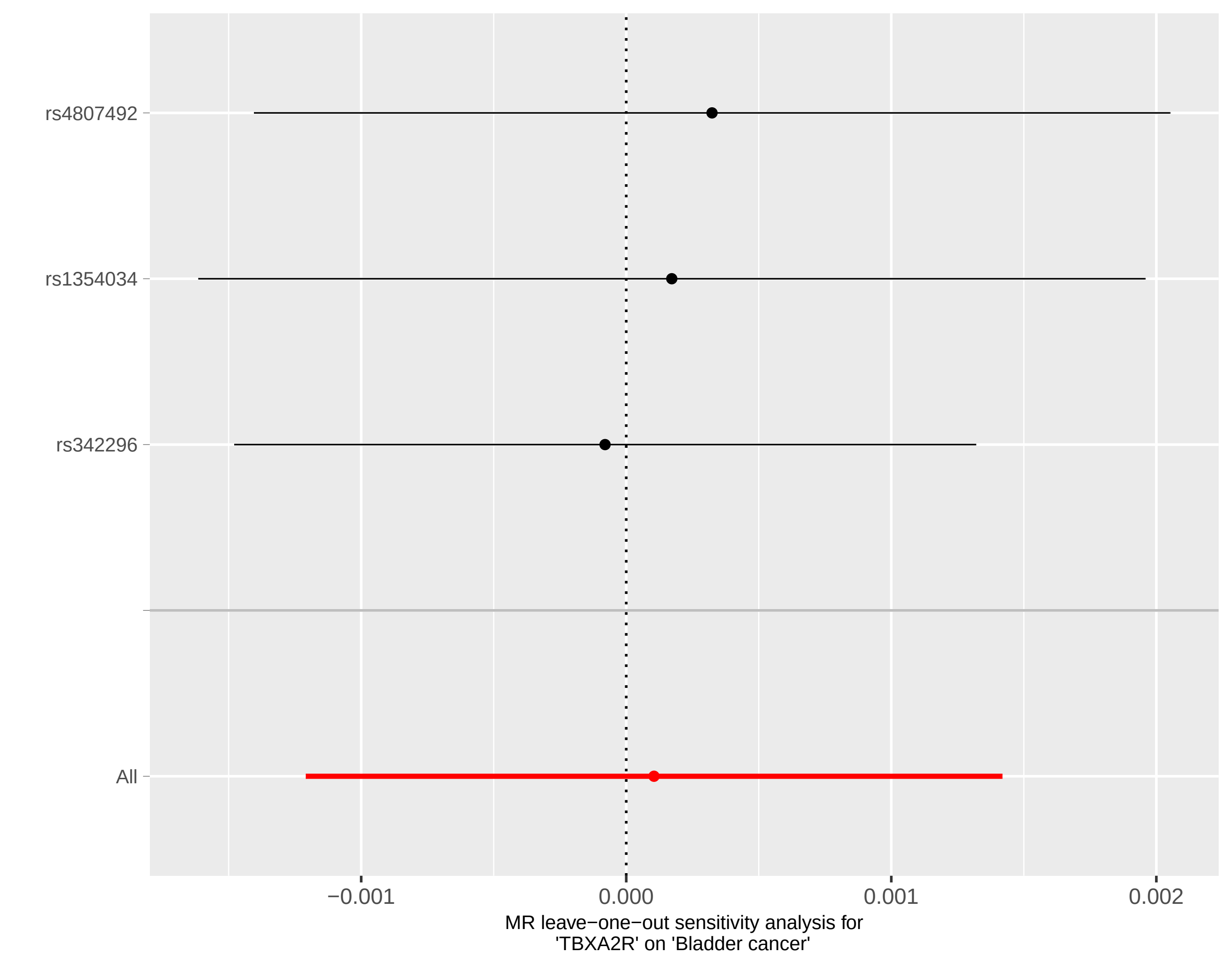
rs4807492
rs1354034
rs342296
All
−0.001
0.000
0.001
0.002
MR leave−one−out sensitivity analysis for
'TBXA2R' on 'Bladder cancer'

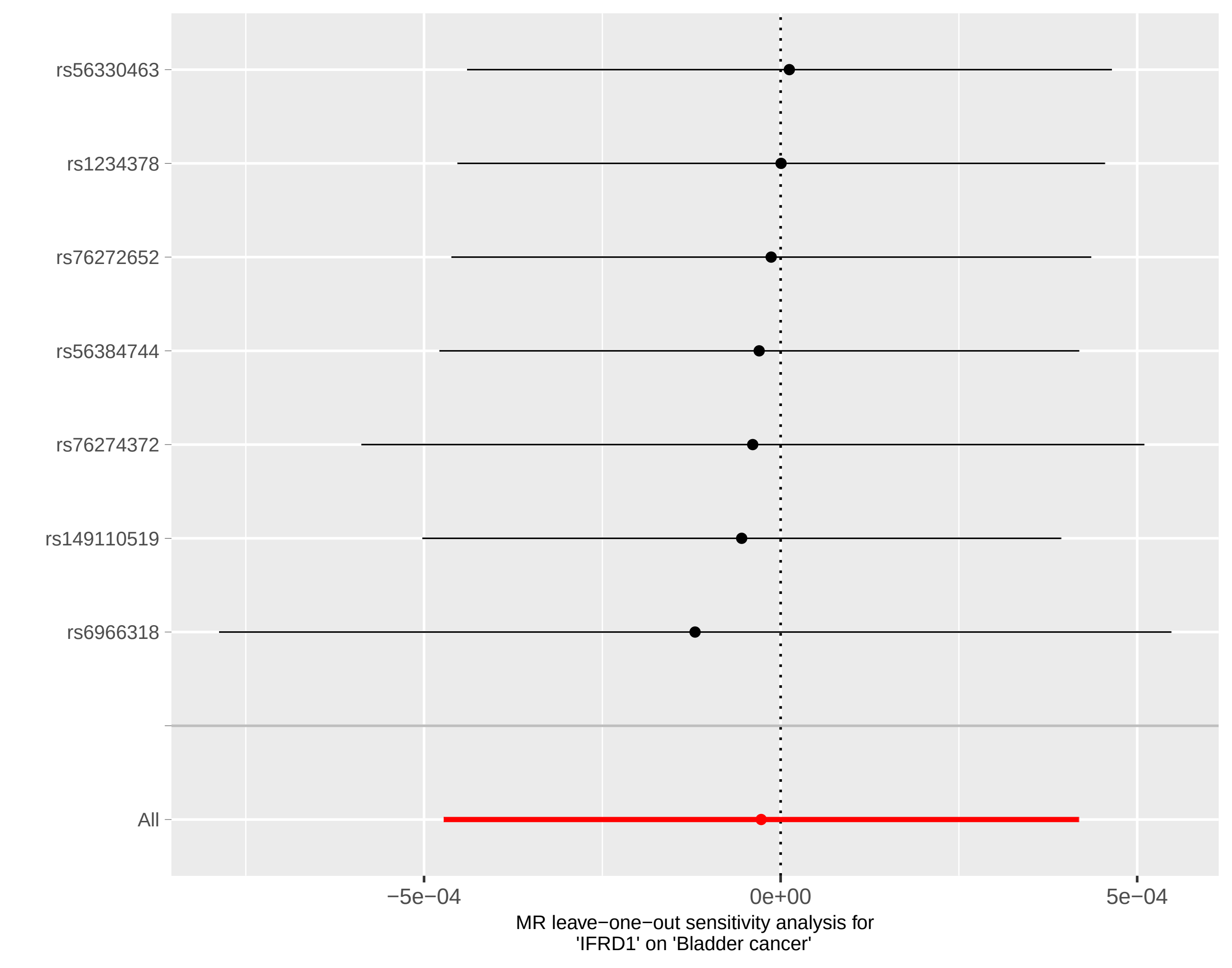
rs56330463
rs1234378
rs76272652
rs56384744
rs76274372
rs149110519
rs6966318
All
−5e−04
0e+00
5e−04
MR leave−one−out sensitivity analysis for
'IFRD1' on 'Bladder cancer'

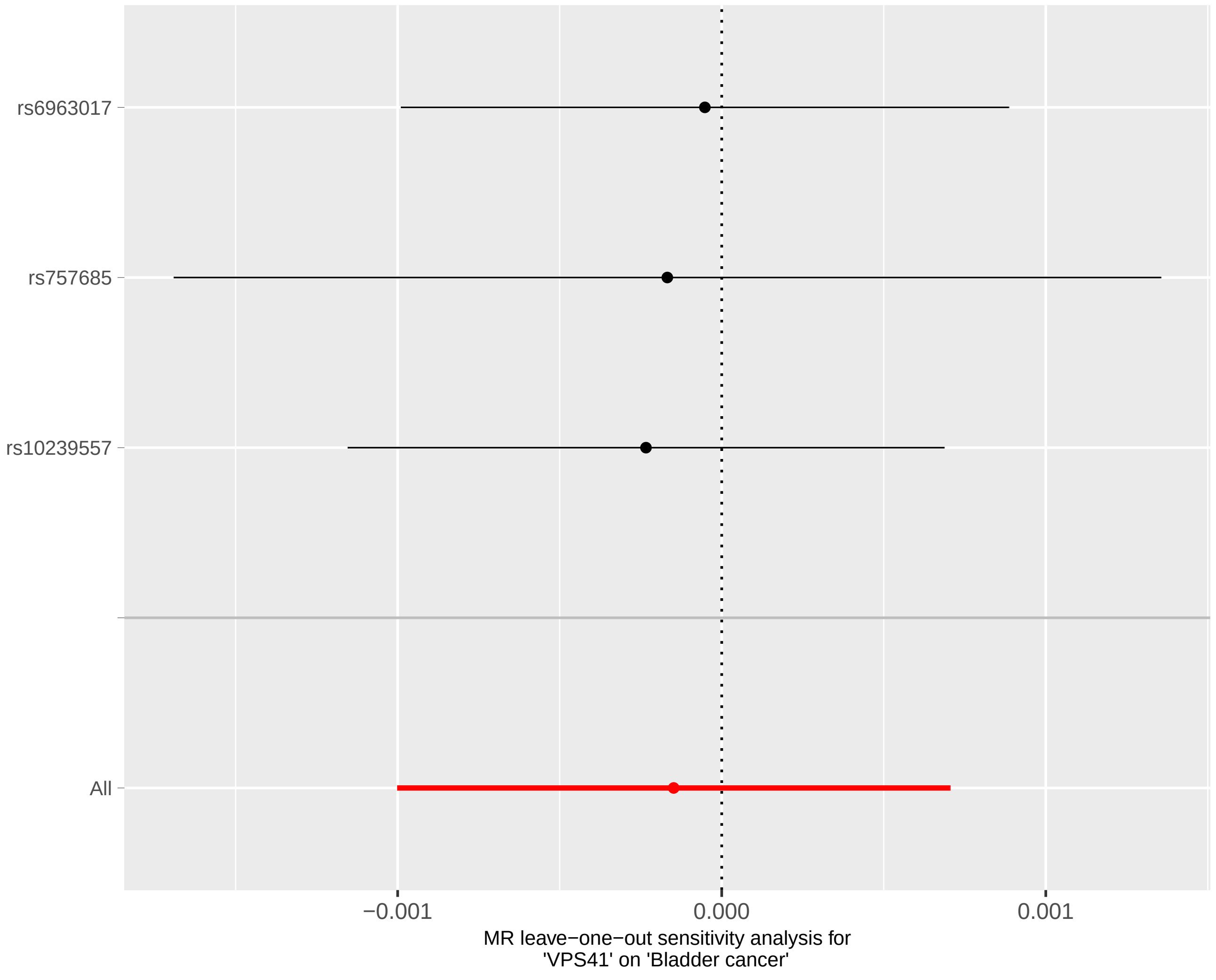
rs6963017
rs757685
rs10239557
All
−0.001
0.000
0.001
MR leave−one−out sensitivity analysis for
'VPS41' on 'Bladder cancer'

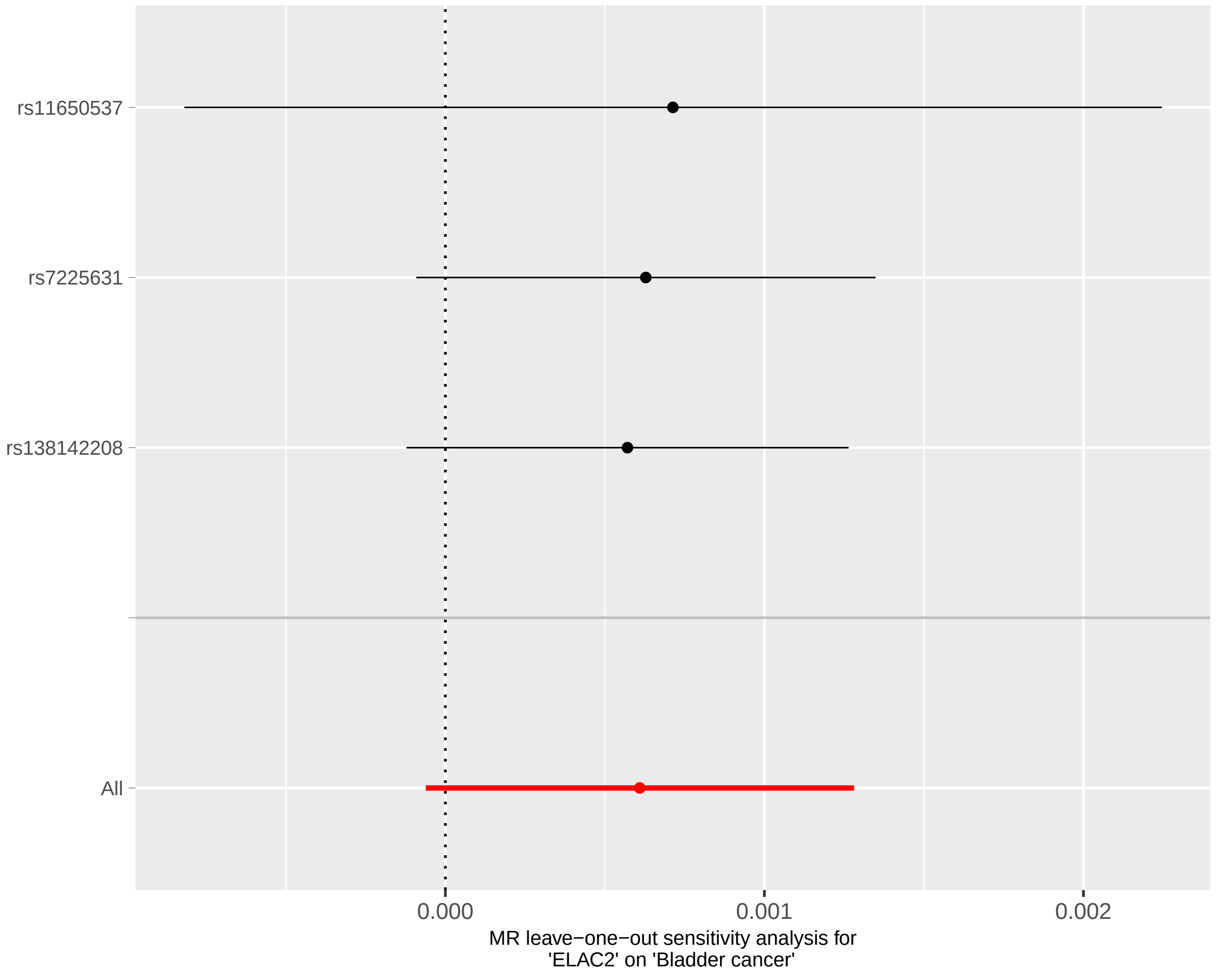
rs11650537
rs7225631
rs138142208
All
0.000
0.001
0.002
MR leave−one−out sensitivity analysis for
'ELAC2' on 'Bladder cancer'

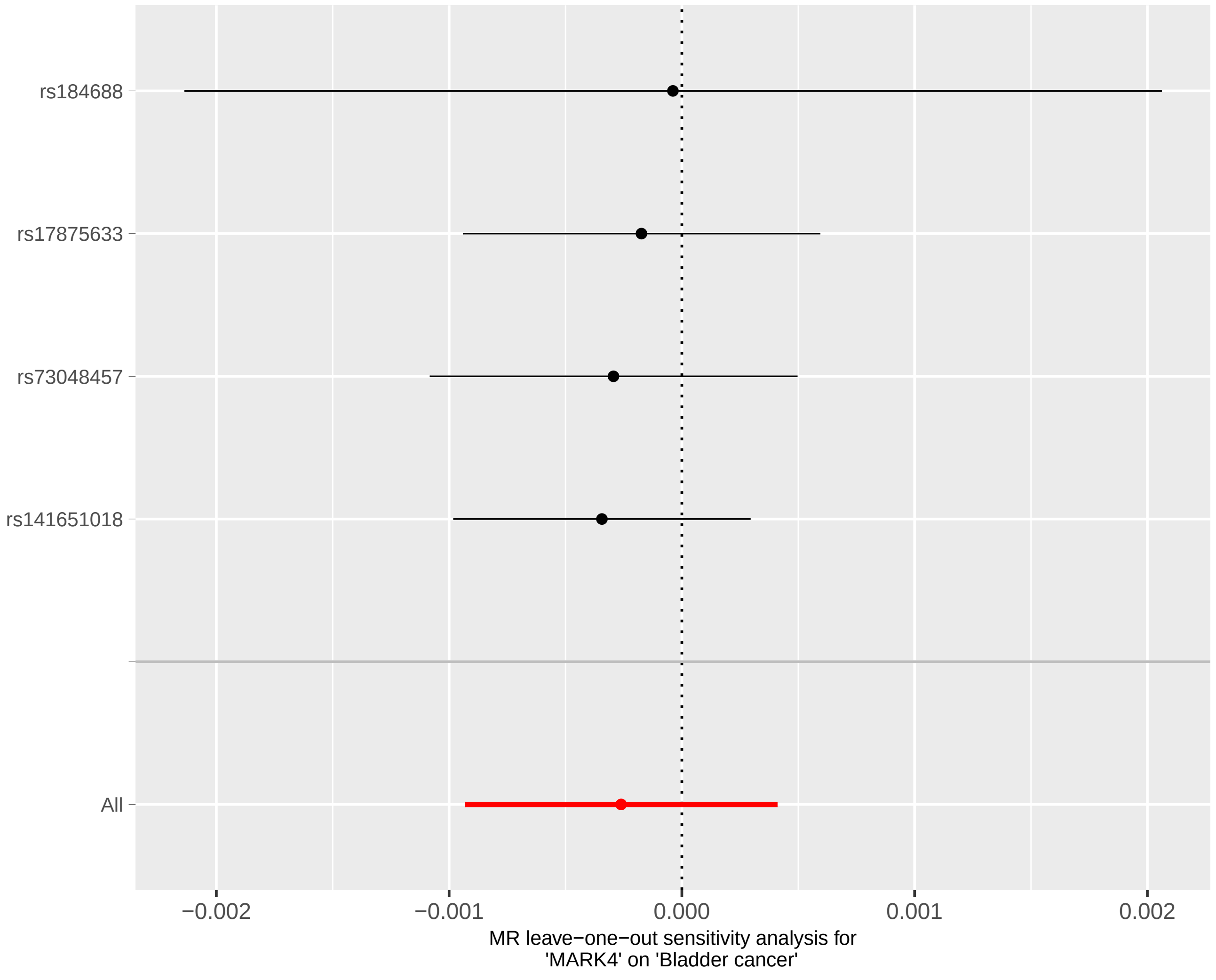

rs184688
rs17875633
rs73048457
rs141651018
All
−0.002
−0.001
0.000
0.001
0.002
MR leave−one−out sensitivity analysis for
'MARK4' on 'Bladder cancer'

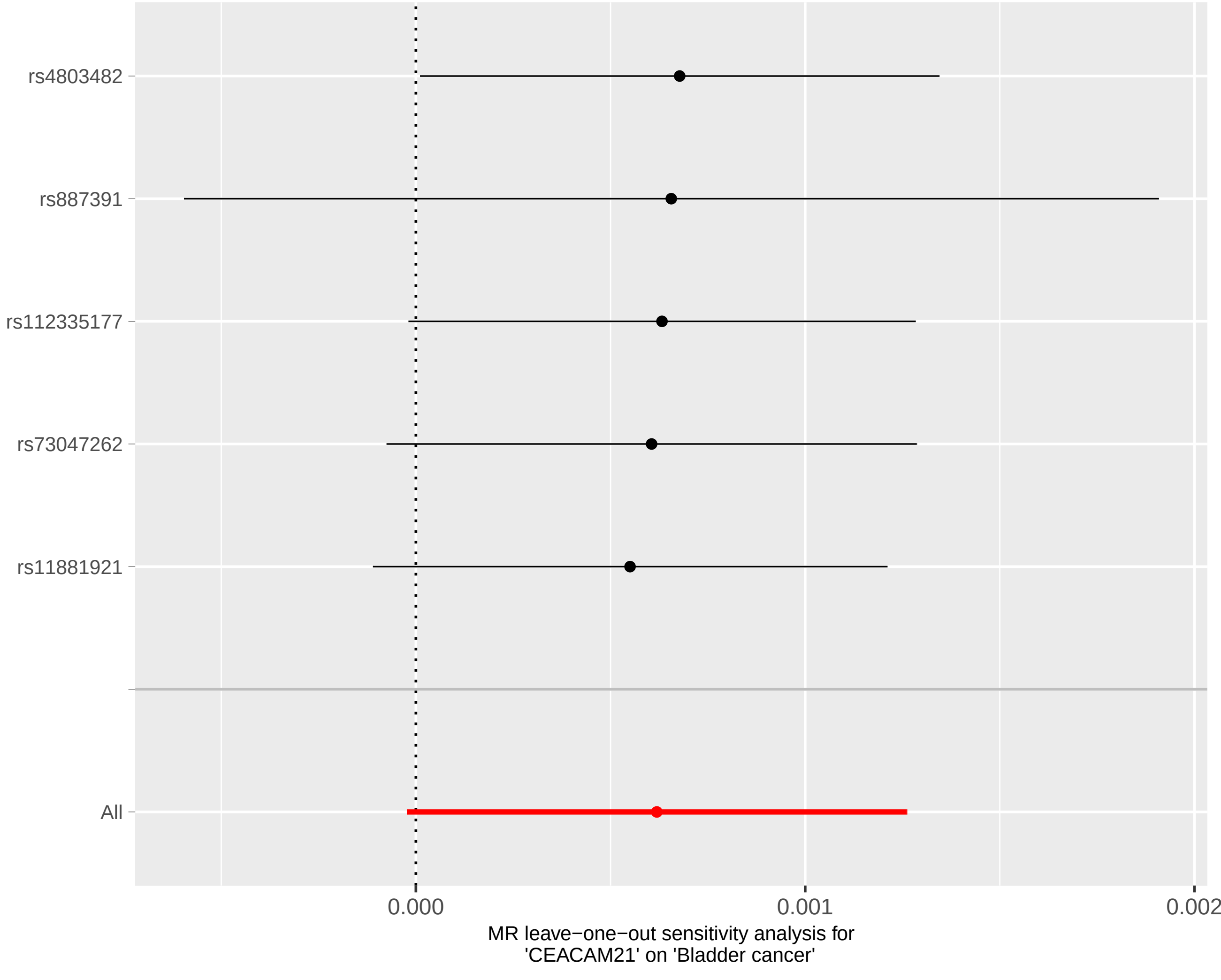
rs4803482
rs887391
rs112335177
rs73047262
rs11881921
All
0.000
0.001
0.002
MR leave−one−out sensitivity analysis for
'CEACAM21' on 'Bladder cancer'

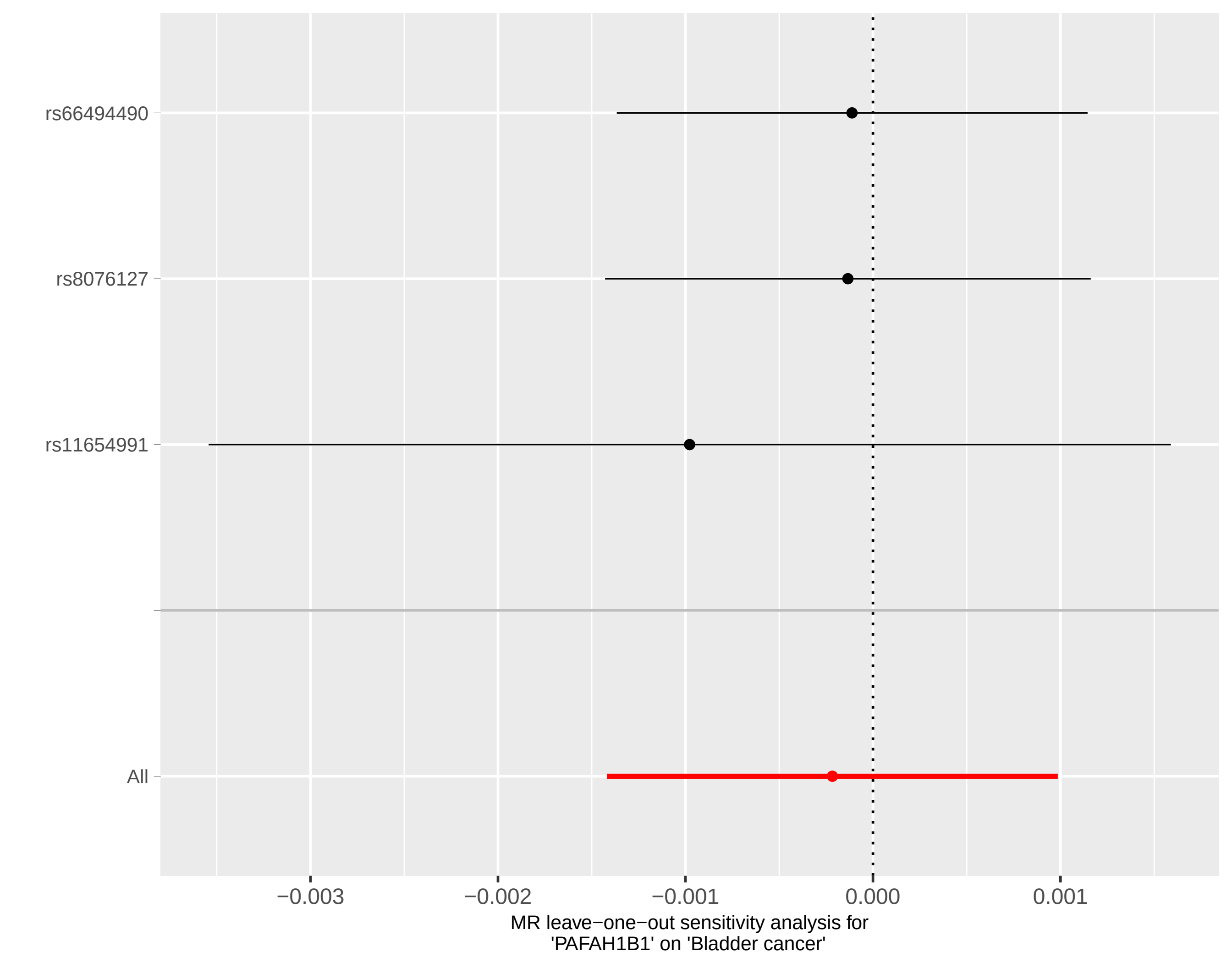
rs66494490
rs8076127
rs11654991
All
−0.003
−0.002
−0.001
0.000
0.001
MR leave−one−out sensitivity analysis for
'PAFAH1B1' on 'Bladder cancer'

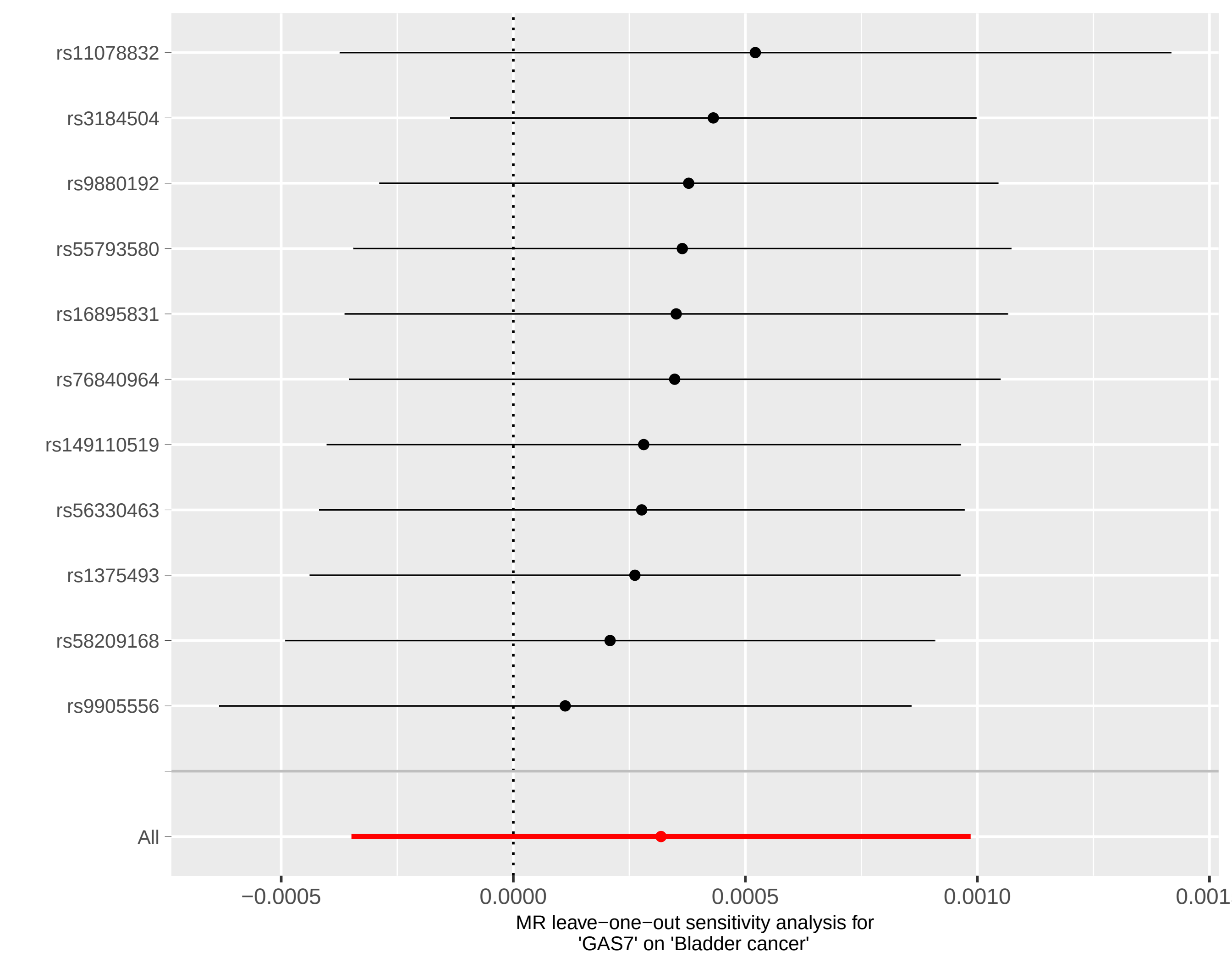
rs11078832
rs3184504
rs9880192
rs55793580
rs16895831
rs76840964
rs149110519
rs56330463
rs1375493
rs58209168
rs9905556
All
−0.0005
0.0000
0.0005
0.0010
MR leave−one−out sensitivity analysis for
'GAS7' on 'Bladder cancer'

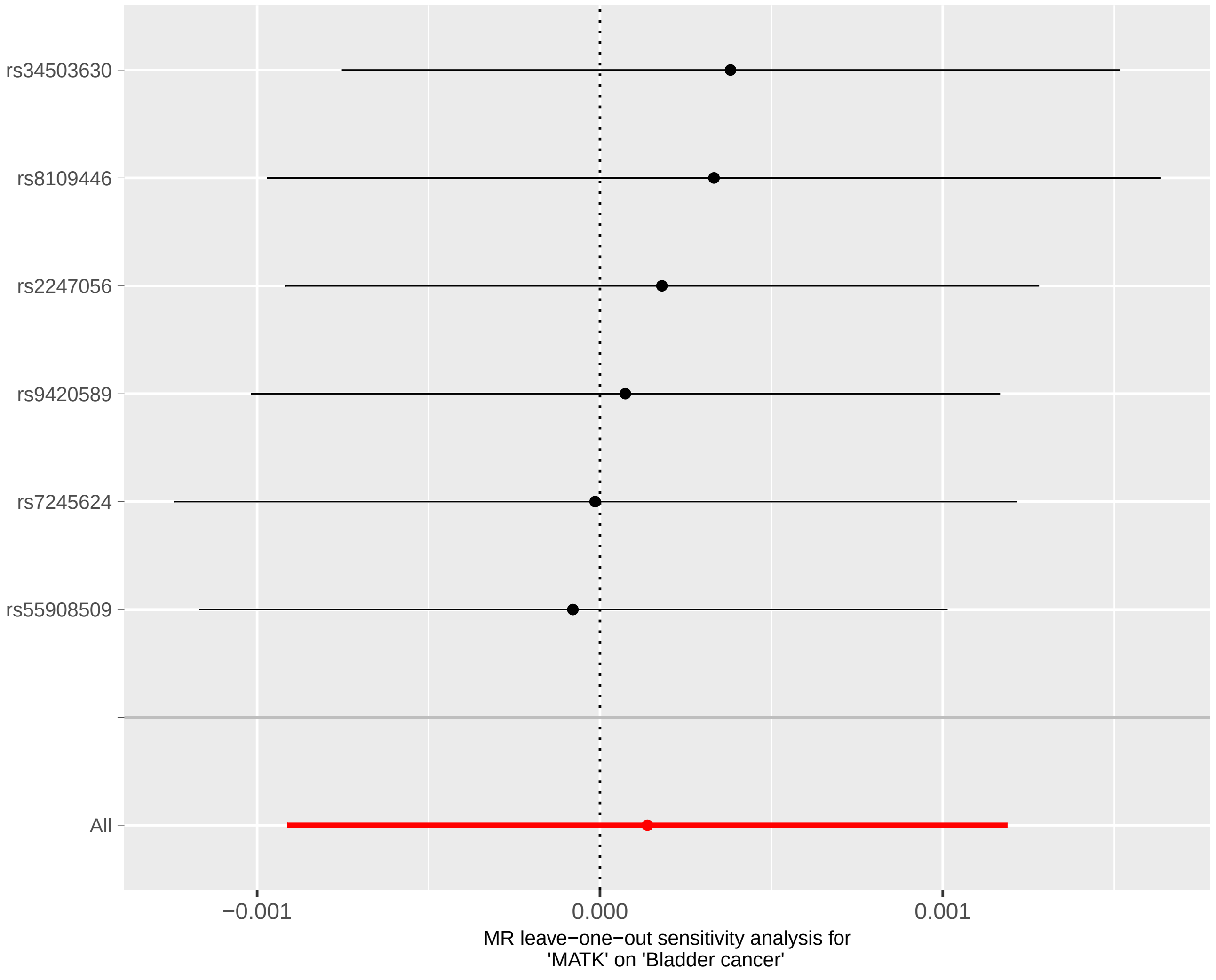
rs34503630
rs8109446
rs2247056
rs9420589
rs7245624
rs55908509
All
−0.001
0.000
0.001
MR leave−one−out sensitivity analysis for
'MATK' on 'Bladder cancer'

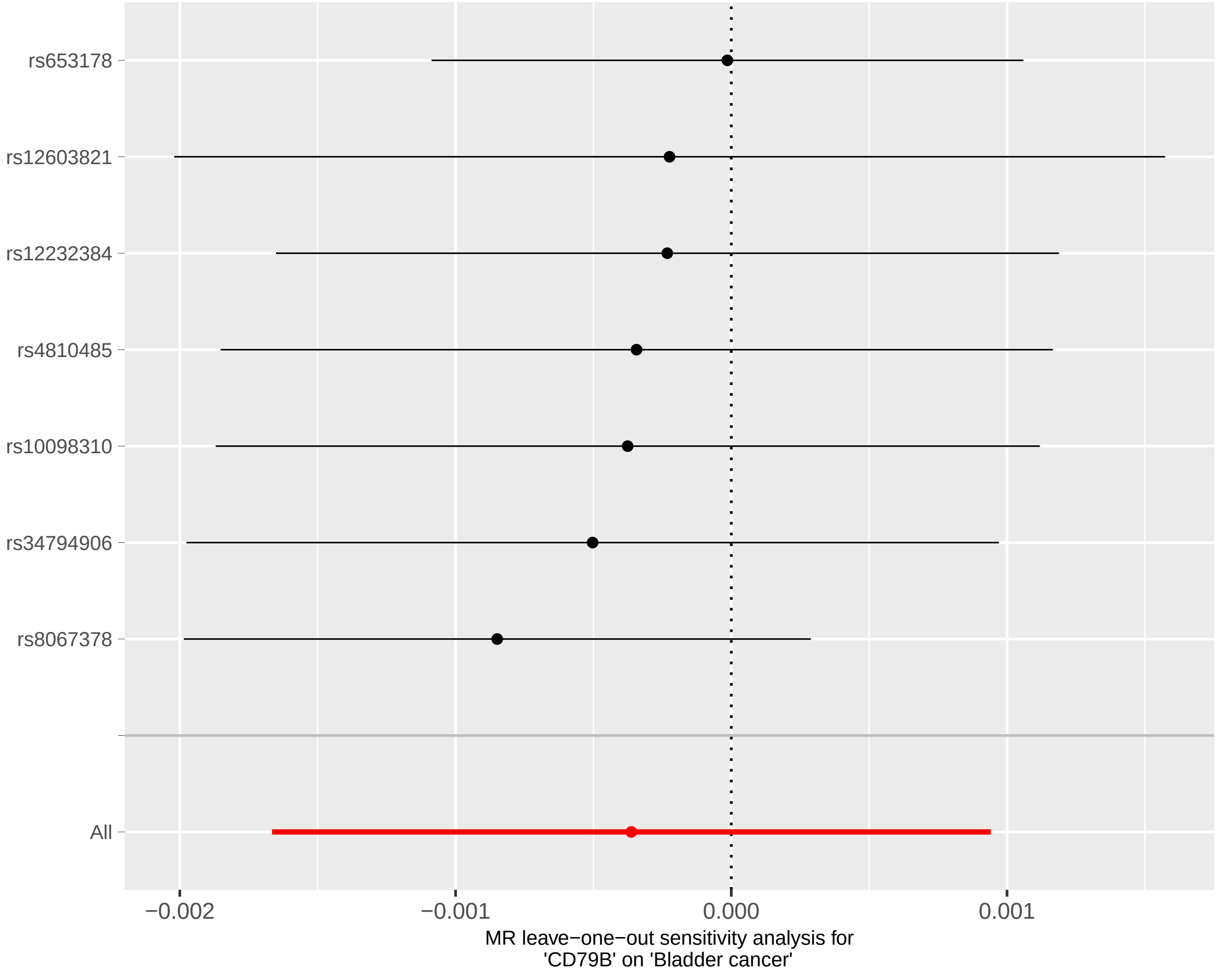
rs653178
rs12603821
rs12232384
rs4810485
rs10098310
rs34794906
rs8067378
All
−0.002
−0.001
0.000
0.001
MR leave−one−out sensitivity analysis for
'CD79B' on 'Bladder cancer'

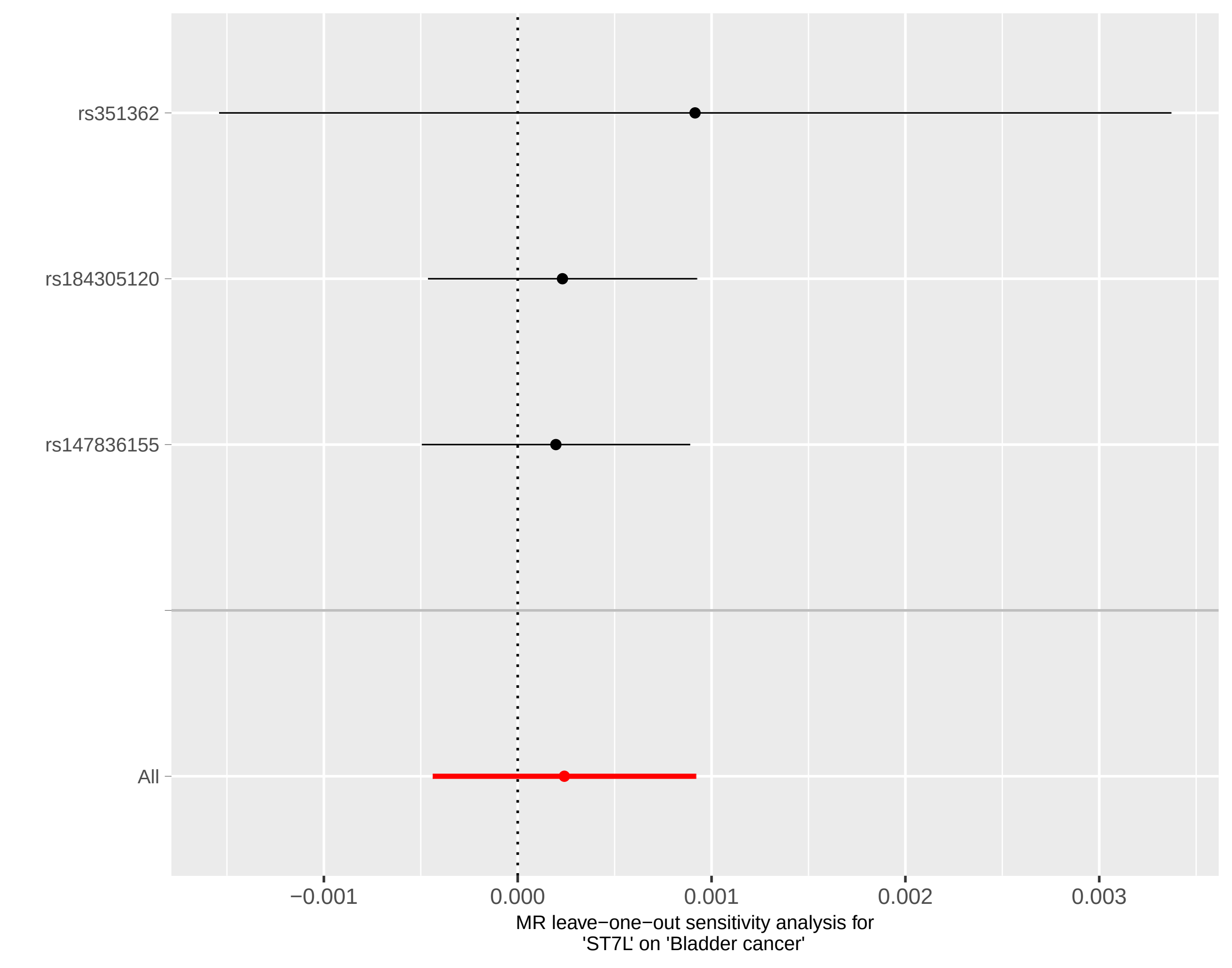

rs351362
rs184305120
rs147836155
All
−0.001
0.000
0.001
0.002
0.003
MR leave−one−out sensitivity analysis for
'ST7L' on 'Bladder cancer'

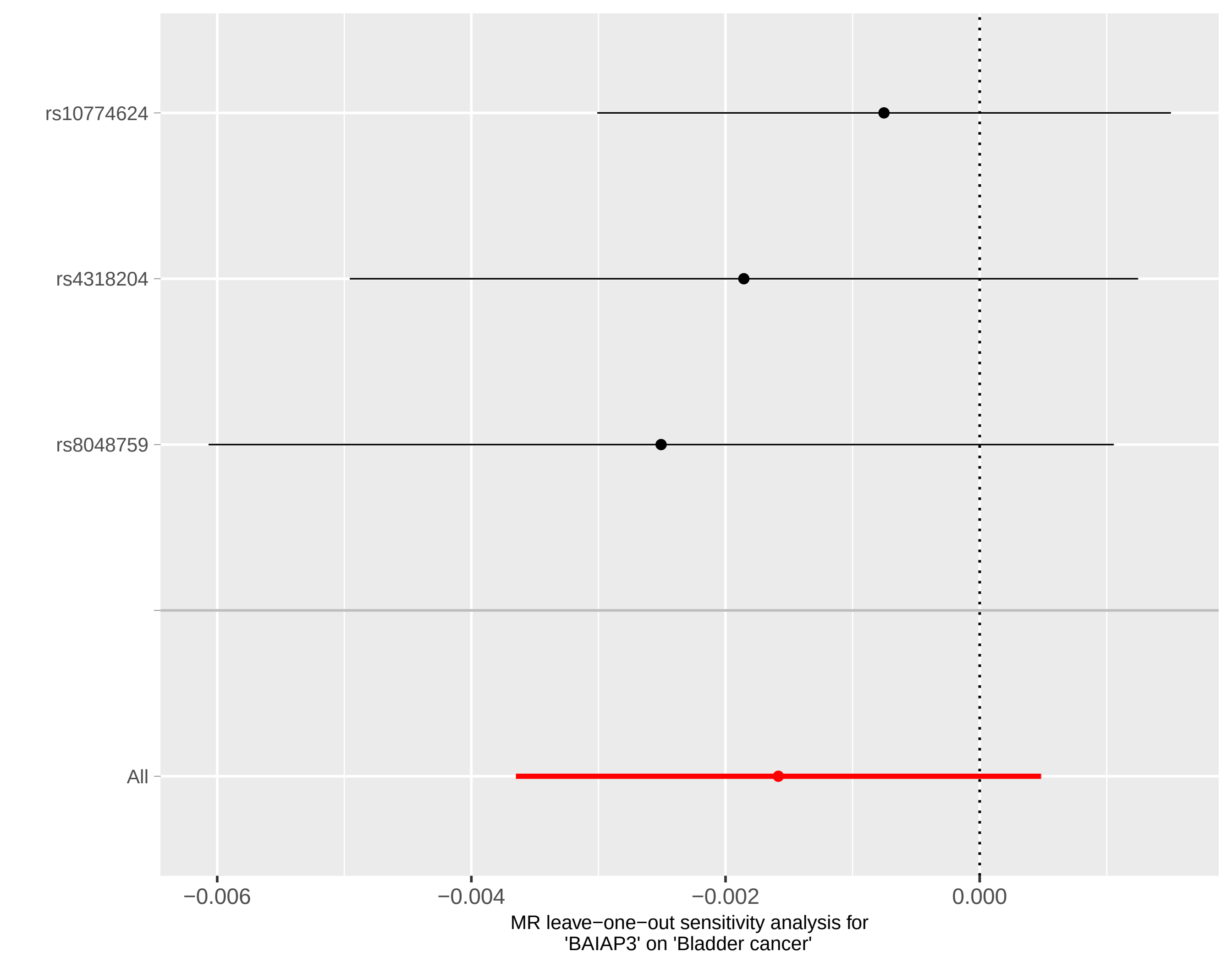

rs10774624
rs4318204
rs8048759
All
−0.006
−0.004
−0.002
0.000
MR leave−one−out sensitivity analysis for
'BAIAP3' on 'Bladder cancer'

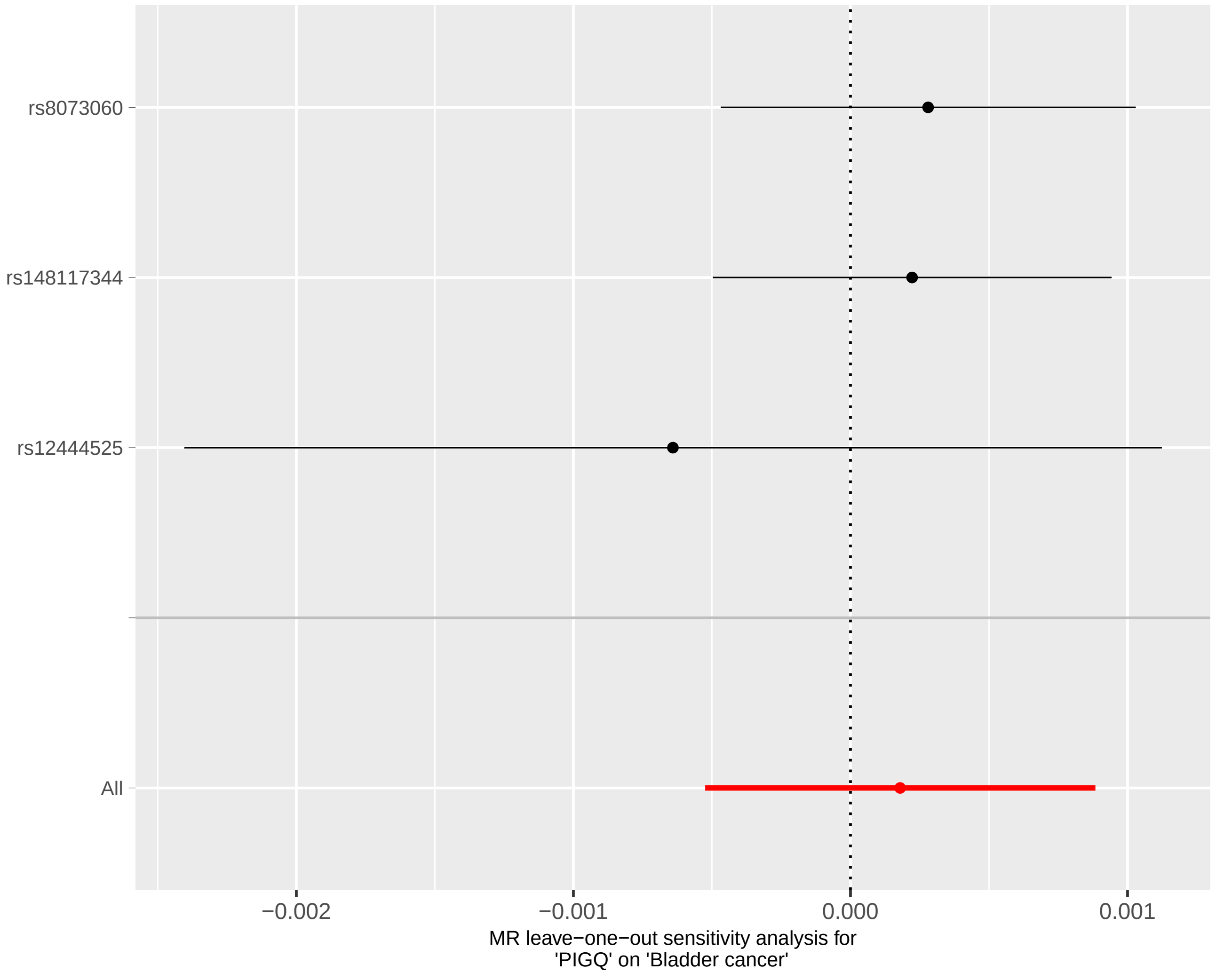

rs8073060
rs148117344
rs12444525
All
−0.002
−0.001
0.000
0.001
MR leave−one−out sensitivity analysis for
'PIGQ' on 'Bladder cancer'

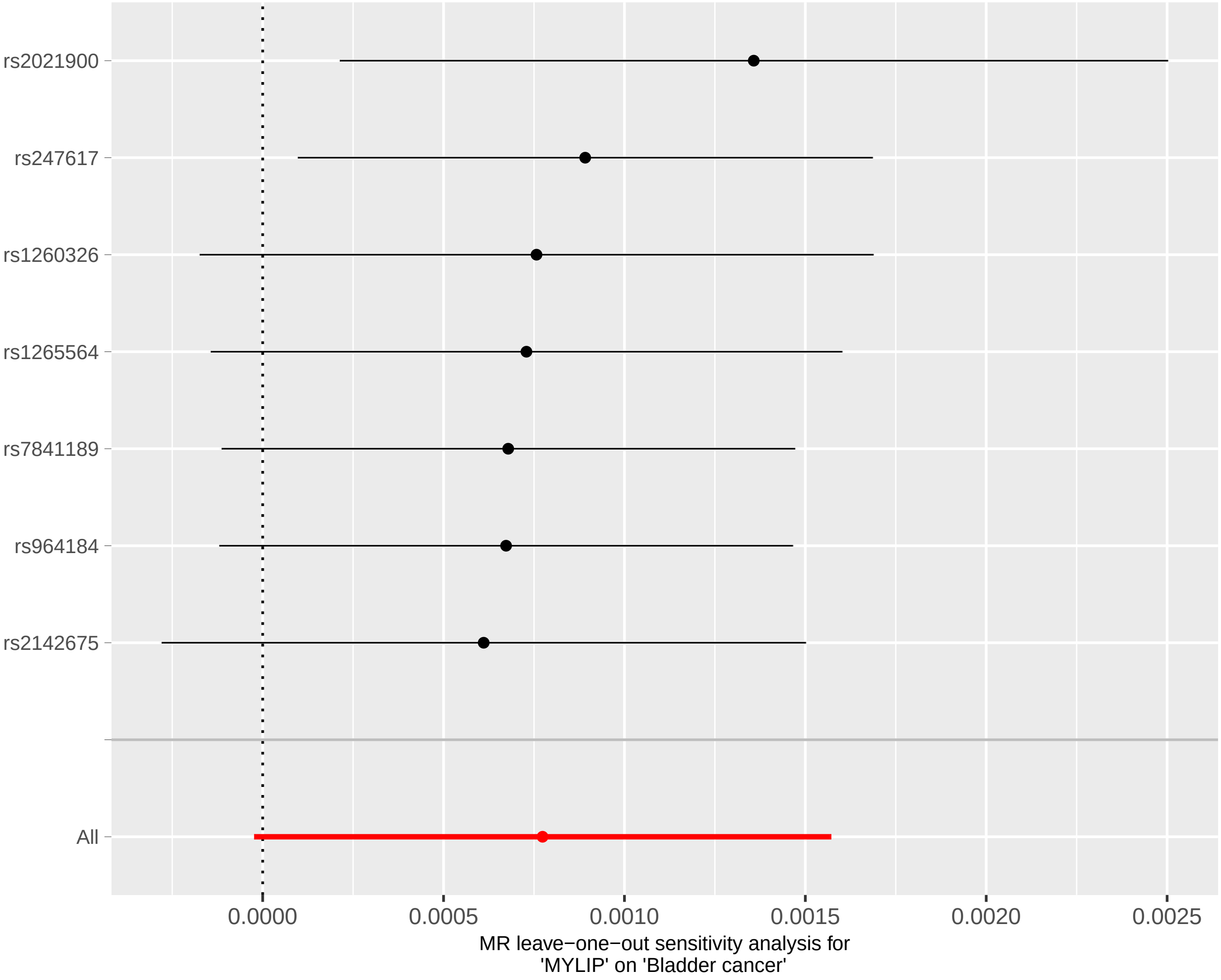
rs2021900
rs247617
rs1260326
rs1265564
rs7841189
rs964184
rs2142675
All
0.0000
0.0005
0.0010
0.0015
0.0020
0.0025
MR leave−one−out sensitivity analysis for
'MYLIP' on 'Bladder cancer'

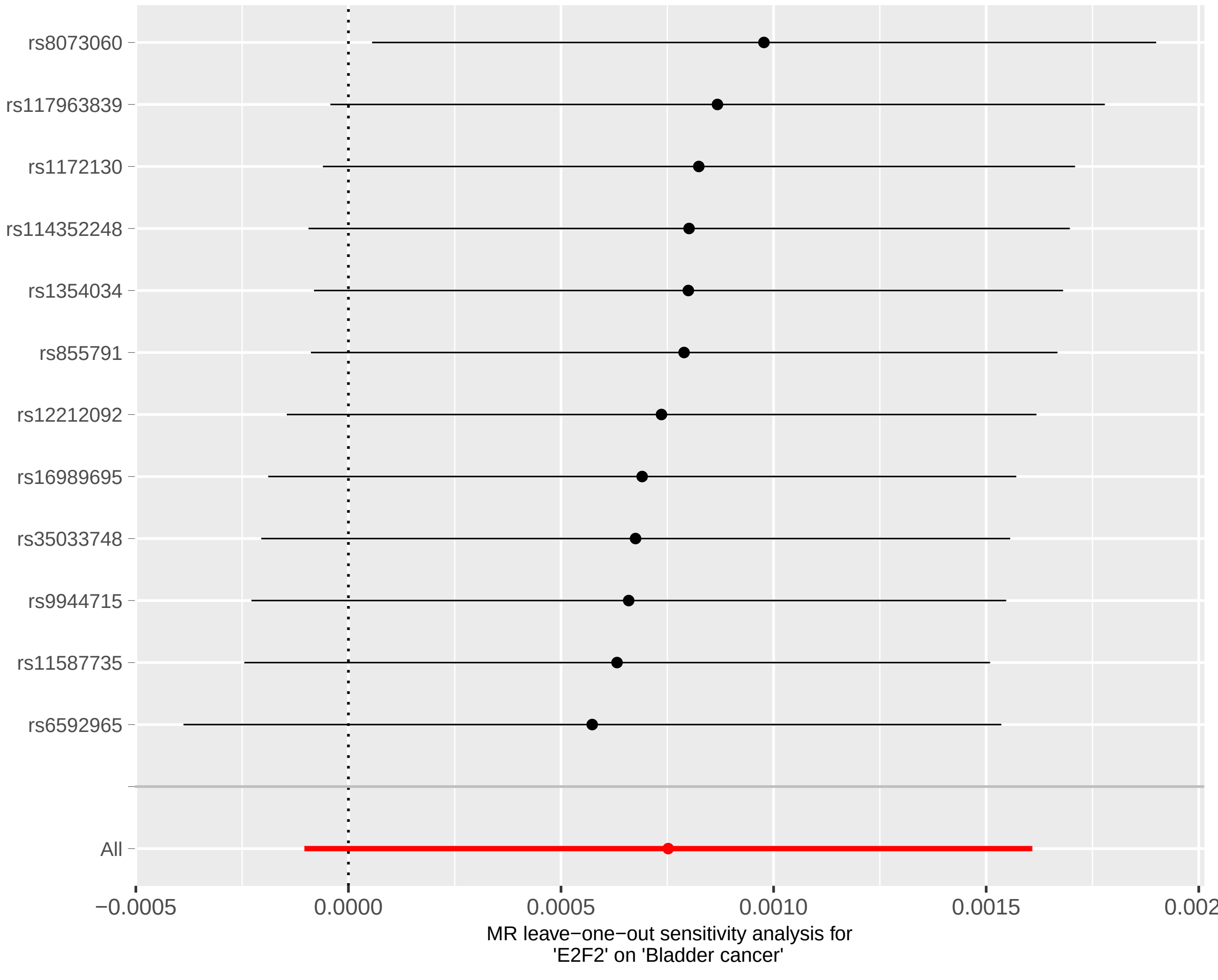
rs8073060
rs117963839
rs1172130
rs114352248
rs1354034
rs855791
rs12212092
rs16989695
rs35033748
rs9944715
rs11587735
rs6592965
All
−0.0005
0.0000
0.0005
0.0010
0.0015
0.002
MR leave−one−out sensitivity analysis for
'E2F2' on 'Bladder cancer'

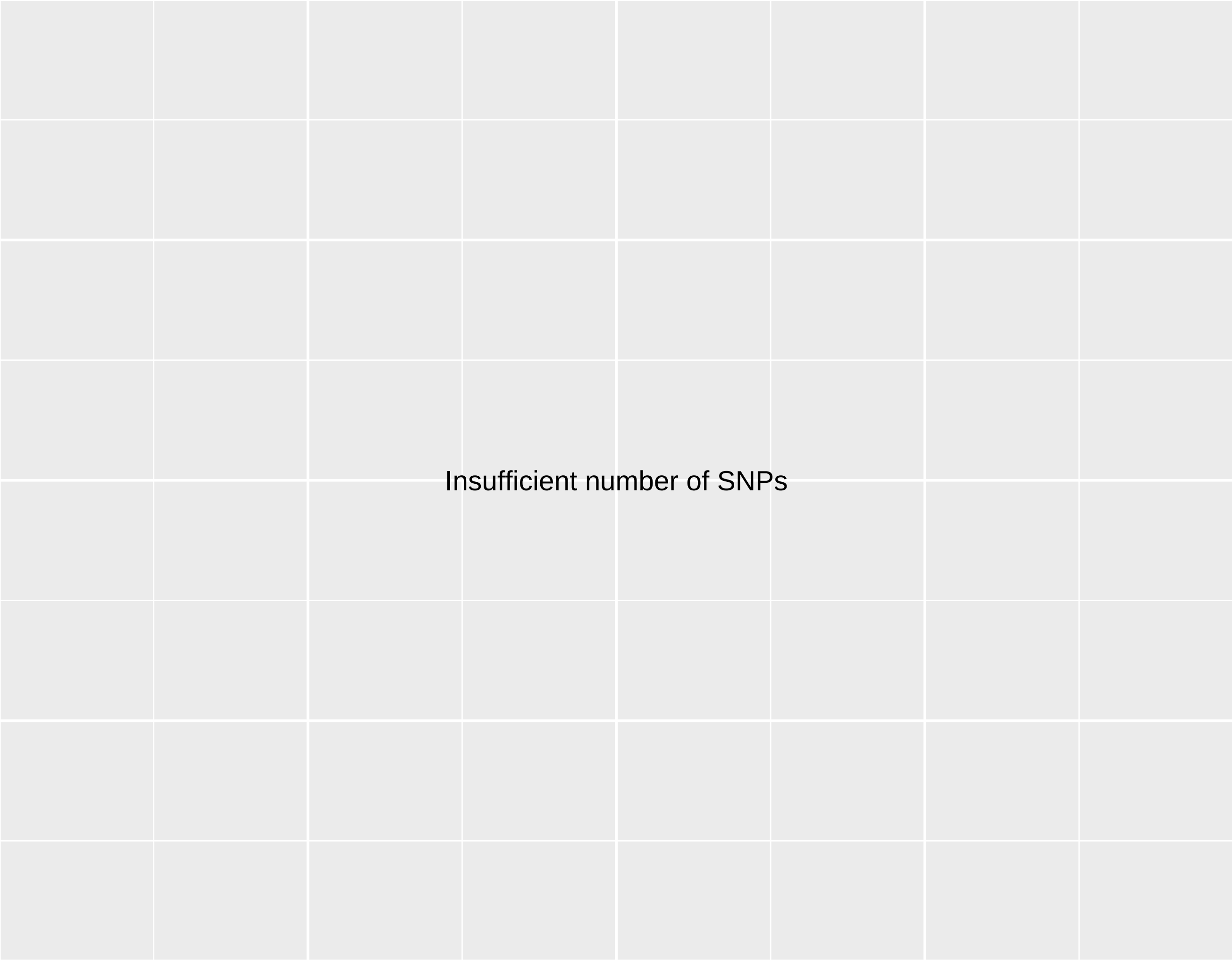
Insufficient number of SNPs

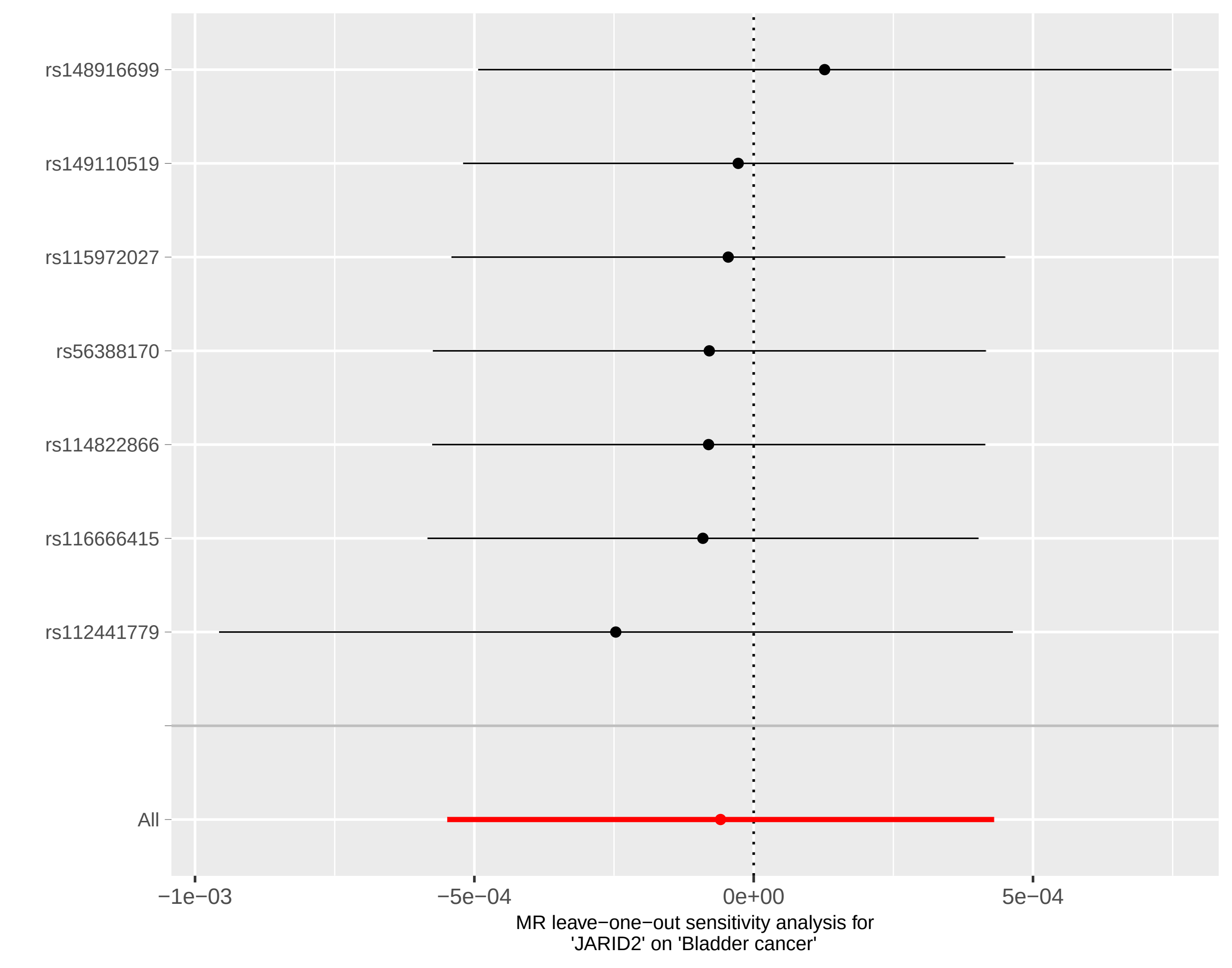

rs148916699
rs149110519
rs115972027
rs56388170
rs114822866
rs116666415
rs112441779
All
−1e−03
−5e−04
0e+00
5e−04
MR leave−one−out sensitivity analysis for
'JARID2' on 'Bladder cancer'

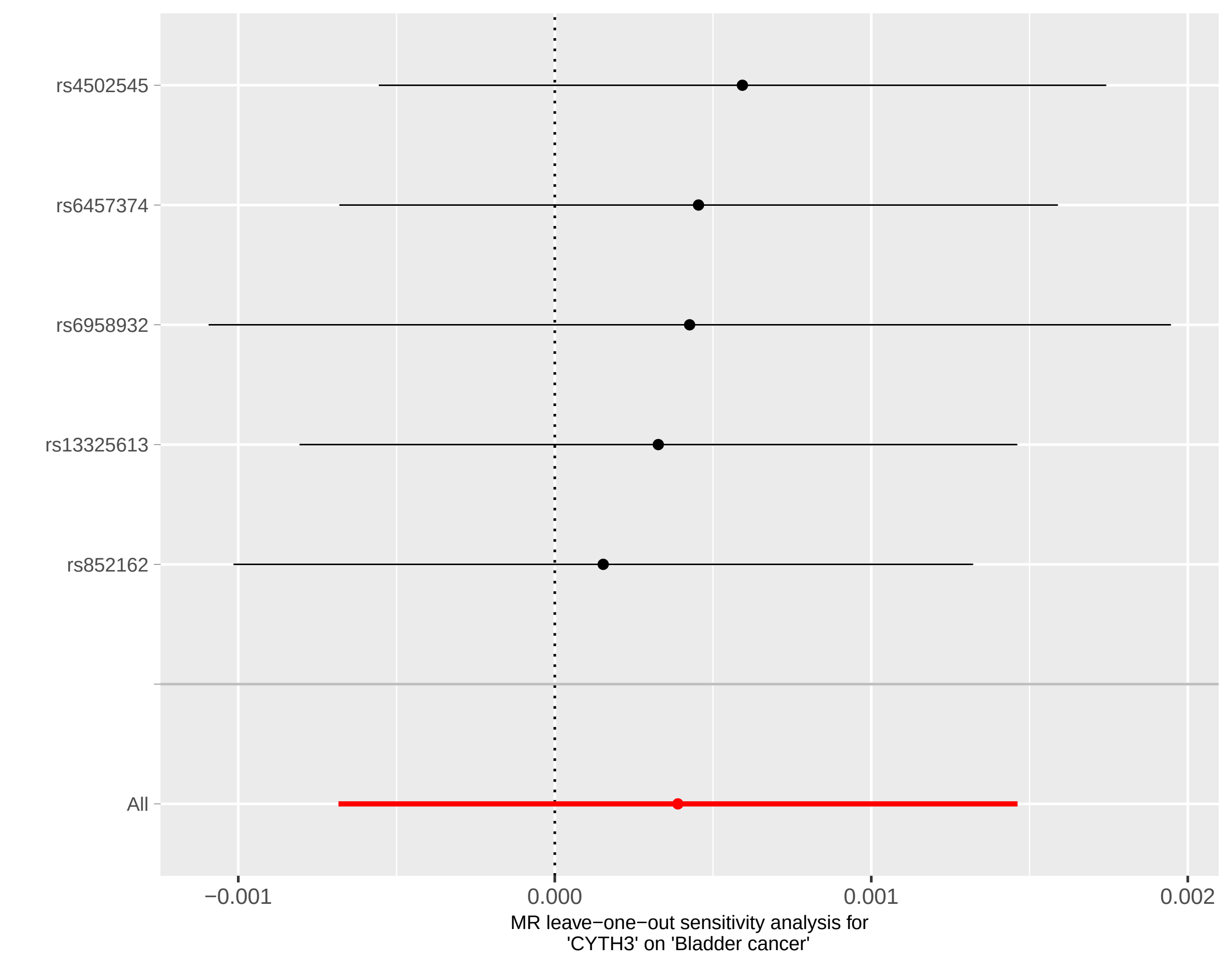
rs4502545
rs6457374
rs6958932
rs13325613
rs852162
All
−0.001
0.000
0.001
0.002
MR leave−one−out sensitivity analysis for
'CYTH3' on 'Bladder cancer'

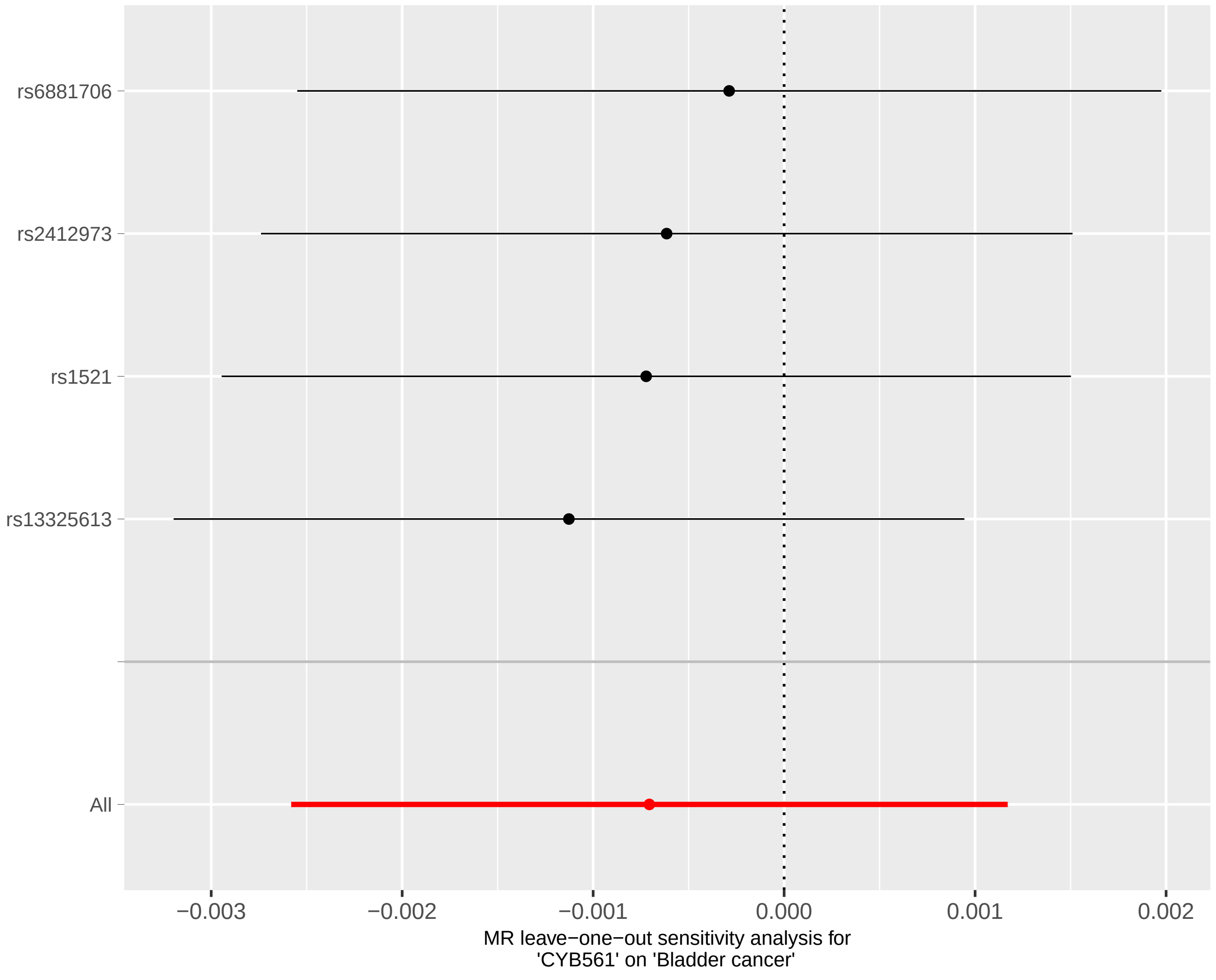
rs6881706
rs2412973
rs1521
rs13325613
All
−0.003
−0.002
−0.001
0.000
0.001
0.002
MR leave−one−out sensitivity analysis for
'CYB561' on 'Bladder cancer'

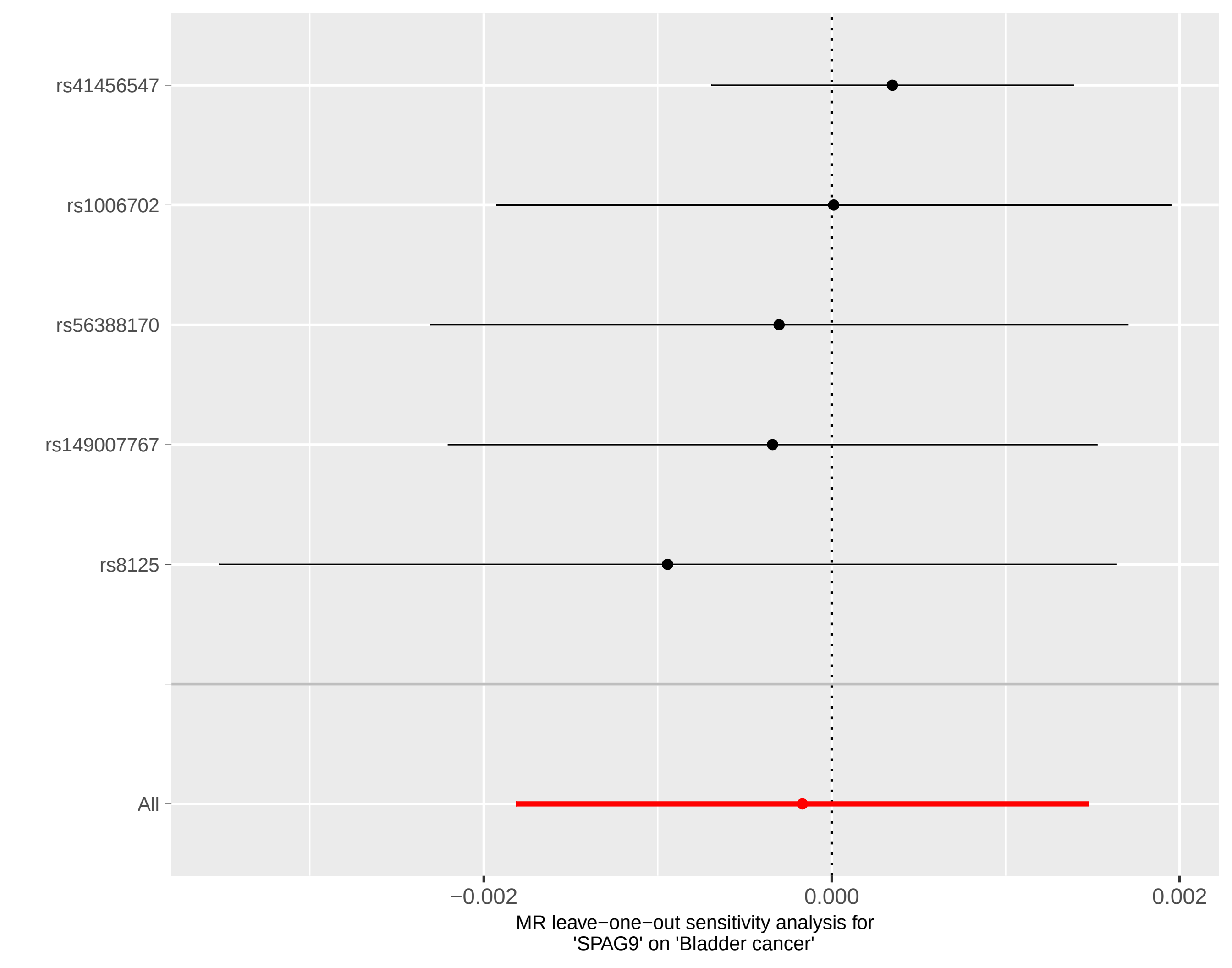
rs41456547
rs1006702
rs56388170
rs149007767
rs8125
All
−0.002
0.000
0.002
MR leave−one−out sensitivity analysis for
'SPAG9' on 'Bladder cancer'

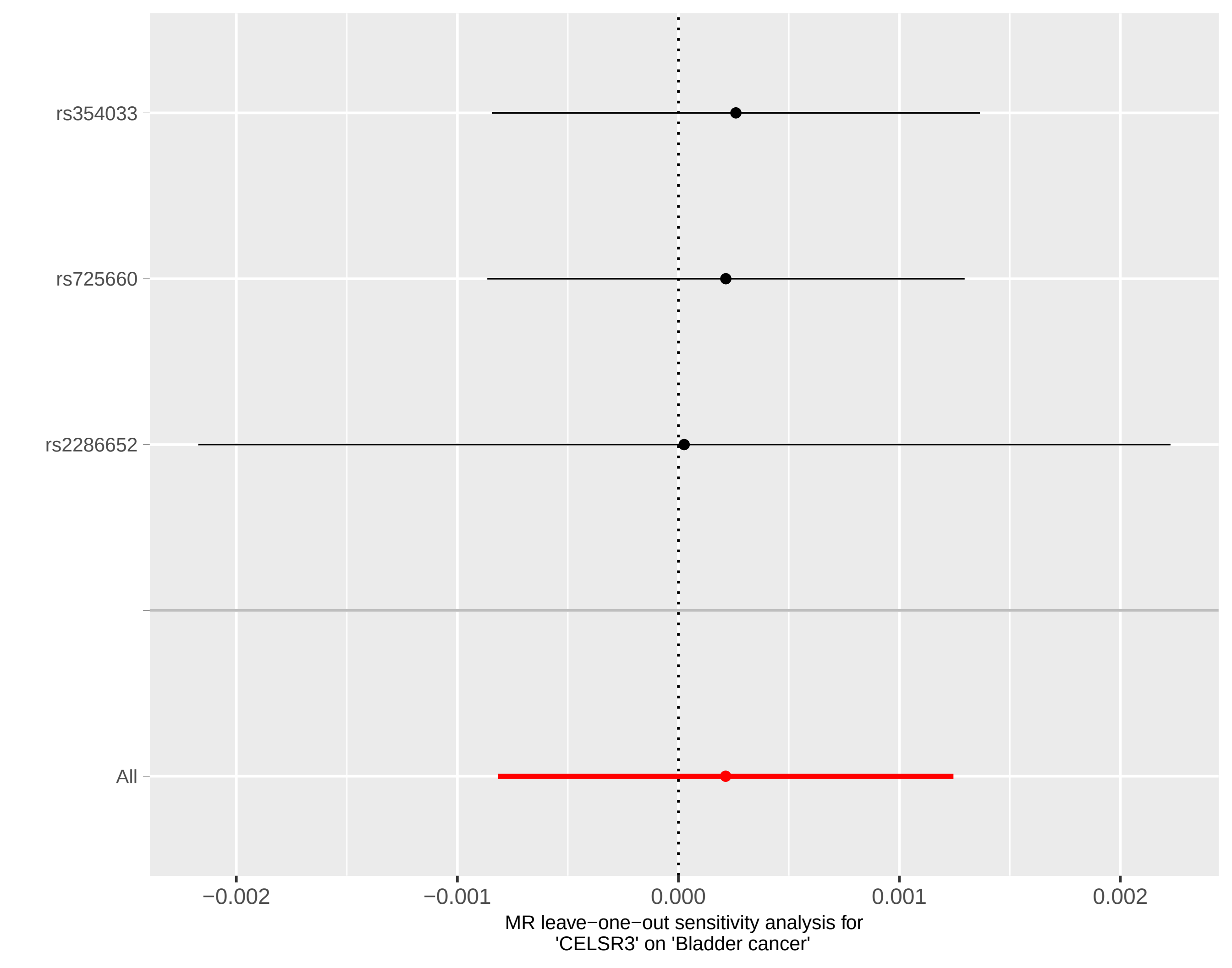
rs354033
rs725660
rs2286652
All
−0.002
−0.001
0.000
0.001
0.002
MR leave−one−out sensitivity analysis for
'CELSR3' on 'Bladder cancer'

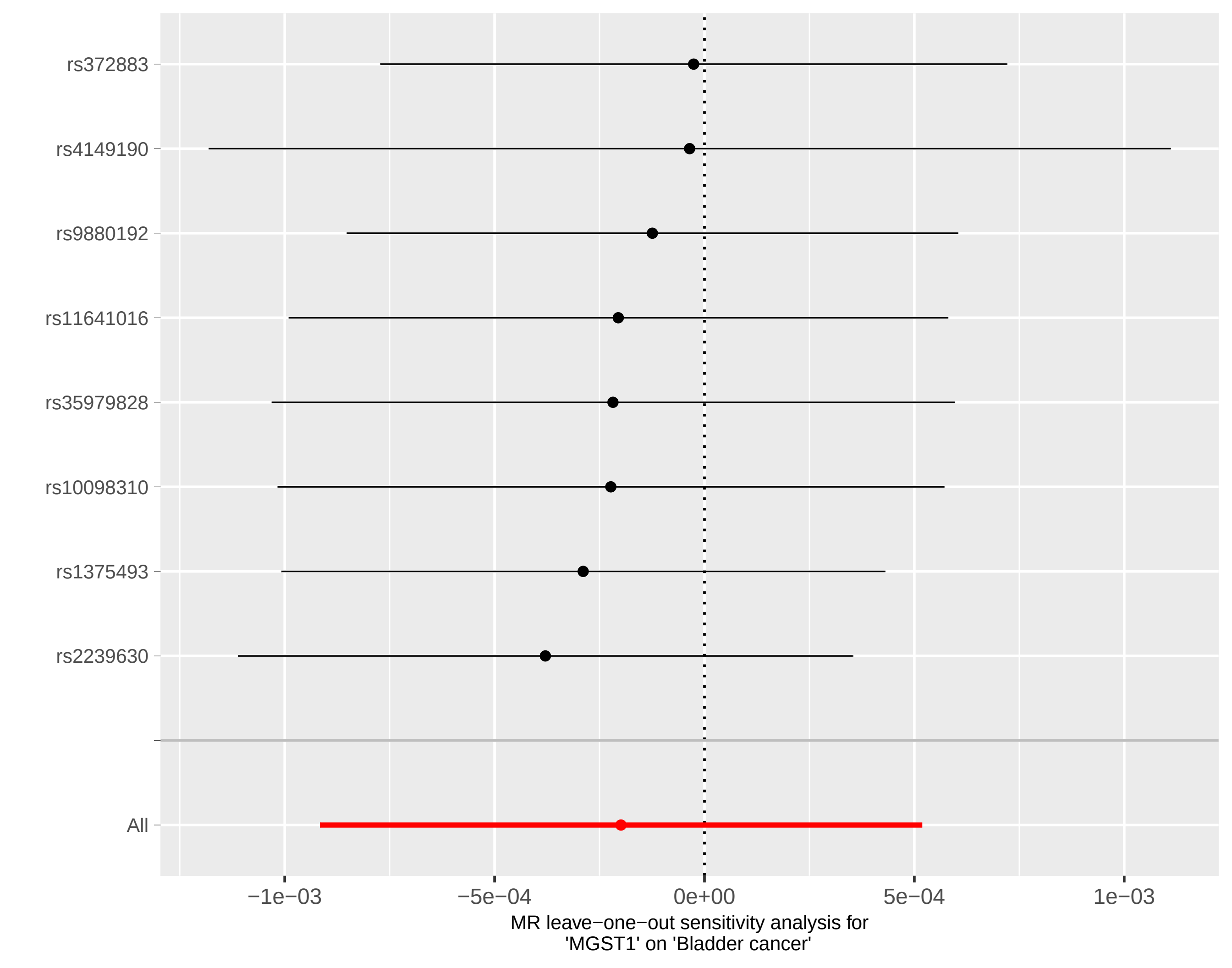
rs372883
rs4149190
rs9880192
rs11641016
rs35979828
rs10098310
rs1375493
rs2239630
All
−1e−03
−5e−04
0e+00
5e−04
1e−03
MR leave−one−out sensitivity analysis for
'MGST1' on 'Bladder cancer'

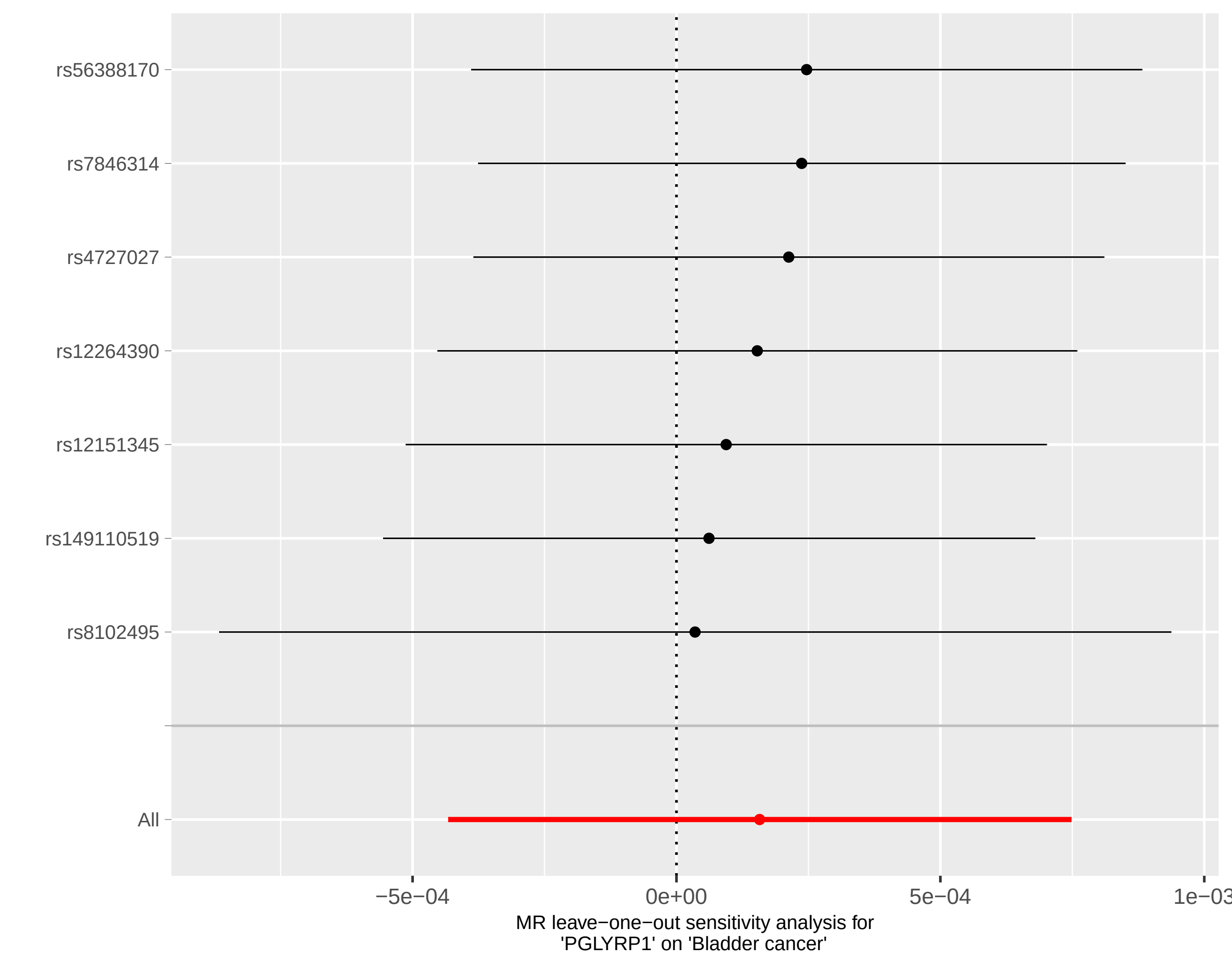
rs56388170
rs7846314
rs4727027
rs12264390
rs12151345
rs149110519
rs8102495
All
−5e−04
0e+00
5e−04
1e−03
MR leave−one−out sensitivity analysis for
'PGLYRP1' on 'Bladder cancer'

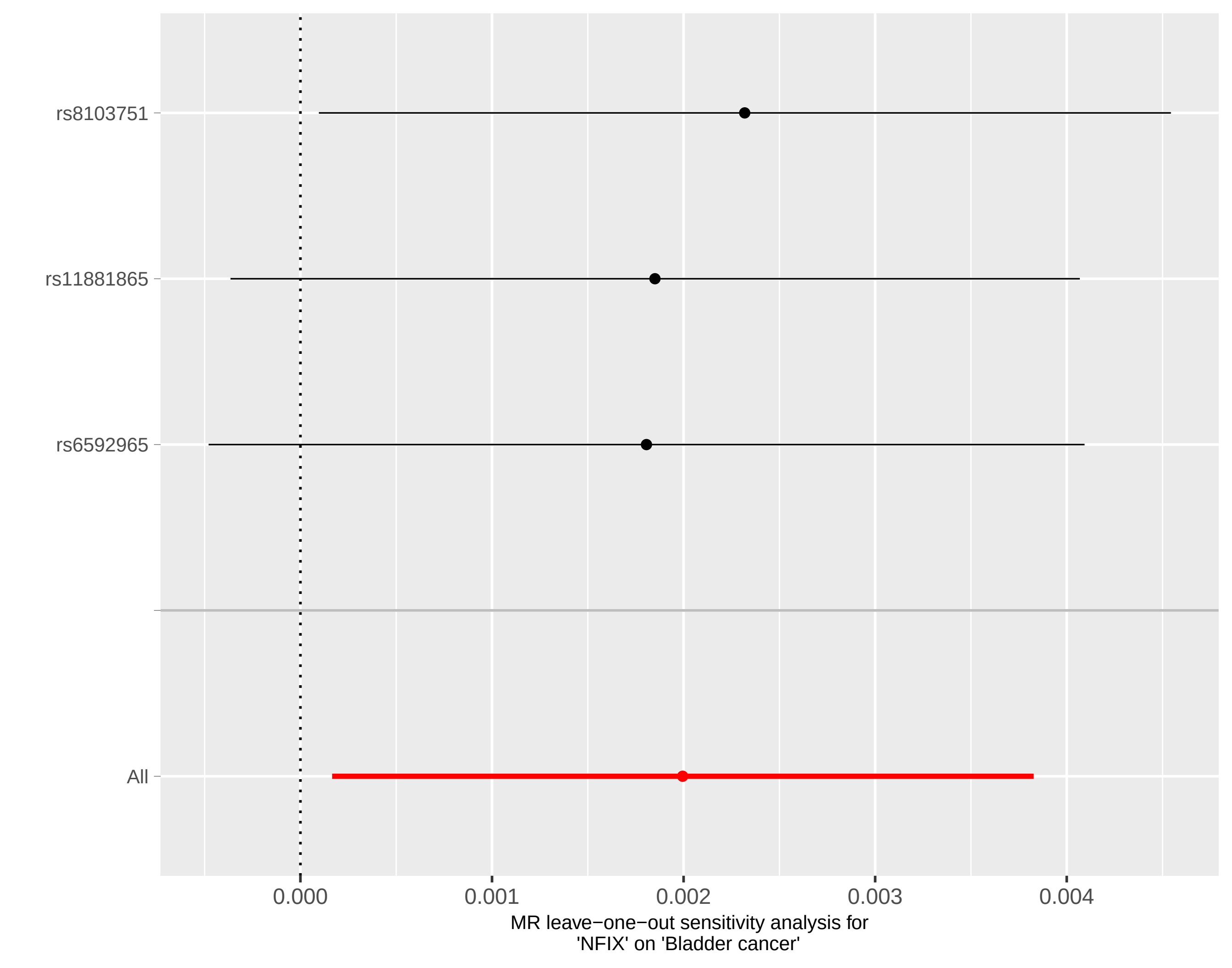
rs8103751
rs11881865
rs6592965
All
0.000
0.001
0.002
0.003
0.004
MR leave−one−out sensitivity analysis for
'NFIX' on 'Bladder cancer'

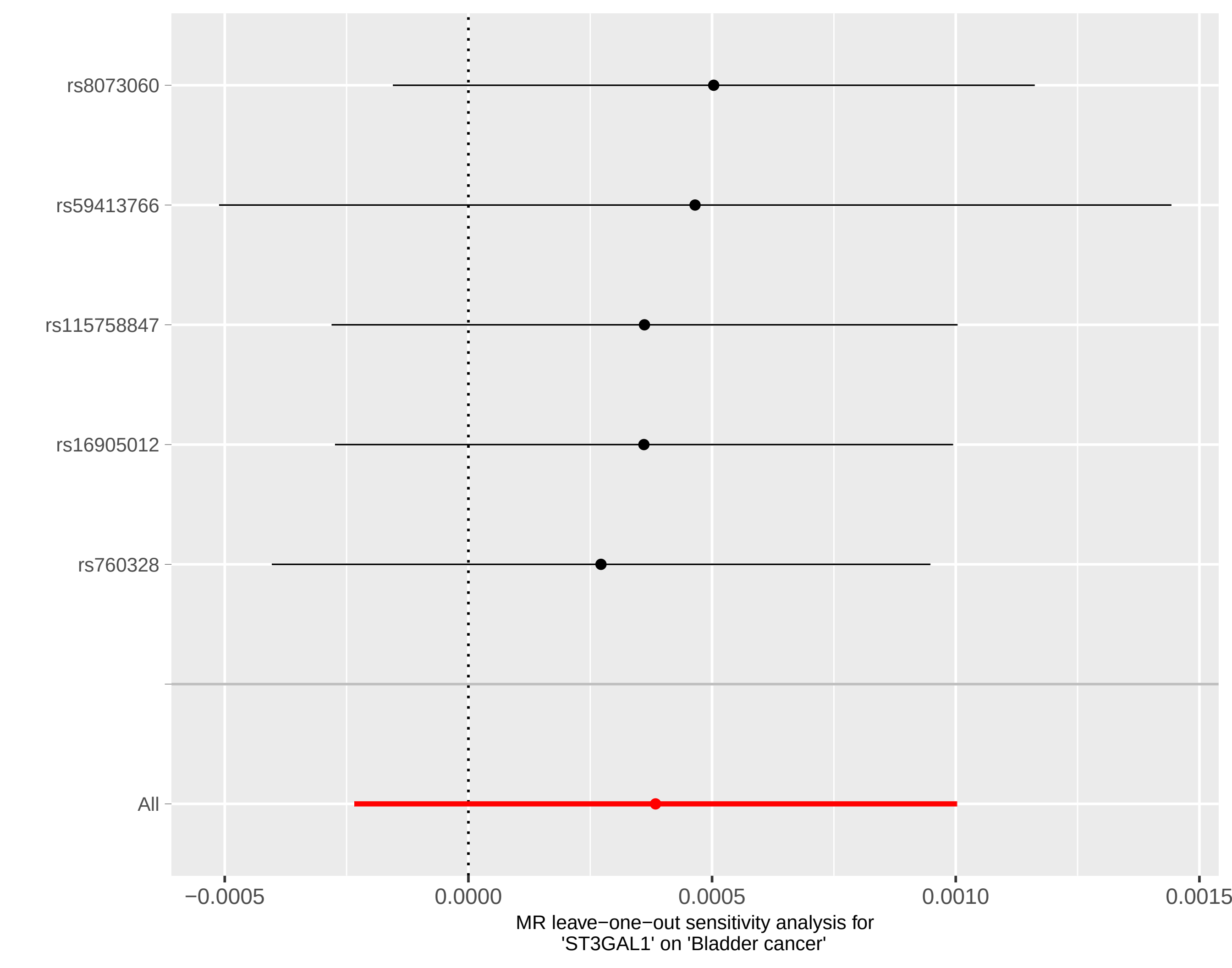
rs8073060
rs59413766
rs115758847
rs16905012
rs760328
All
−0.0005
0.0000
0.0005
0.0010
0.0015
MR leave−one−out sensitivity analysis for
'ST3GAL1' on 'Bladder cancer'

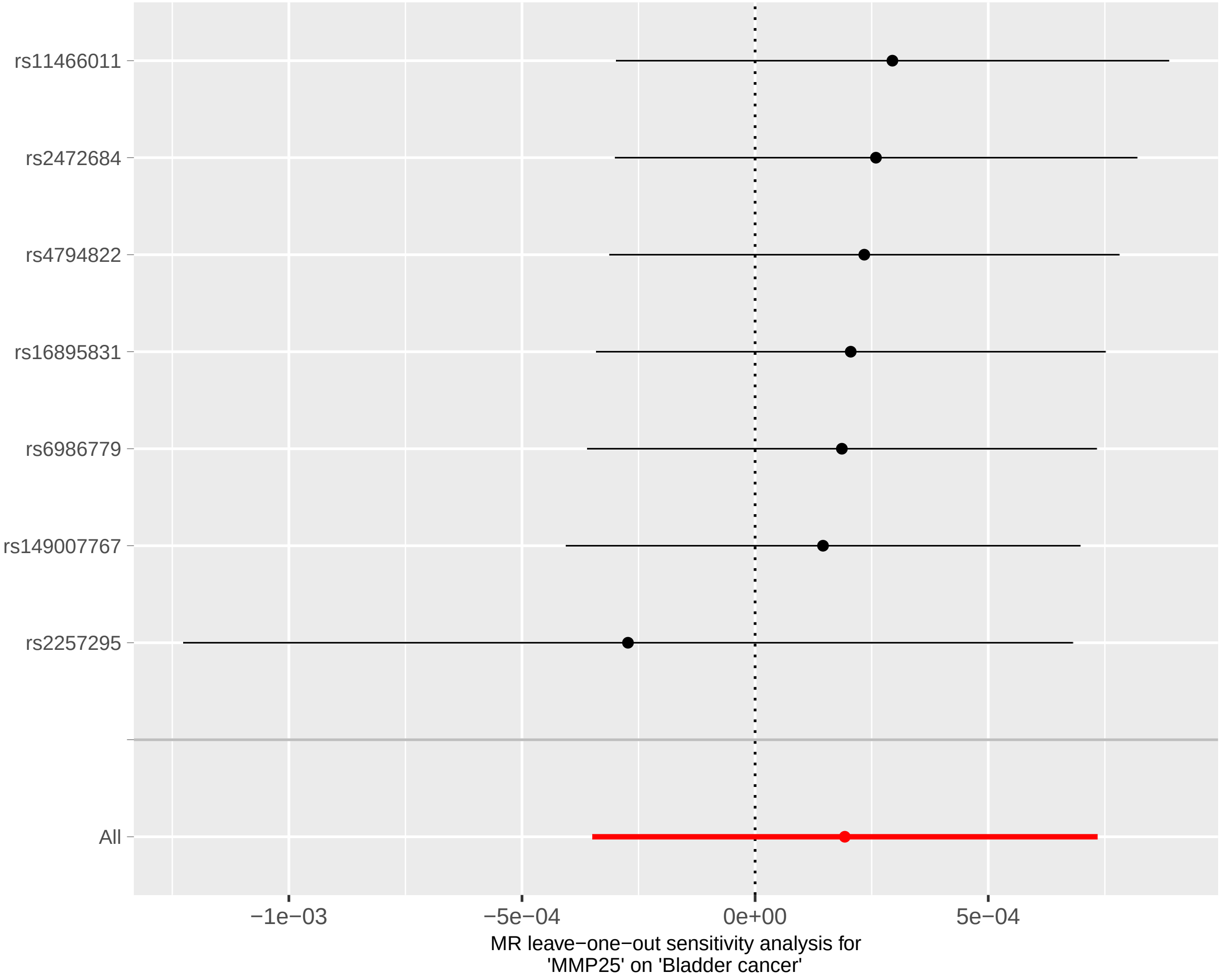
rs11466011
rs2472684
rs4794822
rs16895831
rs6986779
rs149007767
rs2257295
All
−1e−03
−5e−04
0e+00
5e−04
MR leave−one−out sensitivity analysis for
'MMP25' on 'Bladder cancer'

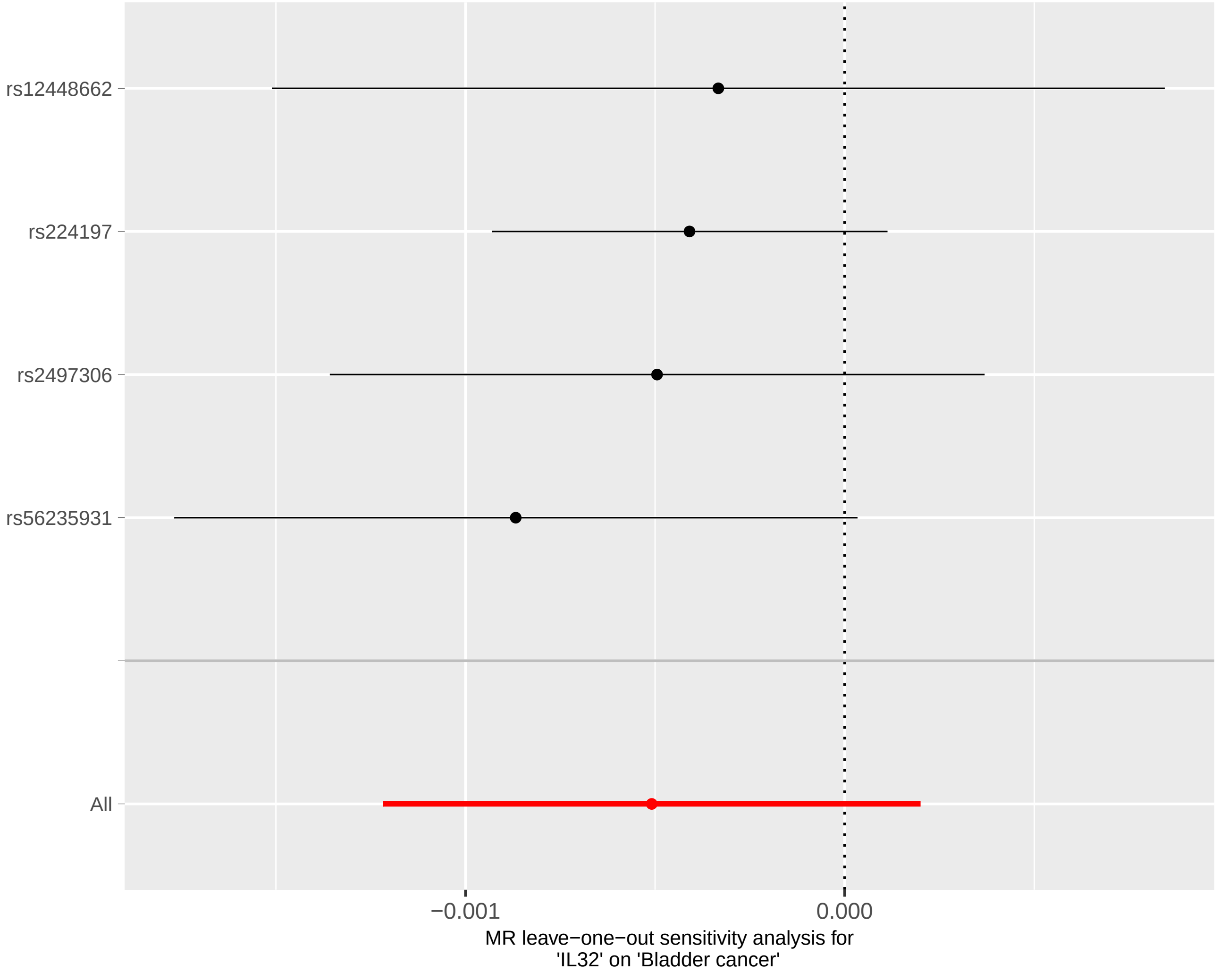
rs12448662
rs224197
rs2497306
rs56235931
All
−0.001
0.000
MR leave−one−out sensitivity analysis for
'IL32' on 'Bladder cancer'

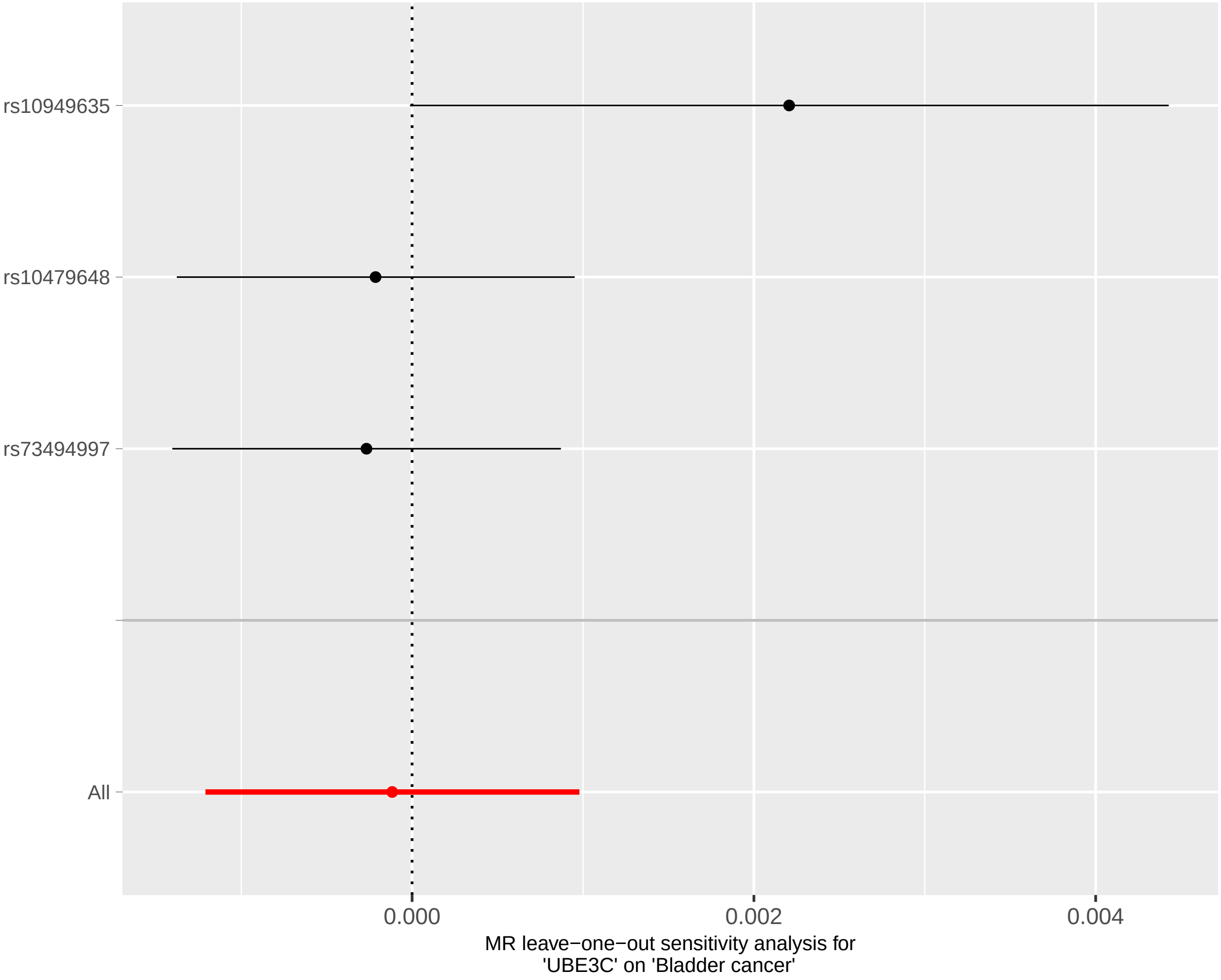
rs10949635
rs10479648
rs73494997
All
0.000
0.002
0.004
MR leave−one−out sensitivity analysis for
'UBE3C' on 'Bladder cancer'

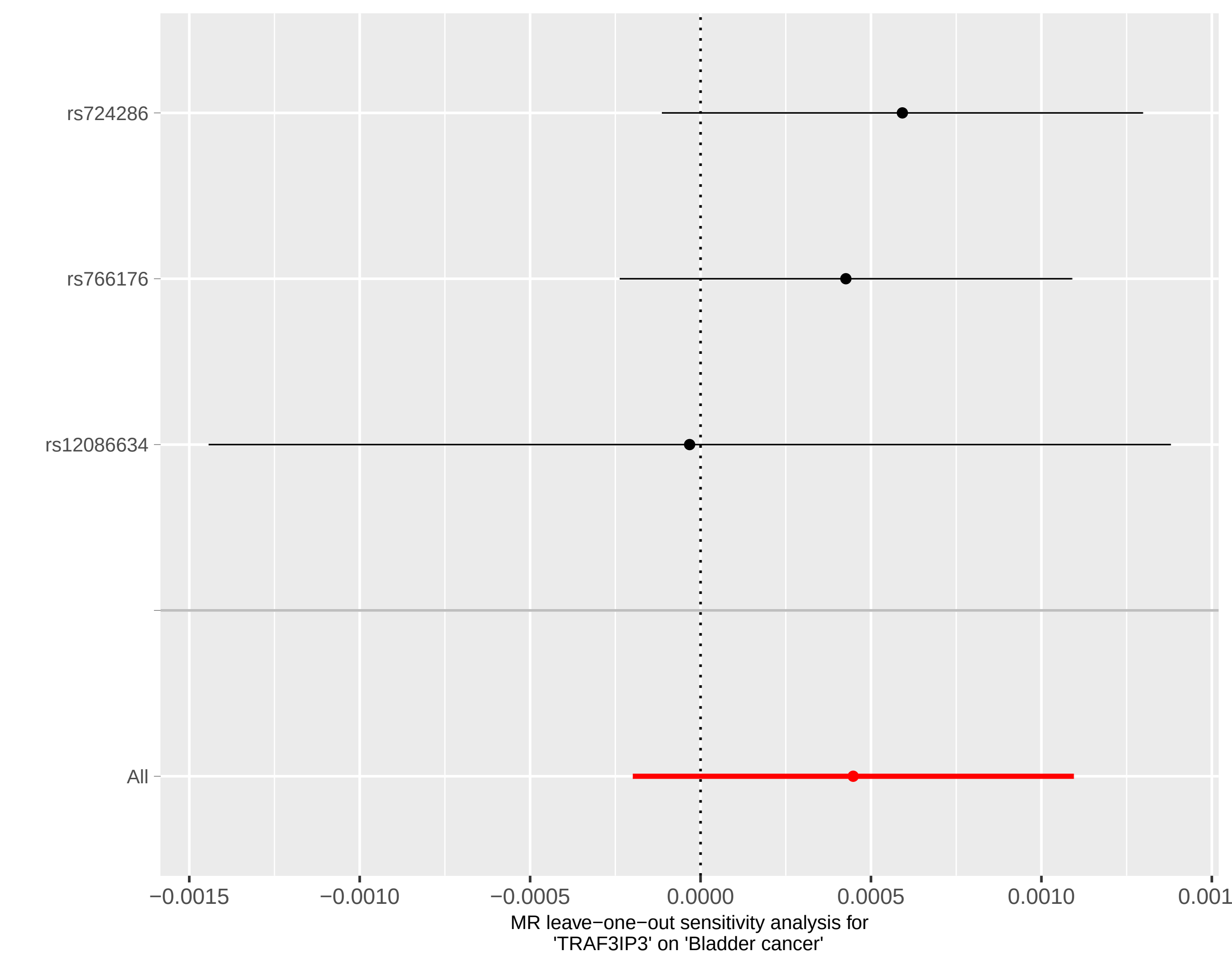

rs724286
rs766176
rs12086634
All
−0.0015
−0.0010
−0.0005
0.0000
0.0005
0.0010
0.001
MR leave−one−out sensitivity analysis for
'TRAF3IP3' on 'Bladder cancer'

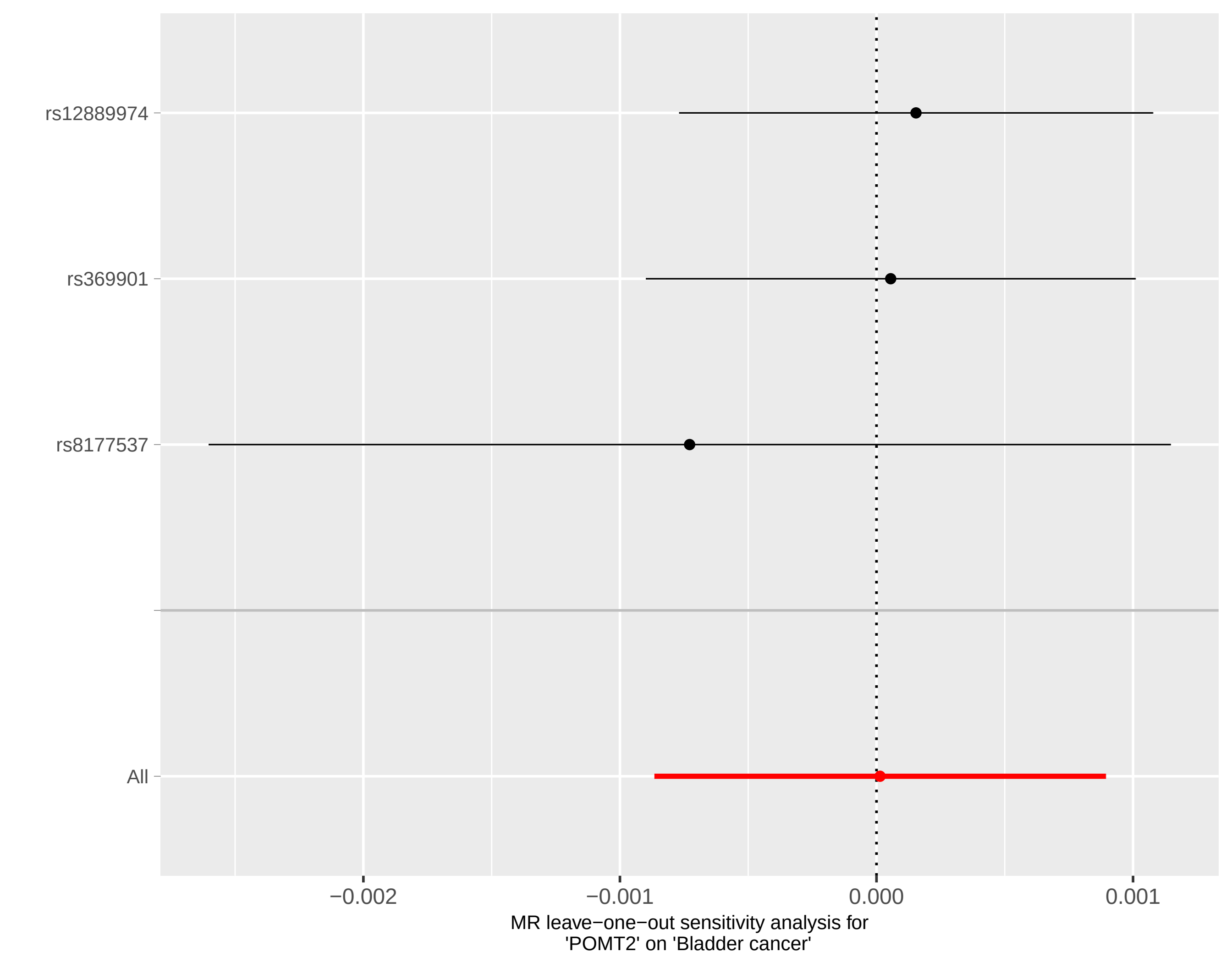
rs12889974
rs369901
rs8177537
All
−0.002
−0.001
0.000
0.001
MR leave−one−out sensitivity analysis for
'POMT2' on 'Bladder cancer'

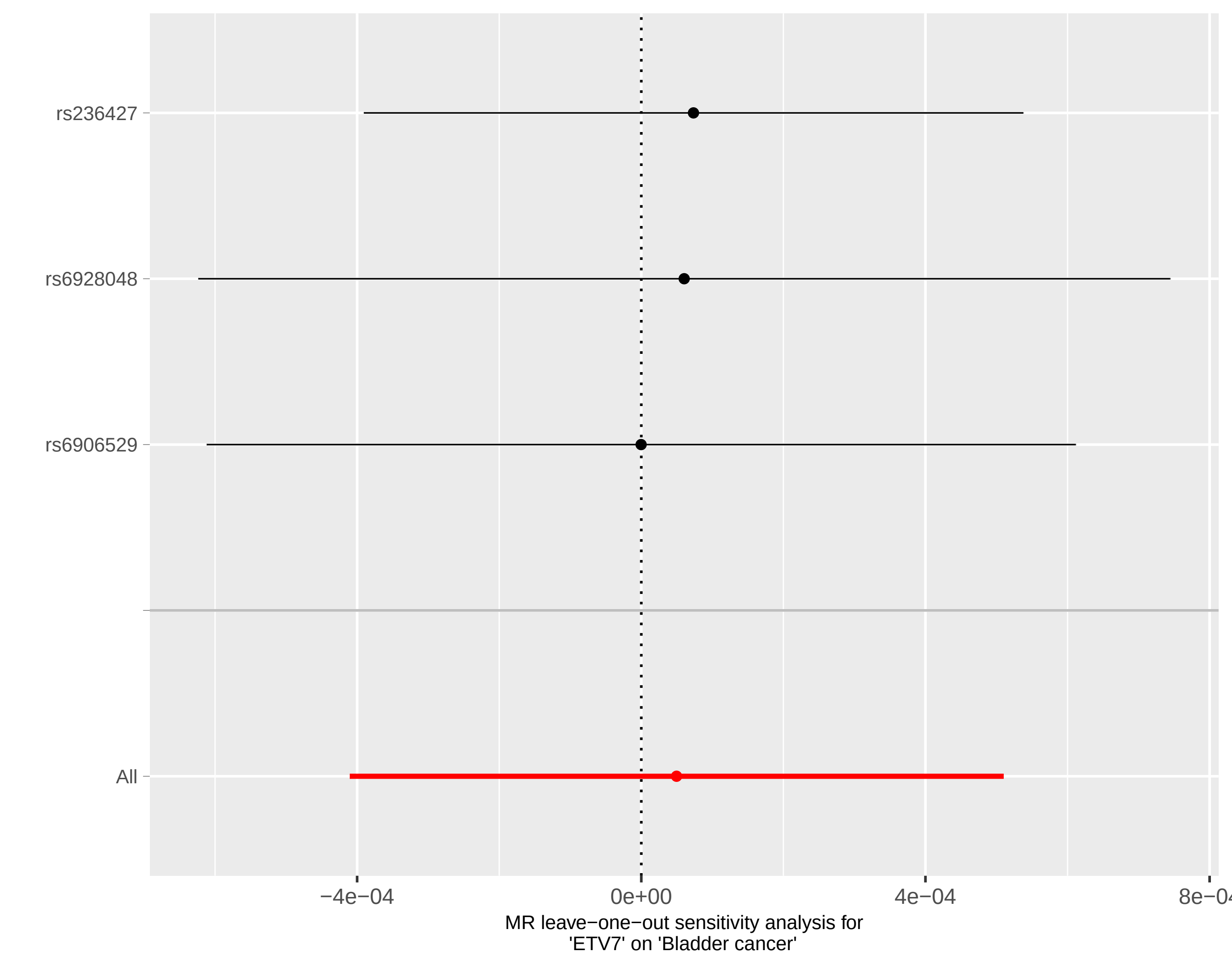
rs236427
rs6928048
rs6906529
All
−4e−04
0e+00
4e−04
8e−04
MR leave−one−out sensitivity analysis for
'ETV7' on 'Bladder cancer'

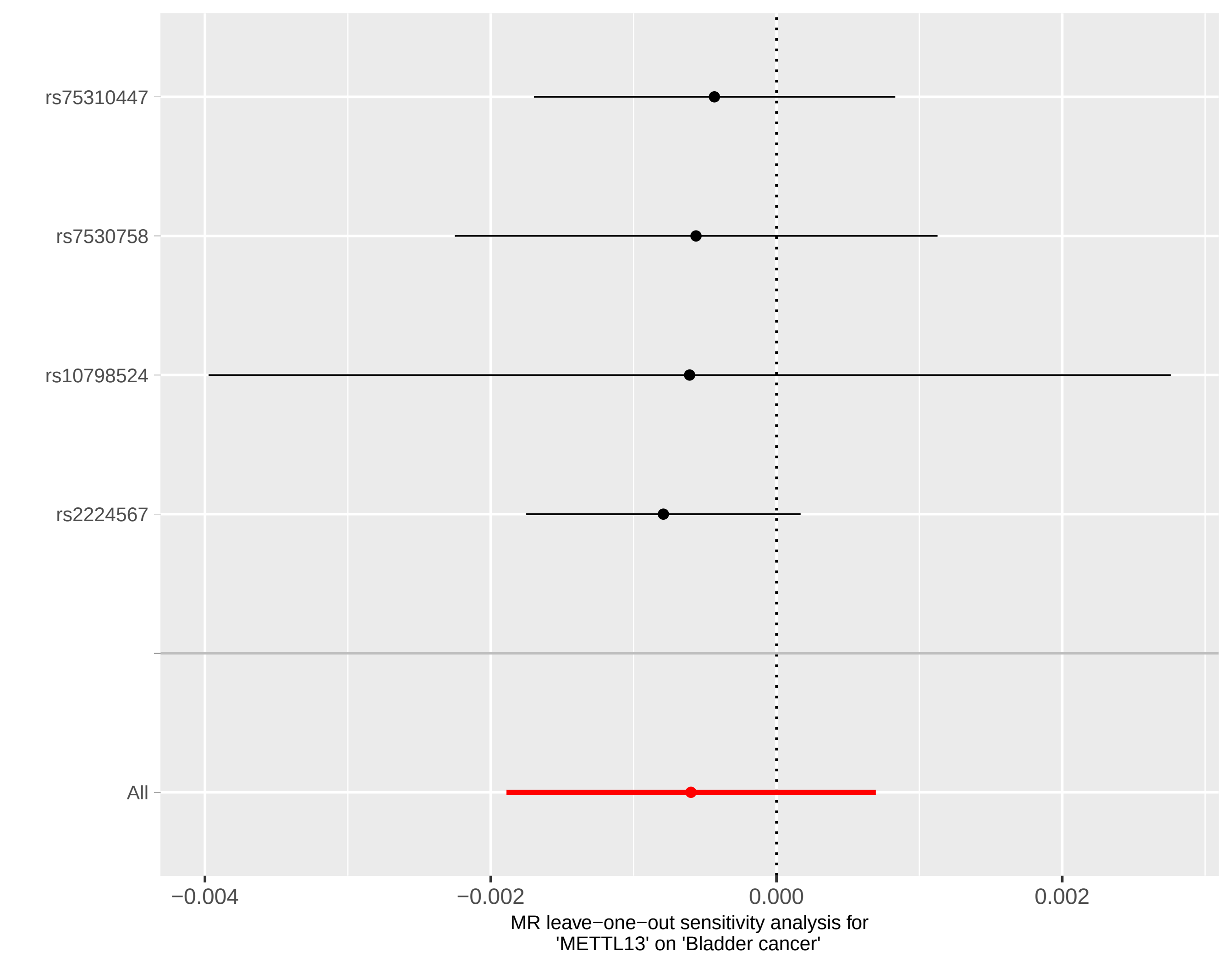
rs75310447
rs7530758
rs10798524
rs2224567
All
−0.004
−0.002
0.000
0.002
MR leave−one−out sensitivity analysis for
'METTL13' on 'Bladder cancer'

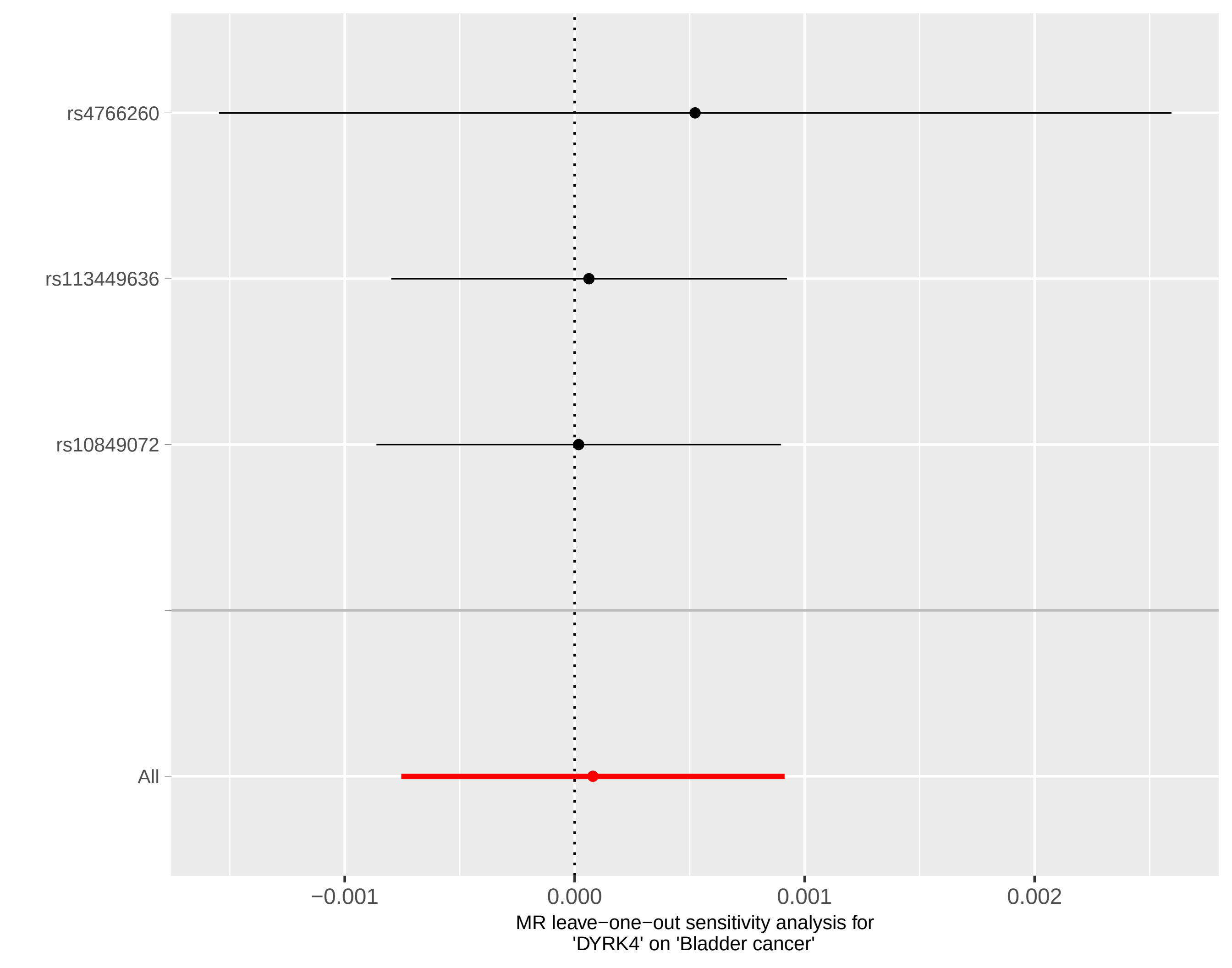
rs4766260
rs113449636
rs10849072
All
−0.001
0.000
0.001
0.002
MR leave−one−out sensitivity analysis for
'DYRK4' on 'Bladder cancer'

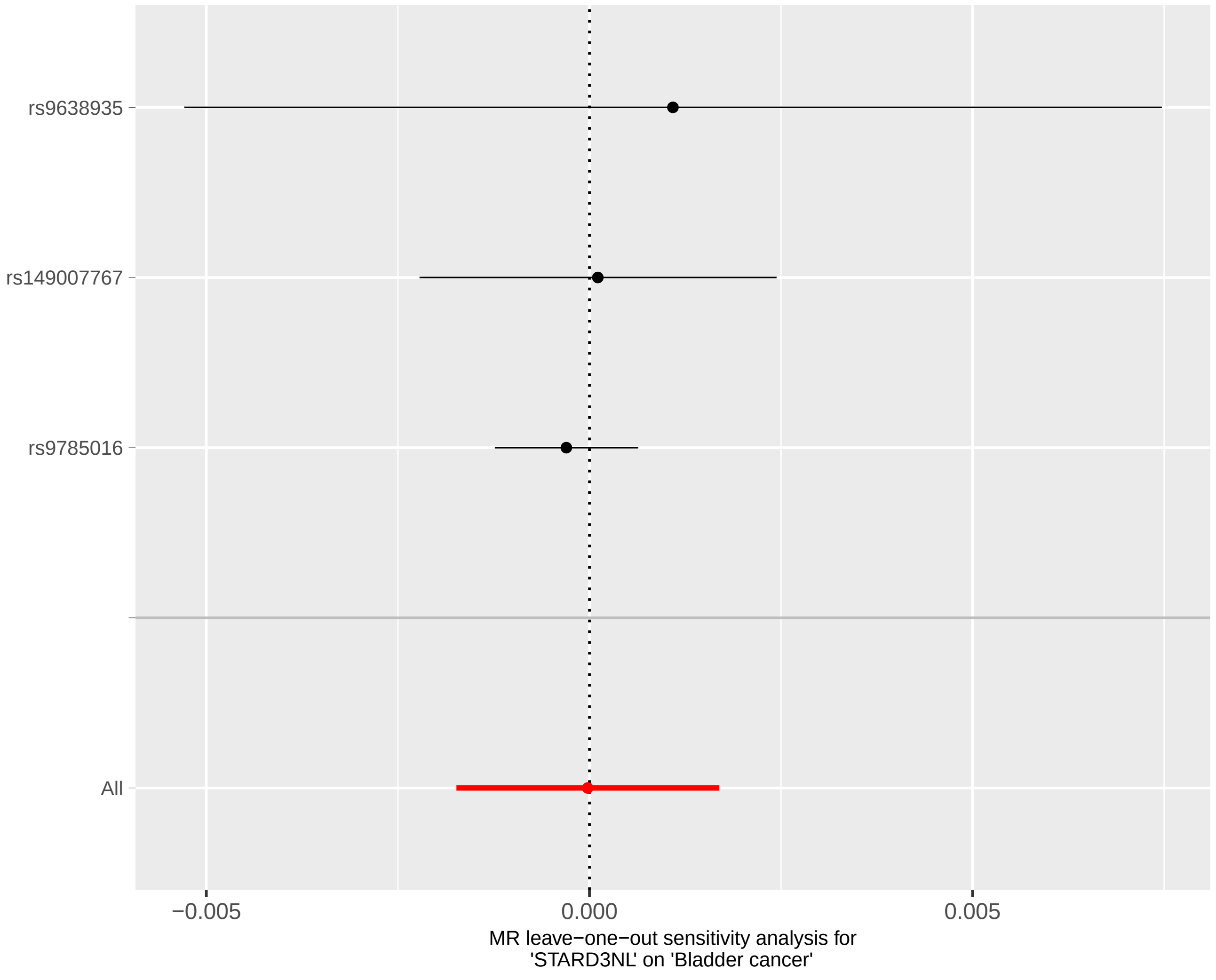
rs9638935
rs149007767
rs9785016
All
−0.005
0.000
0.005
MR leave−one−out sensitivity analysis for
'STARD3NL' on 'Bladder cancer'

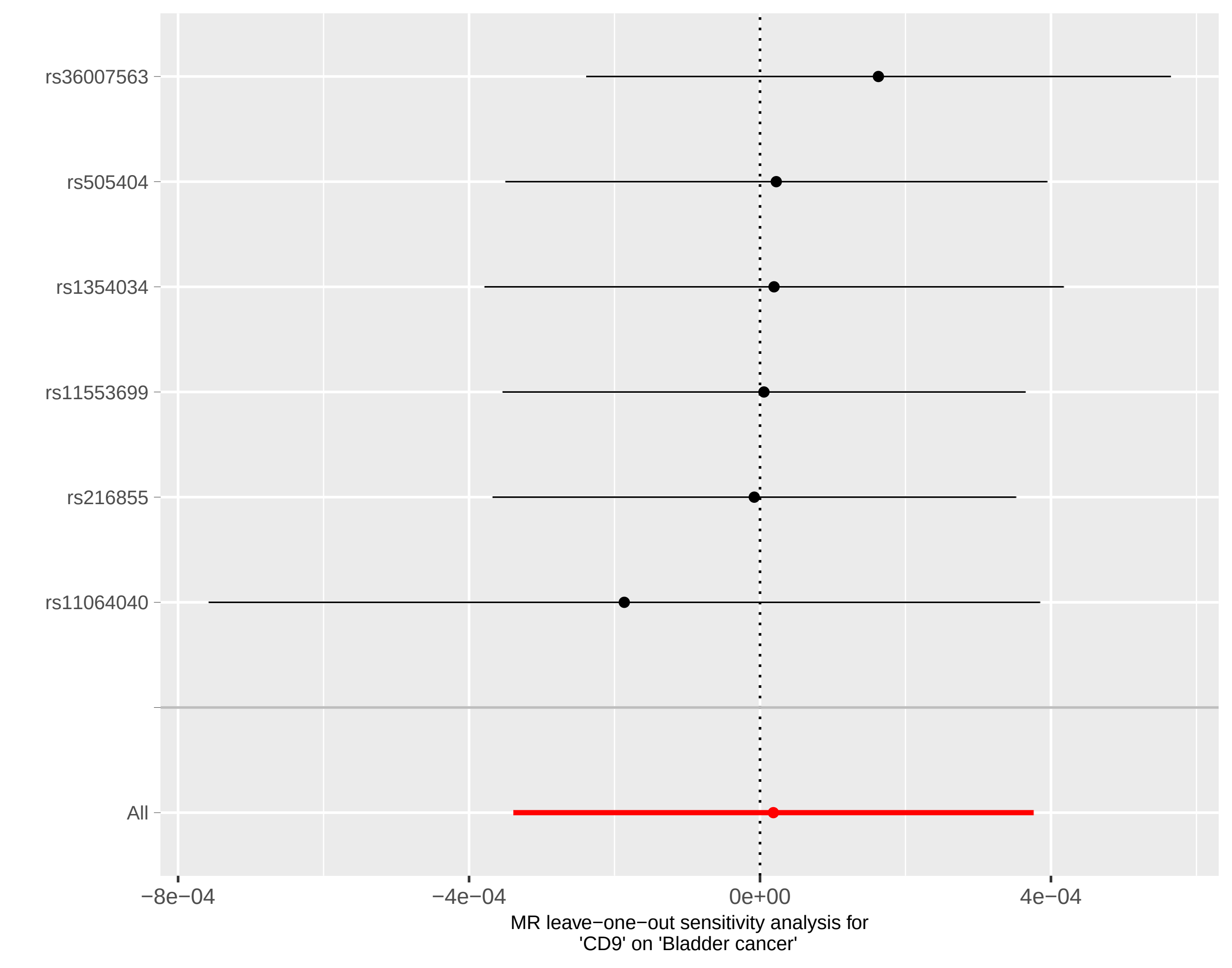
rs36007563
rs505404
rs1354034
rs11553699
rs216855
rs11064040
All
−8e−04
−4e−04
0e+00
4e−04
MR leave−one−out sensitivity analysis for
'CD9' on 'Bladder cancer'

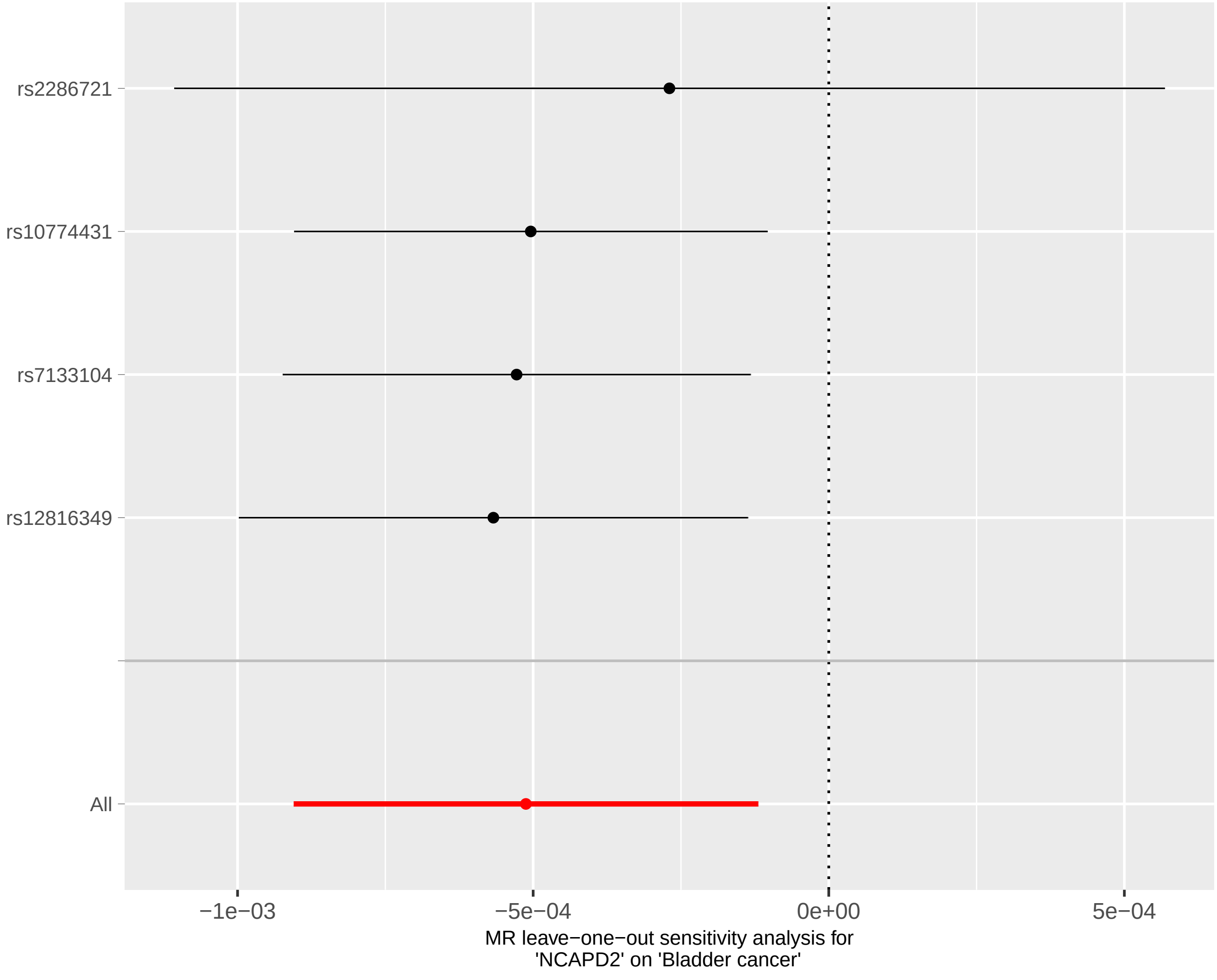
rs2286721
rs10774431
rs7133104
rs12816349
All
−1e−03
−5e−04
0e+00
5e−04
MR leave−one−out sensitivity analysis for
'NCAPD2' on 'Bladder cancer'

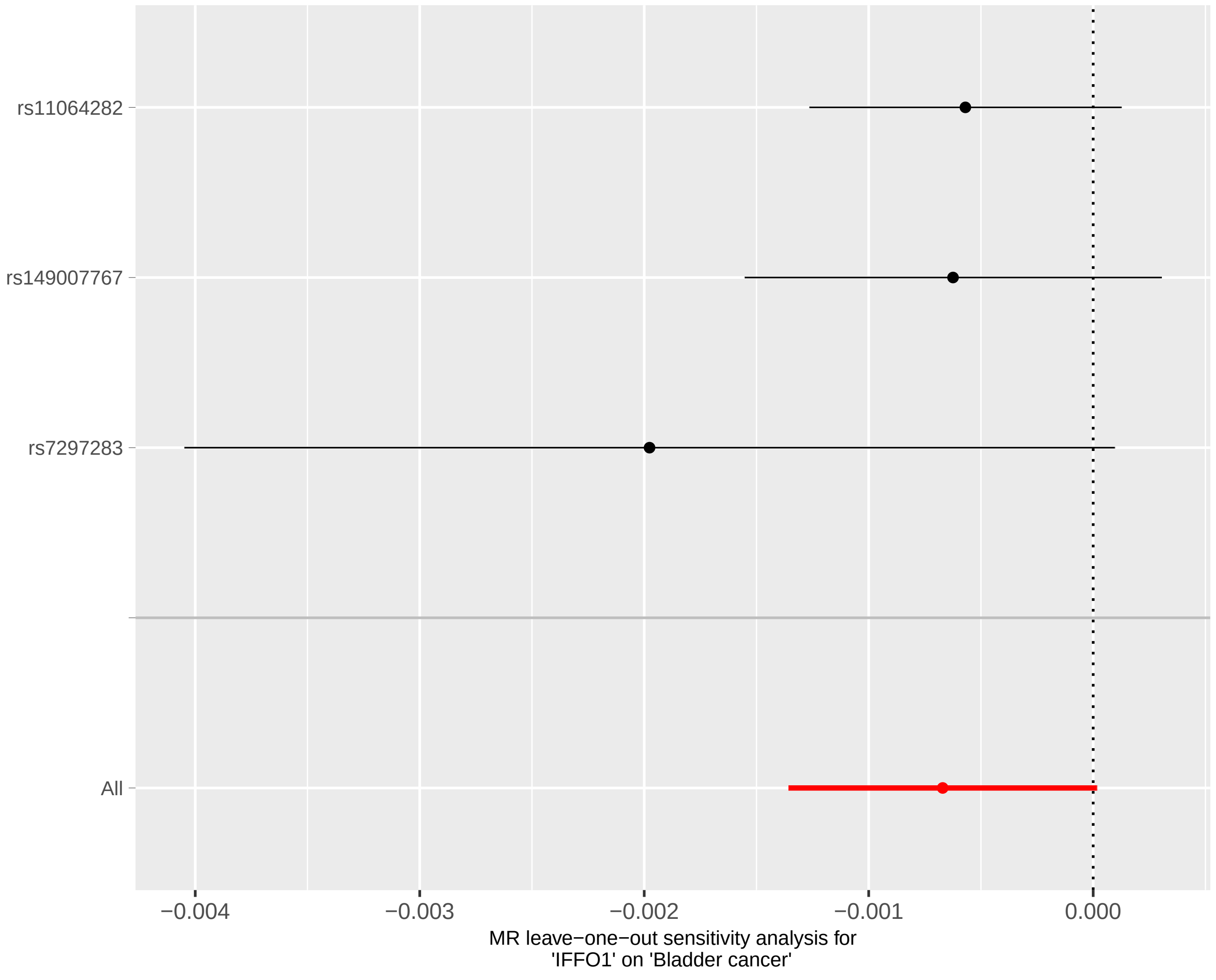

rs11064282
rs149007767
rs7297283
All
−0.004
−0.003
−0.002
−0.001
0.000
MR leave−one−out sensitivity analysis for
'IFFO1' on 'Bladder cancer'

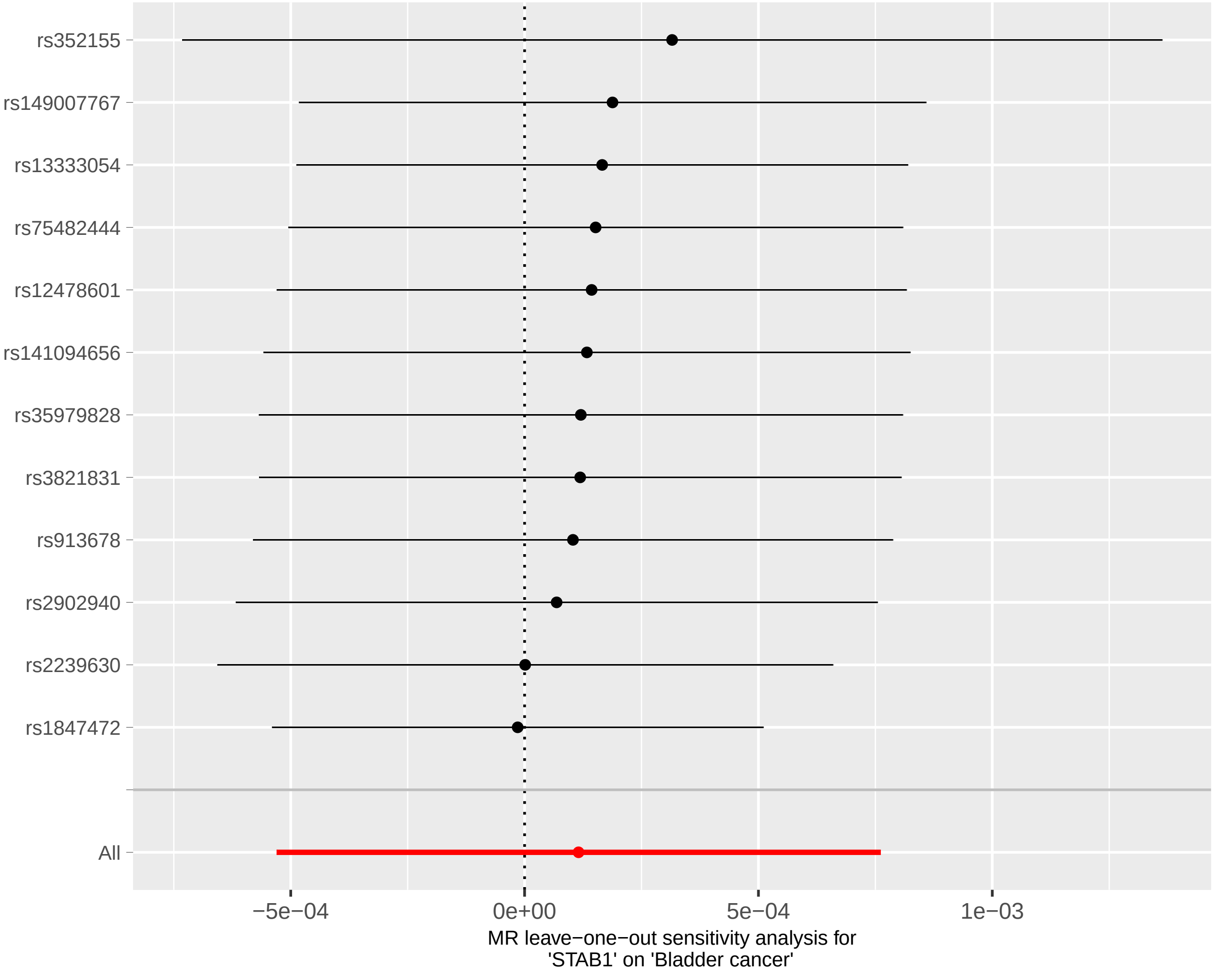
rs352155
rs149007767
rs13333054
rs75482444
rs12478601
rs141094656
rs35979828
rs3821831
rs913678
rs2902940
rs2239630
rs1847472
All
−5e−04
0e+00
5e−04
1e−03
MR leave−one−out sensitivity analysis for
'STAB1' on 'Bladder cancer'

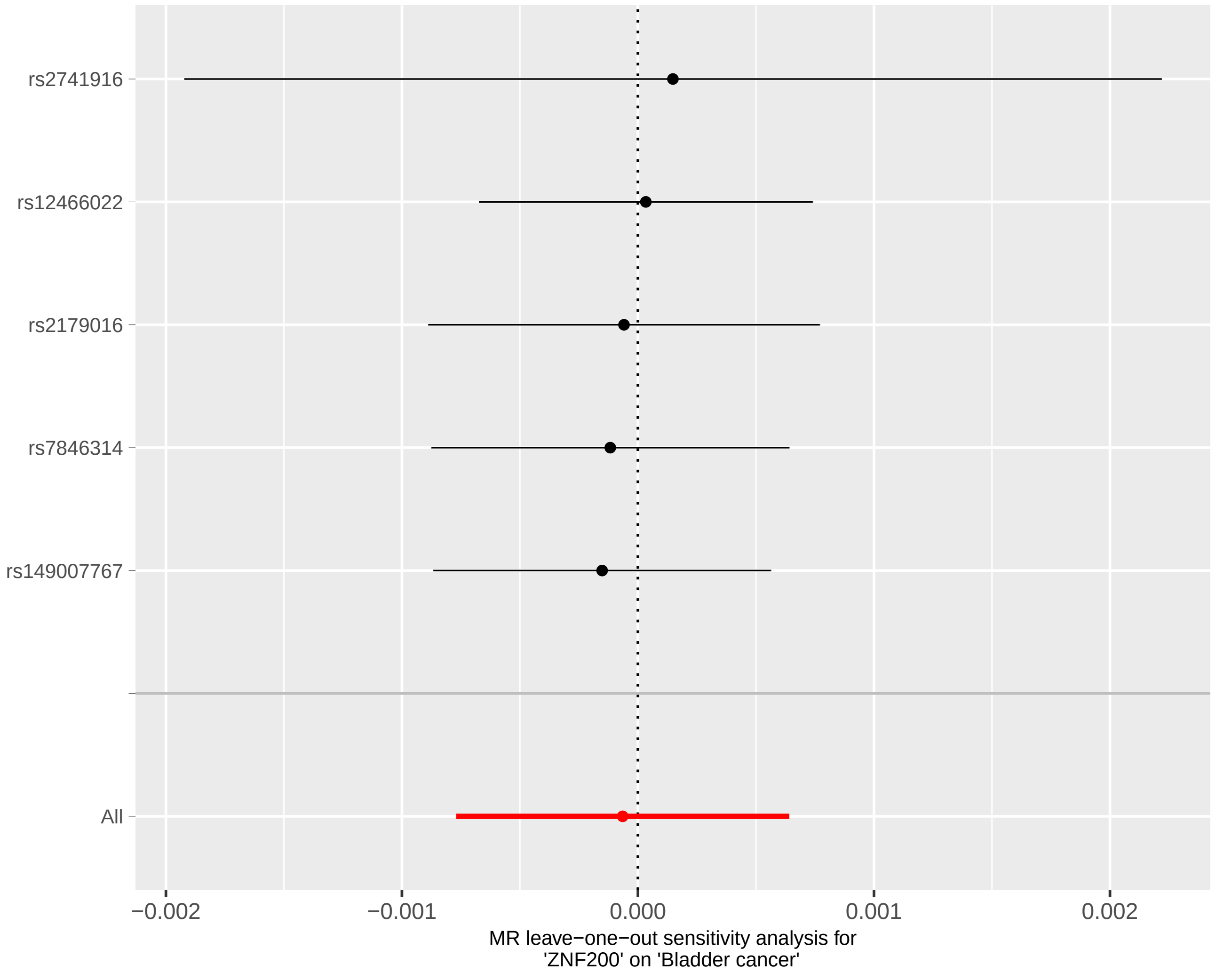
rs2741916
rs12466022
rs2179016
rs7846314
rs149007767
All
−0.002
−0.001
0.000
0.001
0.002
MR leave−one−out sensitivity analysis for
'ZNF200' on 'Bladder cancer'

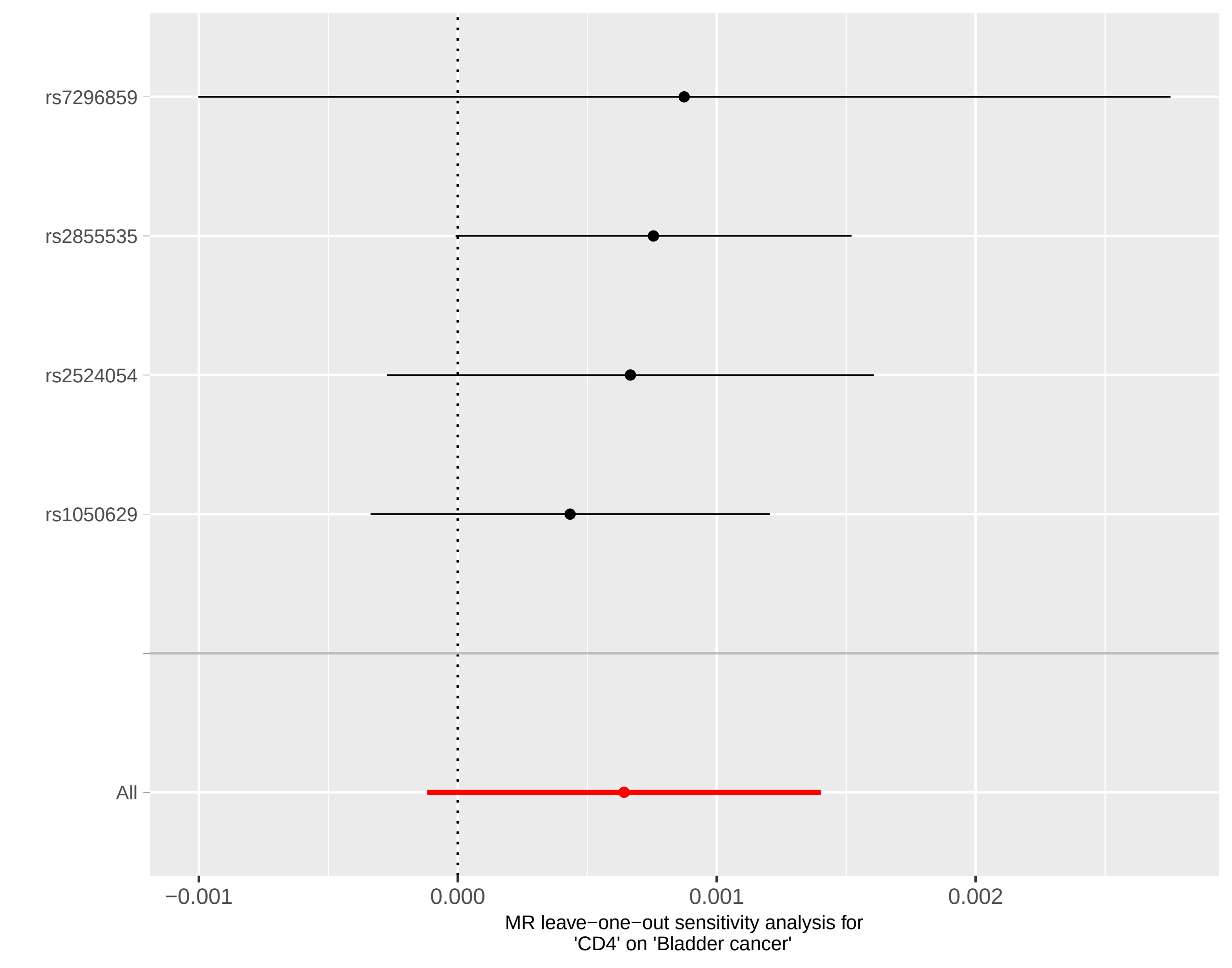
rs7296859
rs2855535
rs2524054
rs1050629
All
−0.001
0.000
0.001
0.002
MR leave−one−out sensitivity analysis for
'CD4' on 'Bladder cancer'

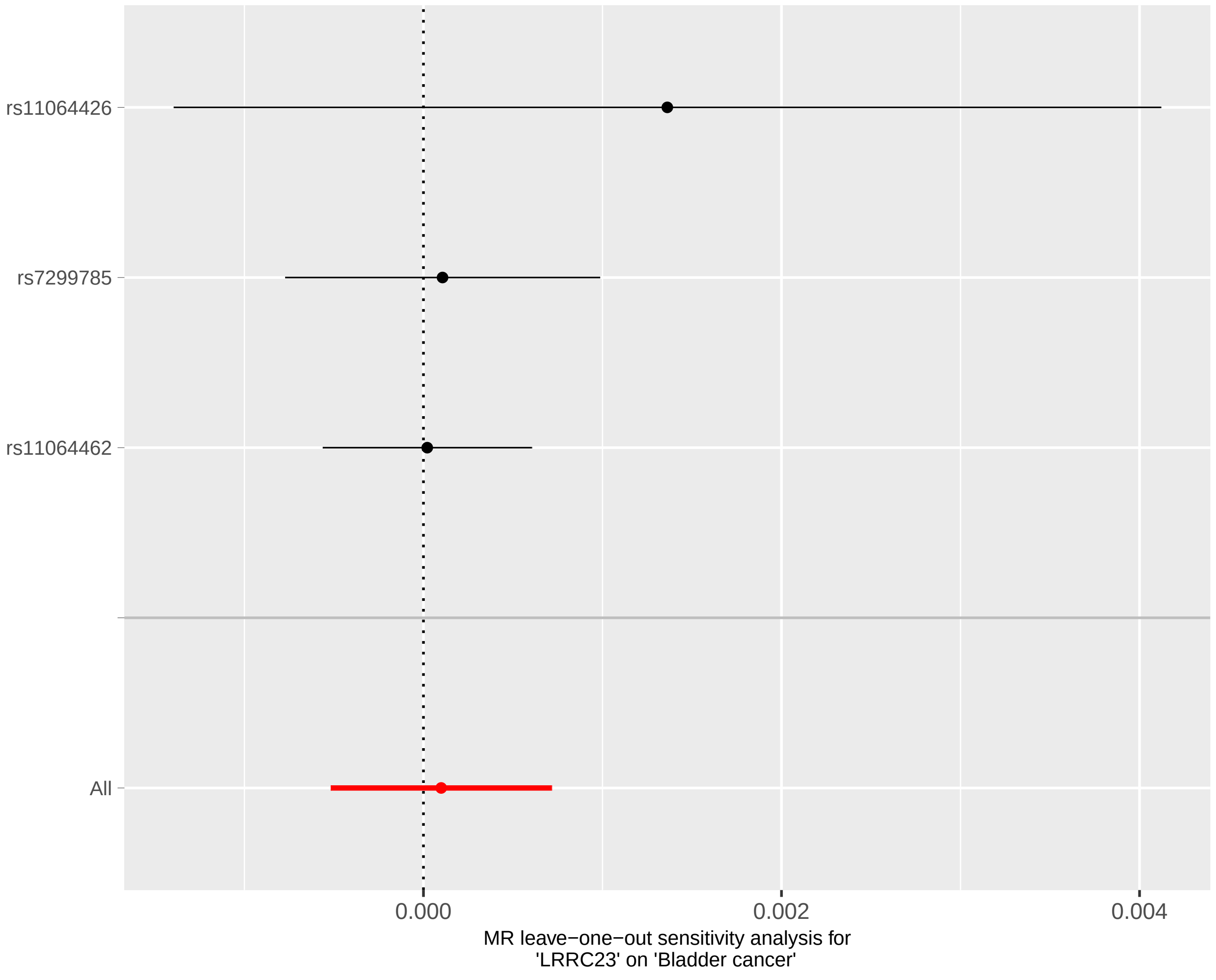

rs11064426
rs7299785
rs11064462
All
0.000
0.002
0.004
MR leave−one−out sensitivity analysis for
'LRRC23' on 'Bladder cancer'

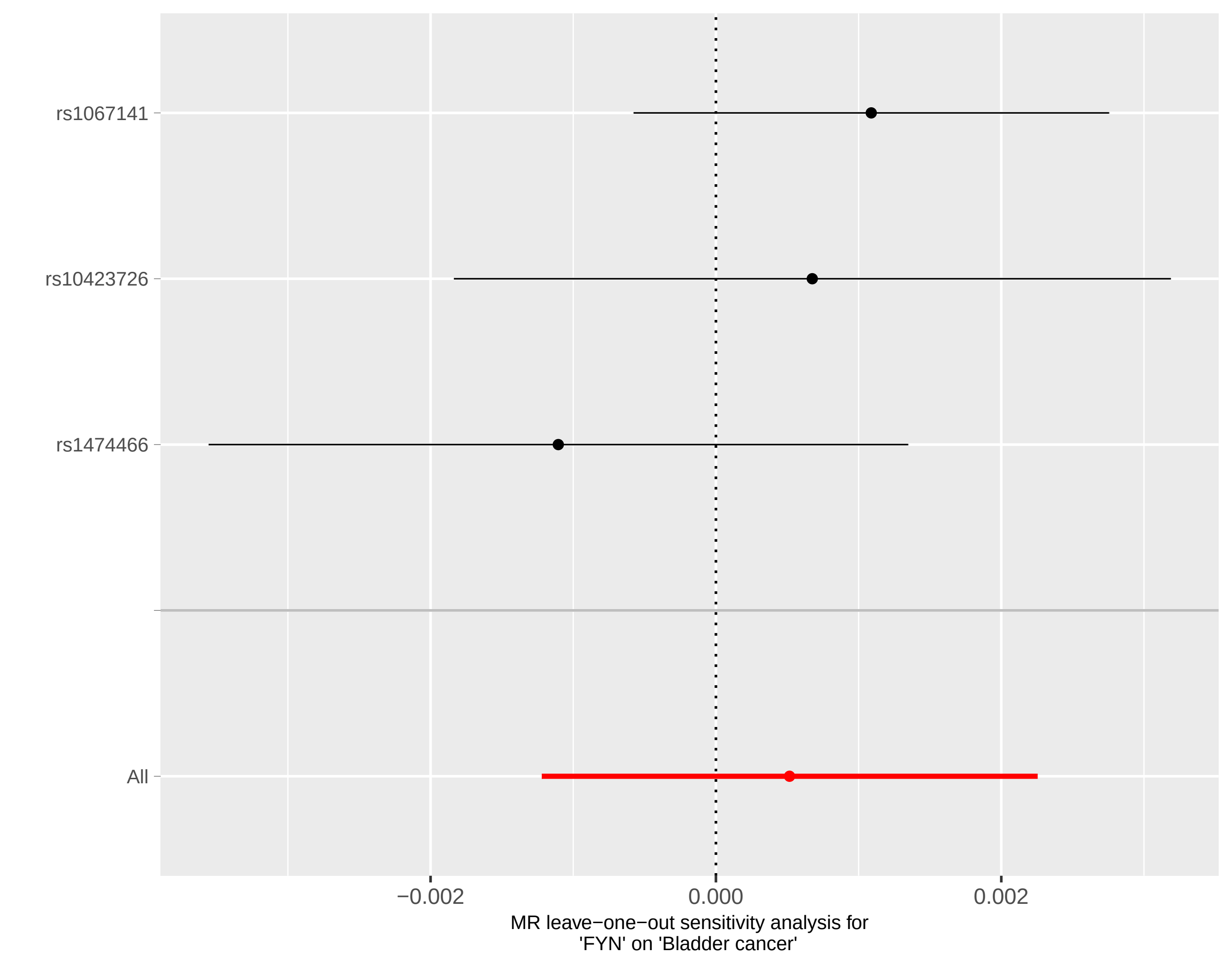
rs1067141
rs10423726
rs1474466
All
−0.002
0.000
0.002
MR leave−one−out sensitivity analysis for
'FYN' on 'Bladder cancer'

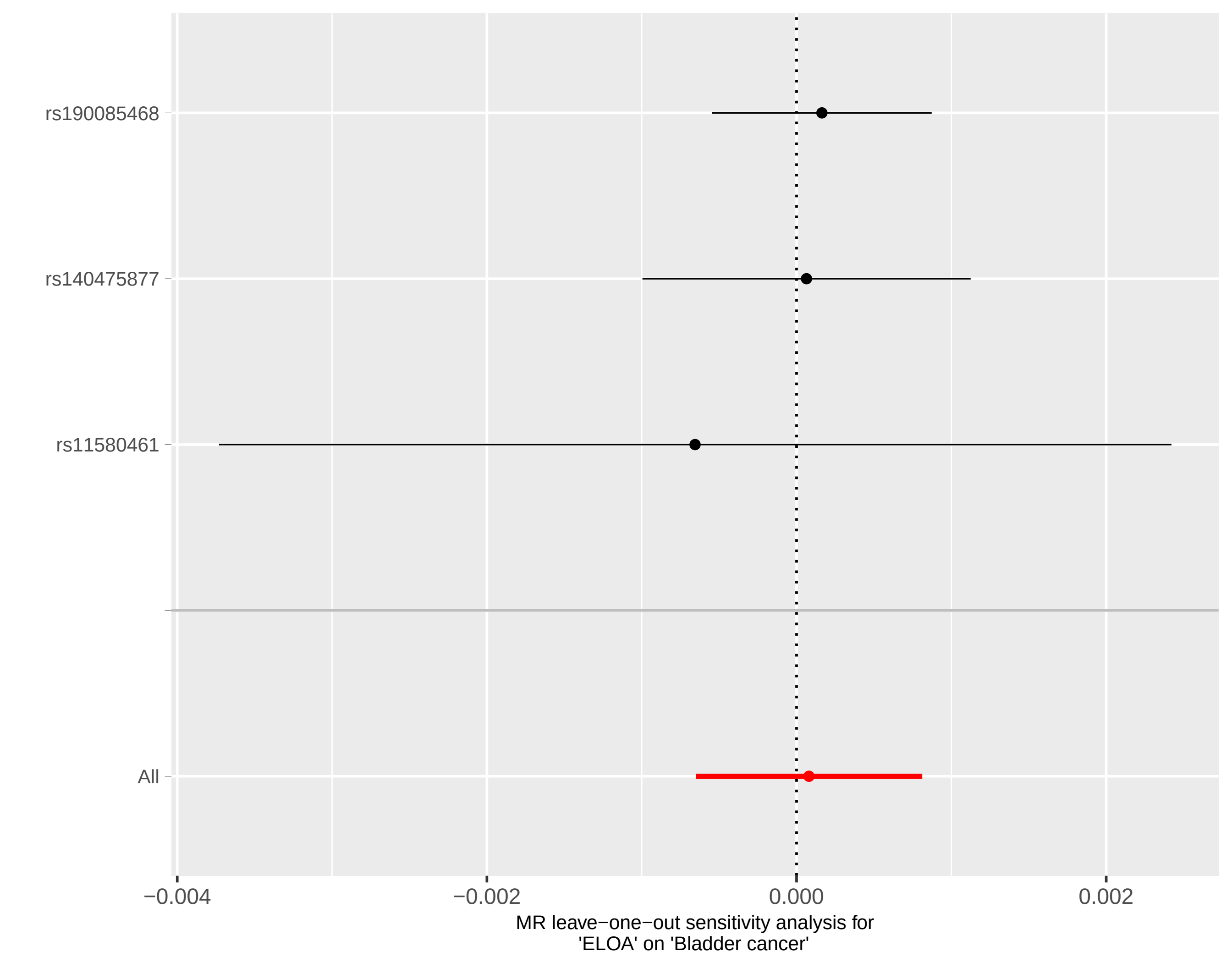

rs190085468
rs140475877
rs11580461
All
−0.004
−0.002
0.000
0.002
MR leave−one−out sensitivity analysis for
'ELOA' on 'Bladder cancer'

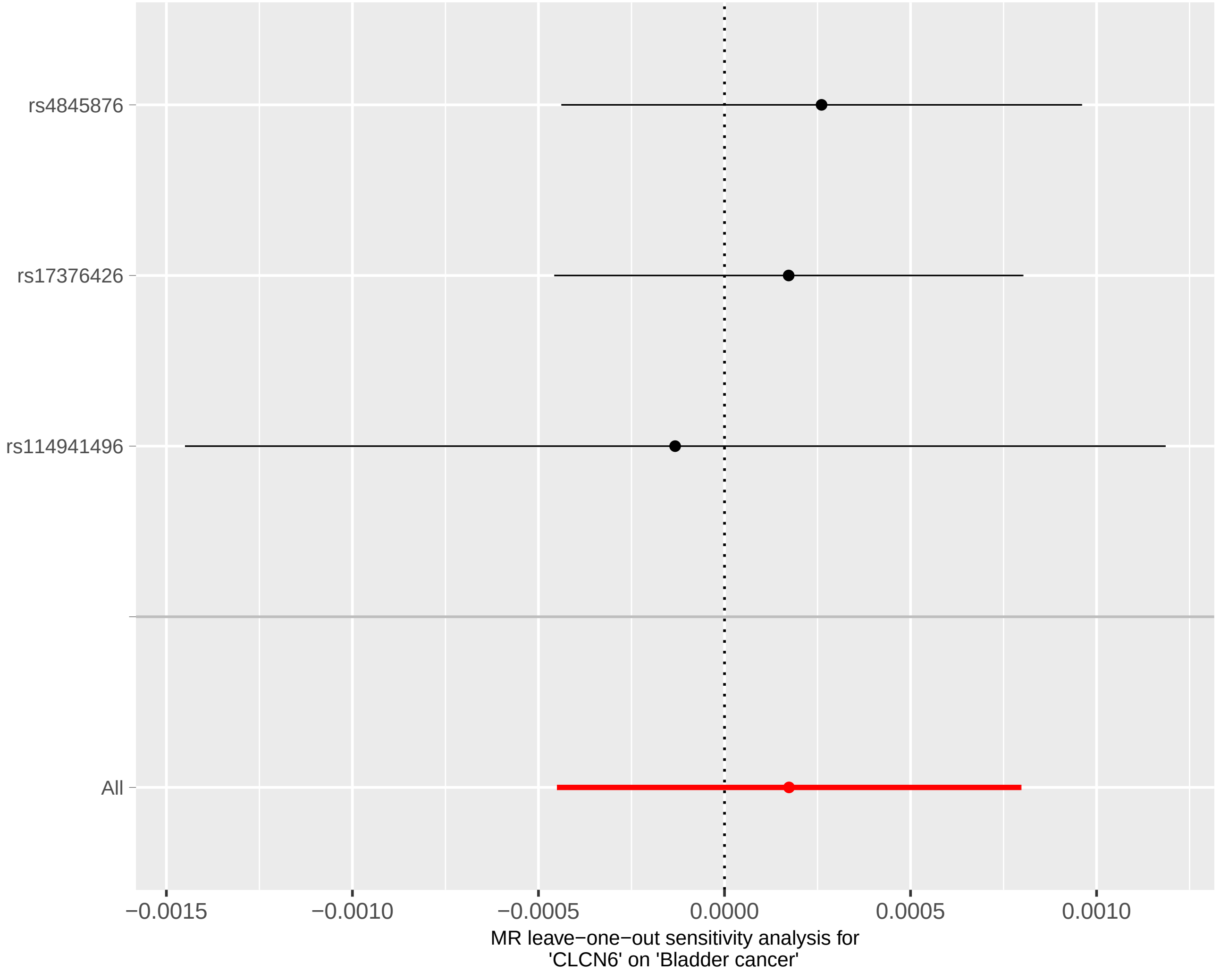

rs4845876
rs17376426
rs114941496
All
−0.0015
−0.0010
−0.0005
0.0000
0.0005
0.0010
MR leave−one−out sensitivity analysis for
'CLCN6' on 'Bladder cancer'

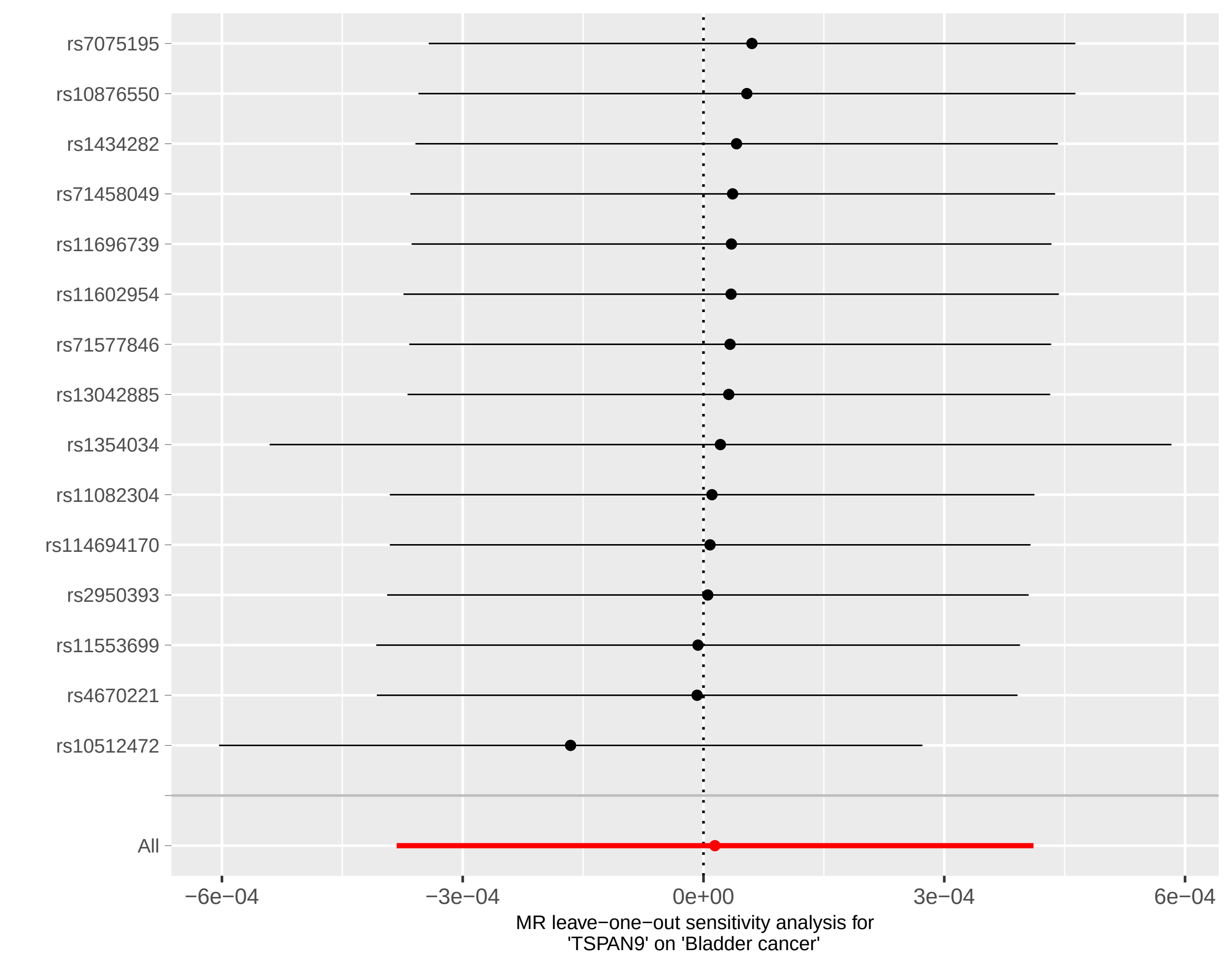
rs7075195
rs10876550
rs1434282
rs71458049
rs11696739
rs11602954
rs71577846
rs13042885
rs1354034
rs11082304
rs114694170
rs2950393
rs11553699
rs4670221
rs10512472
All
−6e−04
−3e−04
0e+00
3e−04
6e−04
MR leave−one−out sensitivity analysis for
'TSPAN9' on 'Bladder cancer'

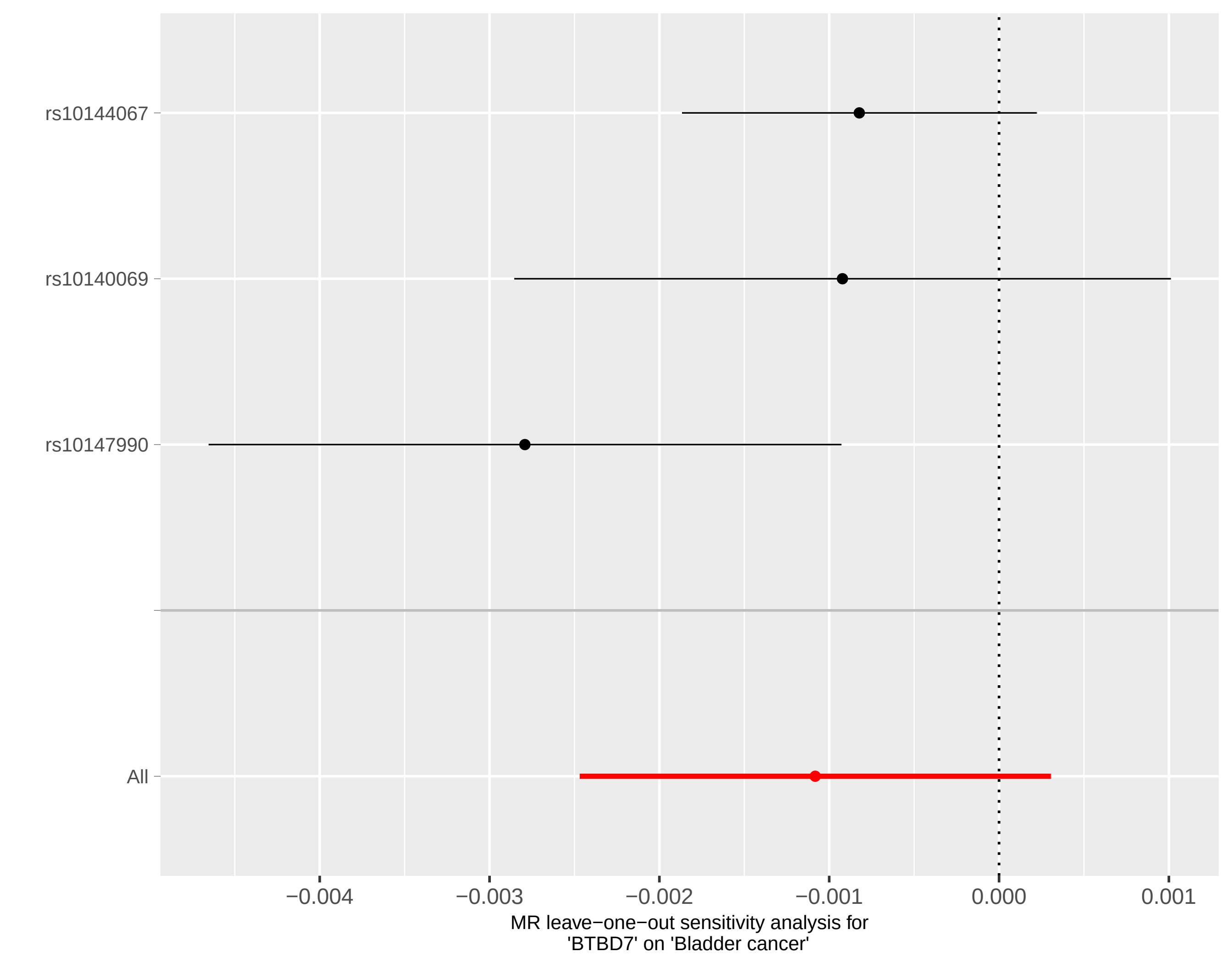
rs10144067
rs10140069
rs10147990
All
−0.004
−0.003
−0.002
−0.001
0.000
0.001
MR leave−one−out sensitivity analysis for
'BTBD7' on 'Bladder cancer'

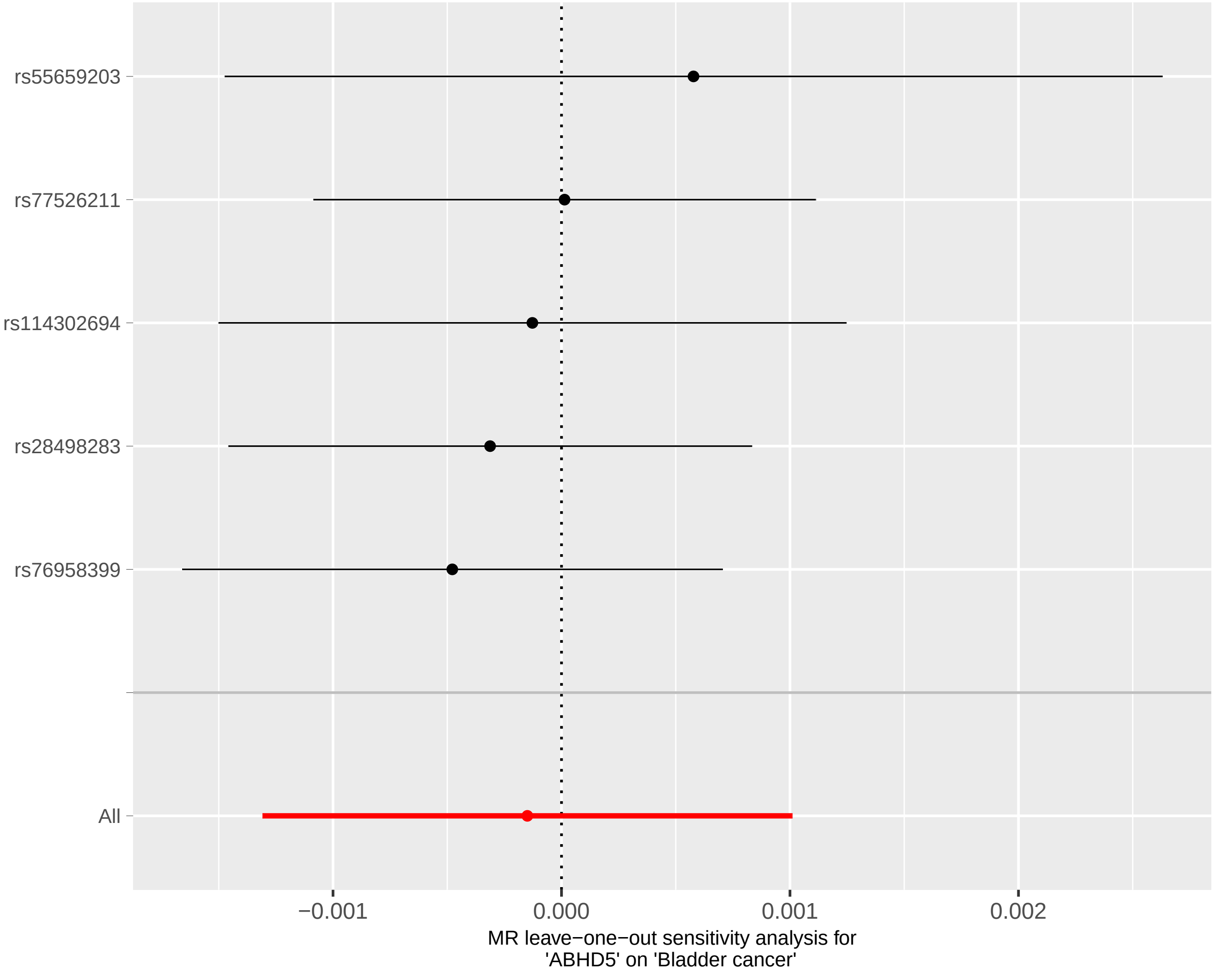
rs55659203
rs77526211
rs114302694
rs28498283
rs76958399
All
−0.001
0.000
0.001
0.002
MR leave−one−out sensitivity analysis for
'ABHD5' on 'Bladder cancer'

Insufficient number of SNPs

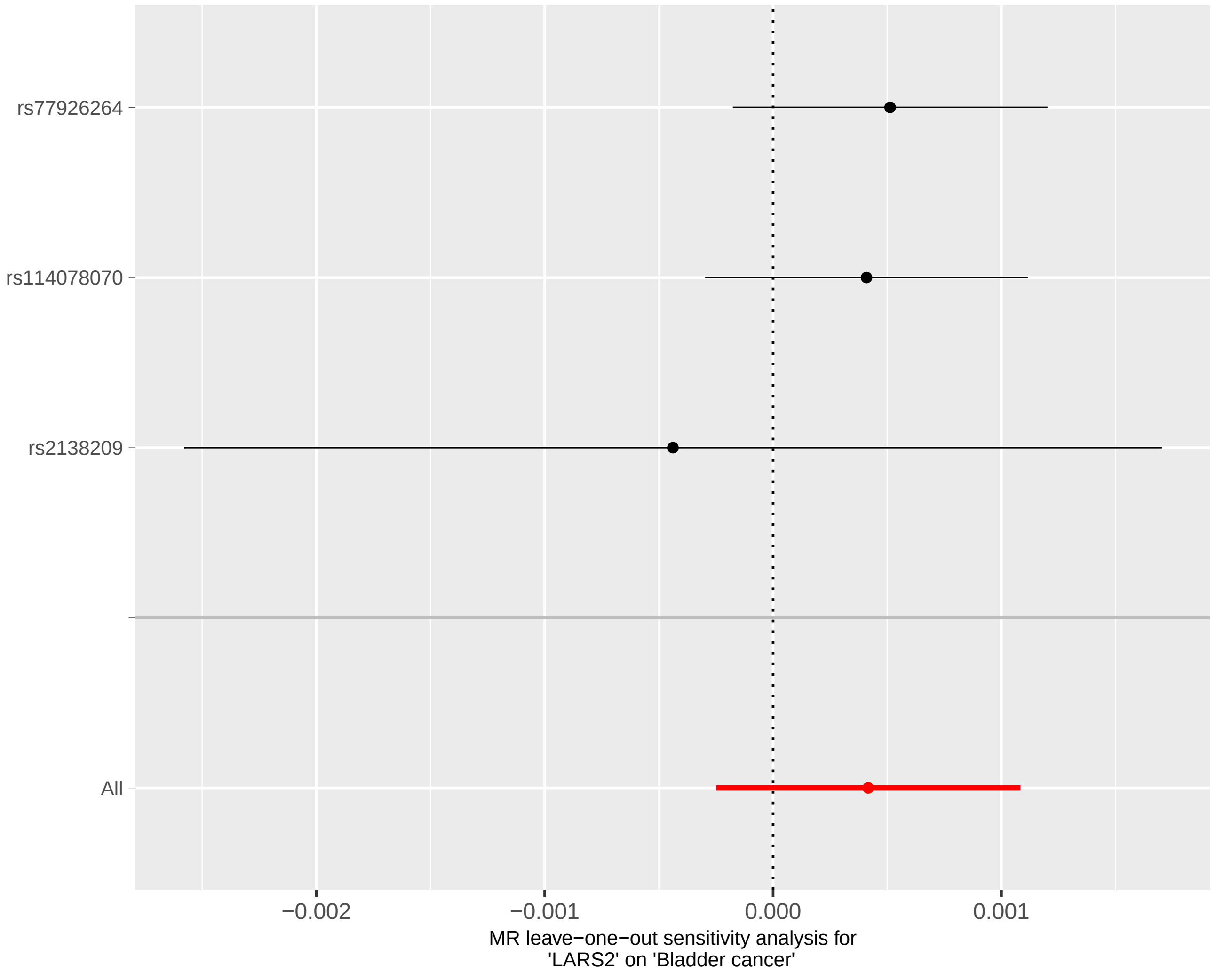
rs77926264
rs114078070
rs2138209
All
−0.002
−0.001
0.000
0.001
MR leave−one−out sensitivity analysis for
'LARS2' on 'Bladder cancer'

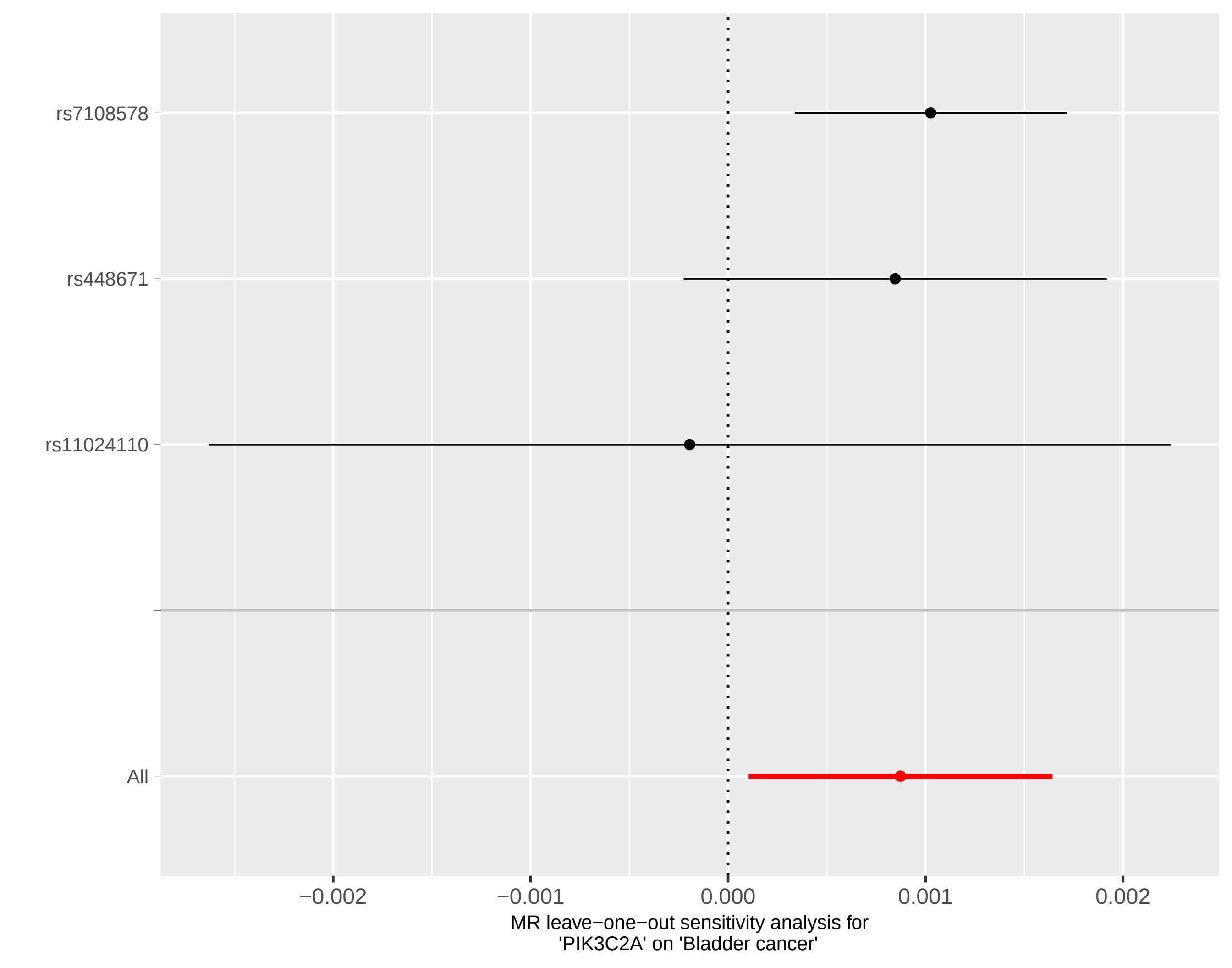
rs7108578
rs448671
rs11024110
All
−0.002
−0.001
0.000
0.001
0.002
MR leave−one−out sensitivity analysis for
'PIK3C2A' on 'Bladder cancer'

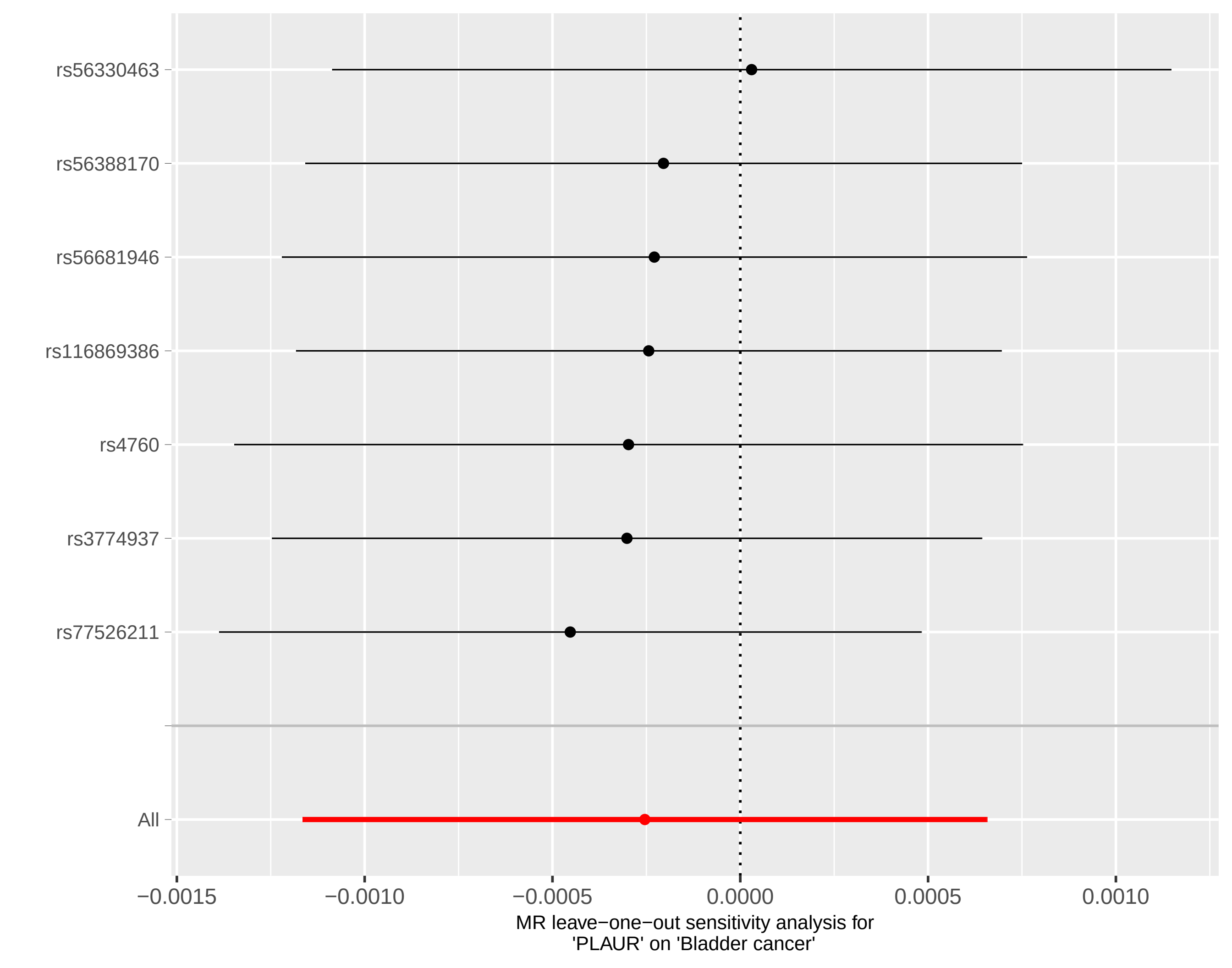
rs56330463
rs56388170
rs56681946
rs116869386
rs4760
rs3774937
rs77526211
All
−0.0015
−0.0010
−0.0005
0.0000
0.0005
0.0010
MR leave−one−out sensitivity analysis for
'PLAUR' on 'Bladder cancer'

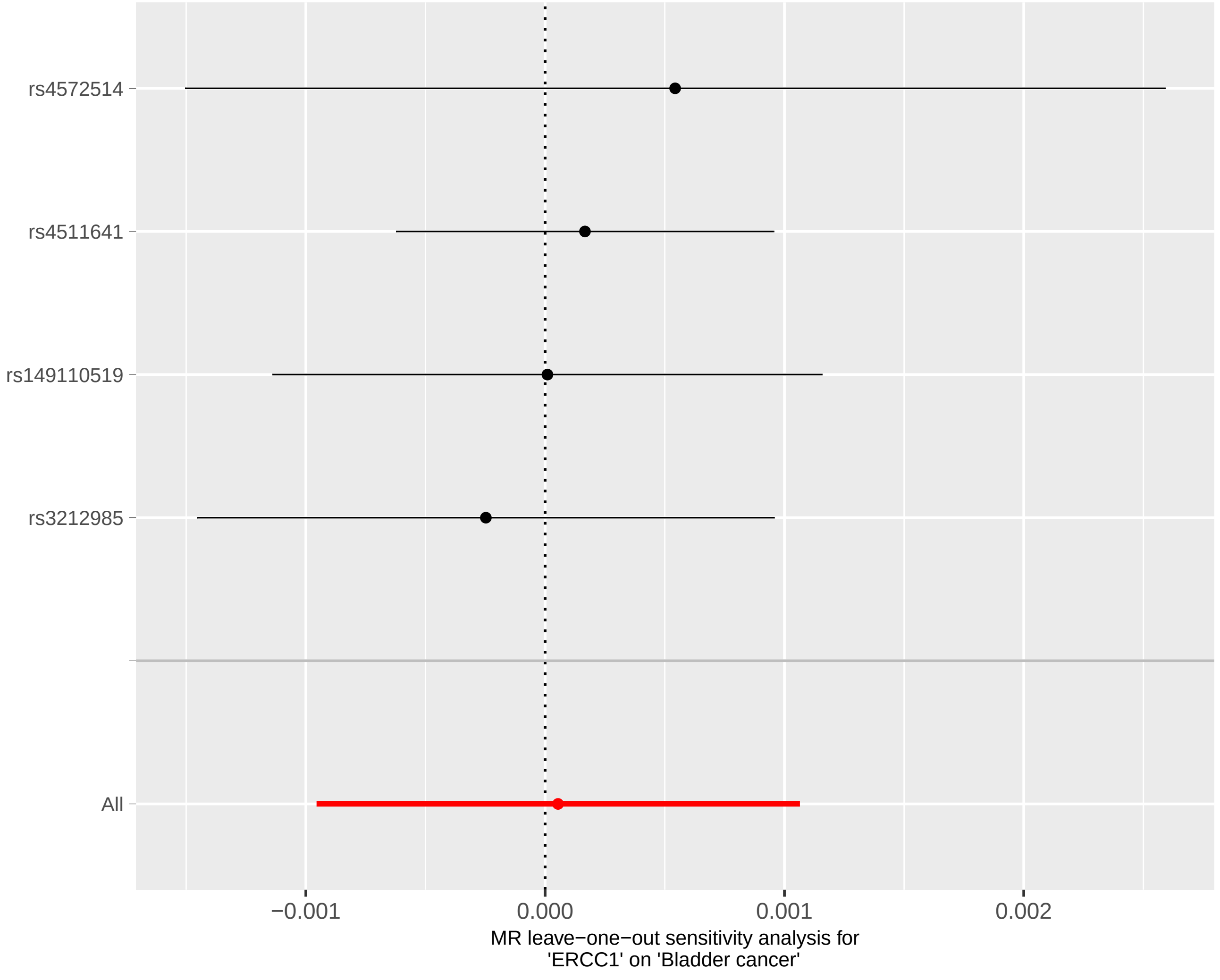
rs4572514
rs4511641
rs149110519
rs3212985
All
−0.001
0.000
0.001
0.002
MR leave−one−out sensitivity analysis for
'ERCC1' on 'Bladder cancer'

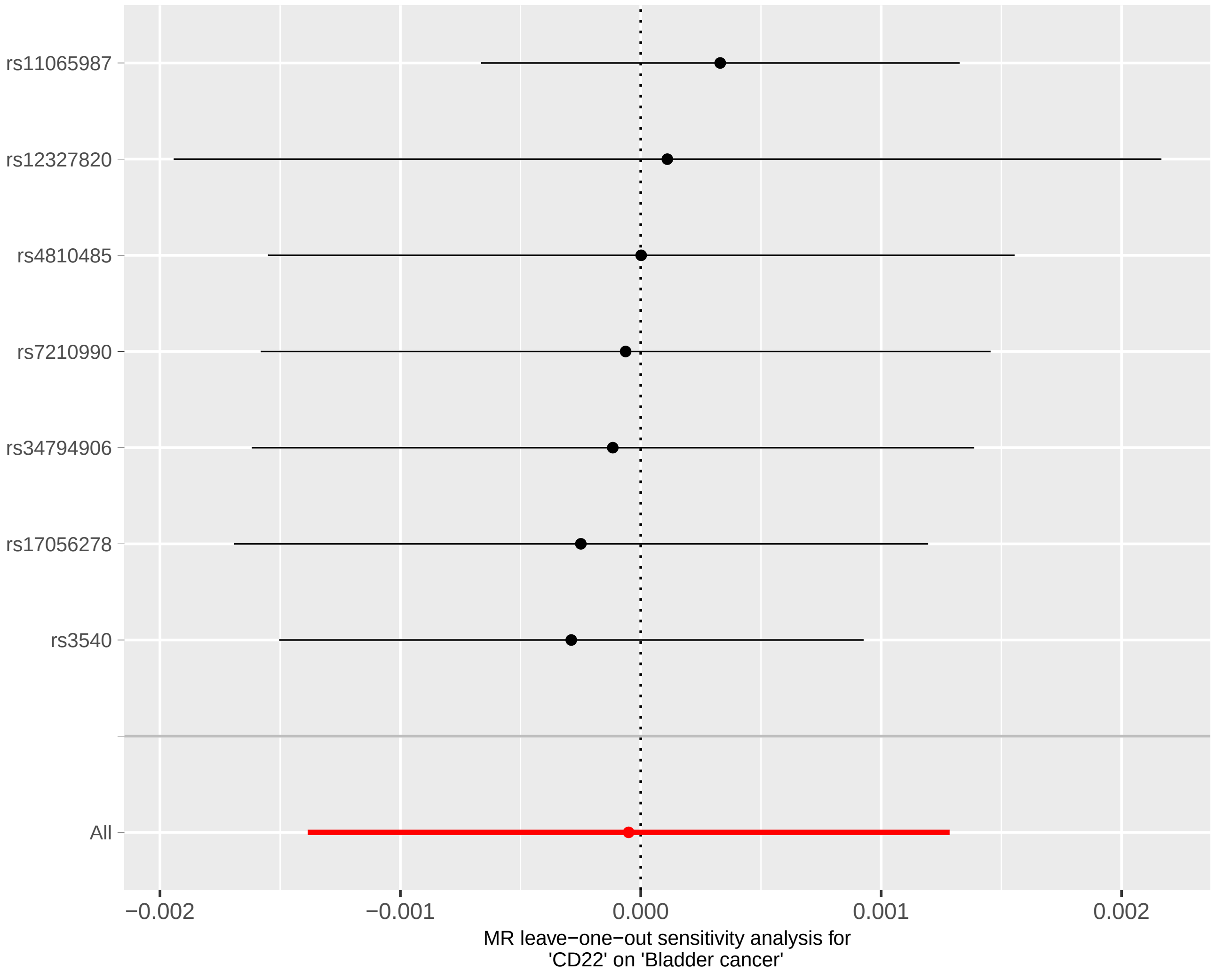

rs11065987
rs12327820
rs4810485
rs7210990
rs34794906
rs17056278
rs3540
All
−0.002
−0.001
0.000
0.001
0.002
MR leave−one−out sensitivity analysis for
'CD22' on 'Bladder cancer'

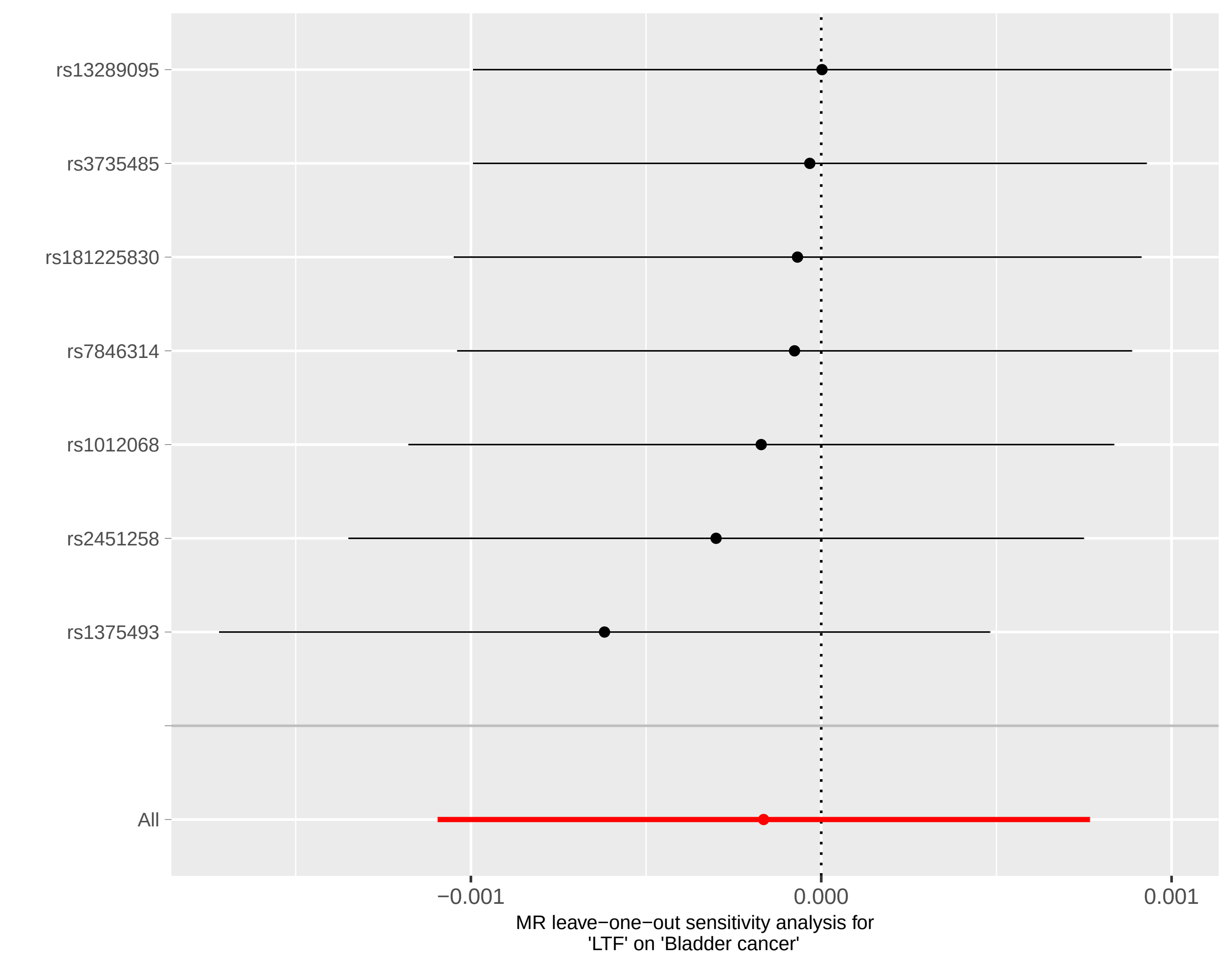
rs13289095
rs3735485
rs181225830
rs7846314
rs1012068
rs2451258
rs1375493
All
−0.001
0.000
0.001
MR leave−one−out sensitivity analysis for
'LTF' on 'Bladder cancer'

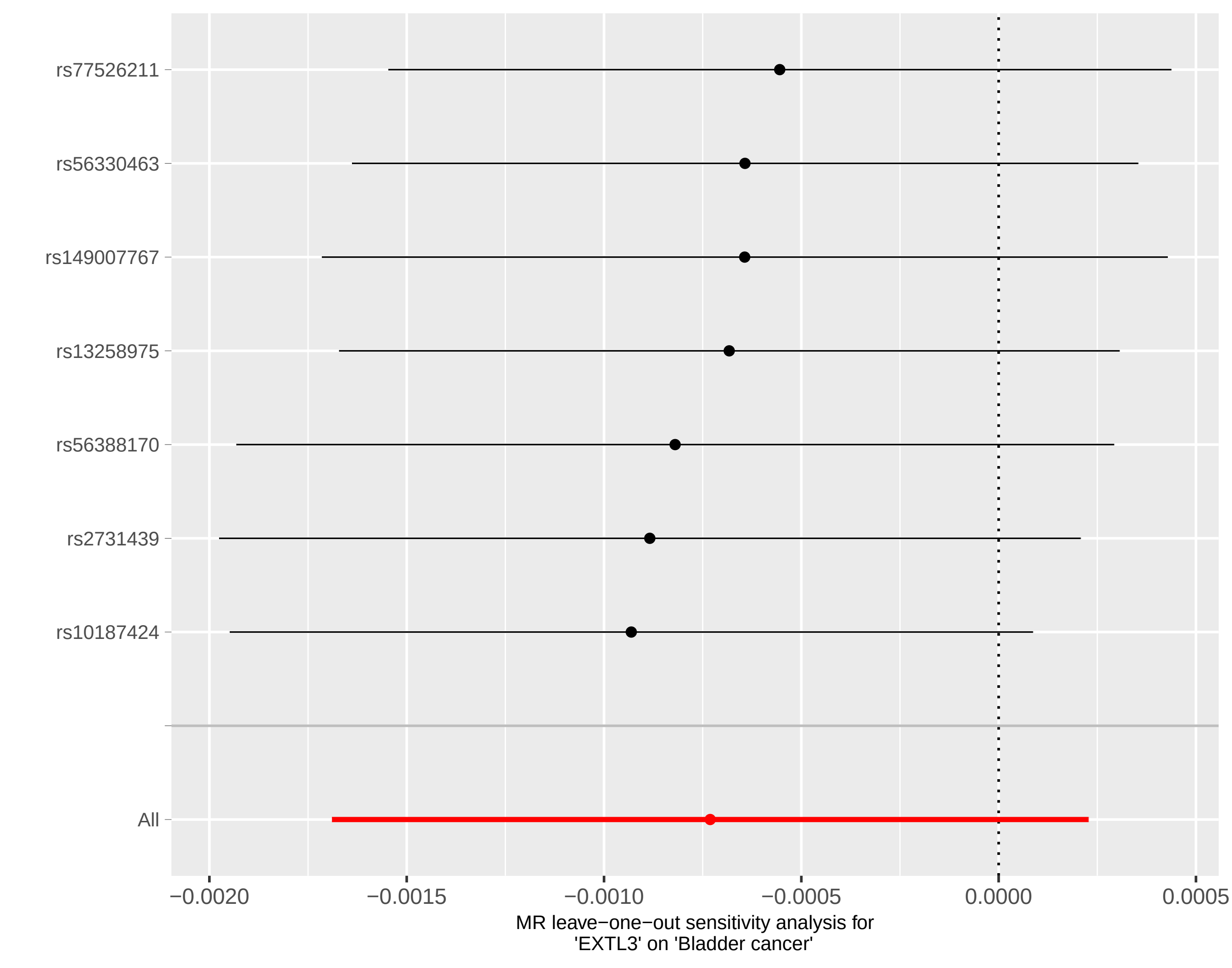
rs77526211
rs56330463
rs149007767
rs13258975
rs56388170
rs2731439
rs10187424
All
−0.0020
−0.0015
−0.0010
−0.0005
0.0000
0.0005
MR leave−one−out sensitivity analysis for
'EXTL3' on 'Bladder cancer'

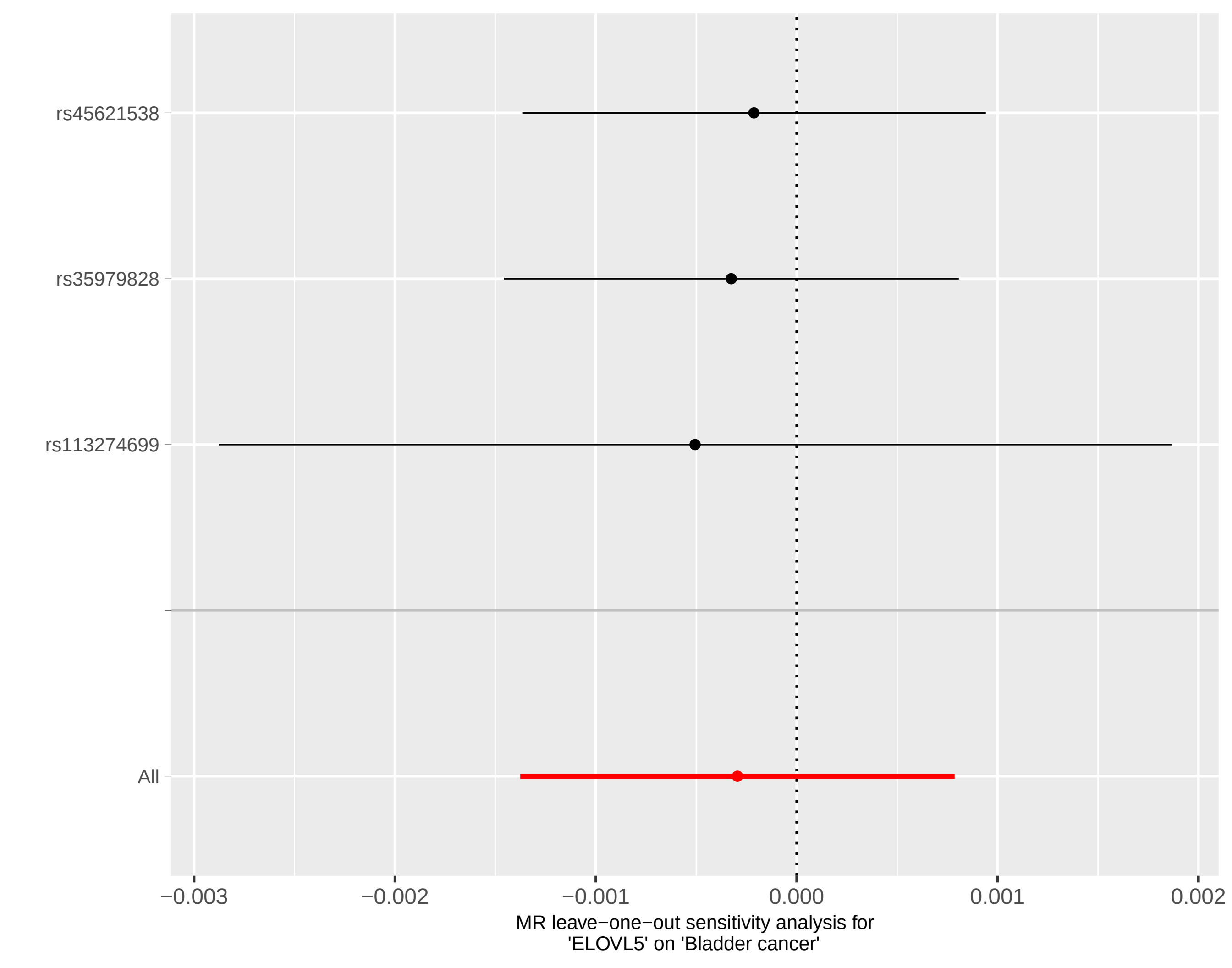
rs45621538
rs35979828
rs113274699
All
−0.003
−0.002
−0.001
0.000
0.001
0.002
MR leave−one−out sensitivity analysis for
'ELOVL5' on 'Bladder cancer'

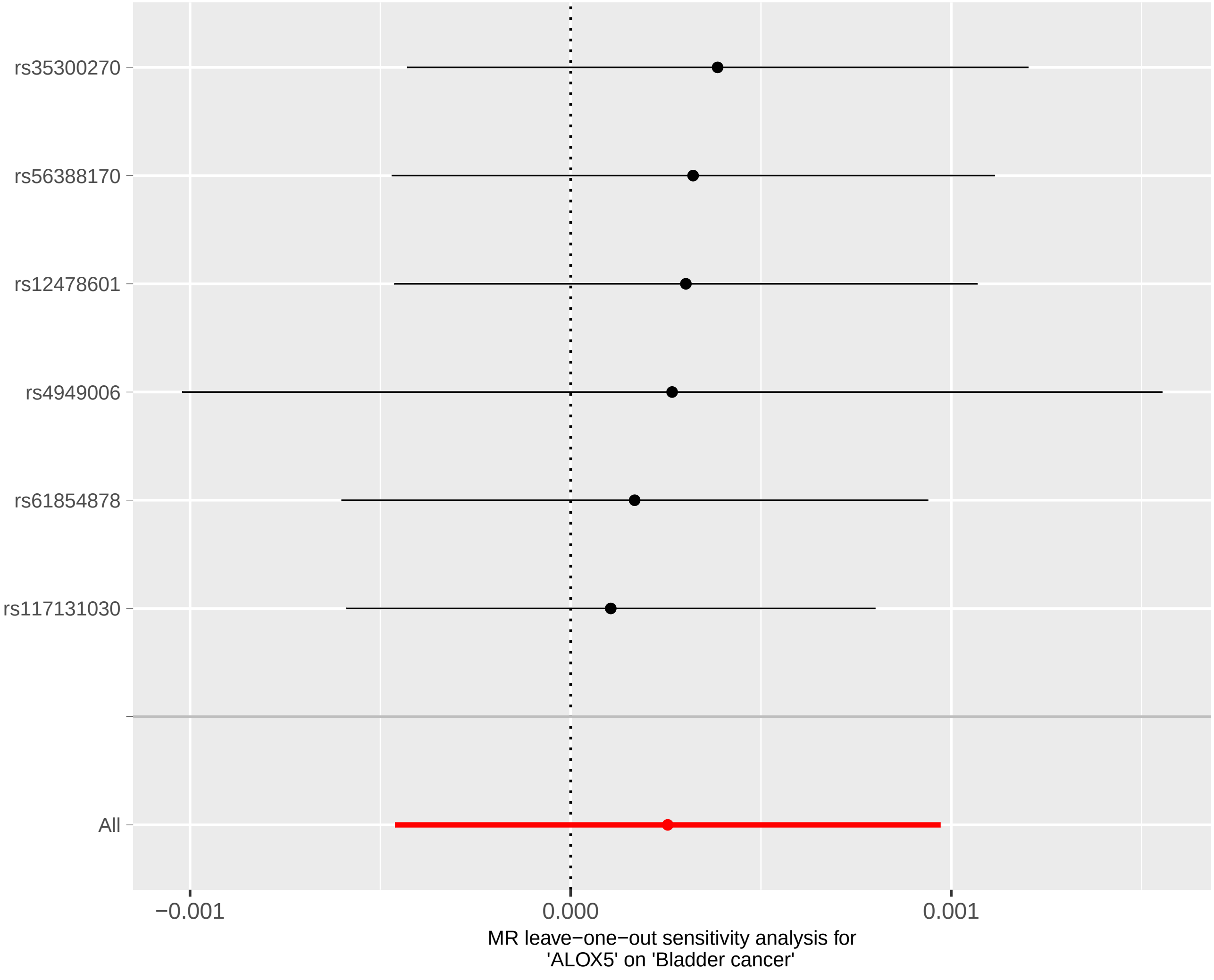
rs35300270
rs56388170
rs12478601
rs4949006
rs61854878
rs117131030
All
−0.001
0.000
0.001
MR leave−one−out sensitivity analysis for
'ALOX5' on 'Bladder cancer'

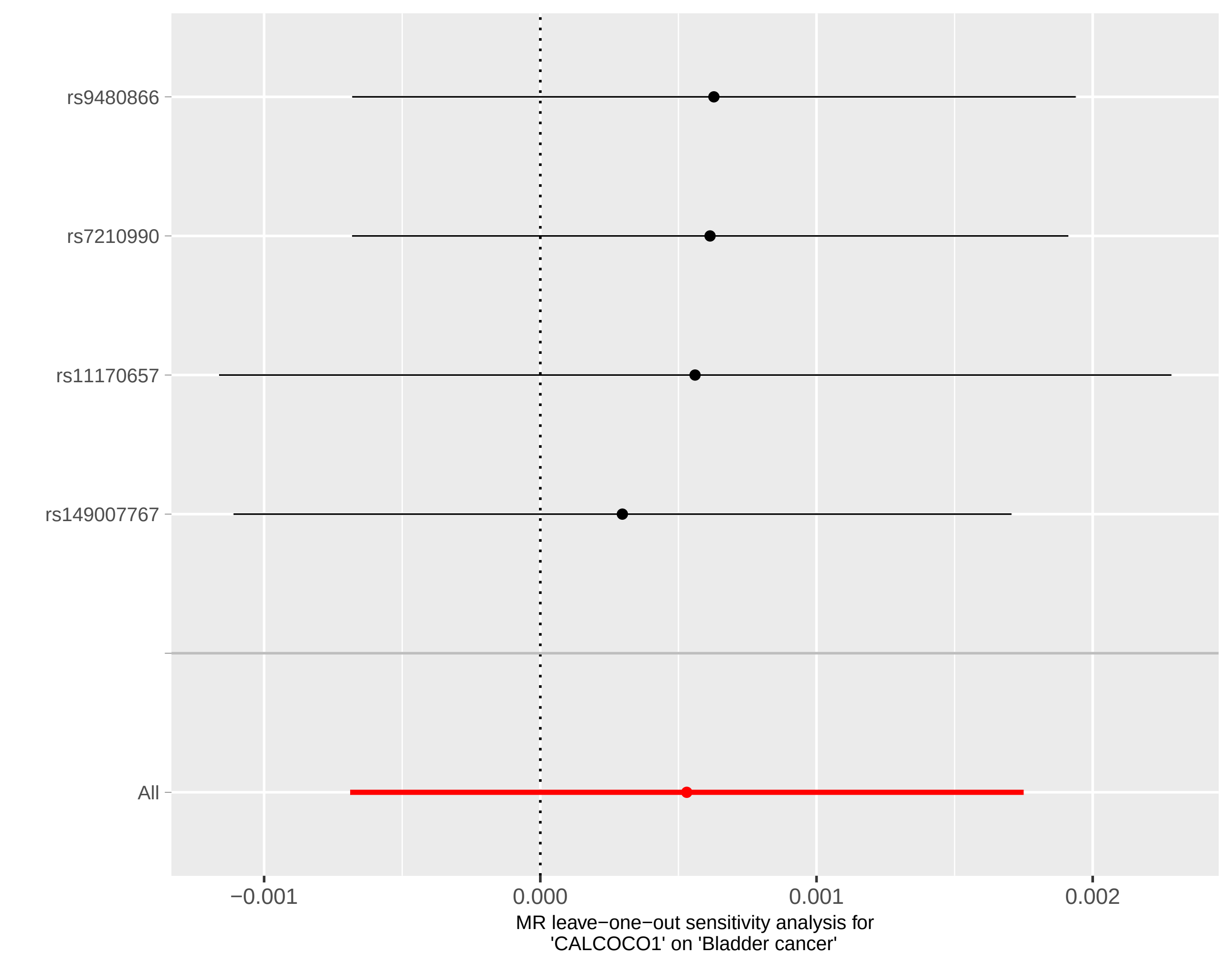
rs9480866
rs7210990
rs11170657
rs149007767
All
−0.001
0.000
0.001
0.002
MR leave−one−out sensitivity analysis for
'CALCOCO1' on 'Bladder cancer'

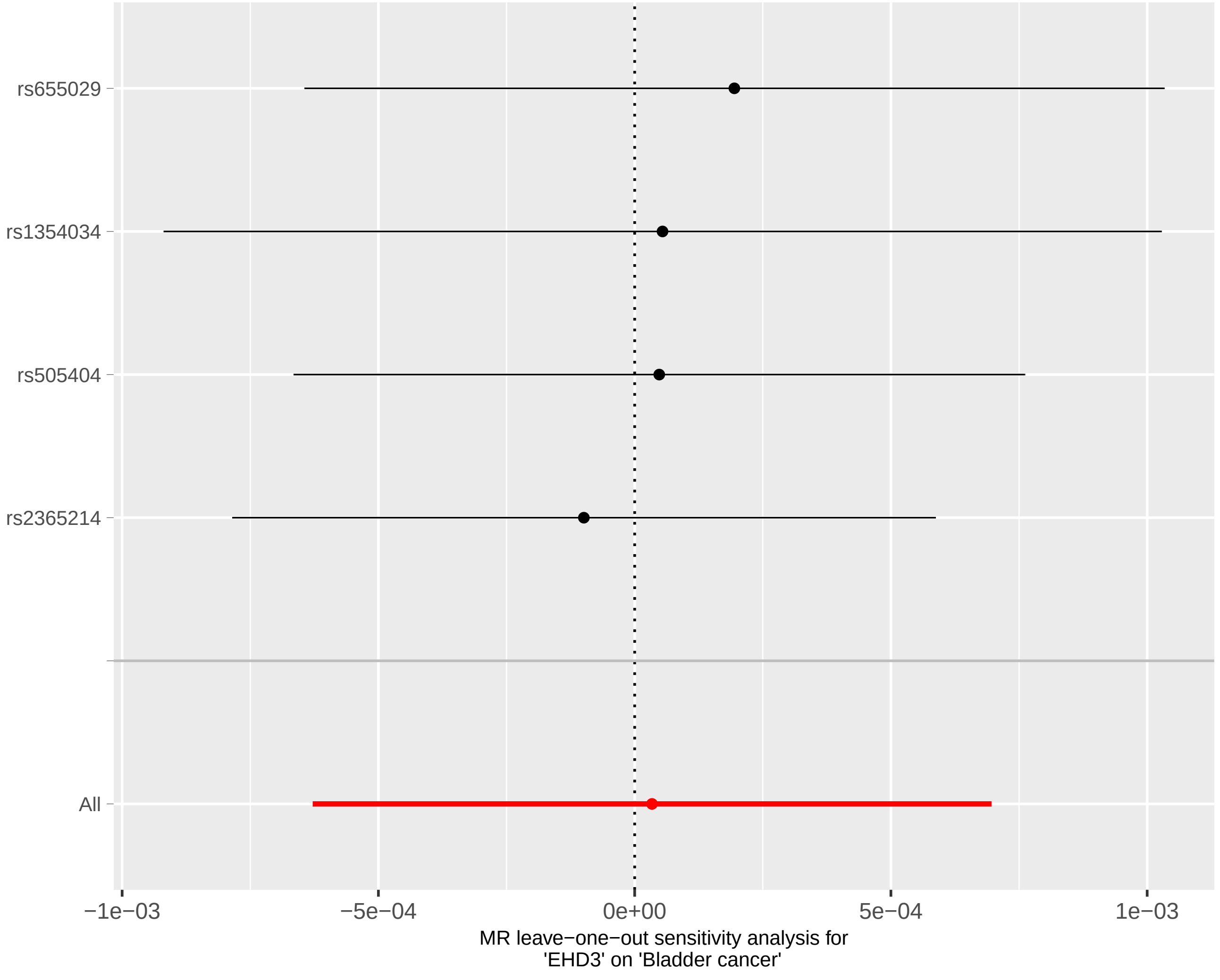
rs655029
rs1354034
rs505404
rs2365214
All
−1e−03
−5e−04
0e+00
5e−04
1e−03
MR leave−one−out sensitivity analysis for
'EHD3' on 'Bladder cancer'

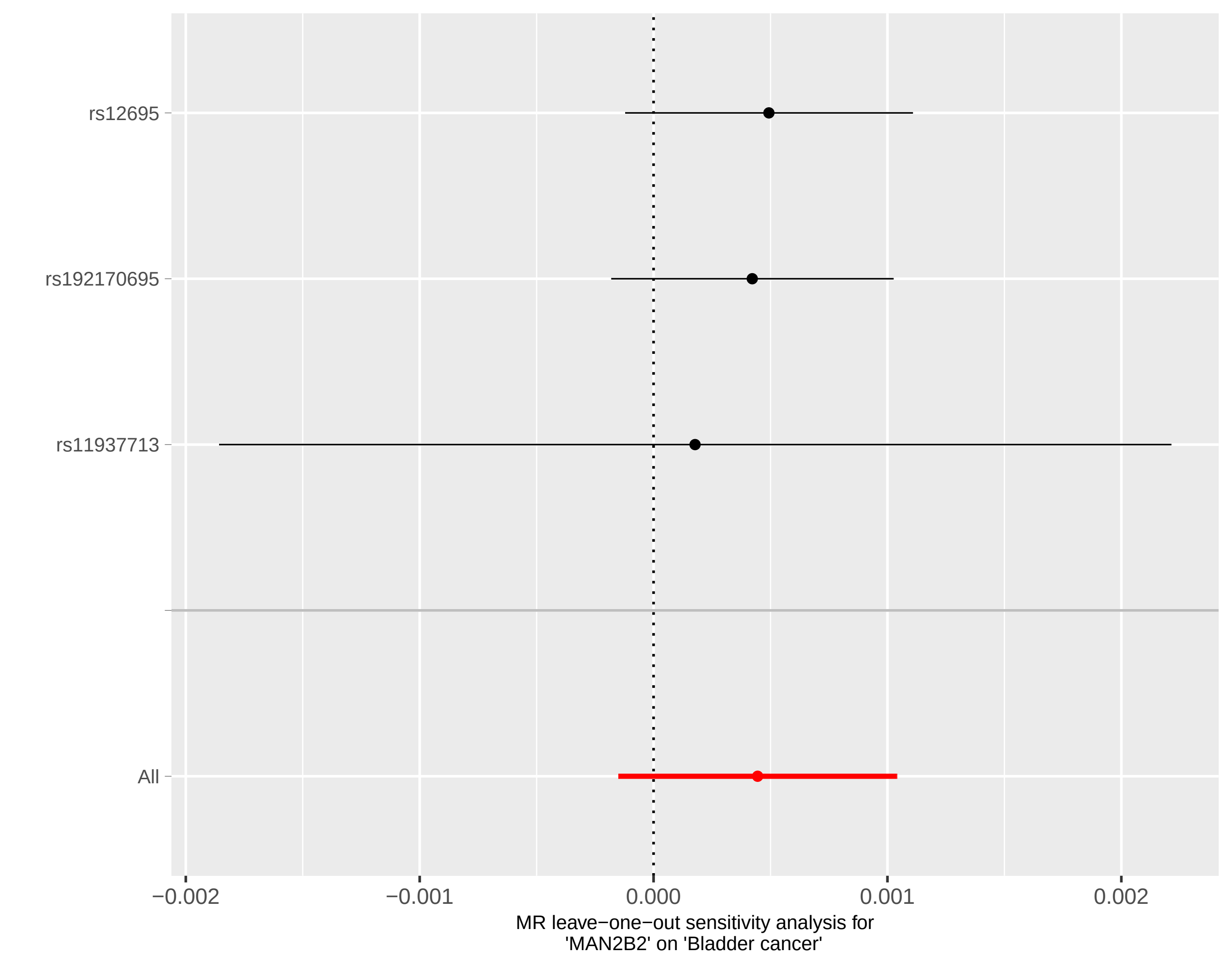
rs12695
rs192170695
rs11937713
All
−0.002
−0.001
0.000
0.001
0.002
MR leave−one−out sensitivity analysis for
'MAN2B2' on 'Bladder cancer'

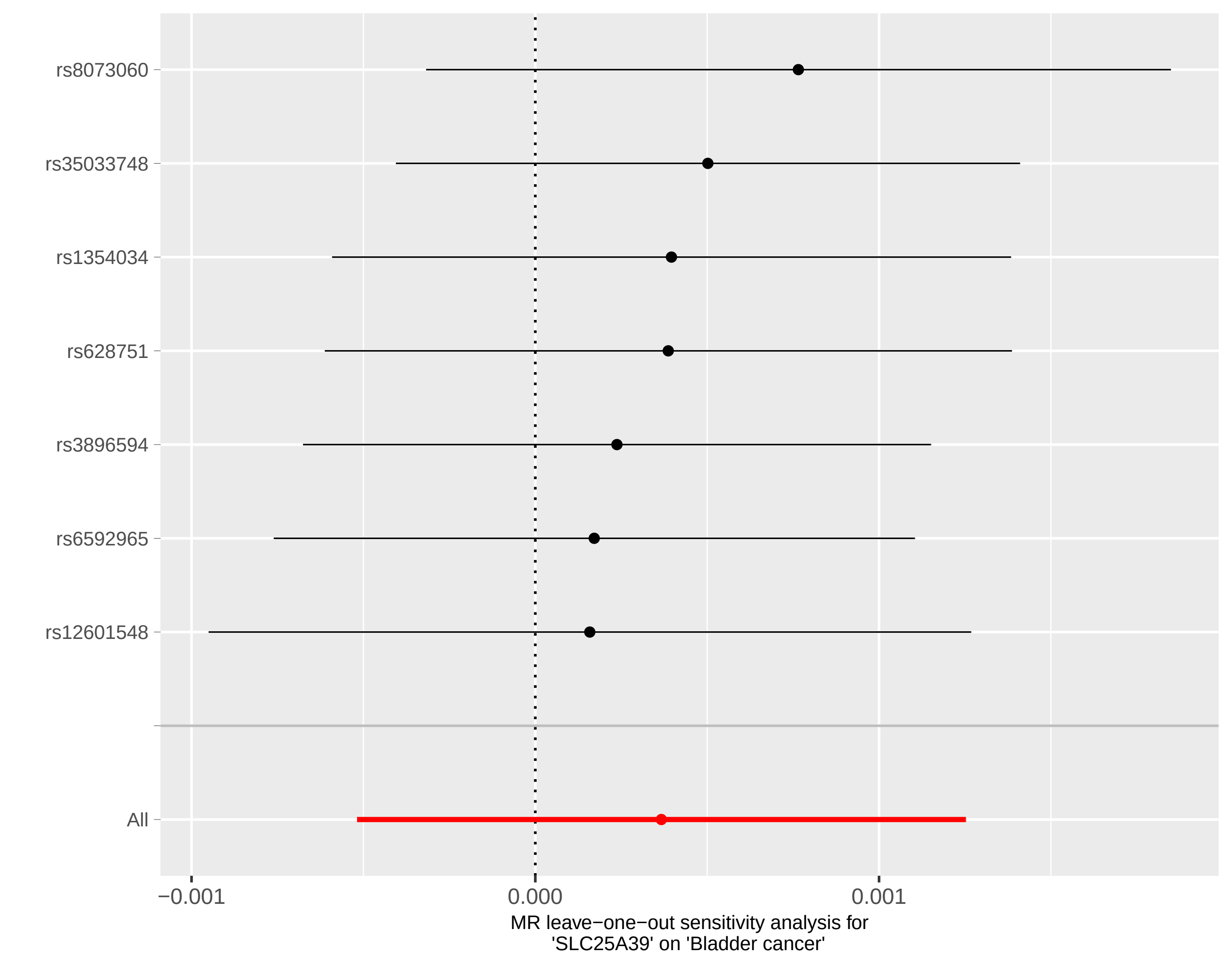
rs8073060
rs35033748
rs1354034
rs628751
rs3896594
rs6592965
rs12601548
All
−0.001
0.000
0.001
MR leave−one−out sensitivity analysis for
'SLC25A39' on 'Bladder cancer'

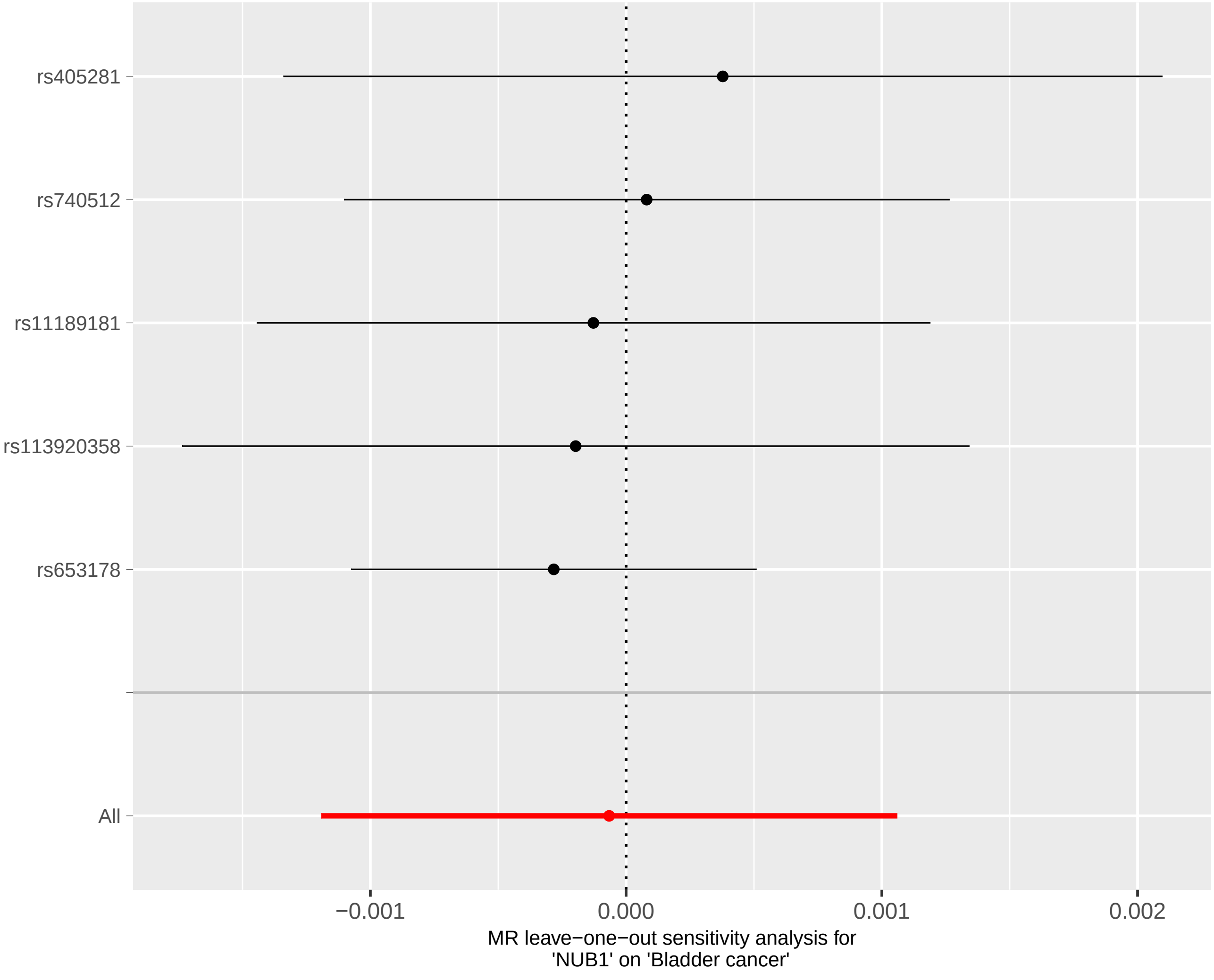
rs405281
rs740512
rs11189181
rs113920358
rs653178
All
−0.001
0.000
0.001
0.002
MR leave−one−out sensitivity analysis for
'NUB1' on 'Bladder cancer'

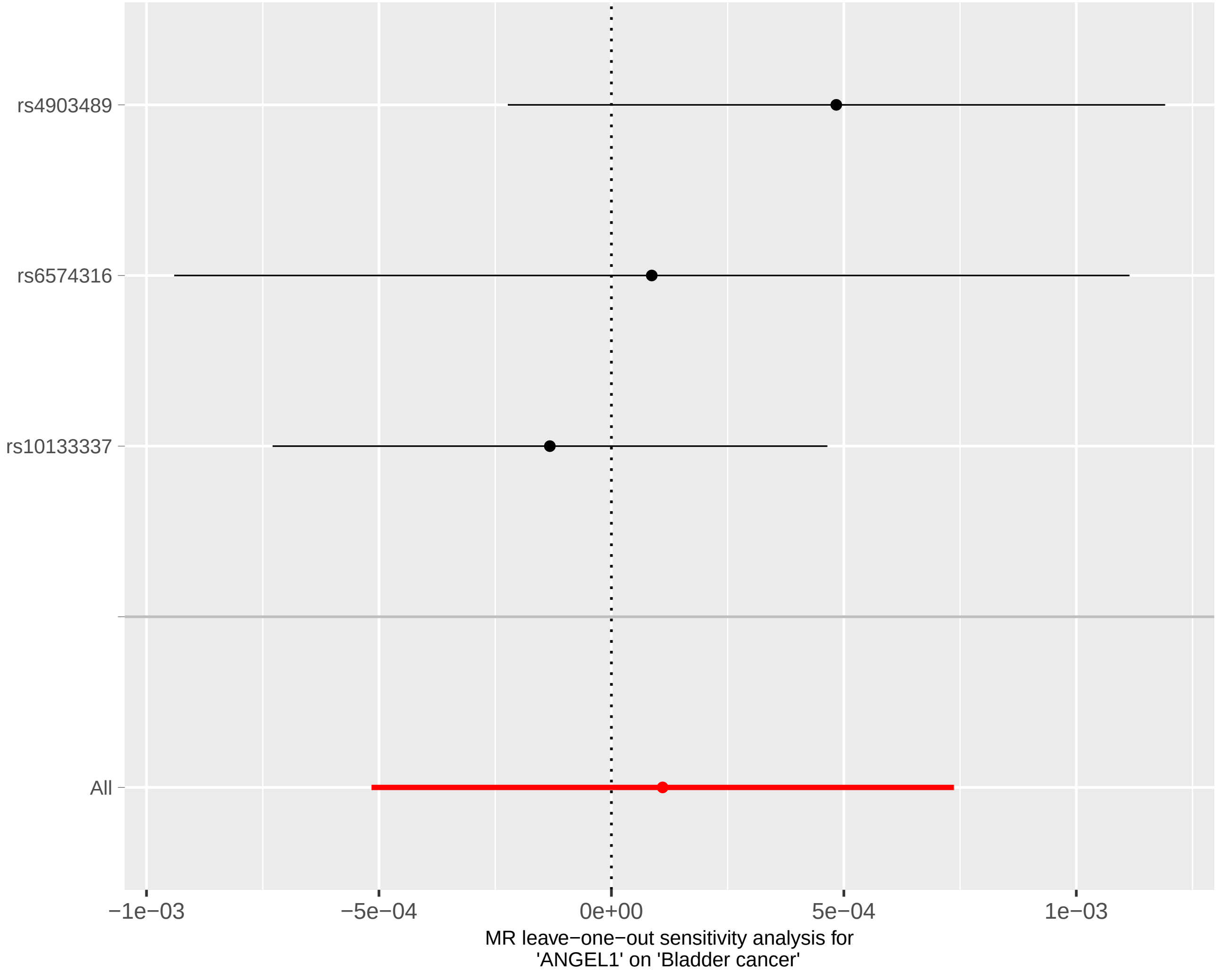
rs4903489
rs6574316
rs10133337
All
−1e−03
−5e−04
0e+00
5e−04
1e−03
MR leave−one−out sensitivity analysis for
'ANGEL1' on 'Bladder cancer'

Insufficient number of SNPs

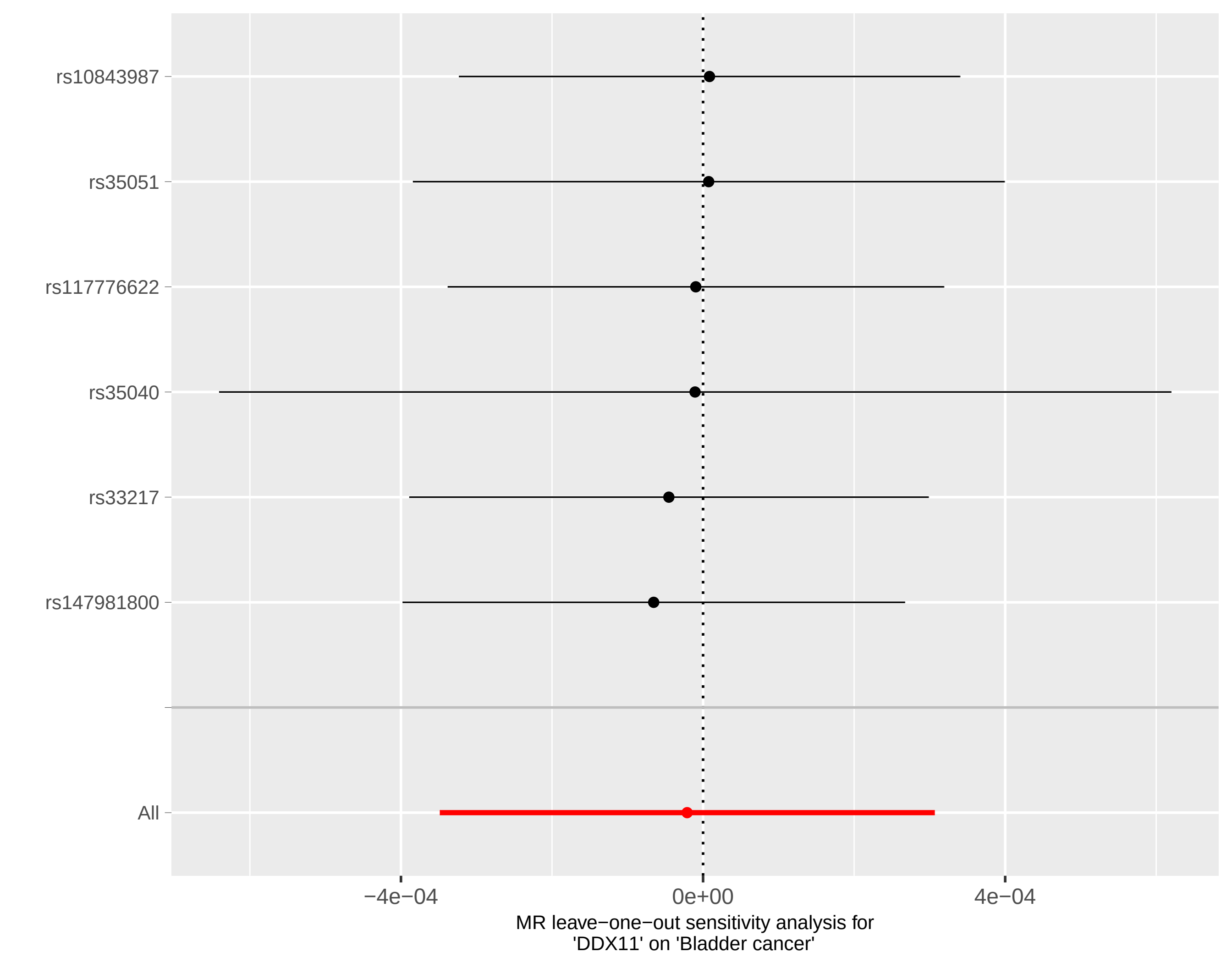
rs10843987
rs35051
rs117776622
rs35040
rs33217
rs147981800
All
−4e−04
0e+00
4e−04
MR leave−one−out sensitivity analysis for
'DDX11' on 'Bladder cancer'

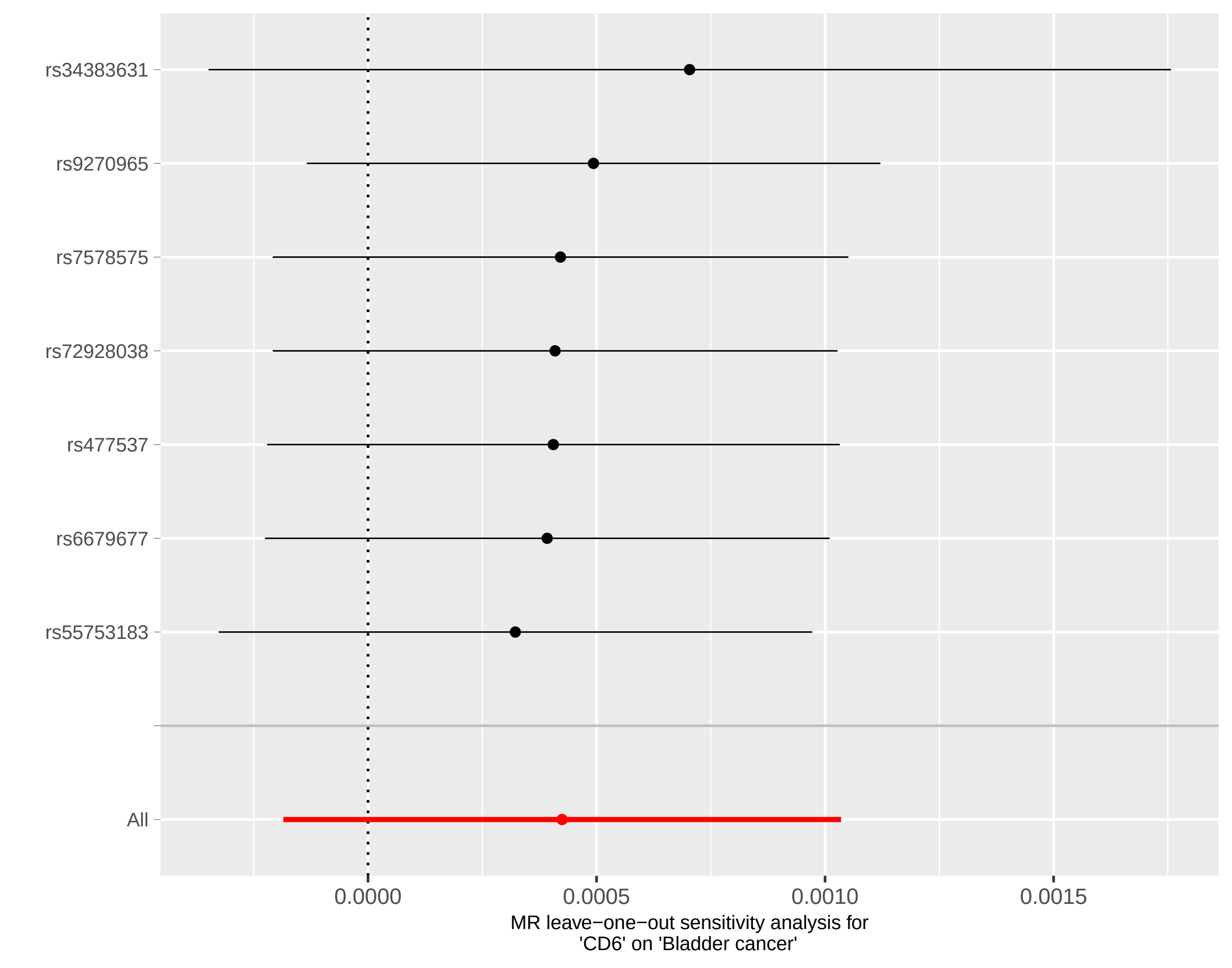
rs34383631
rs9270965
rs7578575
rs72928038
rs477537
rs6679677
rs55753183
All
0.0000
0.0005
0.0010
0.0015
MR leave−one−out sensitivity analysis for
'CD6' on 'Bladder cancer'

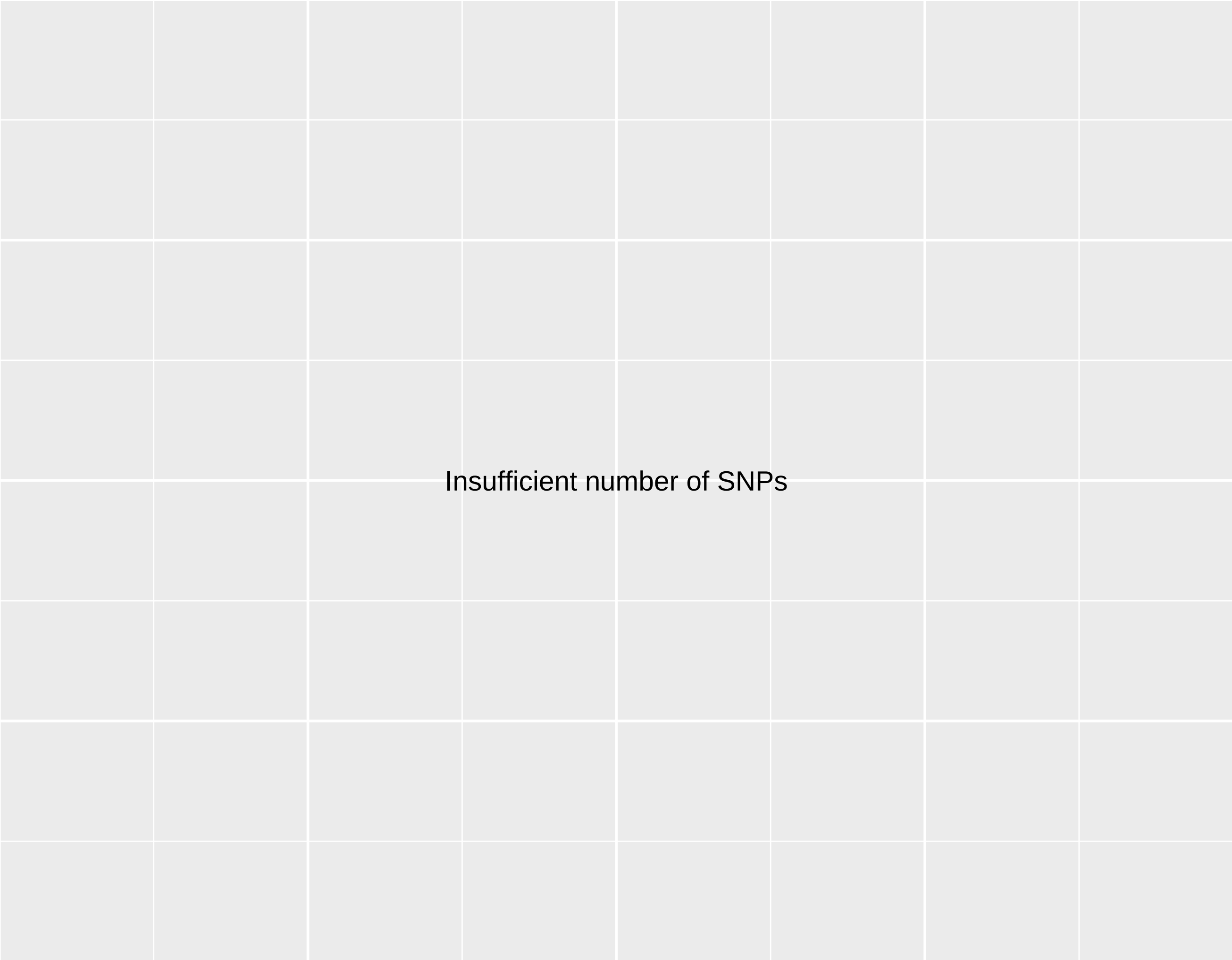
Insufficient number of SNPs

Insufficient number of SNPs

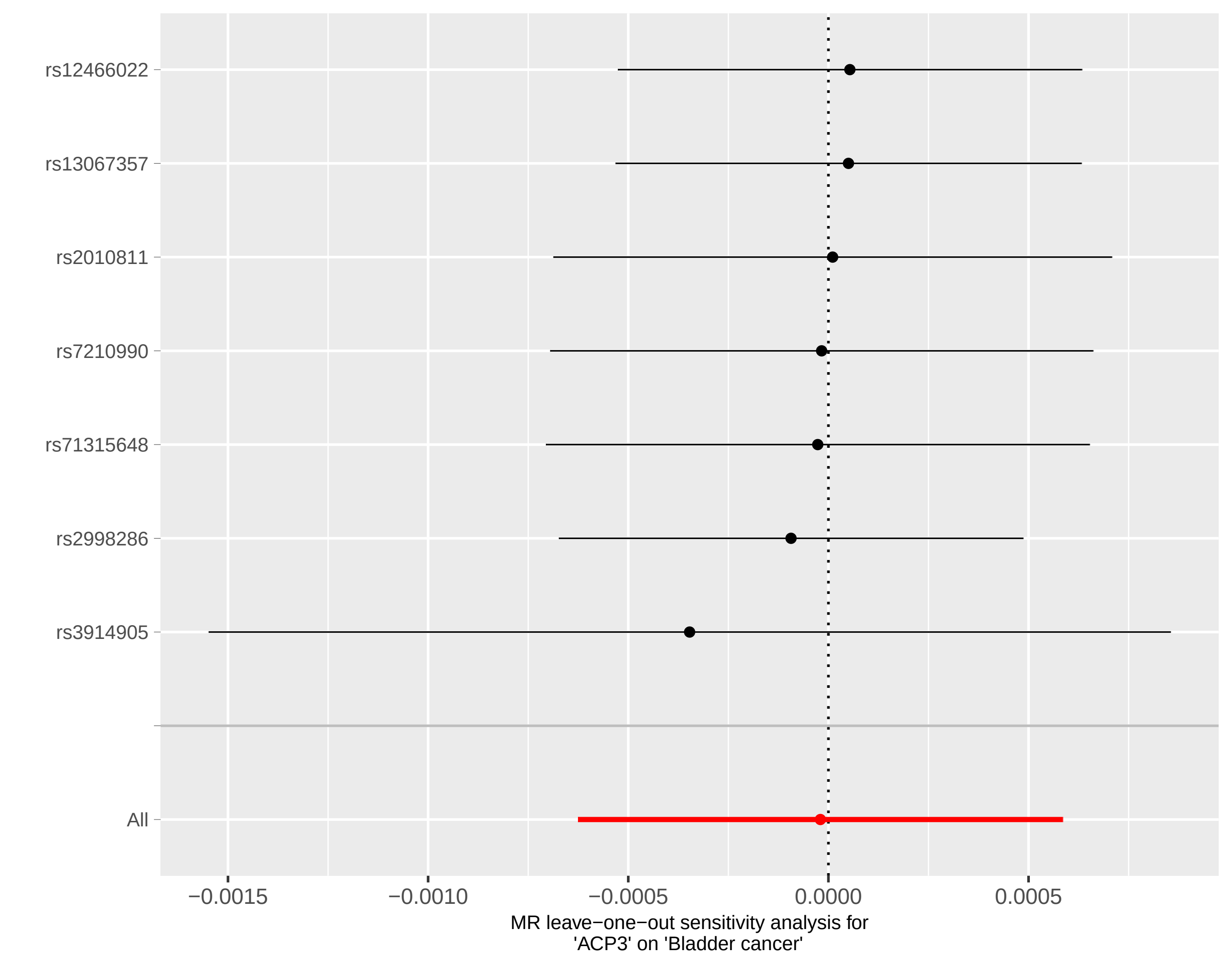
rs12466022
rs13067357
rs2010811
rs7210990
rs71315648
rs2998286
rs3914905
All
−0.0015
−0.0010
−0.0005
0.0000
0.0005
MR leave−one−out sensitivity analysis for
'ACP3' on 'Bladder cancer'

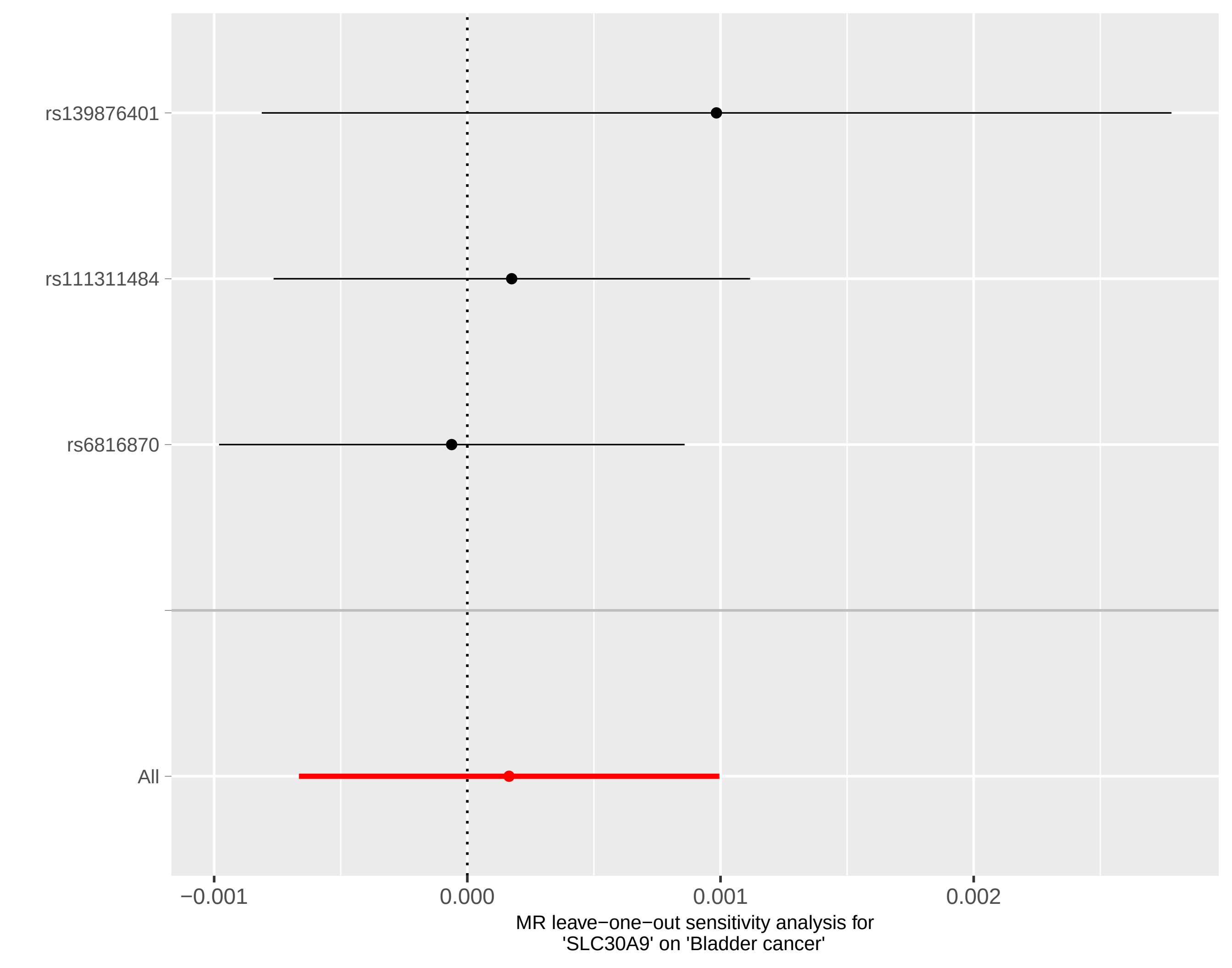

rs139876401
rs111311484
rs6816870
All
−0.001
0.000
0.001
0.002
MR leave−one−out sensitivity analysis for
'SLC30A9' on 'Bladder cancer'

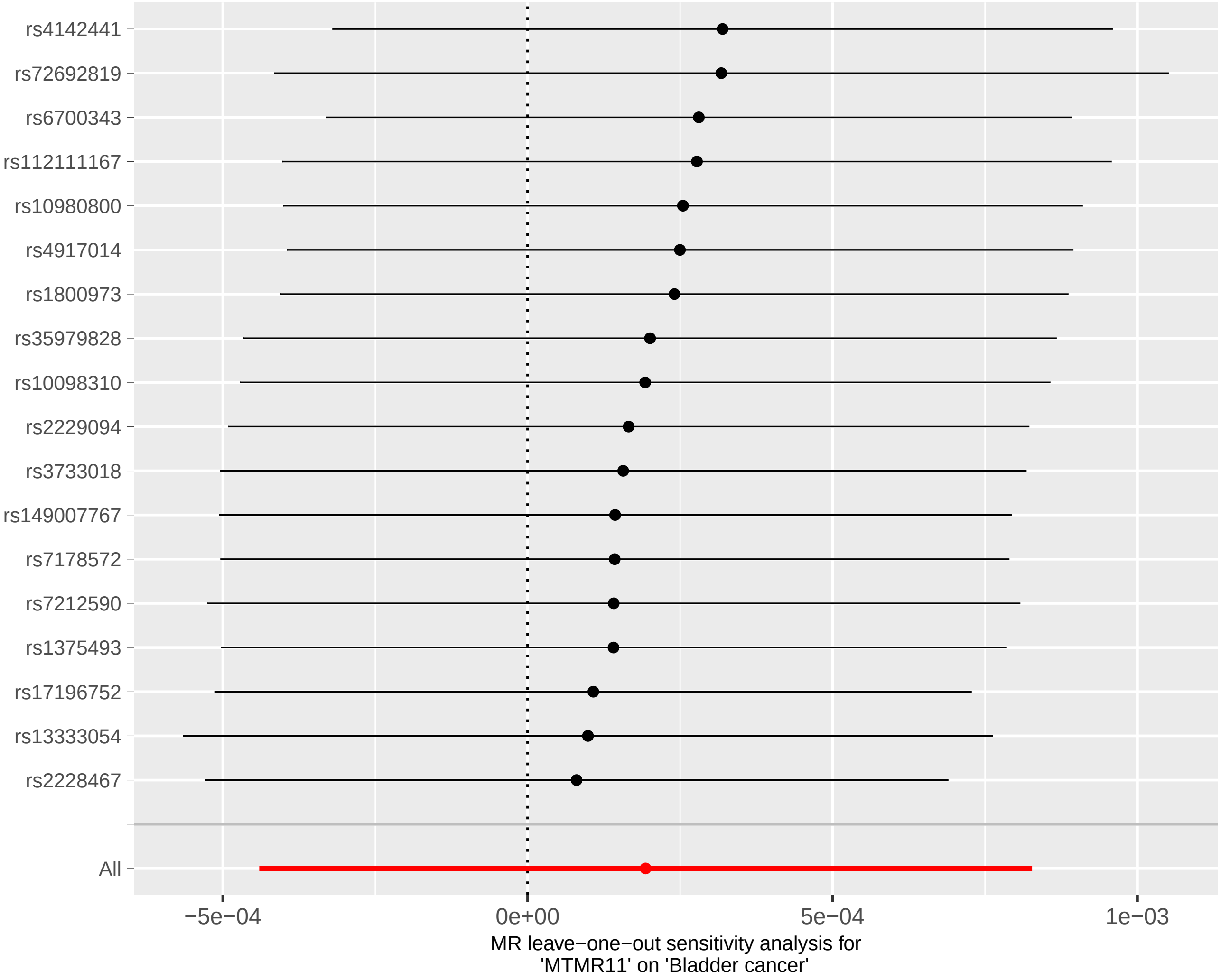

rs4142441
rs72692819
rs6700343
rs112111167
rs10980800
rs4917014
rs1800973
rs35979828
rs10098310
rs2229094
rs3733018
rs149007767
rs7178572
rs7212590
rs1375493
rs17196752
rs13333054
rs2228467
All
−5e−04
0e+00
5e−04
1e−03
MR leave−one−out sensitivity analysis for
'MTMR11' on 'Bladder cancer'

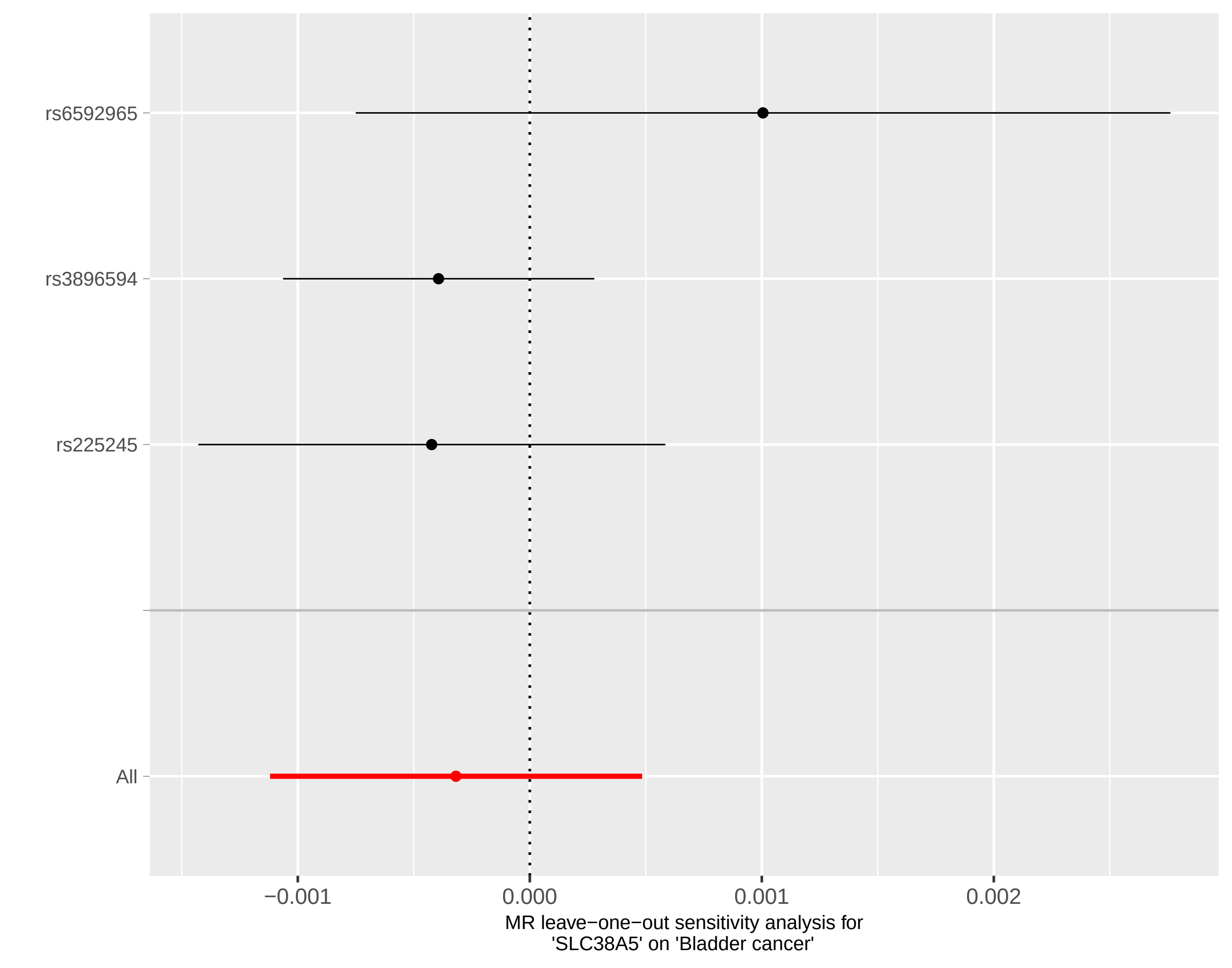
rs6592965
rs3896594
rs225245
All
−0.001
0.000
0.001
0.002
MR leave−one−out sensitivity analysis for
'SLC38A5' on 'Bladder cancer'

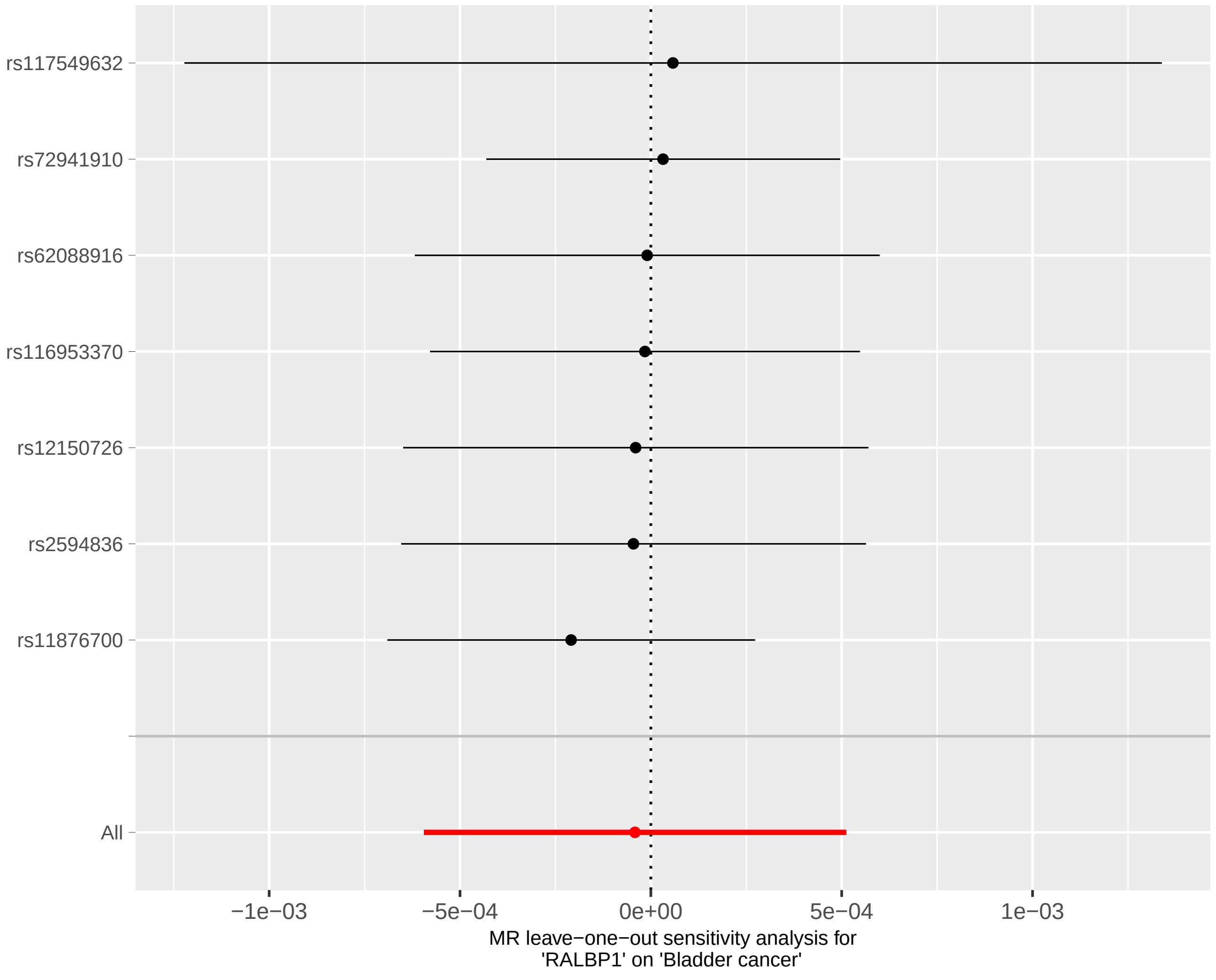
rs117549632
rs72941910
rs62088916
rs116953370
rs12150726
rs2594836
rs11876700
All
−1e−03
−5e−04
0e+00
5e−04
1e−03
MR leave−one−out sensitivity analysis for
'RALBP1' on 'Bladder cancer'

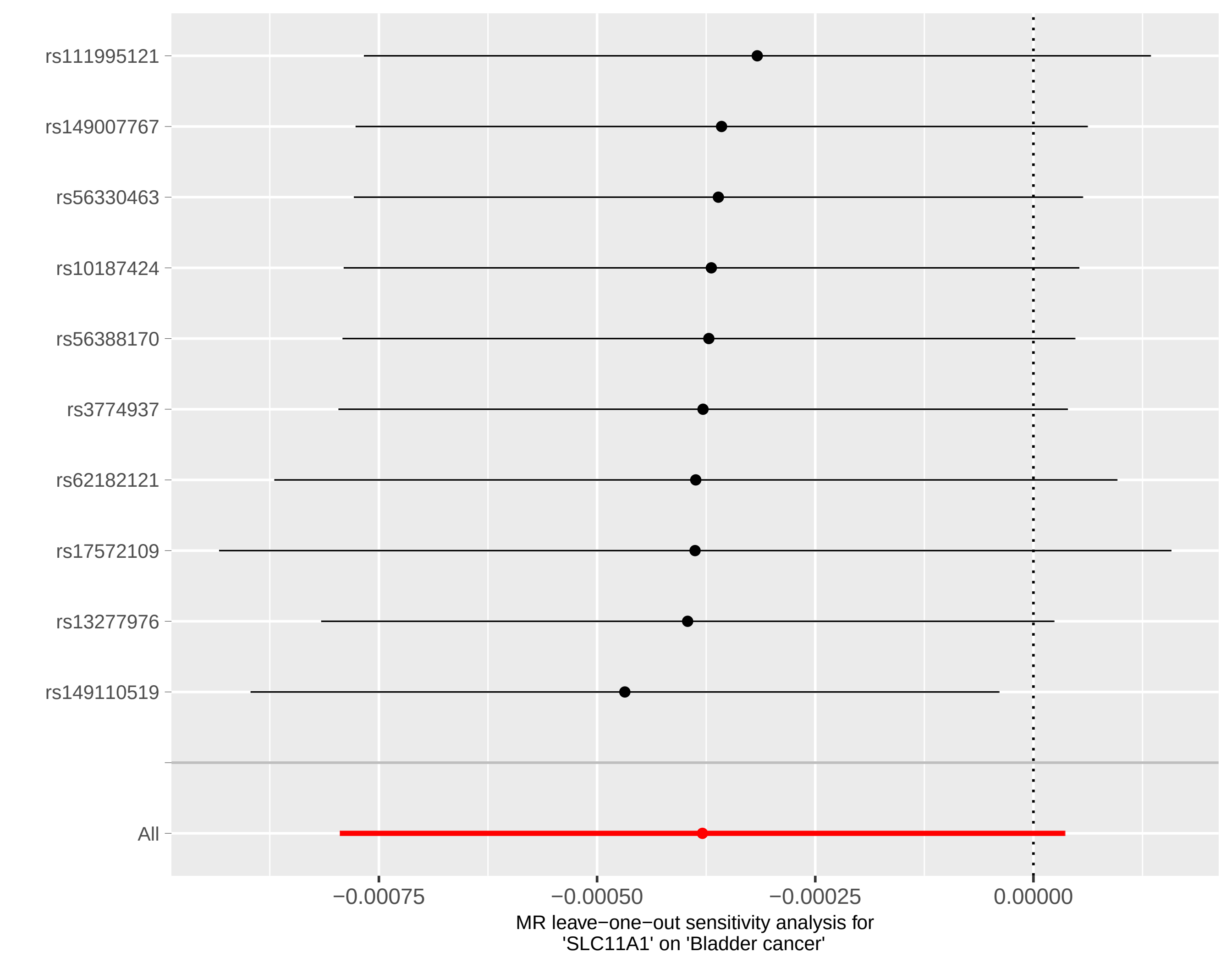
rs111995121
rs149007767
rs56330463
rs10187424
rs56388170
rs3774937
rs62182121
rs17572109
rs13277976
rs149110519
All
−0.00075
−0.00050
−0.00025
0.00000
MR leave−one−out sensitivity analysis for
'SLC11A1' on 'Bladder cancer'

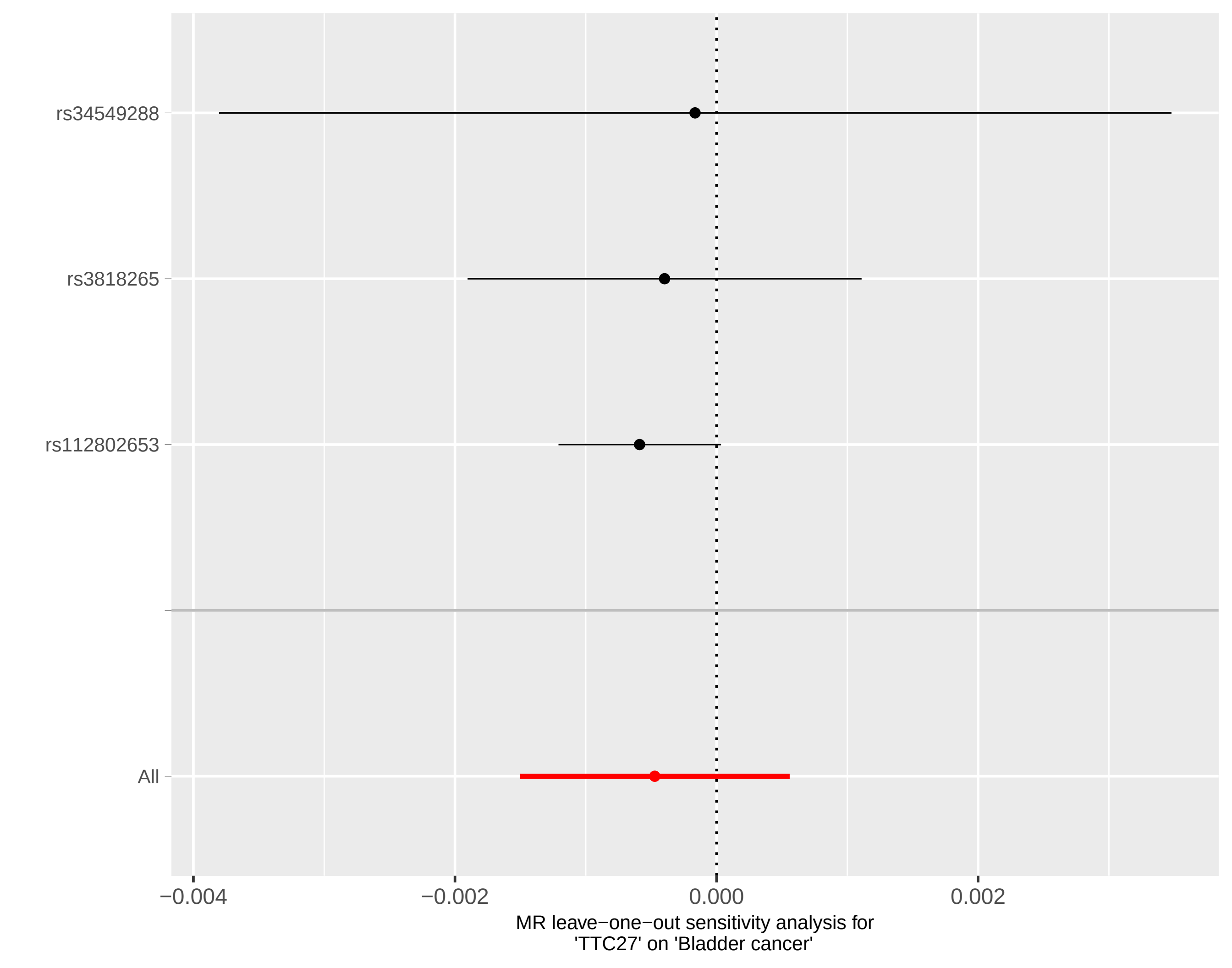
rs34549288
rs3818265
rs112802653
All
−0.004
−0.002
0.000
0.002
MR leave−one−out sensitivity analysis for
'TTC27' on 'Bladder cancer'

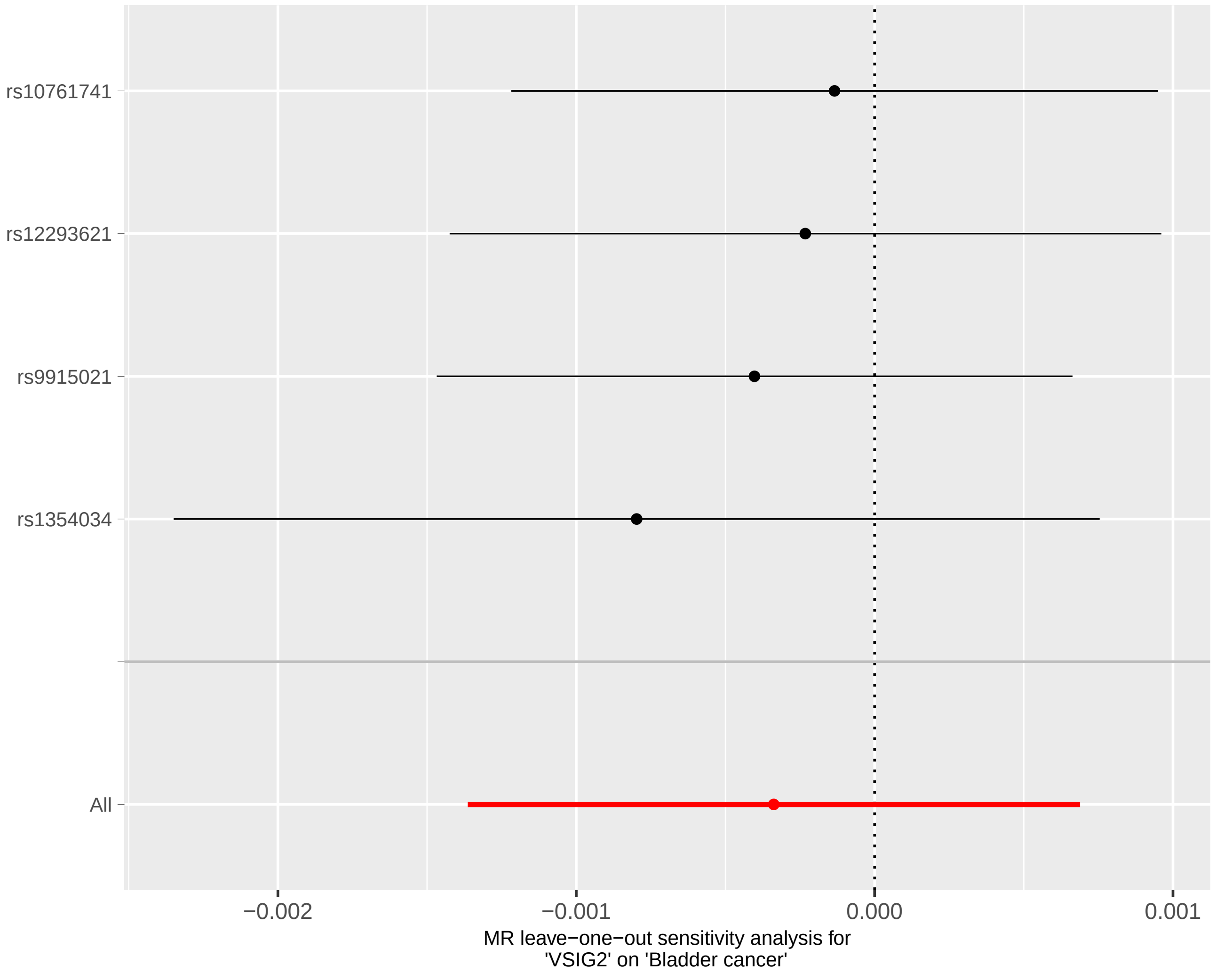
rs10761741
rs12293621
rs9915021
rs1354034
All
−0.002
−0.001
0.000
0.001
MR leave−one−out sensitivity analysis for
'VSIG2' on 'Bladder cancer'

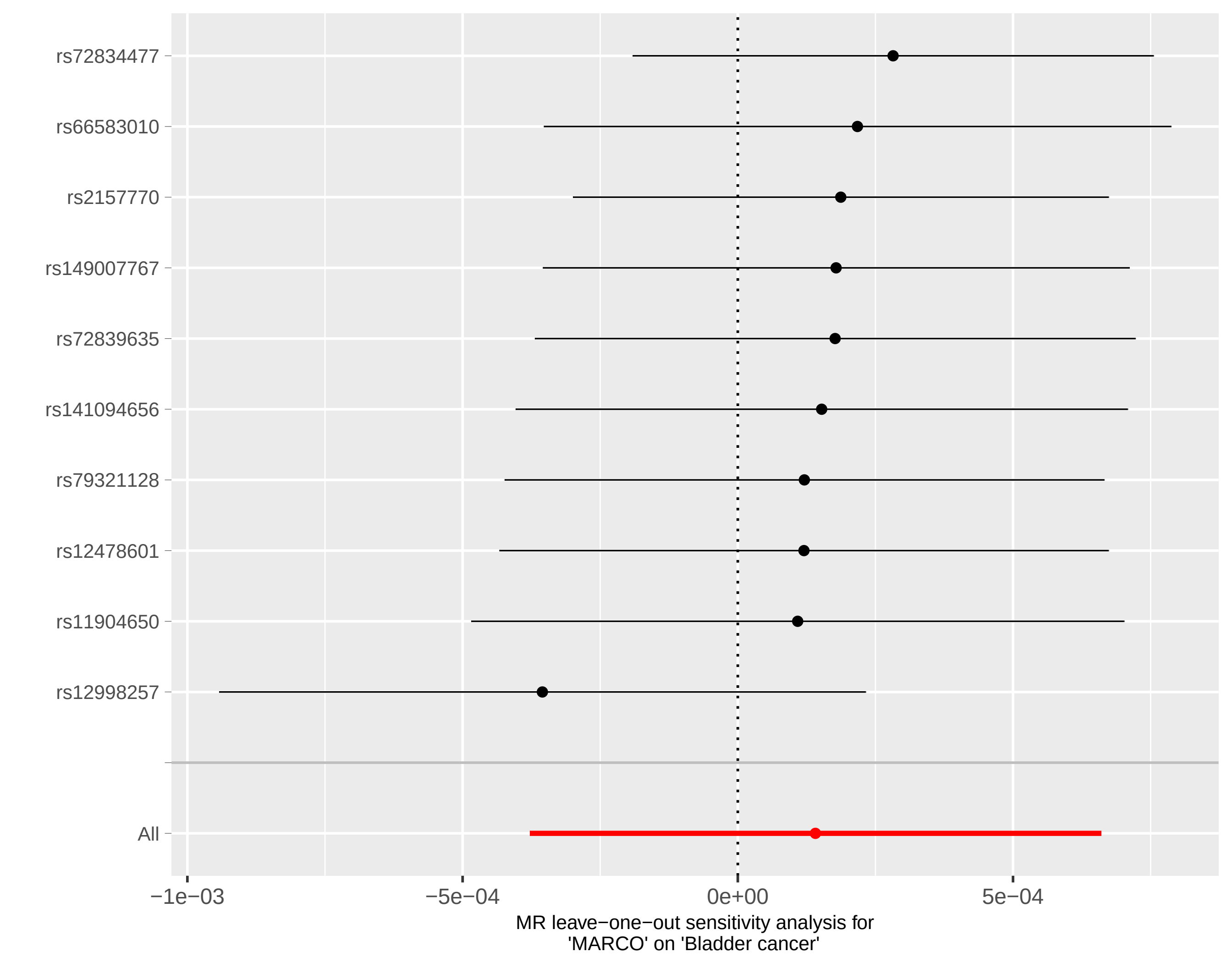
rs72834477
rs66583010
rs2157770
rs149007767
rs72839635
rs141094656
rs79321128
rs12478601
rs11904650
rs12998257
All
−1e−03
−5e−04
0e+00
5e−04
MR leave−one−out sensitivity analysis for
'MARCO' on 'Bladder cancer'

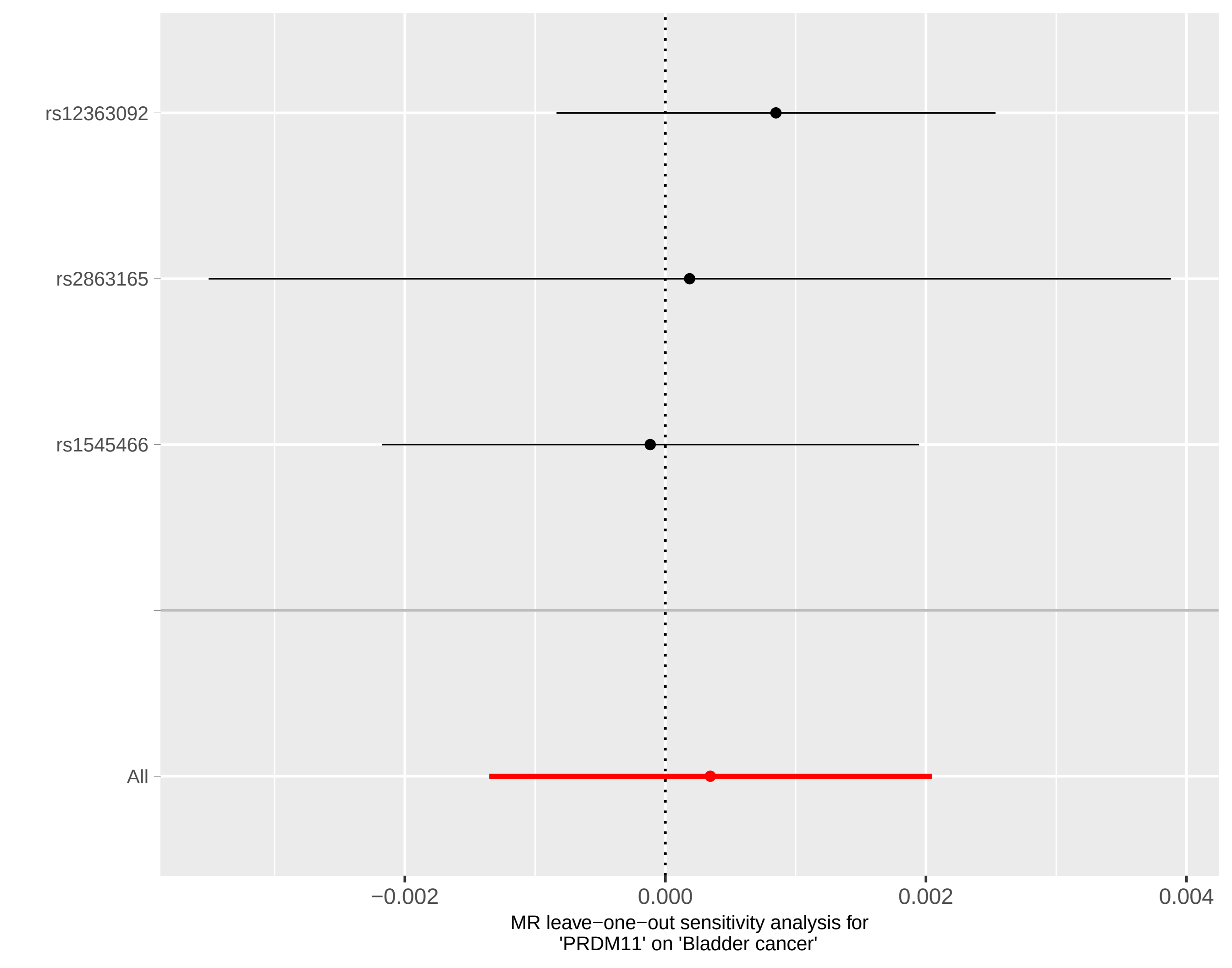
rs12363092
rs2863165
rs1545466
All
−0.002
0.000
0.002
0.004
MR leave−one−out sensitivity analysis for
'PRDM11' on 'Bladder cancer'

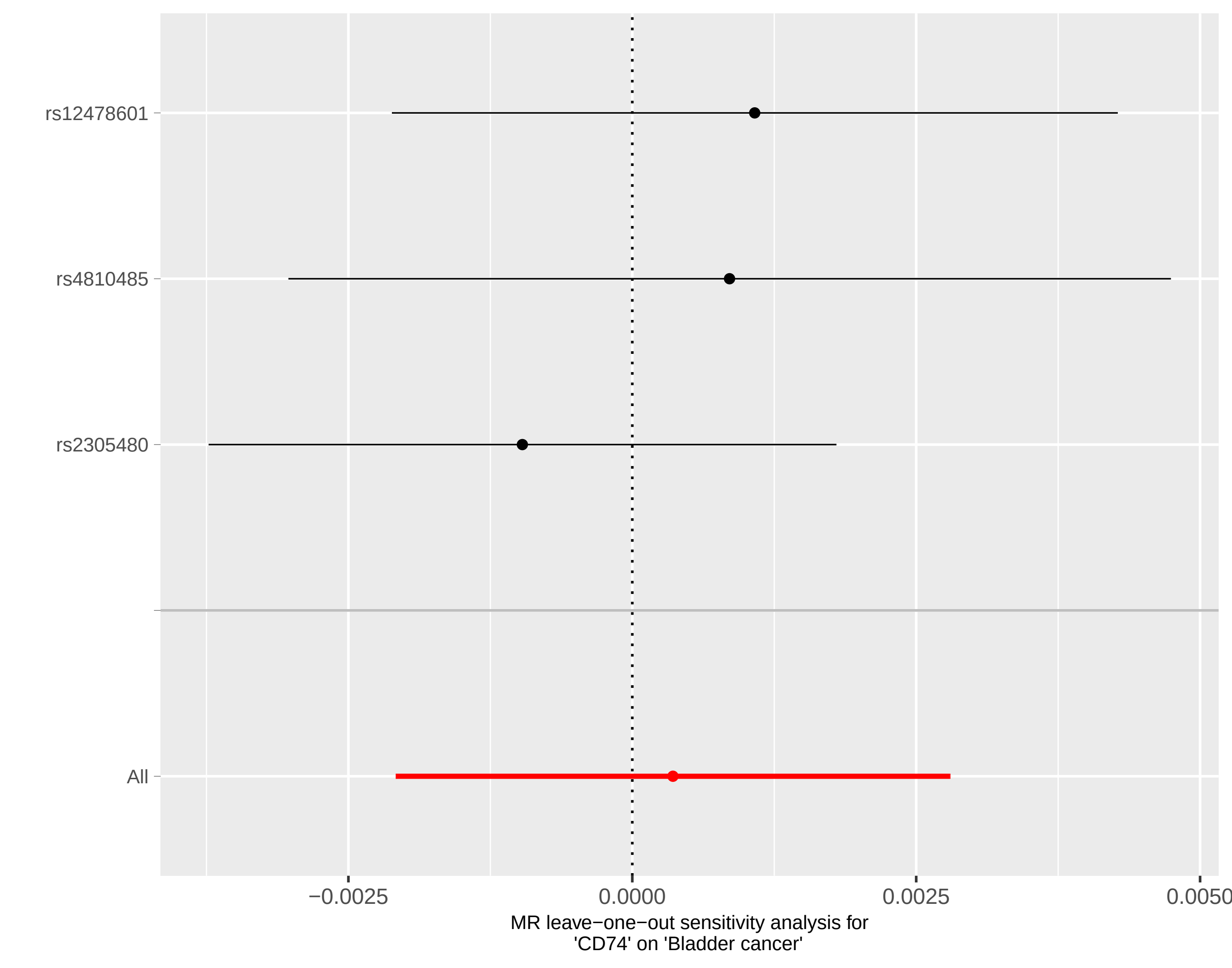
rs12478601
rs4810485
rs2305480
All
−0.0025
0.0000
0.0025
0.0050
MR leave−one−out sensitivity analysis for
'CD74' on 'Bladder cancer'

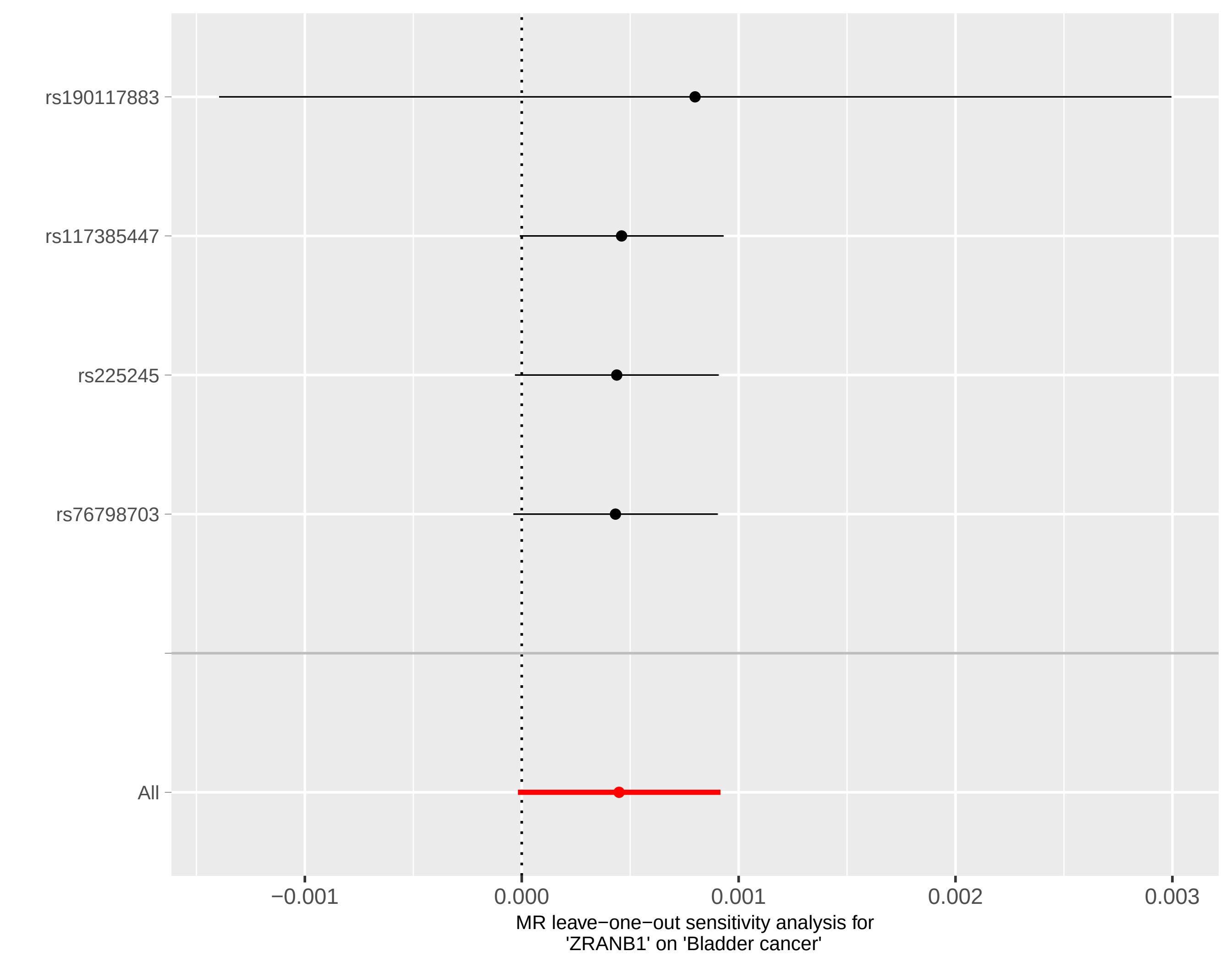
rs190117883
rs117385447
rs225245
rs76798703
All
−0.001
0.000
0.001
0.002
0.003
MR leave−one−out sensitivity analysis for
'ZRANB1' on 'Bladder cancer'

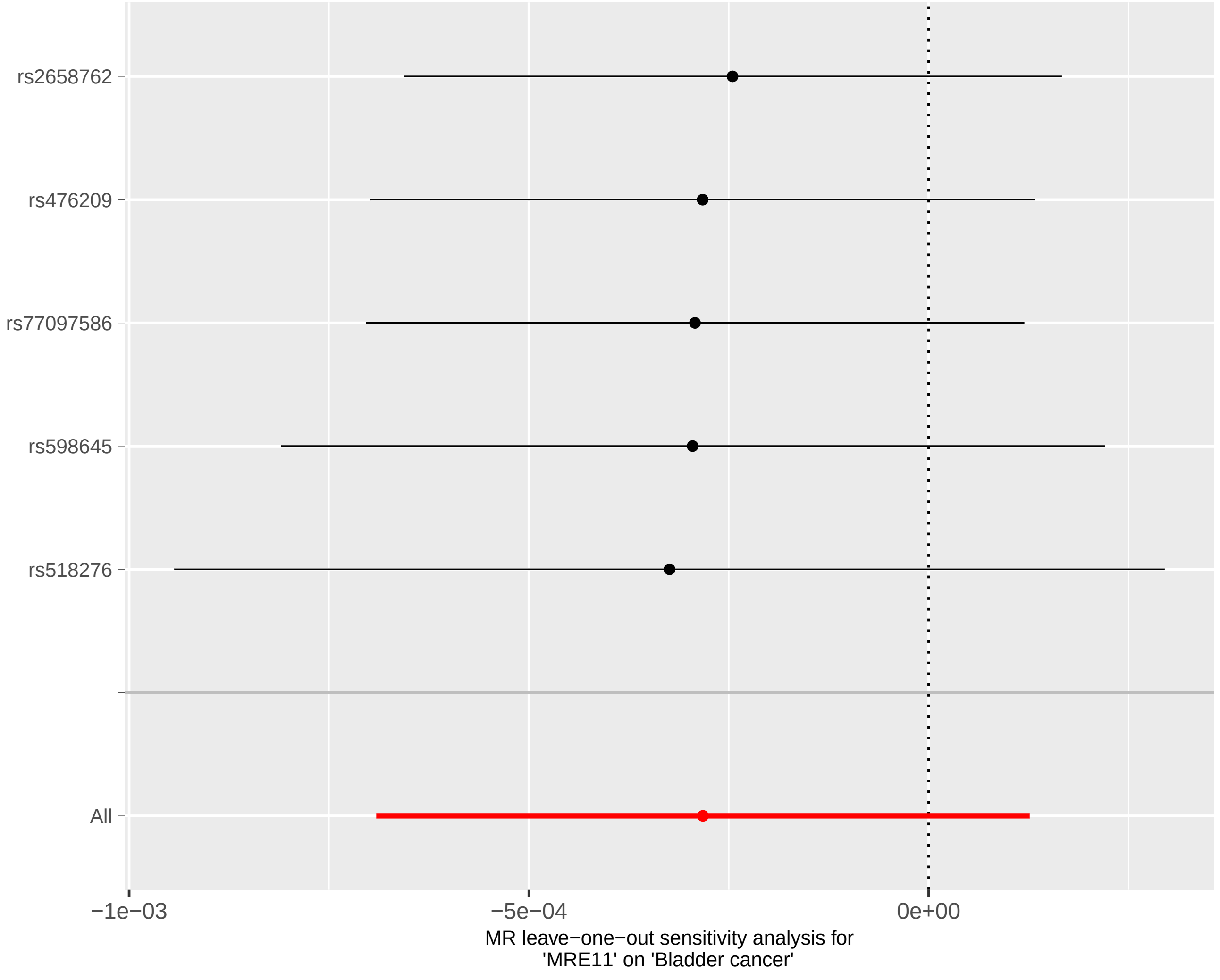
rs2658762
rs476209
rs77097586
rs598645
rs518276
All
−1e−03
−5e−04
0e+00
MR leave−one−out sensitivity analysis for
'MRE11' on 'Bladder cancer'

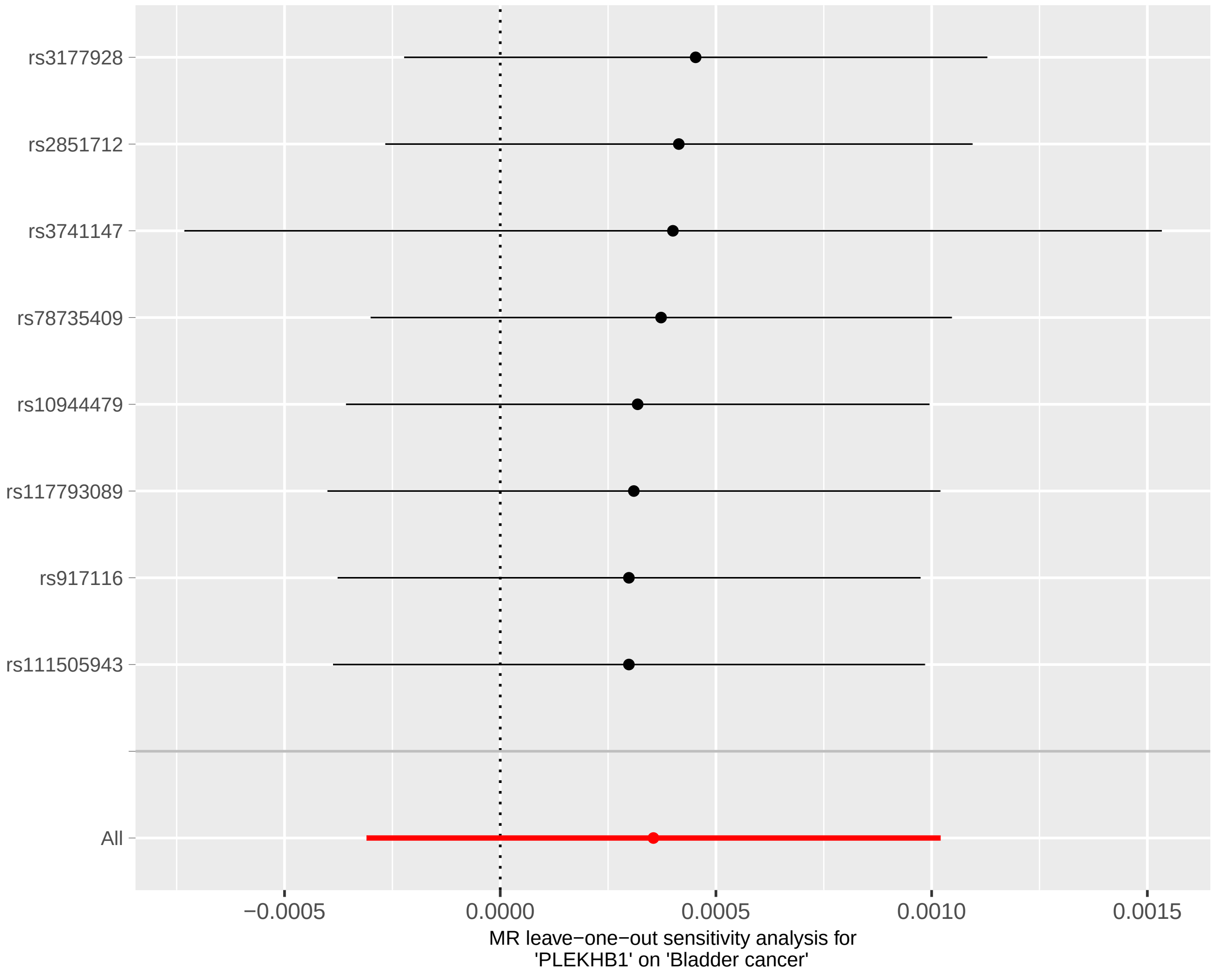
rs3177928
rs2851712
rs3741147
rs78735409
rs10944479
rs117793089
rs917116
rs111505943
All
−0.0005
0.0000
0.0005
0.0010
0.0015
MR leave−one−out sensitivity analysis for
'PLEKHB1' on 'Bladder cancer'

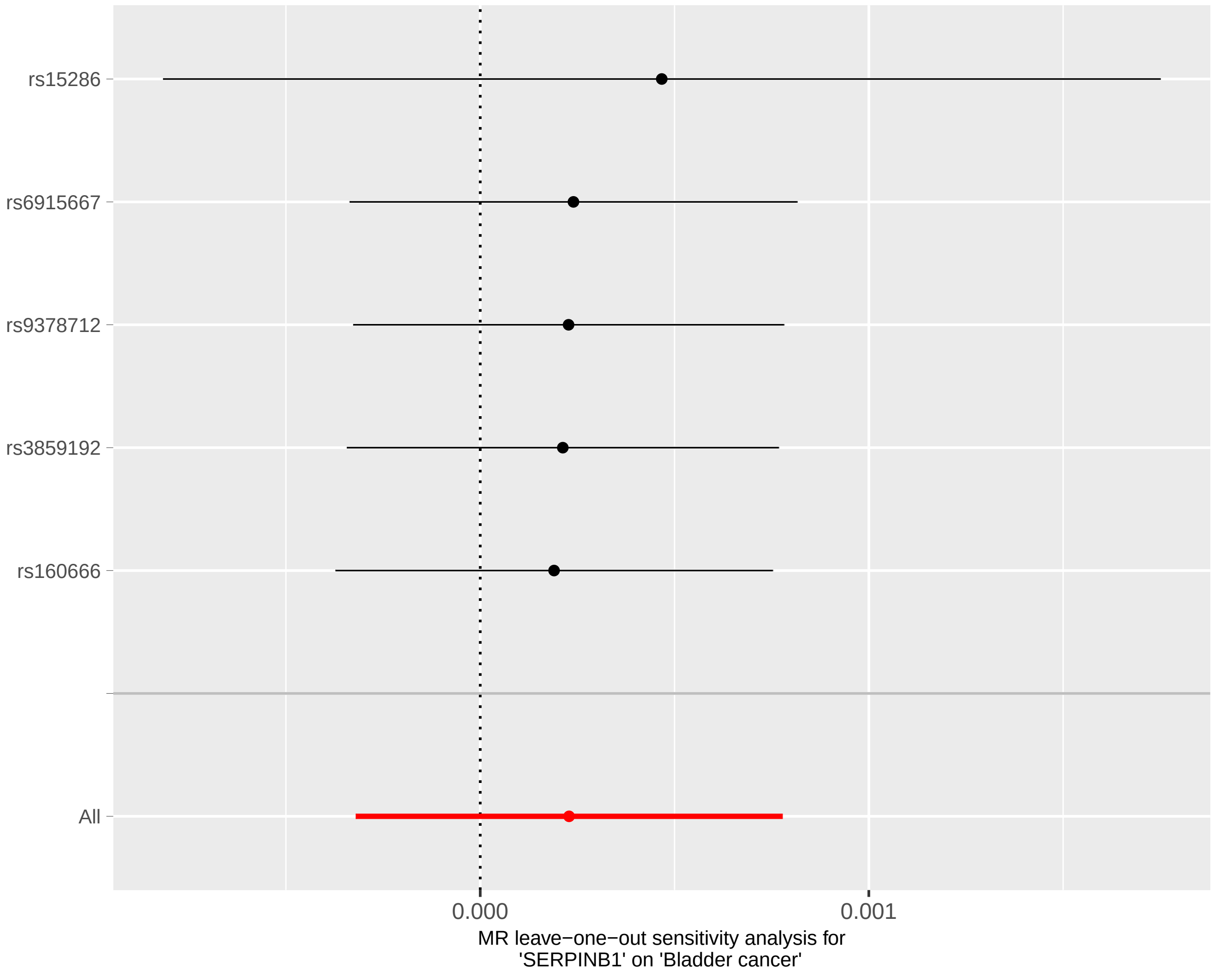
rs15286
rs6915667
rs9378712
rs3859192
rs160666
All
0.000
0.001
MR leave−one−out sensitivity analysis for
'SERPINB1' on 'Bladder cancer'

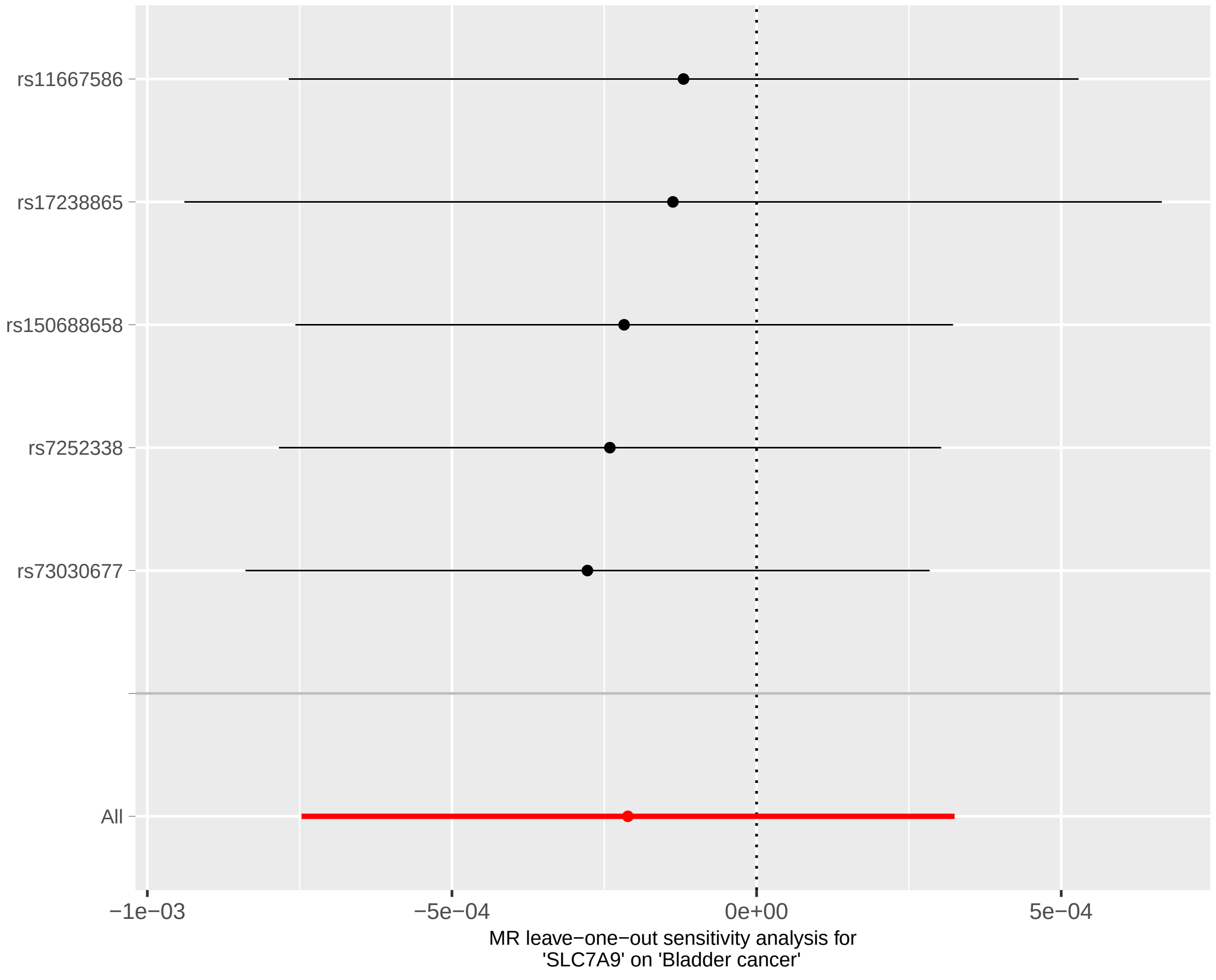
rs11667586
rs17238865
rs150688658
rs7252338
rs73030677
All
−1e−03
−5e−04
0e+00
5e−04
MR leave−one−out sensitivity analysis for
'SLC7A9' on 'Bladder cancer'

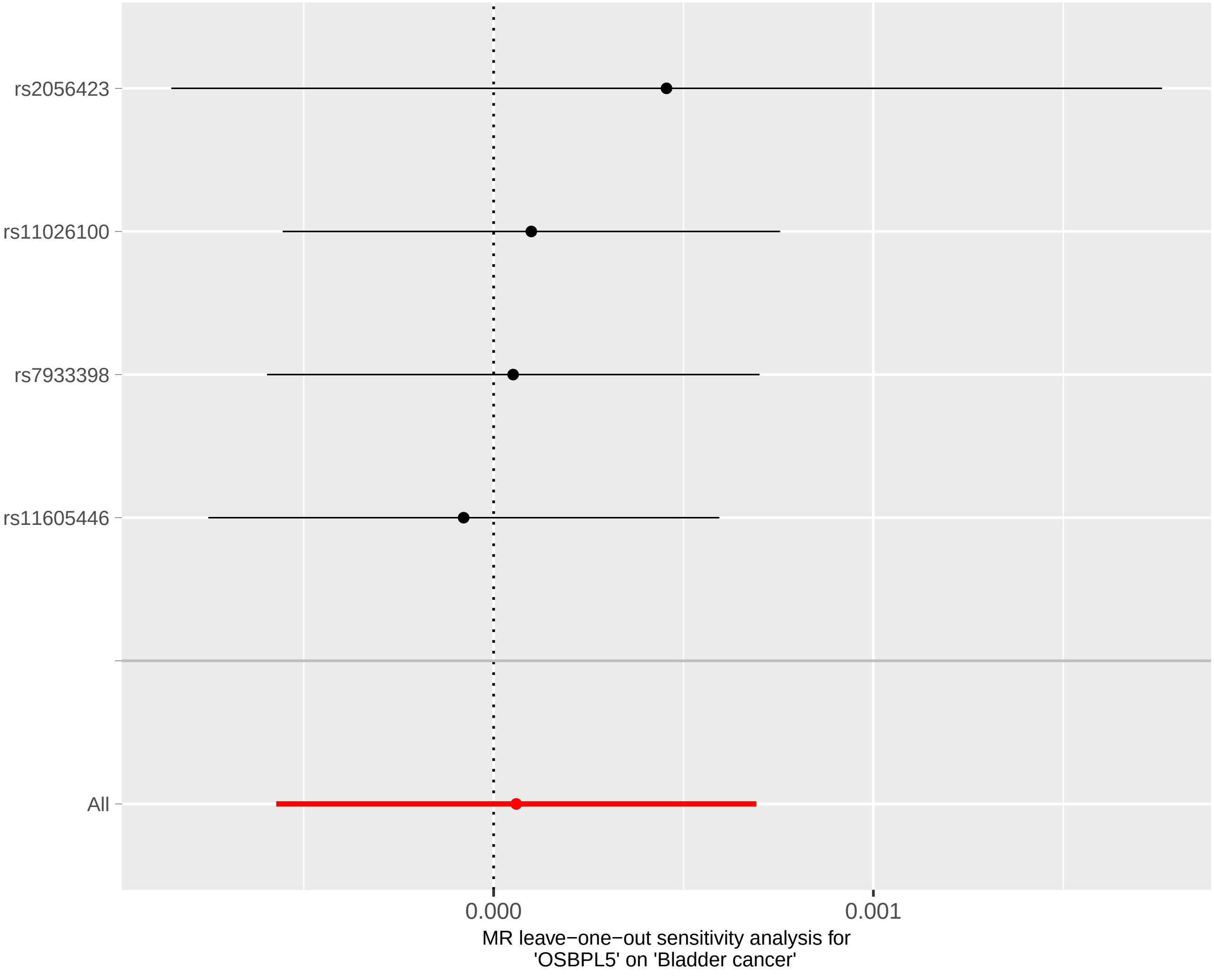

rs2056423
rs11026100
rs7933398
rs11605446
All
0.000
0.001
MR leave−one−out sensitivity analysis for
'OSBPL5' on 'Bladder cancer'

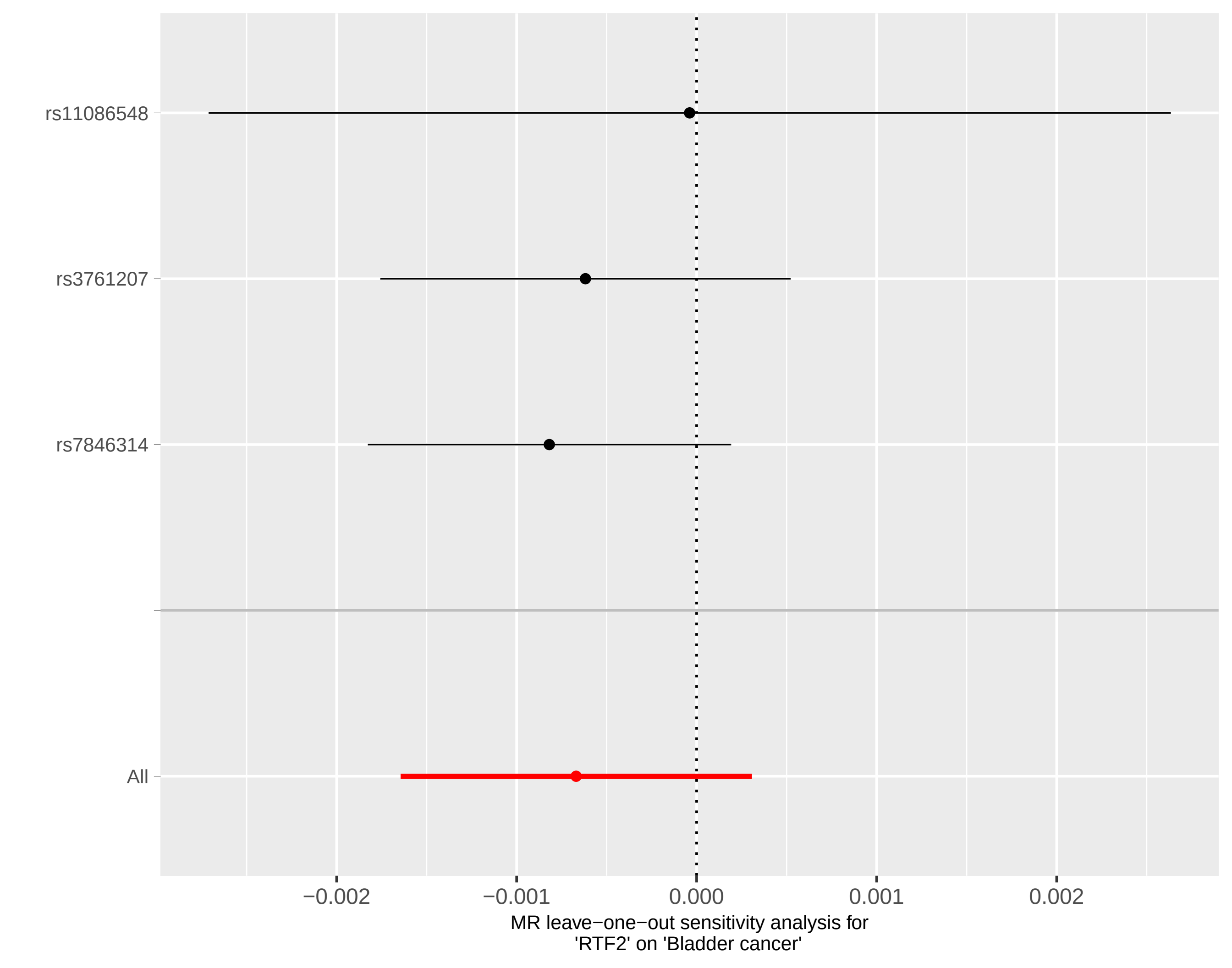

rs11086548
rs3761207
rs7846314
All
−0.002
−0.001
0.000
0.001
0.002
MR leave−one−out sensitivity analysis for
'RTF2' on 'Bladder cancer'

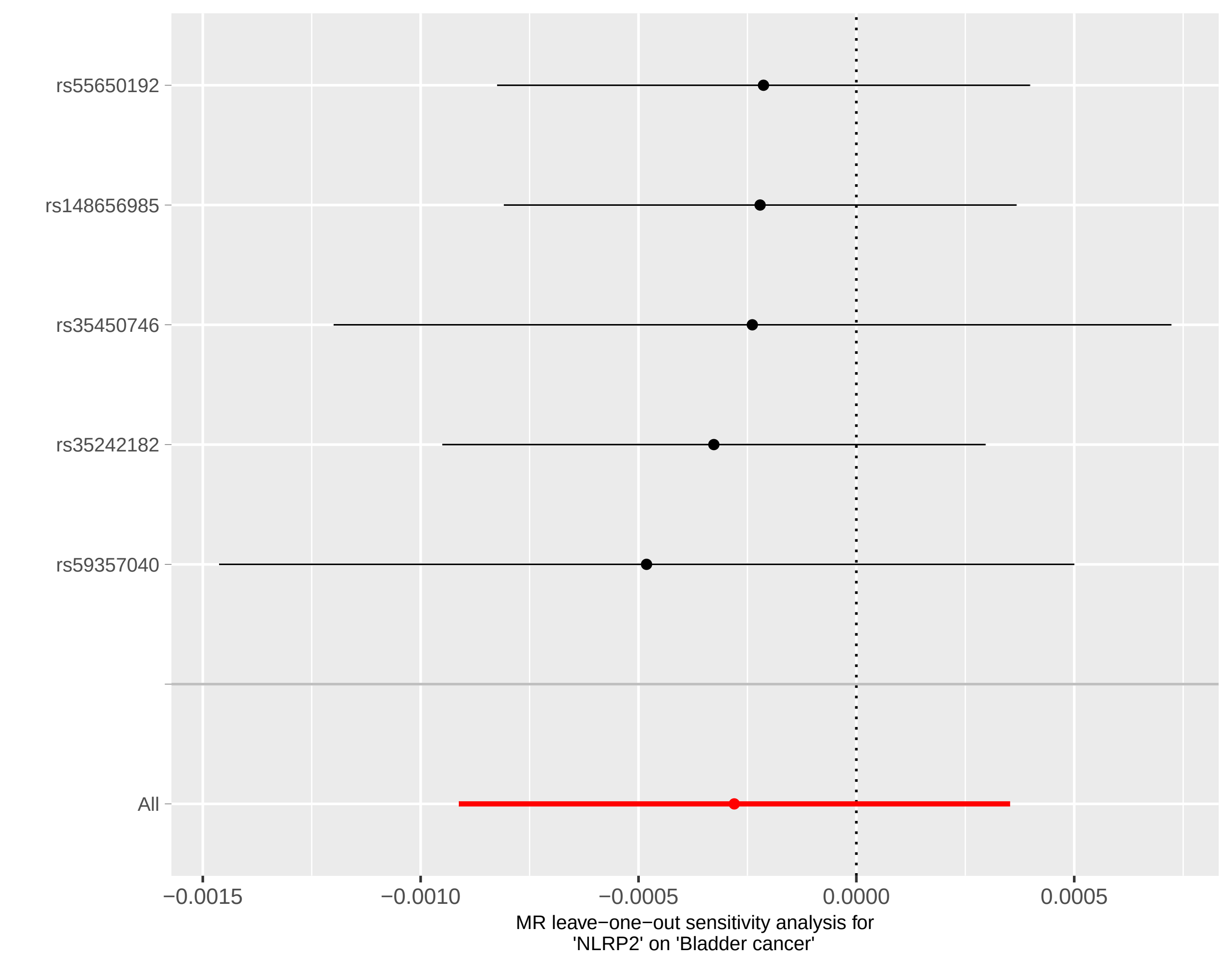
rs55650192
rs148656985
rs35450746
rs35242182
rs59357040
All
−0.0015
−0.0010
−0.0005
0.0000
0.0005
MR leave−one−out sensitivity analysis for
'NLRP2' on 'Bladder cancer'

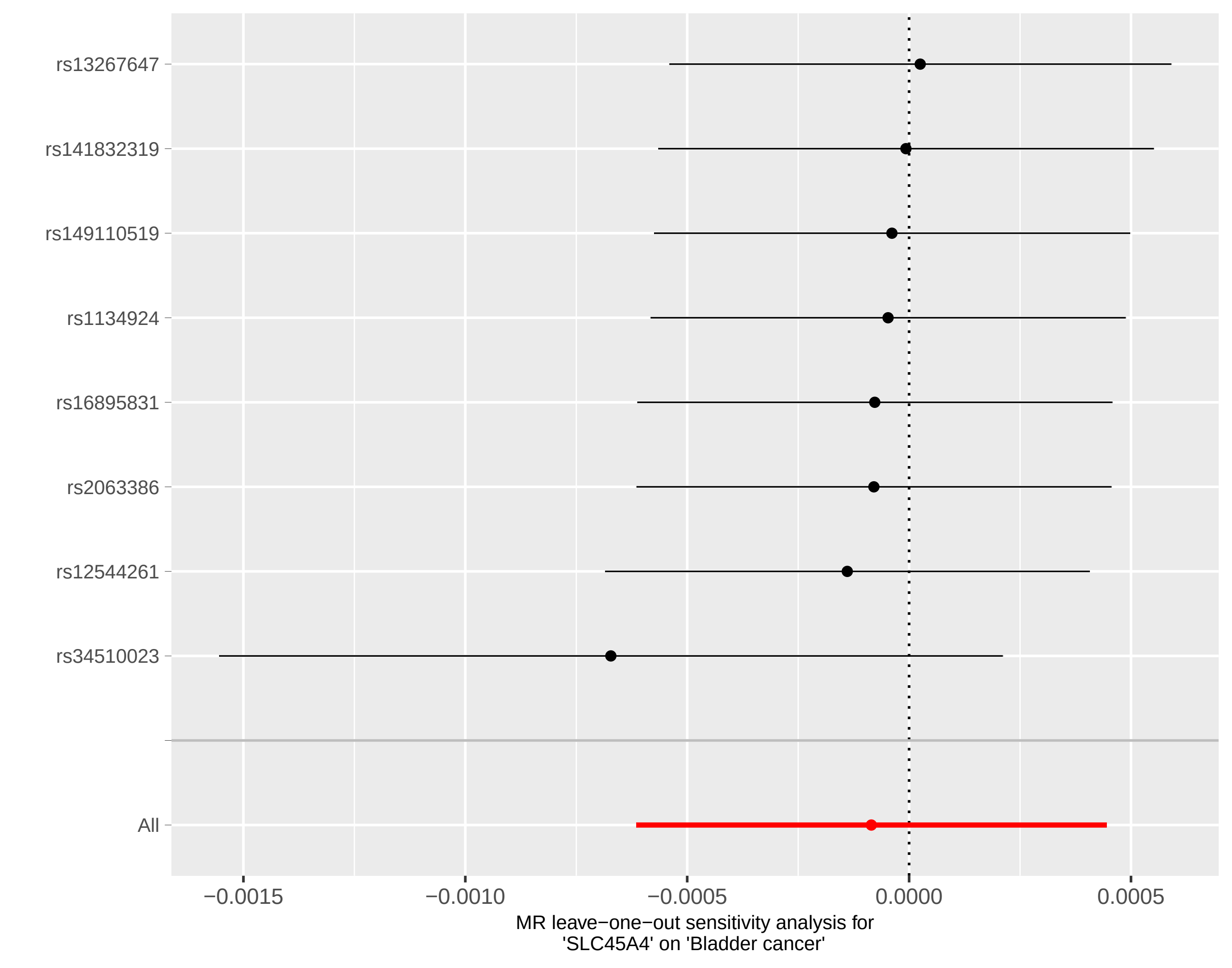
rs13267647
rs141832319
rs149110519
rs1134924
rs16895831
rs2063386
rs12544261
rs34510023
All
−0.0015
−0.0010
−0.0005
0.0000
0.0005
MR leave−one−out sensitivity analysis for
'SLC45A4' on 'Bladder cancer'

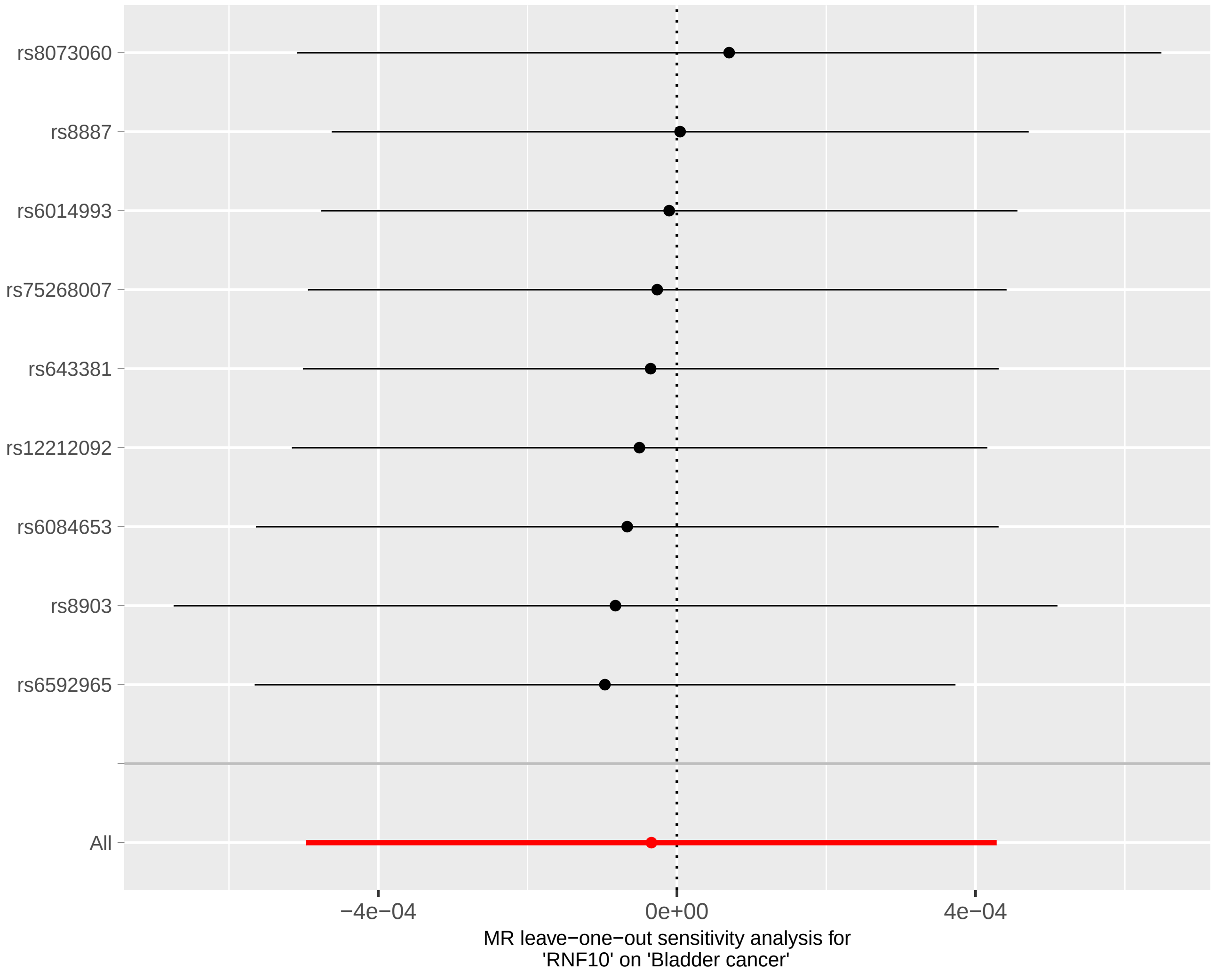
rs8073060
rs8887
rs6014993
rs75268007
rs643381
rs12212092
rs6084653
rs8903
rs6592965
All
−4e−04
0e+00
4e−04
MR leave−one−out sensitivity analysis for
'RNF10' on 'Bladder cancer'

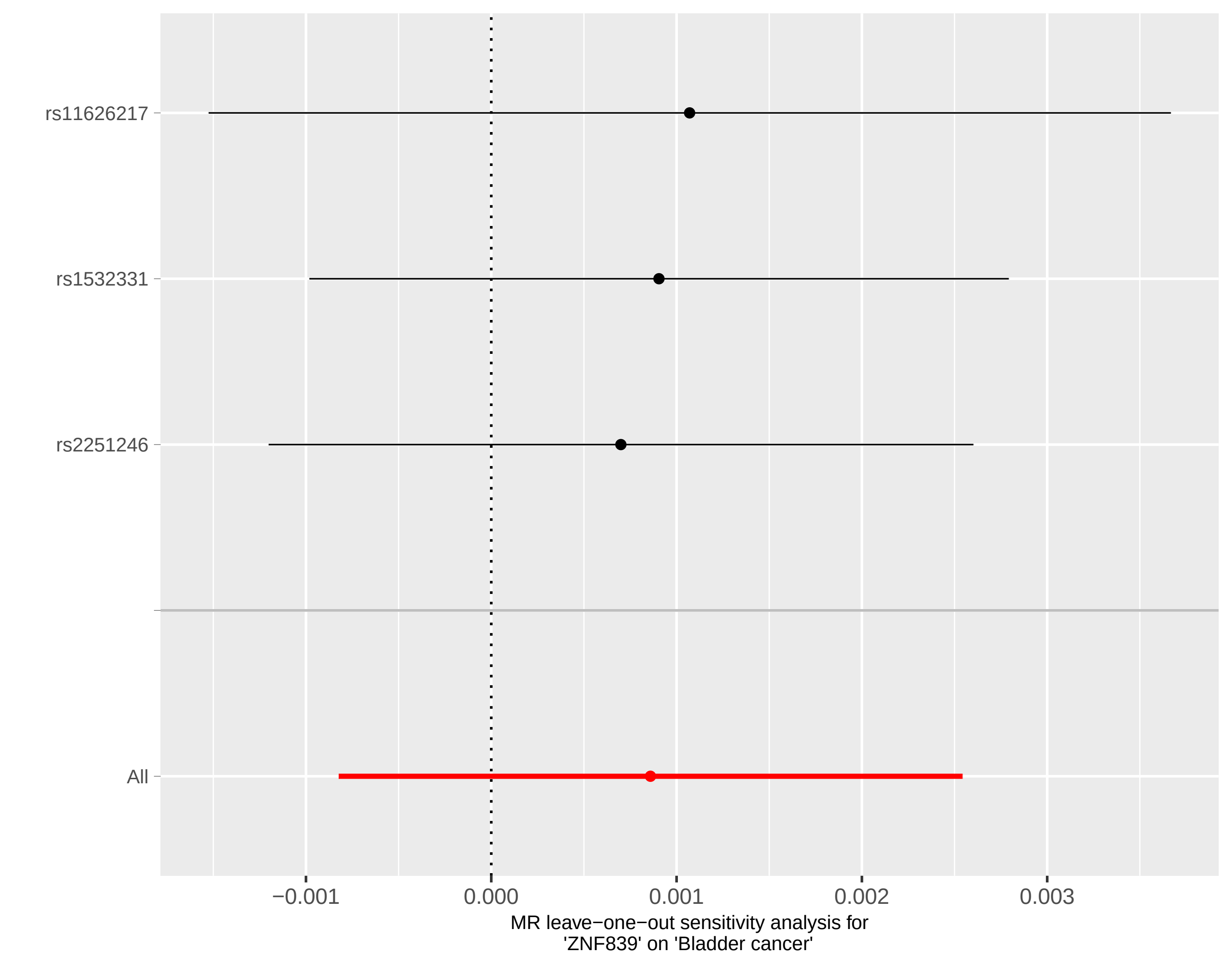
rs11626217
rs1532331
rs2251246
All
−0.001
0.000
0.001
0.002
0.003
MR leave−one−out sensitivity analysis for
'ZNF839' on 'Bladder cancer'

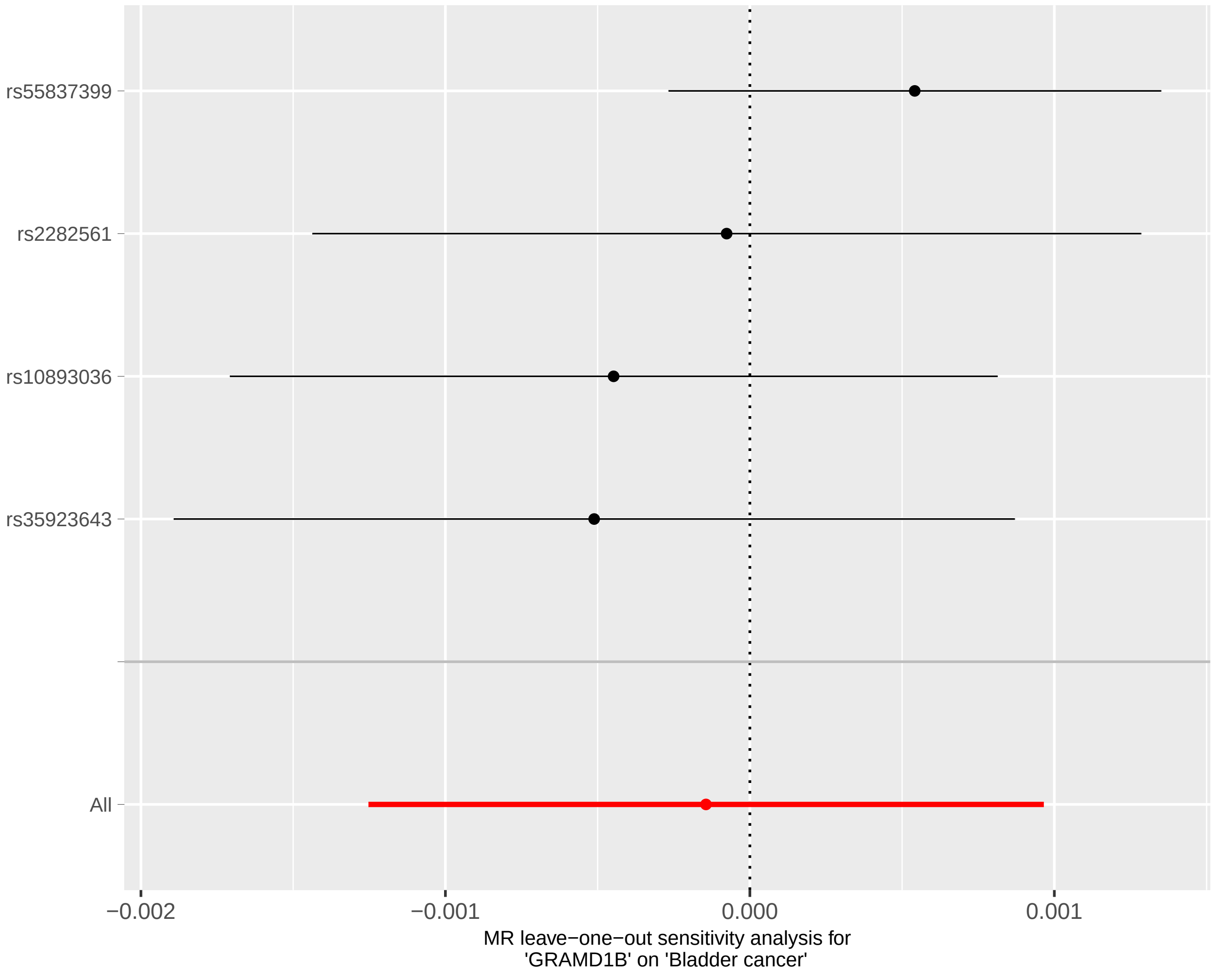
rs55837399
rs2282561
rs10893036
rs35923643
All
−0.002
−0.001
0.000
0.001
MR leave−one−out sensitivity analysis for
'GRAMD1B' on 'Bladder cancer'

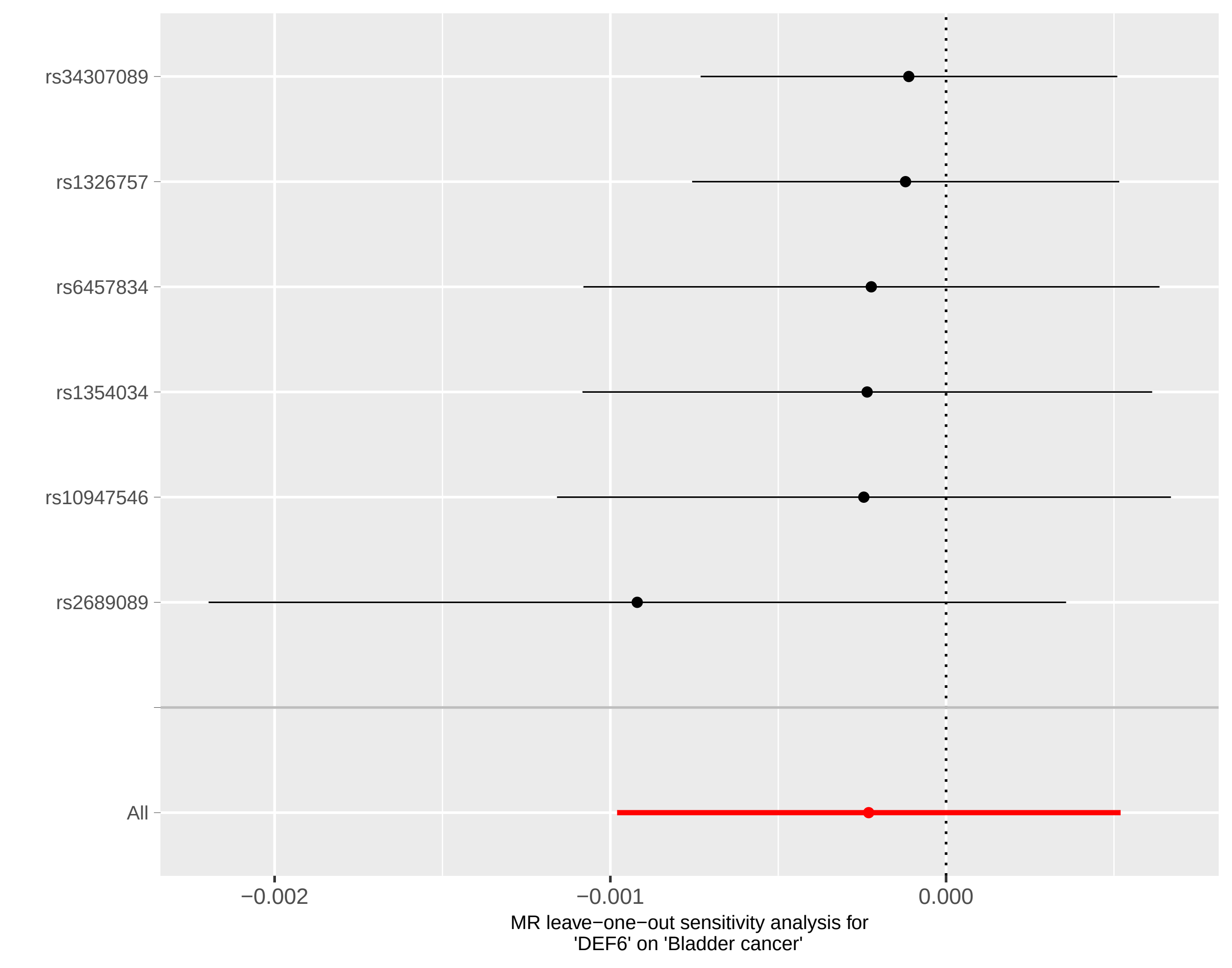
rs34307089
rs1326757
rs6457834
rs1354034
rs10947546
rs2689089
All
−0.002
−0.001
0.000
MR leave−one−out sensitivity analysis for
'DEF6' on 'Bladder cancer'

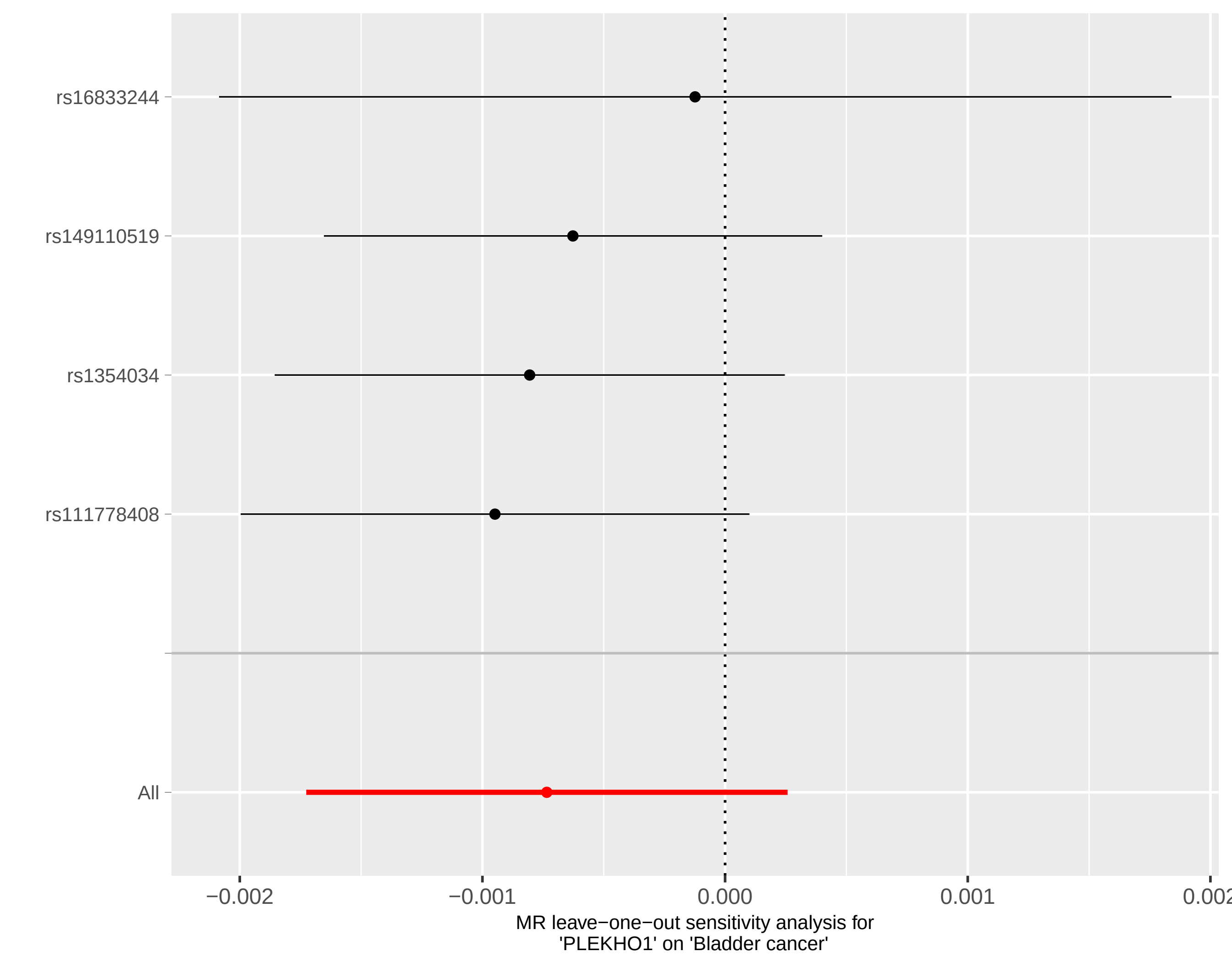
rs16833244
rs149110519
rs1354034
rs111778408
All
−0.002
−0.001
0.000
0.001
0.002
MR leave−one−out sensitivity analysis for
'PLEKHO1' on 'Bladder cancer'

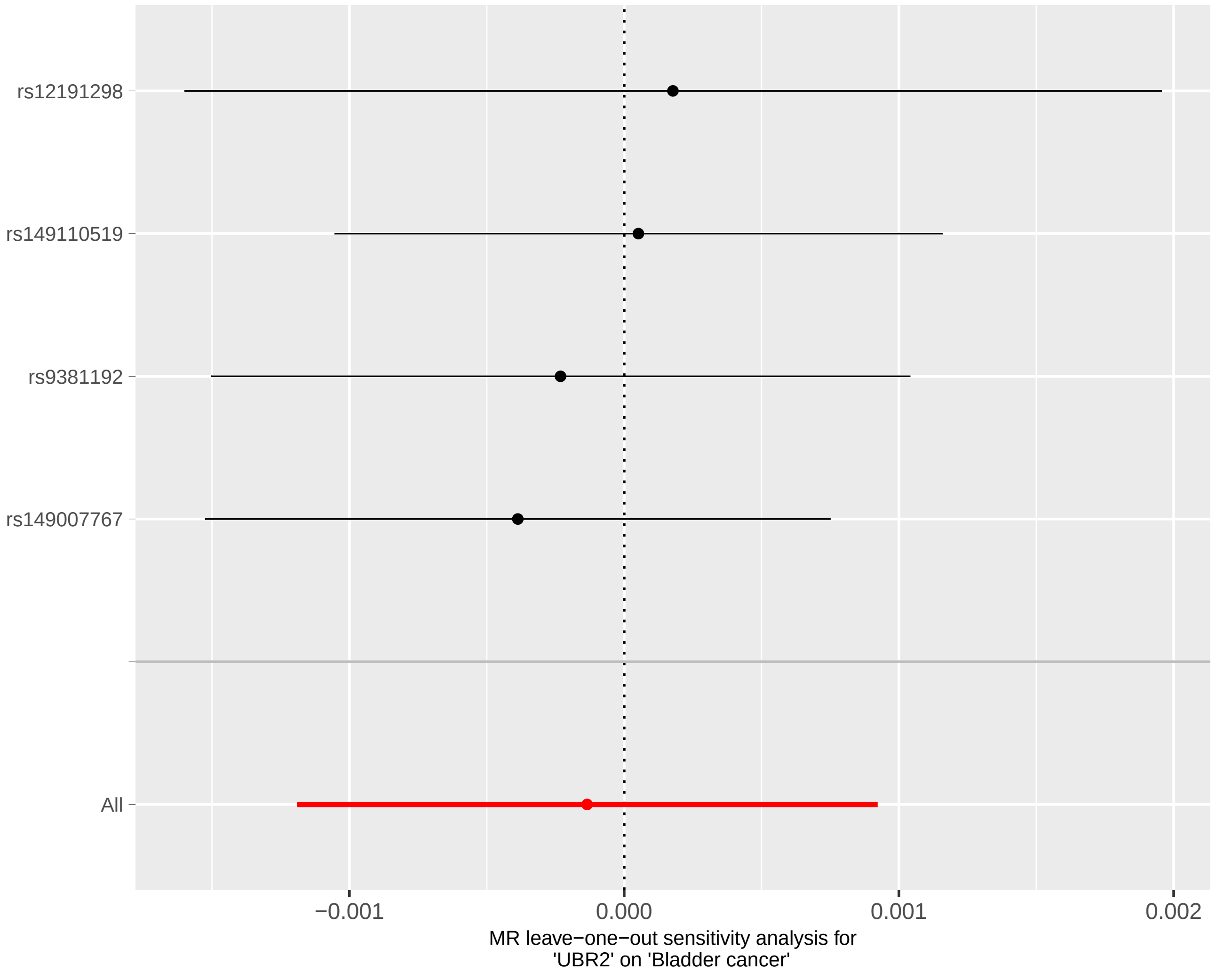
rs12191298
rs149110519
rs9381192
rs149007767
All
−0.001
0.000
0.001
0.002
MR leave−one−out sensitivity analysis for
'UBR2' on 'Bladder cancer'

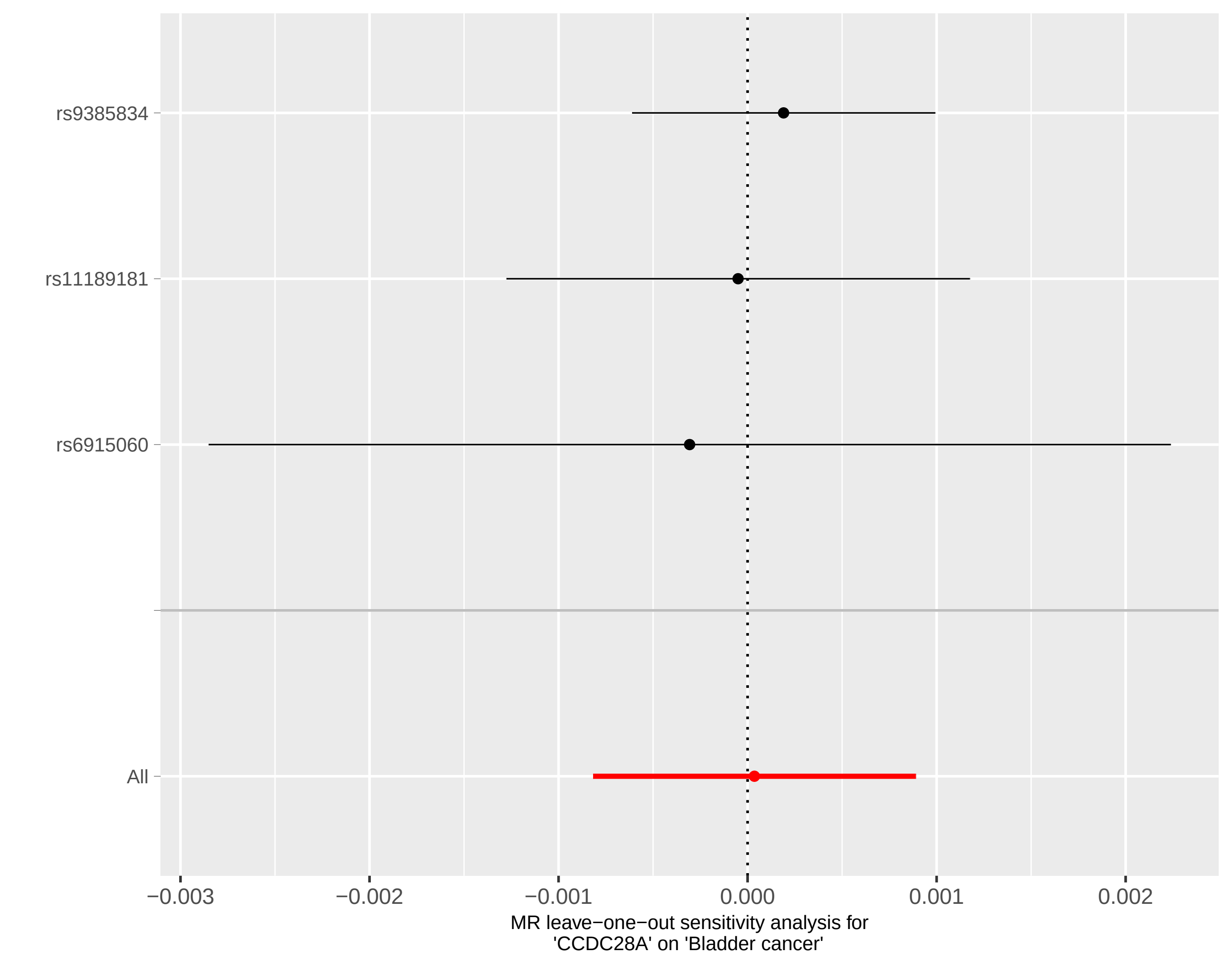
rs9385834
rs11189181
rs6915060
All
−0.003
−0.002
−0.001
0.000
0.001
0.002
MR leave−one−out sensitivity analysis for
'CCDC28A' on 'Bladder cancer'

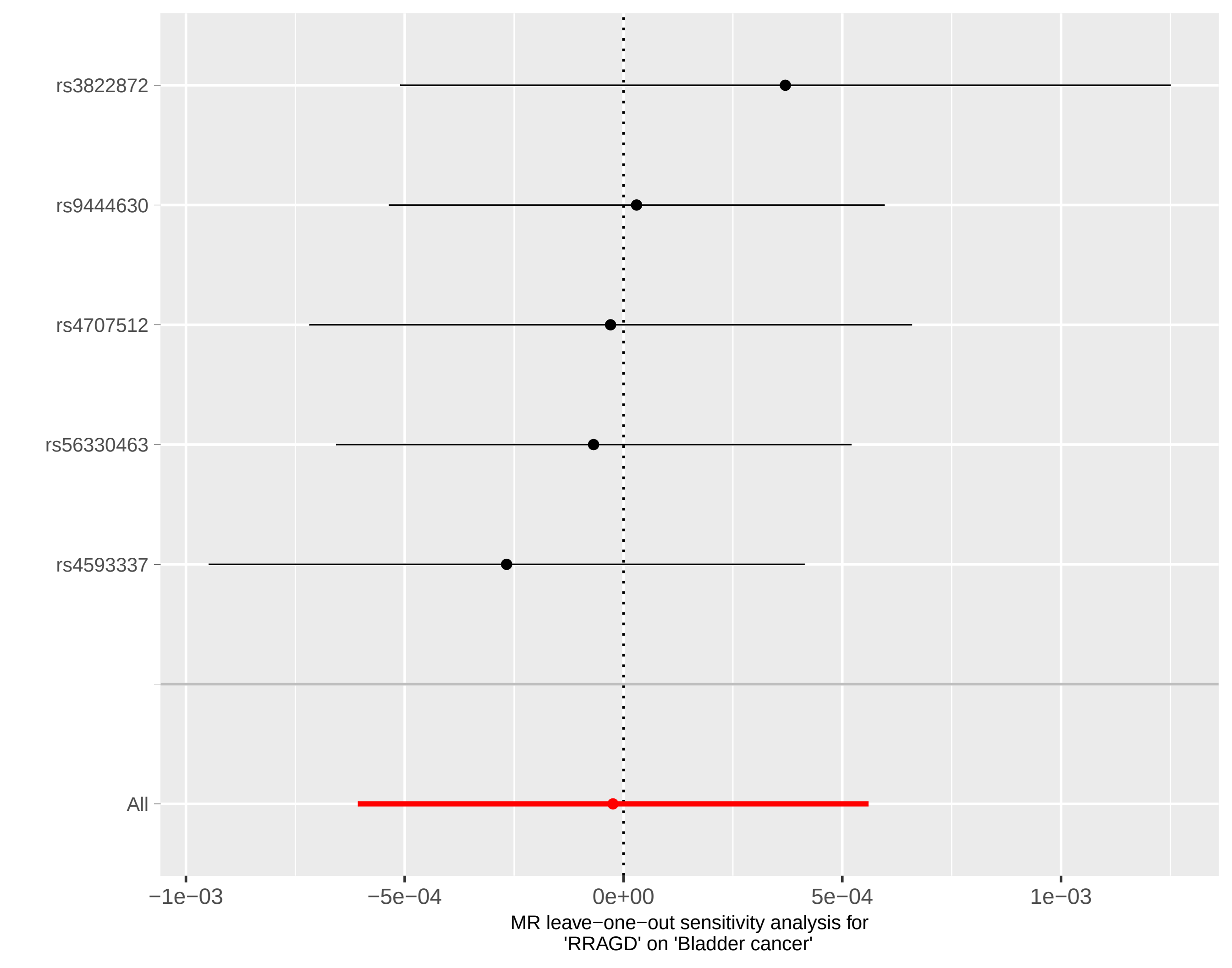
rs3822872
rs9444630
rs4707512
rs56330463
rs4593337
All
−1e−03
−5e−04
0e+00
5e−04
1e−03
MR leave−one−out sensitivity analysis for
'RRAGD' on 'Bladder cancer'

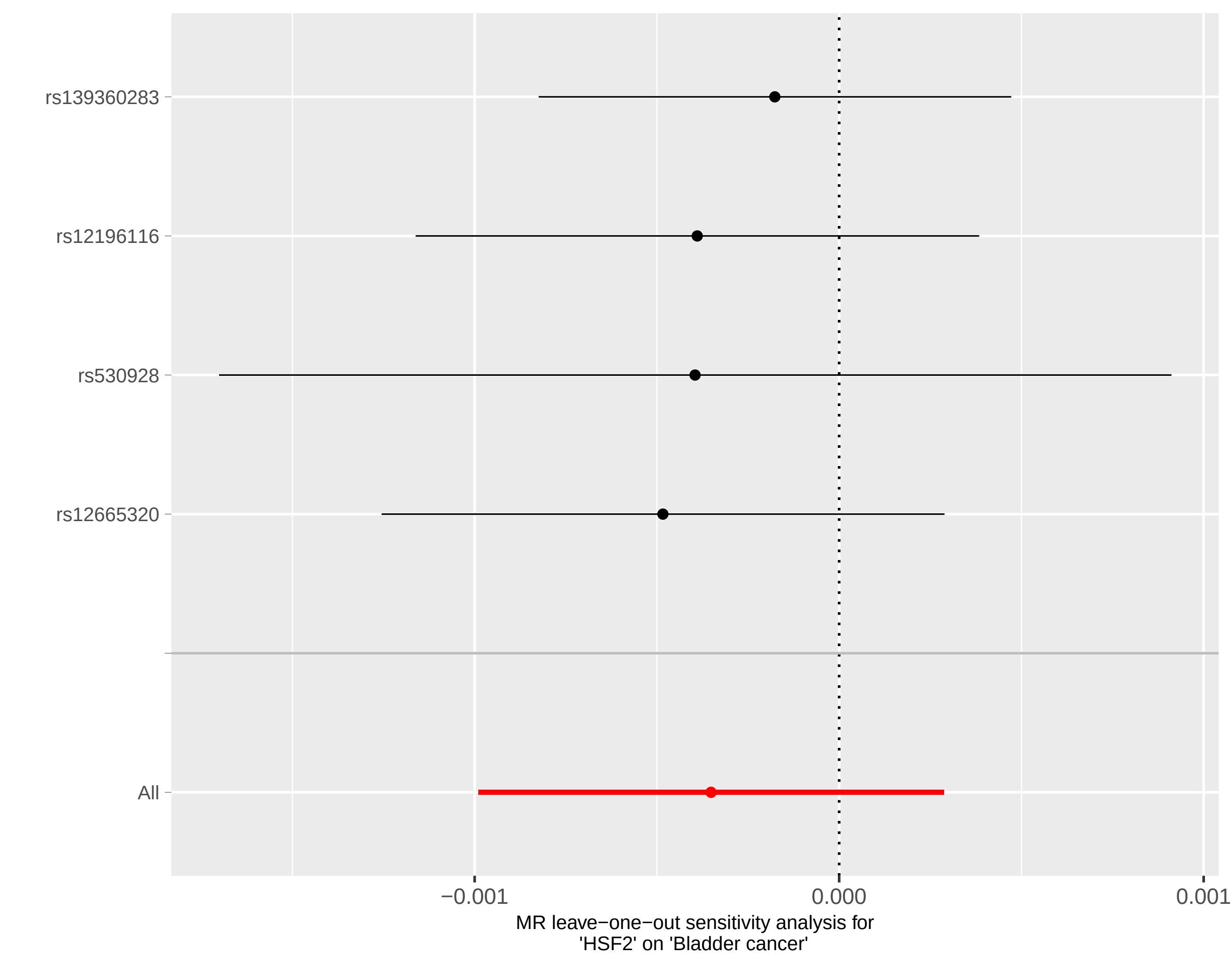
rs139360283
rs12196116
rs530928
rs12665320
All
−0.001
0.000
0.001
MR leave−one−out sensitivity analysis for
'HSF2' on 'Bladder cancer'

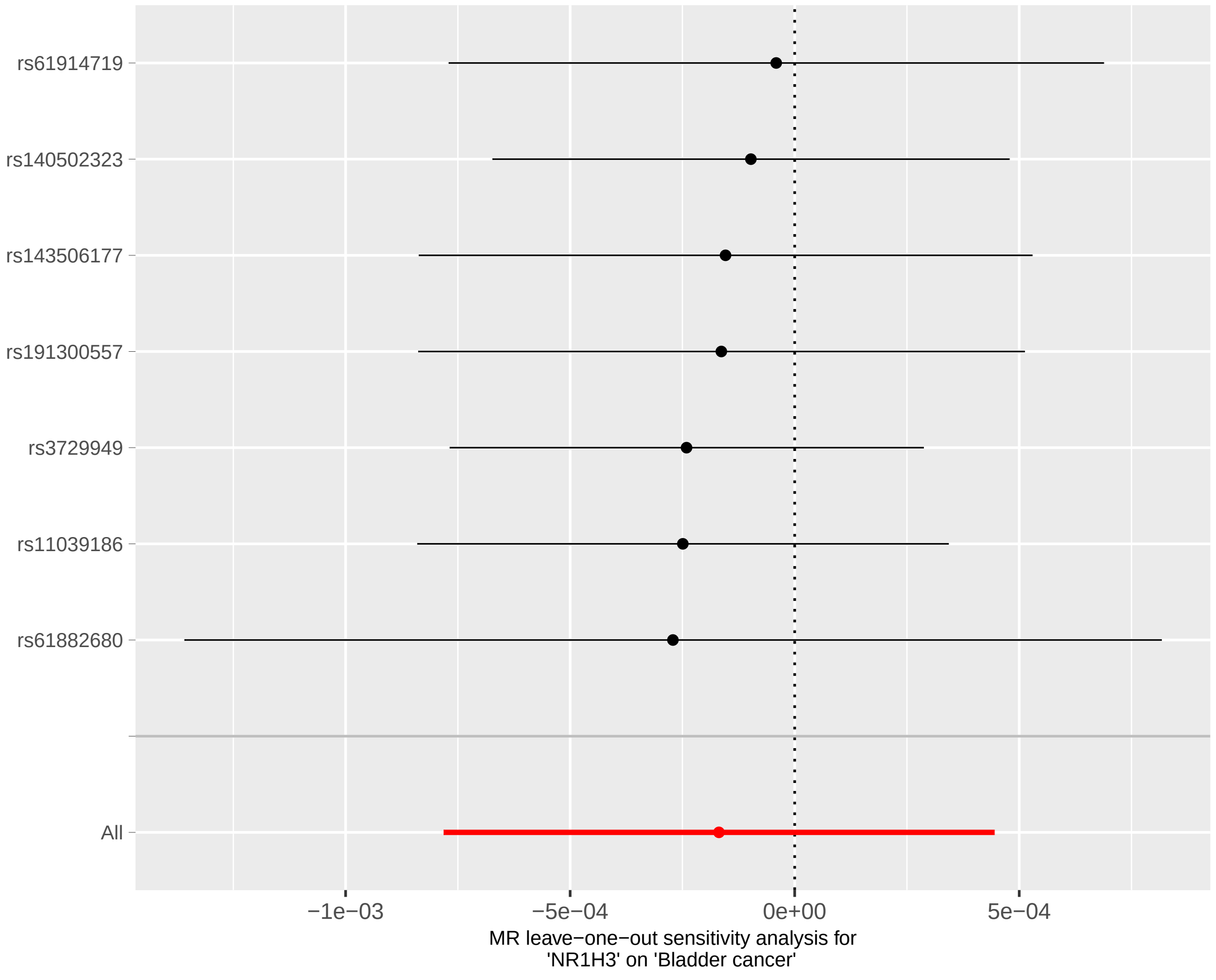
rs61914719
rs140502323
rs143506177
rs191300557
rs3729949
rs11039186
rs61882680
All
−1e−03
−5e−04
0e+00
5e−04
MR leave−one−out sensitivity analysis for
'NR1H3' on 'Bladder cancer'

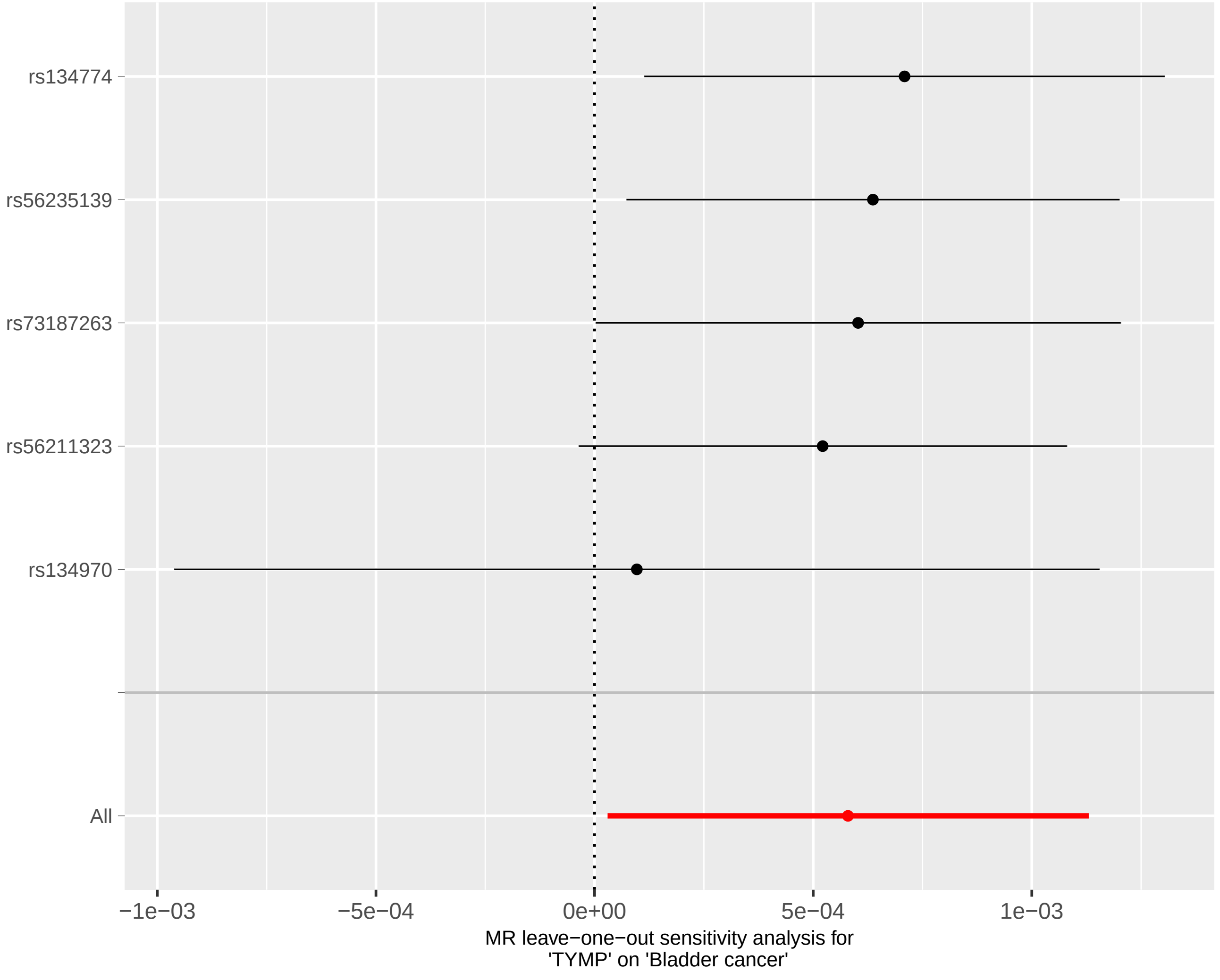
rs134774
rs56235139
rs73187263
rs56211323
rs134970
All
−1e−03
−5e−04
0e+00
5e−04
1e−03
MR leave−one−out sensitivity analysis for
'TYMP' on 'Bladder cancer'

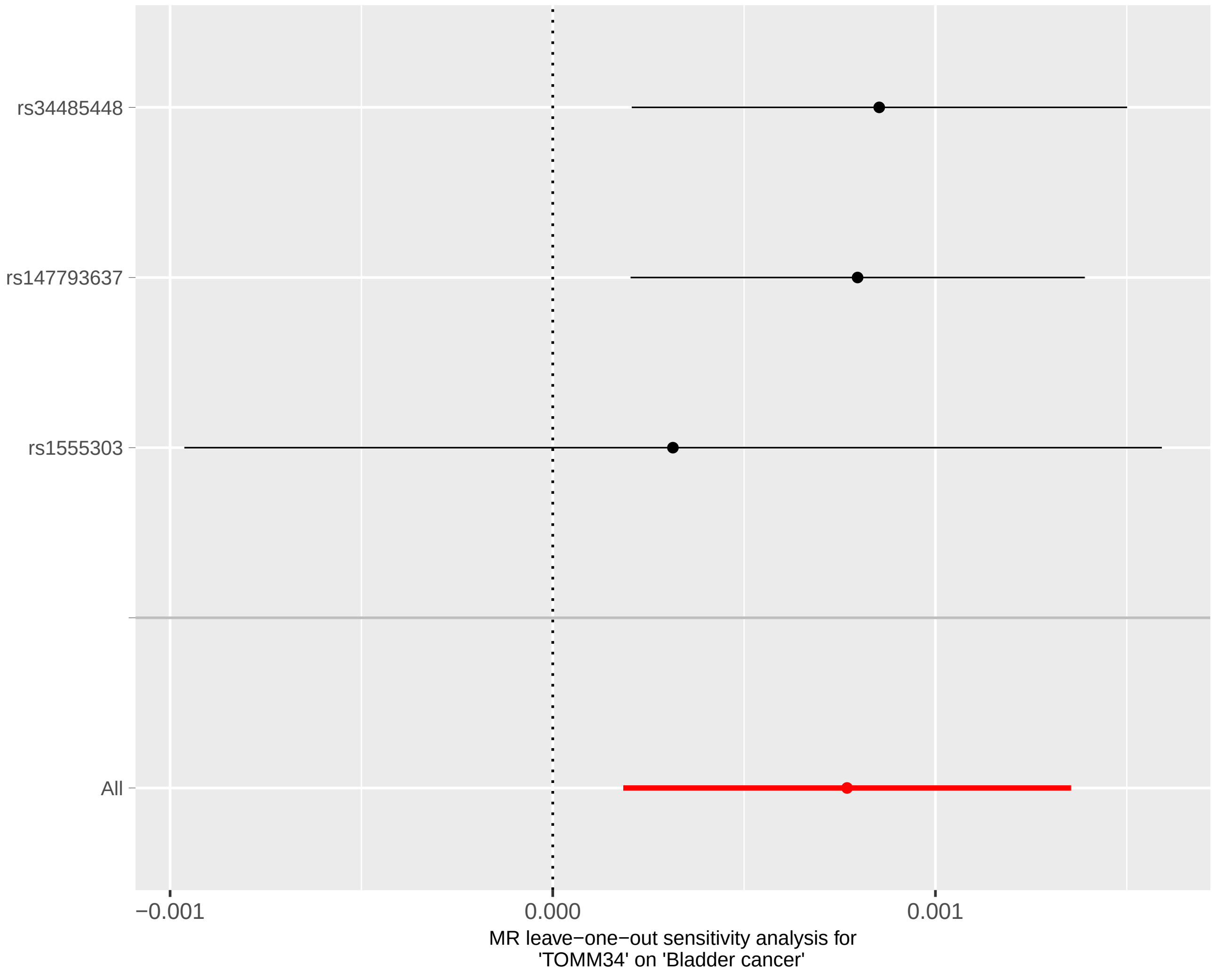
rs34485448
rs147793637
rs1555303
All
−0.001
0.000
0.001
MR leave−one−out sensitivity analysis for
'TOMM34' on 'Bladder cancer'

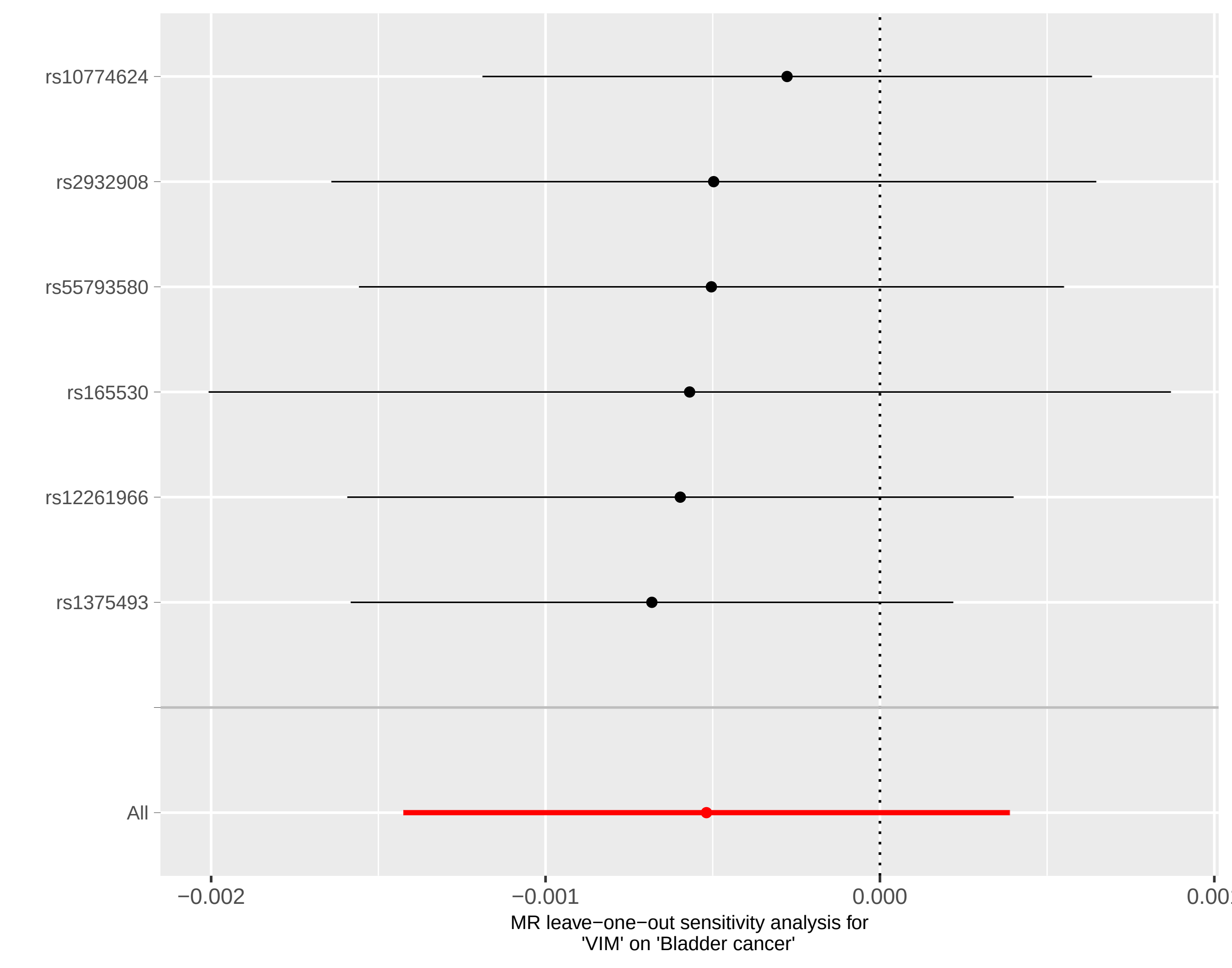
rs10774624
rs2932908
rs55793580
rs165530
rs12261966
rs1375493
All
−0.002
−0.001
0.000
MR leave−one−out sensitivity analysis for
'VIM' on 'Bladder cancer'

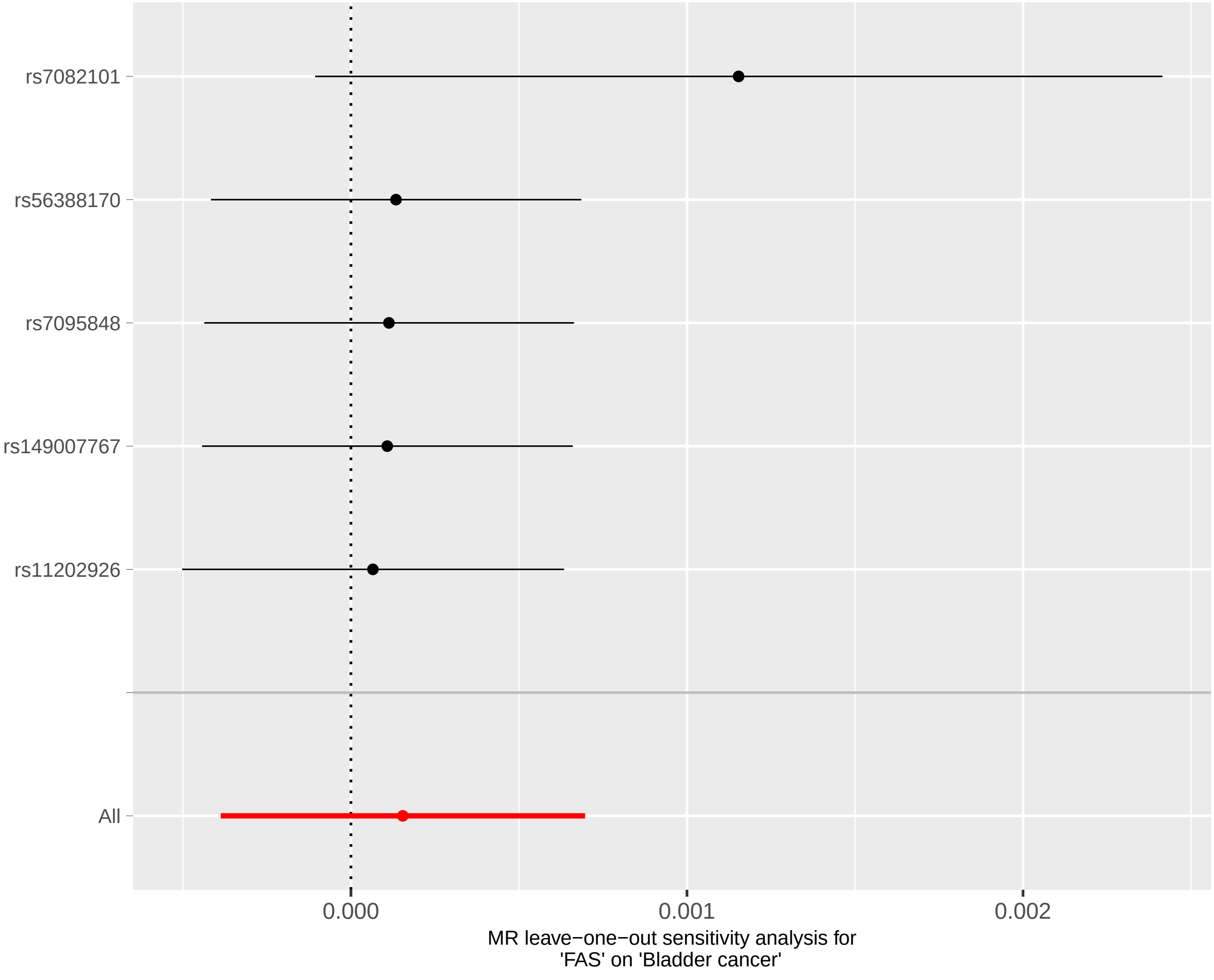
rs7082101
rs56388170
rs7095848
rs149007767
rs11202926
All
0.000
0.001
0.002
MR leave−one−out sensitivity analysis for
'FAS' on 'Bladder cancer'

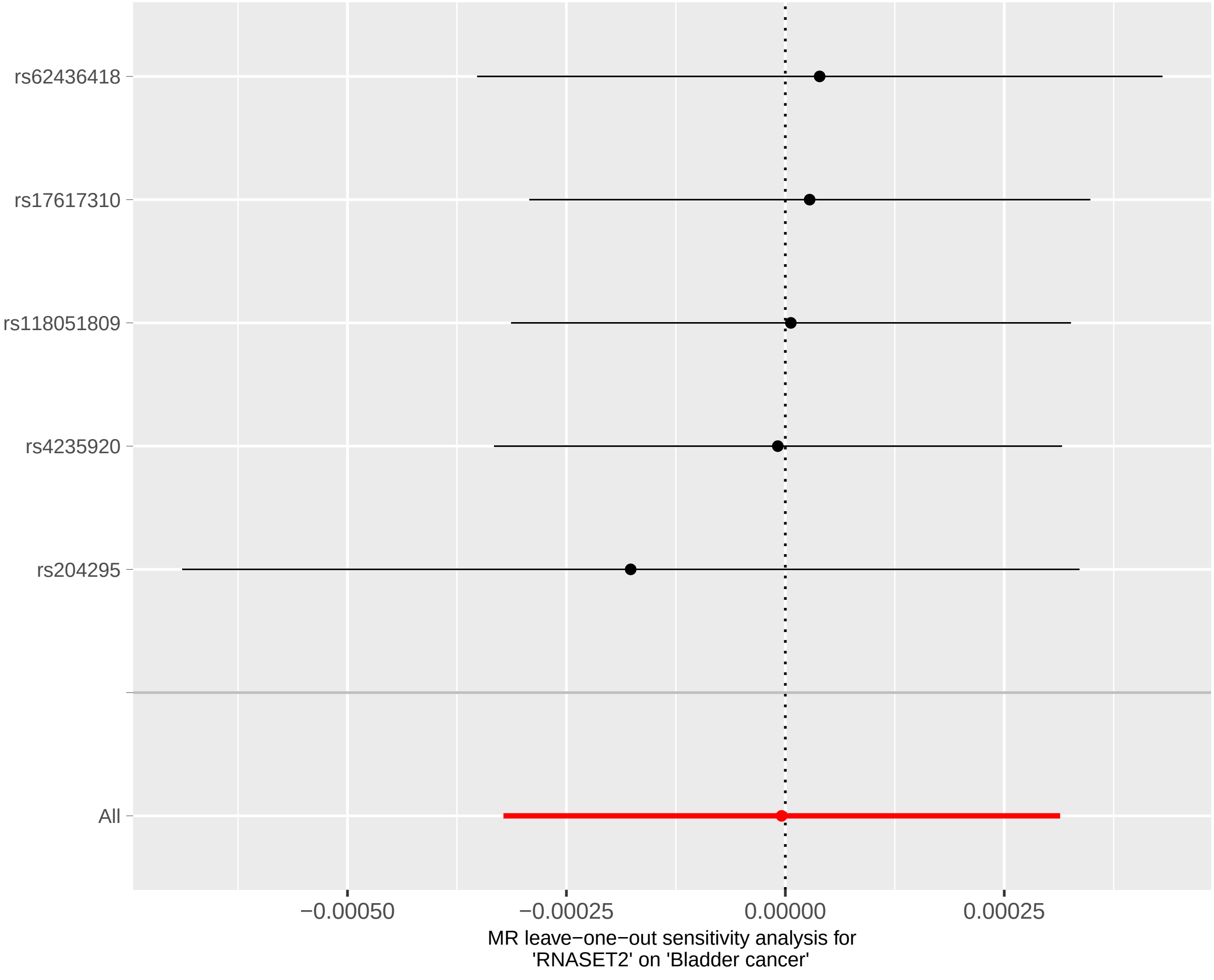

rs62436418
rs17617310
rs118051809
rs4235920
rs204295
All
−0.00050
−0.00025
0.00000
0.00025
MR leave−one−out sensitivity analysis for
'RNASET2' on 'Bladder cancer'

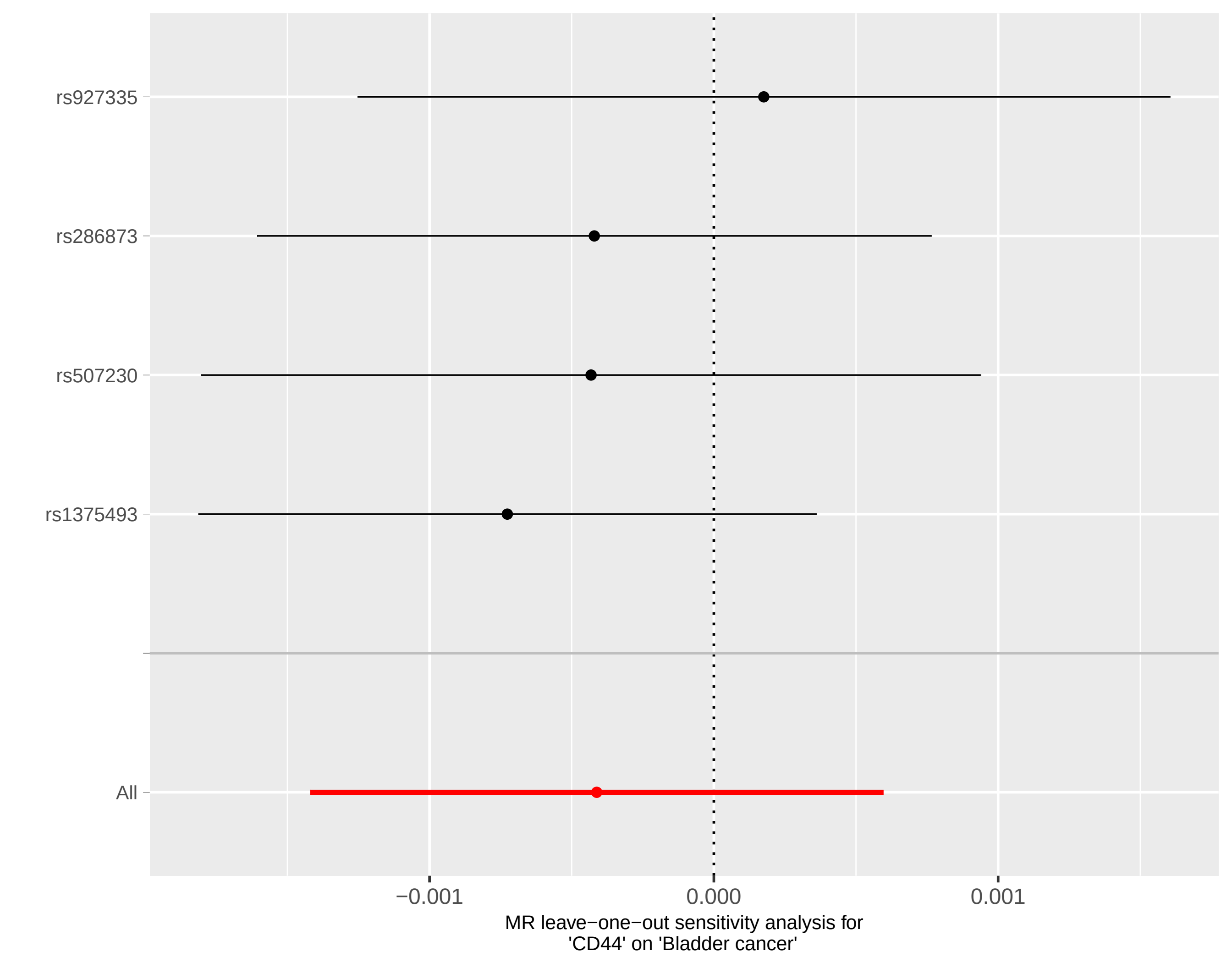
rs927335
rs286873
rs507230
rs1375493
All
−0.001
0.000
0.001
MR leave−one−out sensitivity analysis for
'CD44' on 'Bladder cancer'

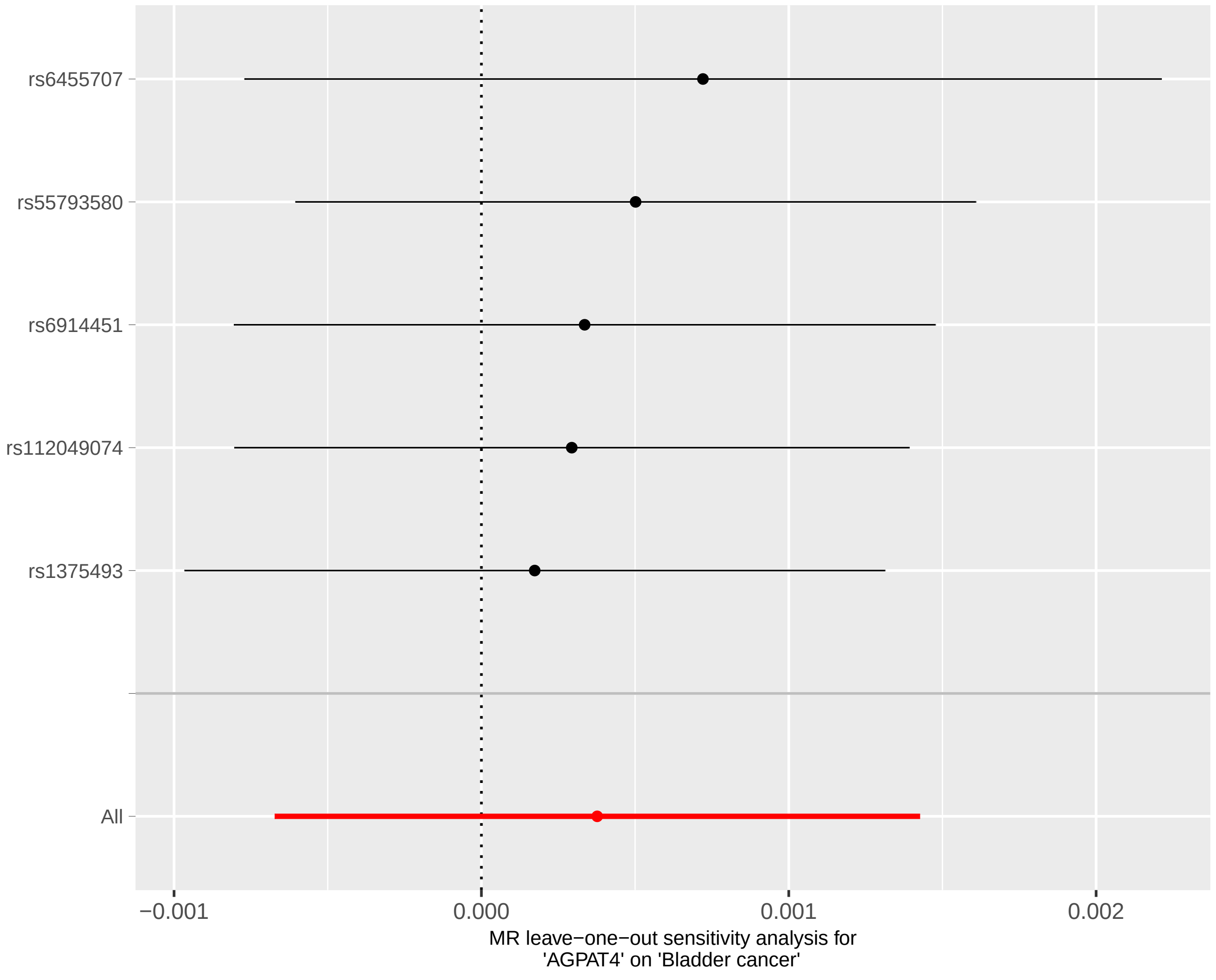
rs6455707
rs55793580
rs6914451
rs112049074
rs1375493
All
−0.001
0.000
0.001
0.002
MR leave−one−out sensitivity analysis for
'AGPAT4' on 'Bladder cancer'

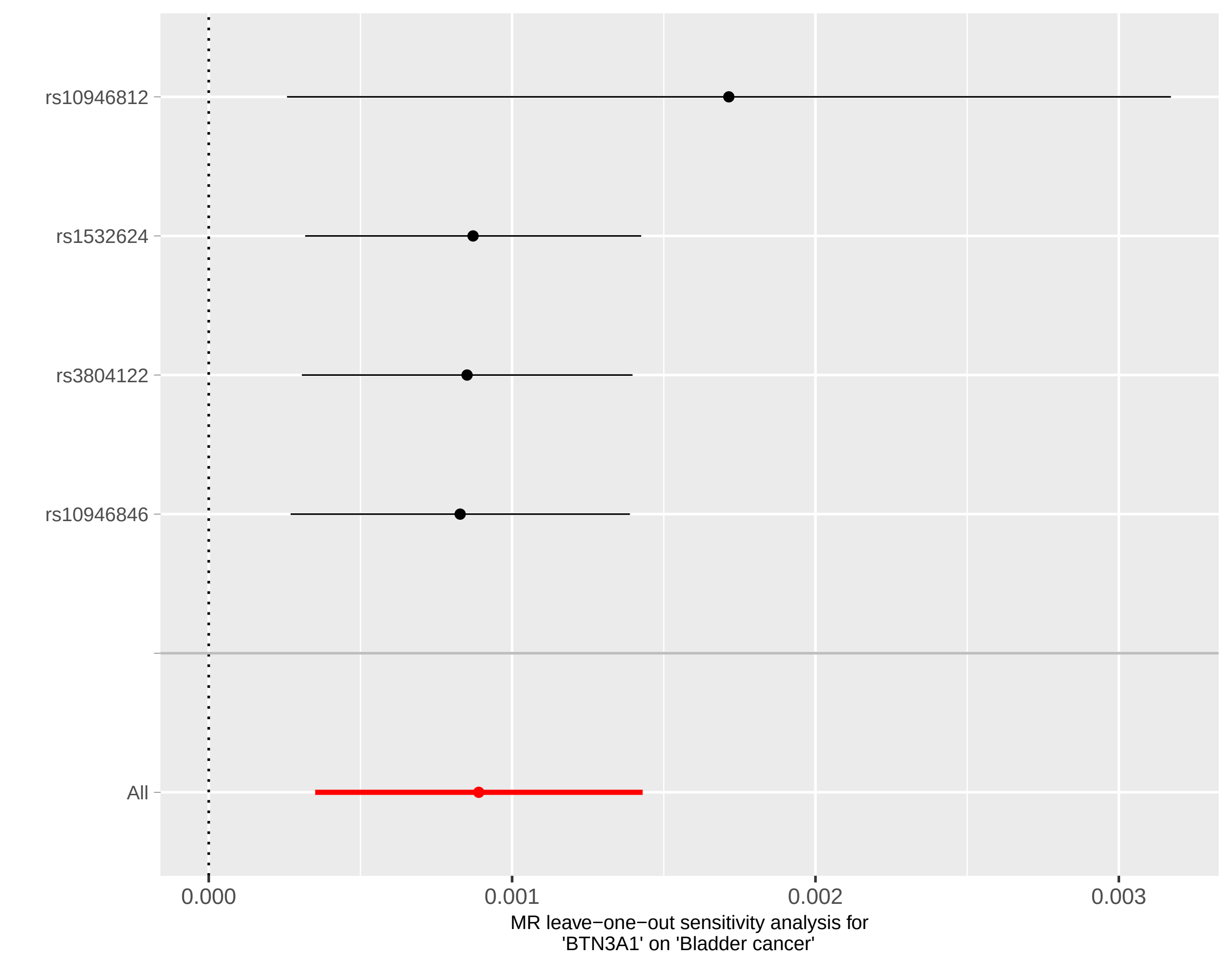
rs10946812
rs1532624
rs3804122
rs10946846
All
0.000
0.001
0.002
0.003
MR leave−one−out sensitivity analysis for
'BTN3A1' on 'Bladder cancer'

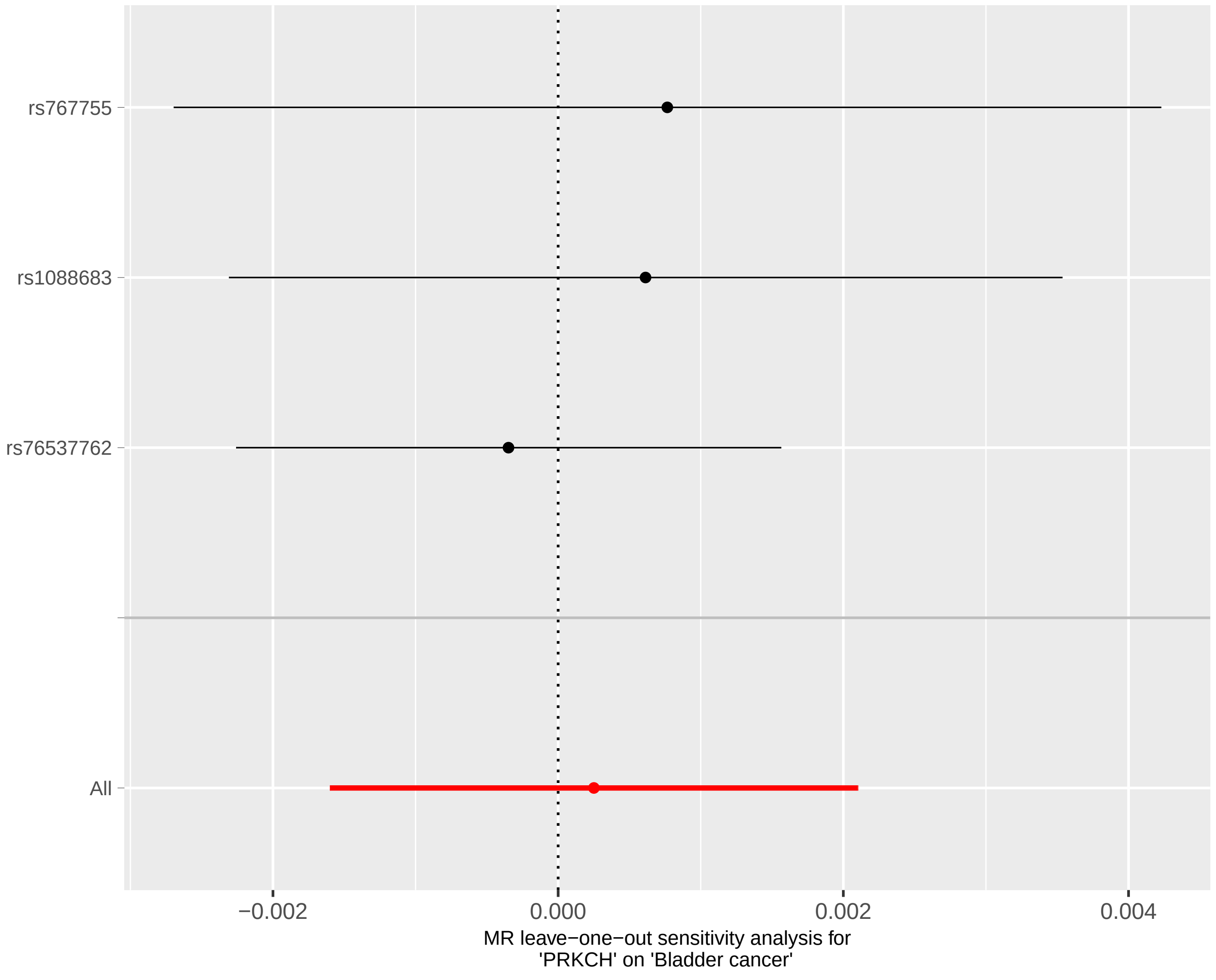
rs767755
rs1088683
rs76537762
All
−0.002
0.000
0.002
0.004
MR leave−one−out sensitivity analysis for
'PRKCH' on 'Bladder cancer'

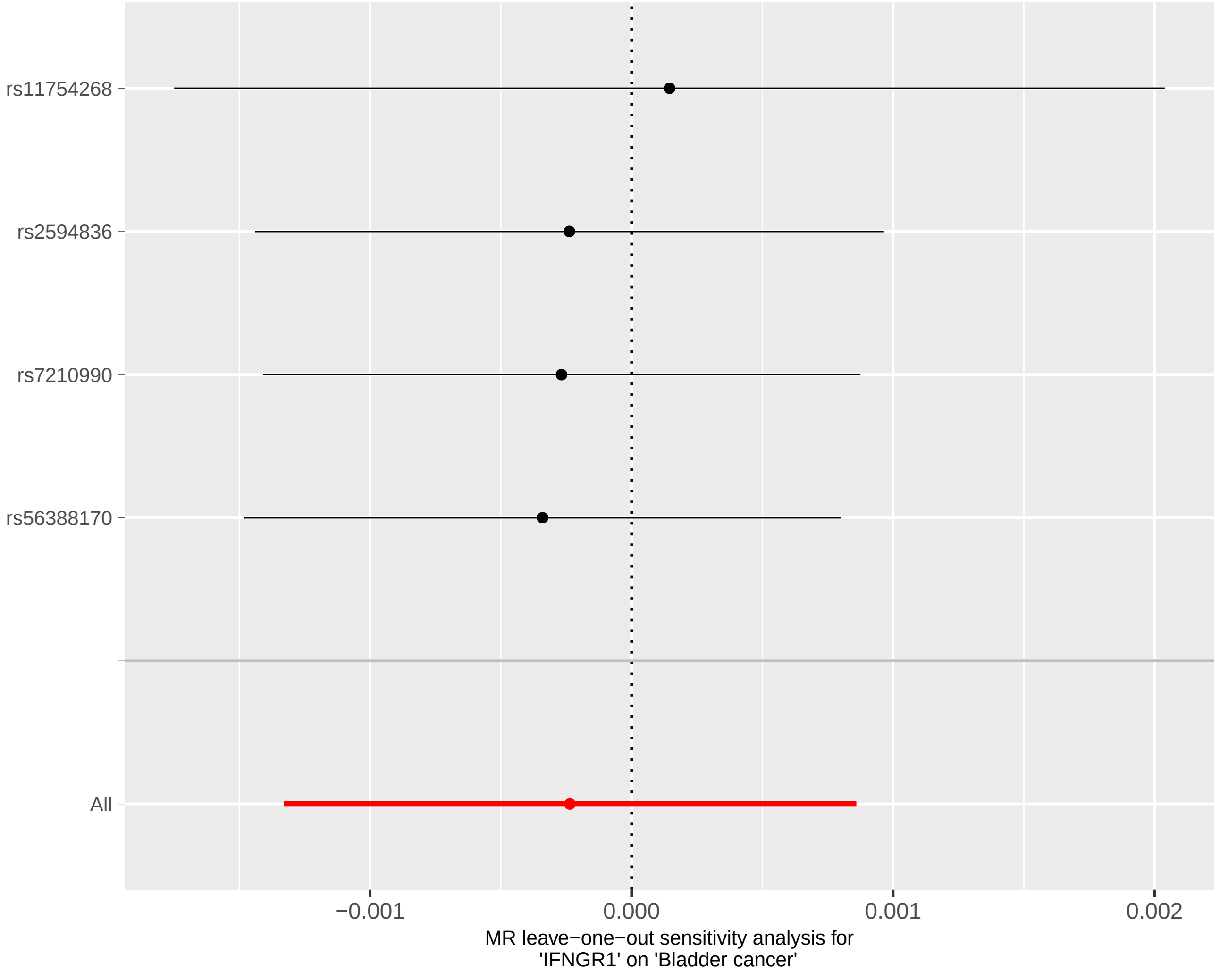
rs11754268
rs2594836
rs7210990
rs56388170
All
−0.001
0.000
0.001
0.002
MR leave−one−out sensitivity analysis for
'IFNGR1' on 'Bladder cancer'

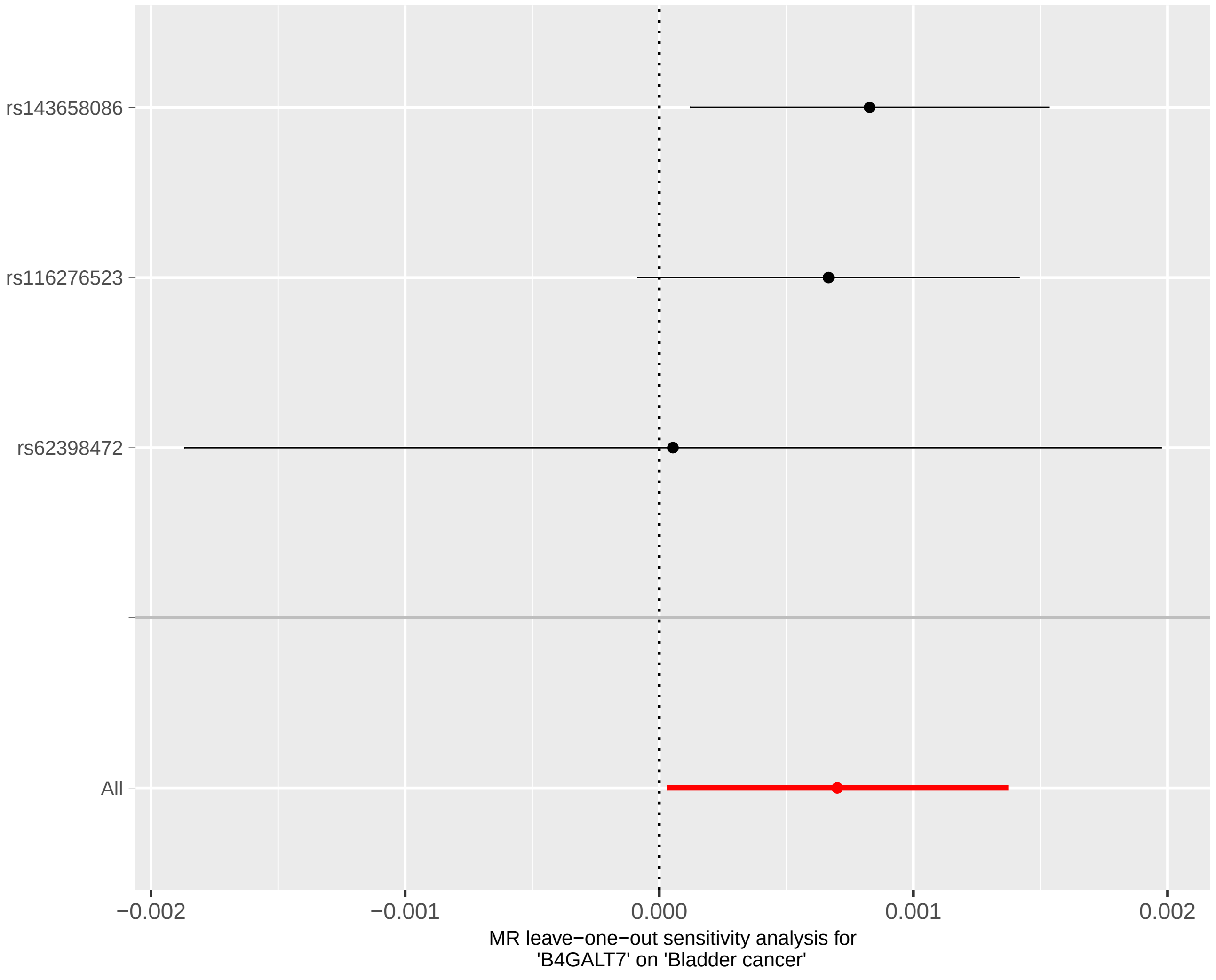
rs143658086
rs116276523
rs62398472
All
−0.002
−0.001
0.000
0.001
0.002
MR leave−one−out sensitivity analysis for
'B4GALT7' on 'Bladder cancer'

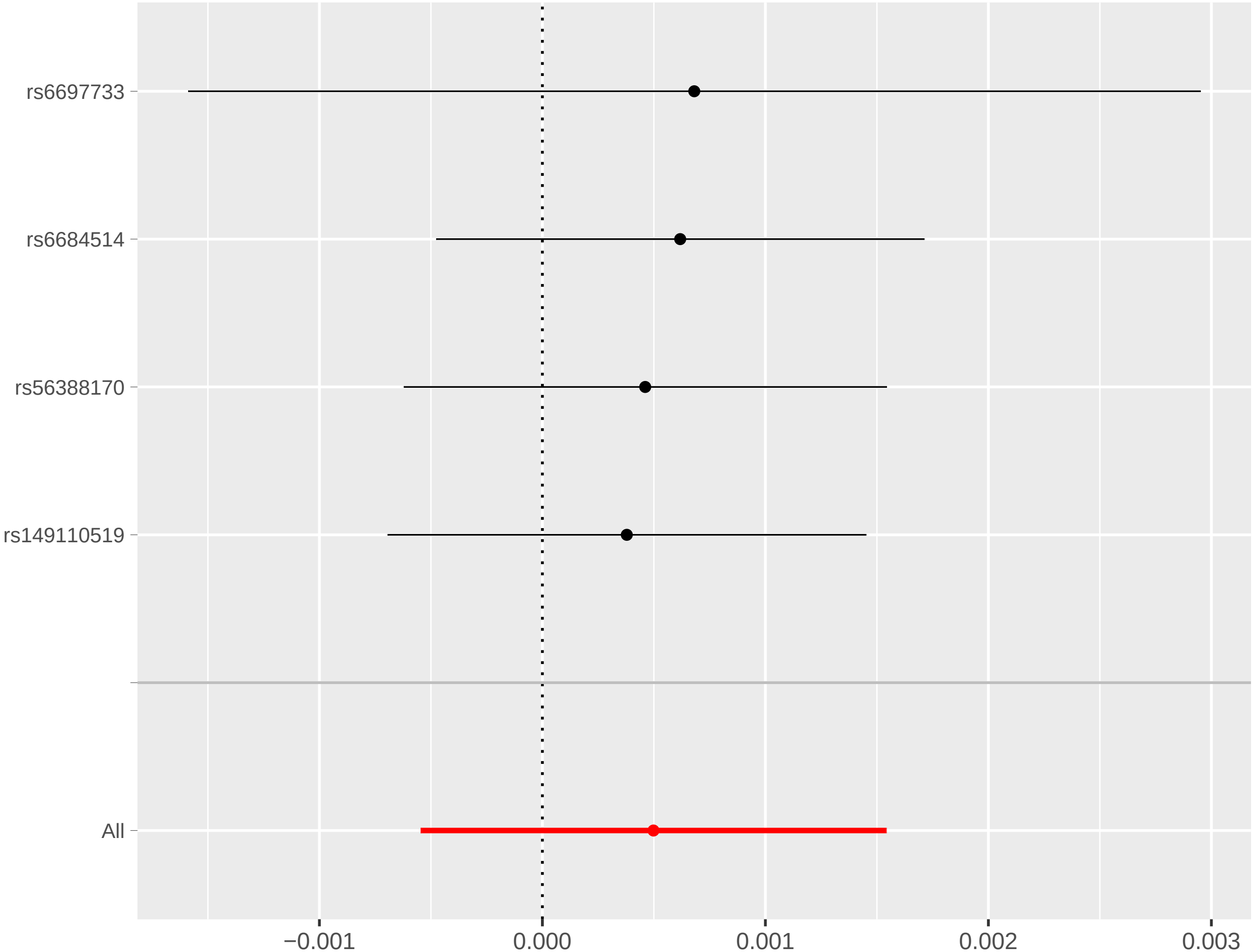

MR leave−one−out sensitivity analysis for
'TNFRSF1B' on 'Bladder cancer'

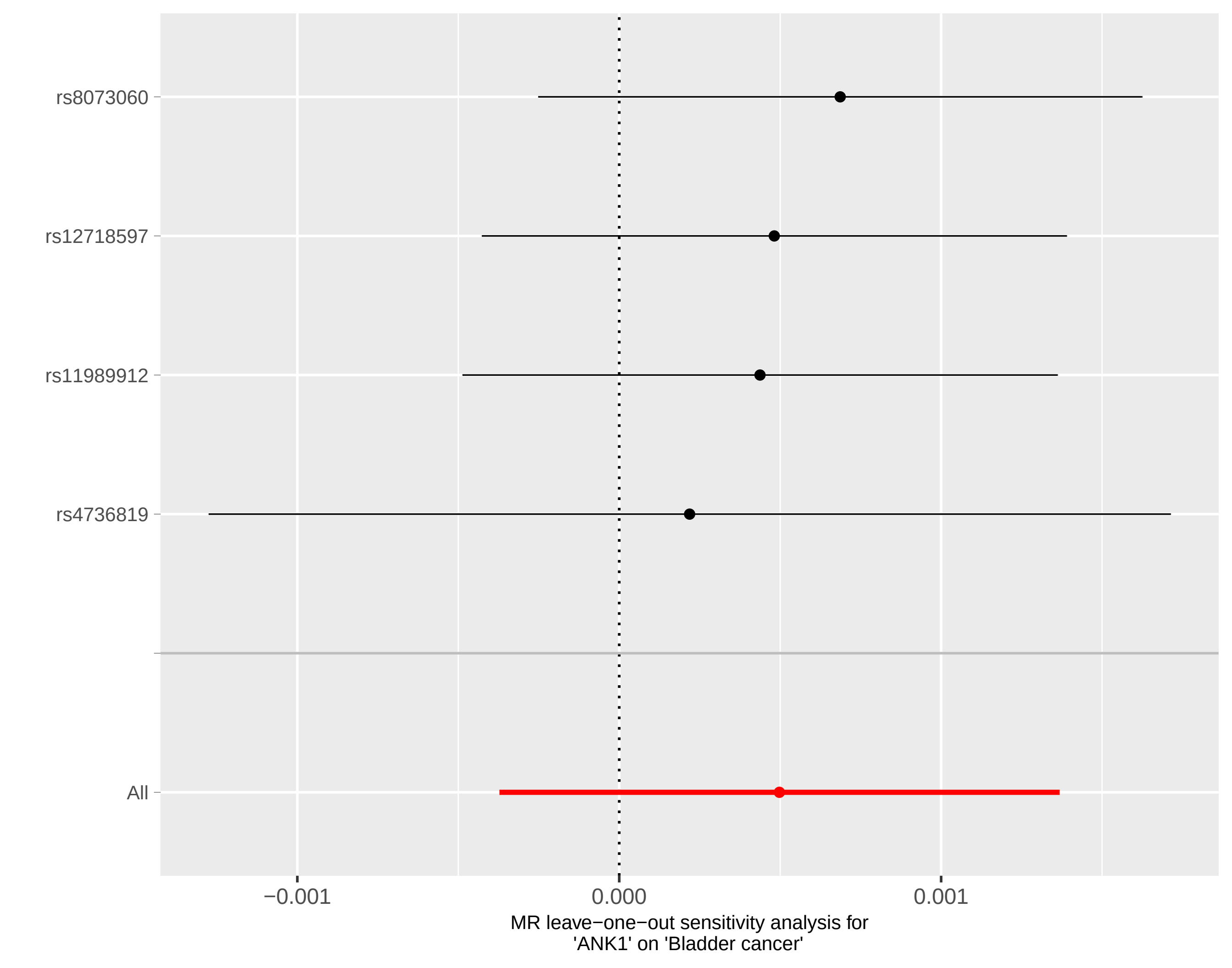
rs8073060
rs12718597
rs11989912
rs4736819
All
−0.001
0.000
0.001
MR leave−one−out sensitivity analysis for
'ANK1' on 'Bladder cancer'

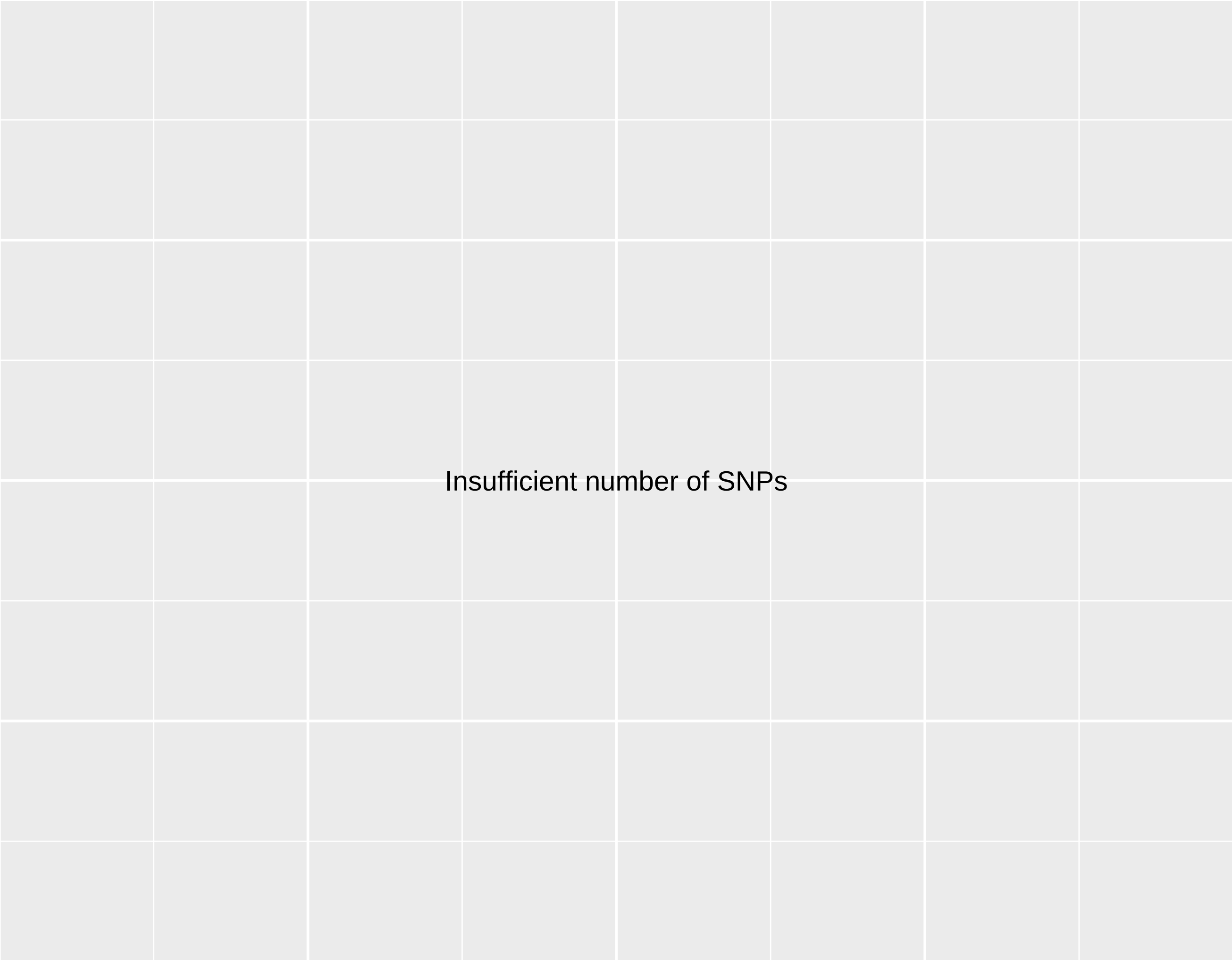
Insufficient number of SNPs

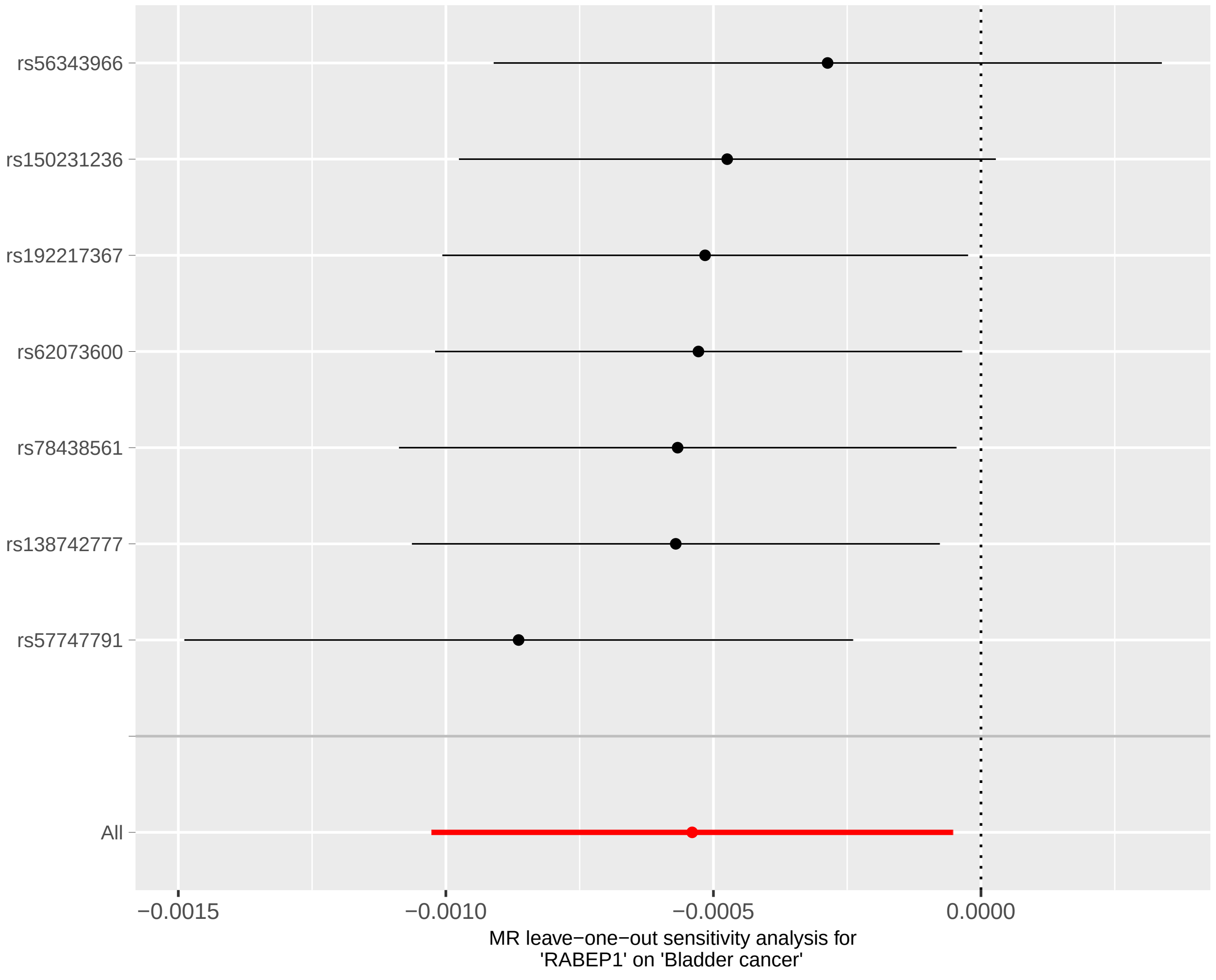
rs56343966
rs150231236
rs192217367
rs62073600
rs78438561
rs138742777
rs57747791
All
−0.0015
−0.0010
−0.0005
0.0000
MR leave−one−out sensitivity analysis for
'RABEP1' on 'Bladder cancer'

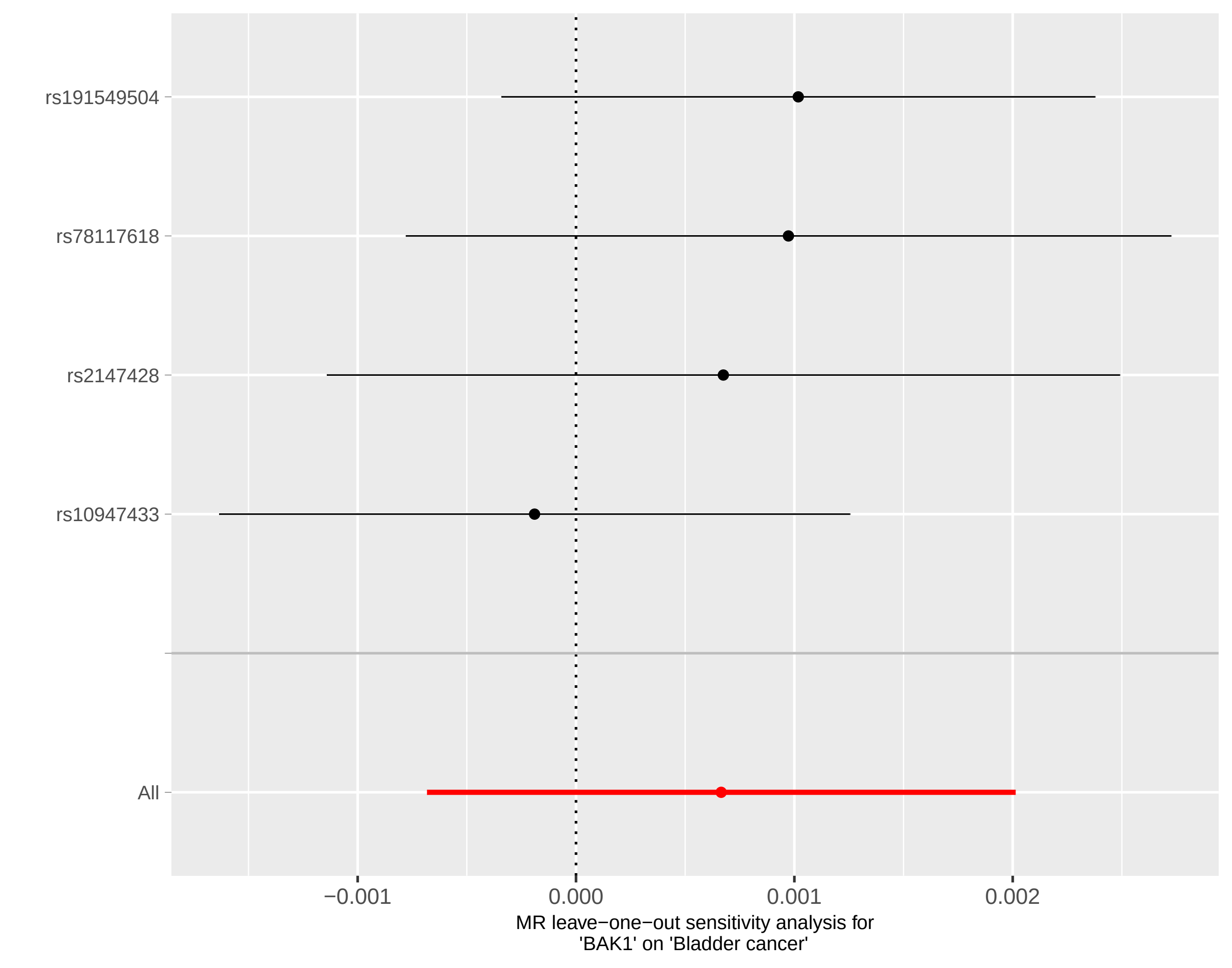
rs191549504
rs78117618
rs2147428
rs10947433
All
−0.001
0.000
0.001
0.002
MR leave−one−out sensitivity analysis for
'BAK1' on 'Bladder cancer'

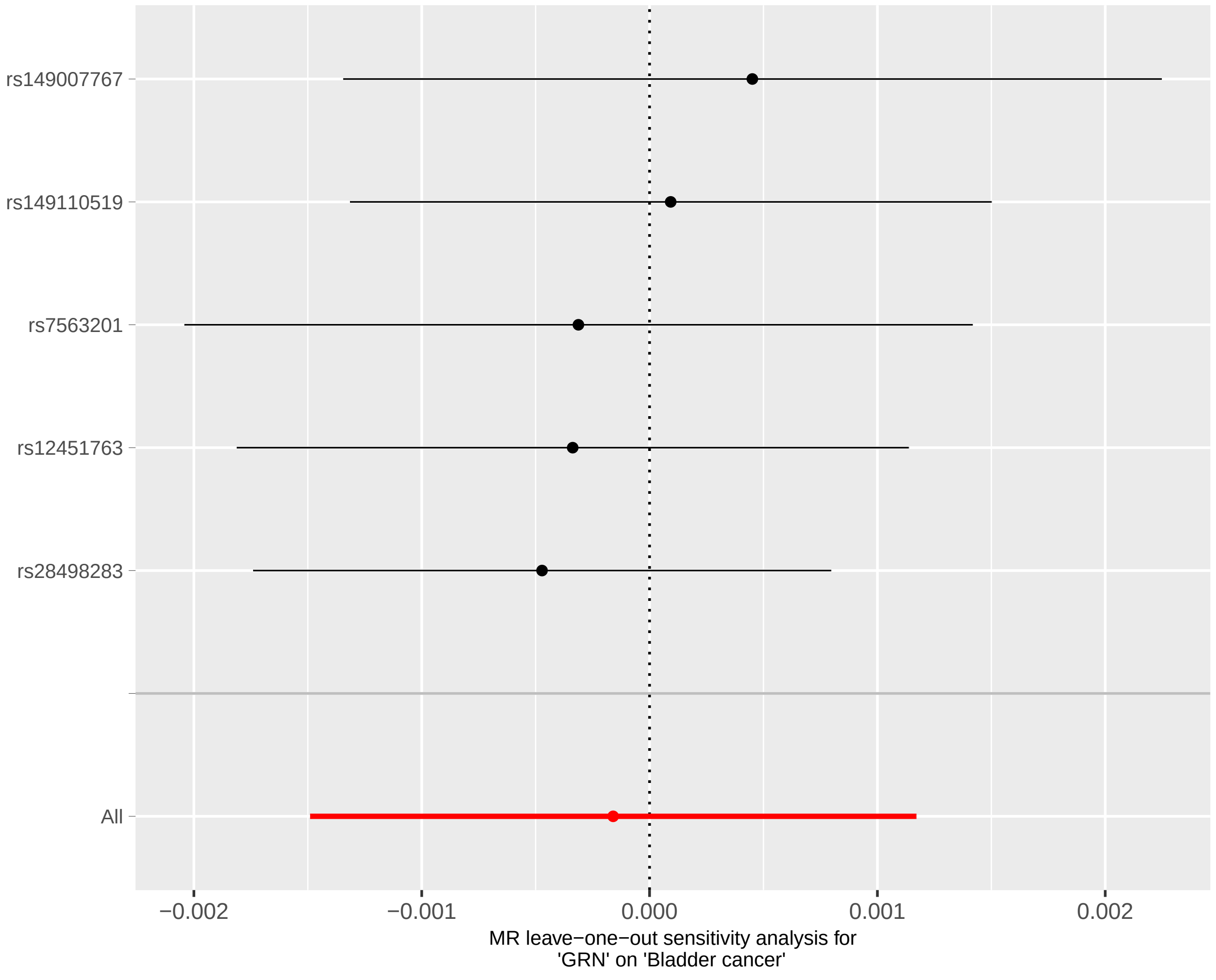
rs149007767
rs149110519
rs7563201
rs12451763
rs28498283
All
−0.002
−0.001
0.000
0.001
0.002
MR leave−one−out sensitivity analysis for
'GRN' on 'Bladder cancer'

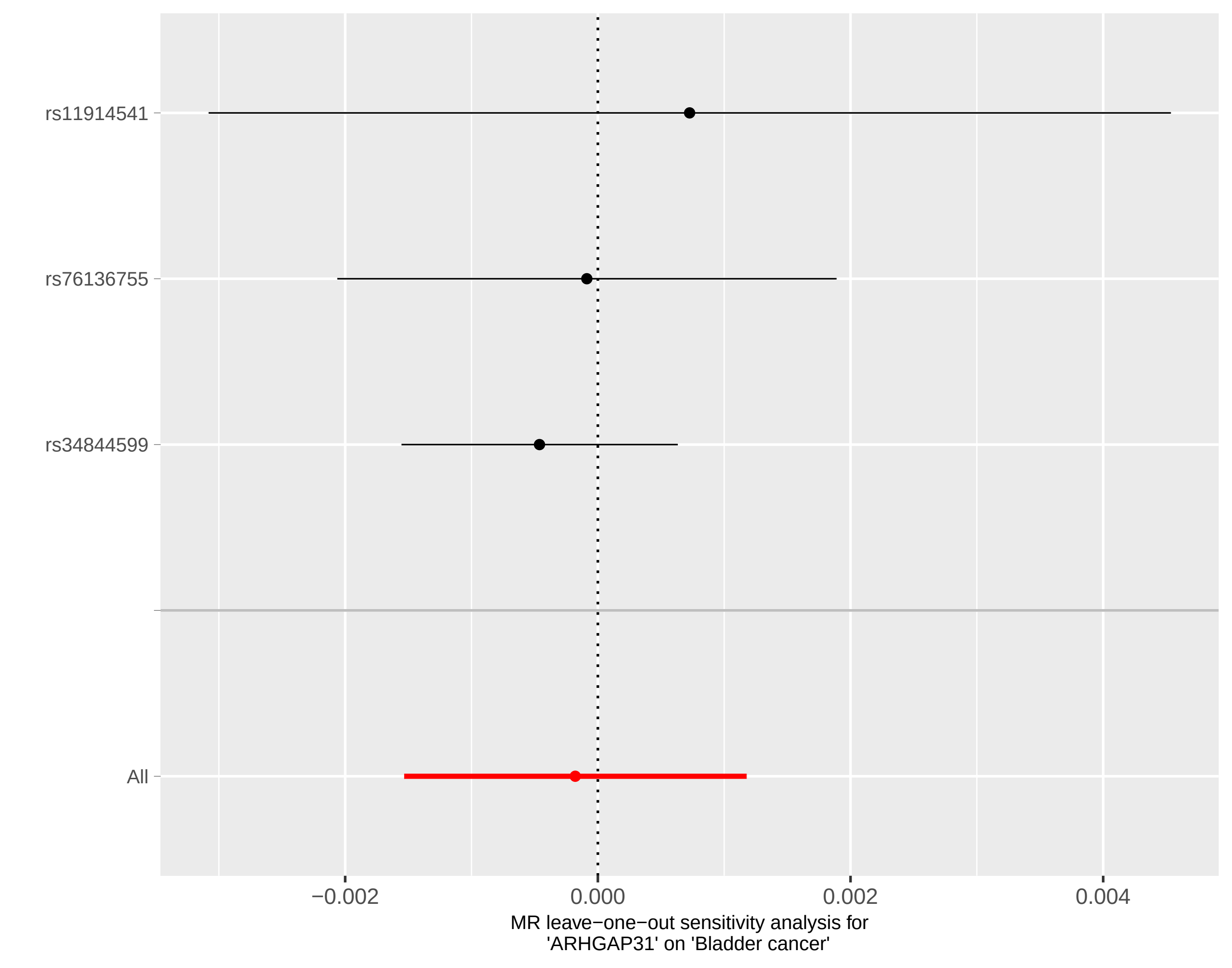
rs11914541
rs76136755
rs34844599
All
−0.002
0.000
0.002
0.004
MR leave−one−out sensitivity analysis for
'ARHGAP31' on 'Bladder cancer'

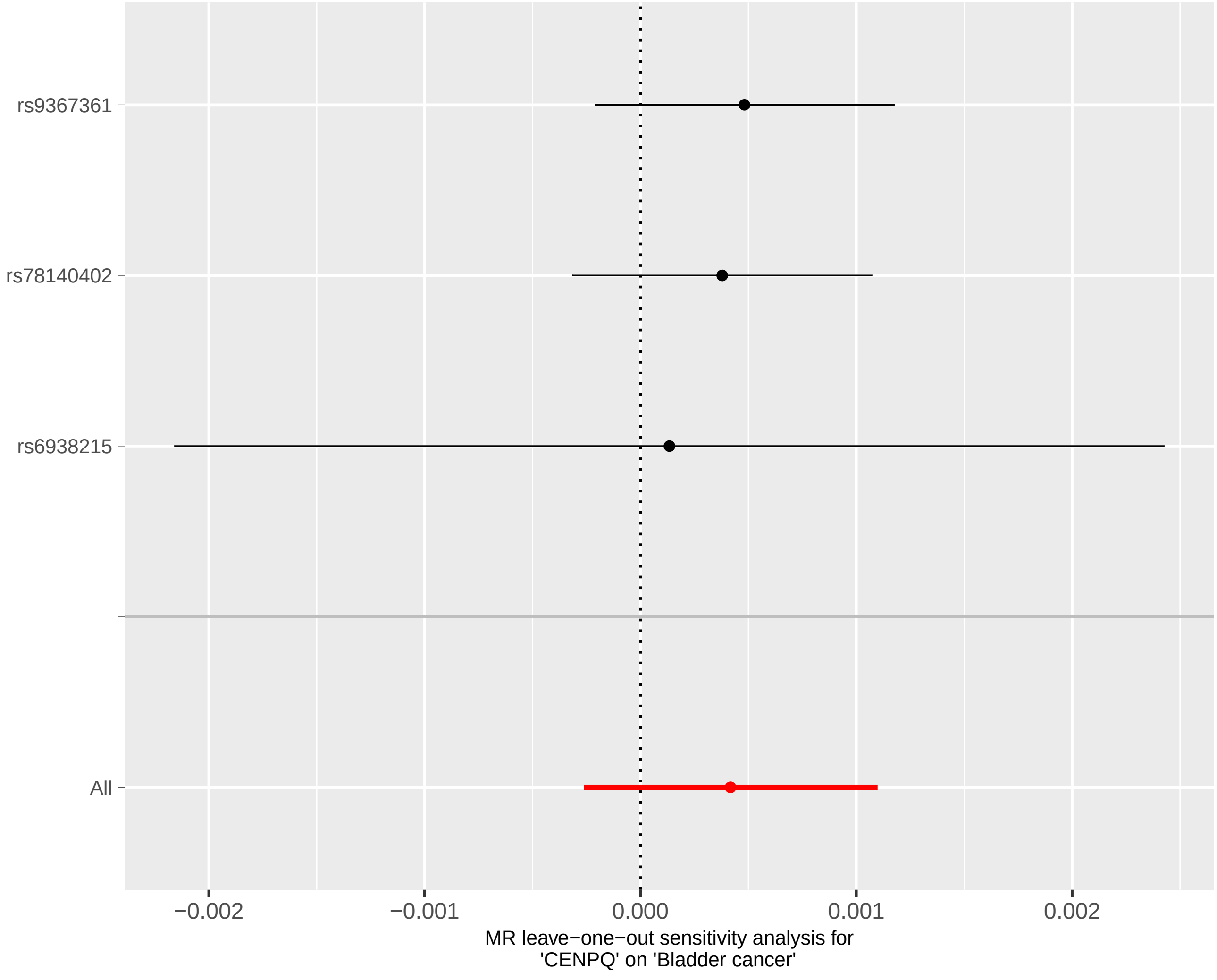
rs9367361
rs78140402
rs6938215
All
−0.002
−0.001
0.000
0.001
0.002
MR leave−one−out sensitivity analysis for
'CENPQ' on 'Bladder cancer'

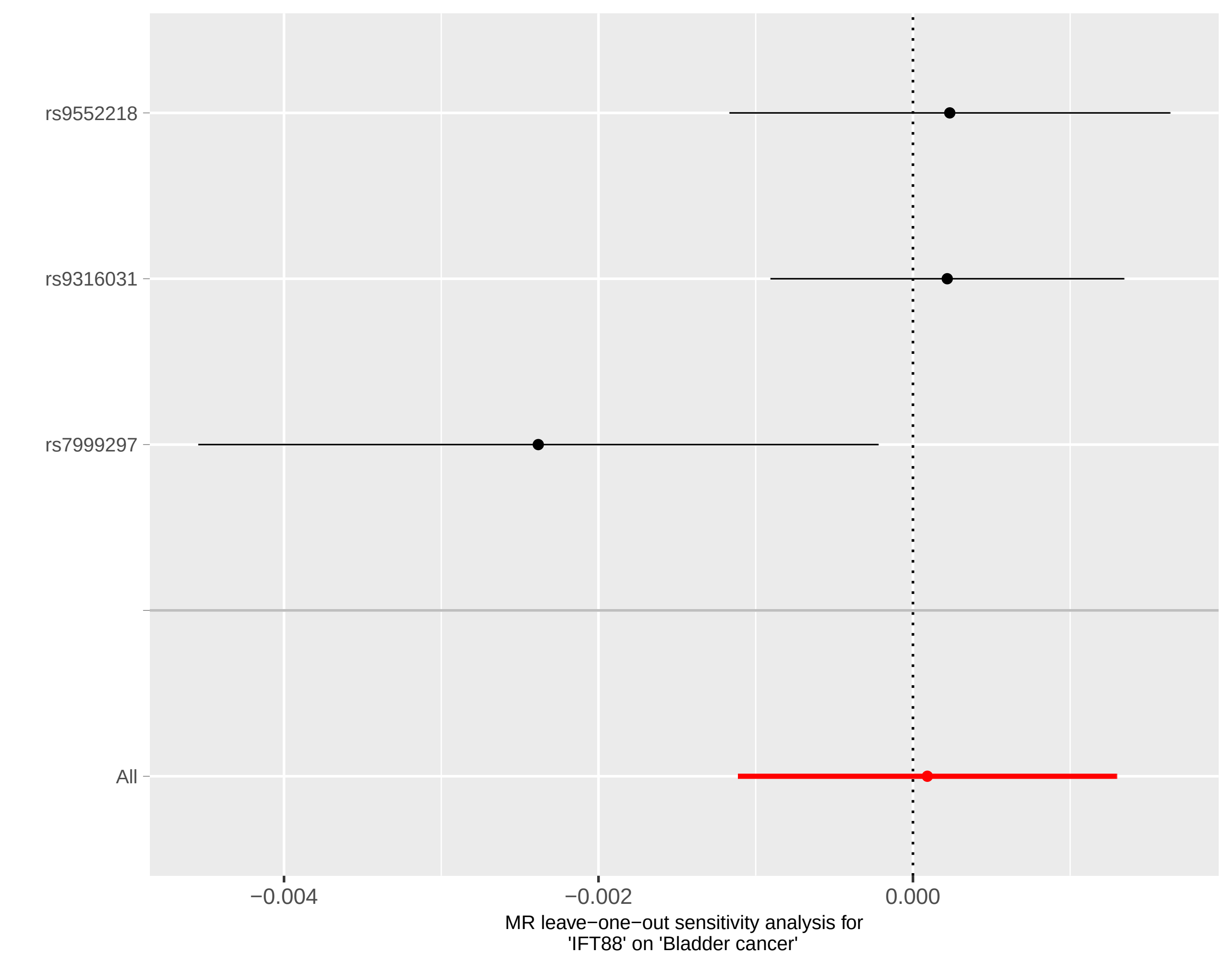
rs9552218
rs9316031
rs7999297
All
−0.004
−0.002
0.000
MR leave−one−out sensitivity analysis for
'IFT88' on 'Bladder cancer'

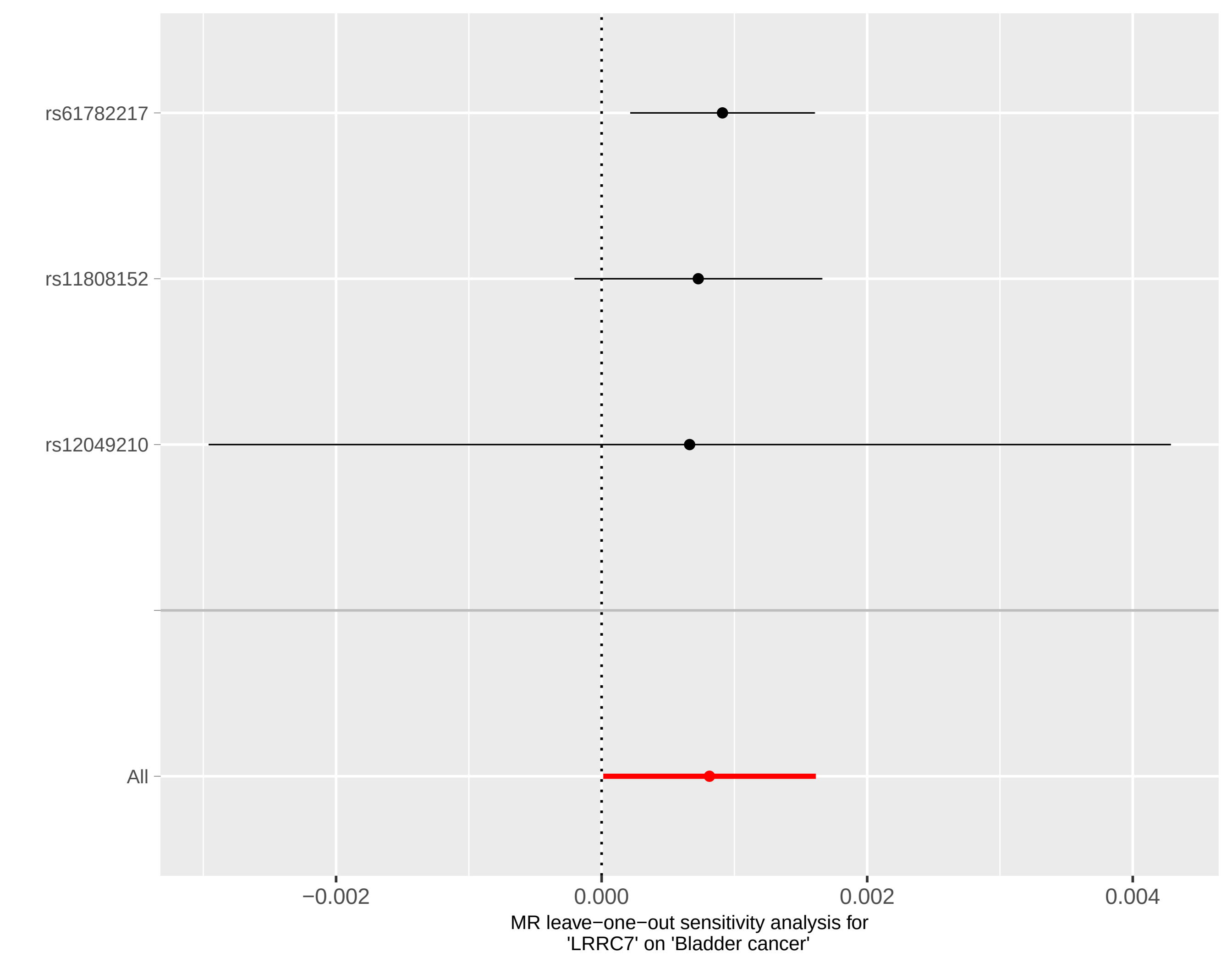
rs61782217
rs11808152
rs12049210
All
−0.002
0.000
0.002
0.004
MR leave−one−out sensitivity analysis for
'LRRC7' on 'Bladder cancer'

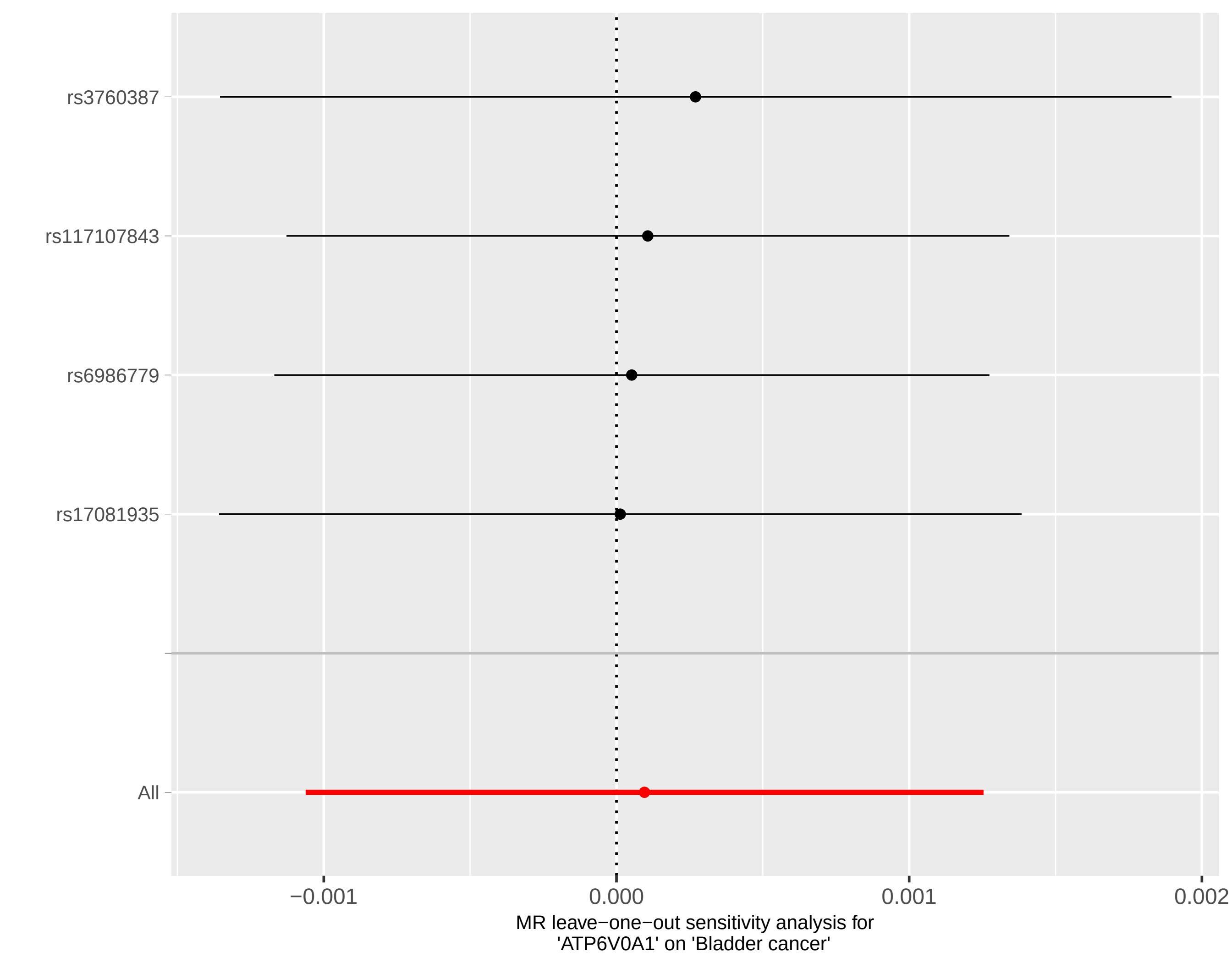
rs3760387
rs117107843
rs6986779
rs17081935
All
−0.001
0.000
0.001
0.002
MR leave−one−out sensitivity analysis for
'ATP6V0A1' on 'Bladder cancer'

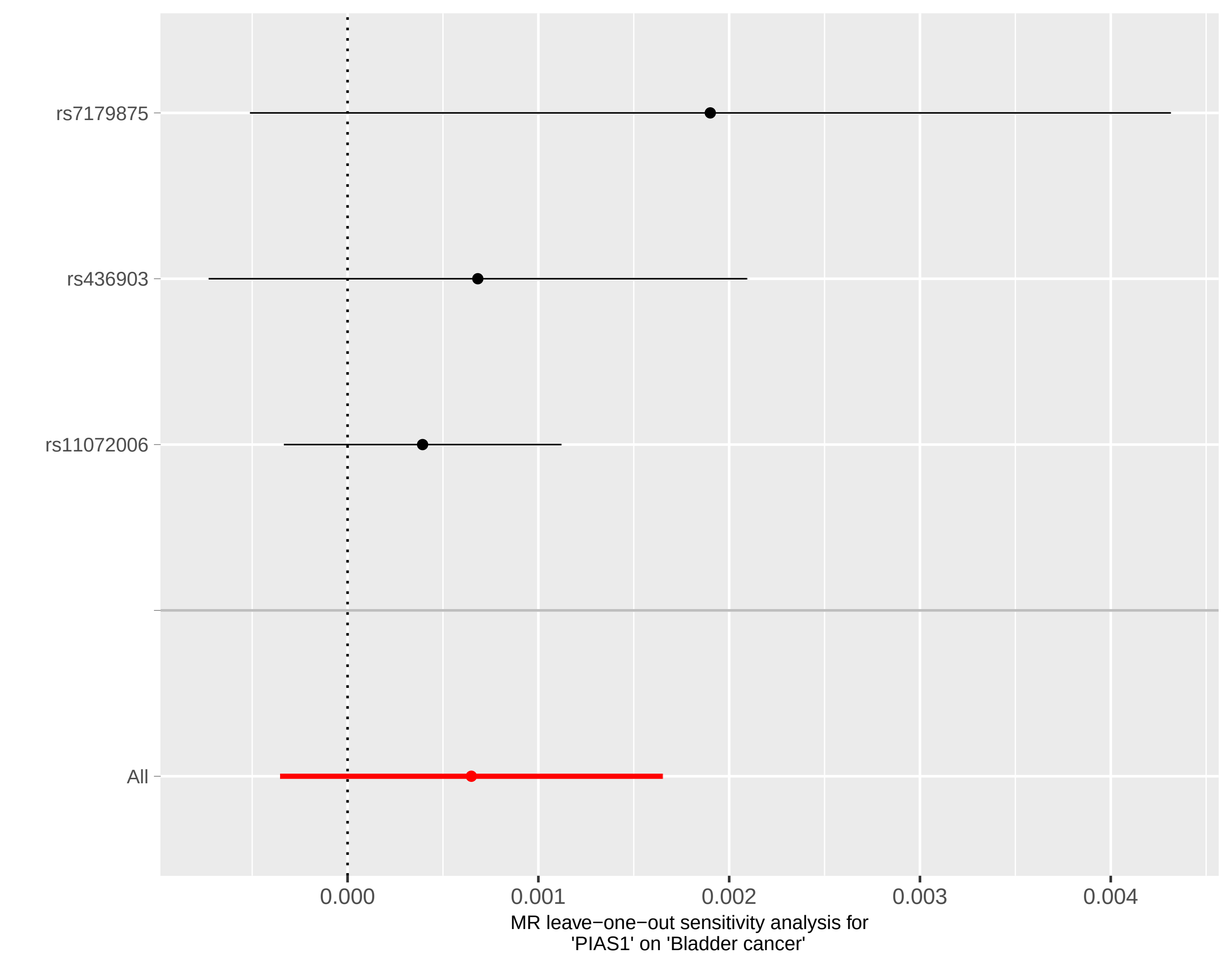
rs7179875
rs436903
rs11072006
All
0.000
0.001
0.002
0.003
0.004
MR leave−one−out sensitivity analysis for
'PIAS1' on 'Bladder cancer'

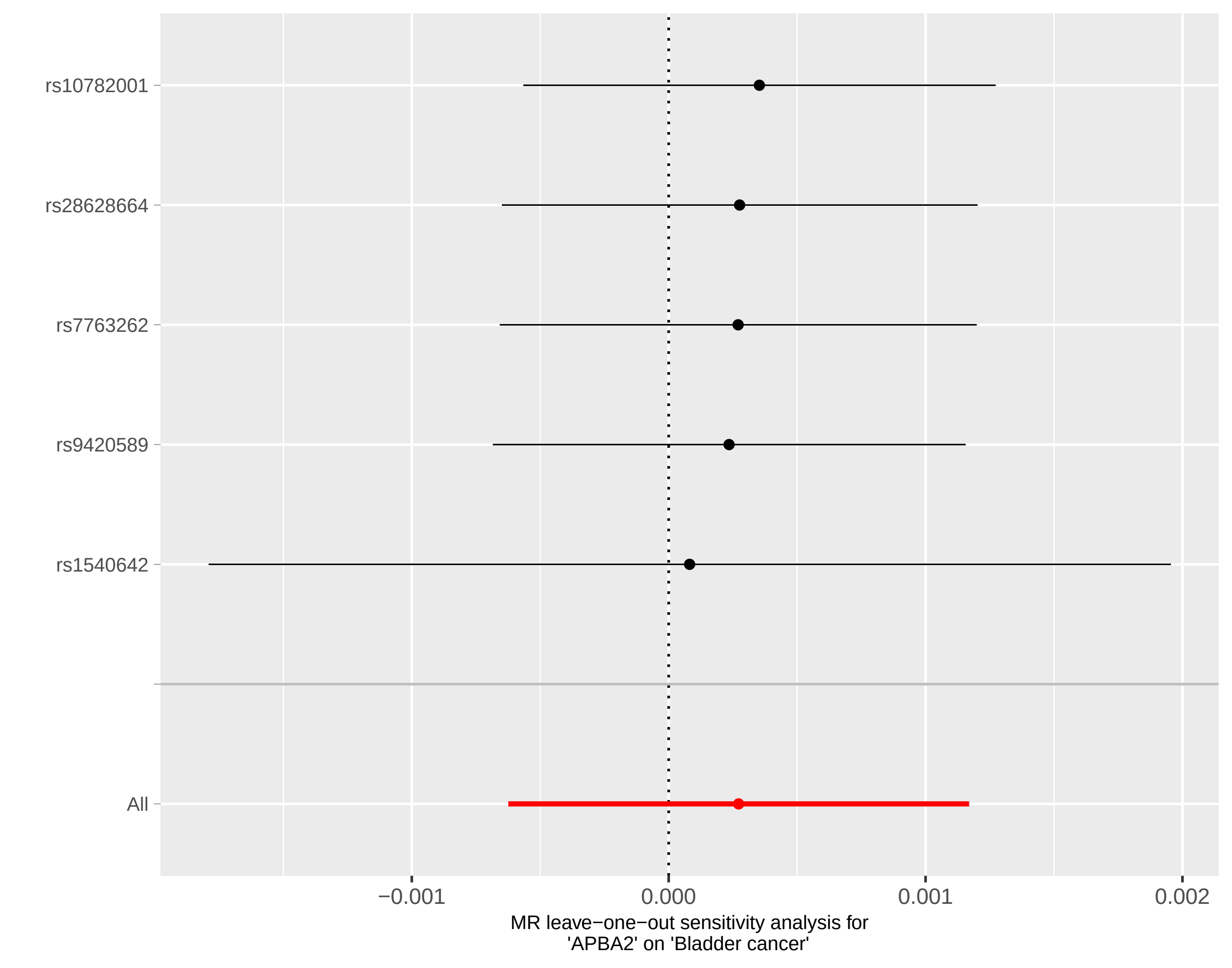
rs10782001
rs28628664
rs7763262
rs9420589
rs1540642
All
−0.001
0.000
0.001
0.002
MR leave−one−out sensitivity analysis for
'APBA2' on 'Bladder cancer'

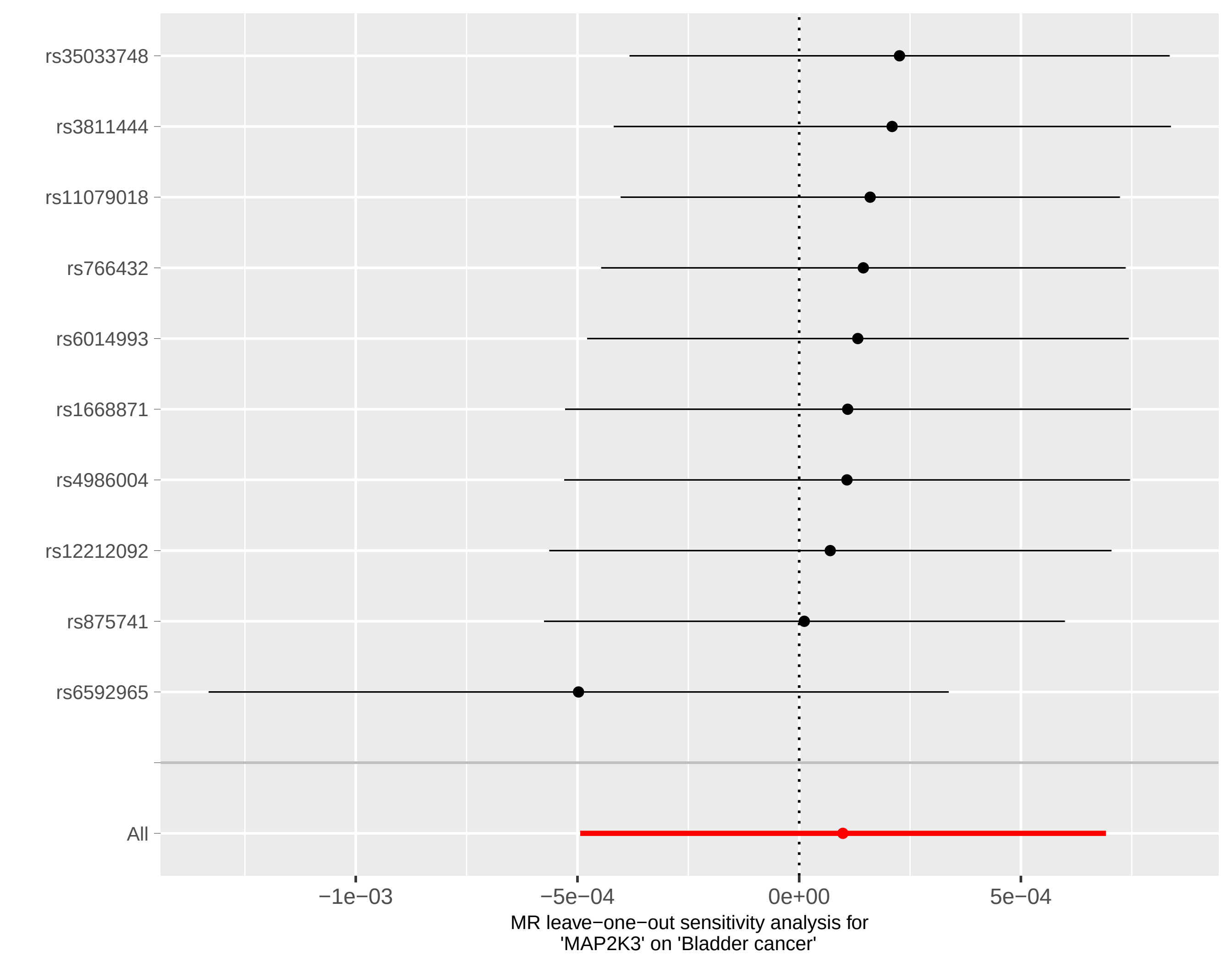
rs35033748
rs3811444
rs11079018
rs766432
rs6014993
rs1668871
rs4986004
rs12212092
rs875741
rs6592965
All
−1e−03
−5e−04
0e+00
5e−04
MR leave−one−out sensitivity analysis for
'MAP2K3' on 'Bladder cancer'

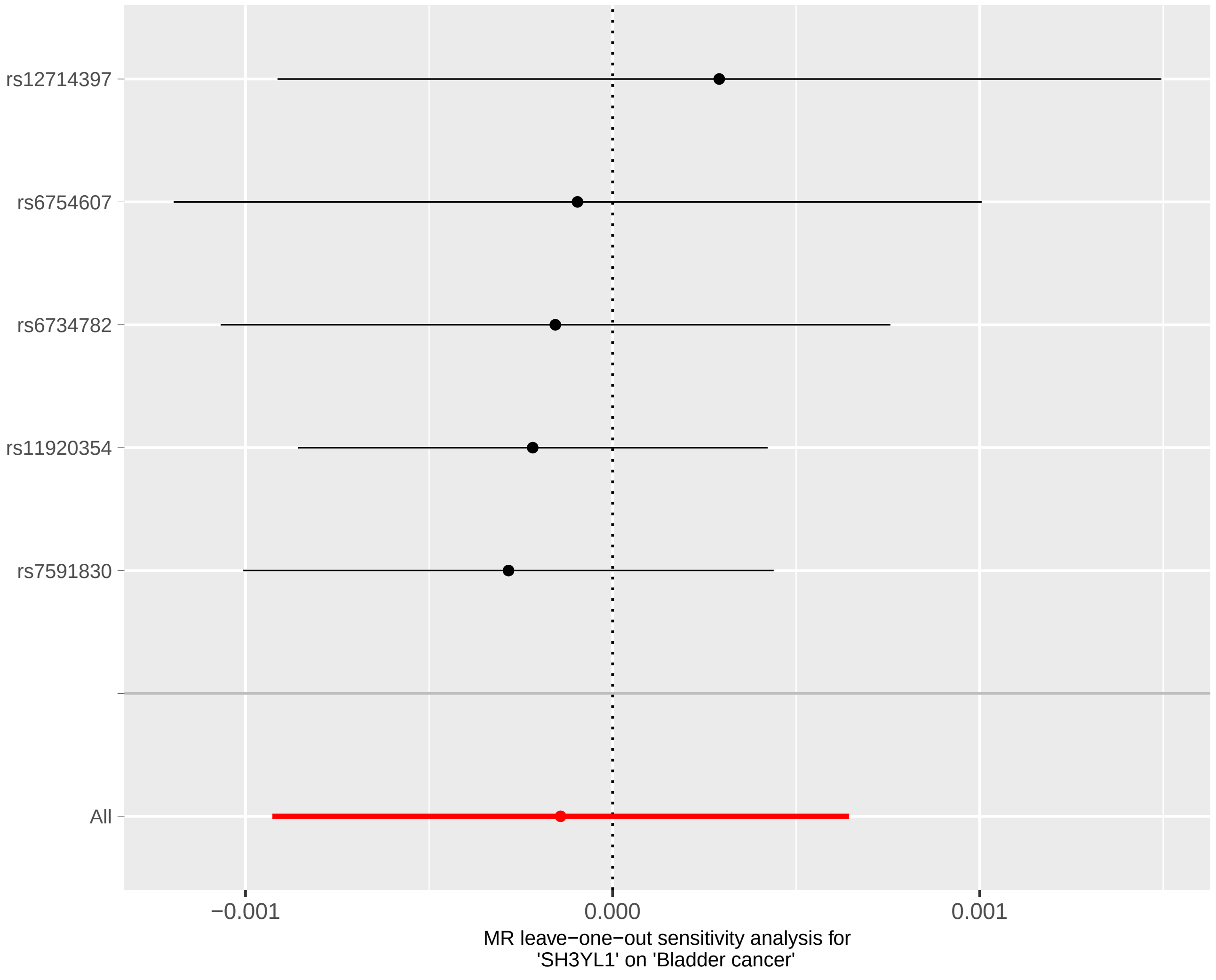

rs12714397
rs6754607
rs6734782
rs11920354
rs7591830
All
−0.001
0.000
0.001
MR leave−one−out sensitivity analysis for
'SH3YL1' on 'Bladder cancer'

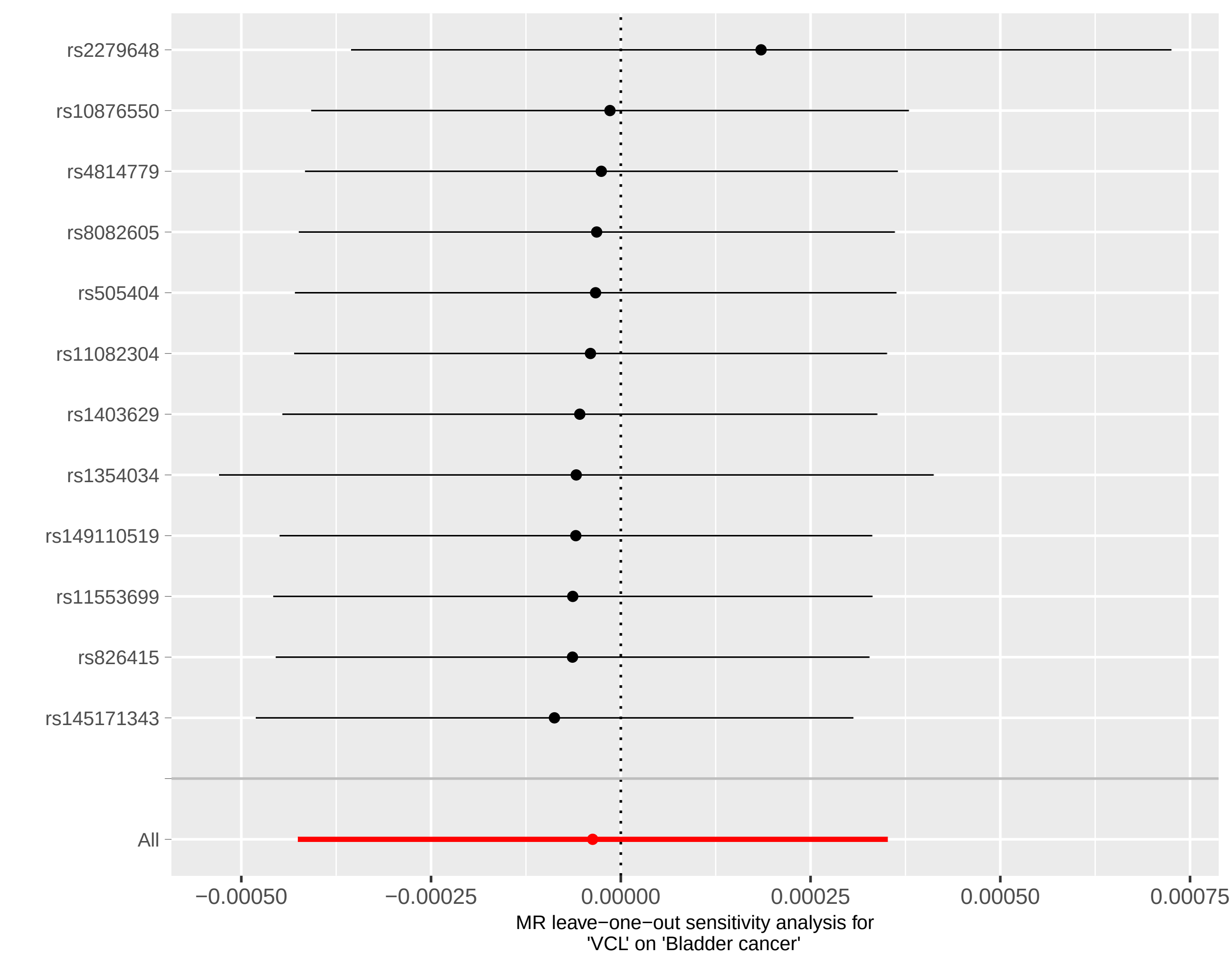
rs2279648
rs10876550
rs4814779
rs8082605
rs505404
rs11082304
rs1403629
rs1354034
rs149110519
rs11553699
rs826415
rs145171343
All
−0.00050
−0.00025
0.00000
0.00025
0.00050
0.00075
MR leave−one−out sensitivity analysis for
'VCL' on 'Bladder cancer'

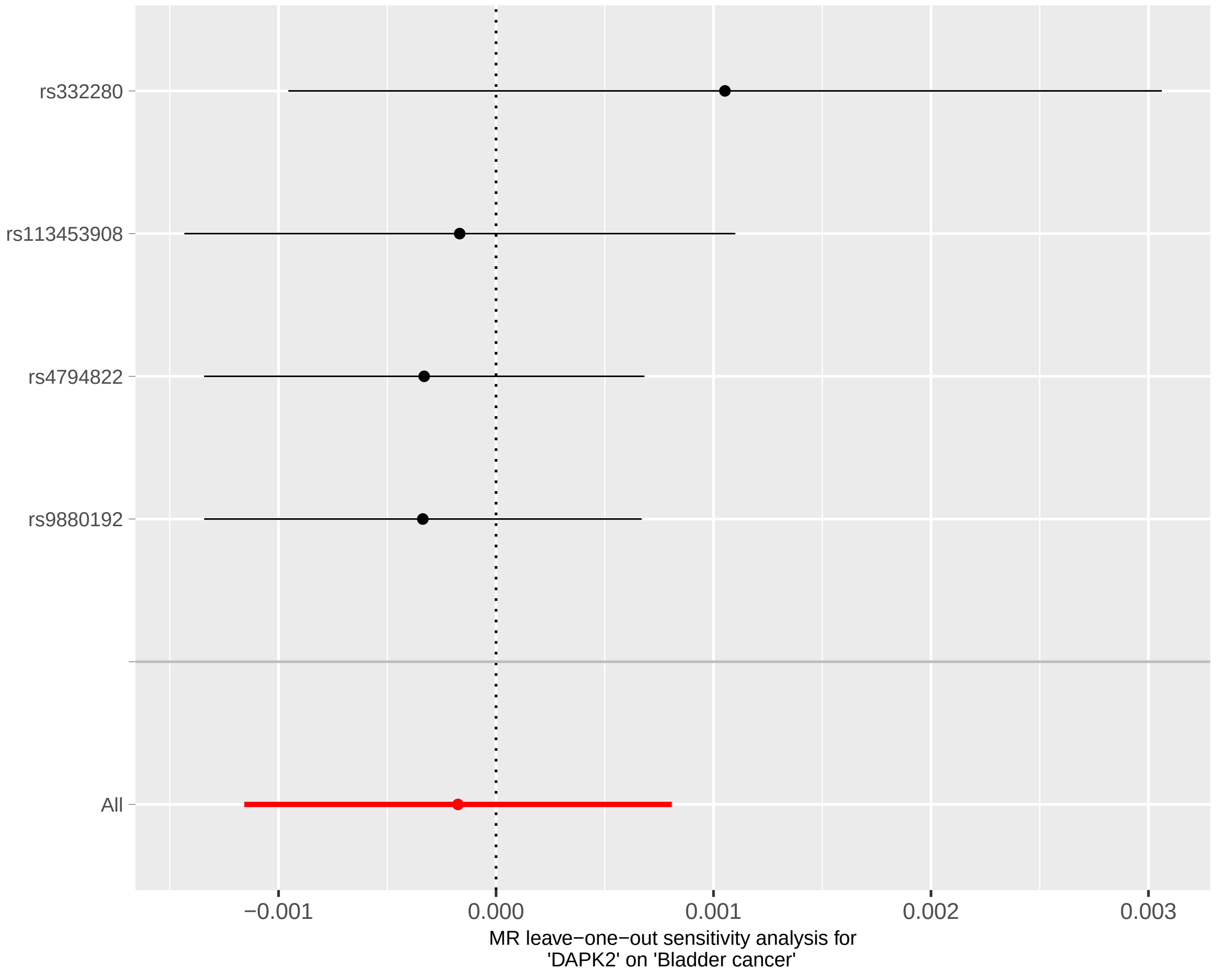
rs332280
rs113453908
rs4794822
rs9880192
All
−0.001
0.000
0.001
0.002
0.003
MR leave−one−out sensitivity analysis for
'DAPK2' on 'Bladder cancer'

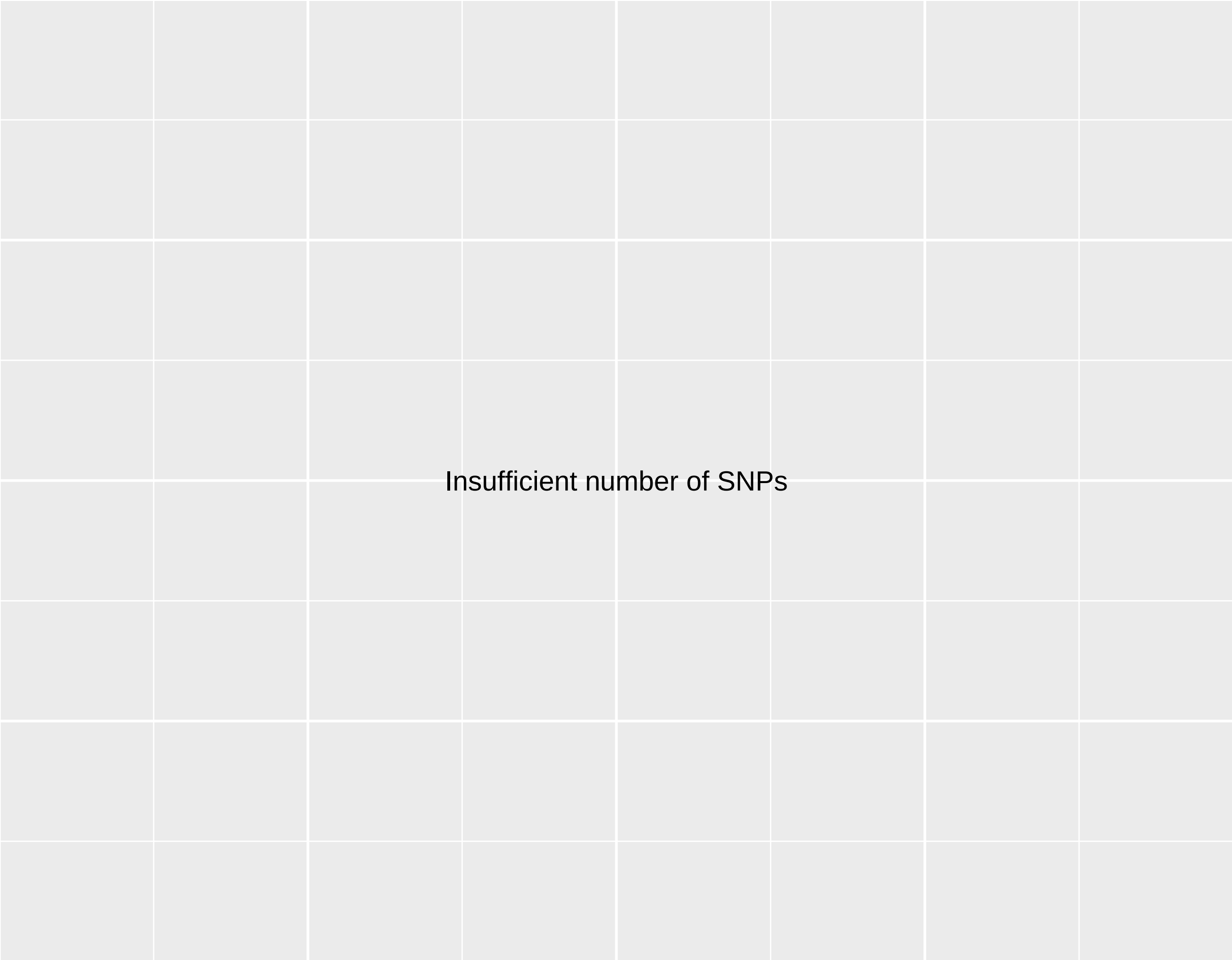
Insufficient number of SNPs

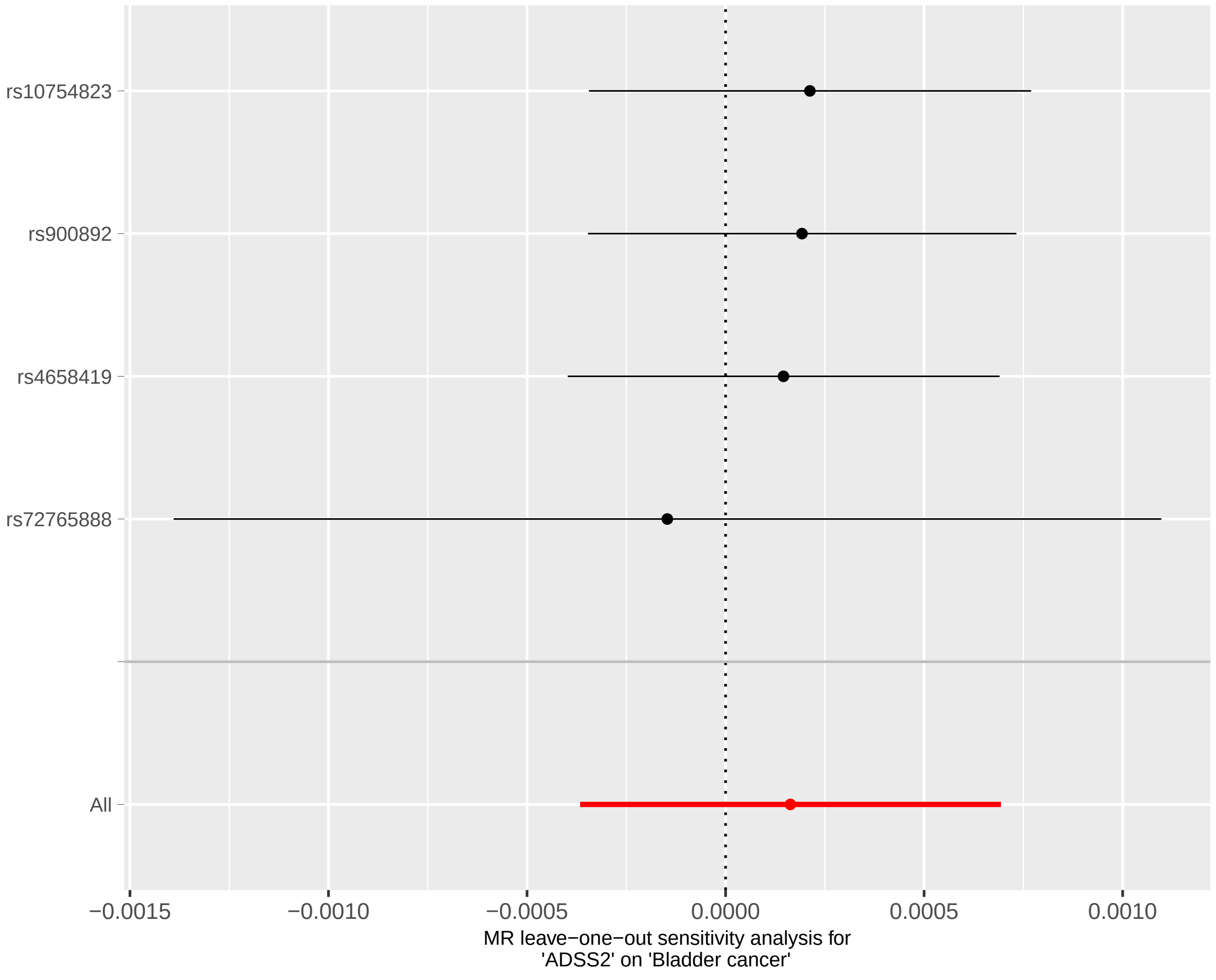
rs10754823
rs900892
rs4658419
rs72765888
All
−0.0015
−0.0010
−0.0005
0.0000
0.0005
0.0010
MR leave−one−out sensitivity analysis for
'ADSS2' on 'Bladder cancer'

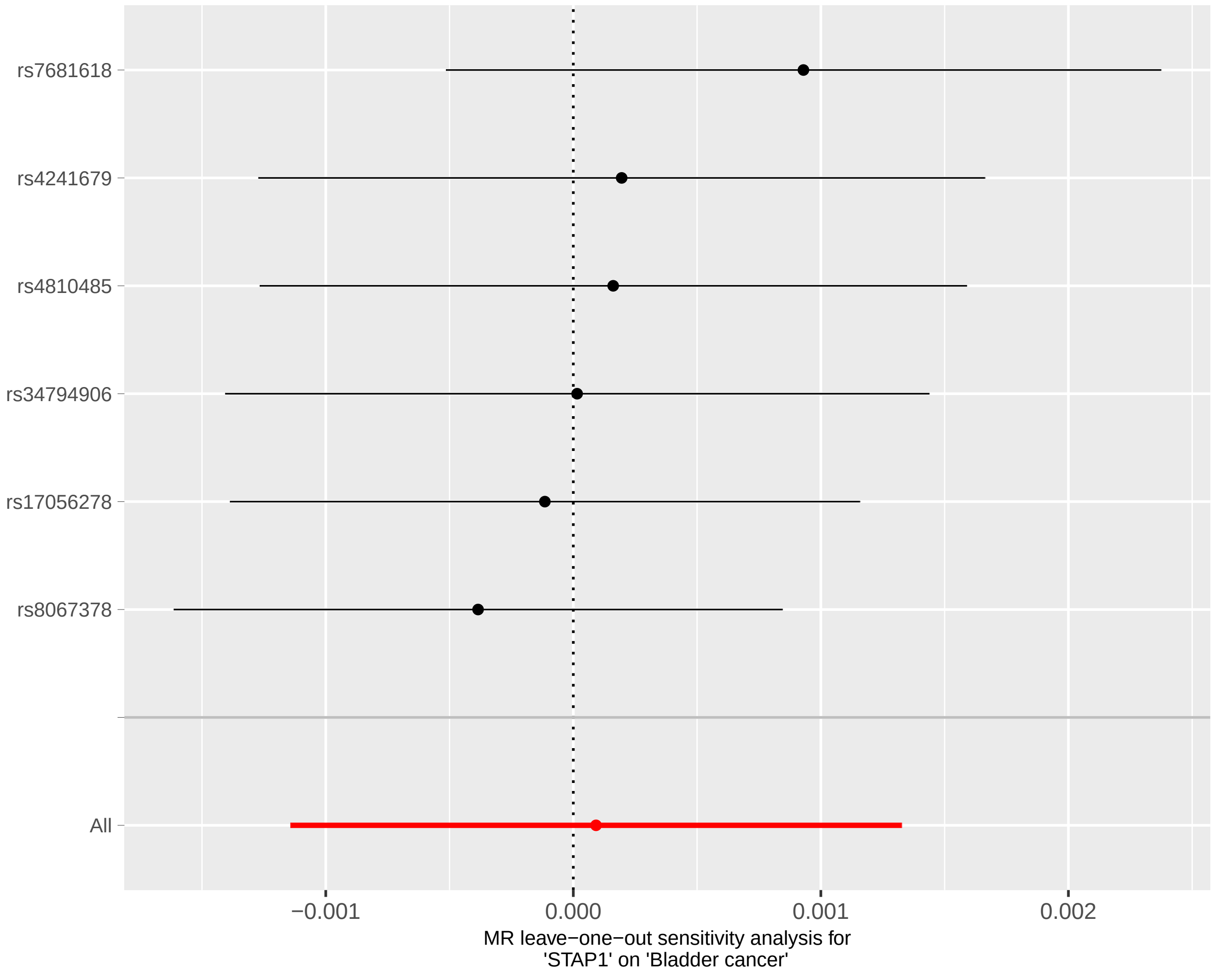
rs7681618
rs4241679
rs4810485
rs34794906
rs17056278
rs8067378
All
−0.001
0.000
0.001
0.002
MR leave−one−out sensitivity analysis for
'STAP1' on 'Bladder cancer'

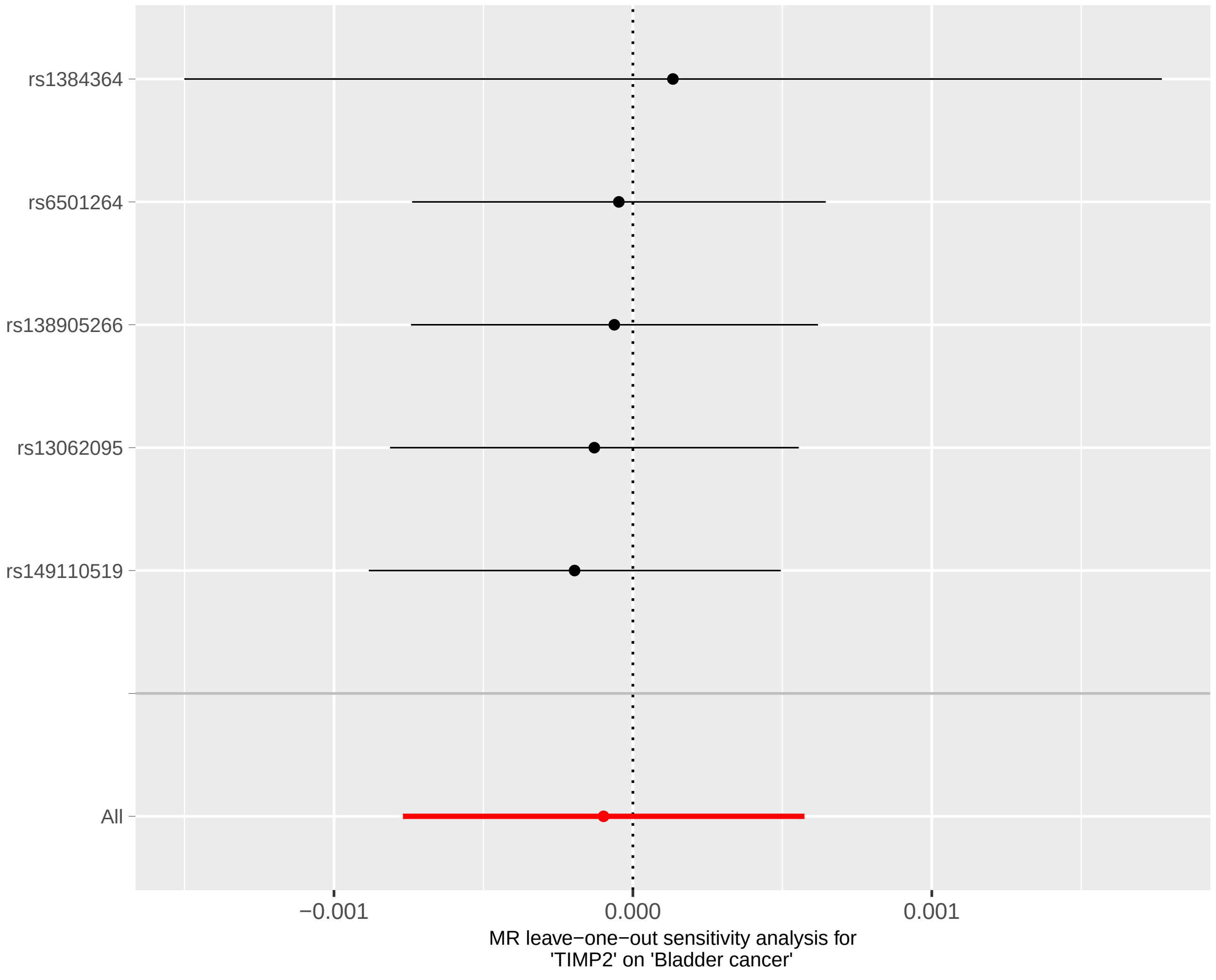
rs1384364
rs6501264
rs138905266
rs13062095
rs149110519
All
−0.001
0.000
0.001
MR leave−one−out sensitivity analysis for
'TIMP2' on 'Bladder cancer'

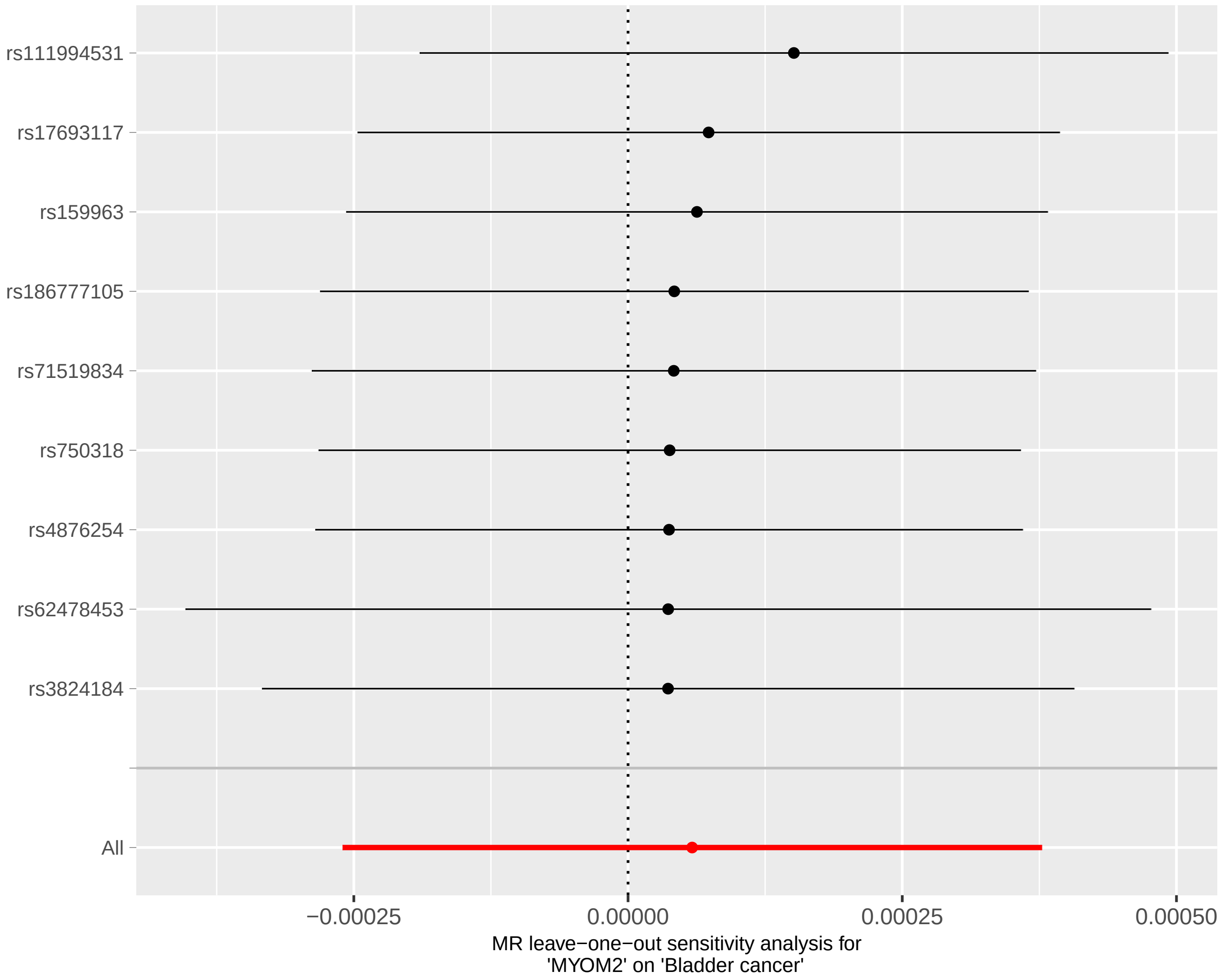
rs111994531
rs17693117
rs159963
rs186777105
rs71519834
rs750318
rs4876254
rs62478453
rs3824184
All
−0.00025
0.00000
0.00025
0.00050
MR leave−one−out sensitivity analysis for
'MYOM2' on 'Bladder cancer'

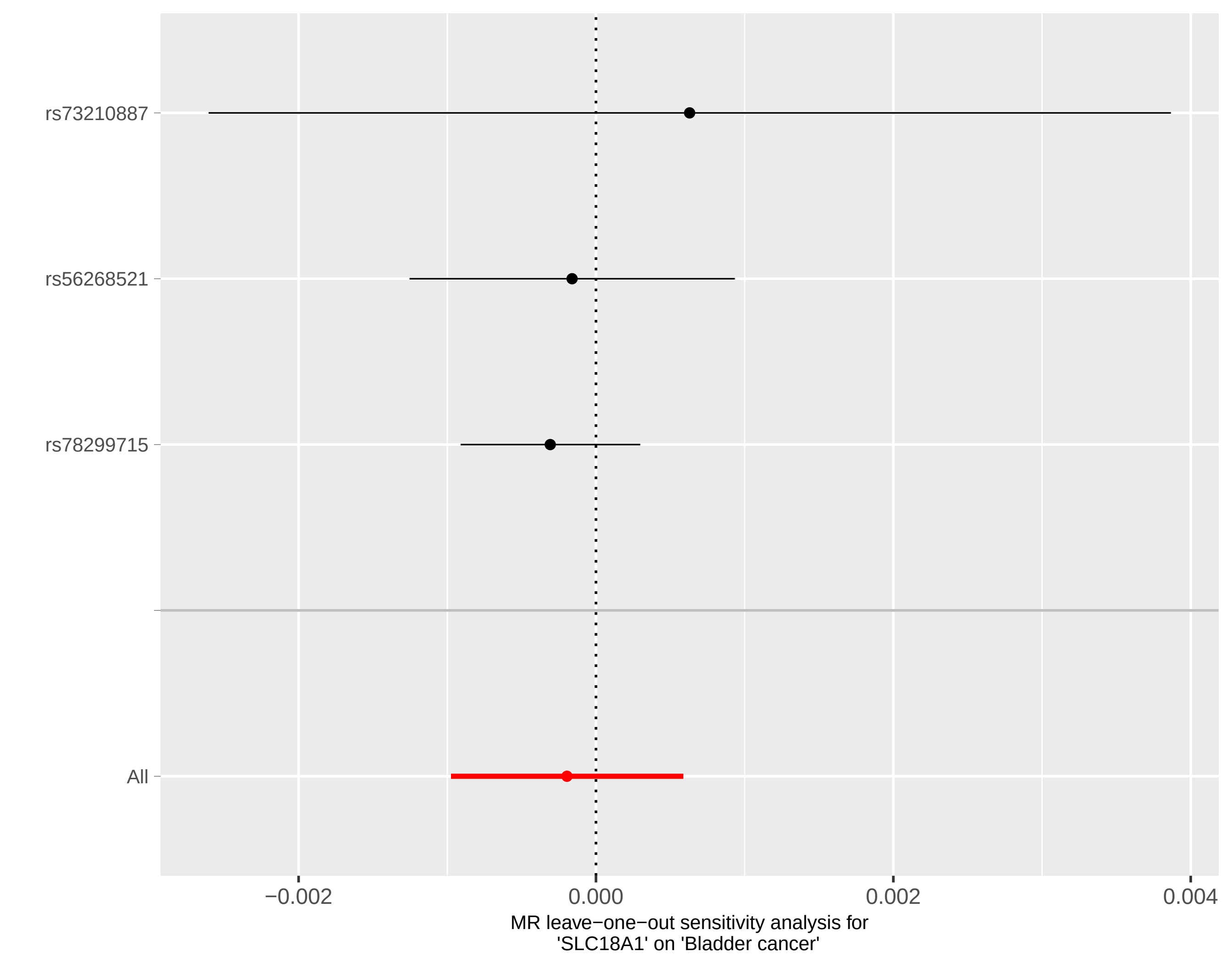
rs73210887
rs56268521
rs78299715
All
−0.002
0.000
0.002
0.004
MR leave−one−out sensitivity analysis for
'SLC18A1' on 'Bladder cancer'

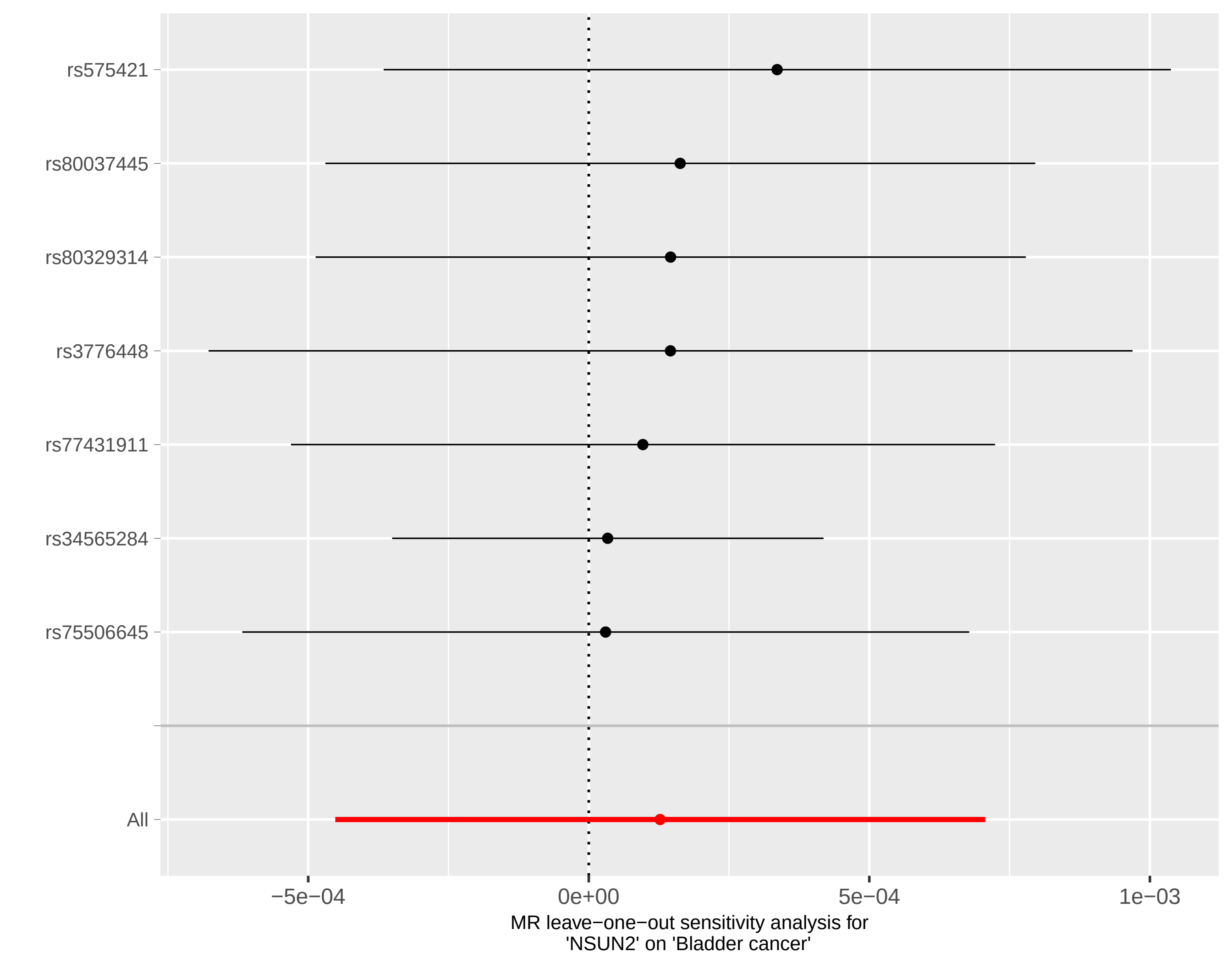
rs575421
rs80037445
rs80329314
rs3776448
rs77431911
rs34565284
rs75506645
All
−5e−04
0e+00
5e−04
1e−03
MR leave−one−out sensitivity analysis for
'NSUN2' on 'Bladder cancer'

Insufficient number of SNPs

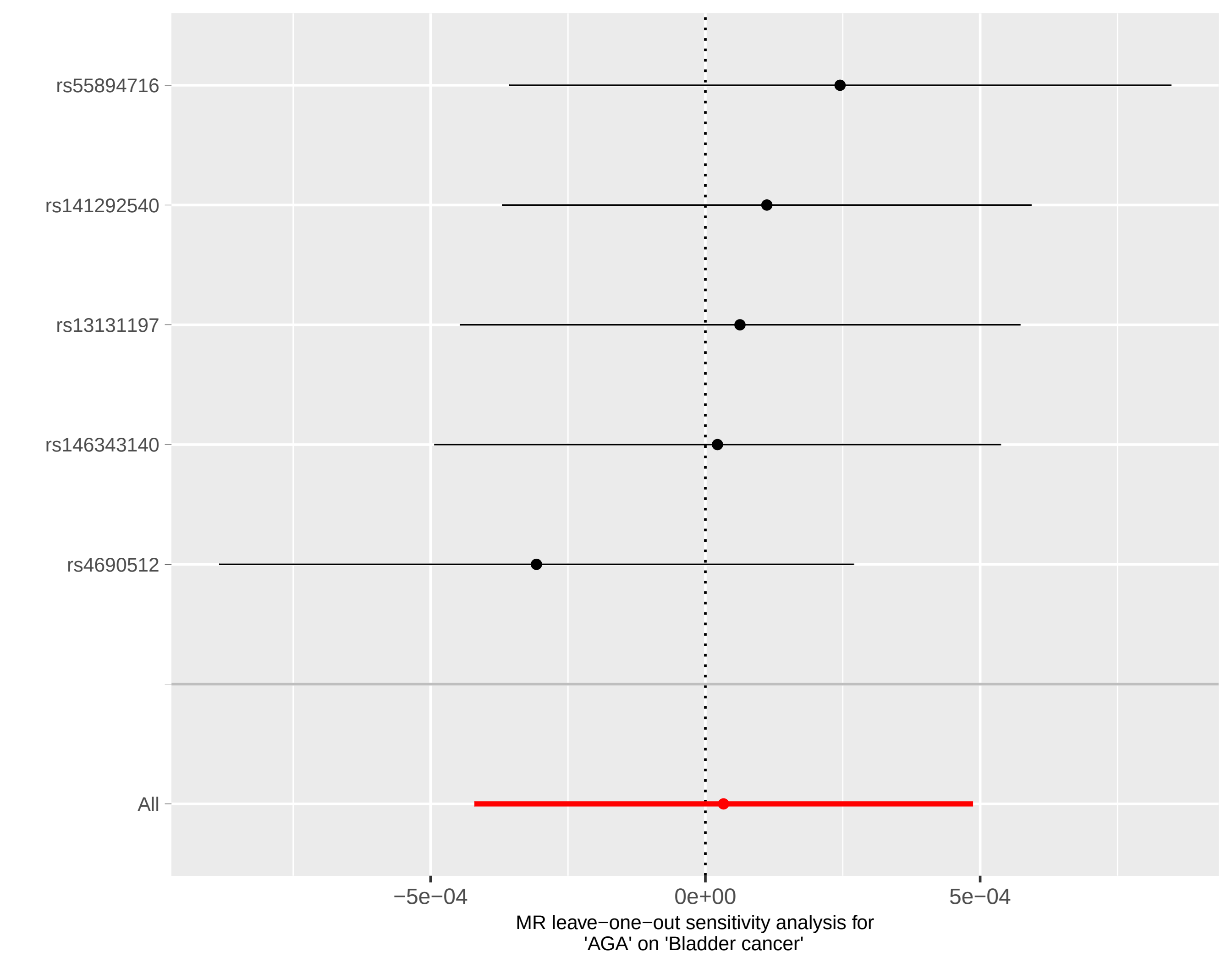
rs55894716
rs141292540
rs13131197
rs146343140
rs4690512
All
−5e−04
0e+00
5e−04
MR leave−one−out sensitivity analysis for
'AGA' on 'Bladder cancer'

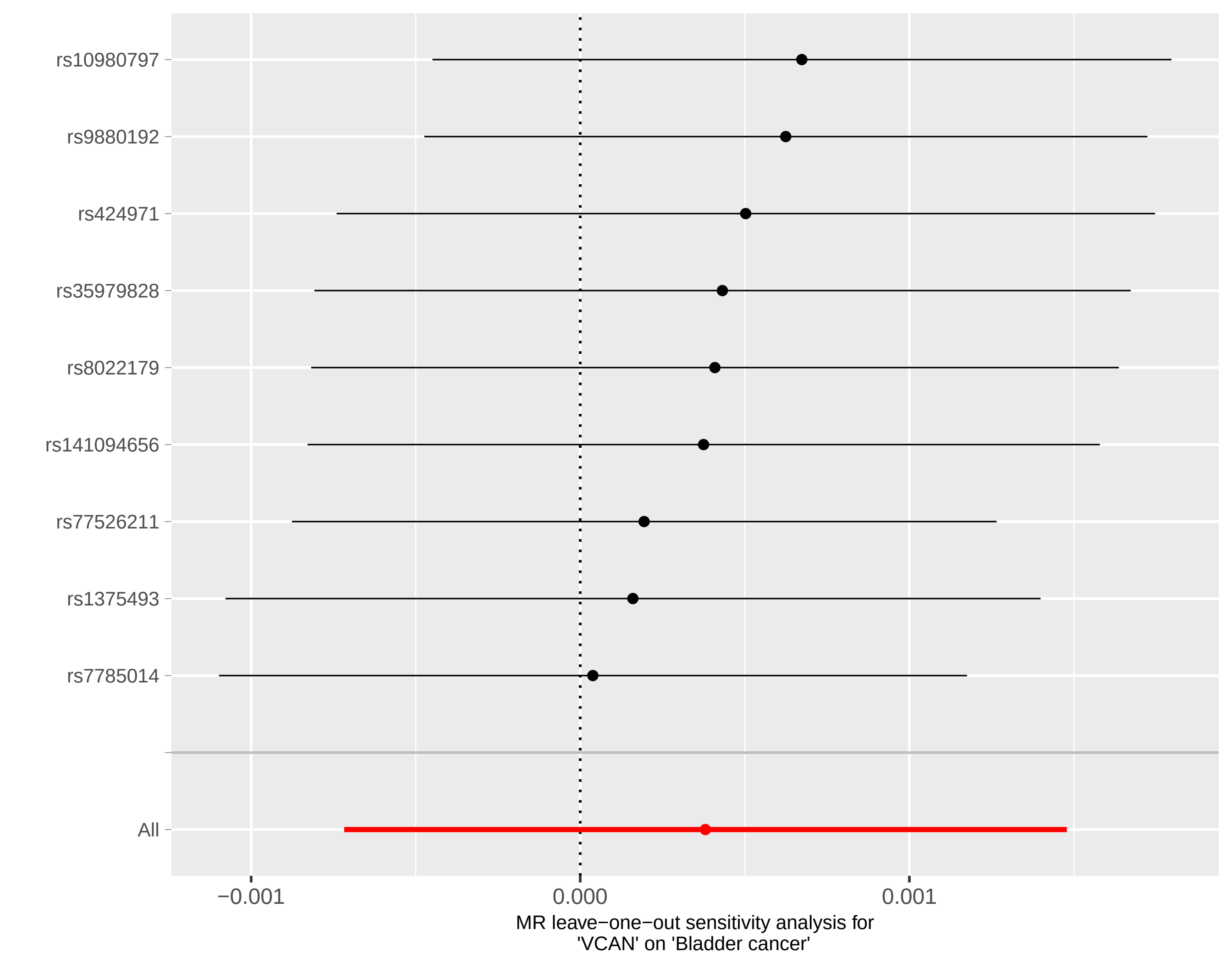

rs10980797
rs9880192
rs424971
rs35979828
rs8022179
rs141094656
rs77526211
rs1375493
rs7785014
All
−0.001
0.000
0.001
MR leave−one−out sensitivity analysis for
'VCAN' on 'Bladder cancer'

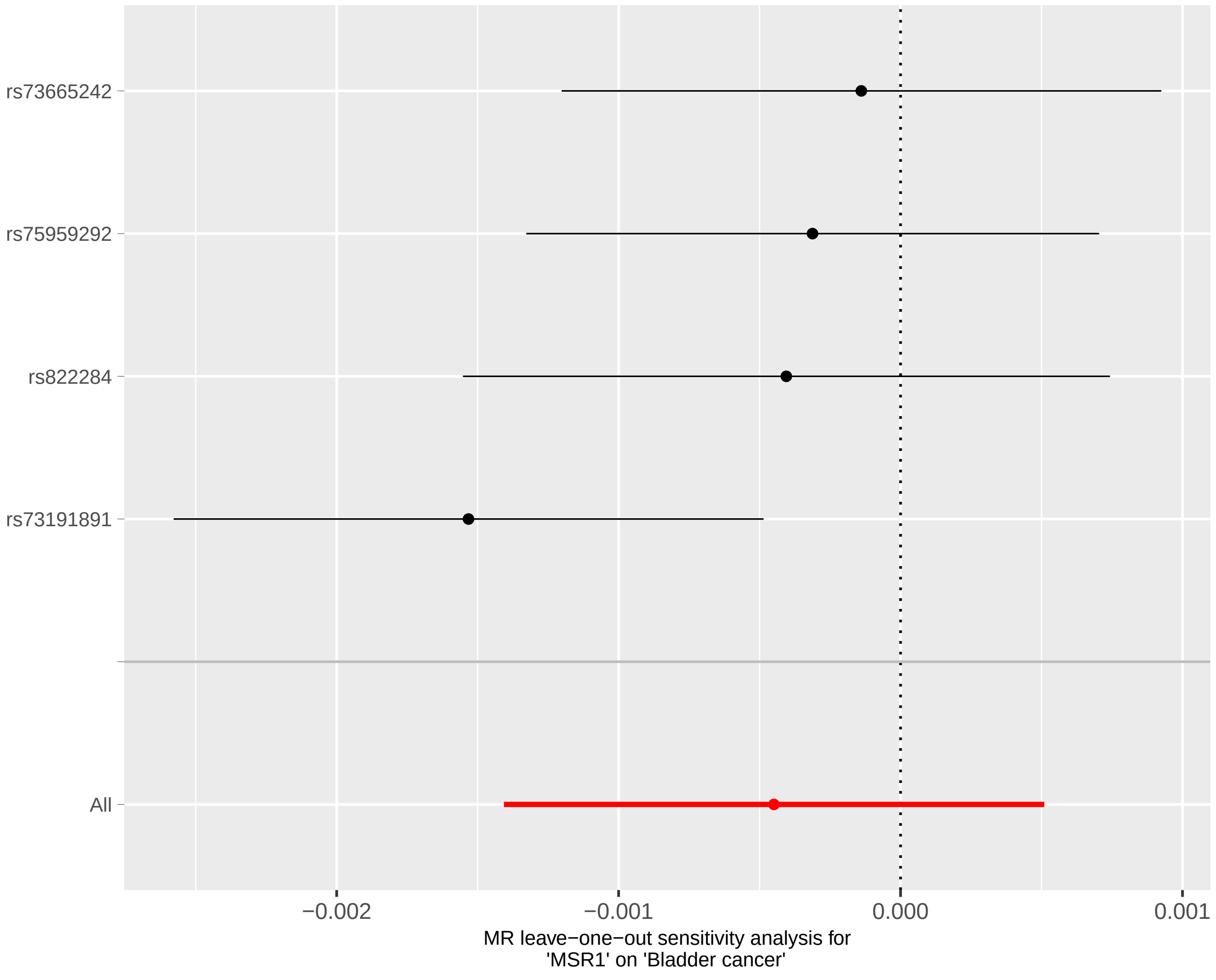

rs73665242
rs75959292
rs822284
rs73191891
All
−0.002
−0.001
0.000
0.001
MR leave−one−out sensitivity analysis for
'MSR1' on 'Bladder cancer'

Insufficient number of SNPs

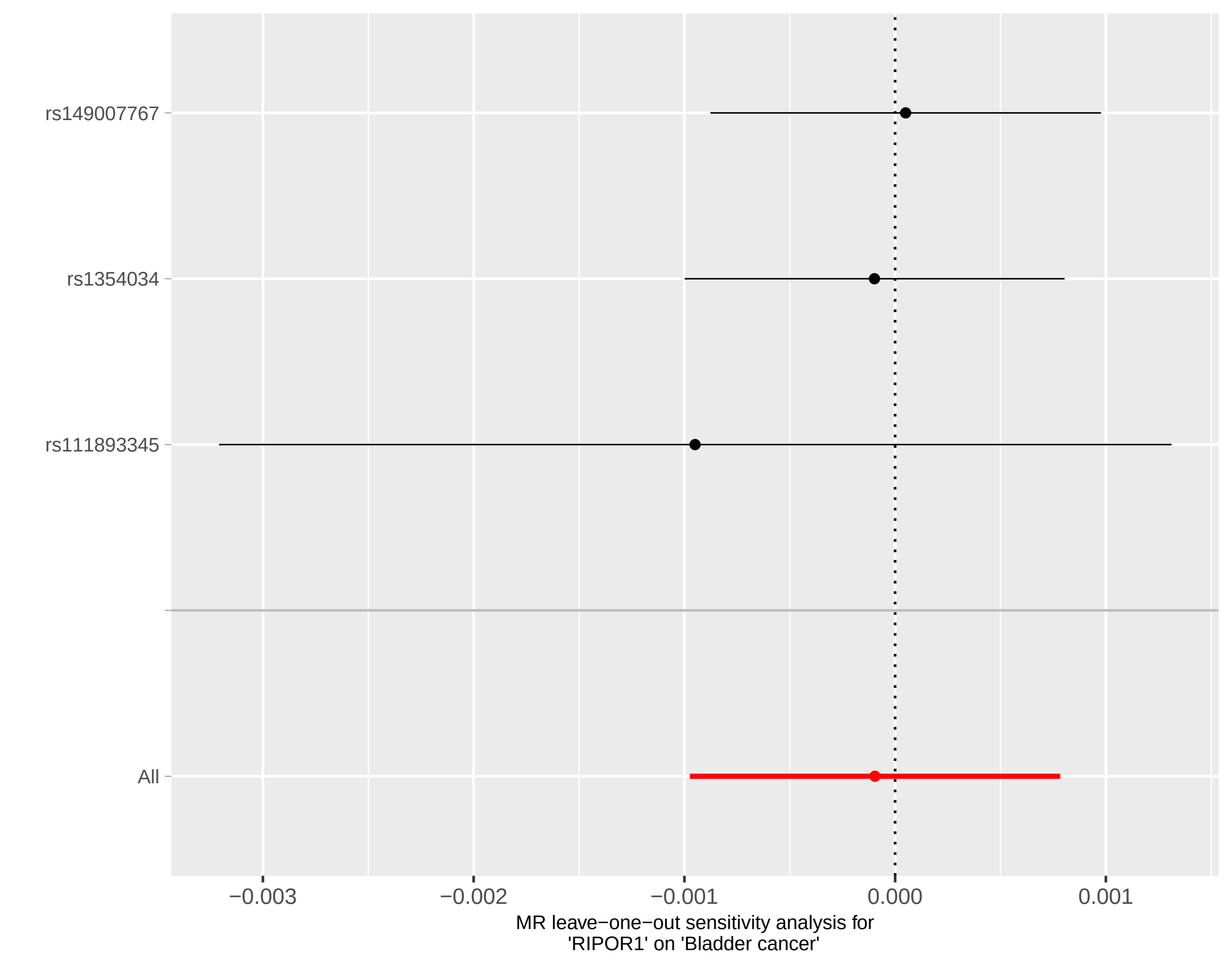
rs149007767
rs1354034
rs111893345
All
−0.003
−0.002
−0.001
0.000
0.001
MR leave−one−out sensitivity analysis for
'RIPOR1' on 'Bladder cancer'

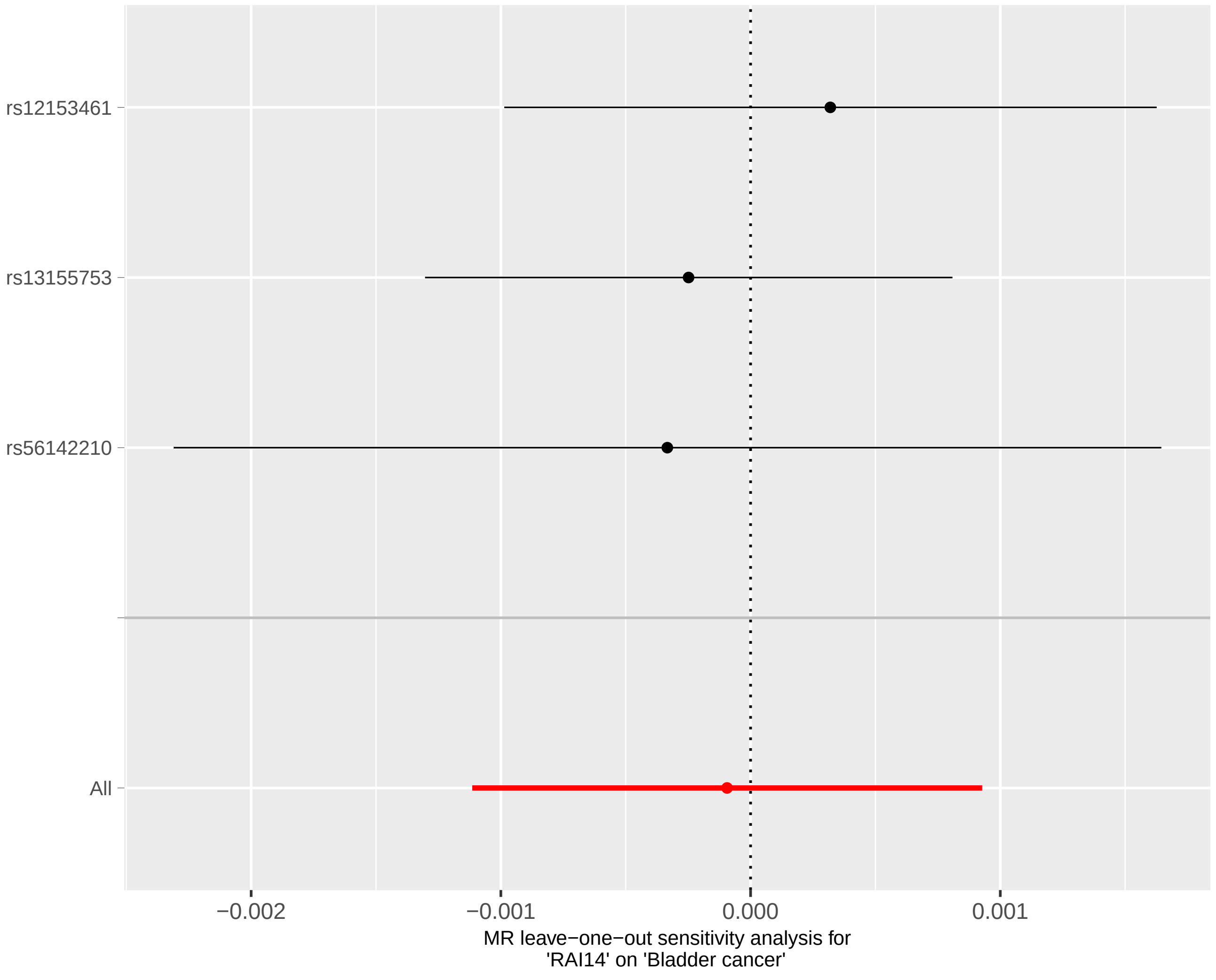
rs12153461
rs13155753
rs56142210
All
−0.002
−0.001
0.000
0.001
MR leave−one−out sensitivity analysis for
'RAI14' on 'Bladder cancer'

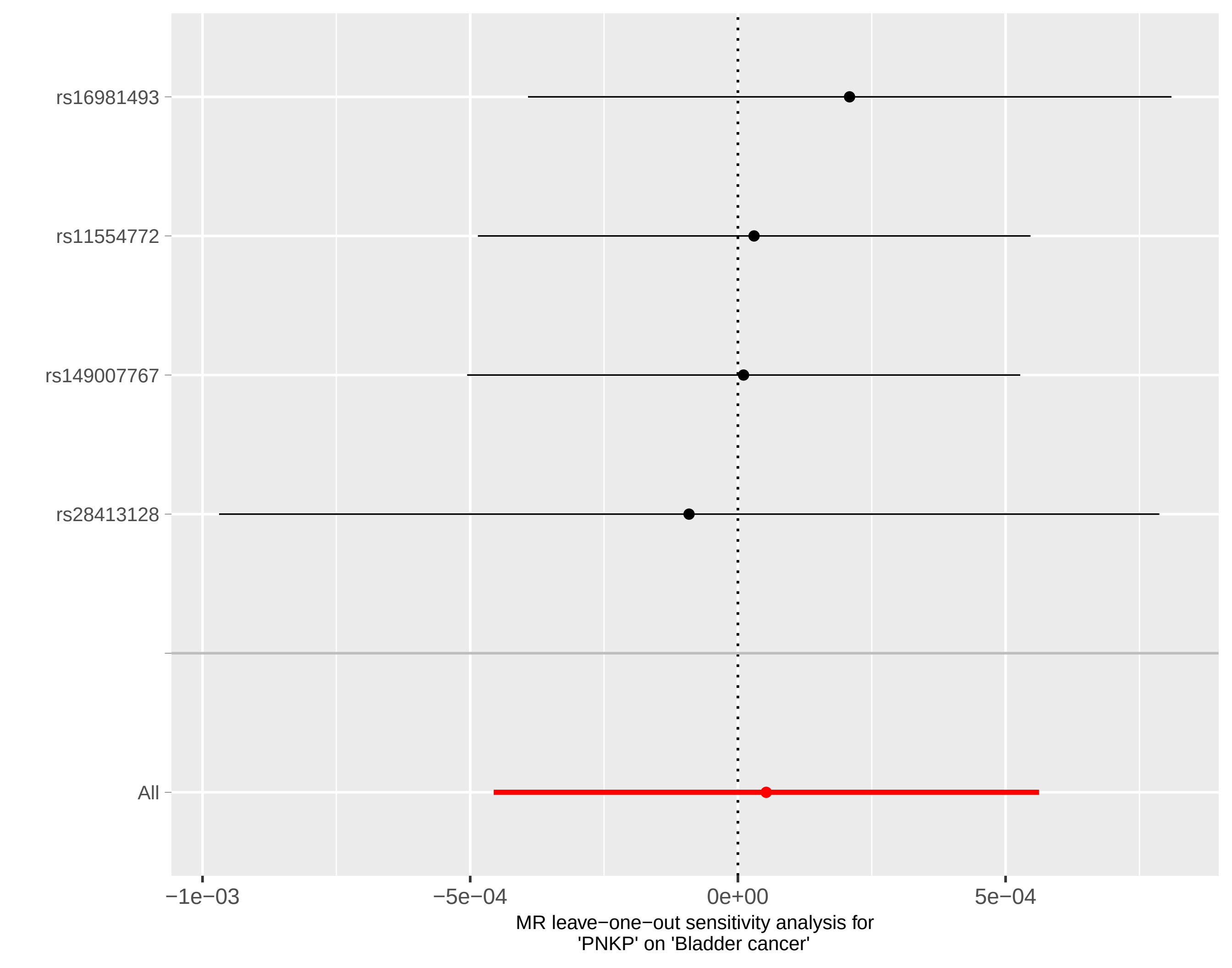
rs16981493
rs11554772
rs149007767
rs28413128
All
−1e−03
−5e−04
0e+00
5e−04
MR leave−one−out sensitivity analysis for
'PNKP' on 'Bladder cancer'

Insufficient number of SNPs

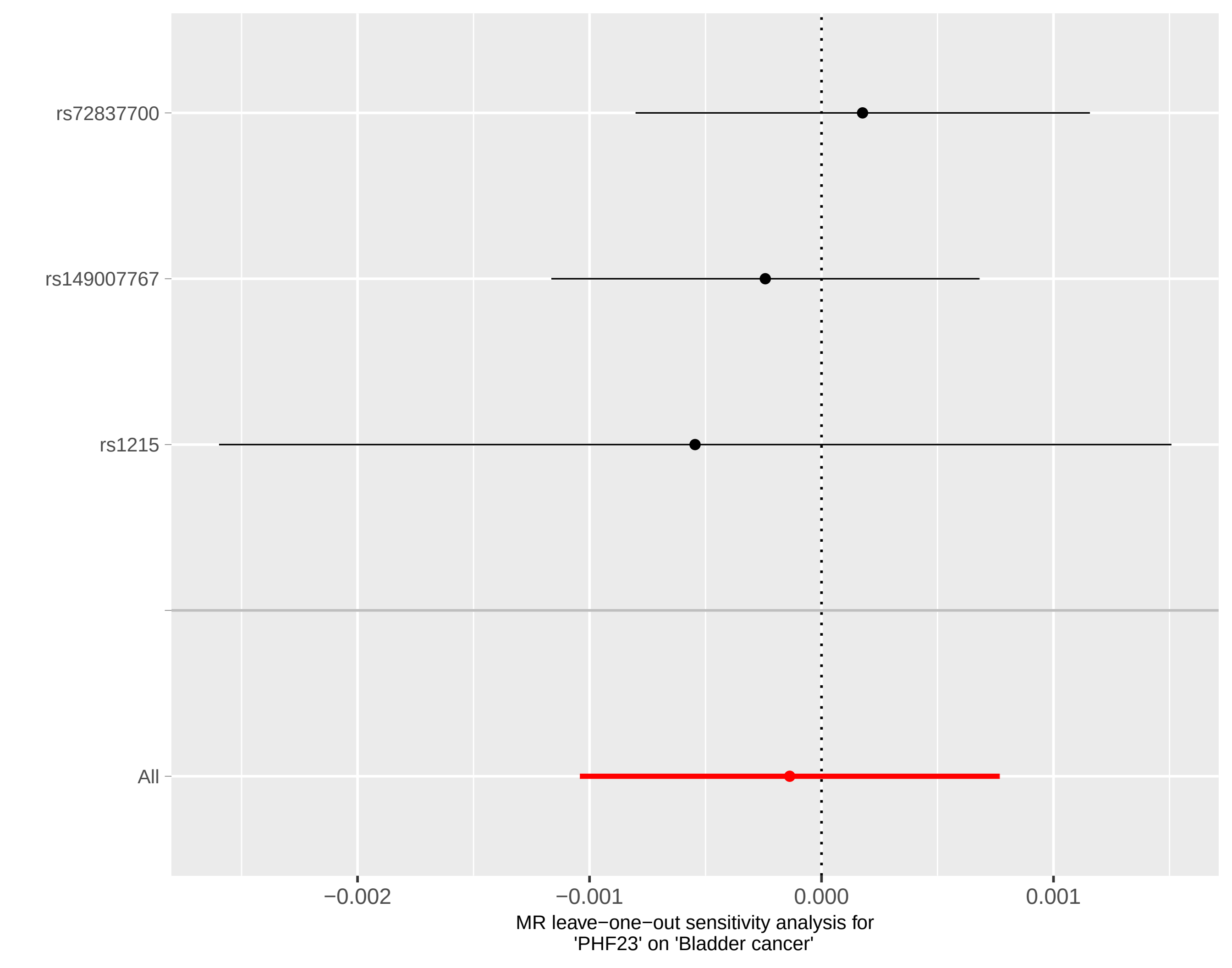
rs72837700
rs149007767
rs1215
All
−0.002
−0.001
0.000
0.001
MR leave−one−out sensitivity analysis for
'PHF23' on 'Bladder cancer'

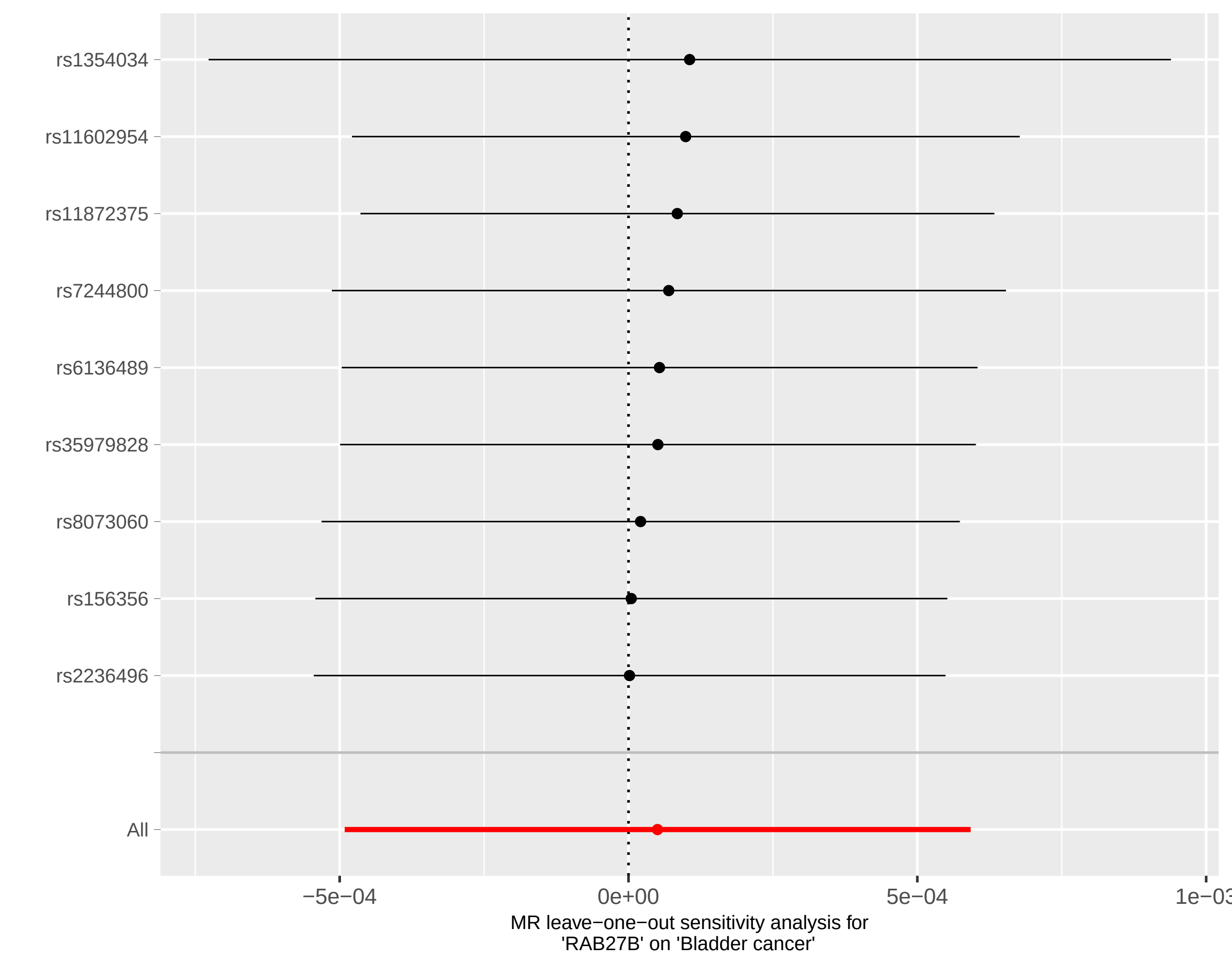
rs1354034
rs11602954
rs11872375
rs7244800
rs6136489
rs35979828
rs8073060
rs156356
rs2236496
All
−5e−04
0e+00
5e−04
1e−03
MR leave−one−out sensitivity analysis for
'RAB27B' on 'Bladder cancer'

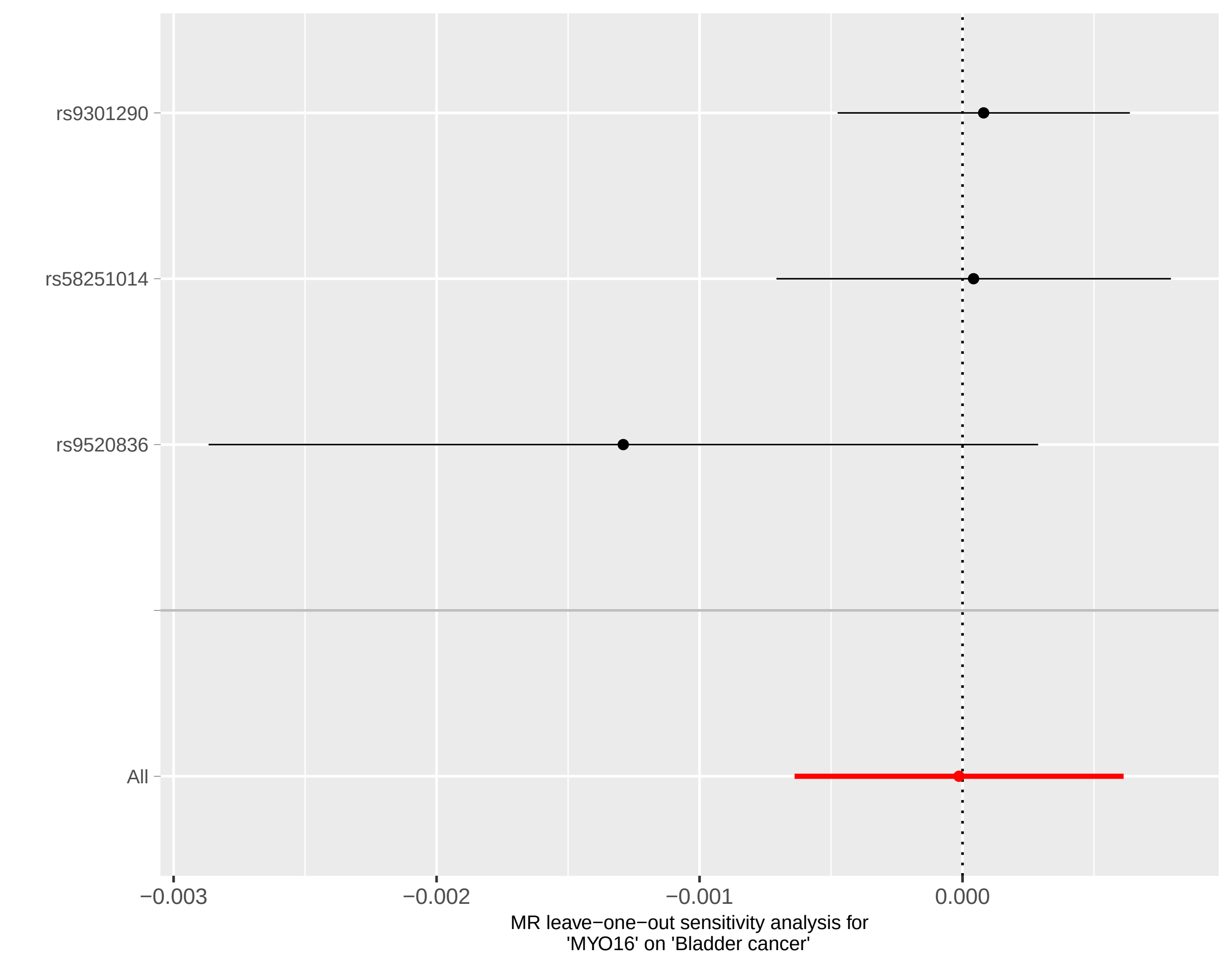
rs9301290
rs58251014
rs9520836
All
−0.003
−0.002
−0.001
0.000
MR leave−one−out sensitivity analysis for
'MYO16' on 'Bladder cancer'

Insufficient number of SNPs

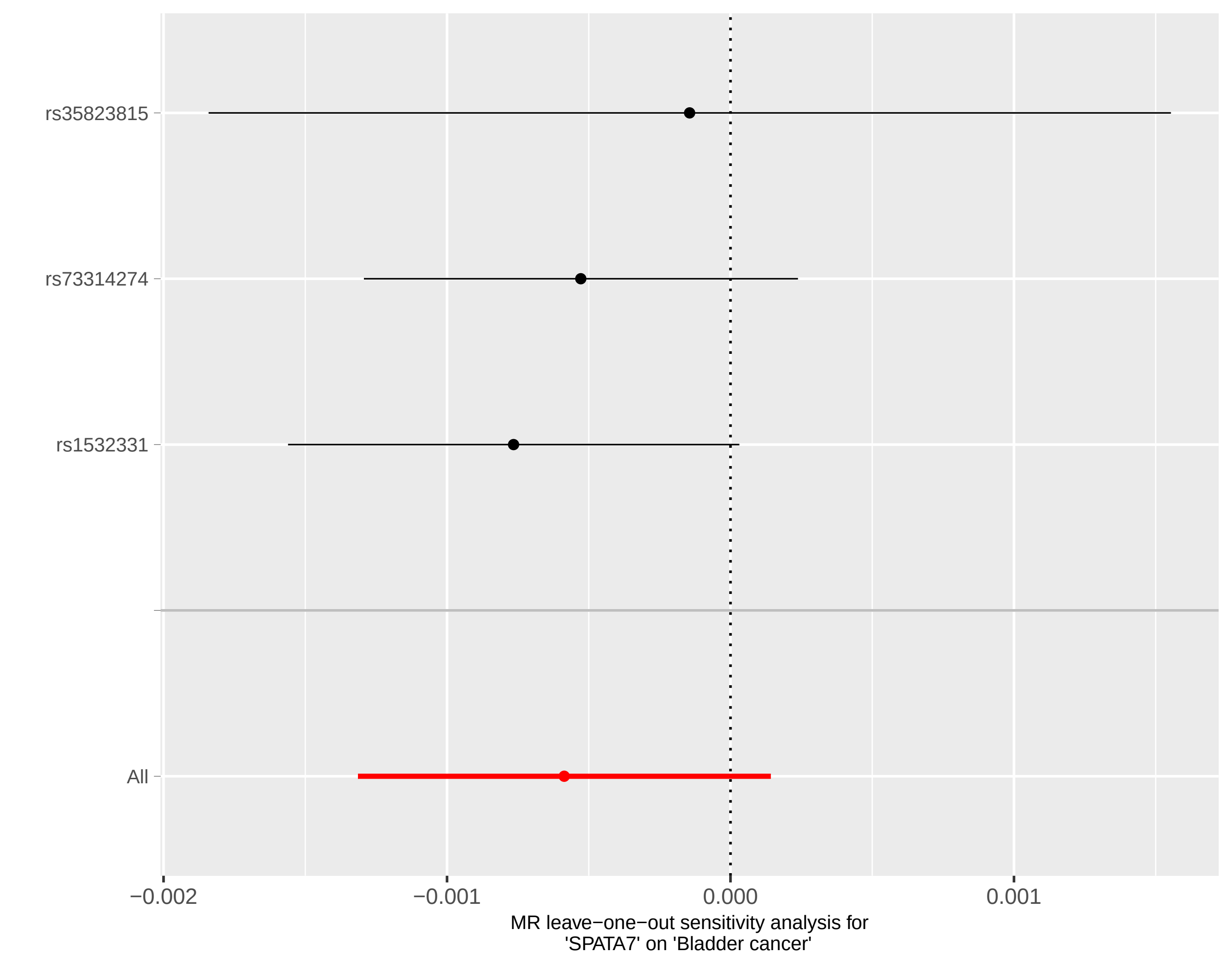
rs35823815
rs73314274
rs1532331
All
−0.002
−0.001
0.000
0.001
MR leave−one−out sensitivity analysis for
'SPATA7' on 'Bladder cancer'

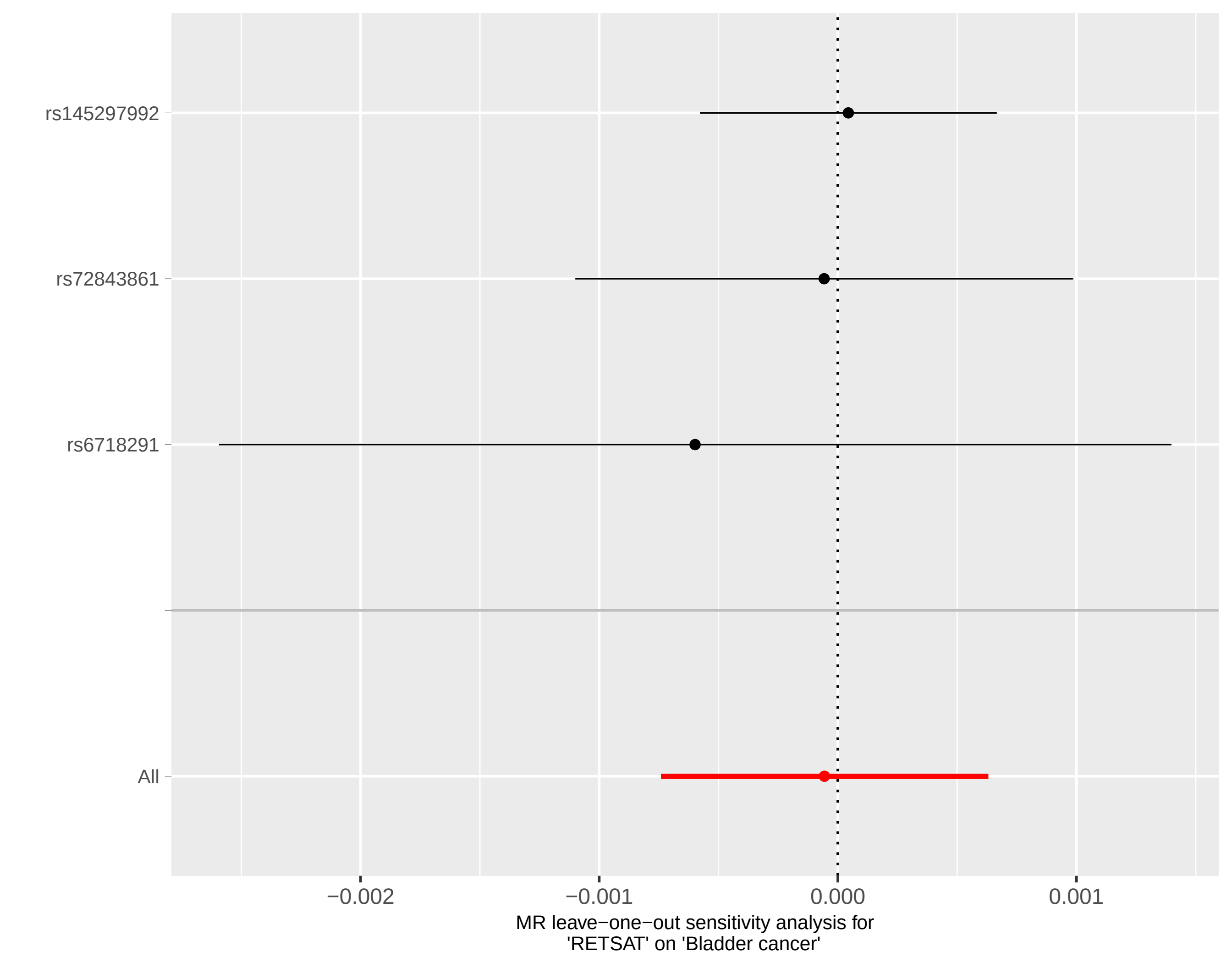
rs145297992
rs72843861
rs6718291
All
−0.002
−0.001
0.000
0.001
MR leave−one−out sensitivity analysis for
'RETSAT' on 'Bladder cancer'

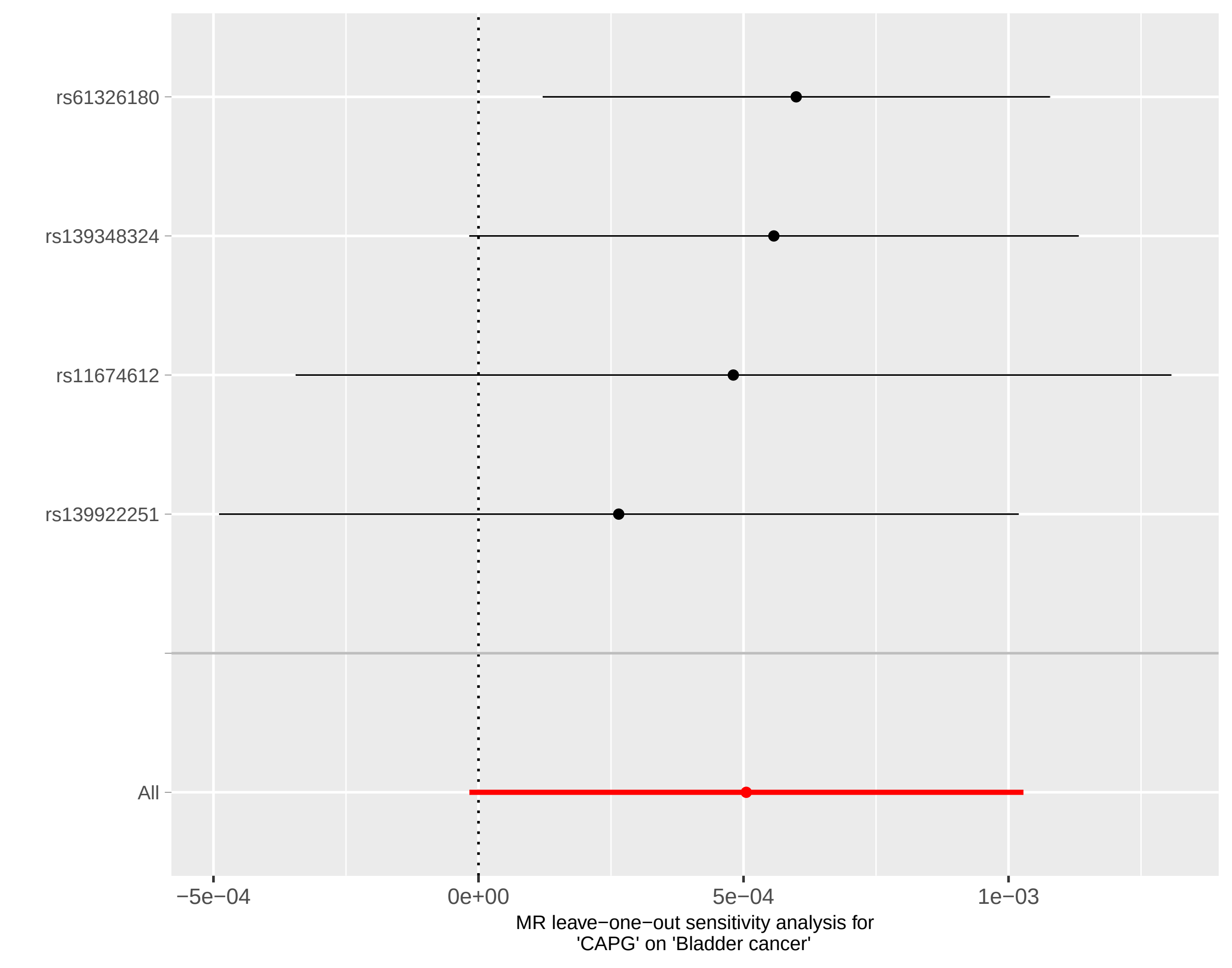
rs61326180
rs139348324
rs11674612
rs139922251
All
−5e−04
0e+00
5e−04
1e−03
MR leave−one−out sensitivity analysis for
'CAPG' on 'Bladder cancer'

Insufficient number of SNPs

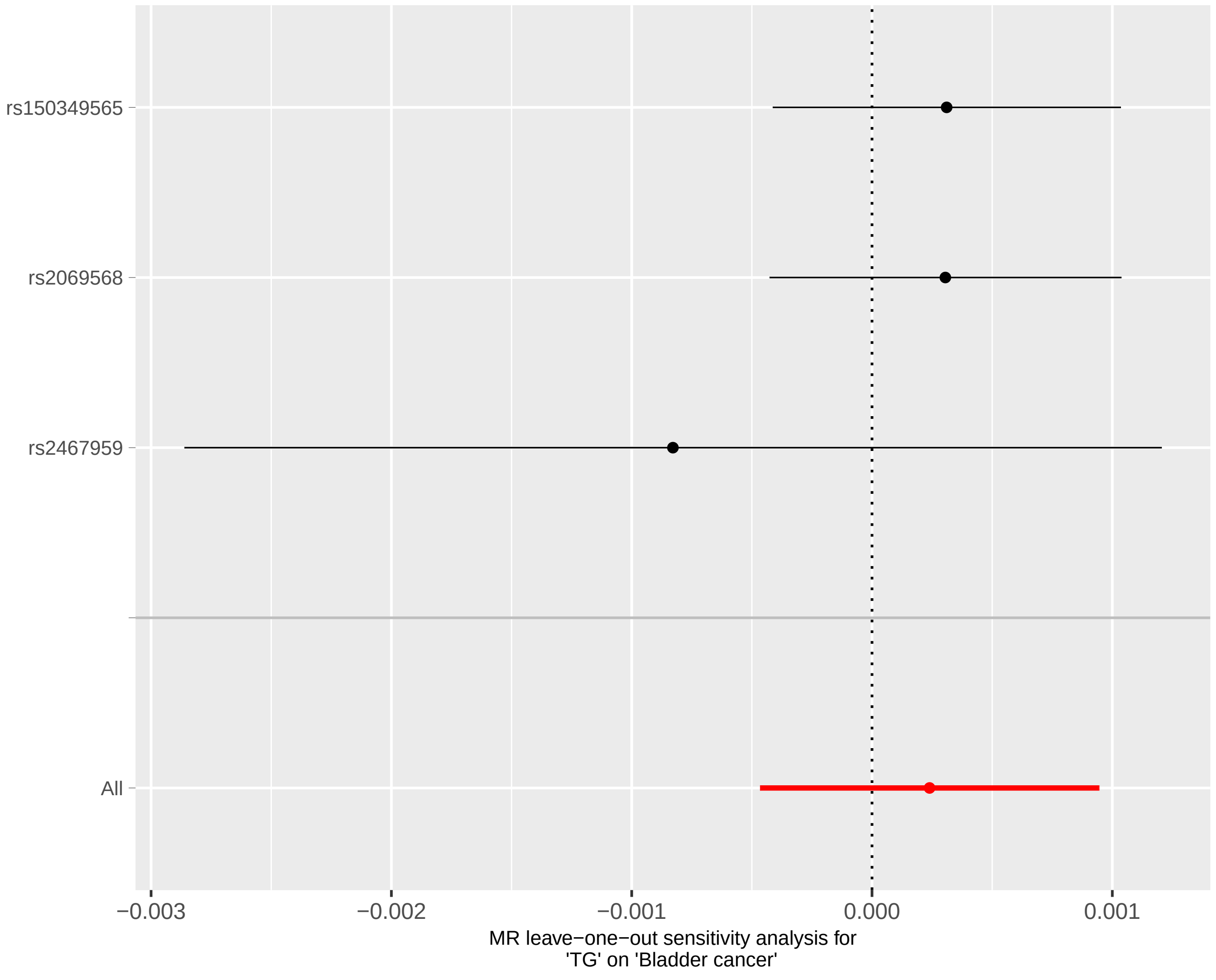
rs150349565
rs2069568
rs2467959
All
−0.003
−0.002
−0.001
0.000
0.001
MR leave−one−out sensitivity analysis for
'TG' on 'Bladder cancer'

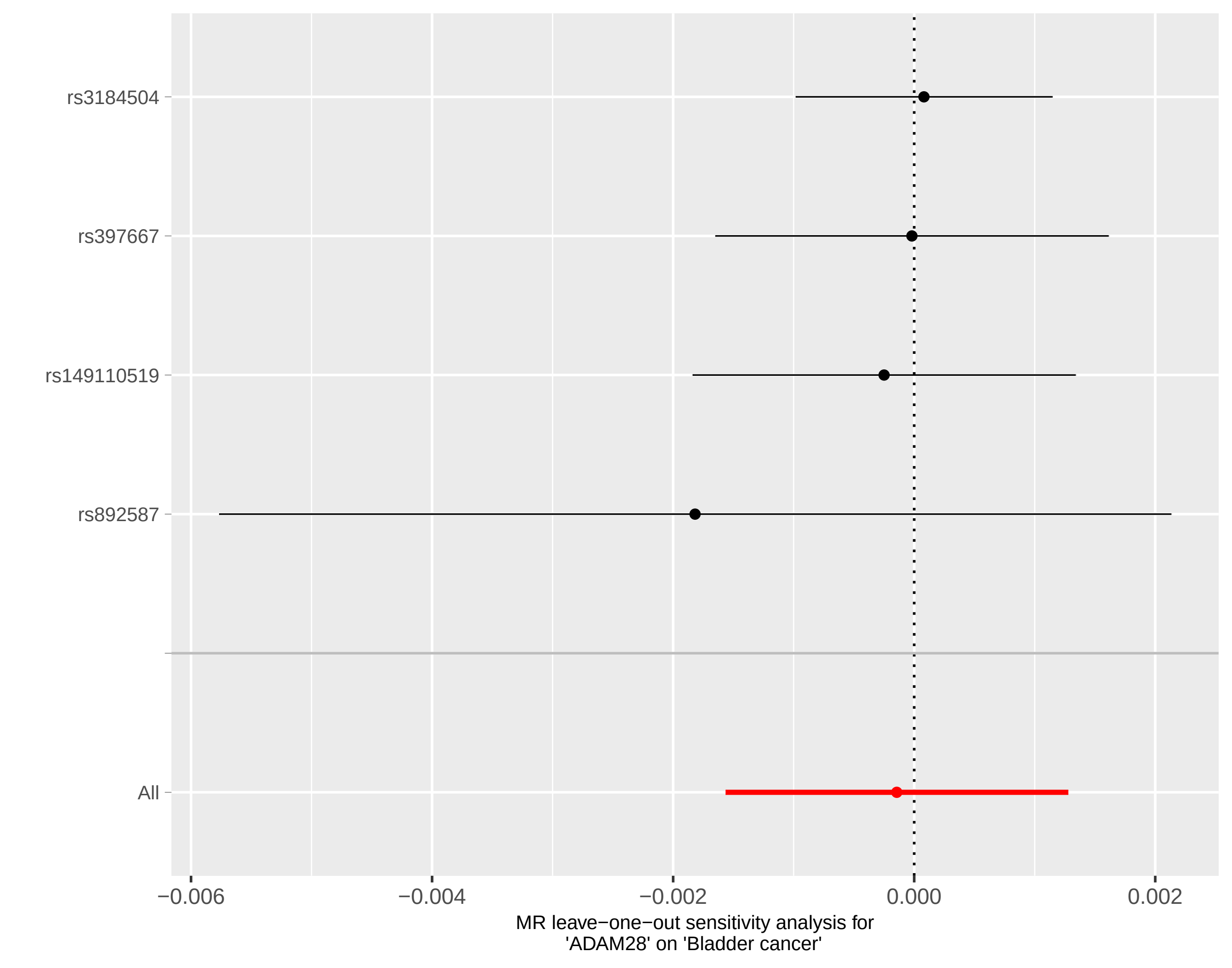
rs3184504
rs397667
rs149110519
rs892587
All
−0.006
−0.004
−0.002
0.000
0.002
MR leave−one−out sensitivity analysis for
'ADAM28' on 'Bladder cancer'

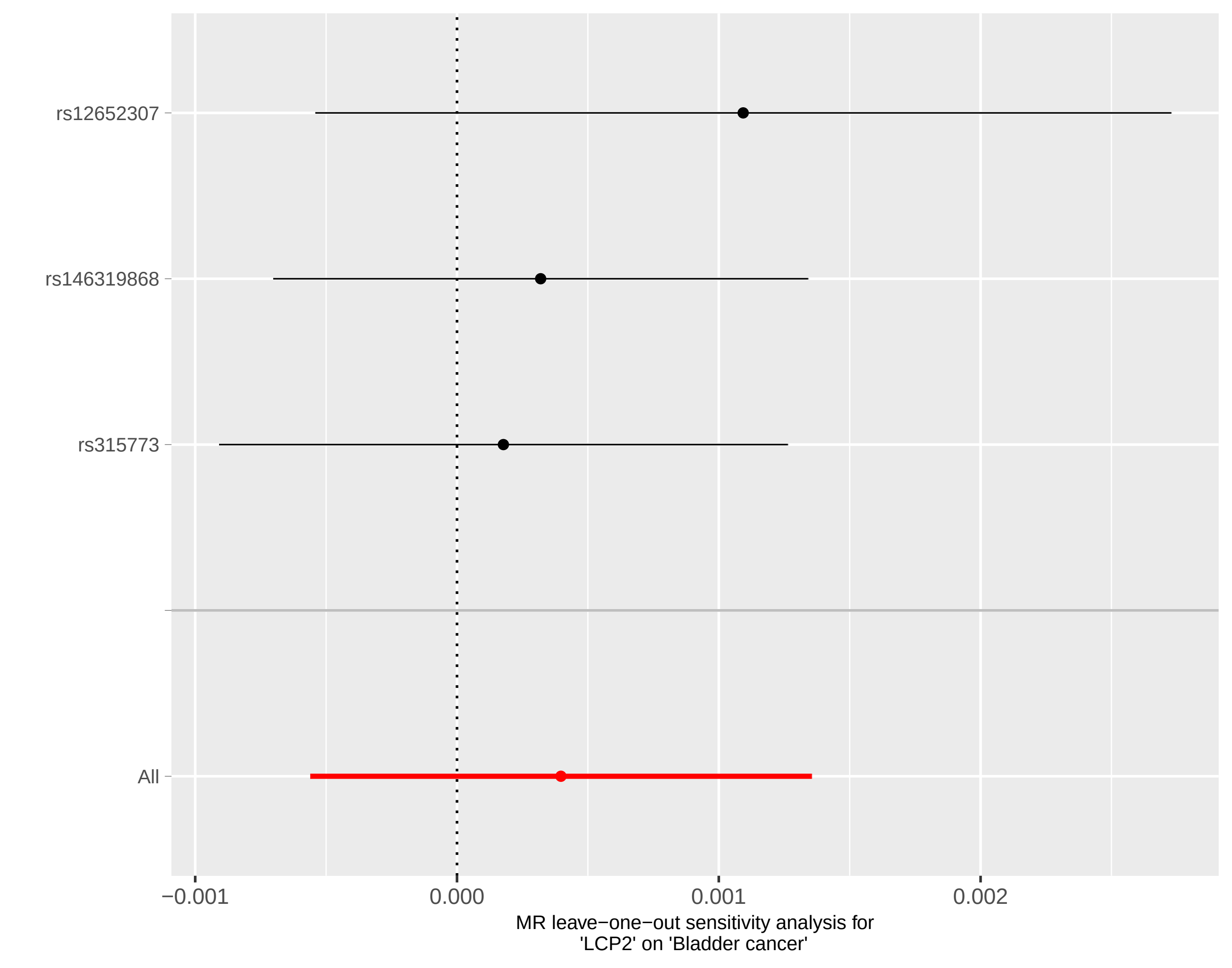
rs12652307
rs146319868
rs315773
All
−0.001
0.000
0.001
0.002
MR leave−one−out sensitivity analysis for
'LCP2' on 'Bladder cancer'

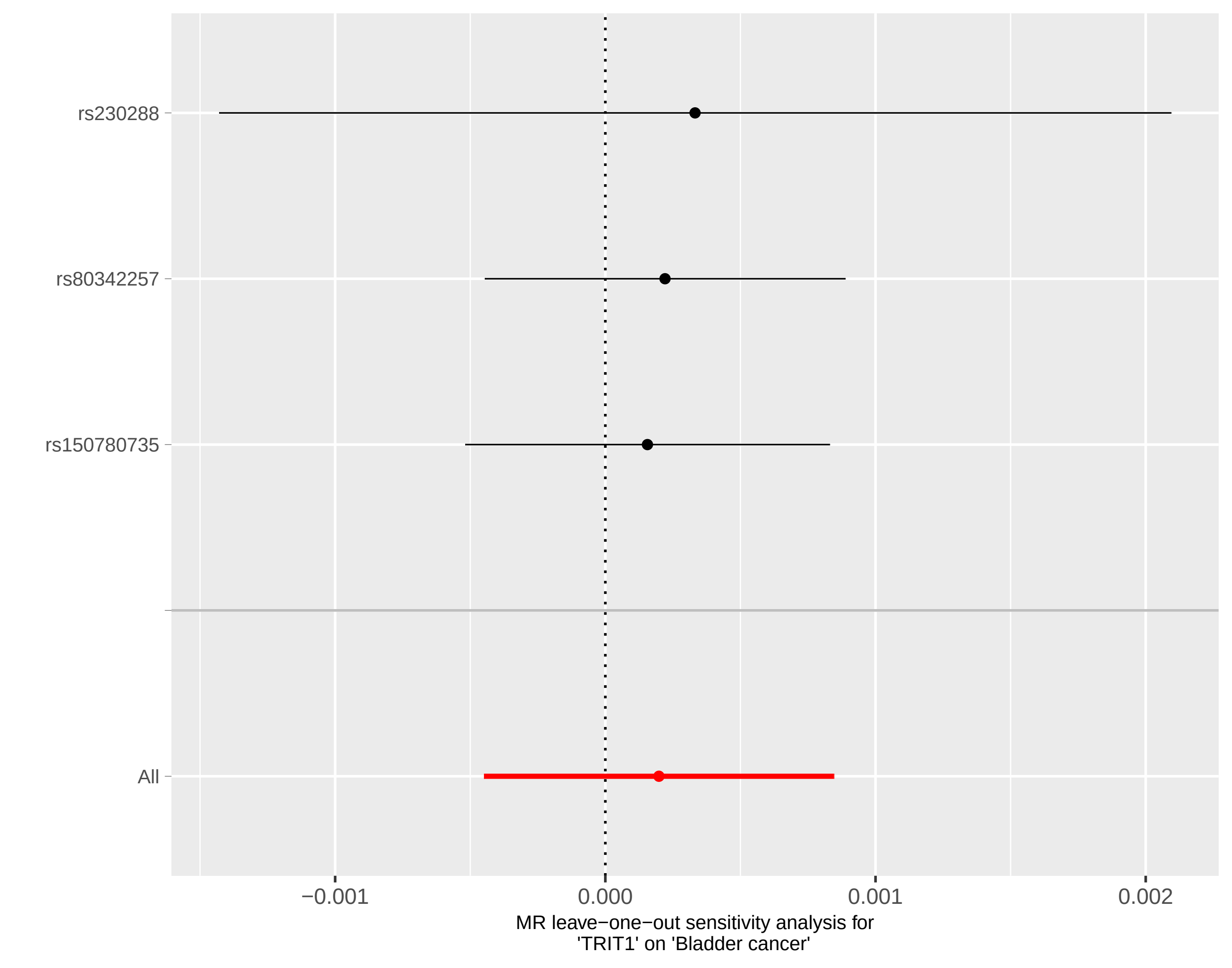
rs230288
rs80342257
rs150780735
All
−0.001
0.000
0.001
0.002
MR leave−one−out sensitivity analysis for
'TRIT1' on 'Bladder cancer'

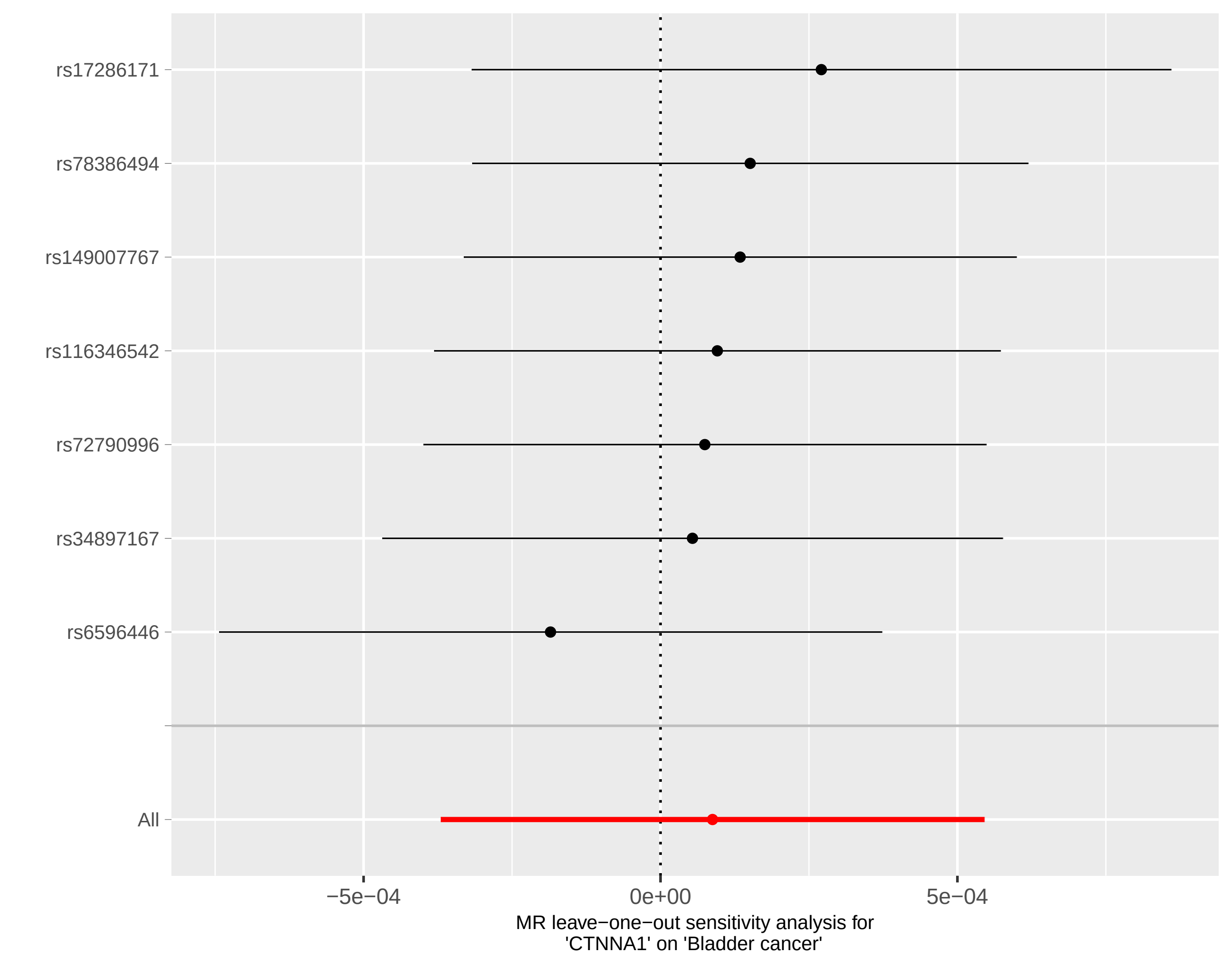
rs17286171
rs78386494
rs149007767
rs116346542
rs72790996
rs34897167
rs6596446
All
−5e−04
0e+00
5e−04
MR leave−one−out sensitivity analysis for
'CTNNA1' on 'Bladder cancer'

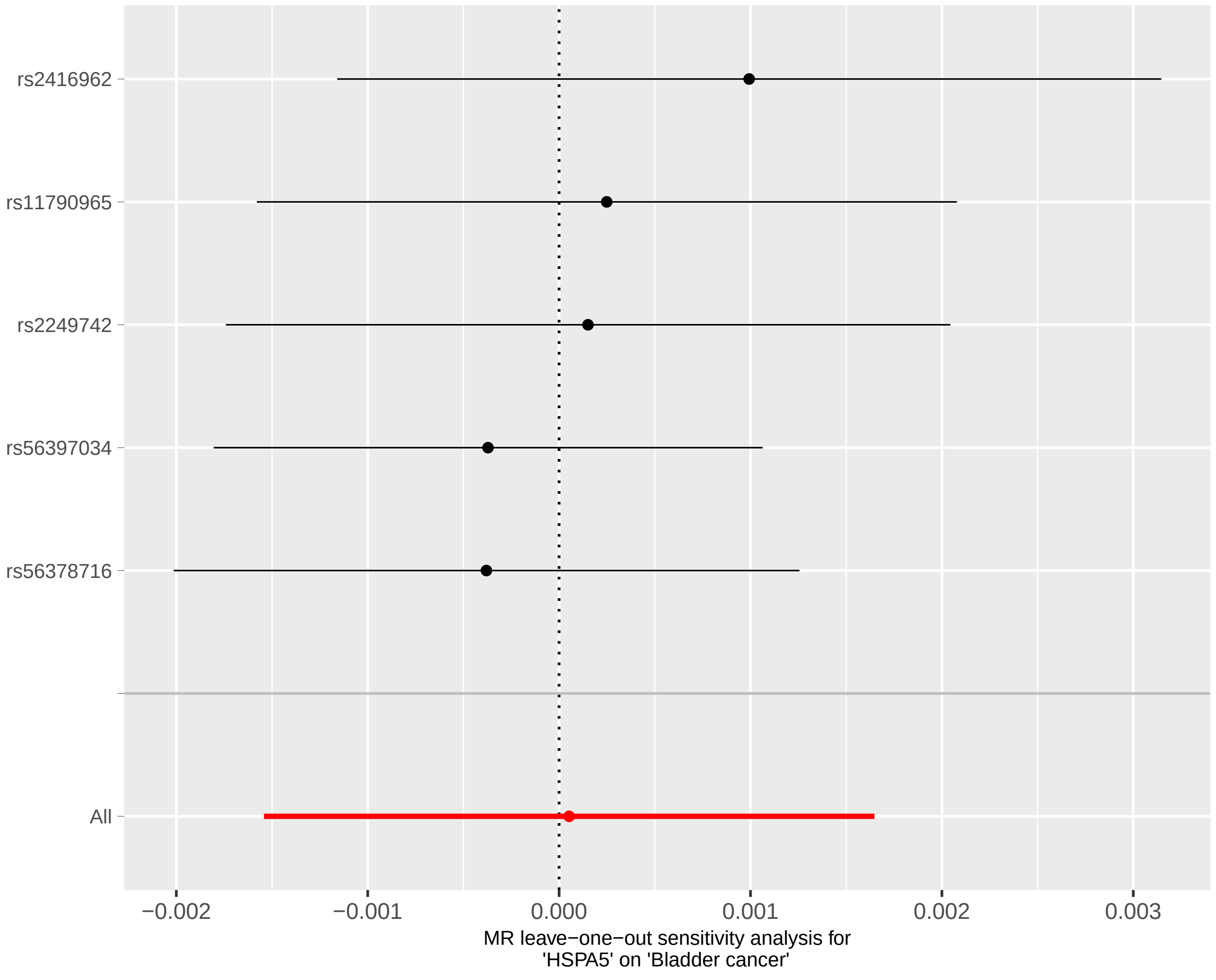
rs2416962
rs11790965
rs2249742
rs56397034
rs56378716
All
−0.002
−0.001
0.000
0.001
0.002
0.003
MR leave−one−out sensitivity analysis for
'HSPA5' on 'Bladder cancer'

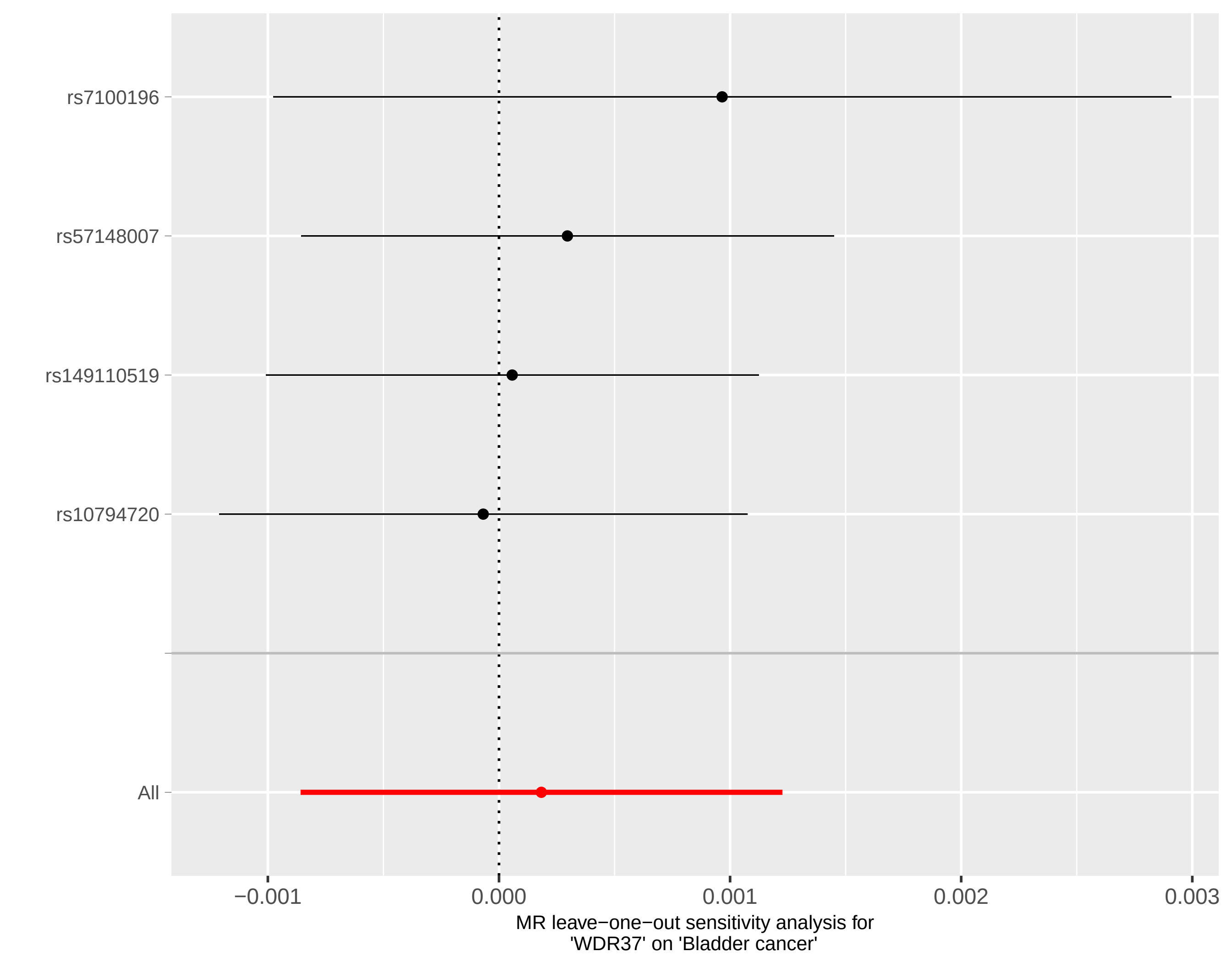
rs7100196
rs57148007
rs149110519
rs10794720
All
−0.001
0.000
0.001
0.002
0.003
MR leave−one−out sensitivity analysis for
'WDR37' on 'Bladder cancer'

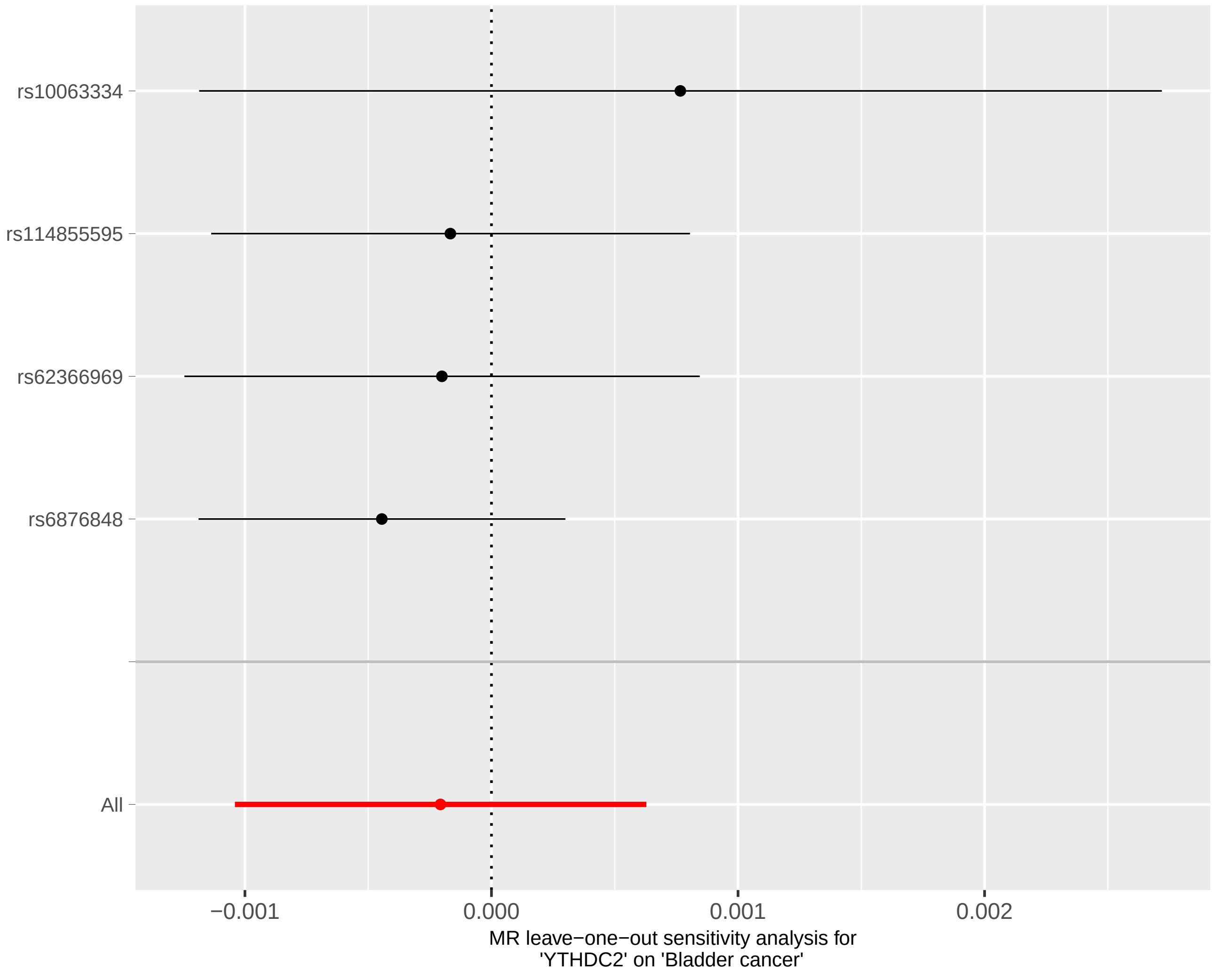
rs10063334
rs114855595
rs62366969
rs6876848
All
−0.001
0.000
0.001
0.002
MR leave−one−out sensitivity analysis for
'YTHDC2' on 'Bladder cancer'

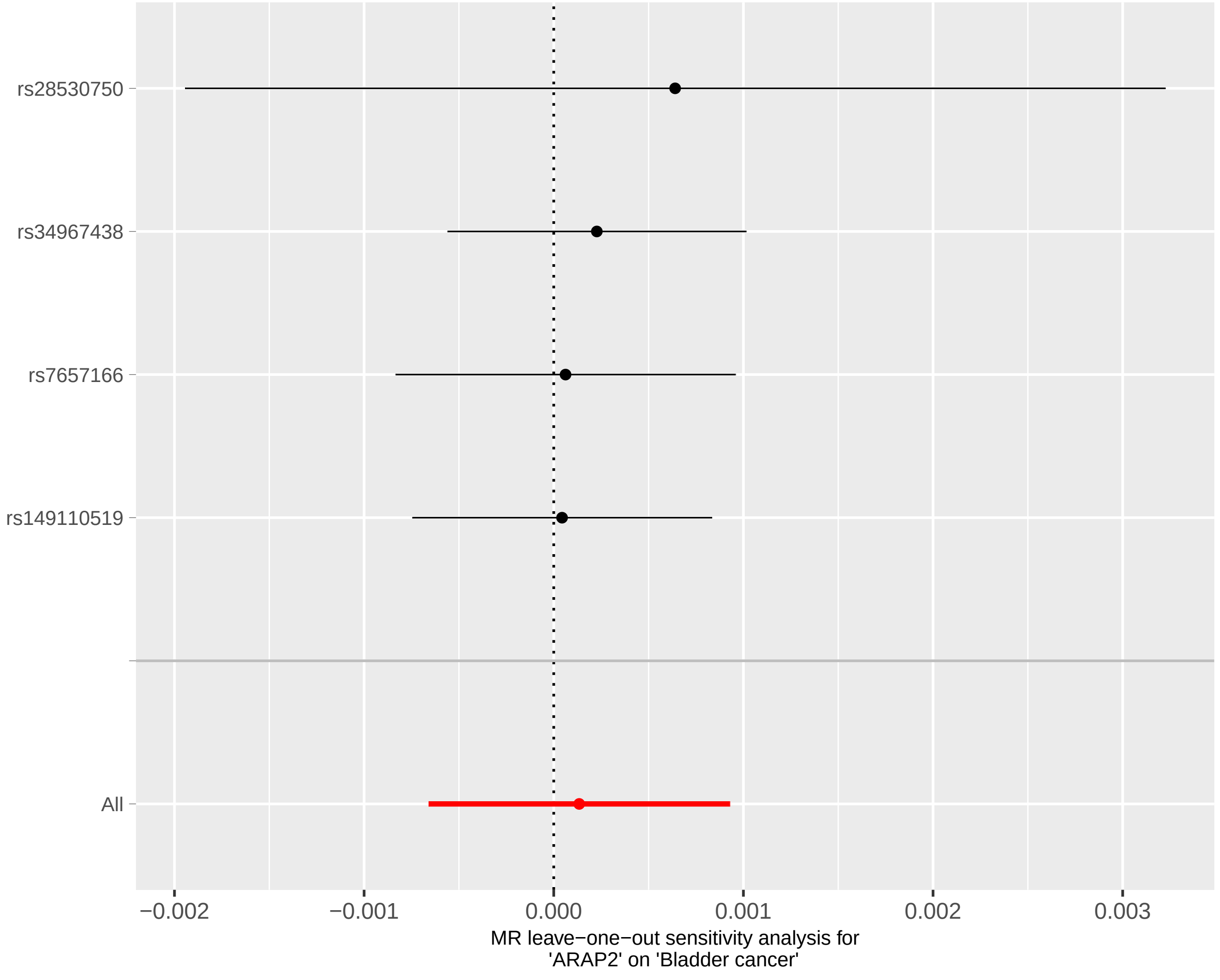
rs28530750
rs34967438
rs7657166
rs149110519
All
−0.002
−0.001
0.000
0.001
0.002
0.003
MR leave−one−out sensitivity analysis for
'ARAP2' on 'Bladder cancer'

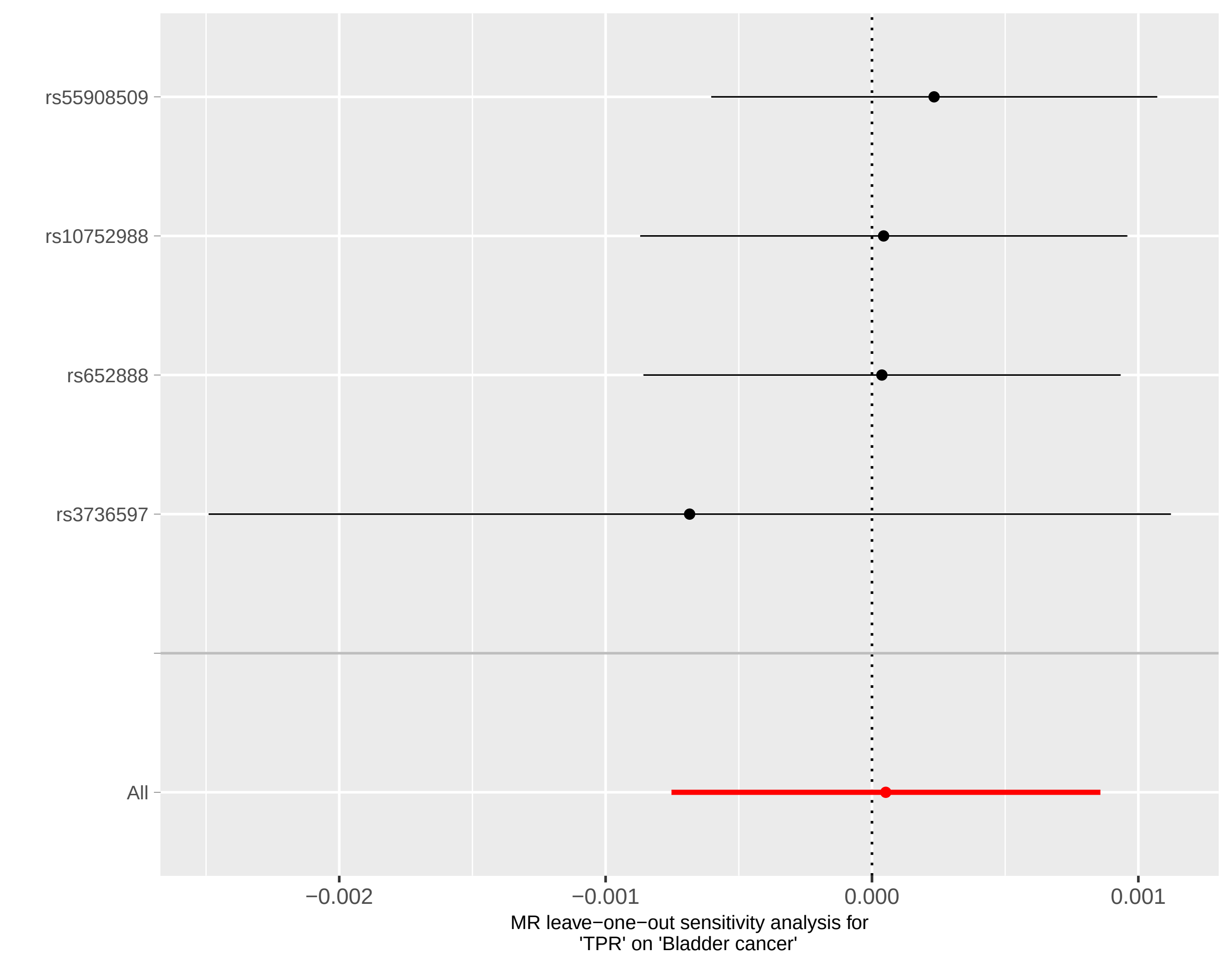

rs55908509
rs10752988
rs652888
rs3736597
All
−0.002
−0.001
0.000
0.001
MR leave−one−out sensitivity analysis for
'TPR' on 'Bladder cancer'

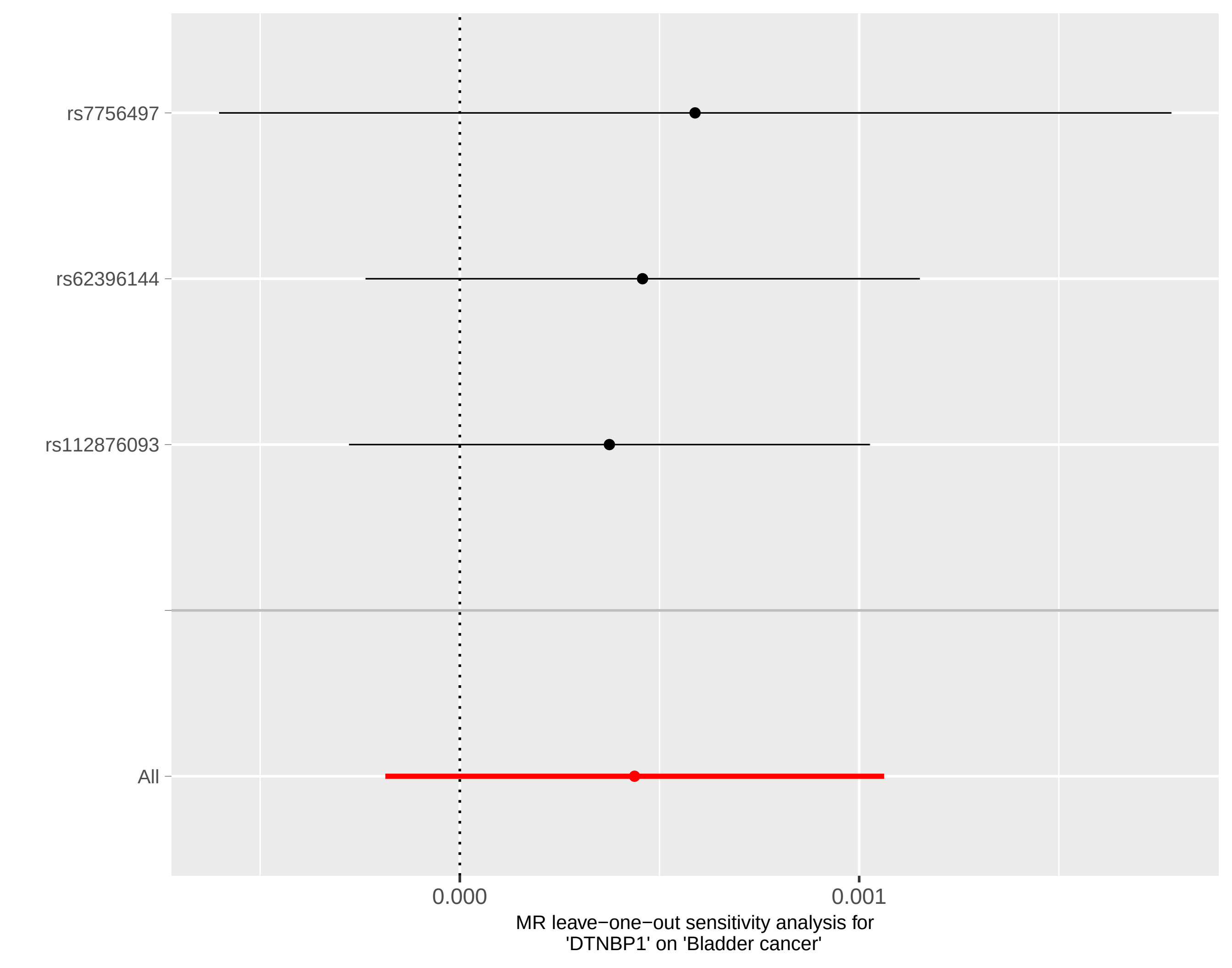
rs7756497
rs62396144
rs112876093
All
0.000
0.001
MR leave−one−out sensitivity analysis for
'DTNBP1' on 'Bladder cancer'

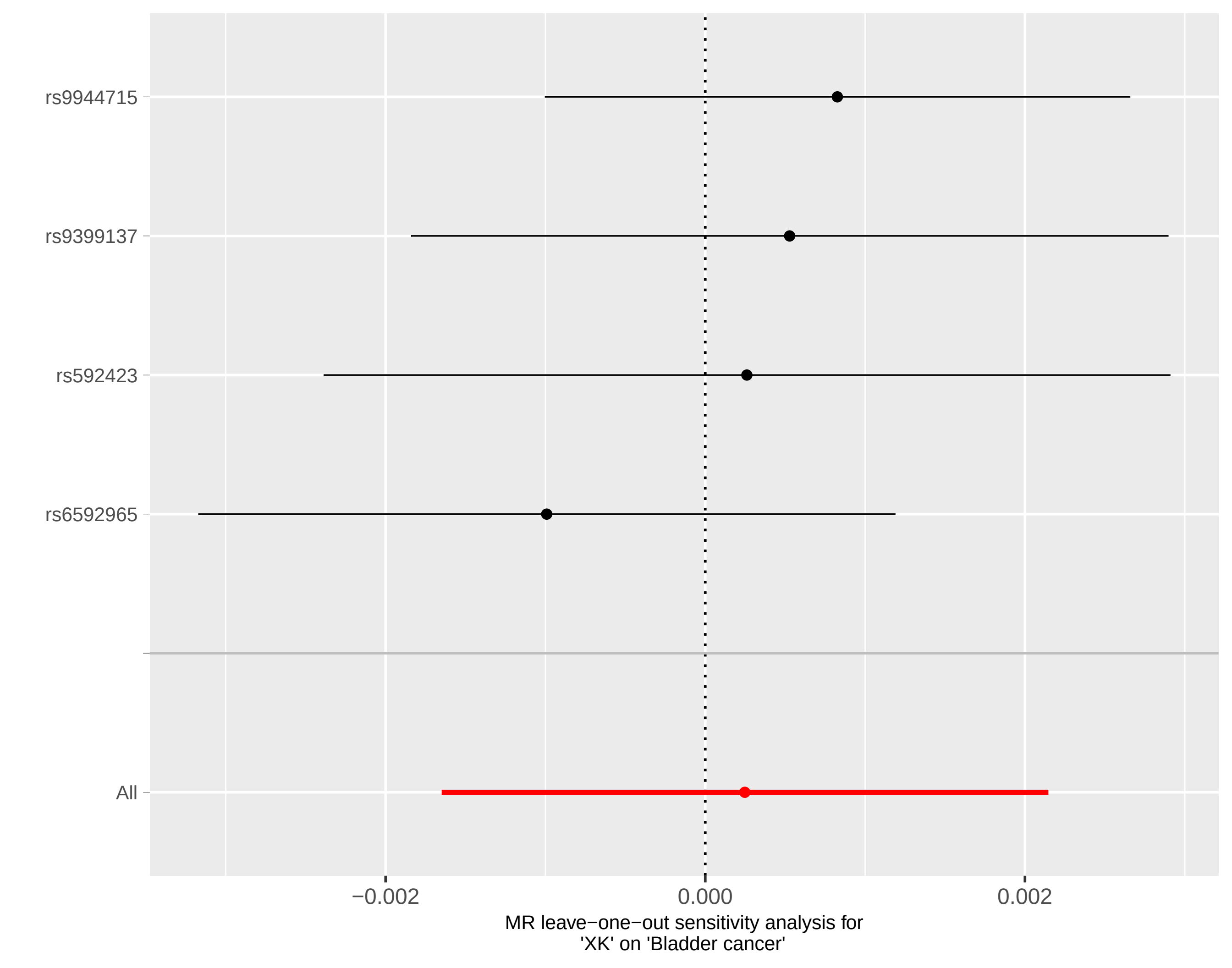
rs9944715
rs9399137
rs592423
rs6592965
All
−0.002
0.000
0.002
MR leave−one−out sensitivity analysis for
'XK' on 'Bladder cancer'

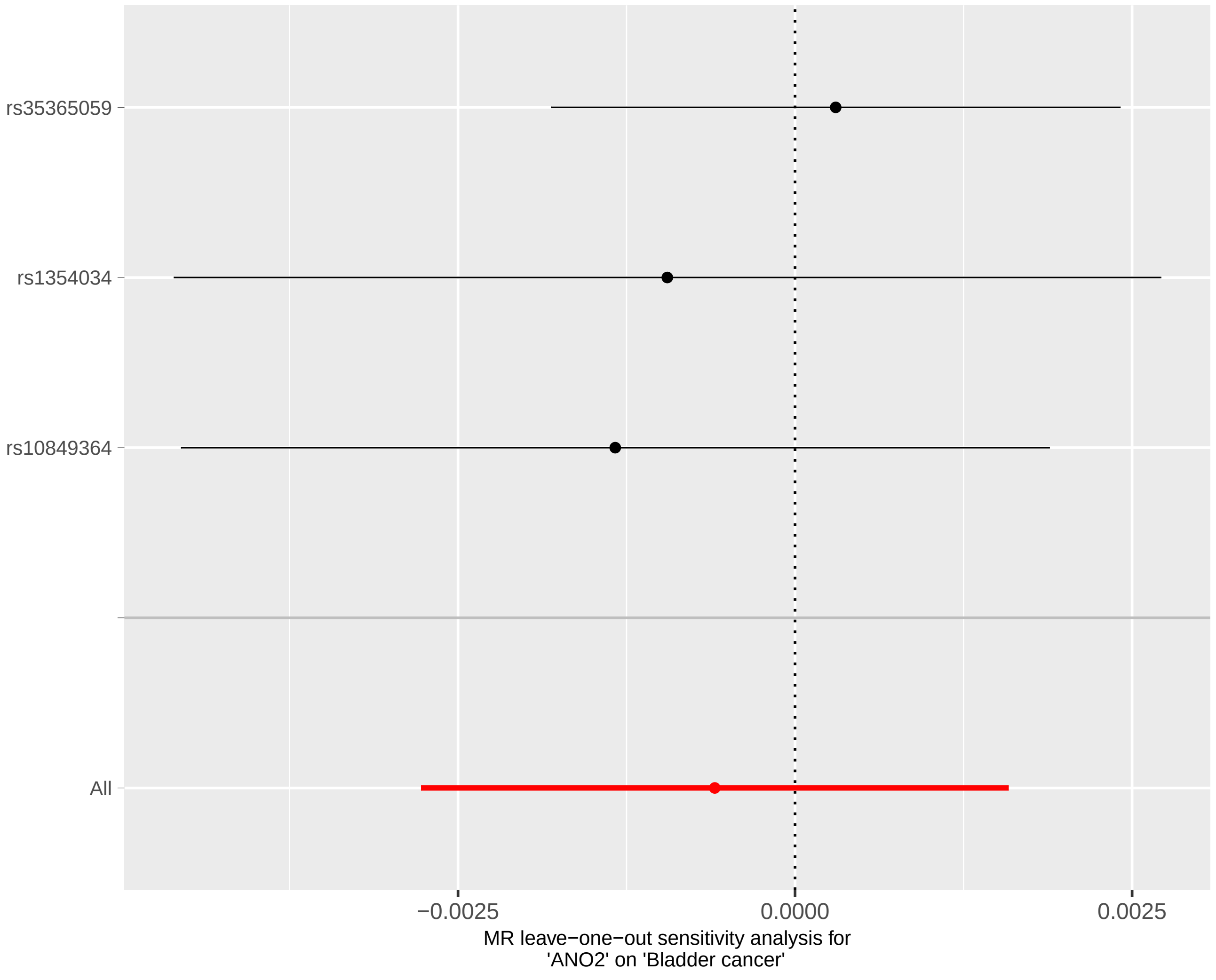
rs35365059
rs1354034
rs10849364
All
−0.0025
0.0000
0.0025
MR leave−one−out sensitivity analysis for
'ANO2' on 'Bladder cancer'

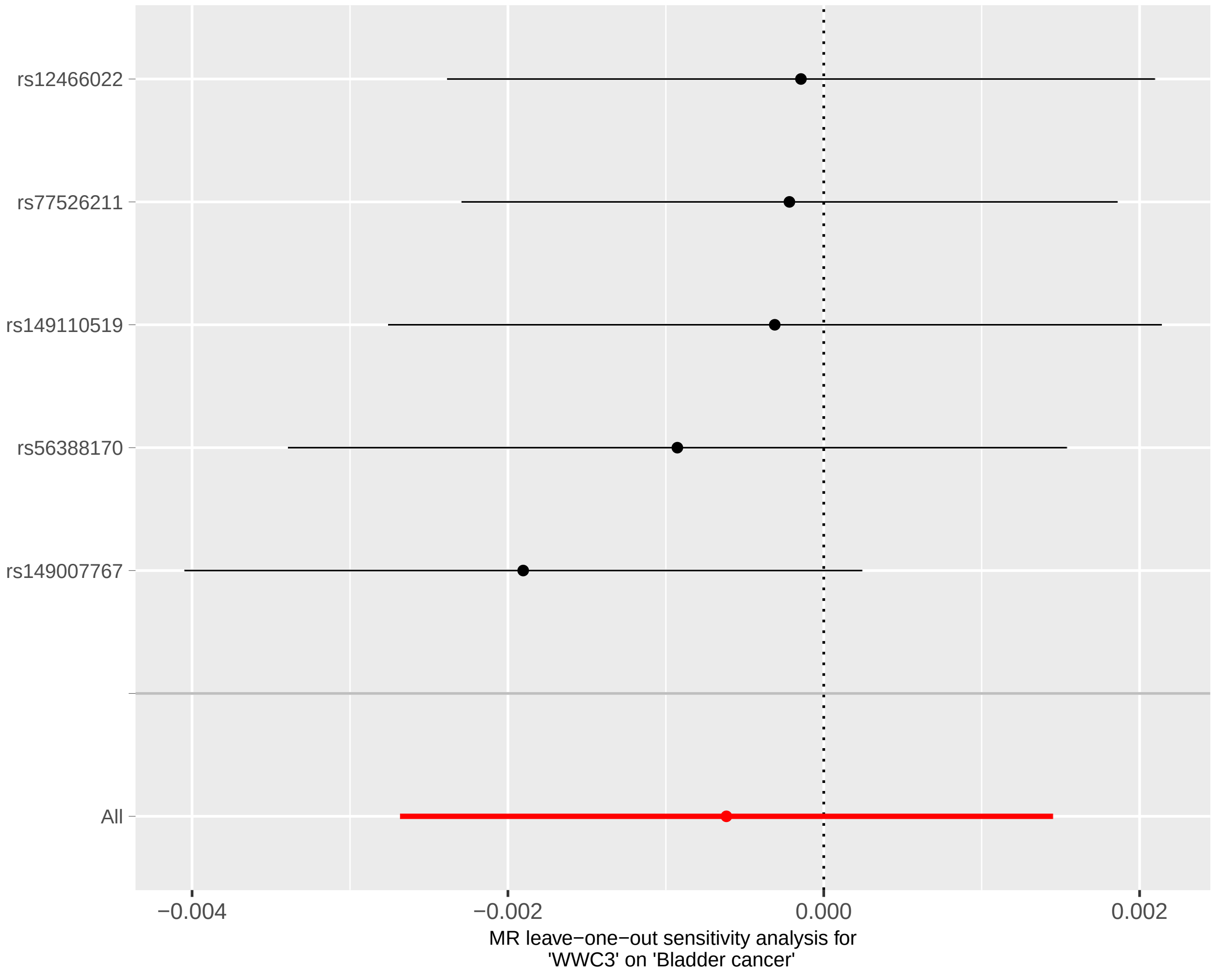
rs12466022
rs77526211
rs149110519
rs56388170
rs149007767
All
−0.004
−0.002
0.000
0.002
MR leave−one−out sensitivity analysis for
'WWC3' on 'Bladder cancer'

Insufficient number of SNPs

Insufficient number of SNPs

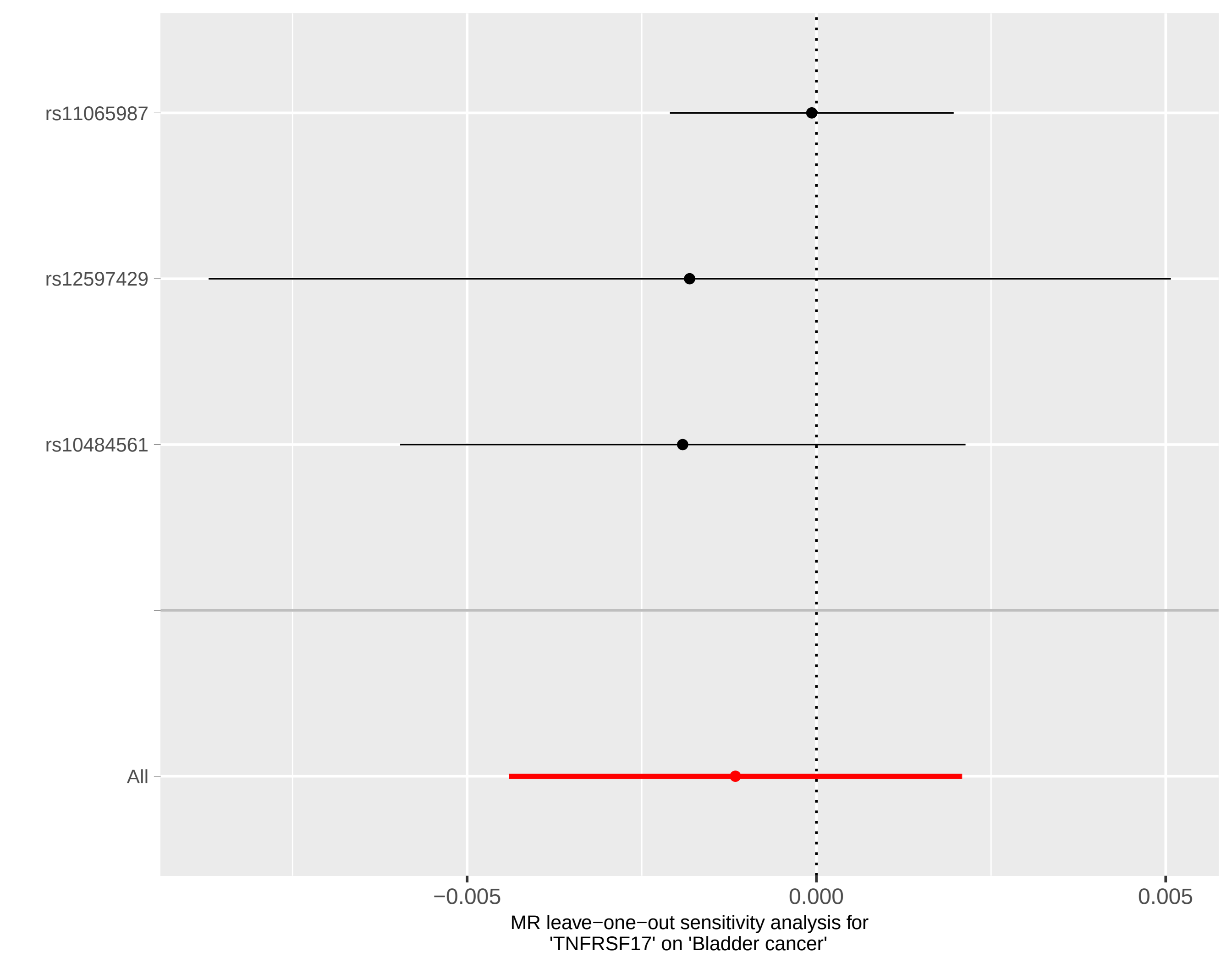
rs11065987
rs12597429
rs10484561
All
−0.005
0.000
0.005
MR leave−one−out sensitivity analysis for
'TNFRSF17' on 'Bladder cancer'

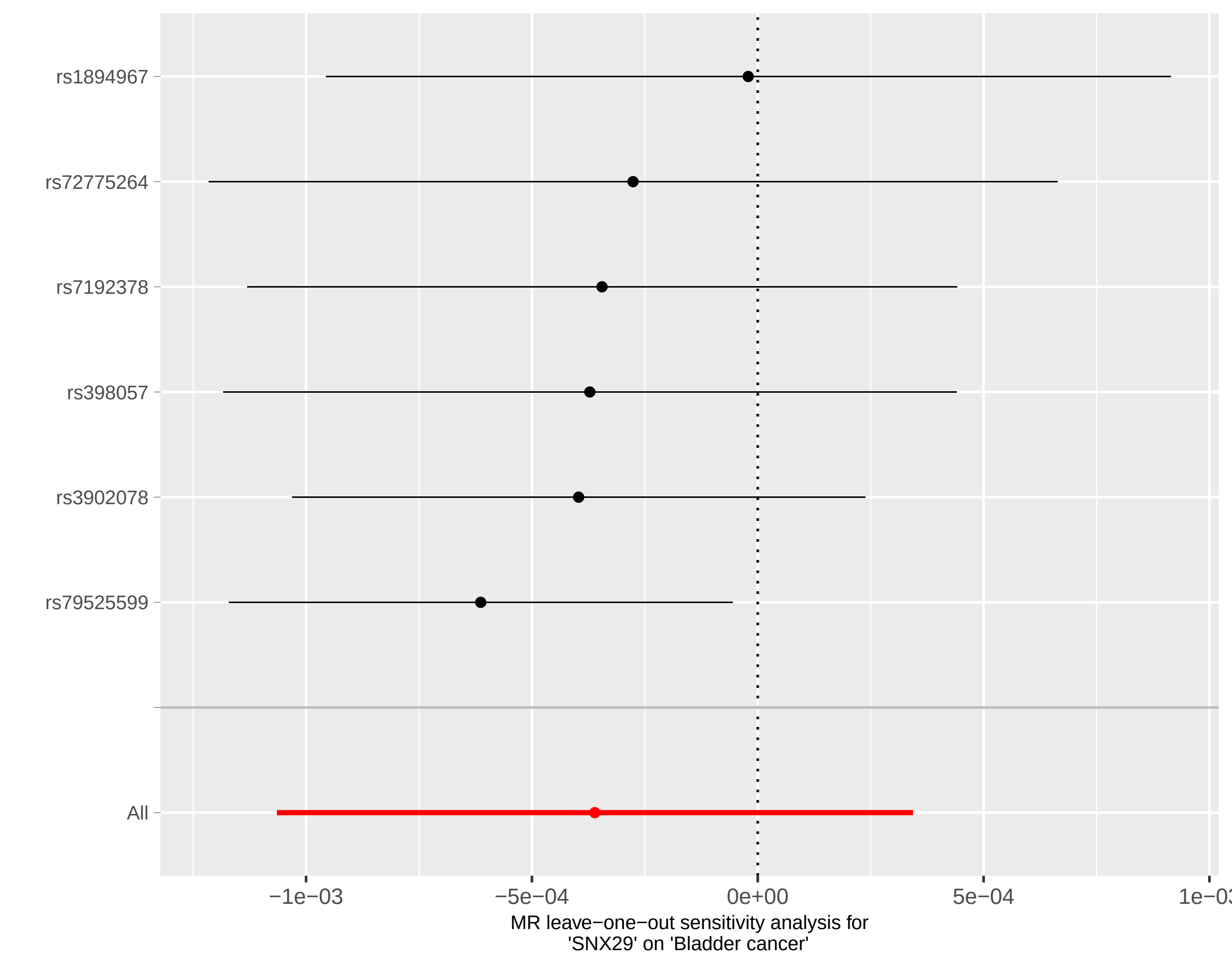
rs1894967
rs72775264
rs7192378
rs398057
rs3902078
rs79525599
All
−1e−03
−5e−04
0e+00
5e−04
1e−03
MR leave−one−out sensitivity analysis for
'SNX29' on 'Bladder cancer'

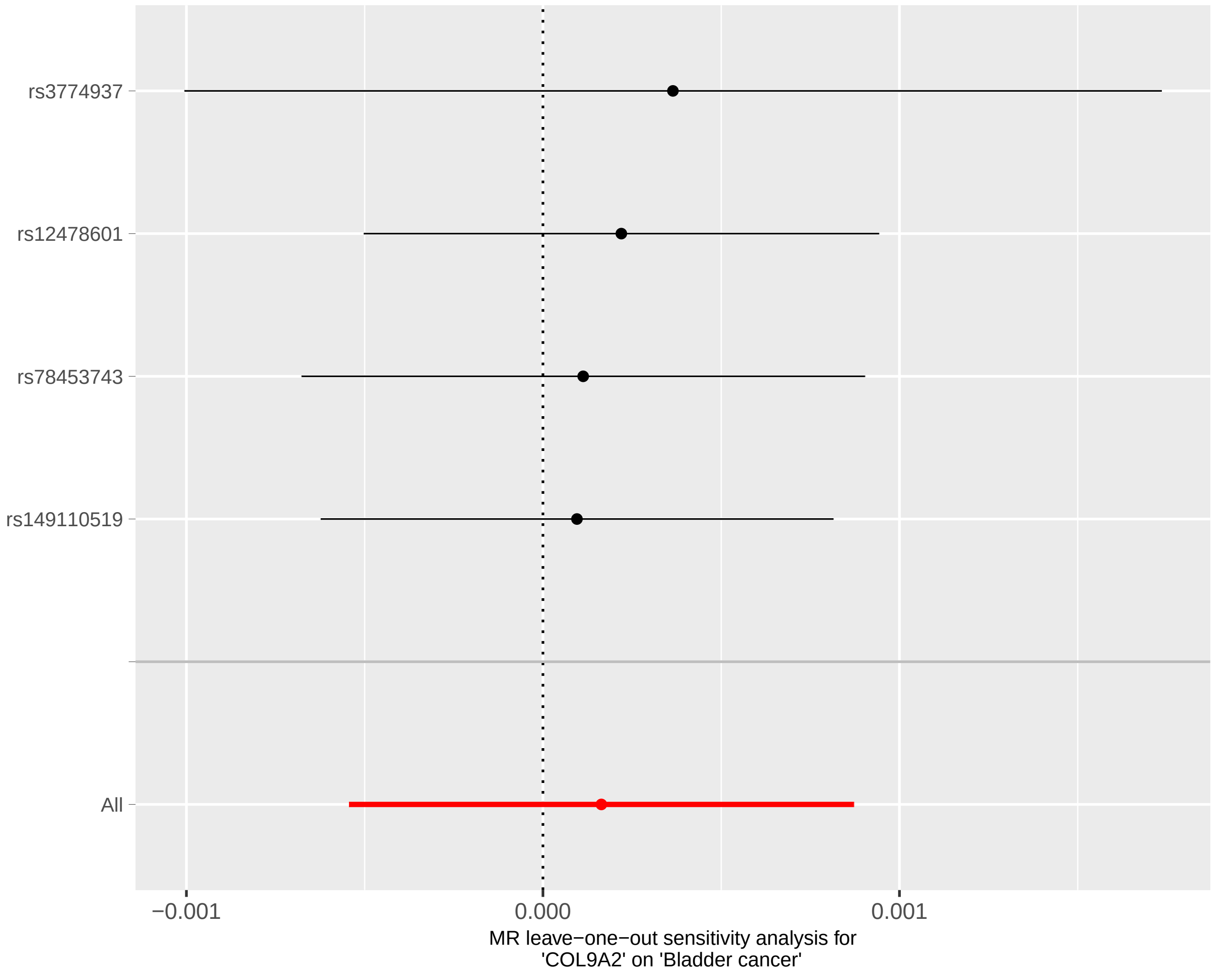
rs3774937
rs12478601
rs78453743
rs149110519
All
−0.001
0.000
0.001
MR leave−one−out sensitivity analysis for
'COL9A2' on 'Bladder cancer'

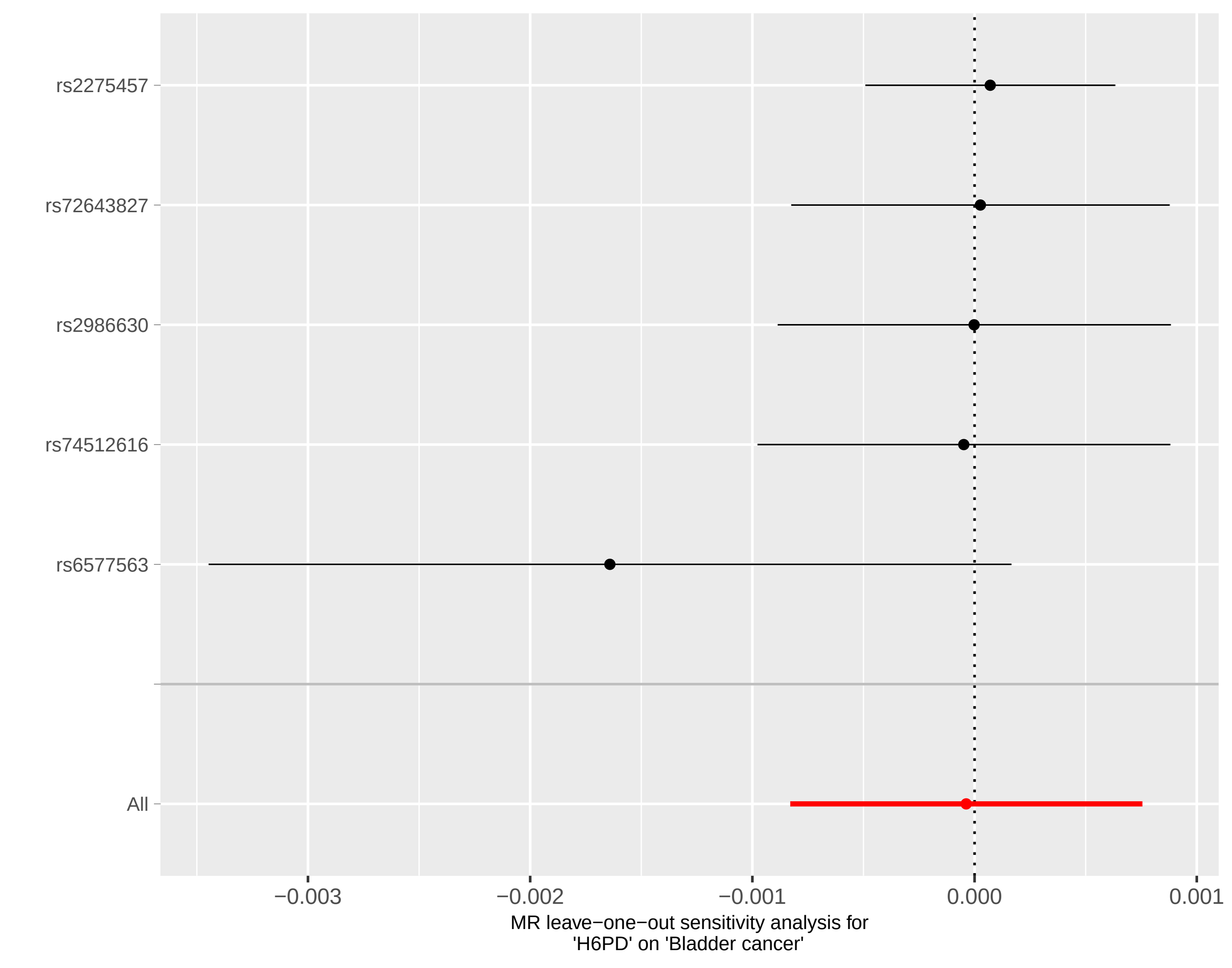

rs2275457
rs72643827
rs2986630
rs74512616
rs6577563
All
−0.003
−0.002
−0.001
0.000
0.001
MR leave−one−out sensitivity analysis for
'H6PD' on 'Bladder cancer'

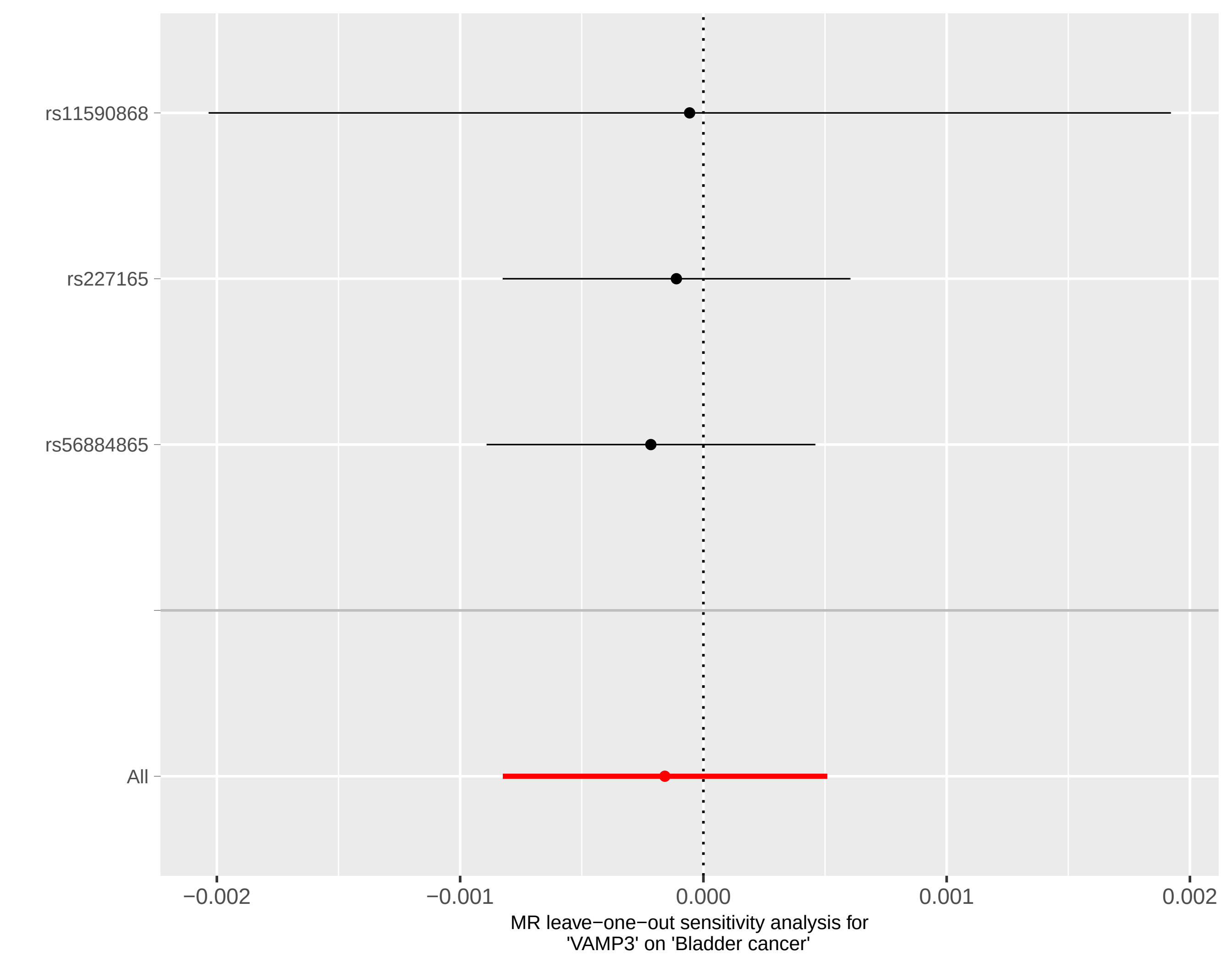
rs11590868
rs227165
rs56884865
All
−0.002
−0.001
0.000
0.001
0.002
MR leave−one−out sensitivity analysis for
'VAMP3' on 'Bladder cancer'

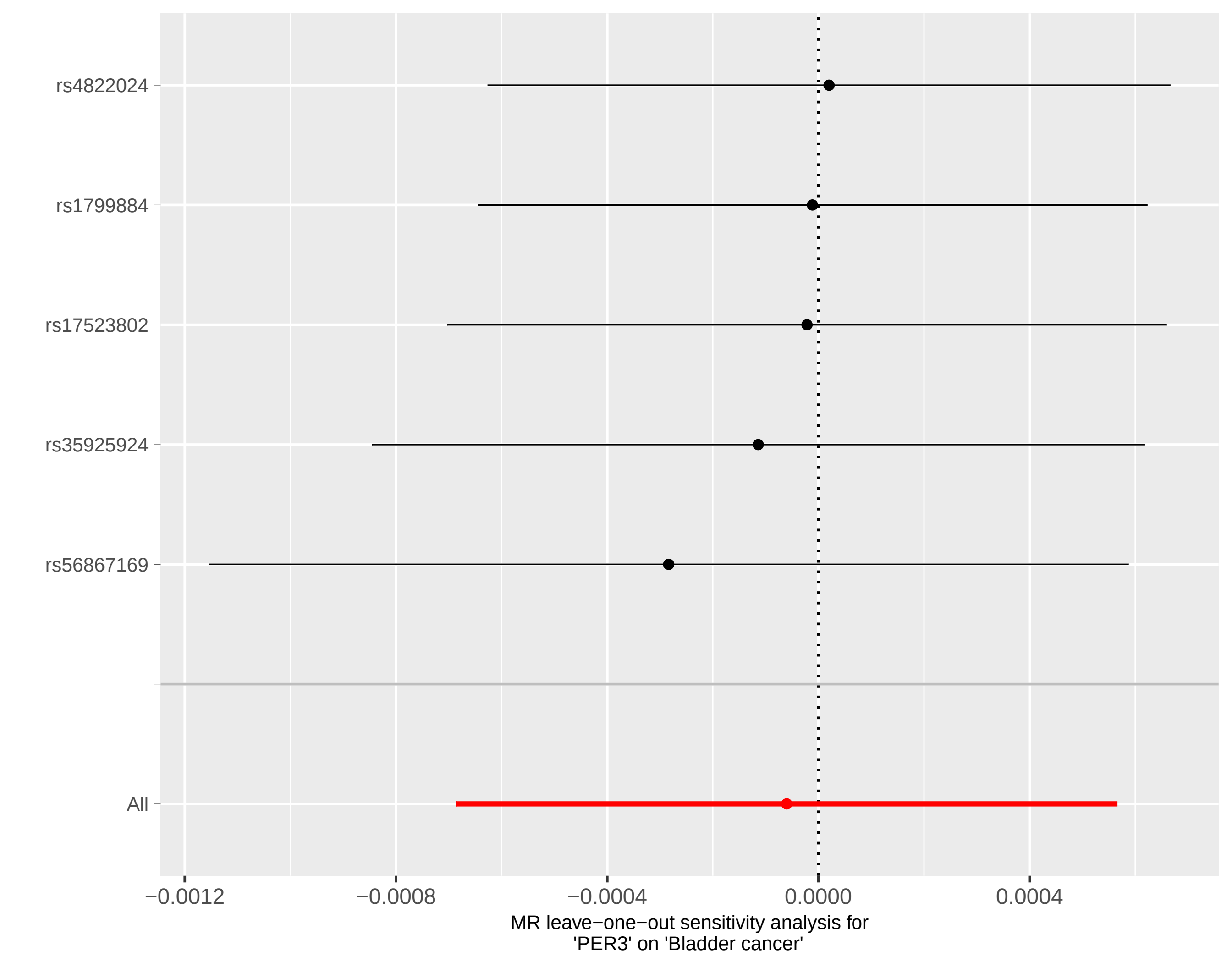
rs4822024
rs1799884
rs17523802
rs35925924
rs56867169
All
−0.0012
−0.0008
−0.0004
0.0000
0.0004
MR leave−one−out sensitivity analysis for
'PER3' on 'Bladder cancer'

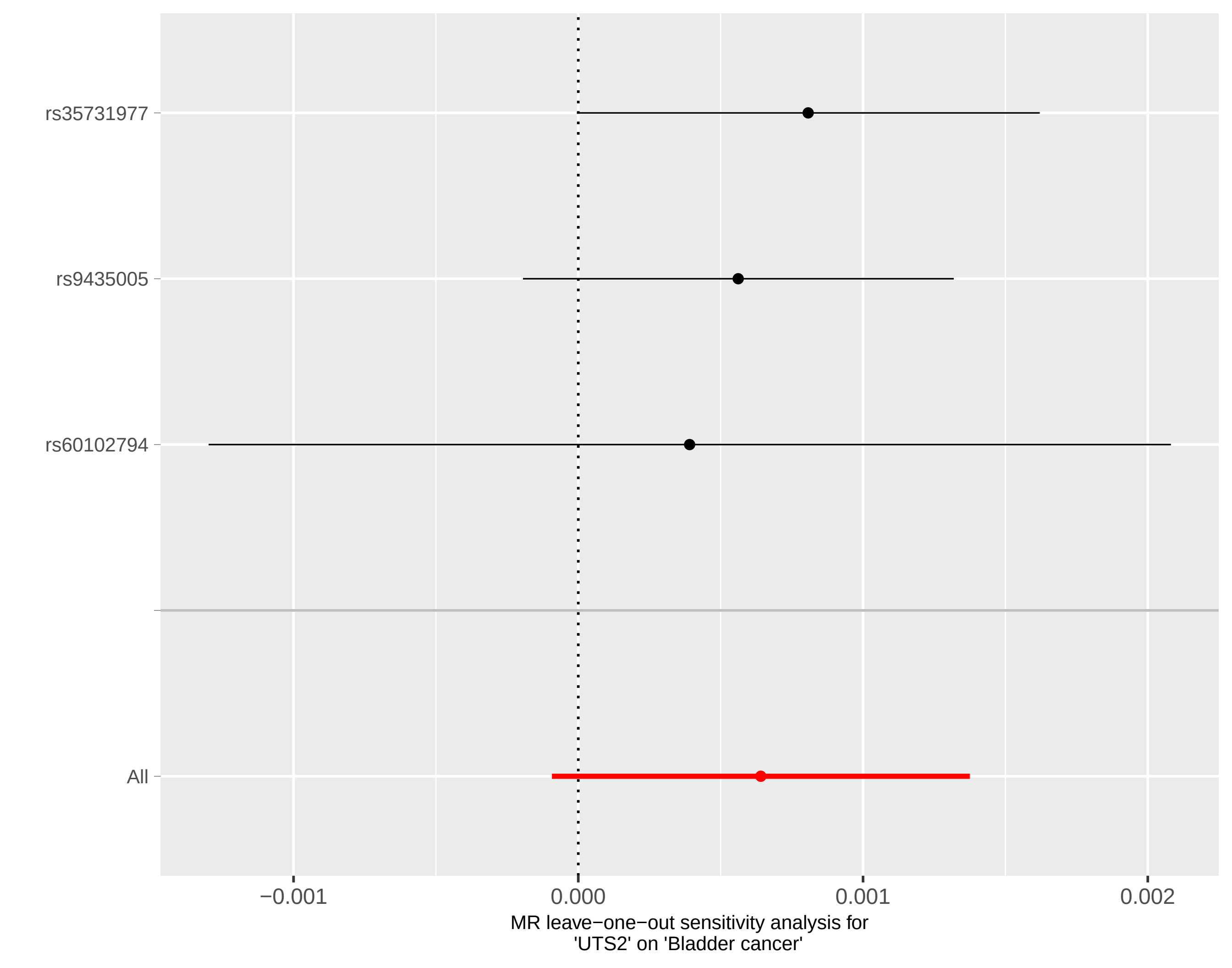

rs35731977
rs9435005
rs60102794
All
−0.001
0.000
0.001
0.002
MR leave−one−out sensitivity analysis for
'UTS2' on 'Bladder cancer'

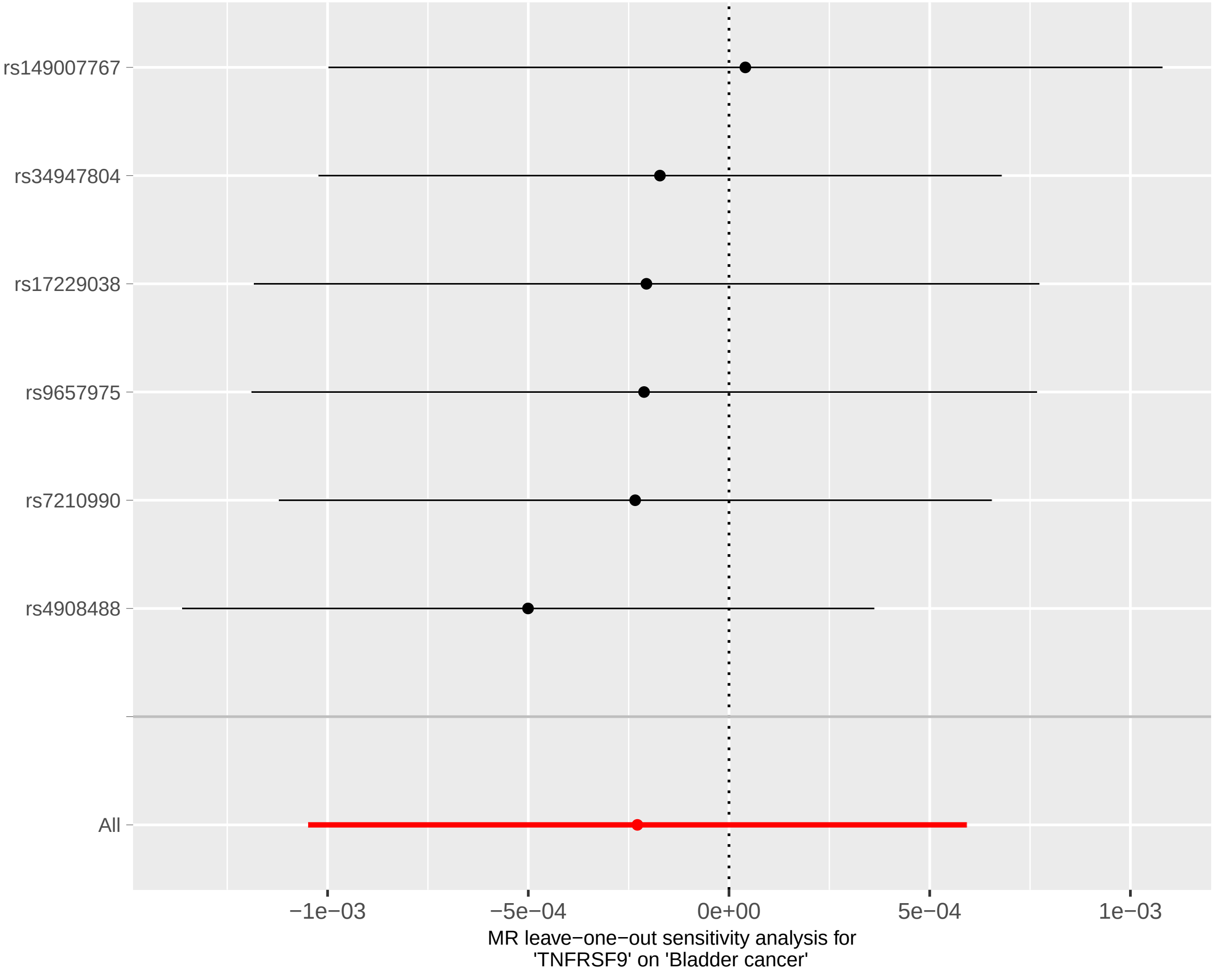
rs149007767
rs34947804
rs17229038
rs9657975
rs7210990
rs4908488
All
−1e−03
−5e−04
0e+00
5e−04
1e−03
MR leave−one−out sensitivity analysis for
'TNFRSF9' on 'Bladder cancer'

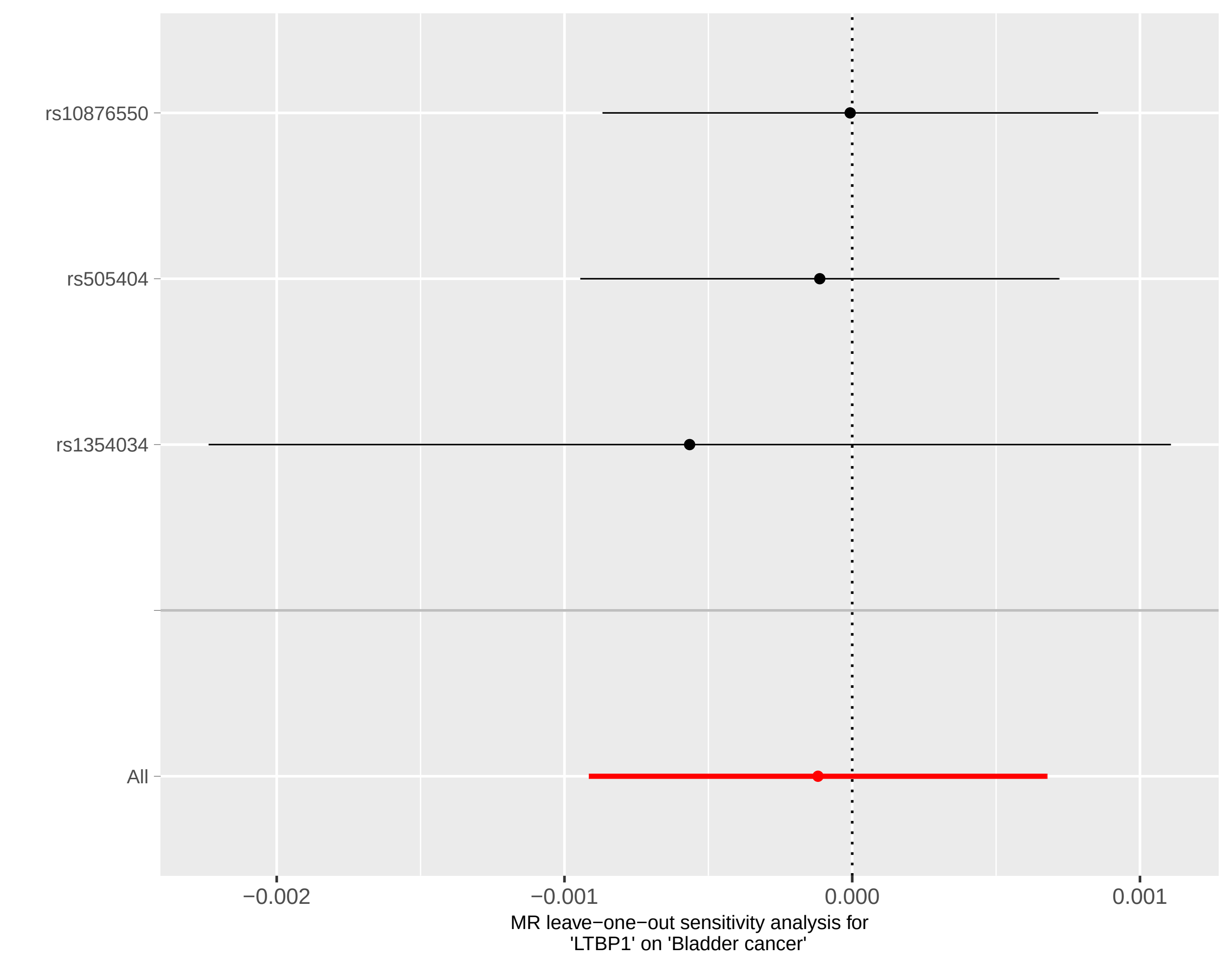
rs10876550
rs505404
rs1354034
All
−0.002
−0.001
0.000
0.001
MR leave−one−out sensitivity analysis for
'LTBP1' on 'Bladder cancer'

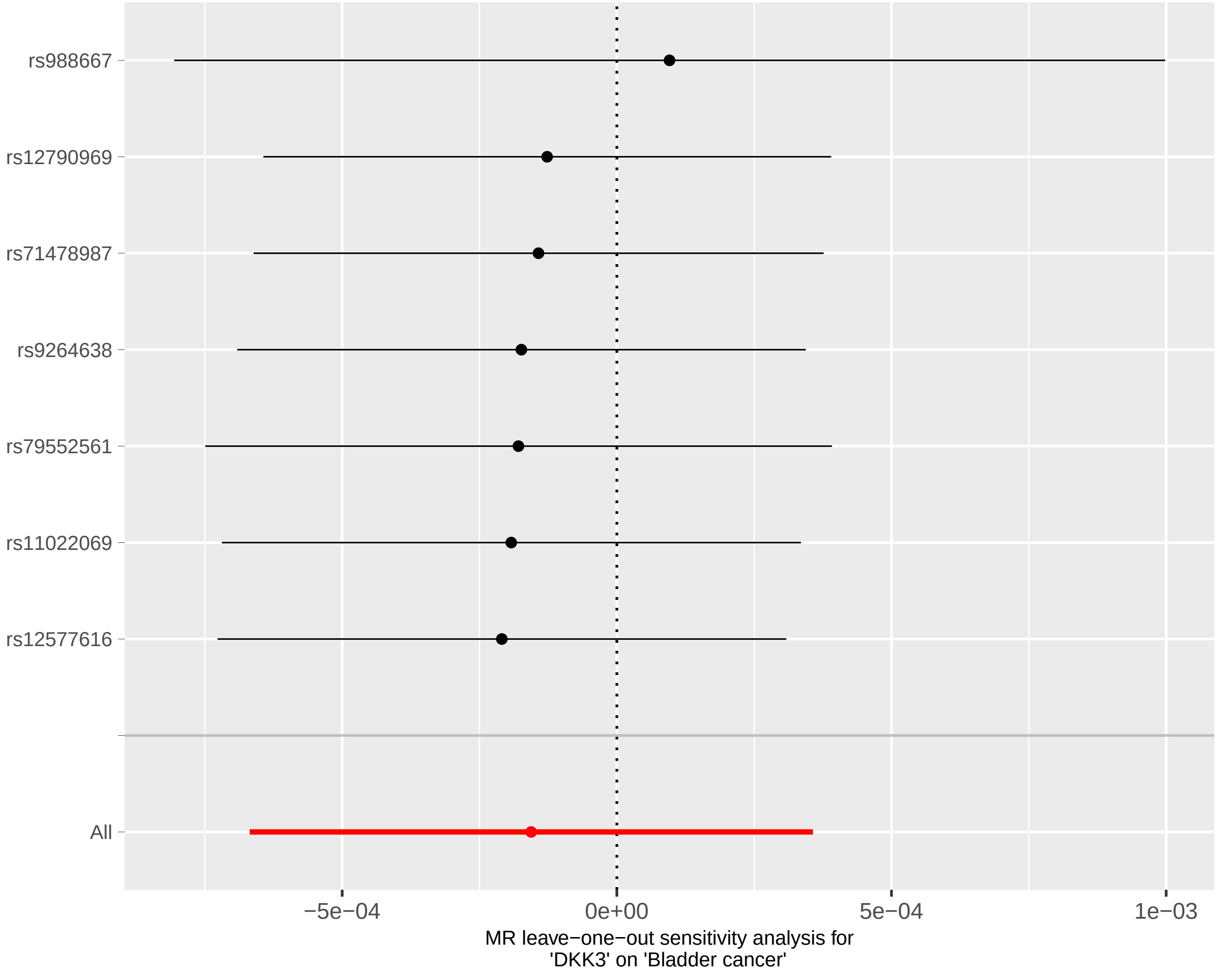

rs988667
rs12790969
rs71478987
rs9264638
rs79552561
rs11022069
rs12577616
All
−5e−04
0e+00
5e−04
1e−03
MR leave−one−out sensitivity analysis for
'DKK3' on 'Bladder cancer'

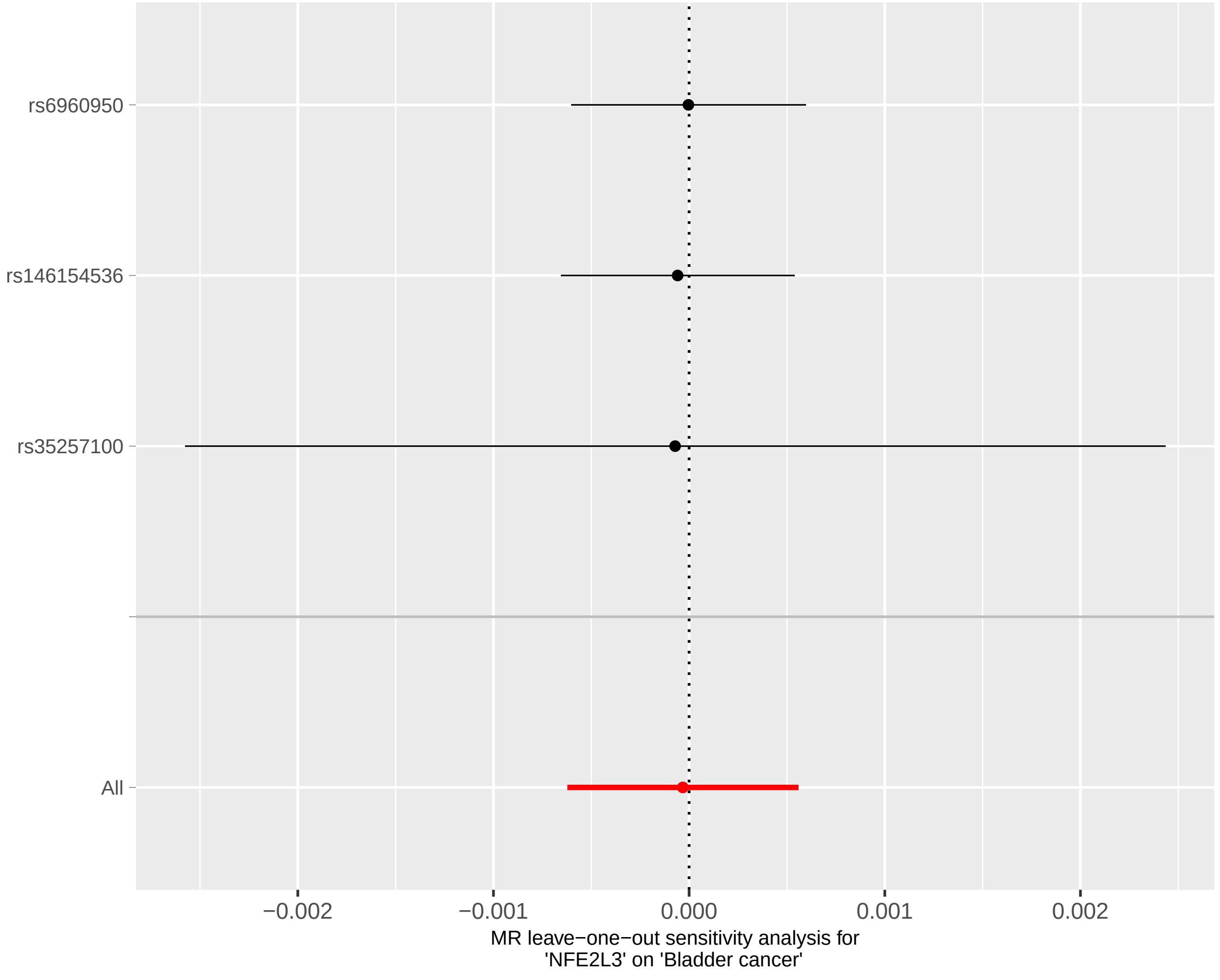

rs6960950
rs146154536
rs35257100
All
−0.002
−0.001
0.000
0.001
0.002
MR leave−one−out sensitivity analysis for
'NFE2L3' on 'Bladder cancer'

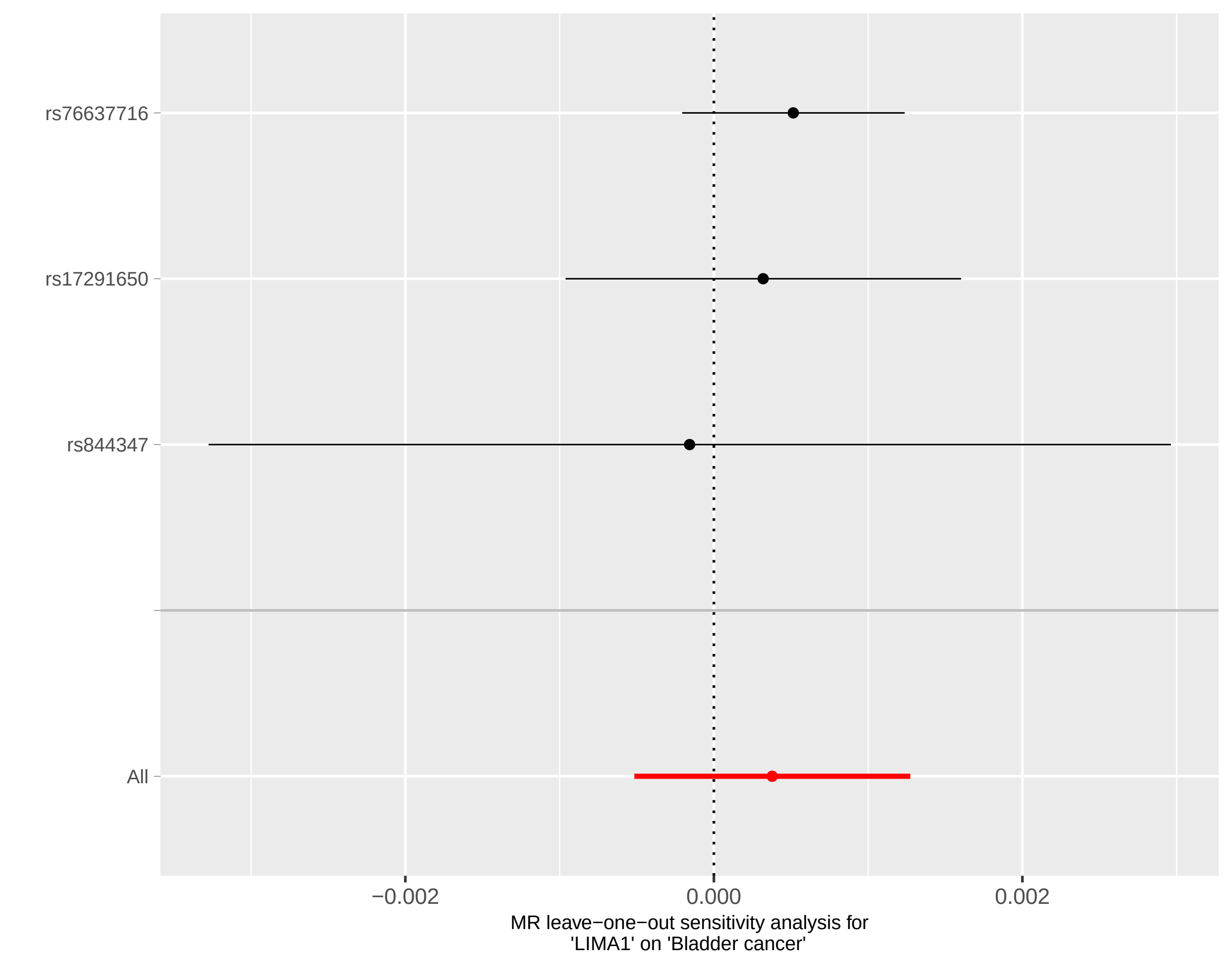
rs76637716
rs17291650
rs844347
All
−0.002
0.000
0.002
MR leave−one−out sensitivity analysis for
'LIMA1' on 'Bladder cancer'

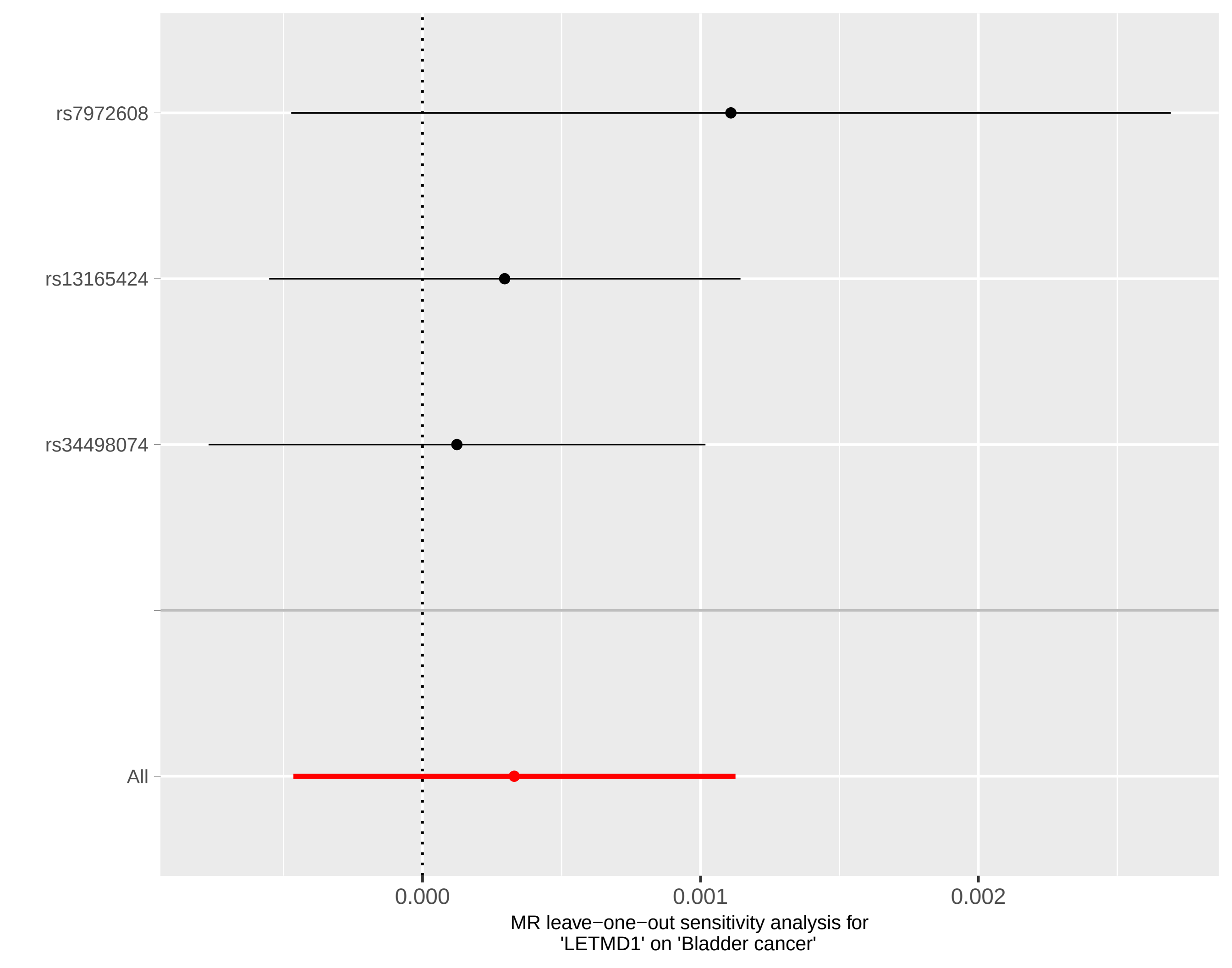
rs7972608
rs13165424
rs34498074
All
0.000
0.001
0.002
MR leave−one−out sensitivity analysis for
'LETMD1' on 'Bladder cancer'

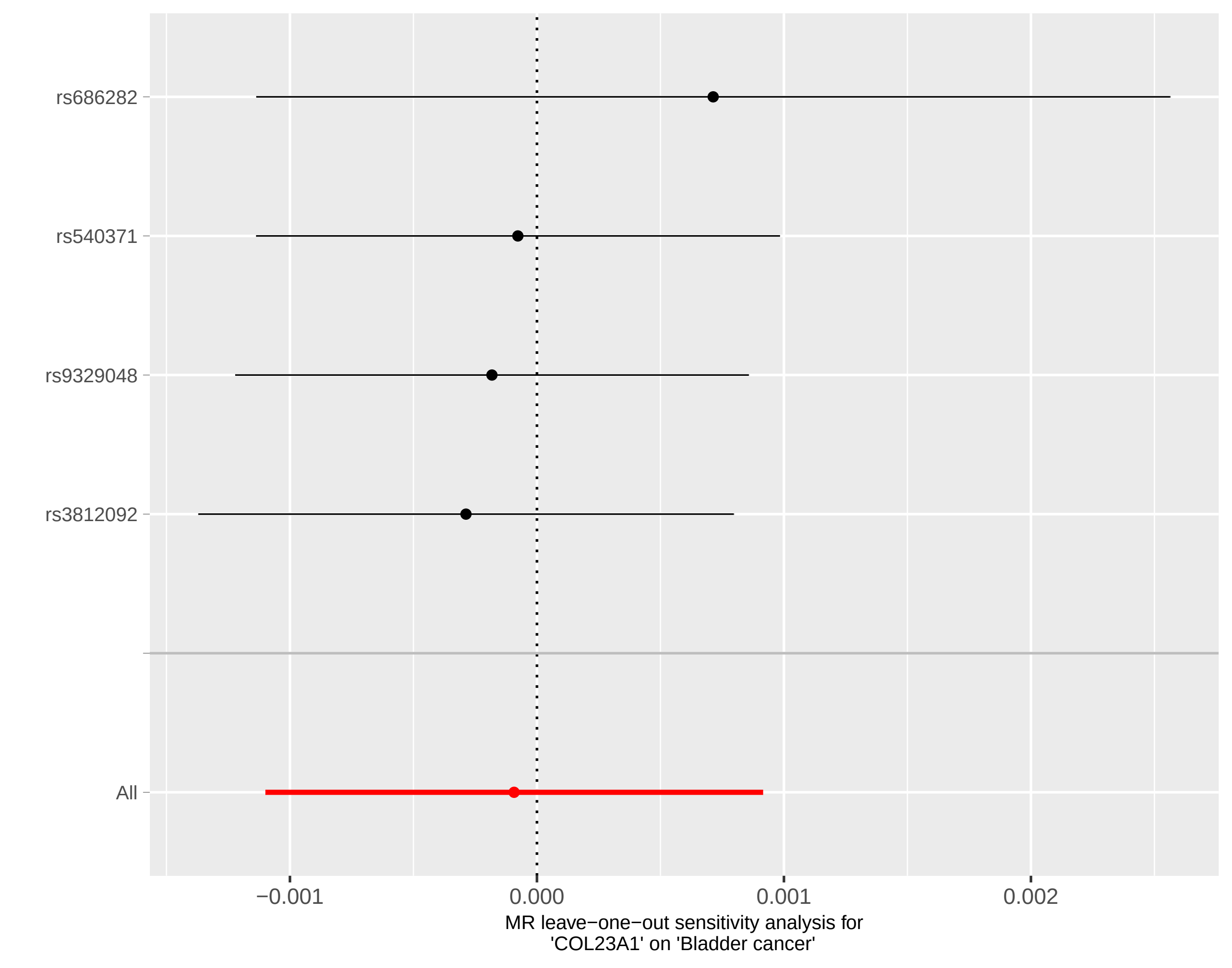
rs686282
rs540371
rs9329048
rs3812092
All
−0.001
0.000
0.001
0.002
MR leave−one−out sensitivity analysis for
'COL23A1' on 'Bladder cancer'

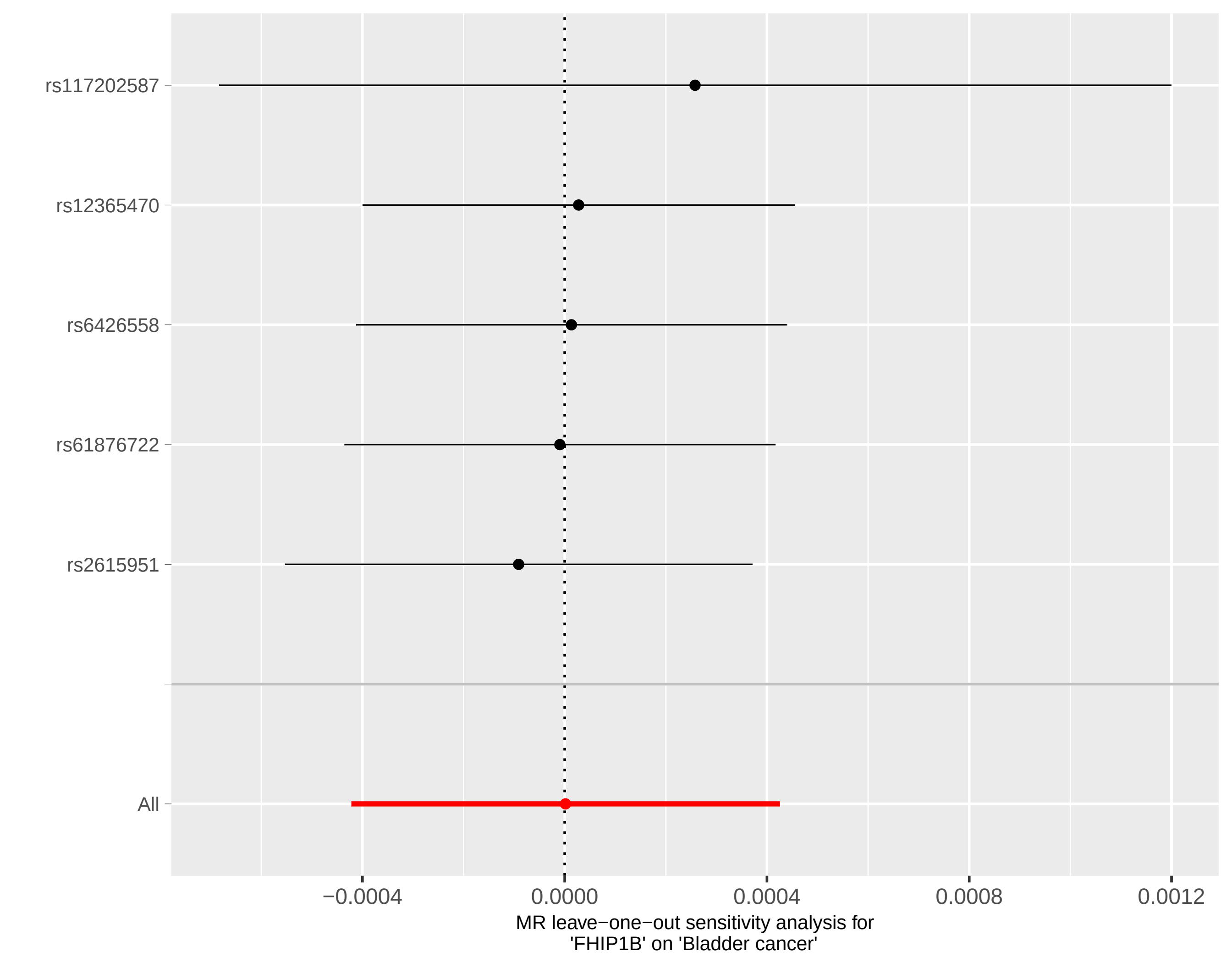
rs117202587
rs12365470
rs6426558
rs61876722
rs2615951
All
−0.0004
0.0000
0.0004
0.0008
0.0012
MR leave−one−out sensitivity analysis for
'FHIP1B' on 'Bladder cancer'

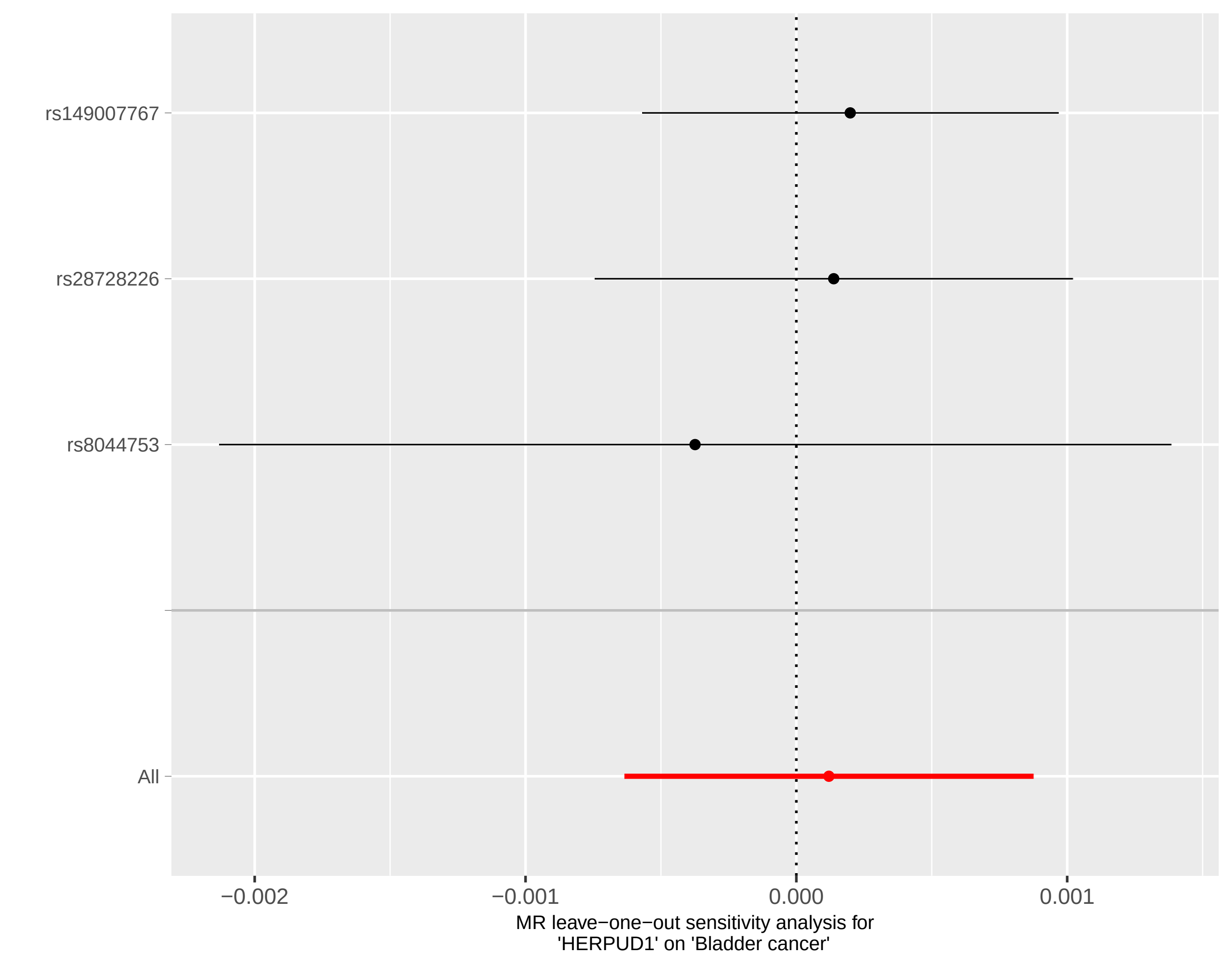
rs149007767
rs28728226
rs8044753
All
−0.002
−0.001
0.000
0.001
MR leave−one−out sensitivity analysis for
'HERPUD1' on 'Bladder cancer'

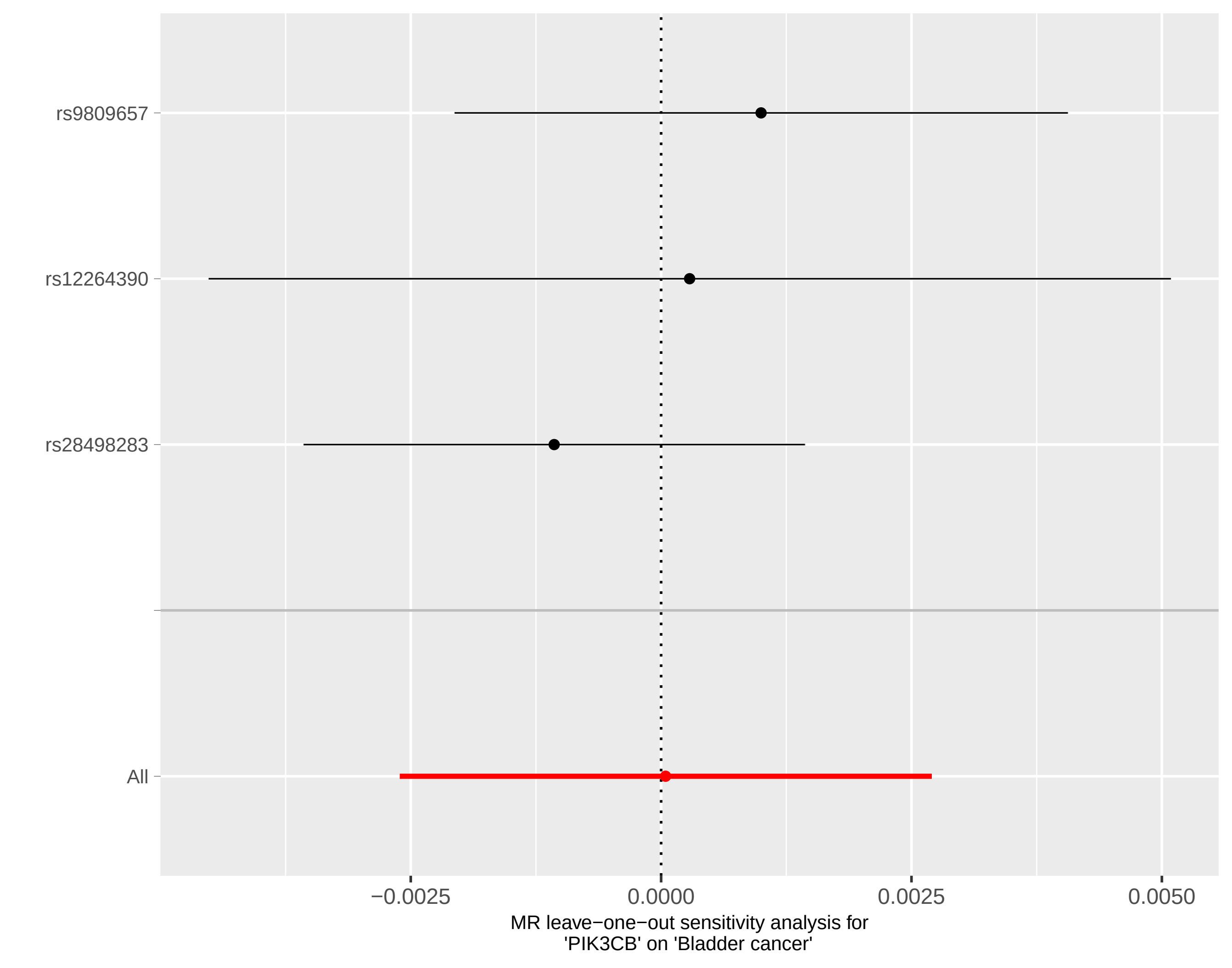
rs9809657
rs12264390
rs28498283
All
−0.0025
0.0000
0.0025
0.0050
MR leave−one−out sensitivity analysis for
'PIK3CB' on 'Bladder cancer'

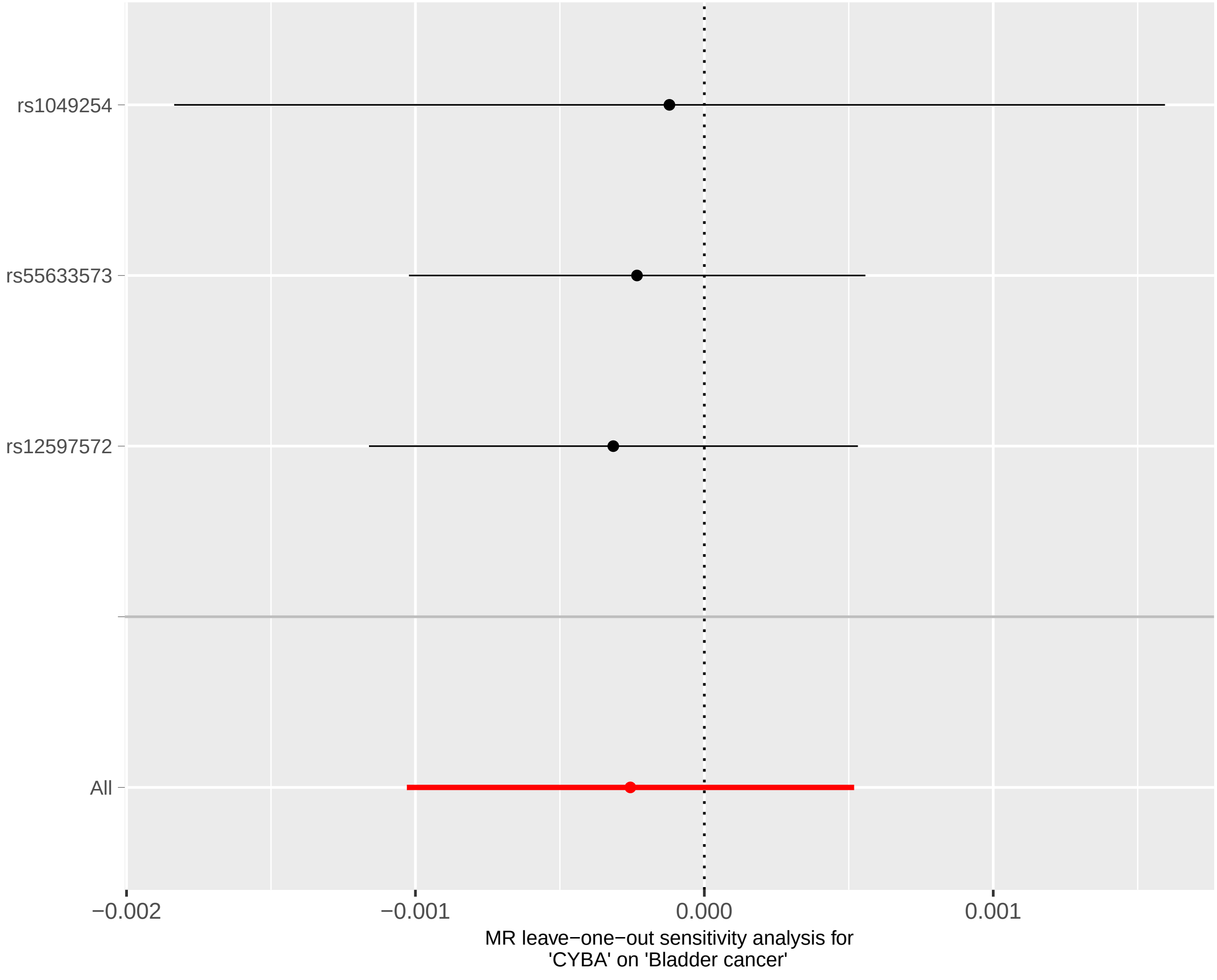
rs1049254
rs55633573
rs12597572
All
−0.002
−0.001
0.000
0.001
MR leave−one−out sensitivity analysis for
'CYBA' on 'Bladder cancer'

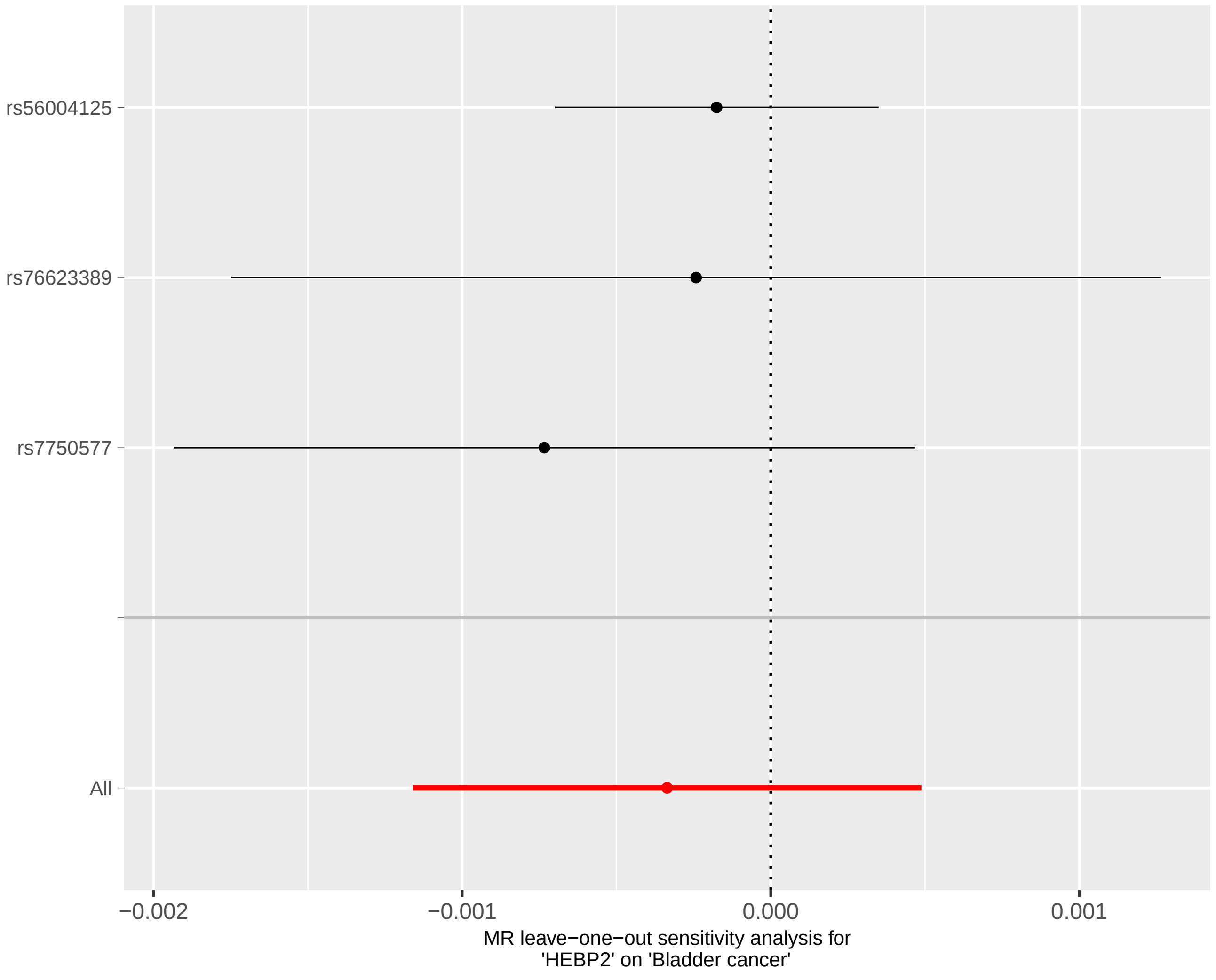
rs56004125
rs76623389
rs7750577
All
−0.002
−0.001
0.000
0.001
MR leave−one−out sensitivity analysis for
'HEBP2' on 'Bladder cancer'

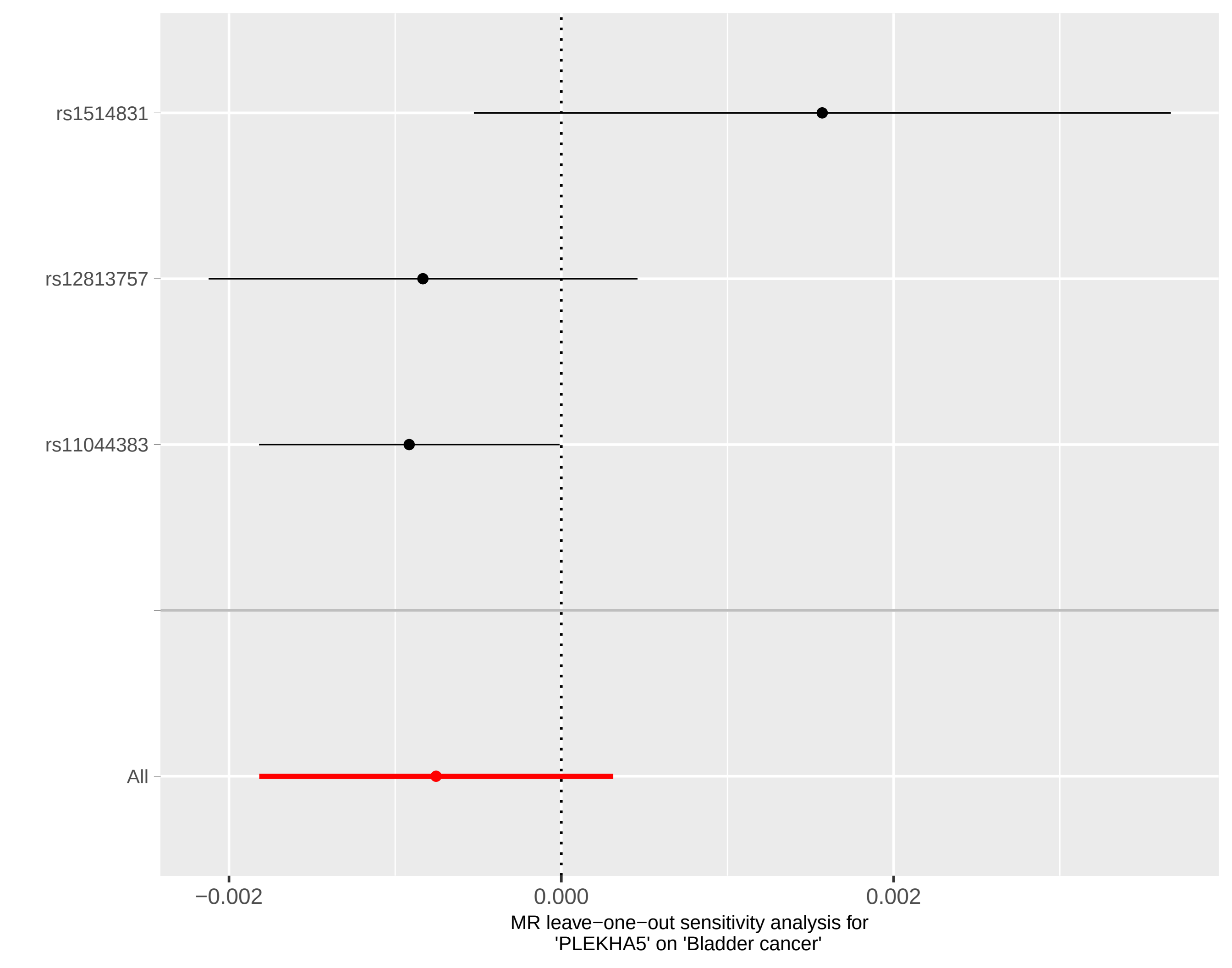
rs1514831
rs12813757
rs11044383
All
−0.002
0.000
0.002
MR leave−one−out sensitivity analysis for
'PLEKHA5' on 'Bladder cancer'

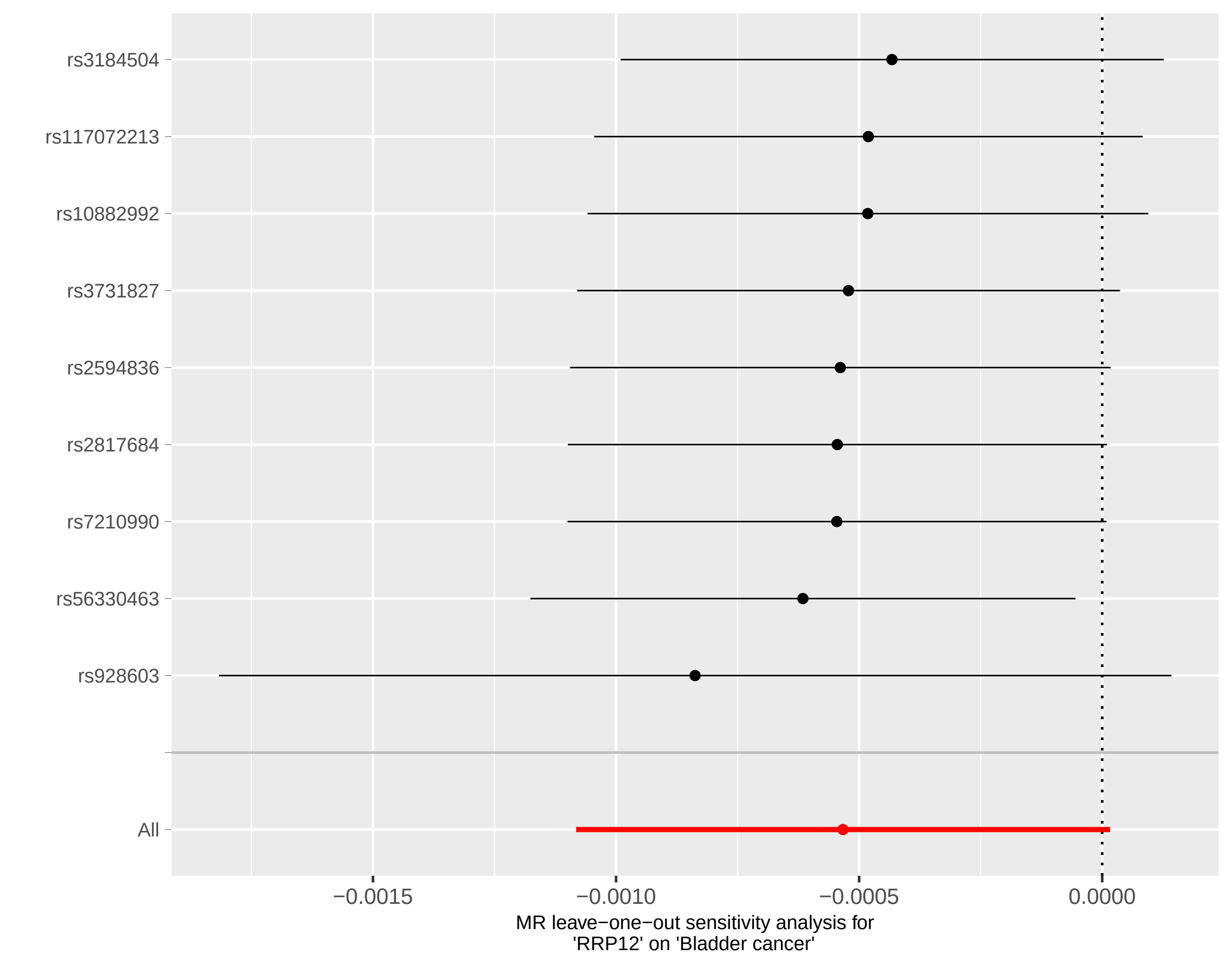
rs3184504
rs117072213
rs10882992
rs3731827
rs2594836
rs2817684
rs7210990
rs56330463
rs928603
All
−0.0015
−0.0010
−0.0005
0.0000
MR leave−one−out sensitivity analysis for
'RRP12' on 'Bladder cancer'

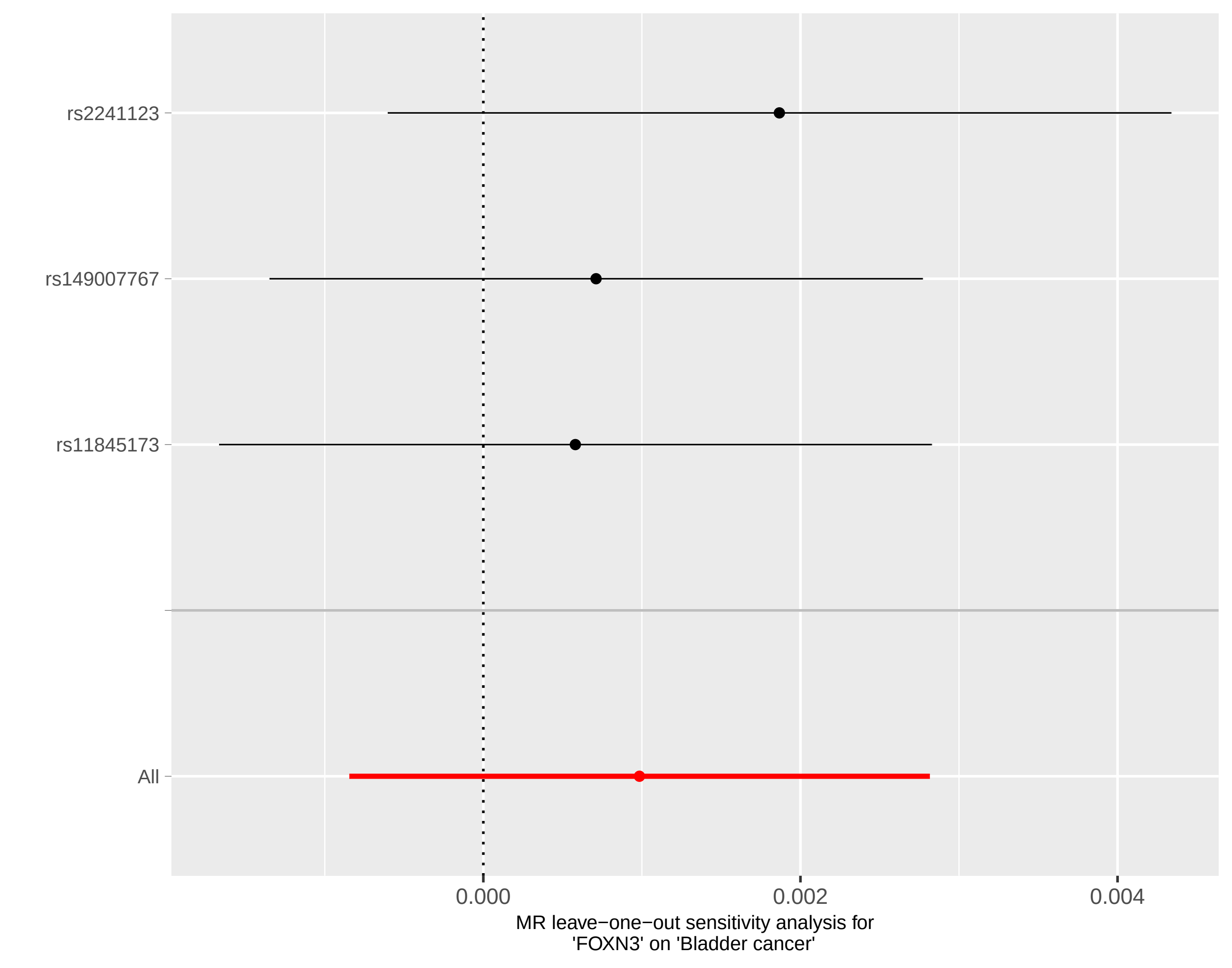
rs2241123
rs149007767
rs11845173
All
0.000
0.002
0.004
MR leave−one−out sensitivity analysis for
'FOXN3' on 'Bladder cancer'

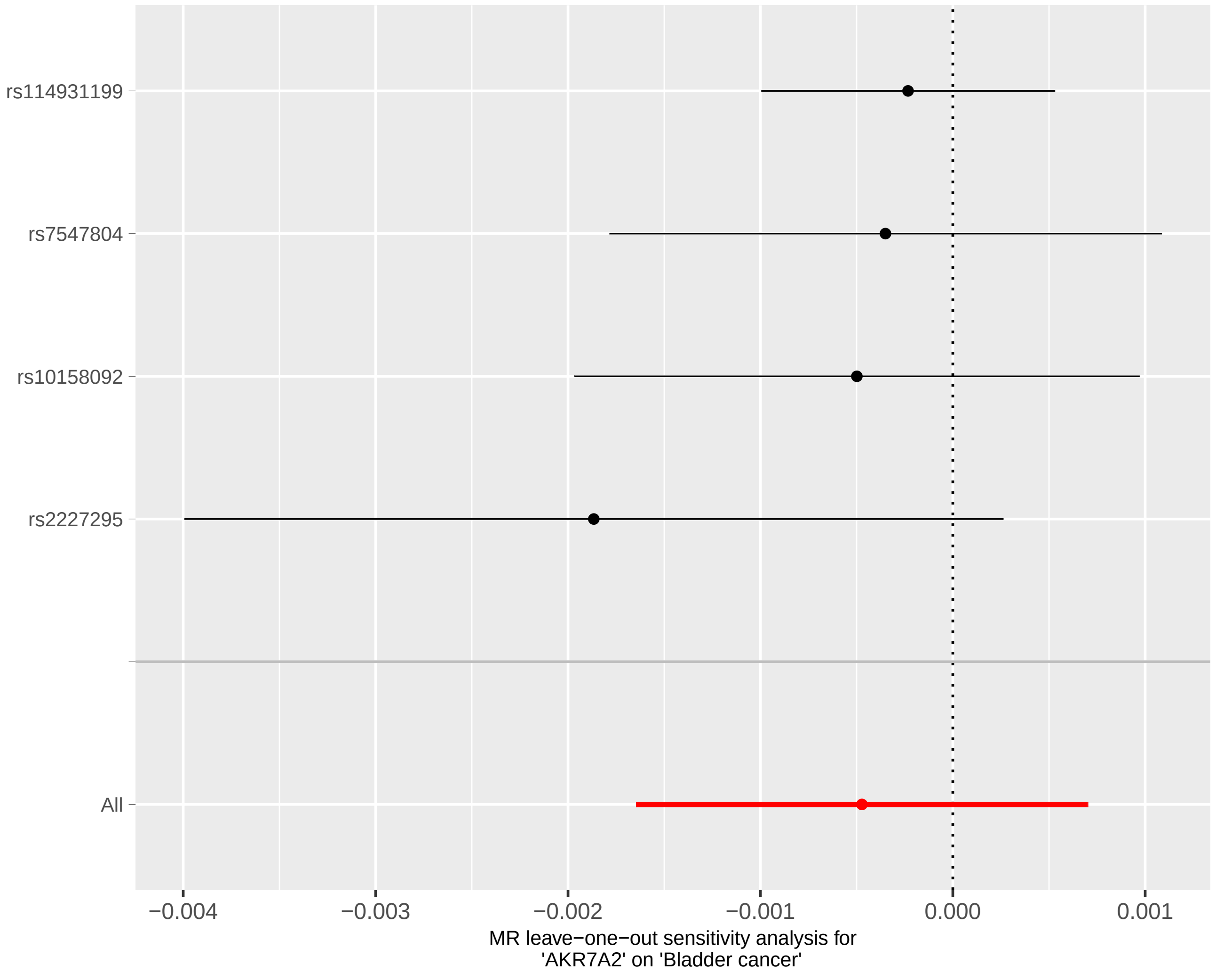
rs114931199
rs7547804
rs10158092
rs2227295
All
−0.004
−0.003
−0.002
−0.001
0.000
0.001
MR leave−one−out sensitivity analysis for
'AKR7A2' on 'Bladder cancer'

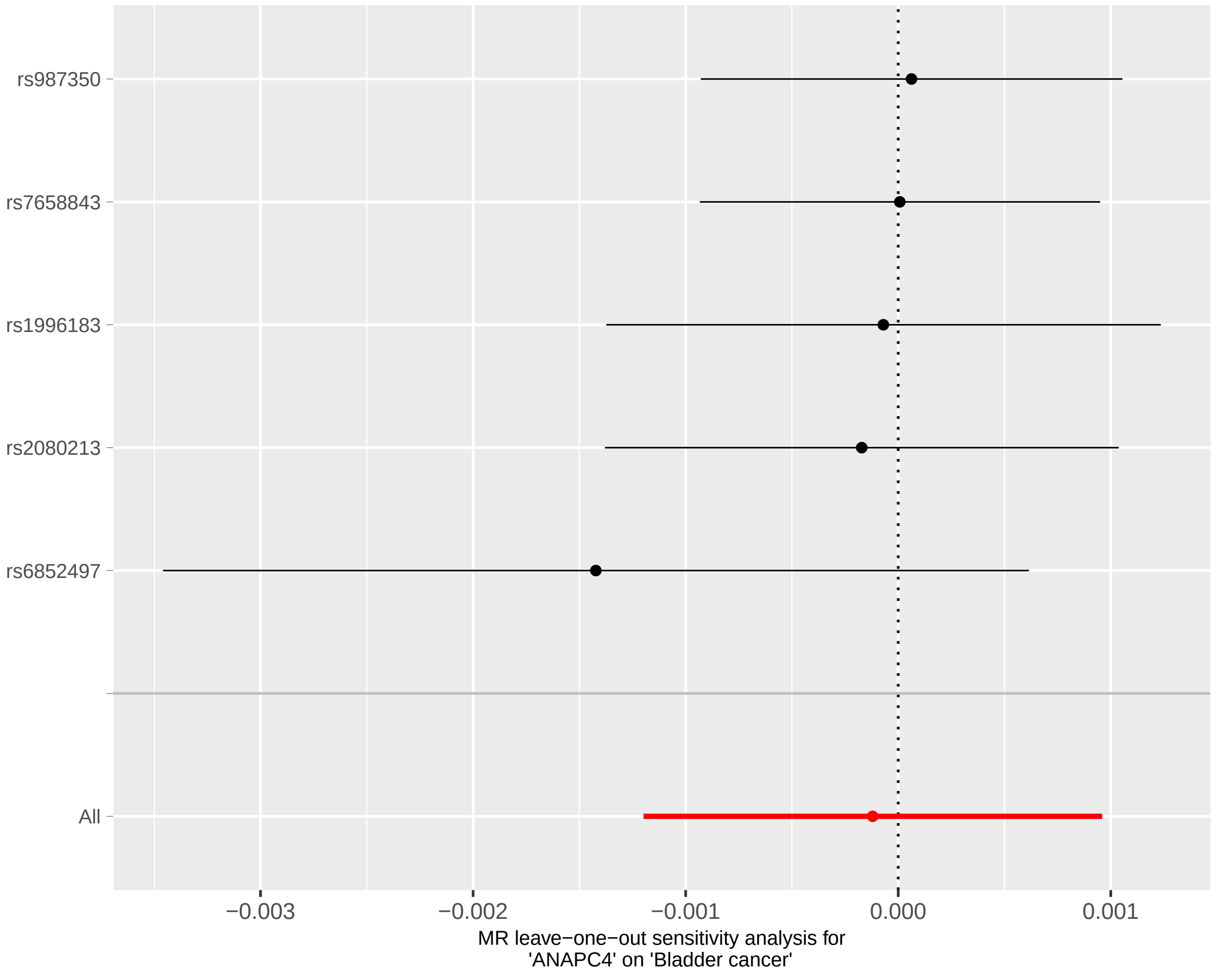
rs987350
rs7658843
rs1996183
rs2080213
rs6852497
All
−0.003
−0.002
−0.001
0.000
0.001
MR leave−one−out sensitivity analysis for
'ANAPC4' on 'Bladder cancer'

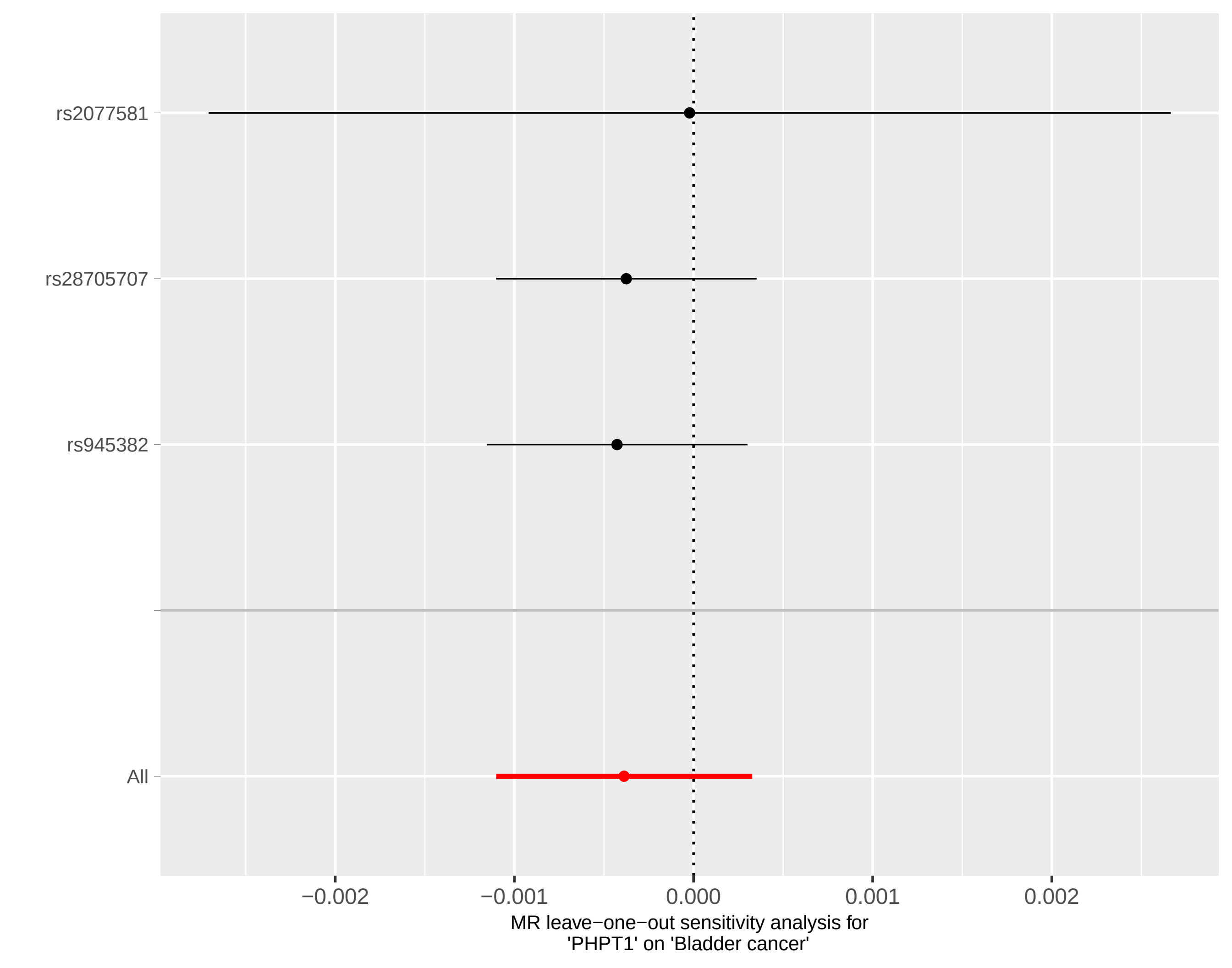
rs2077581
rs28705707
rs945382
All
−0.002
−0.001
0.000
0.001
0.002
MR leave−one−out sensitivity analysis for
'PHPT1' on 'Bladder cancer'

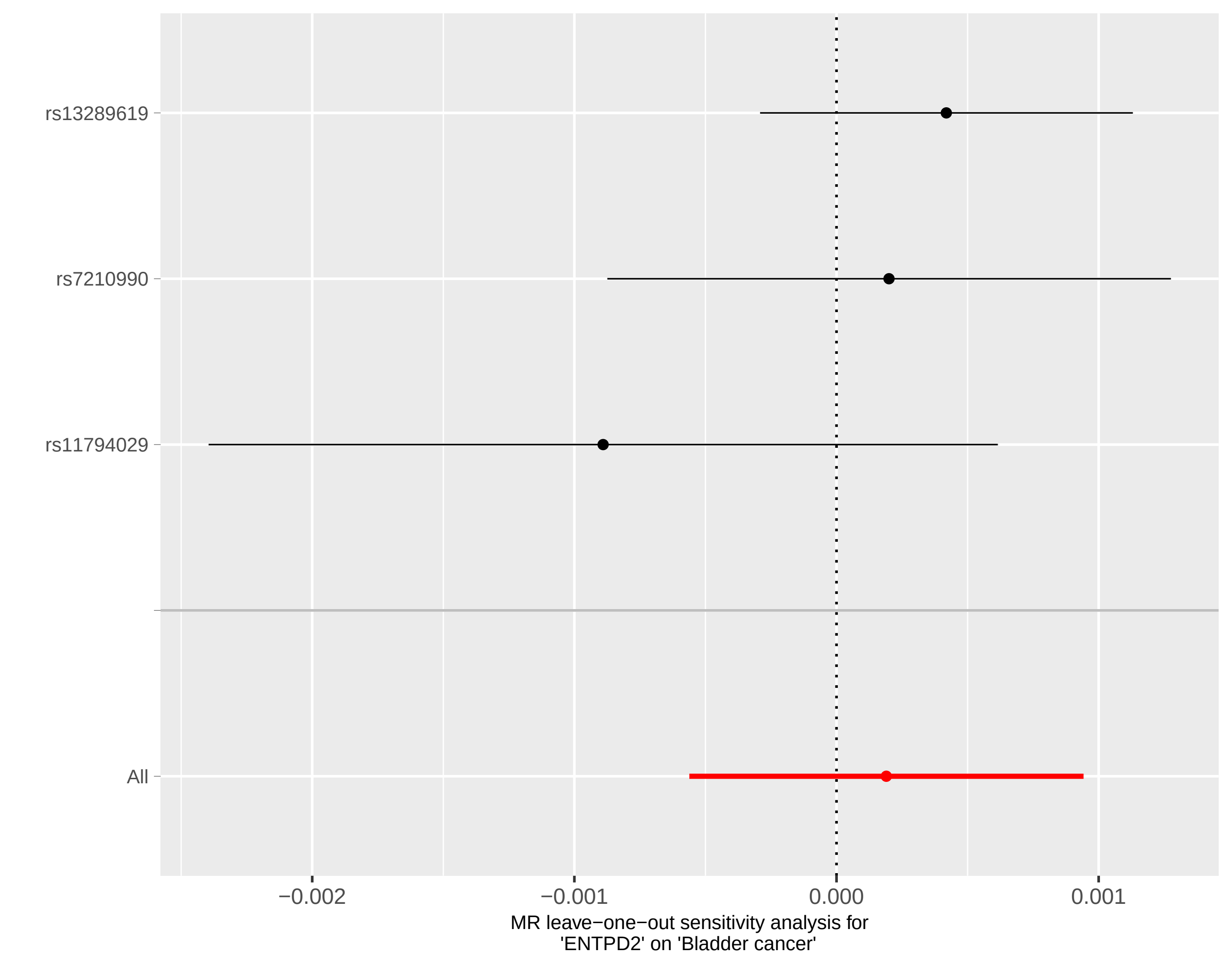
rs13289619
rs7210990
rs11794029
All
−0.002
−0.001
0.000
0.001
MR leave−one−out sensitivity analysis for
'ENTPD2' on 'Bladder cancer'

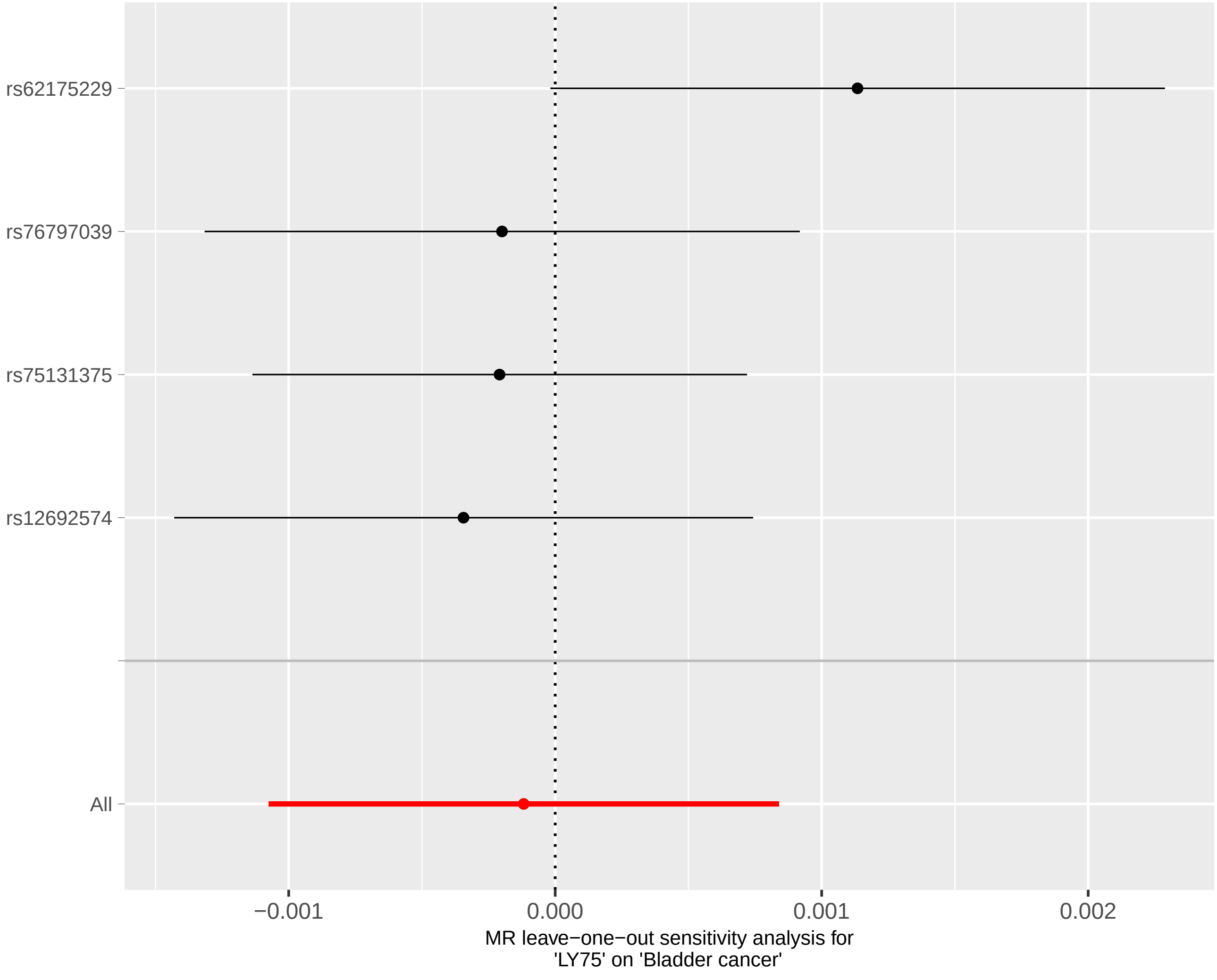
rs62175229
rs76797039
rs75131375
rs12692574
All
−0.001
0.000
0.001
0.002
MR leave−one−out sensitivity analysis for
'LY75' on 'Bladder cancer'

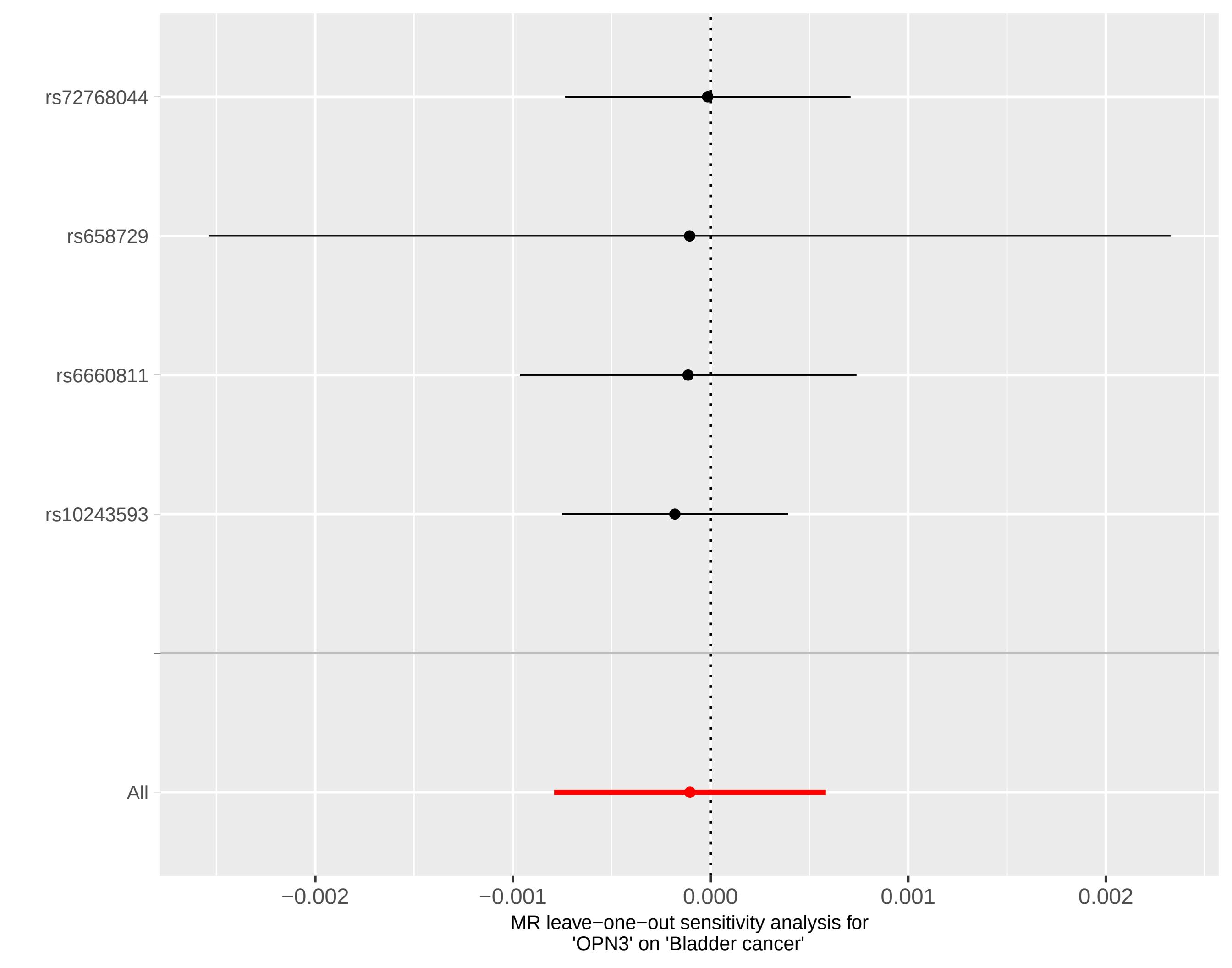
rs72768044
rs658729
rs6660811
rs10243593
All
−0.002
−0.001
0.000
0.001
0.002
MR leave−one−out sensitivity analysis for
'OPN3' on 'Bladder cancer'

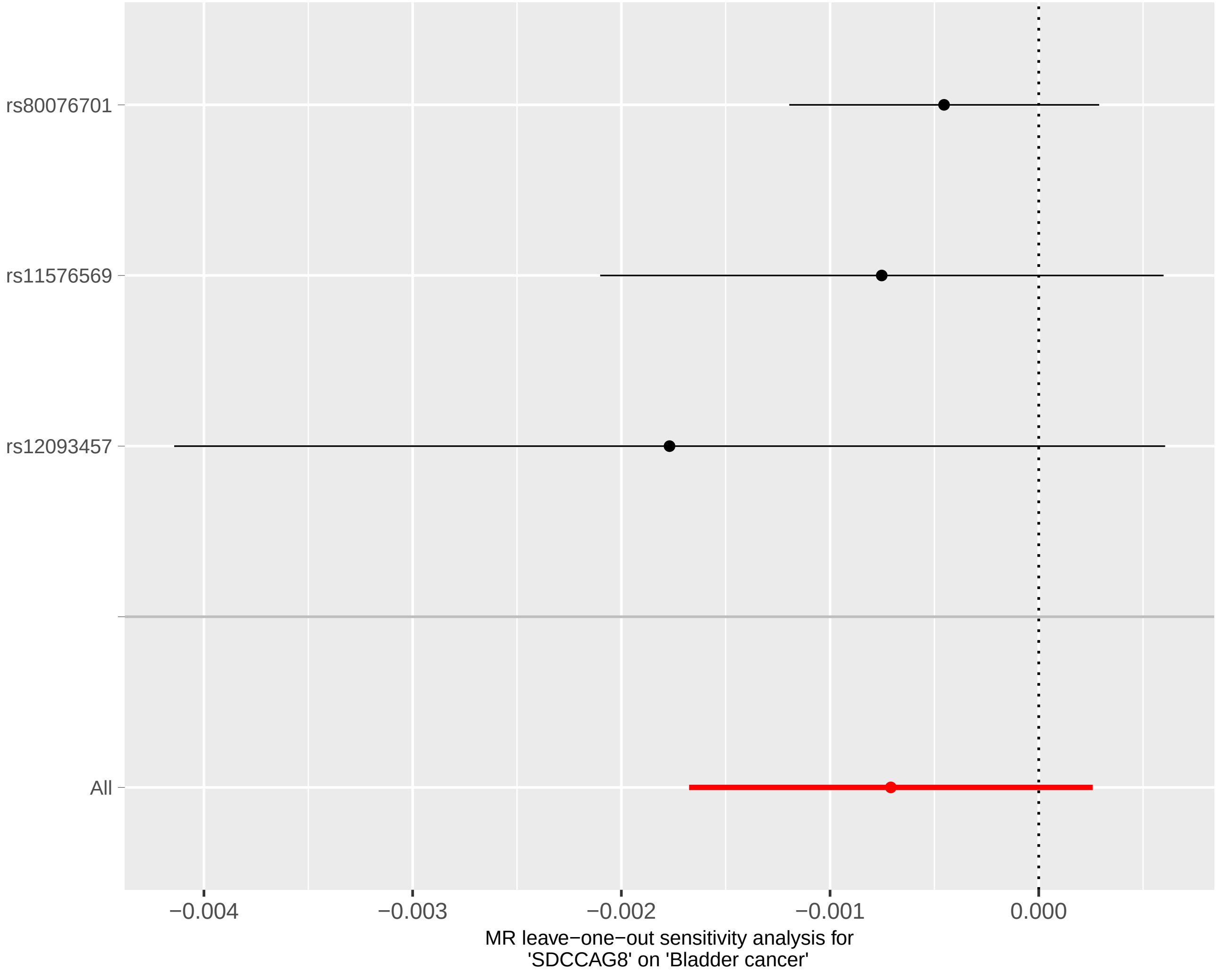
rs80076701
rs11576569
rs12093457
All
−0.004
−0.003
−0.002
−0.001
0.000
MR leave−one−out sensitivity analysis for
'SDCCAG8' on 'Bladder cancer'

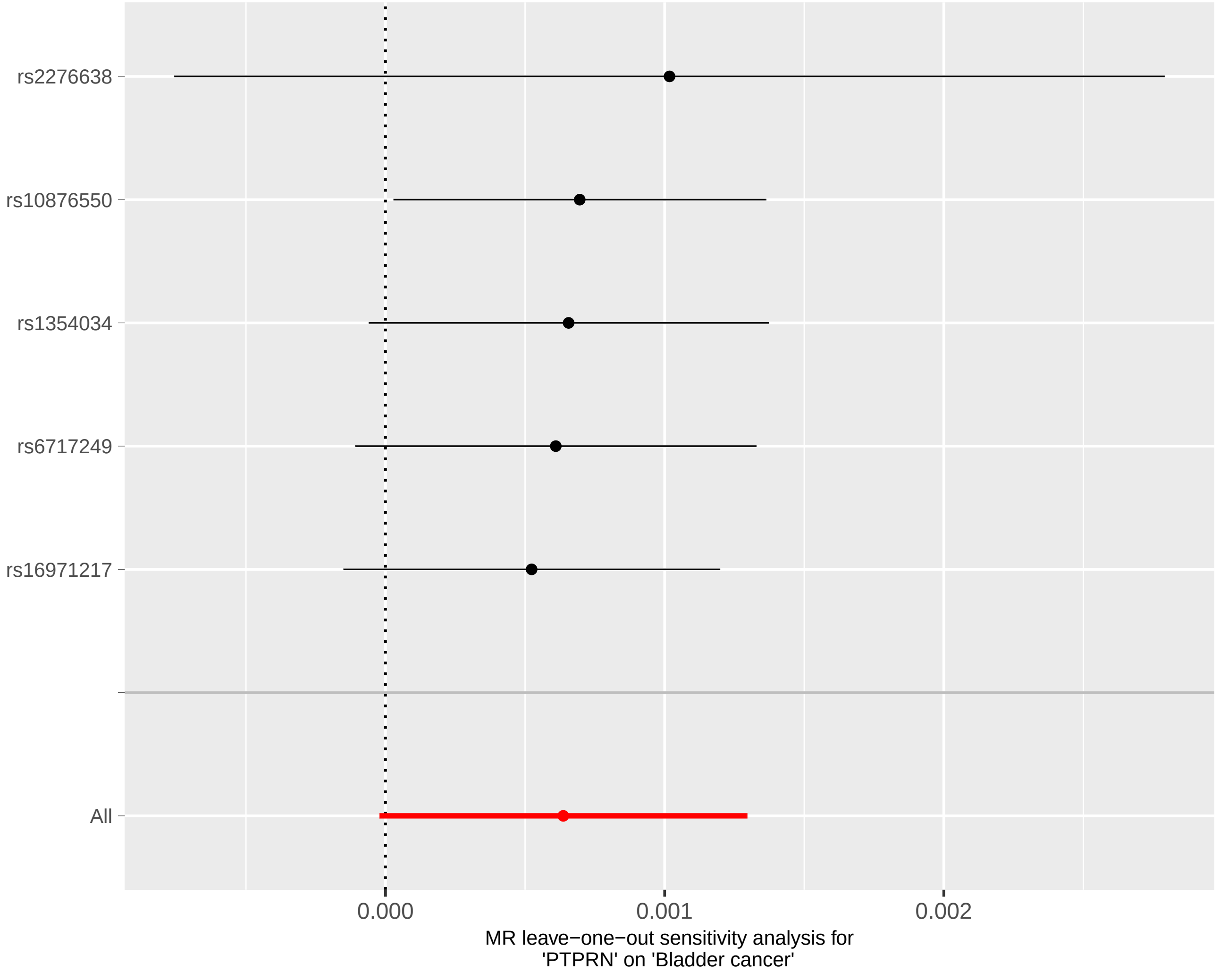

rs2276638
rs10876550
rs1354034
rs6717249
rs16971217
All
0.000
0.001
0.002
MR leave−one−out sensitivity analysis for
'PTPRN' on 'Bladder cancer'

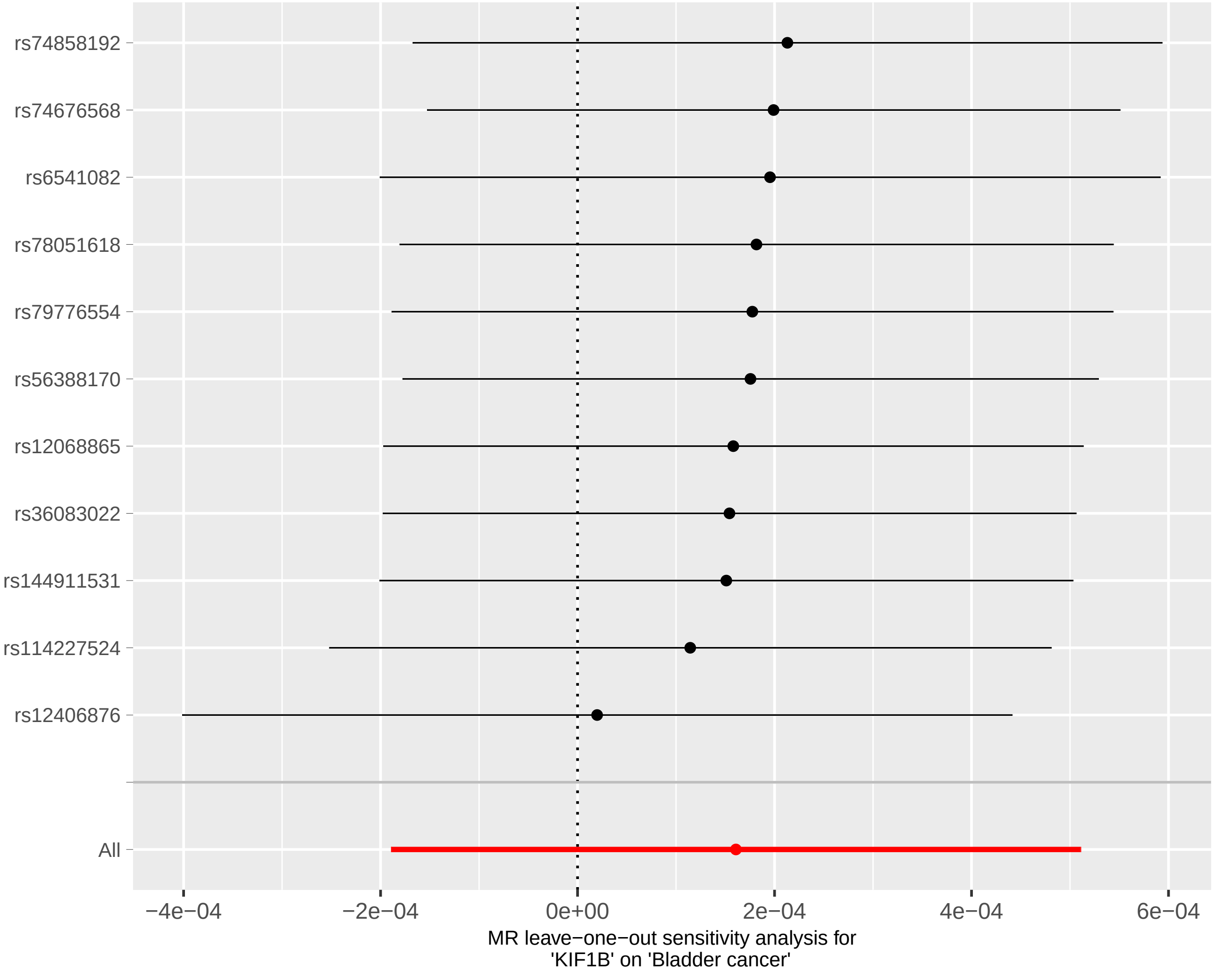
rs74858192
rs74676568
rs6541082
rs78051618
rs79776554
rs56388170
rs12068865
rs36083022
rs144911531
rs114227524
rs12406876
All
−4e−04
−2e−04
0e+00
2e−04
4e−04
6e−04
MR leave−one−out sensitivity analysis for
'KIF1B' on 'Bladder cancer'

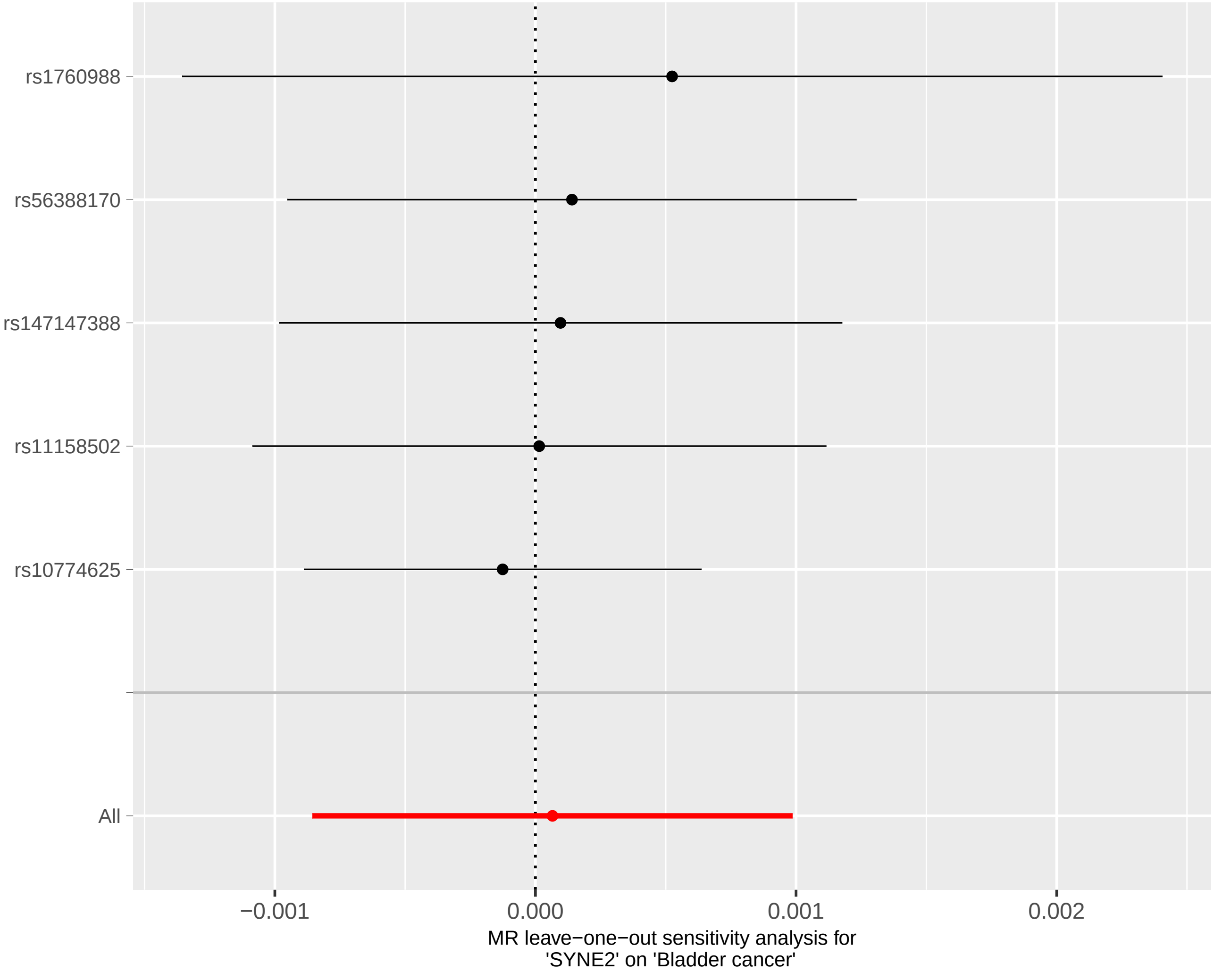
rs1760988
rs56388170
rs147147388
rs11158502
rs10774625
All
−0.001
0.000
0.001
0.002
MR leave−one−out sensitivity analysis for
'SYNE2' on 'Bladder cancer'

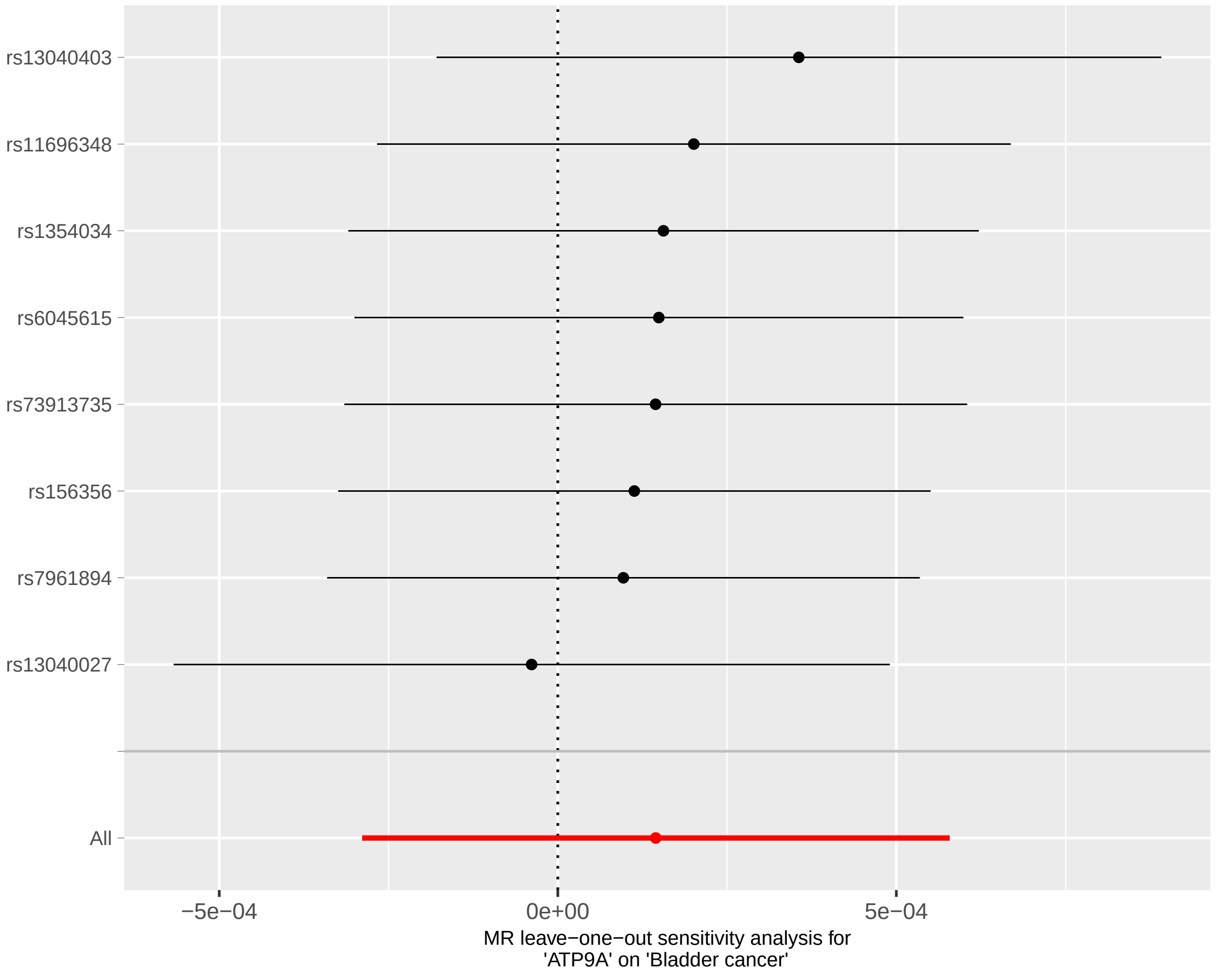
rs13040403
rs11696348
rs1354034
rs6045615
rs73913735
rs156356
rs7961894
rs13040027
All
−5e−04
0e+00
5e−04
MR leave−one−out sensitivity analysis for
'ATP9A' on 'Bladder cancer'

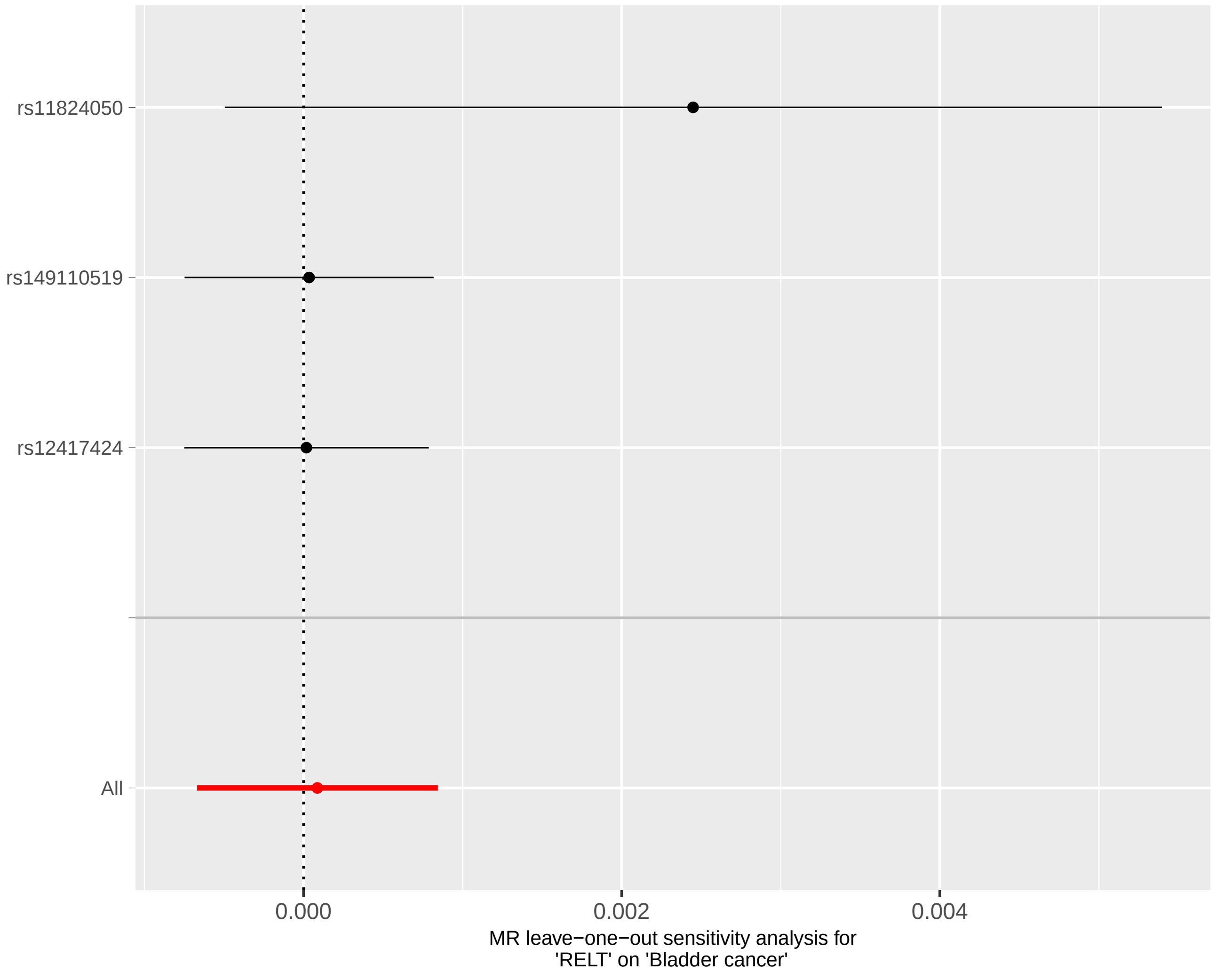
rs11824050
rs149110519
rs12417424
All
0.000
0.002
0.004
MR leave−one−out sensitivity analysis for
'RELT' on 'Bladder cancer'

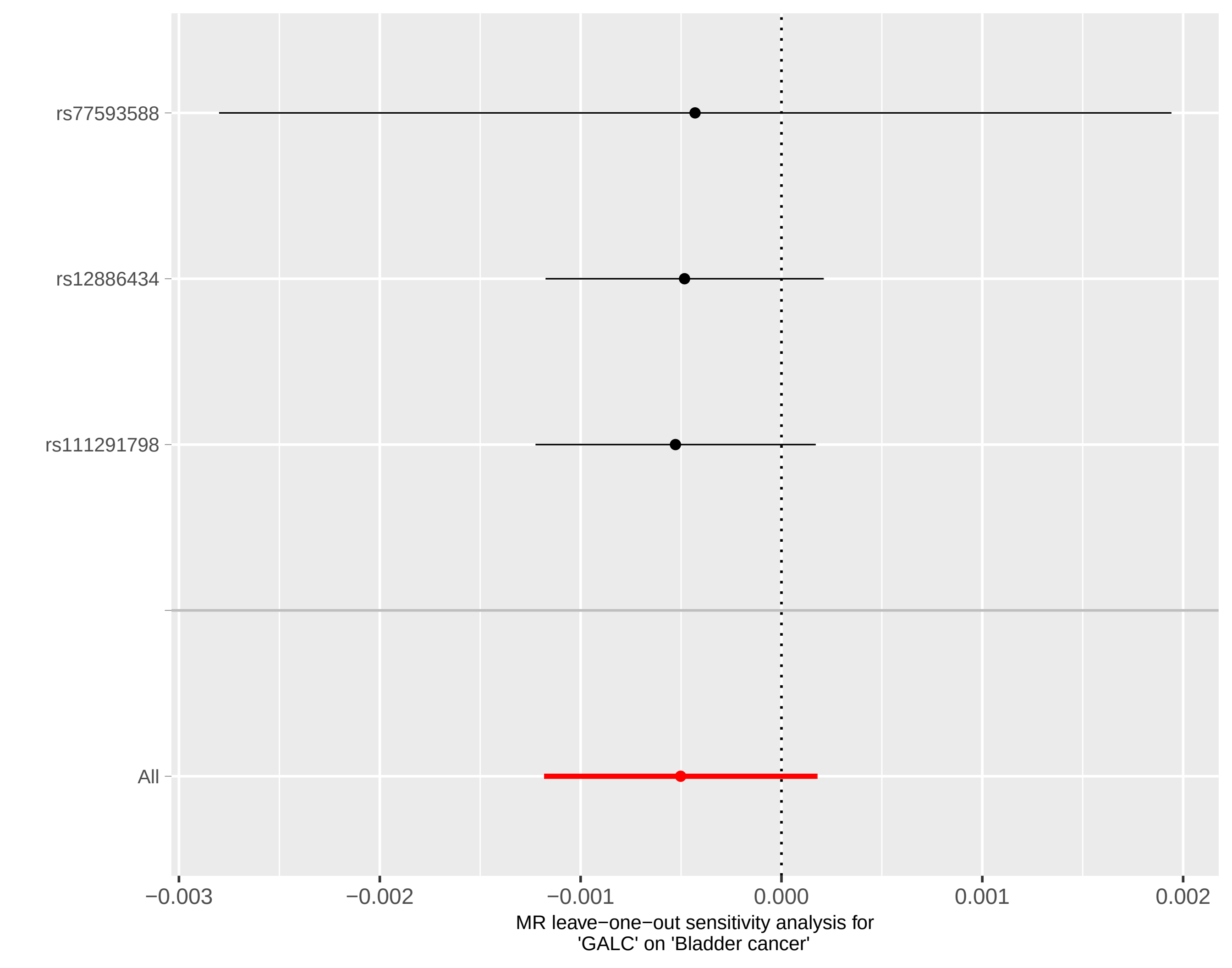
rs77593588
rs12886434
rs111291798
All
−0.003
−0.002
−0.001
0.000
0.001
0.002
MR leave−one−out sensitivity analysis for
'GALC' on 'Bladder cancer'

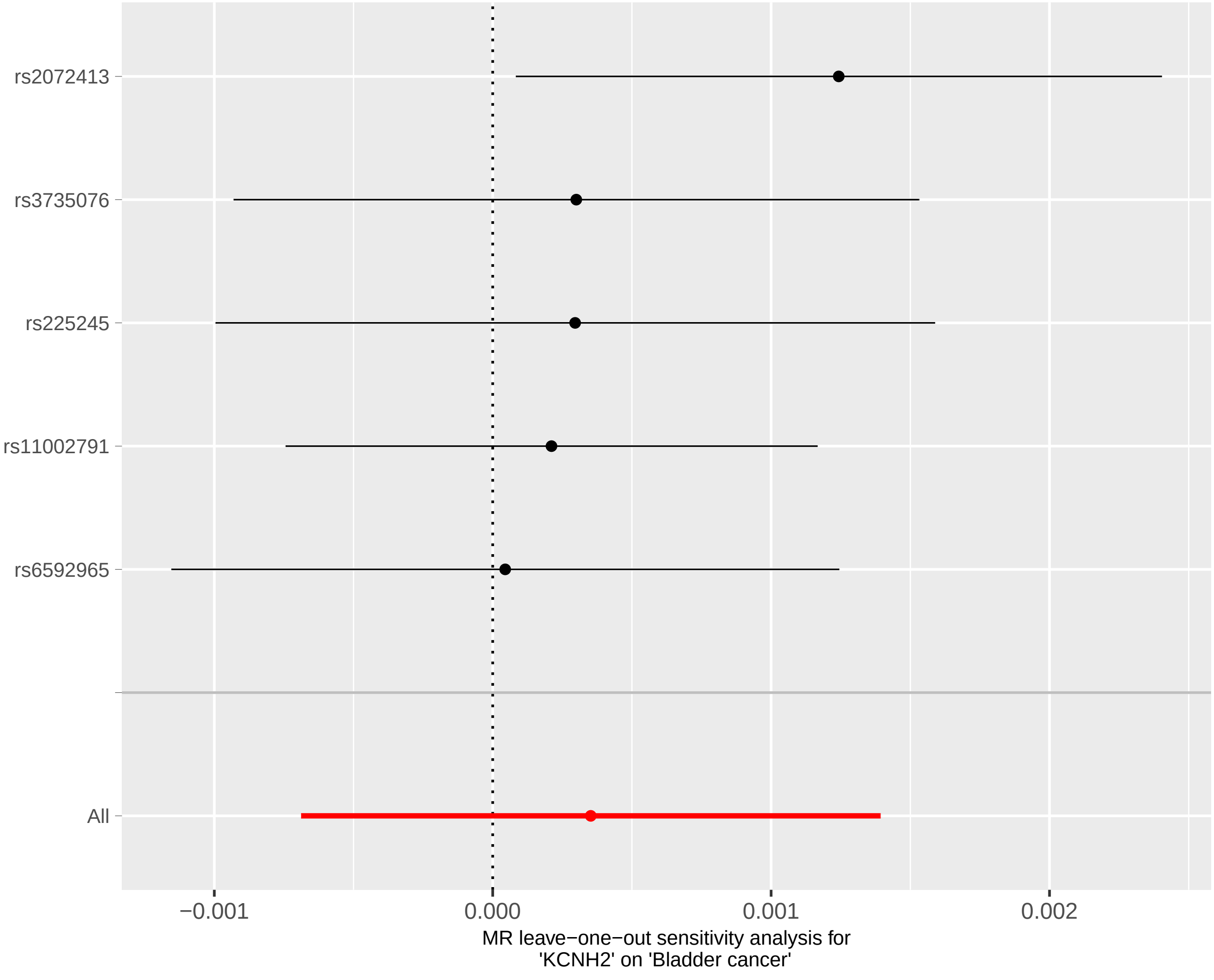
rs2072413
rs3735076
rs225245
rs11002791
rs6592965
All
−0.001
0.000
0.001
0.002
MR leave−one−out sensitivity analysis for
'KCNH2' on 'Bladder cancer'

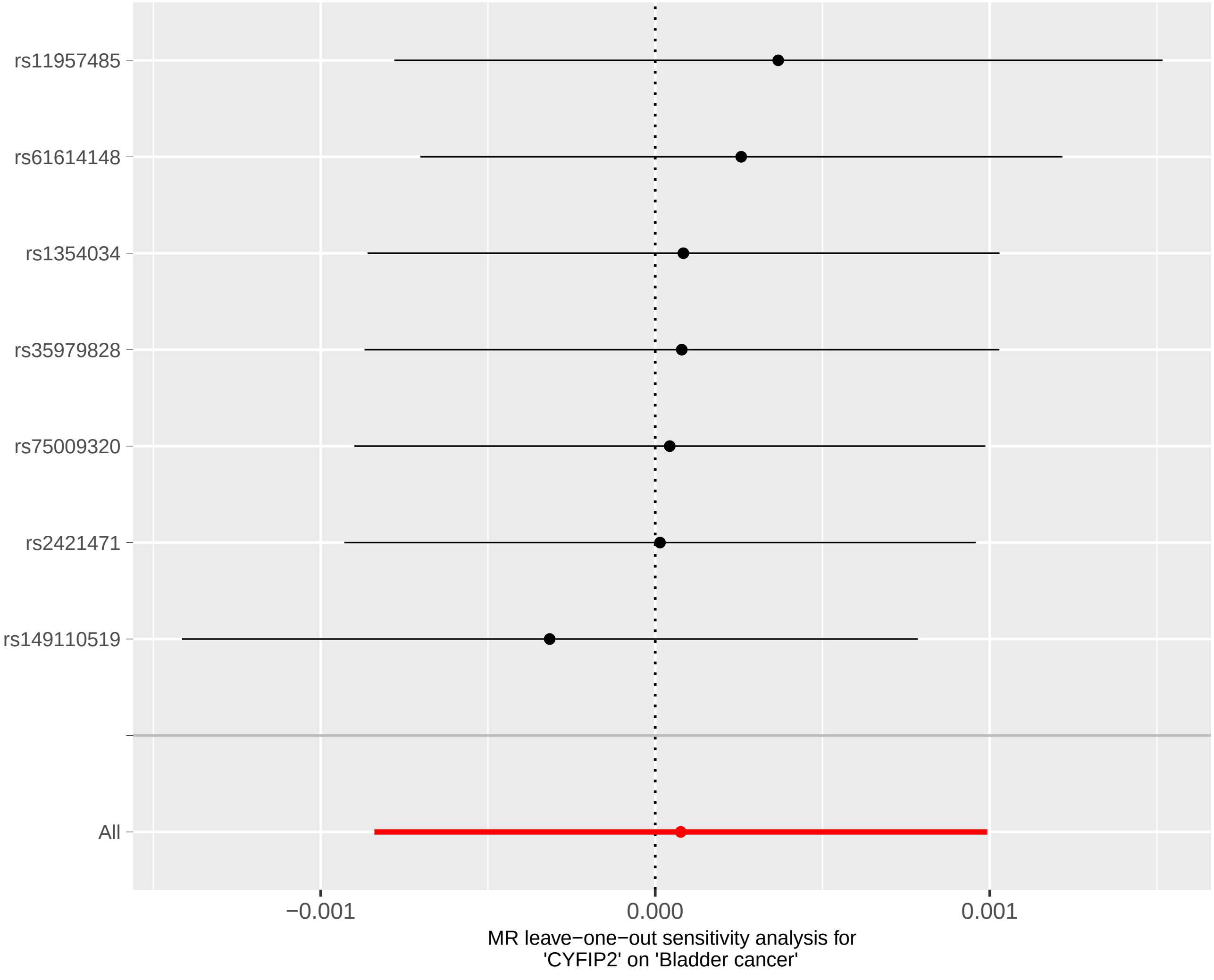
rs11957485
rs61614148
rs1354034
rs35979828
rs75009320
rs2421471
rs149110519
All
−0.001
0.000
0.001
MR leave−one−out sensitivity analysis for
'CYFIP2' on 'Bladder cancer'

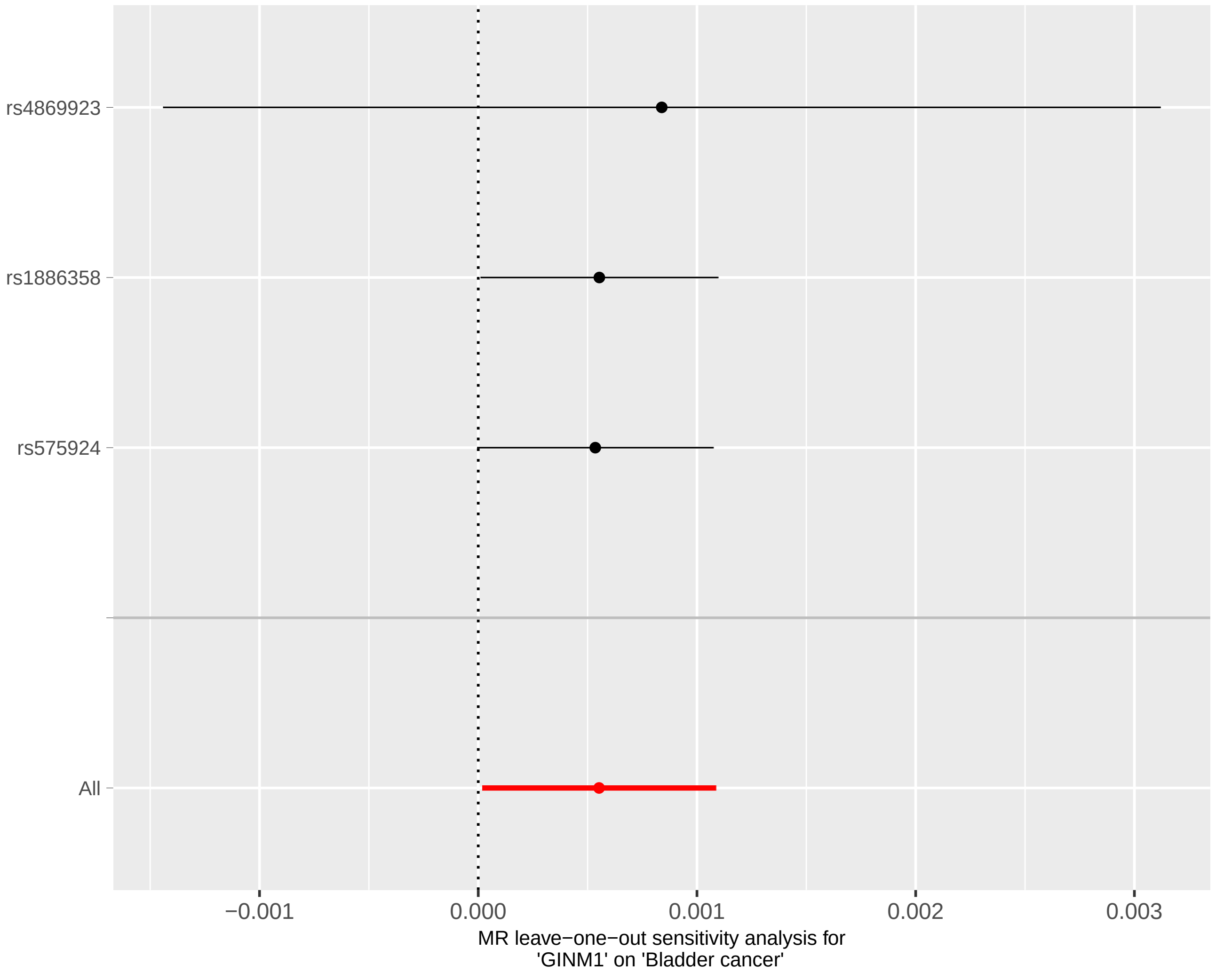

rs4869923
rs1886358
rs575924
All
−0.001
0.000
0.001
0.002
0.003
MR leave−one−out sensitivity analysis for
'GINM1' on 'Bladder cancer'

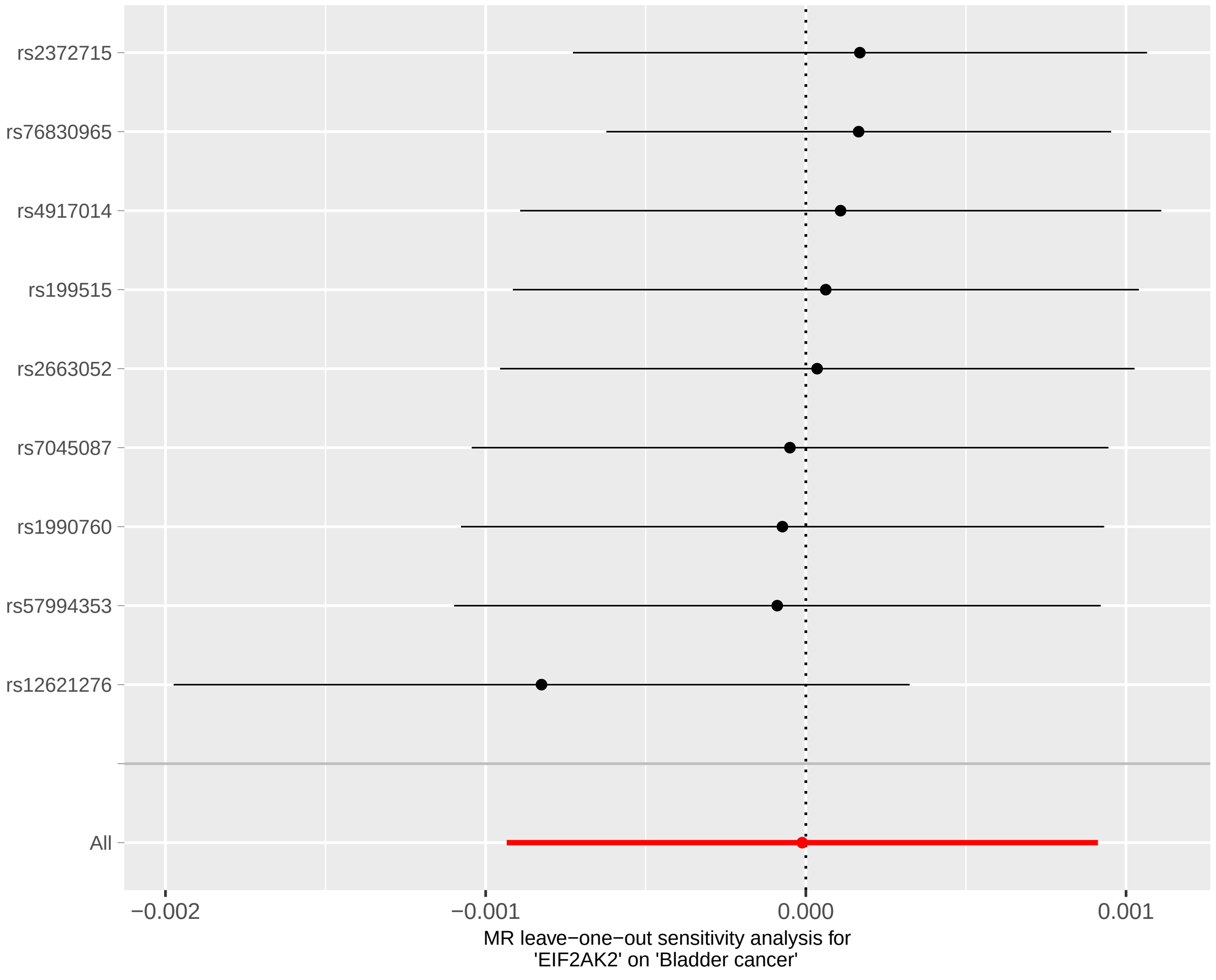
rs2372715
rs76830965
rs4917014
rs199515
rs2663052
rs7045087
rs1990760
rs57994353
rs12621276
All
−0.002
−0.001
0.000
0.001
MR leave−one−out sensitivity analysis for
'EIF2AK2' on 'Bladder cancer'

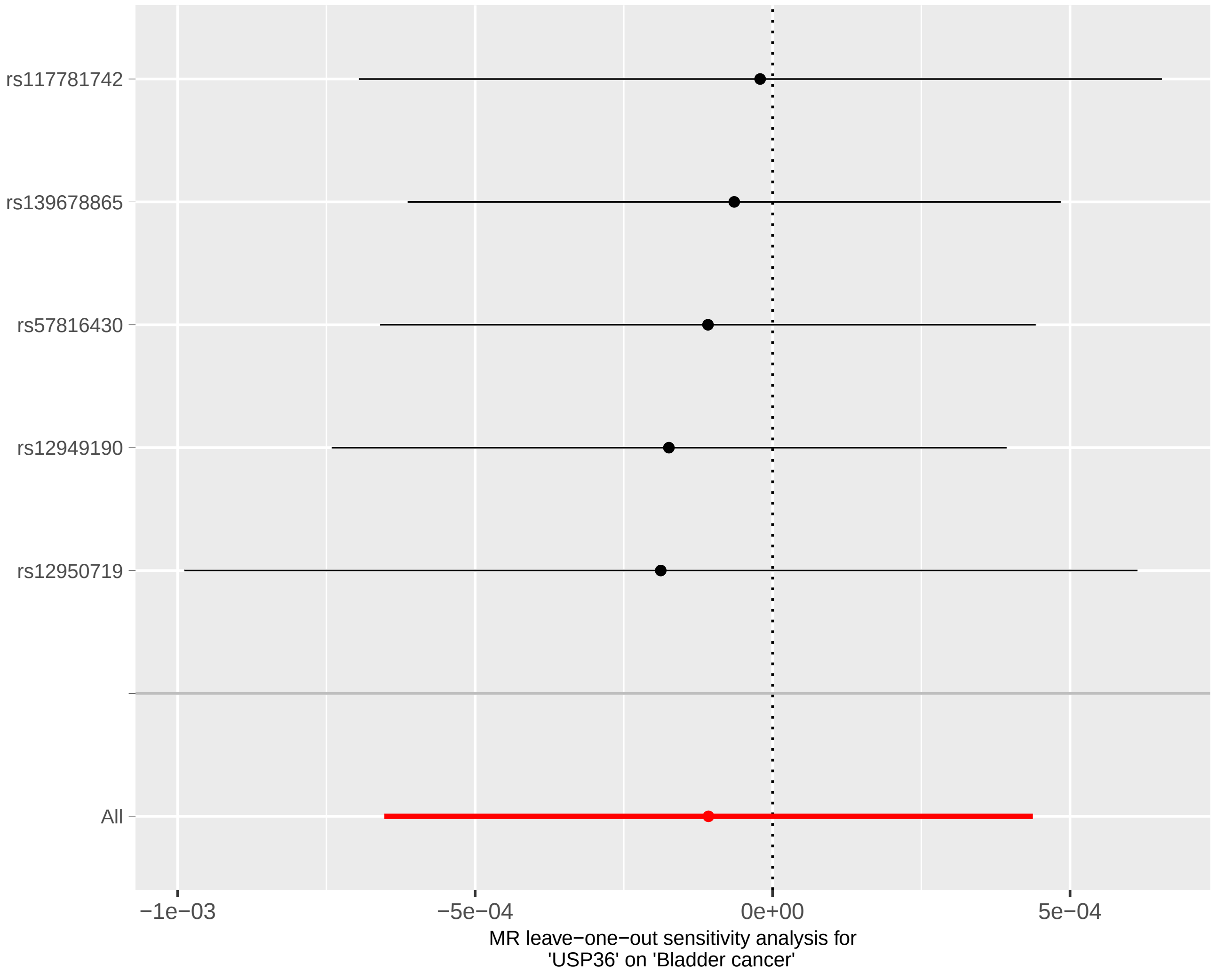

rs117781742
rs139678865
rs57816430
rs12949190
rs12950719
All
−1e−03
−5e−04
0e+00
5e−04
MR leave−one−out sensitivity analysis for
'USP36' on 'Bladder cancer'

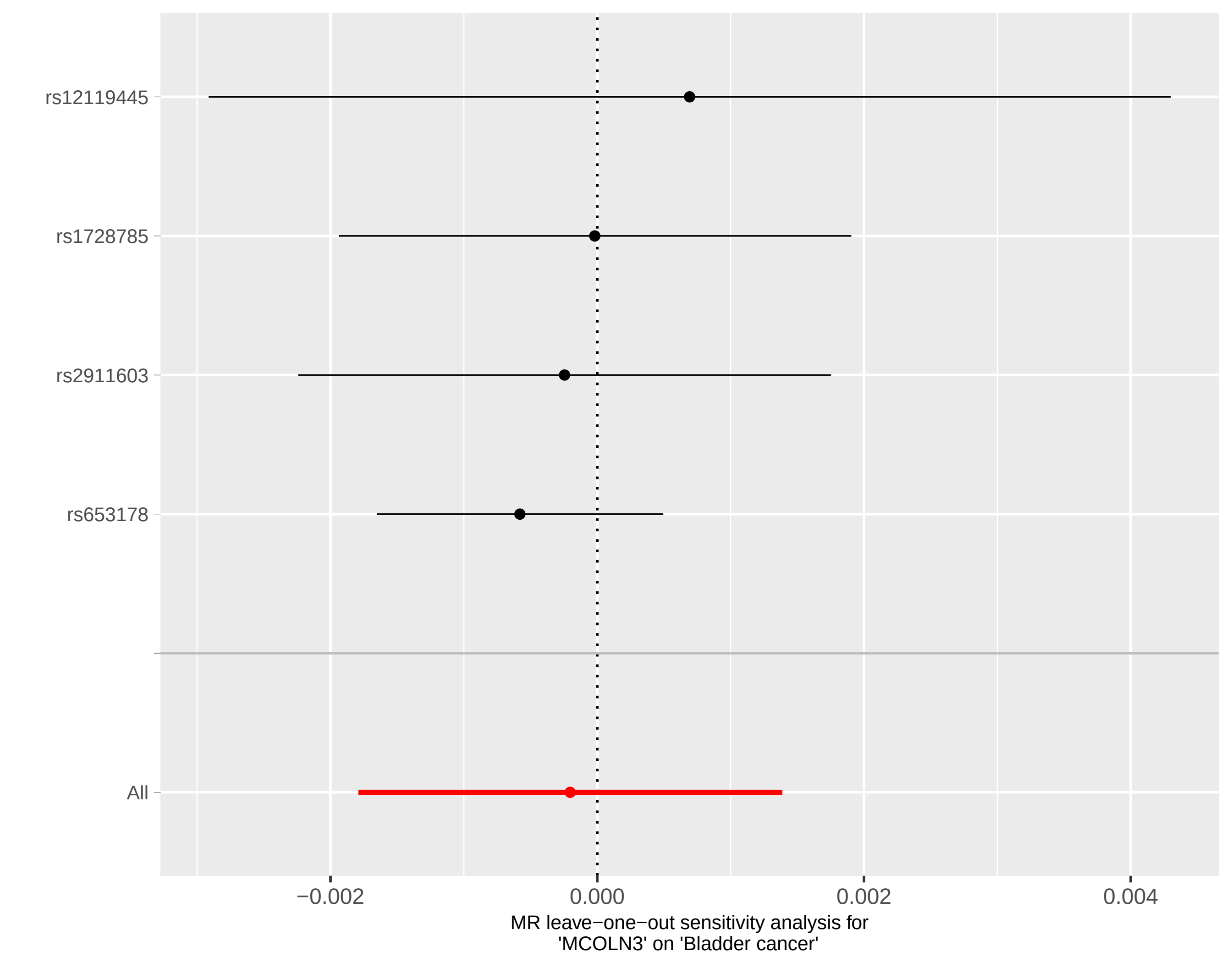
rs12119445
rs1728785
rs2911603
rs653178
All
−0.002
0.000
0.002
0.004
MR leave−one−out sensitivity analysis for
'MCOLN3' on 'Bladder cancer'

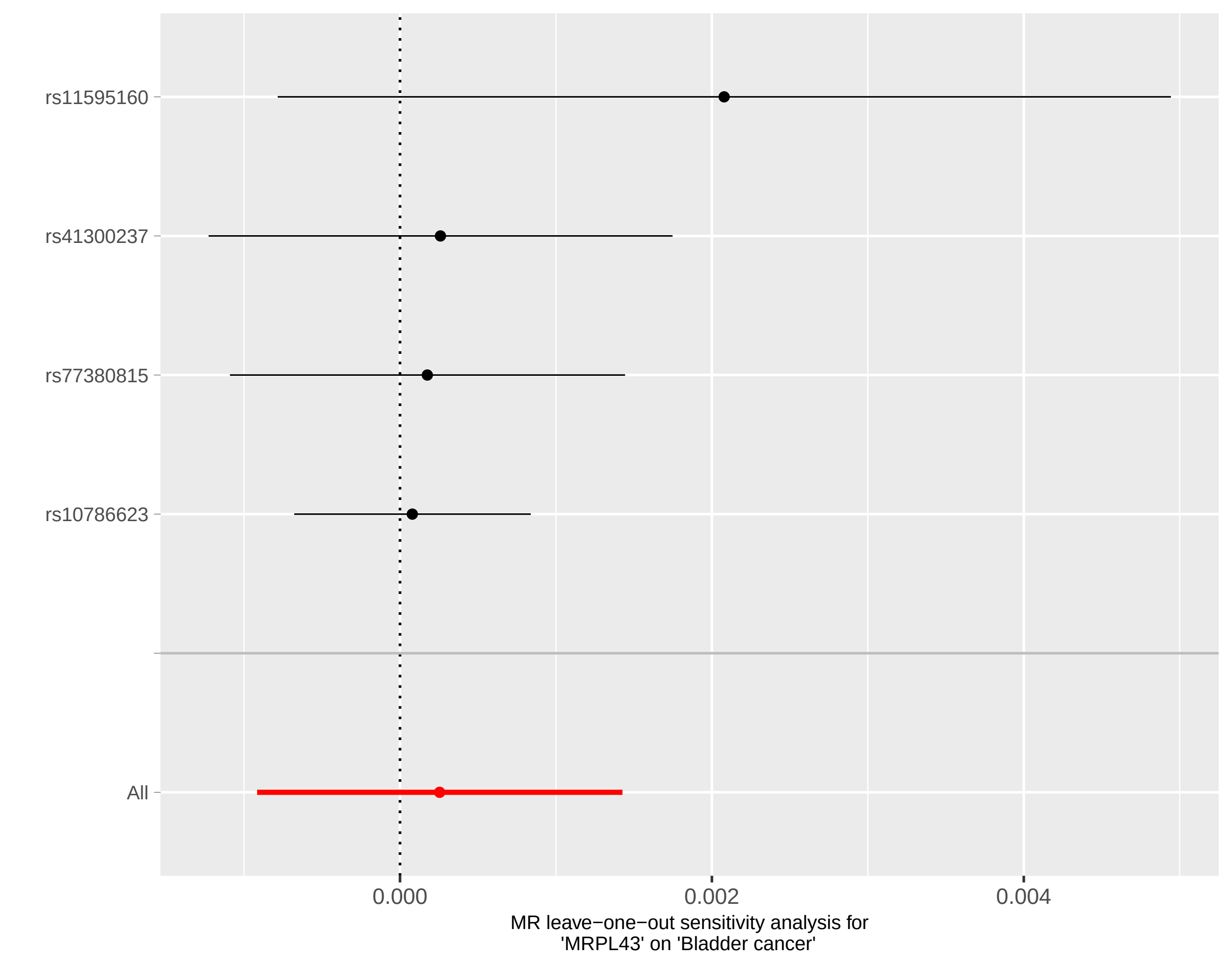
rs11595160
rs41300237
rs77380815
rs10786623
All
0.000
0.002
0.004
MR leave−one−out sensitivity analysis for
'MRPL43' on 'Bladder cancer'

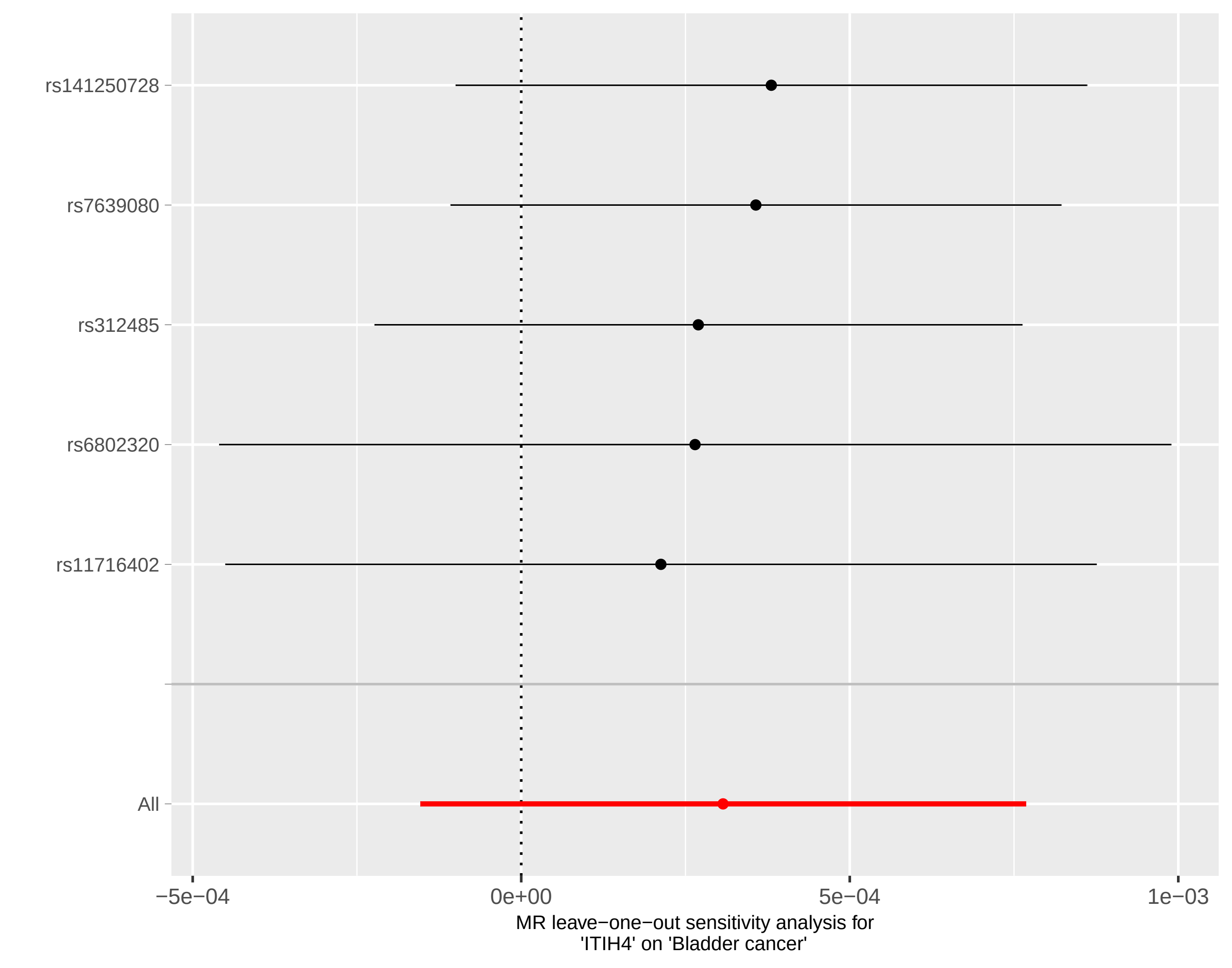
rs141250728
rs7639080
rs312485
rs6802320
rs11716402
All
−5e−04
0e+00
5e−04
1e−03
MR leave−one−out sensitivity analysis for
'ITIH4' on 'Bladder cancer'

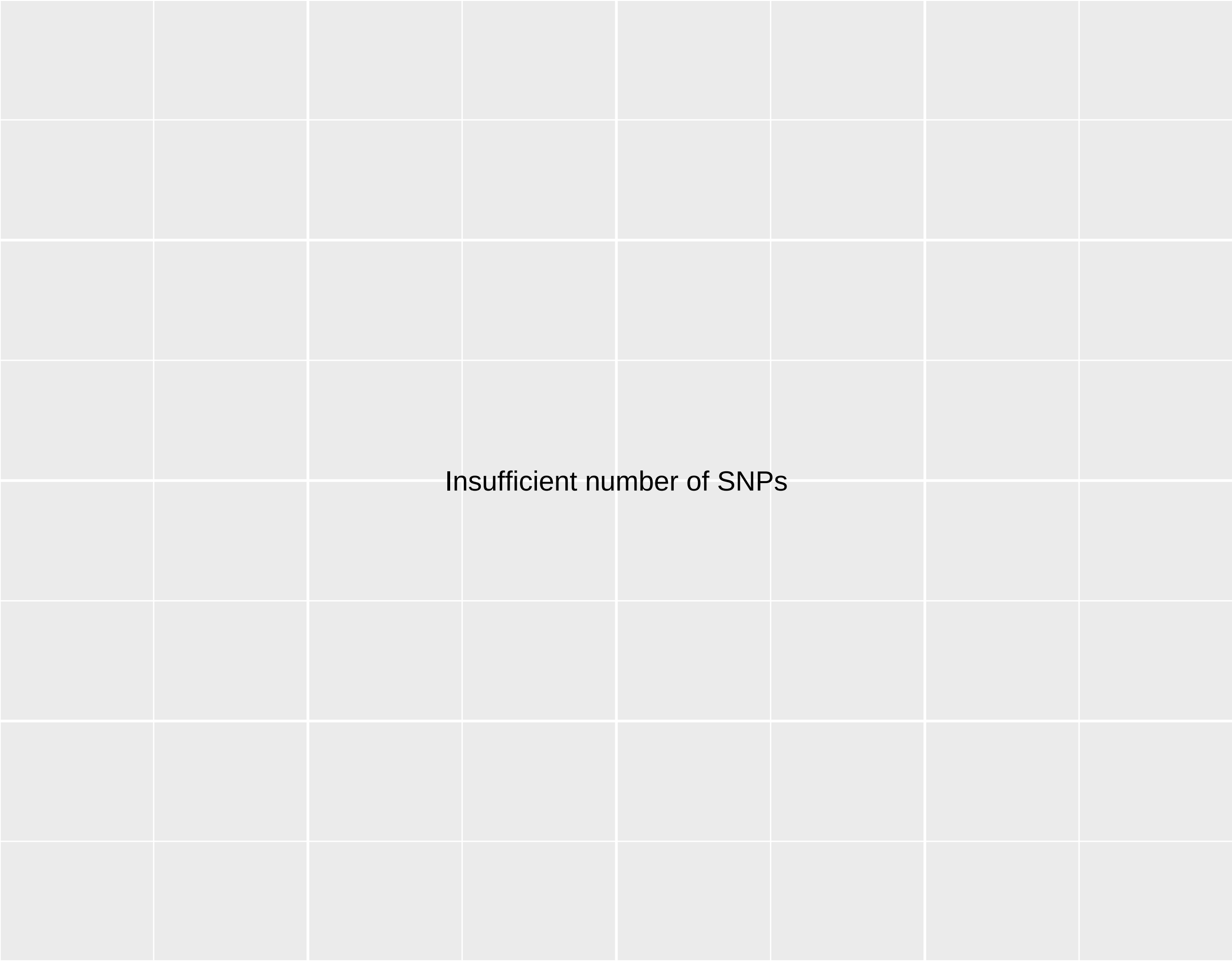
Insufficient number of SNPs

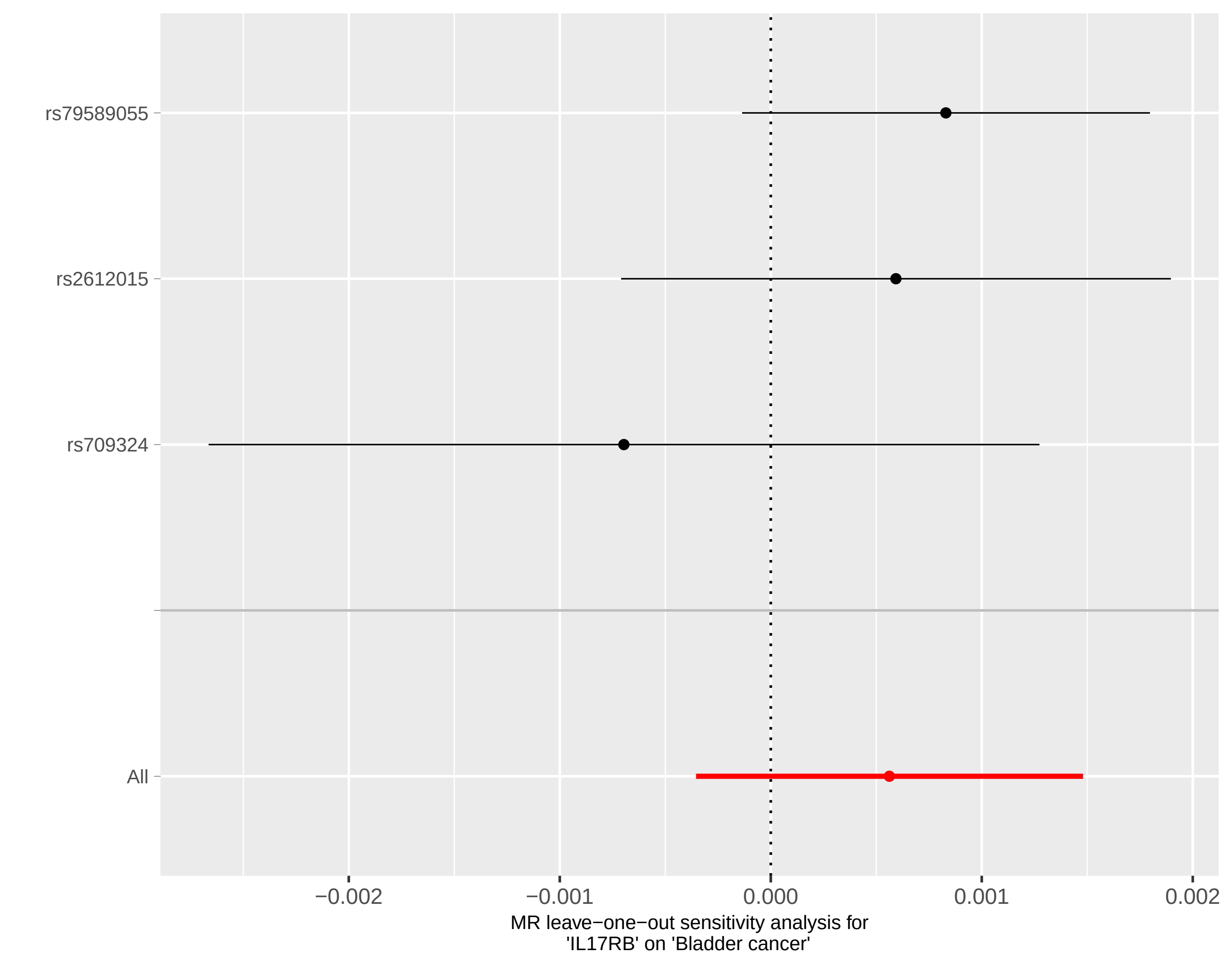
rs79589055
rs2612015
rs709324
All
−0.002
−0.001
0.000
0.001
0.002
MR leave−one−out sensitivity analysis for
'IL17RB' on 'Bladder cancer'

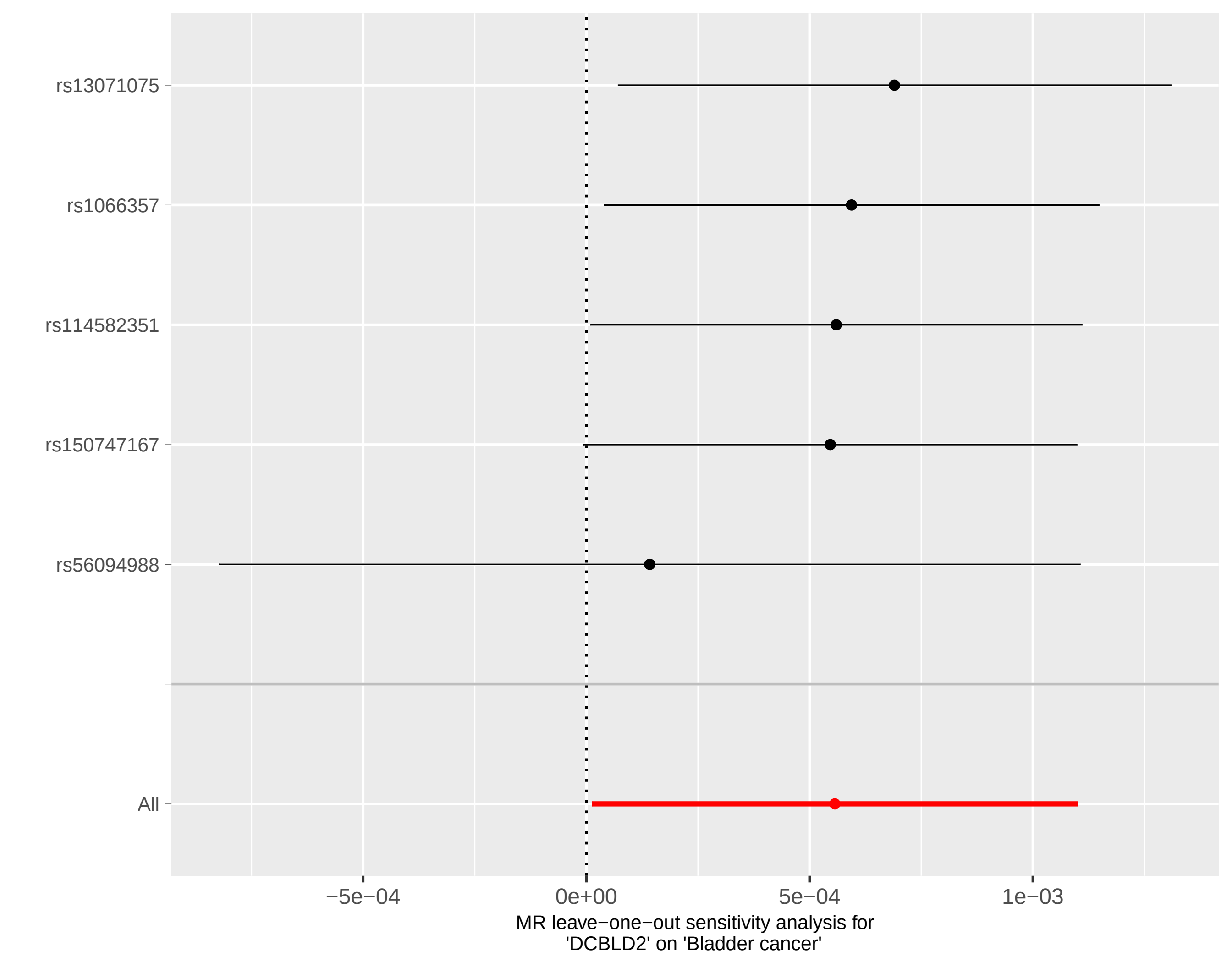
rs13071075
rs1066357
rs114582351
rs150747167
rs56094988
All
−5e−04
0e+00
5e−04
1e−03
MR leave−one−out sensitivity analysis for
'DCBLD2' on 'Bladder cancer'

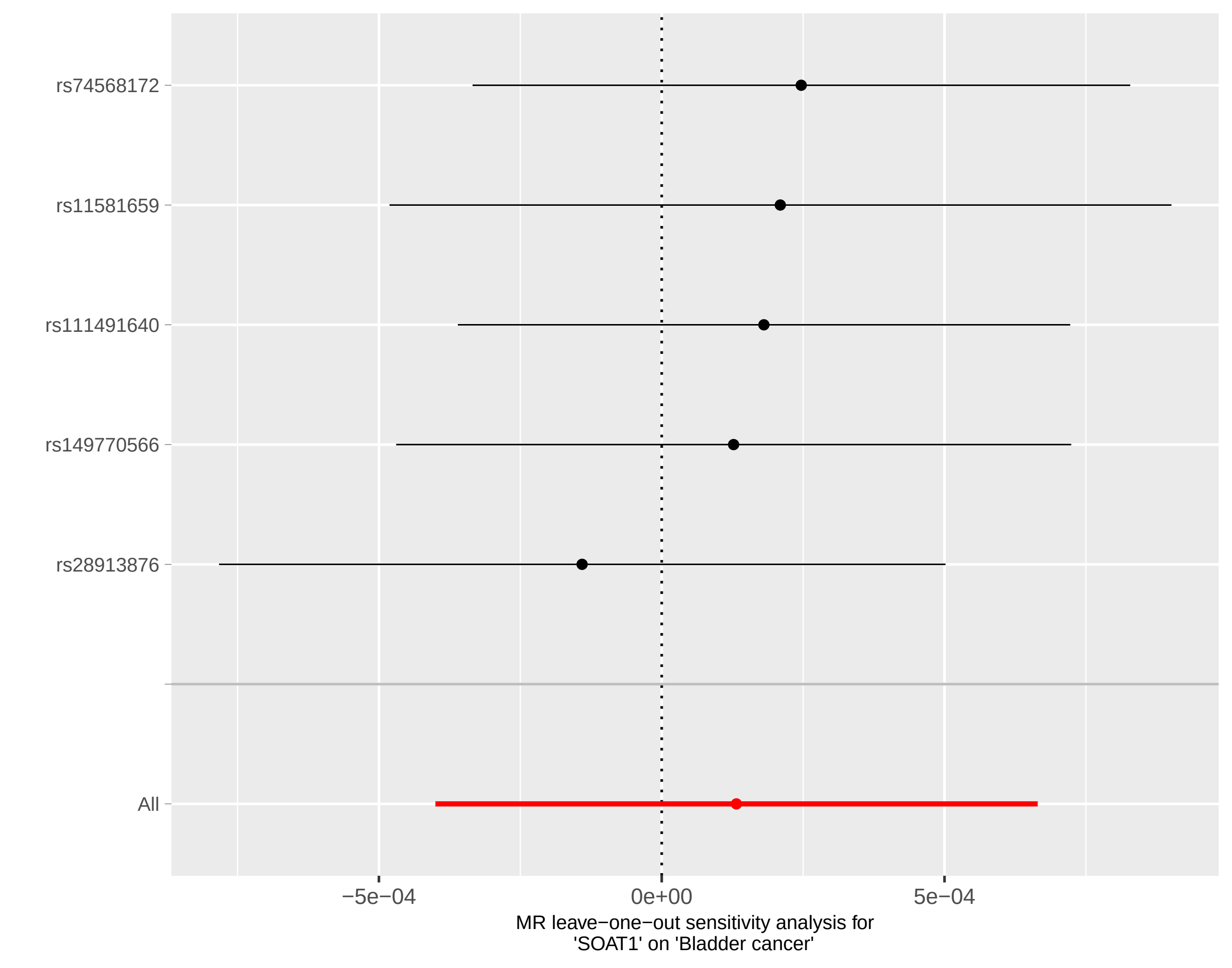
rs74568172
rs11581659
rs111491640
rs149770566
rs28913876
All
−5e−04
0e+00
5e−04
MR leave−one−out sensitivity analysis for
'SOAT1' on 'Bladder cancer'

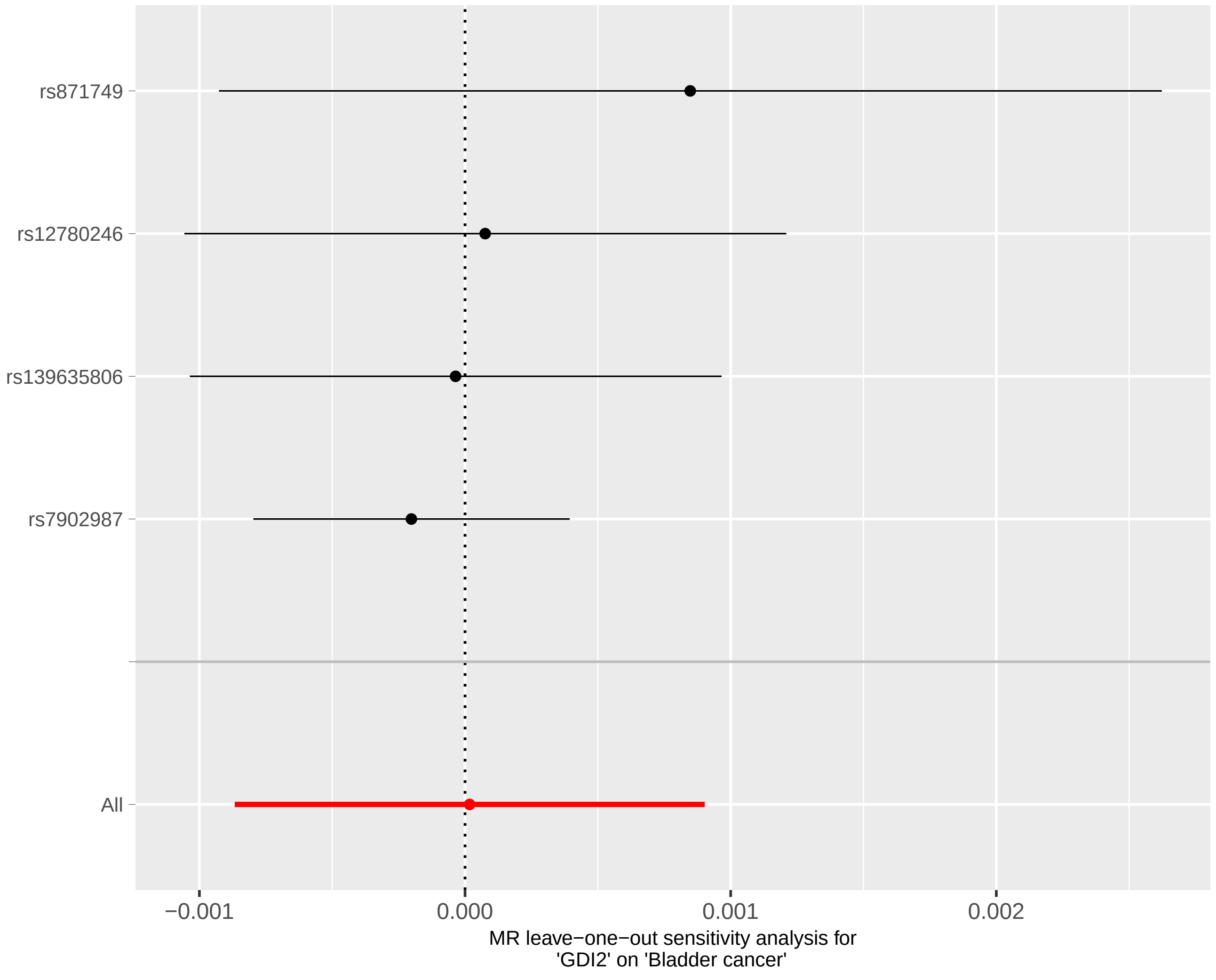
rs871749
rs12780246
rs139635806
rs7902987
All
−0.001
0.000
0.001
0.002
MR leave−one−out sensitivity analysis for
'GDI2' on 'Bladder cancer'

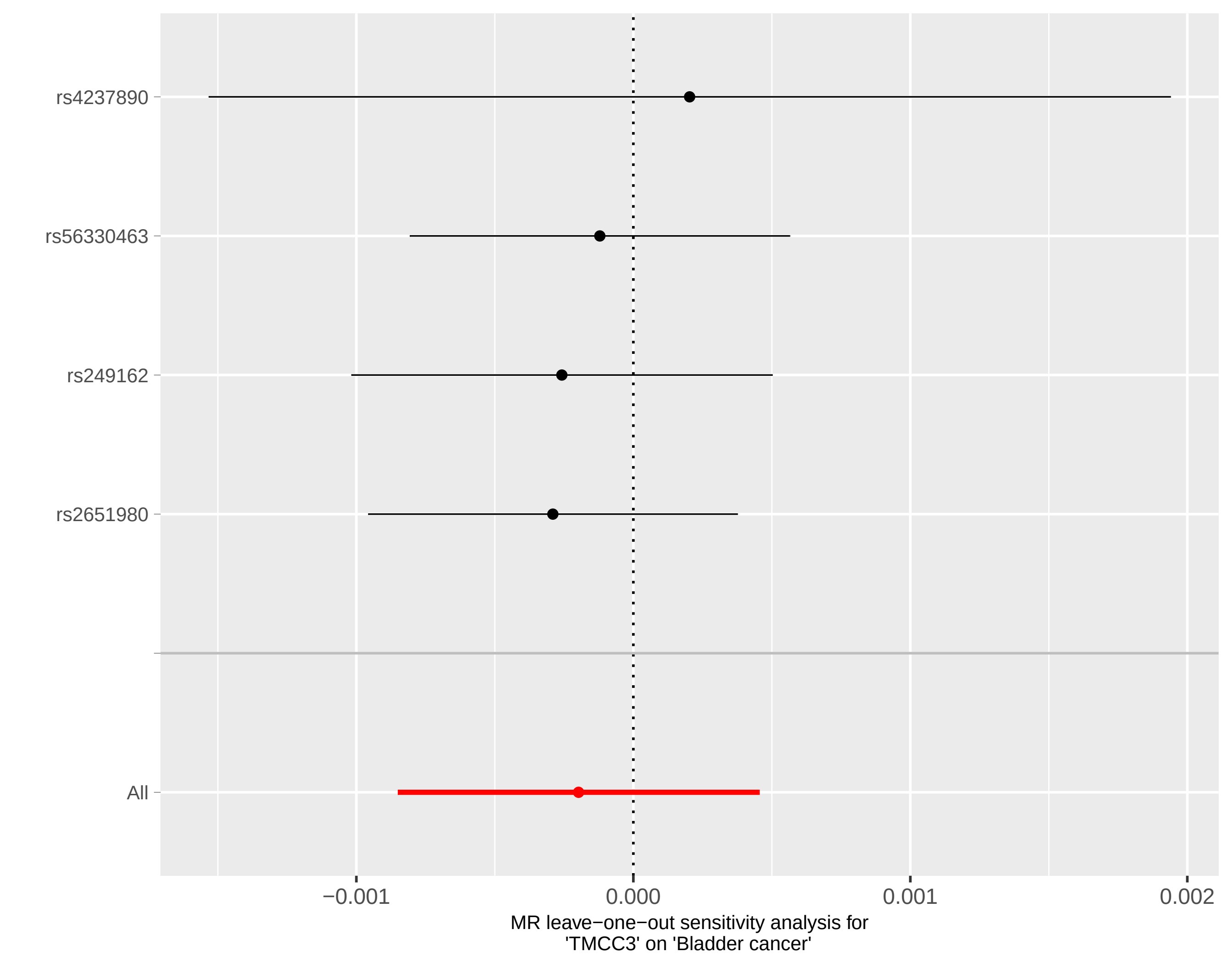
rs4237890
rs56330463
rs249162
rs2651980
All
−0.001
0.000
0.001
0.002
MR leave−one−out sensitivity analysis for
'TMCC3' on 'Bladder cancer'

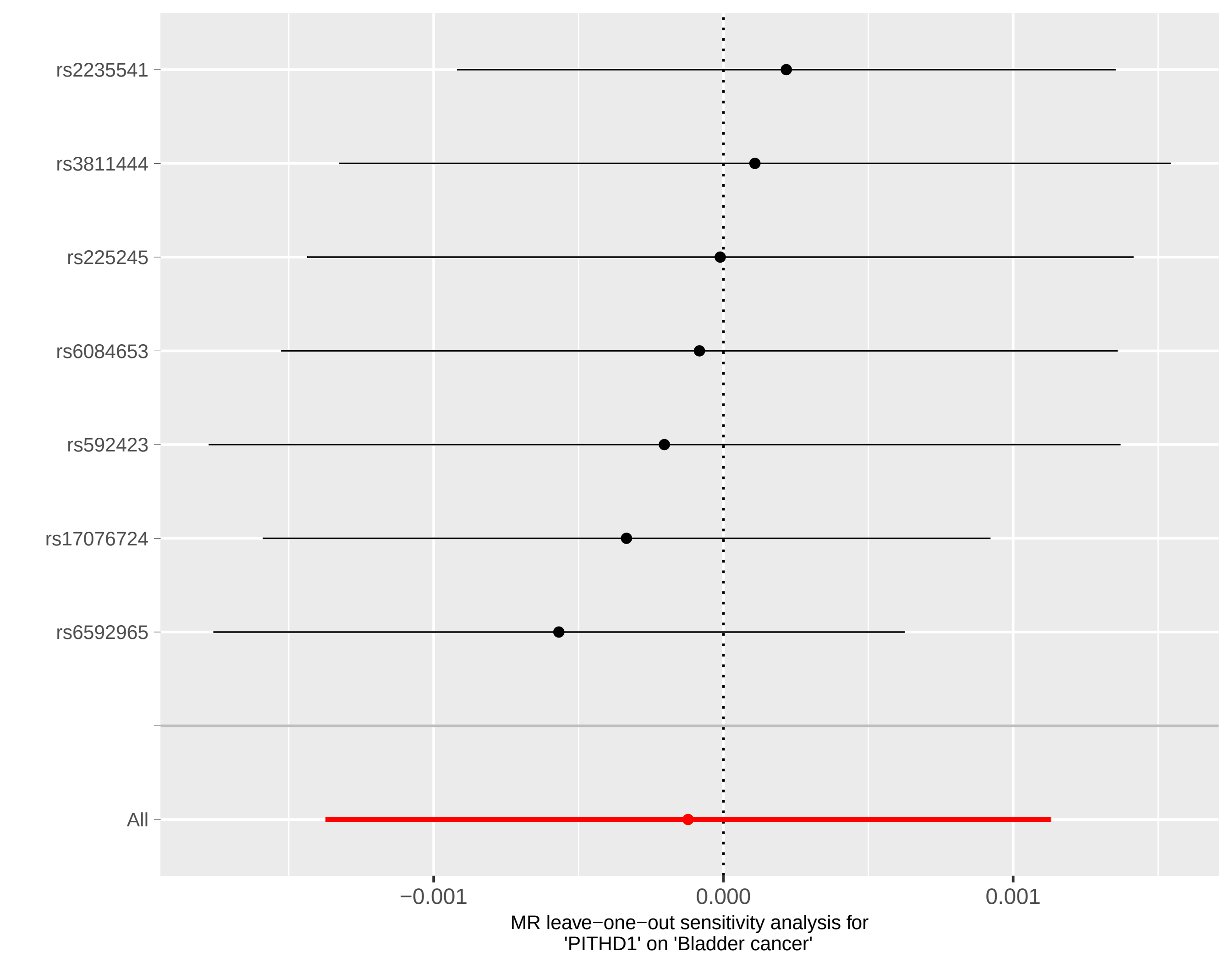
rs2235541
rs3811444
rs225245
rs6084653
rs592423
rs17076724
rs6592965
All
−0.001
0.000
0.001
MR leave−one−out sensitivity analysis for
'PITHD1' on 'Bladder cancer'

Insufficient number of SNPs

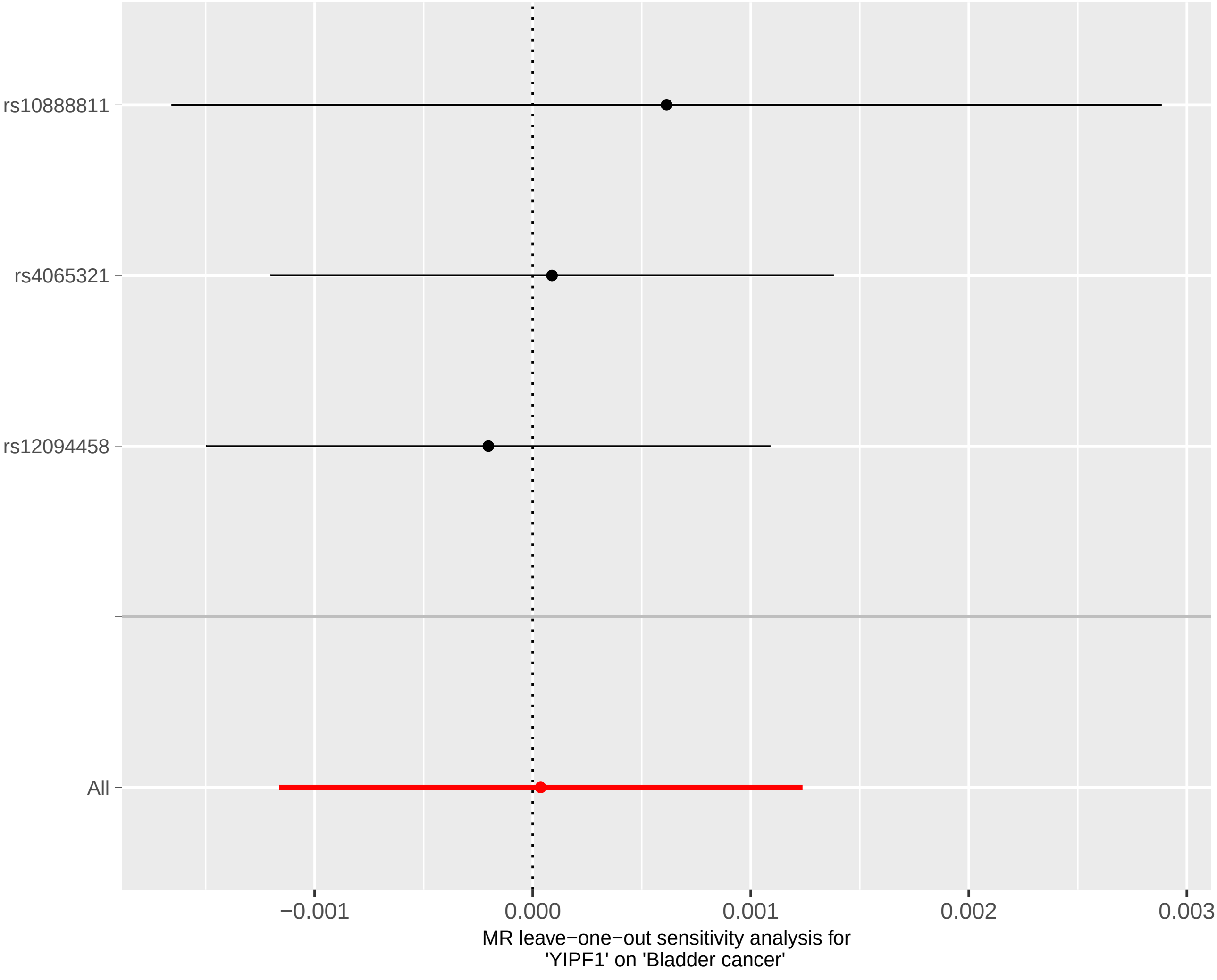
rs10888811
rs4065321
rs12094458
All
−0.001
0.000
0.001
0.002
0.003
MR leave−one−out sensitivity analysis for
'YIPF1' on 'Bladder cancer'

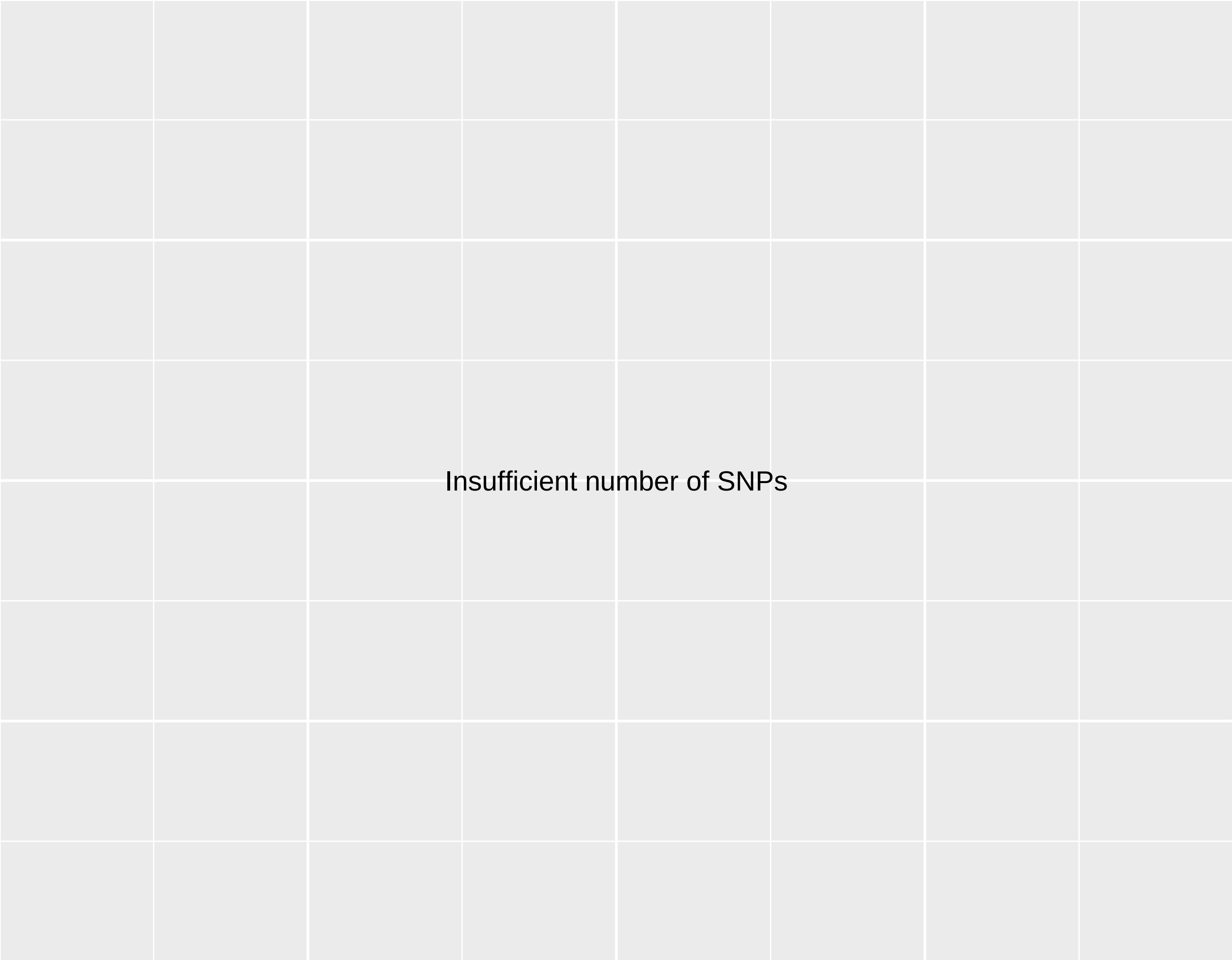
Insufficient number of SNPs

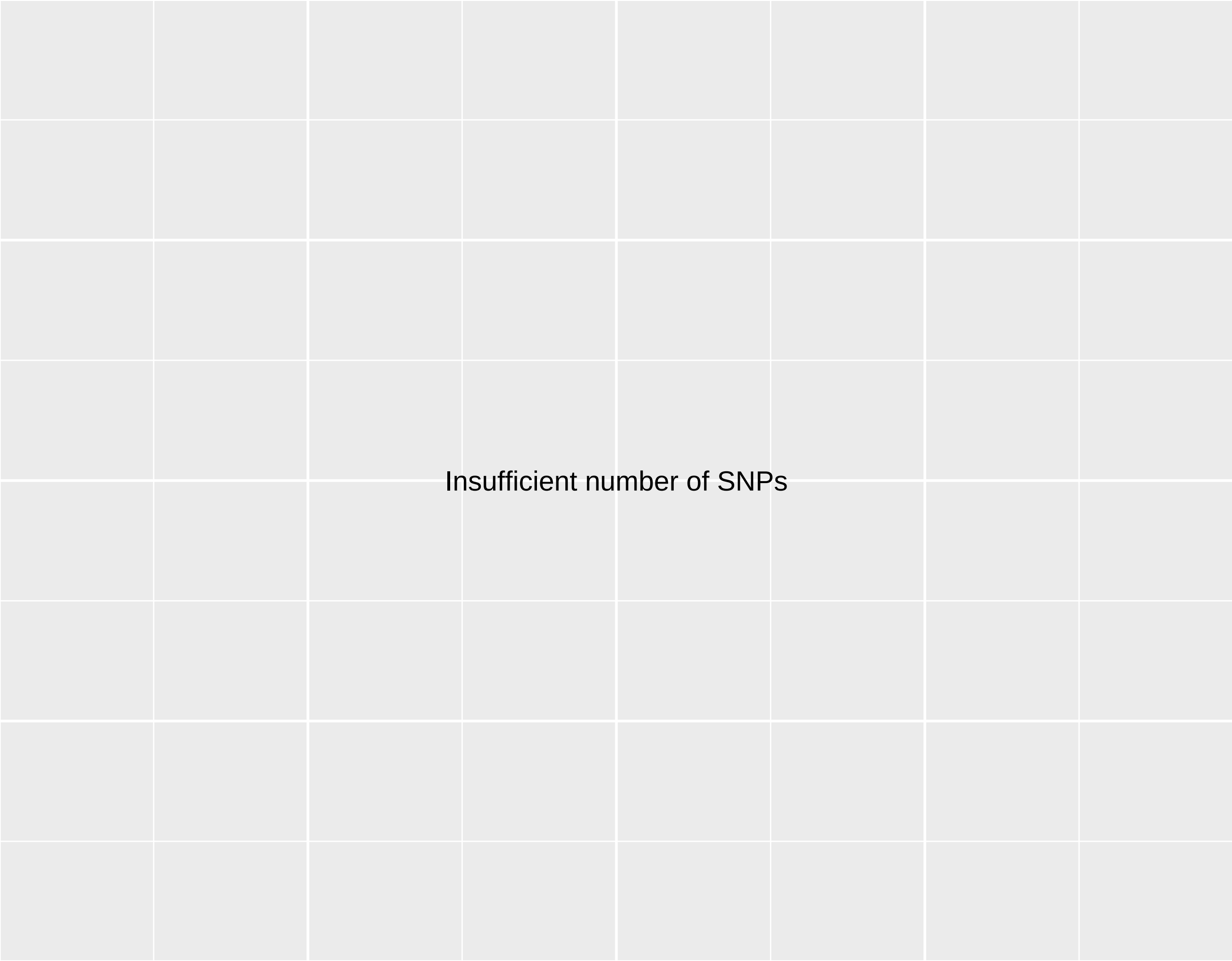
Insufficient number of SNPs

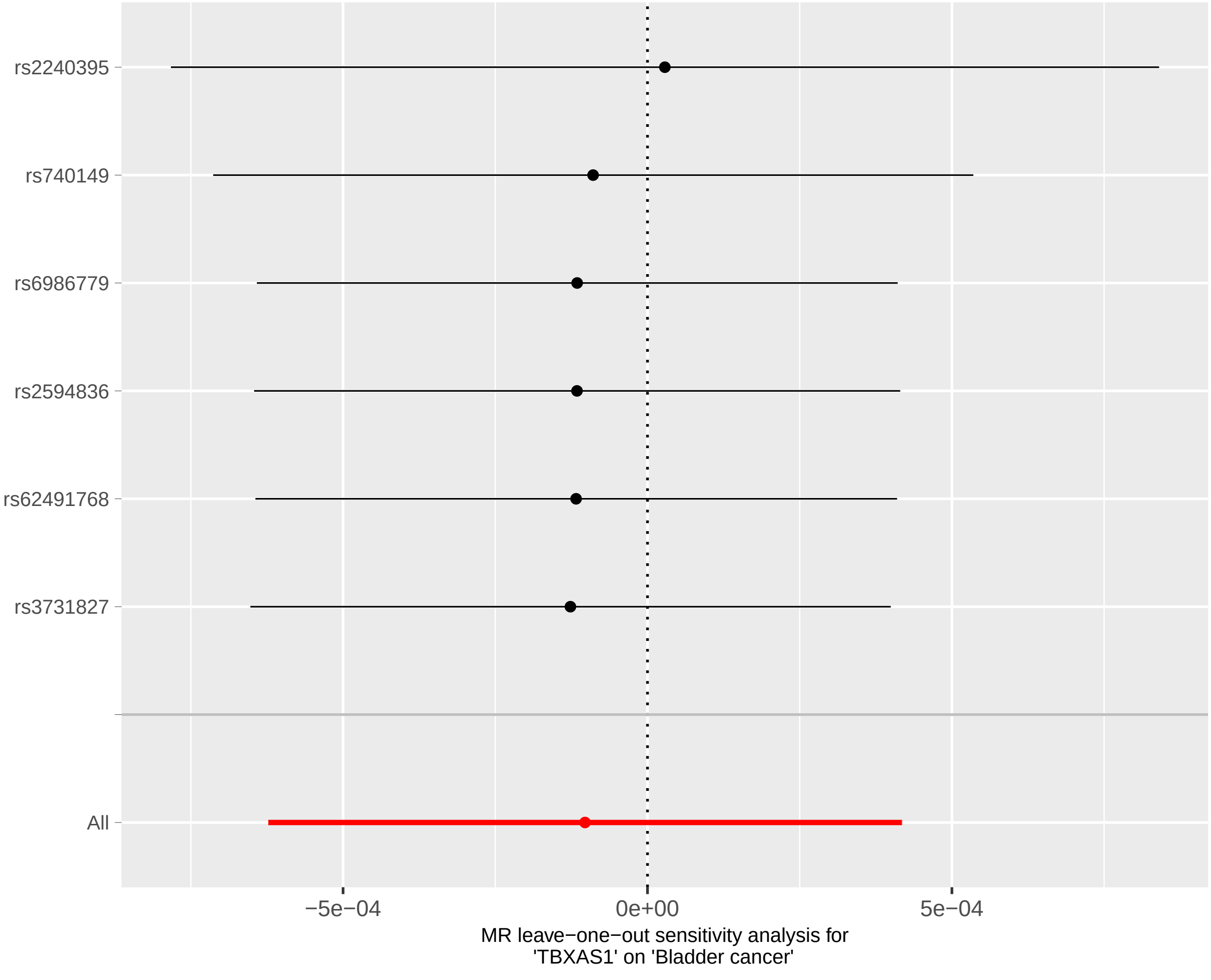
rs2240395
rs740149
rs6986779
rs2594836
rs62491768
rs3731827
All
−5e−04
0e+00
5e−04
MR leave−one−out sensitivity analysis for
'TBXAS1' on 'Bladder cancer'

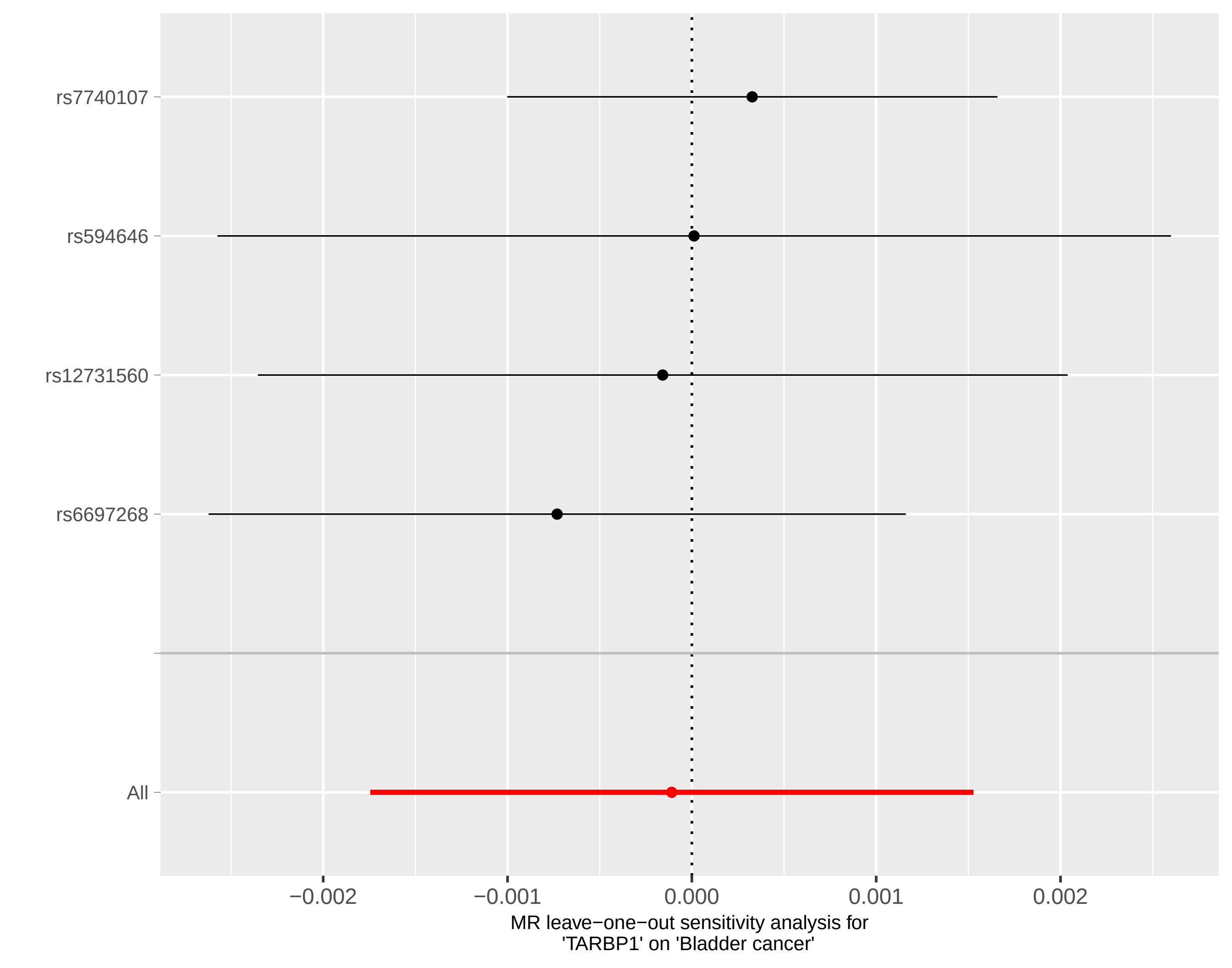
rs7740107
rs594646
rs12731560
rs6697268
All
−0.002
−0.001
0.000
0.001
0.002
MR leave−one−out sensitivity analysis for
'TARBP1' on 'Bladder cancer'

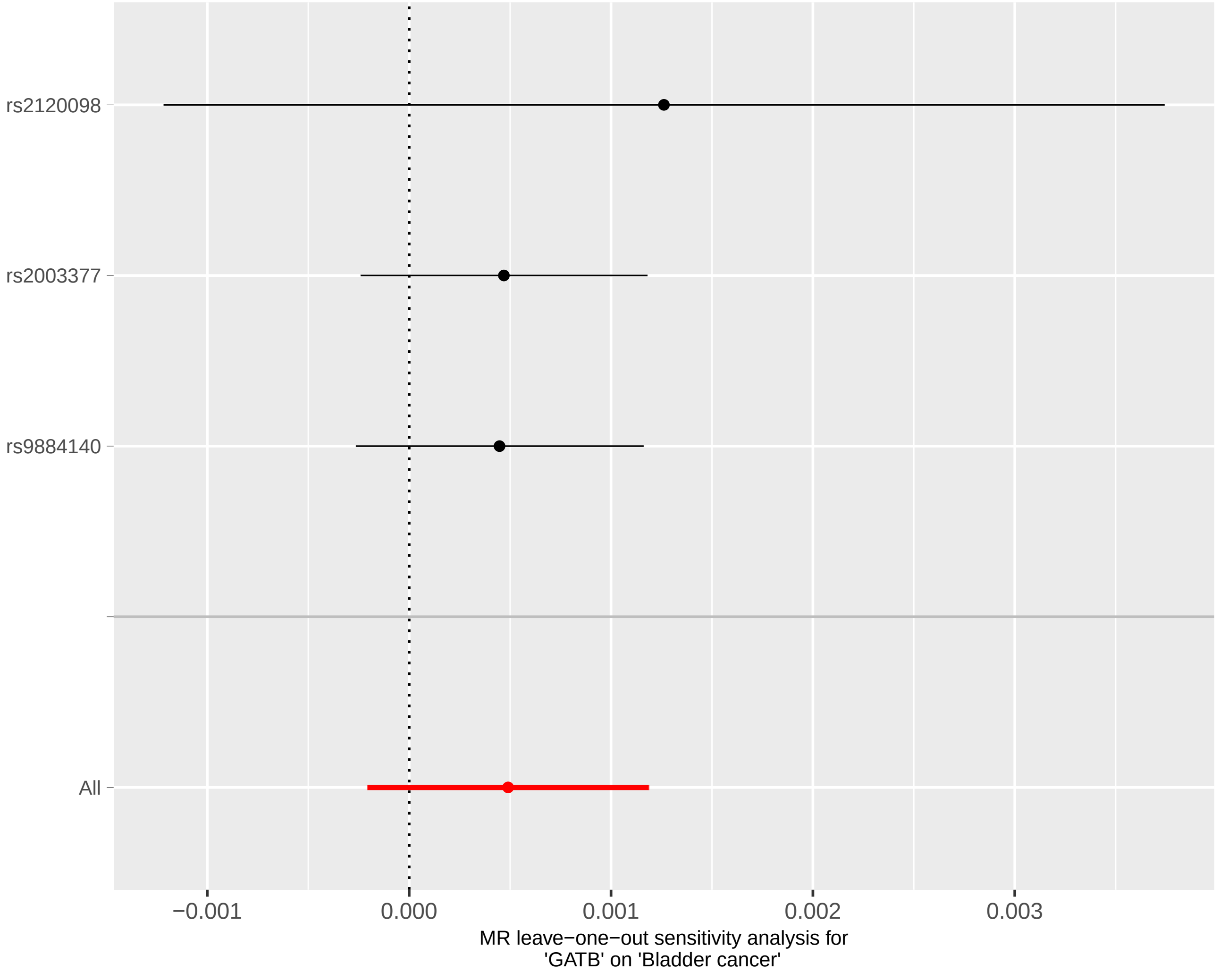
rs2120098
rs2003377
rs9884140
All
−0.001
0.000
0.001
0.002
0.003
MR leave−one−out sensitivity analysis for
'GATB' on 'Bladder cancer'

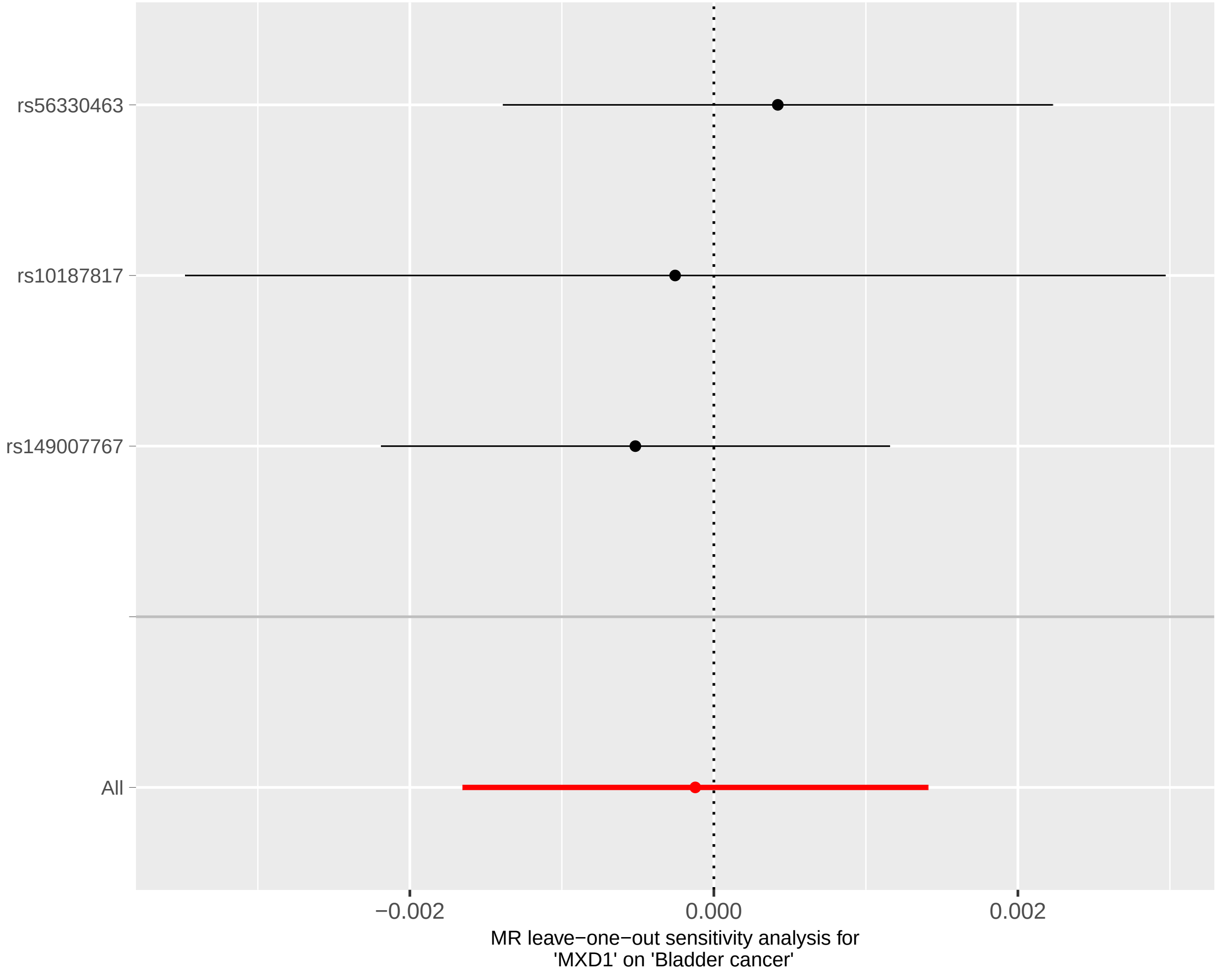

rs56330463
rs10187817
rs149007767
All
−0.002
0.000
0.002
MR leave−one−out sensitivity analysis for
'MXD1' on 'Bladder cancer'

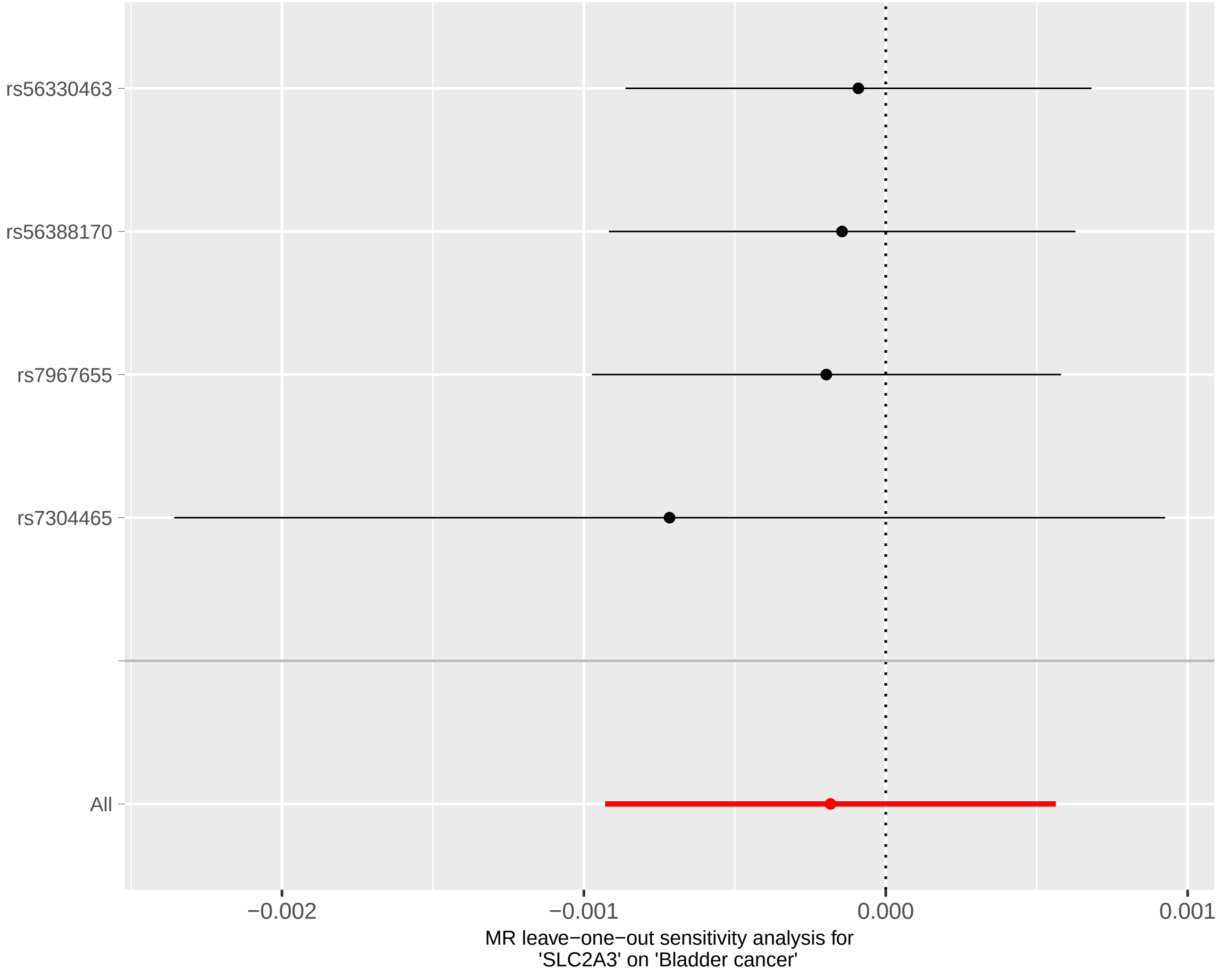
rs56330463
rs56388170
rs7967655
rs7304465
All
−0.002
−0.001
0.000
0.001
MR leave−one−out sensitivity analysis for
'SLC2A3' on 'Bladder cancer'

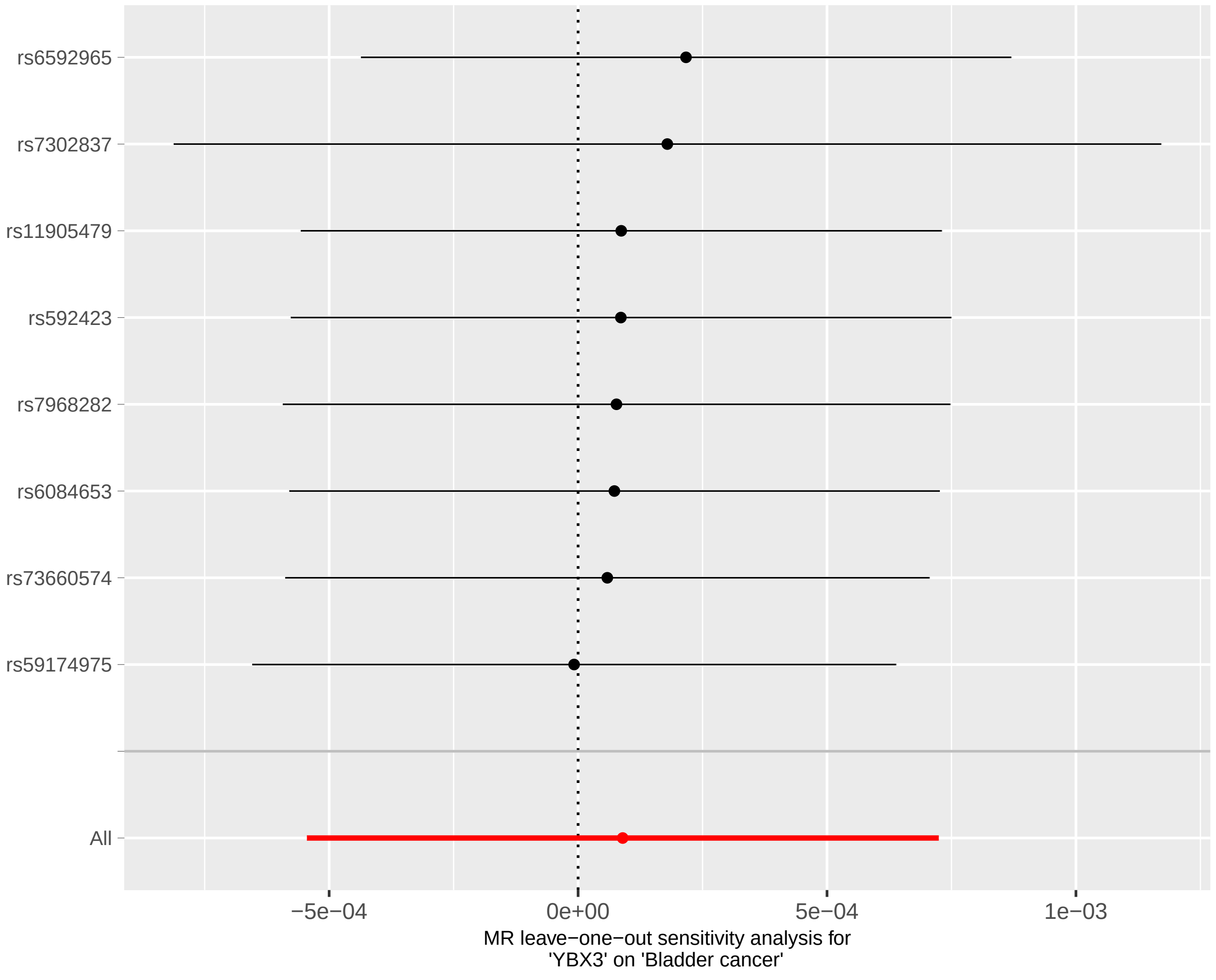

rs6592965
rs7302837
rs11905479
rs592423
rs7968282
rs6084653
rs73660574
rs59174975
All
−5e−04
0e+00
5e−04
1e−03
MR leave−one−out sensitivity analysis for
'YBX3' on 'Bladder cancer'

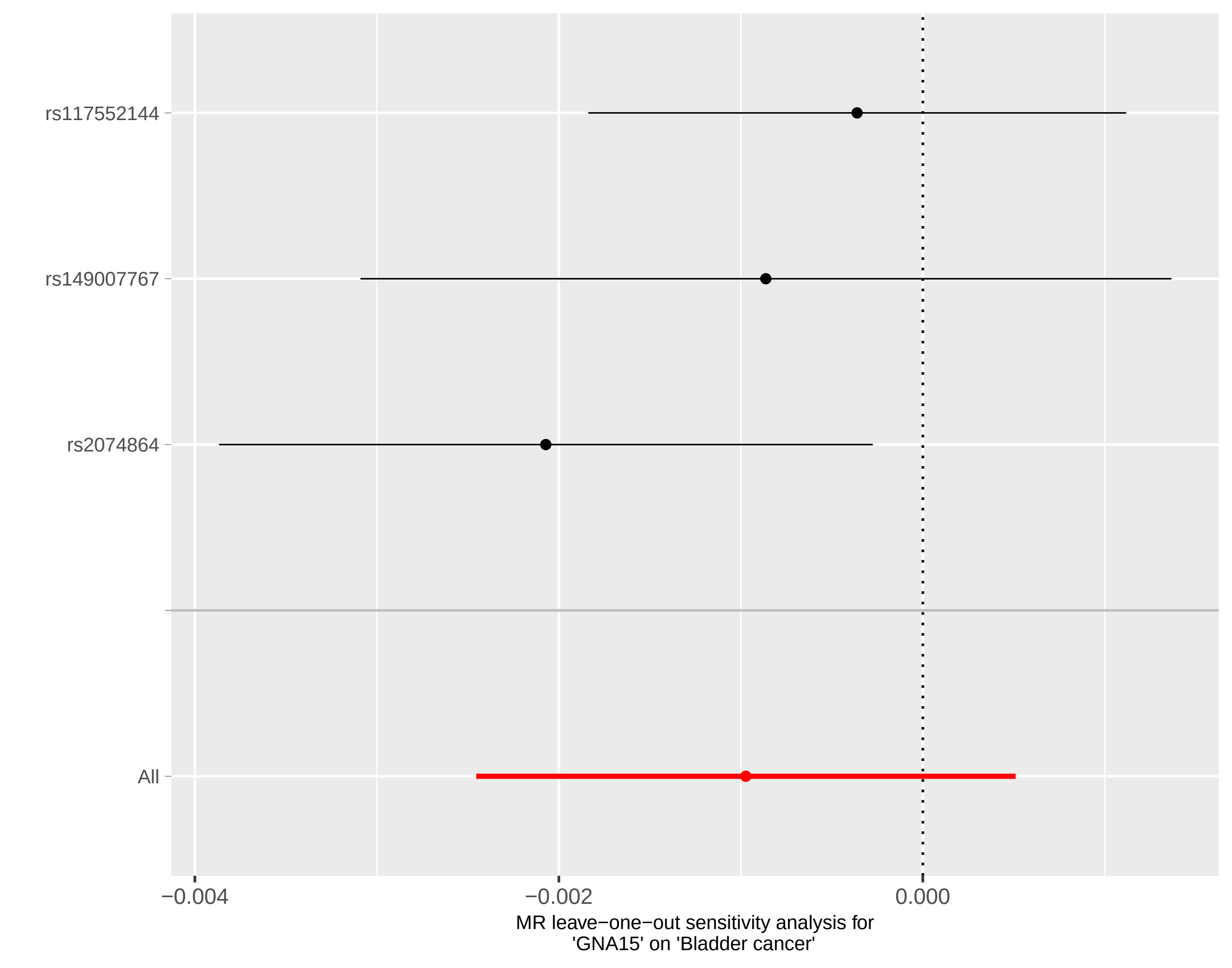

rs117552144
rs149007767
rs2074864
All
−0.004
−0.002
0.000
MR leave−one−out sensitivity analysis for
'GNA15' on 'Bladder cancer'

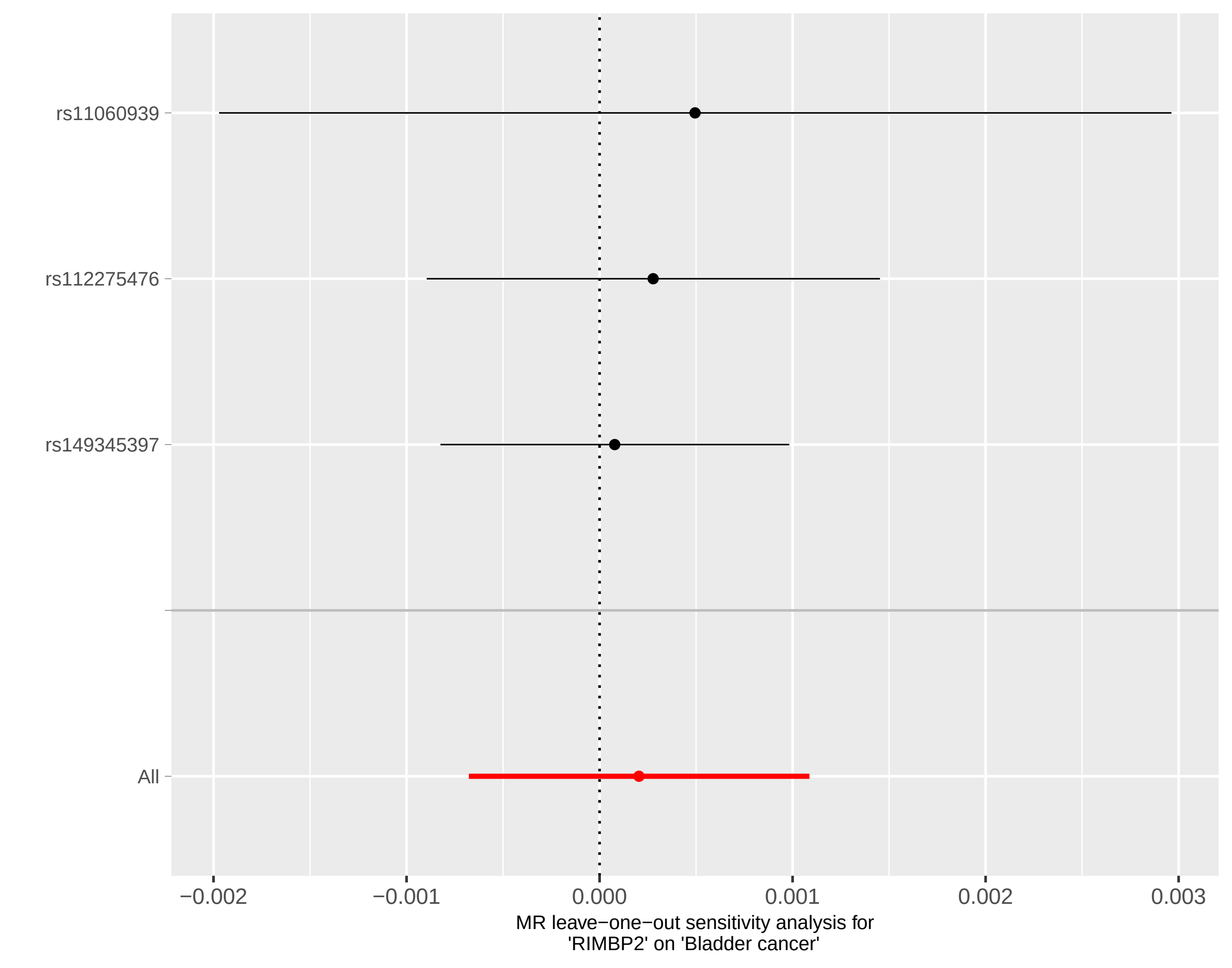
rs11060939
rs112275476
rs149345397
All
−0.002
−0.001
0.000
0.001
0.002
0.003
MR leave−one−out sensitivity analysis for
'RIMBP2' on 'Bladder cancer'

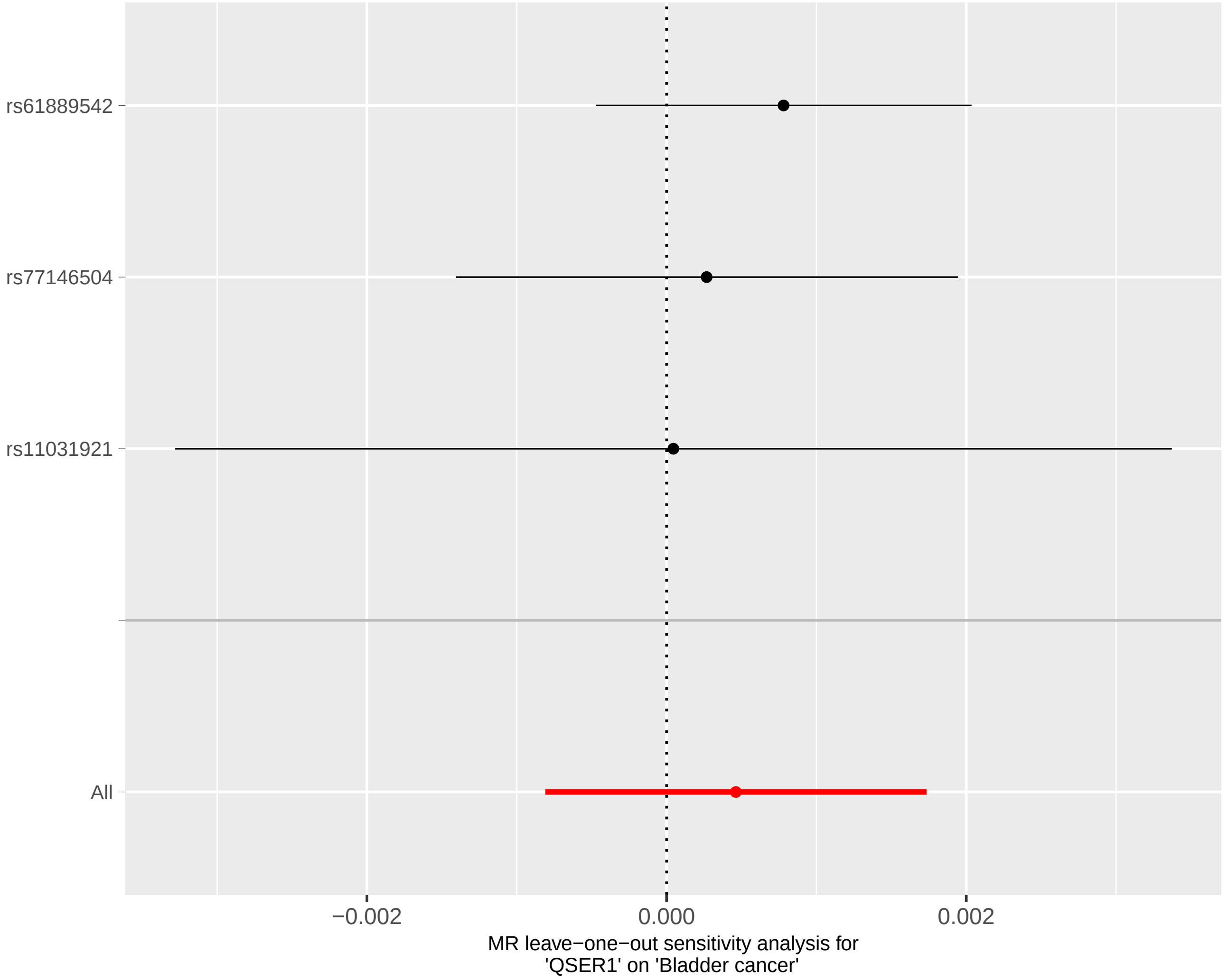

rs61889542
rs77146504
rs11031921
All
−0.002
0.000
0.002
MR leave−one−out sensitivity analysis for
'QSER1' on 'Bladder cancer'

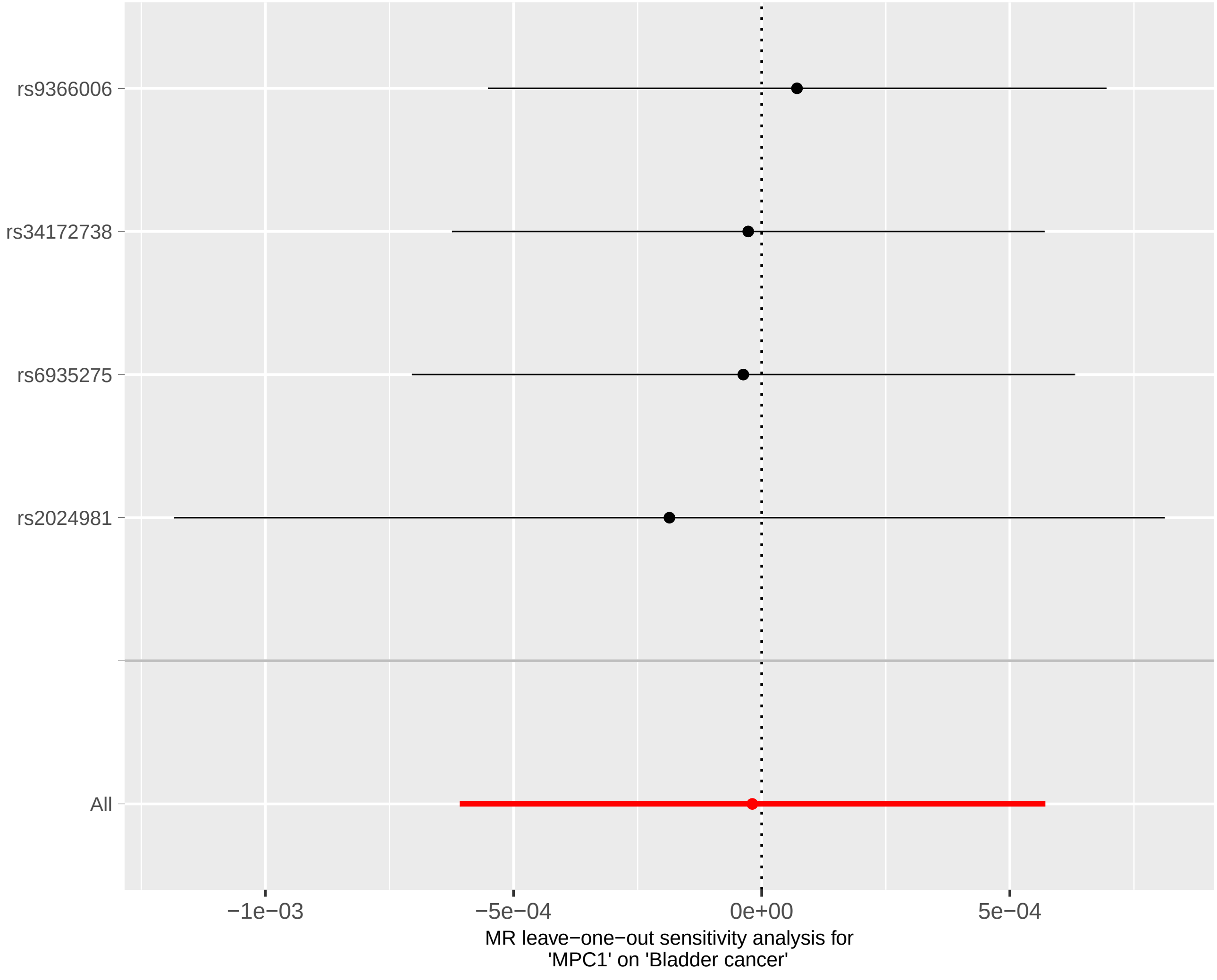

rs9366006
rs34172738
rs6935275
rs2024981
All
−1e−03
−5e−04
0e+00
5e−04
MR leave−one−out sensitivity analysis for
'MPC1' on 'Bladder cancer'

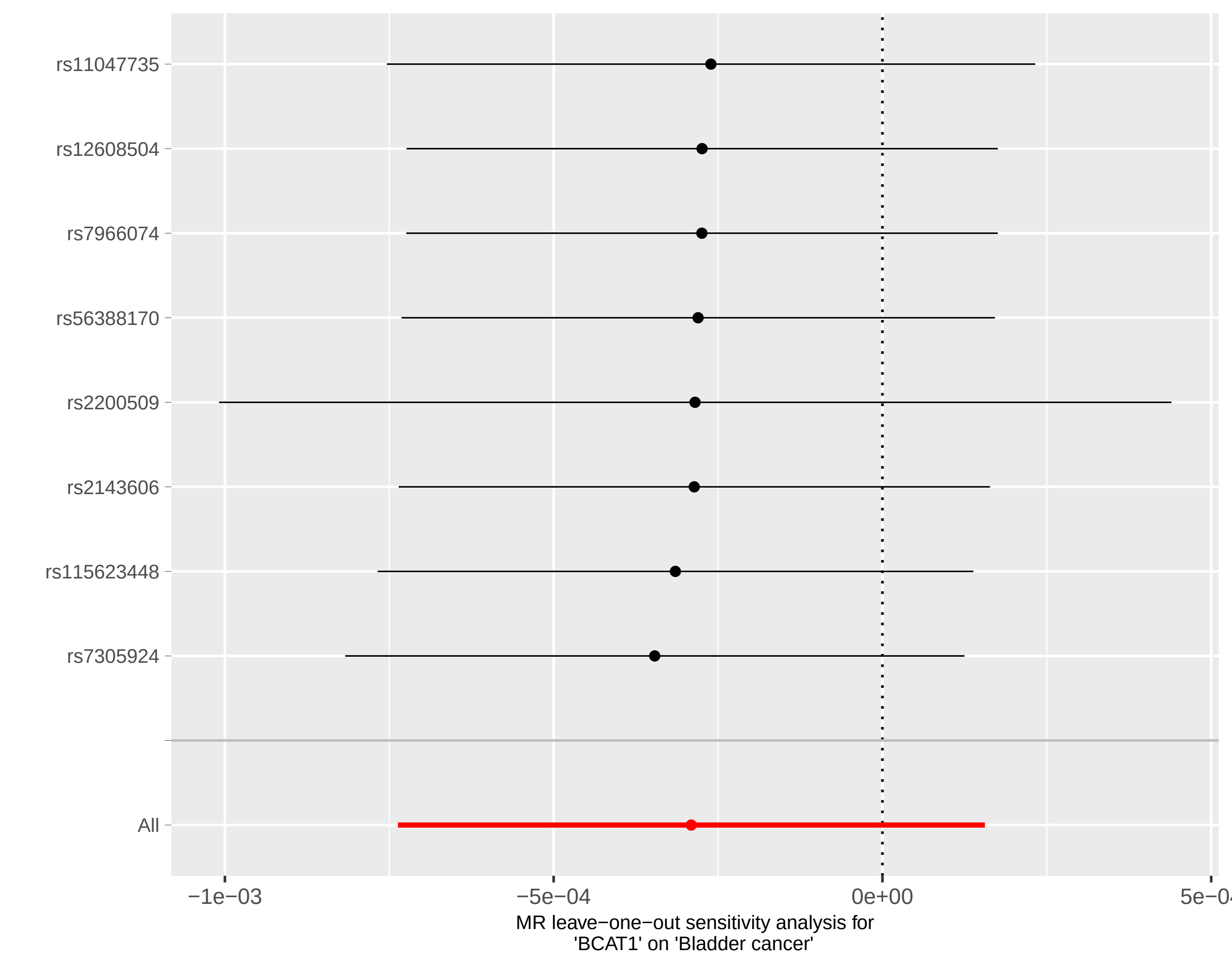
rs11047735
rs12608504
rs7966074
rs56388170
rs2200509
rs2143606
rs115623448
rs7305924
All
−1e−03
−5e−04
0e+00
5e−04
MR leave−one−out sensitivity analysis for
'BCAT1' on 'Bladder cancer'

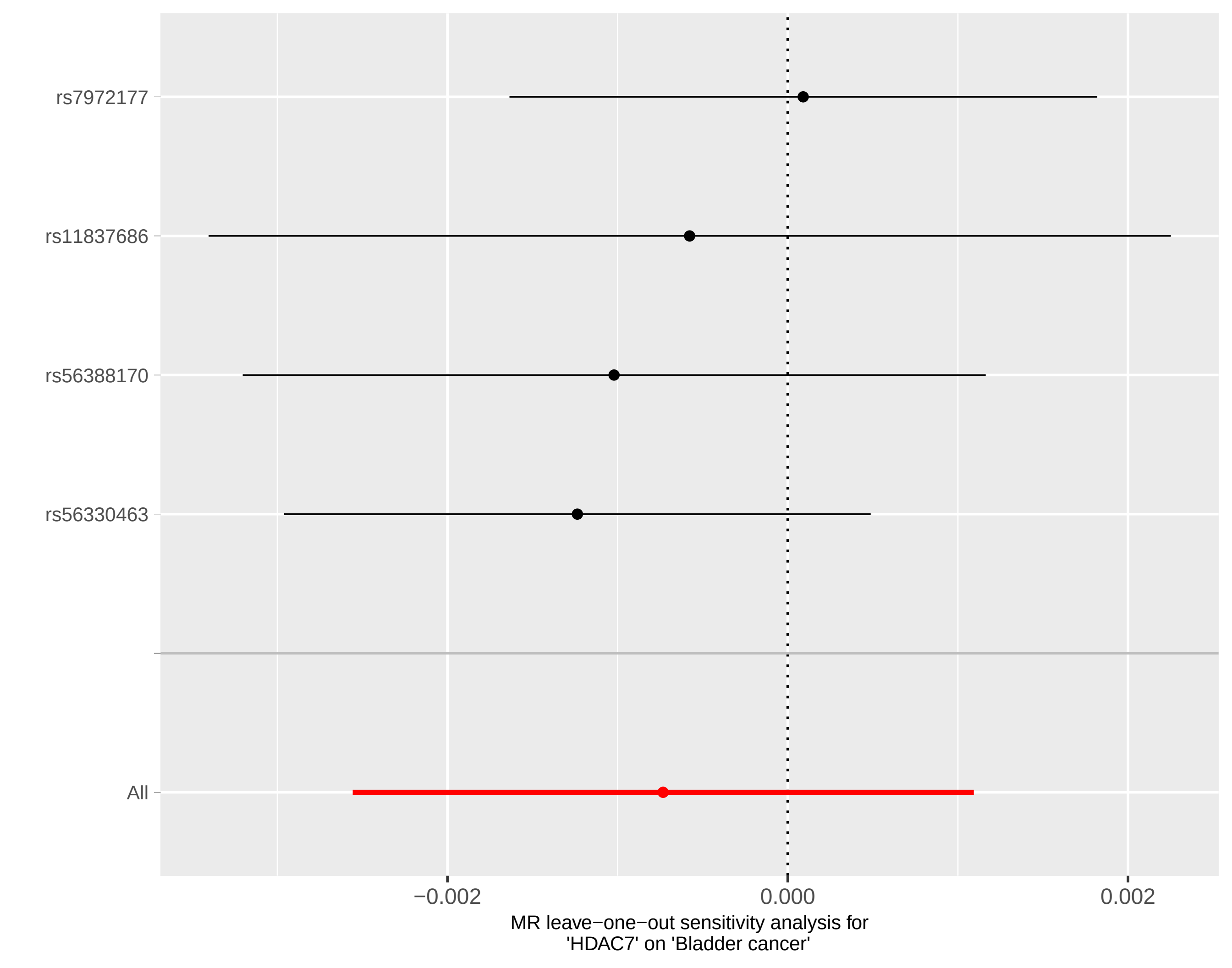

rs7972177
rs11837686
rs56388170
rs56330463
All
−0.002
0.000
0.002
MR leave−one−out sensitivity analysis for
'HDAC7' on 'Bladder cancer'

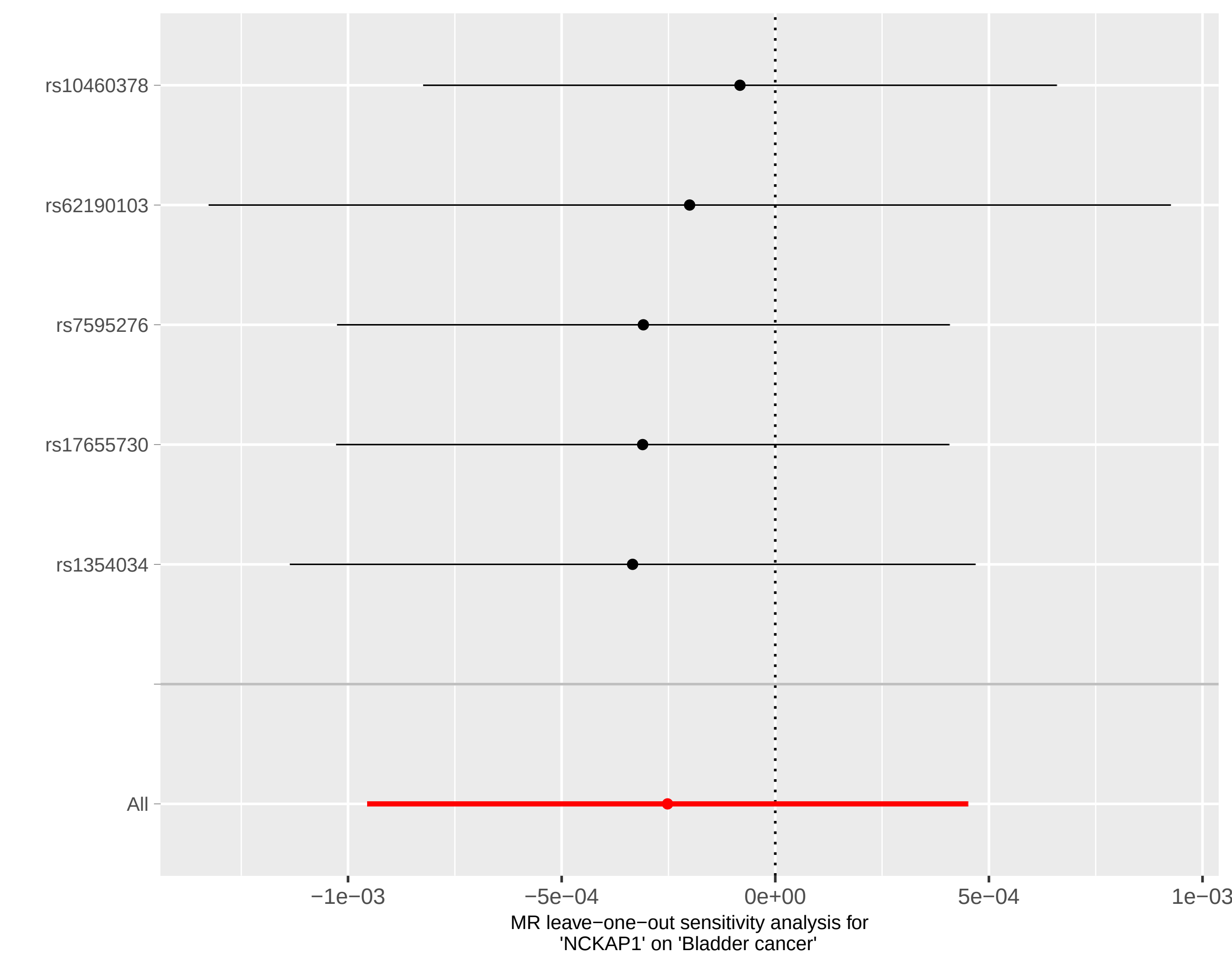
rs10460378
rs62190103
rs7595276
rs17655730
rs1354034
All
−1e−03
−5e−04
0e+00
5e−04
1e−03
MR leave−one−out sensitivity analysis for
'NCKAP1' on 'Bladder cancer'

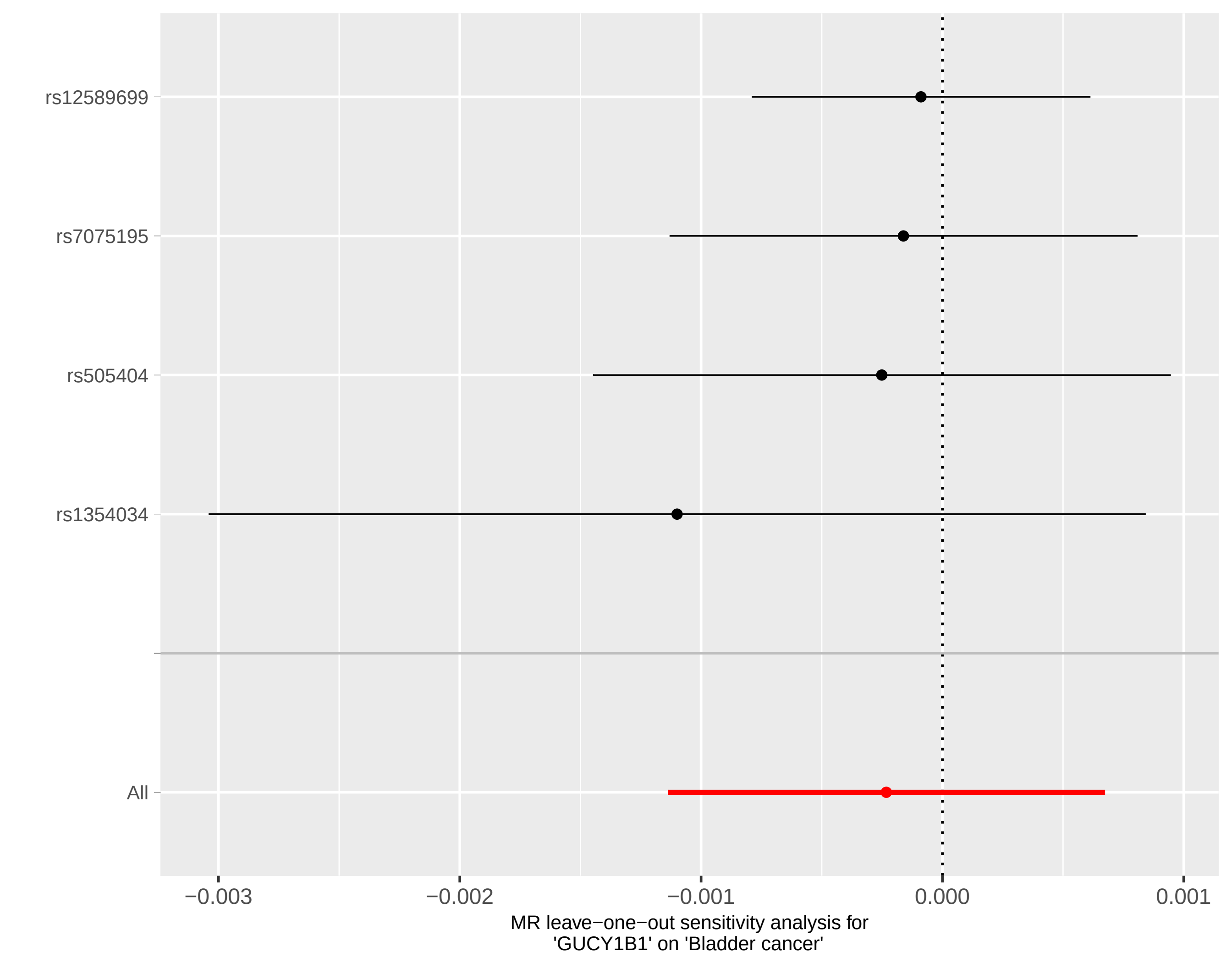
rs12589699
rs7075195
rs505404
rs1354034
All
−0.003
−0.002
−0.001
0.000
0.001
MR leave−one−out sensitivity analysis for
'GUCY1B1' on 'Bladder cancer'

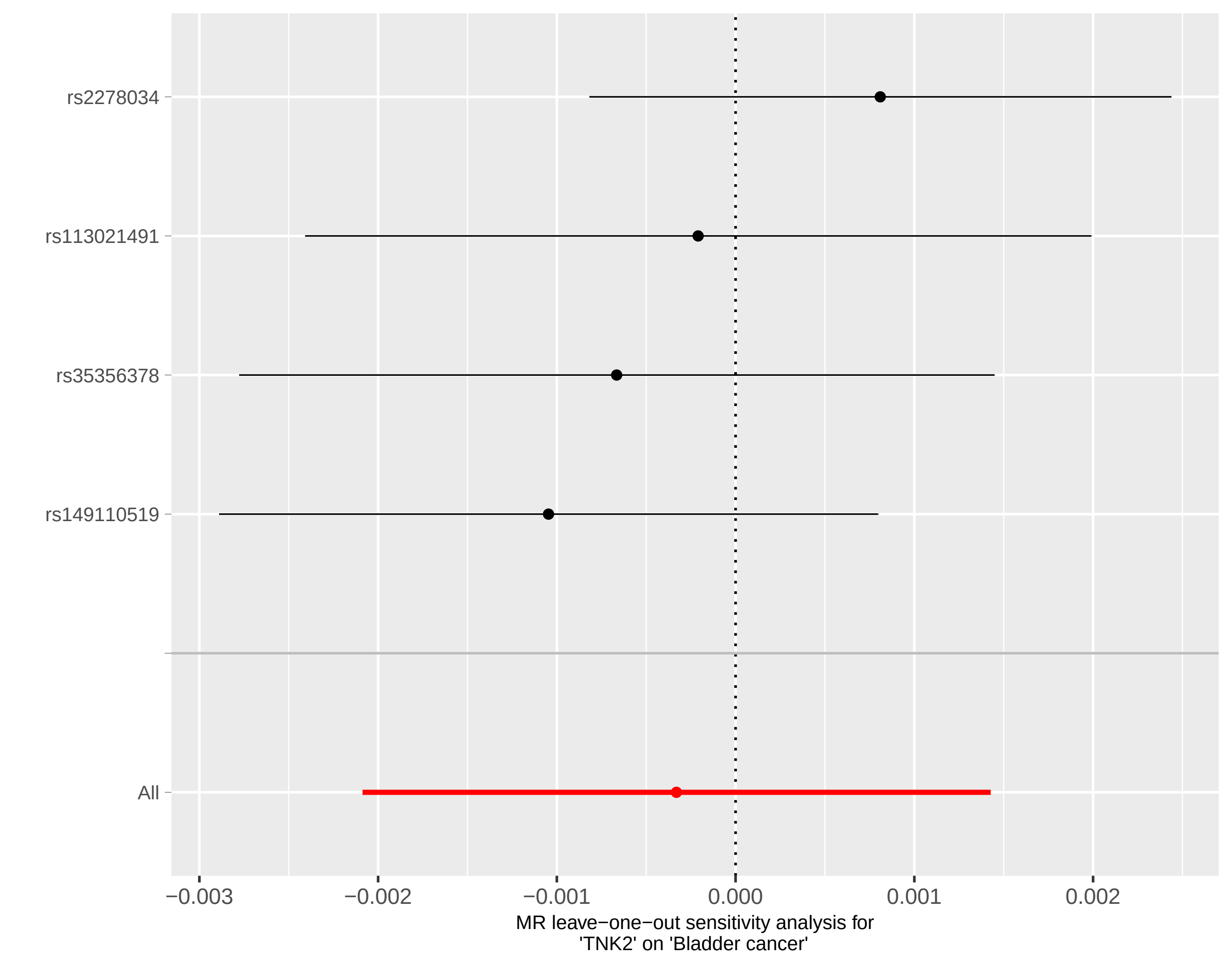
rs2278034
rs113021491
rs35356378
rs149110519
All
−0.003
−0.002
−0.001
0.000
0.001
0.002
MR leave−one−out sensitivity analysis for
'TNK2' on 'Bladder cancer'

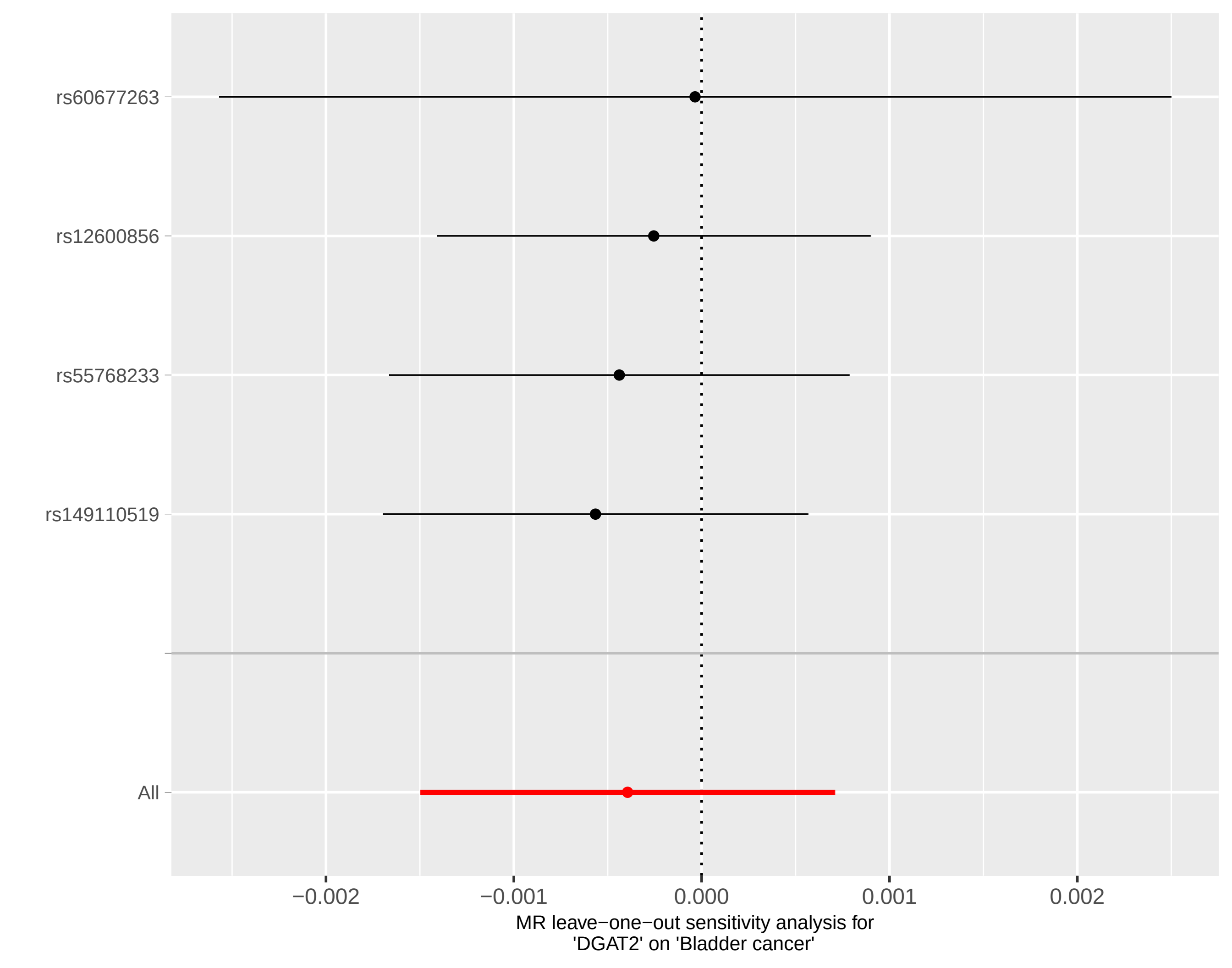

rs60677263
rs12600856
rs55768233
rs149110519
All
−0.002
−0.001
0.000
0.001
0.002
MR leave−one−out sensitivity analysis for
'DGAT2' on 'Bladder cancer'

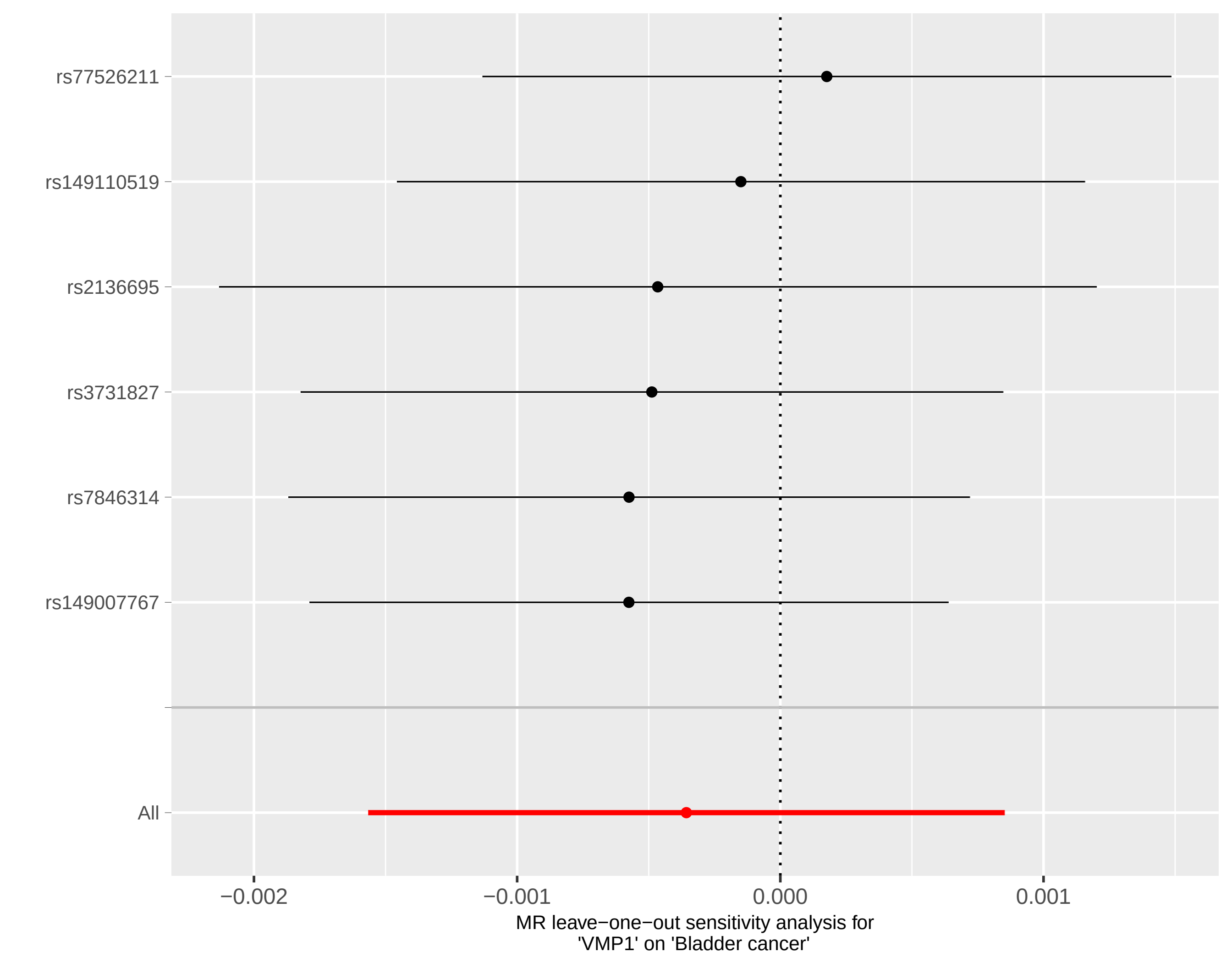

rs77526211
rs149110519
rs2136695
rs3731827
rs7846314
rs149007767
All
−0.002
−0.001
0.000
0.001
MR leave−one−out sensitivity analysis for
'VMP1' on 'Bladder cancer'

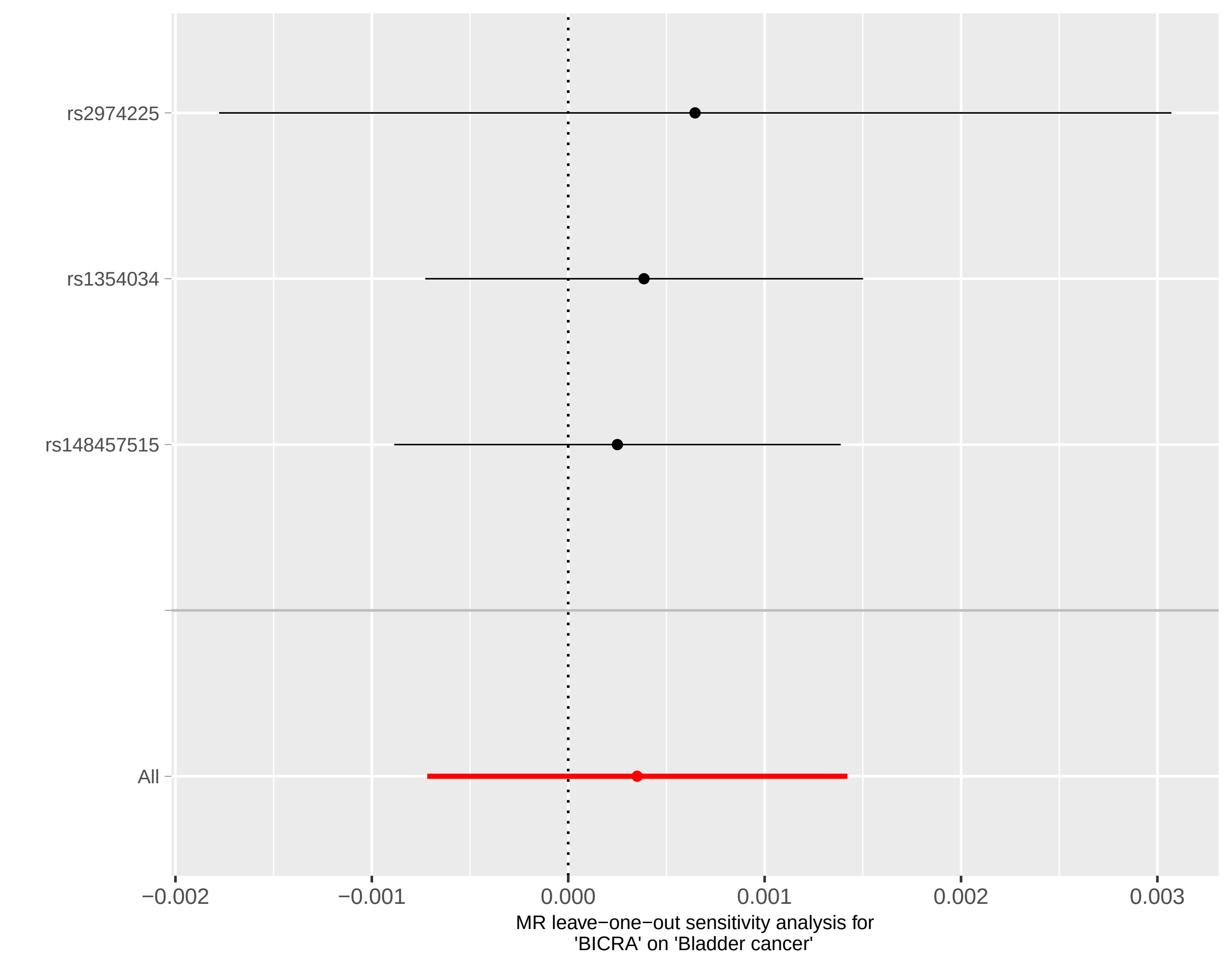
rs2974225
rs1354034
rs148457515
All
−0.002
−0.001
0.000
0.001
0.002
0.003
MR leave−one−out sensitivity analysis for
'BICRA' on 'Bladder cancer'

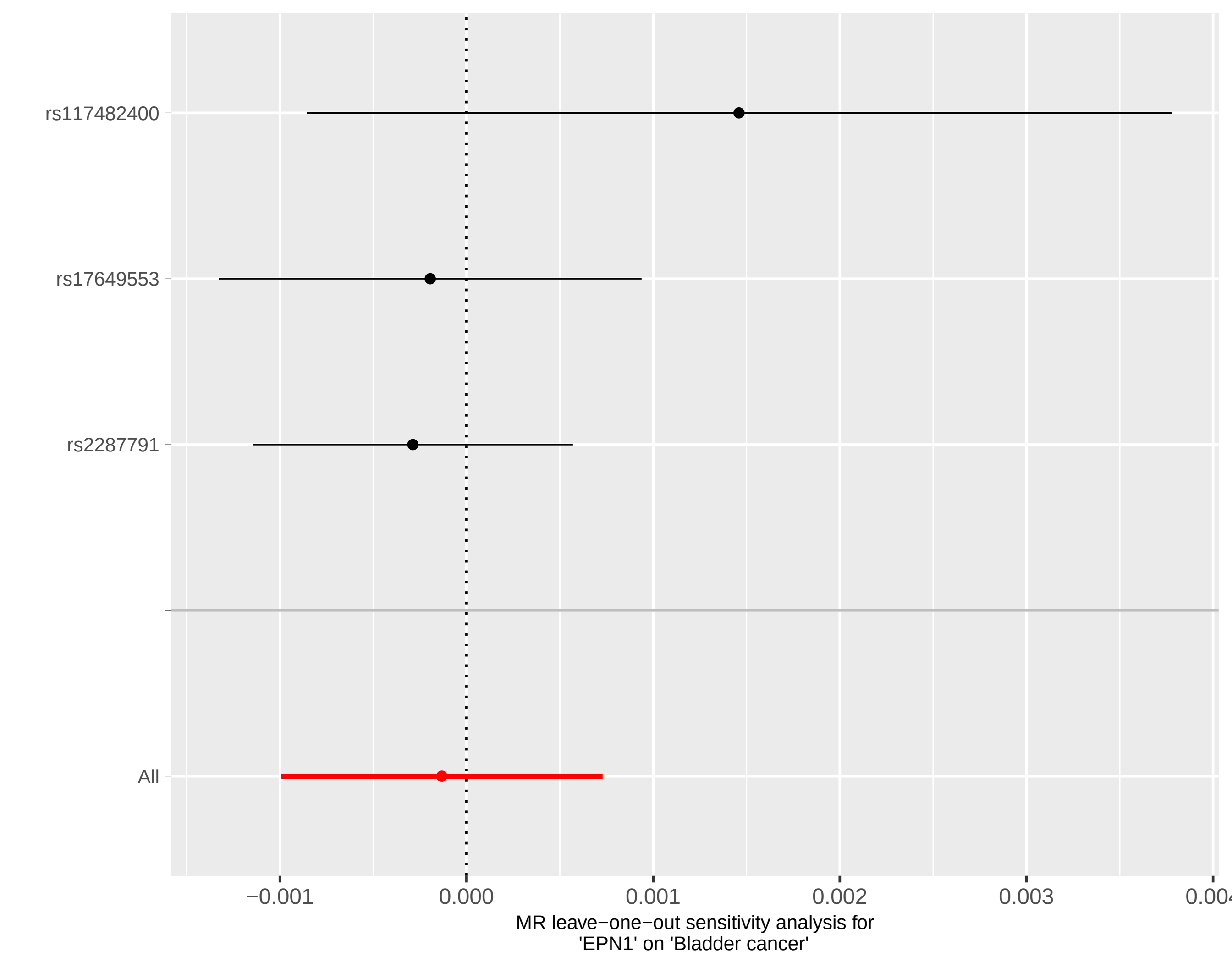

rs117482400
rs17649553
rs2287791
All
−0.001
0.000
0.001
0.002
0.003
0.00
MR leave−one−out sensitivity analysis for
'EPN1' on 'Bladder cancer'

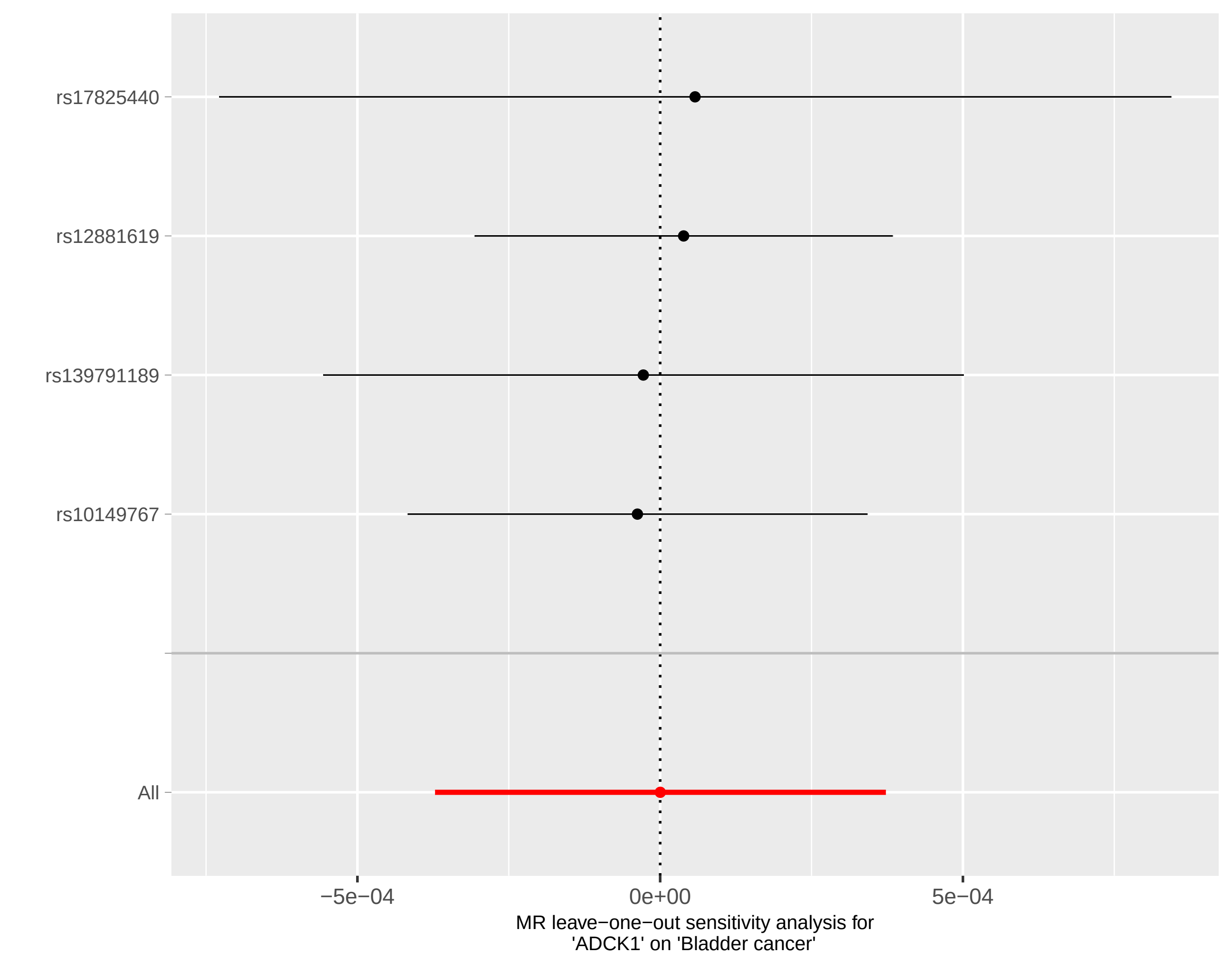
rs17825440
rs12881619
rs139791189
rs10149767
All
−5e−04
0e+00
5e−04
MR leave−one−out sensitivity analysis for
'ADCK1' on 'Bladder cancer'

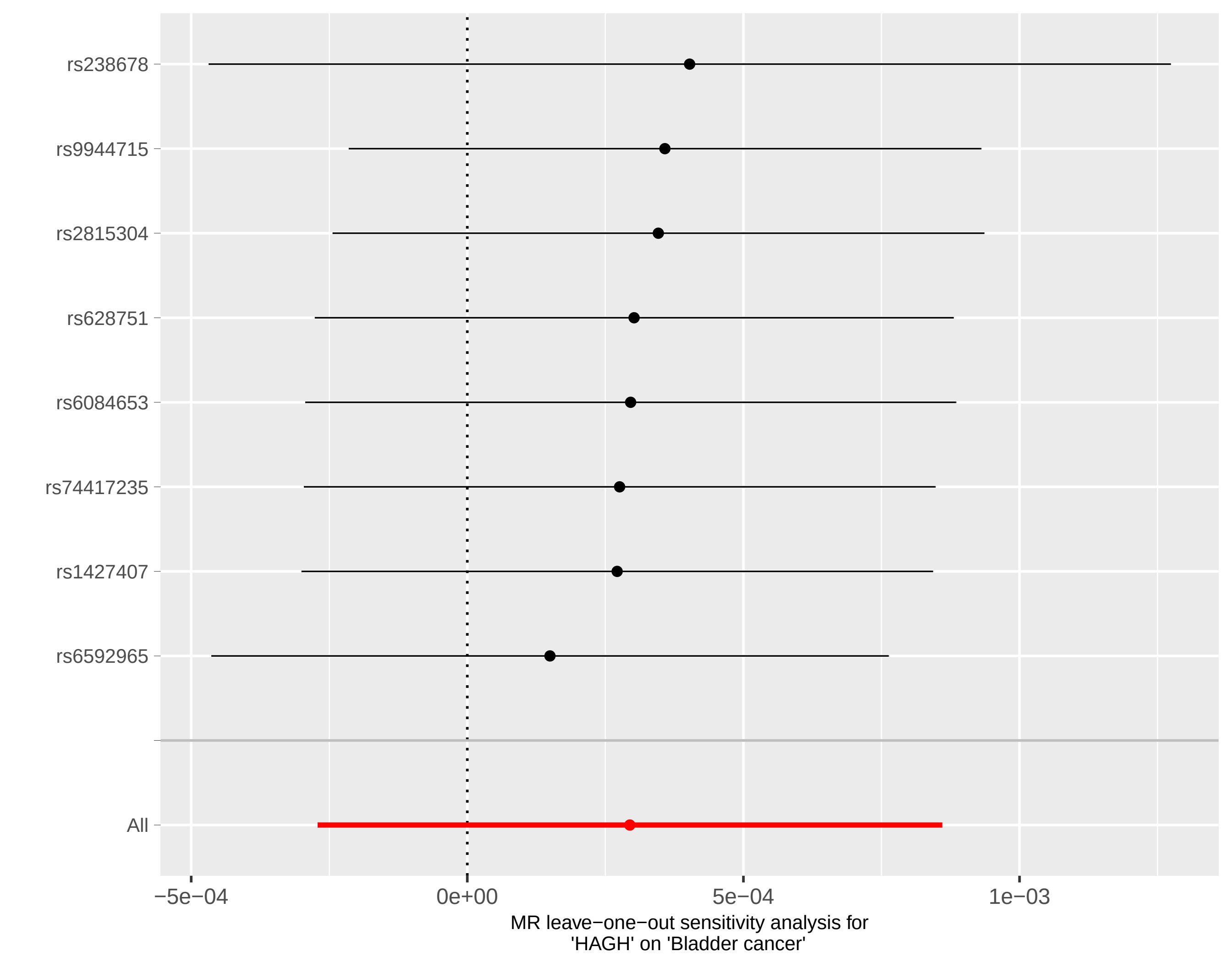

rs238678
rs9944715
rs2815304
rs628751
rs6084653
rs74417235
rs1427407
rs6592965
All
−5e−04
0e+00
5e−04
1e−03
MR leave−one−out sensitivity analysis for
'HAGH' on 'Bladder cancer'

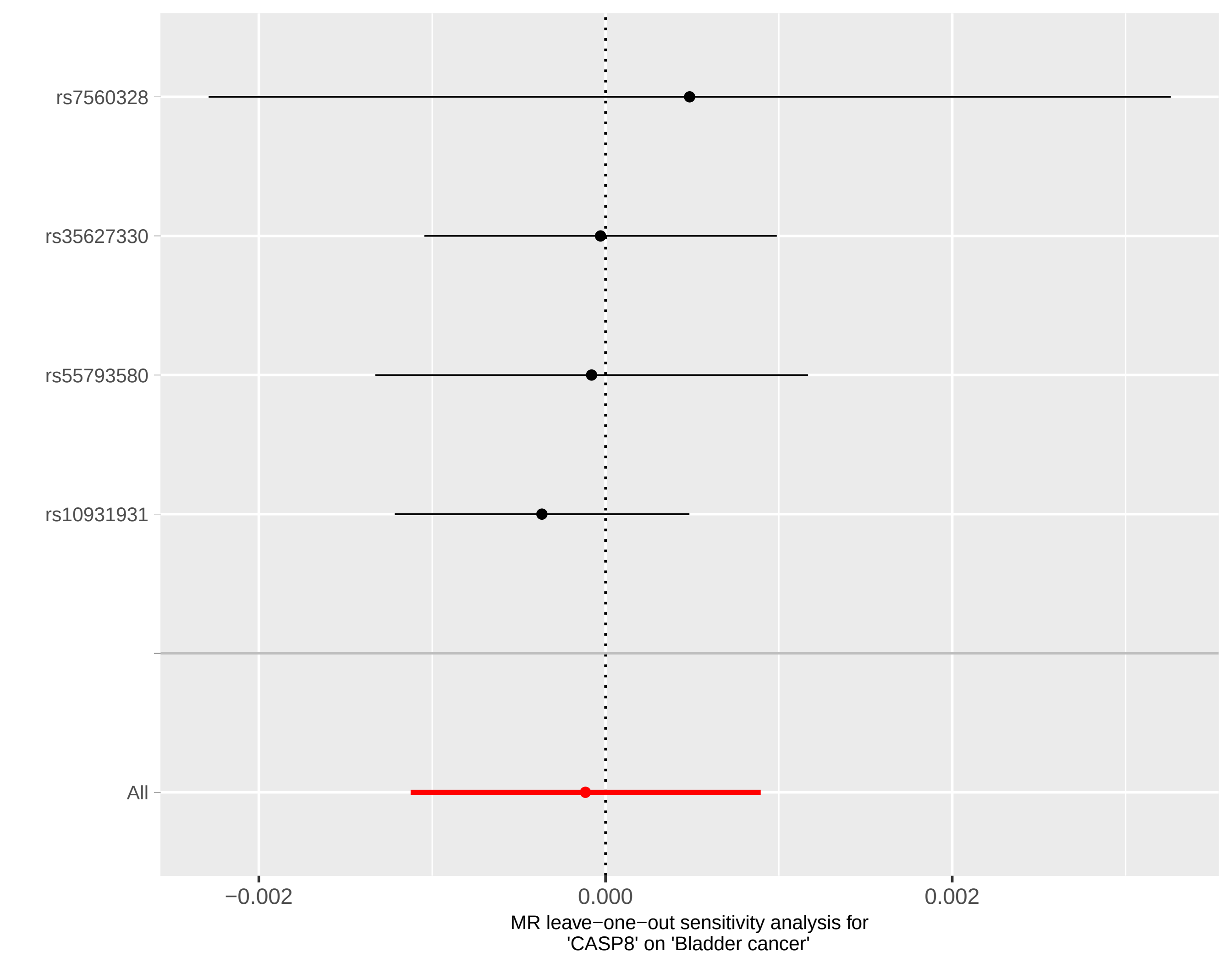
rs7560328
rs35627330
rs55793580
rs10931931
All
−0.002
0.000
0.002
MR leave−one−out sensitivity analysis for
'CASP8' on 'Bladder cancer'

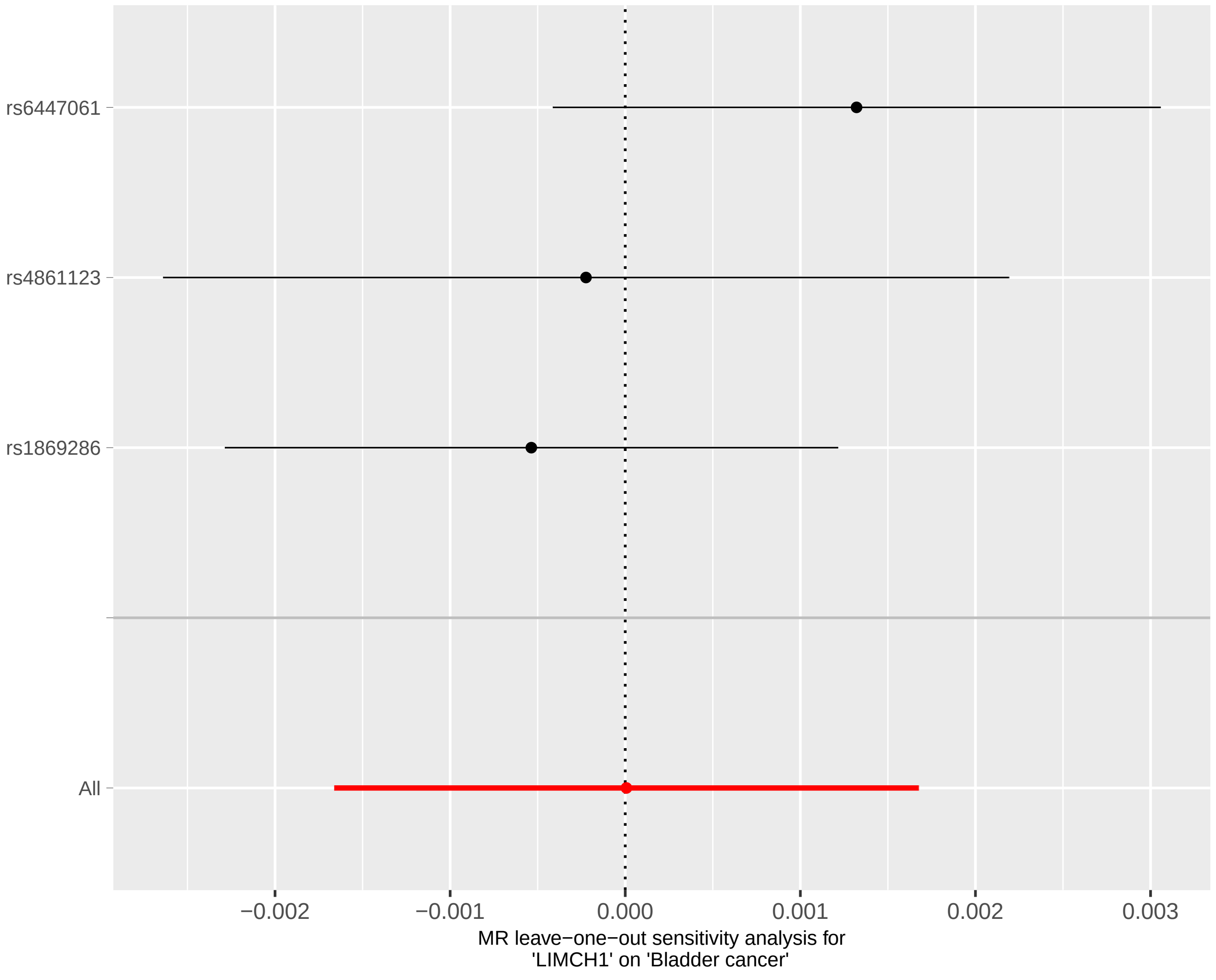
rs6447061
rs4861123
rs1869286
All
−0.002
−0.001
0.000
0.001
0.002
0.003
MR leave−one−out sensitivity analysis for
'LIMCH1' on 'Bladder cancer'

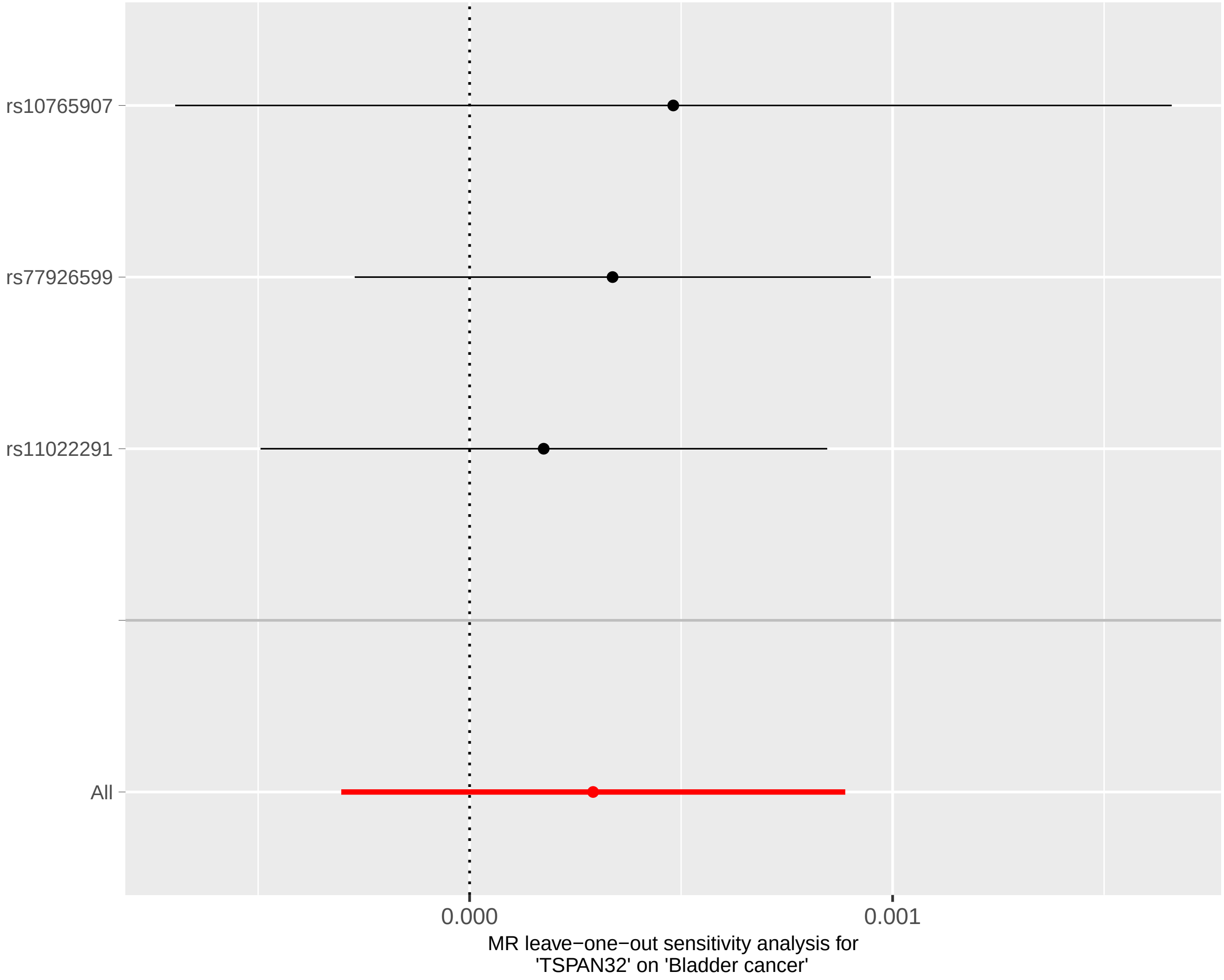
rs10765907
rs77926599
rs11022291
All
0.000
0.001
MR leave−one−out sensitivity analysis for
'TSPAN32' on 'Bladder cancer'

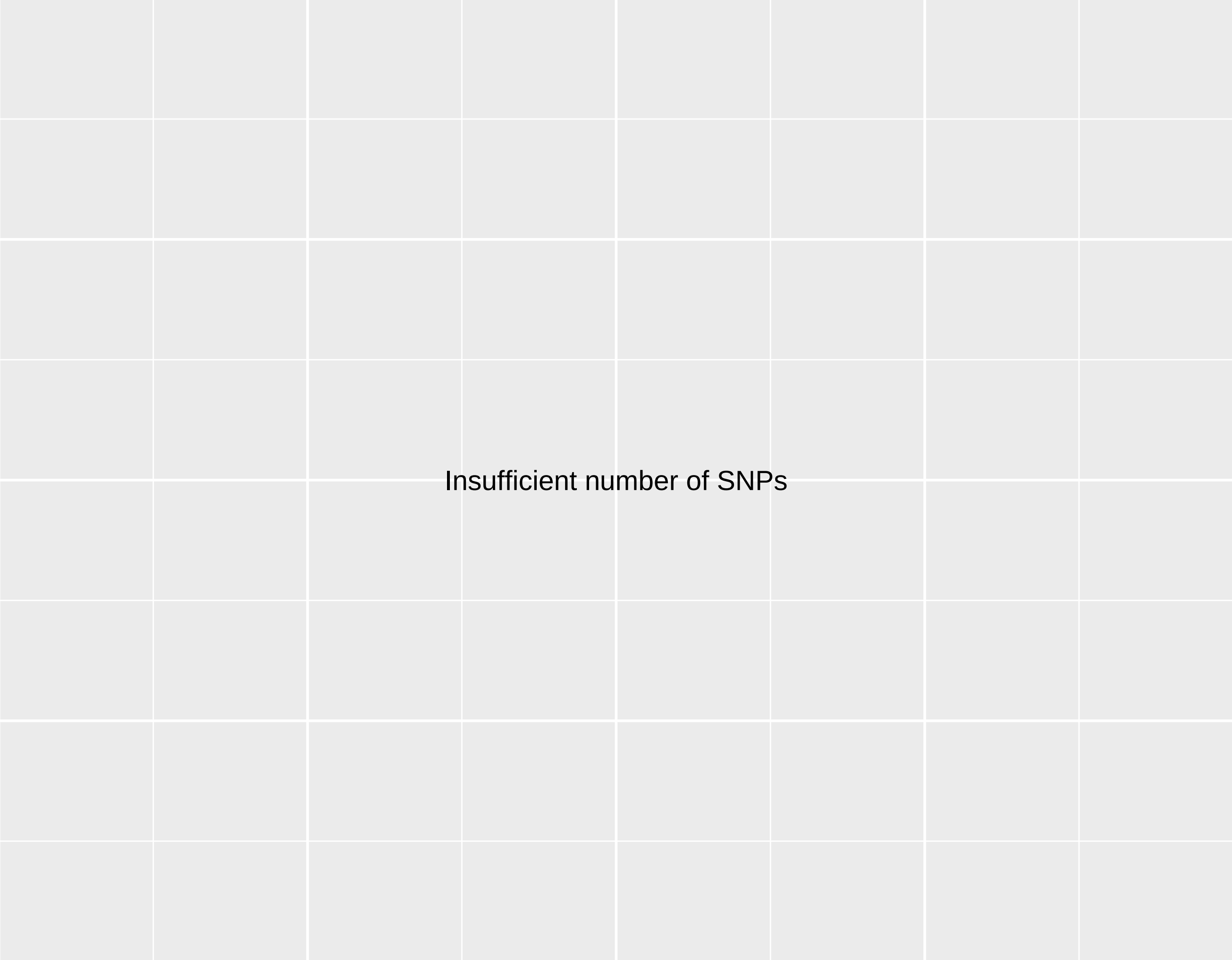
Insufficient number of SNPs

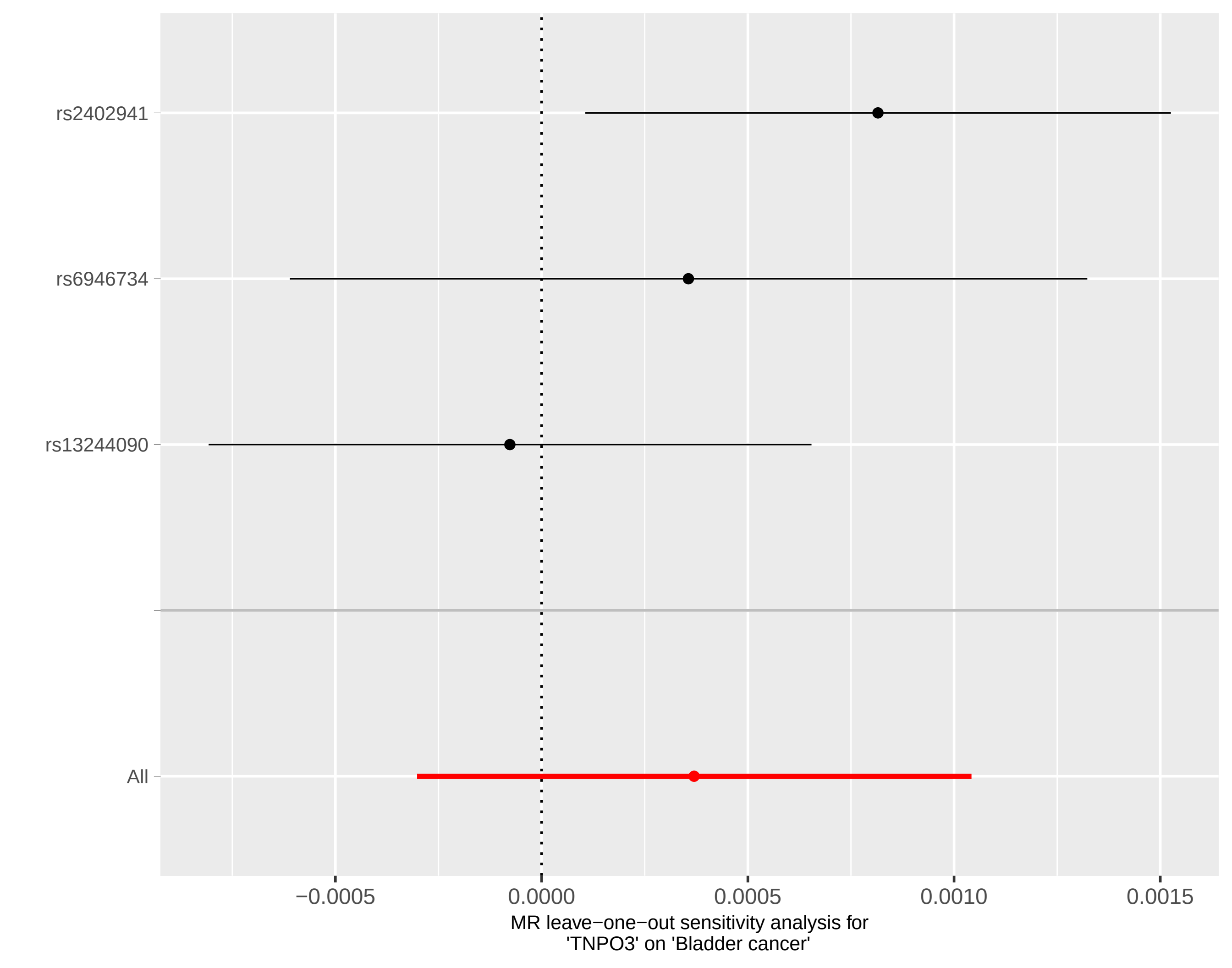
rs2402941
rs6946734
rs13244090
All
−0.0005
0.0000
0.0005
0.0010
0.0015
MR leave−one−out sensitivity analysis for
'TNPO3' on 'Bladder cancer'

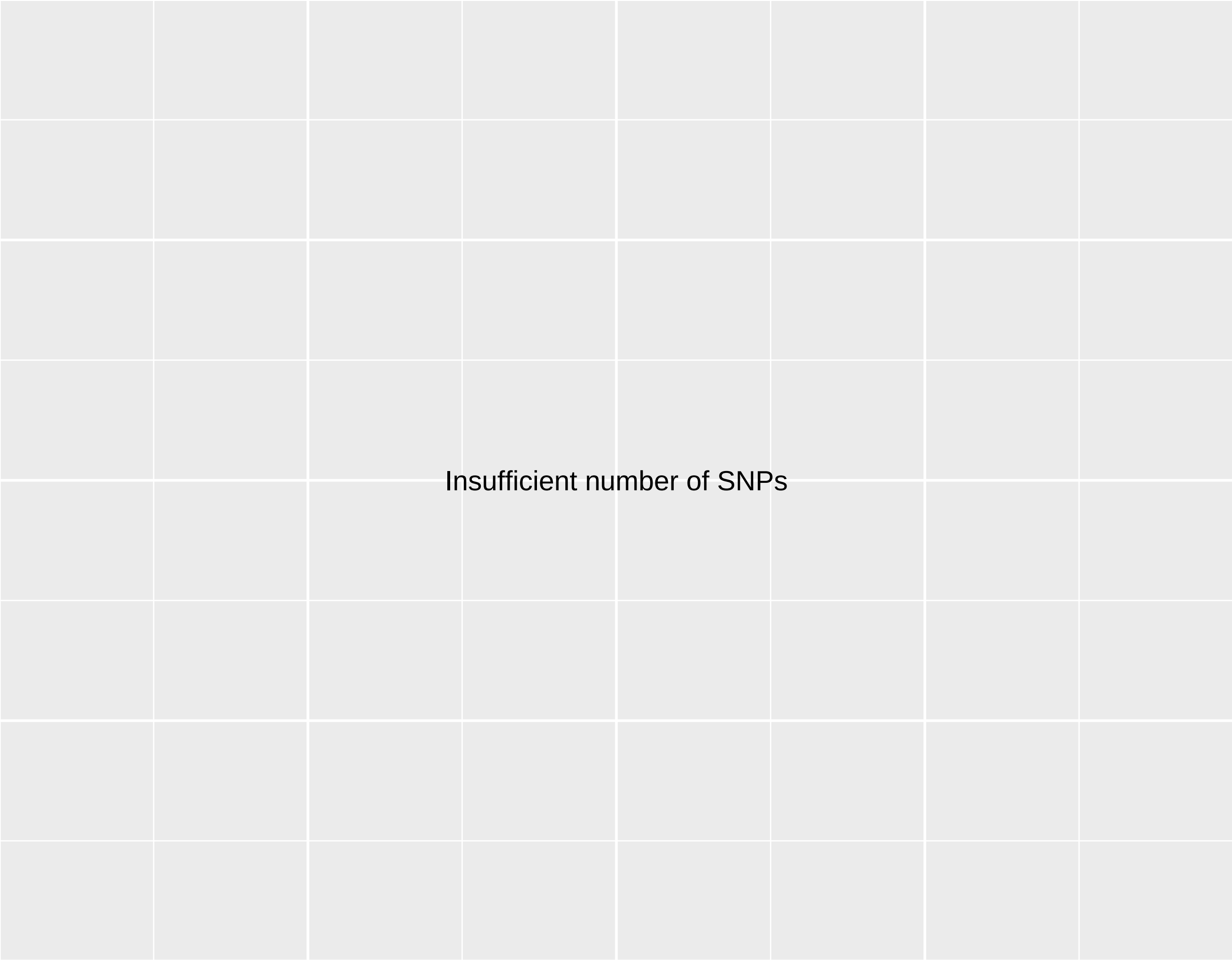
Insufficient number of SNPs

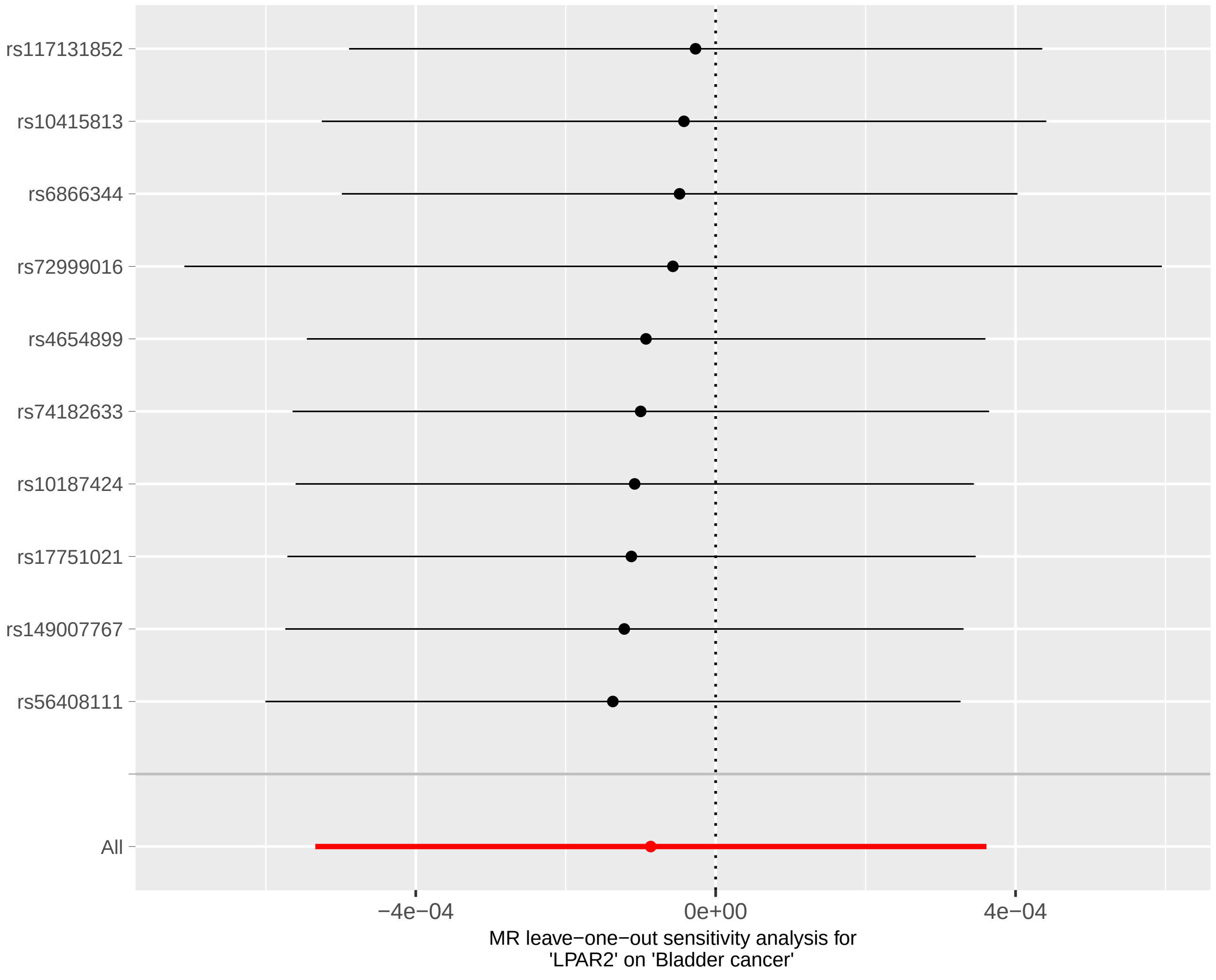
rs117131852
rs10415813
rs6866344
rs72999016
rs4654899
rs74182633
rs10187424
rs17751021
rs149007767
rs56408111
All
−4e−04
0e+00
4e−04
MR leave−one−out sensitivity analysis for
'LPAR2' on 'Bladder cancer'

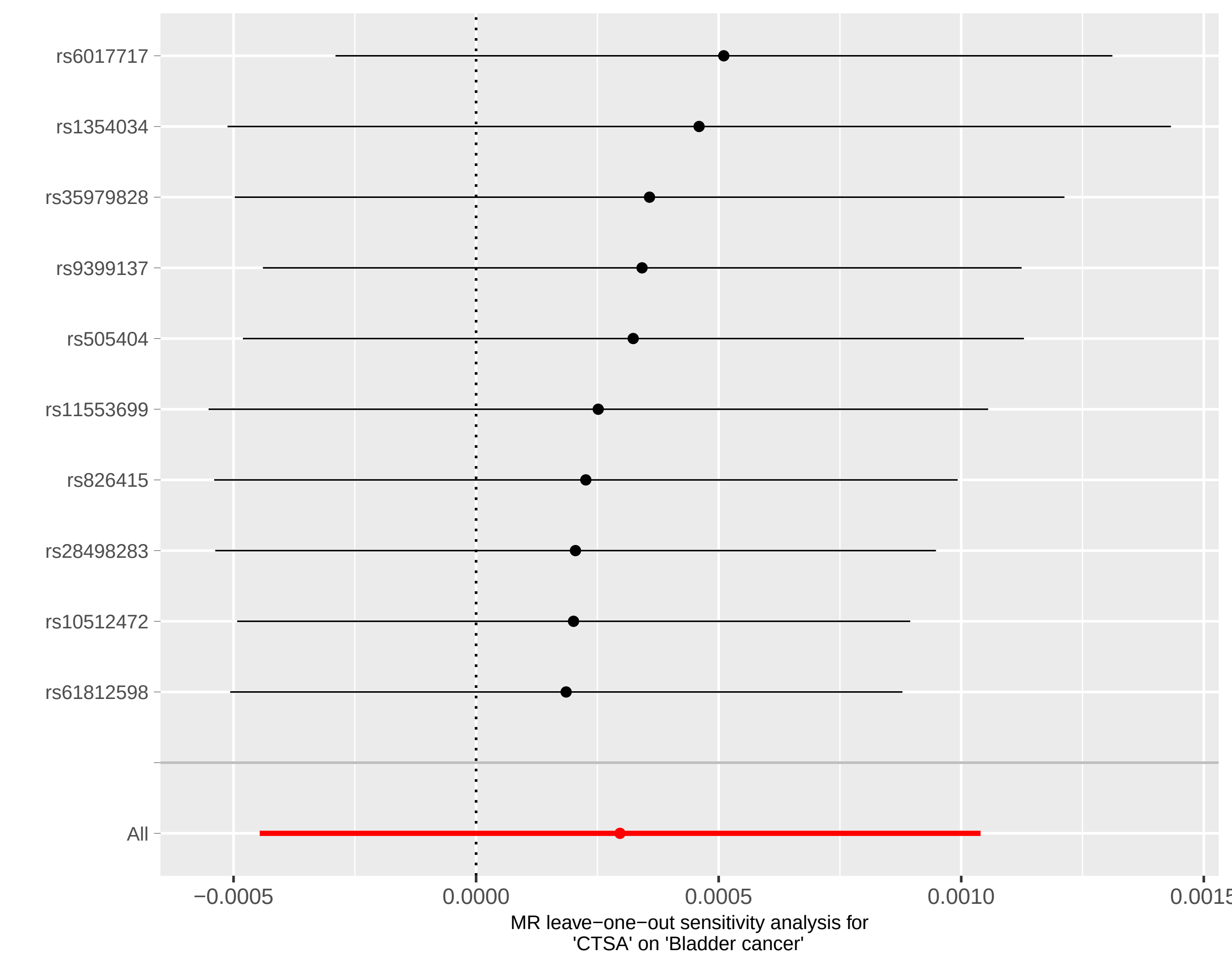

rs6017717
rs1354034
rs35979828
rs9399137
rs505404
rs11553699
rs826415
rs28498283
rs10512472
rs61812598
All
−0.0005
0.0000
0.0005
0.0010
0.0015
MR leave−one−out sensitivity analysis for
'CTSA' on 'Bladder cancer'

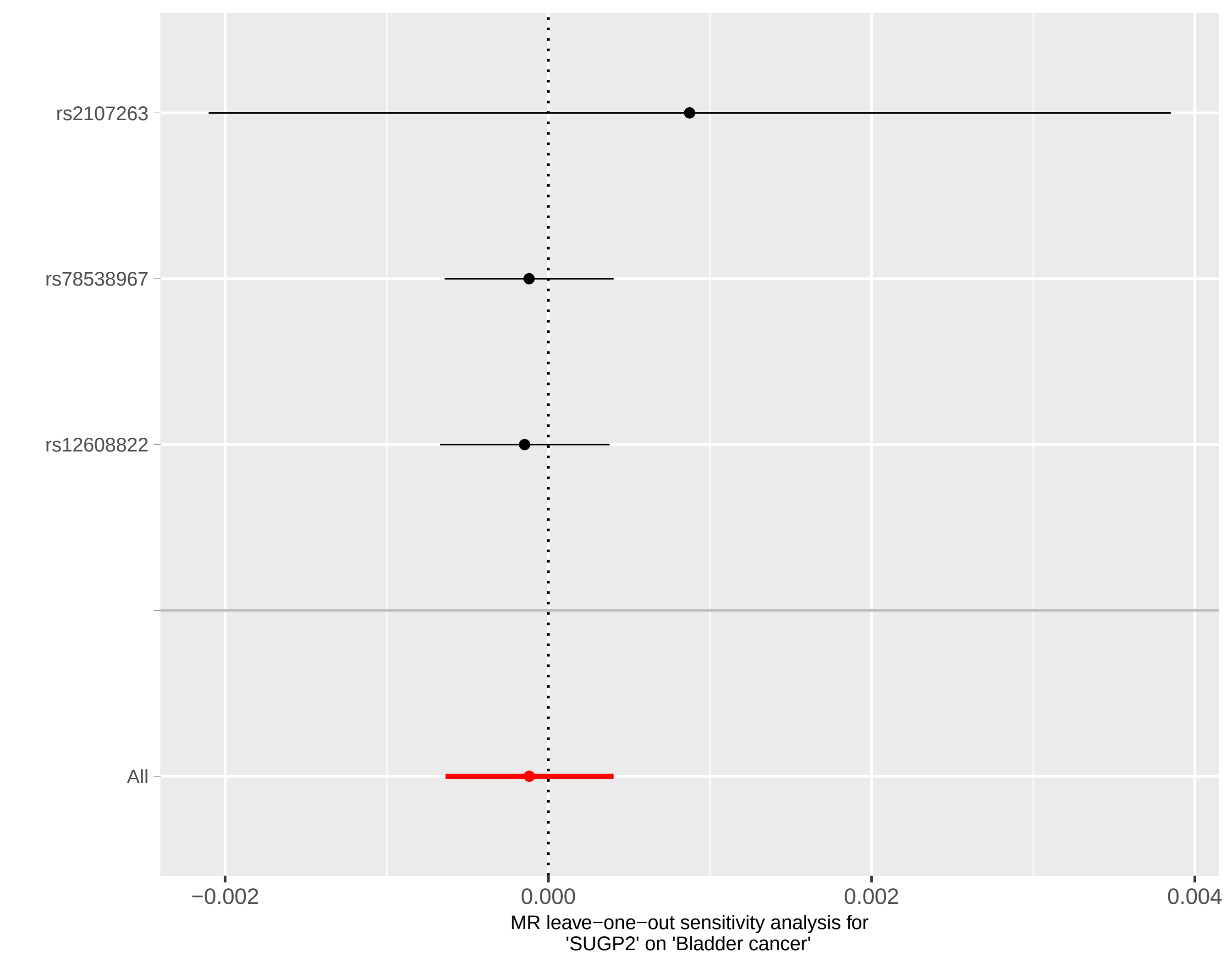
rs2107263
rs78538967
rs12608822
All
−0.002
0.000
0.002
0.004
MR leave−one−out sensitivity analysis for
'SUGP2' on 'Bladder cancer'

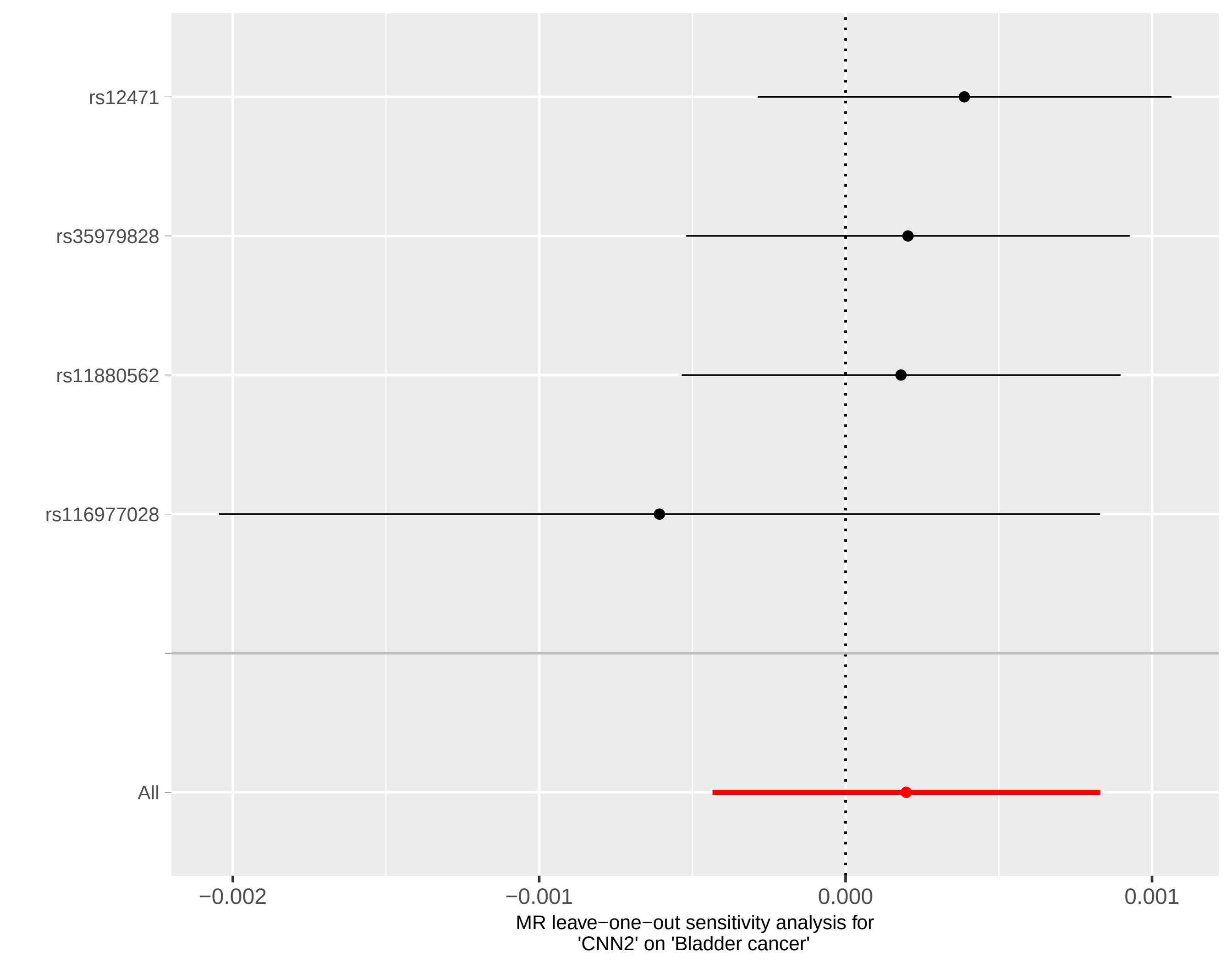

rs12471
rs35979828
rs11880562
rs116977028
All
−0.002
−0.001
0.000
0.001
MR leave−one−out sensitivity analysis for
'CNN2' on 'Bladder cancer'

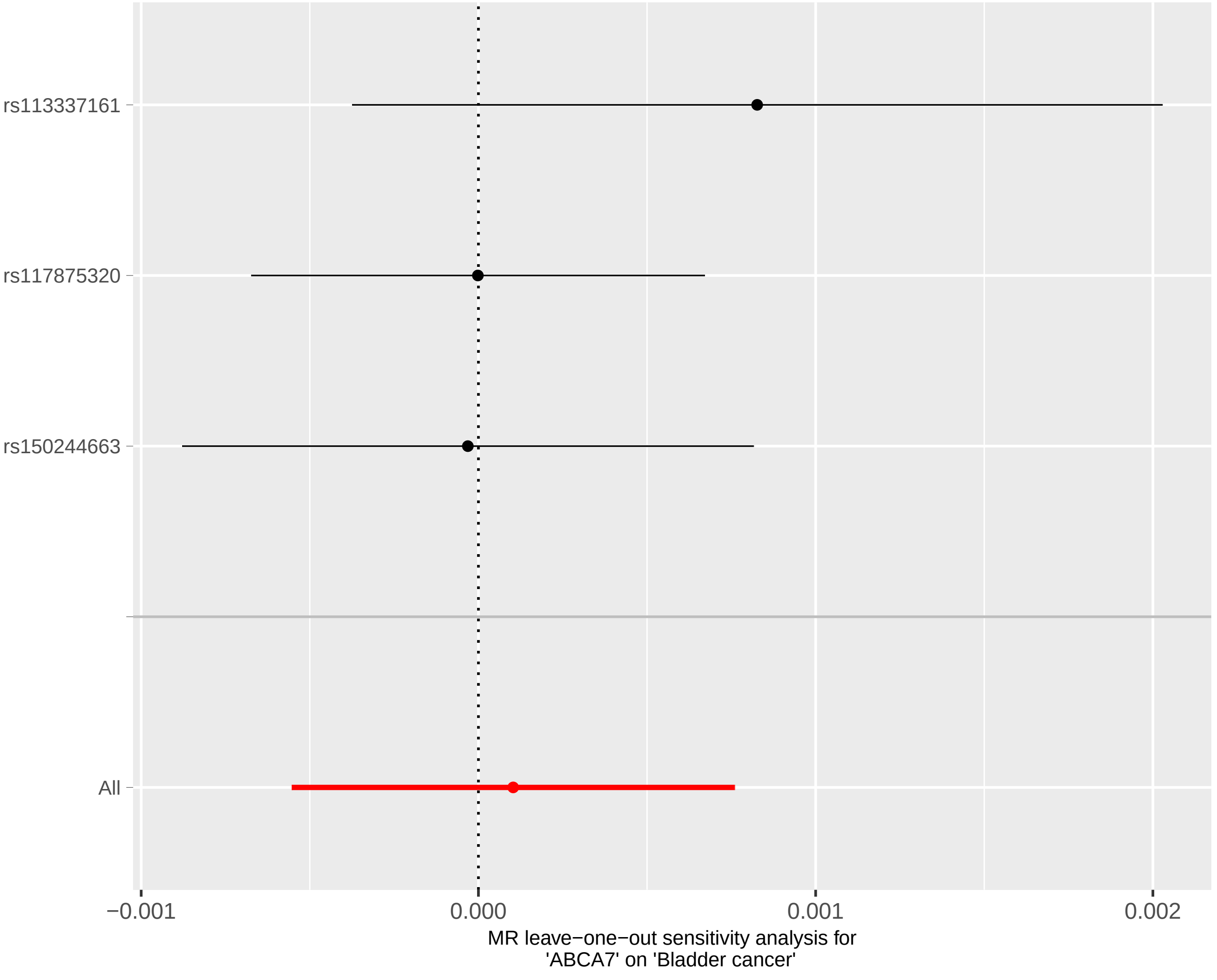
rs113337161
rs117875320
rs150244663
All
−0.001
0.000
0.001
0.002
MR leave−one−out sensitivity analysis for
'ABCA7' on 'Bladder cancer'

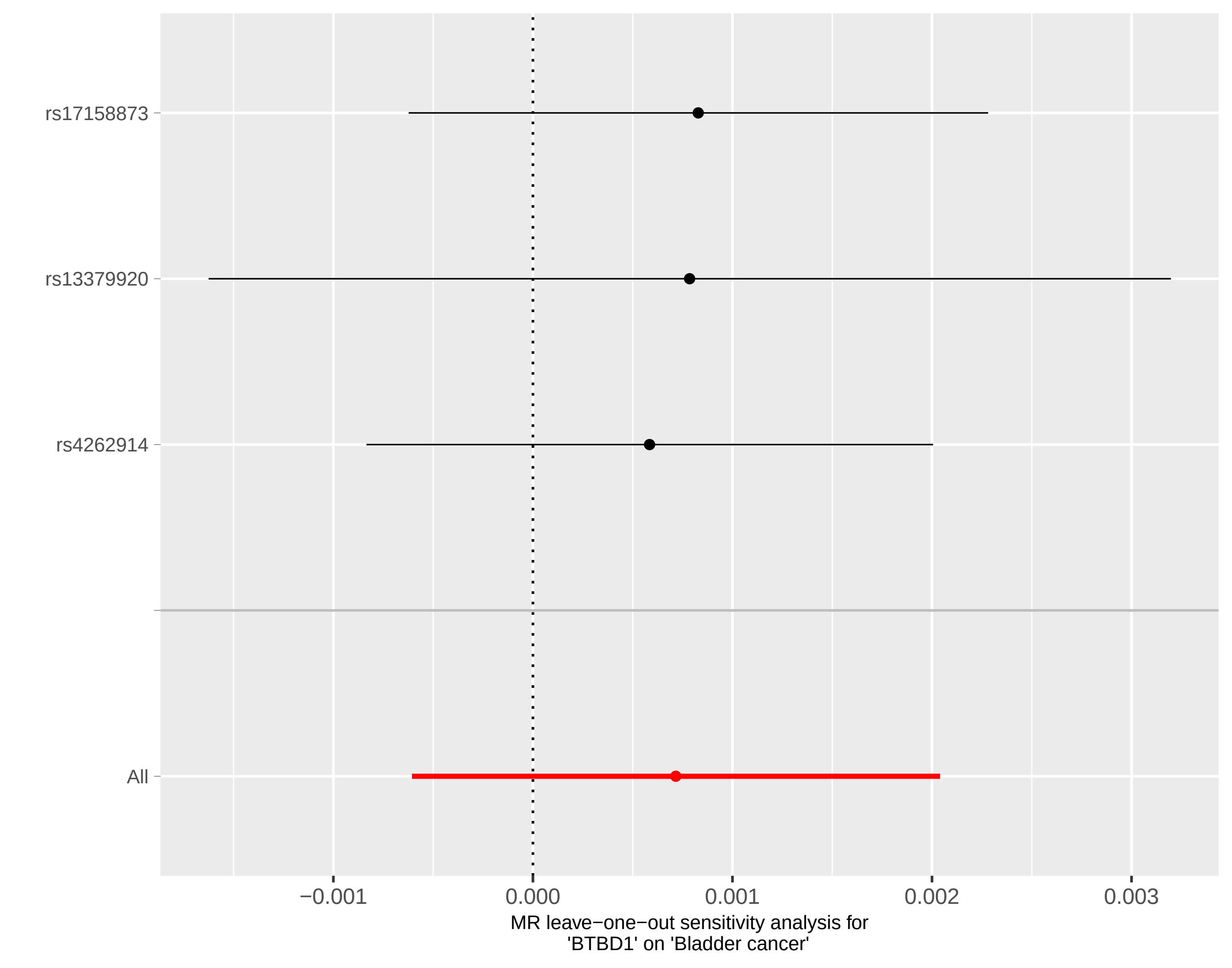
rs17158873
rs13379920
rs4262914
All
−0.001
0.000
0.001
0.002
0.003
MR leave−one−out sensitivity analysis for
'BTBD1' on 'Bladder cancer'

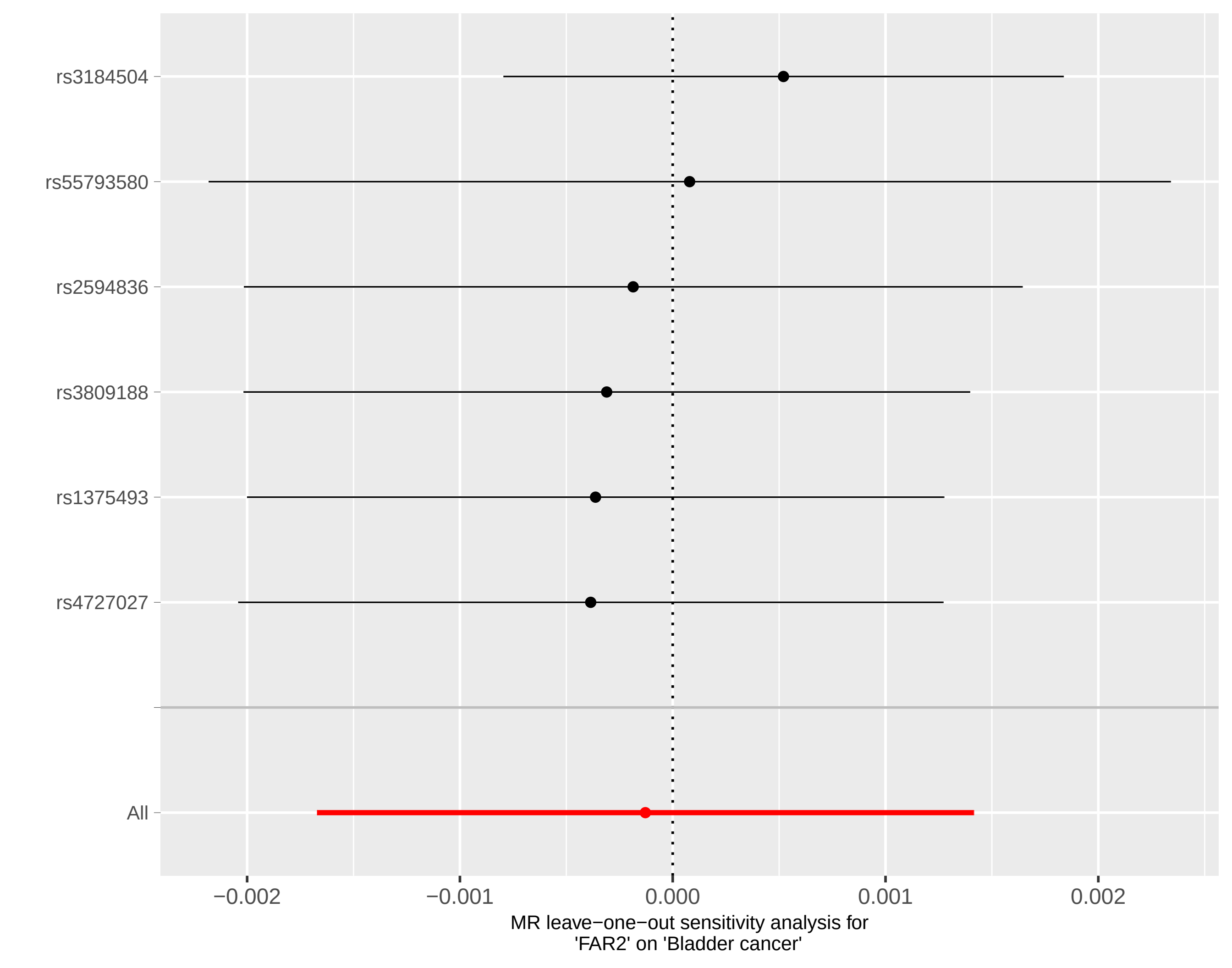
rs3184504
rs55793580
rs2594836
rs3809188
rs1375493
rs4727027
All
−0.002
−0.001
0.000
0.001
0.002
MR leave−one−out sensitivity analysis for
'FAR2' on 'Bladder cancer'

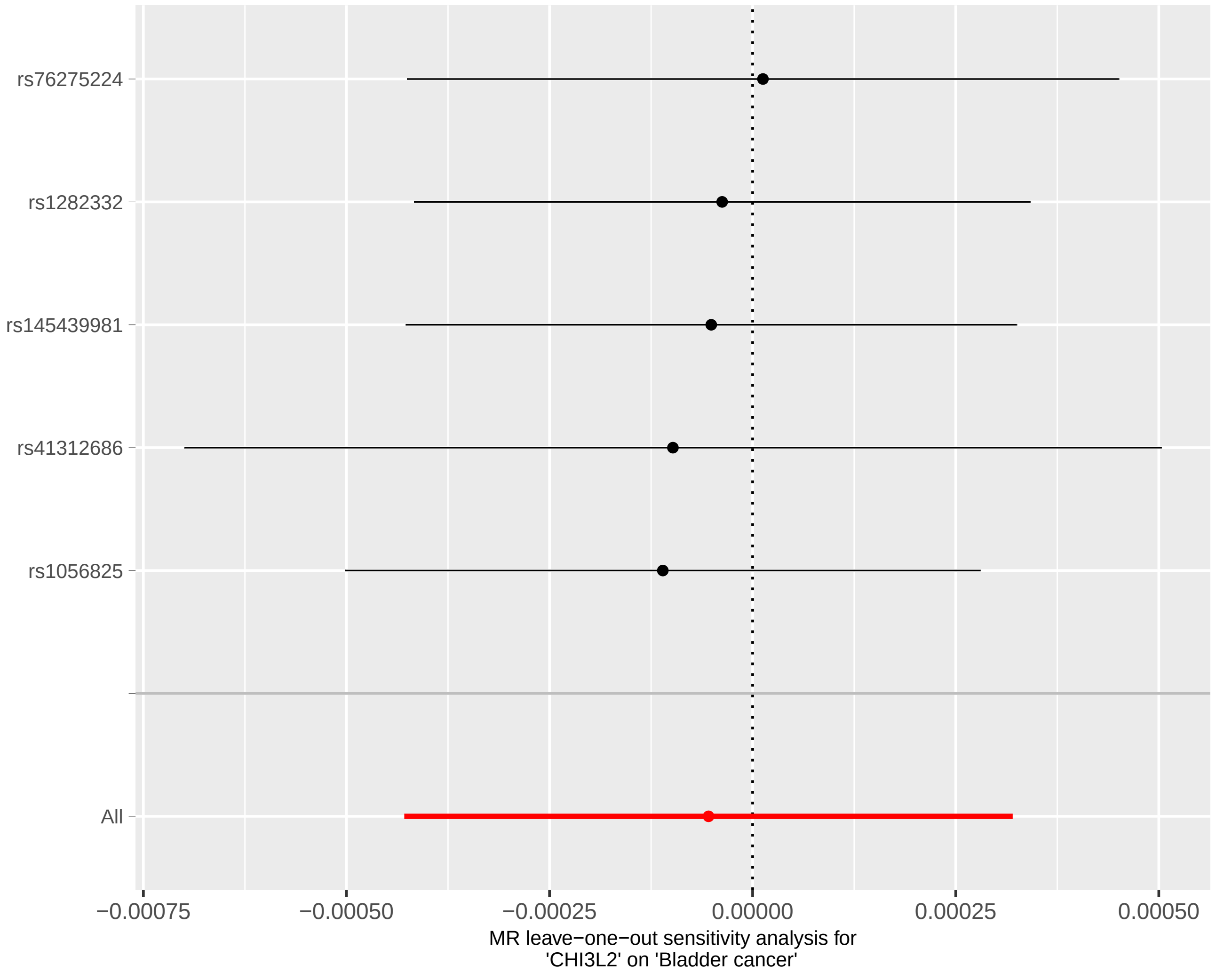
rs76275224
rs1282332
rs145439981
rs41312686
rs1056825
All
−0.00075
−0.00050
−0.00025
0.00000
0.00025
0.00050
MR leave−one−out sensitivity analysis for
'CHI3L2' on 'Bladder cancer'

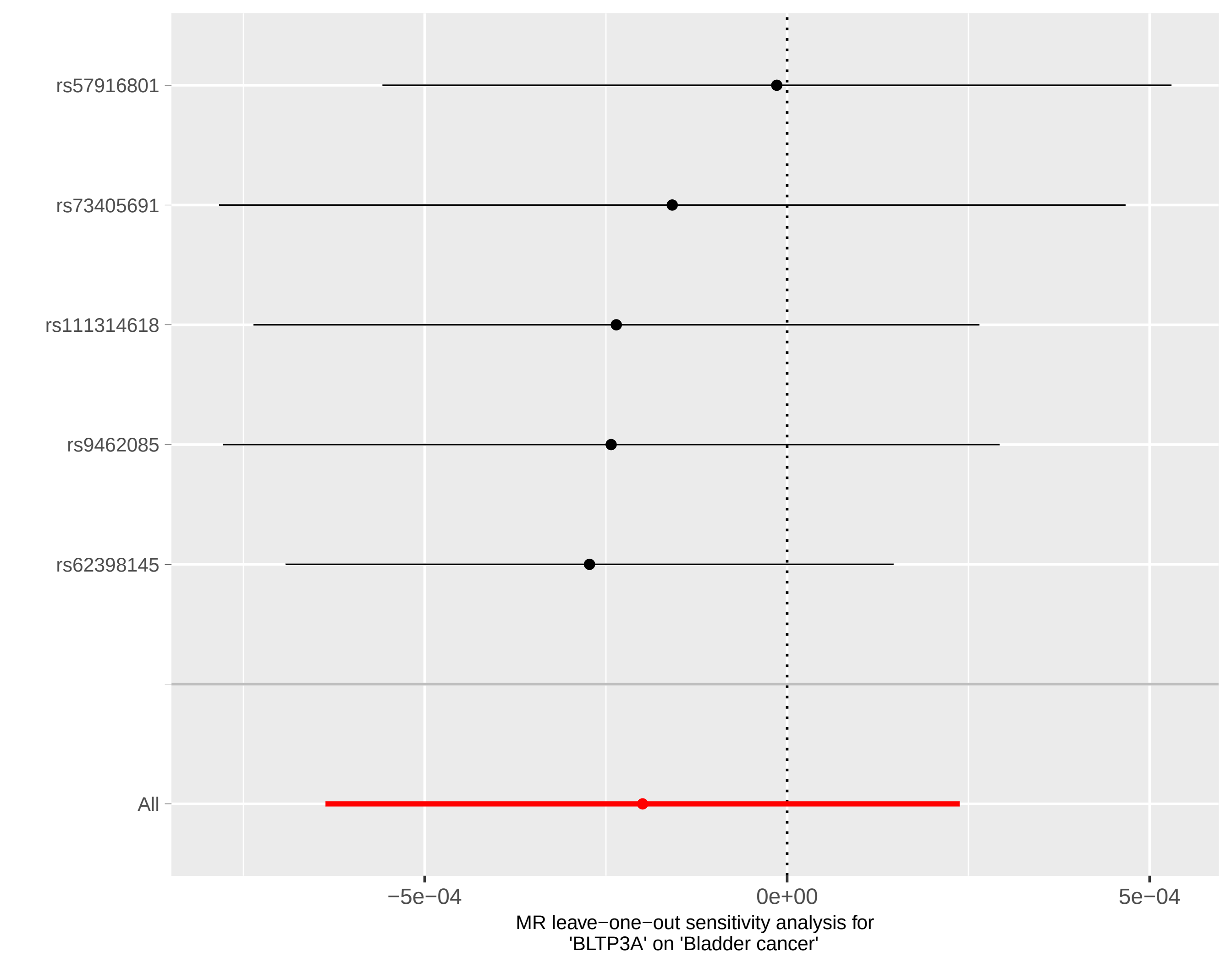
rs57916801
rs73405691
rs111314618
rs9462085
rs62398145
All
−5e−04
0e+00
5e−04
MR leave−one−out sensitivity analysis for
'BLTP3A' on 'Bladder cancer'

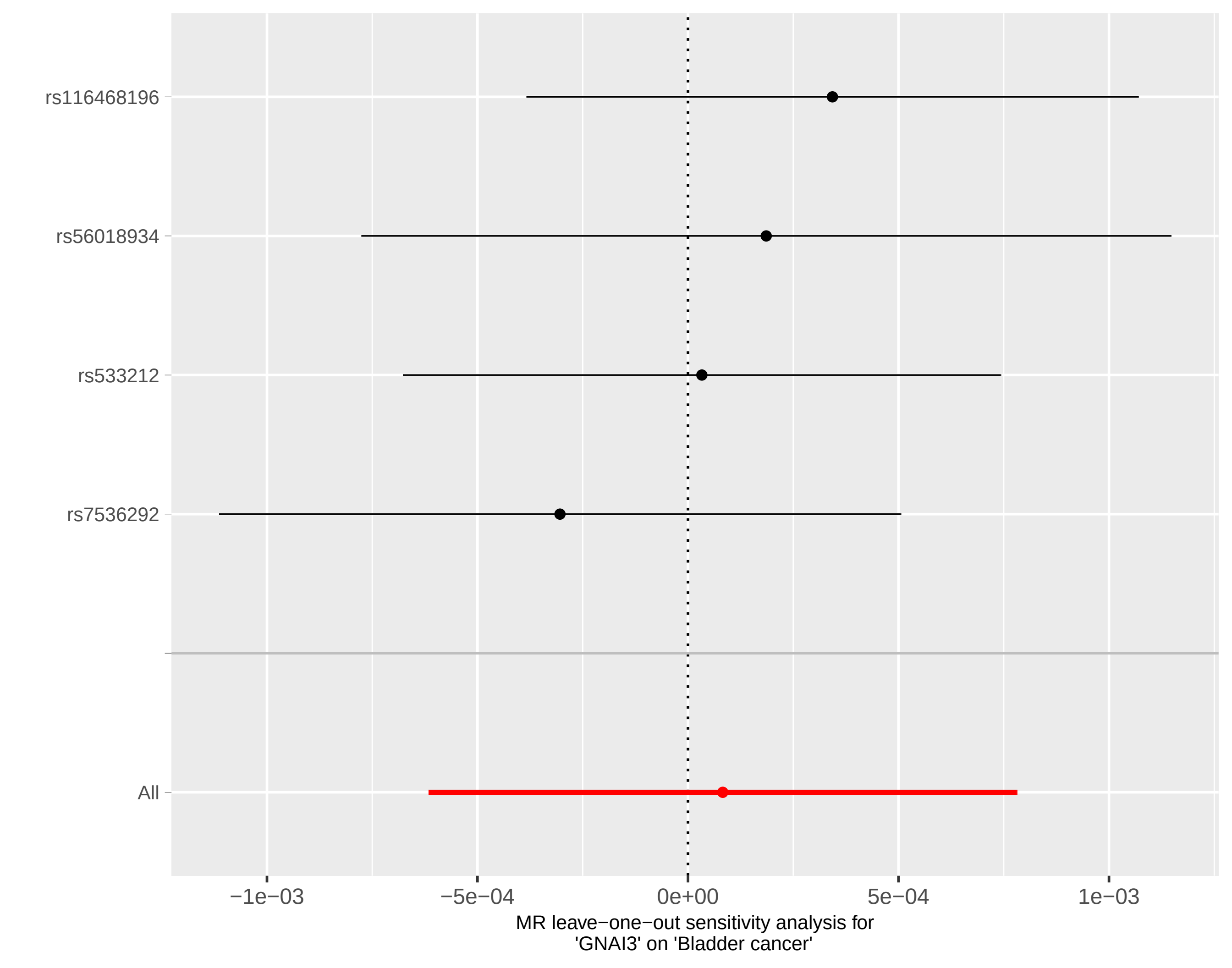

rs116468196
rs56018934
rs533212
rs7536292
All
−1e−03
−5e−04
0e+00
5e−04
1e−03
MR leave−one−out sensitivity analysis for
'GNAI3' on 'Bladder cancer'

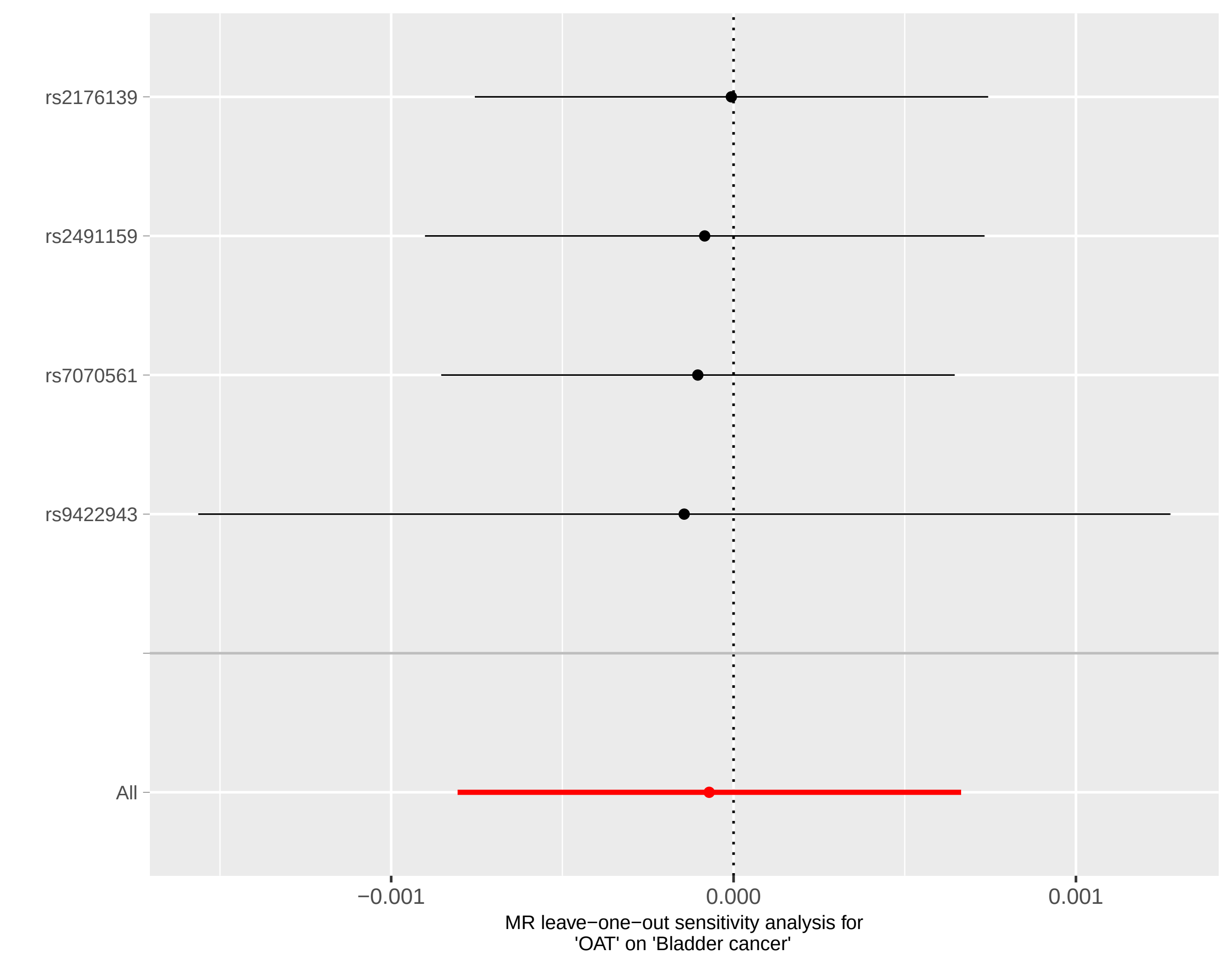
rs2176139
rs2491159
rs7070561
rs9422943
All
−0.001
0.000
0.001
MR leave−one−out sensitivity analysis for
'OAT' on 'Bladder cancer'

Insufficient number of SNPs

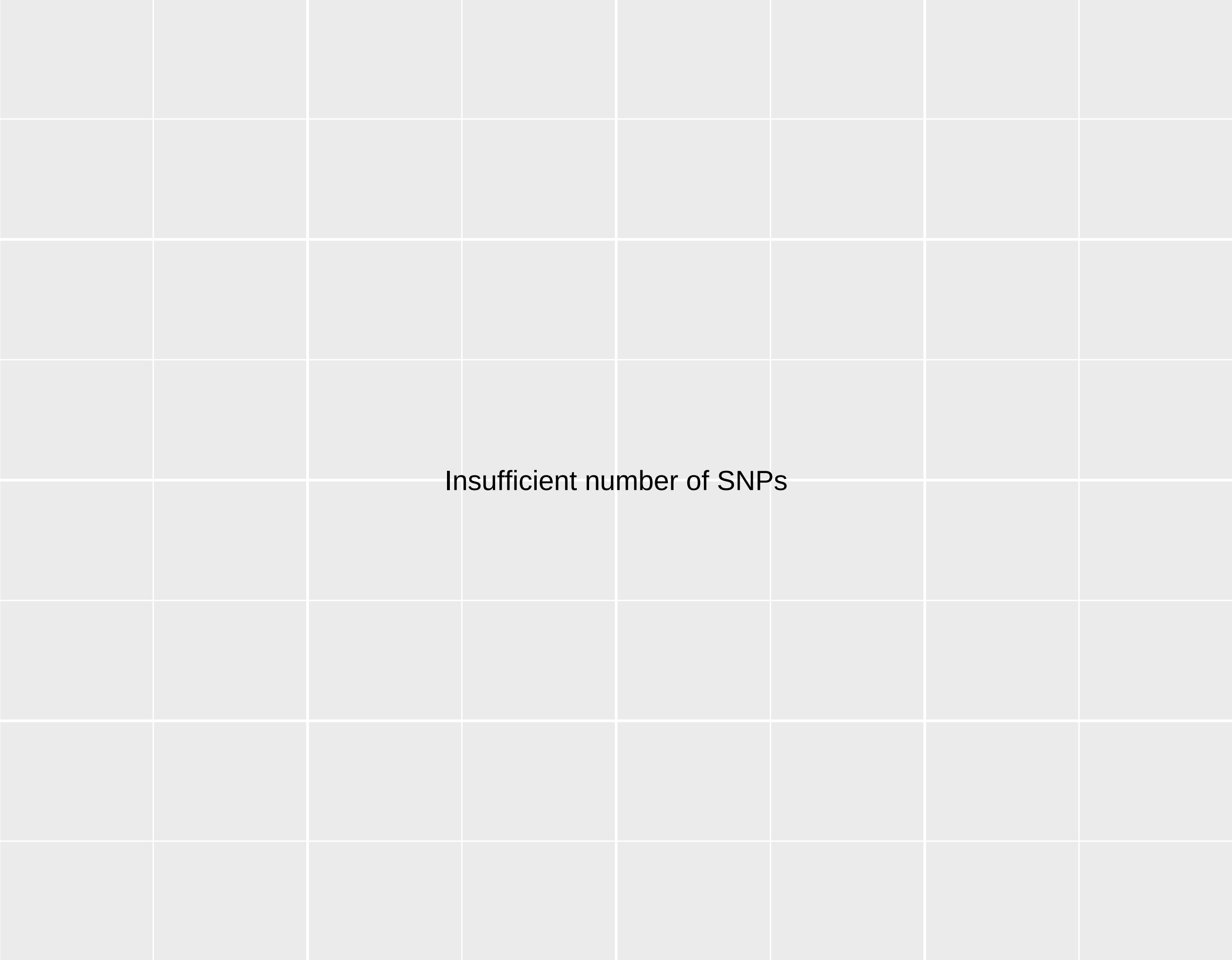
Insufficient number of SNPs

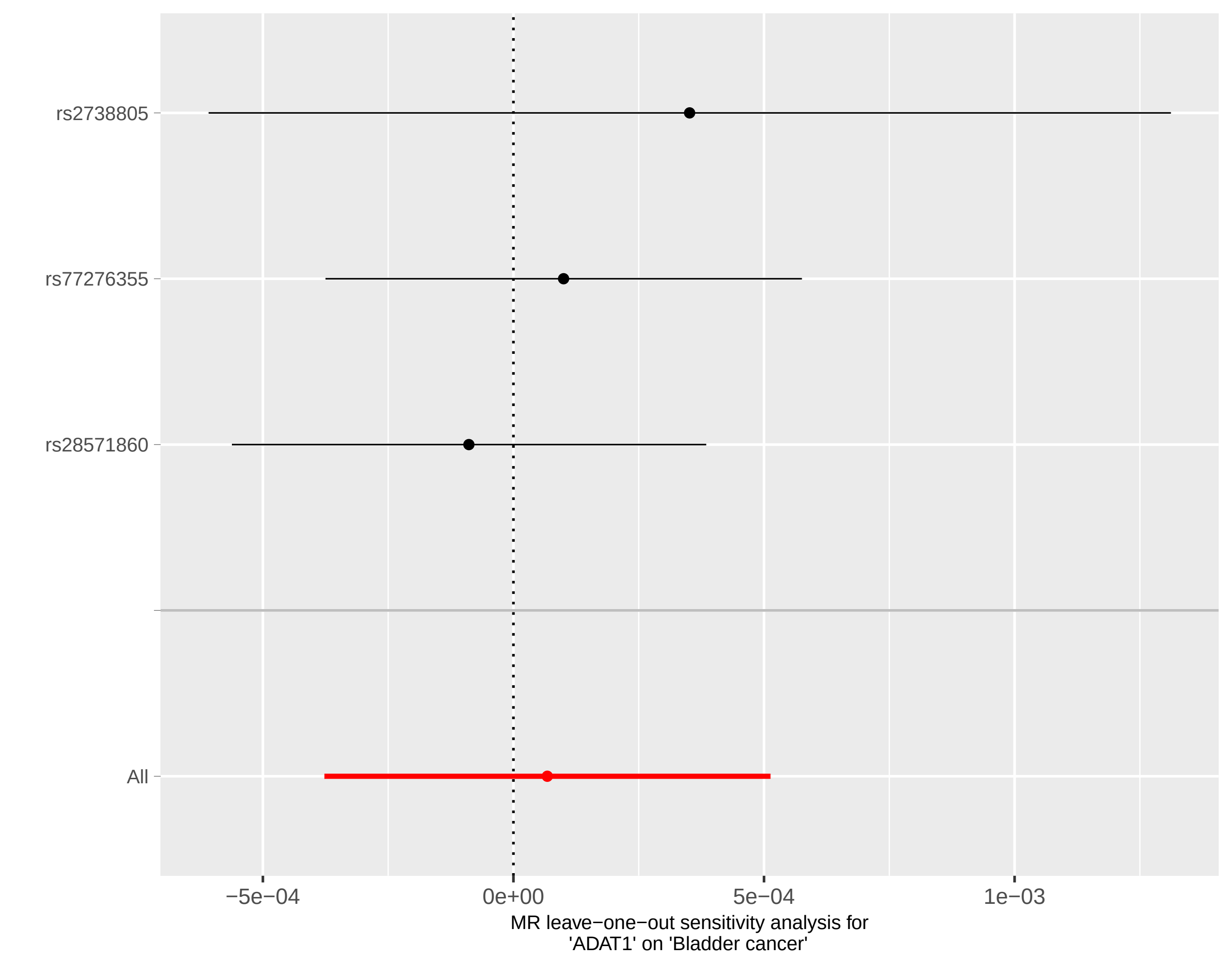
rs2738805
rs77276355
rs28571860
All
−5e−04
0e+00
5e−04
1e−03
MR leave−one−out sensitivity analysis for
'ADAT1' on 'Bladder cancer'

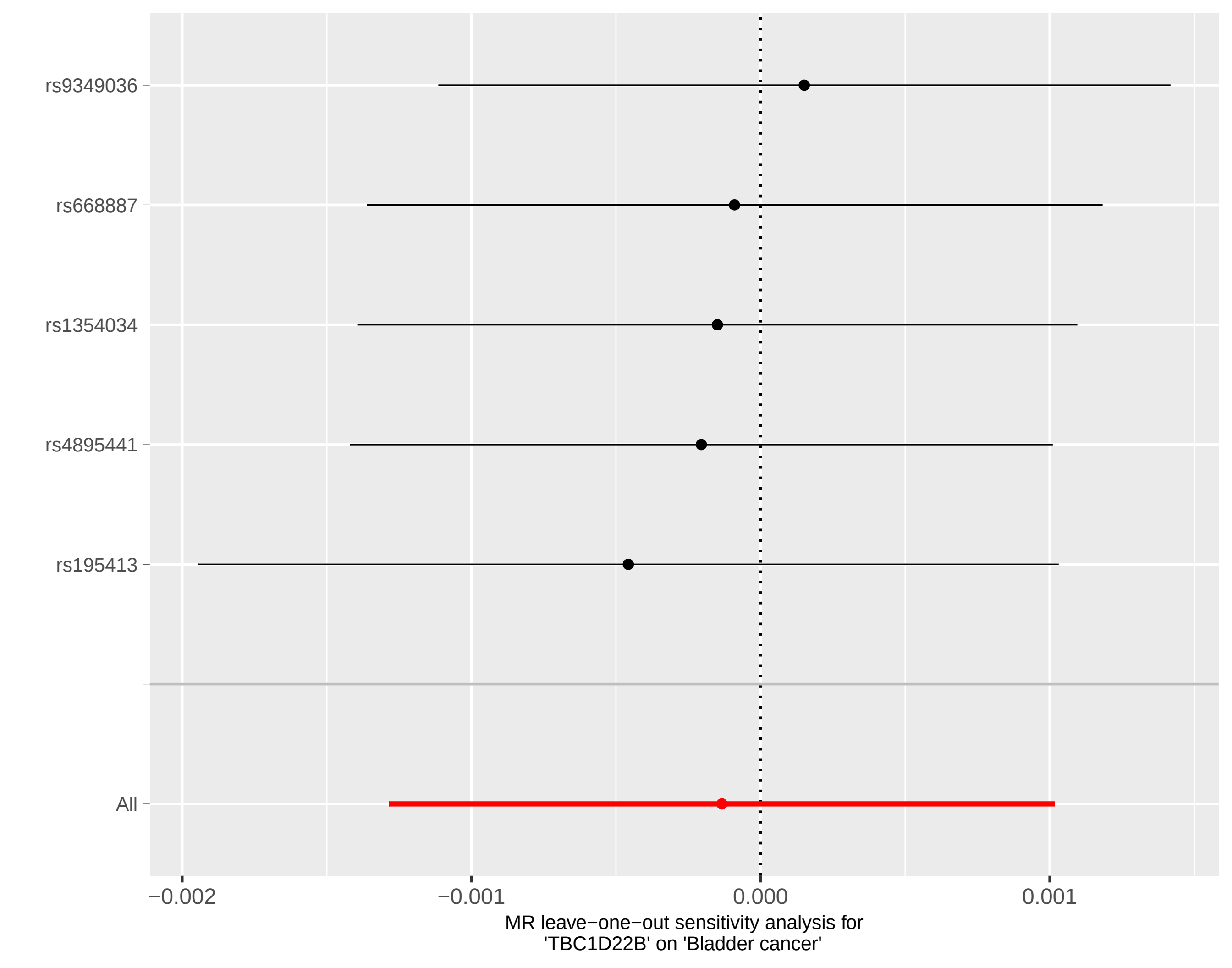
rs9349036
rs668887
rs1354034
rs4895441
rs195413
All
−0.002
−0.001
0.000
0.001
MR leave−one−out sensitivity analysis for
'TBC1D22B' on 'Bladder cancer'

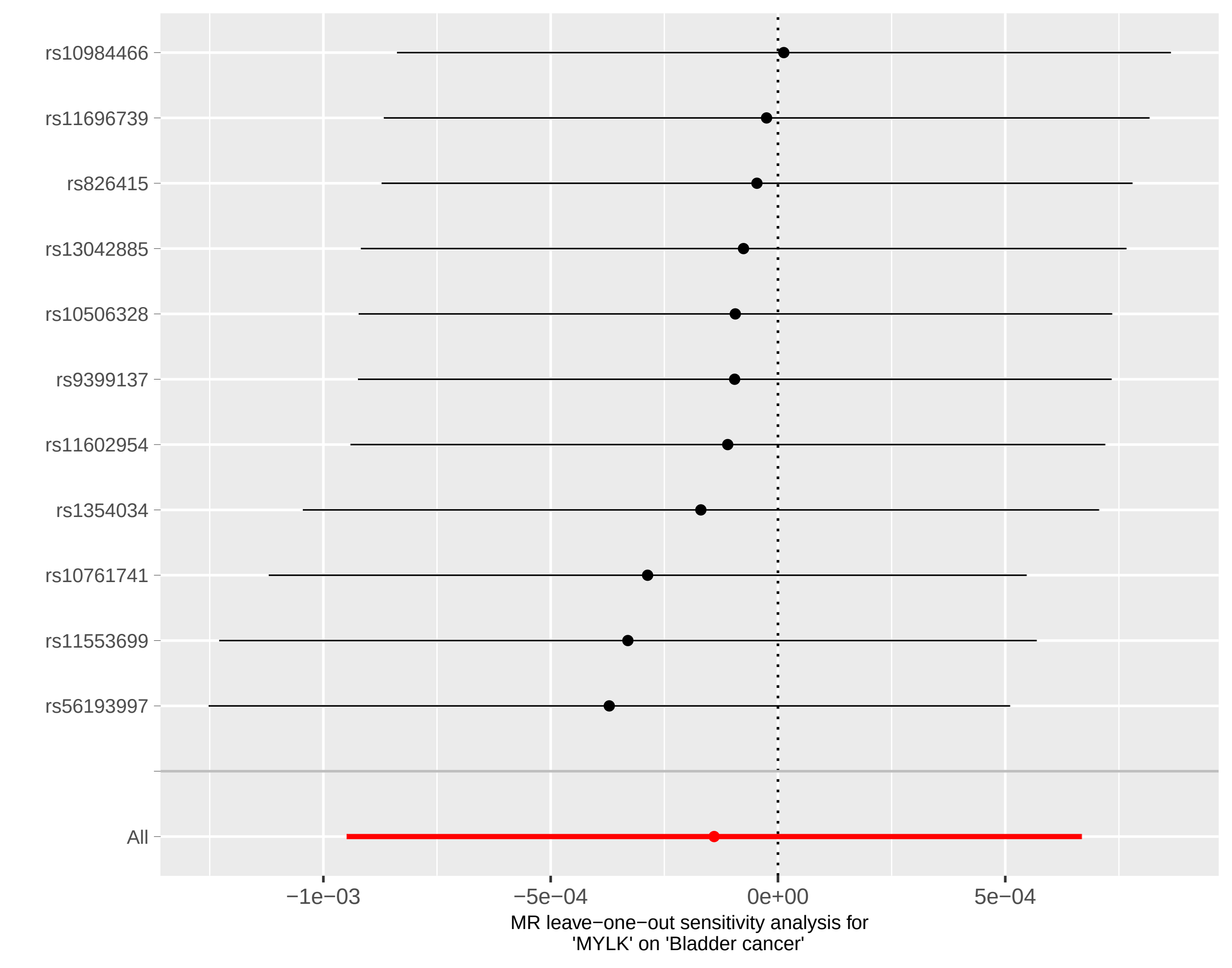
rs10984466
rs11696739
rs826415
rs13042885
rs10506328
rs9399137
rs11602954
rs1354034
rs10761741
rs11553699
rs56193997
All
−1e−03
−5e−04
0e+00
5e−04
MR leave−one−out sensitivity analysis for
'MYLK' on 'Bladder cancer'

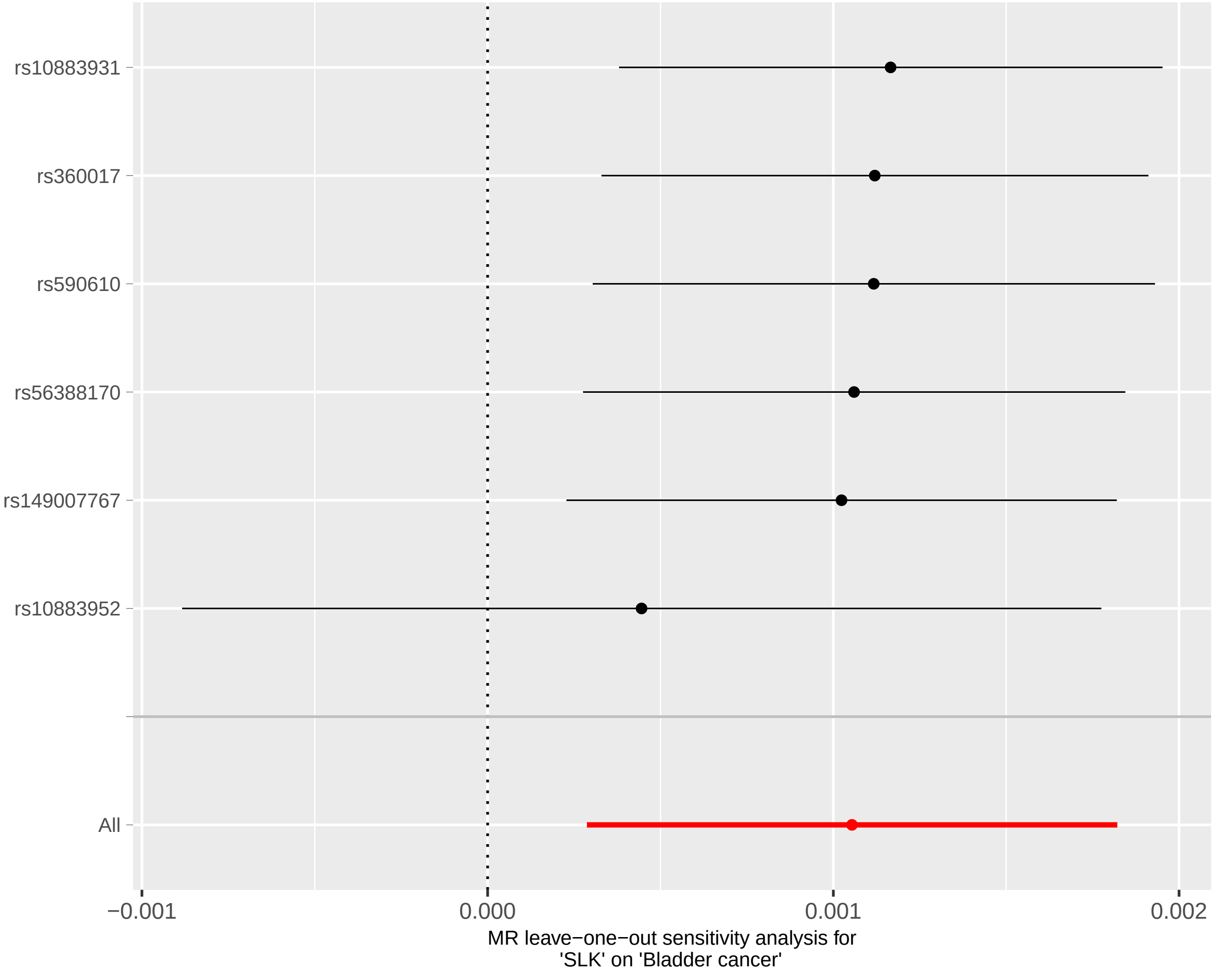

rs10883931
rs360017
rs590610
rs56388170
rs149007767
rs10883952
All
−0.001
0.000
0.001
0.002
MR leave−one−out sensitivity analysis for
'SLK' on 'Bladder cancer'

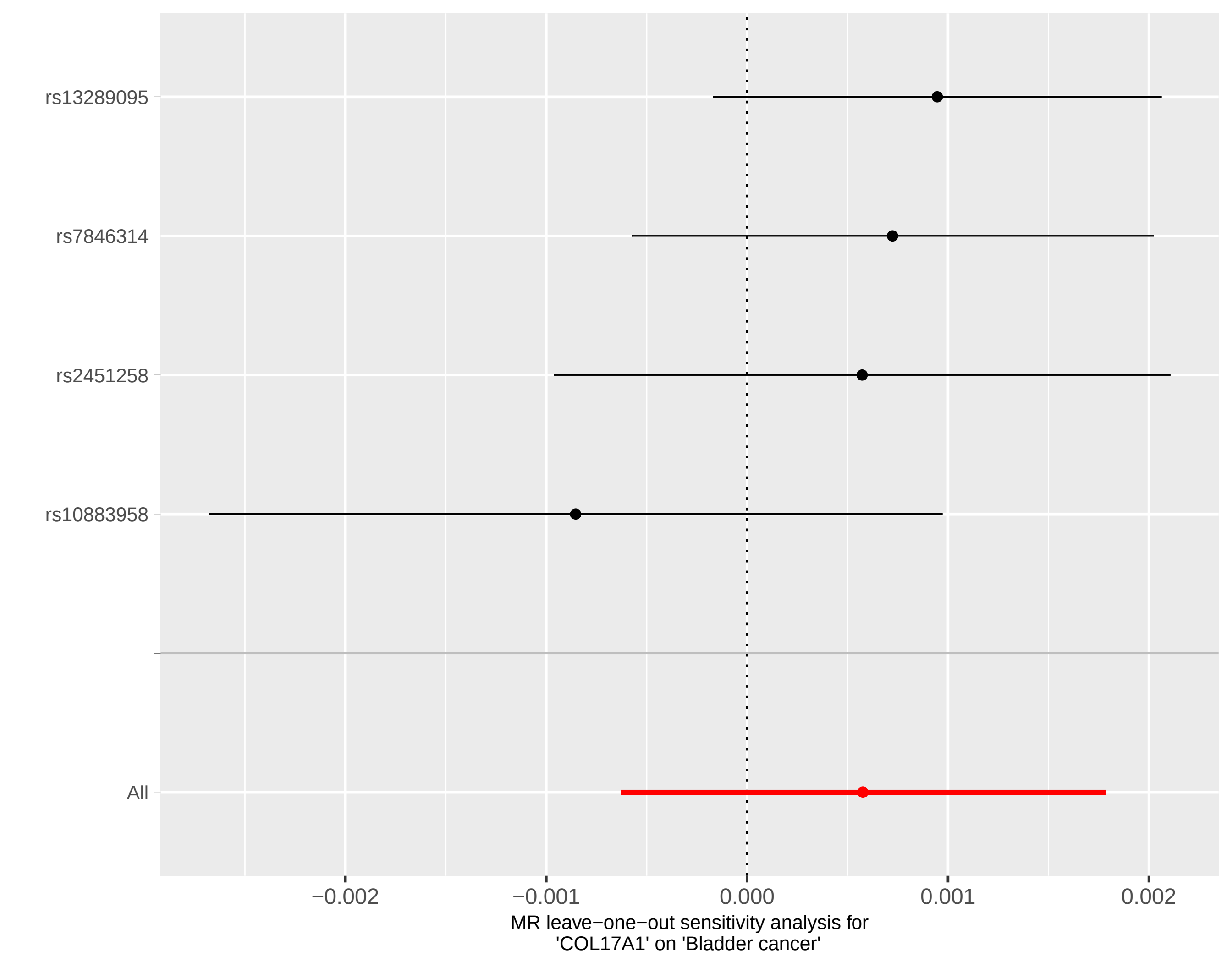
rs13289095
rs7846314
rs2451258
rs10883958
All
−0.002
−0.001
0.000
0.001
0.002
MR leave−one−out sensitivity analysis for
'COL17A1' on 'Bladder cancer'

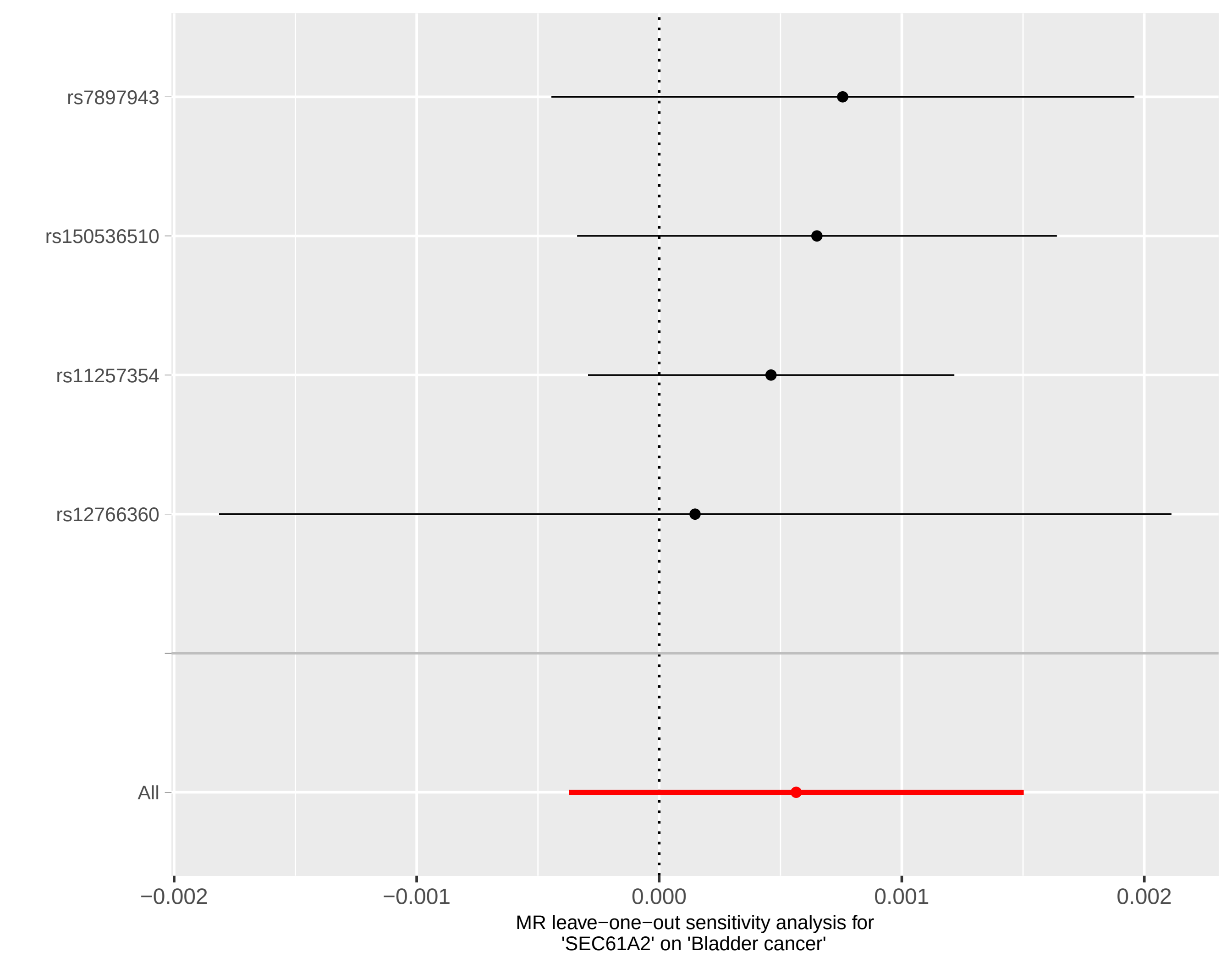
rs7897943
rs150536510
rs11257354
rs12766360
All
−0.002
−0.001
0.000
0.001
0.002
MR leave−one−out sensitivity analysis for
'SEC61A2' on 'Bladder cancer'

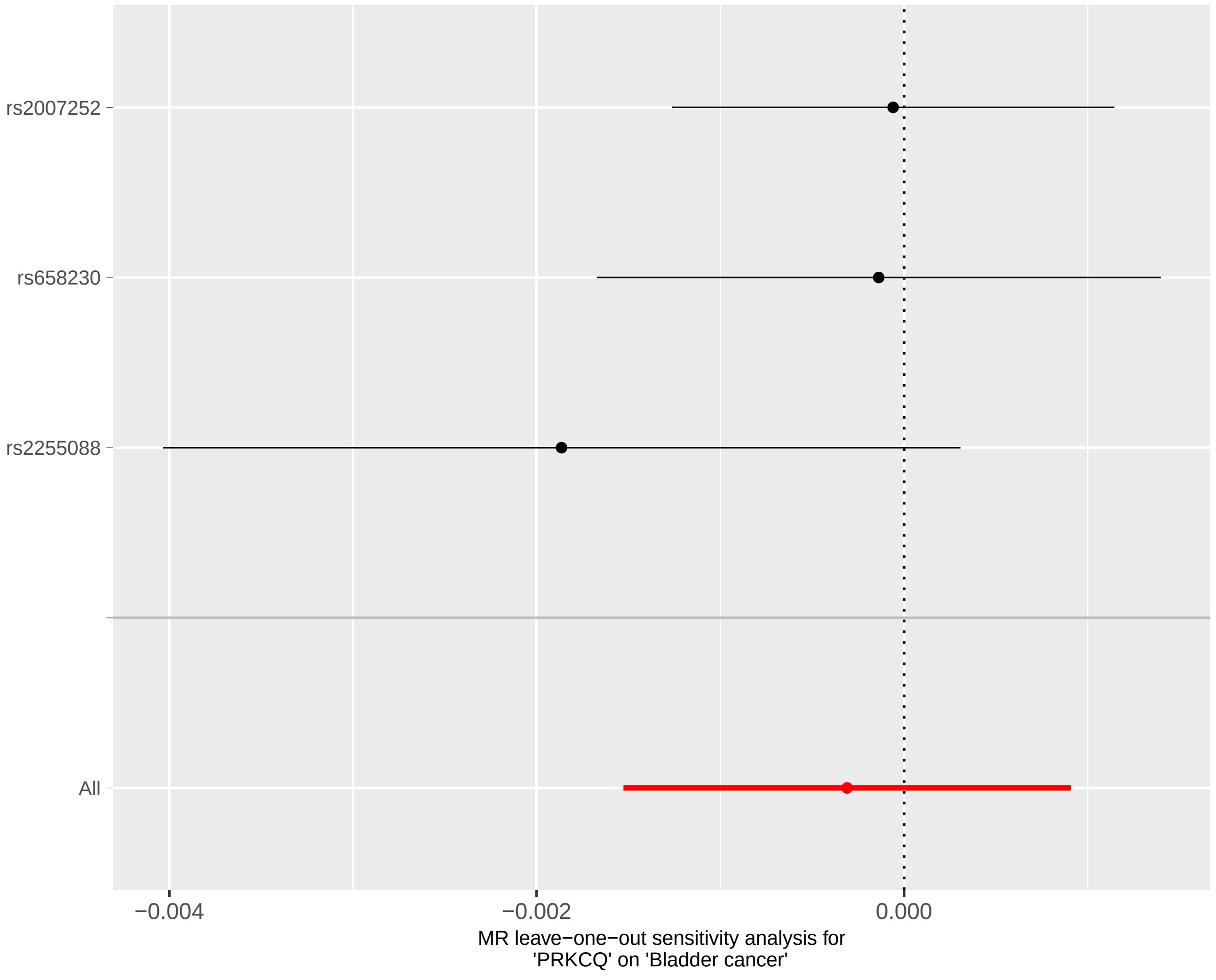
rs2007252
rs658230
rs2255088
All
−0.004
−0.002
0.000
MR leave−one−out sensitivity analysis for
'PRKCQ' on 'Bladder cancer'

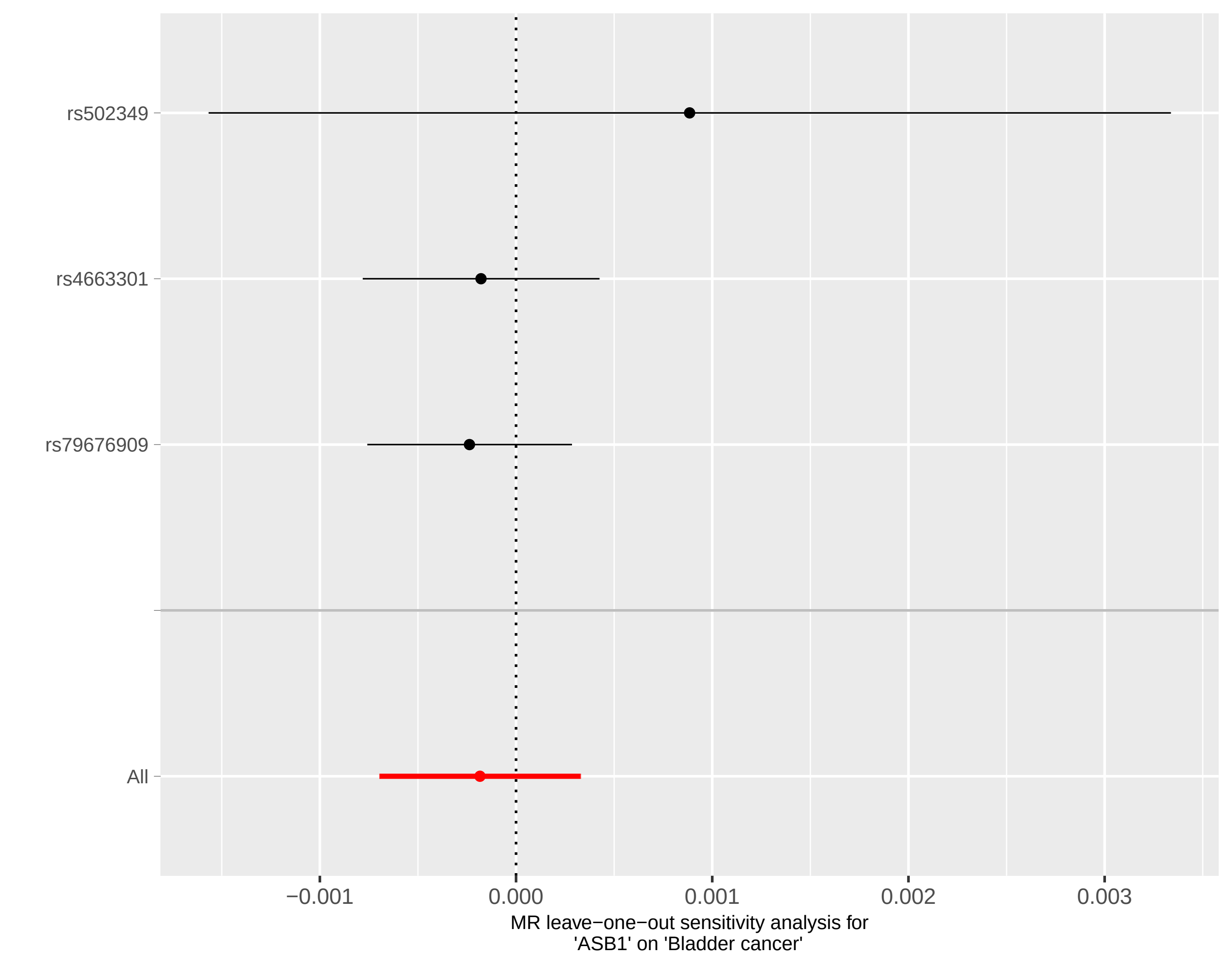
rs502349
rs4663301
rs79676909
All
−0.001
0.000
0.001
0.002
0.003
MR leave−one−out sensitivity analysis for
'ASB1' on 'Bladder cancer'

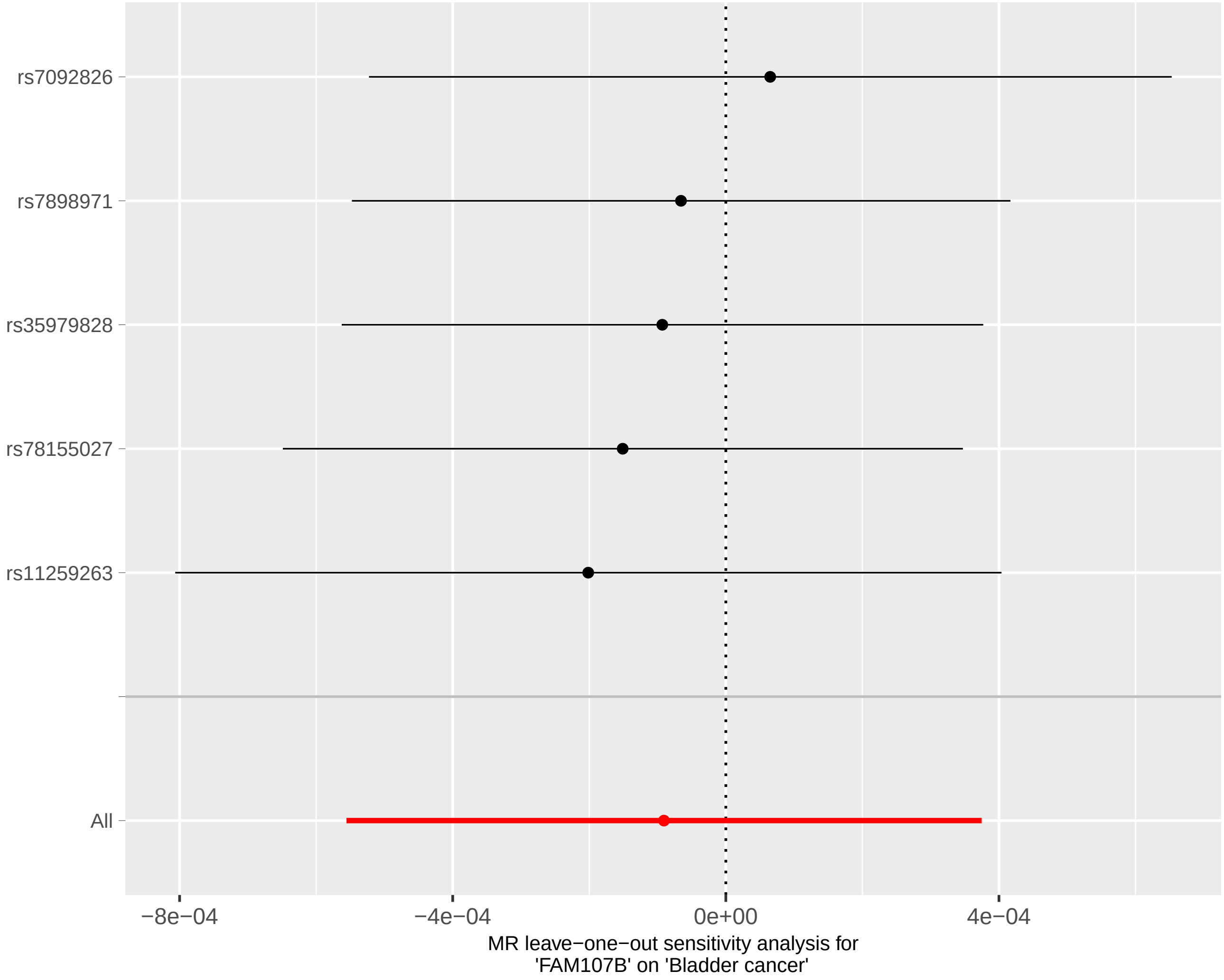

rs7092826
rs7898971
rs35979828
rs78155027
rs11259263
All
−8e−04
−4e−04
0e+00
4e−04
MR leave−one−out sensitivity analysis for
'FAM107B' on 'Bladder cancer'

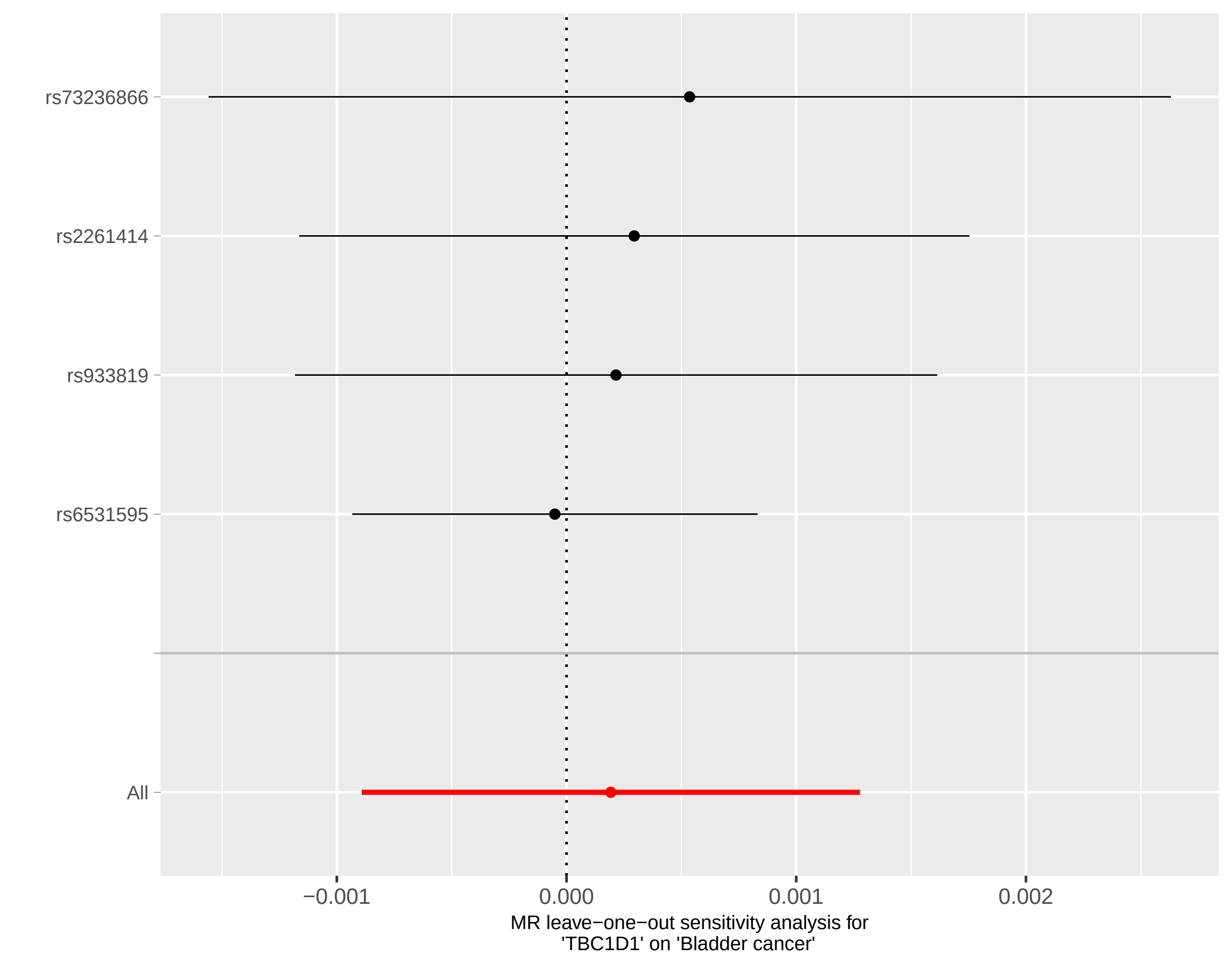
rs73236866
rs2261414
rs933819
rs6531595
All
−0.001
0.000
0.001
0.002
MR leave−one−out sensitivity analysis for
'TBC1D1' on 'Bladder cancer'

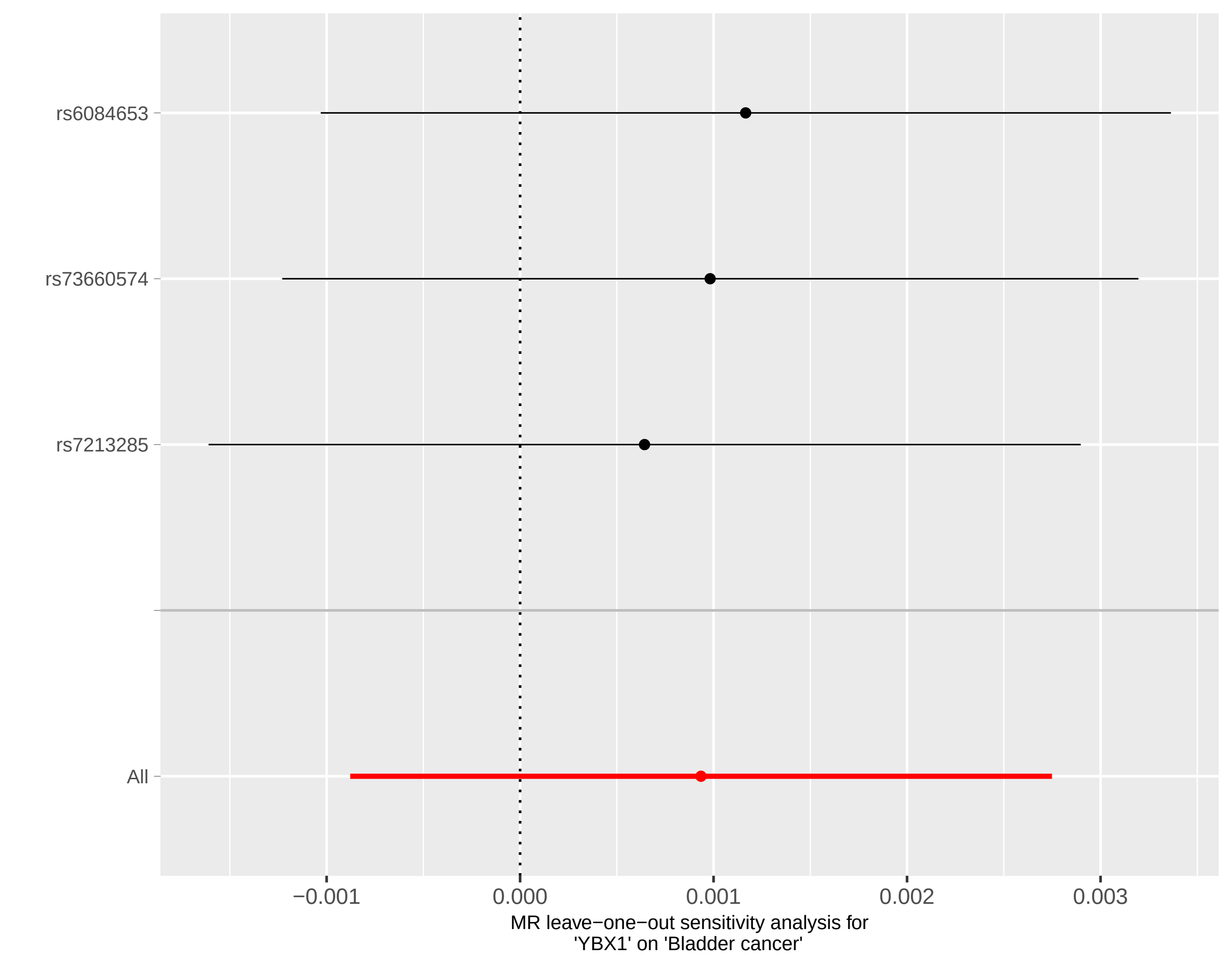
rs6084653
rs73660574
rs7213285
All
−0.001
0.000
0.001
0.002
0.003
MR leave−one−out sensitivity analysis for
'YBX1' on 'Bladder cancer'

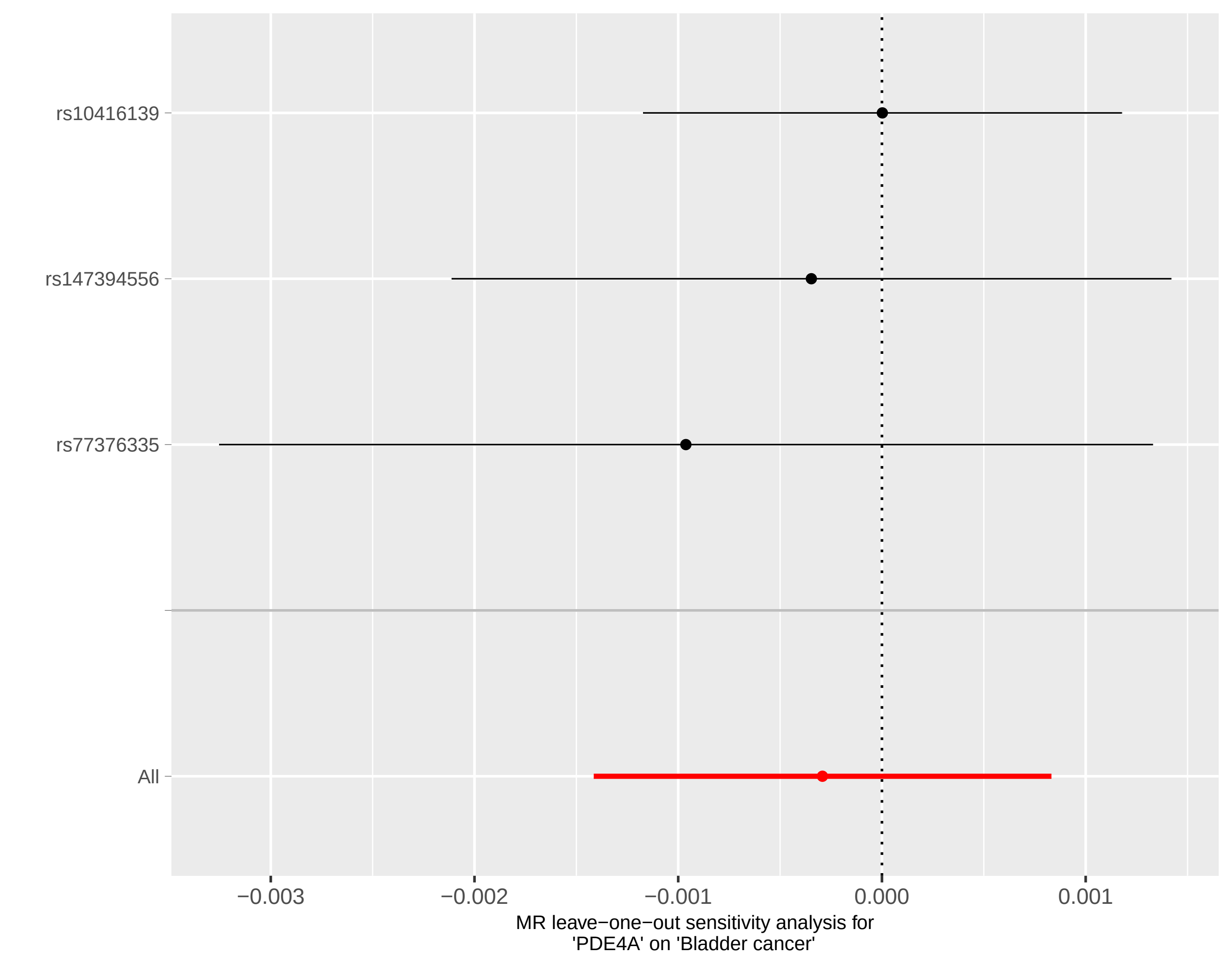
rs10416139
rs147394556
rs77376335
All
−0.003
−0.002
−0.001
0.000
0.001
MR leave−one−out sensitivity analysis for
'PDE4A' on 'Bladder cancer'

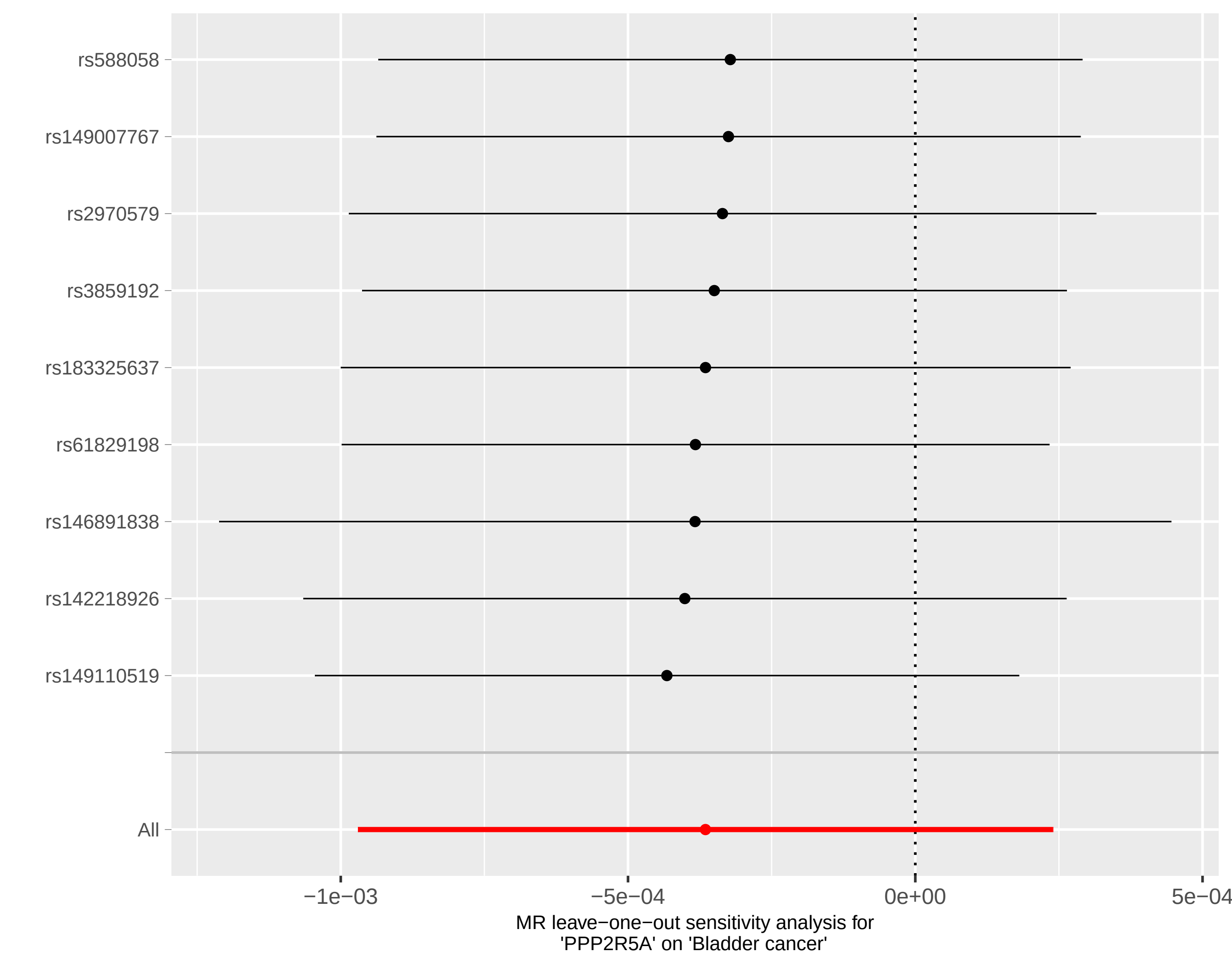
rs588058
rs149007767
rs2970579
rs3859192
rs183325637
rs61829198
rs146891838
rs142218926
rs149110519
All
−1e−03
−5e−04
0e+00
5e−04
MR leave−one−out sensitivity analysis for
'PPP2R5A' on 'Bladder cancer'

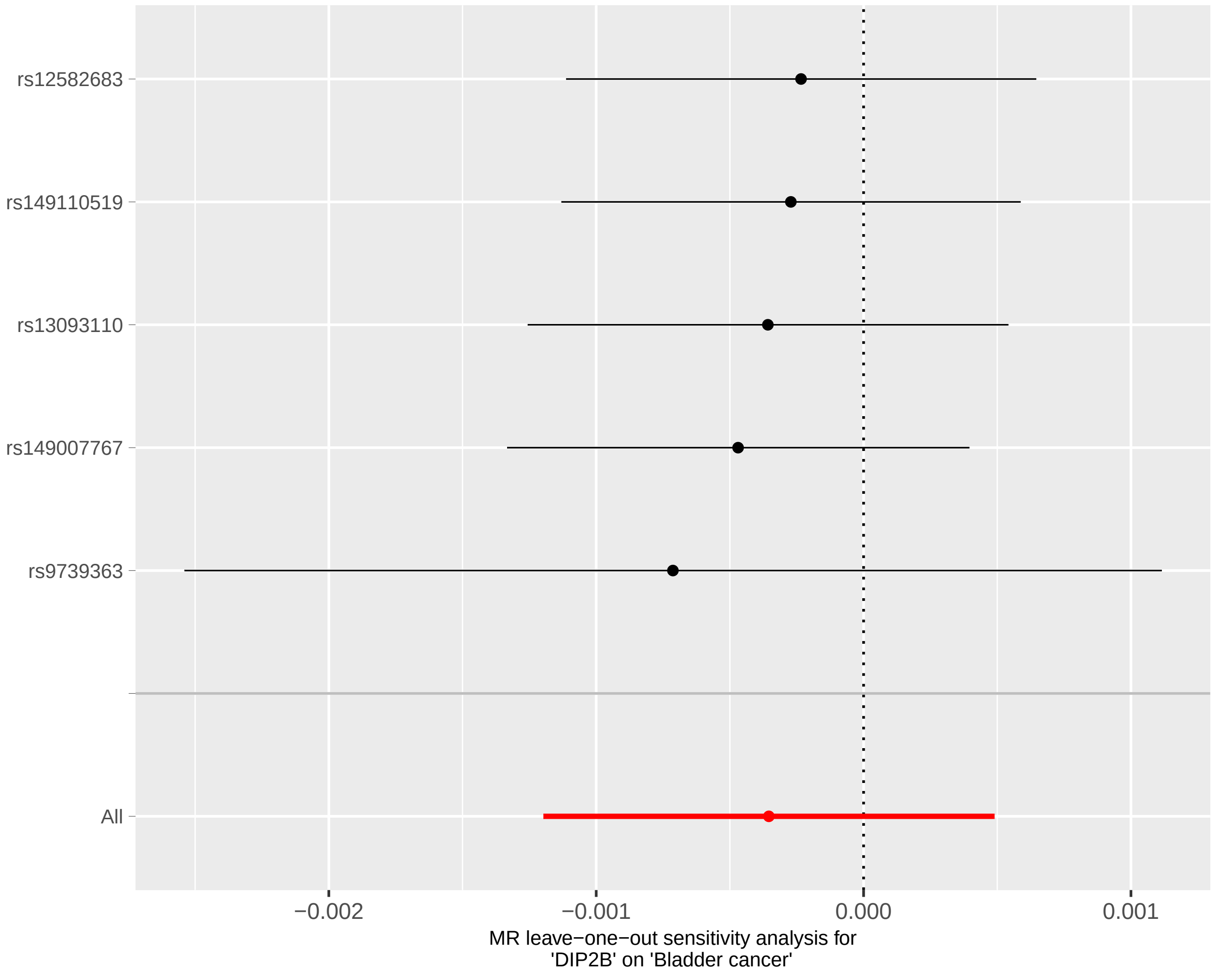

rs12582683
rs149110519
rs13093110
rs149007767
rs9739363
All
−0.002
−0.001
0.000
0.001
MR leave−one−out sensitivity analysis for
'DIP2B' on 'Bladder cancer'

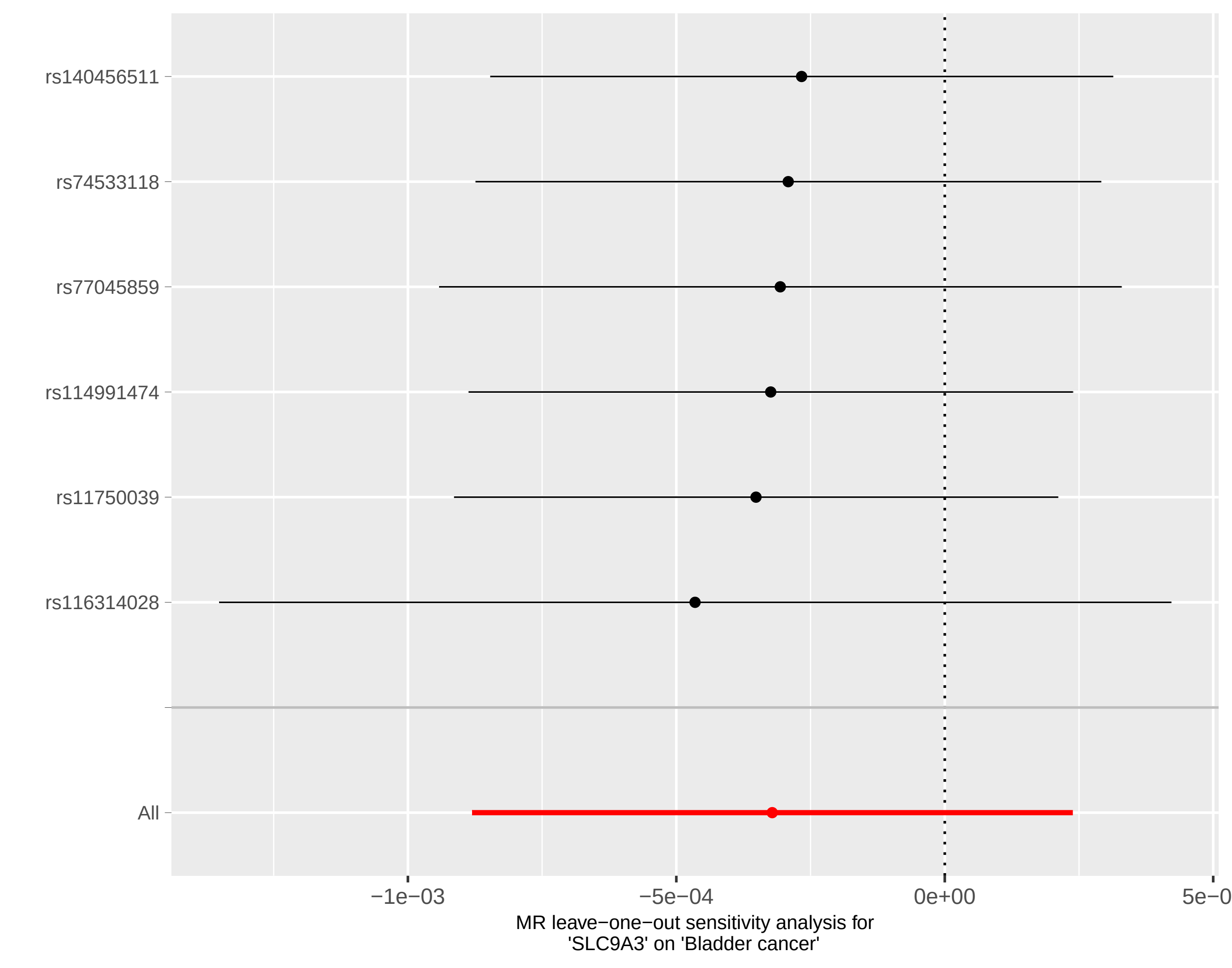
rs140456511
rs74533118
rs77045859
rs114991474
rs11750039
rs116314028
All
−1e−03
−5e−04
0e+00
5e−0
MR leave−one−out sensitivity analysis for
'SLC9A3' on 'Bladder cancer'

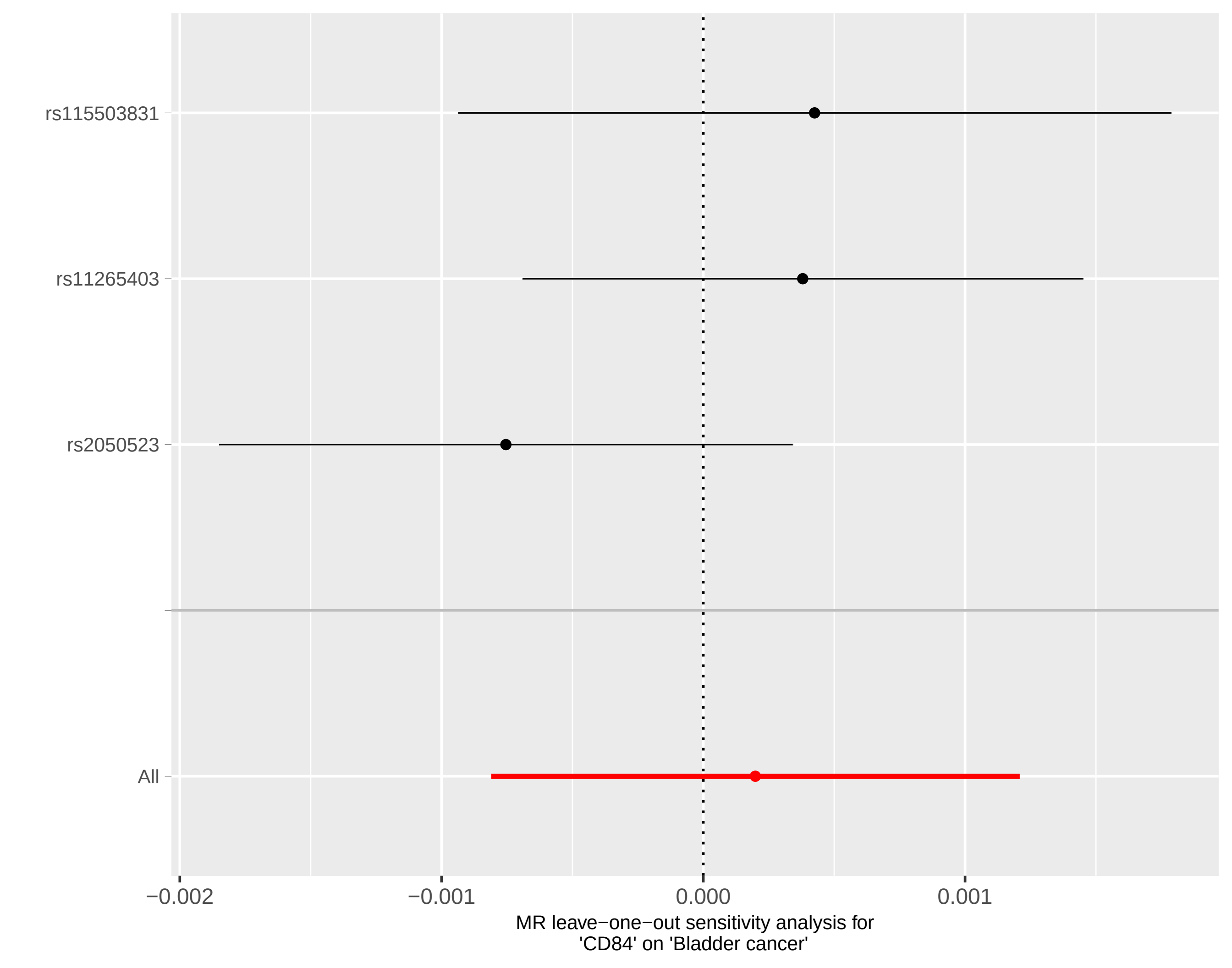
rs115503831
rs11265403
rs2050523
All
−0.002
−0.001
0.000
0.001
MR leave−one−out sensitivity analysis for
'CD84' on 'Bladder cancer'

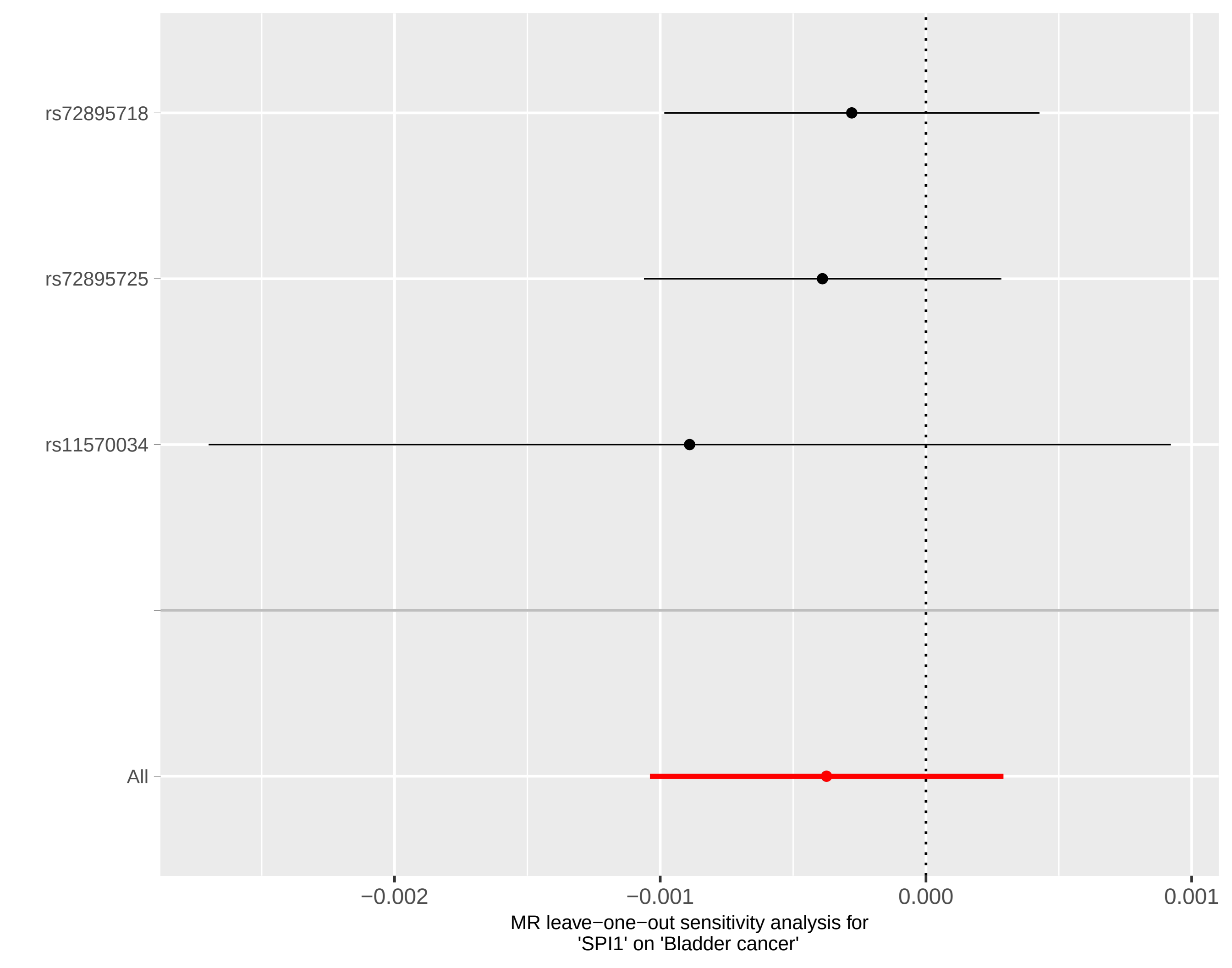
rs72895718
rs72895725
rs11570034
All
−0.002
−0.001
0.000
0.001
MR leave−one−out sensitivity analysis for
'SPI1' on 'Bladder cancer'

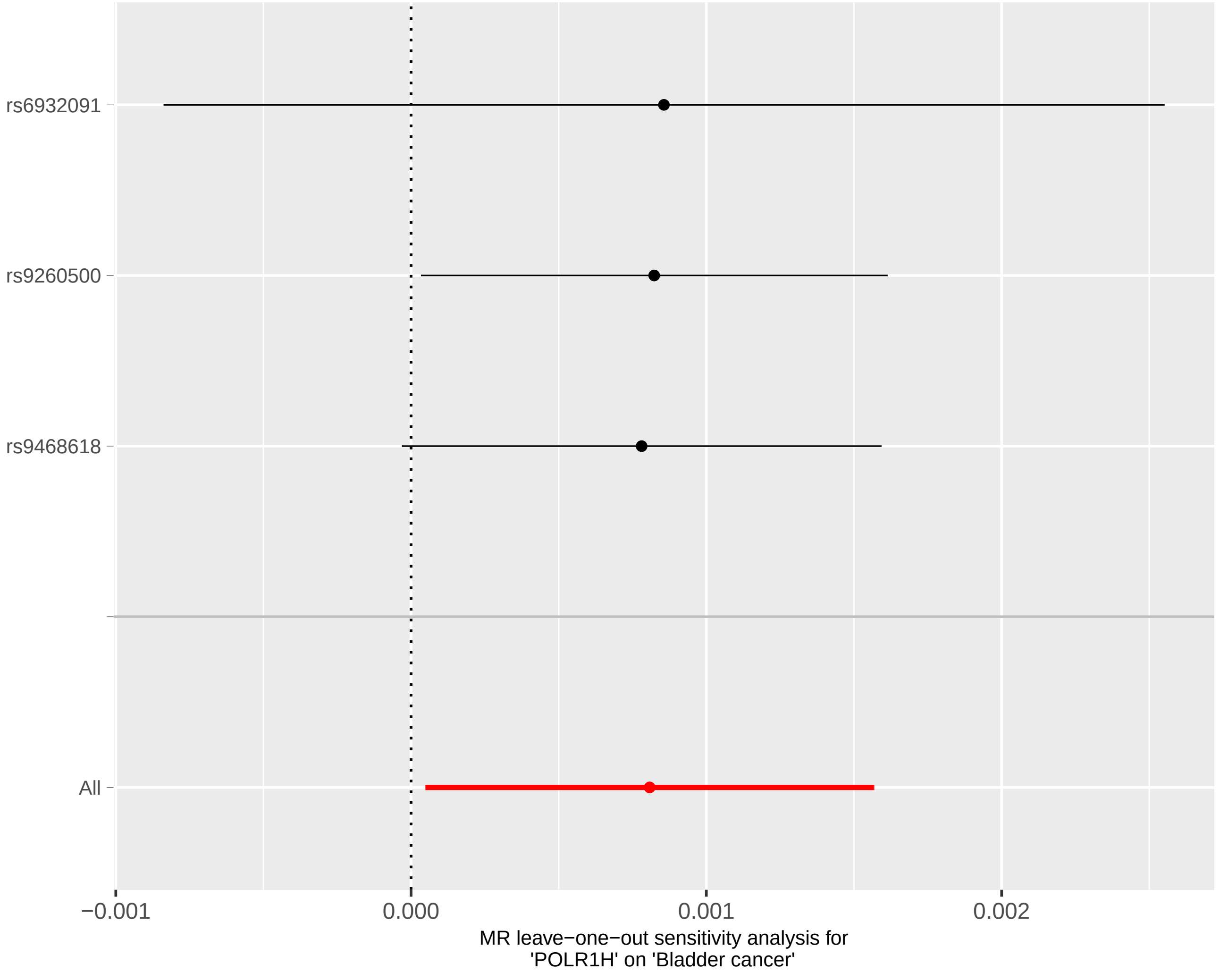
rs6932091
rs9260500
rs9468618
All
−0.001
0.000
0.001
0.002
MR leave−one−out sensitivity analysis for
'POLR1H' on 'Bladder cancer'

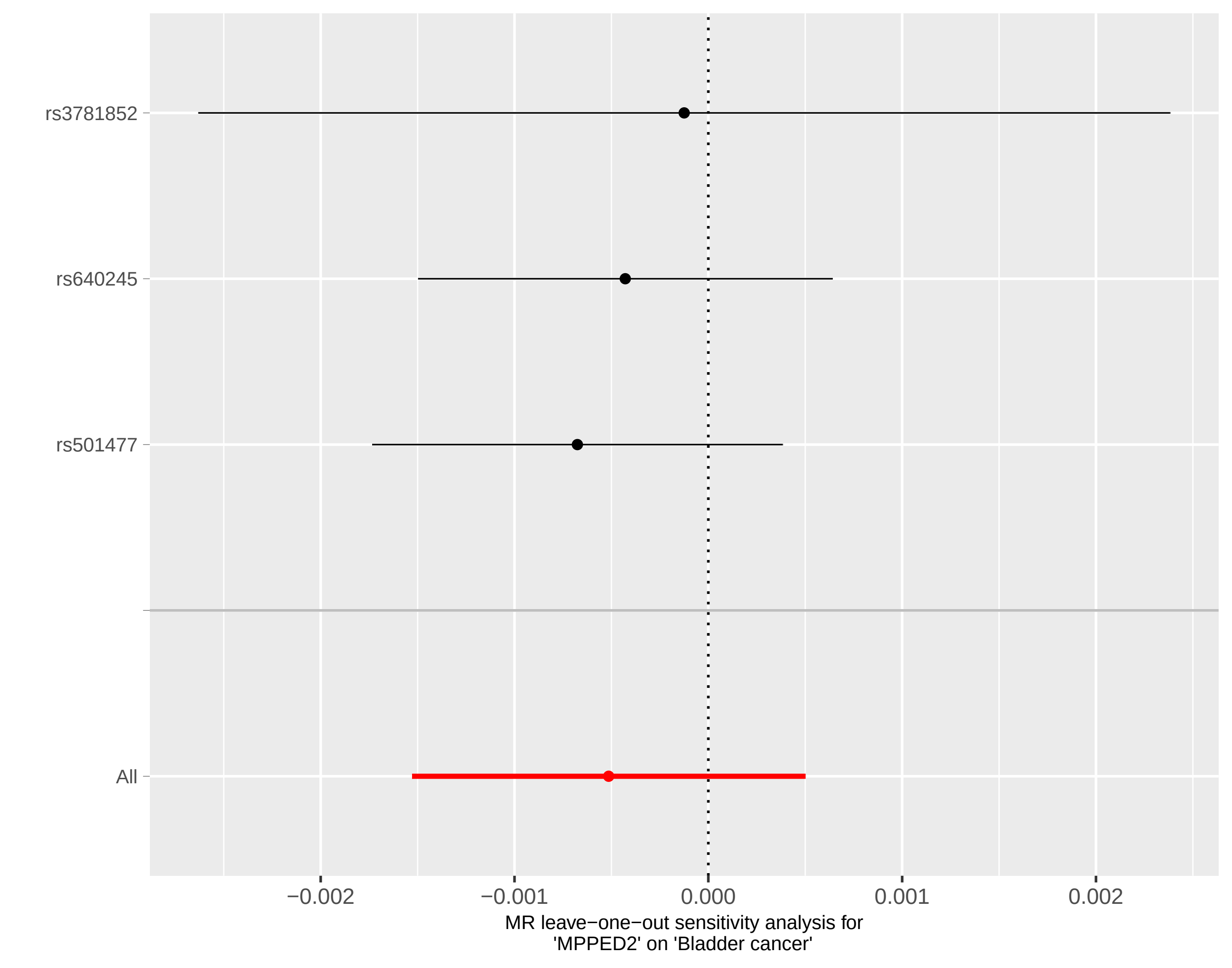
rs3781852
rs640245
rs501477
All
−0.002
−0.001
0.000
0.001
0.002
MR leave−one−out sensitivity analysis for
'MPPED2' on 'Bladder cancer'

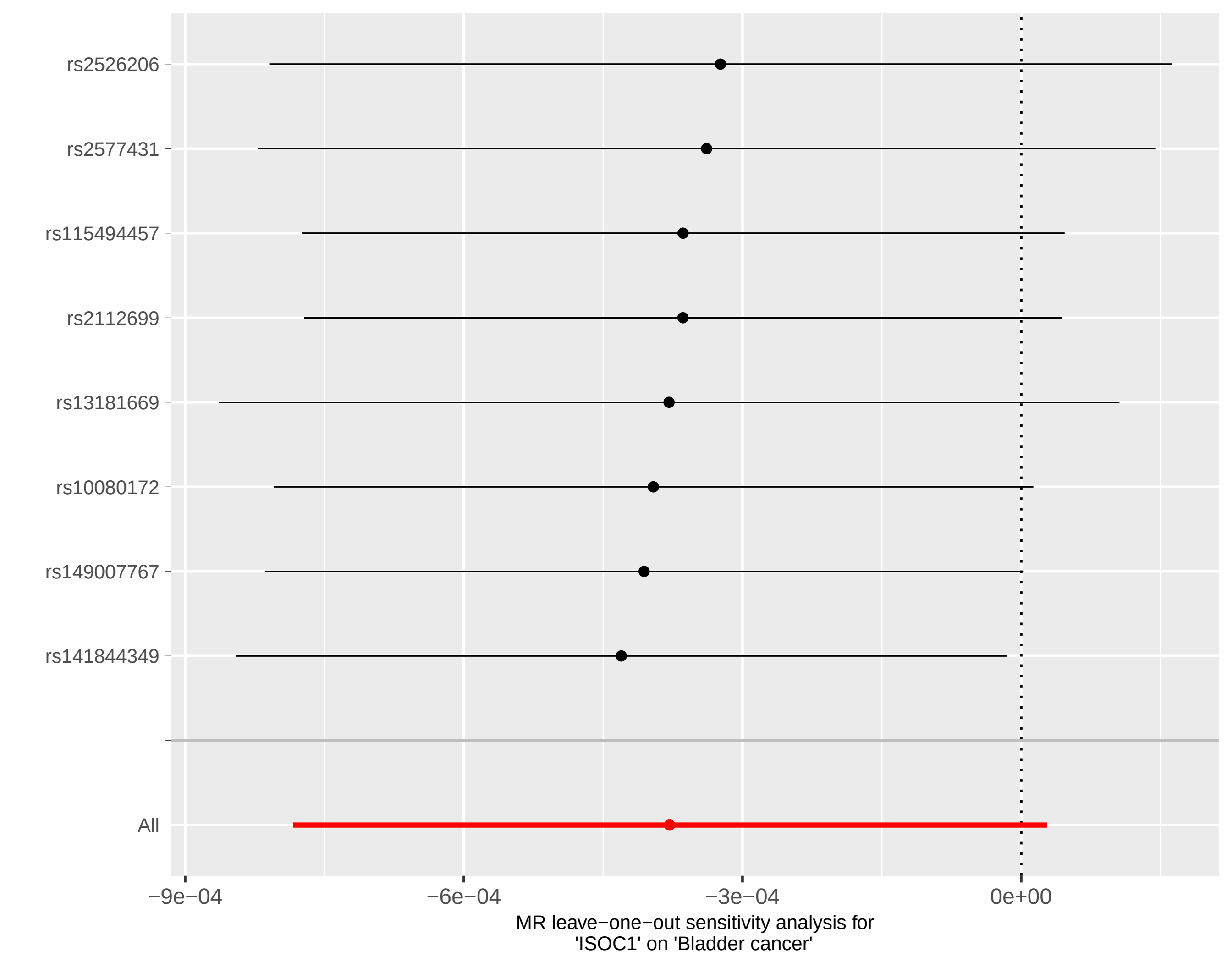
rs2526206
rs2577431
rs115494457
rs2112699
rs13181669
rs10080172
rs149007767
rs141844349
All
−9e−04
−6e−04
−3e−04
0e+00
MR leave−one−out sensitivity analysis for
'ISOC1' on 'Bladder cancer'

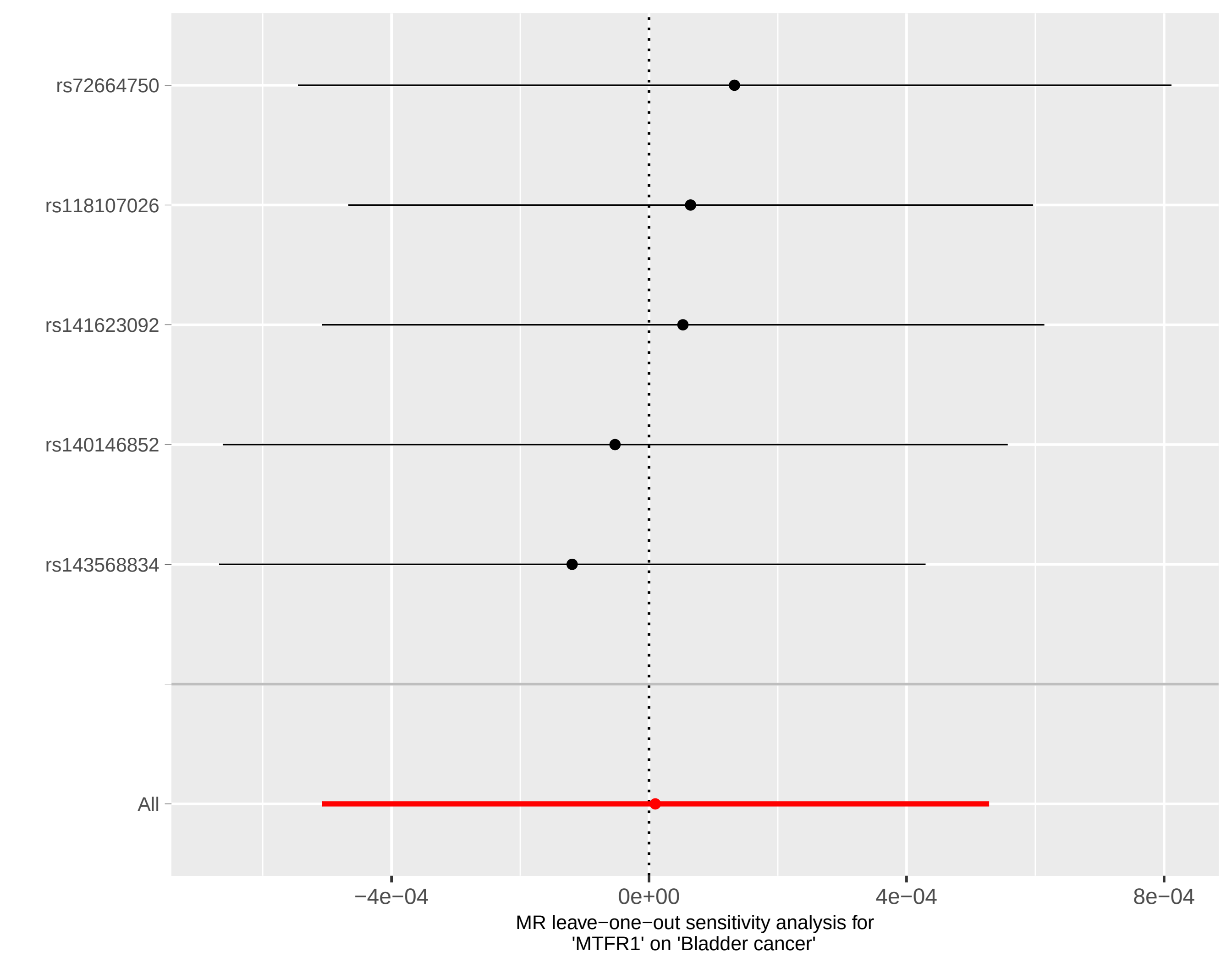
rs72664750
rs118107026
rs141623092
rs140146852
rs143568834
All
−4e−04
0e+00
4e−04
8e−04
MR leave−one−out sensitivity analysis for
'MTFR1' on 'Bladder cancer'

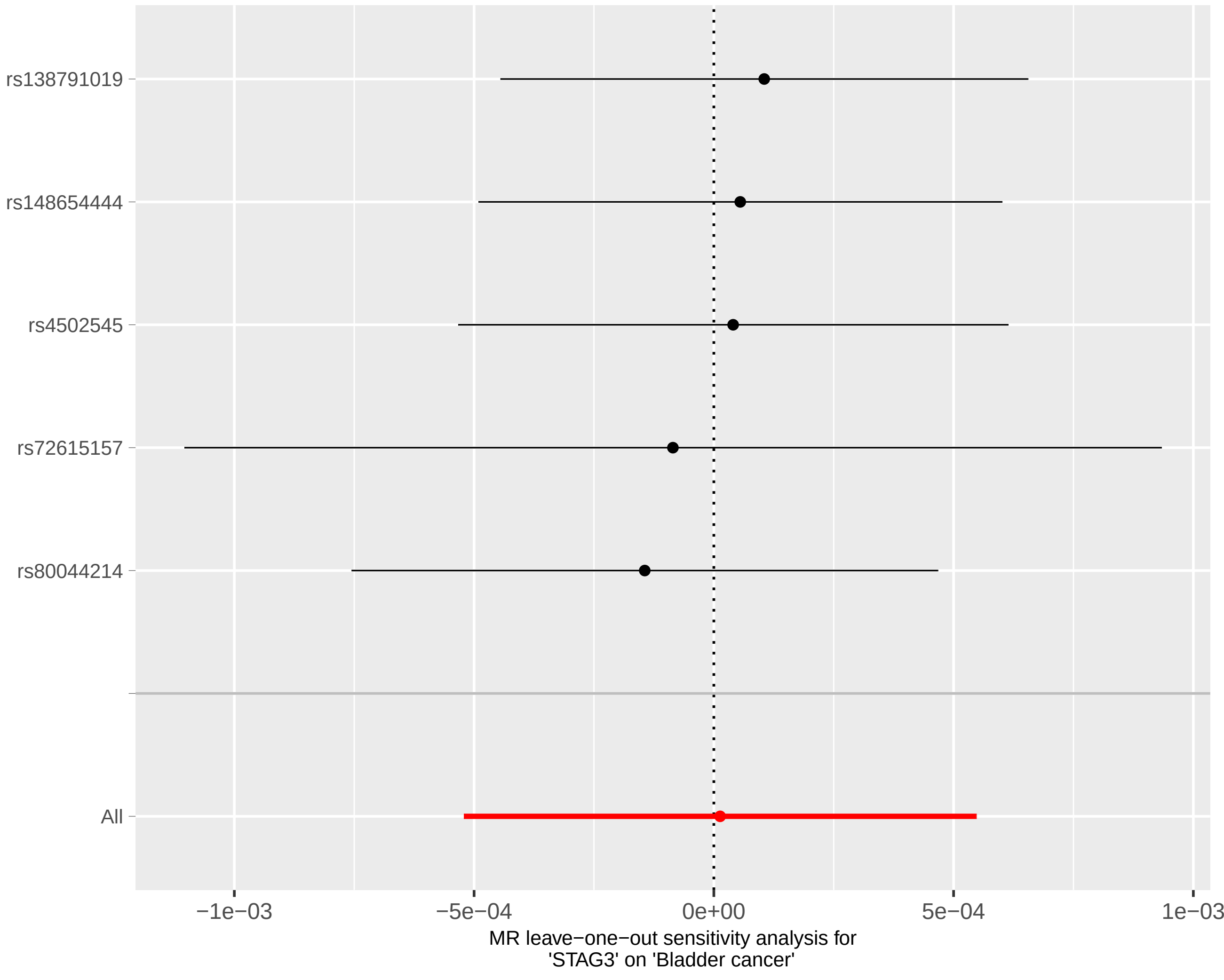

rs138791019
rs148654444
rs4502545
rs72615157
rs80044214
All
−1e−03
−5e−04
0e+00
5e−04
1e−03
MR leave−one−out sensitivity analysis for
'STAG3' on 'Bladder cancer'

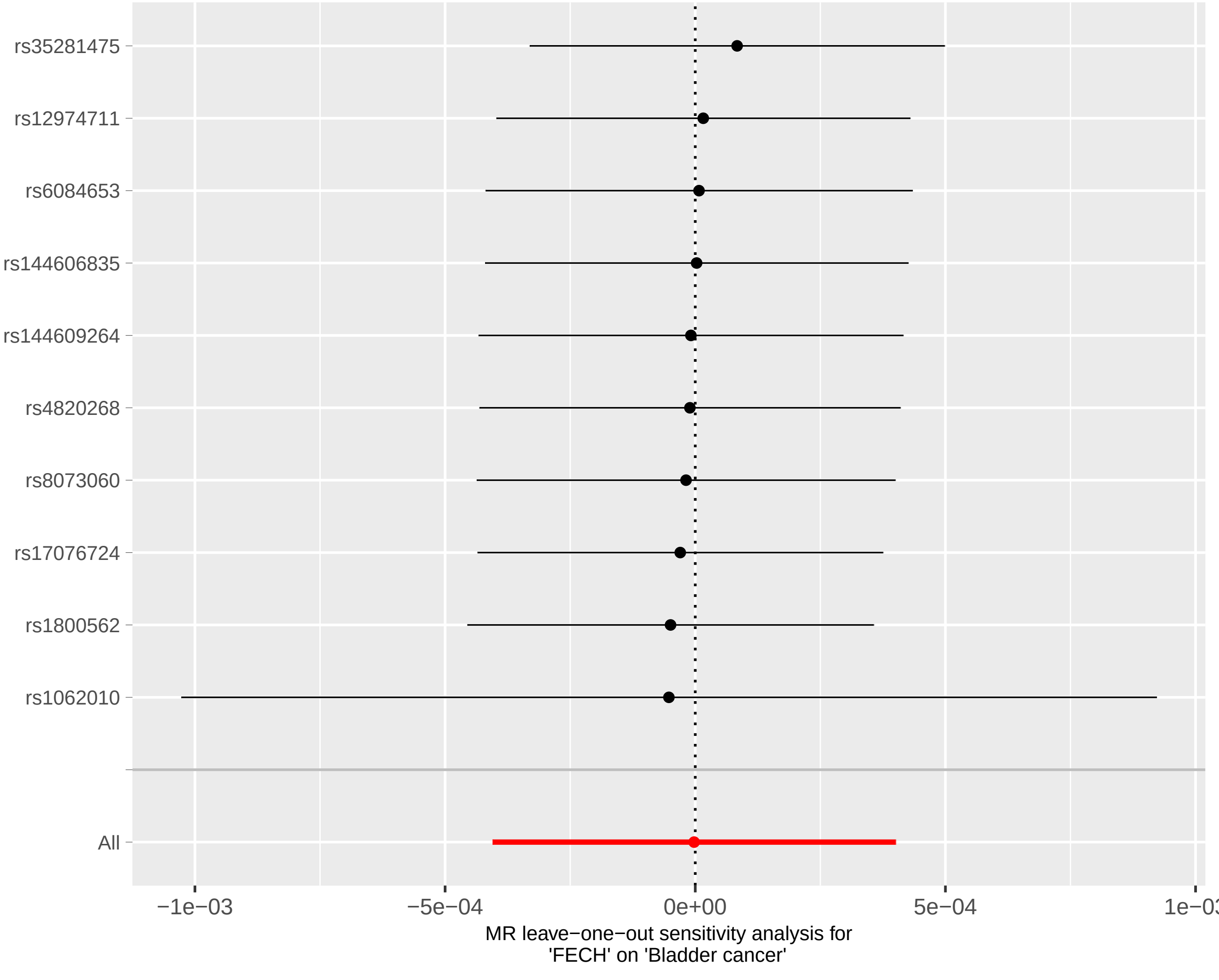

rs35281475
rs12974711
rs6084653
rs144606835
rs144609264
rs4820268
rs8073060
rs17076724
rs1800562
rs1062010
All
−1e−03
−5e−04
0e+00
5e−04
MR leave−one−out sensitivity analysis for
'FECH' on 'Bladder cancer'

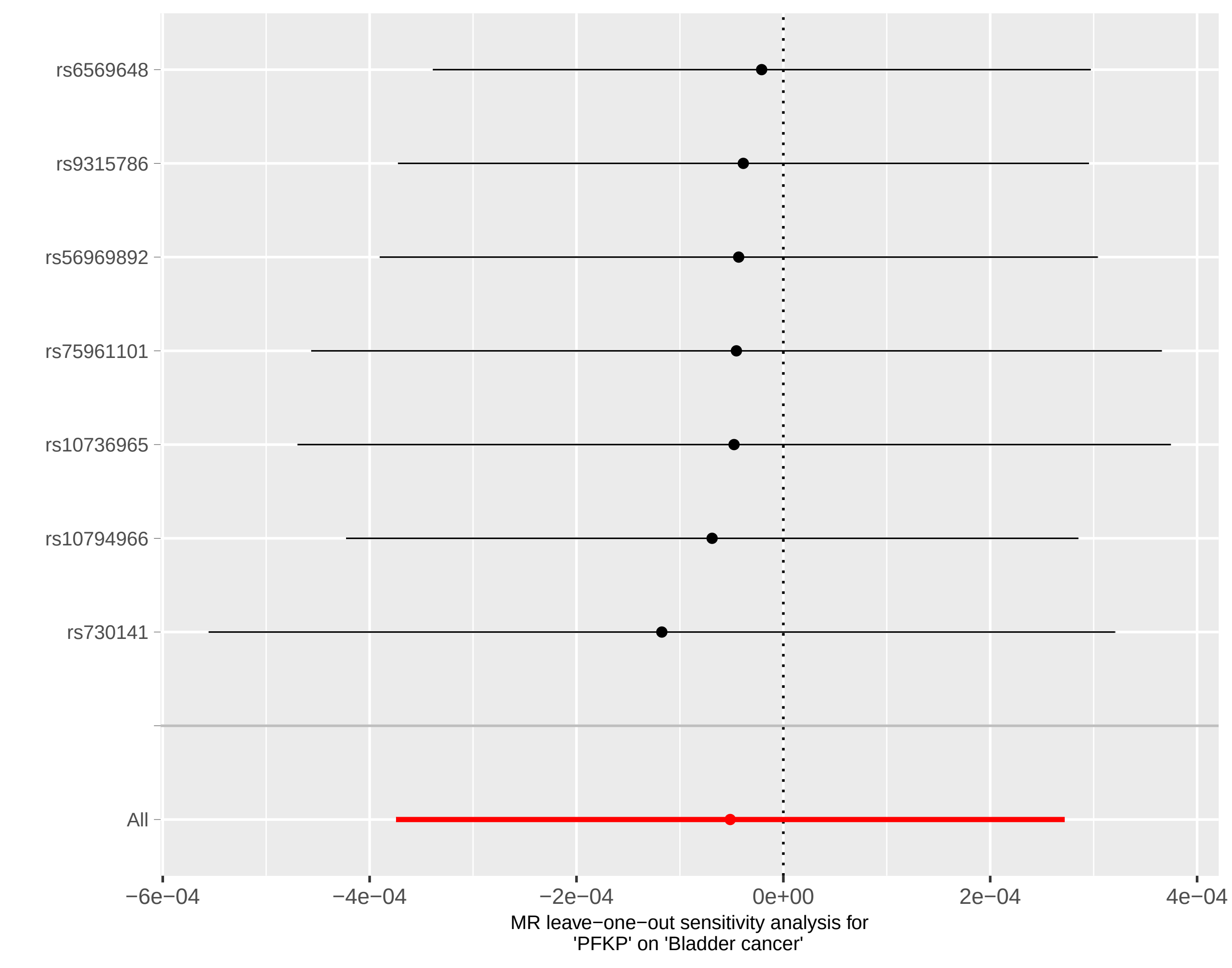
rs6569648
rs9315786
rs56969892
rs75961101
rs10736965
rs10794966
rs730141
All
−6e−04
−4e−04
−2e−04
0e+00
2e−04
4e−04
MR leave−one−out sensitivity analysis for
'PFKP' on 'Bladder cancer'

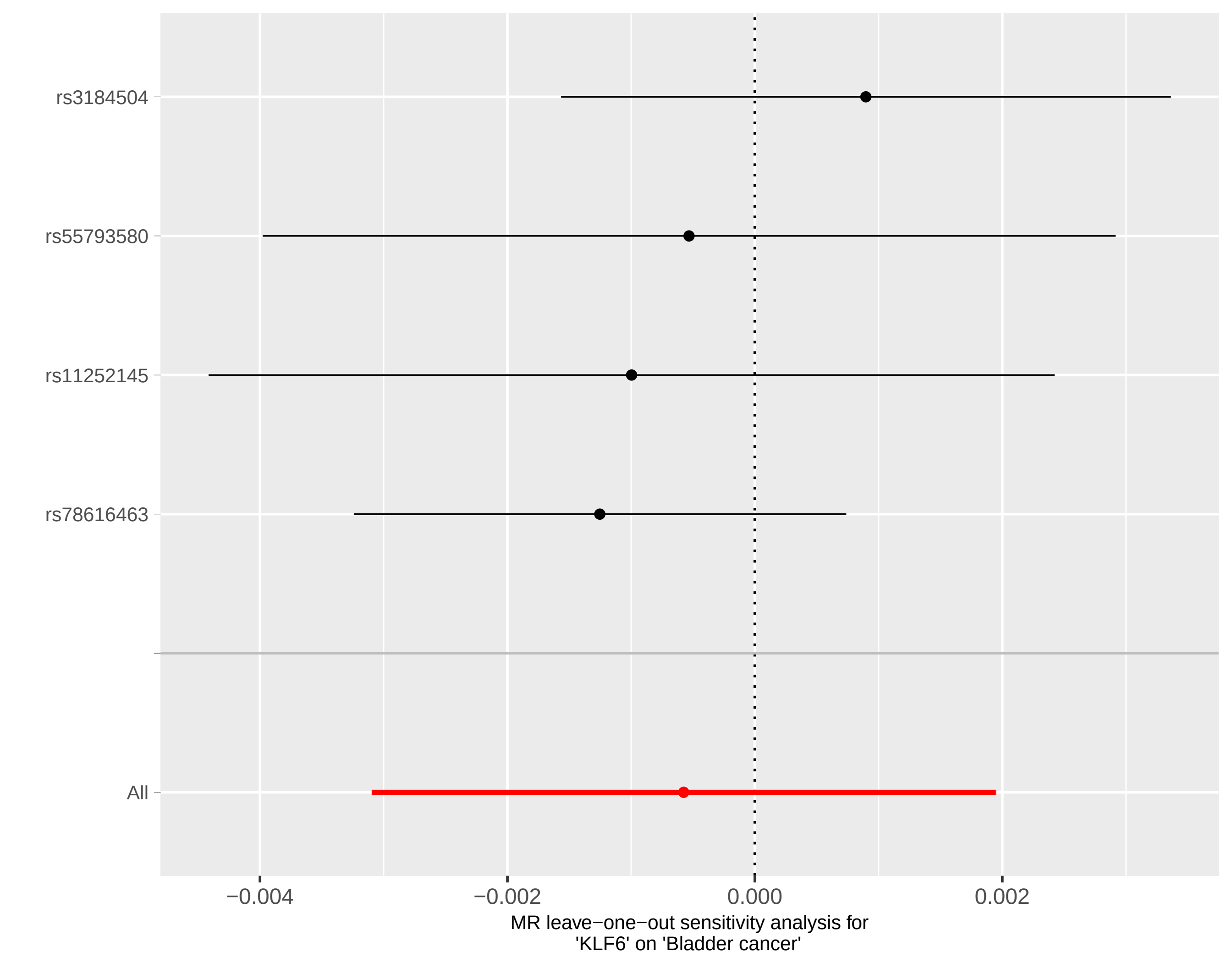
rs3184504
rs55793580
rs11252145
rs78616463
All
−0.004
−0.002
0.000
0.002
MR leave−one−out sensitivity analysis for
'KLF6' on 'Bladder cancer'

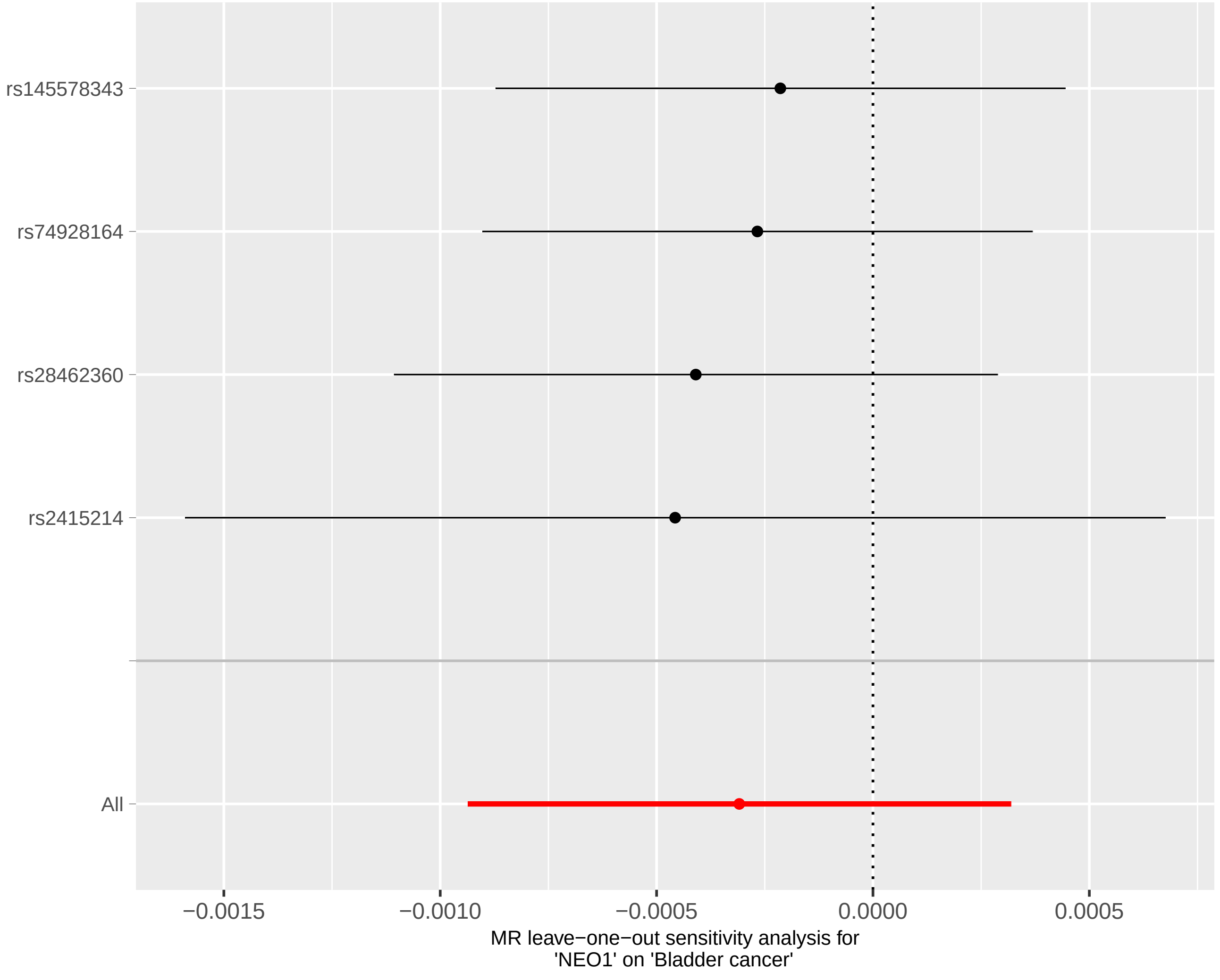
rs145578343
rs74928164
rs28462360
rs2415214
All
−0.0015
−0.0010
−0.0005
0.0000
0.0005
MR leave−one−out sensitivity analysis for
'NEO1' on 'Bladder cancer'

Insufficient number of SNPs

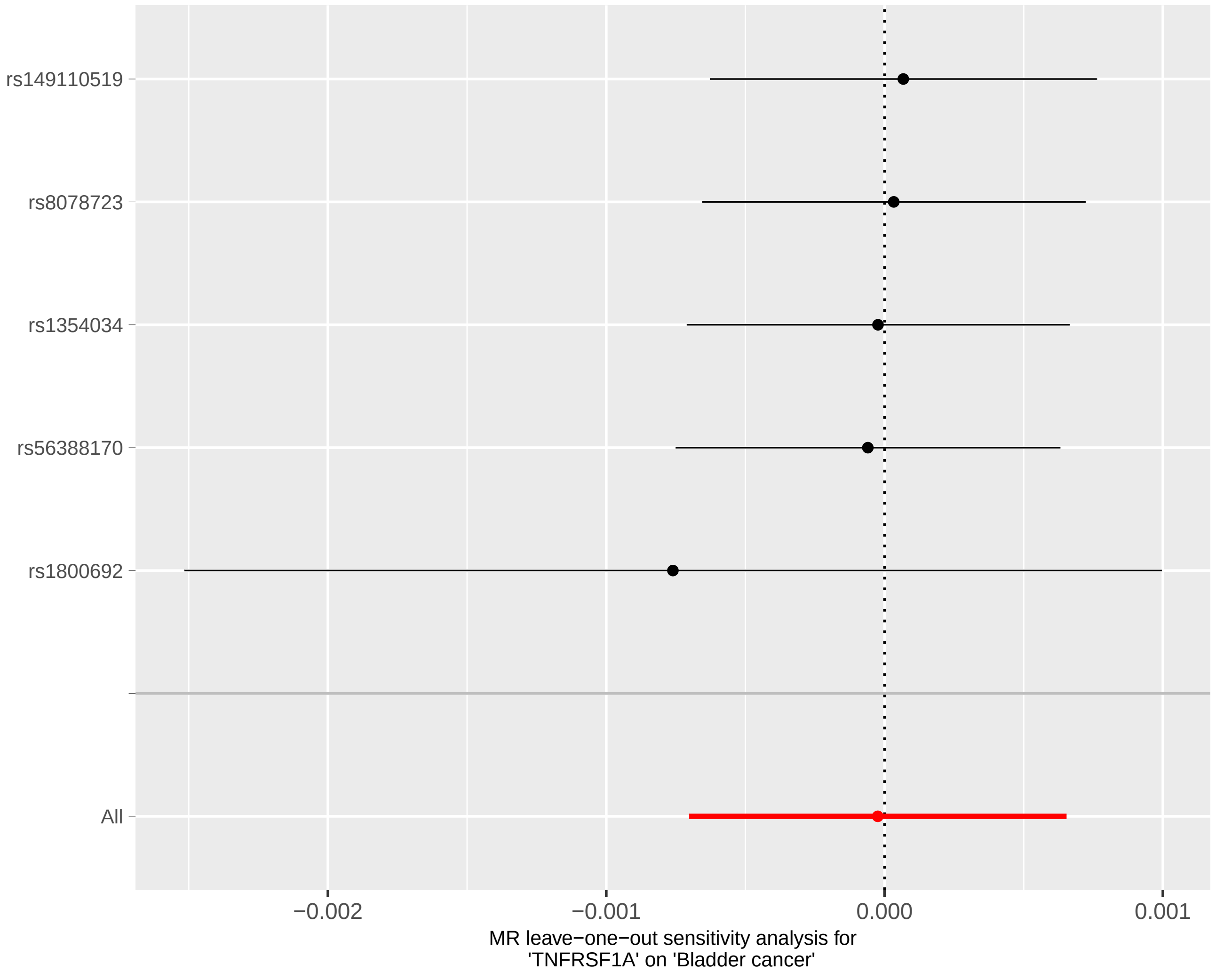

rs149110519
rs8078723
rs1354034
rs56388170
rs1800692
All
−0.002
−0.001
0.000
0.001
MR leave−one−out sensitivity analysis for
'TNFRSF1A' on 'Bladder cancer'

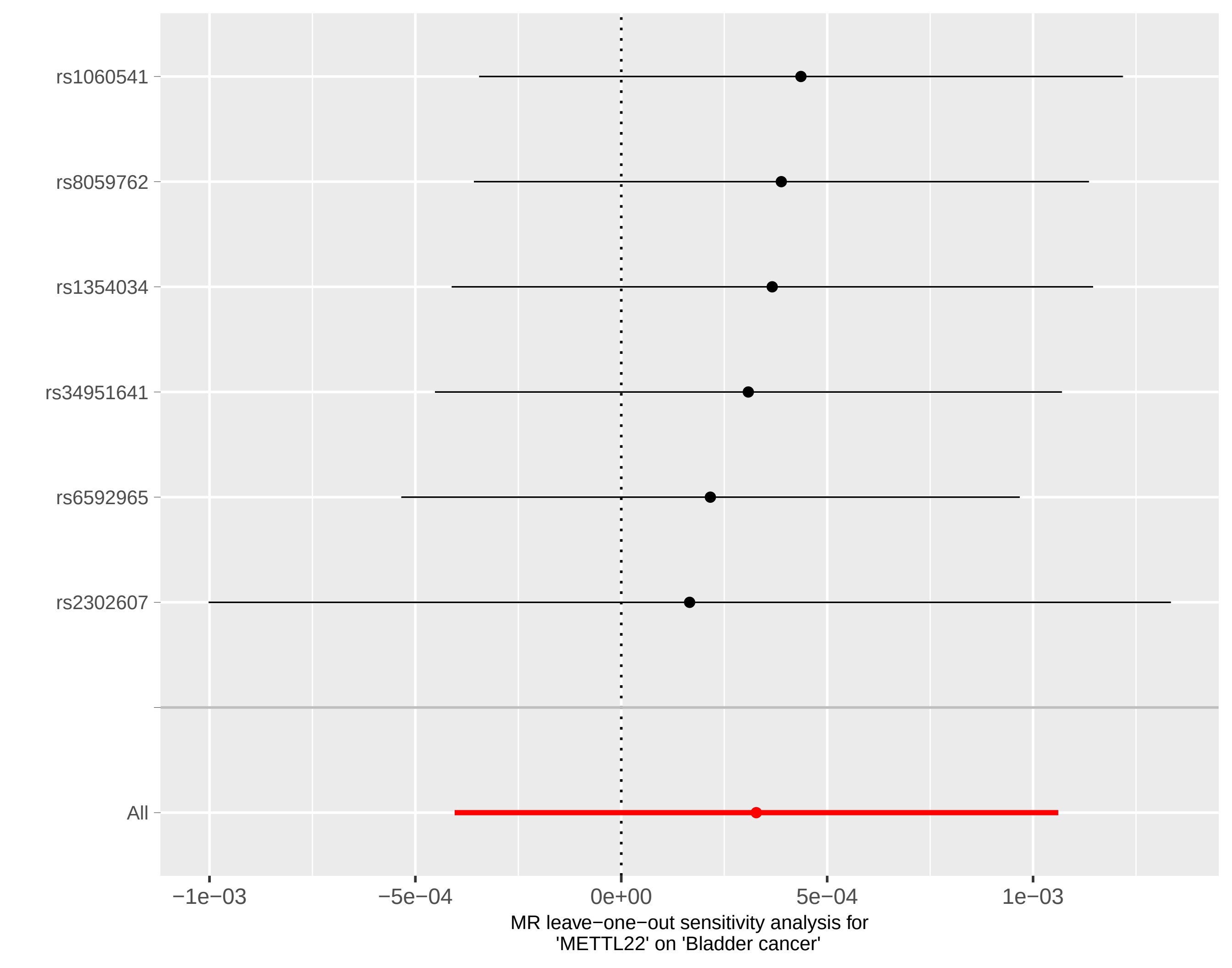
rs1060541
rs8059762
rs1354034
rs34951641
rs6592965
rs2302607
All
−1e−03
−5e−04
0e+00
5e−04
1e−03
MR leave−one−out sensitivity analysis for
'METTL22' on 'Bladder cancer'

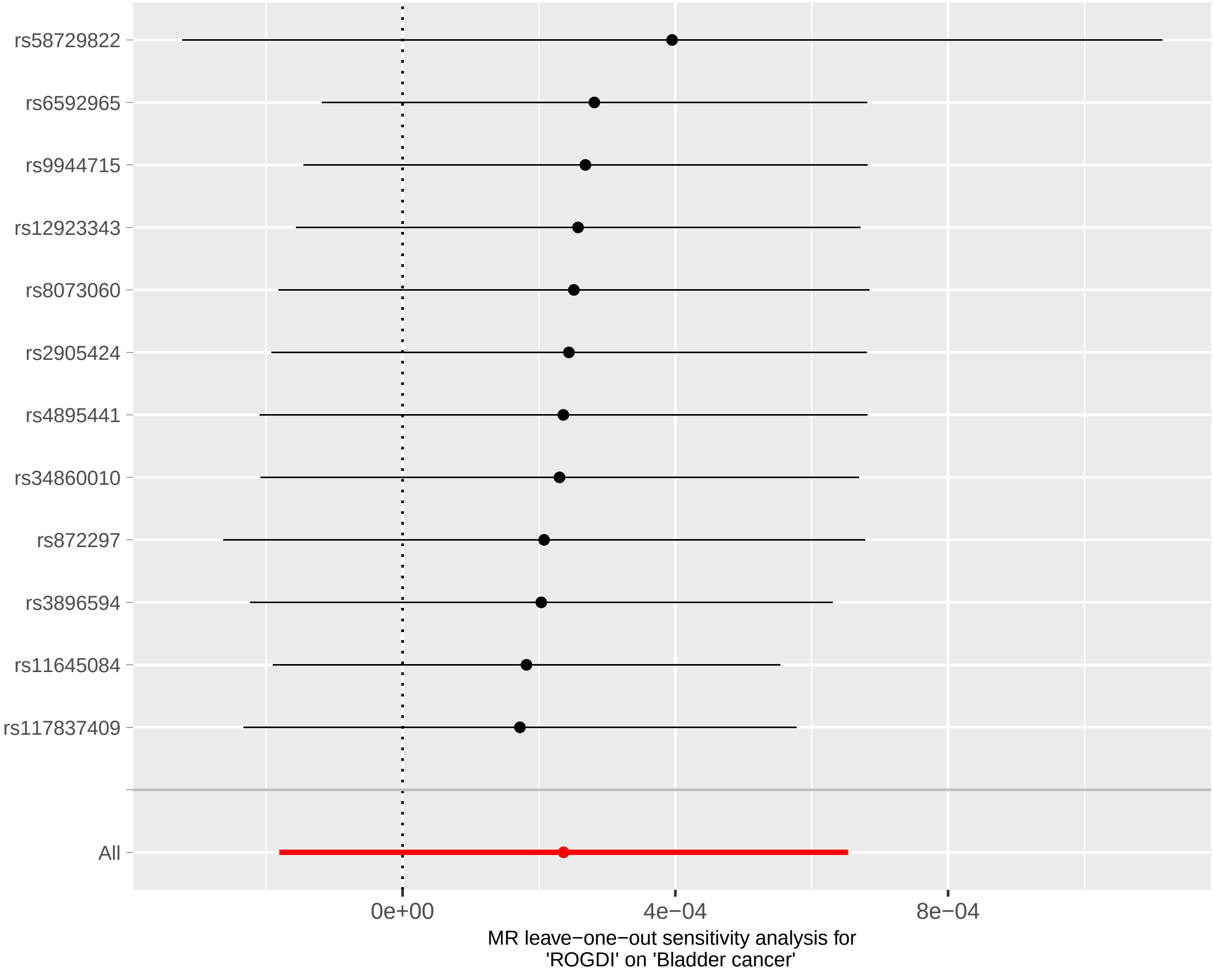
rs58729822
rs6592965
rs9944715
rs12923343
rs8073060
rs2905424
rs4895441
rs34860010
rs872297
rs3896594
rs11645084
rs117837409
All
0e+00
4e−04
8e−04
MR leave−one−out sensitivity analysis for
'ROGDI' on 'Bladder cancer'

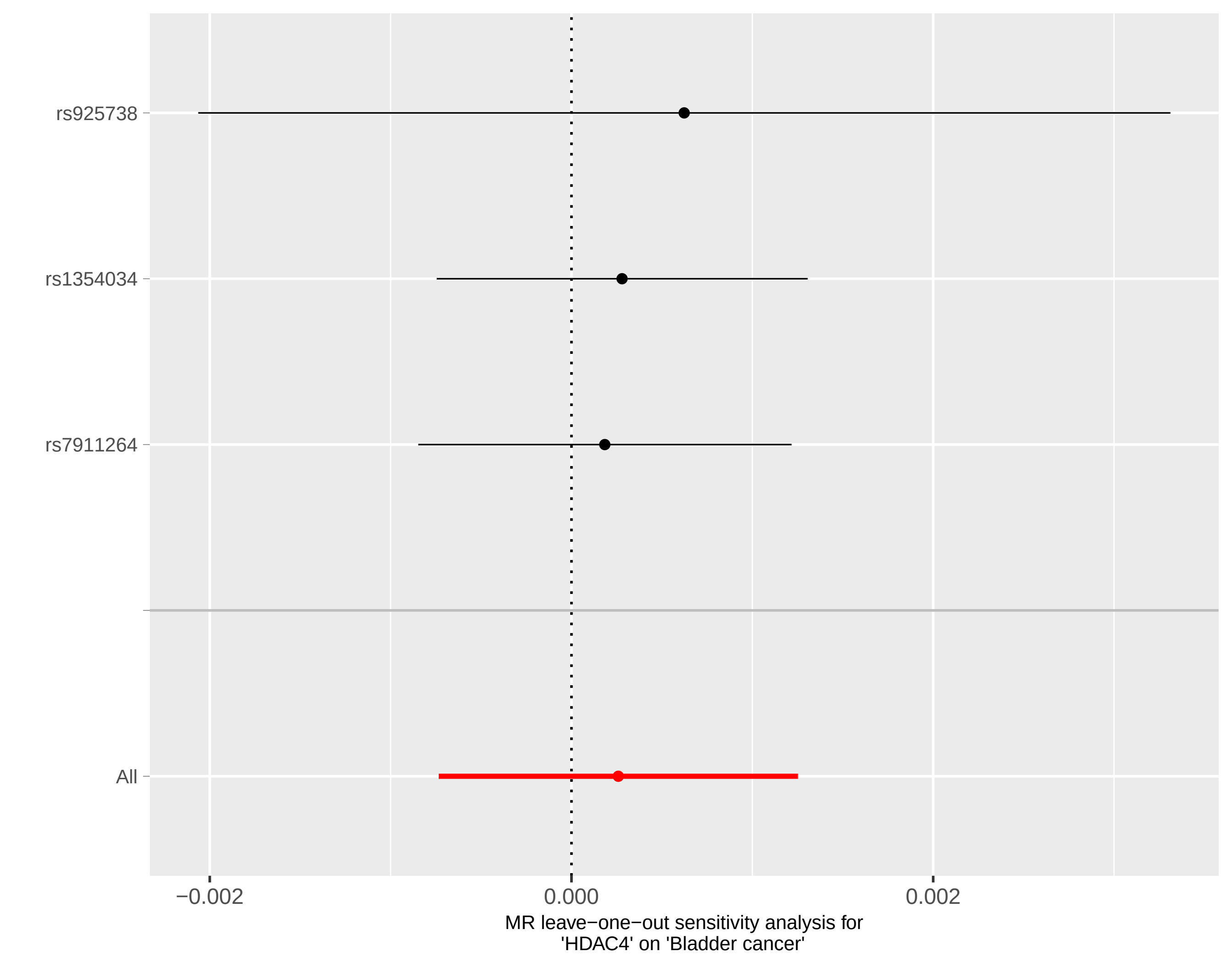
rs925738
rs1354034
rs7911264
All
−0.002
0.000
0.002
MR leave−one−out sensitivity analysis for
'HDAC4' on 'Bladder cancer'

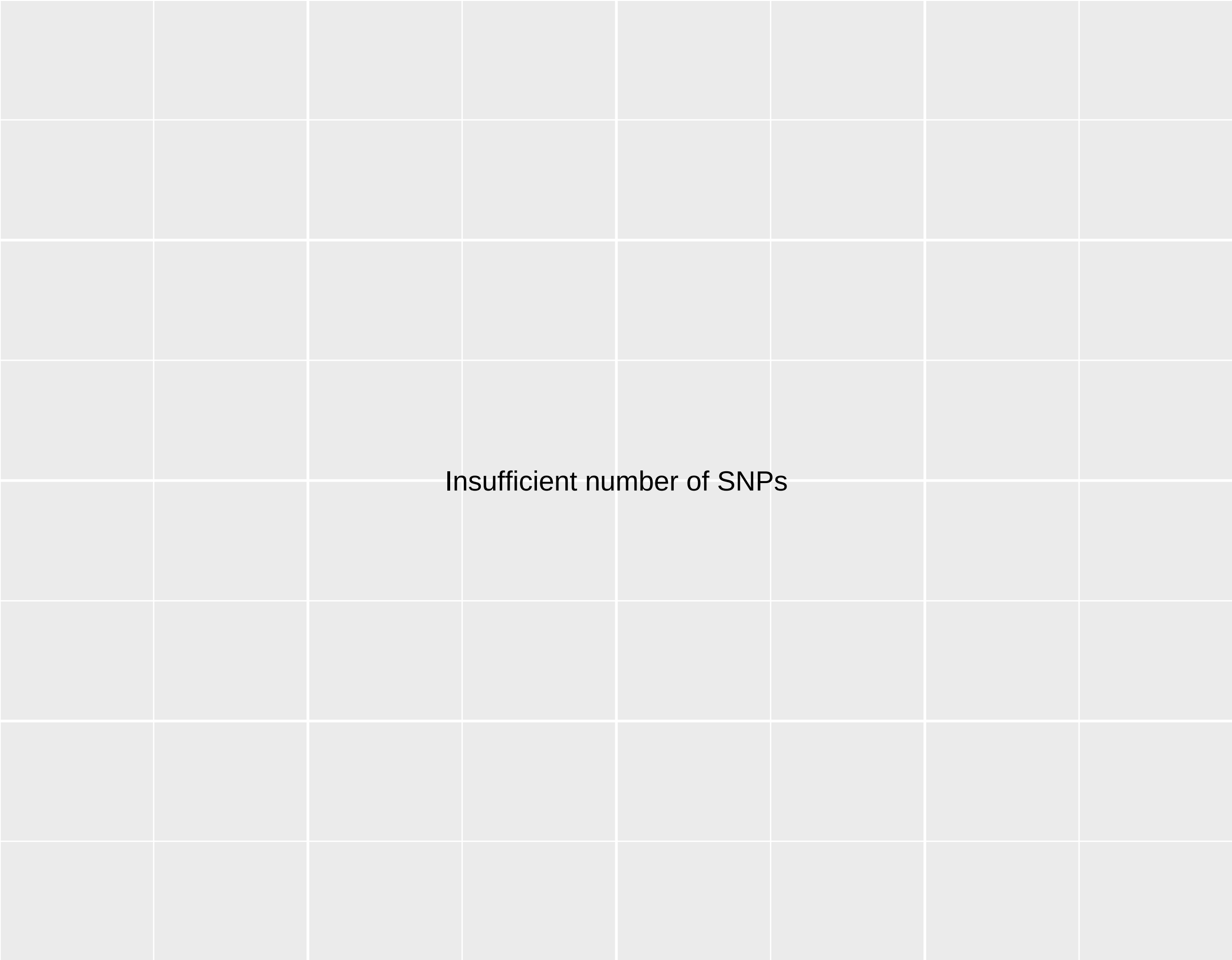
Insufficient number of SNPs

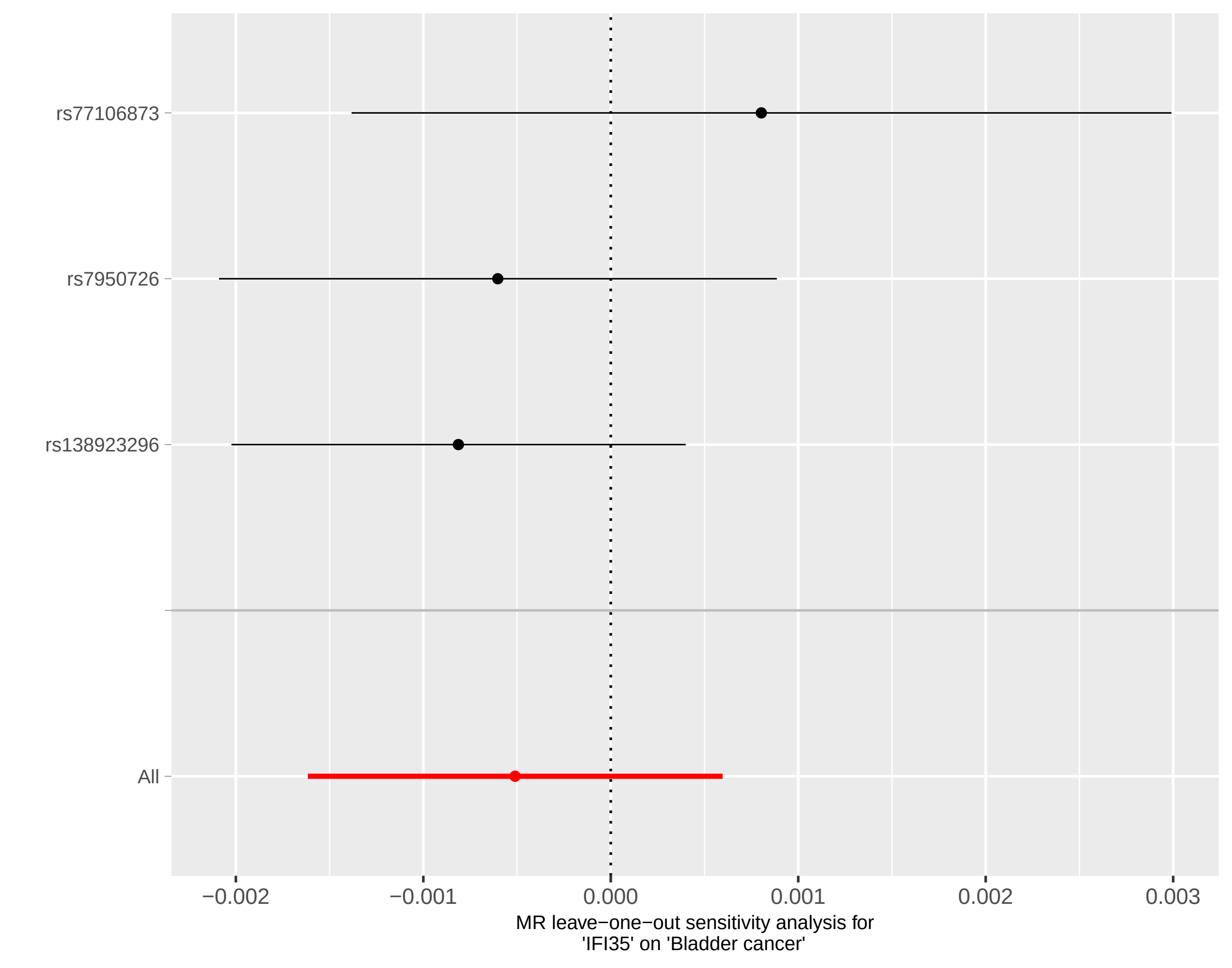
rs77106873
rs7950726
rs138923296
All
−0.002
−0.001
0.000
0.001
0.002
0.003
MR leave−one−out sensitivity analysis for
'IFI35' on 'Bladder cancer'

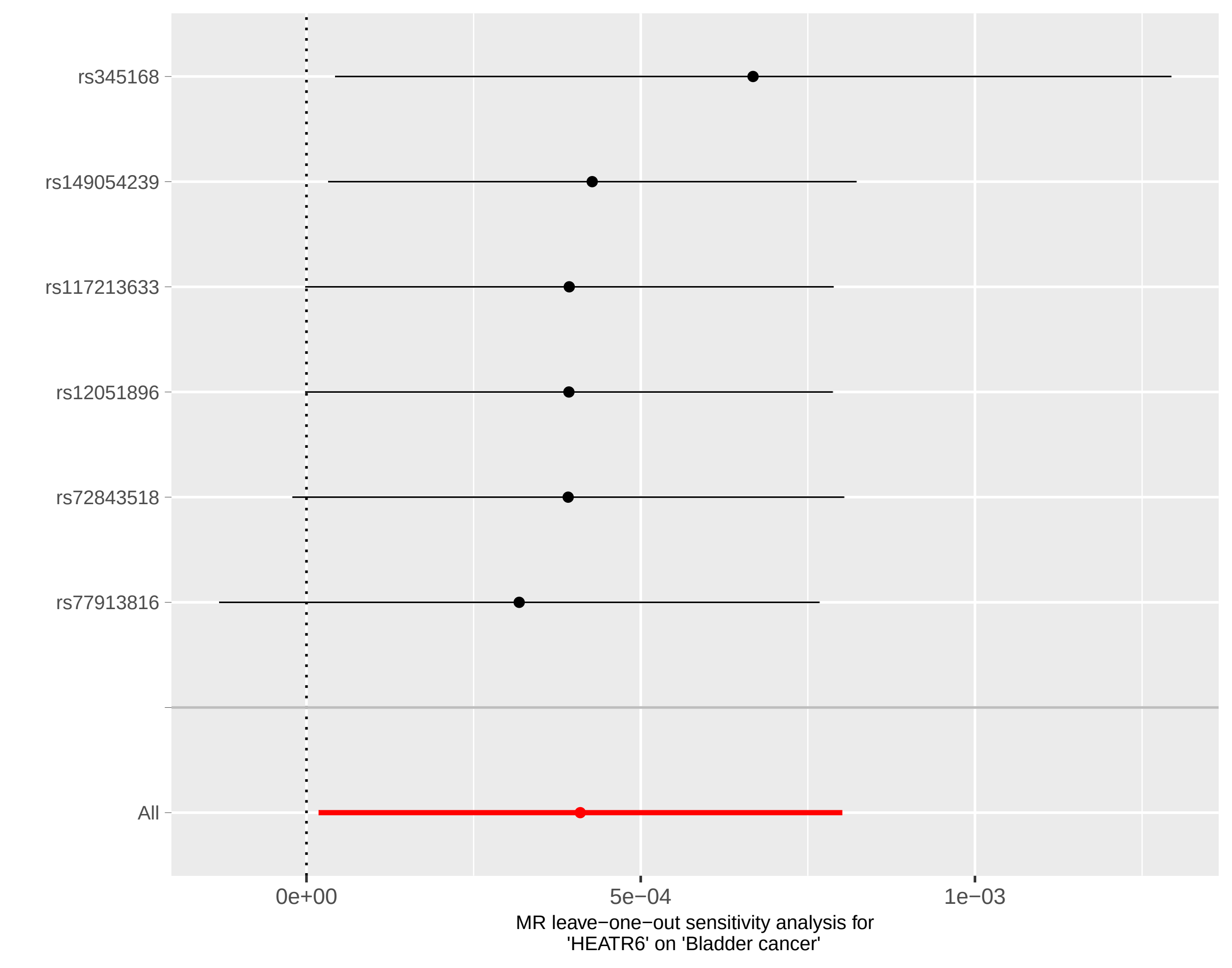
rs345168
rs149054239
rs117213633
rs12051896
rs72843518
rs77913816
All
0e+00
5e−04
1e−03
MR leave−one−out sensitivity analysis for
'HEATR6' on 'Bladder cancer'

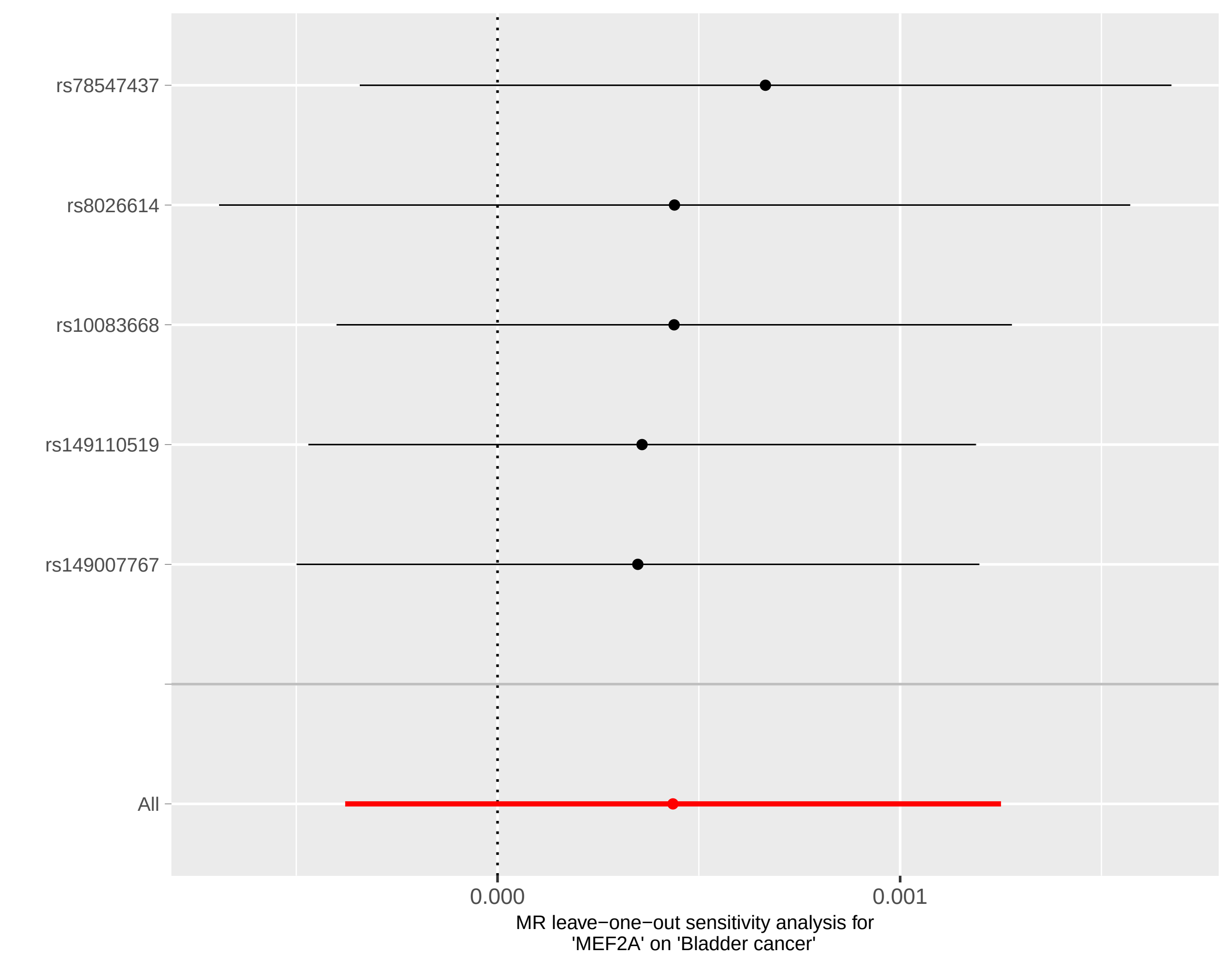
rs78547437
rs8026614
rs10083668
rs149110519
rs149007767
All
0.000
0.001
MR leave−one−out sensitivity analysis for
'MEF2A' on 'Bladder cancer'

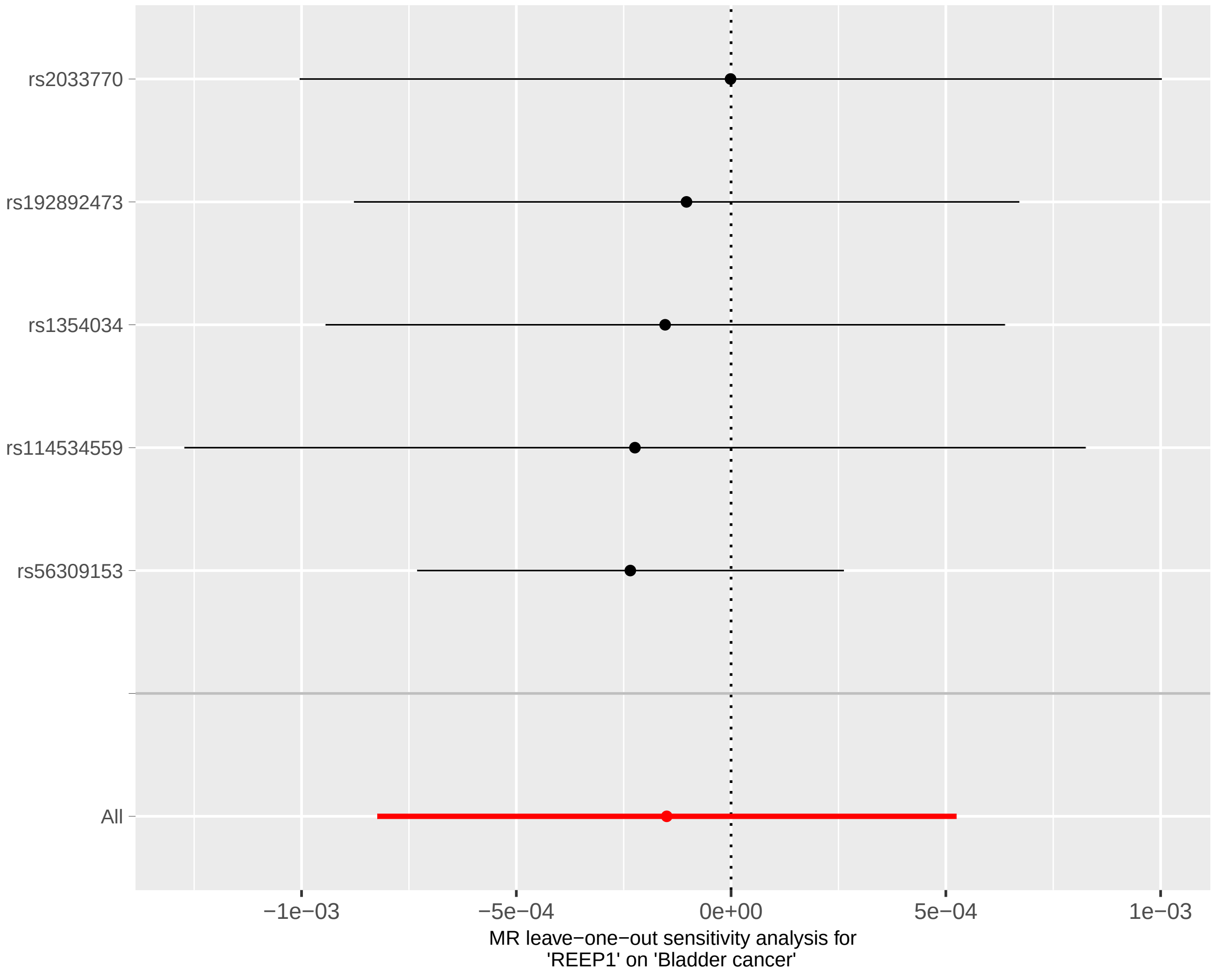
rs2033770
rs192892473
rs1354034
rs114534559
rs56309153
All
−1e−03
−5e−04
0e+00
5e−04
1e−03
MR leave−one−out sensitivity analysis for
'REEP1' on 'Bladder cancer'

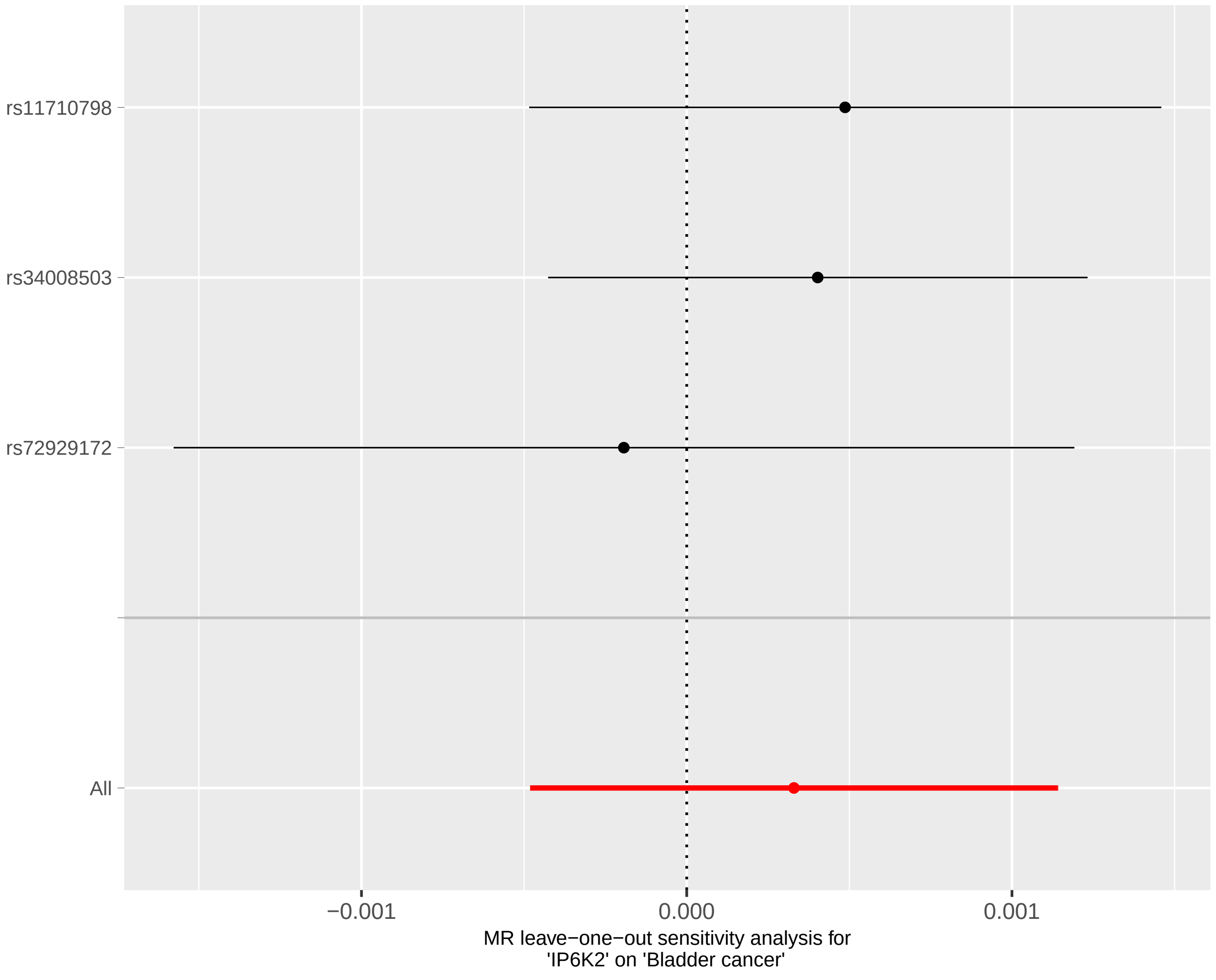
rs11710798
rs34008503
rs72929172
All
−0.001
0.000
0.001
MR leave−one−out sensitivity analysis for
'IP6K2' on 'Bladder cancer'

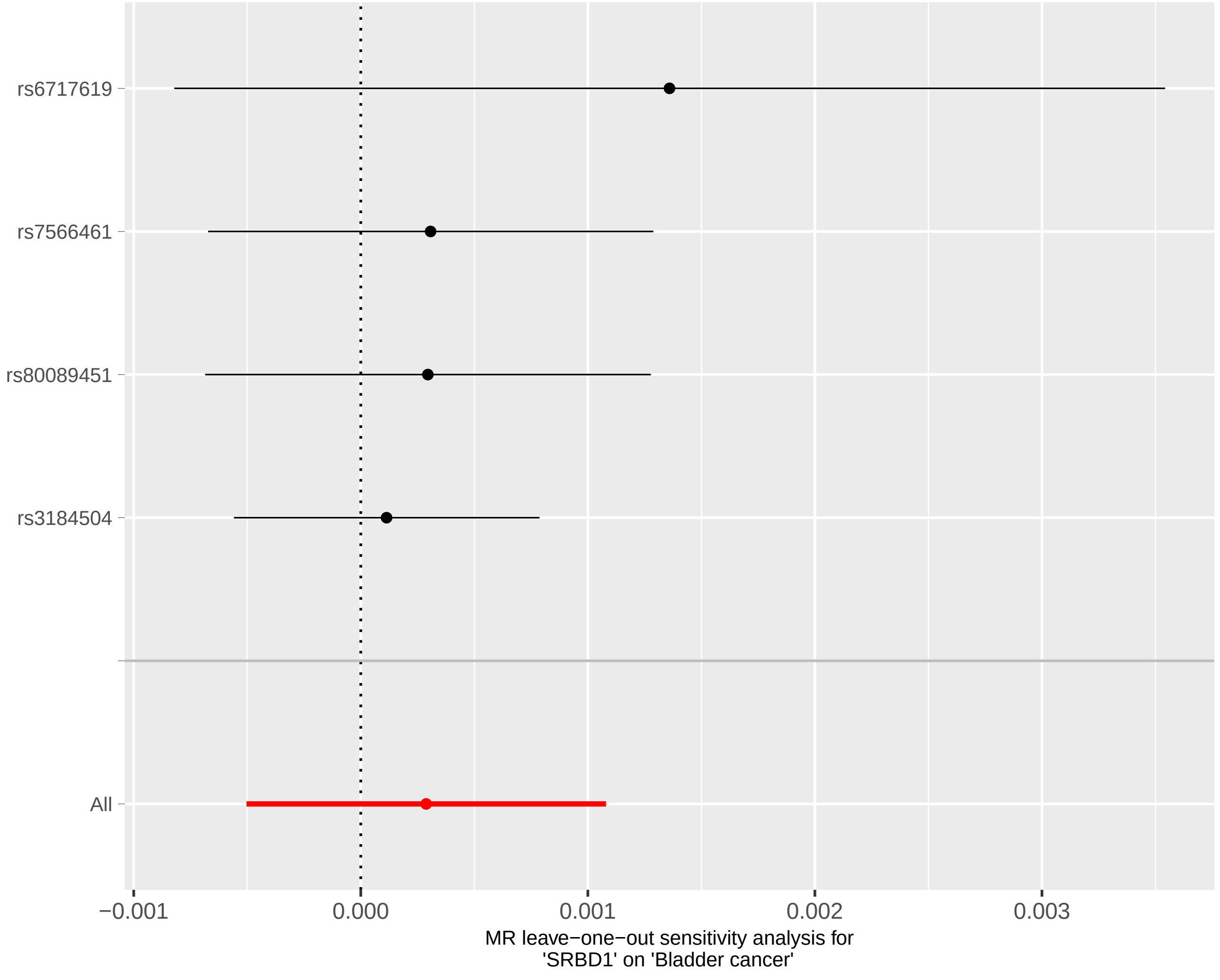
rs6717619
rs7566461
rs80089451
rs3184504
All
−0.001
0.000
0.001
0.002
0.003
MR leave−one−out sensitivity analysis for
'SRBD1' on 'Bladder cancer'

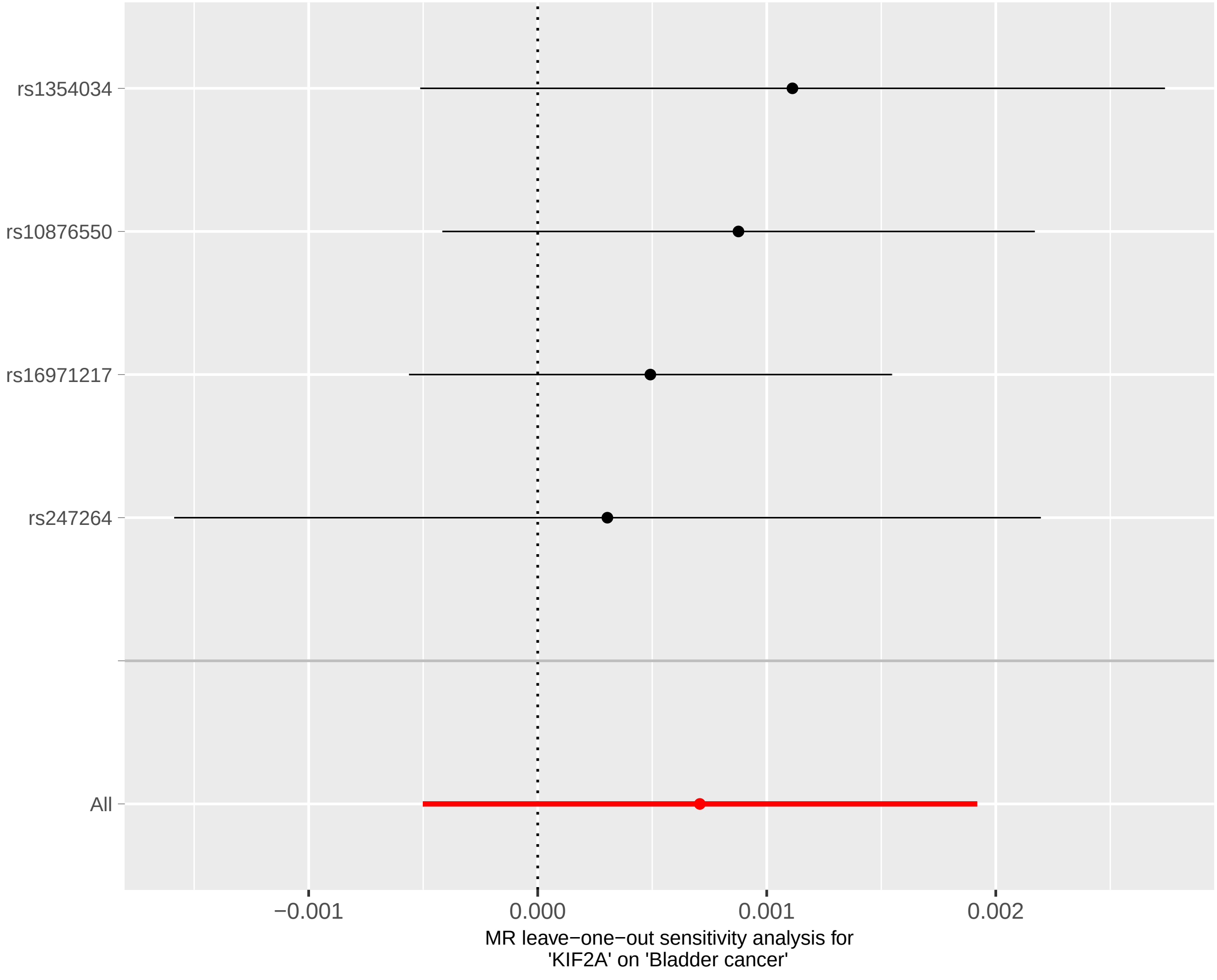

rs1354034
rs10876550
rs16971217
rs247264
All
−0.001
0.000
0.001
0.002
MR leave−one−out sensitivity analysis for
'KIF2A' on 'Bladder cancer'

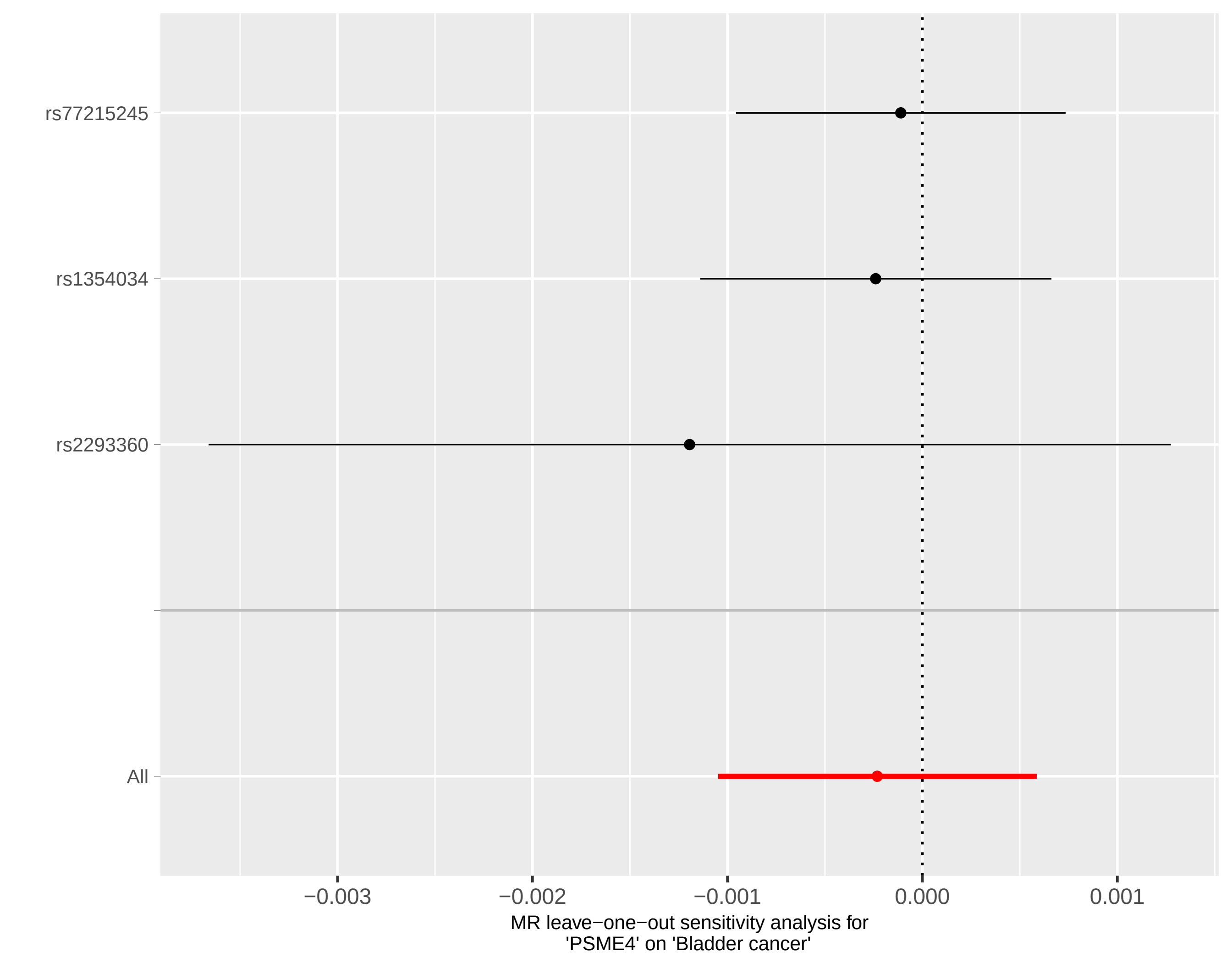
rs77215245
rs1354034
rs2293360
All
−0.003
−0.002
−0.001
0.000
0.001
MR leave−one−out sensitivity analysis for
'PSME4' on 'Bladder cancer'

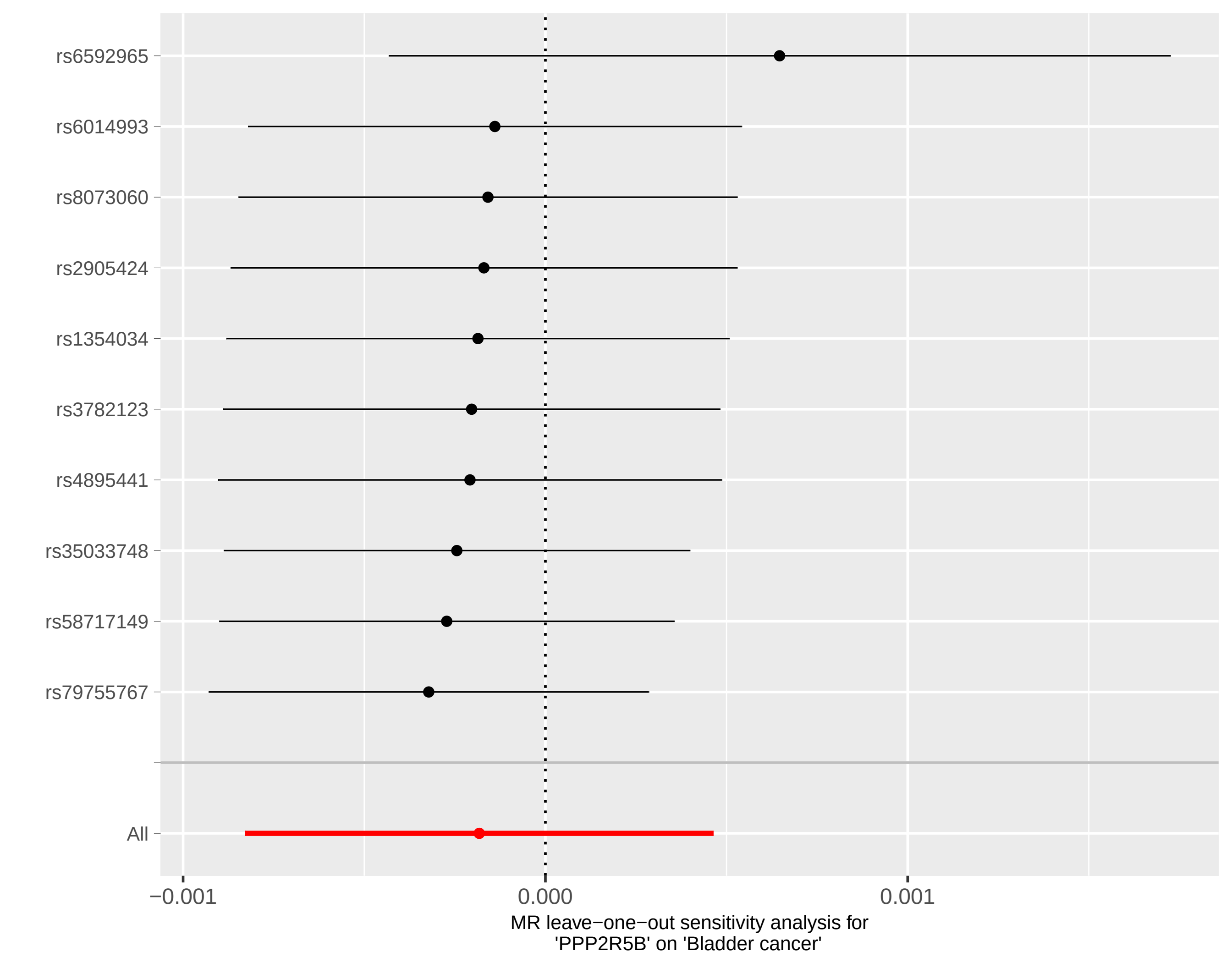
rs6592965
rs6014993
rs8073060
rs2905424
rs1354034
rs3782123
rs4895441
rs35033748
rs58717149
rs79755767
All
−0.001
0.000
0.001
MR leave−one−out sensitivity analysis for
'PPP2R5B' on 'Bladder cancer'

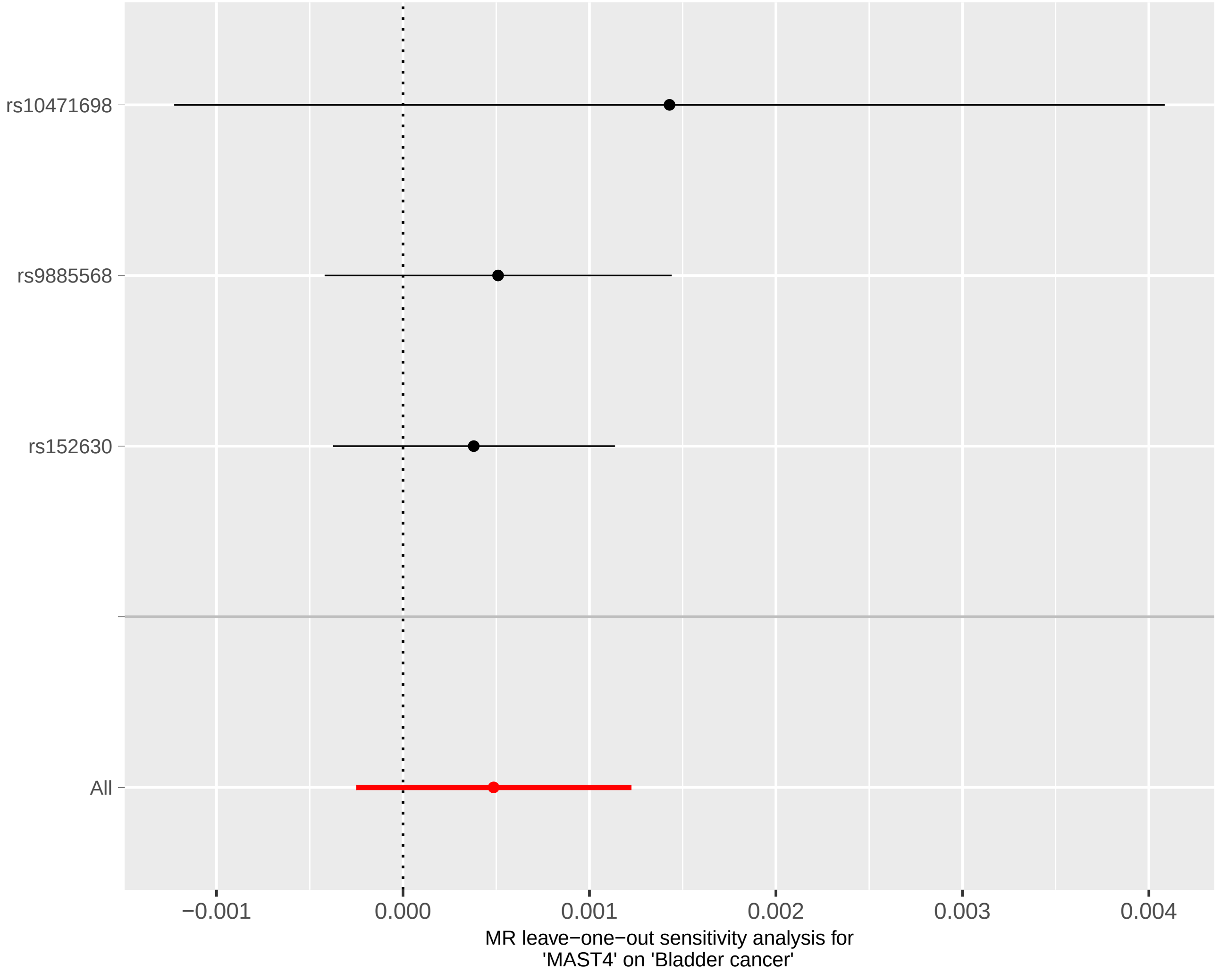
rs10471698
rs9885568
rs152630
All
−0.001
0.000
0.001
0.002
0.003
0.004
MR leave−one−out sensitivity analysis for
'MAST4' on 'Bladder cancer'

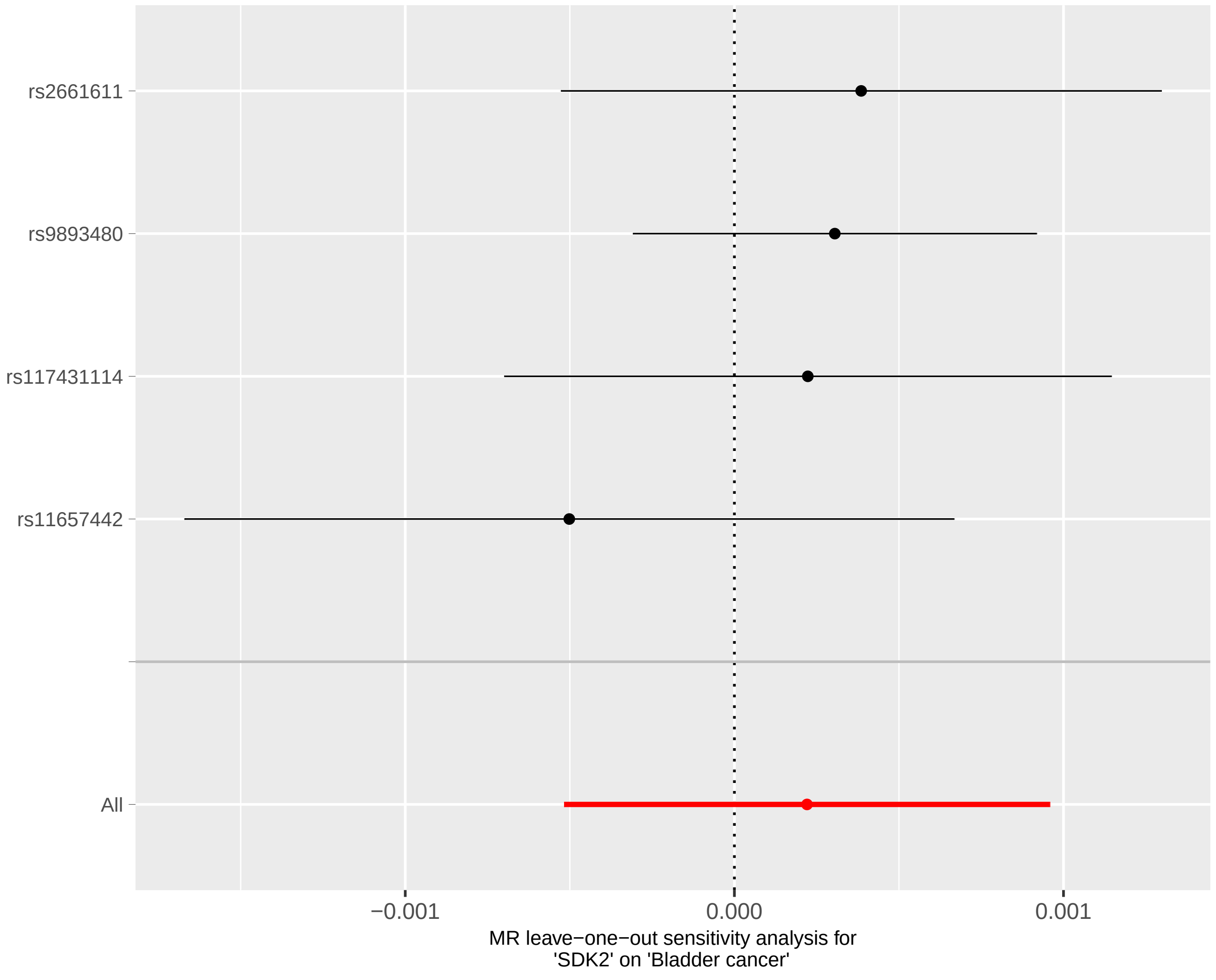
rs2661611
rs9893480
rs117431114
rs11657442
All
−0.001
0.000
0.001
MR leave−one−out sensitivity analysis for
'SDK2' on 'Bladder cancer'

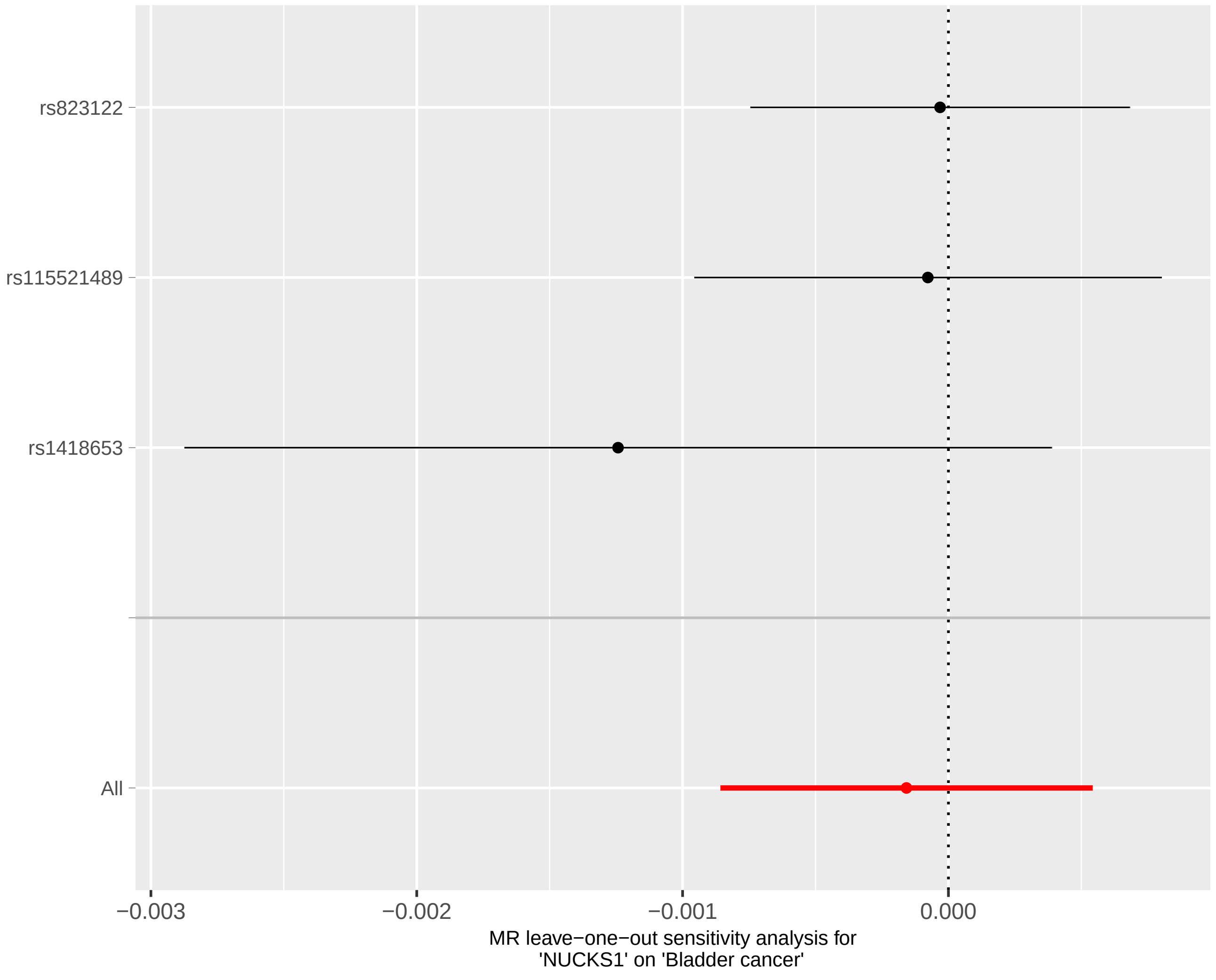
rs823122
rs115521489
rs1418653
All
−0.003
−0.002
−0.001
0.000
MR leave−one−out sensitivity analysis for
'NUCKS1' on 'Bladder cancer'

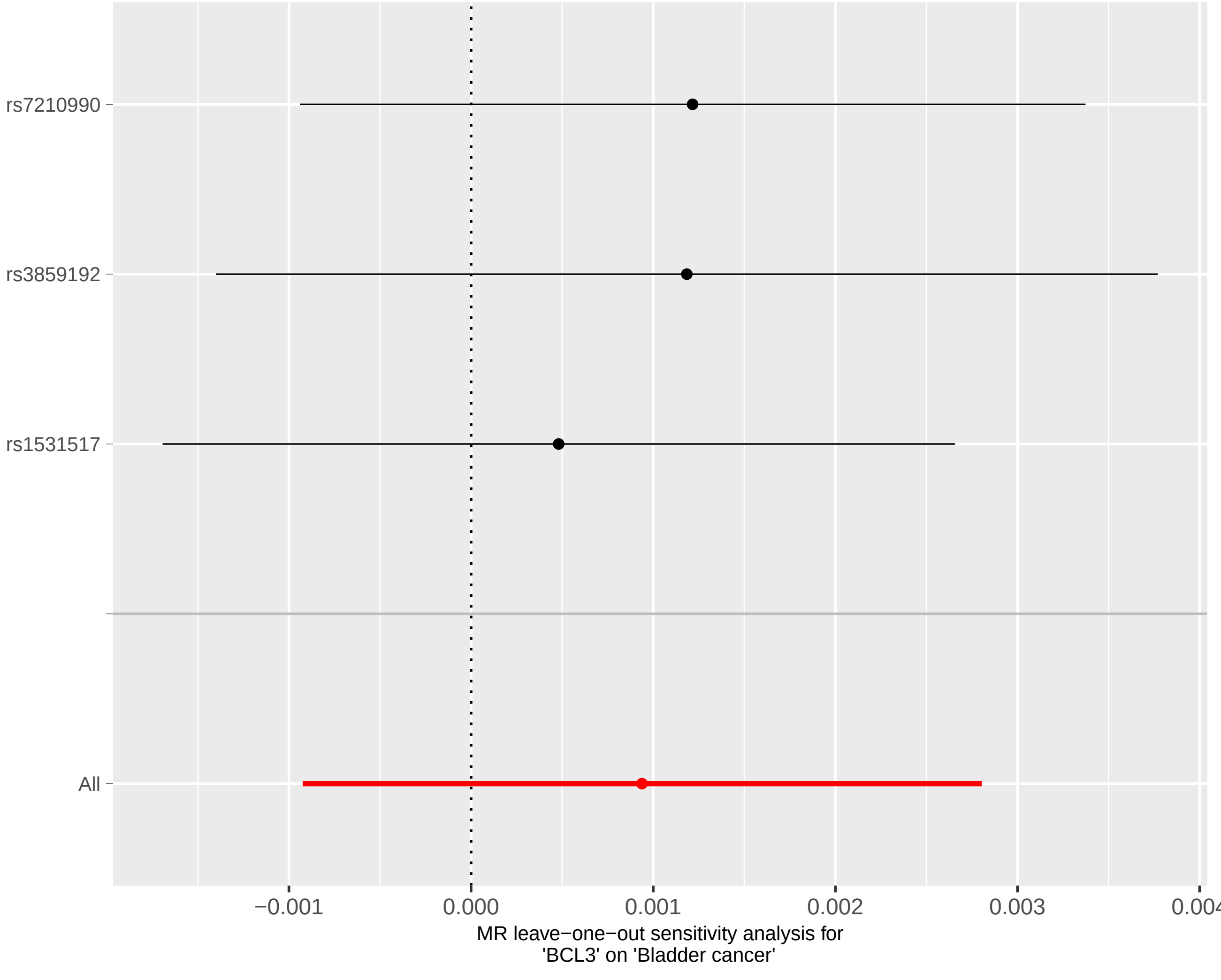
rs7210990
rs3859192
rs1531517
All
−0.001
0.000
0.001
0.002
0.003
0.004
MR leave−one−out sensitivity analysis for
'BCL3' on 'Bladder cancer'

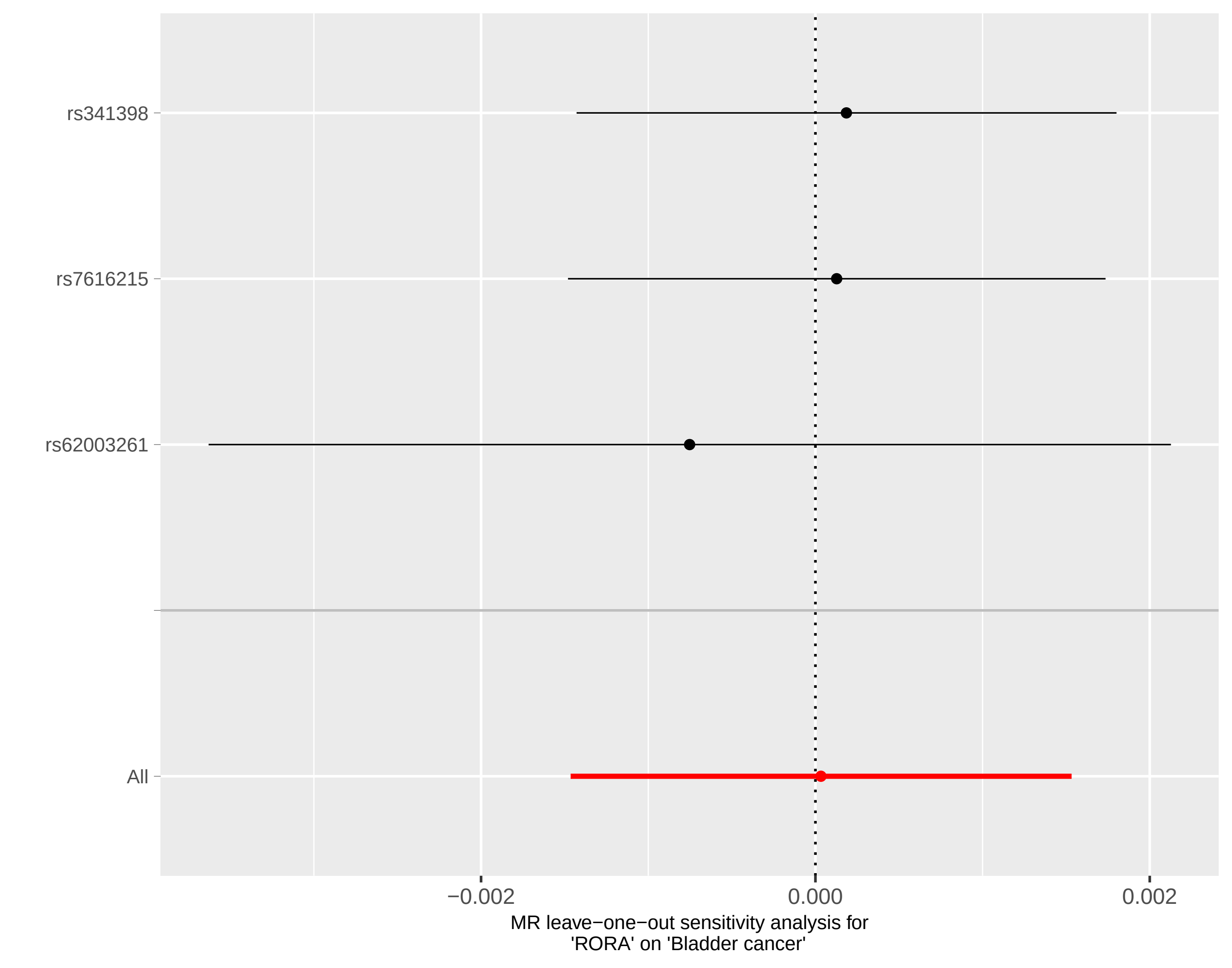
rs341398
rs7616215
rs62003261
All
−0.002
0.000
0.002
MR leave−one−out sensitivity analysis for
'RORA' on 'Bladder cancer'

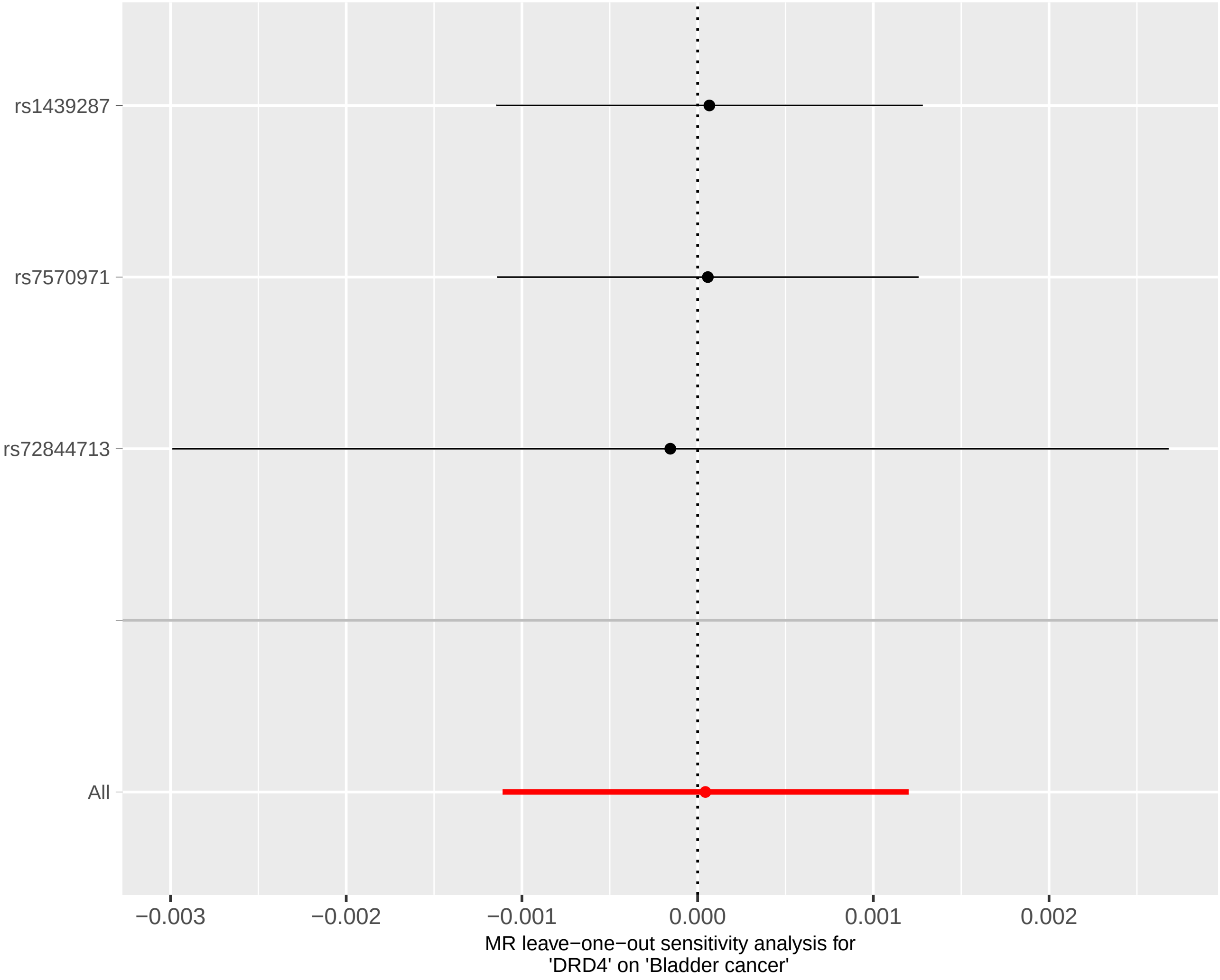
rs1439287
rs7570971
rs72844713
All
−0.003
−0.002
−0.001
0.000
0.001
0.002
MR leave−one−out sensitivity analysis for
'DRD4' on 'Bladder cancer'

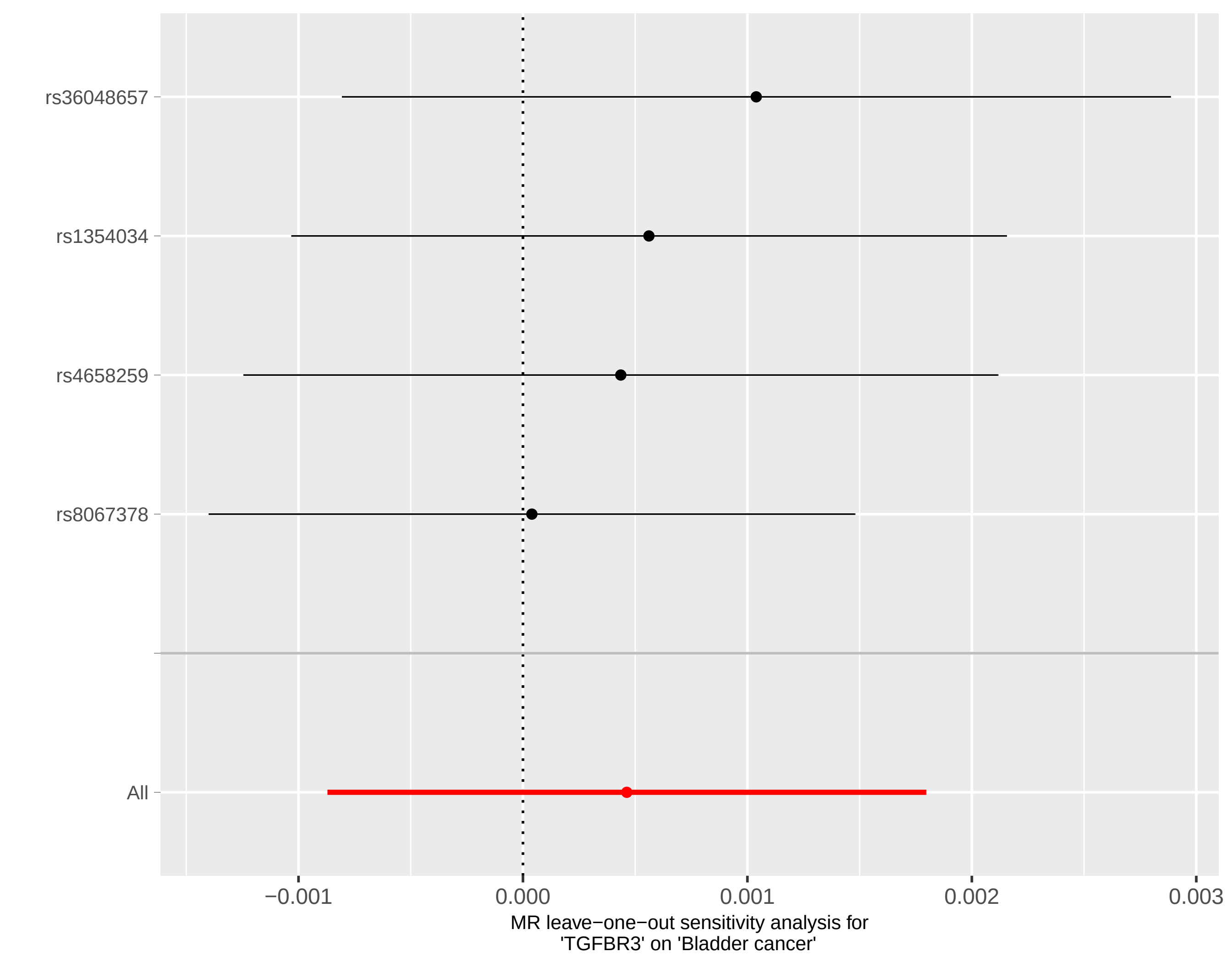
rs36048657
rs1354034
rs4658259
rs8067378
All
−0.001
0.000
0.001
0.002
0.003
MR leave−one−out sensitivity analysis for
'TGFBR3' on 'Bladder cancer'

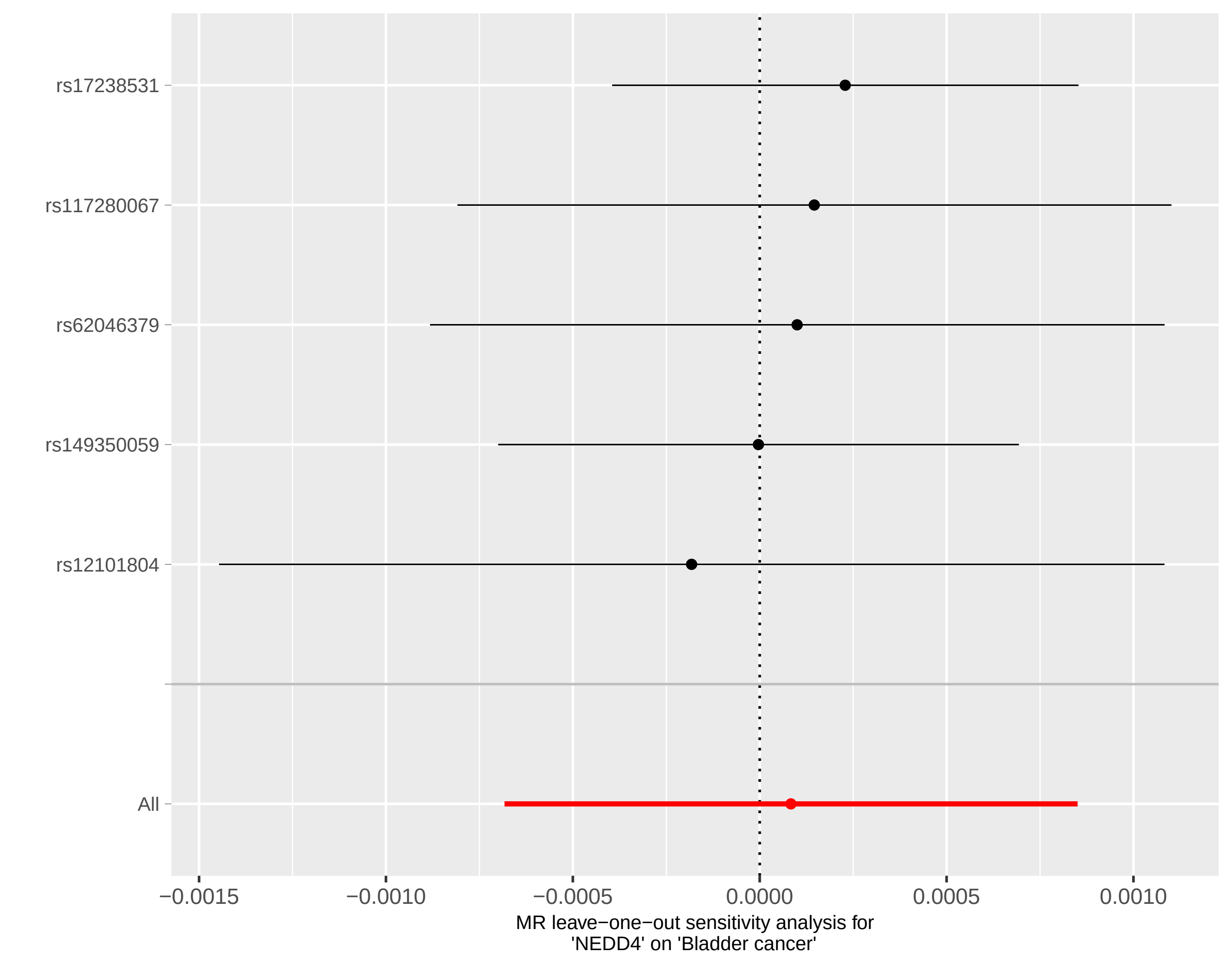

rs17238531
rs117280067
rs62046379
rs149350059
rs12101804
All
−0.0015
−0.0010
−0.0005
0.0000
0.0005
0.0010
MR leave−one−out sensitivity analysis for
'NEDD4' on 'Bladder cancer'

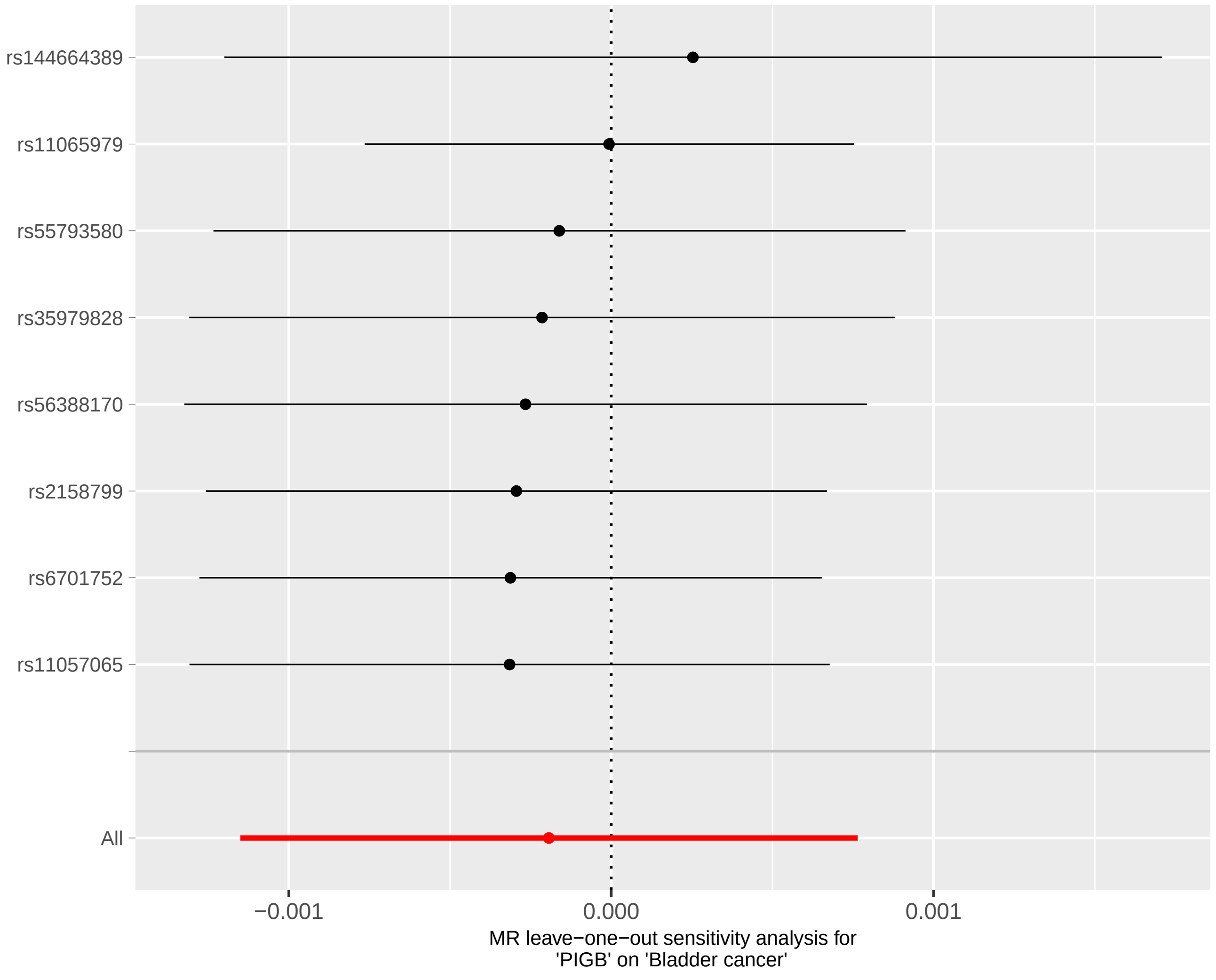
rs144664389
rs11065979
rs55793580
rs35979828
rs56388170
rs2158799
rs6701752
rs11057065
All
−0.001
0.000
0.001
MR leave−one−out sensitivity analysis for
'PIGB' on 'Bladder cancer'

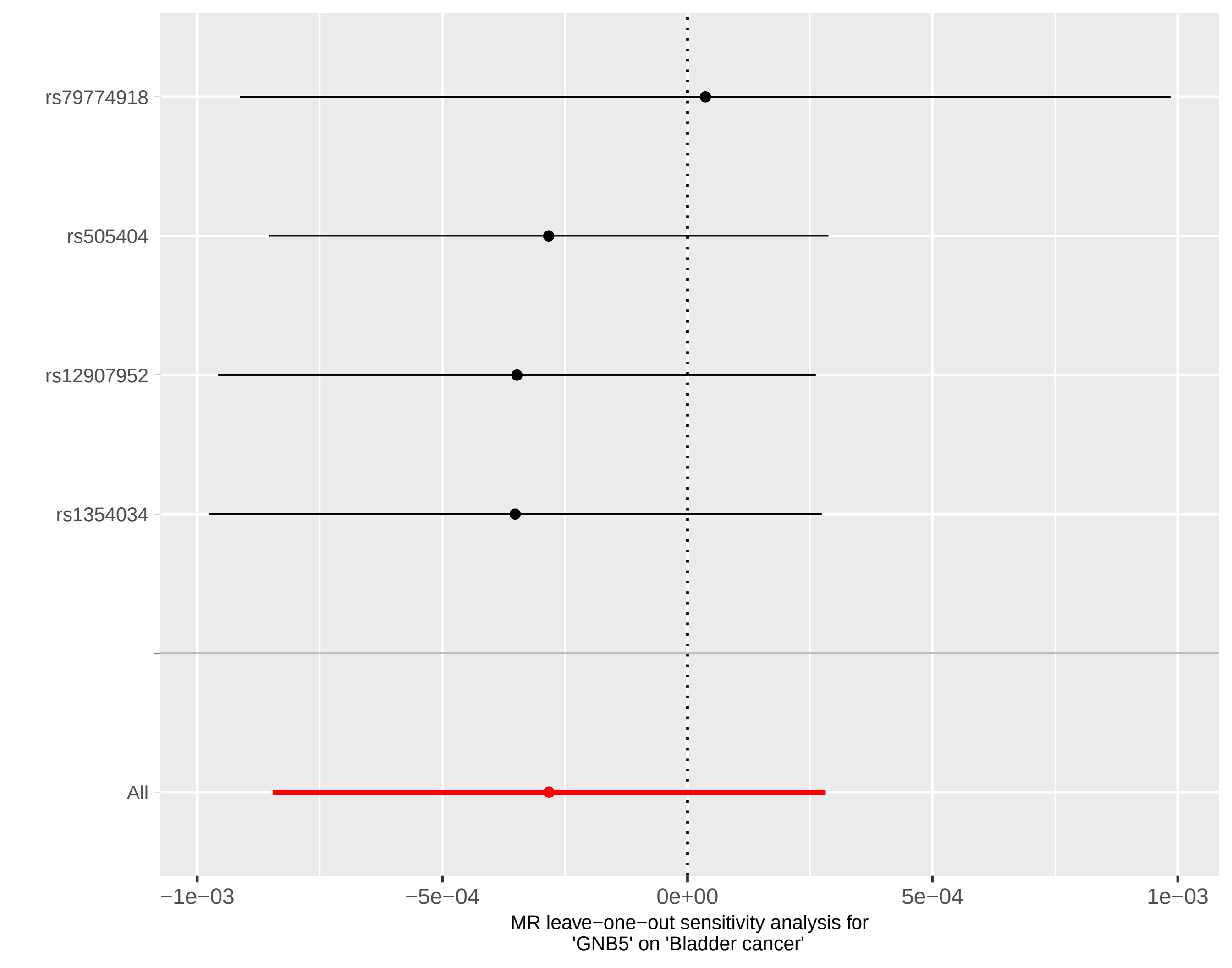
rs79774918
rs505404
rs12907952
rs1354034
All
−1e−03
−5e−04
0e+00
5e−04
1e−03
MR leave−one−out sensitivity analysis for
'GNB5' on 'Bladder cancer'

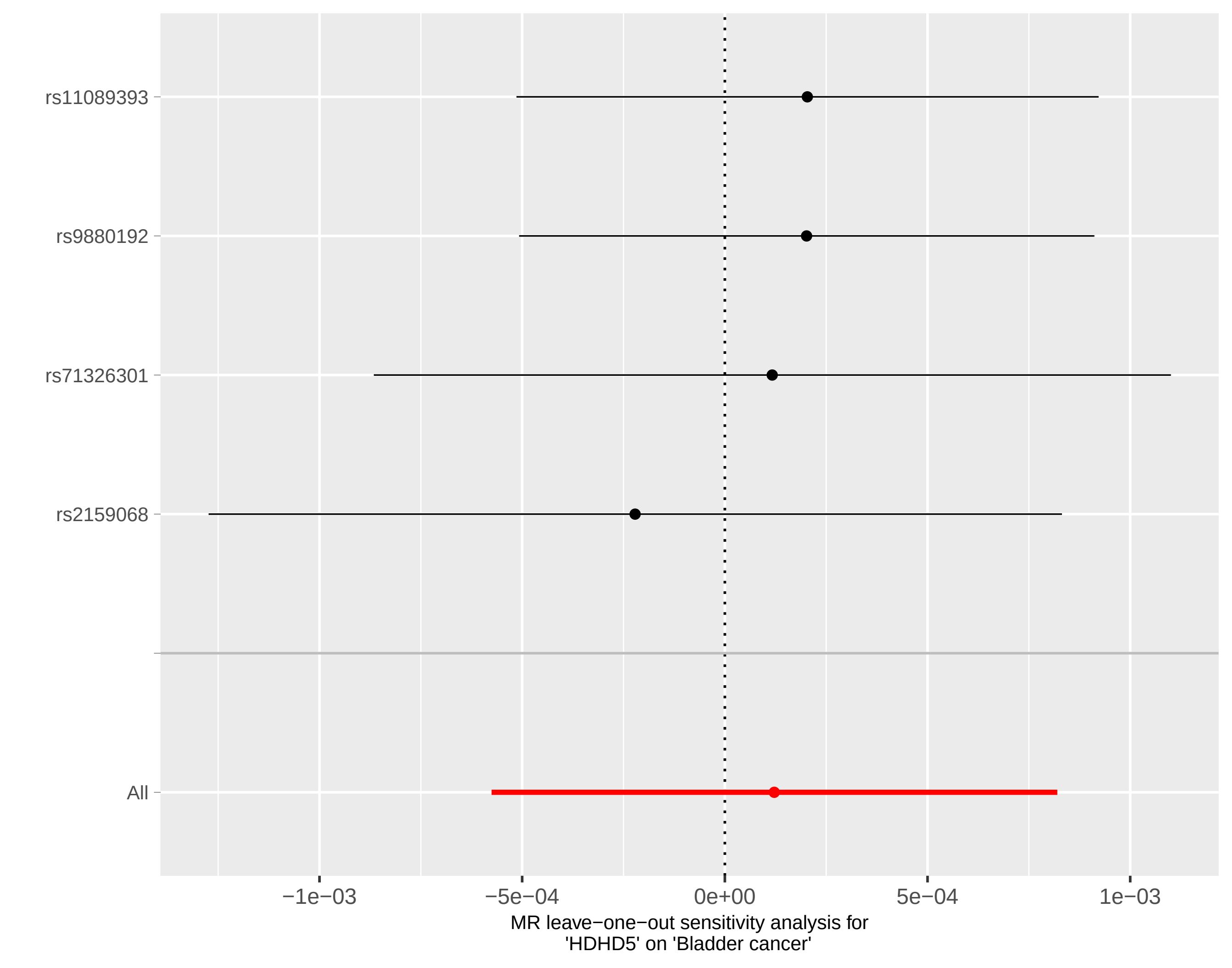
rs11089393
rs9880192
rs71326301
rs2159068
All
−1e−03
−5e−04
0e+00
5e−04
1e−03
MR leave−one−out sensitivity analysis for
'HDHD5' on 'Bladder cancer'

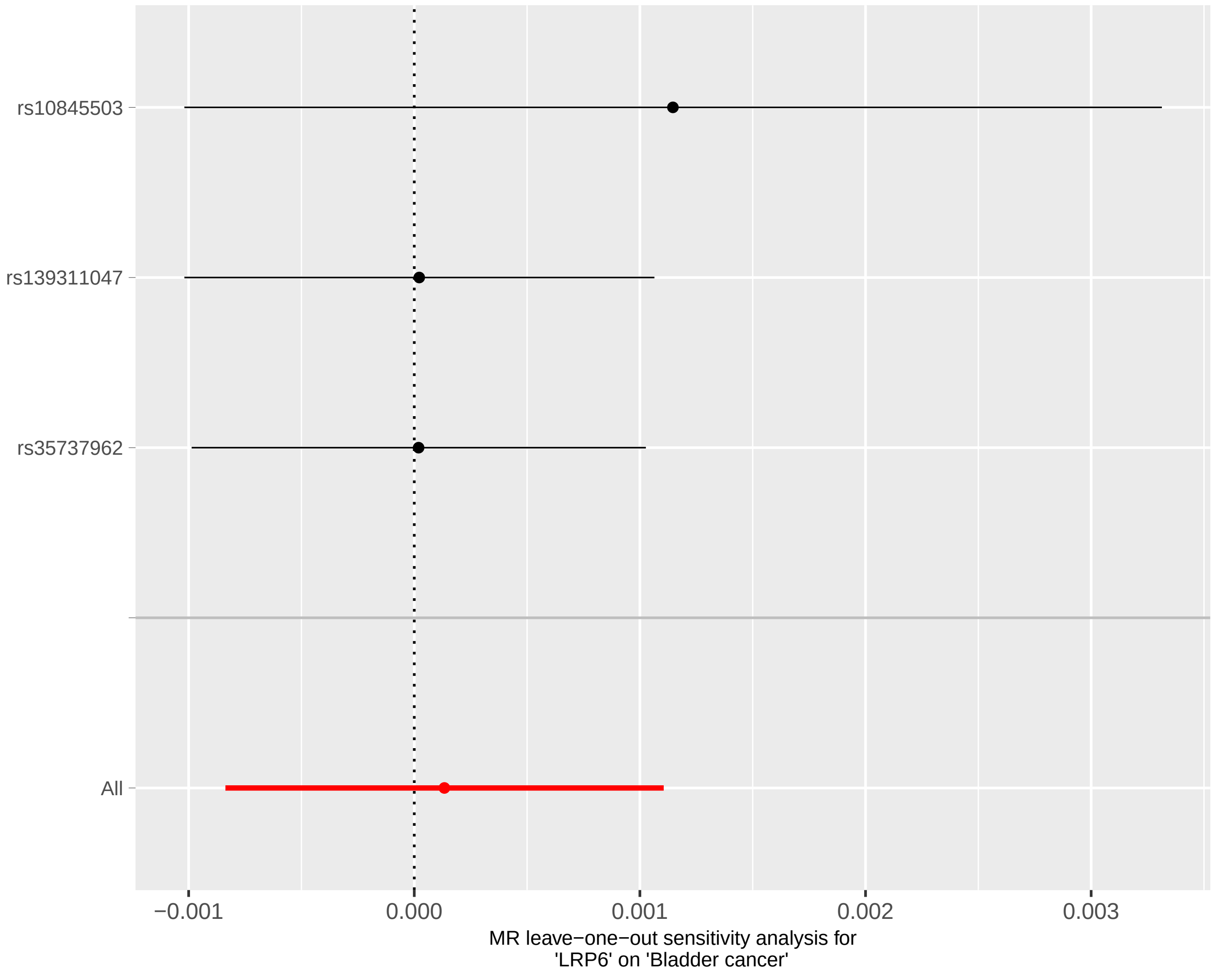

rs10845503
rs139311047
rs35737962
All
−0.001
0.000
0.001
0.002
0.003
MR leave−one−out sensitivity analysis for
'LRP6' on 'Bladder cancer'

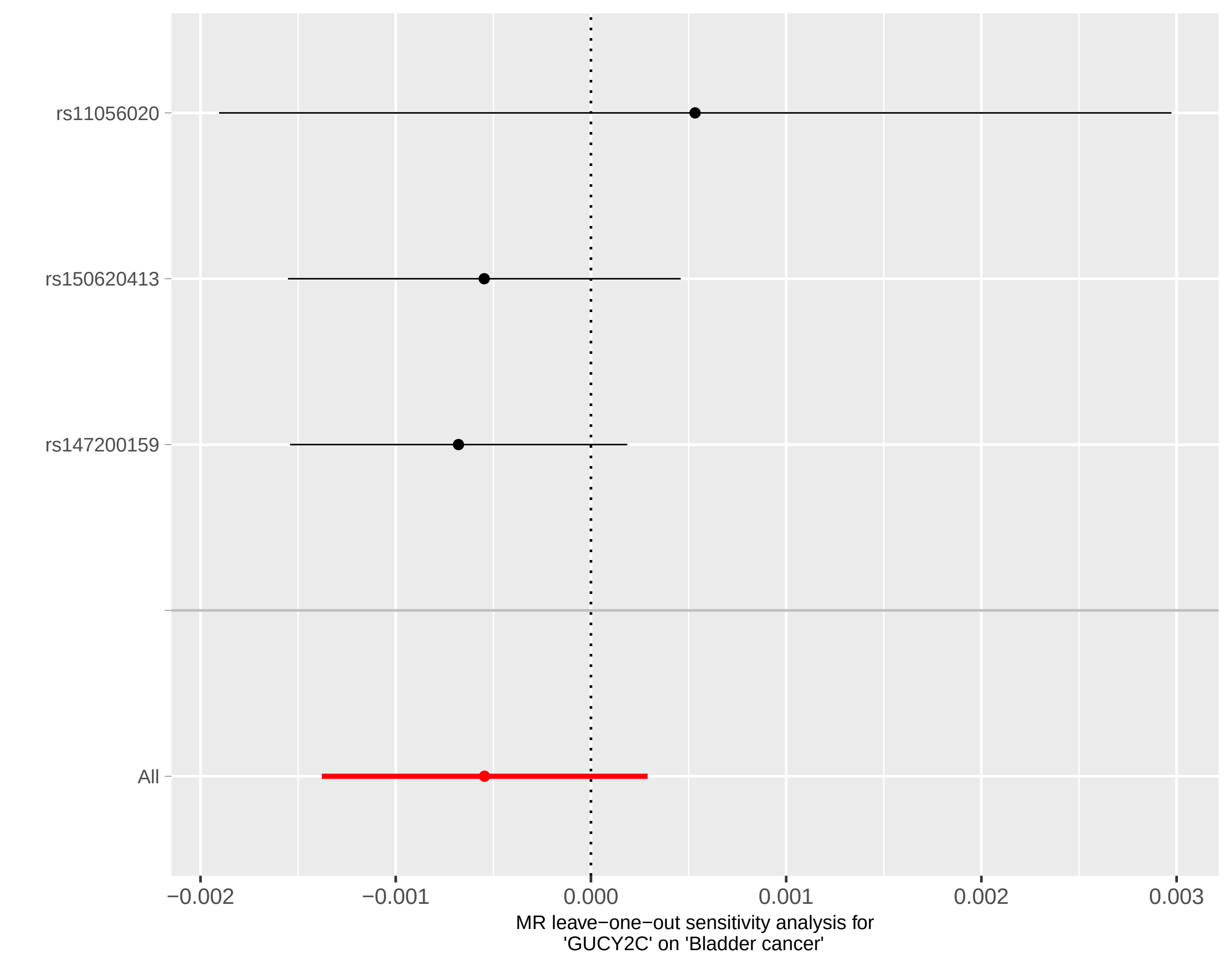
rs11056020
rs150620413
rs147200159
All
−0.002
−0.001
0.000
0.001
0.002
0.003
MR leave−one−out sensitivity analysis for
'GUCY2C' on 'Bladder cancer'

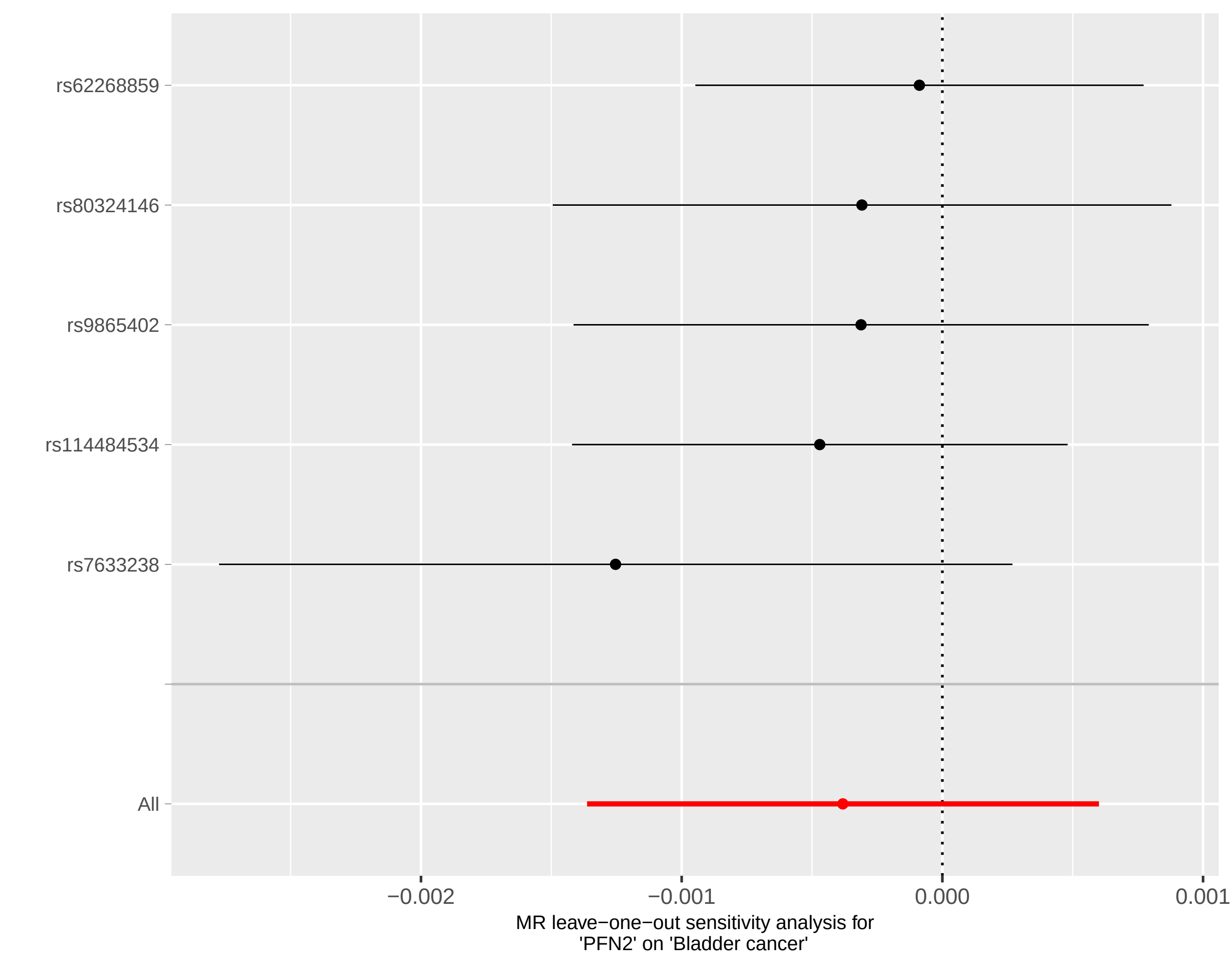

rs62268859
rs80324146
rs9865402
rs114484534
rs7633238
All
−0.002
−0.001
0.000
0.001
MR leave−one−out sensitivity analysis for
'PFN2' on 'Bladder cancer'

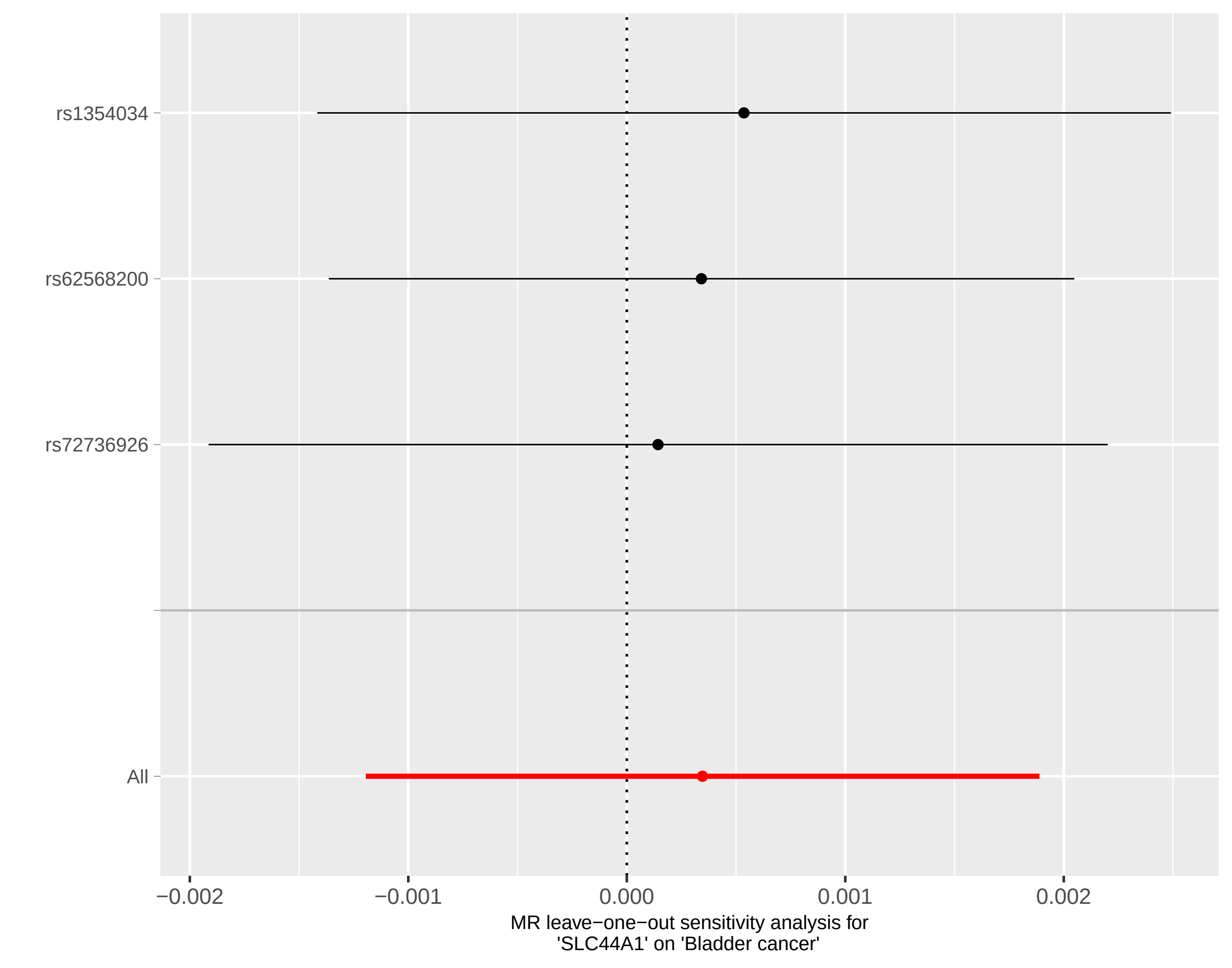
rs1354034
rs62568200
rs72736926
All
−0.002
−0.001
0.000
0.001
0.002
MR leave−one−out sensitivity analysis for
'SLC44A1' on 'Bladder cancer'

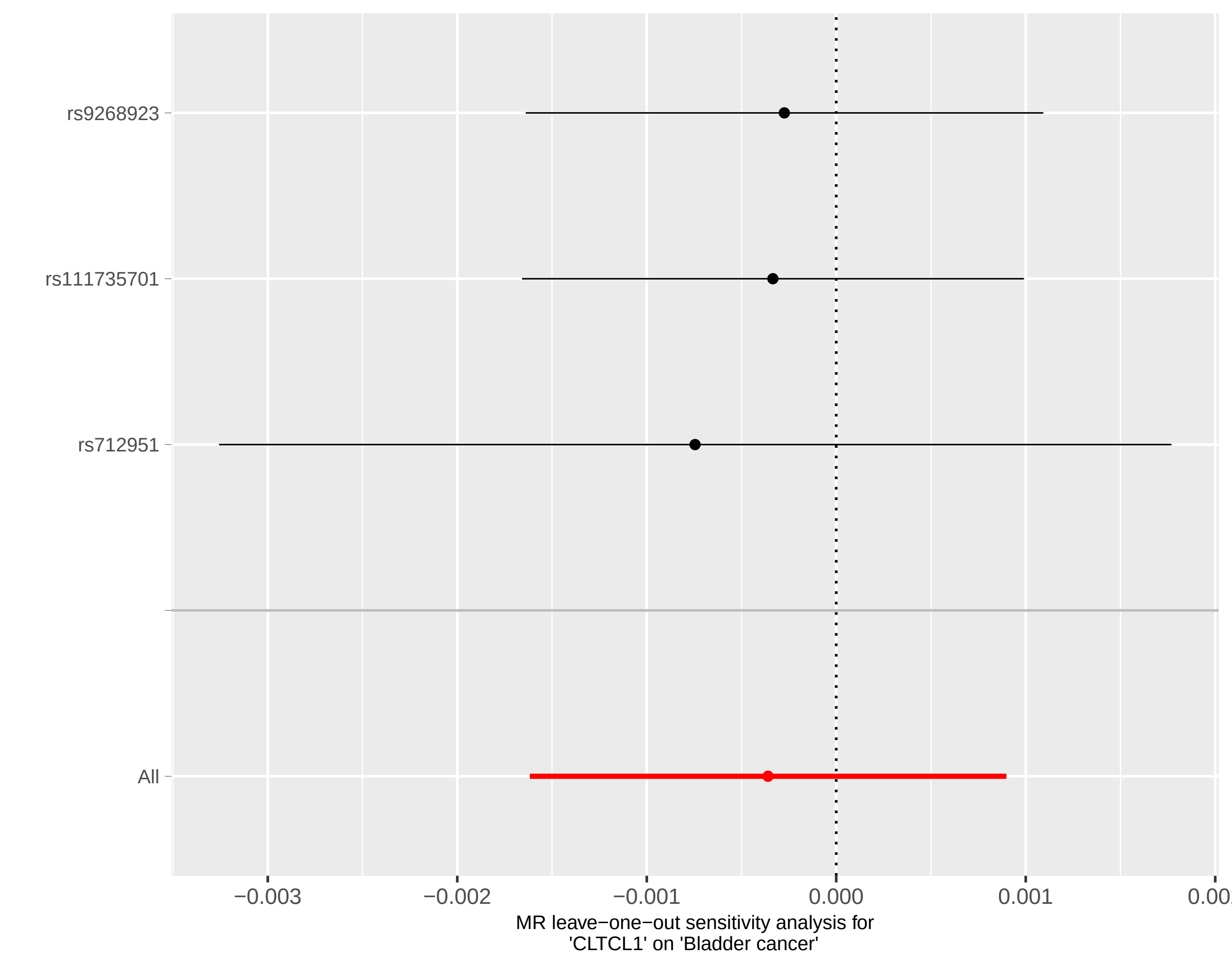
rs9268923
rs111735701
rs712951
All
−0.003
−0.002
−0.001
0.000
0.001
MR leave−one−out sensitivity analysis for
'CLTCL1' on 'Bladder cancer'

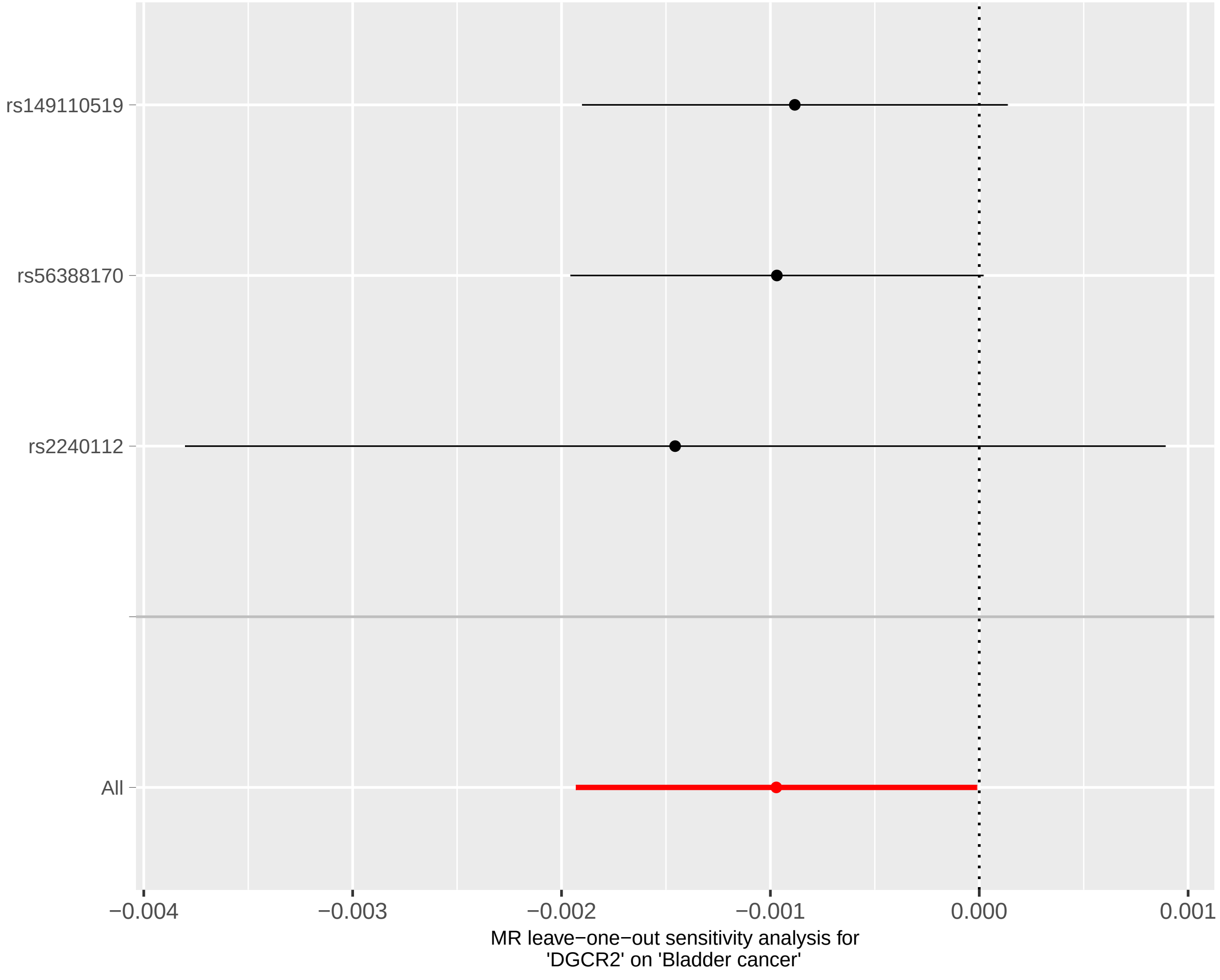
rs149110519
rs56388170
rs2240112
All
−0.004
−0.003
−0.002
−0.001
0.000
0.001
MR leave−one−out sensitivity analysis for
'DGCR2' on 'Bladder cancer'

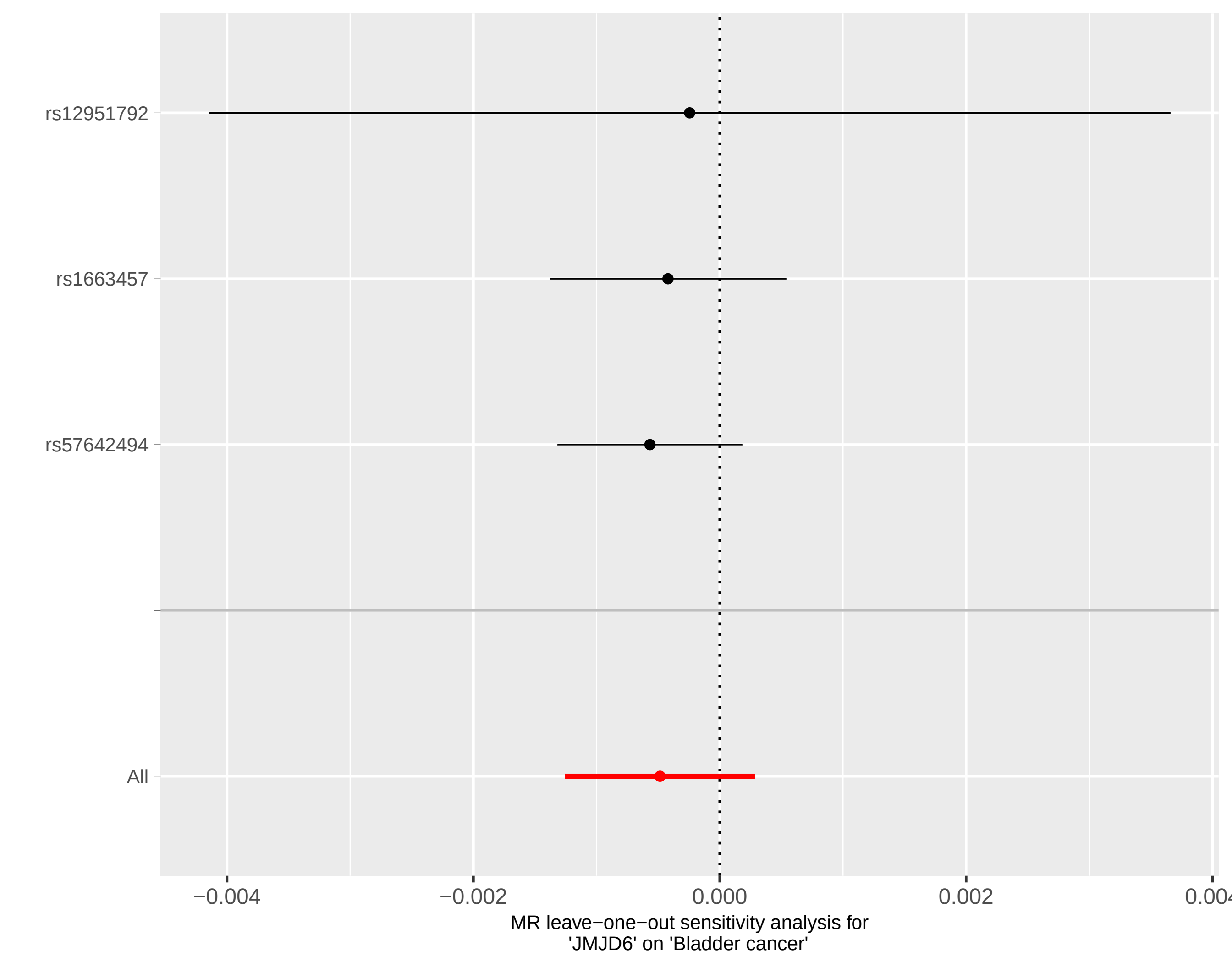

rs12951792
rs1663457
rs57642494
All
−0.004
−0.002
0.000
0.002
0.004
MR leave−one−out sensitivity analysis for
'JMJD6' on 'Bladder cancer'

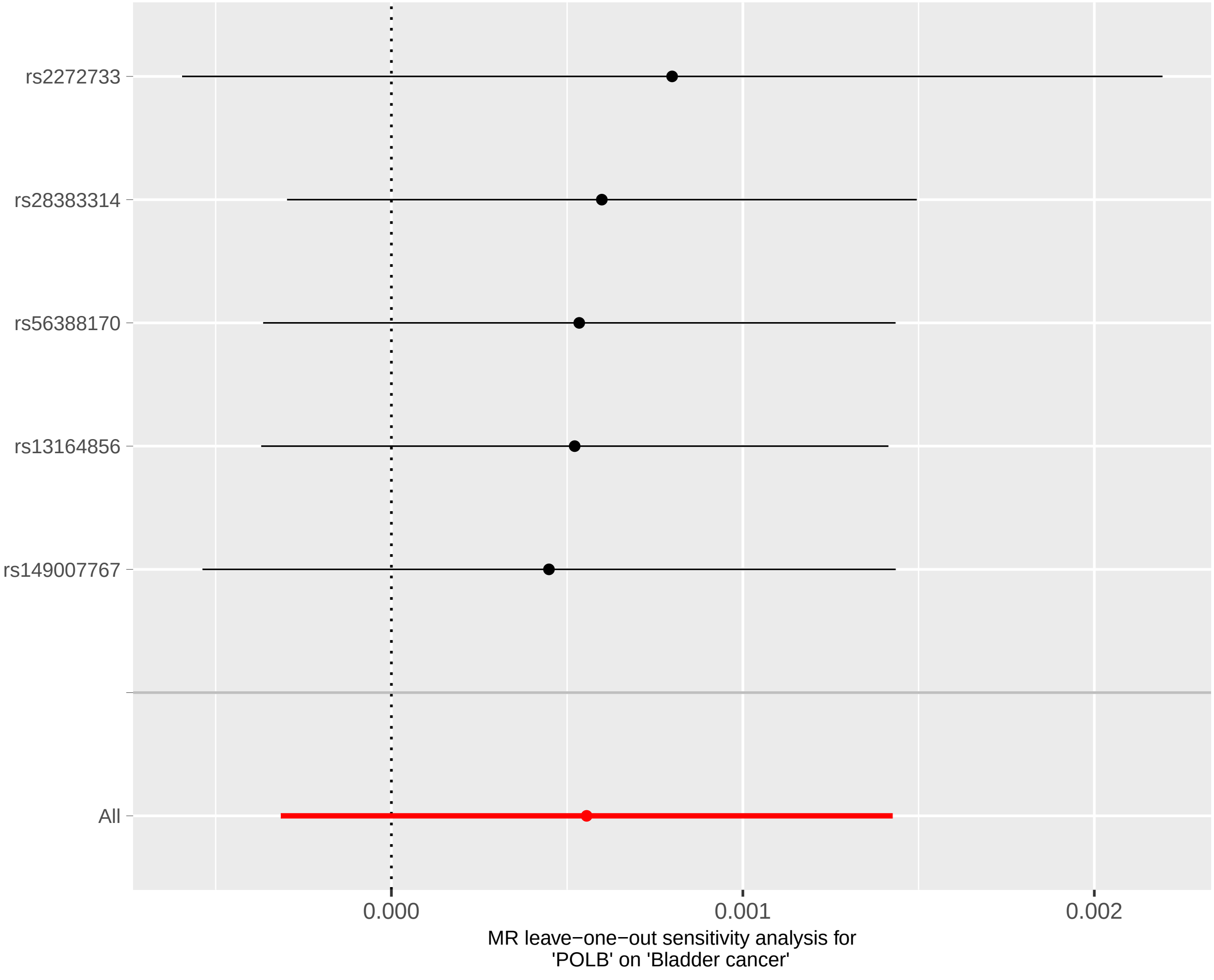
rs2272733
rs28383314
rs56388170
rs13164856
rs149007767
All
0.000
0.001
0.002
MR leave−one−out sensitivity analysis for
'POLB' on 'Bladder cancer'

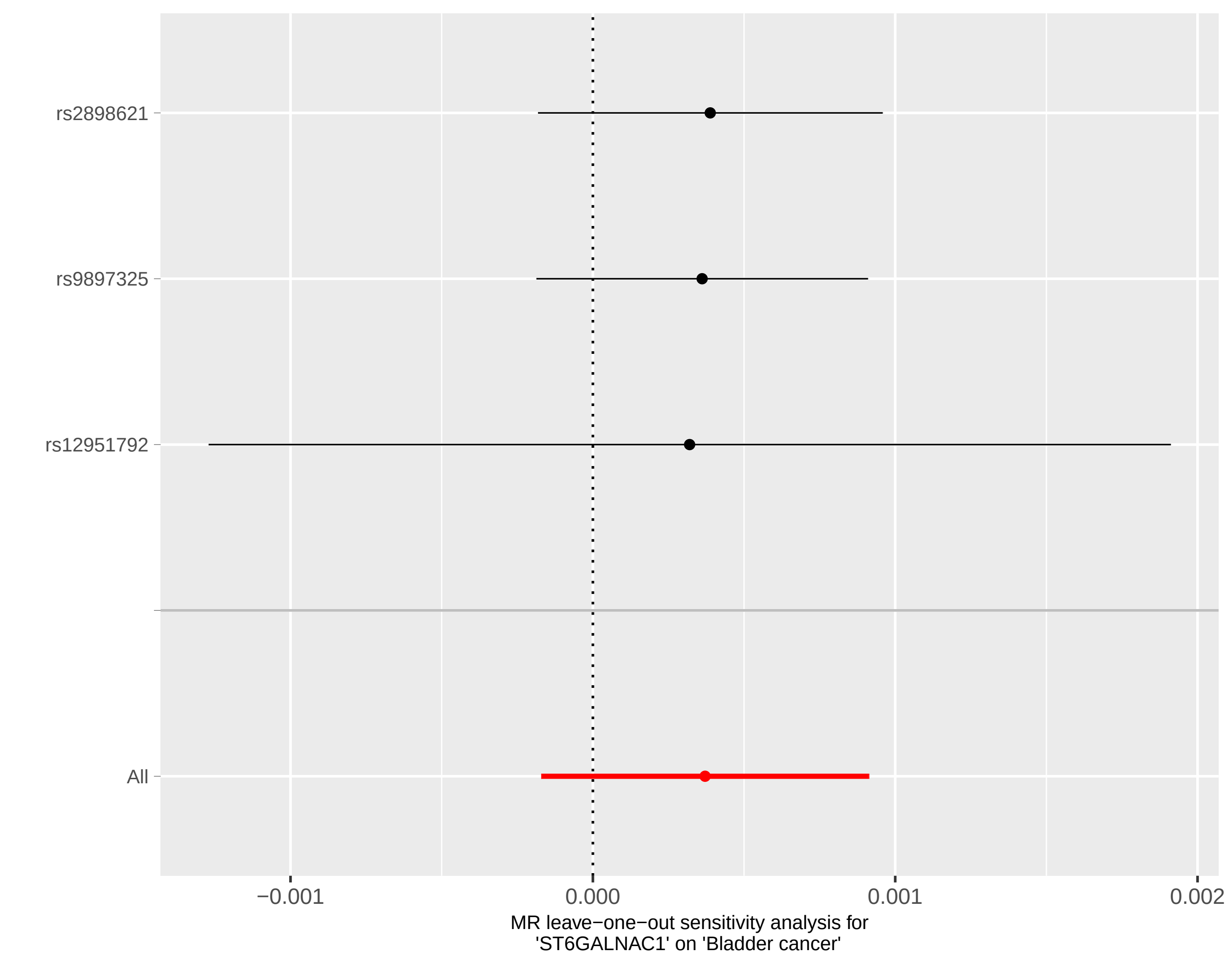
rs2898621
rs9897325
rs12951792
All
−0.001
0.000
0.001
0.002
MR leave−one−out sensitivity analysis for
'ST6GALNAC1' on 'Bladder cancer'

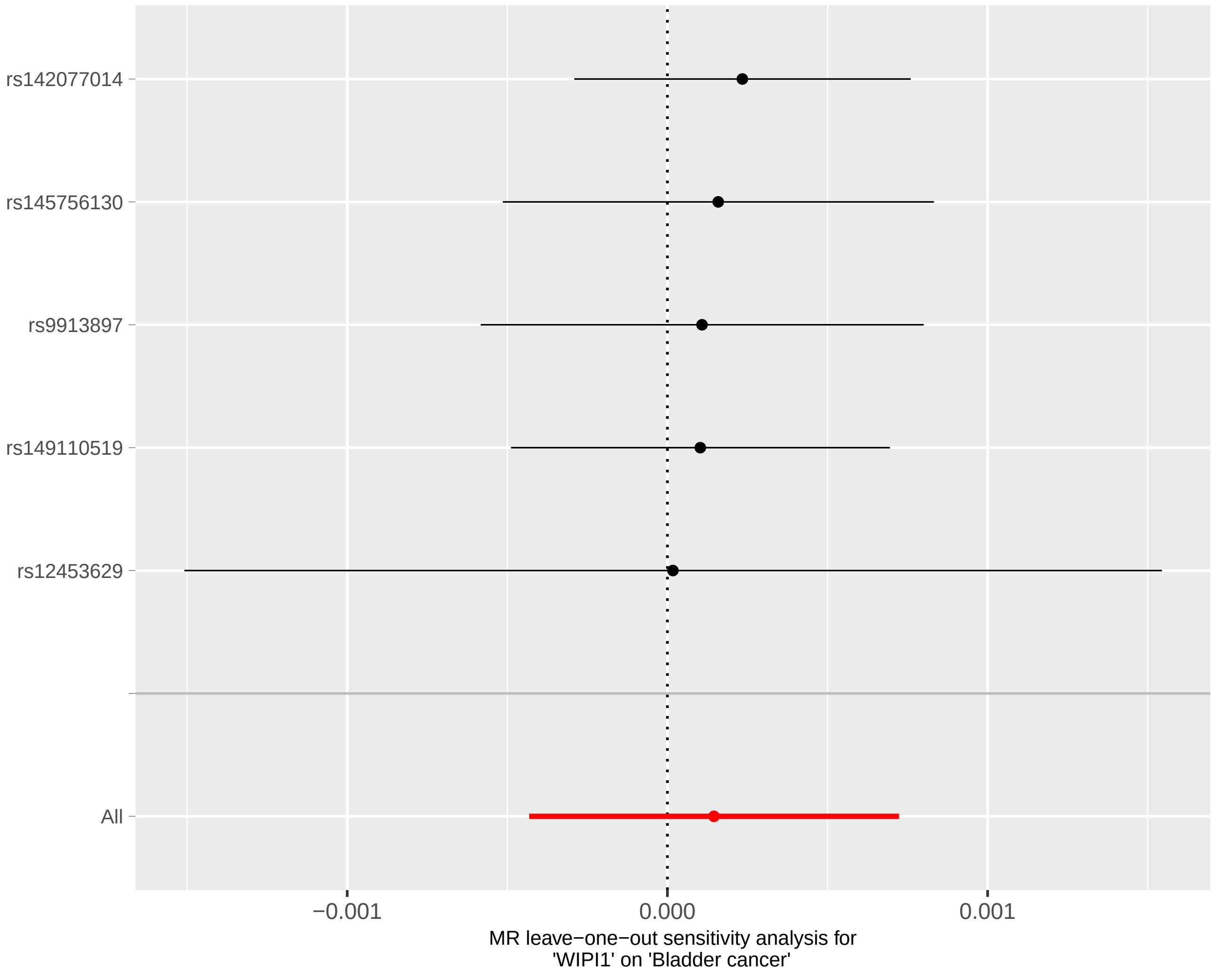
rs142077014
rs145756130
rs9913897
rs149110519
rs12453629
All
−0.001
0.000
0.001
MR leave−one−out sensitivity analysis for
'WIPI1' on 'Bladder cancer'

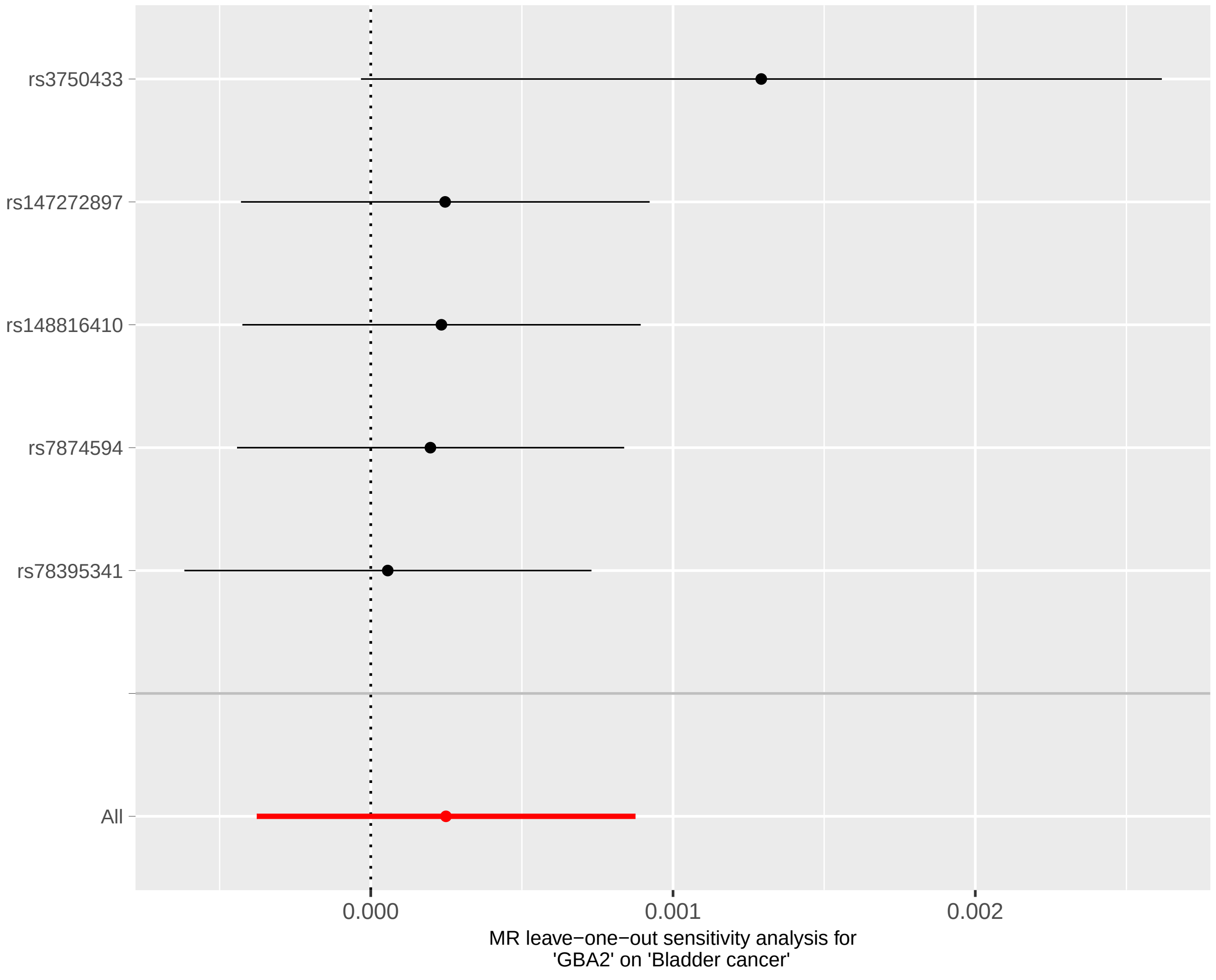
rs3750433
rs147272897
rs148816410
rs7874594
rs78395341
All
0.000
0.001
0.002
MR leave−one−out sensitivity analysis for
'GBA2' on 'Bladder cancer'

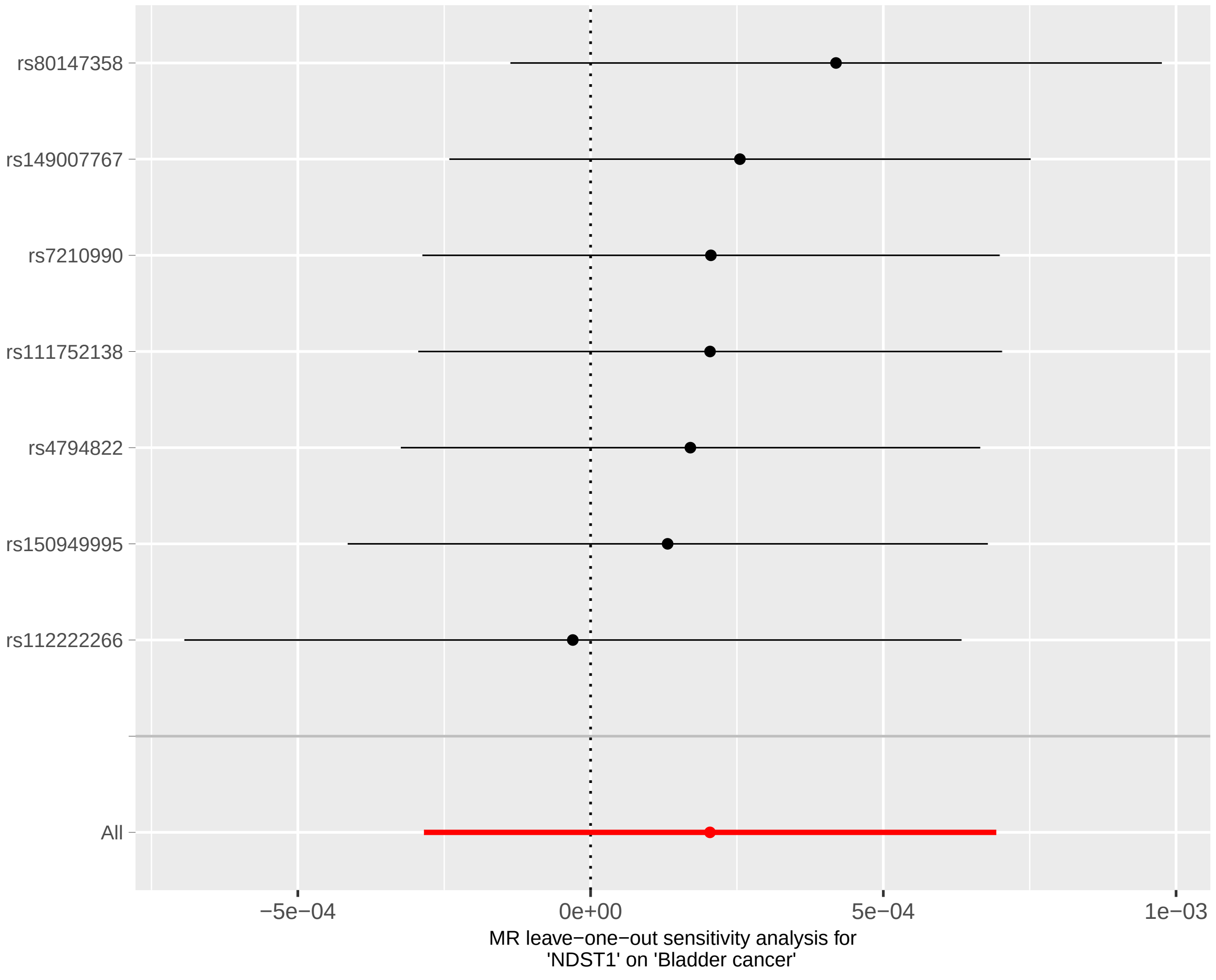
rs80147358
rs149007767
rs7210990
rs111752138
rs4794822
rs150949995
rs112222266
All
−5e−04
0e+00
5e−04
1e−03
MR leave−one−out sensitivity analysis for
'NDST1' on 'Bladder cancer'

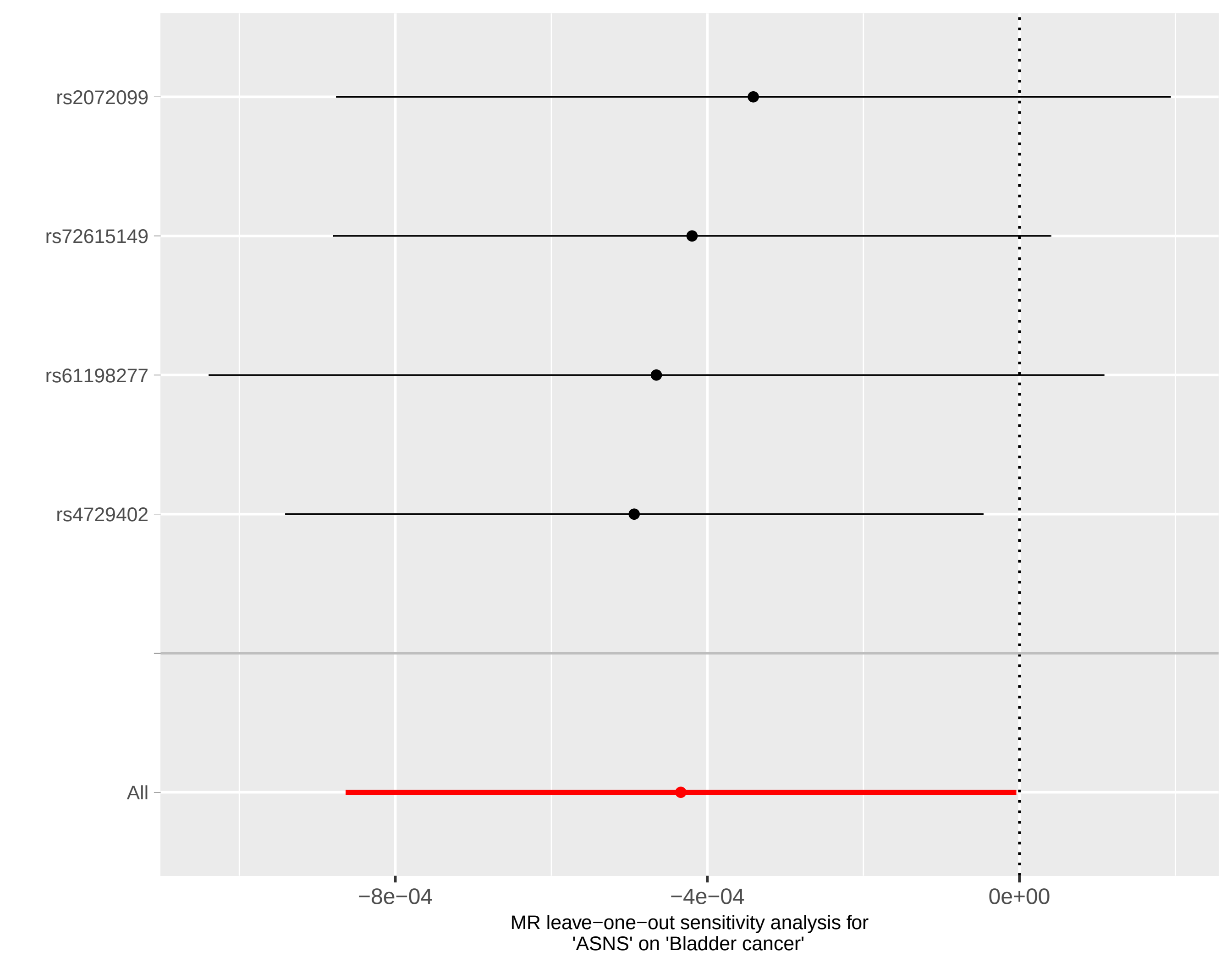
rs2072099
rs72615149
rs61198277
rs4729402
All
−8e−04
−4e−04
0e+00
MR leave−one−out sensitivity analysis for
'ASNS' on 'Bladder cancer'

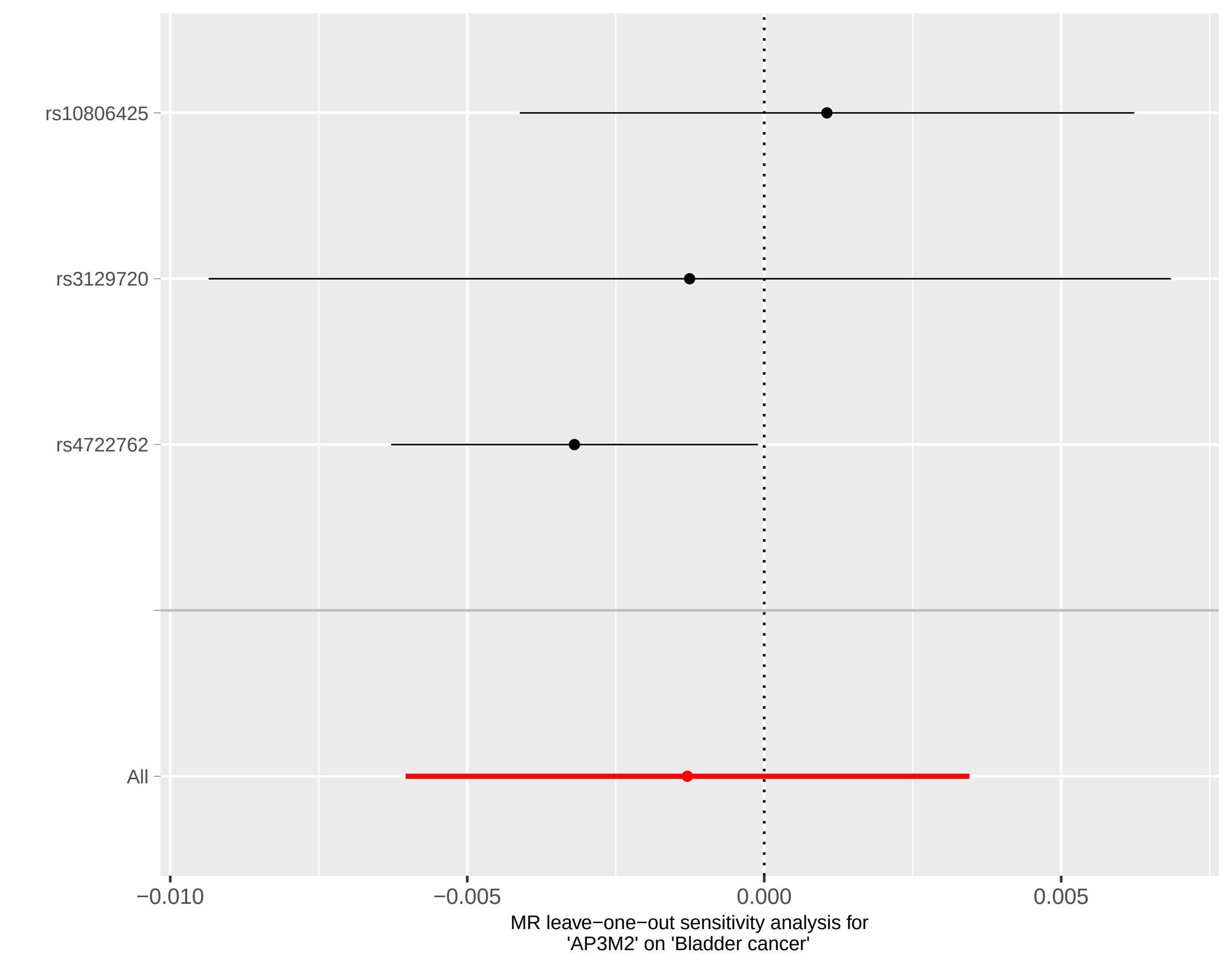
rs10806425
rs3129720
rs4722762
All
−0.010
−0.005
0.000
0.005
MR leave−one−out sensitivity analysis for
'AP3M2' on 'Bladder cancer'

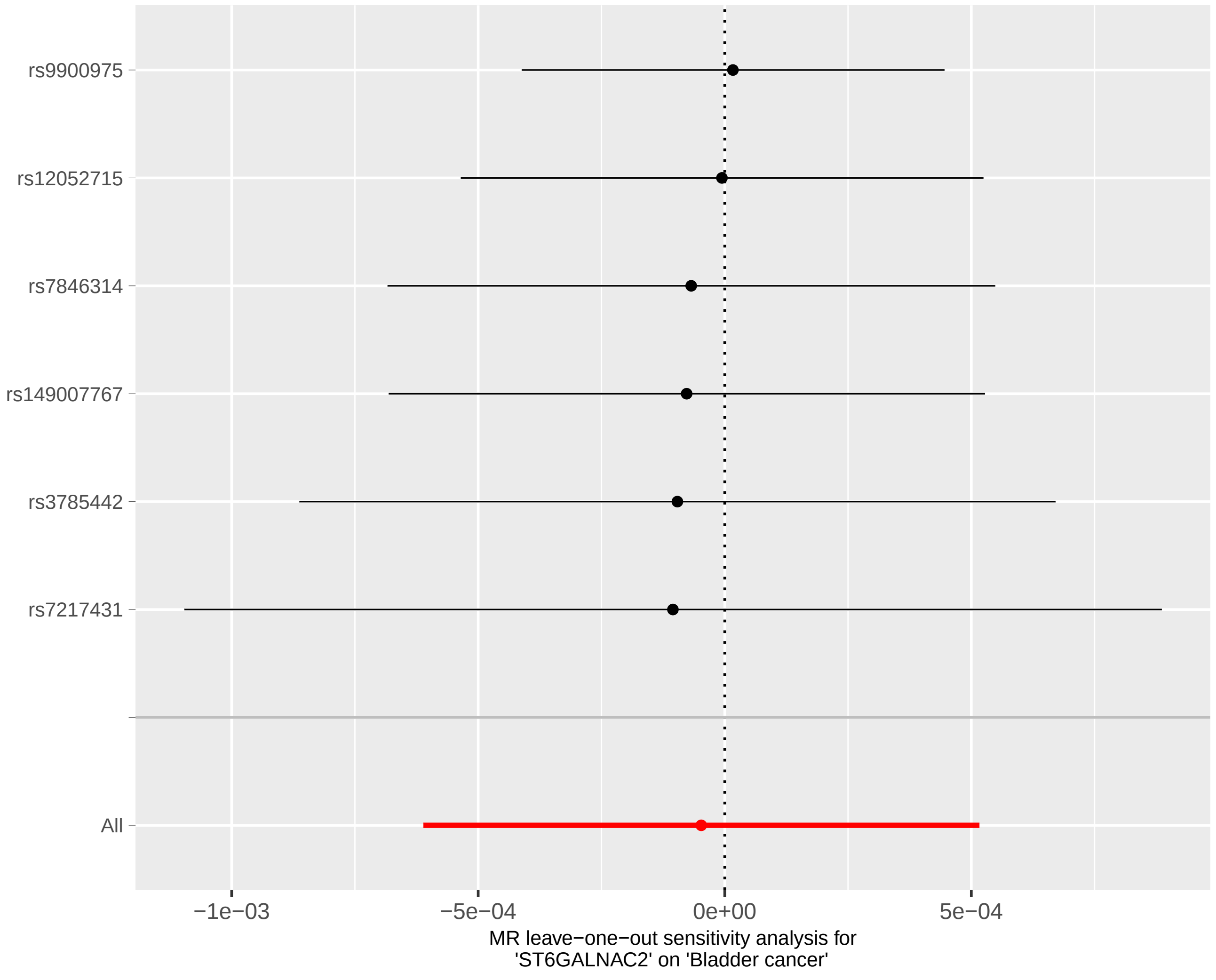
rs9900975
rs12052715
rs7846314
rs149007767
rs3785442
rs7217431
All
−1e−03
−5e−04
0e+00
5e−04
MR leave−one−out sensitivity analysis for
'ST6GALNAC2' on 'Bladder cancer'

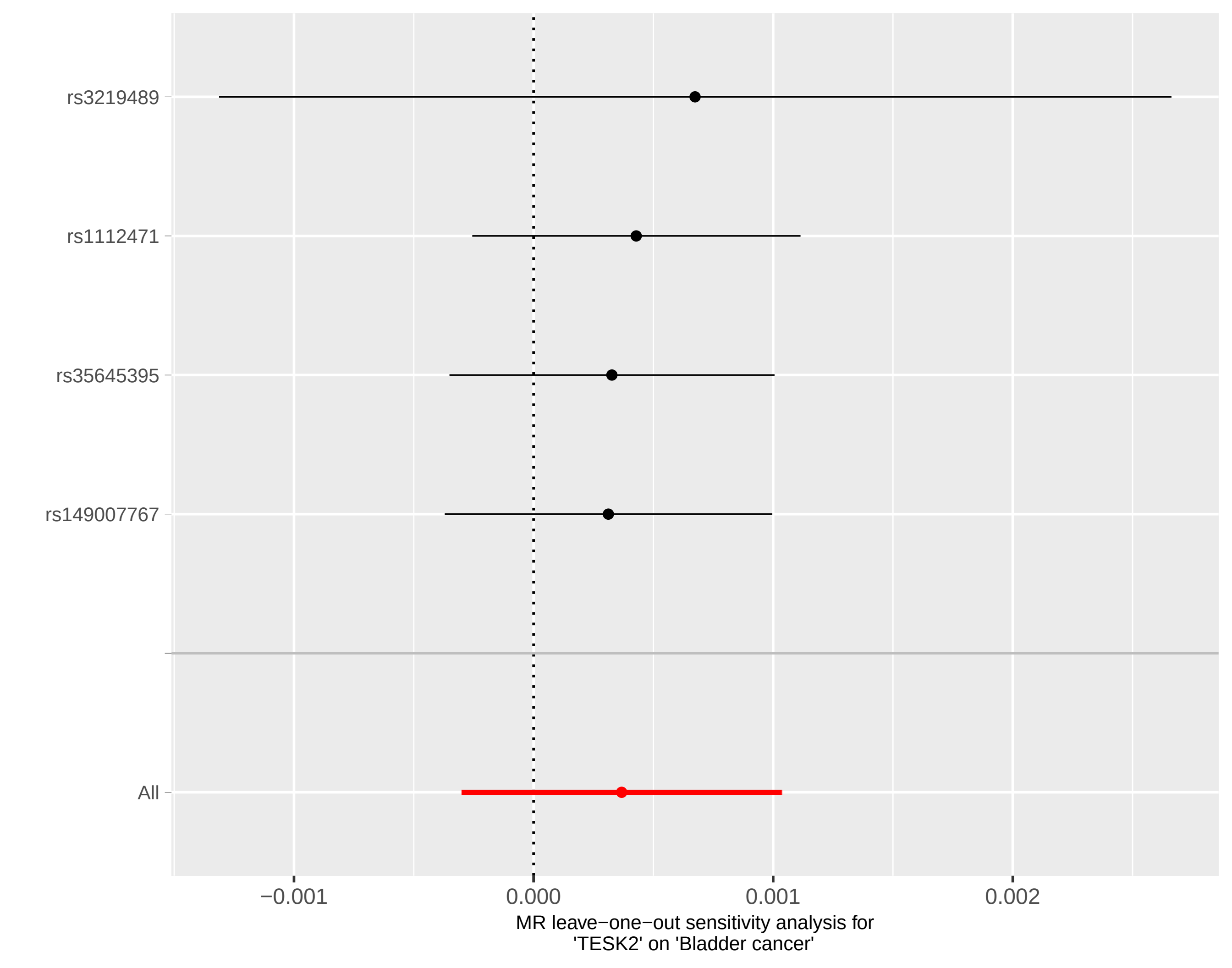
rs3219489
rs1112471
rs35645395
rs149007767
All
−0.001
0.000
0.001
0.002
MR leave−one−out sensitivity analysis for
'TESK2' on 'Bladder cancer'

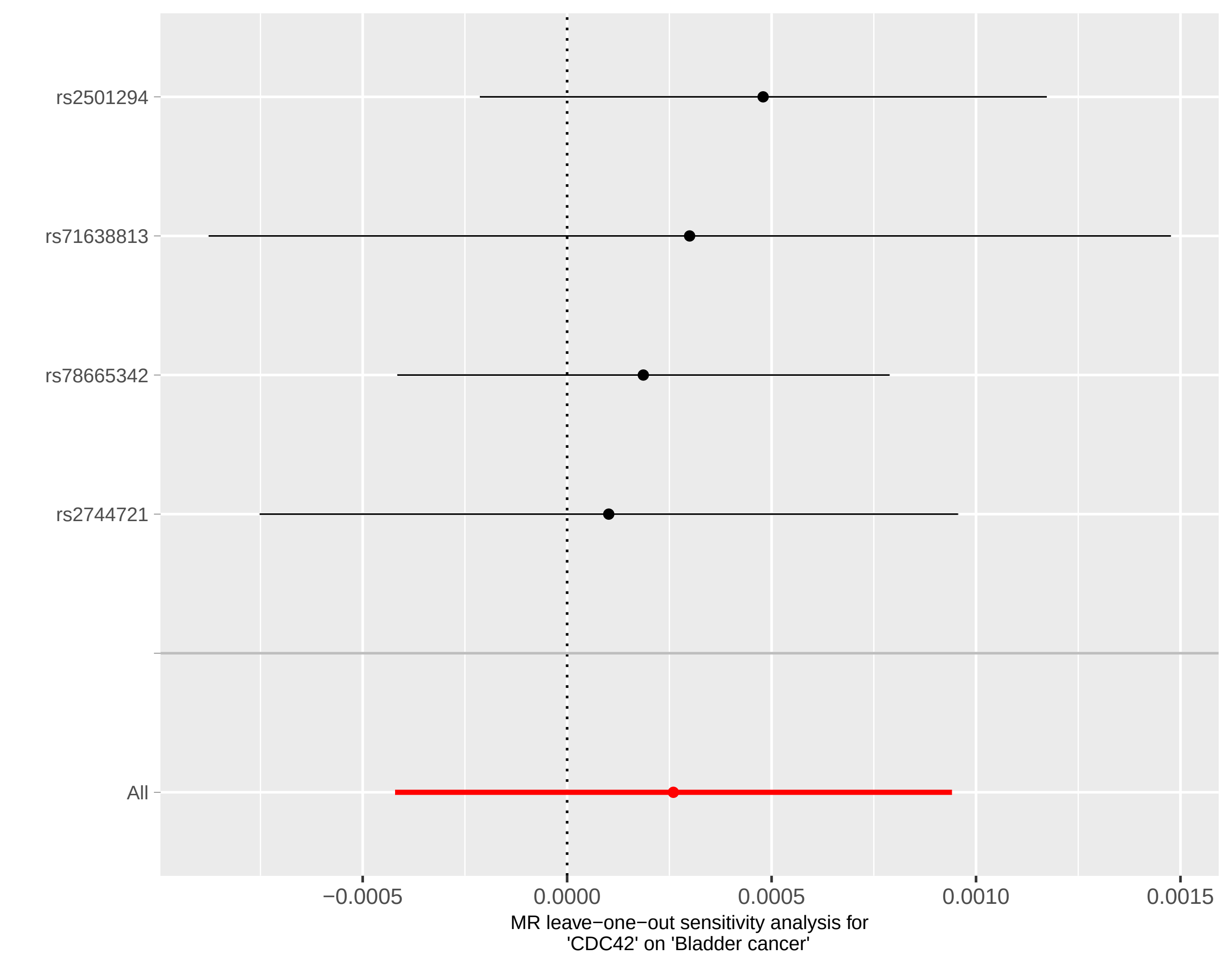
rs2501294
rs71638813
rs78665342
rs2744721
All
−0.0005
0.0000
0.0005
0.0010
0.0015
MR leave−one−out sensitivity analysis for
'CDC42' on 'Bladder cancer'

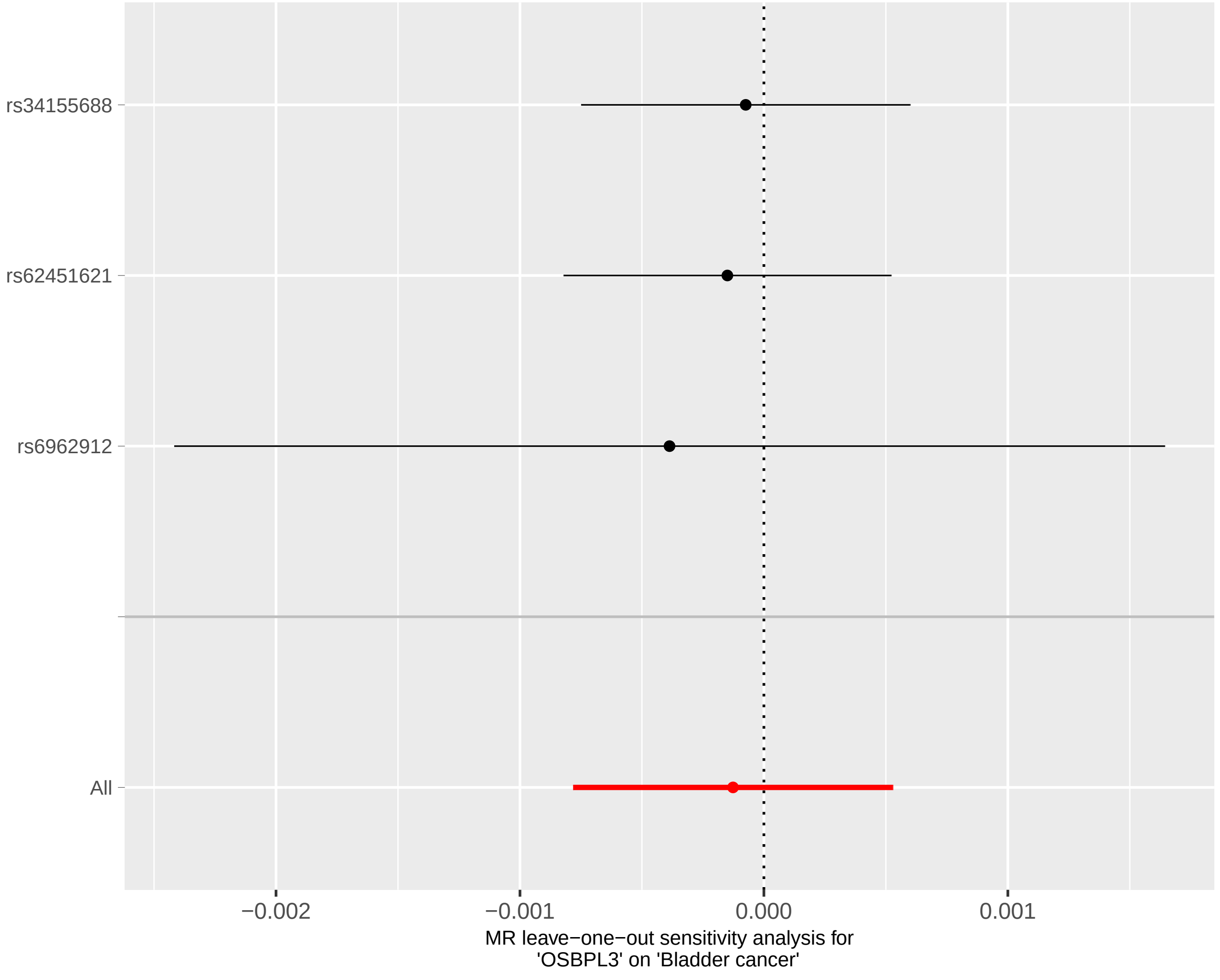
rs34155688
rs62451621
rs6962912
All
−0.002
−0.001
0.000
0.001
MR leave−one−out sensitivity analysis for
'OSBPL3' on 'Bladder cancer'

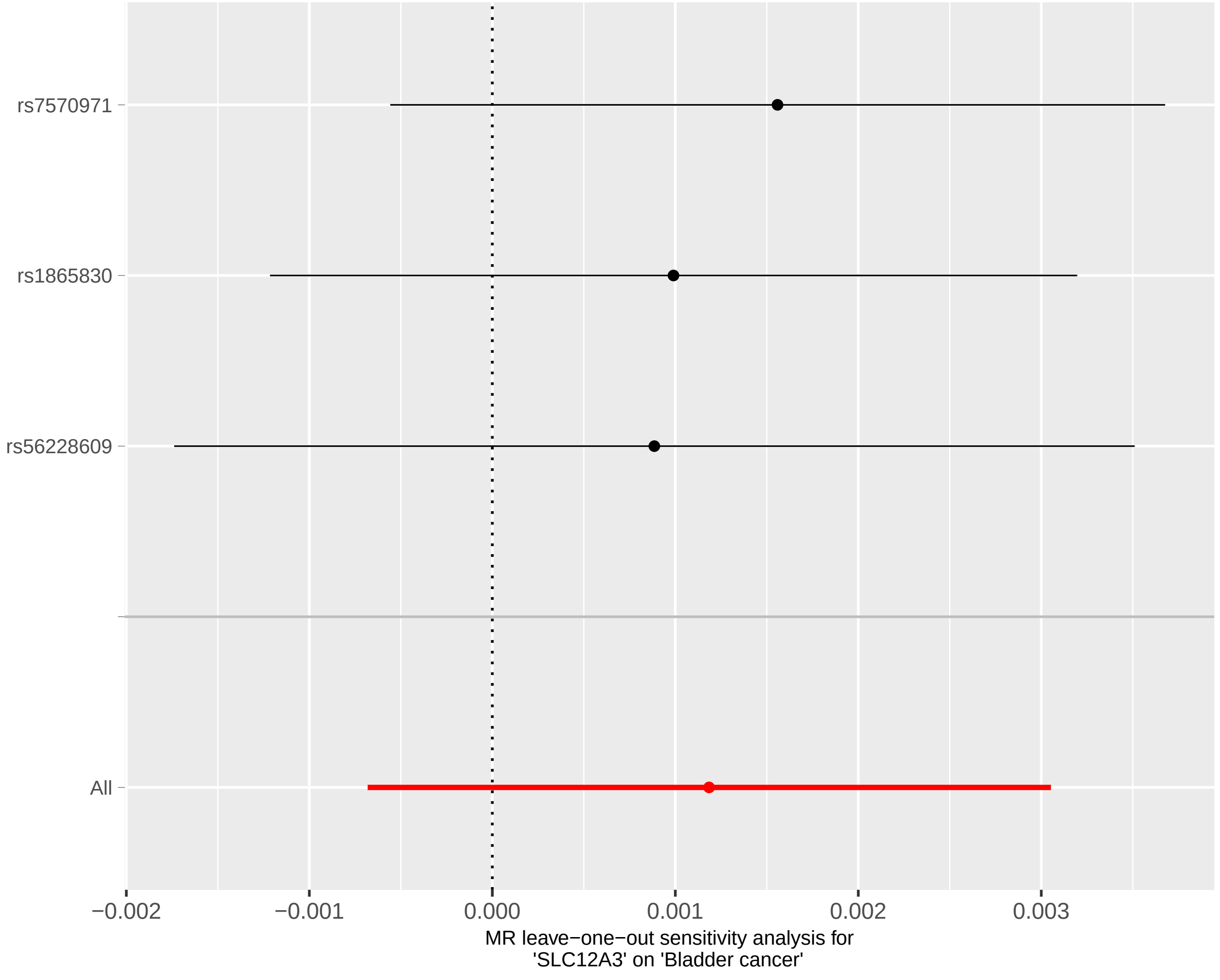
rs7570971
rs1865830
rs56228609
All
−0.002
−0.001
0.000
0.001
0.002
0.003
MR leave−one−out sensitivity analysis for
'SLC12A3' on 'Bladder cancer'

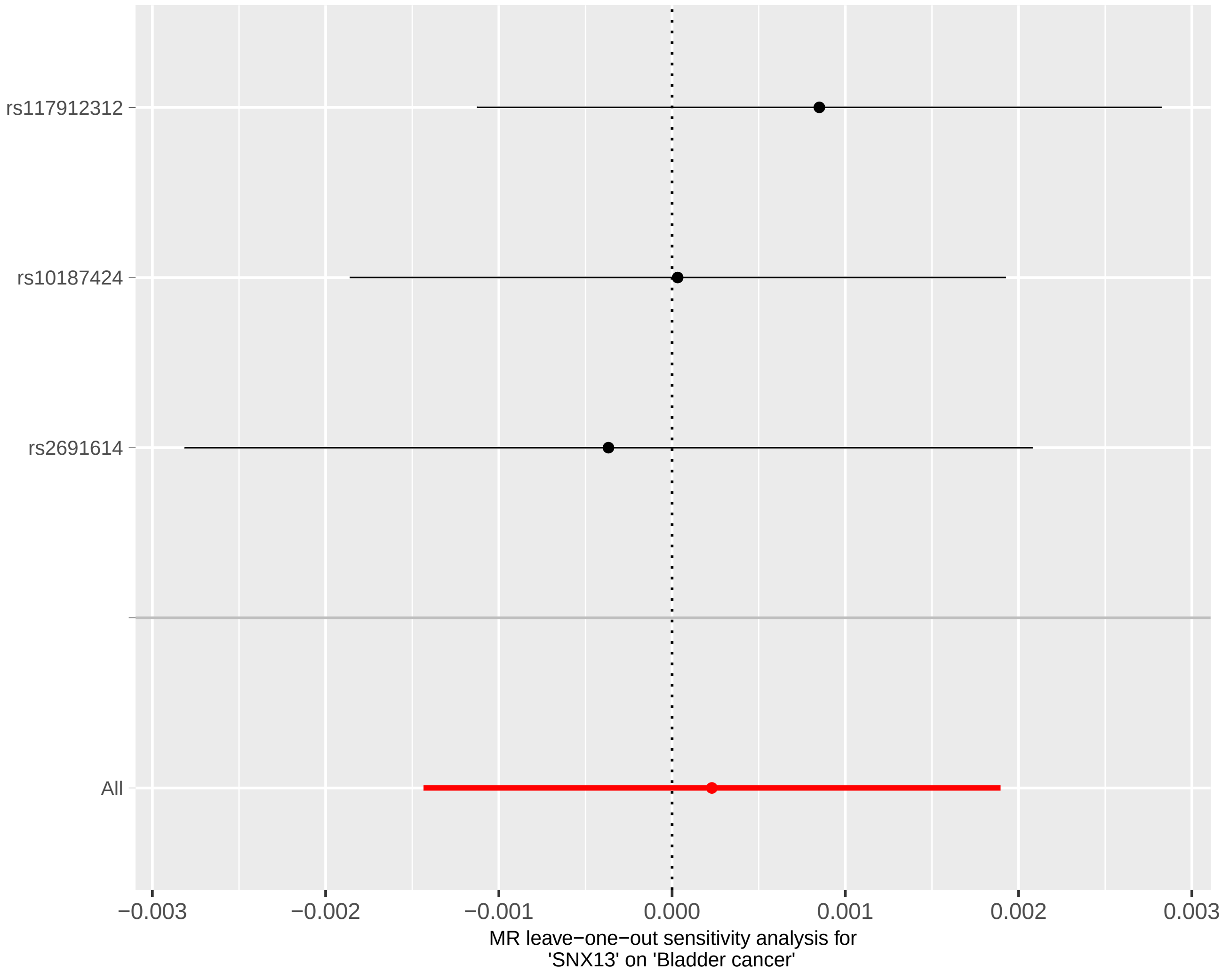
rs117912312
rs10187424
rs2691614
All
−0.003
−0.002
−0.001
0.000
0.001
0.002
0.003
MR leave−one−out sensitivity analysis for
'SNX13' on 'Bladder cancer'

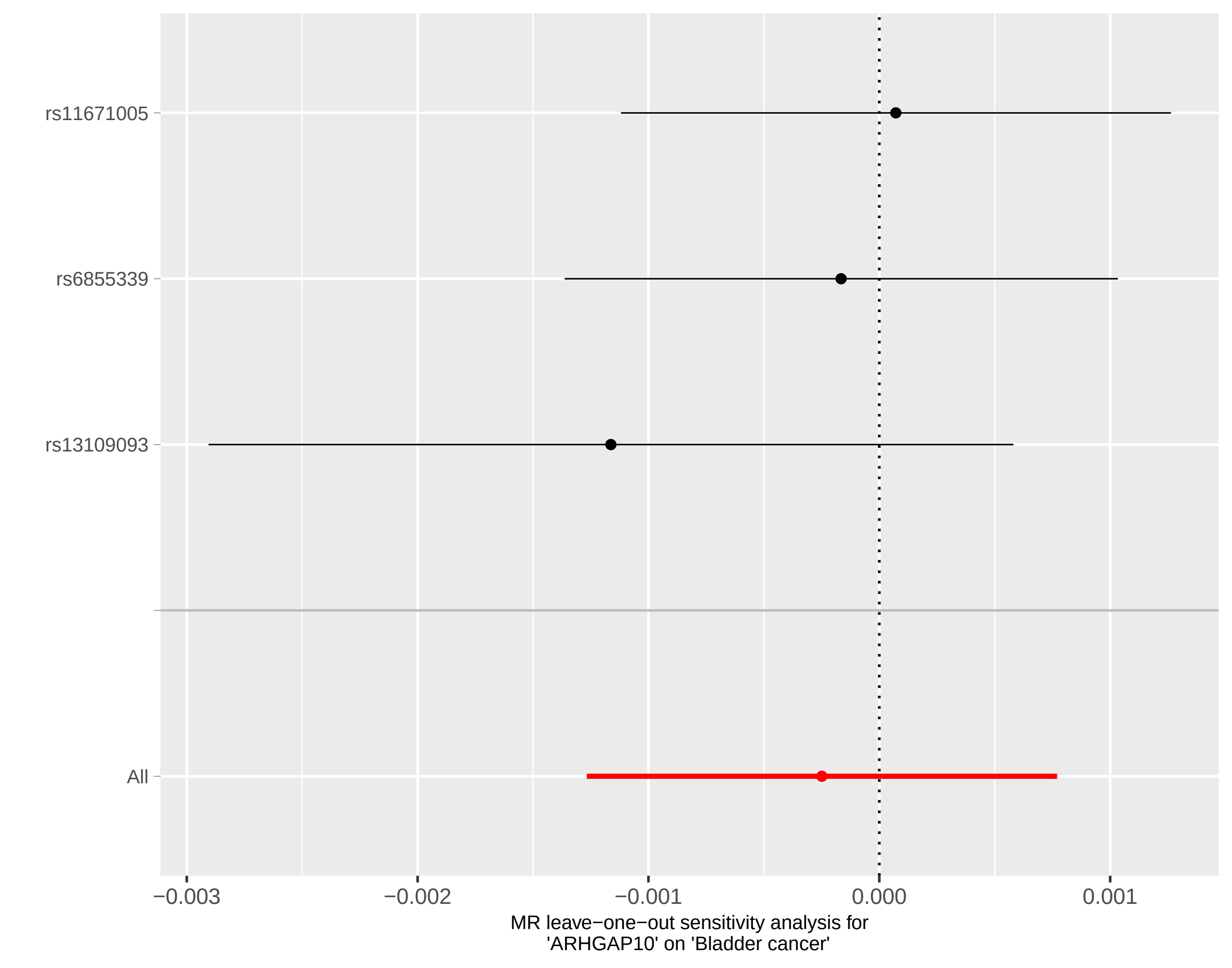
rs11671005
rs6855339
rs13109093
All
−0.003
−0.002
−0.001
0.000
0.001
MR leave−one−out sensitivity analysis for
'ARHGAP10' on 'Bladder cancer'

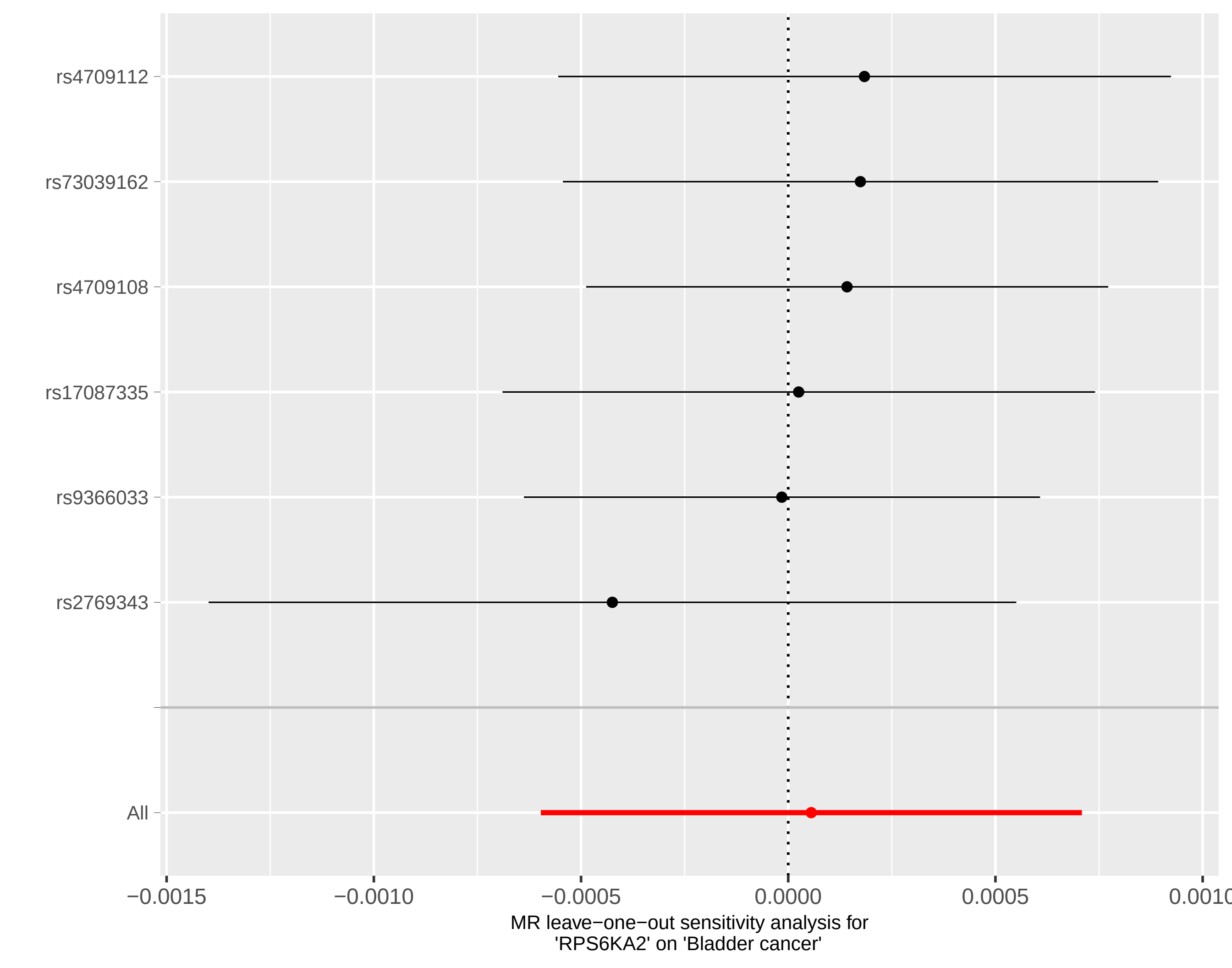

rs4709112
rs73039162
rs4709108
rs17087335
rs9366033
rs2769343
All
−0.0015
−0.0010
−0.0005
0.0000
0.0005
0.0010
MR leave−one−out sensitivity analysis for
'RPS6KA2' on 'Bladder cancer'

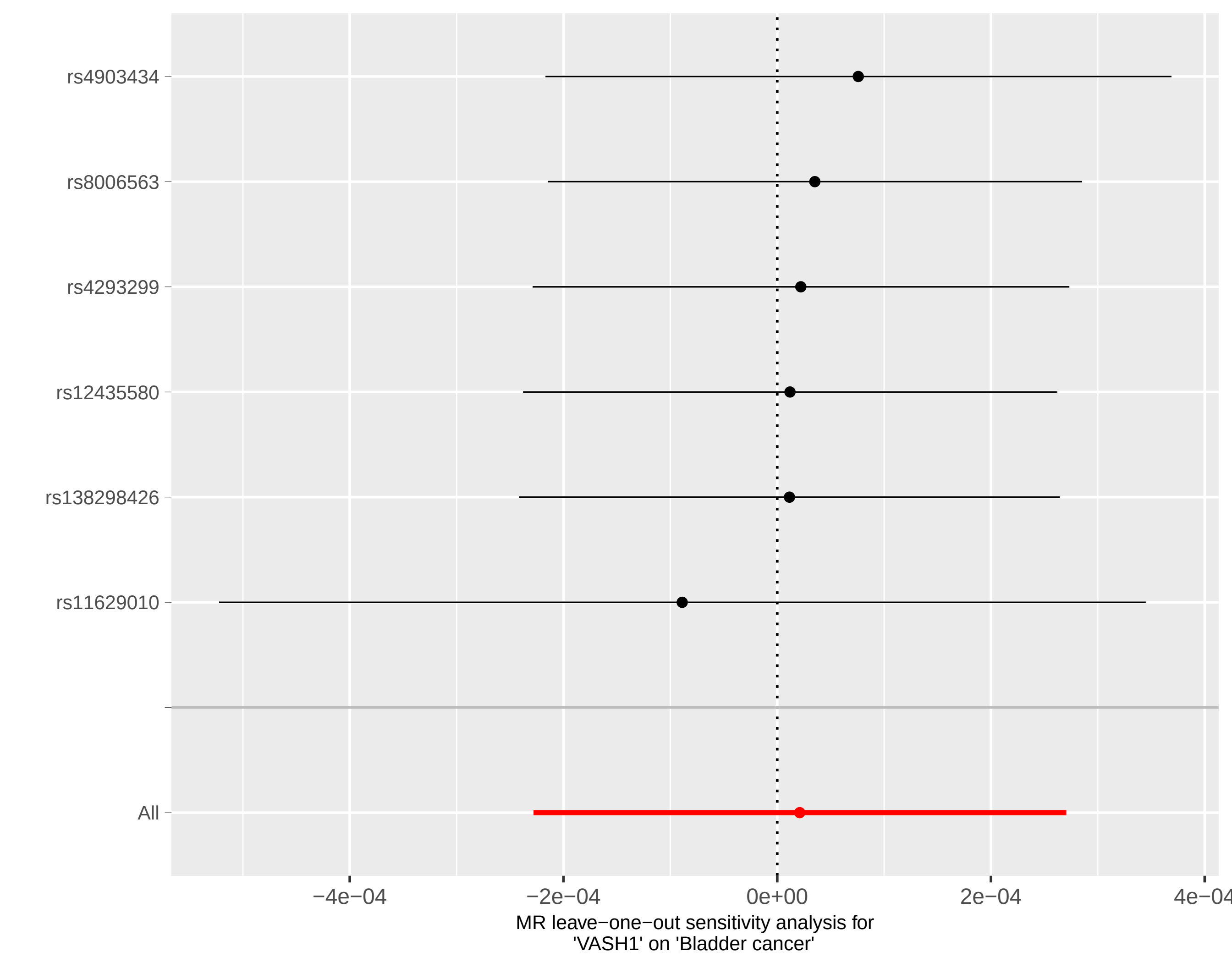
rs4903434
rs8006563
rs4293299
rs12435580
rs138298426
rs11629010
All
−4e−04
−2e−04
0e+00
2e−04
4e−04
MR leave−one−out sensitivity analysis for
'VASH1' on 'Bladder cancer'

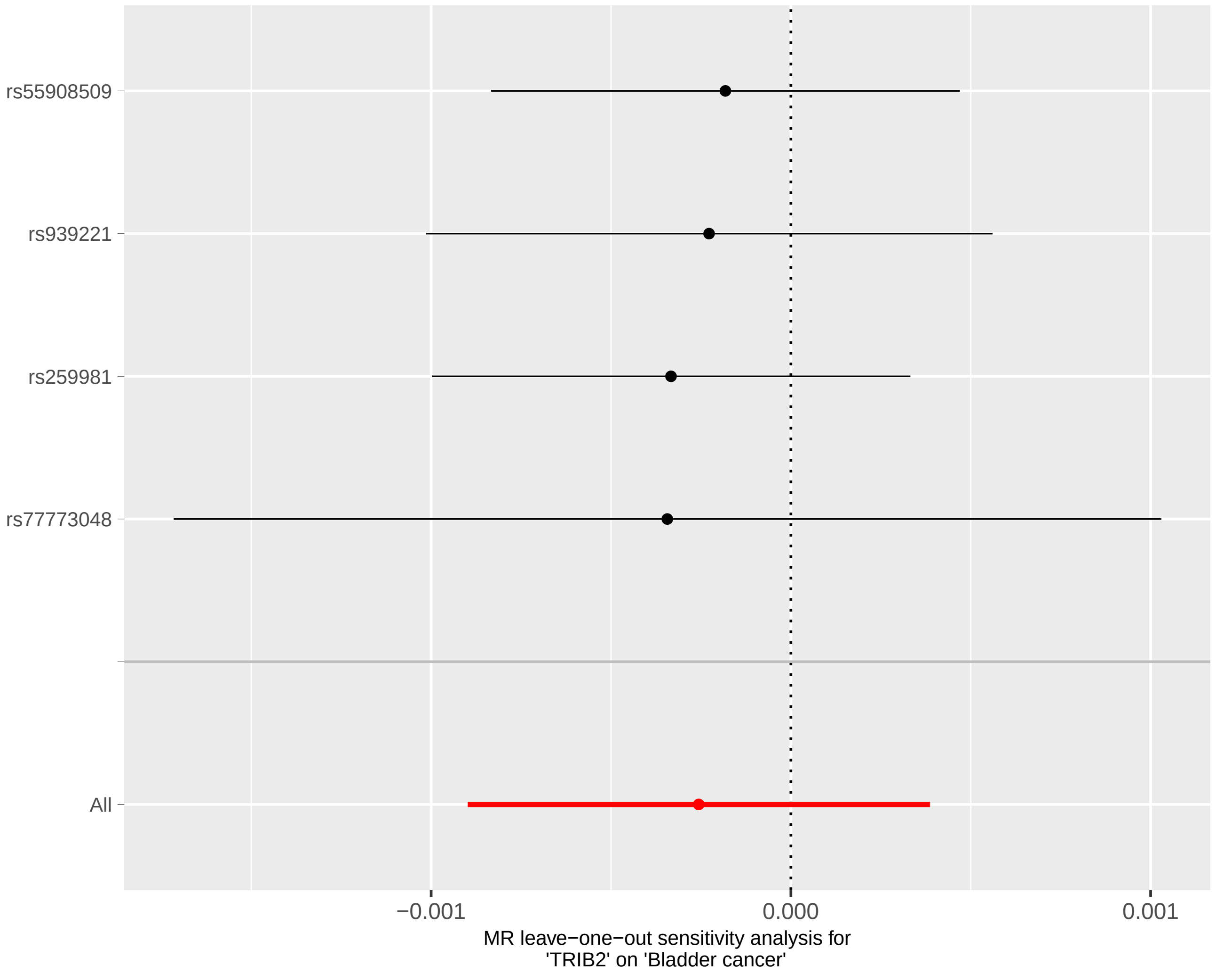

rs55908509
rs939221
rs259981
rs77773048
All
−0.001
0.000
0.001
MR leave−one−out sensitivity analysis for
'TRIB2' on 'Bladder cancer'

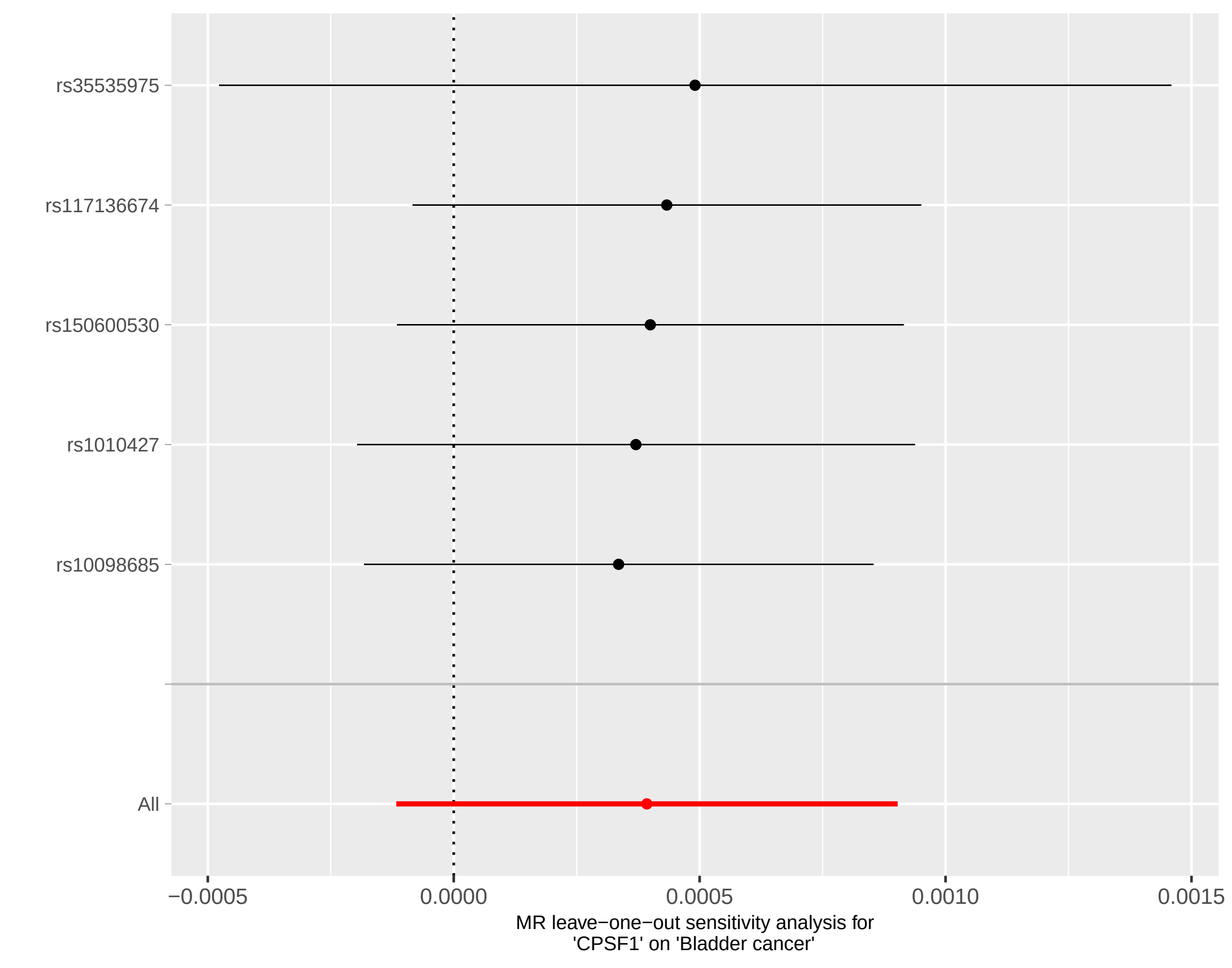
rs35535975
rs117136674
rs150600530
rs1010427
rs10098685
All
−0.0005
0.0000
0.0005
0.0010
0.0015
MR leave−one−out sensitivity analysis for
'CPSF1' on 'Bladder cancer'

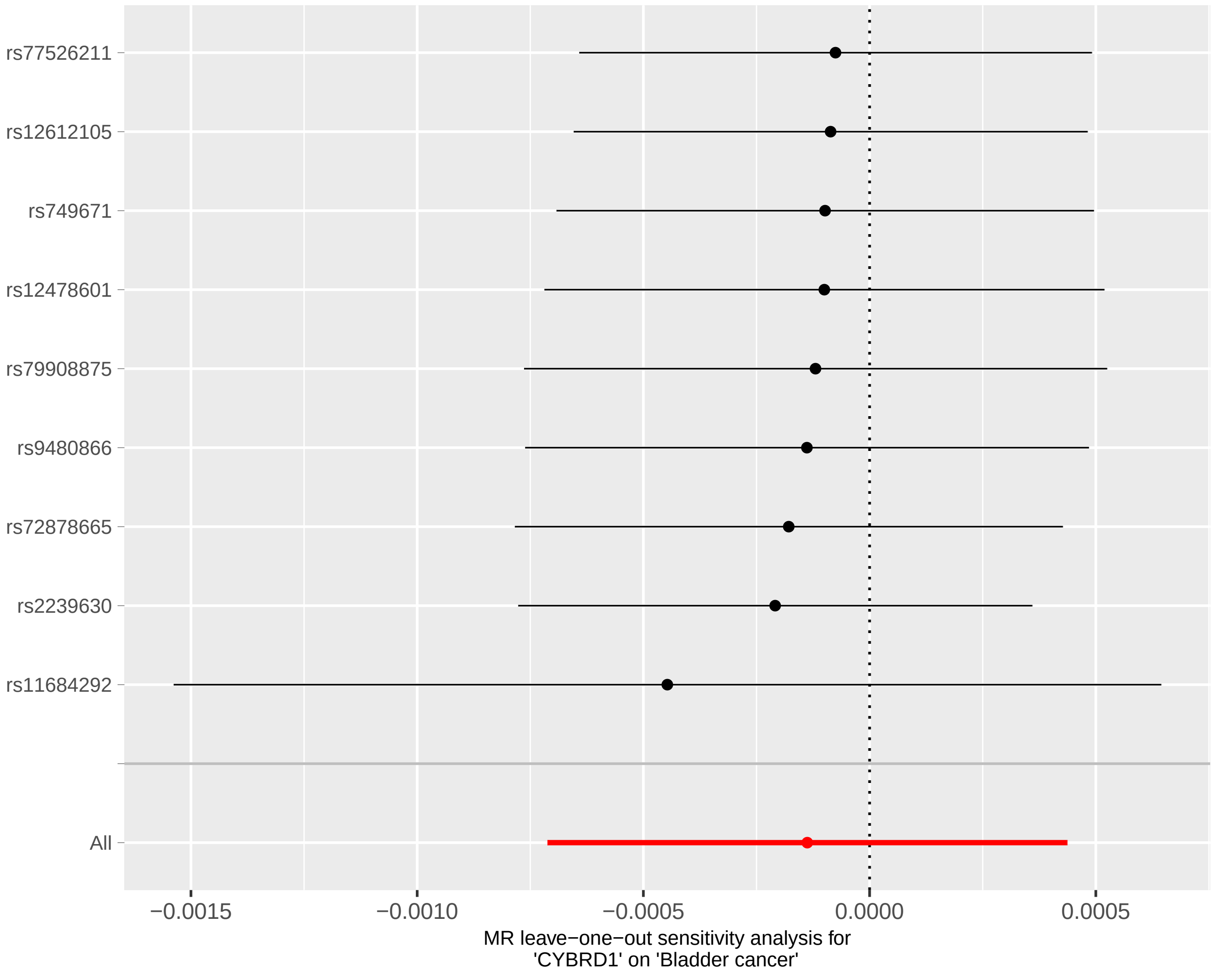
rs77526211
rs12612105
rs749671
rs12478601
rs79908875
rs9480866
rs72878665
rs2239630
rs11684292
All
−0.0015
−0.0010
−0.0005
0.0000
0.0005
MR leave−one−out sensitivity analysis for
'CYBRD1' on 'Bladder cancer'

Insufficient number of SNPs

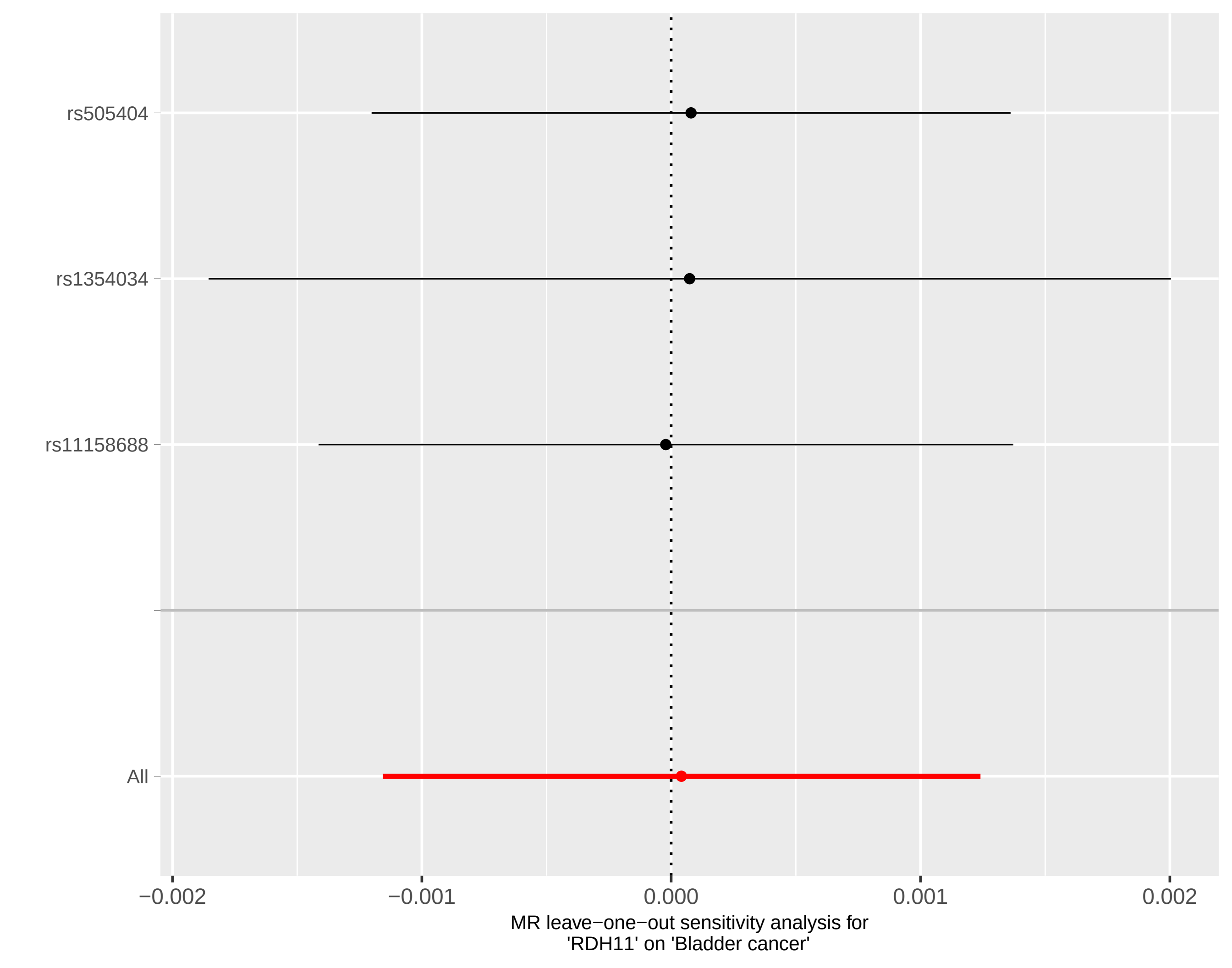
rs505404
rs1354034
rs11158688
All
−0.002
−0.001
0.000
0.001
0.002
MR leave−one−out sensitivity analysis for
'RDH11' on 'Bladder cancer'

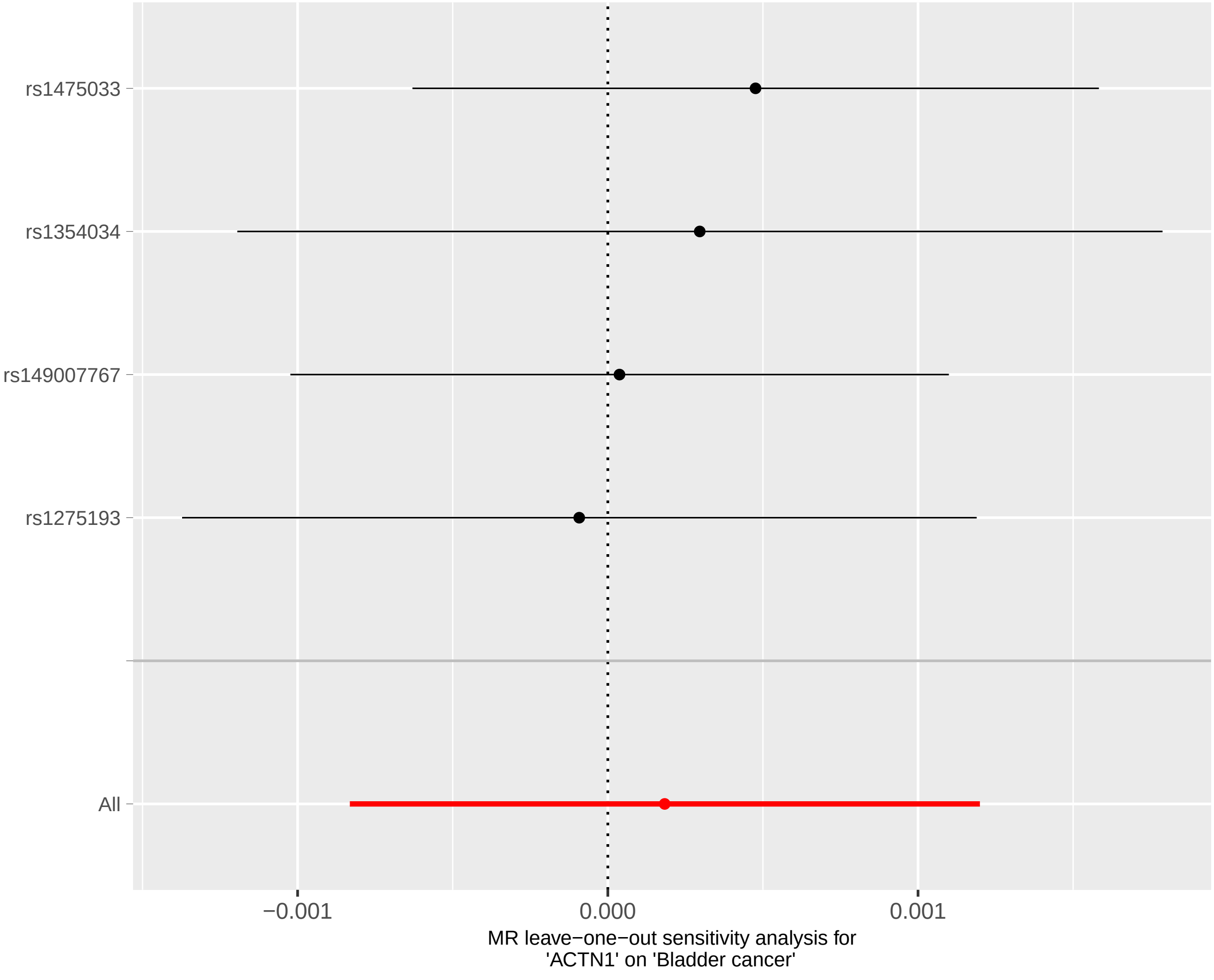
rs1475033
rs1354034
rs149007767
rs1275193
All
−0.001
0.000
0.001
MR leave−one−out sensitivity analysis for
'ACTN1' on 'Bladder cancer'

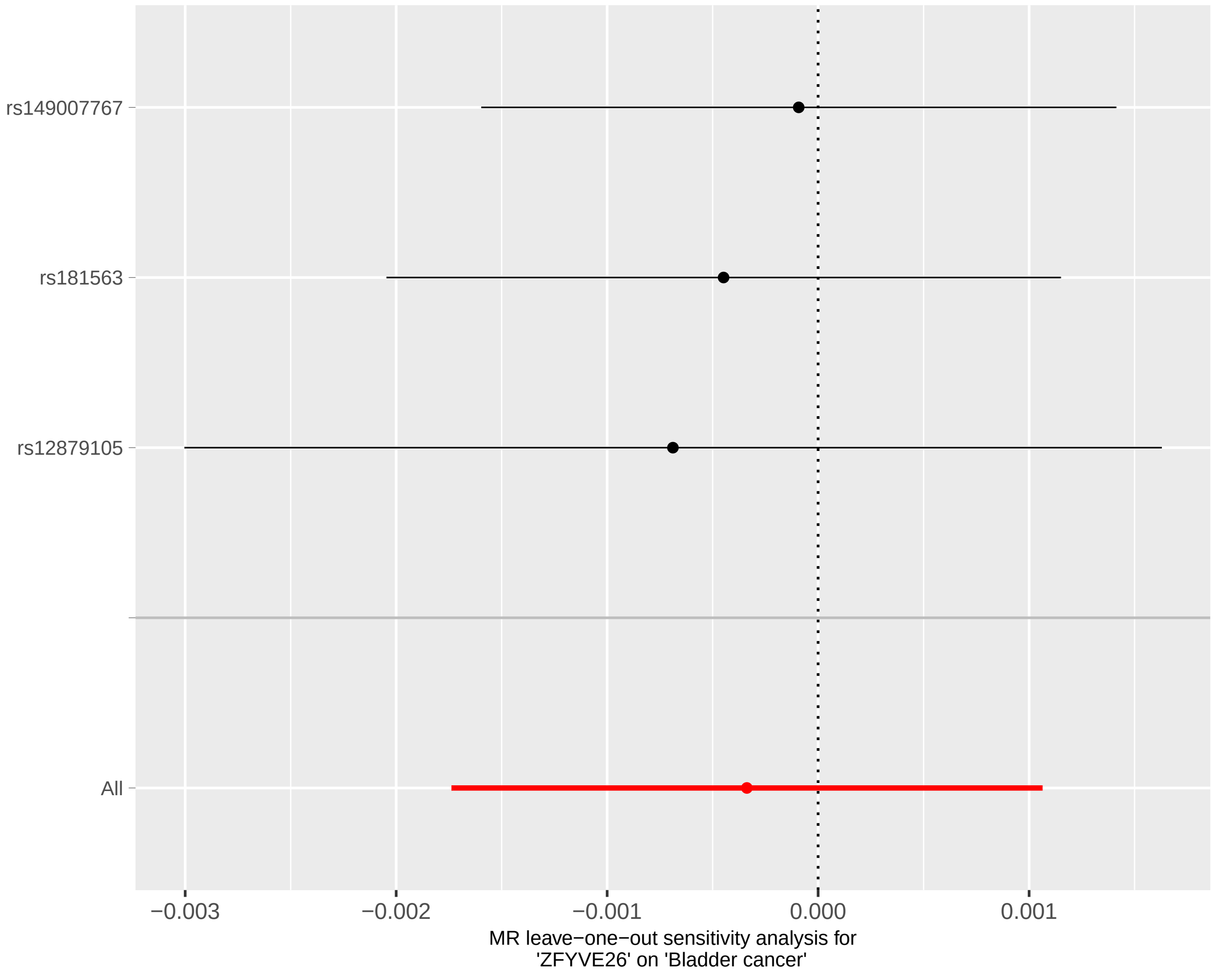
rs149007767
rs181563
rs12879105
All
−0.003
−0.002
−0.001
0.000
0.001
MR leave−one−out sensitivity analysis for
'ZFYVE26' on 'Bladder cancer'

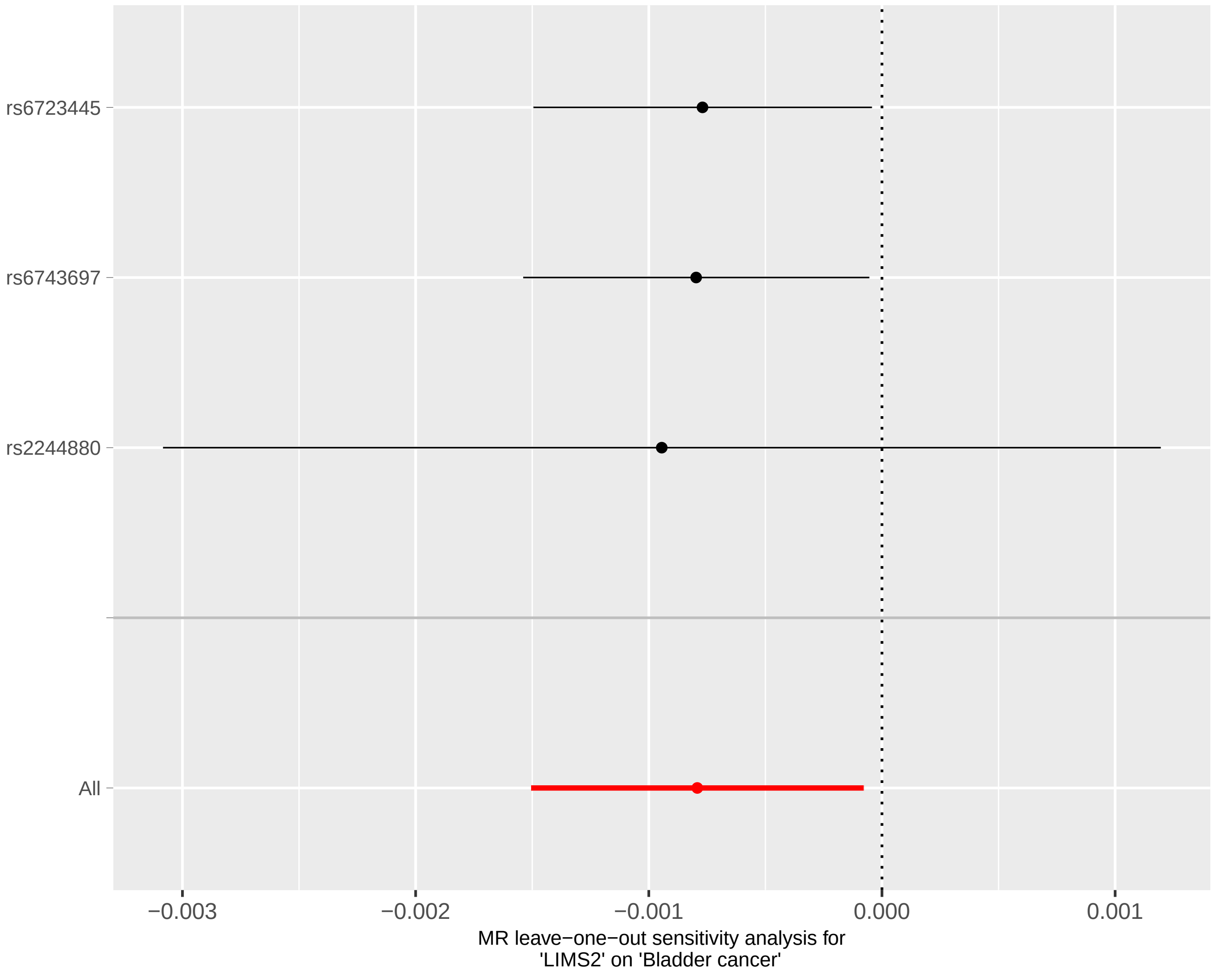
rs6723445
rs6743697
rs2244880
All
−0.003
−0.002
−0.001
0.000
0.001
MR leave−one−out sensitivity analysis for
'LIMS2' on 'Bladder cancer'

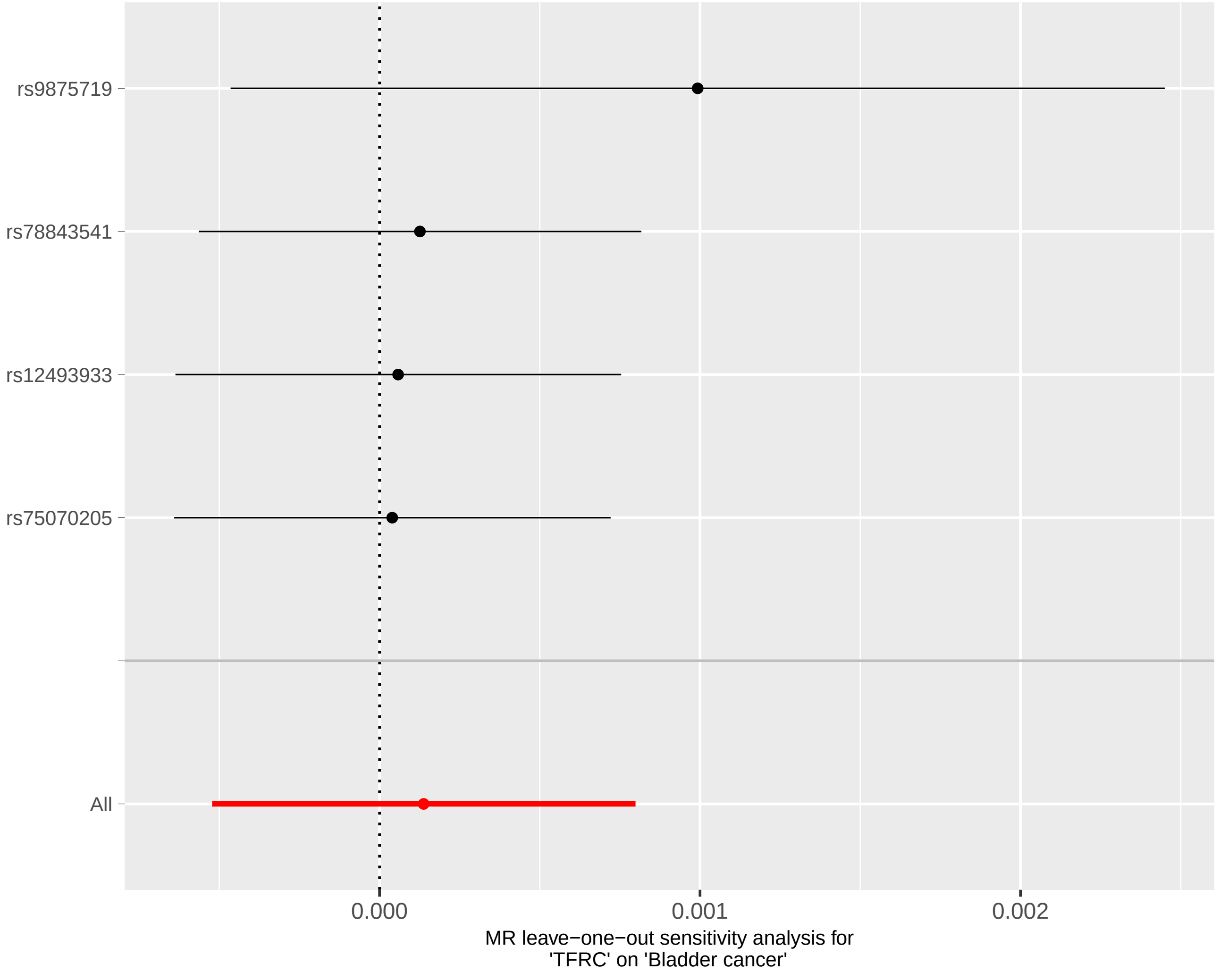
rs9875719
rs78843541
rs12493933
rs75070205
All
0.000
0.001
0.002
MR leave−one−out sensitivity analysis for
'TFRC' on 'Bladder cancer'

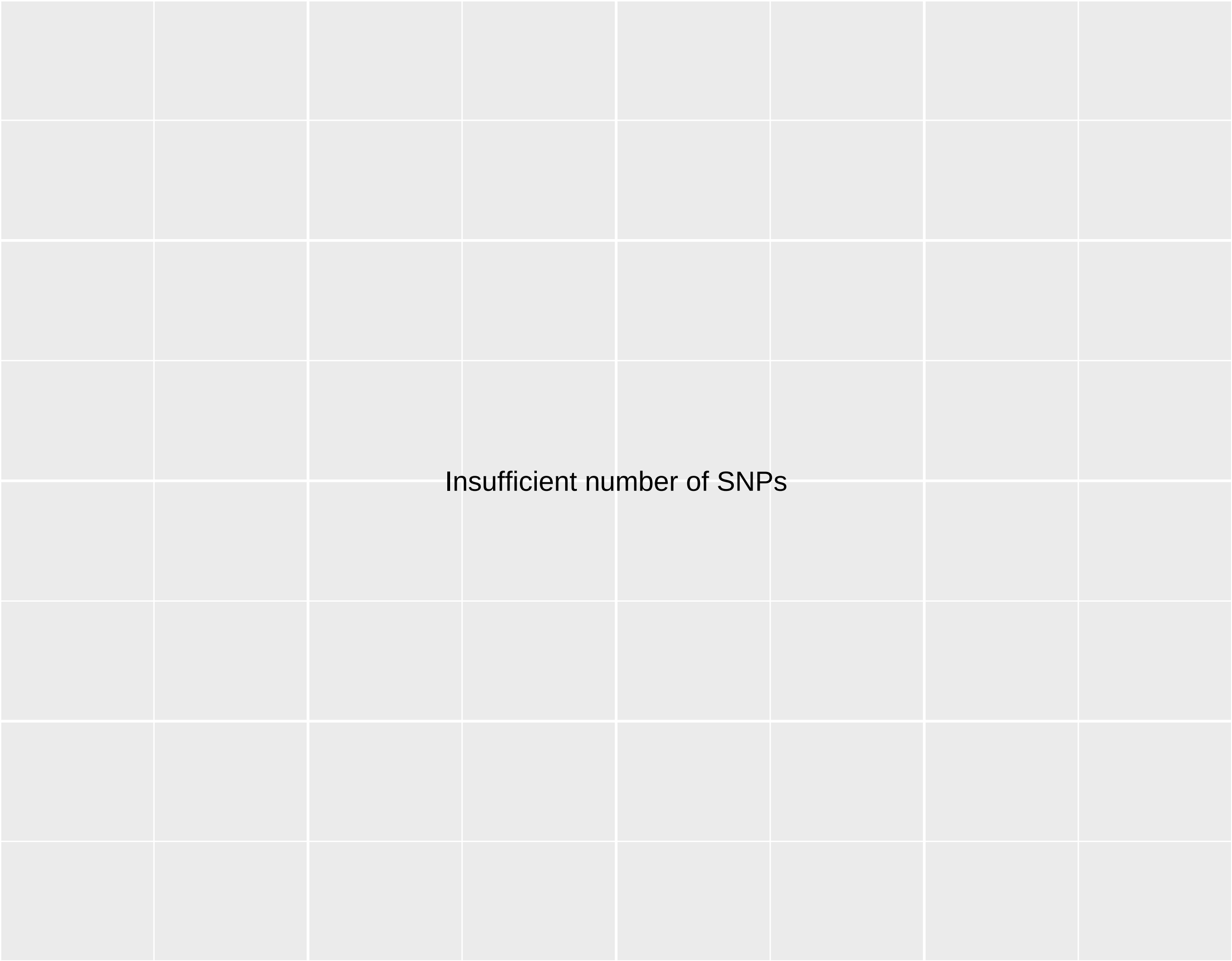
Insufficient number of SNPs

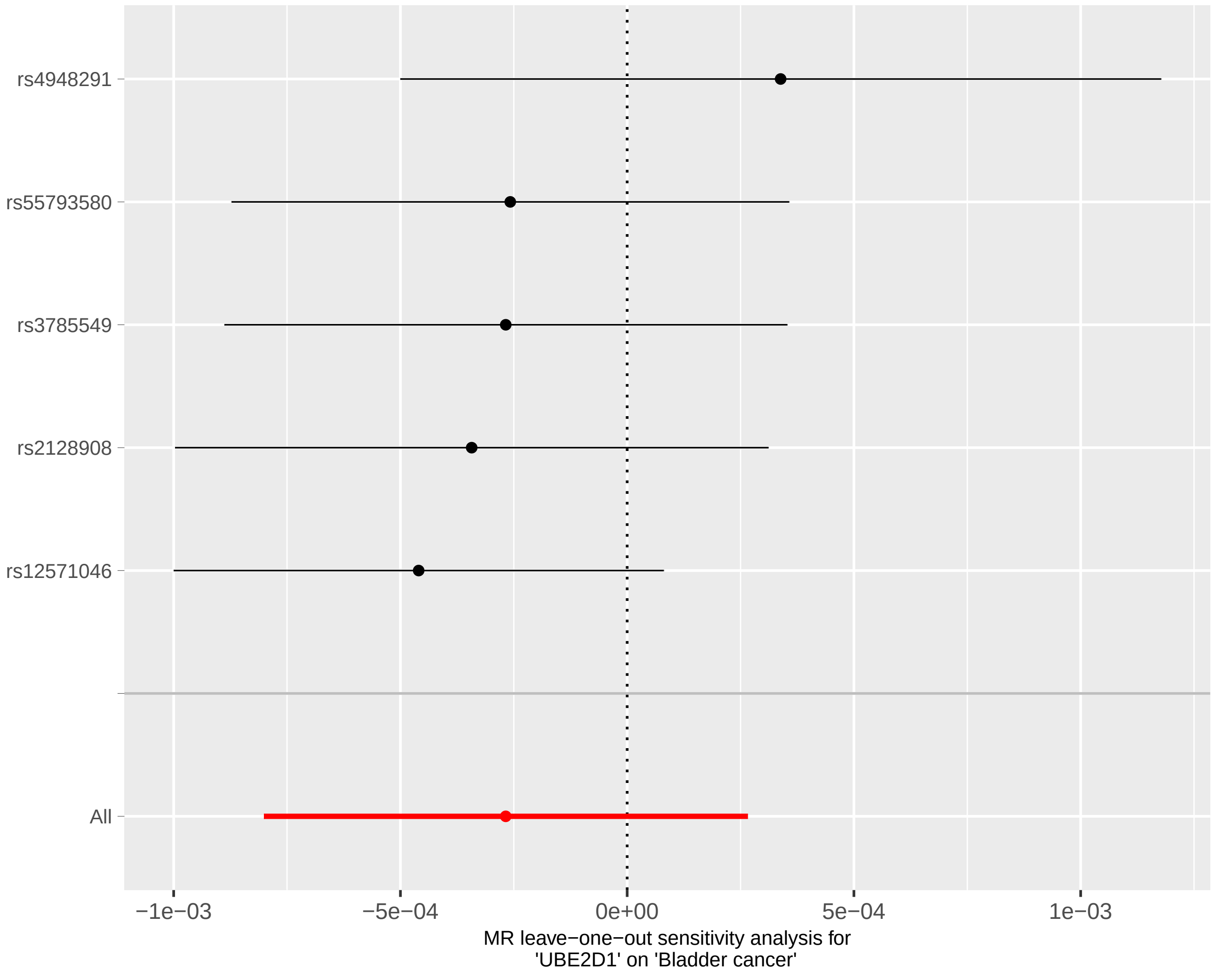
rs4948291
rs55793580
rs3785549
rs2128908
rs12571046
All
−1e−03
−5e−04
0e+00
5e−04
1e−03
MR leave−one−out sensitivity analysis for
'UBE2D1' on 'Bladder cancer'

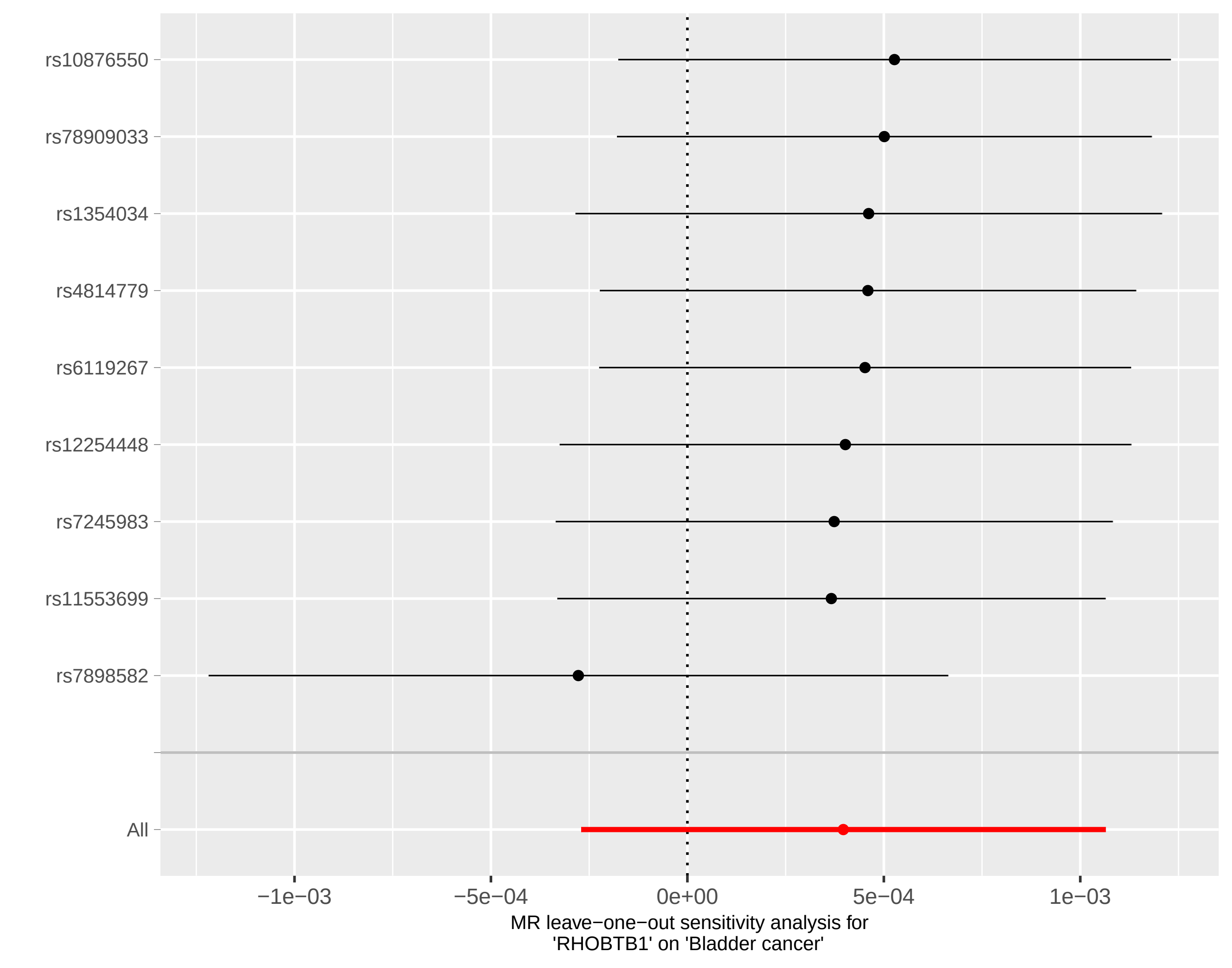
rs10876550
rs78909033
rs1354034
rs4814779
rs6119267
rs12254448
rs7245983
rs11553699
rs7898582
All
−1e−03
−5e−04
0e+00
5e−04
1e−03
MR leave−one−out sensitivity analysis for
'RHOBTB1' on 'Bladder cancer'

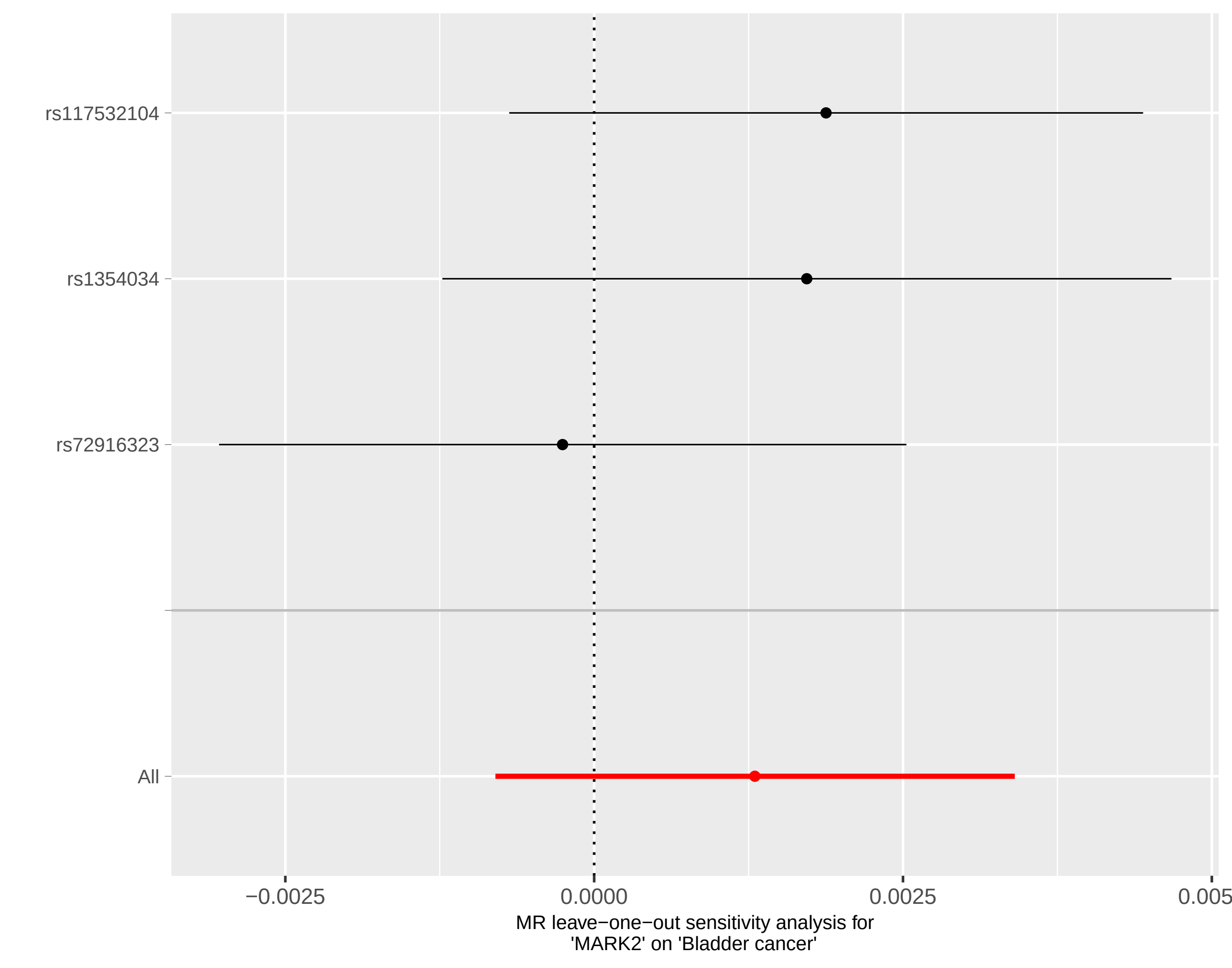

rs117532104
rs1354034
rs72916323
All
−0.0025
0.0000
0.0025
0.005
MR leave−one−out sensitivity analysis for
'MARK2' on 'Bladder cancer'

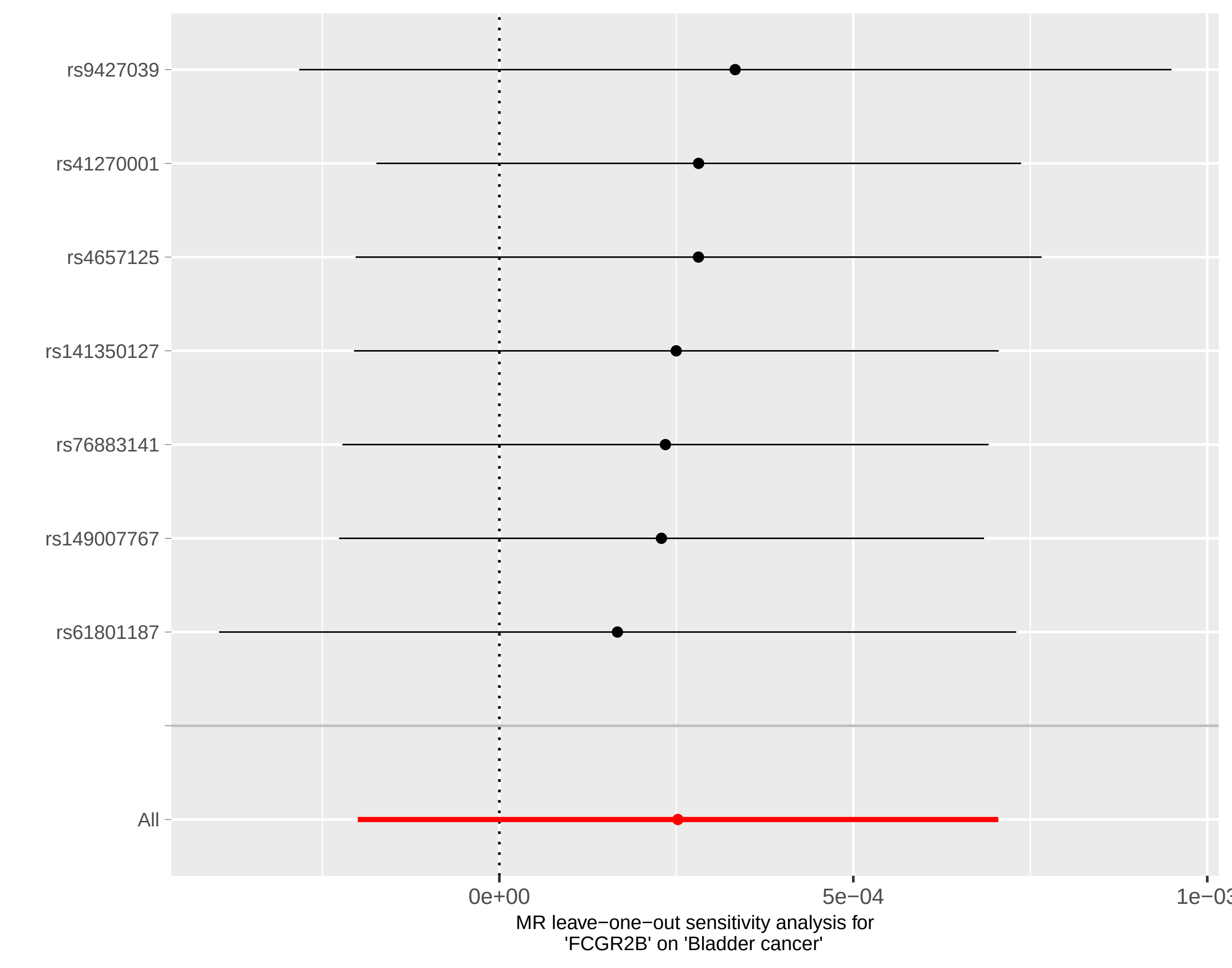
rs9427039
rs41270001
rs4657125
rs141350127
rs76883141
rs149007767
rs61801187
All
0e+00
5e−04
1e−03
MR leave−one−out sensitivity analysis for
'FCGR2B' on 'Bladder cancer'

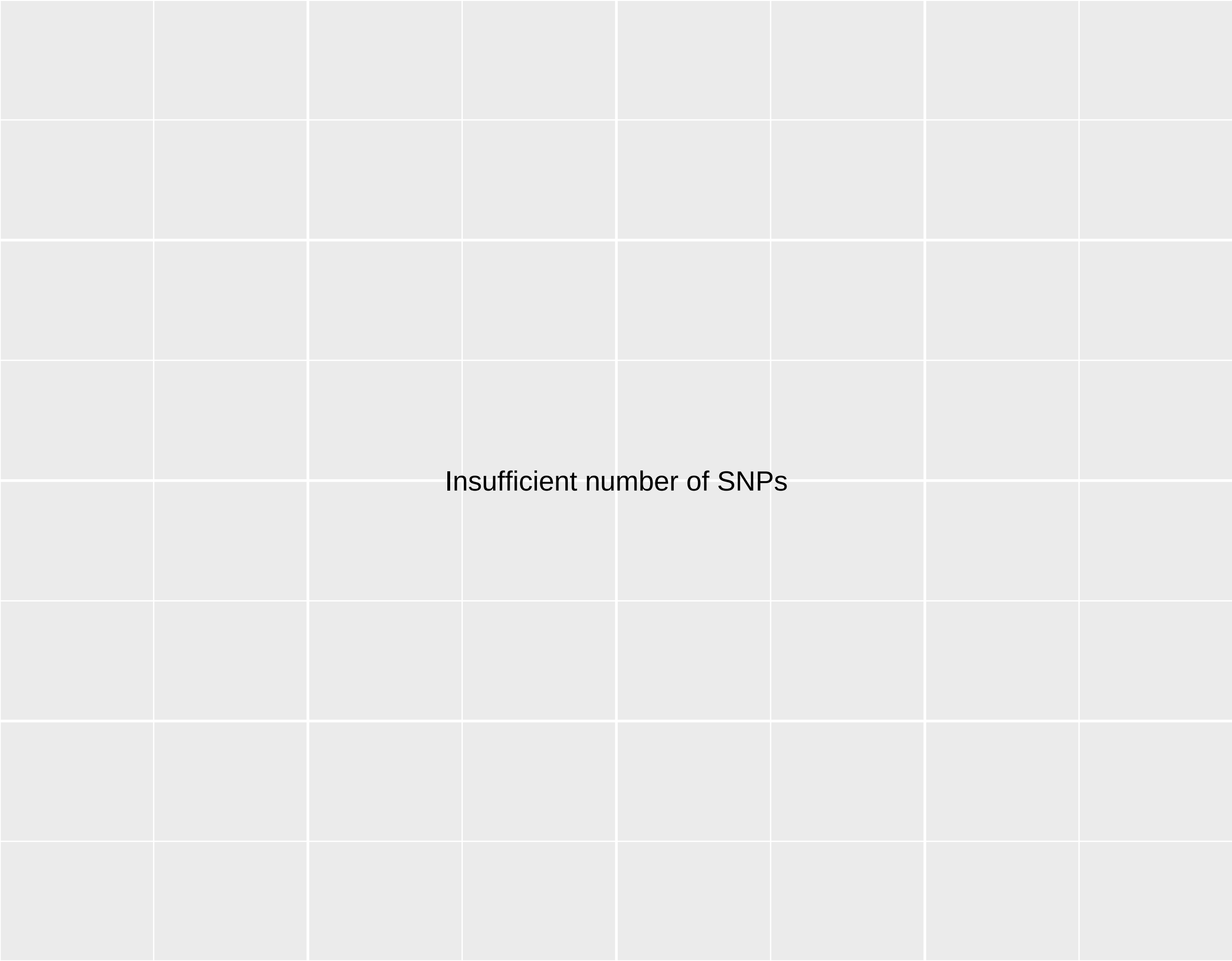
Insufficient number of SNPs

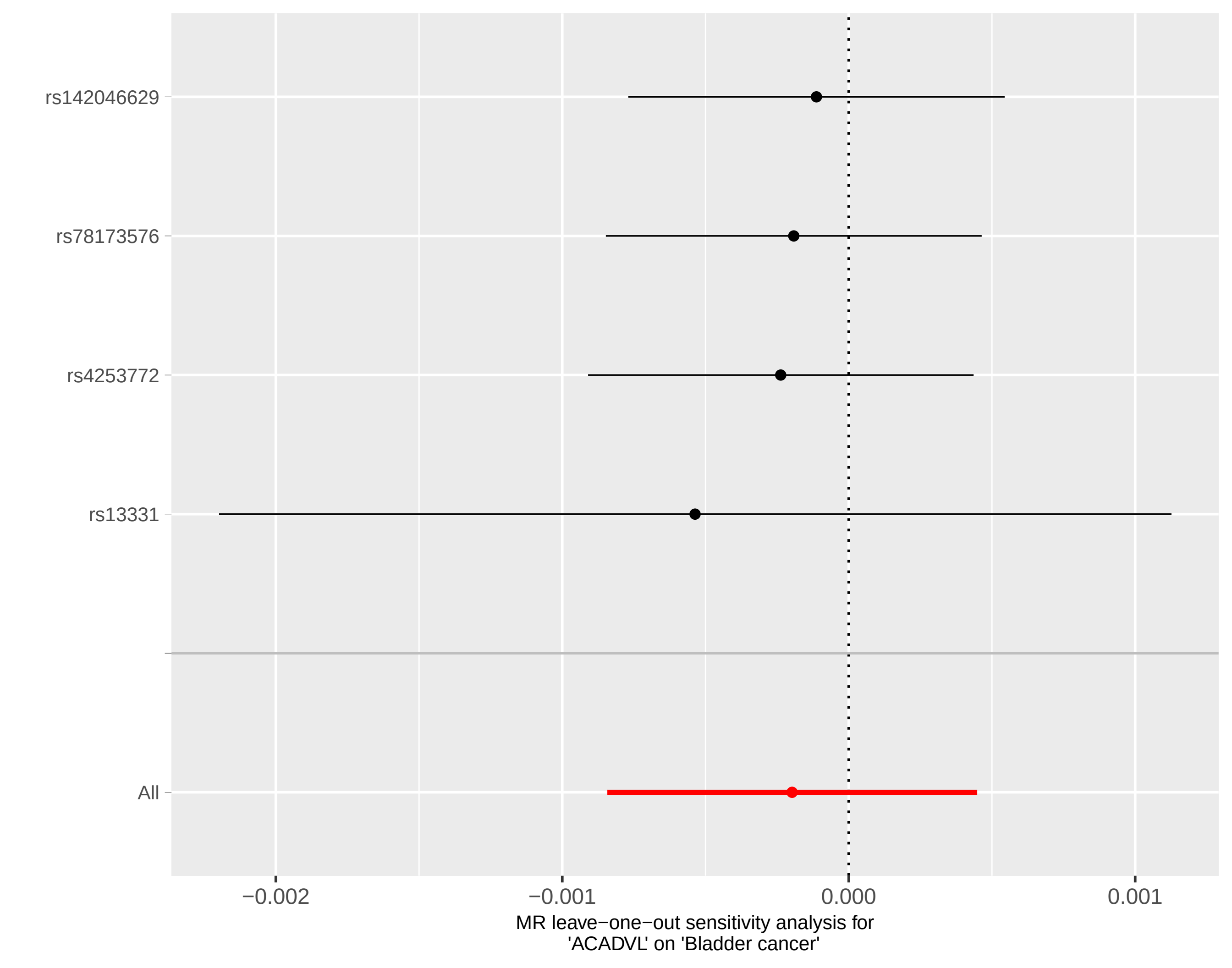
rs142046629
rs78173576
rs4253772
rs13331
All
−0.002
−0.001
0.000
0.001
MR leave−one−out sensitivity analysis for
'ACADVL' on 'Bladder cancer'

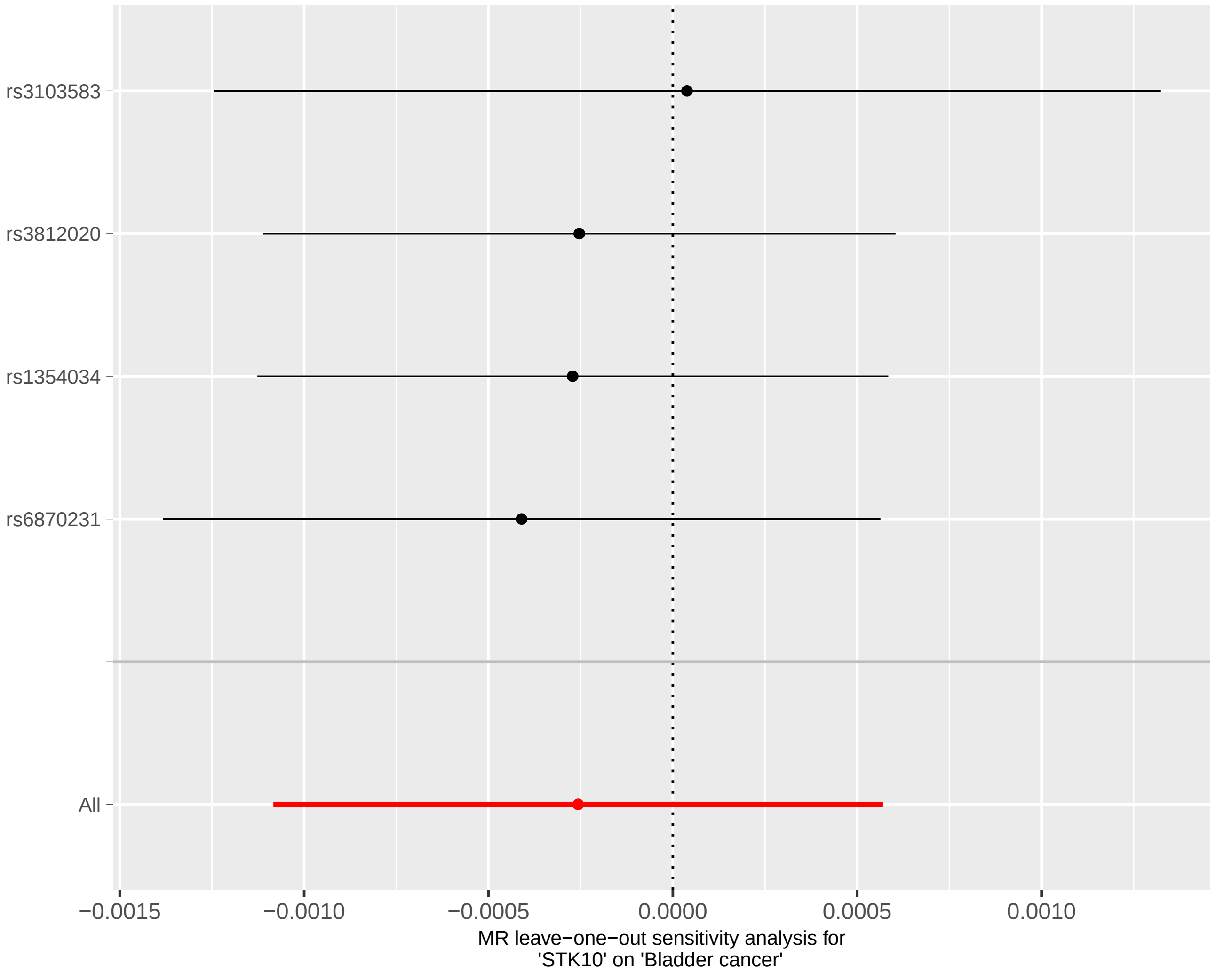
rs3103583
rs3812020
rs1354034
rs6870231
All
−0.0015
−0.0010
−0.0005
0.0000
0.0005
0.0010
MR leave−one−out sensitivity analysis for
'STK10' on 'Bladder cancer'

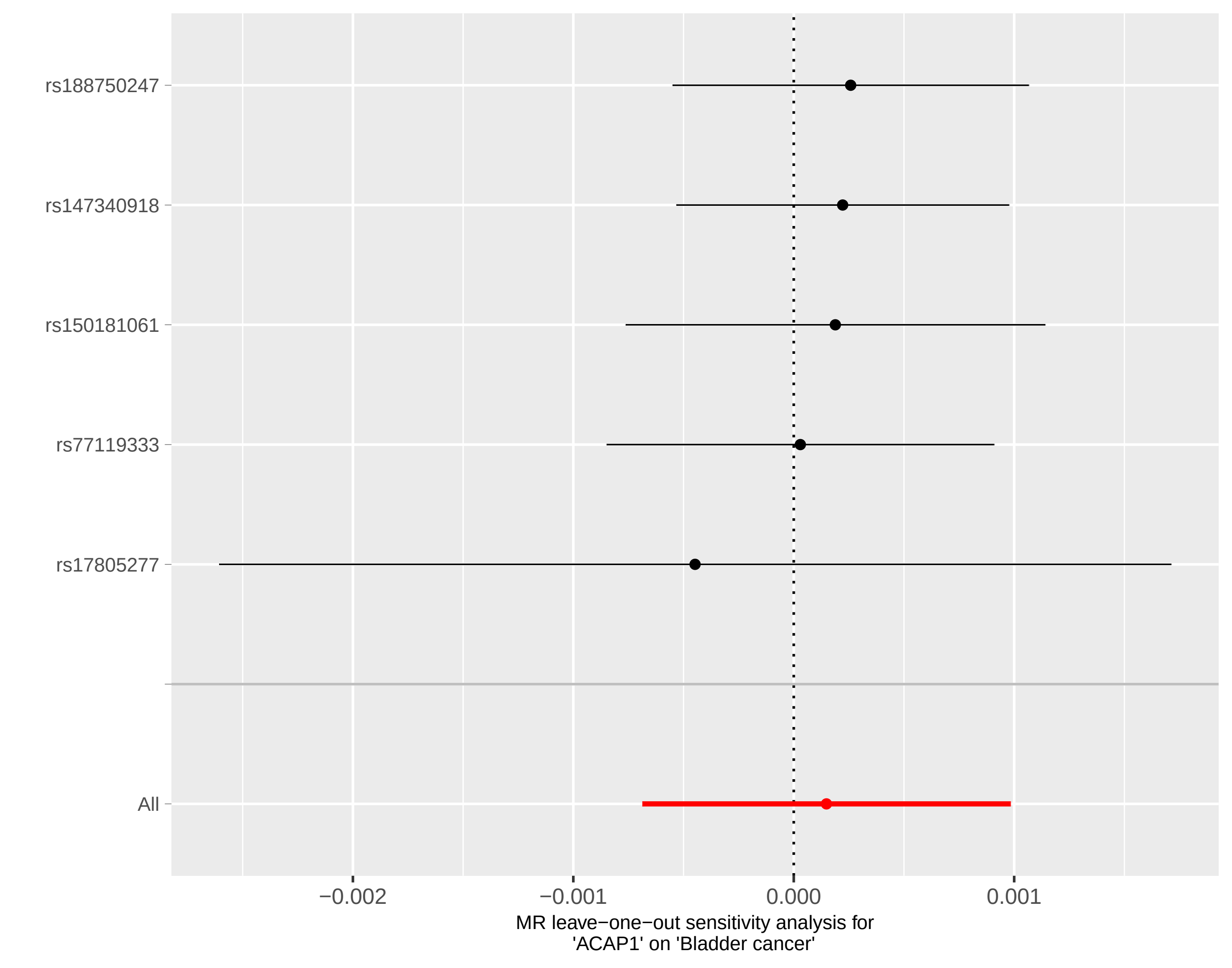

rs188750247
rs147340918
rs150181061
rs77119333
rs17805277
All
−0.002
−0.001
0.000
0.001
MR leave−one−out sensitivity analysis for
'ACAP1' on 'Bladder cancer'

Insufficient number of SNPs

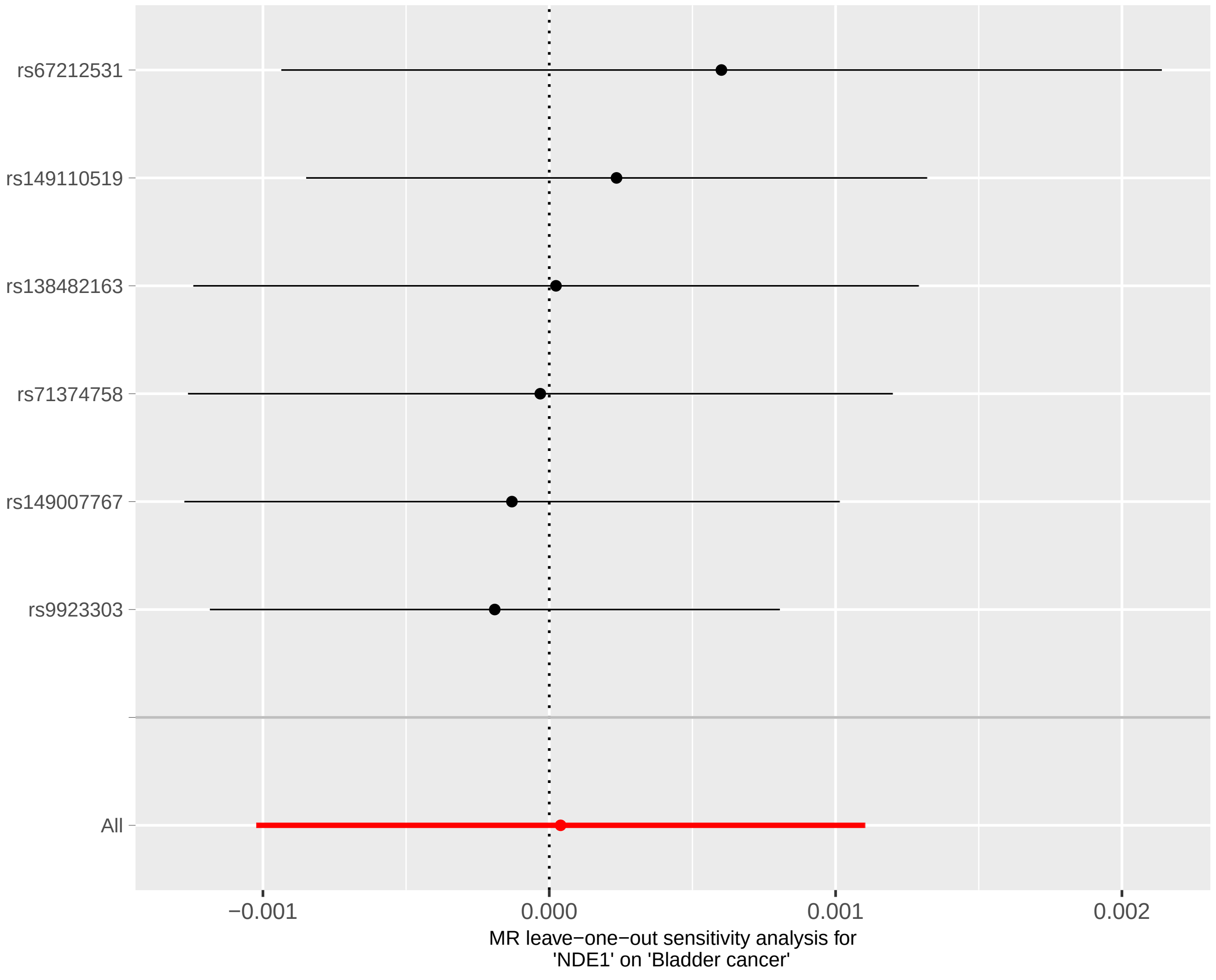

rs67212531
rs149110519
rs138482163
rs71374758
rs149007767
rs9923303
All
−0.001
0.000
0.001
0.002
MR leave−one−out sensitivity analysis for
'NDE1' on 'Bladder cancer'

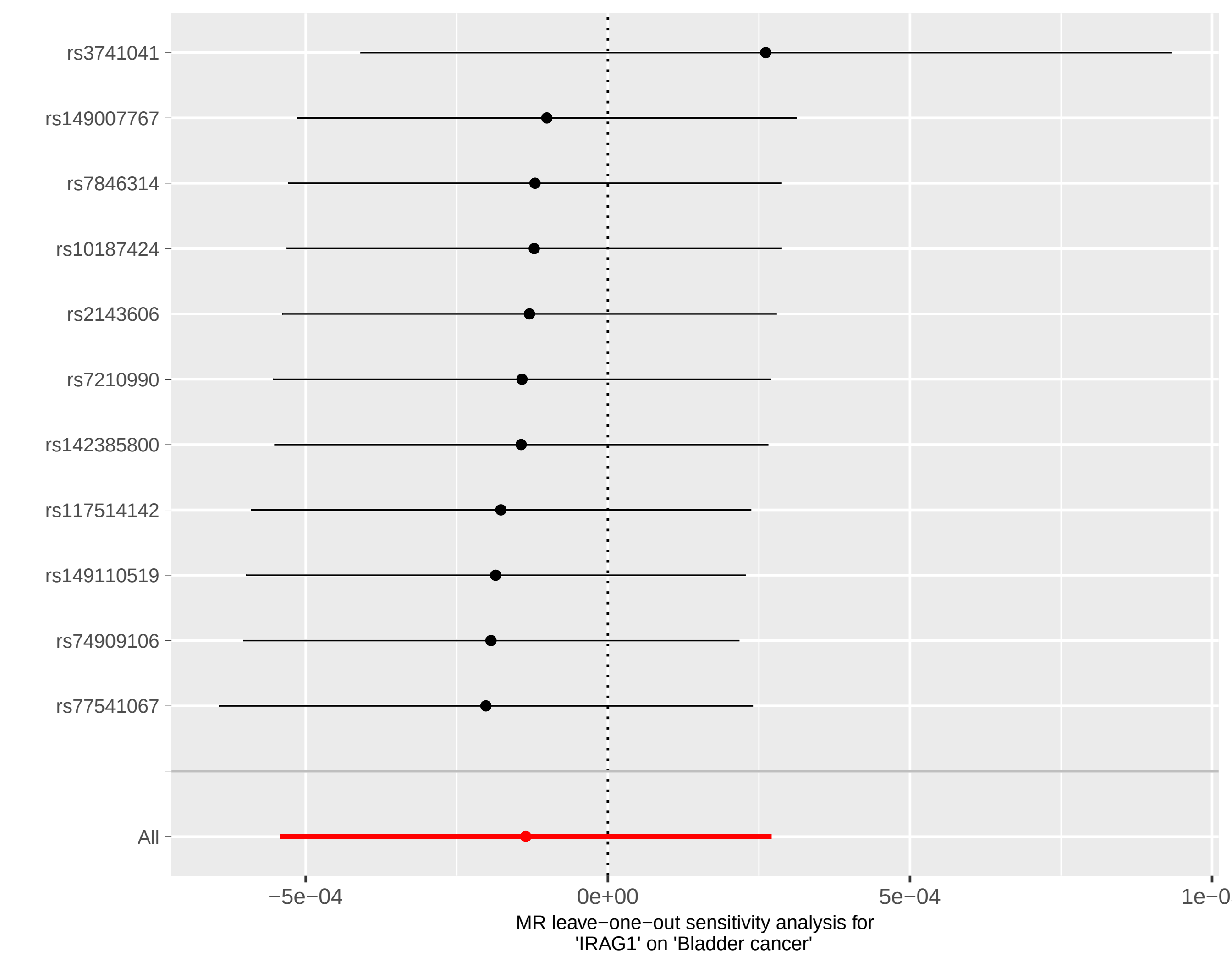
rs3741041
rs149007767
rs7846314
rs10187424
rs2143606
rs7210990
rs142385800
rs117514142
rs149110519
rs74909106
rs77541067
All
−5e−04
0e+00
5e−04
MR leave−one−out sensitivity analysis for
'IRAG1' on 'Bladder cancer'

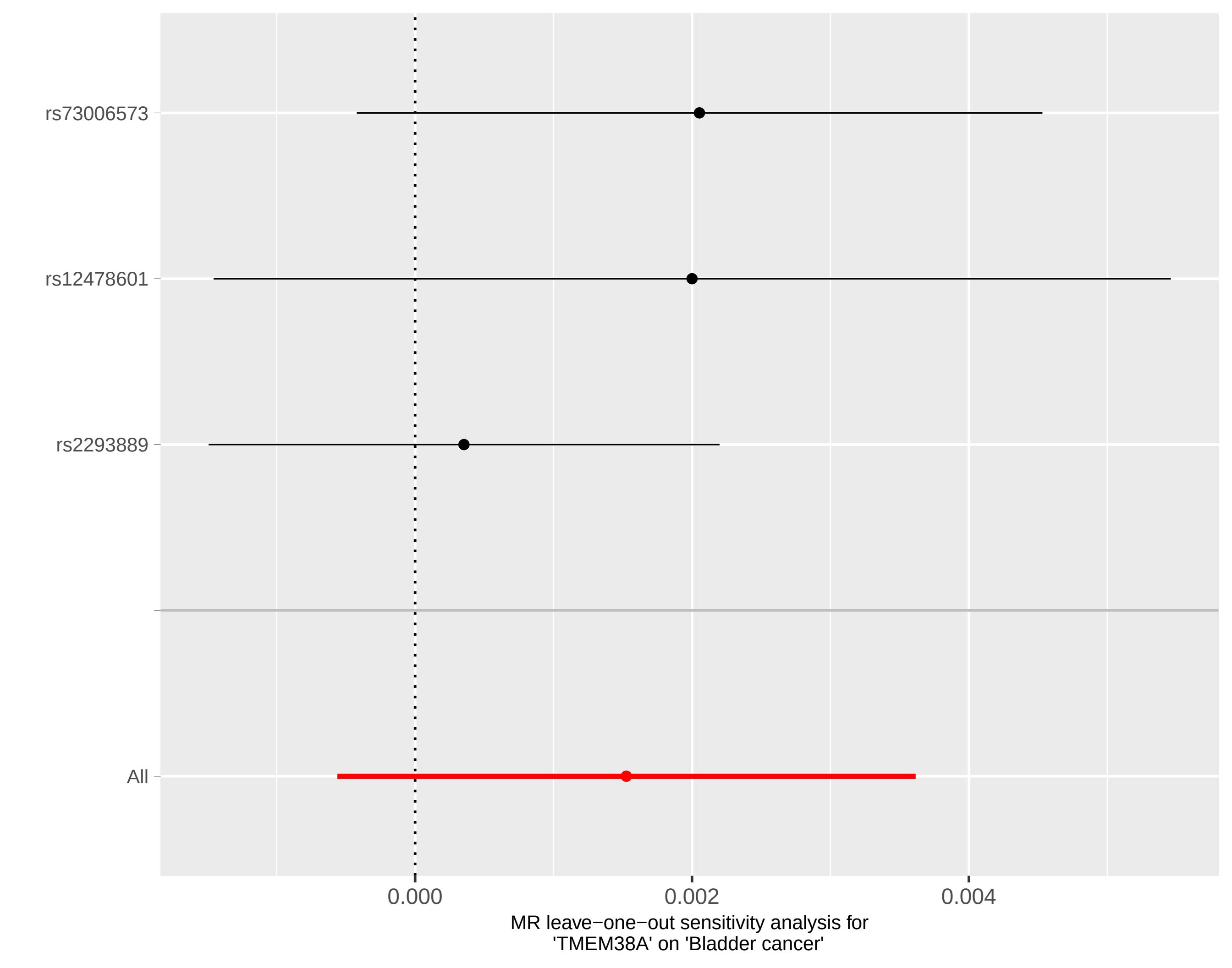
rs73006573
rs12478601
rs2293889
All
0.000
0.002
0.004
MR leave−one−out sensitivity analysis for
'TMEM38A' on 'Bladder cancer'

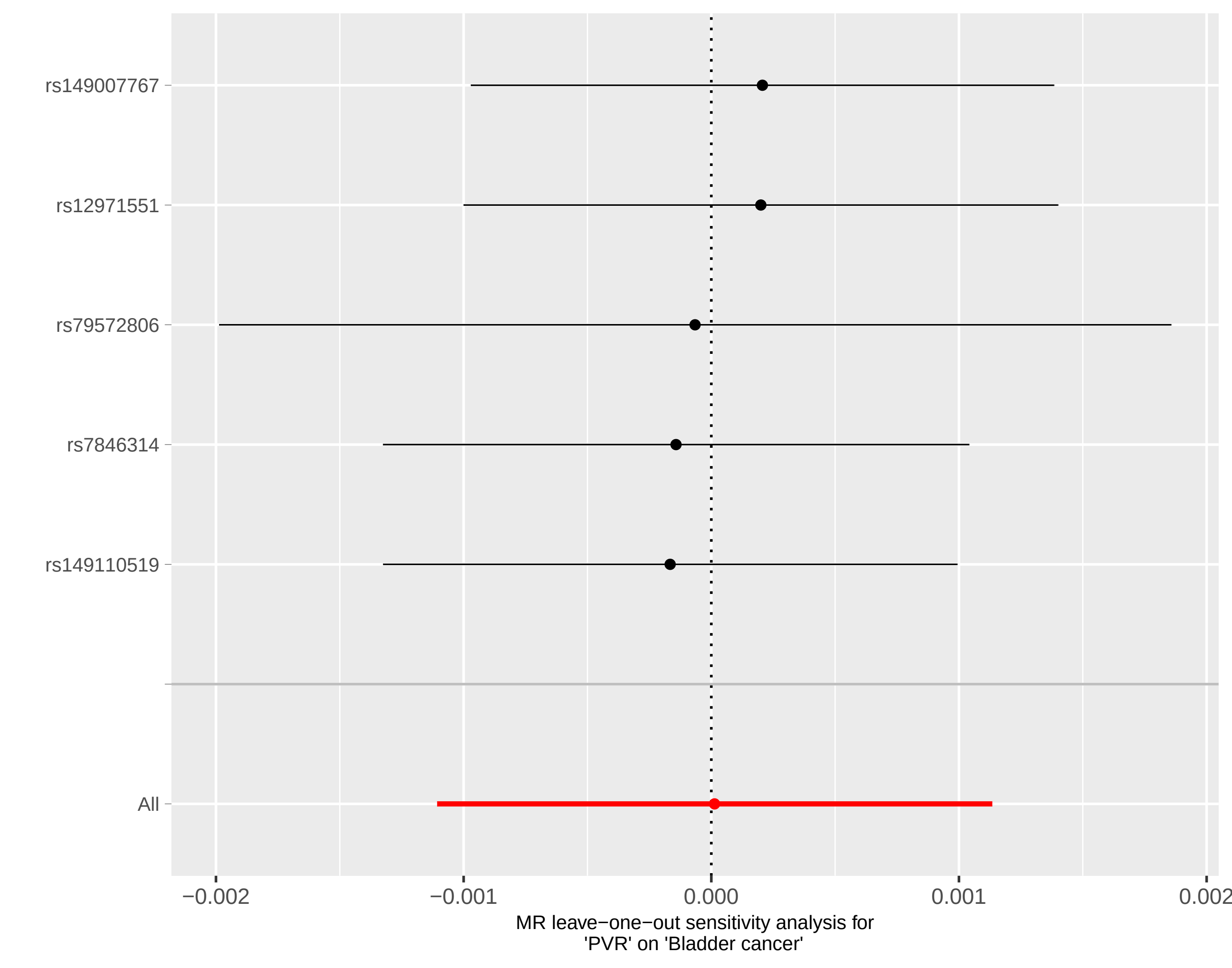
rs149007767
rs12971551
rs79572806
rs7846314
rs149110519
All
−0.002
−0.001
0.000
0.001
0.002
MR leave−one−out sensitivity analysis for
'PVR' on 'Bladder cancer'

Insufficient number of SNPs

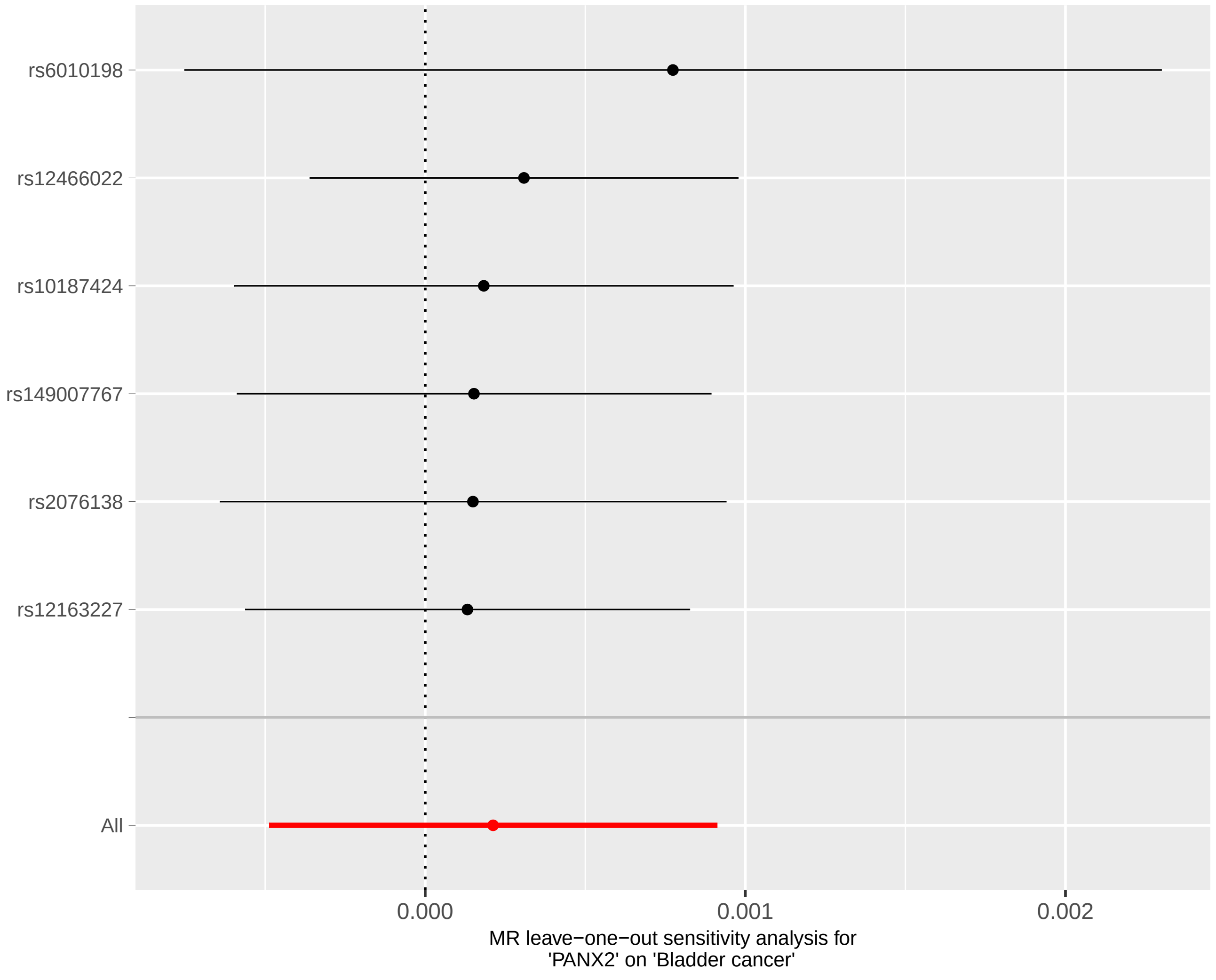
rs6010198
rs12466022
rs10187424
rs149007767
rs2076138
rs12163227
All
0.000
0.001
0.002
MR leave−one−out sensitivity analysis for
'PANX2' on 'Bladder cancer'

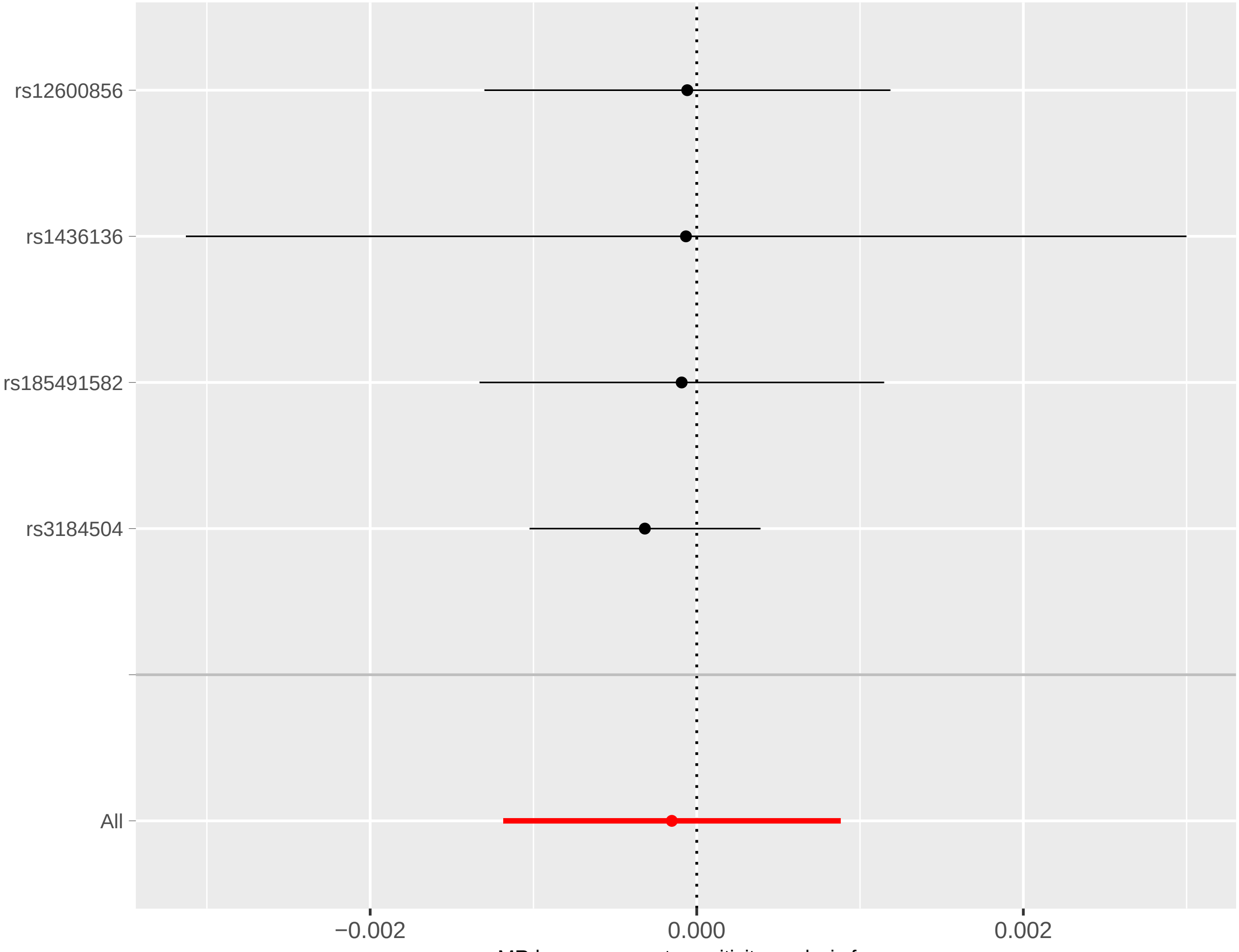

MR leave−one−out sensitivity analysis for
'ALPK1' on 'Bladder cancer'

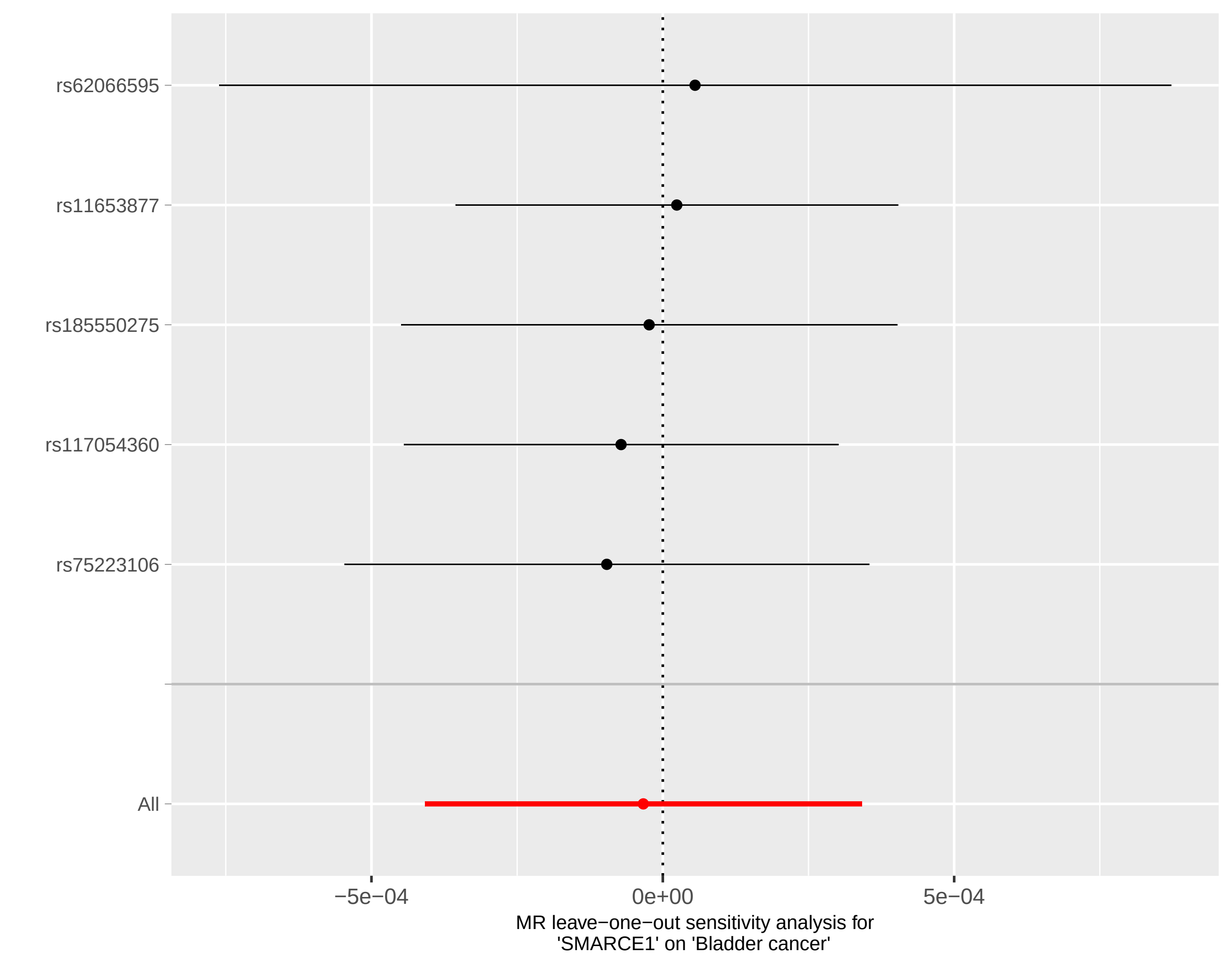
rs62066595
rs11653877
rs185550275
rs117054360
rs75223106
All
−5e−04
0e+00
5e−04
MR leave−one−out sensitivity analysis for
'SMARCE1' on 'Bladder cancer'

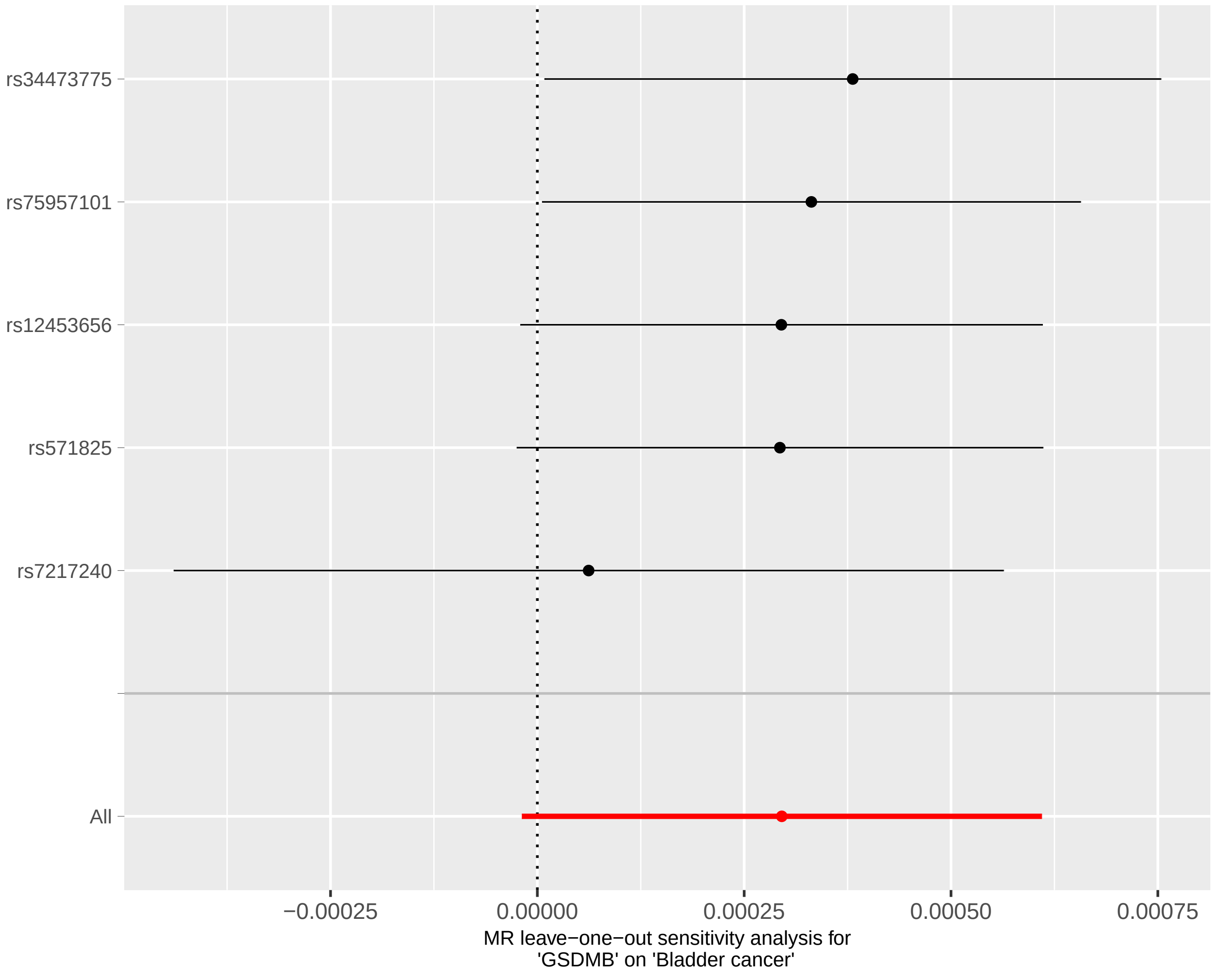
rs34473775
rs75957101
rs12453656
rs571825
rs7217240
All
−0.00025
0.00000
0.00025
0.00050
0.00075
MR leave−one−out sensitivity analysis for
'GSDMB' on 'Bladder cancer'

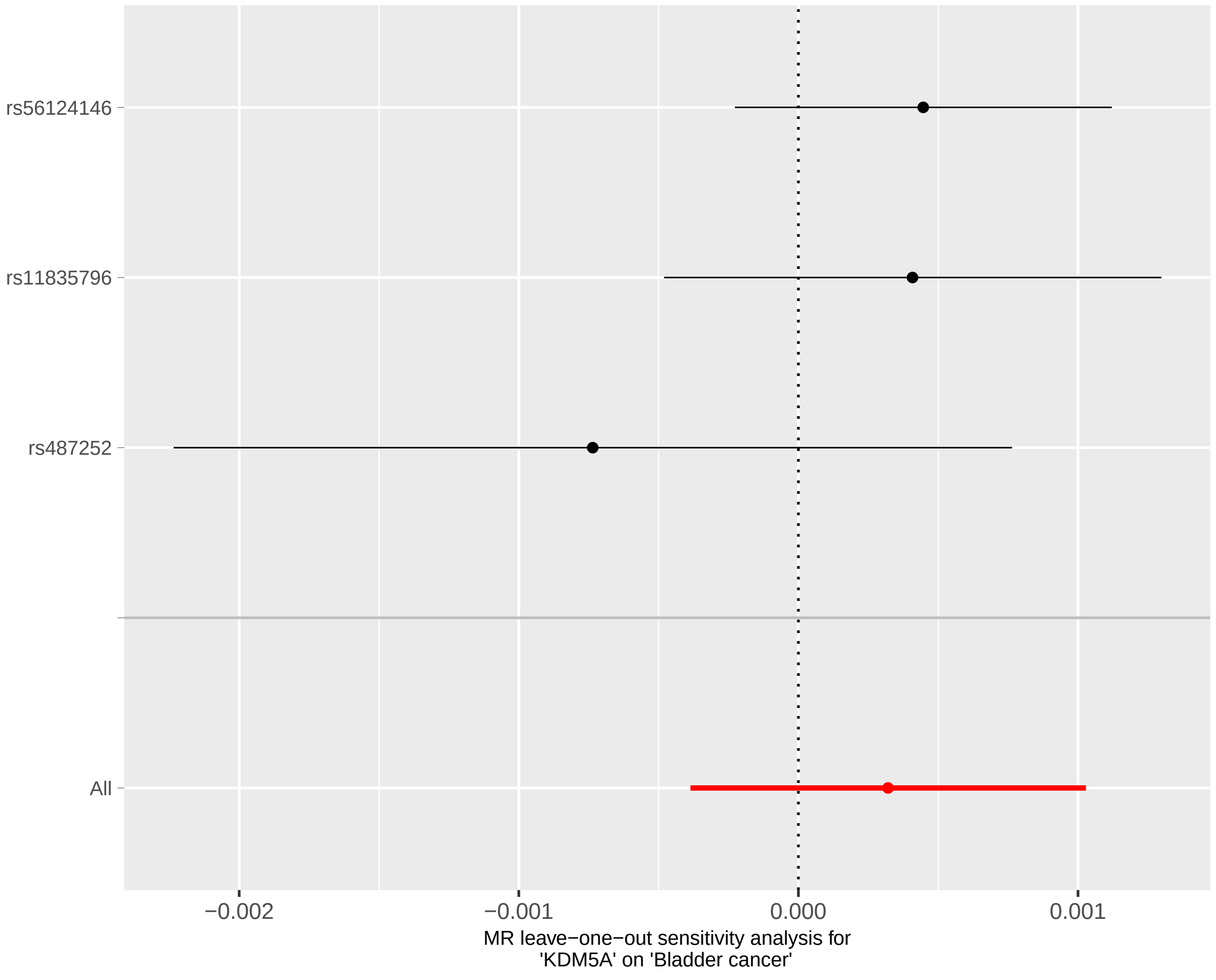
rs56124146
rs11835796
rs487252
All
−0.002
−0.001
0.000
0.001
MR leave−one−out sensitivity analysis for
'KDM5A' on 'Bladder cancer'

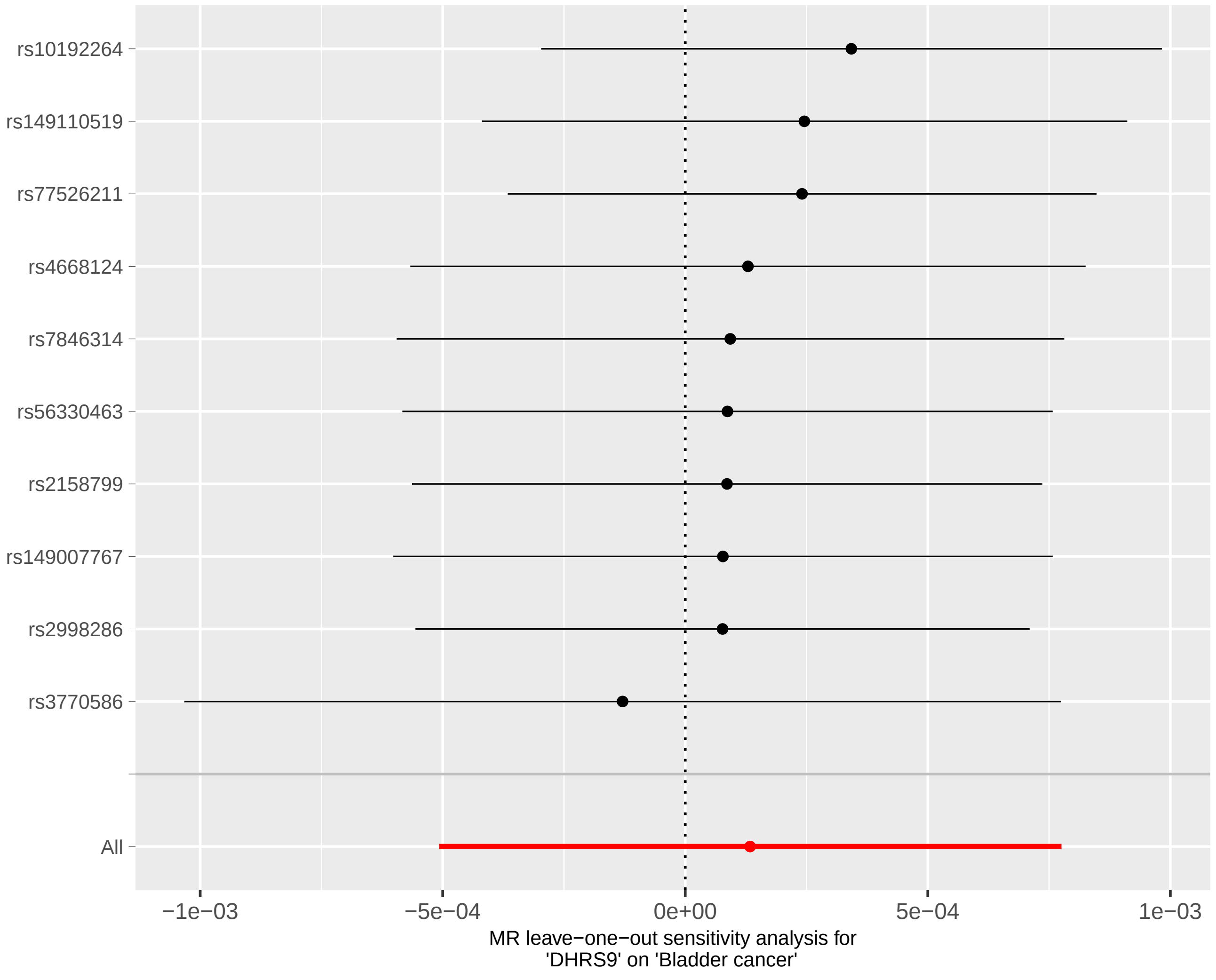
rs10192264
rs149110519
rs77526211
rs4668124
rs7846314
rs56330463
rs2158799
rs149007767
rs2998286
rs3770586
All
−1e−03
−5e−04
0e+00
5e−04
1e−03
MR leave−one−out sensitivity analysis for
'DHRS9' on 'Bladder cancer'

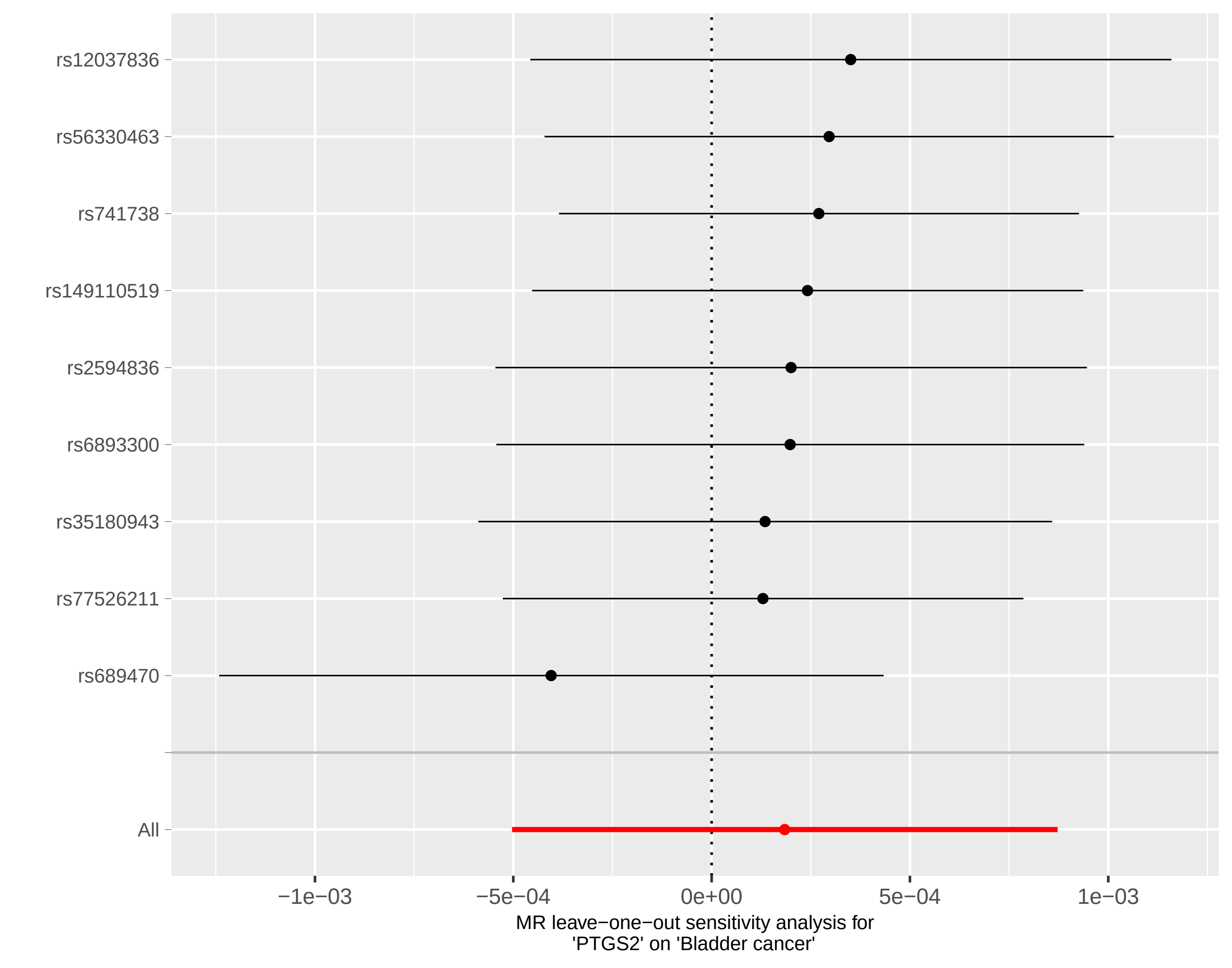
rs12037836
rs56330463
rs741738
rs149110519
rs2594836
rs6893300
rs35180943
rs77526211
rs689470
All
−1e−03
−5e−04
0e+00
5e−04
1e−03
MR leave−one−out sensitivity analysis for
'PTGS2' on 'Bladder cancer'

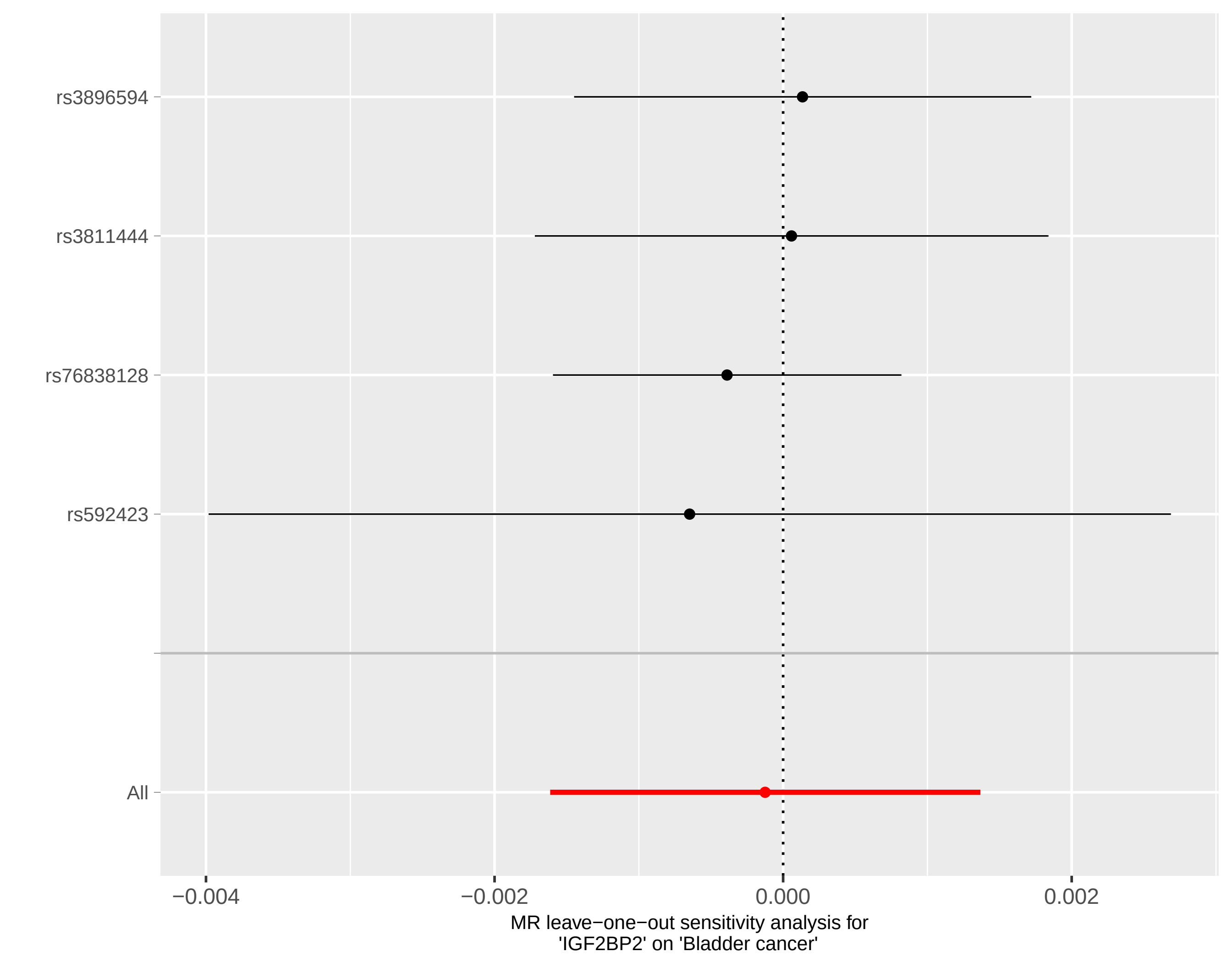
rs3896594
rs3811444
rs76838128
rs592423
All
−0.004
−0.002
0.000
0.002
MR leave−one−out sensitivity analysis for
'IGF2BP2' on 'Bladder cancer'

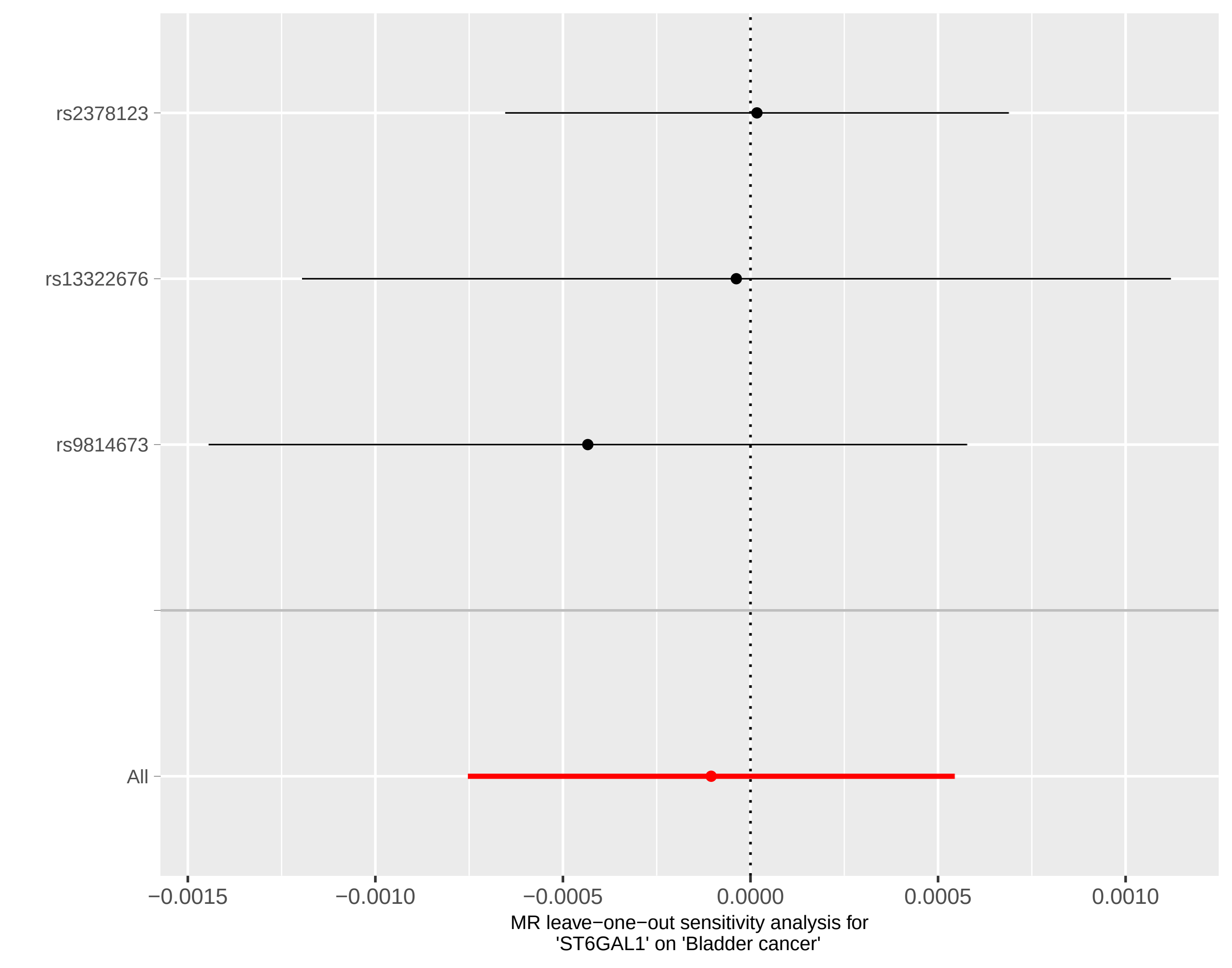
rs2378123
rs13322676
rs9814673
All
−0.0015
−0.0010
−0.0005
0.0000
0.0005
0.0010
MR leave−one−out sensitivity analysis for
'ST6GAL1' on 'Bladder cancer'

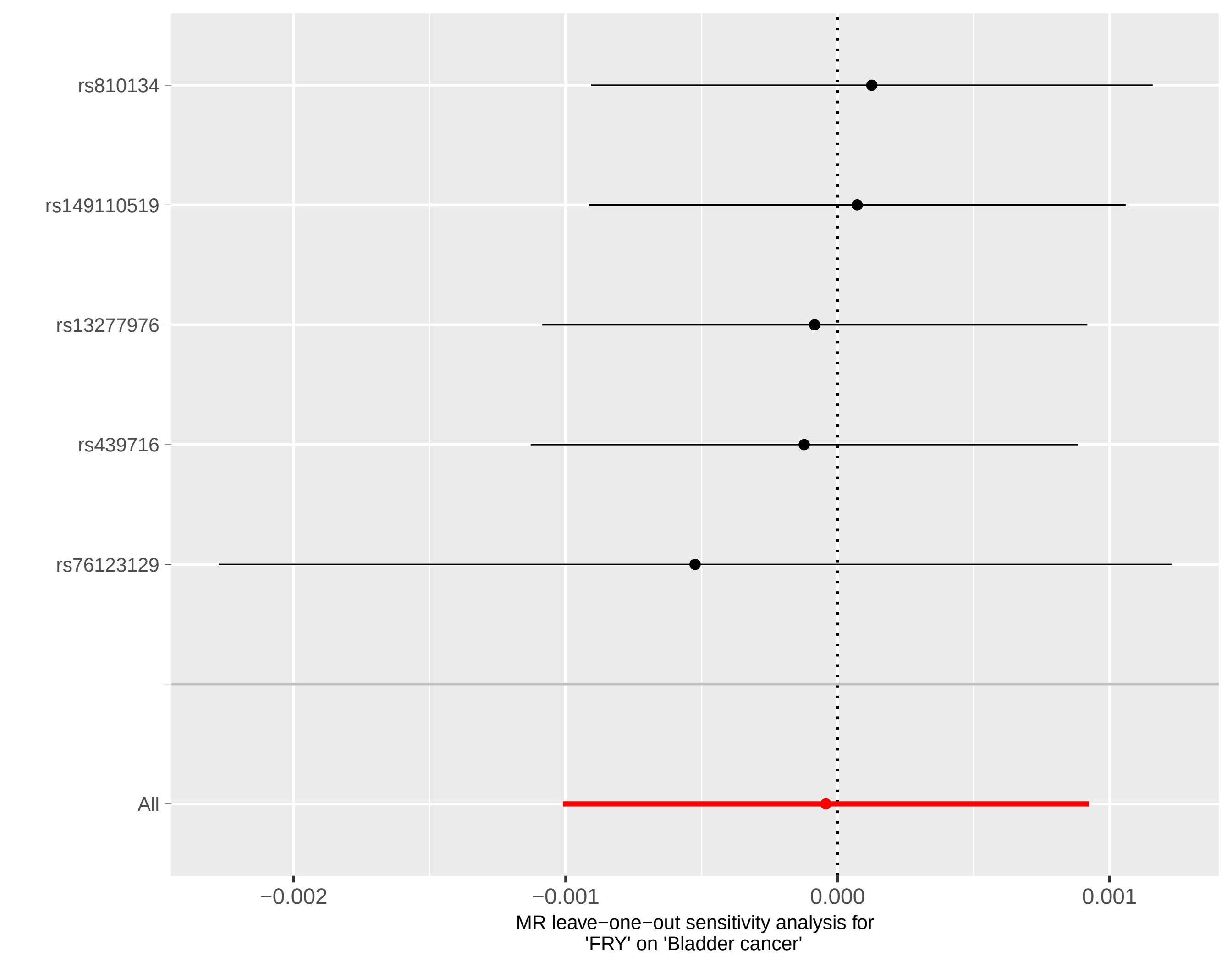

rs810134
rs149110519
rs13277976
rs439716
rs76123129
All
−0.002
−0.001
0.000
0.001
MR leave−one−out sensitivity analysis for
'FRY' on 'Bladder cancer'

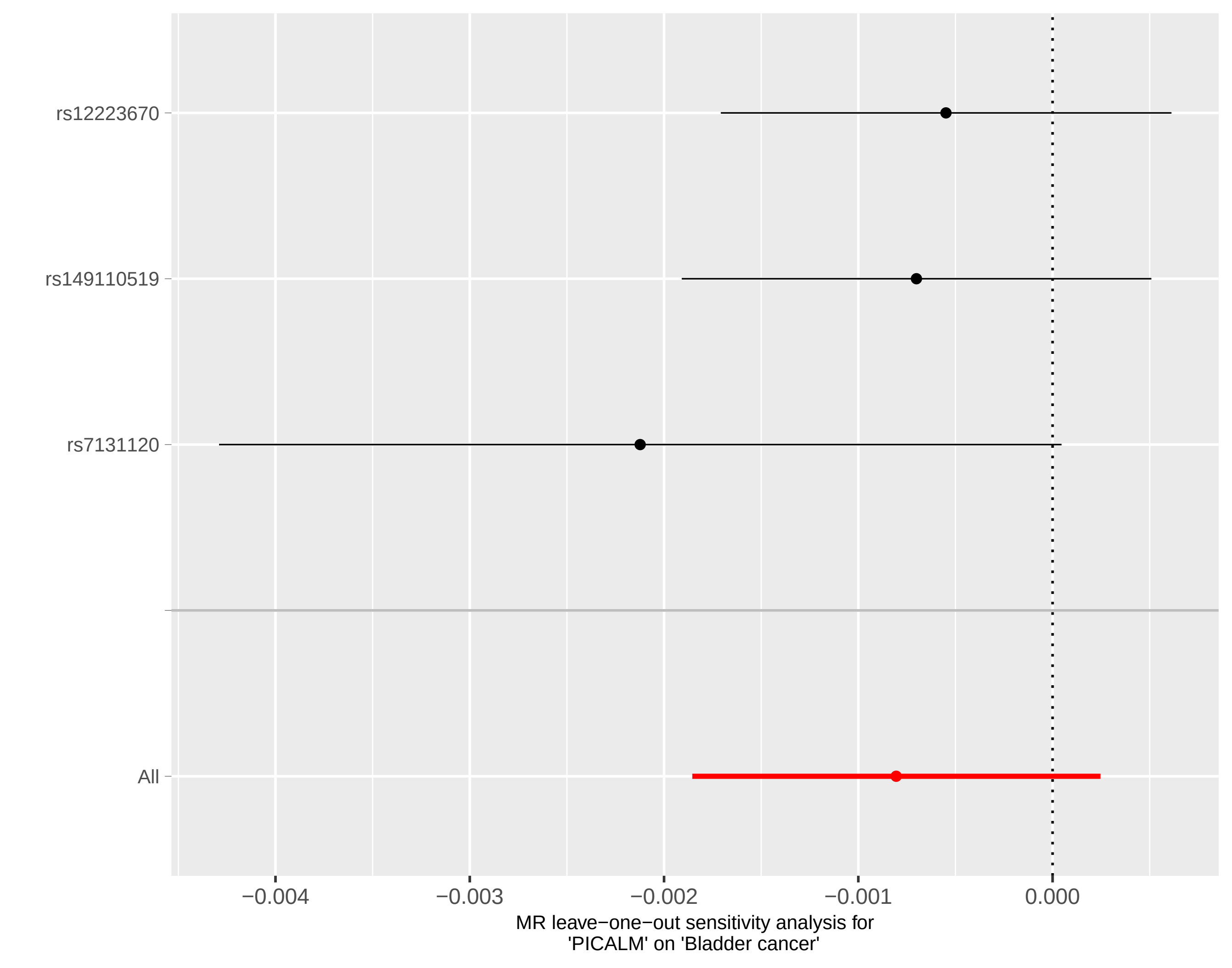
rs12223670
rs149110519
rs7131120
All
−0.004
−0.003
−0.002
−0.001
0.000
MR leave−one−out sensitivity analysis for
'PICALM' on 'Bladder cancer'

Insufficient number of SNPs

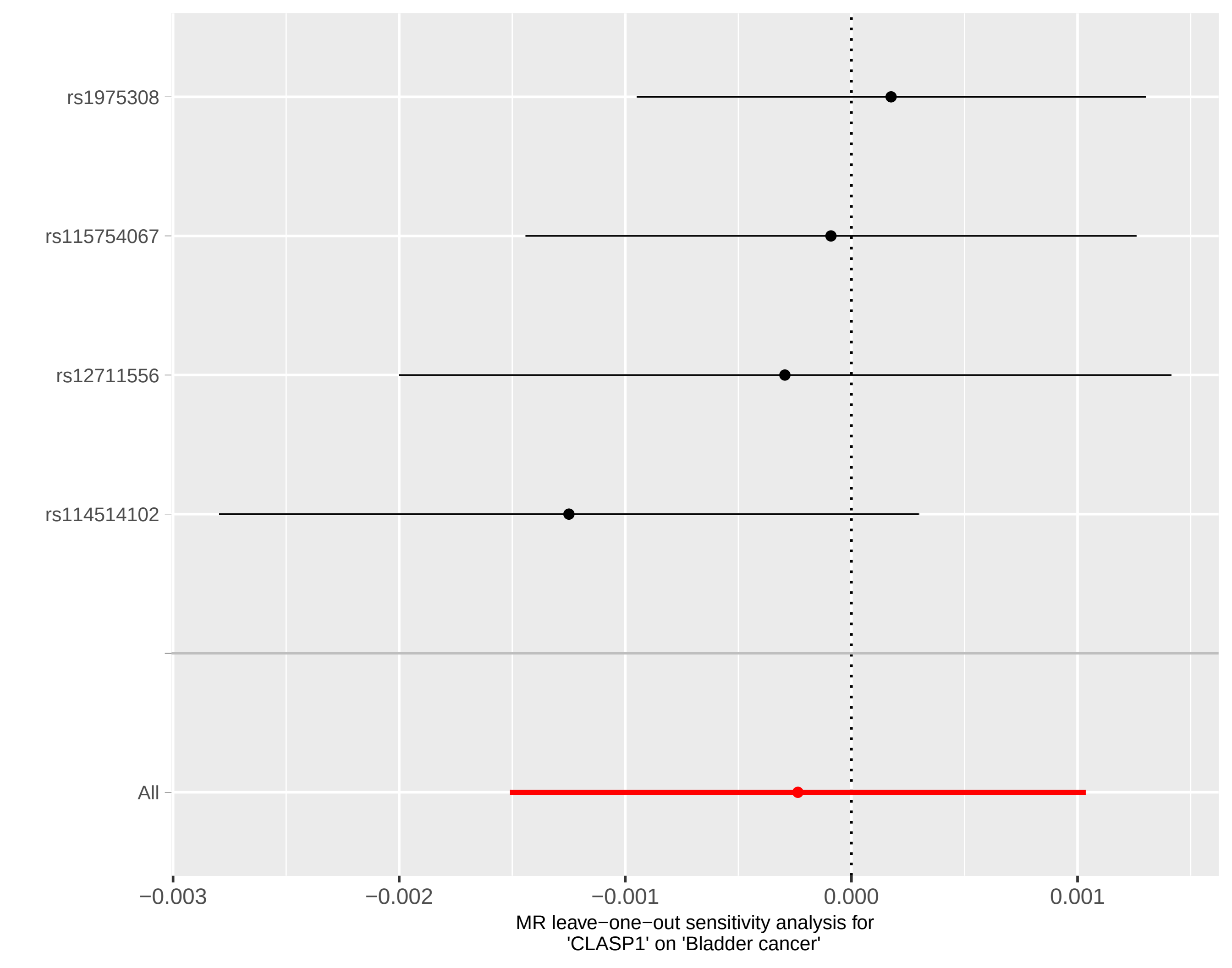
rs1975308
rs115754067
rs12711556
rs114514102
All
−0.003
−0.002
−0.001
0.000
0.001
MR leave−one−out sensitivity analysis for
'CLASP1' on 'Bladder cancer'

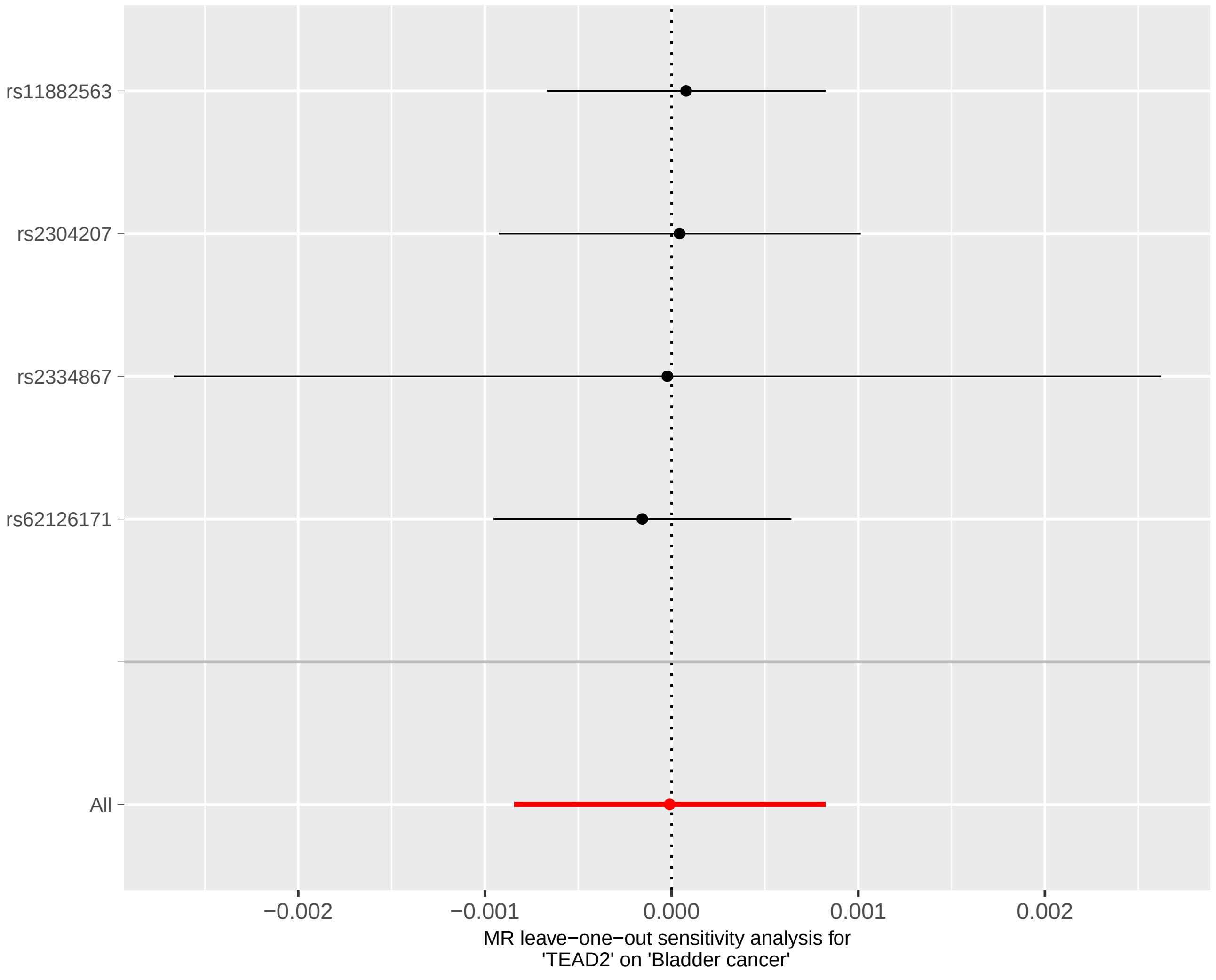
rs11882563
rs2304207
rs2334867
rs62126171
All
−0.002
−0.001
0.000
0.001
0.002
MR leave−one−out sensitivity analysis for
'TEAD2' on 'Bladder cancer'

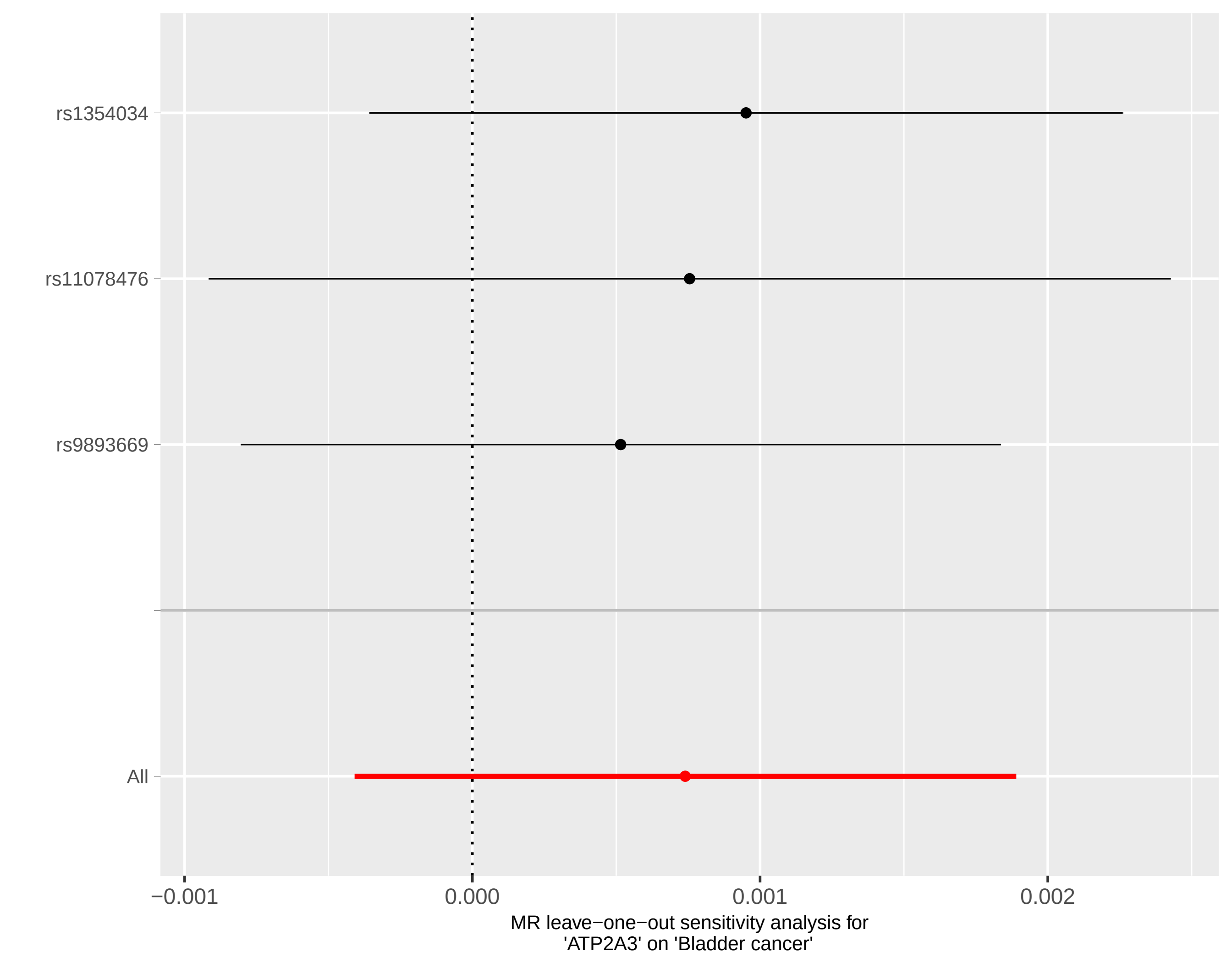
rs1354034
rs11078476
rs9893669
All
−0.001
0.000
0.001
0.002
MR leave−one−out sensitivity analysis for
'ATP2A3' on 'Bladder cancer'

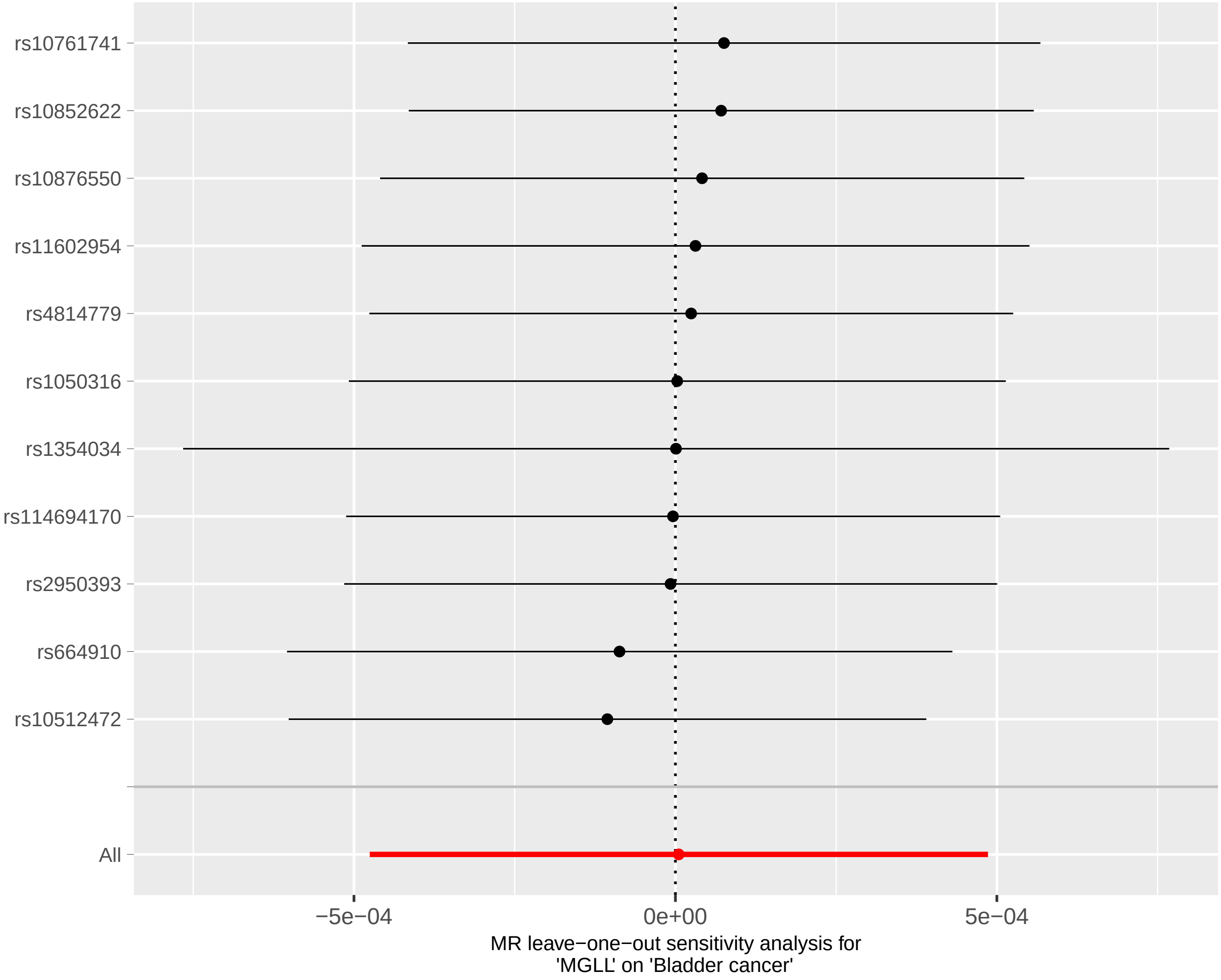
rs10761741
rs10852622
rs10876550
rs11602954
rs4814779
rs1050316
rs1354034
rs114694170
rs2950393
rs664910
rs10512472
All
−5e−04
0e+00
5e−04
MR leave−one−out sensitivity analysis for
'MGLL' on 'Bladder cancer'

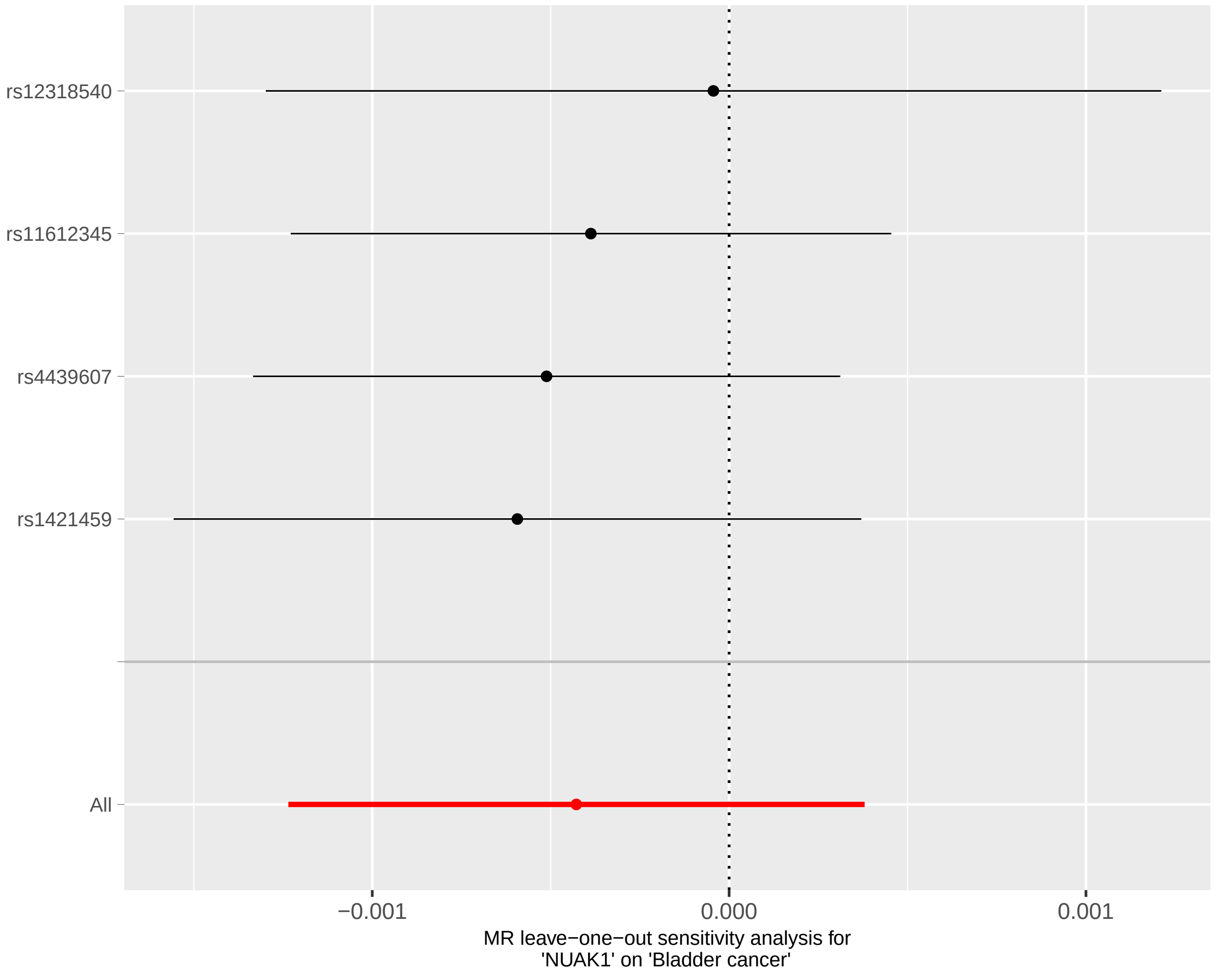
rs12318540
rs11612345
rs4439607
rs1421459
All
−0.001
0.000
0.001
MR leave−one−out sensitivity analysis for
'NUAK1' on 'Bladder cancer'

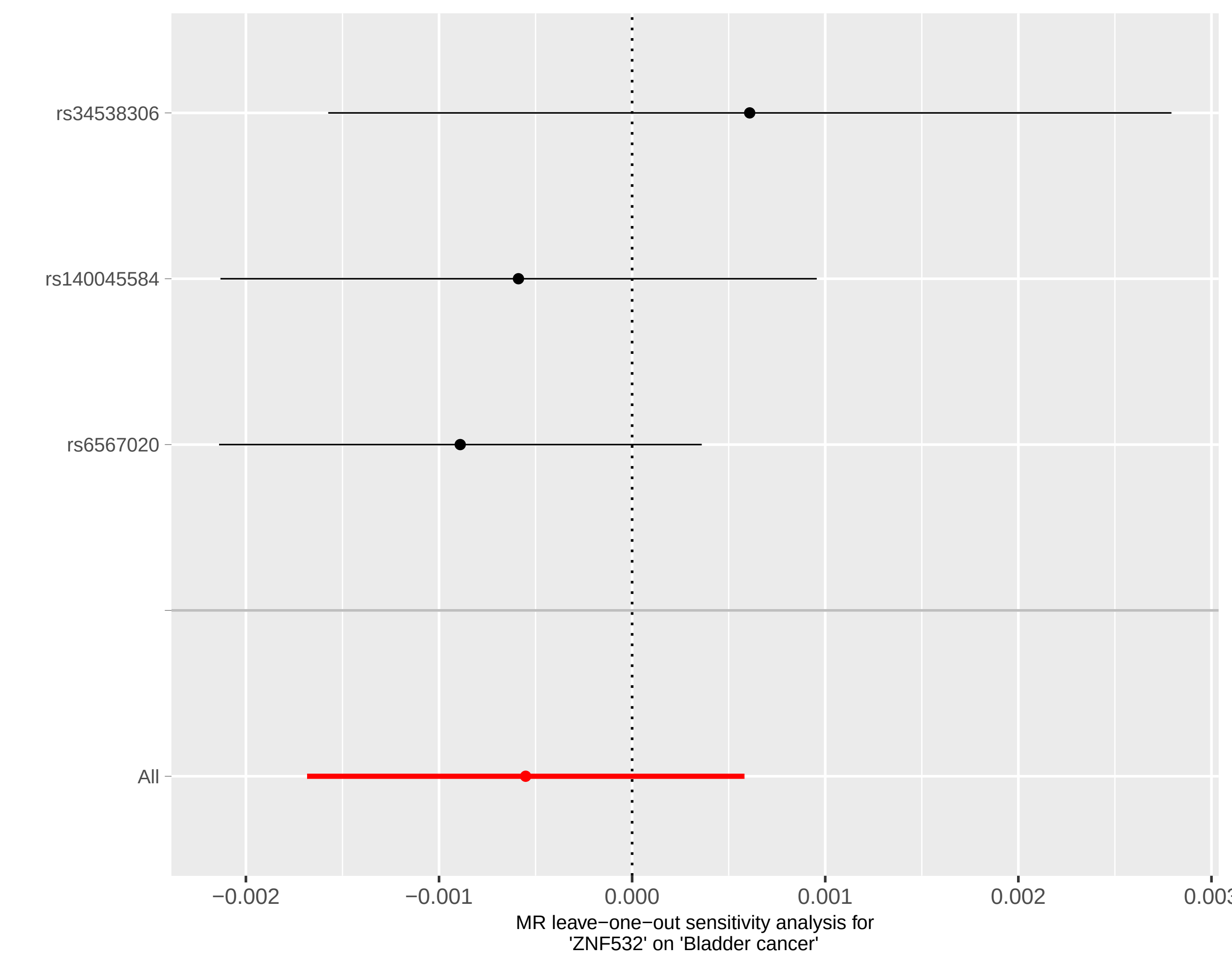

rs34538306
rs140045584
rs6567020
All
−0.002
−0.001
0.000
0.001
0.002
MR leave−one−out sensitivity analysis for
'ZNF532' on 'Bladder cancer'

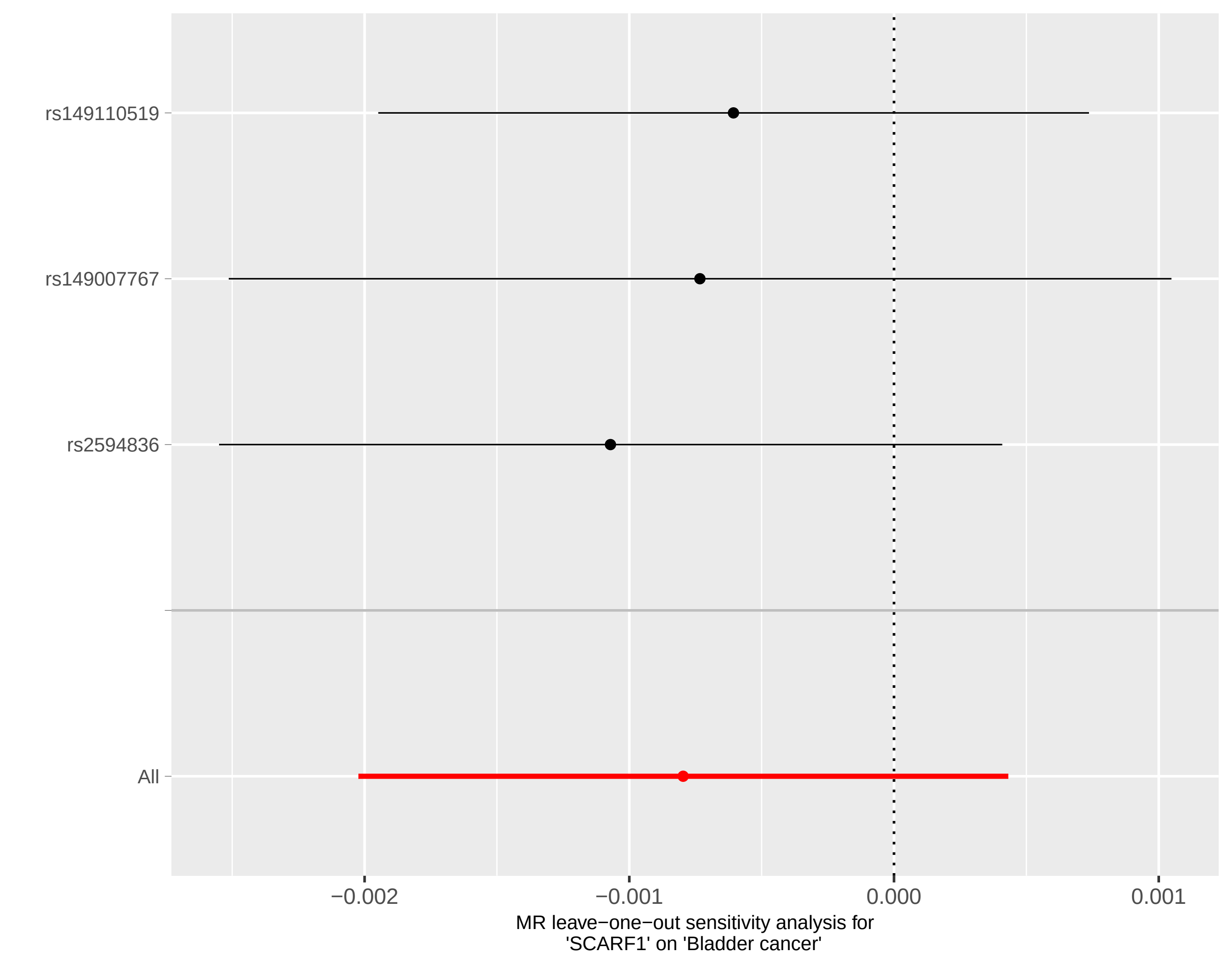
rs149110519
rs149007767
rs2594836
All
−0.002
−0.001
0.000
0.001
MR leave−one−out sensitivity analysis for
'SCARF1' on 'Bladder cancer'

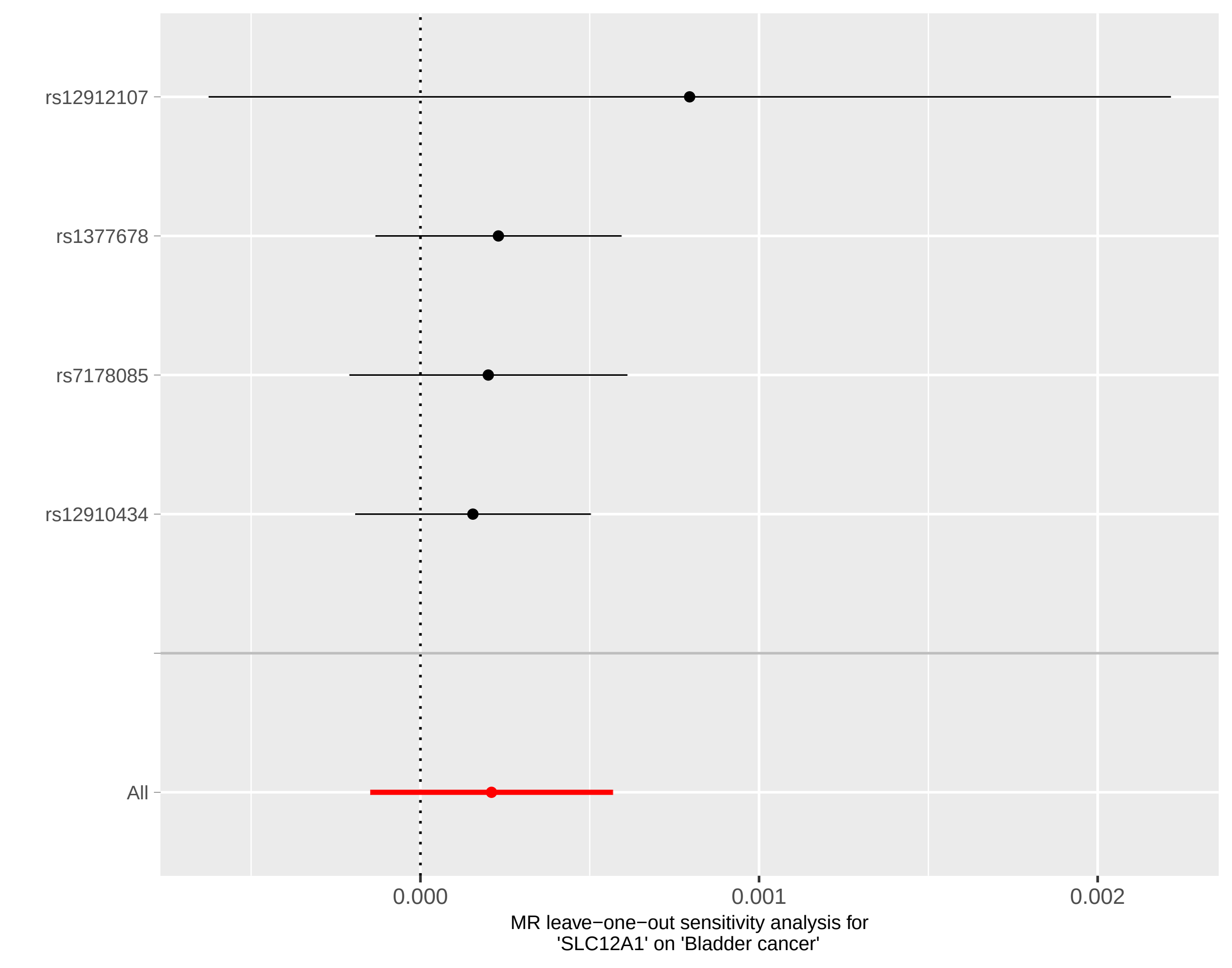
rs12912107
rs1377678
rs7178085
rs12910434
All
0.000
0.001
0.002
MR leave−one−out sensitivity analysis for
'SLC12A1' on 'Bladder cancer'

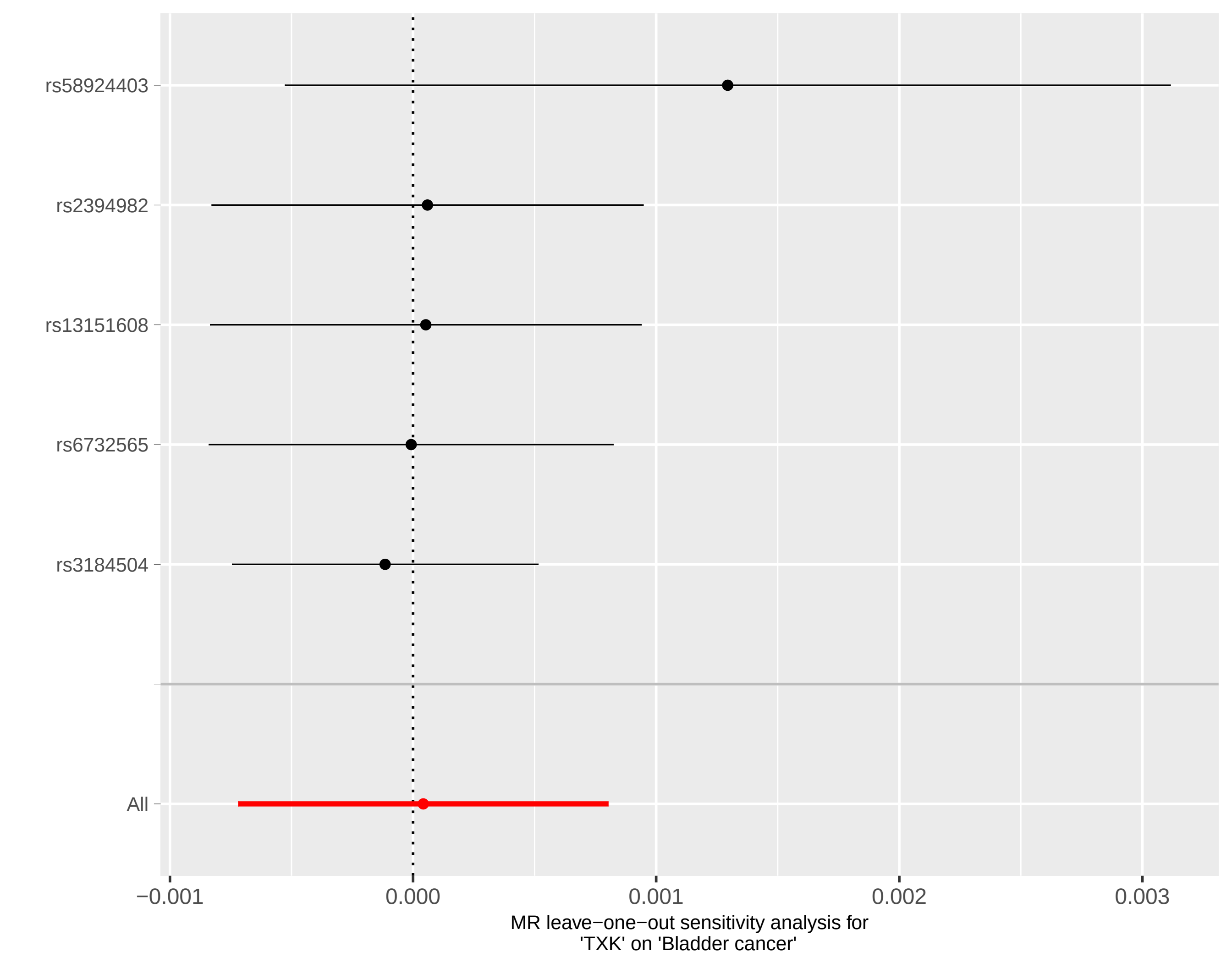
rs58924403
rs2394982
rs13151608
rs6732565
rs3184504
All
−0.001
0.000
0.001
0.002
0.003
MR leave−one−out sensitivity analysis for
'TXK' on 'Bladder cancer'

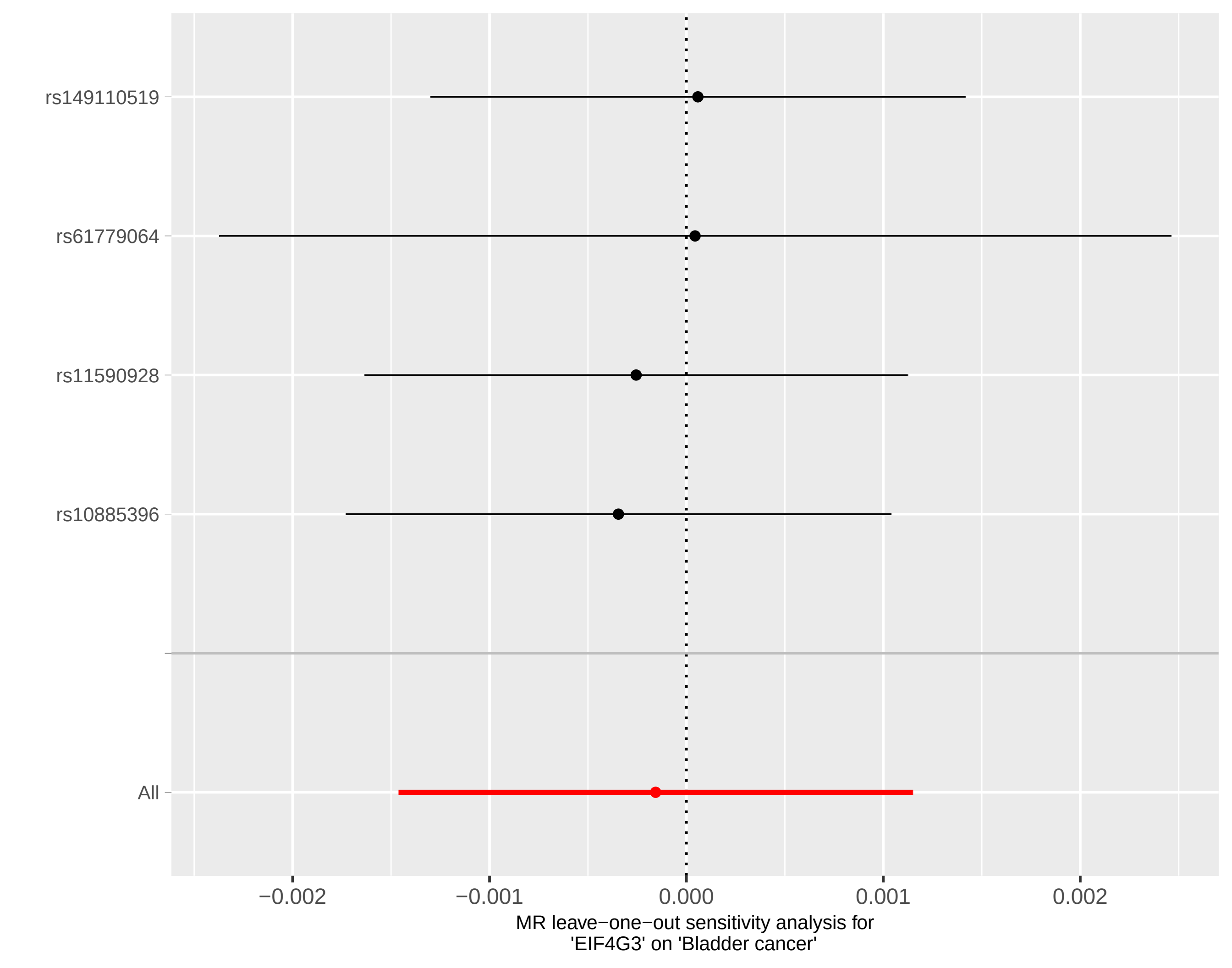

rs149110519
rs61779064
rs11590928
rs10885396
All
−0.002
−0.001
0.000
0.001
0.002
MR leave−one−out sensitivity analysis for
'EIF4G3' on 'Bladder cancer'

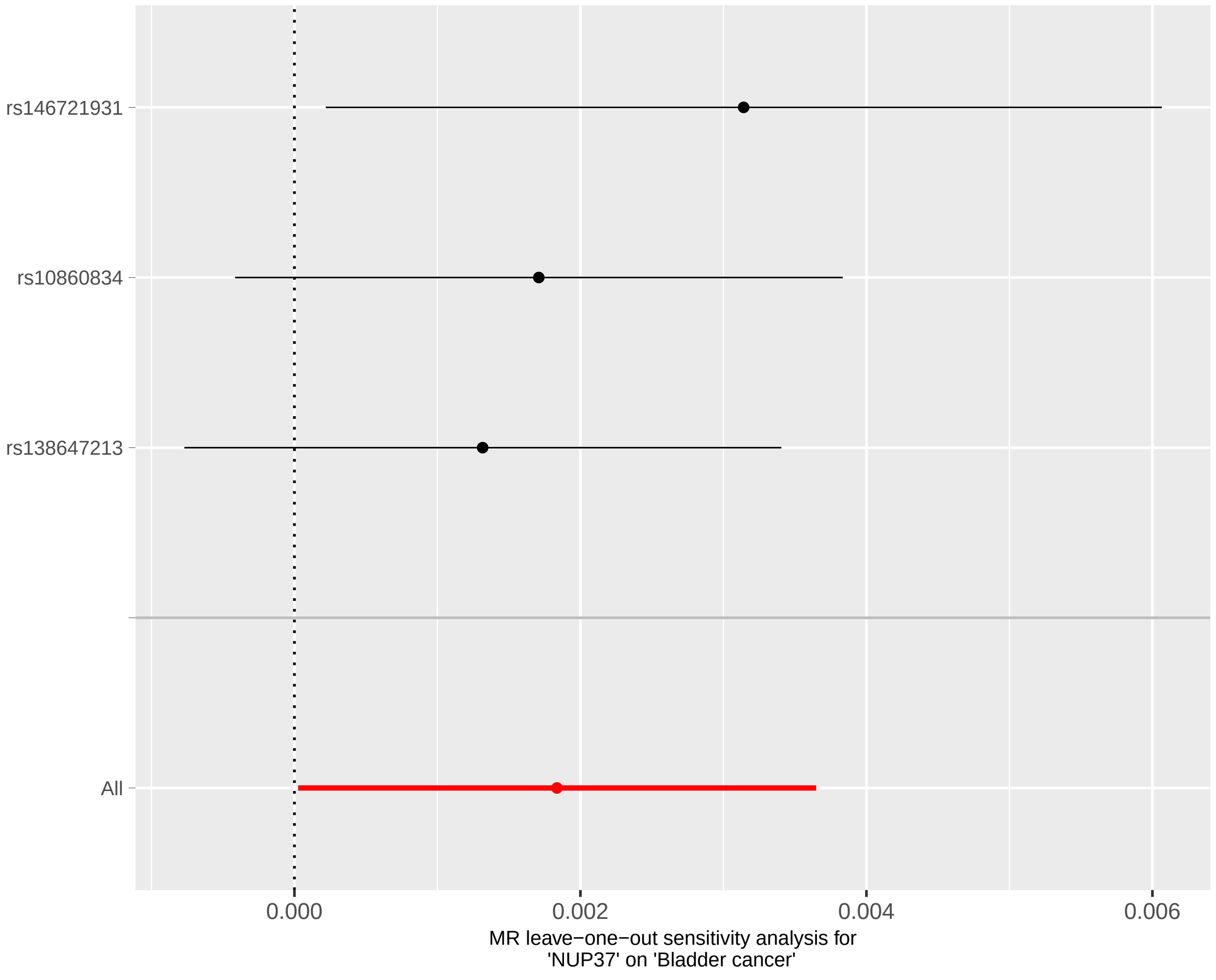
rs146721931
rs10860834
rs138647213
All
0.000
0.002
0.004
0.006
MR leave−one−out sensitivity analysis for
'NUP37' on 'Bladder cancer'

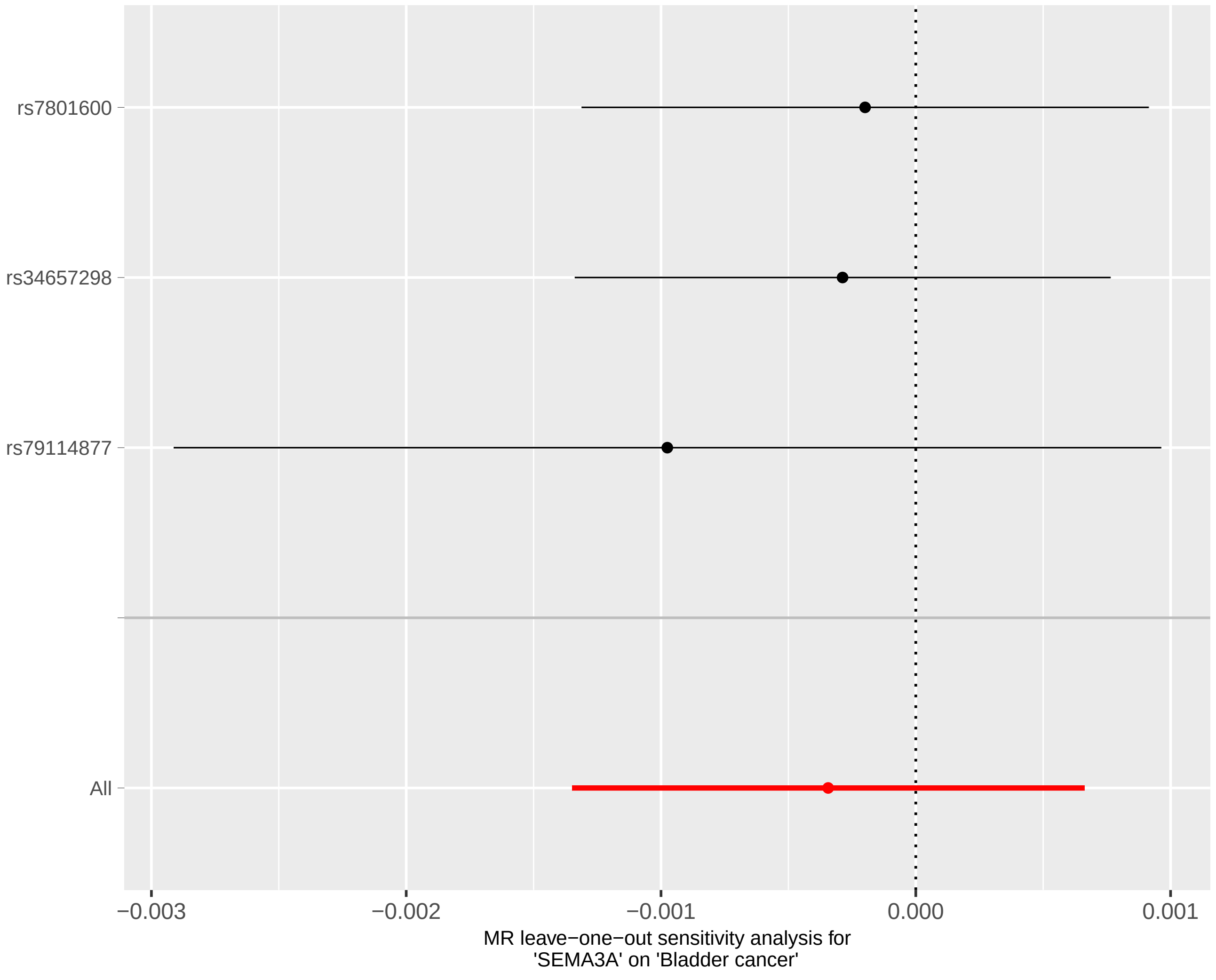
rs7801600
rs34657298
rs79114877
All
−0.003
−0.002
−0.001
0.000
0.001
MR leave−one−out sensitivity analysis for
'SEMA3A' on 'Bladder cancer'

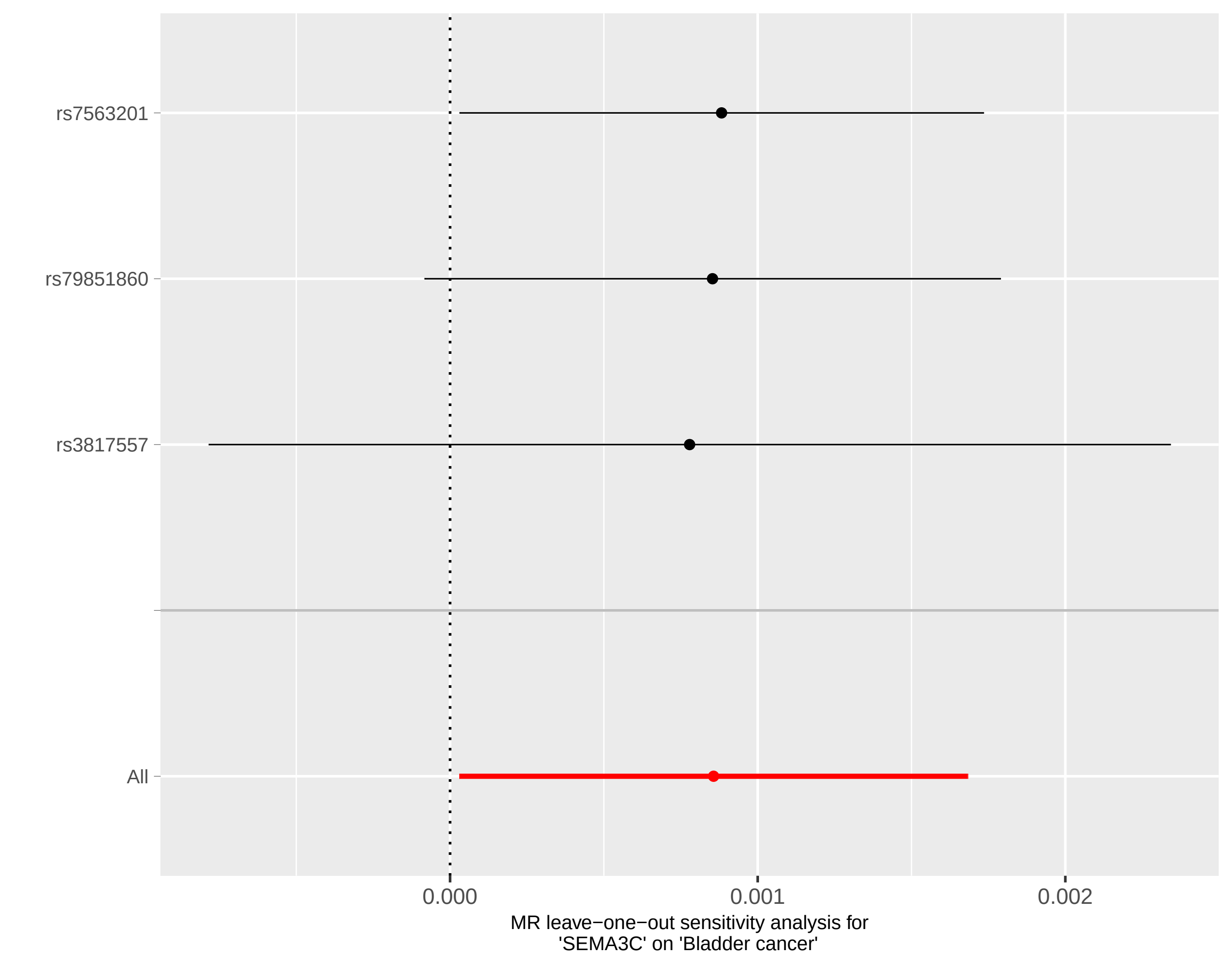
rs7563201
rs79851860
rs3817557
All
0.000
0.001
0.002
MR leave−one−out sensitivity analysis for
'SEMA3C' on 'Bladder cancer'

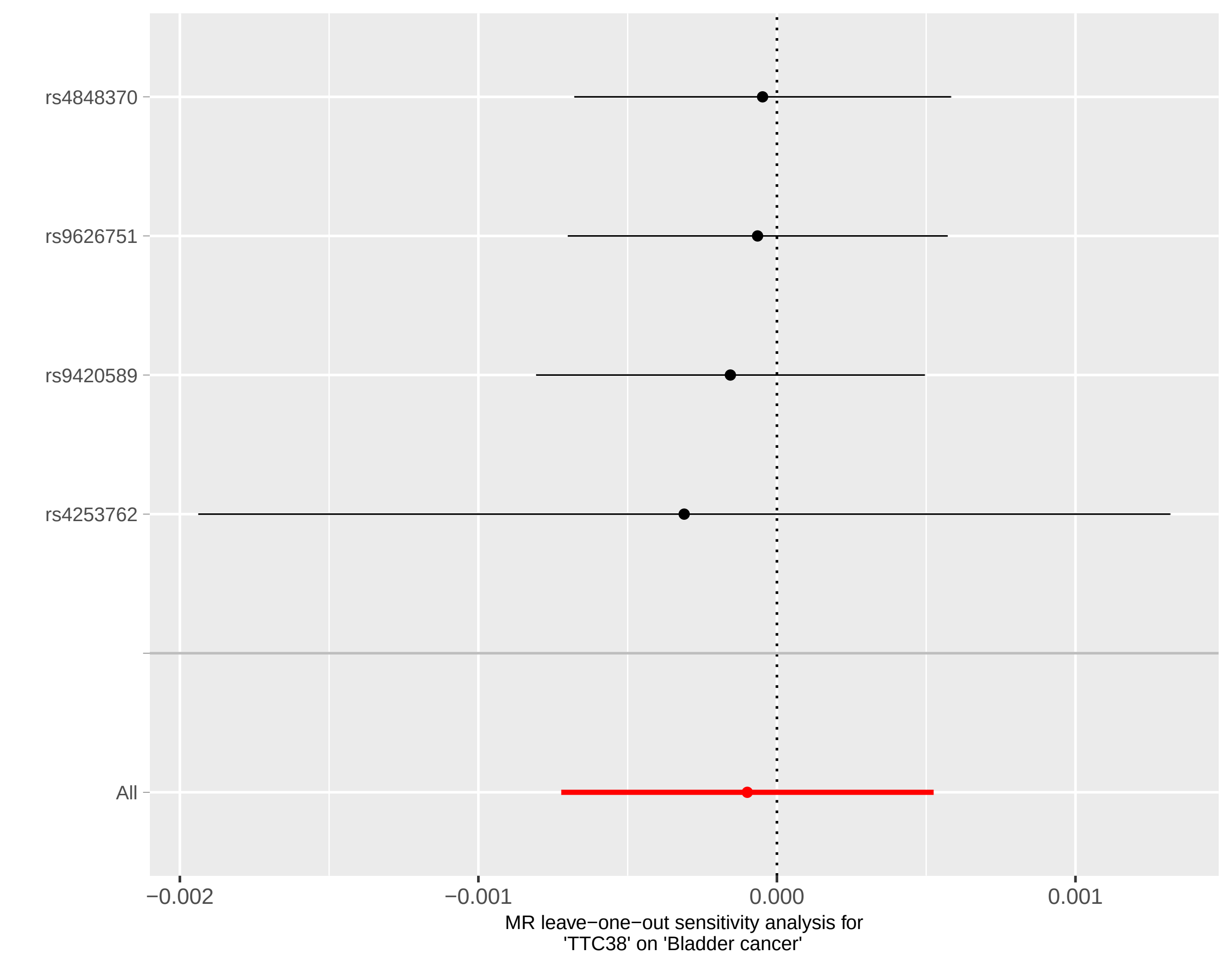

rs4848370
rs9626751
rs9420589
rs4253762
All
−0.002
−0.001
0.000
0.001
MR leave−one−out sensitivity analysis for
'TTC38' on 'Bladder cancer'

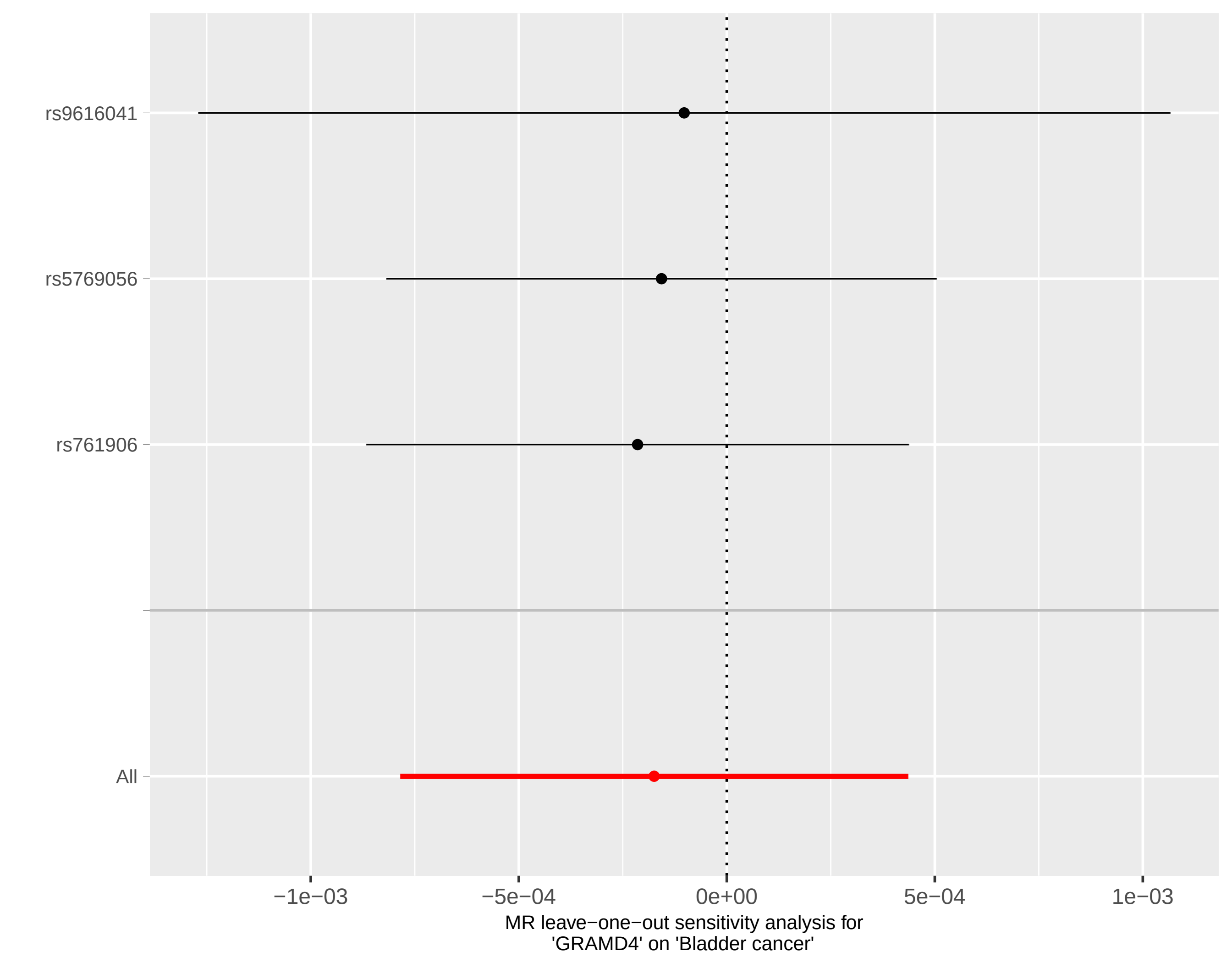
rs9616041
rs5769056
rs761906
All
−1e−03
−5e−04
0e+00
5e−04
1e−03
MR leave−one−out sensitivity analysis for
'GRAMD4' on 'Bladder cancer'

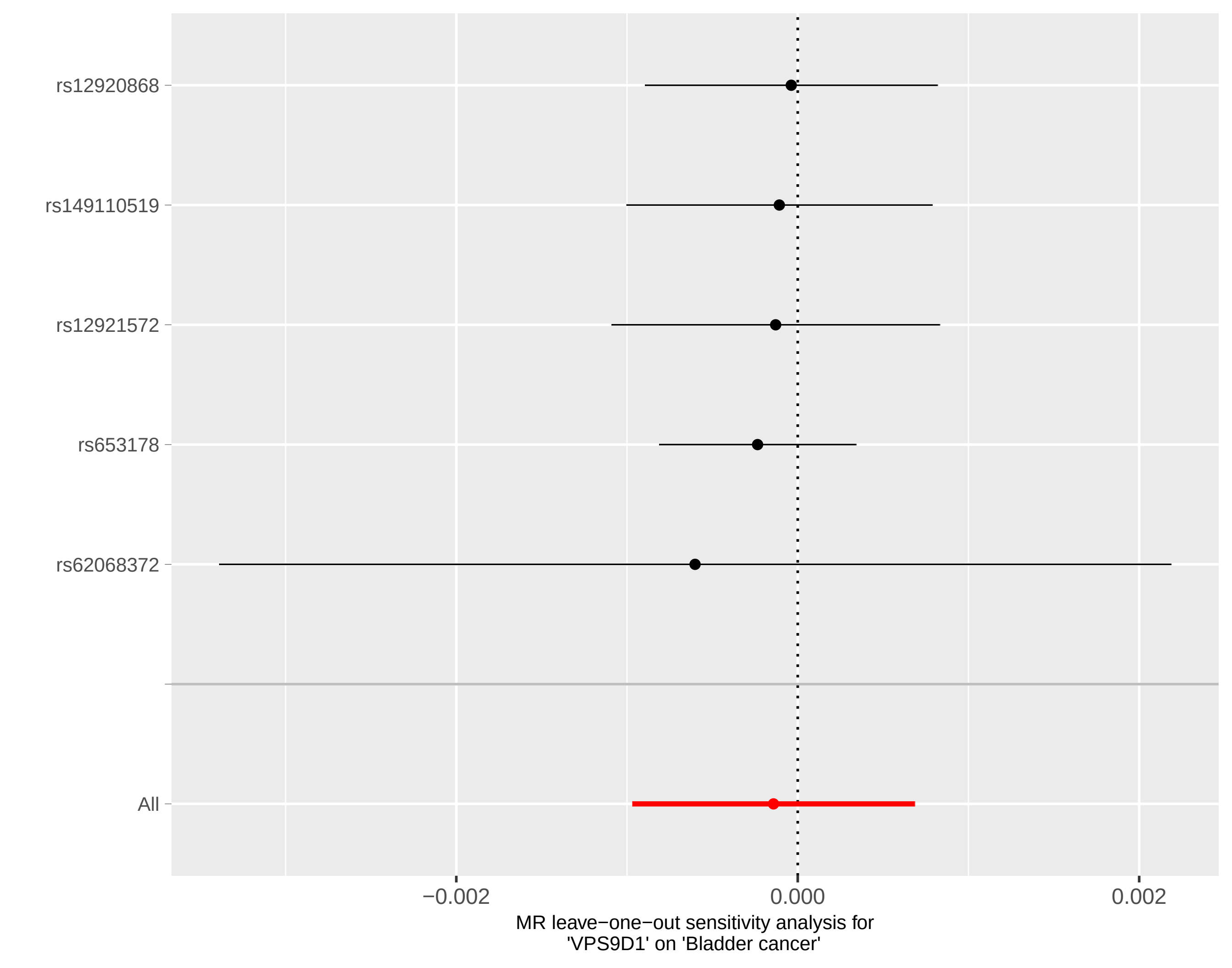

rs12920868
rs149110519
rs12921572
rs653178
rs62068372
All
−0.002
0.000
0.002
MR leave−one−out sensitivity analysis for
'VPS9D1' on 'Bladder cancer'

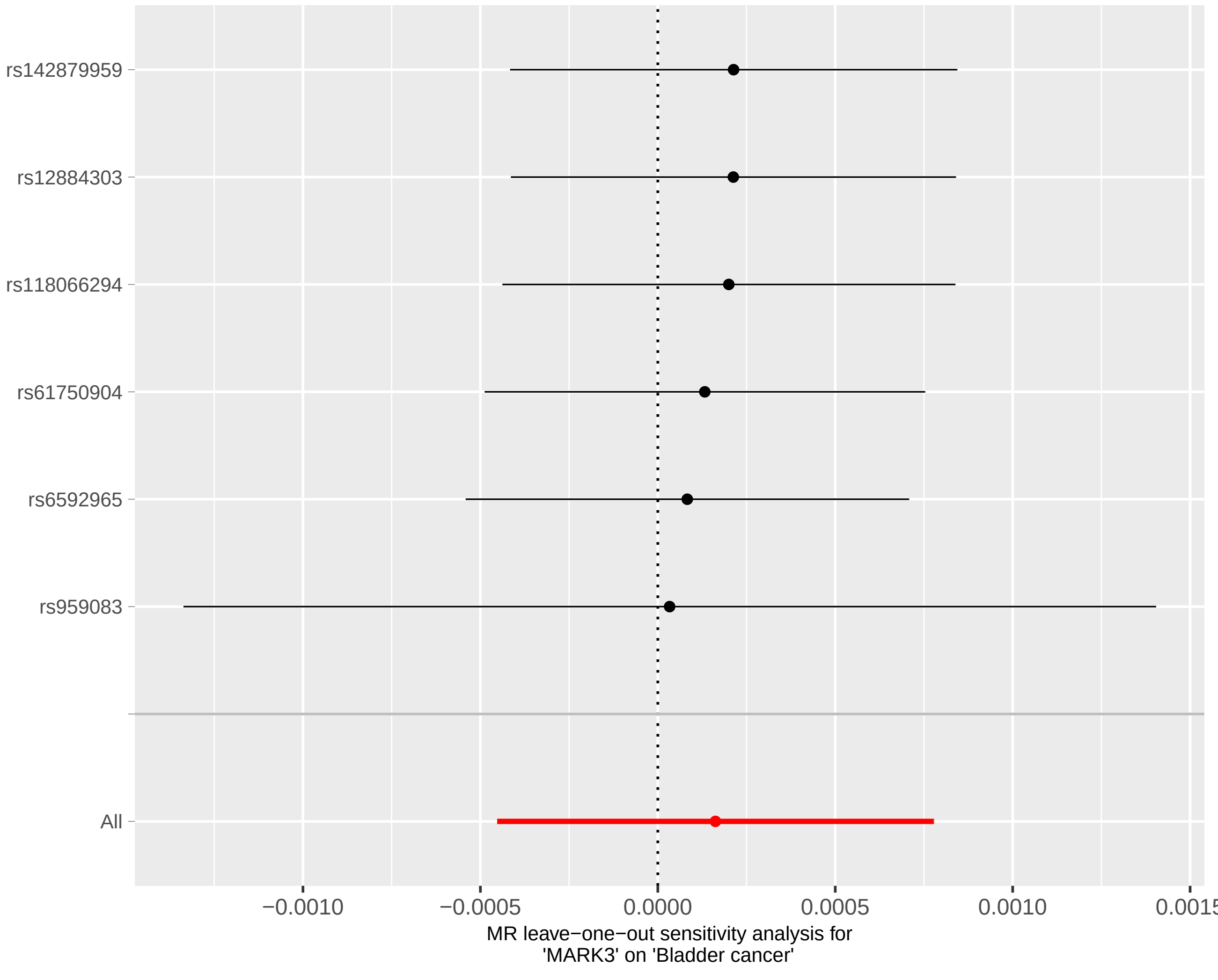
rs142879959
rs12884303
rs118066294
rs61750904
rs6592965
rs959083
All
−0.0010
−0.0005
0.0000
0.0005
0.0010
0.0015
MR leave−one−out sensitivity analysis for
'MARK3' on 'Bladder cancer'

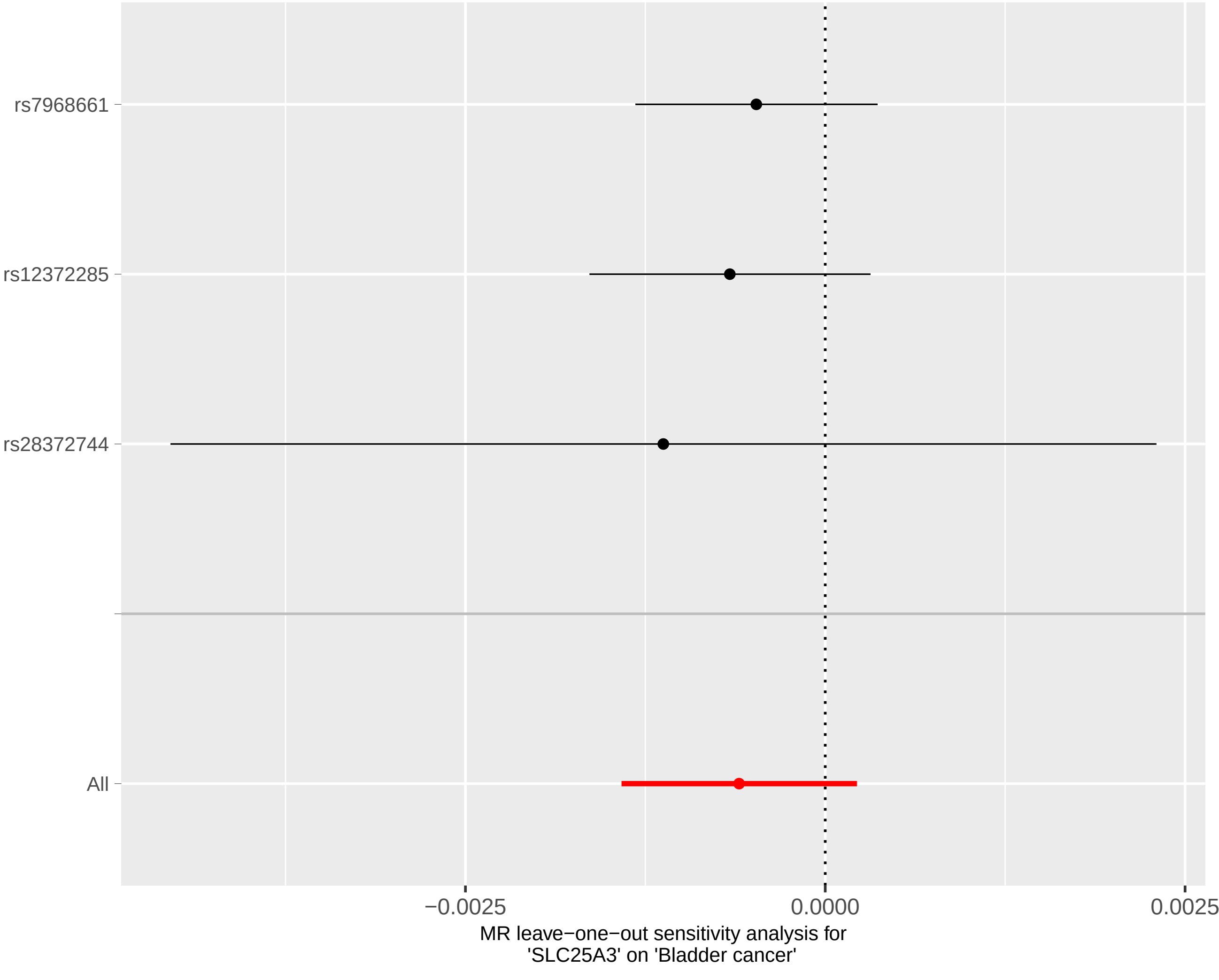
rs7968661
rs12372285
rs28372744
All
−0.0025
0.0000
0.0025
MR leave−one−out sensitivity analysis for
'SLC25A3' on 'Bladder cancer'

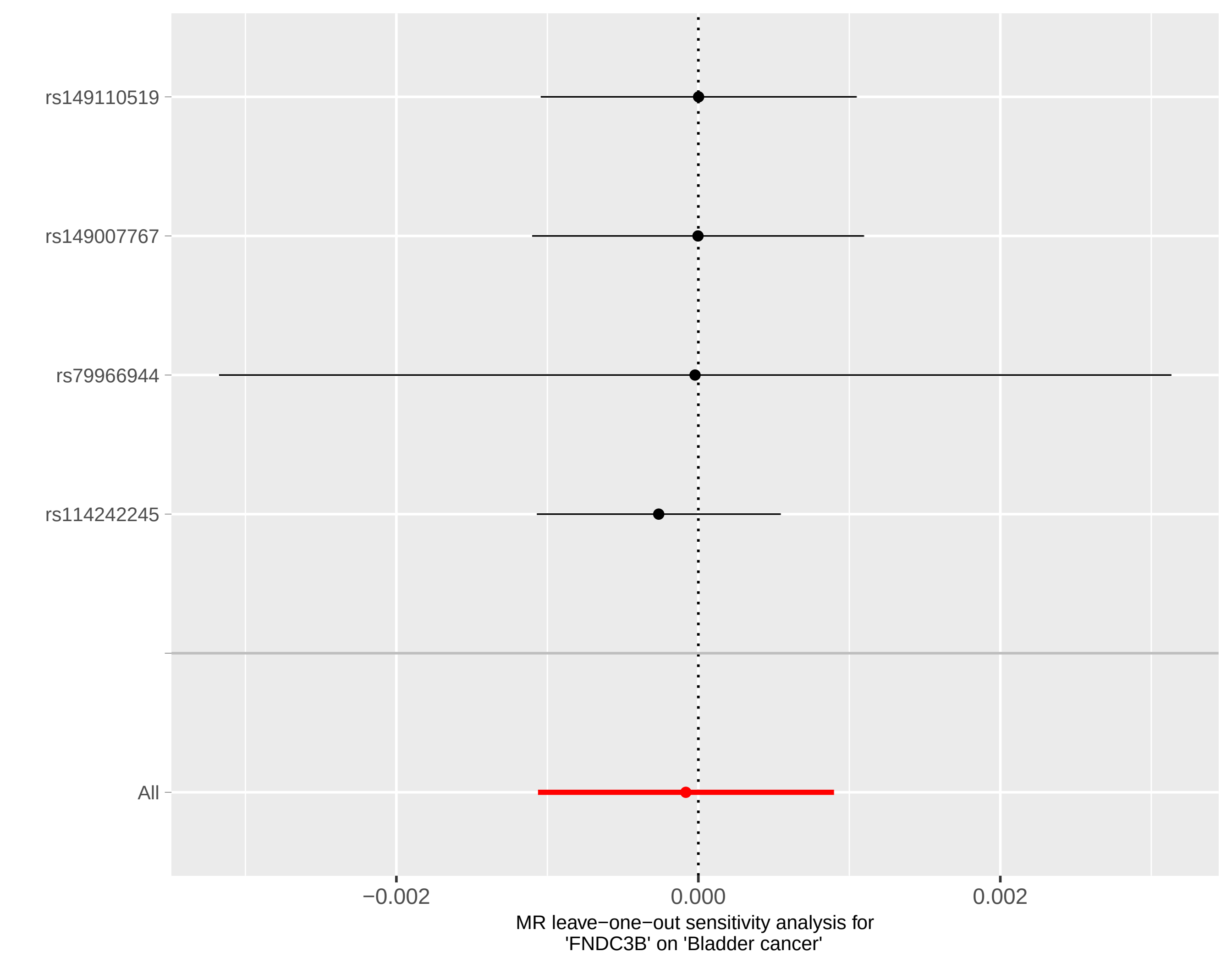
rs149110519
rs149007767
rs79966944
rs114242245
All
−0.002
0.000
0.002
MR leave−one−out sensitivity analysis for
'FNDC3B' on 'Bladder cancer'

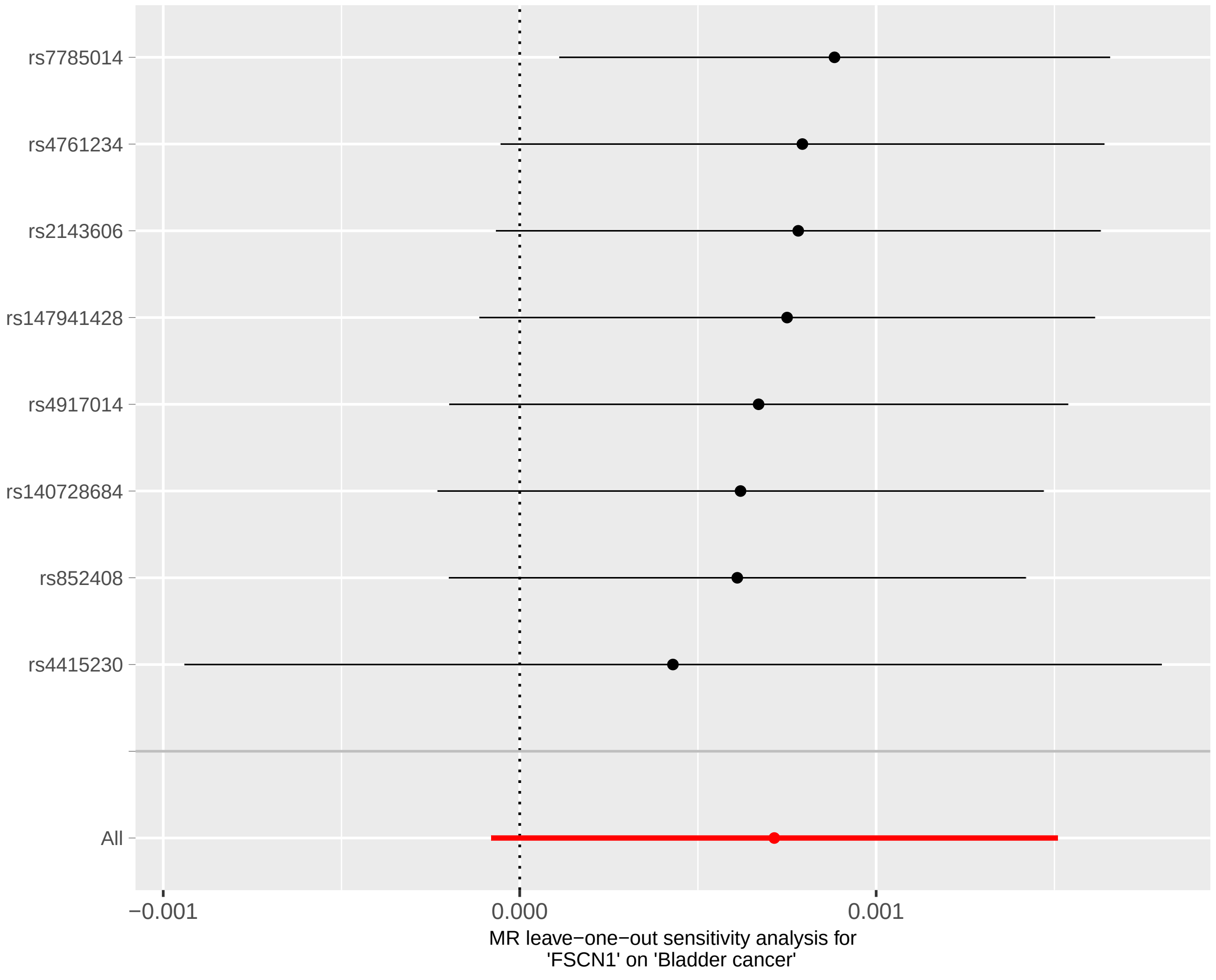
rs7785014
rs4761234
rs2143606
rs147941428
rs4917014
rs140728684
rs852408
rs4415230
All
−0.001
0.000
0.001
MR leave−one−out sensitivity analysis for
'FSCN1' on 'Bladder cancer'

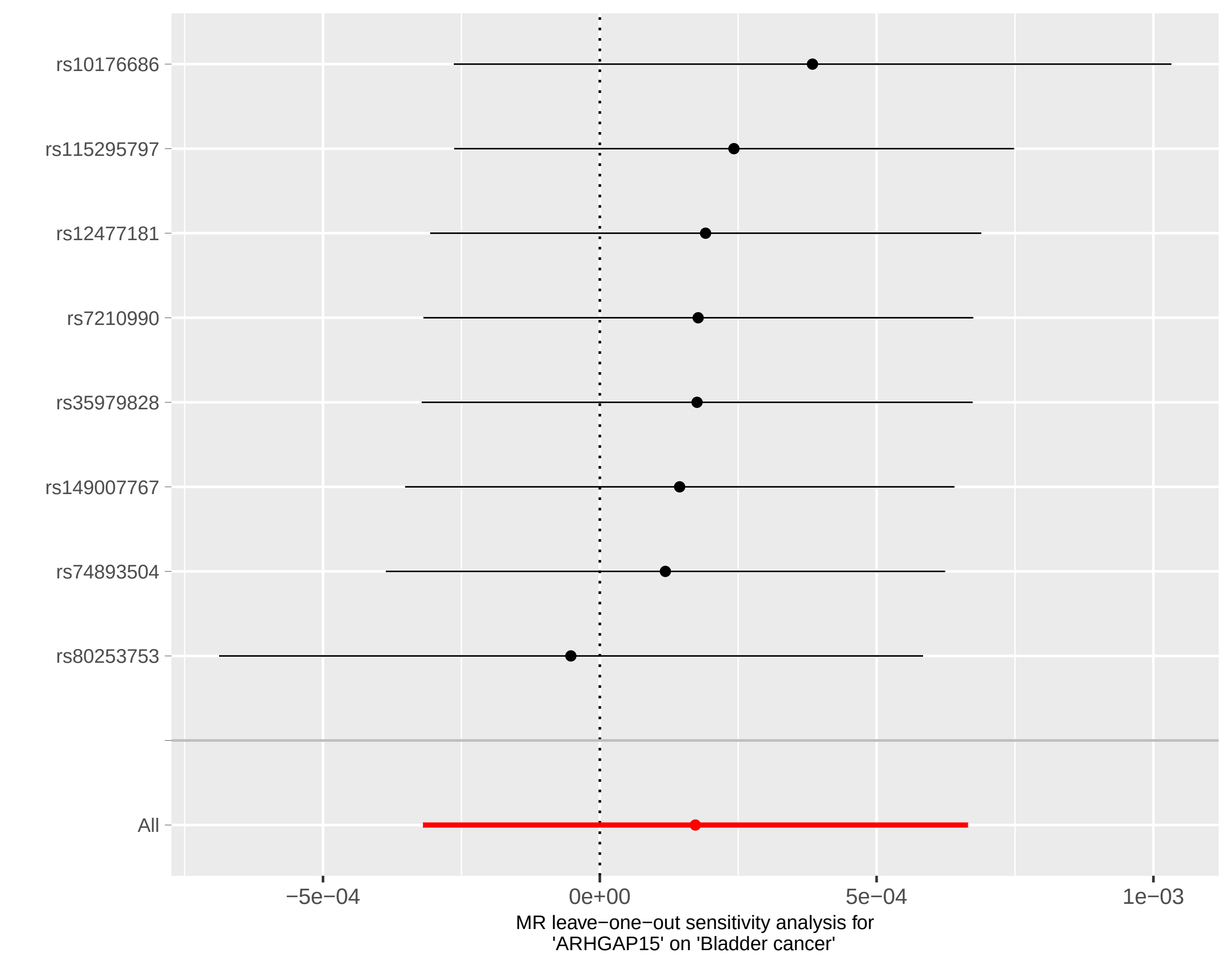
rs10176686
rs115295797
rs12477181
rs7210990
rs35979828
rs149007767
rs74893504
rs80253753
All
−5e−04
0e+00
5e−04
1e−03
MR leave−one−out sensitivity analysis for
'ARHGAP15' on 'Bladder cancer'

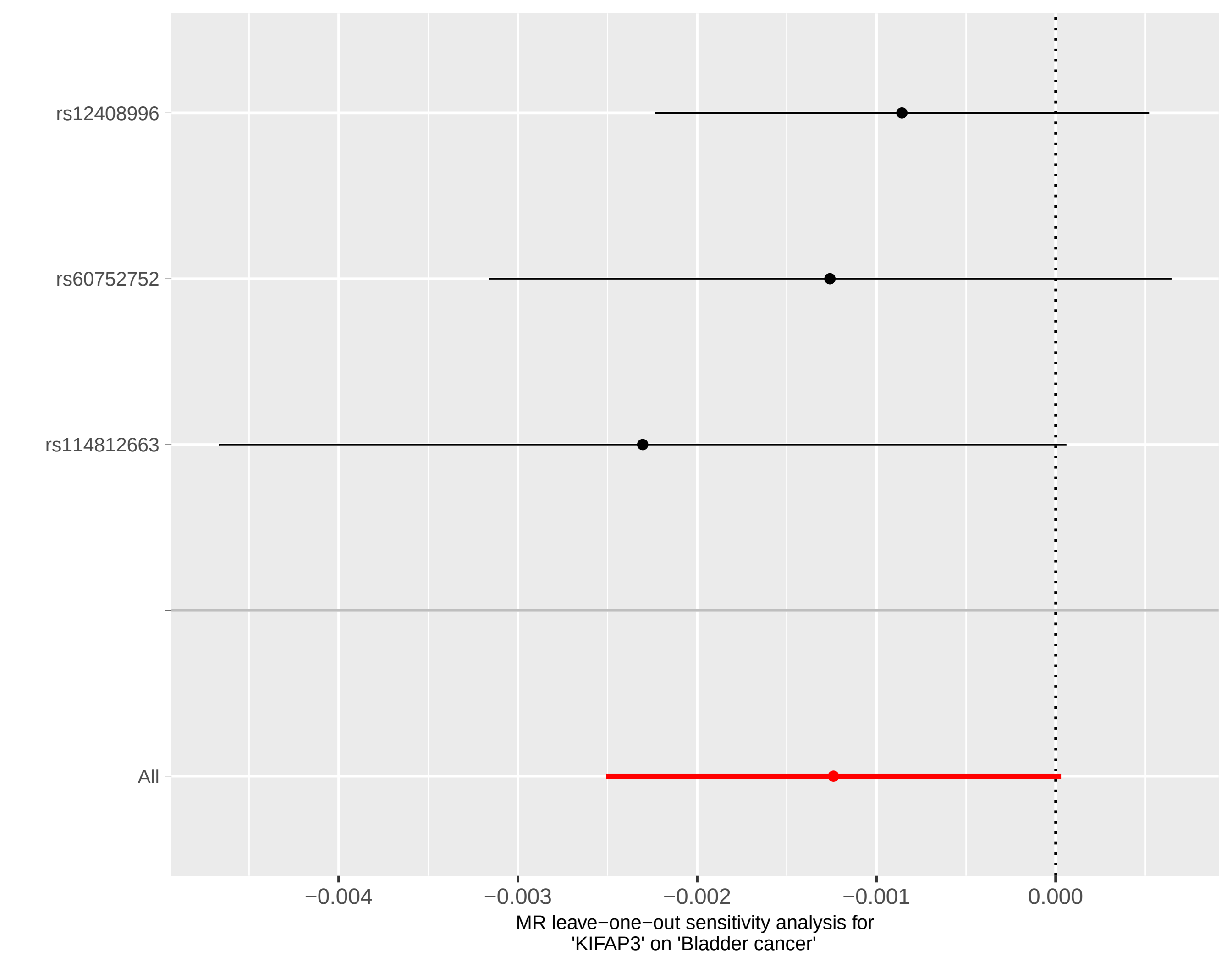
rs12408996
rs60752752
rs114812663
All
−0.004
−0.003
−0.002
−0.001
0.000
MR leave−one−out sensitivity analysis for
'KIFAP3' on 'Bladder cancer'

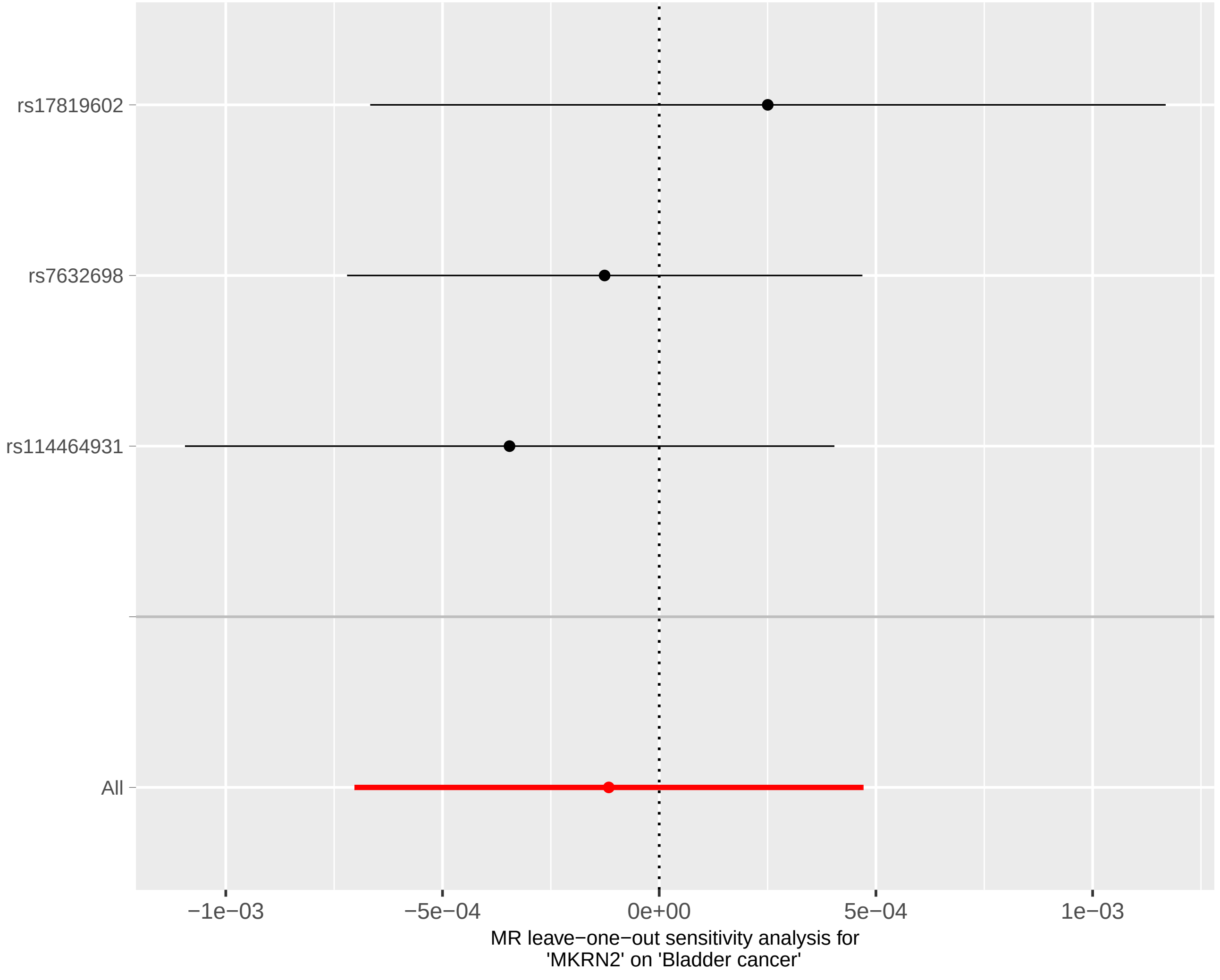

rs17819602
rs7632698
rs114464931
All
−1e−03
−5e−04
0e+00
5e−04
1e−03
MR leave−one−out sensitivity analysis for
'MKRN2' on 'Bladder cancer'

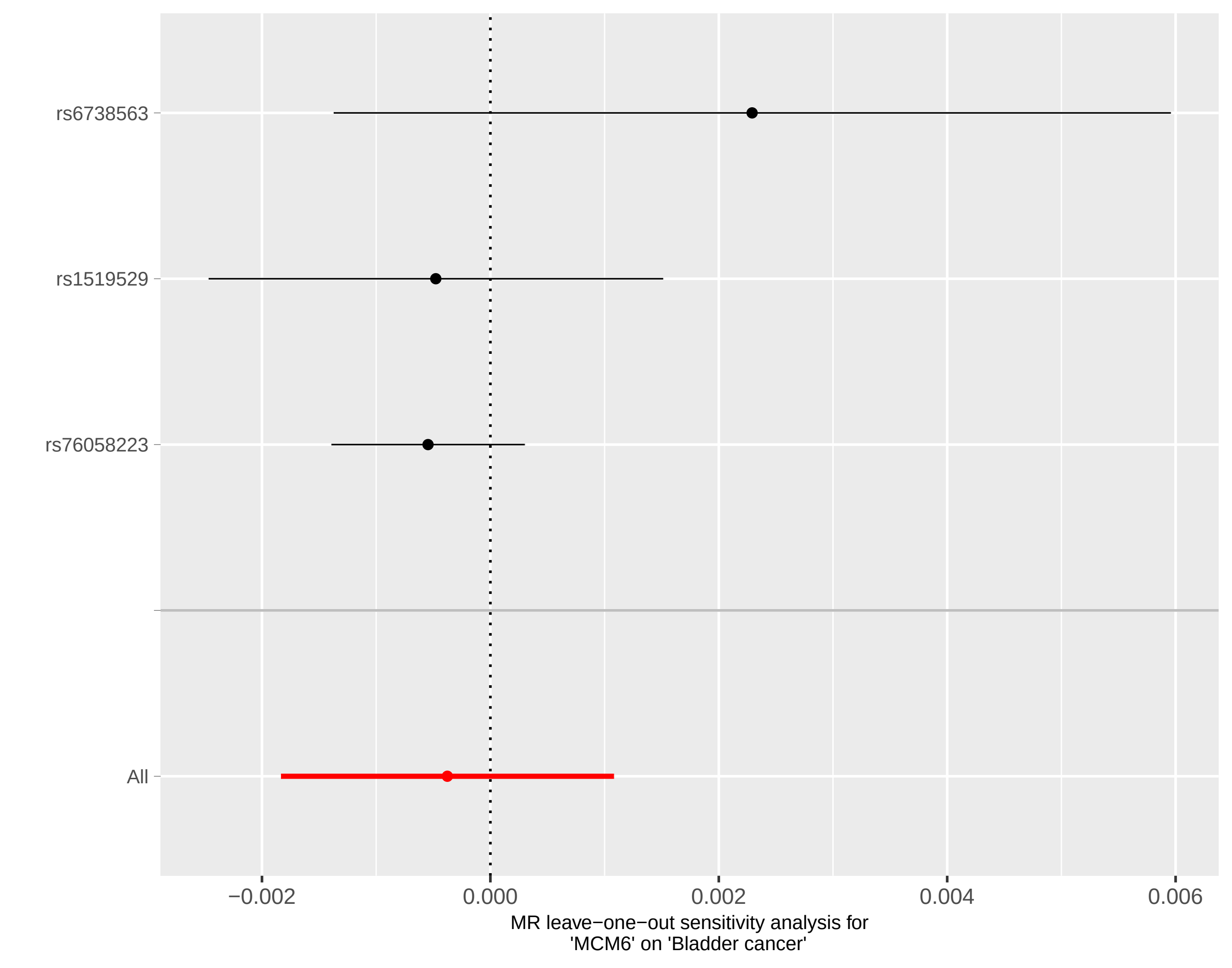
rs6738563
rs1519529
rs76058223
All
−0.002
0.000
0.002
0.004
0.006
MR leave−one−out sensitivity analysis for
'MCM6' on 'Bladder cancer'

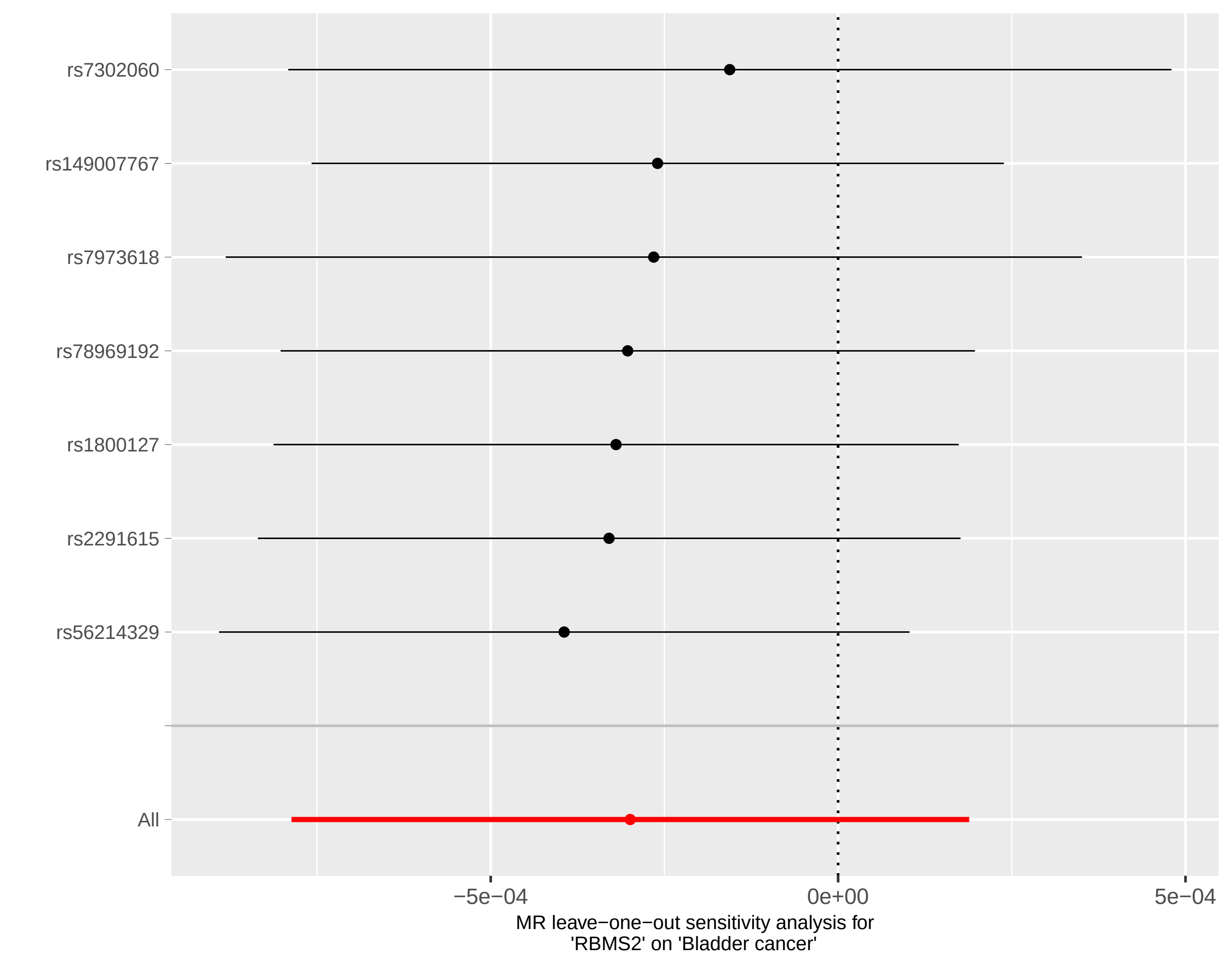

rs7302060
rs149007767
rs7973618
rs78969192
rs1800127
rs2291615
rs56214329
All
−5e−04
0e+00
5e−04
MR leave−one−out sensitivity analysis for
'RBMS2' on 'Bladder cancer'

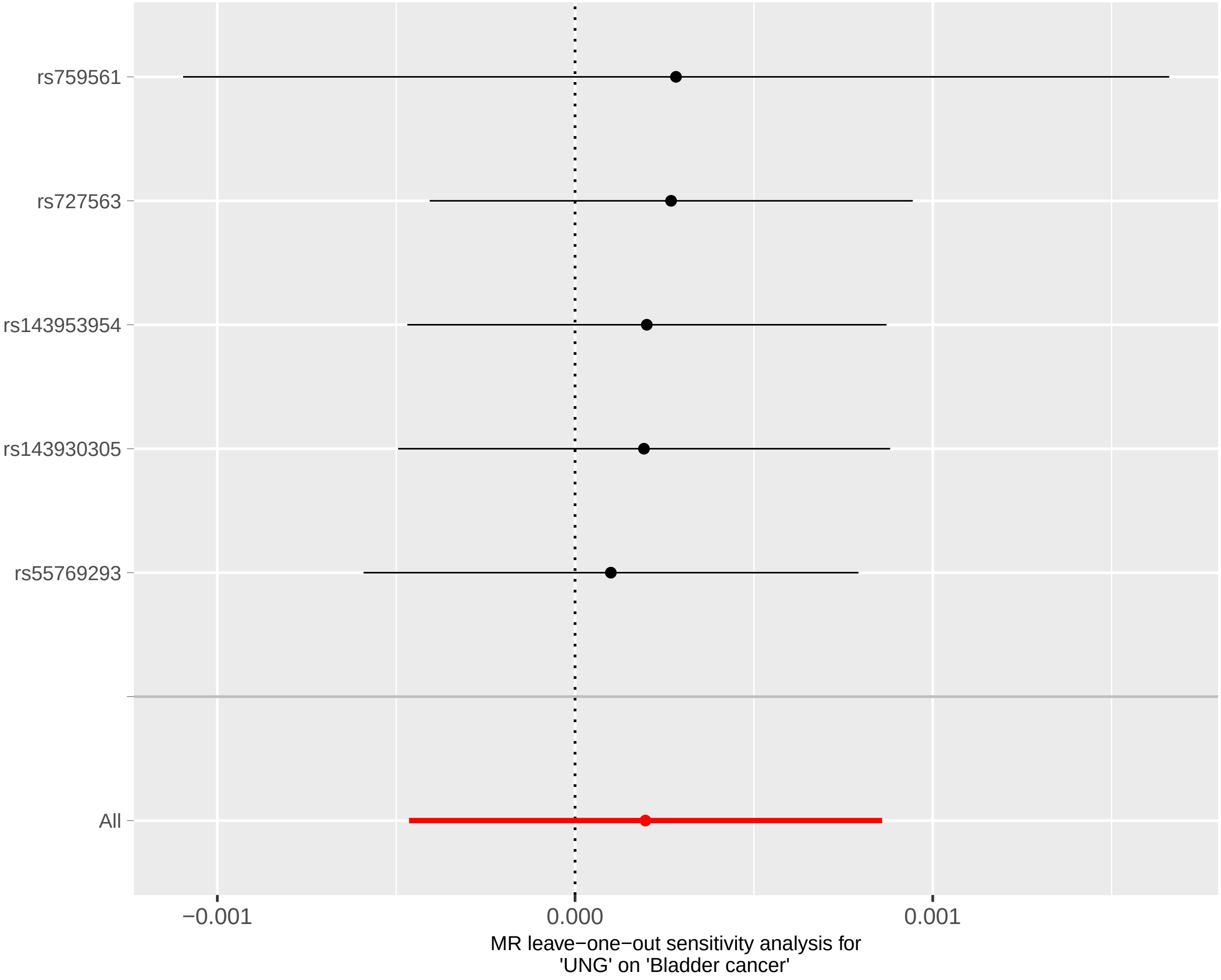
rs759561
rs727563
rs143953954
rs143930305
rs55769293
All
−0.001
0.000
0.001
MR leave−one−out sensitivity analysis for
'UNG' on 'Bladder cancer'

Insufficient number of SNPs

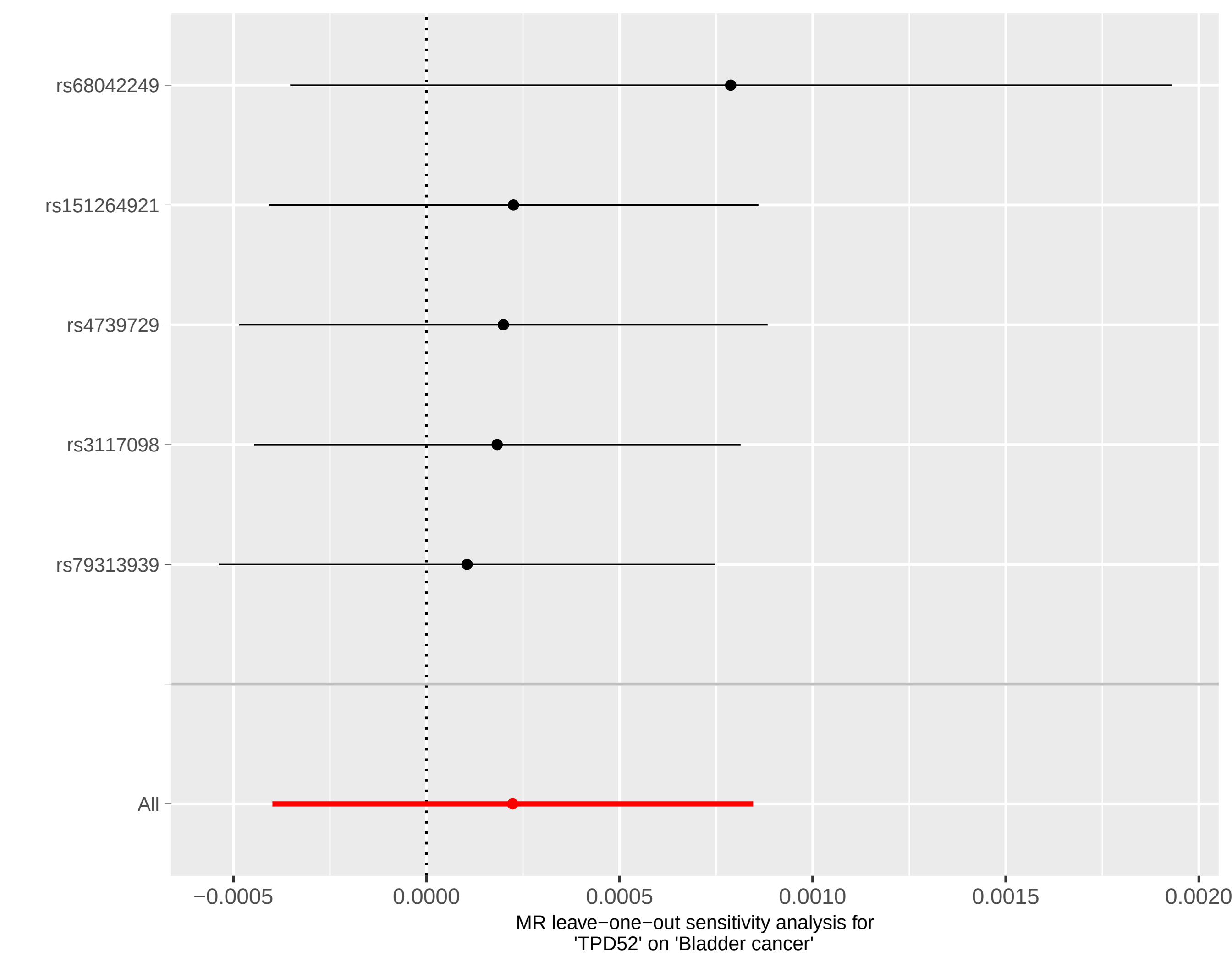
rs68042249
rs151264921
rs4739729
rs3117098
rs79313939
All
−0.0005
0.0000
0.0005
0.0010
0.0015
0.0020
MR leave−one−out sensitivity analysis for
'TPD52' on 'Bladder cancer'

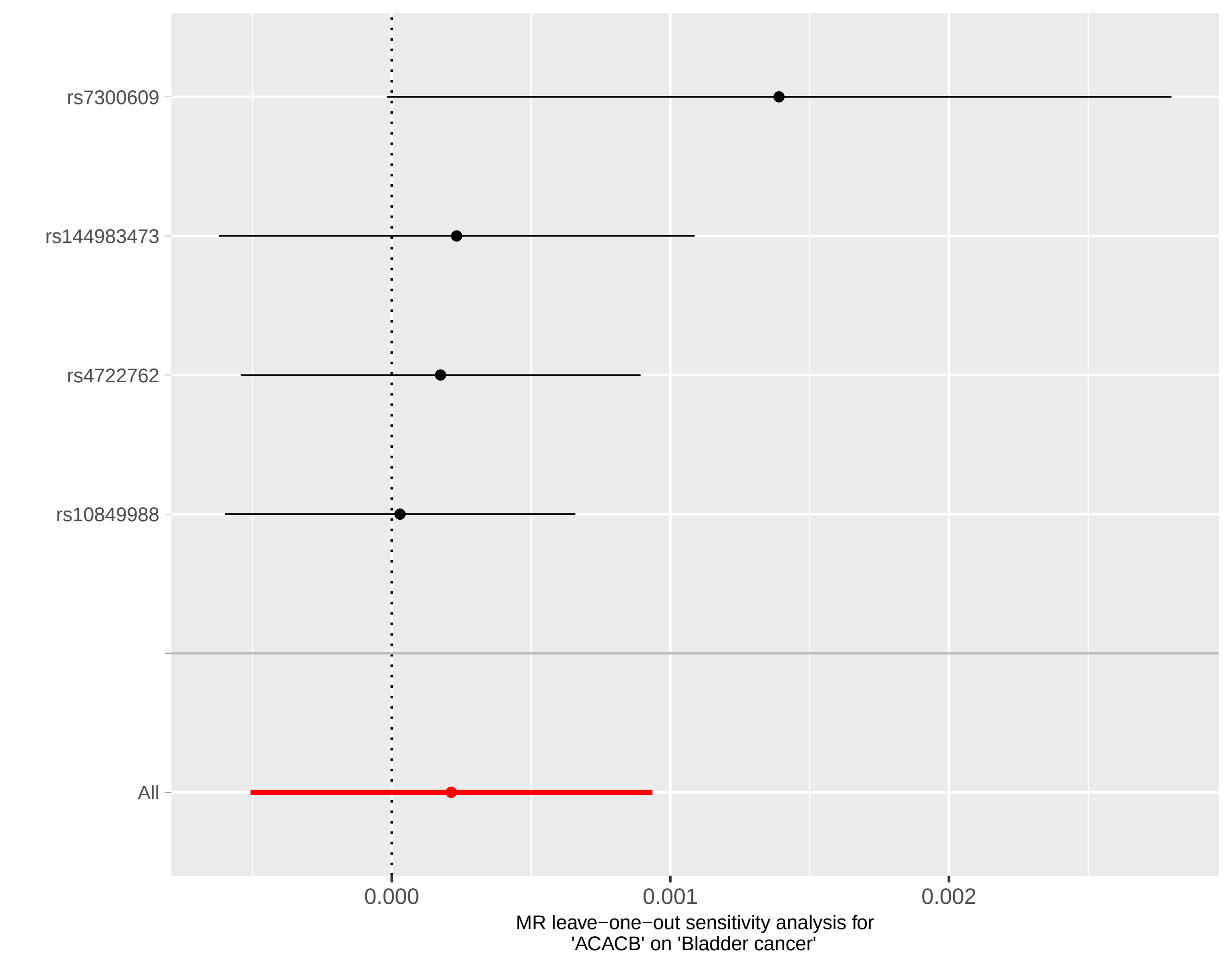
rs7300609
rs144983473
rs4722762
rs10849988
All
0.000
0.001
0.002
MR leave−one−out sensitivity analysis for
'ACACB' on 'Bladder cancer'

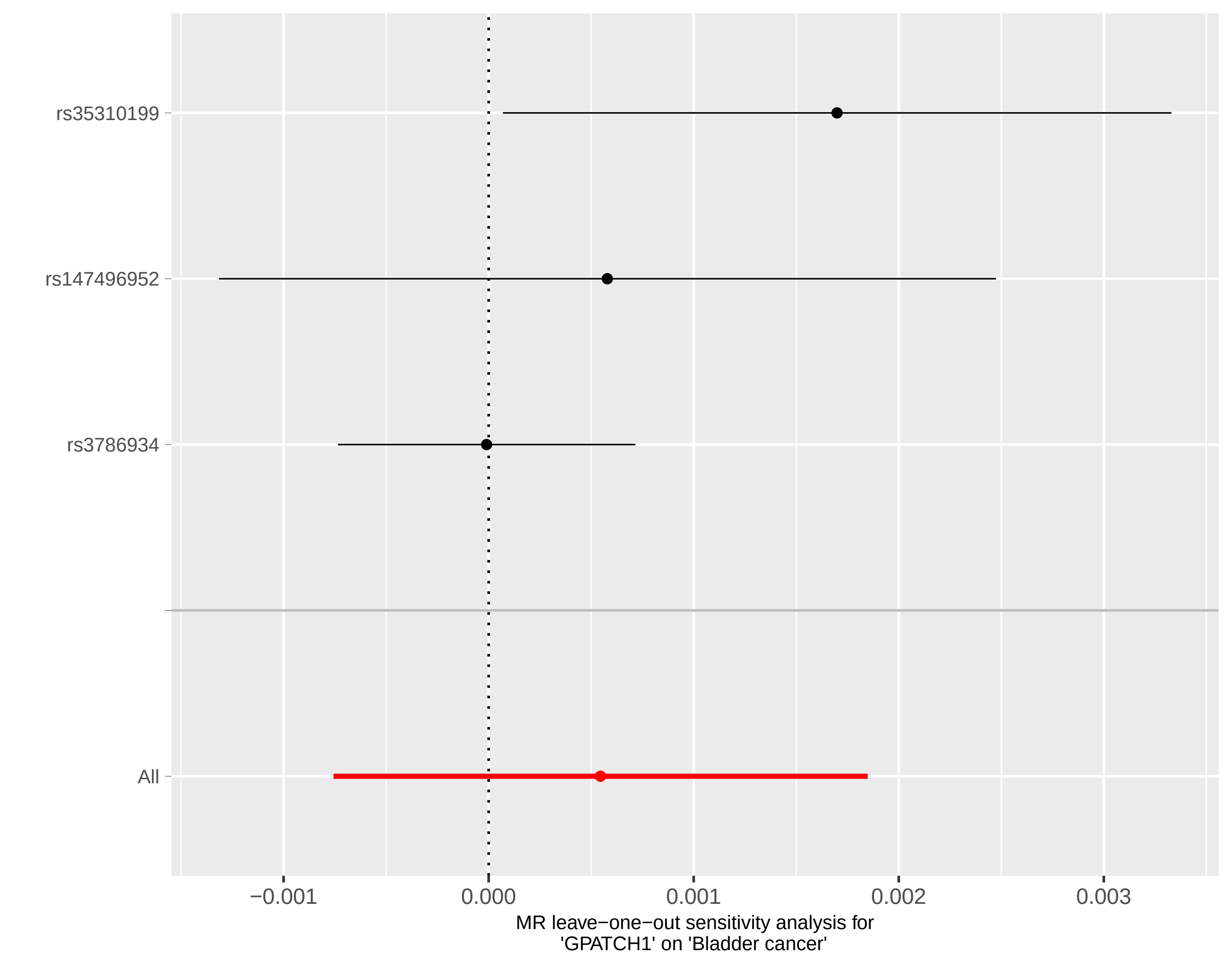
rs35310199
rs147496952
rs3786934
All
−0.001
0.000
0.001
0.002
0.003
MR leave−one−out sensitivity analysis for
'GPATCH1' on 'Bladder cancer'

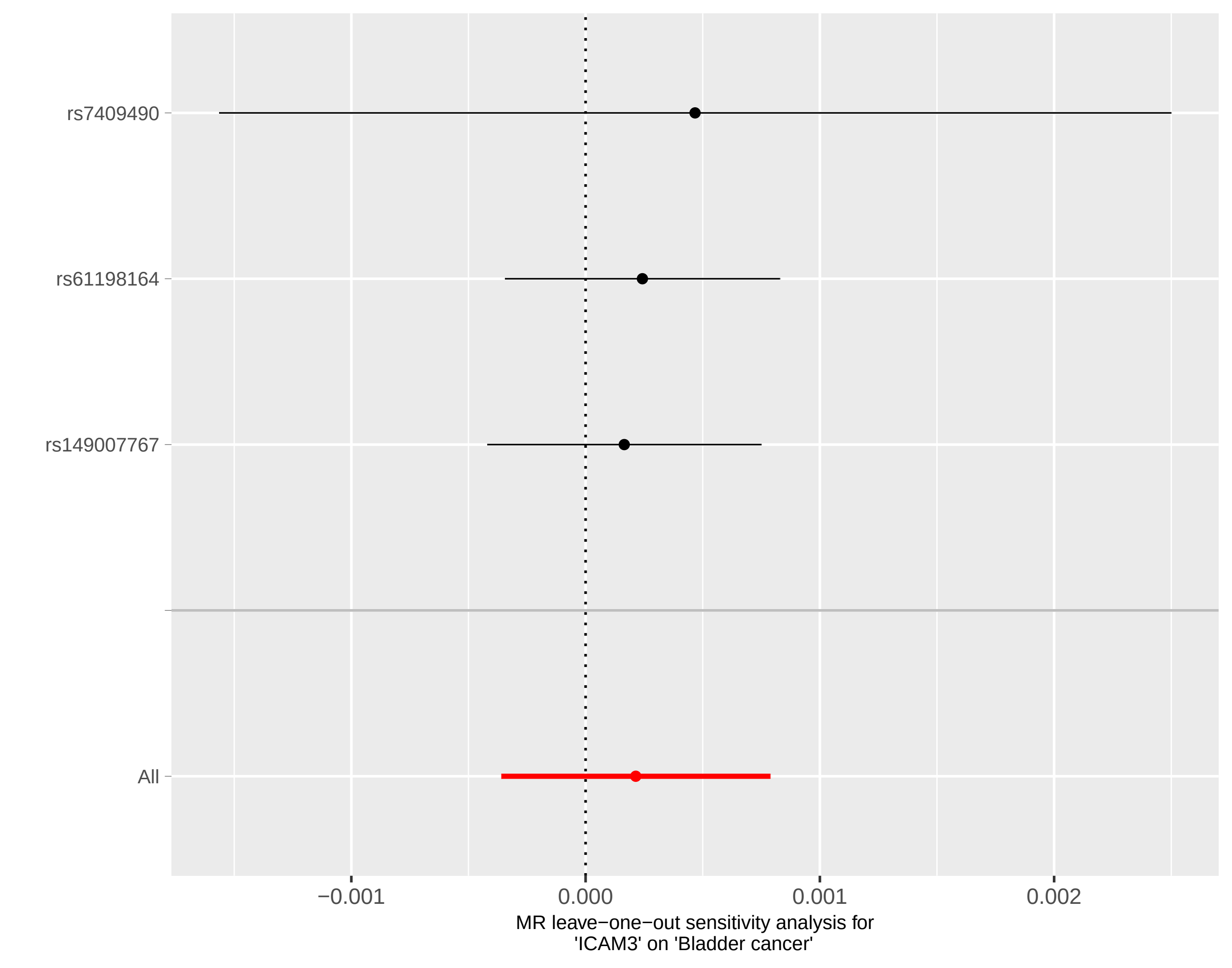

rs7409490
rs61198164
rs149007767
All
−0.001
0.000
0.001
0.002
MR leave−one−out sensitivity analysis for
'ICAM3' on 'Bladder cancer'

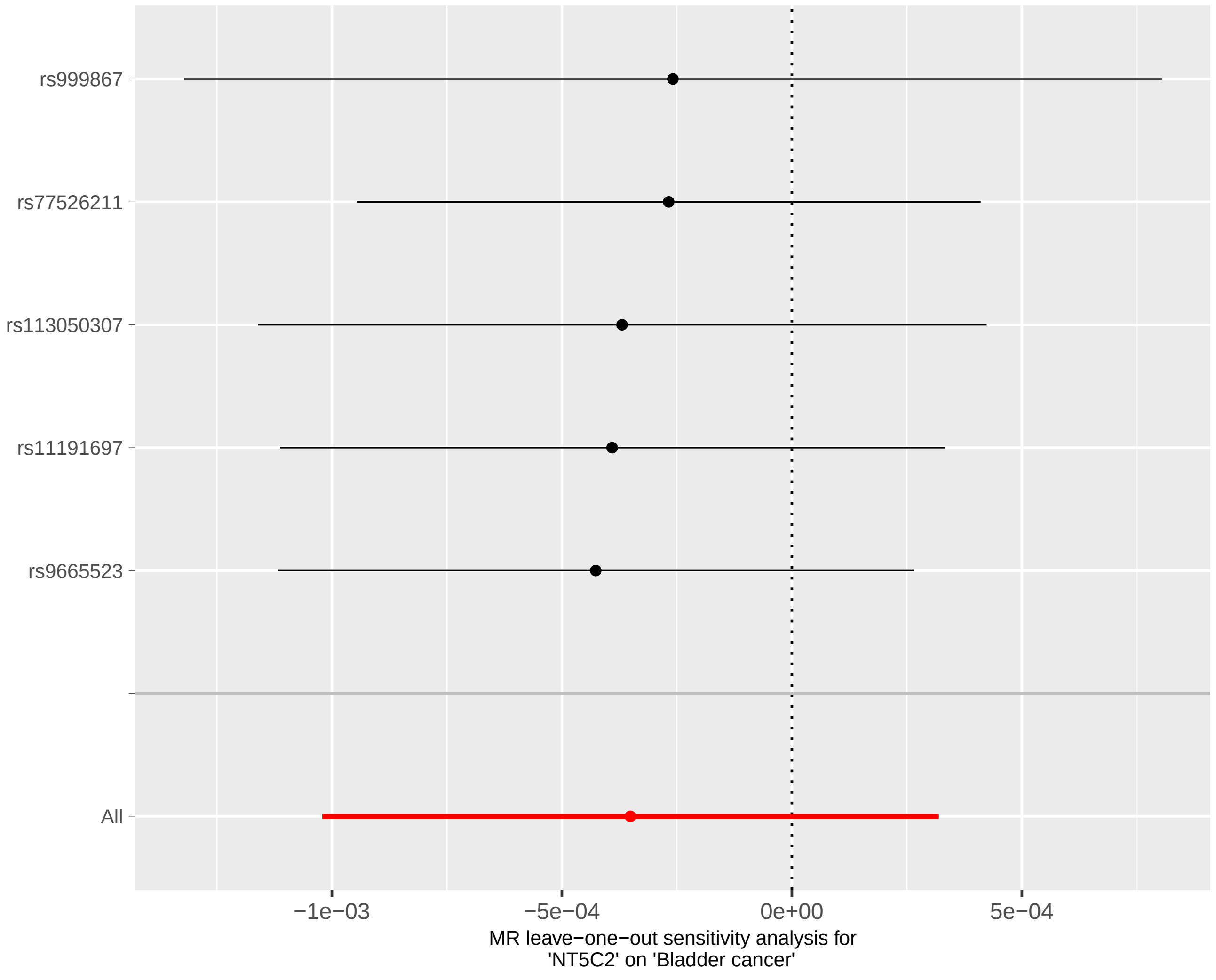

rs999867
rs77526211
rs113050307
rs11191697
rs9665523
All
−1e−03
−5e−04
0e+00
5e−04
MR leave−one−out sensitivity analysis for
'NT5C2' on 'Bladder cancer'

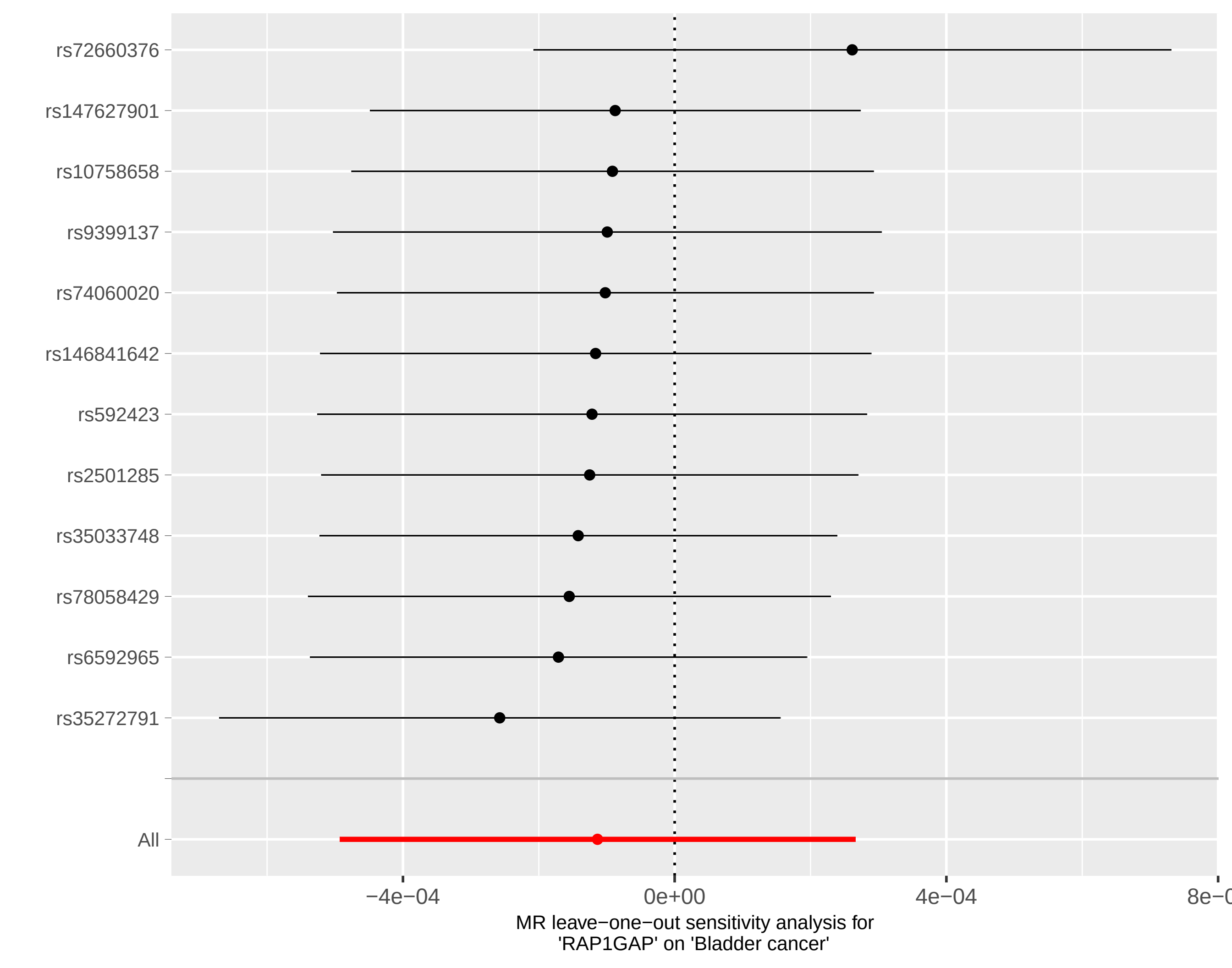
rs72660376
rs147627901
rs10758658
rs9399137
rs74060020
rs146841642
rs592423
rs2501285
rs35033748
rs78058429
rs6592965
rs35272791
All
−4e−04
0e+00
4e−04
MR leave−one−out sensitivity analysis for
'RAP1GAP' on 'Bladder cancer'

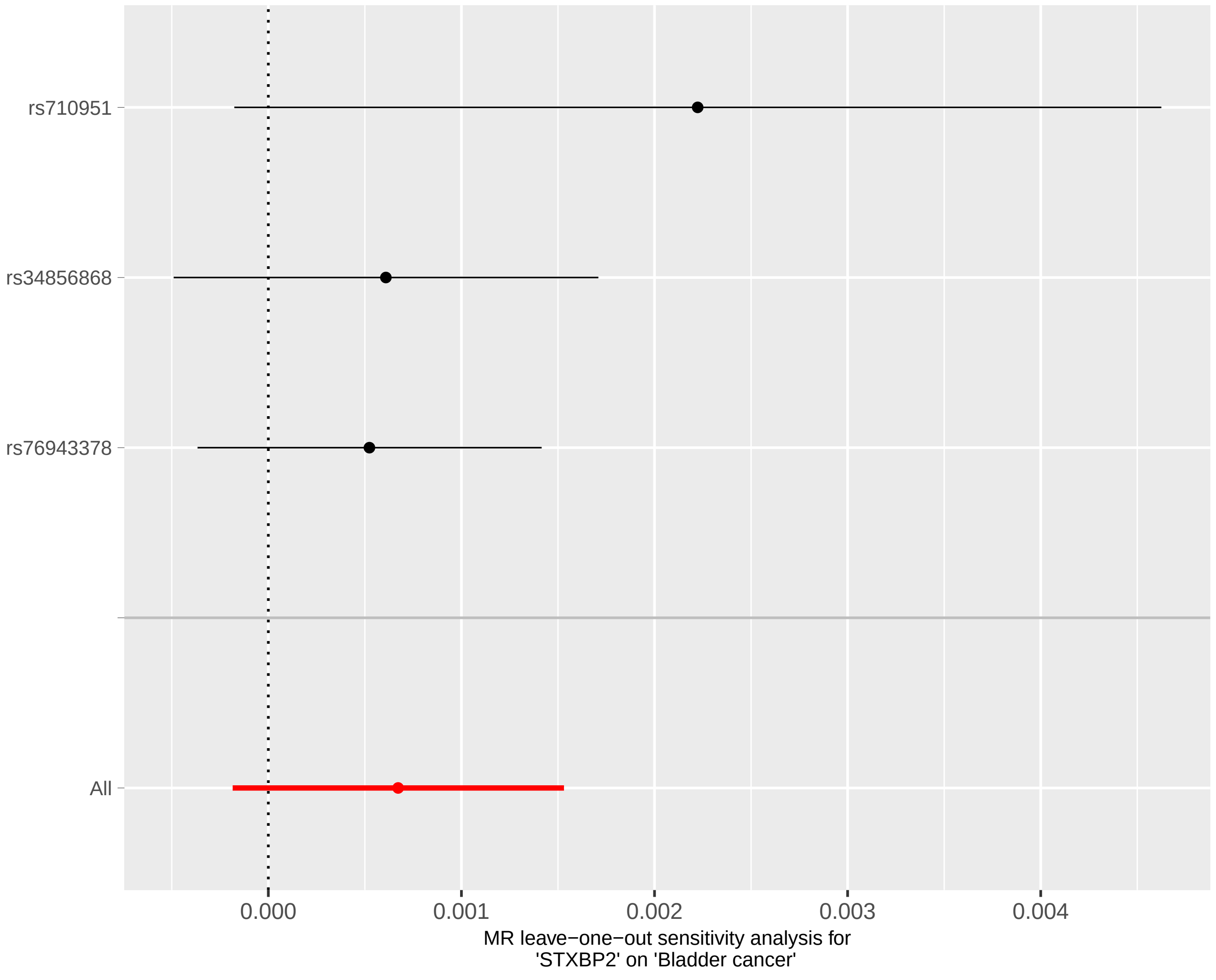
rs710951
rs34856868
rs76943378
All
0.000
0.001
0.002
0.003
0.004
MR leave−one−out sensitivity analysis for
'STXBP2' on 'Bladder cancer'

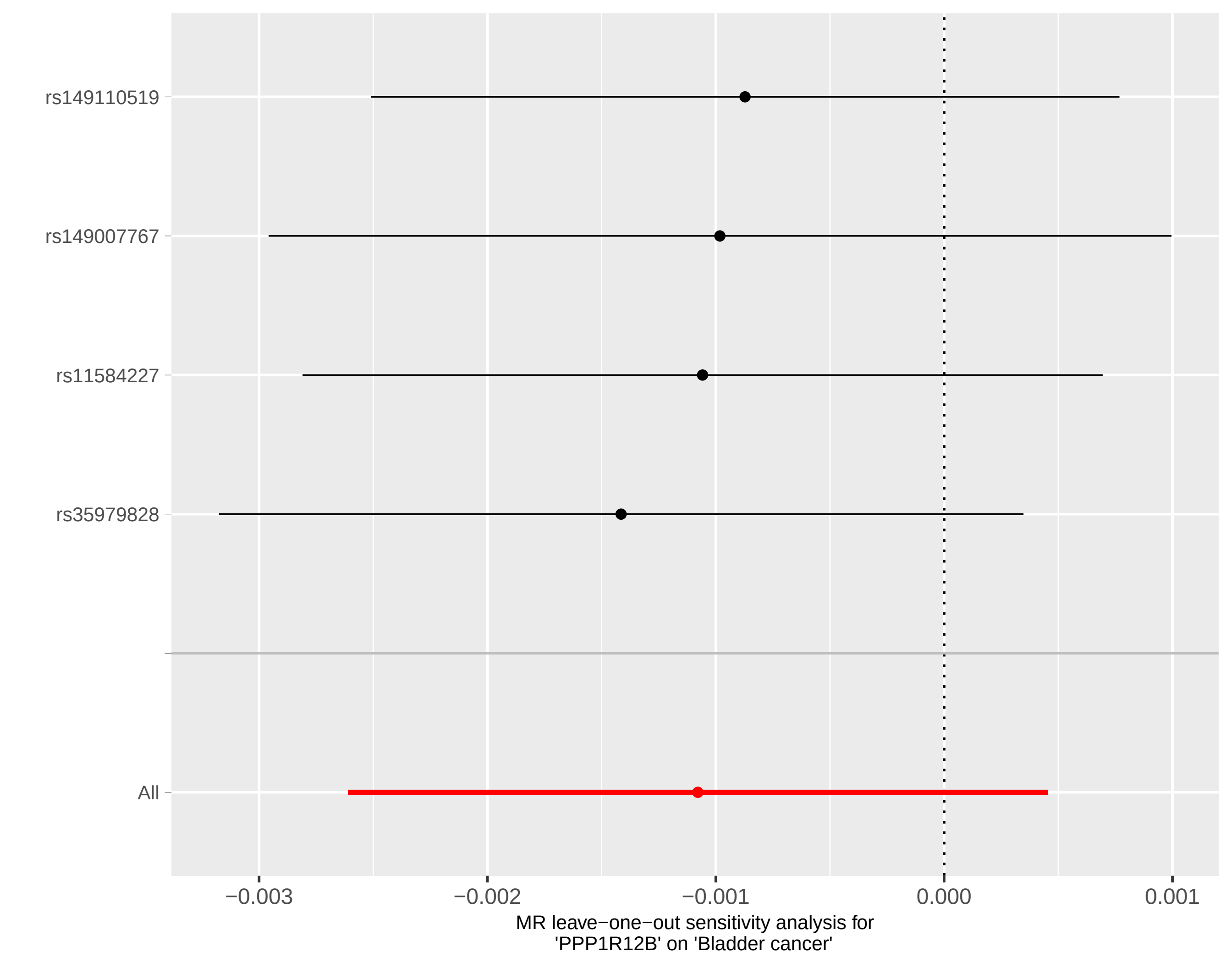

rs149110519
rs149007767
rs11584227
rs35979828
All
−0.003
−0.002
−0.001
0.000
0.001
MR leave−one−out sensitivity analysis for
'PPP1R12B' on 'Bladder cancer'

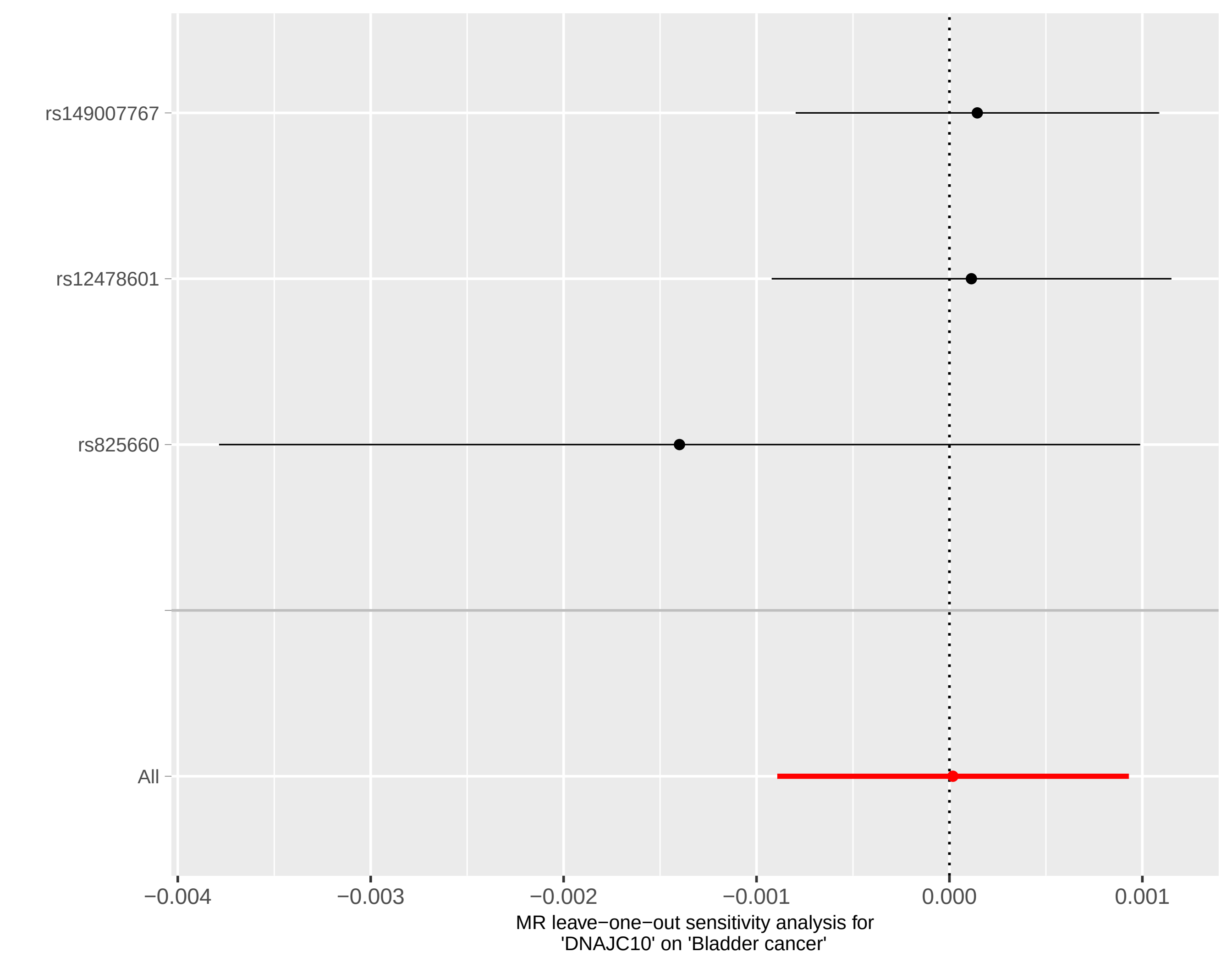
rs149007767
rs12478601
rs825660
All
−0.004
−0.003
−0.002
−0.001
0.000
0.001
MR leave−one−out sensitivity analysis for
'DNAJC10' on 'Bladder cancer'

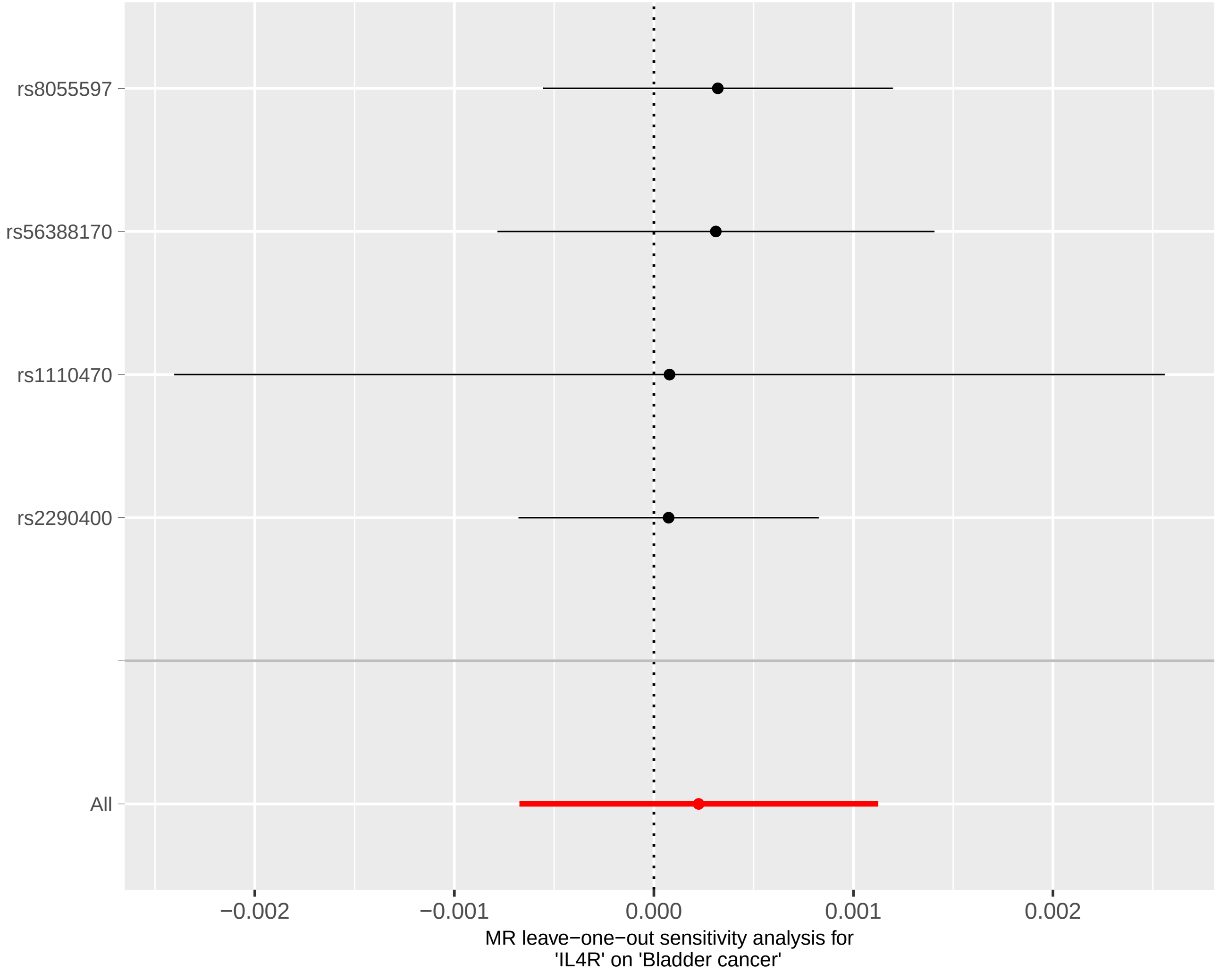
rs8055597
rs56388170
rs1110470
rs2290400
All
−0.002
−0.001
0.000
0.001
0.002
MR leave−one−out sensitivity analysis for
'IL4R' on 'Bladder cancer'

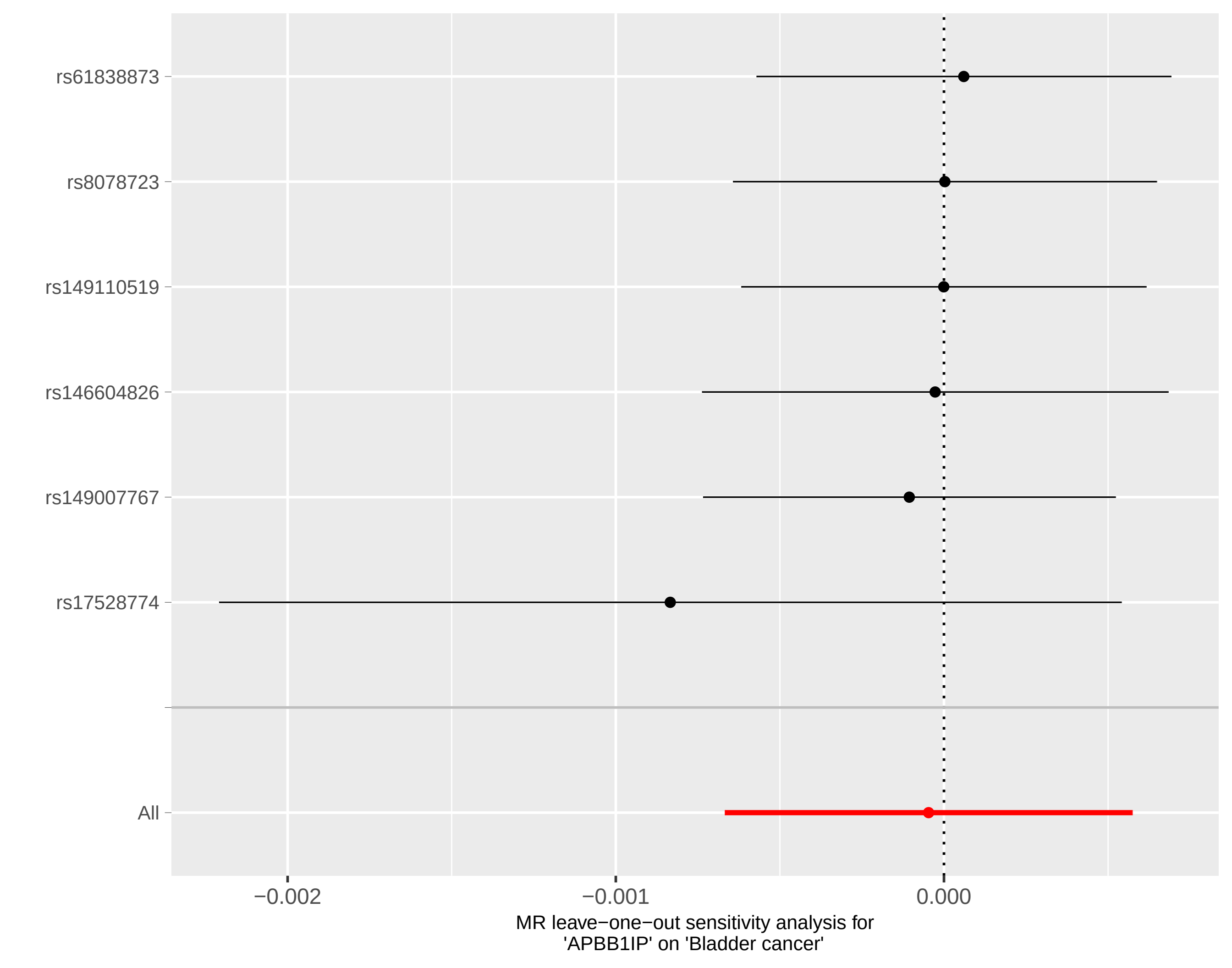
rs61838873
rs8078723
rs149110519
rs146604826
rs149007767
rs17528774
All
−0.002
−0.001
0.000
MR leave−one−out sensitivity analysis for
'APBB1IP' on 'Bladder cancer'

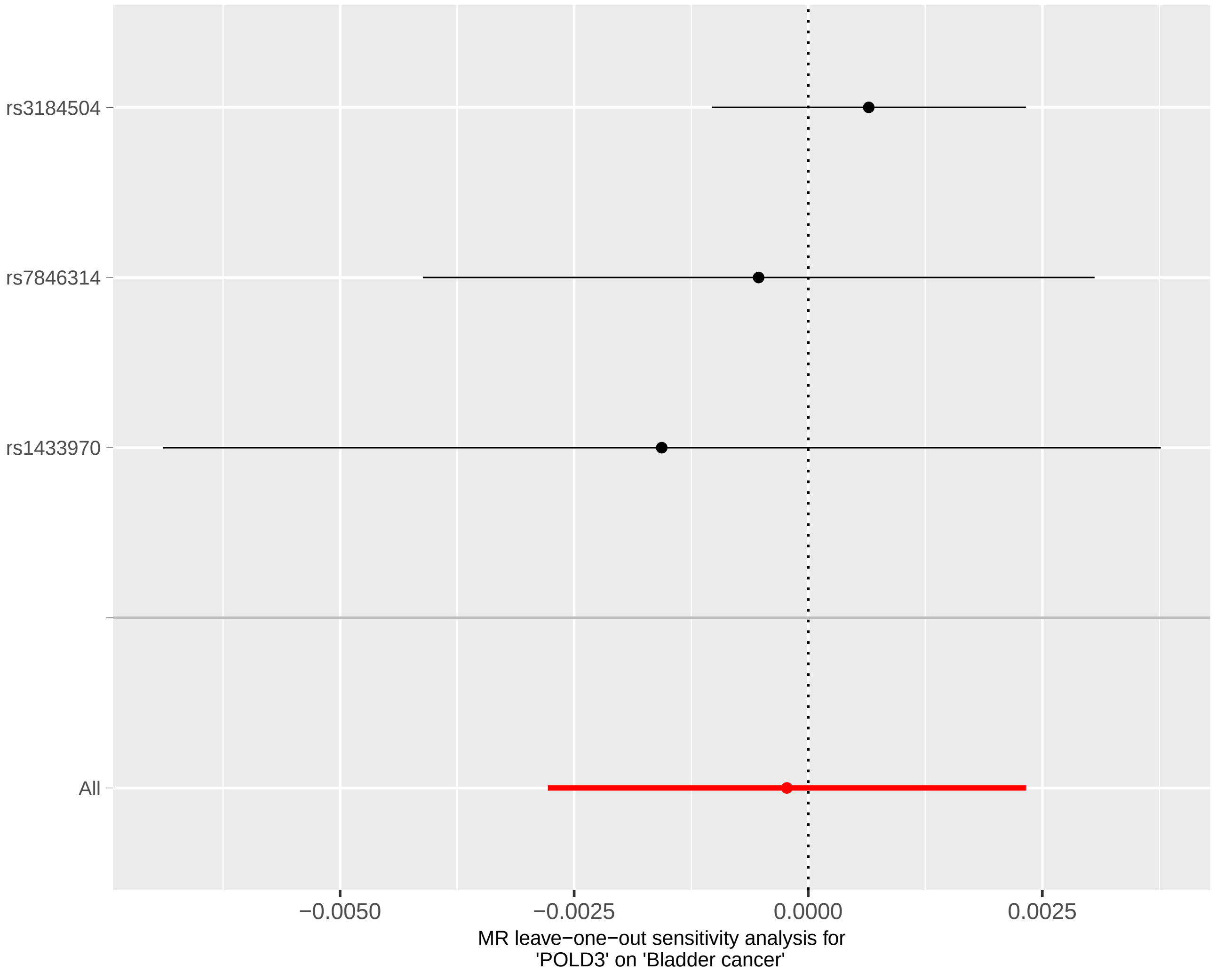

rs3184504
rs7846314
rs1433970
All
−0.0050
−0.0025
0.0000
0.0025
MR leave−one−out sensitivity analysis for
'POLD3' on 'Bladder cancer'

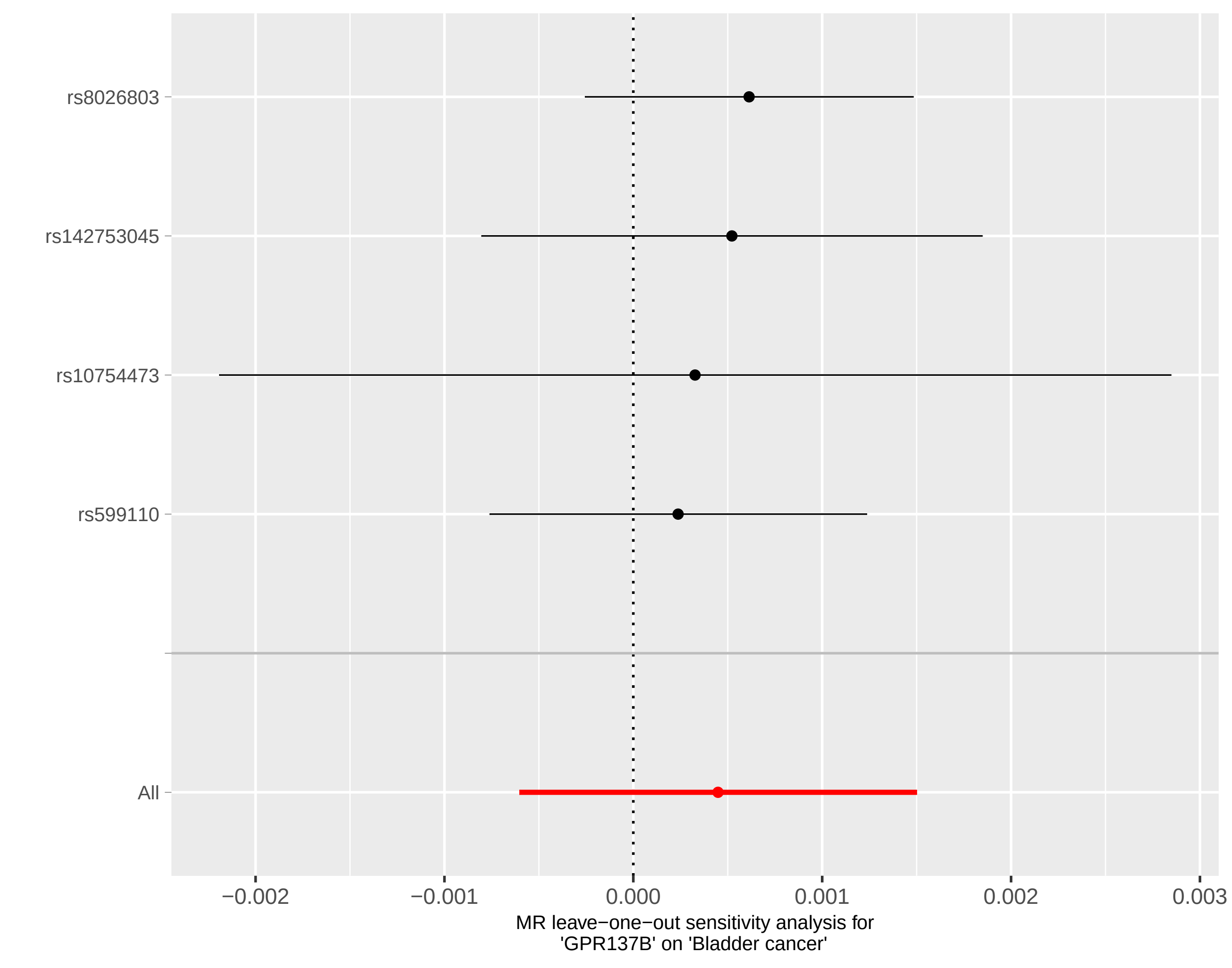

rs8026803
rs142753045
rs10754473
rs599110
All
−0.002
−0.001
0.000
0.001
0.002
0.003
MR leave−one−out sensitivity analysis for
'GPR137B' on 'Bladder cancer'

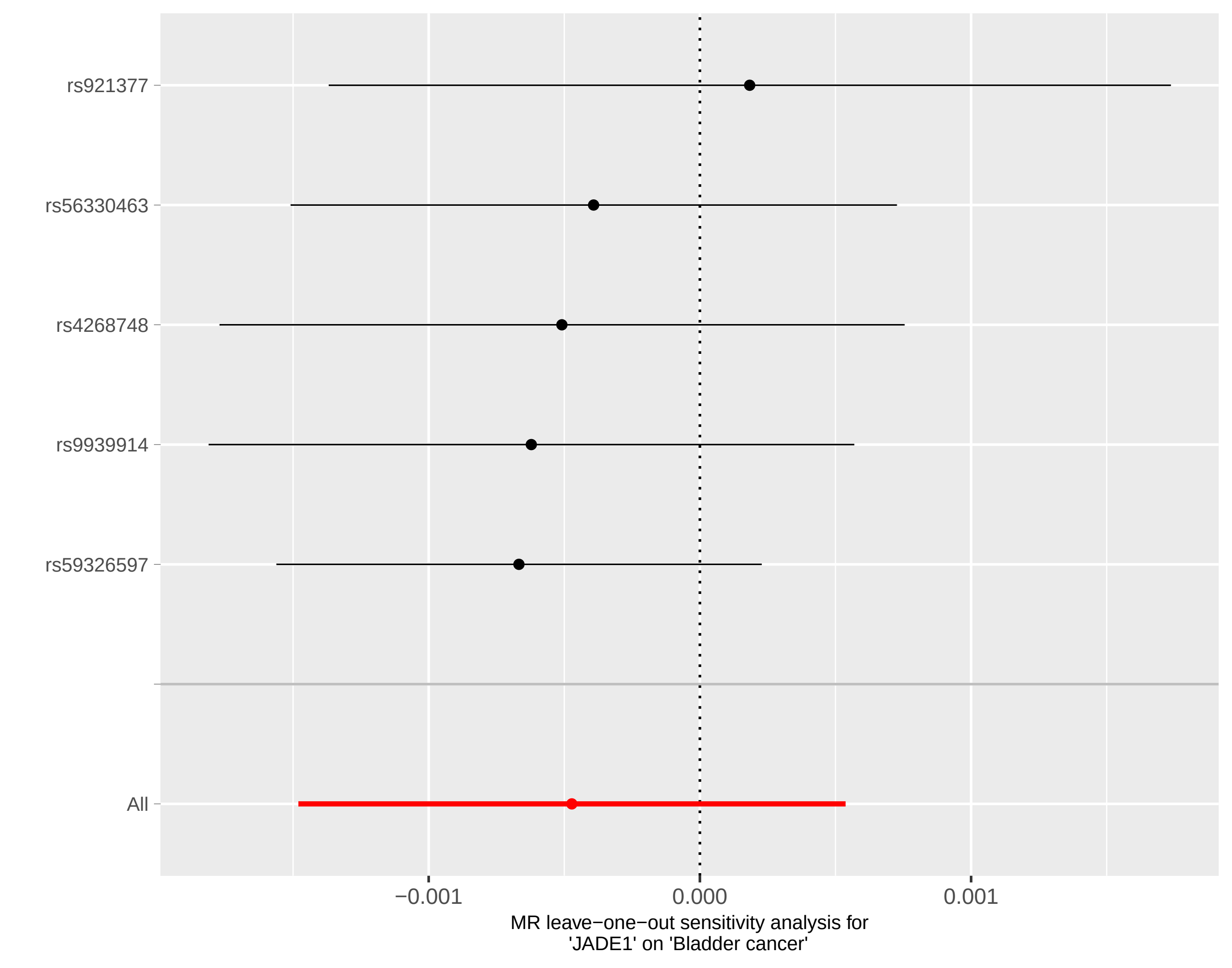
rs921377
rs56330463
rs4268748
rs9939914
rs59326597
All
−0.001
0.000
0.001
MR leave−one−out sensitivity analysis for
'JADE1' on 'Bladder cancer'

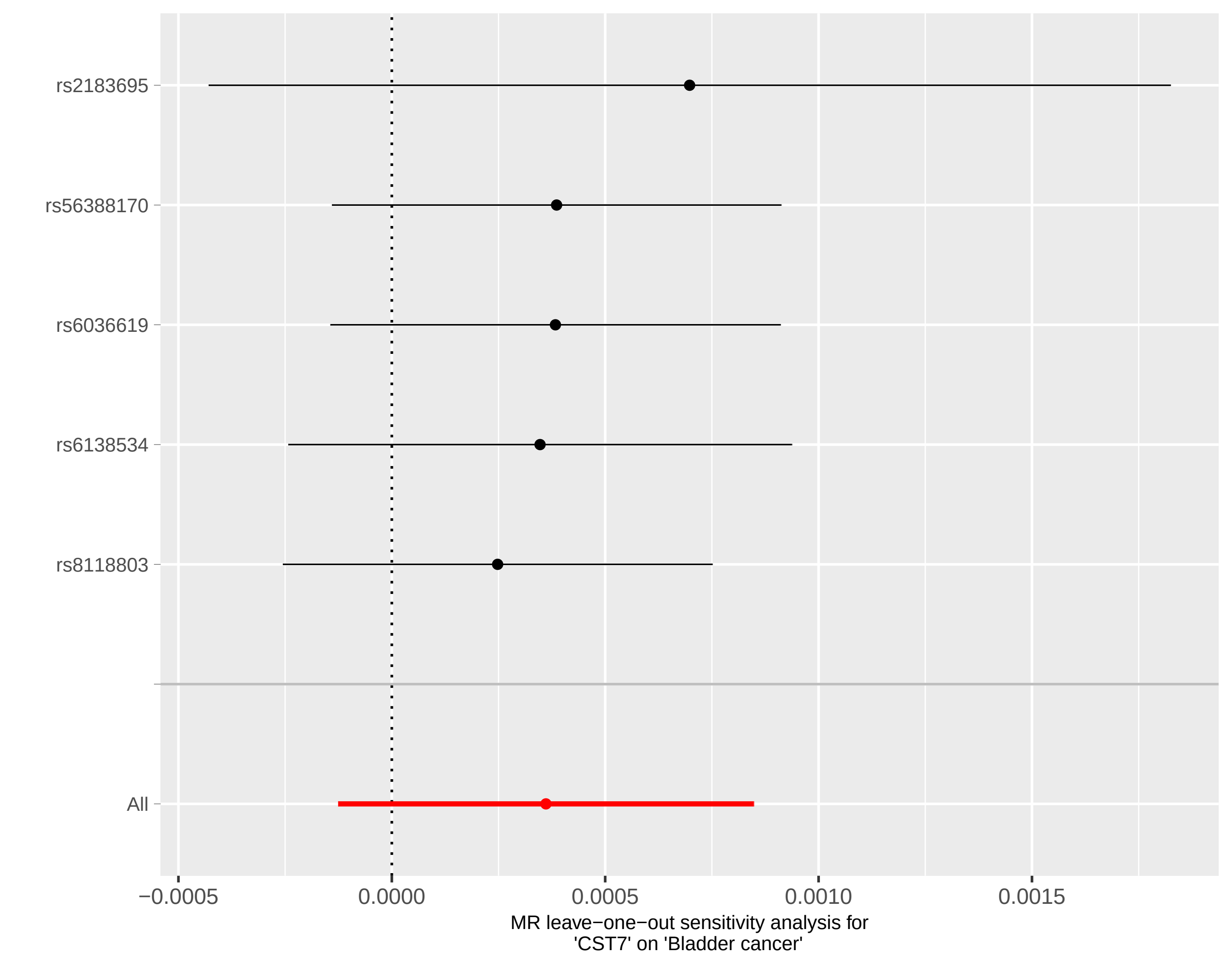
rs2183695
rs56388170
rs6036619
rs6138534
rs8118803
All
−0.0005
0.0000
0.0005
0.0010
0.0015
MR leave−one−out sensitivity analysis for
'CST7' on 'Bladder cancer'

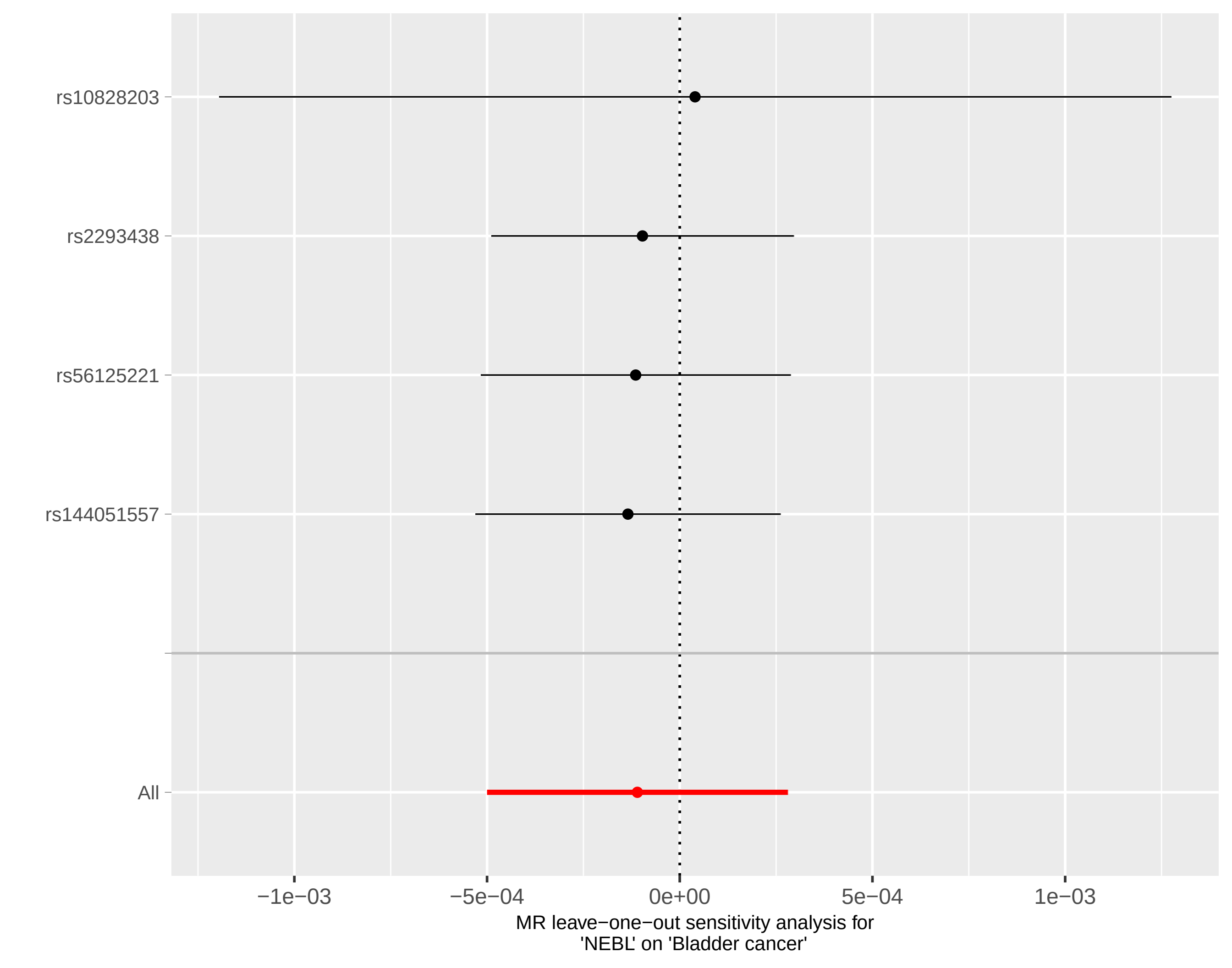
rs10828203
rs2293438
rs56125221
rs144051557
All
−1e−03
−5e−04
0e+00
5e−04
1e−03
MR leave−one−out sensitivity analysis for
'NEBL' on 'Bladder cancer'

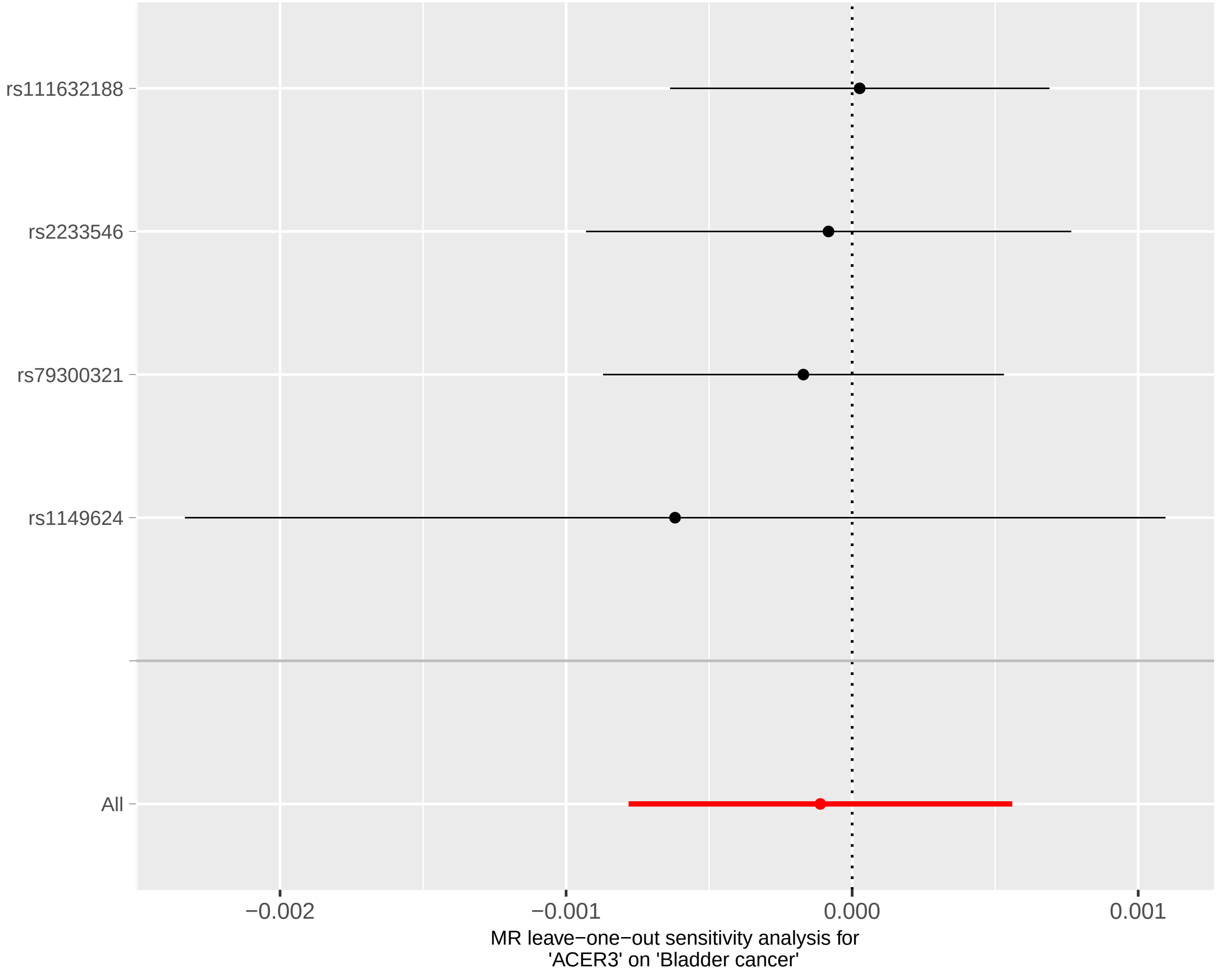
rs111632188
rs2233546
rs79300321
rs1149624
All
−0.002
−0.001
0.000
0.001
MR leave−one−out sensitivity analysis for
'ACER3' on 'Bladder cancer'

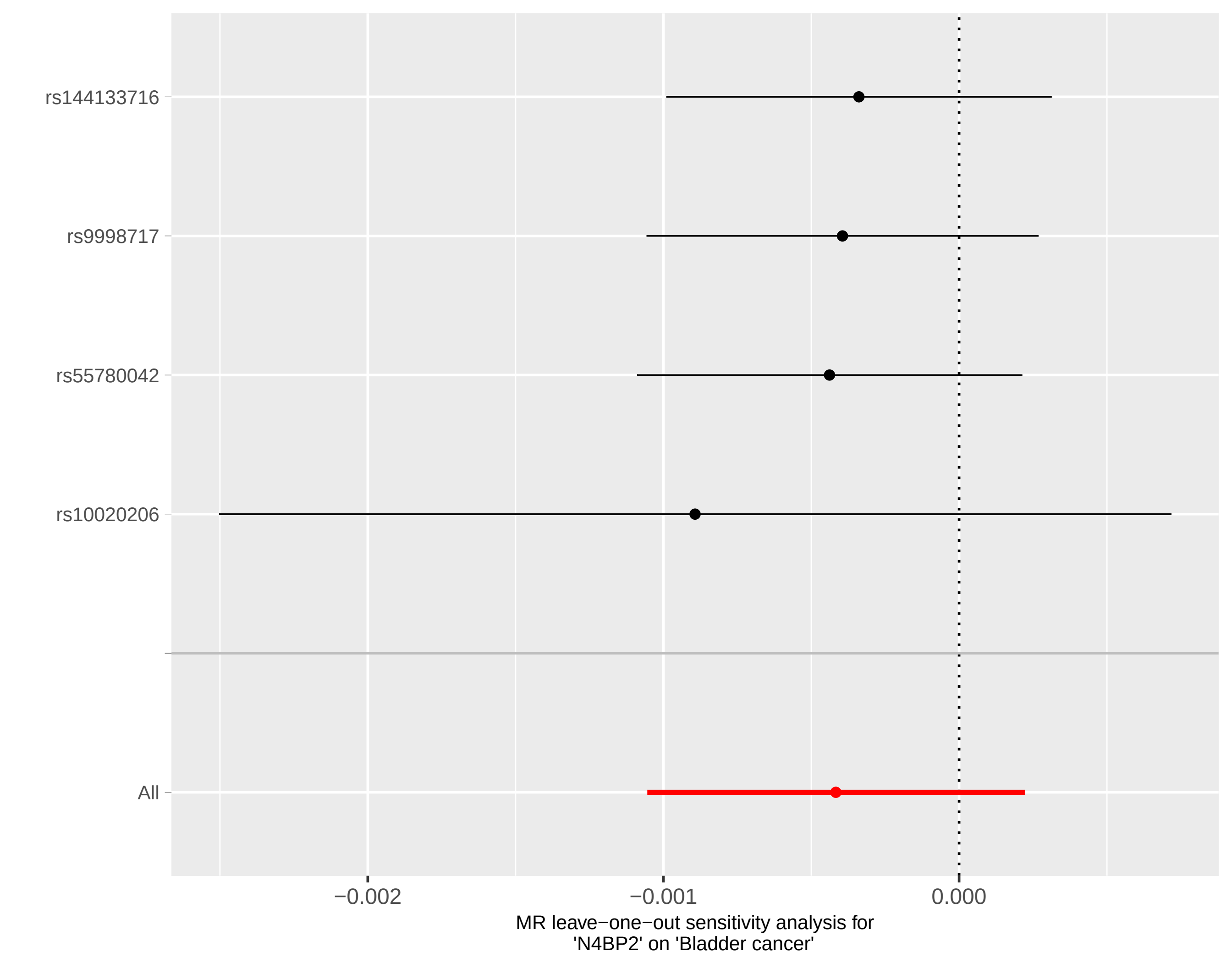

rs144133716
rs9998717
rs55780042
rs10020206
All
−0.002
−0.001
0.000
MR leave−one−out sensitivity analysis for
'N4BP2' on 'Bladder cancer'

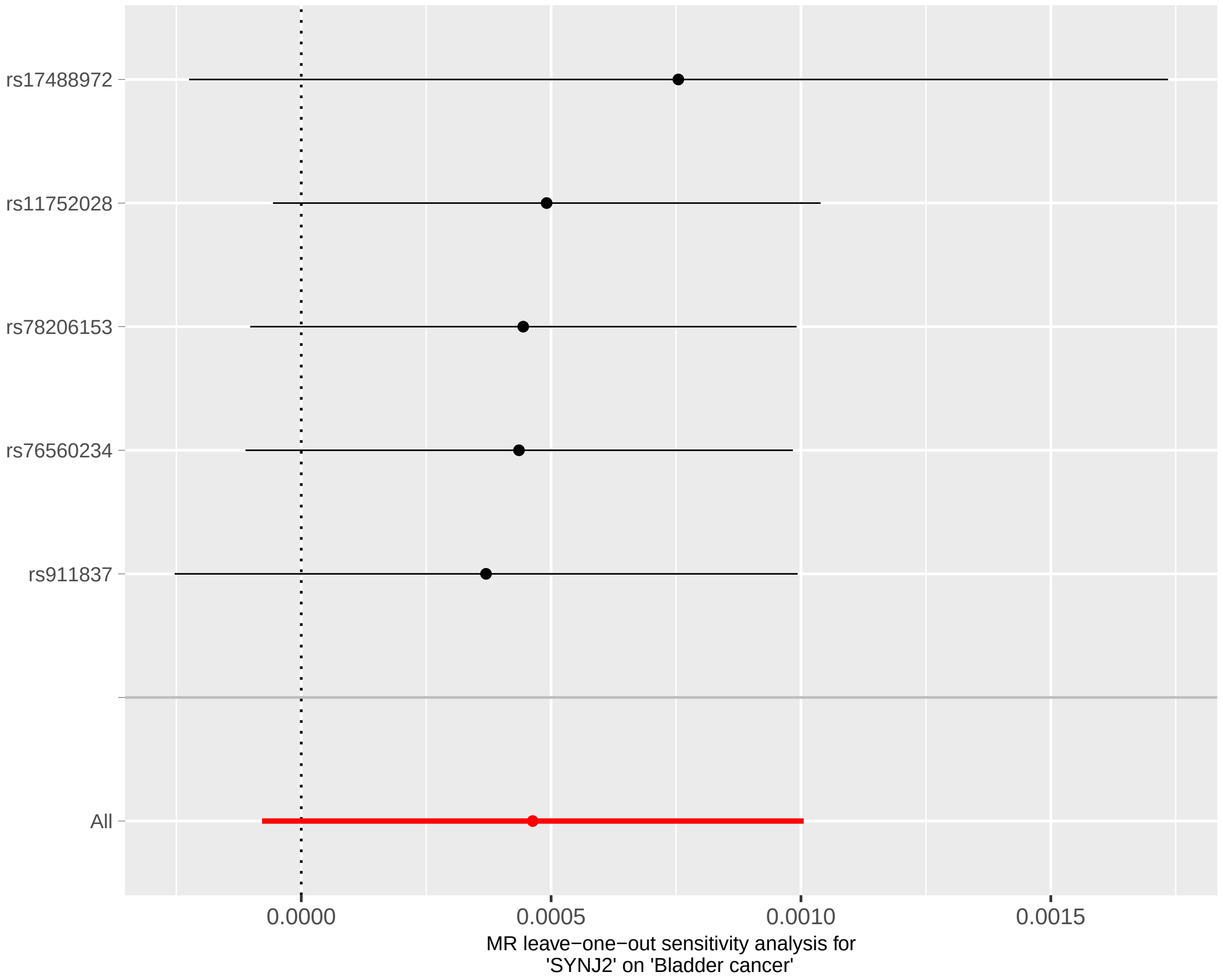
rs17488972
rs11752028
rs78206153
rs76560234
rs911837
All
0.0000
0.0005
0.0010
0.0015
MR leave−one−out sensitivity analysis for
'SYNJ2' on 'Bladder cancer'

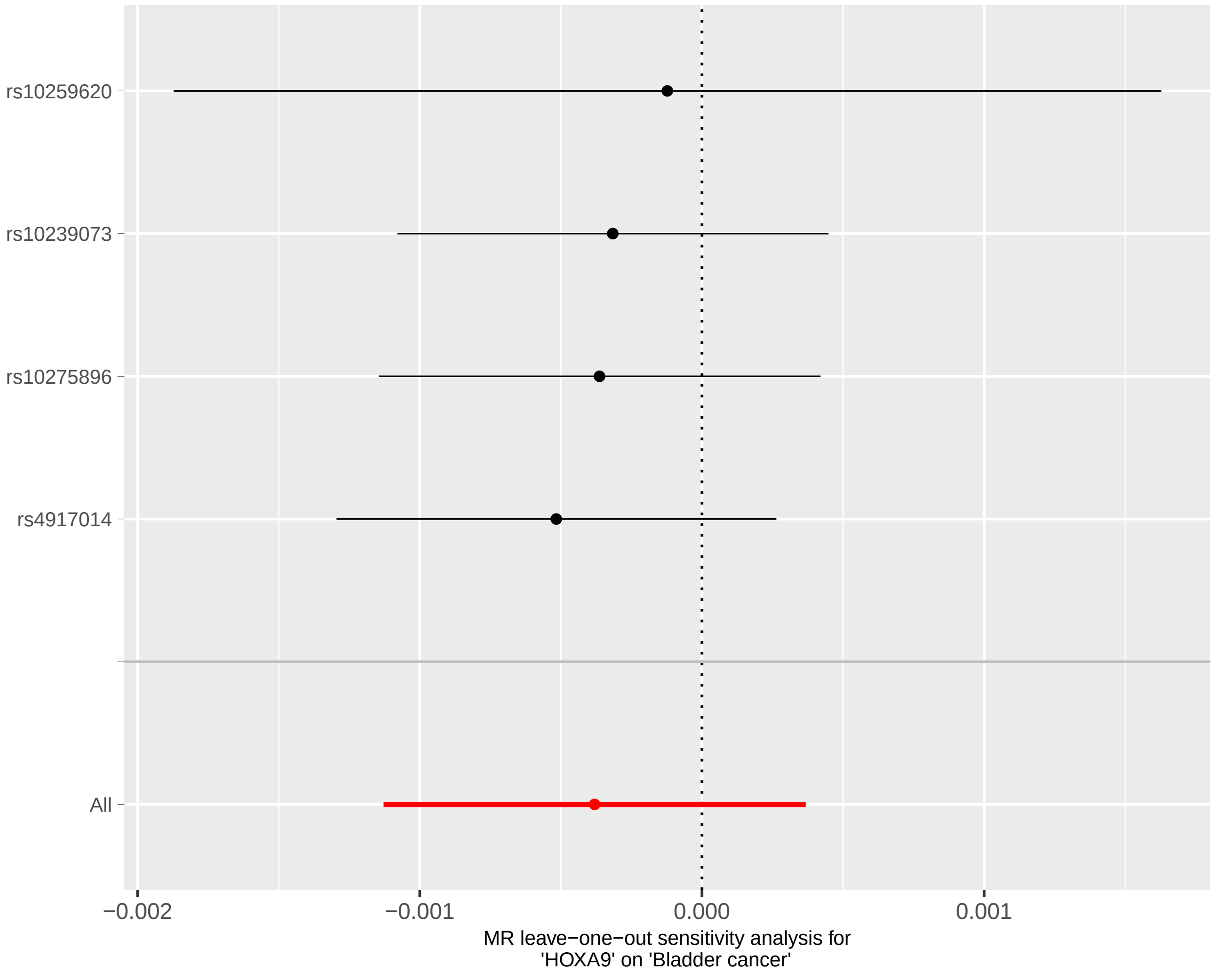
rs10259620
rs10239073
rs10275896
rs4917014
All
−0.002
−0.001
0.000
0.001
MR leave−one−out sensitivity analysis for
'HOXA9' on 'Bladder cancer'

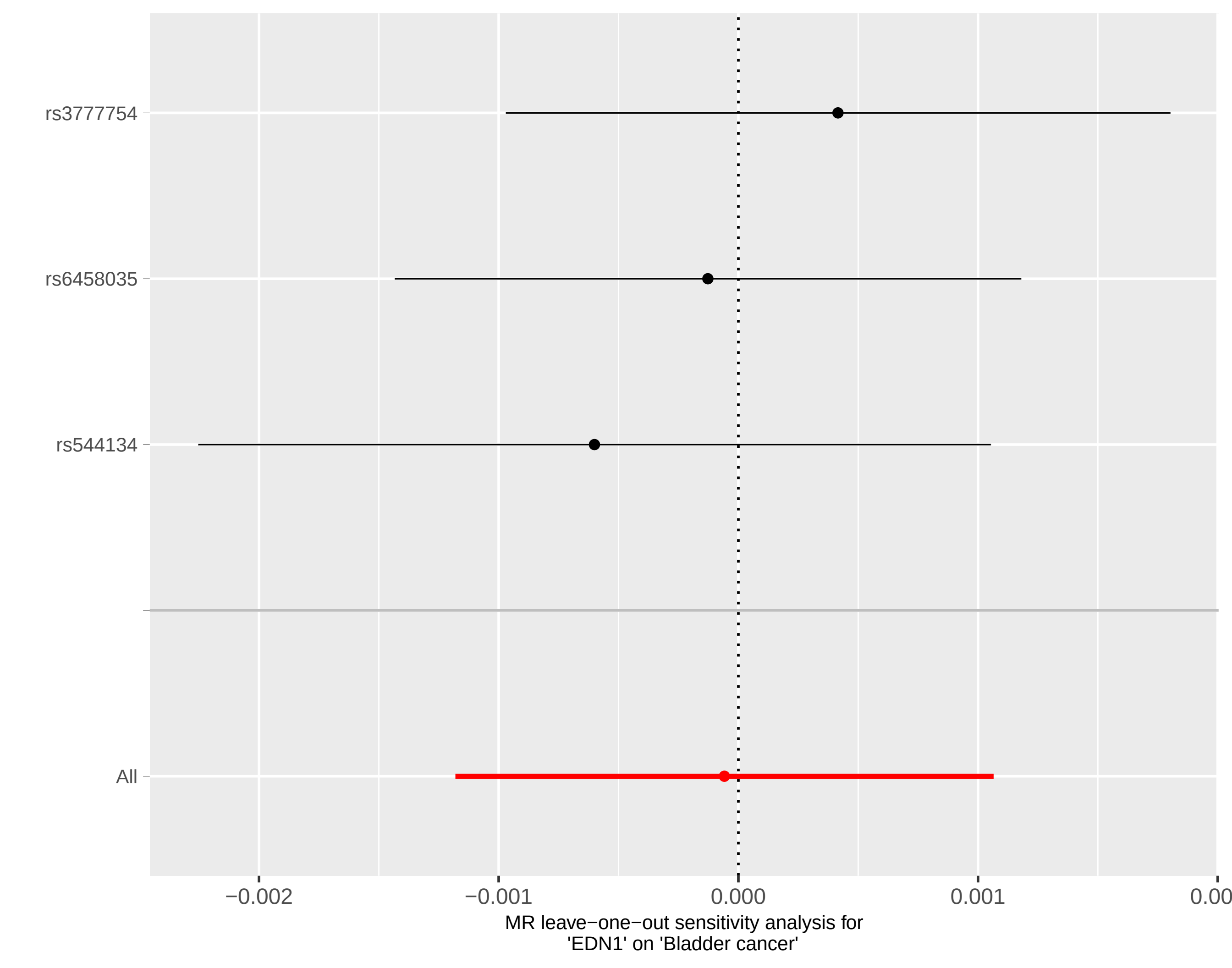
rs3777754
rs6458035
rs544134
All
−0.002
−0.001
0.000
0.001
0.00
MR leave−one−out sensitivity analysis for
'EDN1' on 'Bladder cancer'

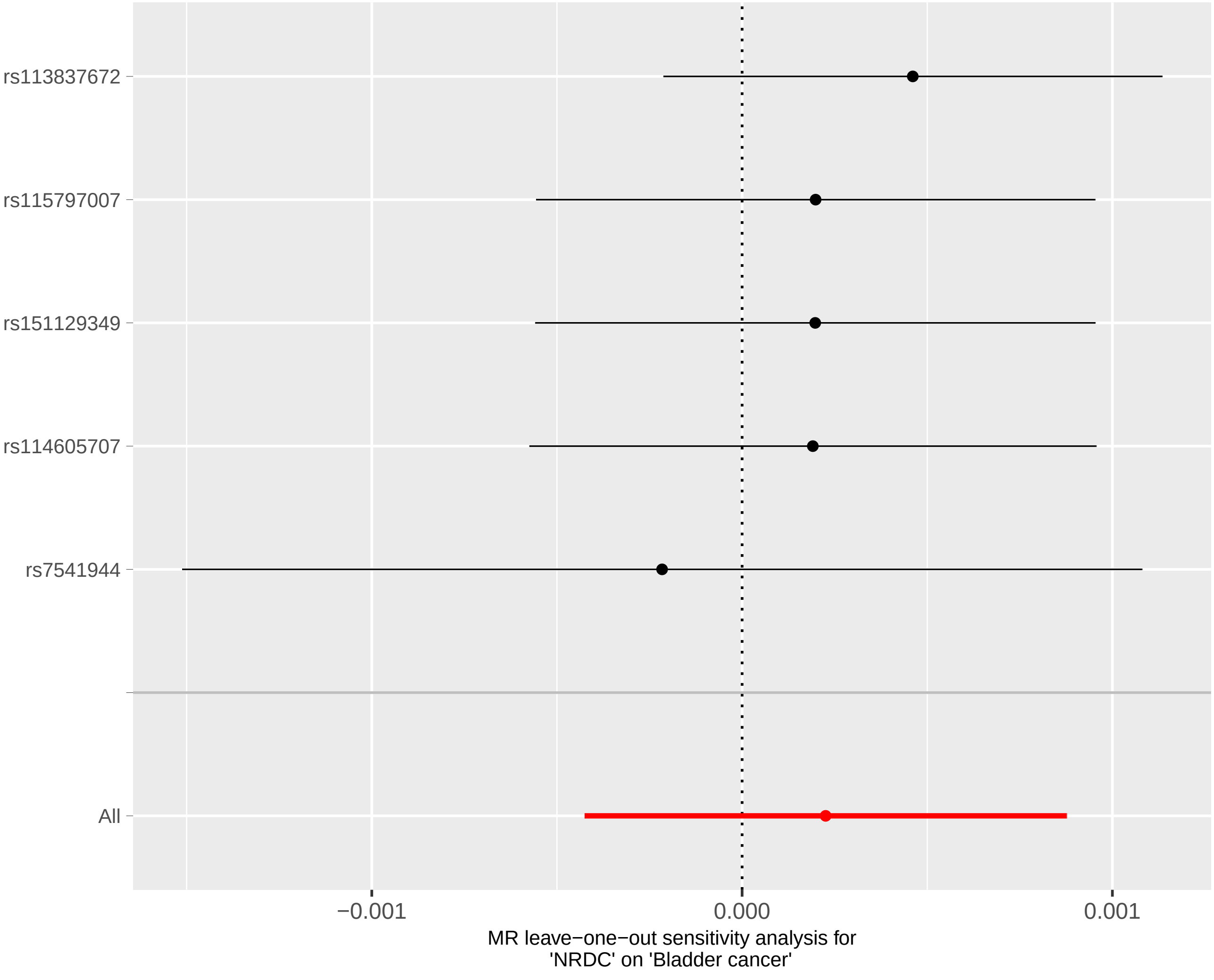
rs113837672
rs115797007
rs151129349
rs114605707
rs7541944
All
−0.001
0.000
0.001
MR leave−one−out sensitivity analysis for
'NRDC' on 'Bladder cancer'

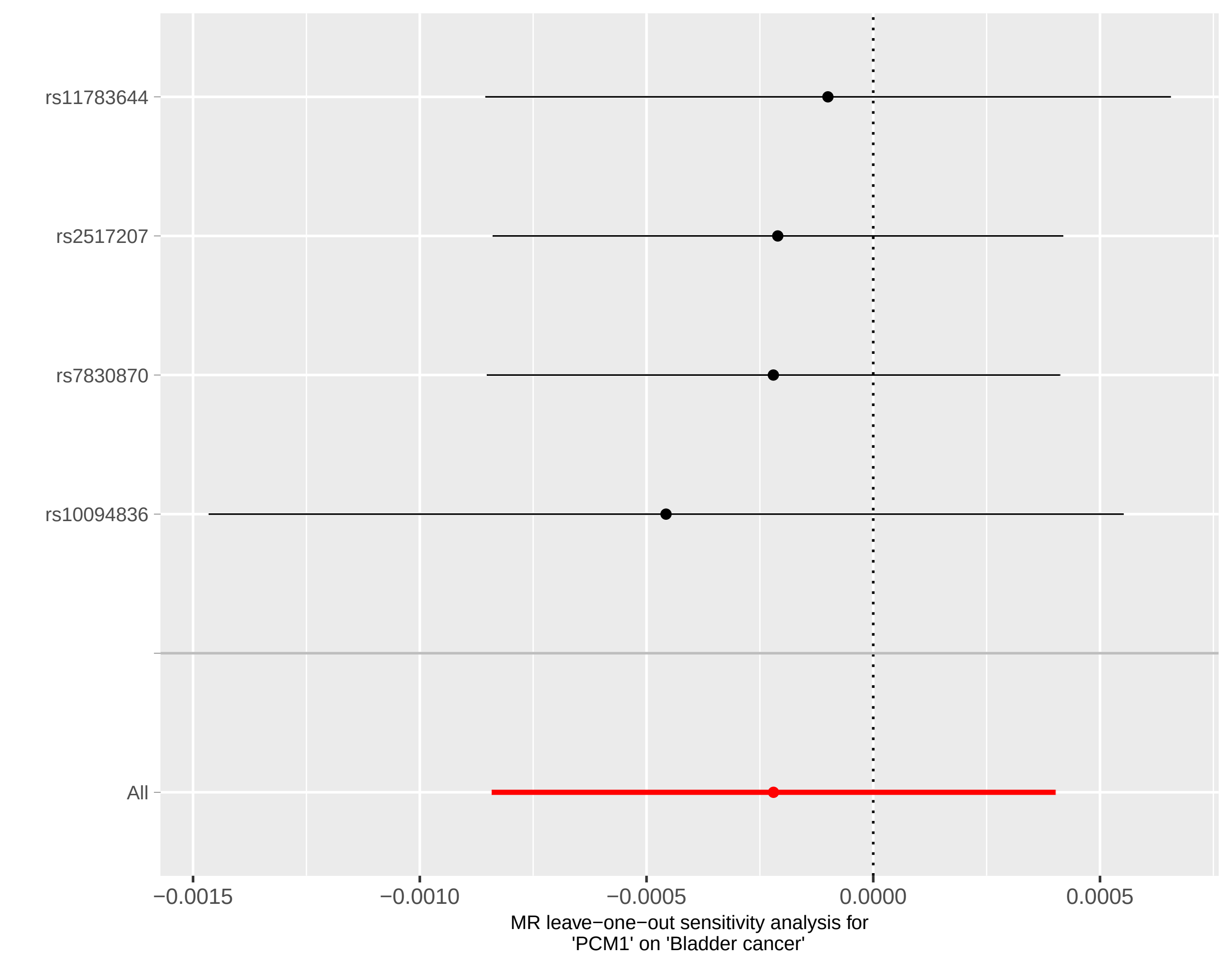
rs11783644
rs2517207
rs7830870
rs10094836
All
−0.0015
−0.0010
−0.0005
0.0000
0.0005
MR leave−one−out sensitivity analysis for
'PCM1' on 'Bladder cancer'

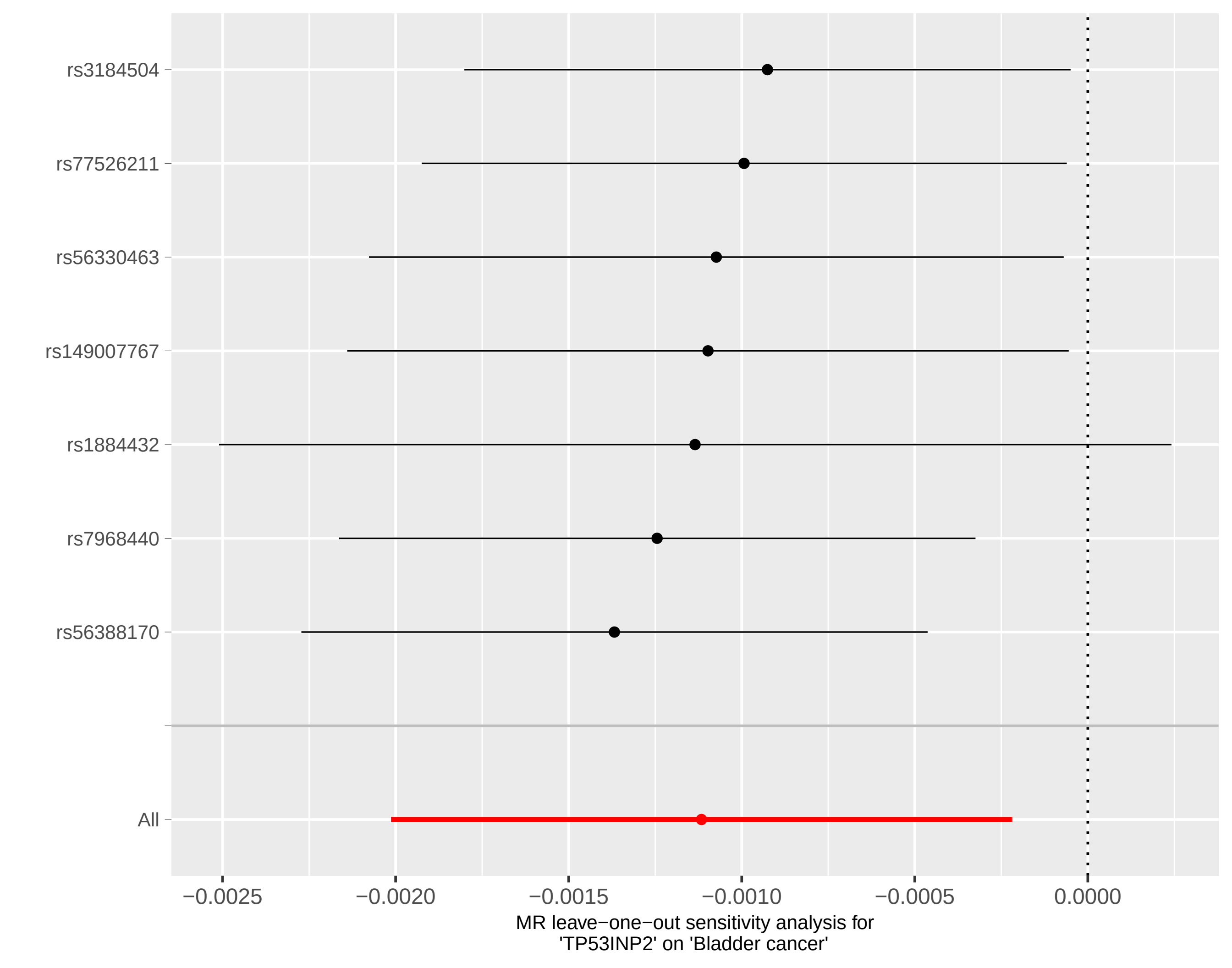

rs3184504
rs77526211
rs56330463
rs149007767
rs1884432
rs7968440
rs56388170
All
−0.0025
−0.0020
−0.0015
−0.0010
−0.0005
0.0000
MR leave−one−out sensitivity analysis for
'TP53INP2' on 'Bladder cancer'

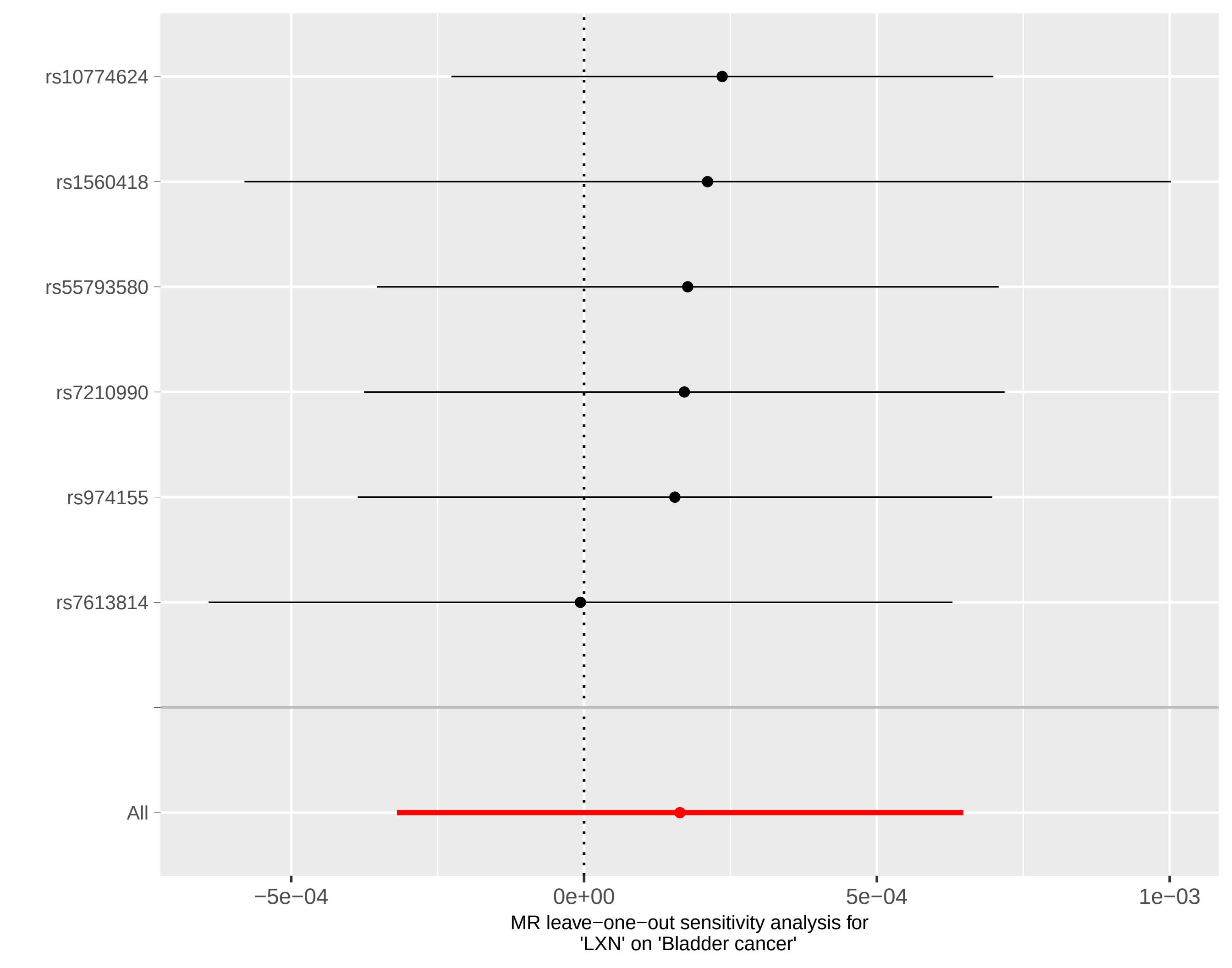
rs10774624
rs1560418
rs55793580
rs7210990
rs974155
rs7613814
All
−5e−04
0e+00
5e−04
1e−03
MR leave−one−out sensitivity analysis for
'LXN' on 'Bladder cancer'

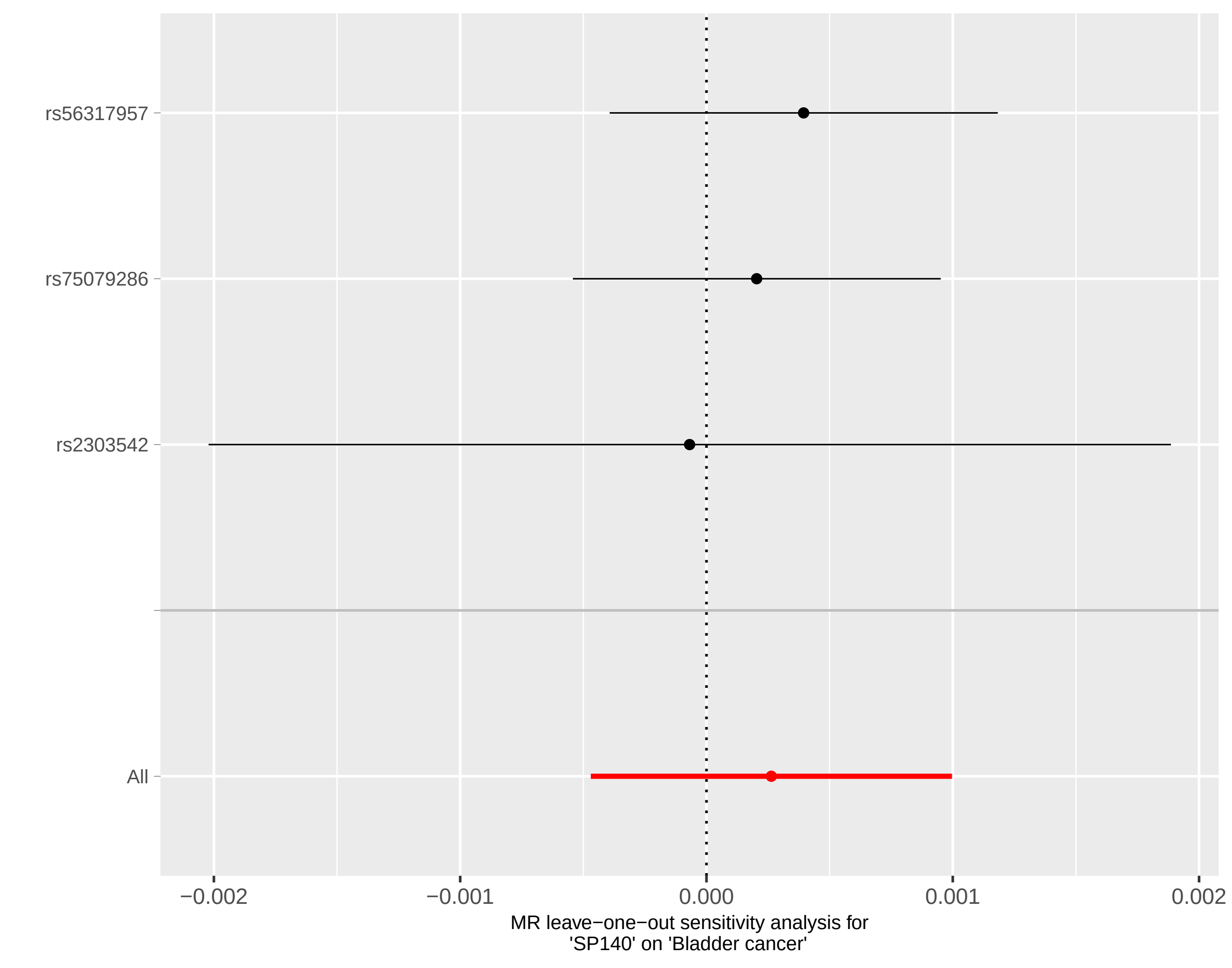
rs56317957
rs75079286
rs2303542
All
−0.002
−0.001
0.000
0.001
0.002
MR leave−one−out sensitivity analysis for
'SP140' on 'Bladder cancer'

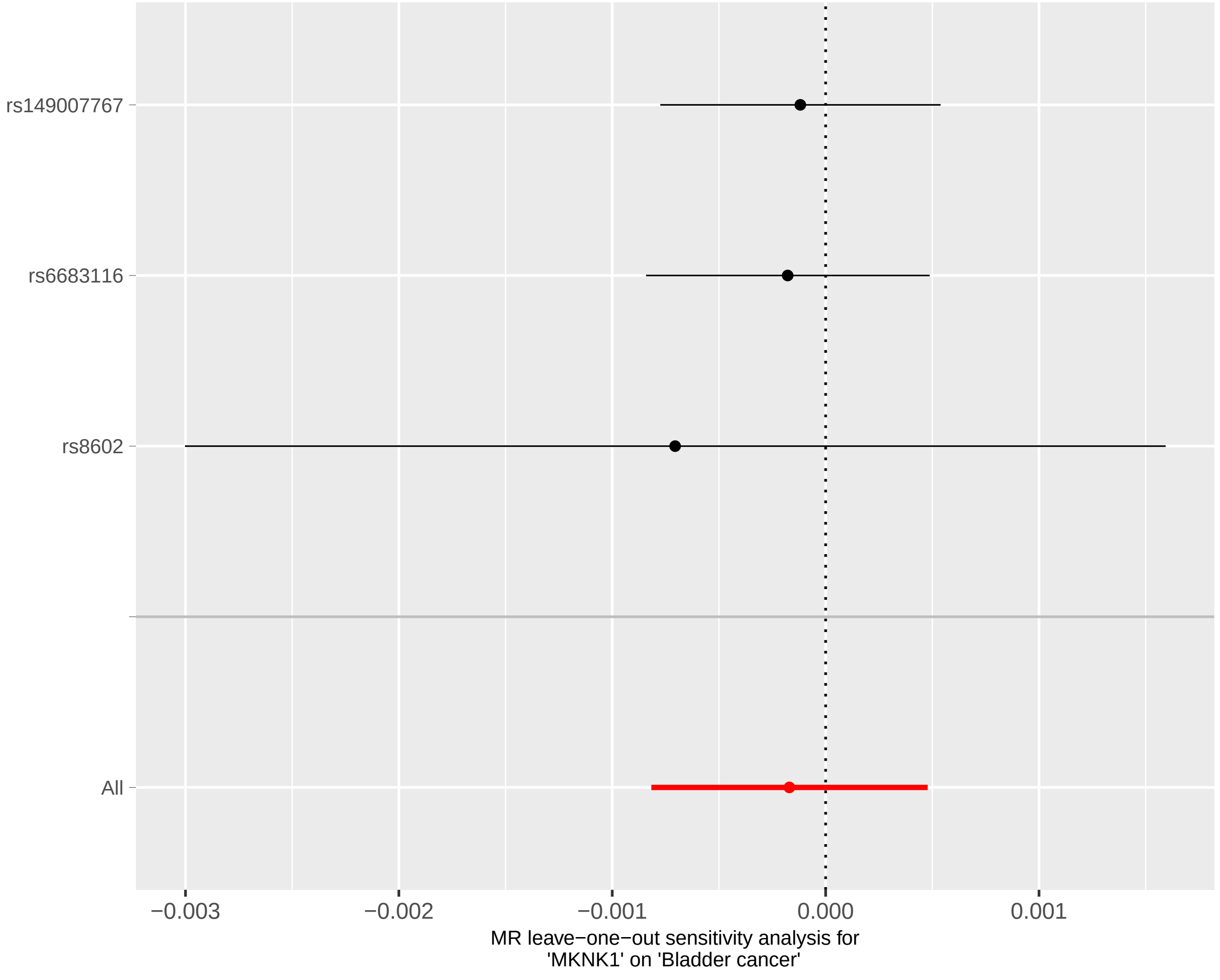
rs149007767
rs6683116
rs8602
All
−0.003
−0.002
−0.001
0.000
0.001
MR leave−one−out sensitivity analysis for
'MKNK1' on 'Bladder cancer'

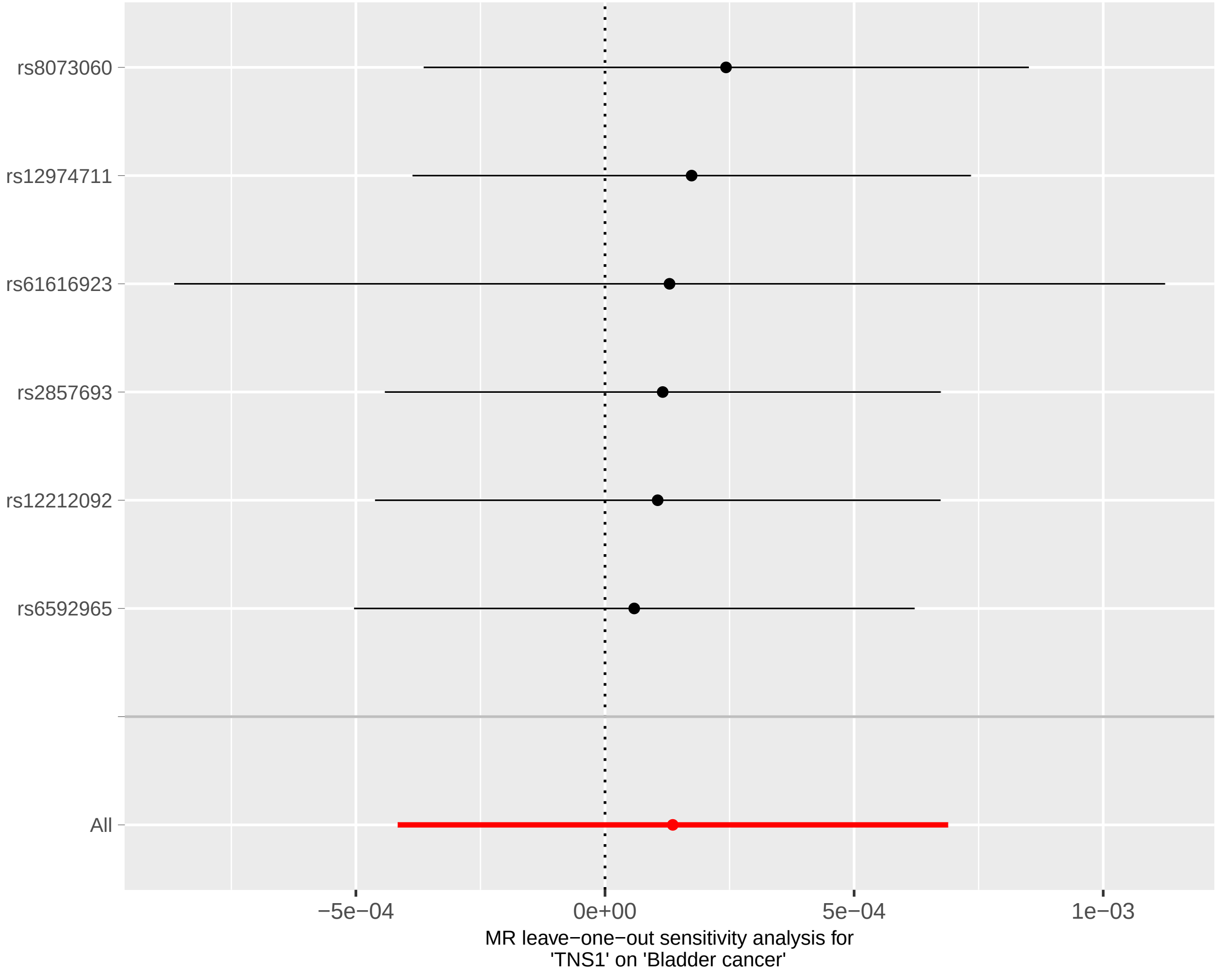
rs8073060
rs12974711
rs61616923
rs2857693
rs12212092
rs6592965
All
−5e−04
0e+00
5e−04
1e−03
MR leave−one−out sensitivity analysis for
'TNS1' on 'Bladder cancer'

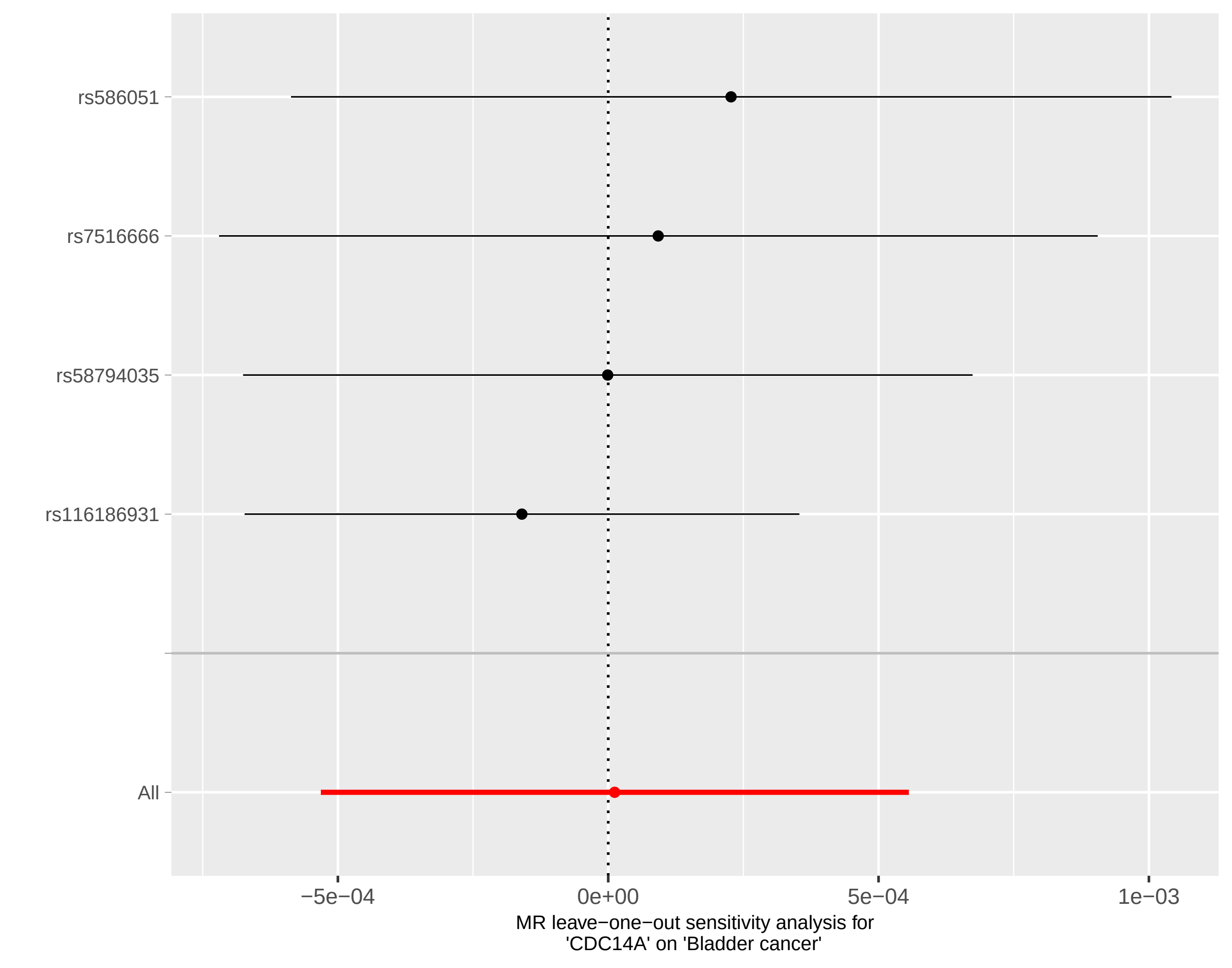
rs586051
rs7516666
rs58794035
rs116186931
All
−5e−04
0e+00
5e−04
1e−03
MR leave−one−out sensitivity analysis for
'CDC14A' on 'Bladder cancer'

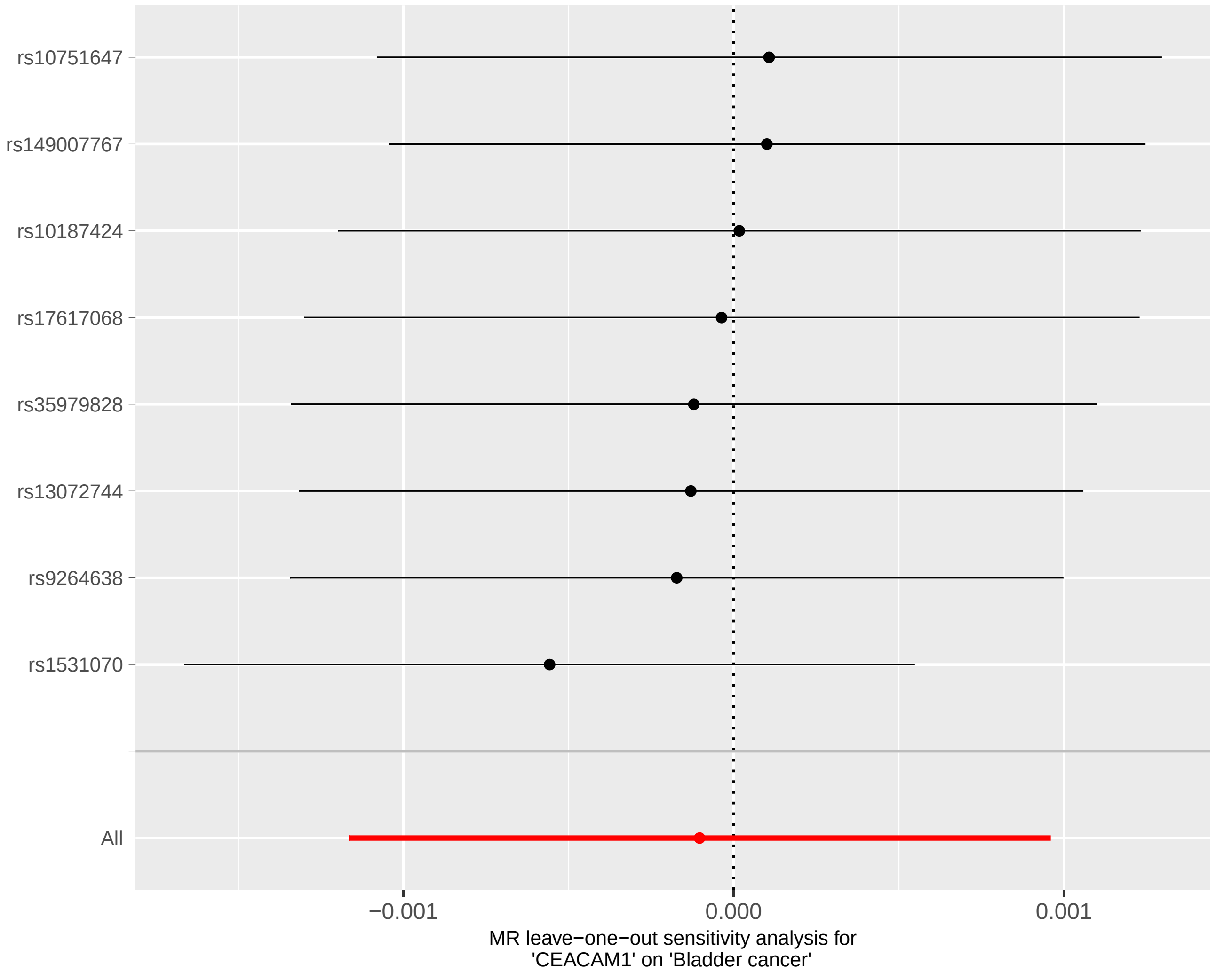
rs10751647
rs149007767
rs10187424
rs17617068
rs35979828
rs13072744
rs9264638
rs1531070
All
−0.001
0.000
0.001
MR leave−one−out sensitivity analysis for
'CEACAM1' on 'Bladder cancer'

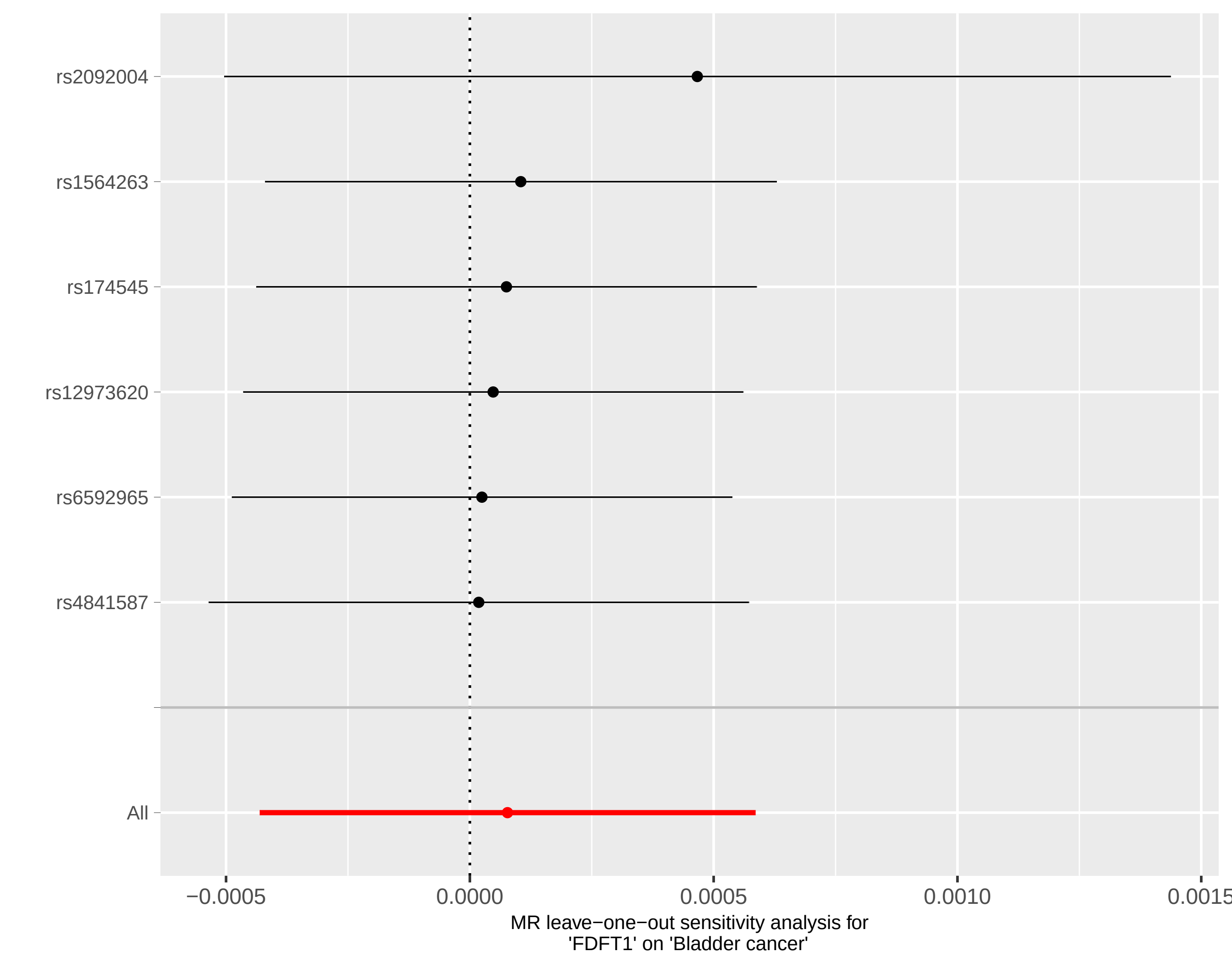
rs2092004
rs1564263
rs174545
rs12973620
rs6592965
rs4841587
All
−0.0005
0.0000
0.0005
0.0010
0.0015
MR leave−one−out sensitivity analysis for
'FDFT1' on 'Bladder cancer'

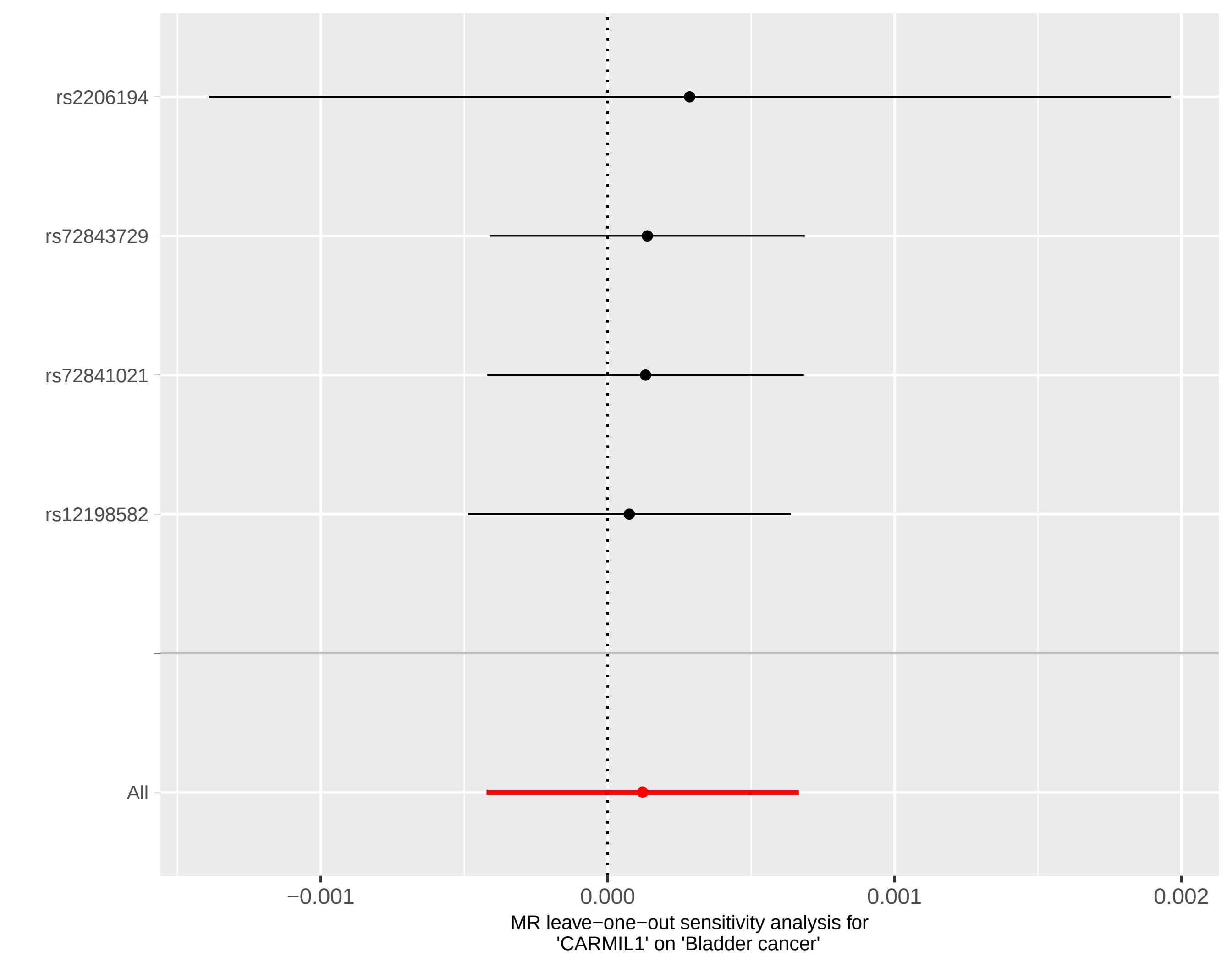
rs2206194
rs72843729
rs72841021
rs12198582
All
−0.001
0.000
0.001
0.002
MR leave−one−out sensitivity analysis for
'CARMIL1' on 'Bladder cancer'

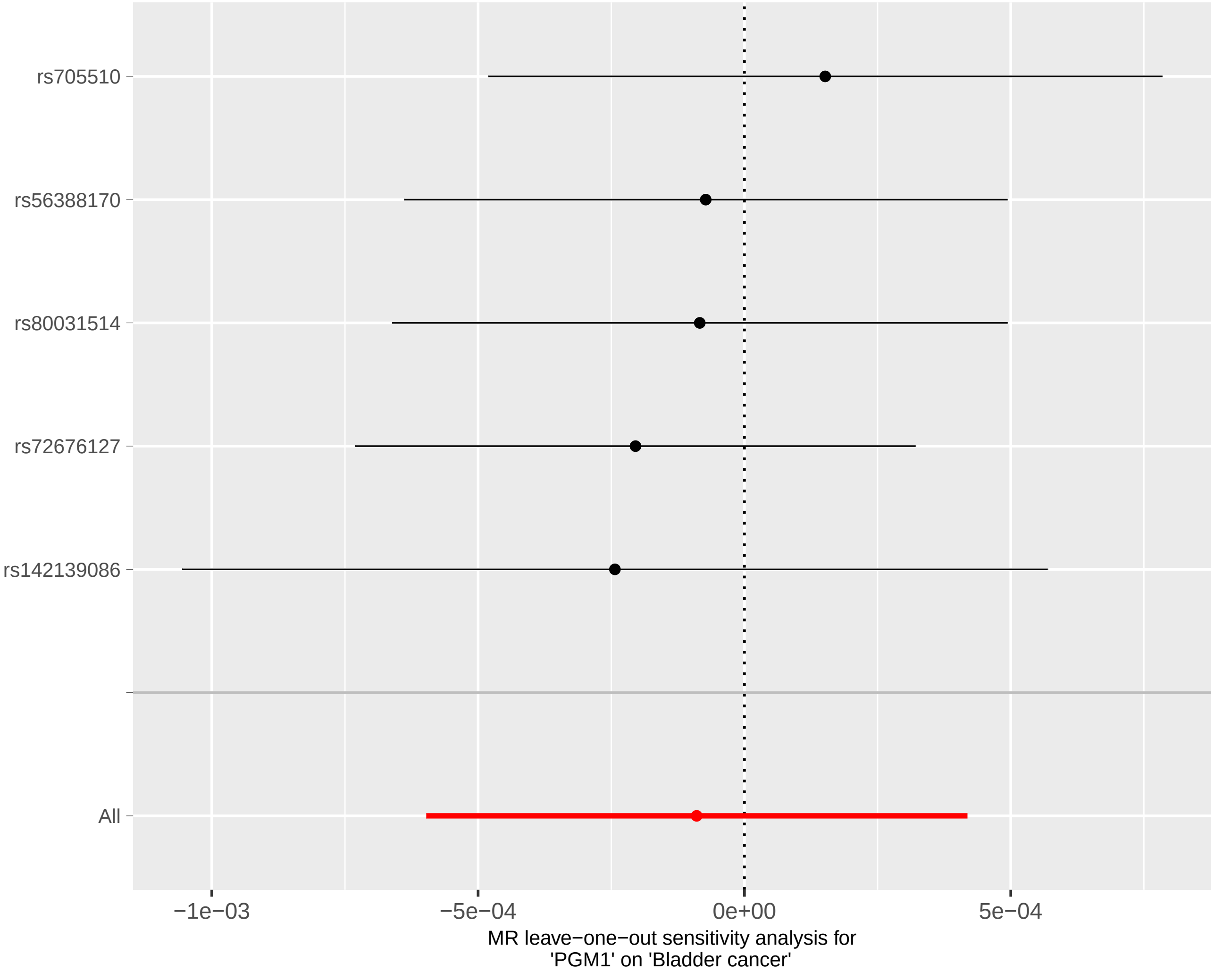
rs705510
rs56388170
rs80031514
rs72676127
rs142139086
All
−1e−03
−5e−04
0e+00
5e−04
MR leave−one−out sensitivity analysis for
'PGM1' on 'Bladder cancer'

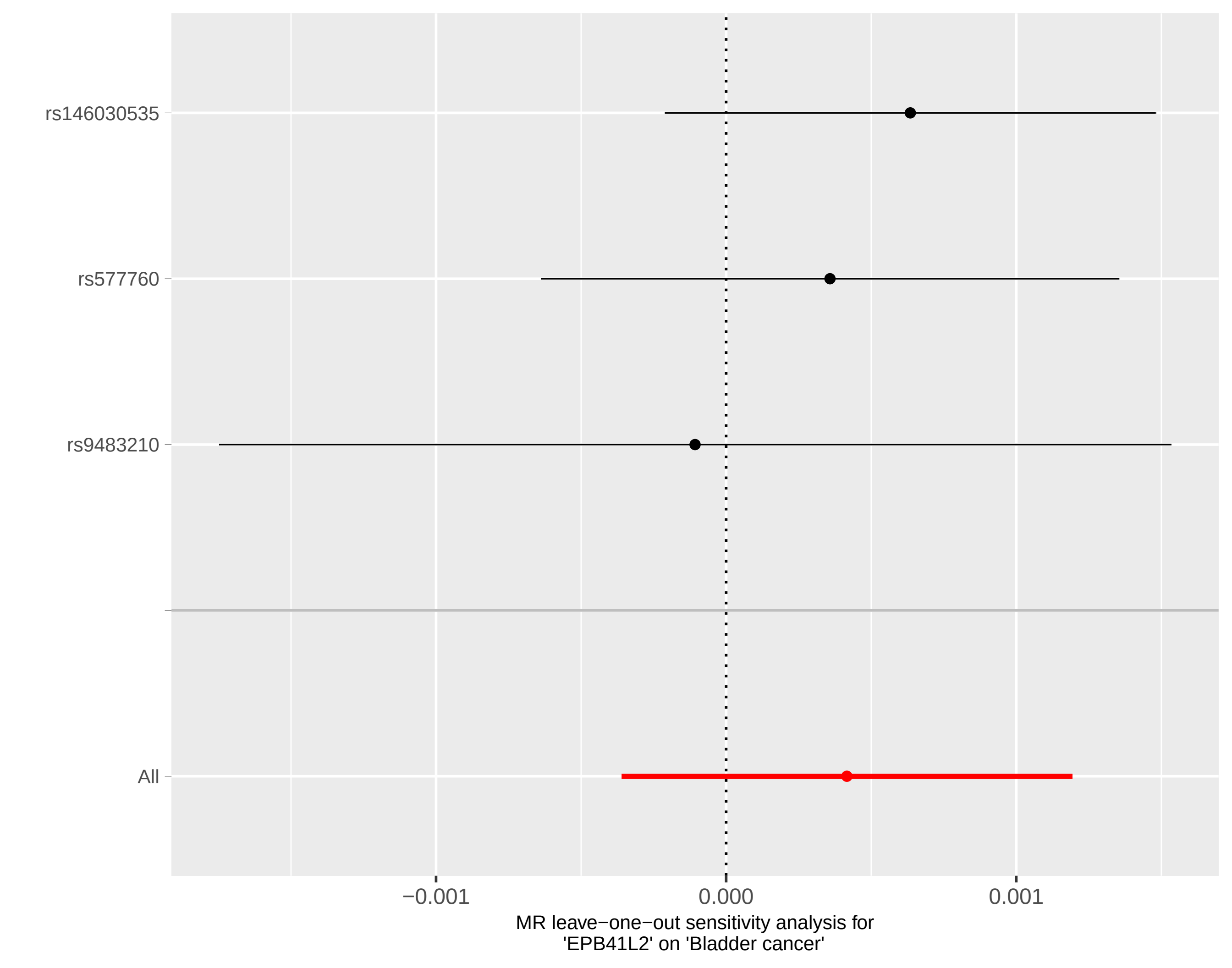
rs146030535
rs577760
rs9483210
All
−0.001
0.000
0.001
MR leave−one−out sensitivity analysis for
'EPB41L2' on 'Bladder cancer'

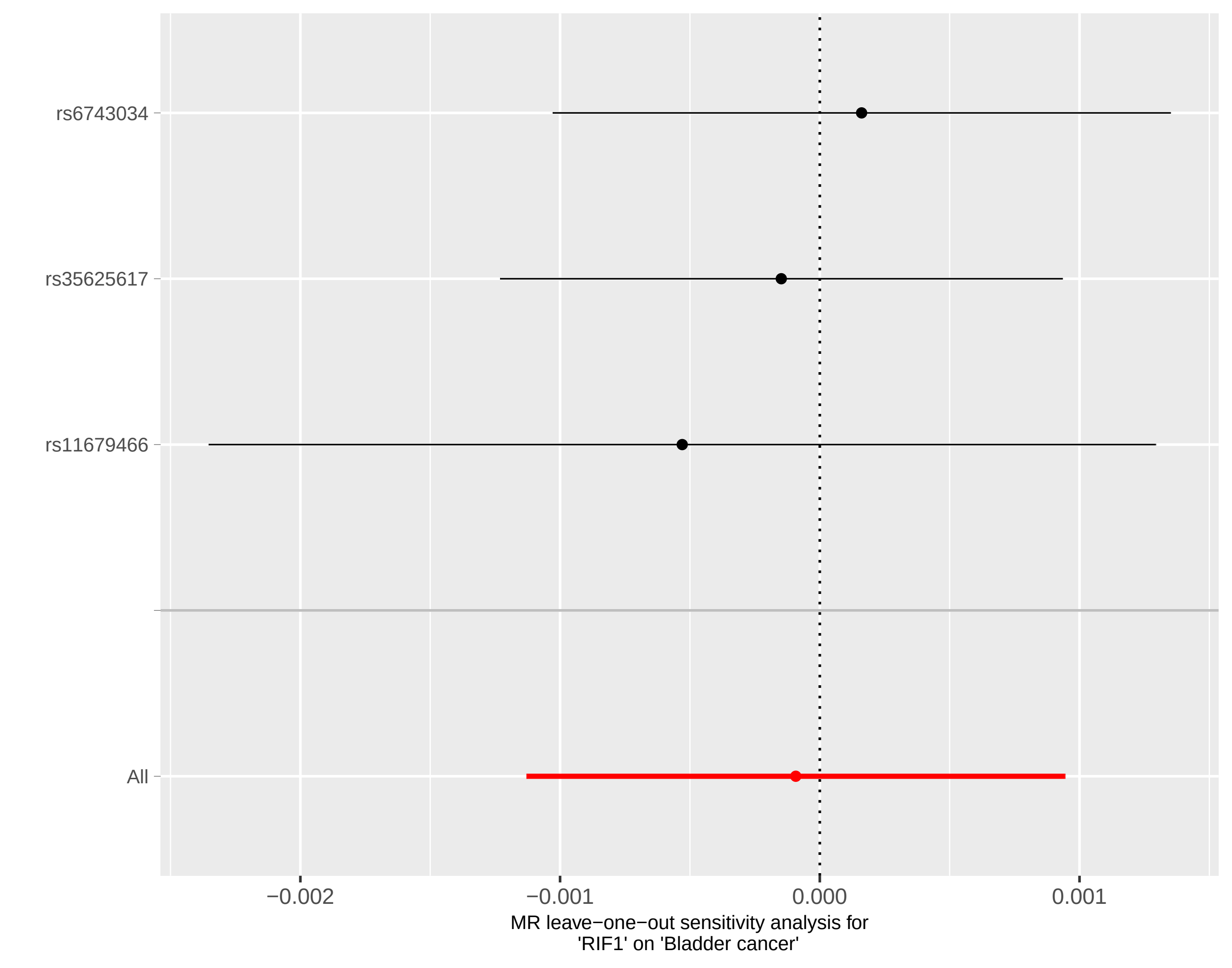
rs6743034
rs35625617
rs11679466
All
−0.002
−0.001
0.000
0.001
MR leave−one−out sensitivity analysis for
'RIF1' on 'Bladder cancer'

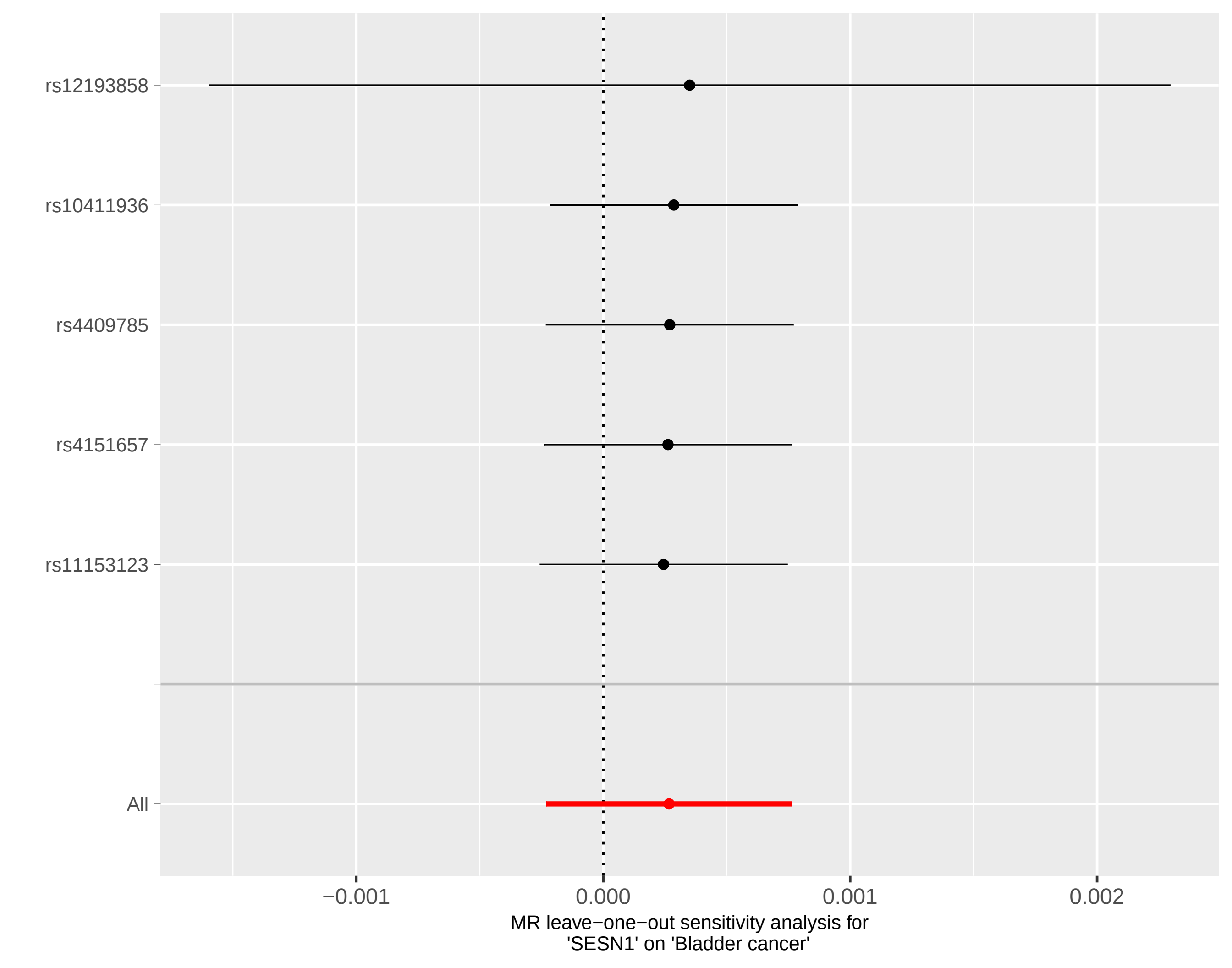
rs12193858
rs10411936
rs4409785
rs4151657
rs11153123
All
−0.001
0.000
0.001
0.002
MR leave−one−out sensitivity analysis for
'SESN1' on 'Bladder cancer'

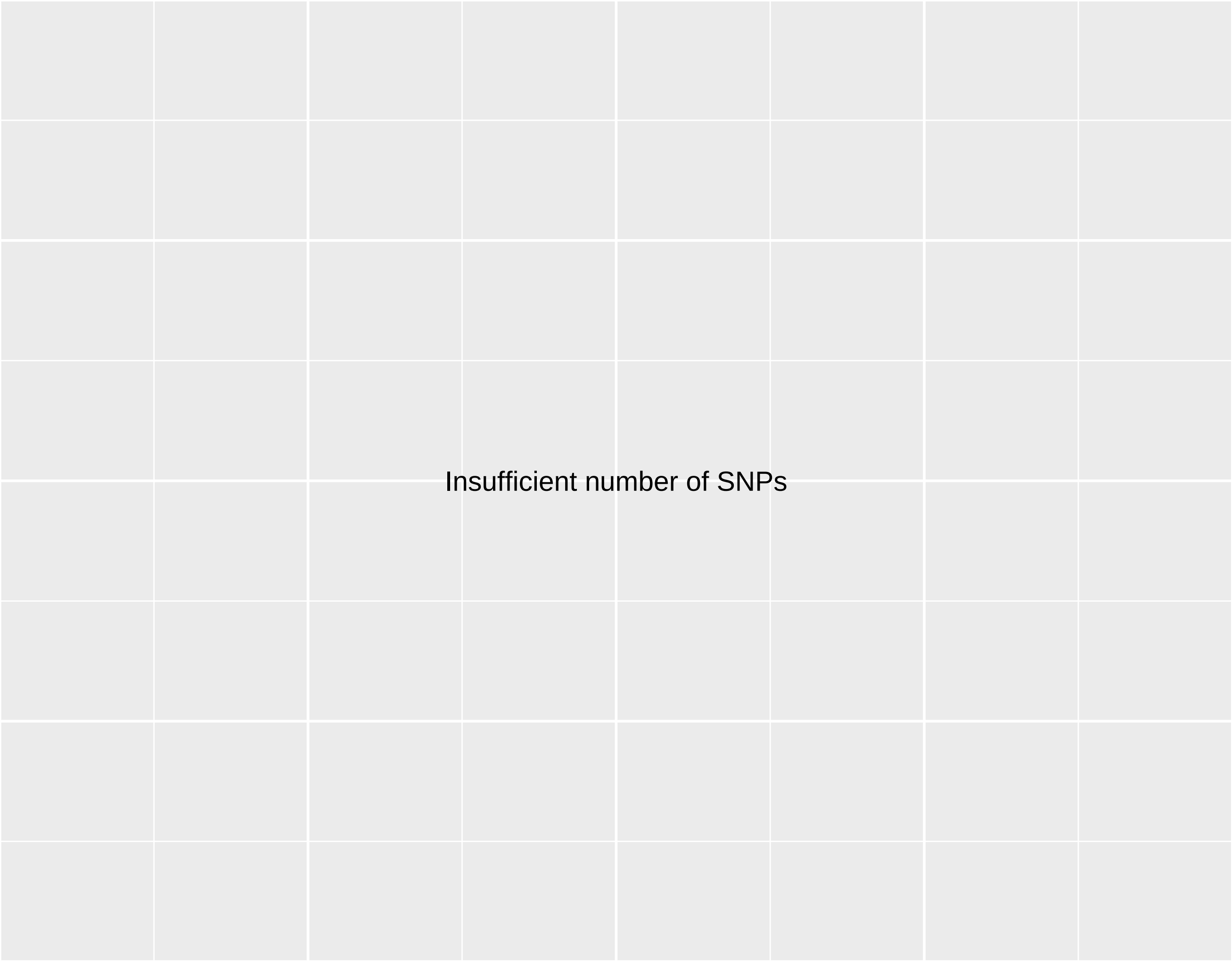
Insufficient number of SNPs

Insufficient number of SNPs

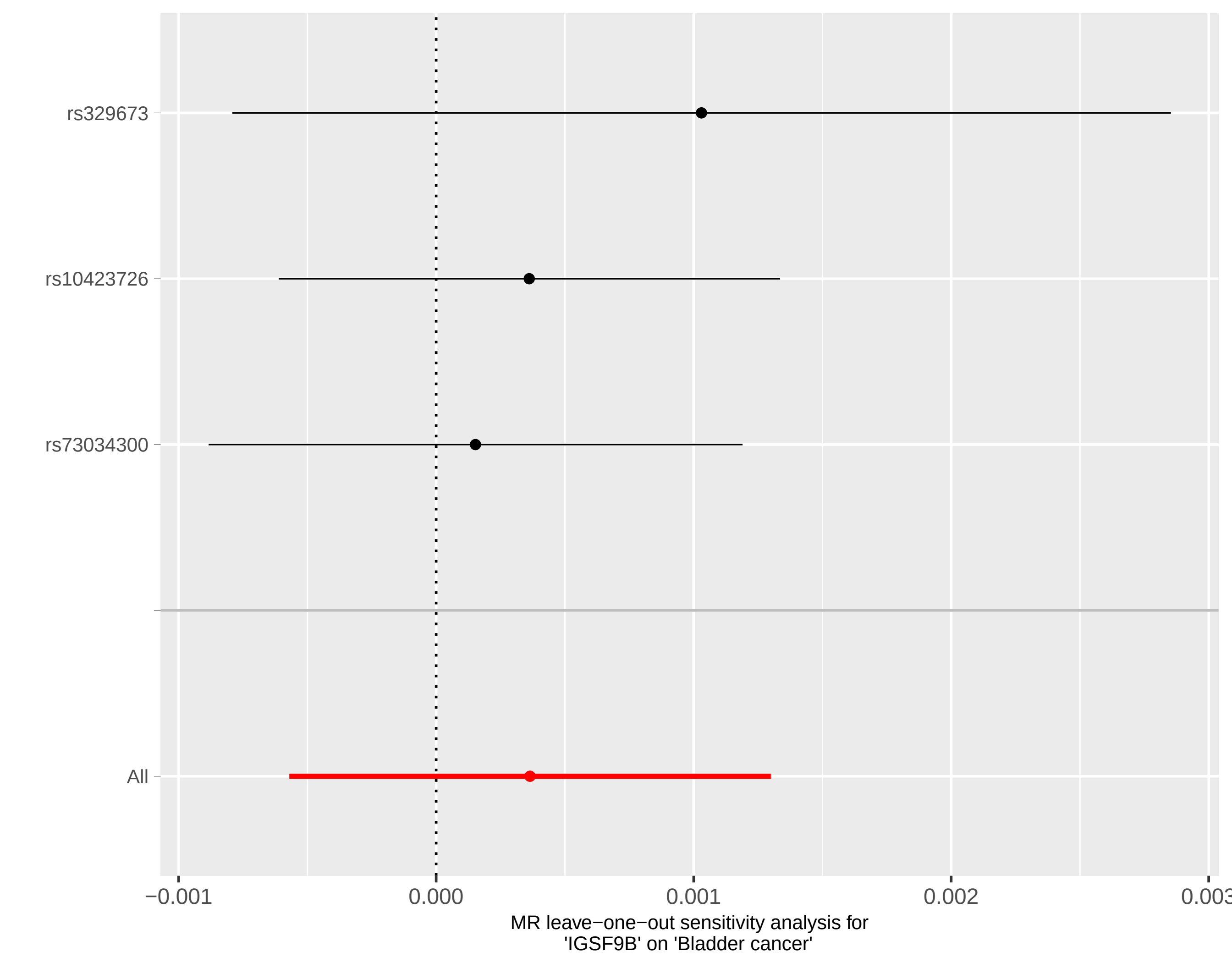
rs329673
rs10423726
rs73034300
All
−0.001
0.000
0.001
0.002
0.003
MR leave−one−out sensitivity analysis for
'IGSF9B' on 'Bladder cancer'

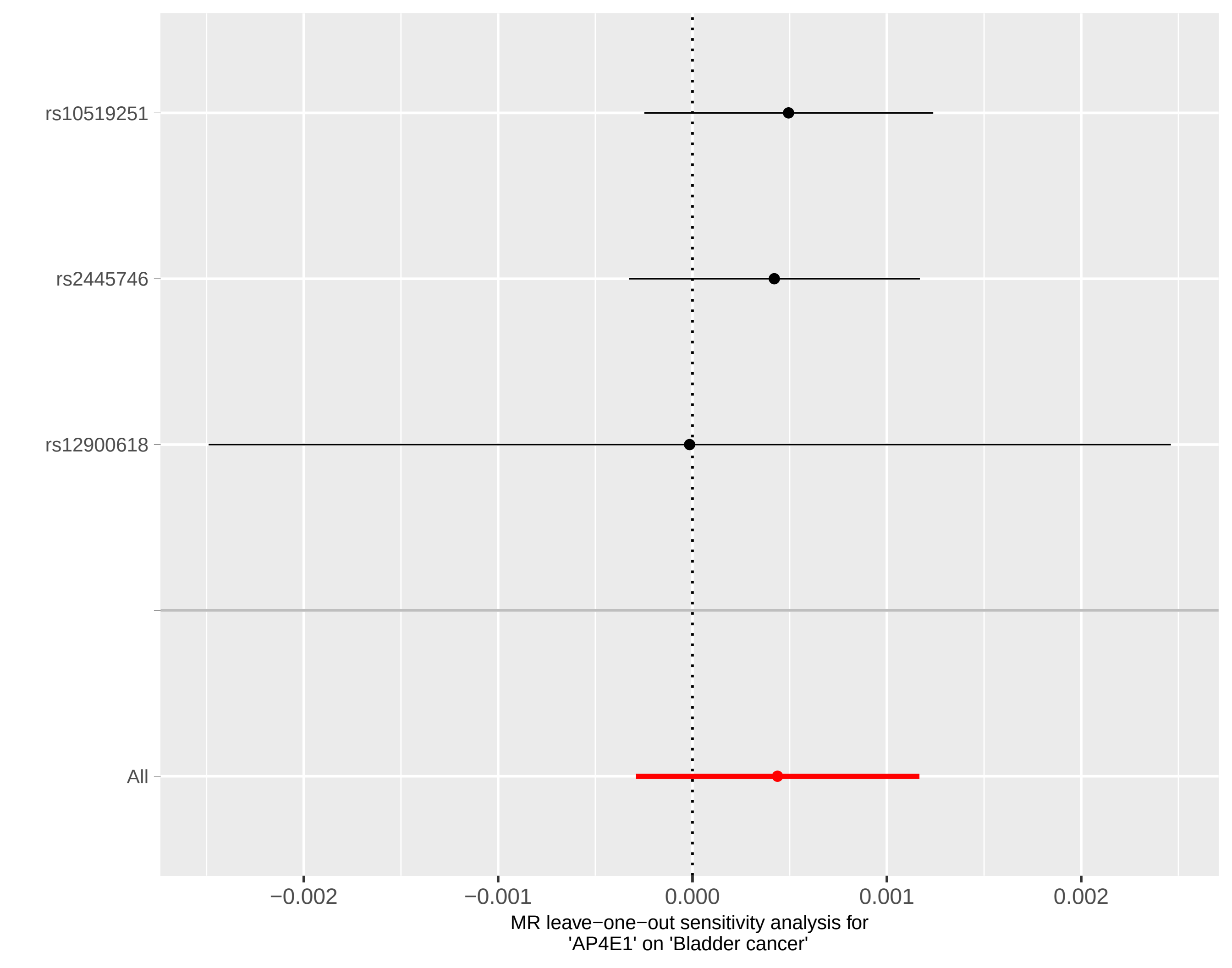
rs10519251
rs2445746
rs12900618
All
−0.002
−0.001
0.000
0.001
0.002
MR leave−one−out sensitivity analysis for
'AP4E1' on 'Bladder cancer'

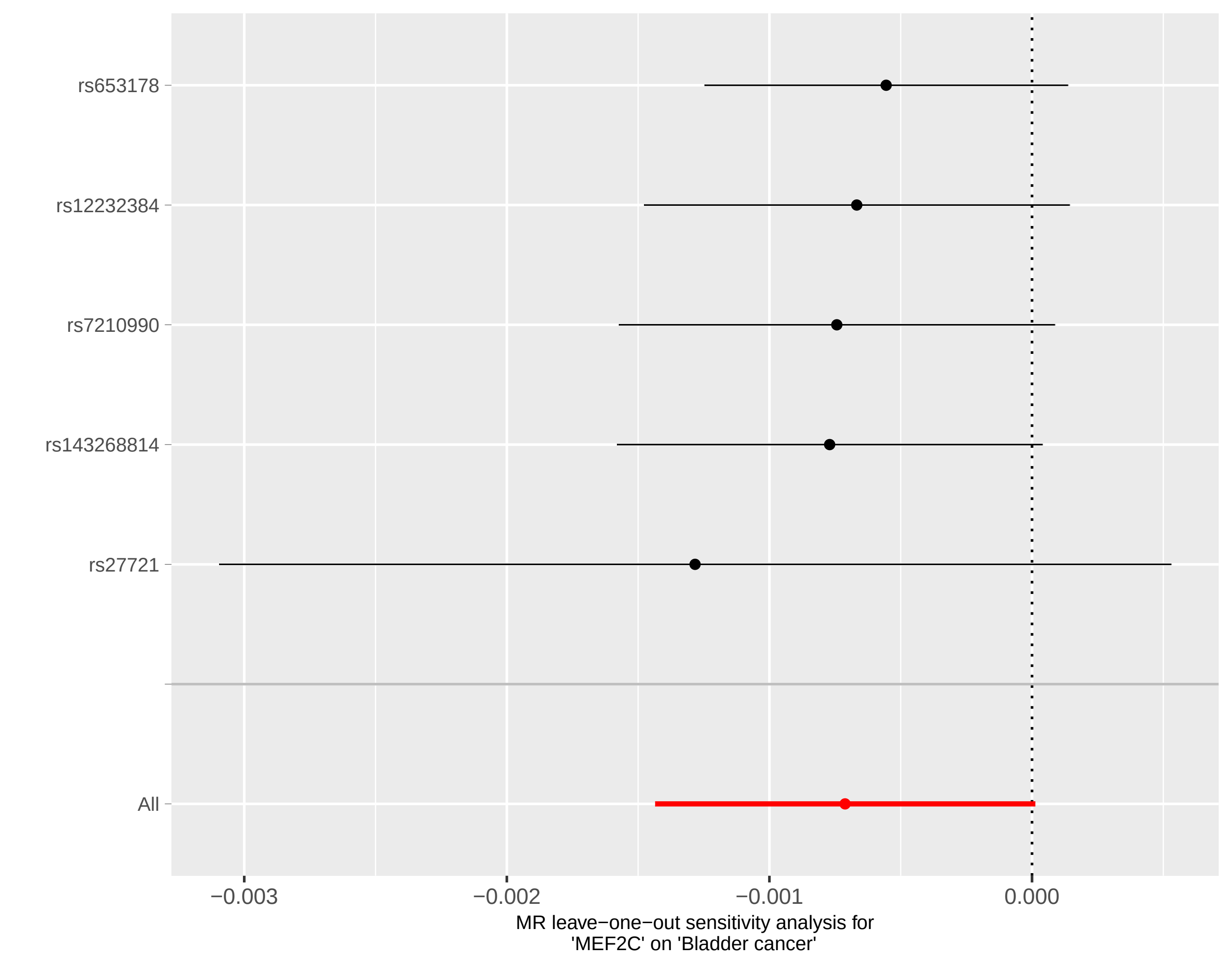
rs653178
rs12232384
rs7210990
rs143268814
rs27721
All
−0.003
−0.002
−0.001
0.000
MR leave−one−out sensitivity analysis for
'MEF2C' on 'Bladder cancer'

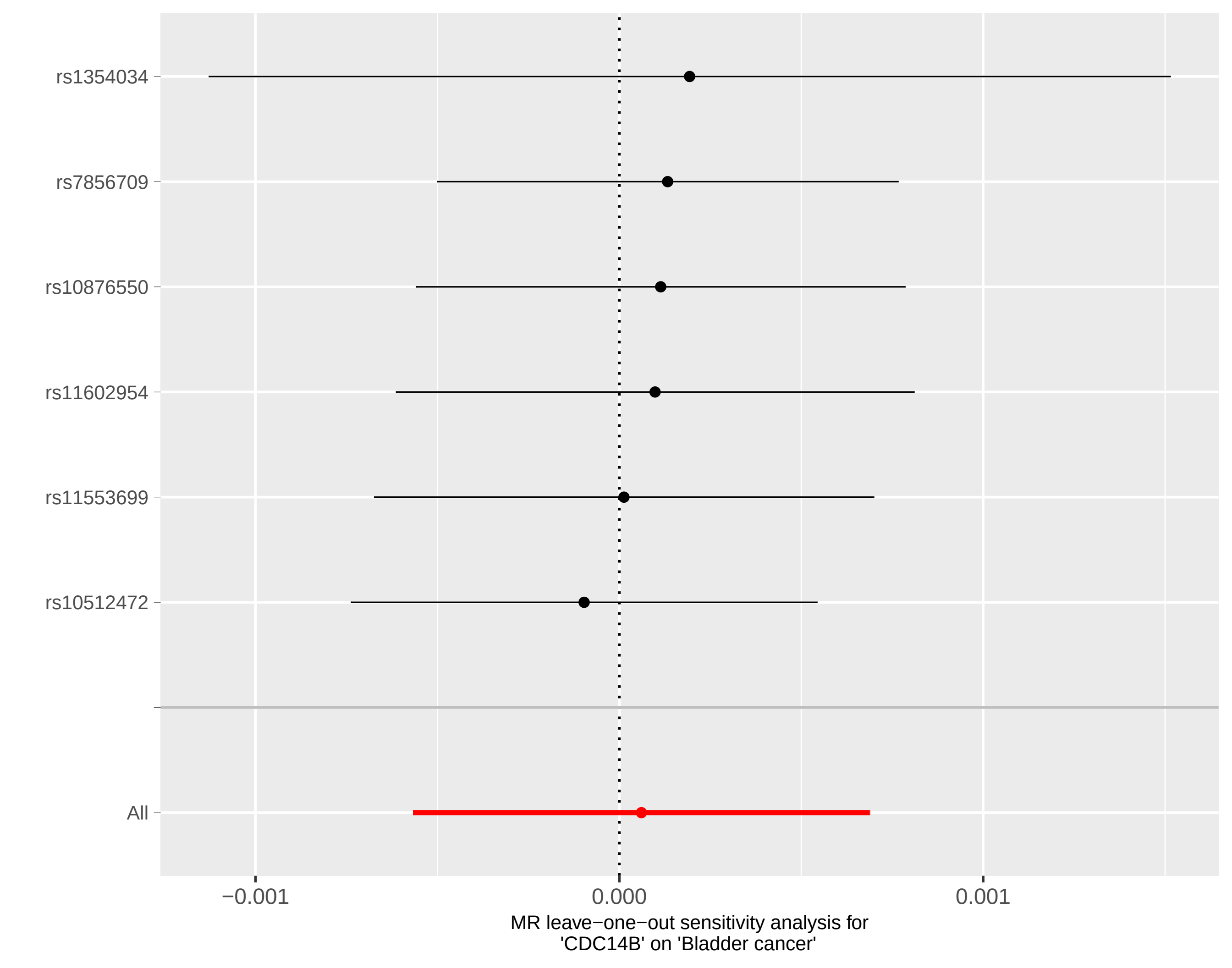
rs1354034
rs7856709
rs10876550
rs11602954
rs11553699
rs10512472
All
−0.001
0.000
0.001
MR leave−one−out sensitivity analysis for
'CDC14B' on 'Bladder cancer'

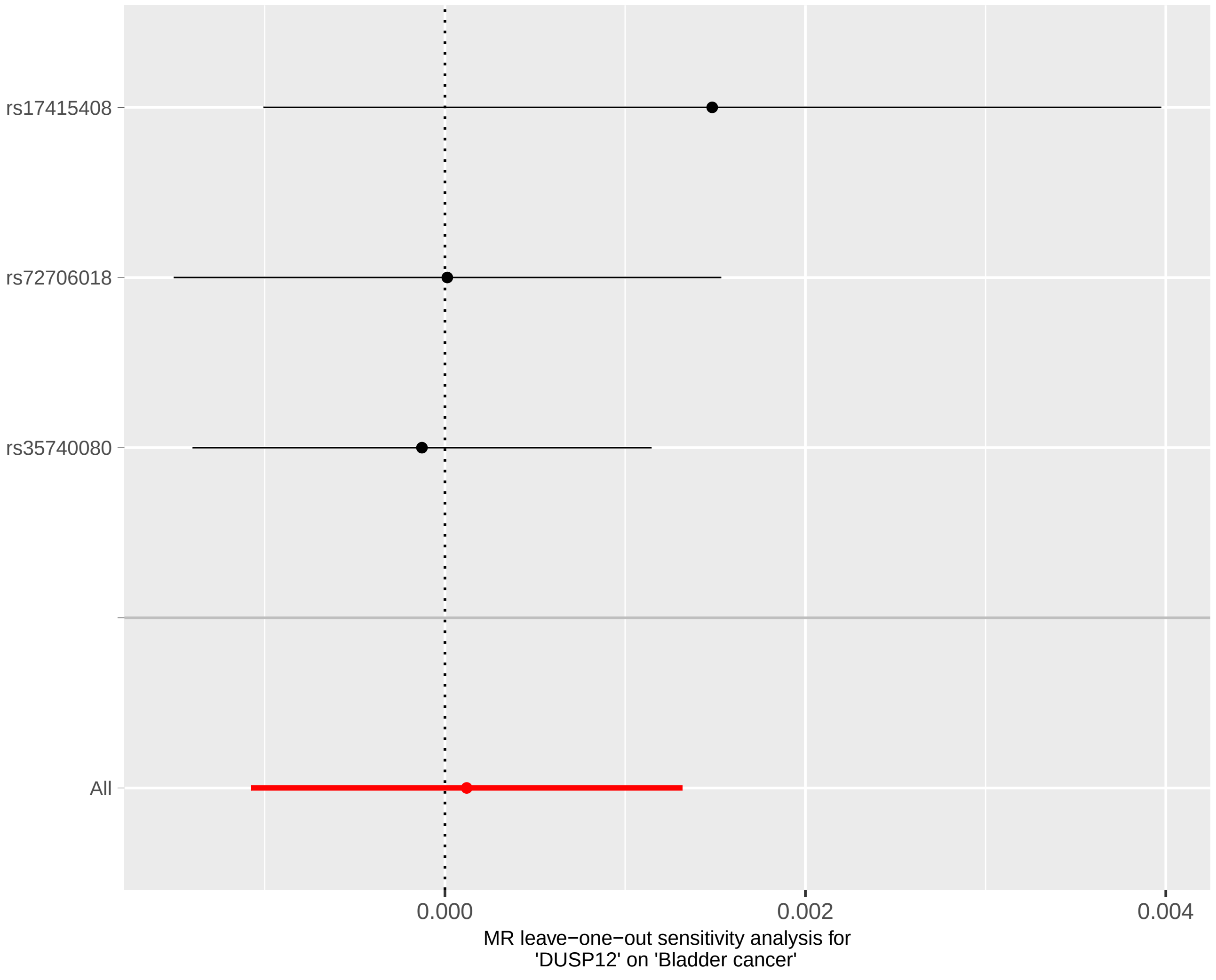
rs17415408
rs72706018
rs35740080
All
0.000
0.002
0.004
MR leave−one−out sensitivity analysis for
'DUSP12' on 'Bladder cancer'

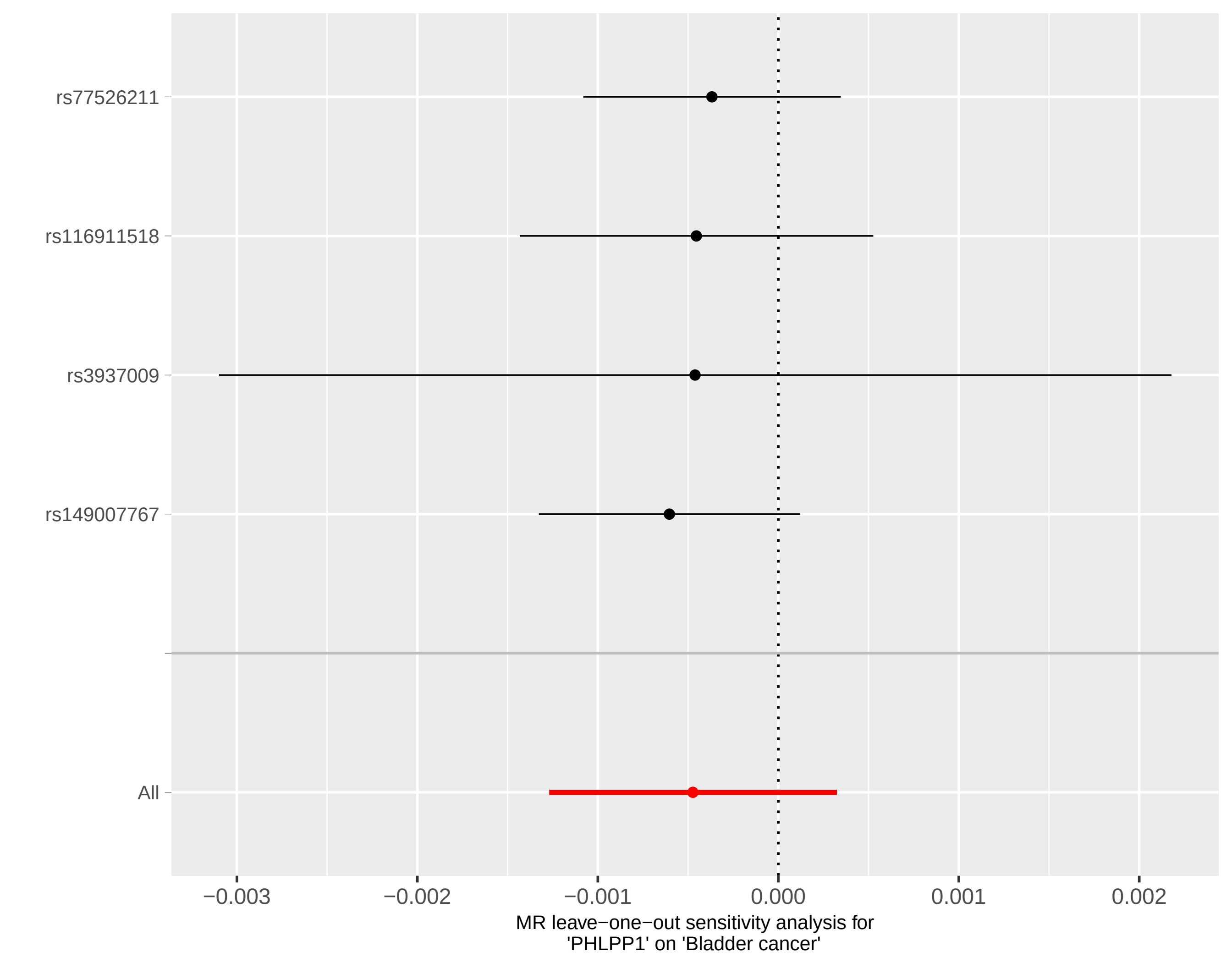
rs77526211
rs116911518
rs3937009
rs149007767
All
−0.003
−0.002
−0.001
0.000
0.001
0.002
MR leave−one−out sensitivity analysis for
'PHLPP1' on 'Bladder cancer'

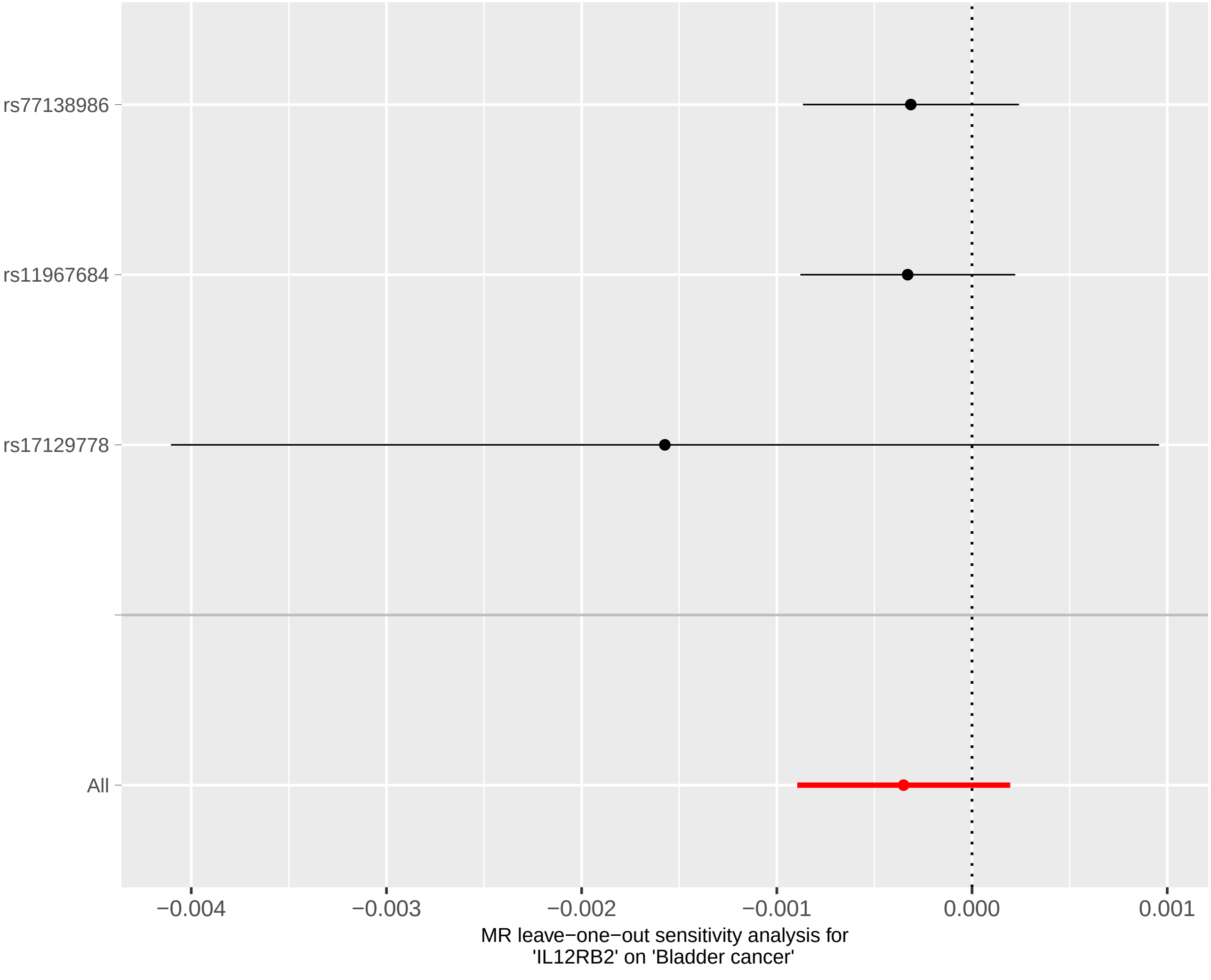
rs77138986
rs11967684
rs17129778
All
−0.004
−0.003
−0.002
−0.001
0.000
0.001
MR leave−one−out sensitivity analysis for
'IL12RB2' on 'Bladder cancer'

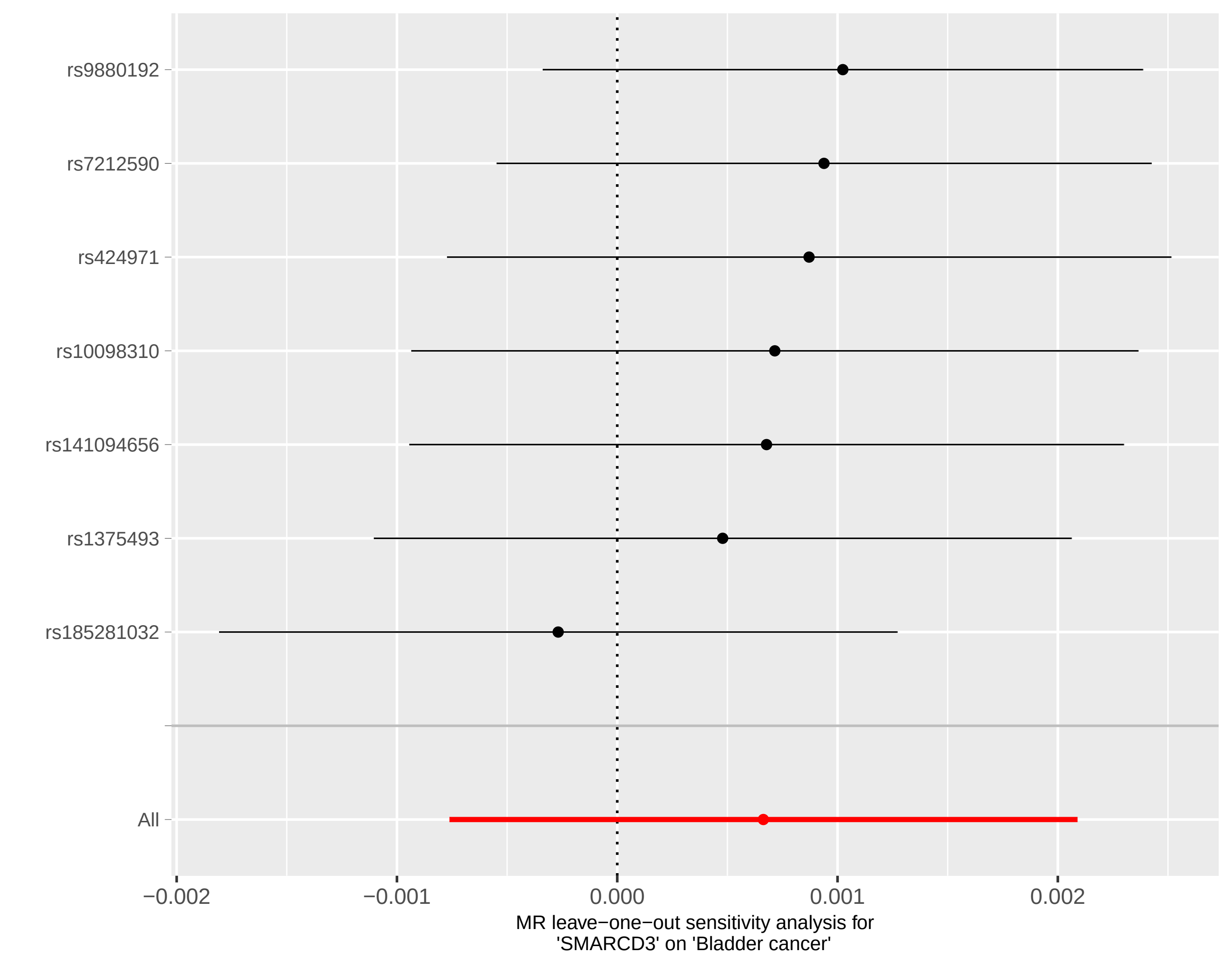
rs9880192
rs7212590
rs424971
rs10098310
rs141094656
rs1375493
rs185281032
All
−0.002
−0.001
0.000
0.001
0.002
MR leave−one−out sensitivity analysis for
'SMARCD3' on 'Bladder cancer'

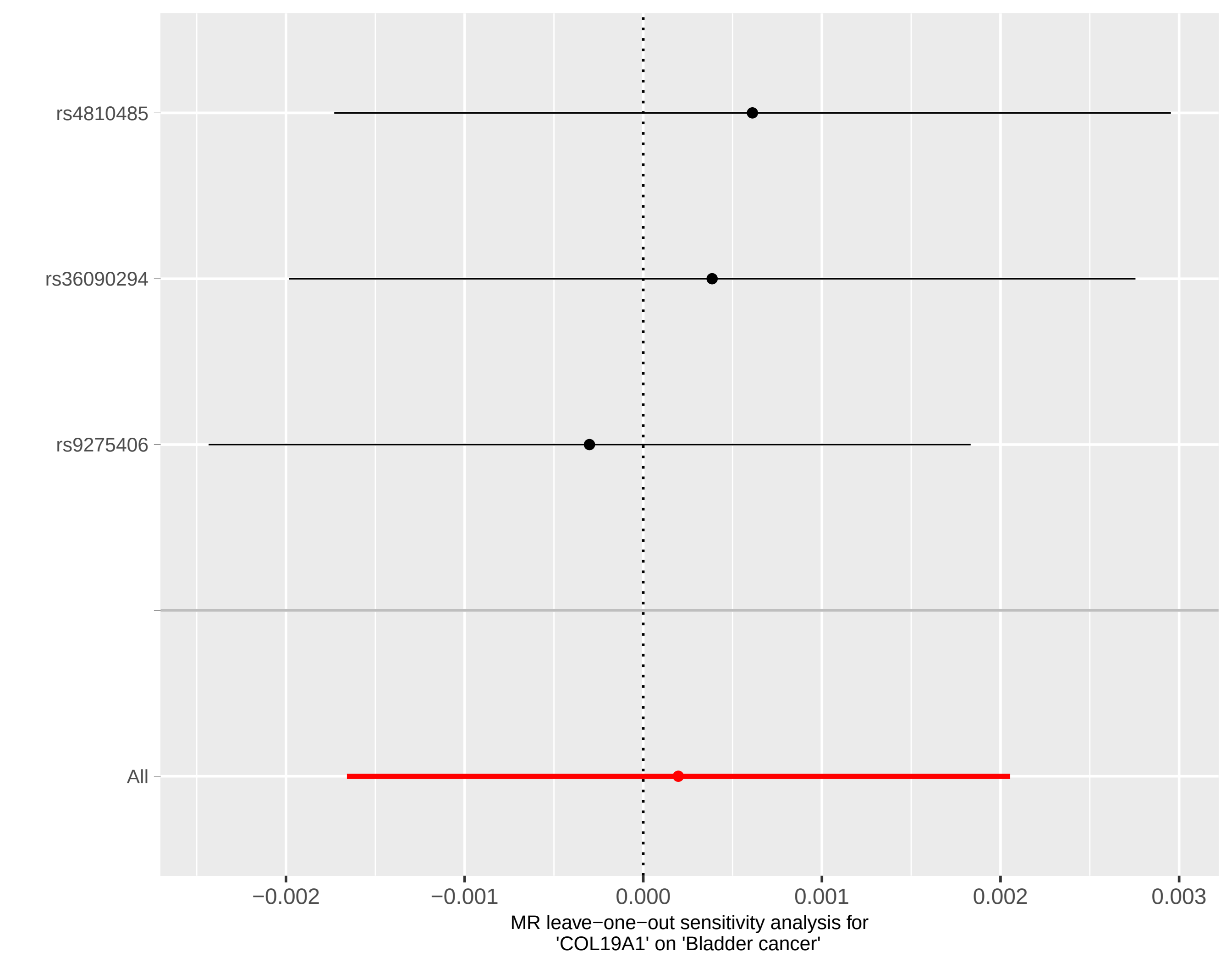

rs4810485
rs36090294
rs9275406
All
−0.002
−0.001
0.000
0.001
0.002
0.003
MR leave−one−out sensitivity analysis for
'COL19A1' on 'Bladder cancer'

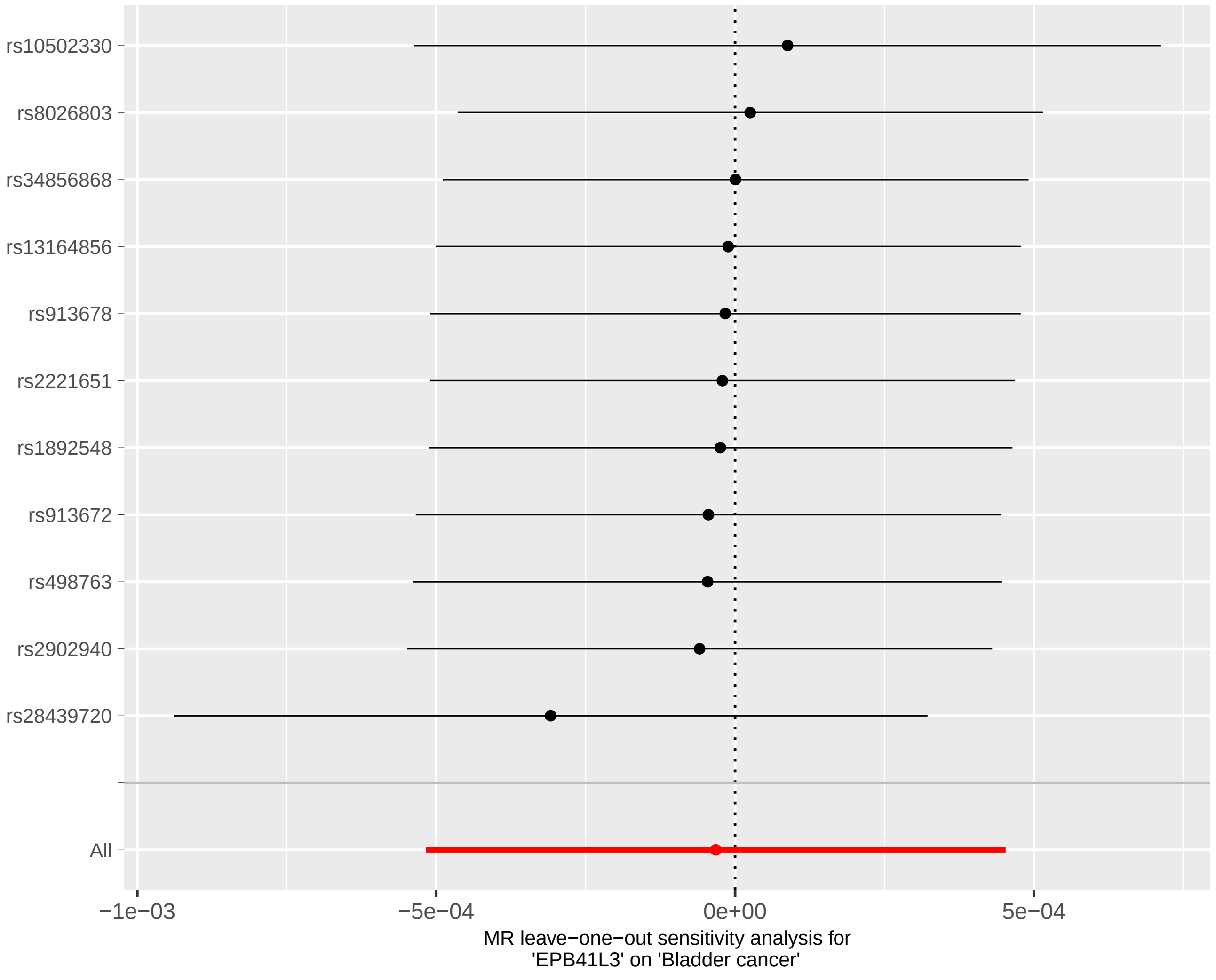

rs10502330
rs8026803
rs34856868
rs13164856
rs913678
rs2221651
rs1892548
rs913672
rs498763
rs2902940
rs28439720
All
−1e−03
−5e−04
0e+00
5e−04
MR leave−one−out sensitivity analysis for
'EPB41L3' on 'Bladder cancer'

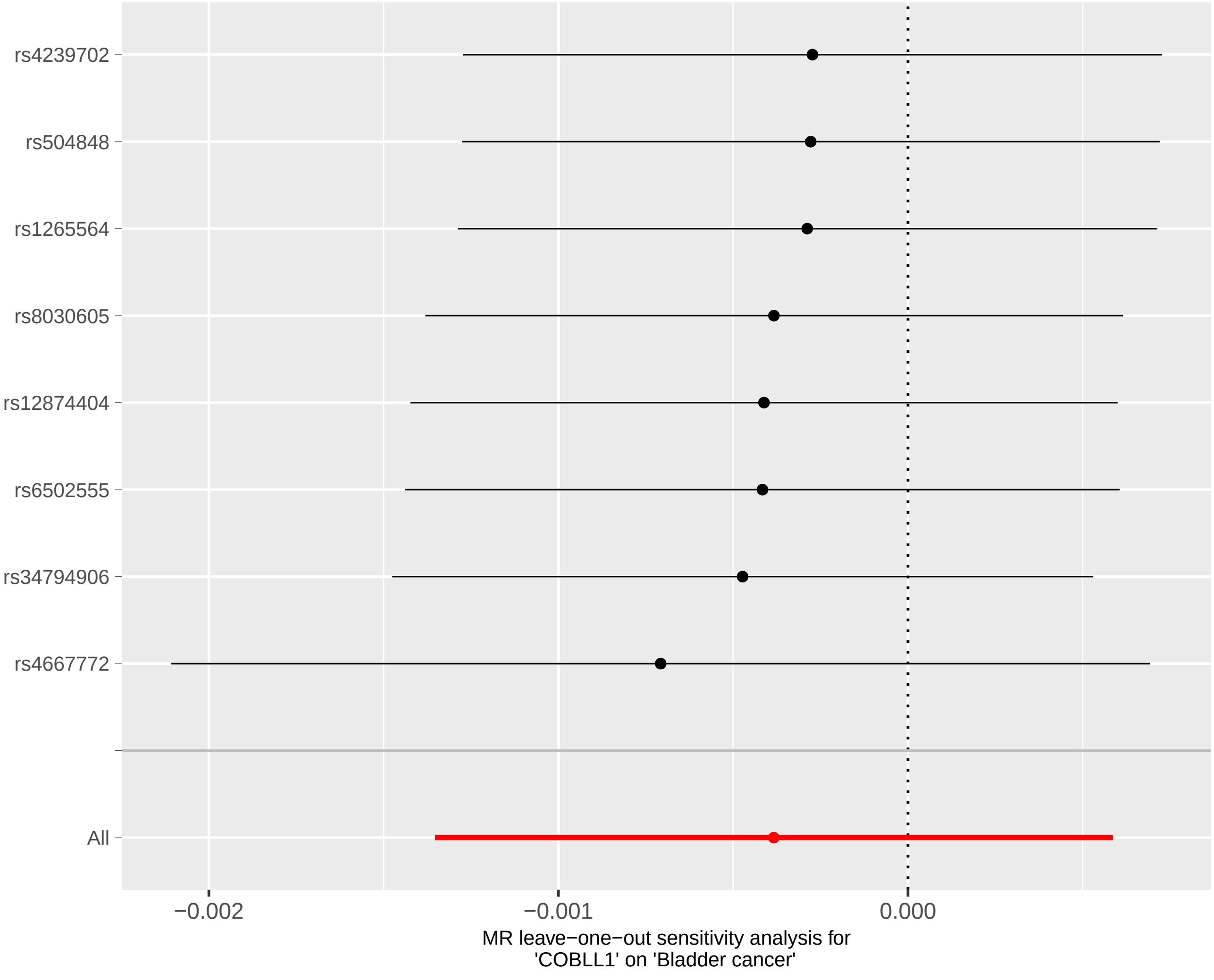
rs4239702
rs504848
rs1265564
rs8030605
rs12874404
rs6502555
rs34794906
rs4667772
All
−0.002
−0.001
0.000
MR leave−one−out sensitivity analysis for
'COBLL1' on 'Bladder cancer'

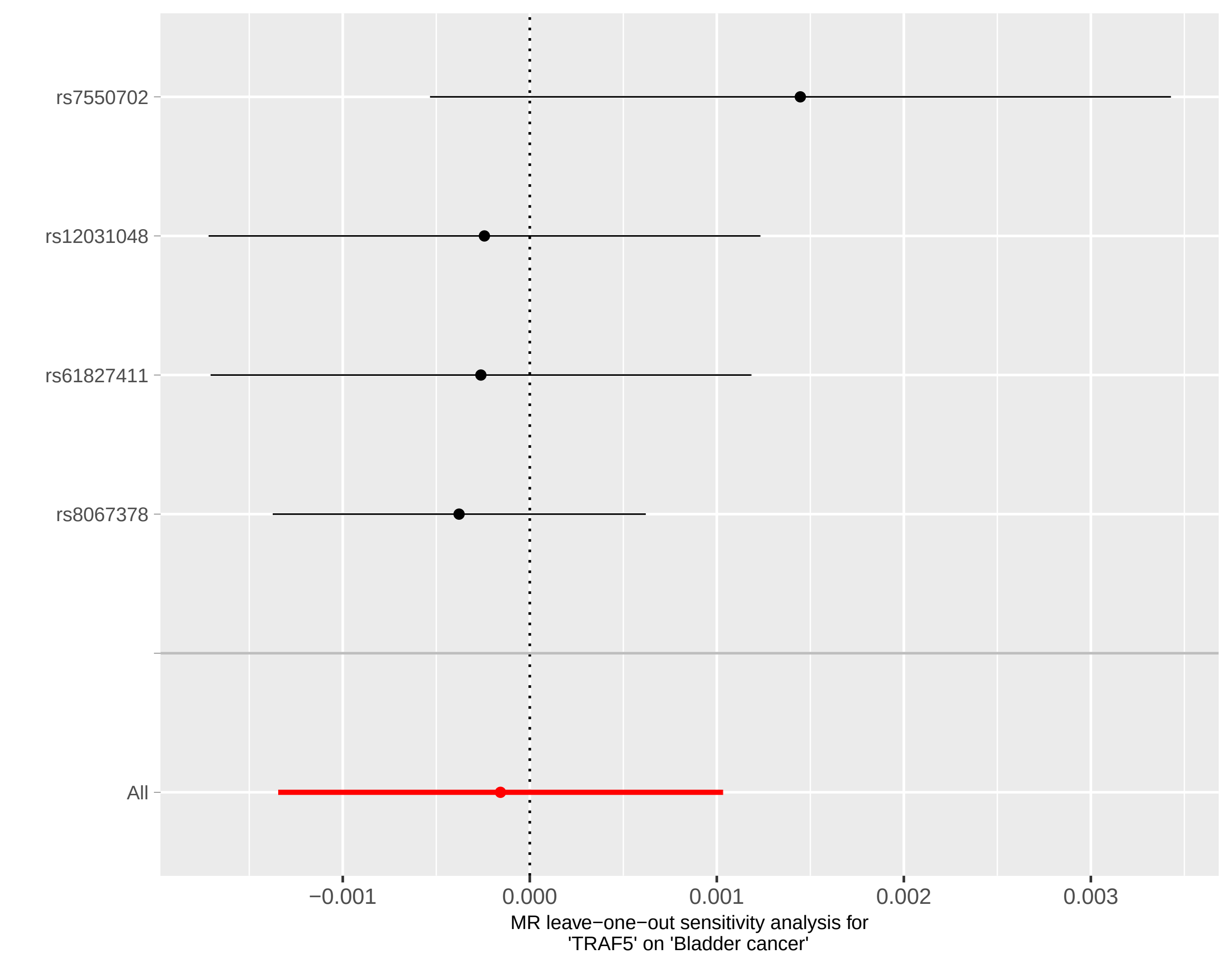
rs7550702
rs12031048
rs61827411
rs8067378
All
−0.001
0.000
0.001
0.002
0.003
MR leave−one−out sensitivity analysis for
'TRAF5' on 'Bladder cancer'

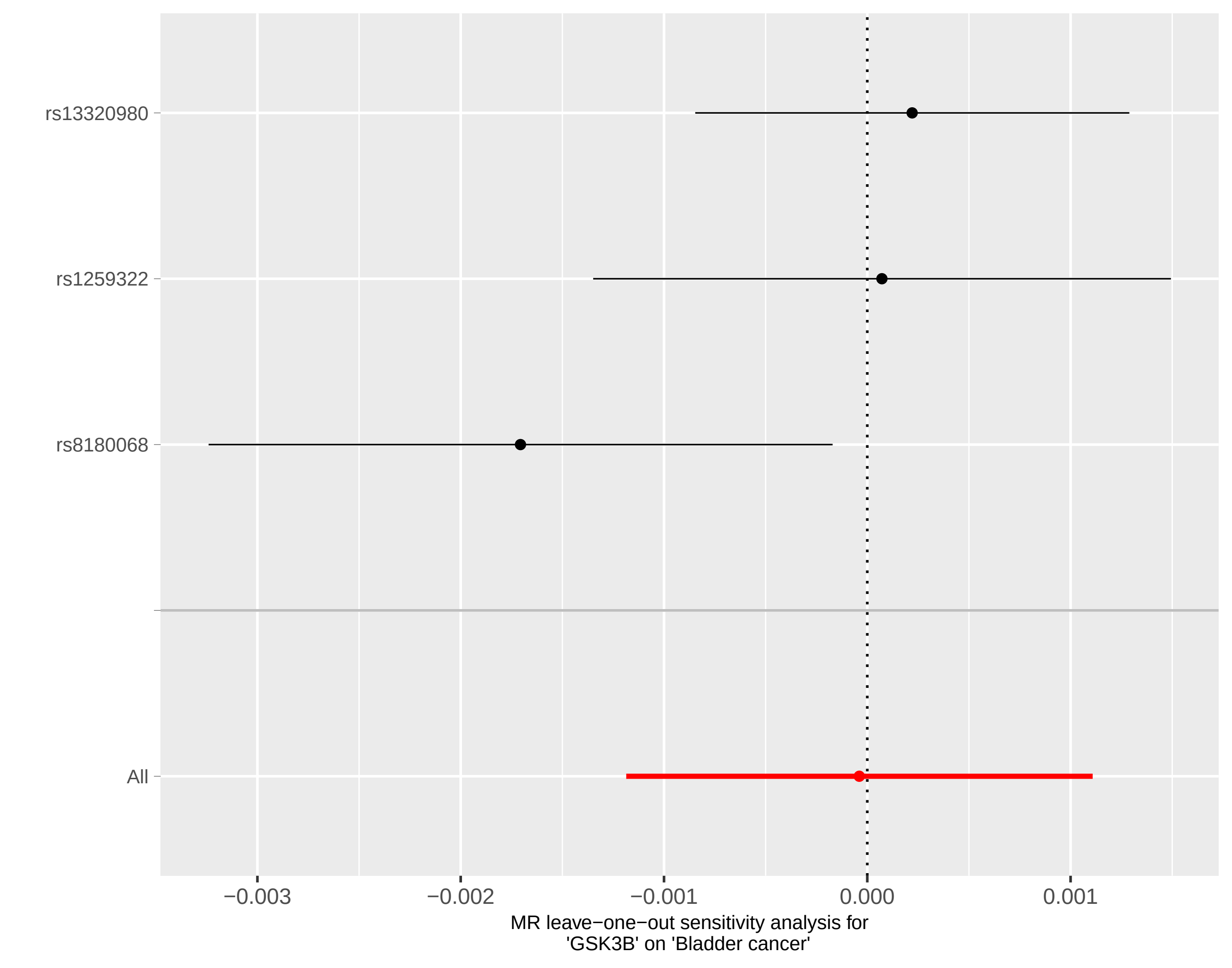
rs13320980
rs1259322
rs8180068
All
−0.003
−0.002
−0.001
0.000
0.001
MR leave−one−out sensitivity analysis for
'GSK3B' on 'Bladder cancer'

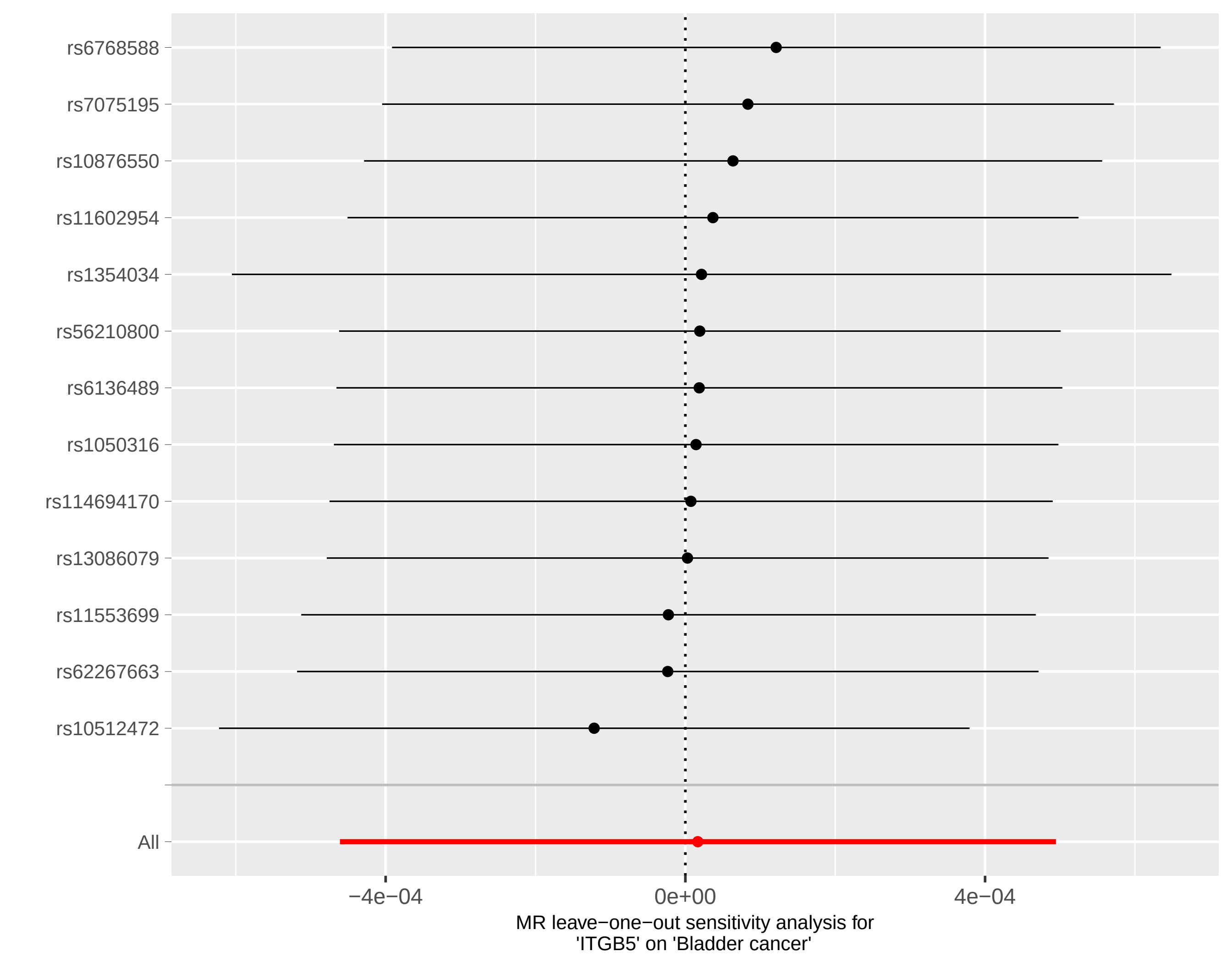
rs6768588
rs7075195
rs10876550
rs11602954
rs1354034
rs56210800
rs6136489
rs1050316
rs114694170
rs13086079
rs11553699
rs62267663
rs10512472
All
−4e−04
0e+00
4e−04
MR leave−one−out sensitivity analysis for
'ITGB5' on 'Bladder cancer'

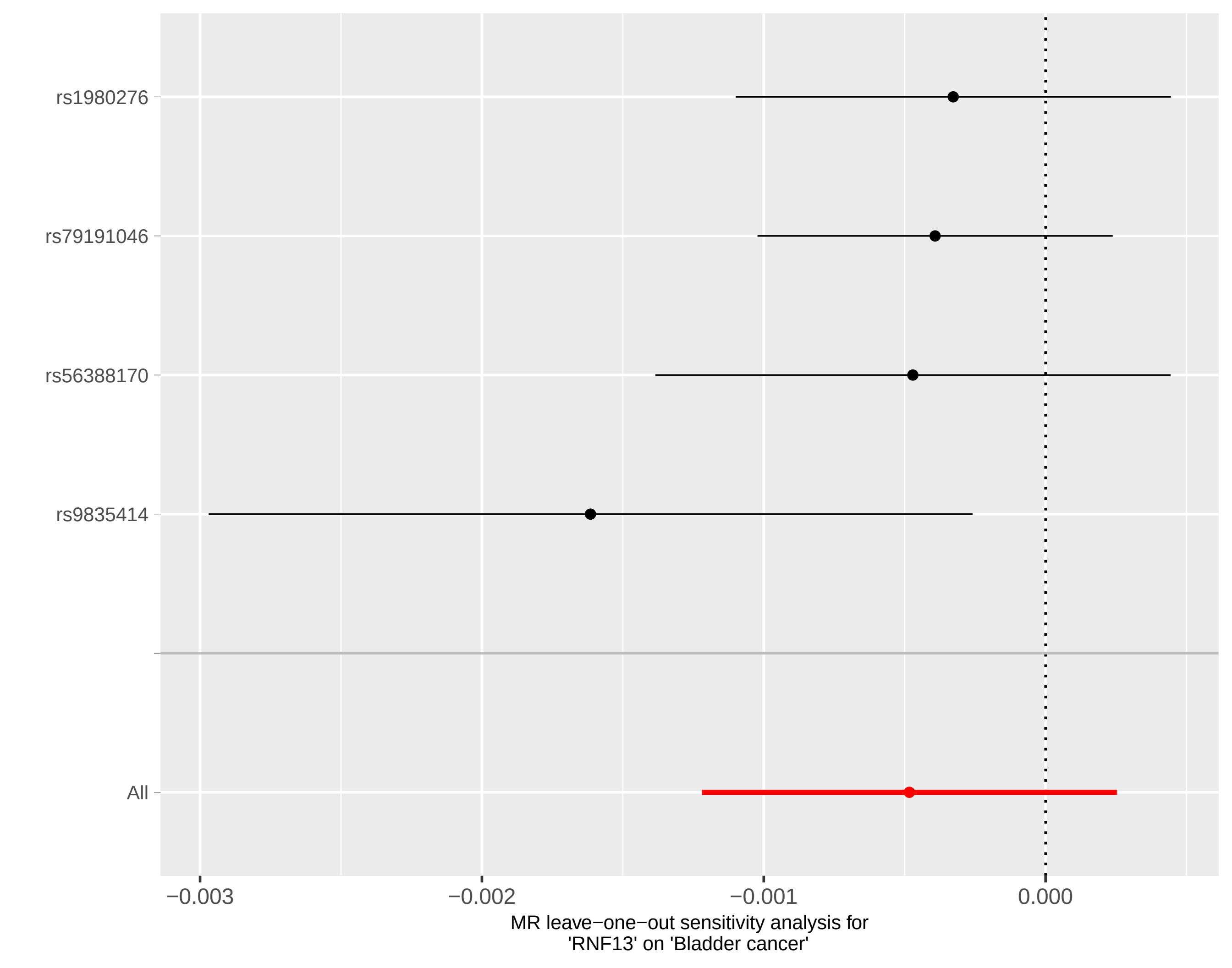
rs1980276
rs79191046
rs56388170
rs9835414
All
−0.003
−0.002
−0.001
0.000
MR leave−one−out sensitivity analysis for
'RNF13' on 'Bladder cancer'

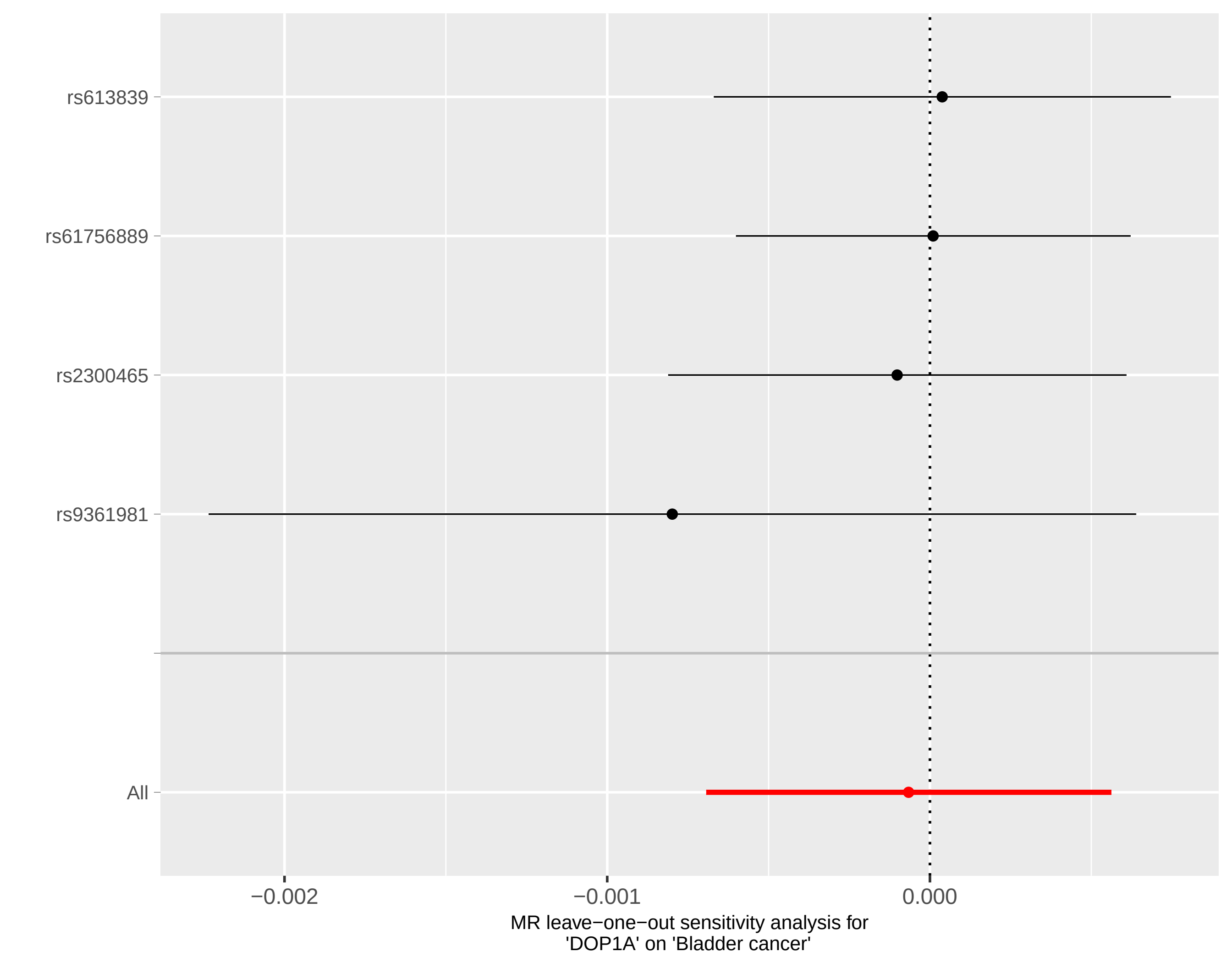
rs613839
rs61756889
rs2300465
rs9361981
All
−0.002
−0.001
0.000
MR leave−one−out sensitivity analysis for
'DOP1A' on 'Bladder cancer'

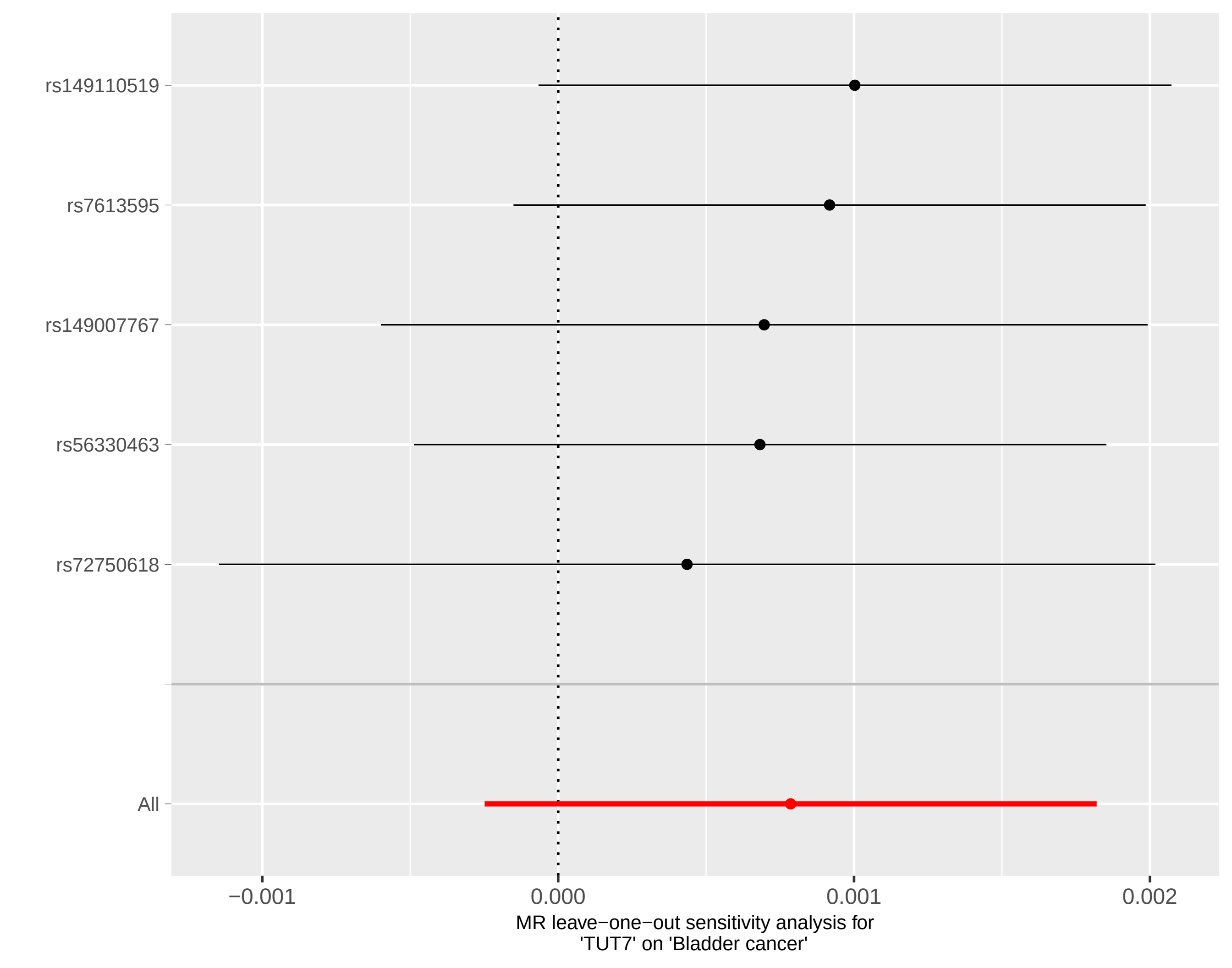
rs149110519
rs7613595
rs149007767
rs56330463
rs72750618
All
−0.001
0.000
0.001
0.002
MR leave−one−out sensitivity analysis for
'TUT7' on 'Bladder cancer'

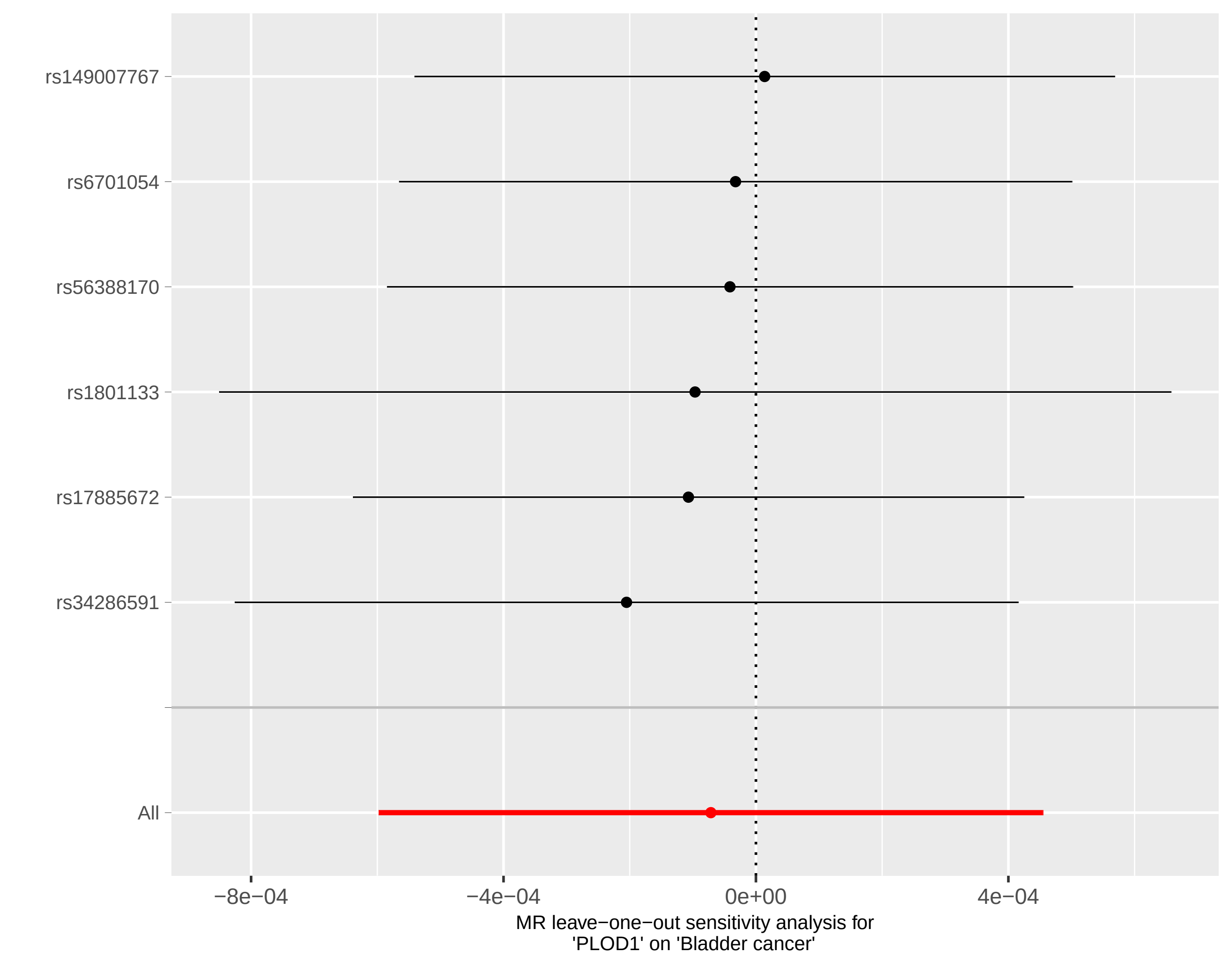
rs149007767
rs6701054
rs56388170
rs1801133
rs17885672
rs34286591
All
−8e−04
−4e−04
0e+00
4e−04
MR leave−one−out sensitivity analysis for
'PLOD1' on 'Bladder cancer'

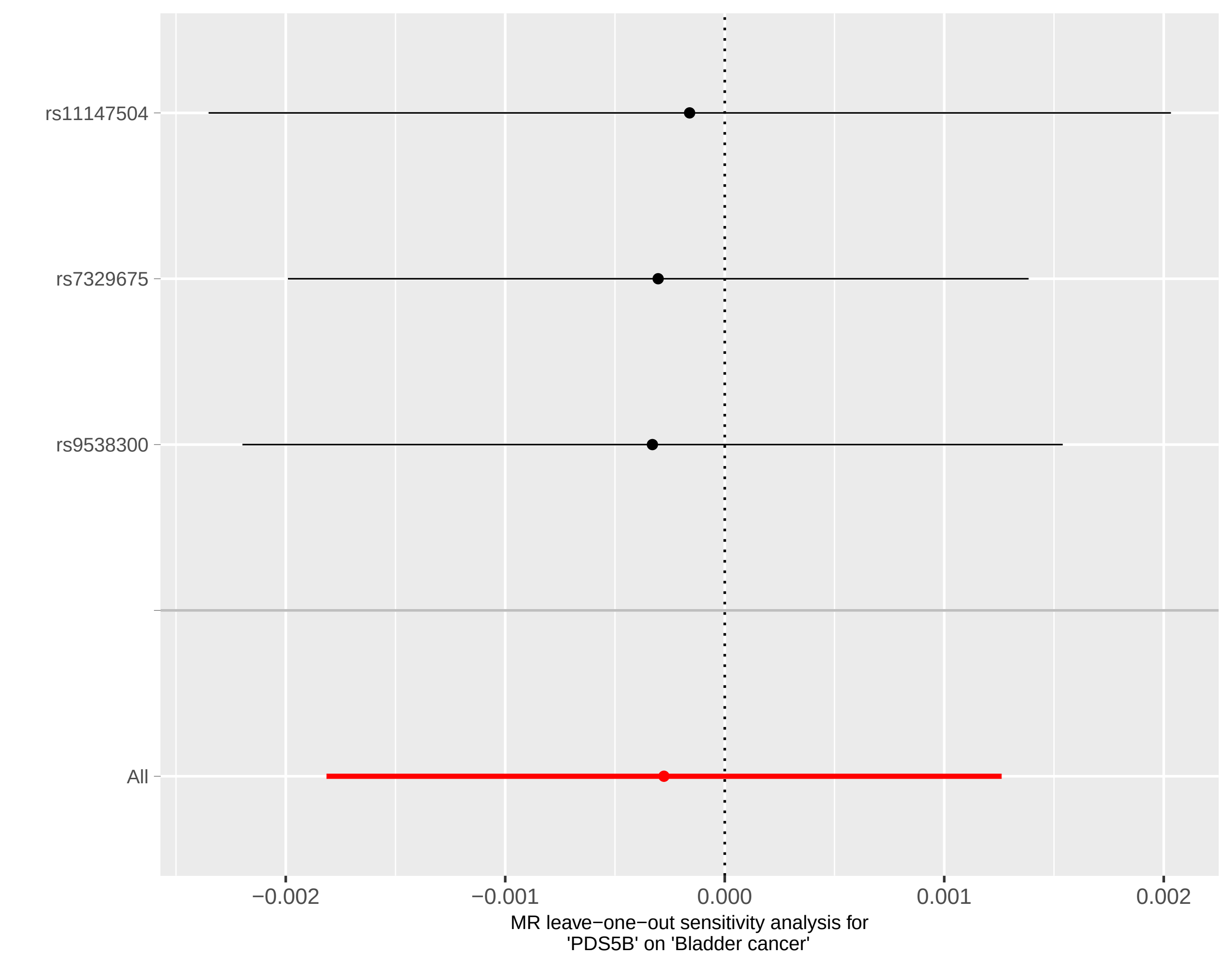
rs11147504
rs7329675
rs9538300
All
−0.002
−0.001
0.000
0.001
0.002
MR leave−one−out sensitivity analysis for
'PDS5B' on 'Bladder cancer'

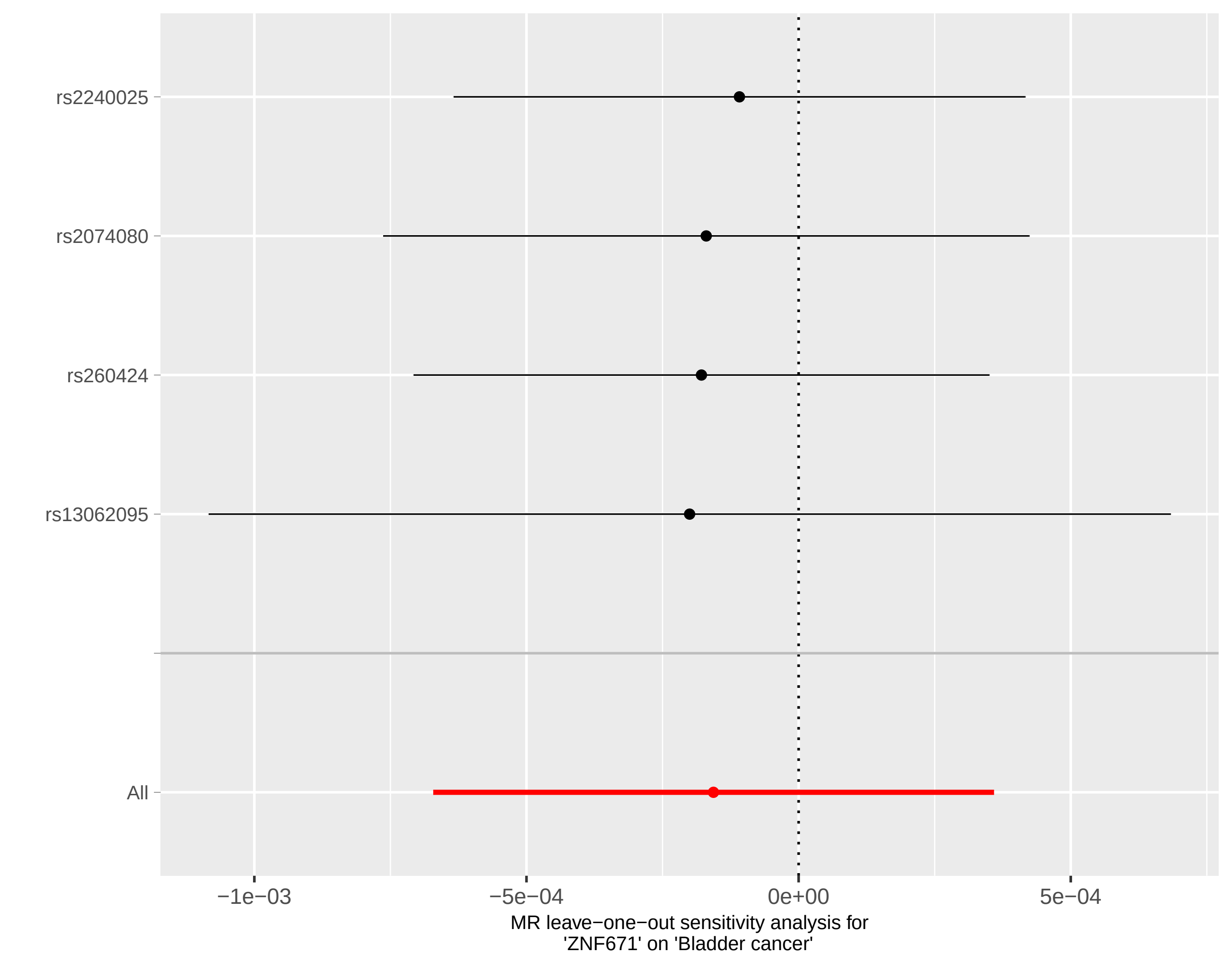
rs2240025
rs2074080
rs260424
rs13062095
All
−1e−03
−5e−04
0e+00
5e−04
MR leave−one−out sensitivity analysis for
'ZNF671' on 'Bladder cancer'

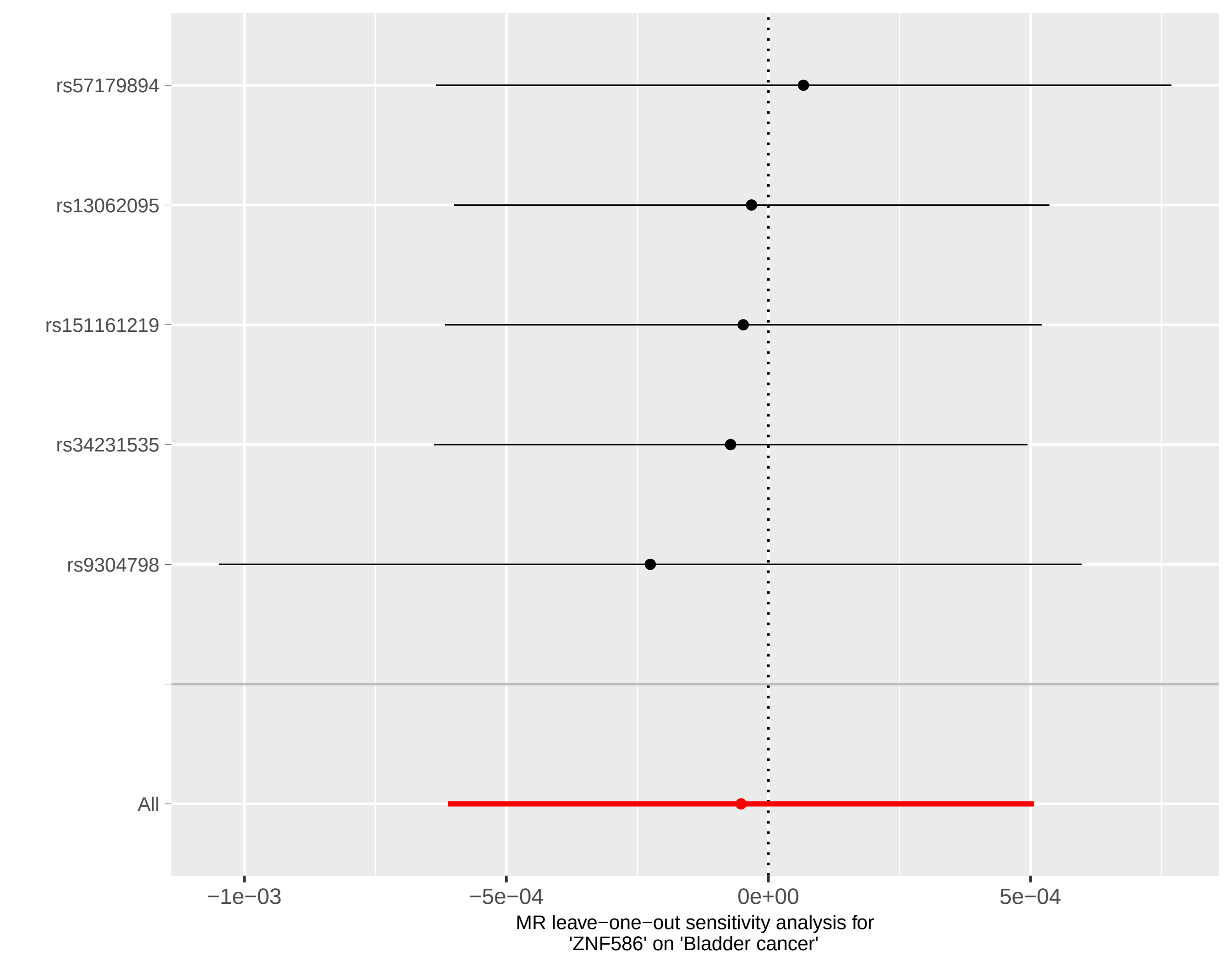
rs57179894
rs13062095
rs151161219
rs34231535
rs9304798
All
−1e−03
−5e−04
0e+00
5e−04
MR leave−one−out sensitivity analysis for
'ZNF586' on 'Bladder cancer'

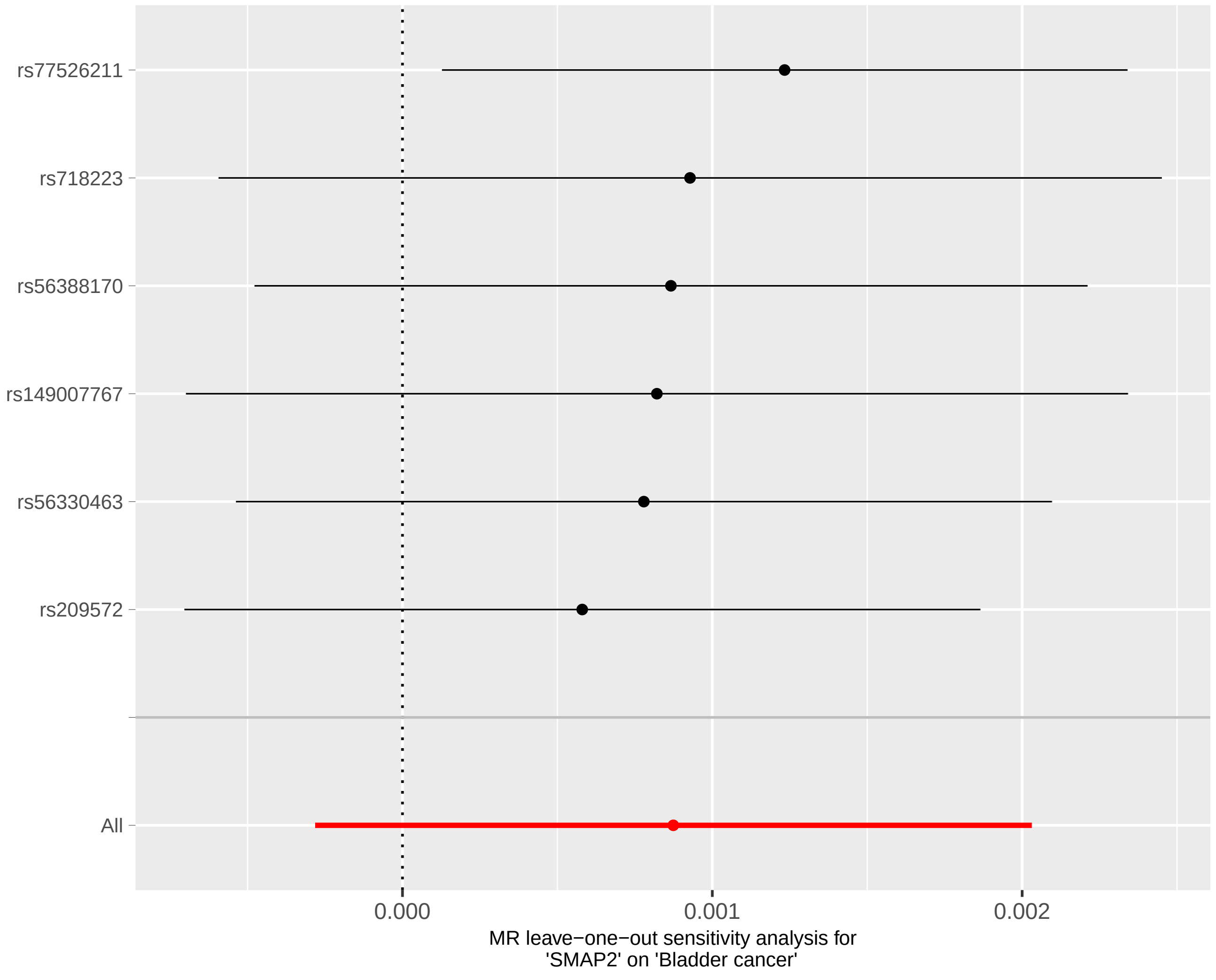
rs77526211
rs718223
rs56388170
rs149007767
rs56330463
rs209572
All
0.000
0.001
0.002
MR leave−one−out sensitivity analysis for
'SMAP2' on 'Bladder cancer'

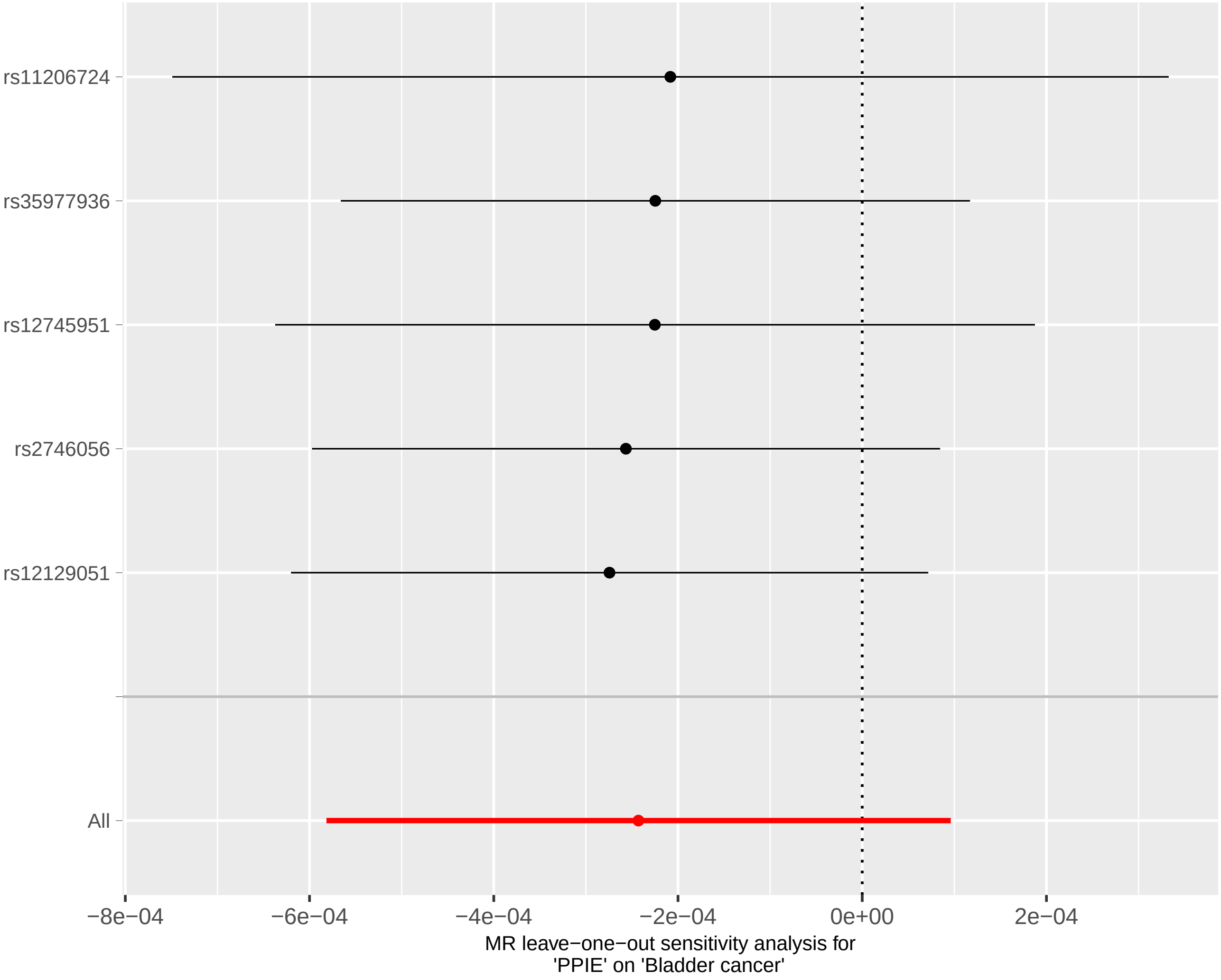
rs11206724
rs35977936
rs12745951
rs2746056
rs12129051
All
−8e−04
−6e−04
−4e−04
−2e−04
0e+00
2e−04
MR leave−one−out sensitivity analysis for
'PPIE' on 'Bladder cancer'

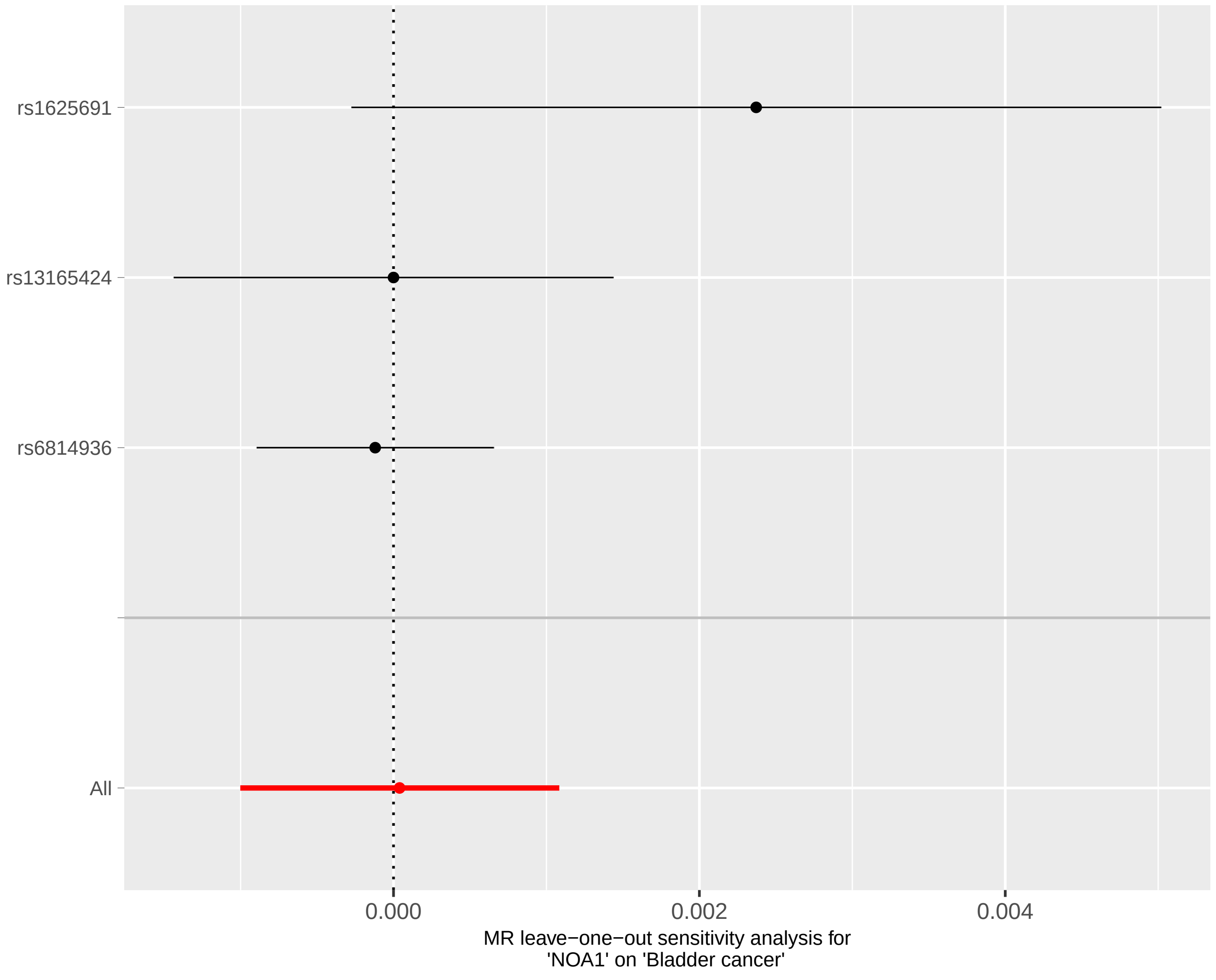

rs1625691
rs13165424
rs6814936
All
0.000
0.002
0.004
MR leave−one−out sensitivity analysis for
'NOA1' on 'Bladder cancer'

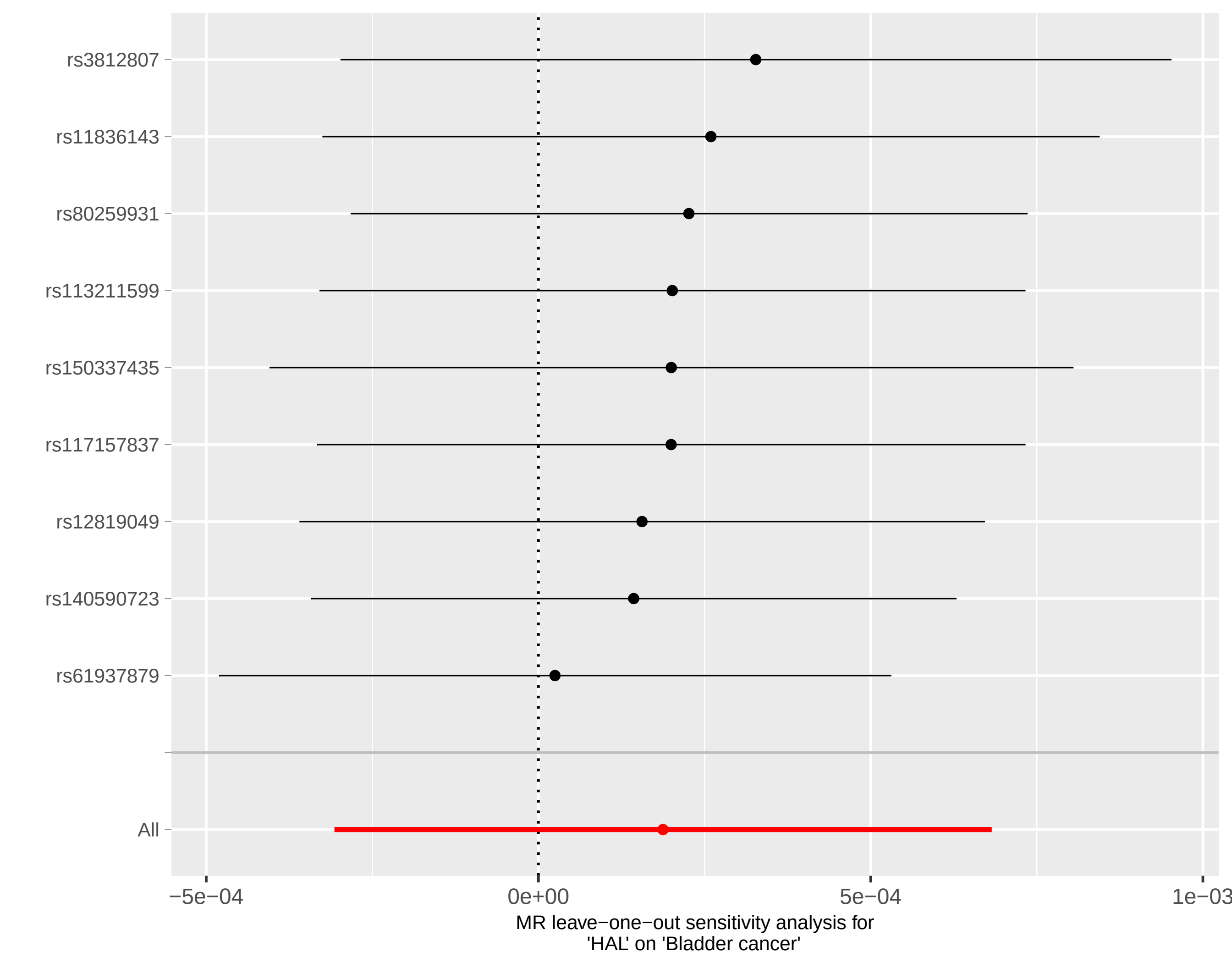
rs3812807
rs11836143
rs80259931
rs113211599
rs150337435
rs117157837
rs12819049
rs140590723
rs61937879
All
−5e−04
0e+00
5e−04
1e−03
MR leave−one−out sensitivity analysis for
'HAL' on 'Bladder cancer'

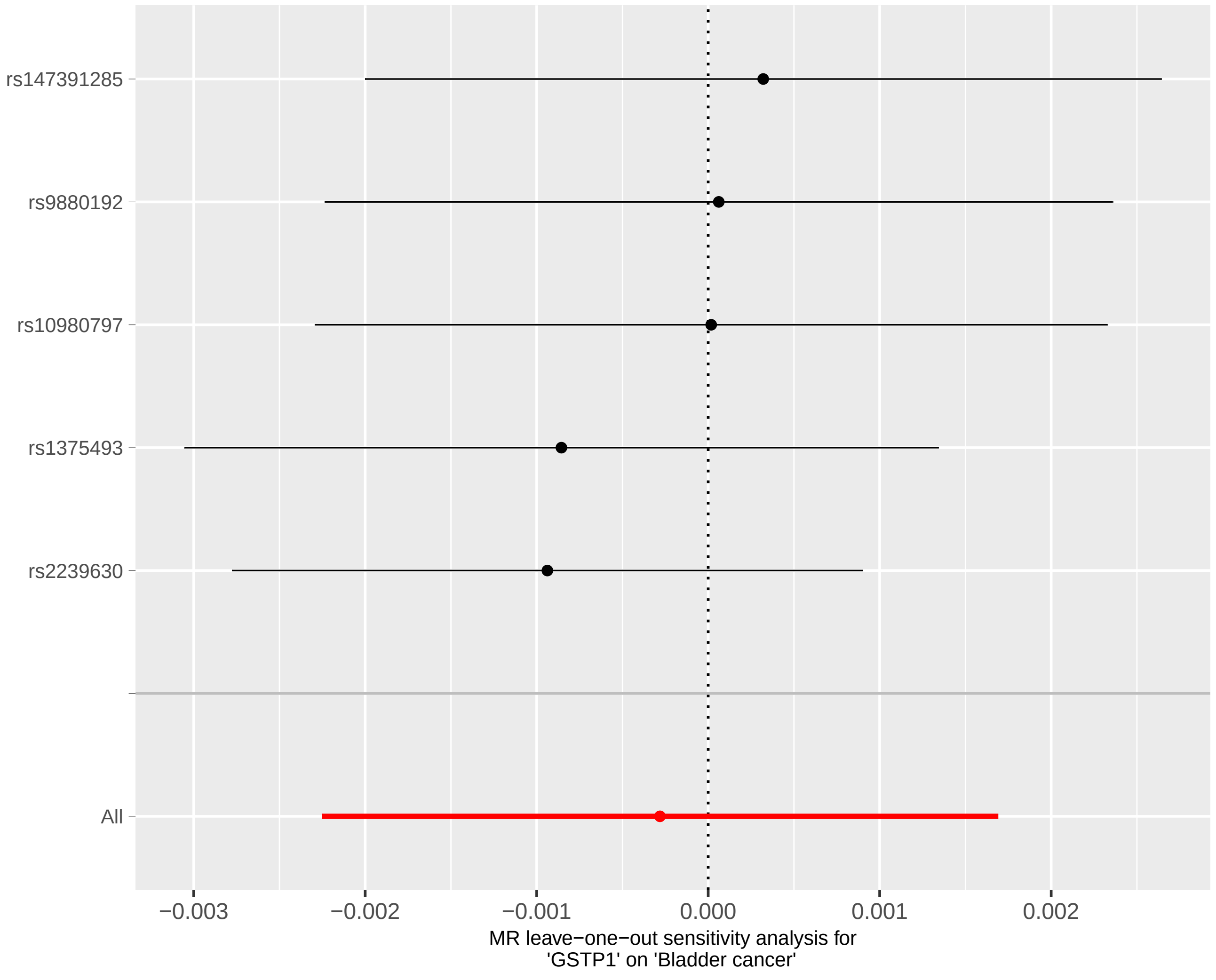

rs147391285
rs9880192
rs10980797
rs1375493
rs2239630
All
−0.003
−0.002
−0.001
0.000
0.001
0.002
MR leave−one−out sensitivity analysis for
'GSTP1' on 'Bladder cancer'

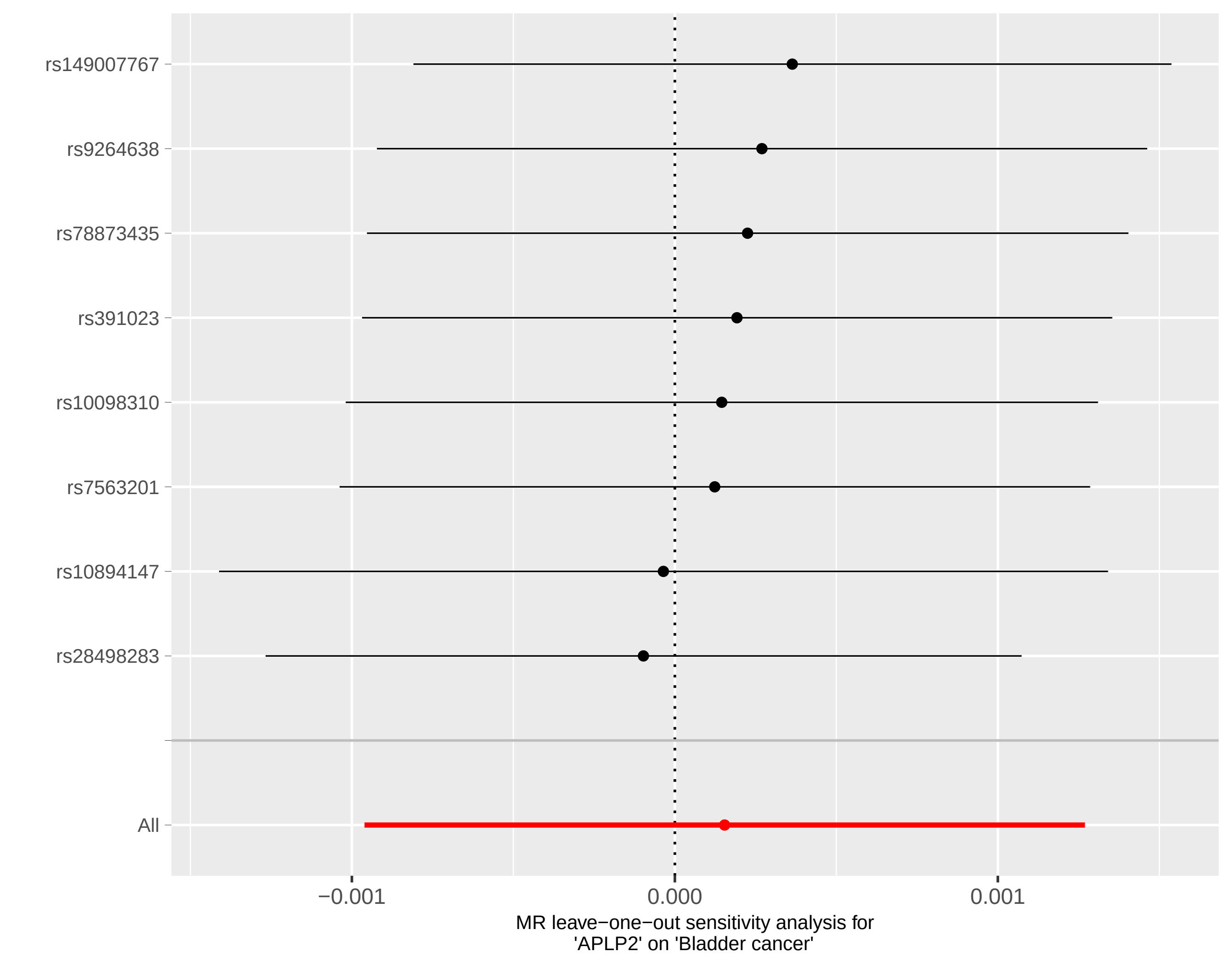
rs149007767
rs9264638
rs78873435
rs391023
rs10098310
rs7563201
rs10894147
rs28498283
All
−0.001
0.000
0.001
MR leave−one−out sensitivity analysis for
'APLP2' on 'Bladder cancer'

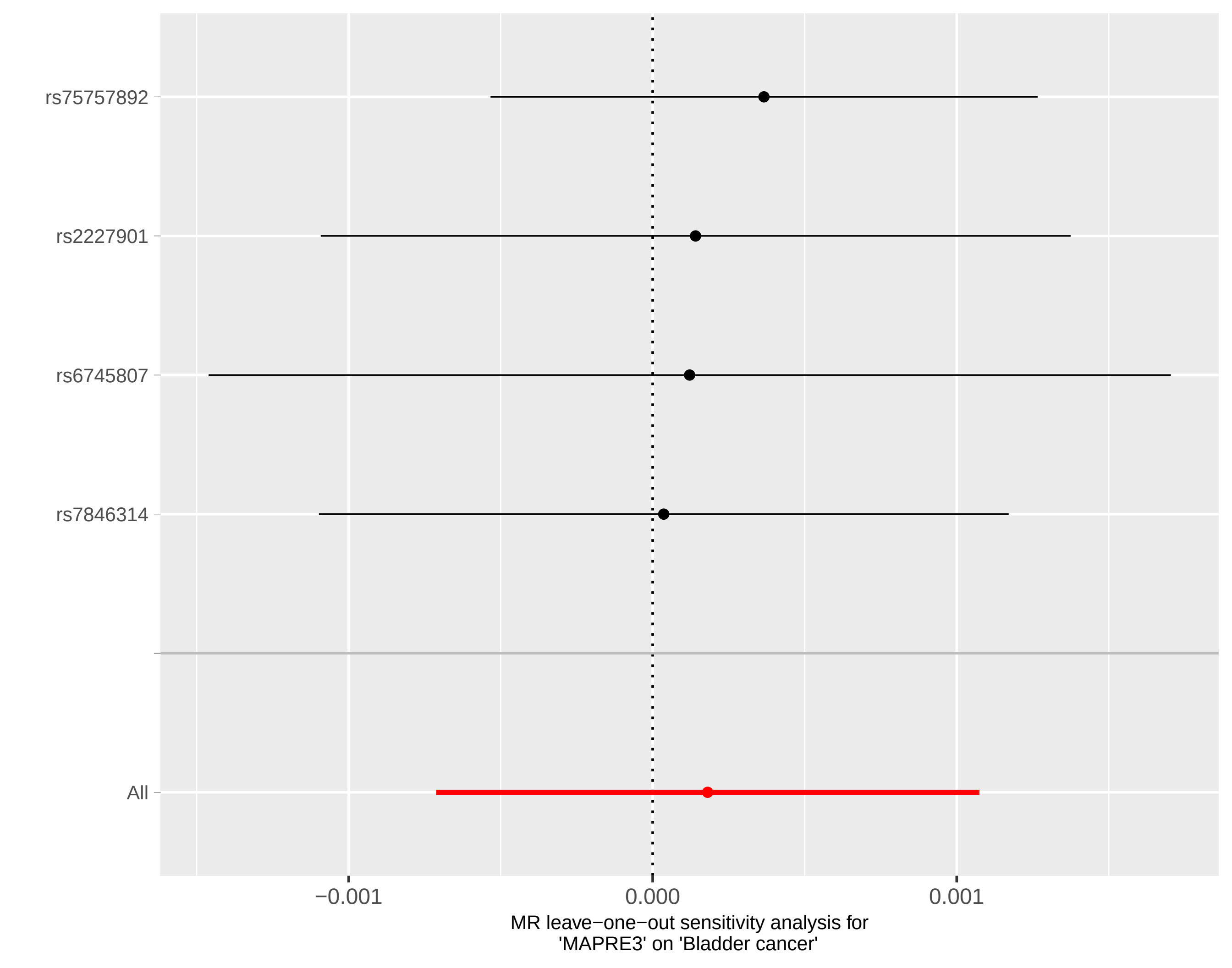
rs75757892
rs2227901
rs6745807
rs7846314
All
−0.001
0.000
0.001
MR leave−one−out sensitivity analysis for
'MAPRE3' on 'Bladder cancer'

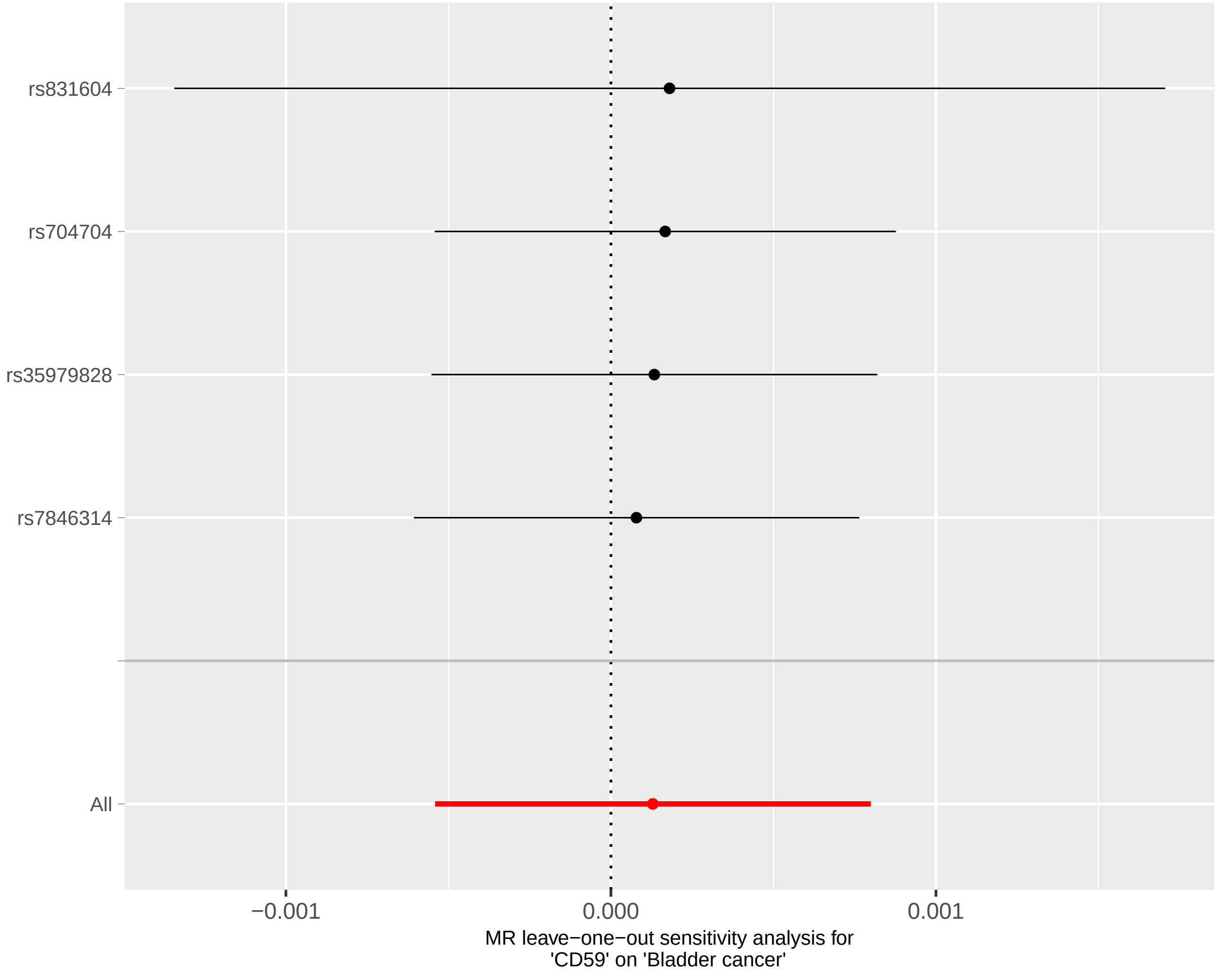
rs831604
rs704704
rs35979828
rs7846314
All
−0.001
0.000
0.001
MR leave−one−out sensitivity analysis for
'CD59' on 'Bladder cancer'

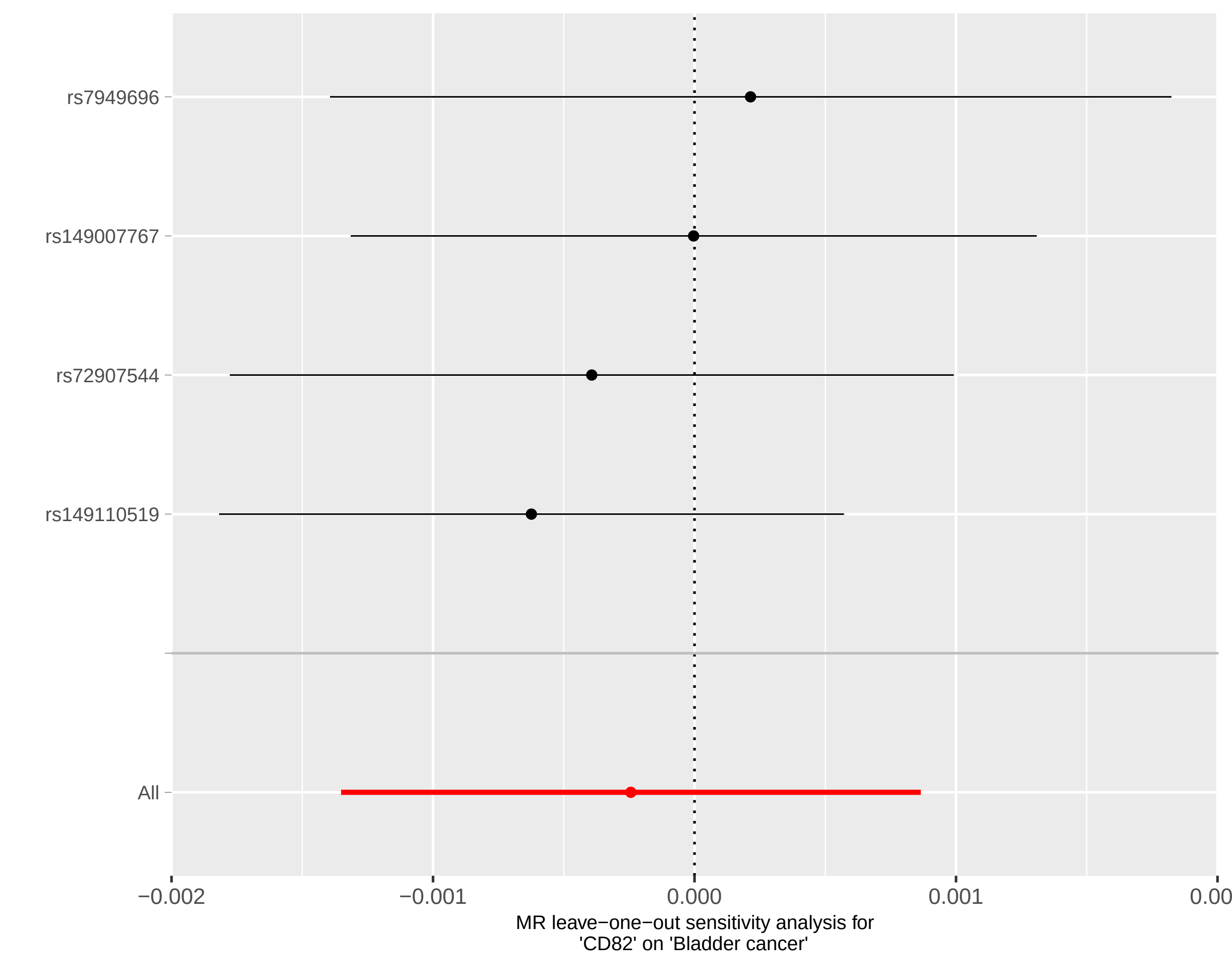
rs7949696
rs149007767
rs72907544
rs149110519
All
−0.002
−0.001
0.000
0.001
0.00
MR leave−one−out sensitivity analysis for
'CD82' on 'Bladder cancer'

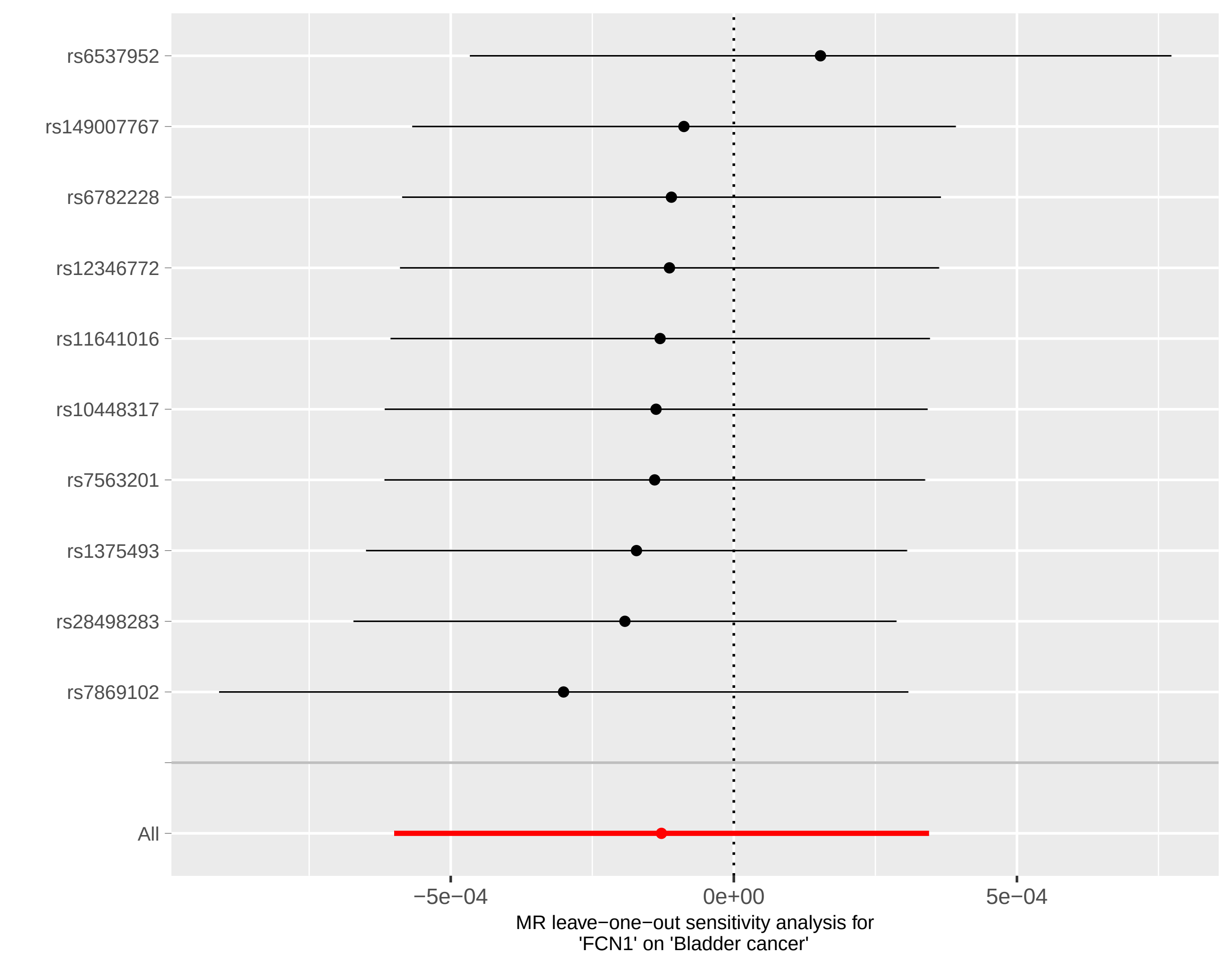
rs6537952
rs149007767
rs6782228
rs12346772
rs11641016
rs10448317
rs7563201
rs1375493
rs28498283
rs7869102
All
−5e−04
0e+00
5e−04
MR leave−one−out sensitivity analysis for
'FCN1' on 'Bladder cancer'

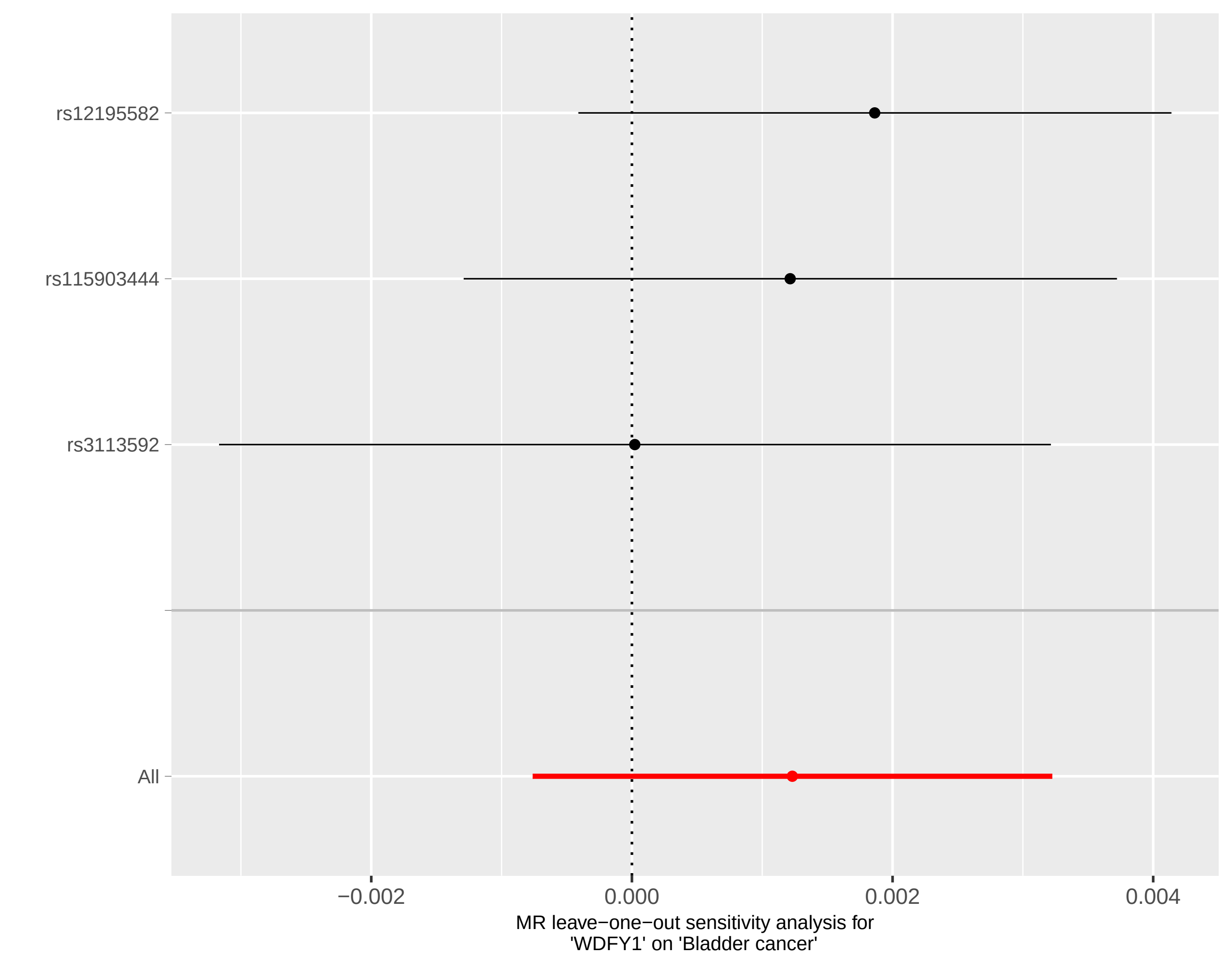

rs12195582
rs115903444
rs3113592
All
−0.002
0.000
0.002
0.004
MR leave−one−out sensitivity analysis for
'WDFY1' on 'Bladder cancer'

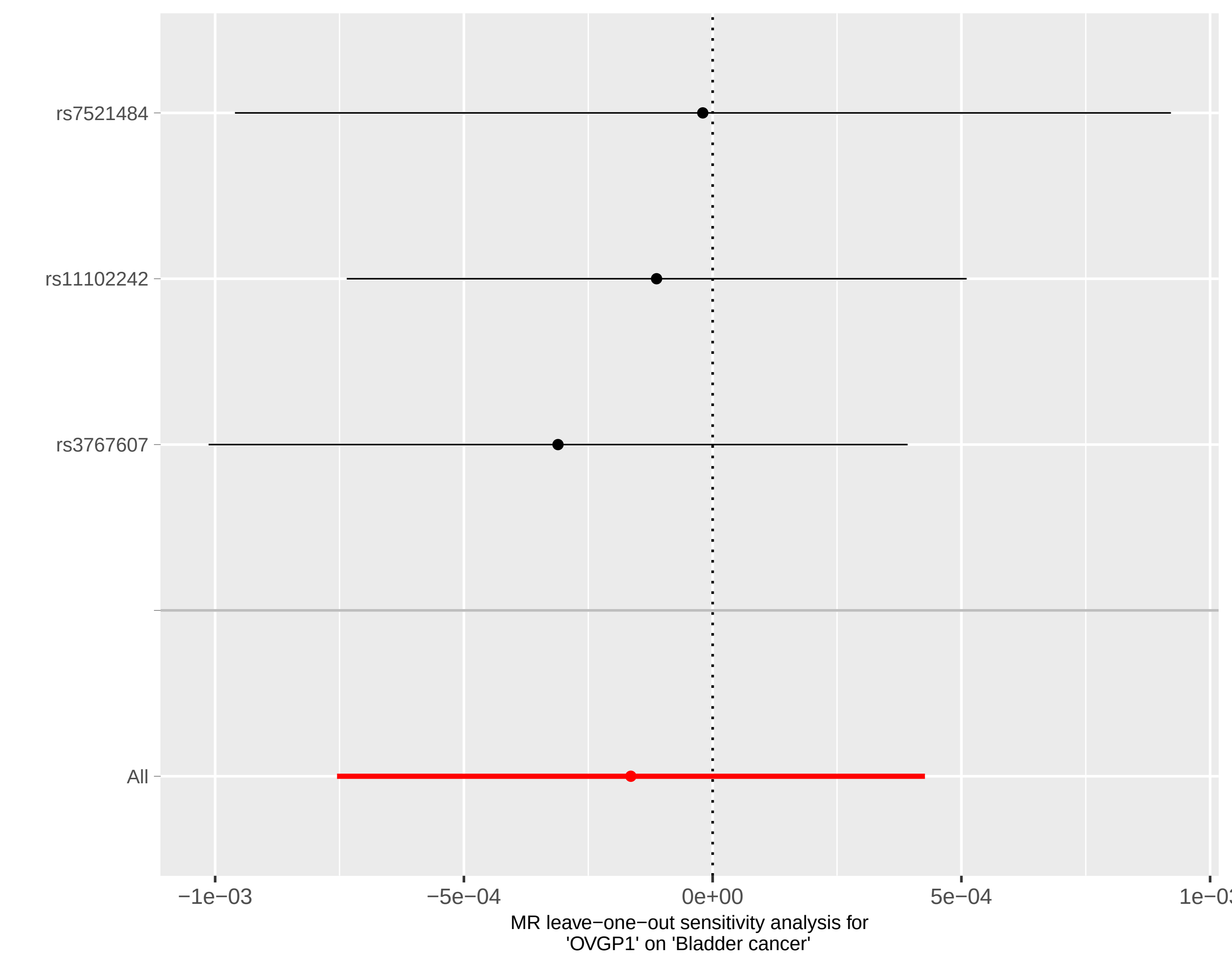
rs7521484
rs11102242
rs3767607
All
−1e−03
−5e−04
0e+00
5e−04
1e−03
MR leave−one−out sensitivity analysis for
'OVGP1' on 'Bladder cancer'

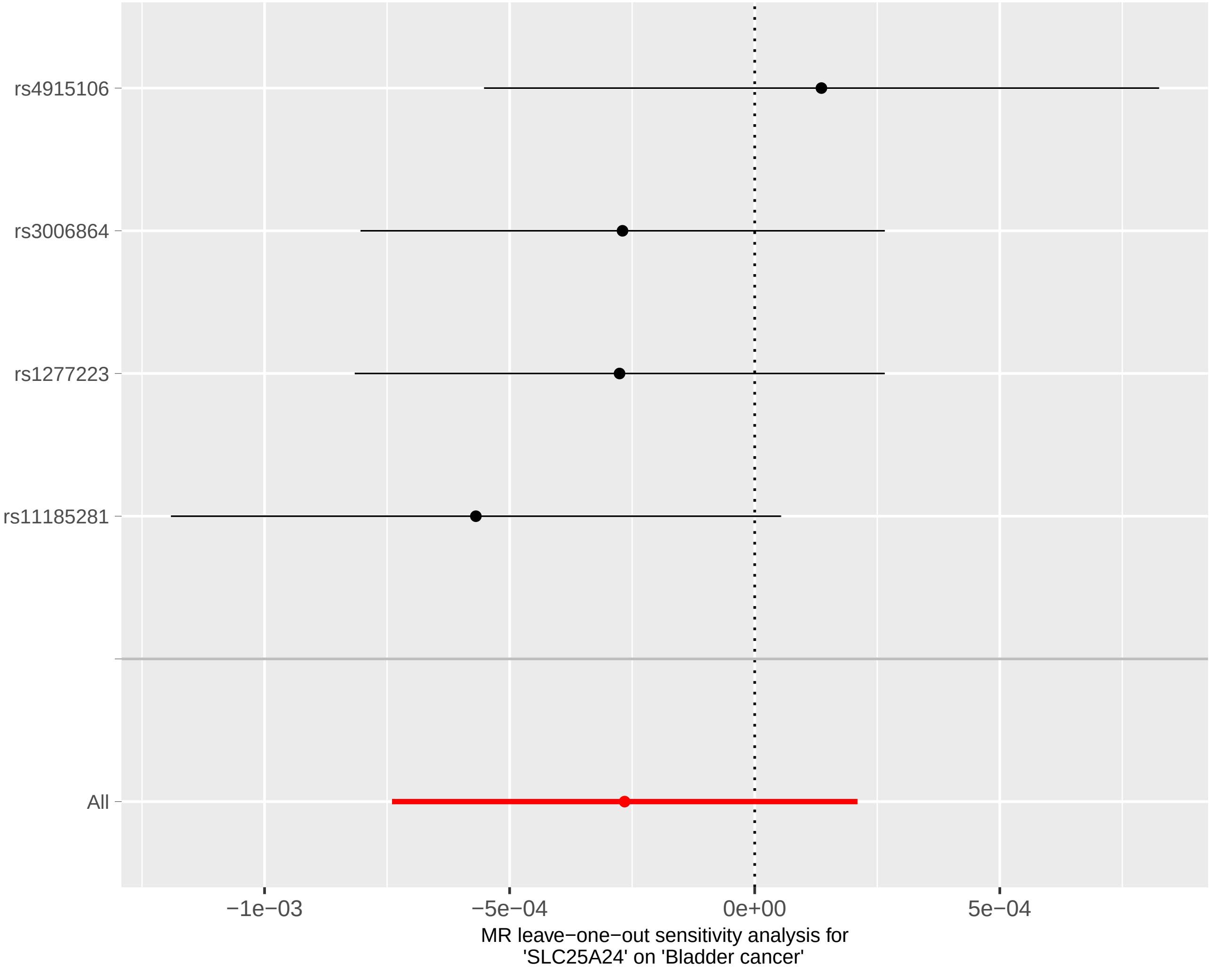
rs4915106
rs3006864
rs1277223
rs11185281
All
−1e−03
−5e−04
0e+00
5e−04
MR leave−one−out sensitivity analysis for
'SLC25A24' on 'Bladder cancer'

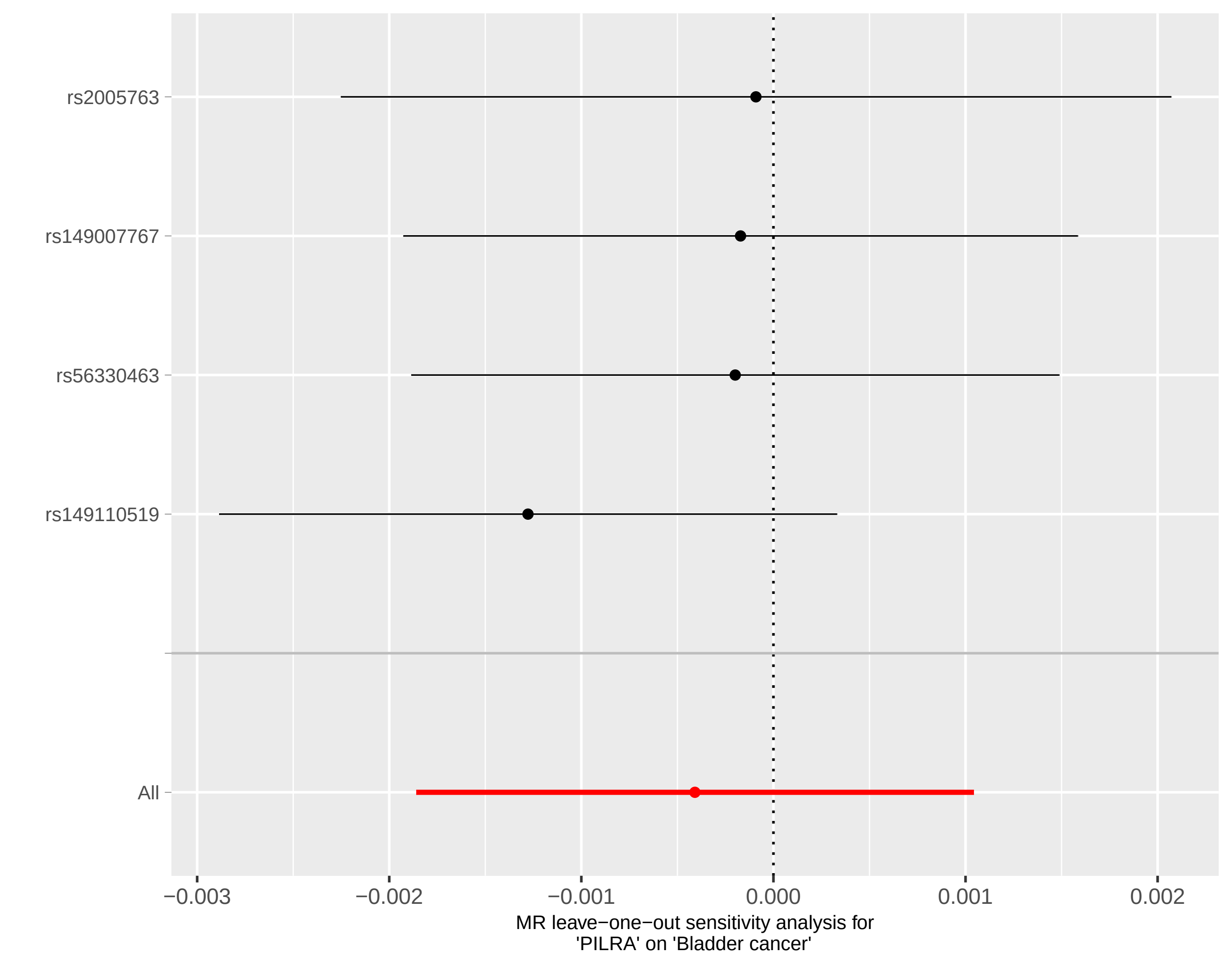
rs2005763
rs149007767
rs56330463
rs149110519
All
−0.003
−0.002
−0.001
0.000
0.001
0.002
MR leave−one−out sensitivity analysis for
'PILRA' on 'Bladder cancer'

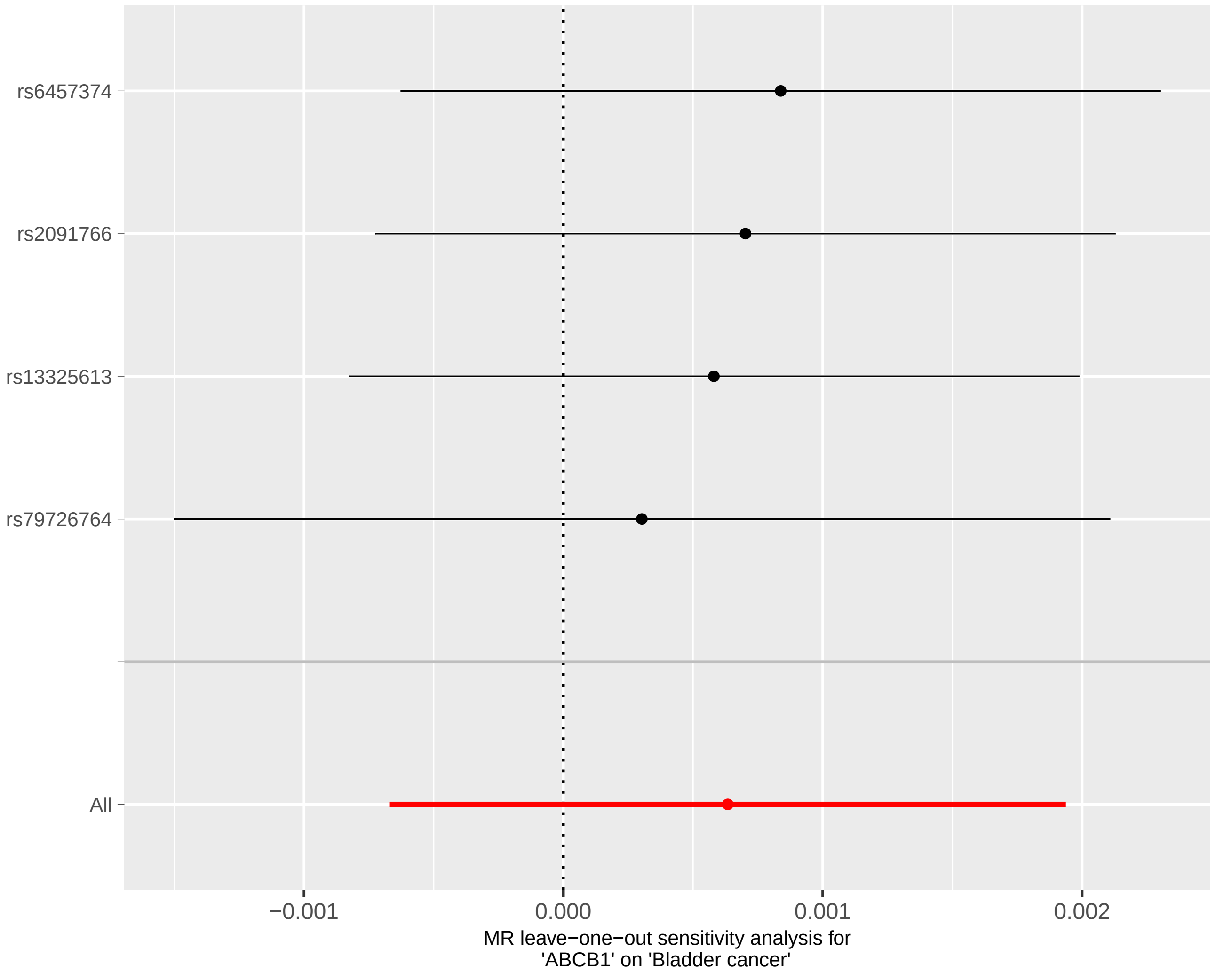

rs6457374
rs2091766
rs13325613
rs79726764
All
−0.001
0.000
0.001
0.002
MR leave−one−out sensitivity analysis for
'ABCB1' on 'Bladder cancer'

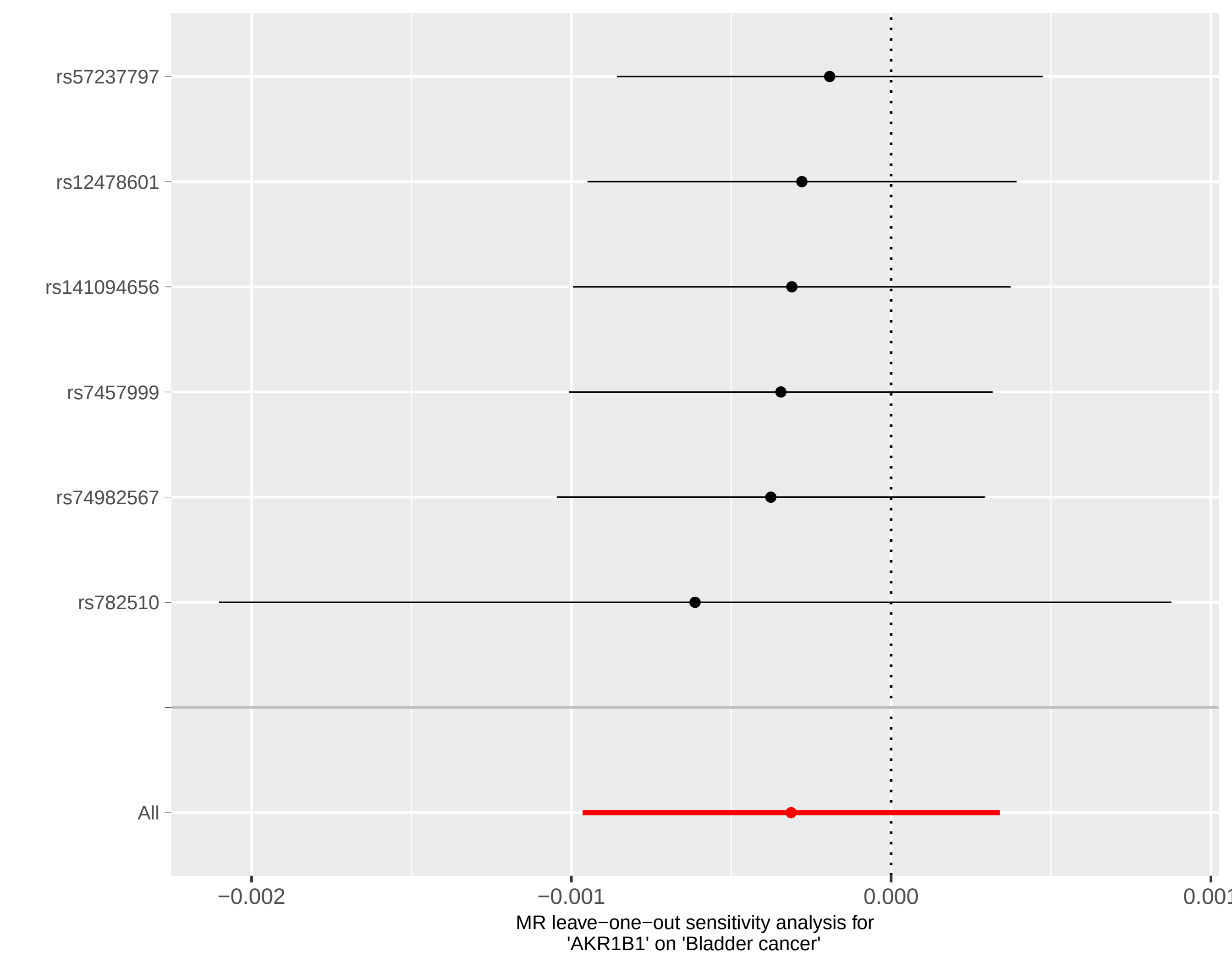
rs57237797
rs12478601
rs141094656
rs7457999
rs74982567
rs782510
All
−0.002
−0.001
0.000
0.001
MR leave−one−out sensitivity analysis for
'AKR1B1' on 'Bladder cancer'

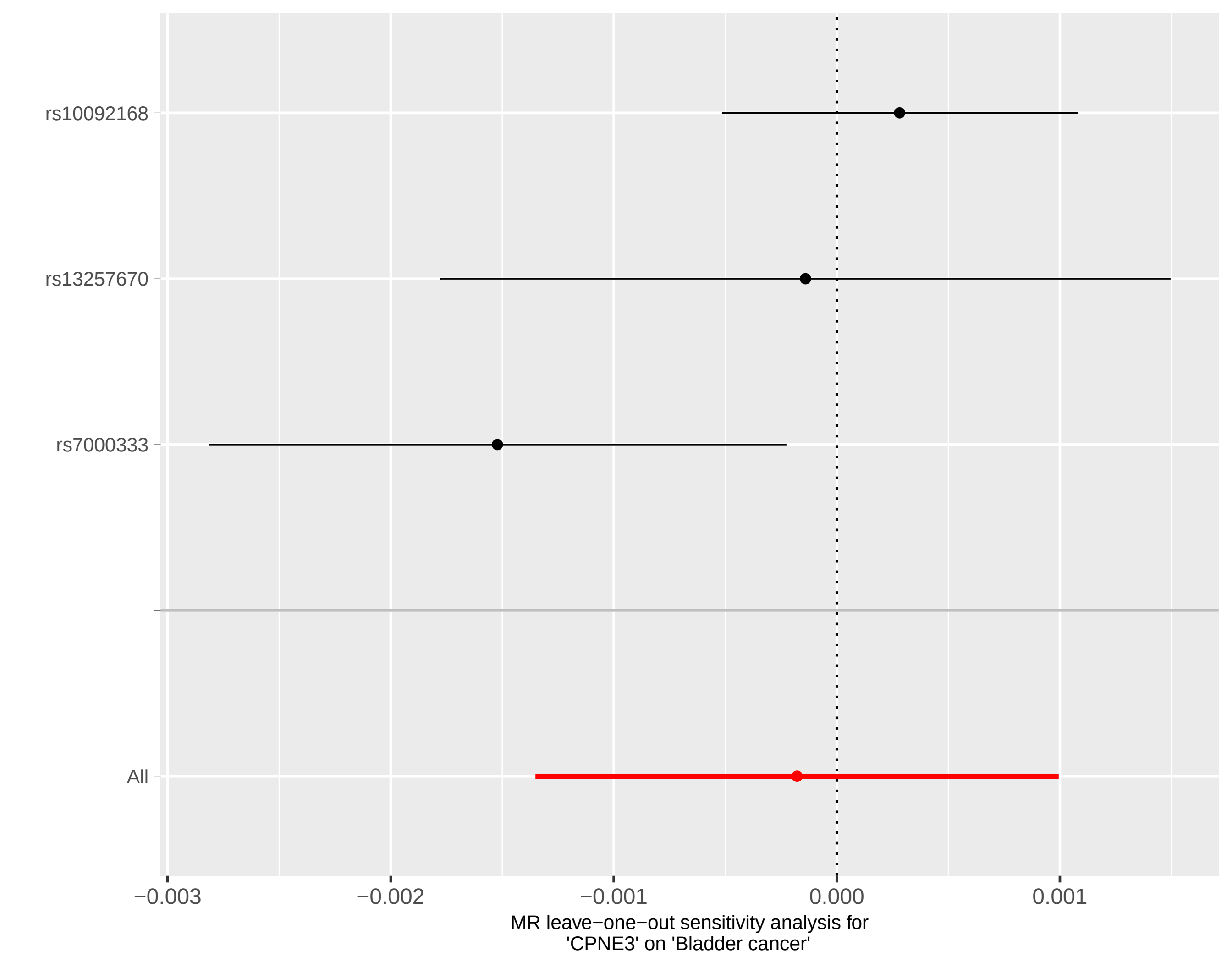
rs10092168
rs13257670
rs7000333
All
−0.003
−0.002
−0.001
0.000
0.001
MR leave−one−out sensitivity analysis for
'CPNE3' on 'Bladder cancer'

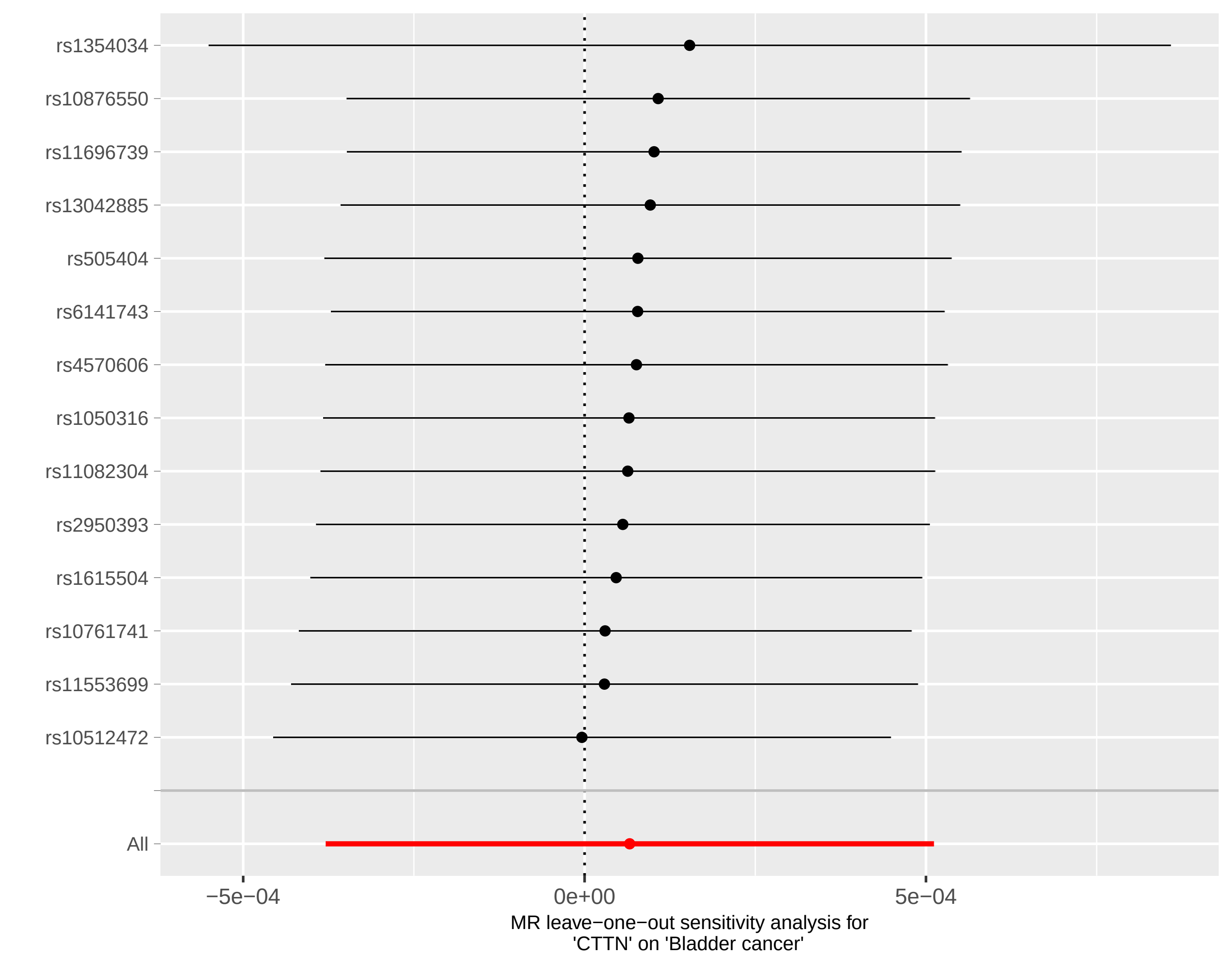
rs1354034
rs10876550
rs11696739
rs13042885
rs505404
rs6141743
rs4570606
rs1050316
rs11082304
rs2950393
rs1615504
rs10761741
rs11553699
rs10512472
All
−5e−04
0e+00
5e−04
MR leave−one−out sensitivity analysis for
'CTTN' on 'Bladder cancer'

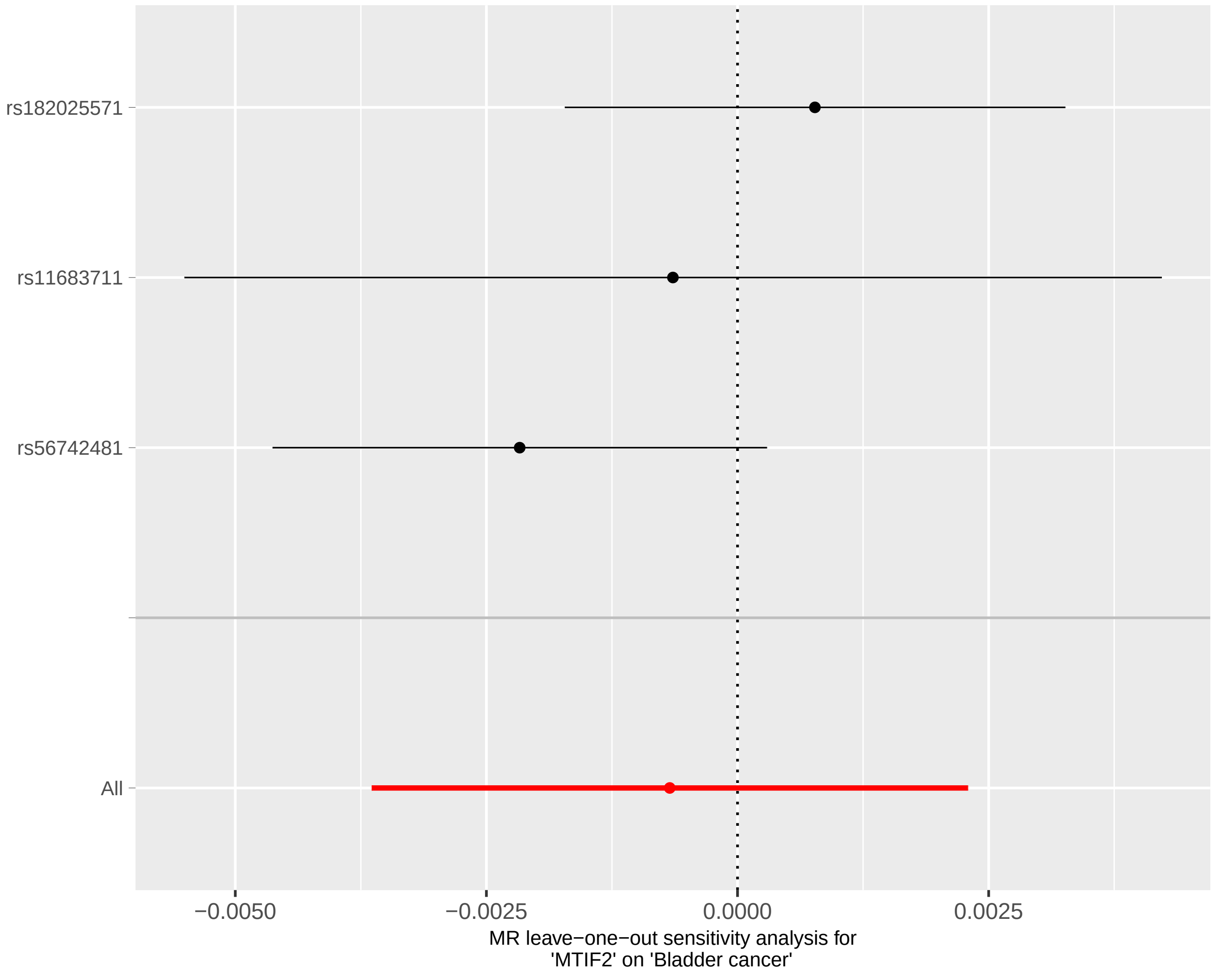
rs182025571
rs11683711
rs56742481
All
−0.0050
−0.0025
0.0000
0.0025
MR leave−one−out sensitivity analysis for
'MTIF2' on 'Bladder cancer'

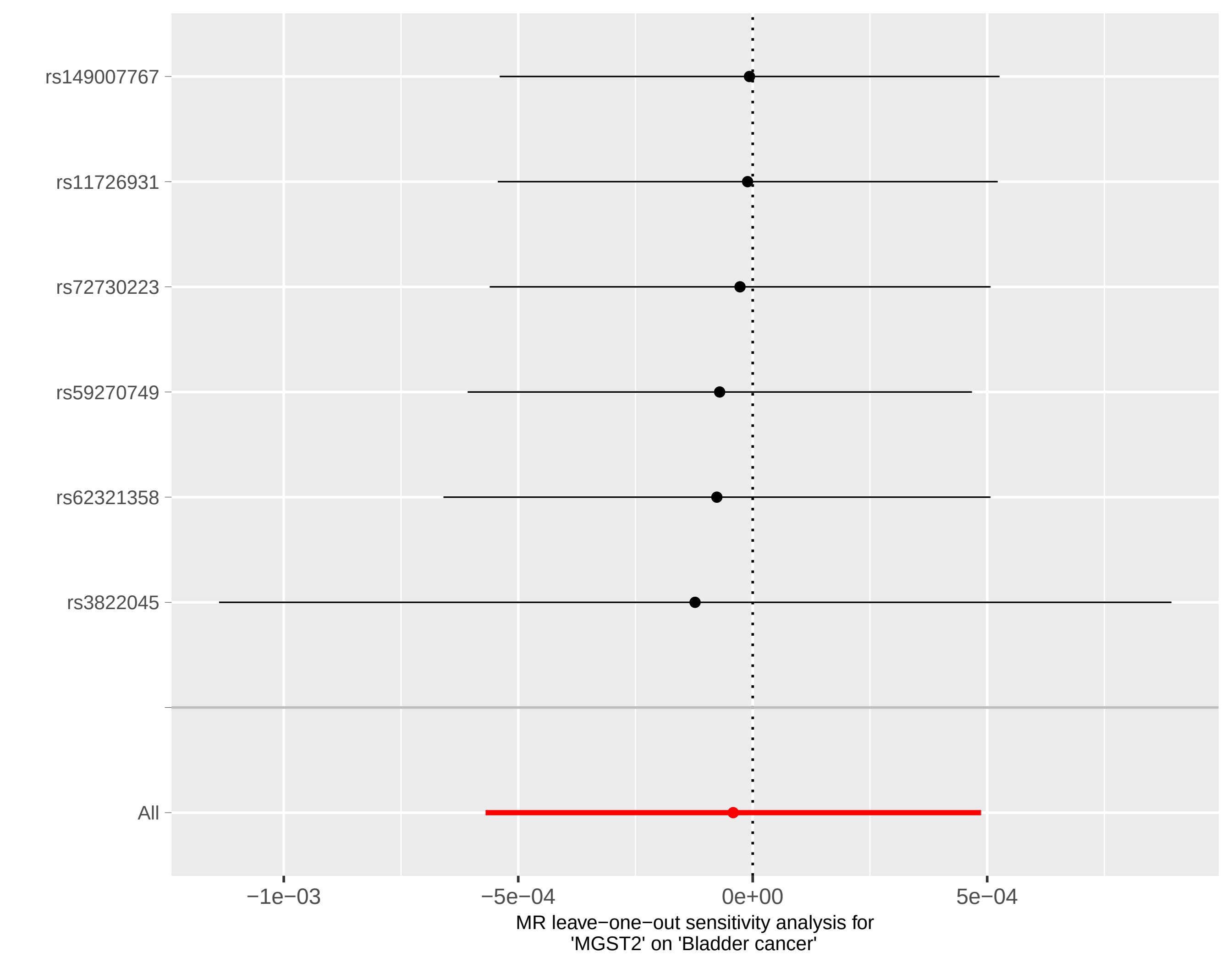

rs149007767
rs11726931
rs72730223
rs59270749
rs62321358
rs3822045
All
−1e−03
−5e−04
0e+00
5e−04
MR leave−one−out sensitivity analysis for
'MGST2' on 'Bladder cancer'

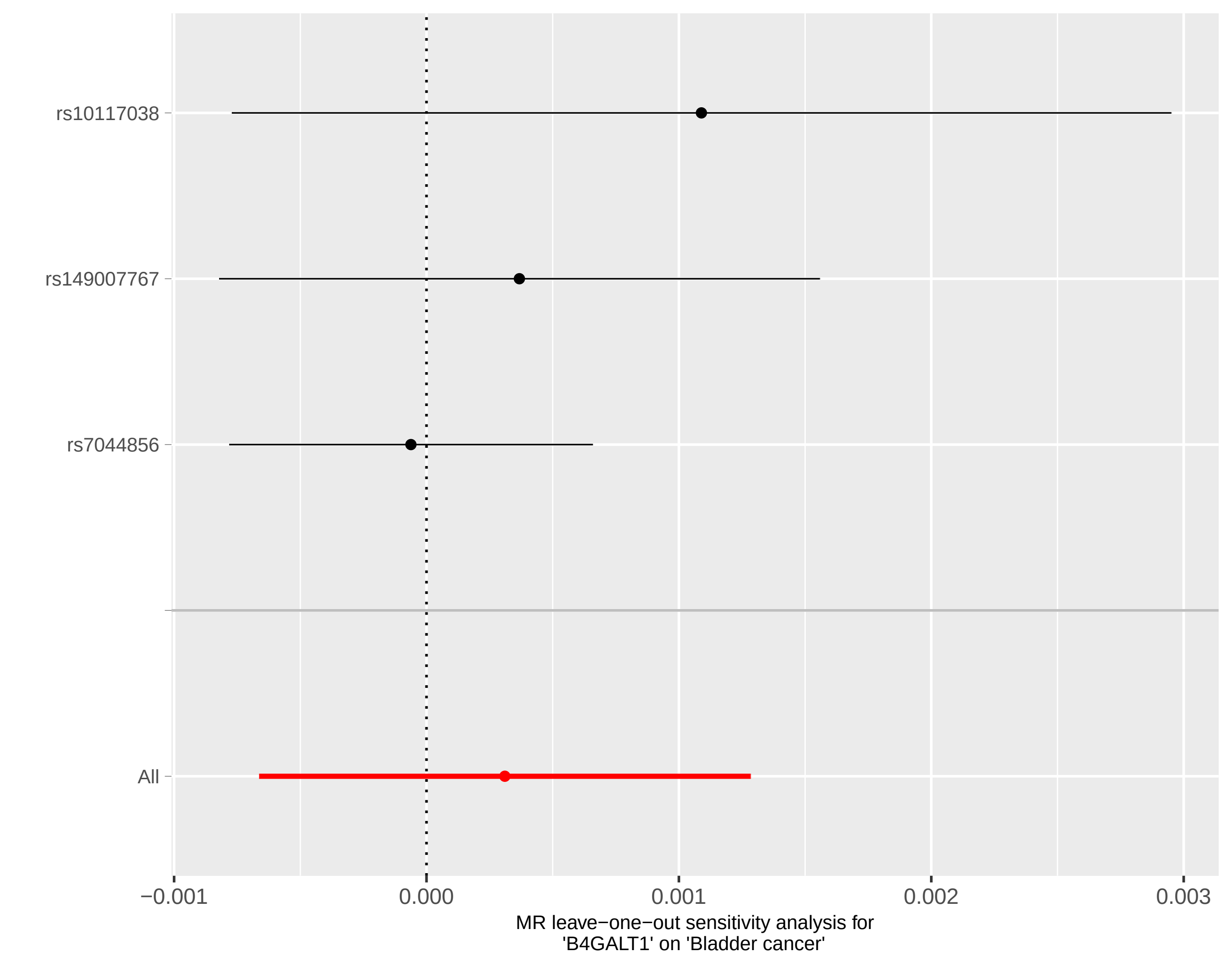

rs10117038
rs149007767
rs7044856
All
−0.001
0.000
0.001
0.002
0.003
MR leave−one−out sensitivity analysis for
'B4GALT1' on 'Bladder cancer'

Insufficient number of SNPs

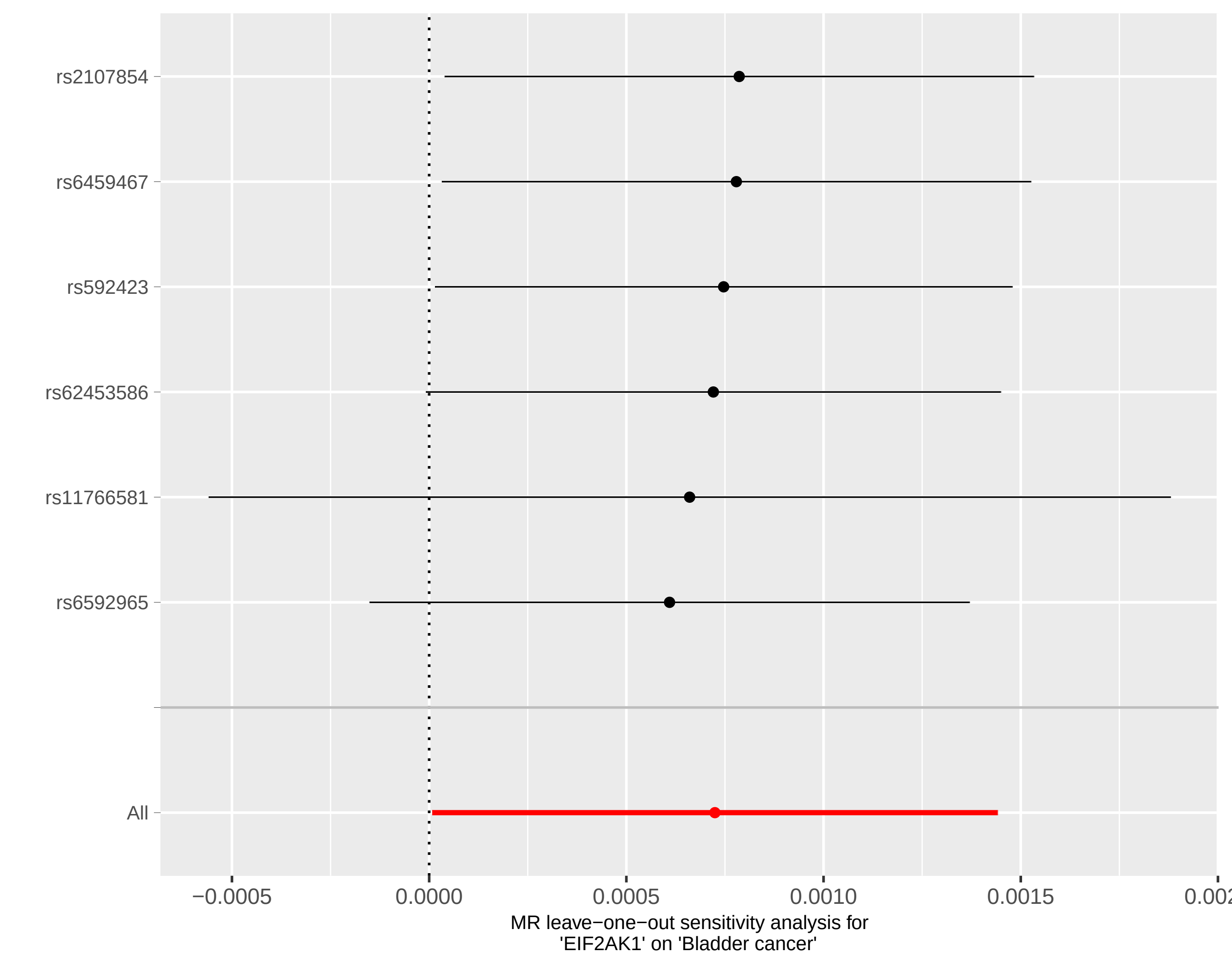
rs2107854
rs6459467
rs592423
rs62453586
rs11766581
rs6592965
All
−0.0005
0.0000
0.0005
0.0010
0.0015
MR leave−one−out sensitivity analysis for
'EIF2AK1' on 'Bladder cancer'

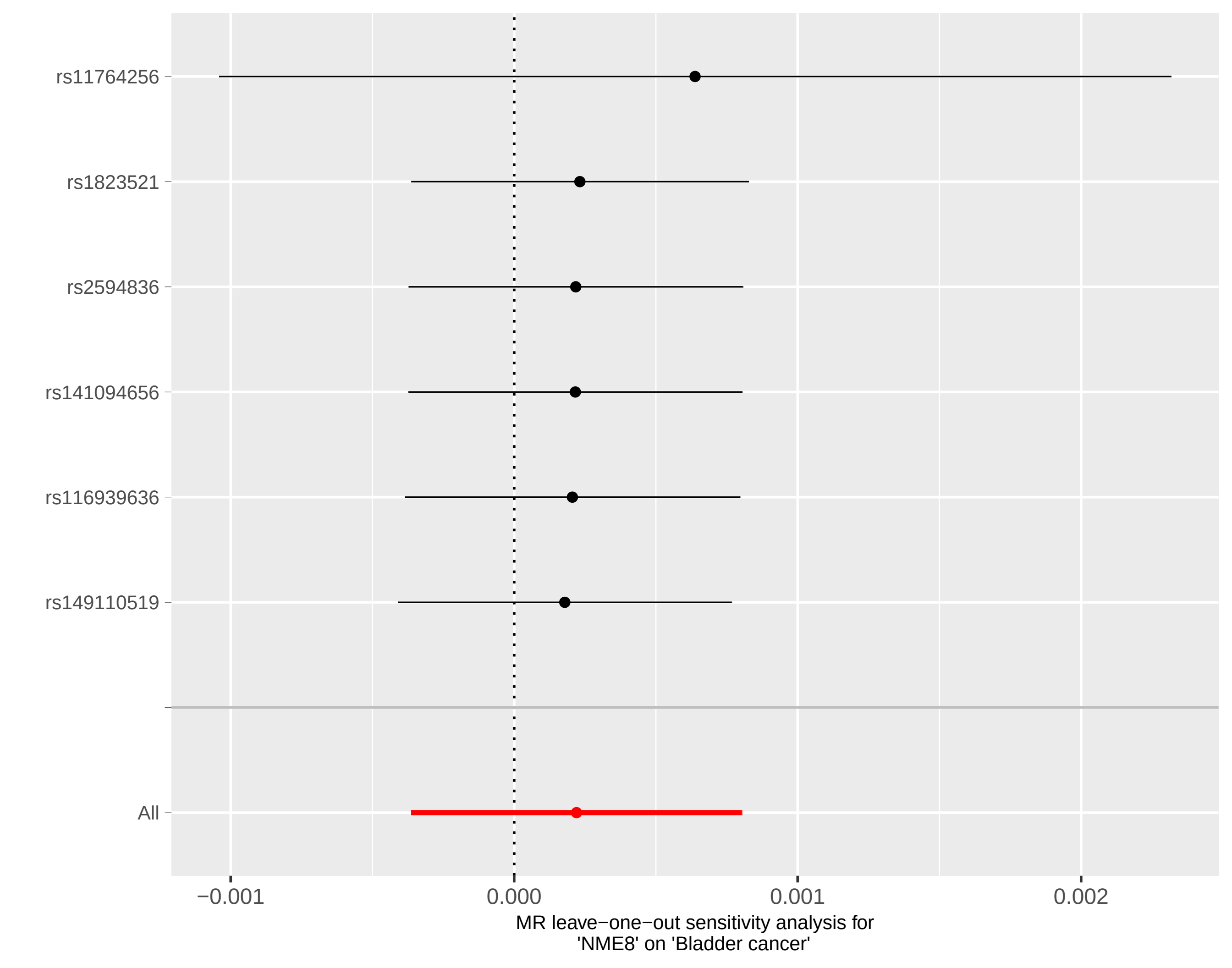
rs11764256
rs1823521
rs2594836
rs141094656
rs116939636
rs149110519
All
−0.001
0.000
0.001
0.002
MR leave−one−out sensitivity analysis for
'NME8' on 'Bladder cancer'

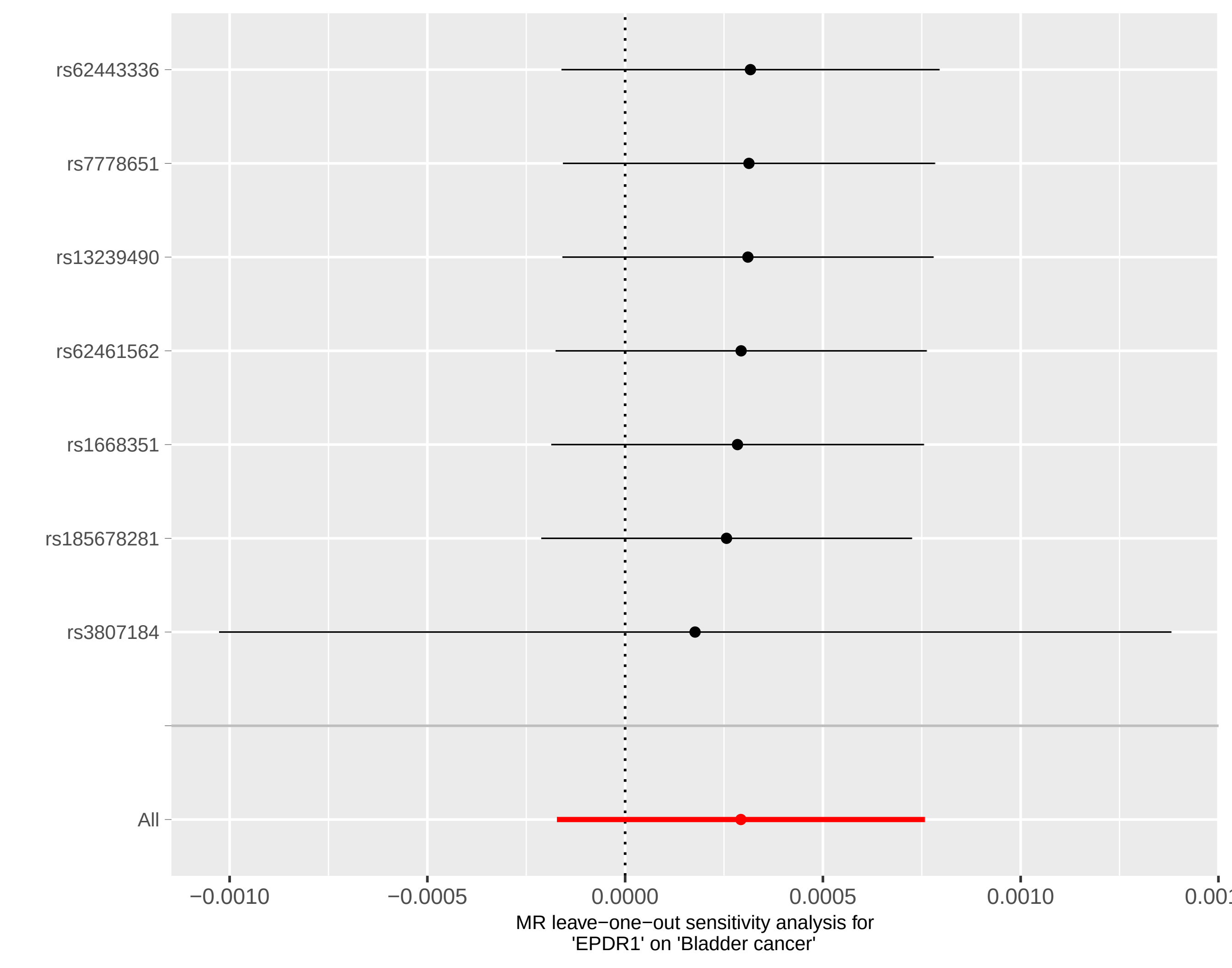
rs62443336
rs7778651
rs13239490
rs62461562
rs1668351
rs185678281
rs3807184
All
−0.0010
−0.0005
0.0000
0.0005
0.0010
0.001
MR leave−one−out sensitivity analysis for
'EPDR1' on 'Bladder cancer'

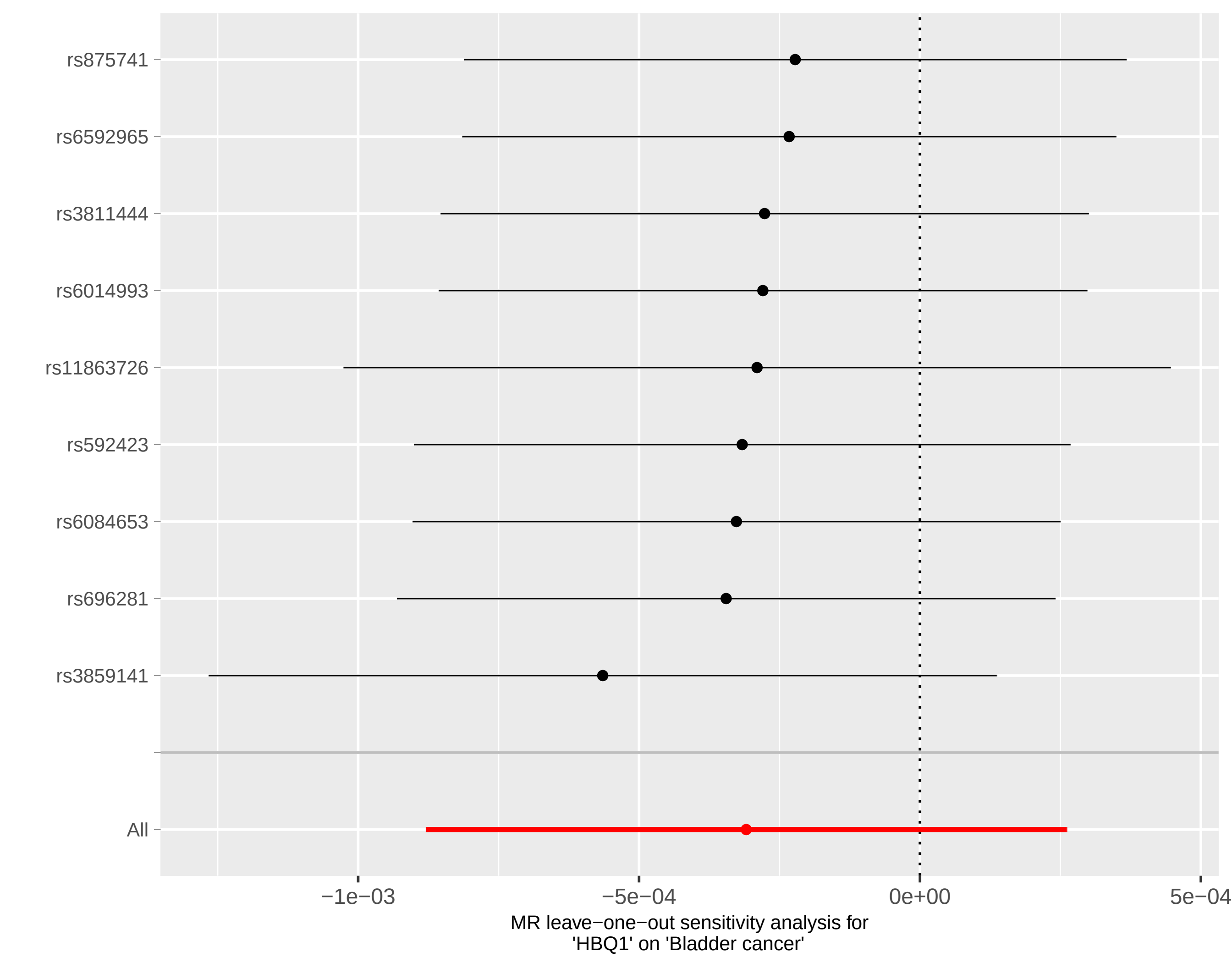

rs875741
rs6592965
rs3811444
rs6014993
rs11863726
rs592423
rs6084653
rs696281
rs3859141
All
−1e−03
−5e−04
0e+00
5e−04
MR leave−one−out sensitivity analysis for
'HBQ1' on 'Bladder cancer'

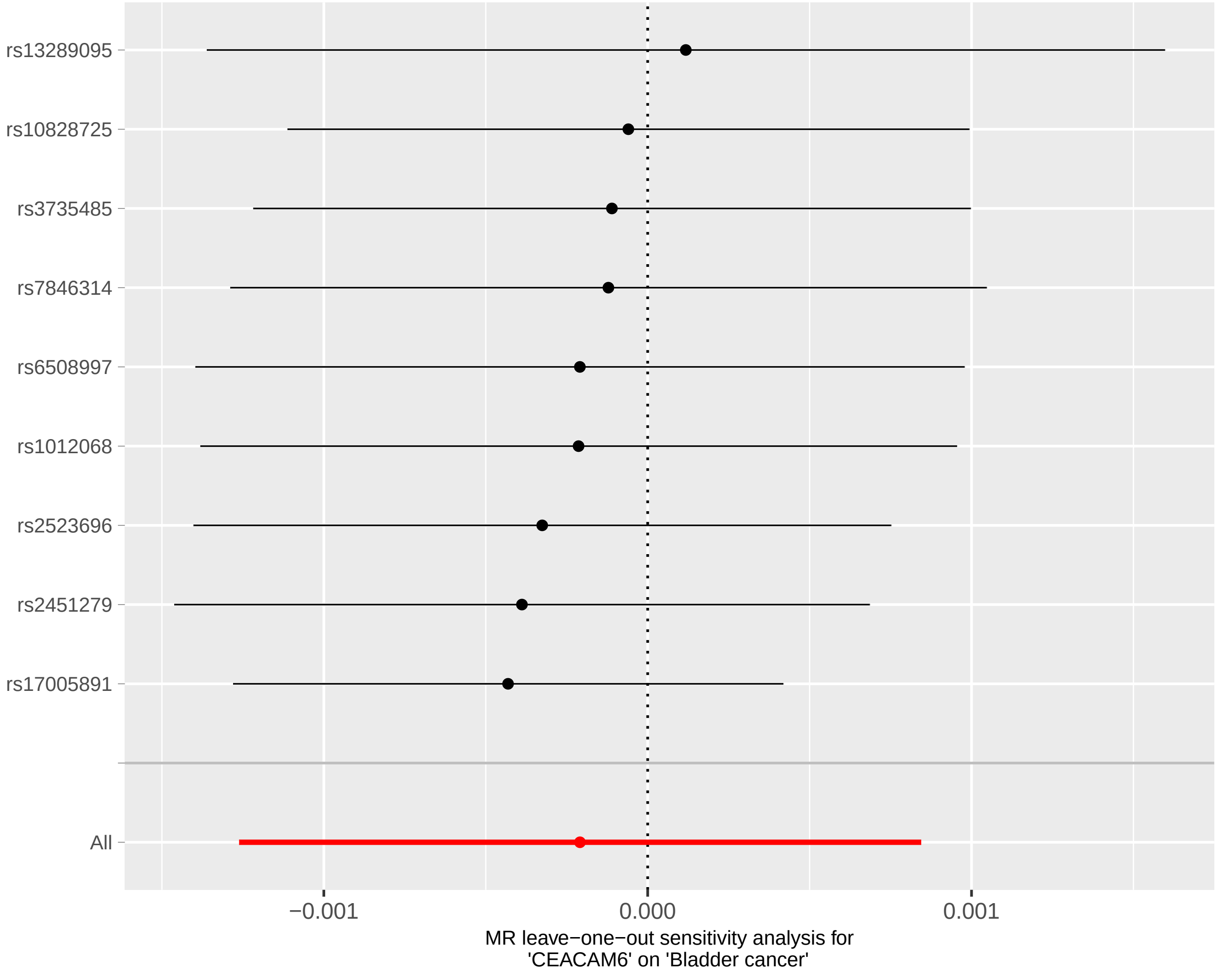

rs13289095
rs10828725
rs3735485
rs7846314
rs6508997
rs1012068
rs2523696
rs2451279
rs17005891
All
−0.001
0.000
0.001
MR leave−one−out sensitivity analysis for
'CEACAM6' on 'Bladder cancer'

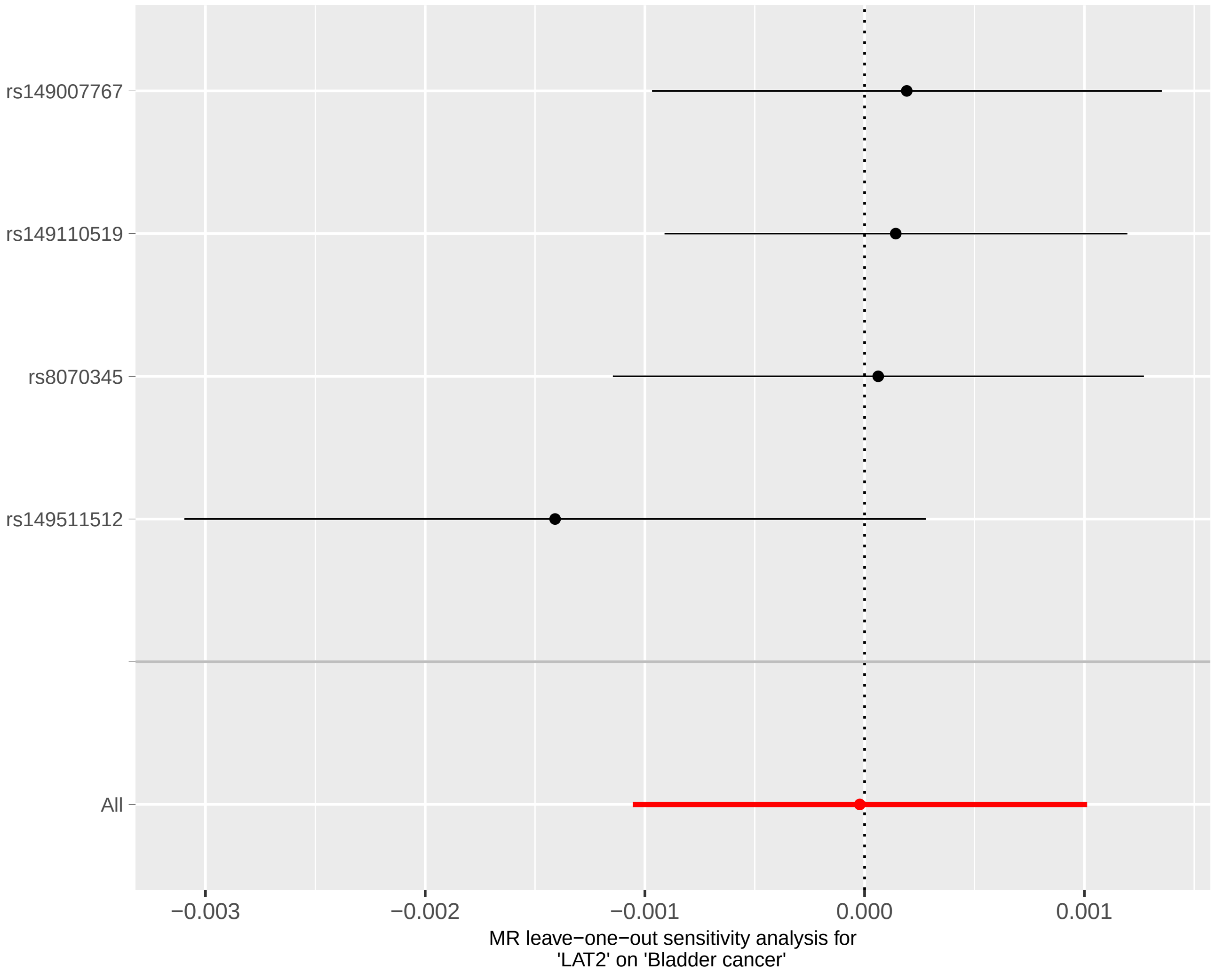
rs149007767
rs149110519
rs8070345
rs149511512
All
−0.003
−0.002
−0.001
0.000
0.001
MR leave−one−out sensitivity analysis for
'LAT2' on 'Bladder cancer'

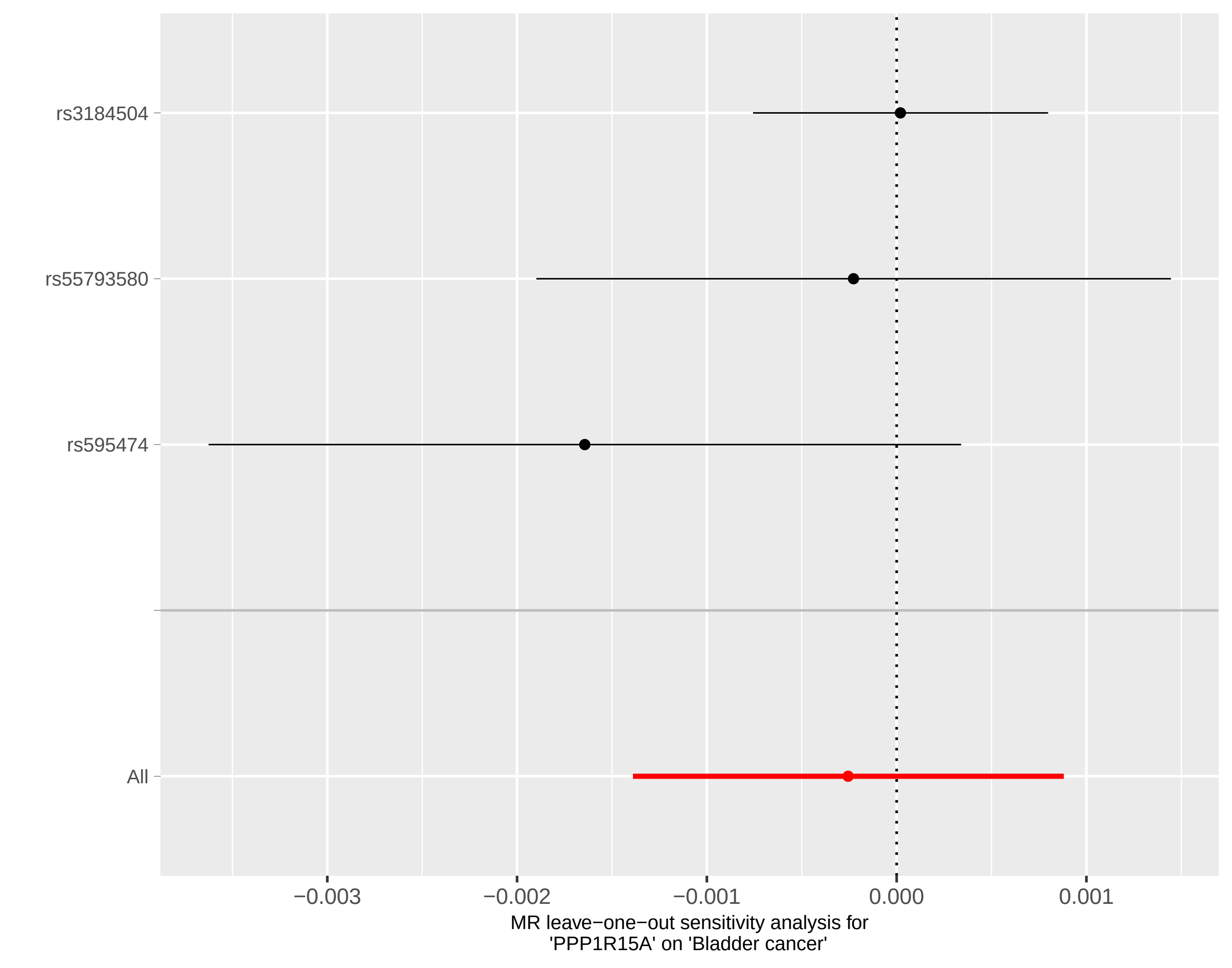
rs3184504
rs55793580
rs595474
All
−0.003
−0.002
−0.001
0.000
0.001
MR leave−one−out sensitivity analysis for
'PPP1R15A' on 'Bladder cancer'

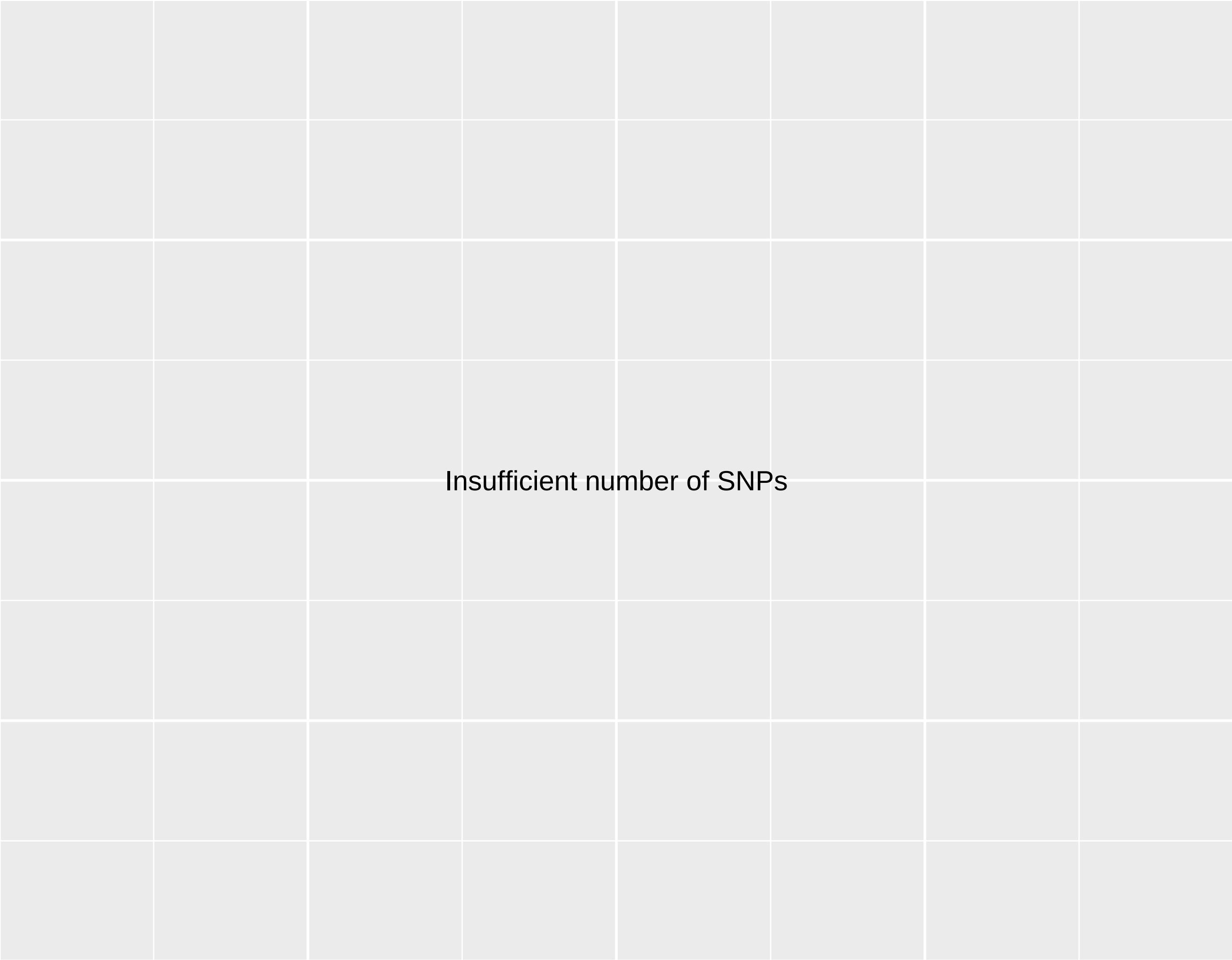
Insufficient number of SNPs

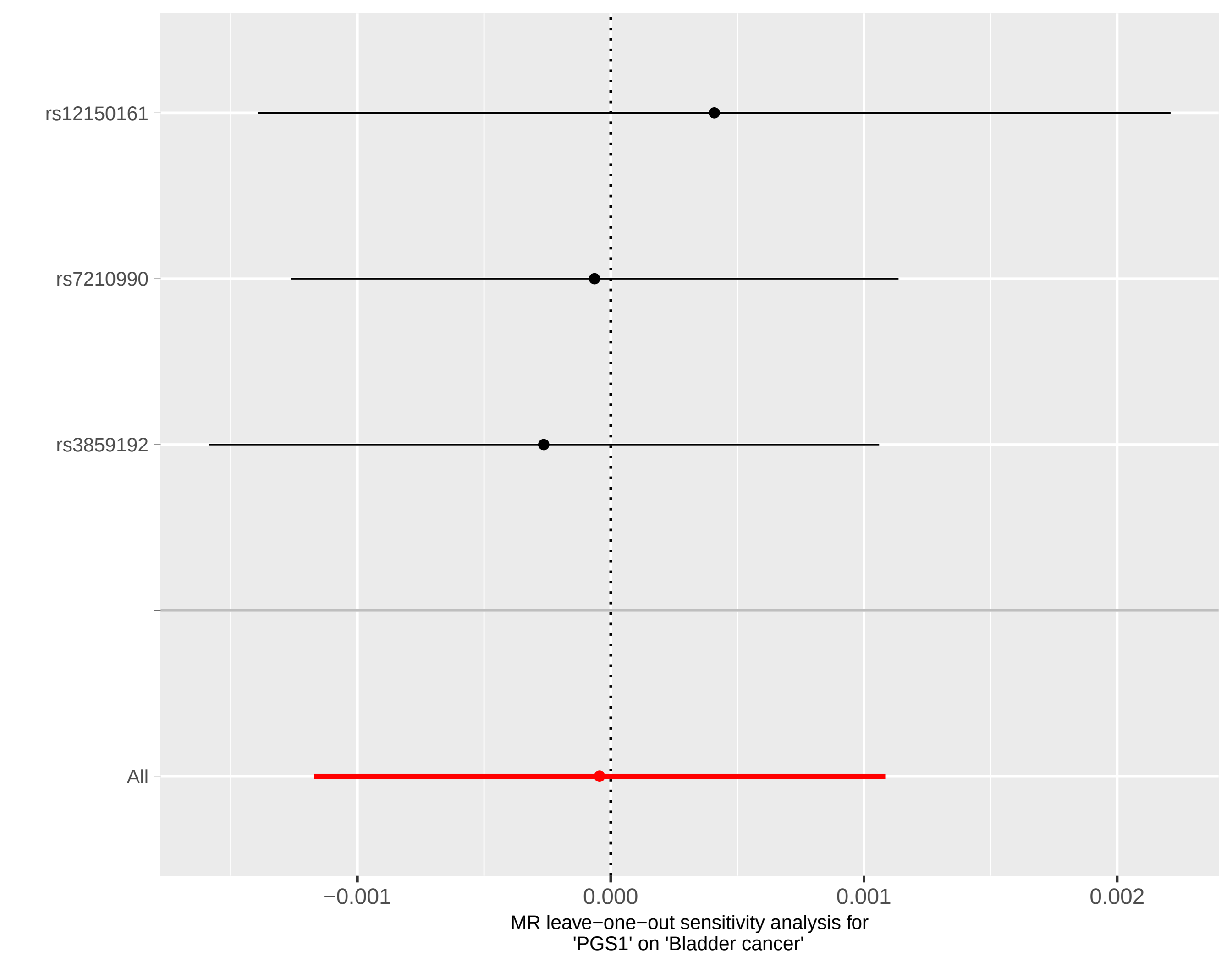

rs12150161
rs7210990
rs3859192
All
−0.001
0.000
0.001
0.002
MR leave−one−out sensitivity analysis for
'PGS1' on 'Bladder cancer'

Insufficient number of SNPs

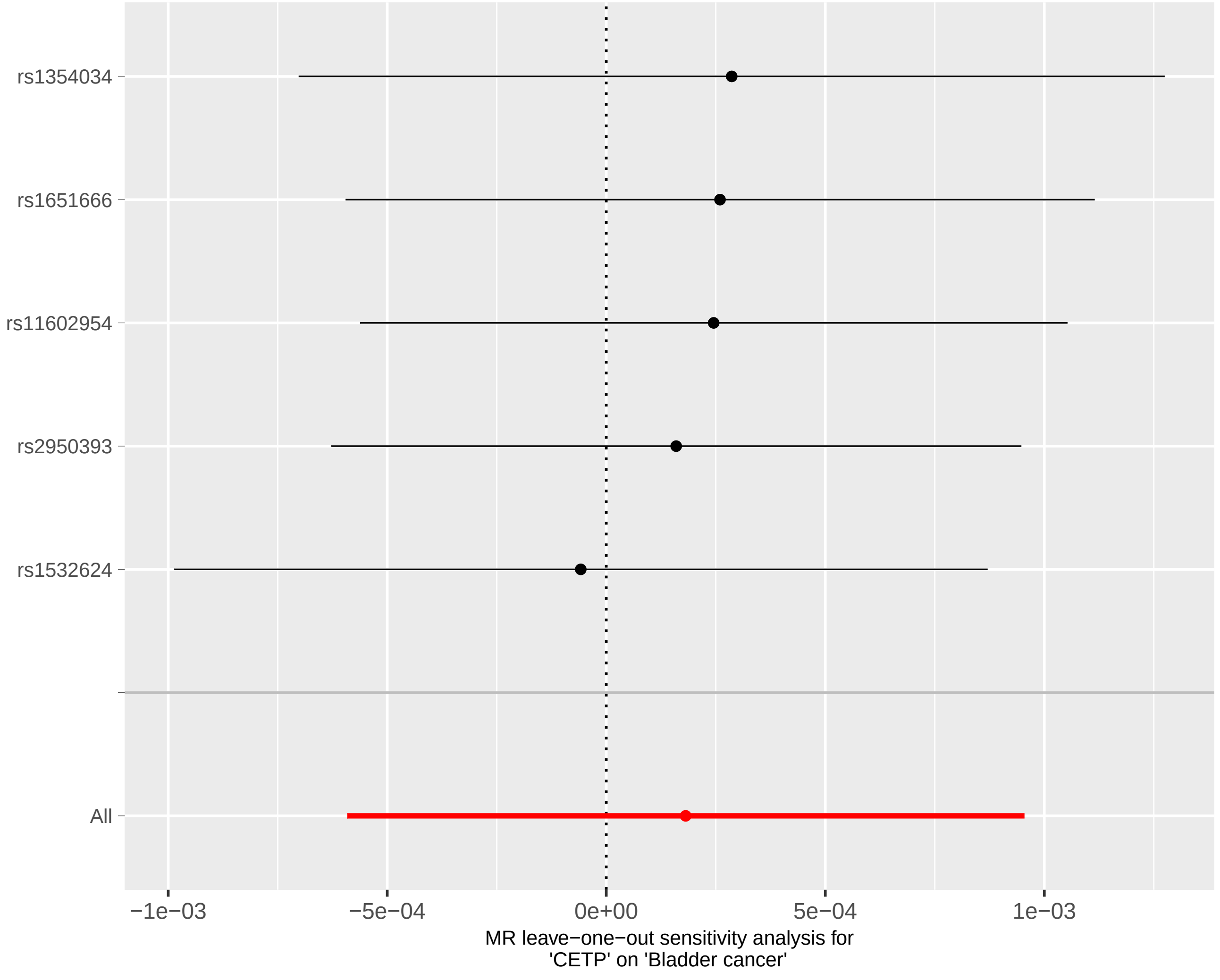

rs1354034
rs1651666
rs11602954
rs2950393
rs1532624
All
−1e−03
−5e−04
0e+00
5e−04
1e−03
MR leave−one−out sensitivity analysis for
'CETP' on 'Bladder cancer'

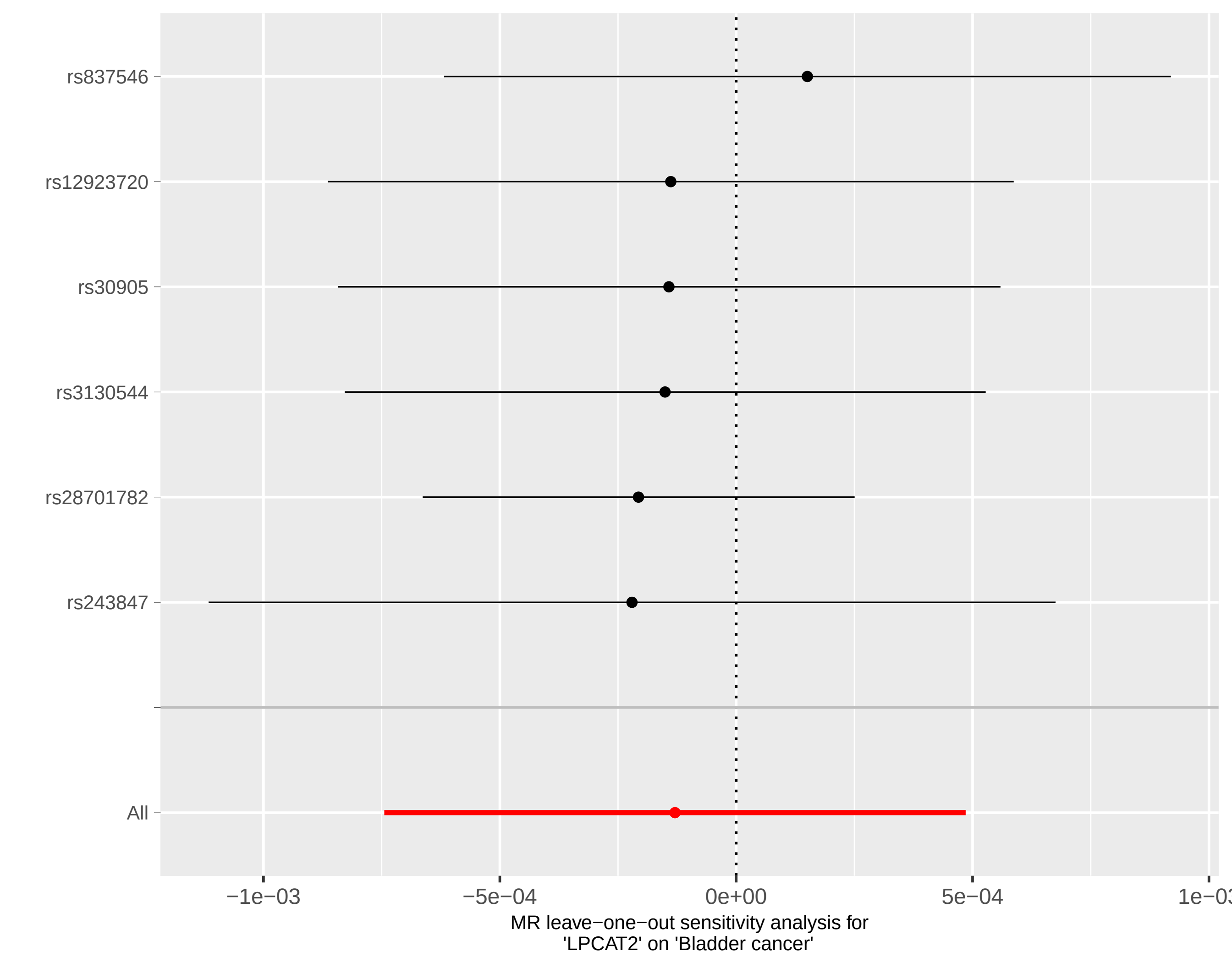
rs837546
rs12923720
rs30905
rs3130544
rs28701782
rs243847
All
−1e−03
−5e−04
0e+00
5e−04
1e−03
MR leave−one−out sensitivity analysis for
'LPCAT2' on 'Bladder cancer'

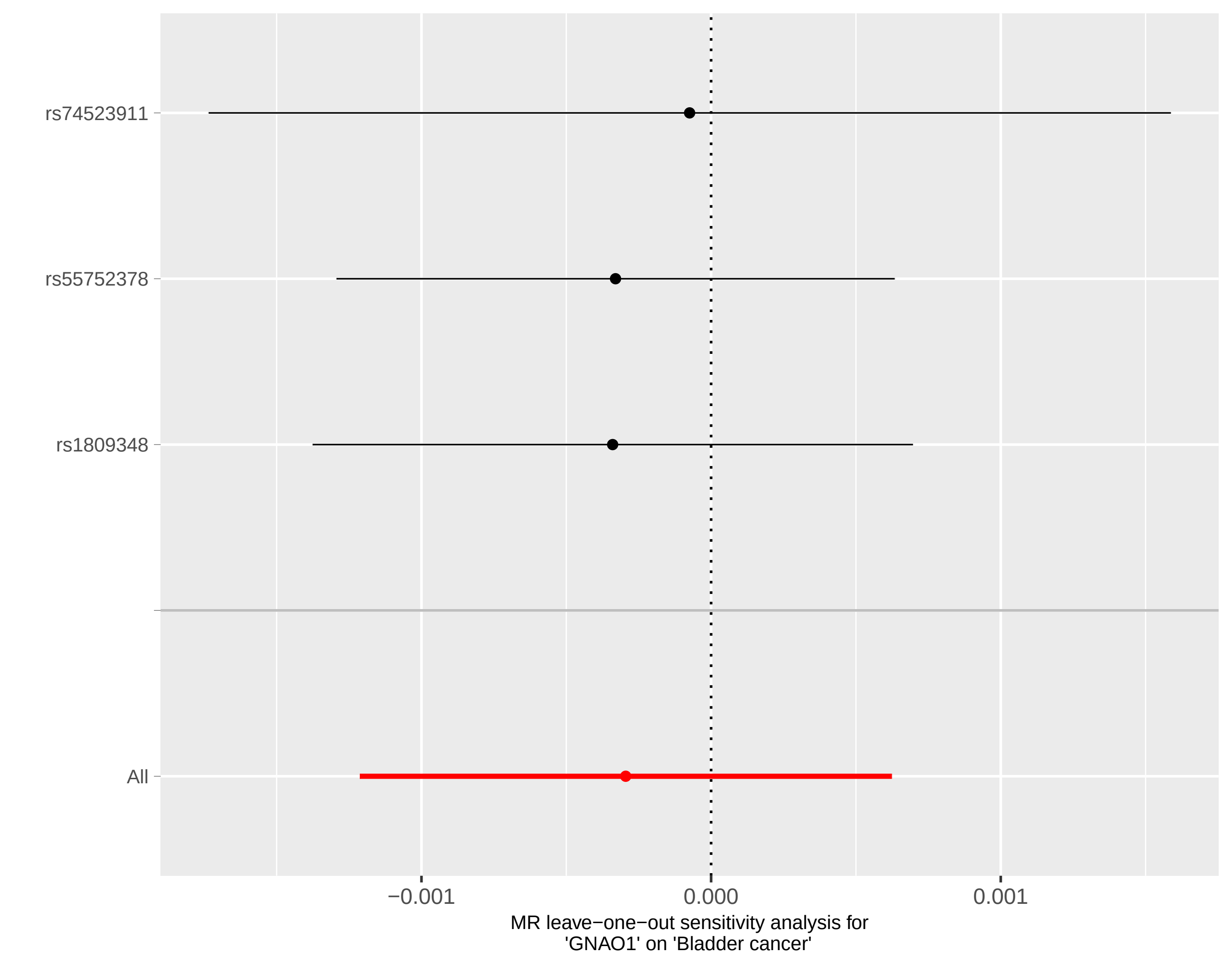
rs74523911
rs55752378
rs1809348
All
−0.001
0.000
0.001
MR leave−one−out sensitivity analysis for
'GNAO1' on 'Bladder cancer'

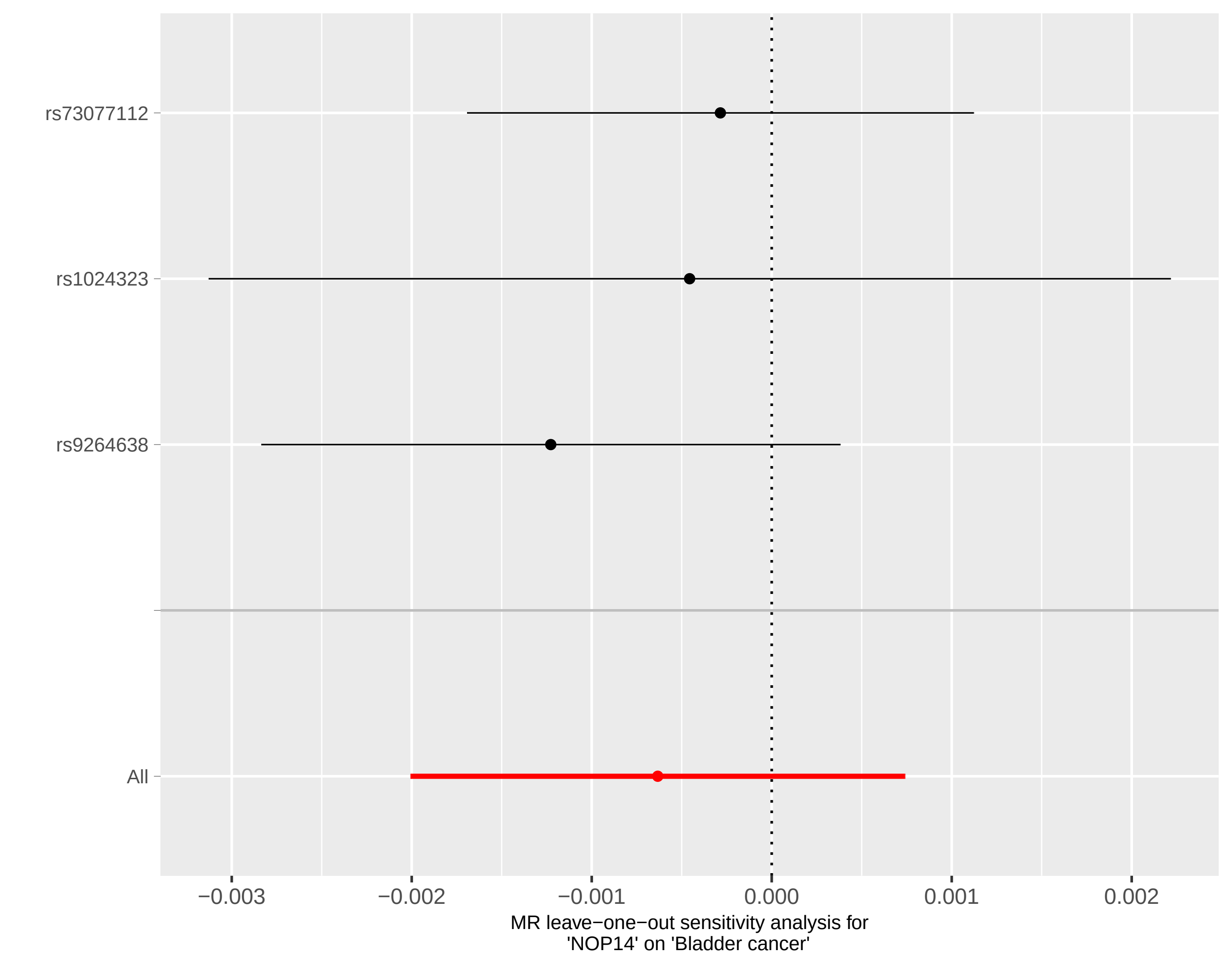
rs73077112
rs1024323
rs9264638
All
−0.003
−0.002
−0.001
0.000
0.001
0.002
MR leave−one−out sensitivity analysis for
'NOP14' on 'Bladder cancer'

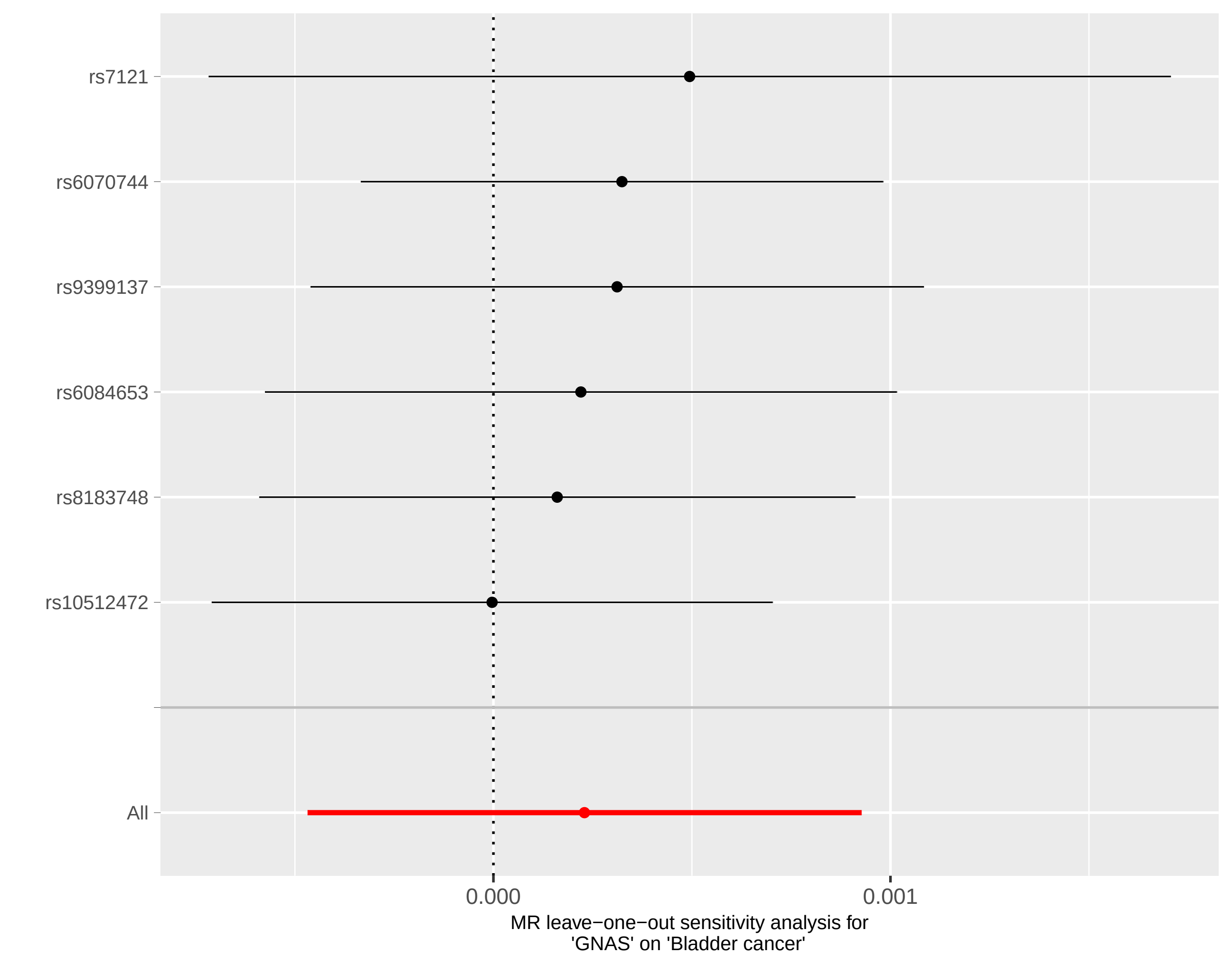

rs7121
rs6070744
rs9399137
rs6084653
rs8183748
rs10512472
All
0.000
0.001
MR leave−one−out sensitivity analysis for
'GNAS' on 'Bladder cancer'

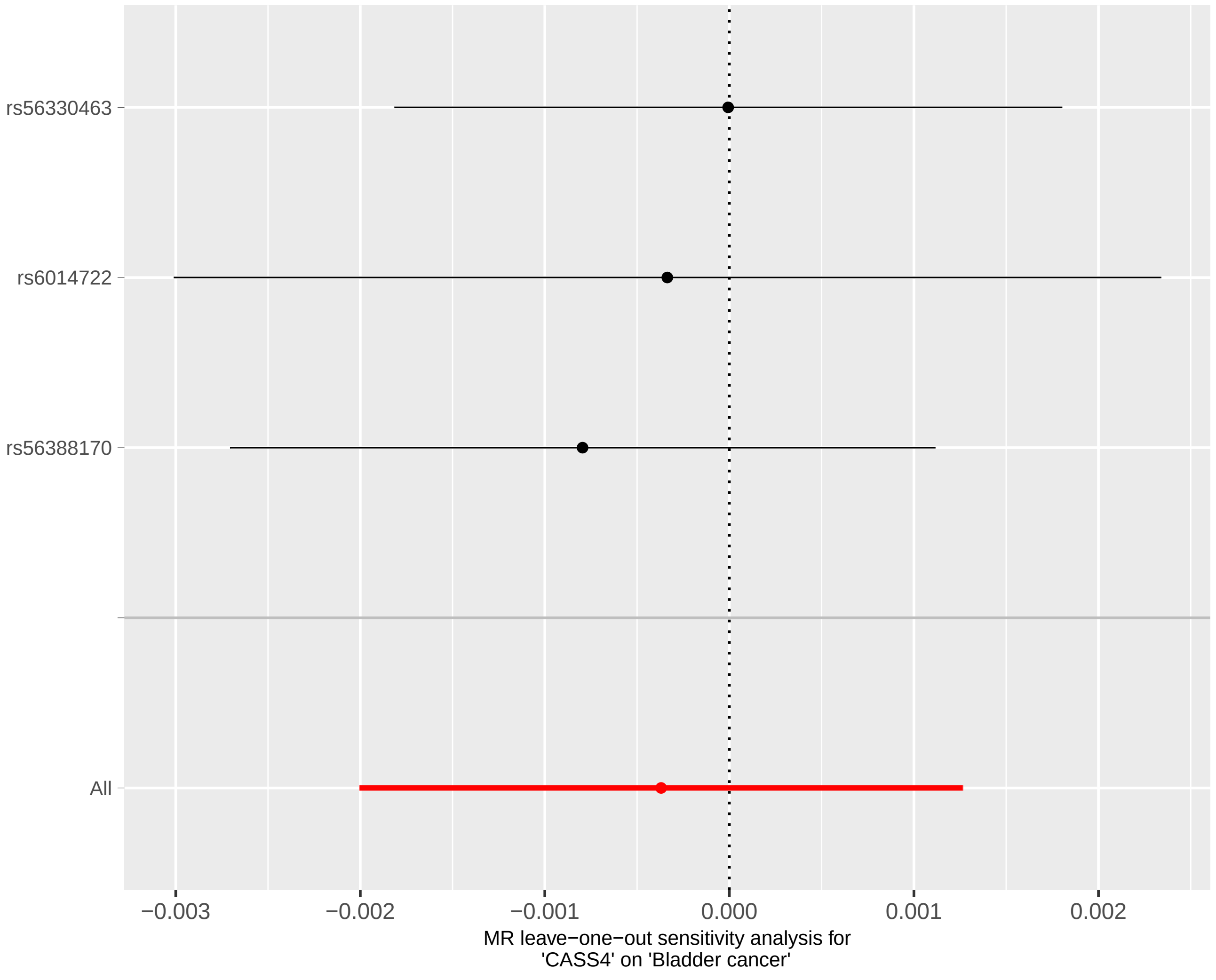
rs56330463
rs6014722
rs56388170
All
−0.003
−0.002
−0.001
0.000
0.001
0.002
MR leave−one−out sensitivity analysis for
'CASS4' on 'Bladder cancer'

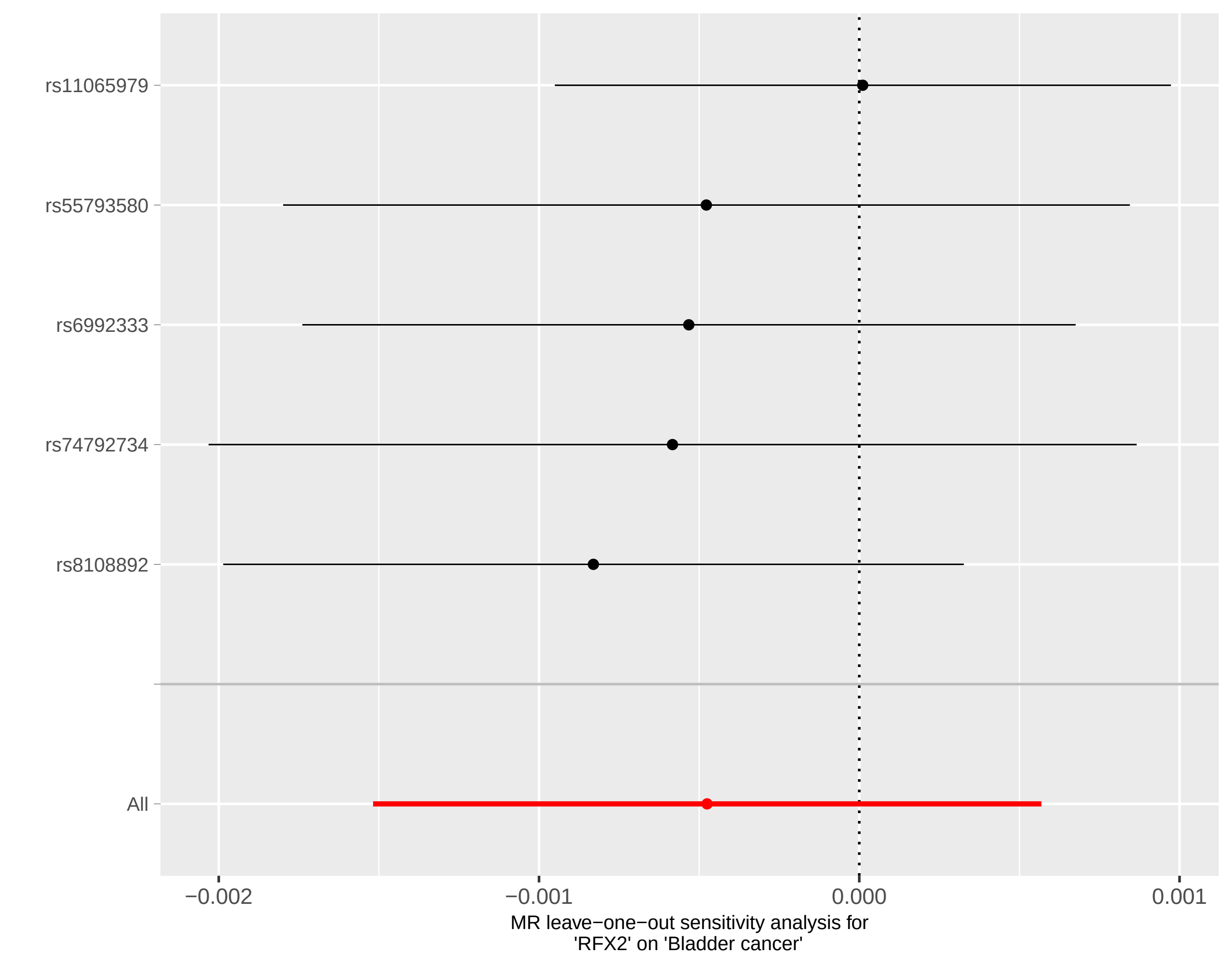
rs11065979
rs55793580
rs6992333
rs74792734
rs8108892
All
−0.002
−0.001
0.000
0.001
MR leave−one−out sensitivity analysis for
'RFX2' on 'Bladder cancer'

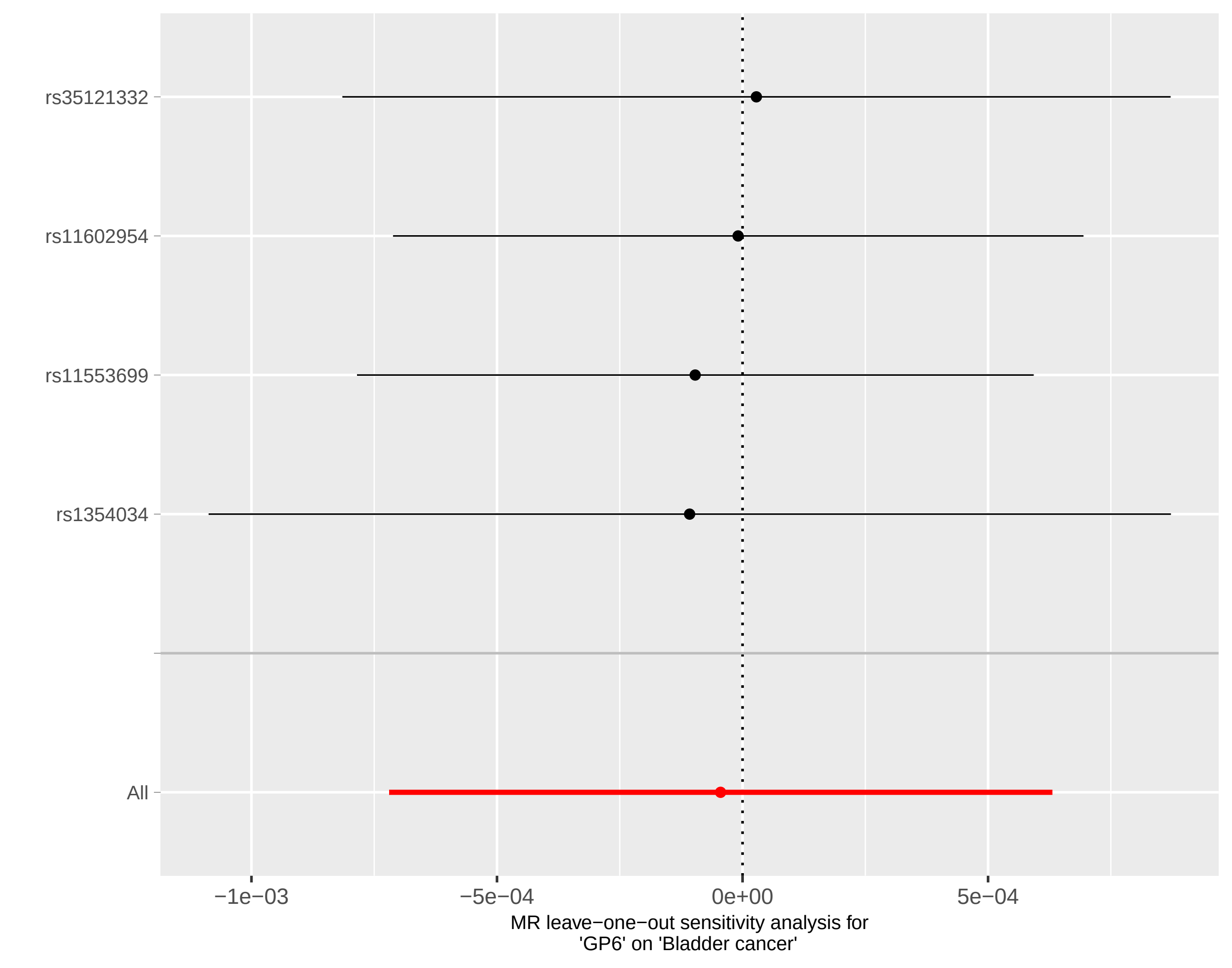
rs35121332
rs11602954
rs11553699
rs1354034
All
−1e−03
−5e−04
0e+00
5e−04
MR leave−one−out sensitivity analysis for
'GP6' on 'Bladder cancer'

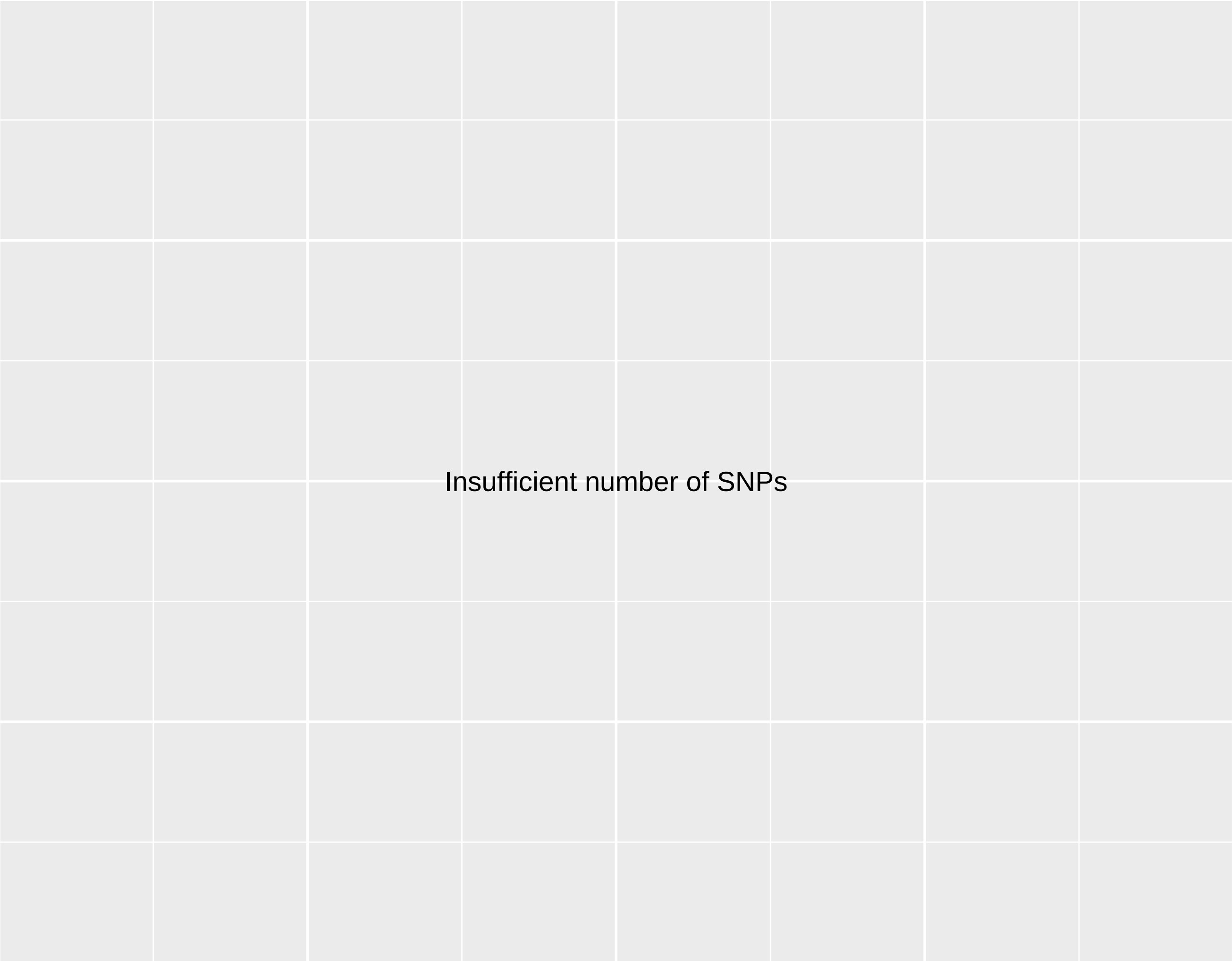
Insufficient number of SNPs

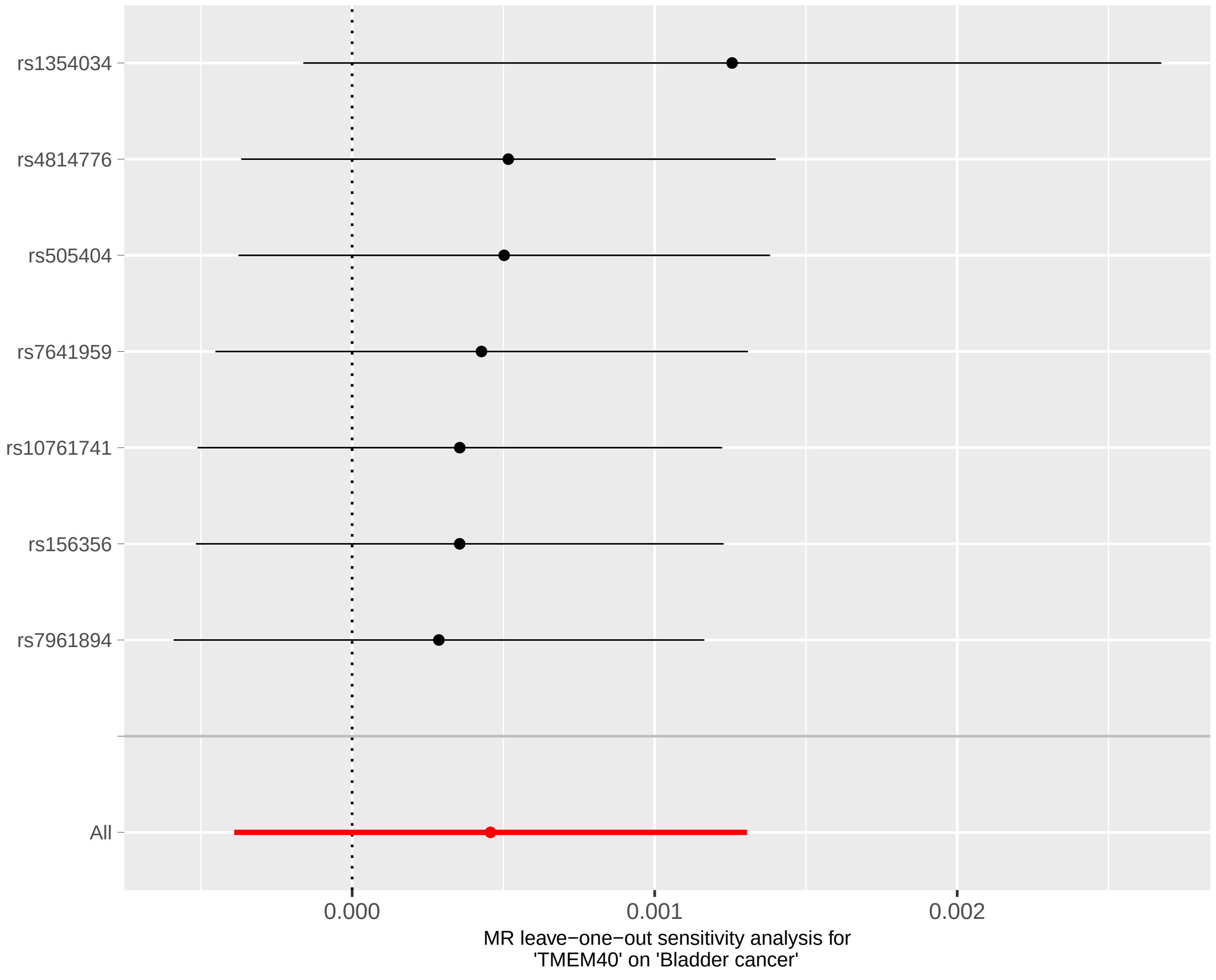

rs1354034
rs4814776
rs505404
rs7641959
rs10761741
rs156356
rs7961894
All
0.000
0.001
0.002
MR leave−one−out sensitivity analysis for
'TMEM40' on 'Bladder cancer'

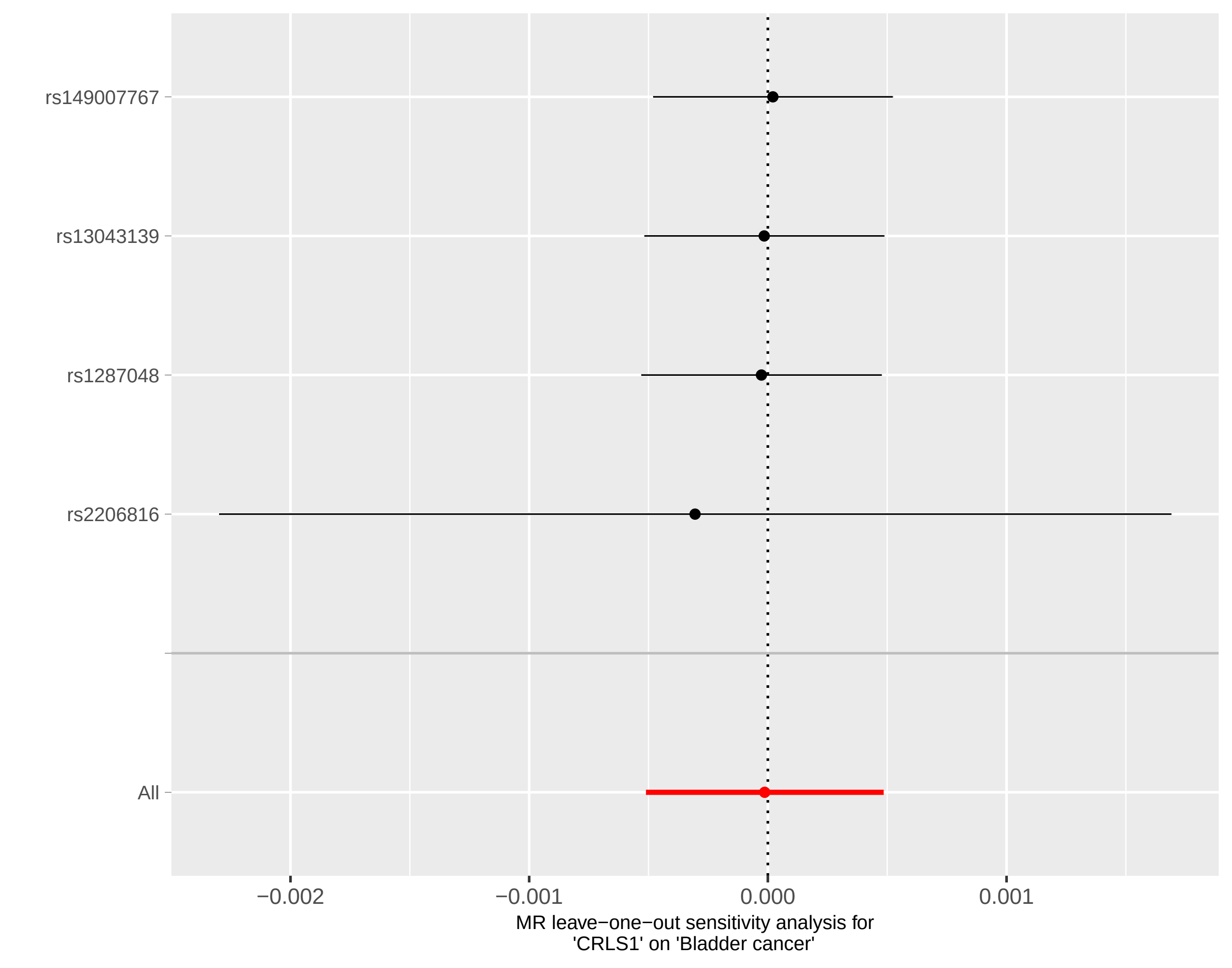
rs149007767
rs13043139
rs1287048
rs2206816
All
−0.002
−0.001
0.000
0.001
MR leave−one−out sensitivity analysis for
'CRLS1' on 'Bladder cancer'

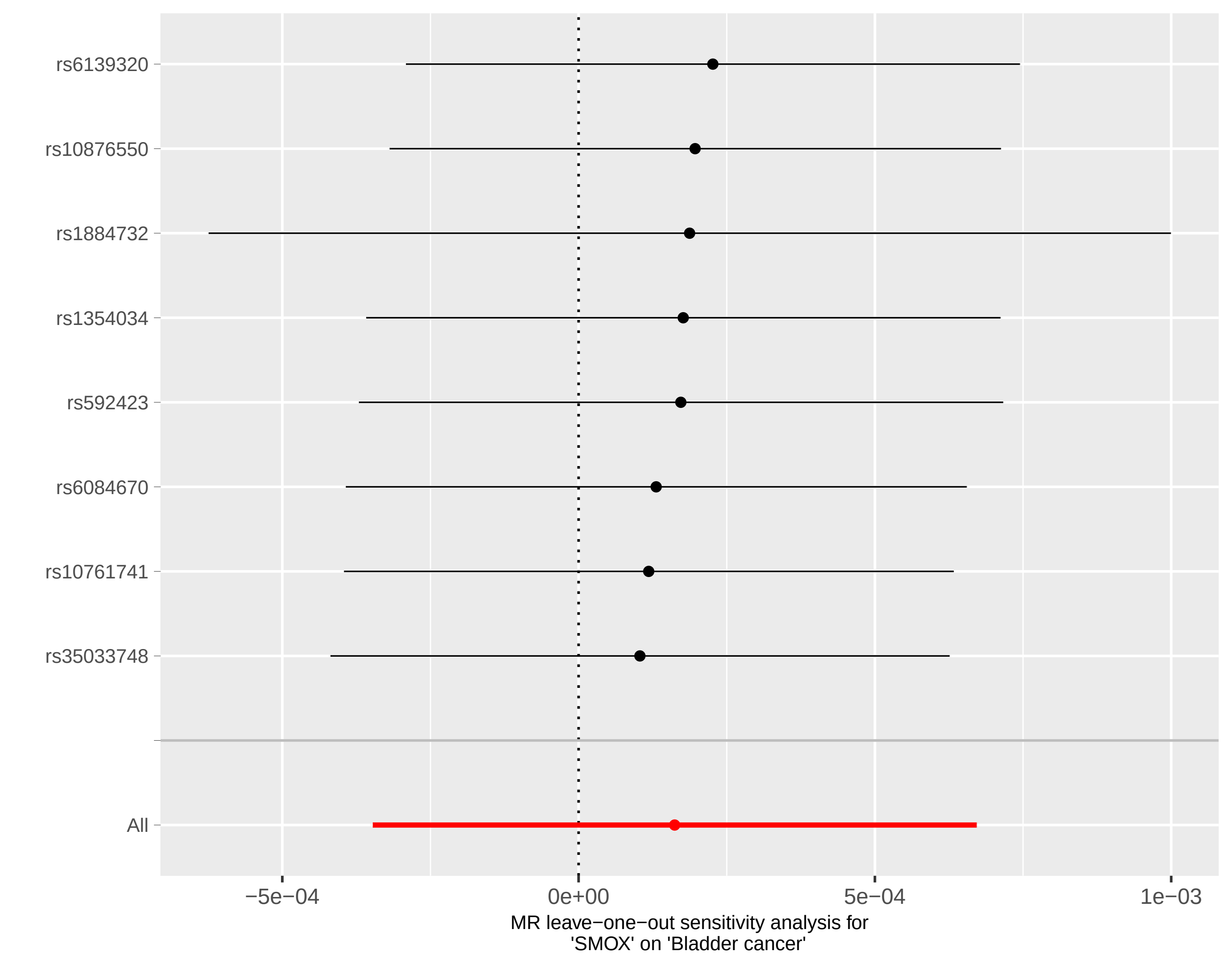
rs6139320
rs10876550
rs1884732
rs1354034
rs592423
rs6084670
rs10761741
rs35033748
All
−5e−04
0e+00
5e−04
1e−03
MR leave−one−out sensitivity analysis for
'SMOX' on 'Bladder cancer'

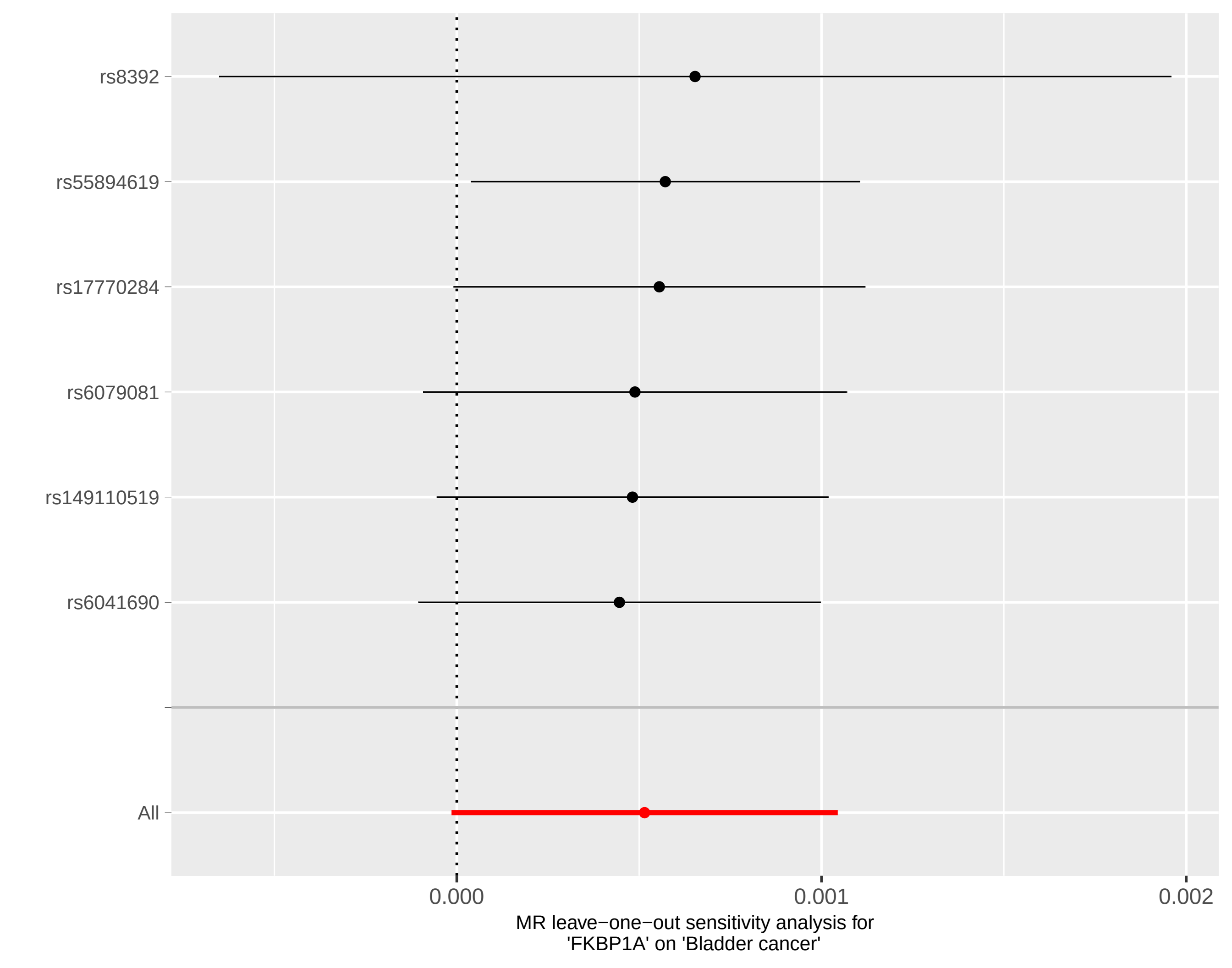
rs8392
rs55894619
rs17770284
rs6079081
rs149110519
rs6041690
All
0.000
0.001
0.002
MR leave−one−out sensitivity analysis for
'FKBP1A' on 'Bladder cancer'

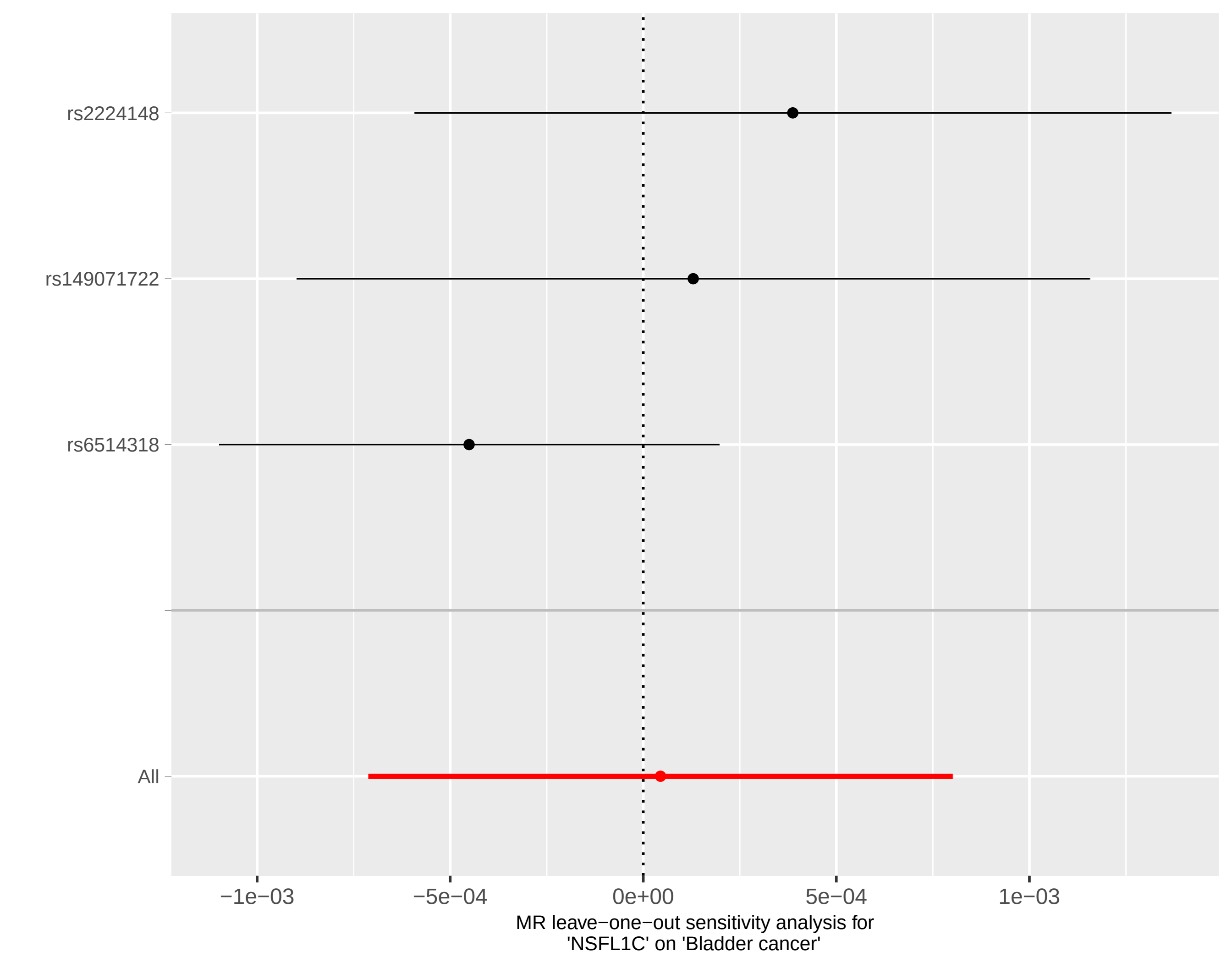
rs2224148
rs149071722
rs6514318
All
−1e−03
−5e−04
0e+00
5e−04
1e−03
MR leave−one−out sensitivity analysis for
'NSFL1C' on 'Bladder cancer'

Insufficient number of SNPs

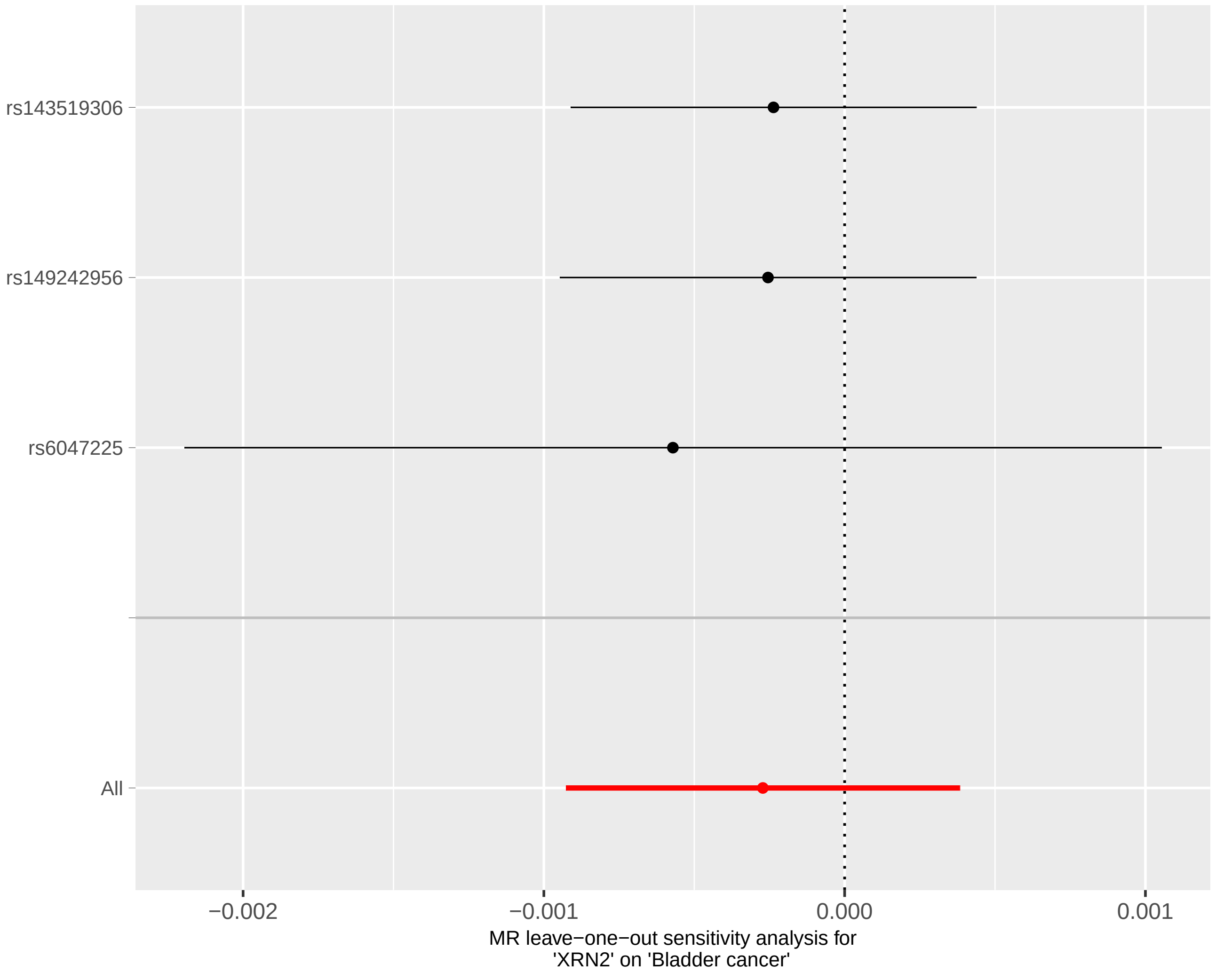
rs143519306
rs149242956
rs6047225
All
−0.002
−0.001
0.000
0.001
MR leave−one−out sensitivity analysis for
'XRN2' on 'Bladder cancer'

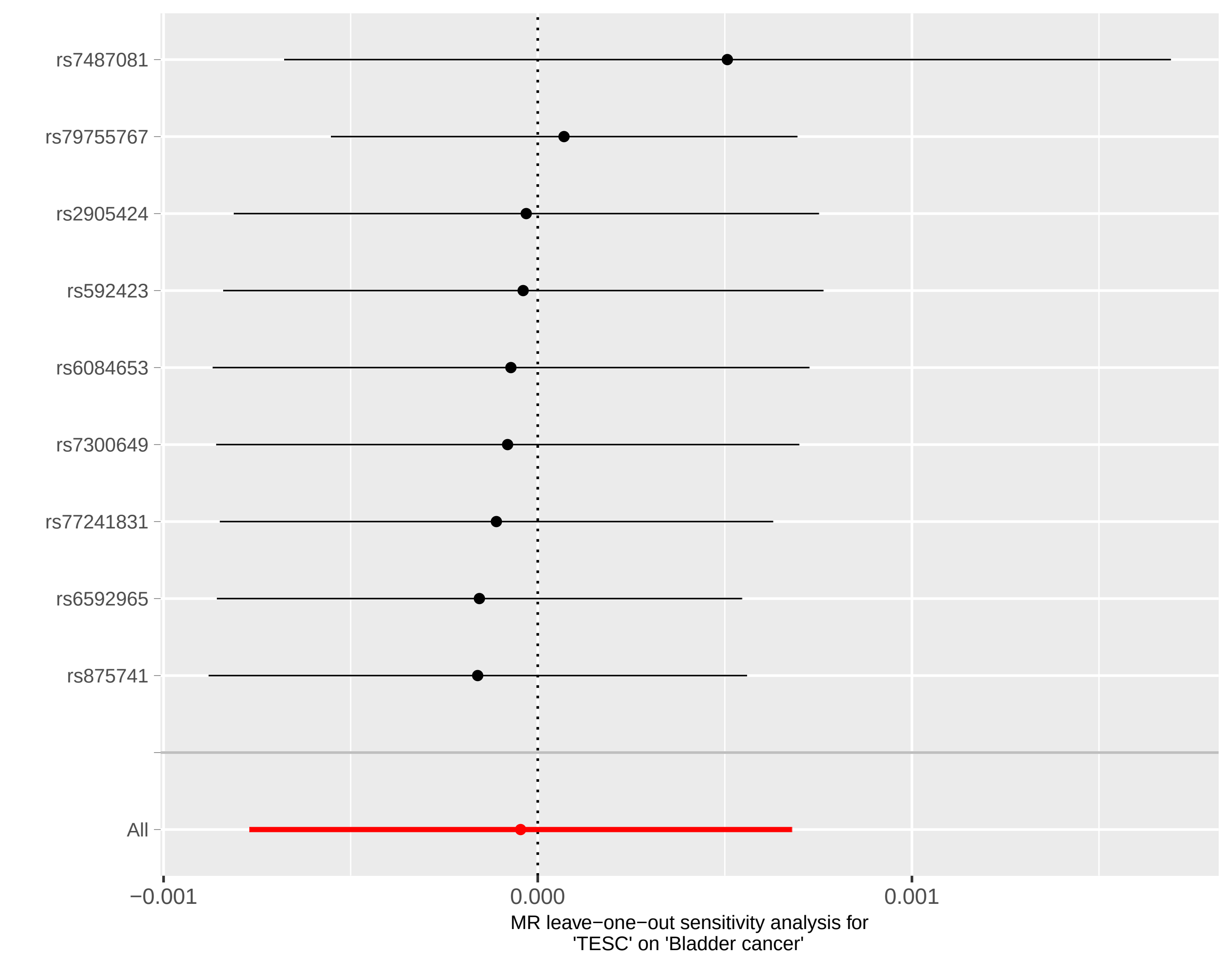

rs7487081
rs79755767
rs2905424
rs592423
rs6084653
rs7300649
rs77241831
rs6592965
rs875741
All
−0.001
0.000
0.001
MR leave−one−out sensitivity analysis for
'TESC' on 'Bladder cancer'

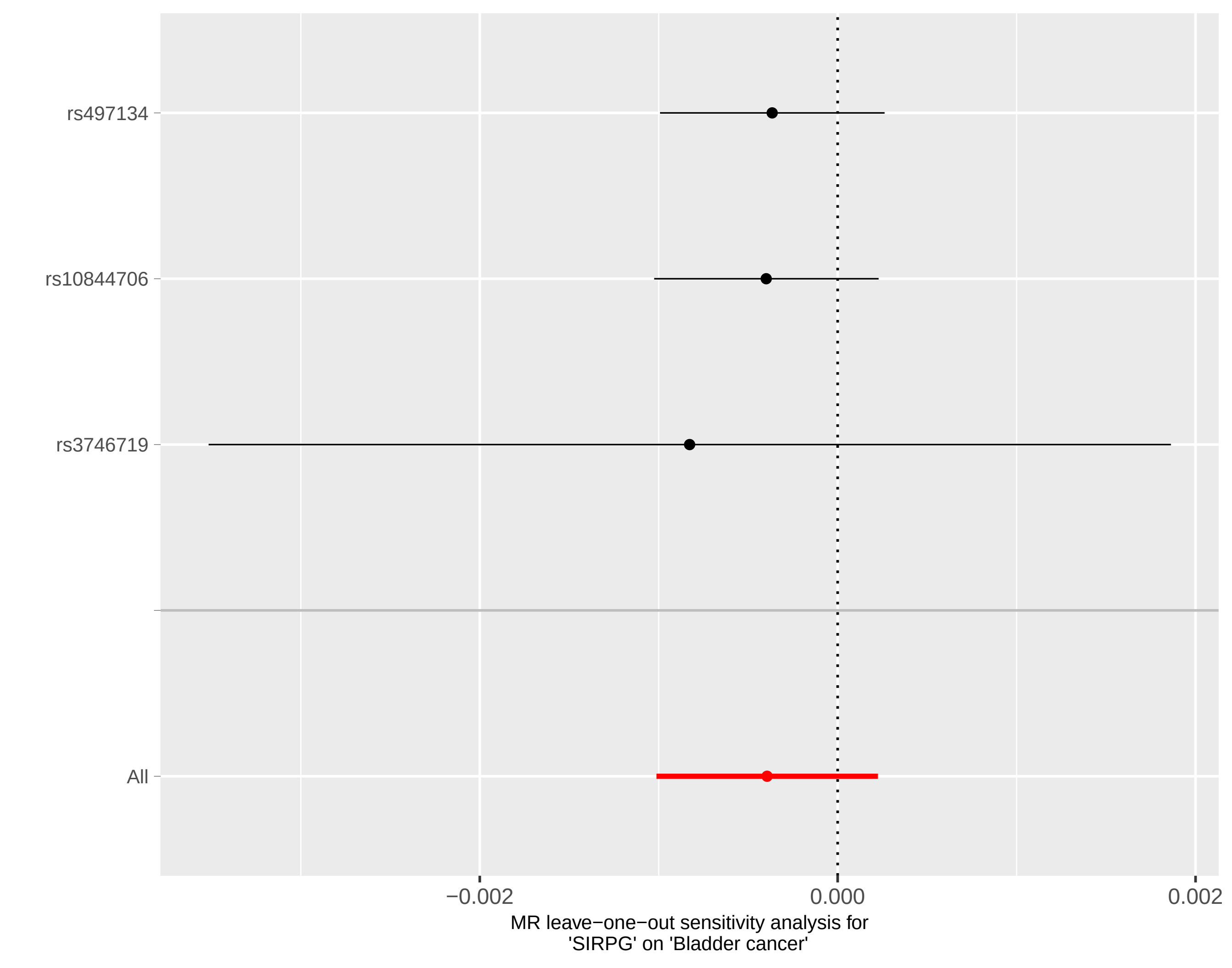
rs497134
rs10844706
rs3746719
All
−0.002
0.000
0.002
MR leave−one−out sensitivity analysis for
'SIRPG' on 'Bladder cancer'

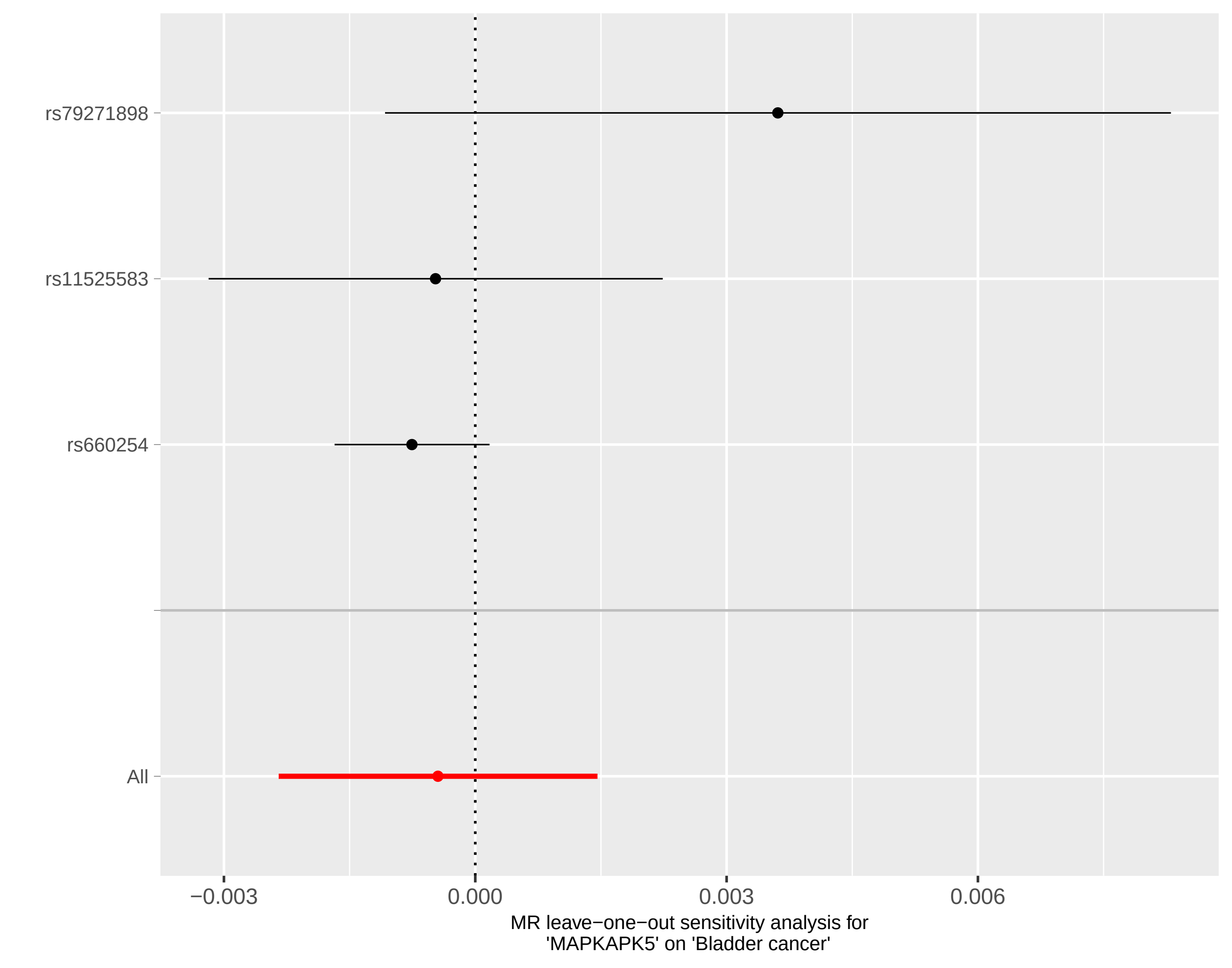

rs79271898
rs11525583
rs660254
All
−0.003
0.000
0.003
0.006
MR leave−one−out sensitivity analysis for
'MAPKAPK5' on 'Bladder cancer'

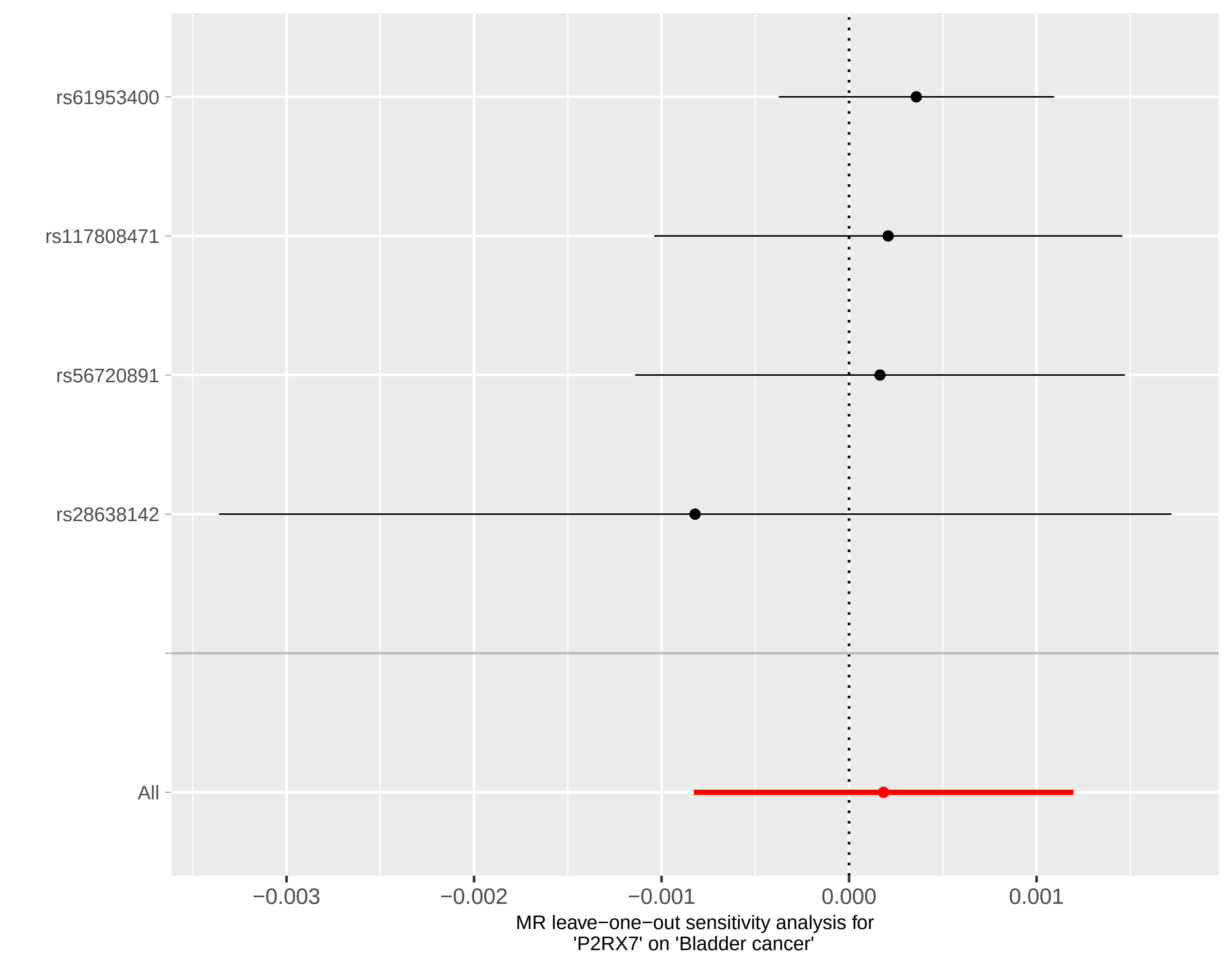
rs61953400
rs117808471
rs56720891
rs28638142
All
−0.003
−0.002
−0.001
0.000
0.001
MR leave−one−out sensitivity analysis for
'P2RX7' on 'Bladder cancer'

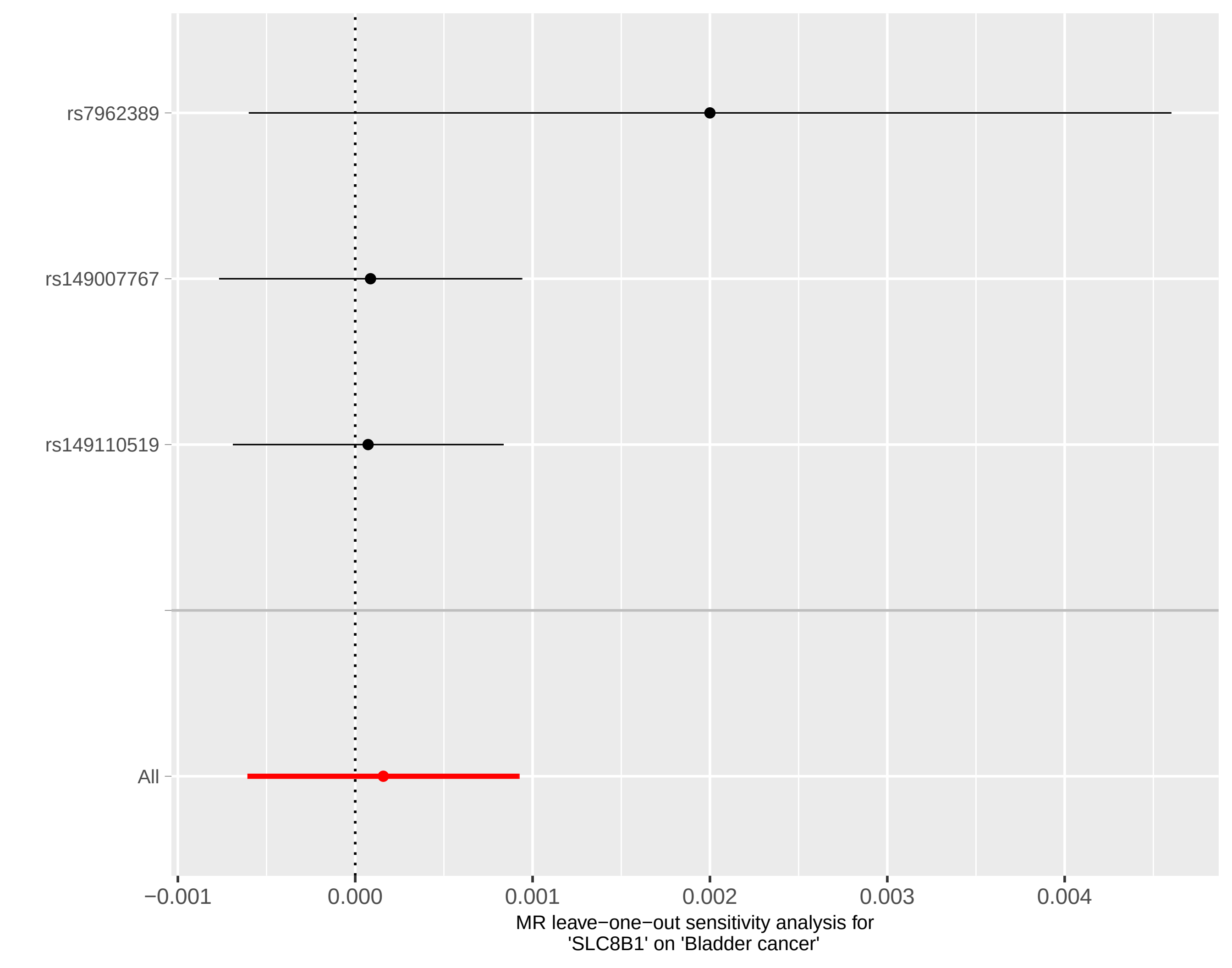
rs7962389
rs149007767
rs149110519
All
−0.001
0.000
0.001
0.002
0.003
0.004
MR leave−one−out sensitivity analysis for
'SLC8B1' on 'Bladder cancer'

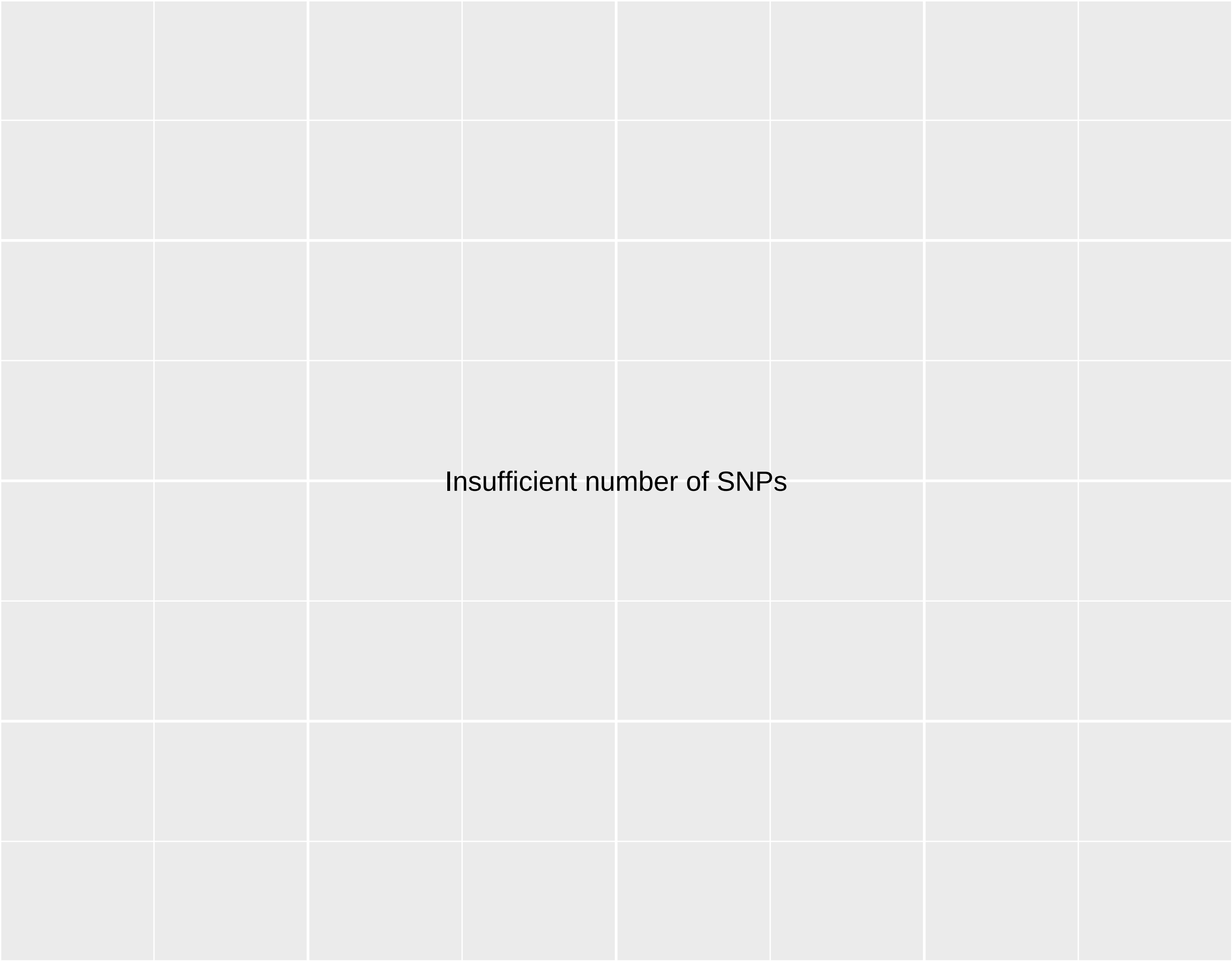
Insufficient number of SNPs

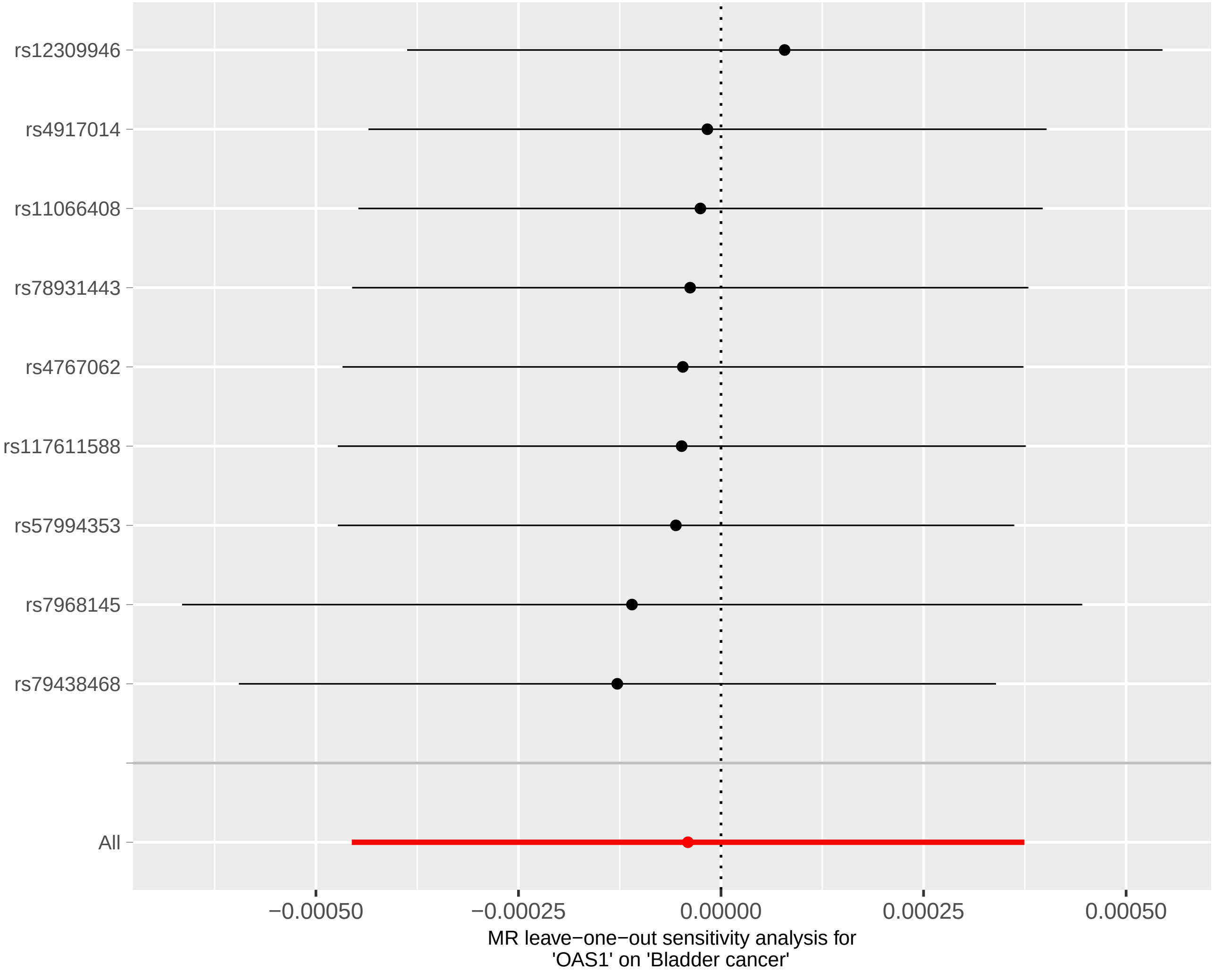

rs12309946
rs4917014
rs11066408
rs78931443
rs4767062
rs117611588
rs57994353
rs7968145
rs79438468
All
−0.00050
−0.00025
0.00000
0.00025
0.00050
MR leave−one−out sensitivity analysis for
'OAS1' on 'Bladder cancer'

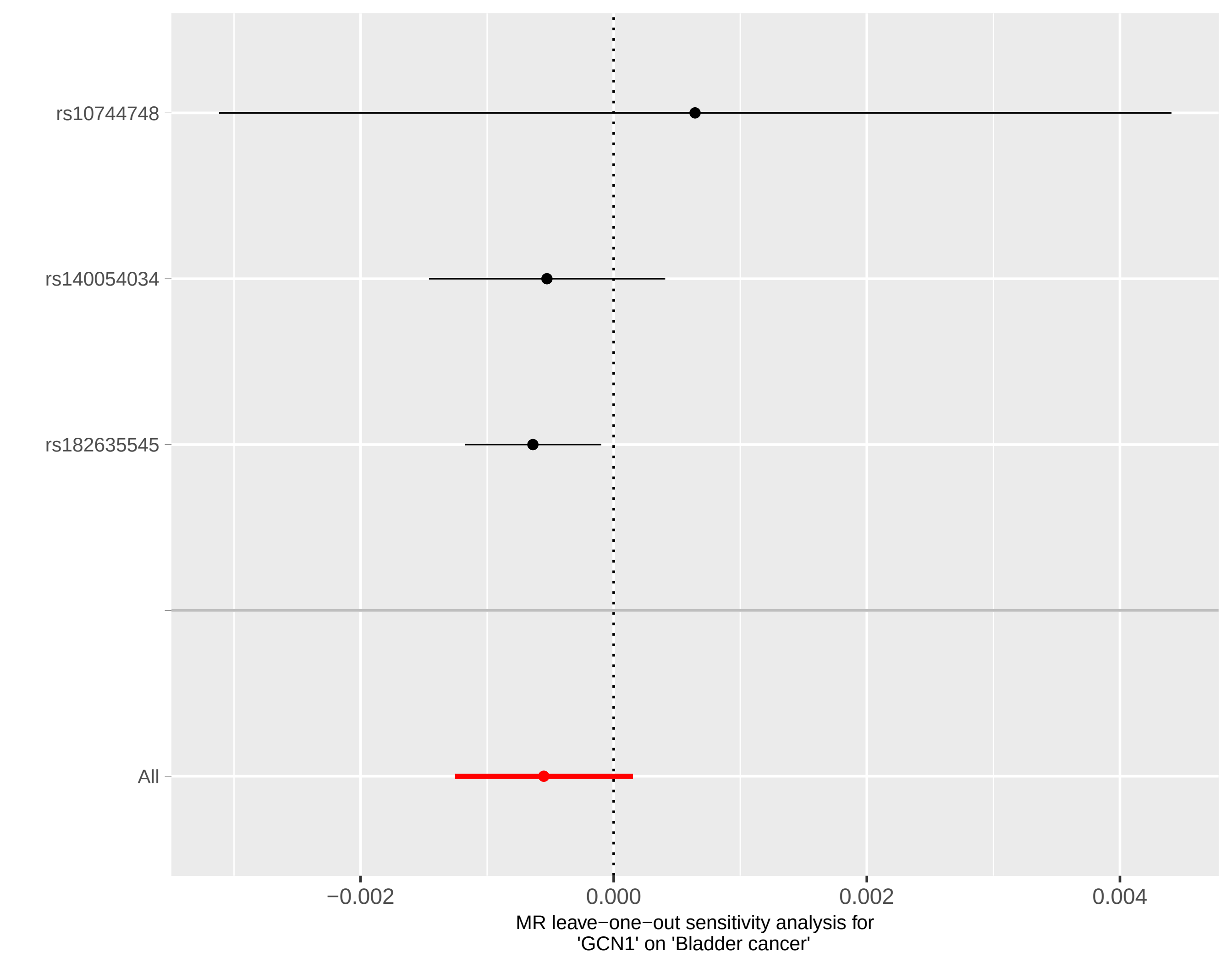
rs10744748
rs140054034
rs182635545
All
−0.002
0.000
0.002
0.004
MR leave−one−out sensitivity analysis for
'GCN1' on 'Bladder cancer'

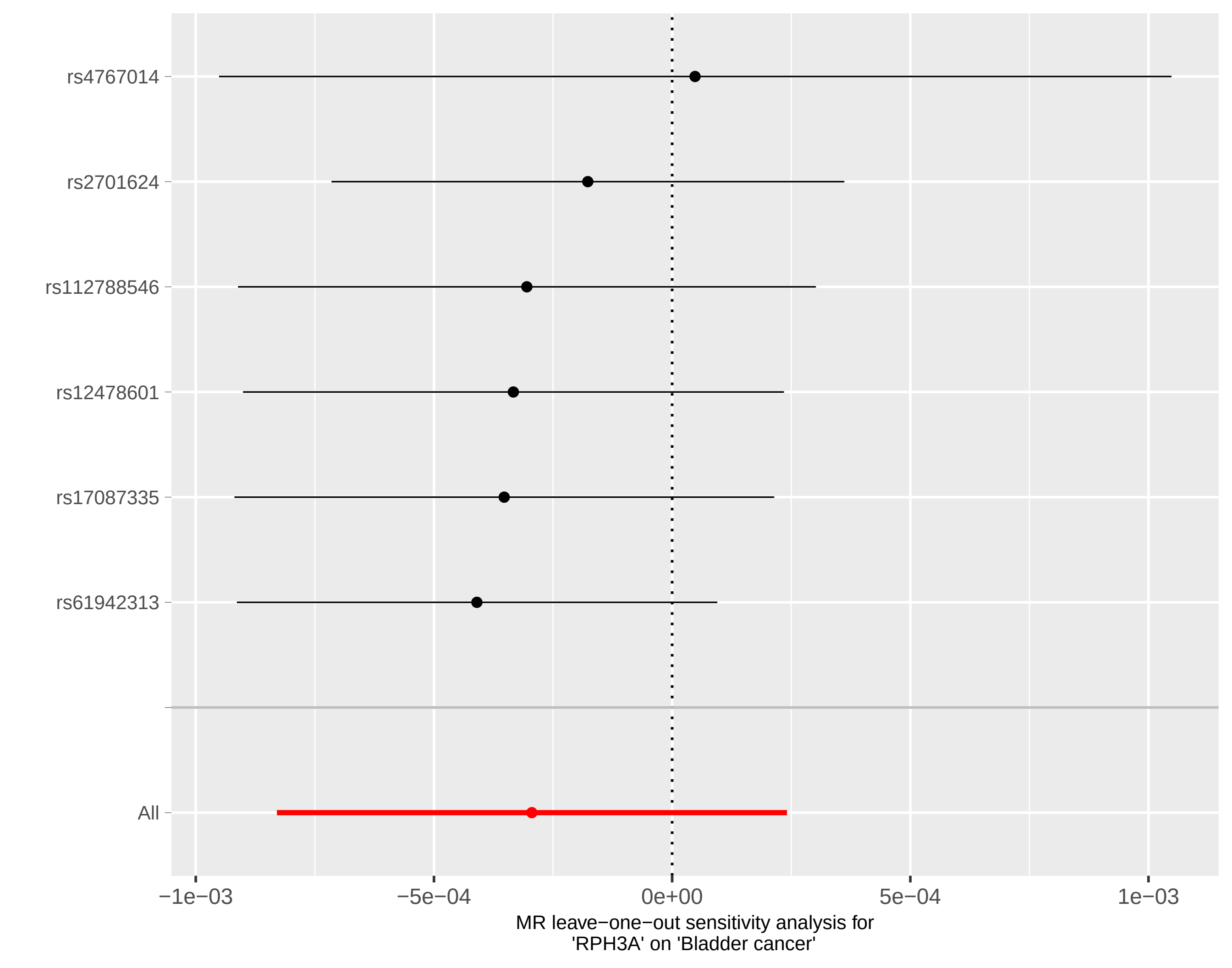

rs4767014
rs2701624
rs112788546
rs12478601
rs17087335
rs61942313
All
−1e−03
−5e−04
0e+00
5e−04
1e−03
MR leave−one−out sensitivity analysis for
'RPH3A' on 'Bladder cancer'

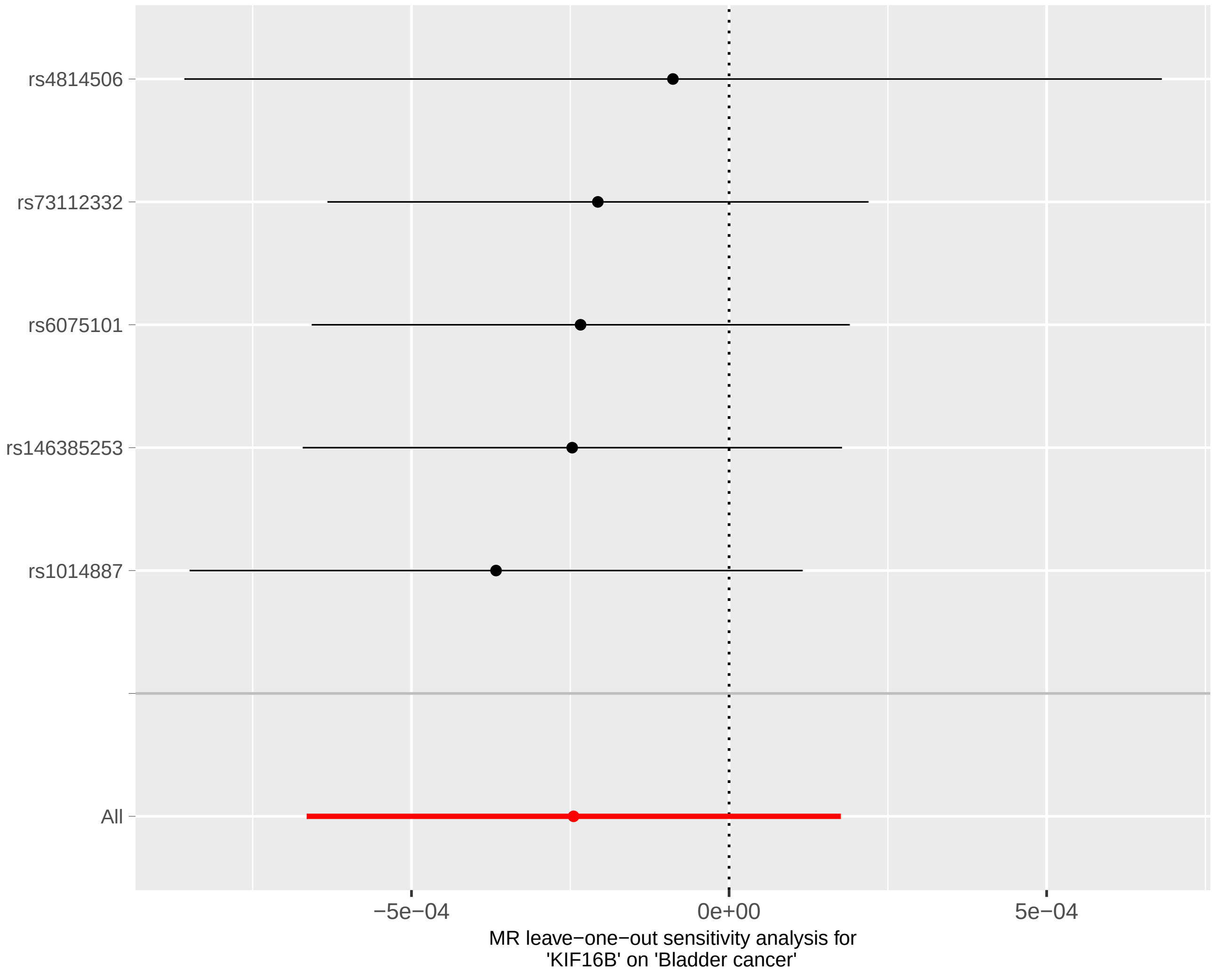
rs4814506
rs73112332
rs6075101
rs146385253
rs1014887
All
−5e−04
0e+00
5e−04
MR leave−one−out sensitivity analysis for
'KIF16B' on 'Bladder cancer'

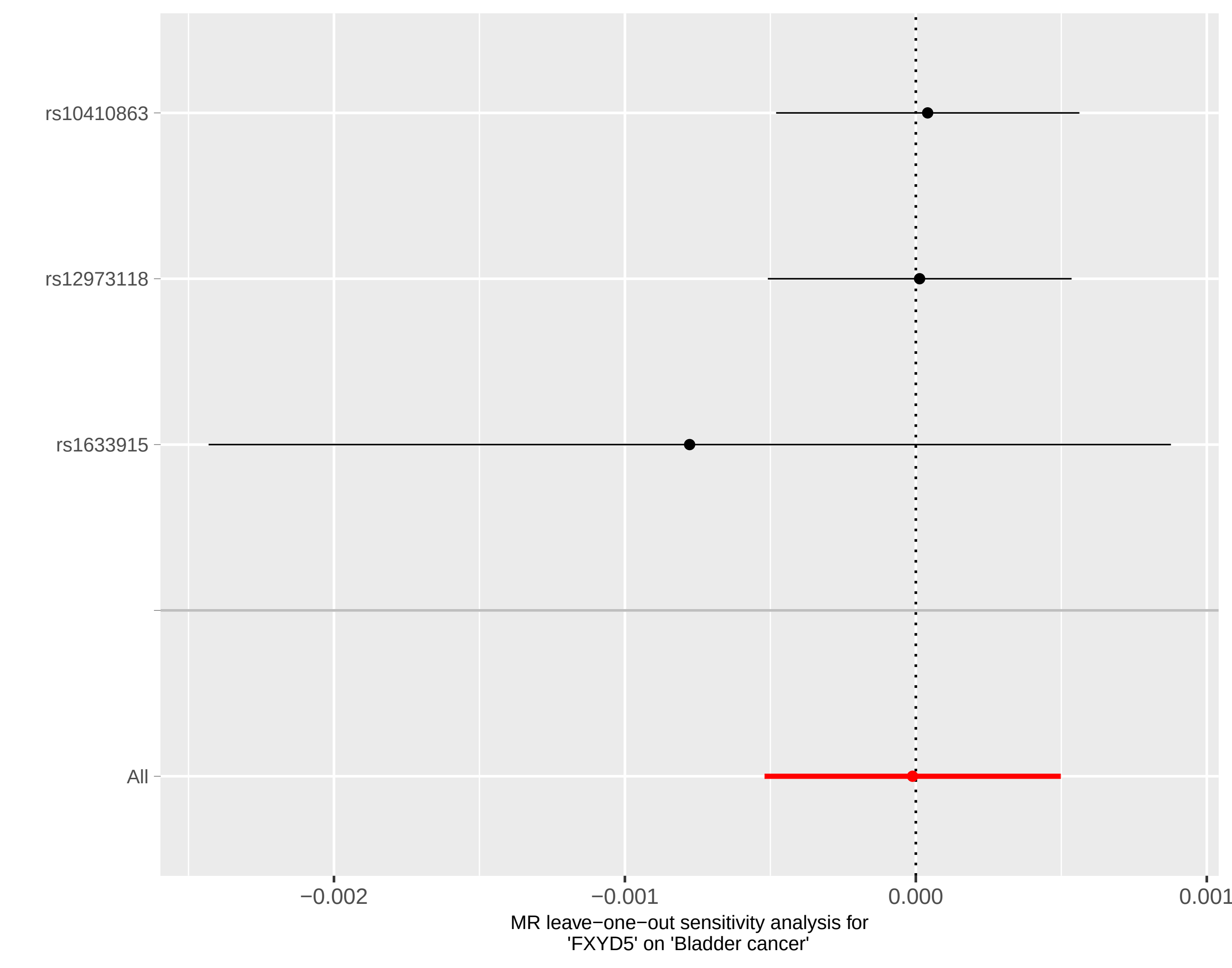
rs10410863
rs12973118
rs1633915
All
−0.002
−0.001
0.000
0.001
MR leave−one−out sensitivity analysis for
'FXYD5' on 'Bladder cancer'

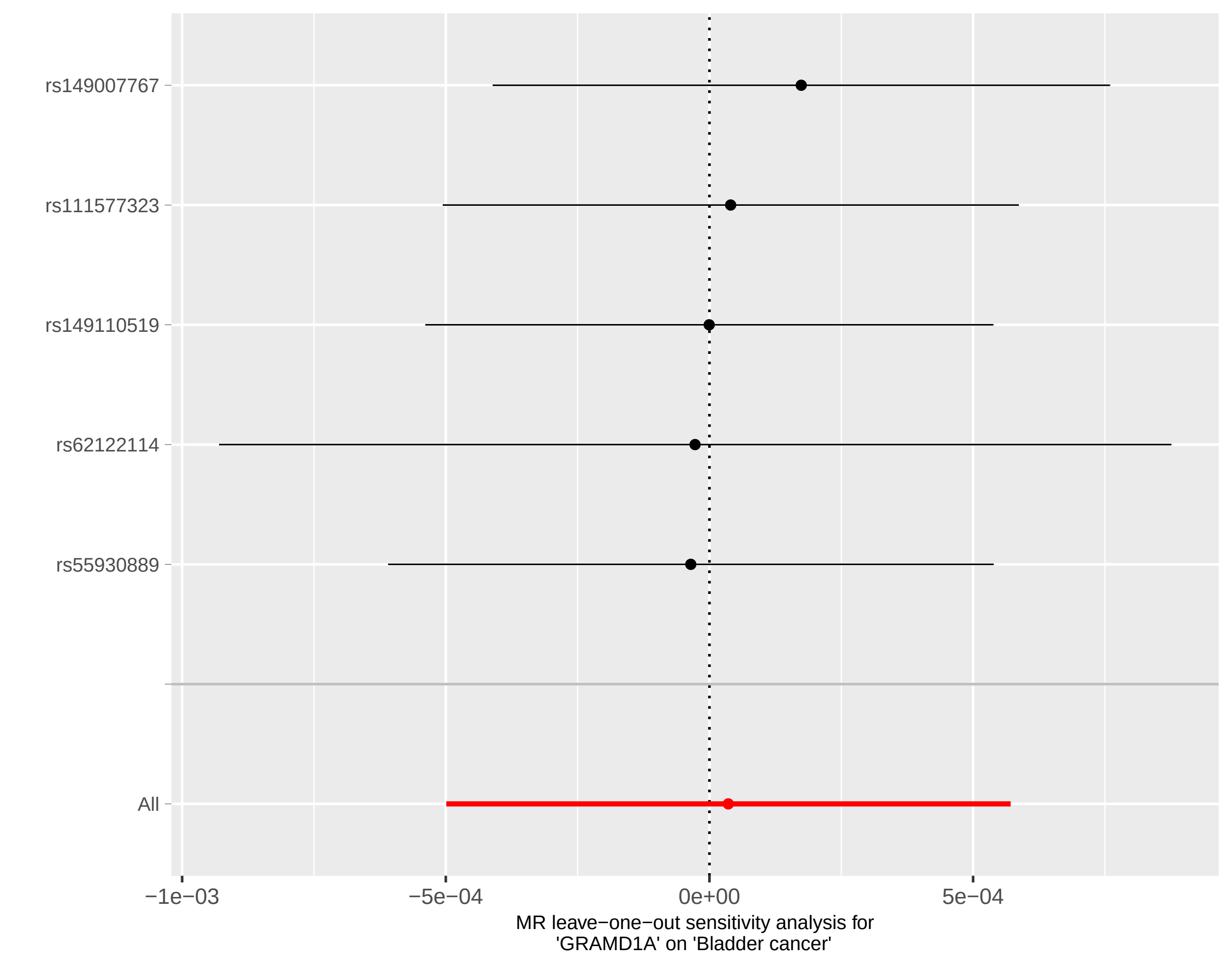
rs149007767
rs111577323
rs149110519
rs62122114
rs55930889
All
−1e−03
−5e−04
0e+00
5e−04
MR leave−one−out sensitivity analysis for
'GRAMD1A' on 'Bladder cancer'

Insufficient number of SNPs

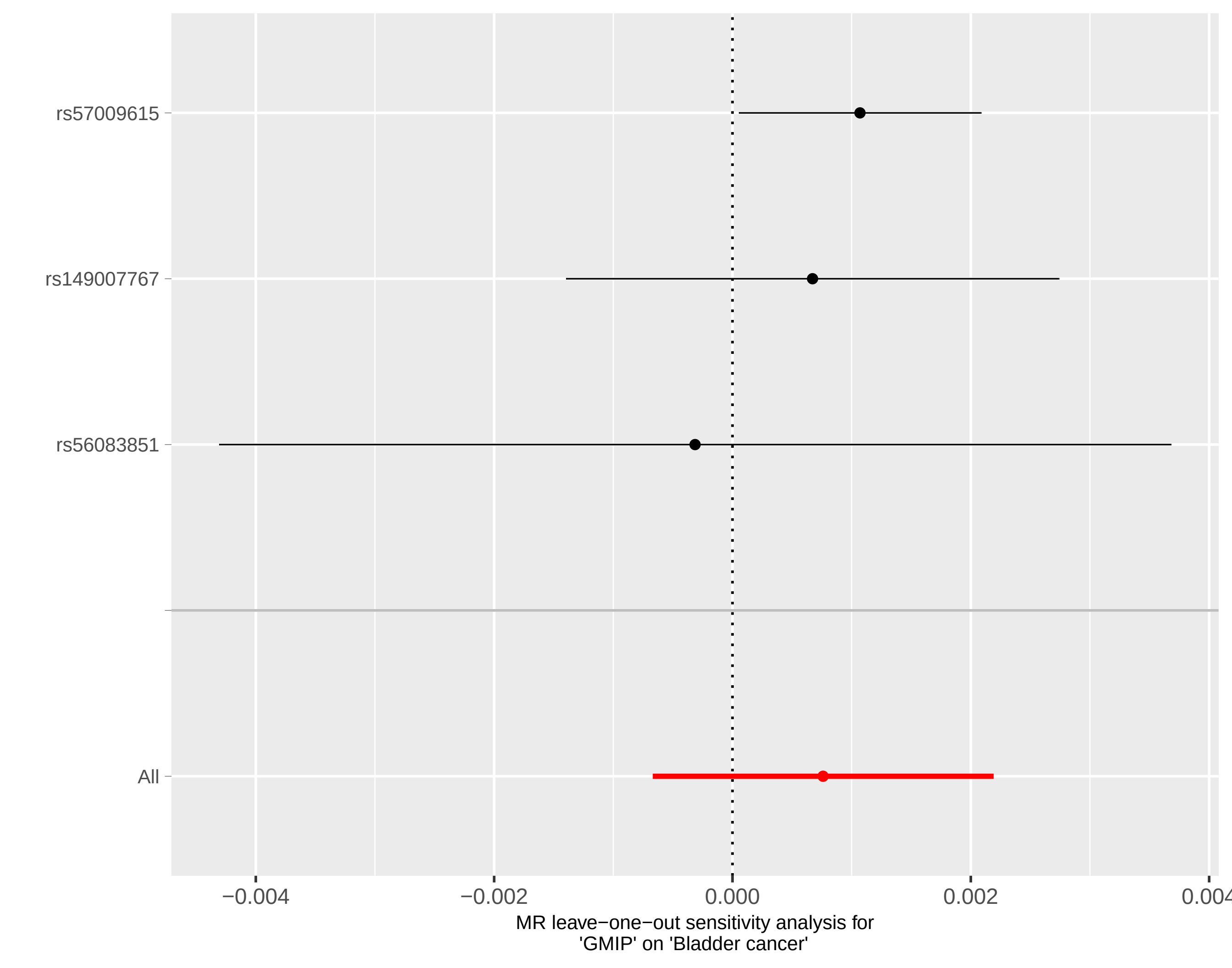

rs57009615
rs149007767
rs56083851
All
−0.004
−0.002
0.000
0.002
0.004
MR leave−one−out sensitivity analysis for
'GMIP' on 'Bladder cancer'

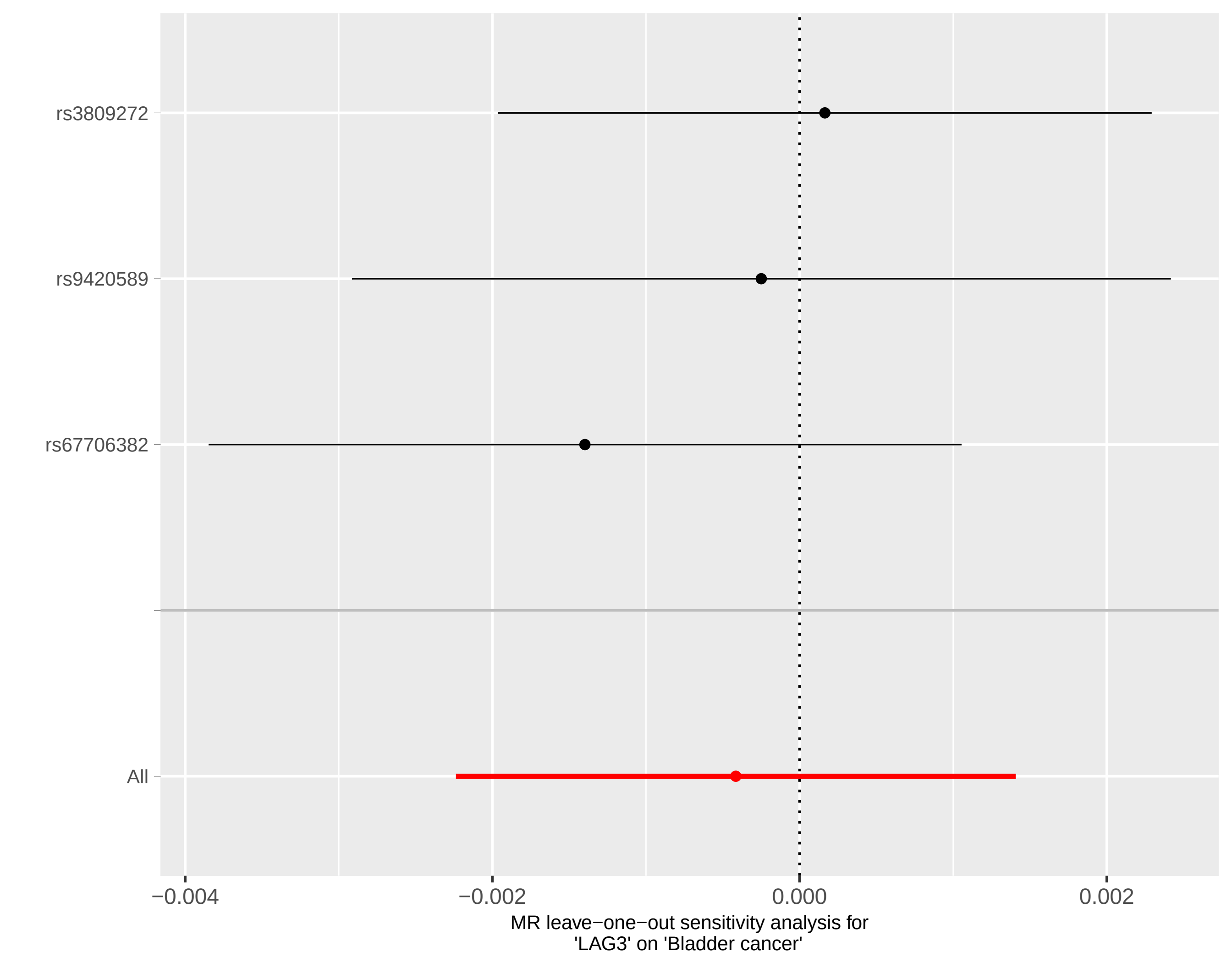
rs3809272
rs9420589
rs67706382
All
−0.004
−0.002
0.000
0.002
MR leave−one−out sensitivity analysis for
'LAG3' on 'Bladder cancer'

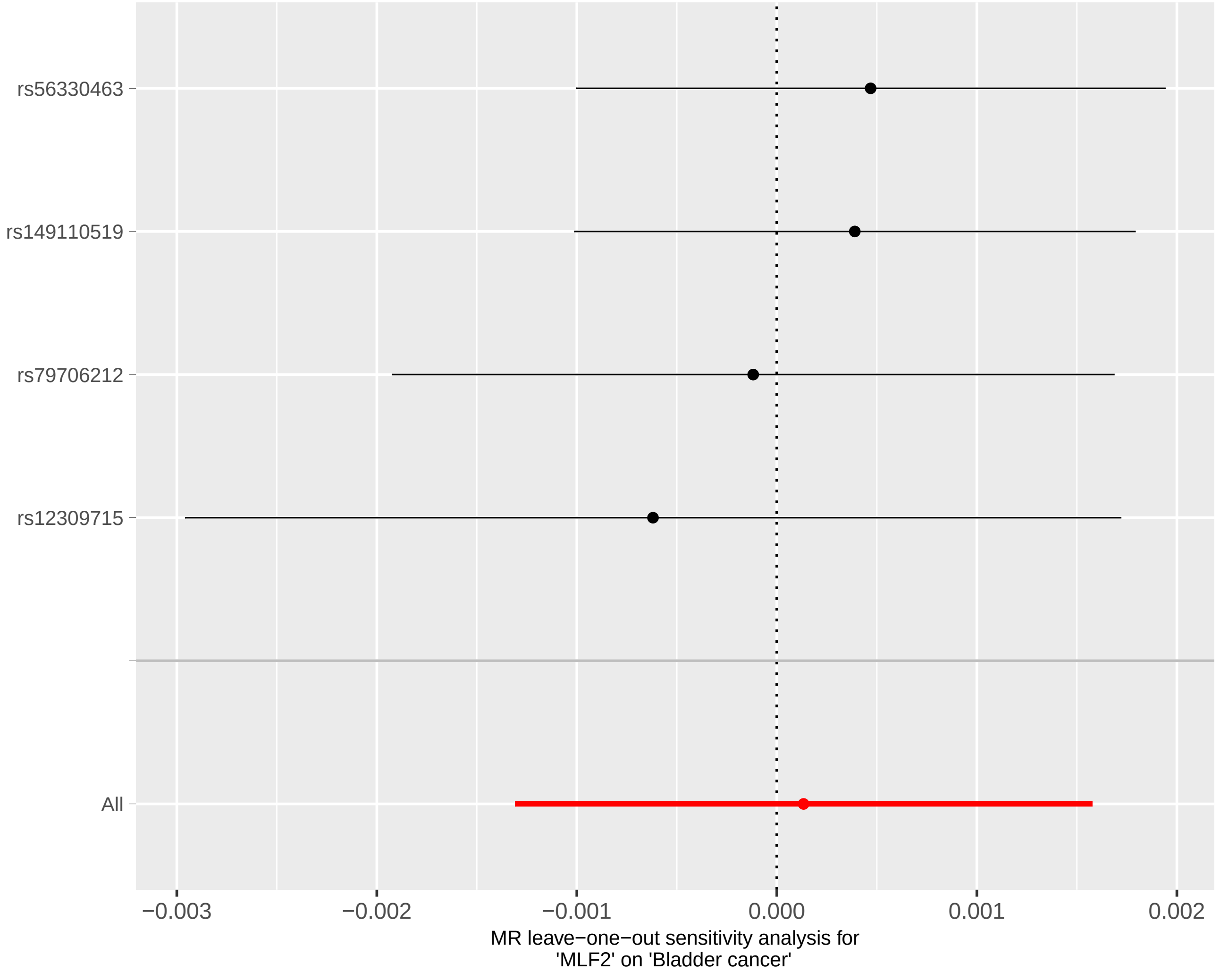
rs56330463
rs149110519
rs79706212
rs12309715
All
−0.003
−0.002
−0.001
0.000
0.001
0.002
MR leave−one−out sensitivity analysis for
'MLF2' on 'Bladder cancer'

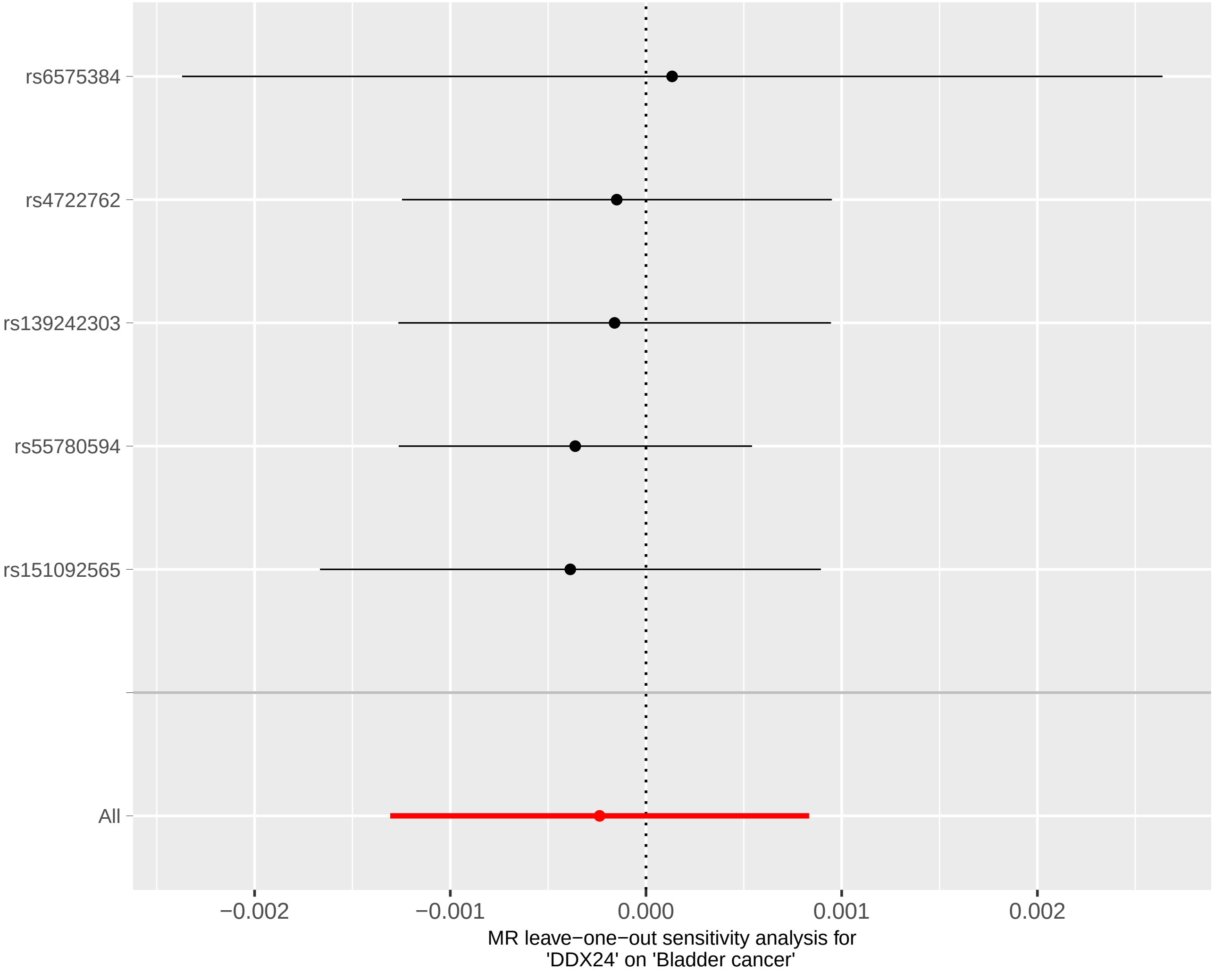

rs6575384
rs4722762
rs139242303
rs55780594
rs151092565
All
−0.002
−0.001
0.000
0.001
0.002
MR leave−one−out sensitivity analysis for
'DDX24' on 'Bladder cancer'

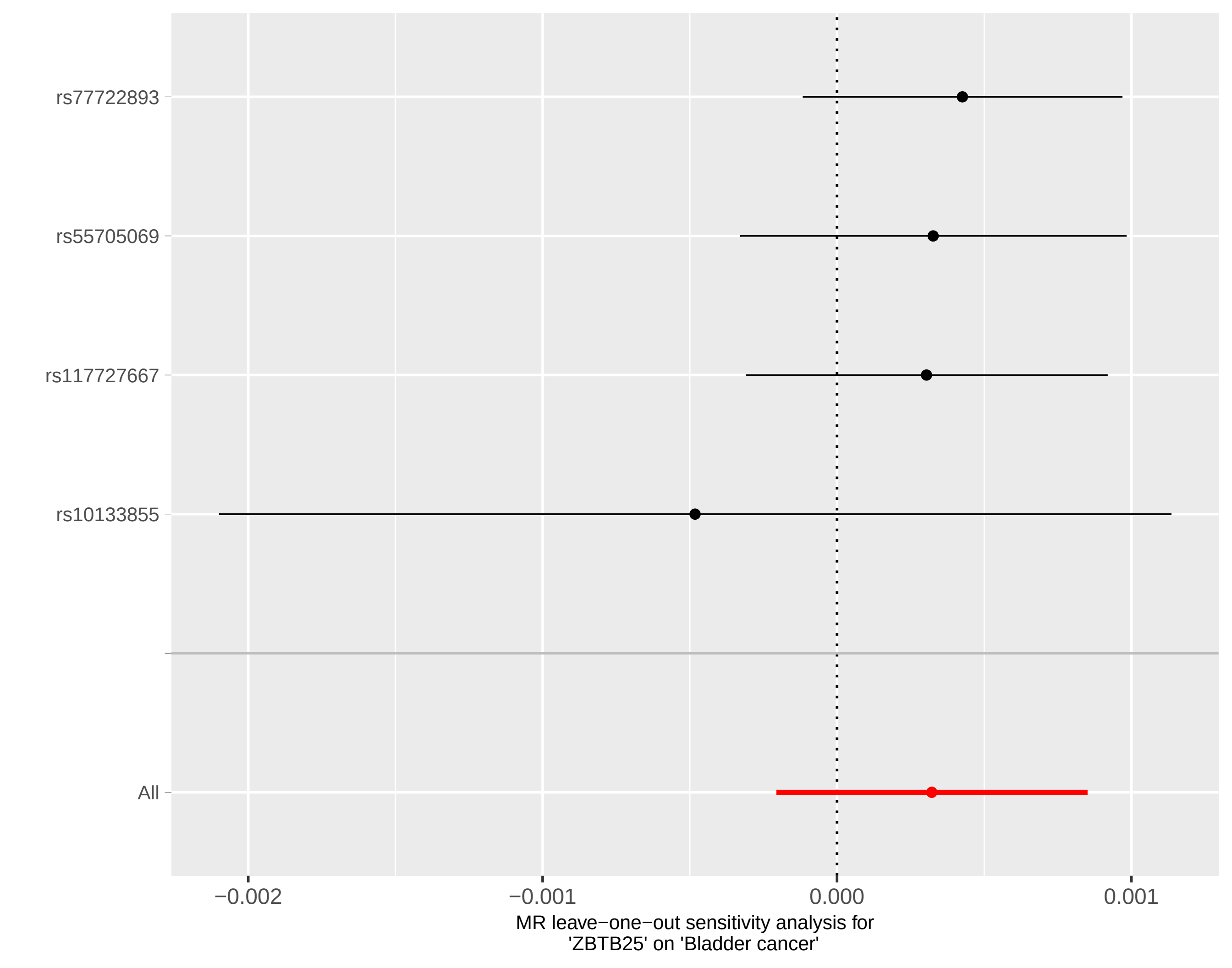
rs77722893
rs55705069
rs117727667
rs10133855
All
−0.002
−0.001
0.000
0.001
MR leave−one−out sensitivity analysis for
'ZBTB25' on 'Bladder cancer'

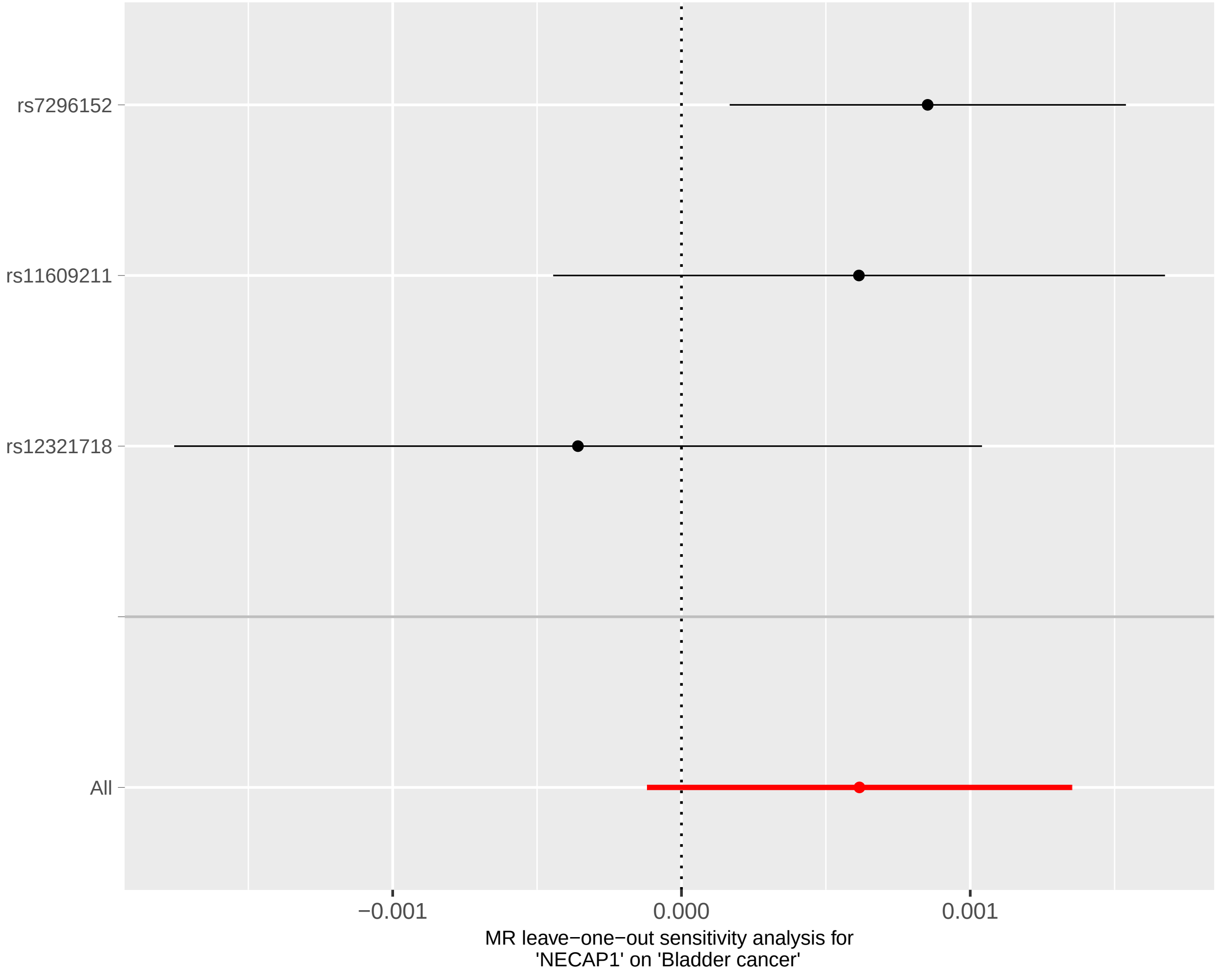

rs7296152
rs11609211
rs12321718
All
−0.001
0.000
0.001
MR leave−one−out sensitivity analysis for
'NECAP1' on 'Bladder cancer'

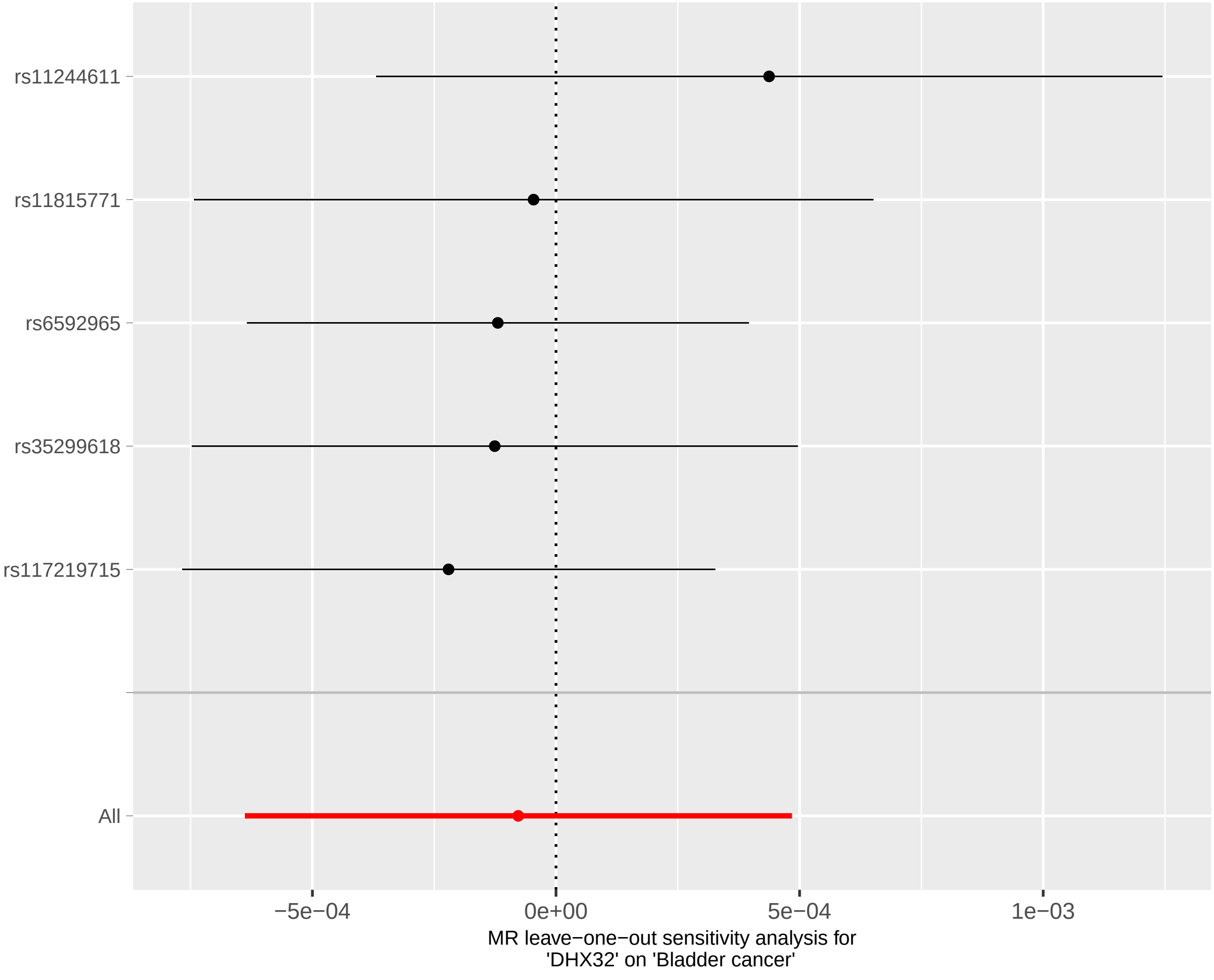
rs11244611
rs11815771
rs6592965
rs35299618
rs117219715
All
−5e−04
0e+00
5e−04
1e−03
MR leave−one−out sensitivity analysis for
'DHX32' on 'Bladder cancer'

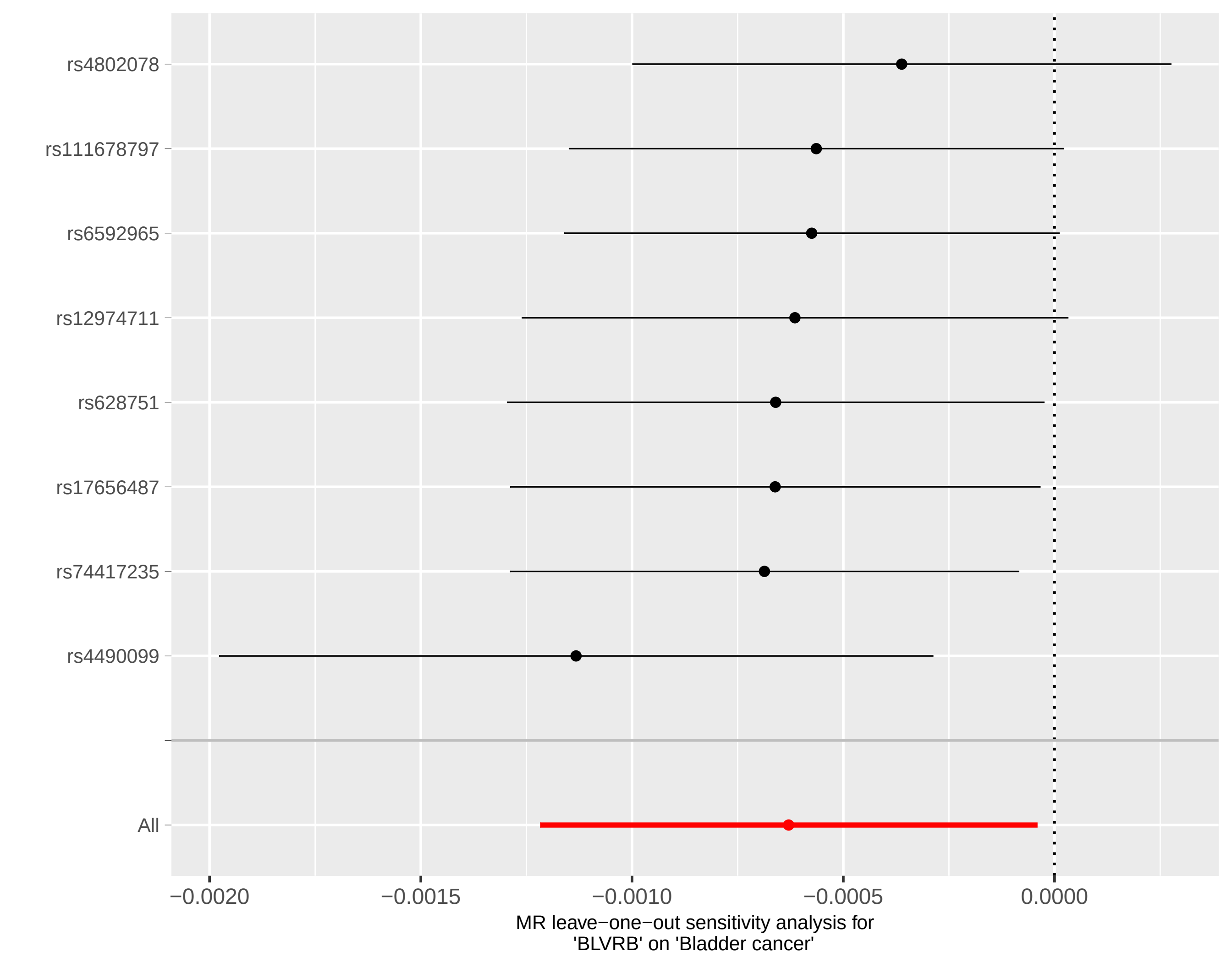
rs4802078
rs111678797
rs6592965
rs12974711
rs628751
rs17656487
rs74417235
rs4490099
All
−0.0020
−0.0015
−0.0010
−0.0005
0.0000
MR leave−one−out sensitivity analysis for
'BLVRB' on 'Bladder cancer'

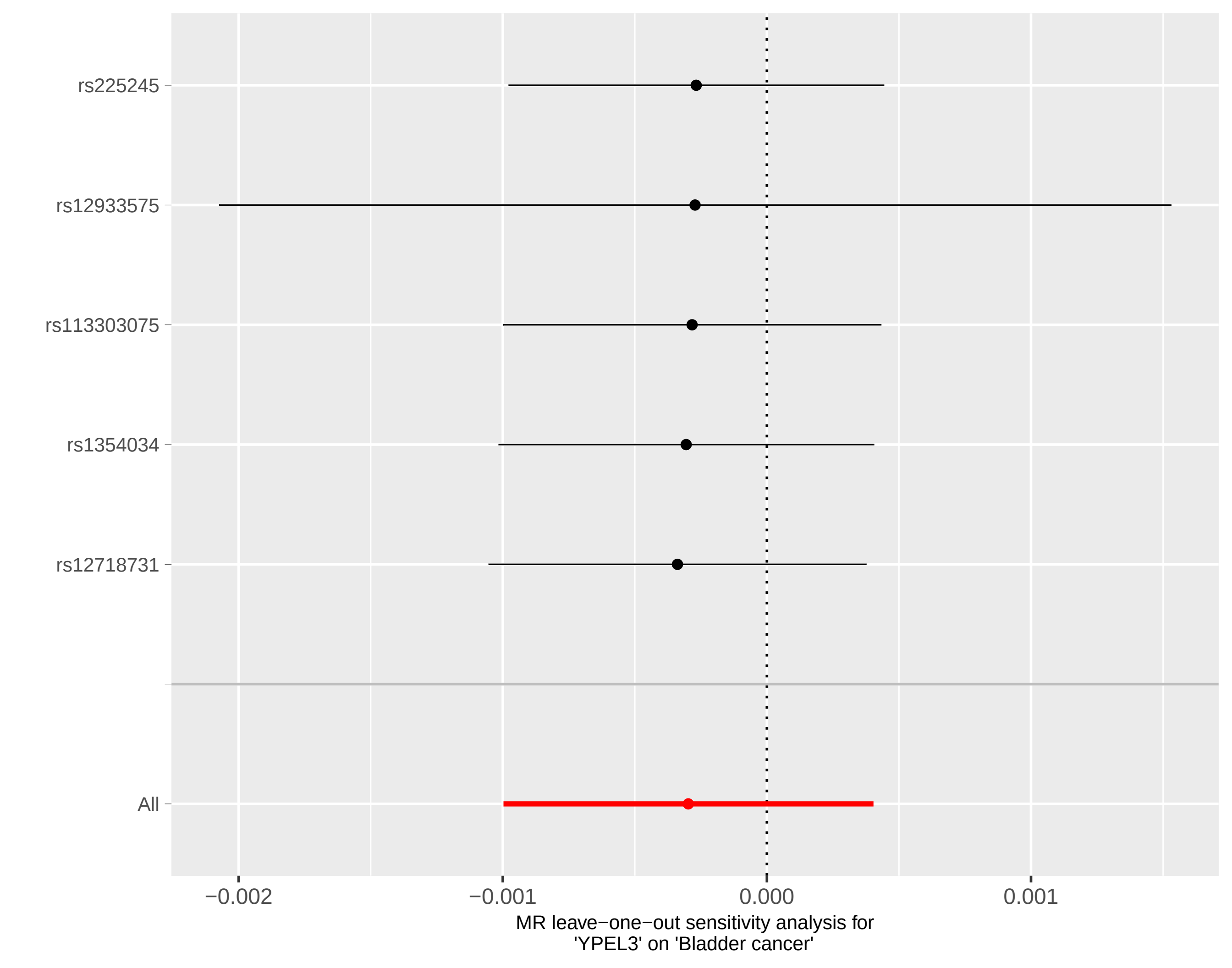
rs225245
rs12933575
rs113303075
rs1354034
rs12718731
All
−0.002
−0.001
0.000
0.001
MR leave−one−out sensitivity analysis for
'YPEL3' on 'Bladder cancer'

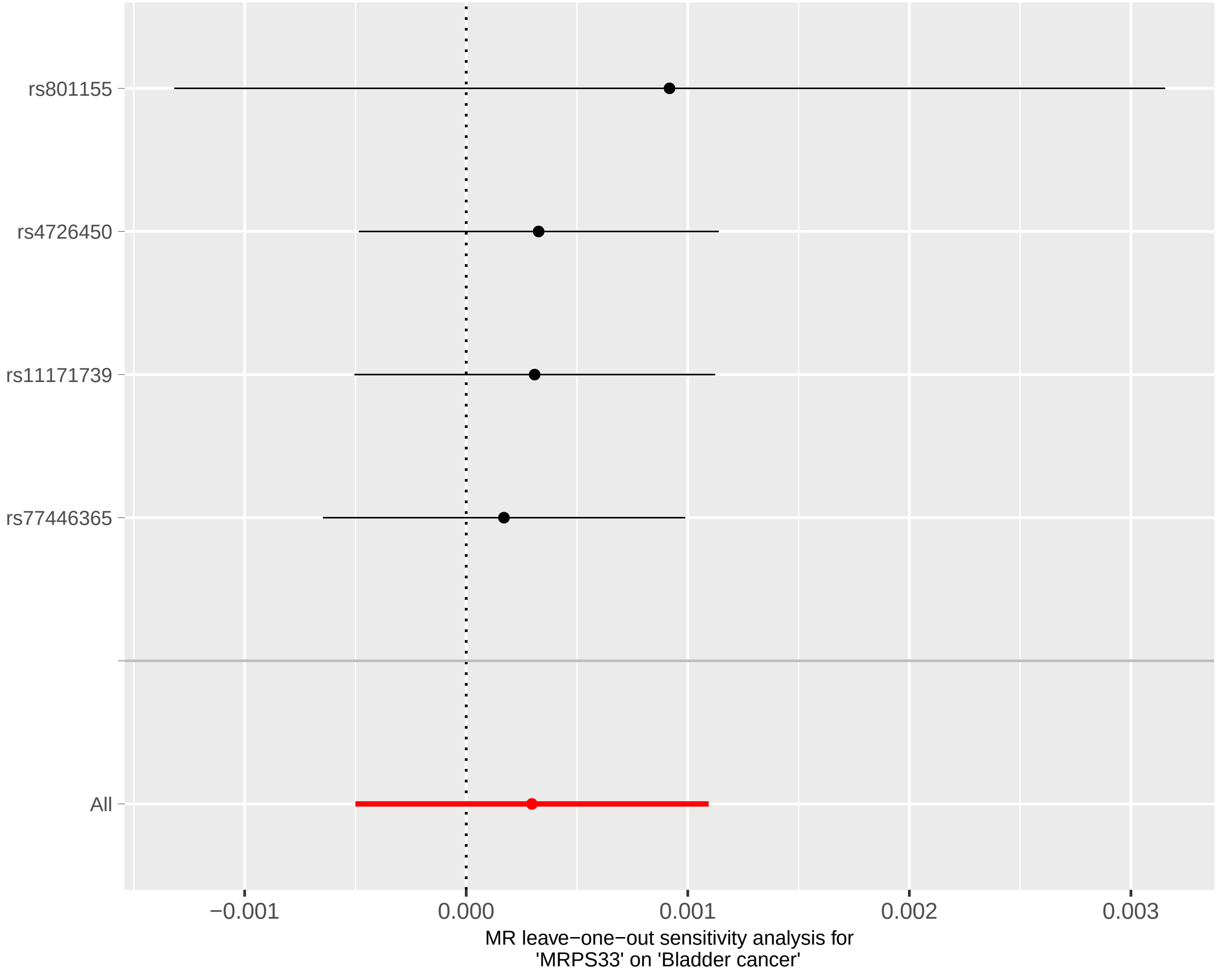
rs801155
rs4726450
rs11171739
rs77446365
All
−0.001
0.000
0.001
0.002
0.003
MR leave−one−out sensitivity analysis for
'MRPS33' on 'Bladder cancer'

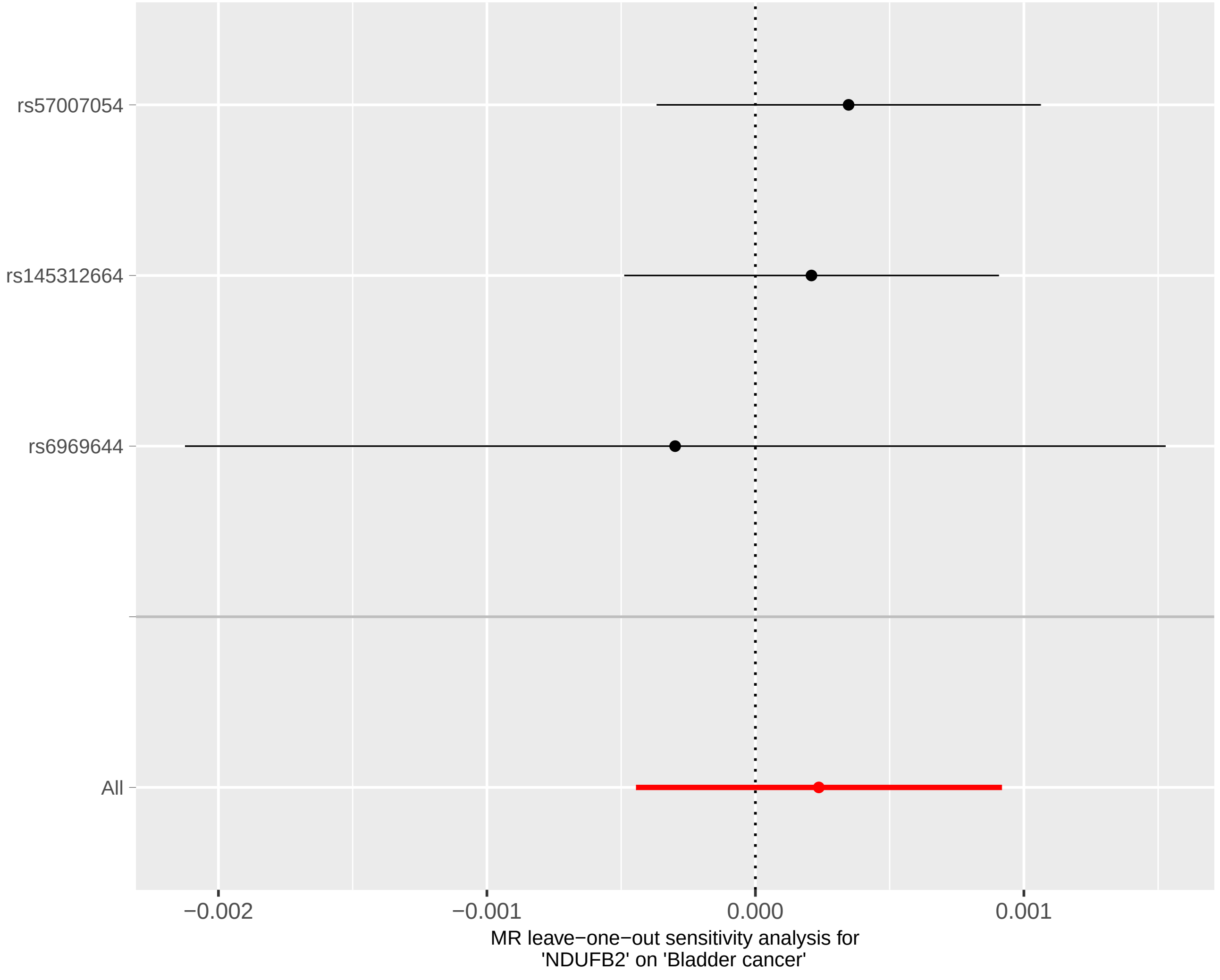

rs57007054
rs145312664
rs6969644
All
−0.002
−0.001
0.000
0.001
MR leave−one−out sensitivity analysis for
'NDUFB2' on 'Bladder cancer'

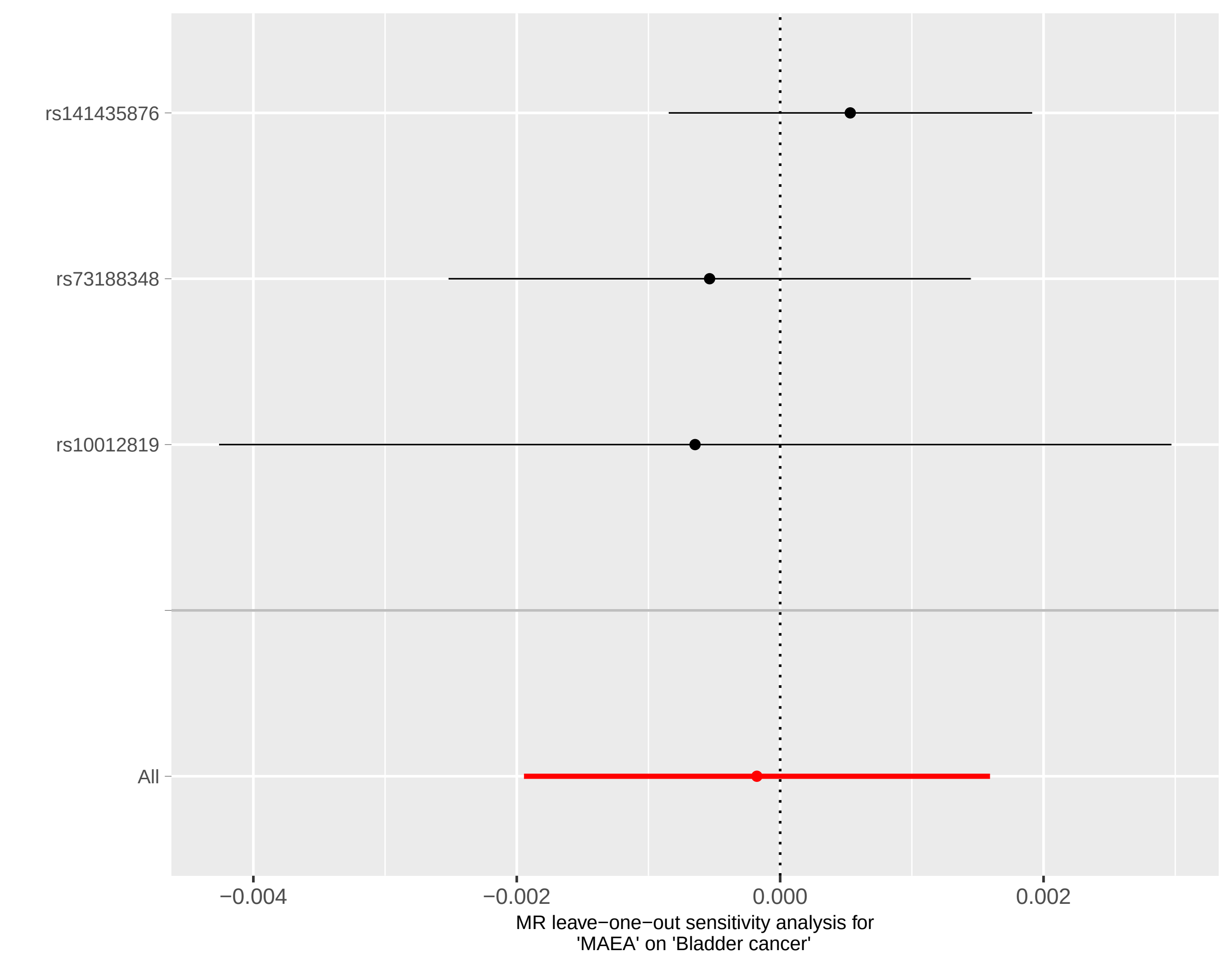
rs141435876
rs73188348
rs10012819
All
−0.004
−0.002
0.000
0.002
MR leave−one−out sensitivity analysis for
'MAEA' on 'Bladder cancer'

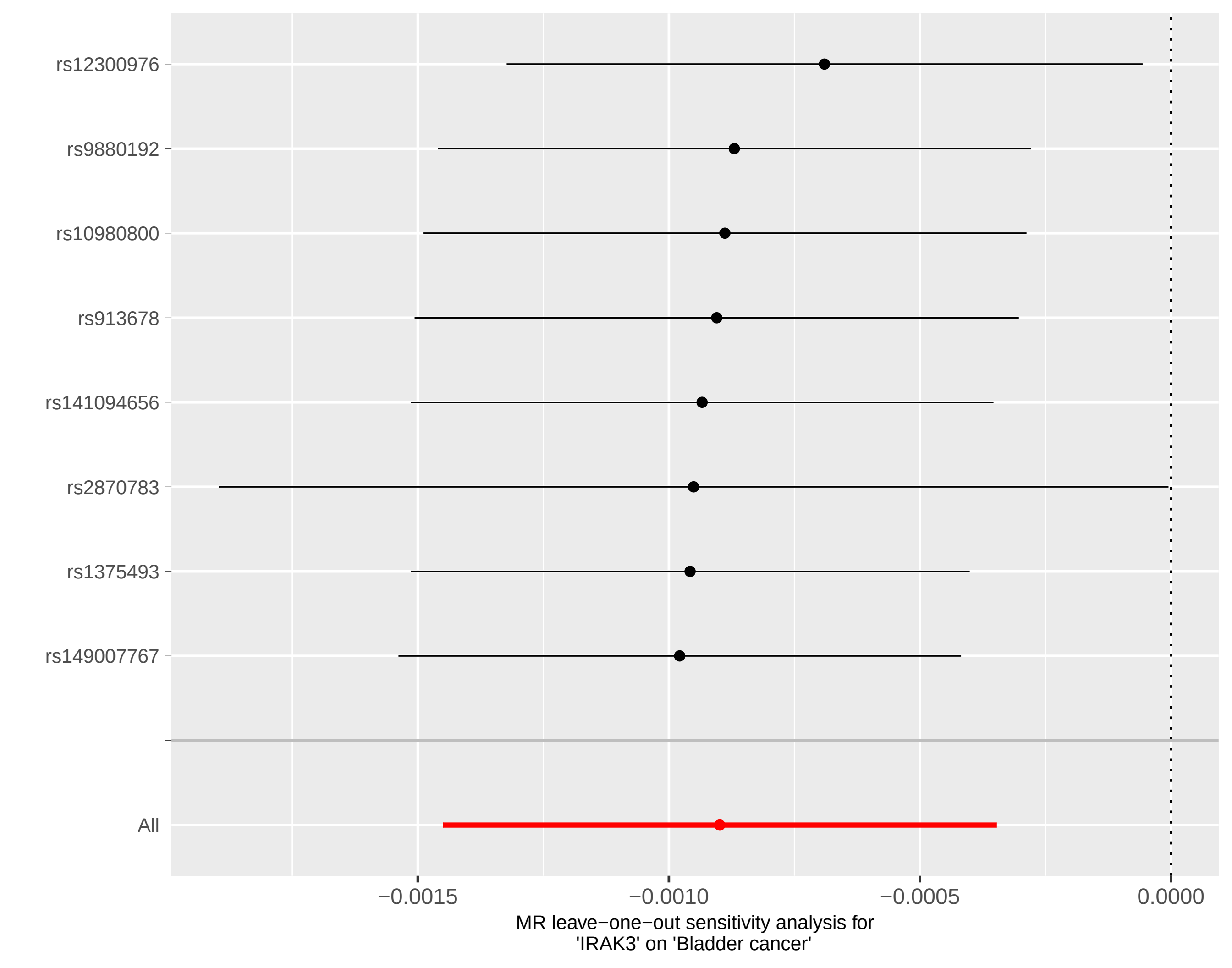
rs12300976
rs9880192
rs10980800
rs913678
rs141094656
rs2870783
rs1375493
rs149007767
All
−0.0015
−0.0010
−0.0005
0.0000
MR leave−one−out sensitivity analysis for
'IRAK3' on 'Bladder cancer'

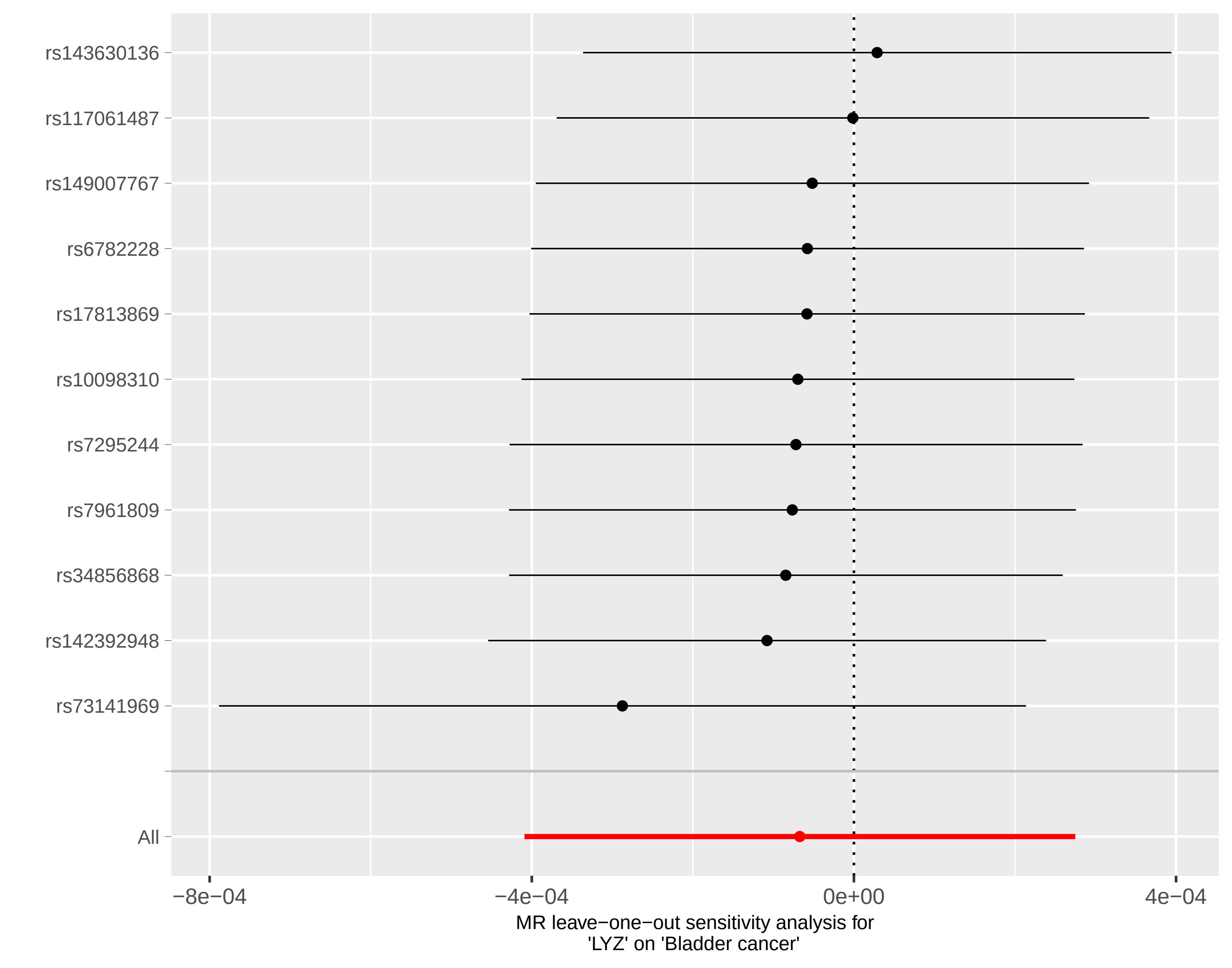
rs143630136
rs117061487
rs149007767
rs6782228
rs17813869
rs10098310
rs7295244
rs7961809
rs34856868
rs142392948
rs73141969
All
−8e−04
−4e−04
0e+00
4e−04
MR leave−one−out sensitivity analysis for
'LYZ' on 'Bladder cancer'

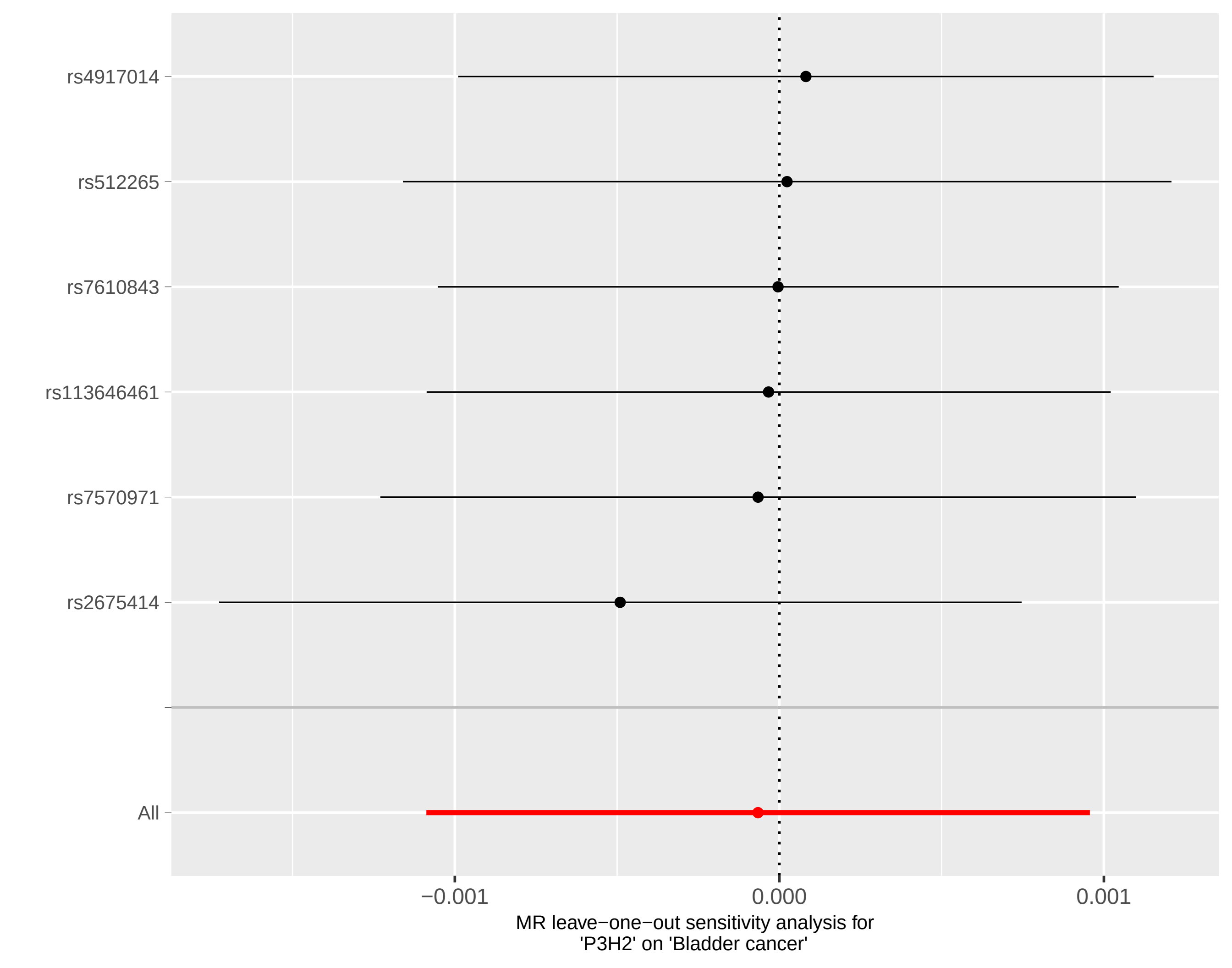
rs4917014
rs512265
rs7610843
rs113646461
rs7570971
rs2675414
All
−0.001
0.000
0.001
MR leave−one−out sensitivity analysis for
'P3H2' on 'Bladder cancer'

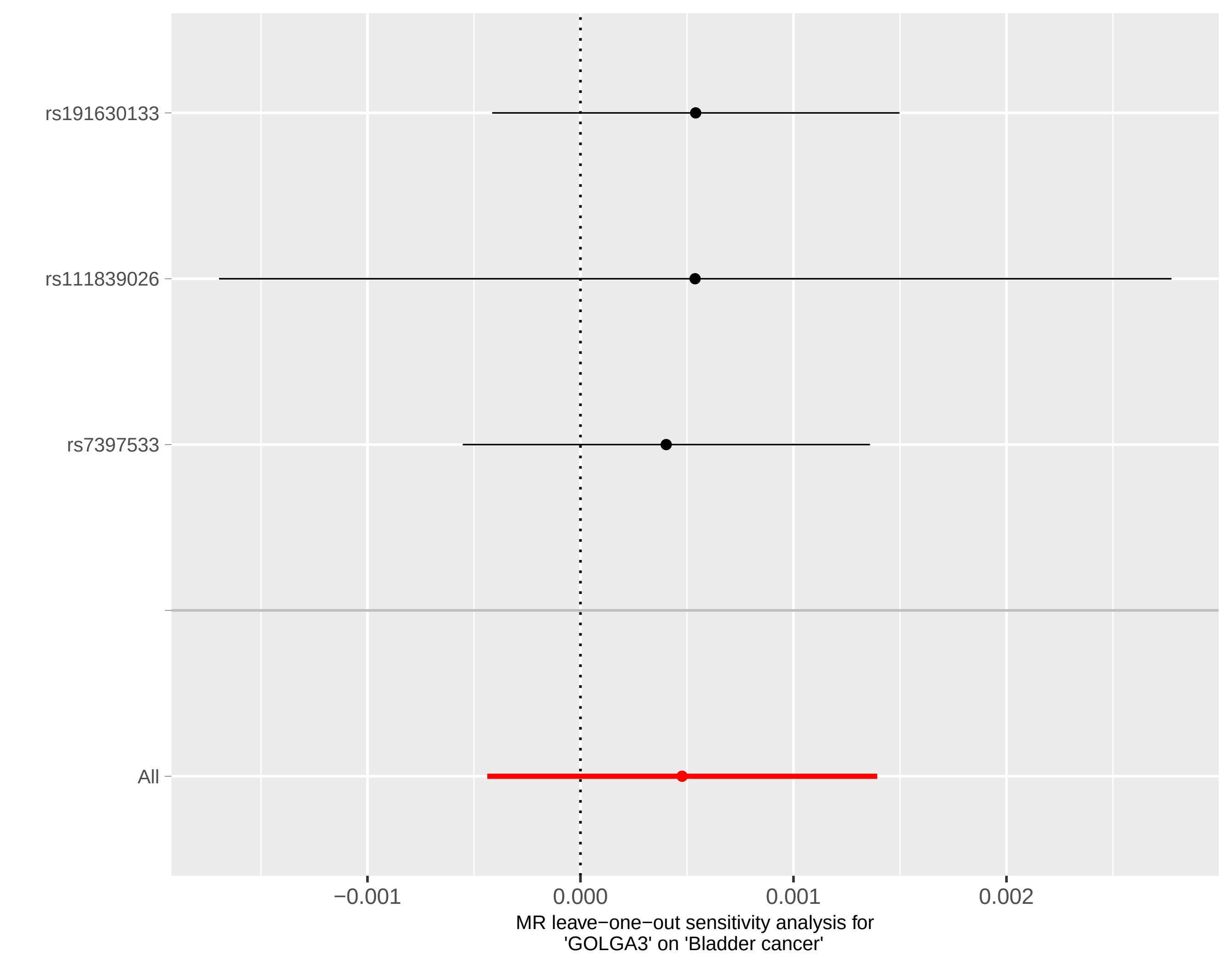
rs191630133
rs111839026
rs7397533
All
−0.001
0.000
0.001
0.002
MR leave−one−out sensitivity analysis for
'GOLGA3' on 'Bladder cancer'

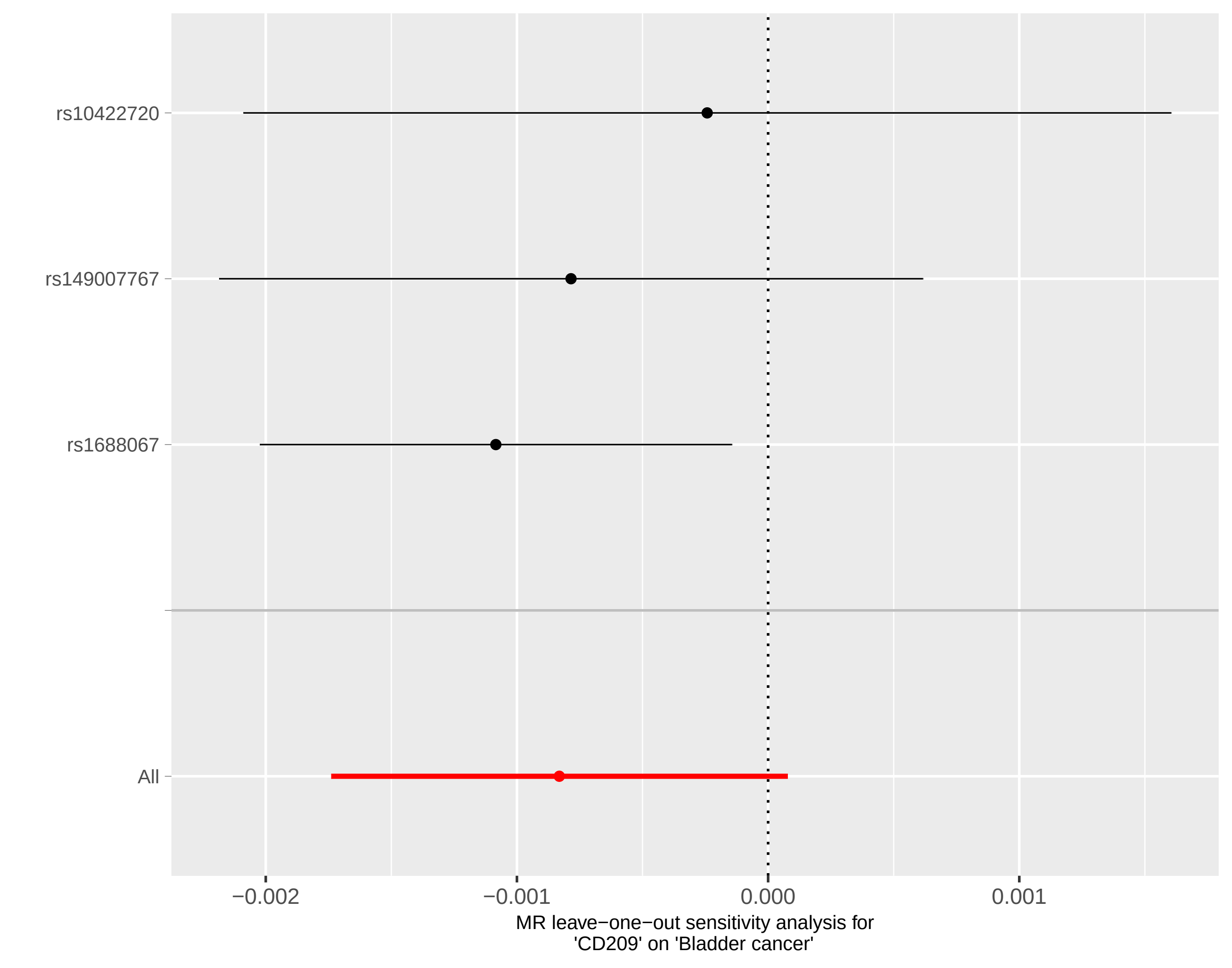
rs10422720
rs149007767
rs1688067
All
−0.002
−0.001
0.000
0.001
MR leave−one−out sensitivity analysis for
'CD209' on 'Bladder cancer'

Insufficient number of SNPs

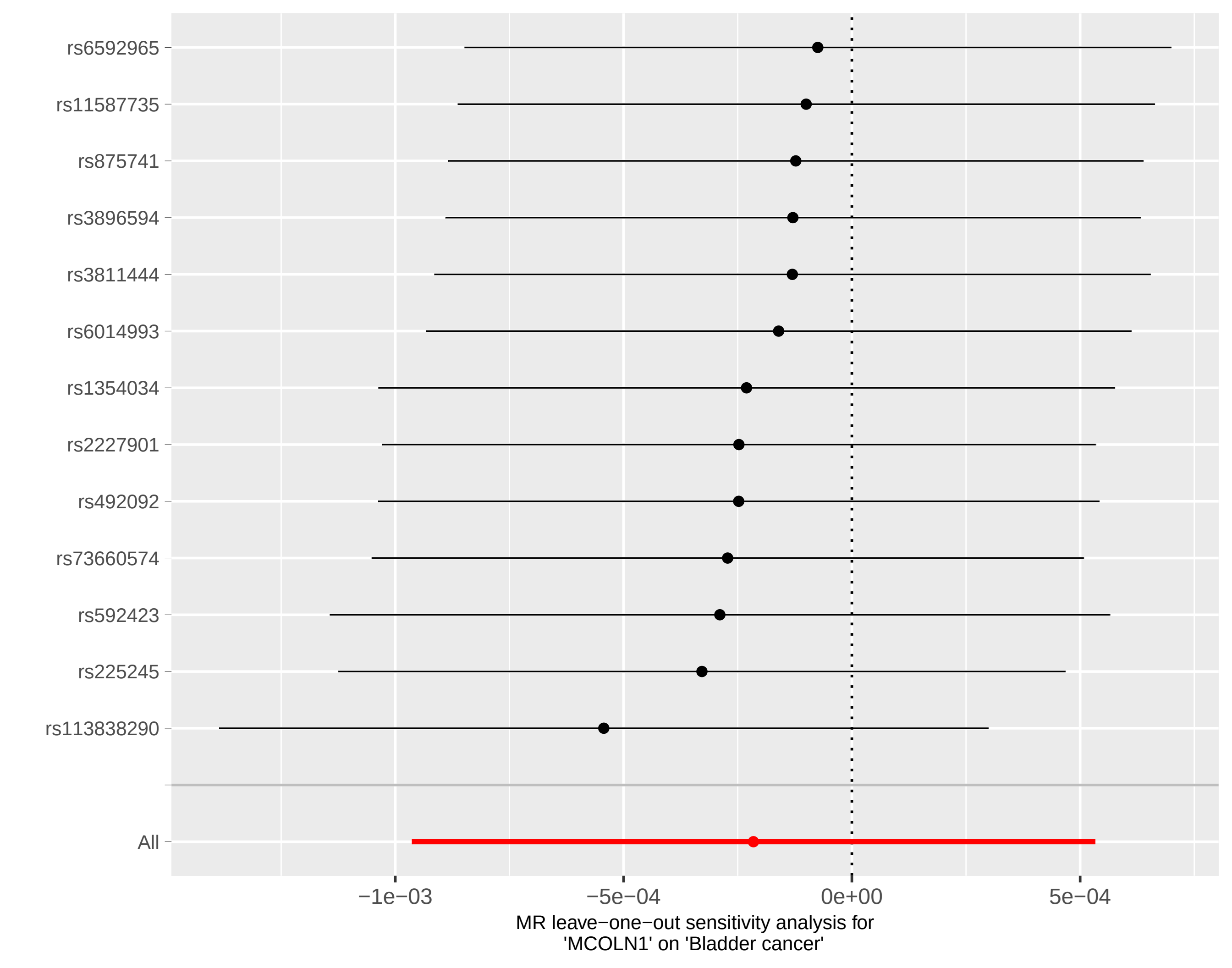
rs6592965
rs11587735
rs875741
rs3896594
rs3811444
rs6014993
rs1354034
rs2227901
rs492092
rs73660574
rs592423
rs225245
rs113838290
All
−1e−03
−5e−04
0e+00
5e−04
MR leave−one−out sensitivity analysis for
'MCOLN1' on 'Bladder cancer'

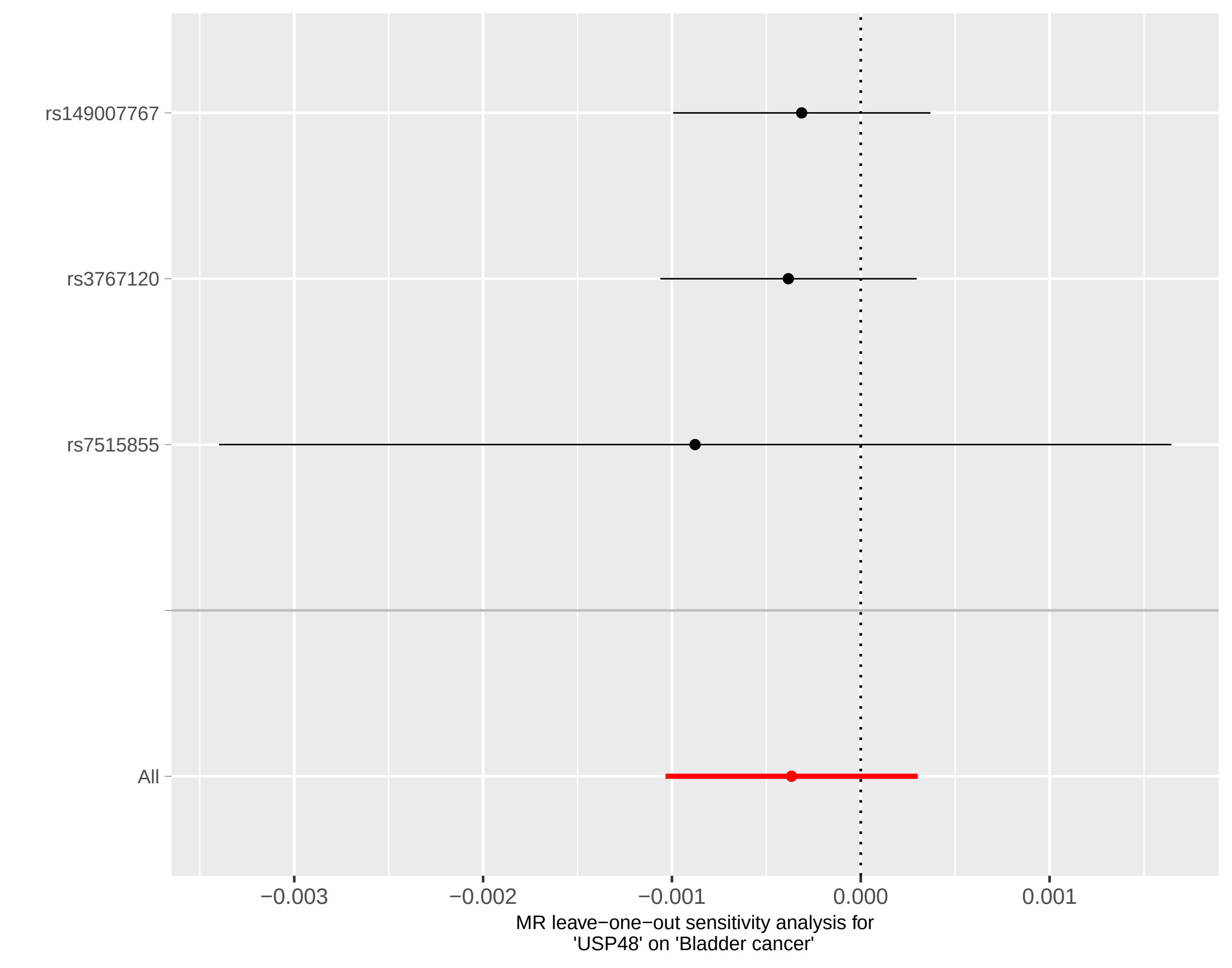
rs149007767
rs3767120
rs7515855
All
−0.003
−0.002
−0.001
0.000
0.001
MR leave−one−out sensitivity analysis for
'USP48' on 'Bladder cancer'

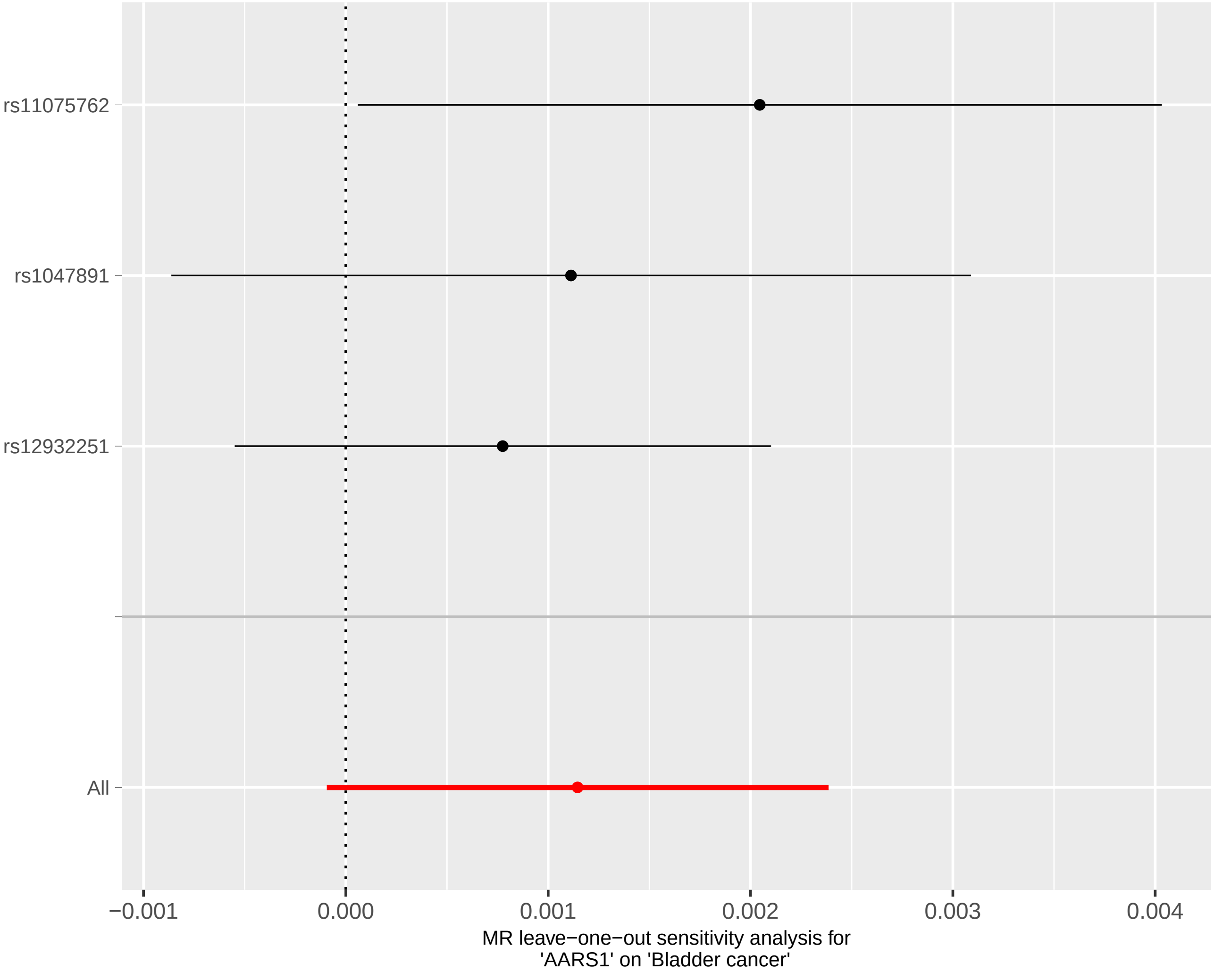

rs11075762
rs1047891
rs12932251
All
−0.001
0.000
0.001
0.002
0.003
0.004
MR leave−one−out sensitivity analysis for
'AARS1' on 'Bladder cancer'

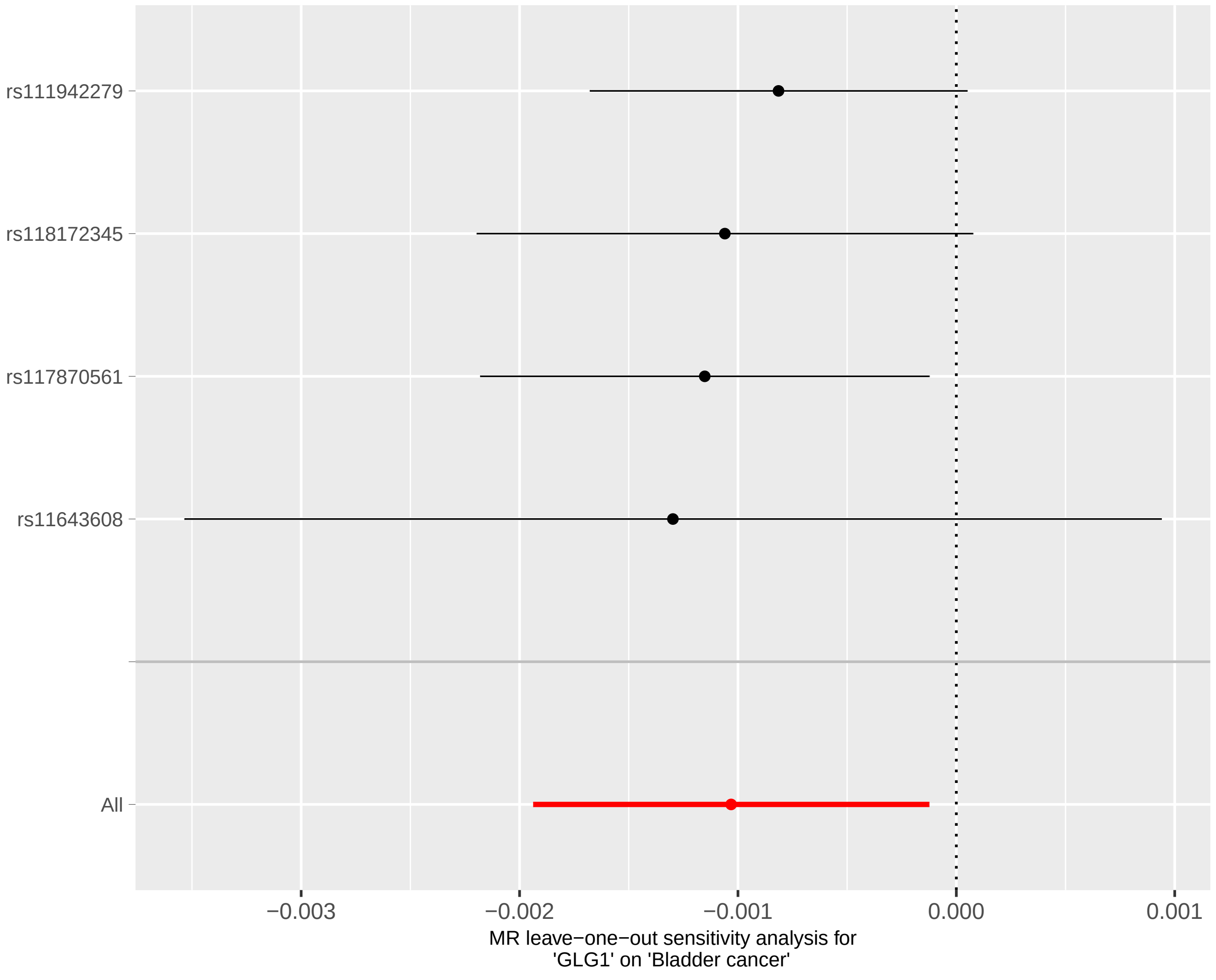
rs111942279
rs118172345
rs117870561
rs11643608
All
−0.003
−0.002
−0.001
0.000
0.001
MR leave−one−out sensitivity analysis for
'GLG1' on 'Bladder cancer'

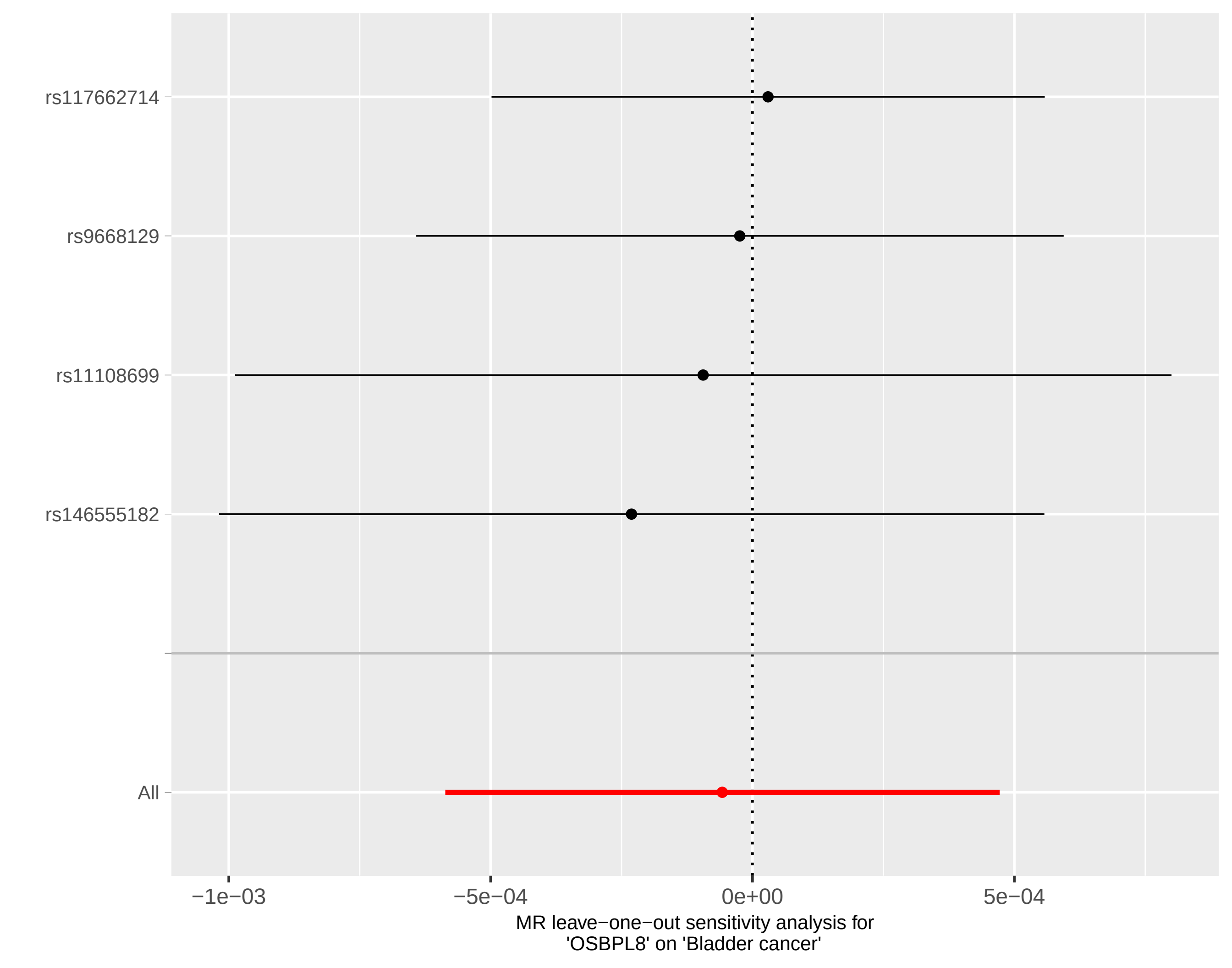
rs117662714
rs9668129
rs11108699
rs146555182
All
−1e−03
−5e−04
0e+00
5e−04
MR leave−one−out sensitivity analysis for
'OSBPL8' on 'Bladder cancer'

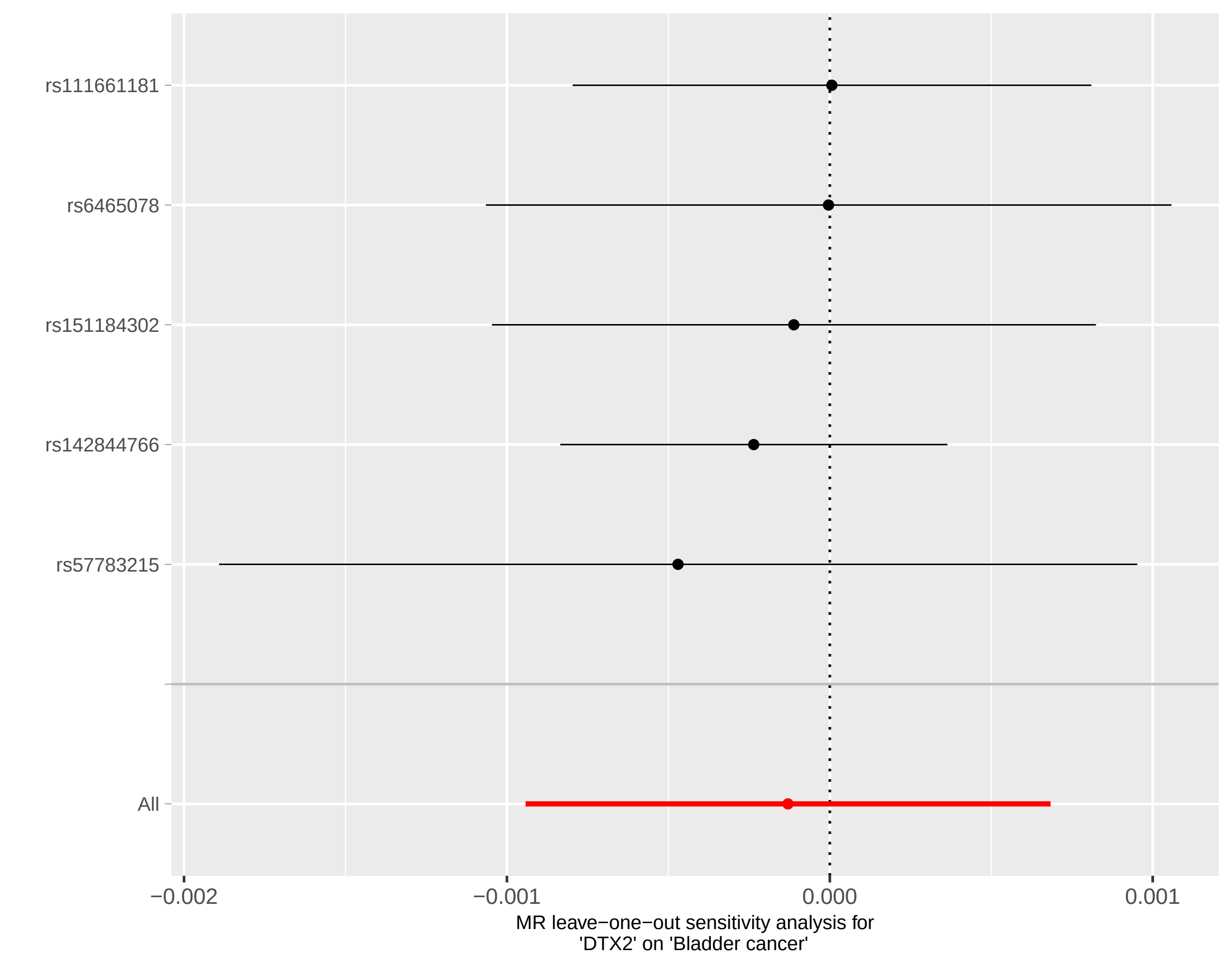
rs111661181
rs6465078
rs151184302
rs142844766
rs57783215
All
−0.002
−0.001
0.000
0.001
MR leave−one−out sensitivity analysis for
'DTX2' on 'Bladder cancer'

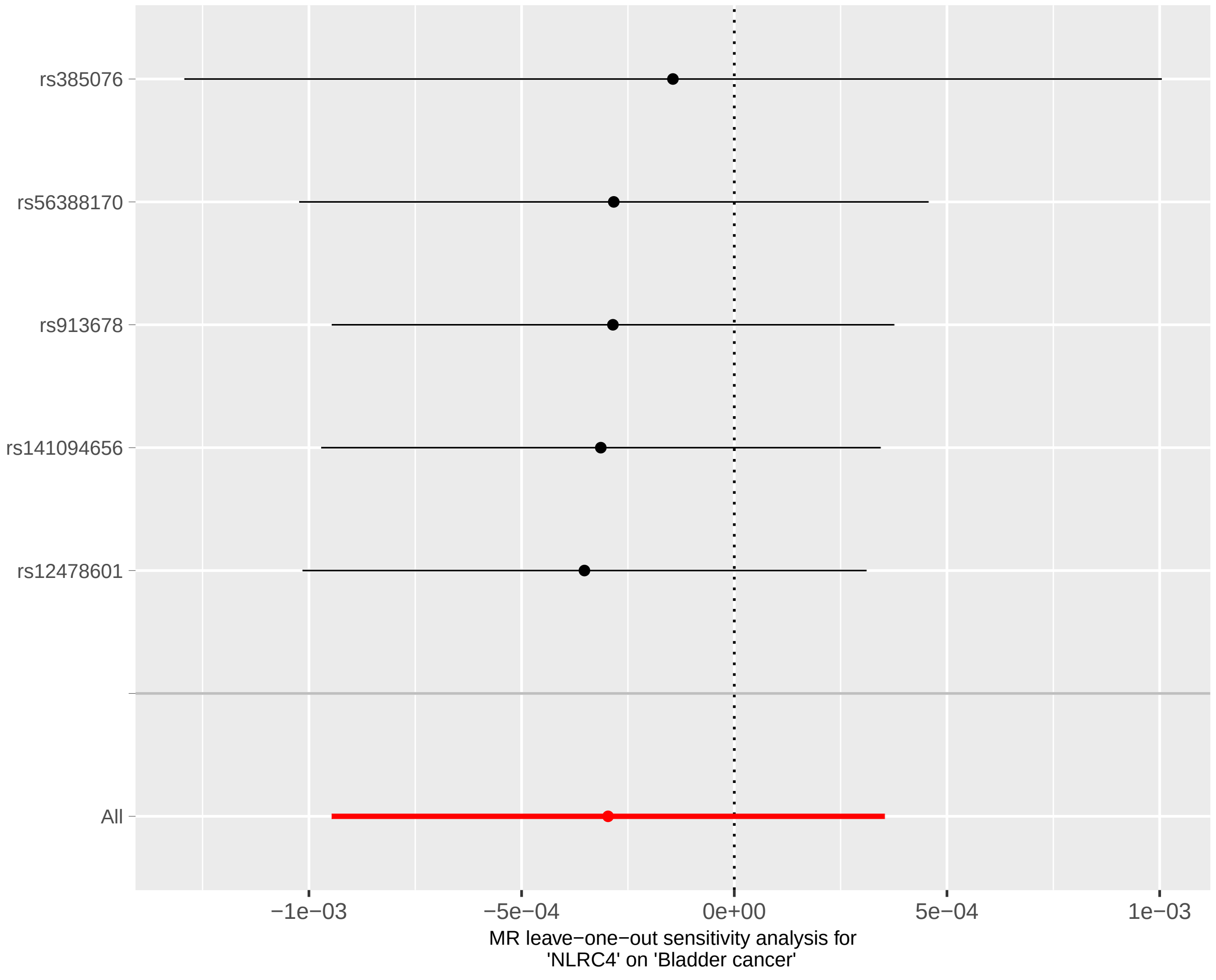
rs385076
rs56388170
rs913678
rs141094656
rs12478601
All
−1e−03
−5e−04
0e+00
5e−04
1e−03
MR leave−one−out sensitivity analysis for
'NLRC4' on 'Bladder cancer'

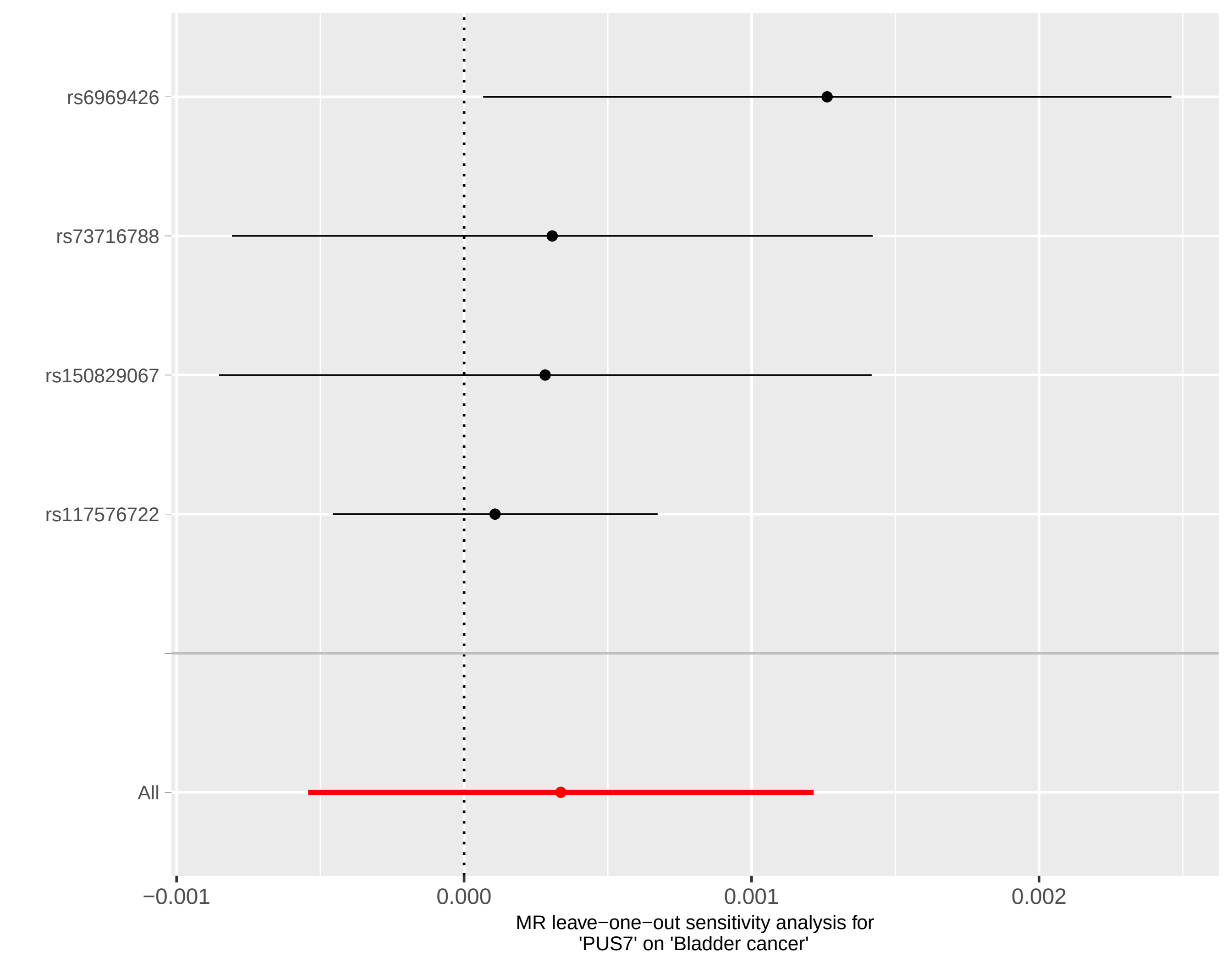
rs6969426
rs73716788
rs150829067
rs117576722
All
−0.001
0.000
0.001
0.002
MR leave−one−out sensitivity analysis for
'PUS7' on 'Bladder cancer'

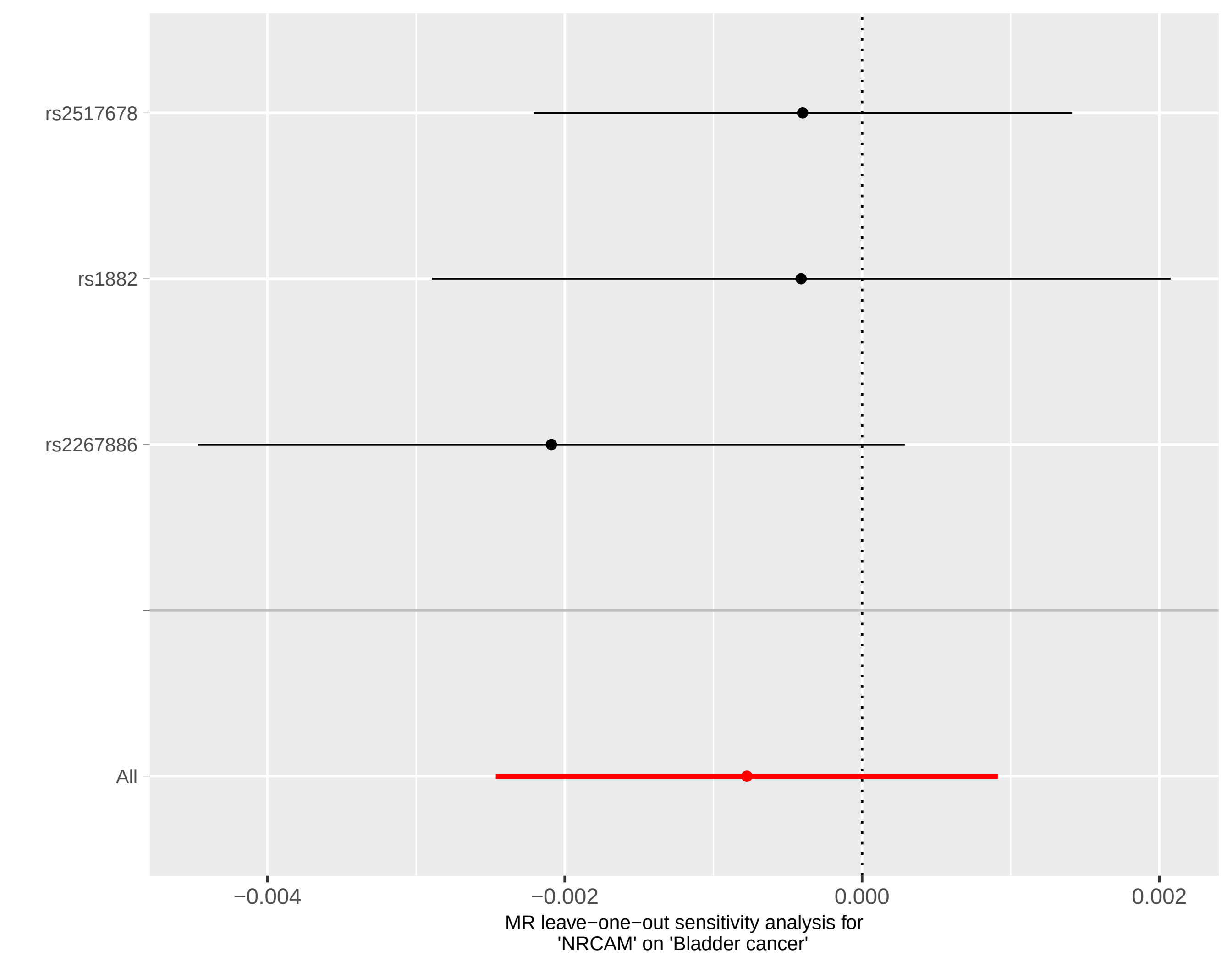
rs2517678
rs1882
rs2267886
All
−0.004
−0.002
0.000
0.002
MR leave−one−out sensitivity analysis for
'NRCAM' on 'Bladder cancer'

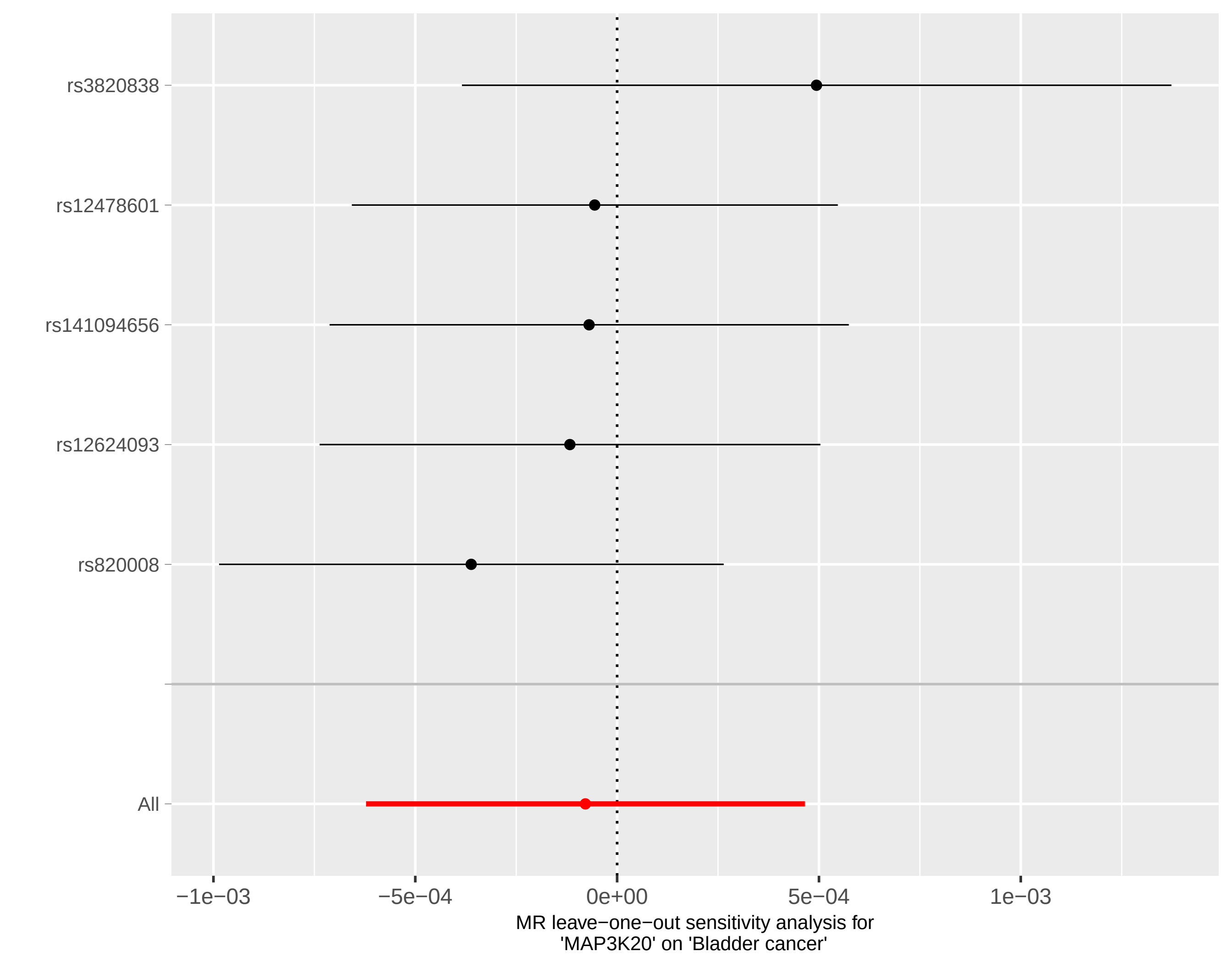
rs3820838
rs12478601
rs141094656
rs12624093
rs820008
All
−1e−03
−5e−04
0e+00
5e−04
1e−03
MR leave−one−out sensitivity analysis for
'MAP3K20' on 'Bladder cancer'

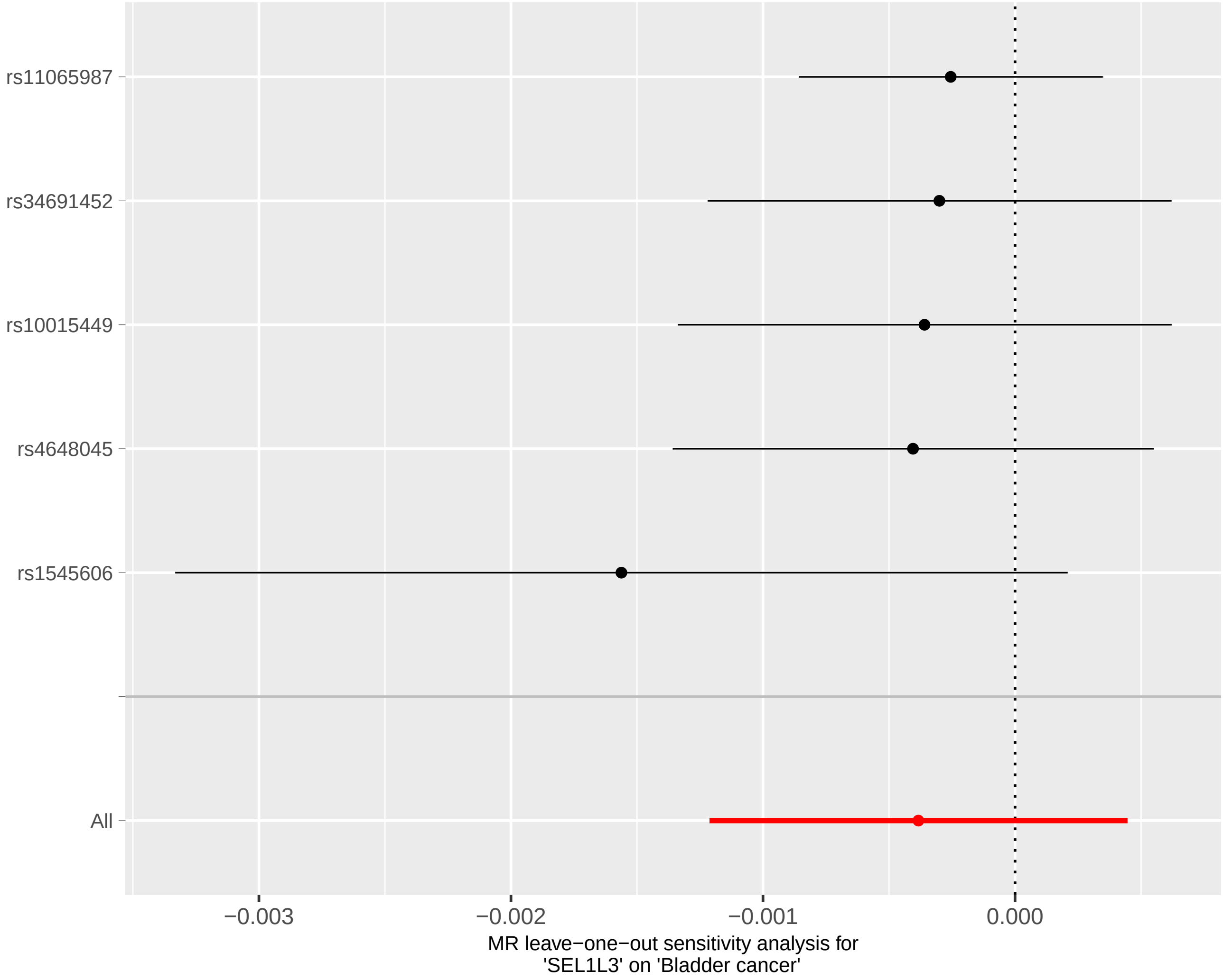
rs11065987
rs34691452
rs10015449
rs4648045
rs1545606
All
−0.003
−0.002
−0.001
0.000
MR leave−one−out sensitivity analysis for
'SEL1L3' on 'Bladder cancer'

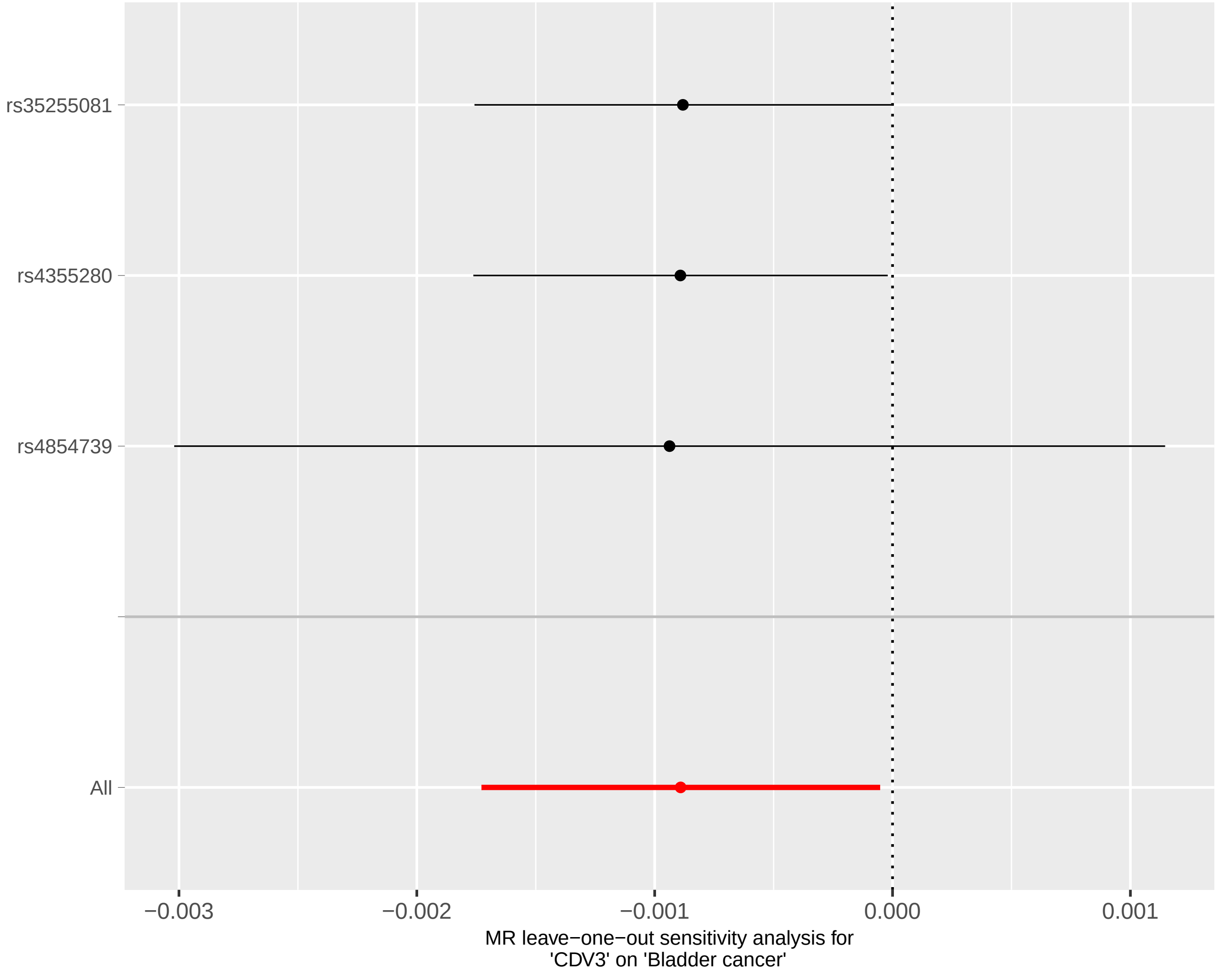
rs35255081
rs4355280
rs4854739
All
−0.003
−0.002
−0.001
0.000
0.001
MR leave−one−out sensitivity analysis for
'CDV3' on 'Bladder cancer'

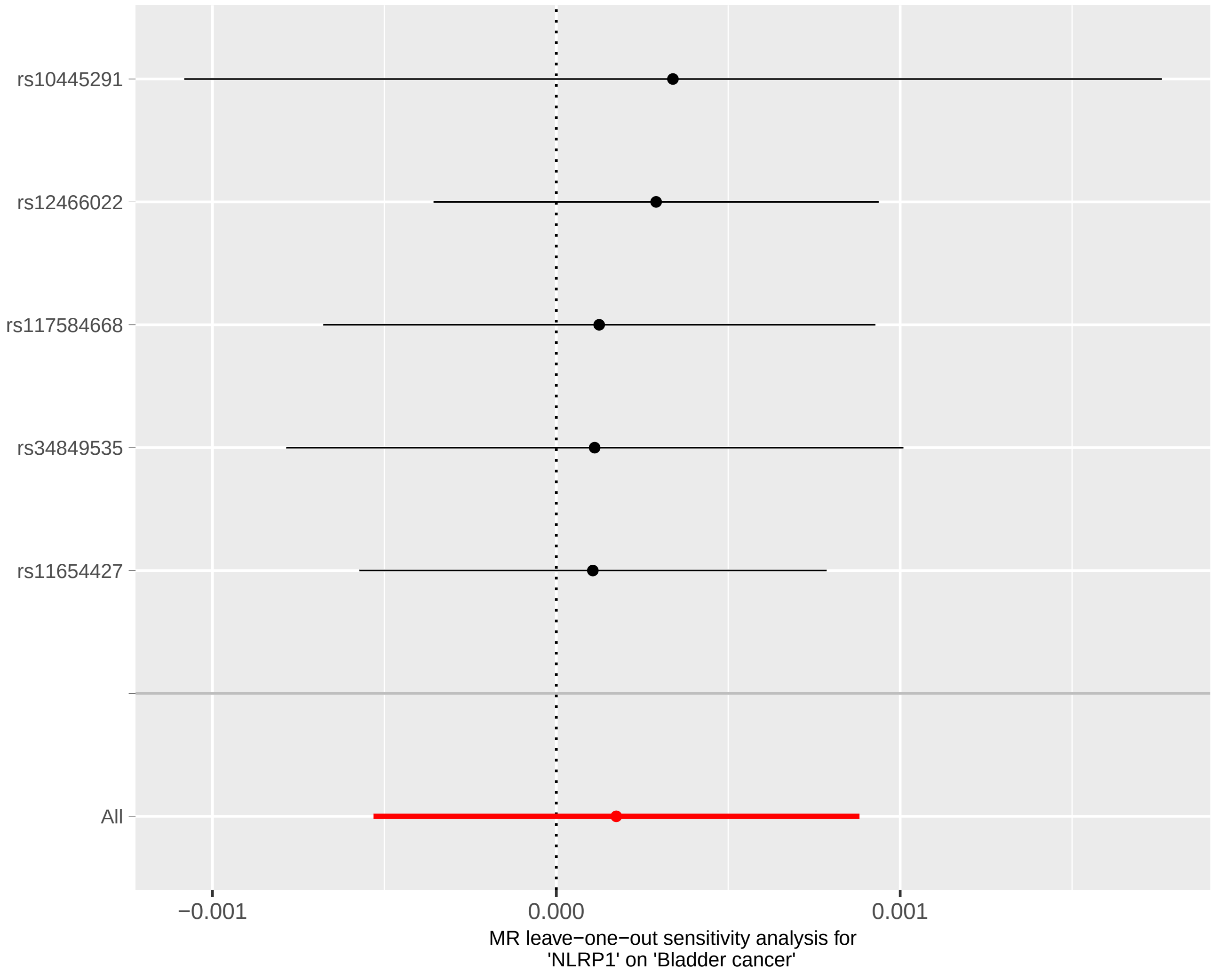
rs10445291
rs12466022
rs117584668
rs34849535
rs11654427
All
−0.001
0.000
0.001
MR leave−one−out sensitivity analysis for
'NLRP1' on 'Bladder cancer'

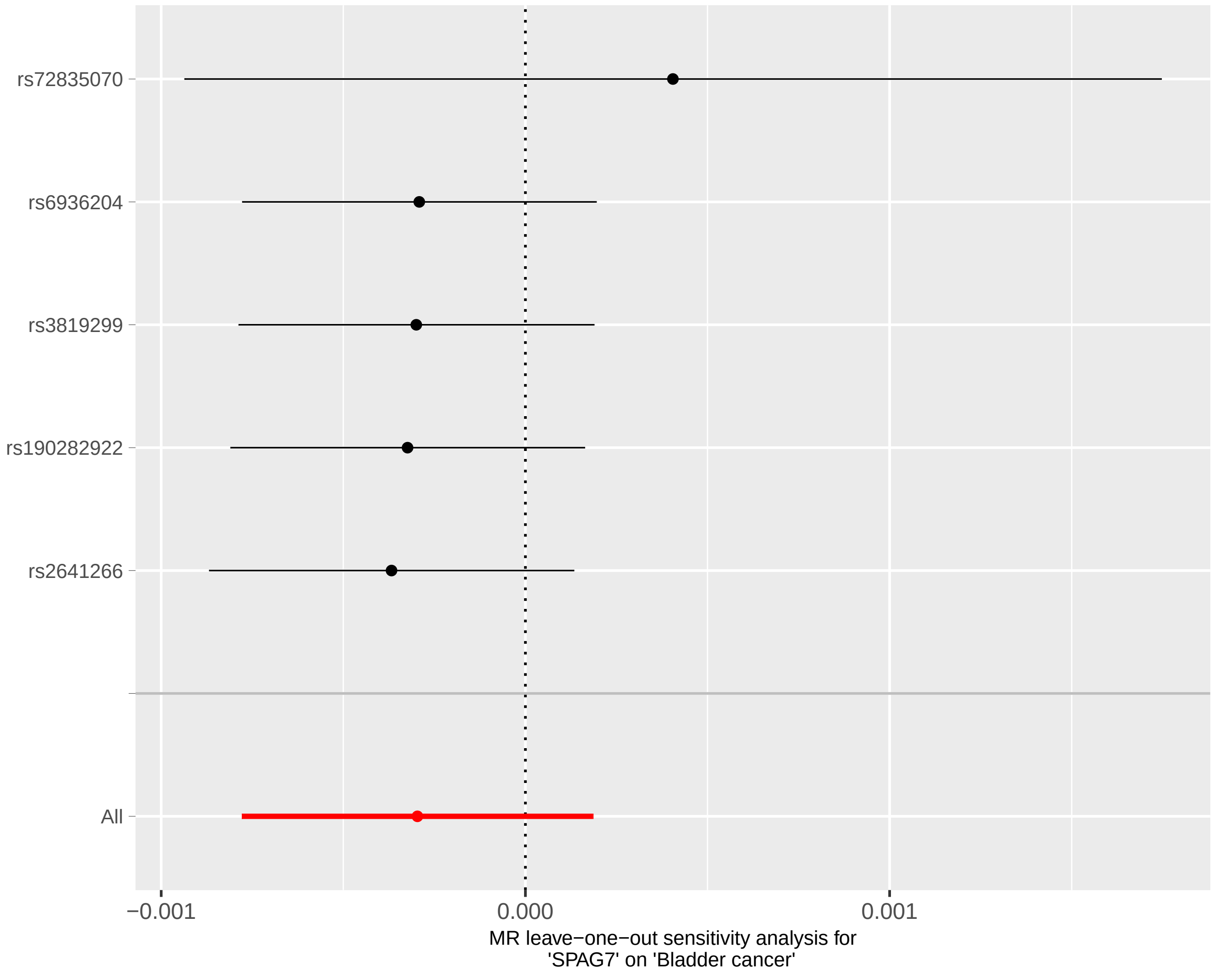
rs72835070
rs6936204
rs3819299
rs190282922
rs2641266
All
−0.001
0.000
0.001
MR leave−one−out sensitivity analysis for
'SPAG7' on 'Bladder cancer'

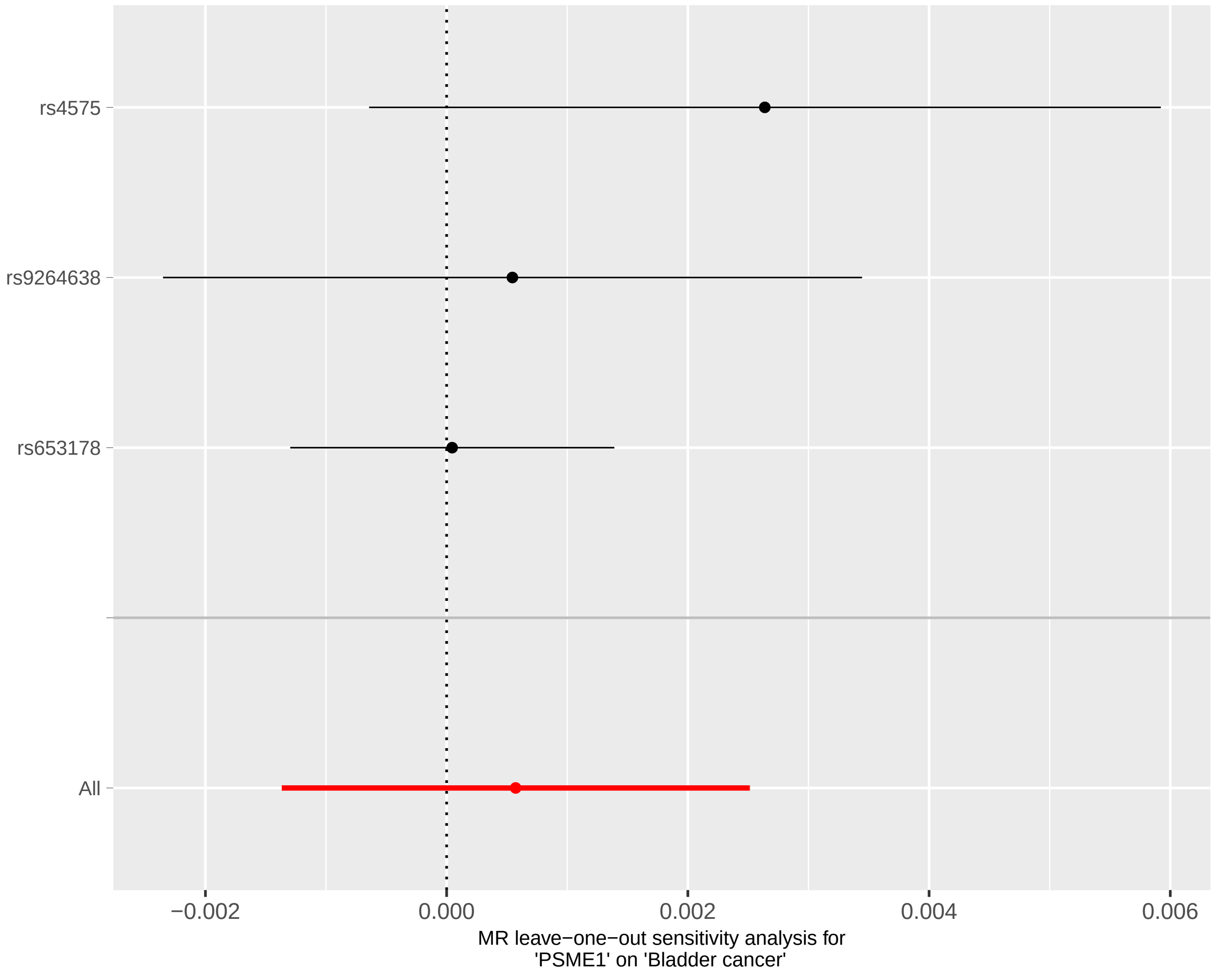
rs4575
rs9264638
rs653178
All
−0.002
0.000
0.002
0.004
0.006
MR leave−one−out sensitivity analysis for
'PSME1' on 'Bladder cancer'

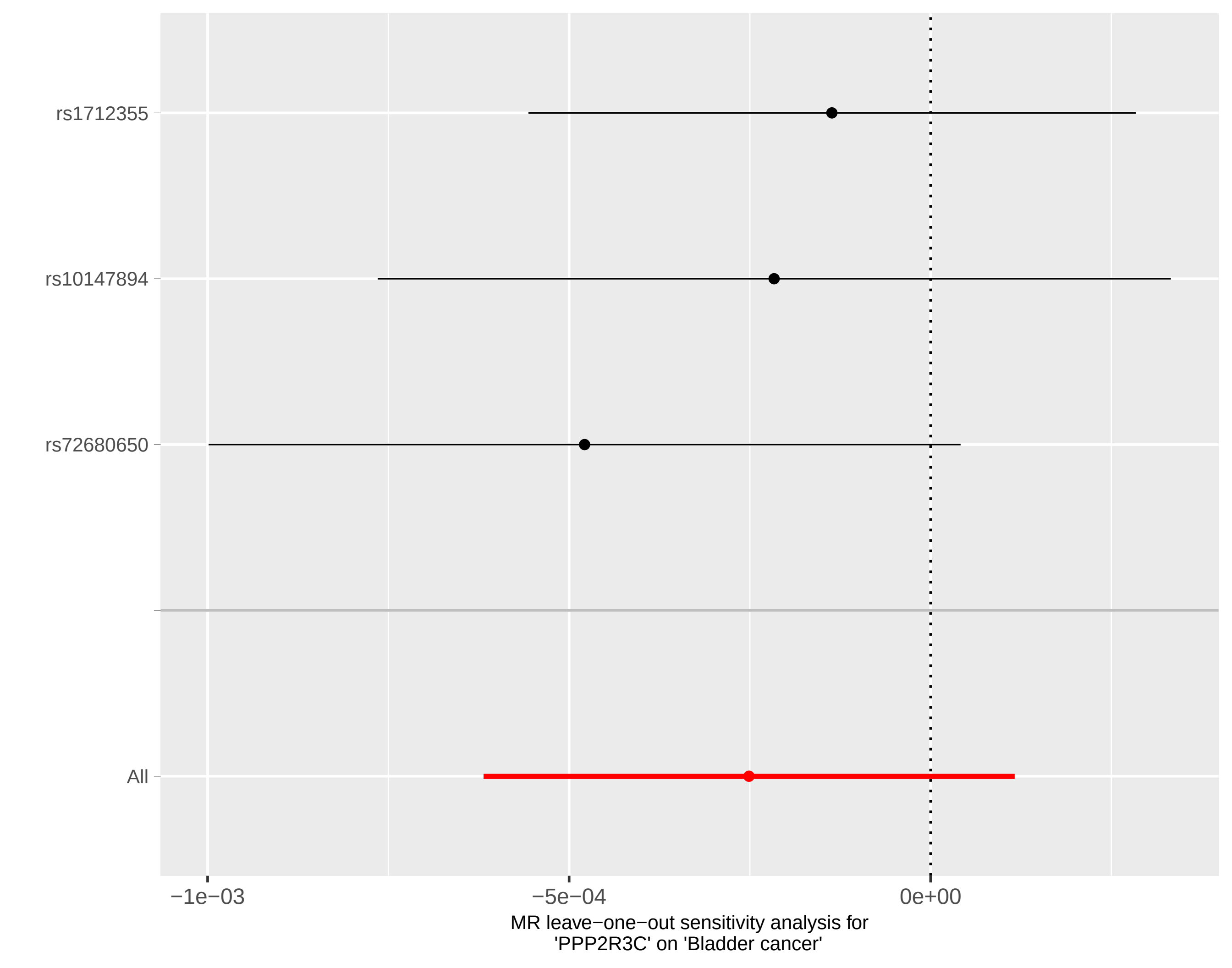
rs1712355
rs10147894
rs72680650
All
−1e−03
−5e−04
0e+00
MR leave−one−out sensitivity analysis for
'PPP2R3C' on 'Bladder cancer'

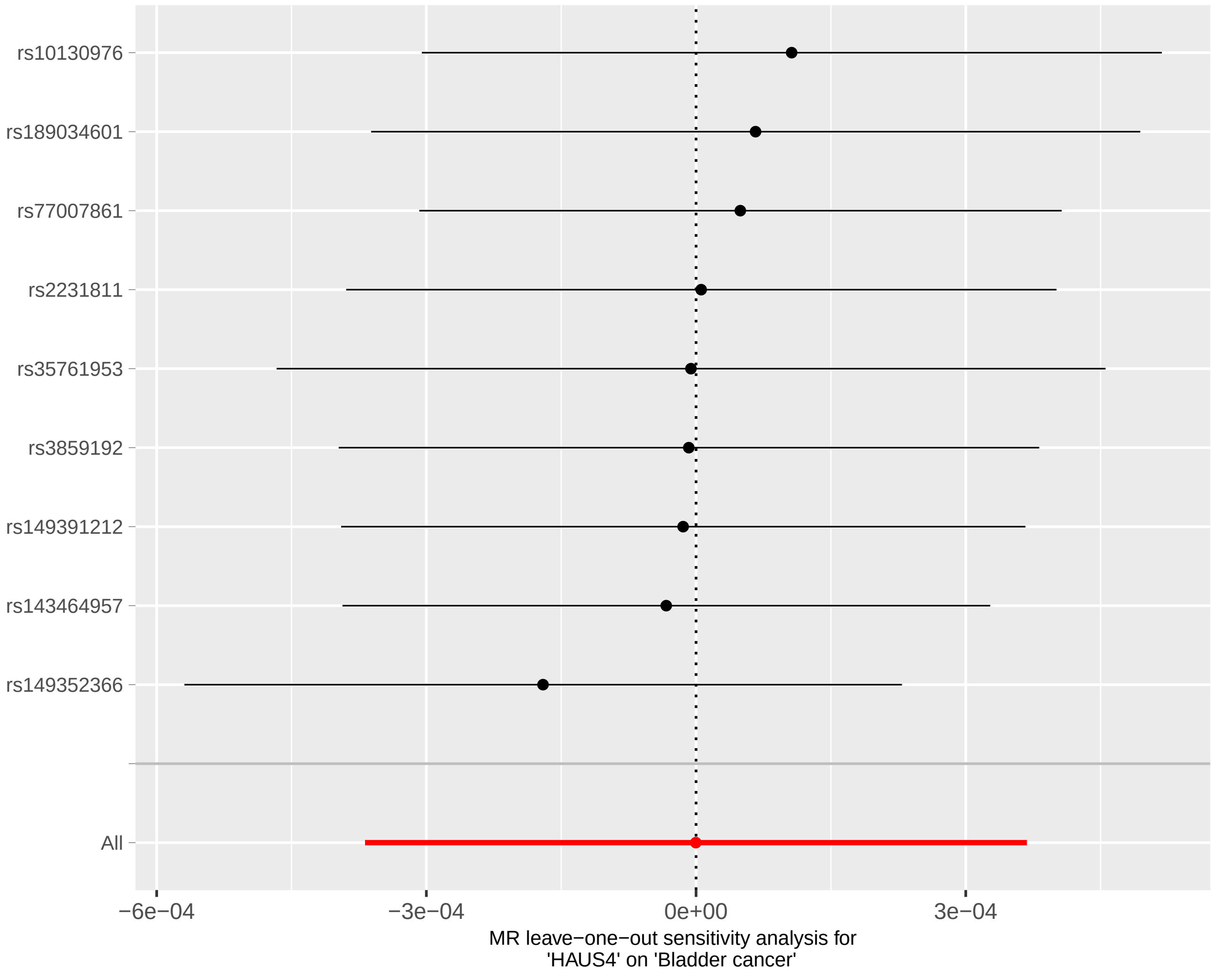
rs10130976
rs189034601
rs77007861
rs2231811
rs35761953
rs3859192
rs149391212
rs143464957
rs149352366
All
−6e−04
−3e−04
0e+00
3e−04
MR leave−one−out sensitivity analysis for
'HAUS4' on 'Bladder cancer'

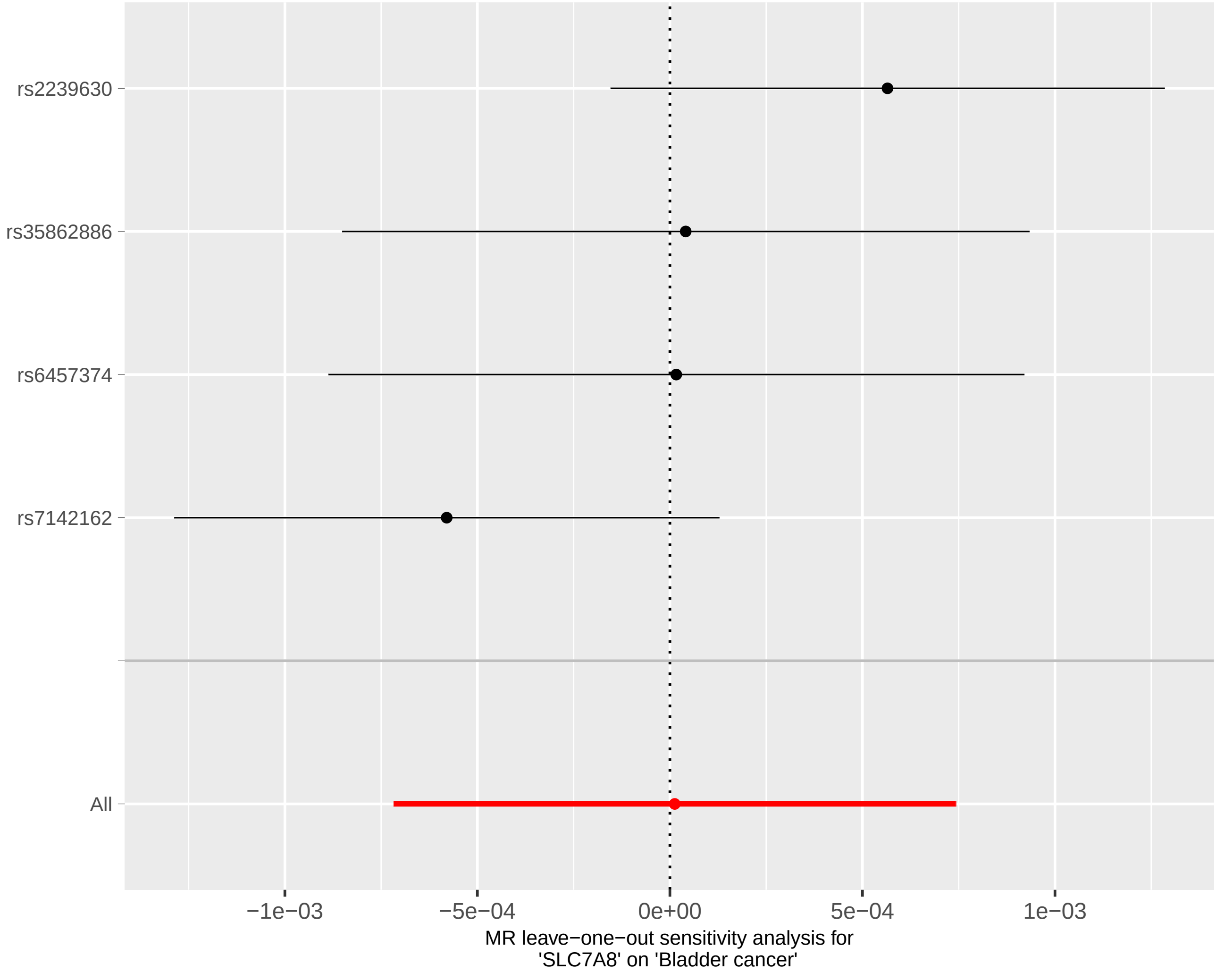
rs2239630
rs35862886
rs6457374
rs7142162
All
−1e−03
−5e−04
0e+00
5e−04
1e−03
MR leave−one−out sensitivity analysis for
'SLC7A8' on 'Bladder cancer'

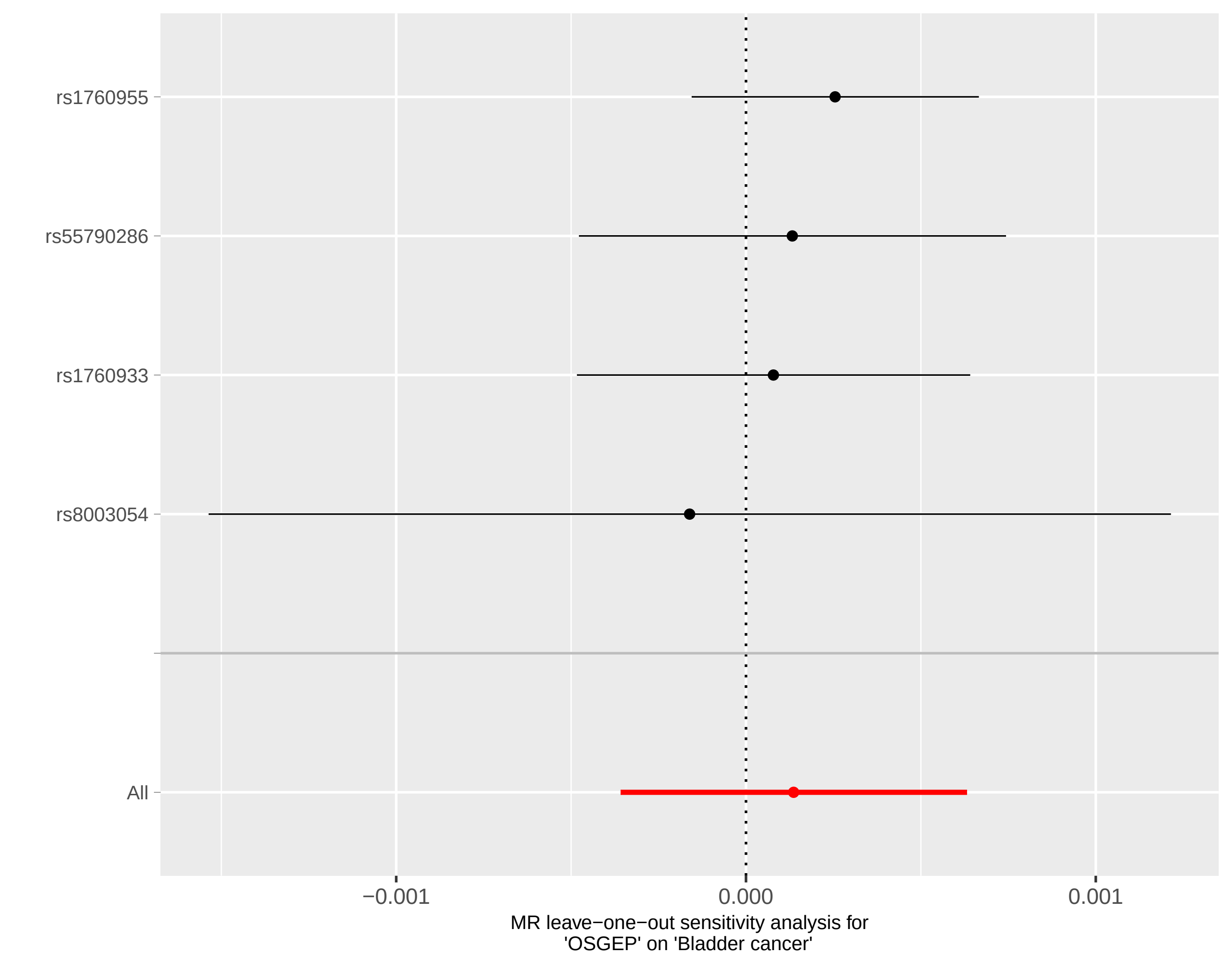
rs1760955
rs55790286
rs1760933
rs8003054
All
−0.001
0.000
0.001
MR leave−one−out sensitivity analysis for
'OSGEP' on 'Bladder cancer'

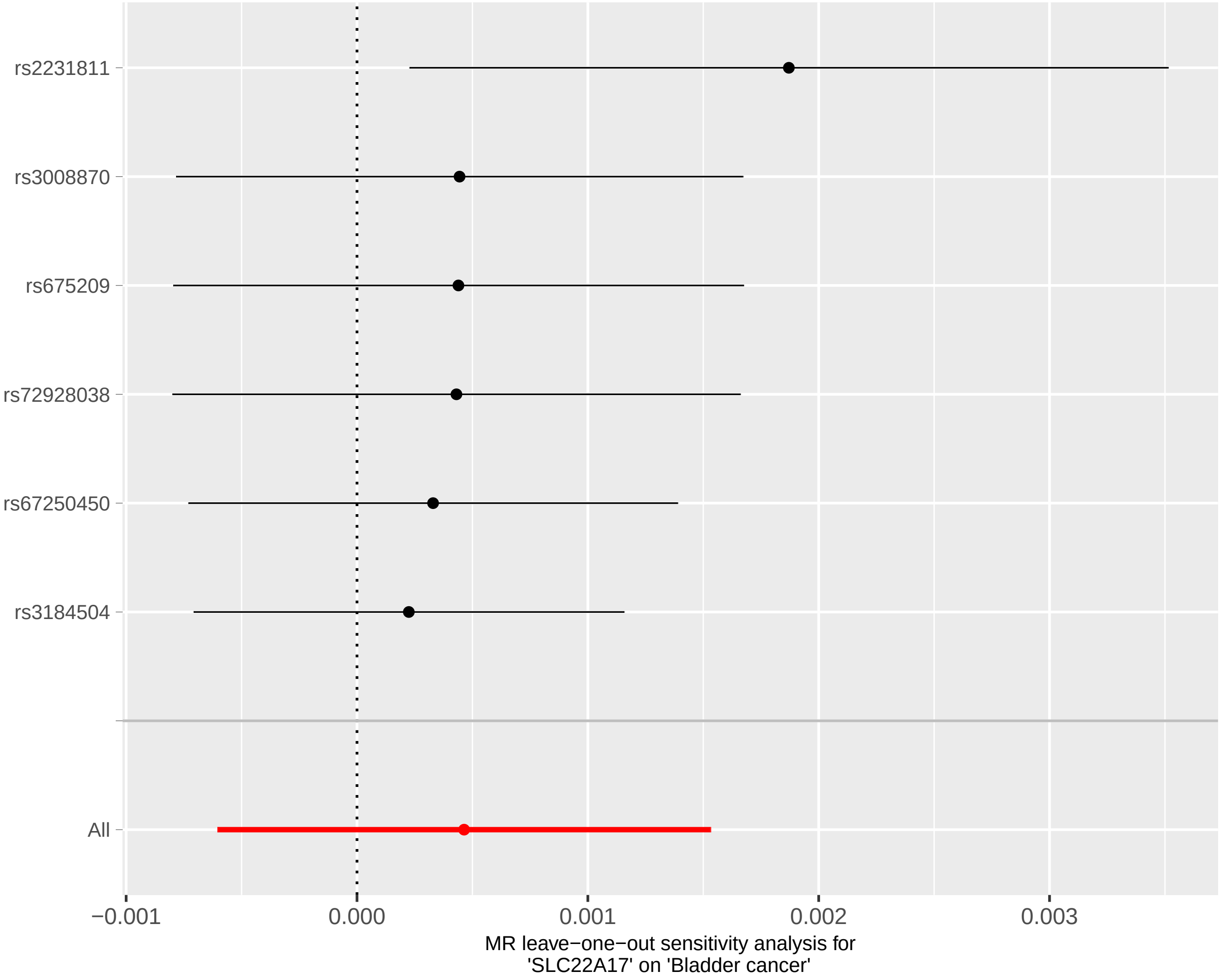
rs2231811
rs3008870
rs675209
rs72928038
rs67250450
rs3184504
All
−0.001
0.000
0.001
0.002
0.003
MR leave−one−out sensitivity analysis for
'SLC22A17' on 'Bladder cancer'

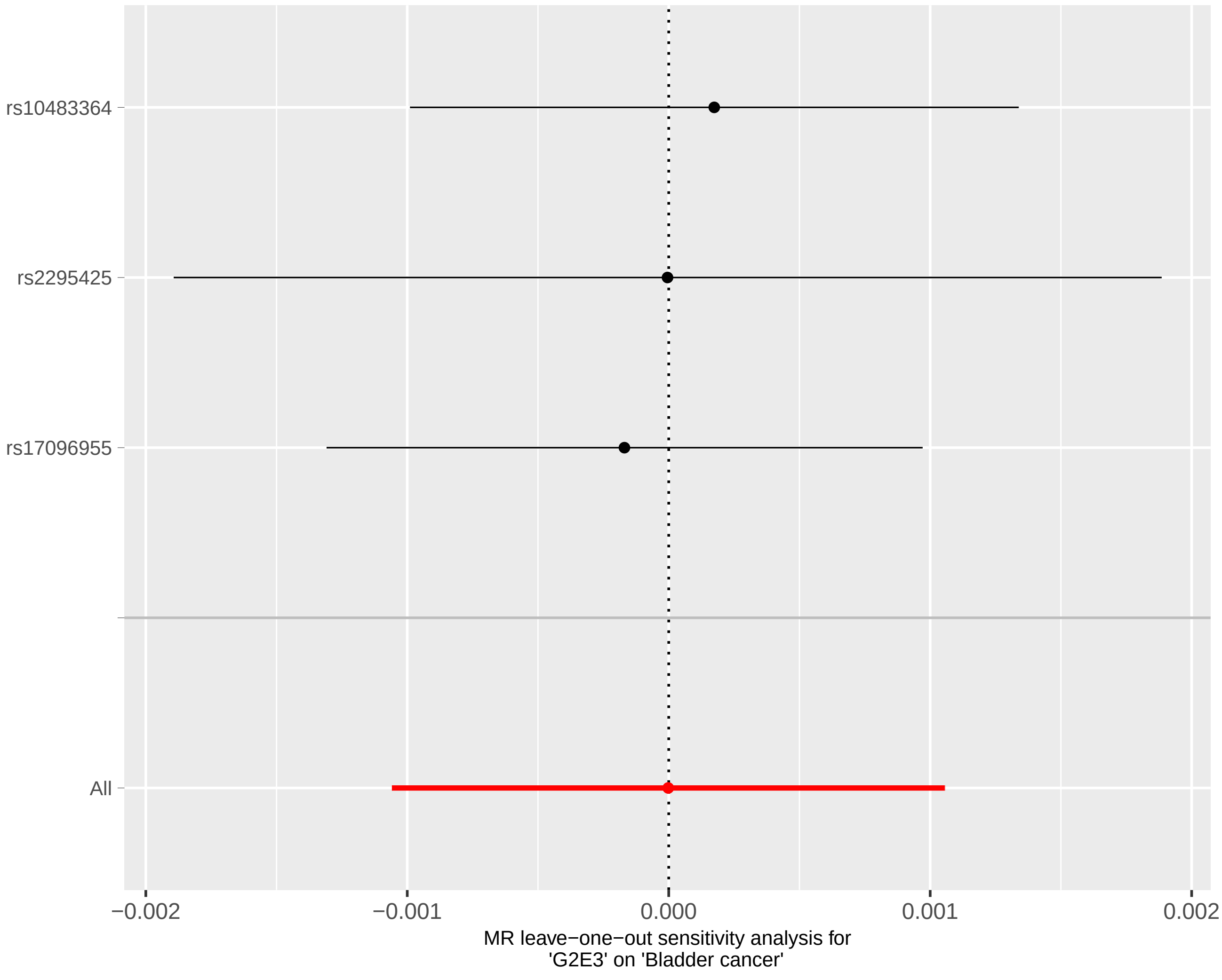
rs10483364
rs2295425
rs17096955
All
−0.002
−0.001
0.000
0.001
0.002
MR leave−one−out sensitivity analysis for
'G2E3' on 'Bladder cancer'

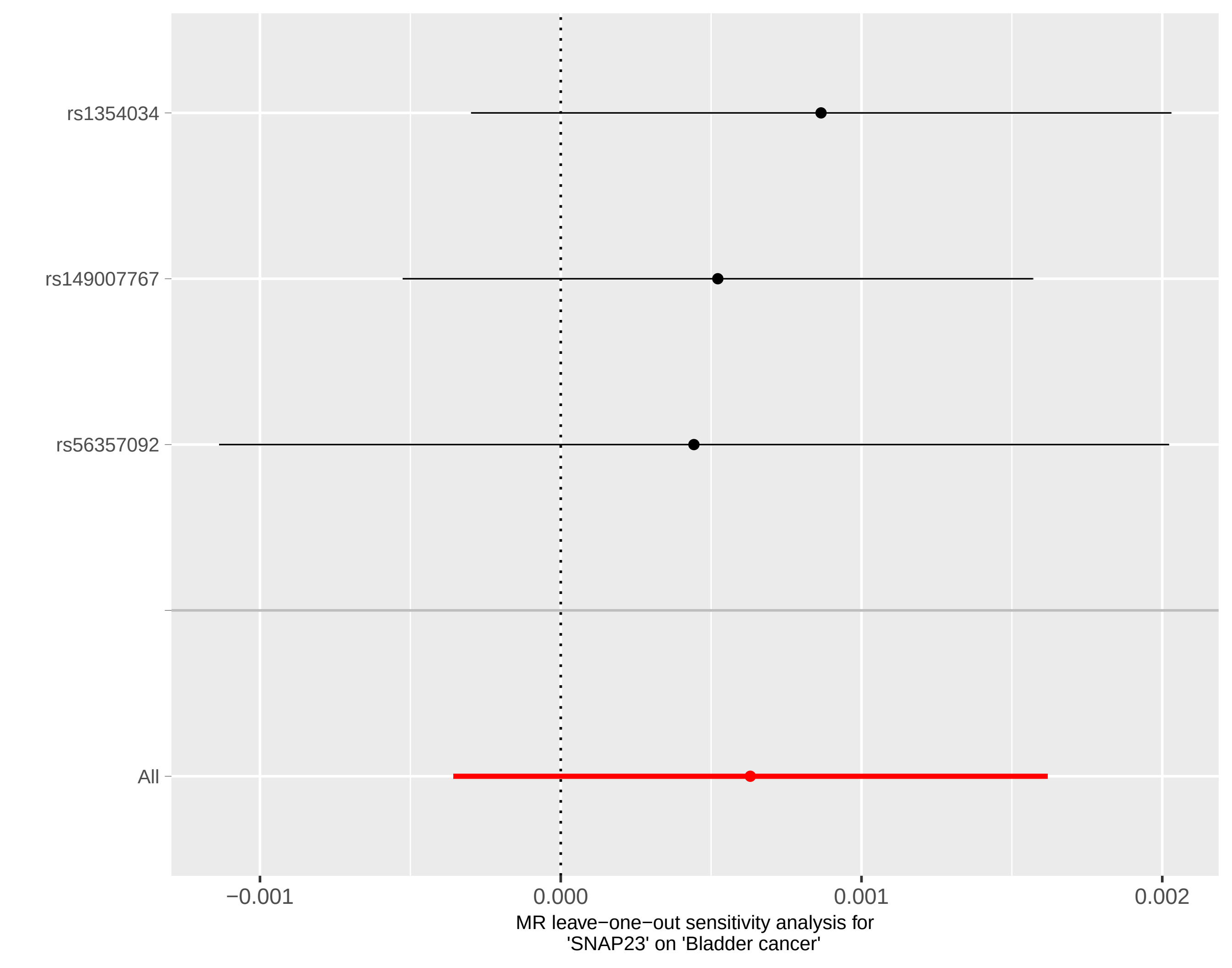
rs1354034
rs149007767
rs56357092
All
−0.001
0.000
0.001
0.002
MR leave−one−out sensitivity analysis for
'SNAP23' on 'Bladder cancer'

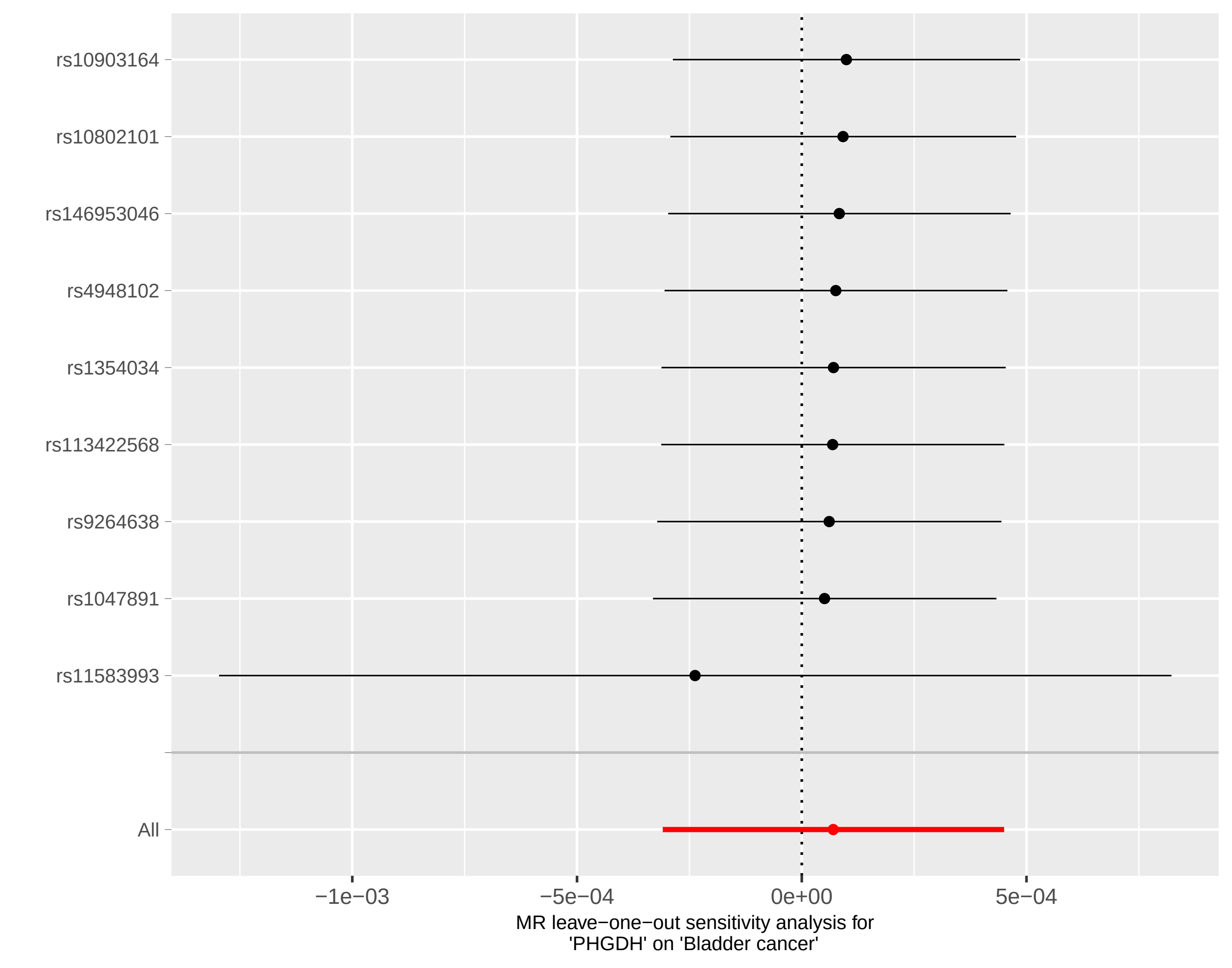

rs10903164
rs10802101
rs146953046
rs4948102
rs1354034
rs113422568
rs9264638
rs1047891
rs11583993
All
−1e−03
−5e−04
0e+00
5e−04
MR leave−one−out sensitivity analysis for
'PHGDH' on 'Bladder cancer'

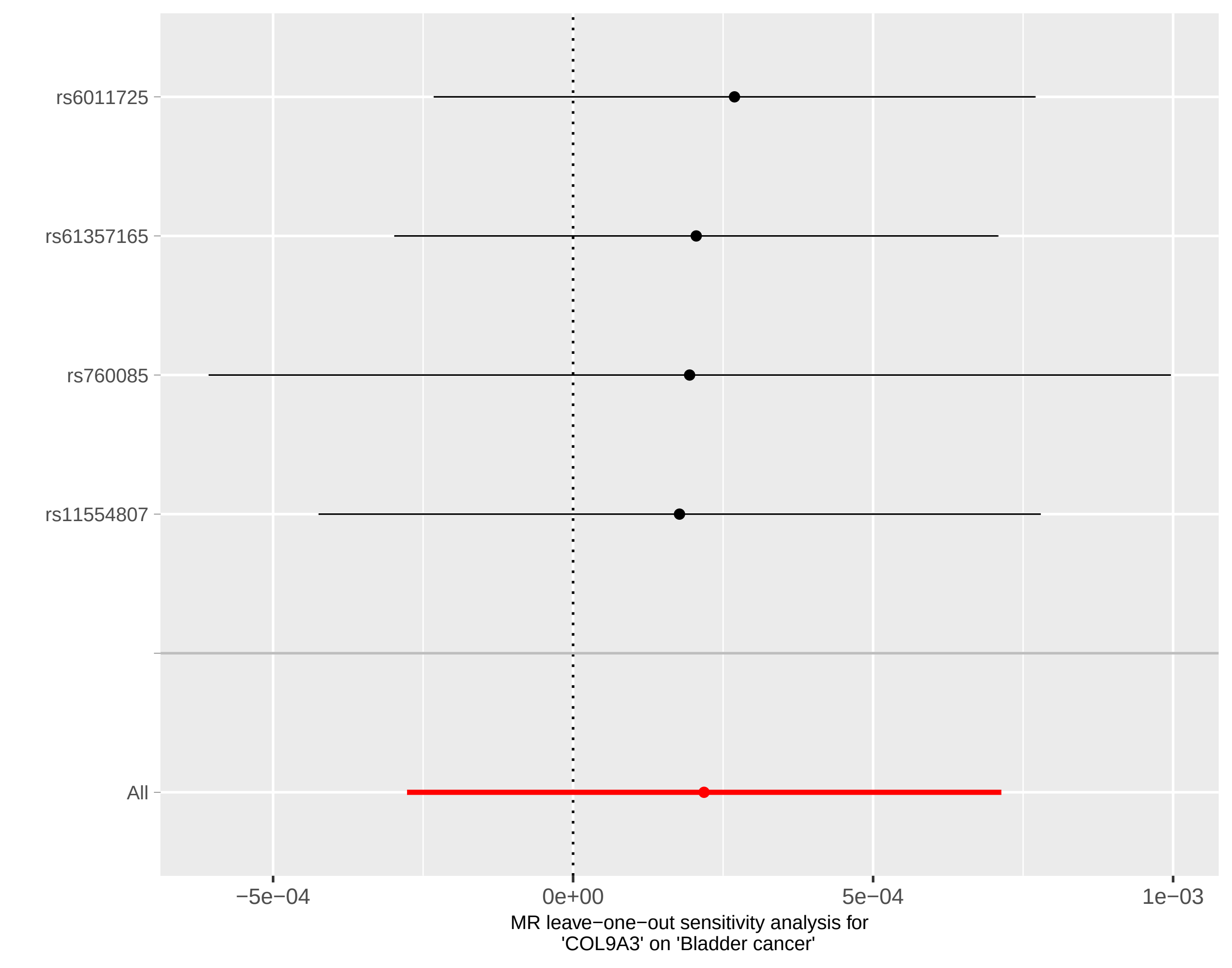
rs6011725
rs61357165
rs760085
rs11554807
All
−5e−04
0e+00
5e−04
1e−03
MR leave−one−out sensitivity analysis for
'COL9A3' on 'Bladder cancer'

Insufficient number of SNPs

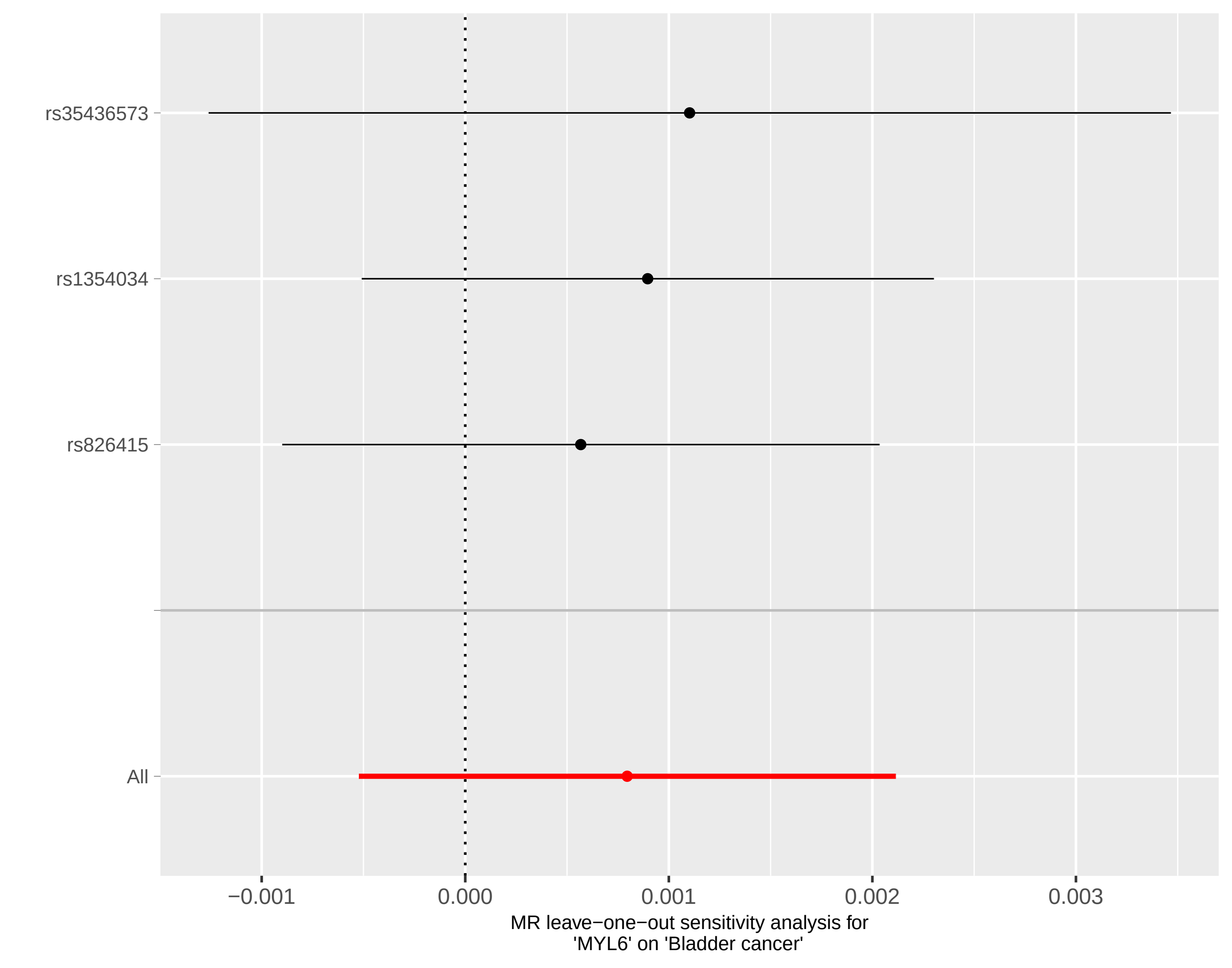

rs35436573
rs1354034
rs826415
All
−0.001
0.000
0.001
0.002
0.003
MR leave−one−out sensitivity analysis for
'MYL6' on 'Bladder cancer'

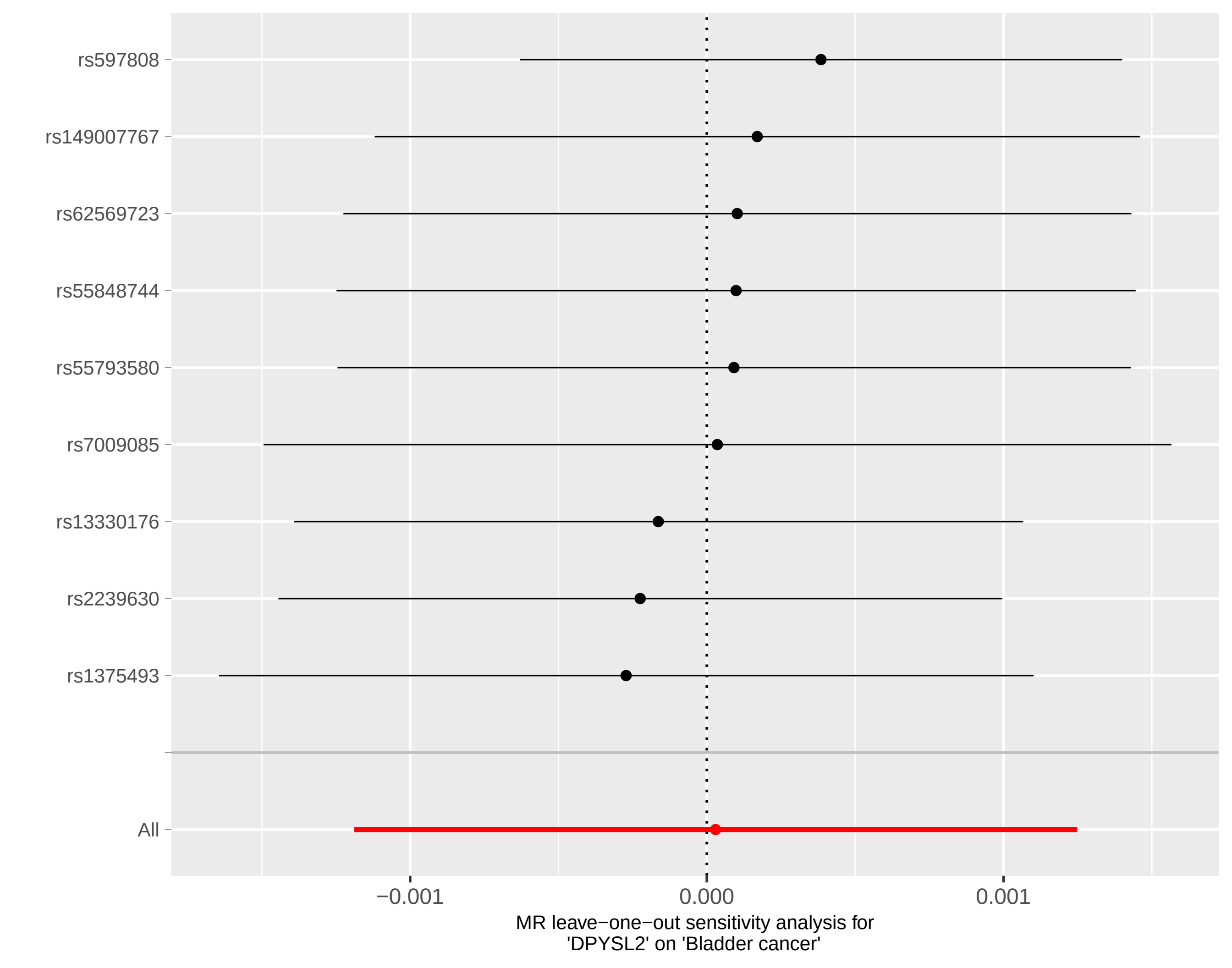
rs597808
rs149007767
rs62569723
rs55848744
rs55793580
rs7009085
rs13330176
rs2239630
rs1375493
All
−0.001
0.000
0.001
MR leave−one−out sensitivity analysis for
'DPYSL2' on 'Bladder cancer'

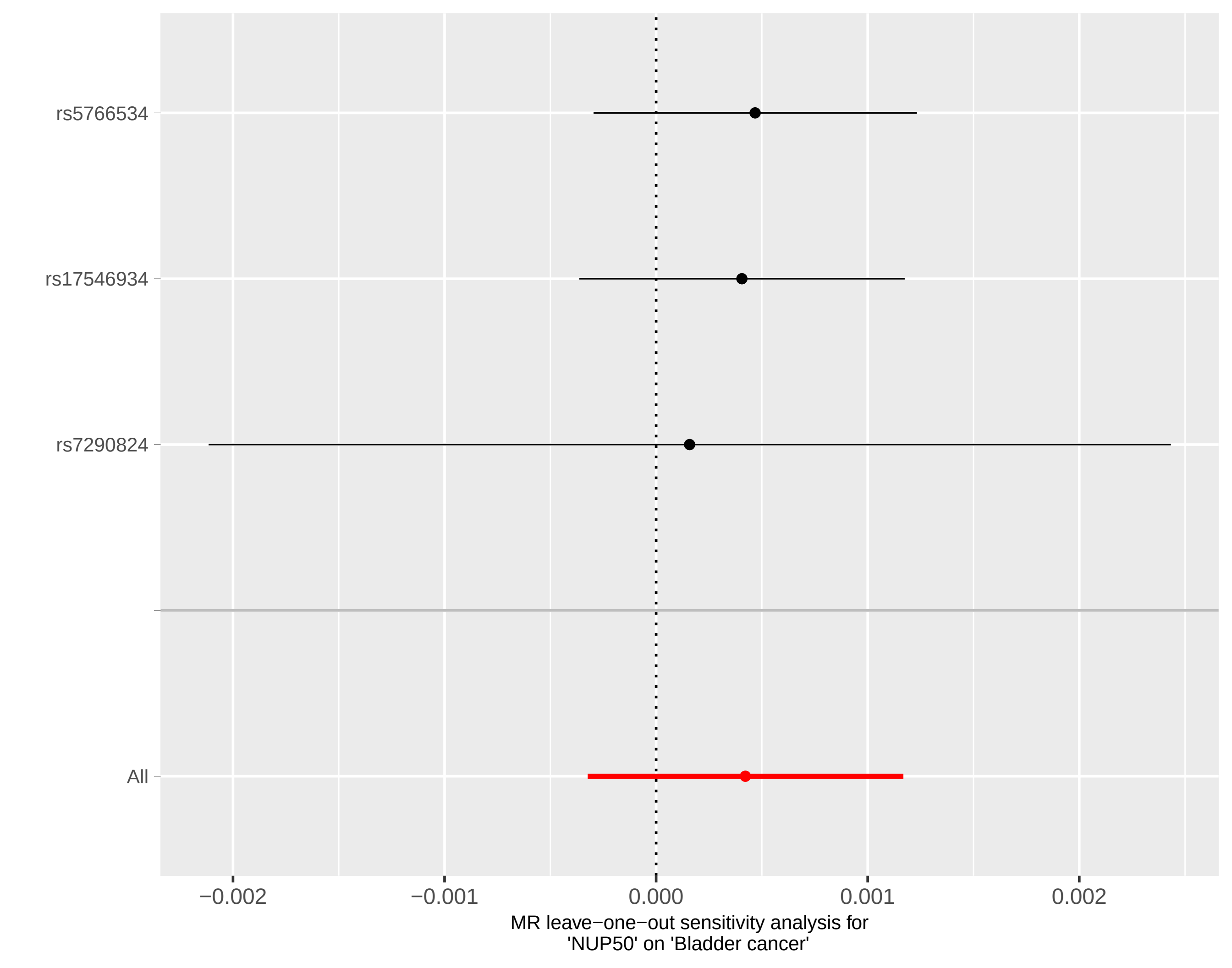
rs5766534
rs17546934
rs7290824
All
−0.002
−0.001
0.000
0.001
0.002
MR leave−one−out sensitivity analysis for
'NUP50' on 'Bladder cancer'

Insufficient number of SNPs

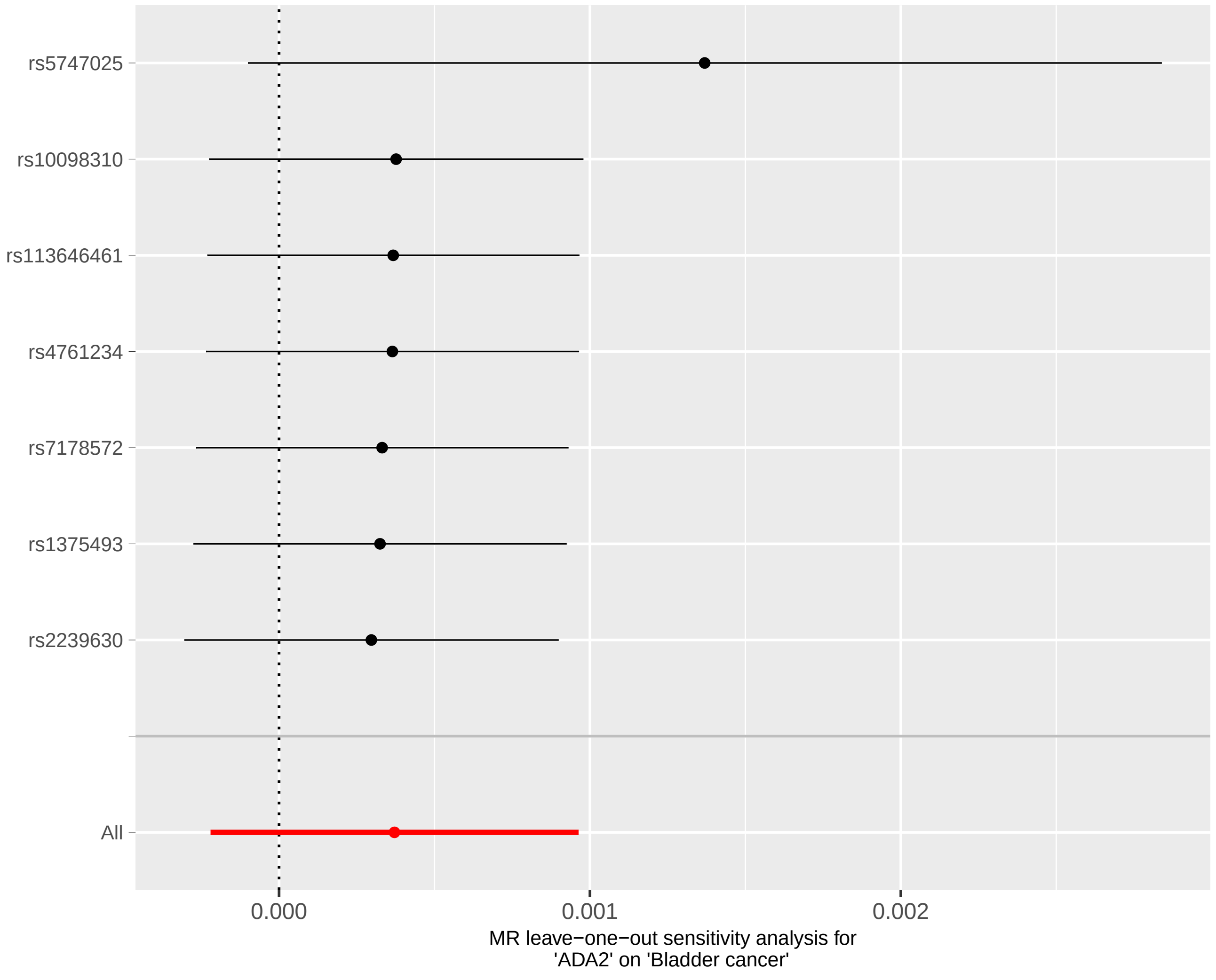
rs5747025
rs10098310
rs113646461
rs4761234
rs7178572
rs1375493
rs2239630
All
0.000
0.001
0.002
MR leave−one−out sensitivity analysis for
'ADA2' on 'Bladder cancer'

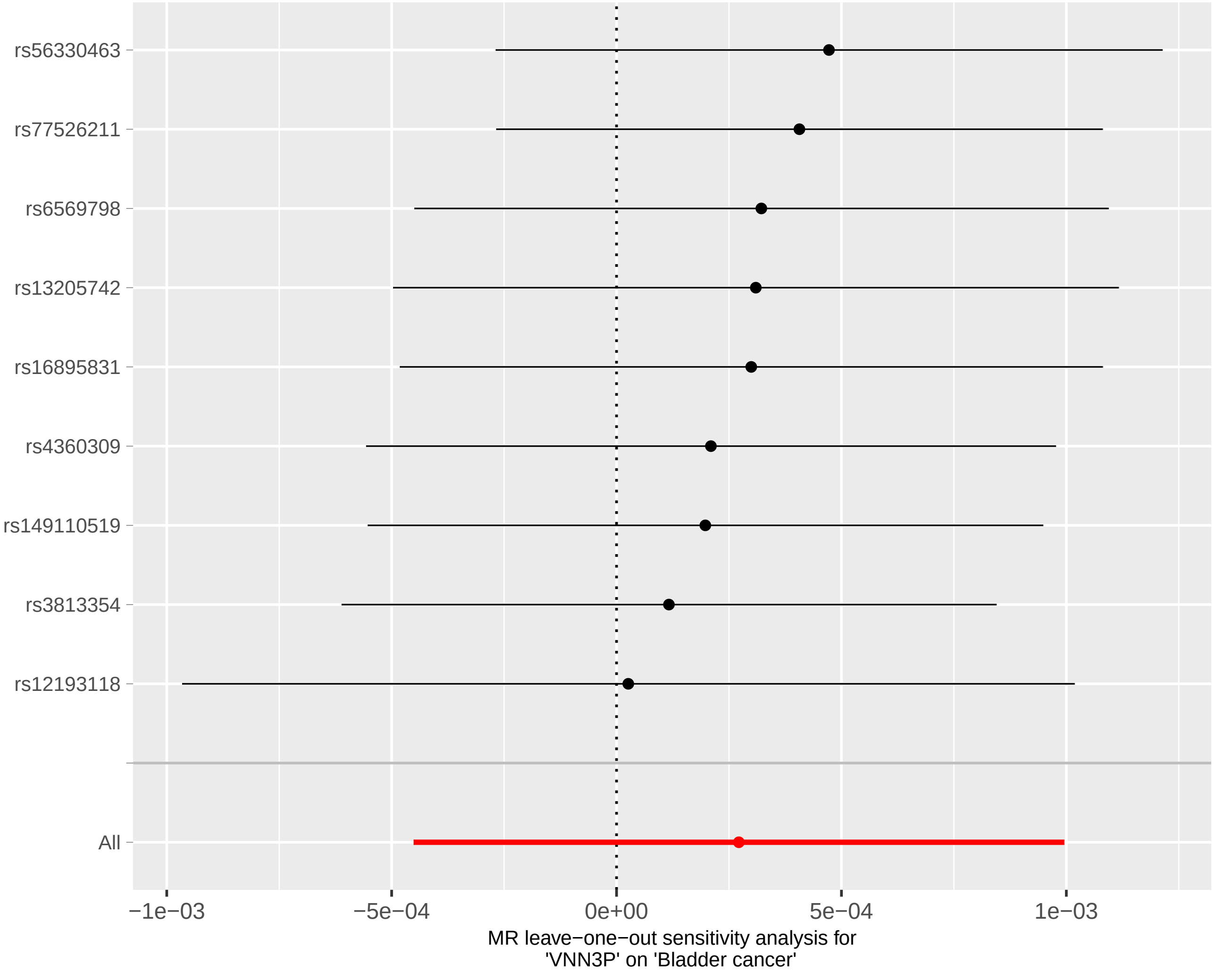

rs56330463
rs77526211
rs6569798
rs13205742
rs16895831
rs4360309
rs149110519
rs3813354
rs12193118
All
−1e−03
−5e−04
0e+00
5e−04
1e−03
MR leave−one−out sensitivity analysis for
'VNN3P' on 'Bladder cancer'

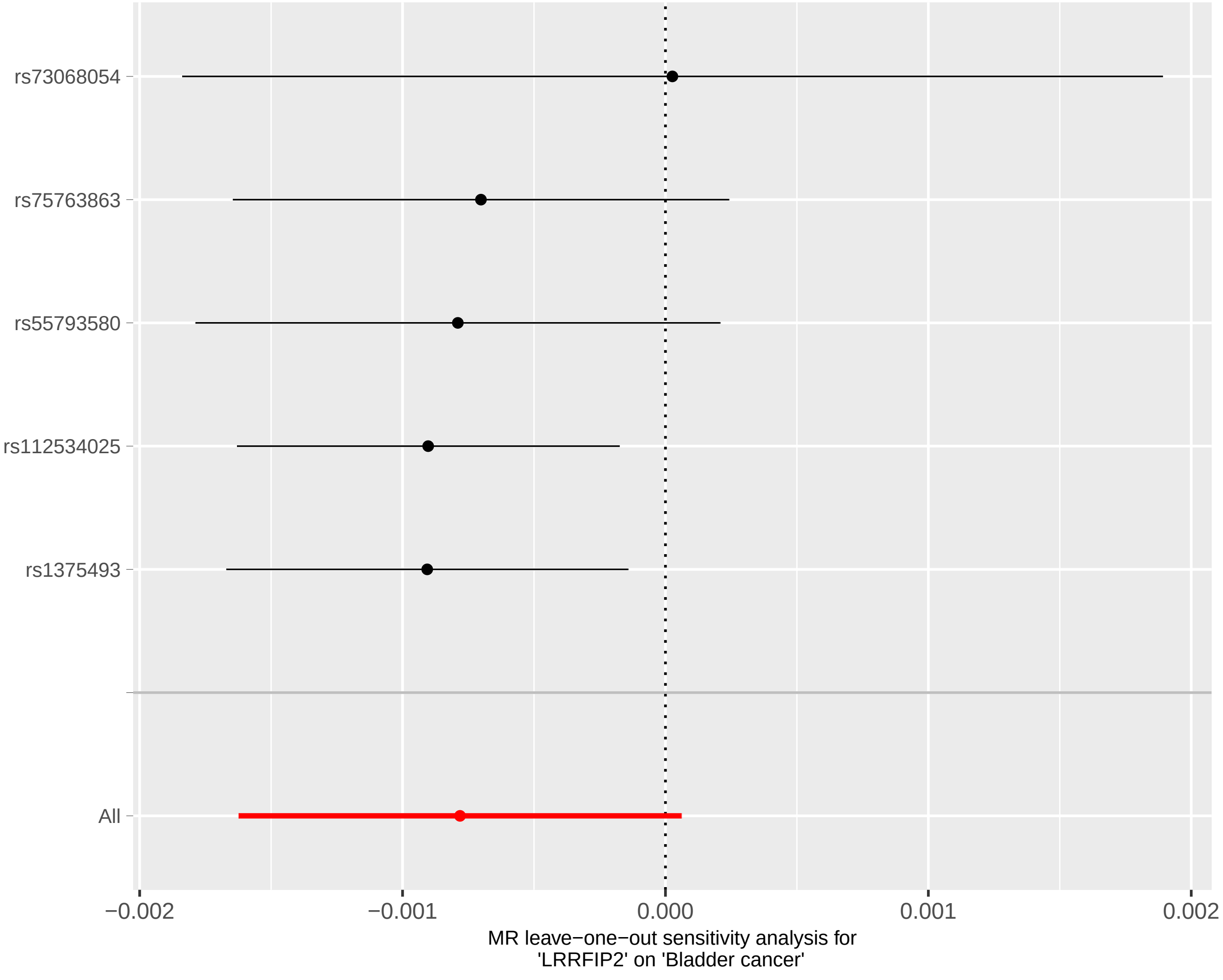

rs73068054
rs75763863
rs55793580
rs112534025
rs1375493
All
−0.002
−0.001
0.000
0.001
0.002
MR leave−one−out sensitivity analysis for
'LRRFIP2' on 'Bladder cancer'

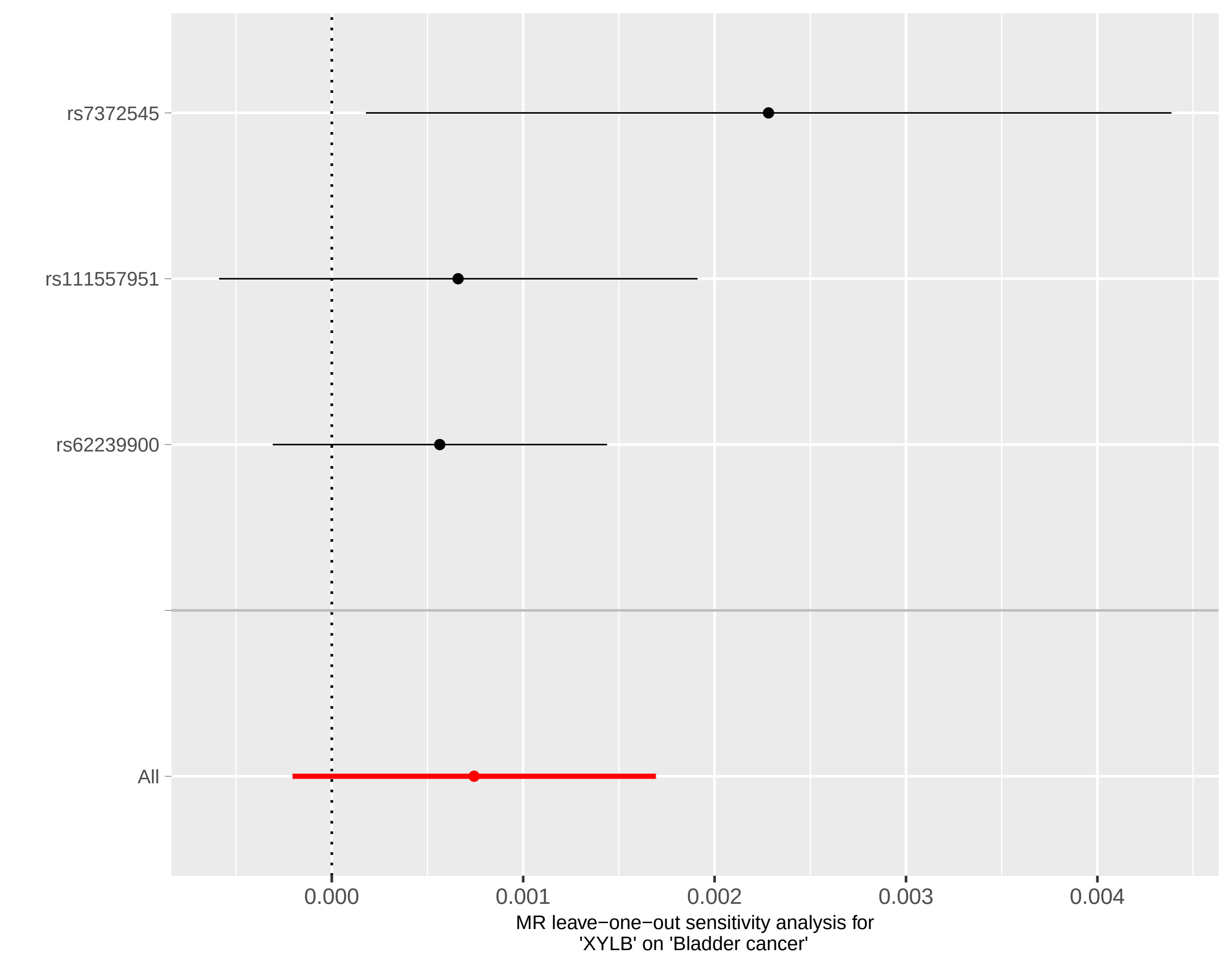

rs7372545
rs111557951
rs62239900
All
0.000
0.001
0.002
0.003
0.004
MR leave−one−out sensitivity analysis for
'XYLB' on 'Bladder cancer'

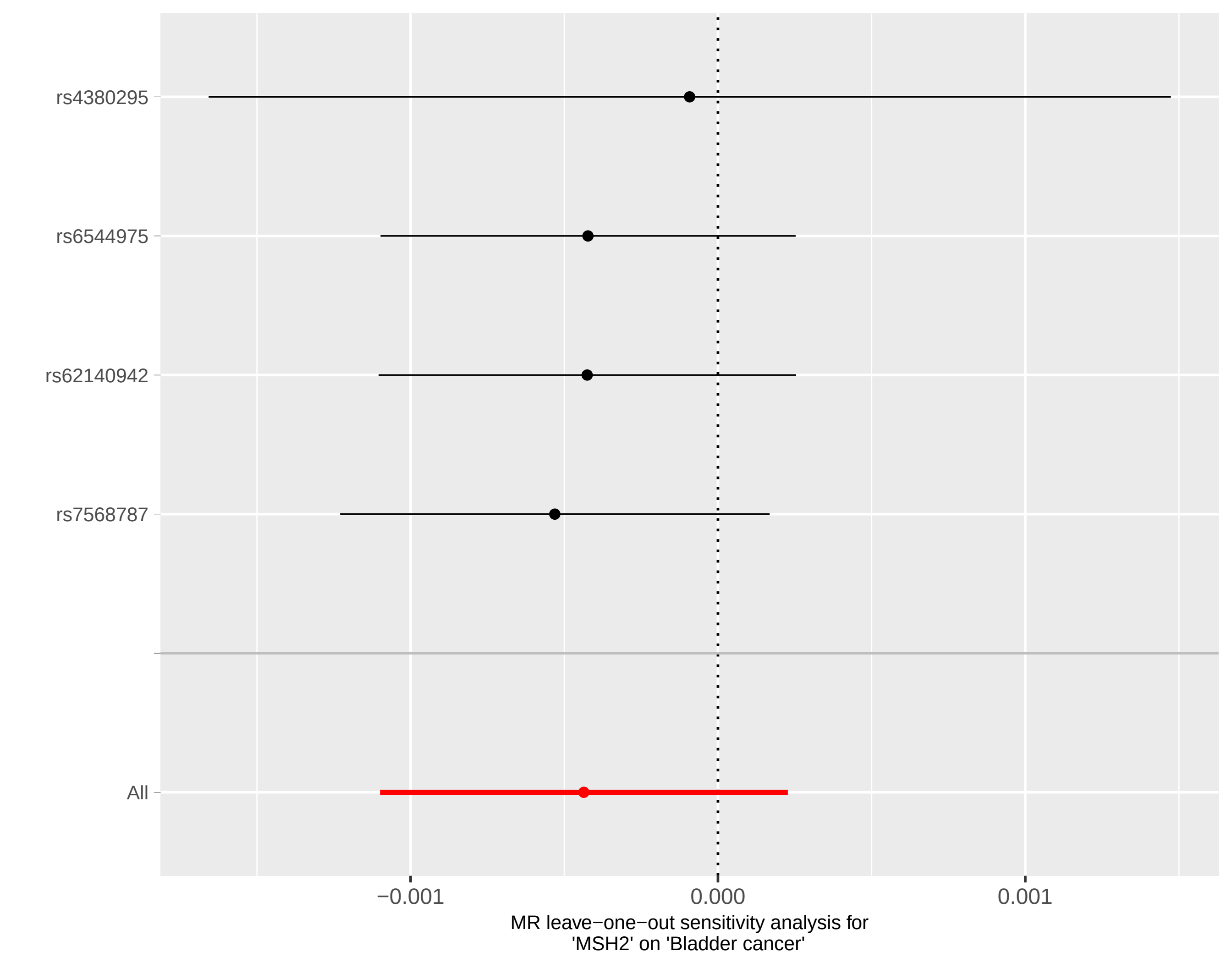
rs4380295
rs6544975
rs62140942
rs7568787
All
−0.001
0.000
0.001
MR leave−one−out sensitivity analysis for
'MSH2' on 'Bladder cancer'

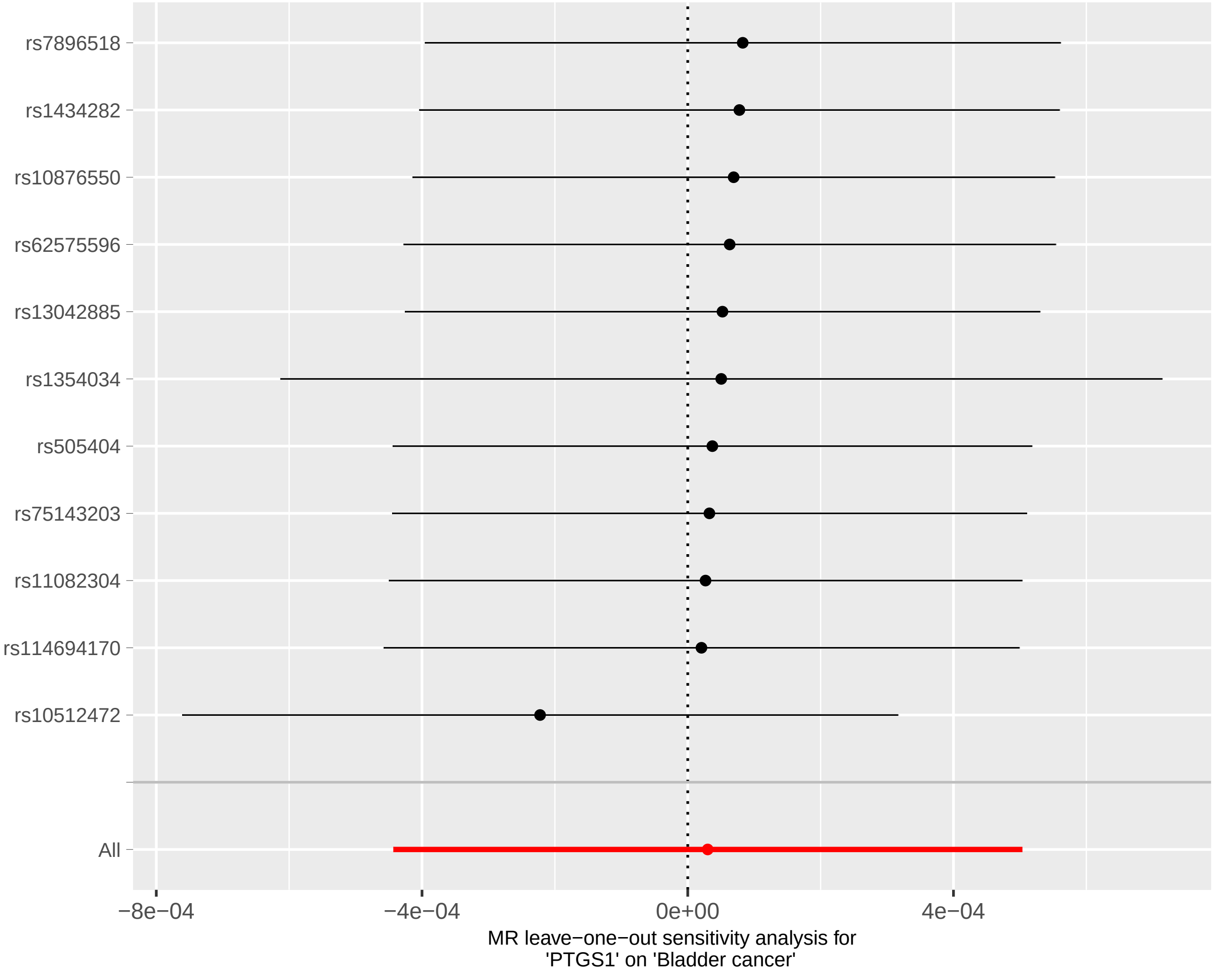
rs7896518
rs1434282
rs10876550
rs62575596
rs13042885
rs1354034
rs505404
rs75143203
rs11082304
rs114694170
rs10512472
All
−8e−04
−4e−04
0e+00
4e−04
MR leave−one−out sensitivity analysis for
'PTGS1' on 'Bladder cancer'

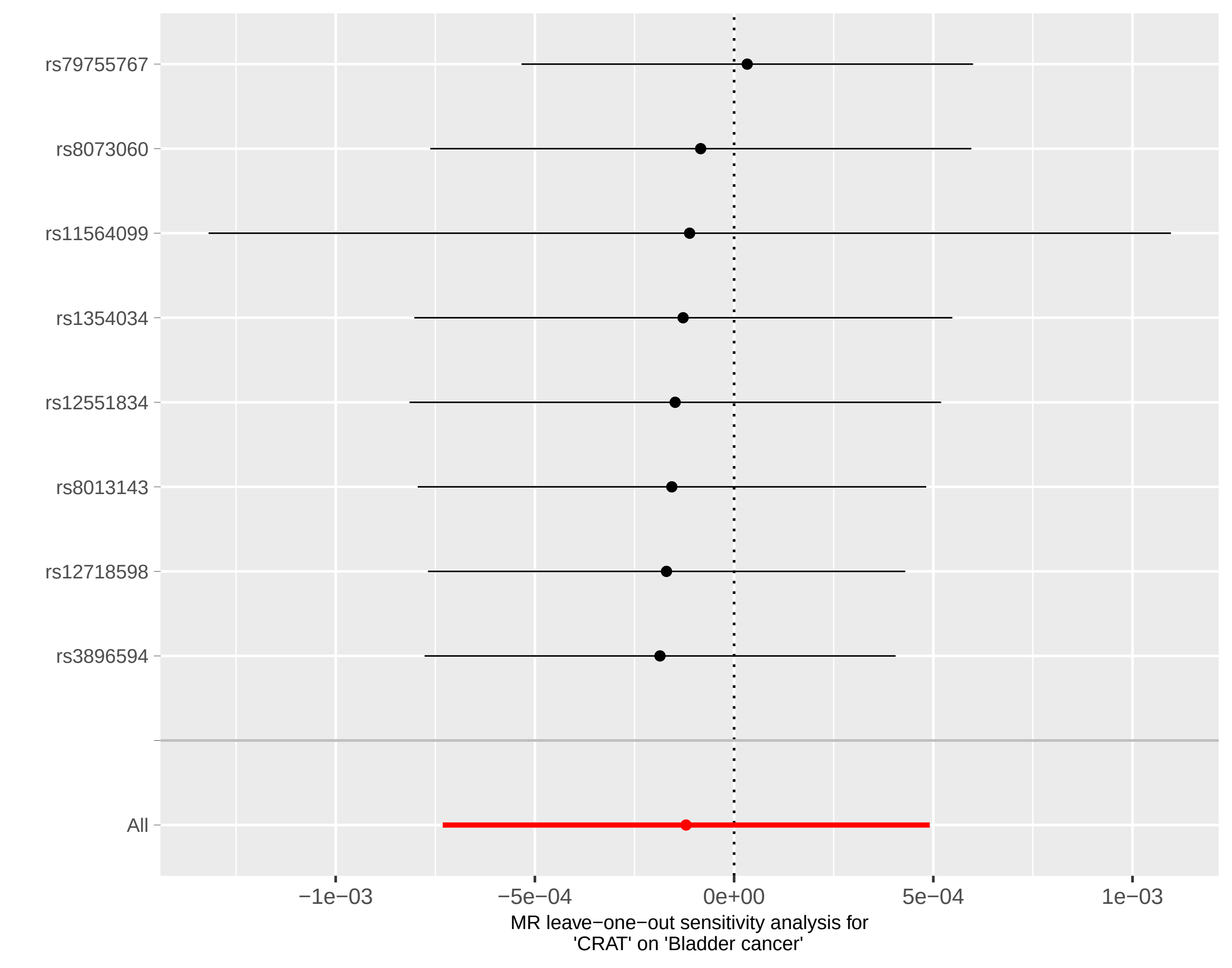

rs79755767
rs8073060
rs11564099
rs1354034
rs12551834
rs8013143
rs12718598
rs3896594
All
−1e−03
−5e−04
0e+00
5e−04
1e−03
MR leave−one−out sensitivity analysis for
'CRAT' on 'Bladder cancer'

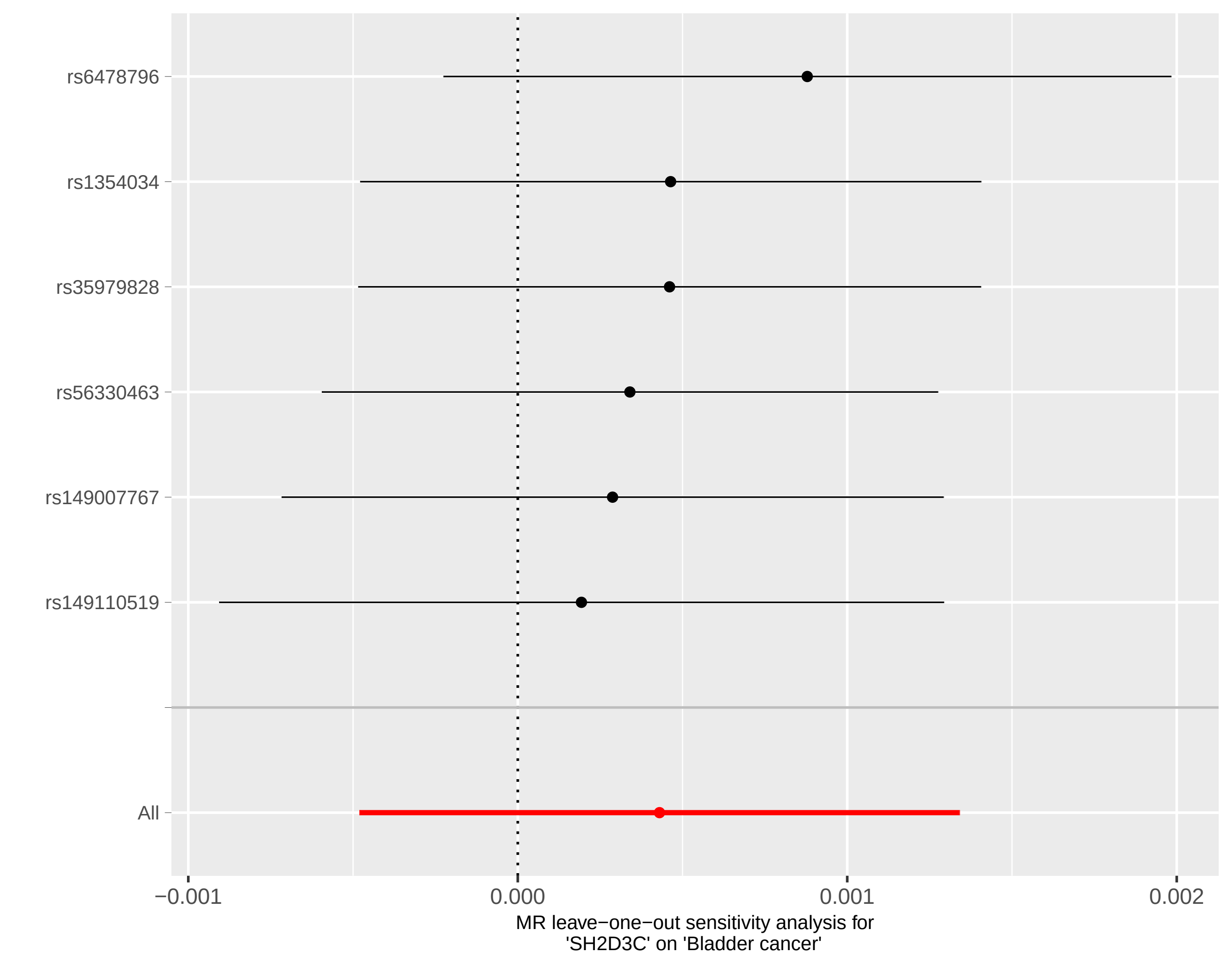
rs6478796
rs1354034
rs35979828
rs56330463
rs149007767
rs149110519
All
−0.001
0.000
0.001
0.002
MR leave−one−out sensitivity analysis for
'SH2D3C' on 'Bladder cancer'

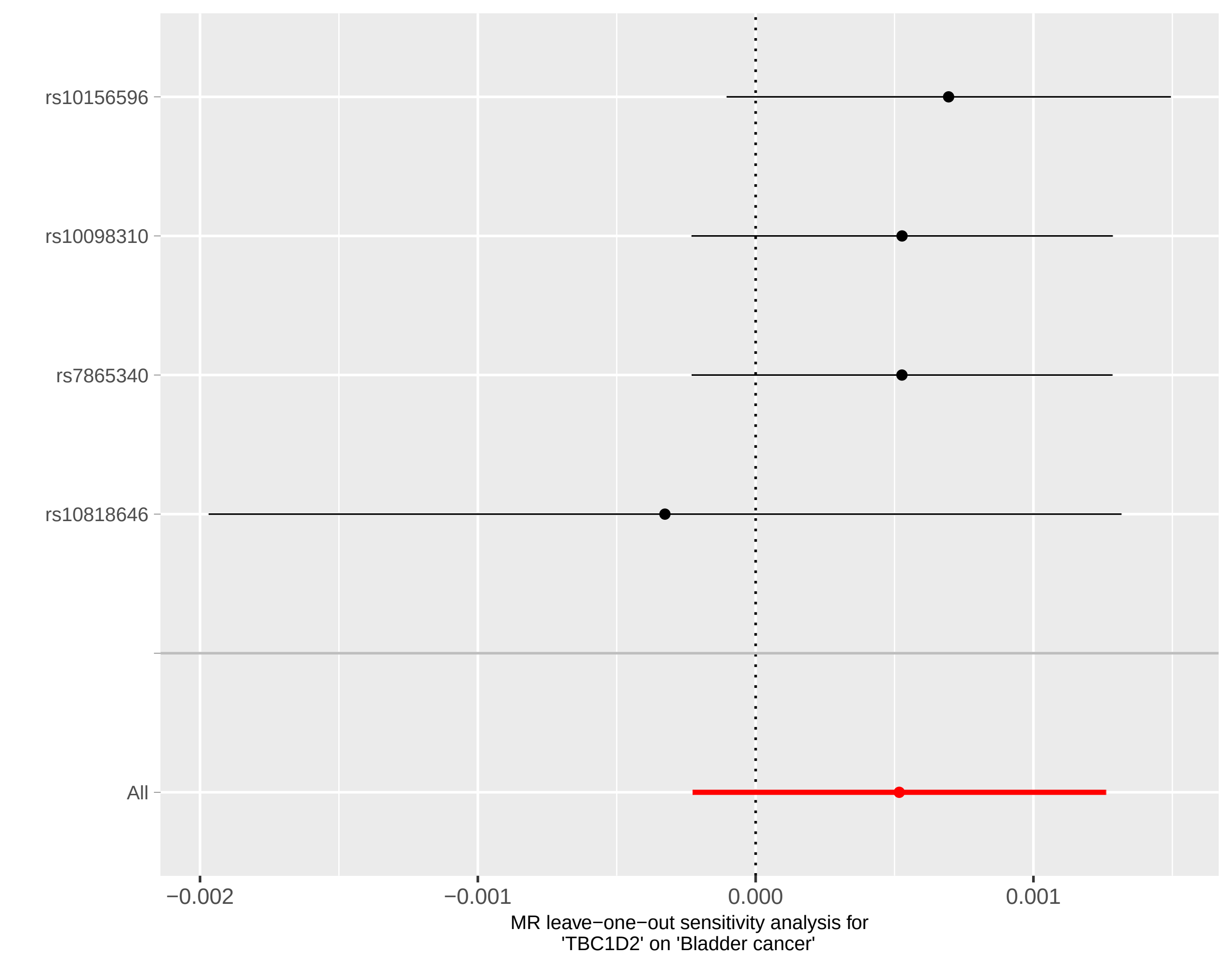
rs10156596
rs10098310
rs7865340
rs10818646
All
−0.002
−0.001
0.000
0.001
MR leave−one−out sensitivity analysis for
'TBC1D2' on 'Bladder cancer'

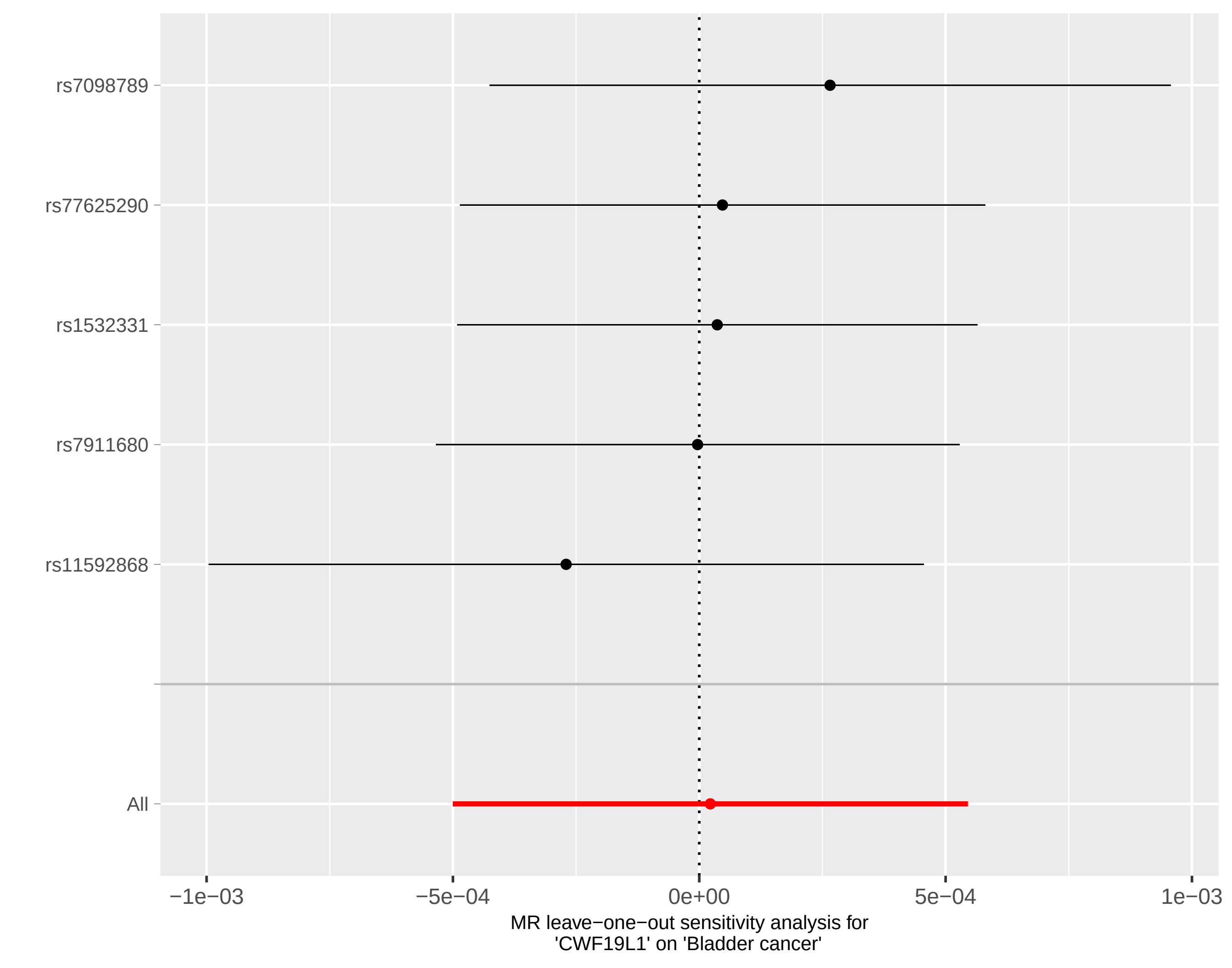
rs7098789
rs77625290
rs1532331
rs7911680
rs11592868
All
−1e−03
−5e−04
0e+00
5e−04
1e−03
MR leave−one−out sensitivity analysis for
'CWF19L1' on 'Bladder cancer'

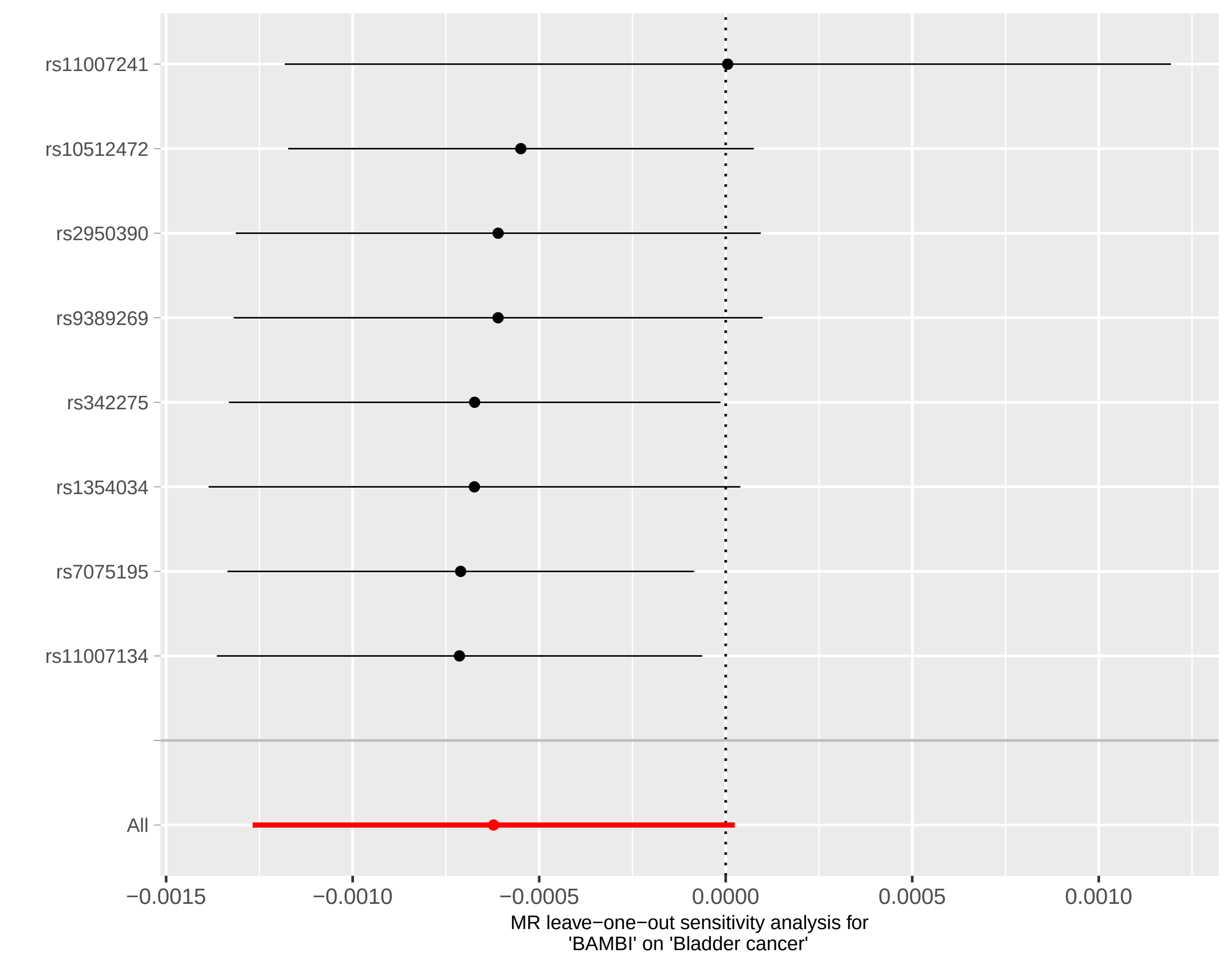
rs11007241
rs10512472
rs2950390
rs9389269
rs342275
rs1354034
rs7075195
rs11007134
All
−0.0015
−0.0010
−0.0005
0.0000
0.0005
0.0010
MR leave−one−out sensitivity analysis for
'BAMBI' on 'Bladder cancer'

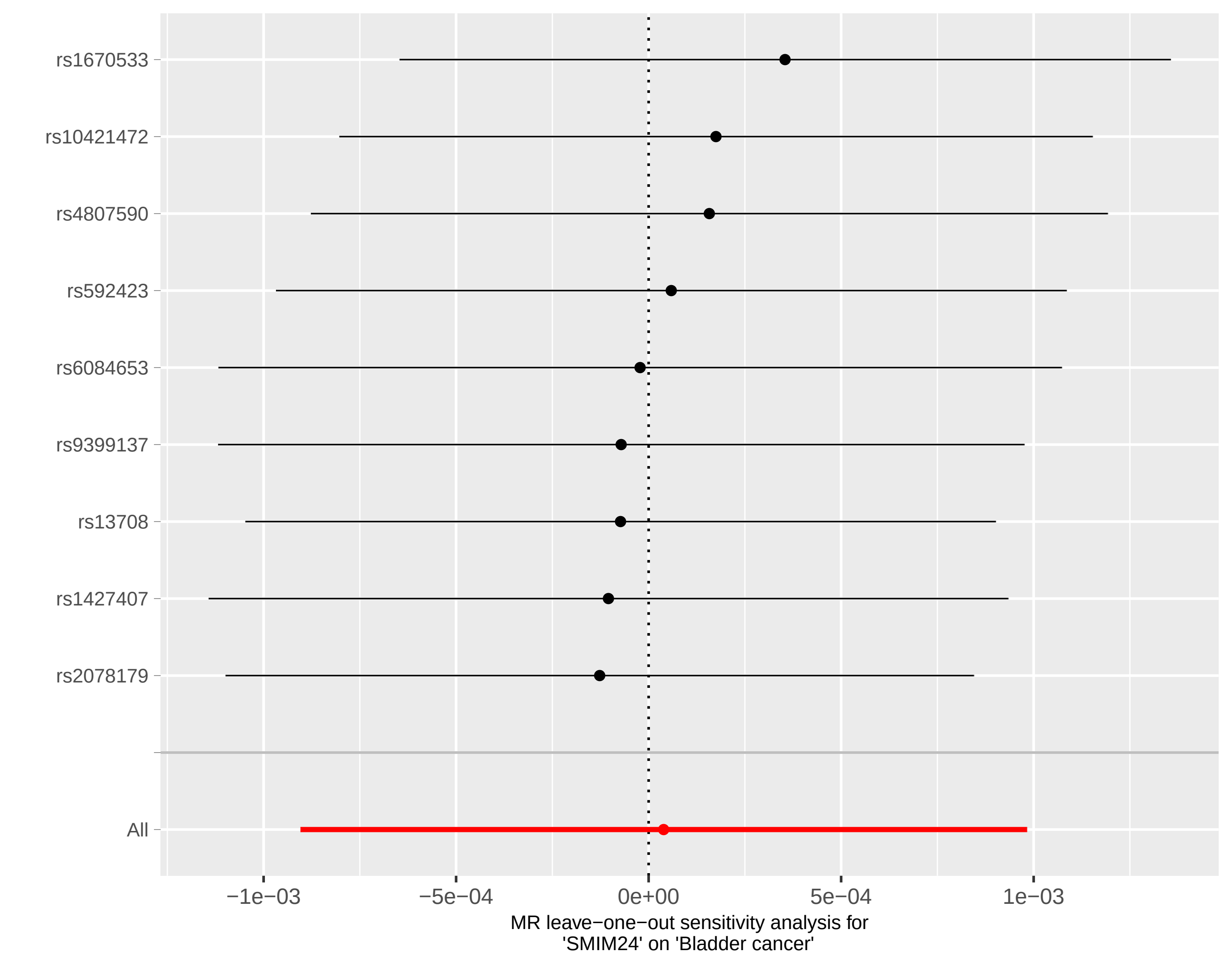
rs1670533
rs10421472
rs4807590
rs592423
rs6084653
rs9399137
rs13708
rs1427407
rs2078179
All
−1e−03
−5e−04
0e+00
5e−04
1e−03
MR leave−one−out sensitivity analysis for
'SMIM24' on 'Bladder cancer'

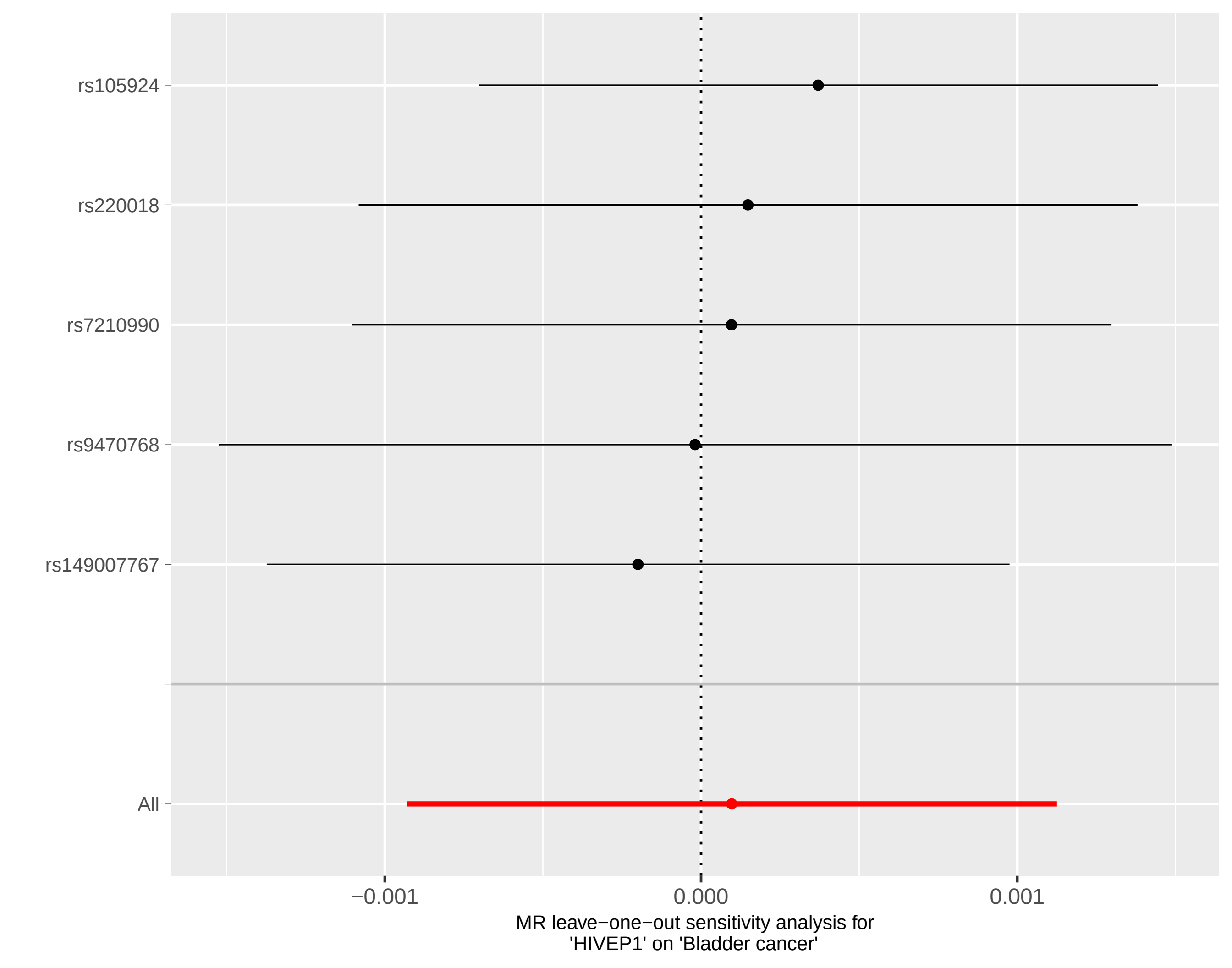
rs105924
rs220018
rs7210990
rs9470768
rs149007767
All
−0.001
0.000
0.001
MR leave−one−out sensitivity analysis for
'HIVEP1' on 'Bladder cancer'

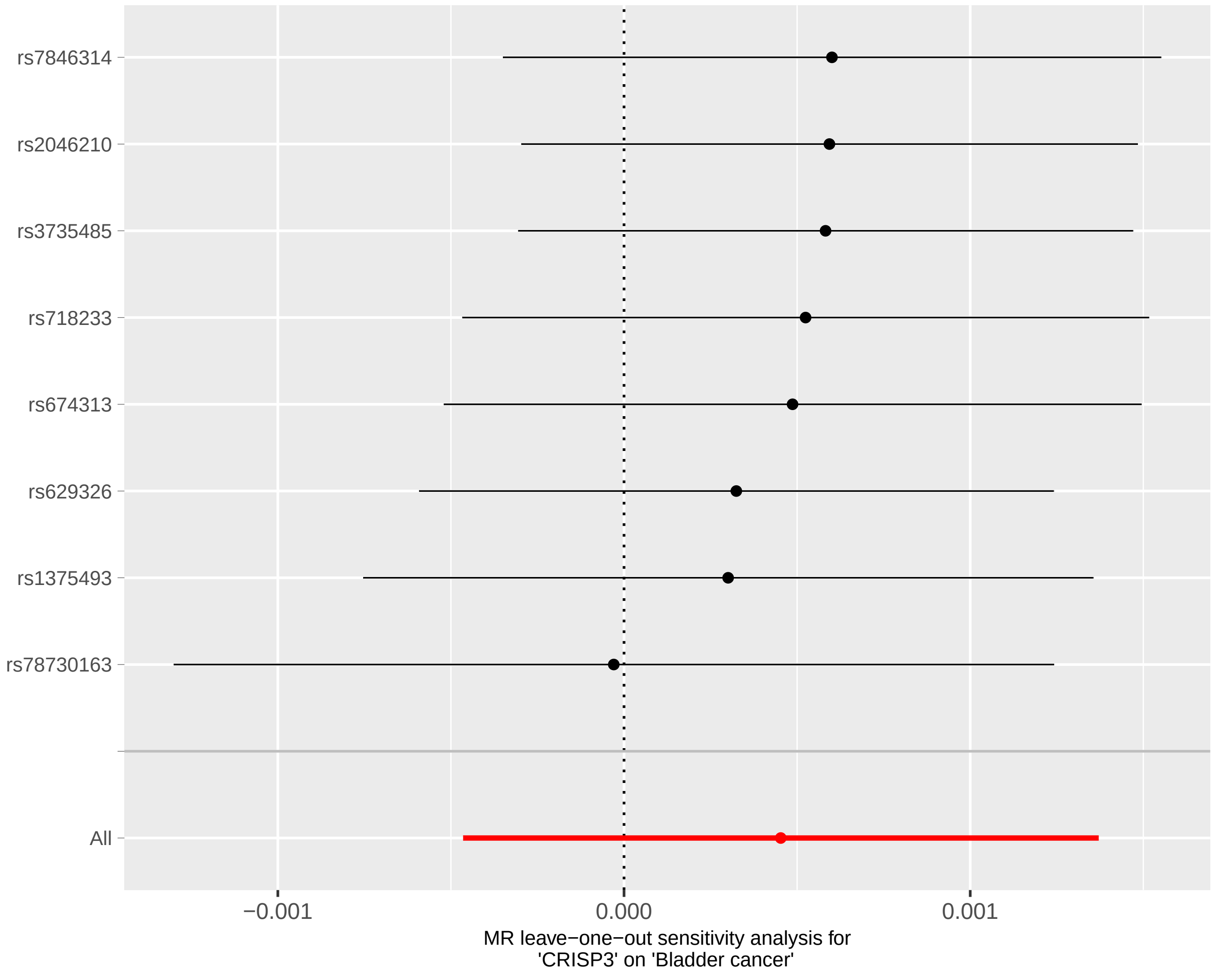
rs7846314
rs2046210
rs3735485
rs718233
rs674313
rs629326
rs1375493
rs78730163
All
−0.001
0.000
0.001
MR leave−one−out sensitivity analysis for
'CRISP3' on 'Bladder cancer'

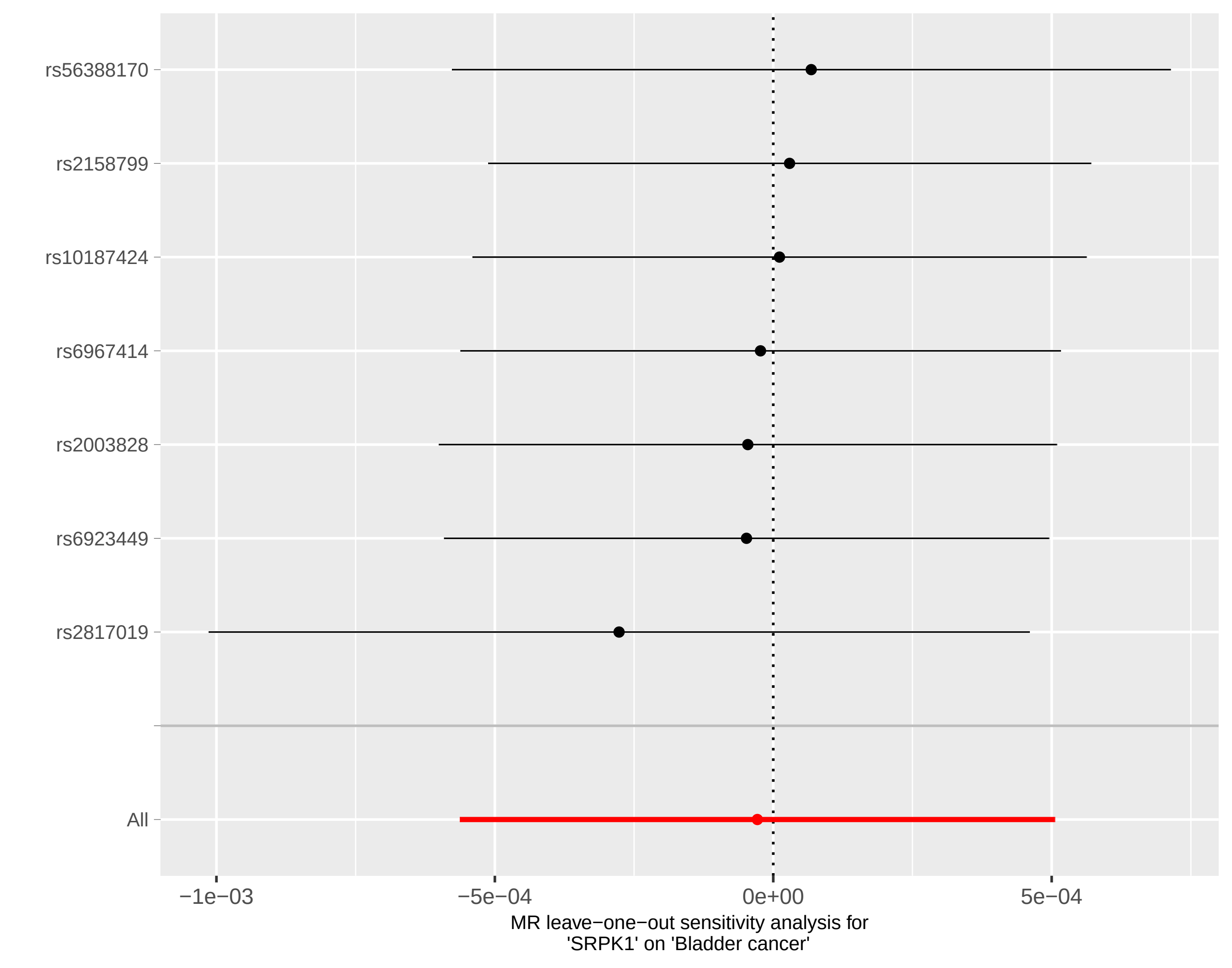
rs56388170
rs2158799
rs10187424
rs6967414
rs2003828
rs6923449
rs2817019
All
−1e−03
−5e−04
0e+00
5e−04
MR leave−one−out sensitivity analysis for
'SRPK1' on 'Bladder cancer'

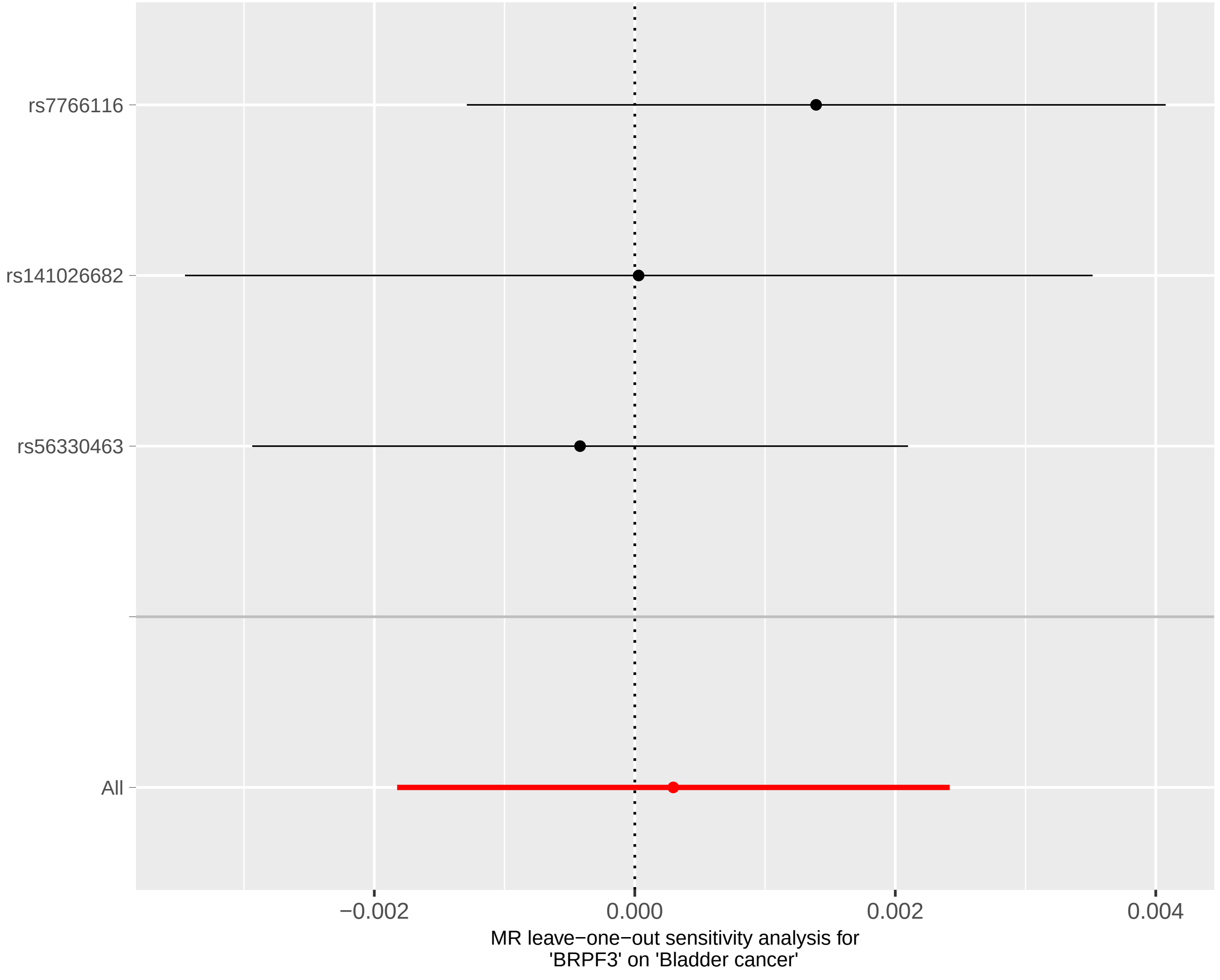

rs7766116
rs141026682
rs56330463
All
−0.002
0.000
0.002
0.004
MR leave−one−out sensitivity analysis for
'BRPF3' on 'Bladder cancer'

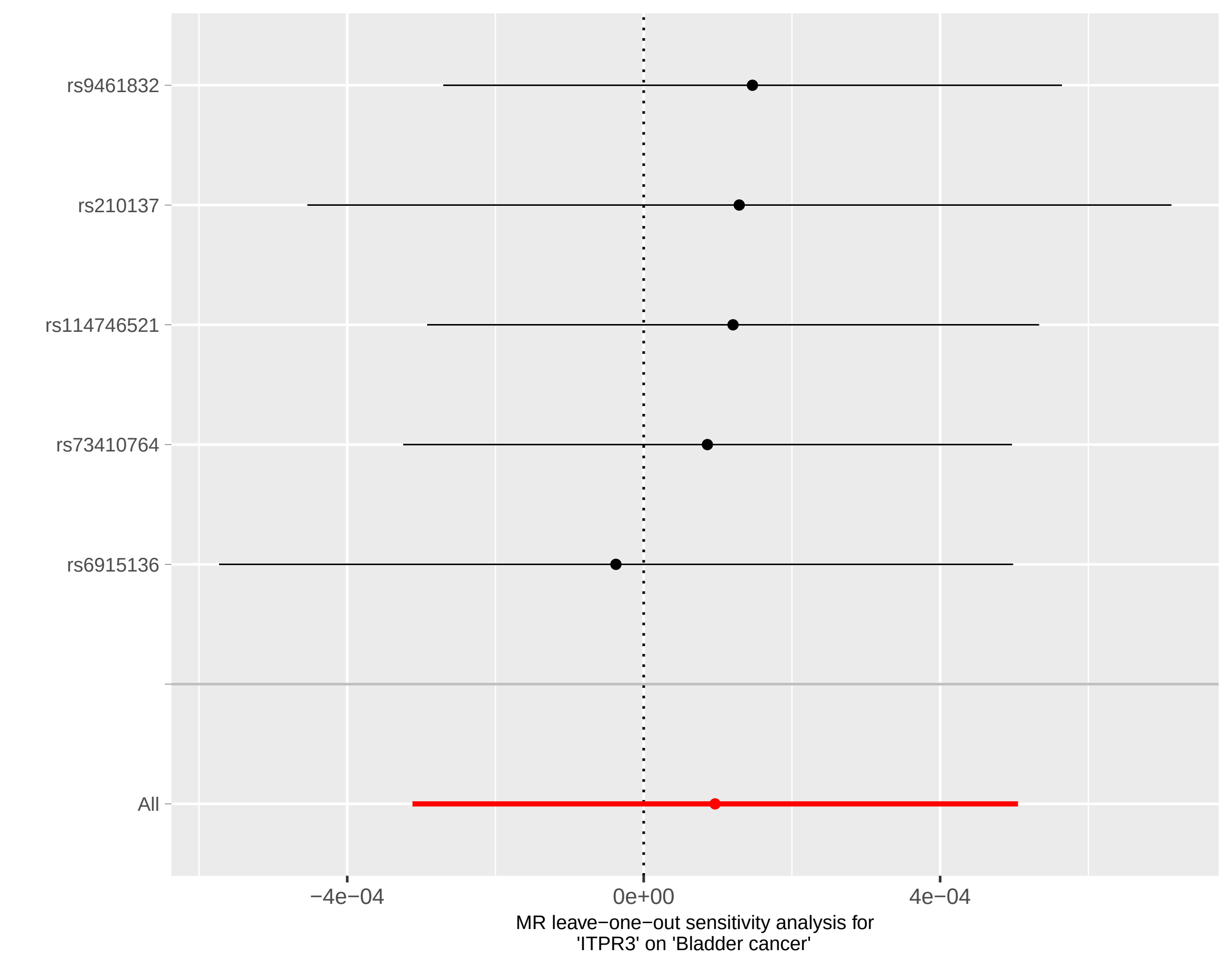
rs9461832
rs210137
rs114746521
rs73410764
rs6915136
All
−4e−04
0e+00
4e−04
MR leave−one−out sensitivity analysis for
'ITPR3' on 'Bladder cancer'

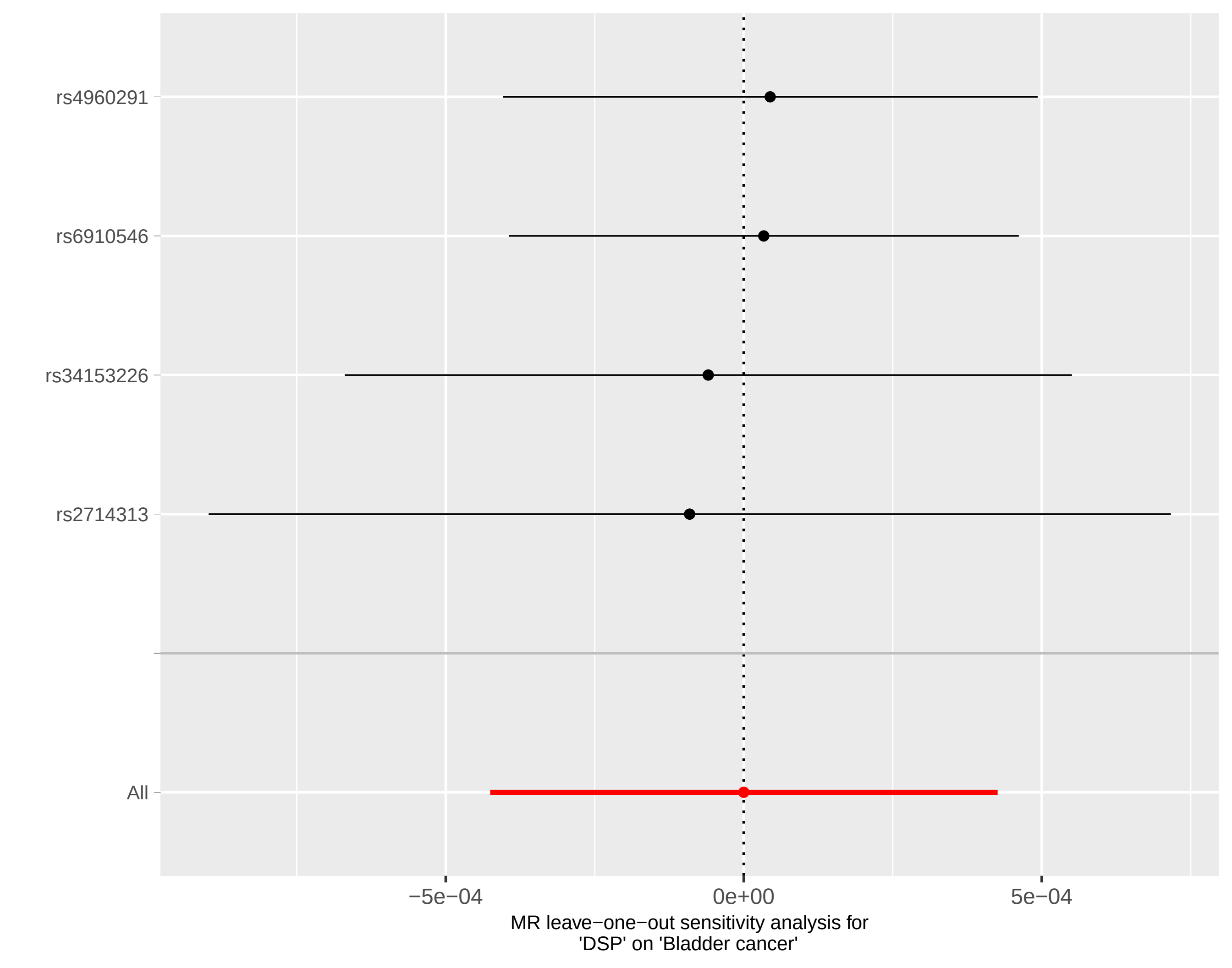

rs4960291
rs6910546
rs34153226
rs2714313
All
−5e−04
0e+00
5e−04
MR leave−one−out sensitivity analysis for
'DSP' on 'Bladder cancer'

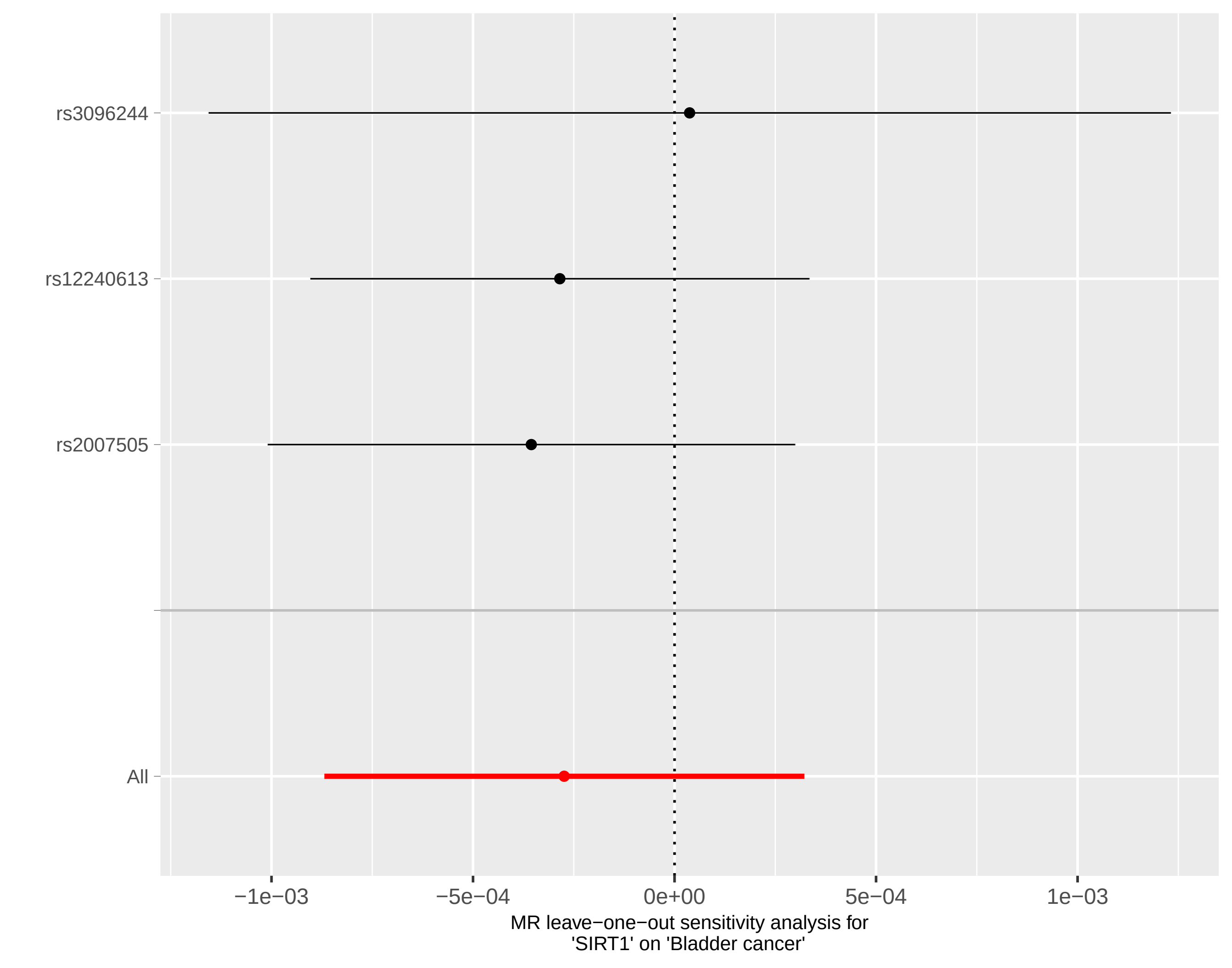
rs3096244
rs12240613
rs2007505
All
−1e−03
−5e−04
0e+00
5e−04
1e−03
MR leave−one−out sensitivity analysis for
'SIRT1' on 'Bladder cancer'

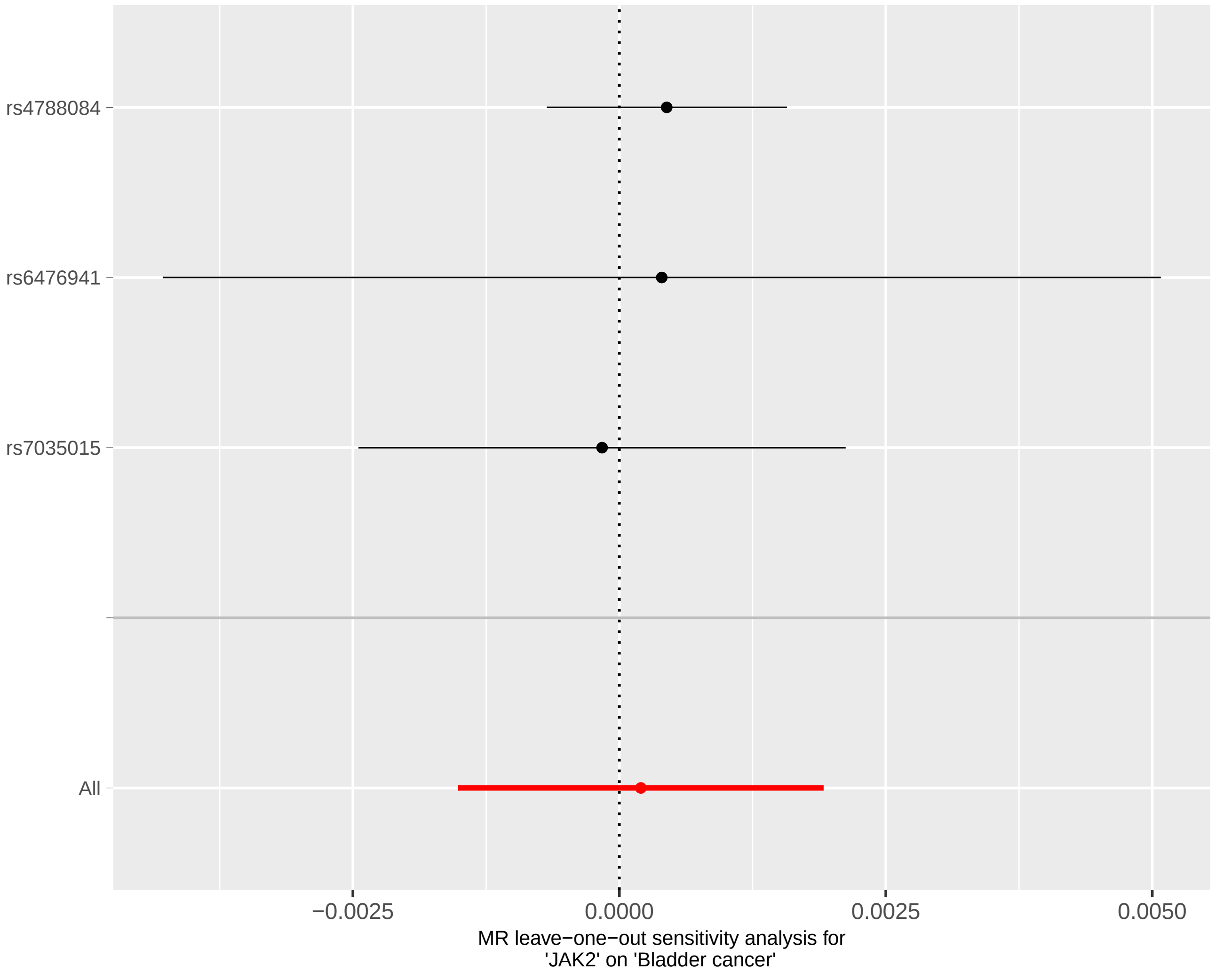
rs4788084
rs6476941
rs7035015
All
−0.0025
0.0000
0.0025
0.0050
MR leave−one−out sensitivity analysis for
'JAK2' on 'Bladder cancer'

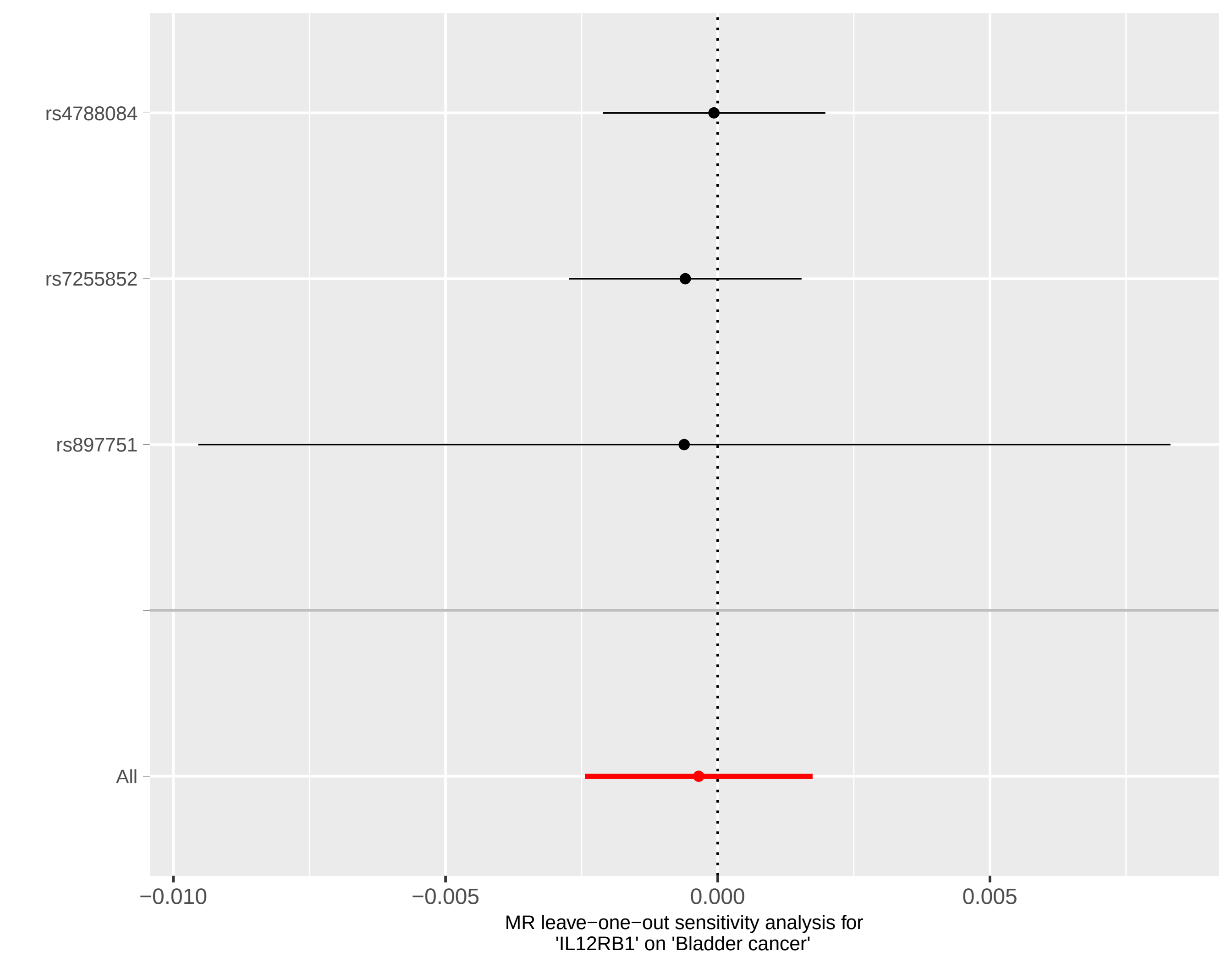
rs4788084
rs7255852
rs897751
All
−0.010
−0.005
0.000
0.005
MR leave−one−out sensitivity analysis for
'IL12RB1' on 'Bladder cancer'

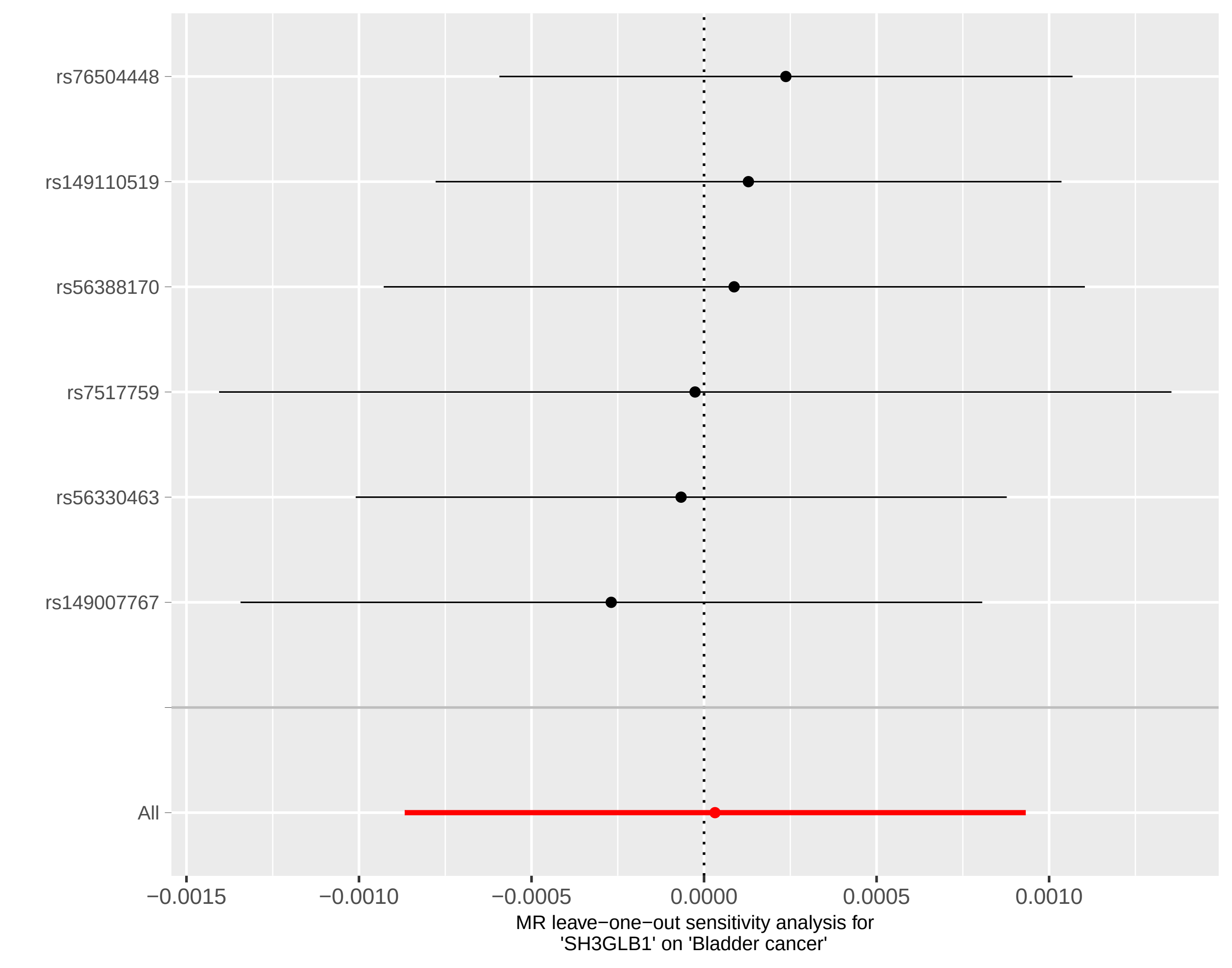
rs76504448
rs149110519
rs56388170
rs7517759
rs56330463
rs149007767
All
−0.0015
−0.0010
−0.0005
0.0000
0.0005
0.0010
MR leave−one−out sensitivity analysis for
'SH3GLB1' on 'Bladder cancer'

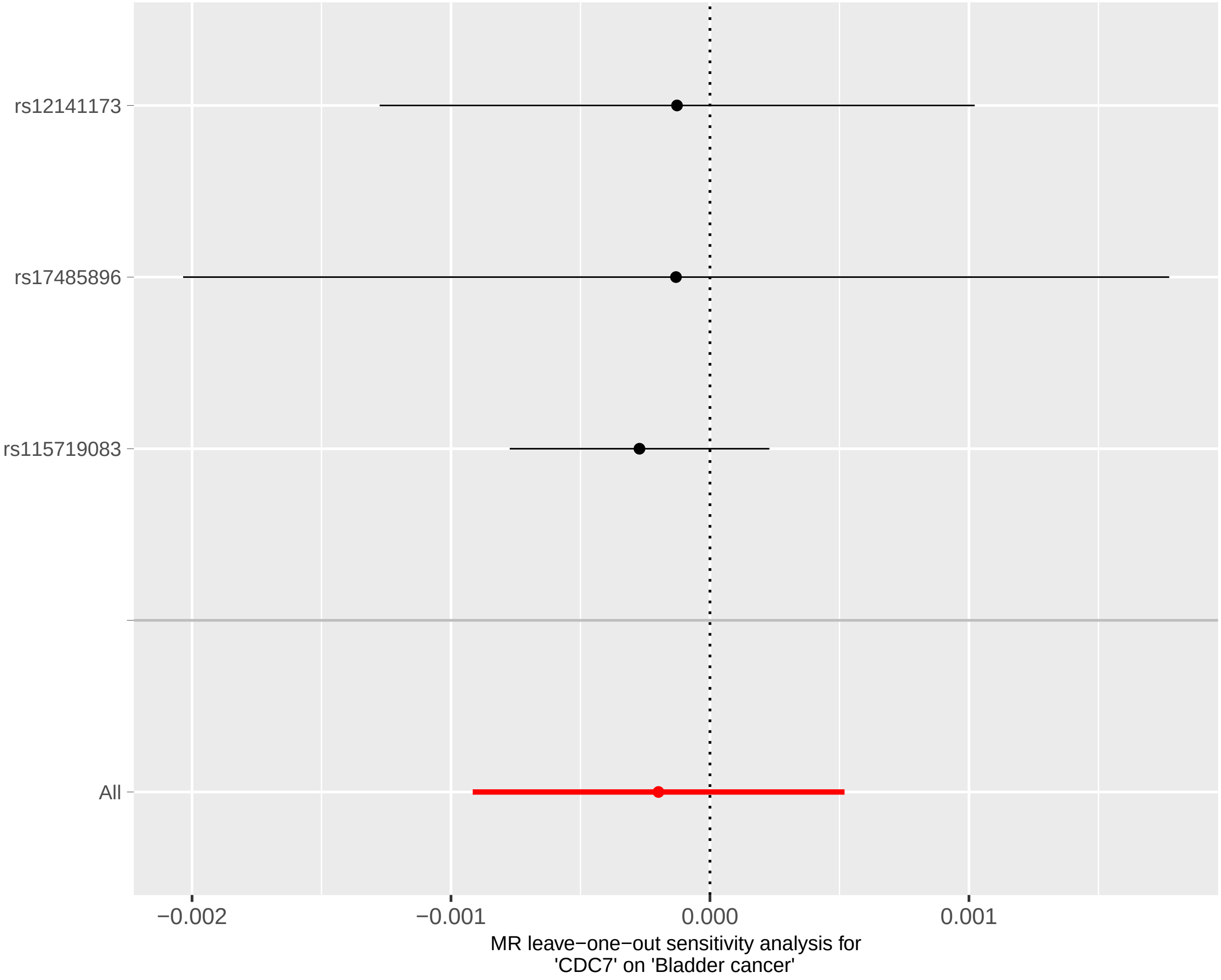
rs12141173
rs17485896
rs115719083
All
−0.002
−0.001
0.000
0.001
MR leave−one−out sensitivity analysis for
'CDC7' on 'Bladder cancer'

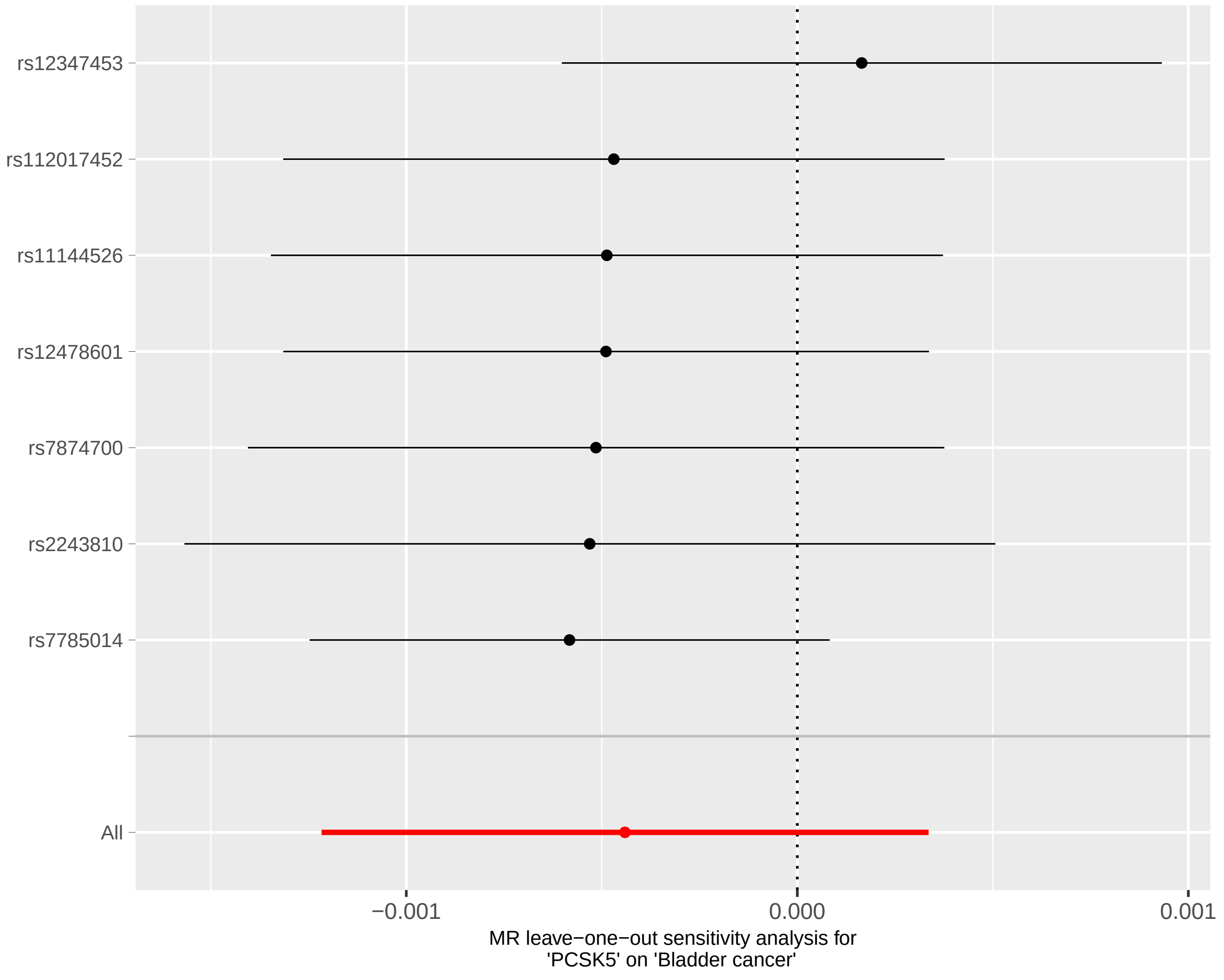
rs12347453
rs112017452
rs11144526
rs12478601
rs7874700
rs2243810
rs7785014
All
−0.001
0.000
0.001
MR leave−one−out sensitivity analysis for
'PCSK5' on 'Bladder cancer'

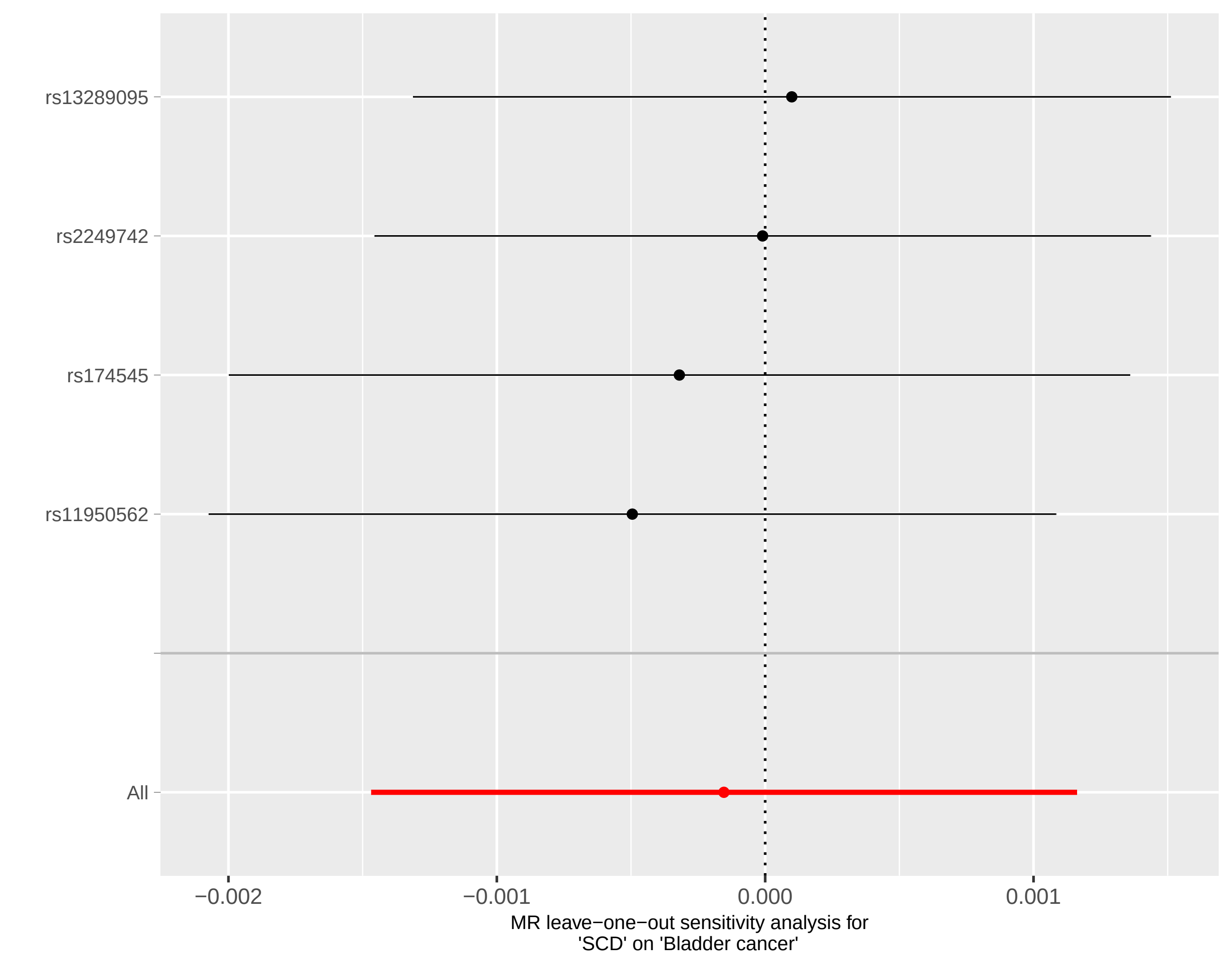
rs13289095
rs2249742
rs174545
rs11950562
All
−0.002
−0.001
0.000
0.001
MR leave−one−out sensitivity analysis for
'SCD' on 'Bladder cancer'

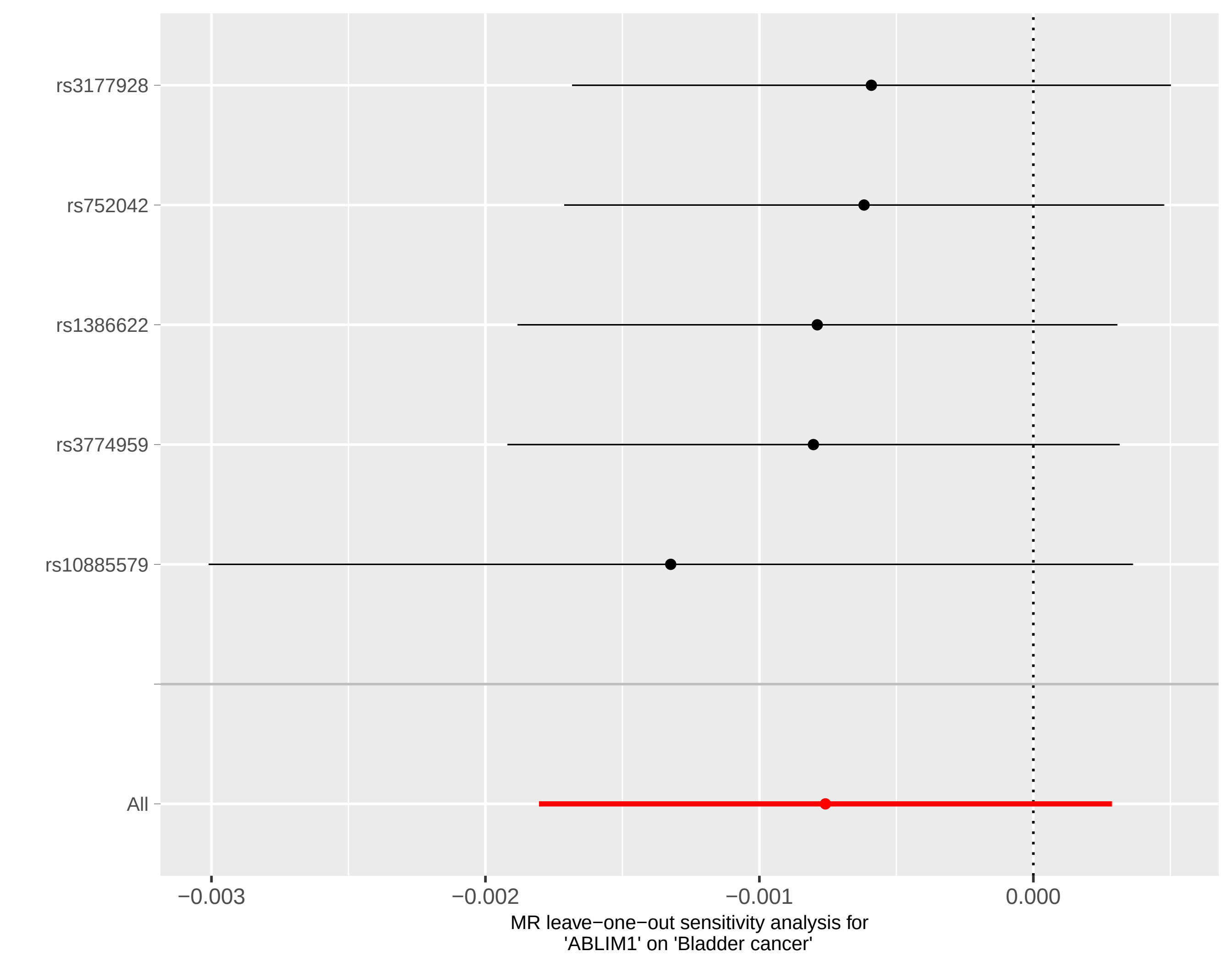
rs3177928
rs752042
rs1386622
rs3774959
rs10885579
All
−0.003
−0.002
−0.001
0.000
MR leave−one−out sensitivity analysis for
'ABLIM1' on 'Bladder cancer'

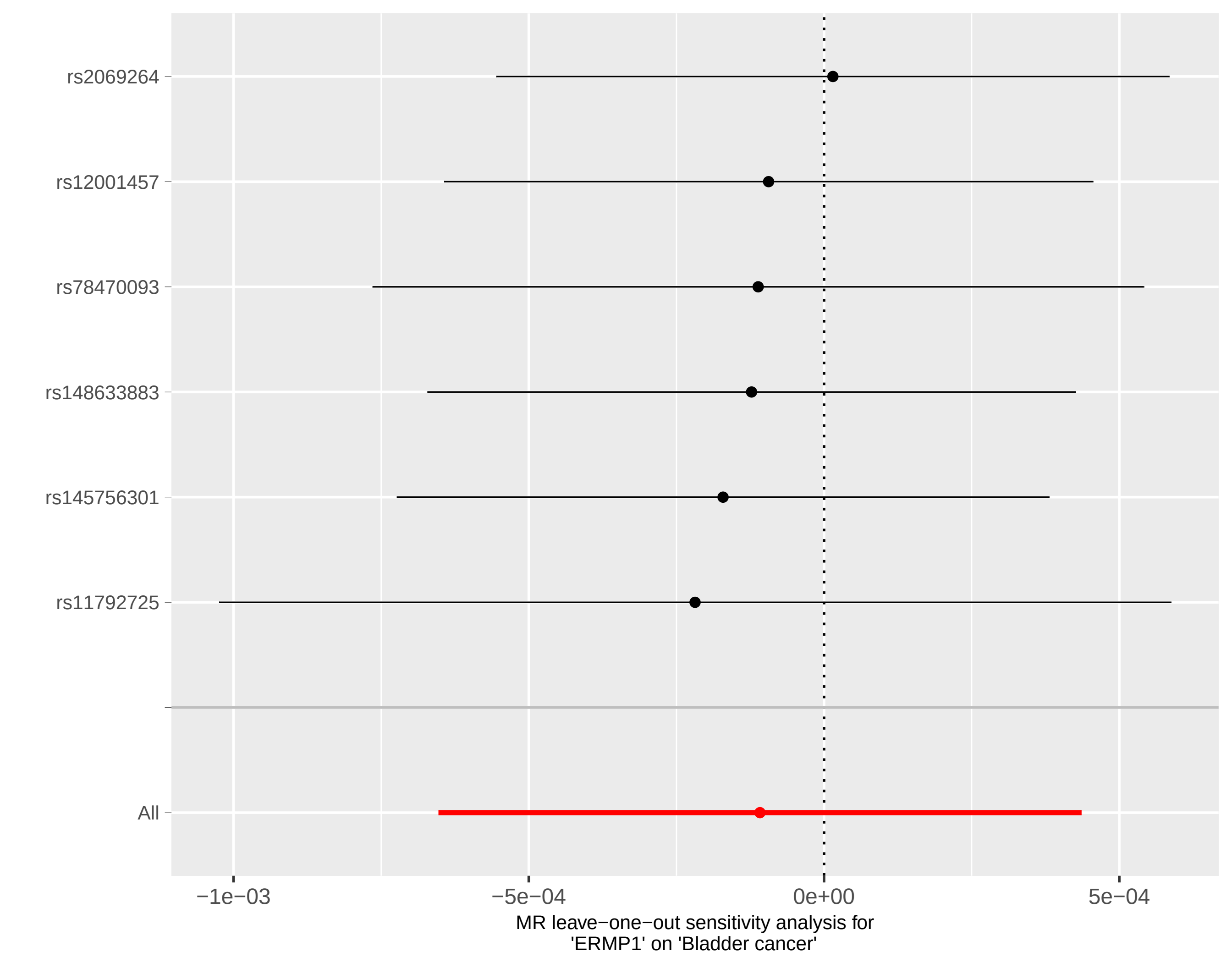

rs2069264
rs12001457
rs78470093
rs148633883
rs145756301
rs11792725
All
−1e−03
−5e−04
0e+00
5e−04
MR leave−one−out sensitivity analysis for
'ERMP1' on 'Bladder cancer'

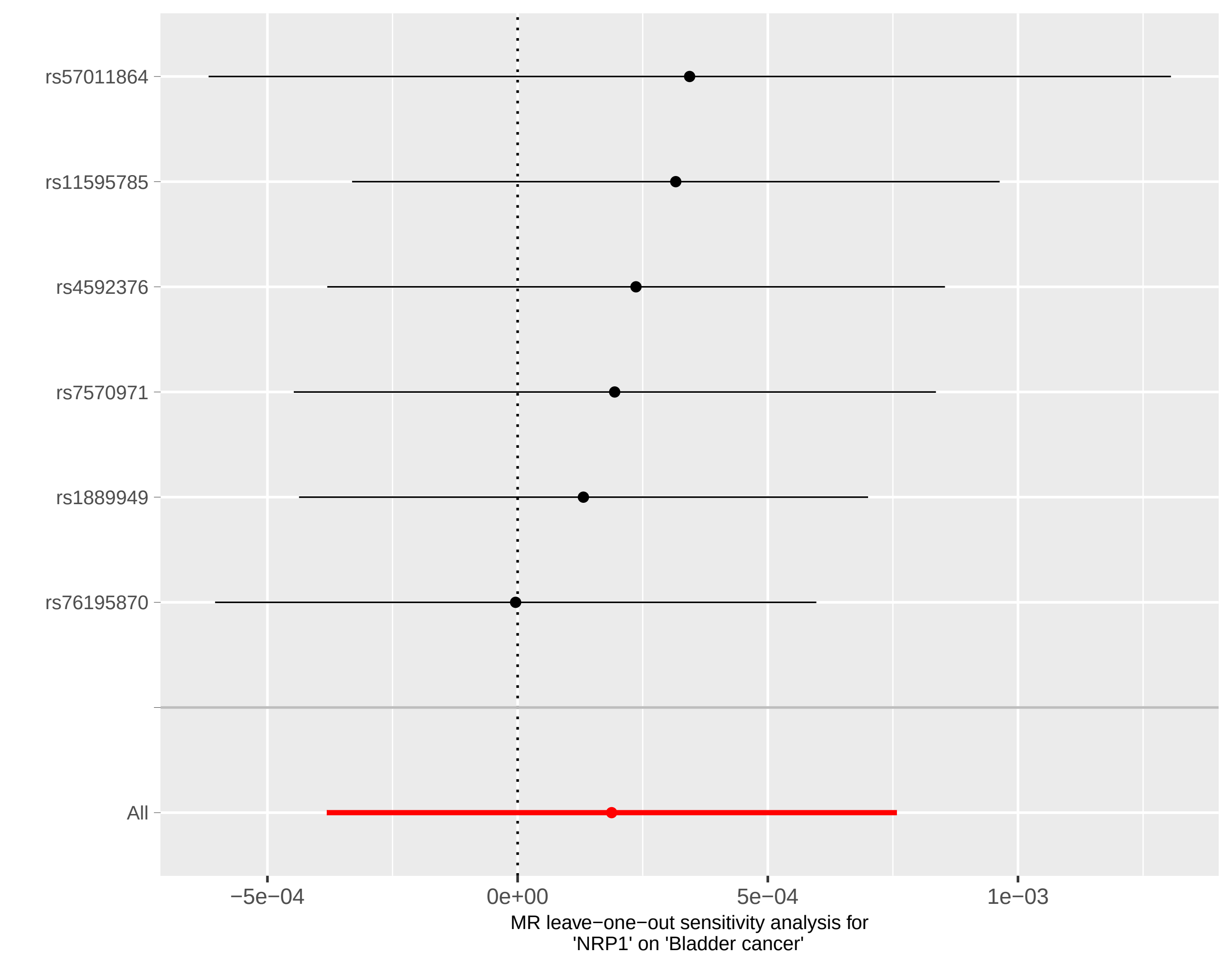
rs57011864
rs11595785
rs4592376
rs7570971
rs1889949
rs76195870
All
−5e−04
0e+00
5e−04
1e−03
MR leave−one−out sensitivity analysis for
'NRP1' on 'Bladder cancer'

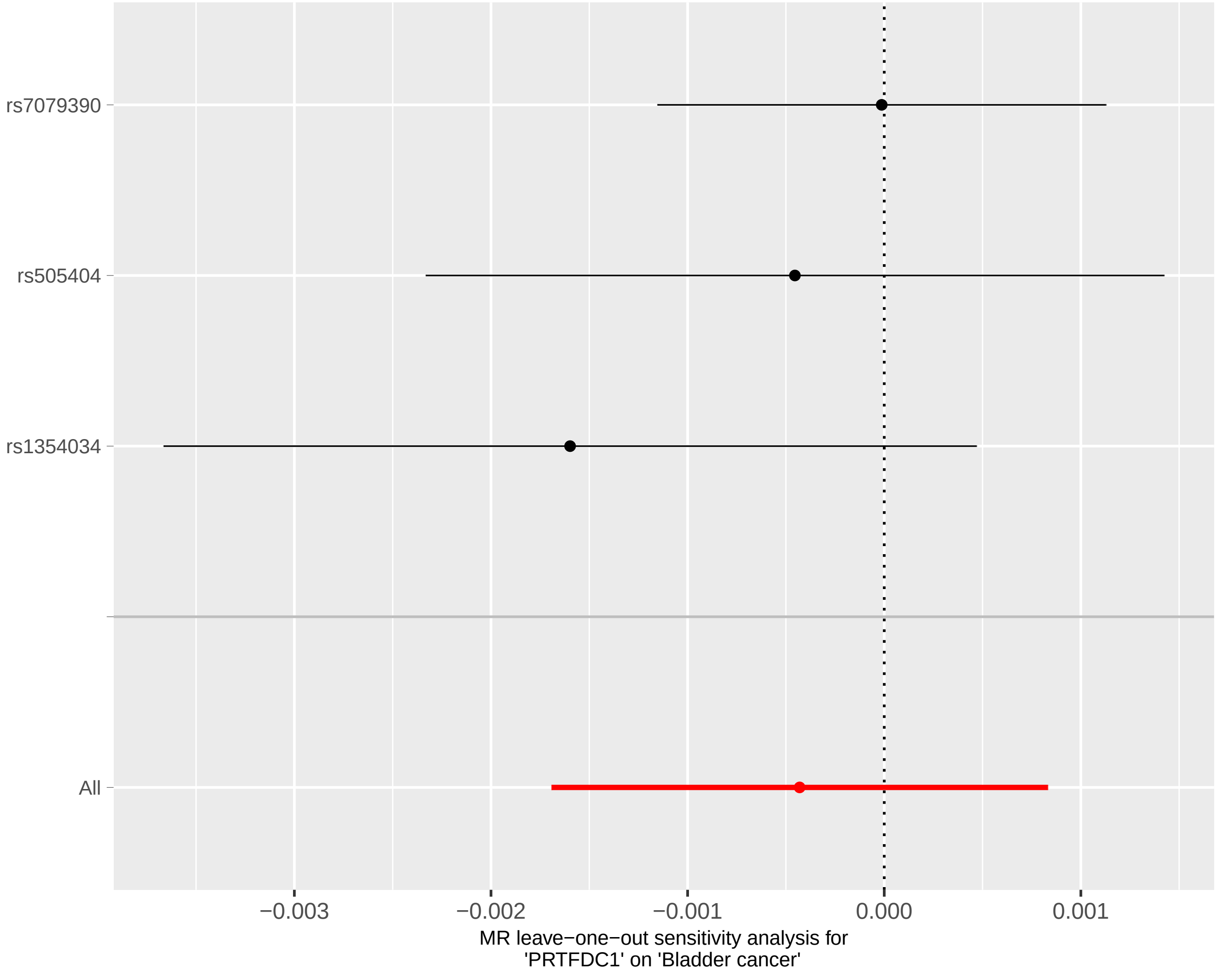
rs7079390
rs505404
rs1354034
All
−0.003
−0.002
−0.001
0.000
0.001
MR leave−one−out sensitivity analysis for
'PRTFDC1' on 'Bladder cancer'

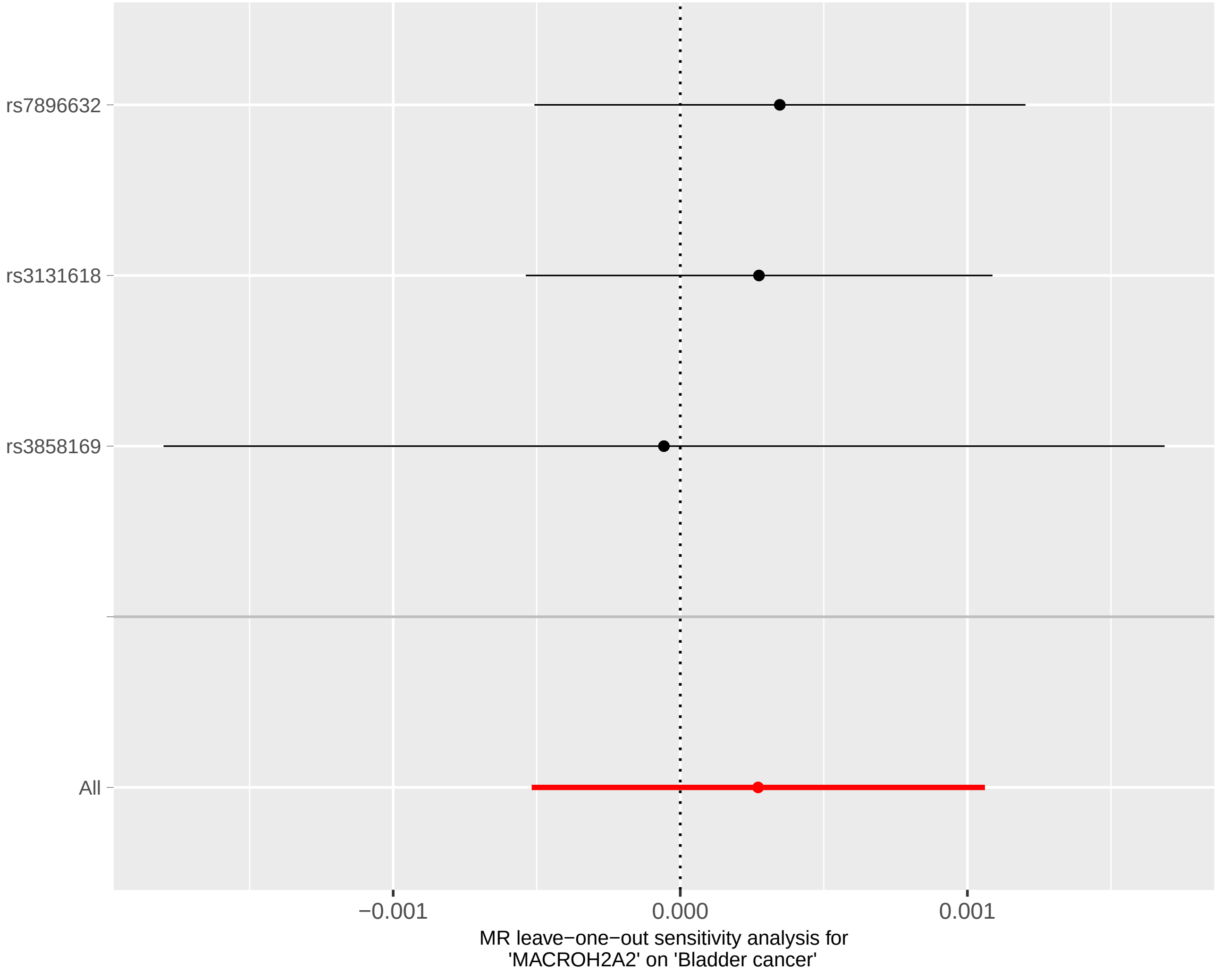
rs7896632
rs3131618
rs3858169
All
−0.001
0.000
0.001
MR leave−one−out sensitivity analysis for
'MACROH2A2' on 'Bladder cancer'

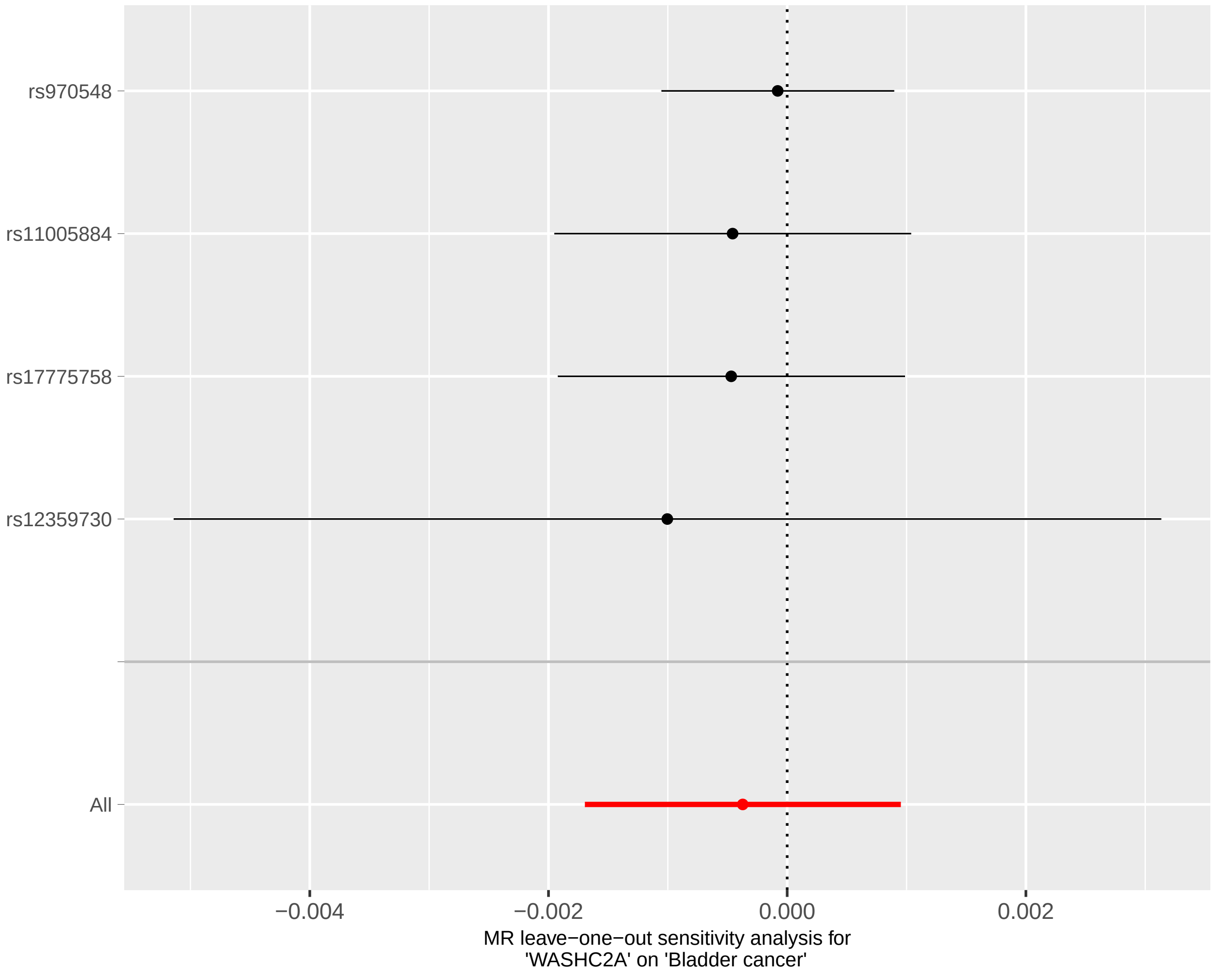
rs970548
rs11005884
rs17775758
rs12359730
All
−0.004
−0.002
0.000
0.002
MR leave−one−out sensitivity analysis for
'WASHC2A' on 'Bladder cancer'

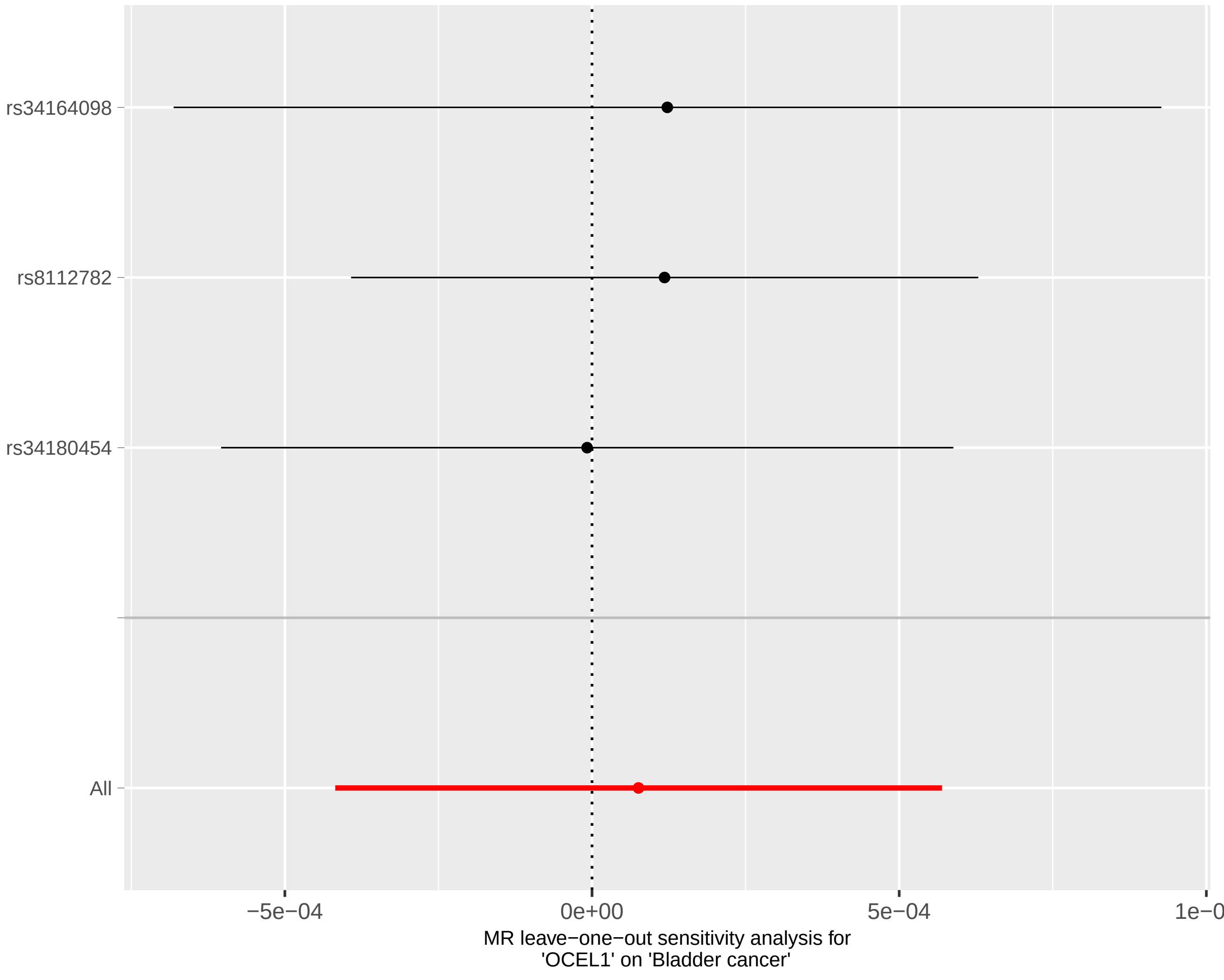

rs34164098
rs8112782
rs34180454
All
−5e−04
0e+00
5e−04
1e−0
MR leave−one−out sensitivity analysis for
'OCEL1' on 'Bladder cancer'

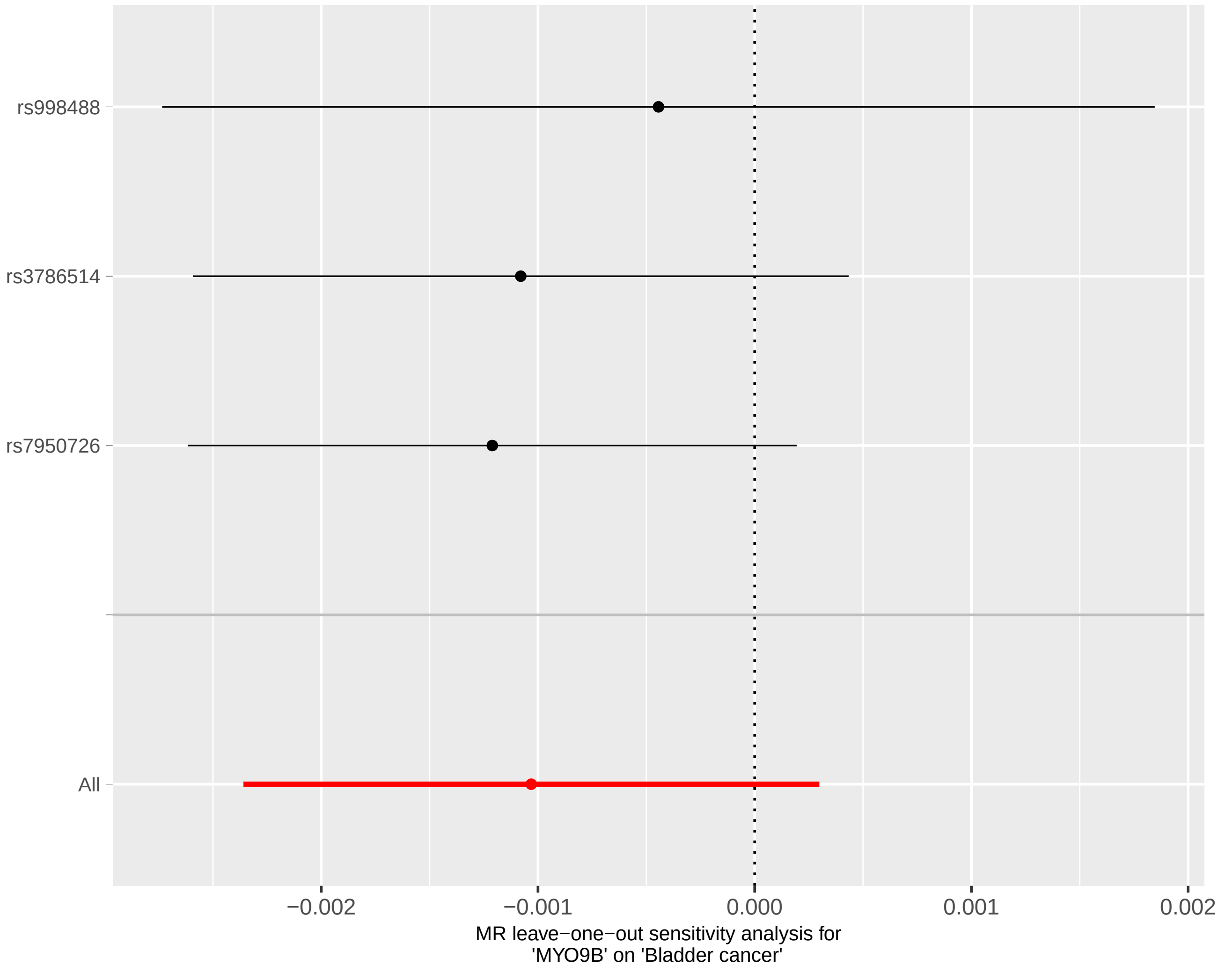

rs998488
rs3786514
rs7950726
All
−0.002
−0.001
0.000
0.001
0.002
MR leave−one−out sensitivity analysis for
'MYO9B' on 'Bladder cancer'

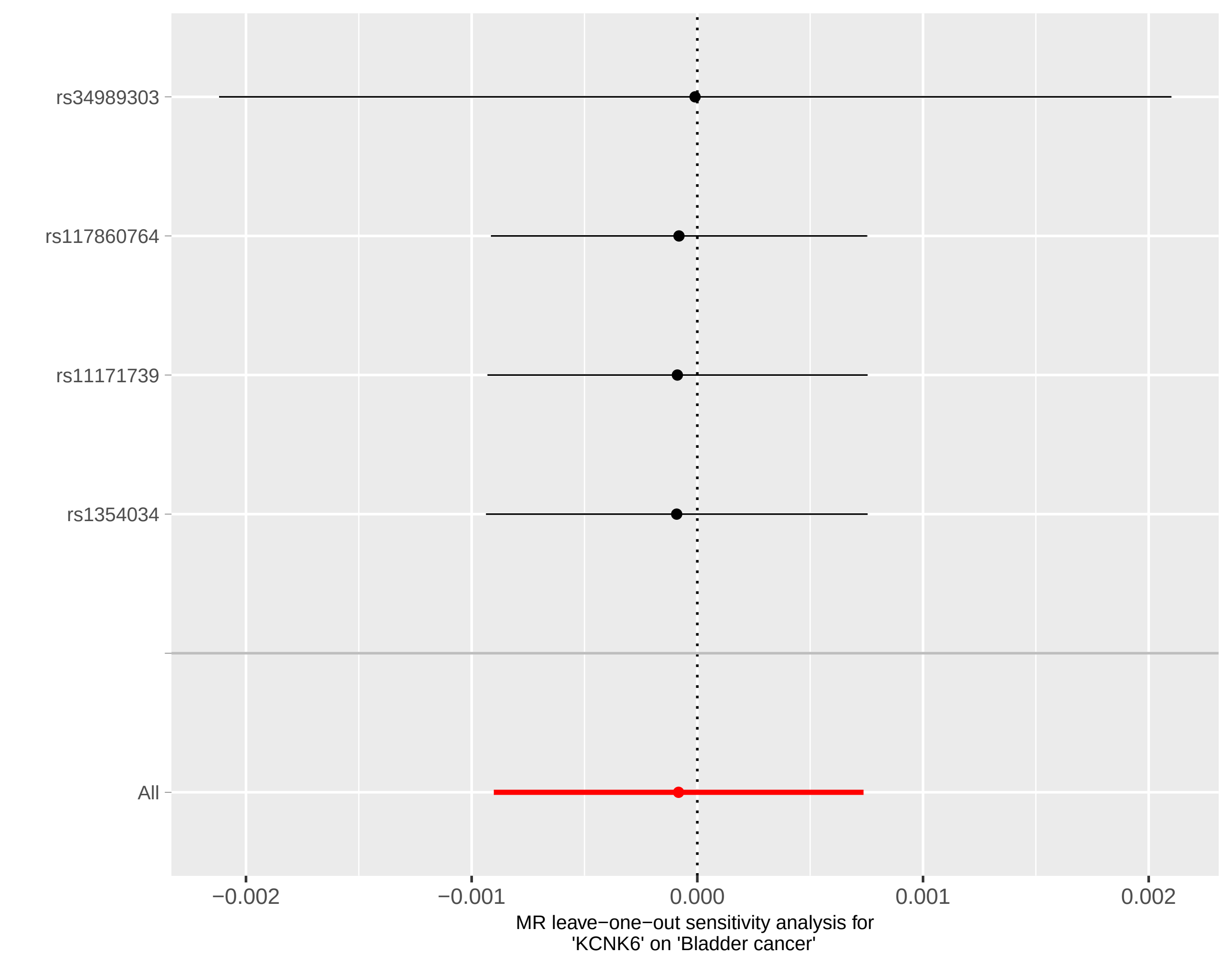
rs34989303
rs117860764
rs11171739
rs1354034
All
−0.002
−0.001
0.000
0.001
0.002
MR leave−one−out sensitivity analysis for
'KCNK6' on 'Bladder cancer'

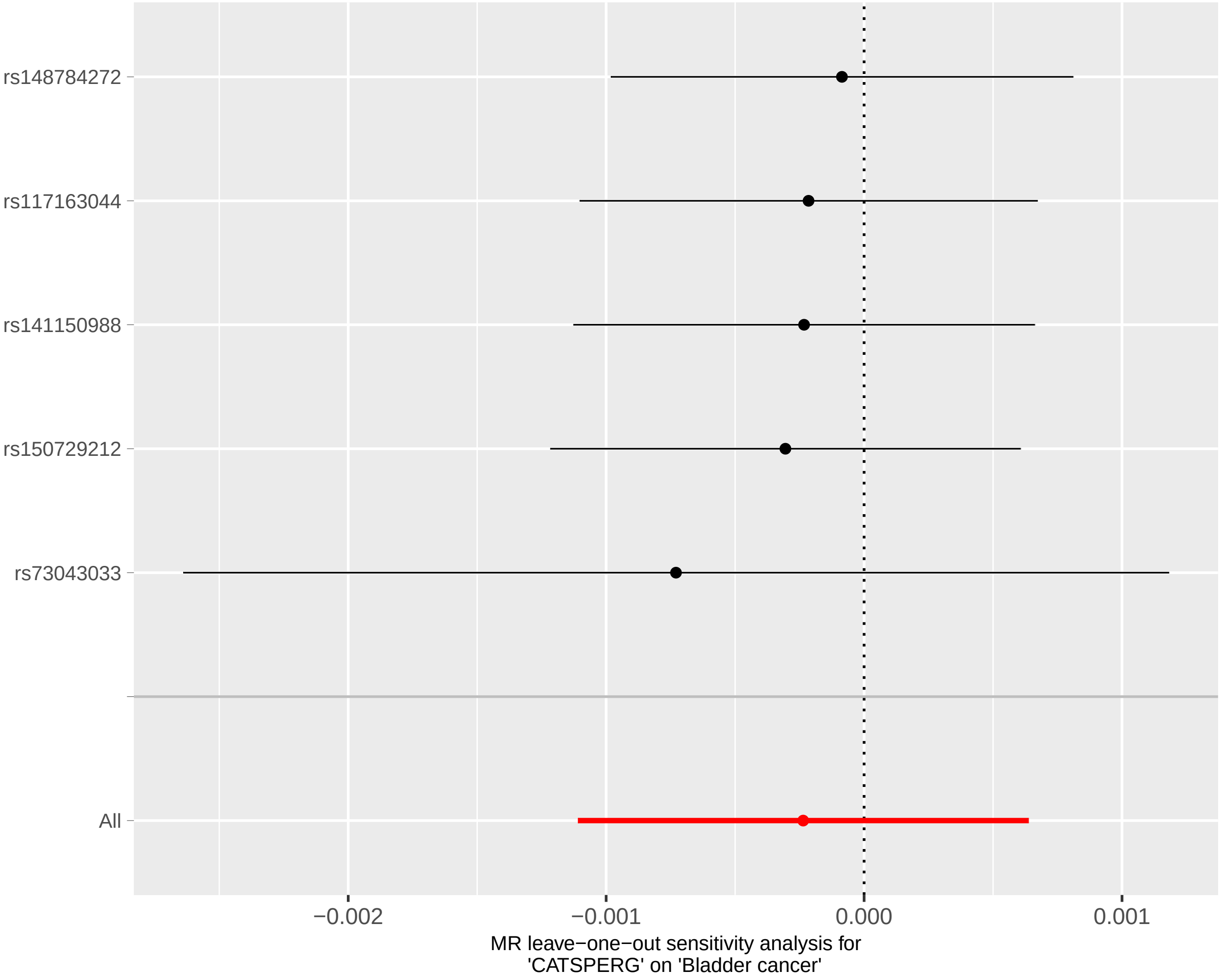
rs148784272
rs117163044
rs141150988
rs150729212
rs73043033
All
−0.002
−0.001
0.000
0.001
MR leave−one−out sensitivity analysis for
'CATSPERG' on 'Bladder cancer'

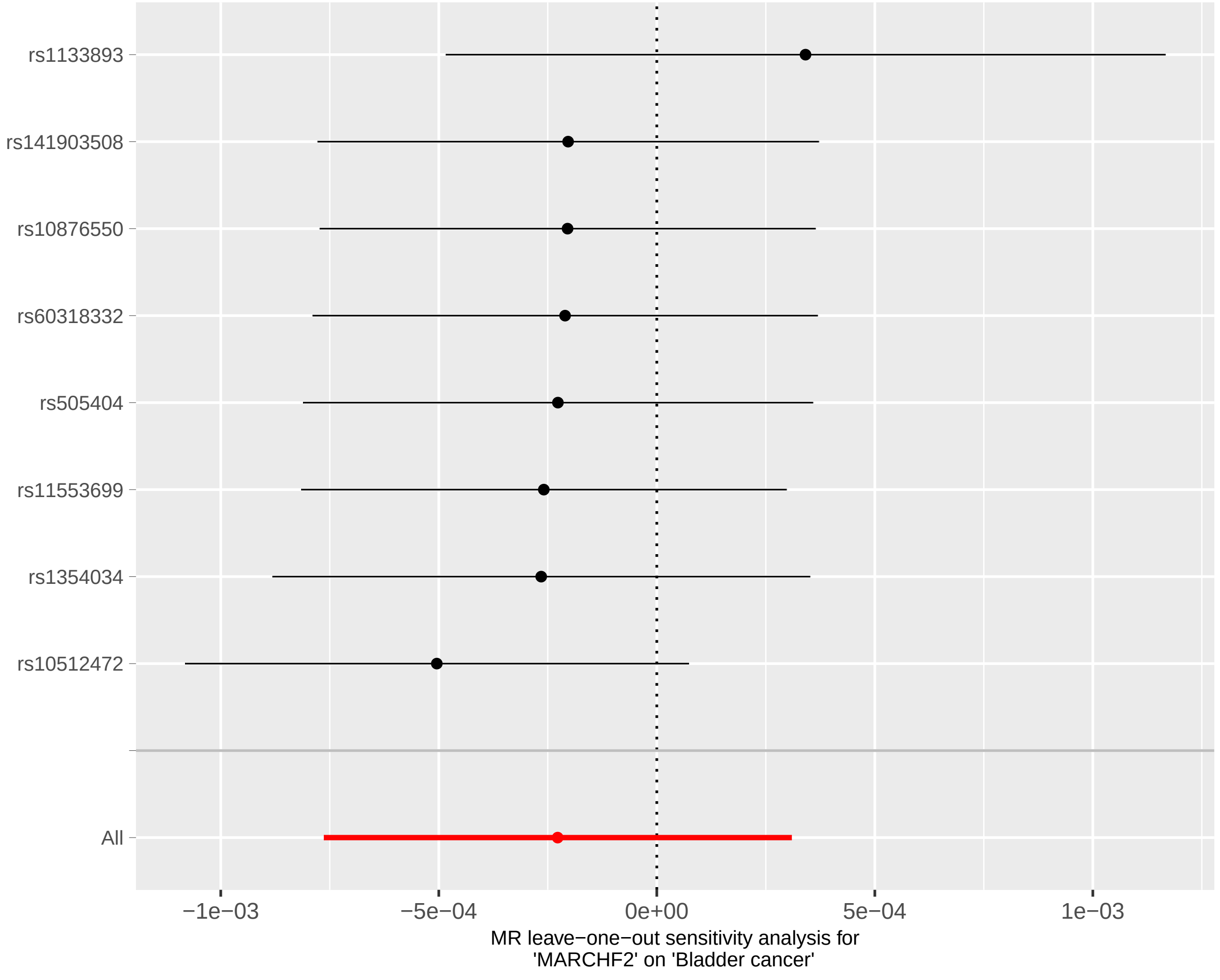
rs1133893
rs141903508
rs10876550
rs60318332
rs505404
rs11553699
rs1354034
rs10512472
All
−1e−03
−5e−04
0e+00
5e−04
1e−03
MR leave−one−out sensitivity analysis for
'MARCHF2' on 'Bladder cancer'

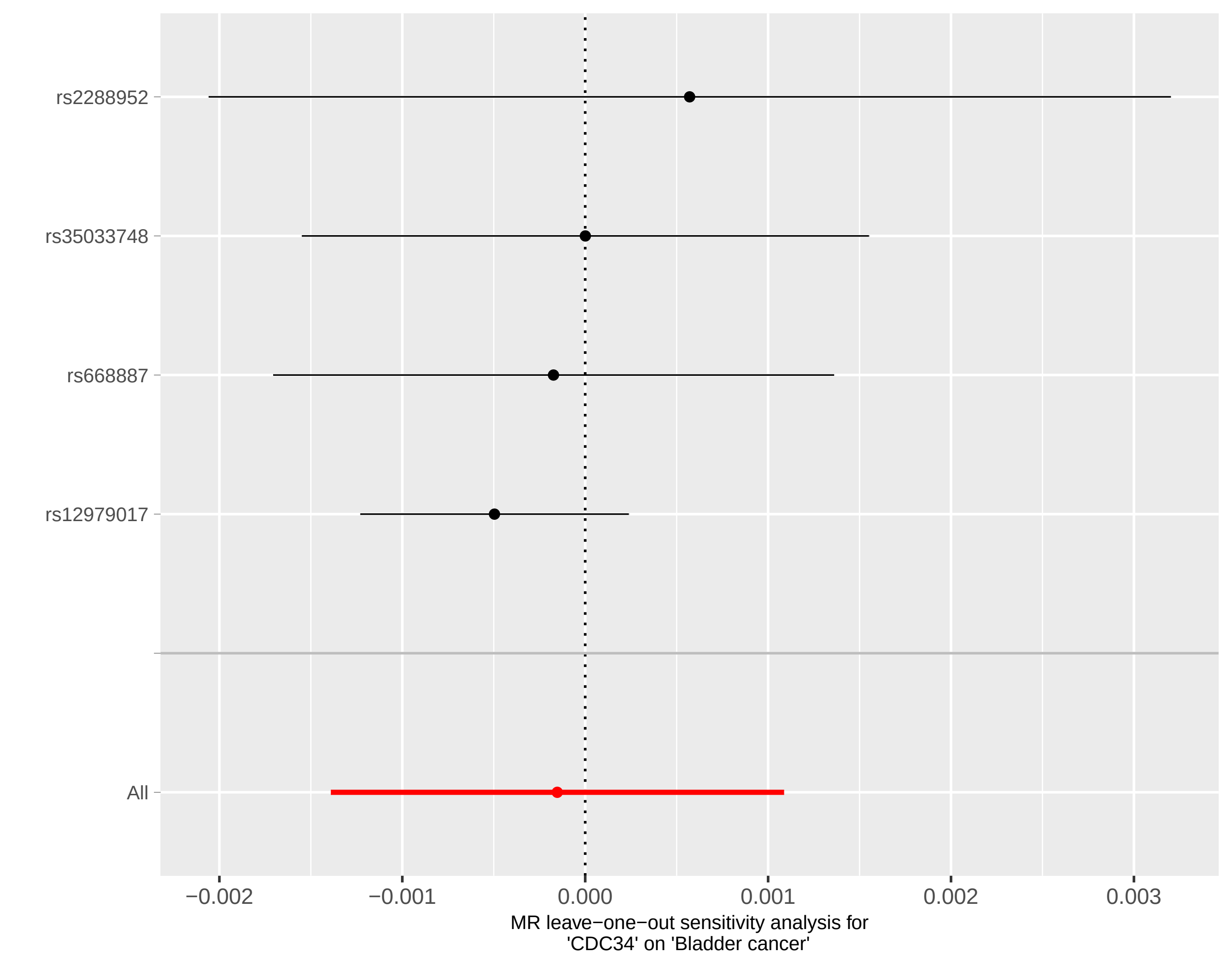
rs2288952
rs35033748
rs668887
rs12979017
All
−0.002
−0.001
0.000
0.001
0.002
0.003
MR leave−one−out sensitivity analysis for
'CDC34' on 'Bladder cancer'

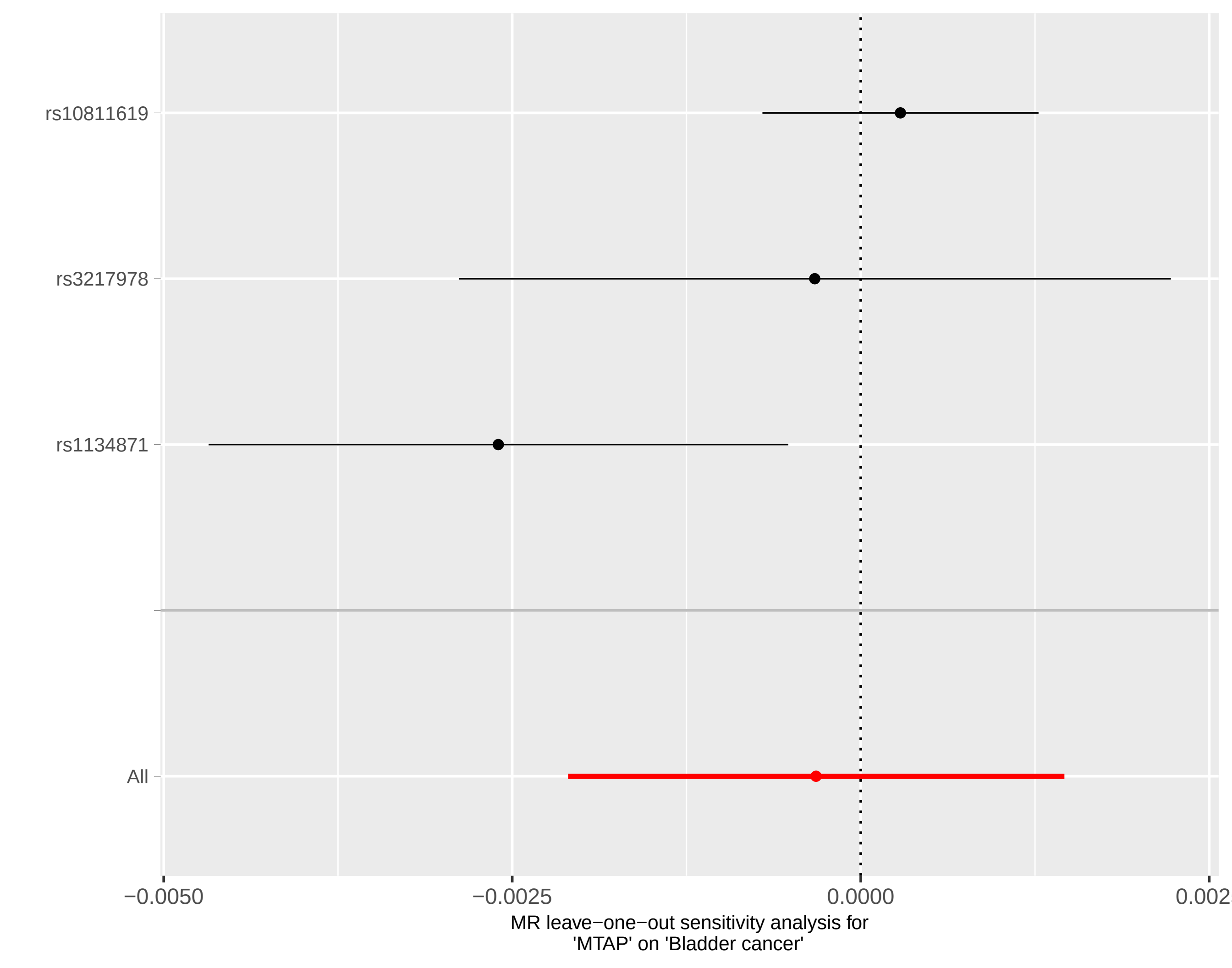
rs10811619
rs3217978
rs1134871
All
−0.0050
−0.0025
0.0000
0.002
MR leave−one−out sensitivity analysis for
'MTAP' on 'Bladder cancer'

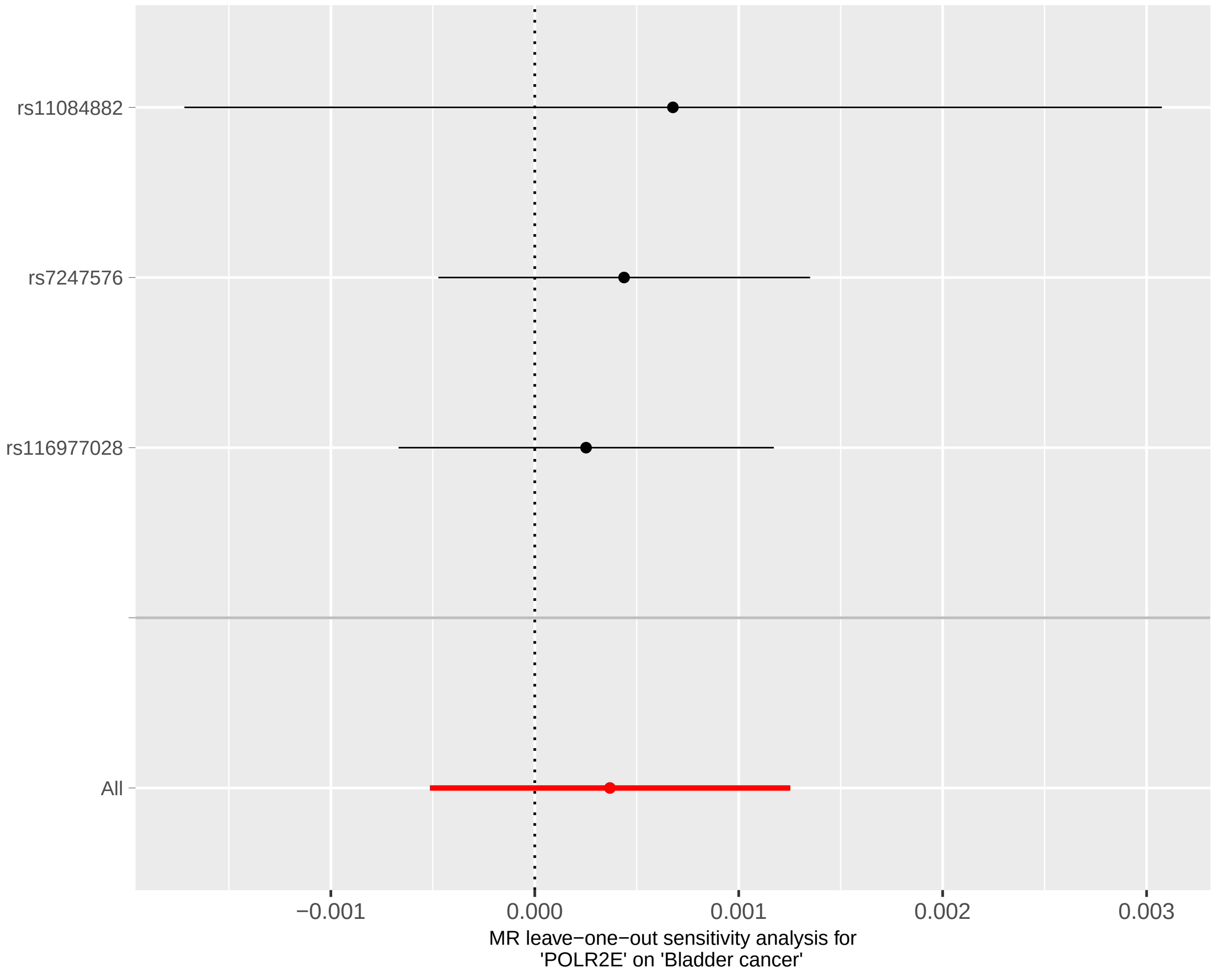
rs11084882
rs7247576
rs116977028
All
−0.001
0.000
0.001
0.002
0.003
MR leave−one−out sensitivity analysis for
'POLR2E' on 'Bladder cancer'

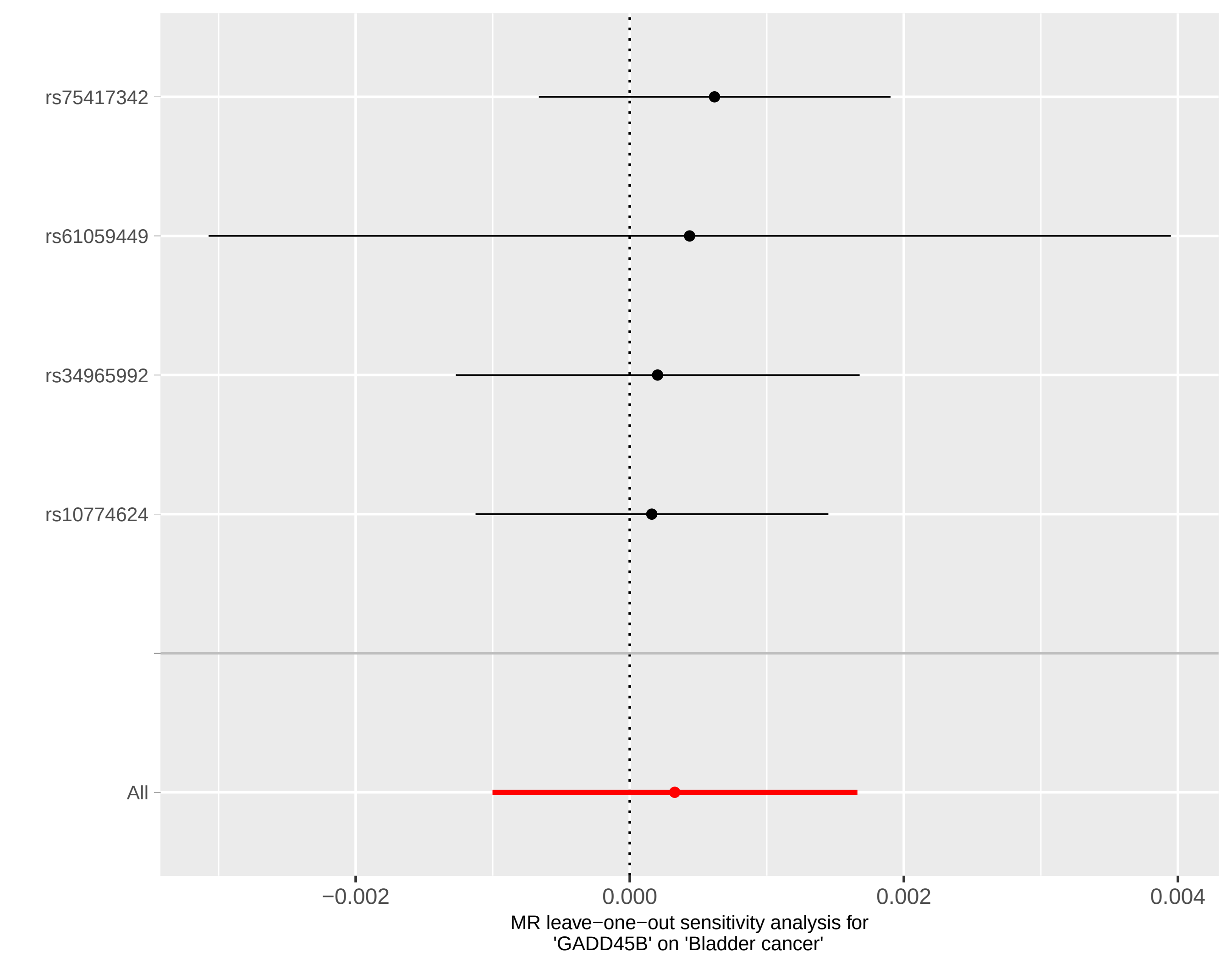
rs75417342
rs61059449
rs34965992
rs10774624
All
−0.002
0.000
0.002
0.004
MR leave−one−out sensitivity analysis for
'GADD45B' on 'Bladder cancer'

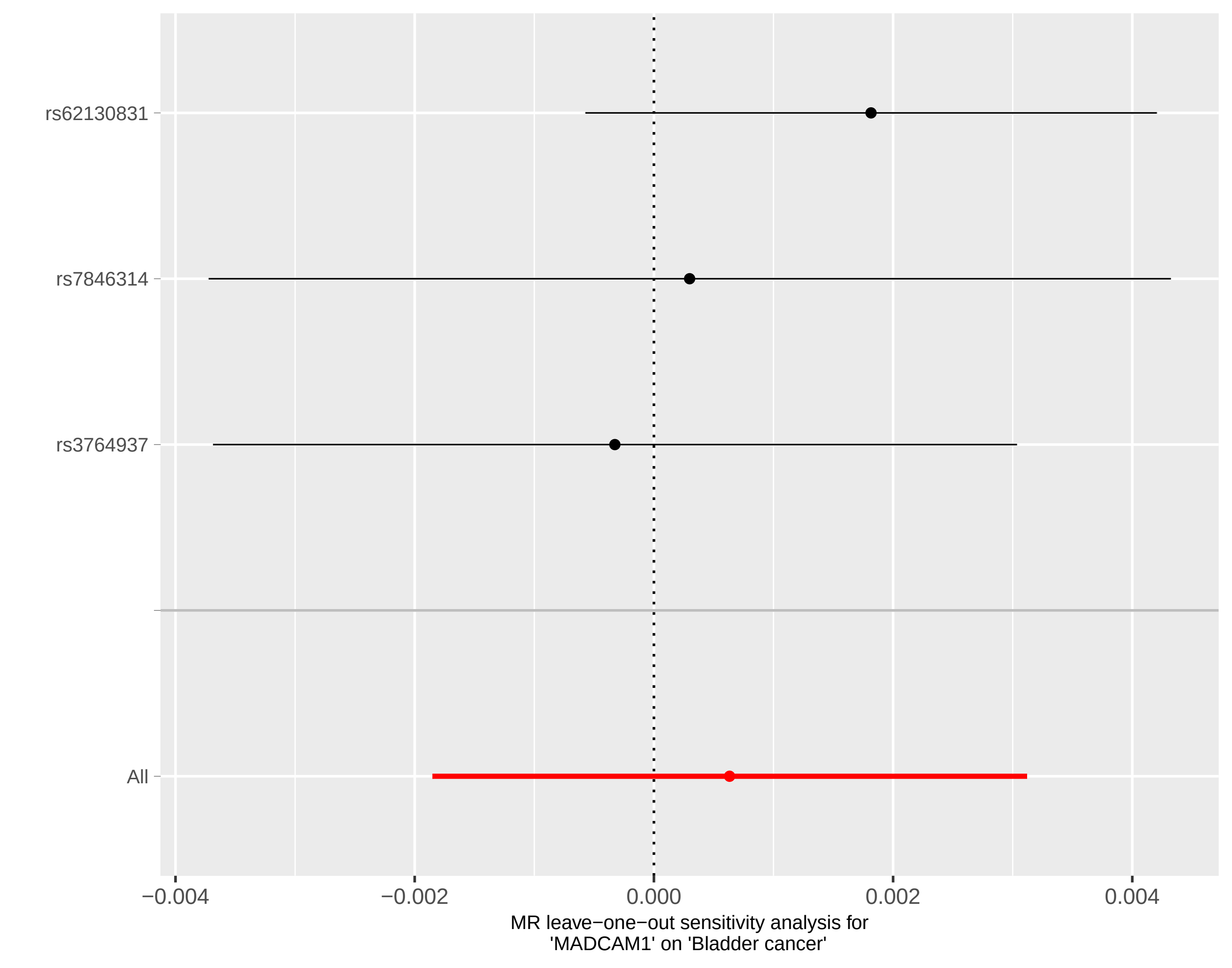

rs62130831
rs7846314
rs3764937
All
−0.004
−0.002
0.000
0.002
0.004
MR leave−one−out sensitivity analysis for
'MADCAM1' on 'Bladder cancer'

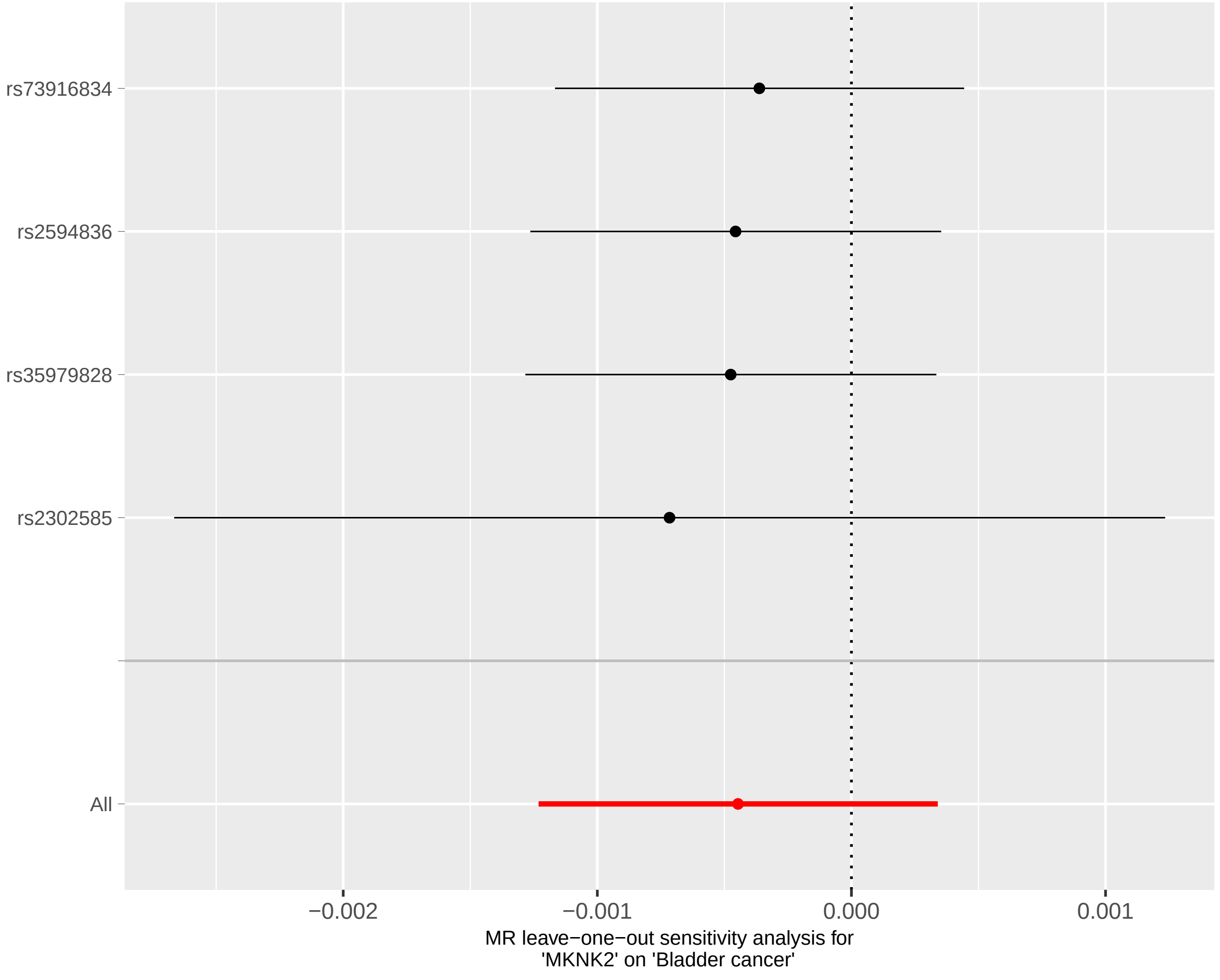
rs73916834
rs2594836
rs35979828
rs2302585
All
−0.002
−0.001
0.000
0.001
MR leave−one−out sensitivity analysis for
'MKNK2' on 'Bladder cancer'

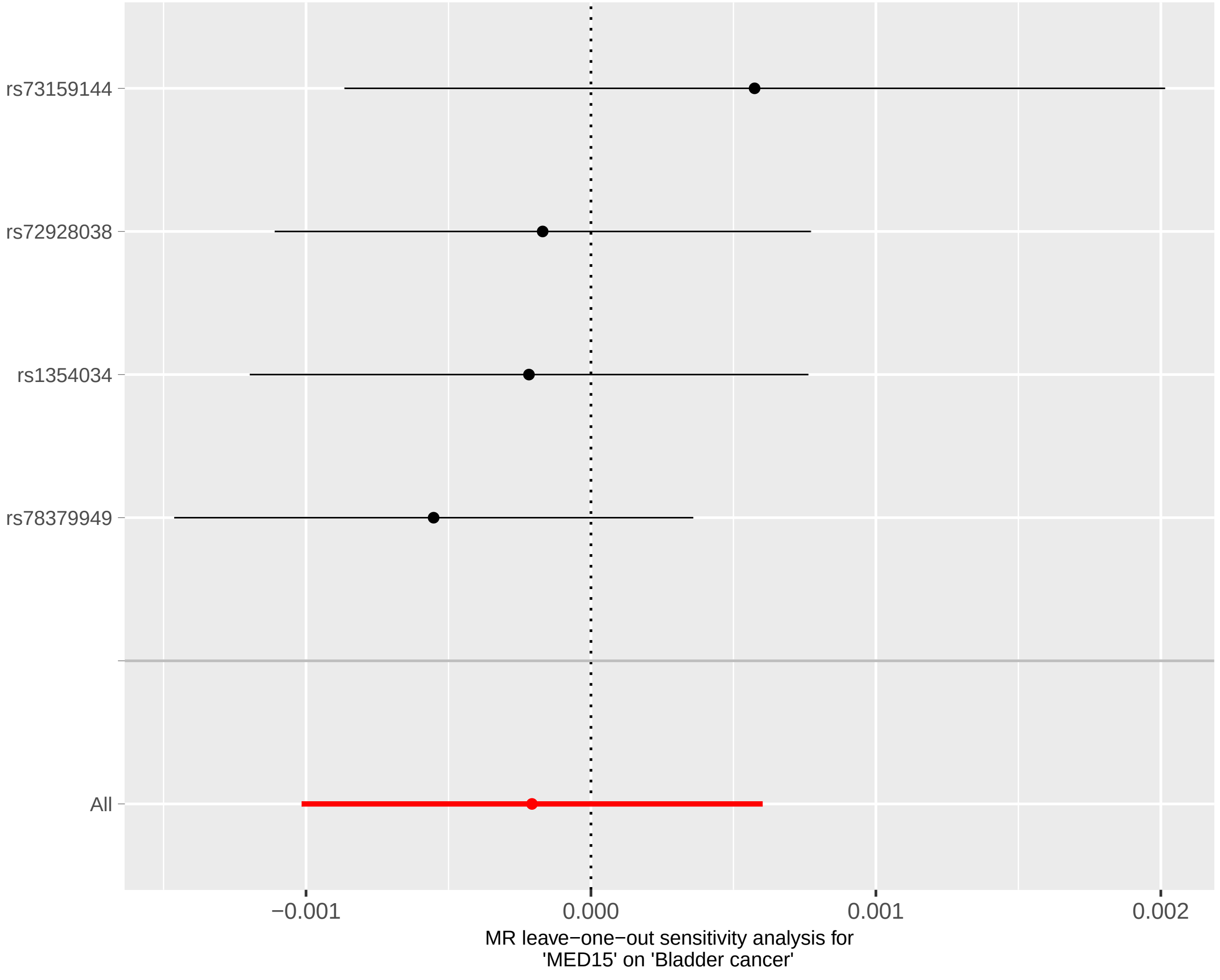
rs73159144
rs72928038
rs1354034
rs78379949
All
−0.001
0.000
0.001
0.002
MR leave−one−out sensitivity analysis for
'MED15' on 'Bladder cancer'

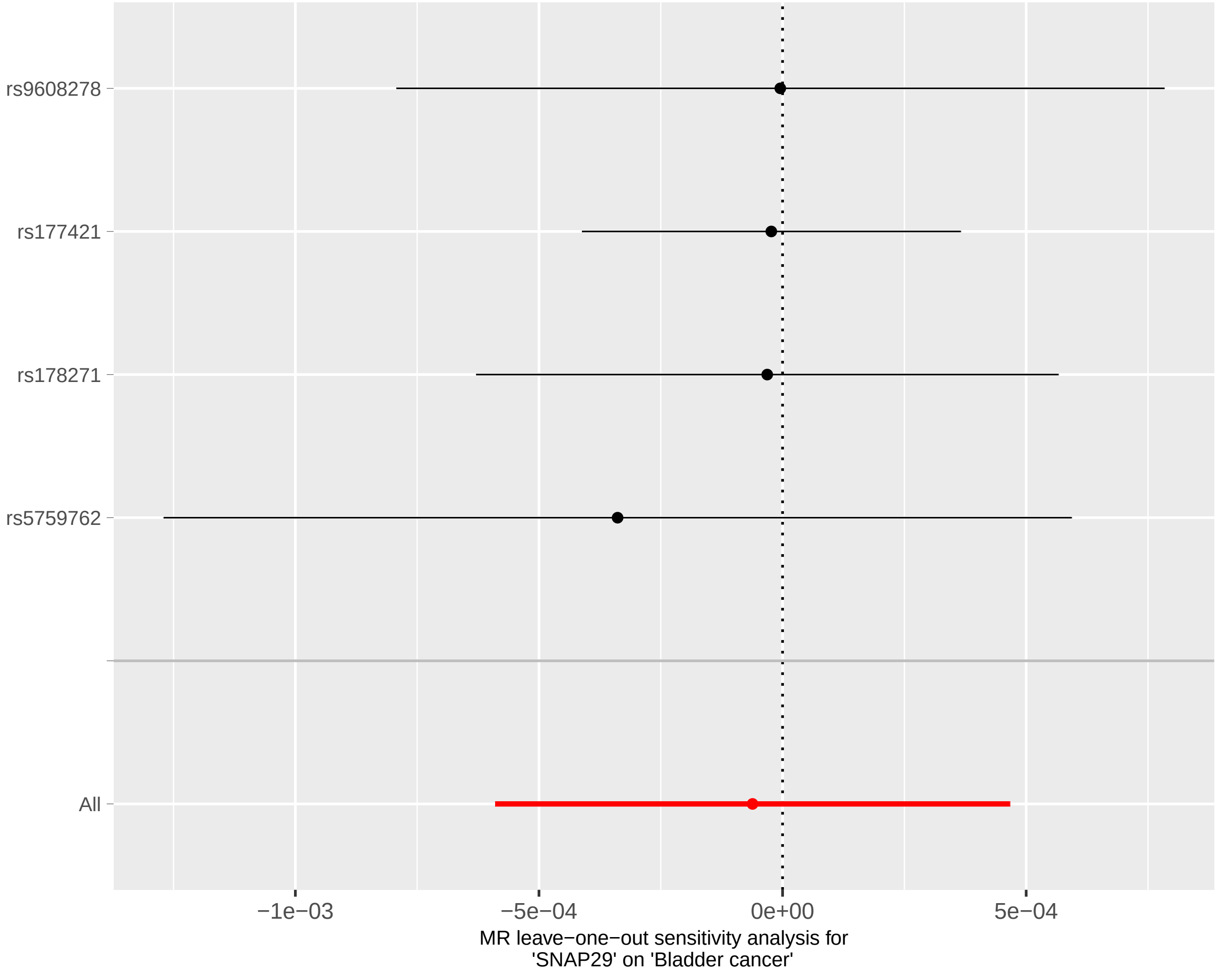
rs9608278
rs177421
rs178271
rs5759762
All
−1e−03
−5e−04
0e+00
5e−04
MR leave−one−out sensitivity analysis for
'SNAP29' on 'Bladder cancer'

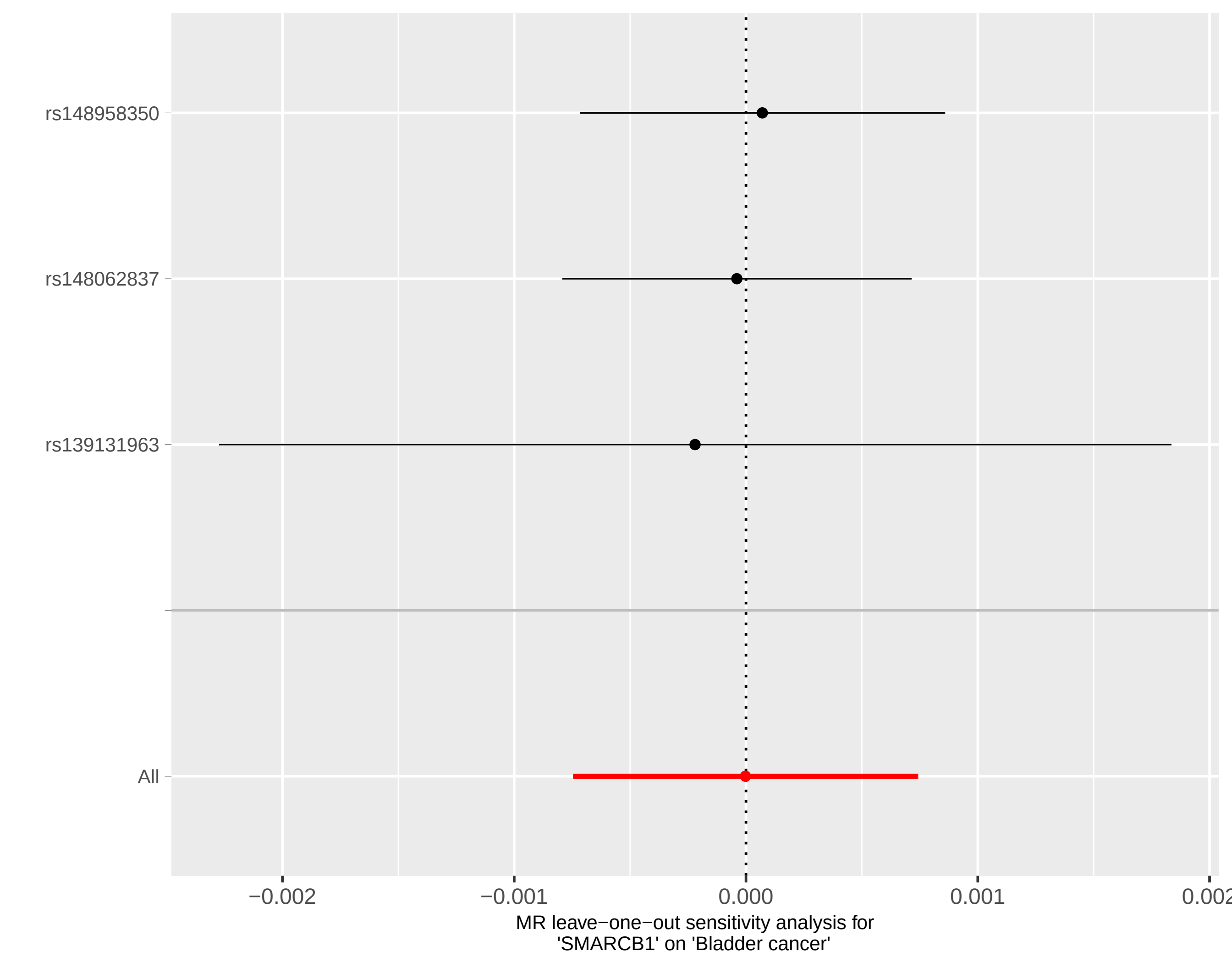
rs148958350
rs148062837
rs139131963
All
−0.002
−0.001
0.000
0.001
0.002
MR leave−one−out sensitivity analysis for
'SMARCB1' on 'Bladder cancer'

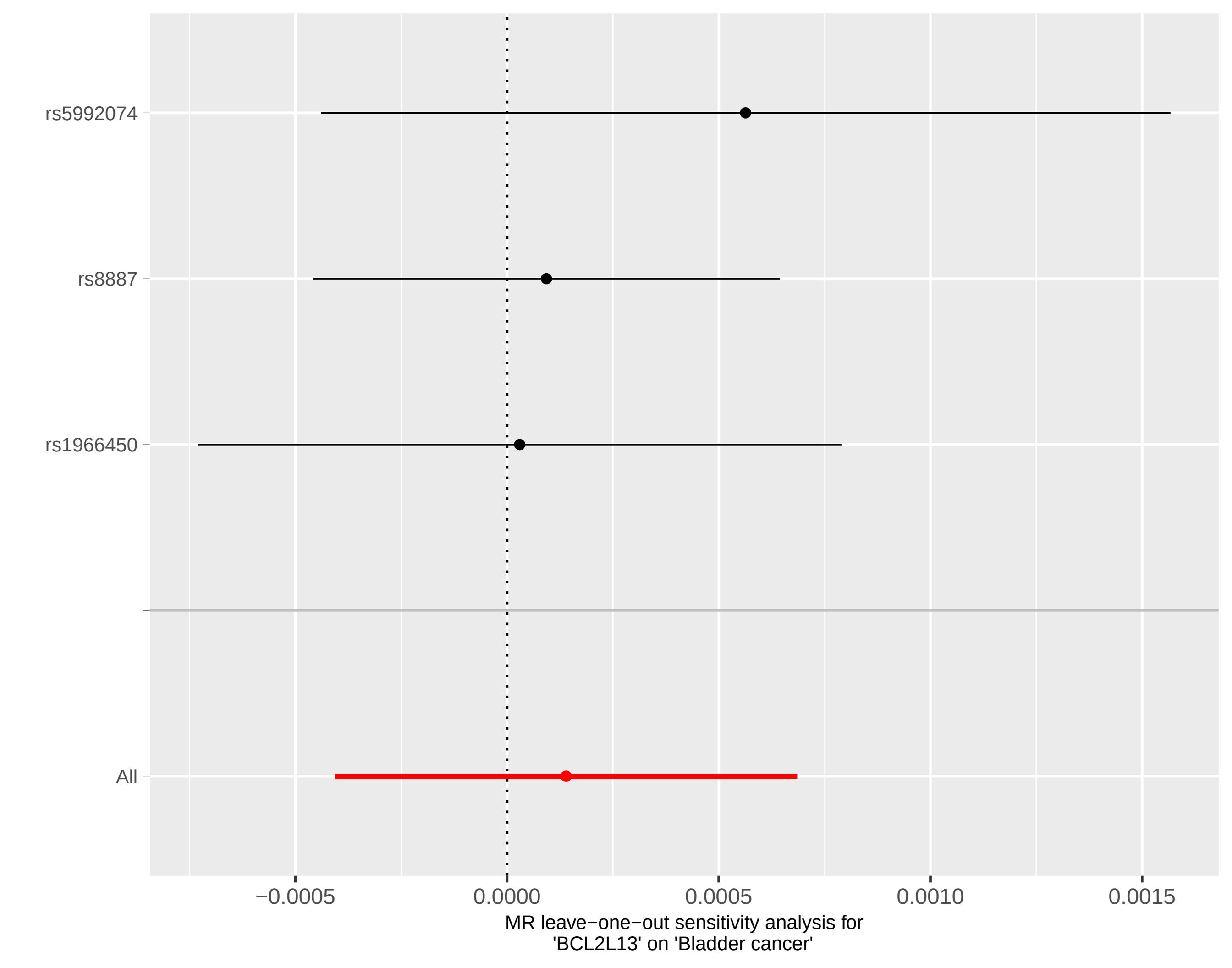
rs5992074
rs8887
rs1966450
All
−0.0005
0.0000
0.0005
0.0010
0.0015
MR leave−one−out sensitivity analysis for
'BCL2L13' on 'Bladder cancer'

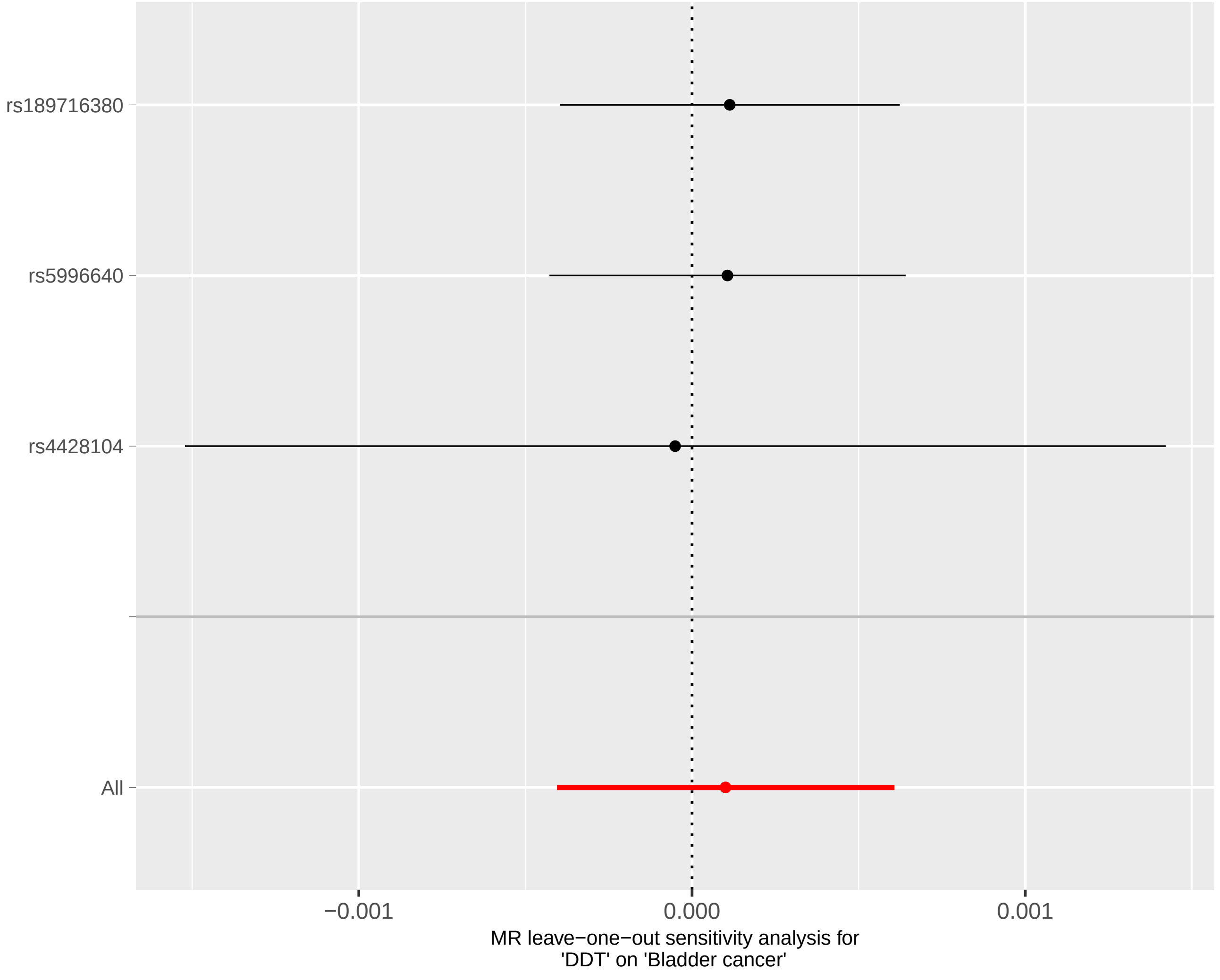
rs189716380
rs5996640
rs4428104
All
−0.001
0.000
0.001
MR leave−one−out sensitivity analysis for
'DDT' on 'Bladder cancer'

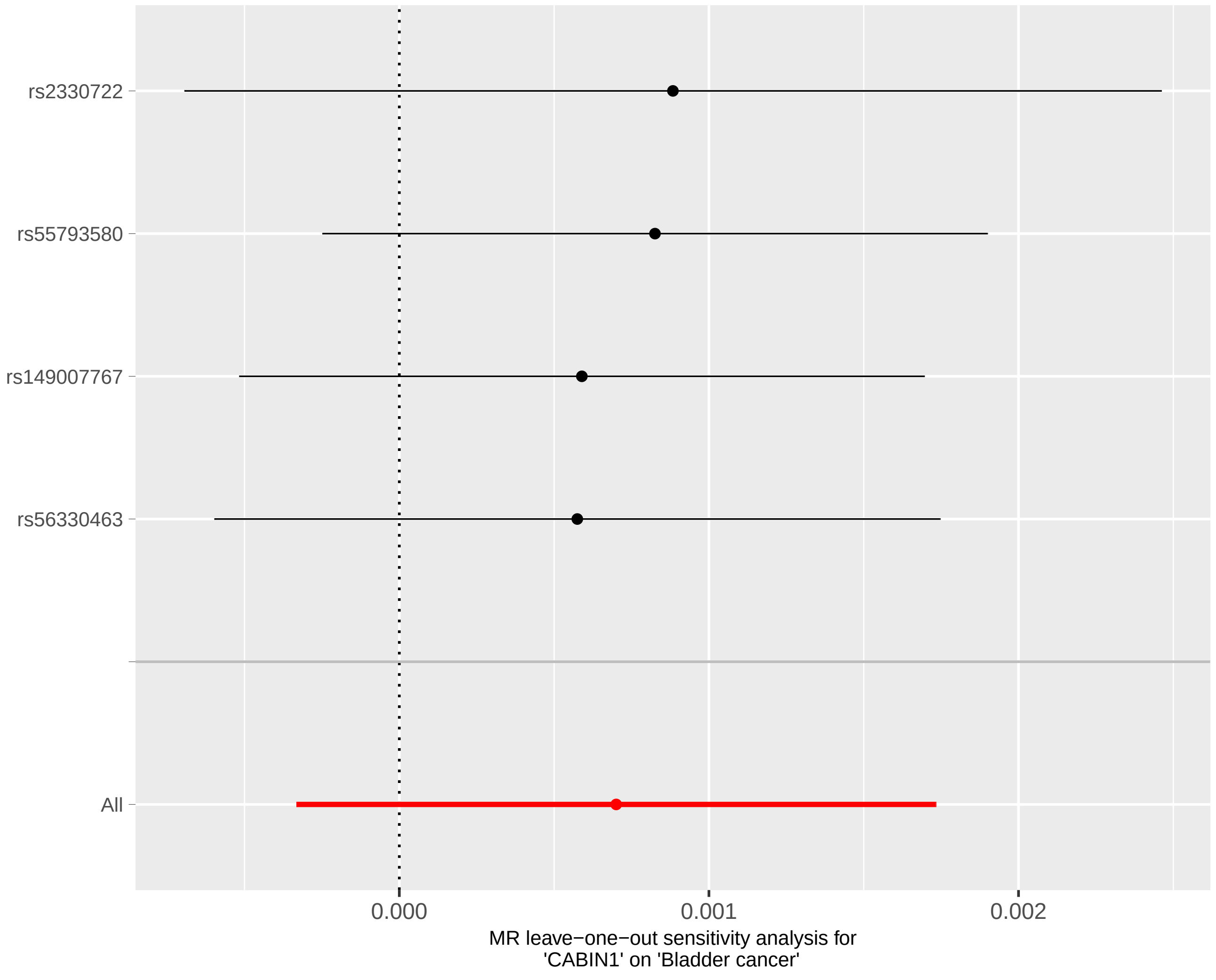
rs2330722
rs55793580
rs149007767
rs56330463
All
0.000
0.001
0.002
MR leave−one−out sensitivity analysis for
'CABIN1' on 'Bladder cancer'

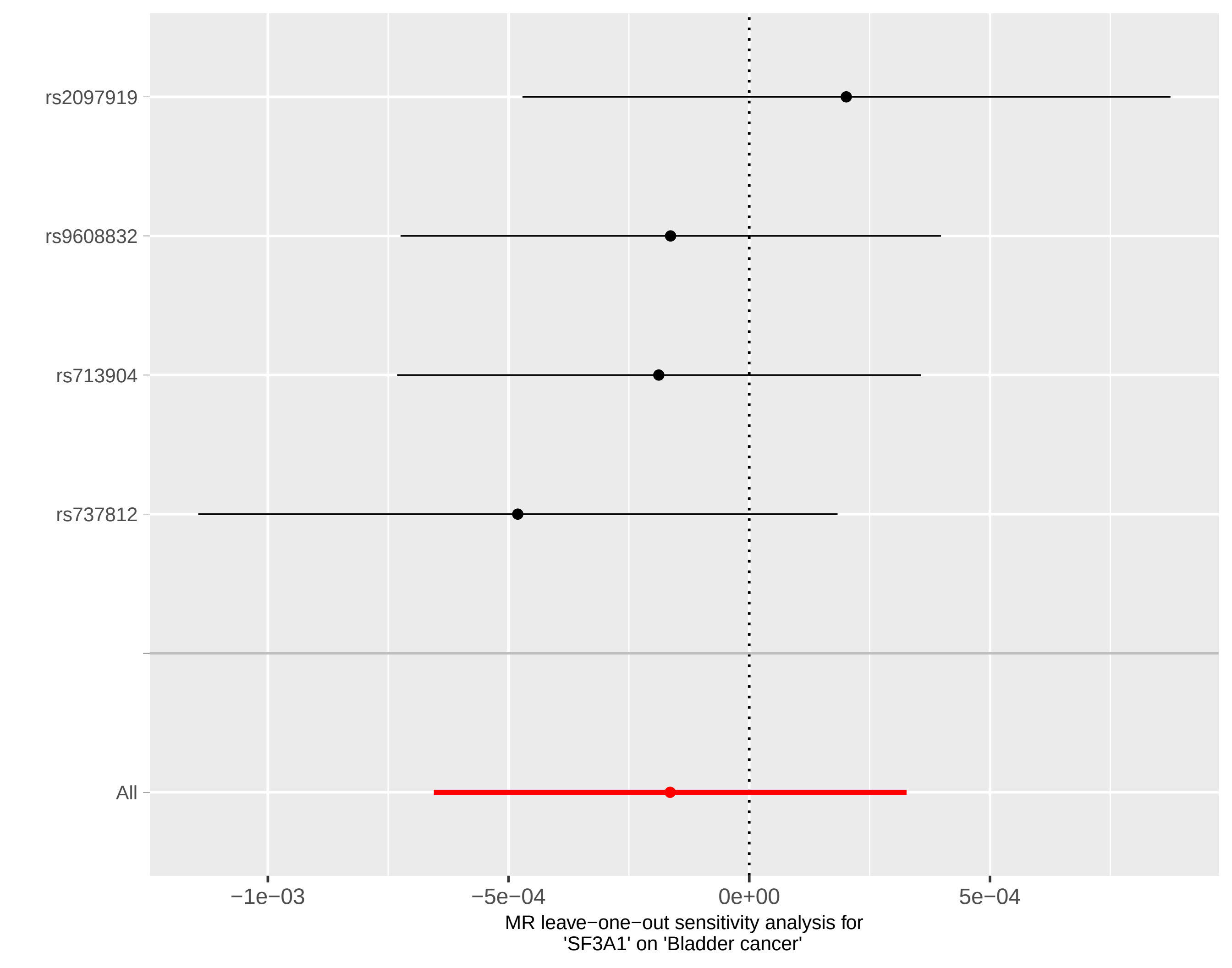
rs2097919
rs9608832
rs713904
rs737812
All
−1e−03
−5e−04
0e+00
5e−04
MR leave−one−out sensitivity analysis for
'SF3A1' on 'Bladder cancer'

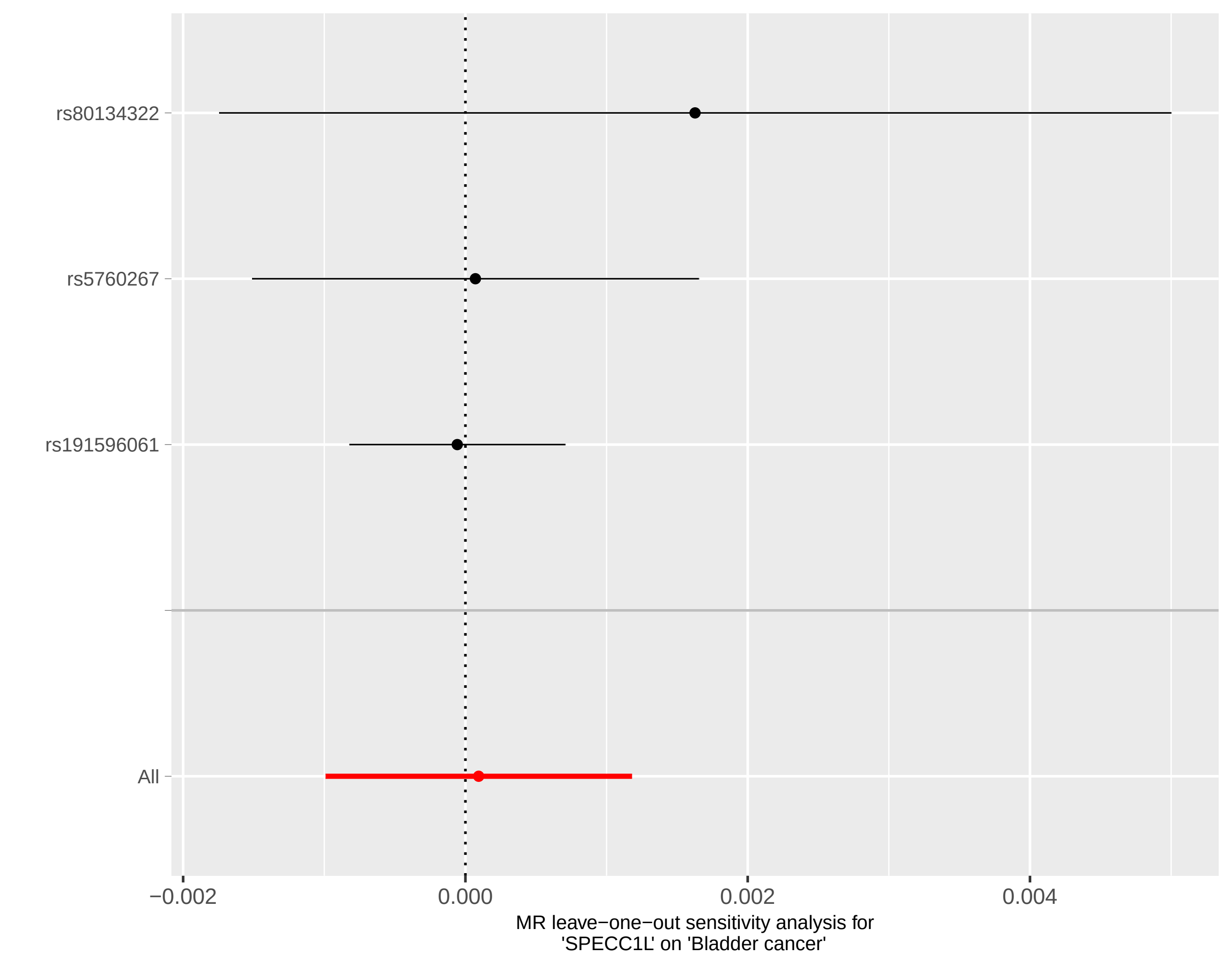
rs80134322
rs5760267
rs191596061
All
−0.002
0.000
0.002
0.004
MR leave−one−out sensitivity analysis for
'SPECC1L' on 'Bladder cancer'

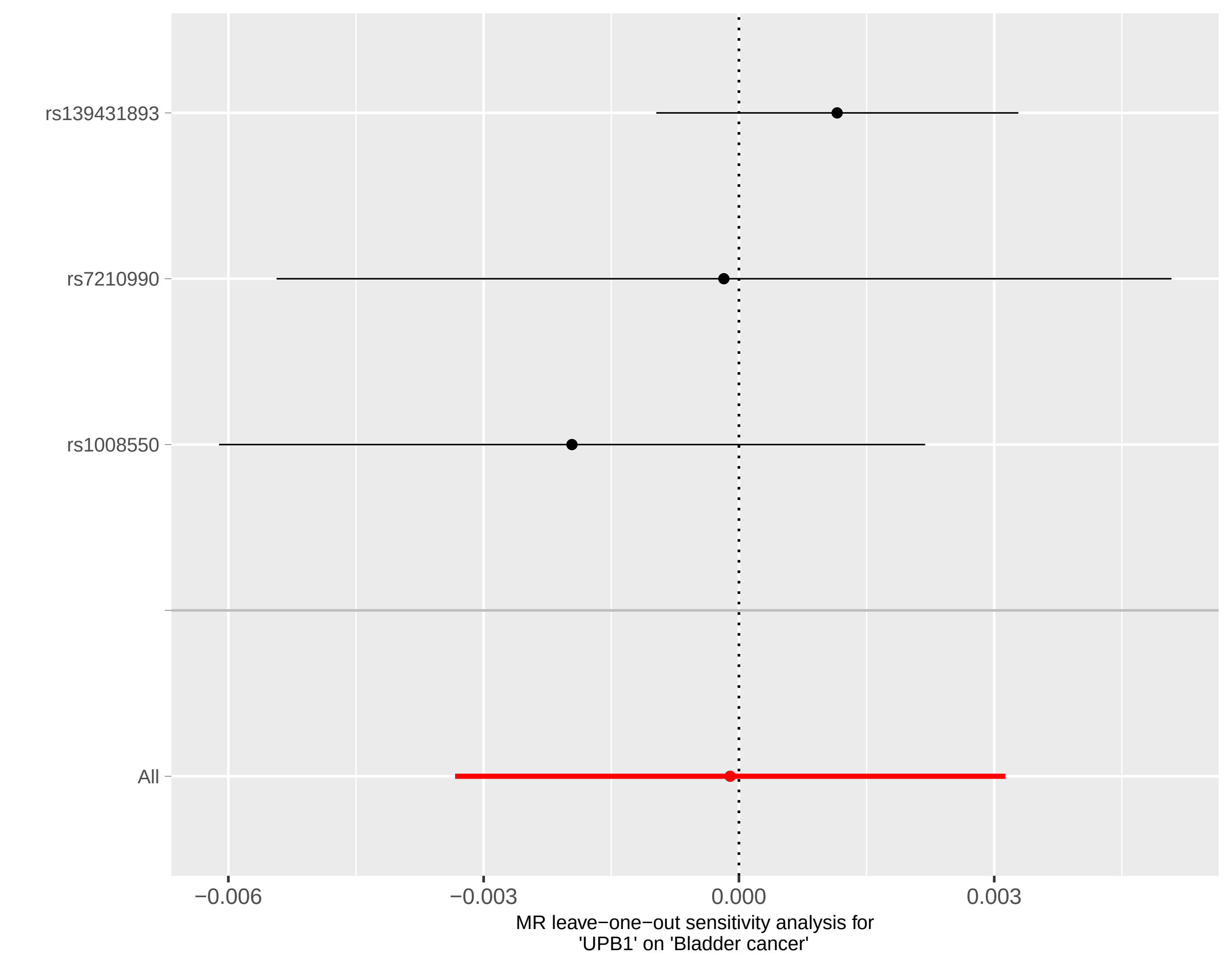
rs139431893
rs7210990
rs1008550
All
−0.006
−0.003
0.000
0.003
MR leave−one−out sensitivity analysis for
'UPB1' on 'Bladder cancer'

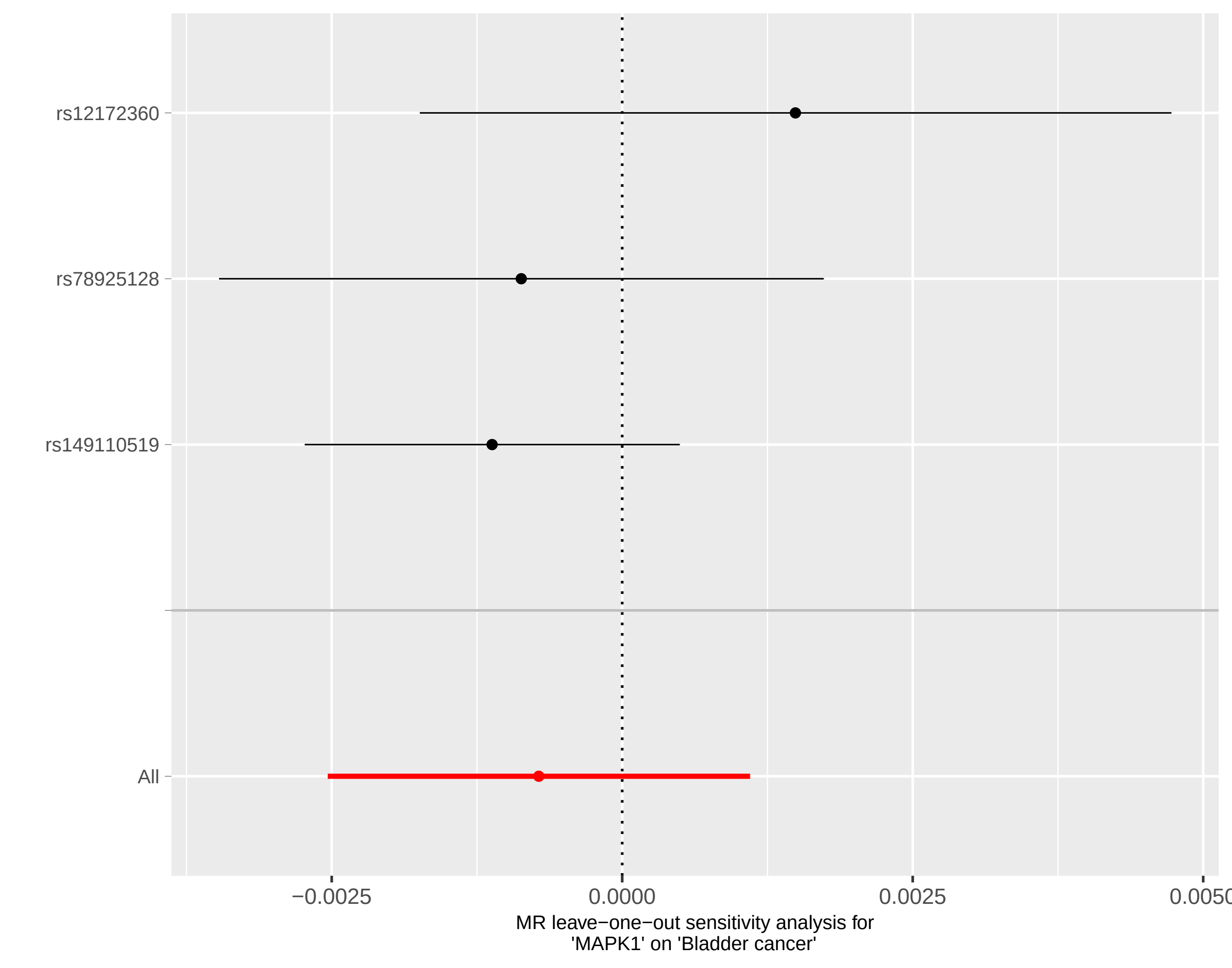

rs12172360
rs78925128
rs149110519
All
−0.0025
0.0000
0.0025
0.0050
MR leave−one−out sensitivity analysis for
'MAPK1' on 'Bladder cancer'

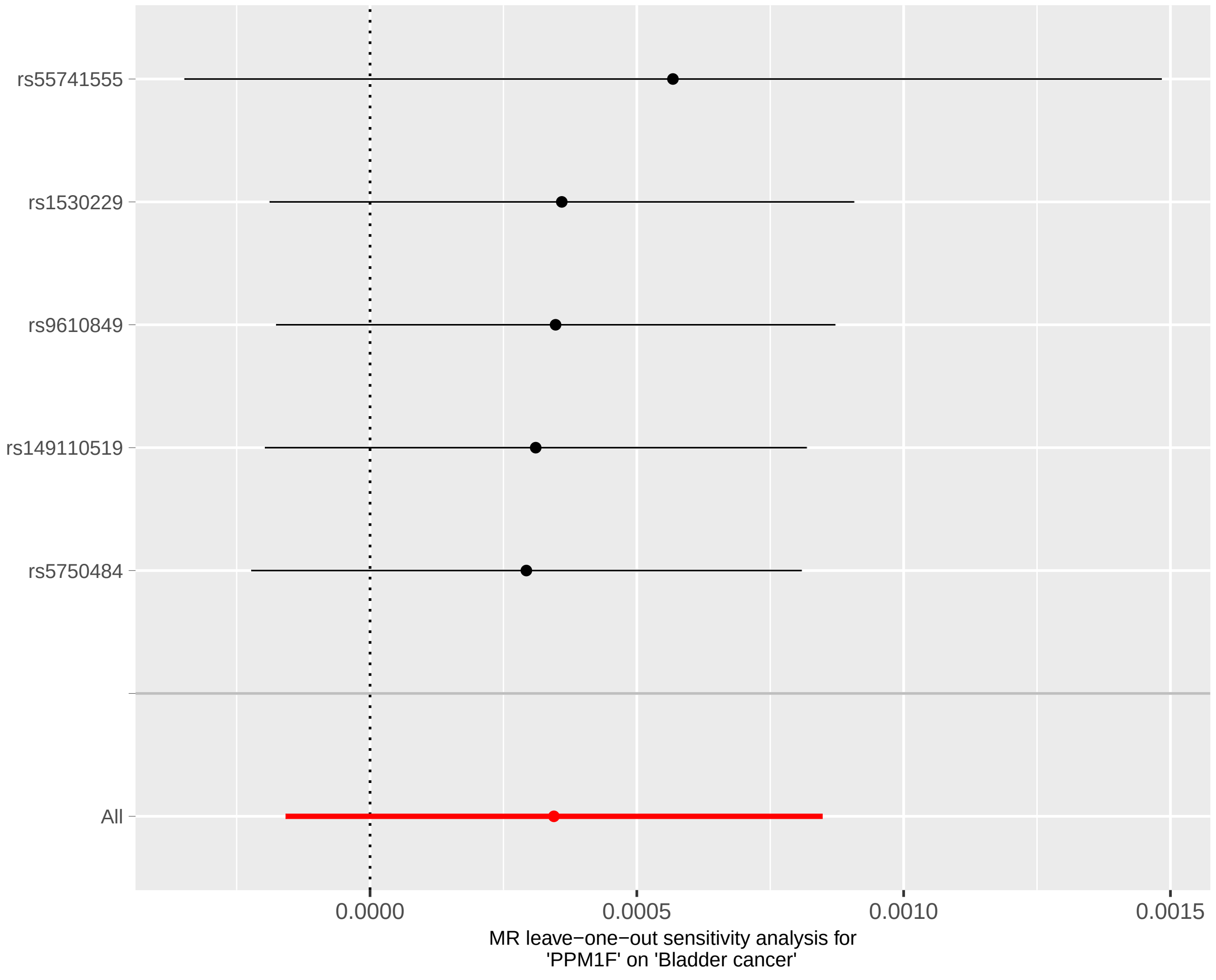

rs55741555
rs1530229
rs9610849
rs149110519
rs5750484
All
0.0000
0.0005
0.0010
0.0015
MR leave−one−out sensitivity analysis for
'PPM1F' on 'Bladder cancer'

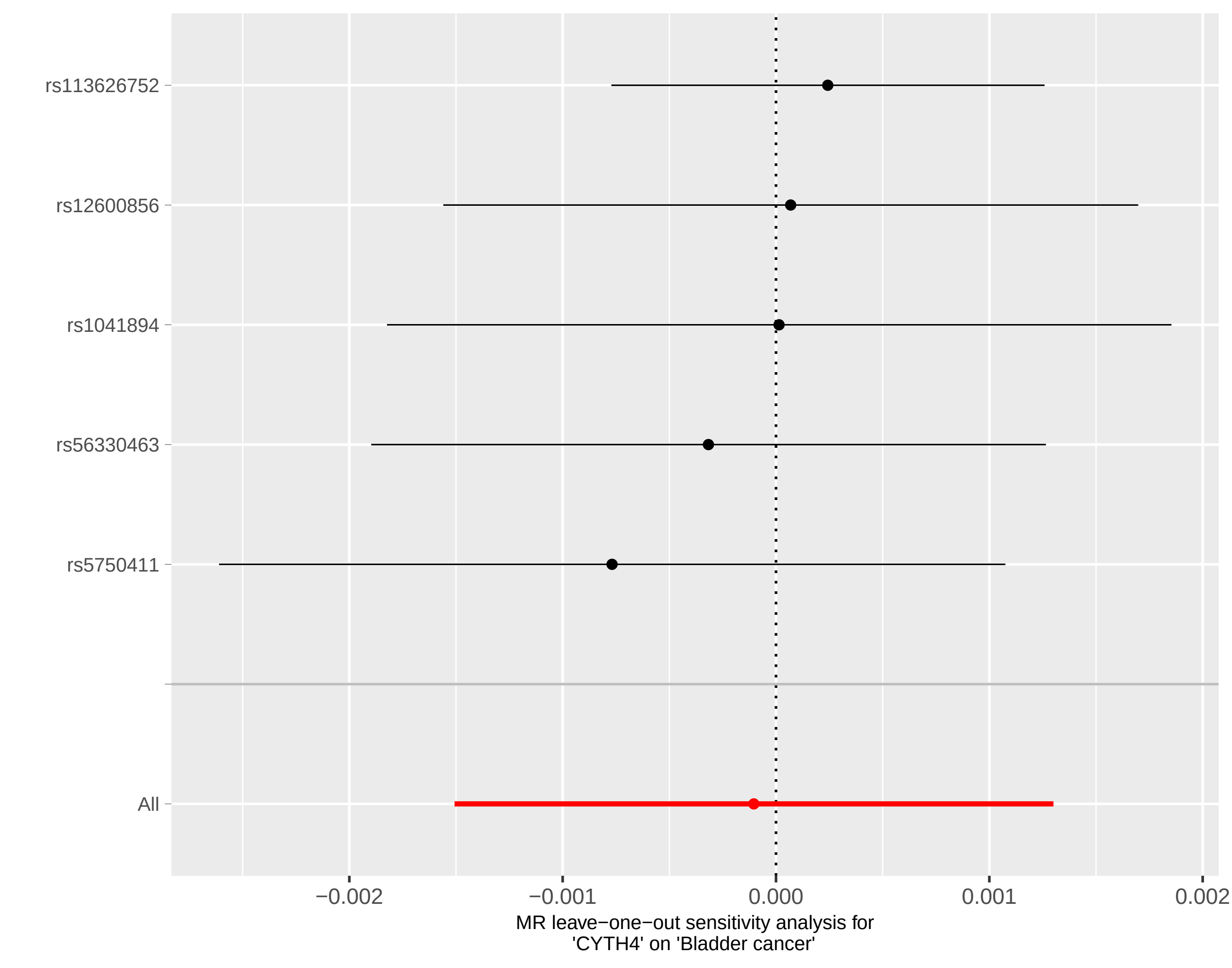
rs113626752
rs12600856
rs1041894
rs56330463
rs5750411
All
−0.002
−0.001
0.000
0.001
0.002
MR leave−one−out sensitivity analysis for
'CYTH4' on 'Bladder cancer'

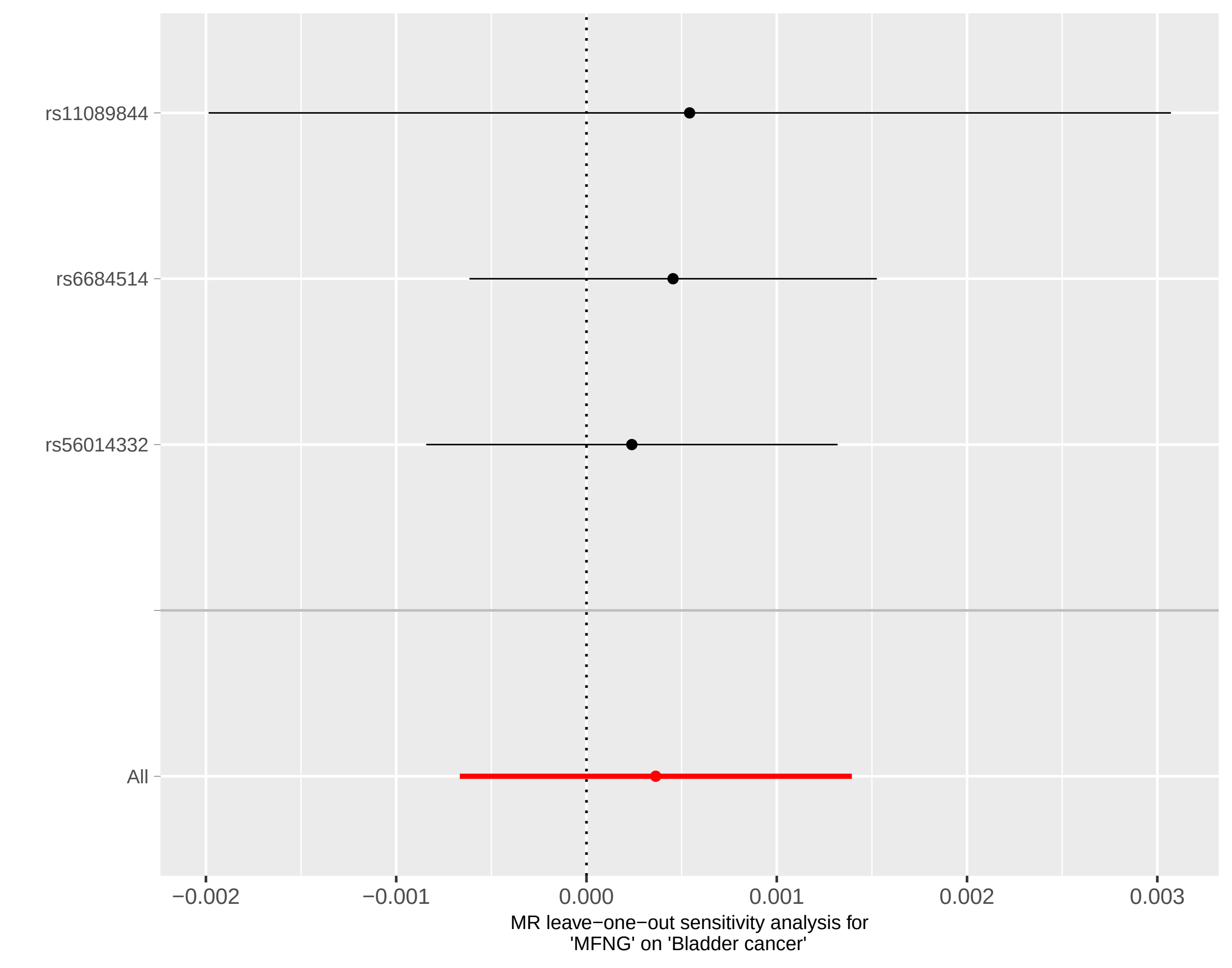
rs11089844
rs6684514
rs56014332
All
−0.002
−0.001
0.000
0.001
0.002
0.003
MR leave−one−out sensitivity analysis for
'MFNG' on 'Bladder cancer'

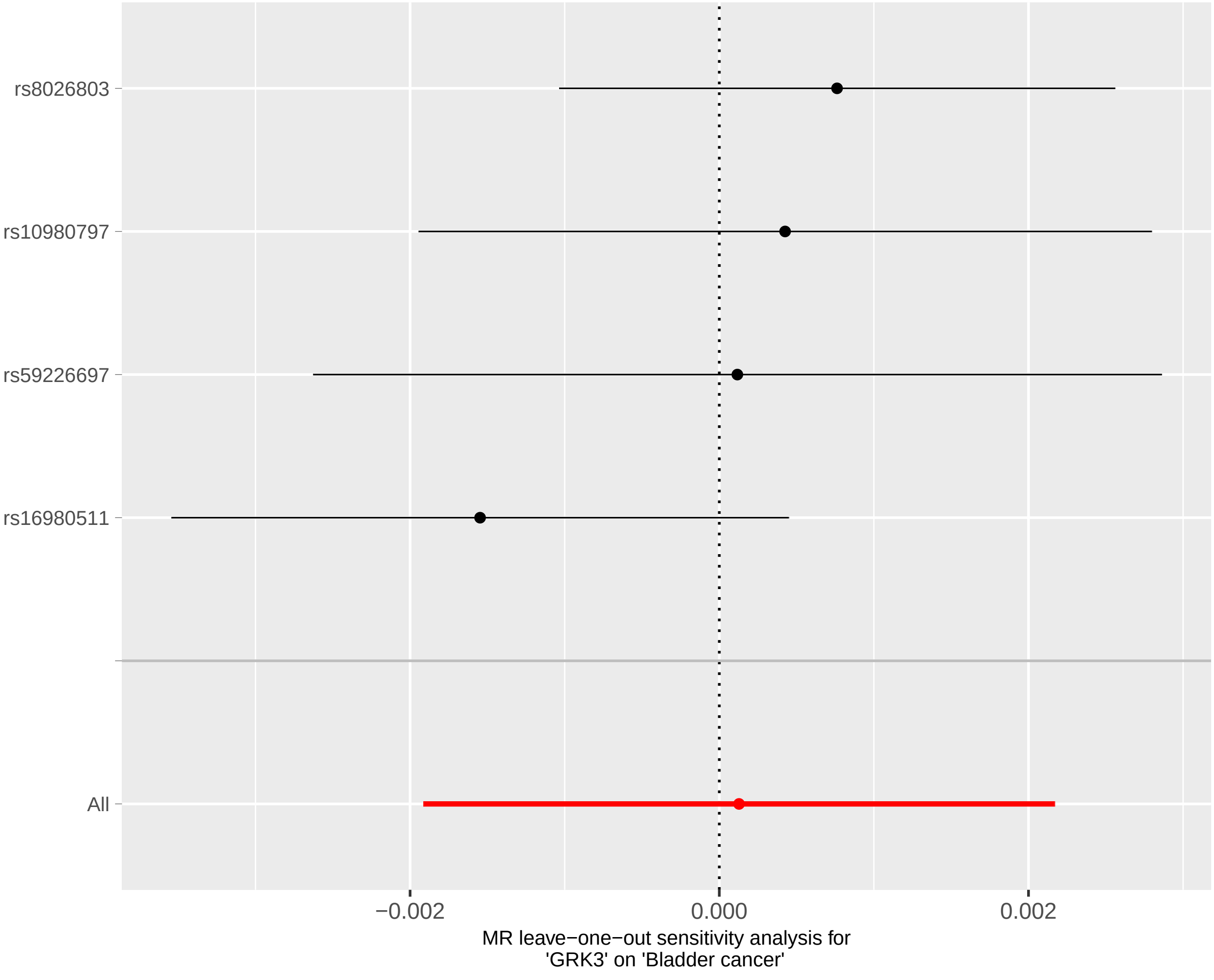
rs8026803
rs10980797
rs59226697
rs16980511
All
−0.002
0.000
0.002
MR leave−one−out sensitivity analysis for
'GRK3' on 'Bladder cancer'

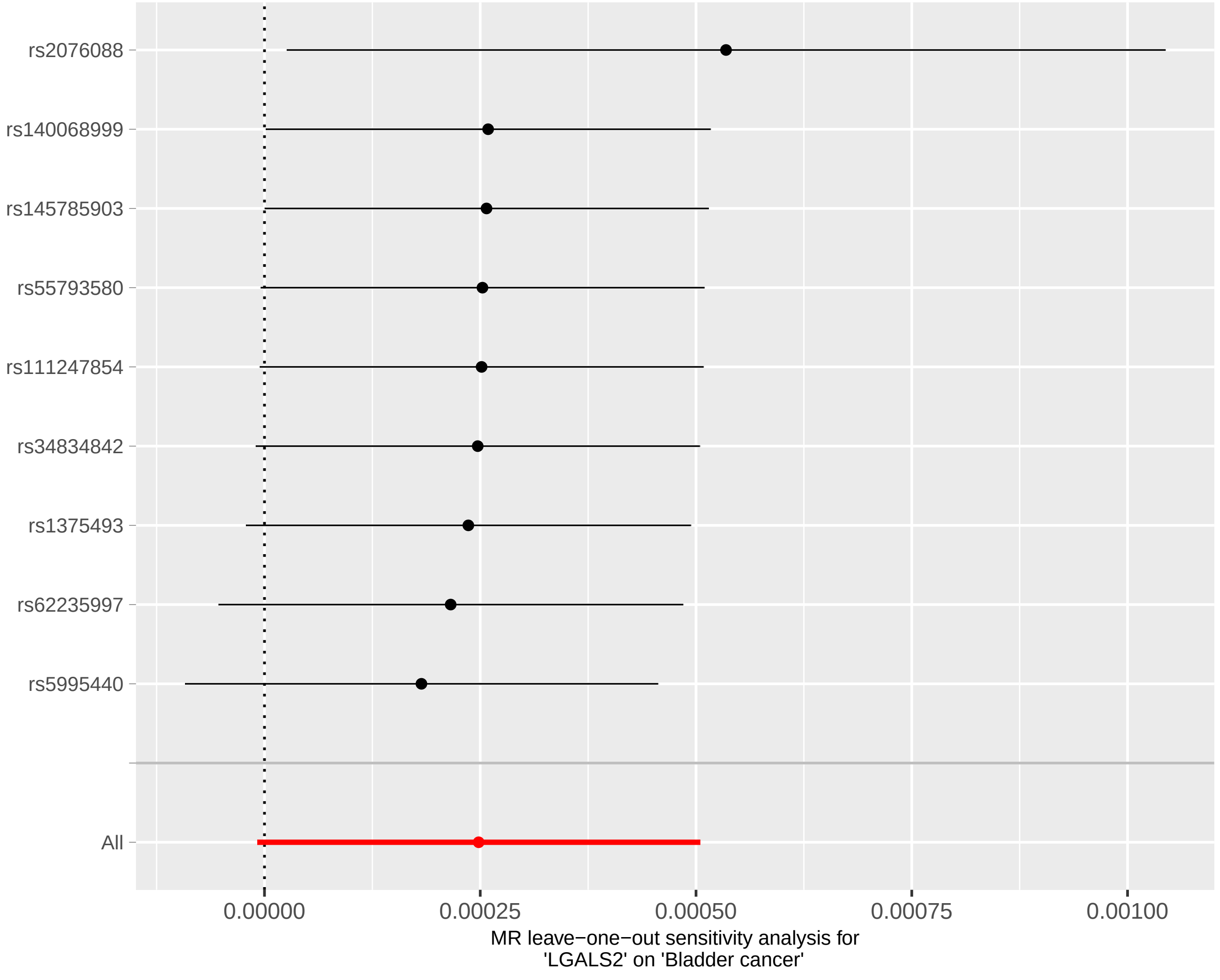

rs2076088
rs140068999
rs145785903
rs55793580
rs111247854
rs34834842
rs1375493
rs62235997
rs5995440
All
0.00000
0.00025
0.00050
0.00075
0.00100
MR leave−one−out sensitivity analysis for
'LGALS2' on 'Bladder cancer'

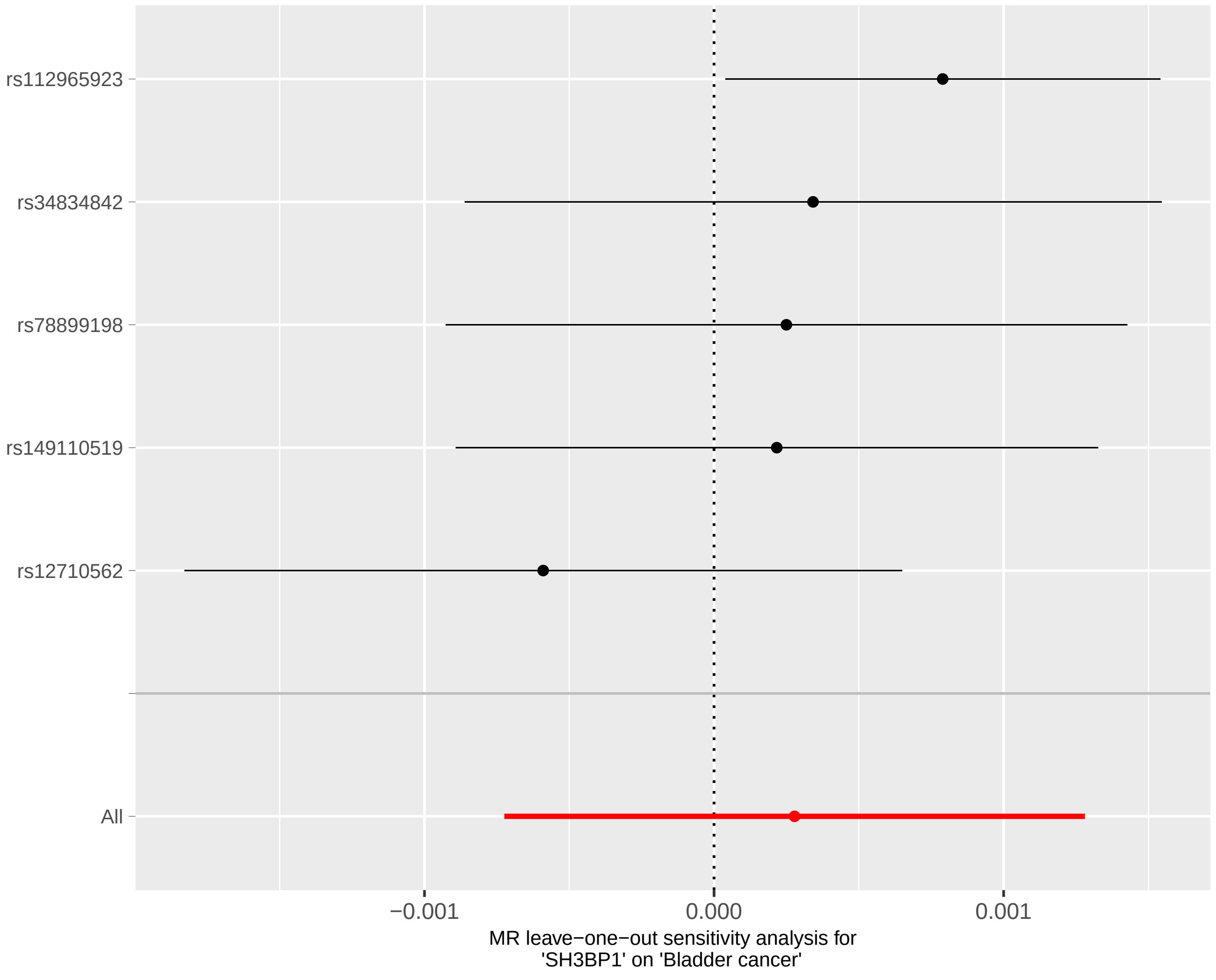
rs112965923
rs34834842
rs78899198
rs149110519
rs12710562
All
−0.001
0.000
0.001
MR leave−one−out sensitivity analysis for
'SH3BP1' on 'Bladder cancer'

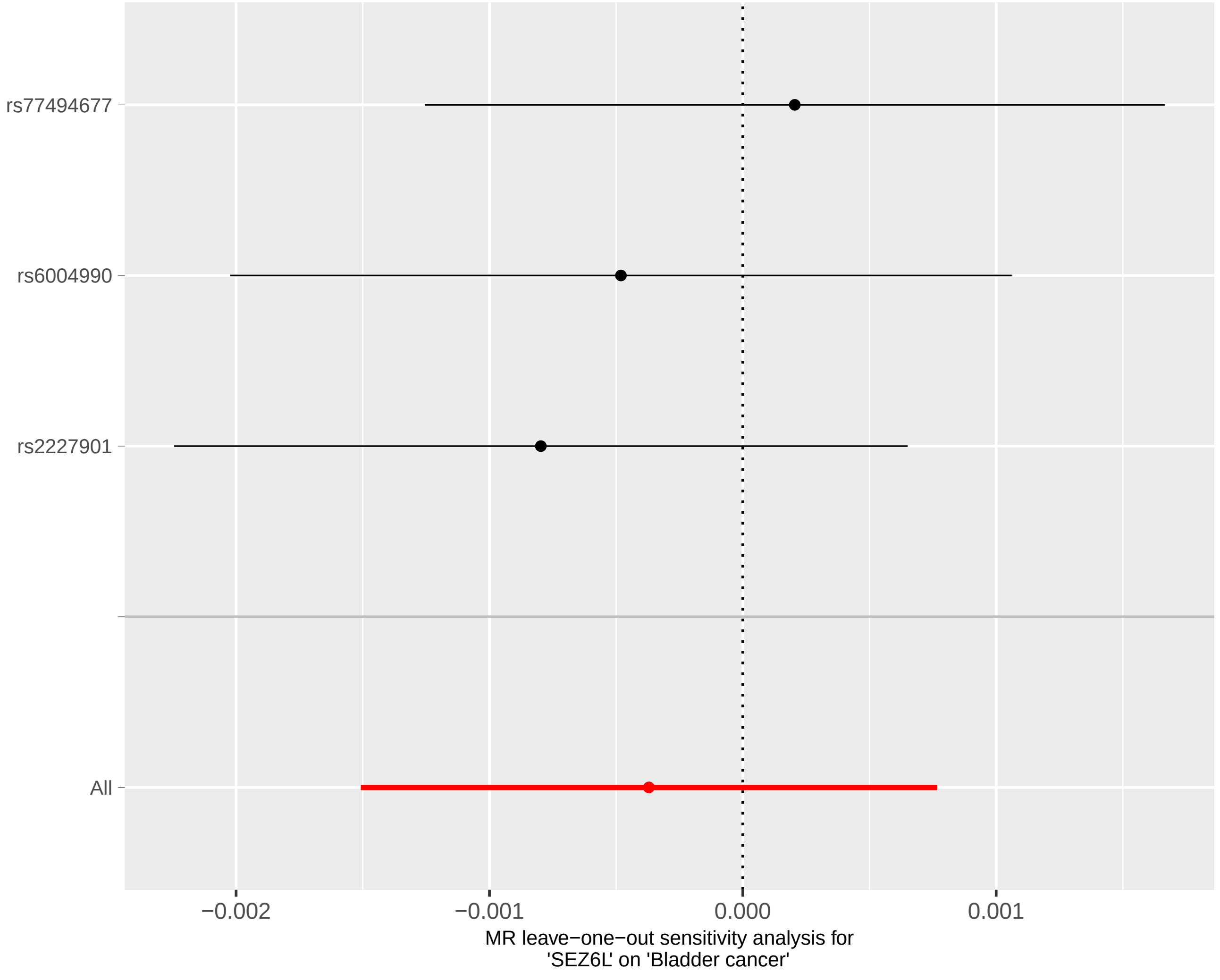
rs77494677
rs6004990
rs2227901
All
−0.002
−0.001
0.000
0.001
MR leave−one−out sensitivity analysis for
'SEZ6L' on 'Bladder cancer'

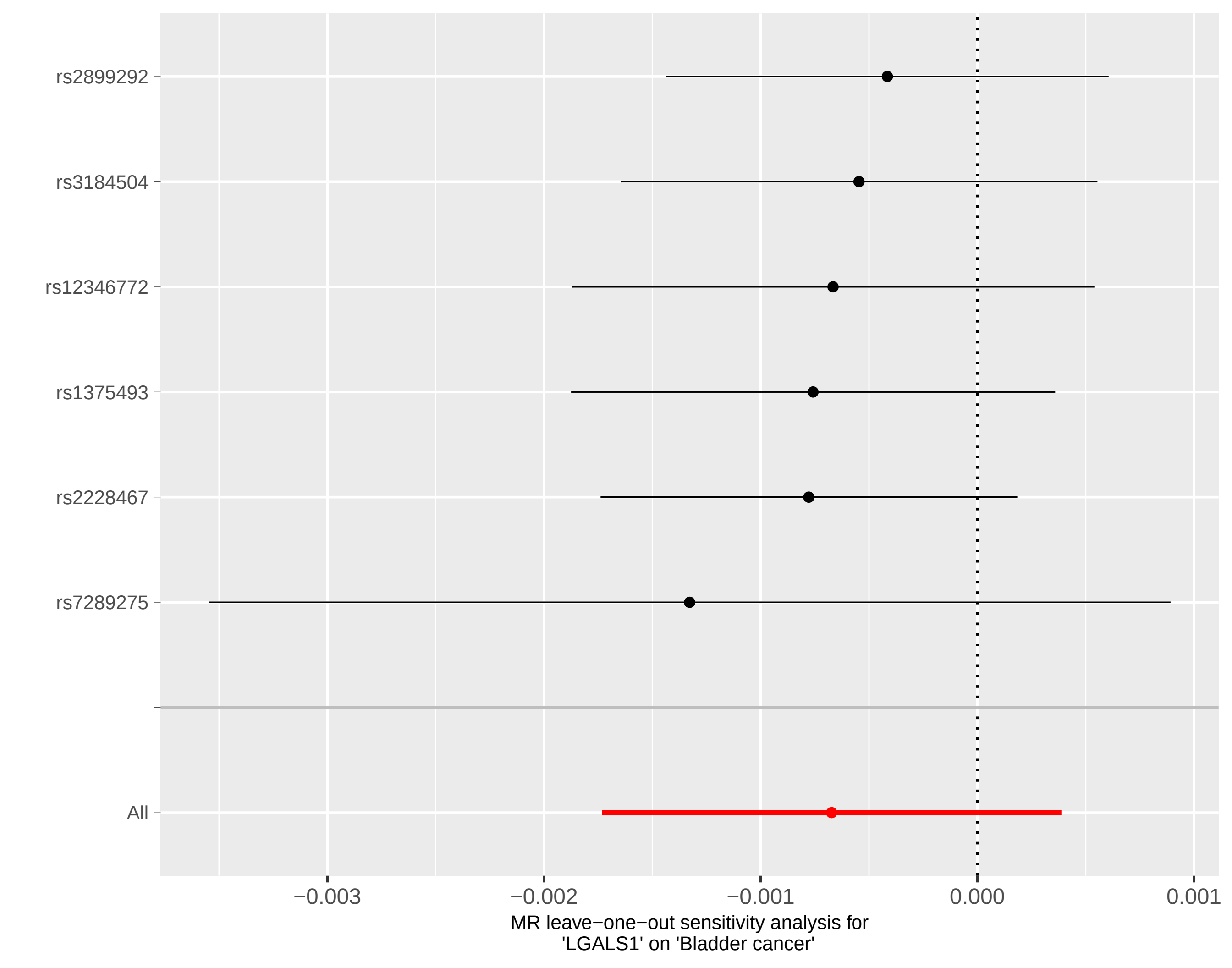

rs2899292
rs3184504
rs12346772
rs1375493
rs2228467
rs7289275
All
−0.003
−0.002
−0.001
0.000
0.001
MR leave−one−out sensitivity analysis for
'LGALS1' on 'Bladder cancer'

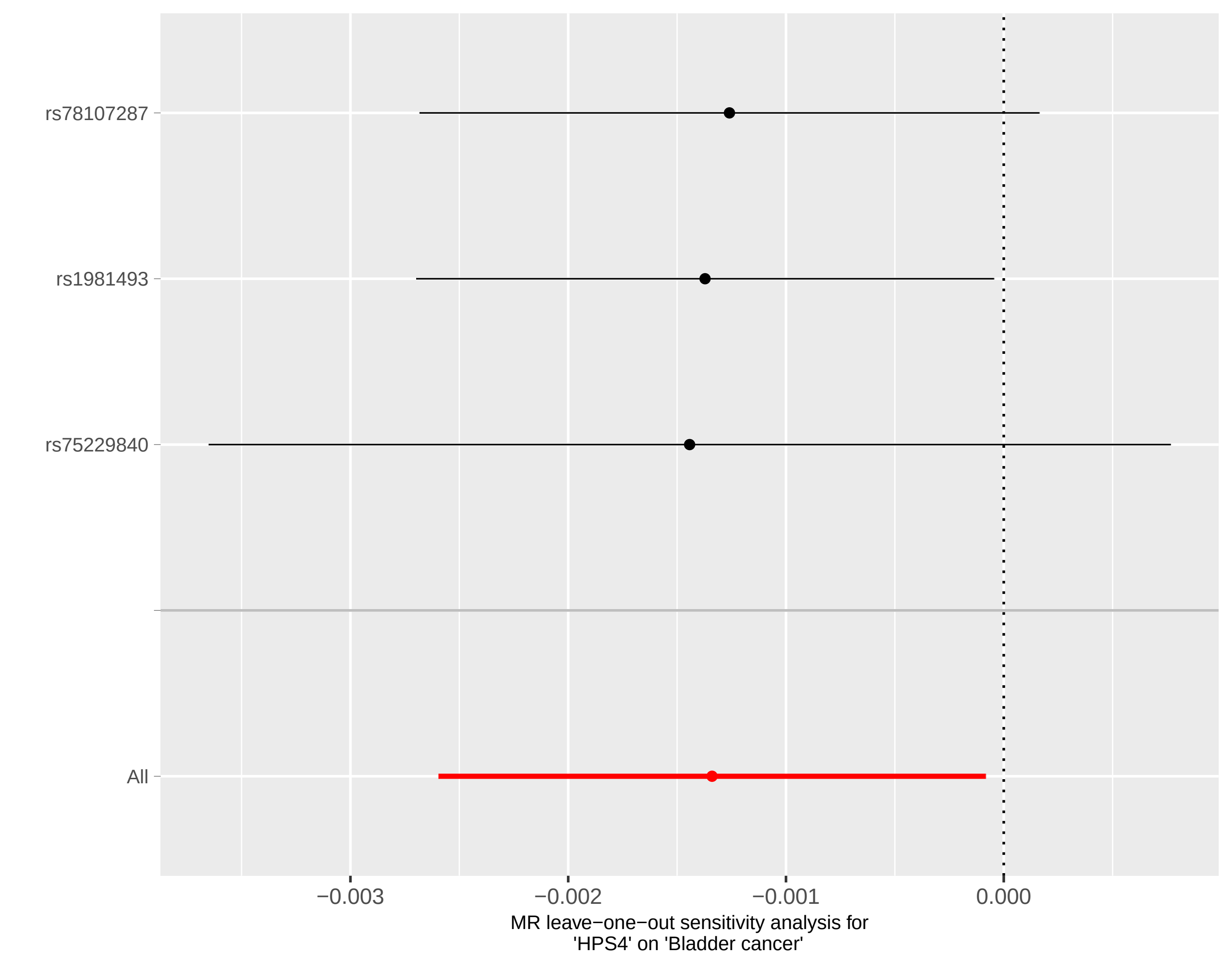

rs78107287
rs1981493
rs75229840
All
−0.003
−0.002
−0.001
0.000
MR leave−one−out sensitivity analysis for
'HPS4' on 'Bladder cancer'

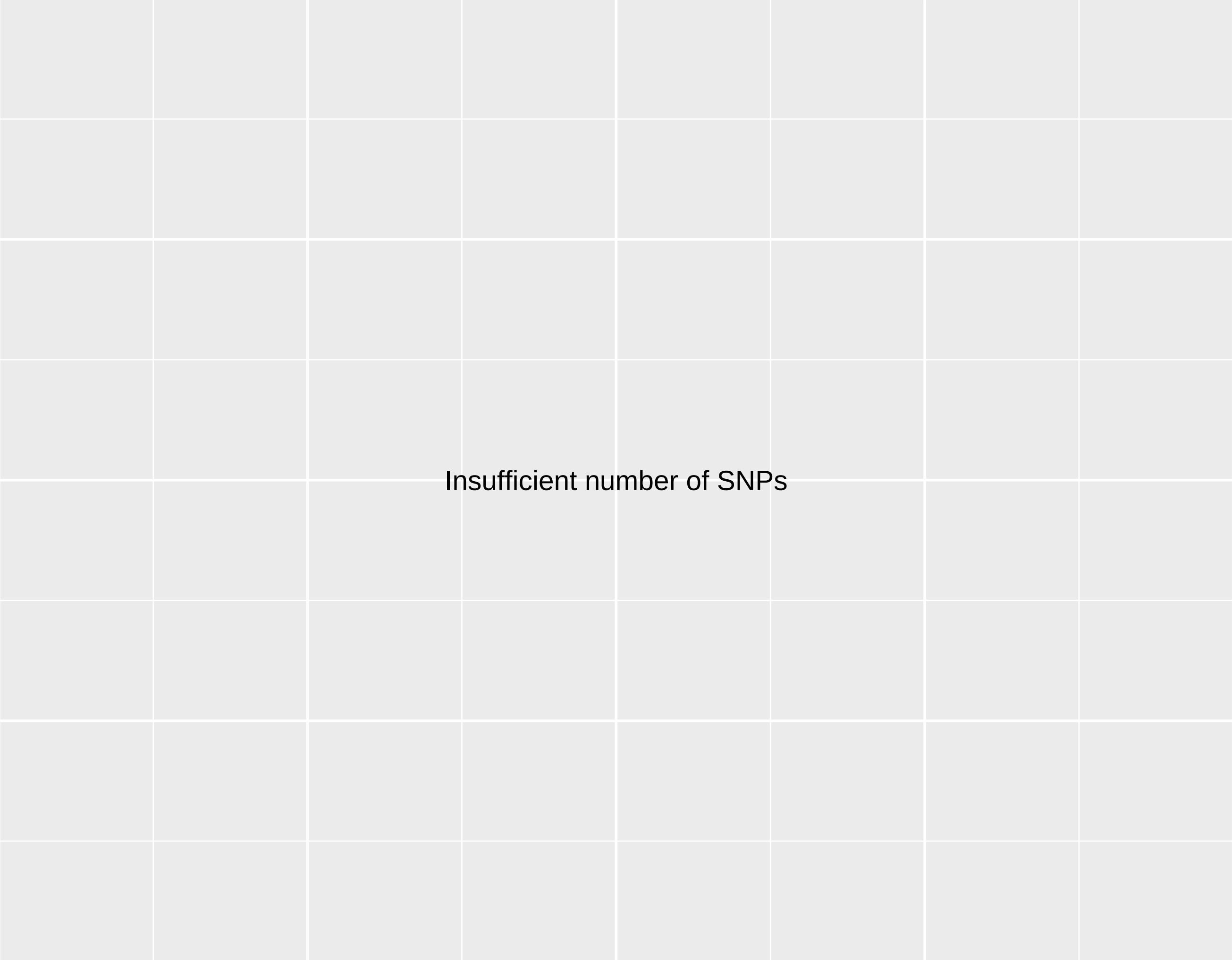
Insufficient number of SNPs

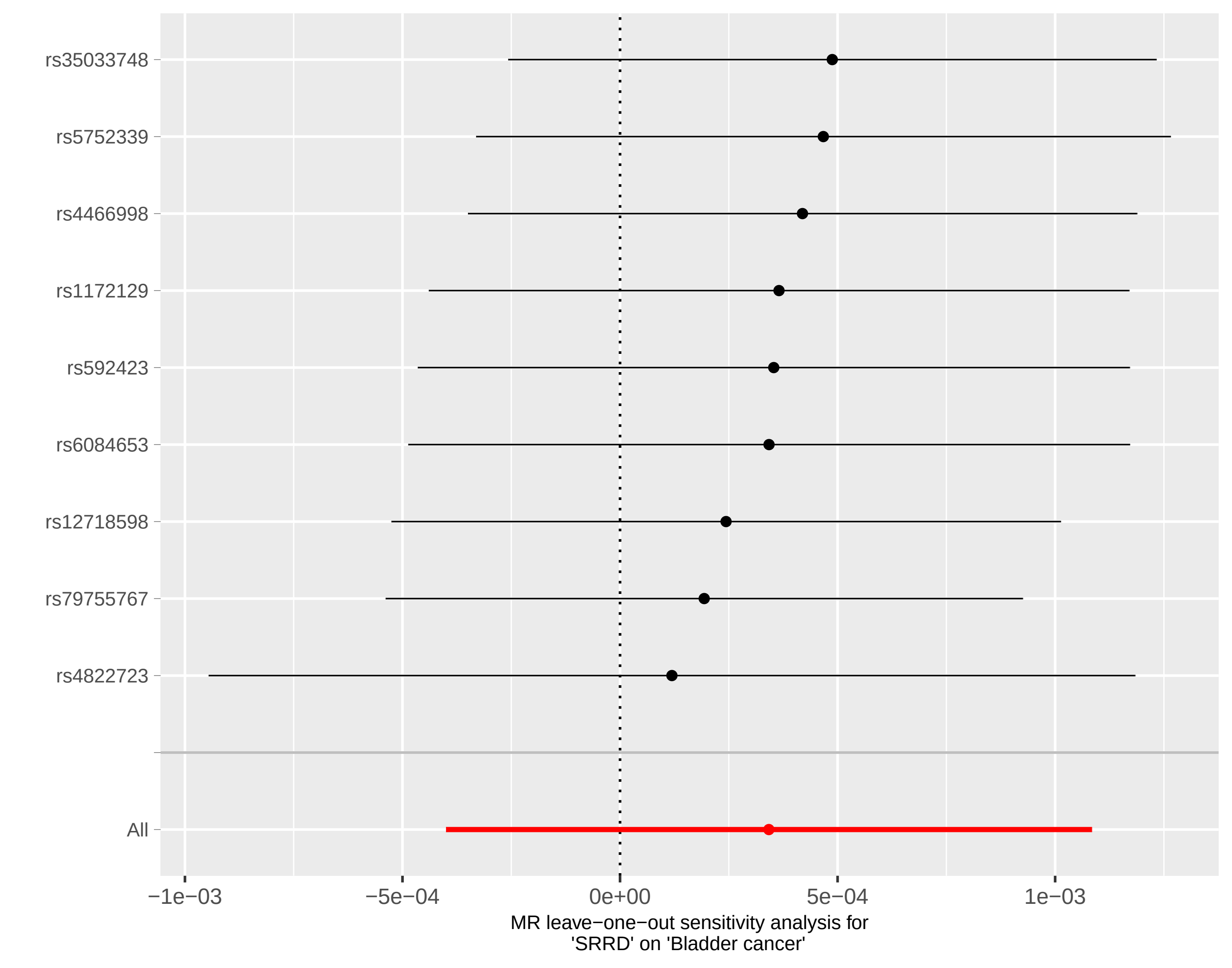
rs35033748
rs5752339
rs4466998
rs1172129
rs592423
rs6084653
rs12718598
rs79755767
rs4822723
All
−1e−03
−5e−04
0e+00
5e−04
1e−03
MR leave−one−out sensitivity analysis for
'SRRD' on 'Bladder cancer'

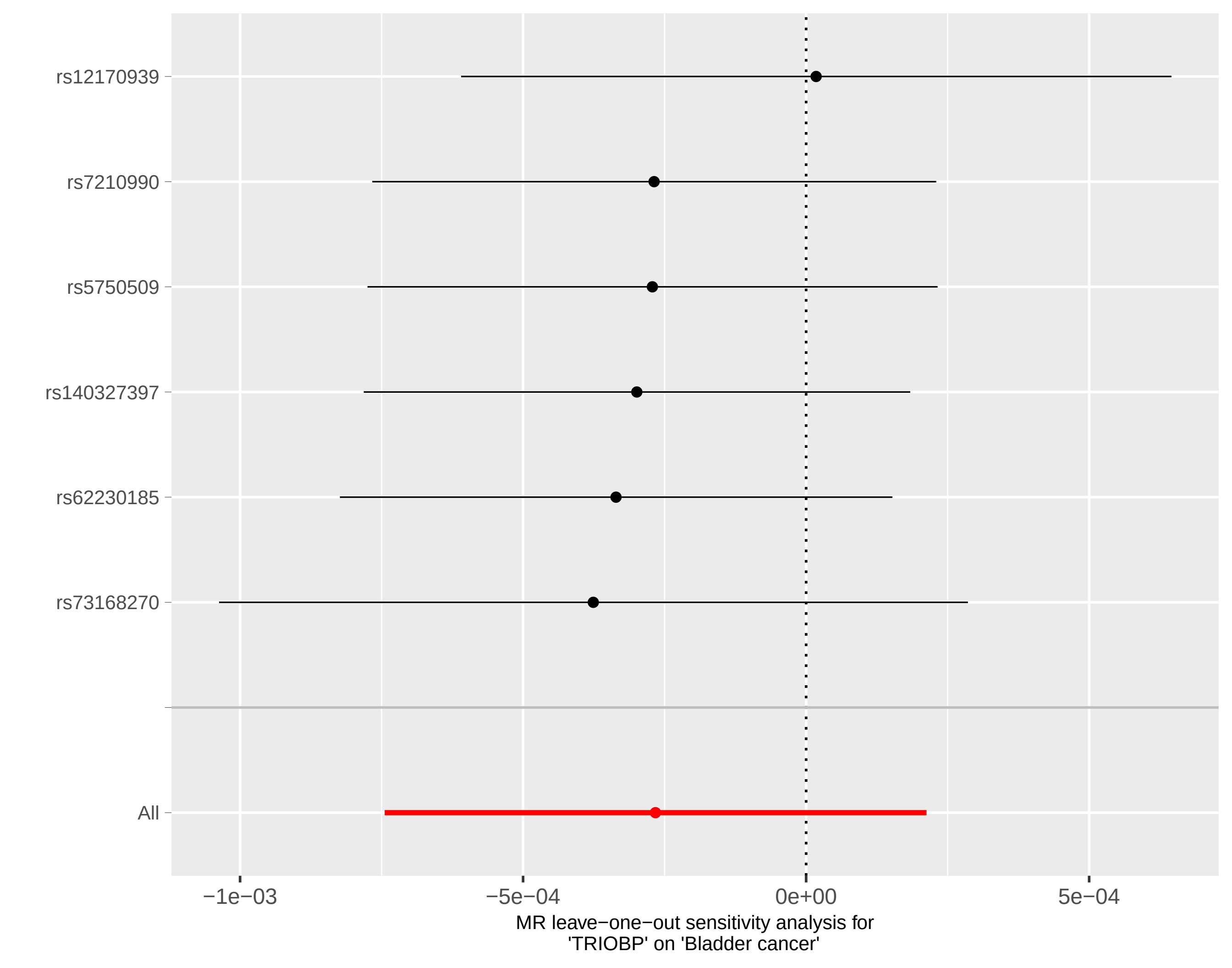

rs12170939
rs7210990
rs5750509
rs140327397
rs62230185
rs73168270
All
−1e−03
−5e−04
0e+00
5e−04
MR leave−one−out sensitivity analysis for
'TRIOBP' on 'Bladder cancer'

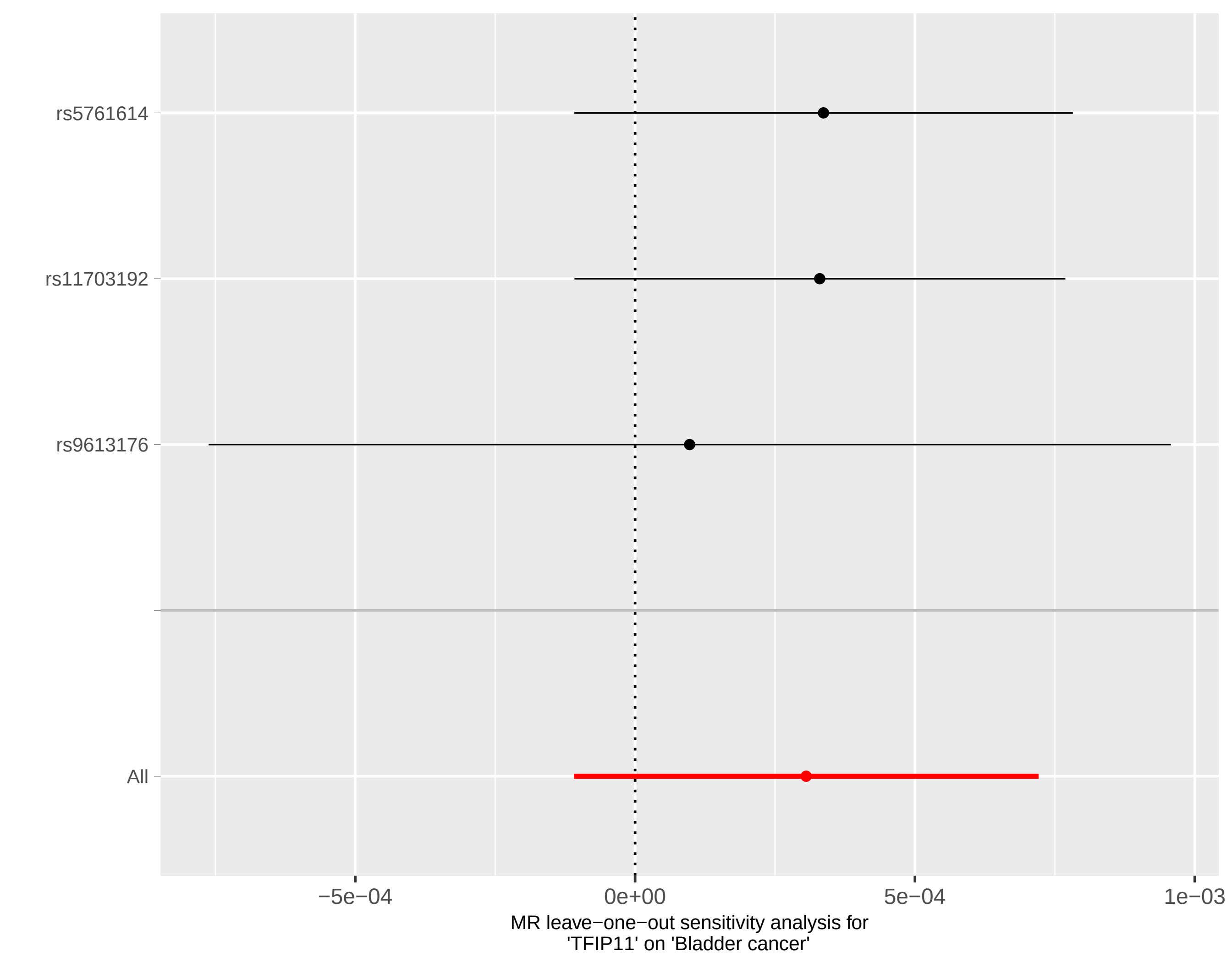
rs5761614
rs11703192
rs9613176
All
−5e−04
0e+00
5e−04
1e−03
MR leave−one−out sensitivity analysis for
'TFIP11' on 'Bladder cancer'

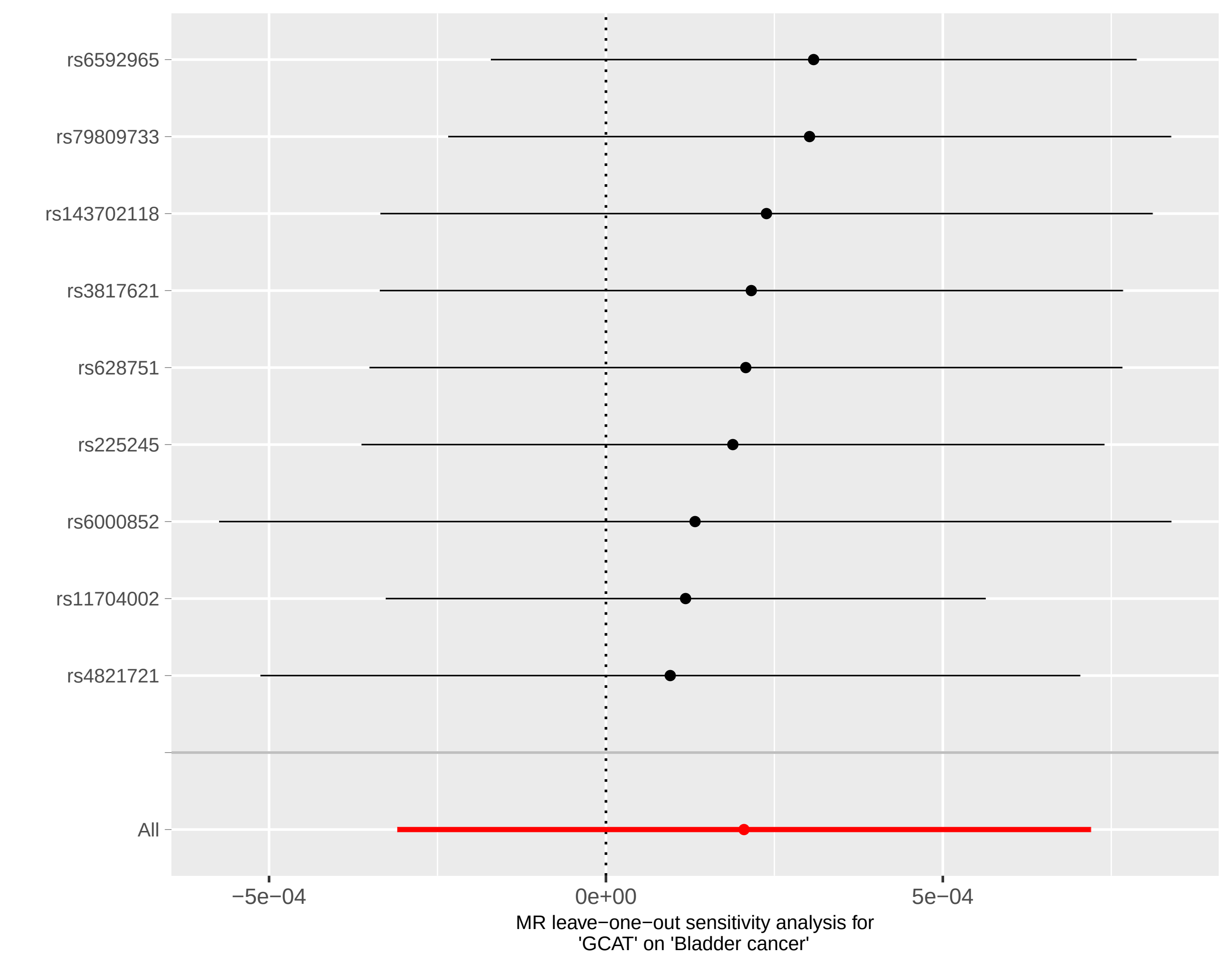
rs6592965
rs79809733
rs143702118
rs3817621
rs628751
rs225245
rs6000852
rs11704002
rs4821721
All
−5e−04
0e+00
5e−04
MR leave−one−out sensitivity analysis for
'GCAT' on 'Bladder cancer'

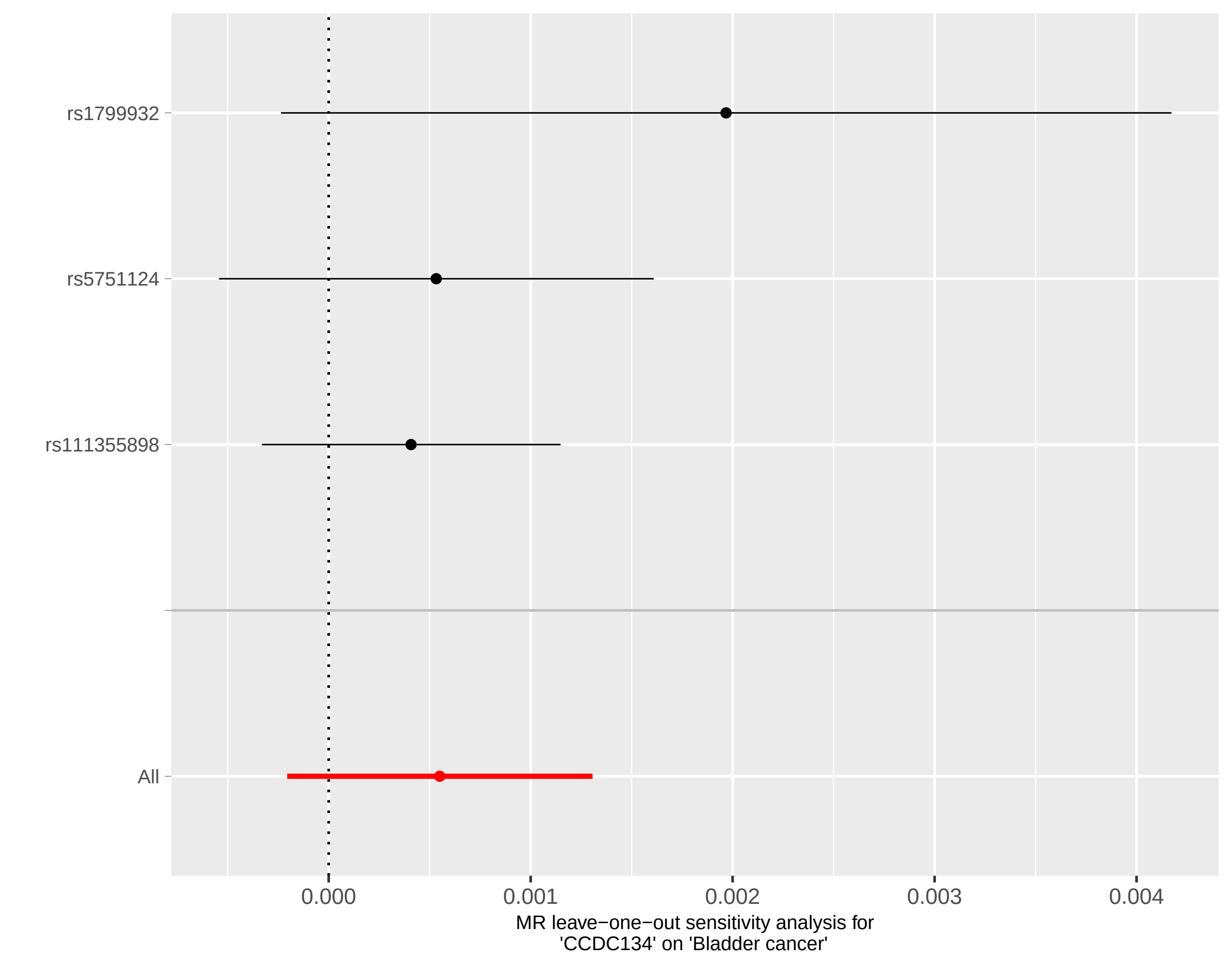
rs1799932
rs5751124
rs111355898
All
0.000
0.001
0.002
0.003
0.004
MR leave−one−out sensitivity analysis for
'CCDC134' on 'Bladder cancer'

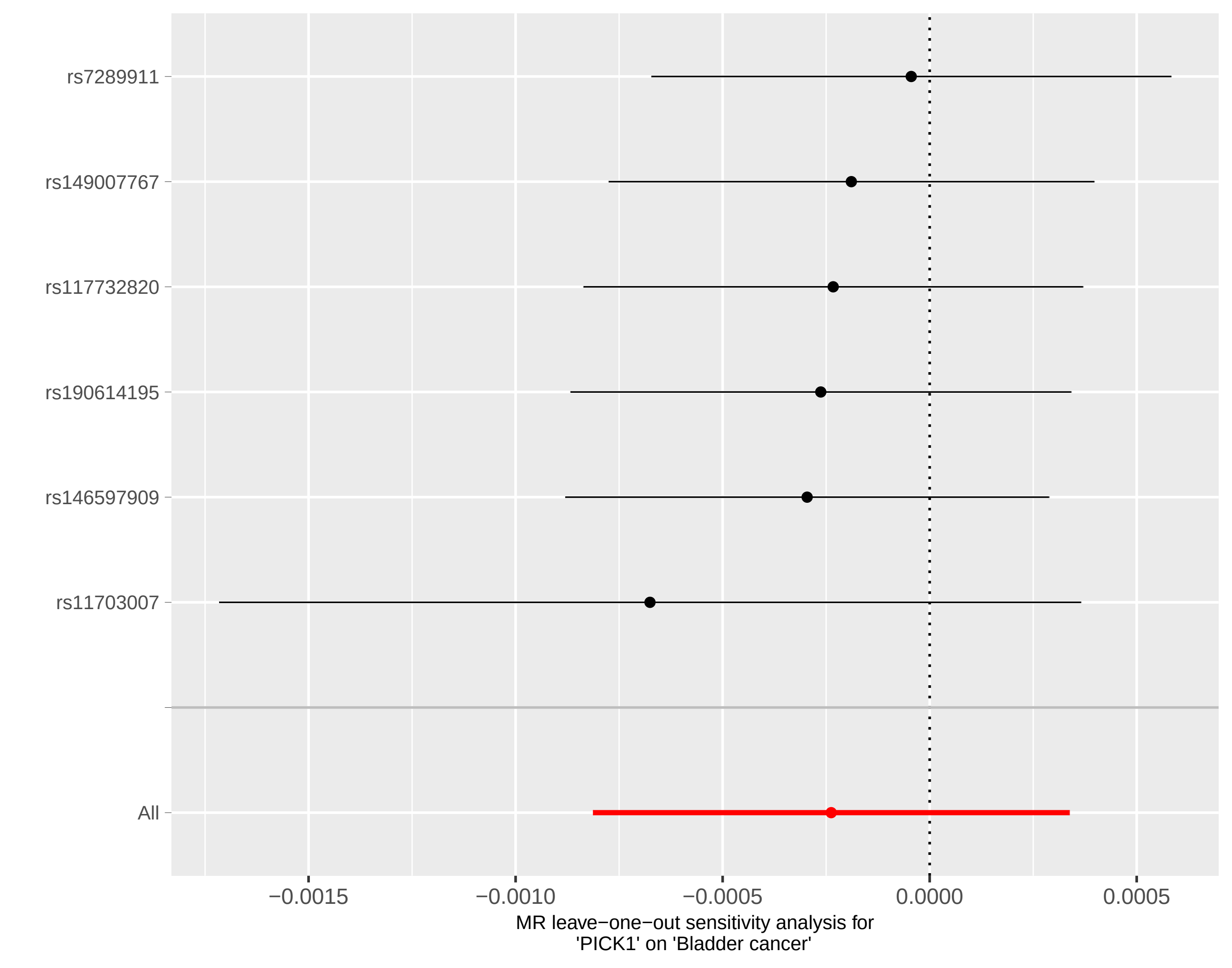
rs7289911
rs149007767
rs117732820
rs190614195
rs146597909
rs11703007
All
−0.0015
−0.0010
−0.0005
0.0000
0.0005
MR leave−one−out sensitivity analysis for
'PICK1' on 'Bladder cancer'

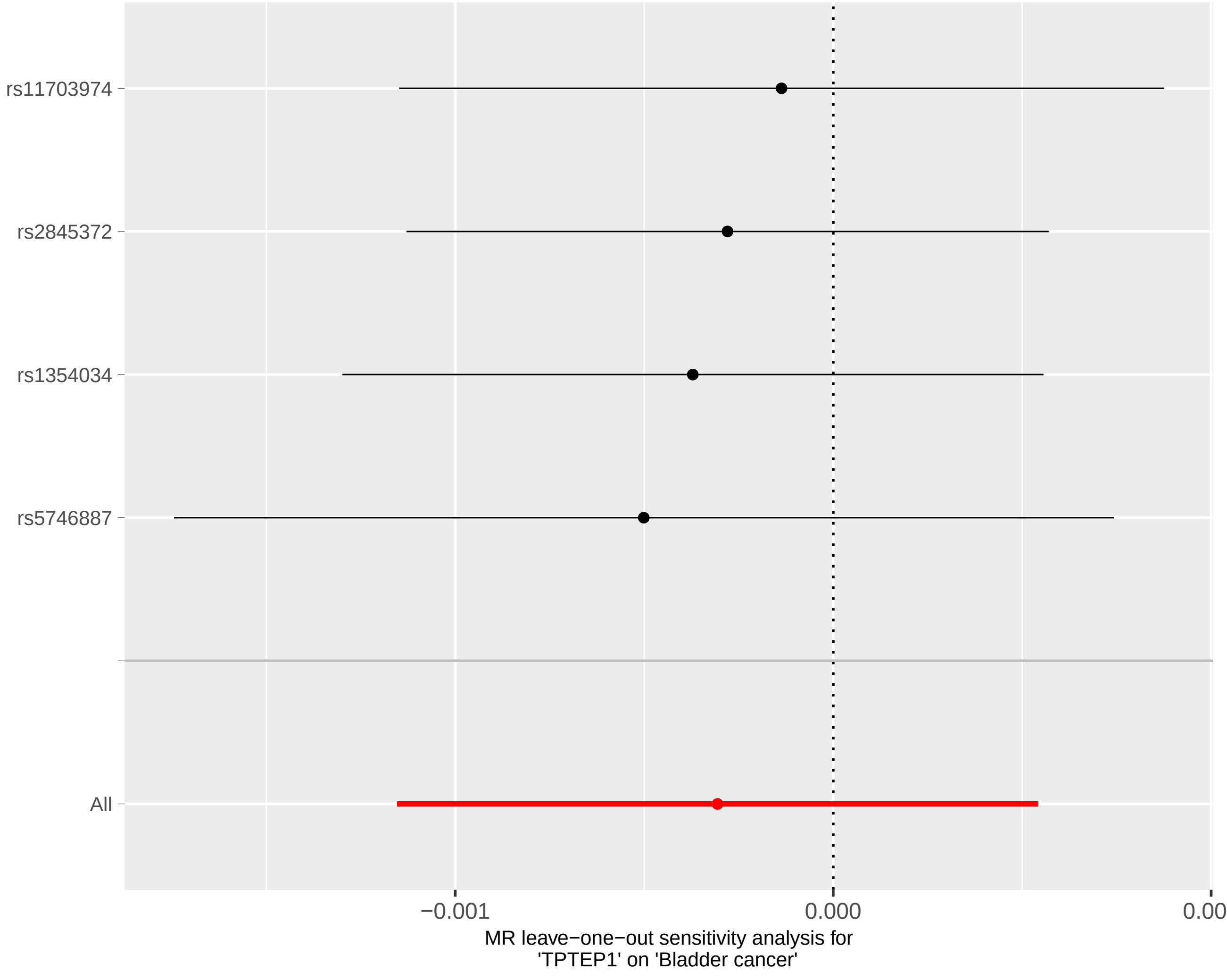
rs11703974
rs2845372
rs1354034
rs5746887
All
−0.001
0.000
0.00
MR leave−one−out sensitivity analysis for
'TPTEP1' on 'Bladder cancer'

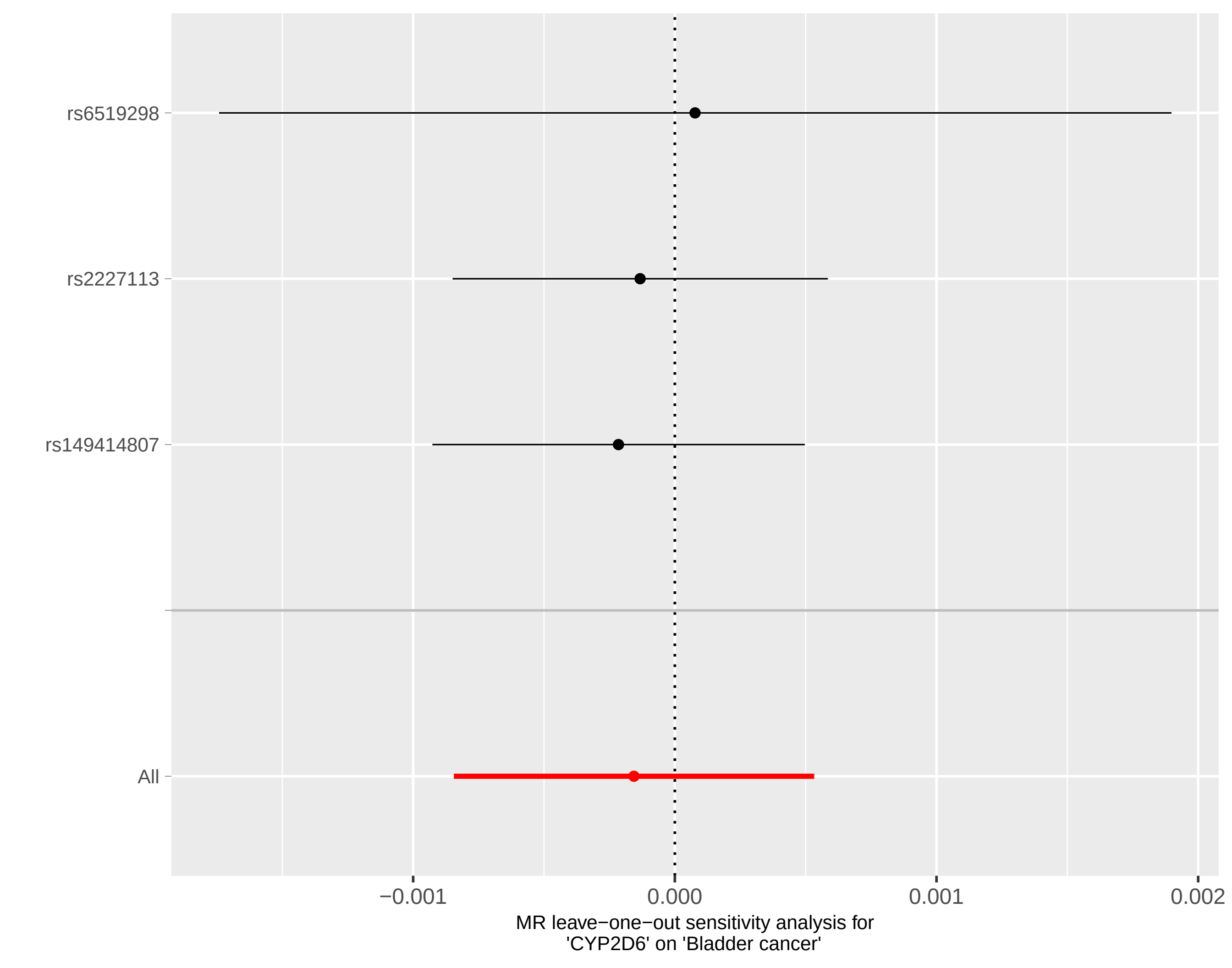
rs6519298
rs2227113
rs149414807
All
−0.001
0.000
0.001
0.002
MR leave−one−out sensitivity analysis for
'CYP2D6' on 'Bladder cancer'

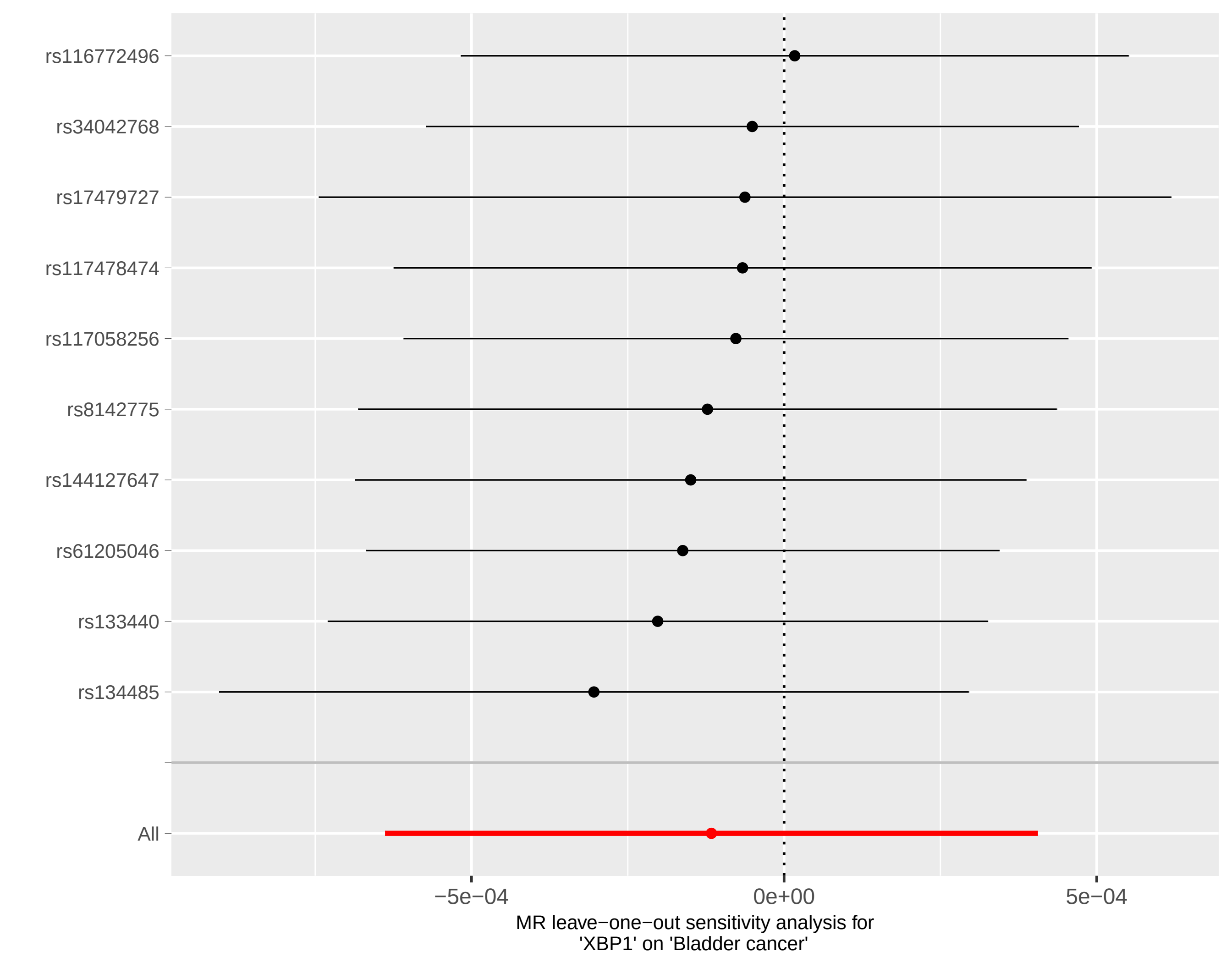
rs116772496
rs34042768
rs17479727
rs117478474
rs117058256
rs8142775
rs144127647
rs61205046
rs133440
rs134485
All
−5e−04
0e+00
5e−04
MR leave−one−out sensitivity analysis for
'XBP1' on 'Bladder cancer'

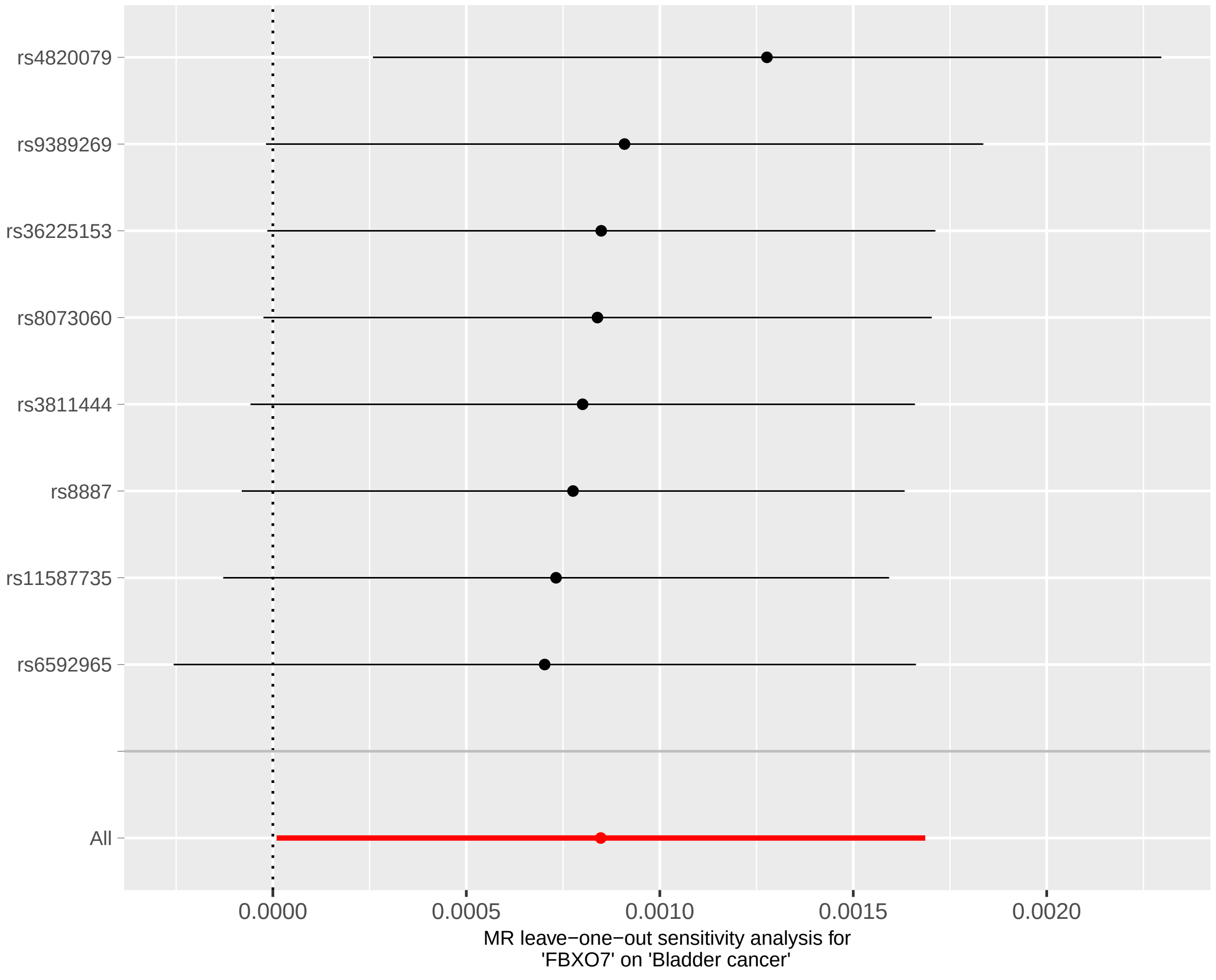
rs4820079
rs9389269
rs36225153
rs8073060
rs3811444
rs8887
rs11587735
rs6592965
All
0.0000
0.0005
0.0010
0.0015
0.0020
MR leave−one−out sensitivity analysis for
'FBXO7' on 'Bladder cancer'

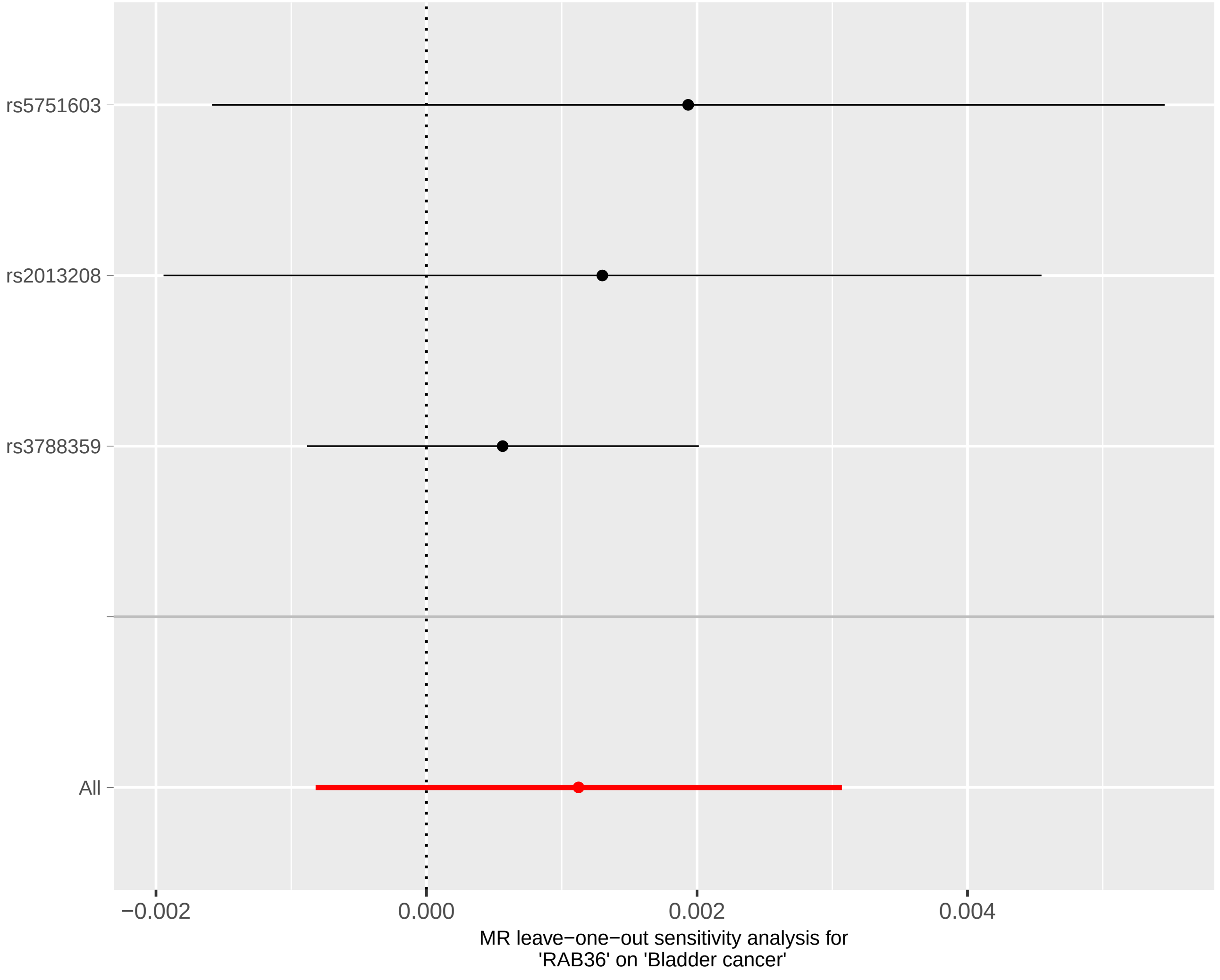
rs5751603
rs2013208
rs3788359
All
−0.002
0.000
0.002
0.004
MR leave−one−out sensitivity analysis for
'RAB36' on 'Bladder cancer'

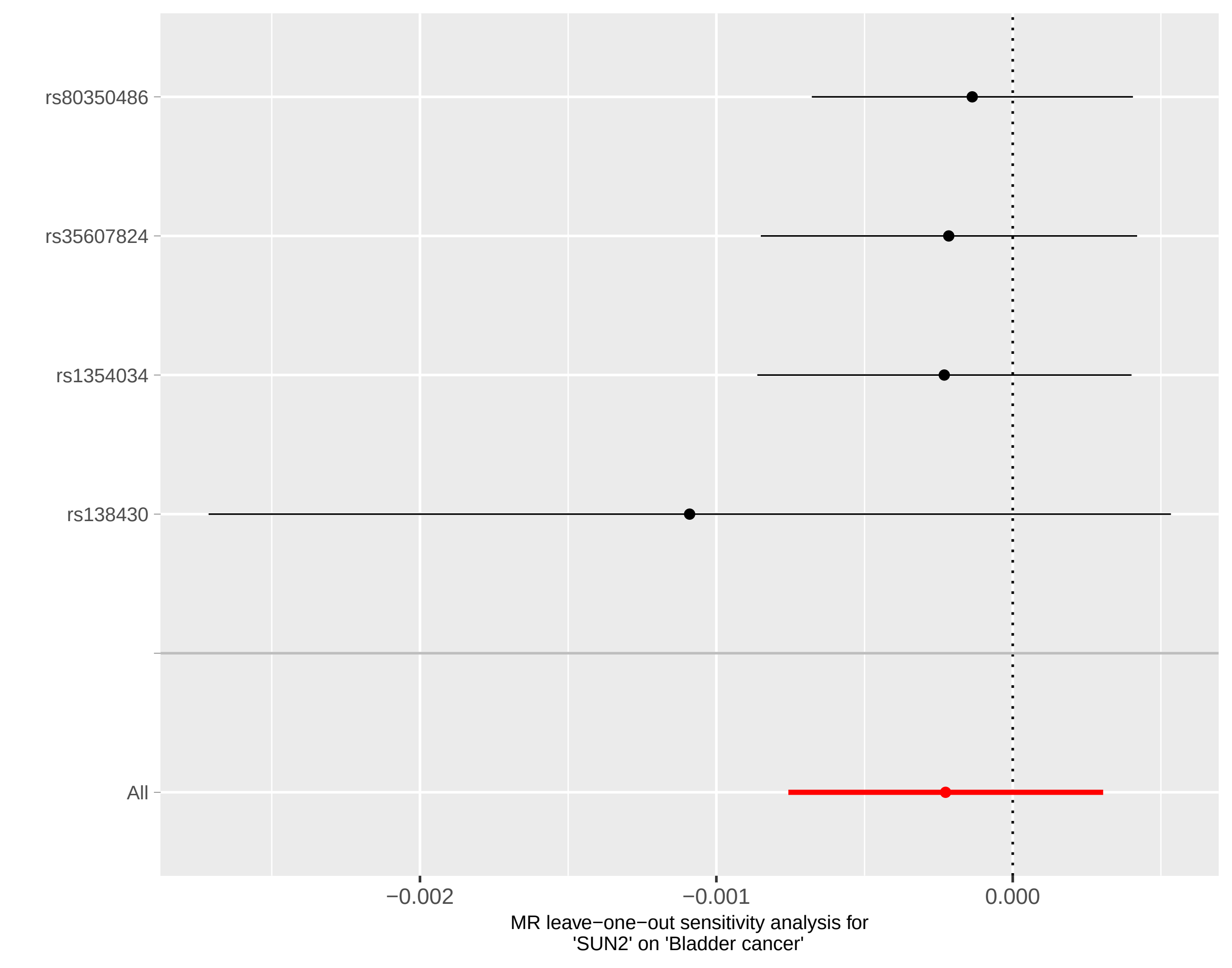
rs80350486
rs35607824
rs1354034
rs138430
All
−0.002
−0.001
0.000
MR leave−one−out sensitivity analysis for
'SUN2' on 'Bladder cancer'

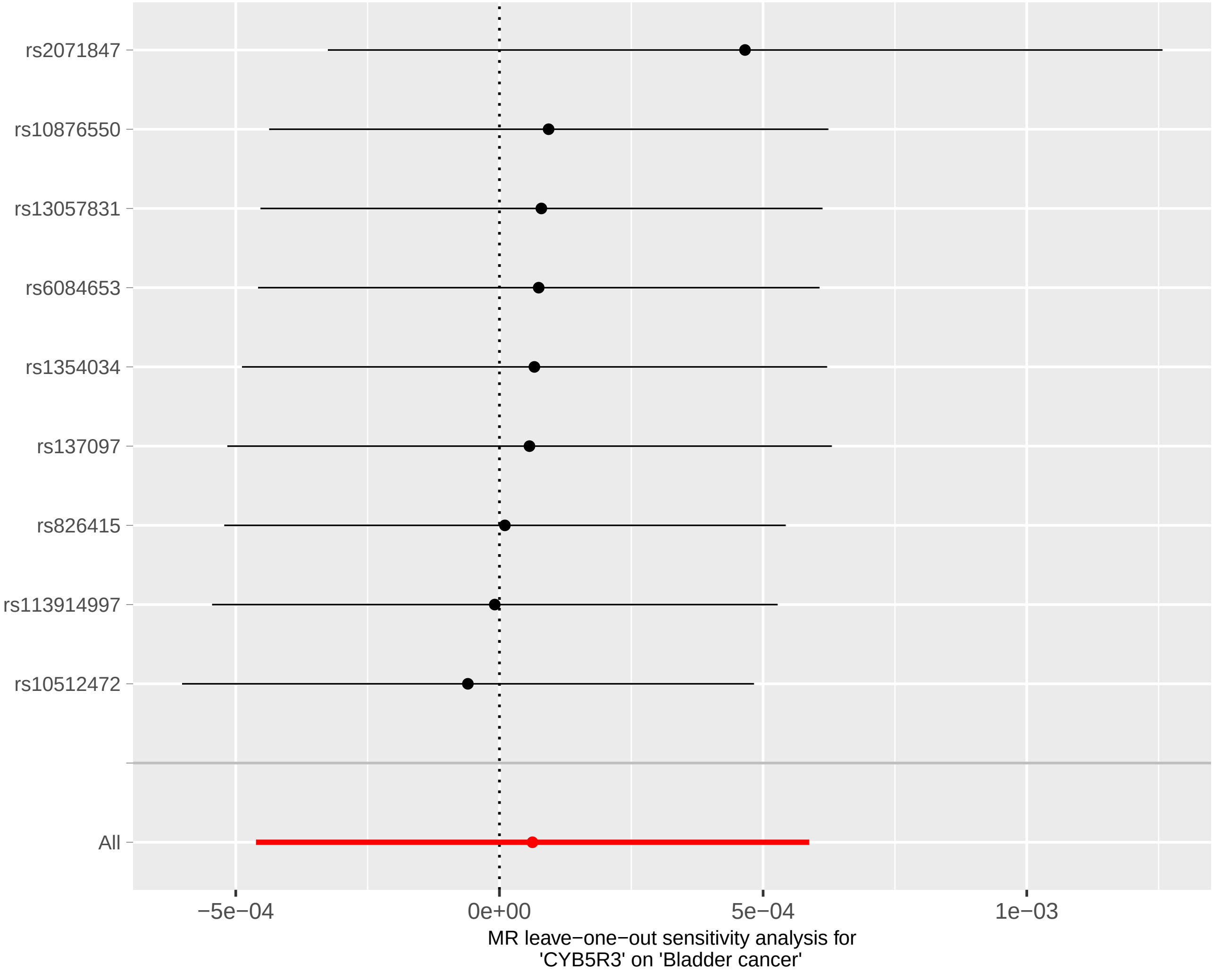
rs2071847
rs10876550
rs13057831
rs6084653
rs1354034
rs137097
rs826415
rs113914997
rs10512472
All
−5e−04
0e+00
5e−04
1e−03
MR leave−one−out sensitivity analysis for
'CYB5R3' on 'Bladder cancer'

Insufficient number of SNPs

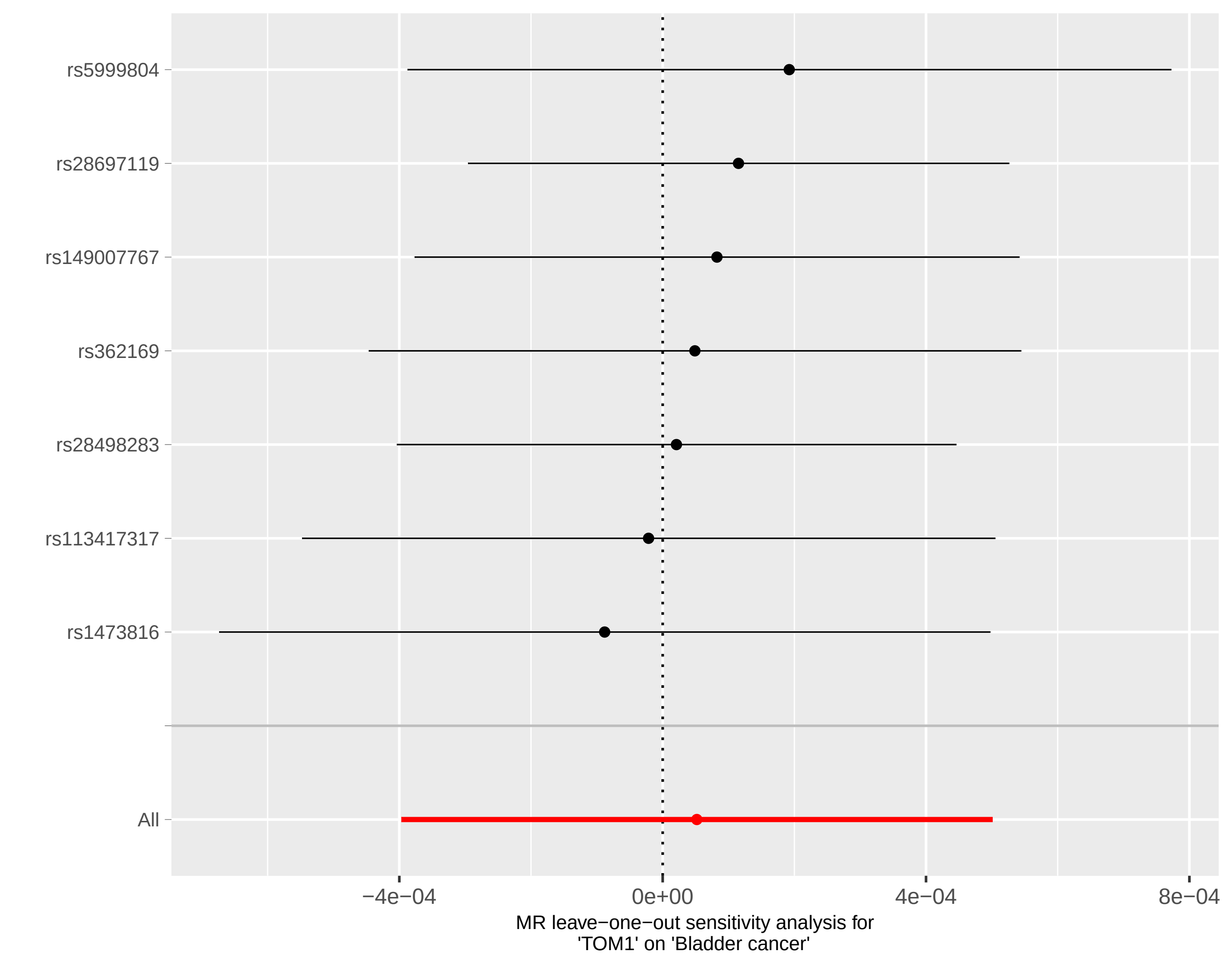
rs5999804
rs28697119
rs149007767
rs362169
rs28498283
rs113417317
rs1473816
All
−4e−04
0e+00
4e−04
8e−04
MR leave−one−out sensitivity analysis for
'TOM1' on 'Bladder cancer'

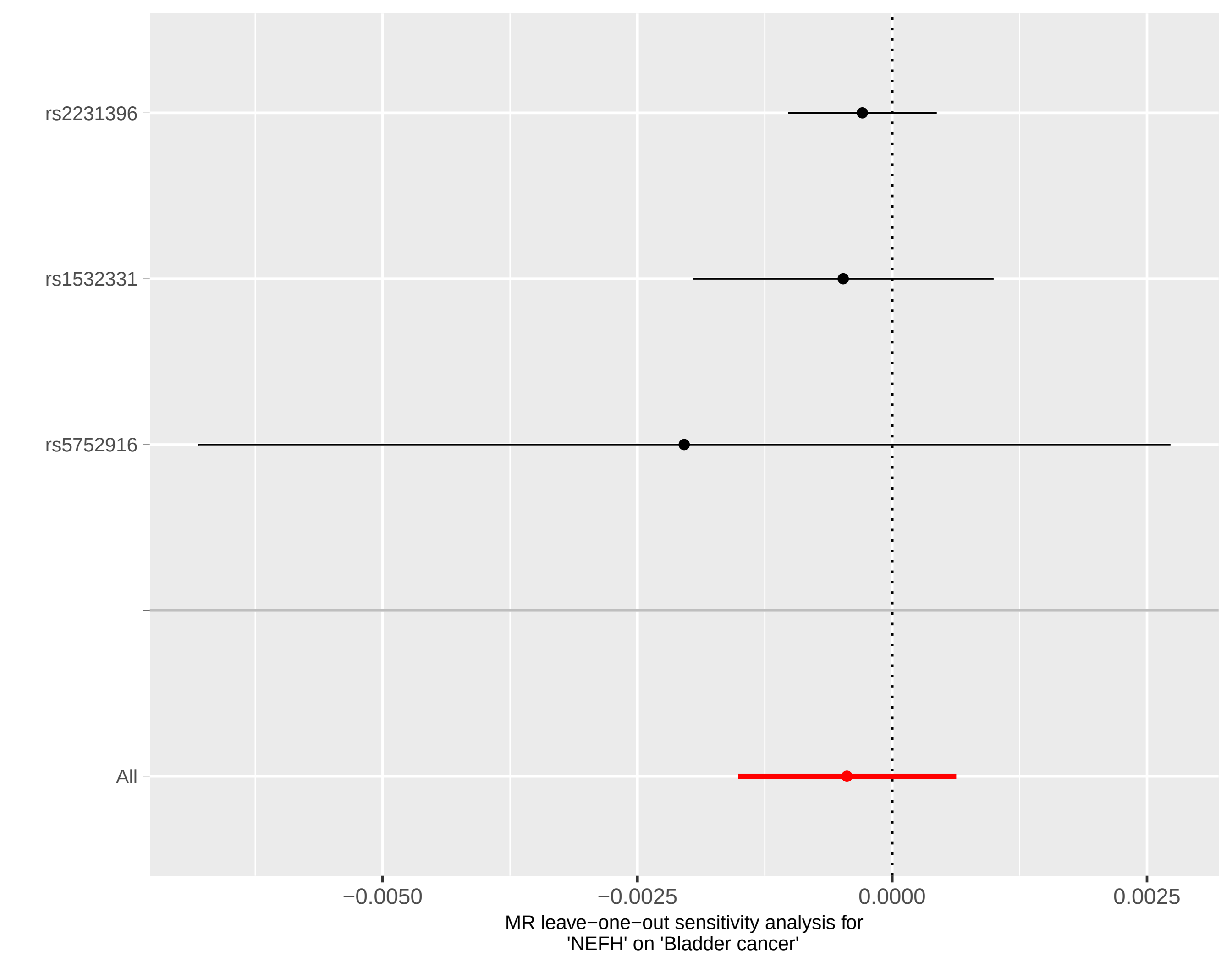
rs2231396
rs1532331
rs5752916
All
−0.0050
−0.0025
0.0000
0.0025
MR leave−one−out sensitivity analysis for
'NEFH' on 'Bladder cancer'

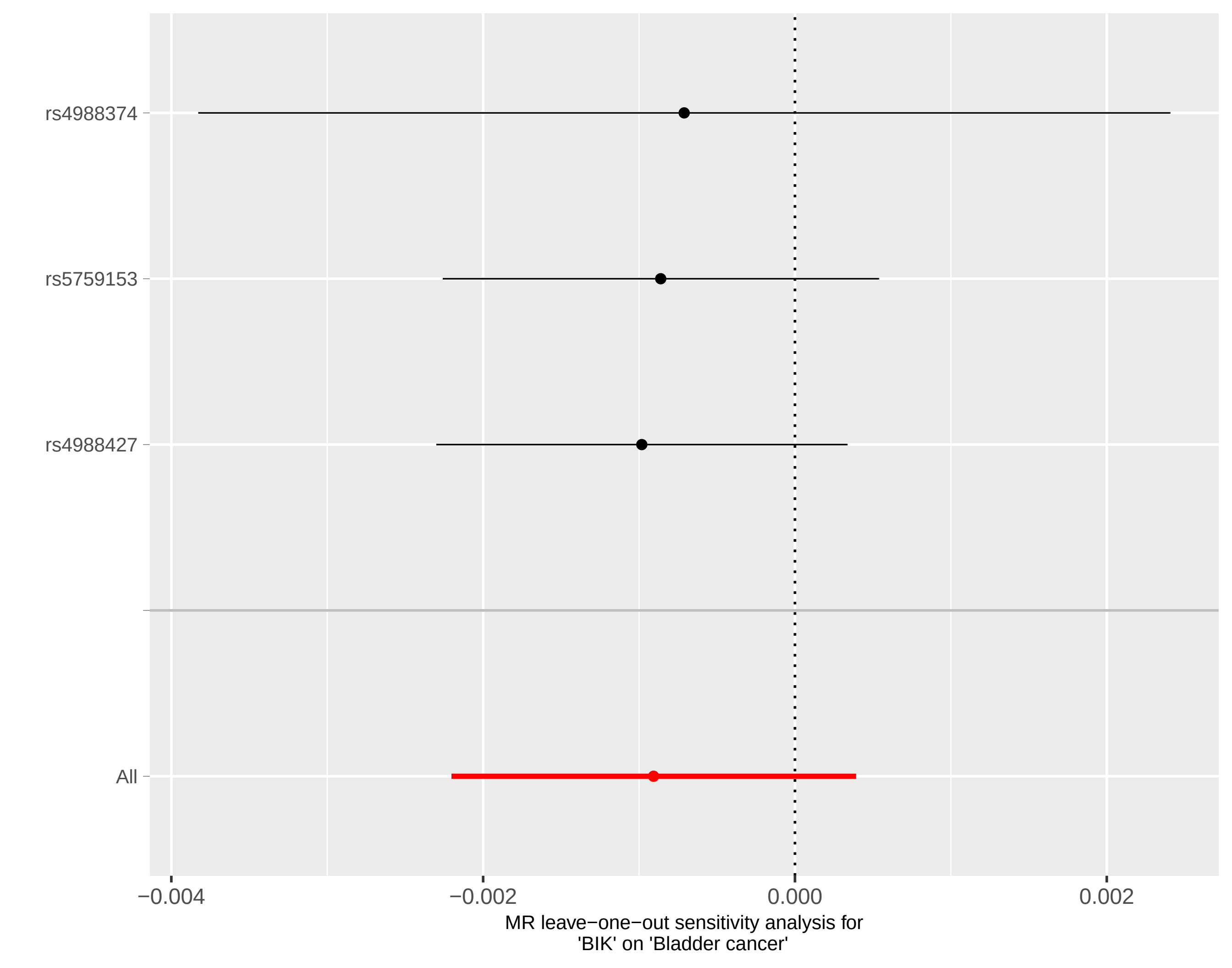
rs4988374
rs5759153
rs4988427
All
−0.004
−0.002
0.000
0.002
MR leave−one−out sensitivity analysis for
'BIK' on 'Bladder cancer'

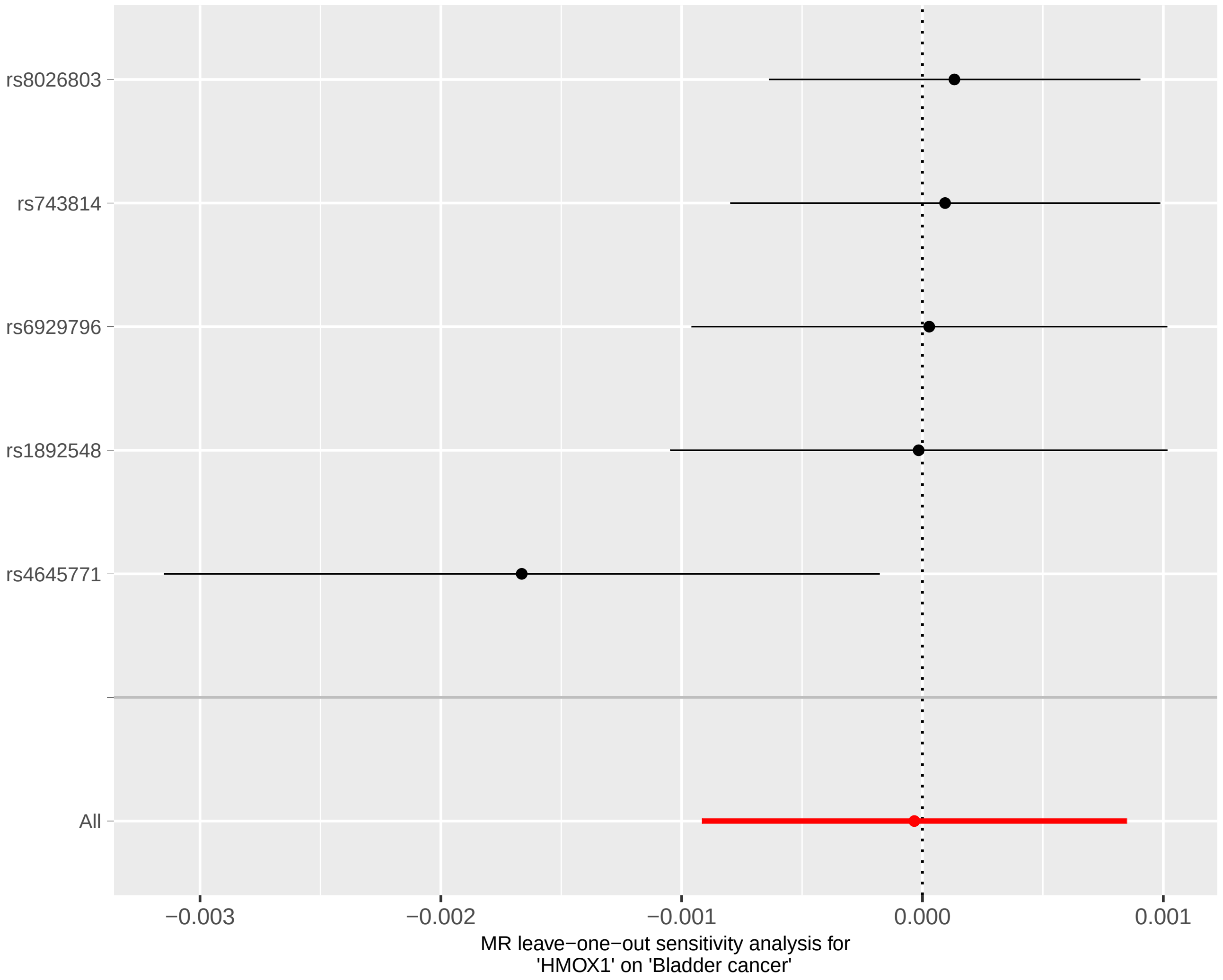

rs8026803
rs743814
rs6929796
rs1892548
rs4645771
All
−0.003
−0.002
−0.001
0.000
0.001
MR leave−one−out sensitivity analysis for
'HMOX1' on 'Bladder cancer'

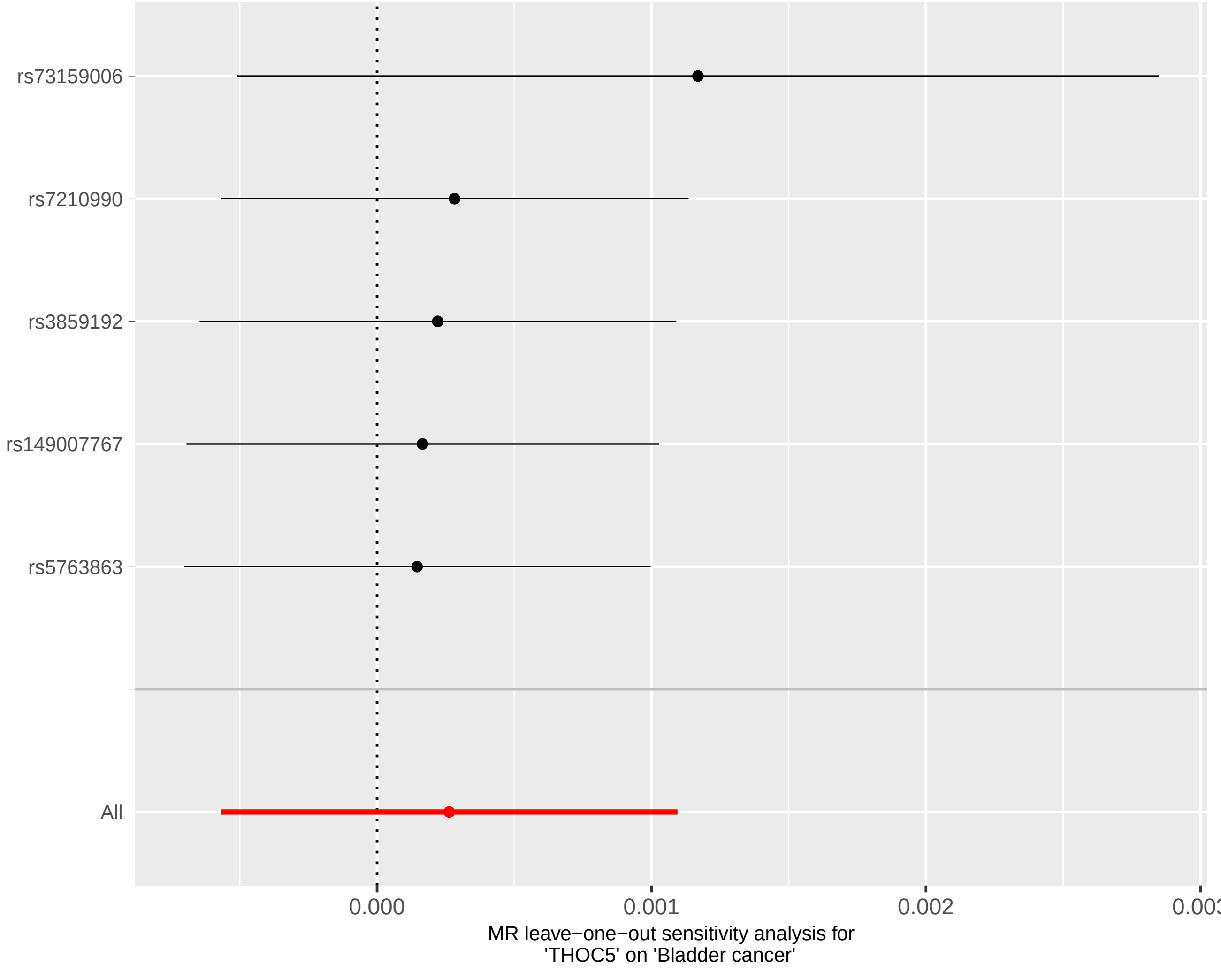
rs73159006
rs7210990
rs3859192
rs149007767
rs5763863
All
0.000
0.001
0.002
0.003
MR leave−one−out sensitivity analysis for
'THOC5' on 'Bladder cancer'

Insufficient number of SNPs

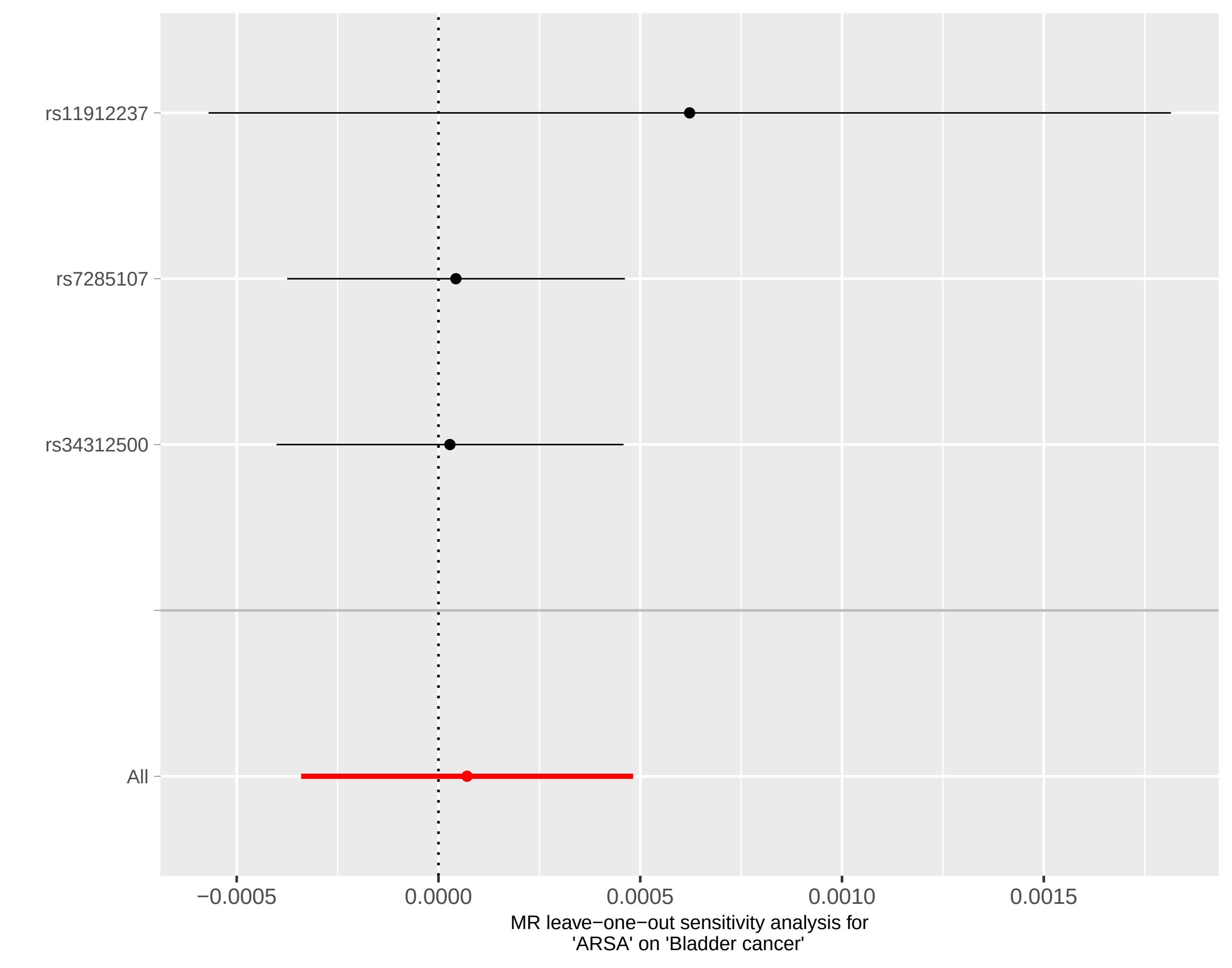
rs11912237
rs7285107
rs34312500
All
−0.0005
0.0000
0.0005
0.0010
0.0015
MR leave−one−out sensitivity analysis for
'ARSA' on 'Bladder cancer'

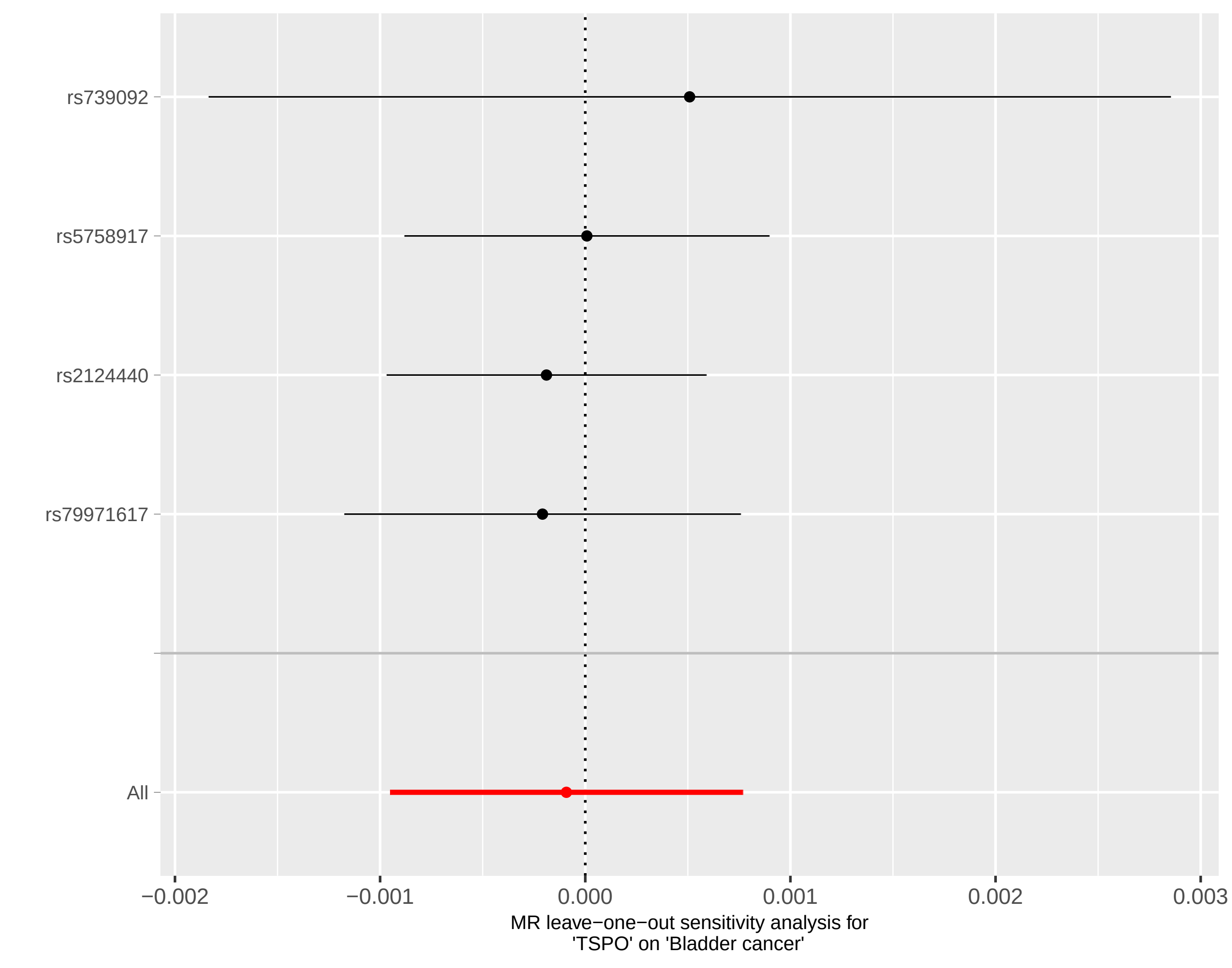
rs739092
rs5758917
rs2124440
rs79971617
All
−0.002
−0.001
0.000
0.001
0.002
0.003
MR leave−one−out sensitivity analysis for
'TSPO' on 'Bladder cancer'

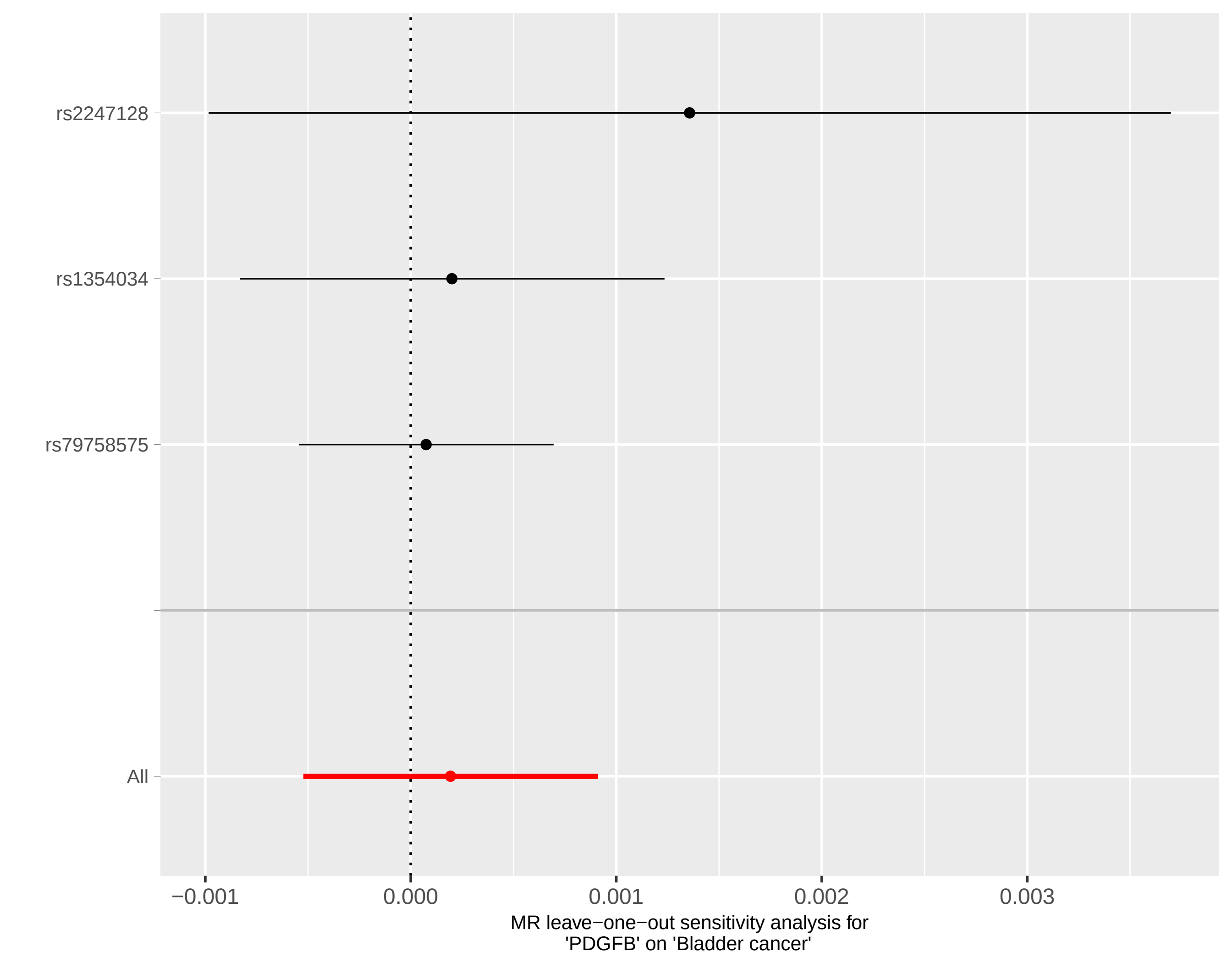
rs2247128
rs1354034
rs79758575
All
−0.001
0.000
0.001
0.002
0.003
MR leave−one−out sensitivity analysis for
'PDGFB' on 'Bladder cancer'

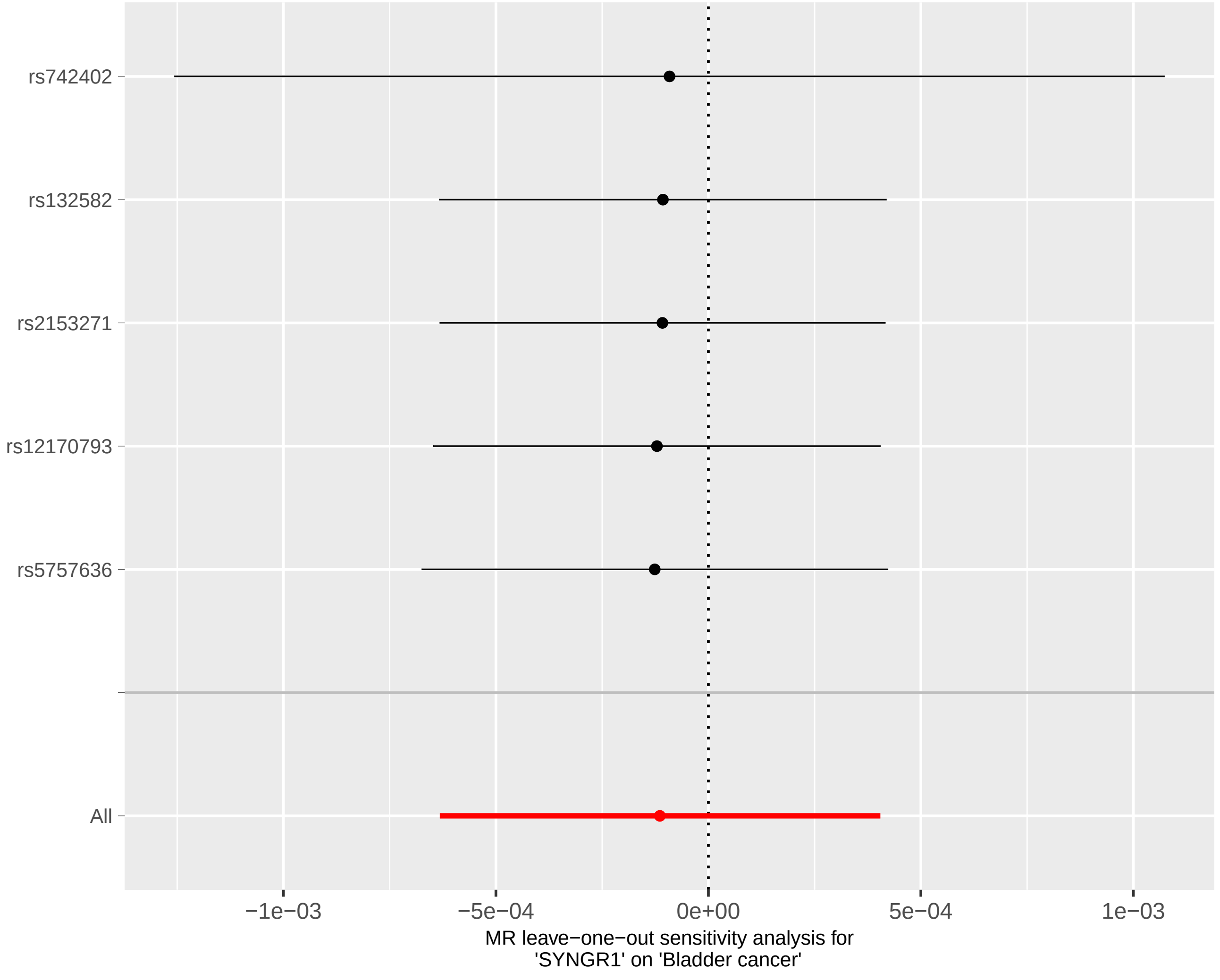
rs742402
rs132582
rs2153271
rs12170793
rs5757636
All
−1e−03
−5e−04
0e+00
5e−04
1e−03
MR leave−one−out sensitivity analysis for
'SYNGR1' on 'Bladder cancer'

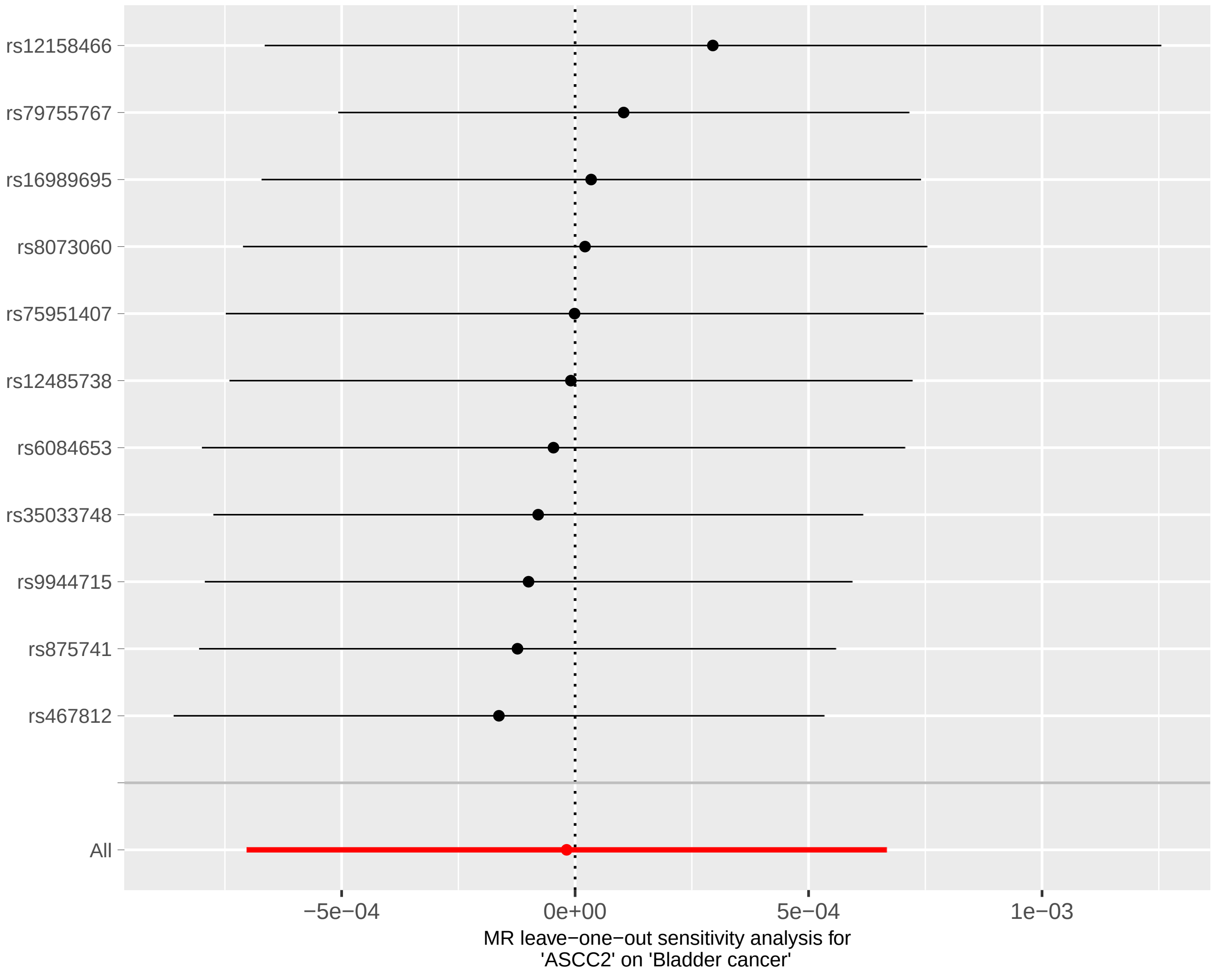
rs12158466
rs79755767
rs16989695
rs8073060
rs75951407
rs12485738
rs6084653
rs35033748
rs9944715
rs875741
rs467812
All
−5e−04
0e+00
5e−04
1e−03
MR leave−one−out sensitivity analysis for
'ASCC2' on 'Bladder cancer'

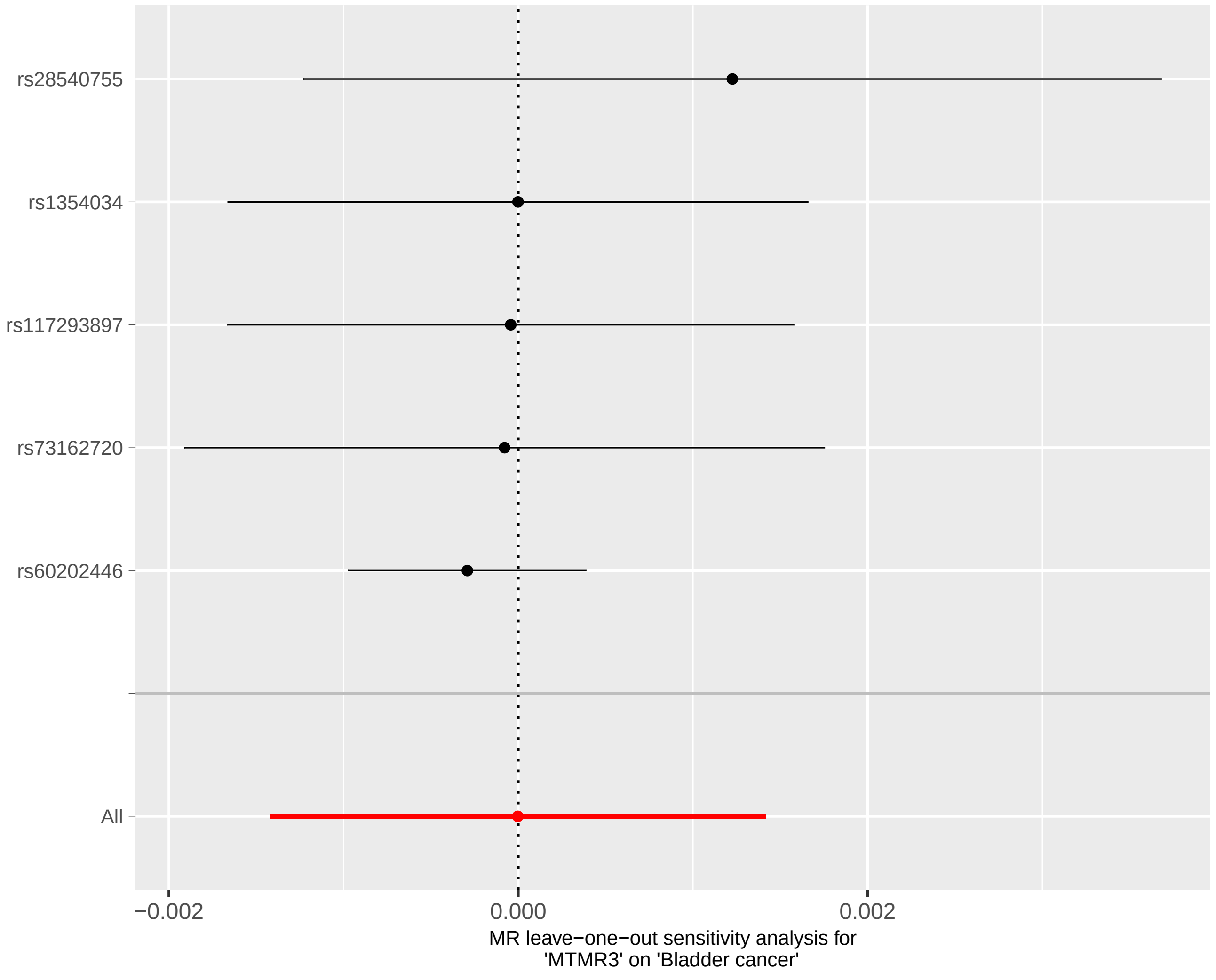
rs28540755
rs1354034
rs117293897
rs73162720
rs60202446
All
−0.002
0.000
0.002
MR leave−one−out sensitivity analysis for
'MTMR3' on 'Bladder cancer'

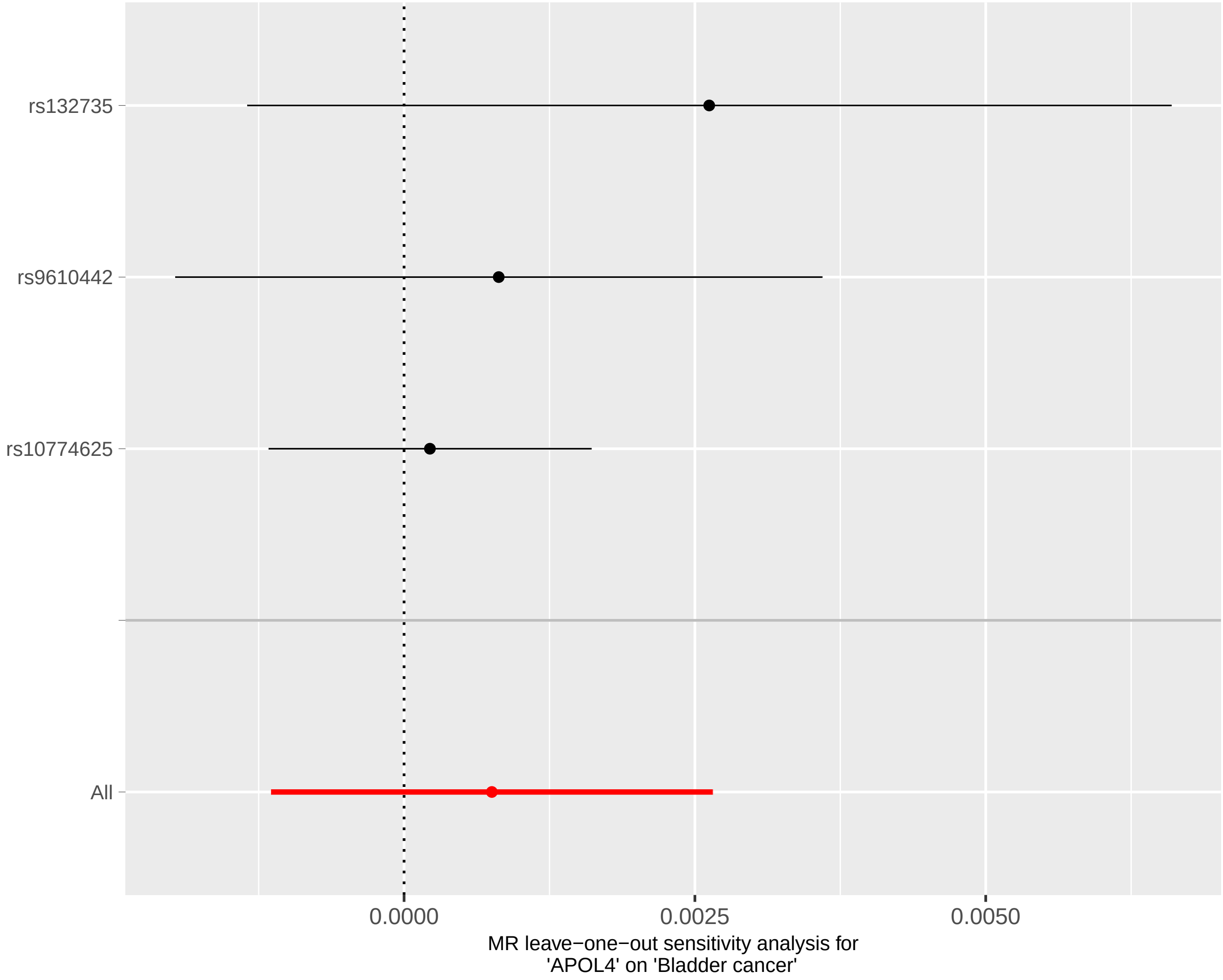
rs132735
rs9610442
rs10774625
All
0.0000
0.0025
0.0050
MR leave−one−out sensitivity analysis for
'APOL4' on 'Bladder cancer'

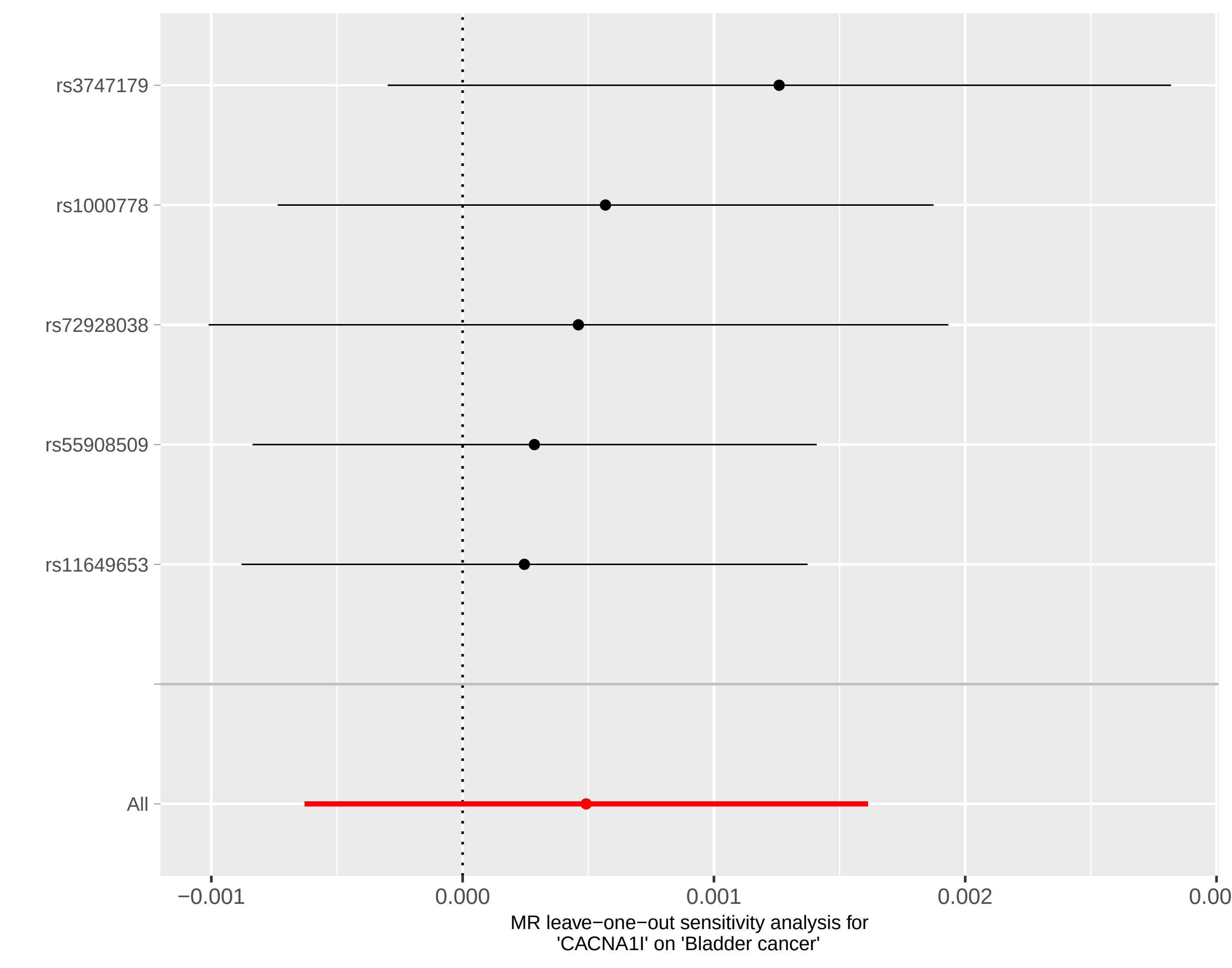

rs3747179
rs1000778
rs72928038
rs55908509
rs11649653
All
−0.001
0.000
0.001
0.002
0.00
MR leave−one−out sensitivity analysis for
'CACNA1I' on 'Bladder cancer'

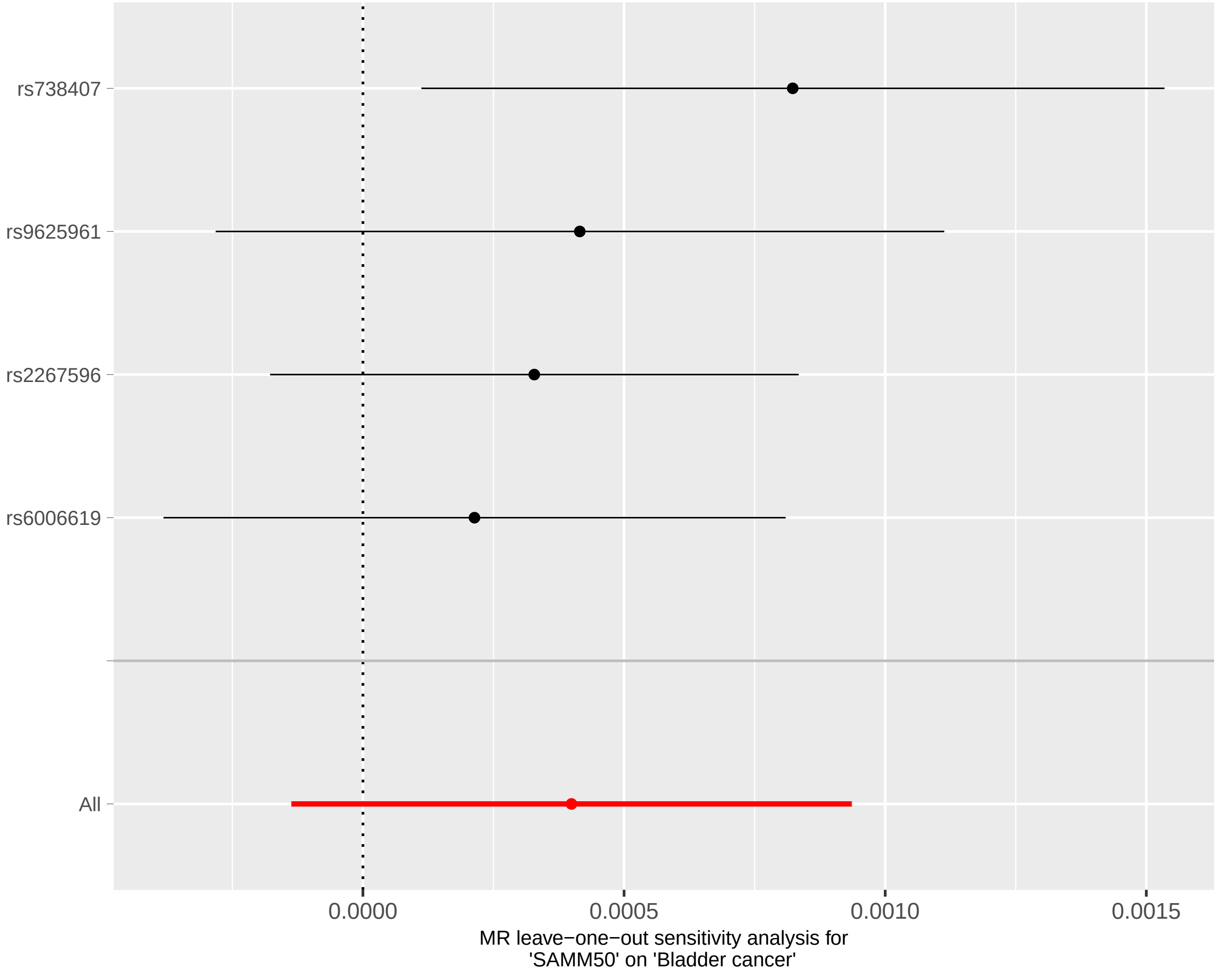
rs738407
rs9625961
rs2267596
rs6006619
All
0.0000
0.0005
0.0010
0.0015
MR leave−one−out sensitivity analysis for
'SAMM50' on 'Bladder cancer'

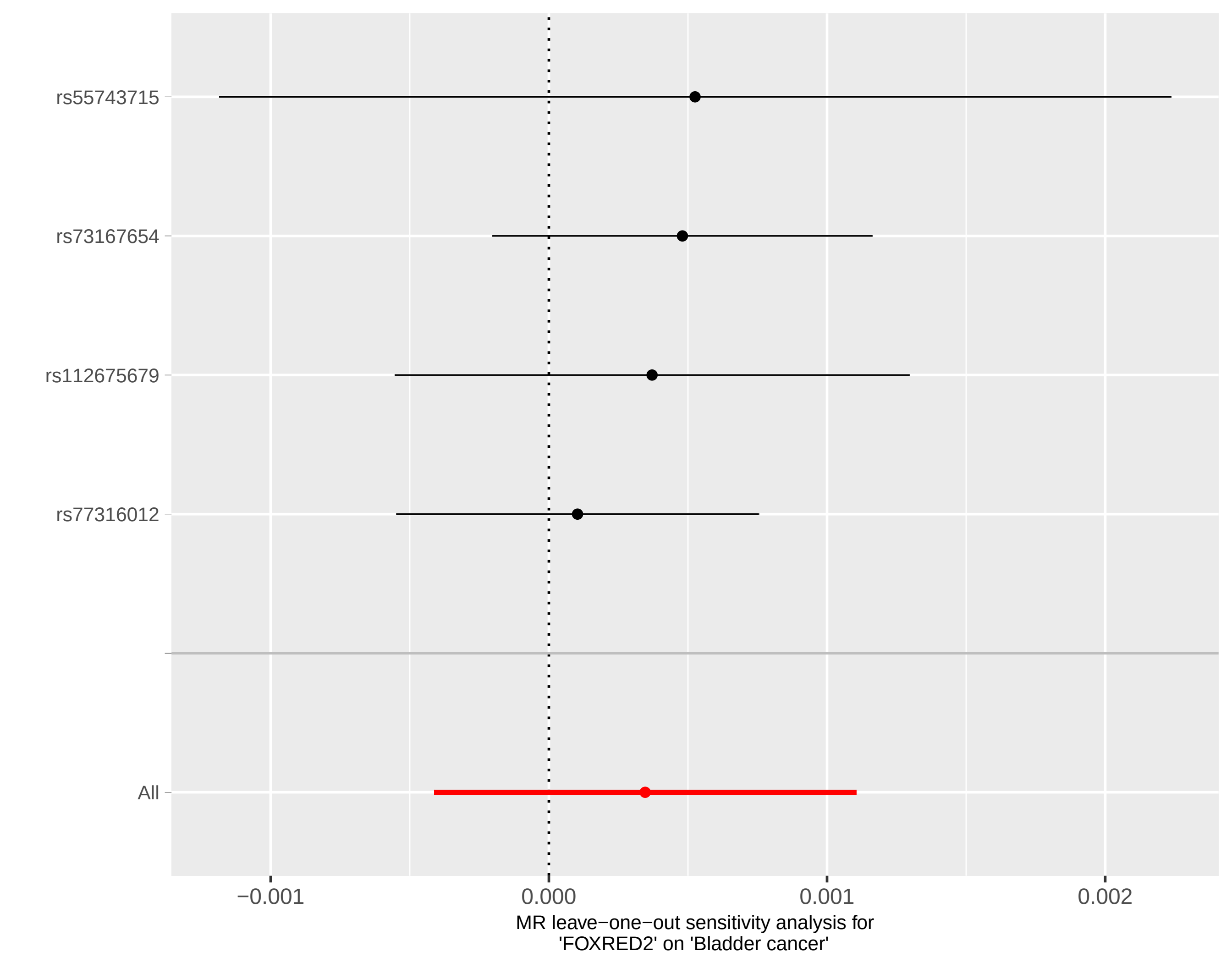

rs55743715
rs73167654
rs112675679
rs77316012
All
−0.001
0.000
0.001
0.002
MR leave−one−out sensitivity analysis for
'FOXRED2' on 'Bladder cancer'

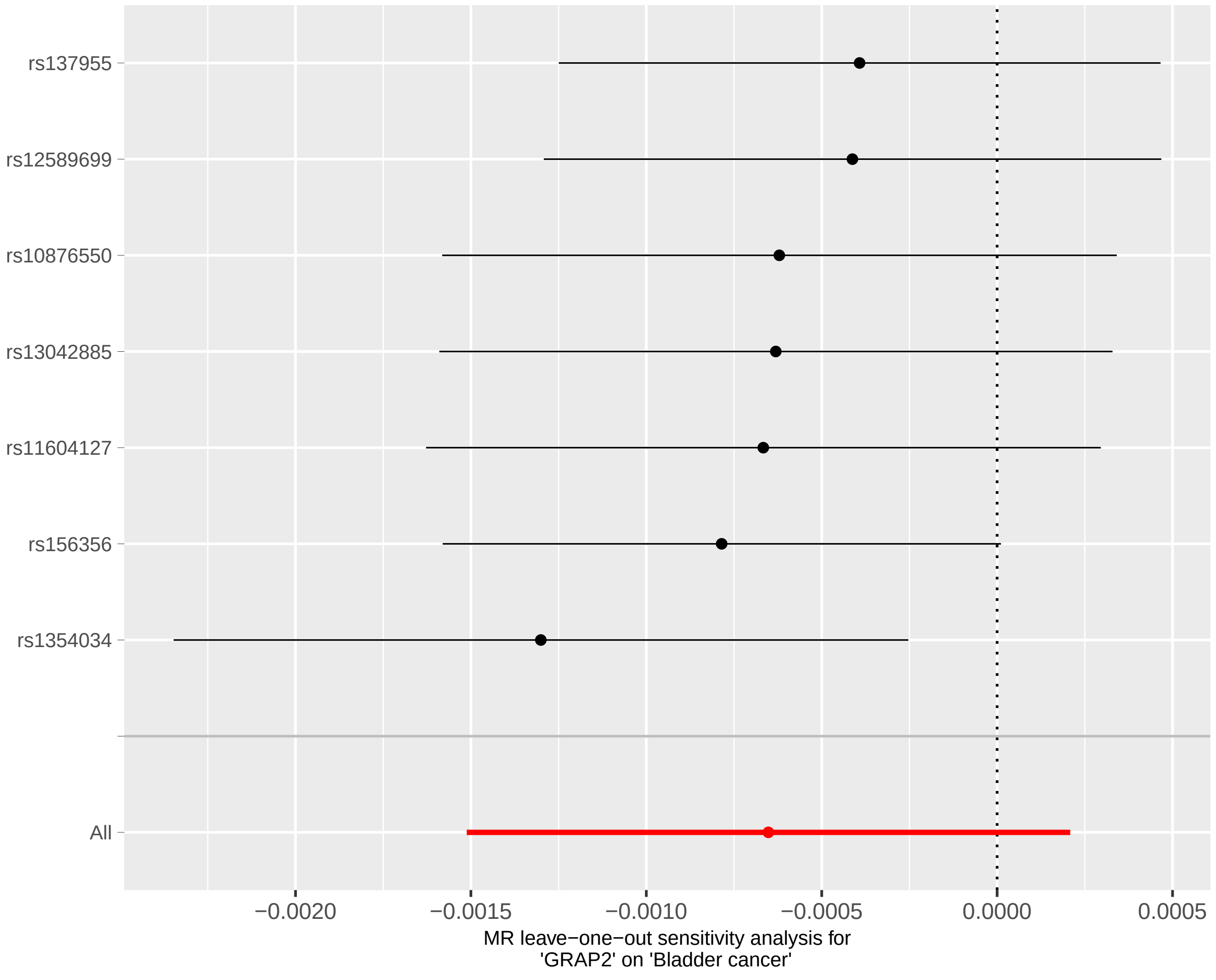

rs137955
rs12589699
rs10876550
rs13042885
rs11604127
rs156356
rs1354034
All
−0.0020
−0.0015
−0.0010
−0.0005
0.0000
0.0005
MR leave−one−out sensitivity analysis for
'GRAP2' on 'Bladder cancer'

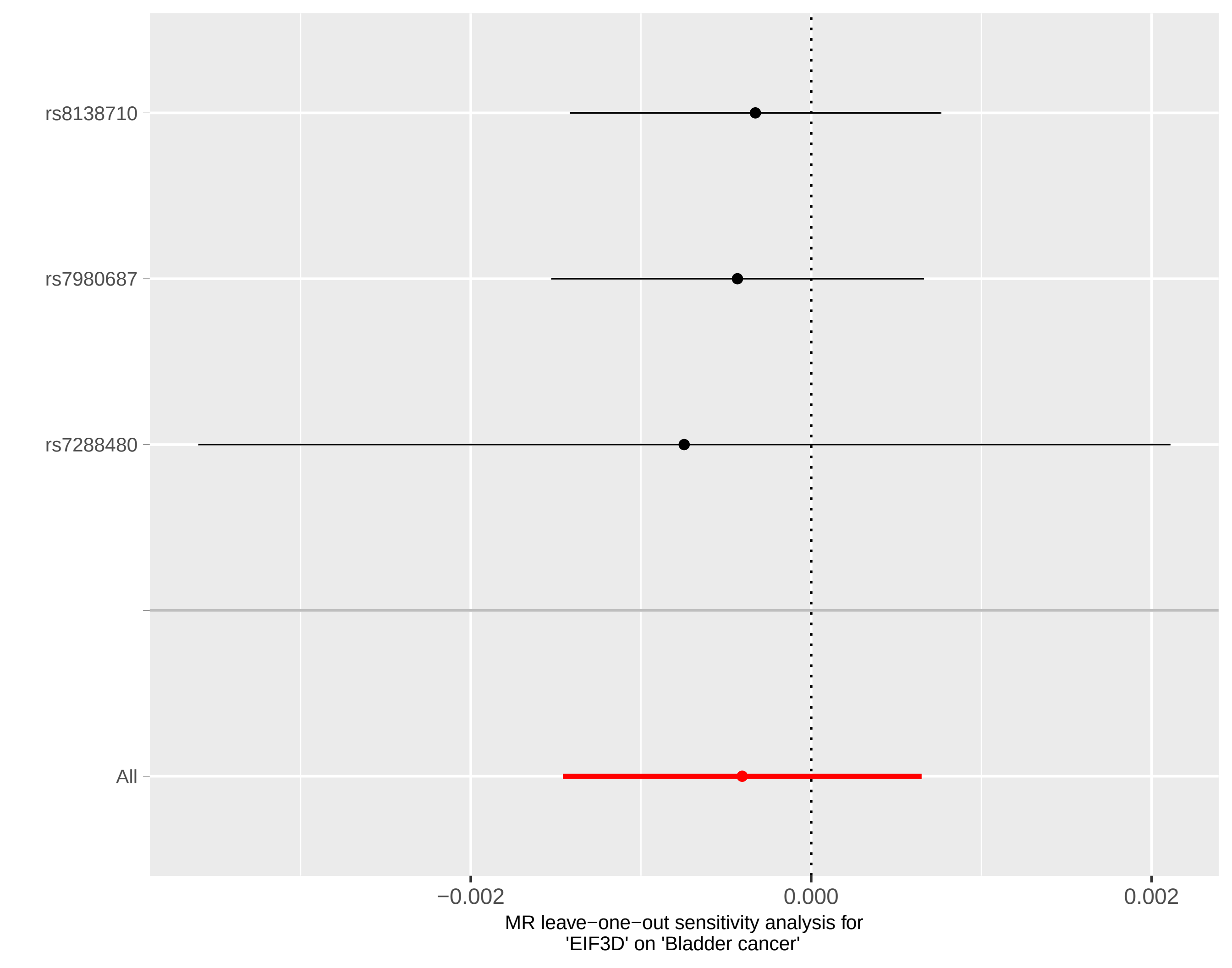
rs8138710
rs7980687
rs7288480
All
−0.002
0.000
0.002
MR leave−one−out sensitivity analysis for
'EIF3D' on 'Bladder cancer'

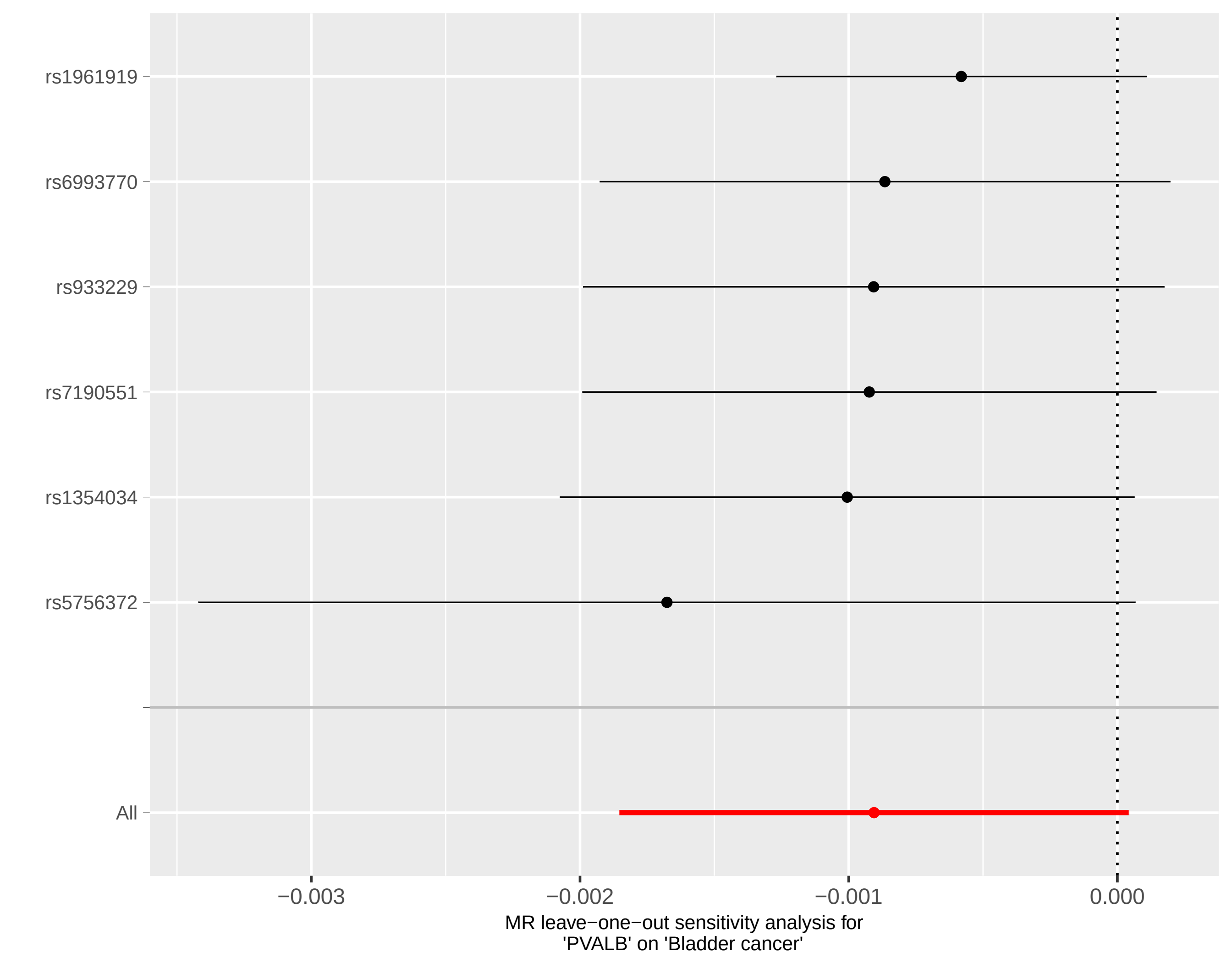

rs1961919
rs6993770
rs933229
rs7190551
rs1354034
rs5756372
All
−0.003
−0.002
−0.001
0.000
MR leave−one−out sensitivity analysis for
'PVALB' on 'Bladder cancer'

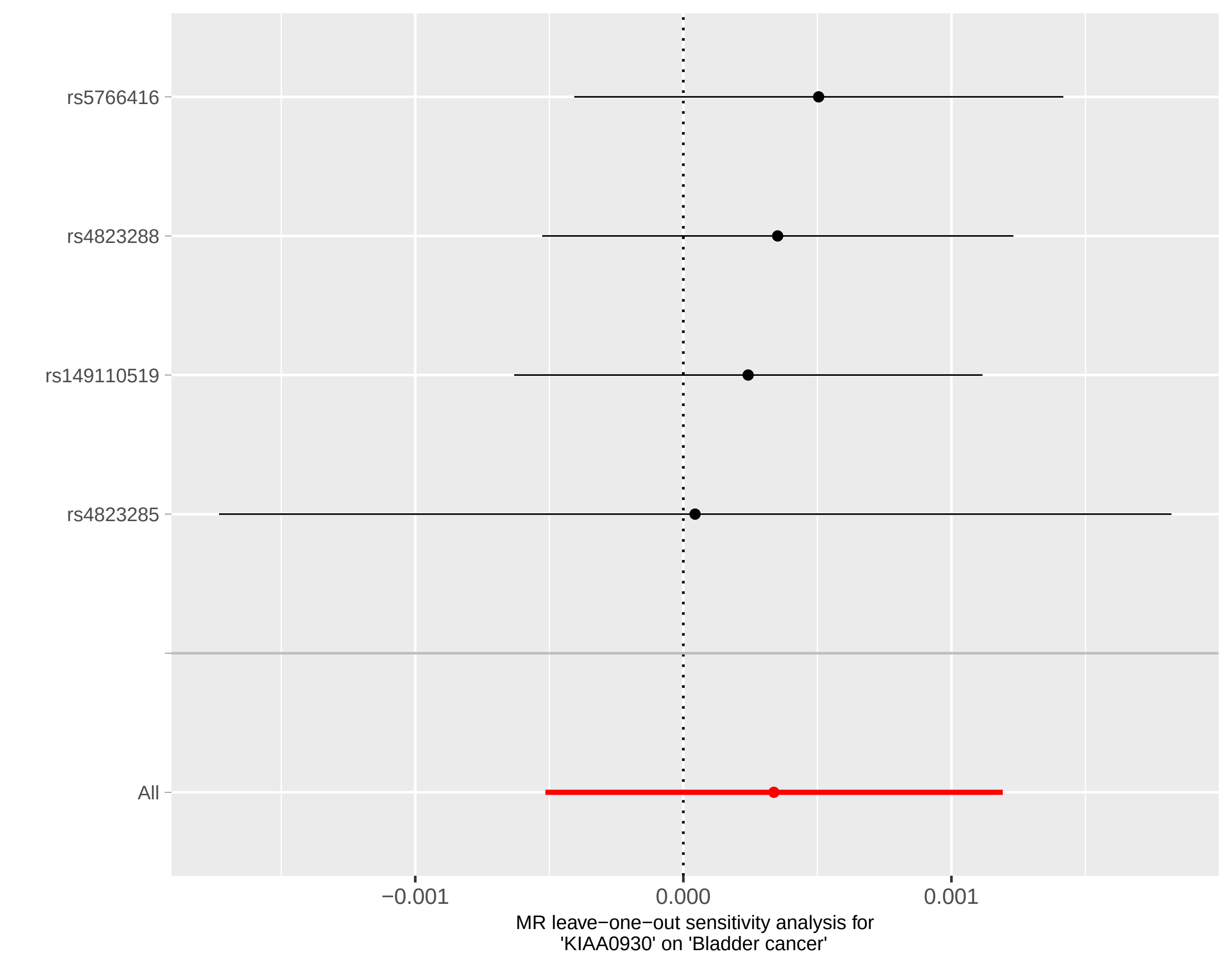
rs5766416
rs4823288
rs149110519
rs4823285
All
−0.001
0.000
0.001
MR leave−one−out sensitivity analysis for
'KIAA0930' on 'Bladder cancer'

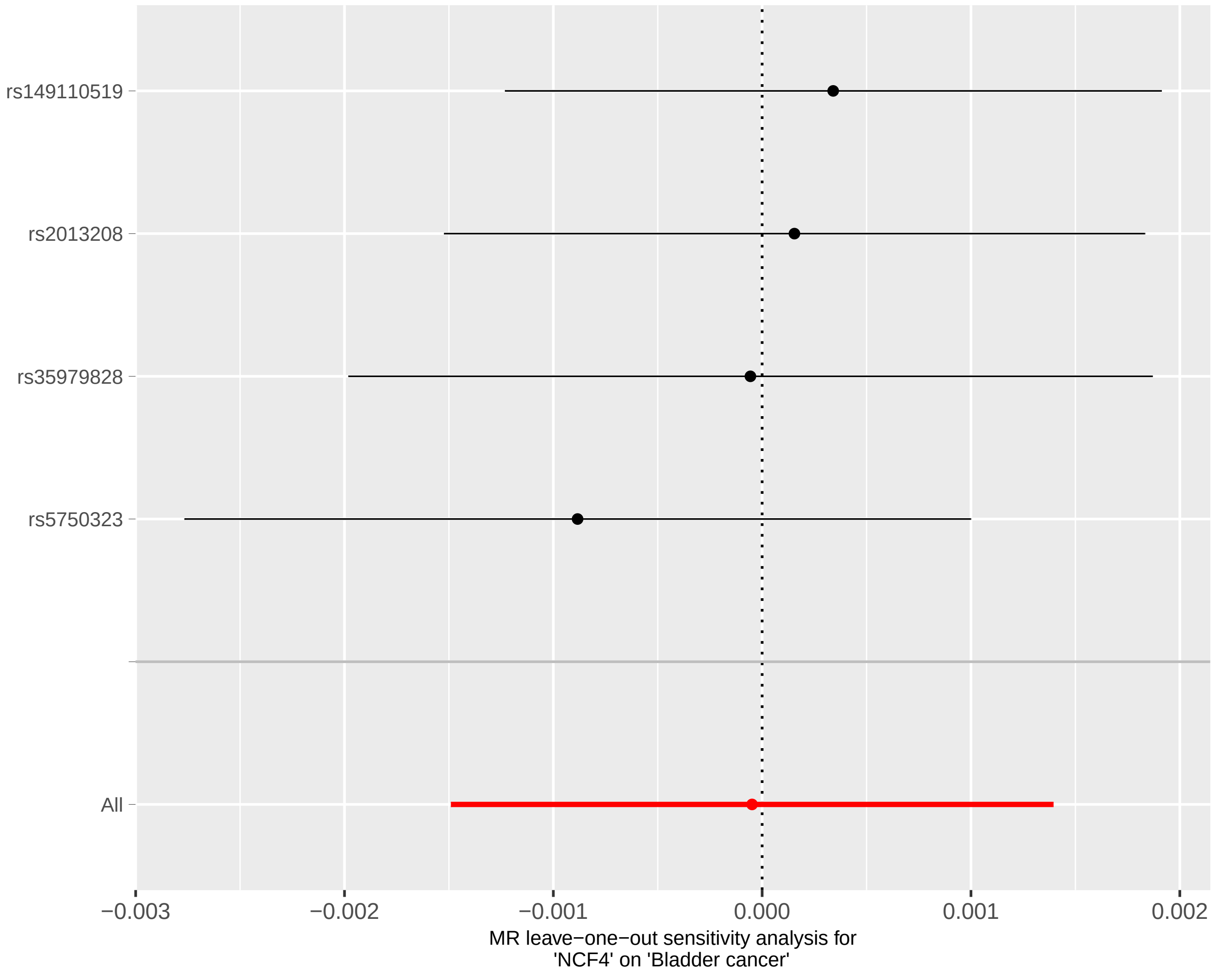

rs149110519
rs2013208
rs35979828
rs5750323
All
−0.003
−0.002
−0.001
0.000
0.001
0.002
MR leave−one−out sensitivity analysis for
'NCF4' on 'Bladder cancer'

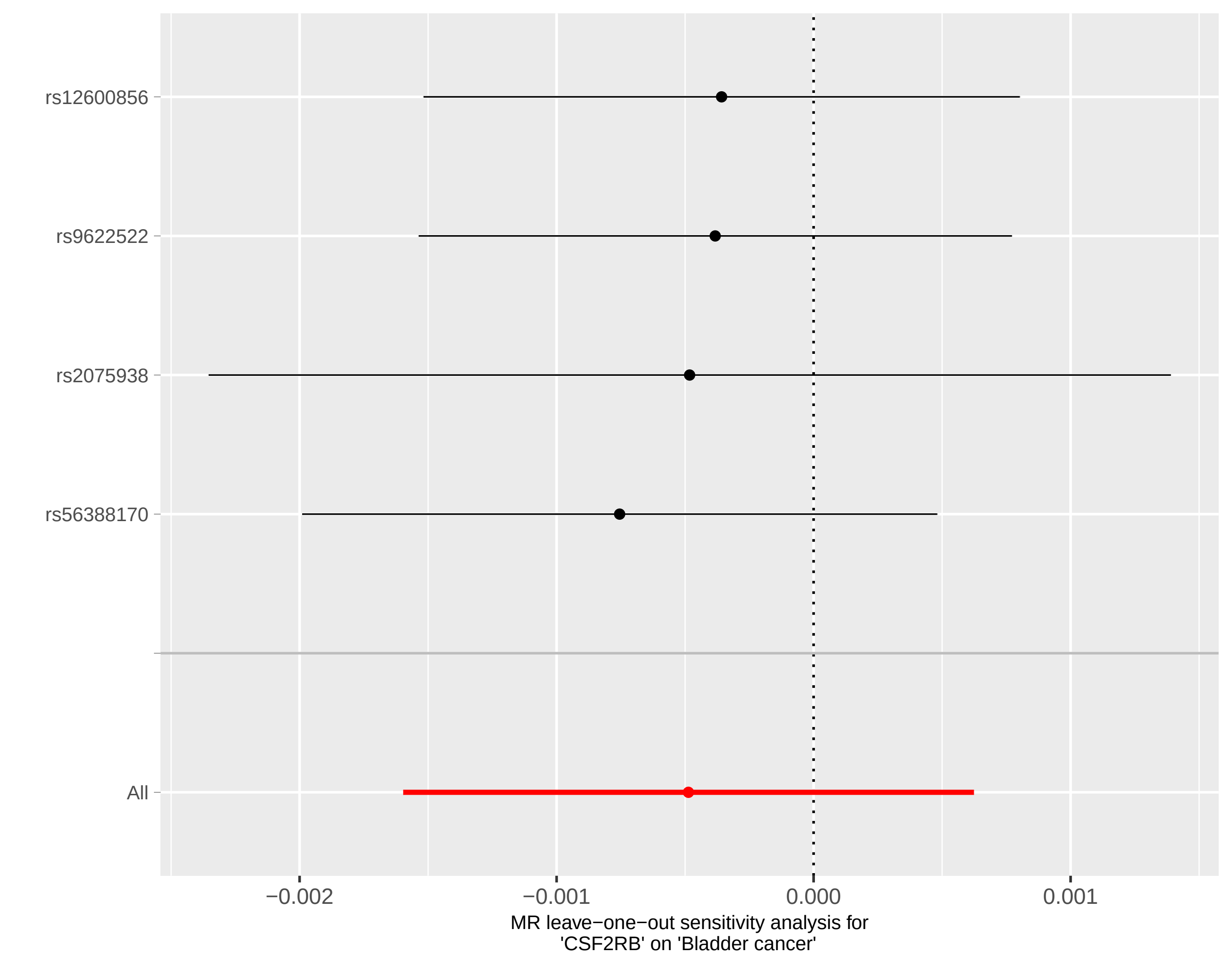

rs12600856
rs9622522
rs2075938
rs56388170
All
−0.002
−0.001
0.000
0.001
MR leave−one−out sensitivity analysis for
'CSF2RB' on 'Bladder cancer'

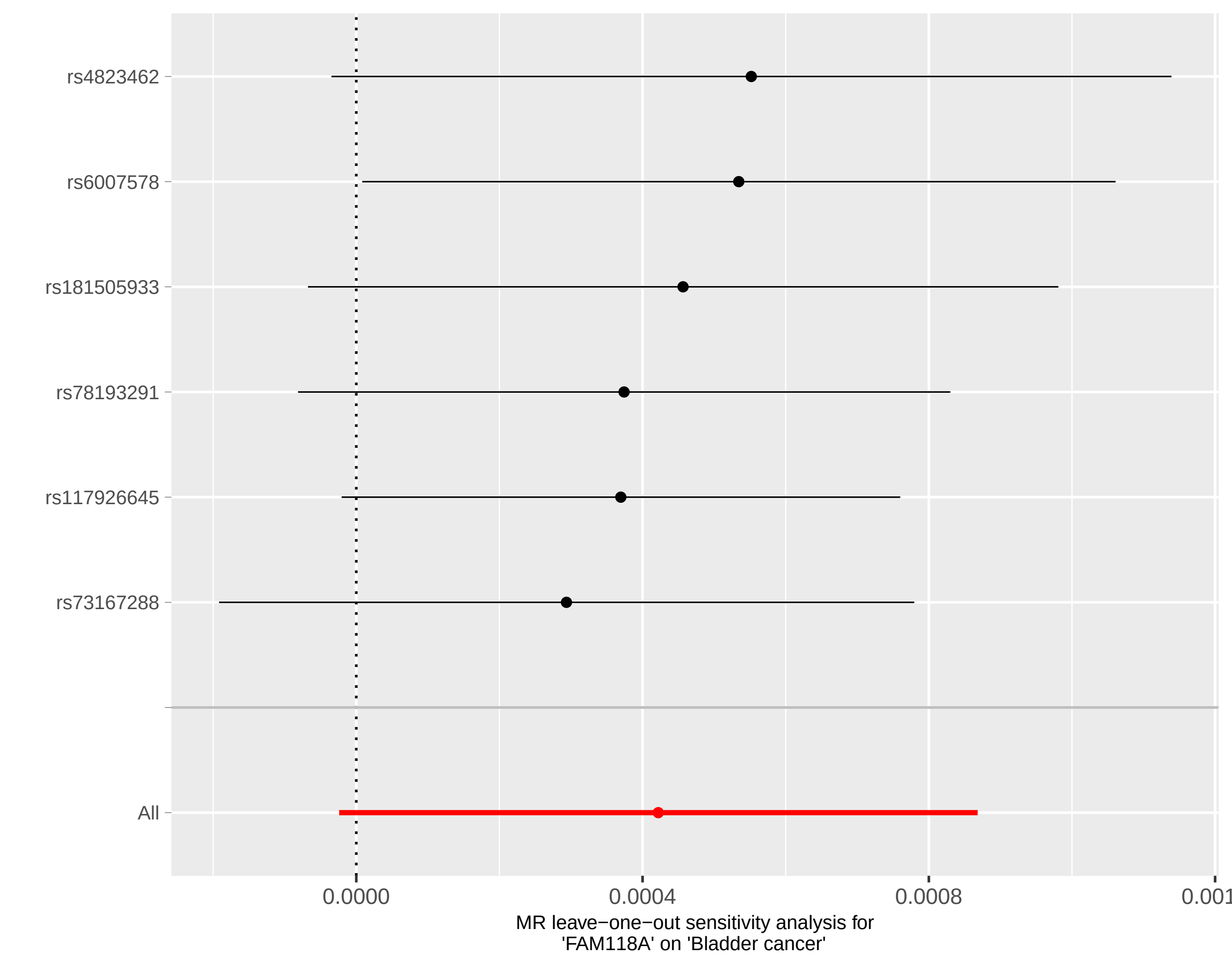

rs4823462
rs6007578
rs181505933
rs78193291
rs117926645
rs73167288
All
0.0000
0.0004
0.0008
0.001
MR leave−one−out sensitivity analysis for
'FAM118A' on 'Bladder cancer'

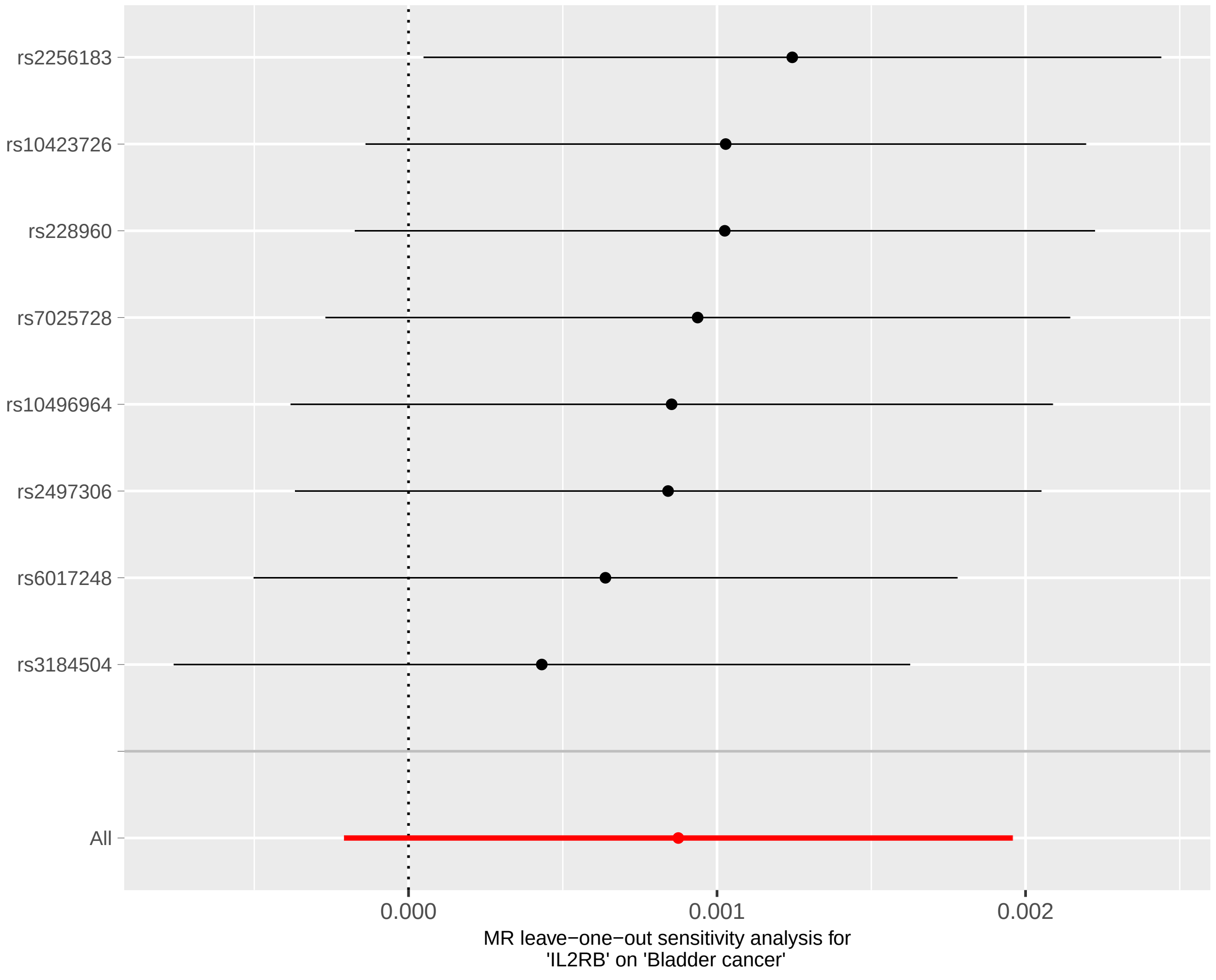
rs2256183
rs10423726
rs228960
rs7025728
rs10496964
rs2497306
rs6017248
rs3184504
All
0.000
0.001
0.002
MR leave−one−out sensitivity analysis for
'IL2RB' on 'Bladder cancer'

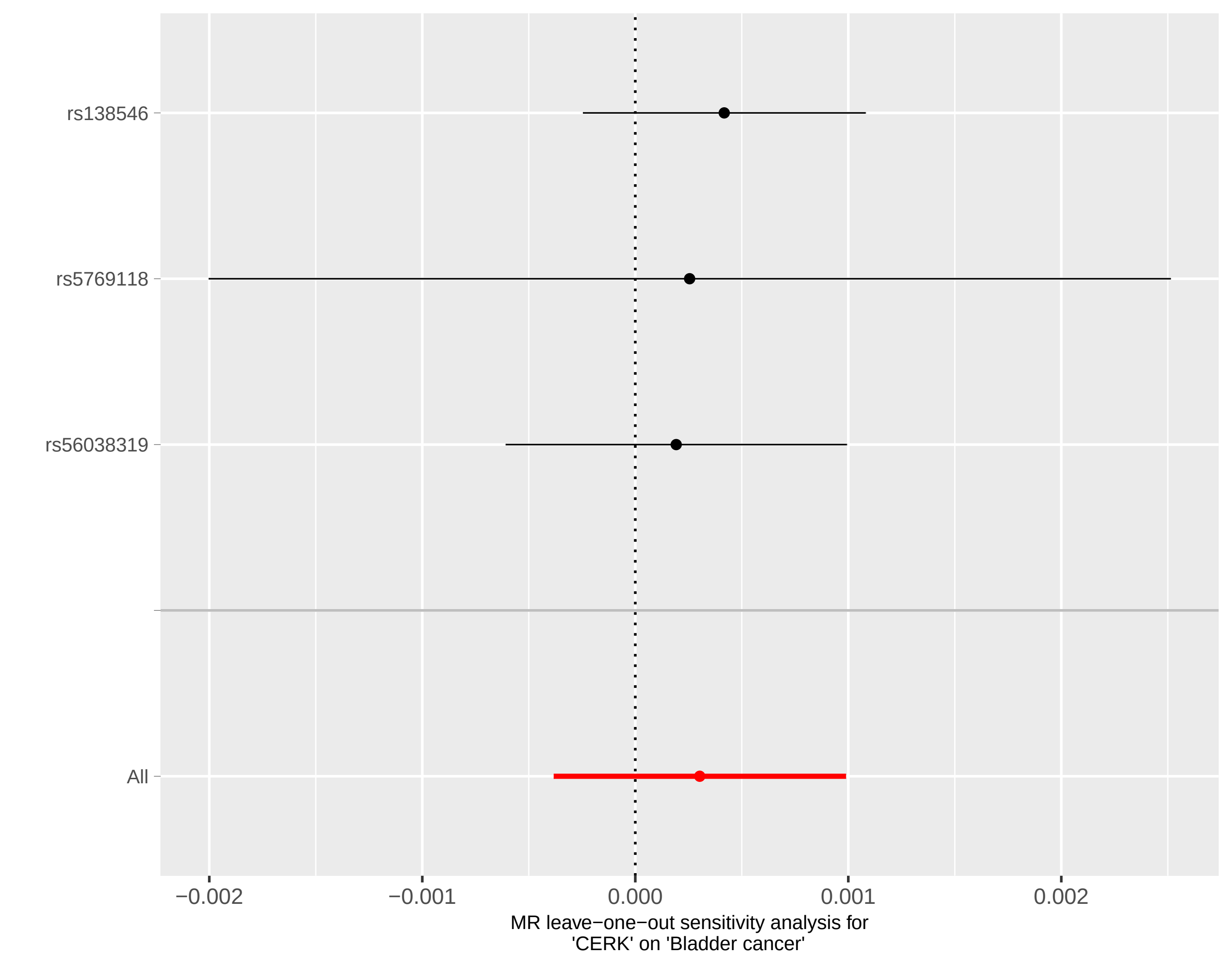

rs138546
rs5769118
rs56038319
All
−0.002
−0.001
0.000
0.001
0.002
MR leave−one−out sensitivity analysis for
'CERK' on 'Bladder cancer'

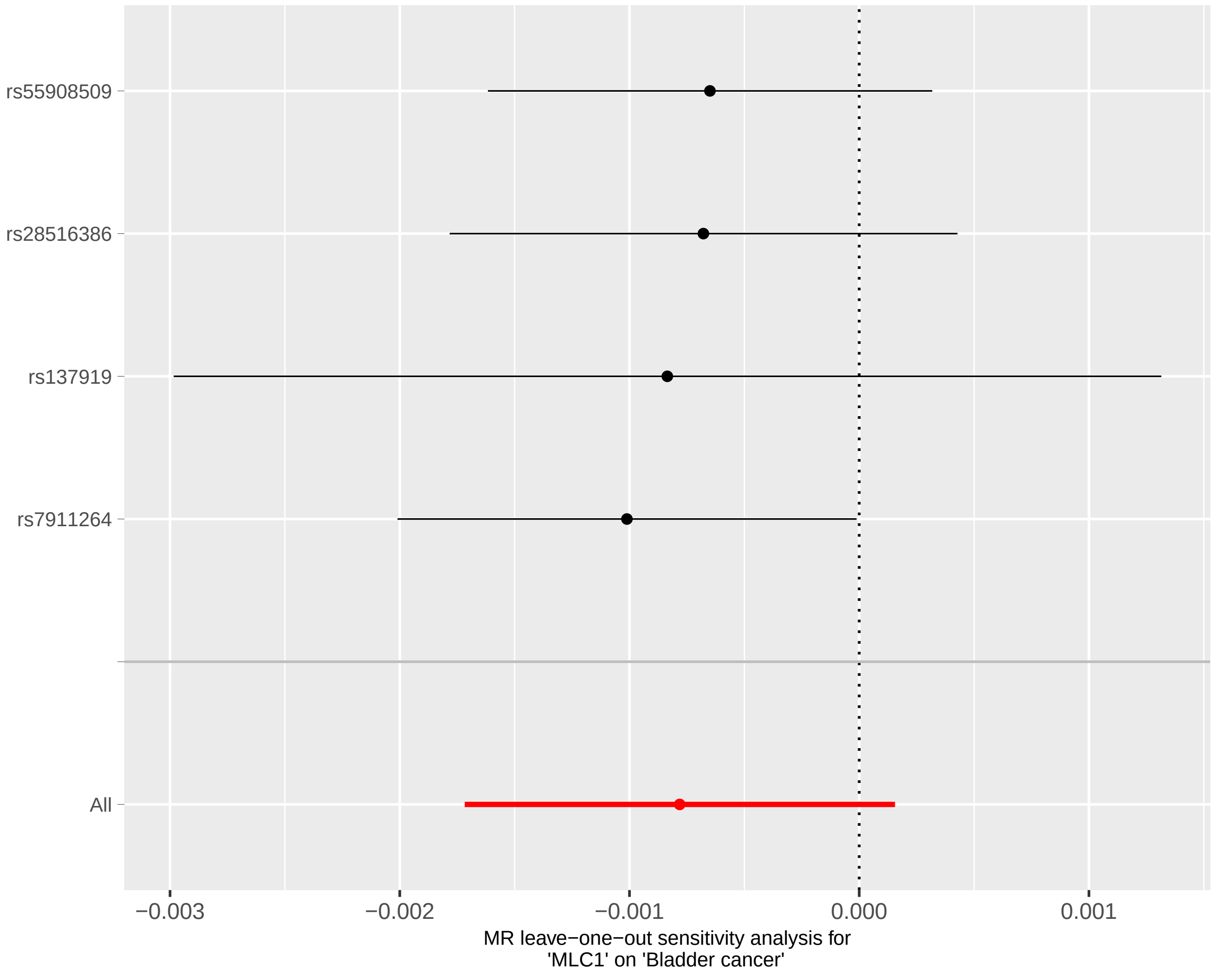
rs55908509
rs28516386
rs137919
rs7911264
All
−0.003
−0.002
−0.001
0.000
0.001
MR leave−one−out sensitivity analysis for
'MLC1' on 'Bladder cancer'

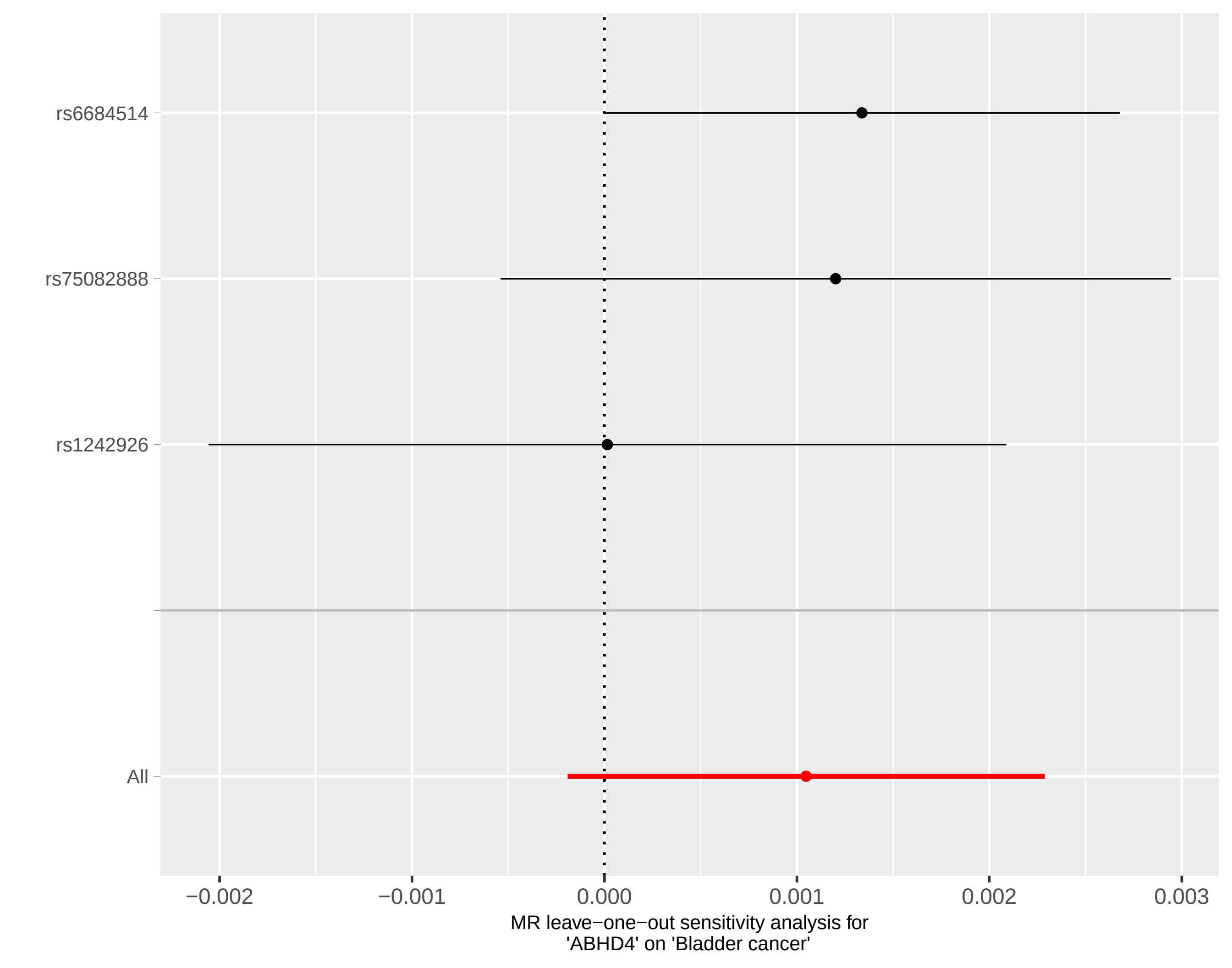
rs6684514
rs75082888
rs1242926
All
−0.002
−0.001
0.000
0.001
0.002
0.003
MR leave−one−out sensitivity analysis for
'ABHD4' on 'Bladder cancer'

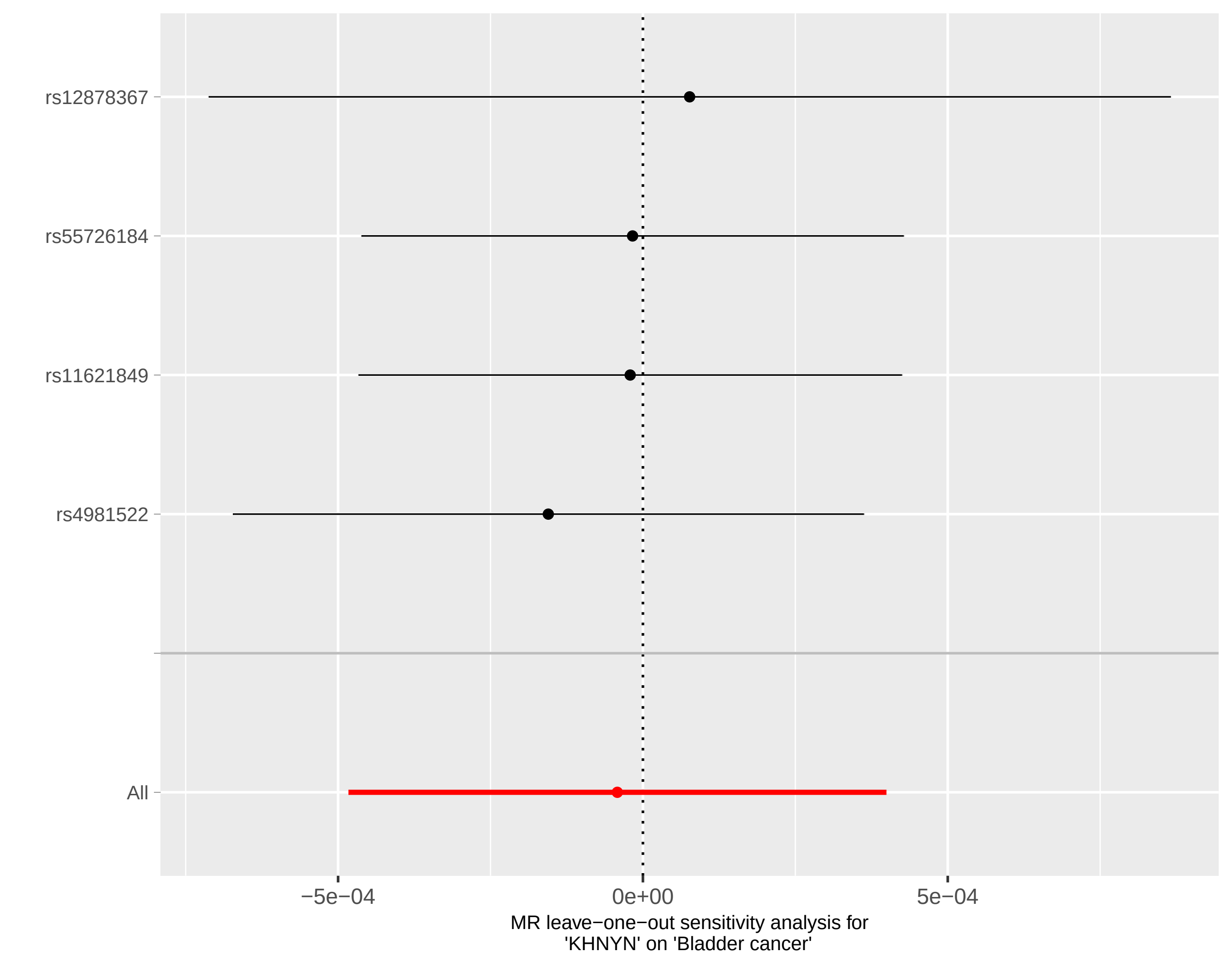

rs12878367
rs55726184
rs11621849
rs4981522
All
−5e−04
0e+00
5e−04
MR leave−one−out sensitivity analysis for
'KHNYN' on 'Bladder cancer'

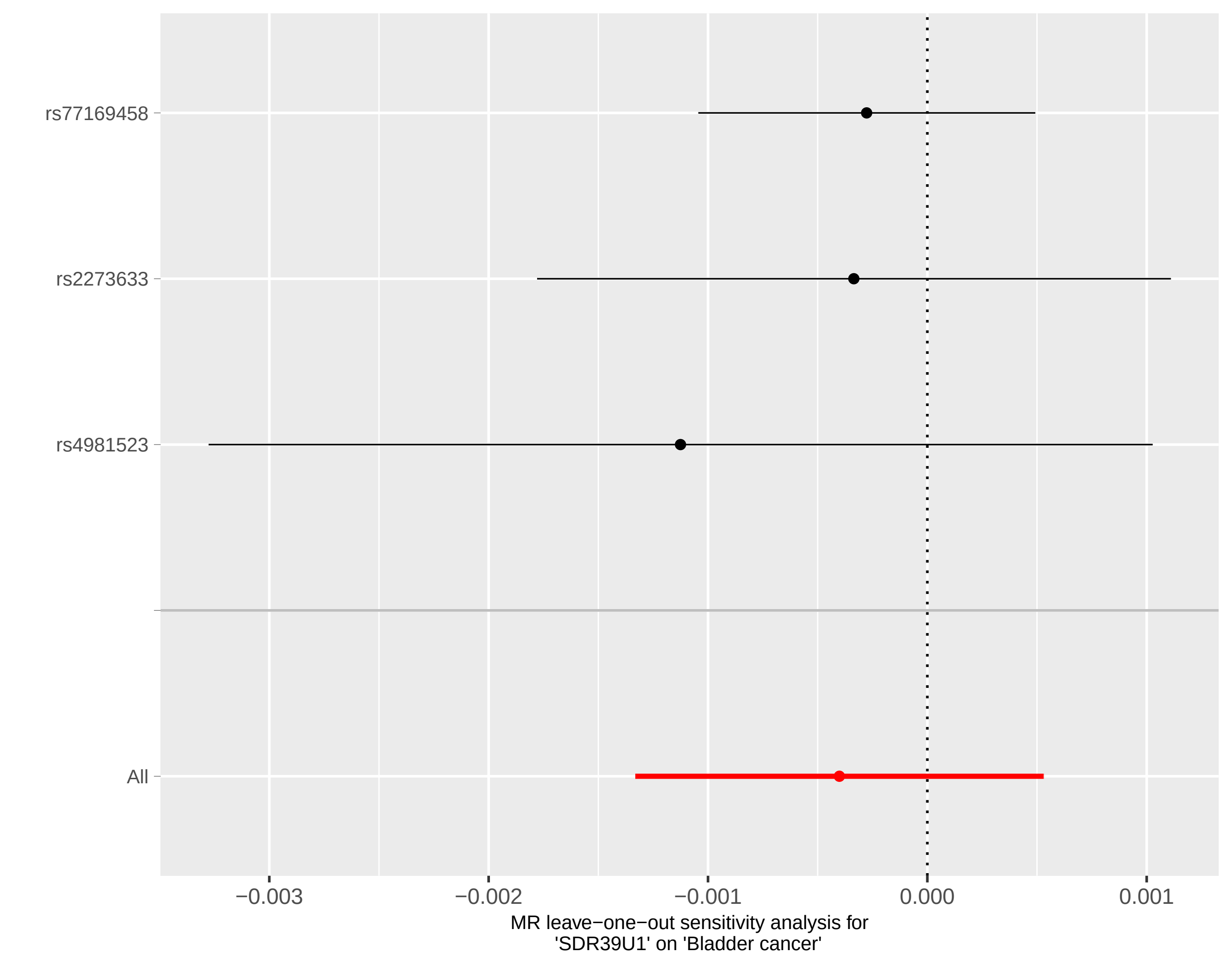
rs77169458
rs2273633
rs4981523
All
−0.003
−0.002
−0.001
0.000
0.001
MR leave−one−out sensitivity analysis for
'SDR39U1' on 'Bladder cancer'

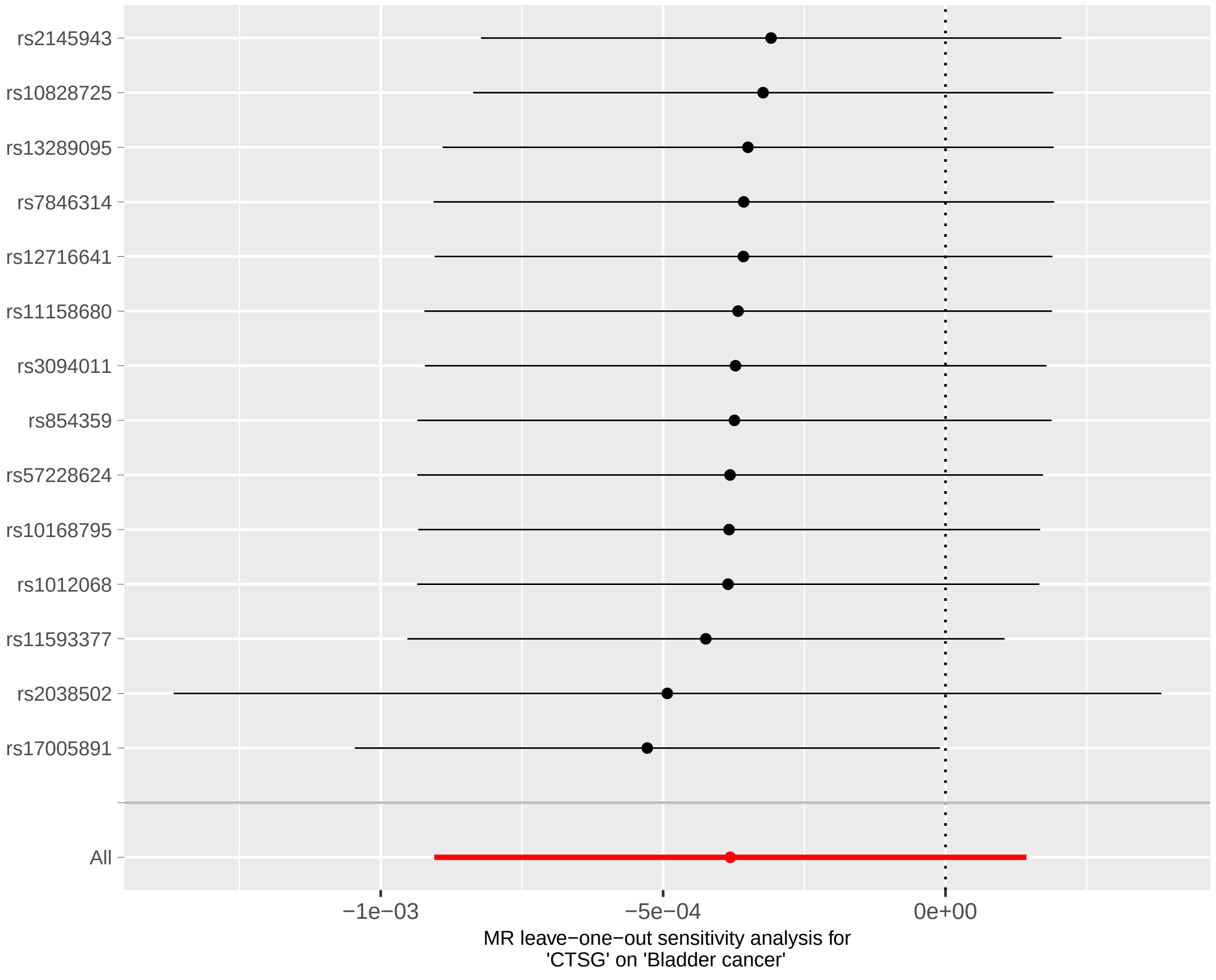
rs2145943
rs10828725
rs13289095
rs7846314
rs12716641
rs11158680
rs3094011
rs854359
rs57228624
rs10168795
rs1012068
rs11593377
rs2038502
rs17005891
All
−1e−03
−5e−04
0e+00
MR leave−one−out sensitivity analysis for
'CTSG' on 'Bladder cancer'

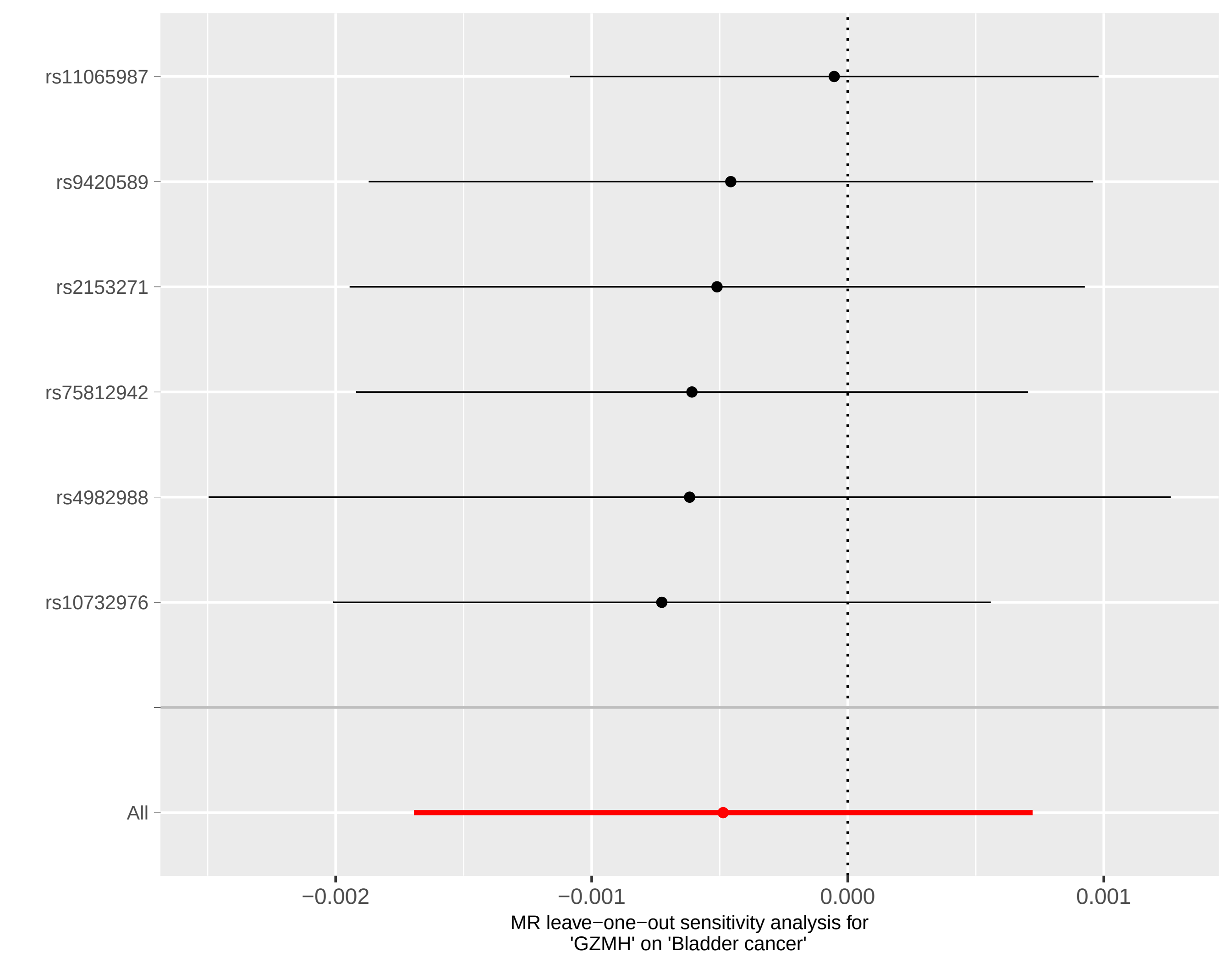
rs11065987
rs9420589
rs2153271
rs75812942
rs4982988
rs10732976
All
−0.002
−0.001
0.000
0.001
MR leave−one−out sensitivity analysis for
'GZMH' on 'Bladder cancer'

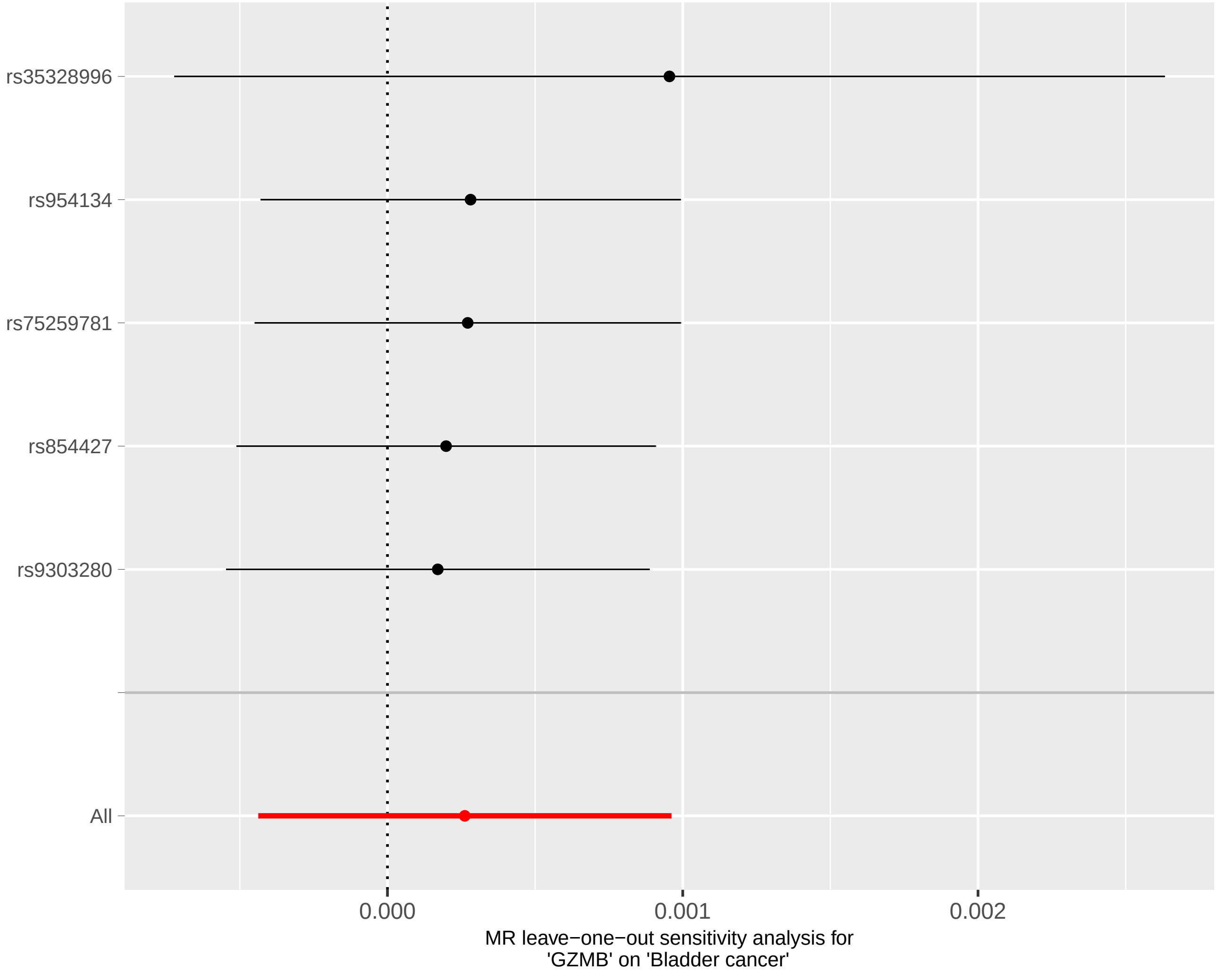
rs35328996
rs954134
rs75259781
rs854427
rs9303280
All
0.000
0.001
0.002
MR leave−one−out sensitivity analysis for
'GZMB' on 'Bladder cancer'

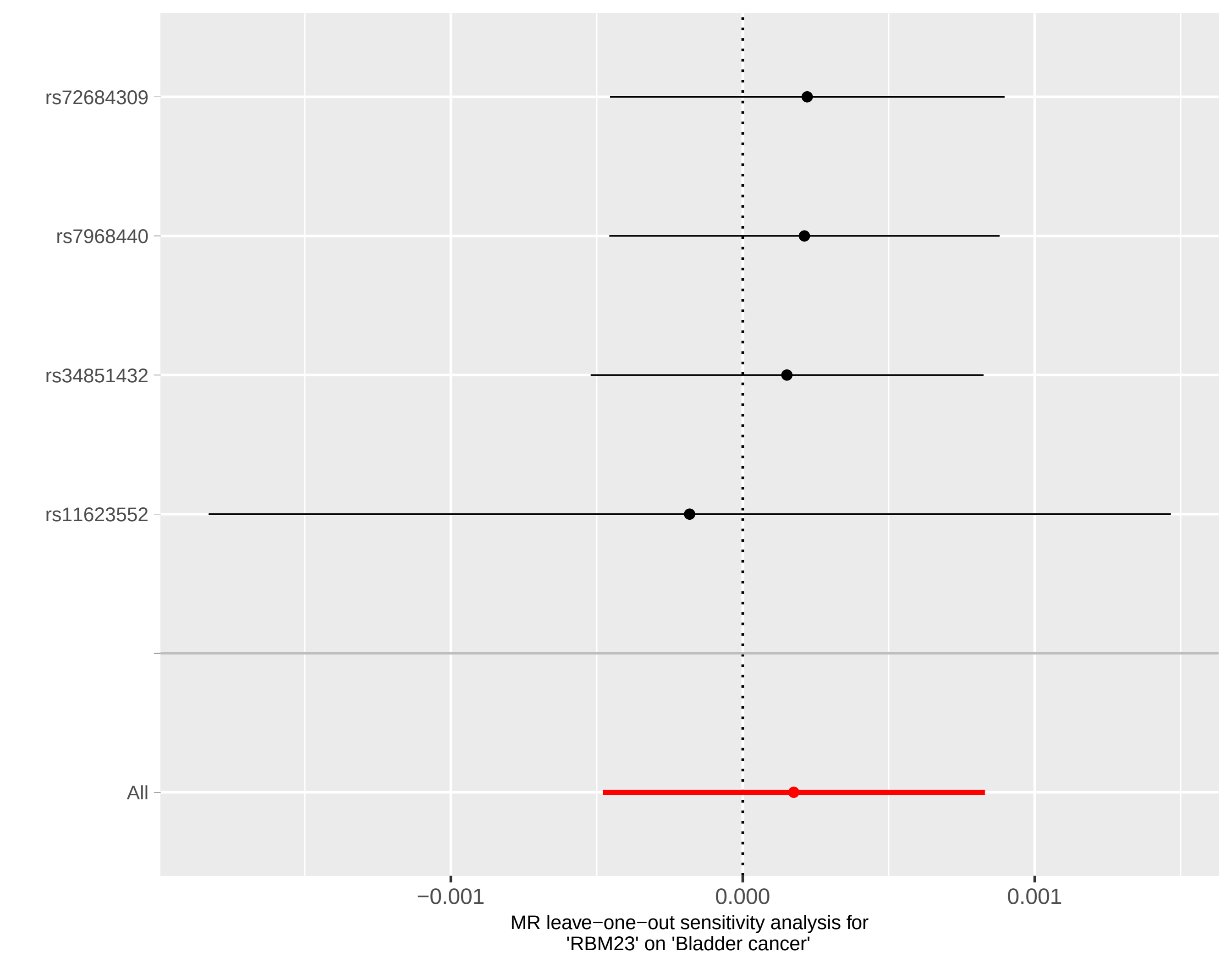
rs72684309
rs7968440
rs34851432
rs11623552
All
−0.001
0.000
0.001
MR leave−one−out sensitivity analysis for
'RBM23' on 'Bladder cancer'

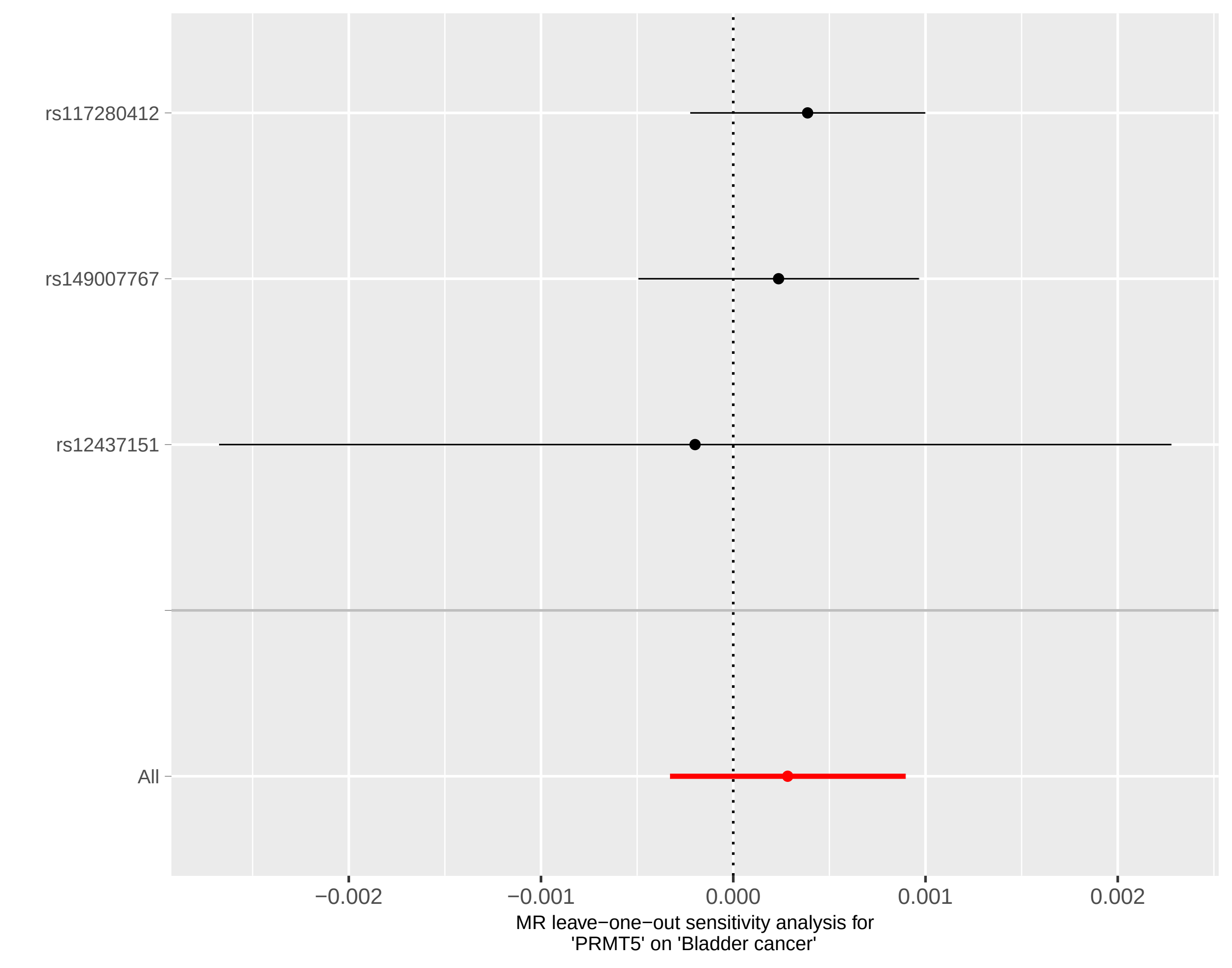

rs117280412
rs149007767
rs12437151
All
−0.002
−0.001
0.000
0.001
0.002
MR leave−one−out sensitivity analysis for
'PRMT5' on 'Bladder cancer'

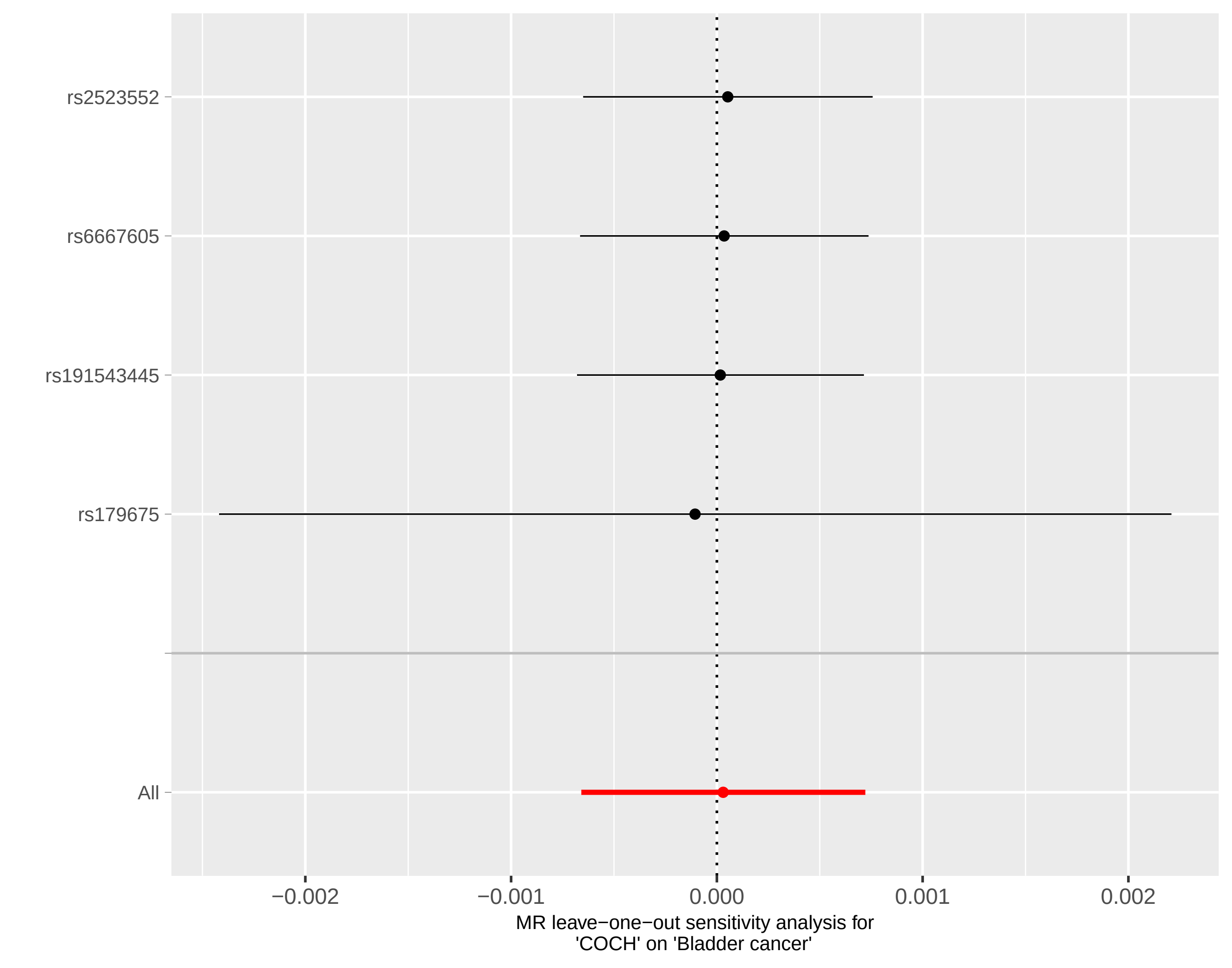
rs2523552
rs6667605
rs191543445
rs179675
All
−0.002
−0.001
0.000
0.001
0.002
MR leave−one−out sensitivity analysis for
'COCH' on 'Bladder cancer'

Insufficient number of SNPs

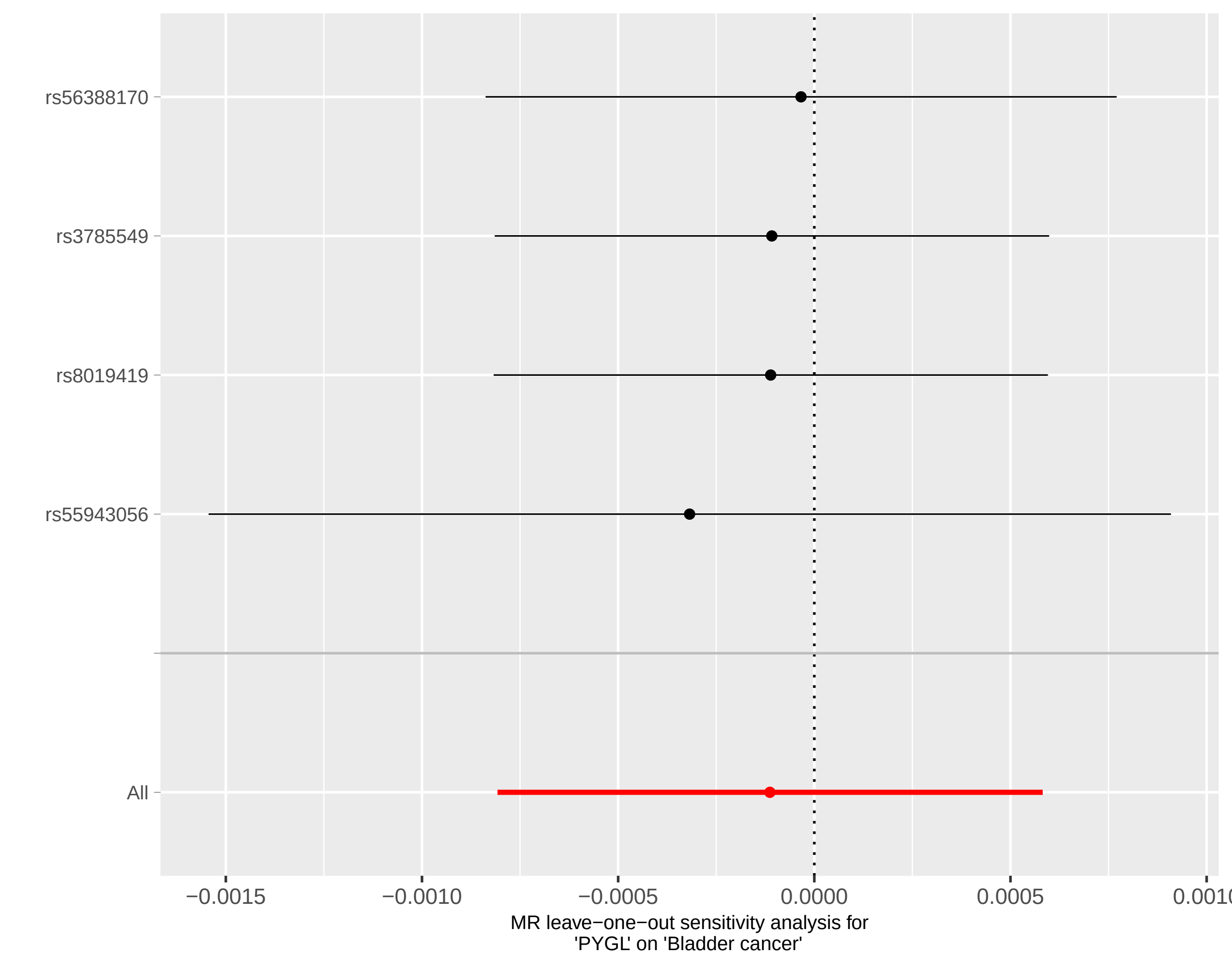

rs56388170
rs3785549
rs8019419
rs55943056
All
−0.0015
−0.0010
−0.0005
0.0000
0.0005
MR leave−one−out sensitivity analysis for
'PYGL' on 'Bladder cancer'

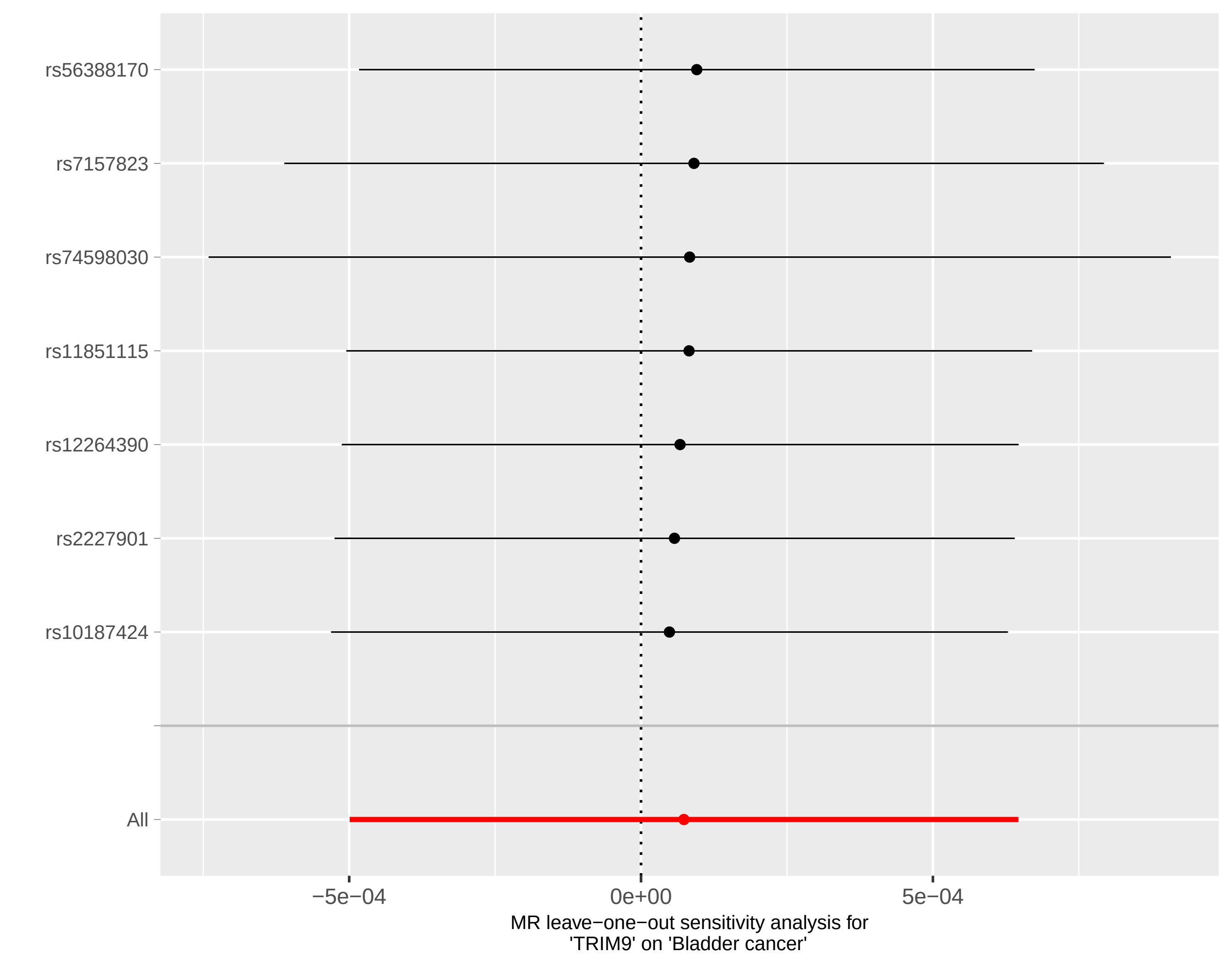
rs56388170
rs7157823
rs74598030
rs11851115
rs12264390
rs2227901
rs10187424
All
−5e−04
0e+00
5e−04
MR leave−one−out sensitivity analysis for
'TRIM9' on 'Bladder cancer'

Insufficient number of SNPs

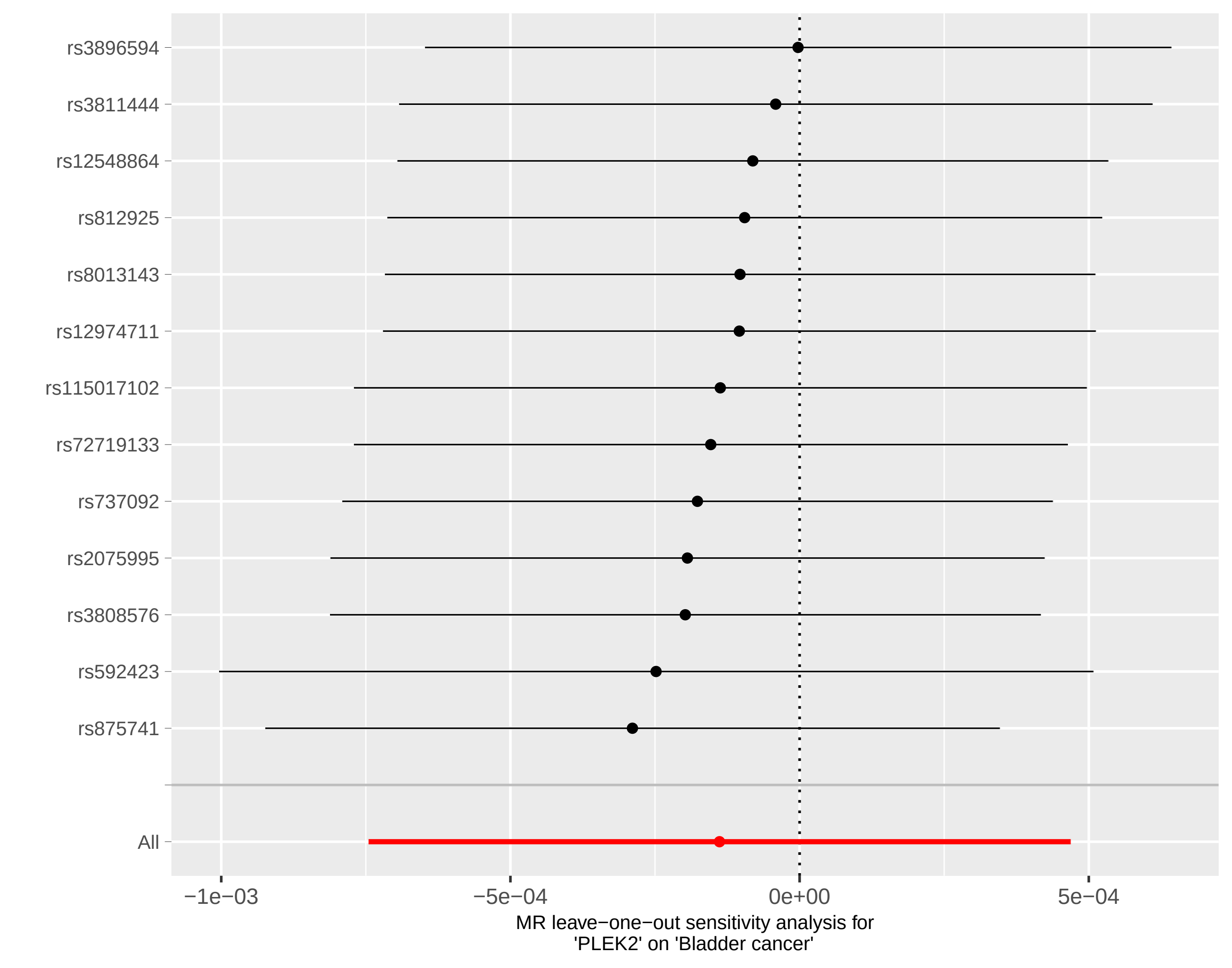
rs3896594
rs3811444
rs12548864
rs812925
rs8013143
rs12974711
rs115017102
rs72719133
rs737092
rs2075995
rs3808576
rs592423
rs875741
All
−1e−03
−5e−04
0e+00
5e−04
MR leave−one−out sensitivity analysis for
'PLEK2' on 'Bladder cancer'

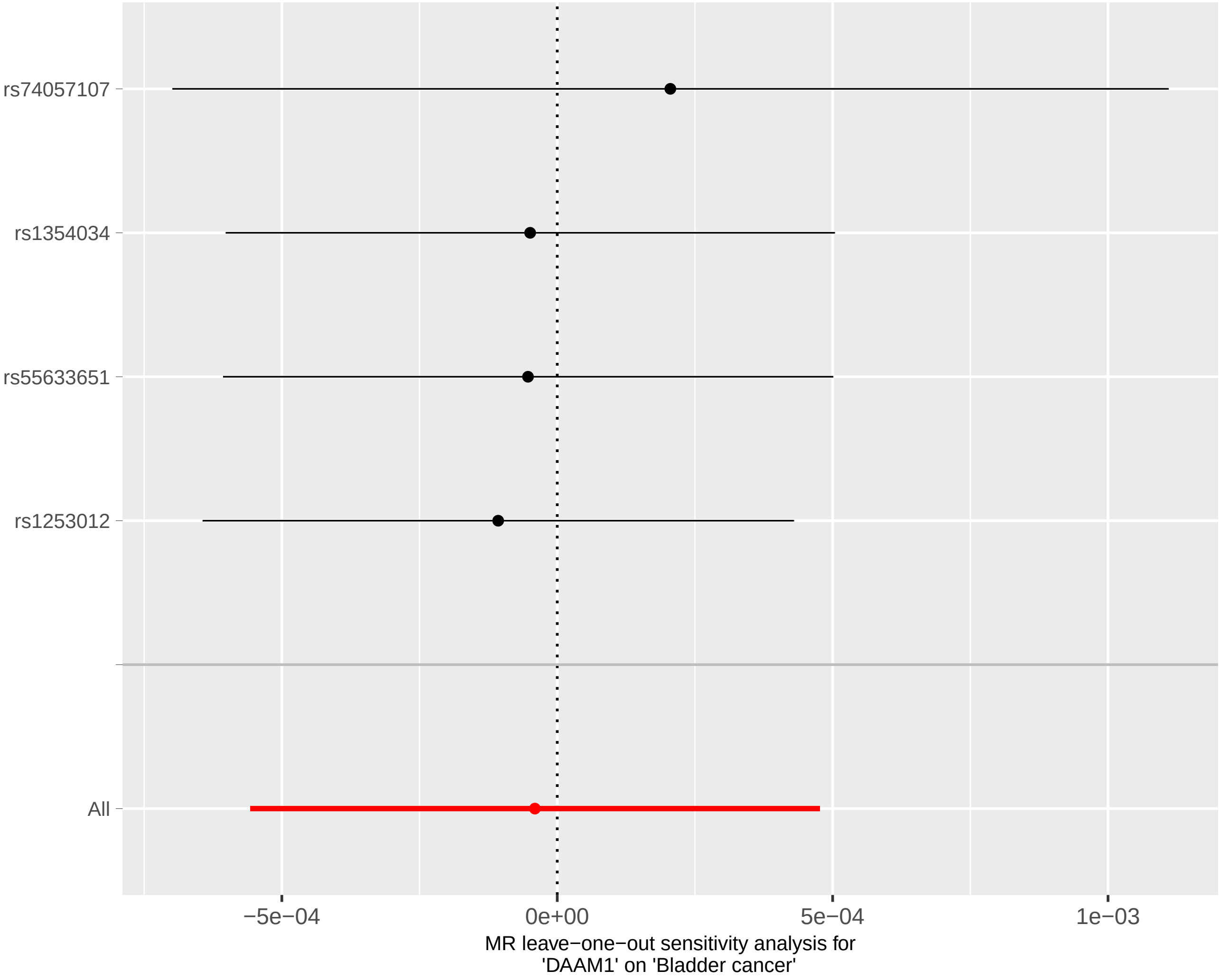
rs74057107
rs1354034
rs55633651
rs1253012
All
−5e−04
0e+00
5e−04
1e−03
MR leave−one−out sensitivity analysis for
'DAAM1' on 'Bladder cancer'

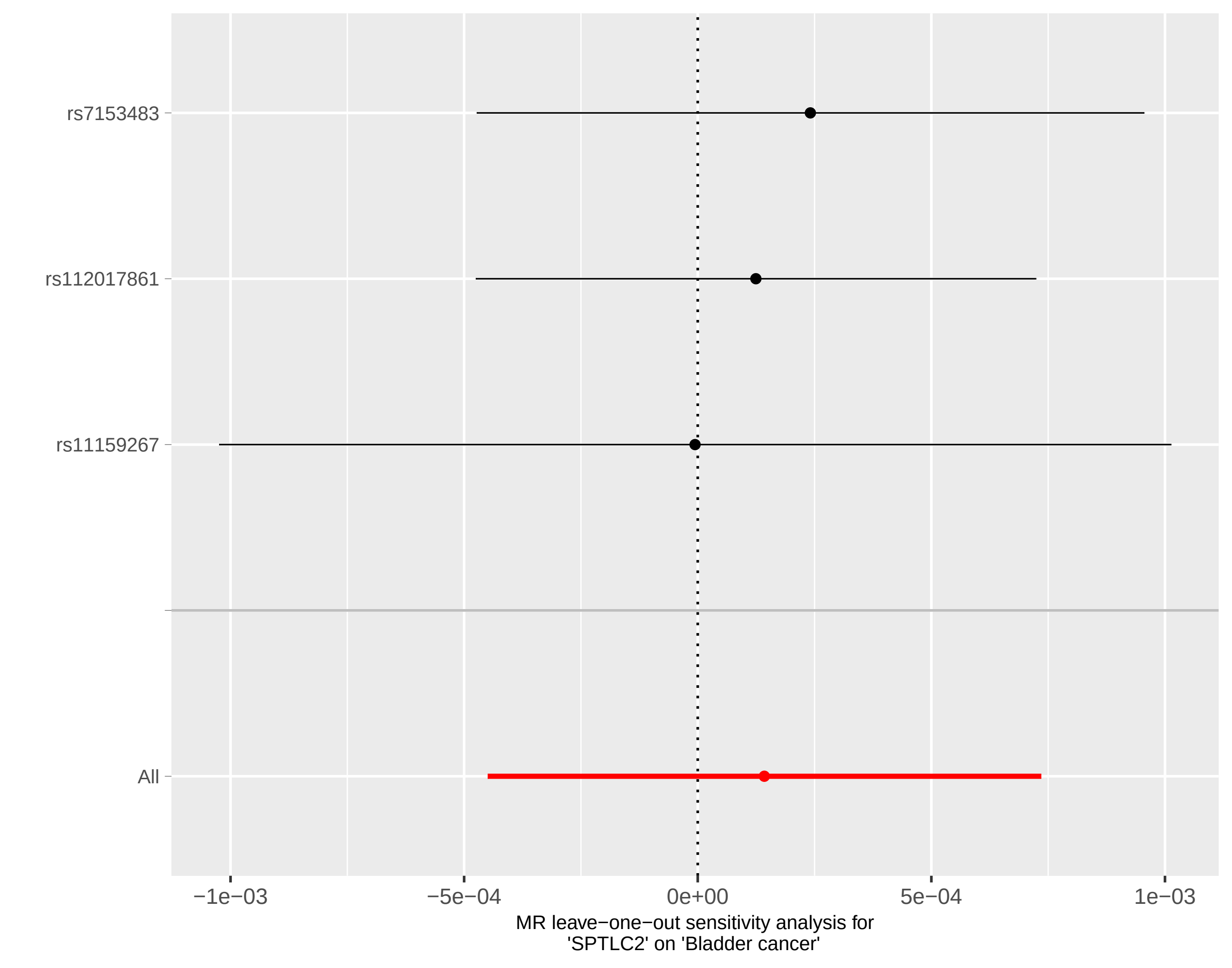
rs7153483
rs112017861
rs11159267
All
−1e−03
−5e−04
0e+00
5e−04
1e−03
MR leave−one−out sensitivity analysis for
'SPTLC2' on 'Bladder cancer'

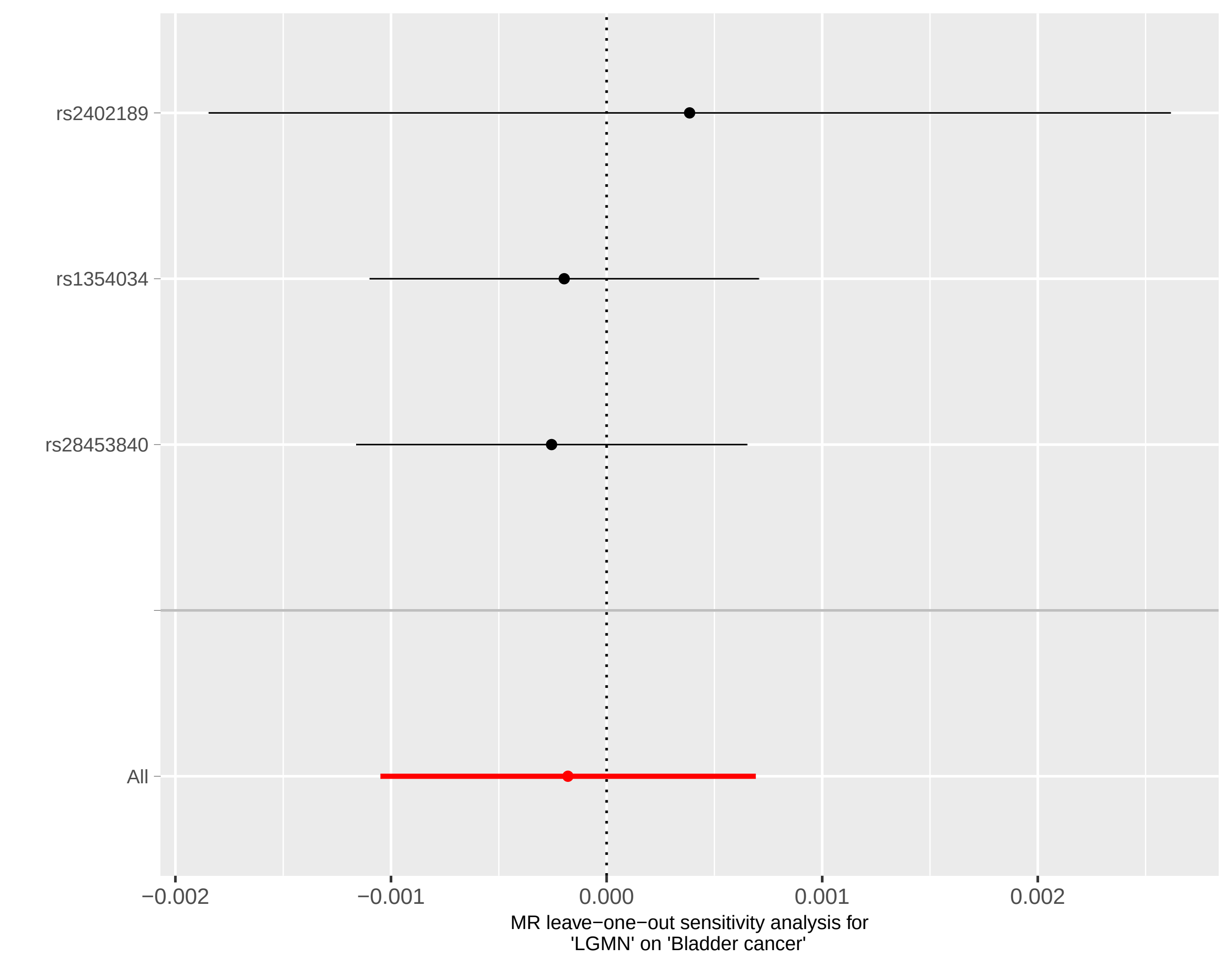
rs2402189
rs1354034
rs28453840
All
−0.002
−0.001
0.000
0.001
0.002
MR leave−one−out sensitivity analysis for
'LGMN' on 'Bladder cancer'

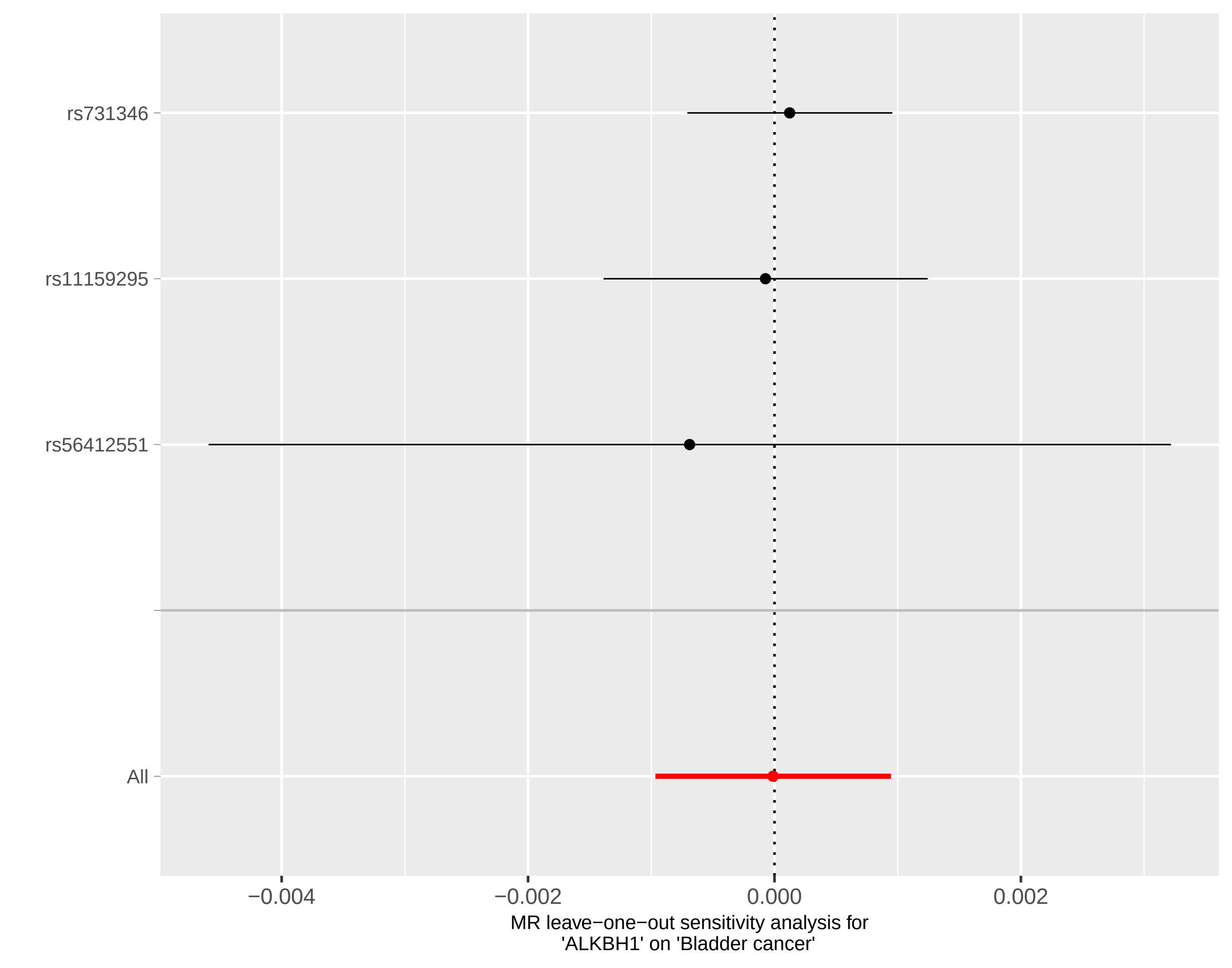
rs731346
rs11159295
rs56412551
All
−0.004
−0.002
0.000
0.002
MR leave−one−out sensitivity analysis for
'ALKBH1' on 'Bladder cancer'

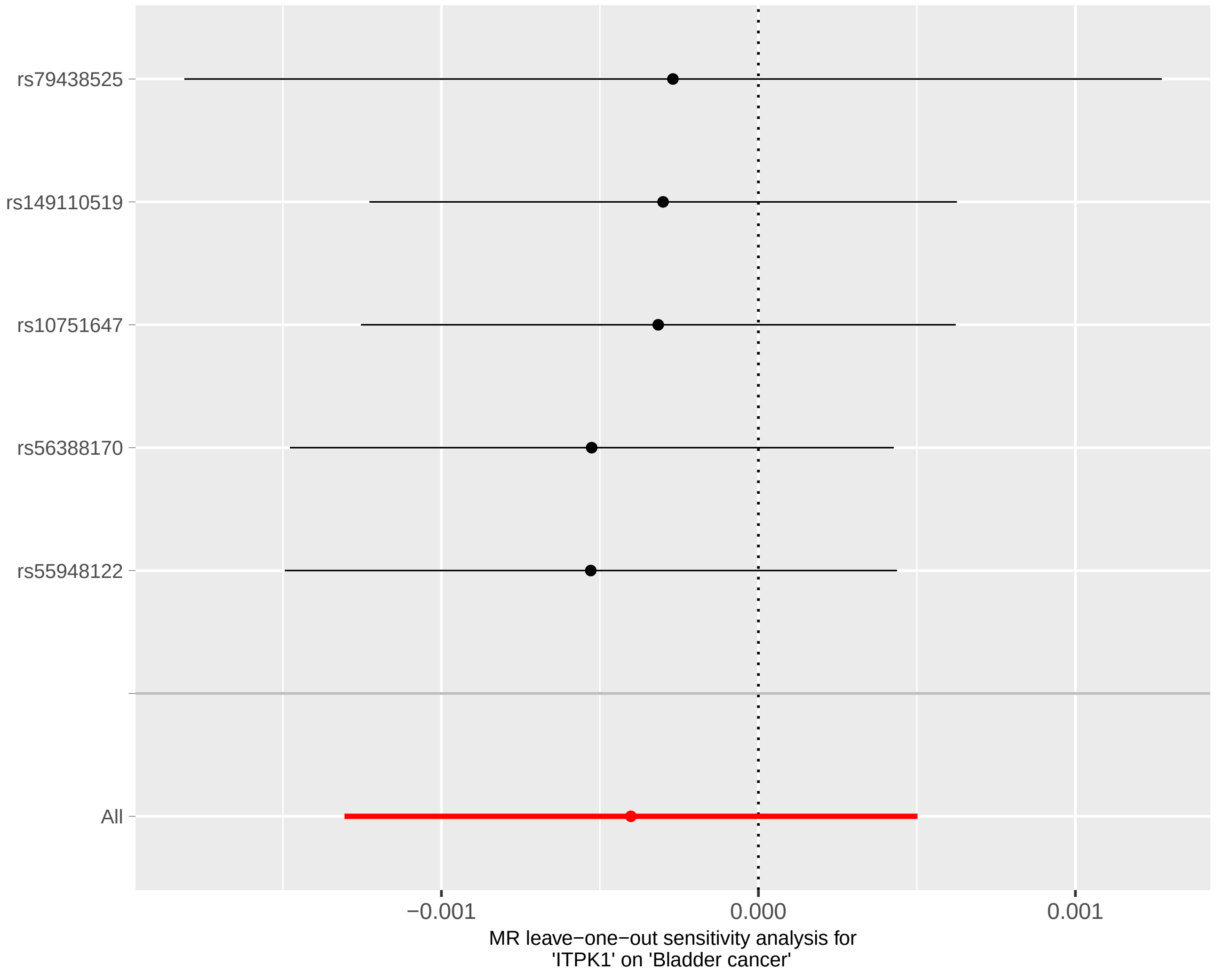
rs79438525
rs149110519
rs10751647
rs56388170
rs55948122
All
−0.001
0.000
0.001
MR leave−one−out sensitivity analysis for
'ITPK1' on 'Bladder cancer'

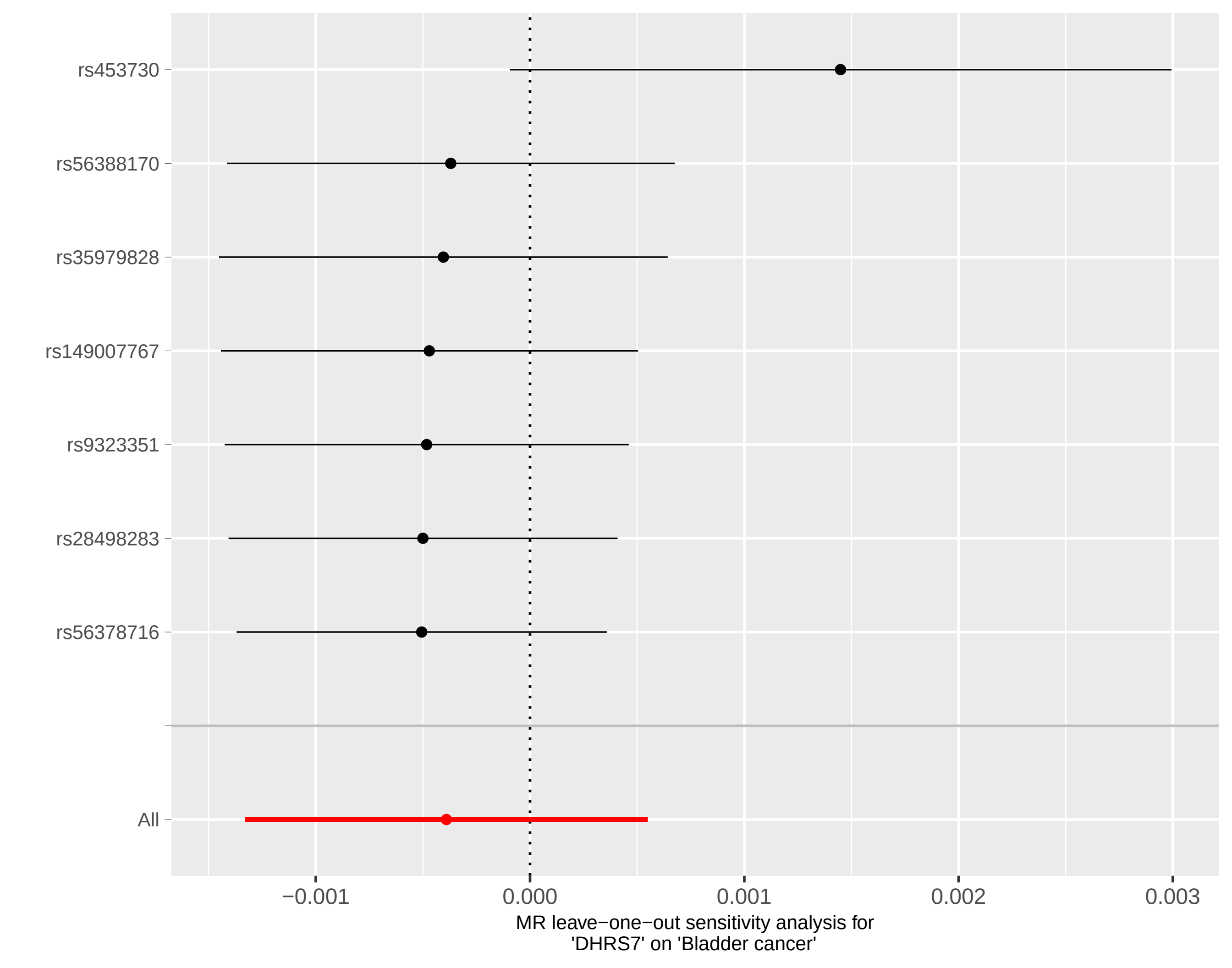

rs453730
rs56388170
rs35979828
rs149007767
rs9323351
rs28498283
rs56378716
All
−0.001
0.000
0.001
0.002
0.003
MR leave−one−out sensitivity analysis for
'DHRS7' on 'Bladder cancer'

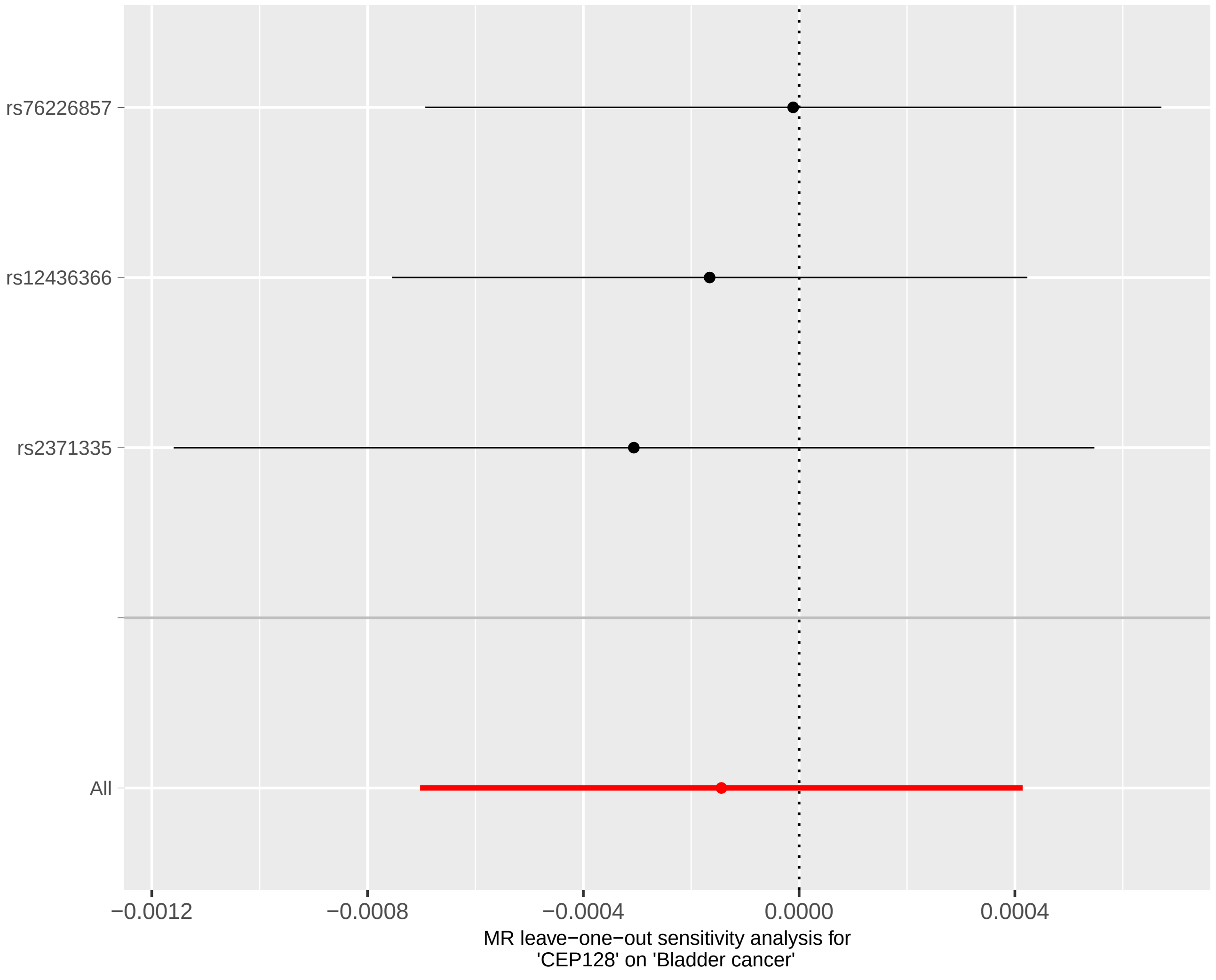
rs76226857
rs12436366
rs2371335
All
−0.0012
−0.0008
−0.0004
0.0000
0.0004
MR leave−one−out sensitivity analysis for
'CEP128' on 'Bladder cancer'

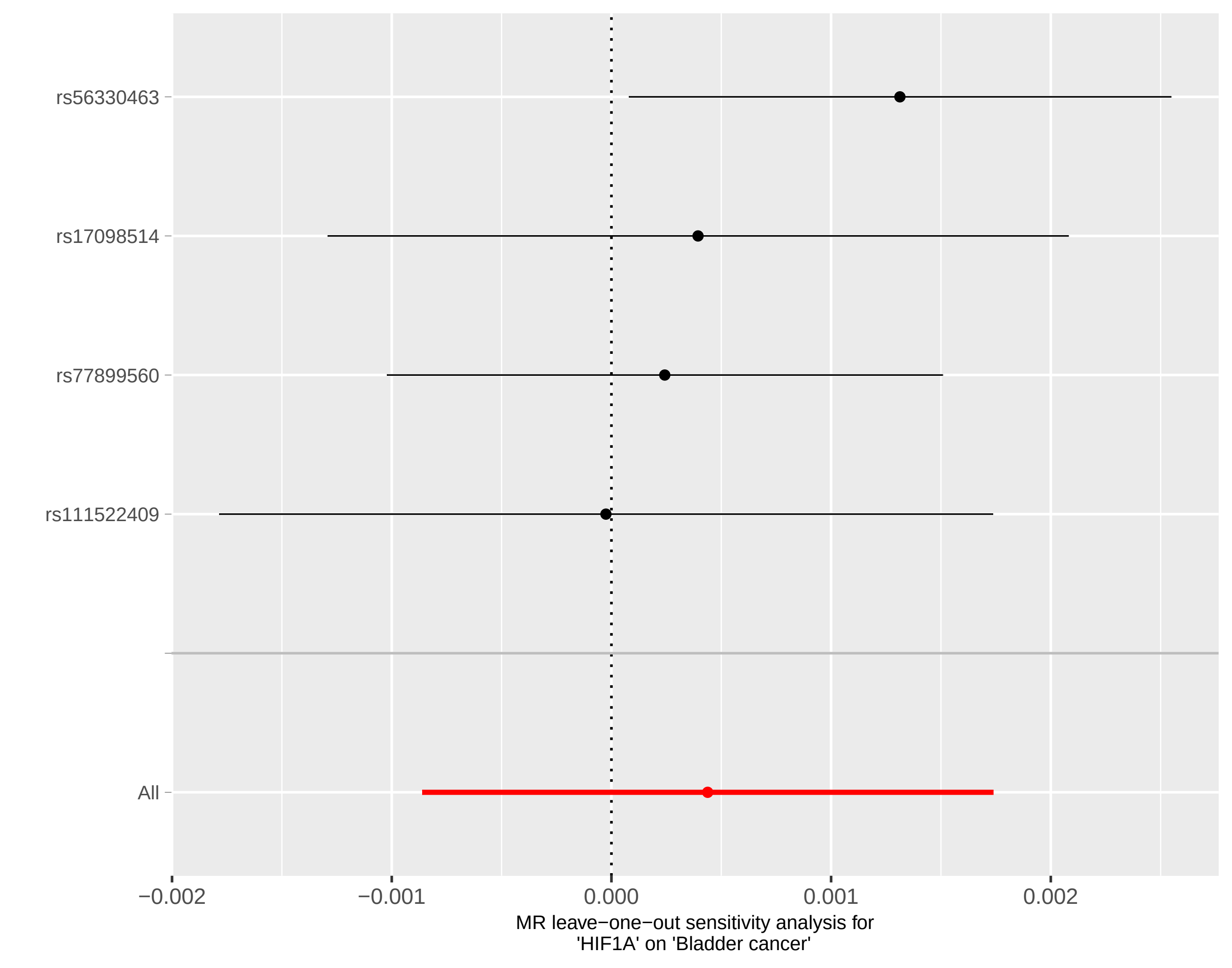
rs56330463
rs17098514
rs77899560
rs111522409
All
−0.002
−0.001
0.000
0.001
0.002
MR leave−one−out sensitivity analysis for
'HIF1A' on 'Bladder cancer'

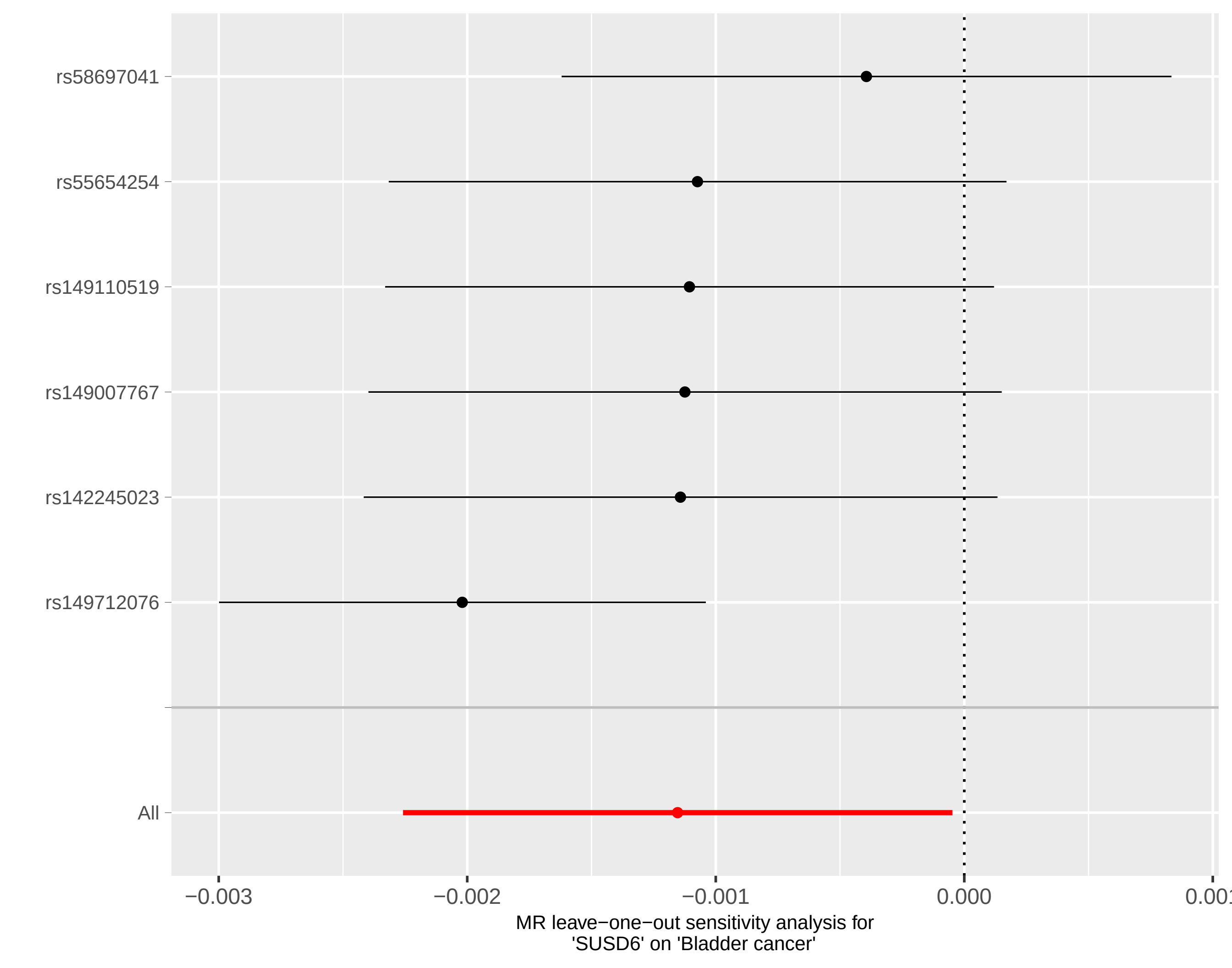
rs58697041
rs55654254
rs149110519
rs149007767
rs142245023
rs149712076
All
−0.003
−0.002
−0.001
0.000
MR leave−one−out sensitivity analysis for
'SUSD6' on 'Bladder cancer'

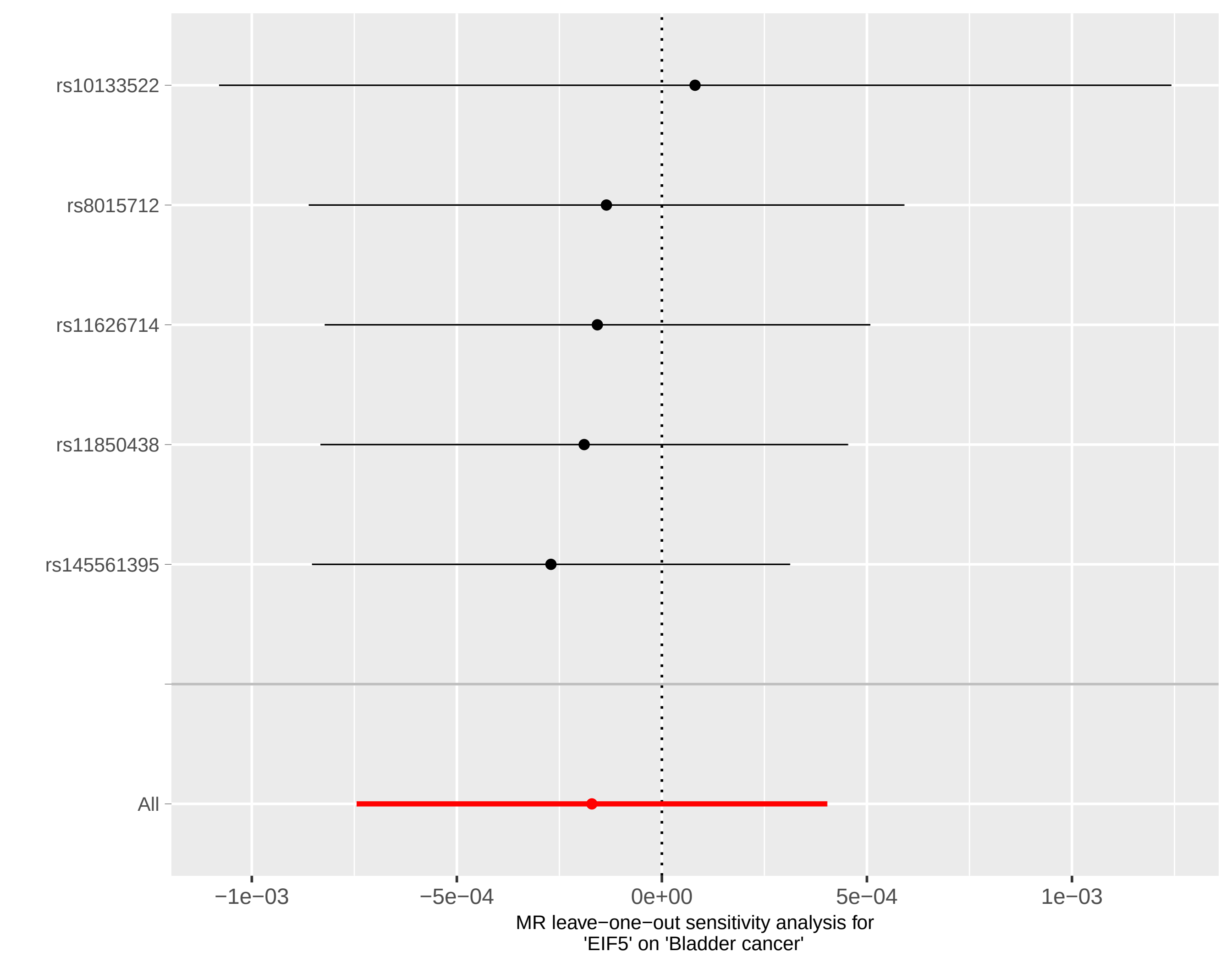
rs10133522
rs8015712
rs11626714
rs11850438
rs145561395
All
−1e−03
−5e−04
0e+00
5e−04
1e−03
MR leave−one−out sensitivity analysis for
'EIF5' on 'Bladder cancer'

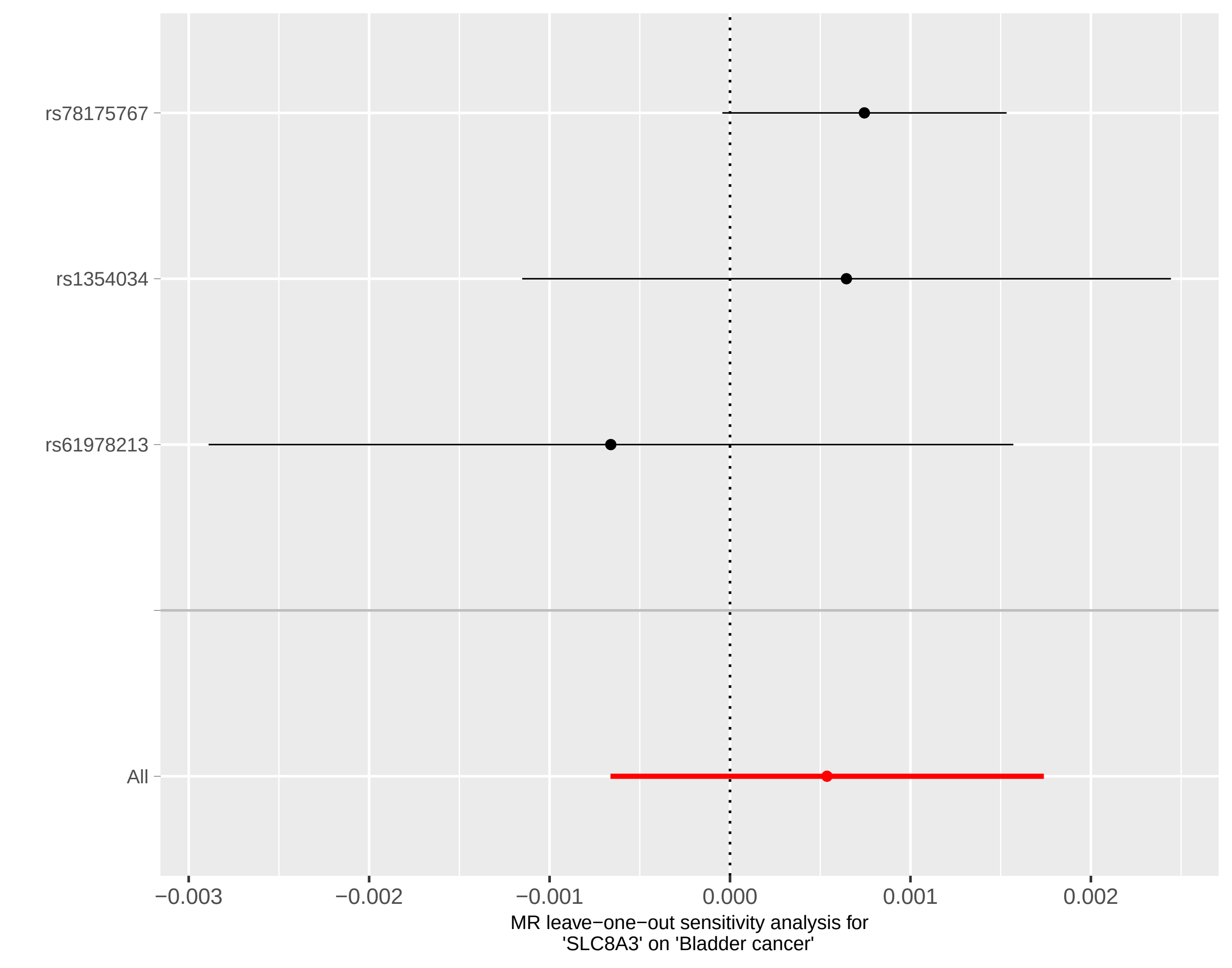
rs78175767
rs1354034
rs61978213
All
−0.003
−0.002
−0.001
0.000
0.001
0.002
MR leave−one−out sensitivity analysis for
'SLC8A3' on 'Bladder cancer'

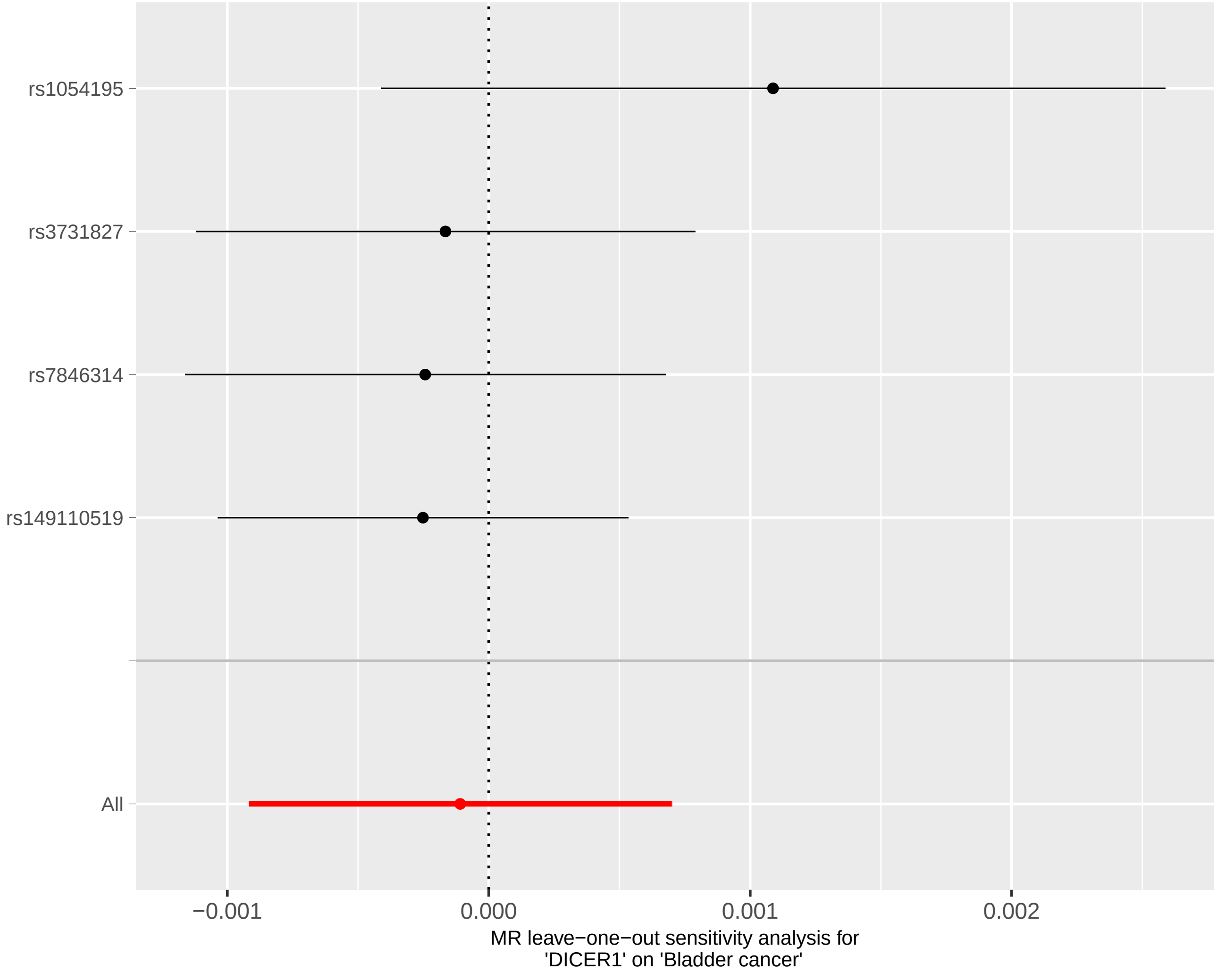
rs1054195
rs3731827
rs7846314
rs149110519
All
−0.001
0.000
0.001
0.002
MR leave−one−out sensitivity analysis for
'DICER1' on 'Bladder cancer'

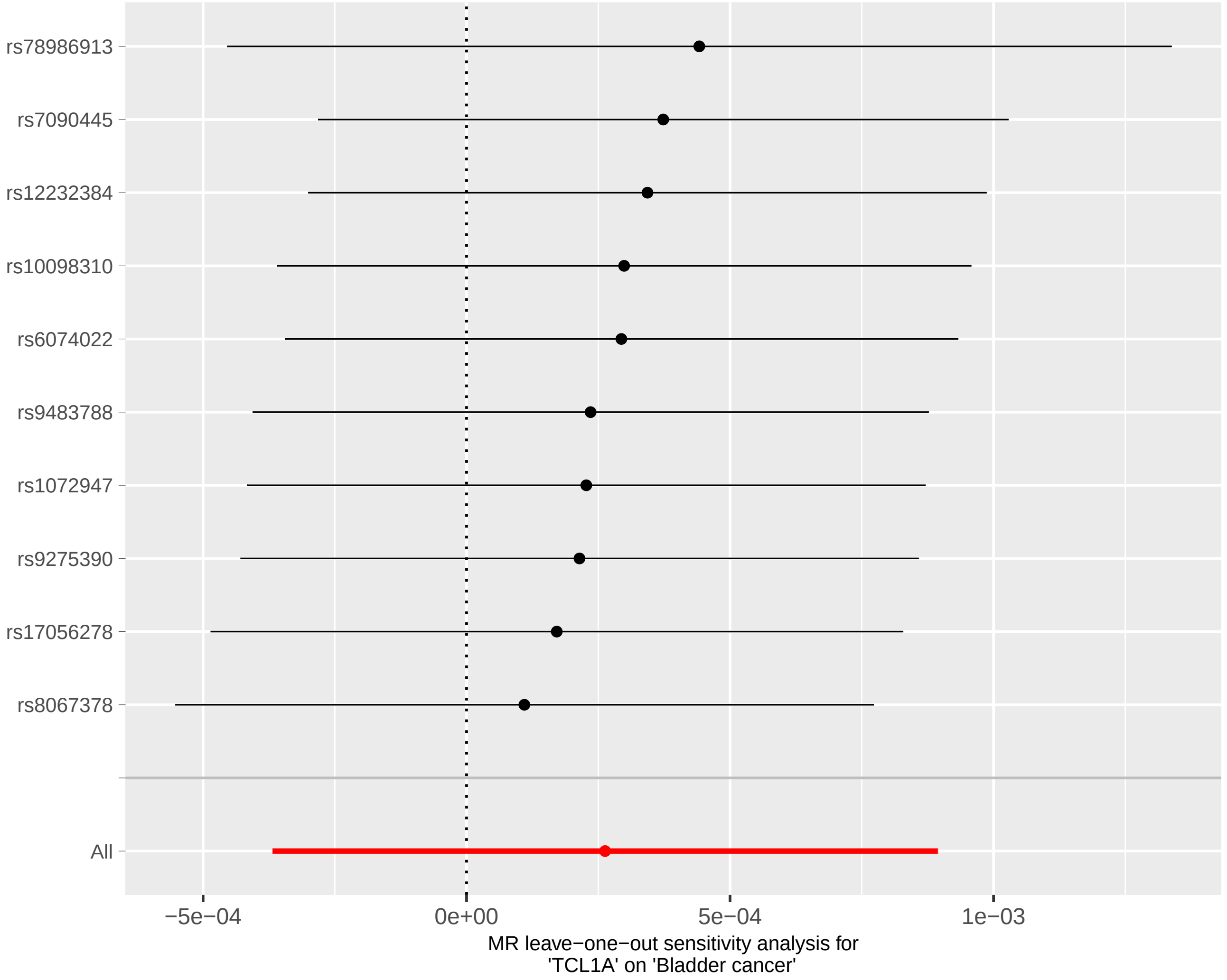

rs78986913
rs7090445
rs12232384
rs10098310
rs6074022
rs9483788
rs1072947
rs9275390
rs17056278
rs8067378
All
−5e−04
0e+00
5e−04
1e−03
MR leave−one−out sensitivity analysis for
'TCL1A' on 'Bladder cancer'

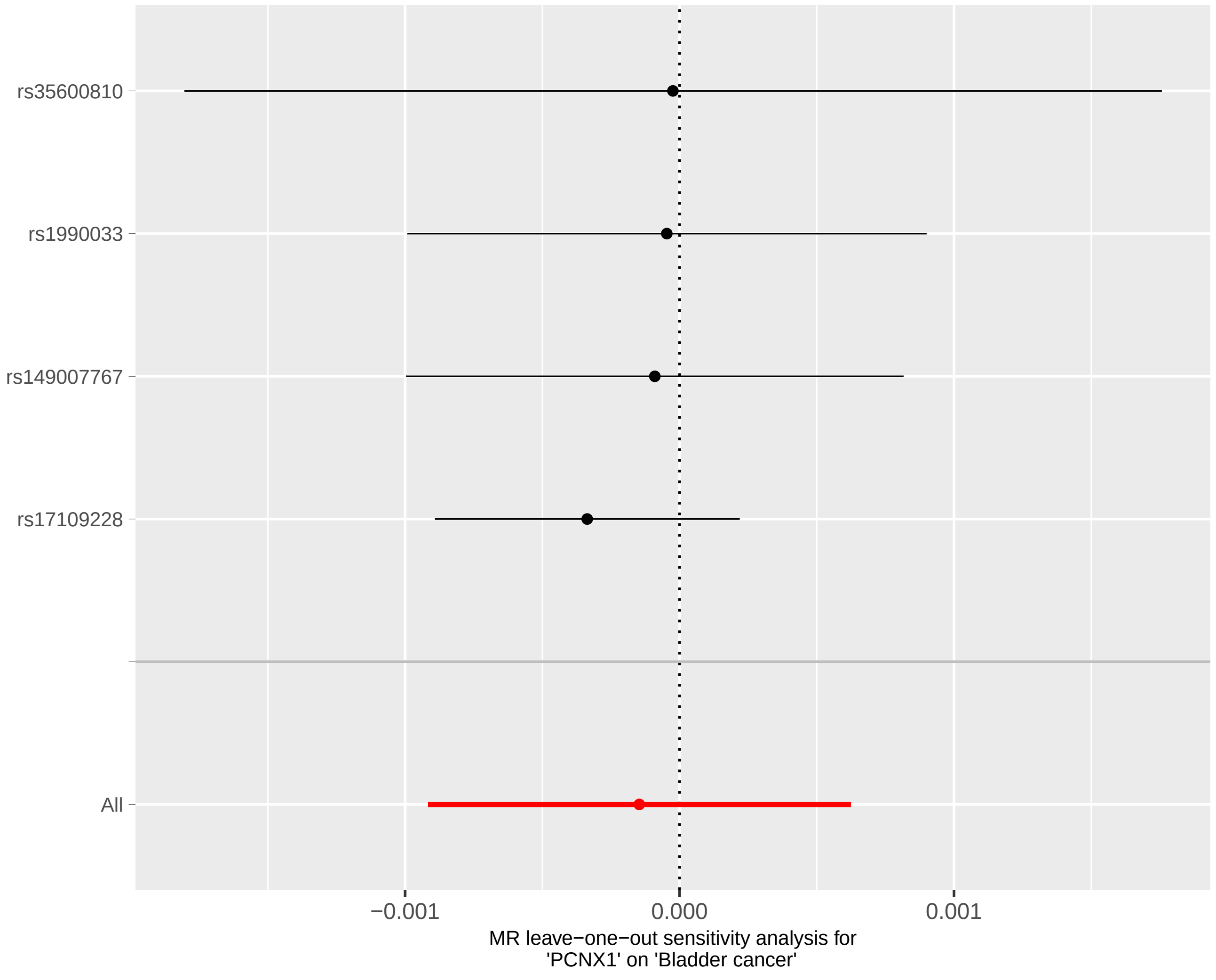
rs35600810
rs1990033
rs149007767
rs17109228
All
−0.001
0.000
0.001
MR leave−one−out sensitivity analysis for
'PCNX1' on 'Bladder cancer'

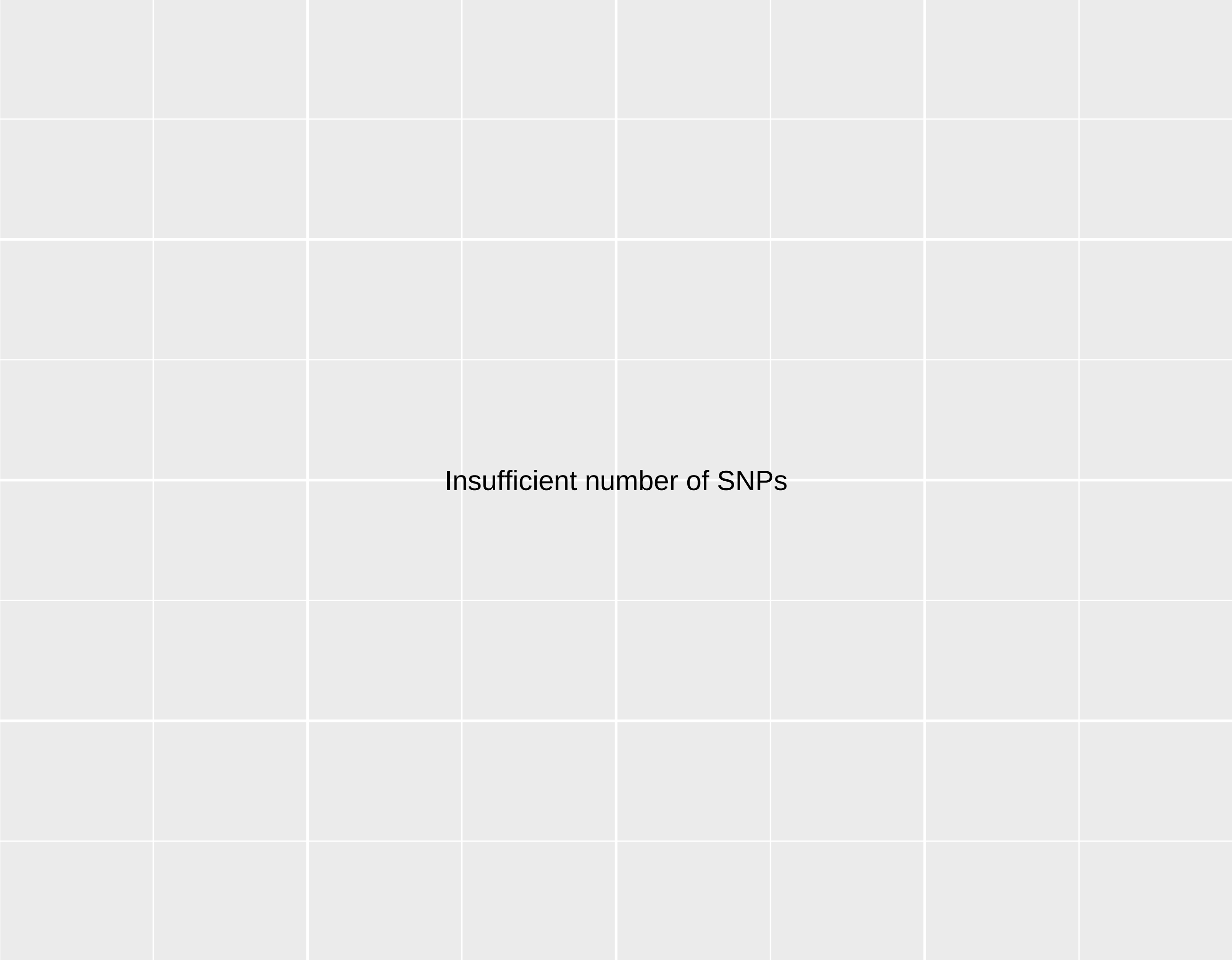
Insufficient number of SNPs

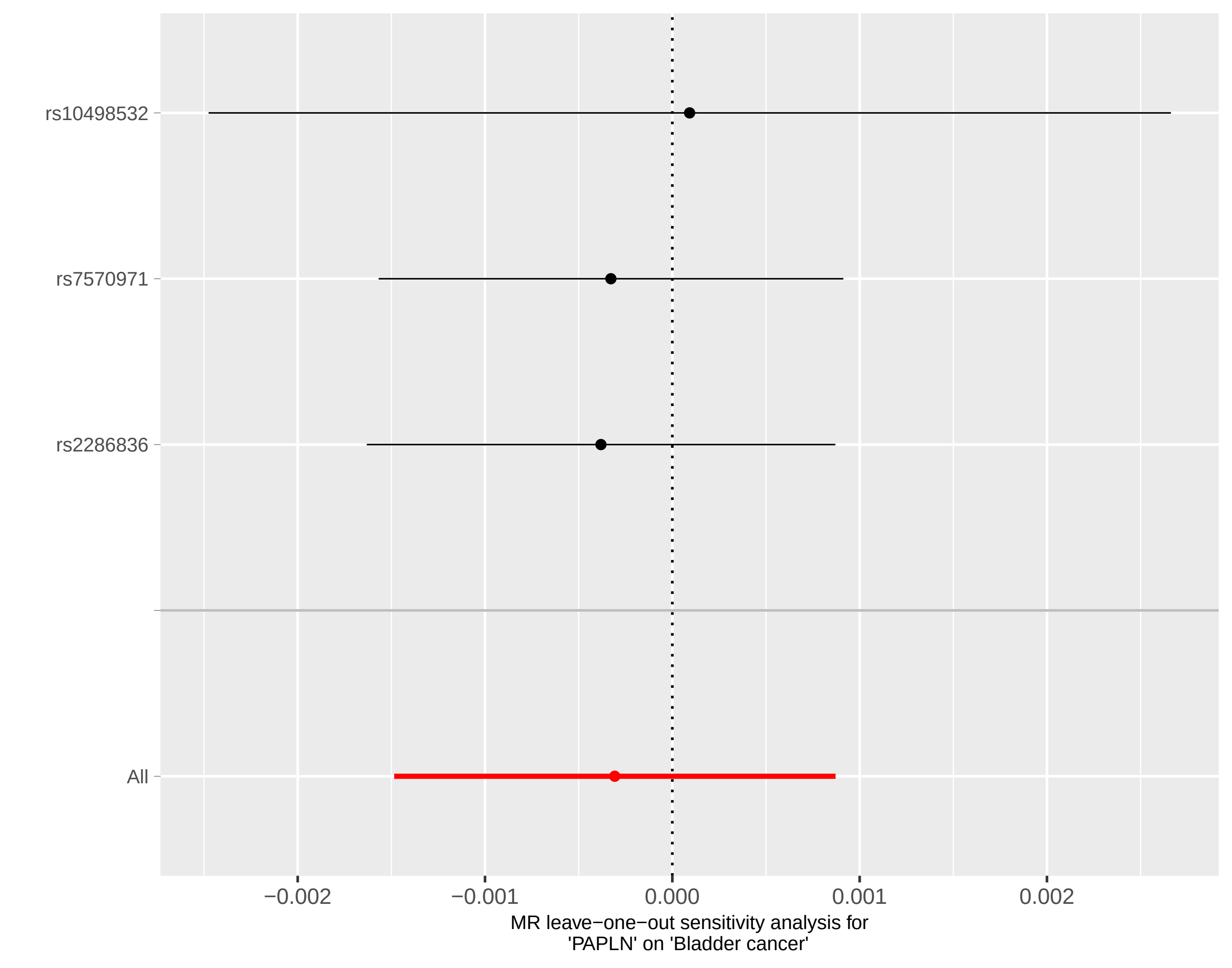
rs10498532
rs7570971
rs2286836
All
−0.002
−0.001
0.000
0.001
0.002
MR leave−one−out sensitivity analysis for
'PAPLN' on 'Bladder cancer'

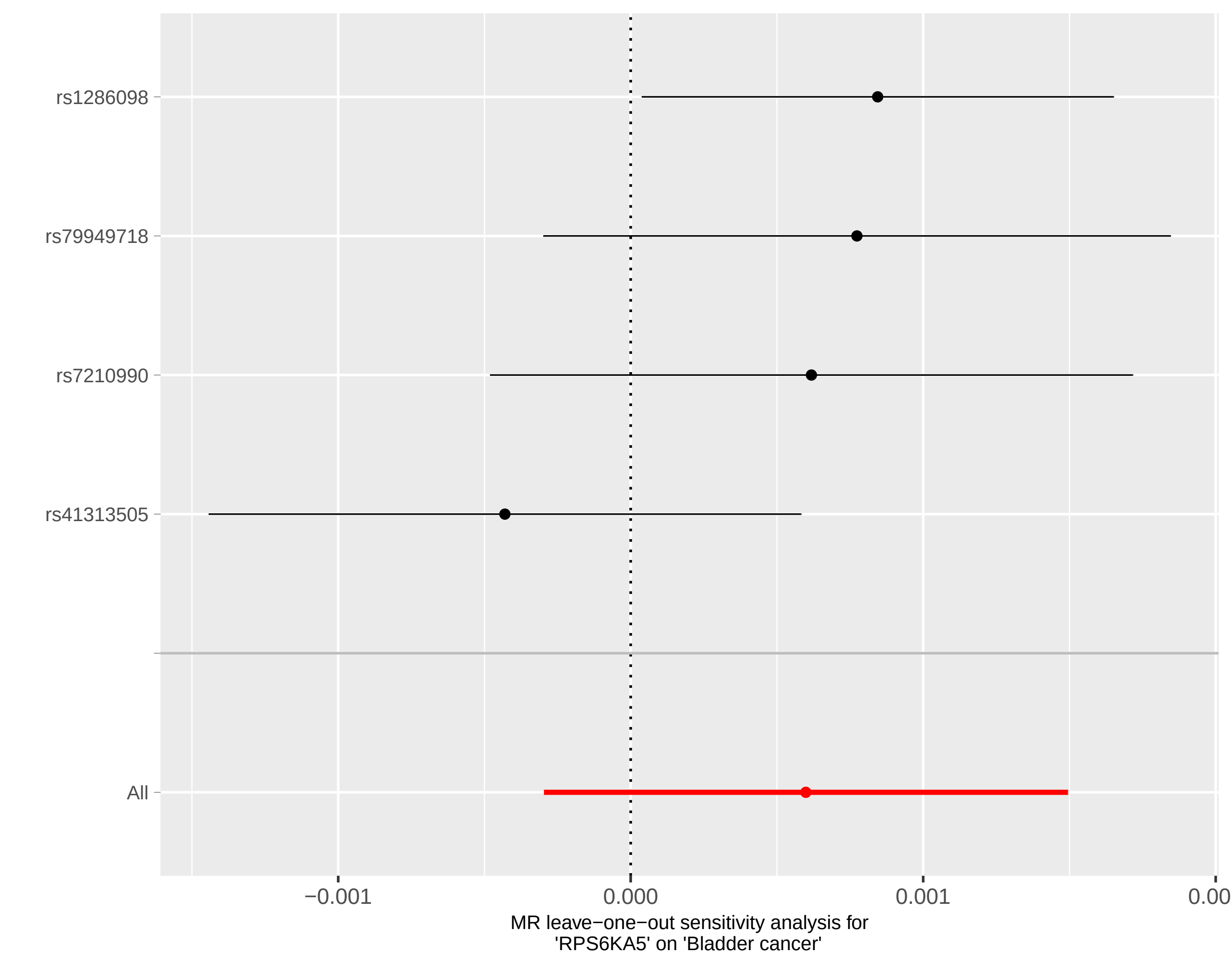
rs1286098
rs79949718
rs7210990
rs41313505
All
−0.001
0.000
0.001
0.00
MR leave−one−out sensitivity analysis for
'RPS6KA5' on 'Bladder cancer'

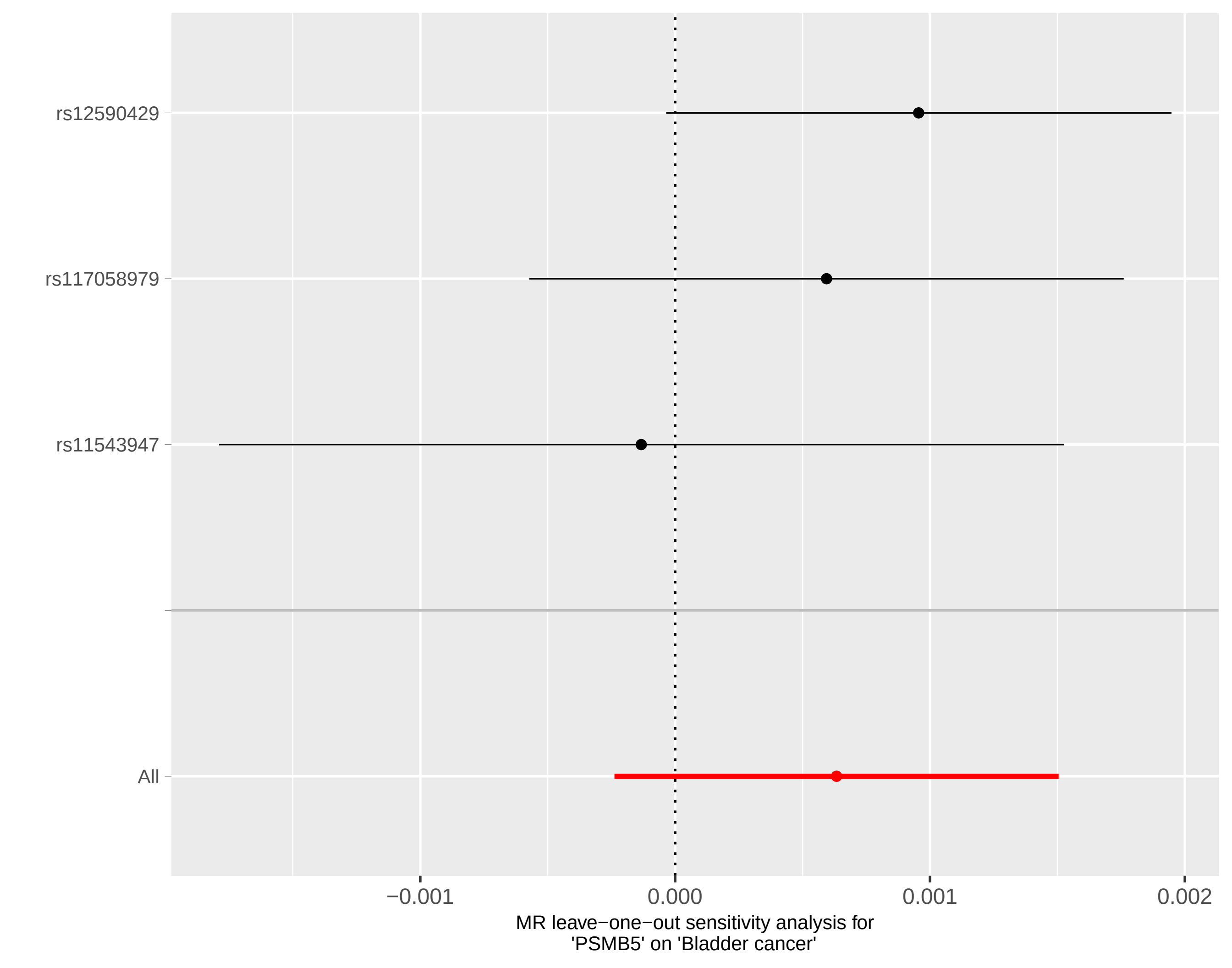
rs12590429
rs117058979
rs11543947
All
−0.001
0.000
0.001
0.002
MR leave−one−out sensitivity analysis for
'PSMB5' on 'Bladder cancer'

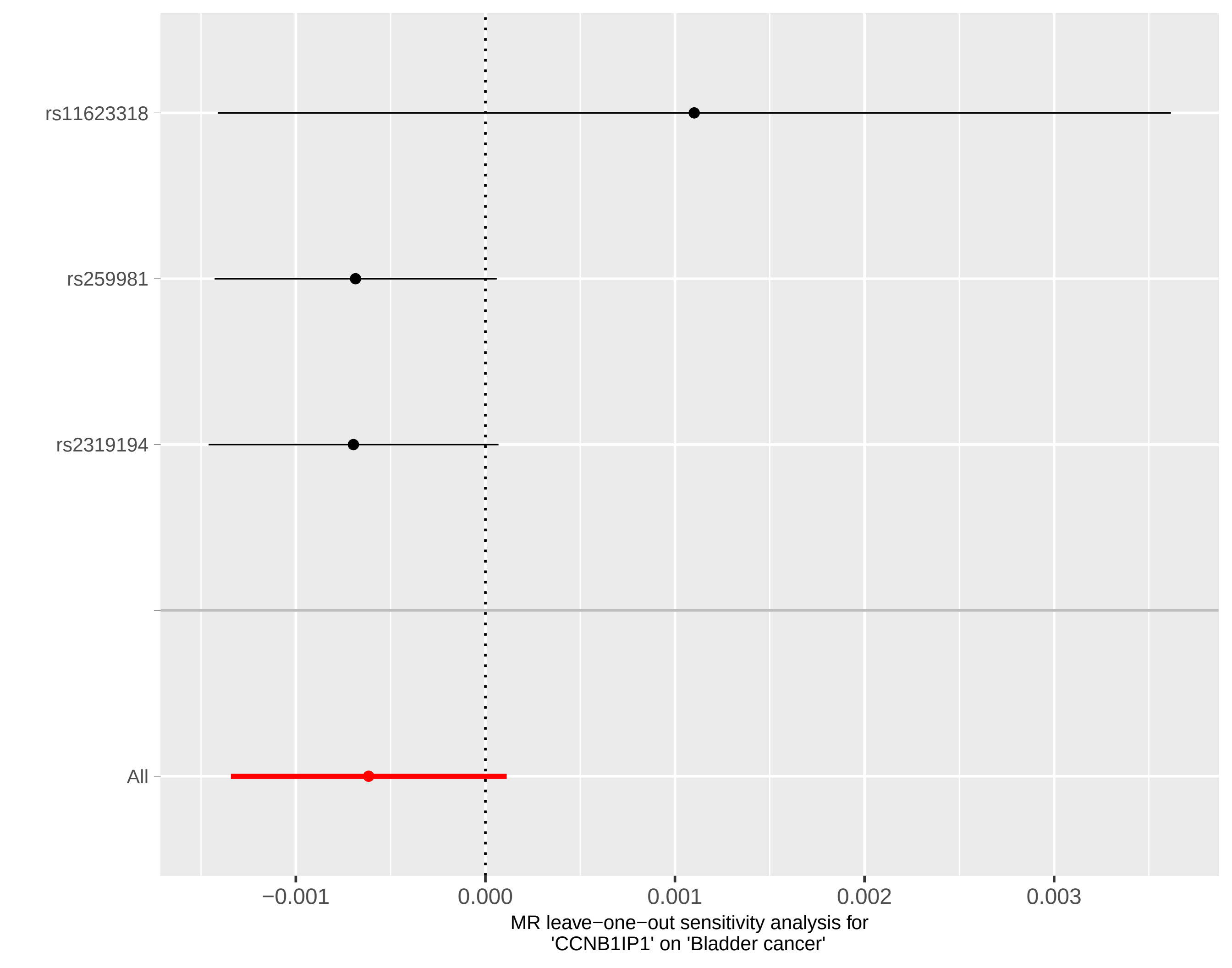
rs11623318
rs259981
rs2319194
All
−0.001
0.000
0.001
0.002
0.003
MR leave−one−out sensitivity analysis for
'CCNB1IP1' on 'Bladder cancer'

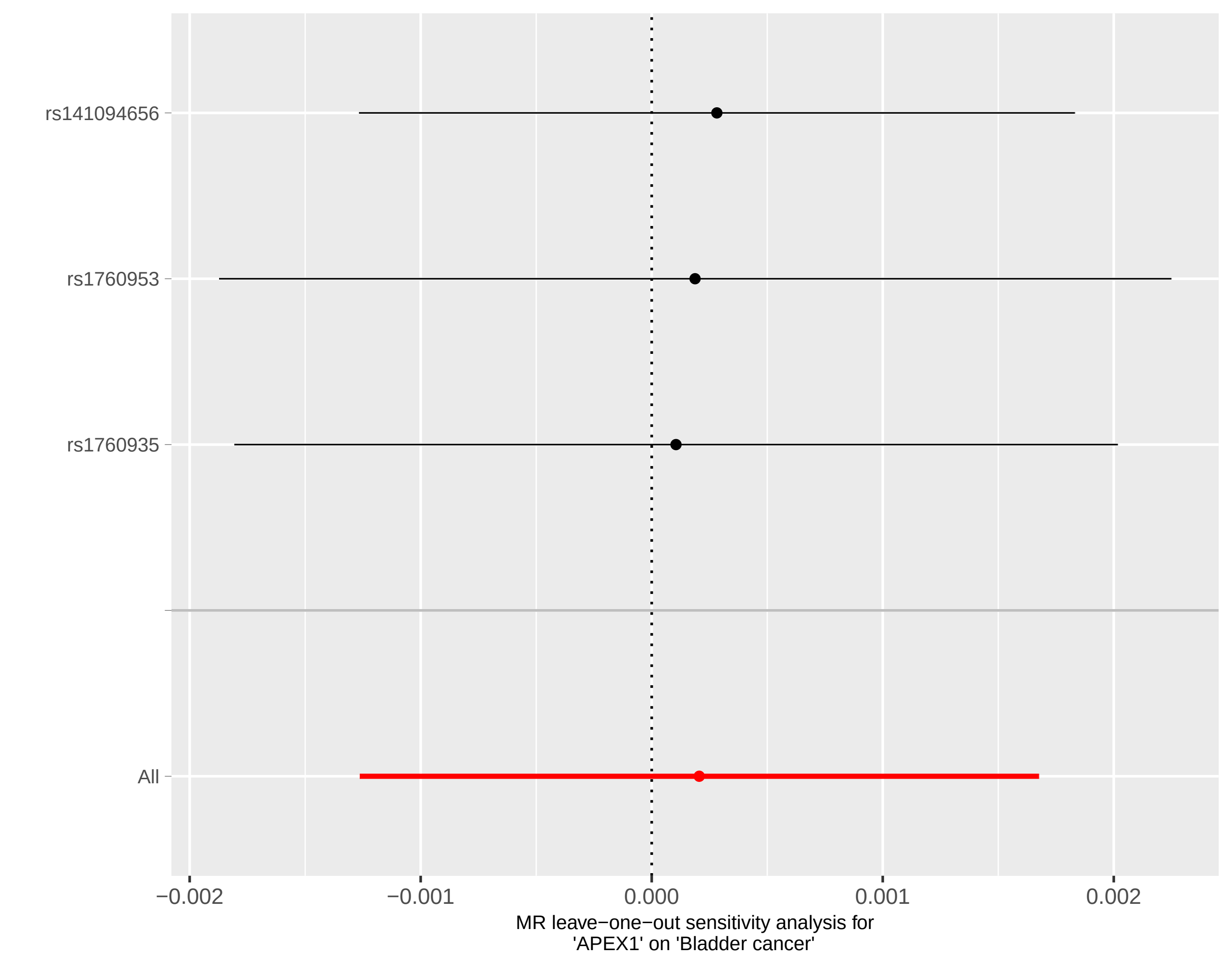
rs141094656
rs1760953
rs1760935
All
−0.002
−0.001
0.000
0.001
0.002
MR leave−one−out sensitivity analysis for
'APEX1' on 'Bladder cancer'

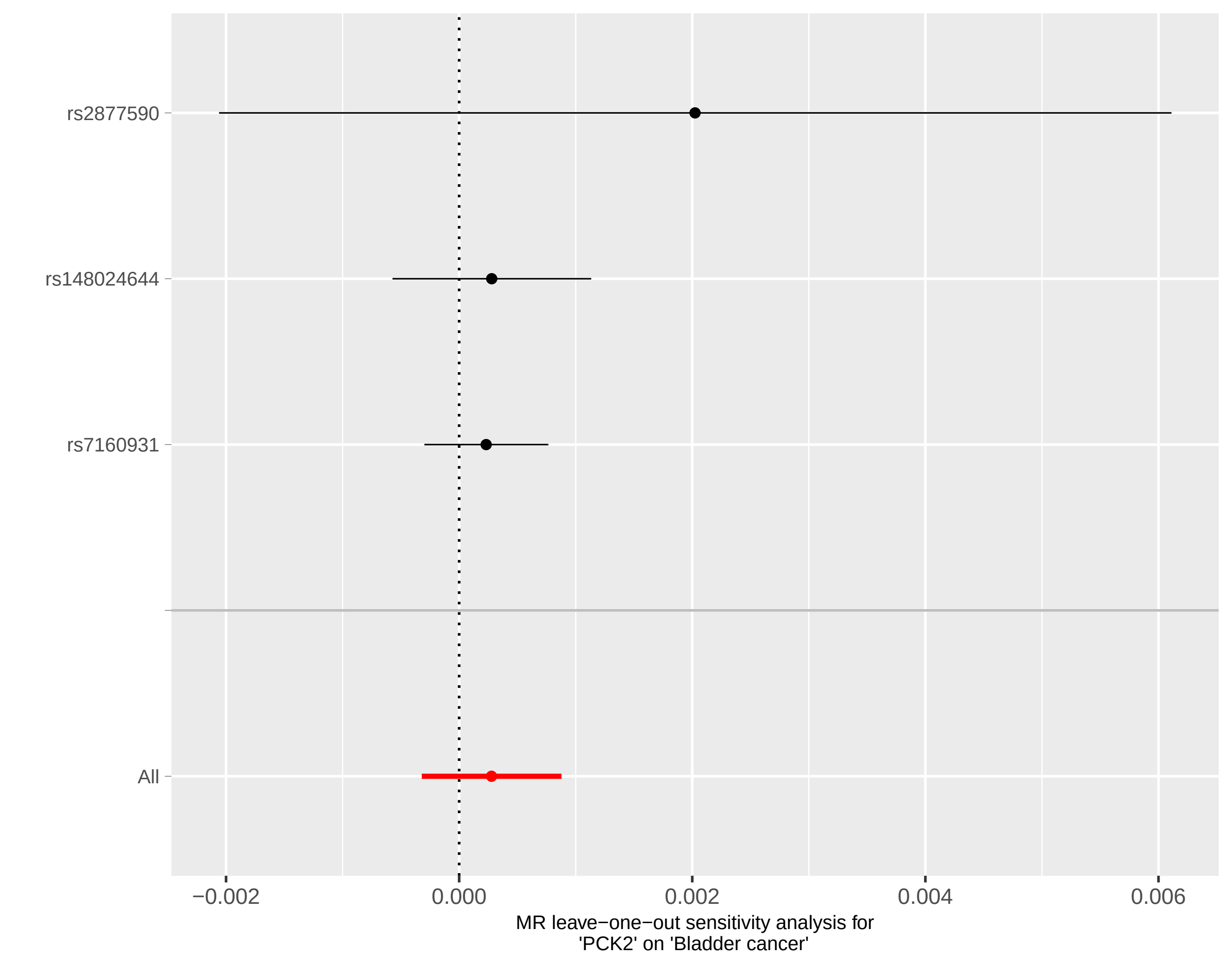
rs2877590
rs148024644
rs7160931
All
−0.002
0.000
0.002
0.004
0.006
MR leave−one−out sensitivity analysis for
'PCK2' on 'Bladder cancer'

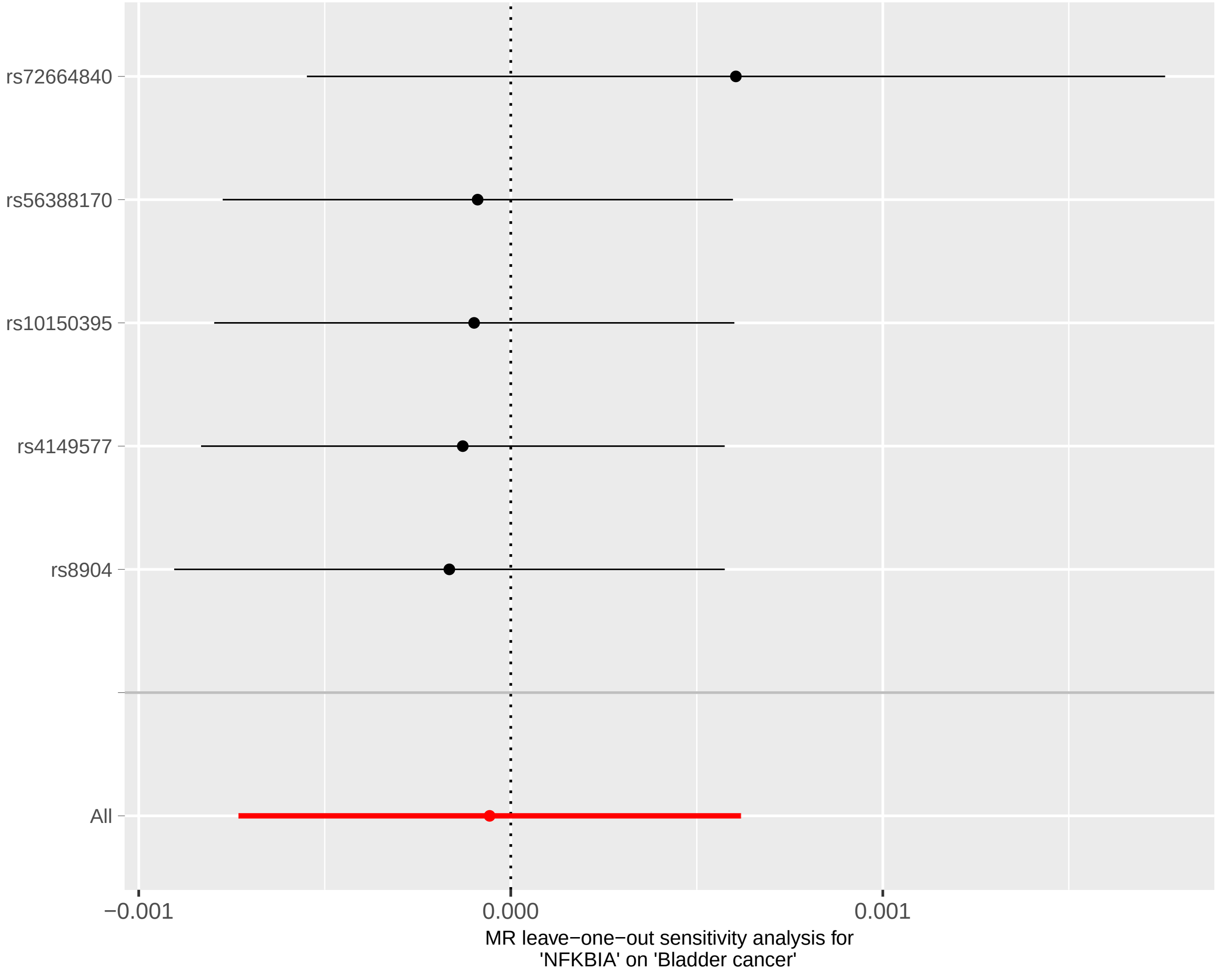
rs72664840
rs56388170
rs10150395
rs4149577
rs8904
All
−0.001
0.000
0.001
MR leave−one−out sensitivity analysis for
'NFKBIA' on 'Bladder cancer'

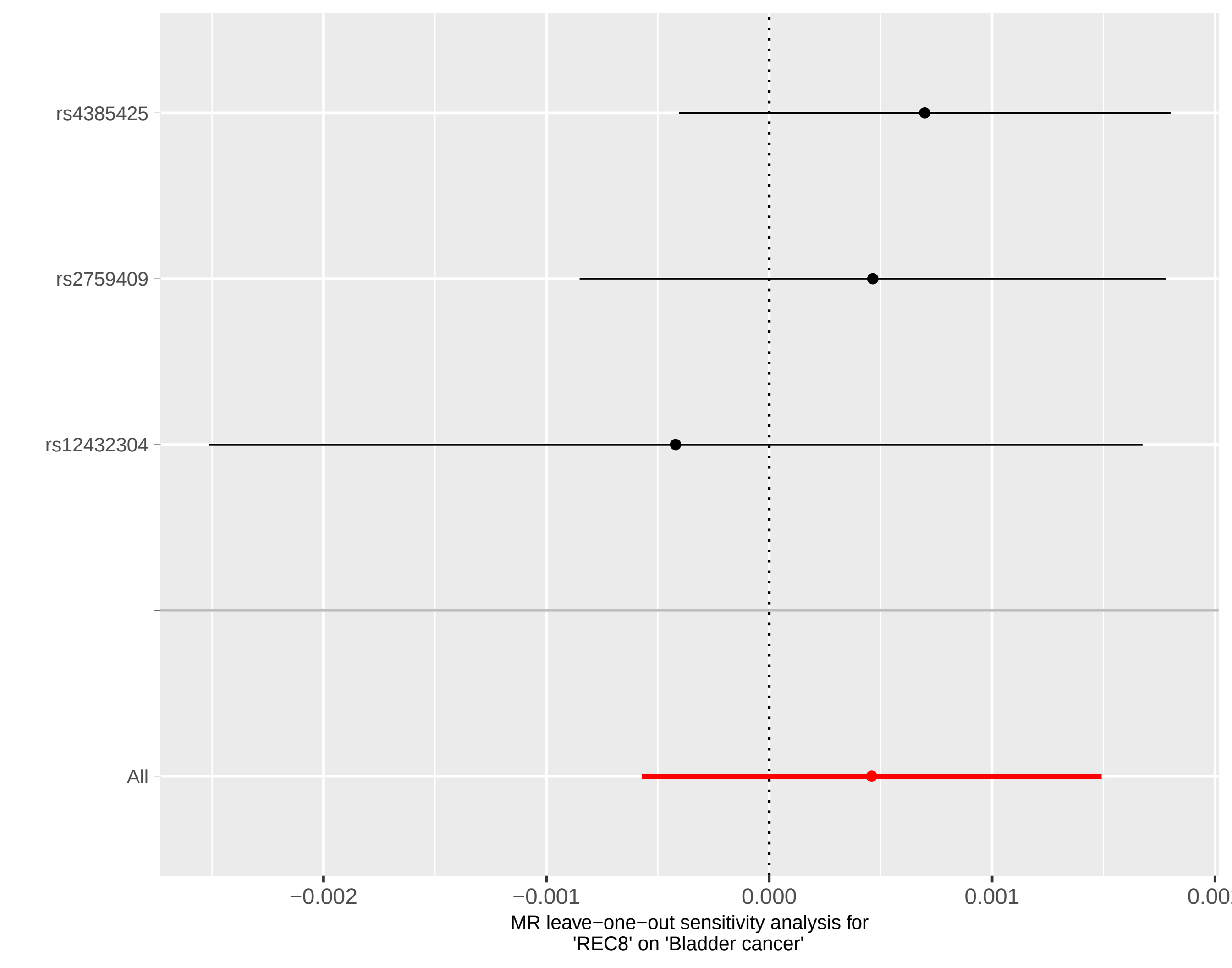
rs4385425
rs2759409
rs12432304
All
−0.002
−0.001
0.000
0.001
MR leave−one−out sensitivity analysis for
'REC8' on 'Bladder cancer'

Insufficient number of SNPs

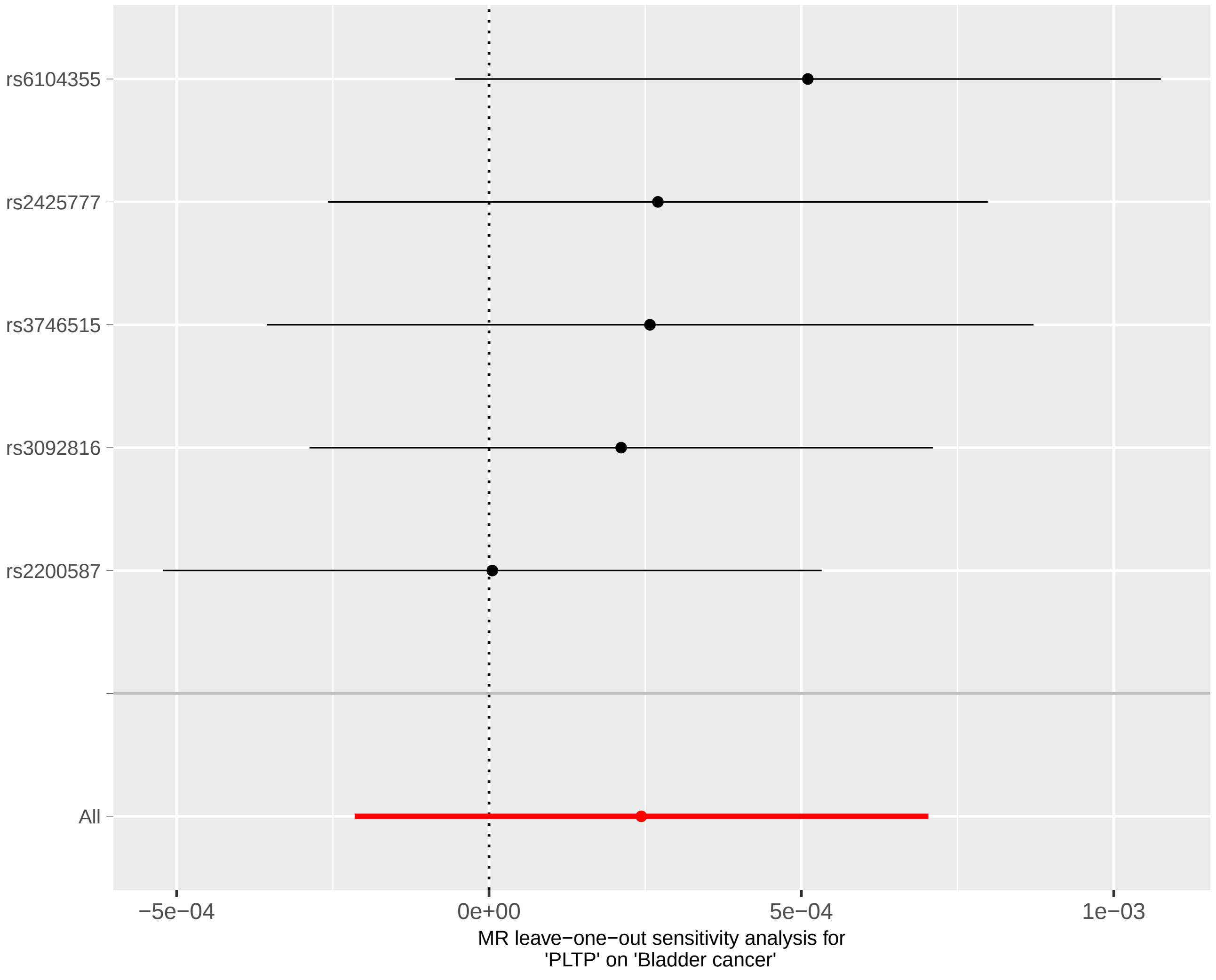
rs6104355
rs2425777
rs3746515
rs3092816
rs2200587
All
−5e−04
0e+00
5e−04
1e−03
MR leave−one−out sensitivity analysis for
'PLTP' on 'Bladder cancer'

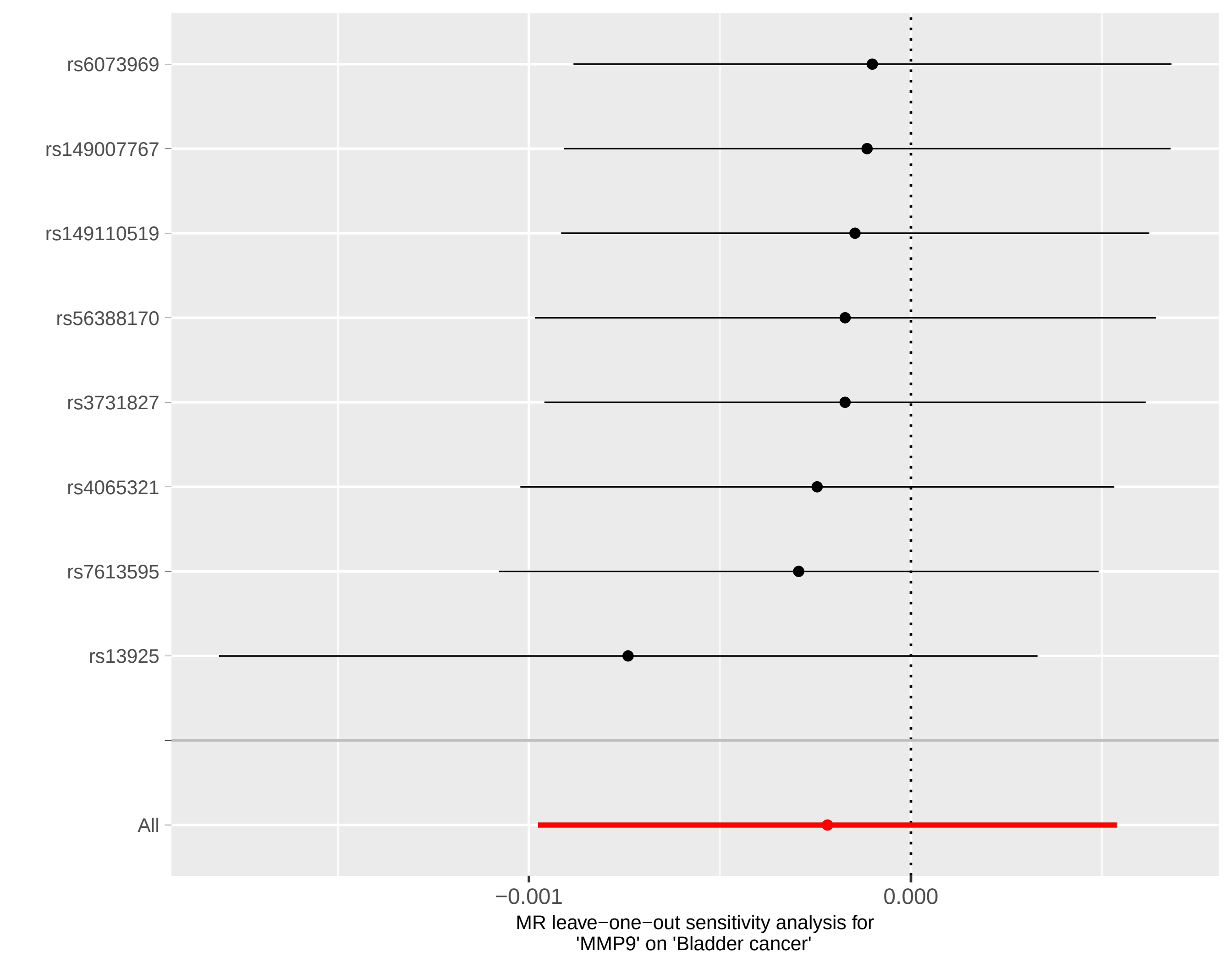

rs6073969
rs149007767
rs149110519
rs56388170
rs3731827
rs4065321
rs7613595
rs13925
All
−0.001
0.000
MR leave−one−out sensitivity analysis for
'MMP9' on 'Bladder cancer'

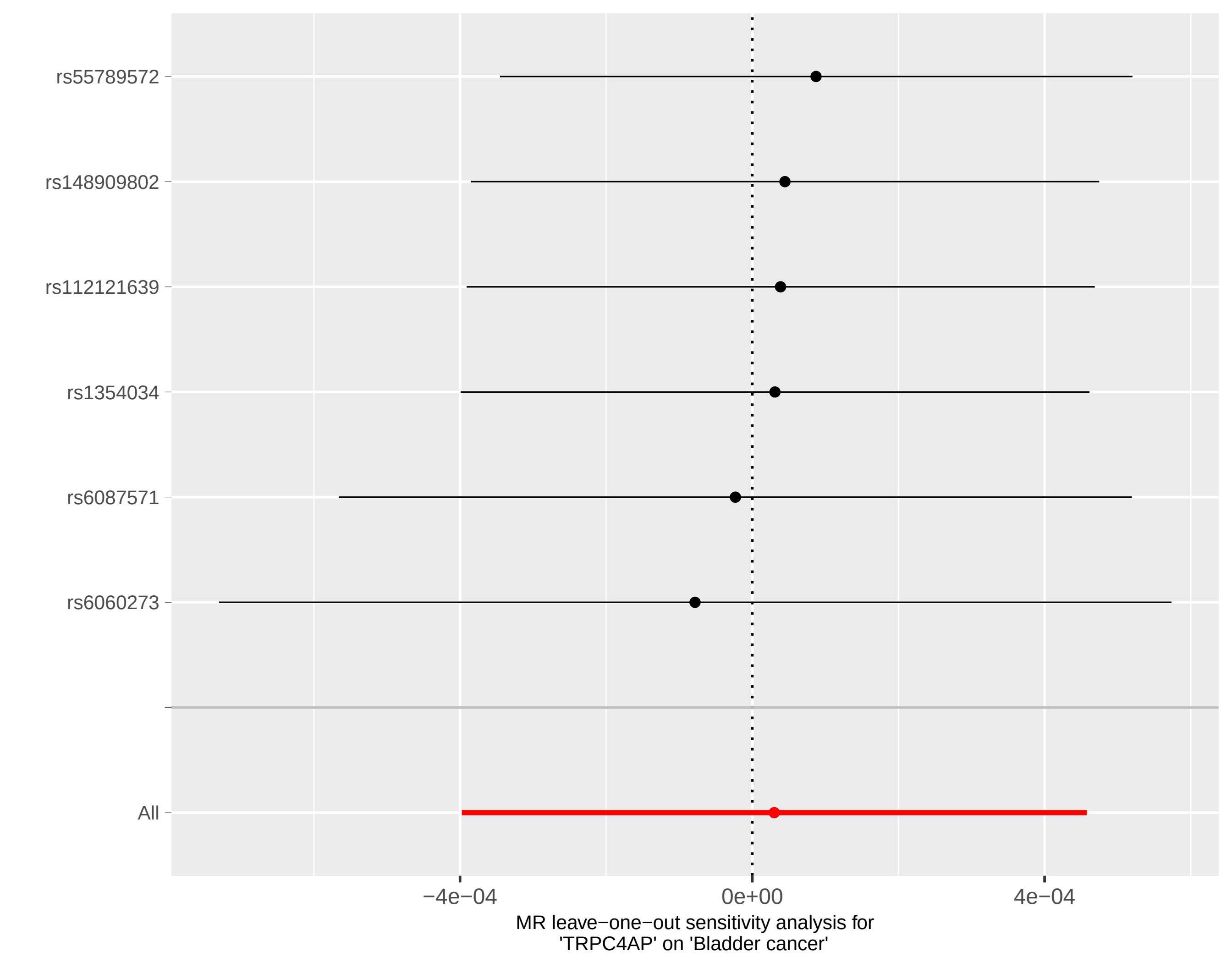
rs55789572
rs148909802
rs112121639
rs1354034
rs6087571
rs6060273
All
−4e−04
0e+00
4e−04
MR leave−one−out sensitivity analysis for
'TRPC4AP' on 'Bladder cancer'

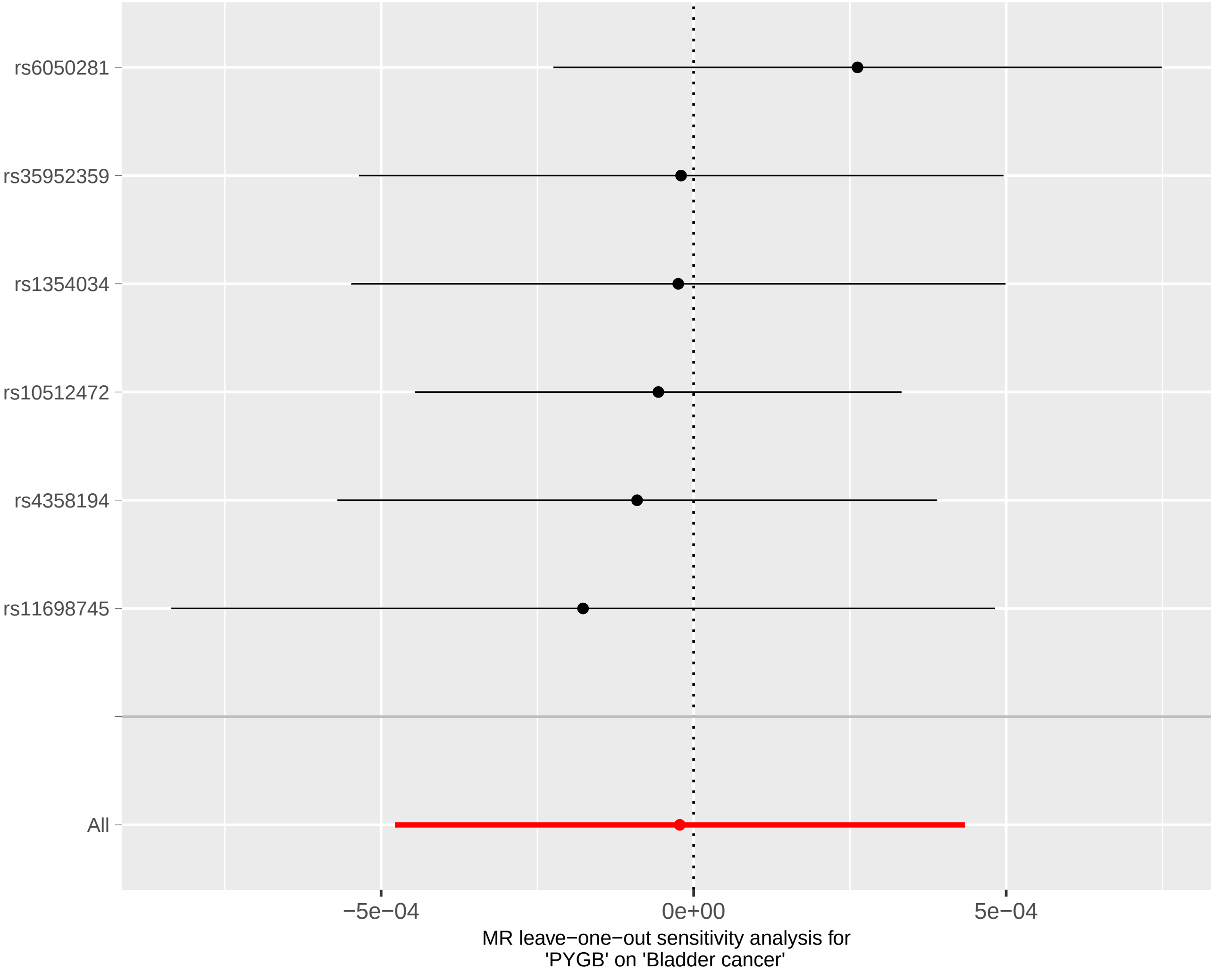
rs6050281
rs35952359
rs1354034
rs10512472
rs4358194
rs11698745
All
−5e−04
0e+00
5e−04
MR leave−one−out sensitivity analysis for
'PYGB' on 'Bladder cancer'

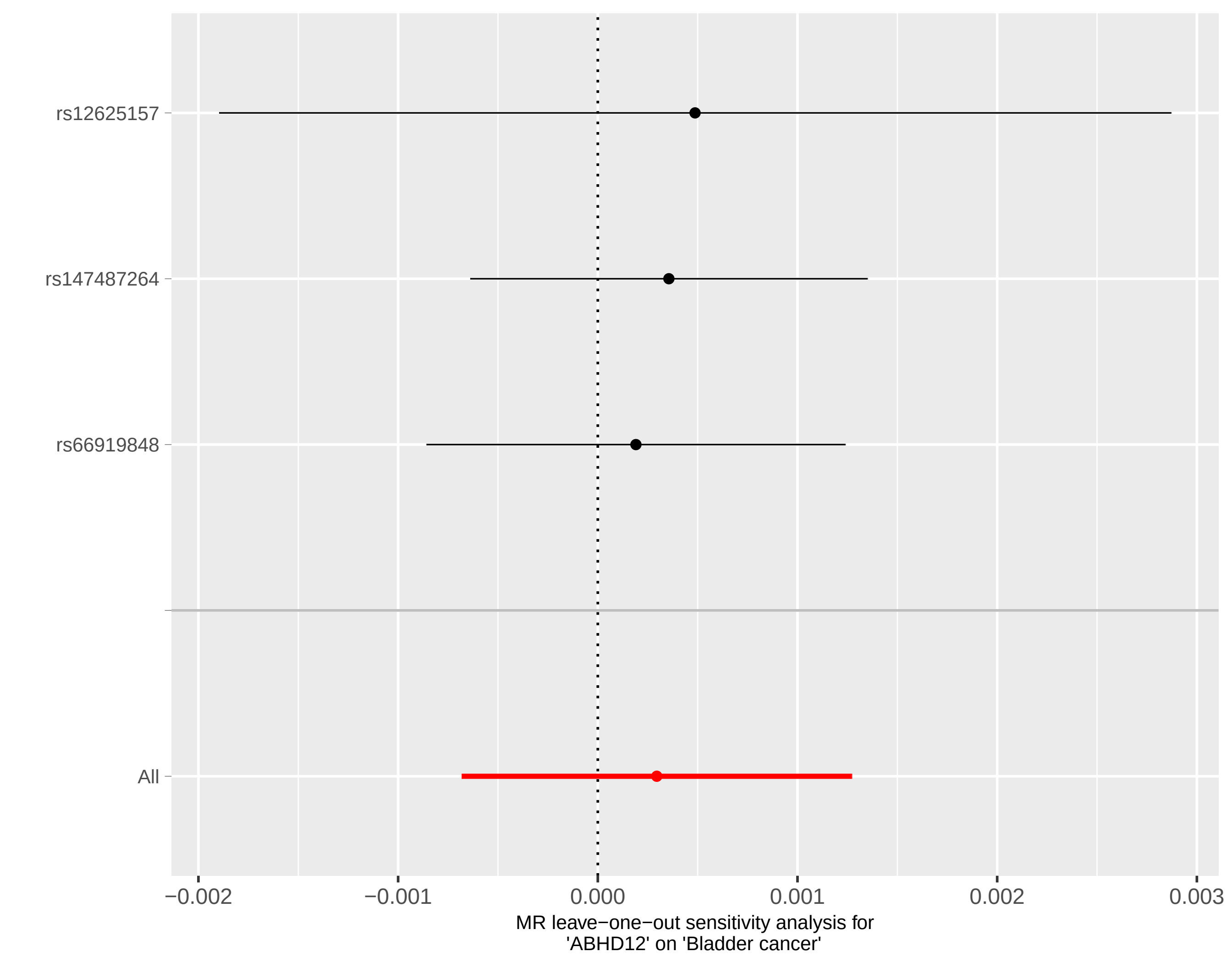
rs12625157
rs147487264
rs66919848
All
−0.002
−0.001
0.000
0.001
0.002
0.003
MR leave−one−out sensitivity analysis for
'ABHD12' on 'Bladder cancer'

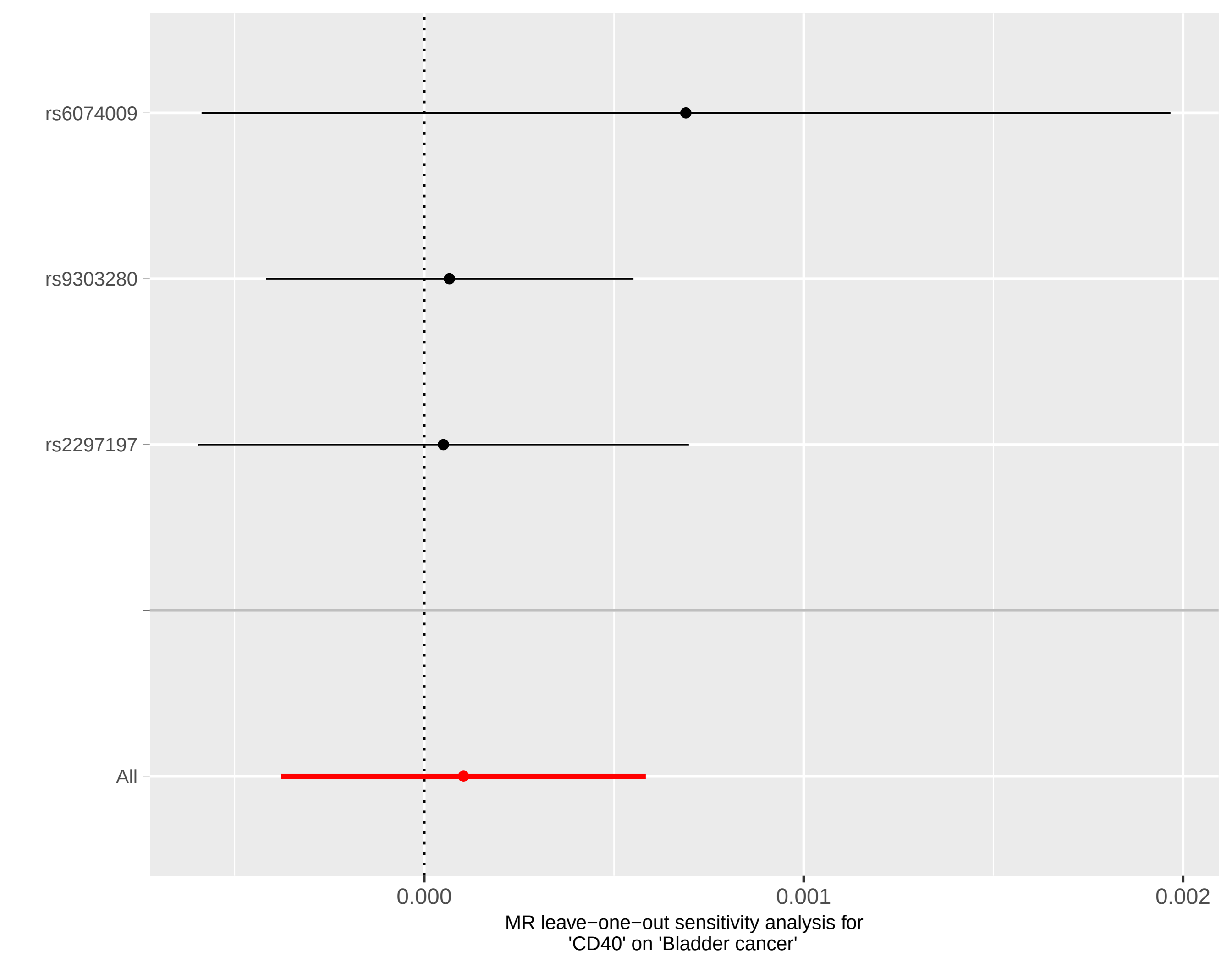
rs6074009
rs9303280
rs2297197
All
0.000
0.001
0.002
MR leave−one−out sensitivity analysis for
'CD40' on 'Bladder cancer'

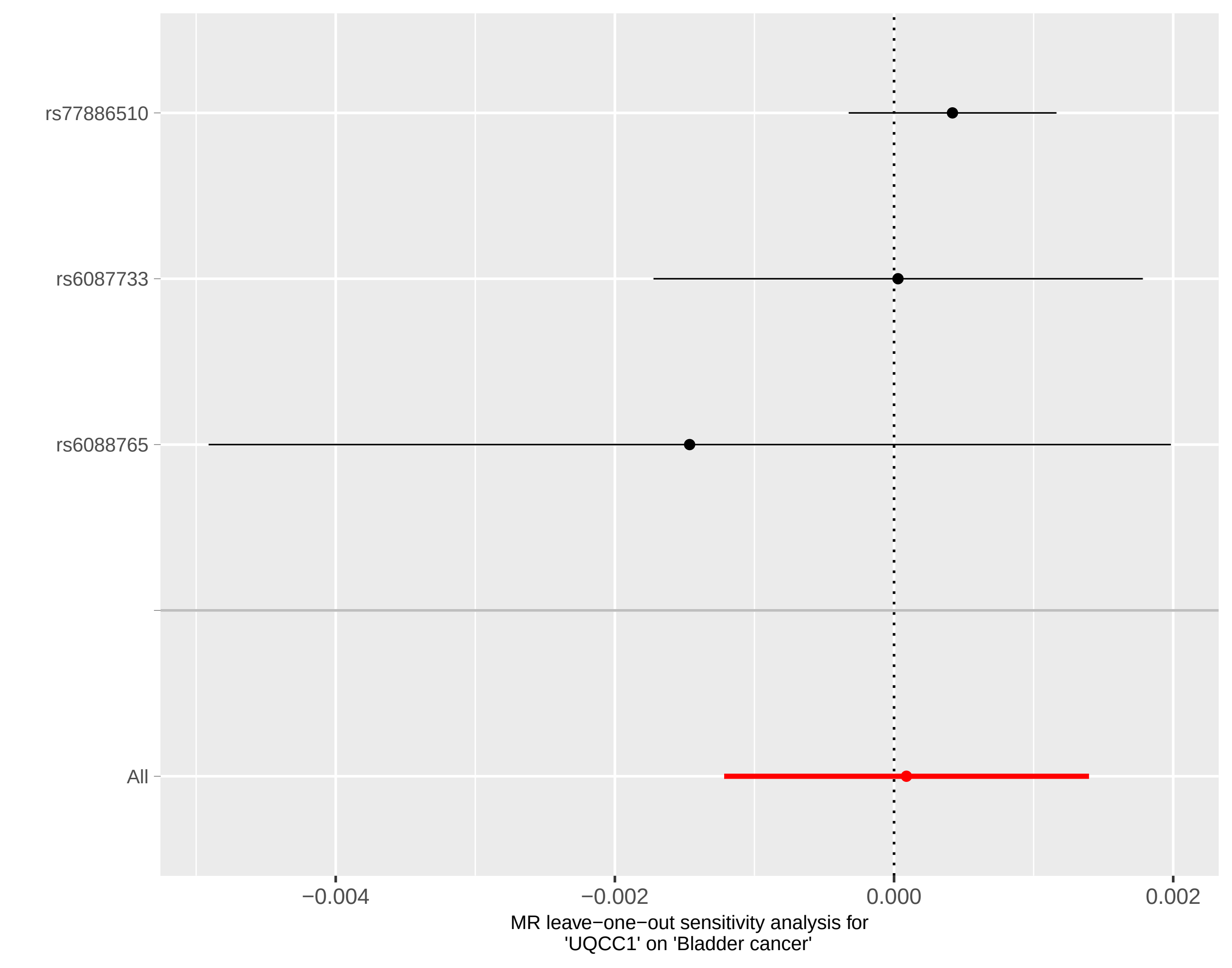
rs77886510
rs6087733
rs6088765
All
−0.004
−0.002
0.000
0.002
MR leave−one−out sensitivity analysis for
'UQCC1' on 'Bladder cancer'

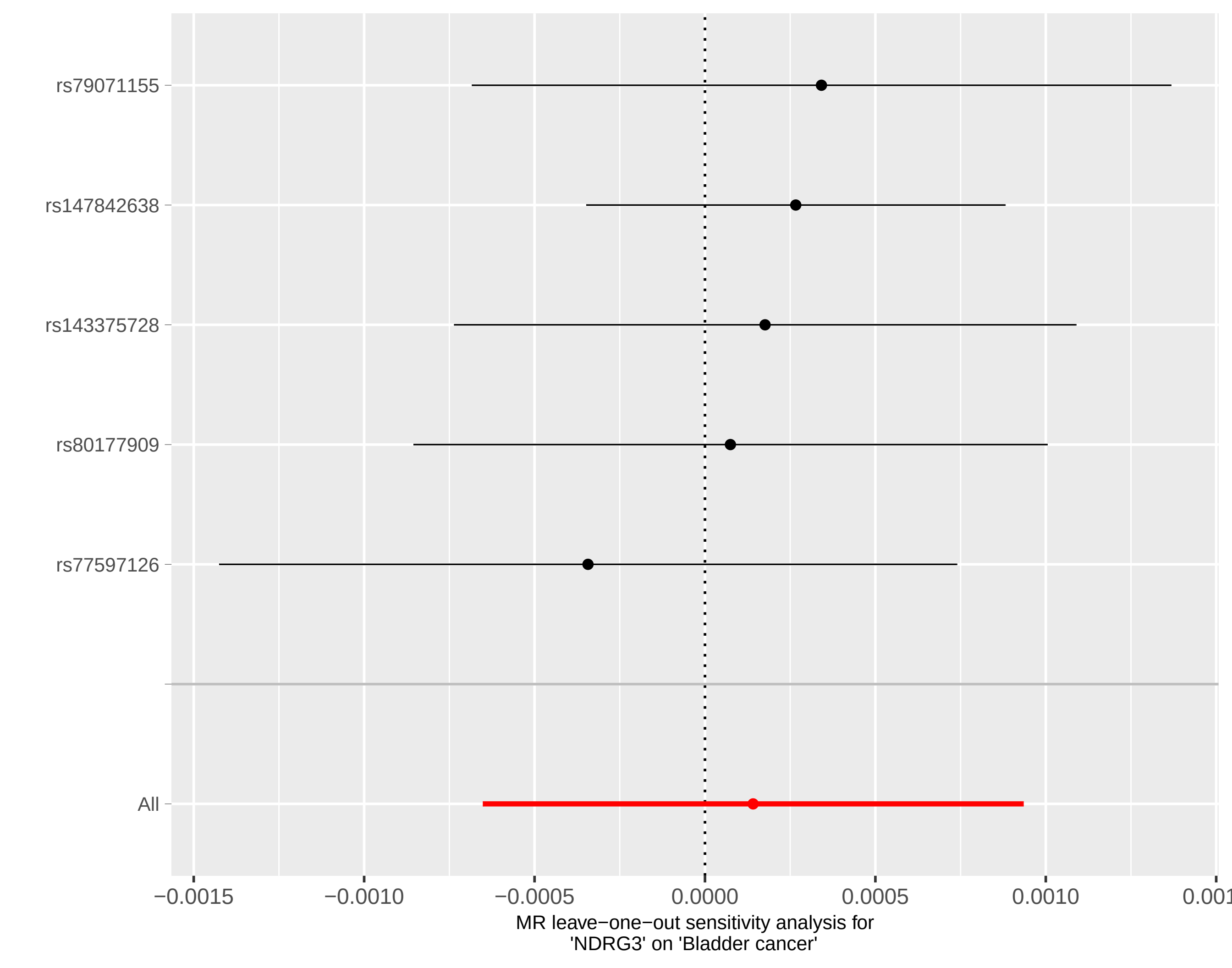
rs79071155
rs147842638
rs143375728
rs80177909
rs77597126
All
−0.0015
−0.0010
−0.0005
0.0000
0.0005
0.0010
MR leave−one−out sensitivity analysis for
'NDRG3' on 'Bladder cancer'

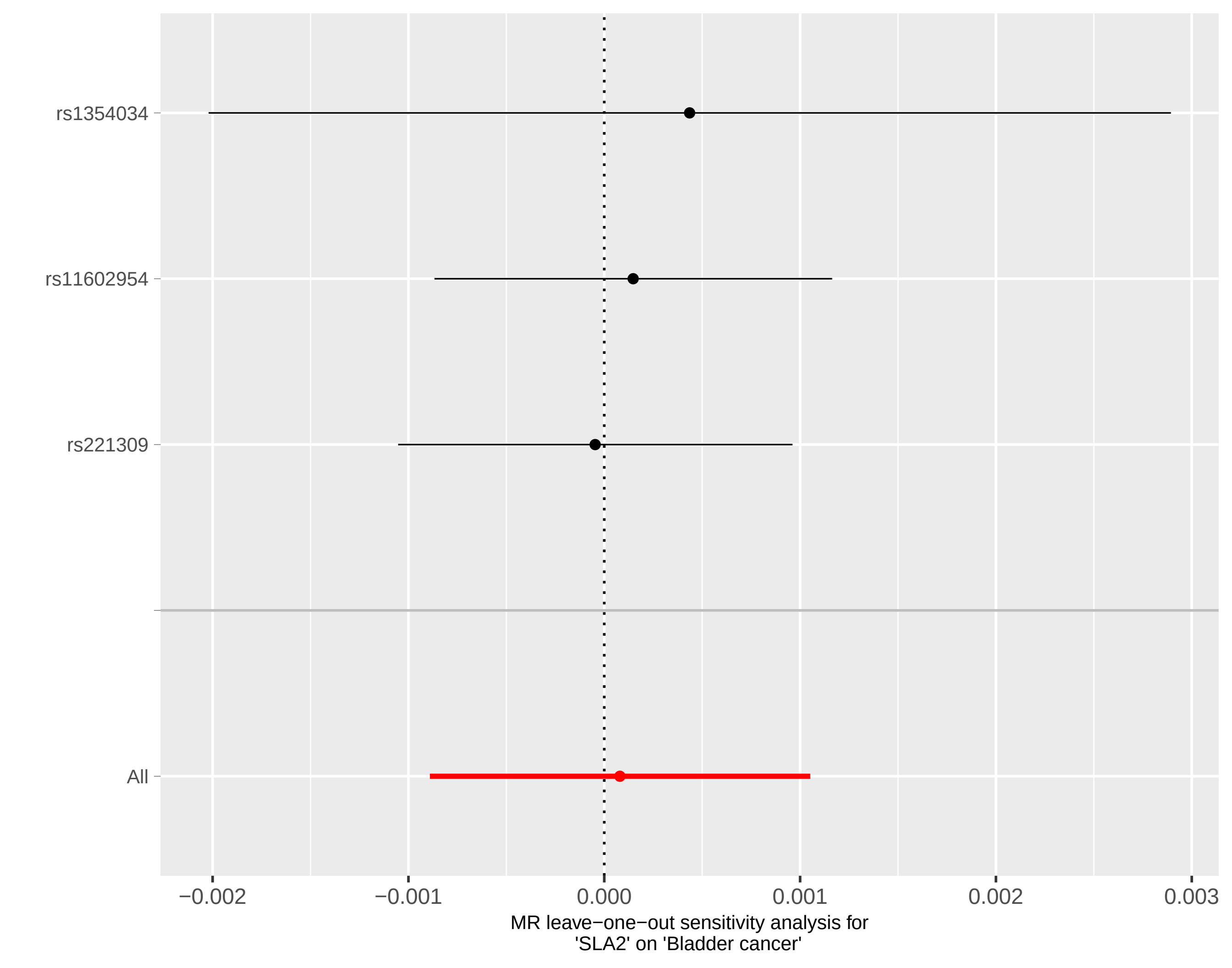
rs1354034
rs11602954
rs221309
All
−0.002
−0.001
0.000
0.001
0.002
0.003
MR leave−one−out sensitivity analysis for
'SLA2' on 'Bladder cancer'

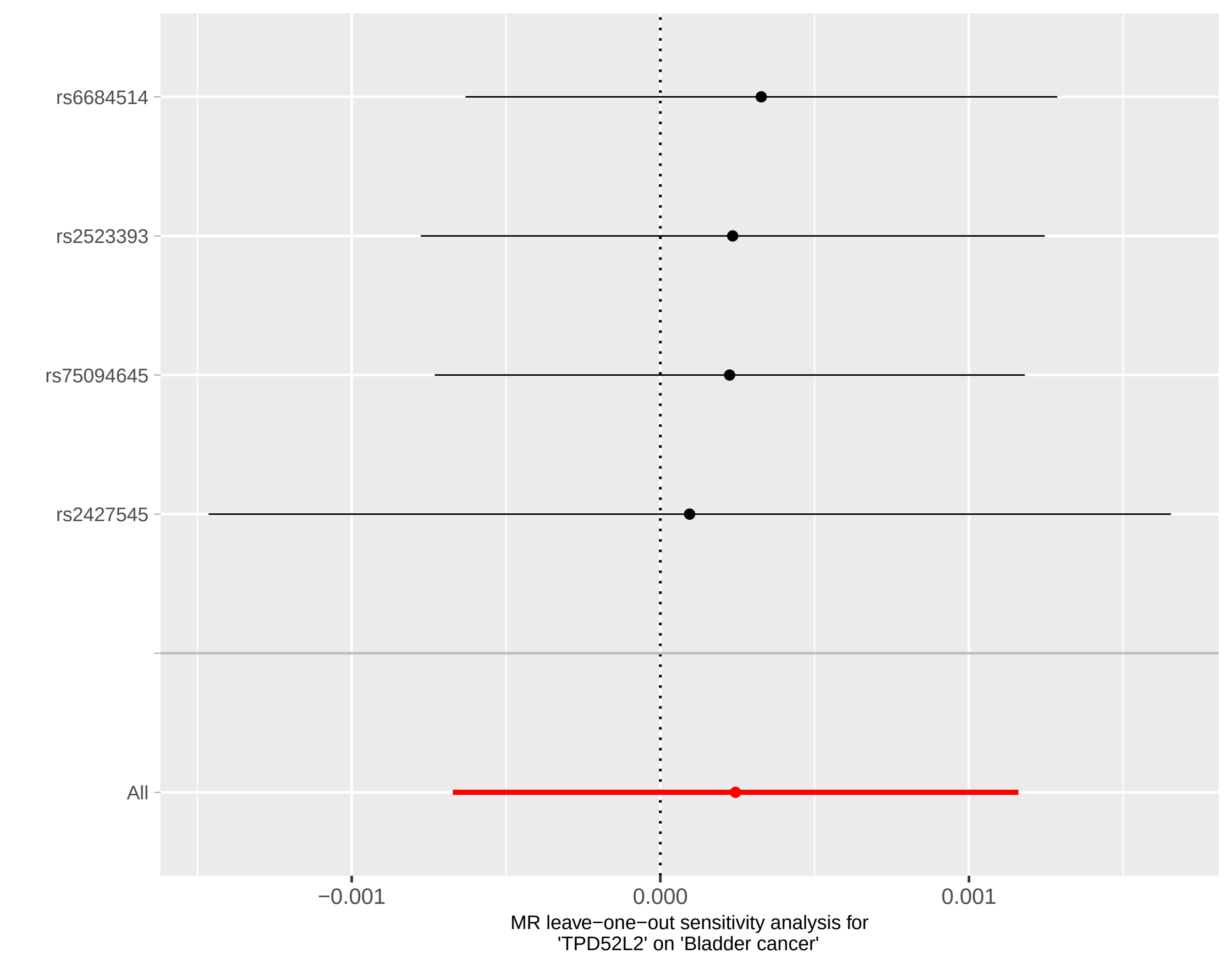
rs6684514
rs2523393
rs75094645
rs2427545
All
−0.001
0.000
0.001
MR leave−one−out sensitivity analysis for
'TPD52L2' on 'Bladder cancer'

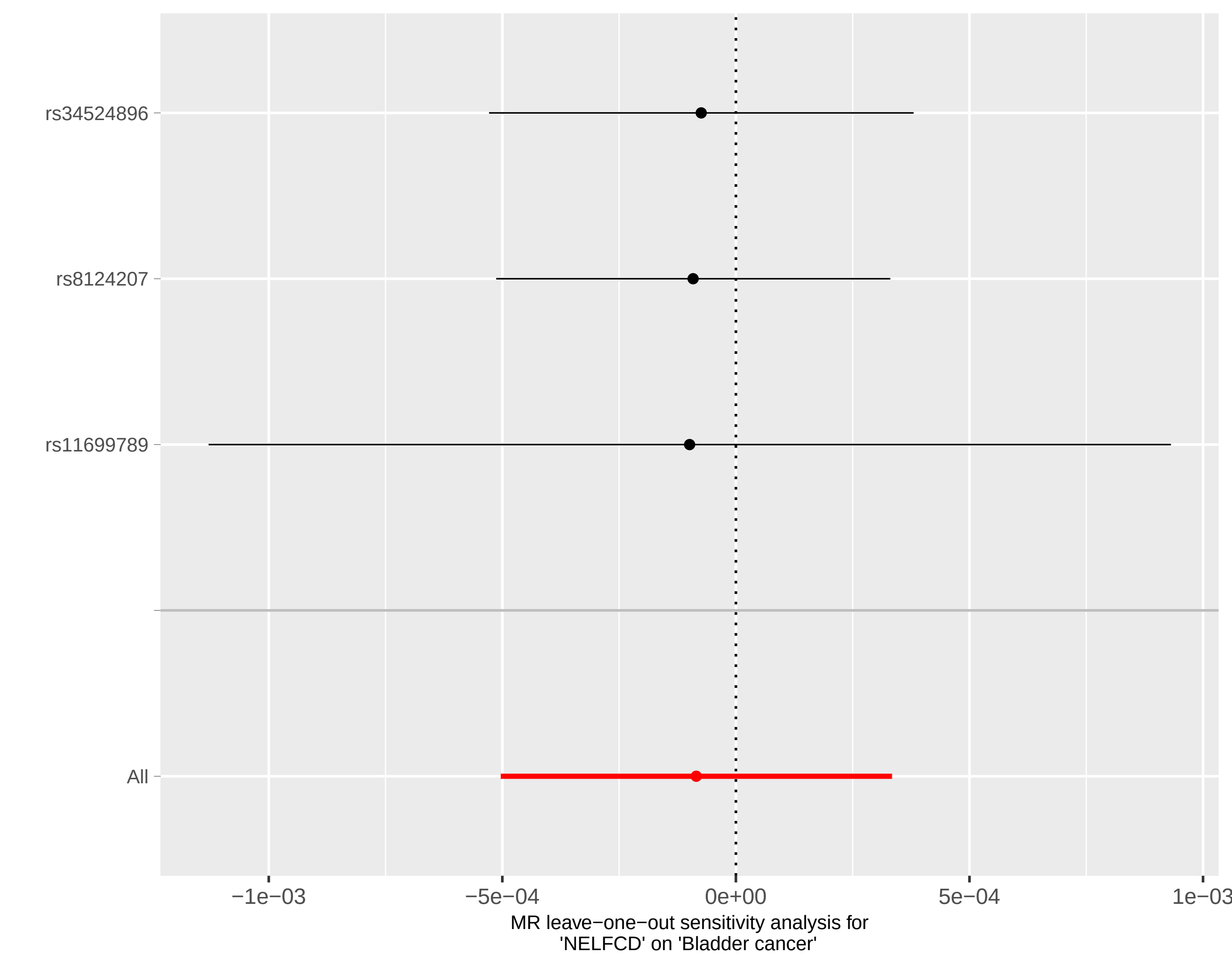
rs34524896
rs8124207
rs11699789
All
−1e−03
−5e−04
0e+00
5e−04
1e−03
MR leave−one−out sensitivity analysis for
'NELFCD' on 'Bladder cancer'

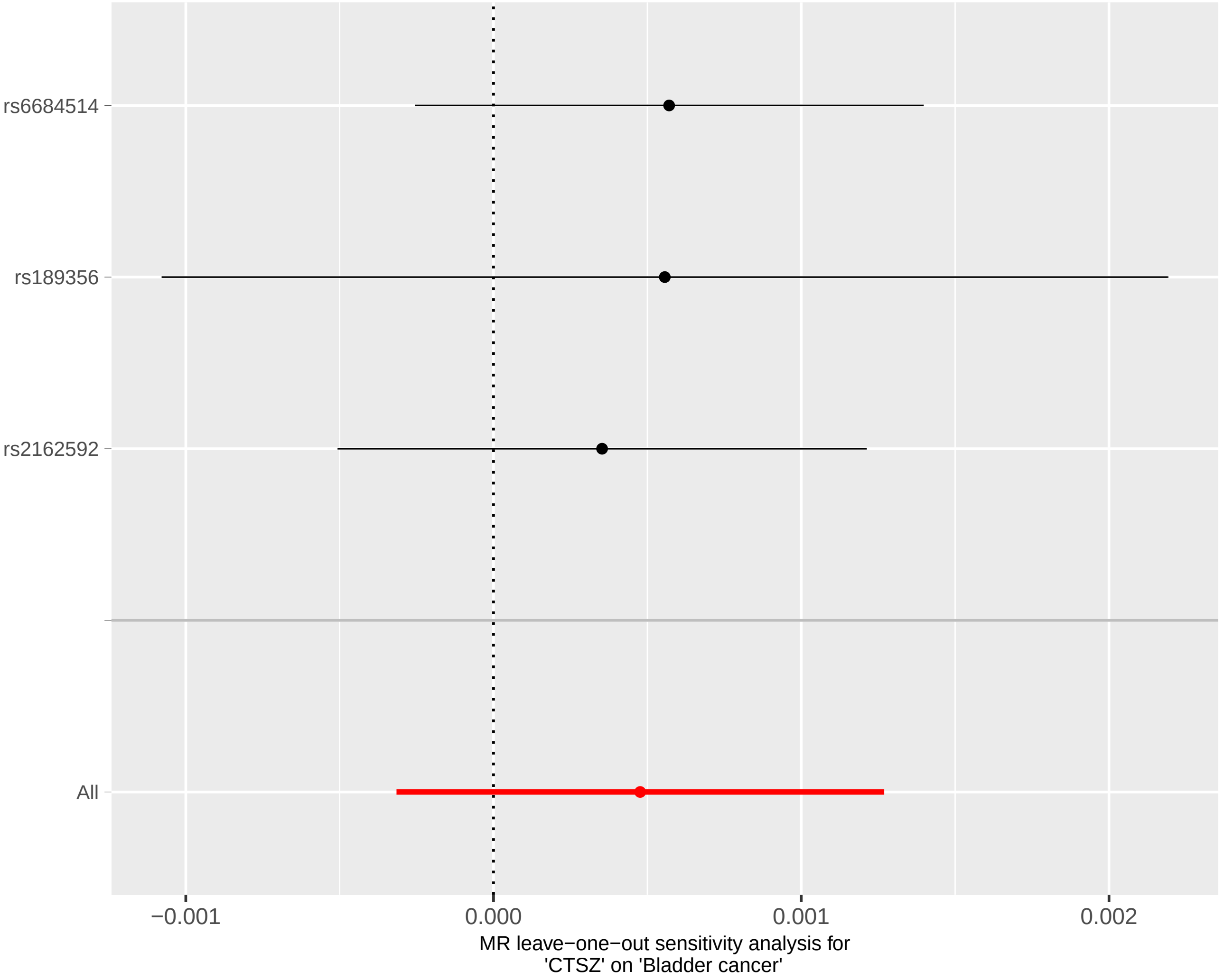
rs6684514
rs189356
rs2162592
All
−0.001
0.000
0.001
0.002
MR leave−one−out sensitivity analysis for
'CTSZ' on 'Bladder cancer'

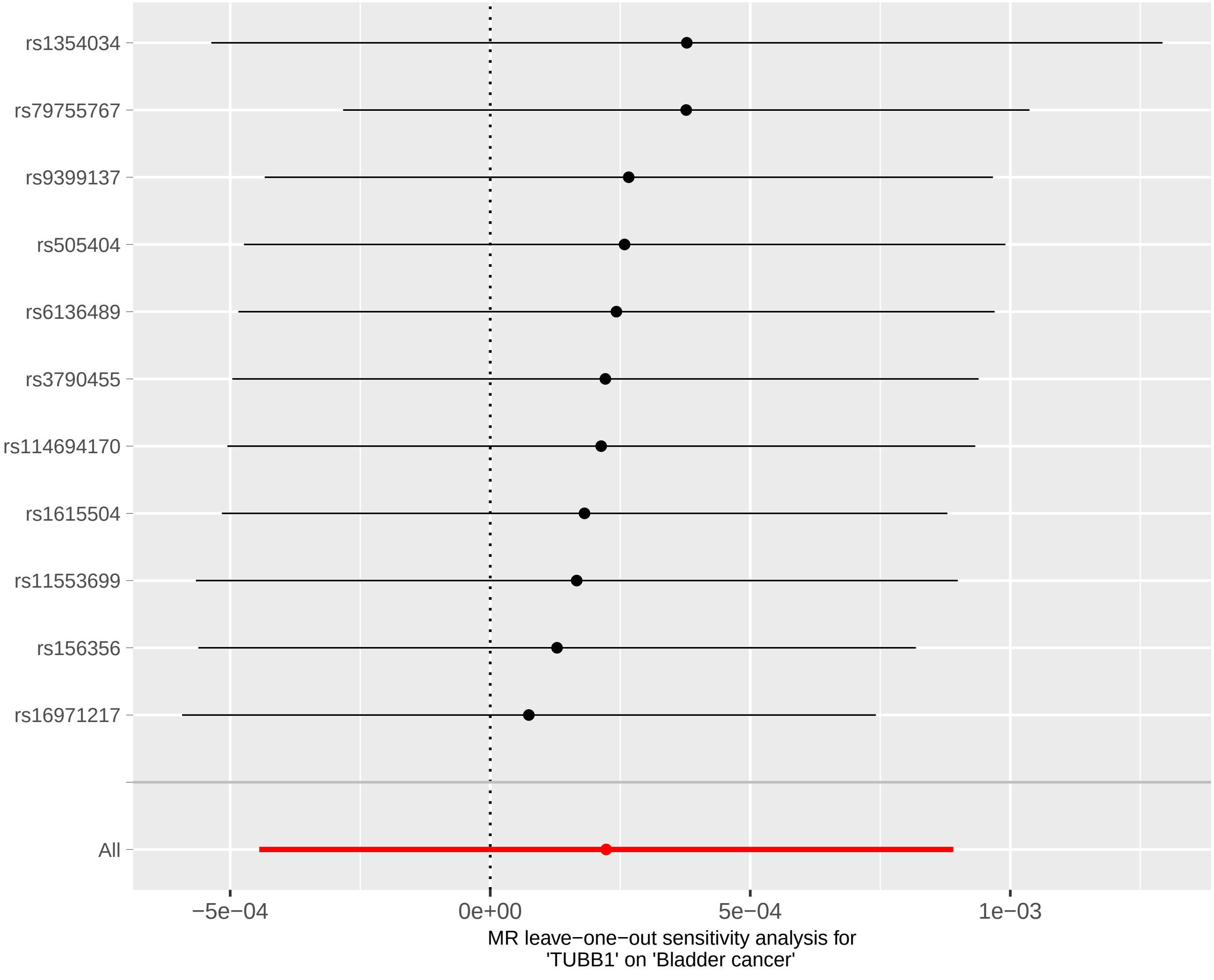
rs1354034
rs79755767
rs9399137
rs505404
rs6136489
rs3790455
rs114694170
rs1615504
rs11553699
rs156356
rs16971217
All
−5e−04
0e+00
5e−04
1e−03
MR leave−one−out sensitivity analysis for
'TUBB1' on 'Bladder cancer'

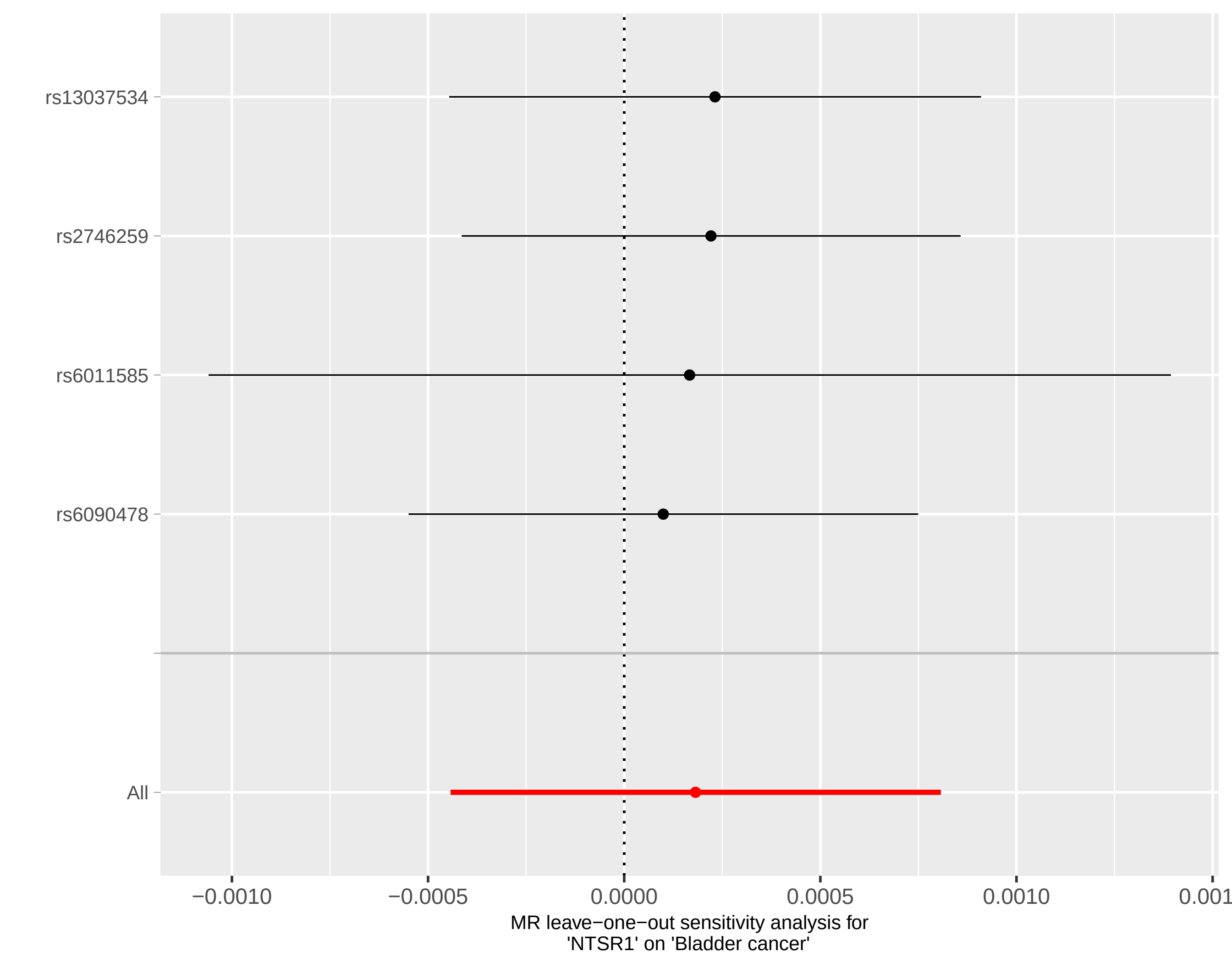
rs13037534
rs2746259
rs6011585
rs6090478
All
−0.0010
−0.0005
0.0000
0.0005
0.0010
0.001
MR leave−one−out sensitivity analysis for
'NTSR1' on 'Bladder cancer'

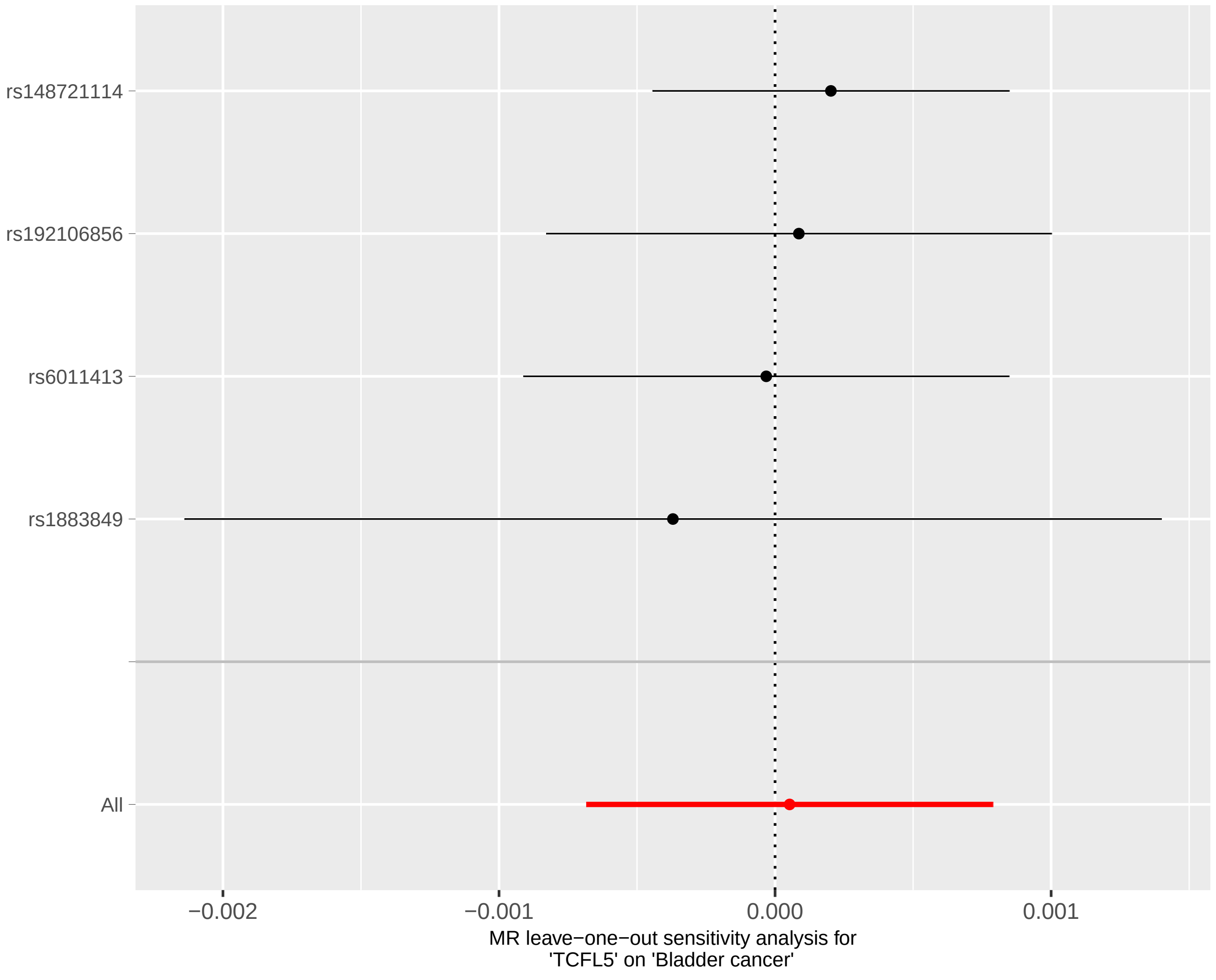
rs148721114
rs192106856
rs6011413
rs1883849
All
−0.002
−0.001
0.000
0.001
MR leave−one−out sensitivity analysis for
'TCFL5' on 'Bladder cancer'

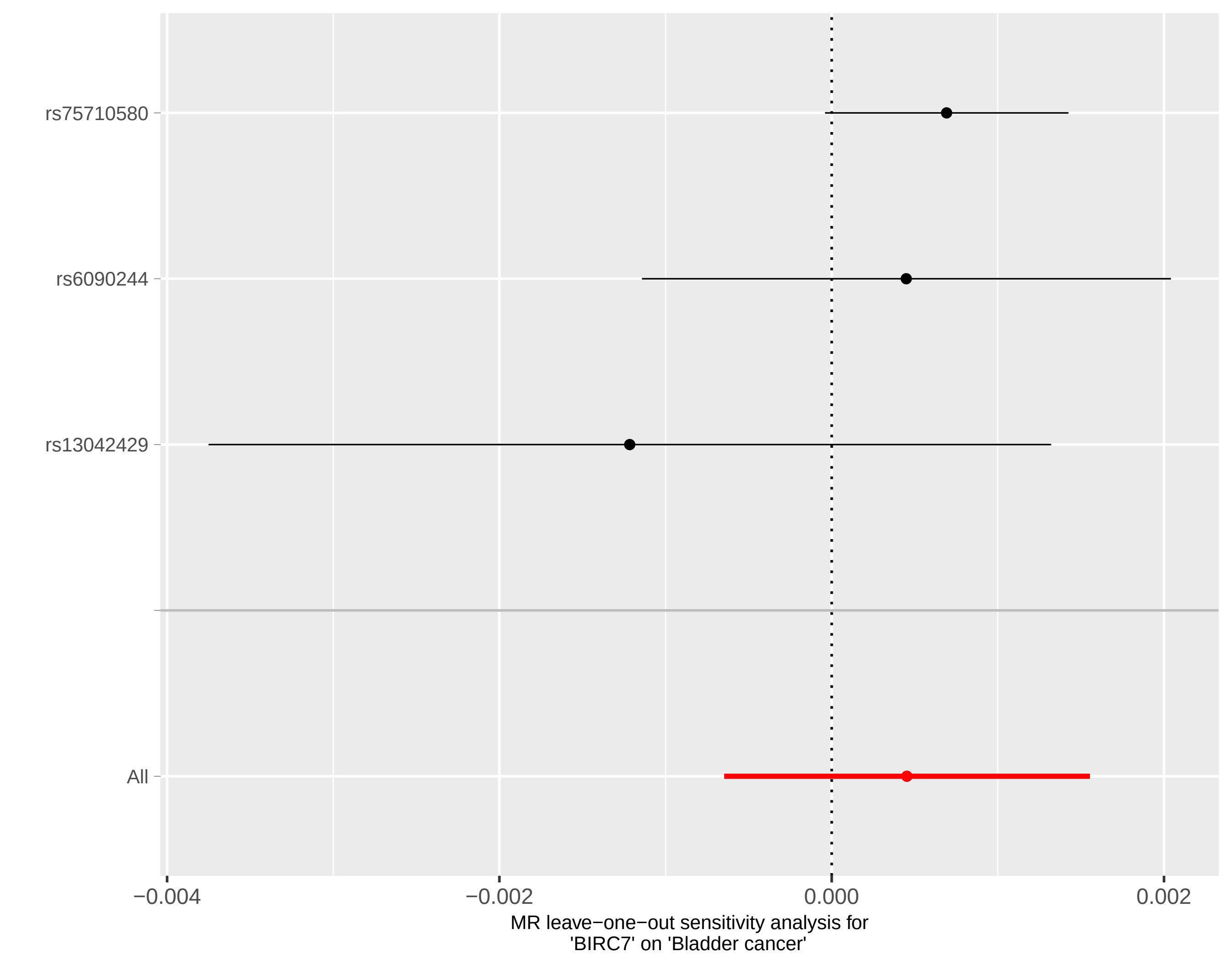
rs75710580
rs6090244
rs13042429
All
−0.004
−0.002
0.000
0.002
MR leave−one−out sensitivity analysis for
'BIRC7' on 'Bladder cancer'

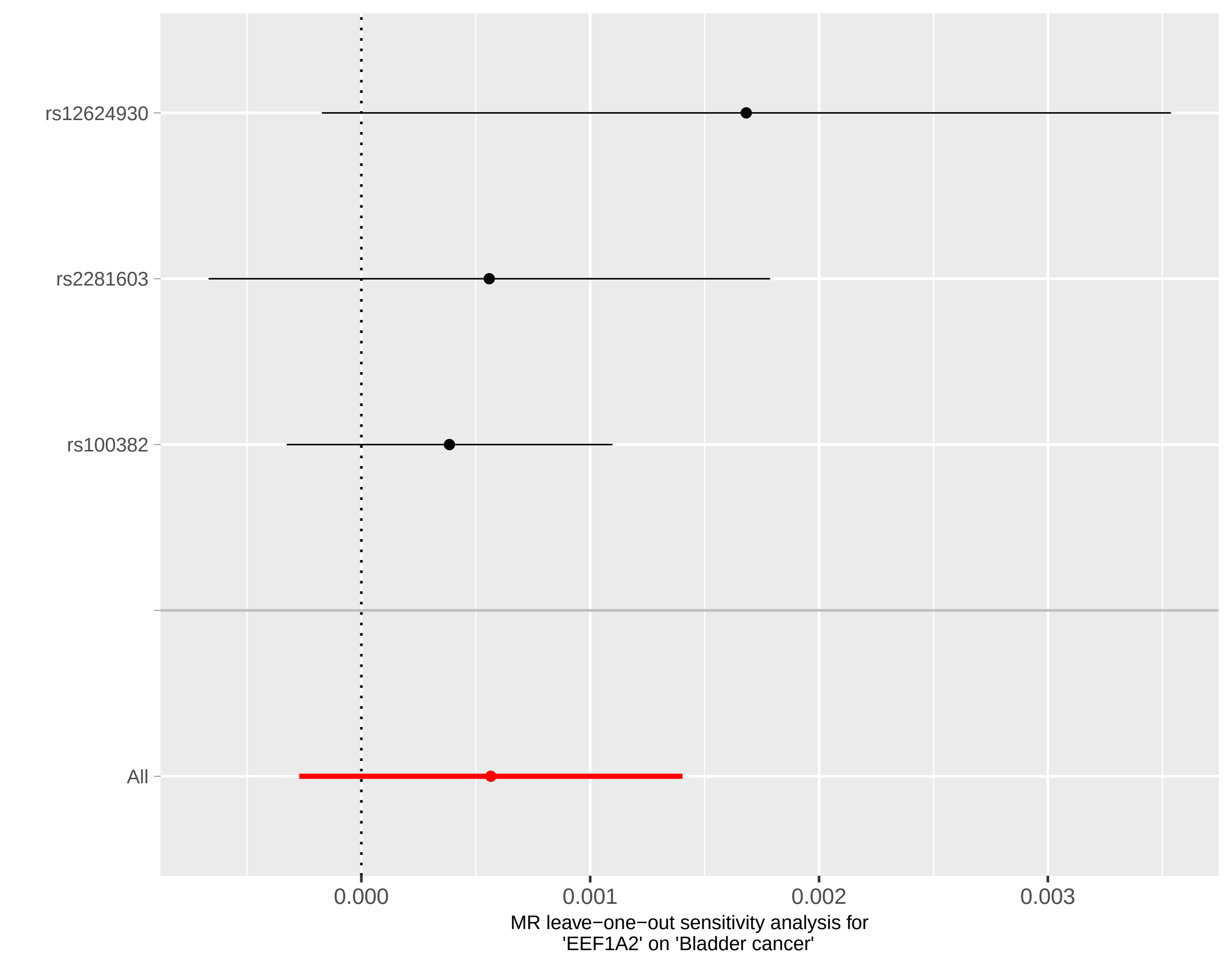
rs12624930
rs2281603
rs100382
All
0.000
0.001
0.002
0.003
MR leave−one−out sensitivity analysis for
'EEF1A2' on 'Bladder cancer'

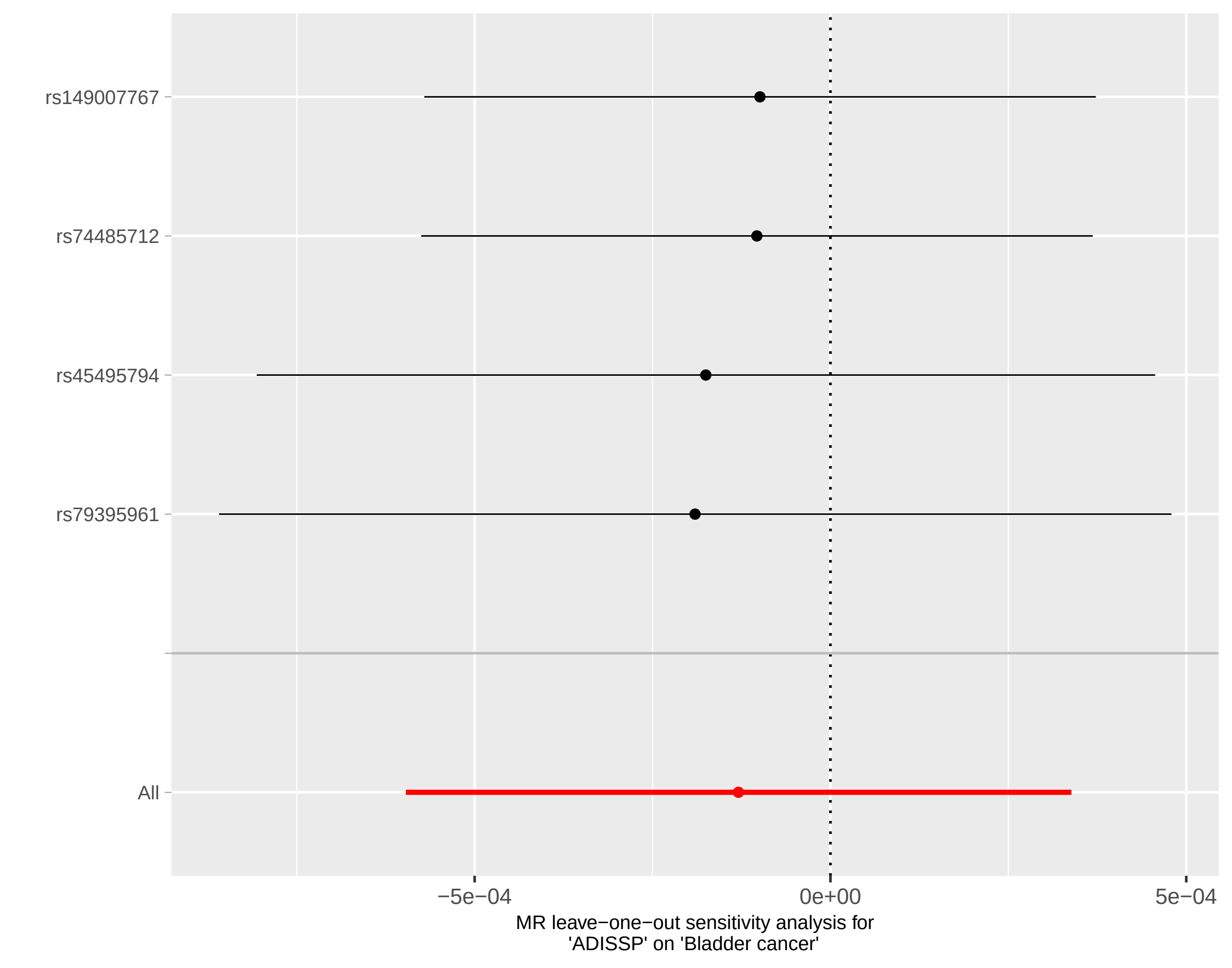

rs149007767
rs74485712
rs45495794
rs79395961
All
−5e−04
0e+00
5e−04
MR leave−one−out sensitivity analysis for
'ADISSP' on 'Bladder cancer'

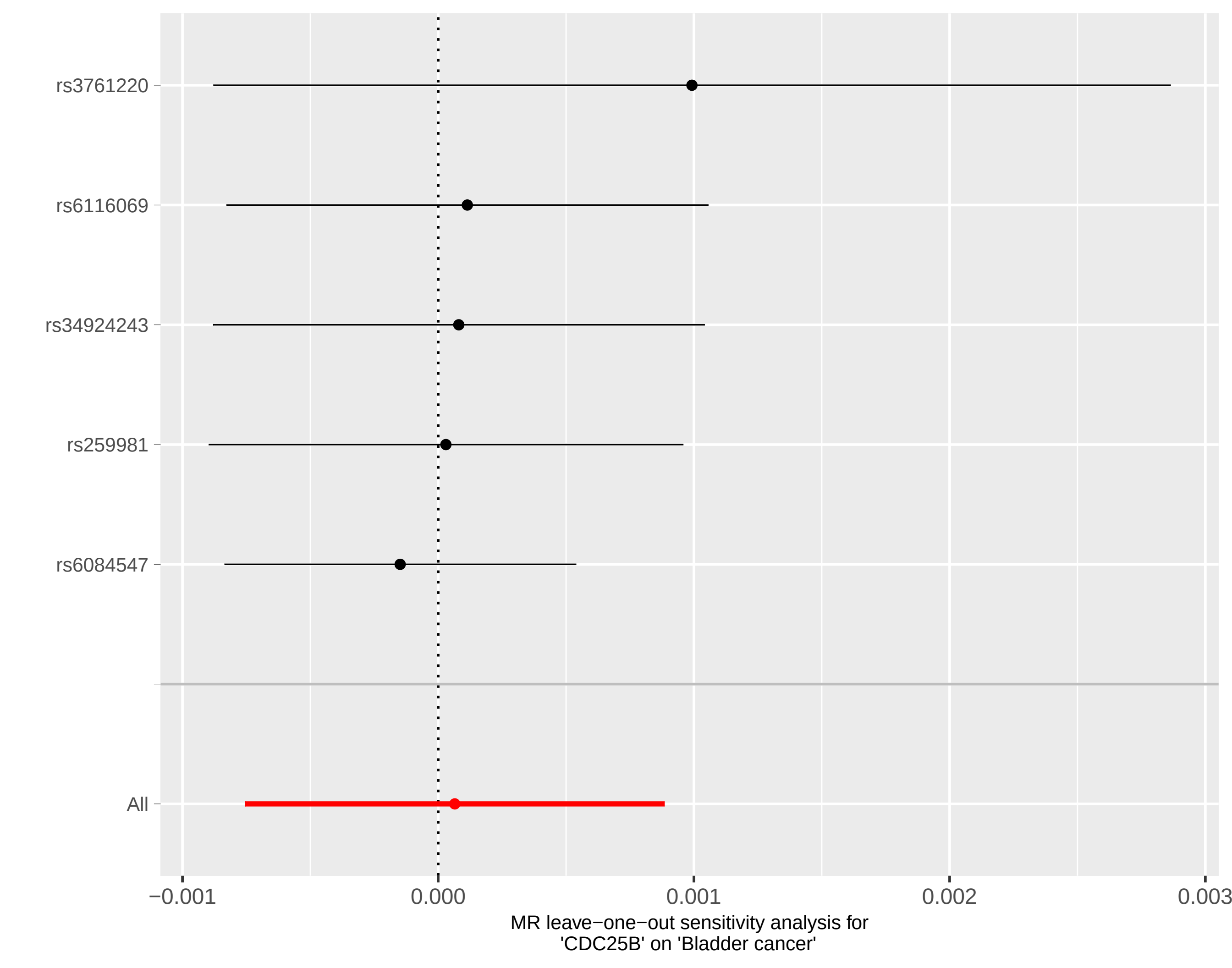
rs3761220
rs6116069
rs34924243
rs259981
rs6084547
All
−0.001
0.000
0.001
0.002
0.003
MR leave−one−out sensitivity analysis for
'CDC25B' on 'Bladder cancer'

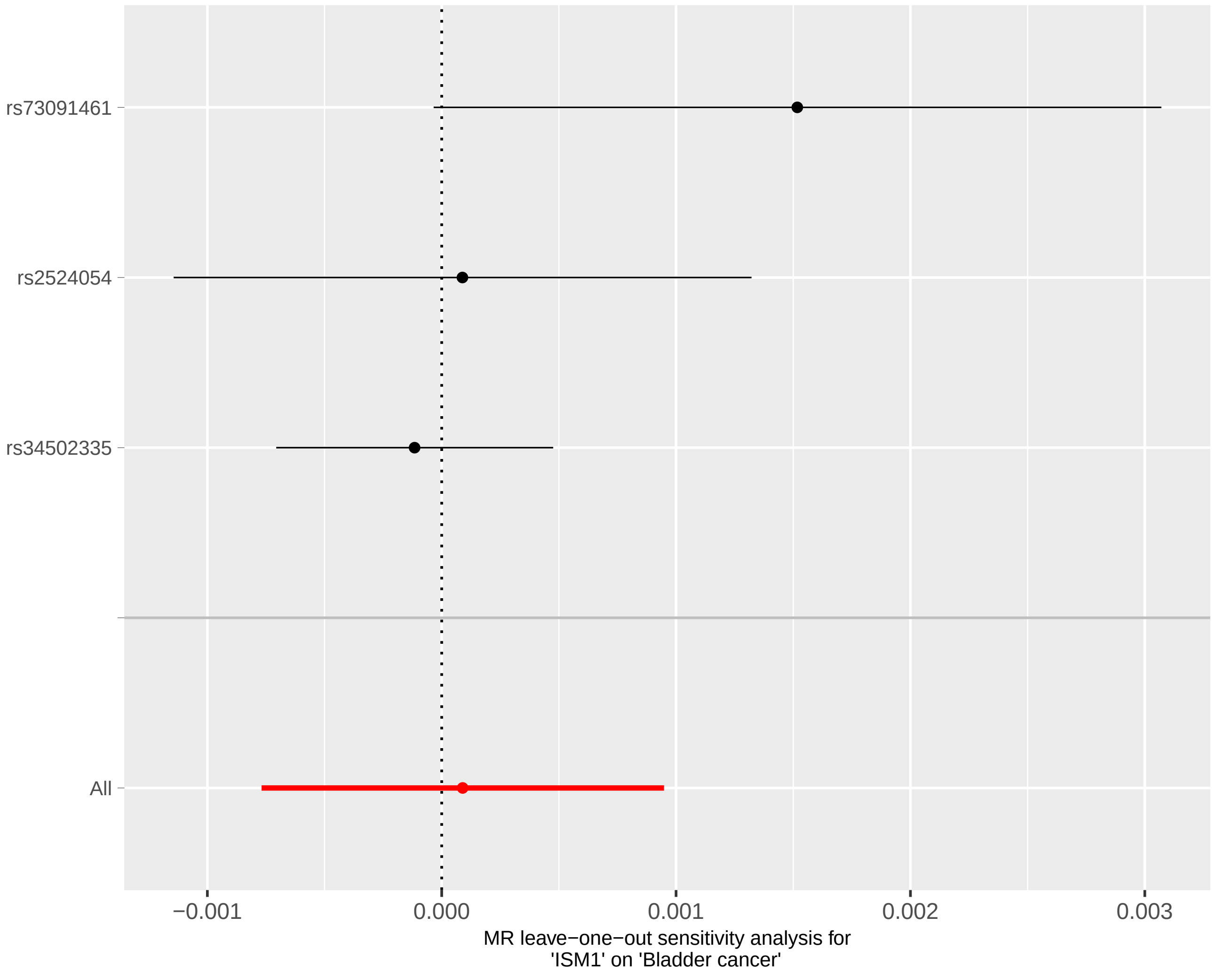
rs73091461
rs2524054
rs34502335
All
−0.001
0.000
0.001
0.002
0.003
MR leave−one−out sensitivity analysis for
'ISM1' on 'Bladder cancer'

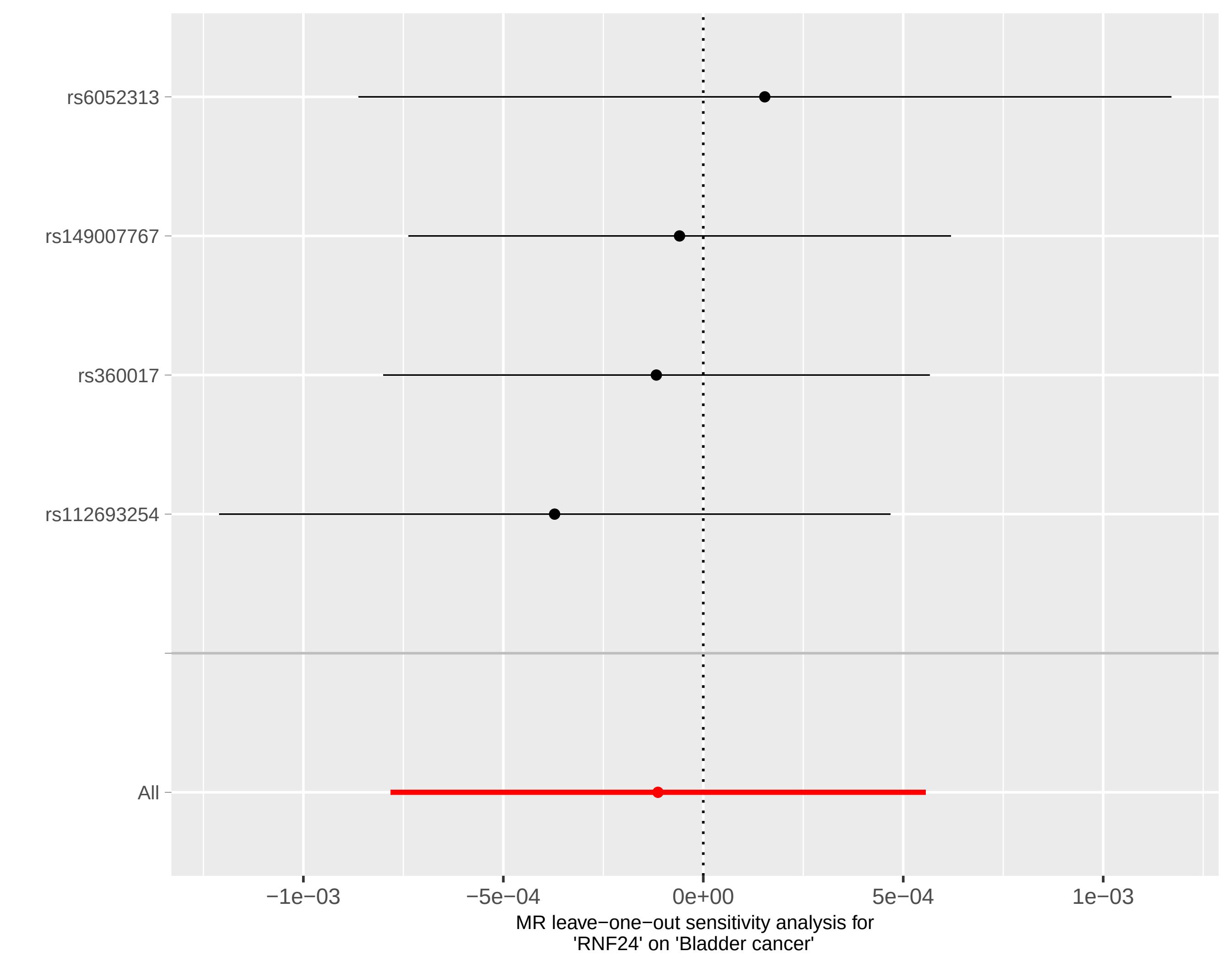
rs6052313
rs149007767
rs360017
rs112693254
All
−1e−03
−5e−04
0e+00
5e−04
1e−03
MR leave−one−out sensitivity analysis for
'RNF24' on 'Bladder cancer'

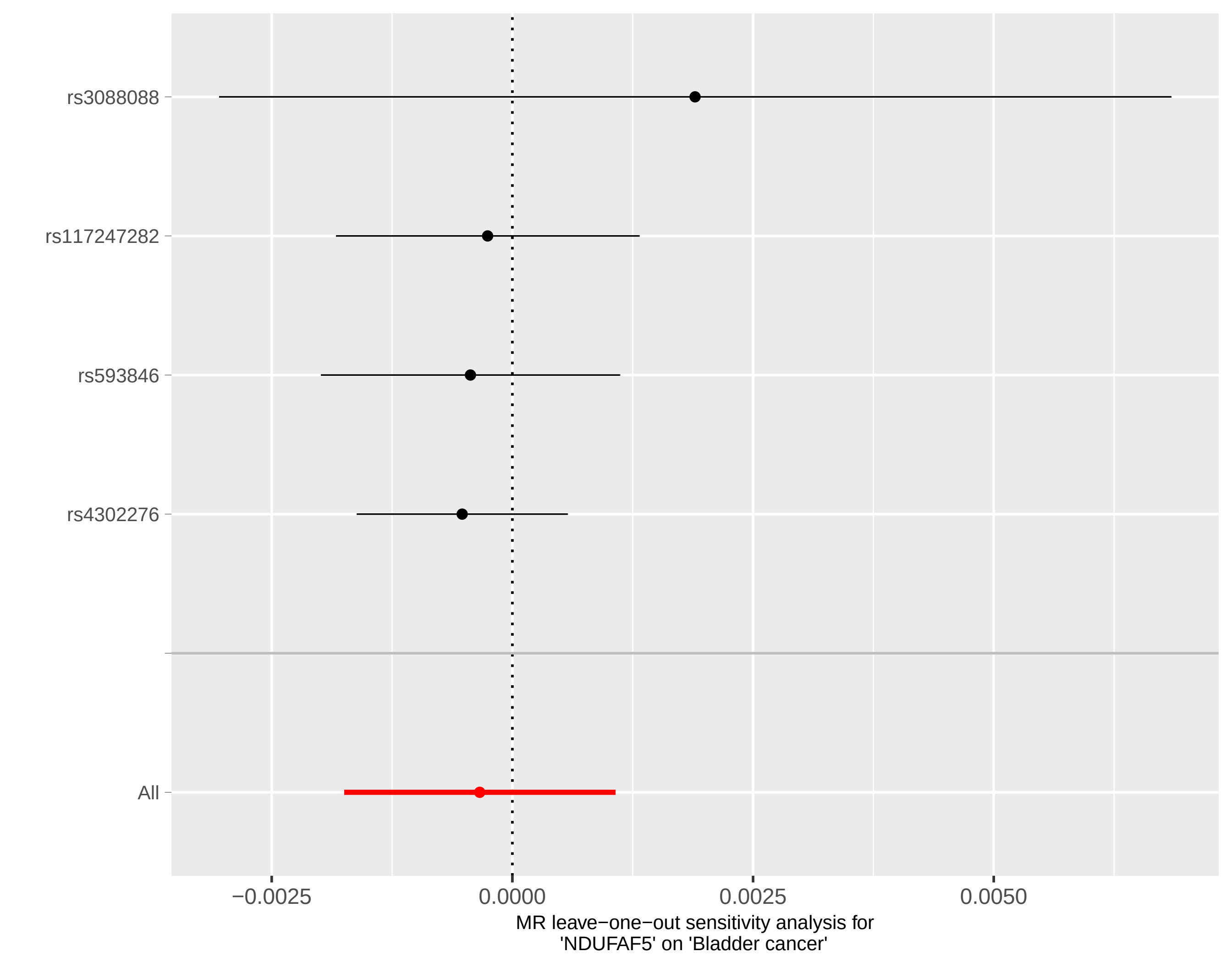
rs3088088
rs117247282
rs593846
rs4302276
All
−0.0025
0.0000
0.0025
0.0050
MR leave−one−out sensitivity analysis for
'NDUFAF5' on 'Bladder cancer'

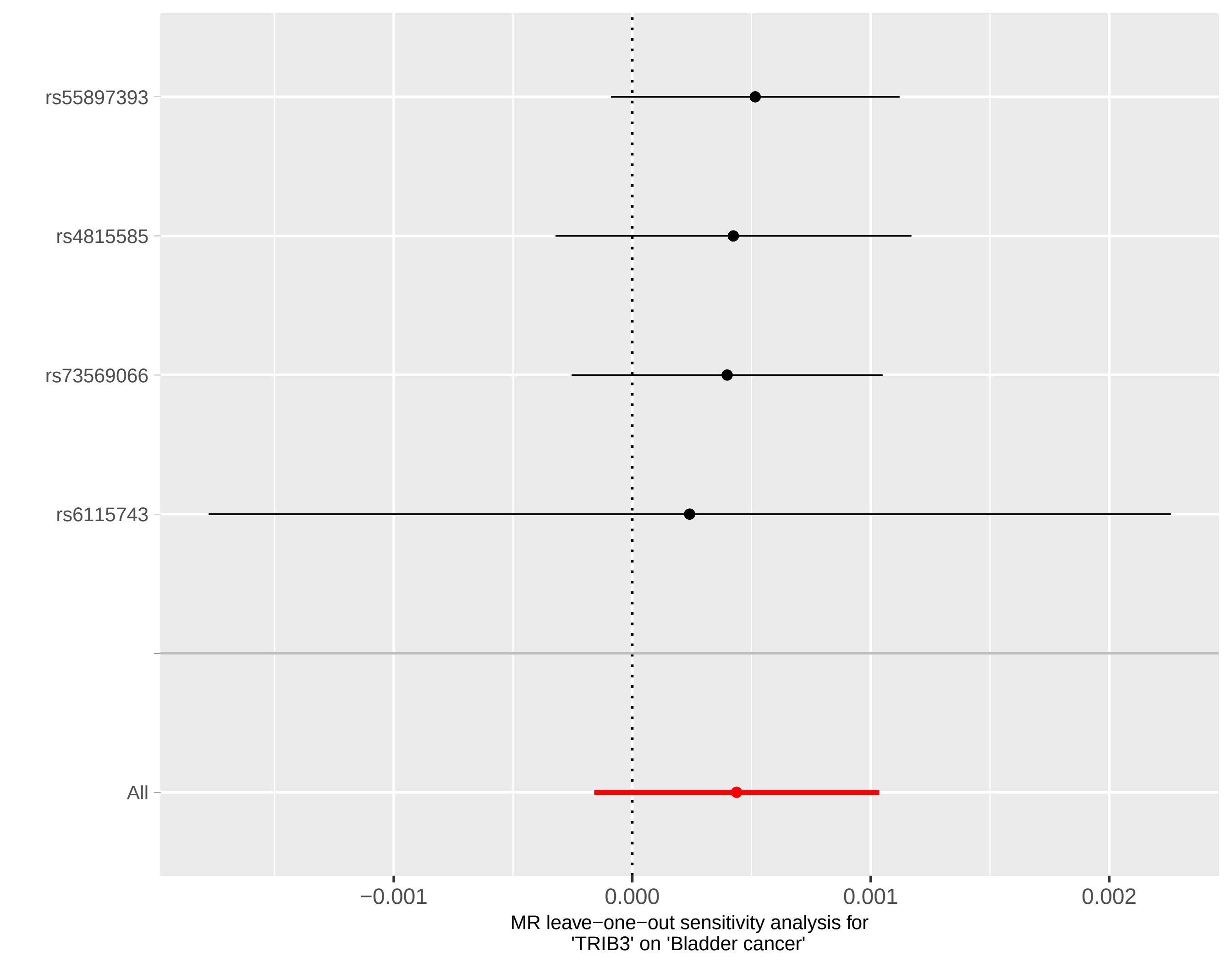
rs55897393
rs4815585
rs73569066
rs6115743
All
−0.001
0.000
0.001
0.002
MR leave−one−out sensitivity analysis for
'TRIB3' on 'Bladder cancer'

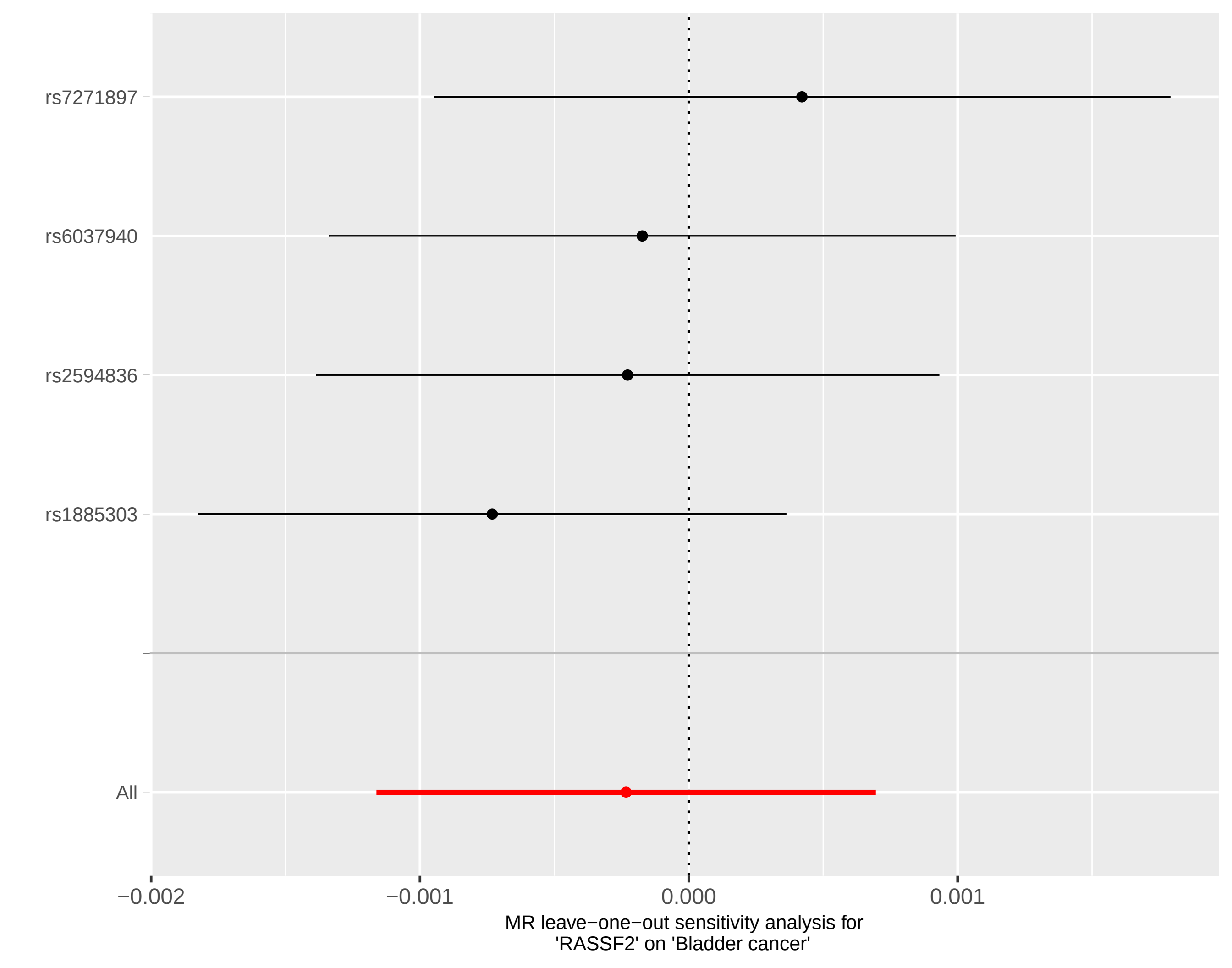
rs7271897
rs6037940
rs2594836
rs1885303
All
−0.002
−0.001
0.000
0.001
MR leave−one−out sensitivity analysis for
'RASSF2' on 'Bladder cancer'

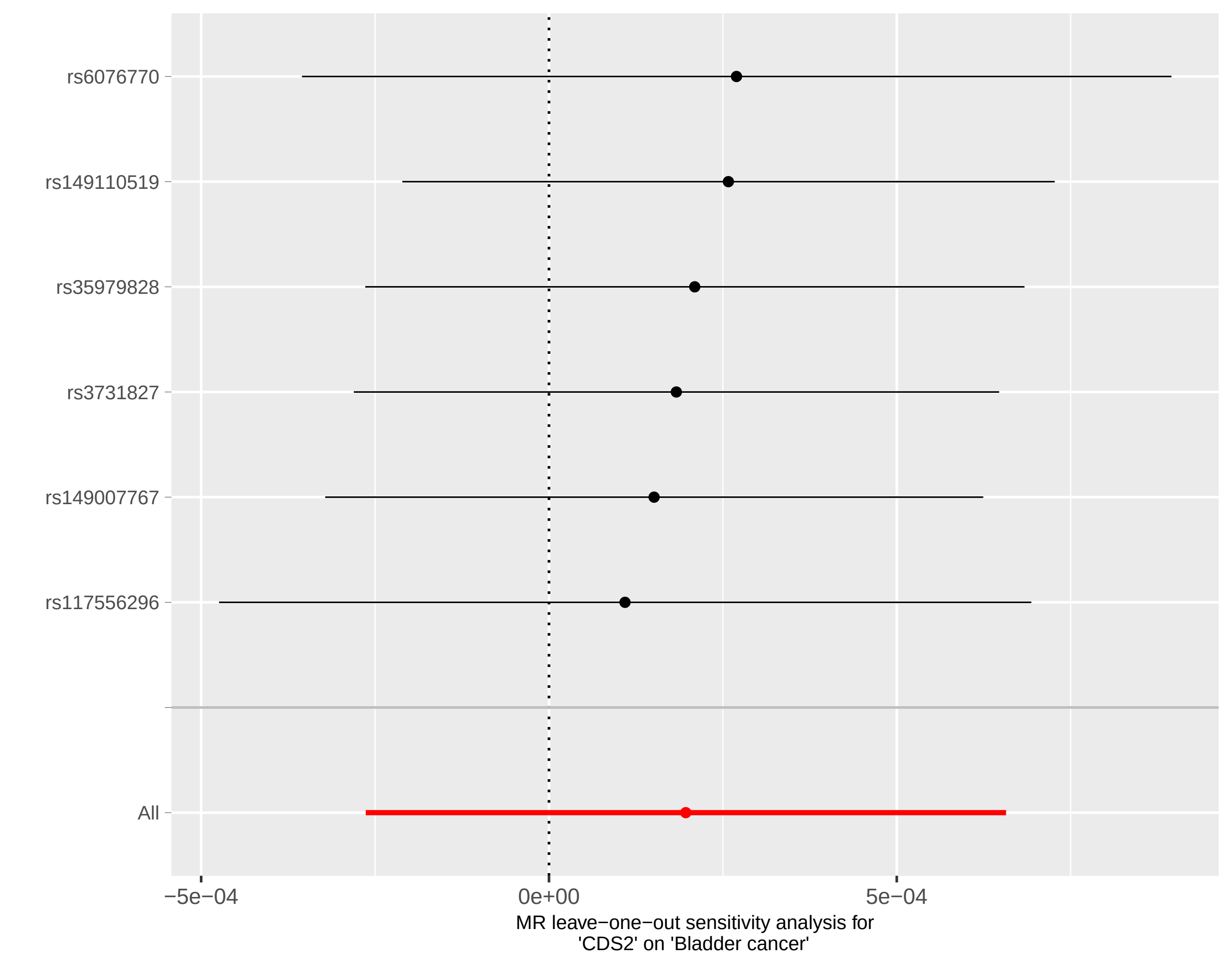
rs6076770
rs149110519
rs35979828
rs3731827
rs149007767
rs117556296
All
−5e−04
0e+00
5e−04
MR leave−one−out sensitivity analysis for
'CDS2' on 'Bladder cancer'

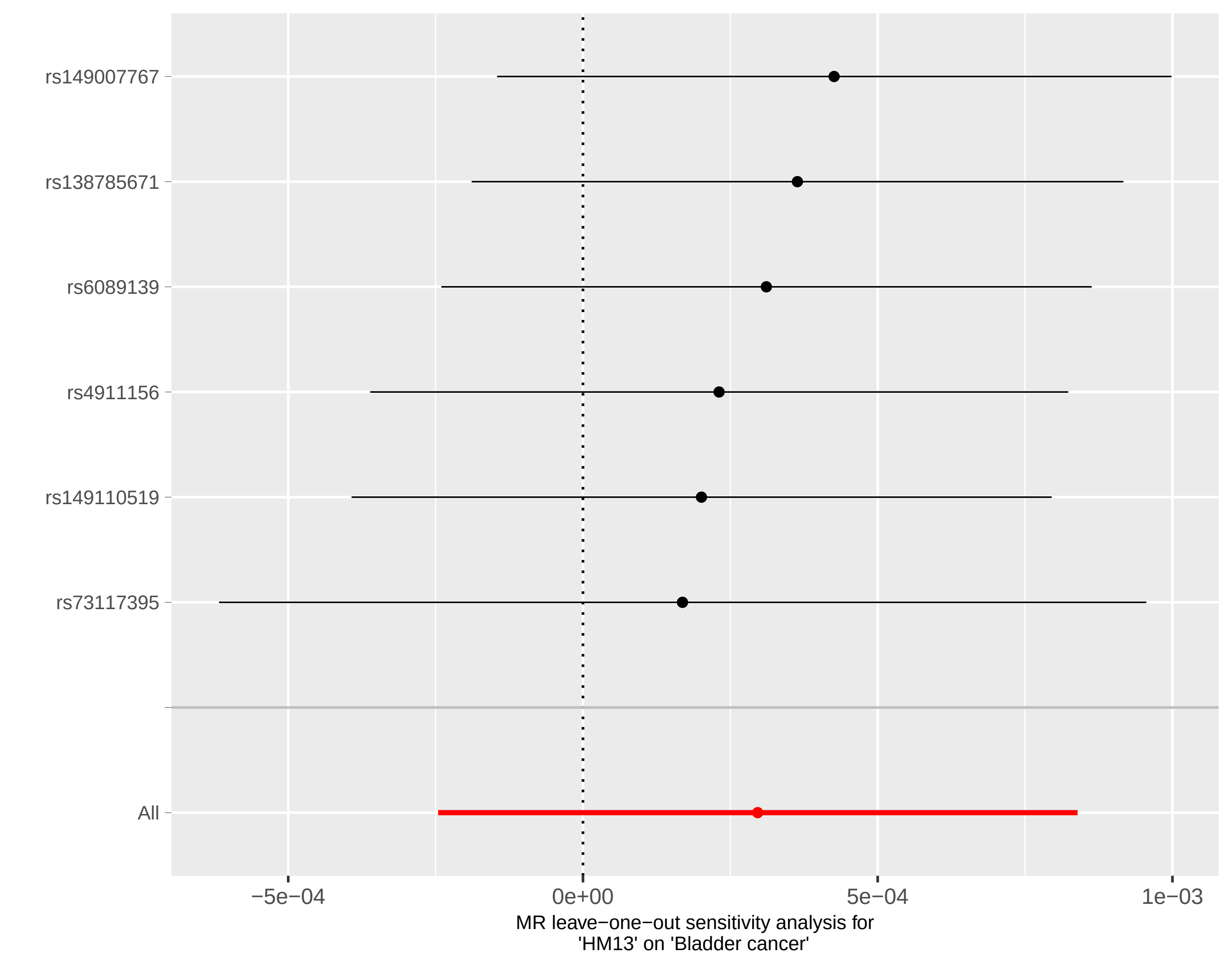
rs149007767
rs138785671
rs6089139
rs4911156
rs149110519
rs73117395
All
−5e−04
0e+00
5e−04
1e−03
MR leave−one−out sensitivity analysis for
'HM13' on 'Bladder cancer'

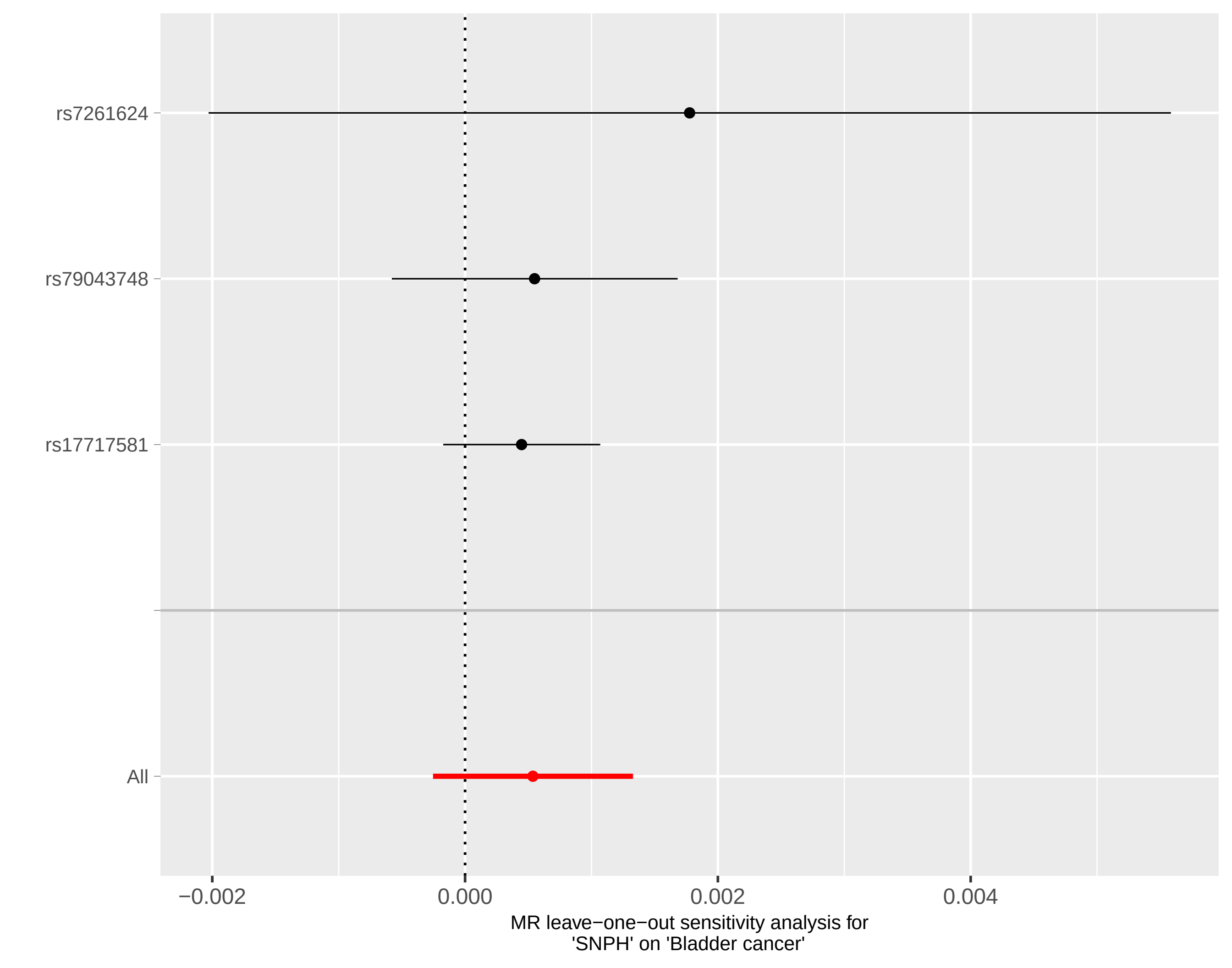
rs7261624
rs79043748
rs17717581
All
−0.002
0.000
0.002
0.004
MR leave−one−out sensitivity analysis for
'SNPH' on 'Bladder cancer'

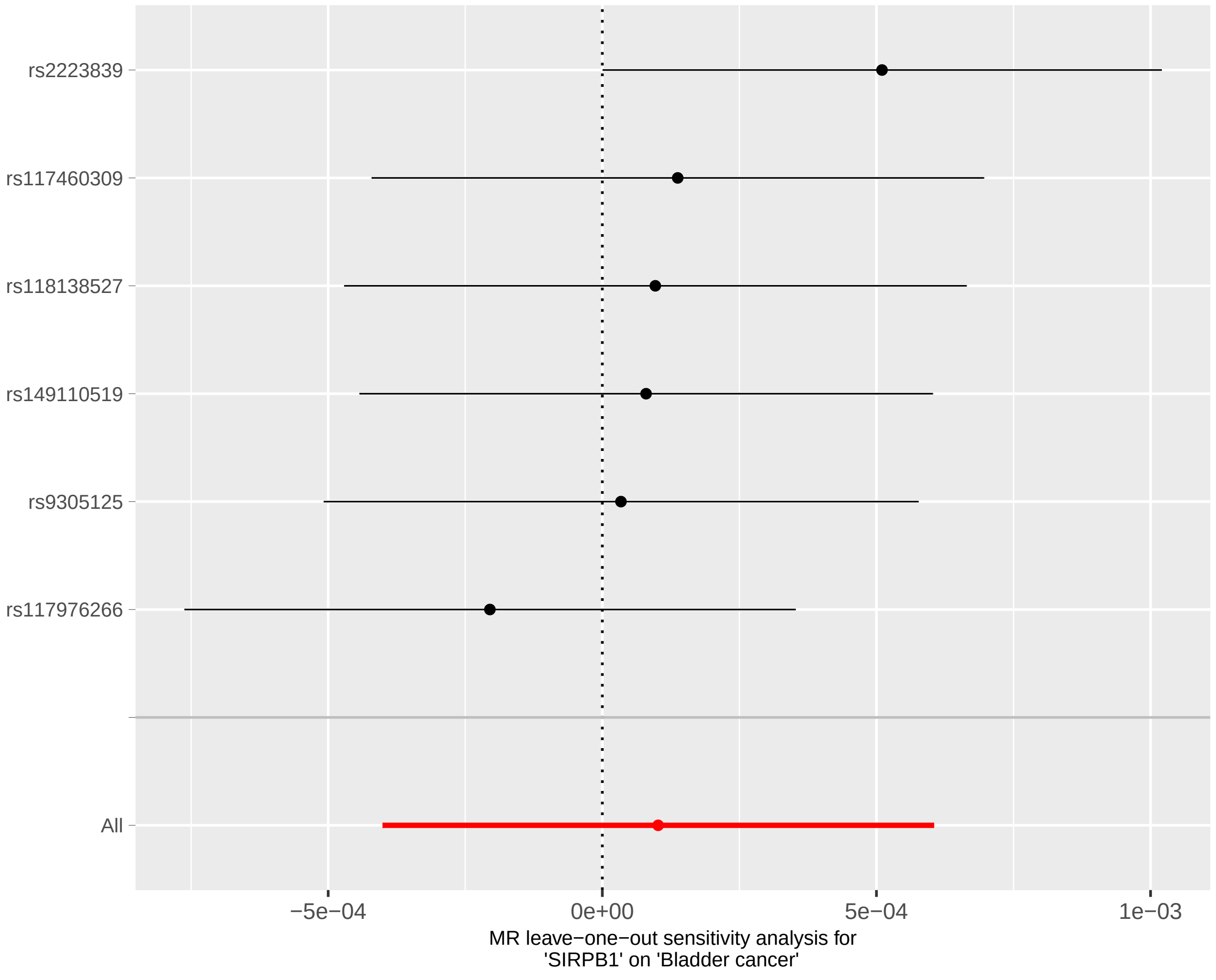
rs2223839
rs117460309
rs118138527
rs149110519
rs9305125
rs117976266
All
−5e−04
0e+00
5e−04
1e−03
MR leave−one−out sensitivity analysis for
'SIRPB1' on 'Bladder cancer'

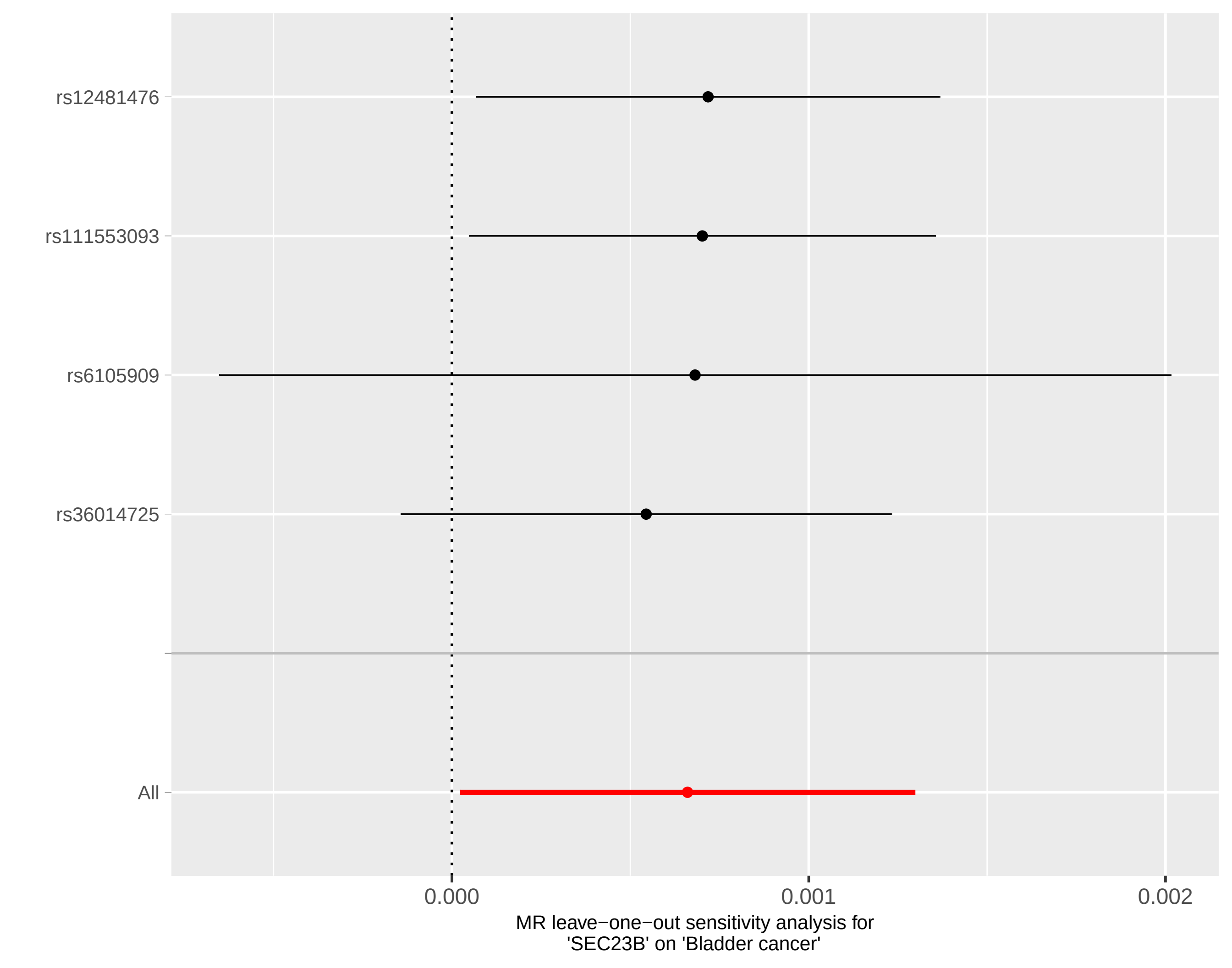
rs12481476
rs111553093
rs6105909
rs36014725
All
0.000
0.001
0.002
MR leave−one−out sensitivity analysis for
'SEC23B' on 'Bladder cancer'

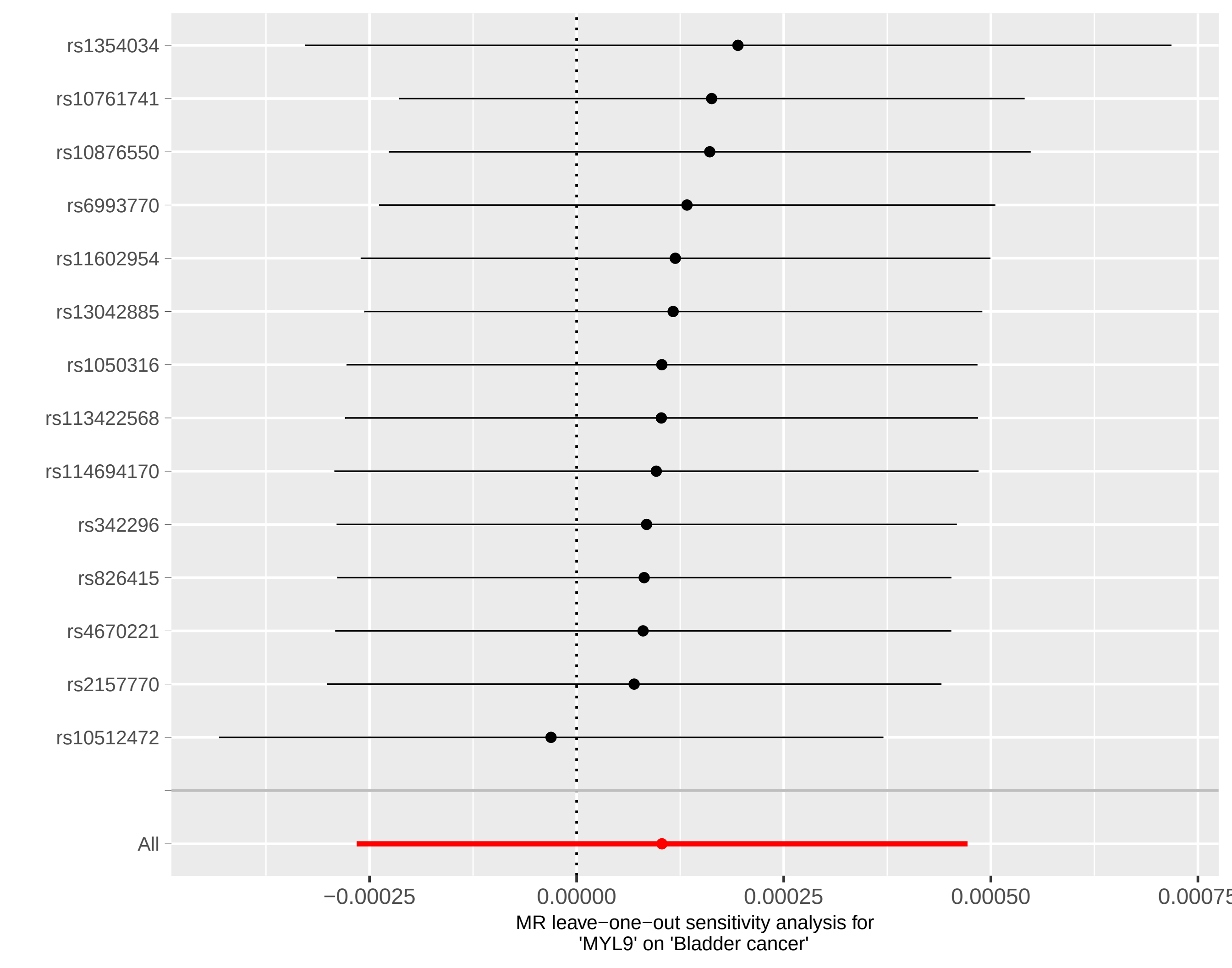
rs1354034
rs10761741
rs10876550
rs6993770
rs11602954
rs13042885
rs1050316
rs113422568
rs114694170
rs342296
rs826415
rs4670221
rs2157770
rs10512472
All
−0.00025
0.00000
0.00025
0.00050
MR leave−one−out sensitivity analysis for
'MYL9' on 'Bladder cancer'

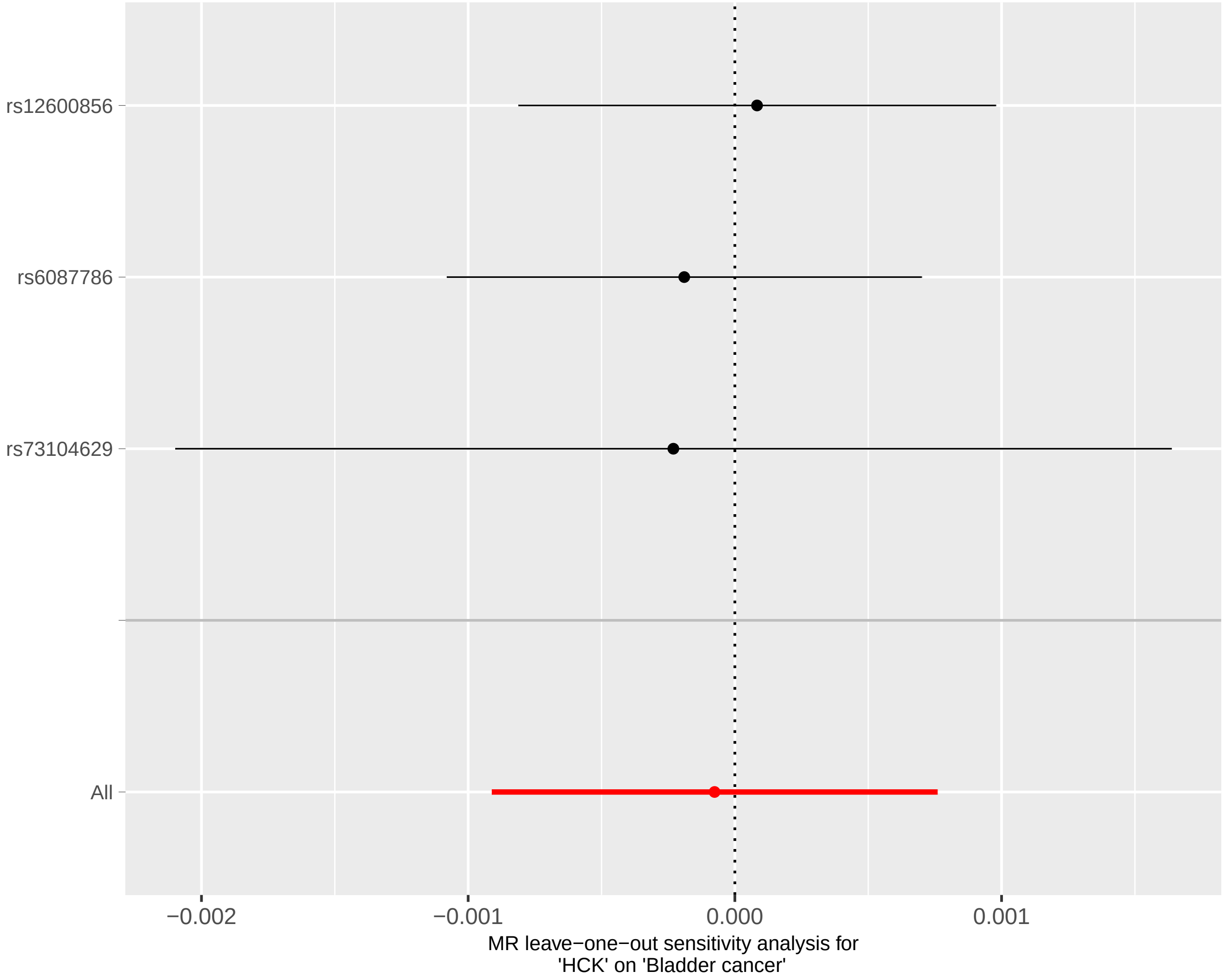
rs12600856
rs6087786
rs73104629
All
−0.002
−0.001
0.000
0.001
MR leave−one−out sensitivity analysis for
'HCK' on 'Bladder cancer'

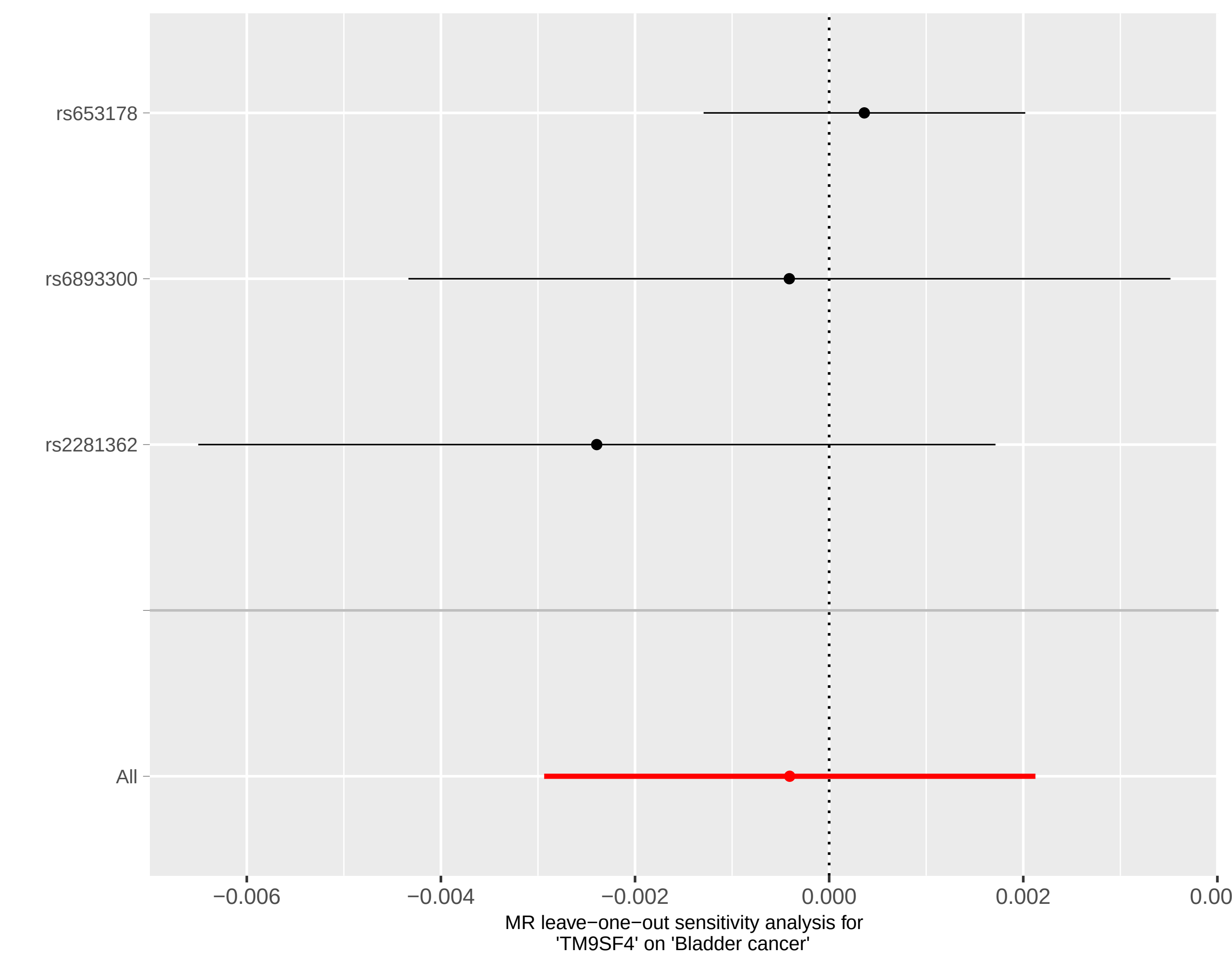
rs653178
rs6893300
rs2281362
All
−0.006
−0.004
−0.002
0.000
0.002
0.00
MR leave−one−out sensitivity analysis for
'TM9SF4' on 'Bladder cancer'

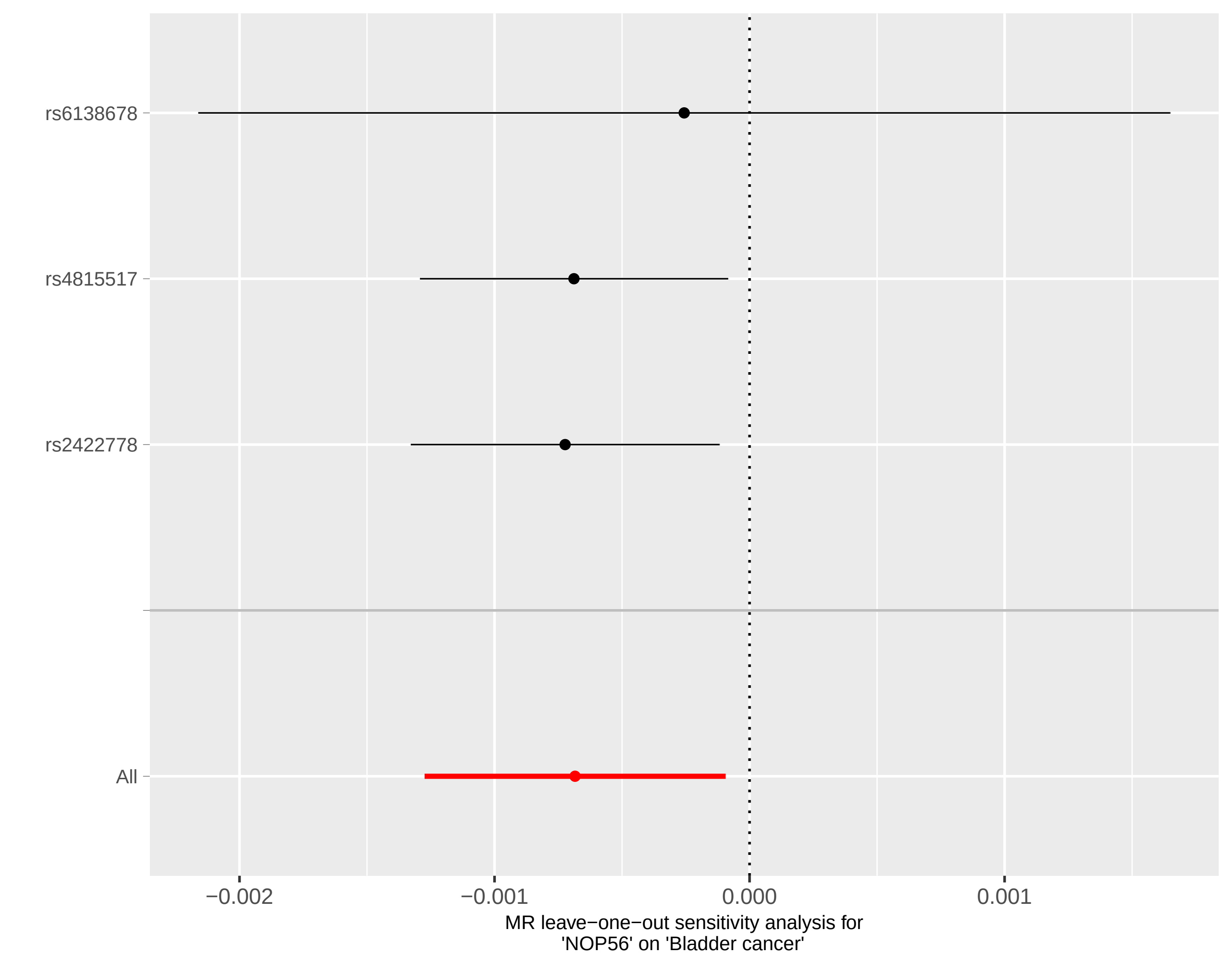
rs6138678
rs4815517
rs2422778
All
−0.002
−0.001
0.000
0.001
MR leave−one−out sensitivity analysis for
'NOP56' on 'Bladder cancer'

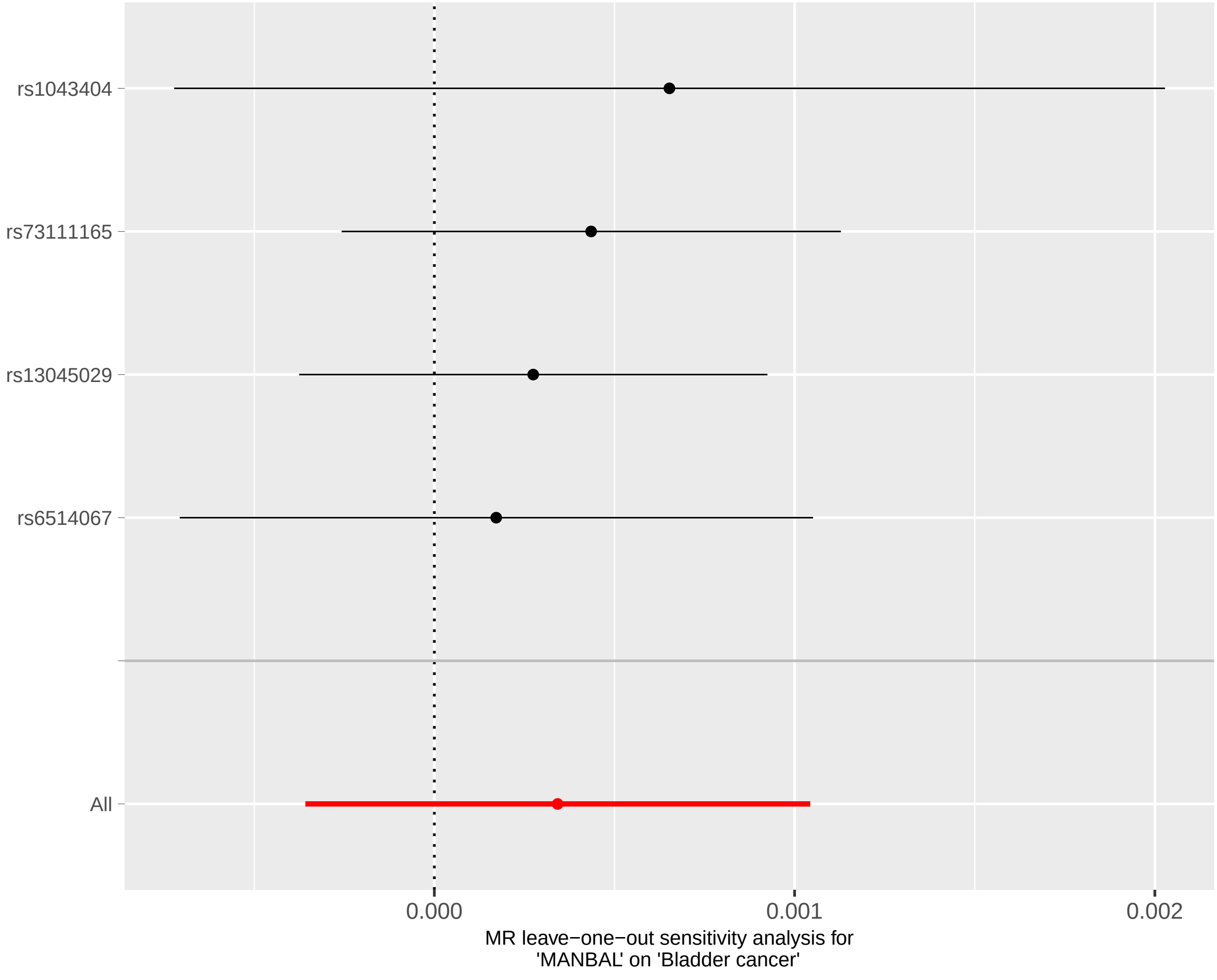
rs1043404
rs73111165
rs13045029
rs6514067
All
0.000
0.001
0.002
MR leave−one−out sensitivity analysis for
'MANBAL' on 'Bladder cancer'

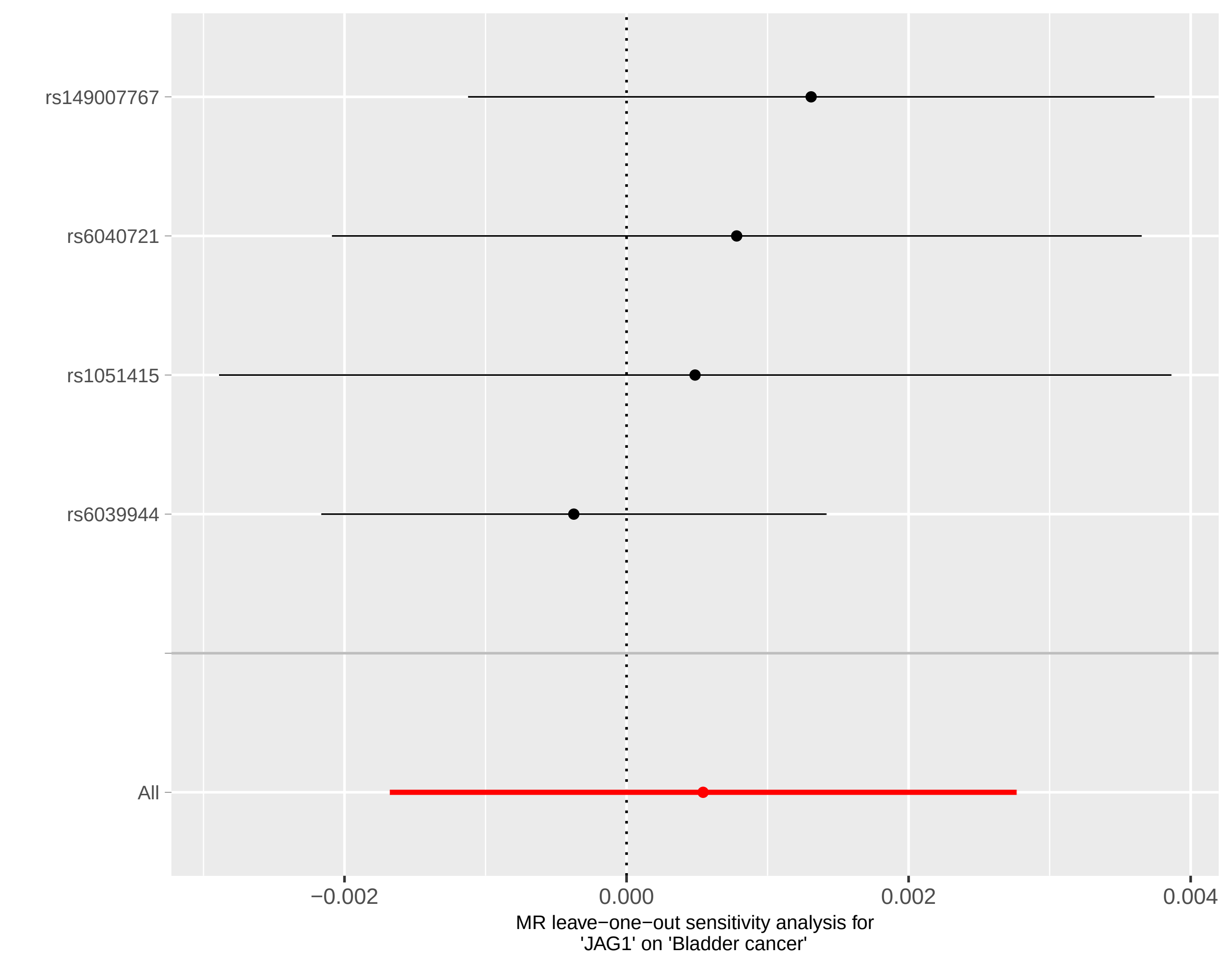
rs149007767
rs6040721
rs1051415
rs6039944
All
−0.002
0.000
0.002
0.004
MR leave−one−out sensitivity analysis for
'JAG1' on 'Bladder cancer'

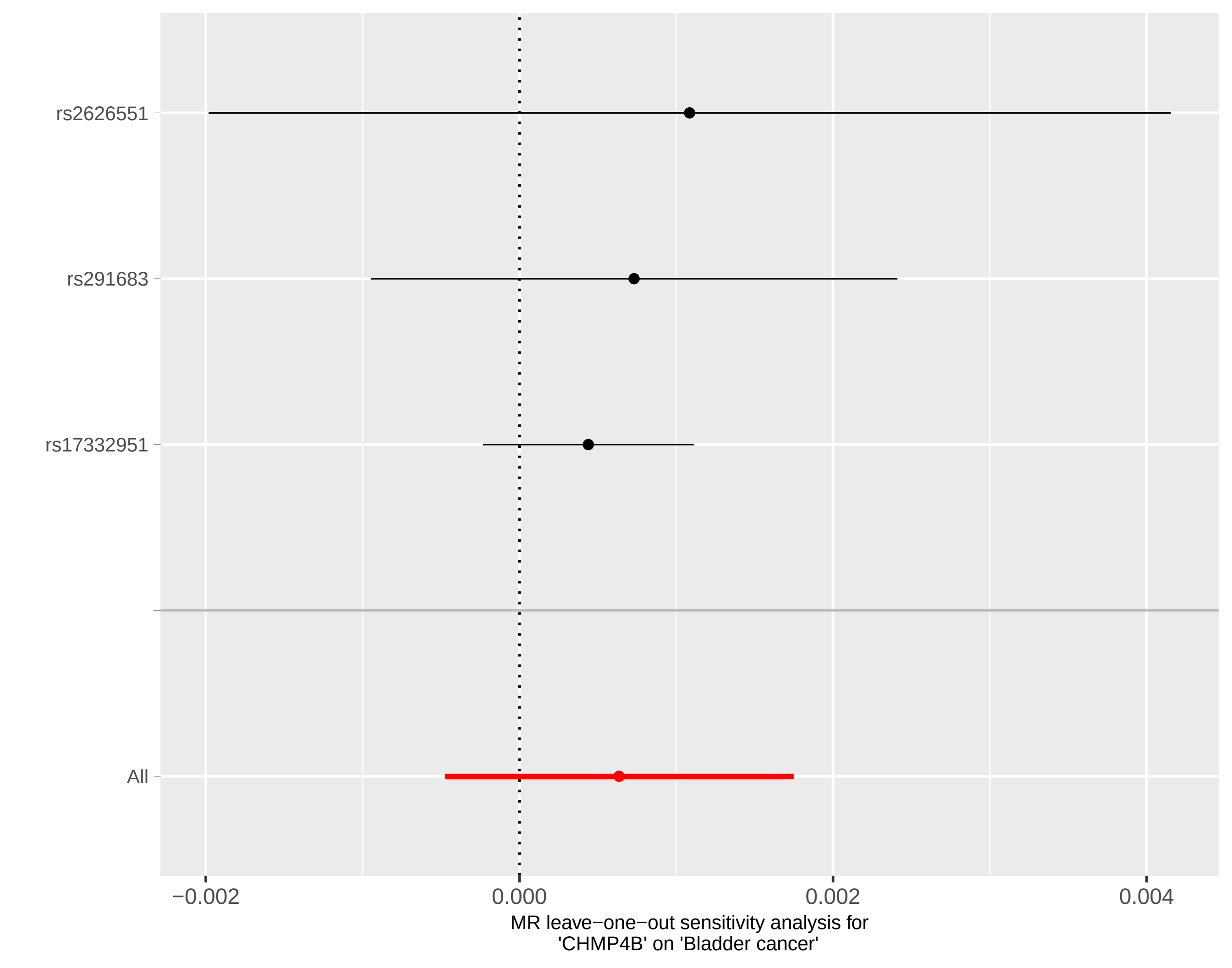
rs2626551
rs291683
rs17332951
All
−0.002
0.000
0.002
0.004
MR leave−one−out sensitivity analysis for
'CHMP4B' on 'Bladder cancer'

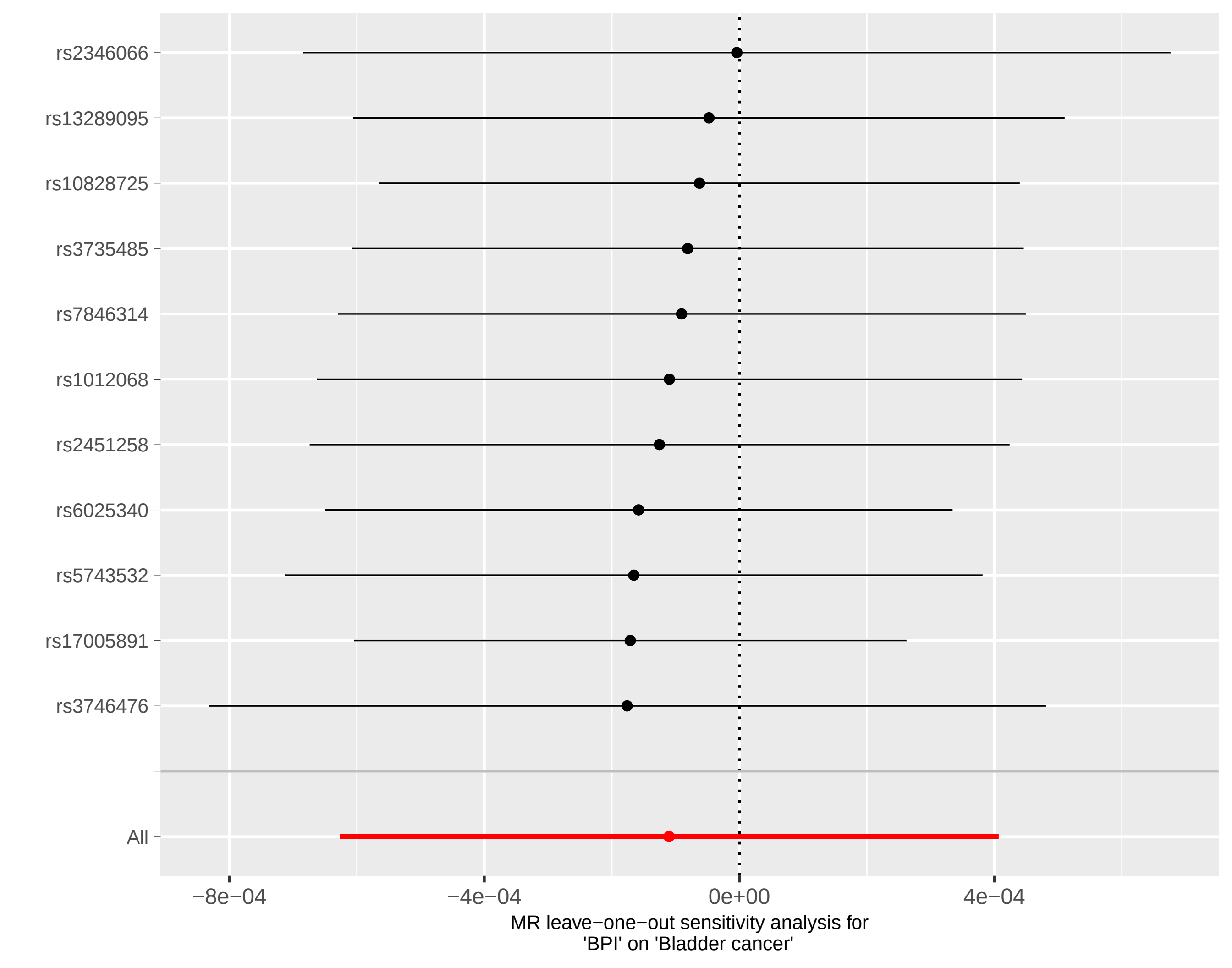
rs2346066
rs13289095
rs10828725
rs3735485
rs7846314
rs1012068
rs2451258
rs6025340
rs5743532
rs17005891
rs3746476
All
−8e−04
−4e−04
0e+00
4e−04
MR leave−one−out sensitivity analysis for
'BPI' on 'Bladder cancer'

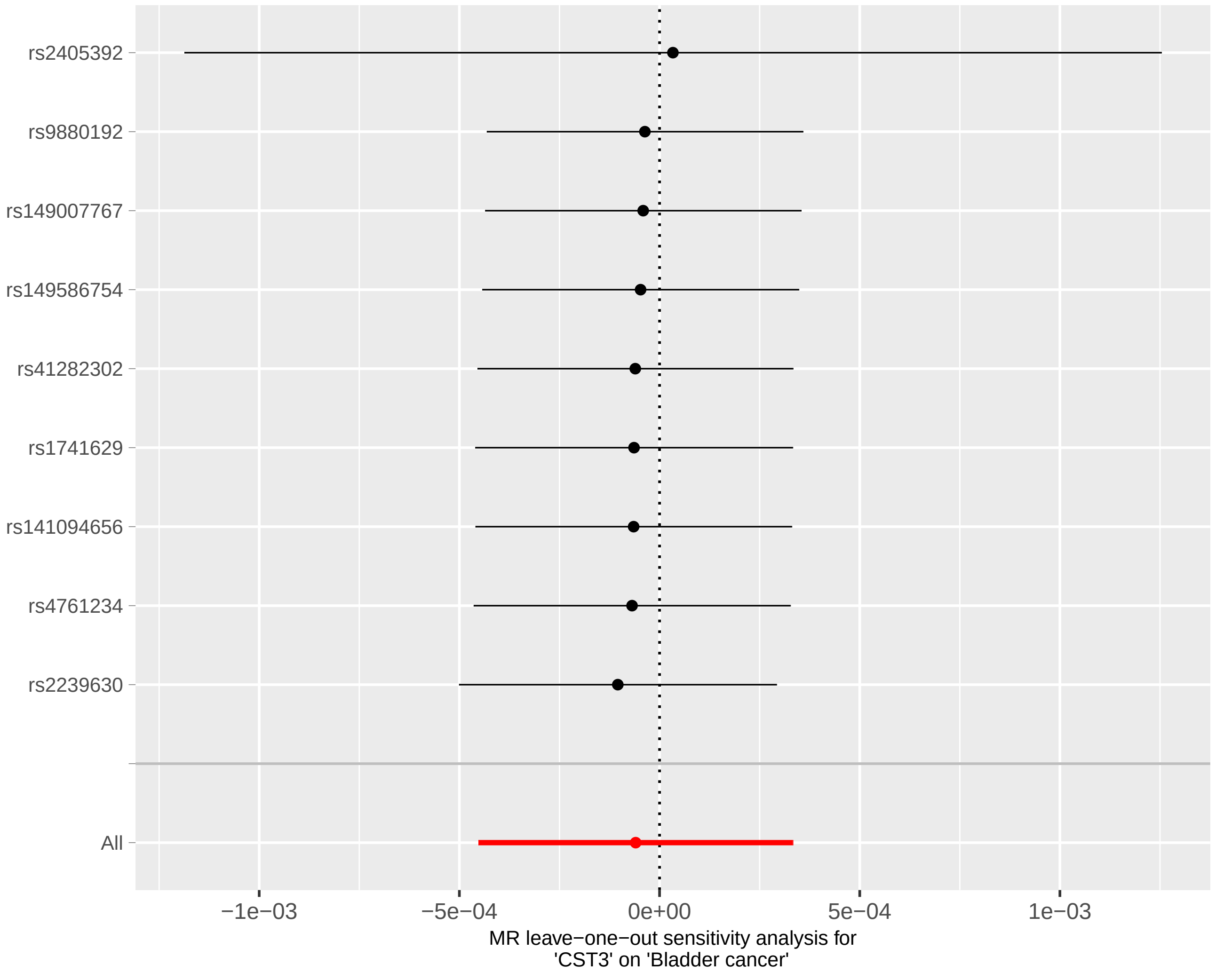
rs2405392
rs9880192
rs149007767
rs149586754
rs41282302
rs1741629
rs141094656
rs4761234
rs2239630
All
−1e−03
−5e−04
0e+00
5e−04
1e−03
MR leave−one−out sensitivity analysis for
'CST3' on 'Bladder cancer'

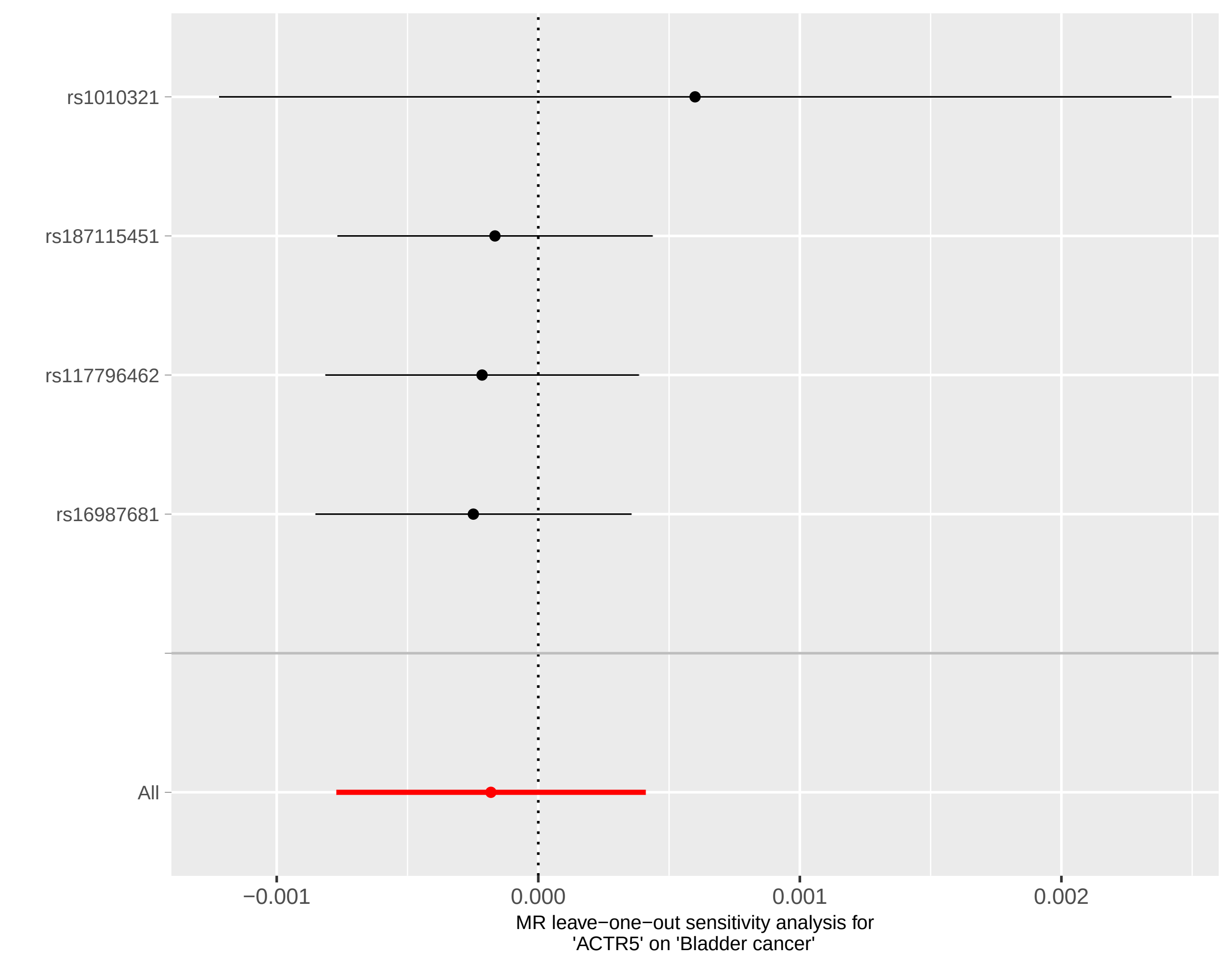
rs1010321
rs187115451
rs117796462
rs16987681
All
−0.001
0.000
0.001
0.002
MR leave−one−out sensitivity analysis for
'ACTR5' on 'Bladder cancer'

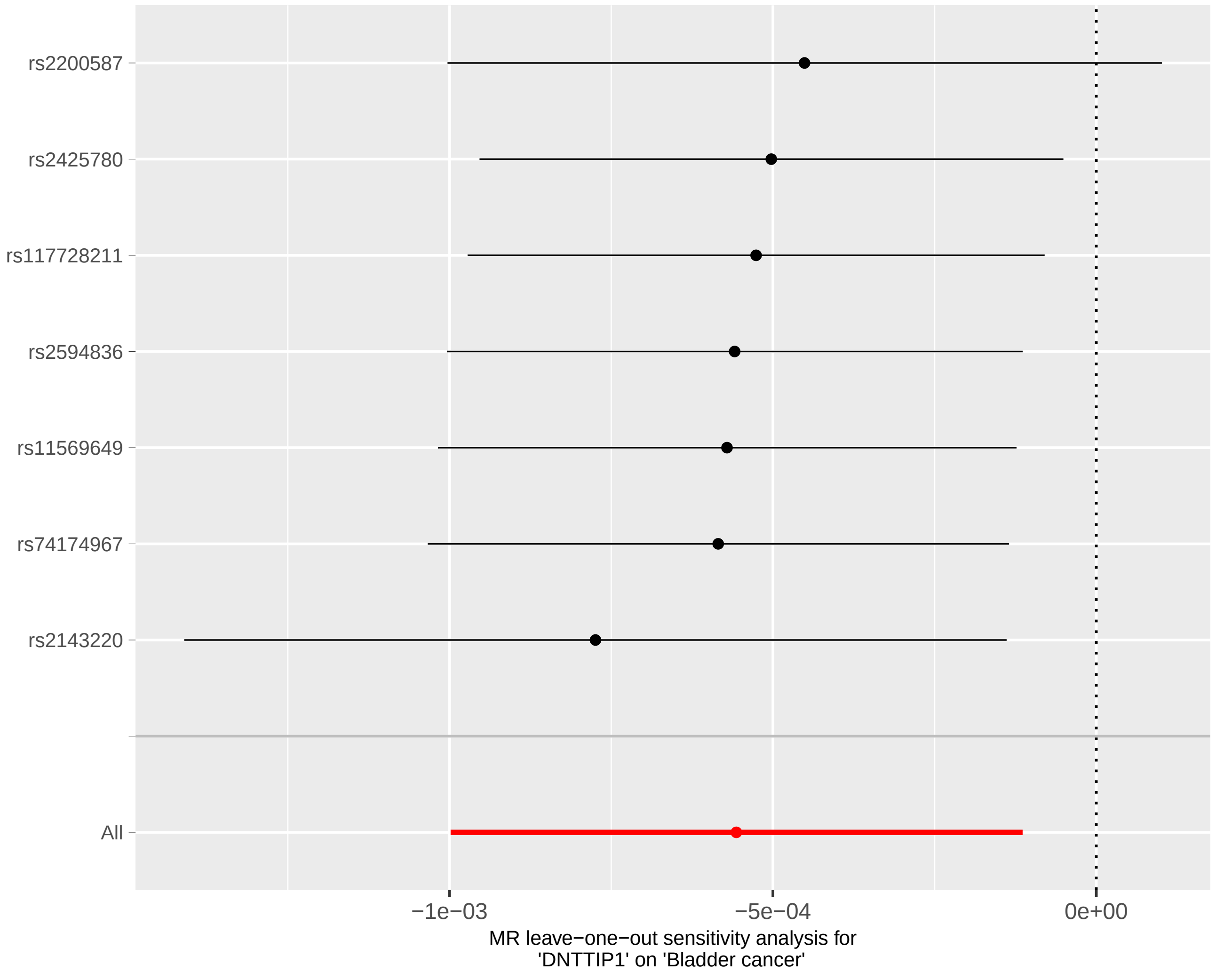
rs2200587
rs2425780
rs117728211
rs2594836
rs11569649
rs74174967
rs2143220
All
−1e−03
−5e−04
0e+00
MR leave−one−out sensitivity analysis for
'DNTTIP1' on 'Bladder cancer'

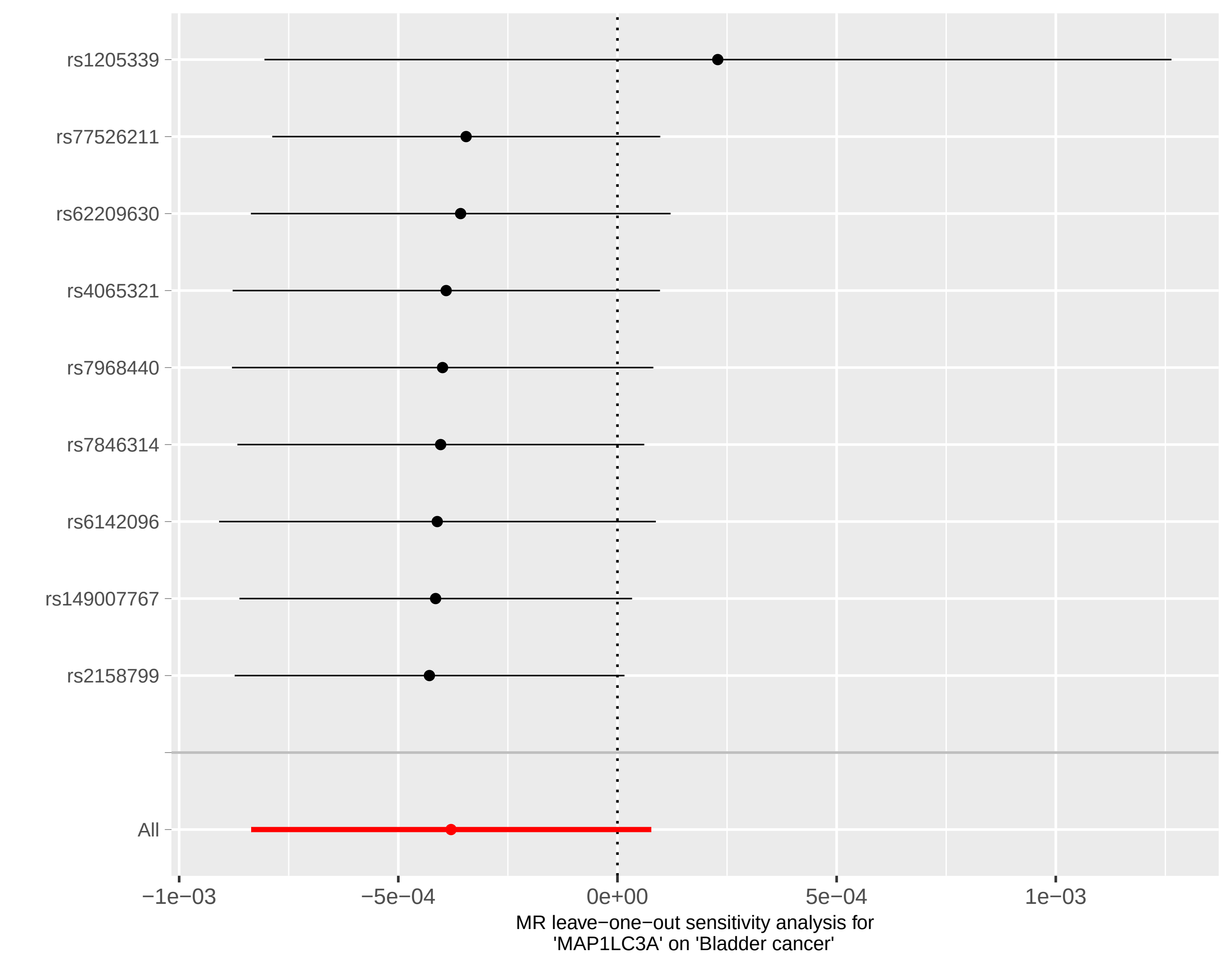
rs1205339
rs77526211
rs62209630
rs4065321
rs7968440
rs7846314
rs6142096
rs149007767
rs2158799
All
−1e−03
−5e−04
0e+00
5e−04
1e−03
MR leave−one−out sensitivity analysis for
'MAP1LC3A' on 'Bladder cancer'

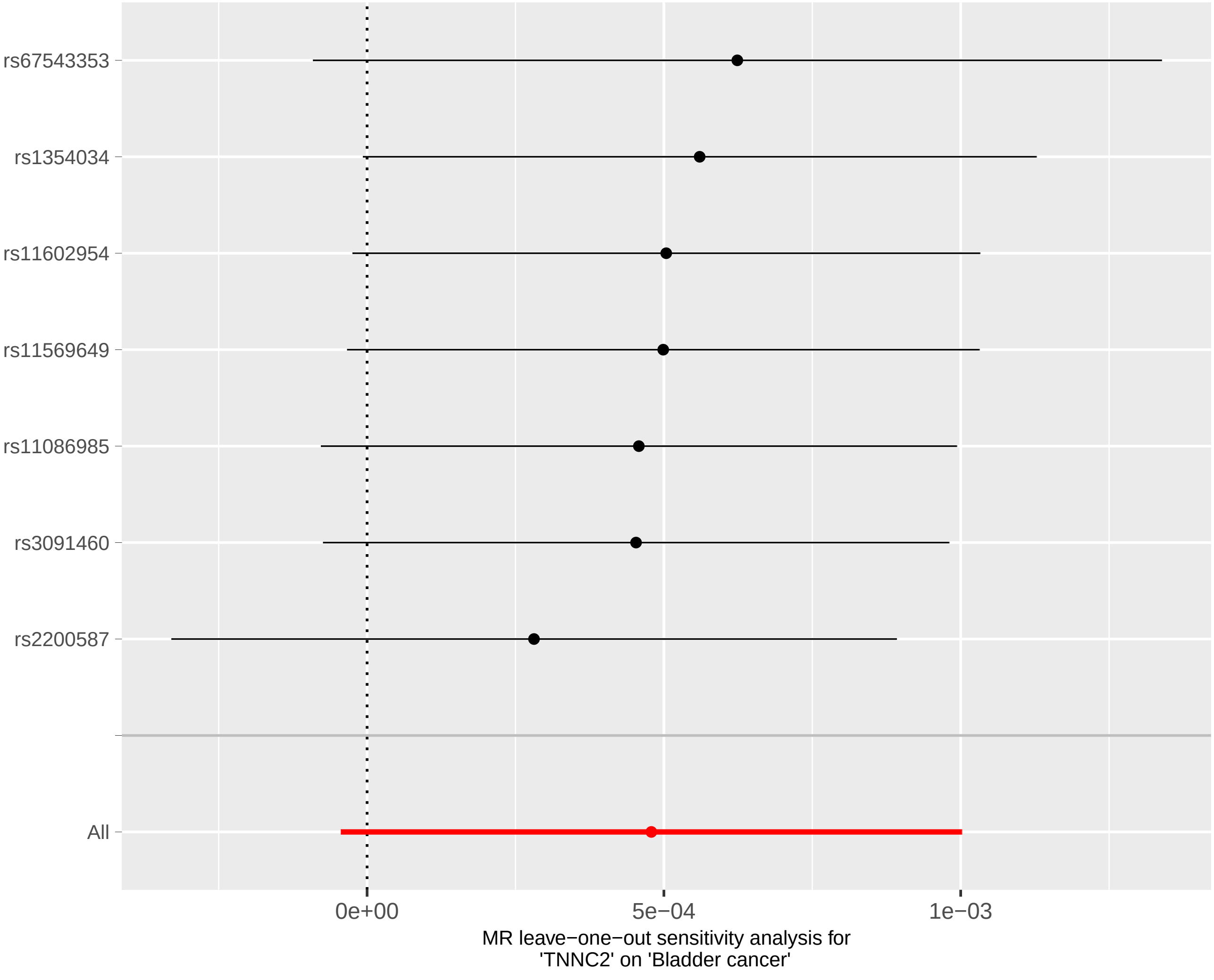
rs67543353
rs1354034
rs11602954
rs11569649
rs11086985
rs3091460
rs2200587
All
0e+00
5e−04
1e−03
MR leave−one−out sensitivity analysis for
'TNNC2' on 'Bladder cancer'

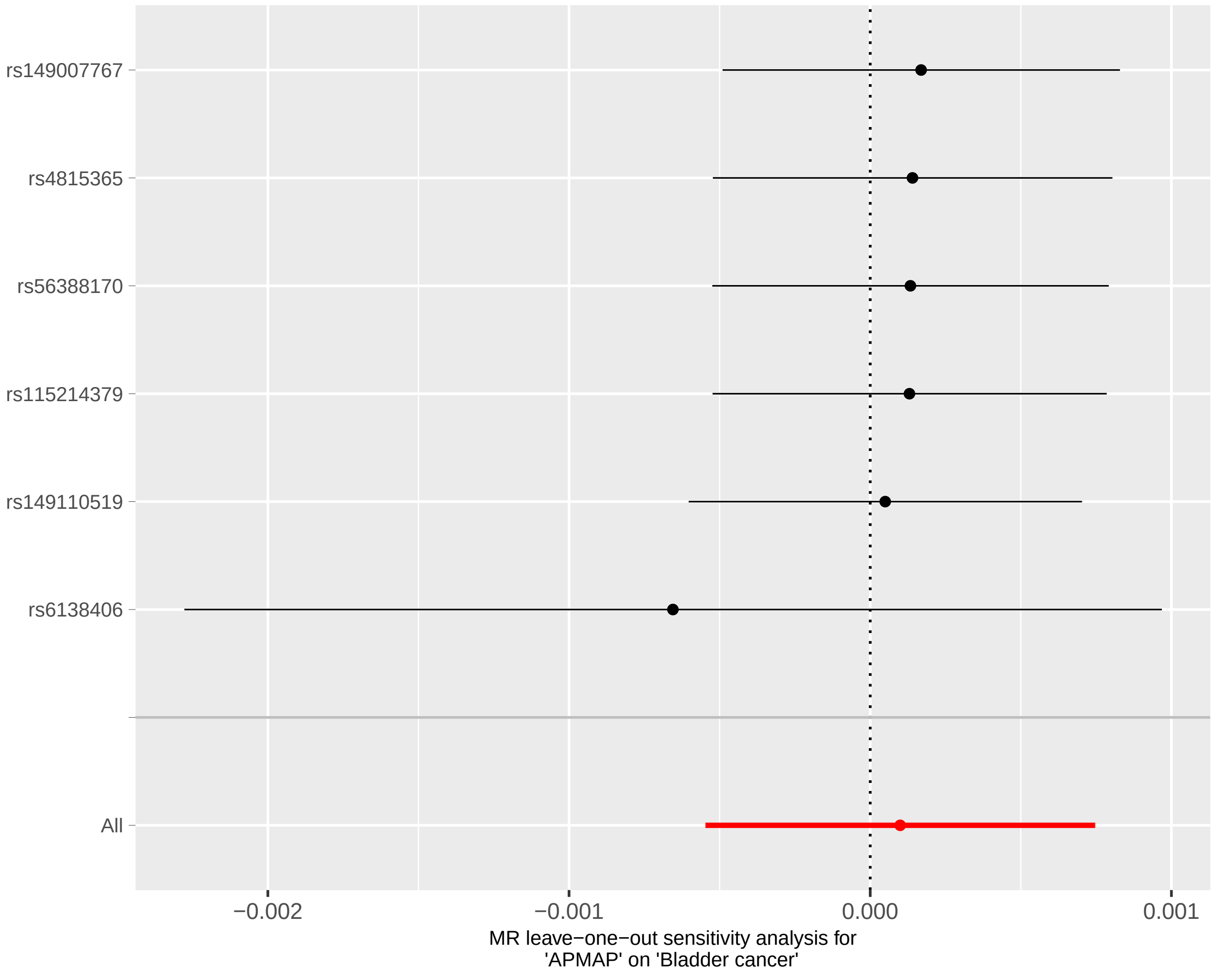
rs149007767
rs4815365
rs56388170
rs115214379
rs149110519
rs6138406
All
−0.002
−0.001
0.000
0.001
MR leave−one−out sensitivity analysis for
'APMAP' on 'Bladder cancer'

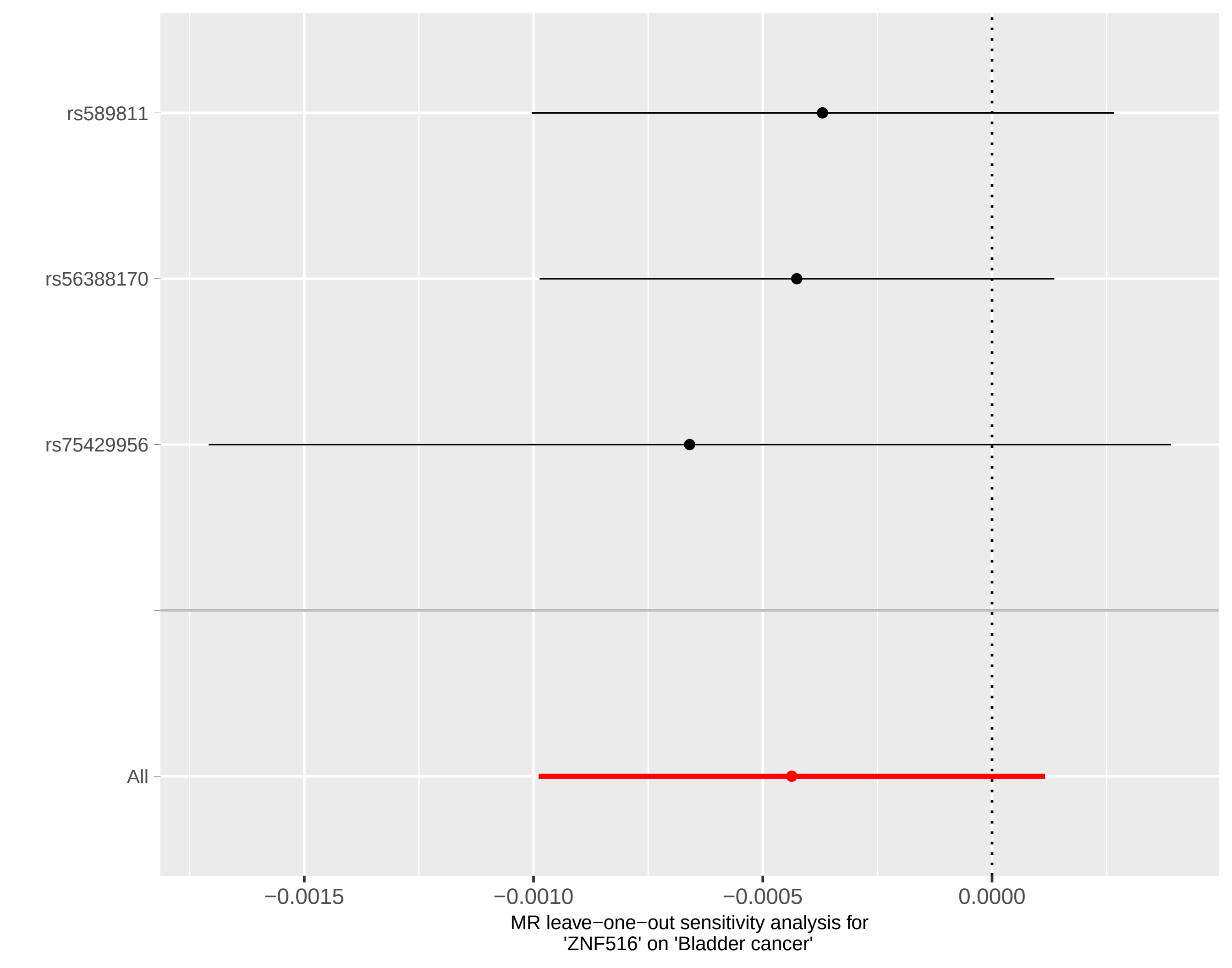
rs589811
rs56388170
rs75429956
All
−0.0015
−0.0010
−0.0005
0.0000
MR leave−one−out sensitivity analysis for
'ZNF516' on 'Bladder cancer'

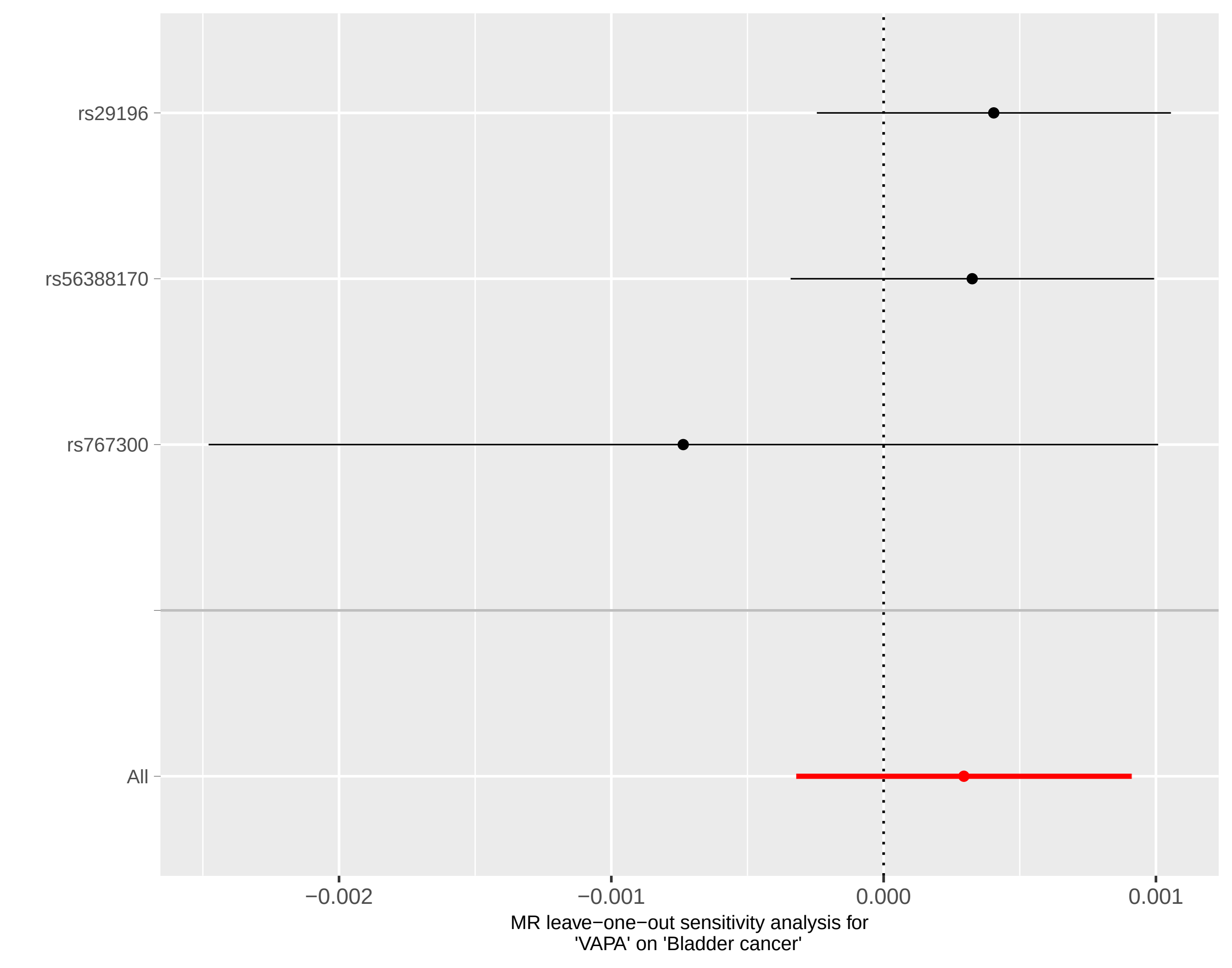
rs29196
rs56388170
rs767300
All
−0.002
−0.001
0.000
0.001
MR leave−one−out sensitivity analysis for
'VAPA' on 'Bladder cancer'

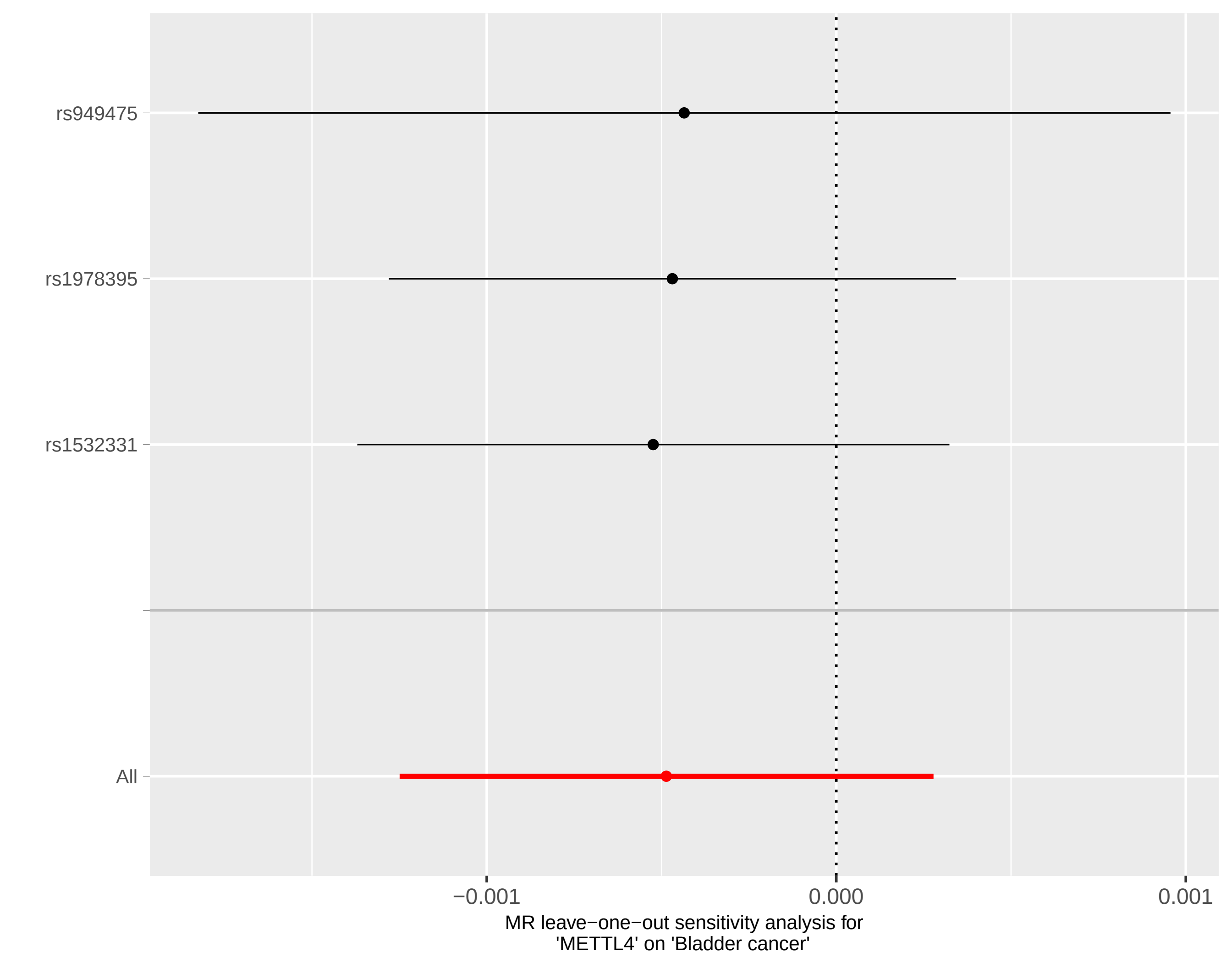

rs949475
rs1978395
rs1532331
All
−0.001
0.000
0.001
MR leave−one−out sensitivity analysis for
'METTL4' on 'Bladder cancer'

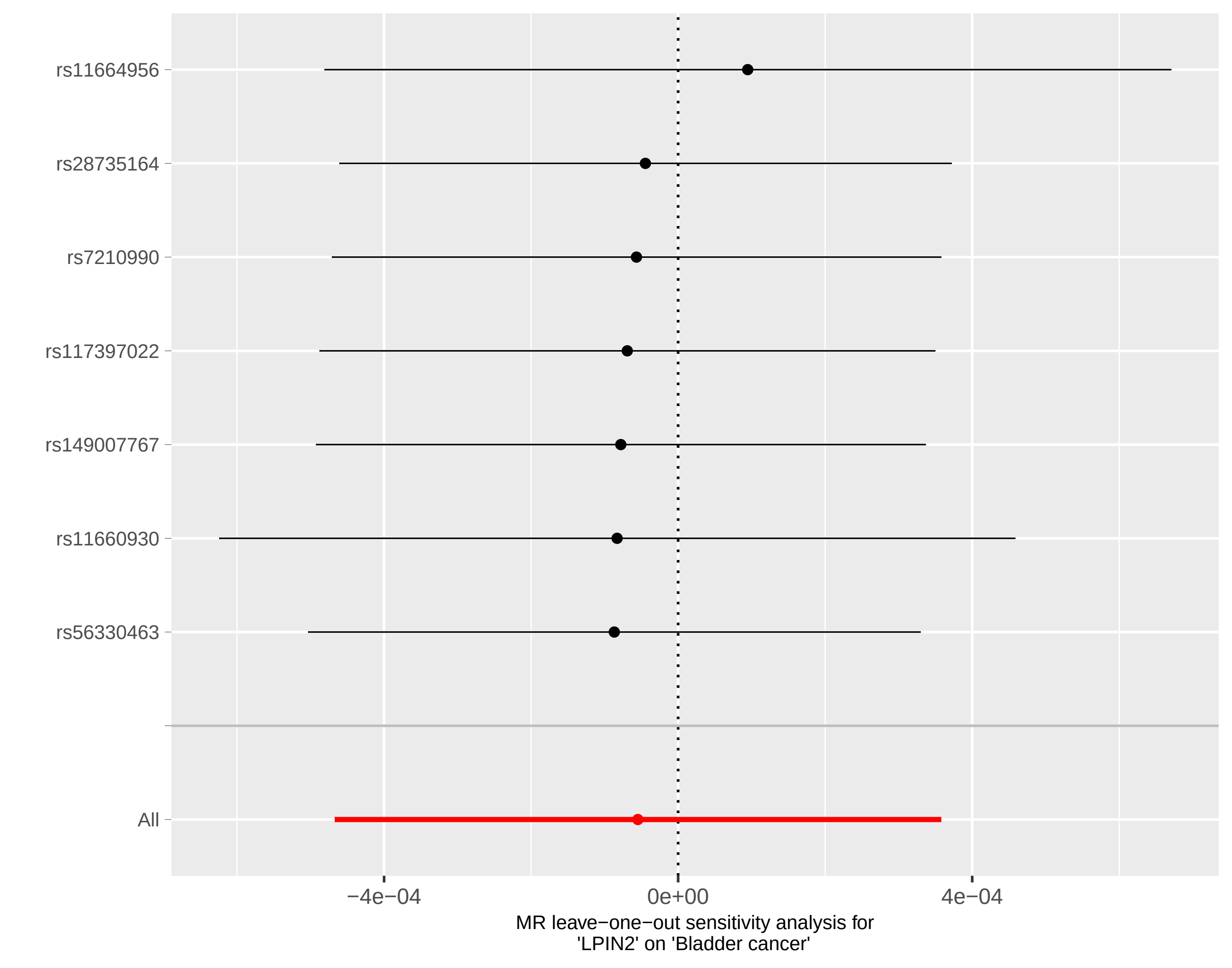
rs11664956
rs28735164
rs7210990
rs117397022
rs149007767
rs11660930
rs56330463
All
−4e−04
0e+00
4e−04
MR leave−one−out sensitivity analysis for
'LPIN2' on 'Bladder cancer'

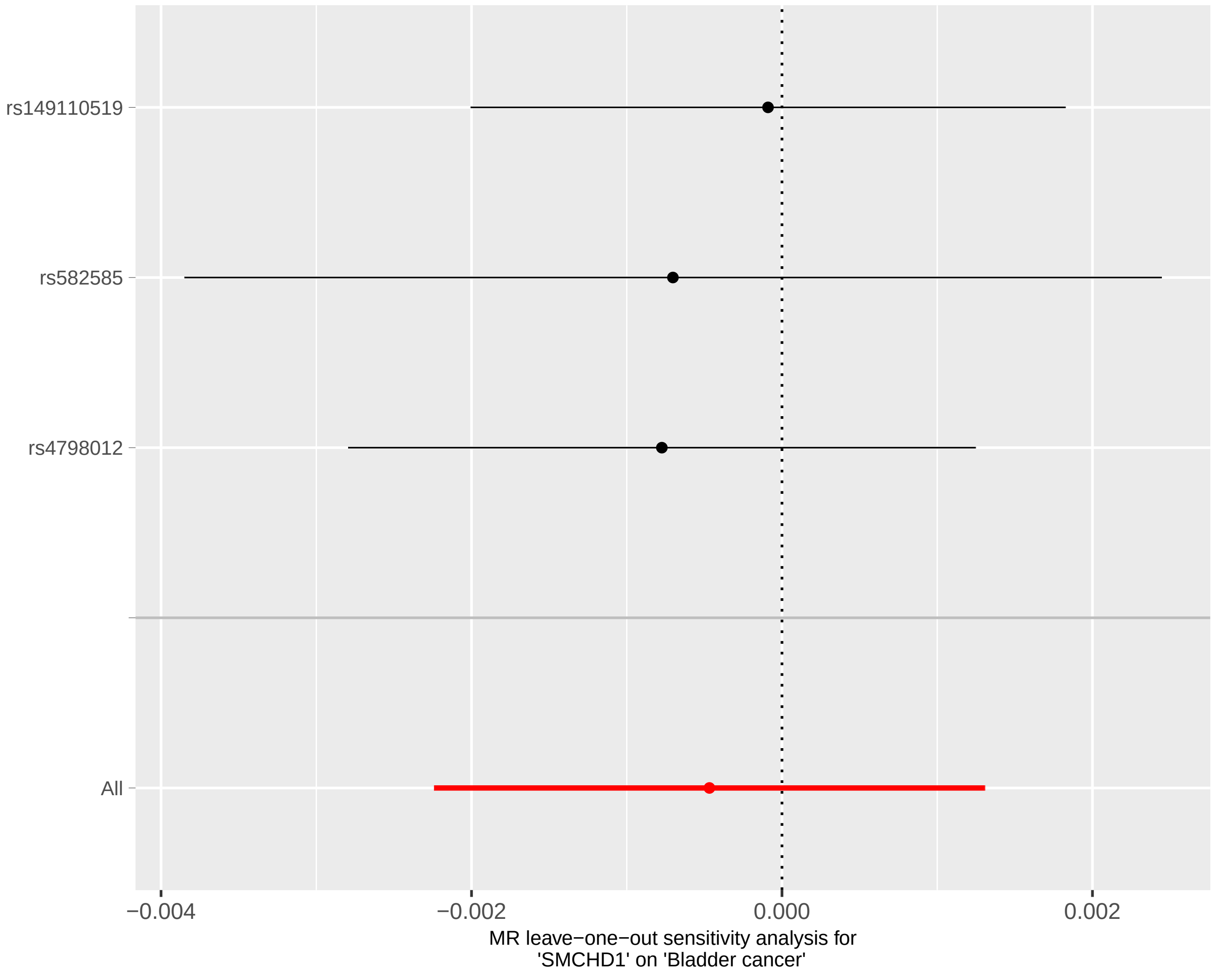
rs149110519
rs582585
rs4798012
All
−0.004
−0.002
0.000
0.002
MR leave−one−out sensitivity analysis for
'SMCHD1' on 'Bladder cancer'

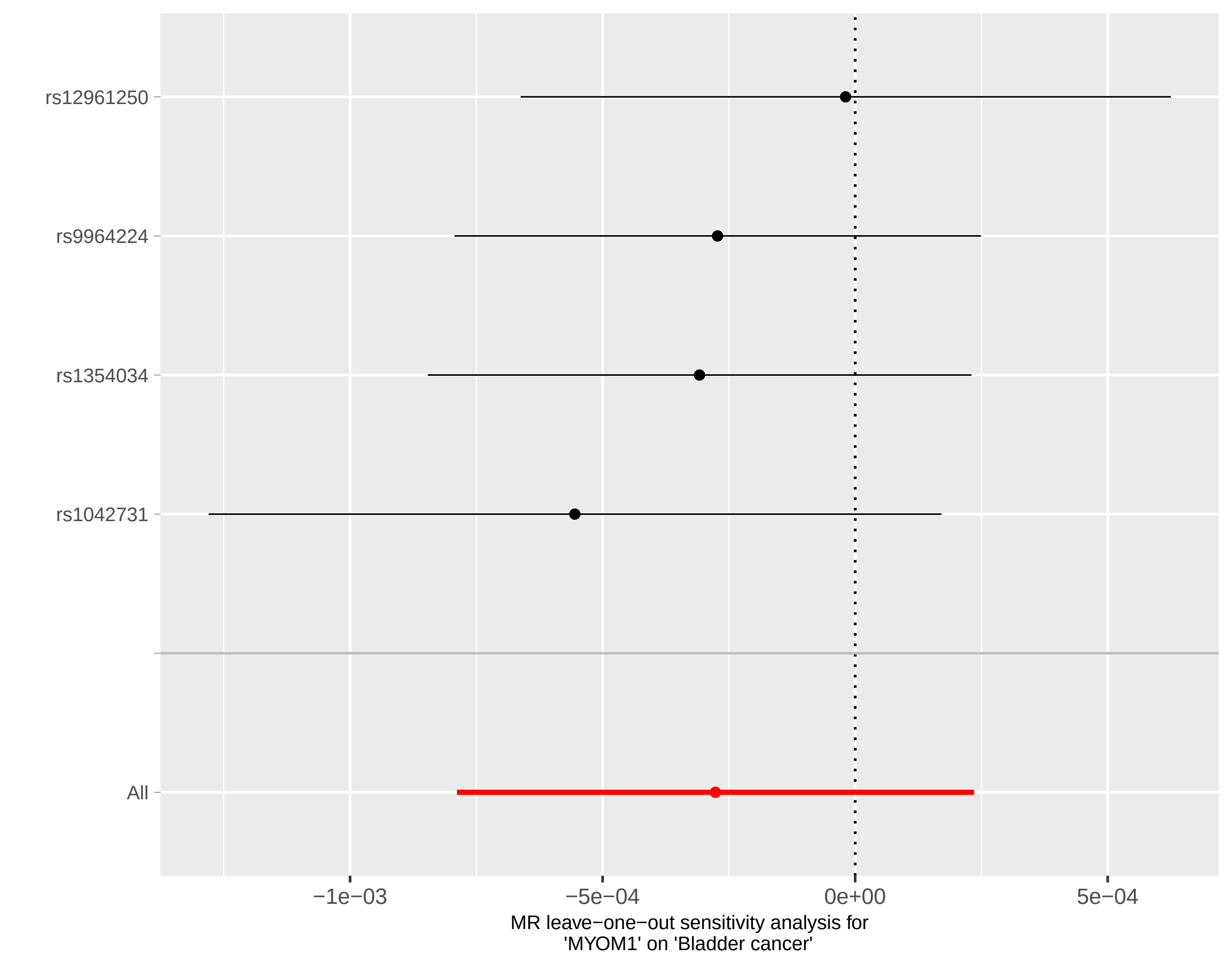
rs12961250
rs9964224
rs1354034
rs1042731
All
−1e−03
−5e−04
0e+00
5e−04
MR leave−one−out sensitivity analysis for
'MYOM1' on 'Bladder cancer'

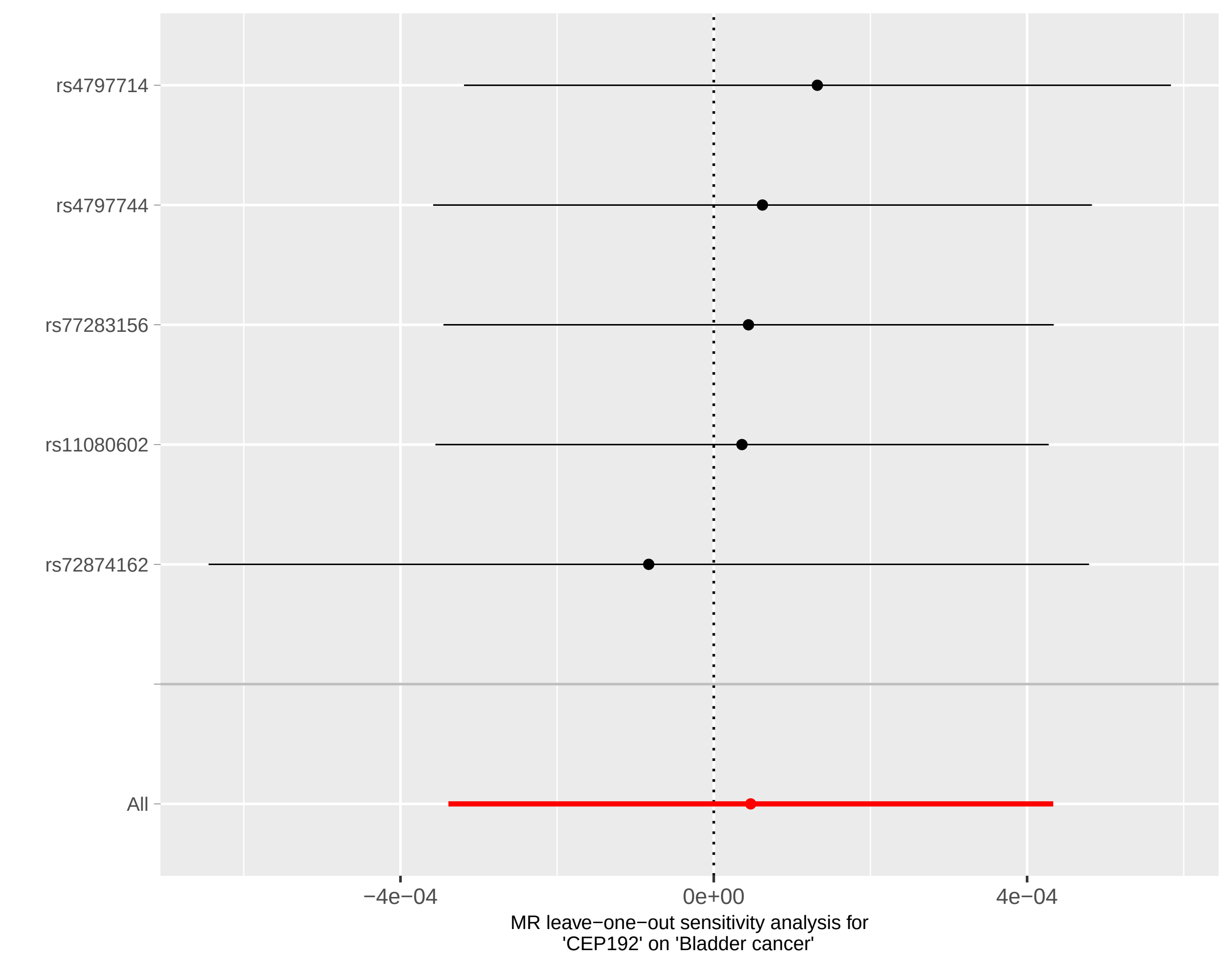
rs4797714
rs4797744
rs77283156
rs11080602
rs72874162
All
−4e−04
0e+00
4e−04
MR leave−one−out sensitivity analysis for
'CEP192' on 'Bladder cancer'

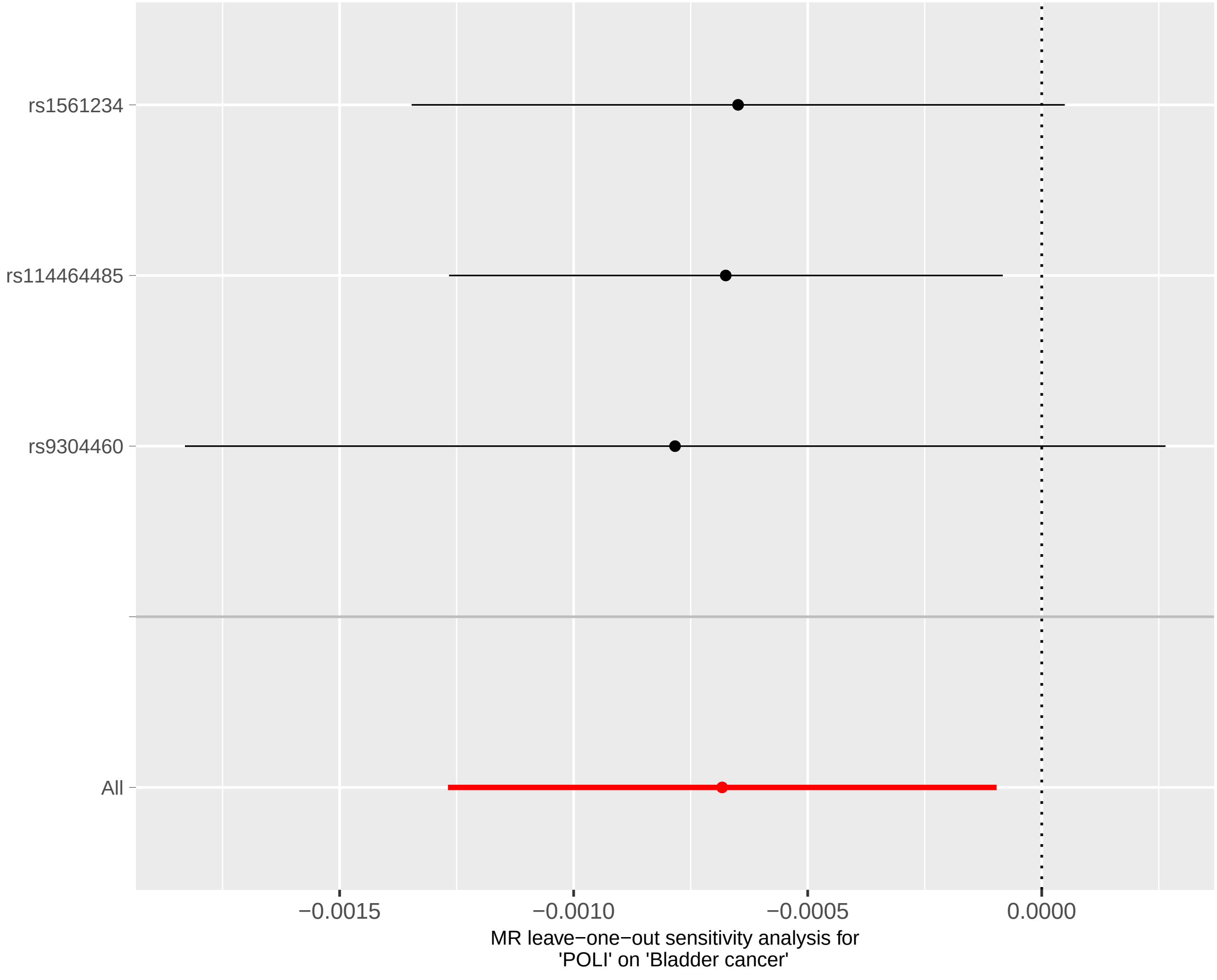
rs1561234
rs114464485
rs9304460
All
−0.0015
−0.0010
−0.0005
0.0000
MR leave−one−out sensitivity analysis for
'POLI' on 'Bladder cancer'

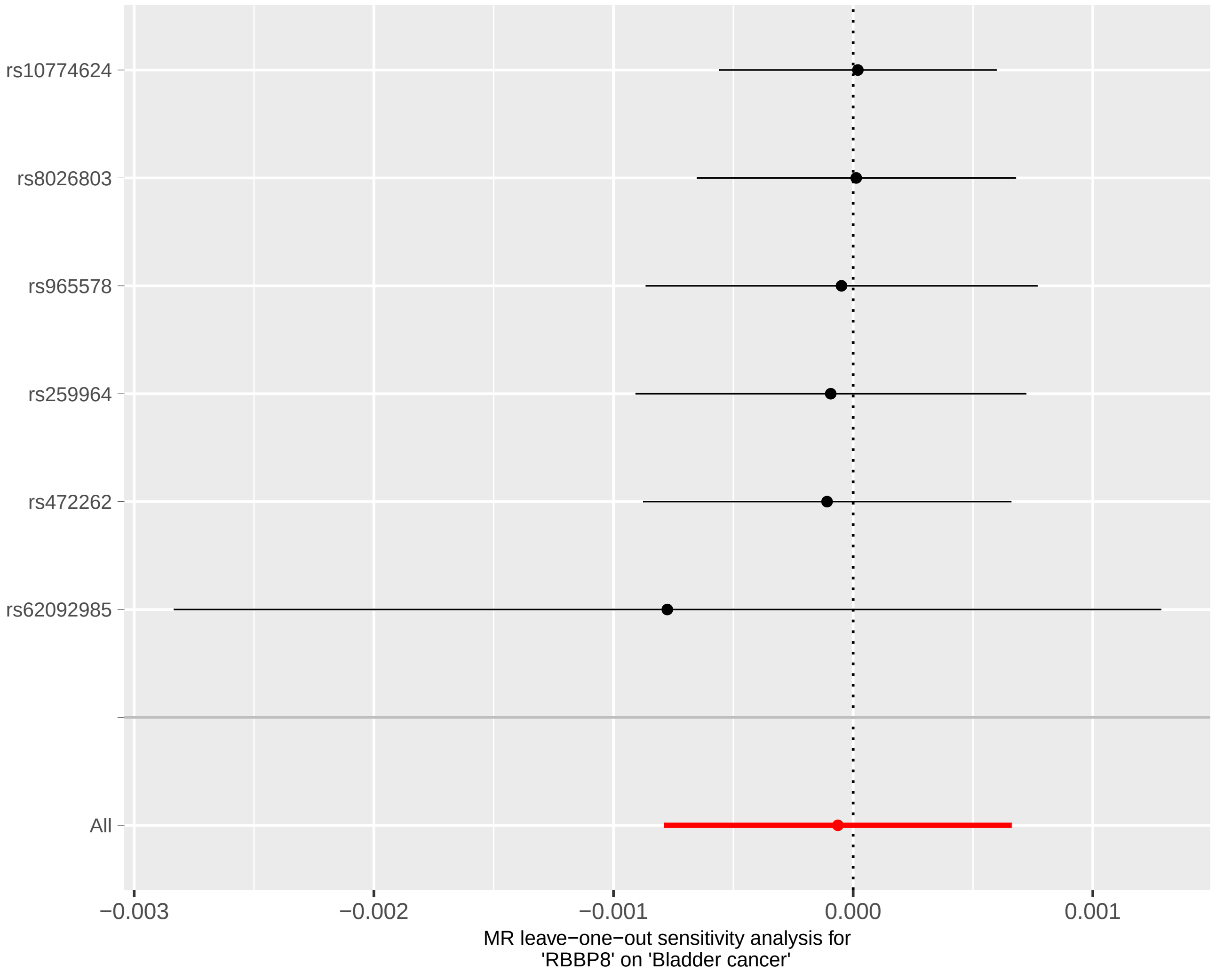
rs10774624
rs8026803
rs965578
rs259964
rs472262
rs62092985
All
−0.003
−0.002
−0.001
0.000
0.001
MR leave−one−out sensitivity analysis for
'RBBP8' on 'Bladder cancer'

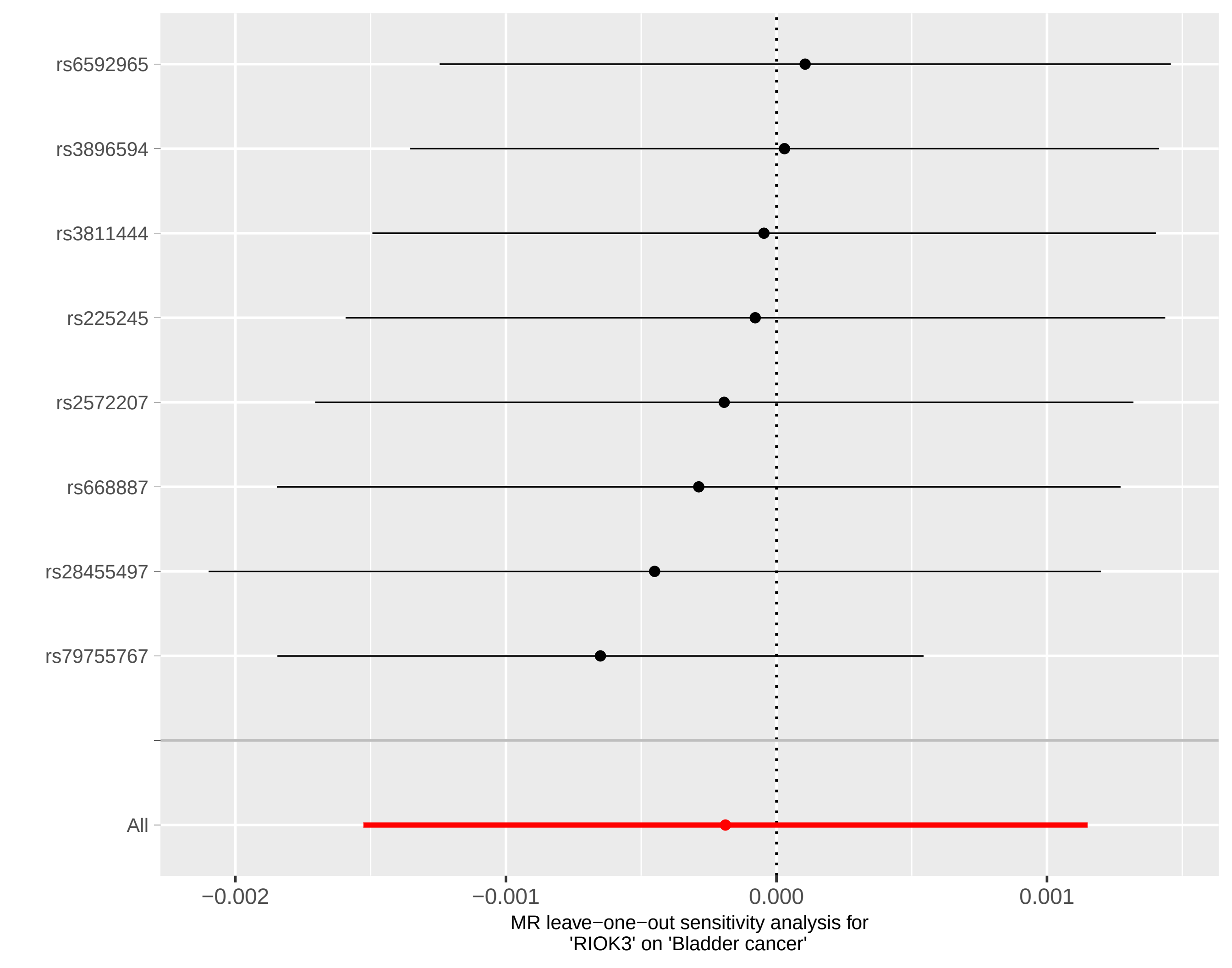
rs6592965
rs3896594
rs3811444
rs225245
rs2572207
rs668887
rs28455497
rs79755767
All
−0.002
−0.001
0.000
0.001
MR leave−one−out sensitivity analysis for
'RIOK3' on 'Bladder cancer'

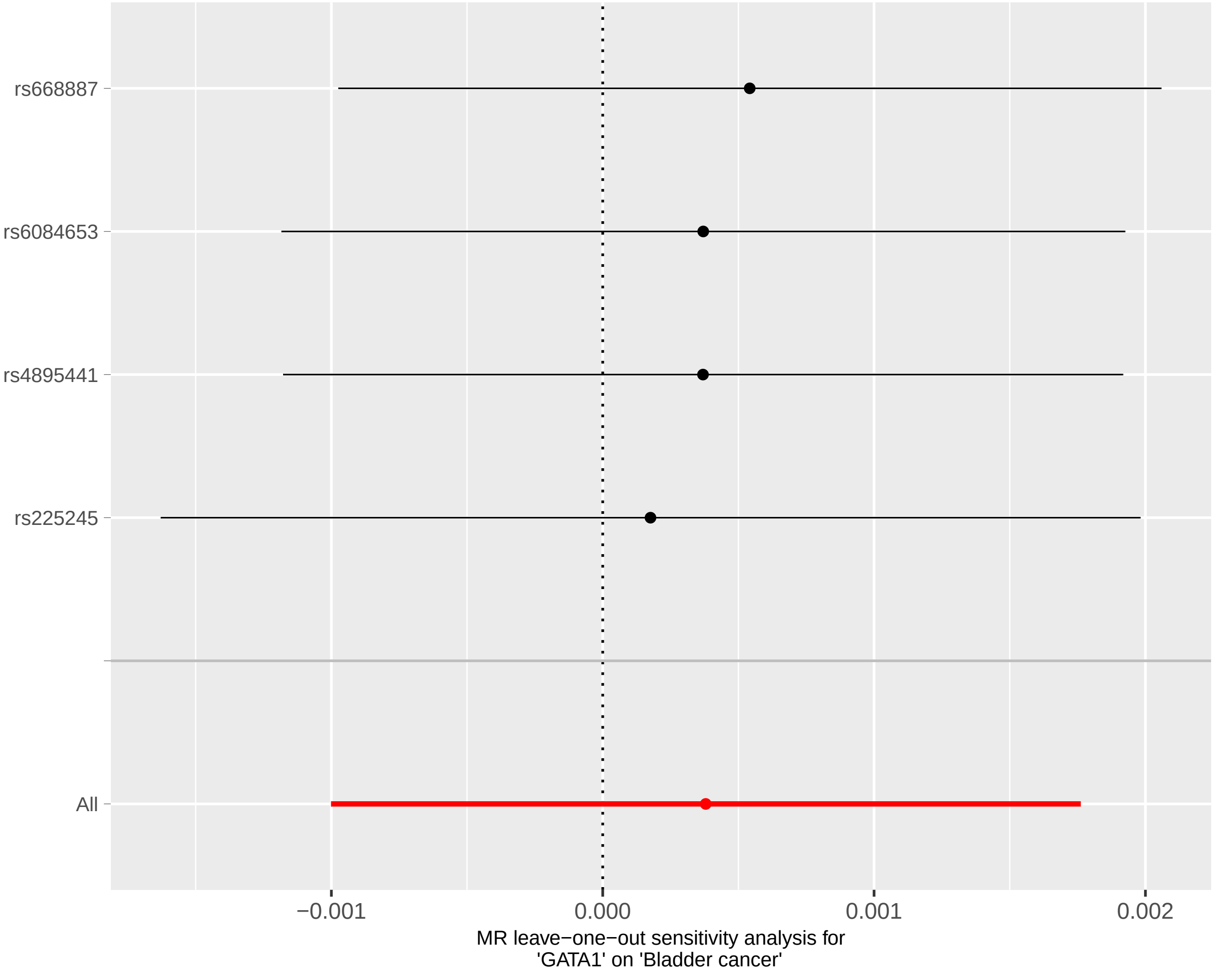
rs668887
rs6084653
rs4895441
rs225245
All
−0.001
0.000
0.001
0.002
MR leave−one−out sensitivity analysis for
'GATA1' on 'Bladder cancer'

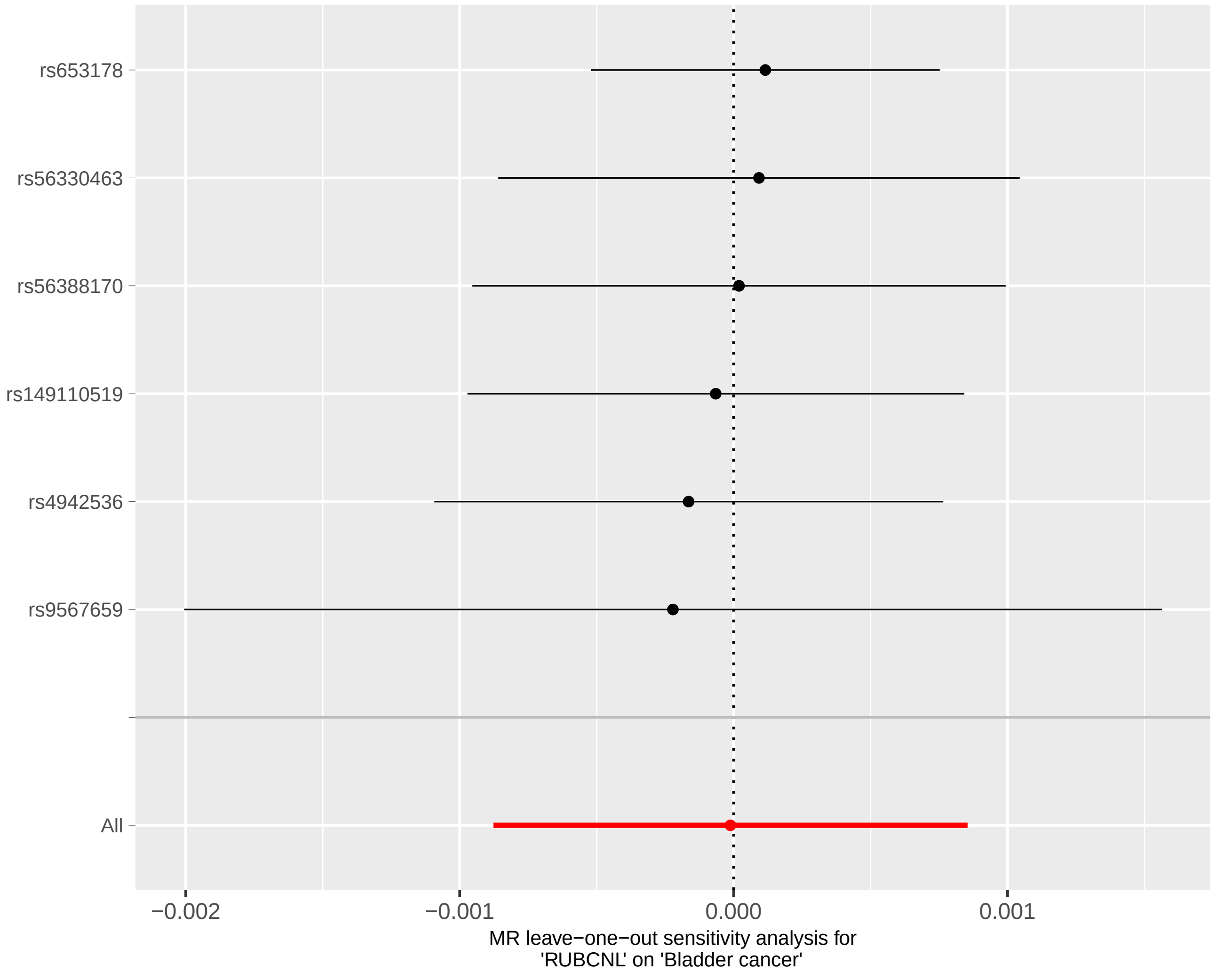
rs653178
rs56330463
rs56388170
rs149110519
rs4942536
rs9567659
All
−0.002
−0.001
0.000
0.001
MR leave−one−out sensitivity analysis for
'RUBCNL' on 'Bladder cancer'

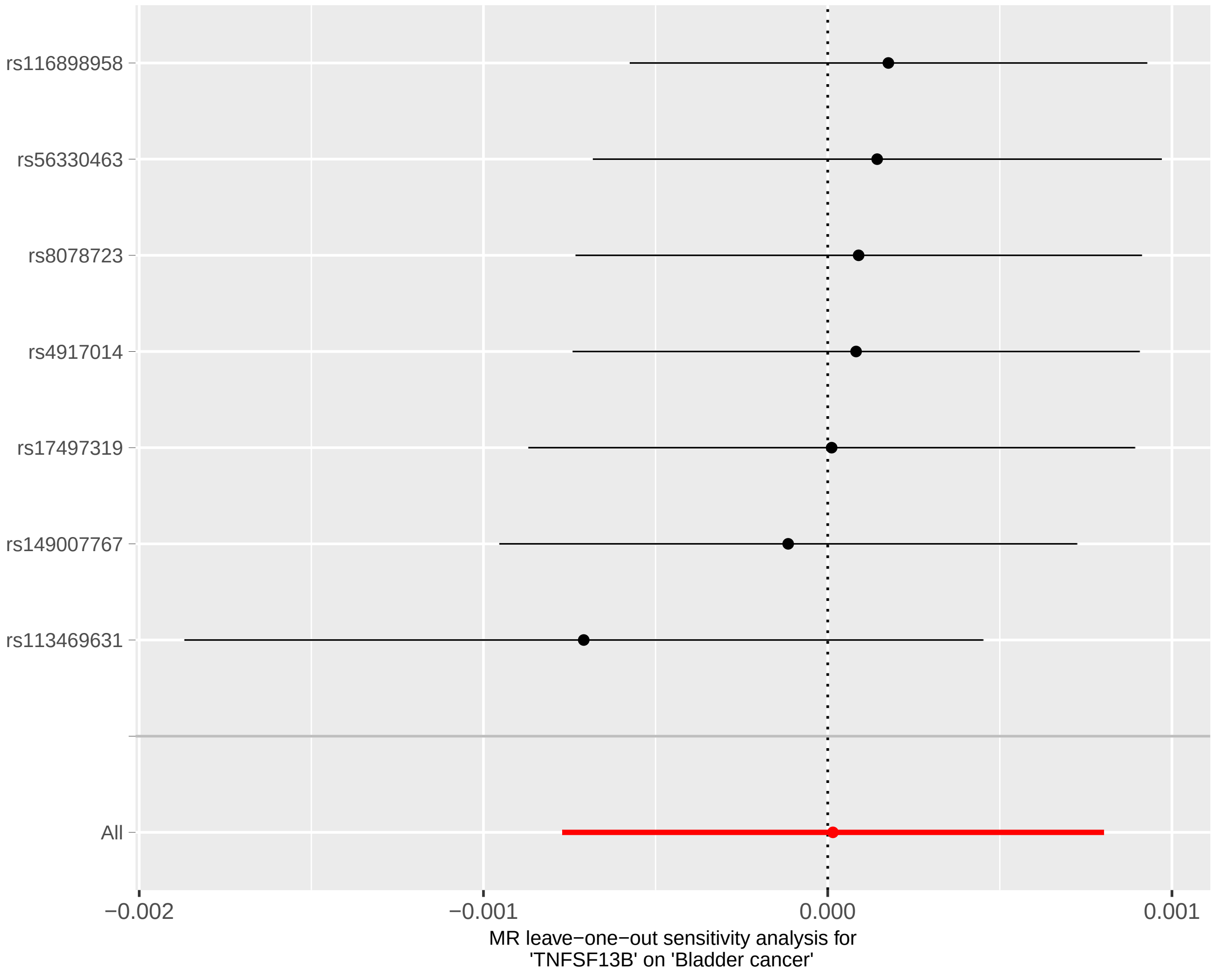
rs116898958
rs56330463
rs8078723
rs4917014
rs17497319
rs149007767
rs113469631
All
−0.002
−0.001
0.000
0.001
MR leave−one−out sensitivity analysis for
'TNFSF13B' on 'Bladder cancer'

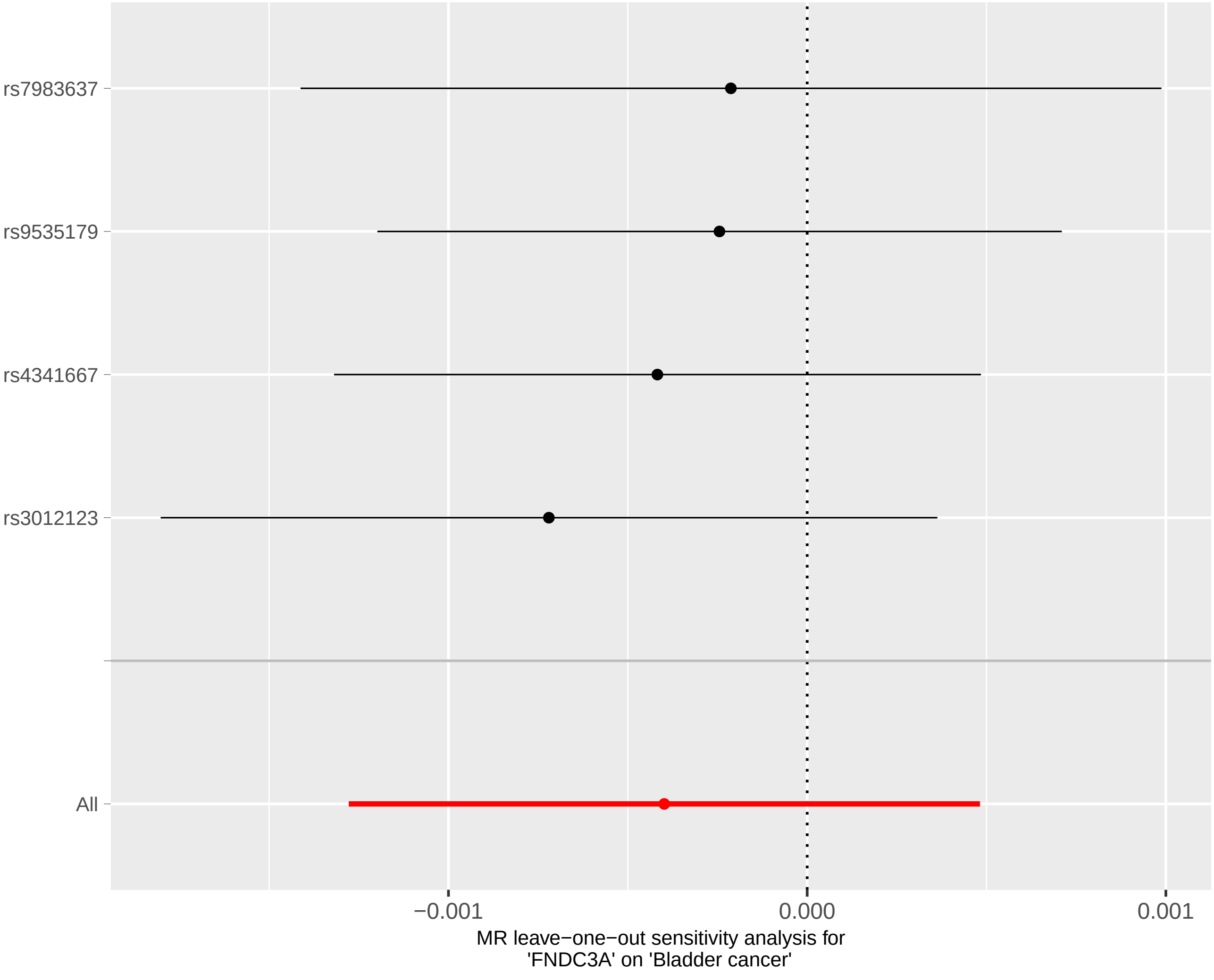
rs7983637
rs9535179
rs4341667
rs3012123
All
−0.001
0.000
0.001
MR leave−one−out sensitivity analysis for
'FNDC3A' on 'Bladder cancer'

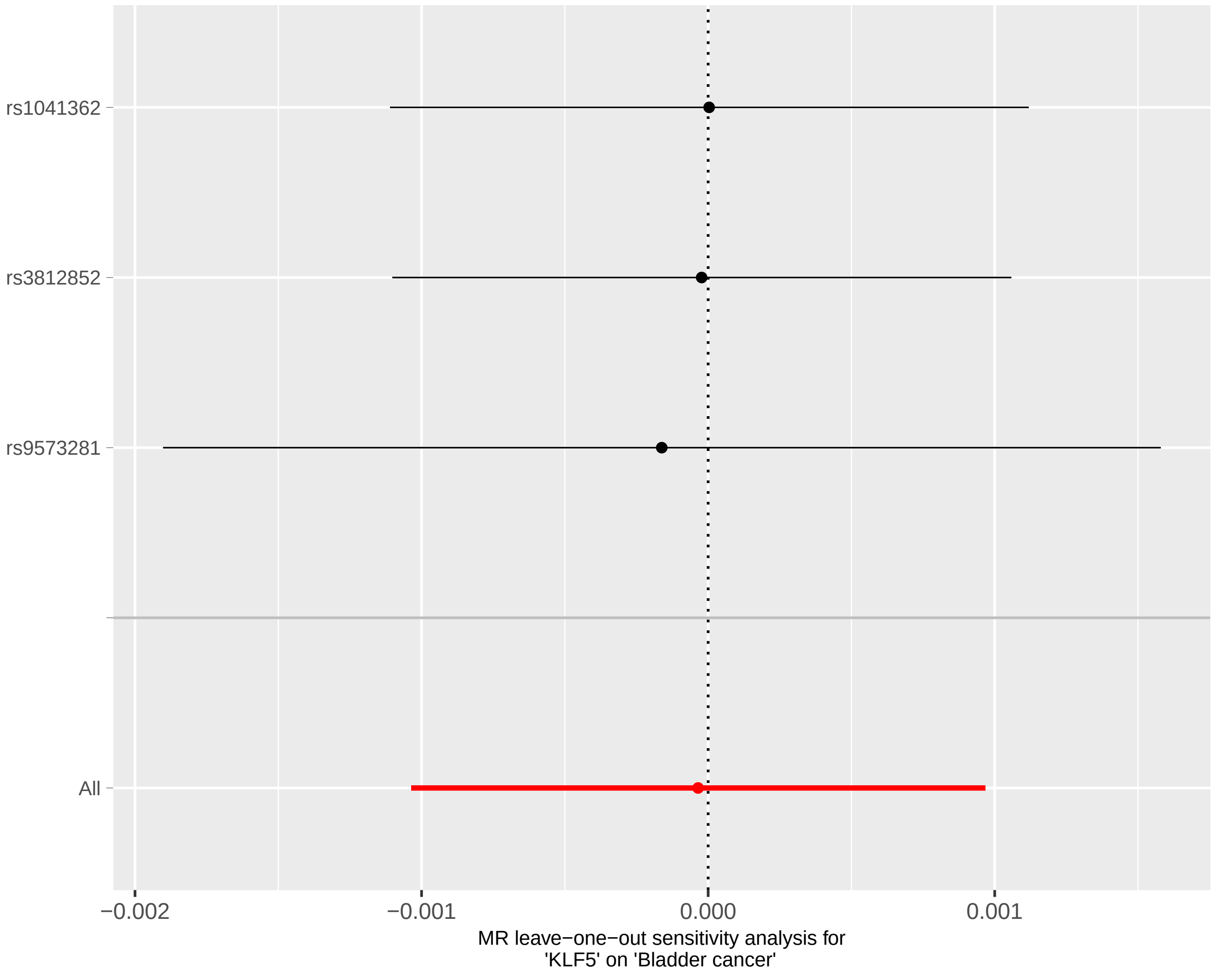
rs1041362
rs3812852
rs9573281
All
−0.002
−0.001
0.000
0.001
MR leave−one−out sensitivity analysis for
'KLF5' on 'Bladder cancer'

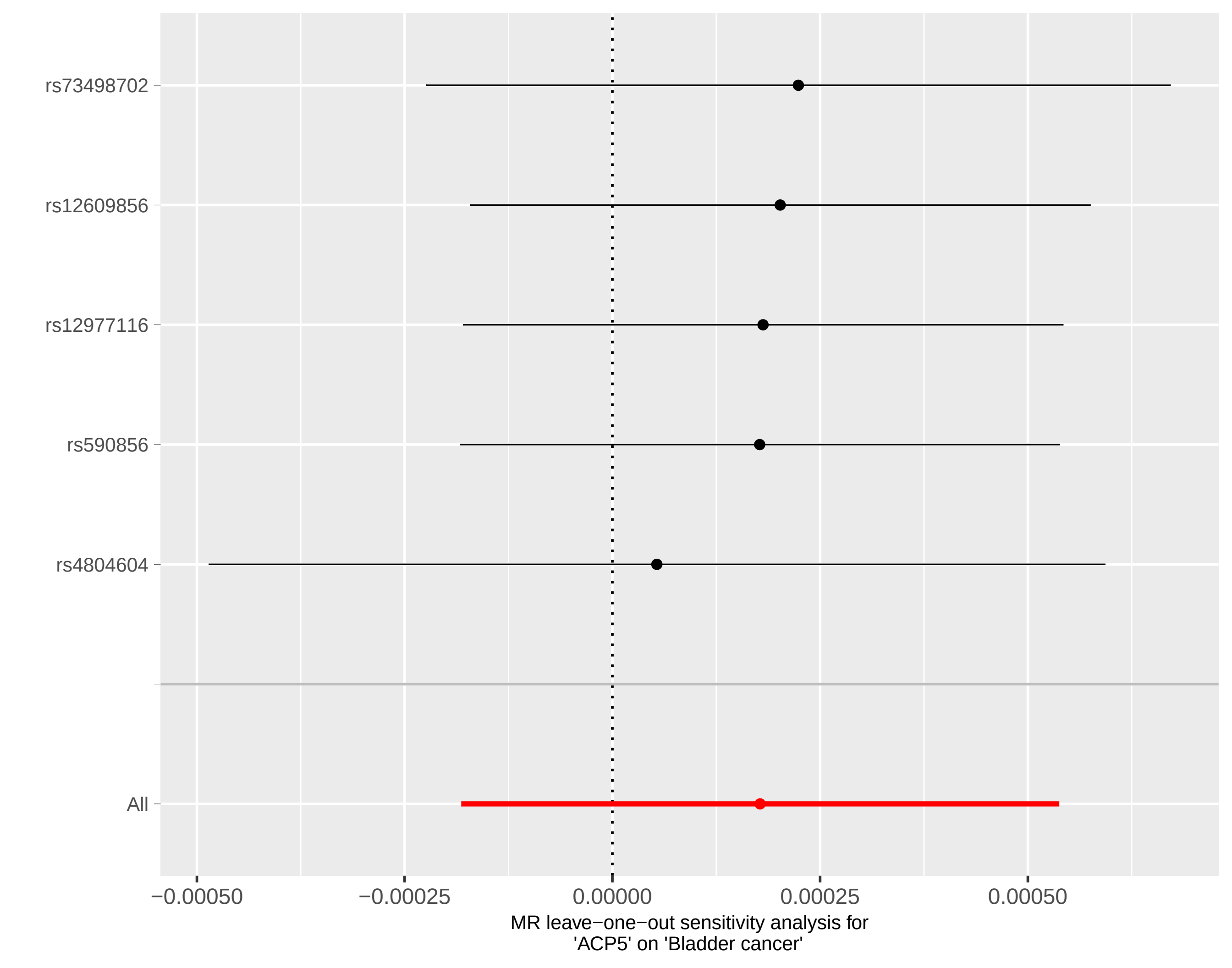

rs73498702
rs12609856
rs12977116
rs590856
rs4804604
All
−0.00050
−0.00025
0.00000
0.00025
0.00050
MR leave−one−out sensitivity analysis for
'ACP5' on 'Bladder cancer'

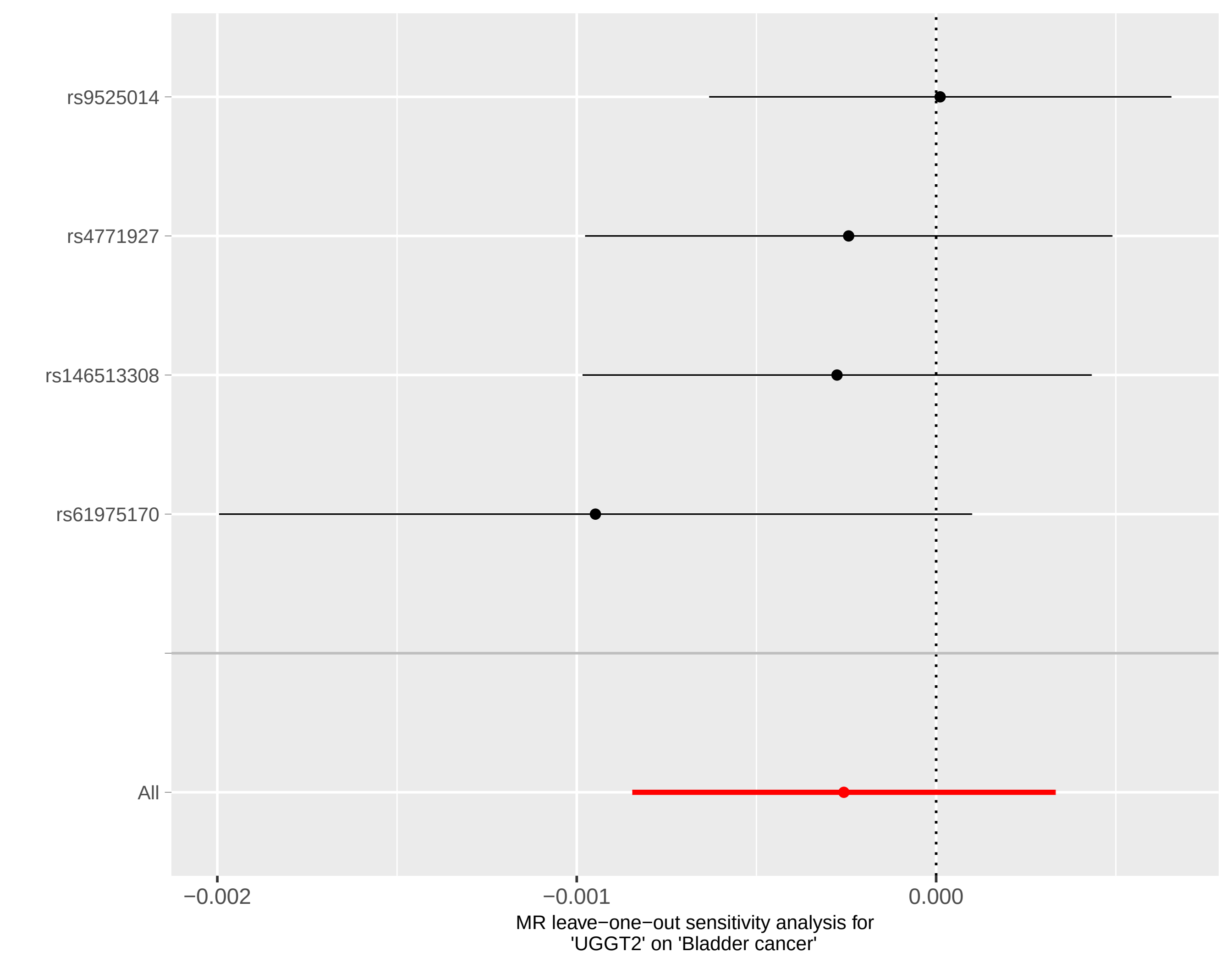
rs9525014
rs4771927
rs146513308
rs61975170
All
−0.002
−0.001
0.000
MR leave−one−out sensitivity analysis for
'UGGT2' on 'Bladder cancer'

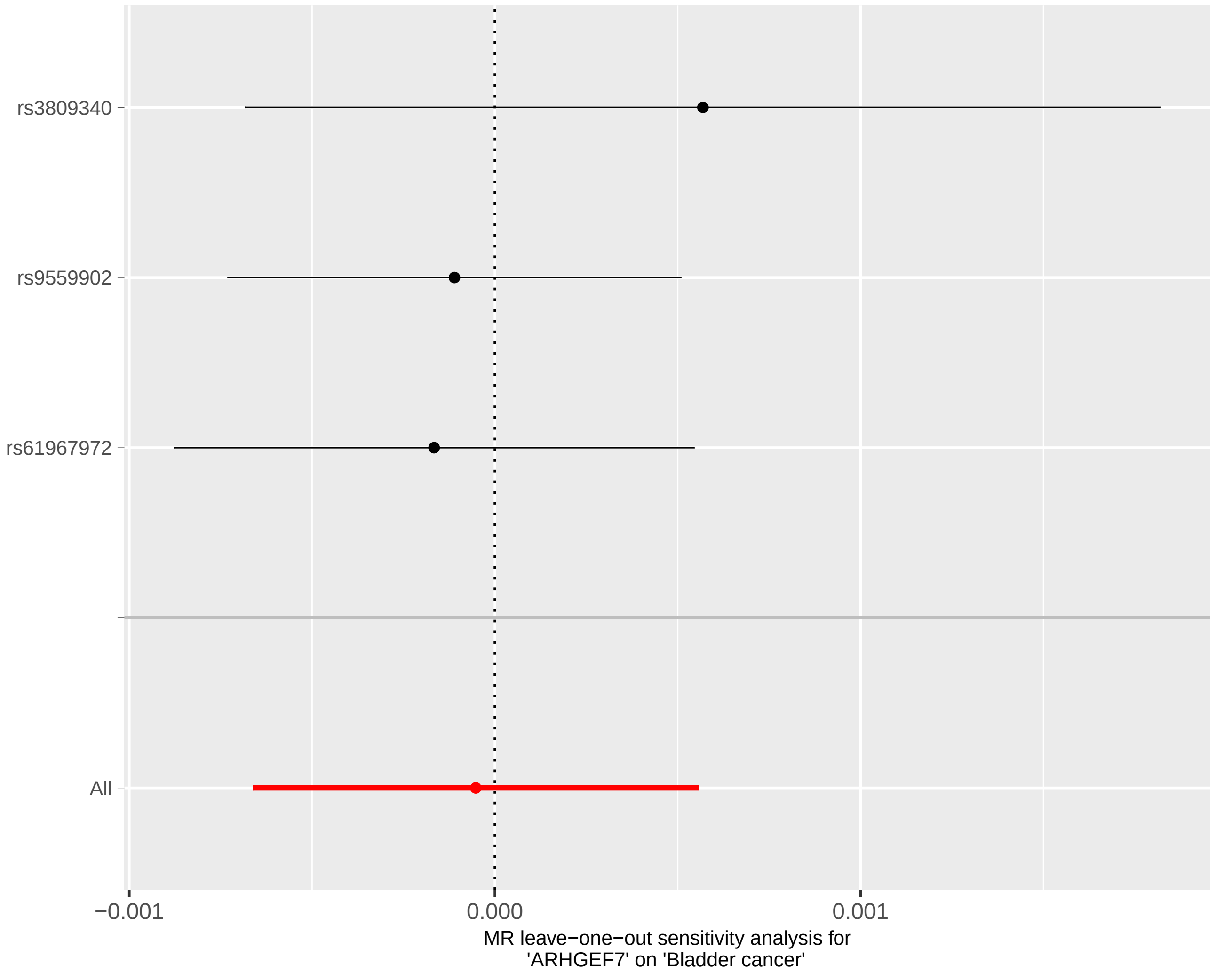

rs3809340
rs9559902
rs61967972
All
−0.001
0.000
0.001
MR leave−one−out sensitivity analysis for
'ARHGEF7' on 'Bladder cancer'

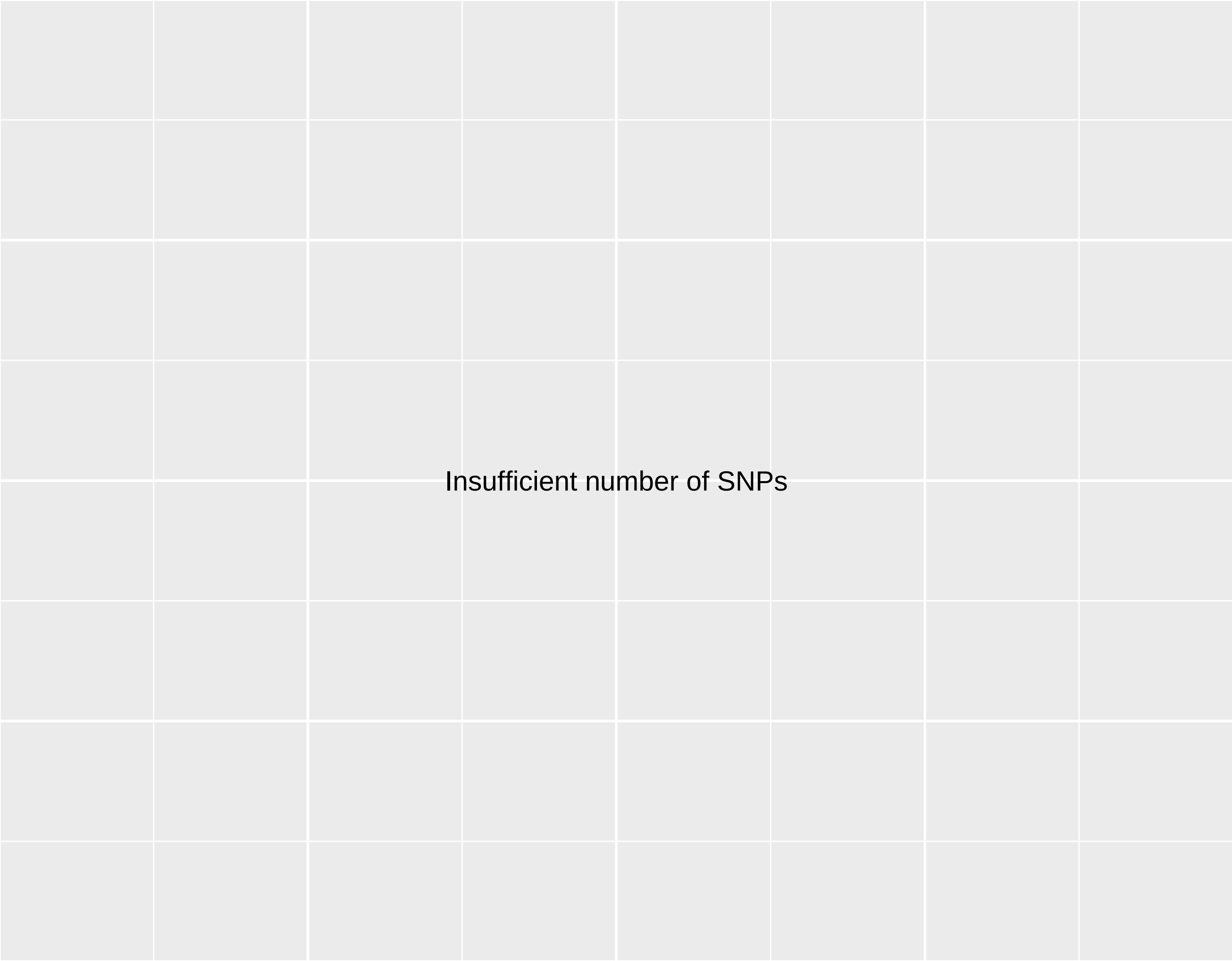
Insufficient number of SNPs

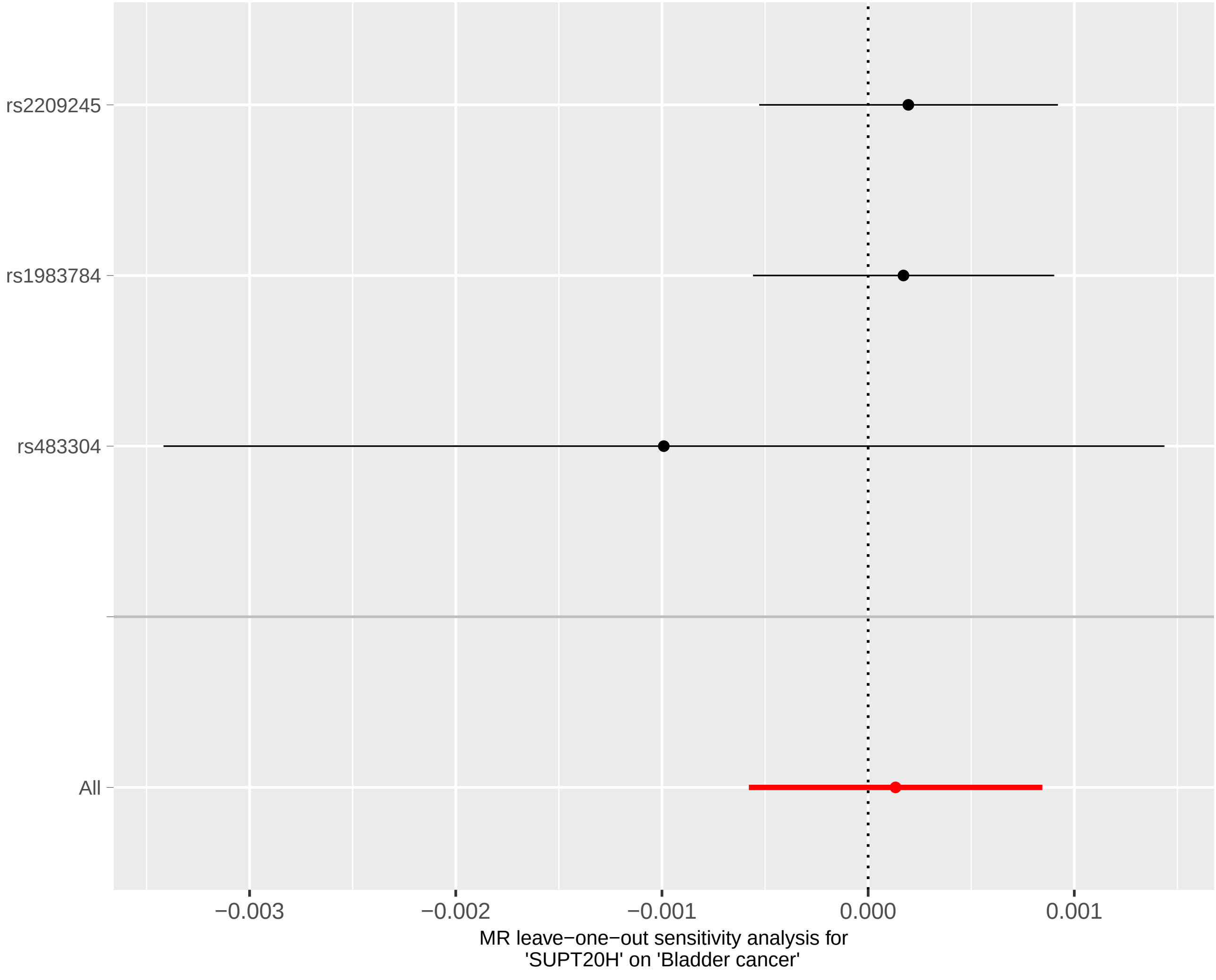
rs2209245
rs1983784
rs483304
All
−0.003
−0.002
−0.001
0.000
0.001
MR leave−one−out sensitivity analysis for
'SUPT20H' on 'Bladder cancer'

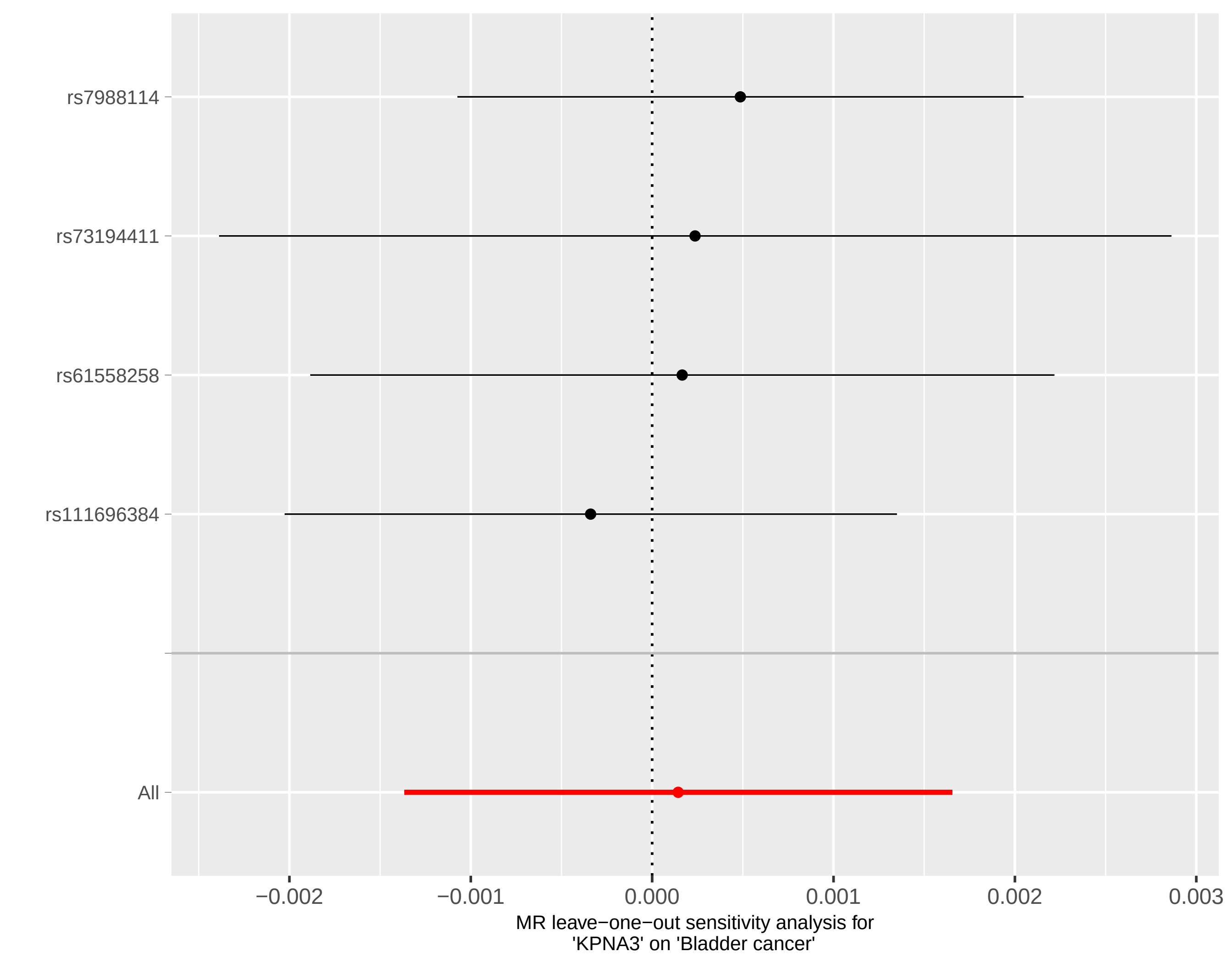
rs7988114
rs73194411
rs61558258
rs111696384
All
−0.002
−0.001
0.000
0.001
0.002
0.003
MR leave−one−out sensitivity analysis for
'KPNA3' on 'Bladder cancer'

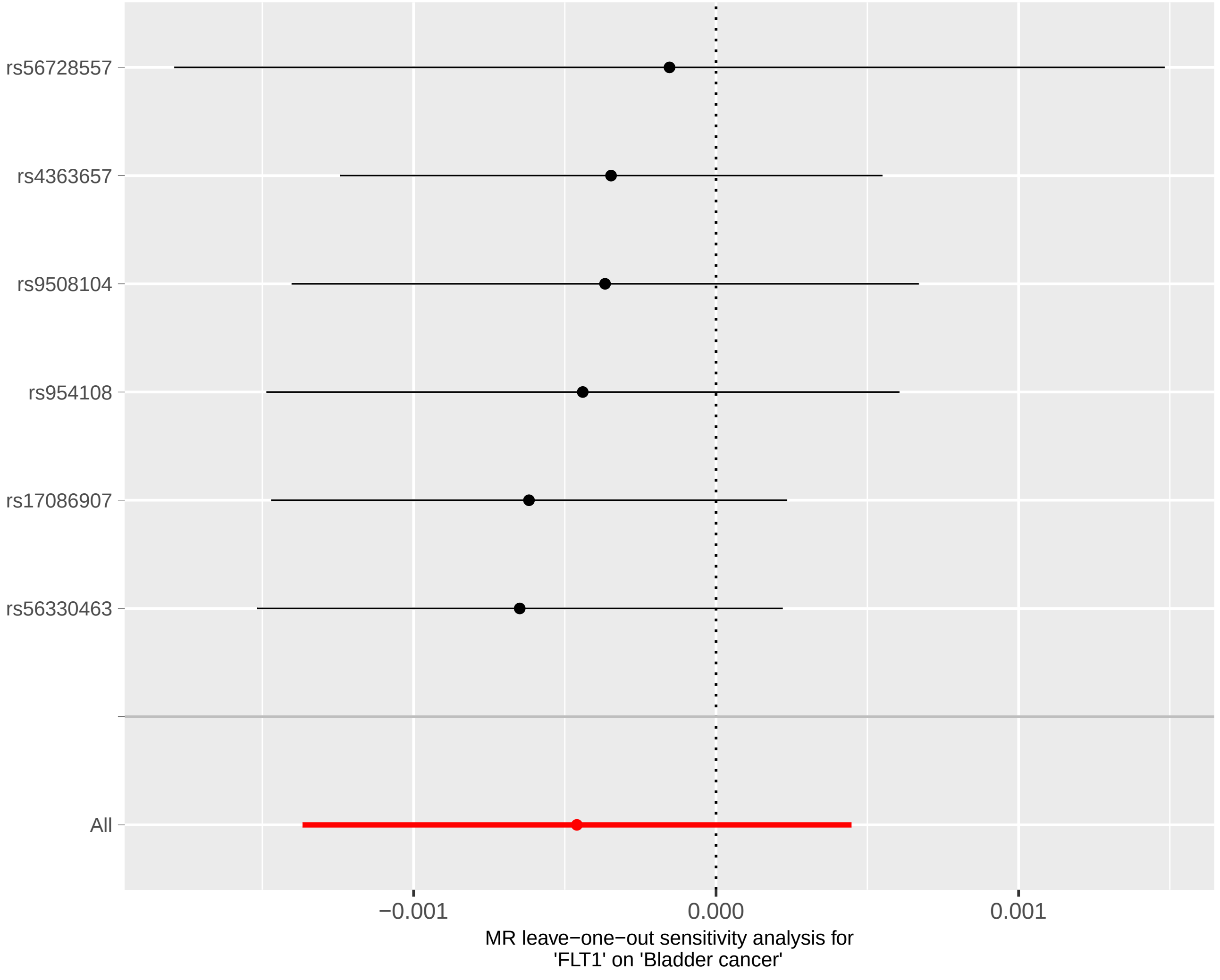
rs56728557
rs4363657
rs9508104
rs954108
rs17086907
rs56330463
All
−0.001
0.000
0.001
MR leave−one−out sensitivity analysis for
'FLT1' on 'Bladder cancer'

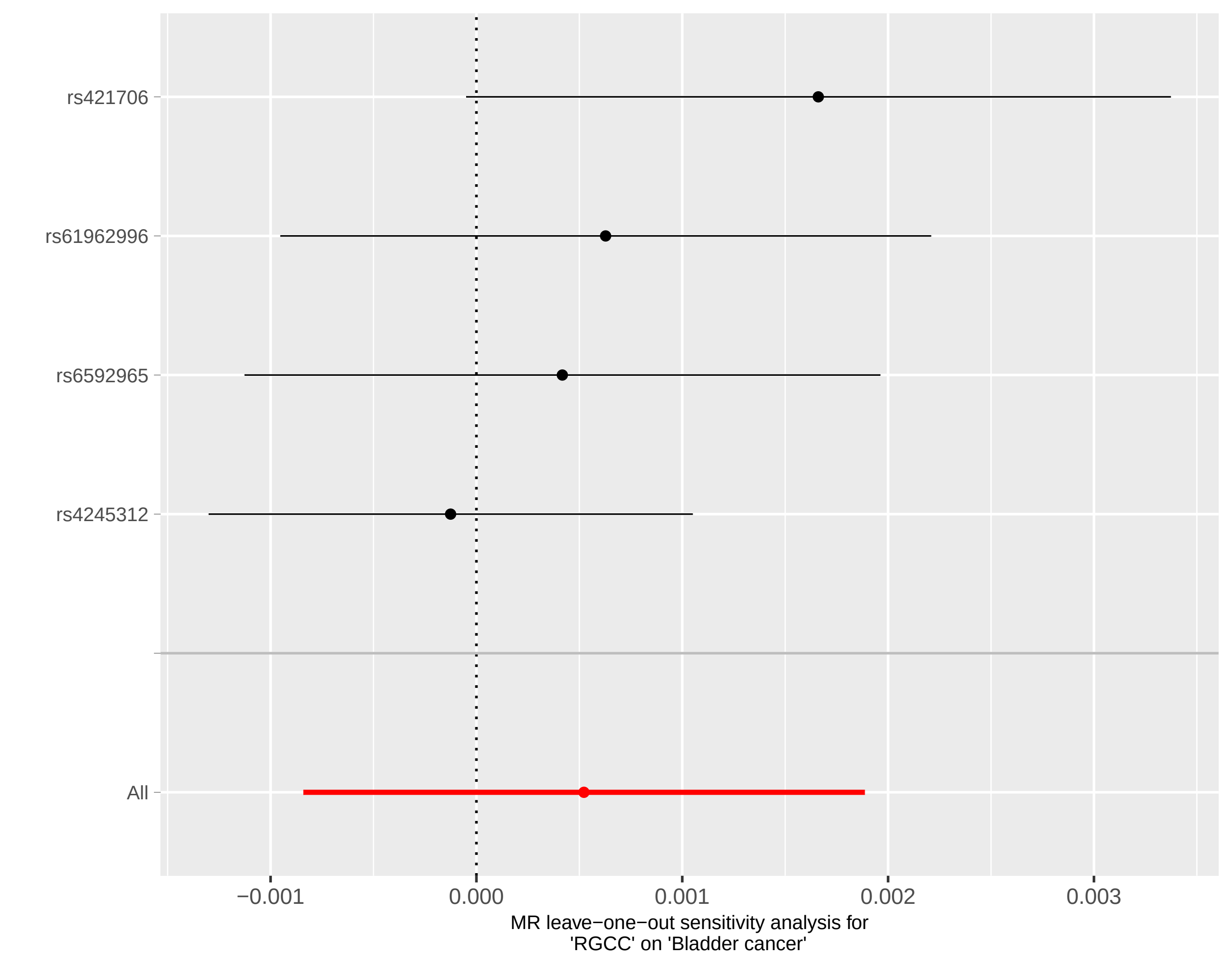
rs421706
rs61962996
rs6592965
rs4245312
All
−0.001
0.000
0.001
0.002
0.003
MR leave−one−out sensitivity analysis for
'RGCC' on 'Bladder cancer'

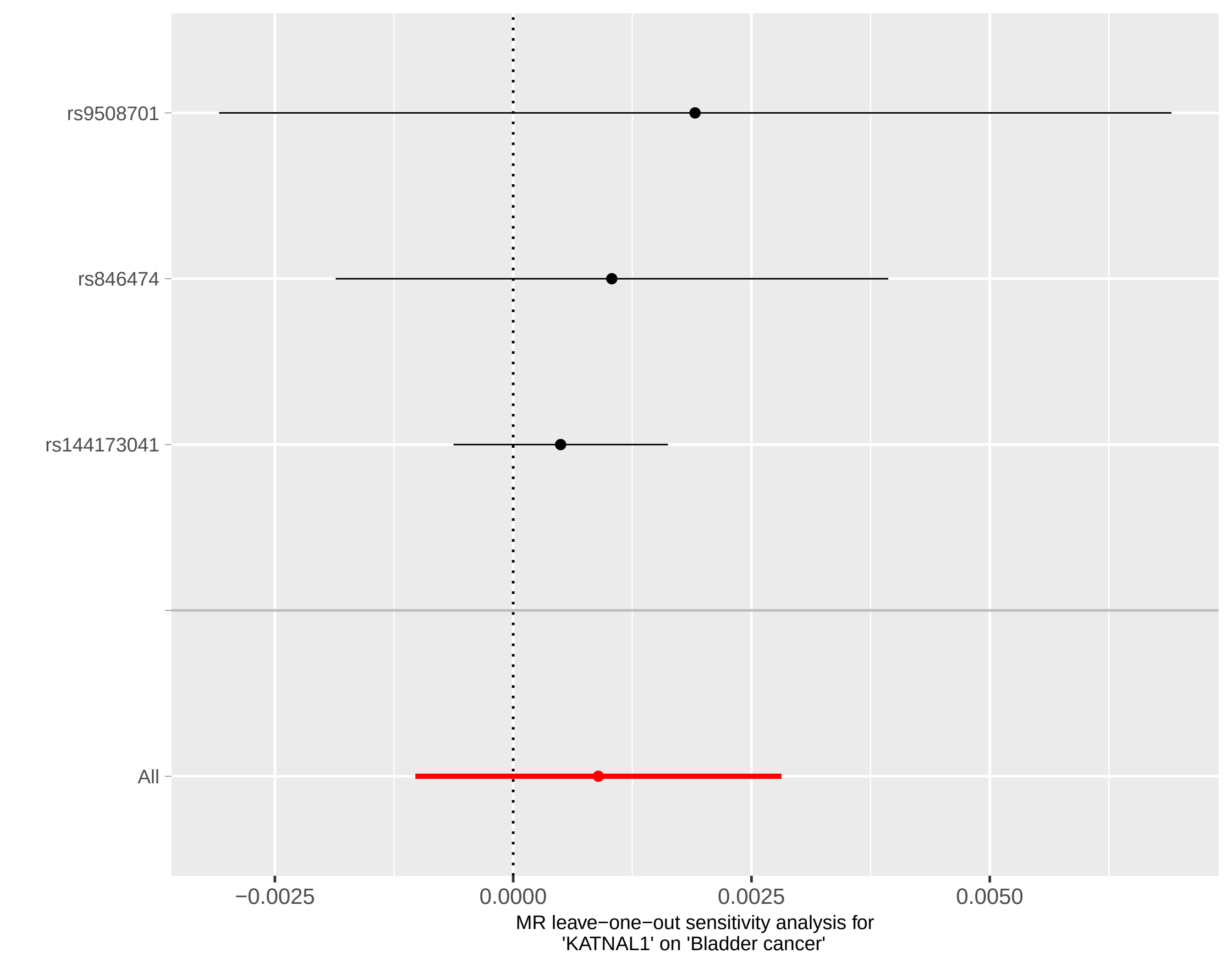
rs9508701
rs846474
rs144173041
All
−0.0025
0.0000
0.0025
0.0050
MR leave−one−out sensitivity analysis for
'KATNAL1' on 'Bladder cancer'

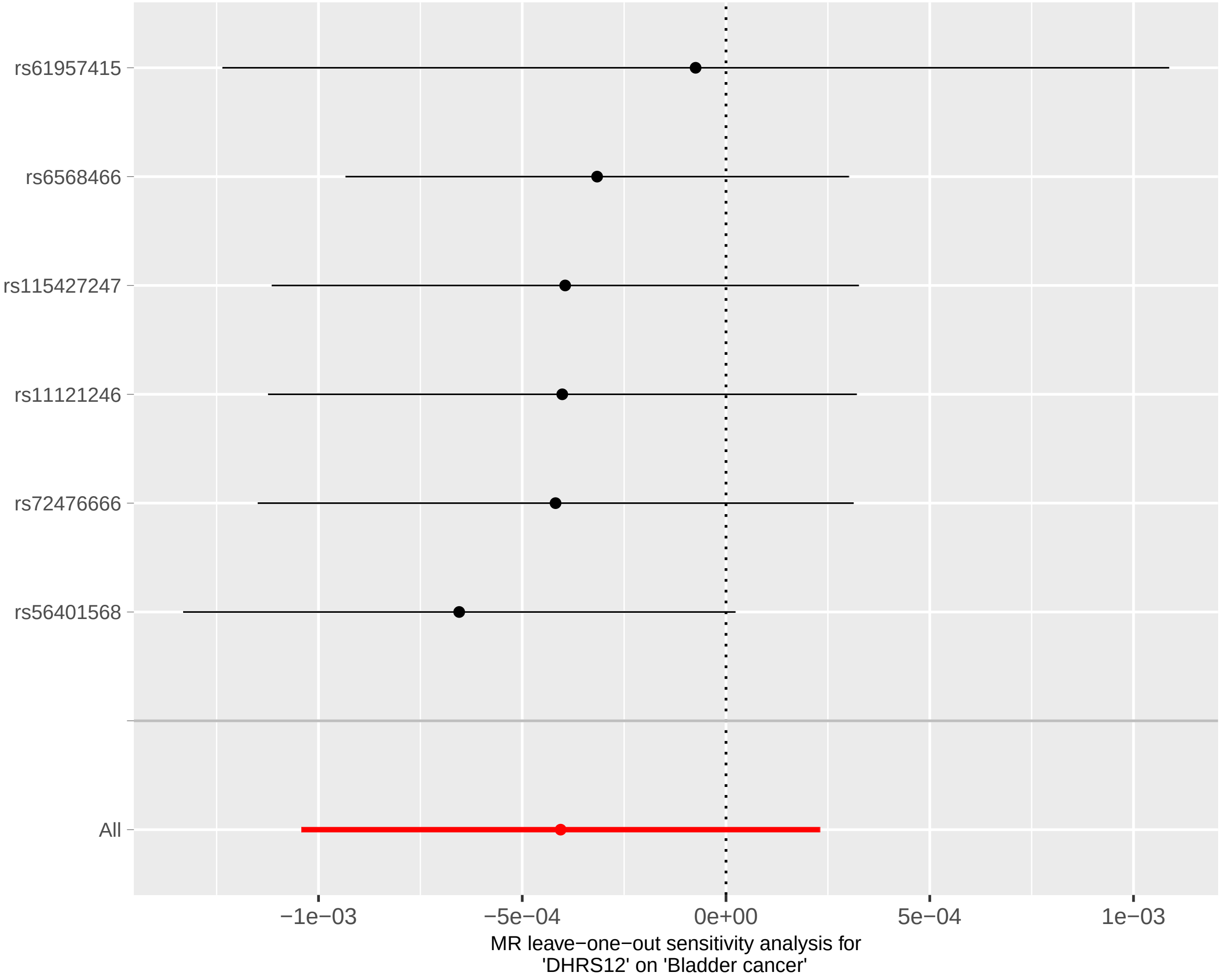
rs61957415
rs6568466
rs115427247
rs11121246
rs72476666
rs56401568
All
−1e−03
−5e−04
0e+00
5e−04
1e−03
MR leave−one−out sensitivity analysis for
'DHRS12' on 'Bladder cancer'

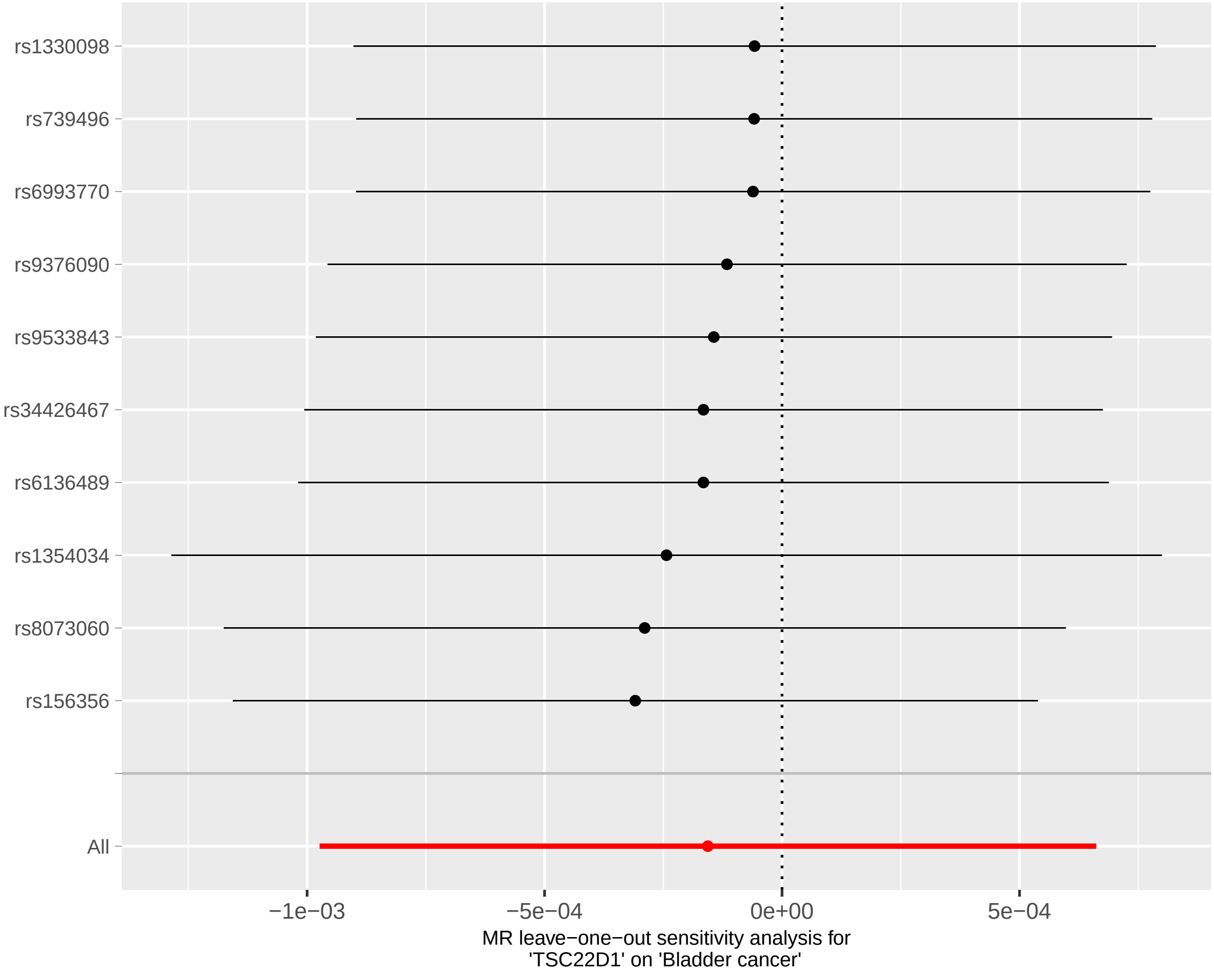
rs1330098
rs739496
rs6993770
rs9376090
rs9533843
rs34426467
rs6136489
rs1354034
rs8073060
rs156356
All
−1e−03
−5e−04
0e+00
5e−04
MR leave−one−out sensitivity analysis for
'TSC22D1' on 'Bladder cancer'

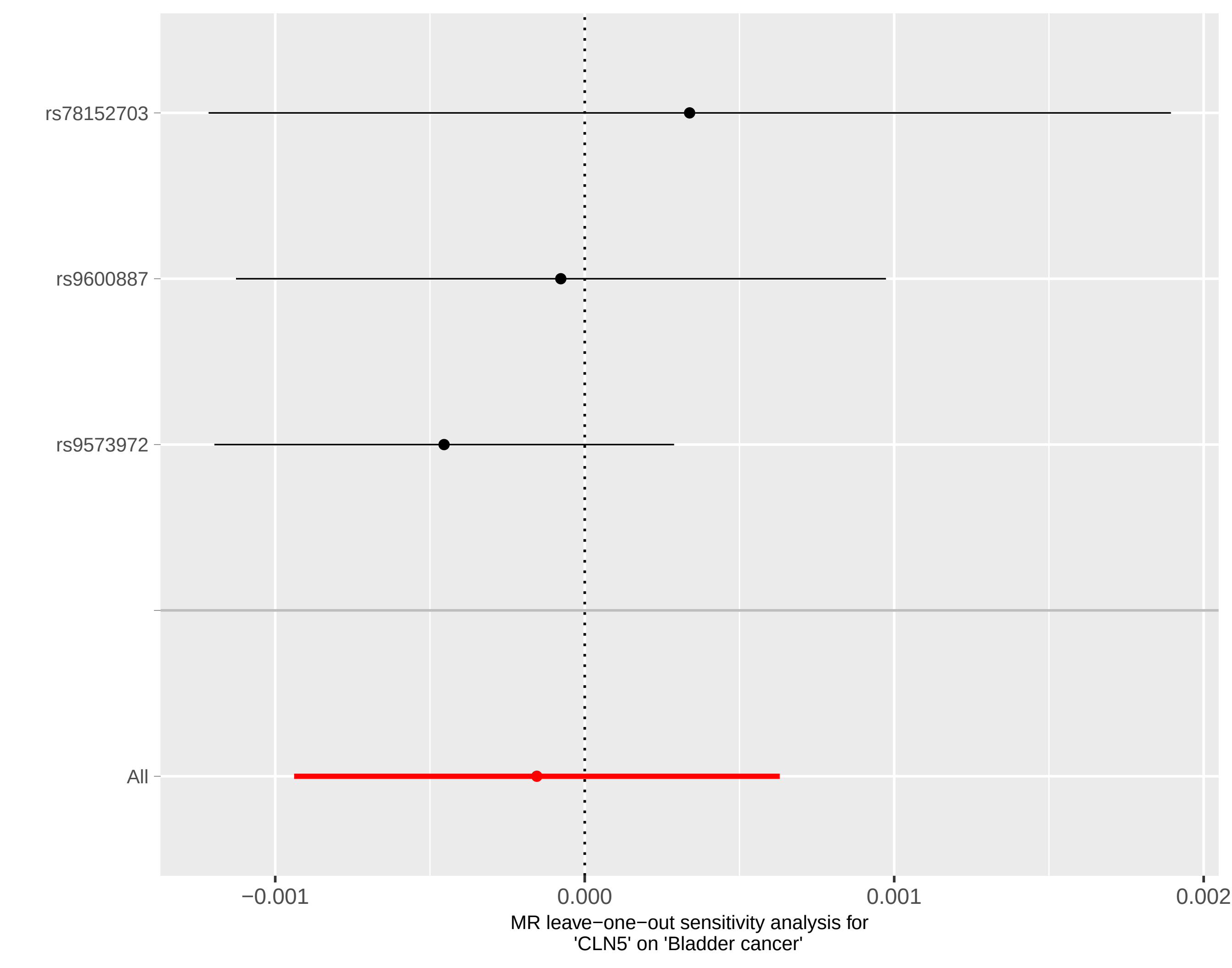
rs78152703
rs9600887
rs9573972
All
−0.001
0.000
0.001
0.002
MR leave−one−out sensitivity analysis for
'CLN5' on 'Bladder cancer'

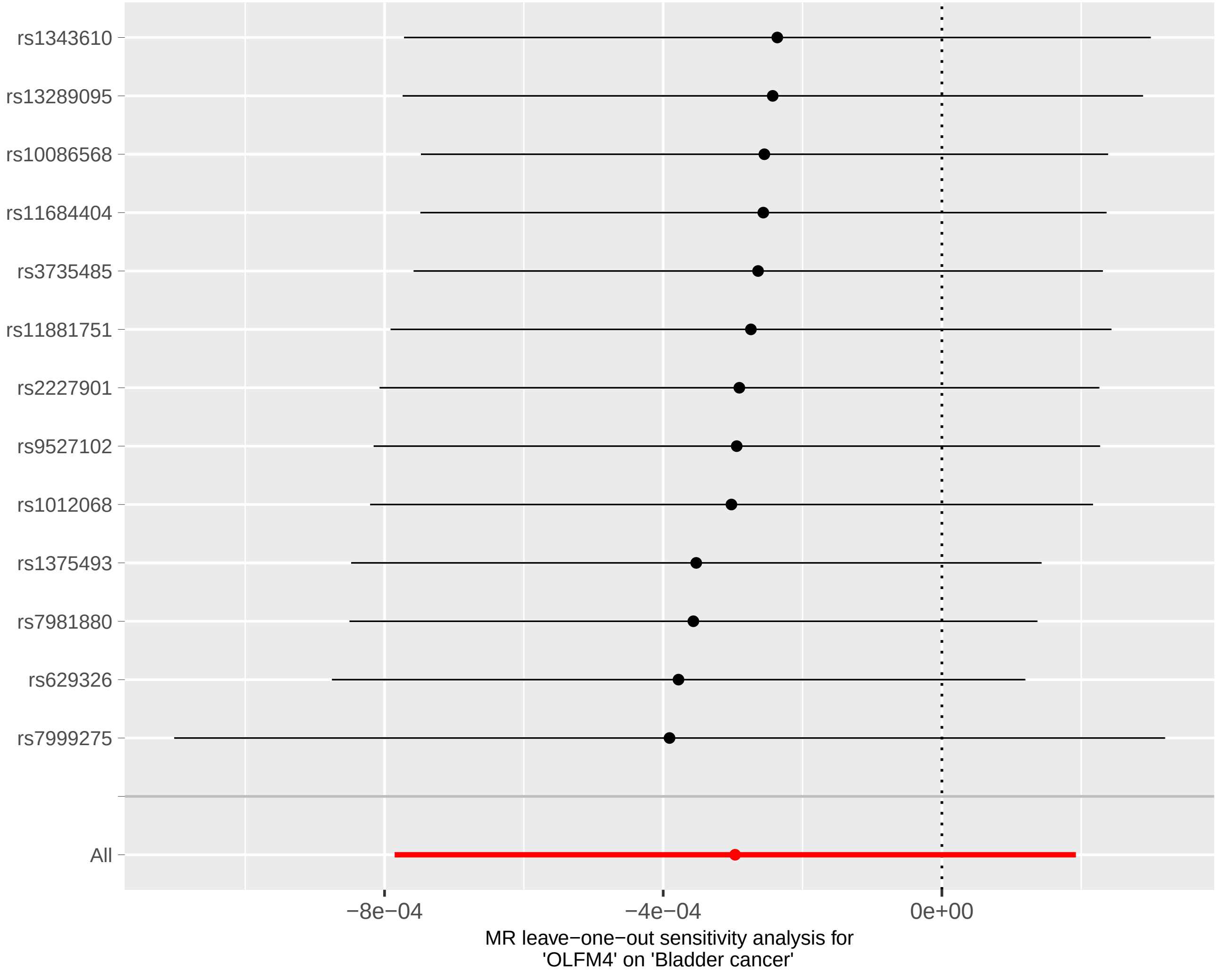
rs1343610
rs13289095
rs10086568
rs11684404
rs3735485
rs11881751
rs2227901
rs9527102
rs1012068
rs1375493
rs7981880
rs629326
rs7999275
All
−8e−04
−4e−04
0e+00
MR leave−one−out sensitivity analysis for
'OLFM4' on 'Bladder cancer'

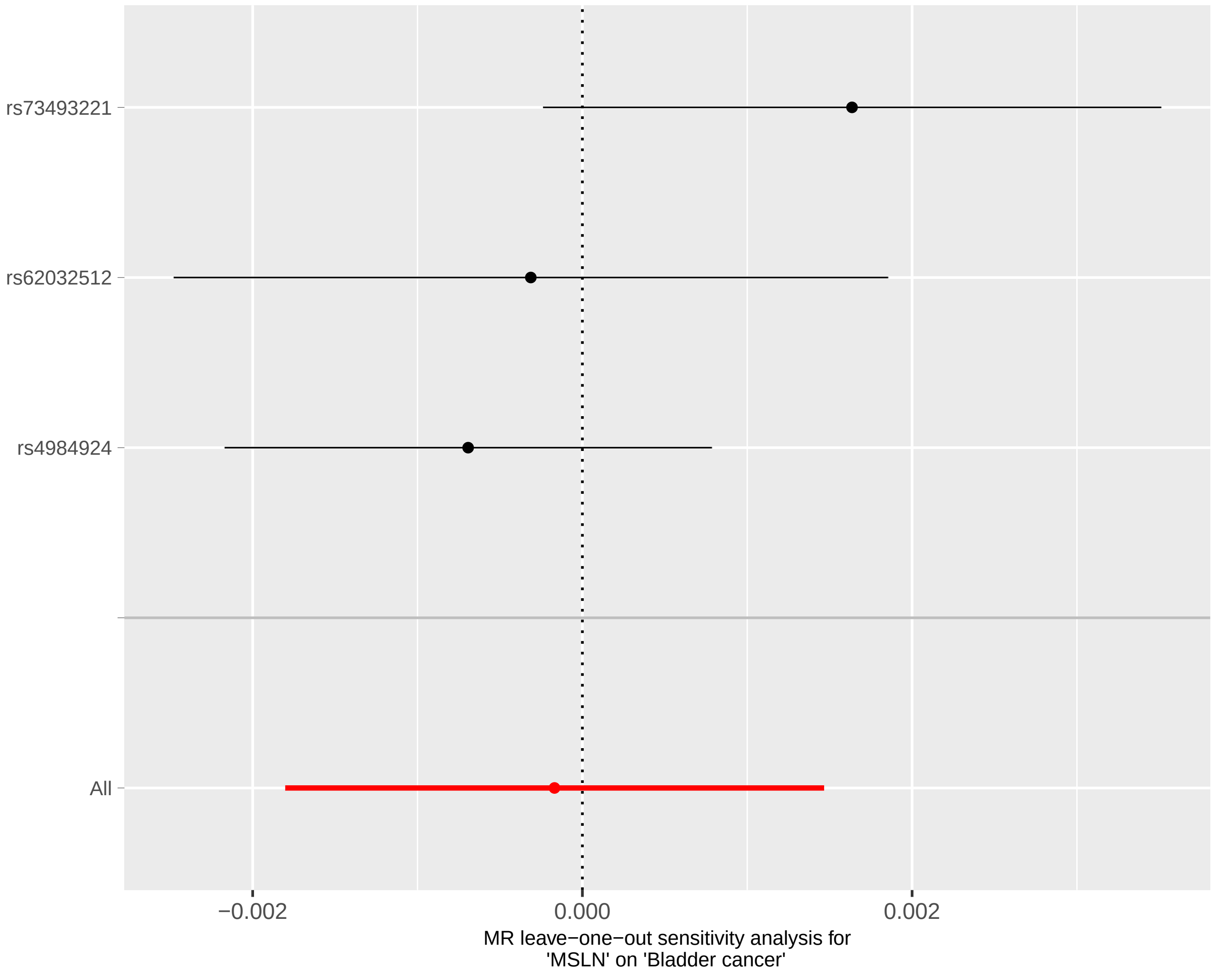
rs73493221
rs62032512
rs4984924
All
−0.002
0.000
0.002
MR leave−one−out sensitivity analysis for
'MSLN' on 'Bladder cancer'

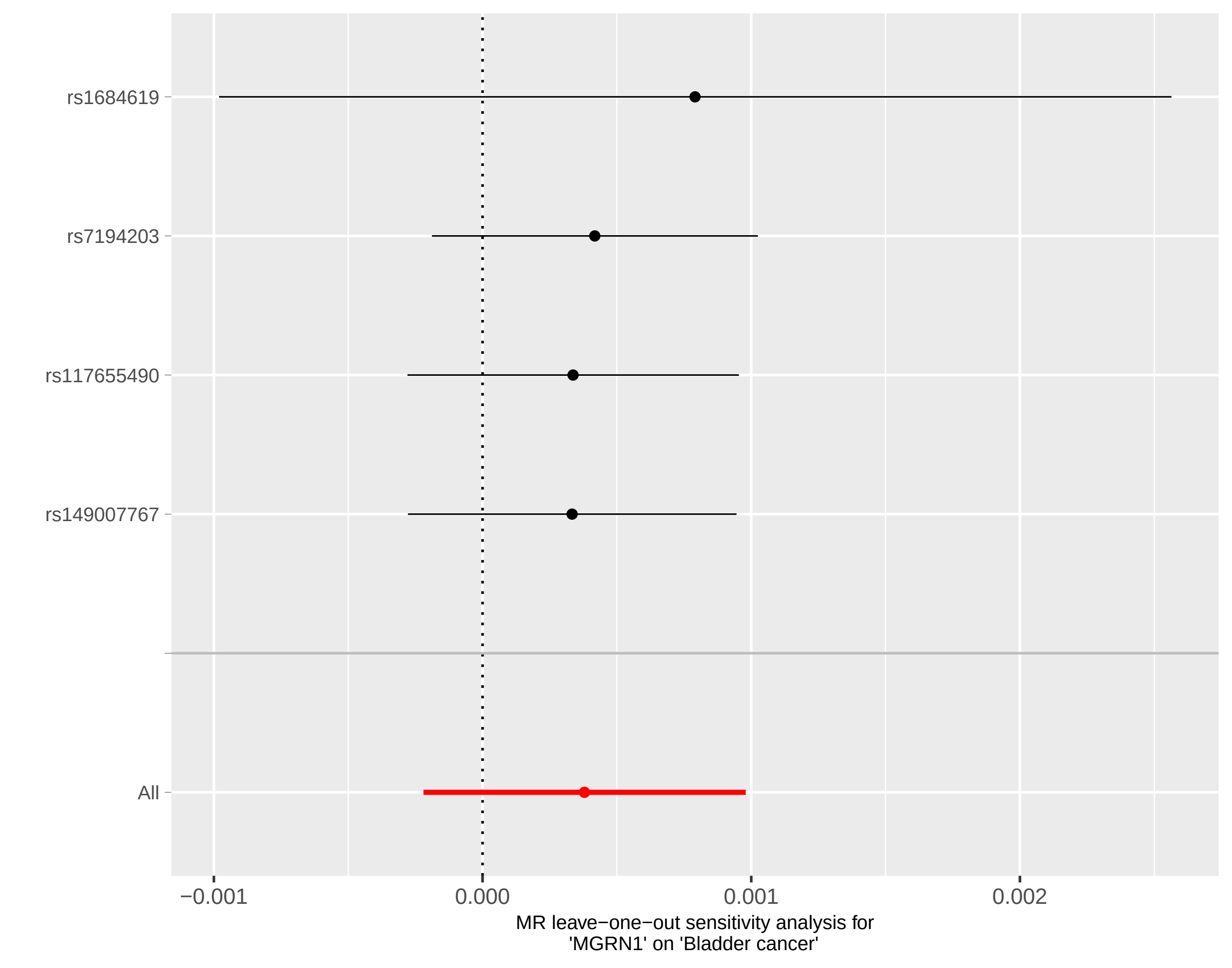
rs1684619
rs7194203
rs117655490
rs149007767
All
−0.001
0.000
0.001
0.002
MR leave−one−out sensitivity analysis for
'MGRN1' on 'Bladder cancer'

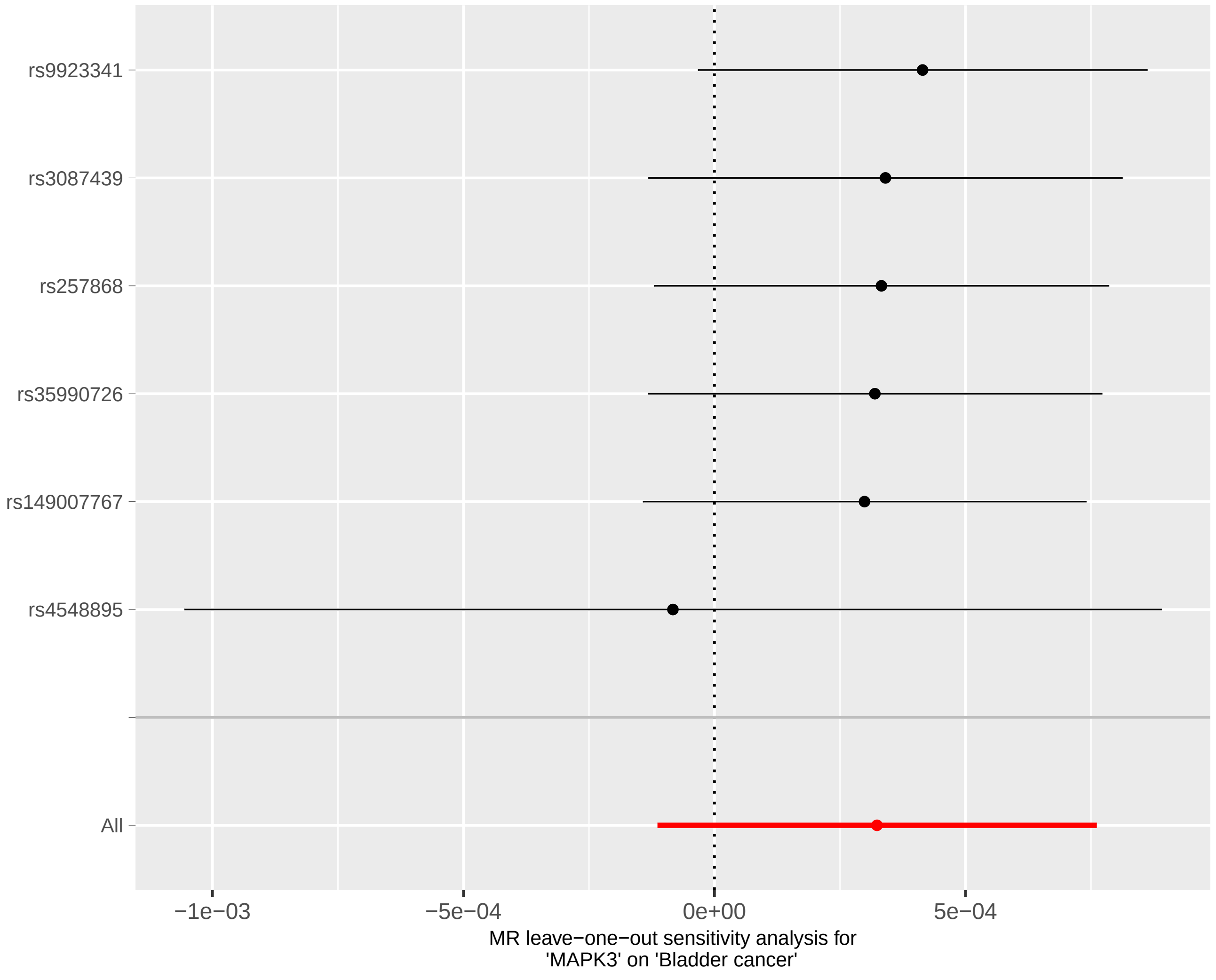

rs9923341
rs3087439
rs257868
rs35990726
rs149007767
rs4548895
All
−1e−03
−5e−04
0e+00
5e−04
MR leave−one−out sensitivity analysis for
'MAPK3' on 'Bladder cancer'

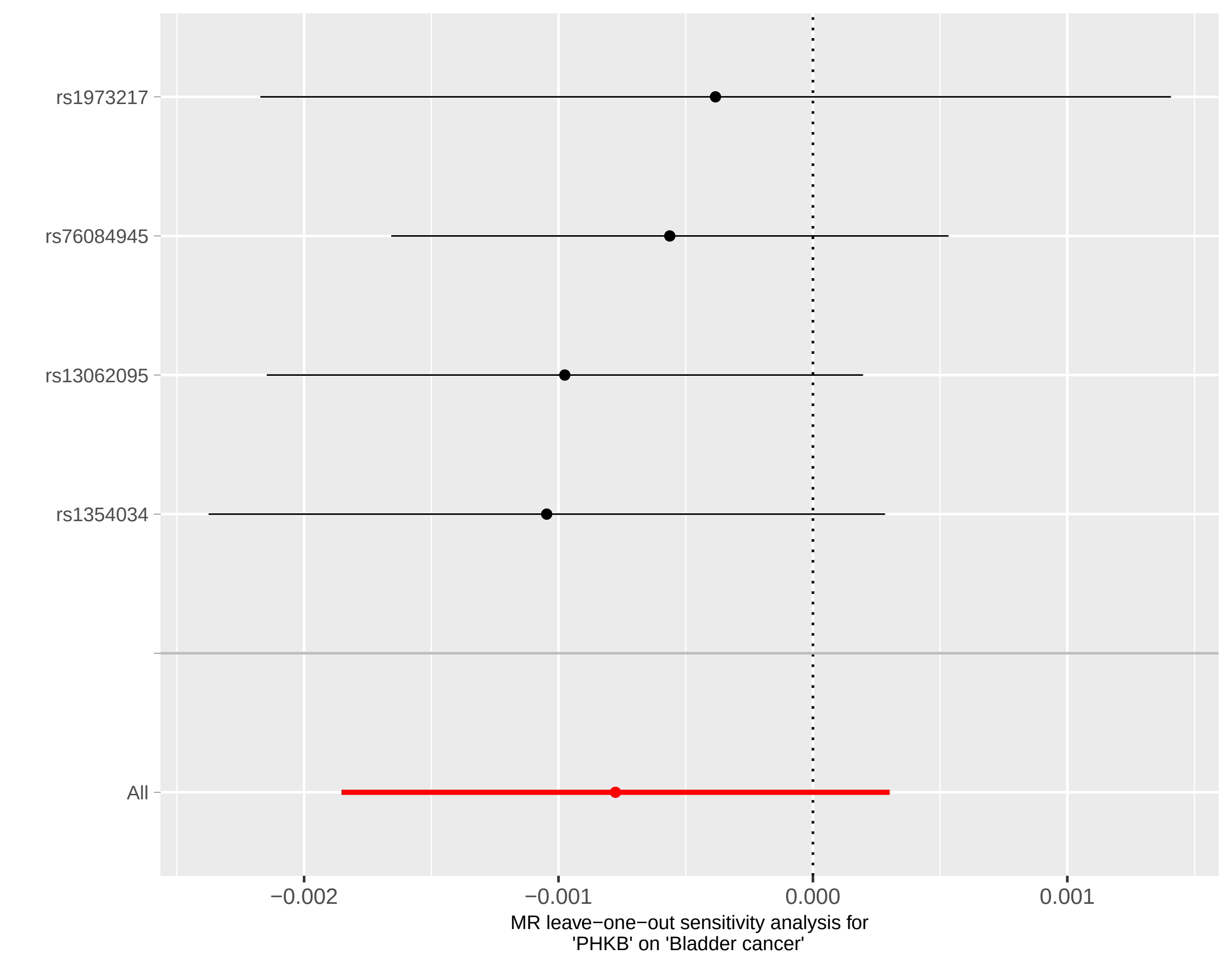
rs1973217
rs76084945
rs13062095
rs1354034
All
−0.002
−0.001
0.000
0.001
MR leave−one−out sensitivity analysis for
'PHKB' on 'Bladder cancer'

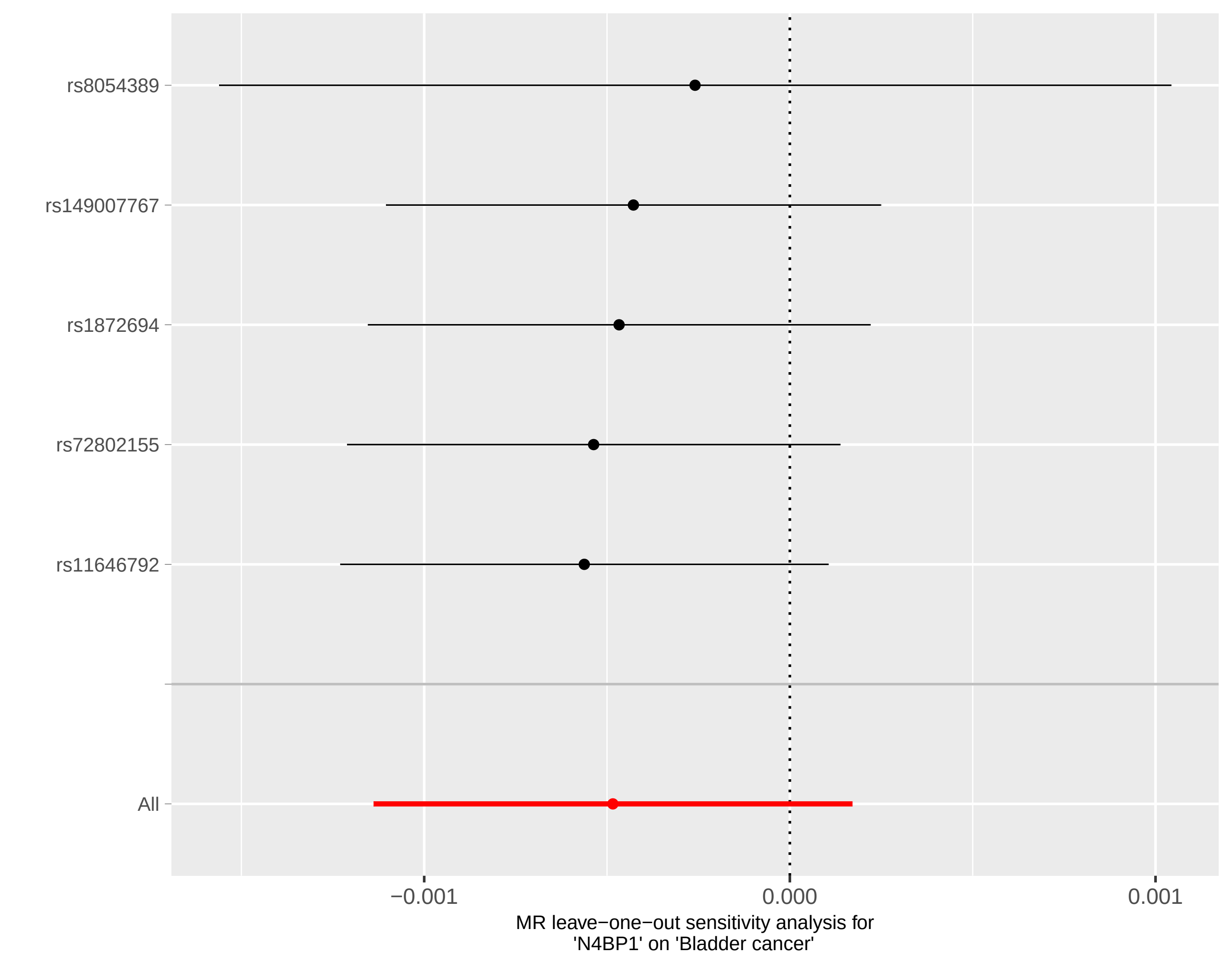
rs8054389
rs149007767
rs1872694
rs72802155
rs11646792
All
−0.001
0.000
0.001
MR leave−one−out sensitivity analysis for
'N4BP1' on 'Bladder cancer'

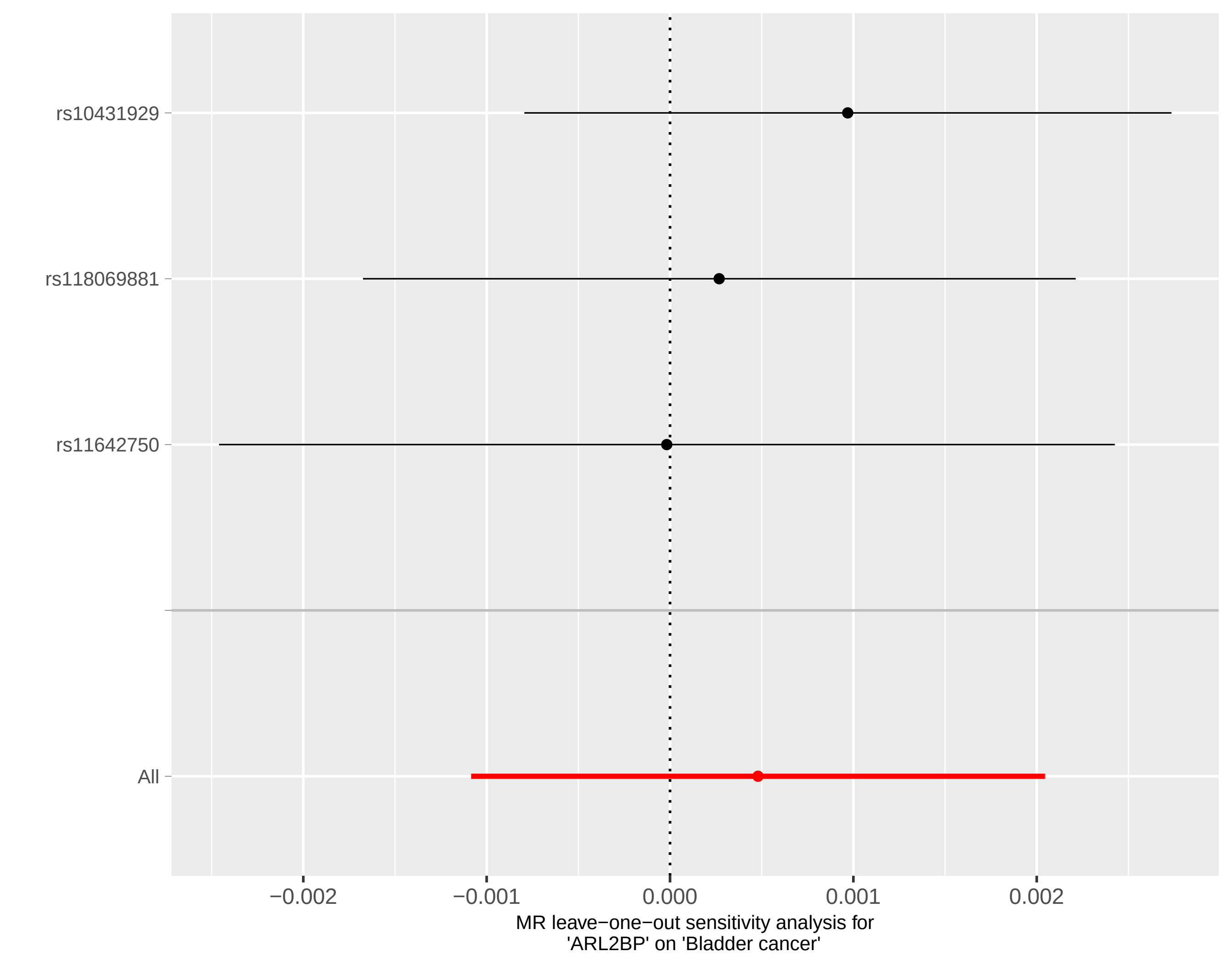
rs10431929
rs118069881
rs11642750
All
−0.002
−0.001
0.000
0.001
0.002
MR leave−one−out sensitivity analysis for
'ARL2BP' on 'Bladder cancer'

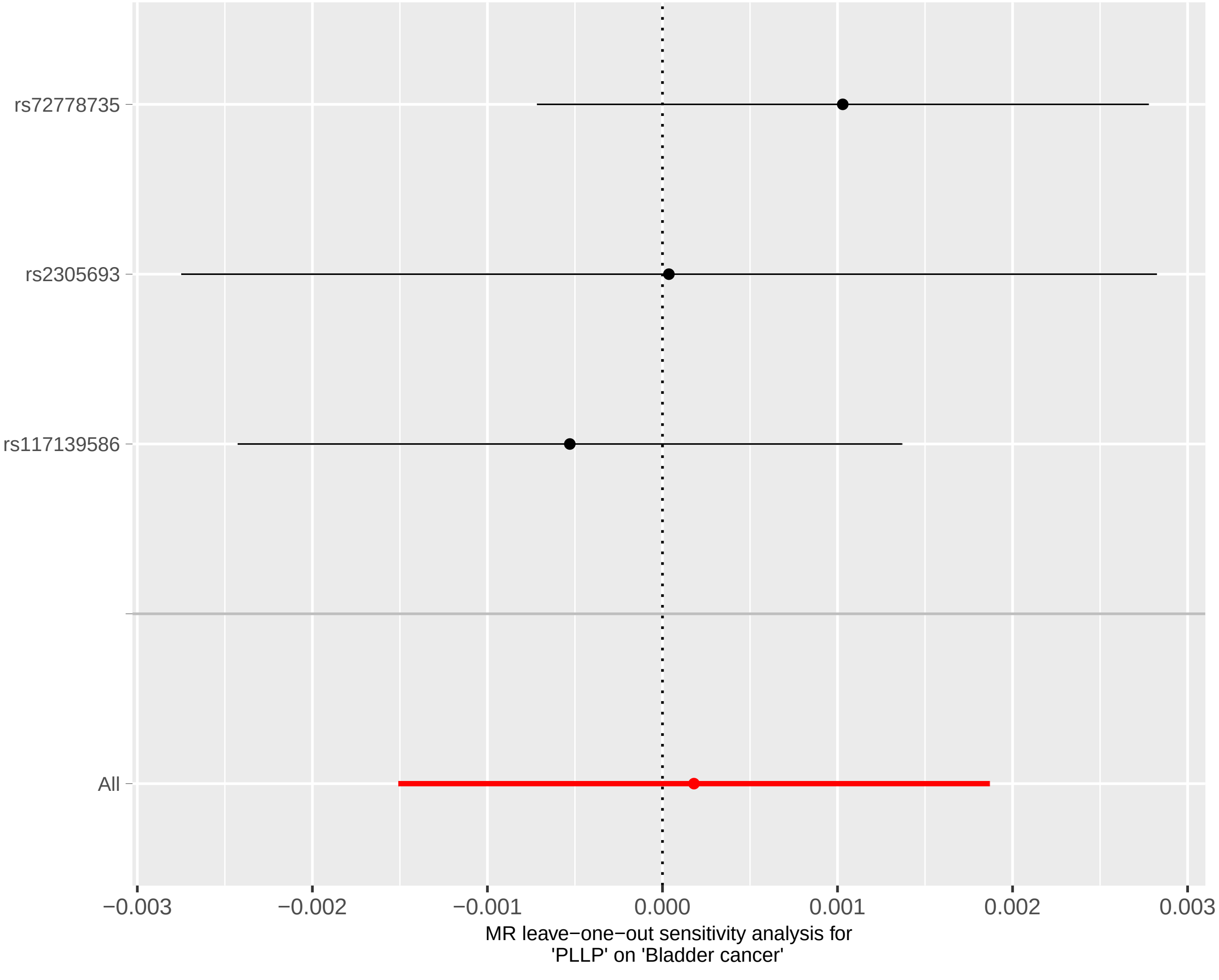
rs72778735
rs2305693
rs117139586
All
−0.003
−0.002
−0.001
0.000
0.001
0.002
0.003
MR leave−one−out sensitivity analysis for
'PLLP' on 'Bladder cancer'

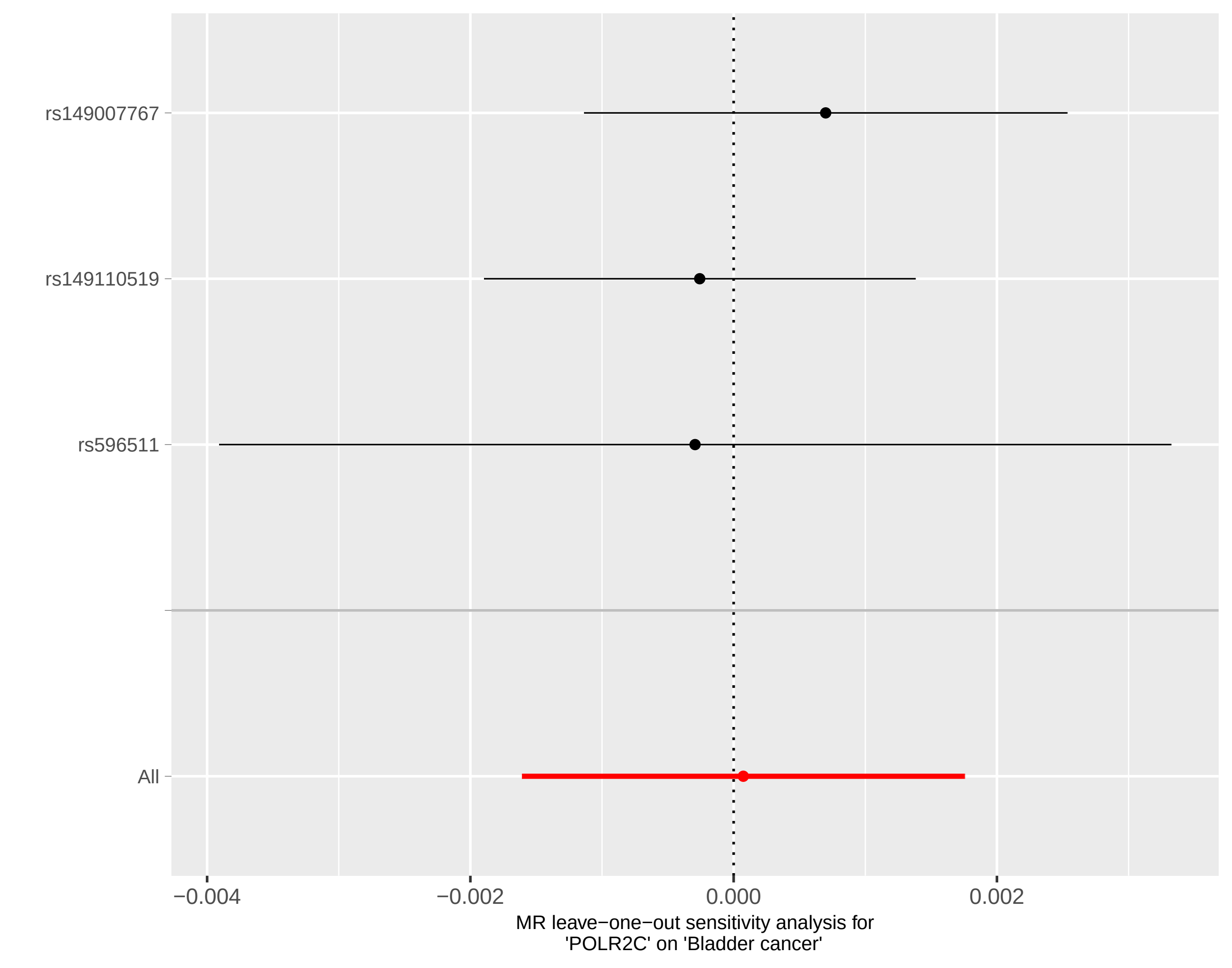
rs149007767
rs149110519
rs596511
All
−0.004
−0.002
0.000
0.002
MR leave−one−out sensitivity analysis for
'POLR2C' on 'Bladder cancer'

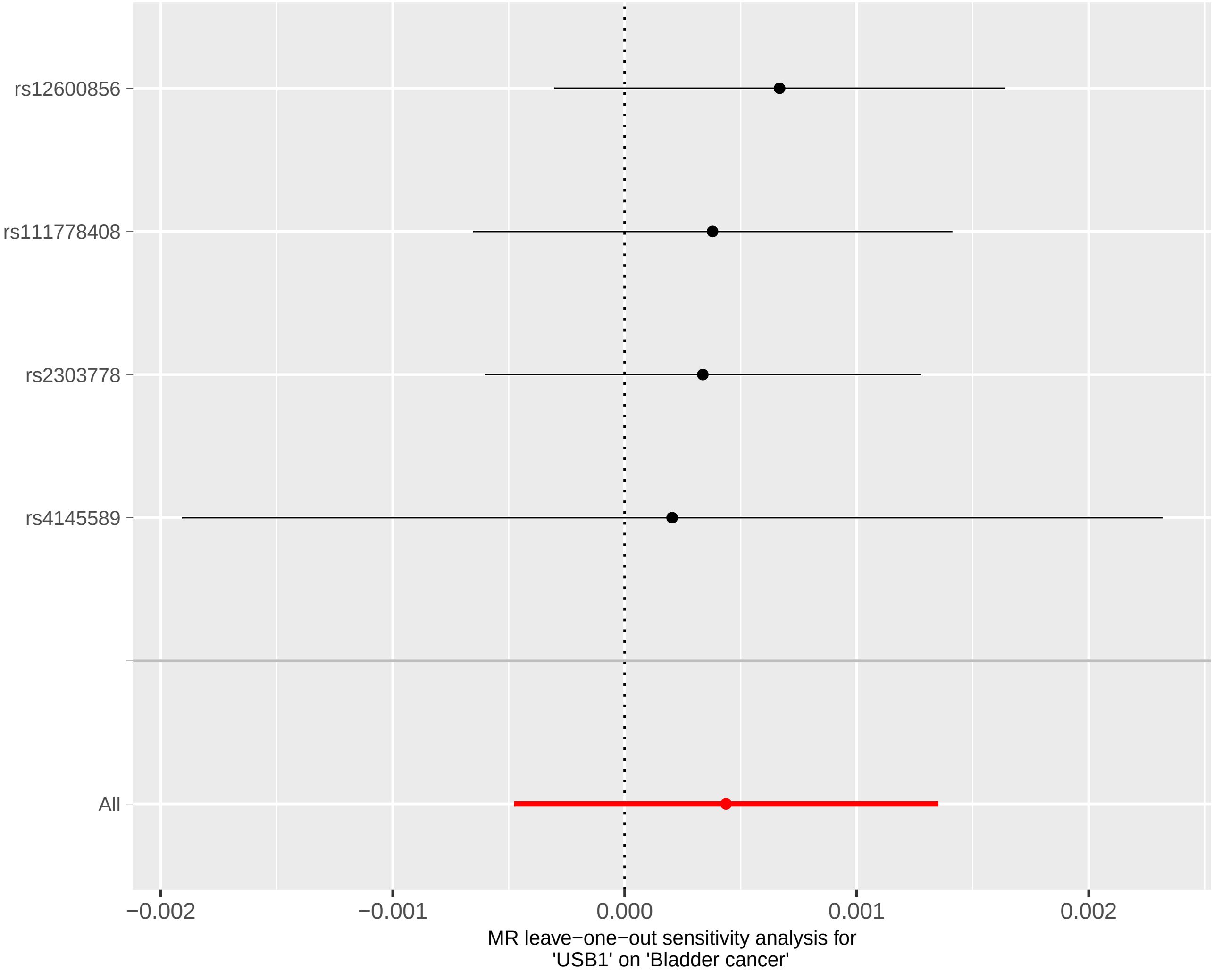
rs12600856
rs111778408
rs2303778
rs4145589
All
−0.002
−0.001
0.000
0.001
0.002
MR leave−one−out sensitivity analysis for
'USB1' on 'Bladder cancer'

Insufficient number of SNPs

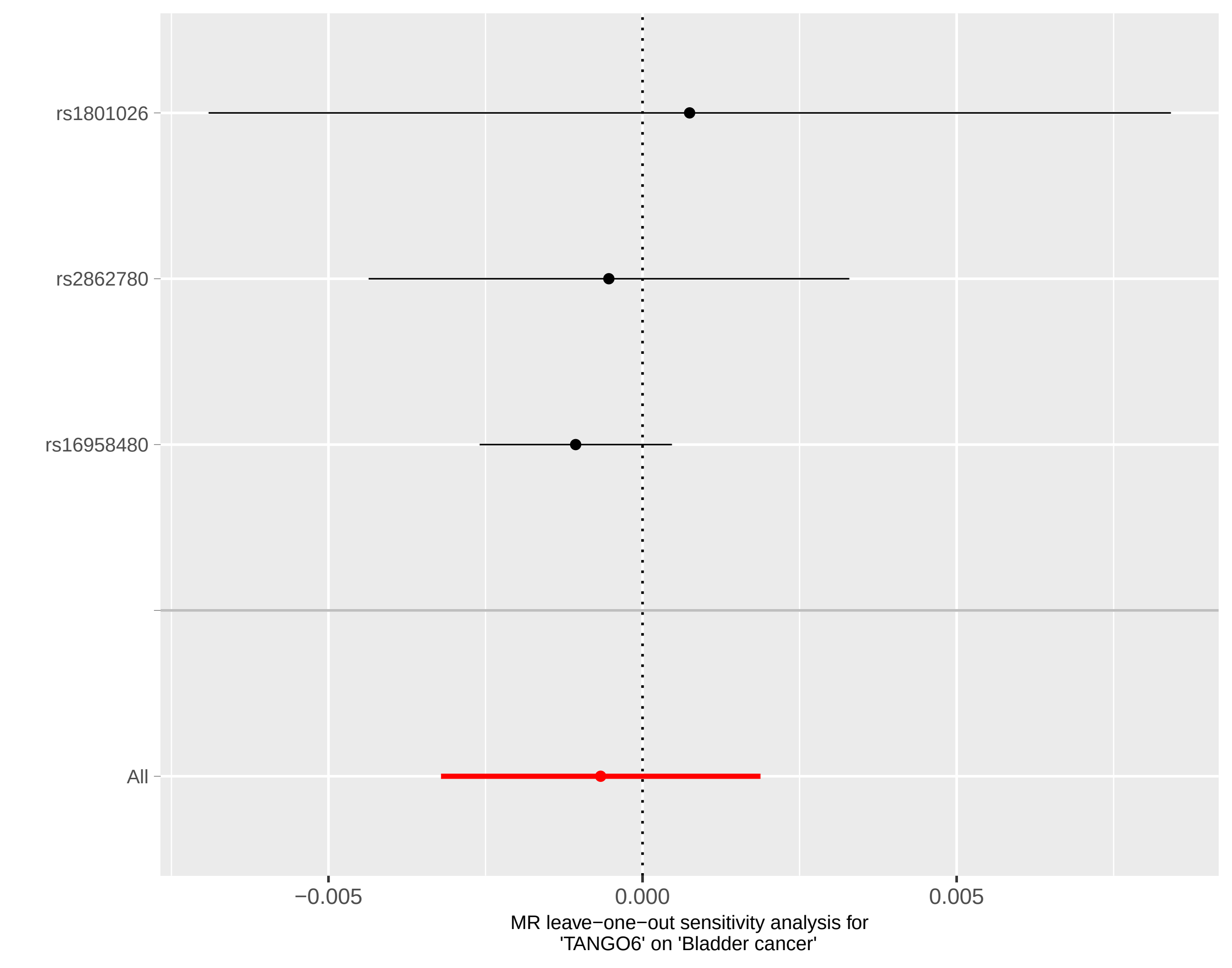
rs1801026
rs2862780
rs16958480
All
−0.005
0.000
0.005
MR leave−one−out sensitivity analysis for
'TANGO6' on 'Bladder cancer'

Insufficient number of SNPs

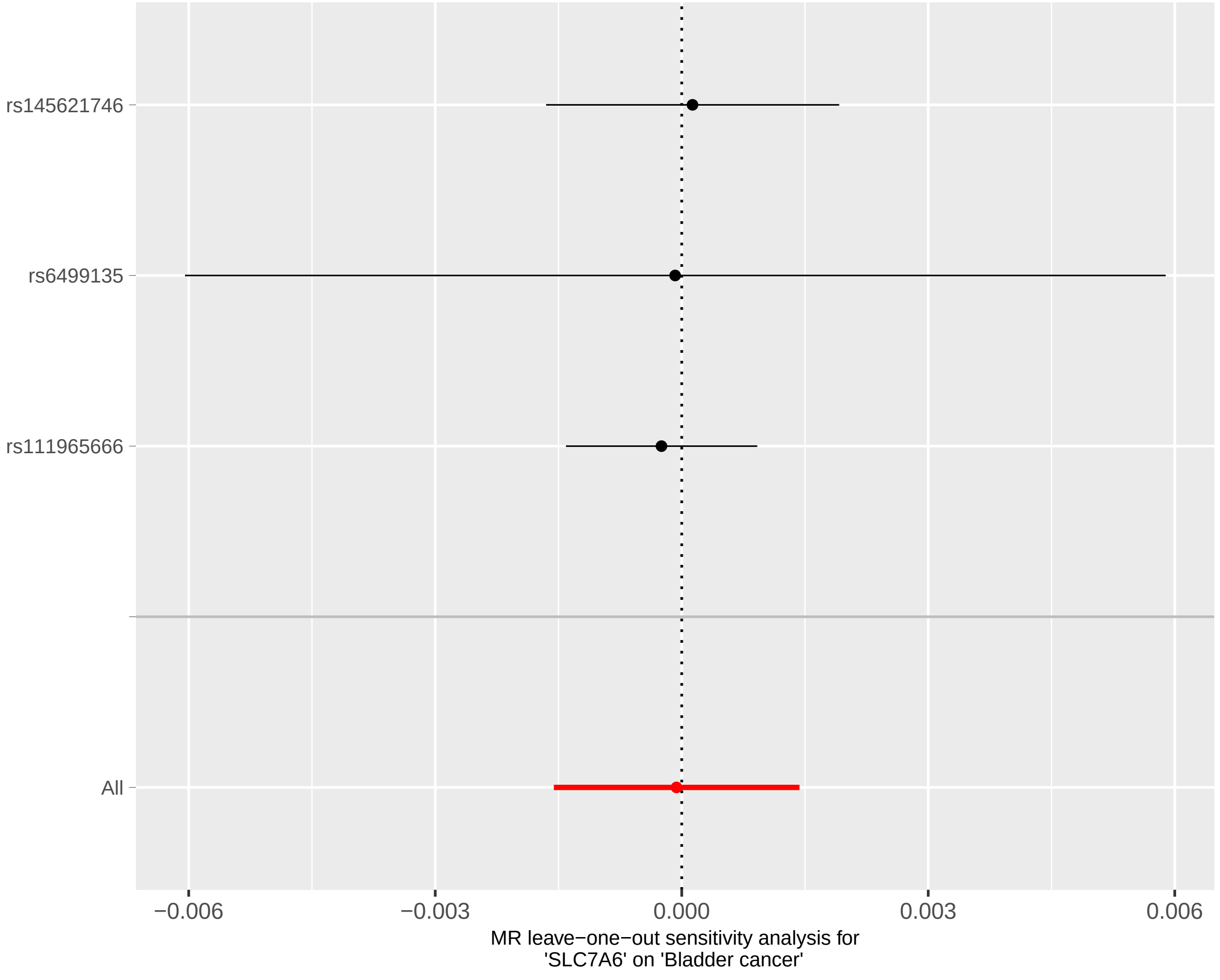
rs145621746
rs6499135
rs111965666
All
−0.006
−0.003
0.000
0.003
0.006
MR leave−one−out sensitivity analysis for
'SLC7A6' on 'Bladder cancer'

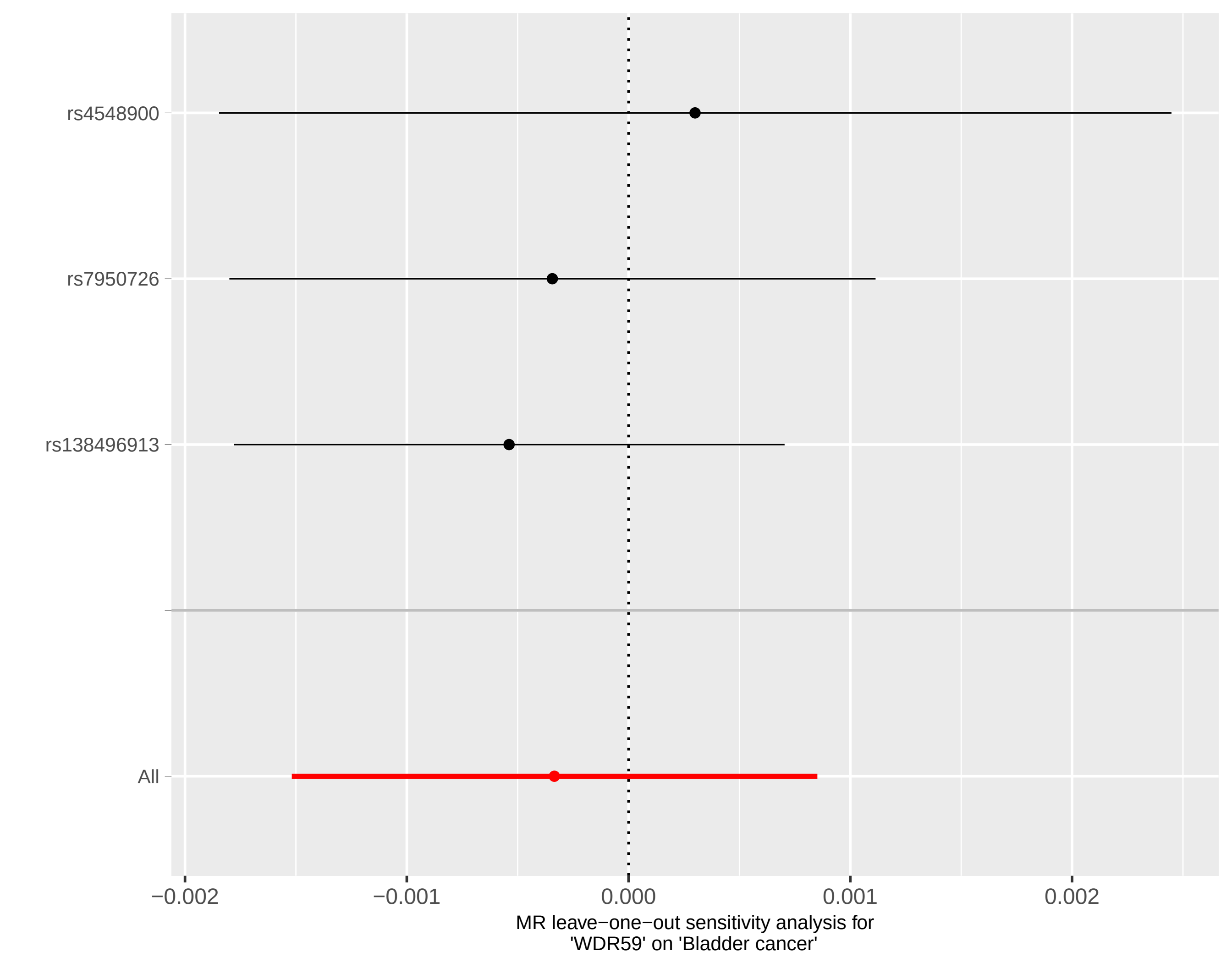
rs4548900
rs7950726
rs138496913
All
−0.002
−0.001
0.000
0.001
0.002
MR leave−one−out sensitivity analysis for
'WDR59' on 'Bladder cancer'

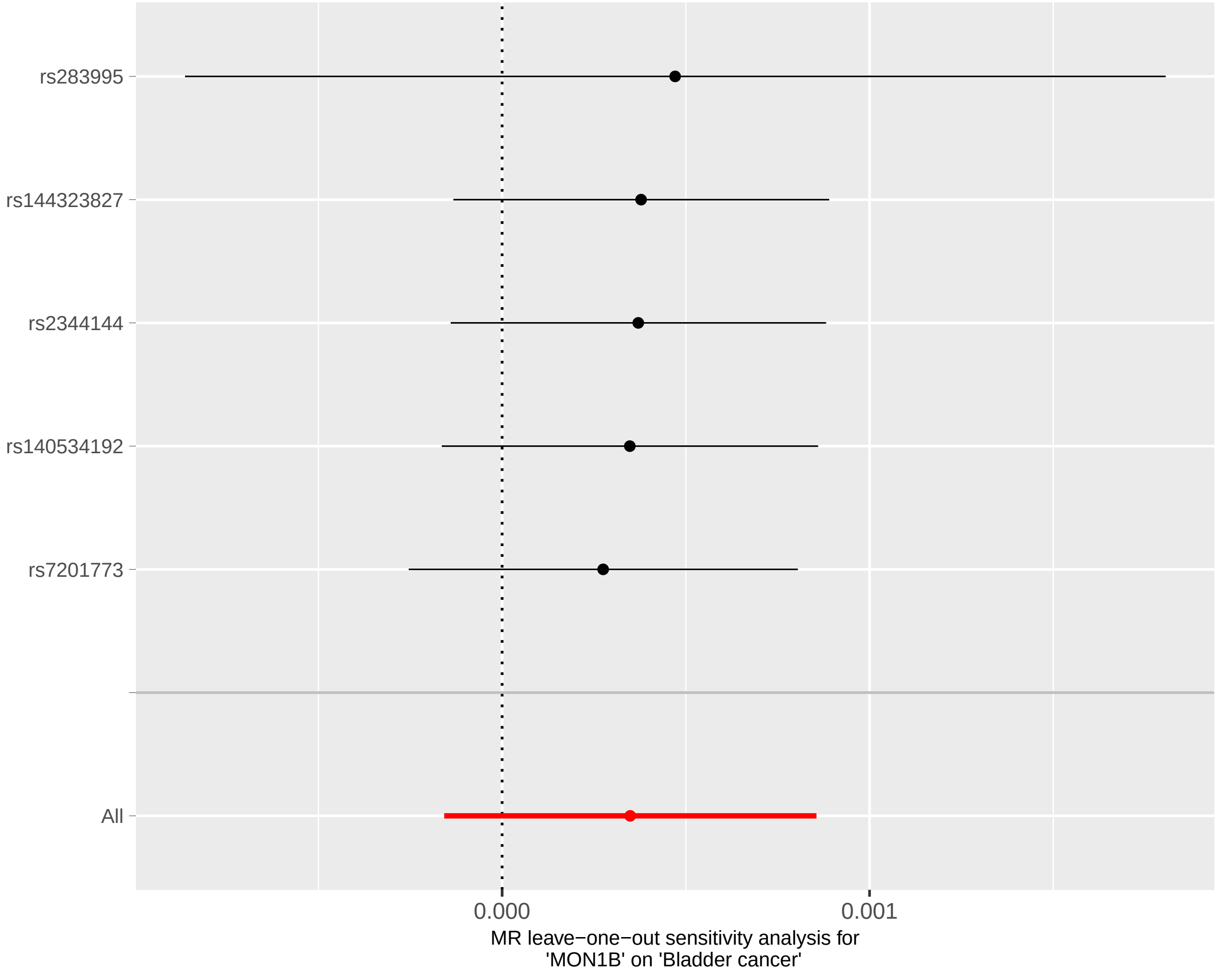
rs283995
rs144323827
rs2344144
rs140534192
rs7201773
All
0.000
0.001
MR leave−one−out sensitivity analysis for
'MON1B' on 'Bladder cancer'

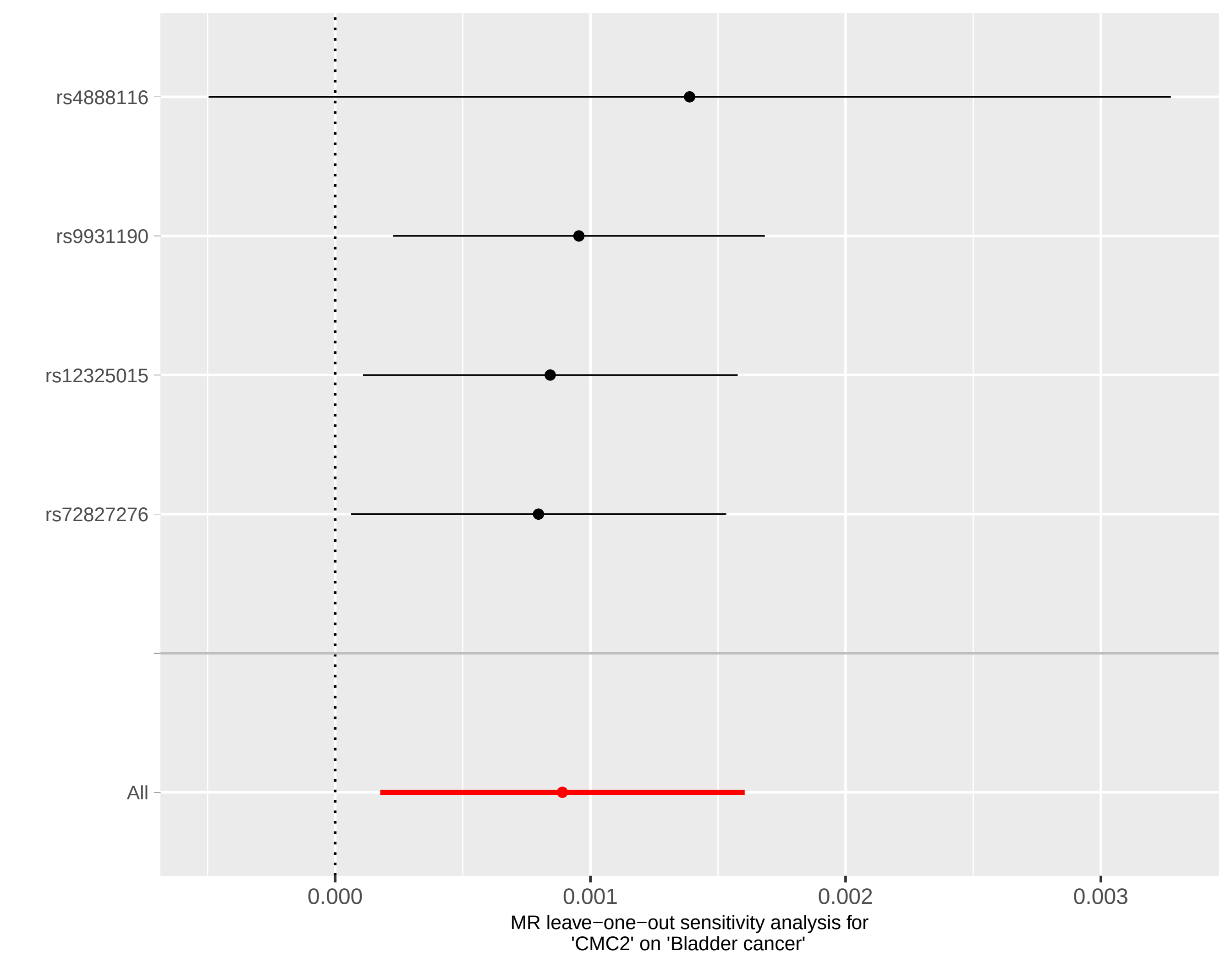

rs4888116
rs9931190
rs12325015
rs72827276
All
0.000
0.001
0.002
0.003
MR leave−one−out sensitivity analysis for
'CMC2' on 'Bladder cancer'

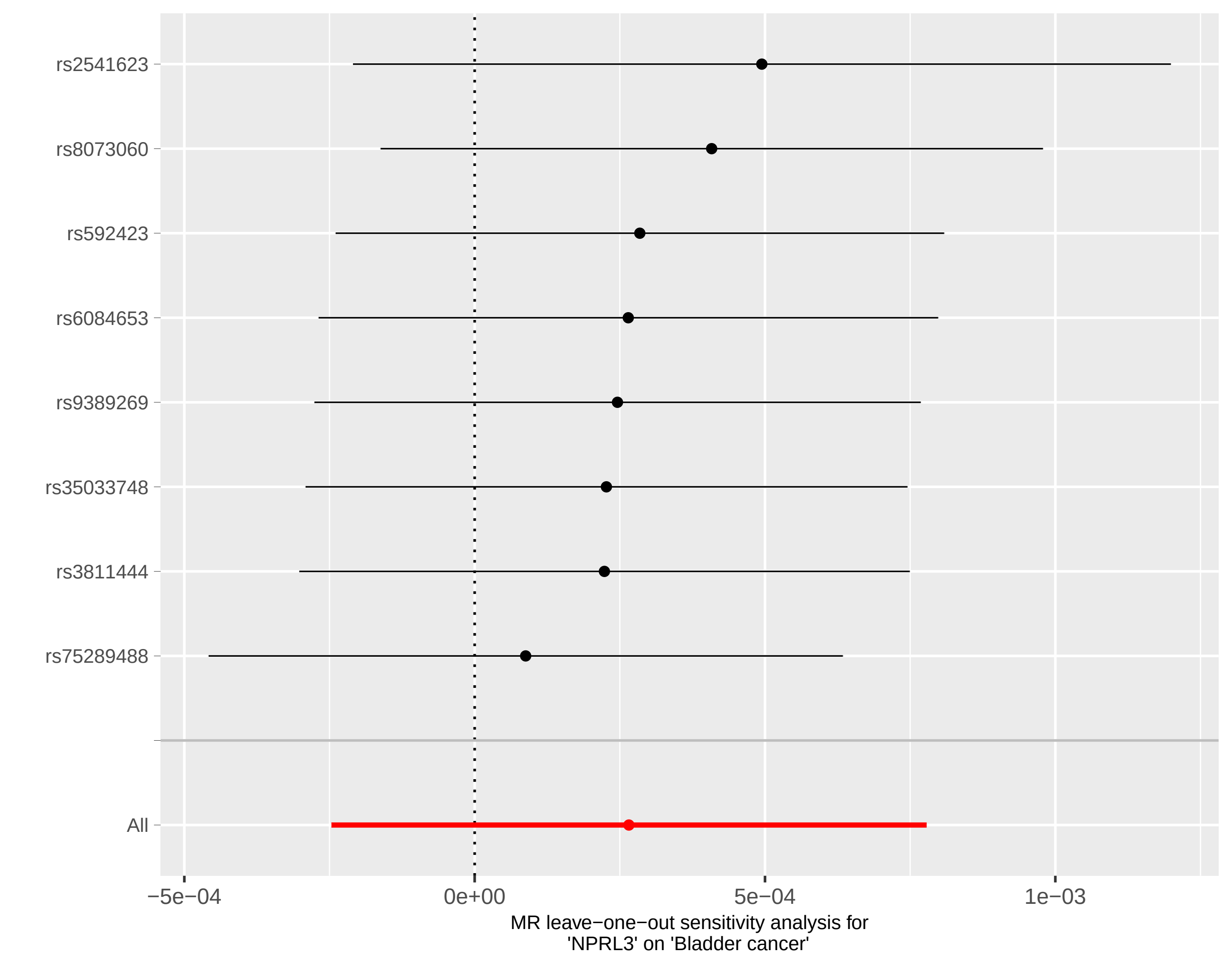
rs2541623
rs8073060
rs592423
rs6084653
rs9389269
rs35033748
rs3811444
rs75289488
All
−5e−04
0e+00
5e−04
1e−03
MR leave−one−out sensitivity analysis for
'NPRL3' on 'Bladder cancer'

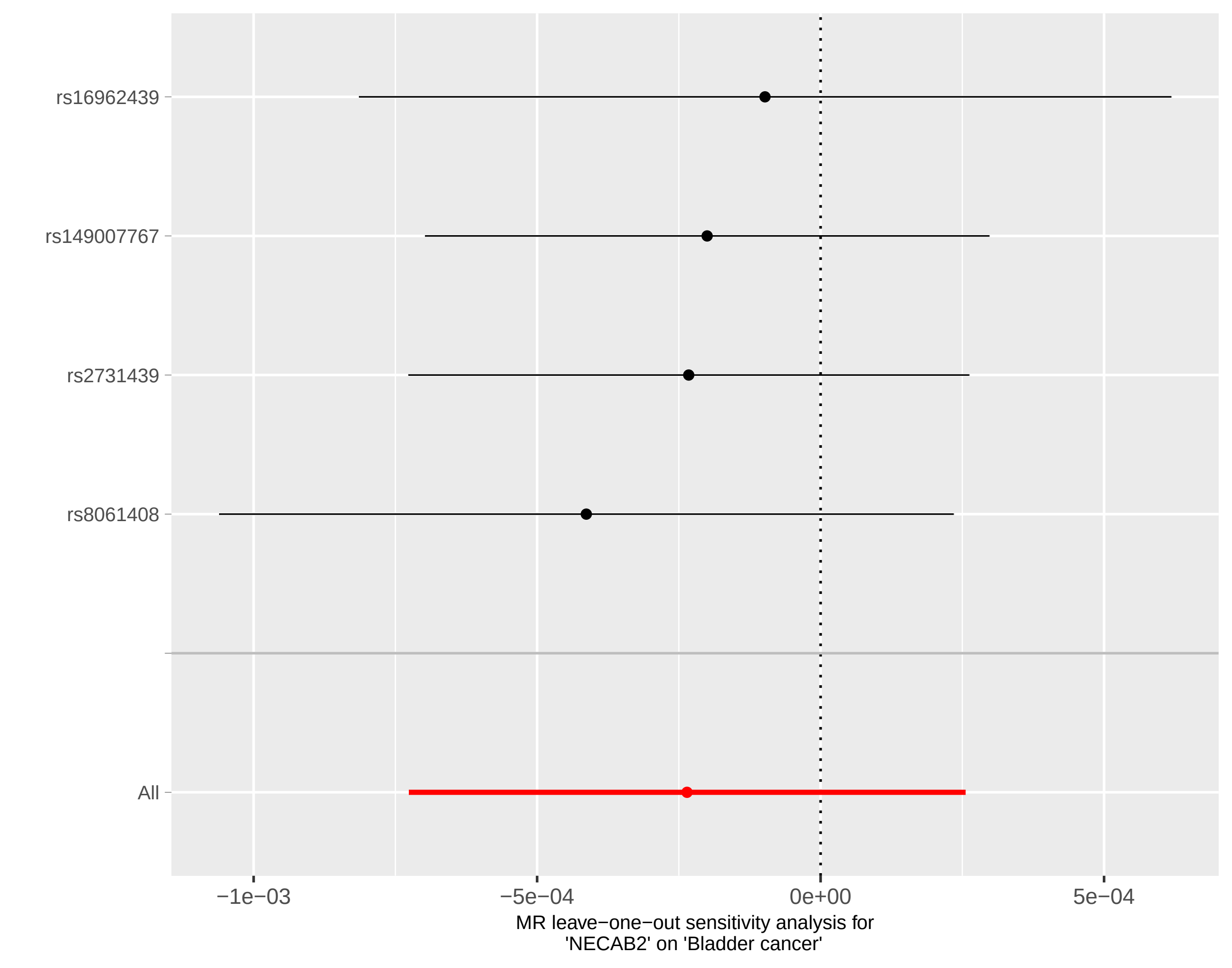
rs16962439
rs149007767
rs2731439
rs8061408
All
−1e−03
−5e−04
0e+00
5e−04
MR leave−one−out sensitivity analysis for
'NECAB2' on 'Bladder cancer'

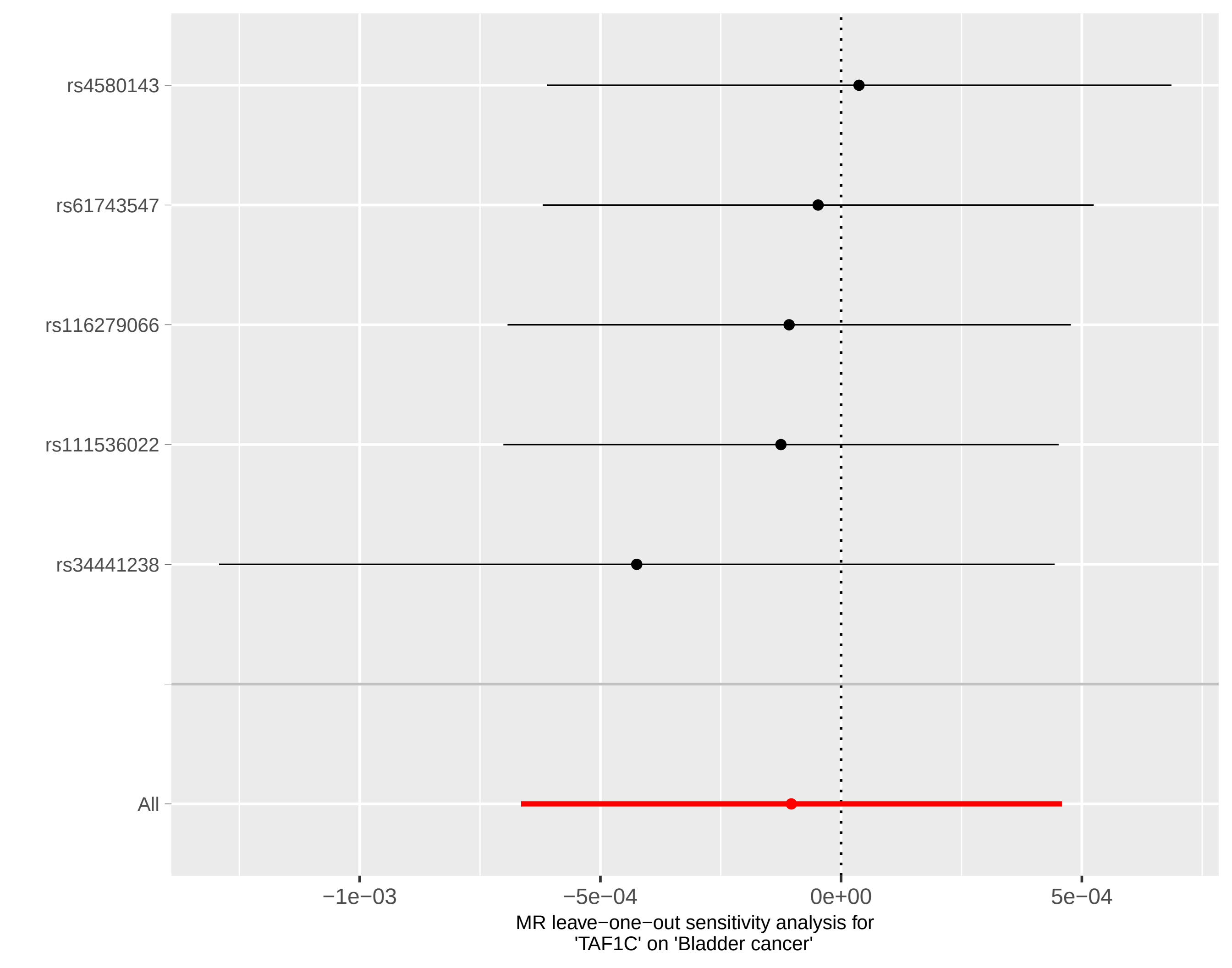
rs4580143
rs61743547
rs116279066
rs111536022
rs34441238
All
−1e−03
−5e−04
0e+00
5e−04
MR leave−one−out sensitivity analysis for
'TAF1C' on 'Bladder cancer'

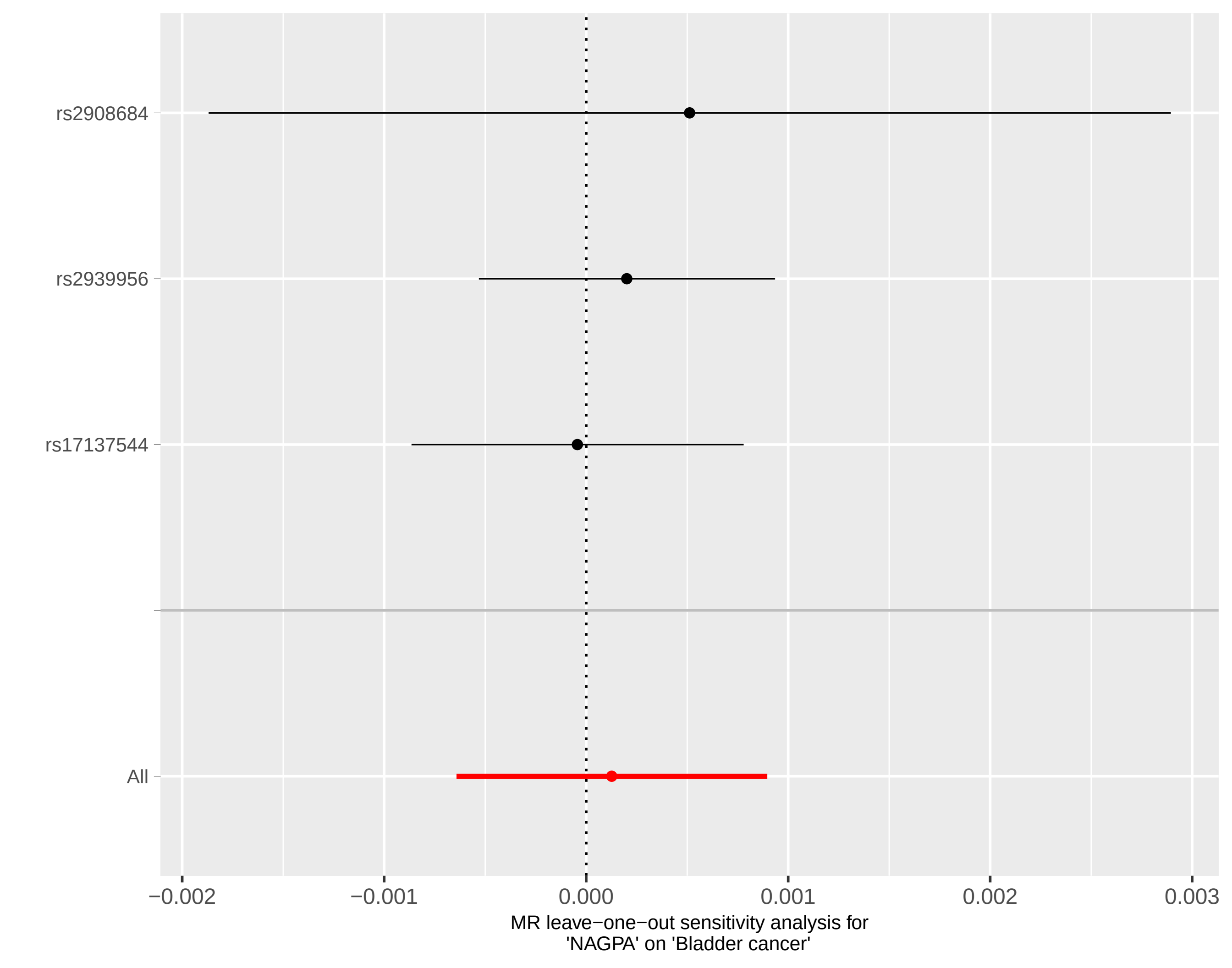
rs2908684
rs2939956
rs17137544
All
−0.002
−0.001
0.000
0.001
0.002
0.003
MR leave−one−out sensitivity analysis for
'NAGPA' on 'Bladder cancer'

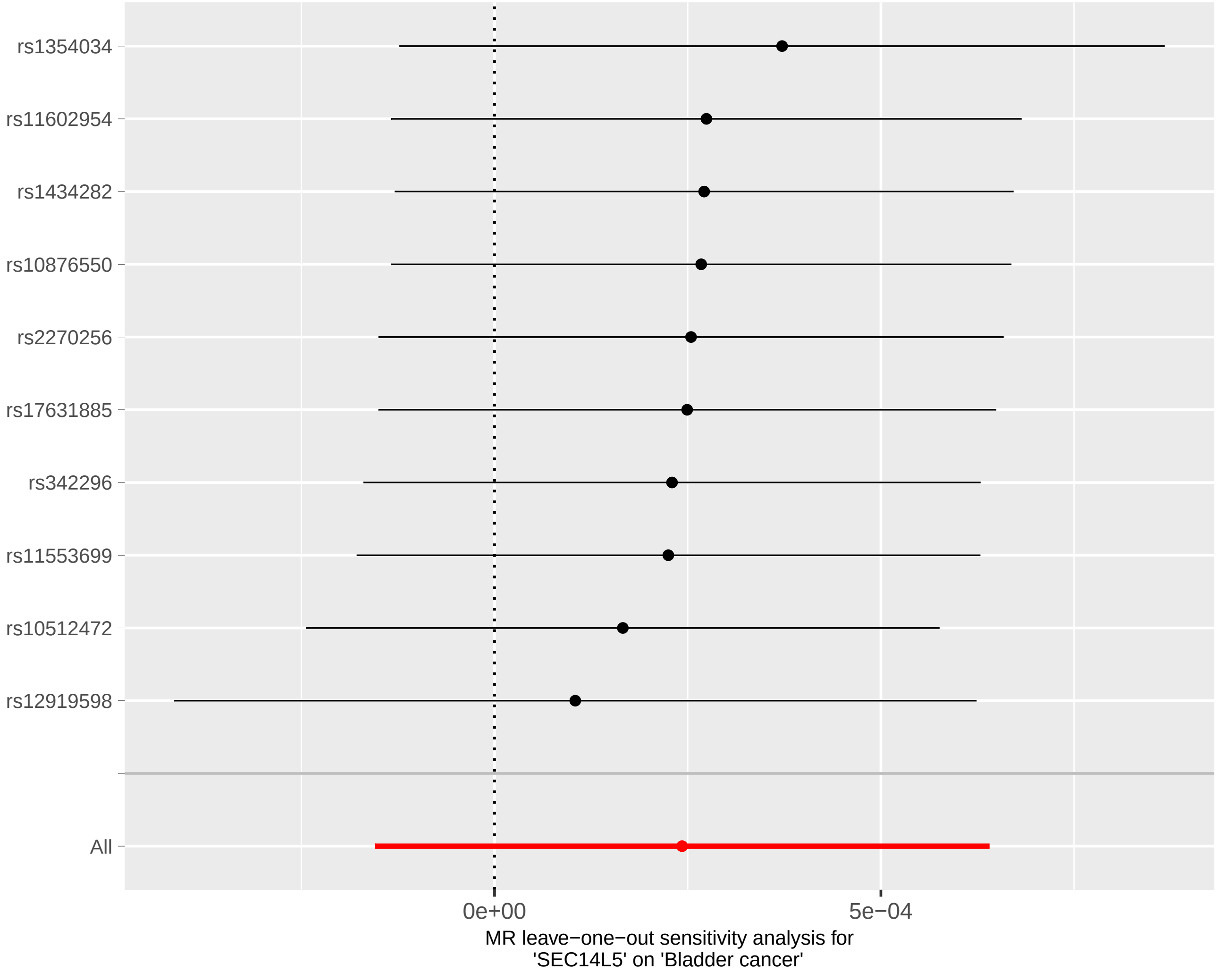
rs1354034
rs11602954
rs1434282
rs10876550
rs2270256
rs17631885
rs342296
rs11553699
rs10512472
rs12919598
All
0e+00
5e−04
MR leave−one−out sensitivity analysis for
'SEC14L5' on 'Bladder cancer'

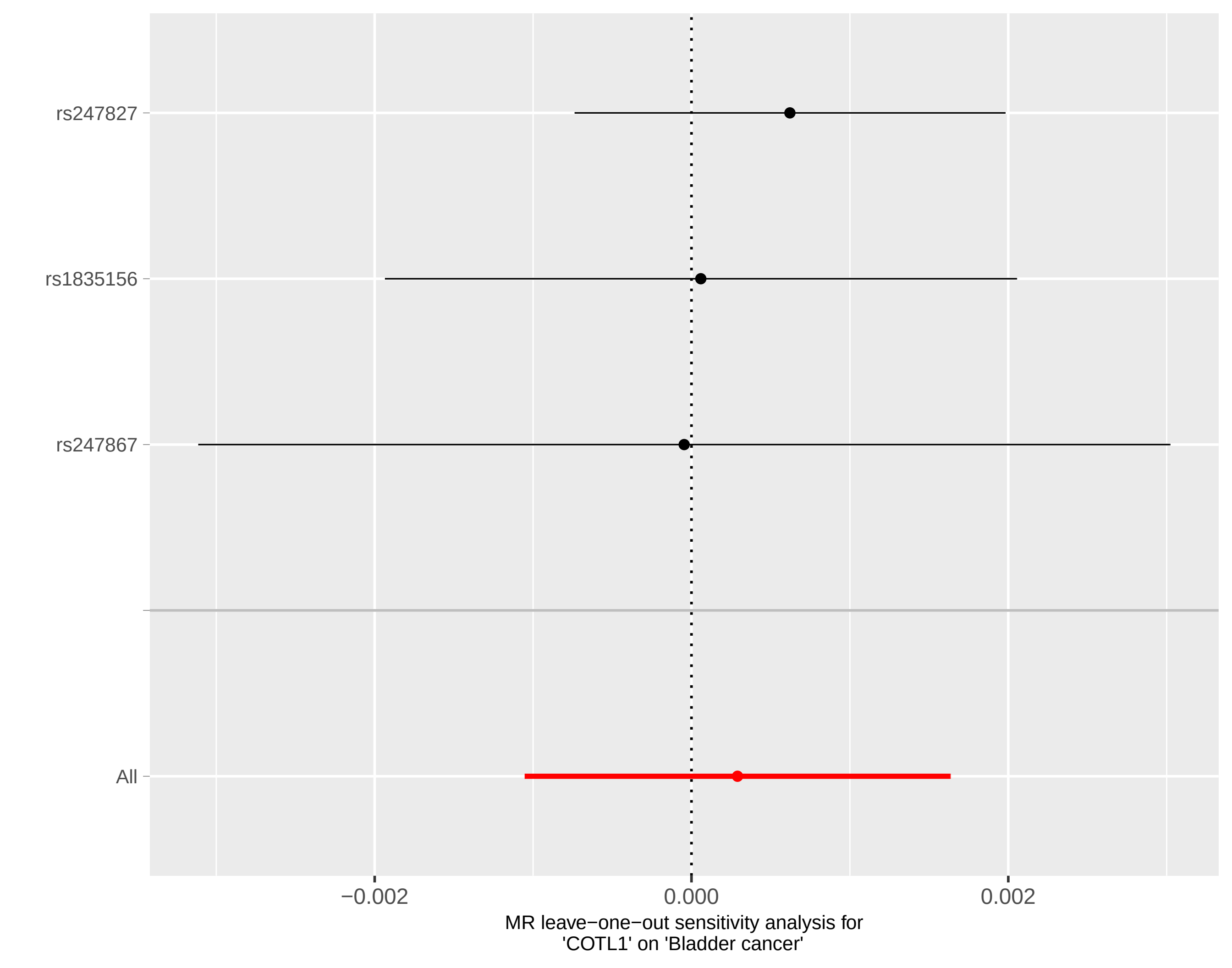
rs247827
rs1835156
rs247867
All
−0.002
0.000
0.002
MR leave−one−out sensitivity analysis for
'COTL1' on 'Bladder cancer'

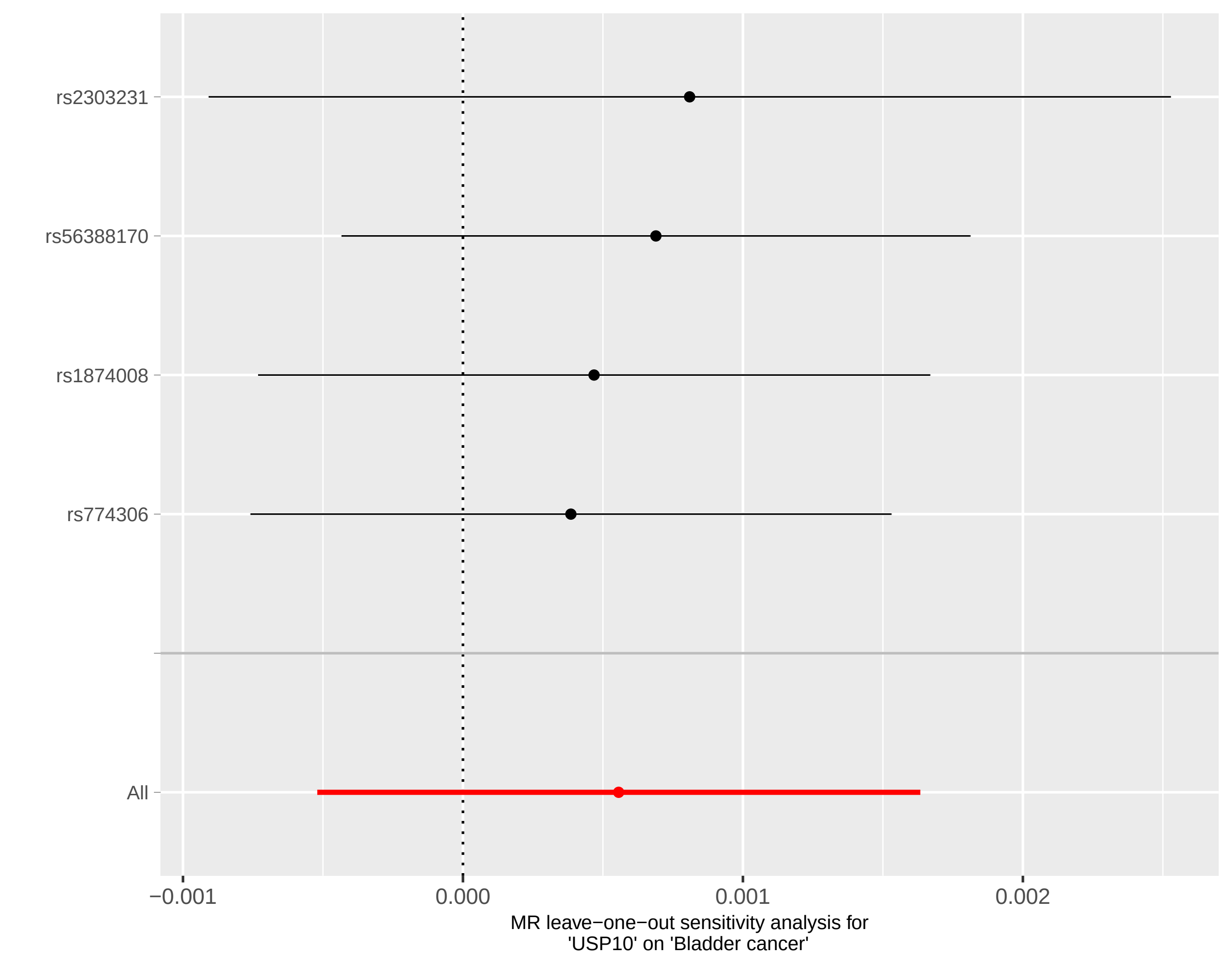
rs2303231
rs56388170
rs1874008
rs774306
All
−0.001
0.000
0.001
0.002
MR leave−one−out sensitivity analysis for
'USP10' on 'Bladder cancer'

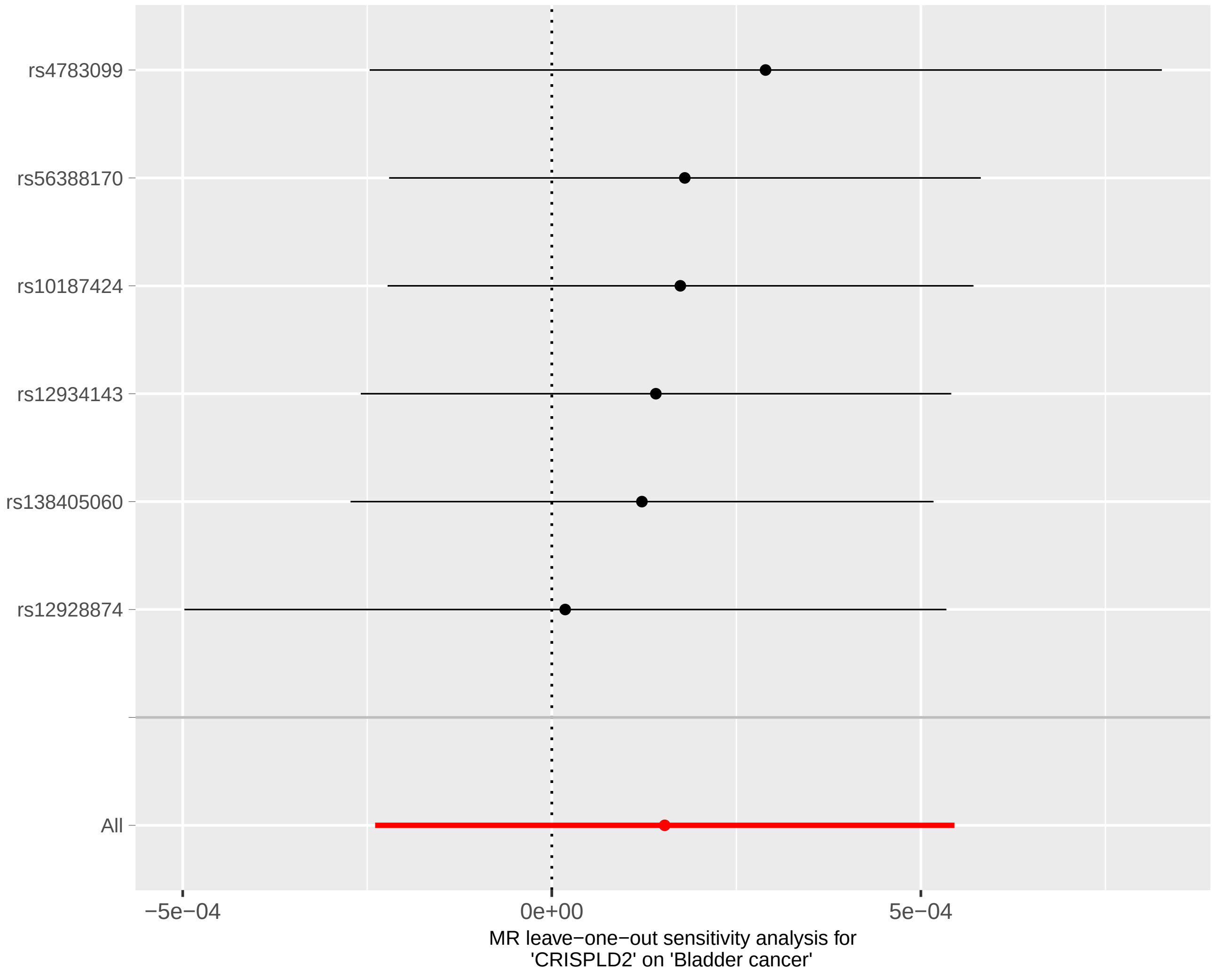

rs4783099
rs56388170
rs10187424
rs12934143
rs138405060
rs12928874
All
−5e−04
0e+00
5e−04
MR leave−one−out sensitivity analysis for
'CRISPLD2' on 'Bladder cancer'

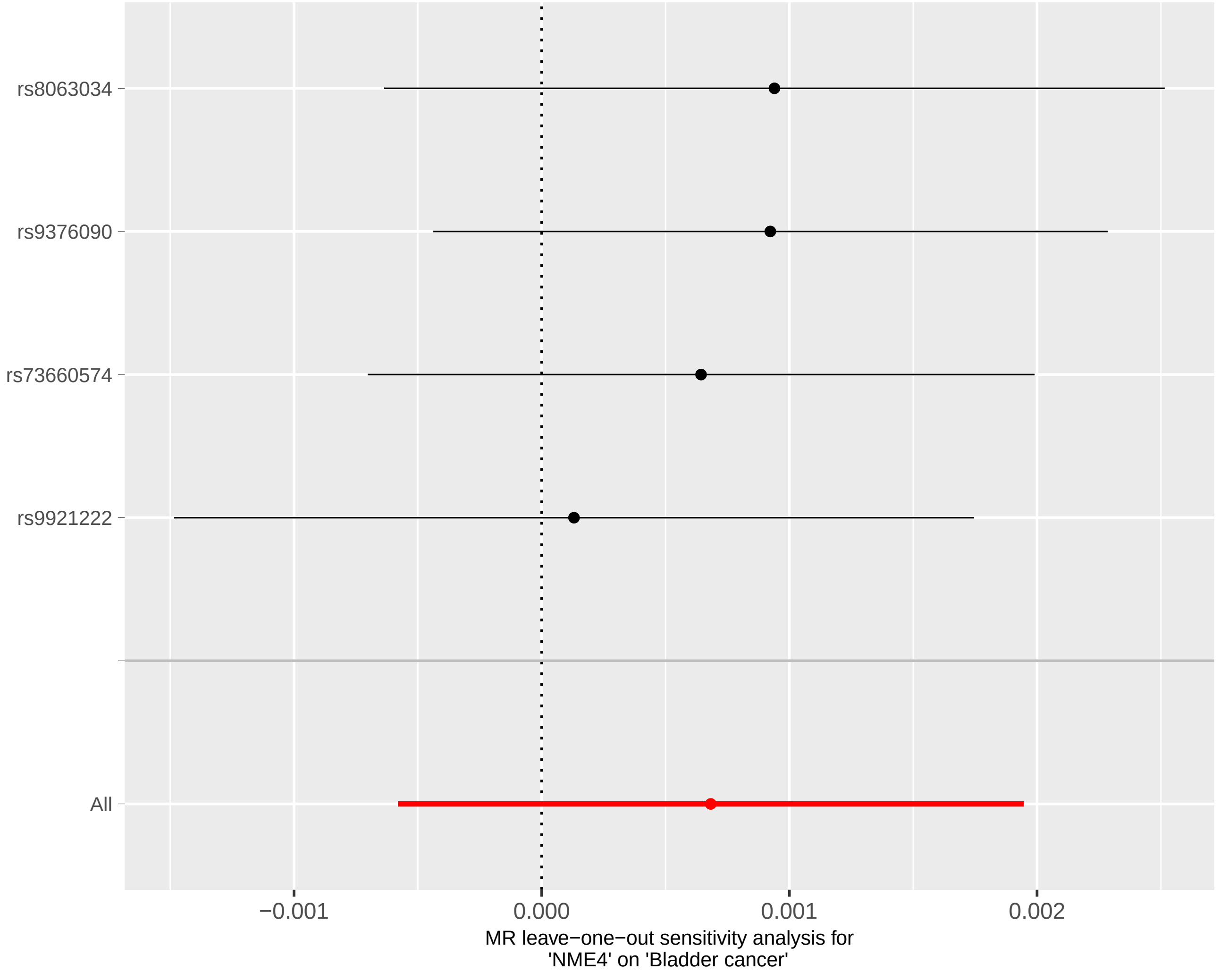
rs8063034
rs9376090
rs73660574
rs9921222
All
−0.001
0.000
0.001
0.002
MR leave−one−out sensitivity analysis for
'NME4' on 'Bladder cancer'

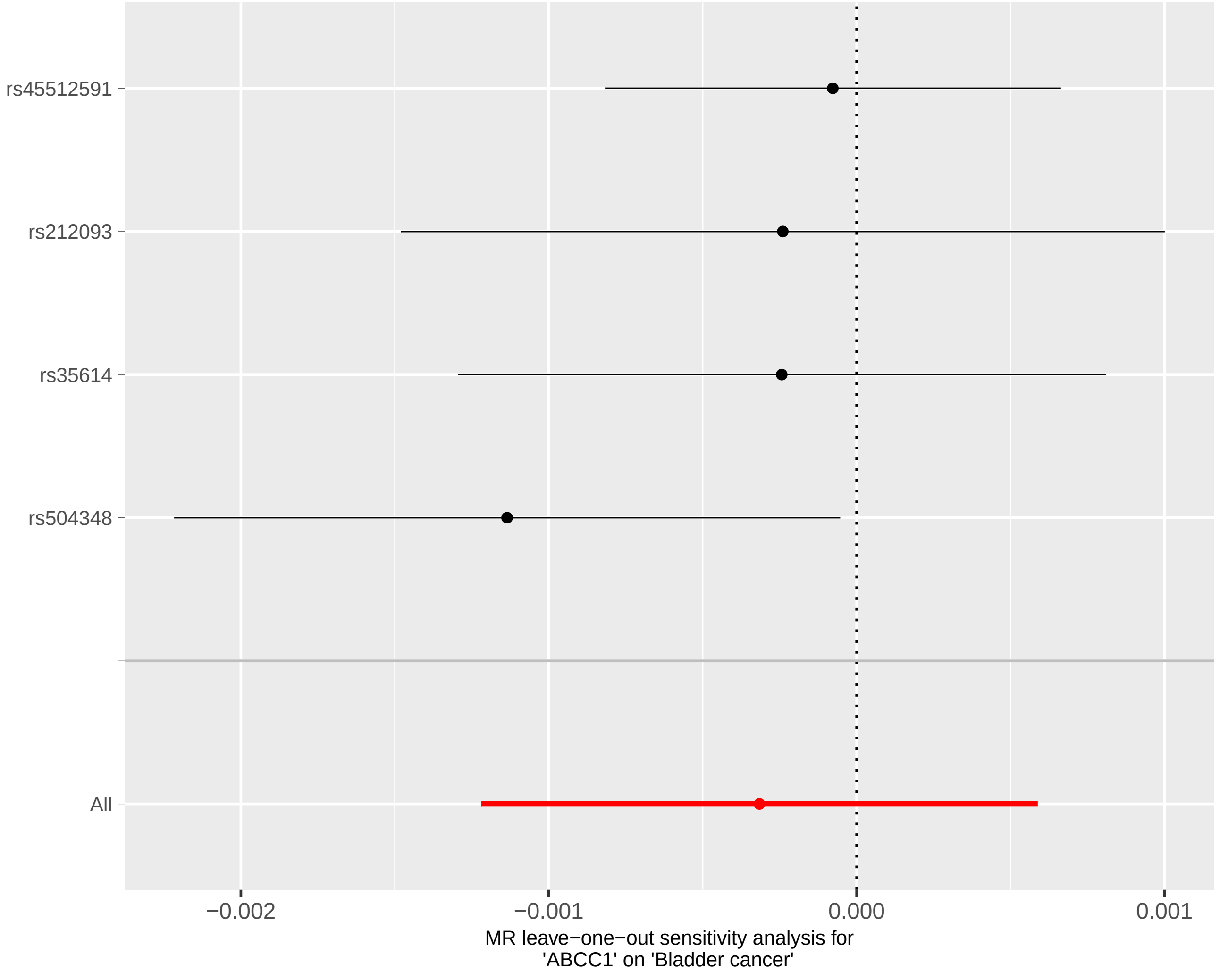
rs45512591
rs212093
rs35614
rs504348
All
−0.002
−0.001
0.000
0.001
MR leave−one−out sensitivity analysis for
'ABCC1' on 'Bladder cancer'

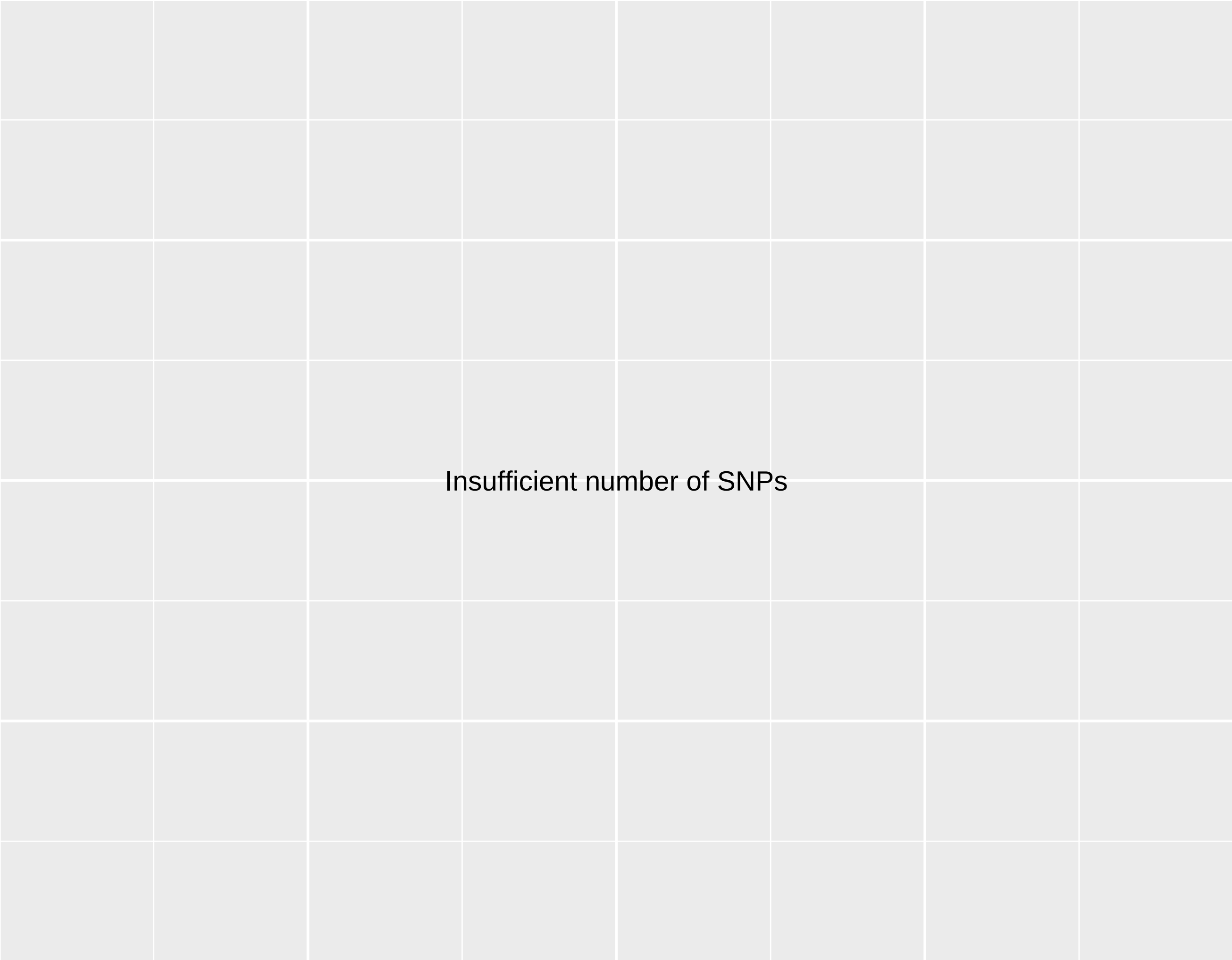
Insufficient number of SNPs

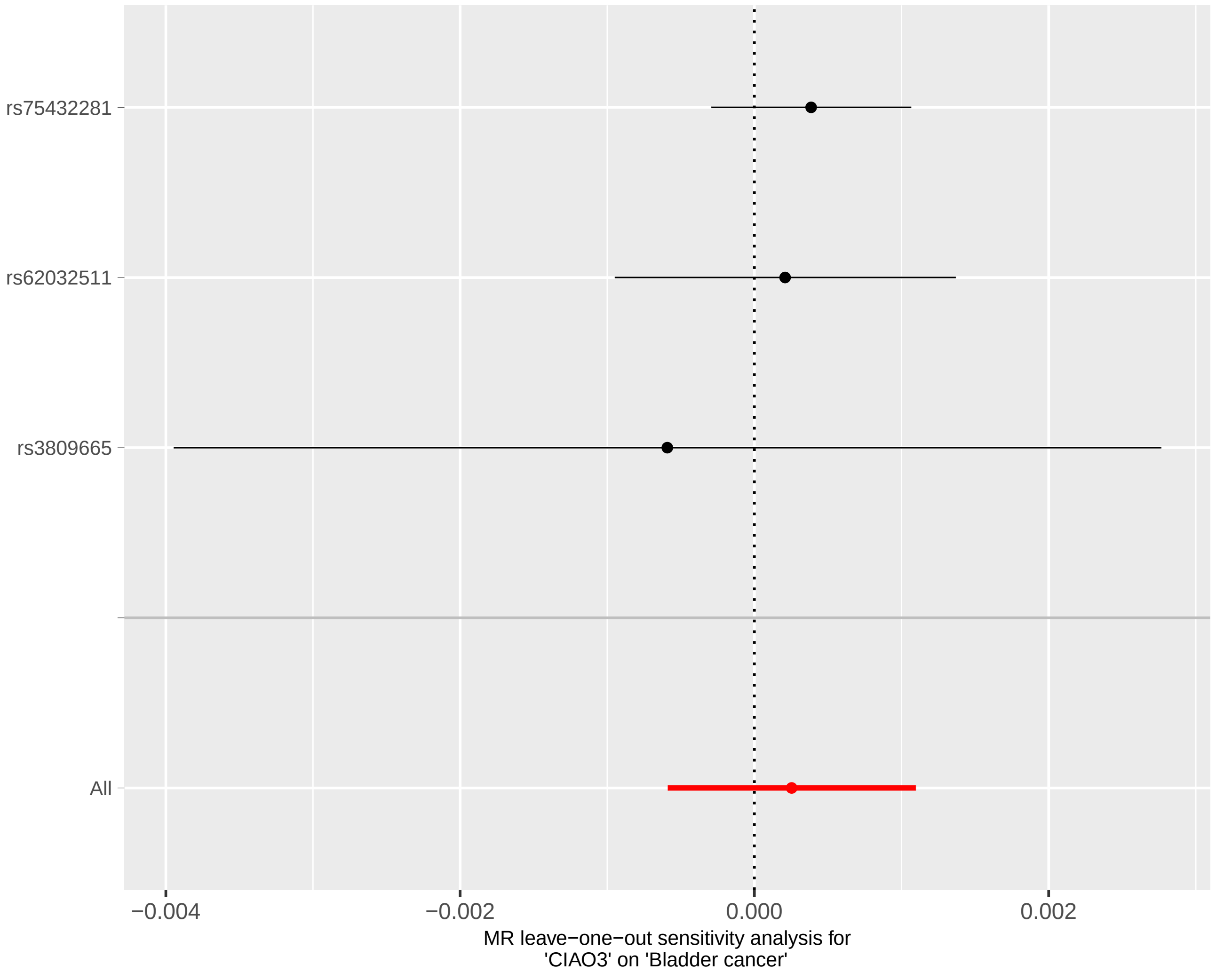
rs75432281
rs62032511
rs3809665
All
−0.004
−0.002
0.000
0.002
MR leave−one−out sensitivity analysis for
'CIAO3' on 'Bladder cancer'

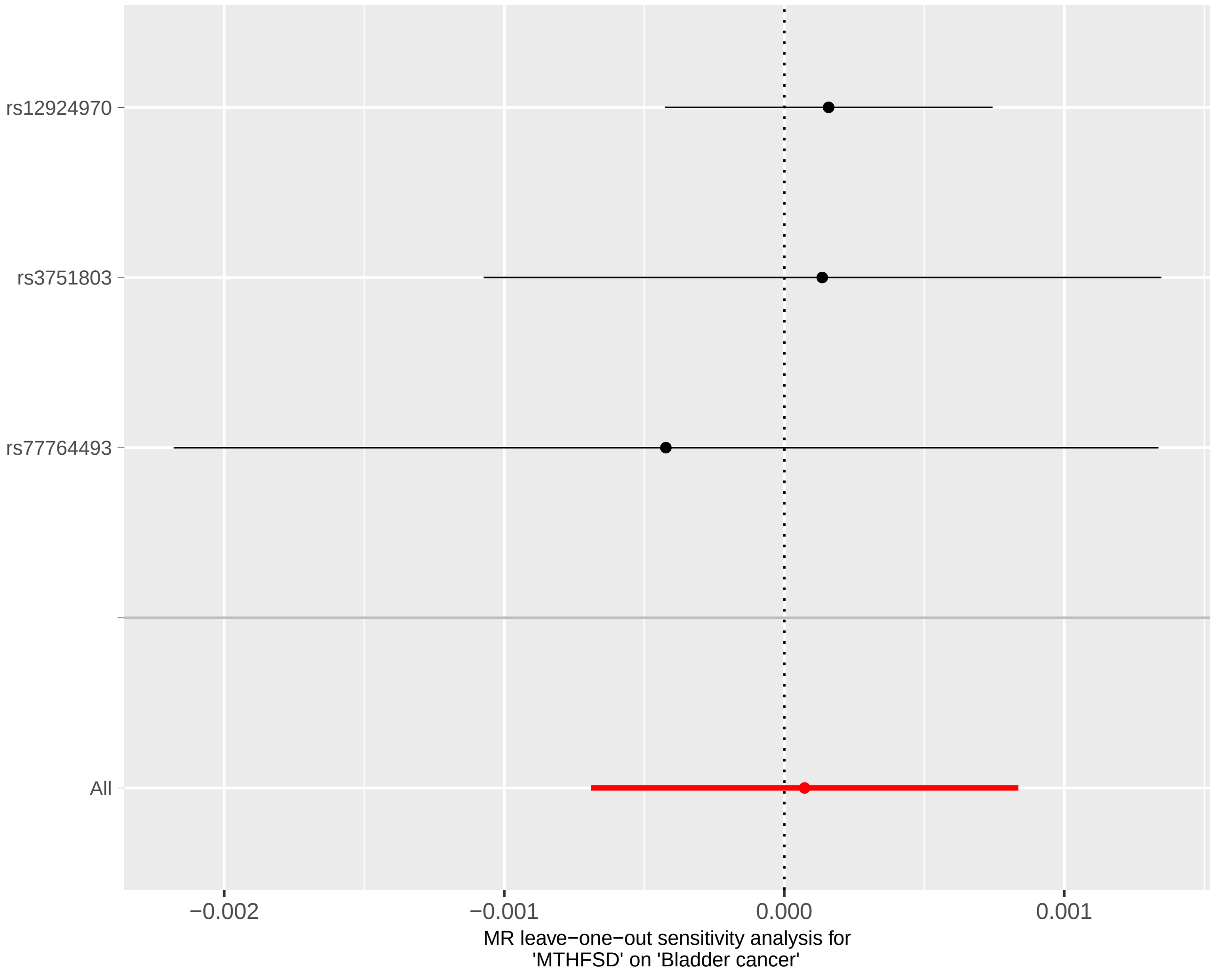
rs12924970
rs3751803
rs77764493
All
−0.002
−0.001
0.000
0.001
MR leave−one−out sensitivity analysis for
'MTHFSD' on 'Bladder cancer'

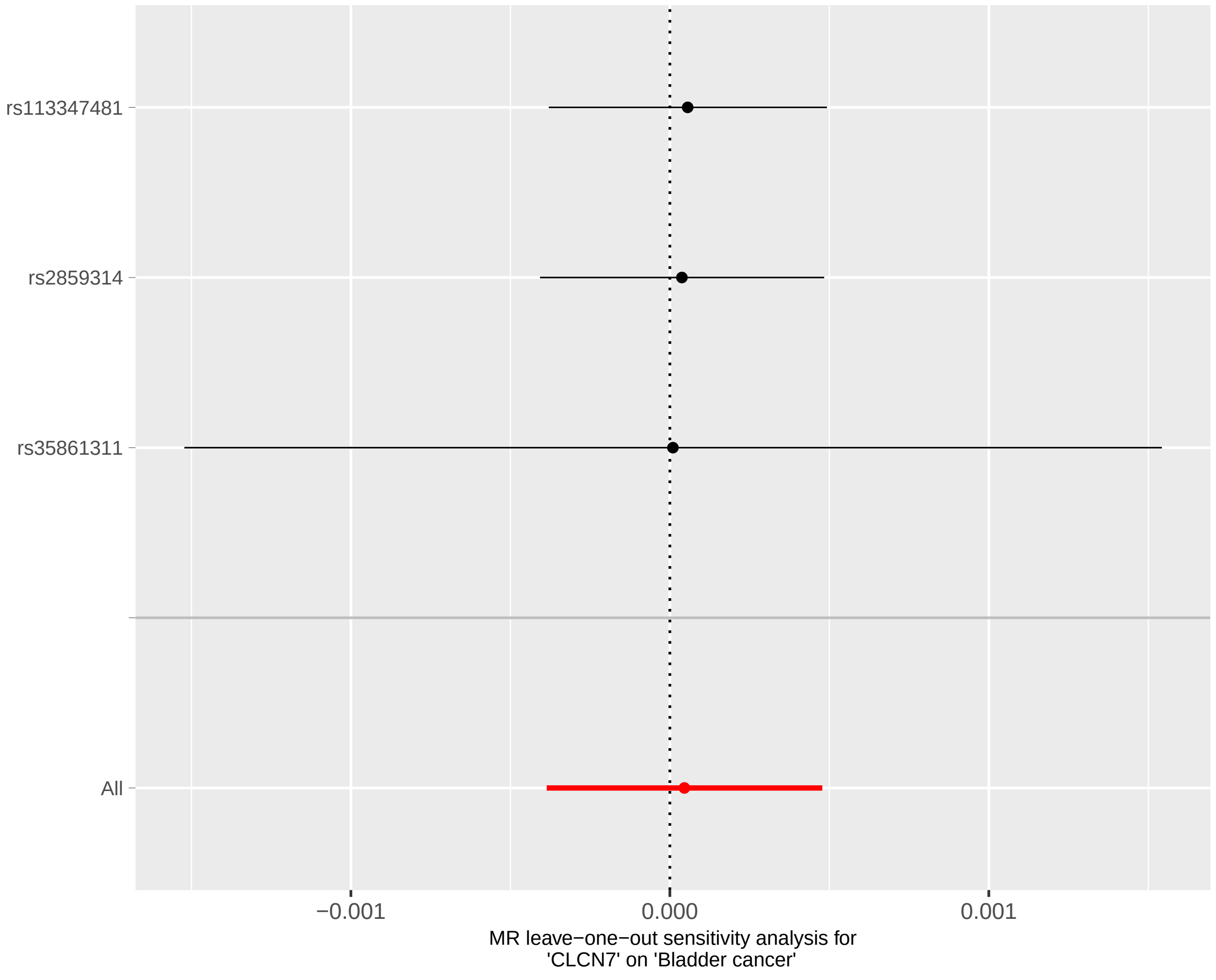
rs113347481
rs2859314
rs35861311
All
−0.001
0.000
0.001
MR leave−one−out sensitivity analysis for
'CLCN7' on 'Bladder cancer'

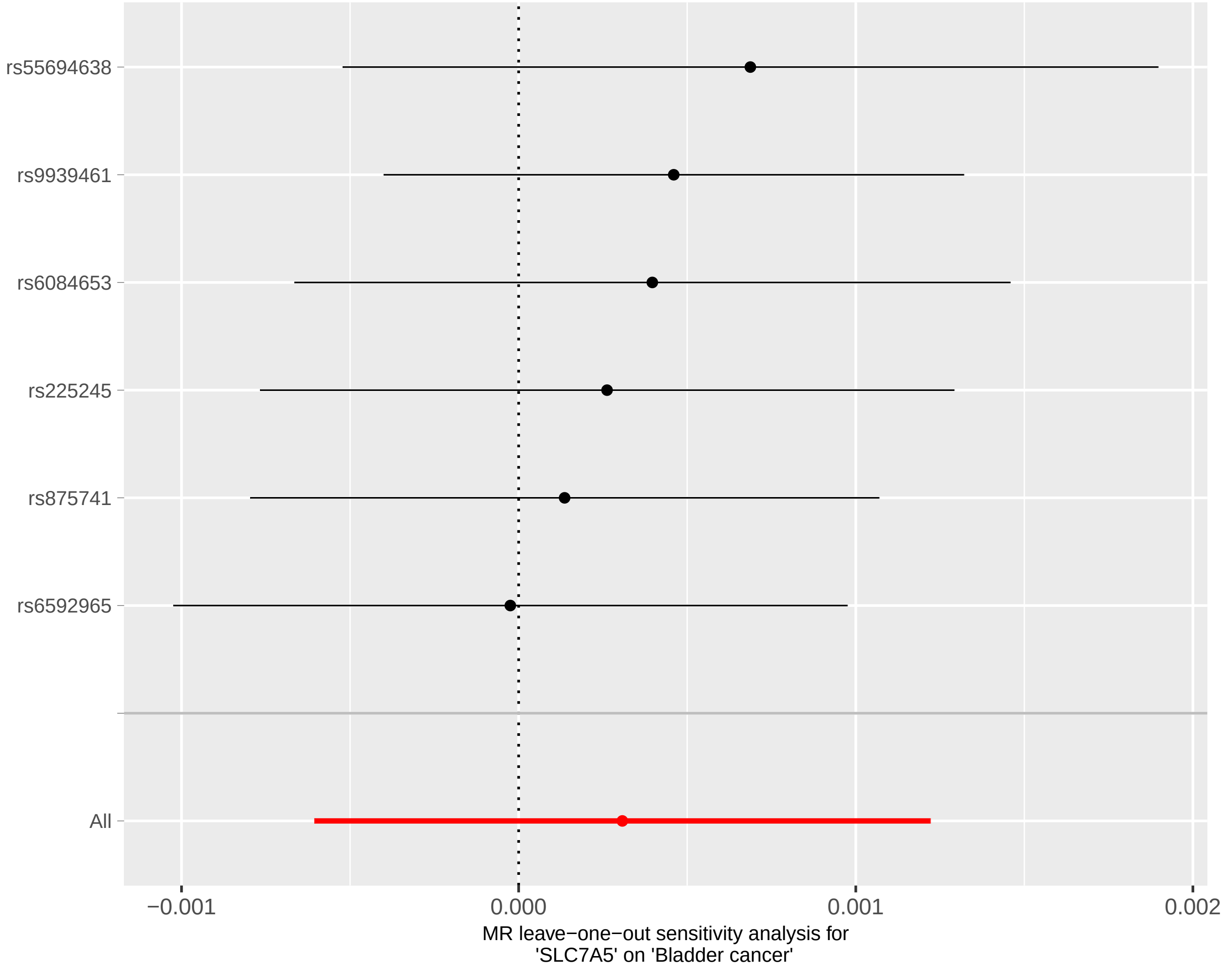
rs55694638
rs9939461
rs6084653
rs225245
rs875741
rs6592965
All
−0.001
0.000
0.001
0.002
MR leave−one−out sensitivity analysis for
'SLC7A5' on 'Bladder cancer'

Insufficient number of SNPs

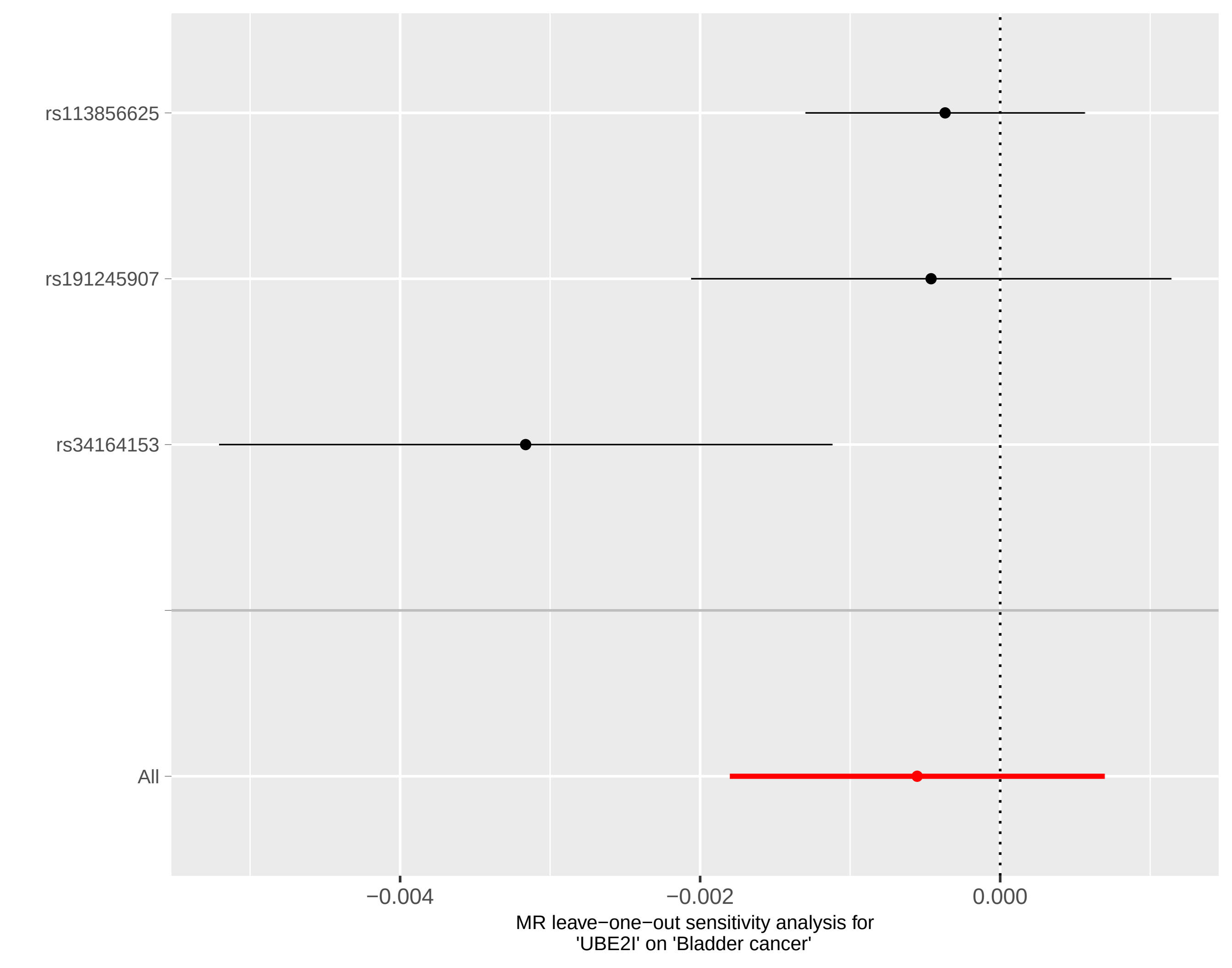
rs113856625
rs191245907
rs34164153
All
−0.004
−0.002
0.000
MR leave−one−out sensitivity analysis for
'UBE2I' on 'Bladder cancer'

Insufficient number of SNPs

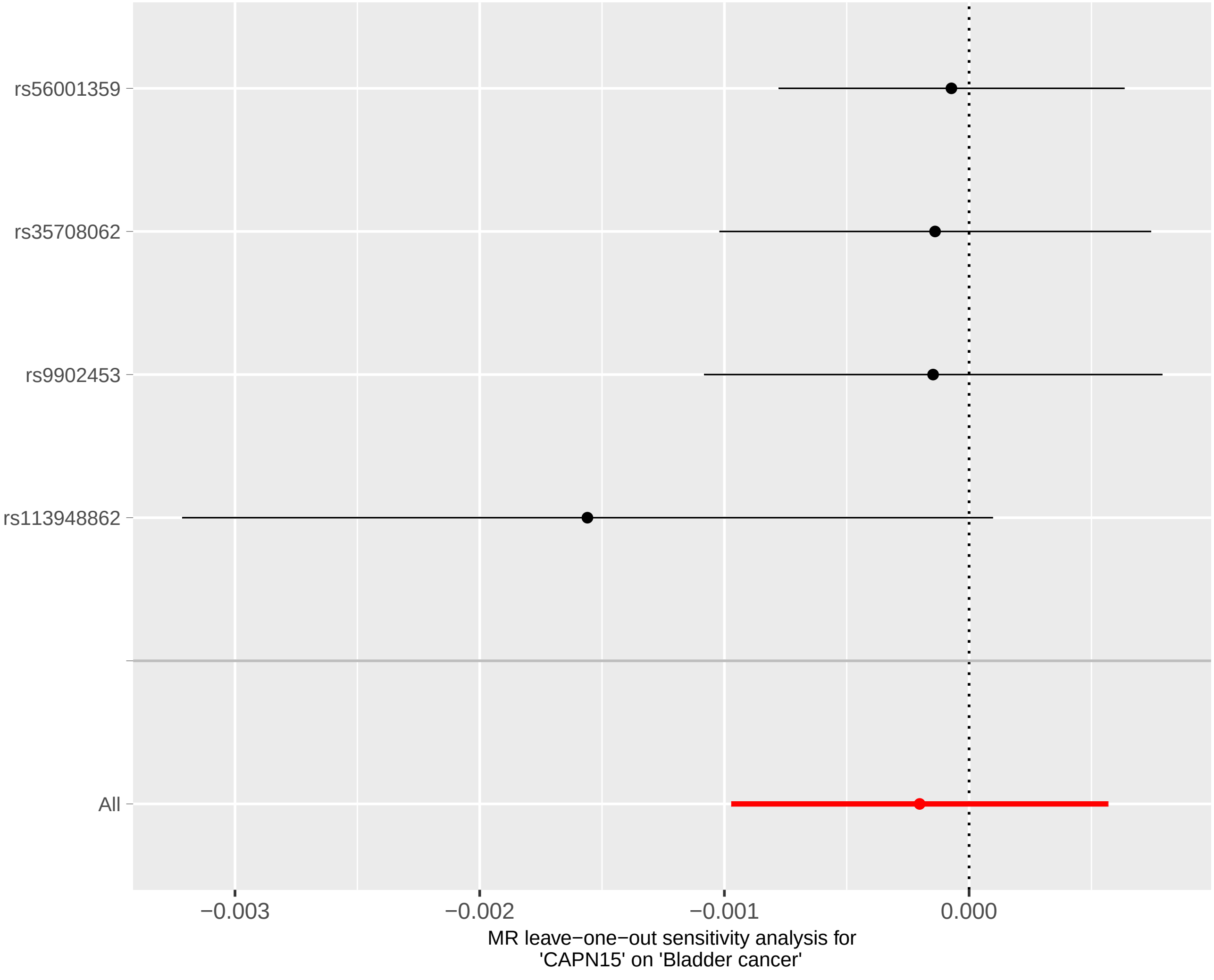
rs56001359
rs35708062
rs9902453
rs113948862
All
−0.003
−0.002
−0.001
0.000
MR leave−one−out sensitivity analysis for
'CAPN15' on 'Bladder cancer'

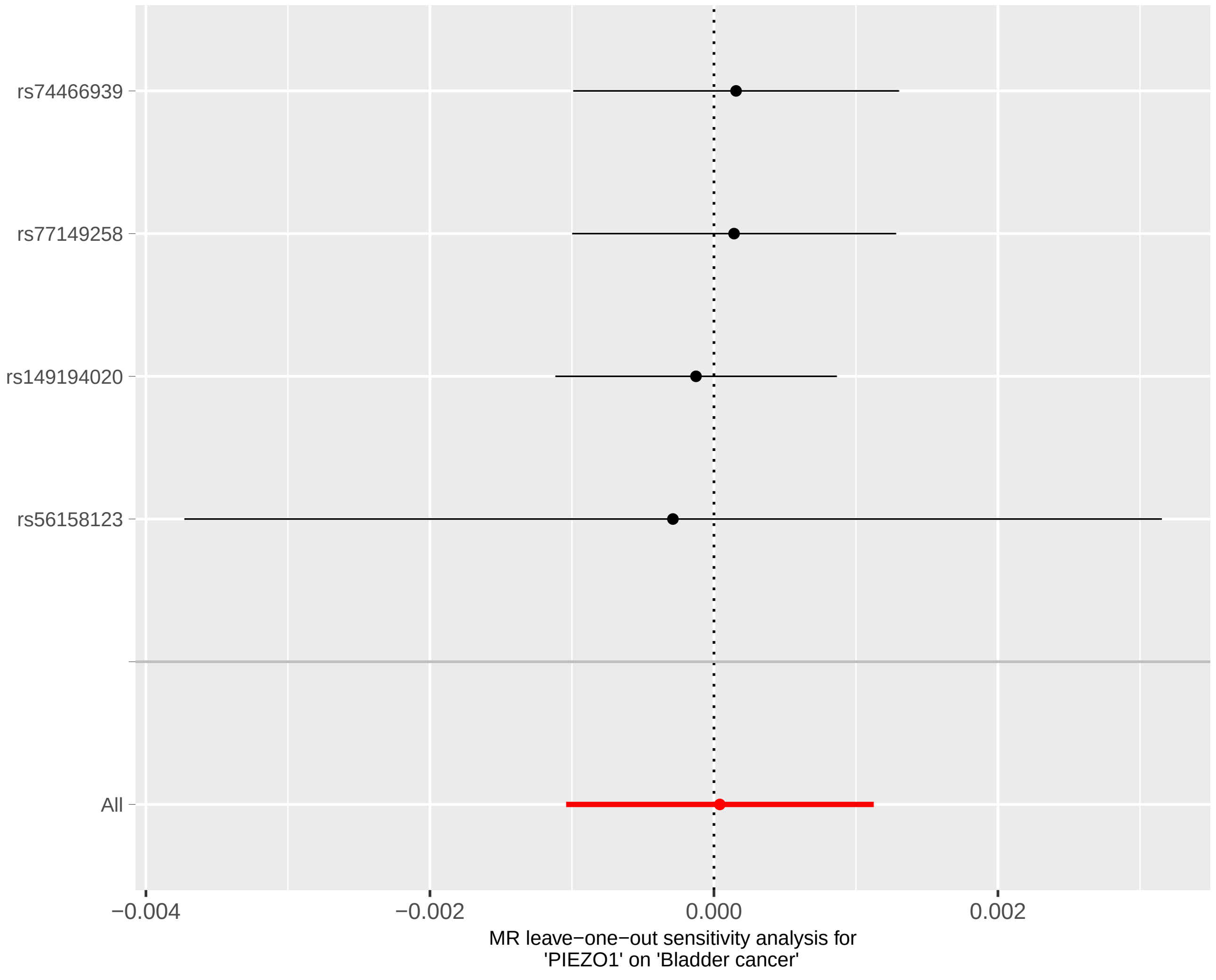
rs74466939
rs77149258
rs149194020
rs56158123
All
−0.004
−0.002
0.000
0.002
MR leave−one−out sensitivity analysis for
'PIEZO1' on 'Bladder cancer'

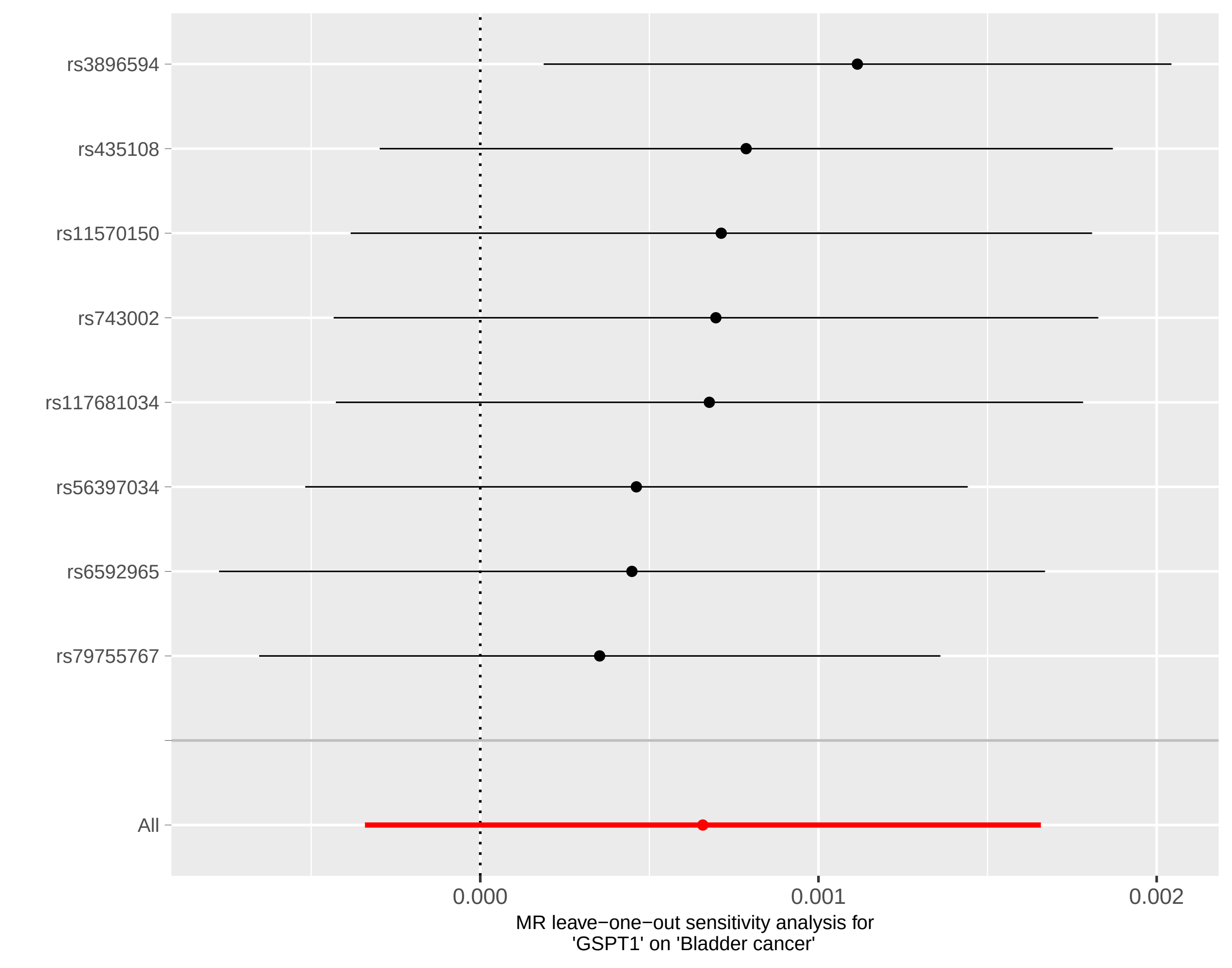
rs3896594
rs435108
rs11570150
rs743002
rs117681034
rs56397034
rs6592965
rs79755767
All
0.000
0.001
0.002
MR leave−one−out sensitivity analysis for
'GSPT1' on 'Bladder cancer'

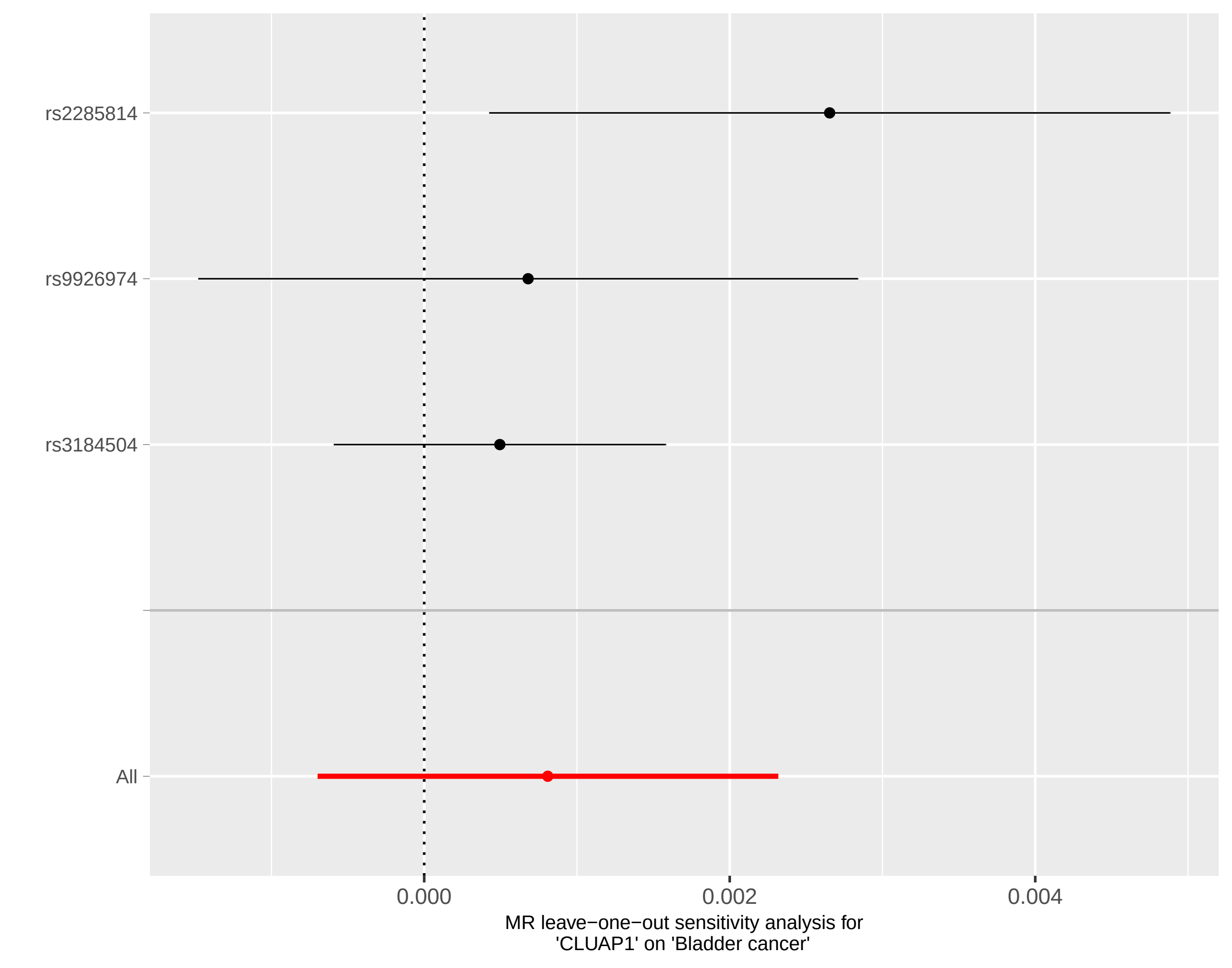
rs2285814
rs9926974
rs3184504
All
0.000
0.002
0.004
MR leave−one−out sensitivity analysis for
'CLUAP1' on 'Bladder cancer'

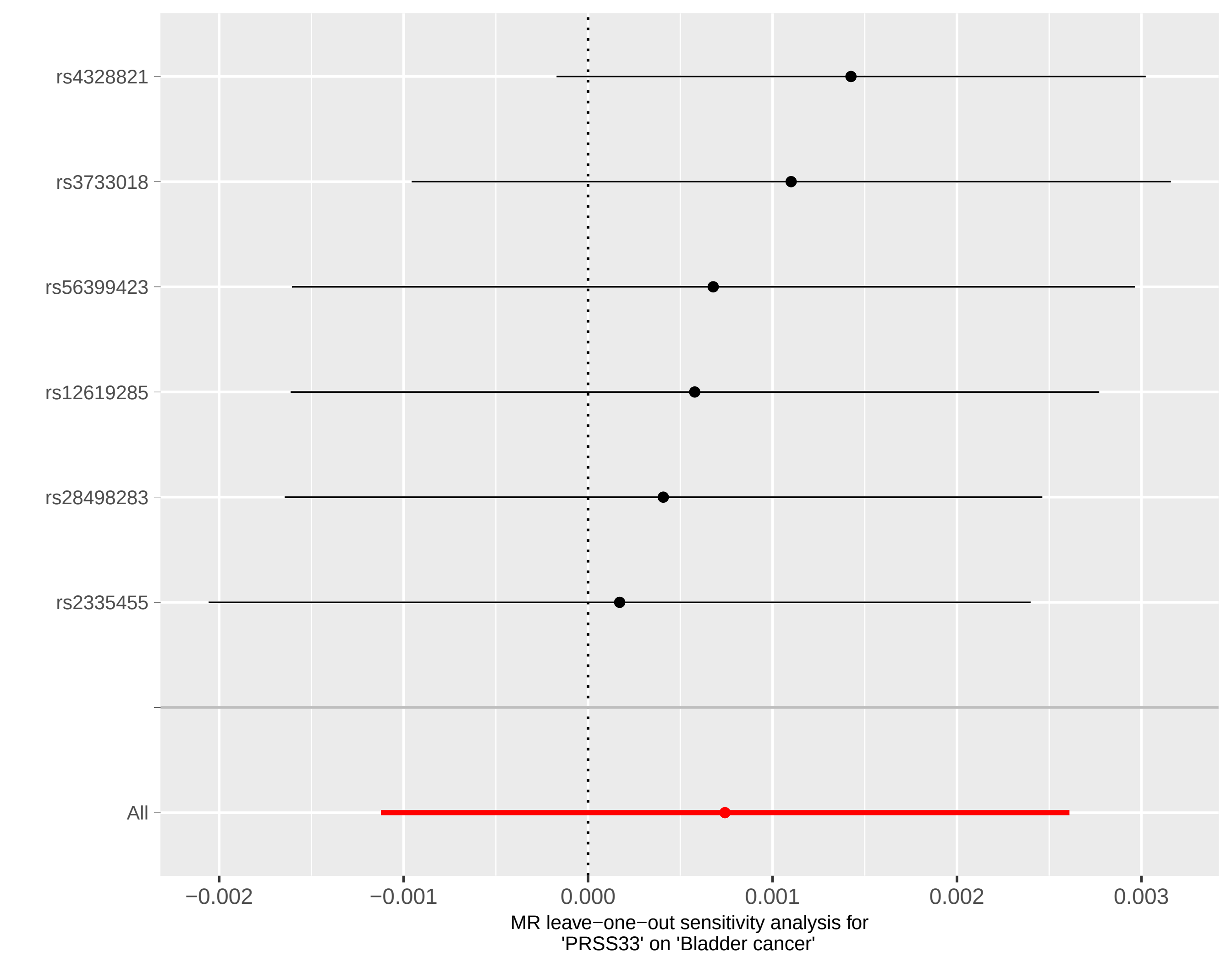
rs4328821
rs3733018
rs56399423
rs12619285
rs28498283
rs2335455
All
−0.002
−0.001
0.000
0.001
0.002
0.003
MR leave−one−out sensitivity analysis for
'PRSS33' on 'Bladder cancer'

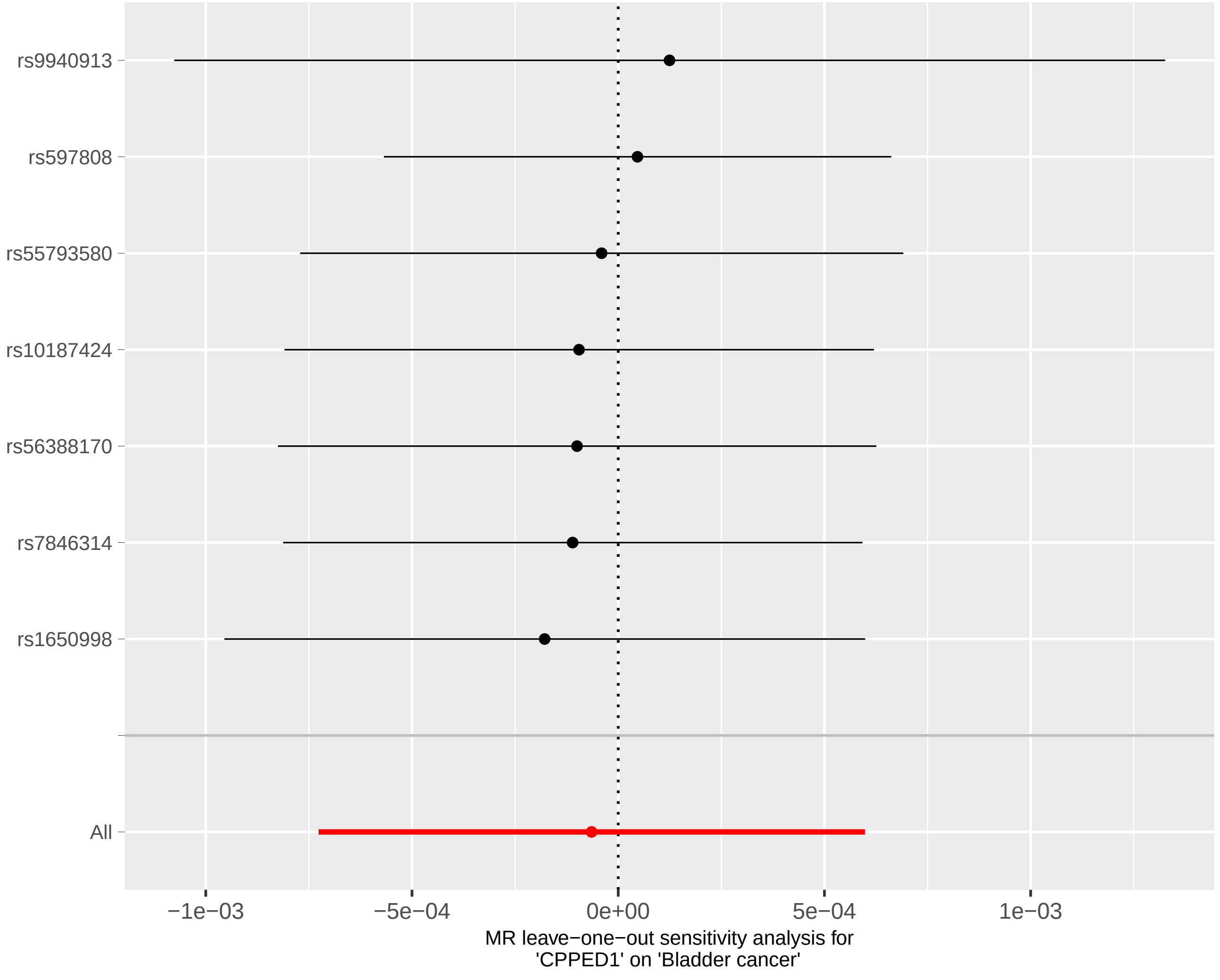
rs9940913
rs597808
rs55793580
rs10187424
rs56388170
rs7846314
rs1650998
All
−1e−03
−5e−04
0e+00
5e−04
1e−03
MR leave−one−out sensitivity analysis for
'CPPED1' on 'Bladder cancer'

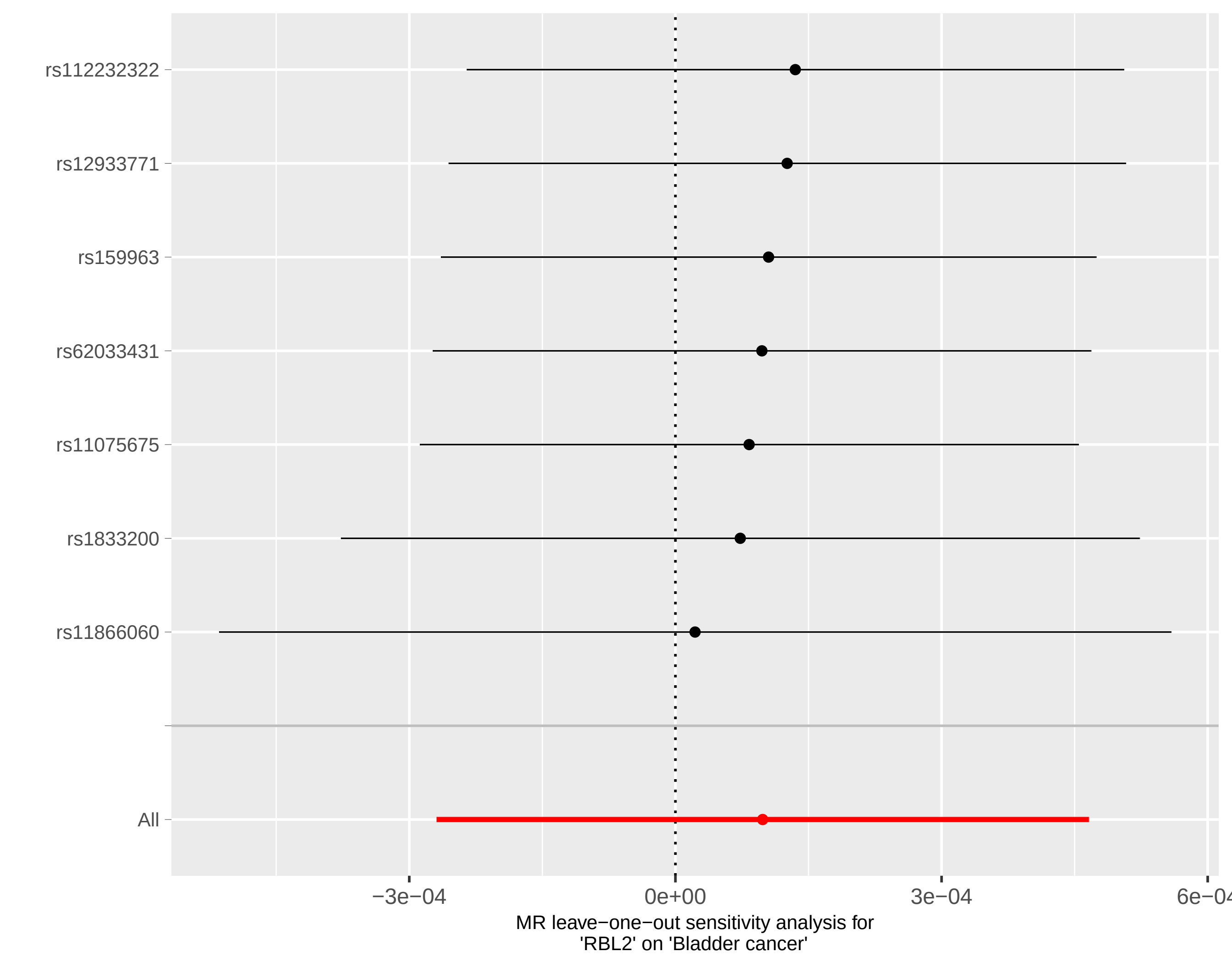
rs112232322
rs12933771
rs159963
rs62033431
rs11075675
rs1833200
rs11866060
All
−3e−04
0e+00
3e−04
MR leave−one−out sensitivity analysis for
'RBL2' on 'Bladder cancer'

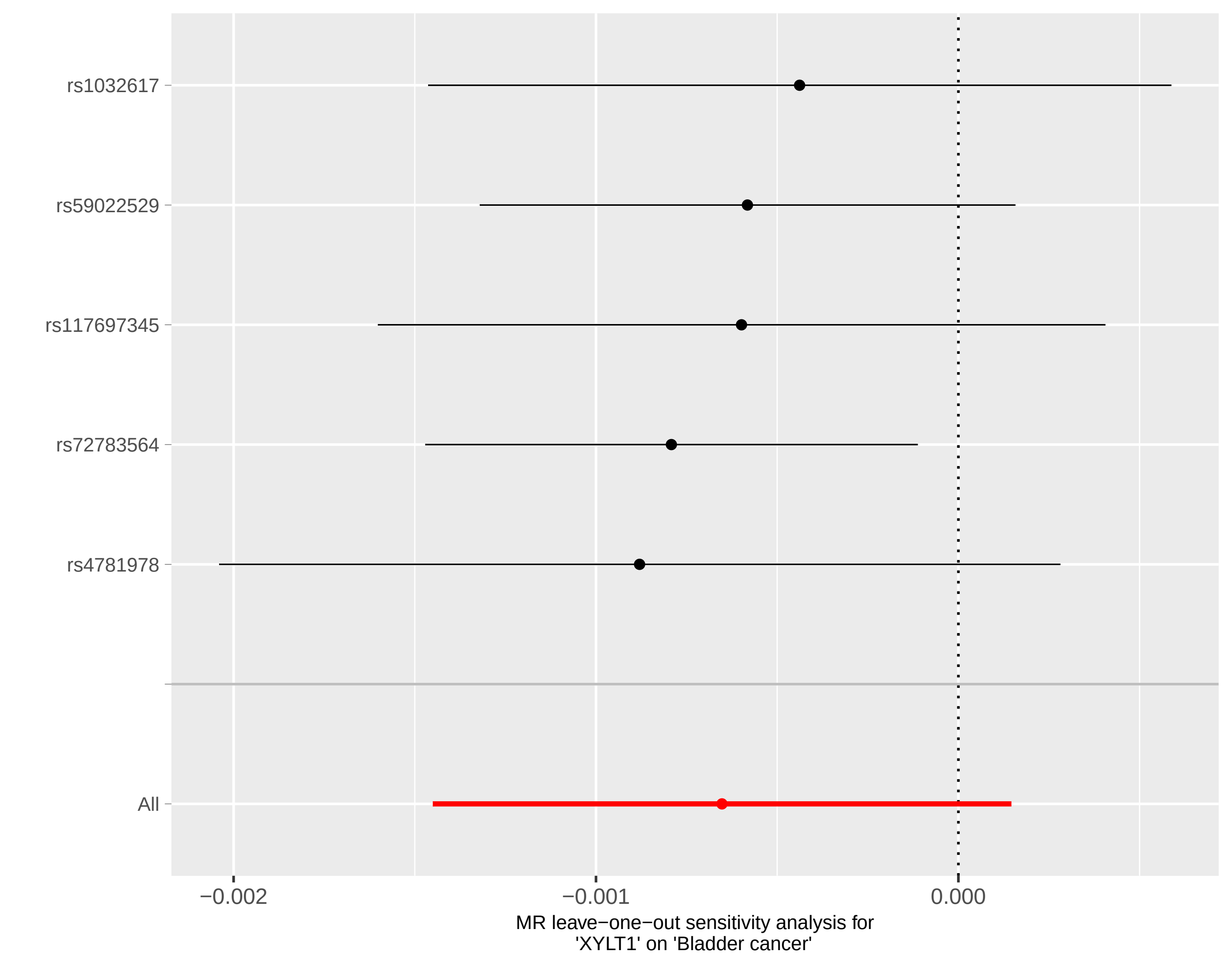
rs1032617
rs59022529
rs117697345
rs72783564
rs4781978
All
−0.002
−0.001
0.000
MR leave−one−out sensitivity analysis for
'XYLT1' on 'Bladder cancer'

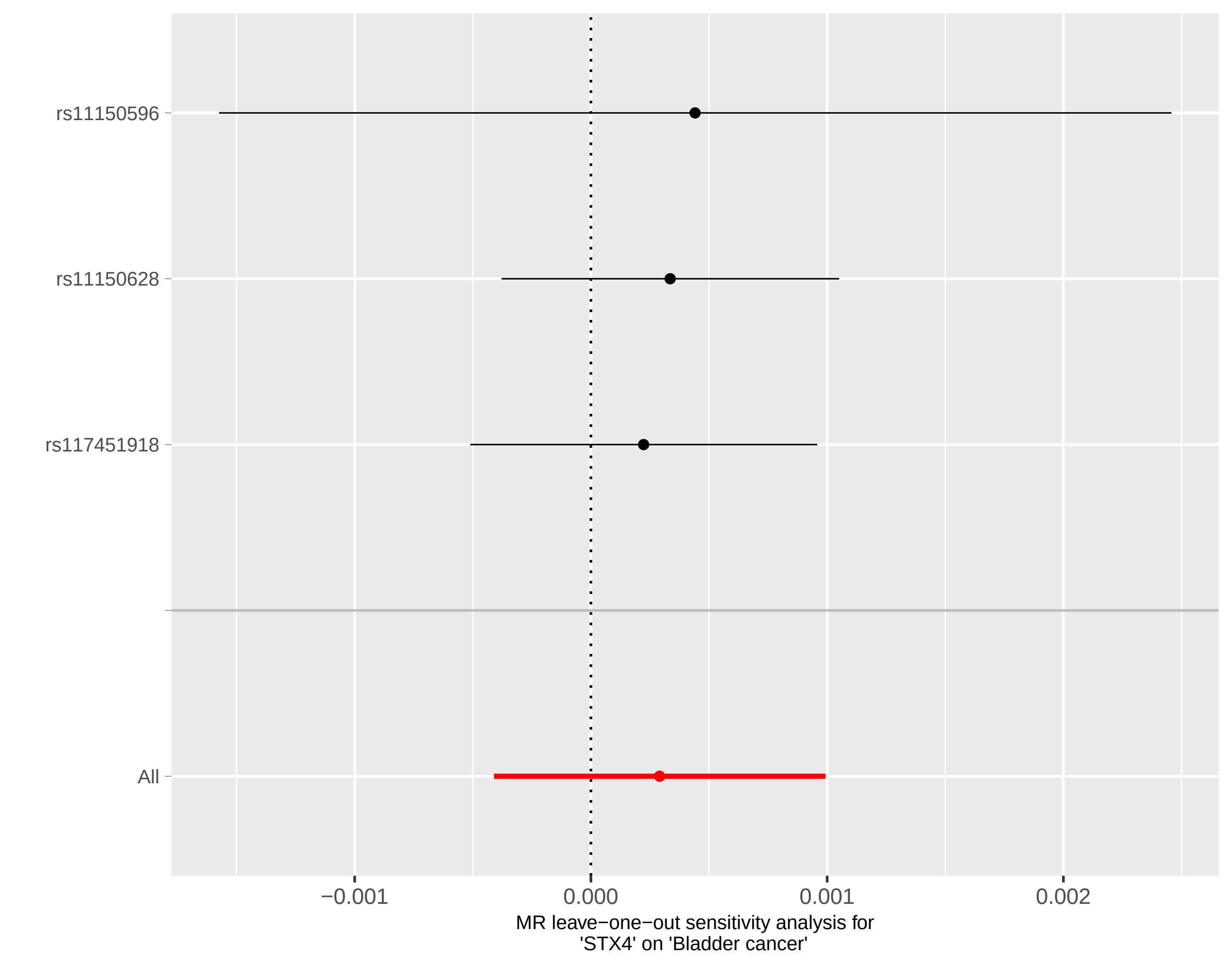
rs11150596
rs11150628
rs117451918
All
−0.001
0.000
0.001
0.002
MR leave−one−out sensitivity analysis for
'STX4' on 'Bladder cancer'

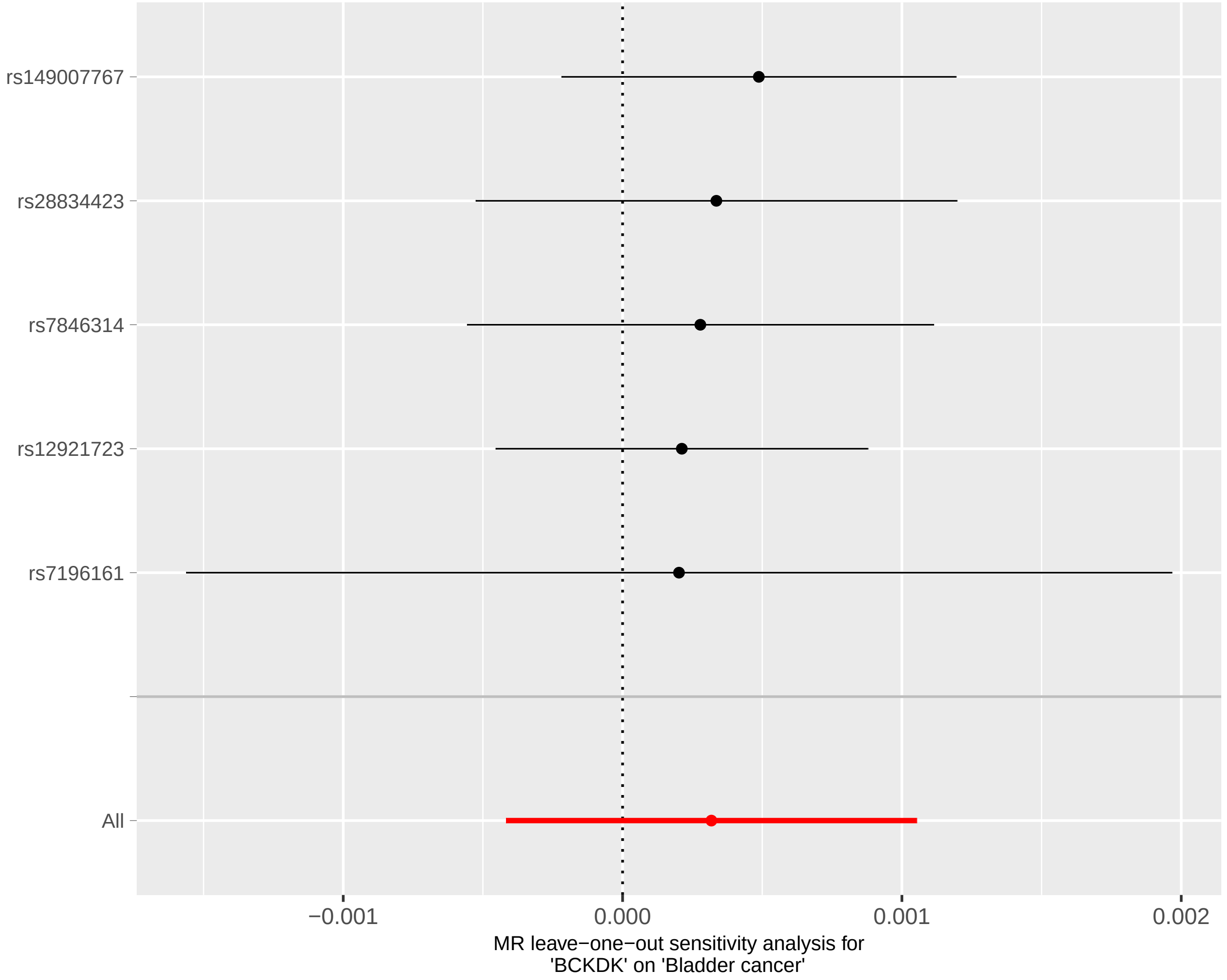
rs149007767
rs28834423
rs7846314
rs12921723
rs7196161
All
−0.001
0.000
0.001
0.002
MR leave−one−out sensitivity analysis for
'BCKDK' on 'Bladder cancer'

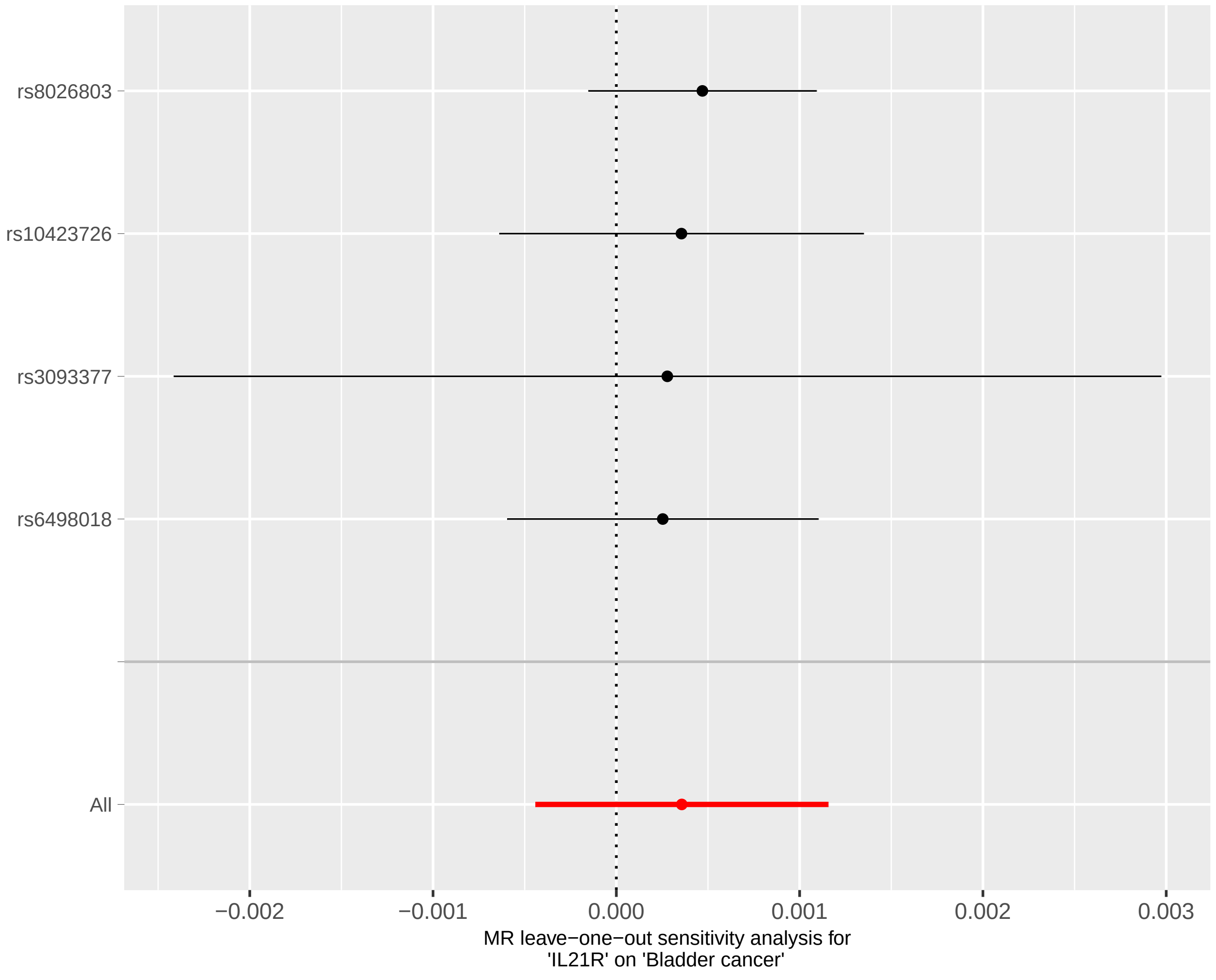
rs8026803
rs10423726
rs3093377
rs6498018
All
−0.002
−0.001
0.000
0.001
0.002
0.003
MR leave−one−out sensitivity analysis for
'IL21R' on 'Bladder cancer'

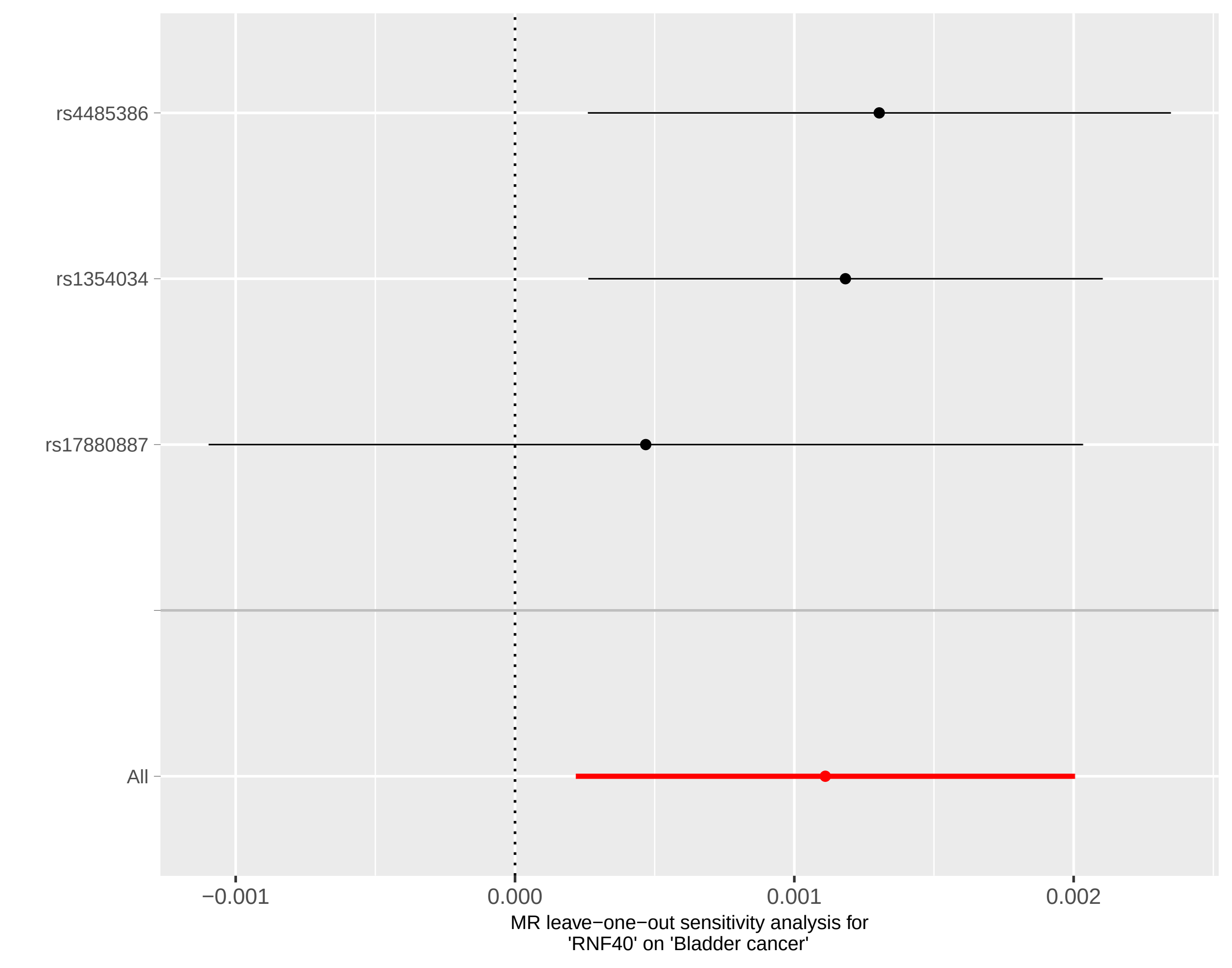
rs4485386
rs1354034
rs17880887
All
−0.001
0.000
0.001
0.002
MR leave−one−out sensitivity analysis for
'RNF40' on 'Bladder cancer'

Insufficient number of SNPs

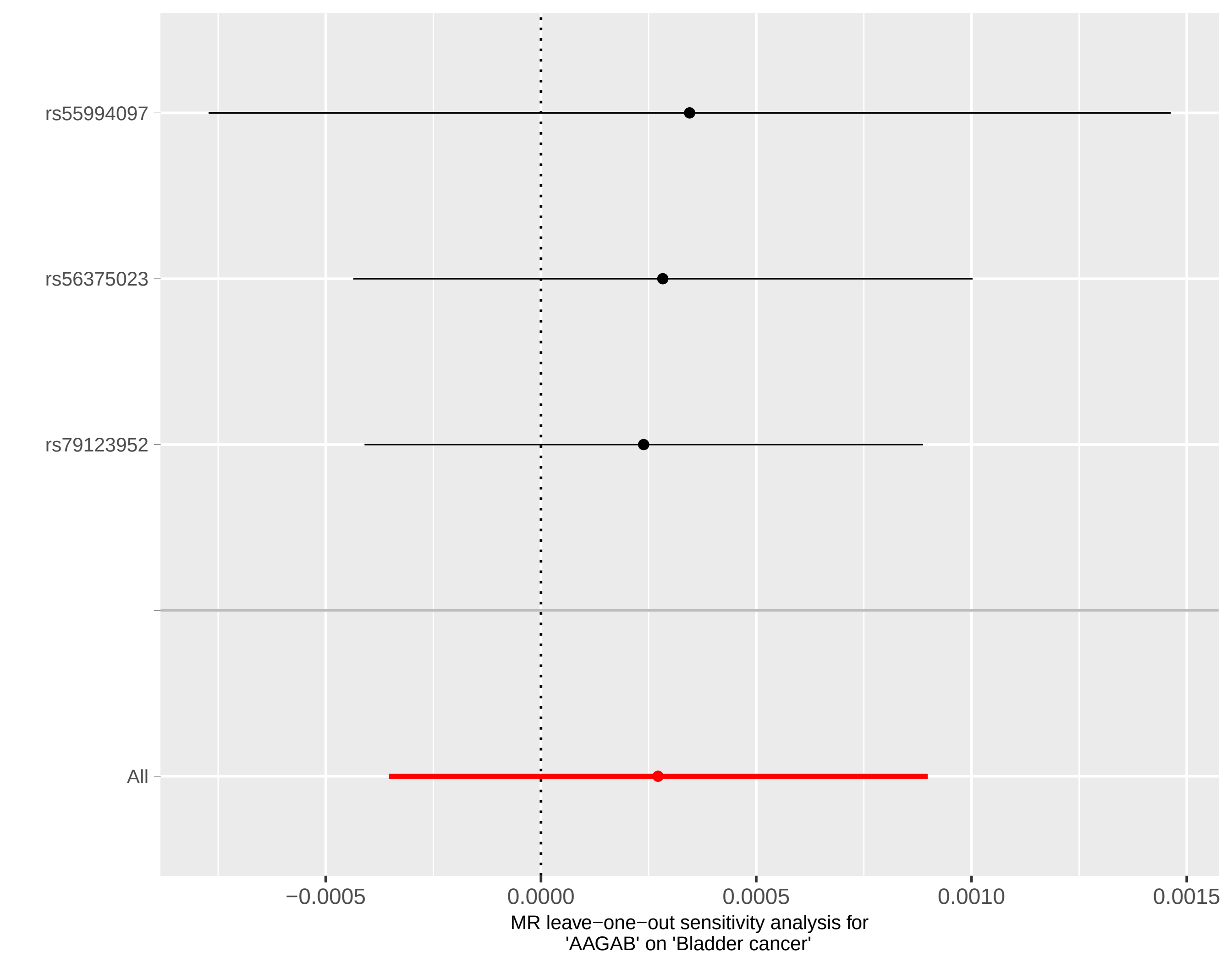
rs55994097
rs56375023
rs79123952
All
−0.0005
0.0000
0.0005
0.0010
0.0015
MR leave−one−out sensitivity analysis for
'AAGAB' on 'Bladder cancer'

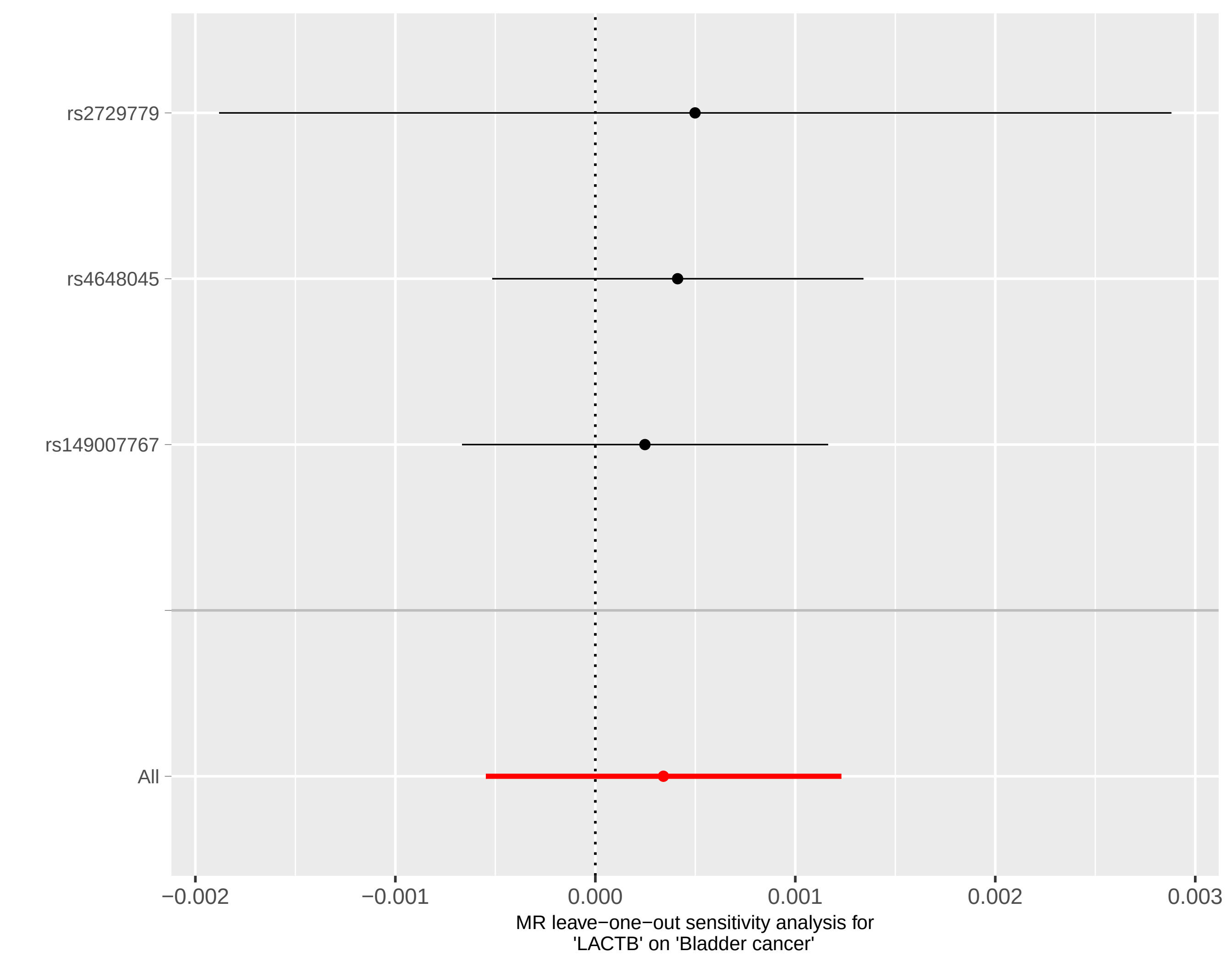

rs2729779
rs4648045
rs149007767
All
−0.002
−0.001
0.000
0.001
0.002
0.003
MR leave−one−out sensitivity analysis for
'LACTB' on 'Bladder cancer'

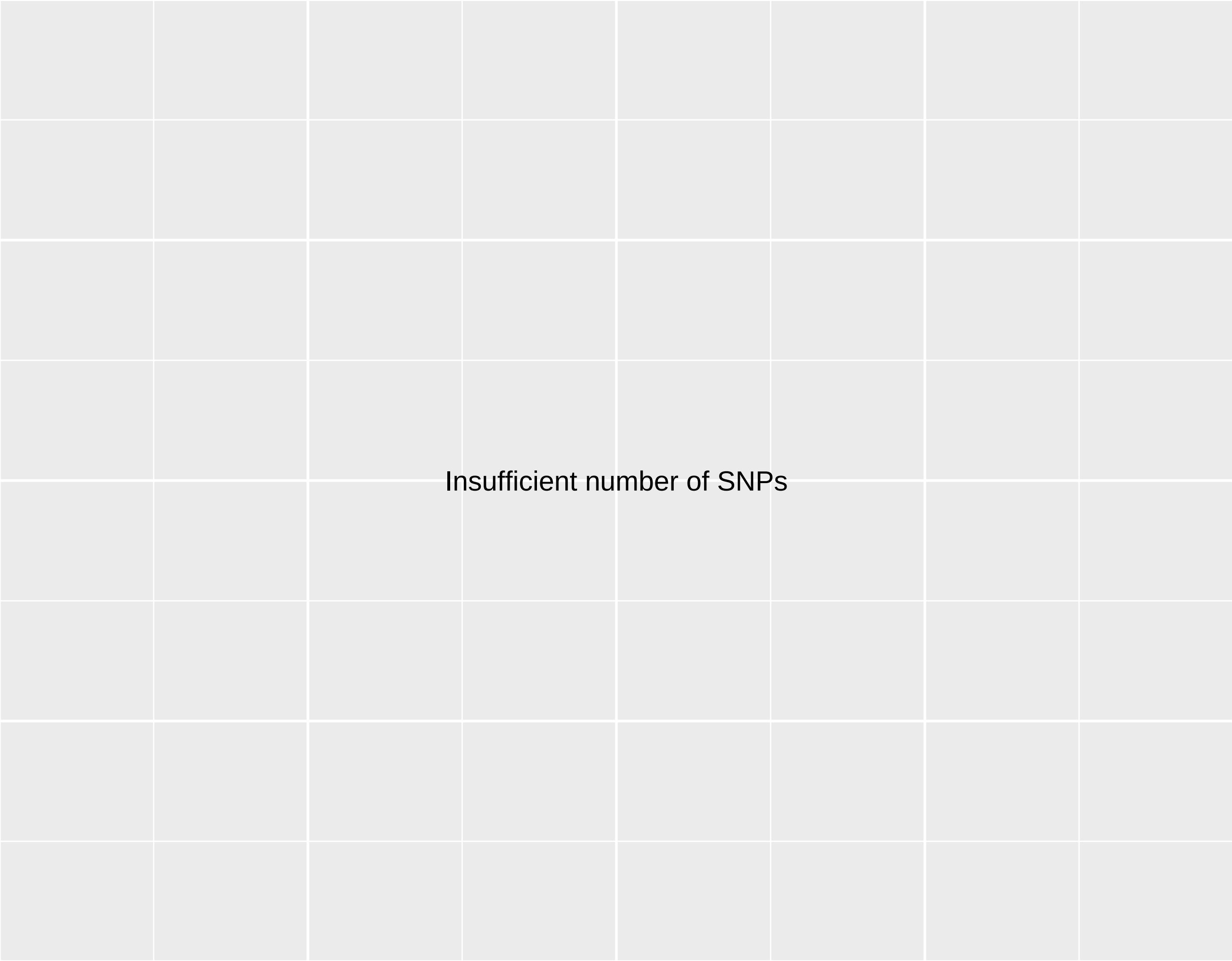
Insufficient number of SNPs

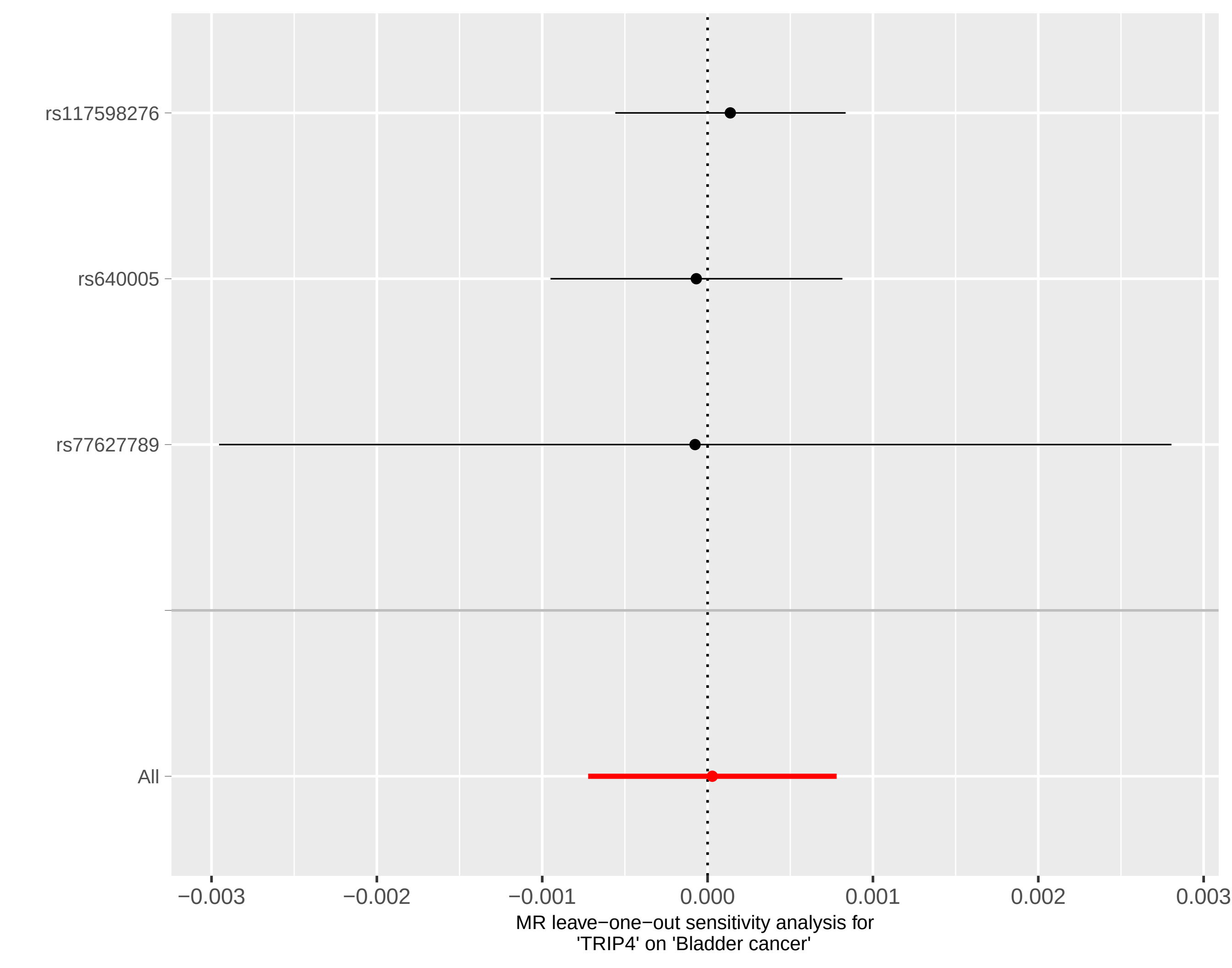
rs117598276
rs640005
rs77627789
All
−0.003
−0.002
−0.001
0.000
0.001
0.002
0.003
MR leave−one−out sensitivity analysis for
'TRIP4' on 'Bladder cancer'

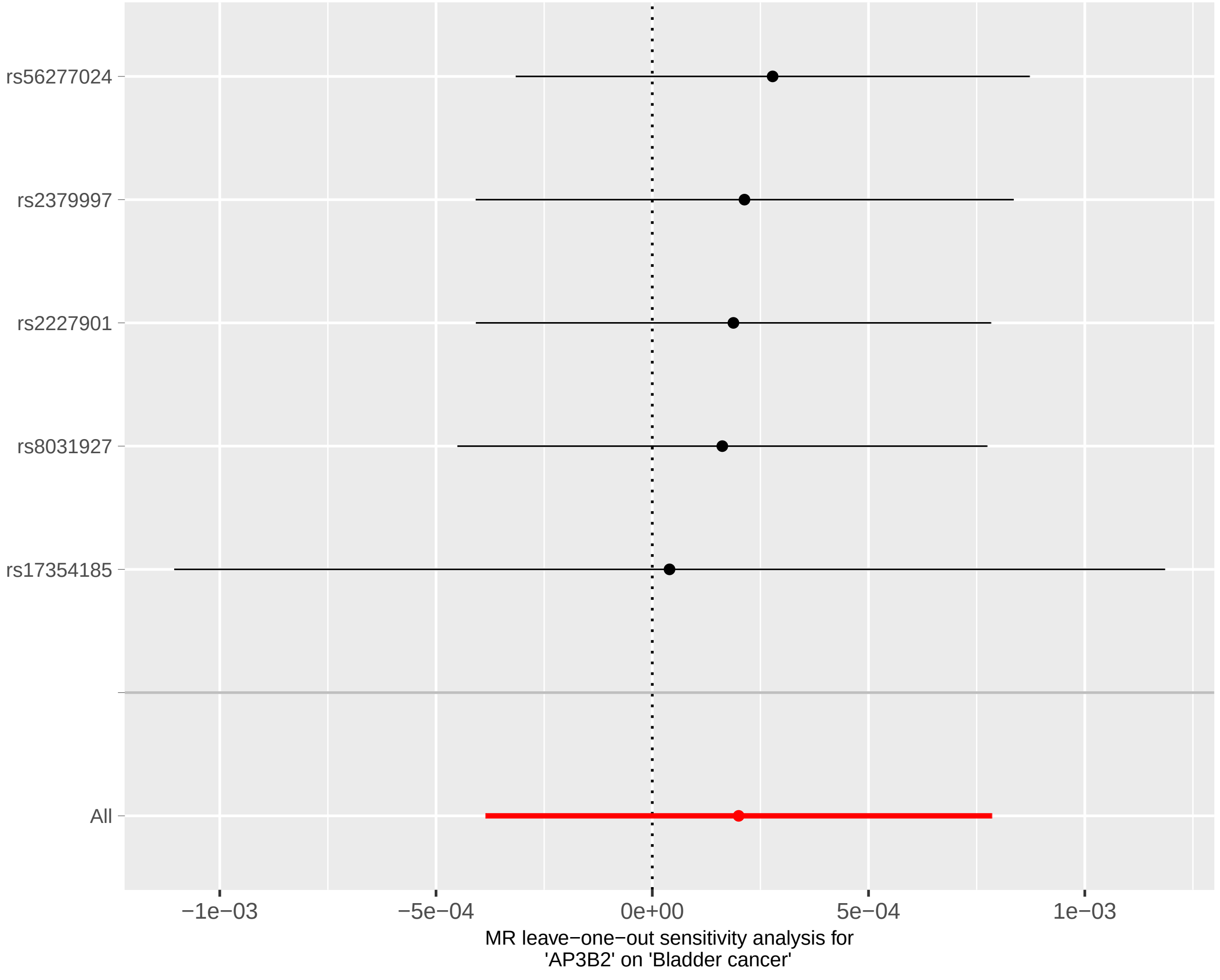

rs56277024
rs2379997
rs2227901
rs8031927
rs17354185
All
−1e−03
−5e−04
0e+00
5e−04
1e−03
MR leave−one−out sensitivity analysis for
'AP3B2' on 'Bladder cancer'

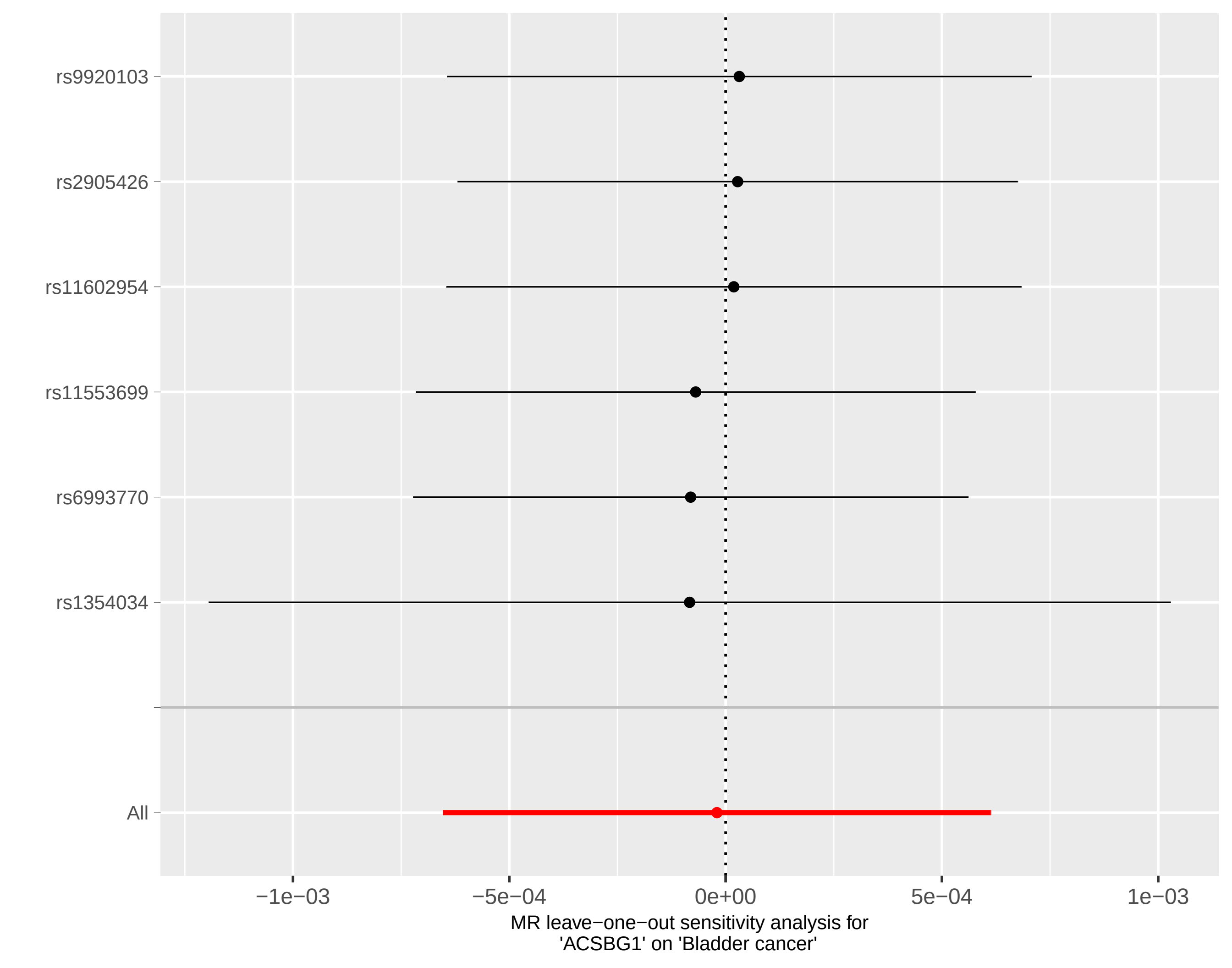
rs9920103
rs2905426
rs11602954
rs11553699
rs6993770
rs1354034
All
−1e−03
−5e−04
0e+00
5e−04
1e−03
MR leave−one−out sensitivity analysis for
'ACSBG1' on 'Bladder cancer'

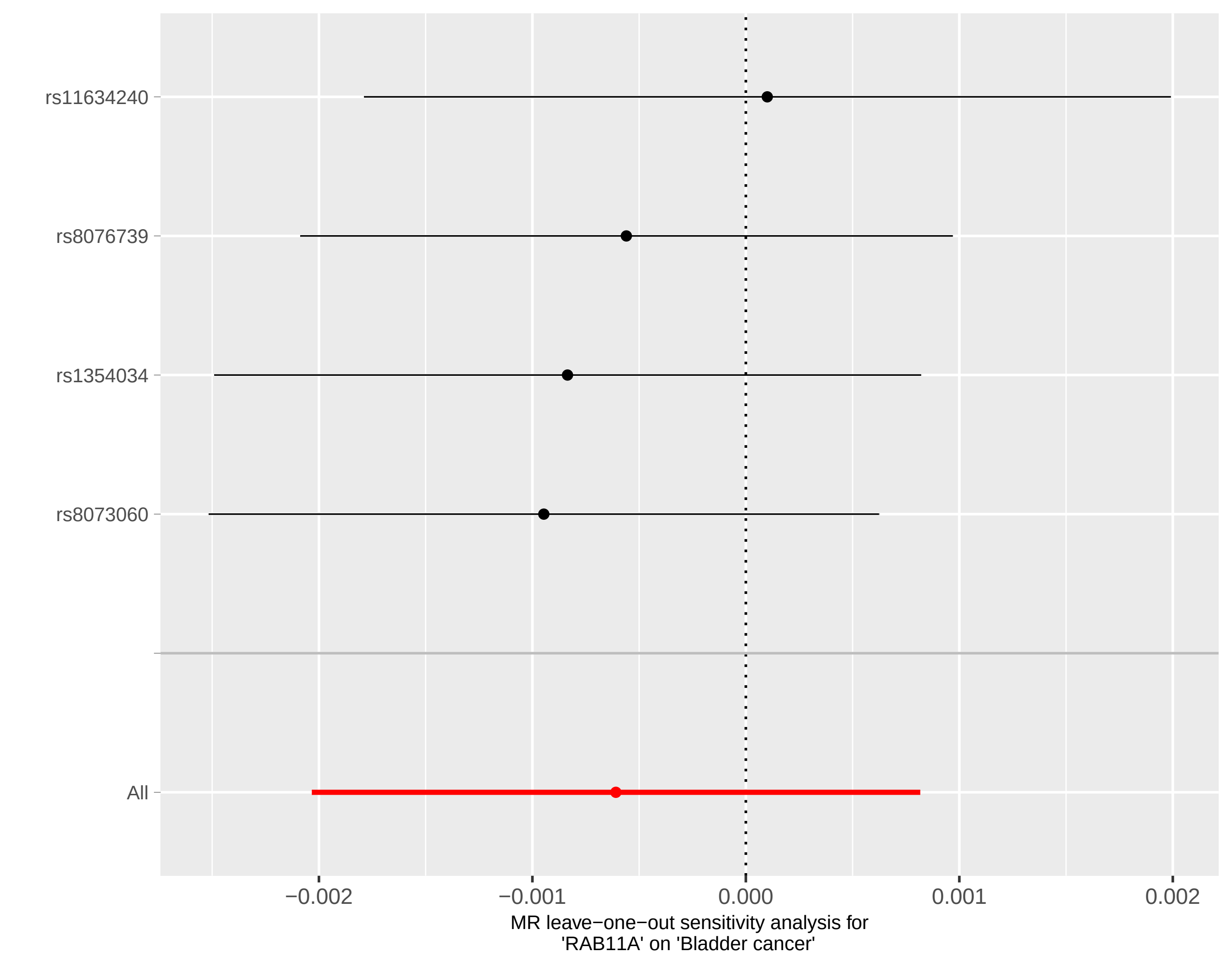
rs11634240
rs8076739
rs1354034
rs8073060
All
−0.002
−0.001
0.000
0.001
0.002
MR leave−one−out sensitivity analysis for
'RAB11A' on 'Bladder cancer'

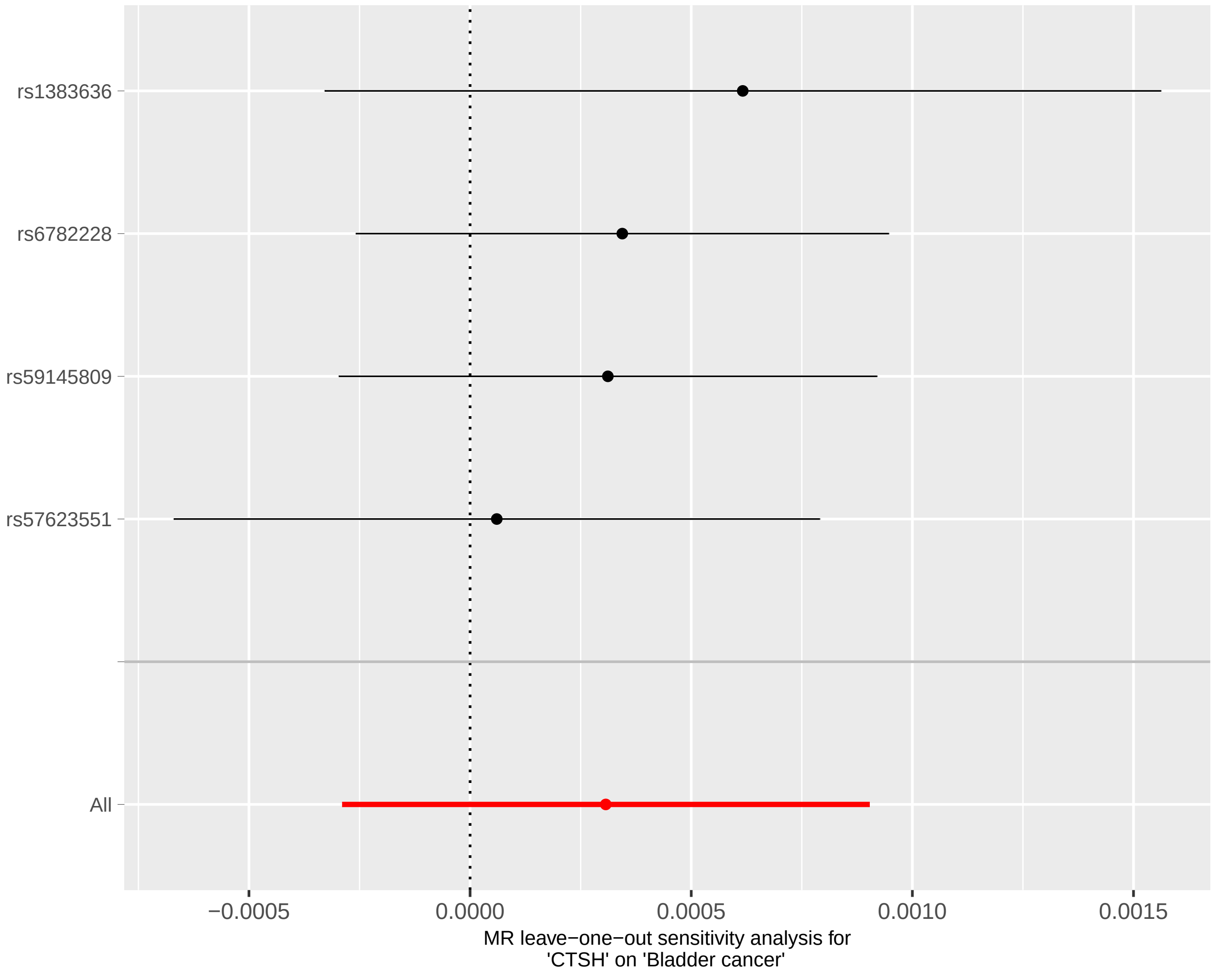
rs1383636
rs6782228
rs59145809
rs57623551
All
−0.0005
0.0000
0.0005
0.0010
0.0015
MR leave−one−out sensitivity analysis for
'CTSH' on 'Bladder cancer'

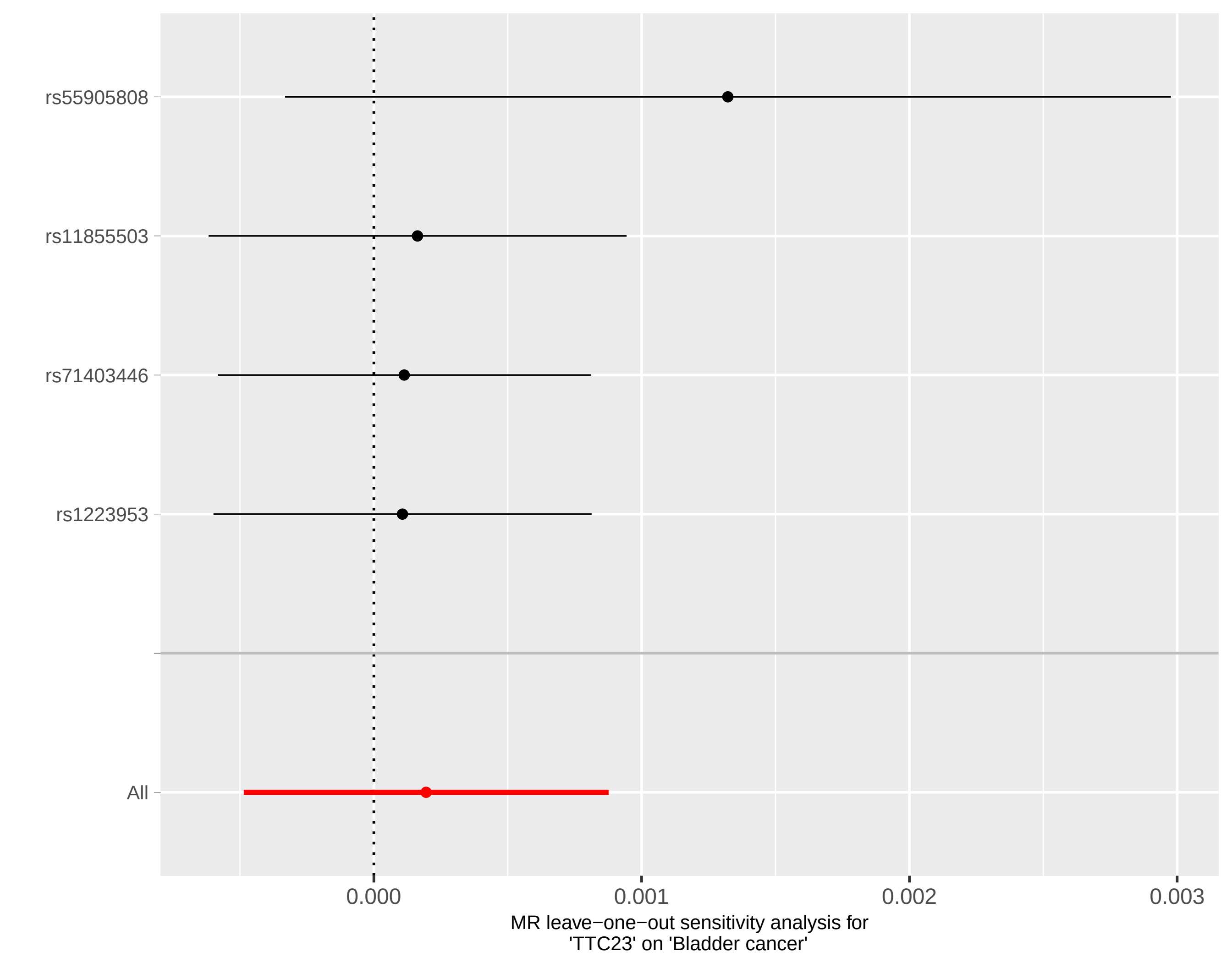
rs55905808
rs11855503
rs71403446
rs1223953
All
0.000
0.001
0.002
0.003
MR leave−one−out sensitivity analysis for
'TTC23' on 'Bladder cancer'

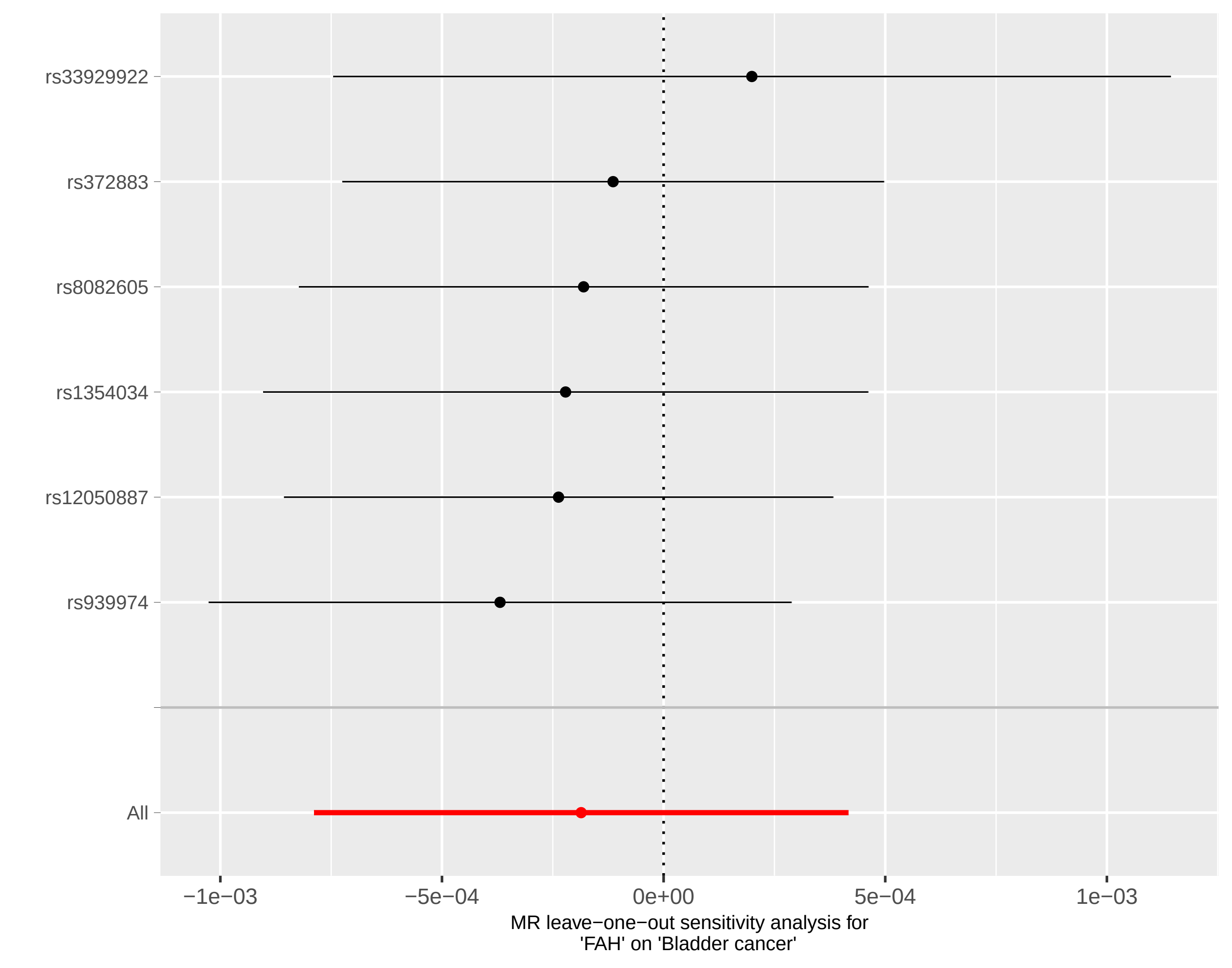
rs33929922
rs372883
rs8082605
rs1354034
rs12050887
rs939974
All
−1e−03
−5e−04
0e+00
5e−04
1e−03
MR leave−one−out sensitivity analysis for
'FAH' on 'Bladder cancer'

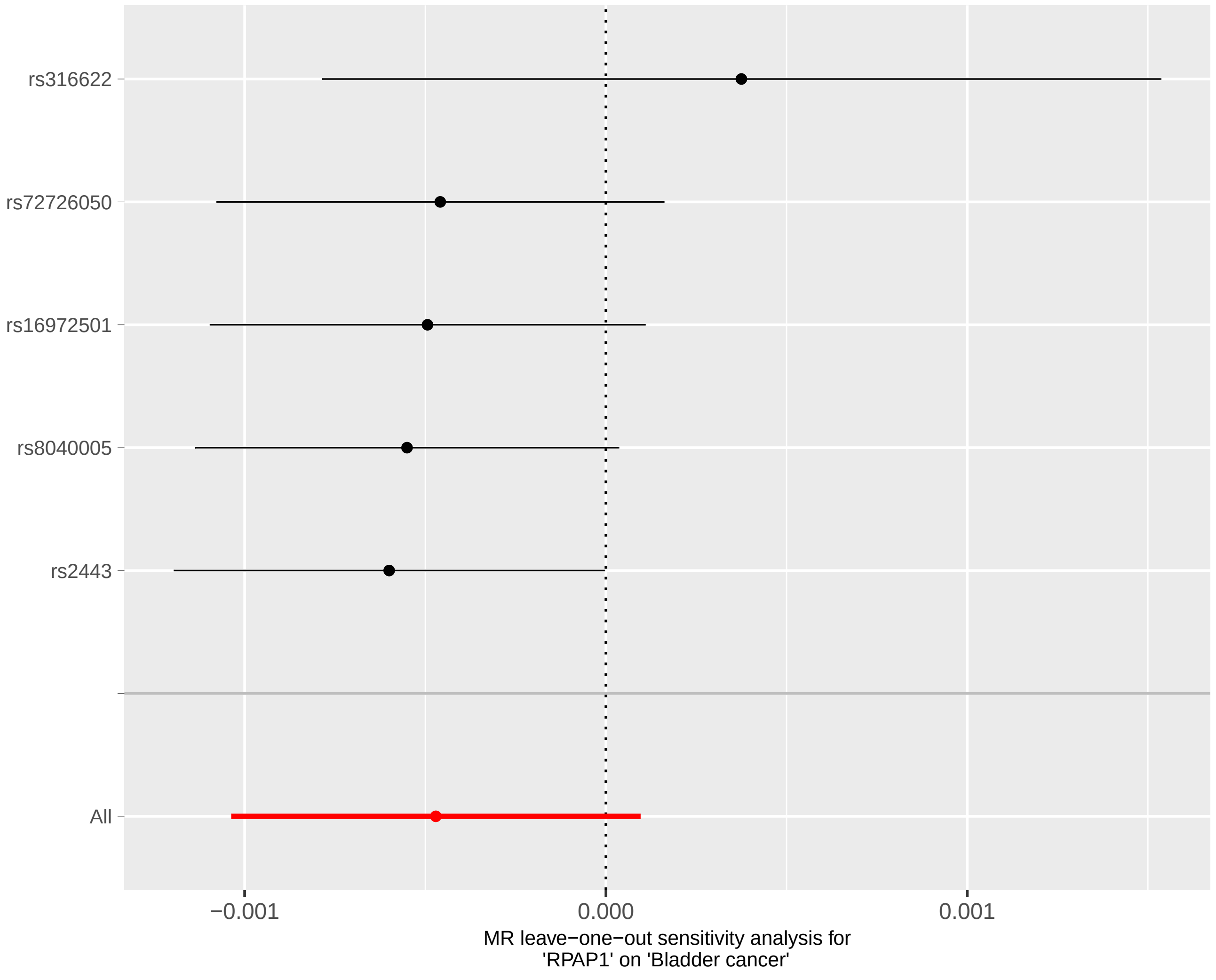
rs316622
rs72726050
rs16972501
rs8040005
rs2443
All
−0.001
0.000
0.001
MR leave−one−out sensitivity analysis for
'RPAP1' on 'Bladder cancer'

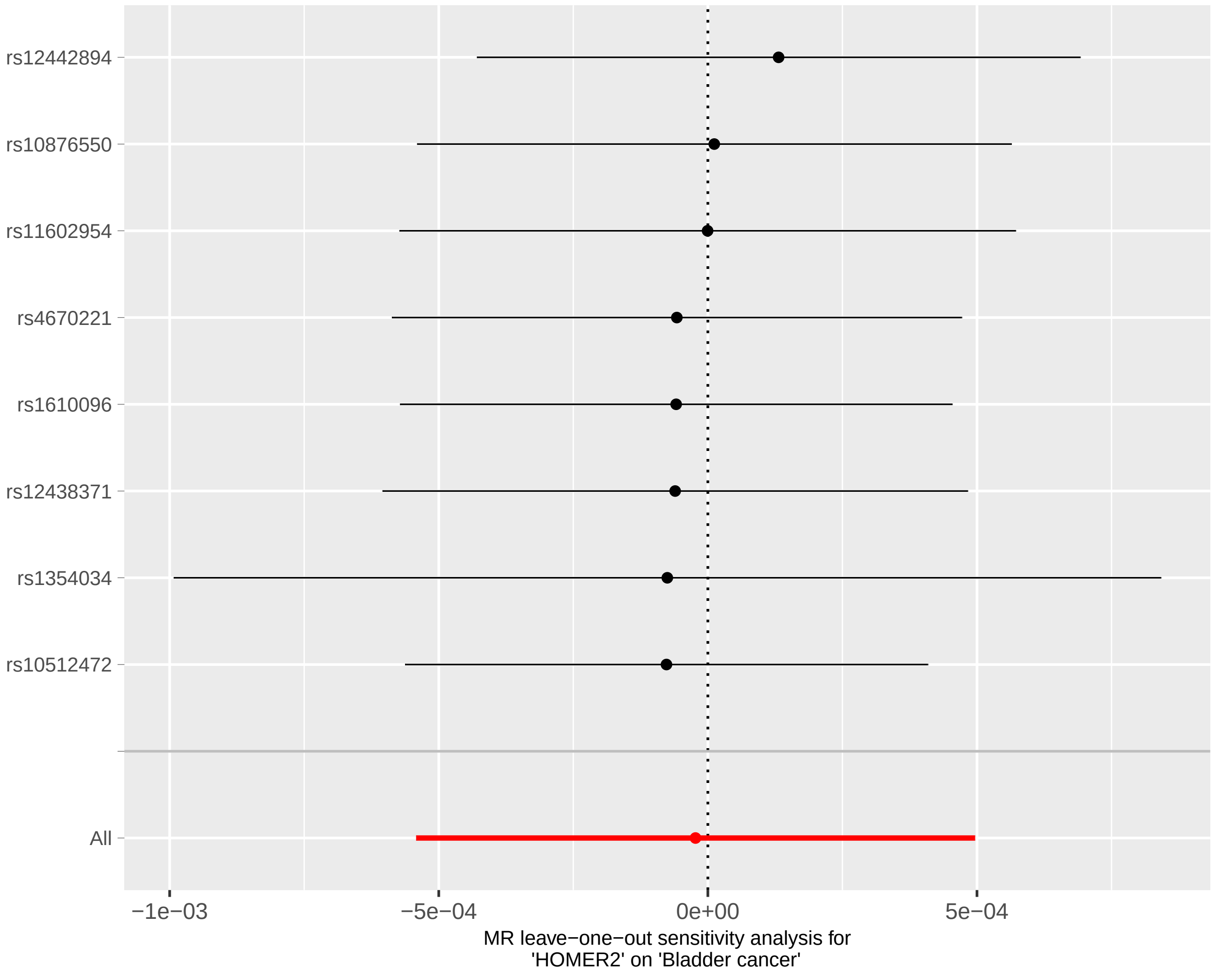

rs12442894
rs10876550
rs11602954
rs4670221
rs1610096
rs12438371
rs1354034
rs10512472
All
−1e−03
−5e−04
0e+00
5e−04
MR leave−one−out sensitivity analysis for
'HOMER2' on 'Bladder cancer'

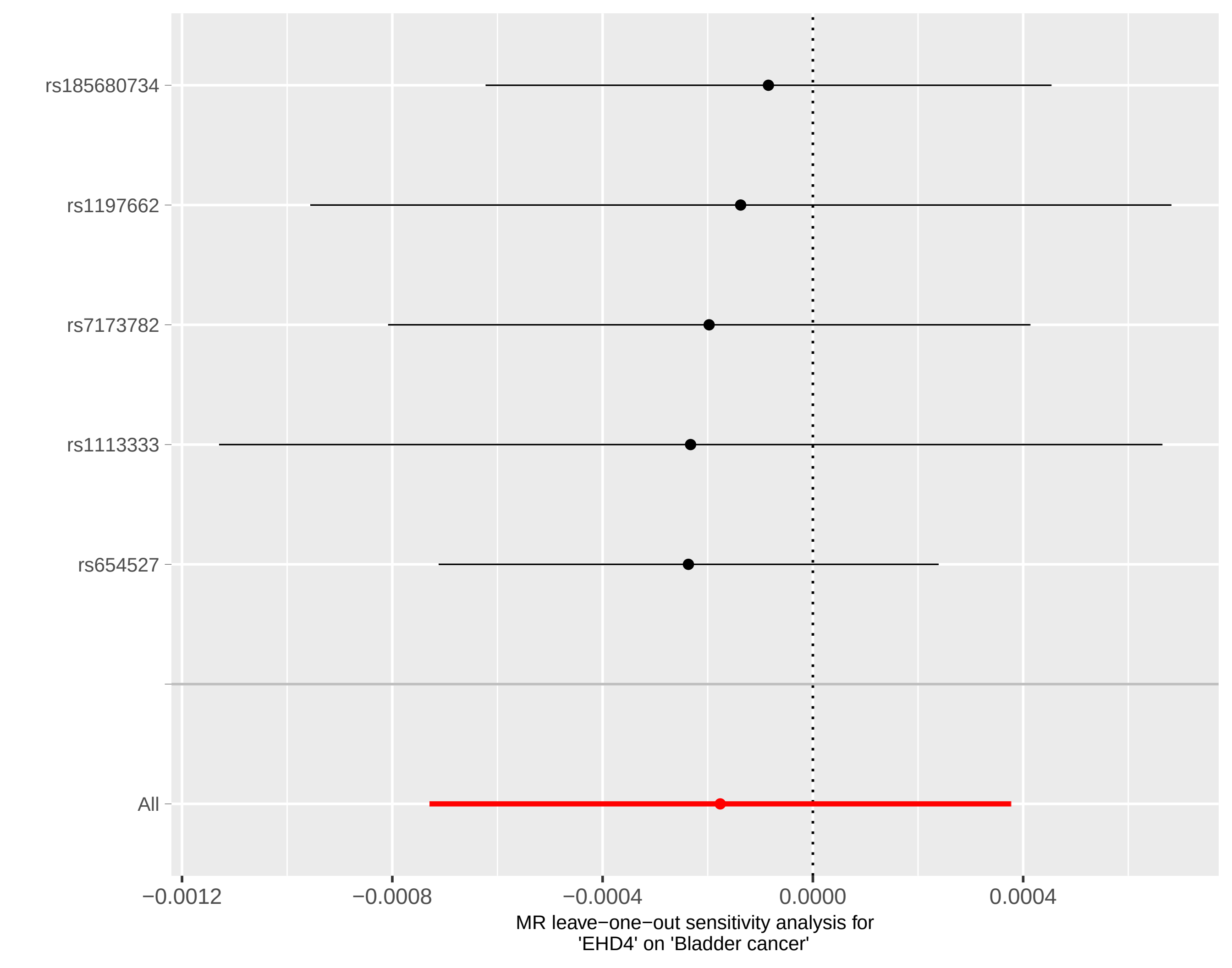

rs185680734
rs1197662
rs7173782
rs1113333
rs654527
All
−0.0012
−0.0008
−0.0004
0.0000
0.0004
MR leave−one−out sensitivity analysis for
'EHD4' on 'Bladder cancer'

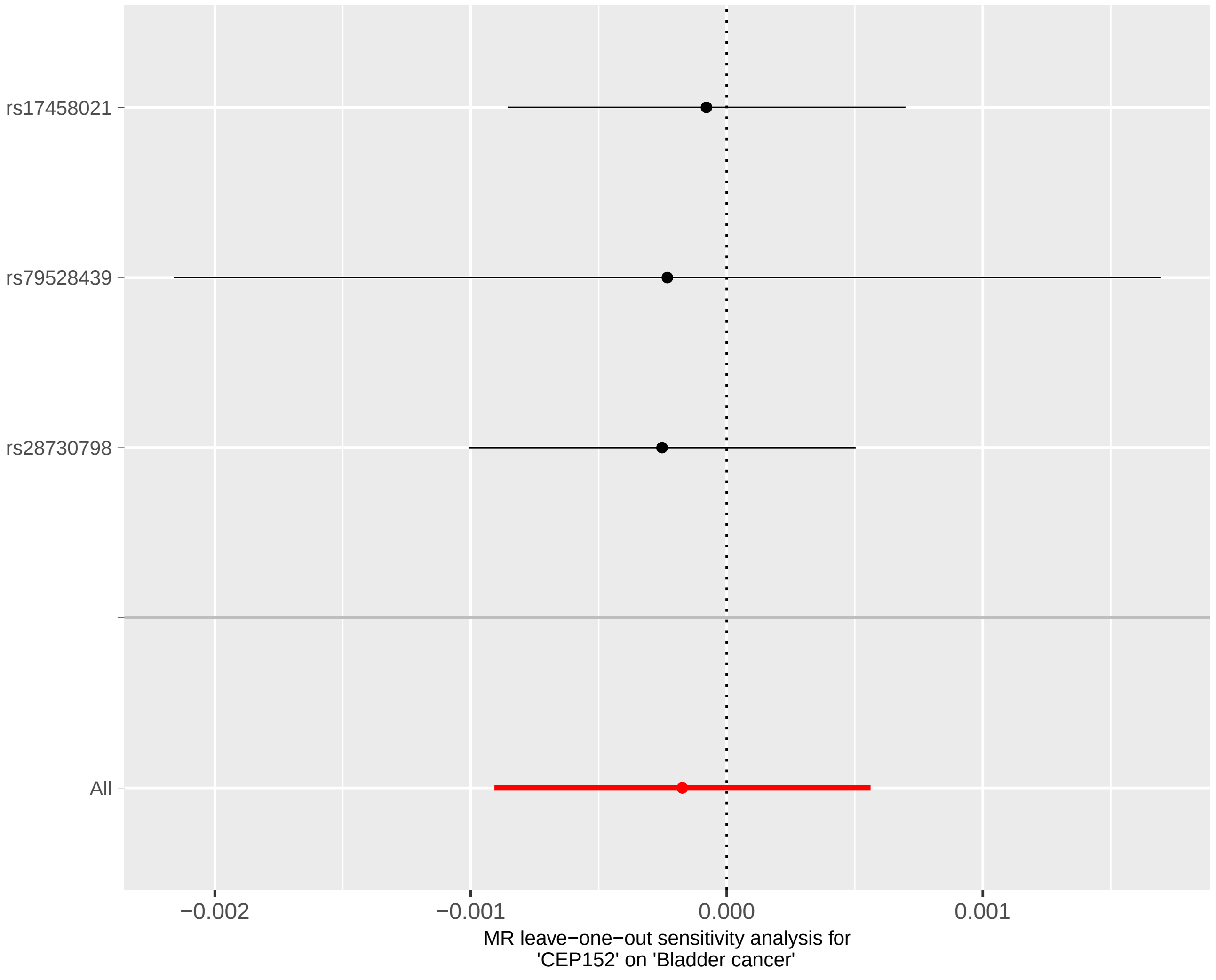
rs17458021
rs79528439
rs28730798
All
−0.002
−0.001
0.000
0.001
MR leave−one−out sensitivity analysis for
'CEP152' on 'Bladder cancer'

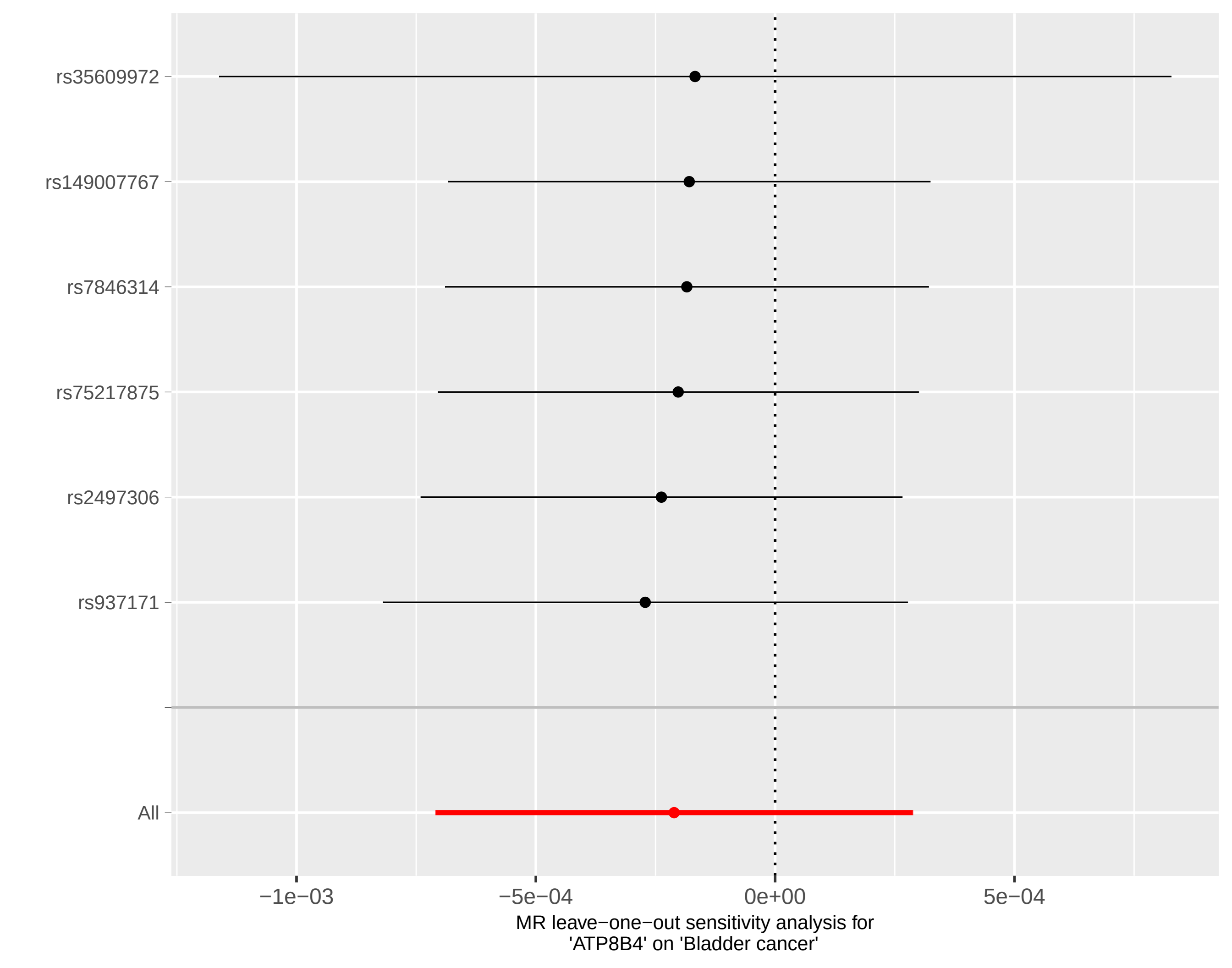
rs35609972
rs149007767
rs7846314
rs75217875
rs2497306
rs937171
All
−1e−03
−5e−04
0e+00
5e−04
MR leave−one−out sensitivity analysis for
'ATP8B4' on 'Bladder cancer'

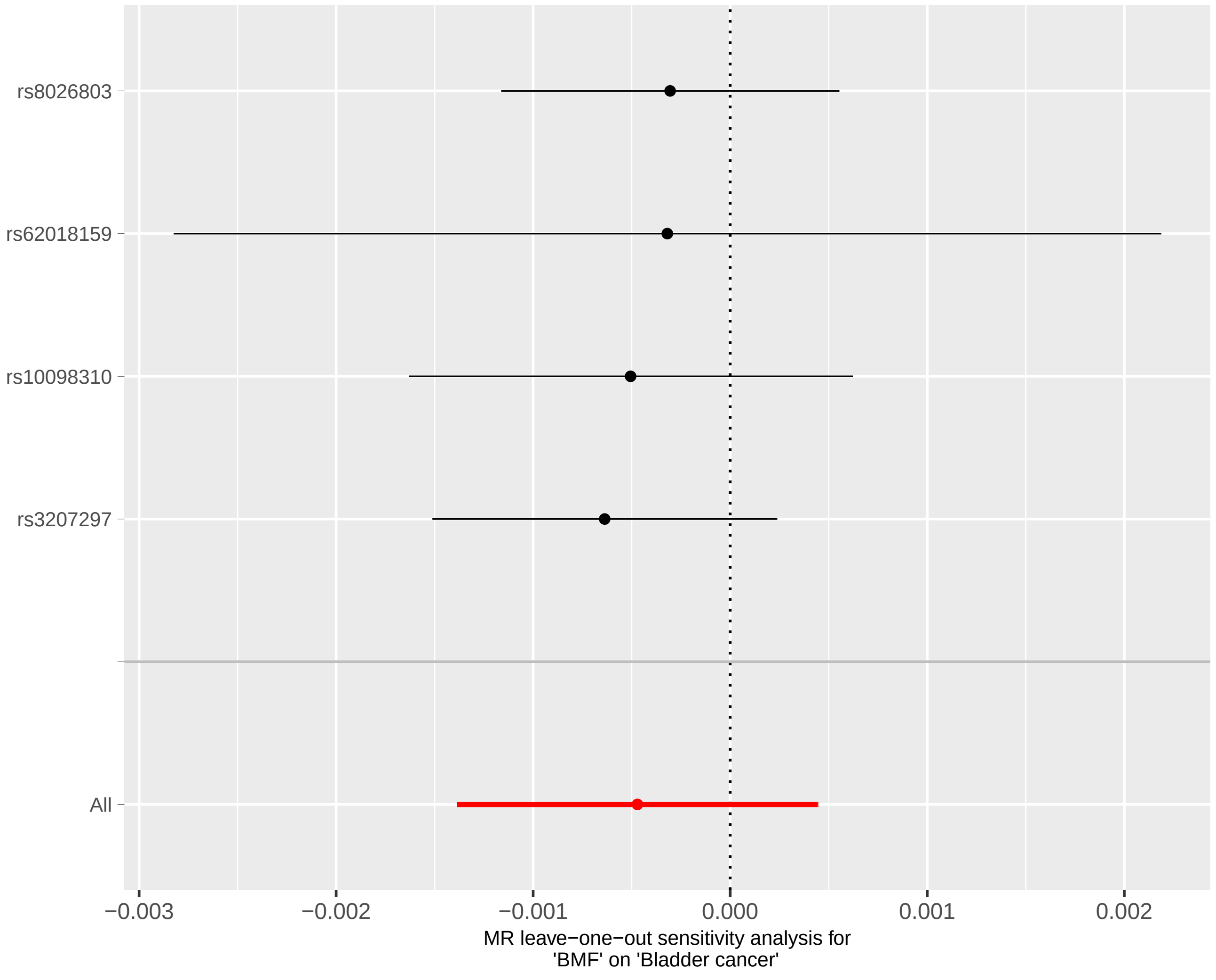
rs8026803
rs62018159
rs10098310
rs3207297
All
−0.003
−0.002
−0.001
0.000
0.001
0.002
MR leave−one−out sensitivity analysis for
'BMF' on 'Bladder cancer'

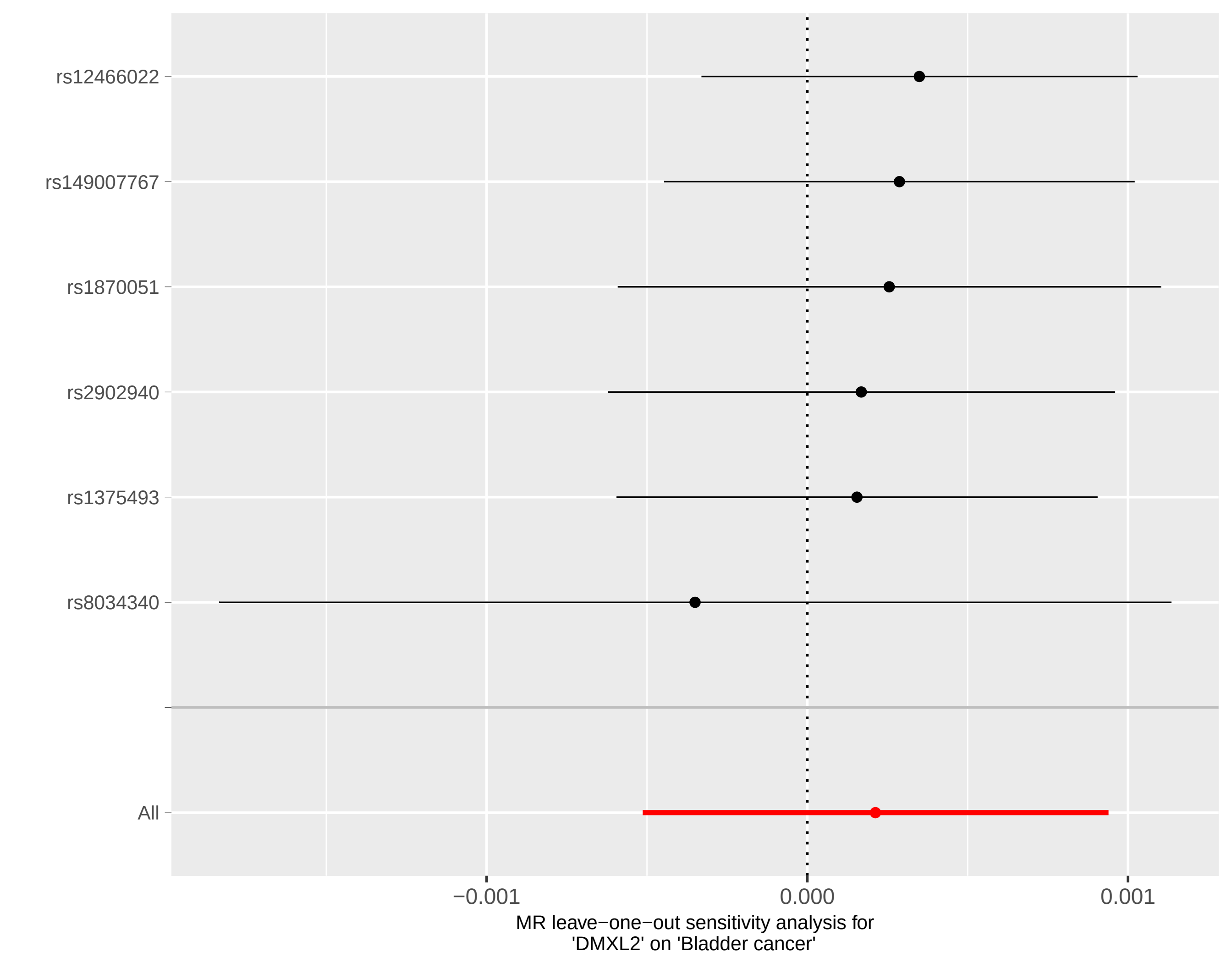
rs12466022
rs149007767
rs1870051
rs2902940
rs1375493
rs8034340
All
−0.001
0.000
0.001
MR leave−one−out sensitivity analysis for
'DMXL2' on 'Bladder cancer'

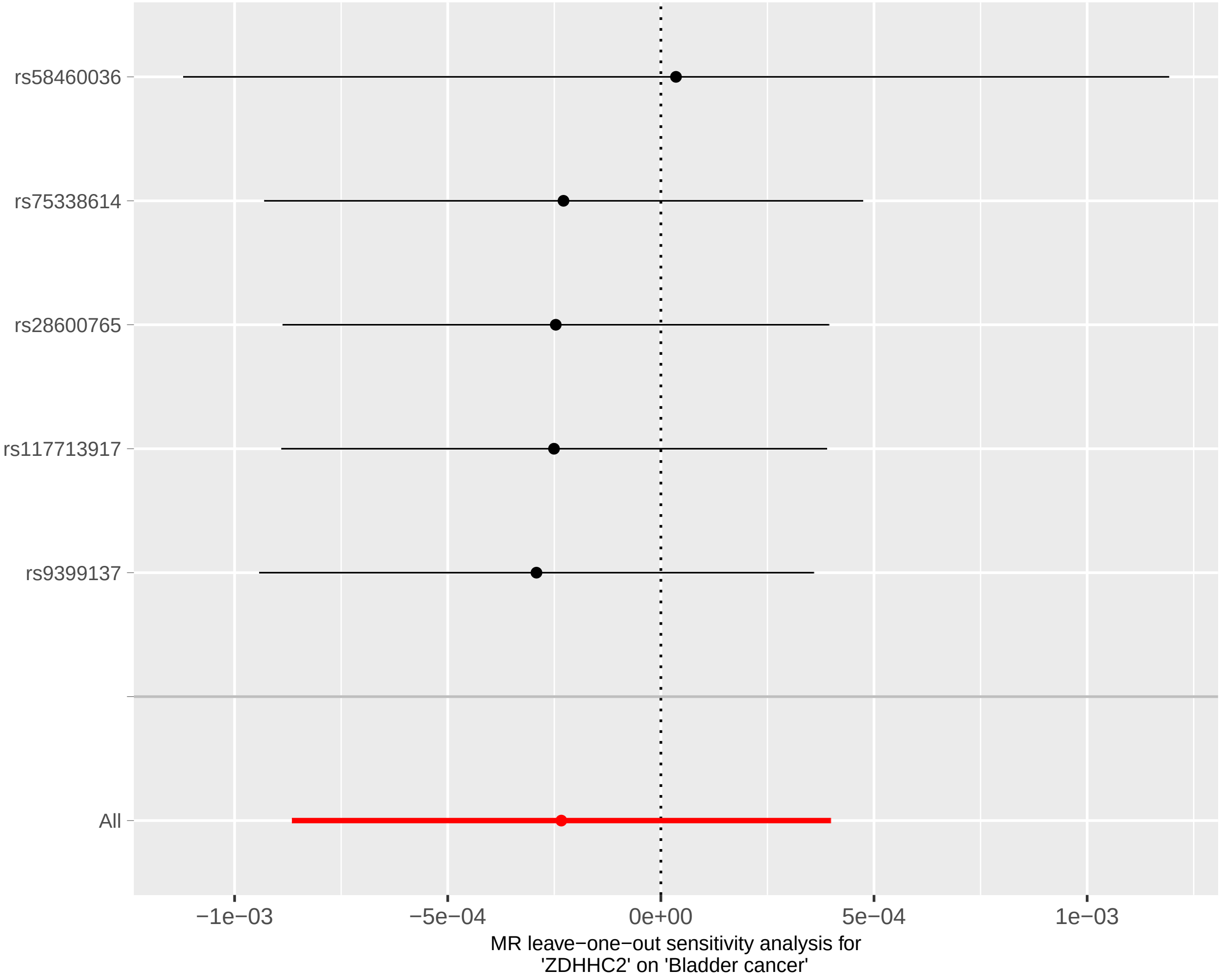
rs58460036
rs75338614
rs28600765
rs117713917
rs9399137
All
−1e−03
−5e−04
0e+00
5e−04
1e−03
MR leave−one−out sensitivity analysis for
'ZDHHC2' on 'Bladder cancer'

Insufficient number of SNPs

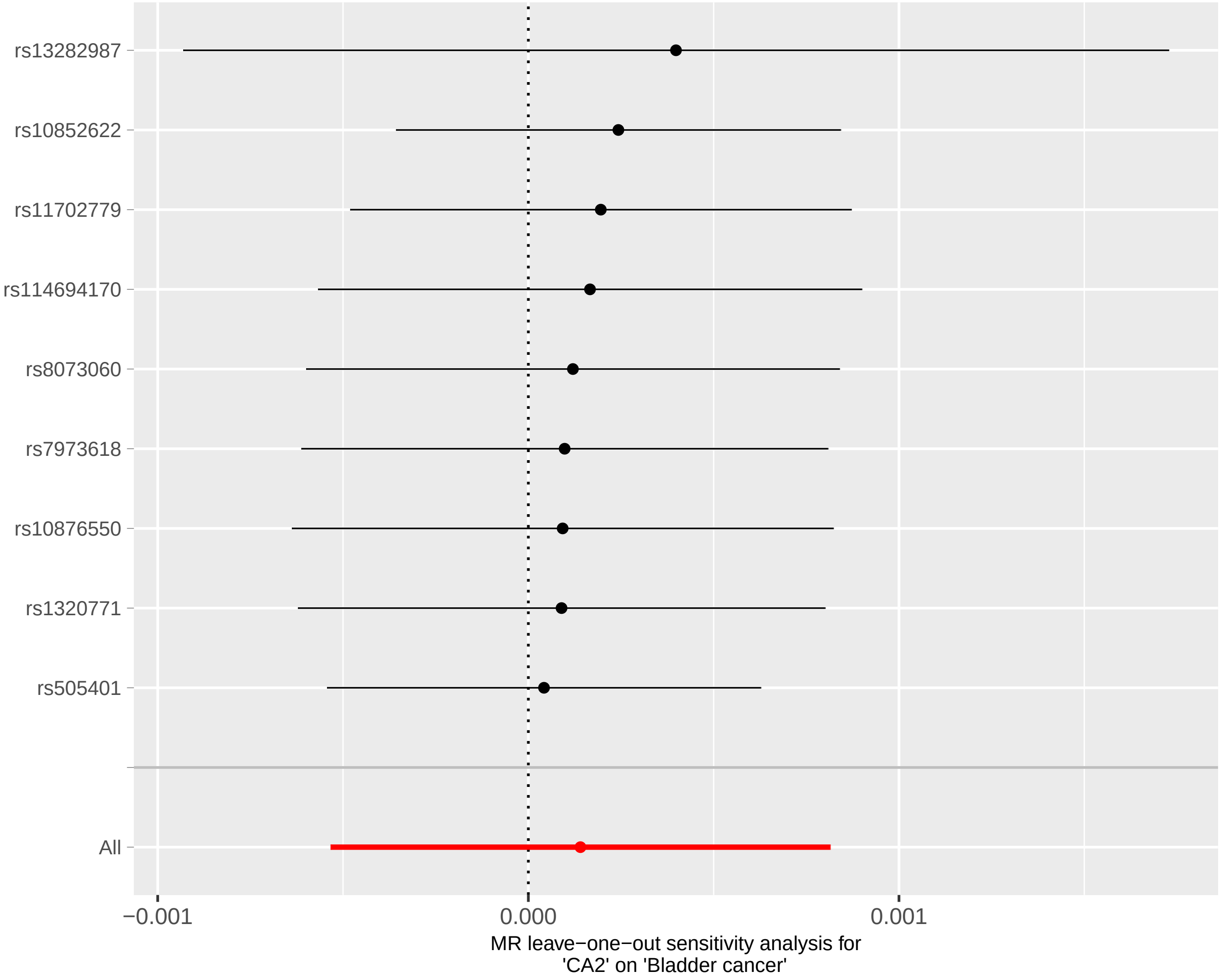
rs13282987
rs10852622
rs11702779
rs114694170
rs8073060
rs7973618
rs10876550
rs1320771
rs505401
All
−0.001
0.000
0.001
MR leave−one−out sensitivity analysis for
'CA2' on 'Bladder cancer'

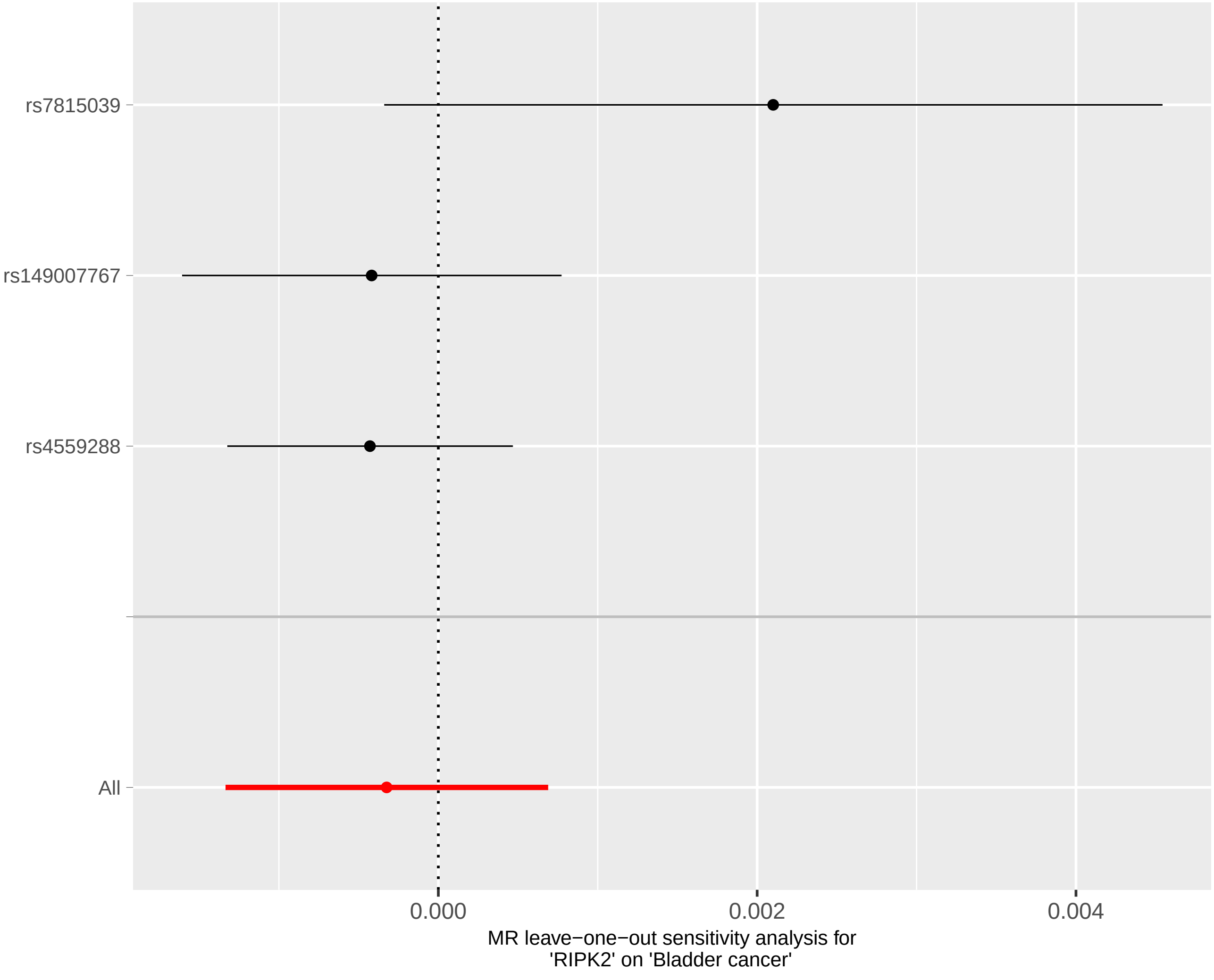
rs7815039
rs149007767
rs4559288
All
0.000
0.002
0.004
MR leave−one−out sensitivity analysis for
'RIPK2' on 'Bladder cancer'

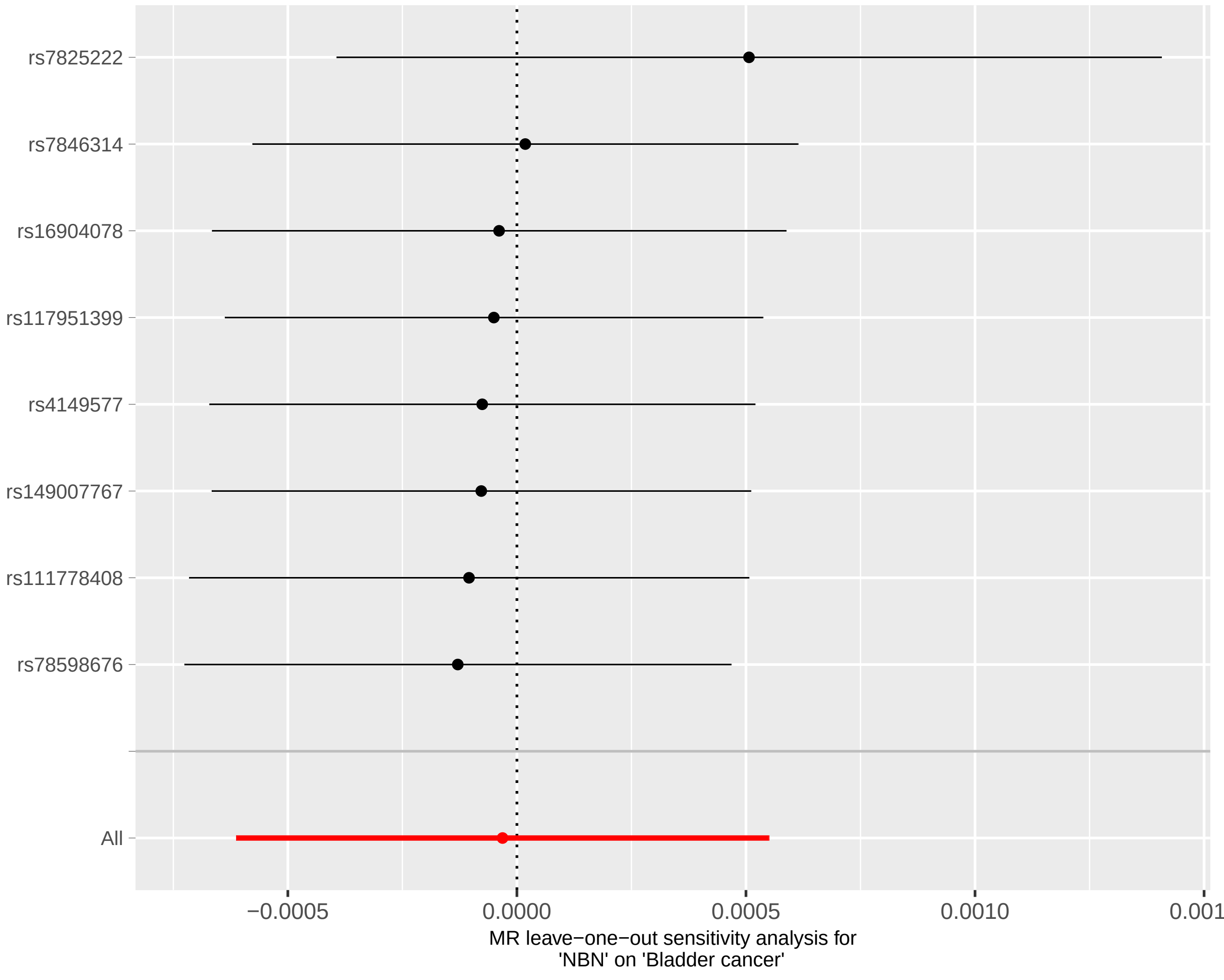
rs7825222
rs7846314
rs16904078
rs117951399
rs4149577
rs149007767
rs111778408
rs78598676
All
−0.0005
0.0000
0.0005
0.0010
0.001
MR leave−one−out sensitivity analysis for
'NBN' on 'Bladder cancer'

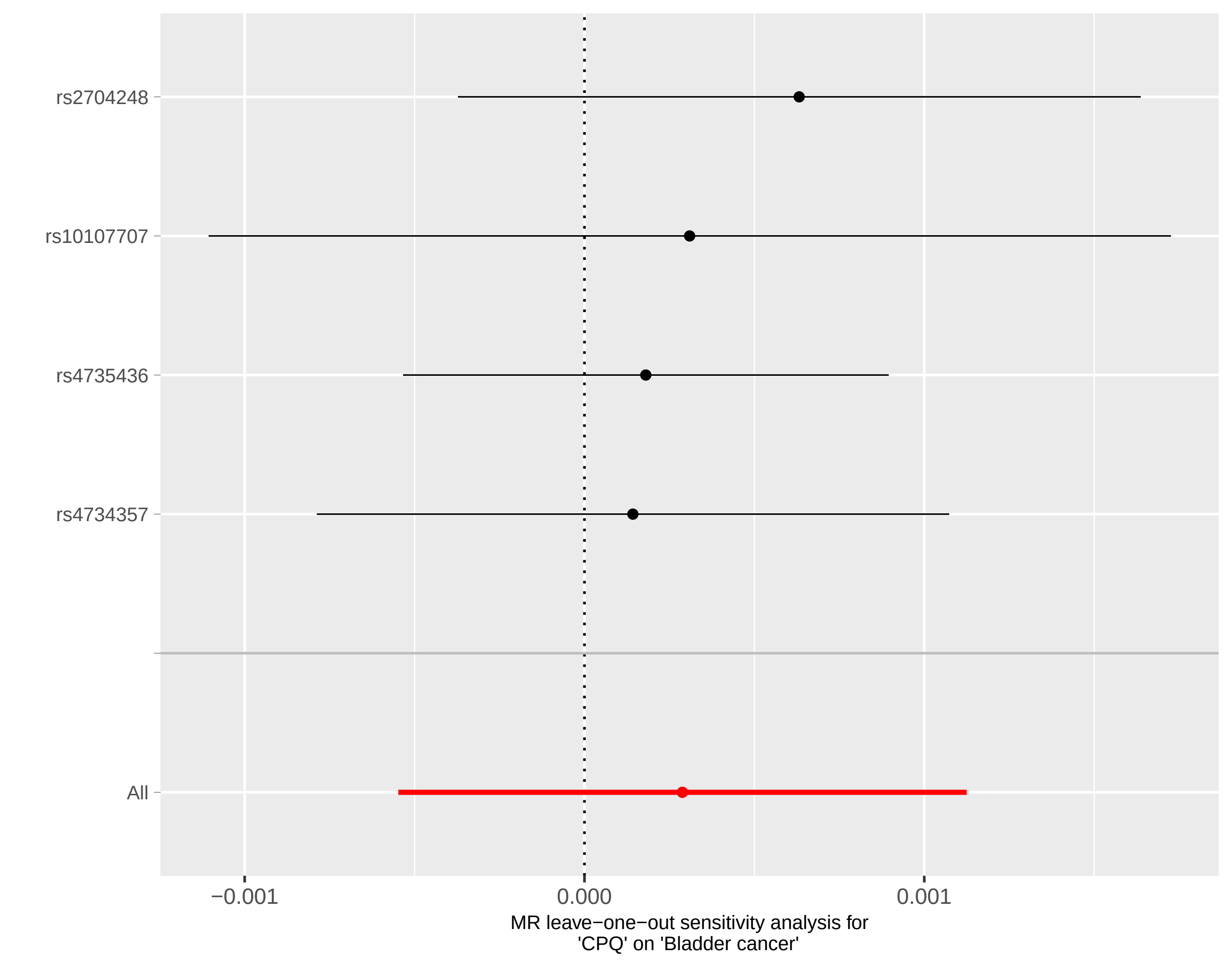

rs2704248
rs10107707
rs4735436
rs4734357
All
−0.001
0.000
0.001
MR leave−one−out sensitivity analysis for
'CPQ' on 'Bladder cancer'

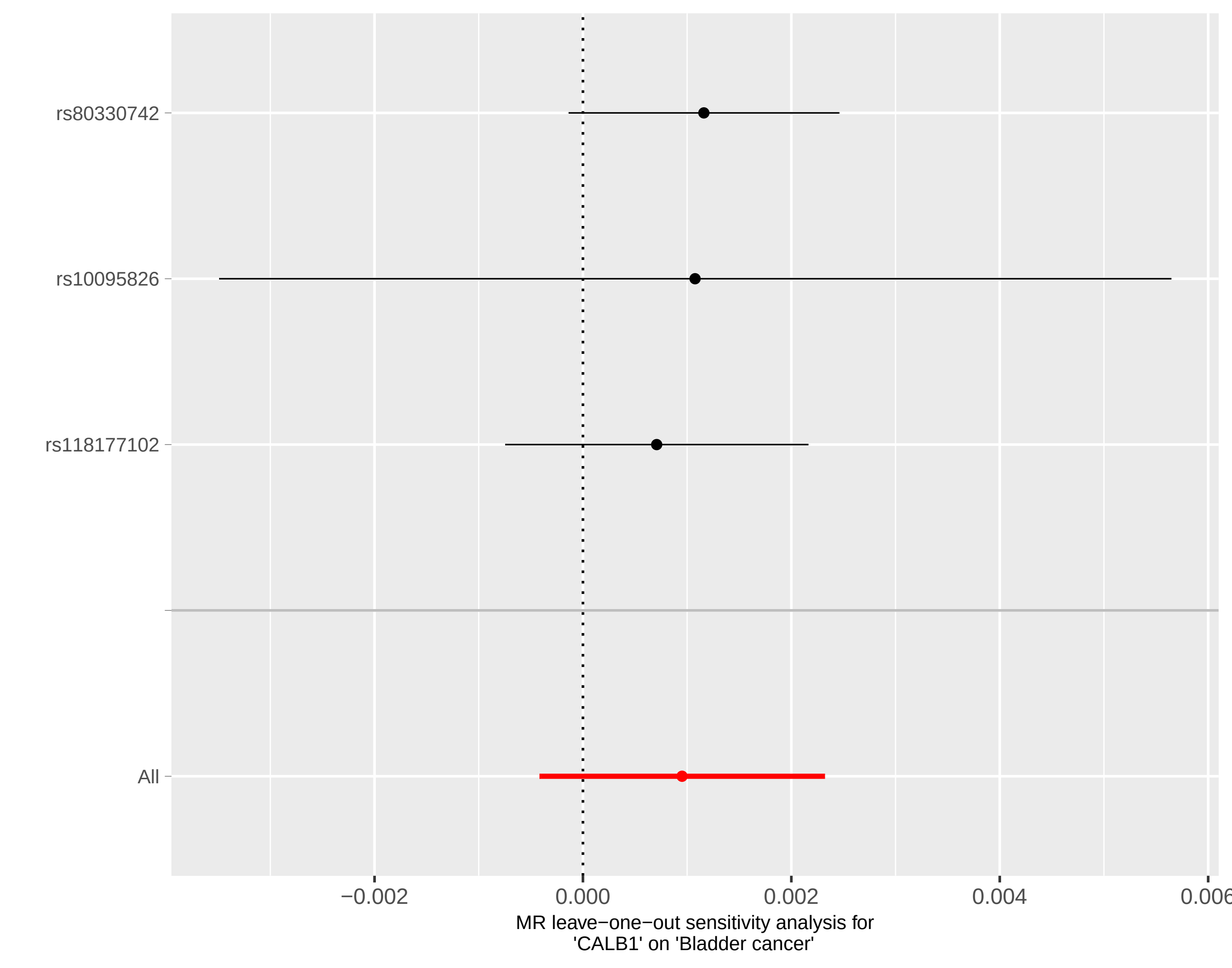
rs80330742
rs10095826
rs118177102
All
−0.002
0.000
0.002
0.004
0.006
MR leave−one−out sensitivity analysis for
'CALB1' on 'Bladder cancer'

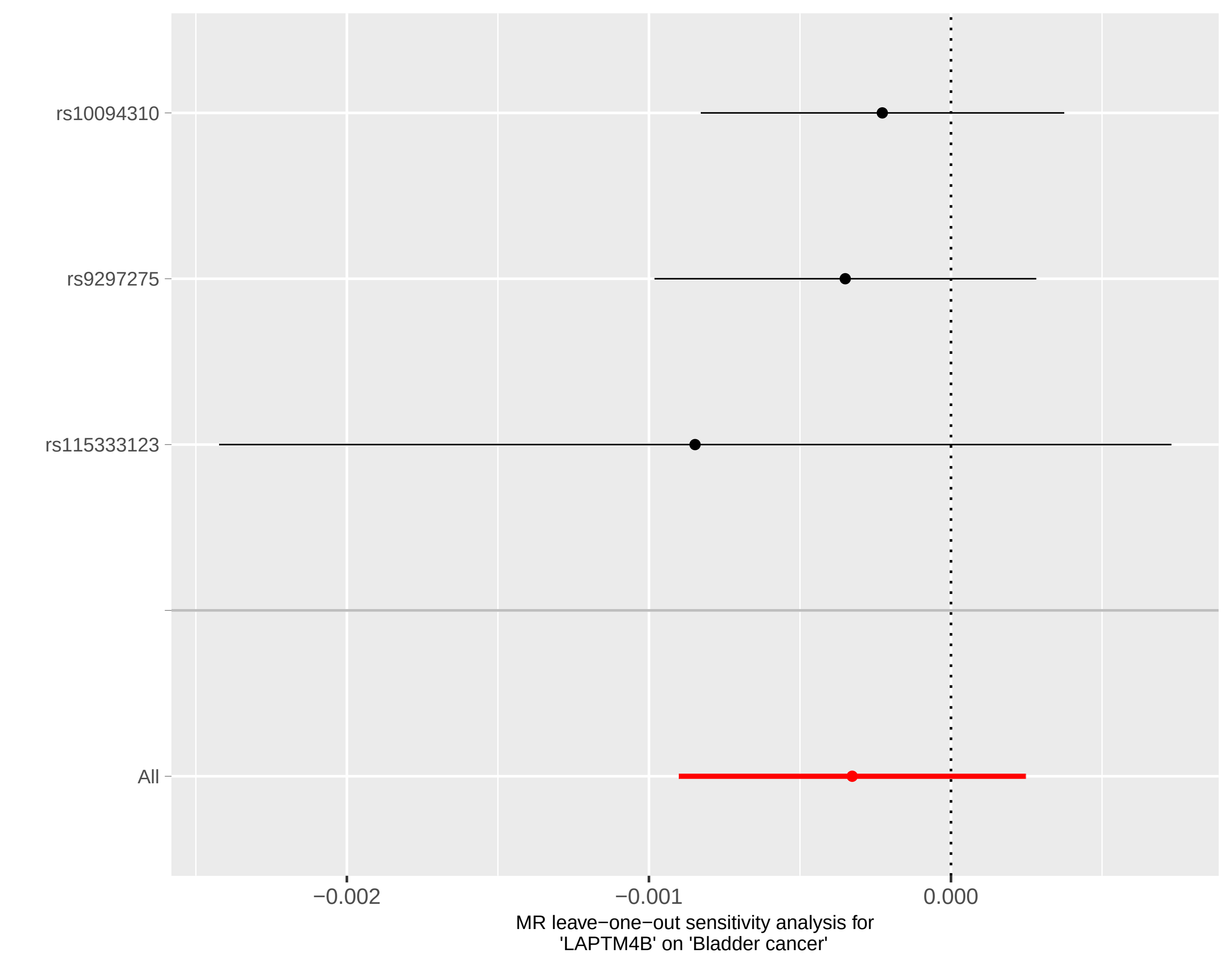
rs10094310
rs9297275
rs115333123
All
−0.002
−0.001
0.000
MR leave−one−out sensitivity analysis for
'LAPTM4B' on 'Bladder cancer'

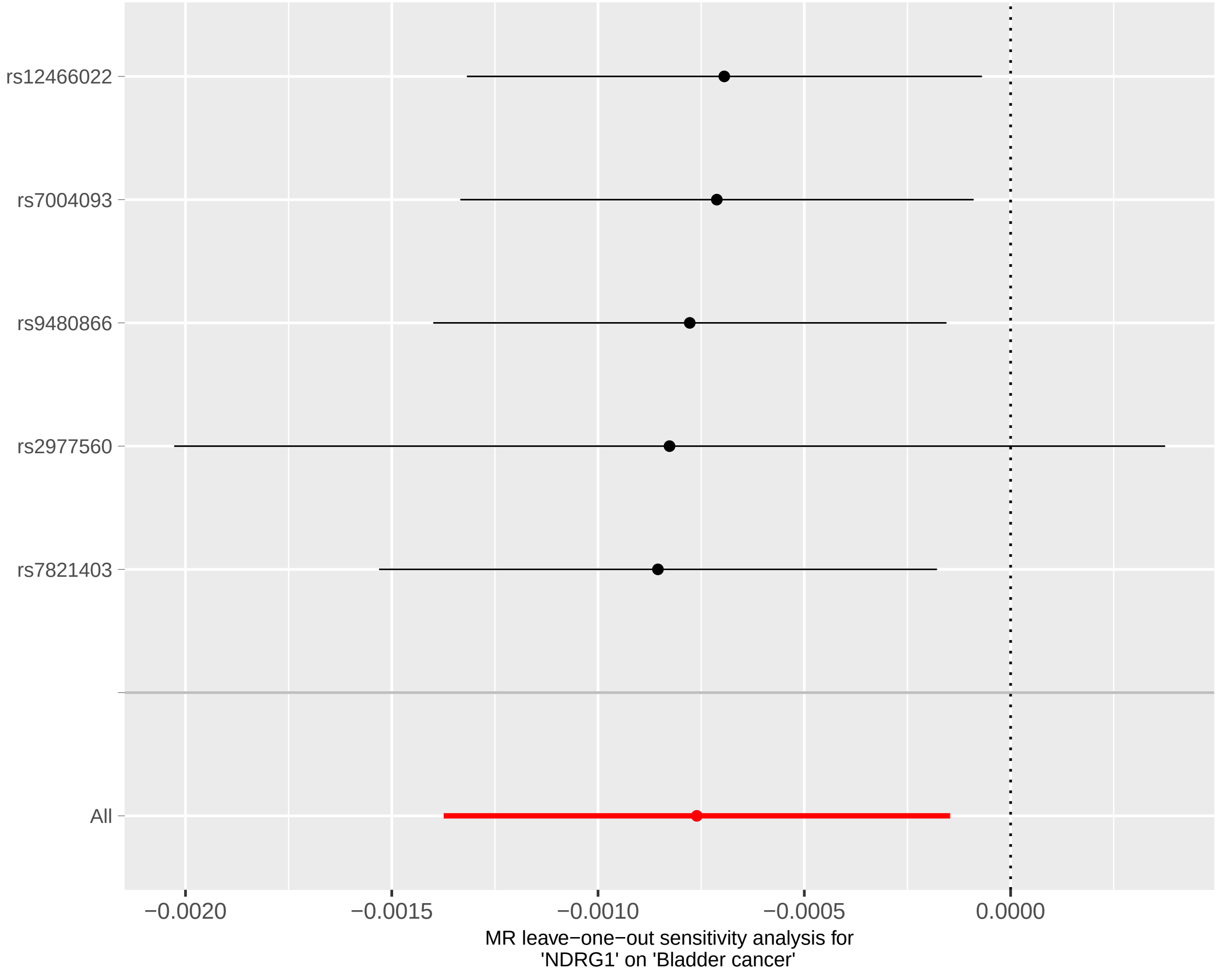
rs12466022
rs7004093
rs9480866
rs2977560
rs7821403
All
−0.0020
−0.0015
−0.0010
−0.0005
0.0000
MR leave−one−out sensitivity analysis for
'NDRG1' on 'Bladder cancer'

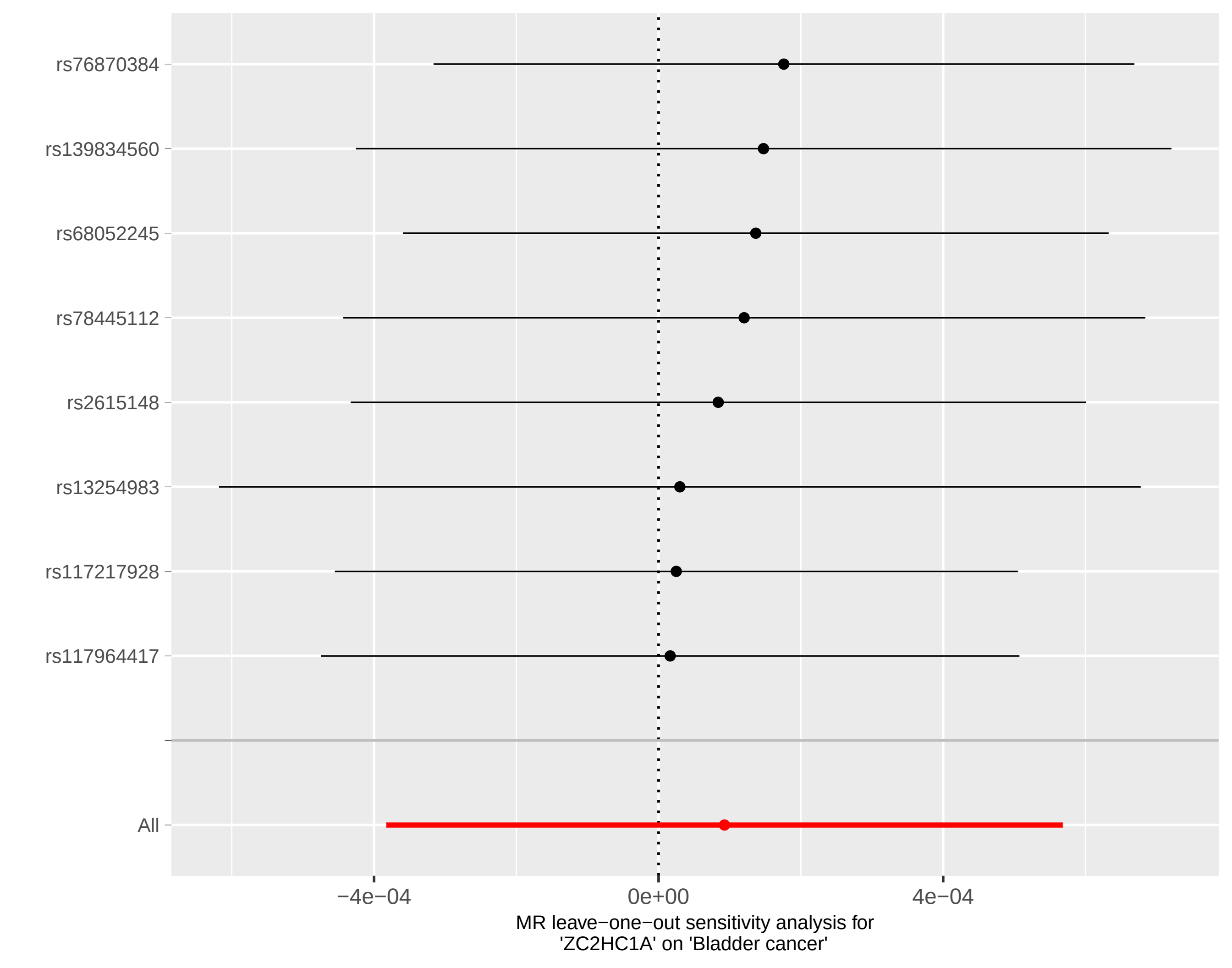
rs76870384
rs139834560
rs68052245
rs78445112
rs2615148
rs13254983
rs117217928
rs117964417
All
−4e−04
0e+00
4e−04
MR leave−one−out sensitivity analysis for
'ZC2HC1A' on 'Bladder cancer'

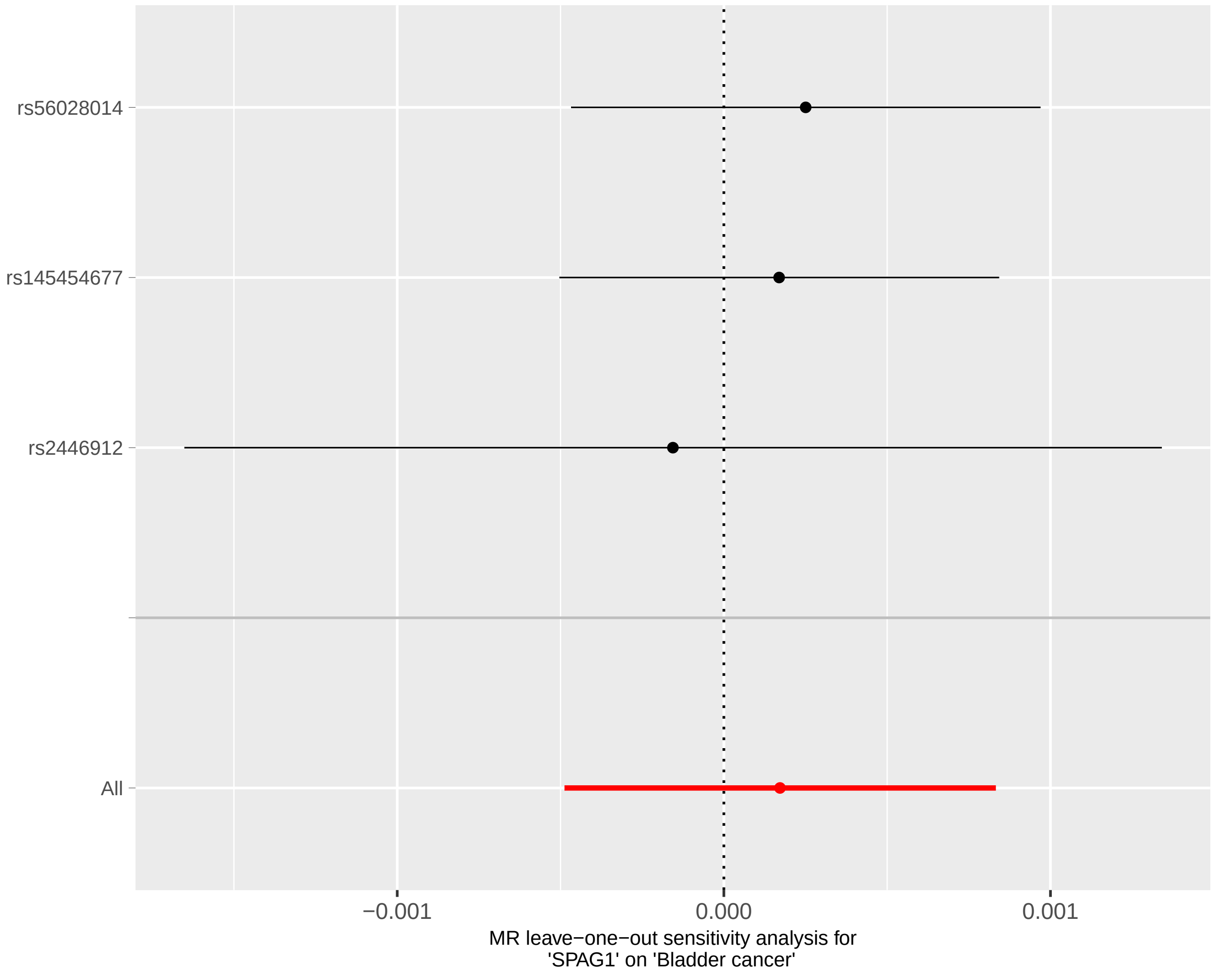
rs56028014
rs145454677
rs2446912
All
−0.001
0.000
0.001
MR leave−one−out sensitivity analysis for
'SPAG1' on 'Bladder cancer'

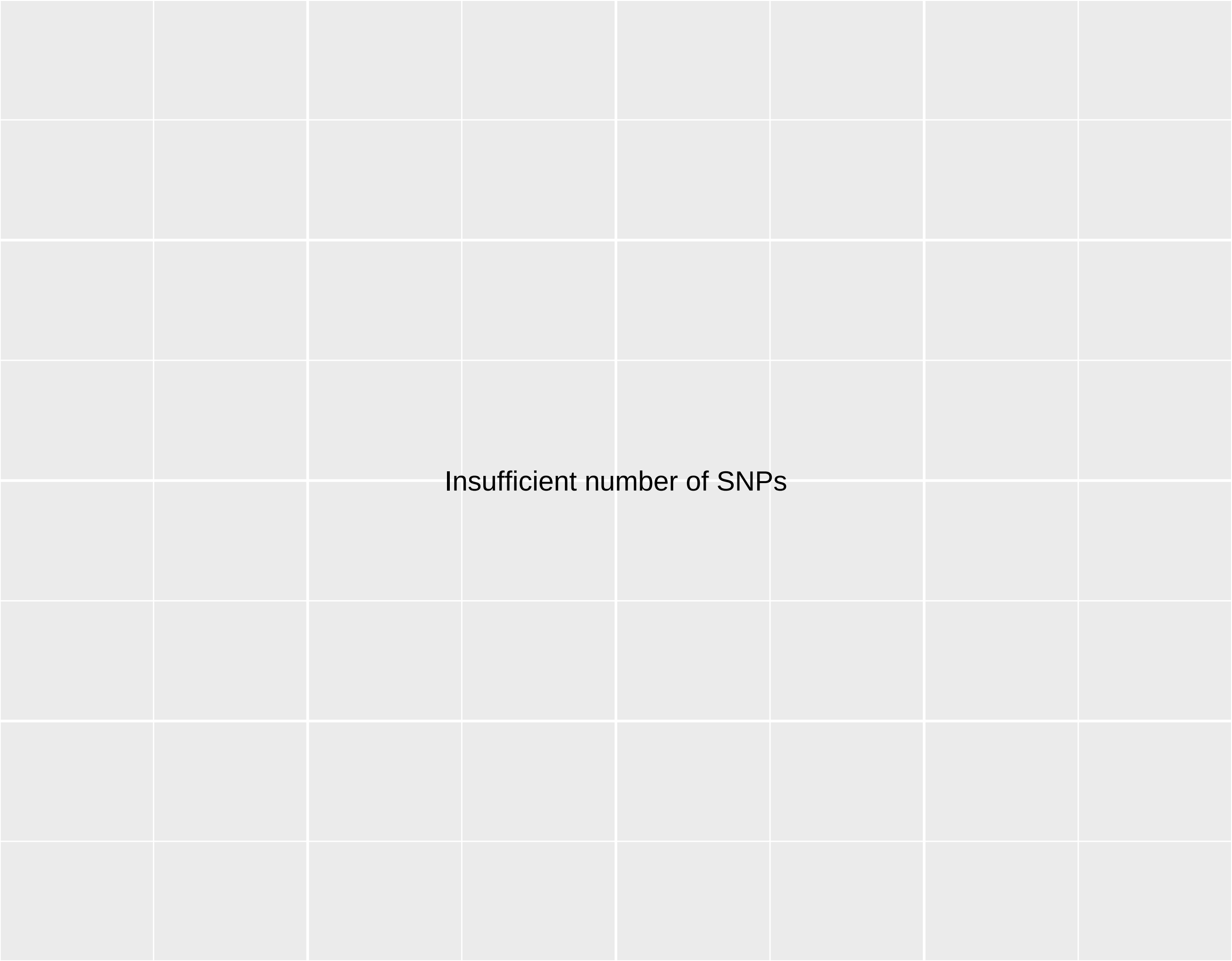
Insufficient number of SNPs

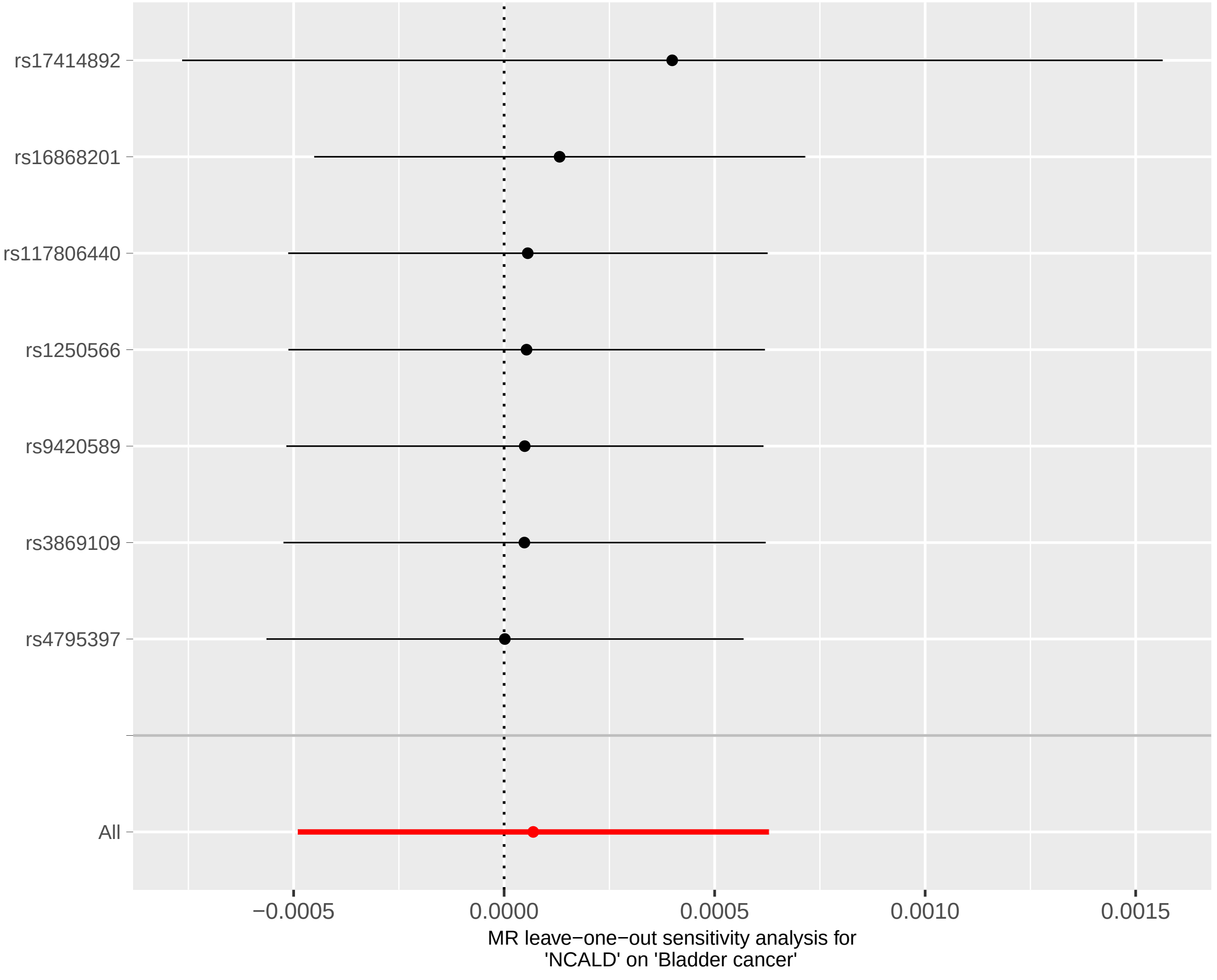

rs17414892
rs16868201
rs117806440
rs1250566
rs9420589
rs3869109
rs4795397
All
−0.0005
0.0000
0.0005
0.0010
0.0015
MR leave−one−out sensitivity analysis for
'NCALD' on 'Bladder cancer'

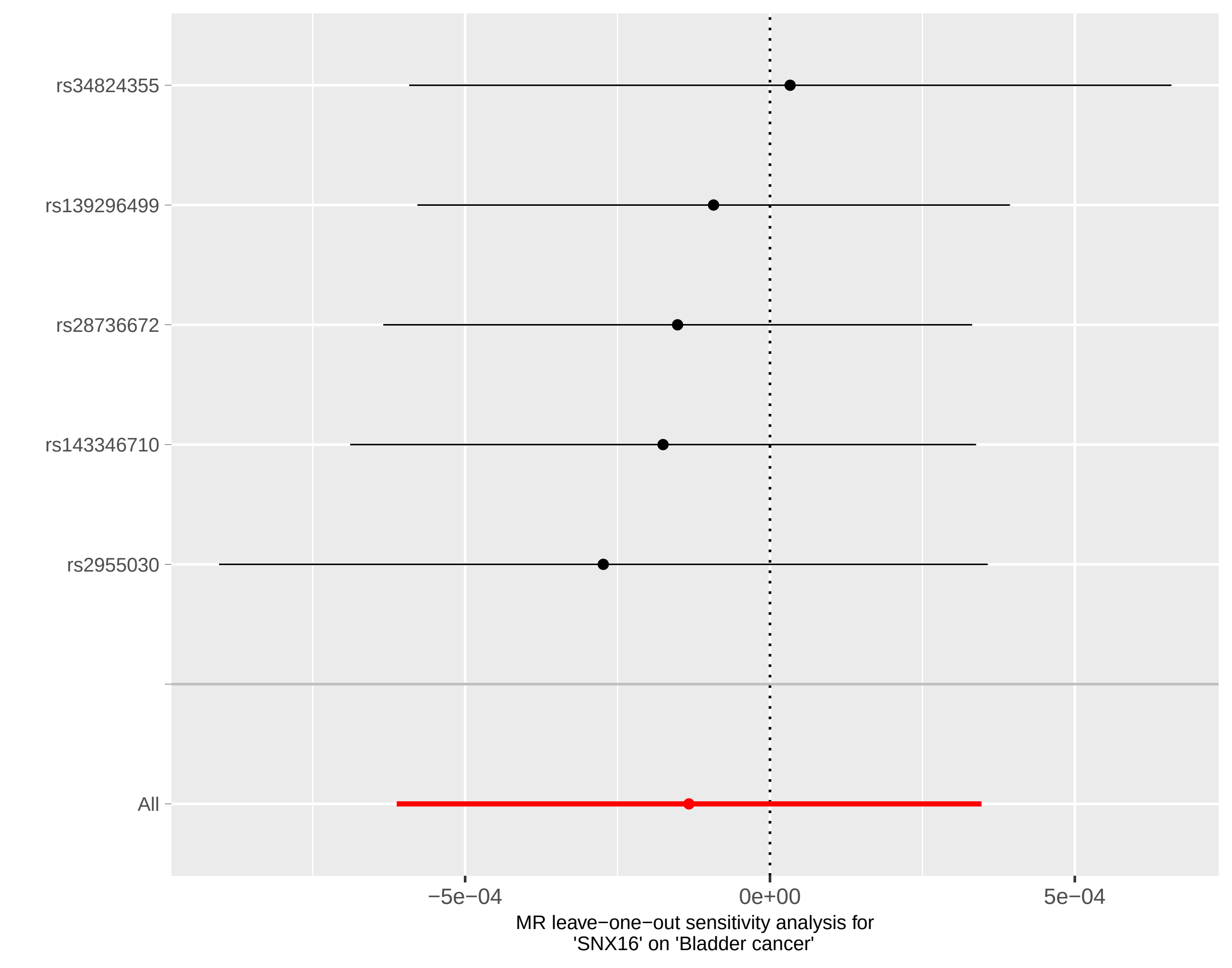

rs34824355
rs139296499
rs28736672
rs143346710
rs2955030
All
−5e−04
0e+00
5e−04
MR leave−one−out sensitivity analysis for
'SNX16' on 'Bladder cancer'

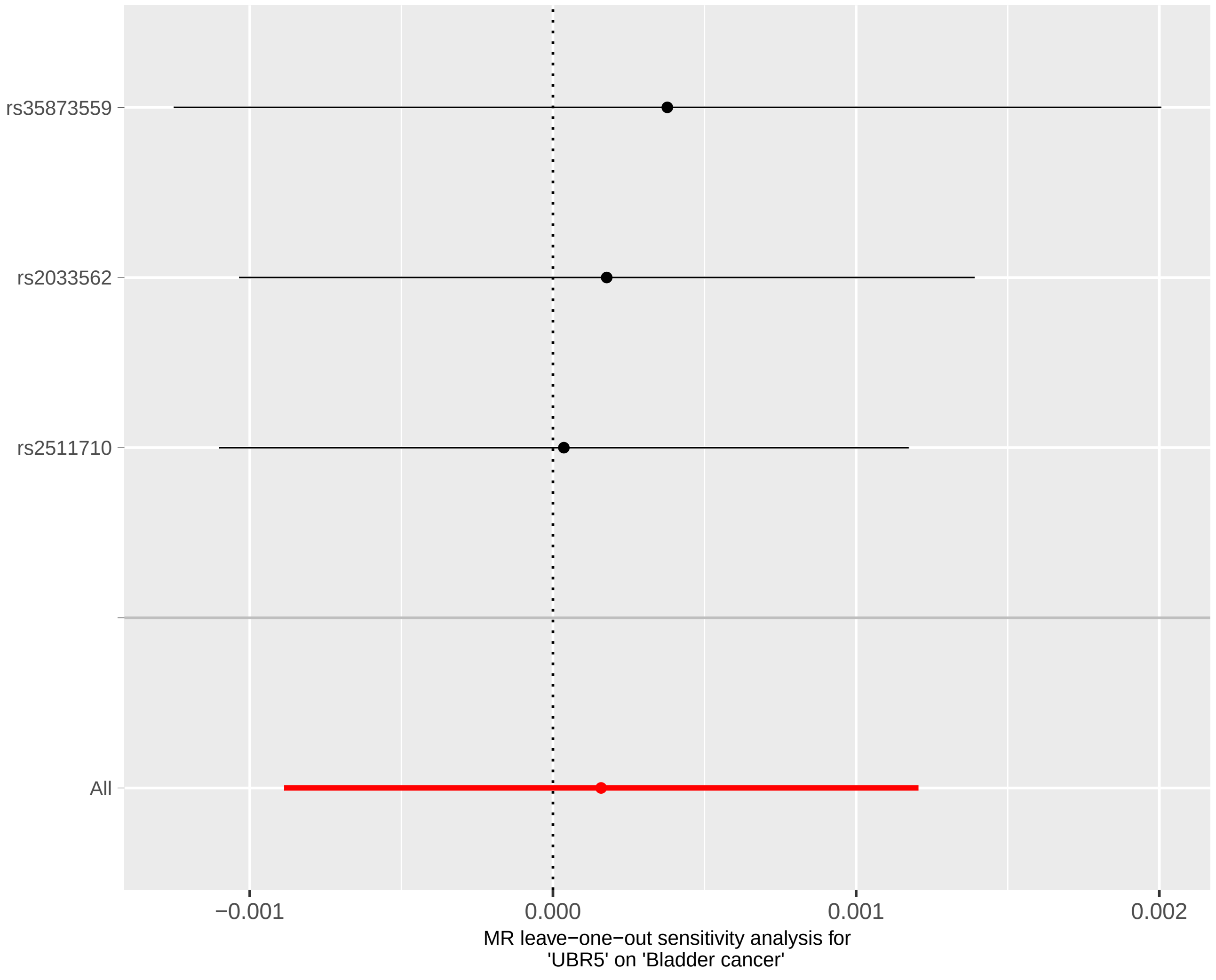

rs35873559
rs2033562
rs2511710
All
−0.001
0.000
0.001
0.002
MR leave−one−out sensitivity analysis for
'UBR5' on 'Bladder cancer'

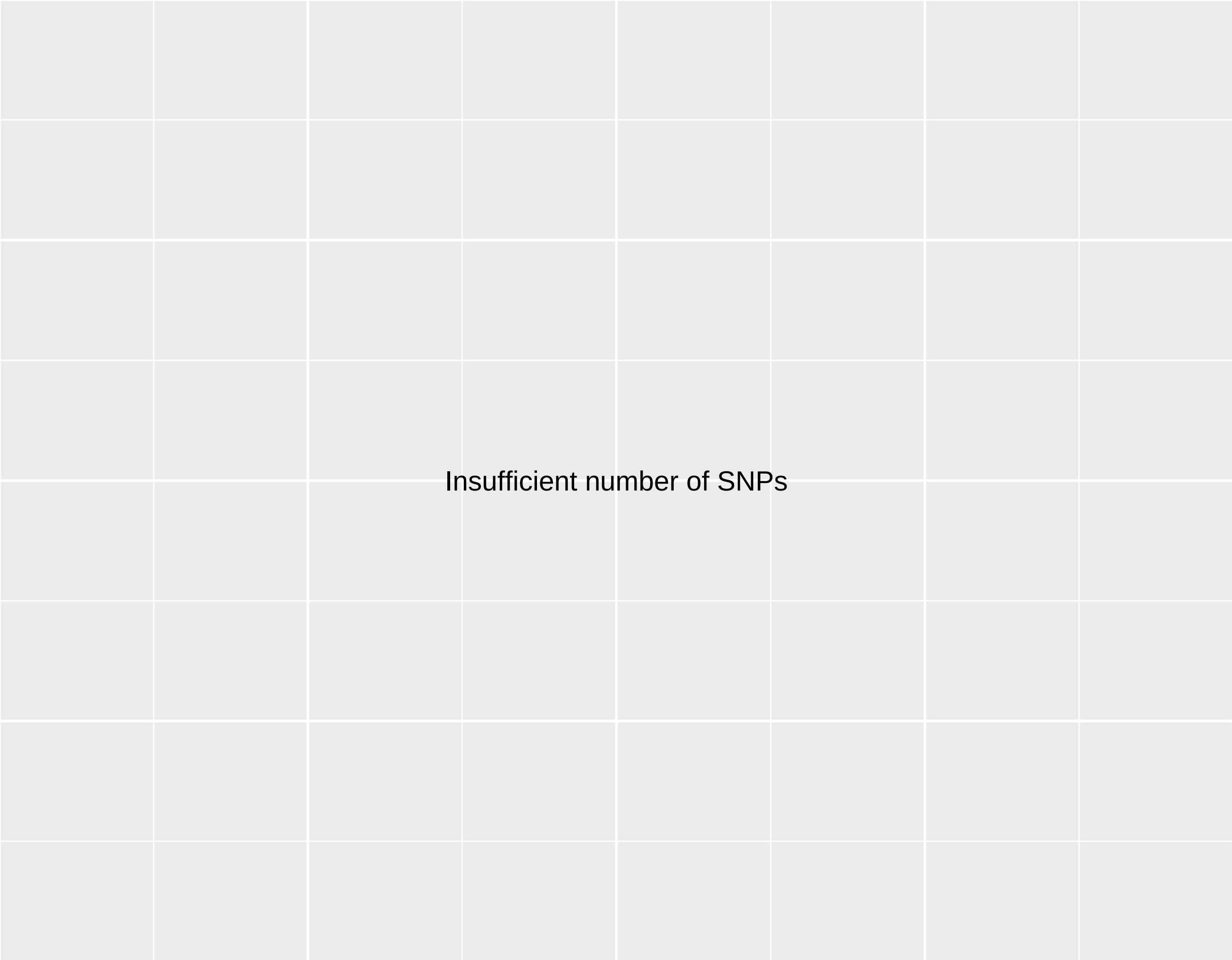
Insufficient number of SNPs

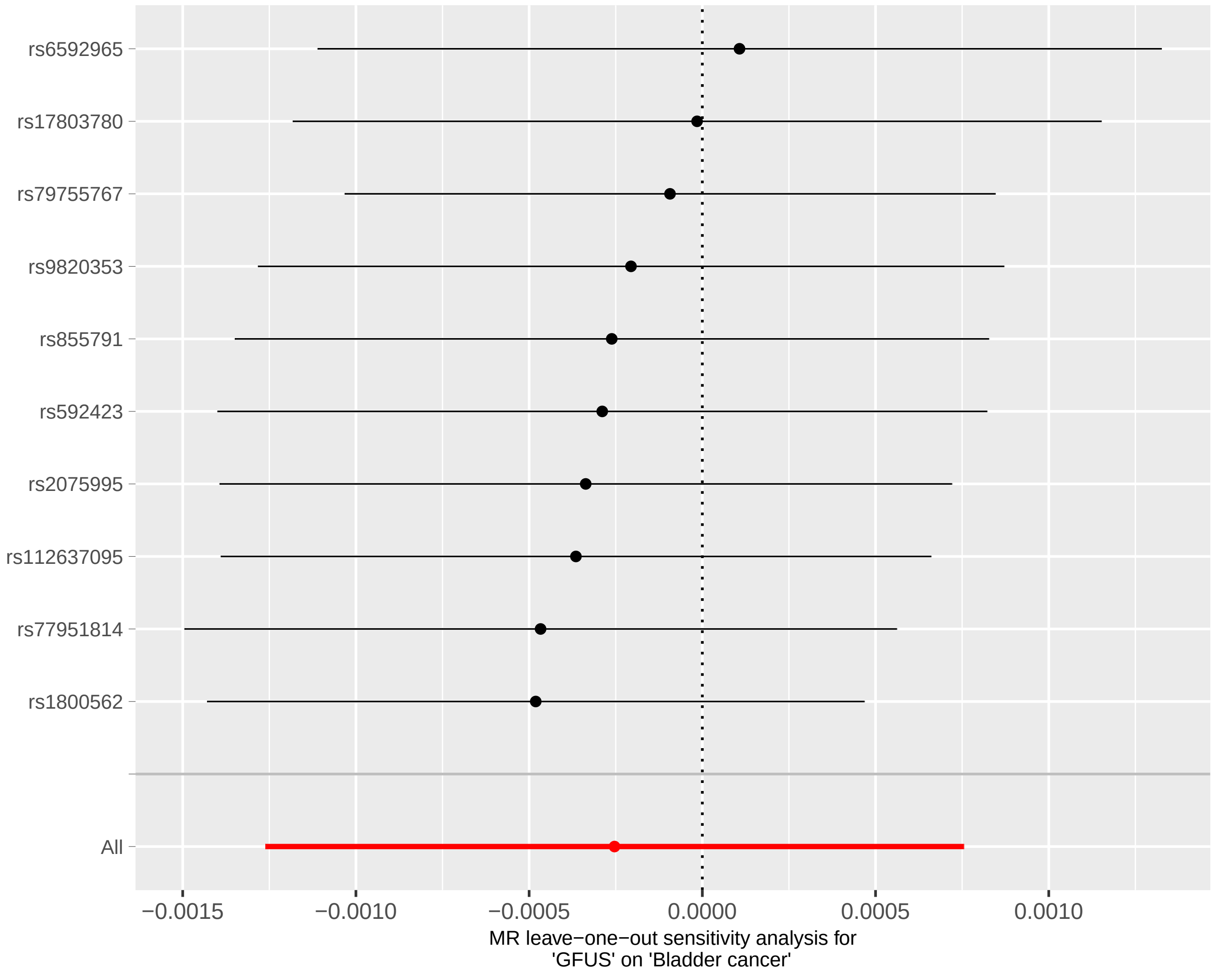

rs6592965
rs17803780
rs79755767
rs9820353
rs855791
rs592423
rs2075995
rs112637095
rs77951814
rs1800562
All
−0.0015
−0.0010
−0.0005
0.0000
0.0005
0.0010
MR leave−one−out sensitivity analysis for
'GFUS' on 'Bladder cancer'

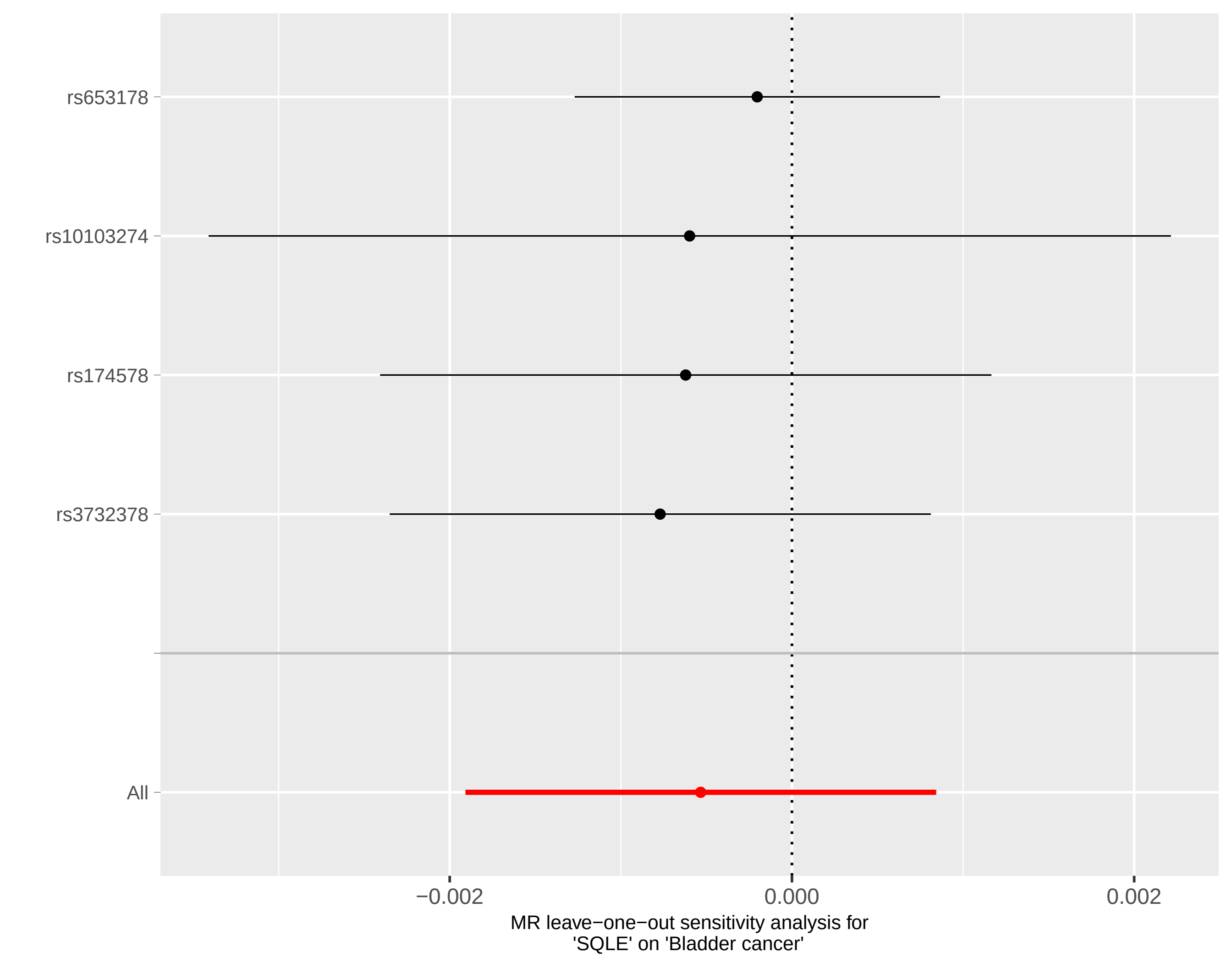
rs653178
rs10103274
rs174578
rs3732378
All
−0.002
0.000
0.002
MR leave−one−out sensitivity analysis for
'SQLE' on 'Bladder cancer'

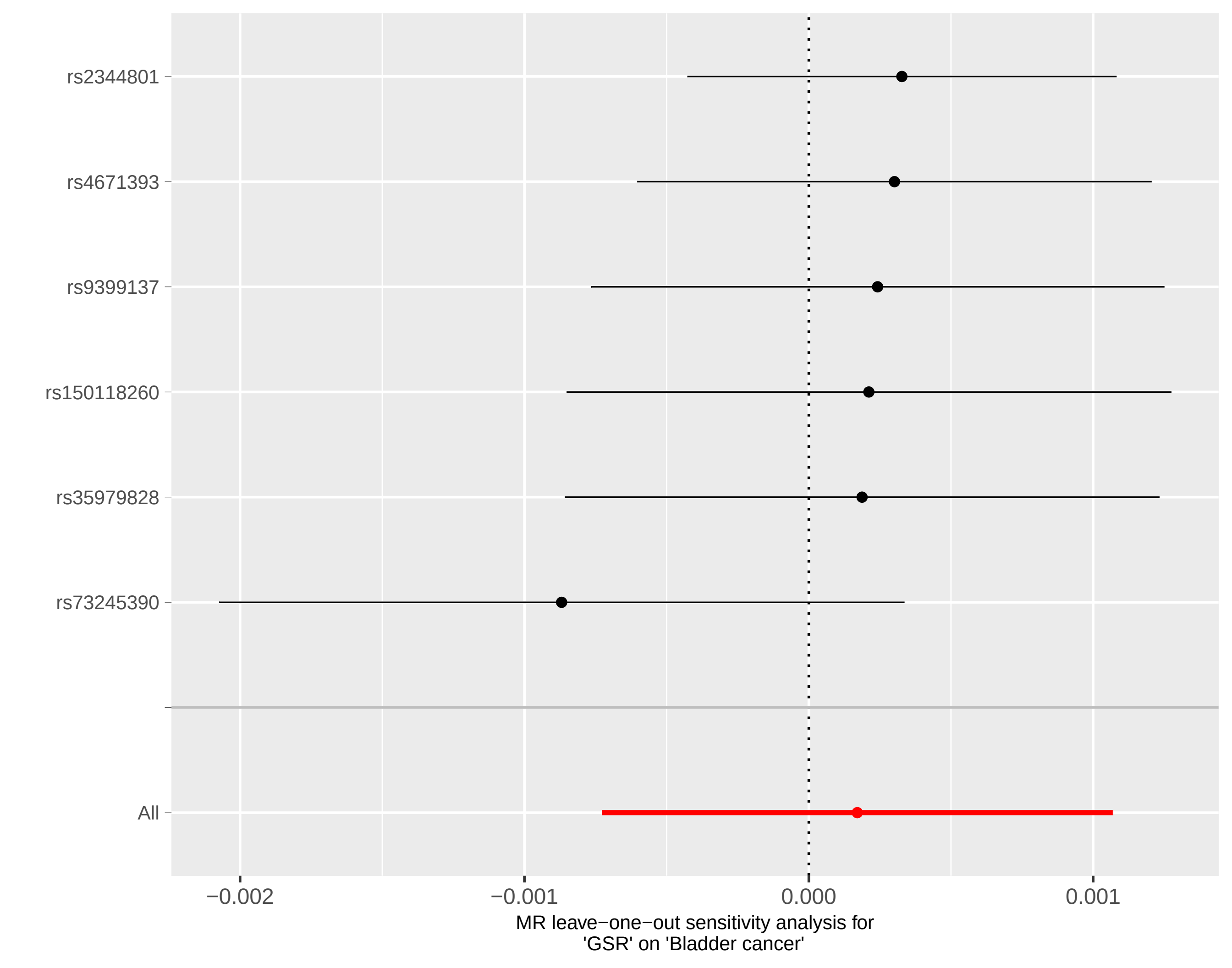

rs2344801
rs4671393
rs9399137
rs150118260
rs35979828
rs73245390
All
−0.002
−0.001
0.000
0.001
MR leave−one−out sensitivity analysis for
'GSR' on 'Bladder cancer'

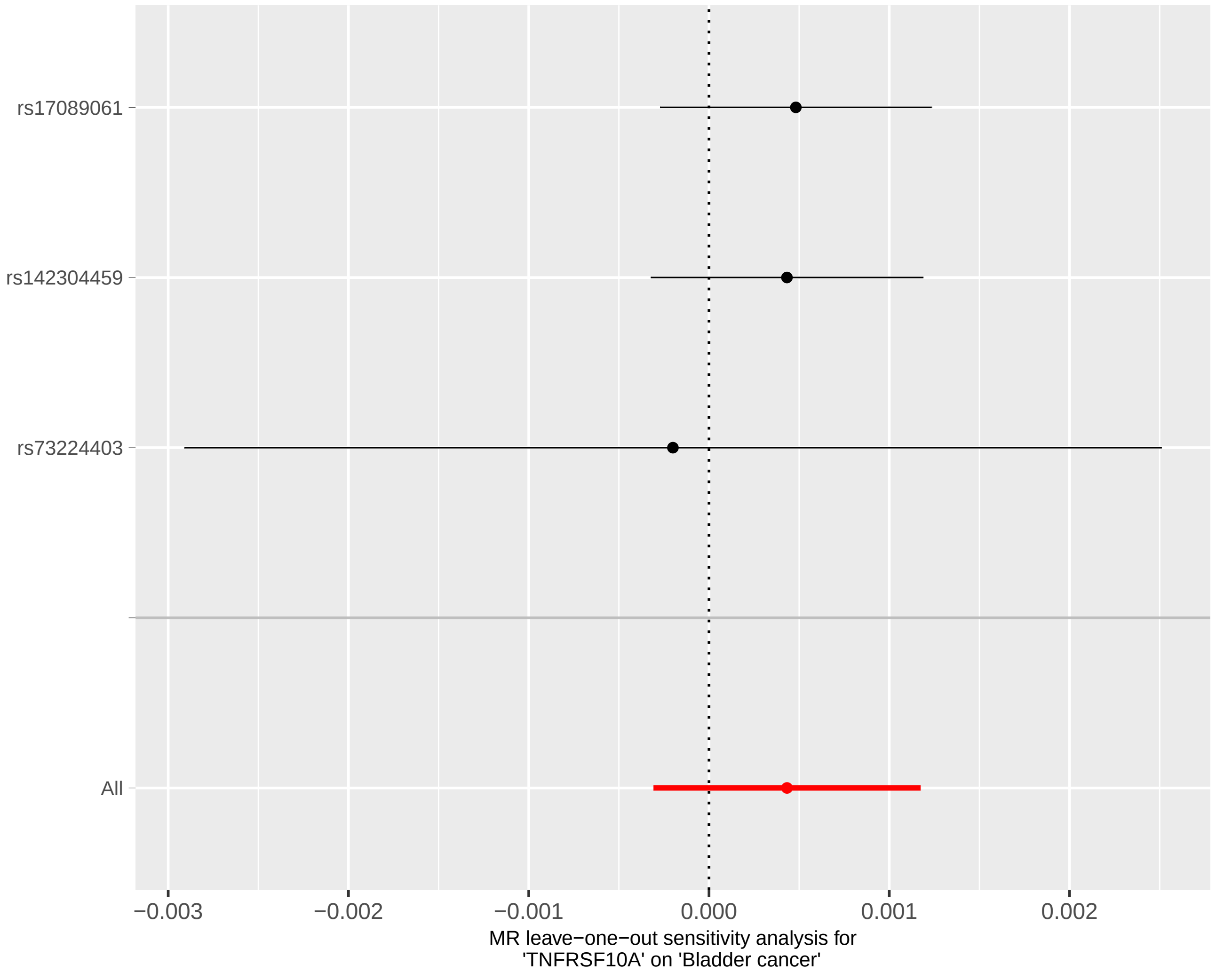
rs17089061
rs142304459
rs73224403
All
−0.003
−0.002
−0.001
0.000
0.001
0.002
MR leave−one−out sensitivity analysis for
'TNFRSF10A' on 'Bladder cancer'

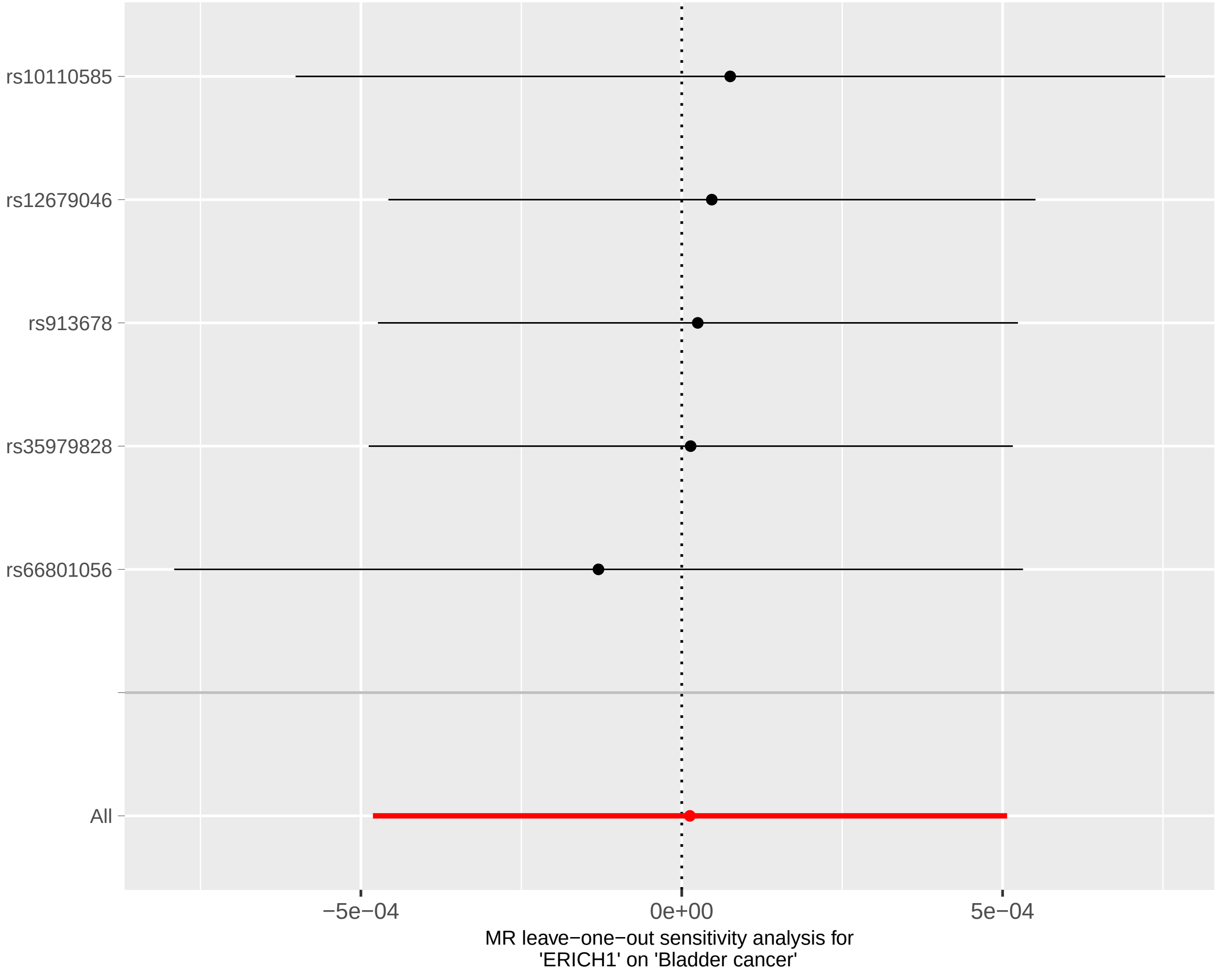
rs10110585
rs12679046
rs913678
rs35979828
rs66801056
All
−5e−04
0e+00
5e−04
MR leave−one−out sensitivity analysis for
'ERICH1' on 'Bladder cancer'

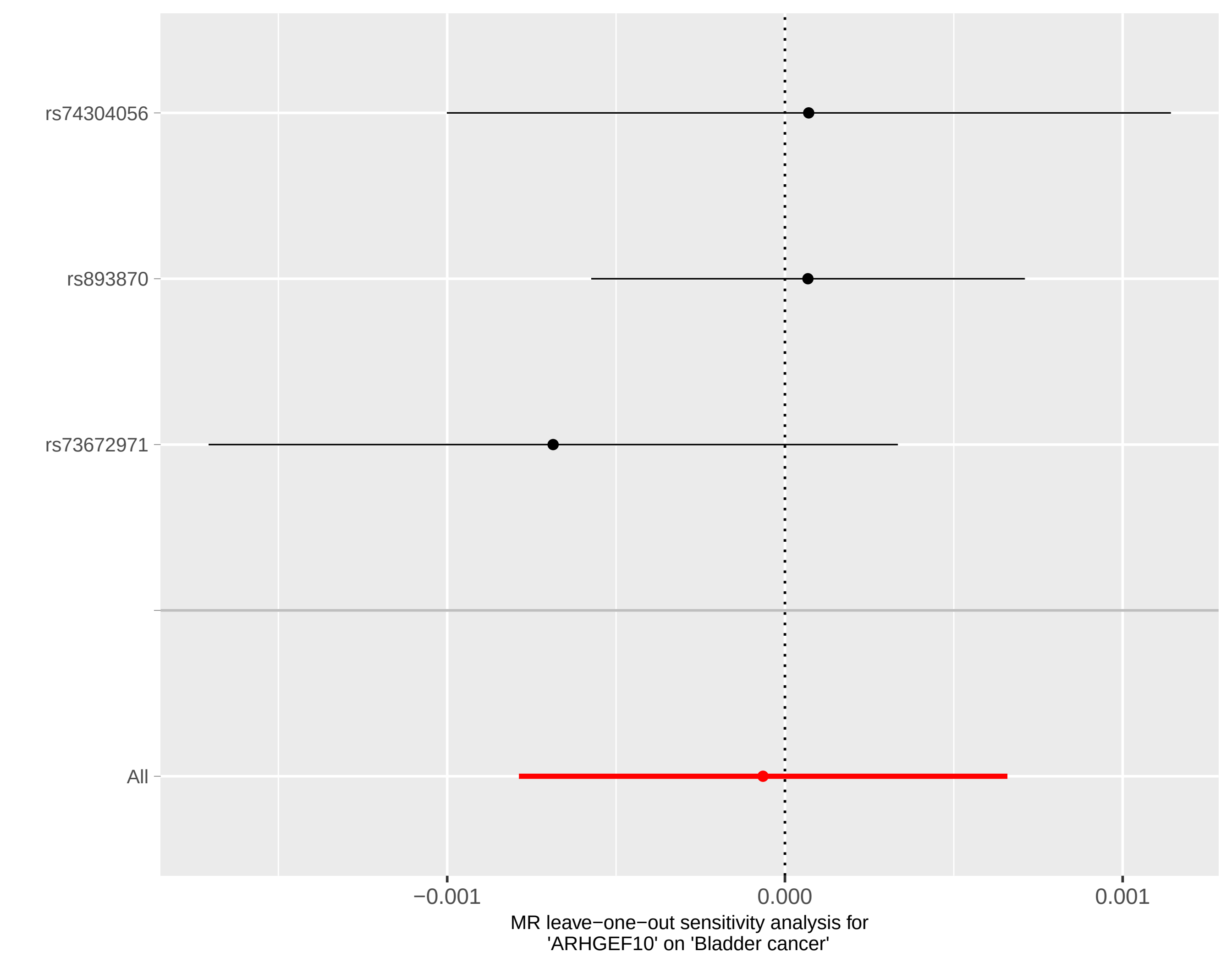

rs74304056
rs893870
rs73672971
All
−0.001
0.000
0.001
MR leave−one−out sensitivity analysis for
'ARHGEF10' on 'Bladder cancer'

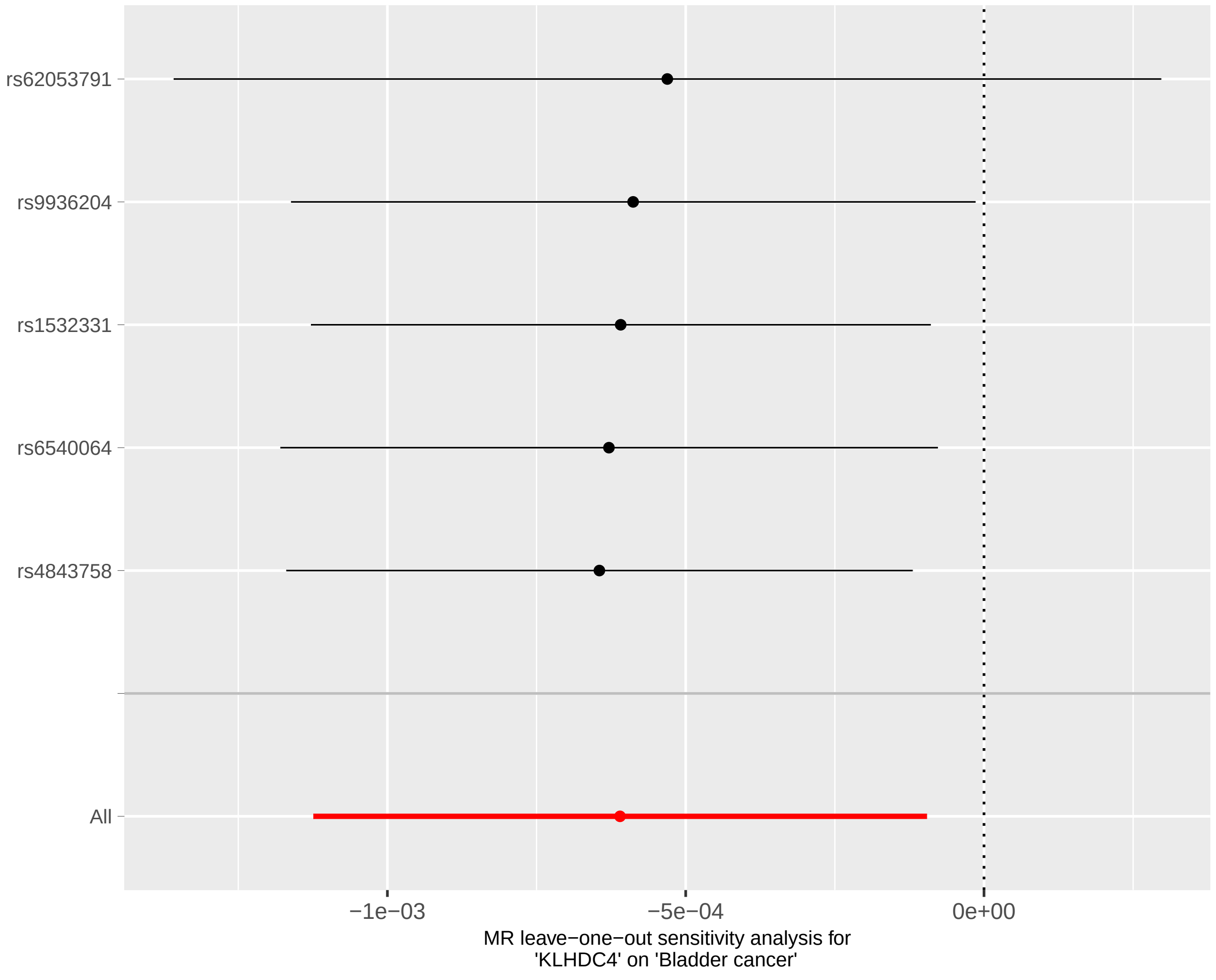

rs62053791
rs9936204
rs1532331
rs6540064
rs4843758
All
−1e−03
−5e−04
0e+00
MR leave−one−out sensitivity analysis for
'KLHDC4' on 'Bladder cancer'

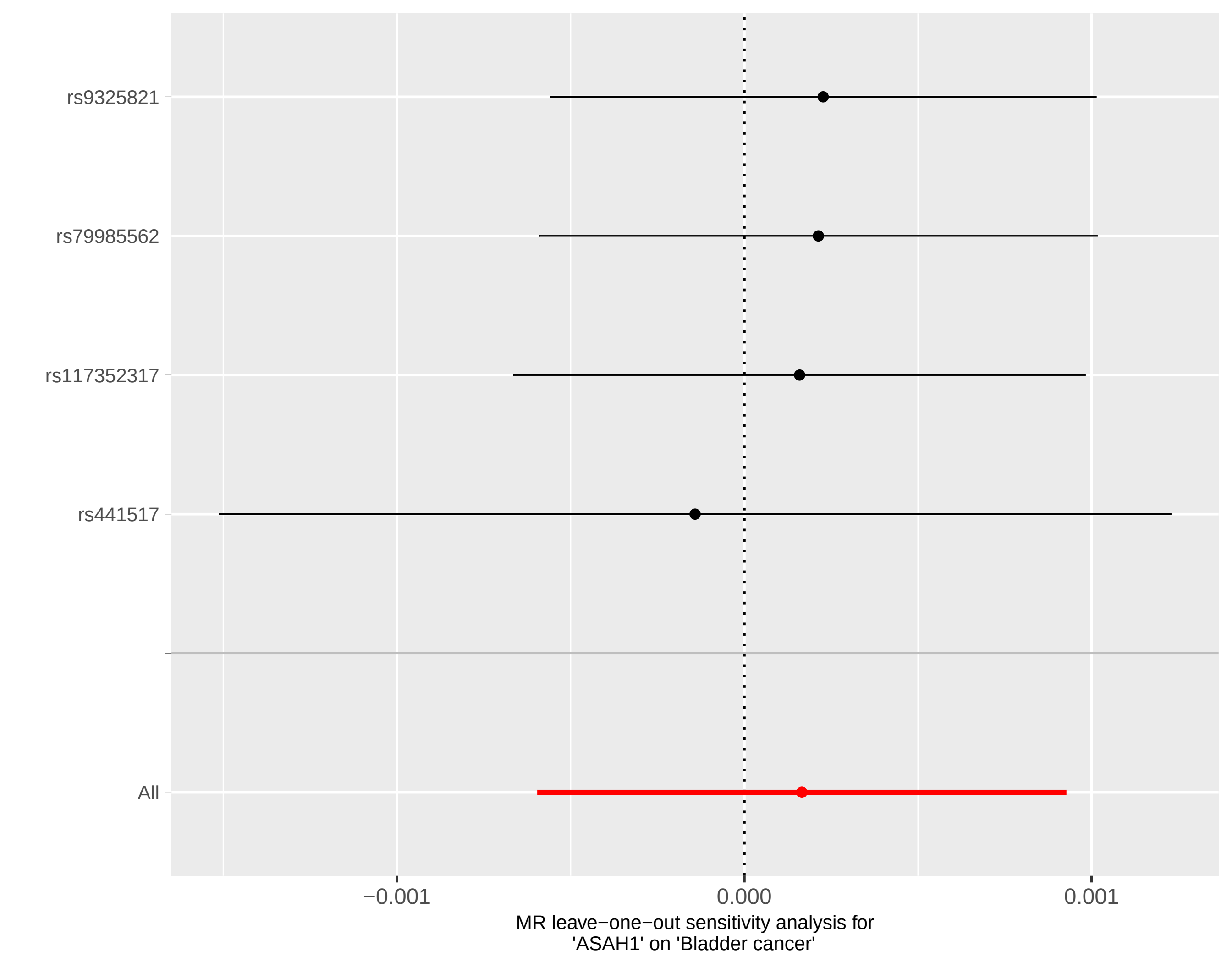
rs9325821
rs79985562
rs117352317
rs441517
All
−0.001
0.000
0.001
MR leave−one−out sensitivity analysis for
'ASAH1' on 'Bladder cancer'

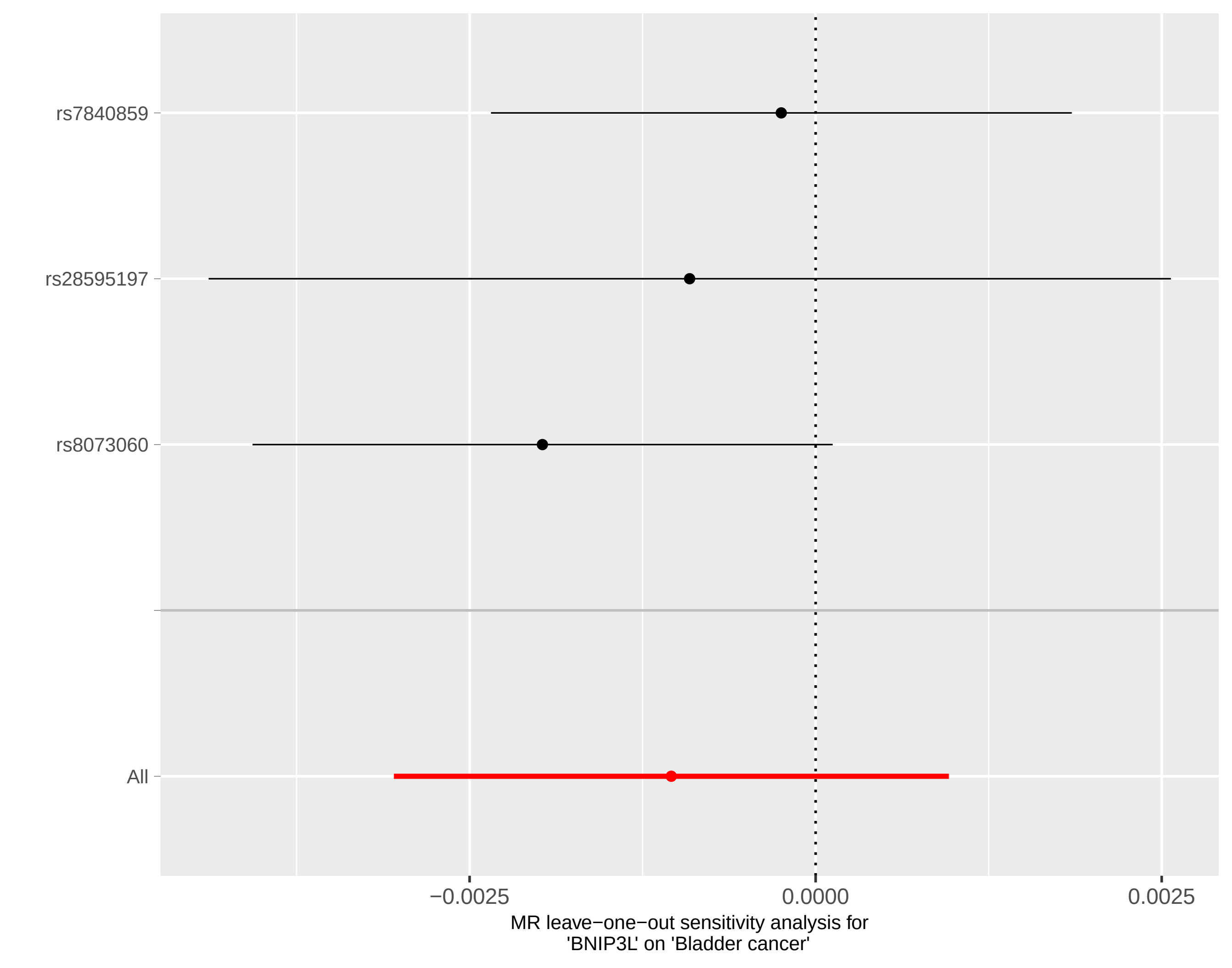
rs7840859
rs28595197
rs8073060
All
−0.0025
0.0000
0.0025
MR leave−one−out sensitivity analysis for
'BNIP3L' on 'Bladder cancer'

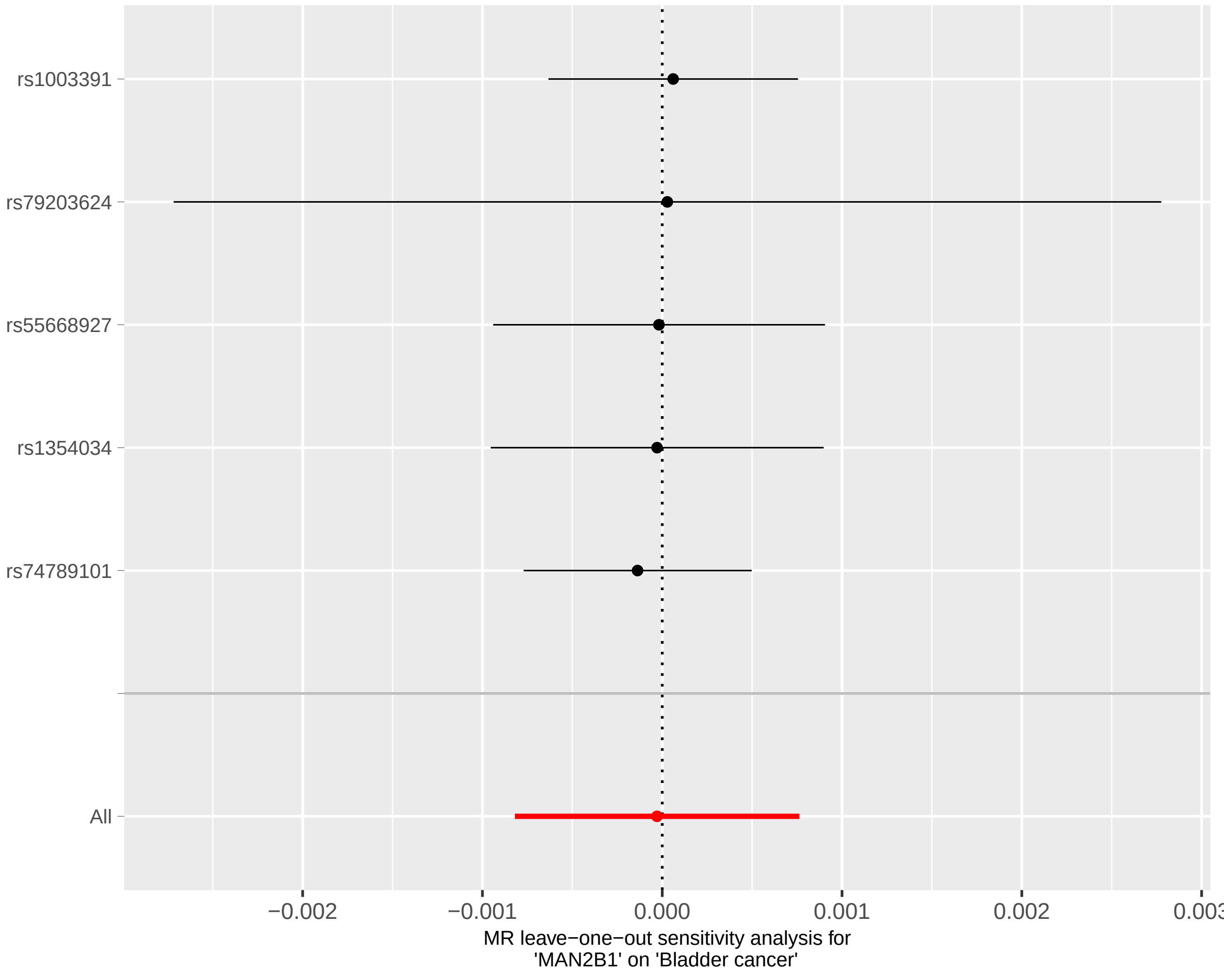
rs1003391
rs79203624
rs55668927
rs1354034
rs74789101
All
−0.002
−0.001
0.000
0.001
0.002
0.003
MR leave−one−out sensitivity analysis for
'MAN2B1' on 'Bladder cancer'

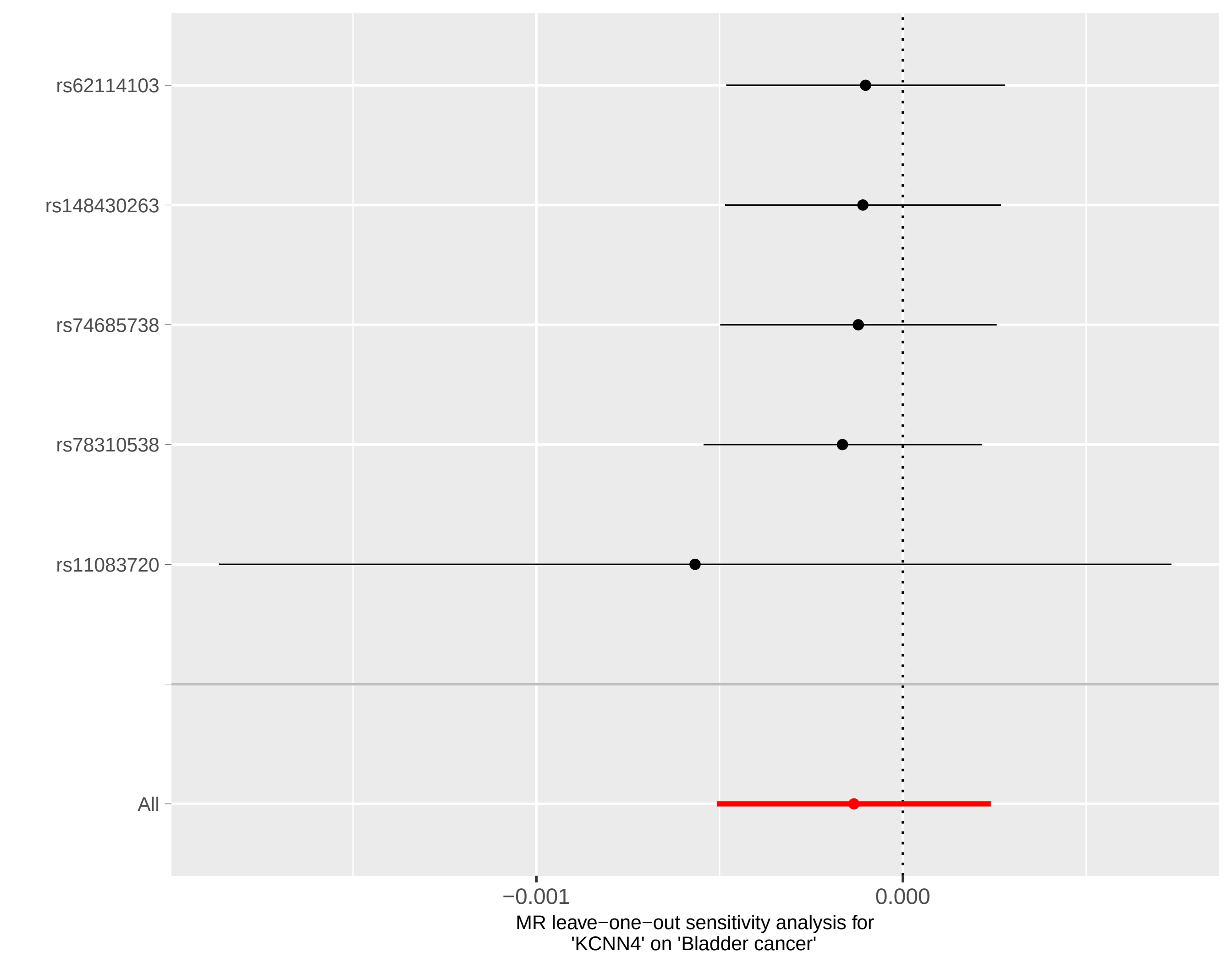
rs62114103
rs148430263
rs74685738
rs78310538
rs11083720
All
−0.001
0.000
MR leave−one−out sensitivity analysis for
'KCNN4' on 'Bladder cancer'

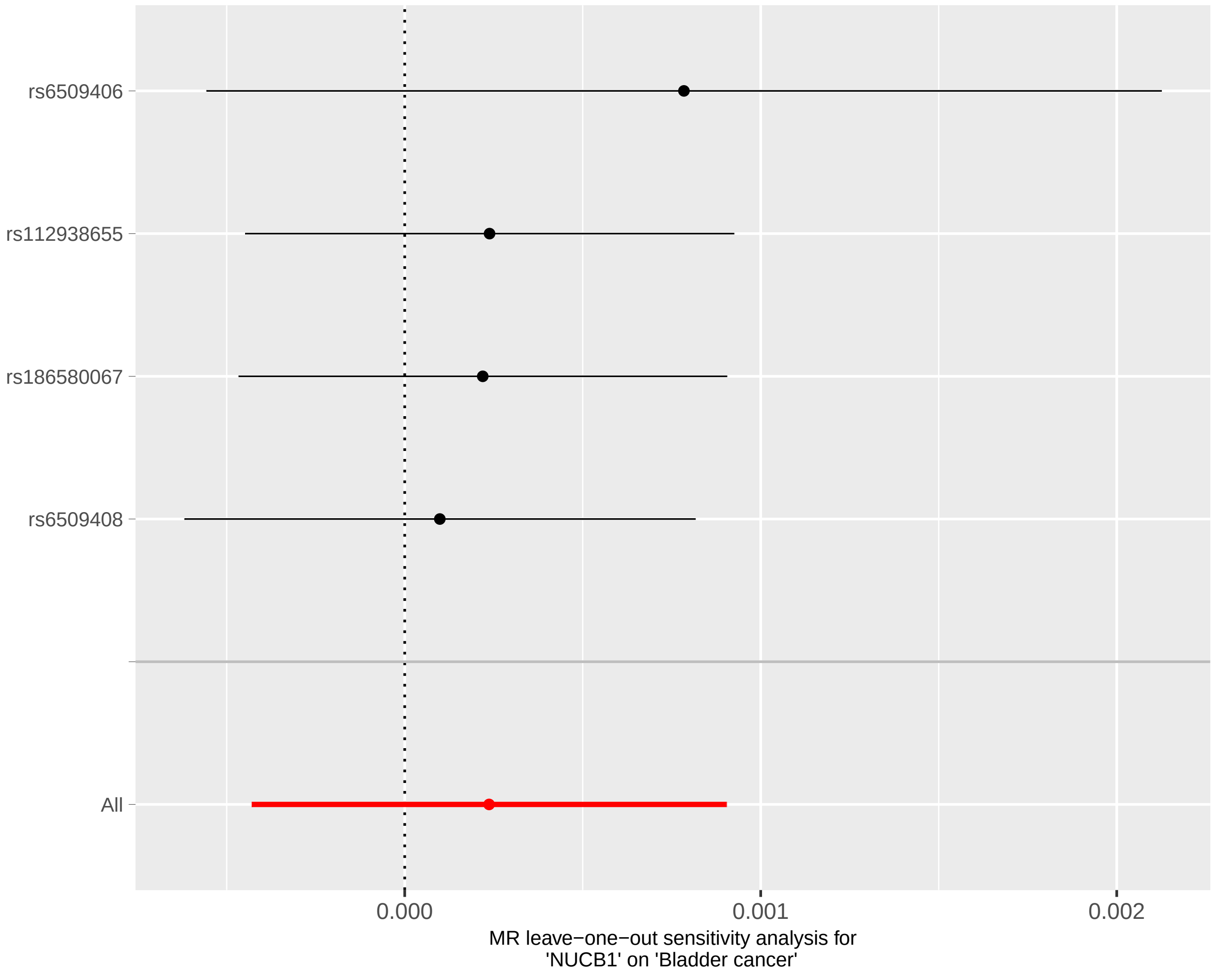
rs6509406
rs112938655
rs186580067
rs6509408
All
0.000
0.001
0.002
MR leave−one−out sensitivity analysis for
'NUCB1' on 'Bladder cancer'

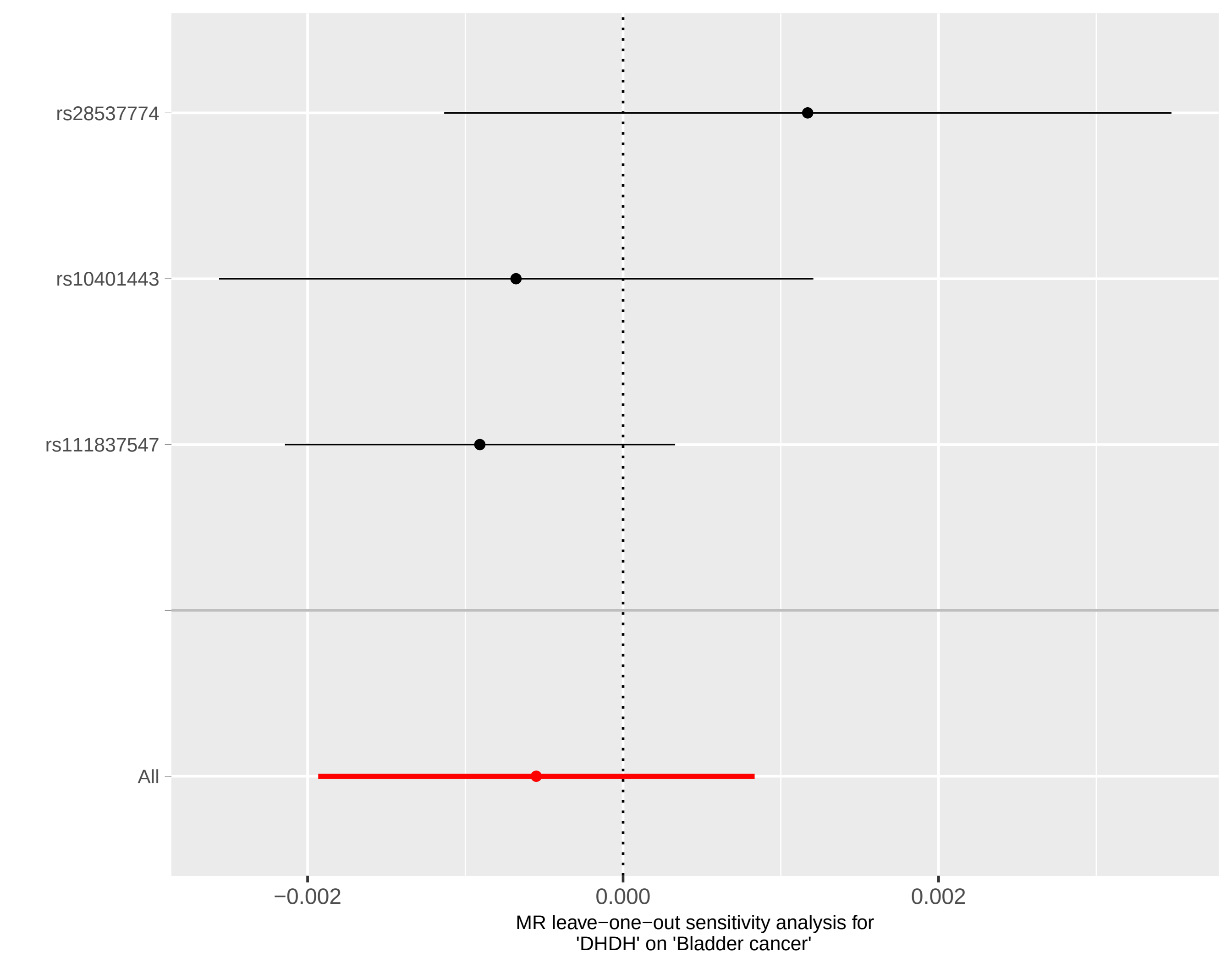
rs28537774
rs10401443
rs111837547
All
−0.002
0.000
0.002
MR leave−one−out sensitivity analysis for
'DHDH' on 'Bladder cancer'

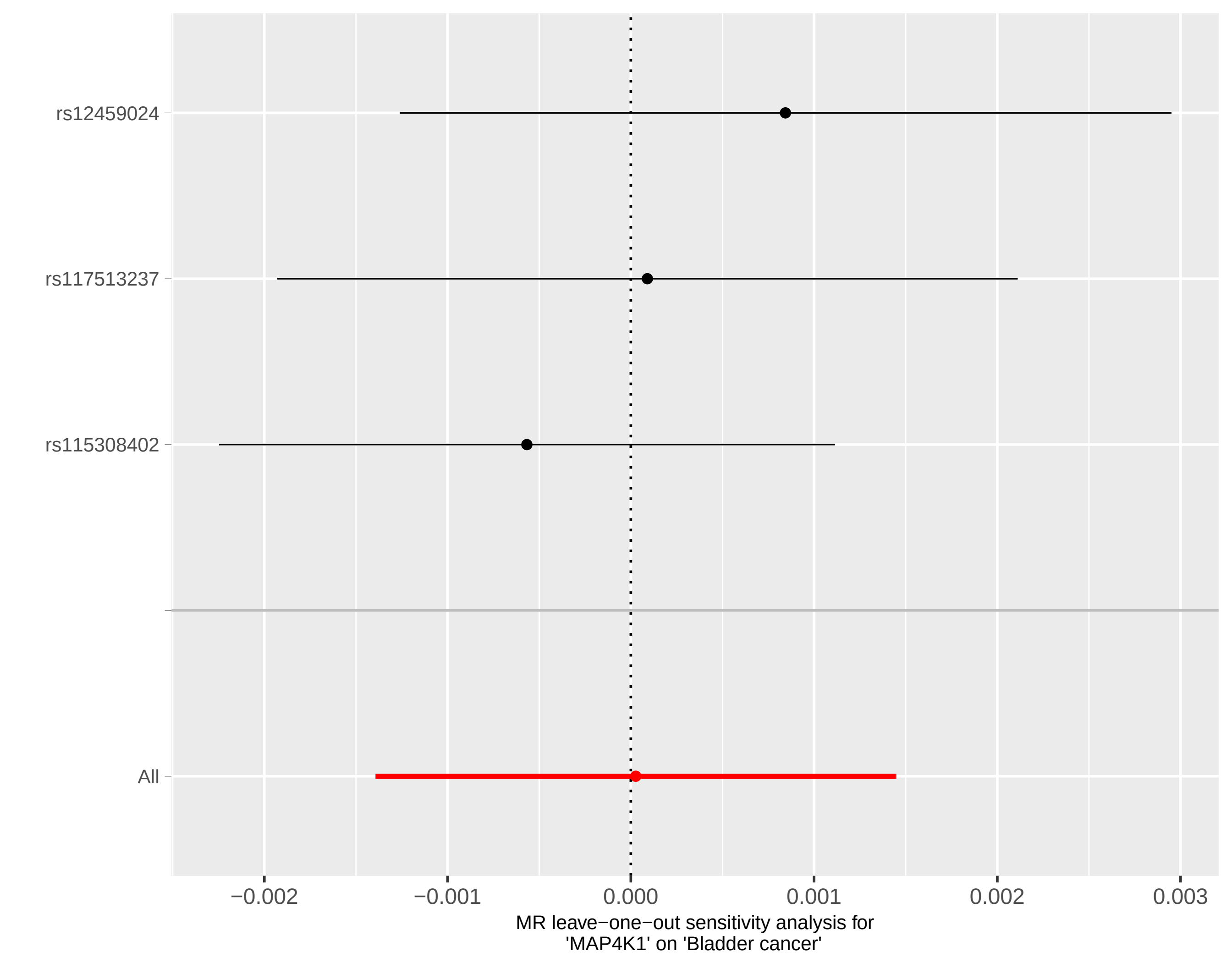
rs12459024
rs117513237
rs115308402
All
−0.002
−0.001
0.000
0.001
0.002
0.003
MR leave−one−out sensitivity analysis for
'MAP4K1' on 'Bladder cancer'

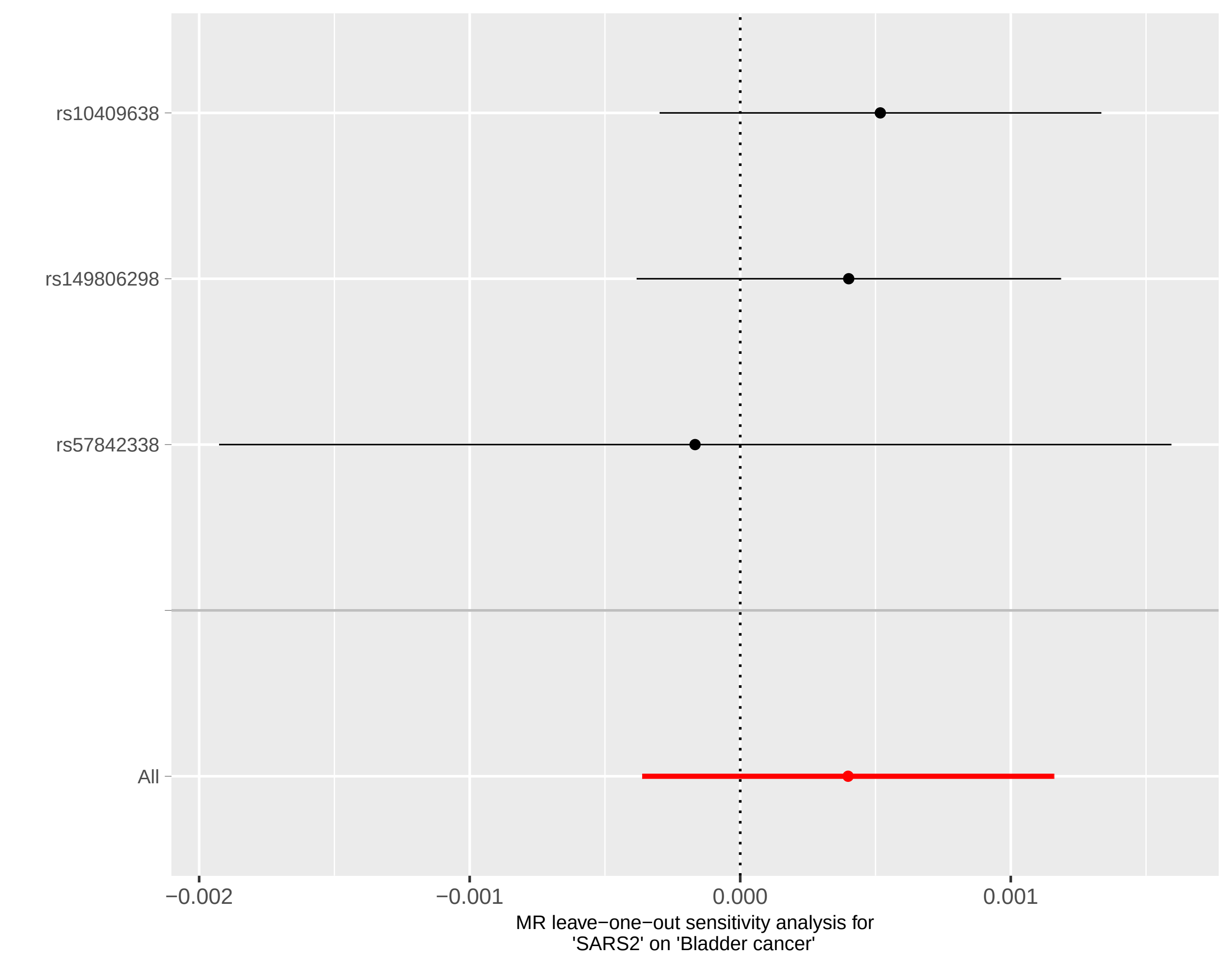
rs10409638
rs149806298
rs57842338
All
−0.002
−0.001
0.000
0.001
MR leave−one−out sensitivity analysis for
'SARS2' on 'Bladder cancer'

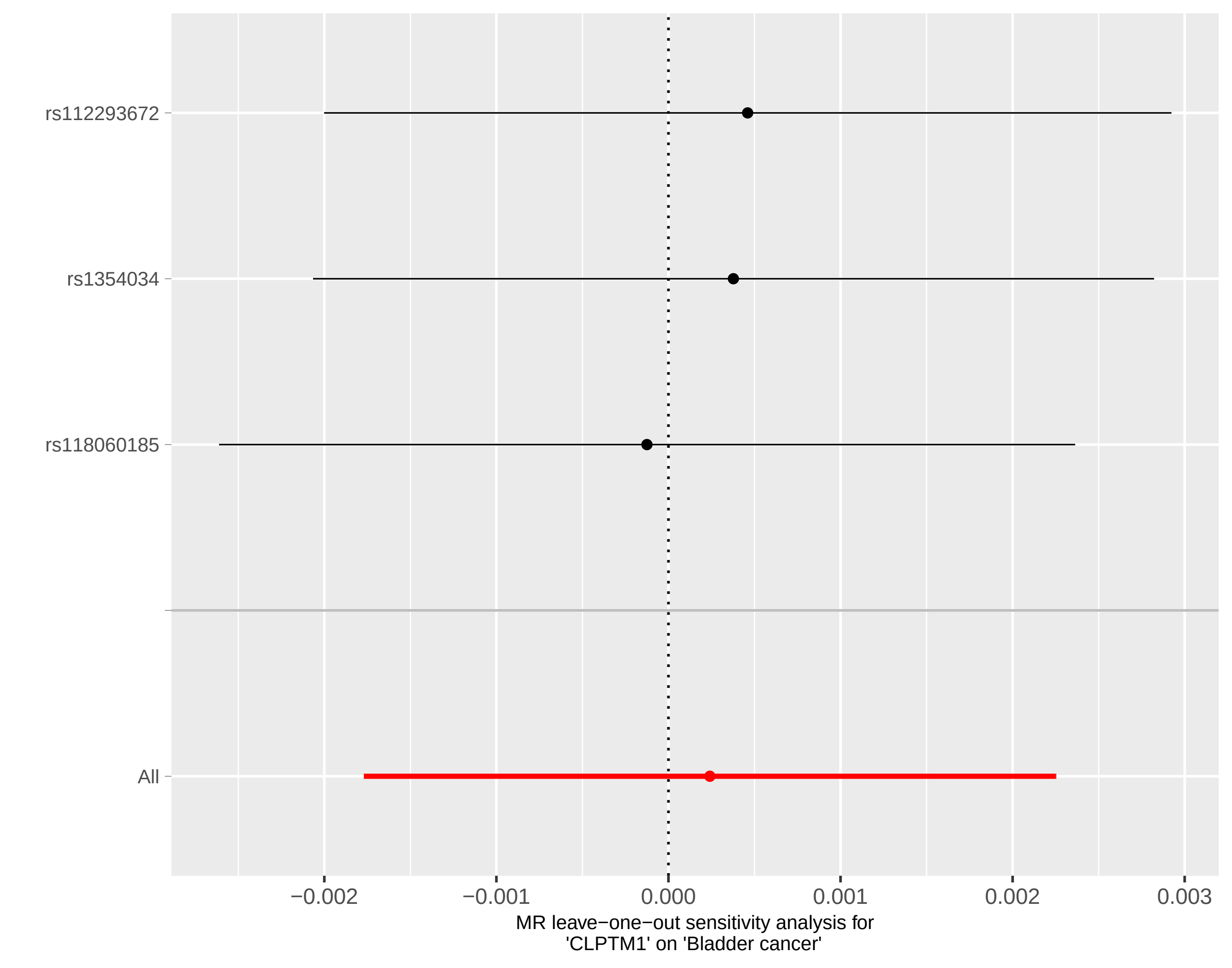
rs112293672
rs1354034
rs118060185
All
−0.002
−0.001
0.000
0.001
0.002
0.003
MR leave−one−out sensitivity analysis for
'CLPTM1' on 'Bladder cancer'

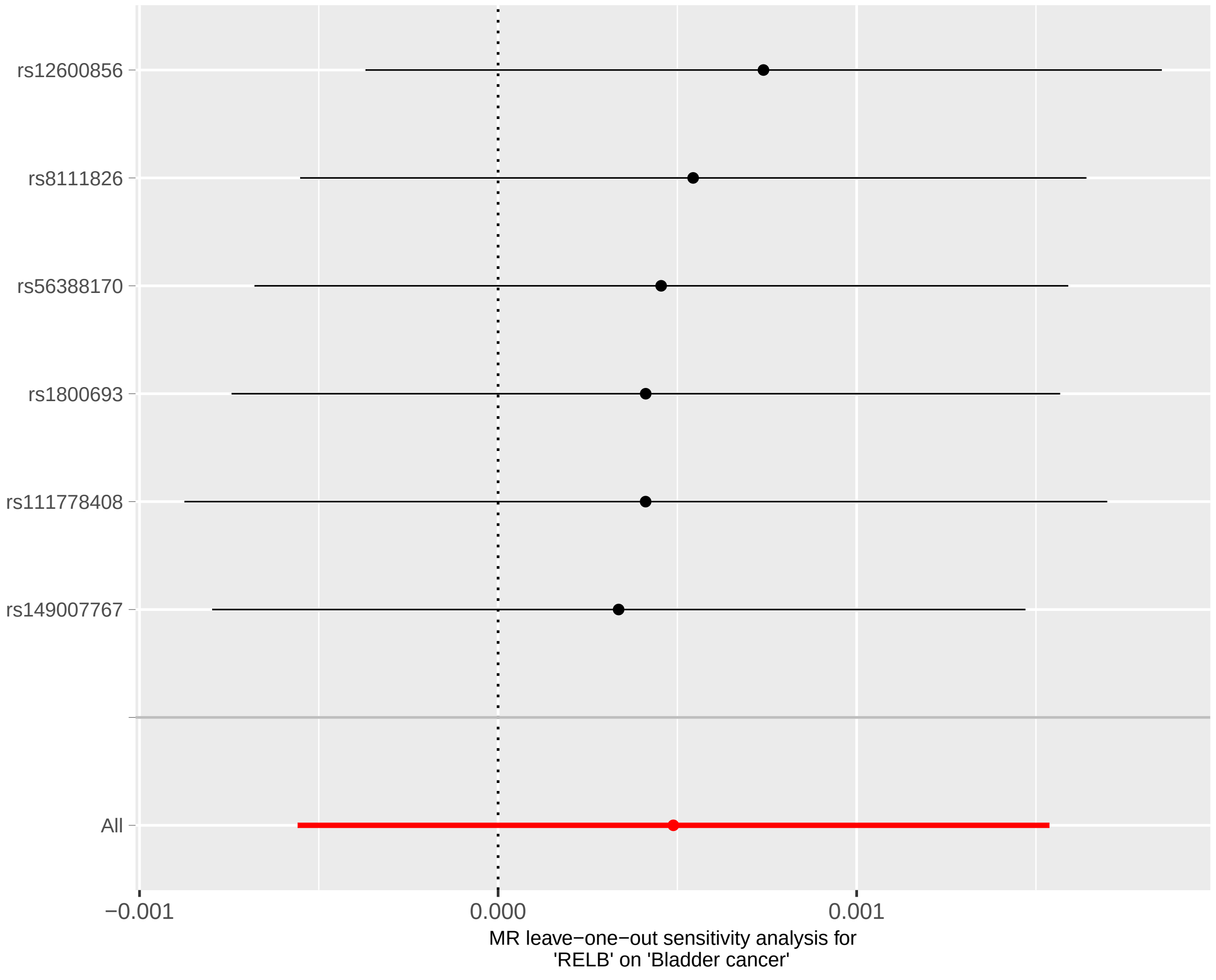
rs12600856
rs8111826
rs56388170
rs1800693
rs111778408
rs149007767
All
−0.001
0.000
0.001
MR leave−one−out sensitivity analysis for
'RELB' on 'Bladder cancer'

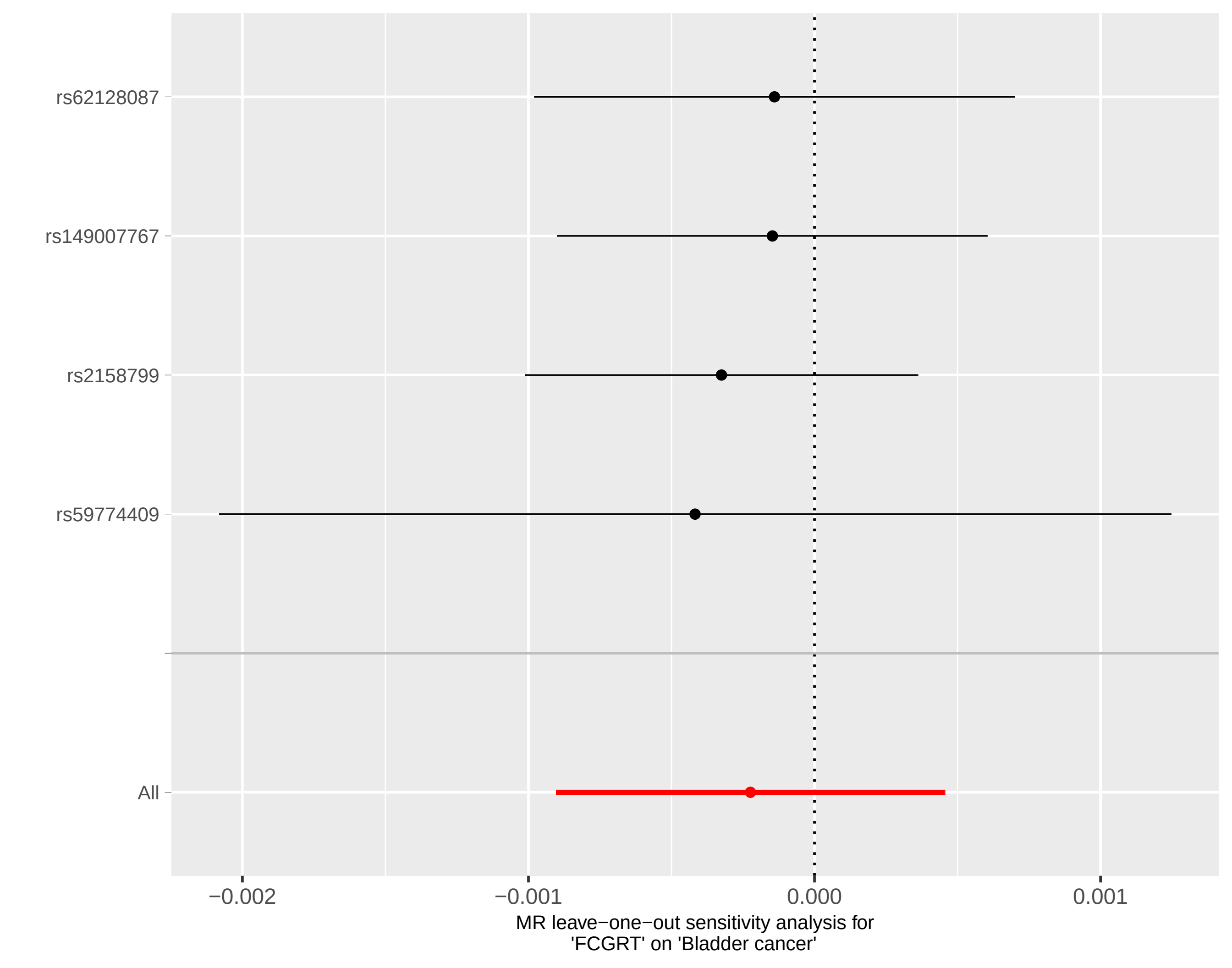
rs62128087
rs149007767
rs2158799
rs59774409
All
−0.002
−0.001
0.000
0.001
MR leave−one−out sensitivity analysis for
'FCGRT' on 'Bladder cancer'

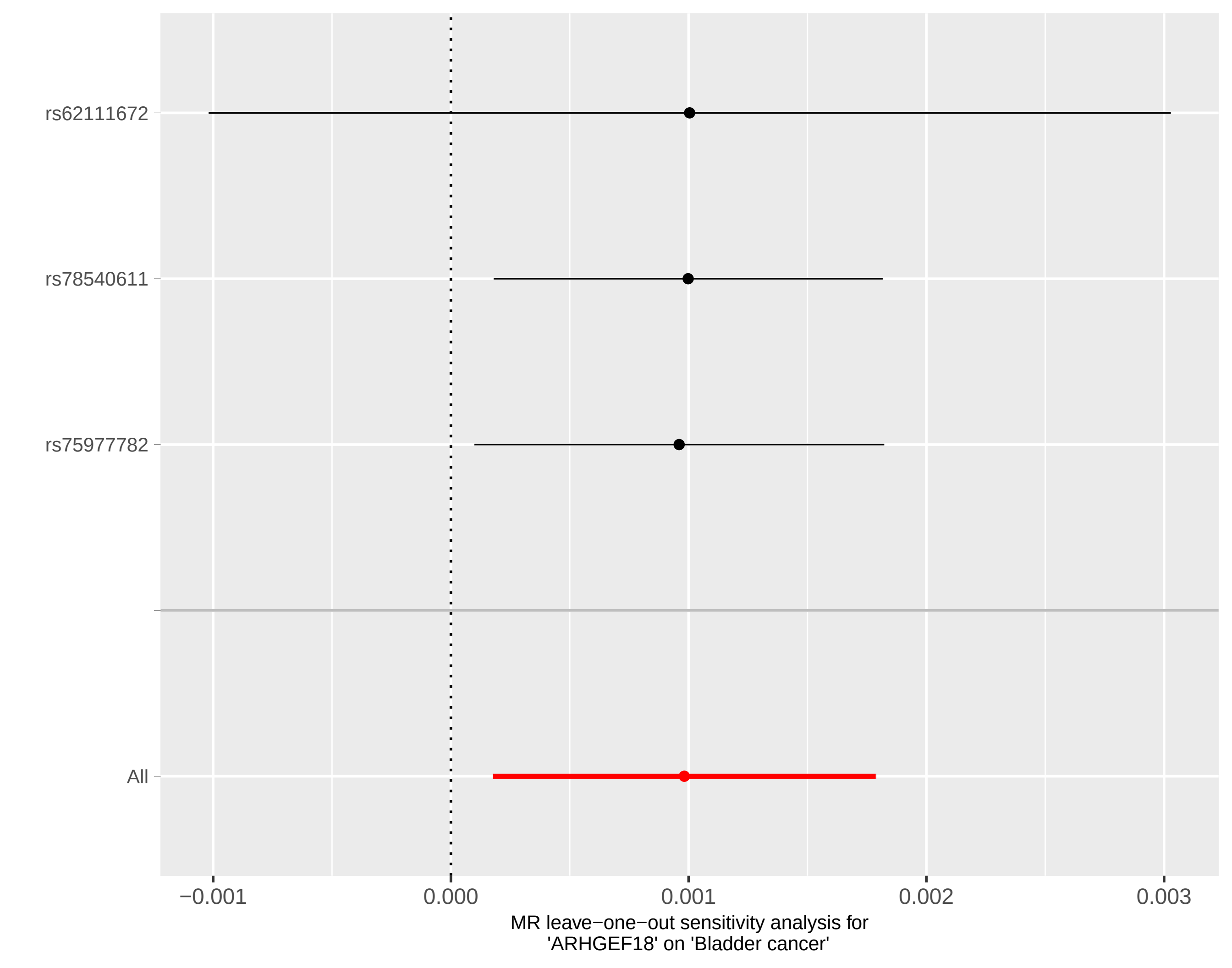
rs62111672
rs78540611
rs75977782
All
−0.001
0.000
0.001
0.002
0.003
MR leave−one−out sensitivity analysis for
'ARHGEF18' on 'Bladder cancer'

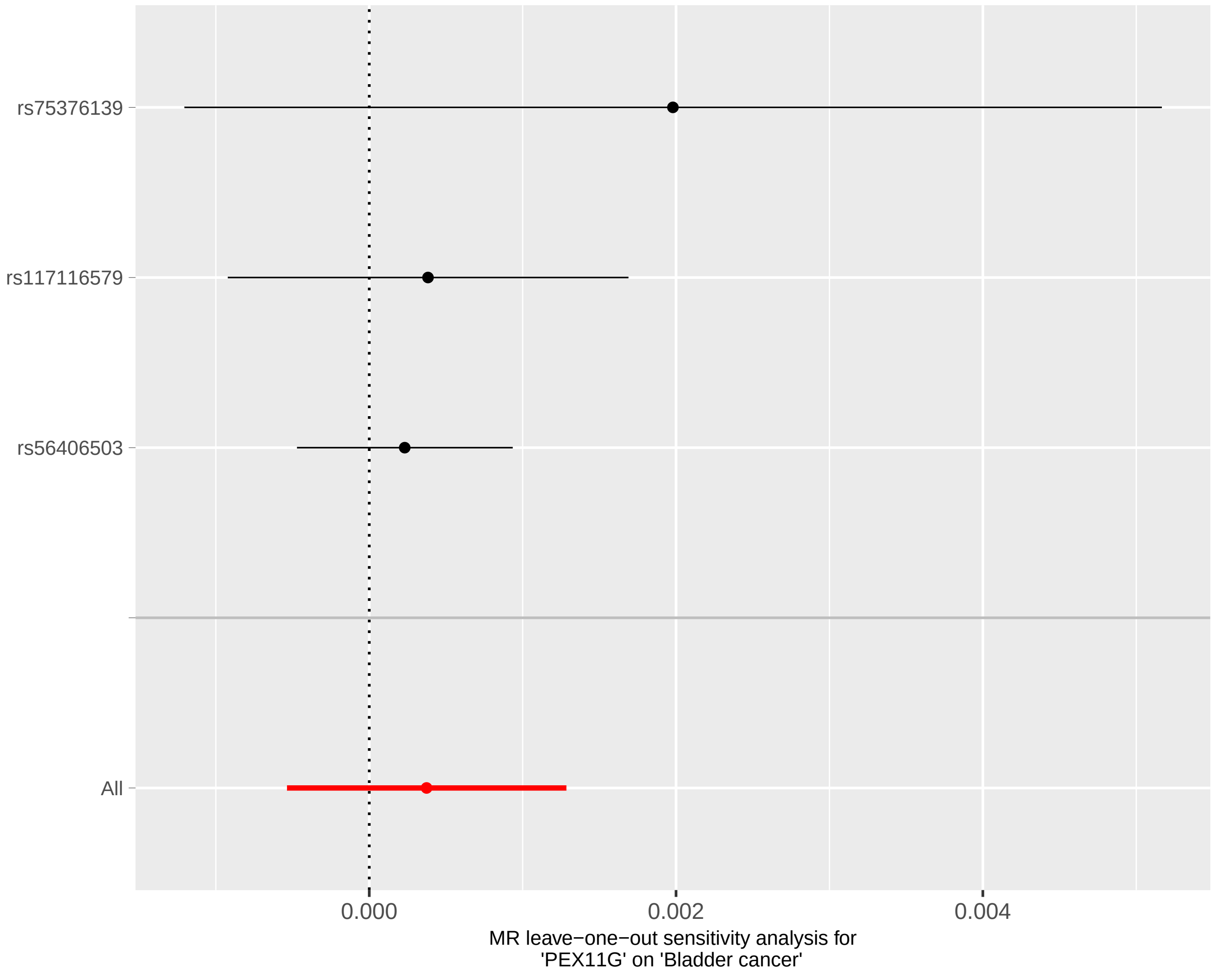
rs75376139
rs117116579
rs56406503
All
0.000
0.002
0.004
MR leave−one−out sensitivity analysis for
'PEX11G' on 'Bladder cancer'

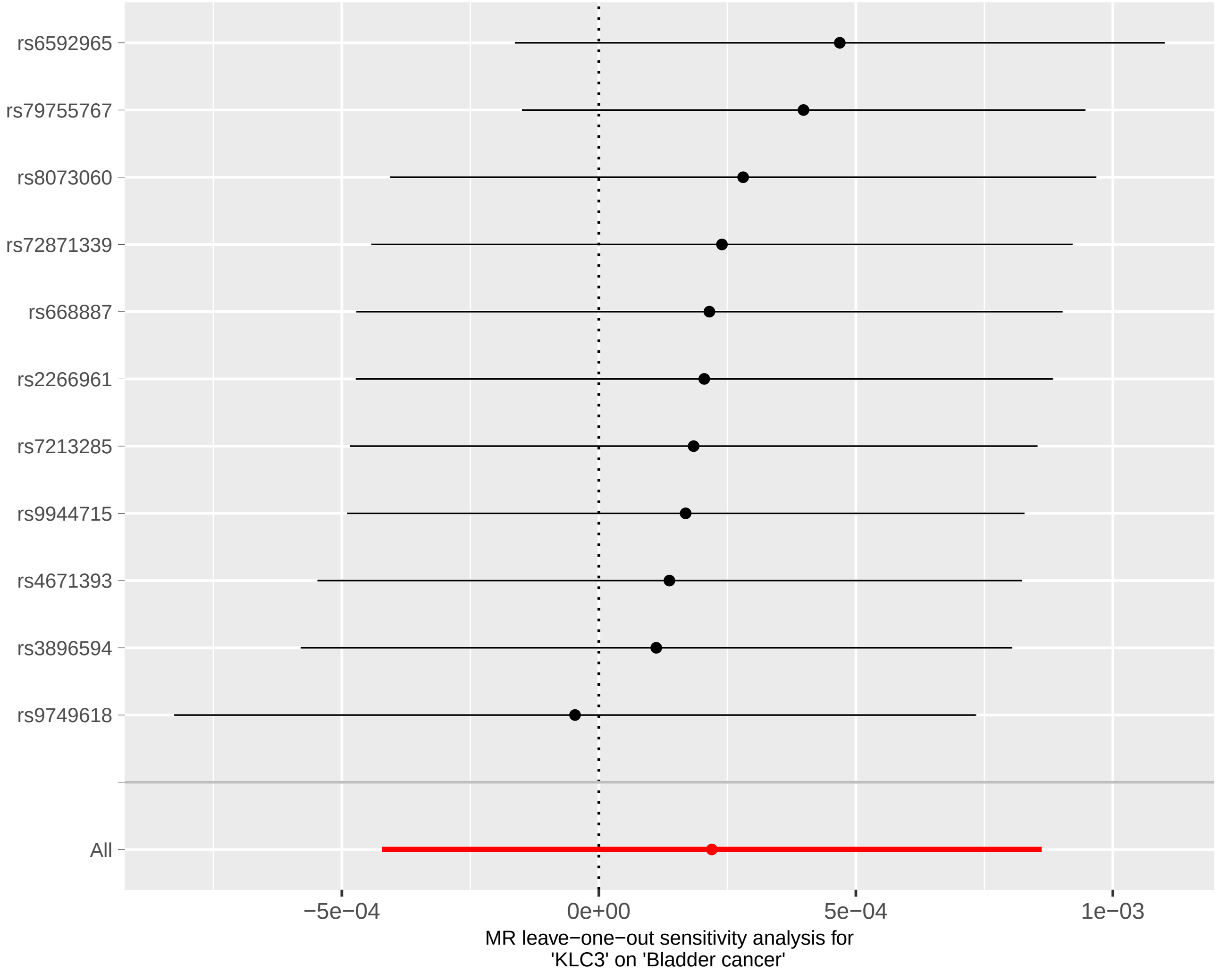

rs6592965
rs79755767
rs8073060
rs72871339
rs668887
rs2266961
rs7213285
rs9944715
rs4671393
rs3896594
rs9749618
All
−5e−04
0e+00
5e−04
1e−03
MR leave−one−out sensitivity analysis for
'KLC3' on 'Bladder cancer'

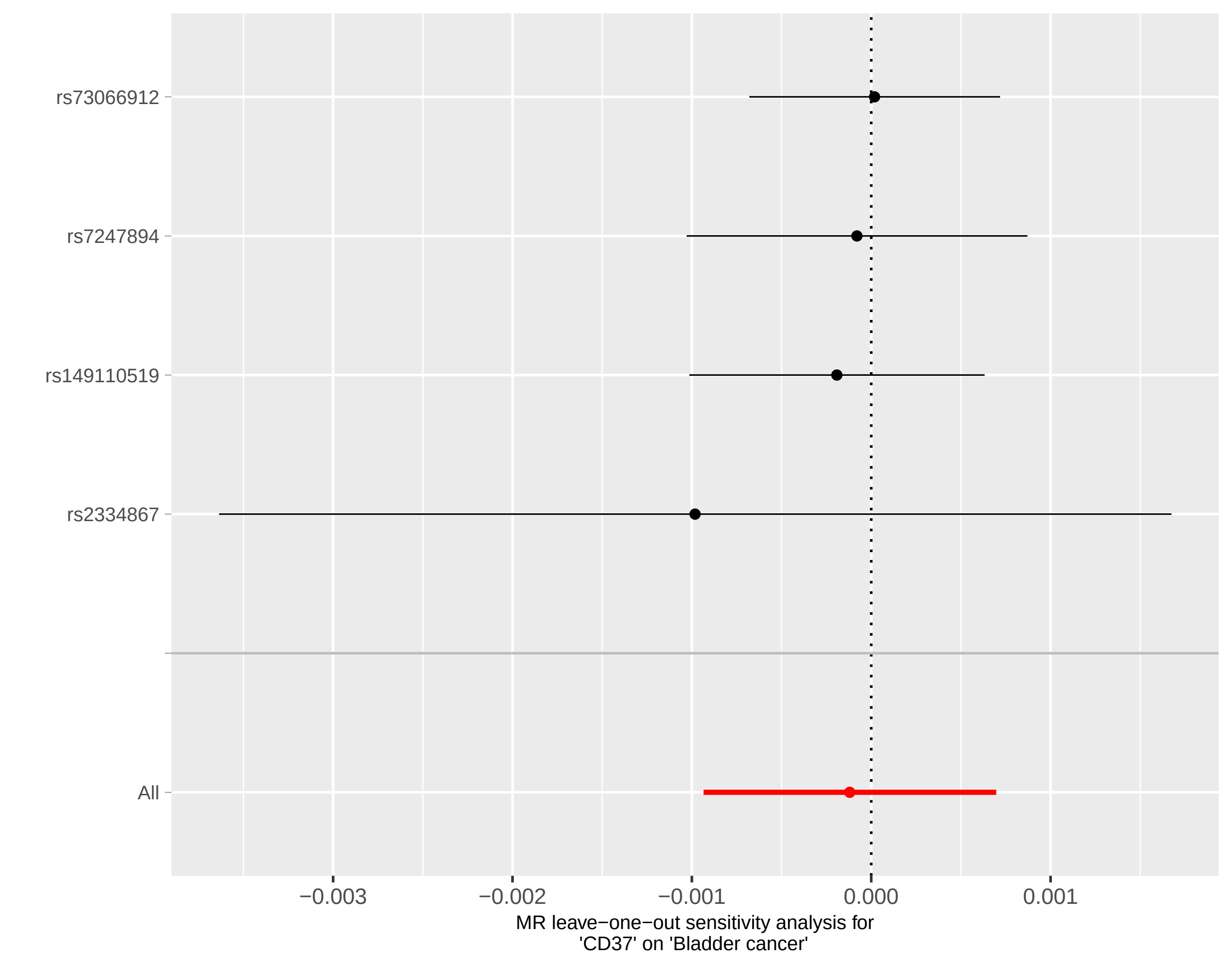
rs73066912
rs7247894
rs149110519
rs2334867
All
−0.003
−0.002
−0.001
0.000
0.001
MR leave−one−out sensitivity analysis for
'CD37' on 'Bladder cancer'

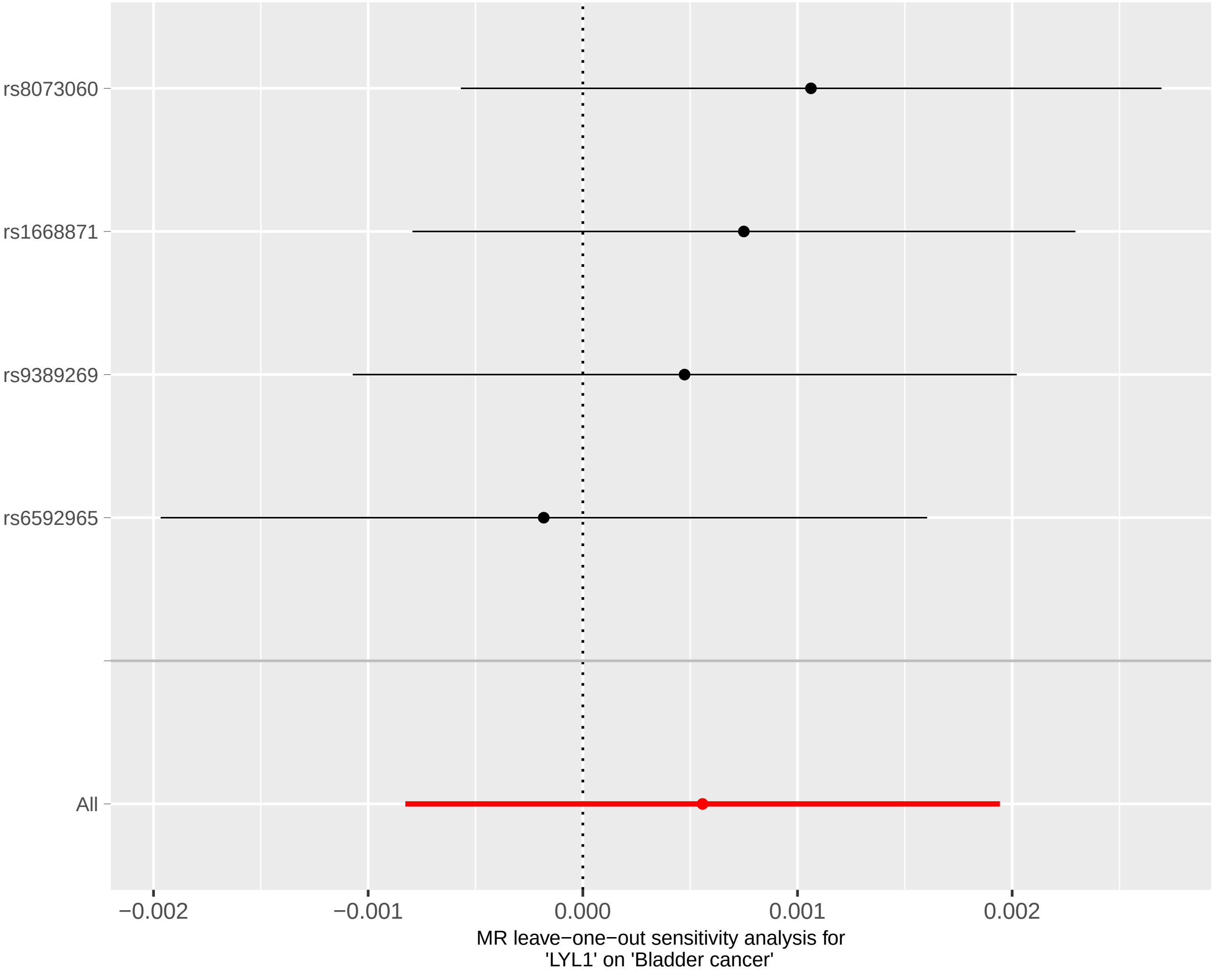
rs8073060
rs1668871
rs9389269
rs6592965
All
−0.002
−0.001
0.000
0.001
0.002
MR leave−one−out sensitivity analysis for
'LYL1' on 'Bladder cancer'

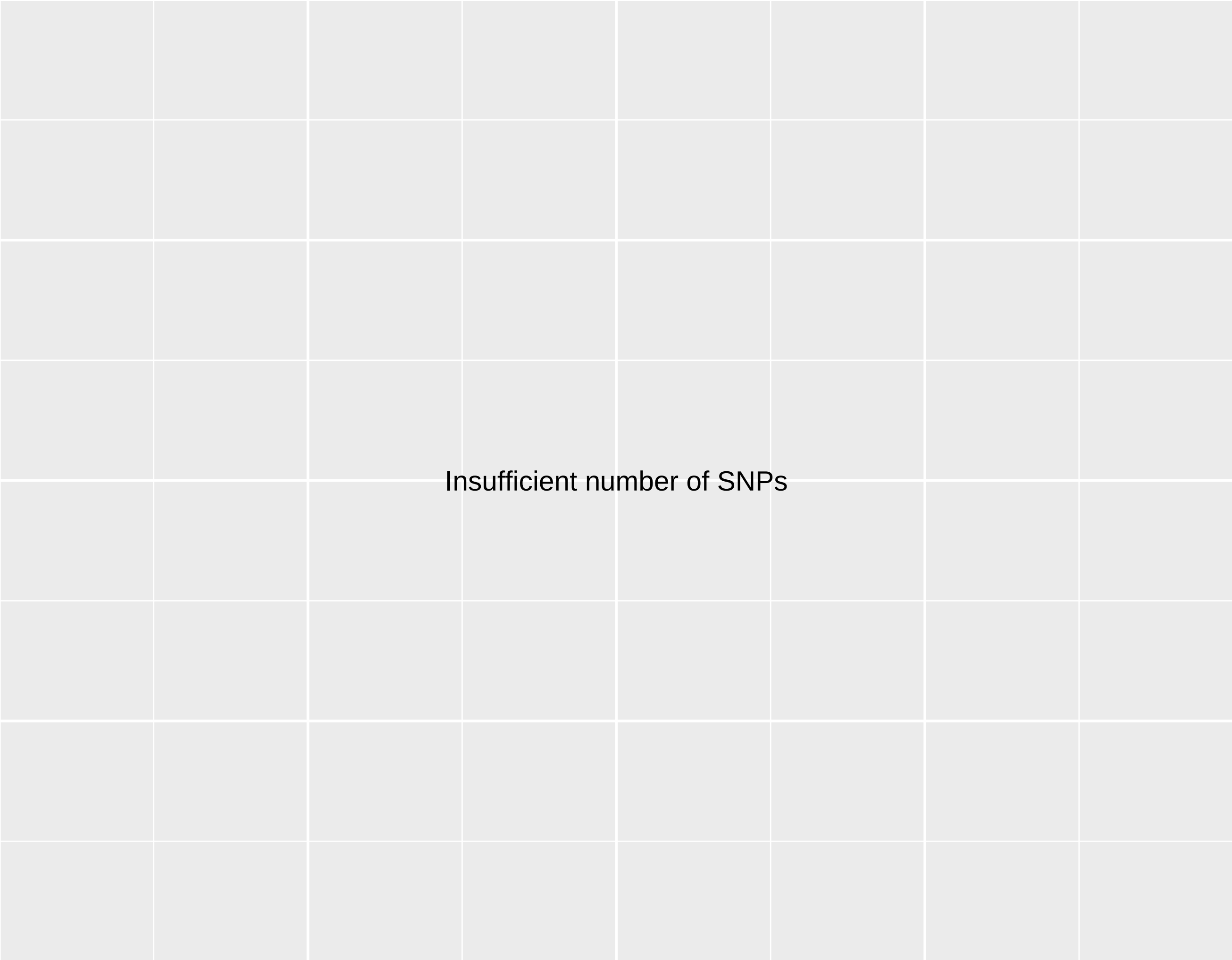
Insufficient number of SNPs

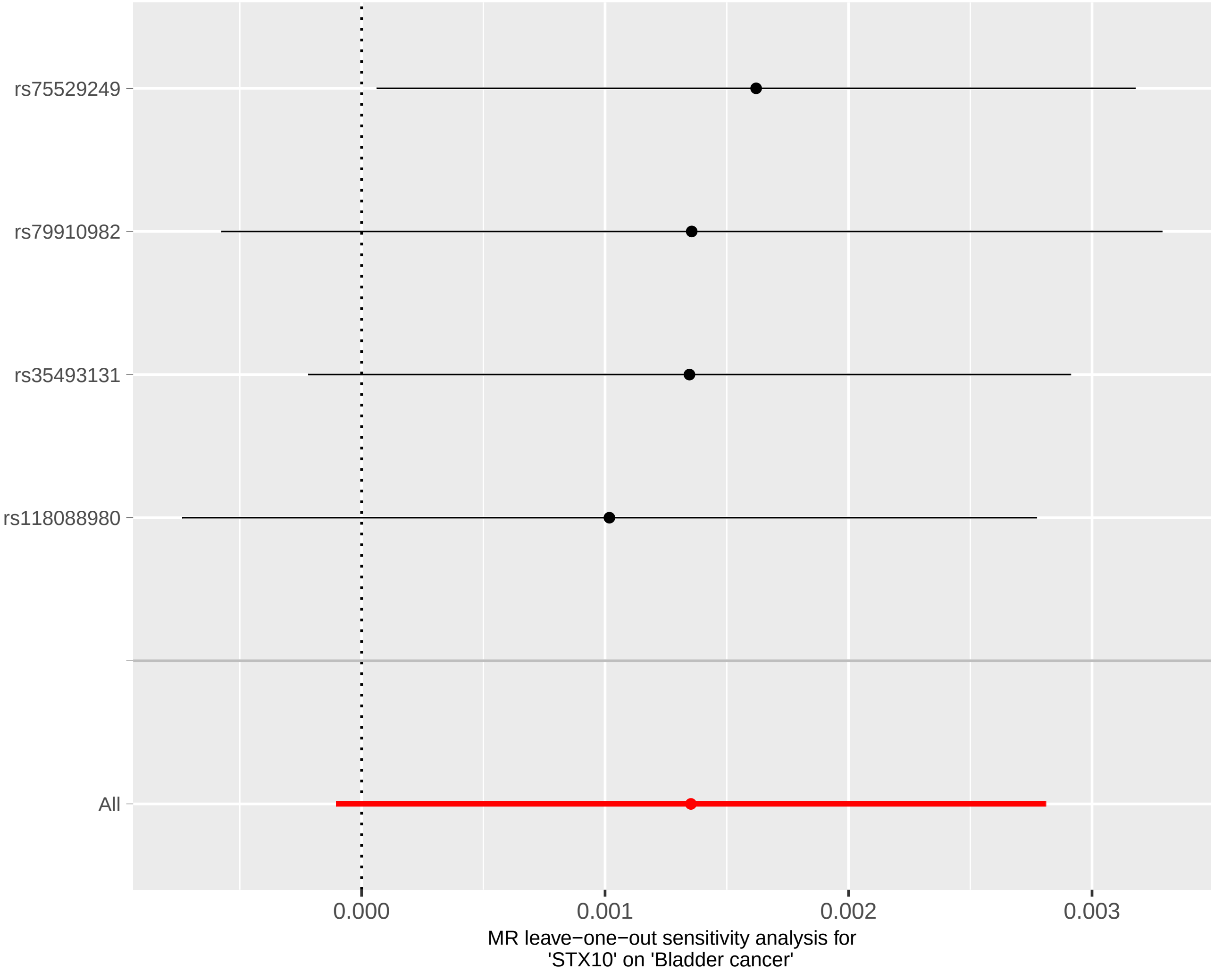

rs75529249
rs79910982
rs35493131
rs118088980
All
0.000
0.001
0.002
0.003
MR leave−one−out sensitivity analysis for
'STX10' on 'Bladder cancer'

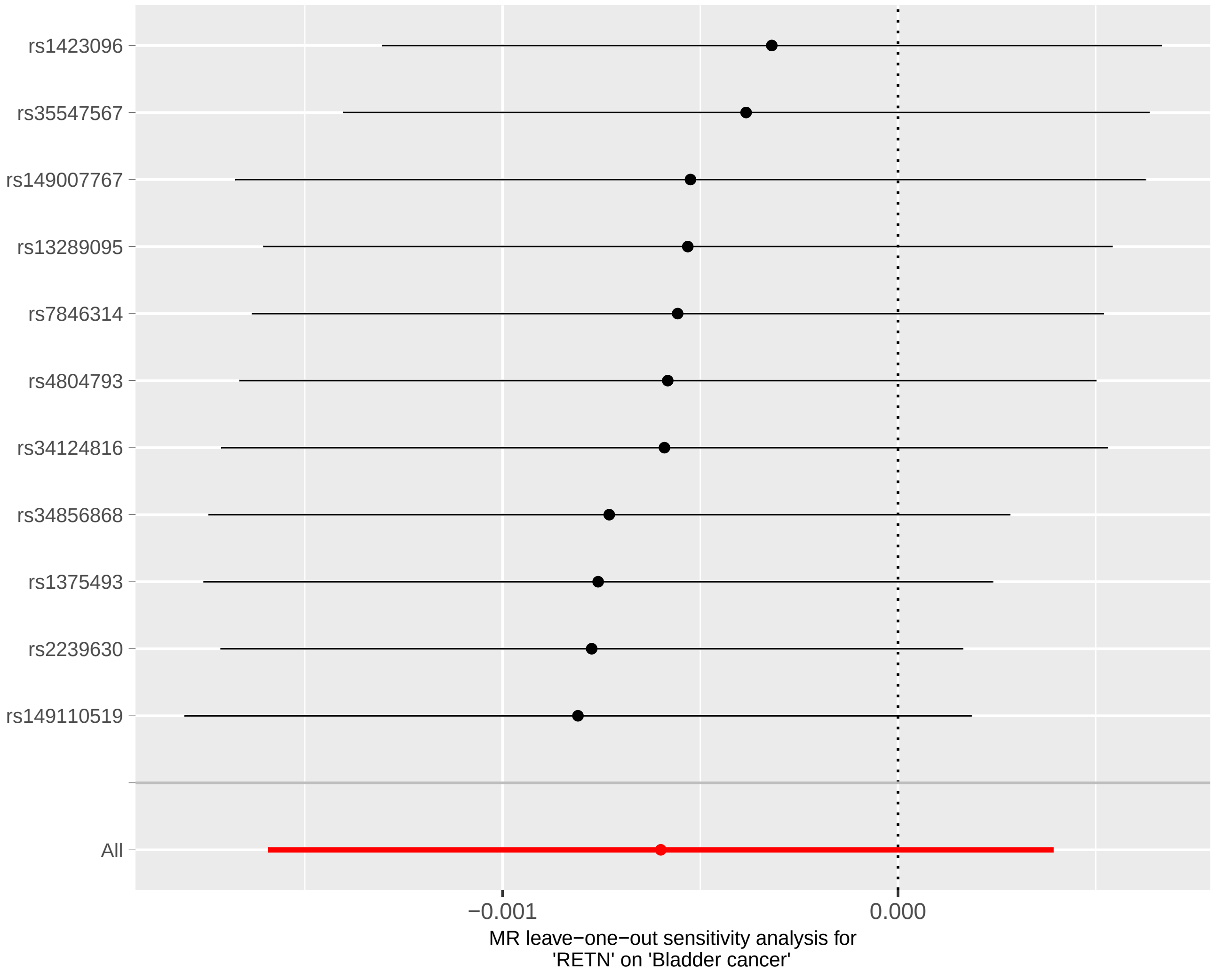

rs1423096
rs35547567
rs149007767
rs13289095
rs7846314
rs4804793
rs34124816
rs34856868
rs1375493
rs2239630
rs149110519
All
−0.001
0.000
MR leave−one−out sensitivity analysis for
'RETN' on 'Bladder cancer'

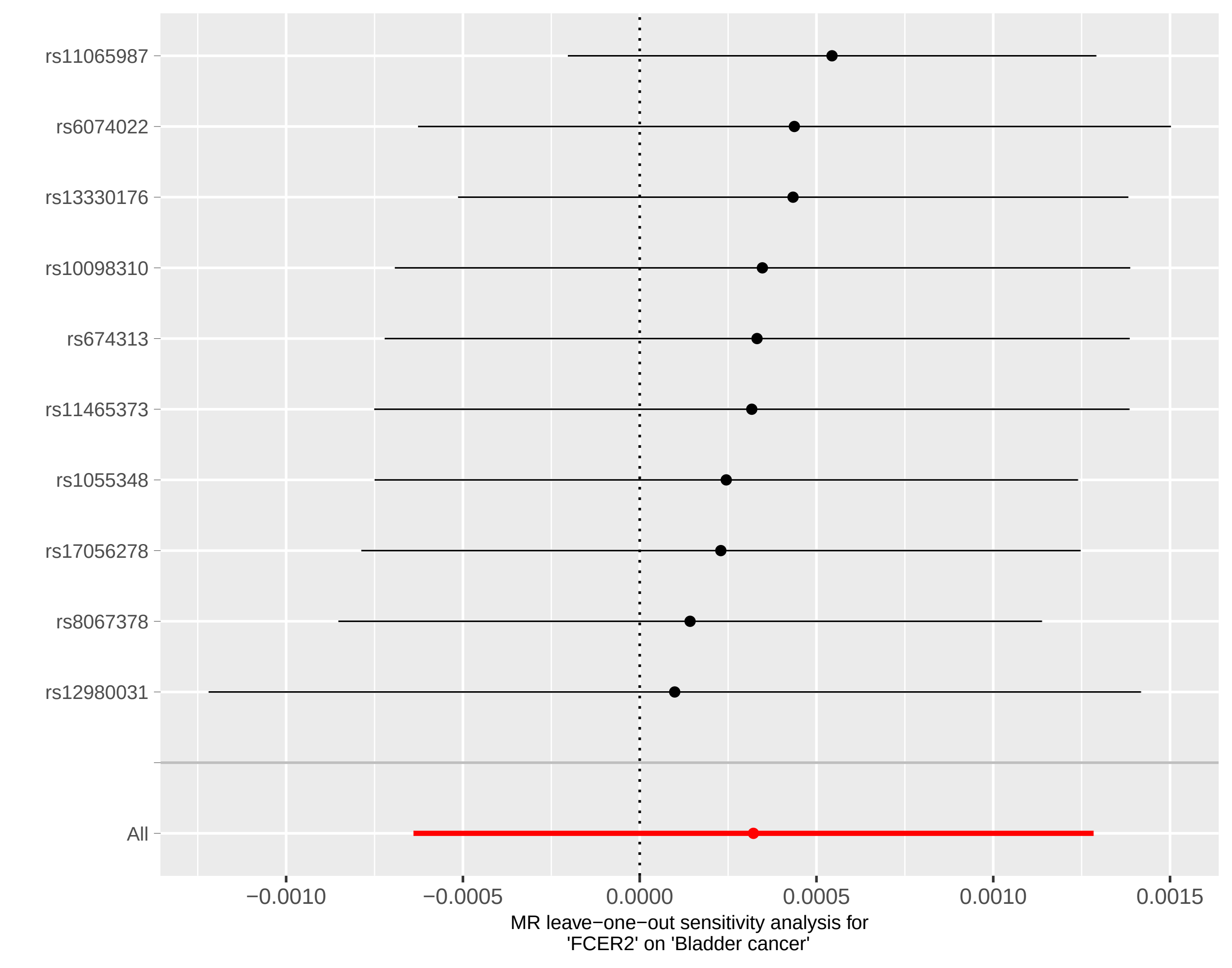

rs11065987
rs6074022
rs13330176
rs10098310
rs674313
rs11465373
rs1055348
rs17056278
rs8067378
rs12980031
All
−0.0010
−0.0005
0.0000
0.0005
0.0010
0.0015
MR leave−one−out sensitivity analysis for
'FCER2' on 'Bladder cancer'

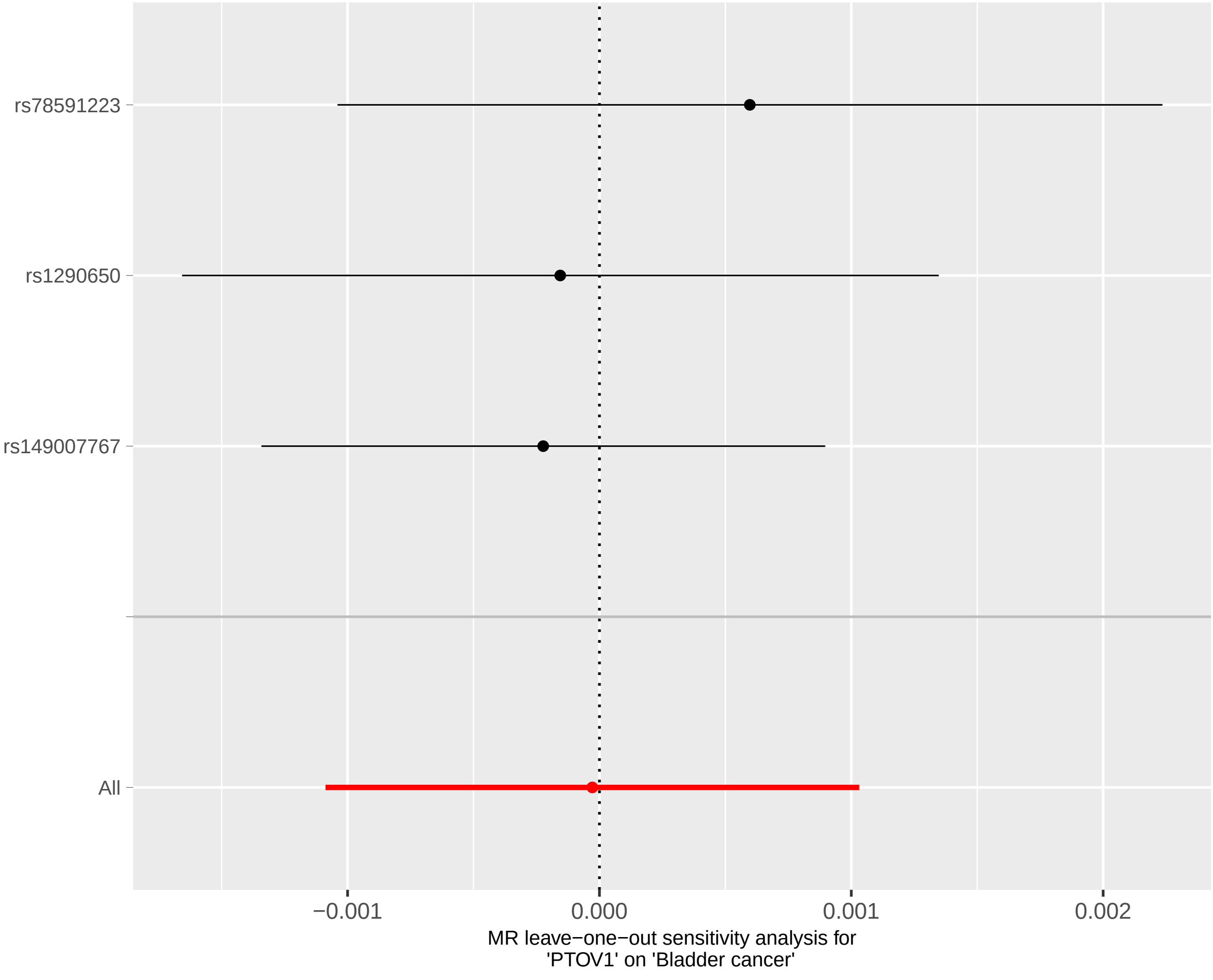
rs78591223
rs1290650
rs149007767
All
−0.001
0.000
0.001
0.002
MR leave−one−out sensitivity analysis for
'PTOV1' on 'Bladder cancer'

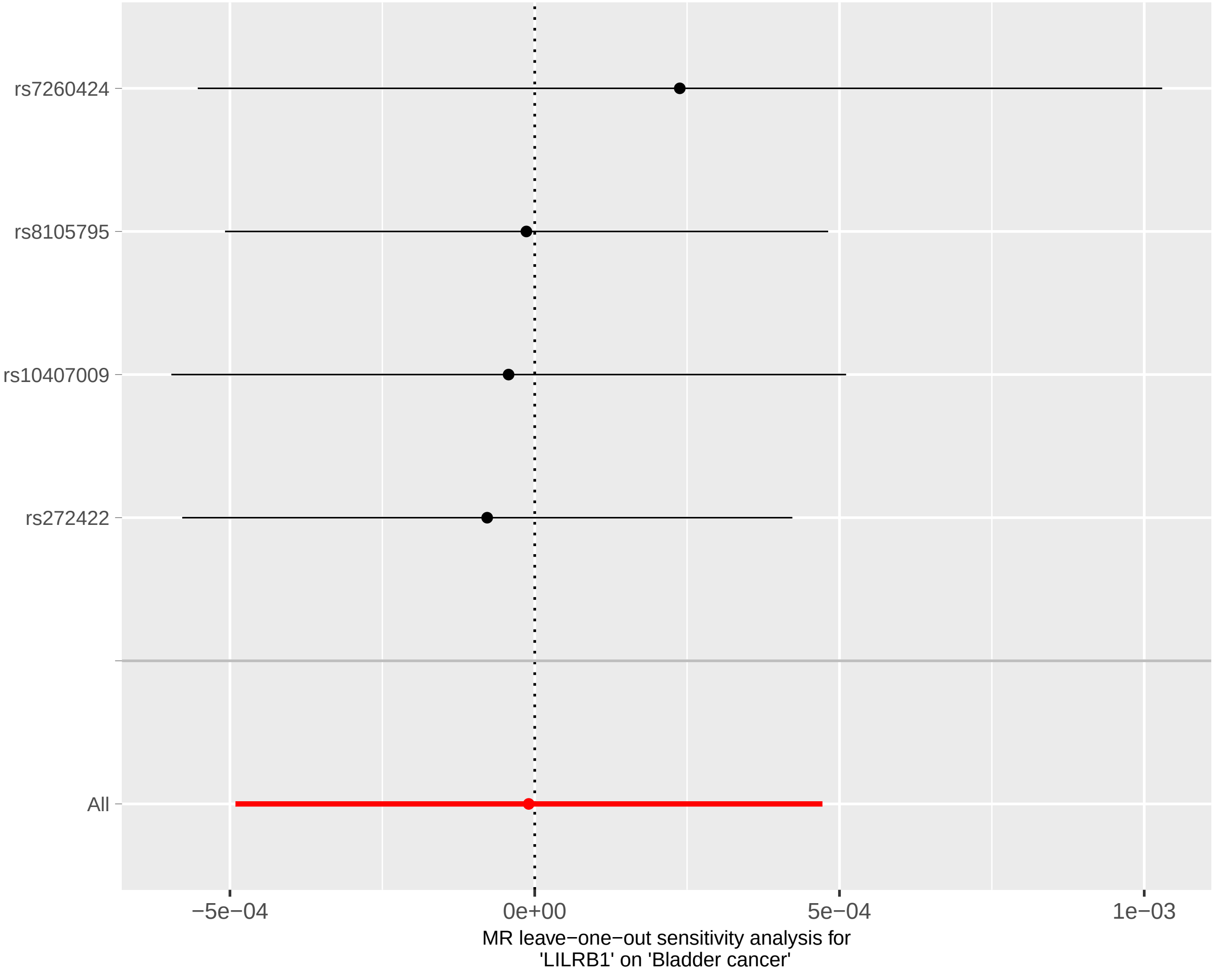

rs7260424
rs8105795
rs10407009
rs272422
All
−5e−04
0e+00
5e−04
1e−03
MR leave−one−out sensitivity analysis for
'LILRB1' on 'Bladder cancer'

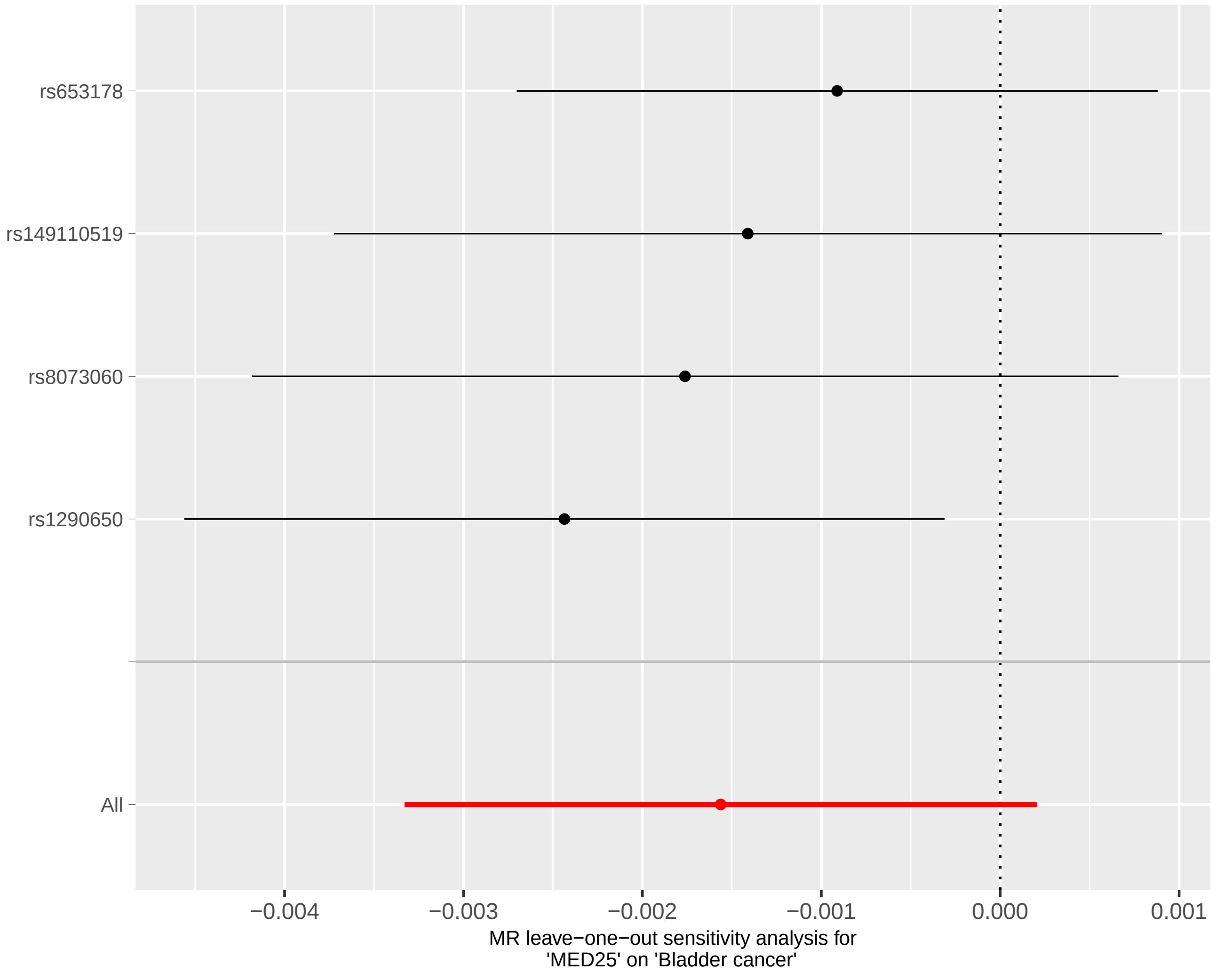
rs653178
rs149110519
rs8073060
rs1290650
All
−0.004
−0.003
−0.002
−0.001
0.000
0.001
MR leave−one−out sensitivity analysis for
'MED25' on 'Bladder cancer'

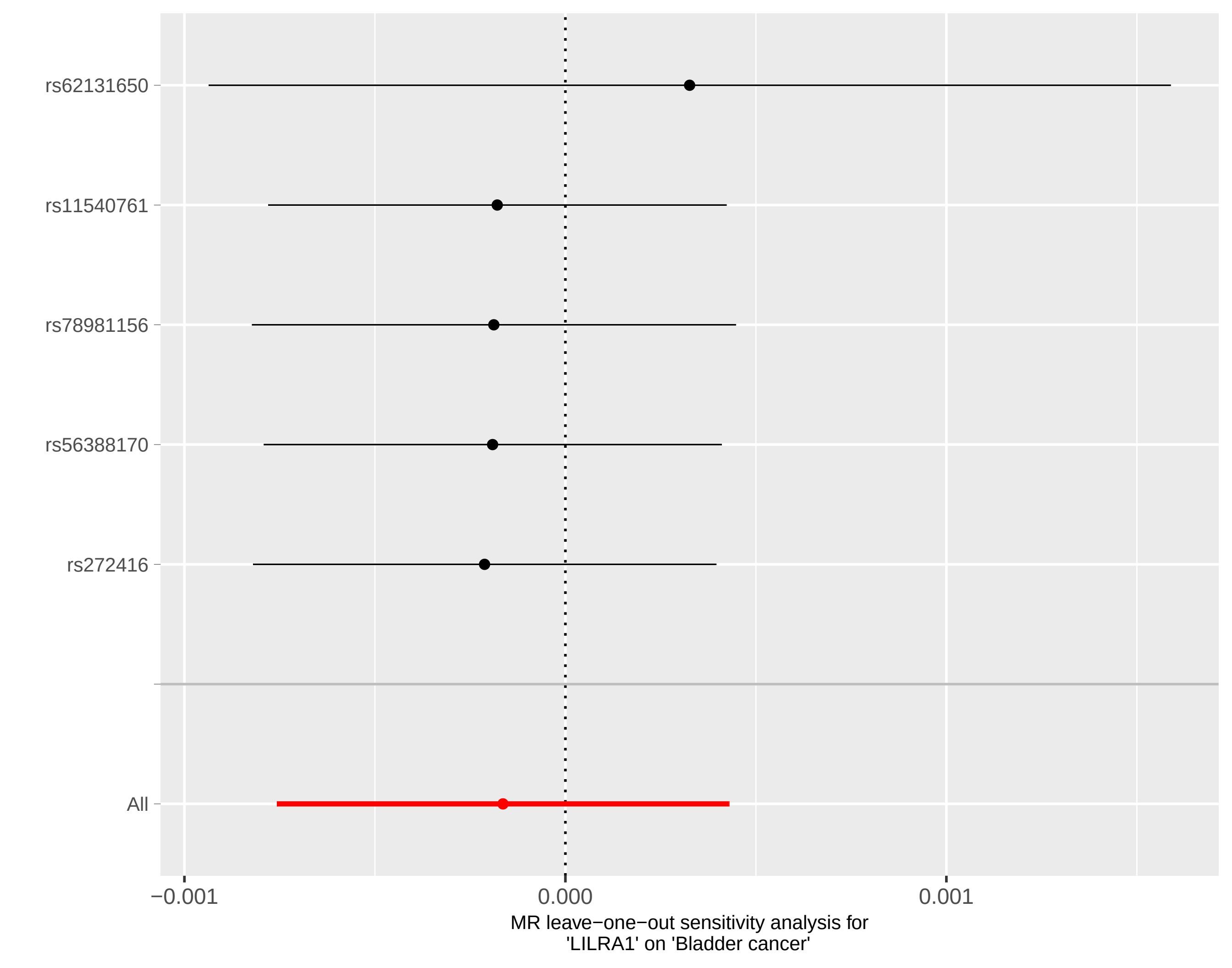
rs62131650
rs11540761
rs78981156
rs56388170
rs272416
All
−0.001
0.000
0.001
MR leave−one−out sensitivity analysis for
'LILRA1' on 'Bladder cancer'

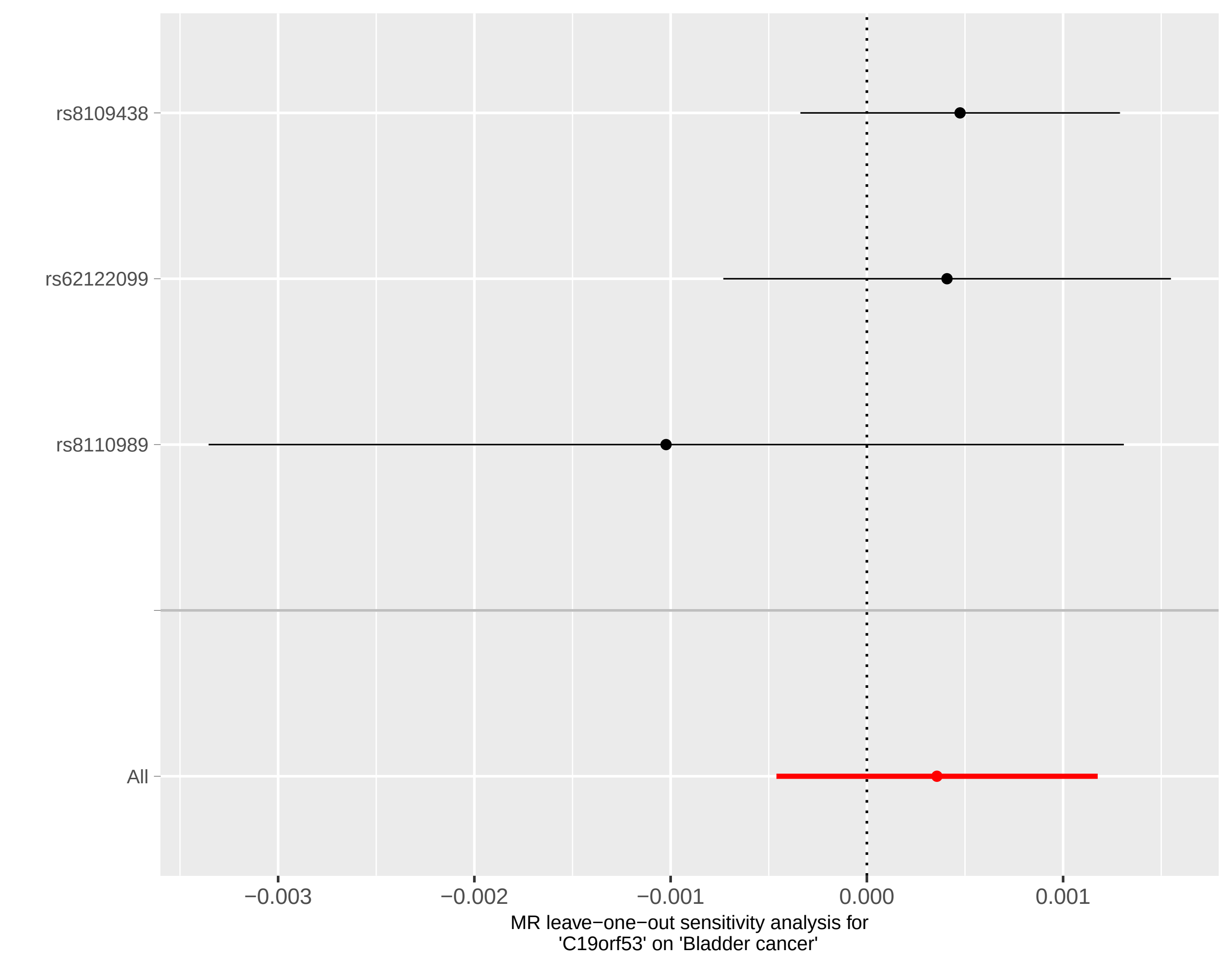
rs8109438
rs62122099
rs8110989
All
−0.003
−0.002
−0.001
0.000
0.001
MR leave−one−out sensitivity analysis for
'C19orf53' on 'Bladder cancer'

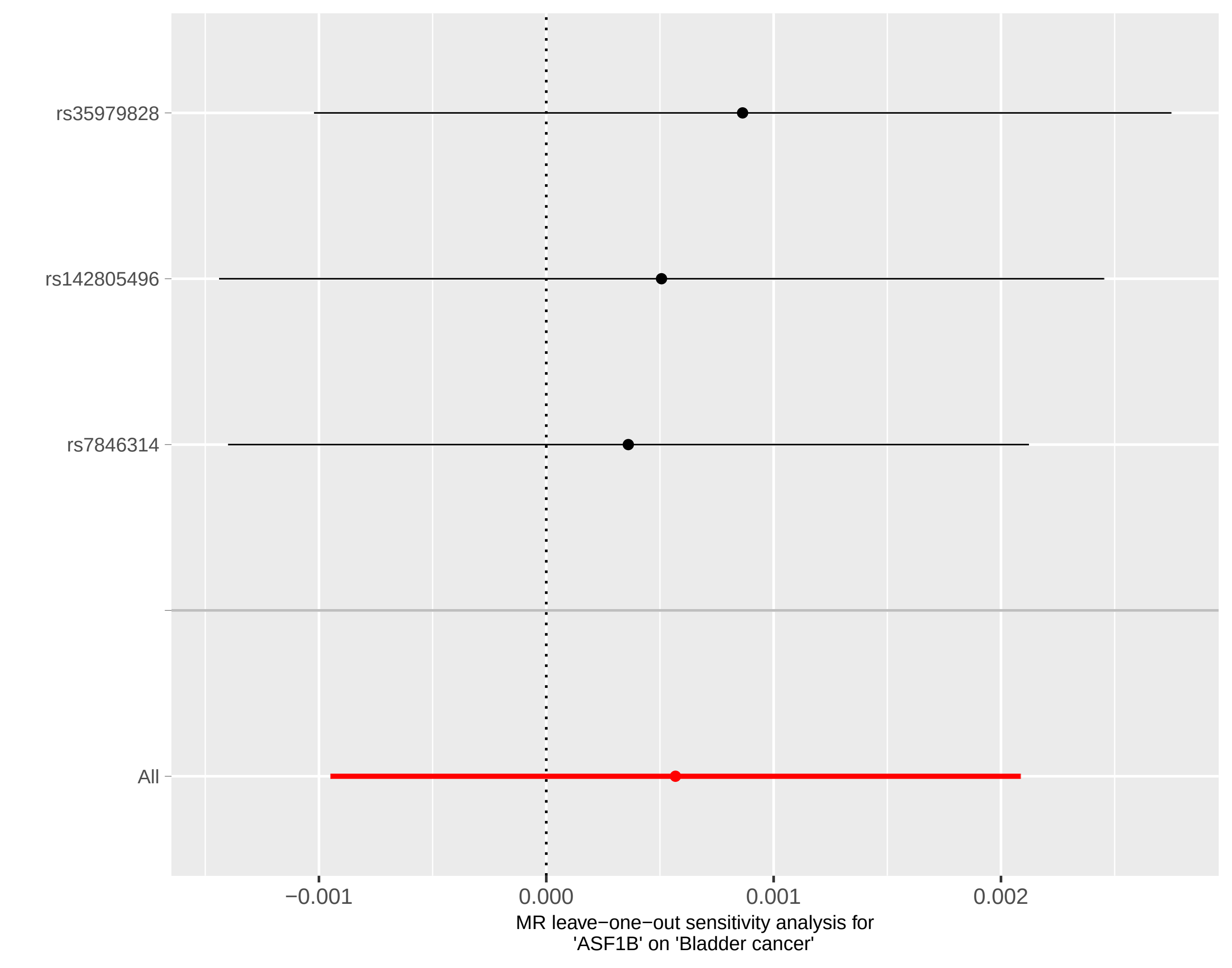

rs35979828
rs142805496
rs7846314
All
−0.001
0.000
0.001
0.002
MR leave−one−out sensitivity analysis for
'ASF1B' on 'Bladder cancer'

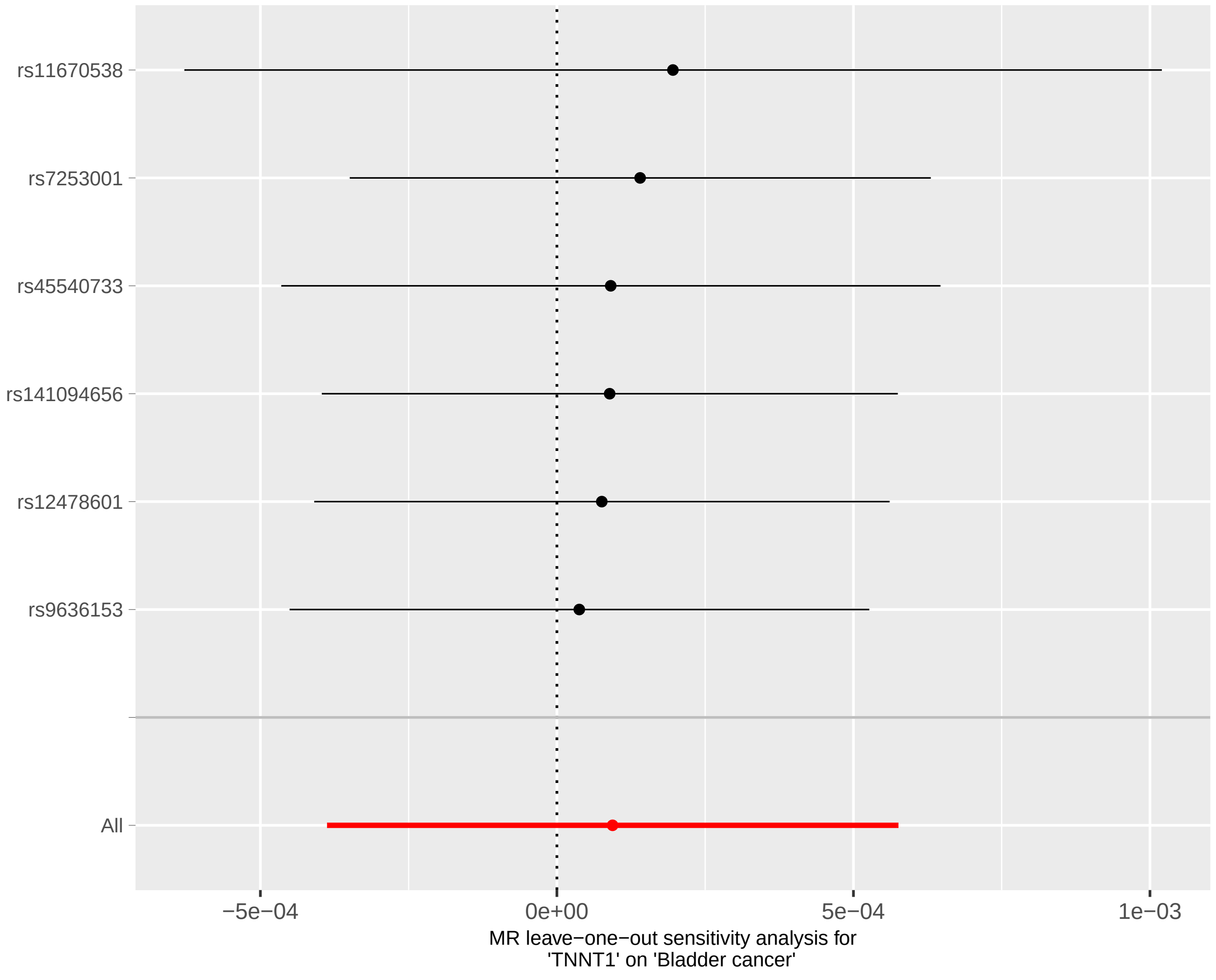
rs11670538
rs7253001
rs45540733
rs141094656
rs12478601
rs9636153
All
−5e−04
0e+00
5e−04
1e−03
MR leave−one−out sensitivity analysis for
'TNNT1' on 'Bladder cancer'

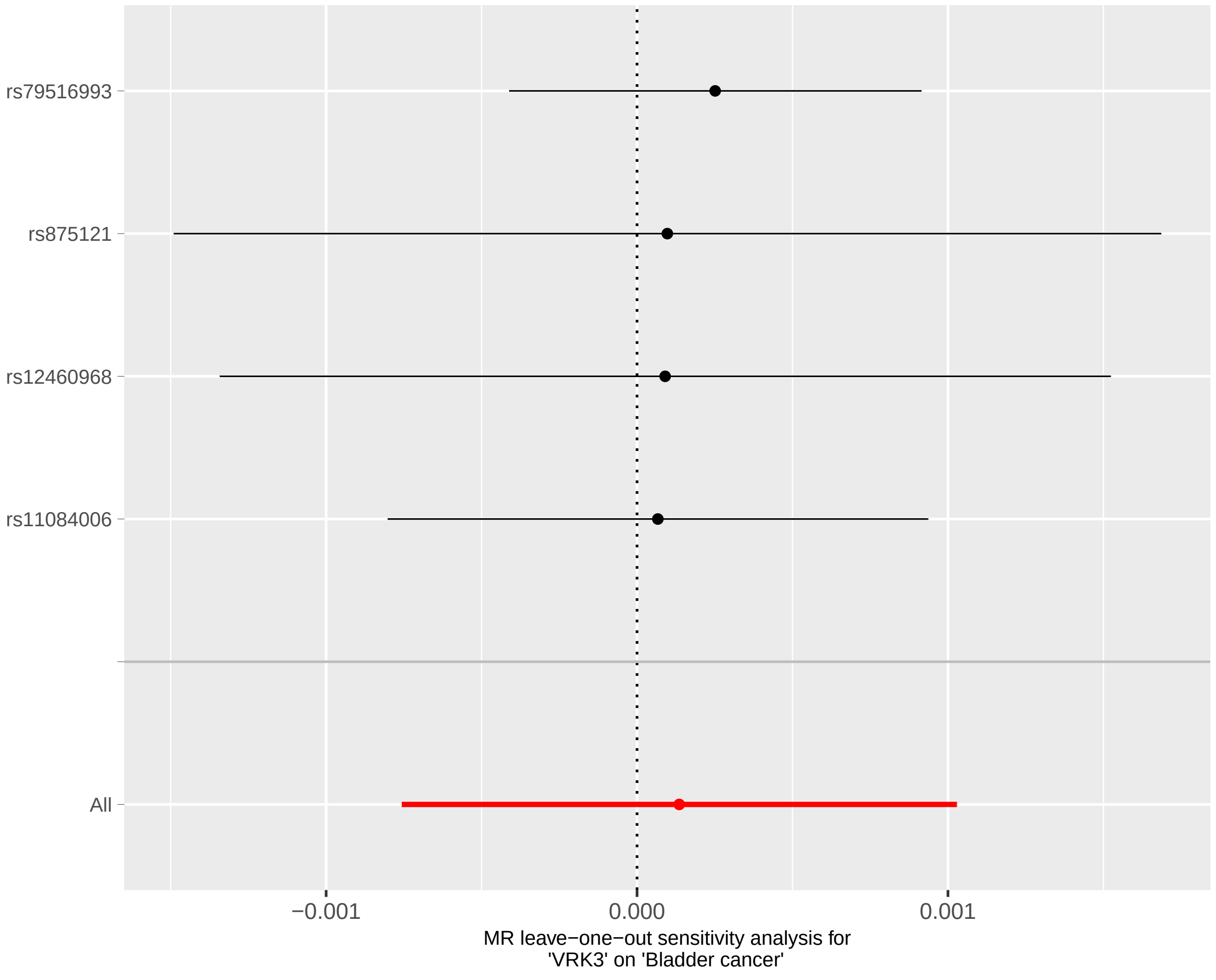
rs79516993
rs875121
rs12460968
rs11084006
All
−0.001
0.000
0.001
MR leave−one−out sensitivity analysis for
'VRK3' on 'Bladder cancer'

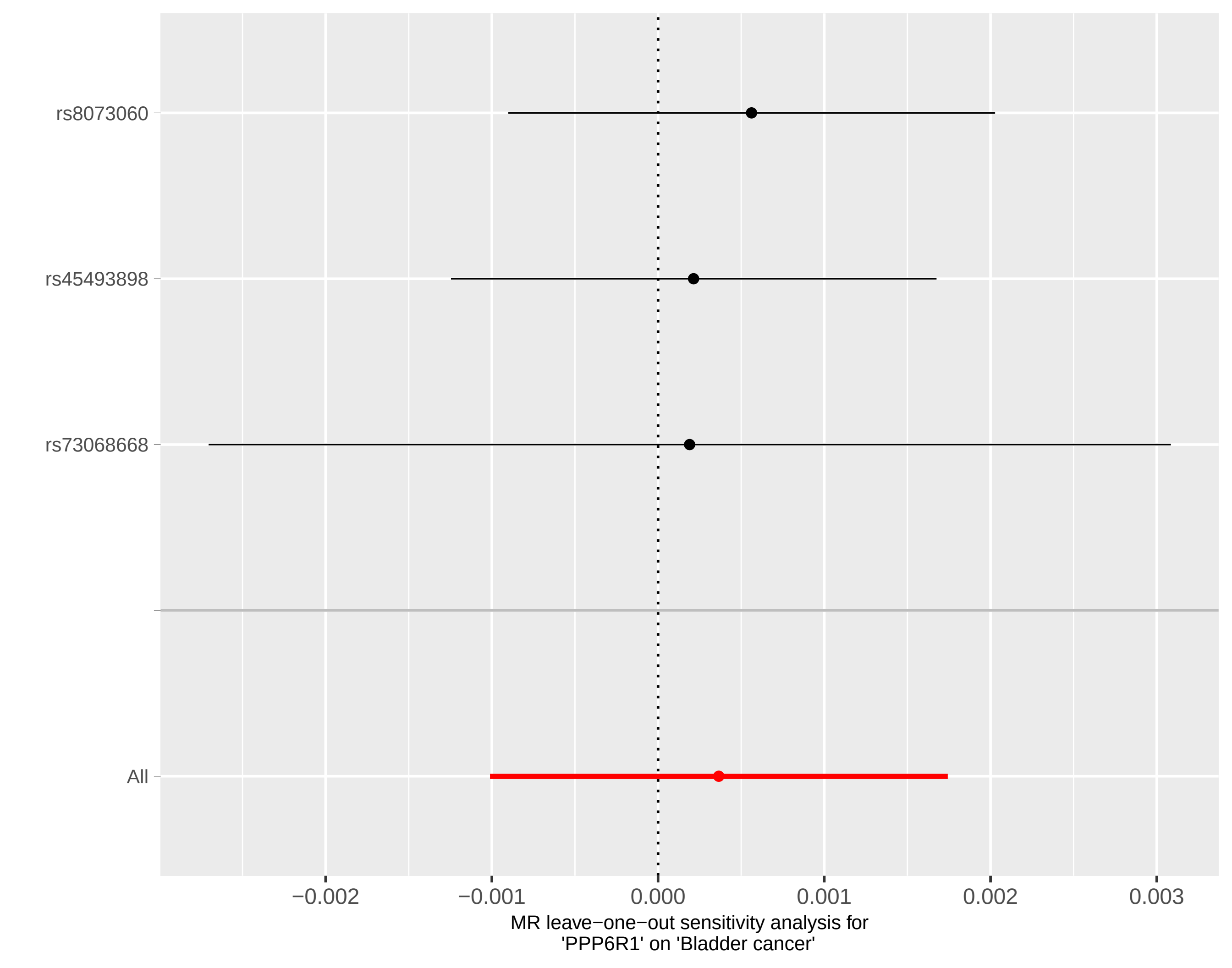
rs8073060
rs45493898
rs73068668
All
−0.002
−0.001
0.000
0.001
0.002
0.003
MR leave−one−out sensitivity analysis for
'PPP6R1' on 'Bladder cancer'

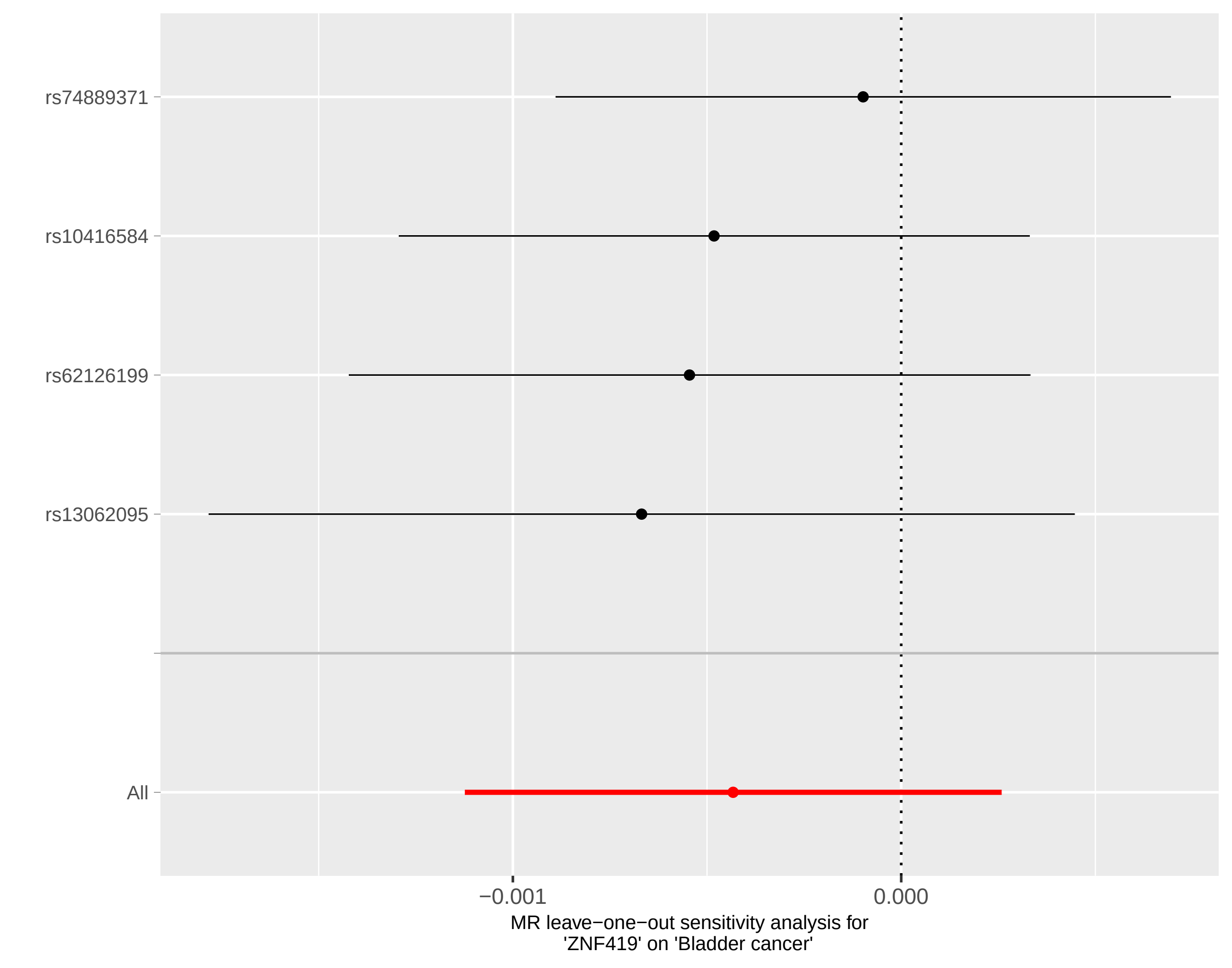
rs74889371
rs10416584
rs62126199
rs13062095
All
−0.001
0.000
MR leave−one−out sensitivity analysis for
'ZNF419' on 'Bladder cancer'

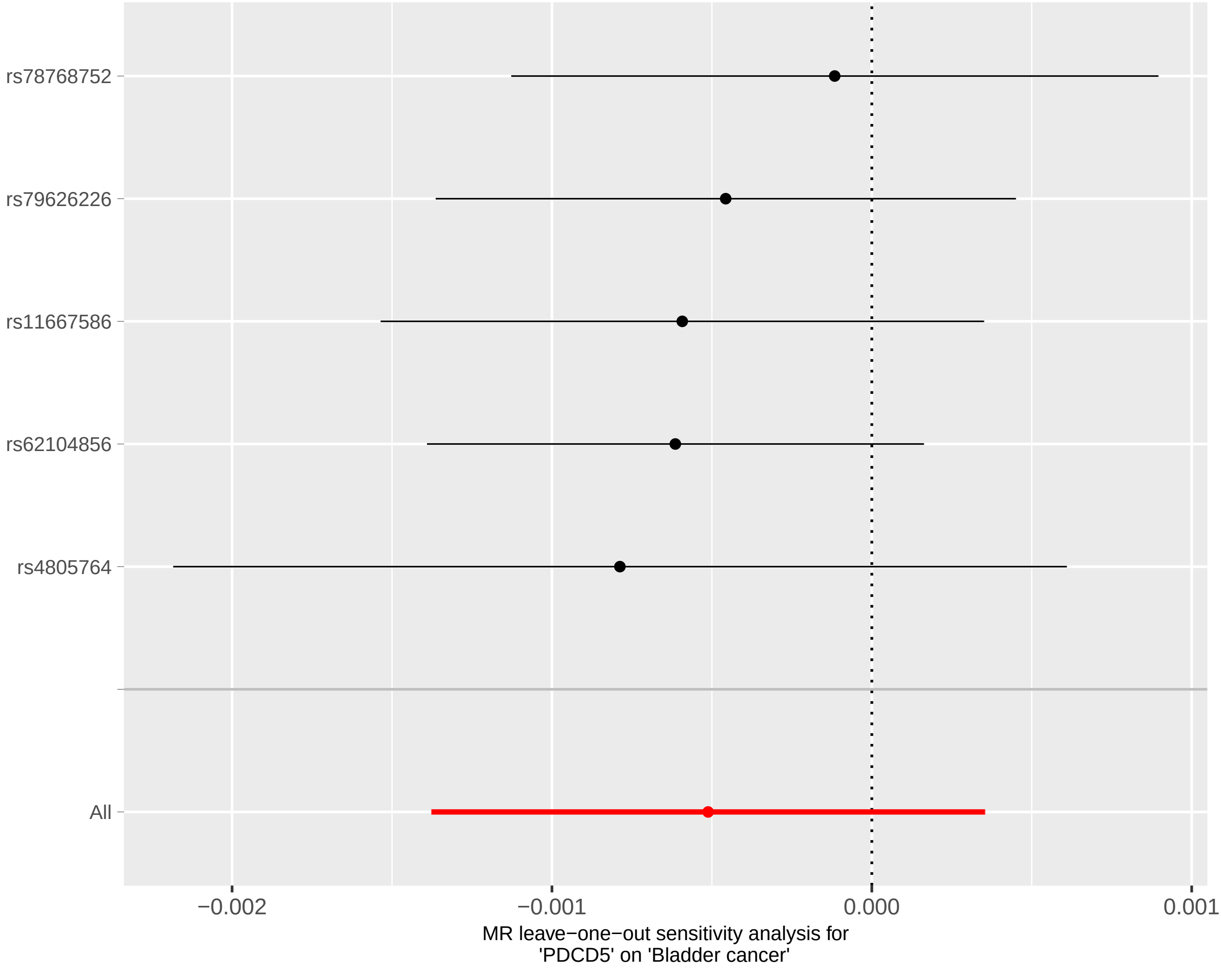

rs78768752
rs79626226
rs11667586
rs62104856
rs4805764
All
−0.002
−0.001
0.000
0.001
MR leave−one−out sensitivity analysis for
'PDCD5' on 'Bladder cancer'

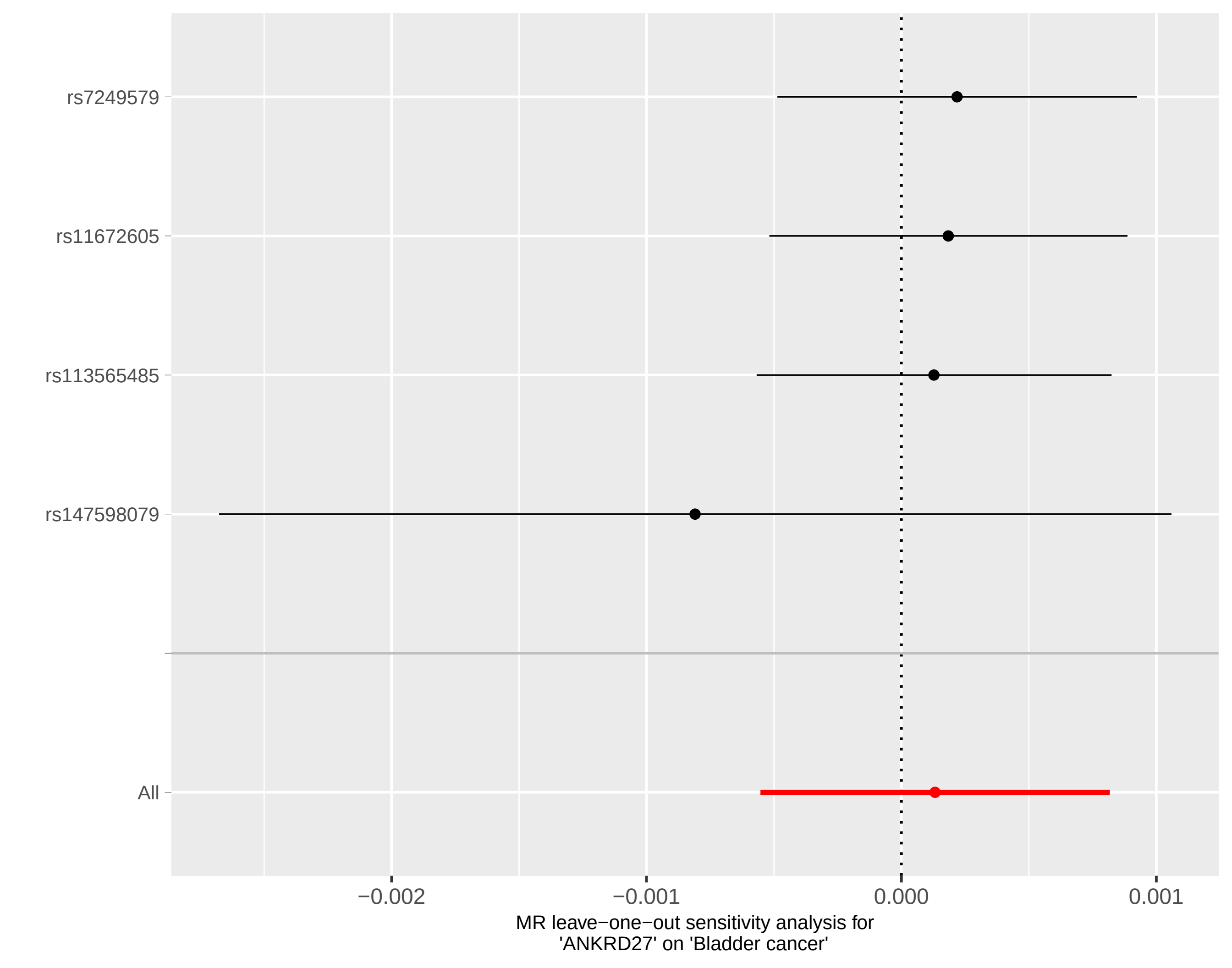
rs7249579
rs11672605
rs113565485
rs147598079
All
−0.002
−0.001
0.000
0.001
MR leave−one−out sensitivity analysis for
'ANKRD27' on 'Bladder cancer'

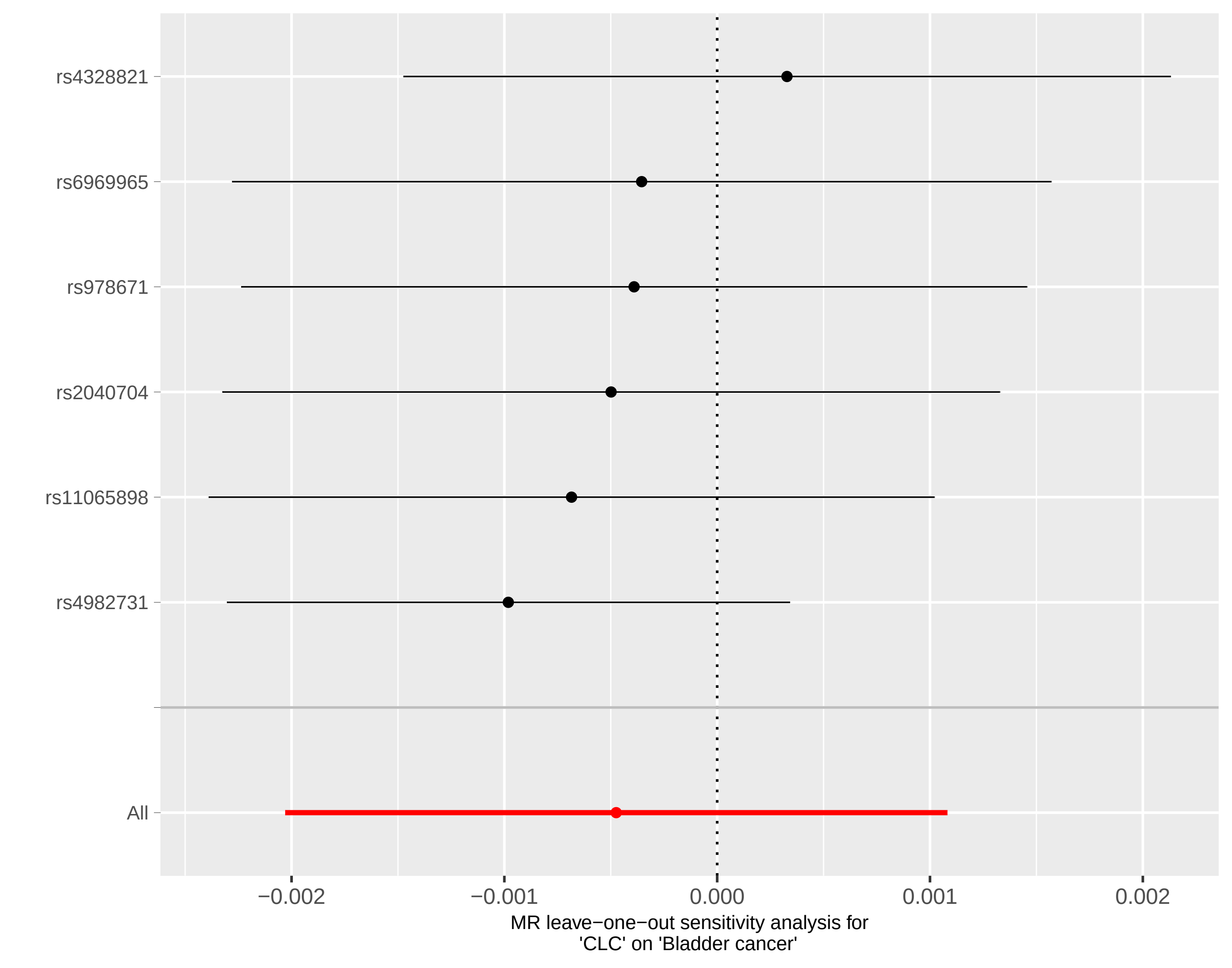

rs4328821
rs6969965
rs978671
rs2040704
rs11065898
rs4982731
All
−0.002
−0.001
0.000
0.001
0.002
MR leave−one−out sensitivity analysis for
'CLC' on 'Bladder cancer'

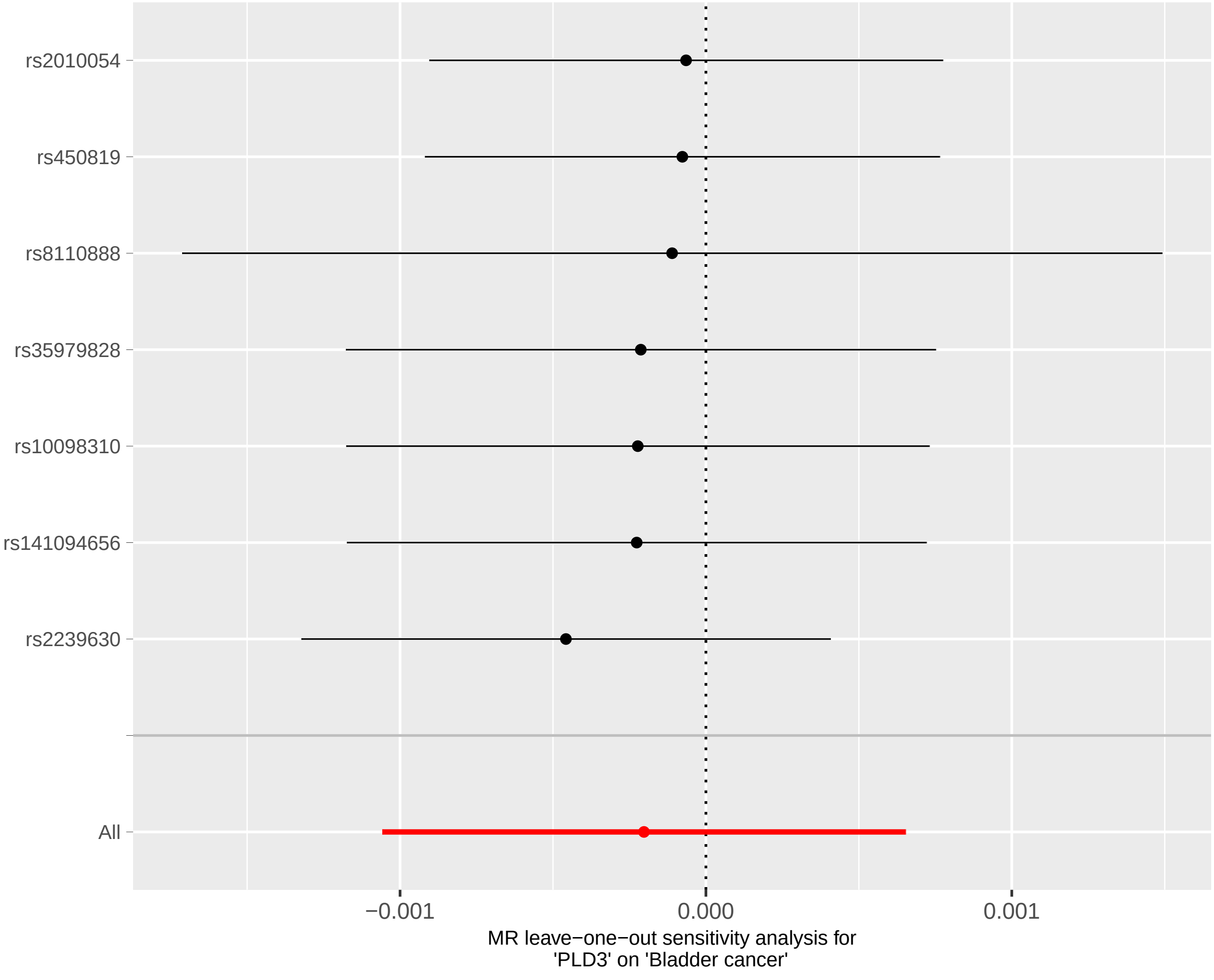
rs2010054
rs450819
rs8110888
rs35979828
rs10098310
rs141094656
rs2239630
All
−0.001
0.000
0.001
MR leave−one−out sensitivity analysis for
'PLD3' on 'Bladder cancer'

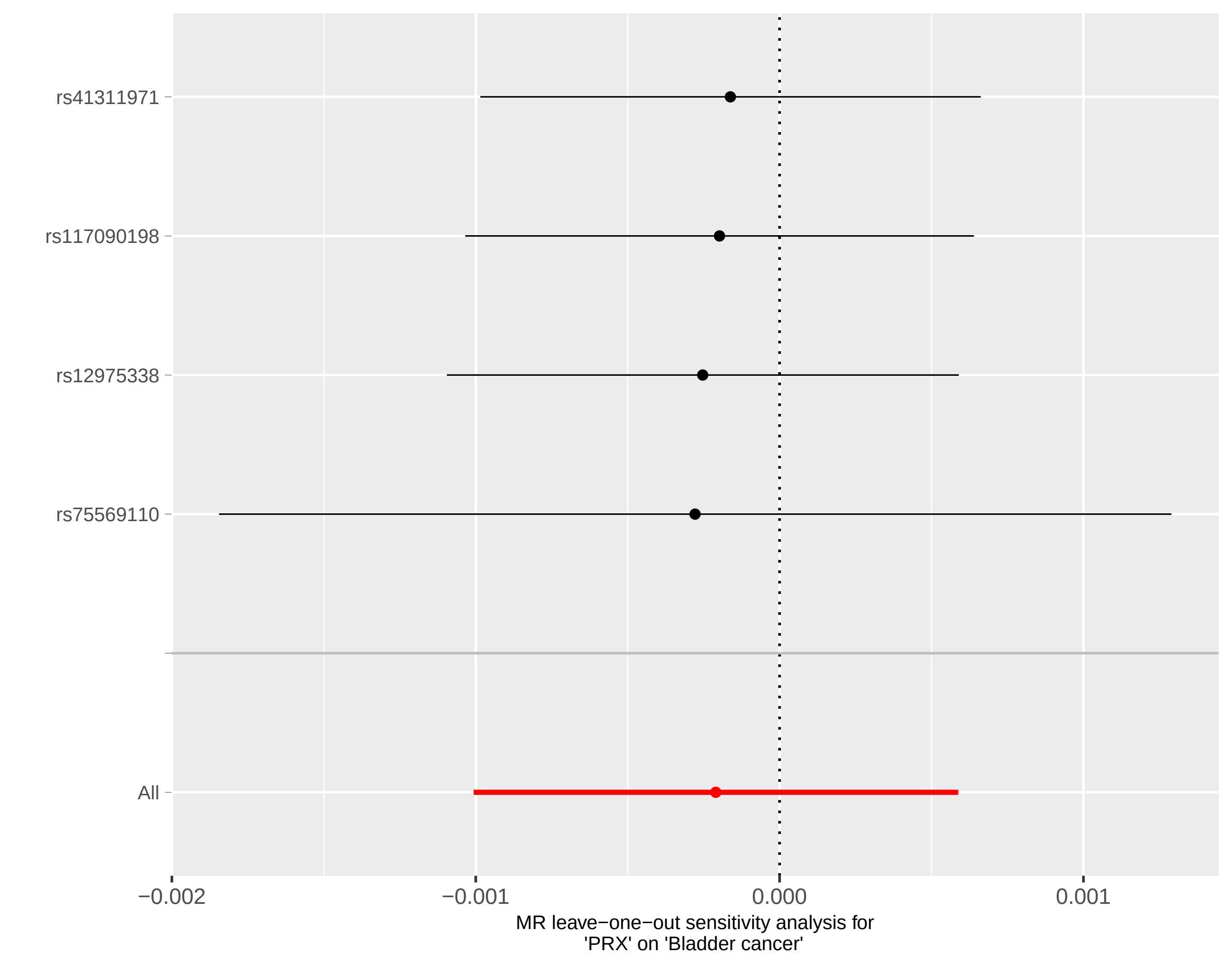
rs41311971
rs117090198
rs12975338
rs75569110
All
−0.002
−0.001
0.000
0.001
MR leave−one−out sensitivity analysis for
'PRX' on 'Bladder cancer'

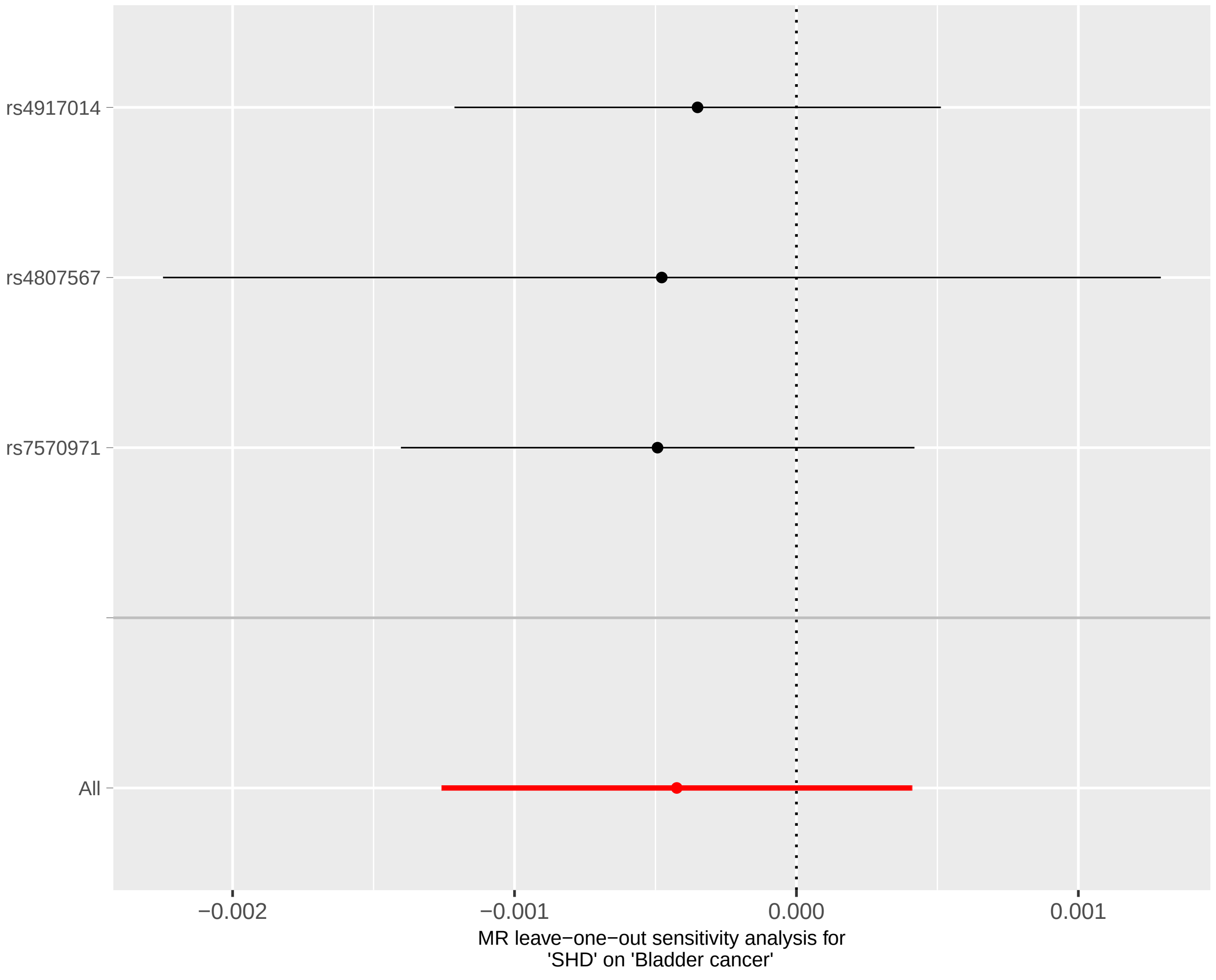
rs4917014
rs4807567
rs7570971
All
−0.002
−0.001
0.000
0.001
MR leave−one−out sensitivity analysis for
'SHD' on 'Bladder cancer'

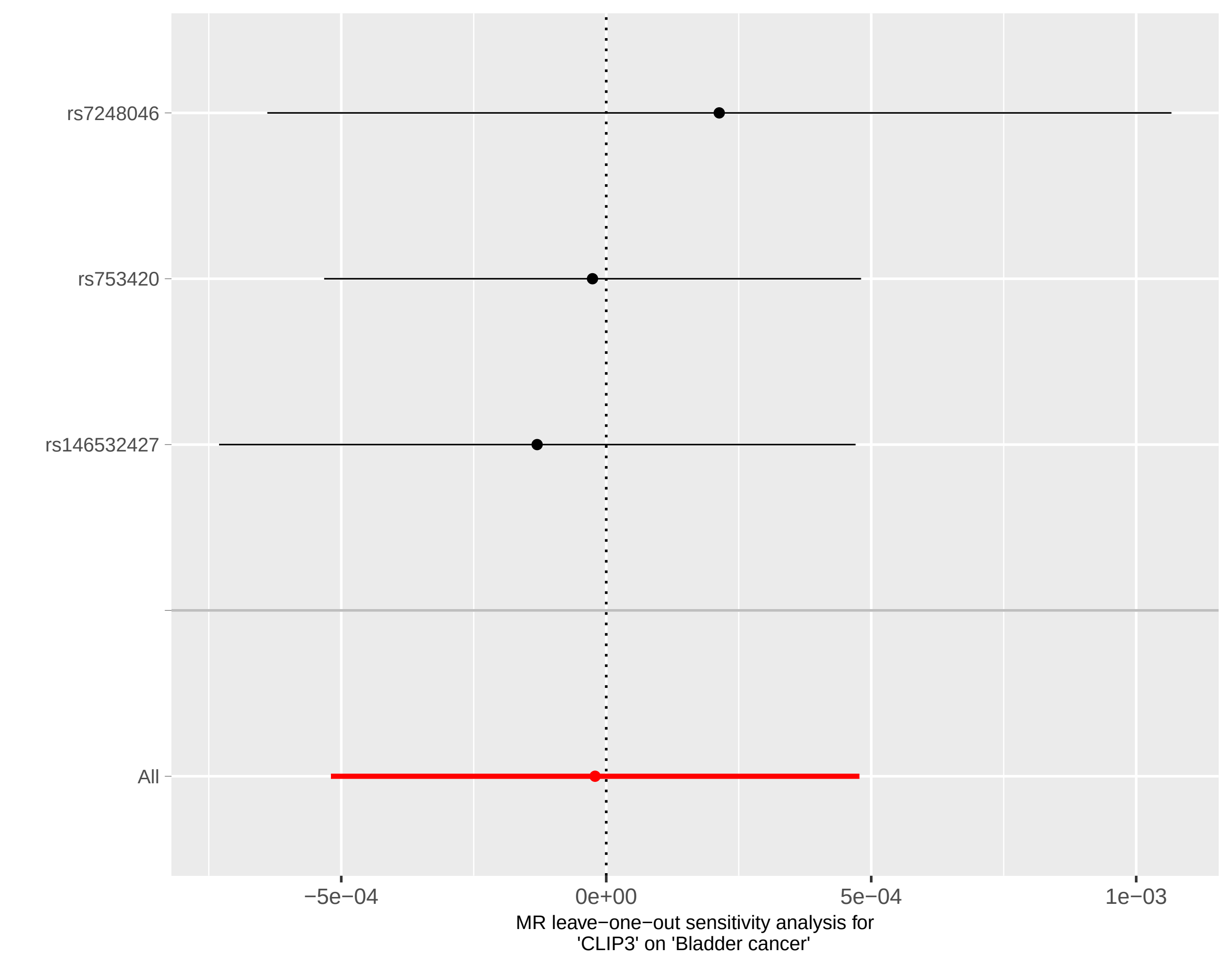
rs7248046
rs753420
rs146532427
All
−5e−04
0e+00
5e−04
1e−03
MR leave−one−out sensitivity analysis for
'CLIP3' on 'Bladder cancer'

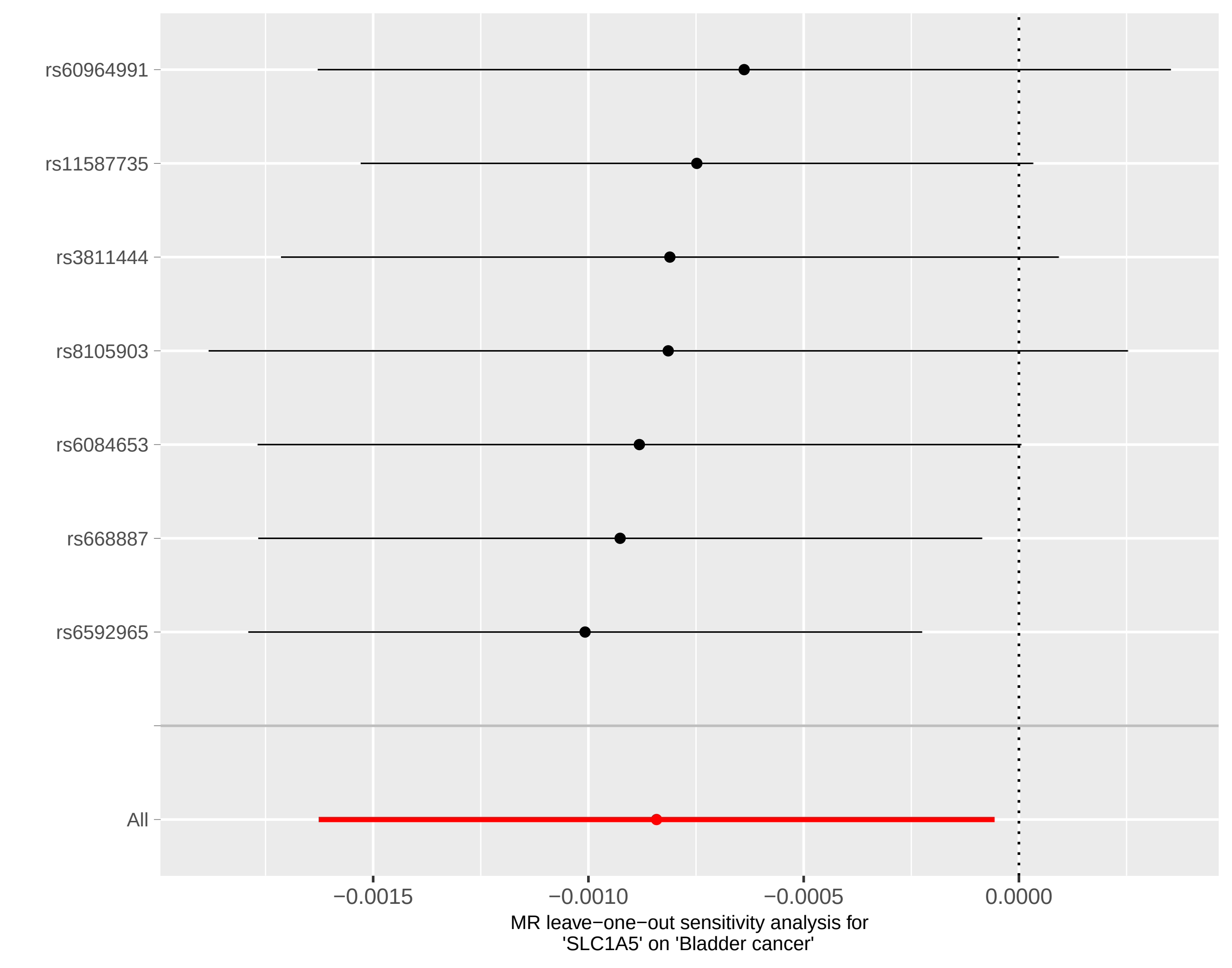
rs60964991
rs11587735
rs3811444
rs8105903
rs6084653
rs668887
rs6592965
All
−0.0015
−0.0010
−0.0005
0.0000
MR leave−one−out sensitivity analysis for
'SLC1A5' on 'Bladder cancer'

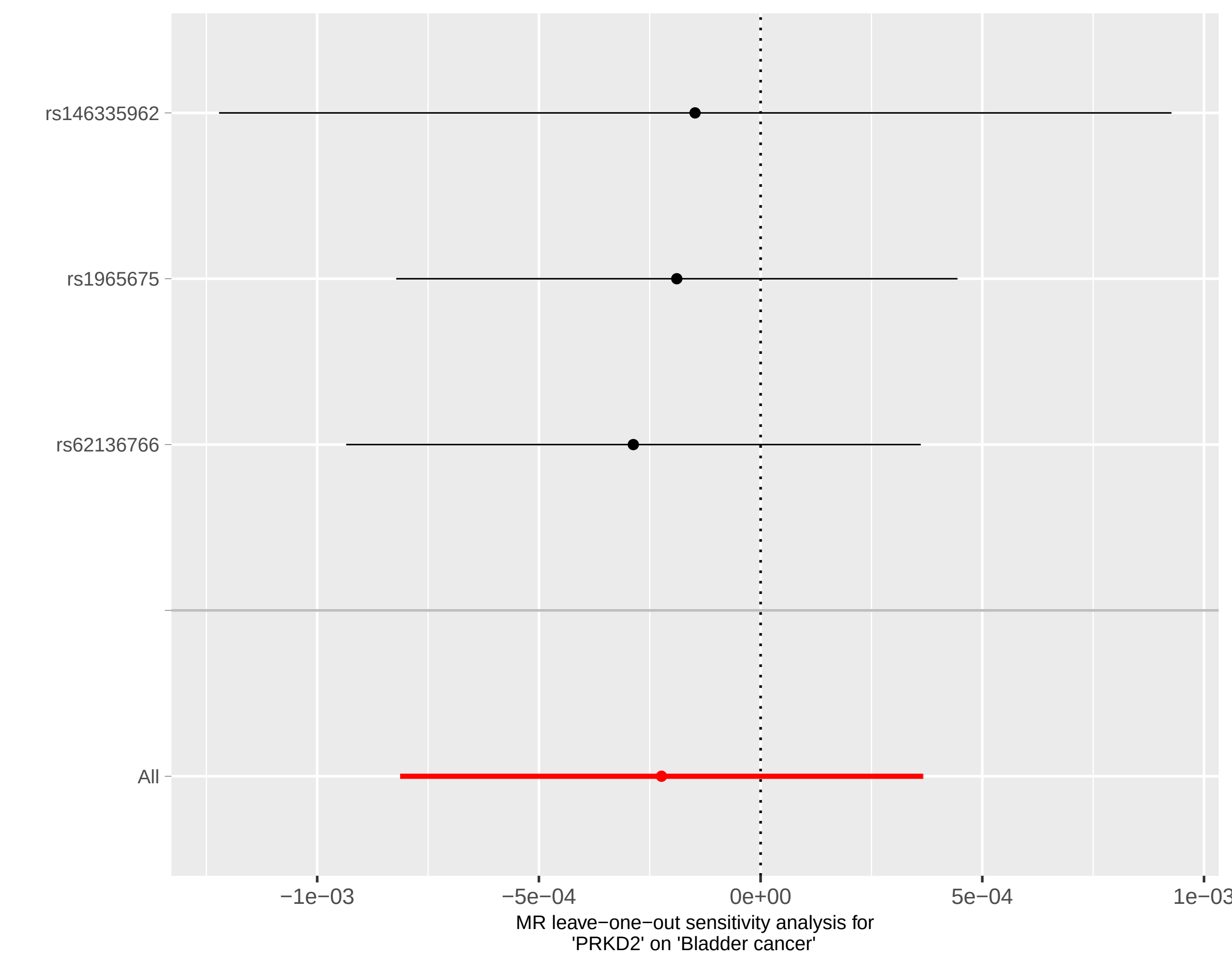
rs146335962
rs1965675
rs62136766
All
−1e−03
−5e−04
0e+00
5e−04
1e−03
MR leave−one−out sensitivity analysis for
'PRKD2' on 'Bladder cancer'

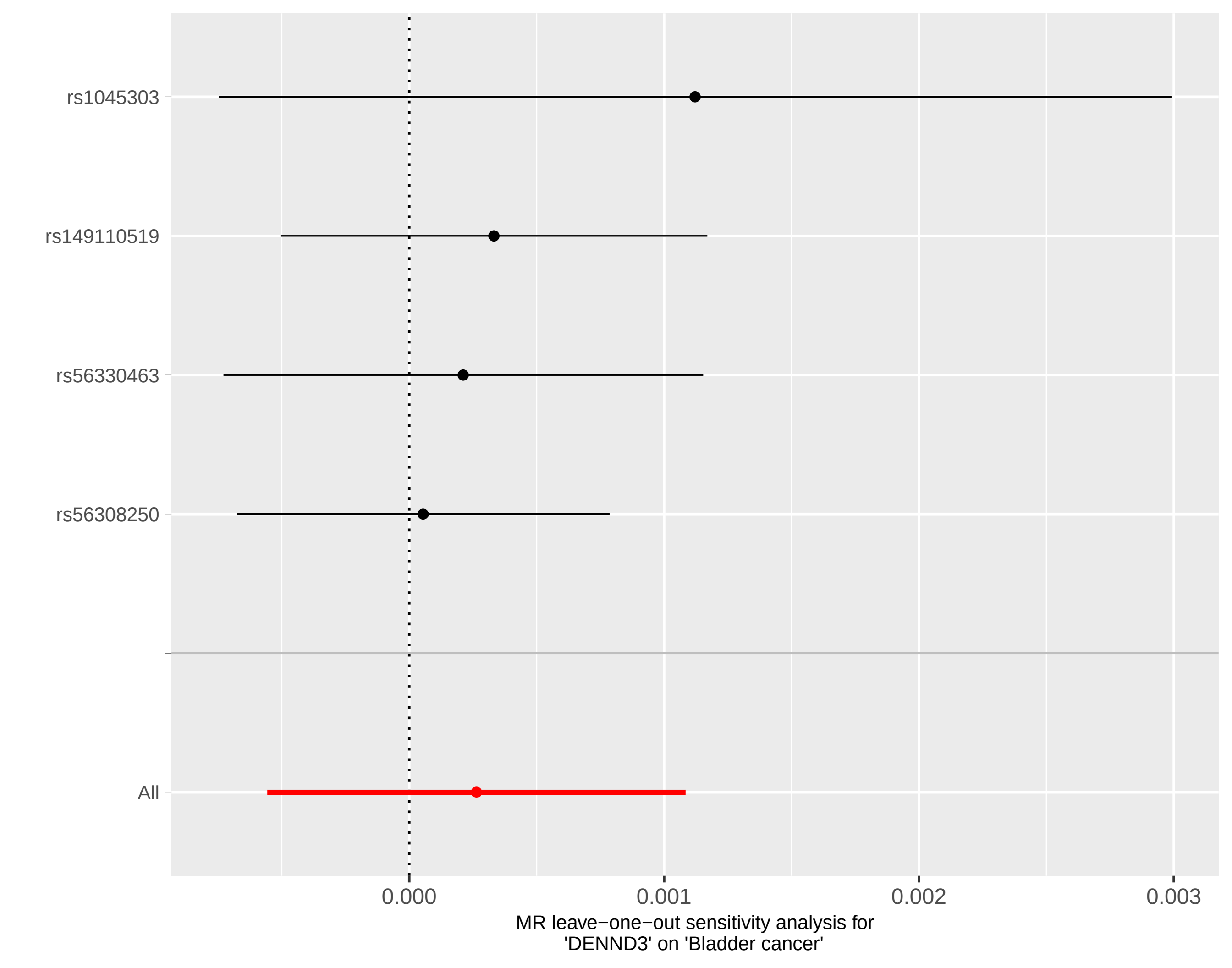
rs1045303
rs149110519
rs56330463
rs56308250
All
0.000
0.001
0.002
0.003
MR leave−one−out sensitivity analysis for
'DENND3' on 'Bladder cancer'

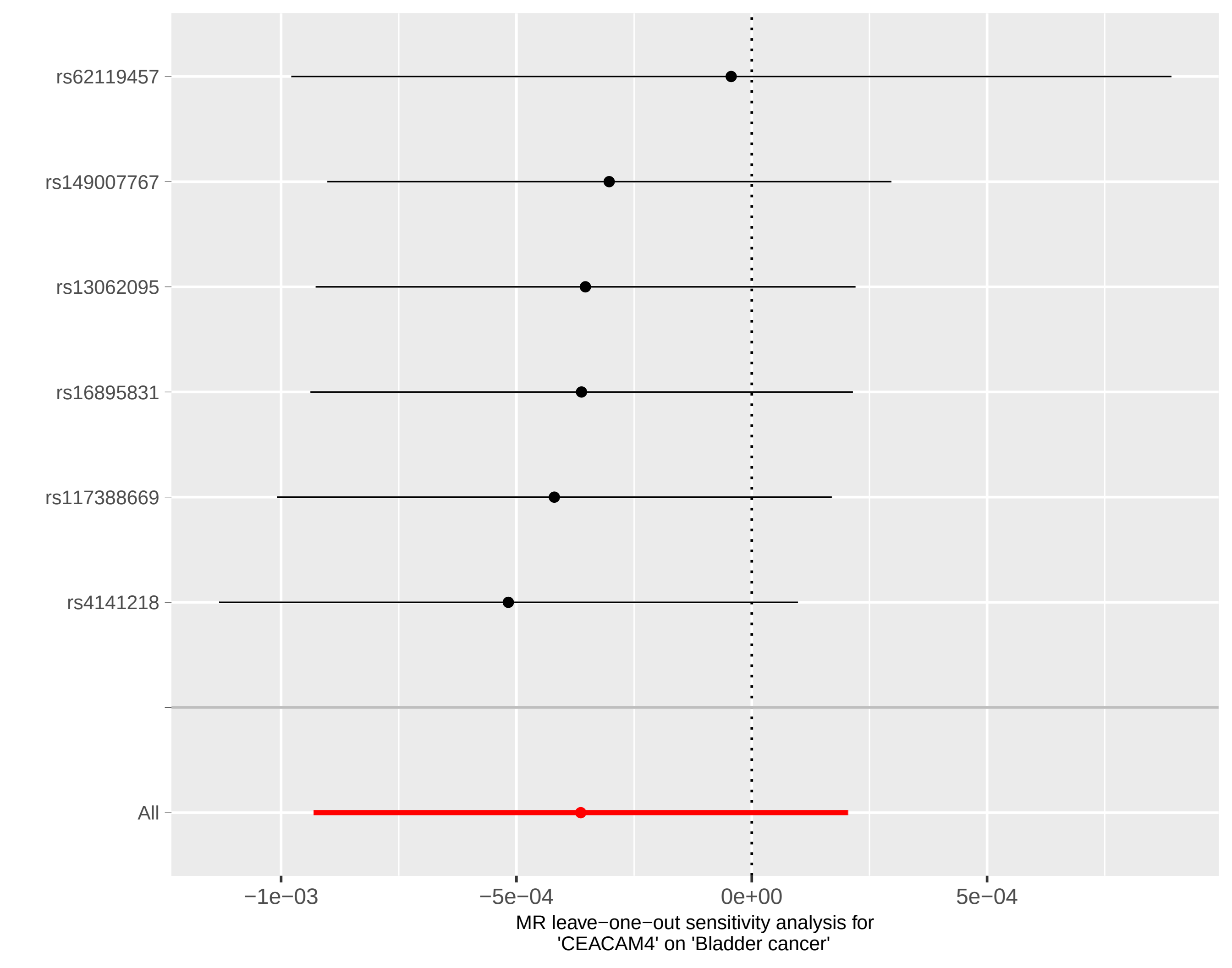
rs62119457
rs149007767
rs13062095
rs16895831
rs117388669
rs4141218
All
−1e−03
−5e−04
0e+00
5e−04
MR leave−one−out sensitivity analysis for
'CEACAM4' on 'Bladder cancer'

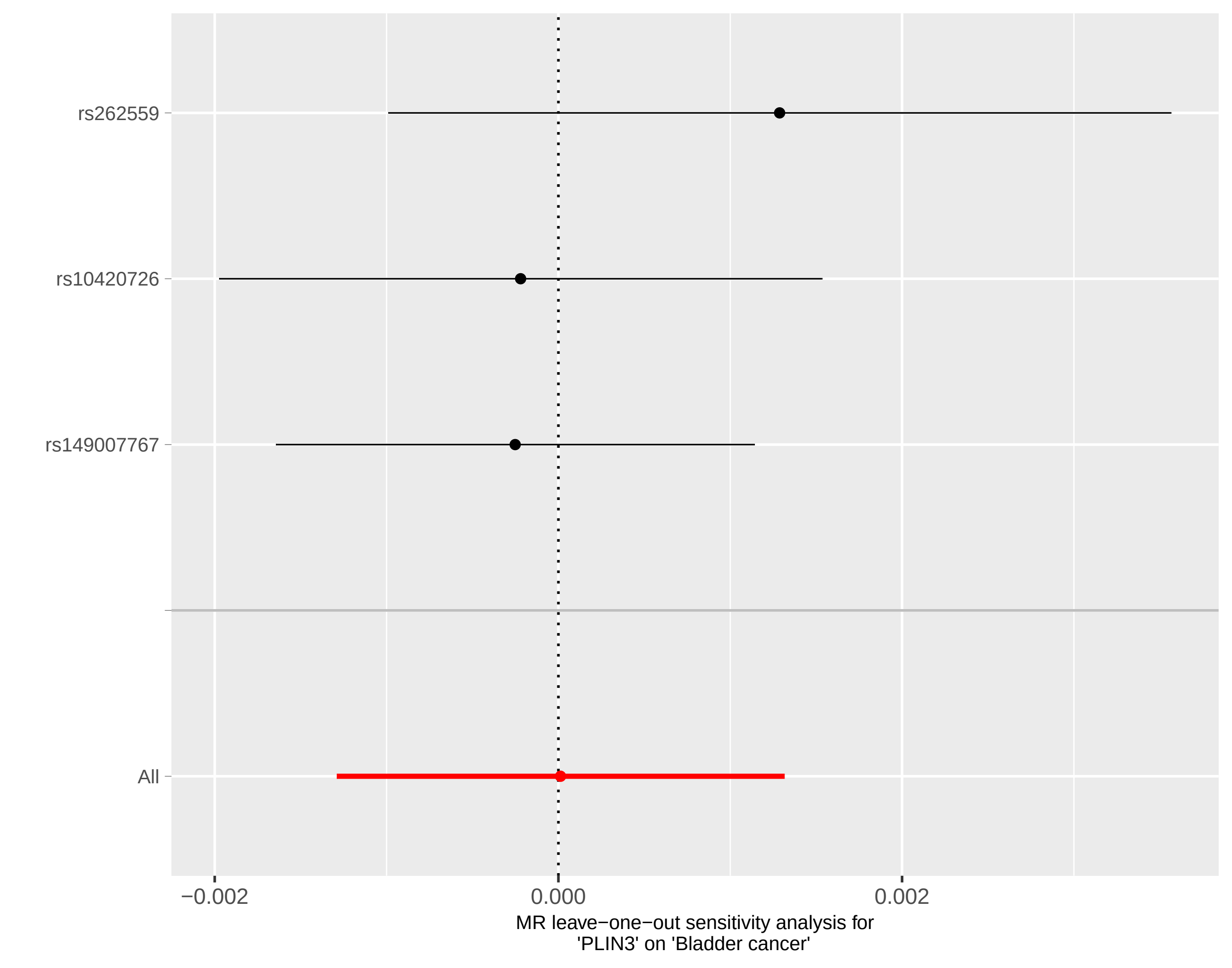
rs262559
rs10420726
rs149007767
All
−0.002
0.000
0.002
MR leave−one−out sensitivity analysis for
'PLIN3' on 'Bladder cancer'

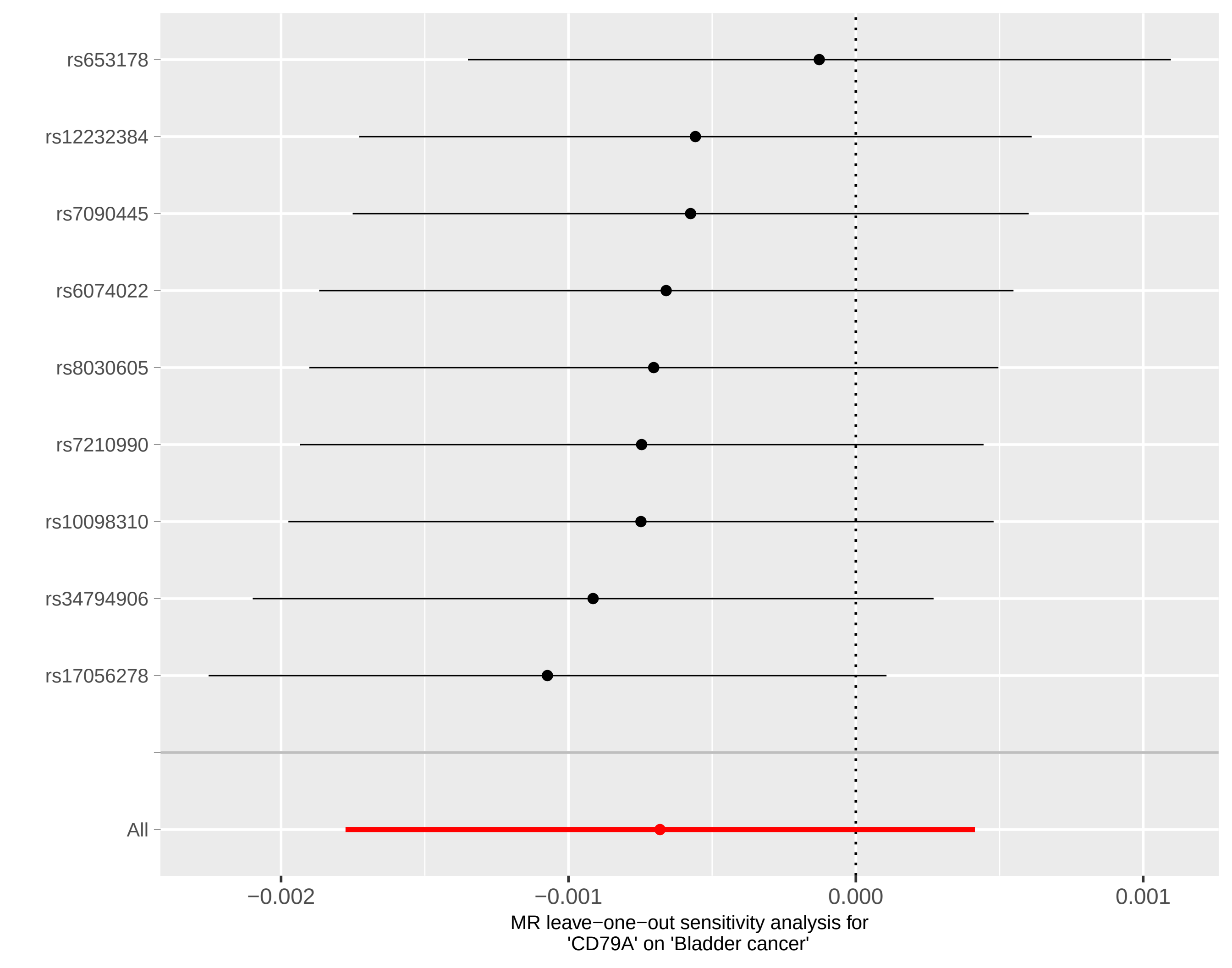
rs653178
rs12232384
rs7090445
rs6074022
rs8030605
rs7210990
rs10098310
rs34794906
rs17056278
All
−0.002
−0.001
0.000
0.001
MR leave−one−out sensitivity analysis for
'CD79A' on 'Bladder cancer'

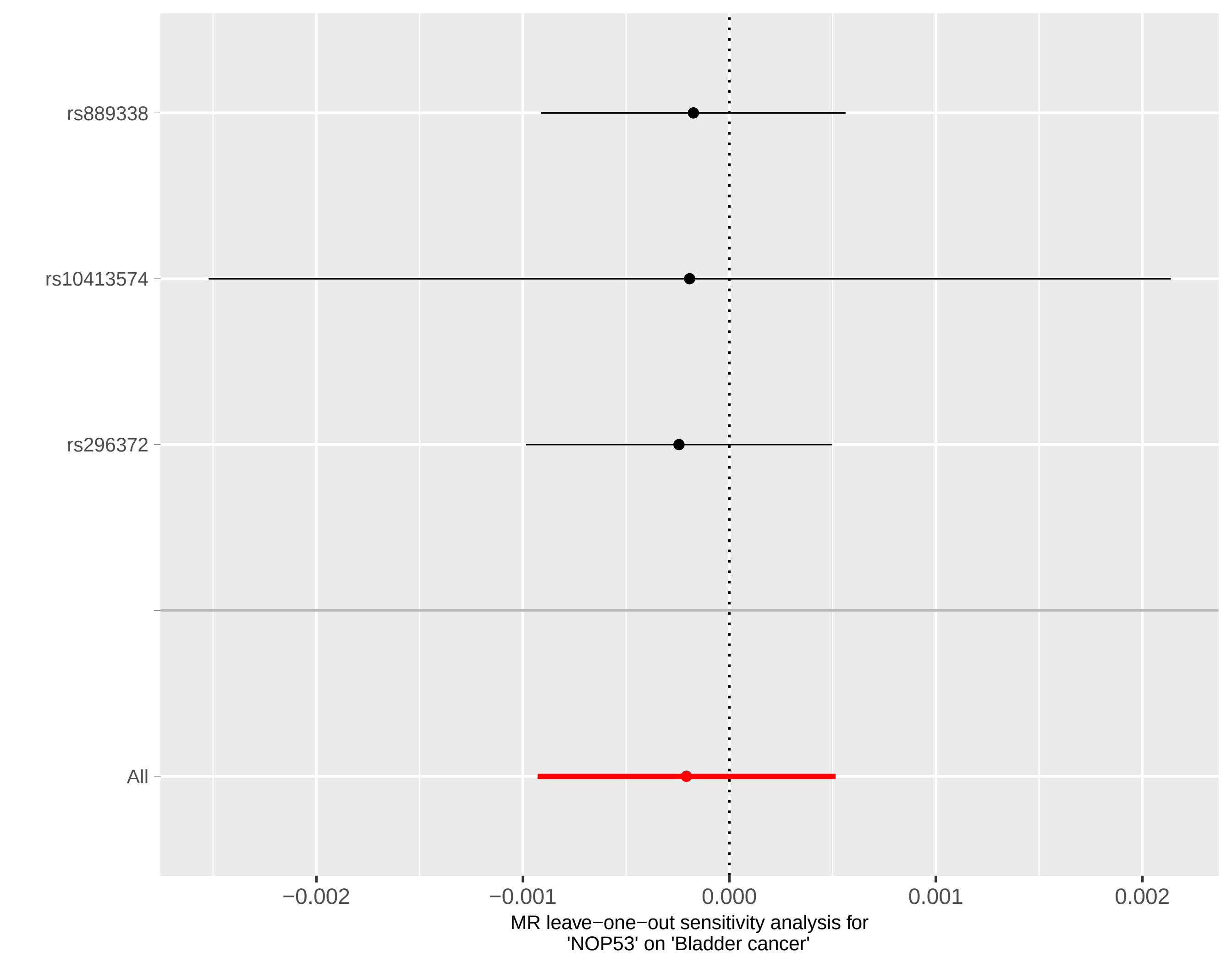
rs889338
rs10413574
rs296372
All
−0.002
−0.001
0.000
0.001
0.002
MR leave−one−out sensitivity analysis for
'NOP53' on 'Bladder cancer'

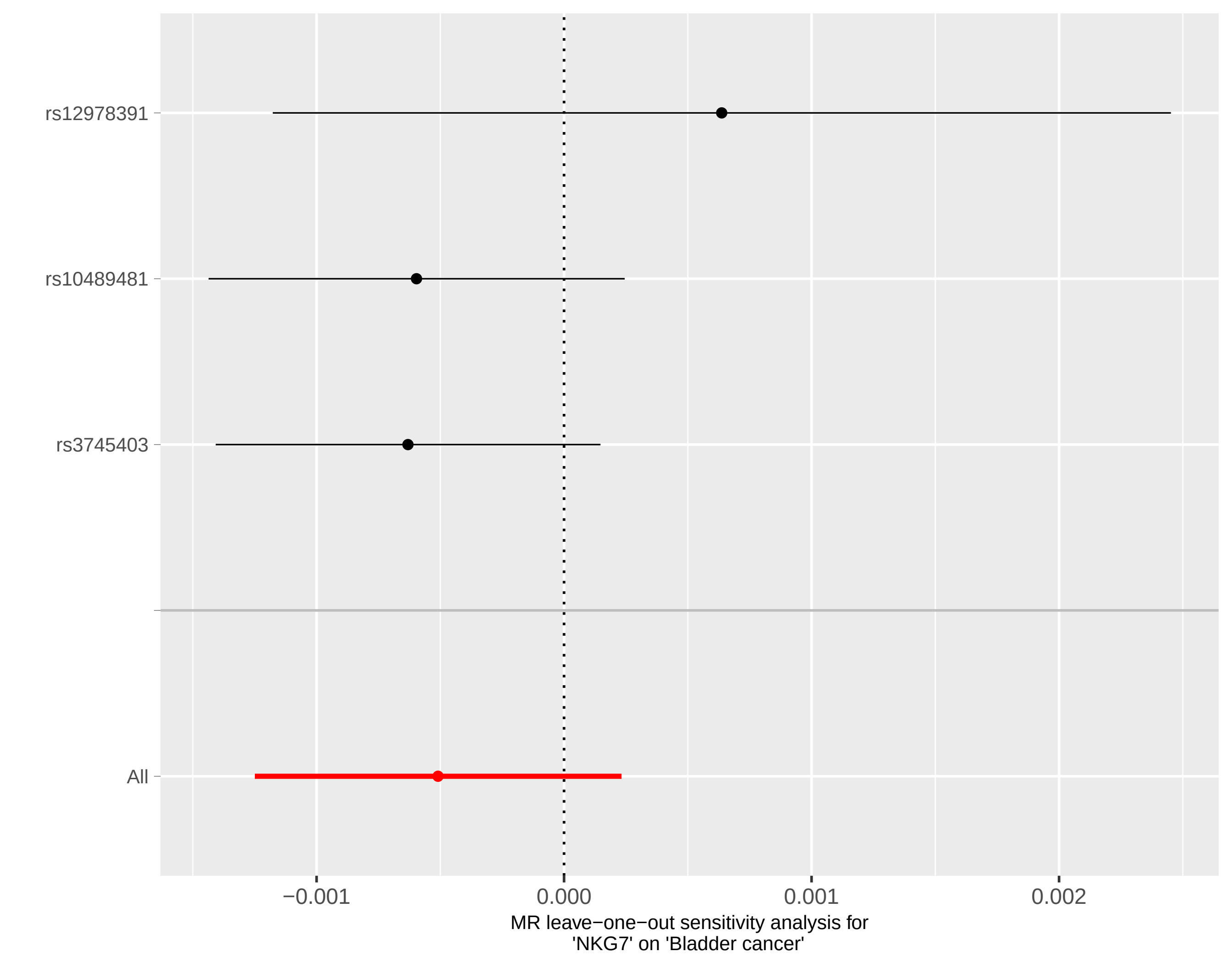
rs12978391
rs10489481
rs3745403
All
−0.001
0.000
0.001
0.002
MR leave−one−out sensitivity analysis for
'NKG7' on 'Bladder cancer'

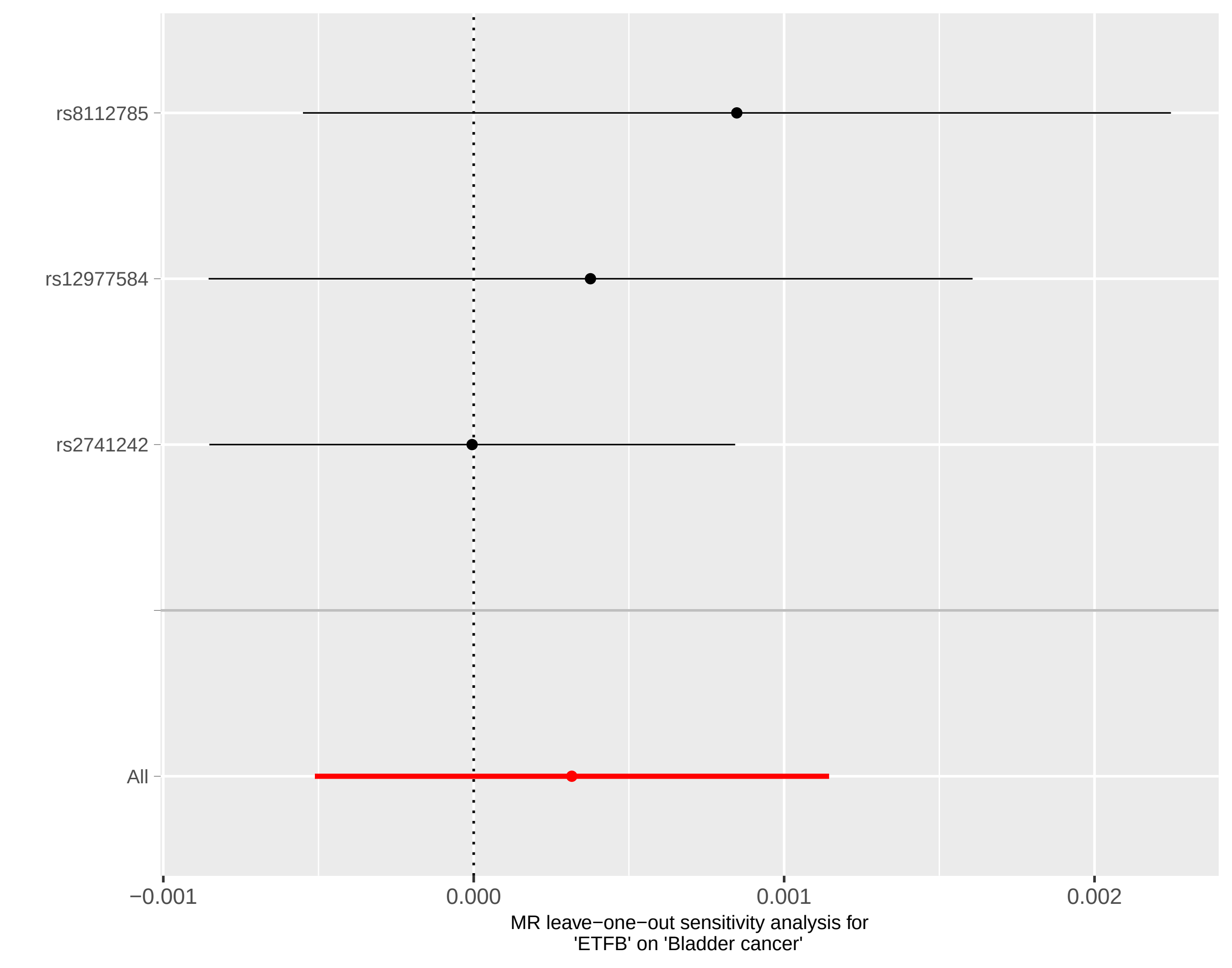
rs8112785
rs12977584
rs2741242
All
−0.001
0.000
0.001
0.002
MR leave−one−out sensitivity analysis for
'ETFB' on 'Bladder cancer'

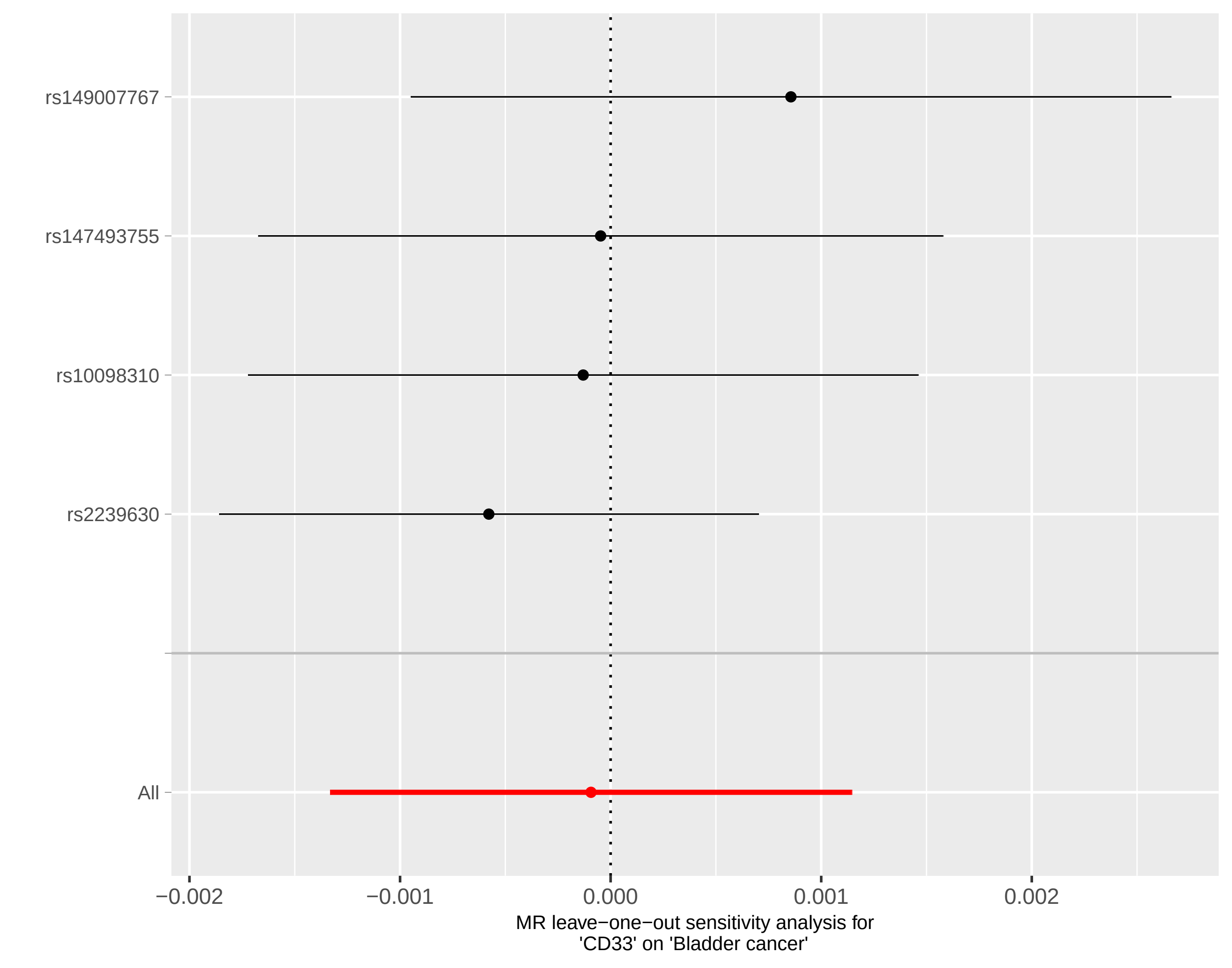
rs149007767
rs147493755
rs10098310
rs2239630
All
−0.002
−0.001
0.000
0.001
0.002
MR leave−one−out sensitivity analysis for
'CD33' on 'Bladder cancer'

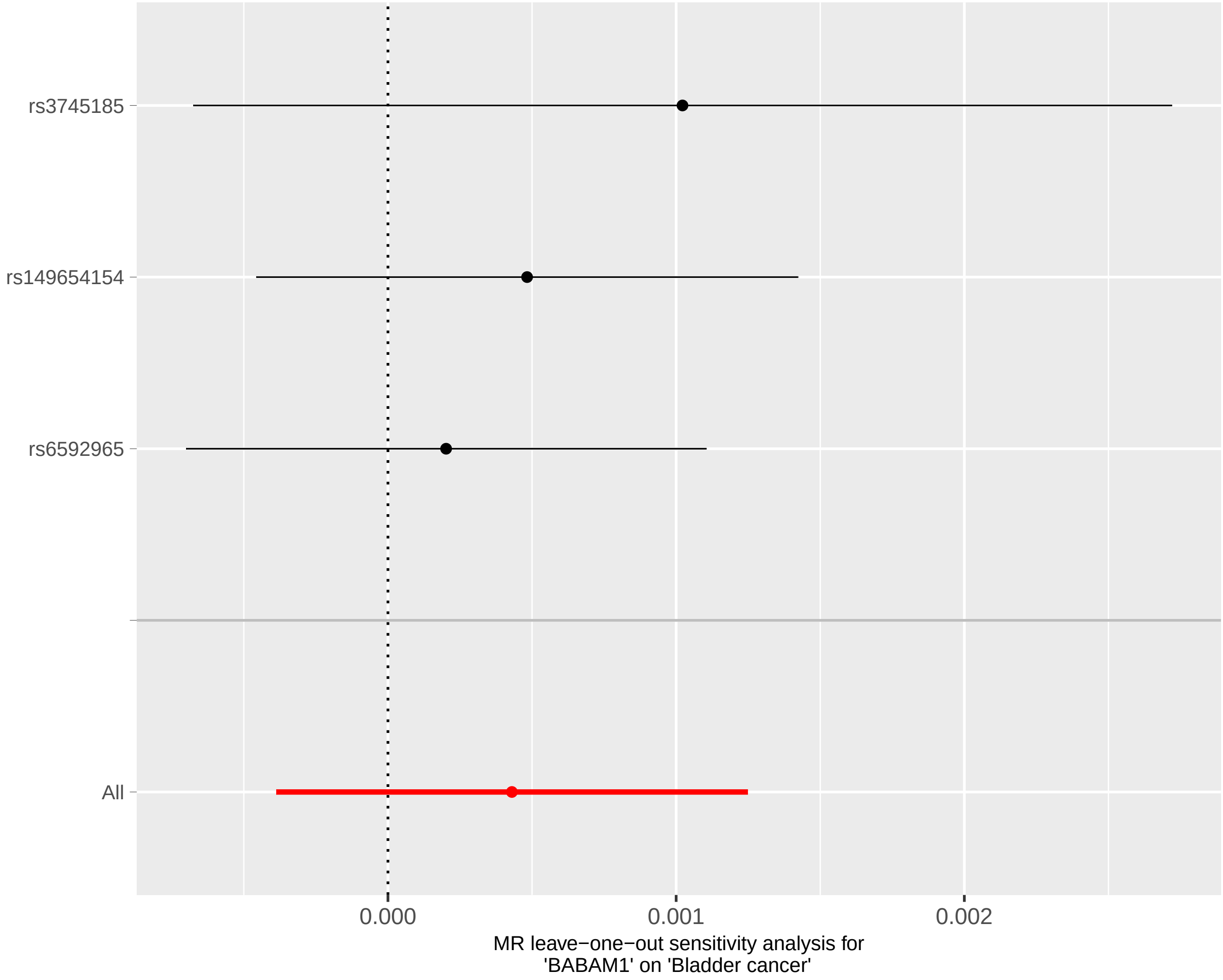
rs3745185
rs149654154
rs6592965
All
0.000
0.001
0.002
MR leave−one−out sensitivity analysis for
'BABAM1' on 'Bladder cancer'

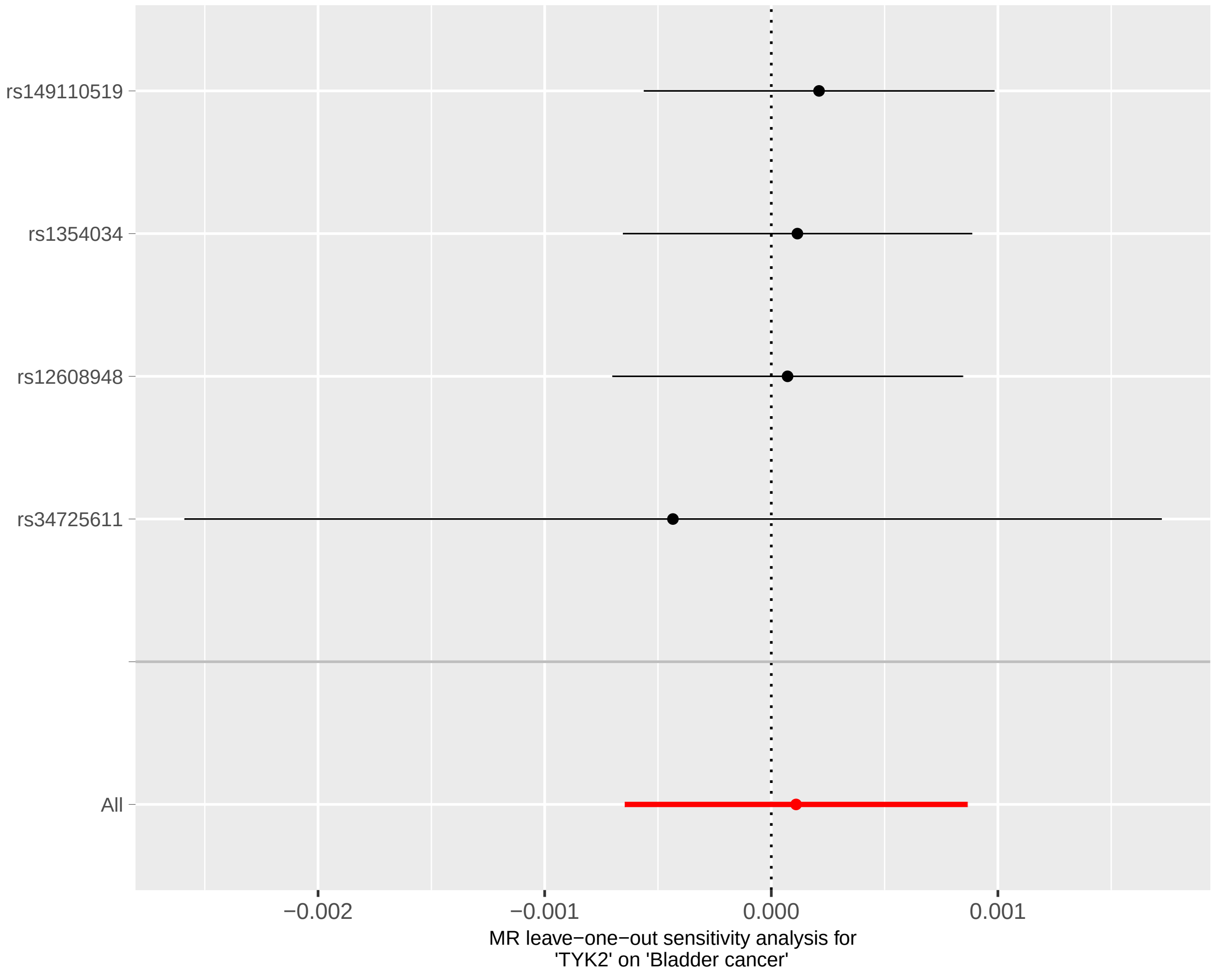
rs149110519
rs1354034
rs12608948
rs34725611
All
−0.002
−0.001
0.000
0.001
MR leave−one−out sensitivity analysis for
'TYK2' on 'Bladder cancer'

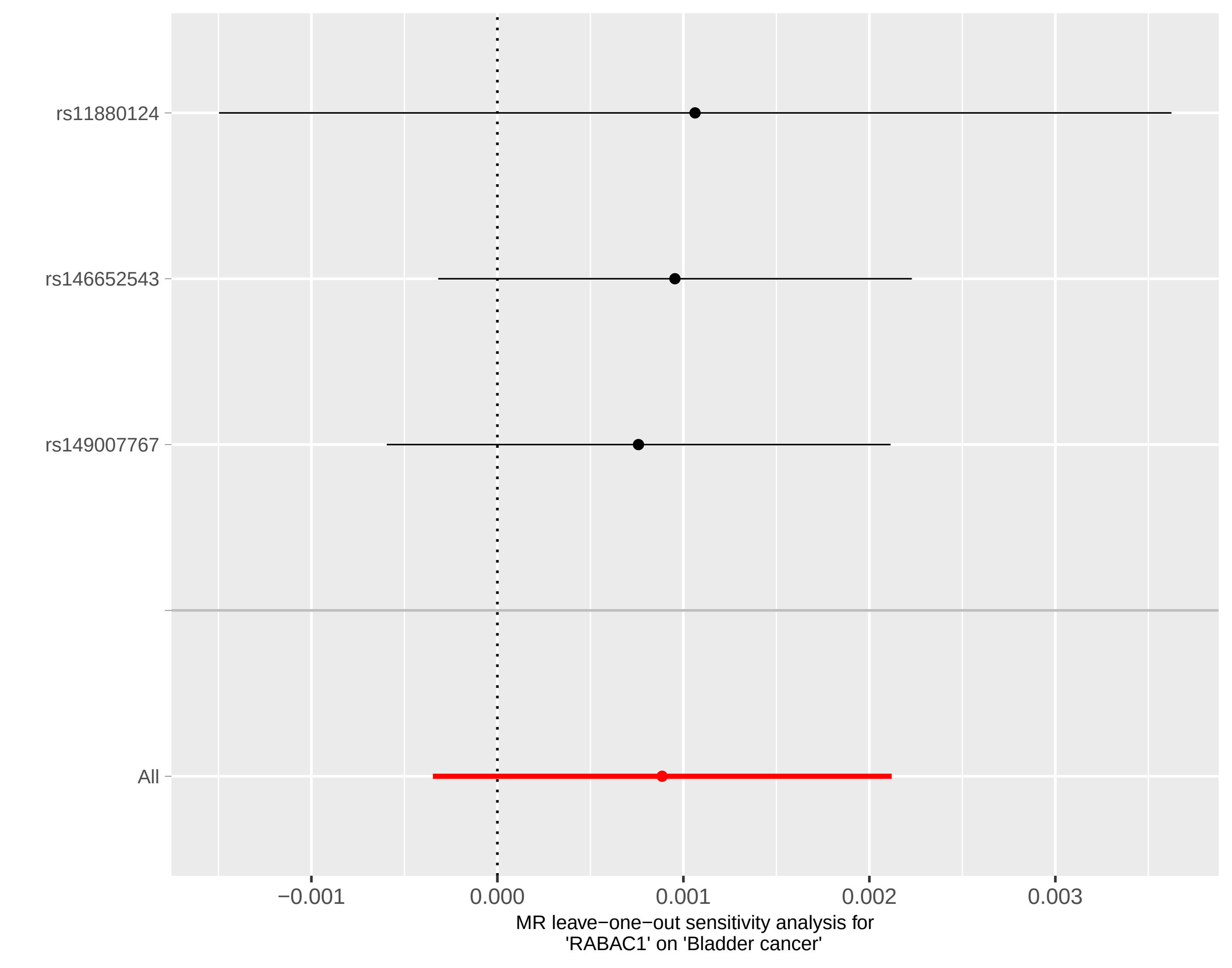
rs11880124
rs146652543
rs149007767
All
−0.001
0.000
0.001
0.002
0.003
MR leave−one−out sensitivity analysis for
'RABAC1' on 'Bladder cancer'

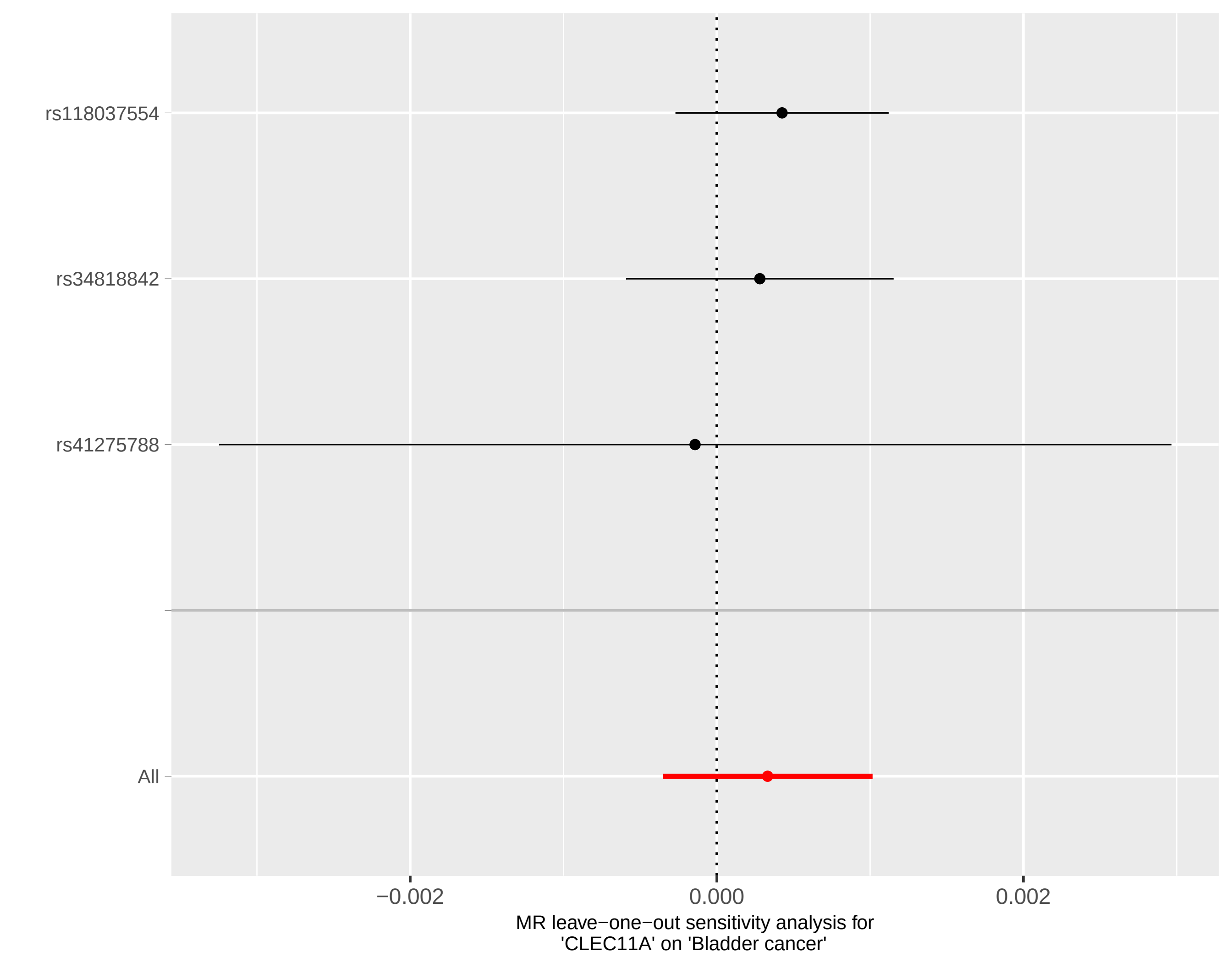
rs118037554
rs34818842
rs41275788
All
−0.002
0.000
0.002
MR leave−one−out sensitivity analysis for
'CLEC11A' on 'Bladder cancer'

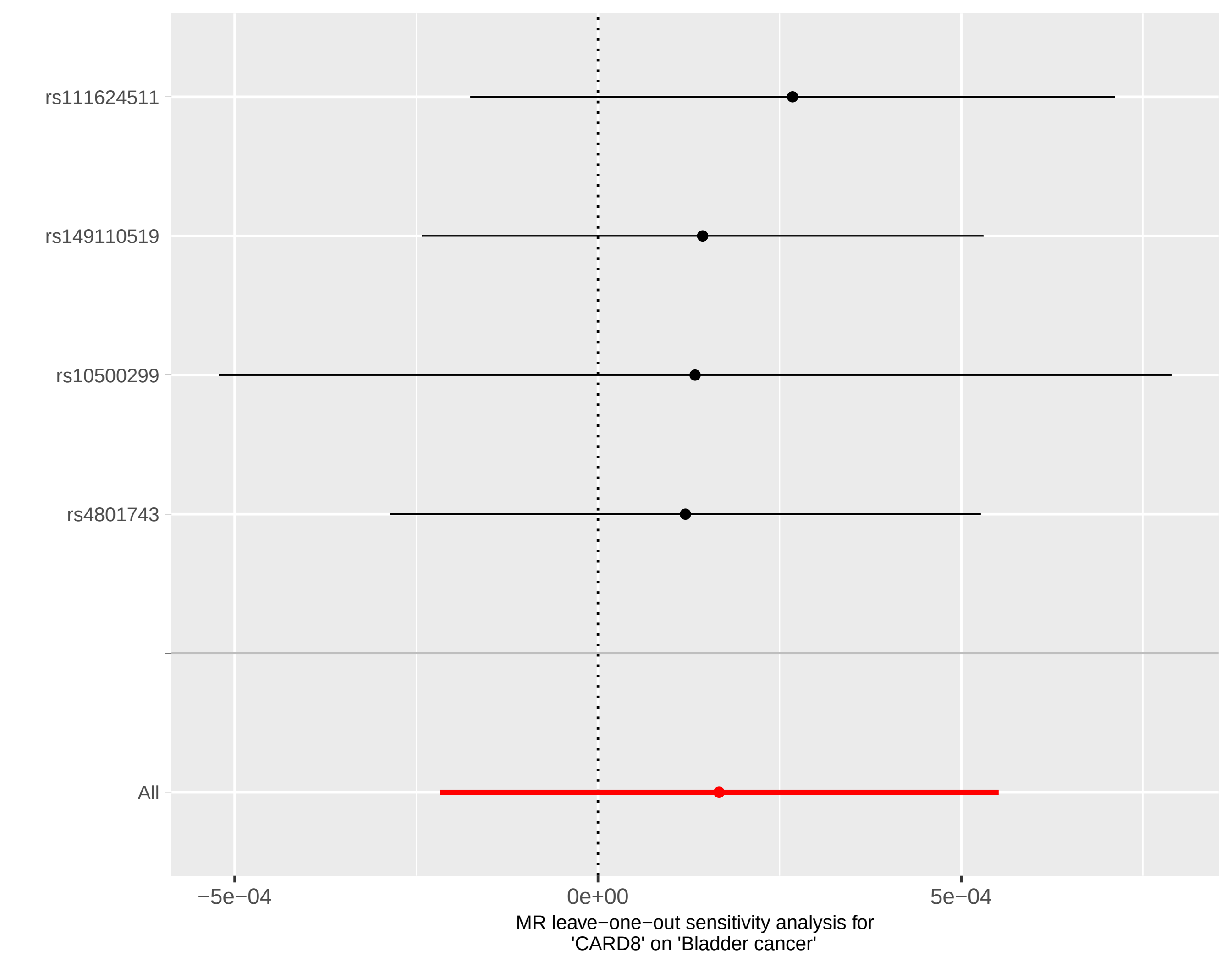
rs111624511
rs149110519
rs10500299
rs4801743
All
−5e−04
0e+00
5e−04
MR leave−one−out sensitivity analysis for
'CARD8' on 'Bladder cancer'

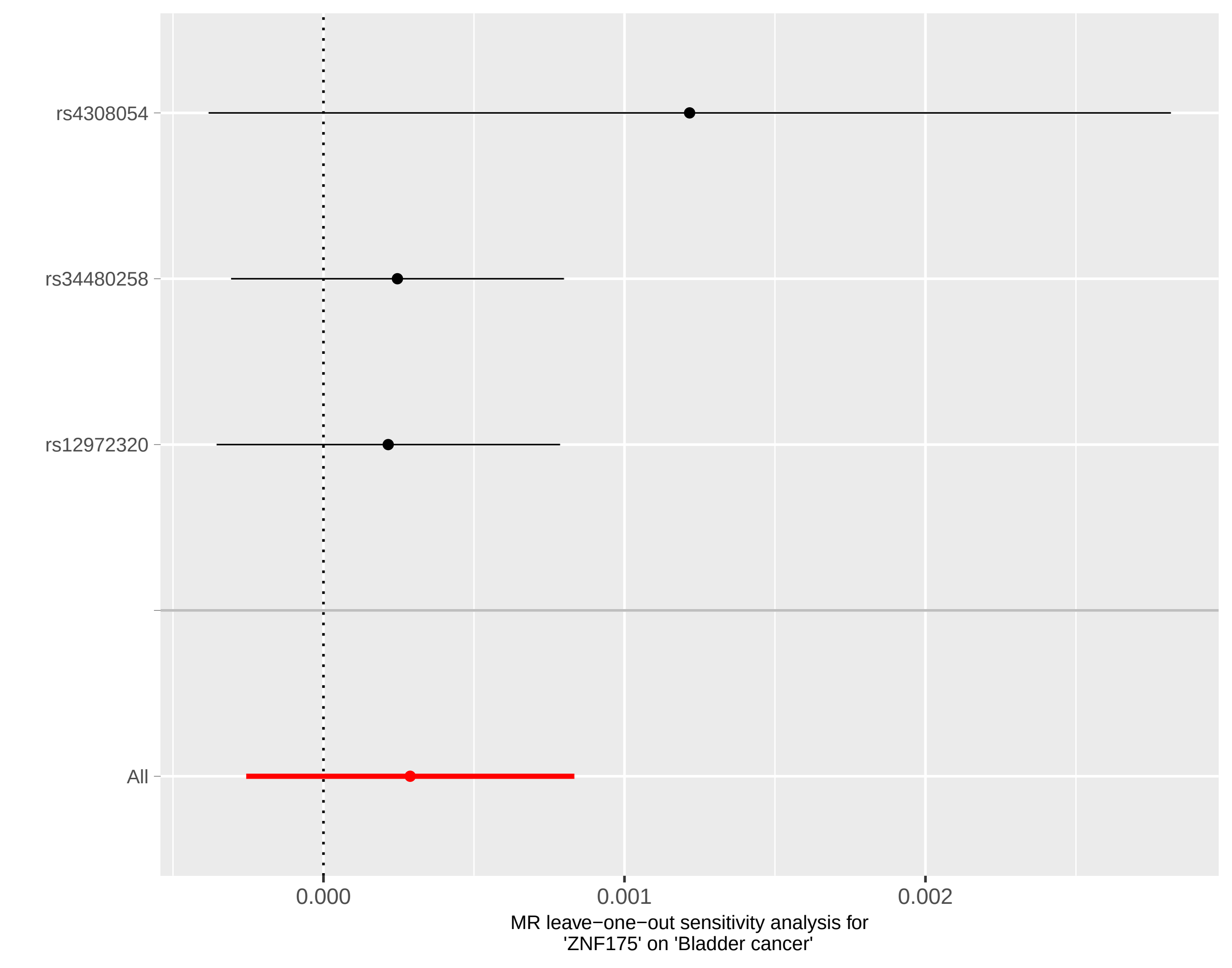
rs4308054
rs34480258
rs12972320
All
0.000
0.001
0.002
MR leave−one−out sensitivity analysis for
'ZNF175' on 'Bladder cancer'

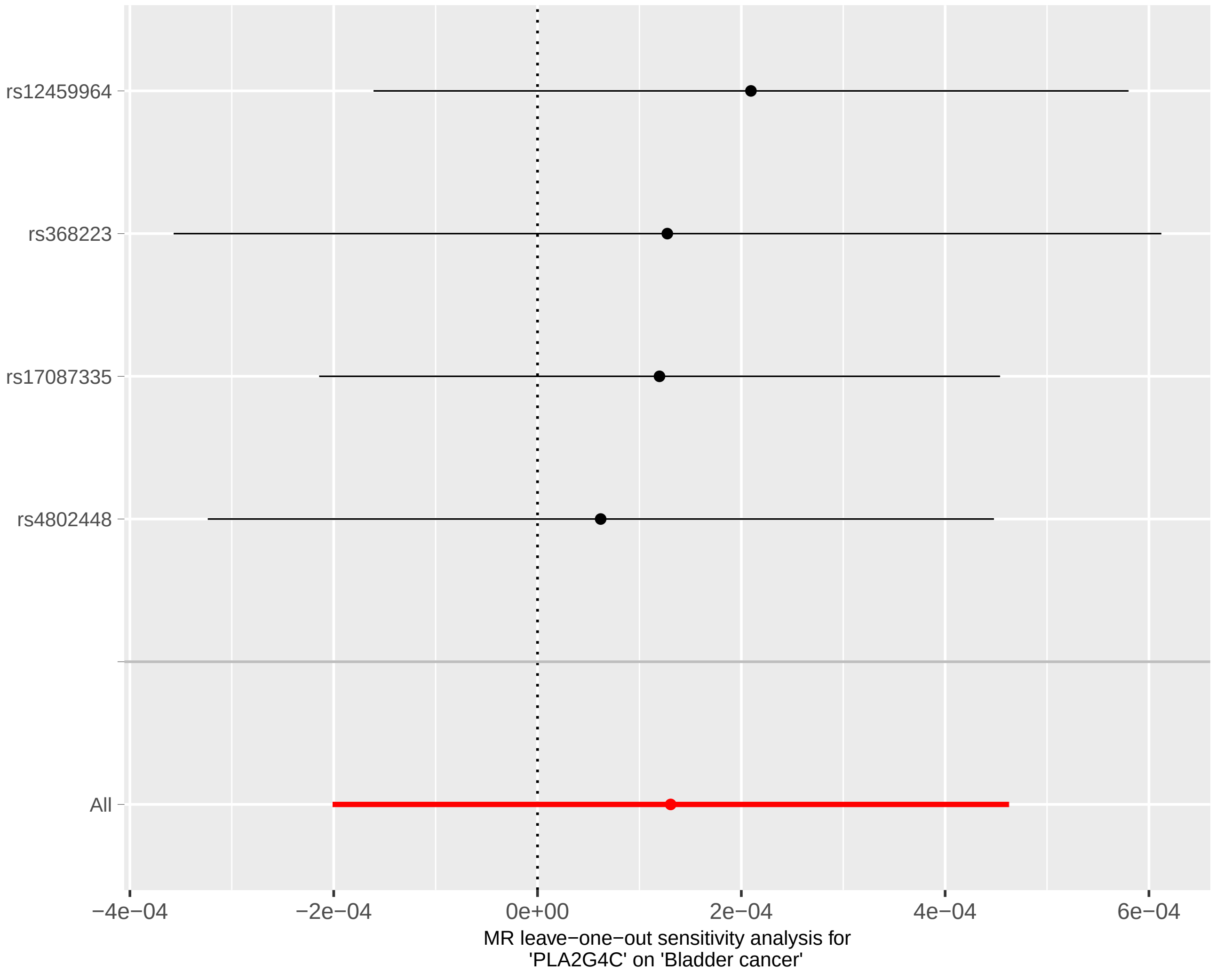
rs12459964
rs368223
rs17087335
rs4802448
All
−4e−04
−2e−04
0e+00
2e−04
4e−04
6e−04
MR leave−one−out sensitivity analysis for
'PLA2G4C' on 'Bladder cancer'

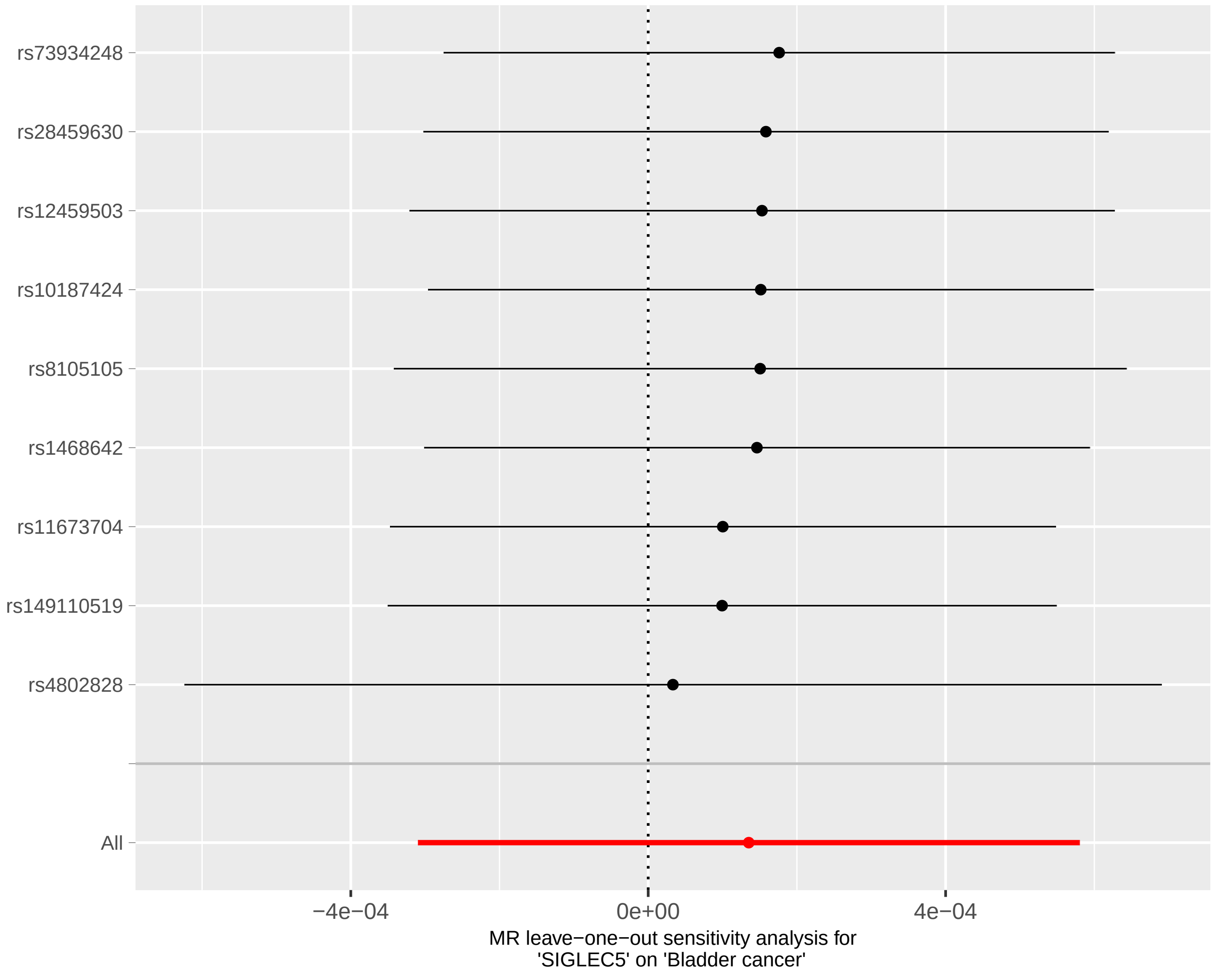
rs73934248
rs28459630
rs12459503
rs10187424
rs8105105
rs1468642
rs11673704
rs149110519
rs4802828
All
−4e−04
0e+00
4e−04
MR leave−one−out sensitivity analysis for
'SIGLEC5' on 'Bladder cancer'

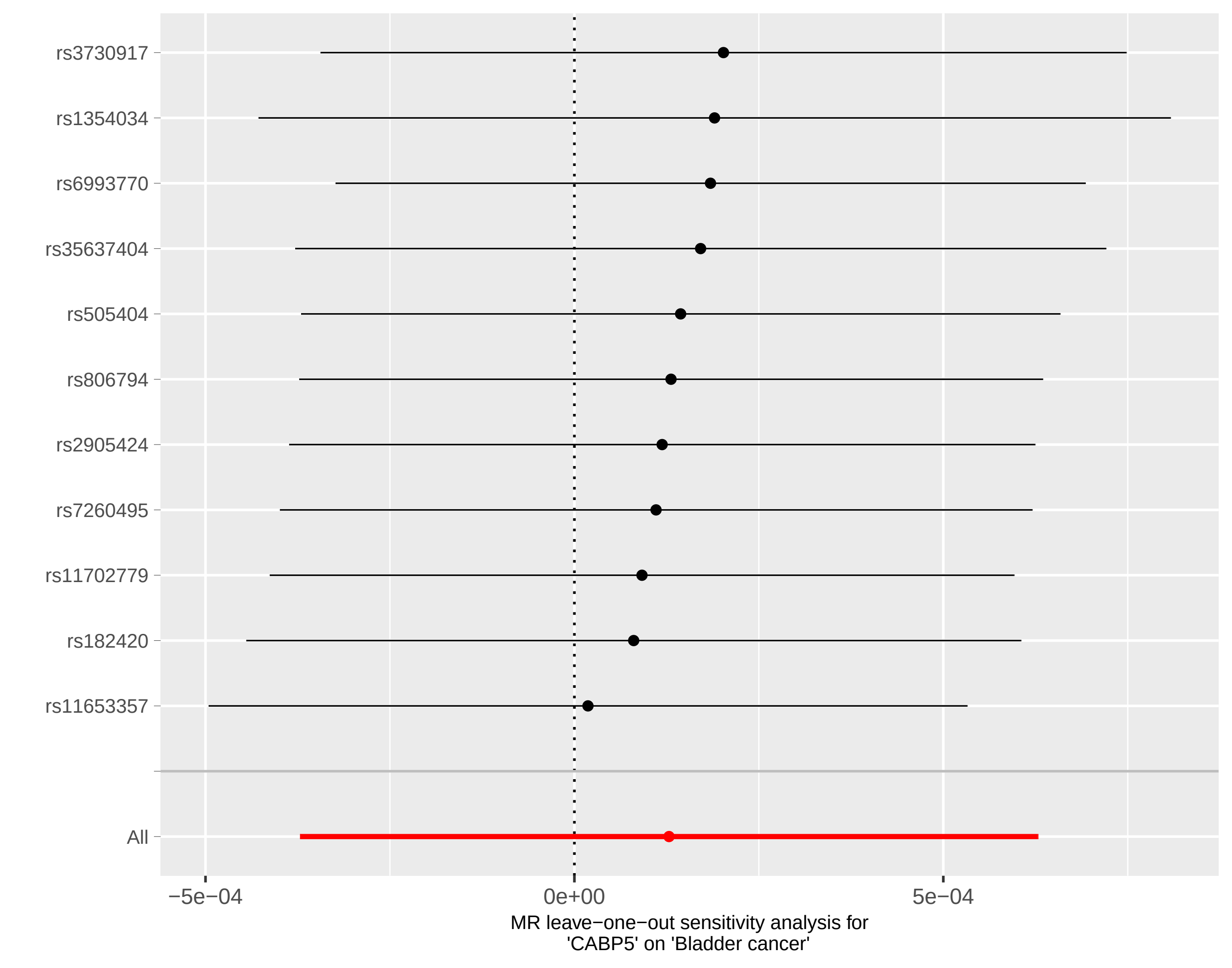

rs3730917
rs1354034
rs6993770
rs35637404
rs505404
rs806794
rs2905424
rs7260495
rs11702779
rs182420
rs11653357
All
−5e−04
0e+00
5e−04
MR leave−one−out sensitivity analysis for
'CABP5' on 'Bladder cancer'

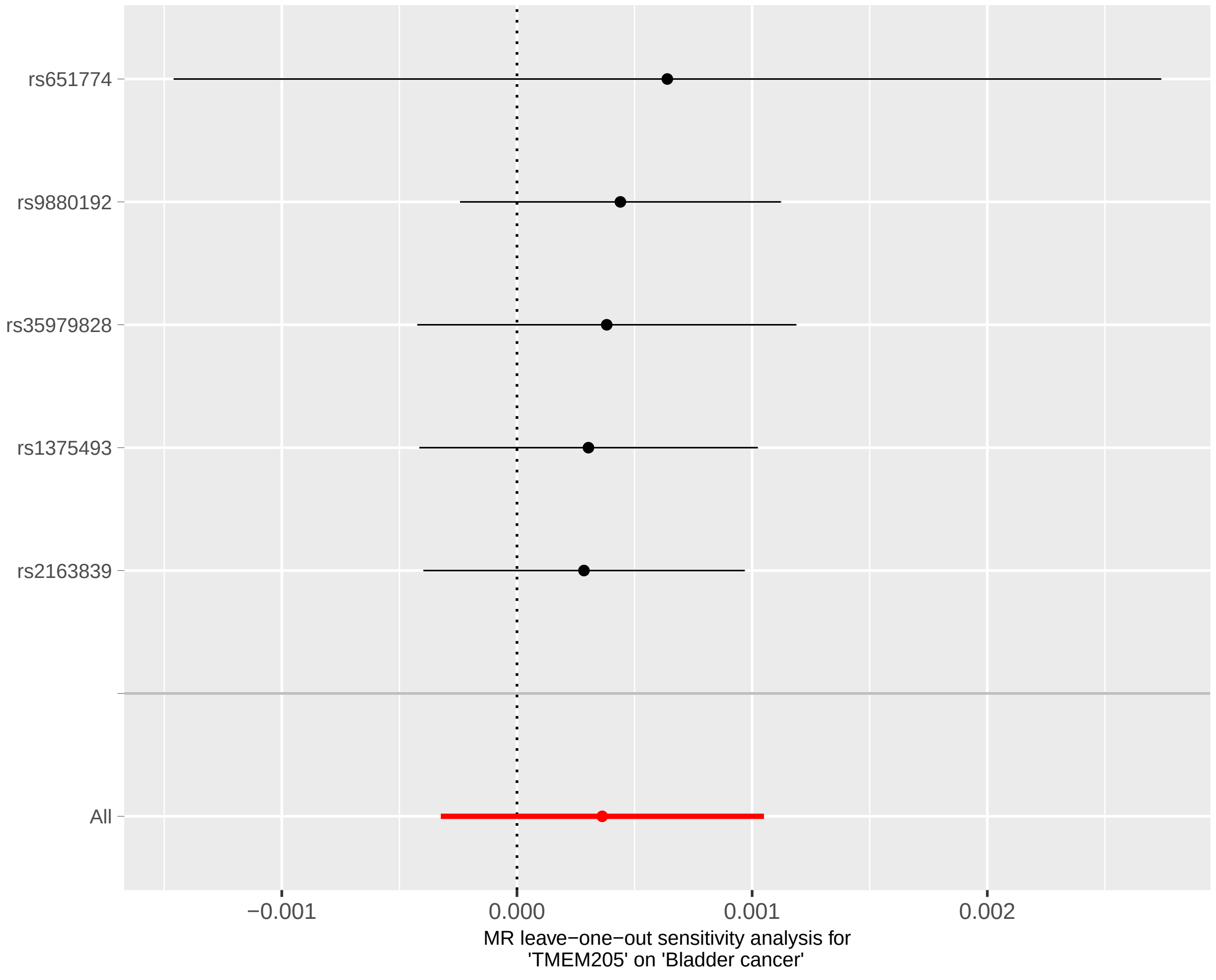
rs651774
rs9880192
rs35979828
rs1375493
rs2163839
All
−0.001
0.000
0.001
0.002
MR leave−one−out sensitivity analysis for
'TMEM205' on 'Bladder cancer'

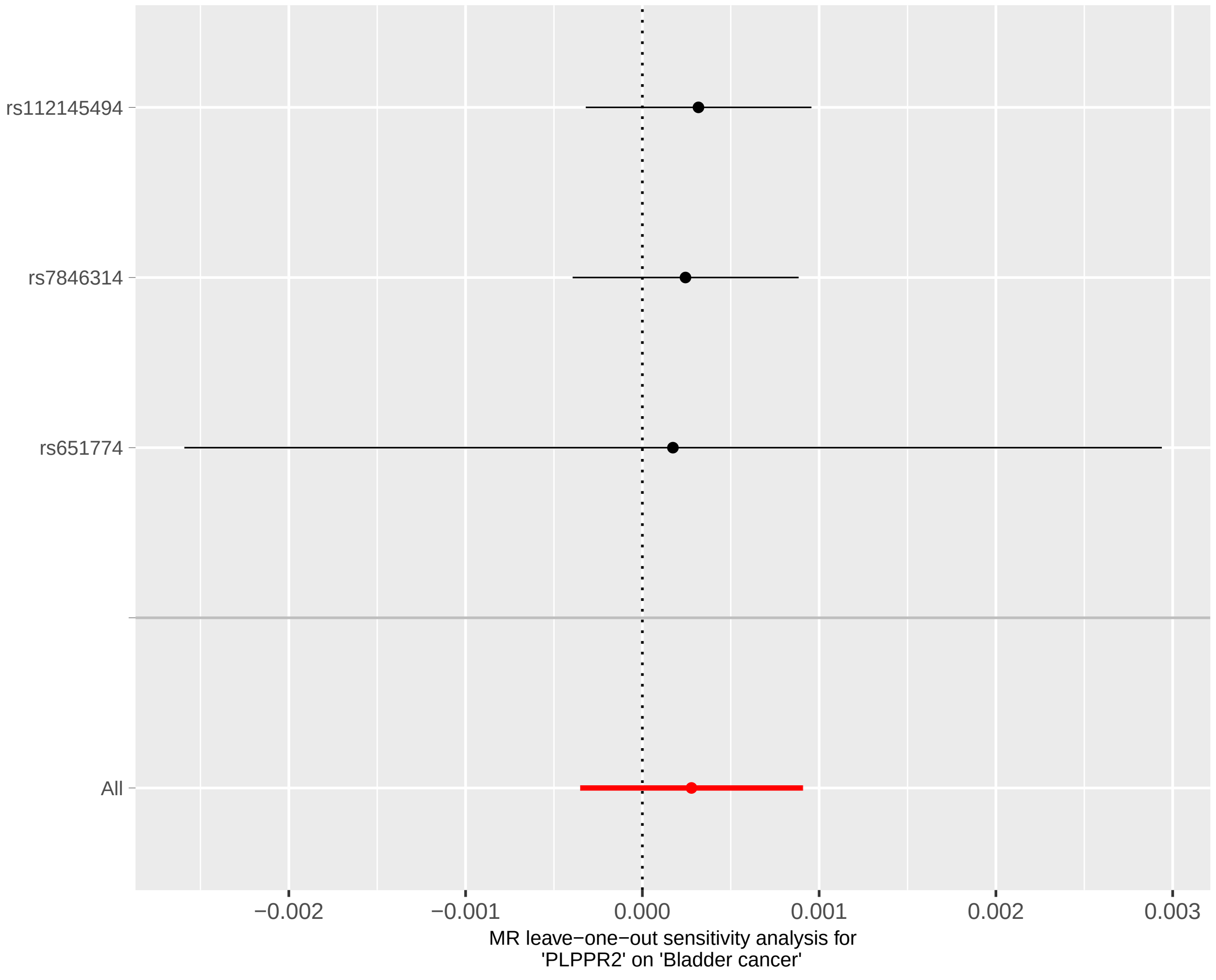
rs112145494
rs7846314
rs651774
All
−0.002
−0.001
0.000
0.001
0.002
0.003
MR leave−one−out sensitivity analysis for
'PLPPR2' on 'Bladder cancer'

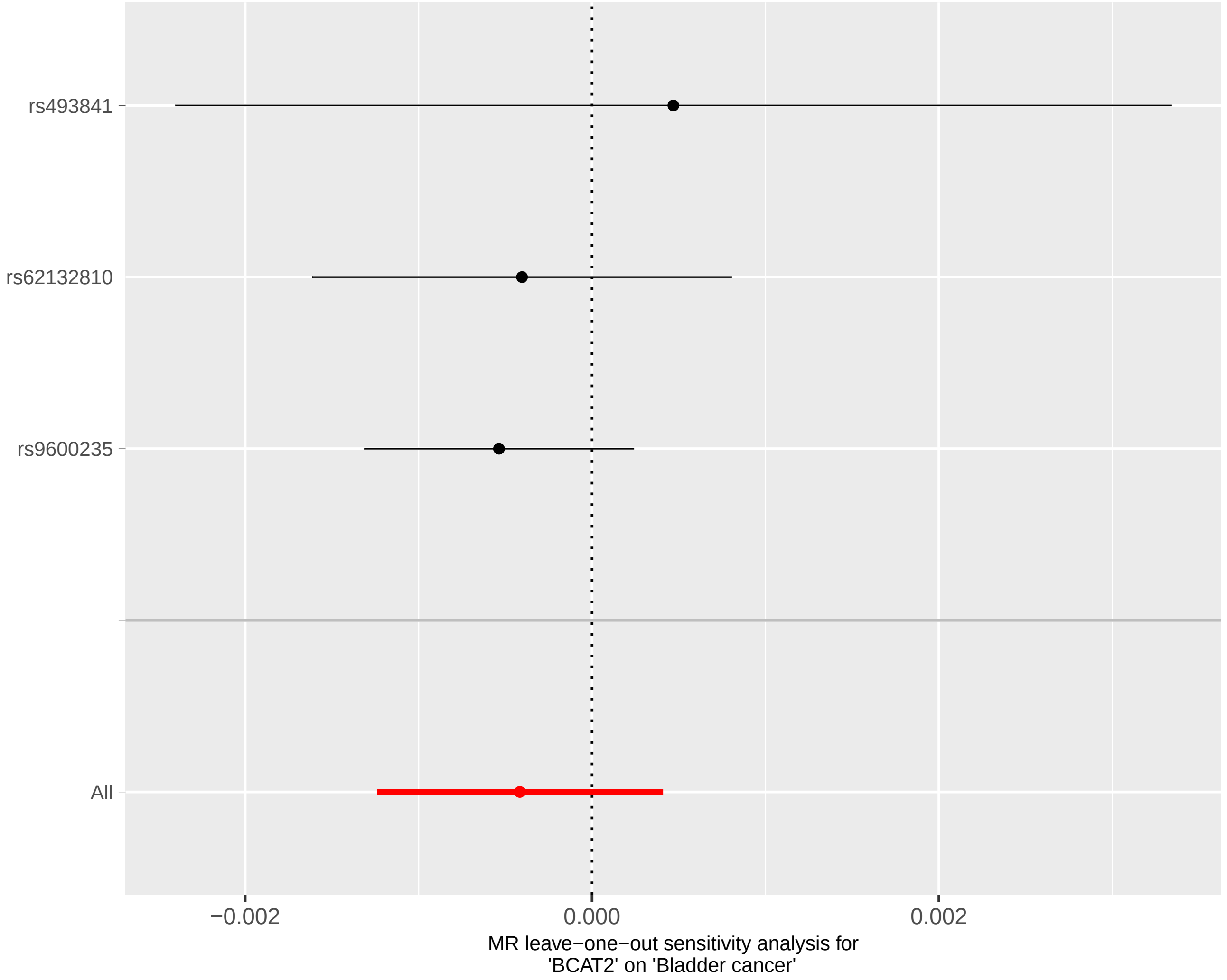
rs493841
rs62132810
rs9600235
All
−0.002
0.000
0.002
MR leave−one−out sensitivity analysis for
'BCAT2' on 'Bladder cancer'

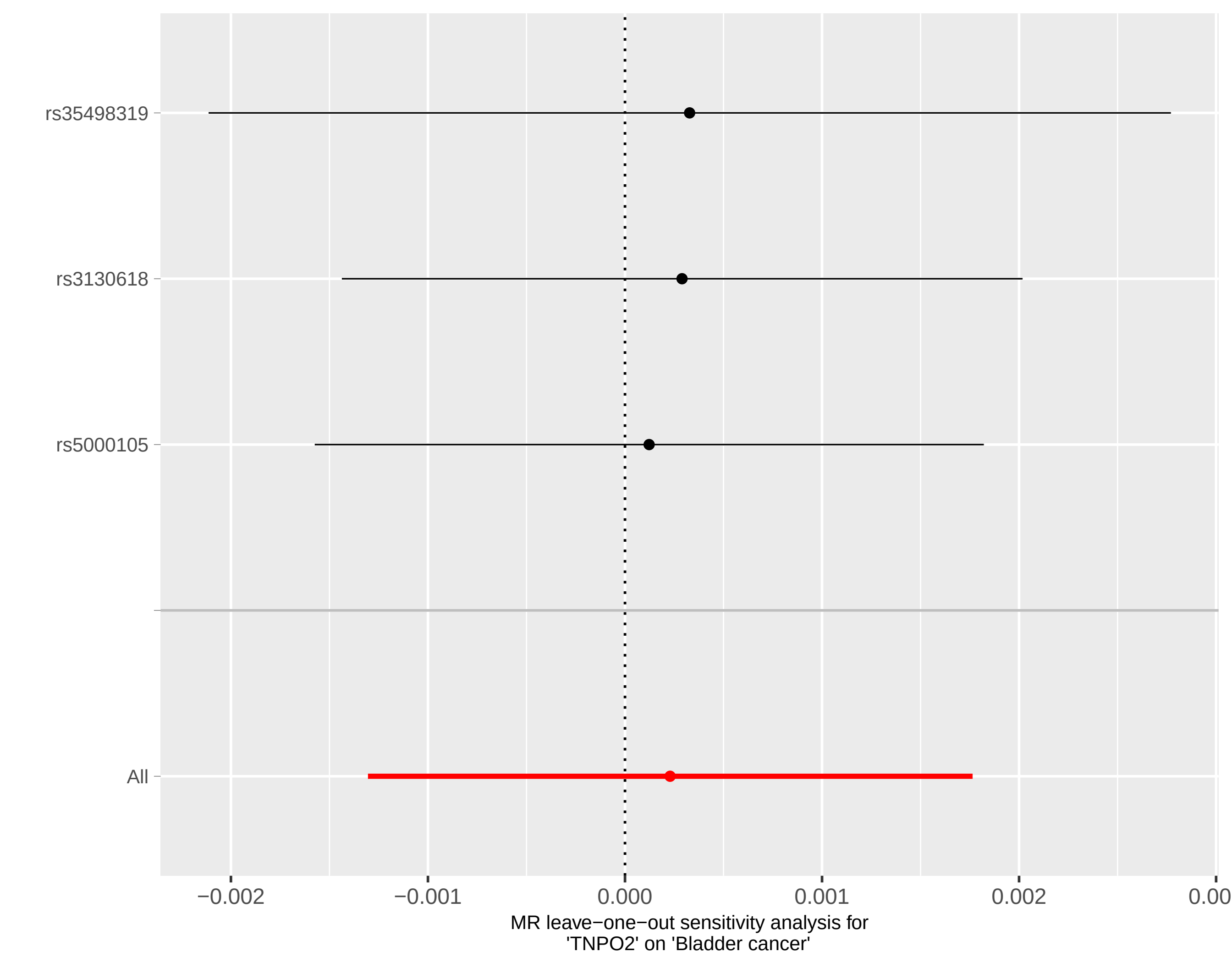
rs35498319
rs3130618
rs5000105
All
−0.002
−0.001
0.000
0.001
0.002
0.00
MR leave−one−out sensitivity analysis for
'TNPO2' on 'Bladder cancer'

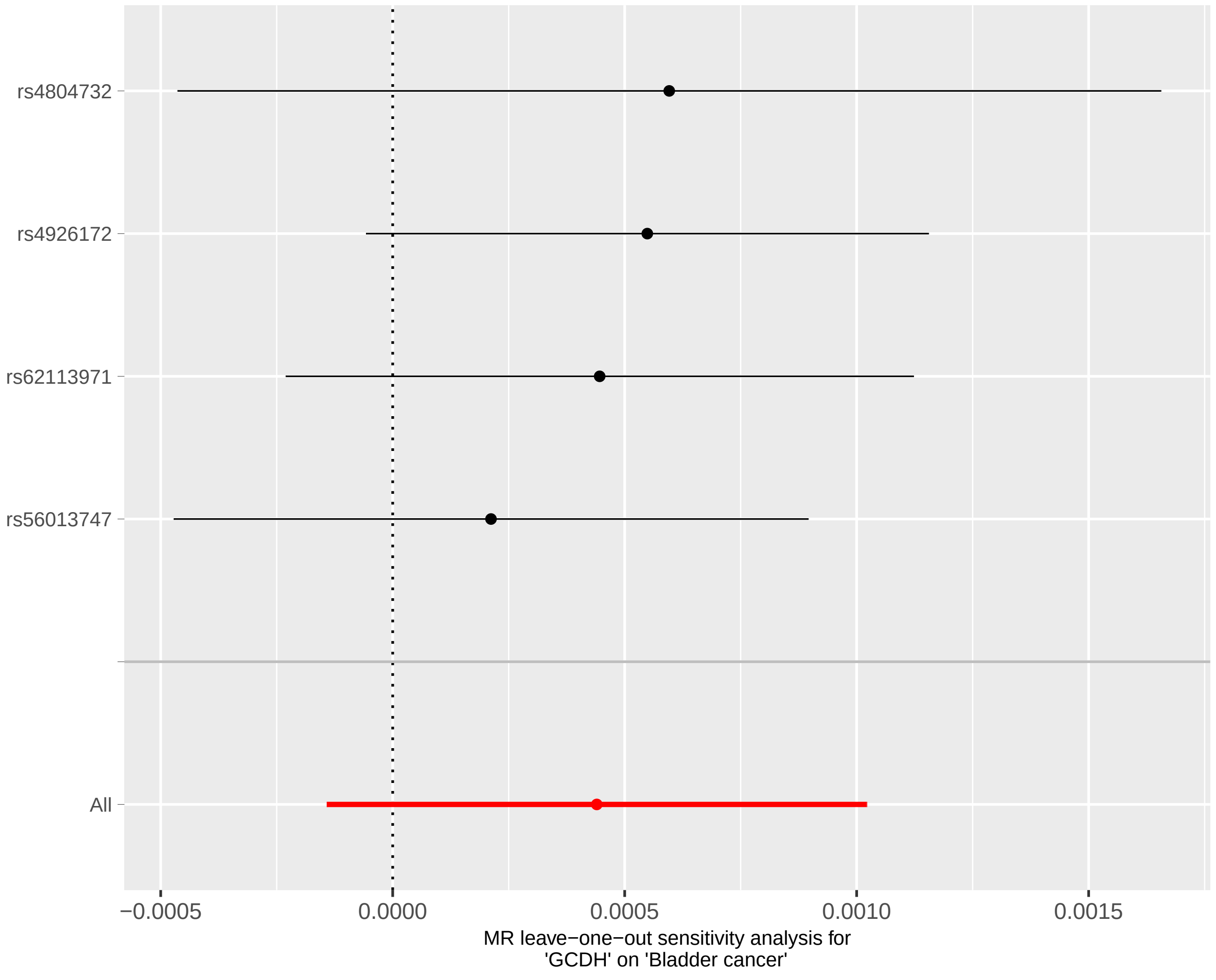
rs4804732
rs4926172
rs62113971
rs56013747
All
−0.0005
0.0000
0.0005
0.0010
0.0015
MR leave−one−out sensitivity analysis for
'GCDH' on 'Bladder cancer'

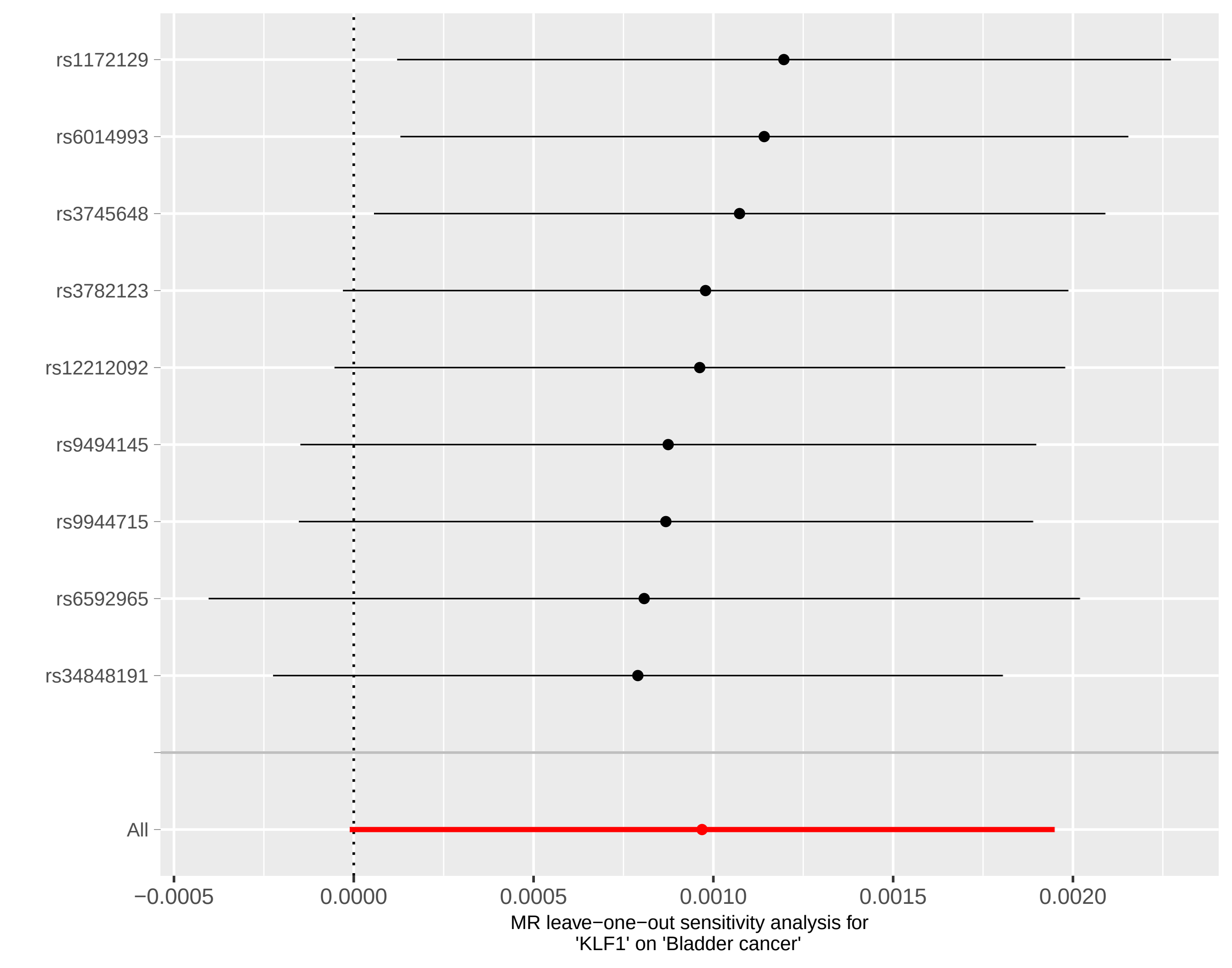
rs1172129
rs6014993
rs3745648
rs3782123
rs12212092
rs9494145
rs9944715
rs6592965
rs34848191
All
−0.0005
0.0000
0.0005
0.0010
0.0015
0.0020
MR leave−one−out sensitivity analysis for
'KLF1' on 'Bladder cancer'

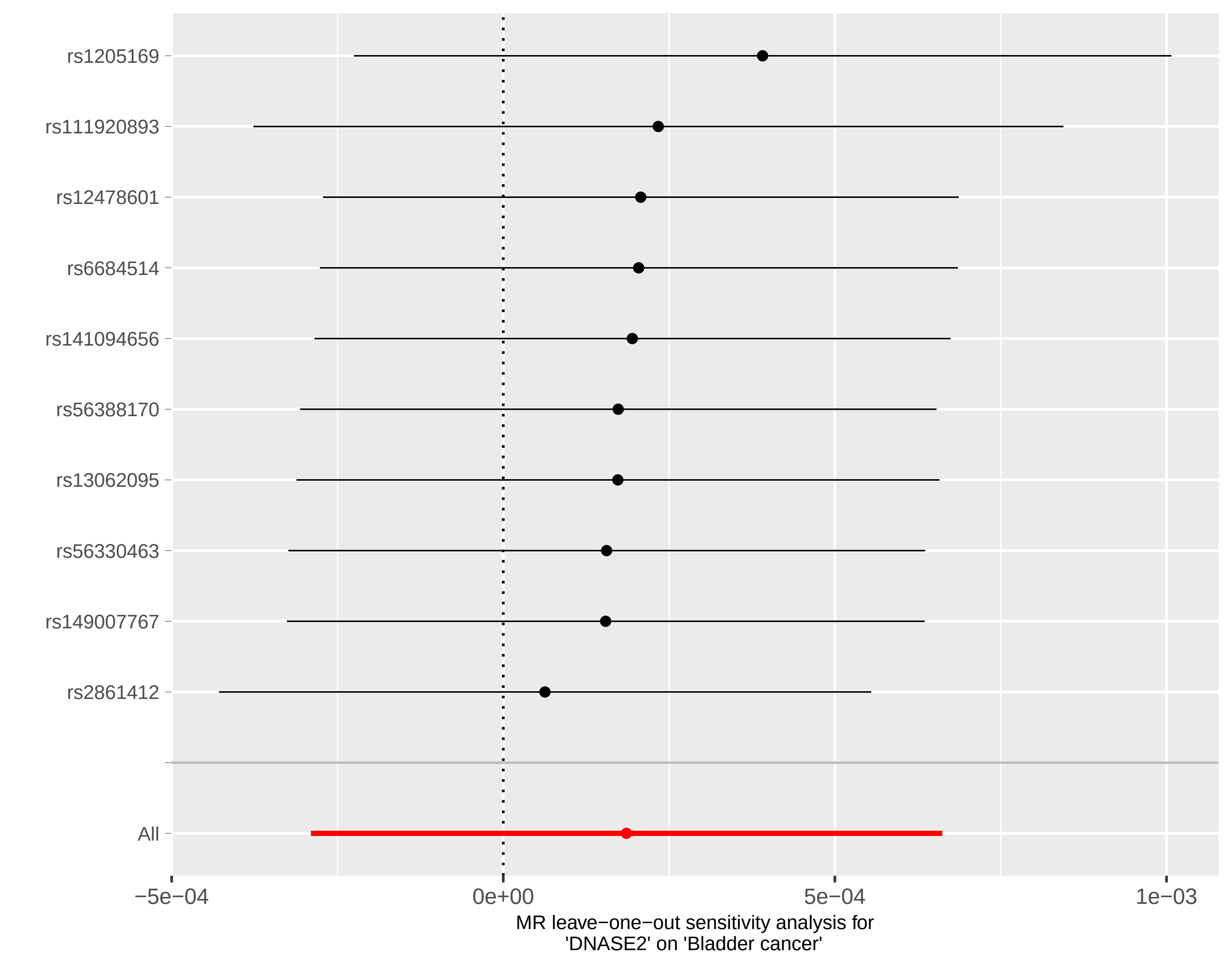

rs1205169
rs111920893
rs12478601
rs6684514
rs141094656
rs56388170
rs13062095
rs56330463
rs149007767
rs2861412
All
−5e−04
0e+00
5e−04
1e−03
MR leave−one−out sensitivity analysis for
'DNASE2' on 'Bladder cancer'

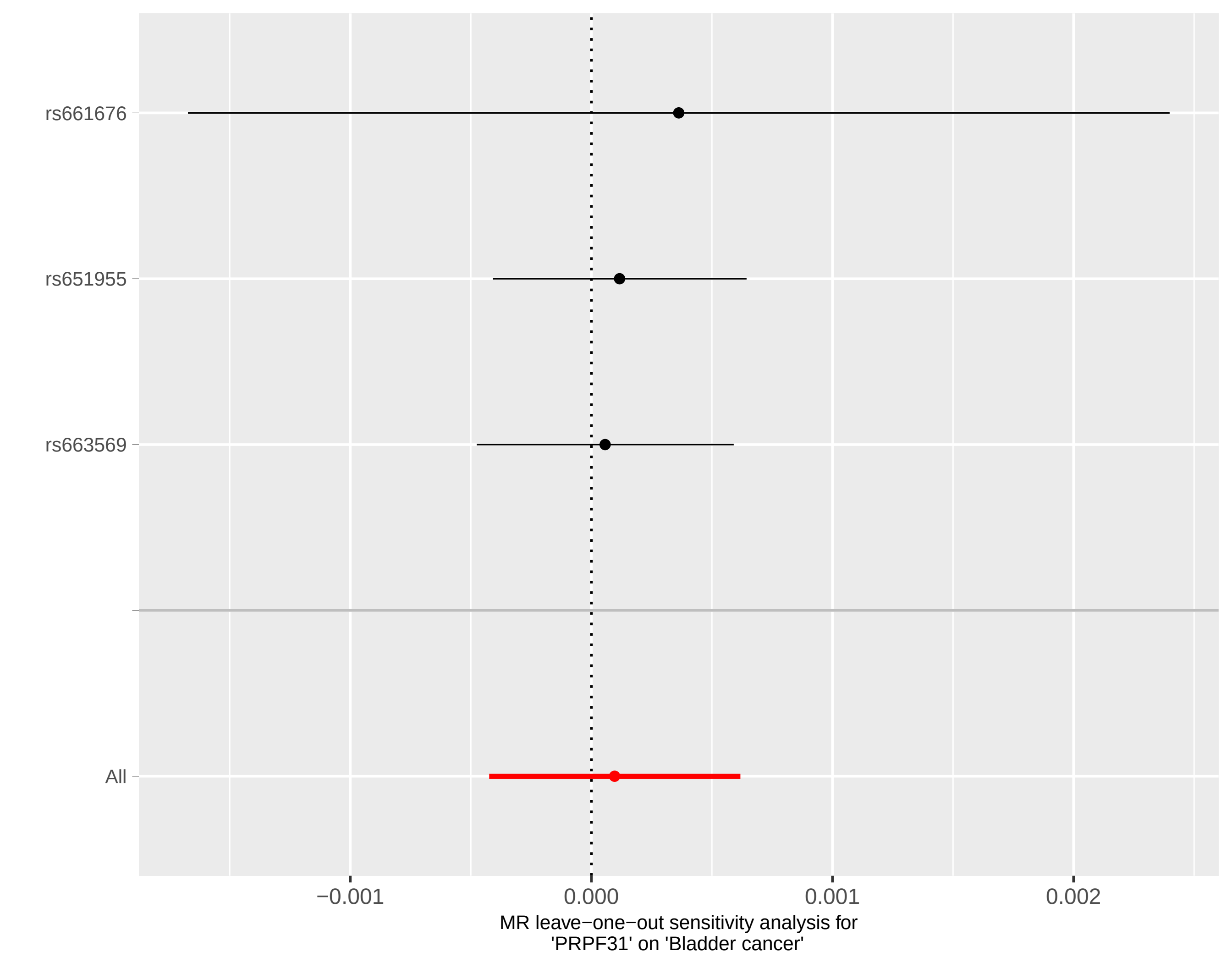
rs661676
rs651955
rs663569
All
−0.001
0.000
0.001
0.002
MR leave−one−out sensitivity analysis for
'PRPF31' on 'Bladder cancer'

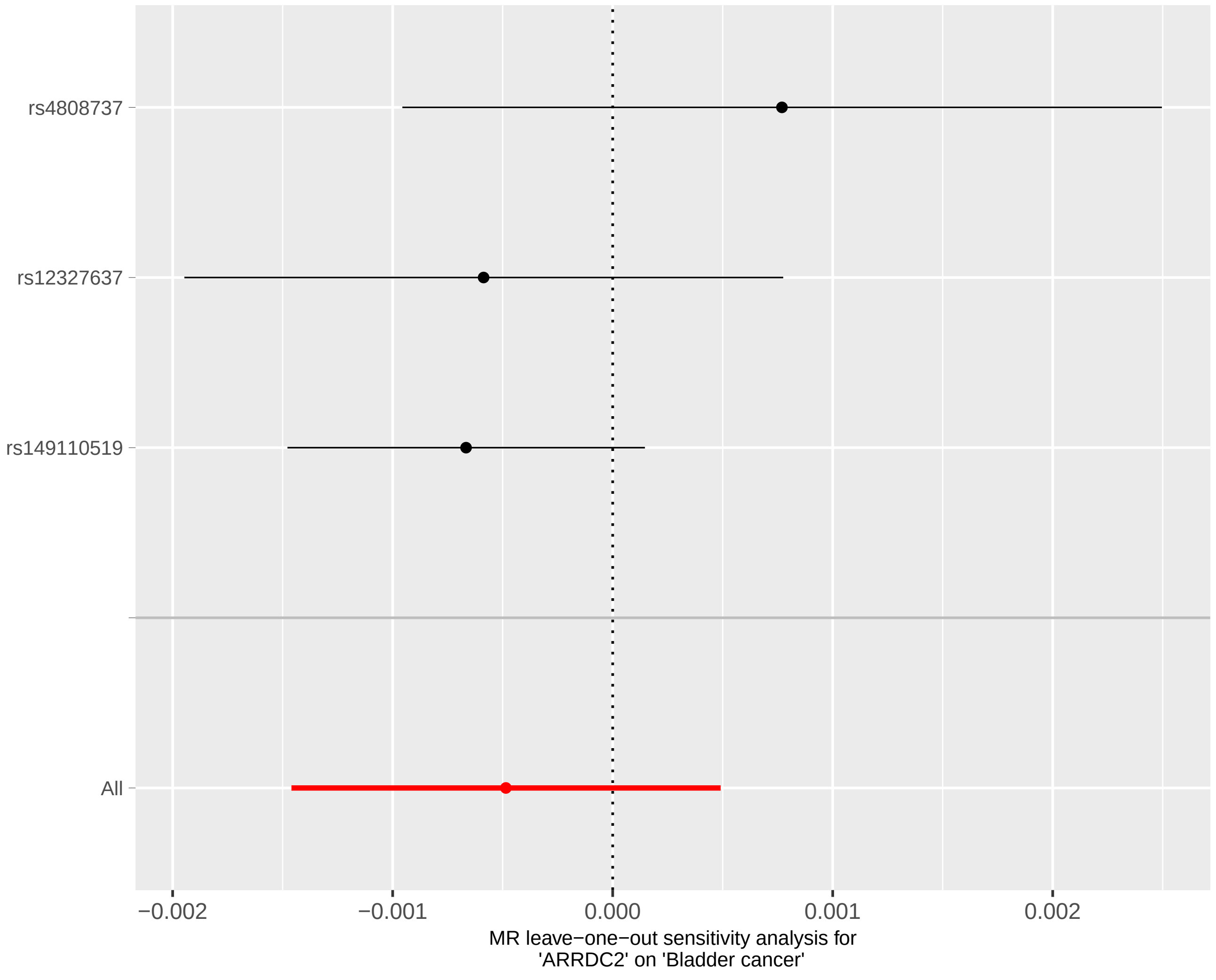
rs4808737
rs12327637
rs149110519
All
−0.002
−0.001
0.000
0.001
0.002
MR leave−one−out sensitivity analysis for
'ARRDC2' on 'Bladder cancer'

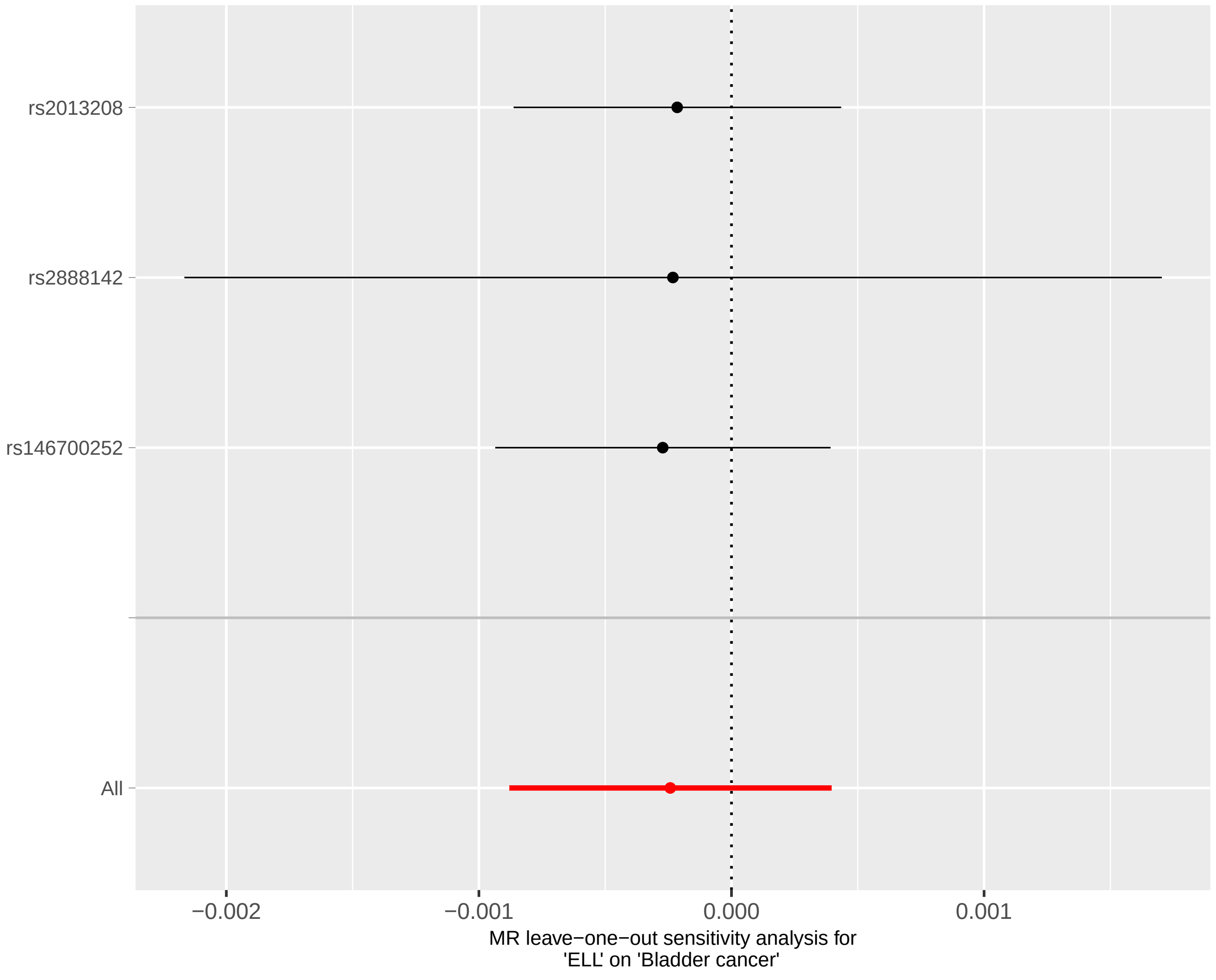
rs2013208
rs2888142
rs146700252
All
−0.002
−0.001
0.000
0.001
MR leave−one−out sensitivity analysis for
'ELL' on 'Bladder cancer'

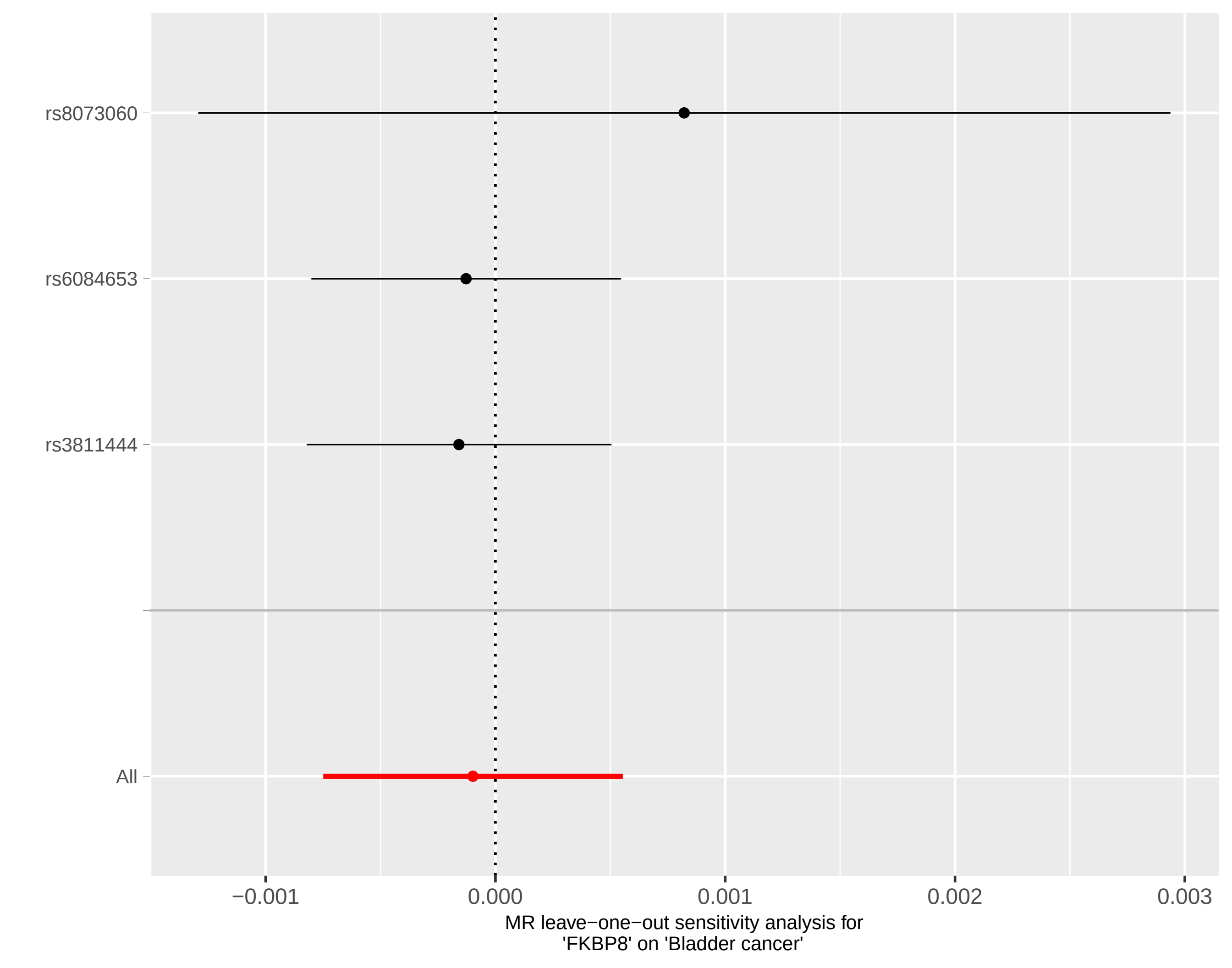

rs8073060
rs6084653
rs3811444
All
−0.001
0.000
0.001
0.002
0.003
MR leave−one−out sensitivity analysis for
'FKBP8' on 'Bladder cancer'

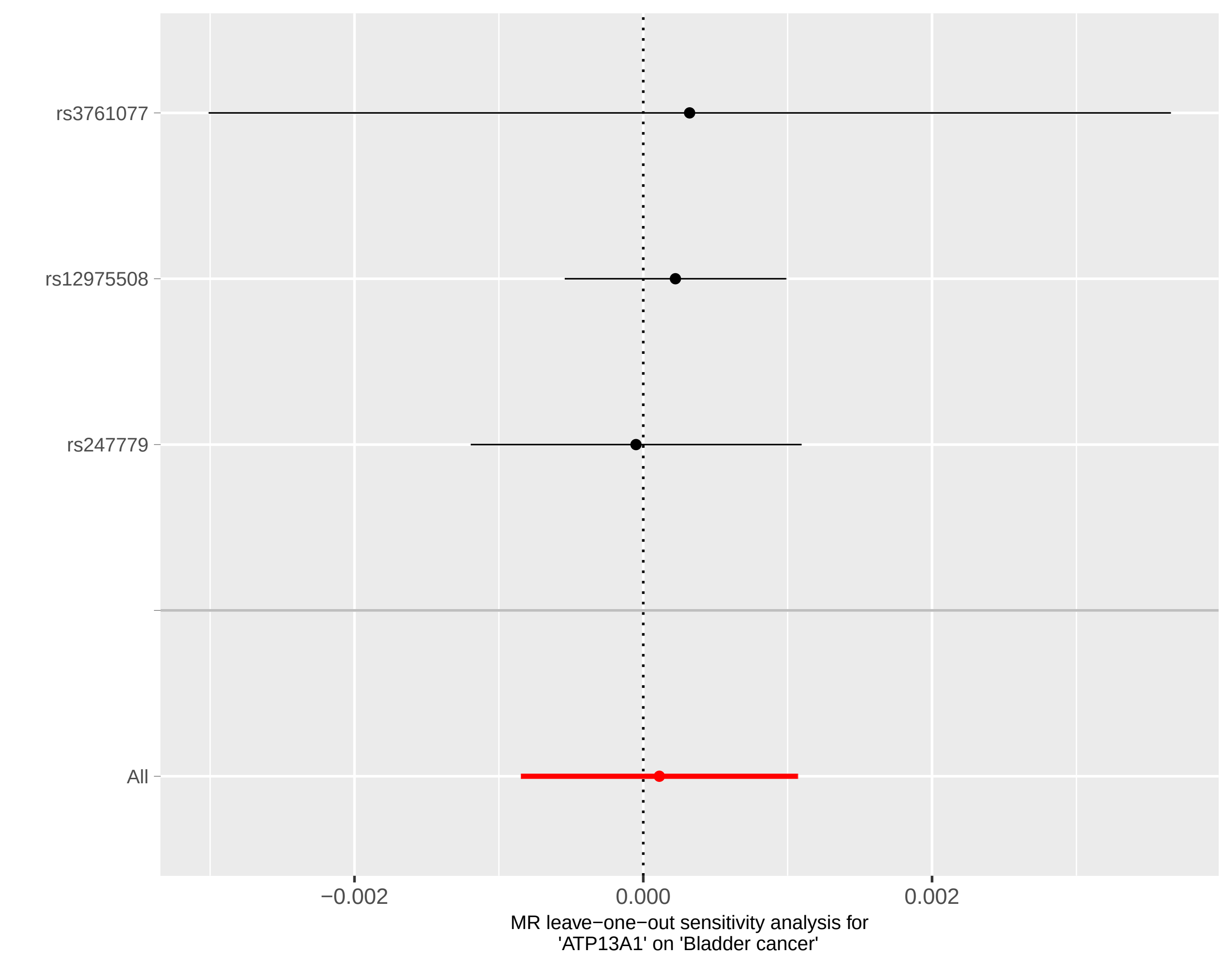
rs3761077
rs12975508
rs247779
All
−0.002
0.000
0.002
MR leave−one−out sensitivity analysis for
'ATP13A1' on 'Bladder cancer'

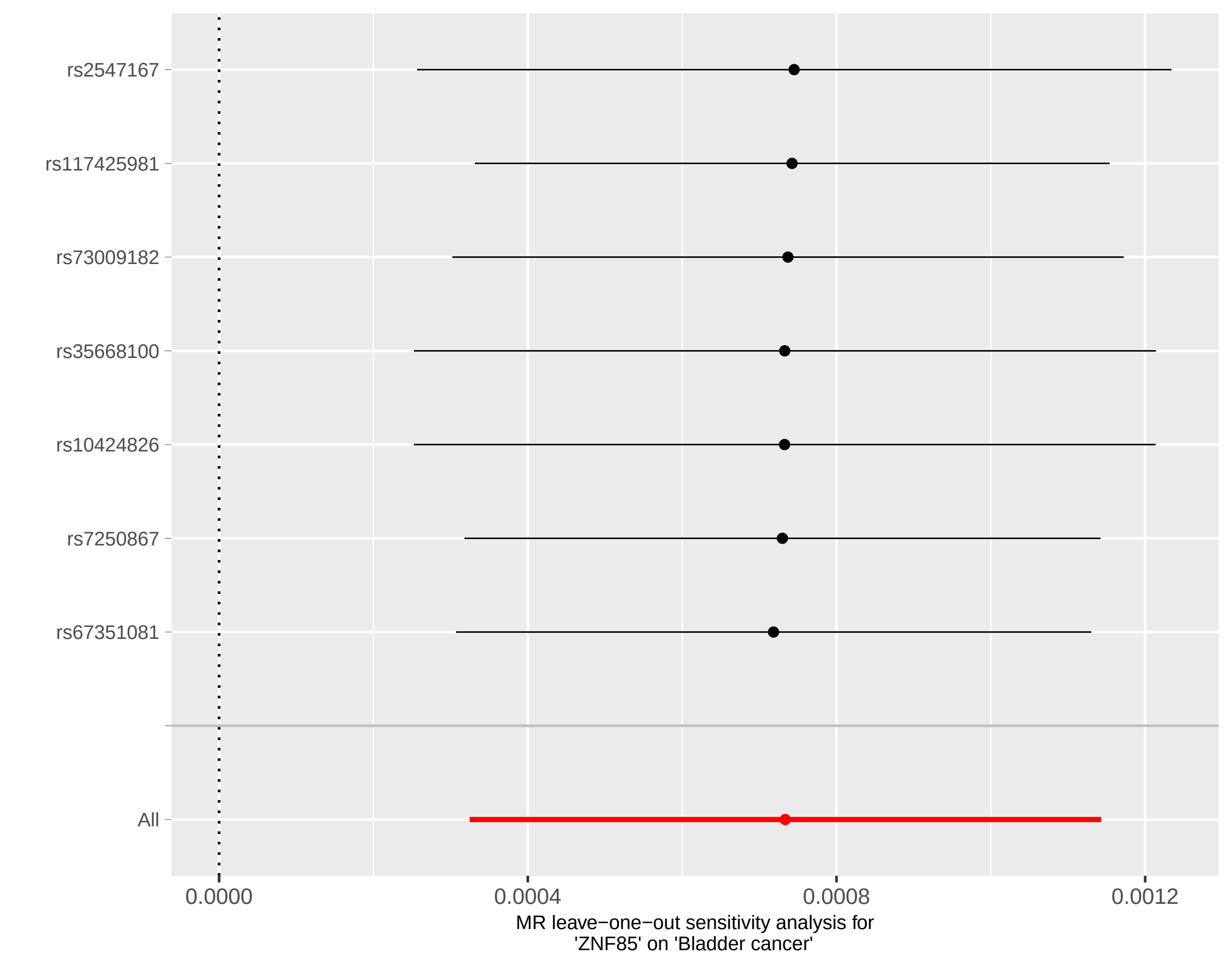

rs2547167
rs117425981
rs73009182
rs35668100
rs10424826
rs7250867
rs67351081
All
0.0000
0.0004
0.0008
0.0012
MR leave−one−out sensitivity analysis for
'ZNF85' on 'Bladder cancer'

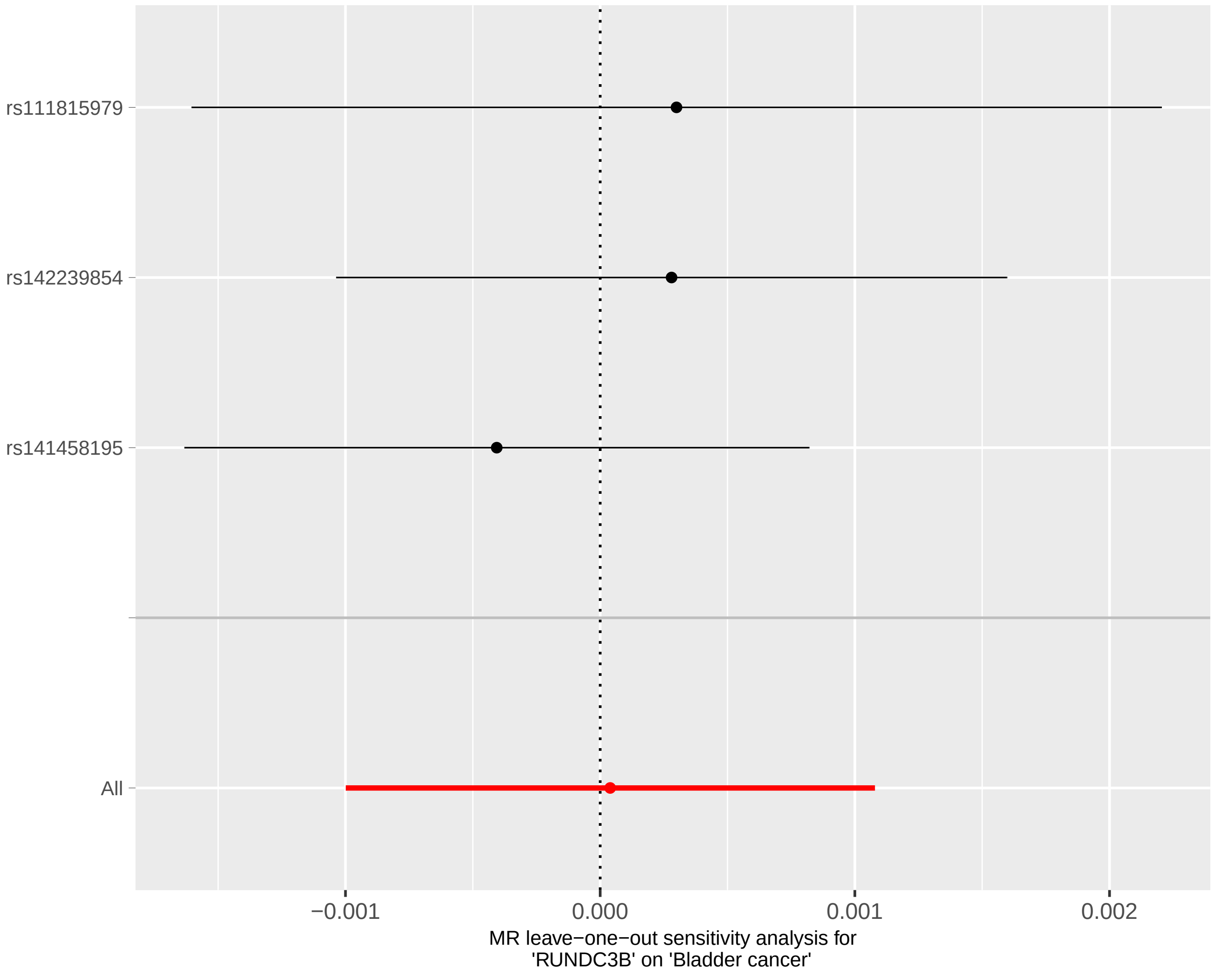
rs111815979
rs142239854
rs141458195
All
−0.001
0.000
0.001
0.002
MR leave−one−out sensitivity analysis for
'RUNDC3B' on 'Bladder cancer'

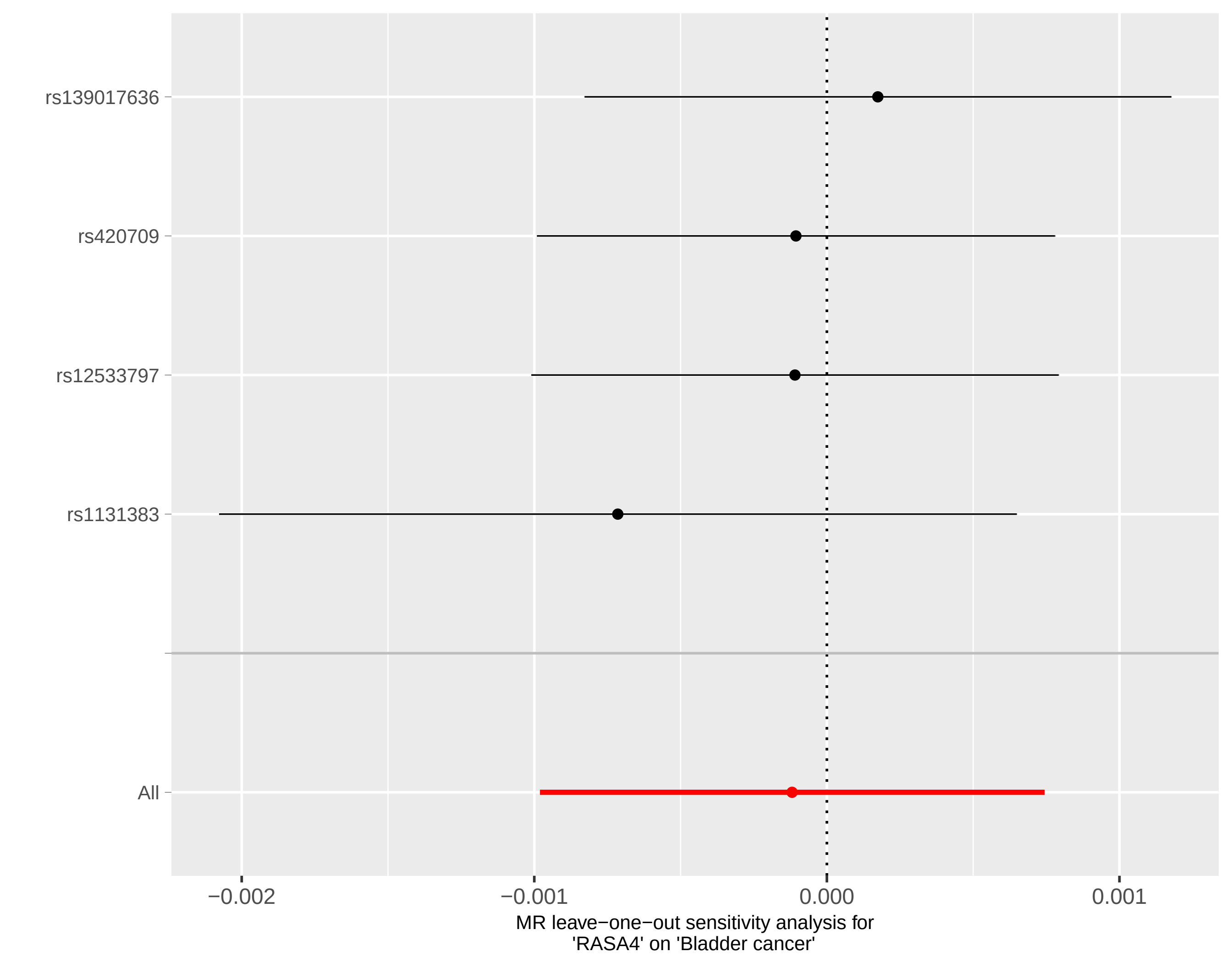
rs139017636
rs420709
rs12533797
rs1131383
All
−0.002
−0.001
0.000
0.001
MR leave−one−out sensitivity analysis for
'RASA4' on 'Bladder cancer'

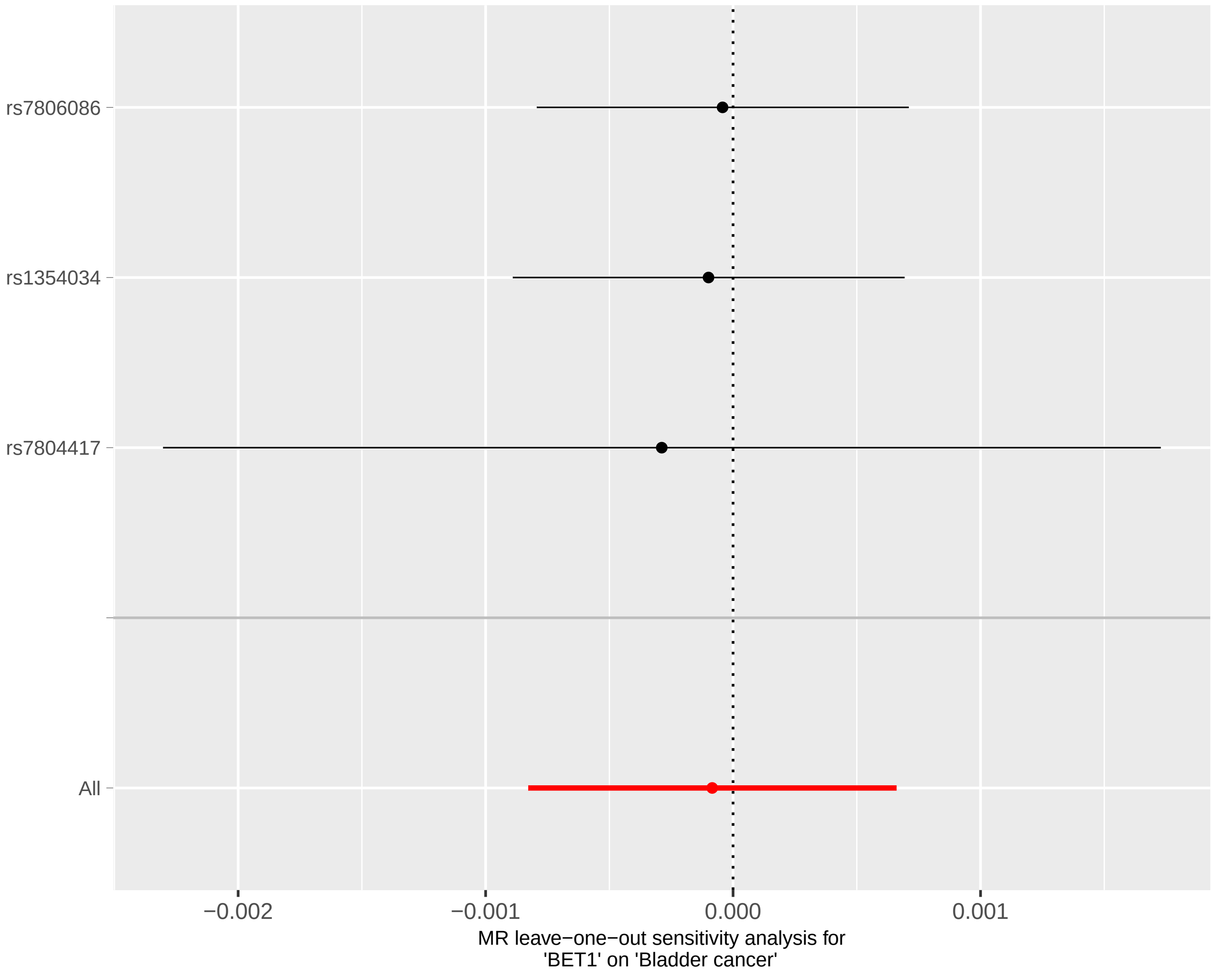
rs7806086
rs1354034
rs7804417
All
−0.002
−0.001
0.000
0.001
MR leave−one−out sensitivity analysis for
'BET1' on 'Bladder cancer'

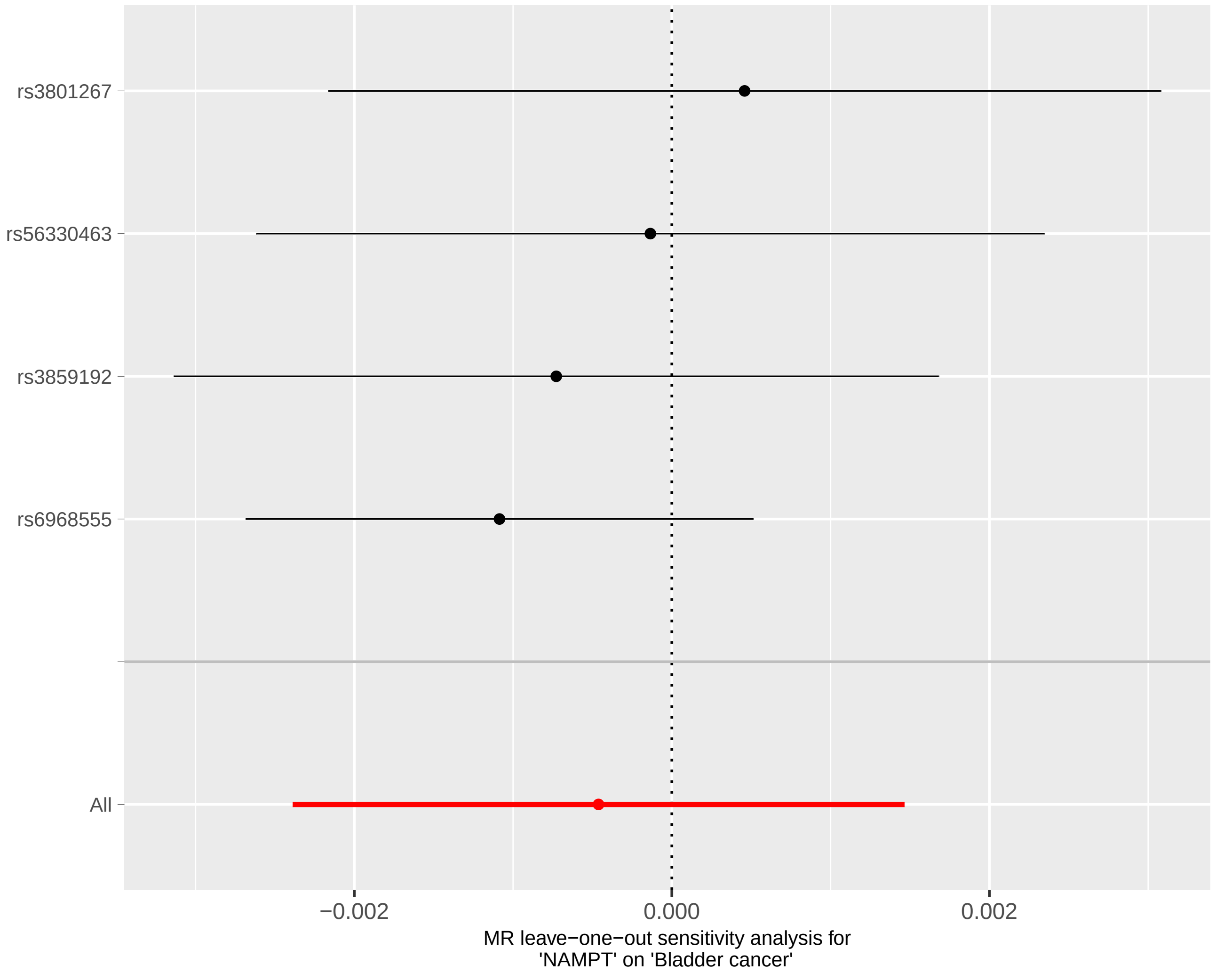
rs3801267
rs56330463
rs3859192
rs6968555
All
−0.002
0.000
0.002
MR leave−one−out sensitivity analysis for
'NAMPT' on 'Bladder cancer'

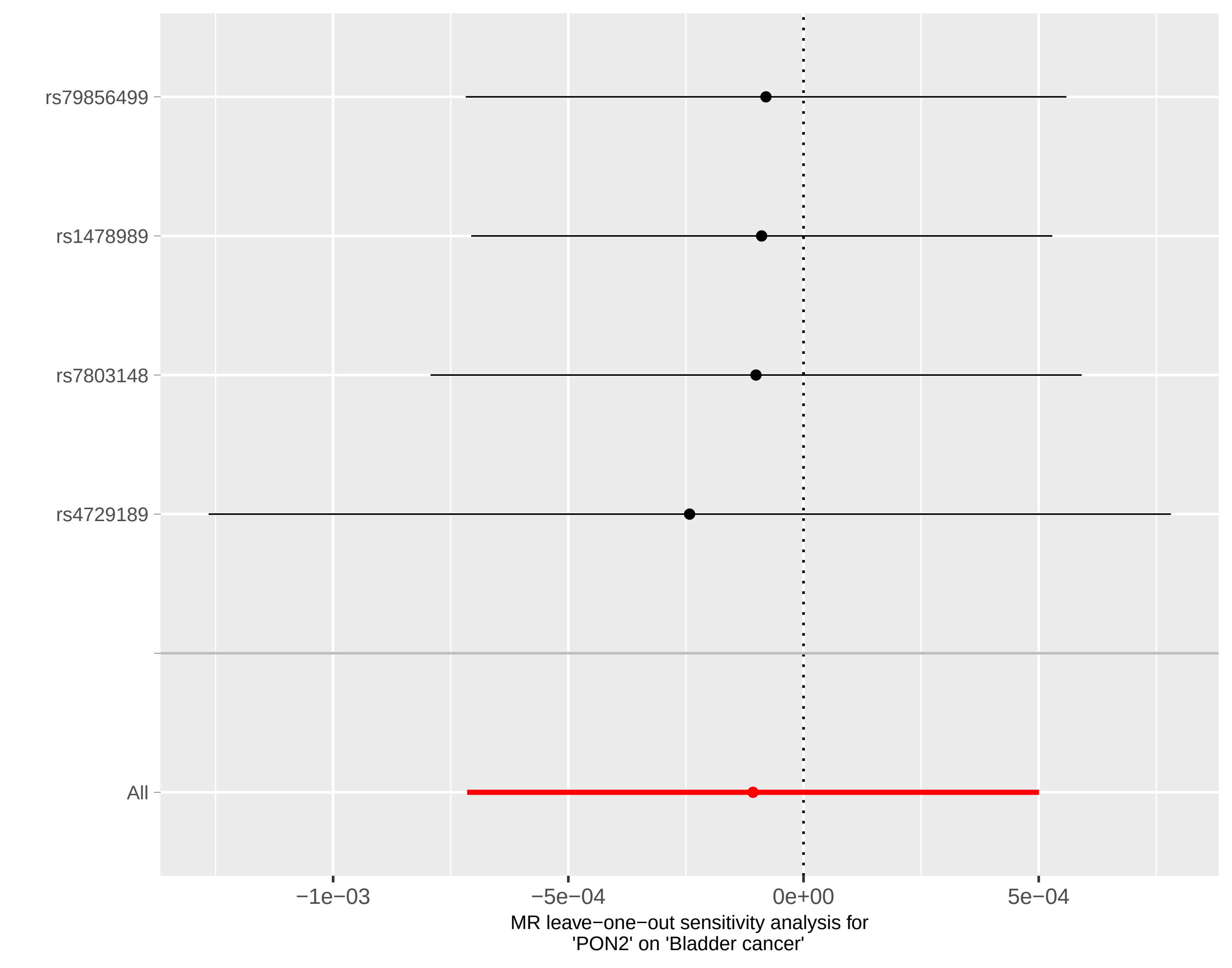
rs79856499
rs1478989
rs7803148
rs4729189
All
−1e−03
−5e−04
0e+00
5e−04
MR leave−one−out sensitivity analysis for
'PON2' on 'Bladder cancer'

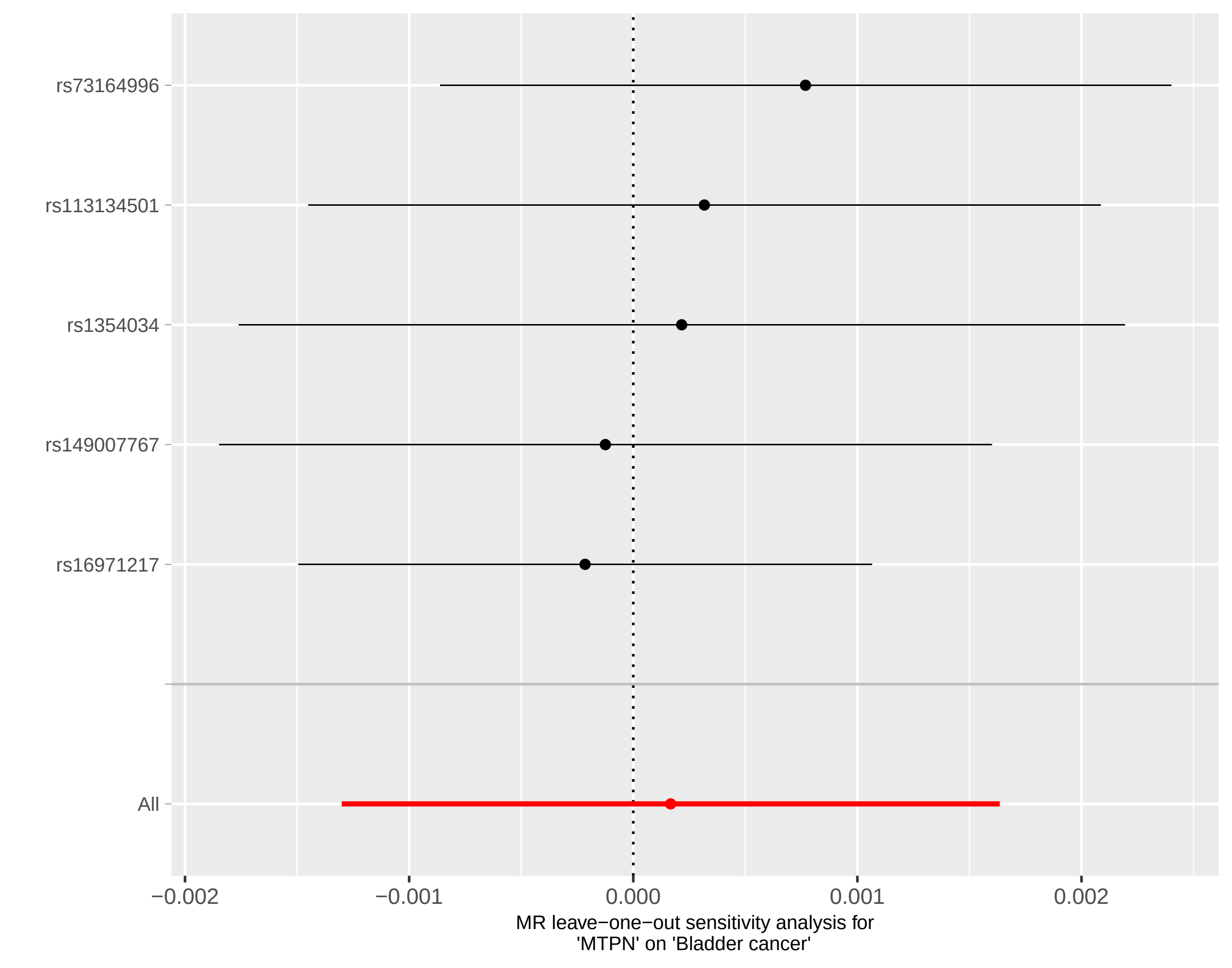

rs73164996
rs113134501
rs1354034
rs149007767
rs16971217
All
−0.002
−0.001
0.000
0.001
0.002
MR leave−one−out sensitivity analysis for
'MTPN' on 'Bladder cancer'

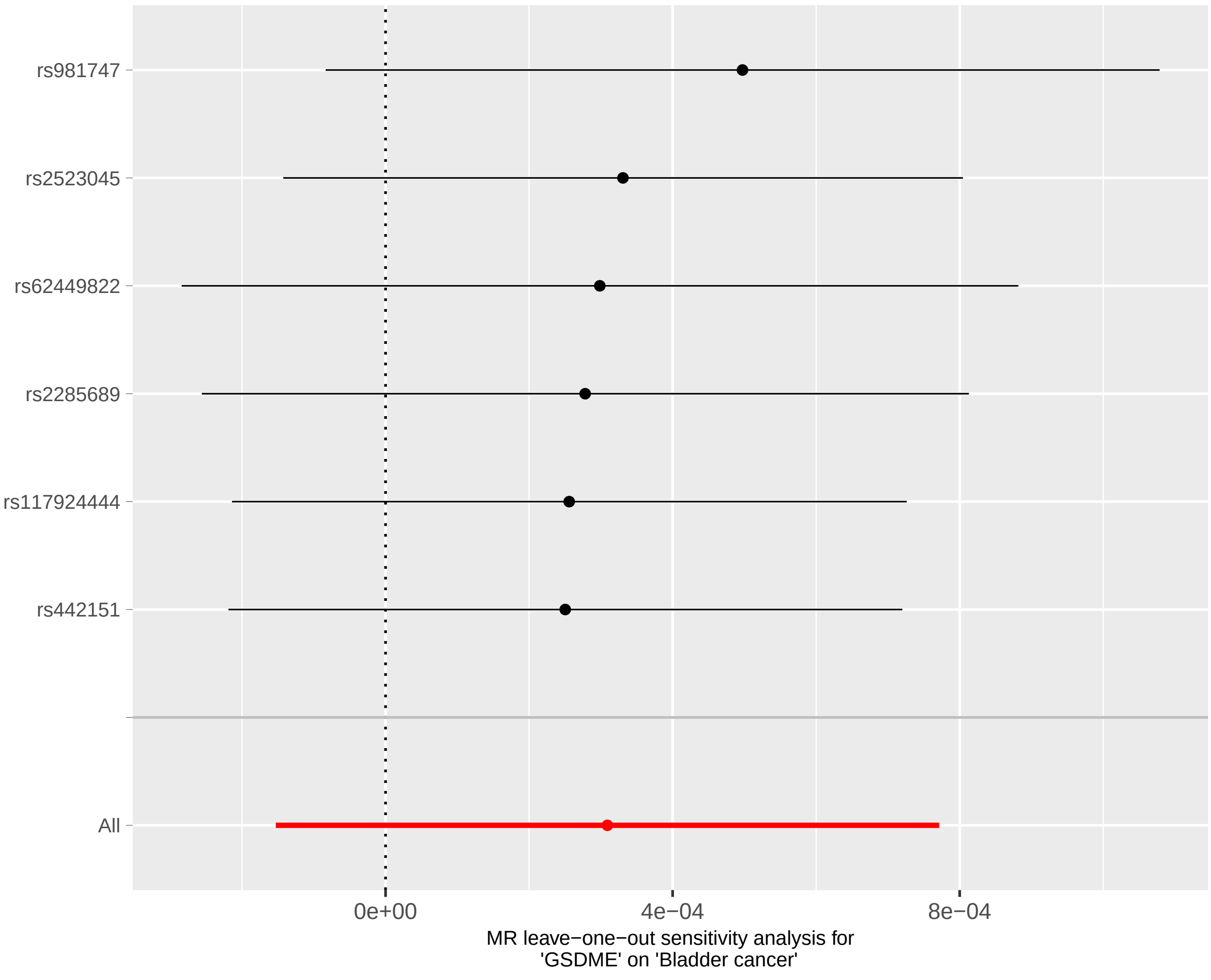
rs981747
rs2523045
rs62449822
rs2285689
rs117924444
rs442151
All
0e+00
4e−04
8e−04
MR leave−one−out sensitivity analysis for
'GSDME' on 'Bladder cancer'

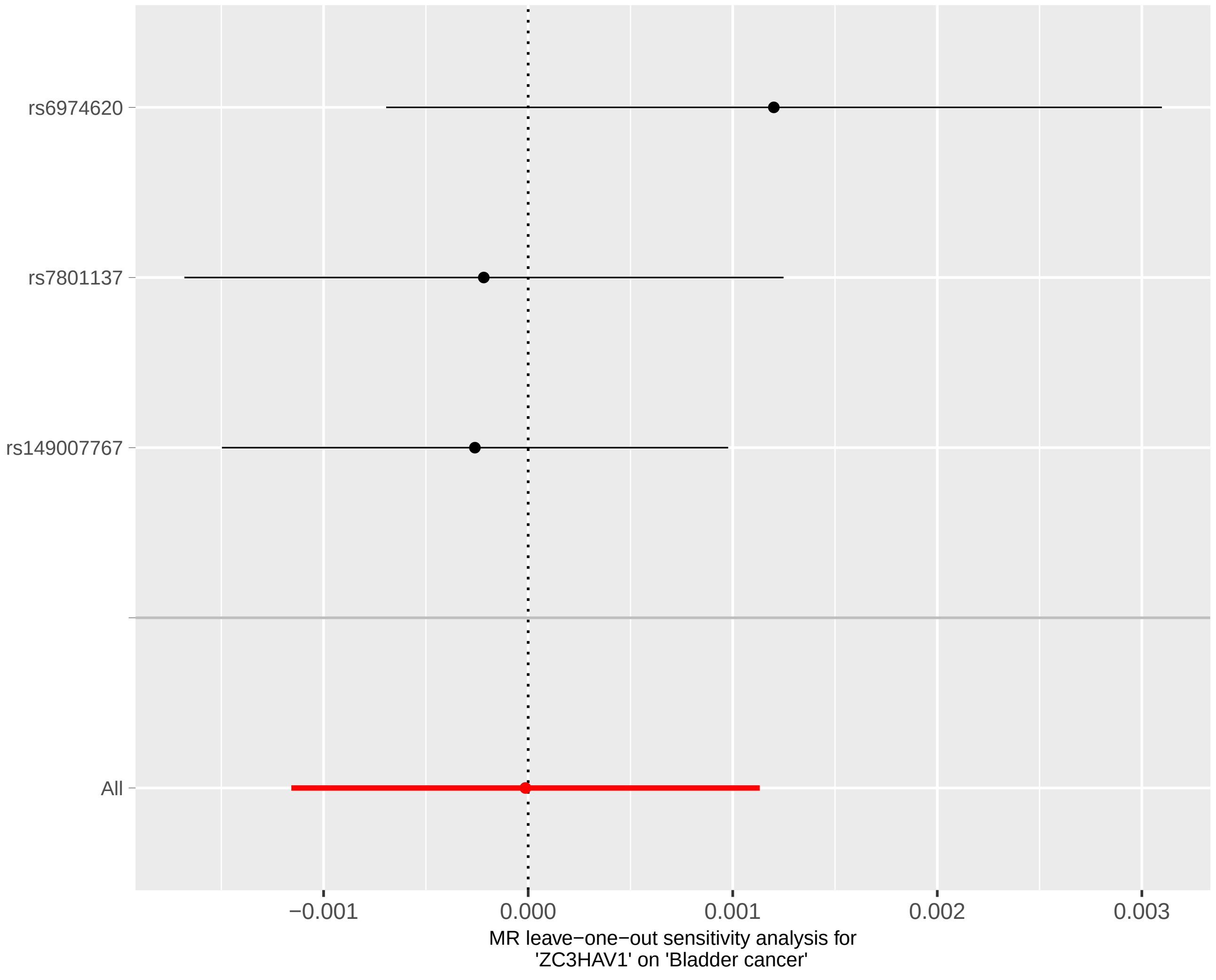
rs6974620
rs7801137
rs149007767
All
−0.001
0.000
0.001
0.002
0.003
MR leave−one−out sensitivity analysis for
'ZC3HAV1' on 'Bladder cancer'

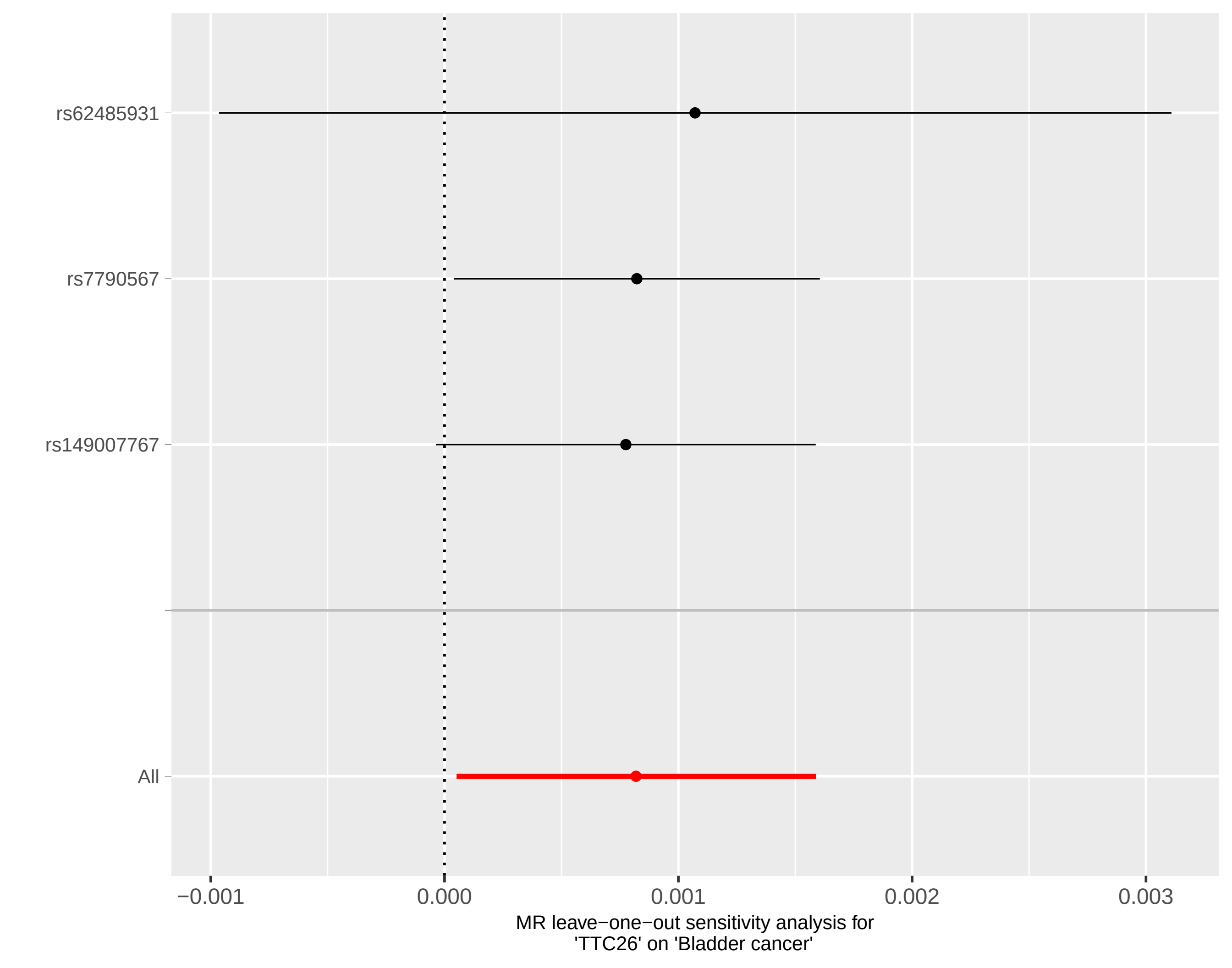
rs62485931
rs7790567
rs149007767
All
−0.001
0.000
0.001
0.002
0.003
MR leave−one−out sensitivity analysis for
'TTC26' on 'Bladder cancer'

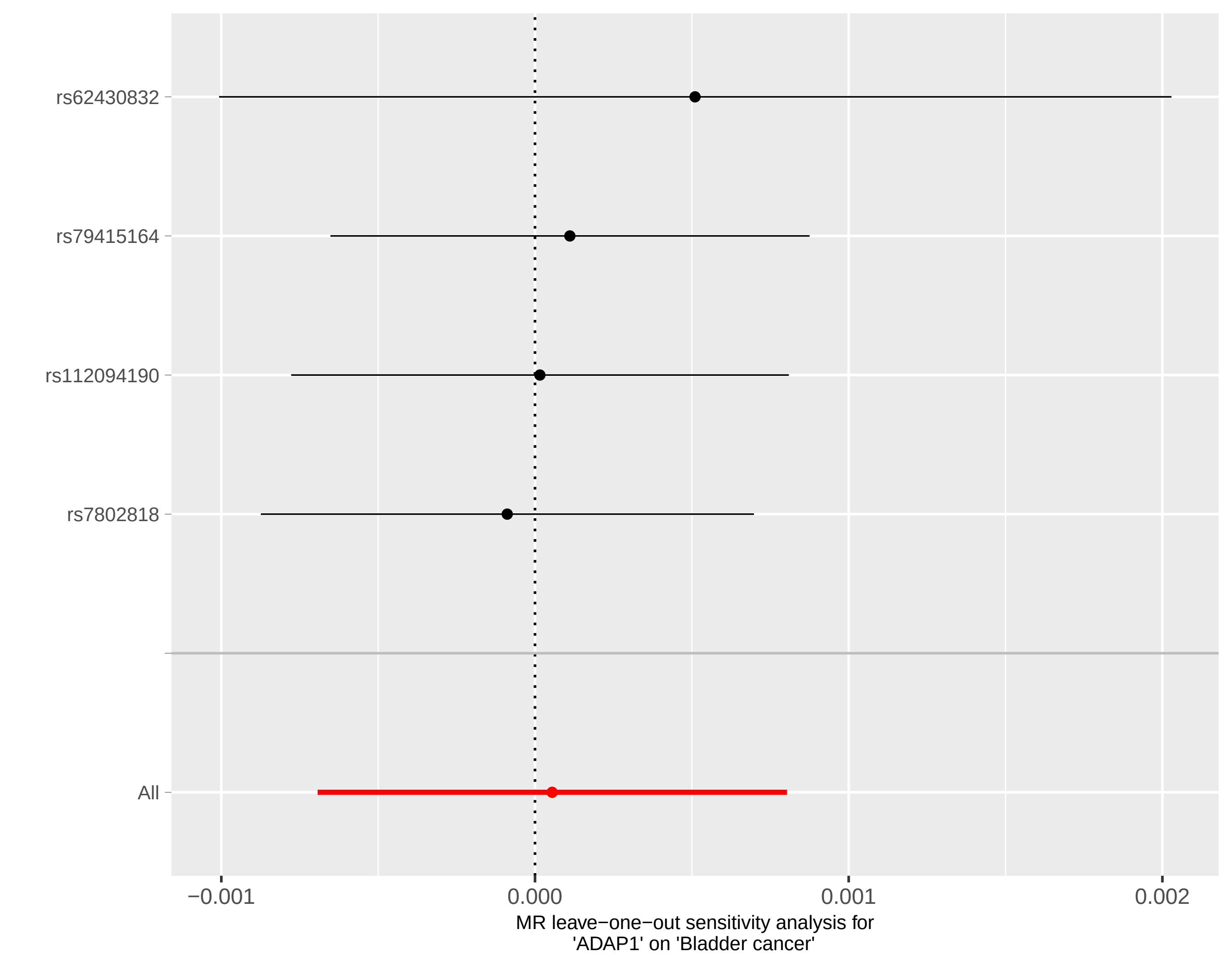
rs62430832
rs79415164
rs112094190
rs7802818
All
−0.001
0.000
0.001
0.002
MR leave−one−out sensitivity analysis for
'ADAP1' on 'Bladder cancer'

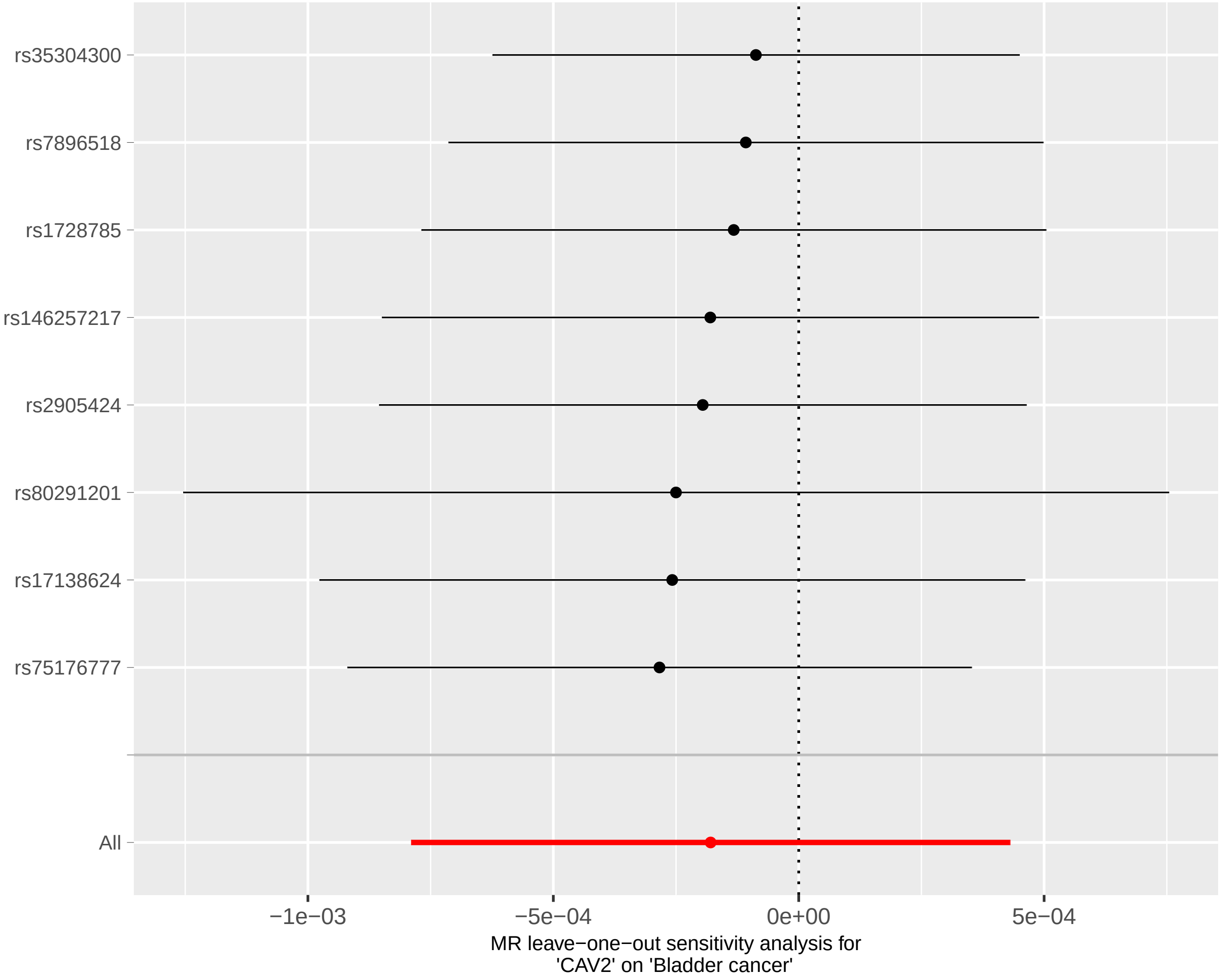
rs35304300
rs7896518
rs1728785
rs146257217
rs2905424
rs80291201
rs17138624
rs75176777
All
−1e−03
−5e−04
0e+00
5e−04
MR leave−one−out sensitivity analysis for
'CAV2' on 'Bladder cancer'

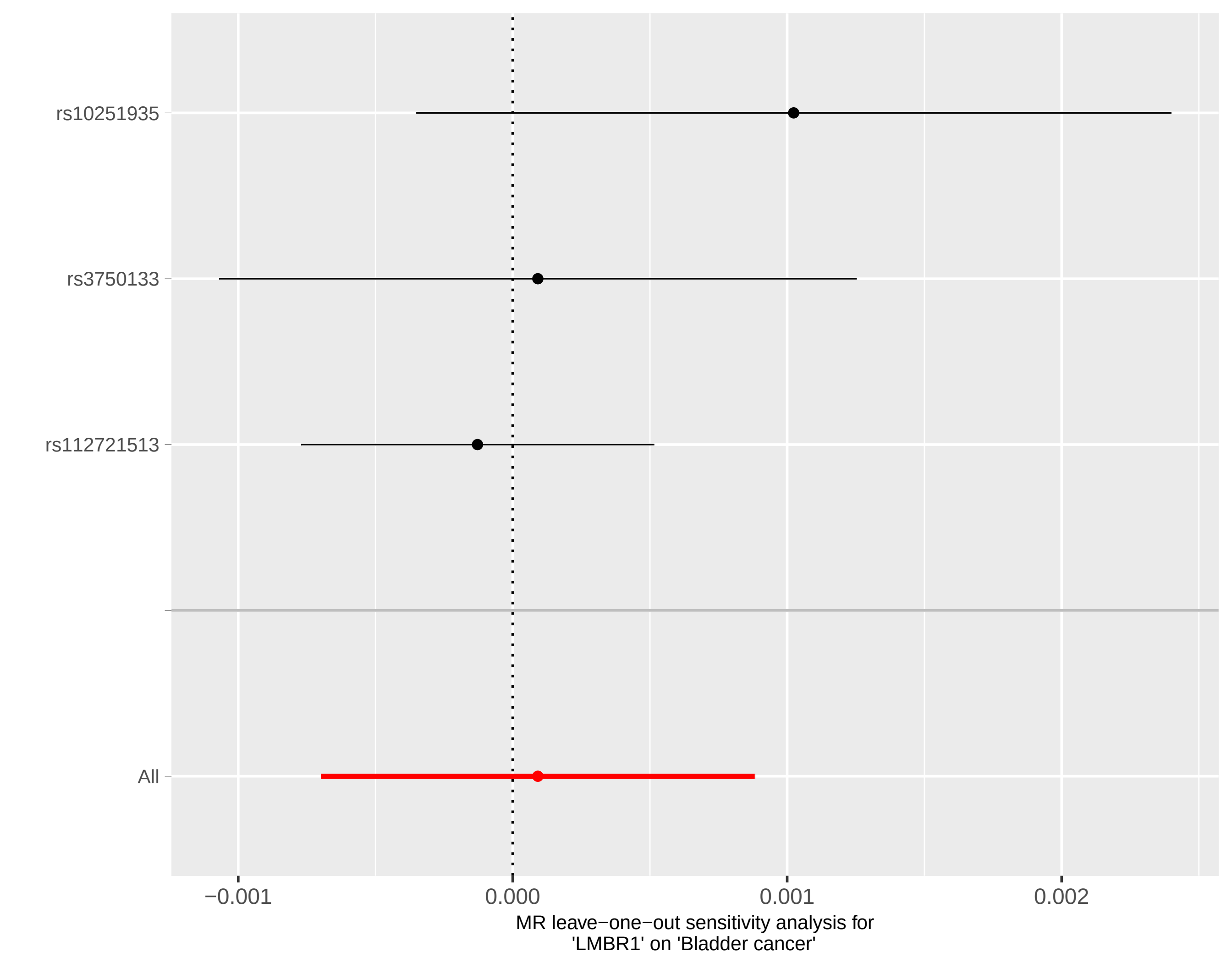
rs10251935
rs3750133
rs112721513
All
−0.001
0.000
0.001
0.002
MR leave−one−out sensitivity analysis for
'LMBR1' on 'Bladder cancer'

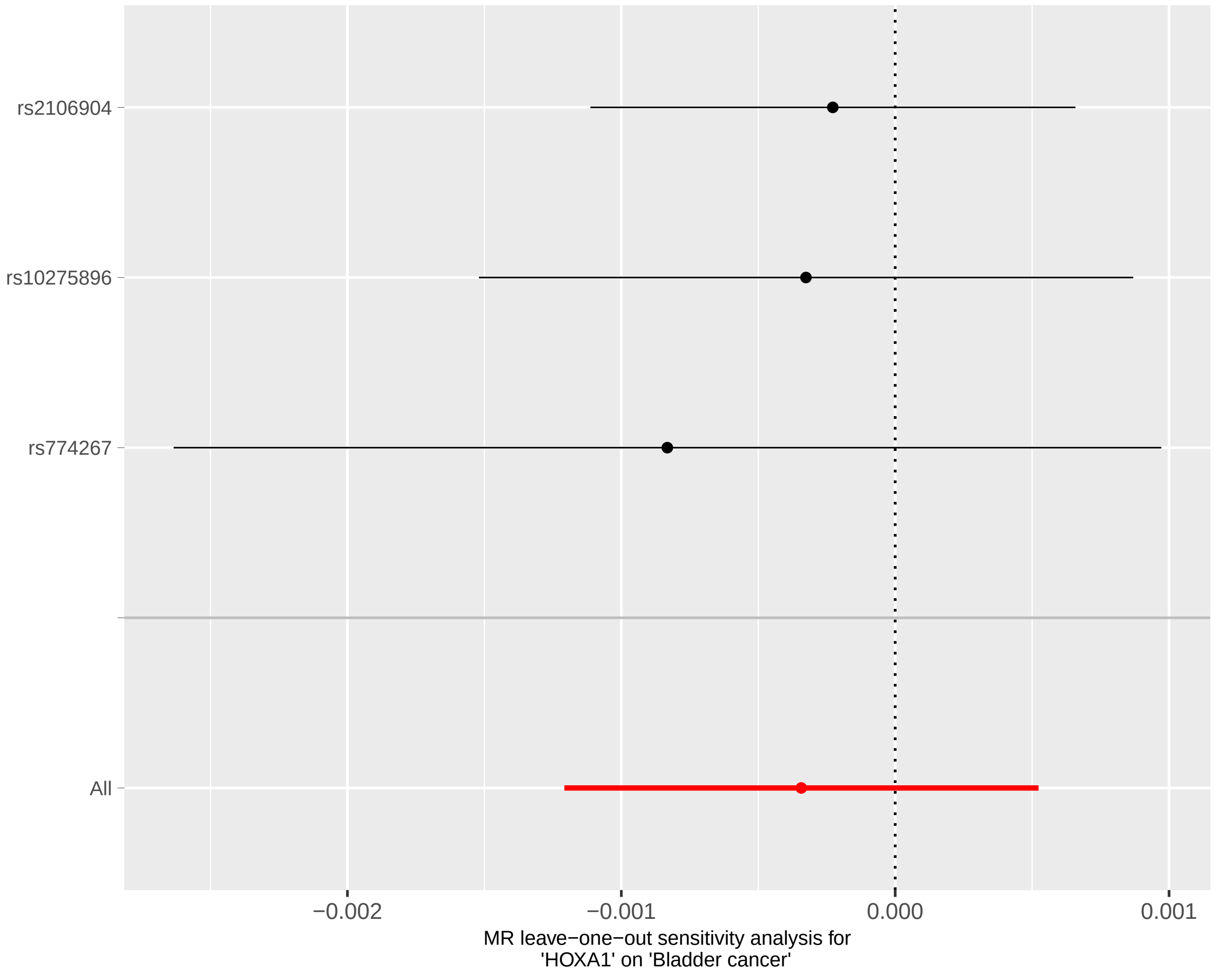
rs2106904
rs10275896
rs774267
All
−0.002
−0.001
0.000
0.001
MR leave−one−out sensitivity analysis for
'HOXA1' on 'Bladder cancer'

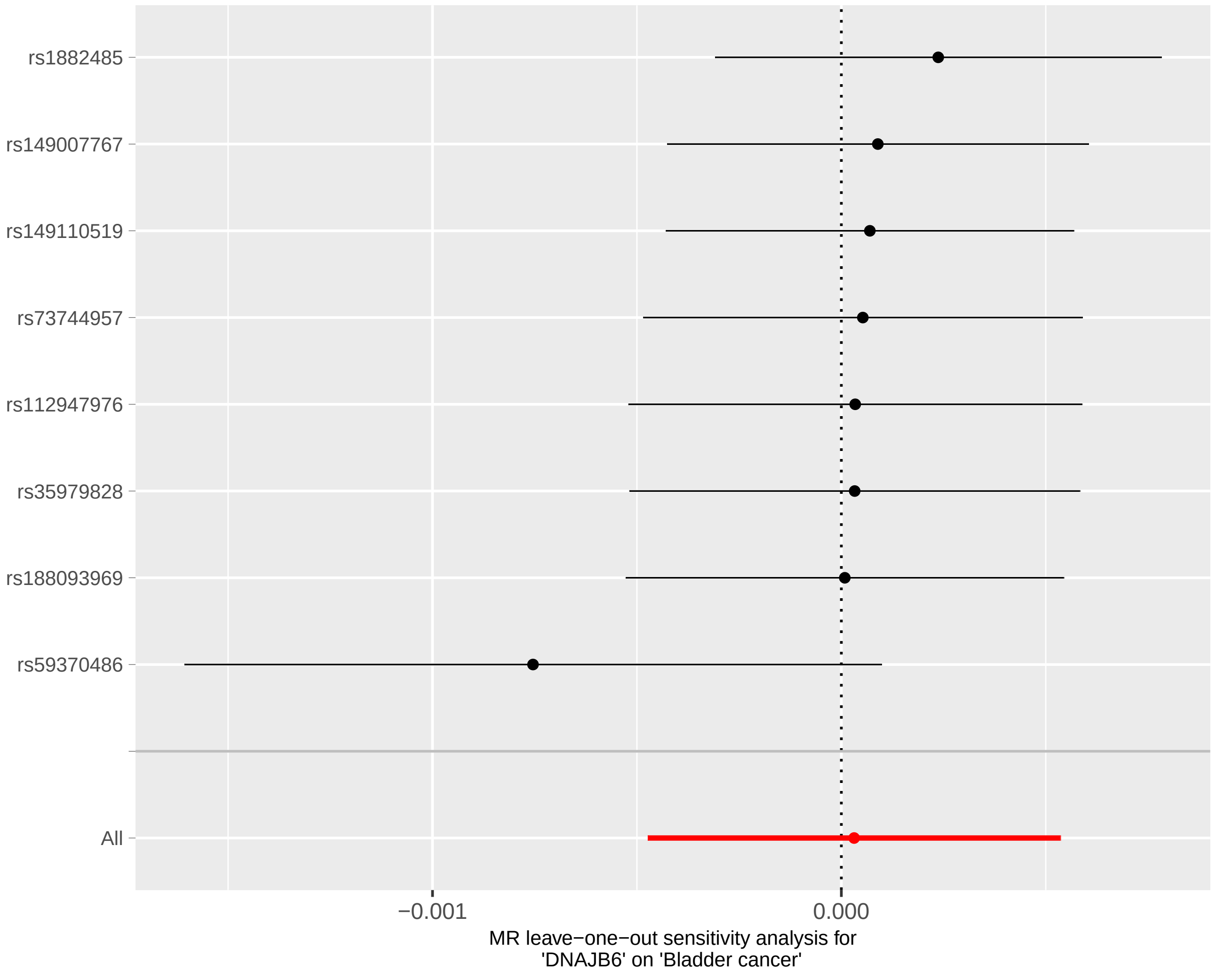
rs1882485
rs149007767
rs149110519
rs73744957
rs112947976
rs35979828
rs188093969
rs59370486
All
−0.001
0.000
MR leave−one−out sensitivity analysis for
'DNAJB6' on 'Bladder cancer'

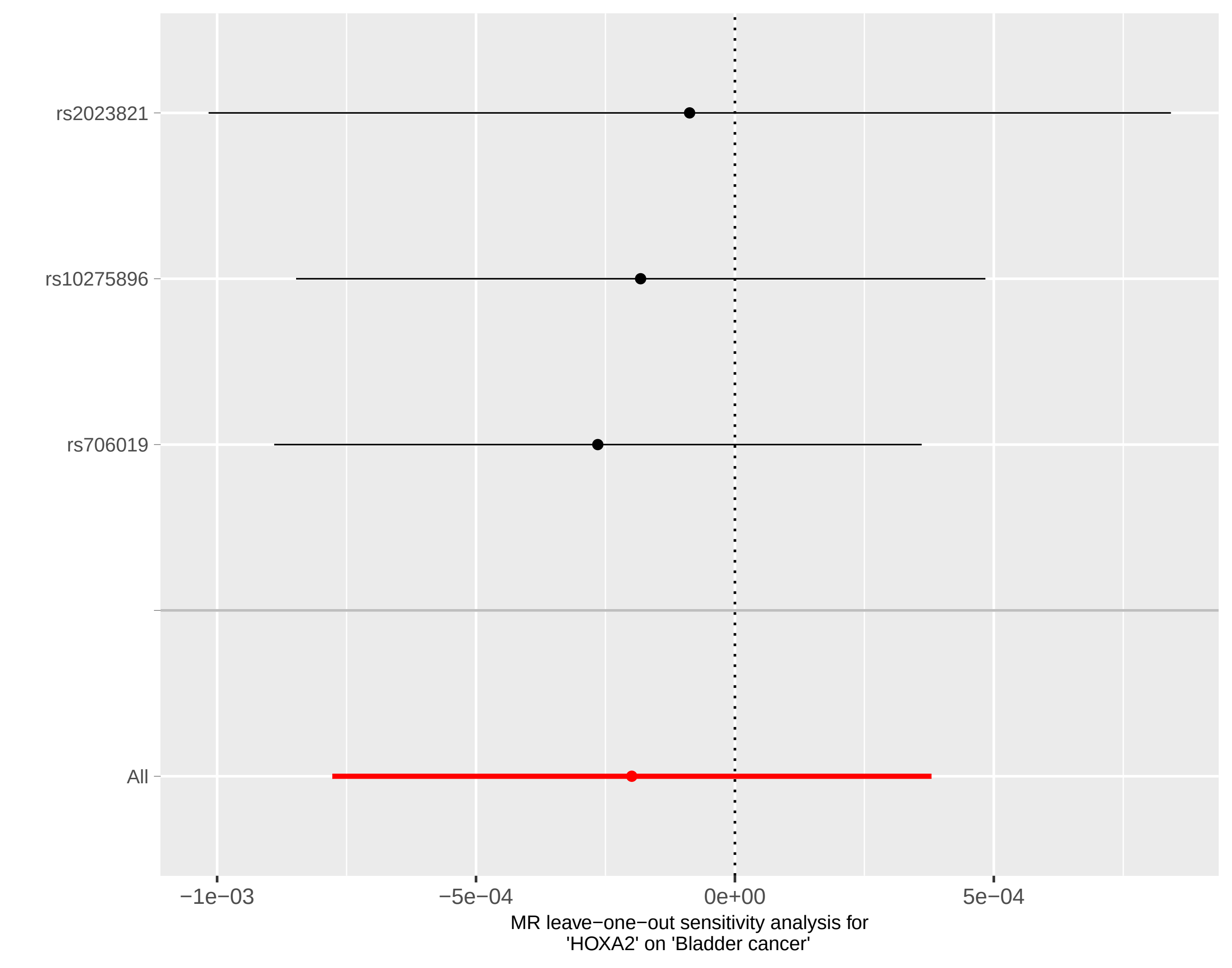
rs2023821
rs10275896
rs706019
All
−1e−03
−5e−04
0e+00
5e−04
MR leave−one−out sensitivity analysis for
'HOXA2' on 'Bladder cancer'

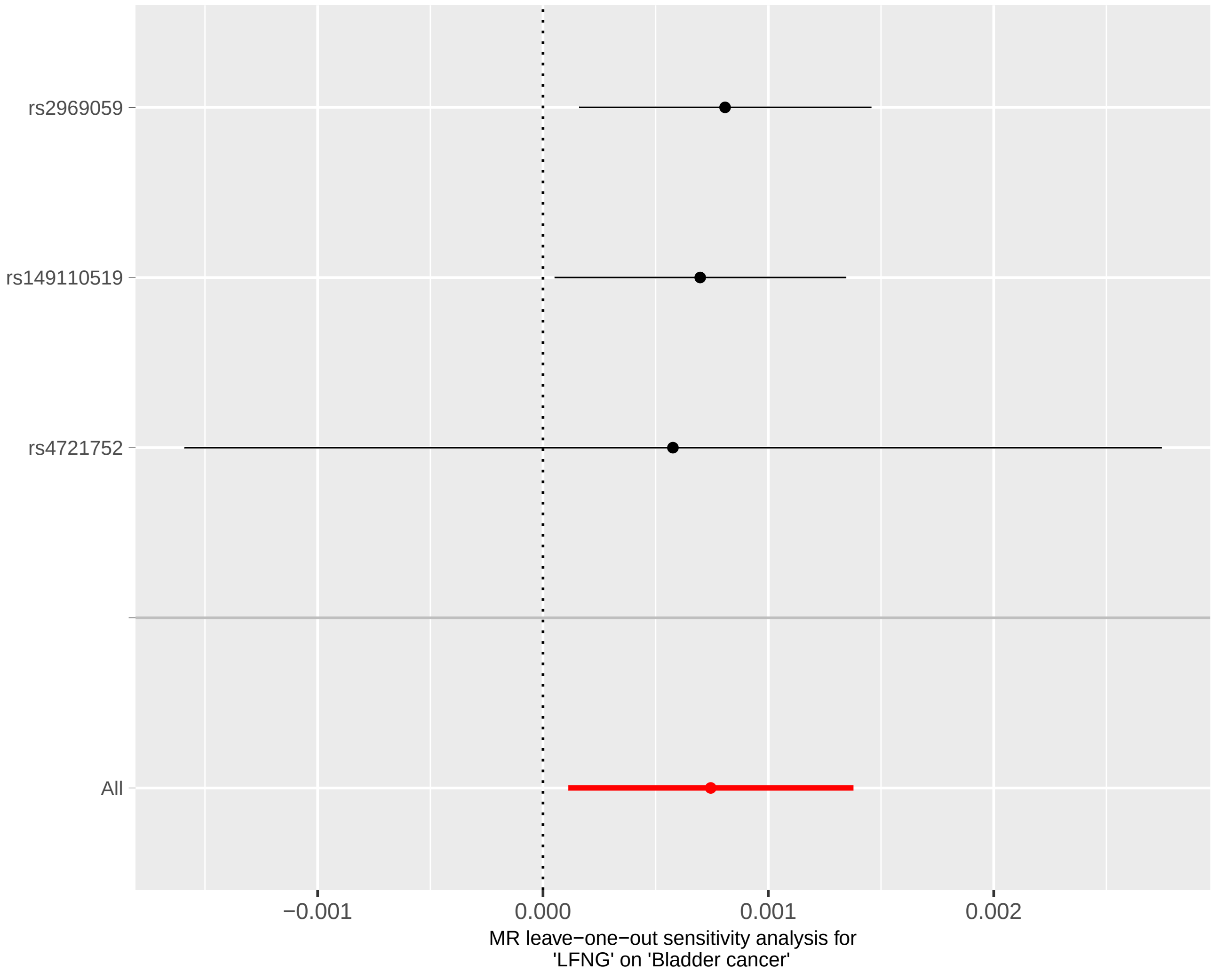
rs2969059
rs149110519
rs4721752
All
−0.001
0.000
0.001
0.002
MR leave−one−out sensitivity analysis for
'LFNG' on 'Bladder cancer'

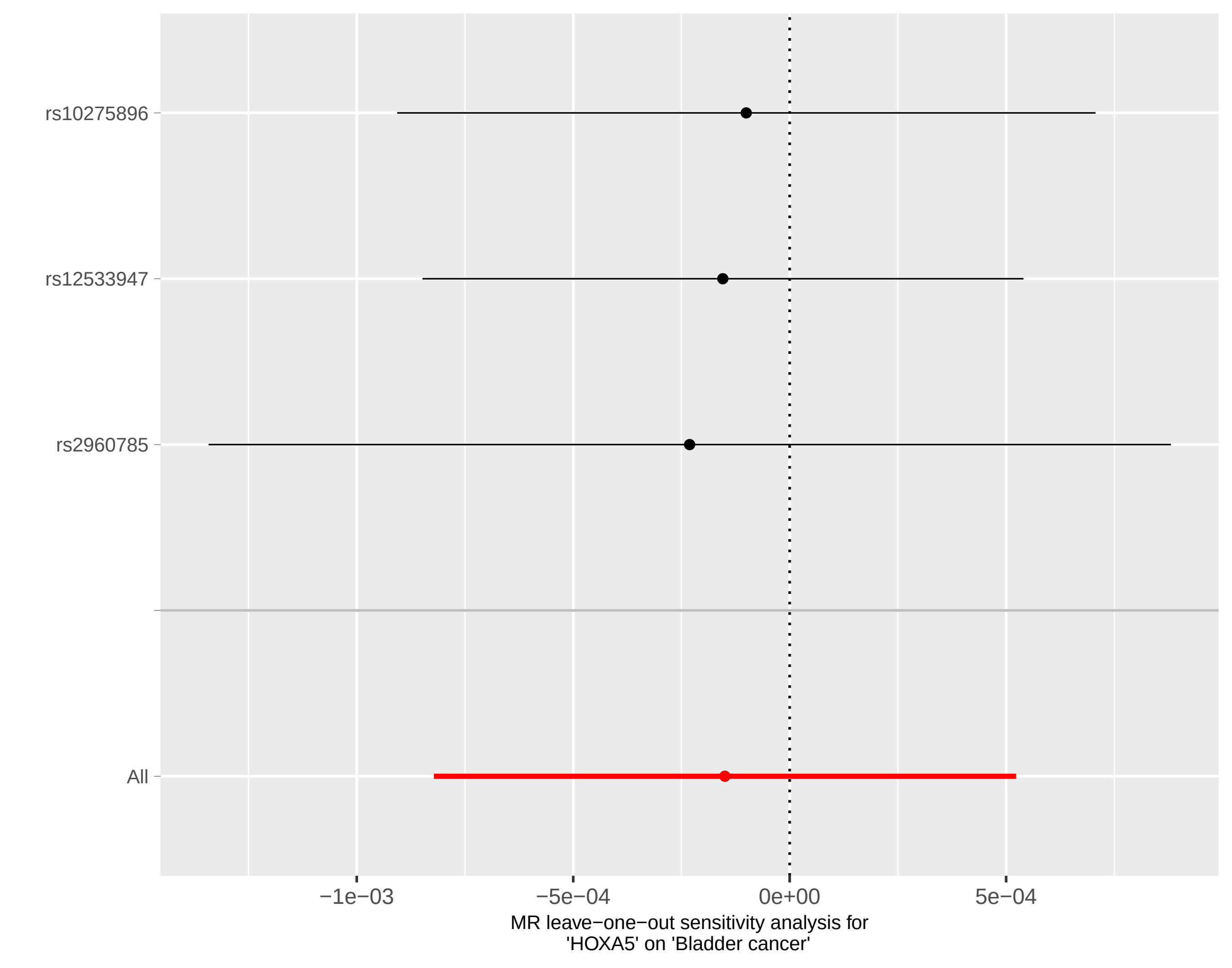
rs10275896
rs12533947
rs2960785
All
−1e−03
−5e−04
0e+00
5e−04
MR leave−one−out sensitivity analysis for
'HOXA5' on 'Bladder cancer'

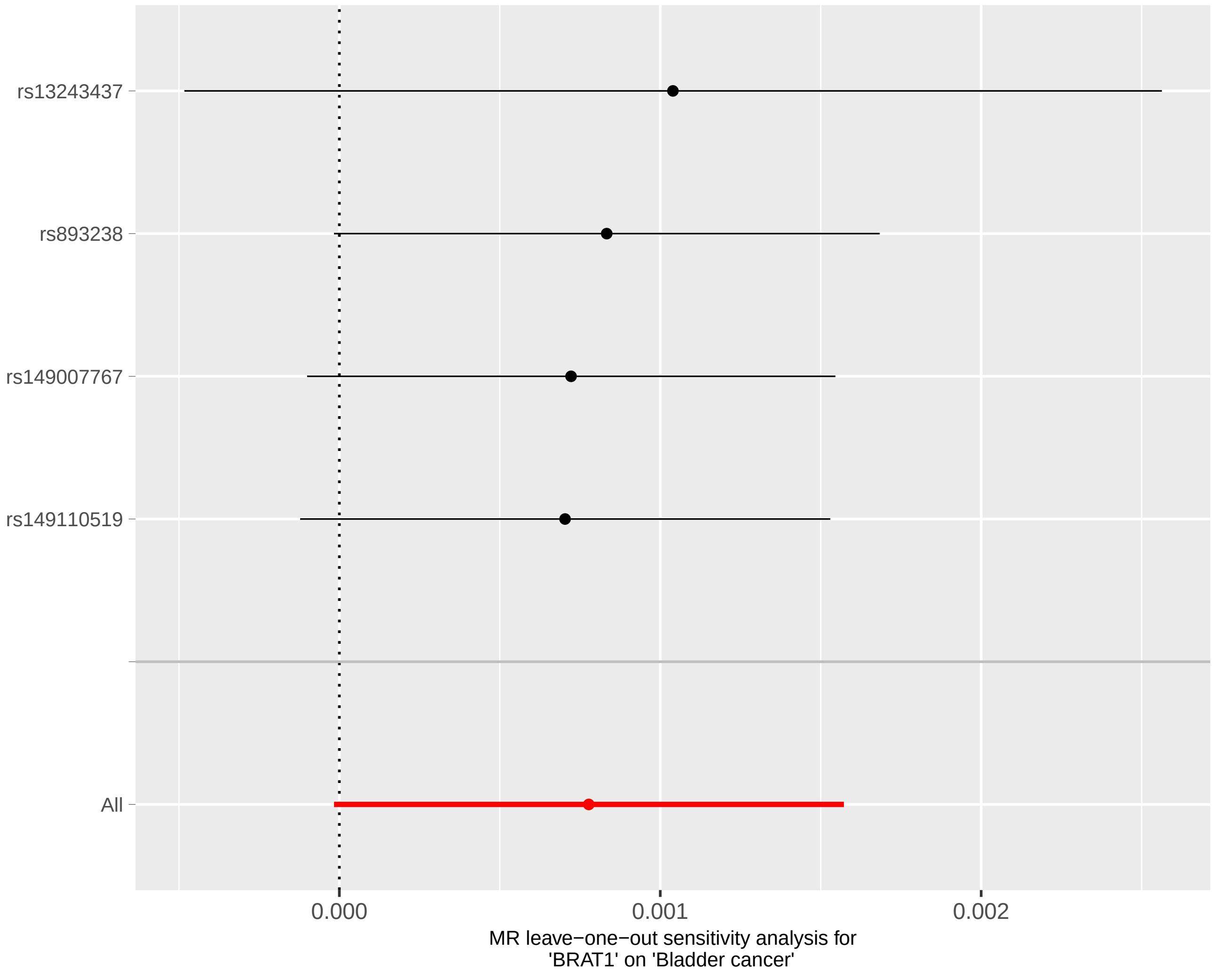

rs13243437
rs893238
rs149007767
rs149110519
All
0.000
0.001
0.002
MR leave−one−out sensitivity analysis for
'BRAT1' on 'Bladder cancer'

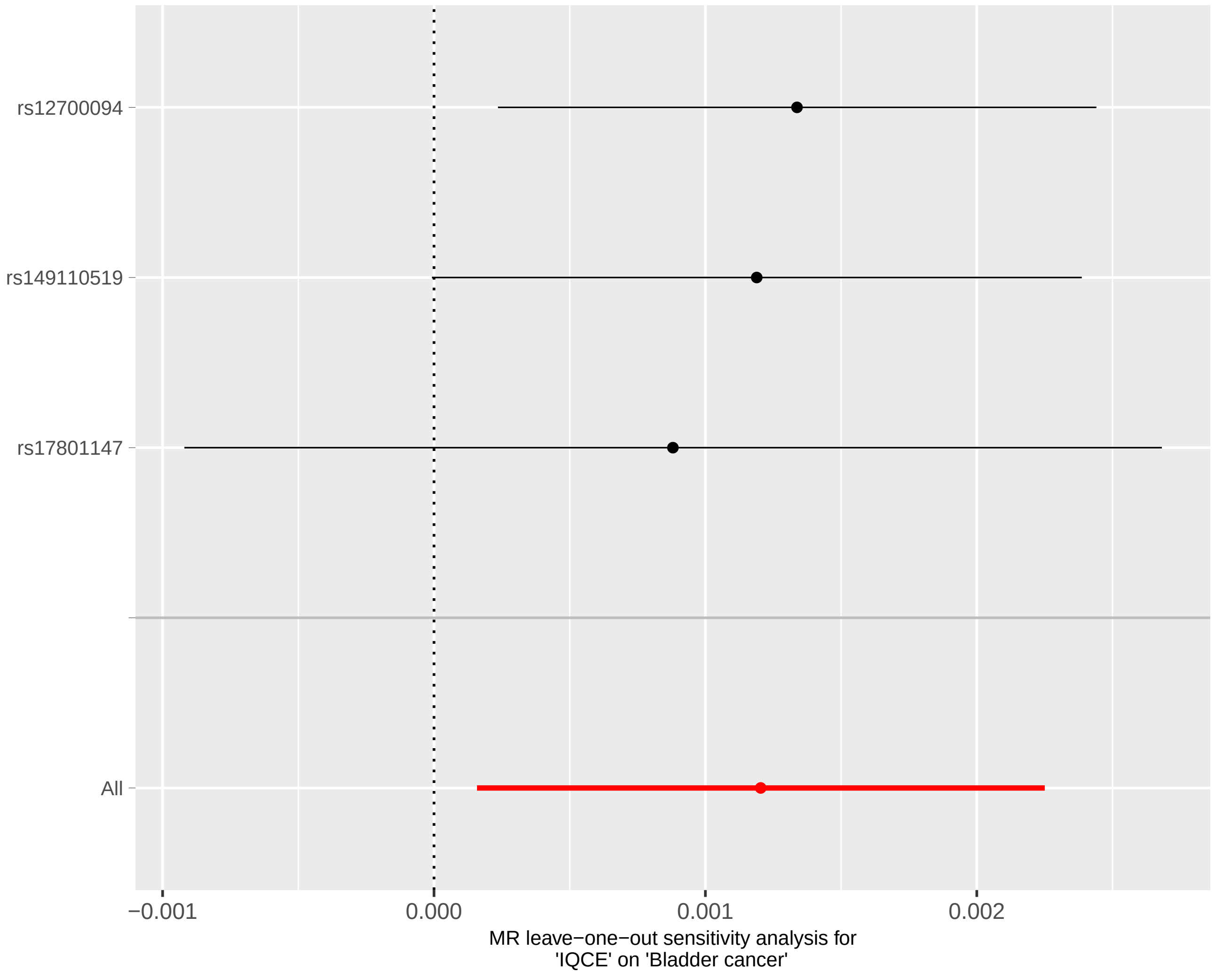
rs12700094
rs149110519
rs17801147
All
−0.001
0.000
0.001
0.002
MR leave−one−out sensitivity analysis for
'IQCE' on 'Bladder cancer'

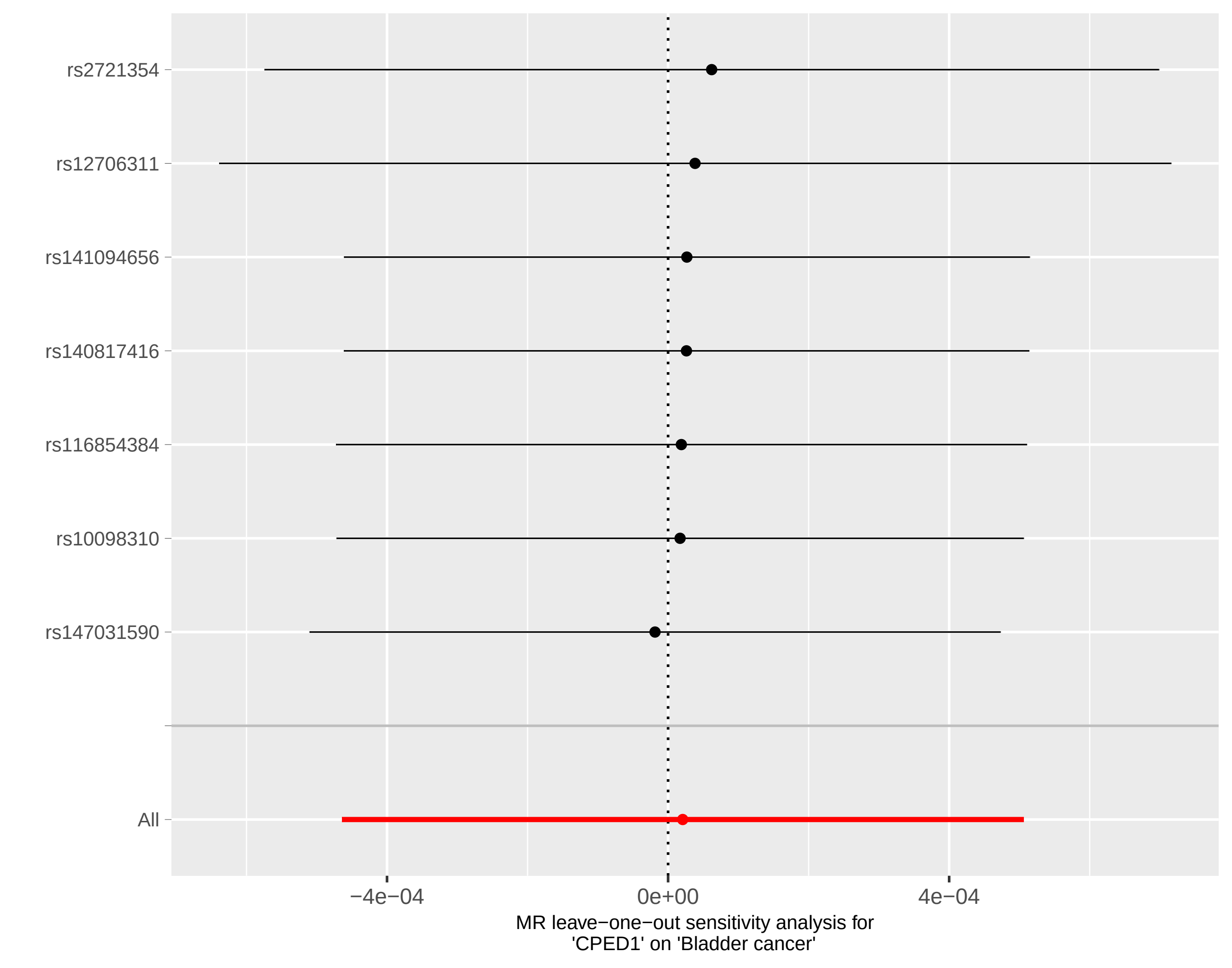
rs2721354
rs12706311
rs141094656
rs140817416
rs116854384
rs10098310
rs147031590
All
−4e−04
0e+00
4e−04
MR leave−one−out sensitivity analysis for
'CPED1' on 'Bladder cancer'

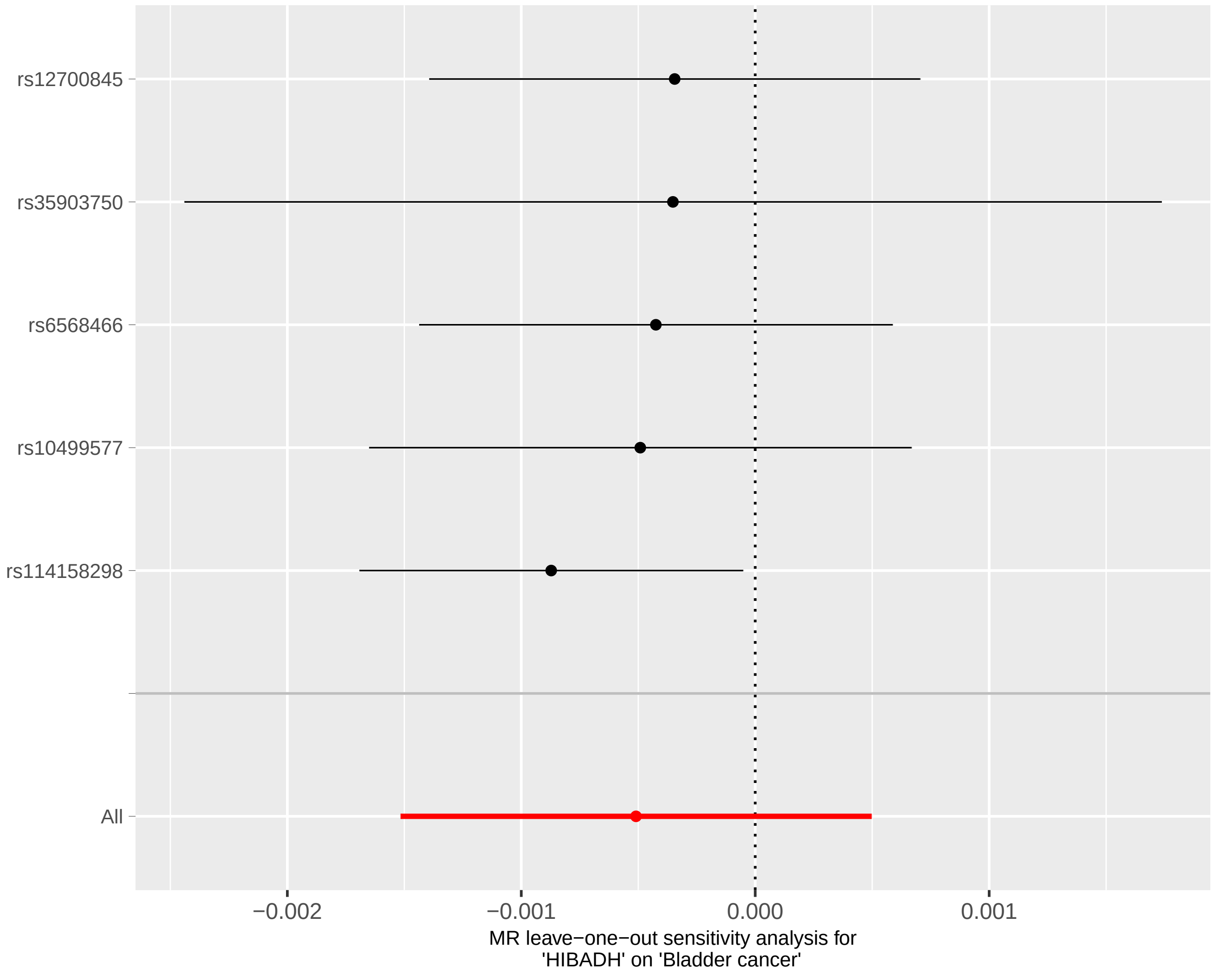
rs12700845
rs35903750
rs6568466
rs10499577
rs114158298
All
−0.002
−0.001
0.000
0.001
MR leave−one−out sensitivity analysis for
'HIBADH' on 'Bladder cancer'

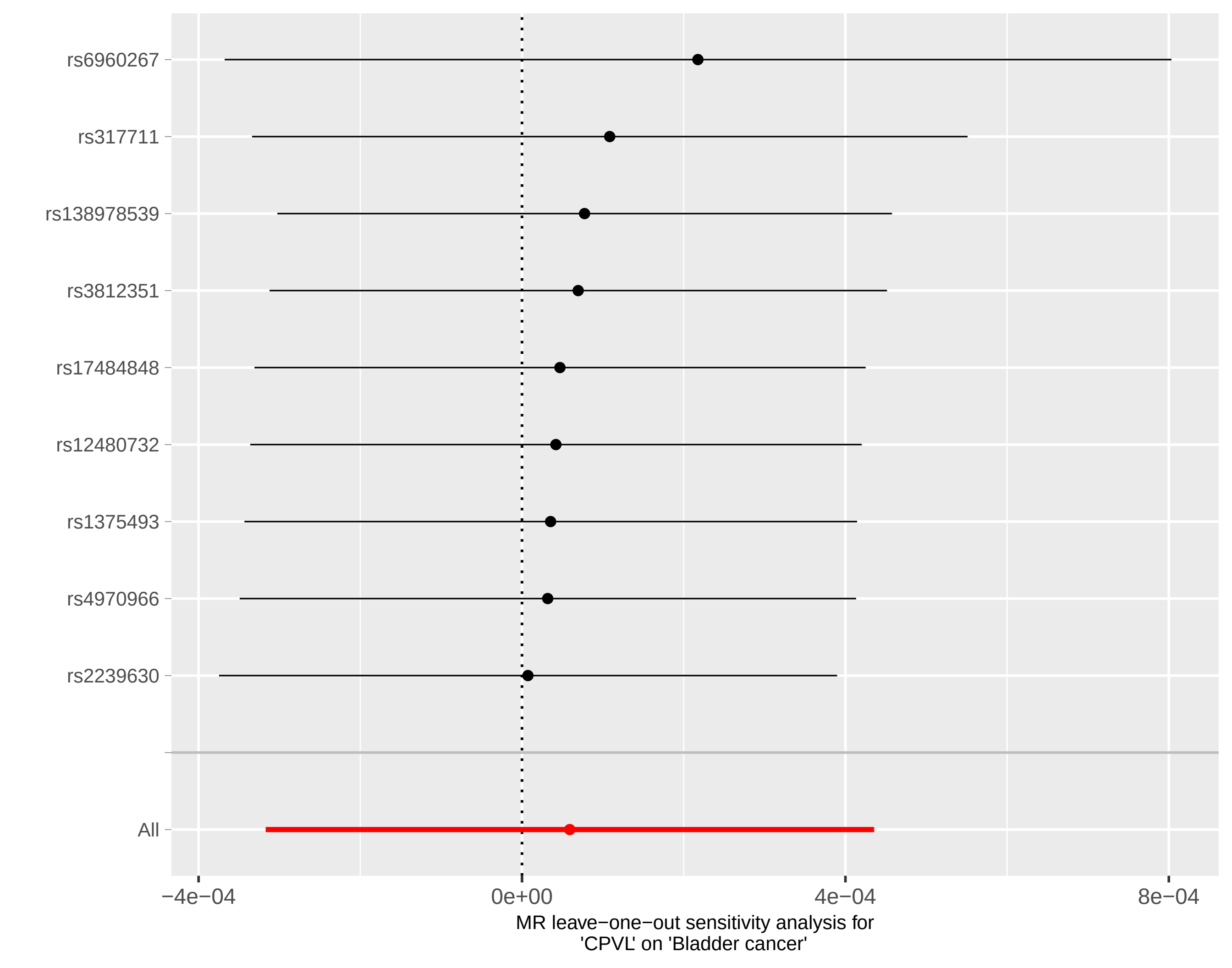
rs6960267
rs317711
rs138978539
rs3812351
rs17484848
rs12480732
rs1375493
rs4970966
rs2239630
All
−4e−04
0e+00
4e−04
8e−04
MR leave−one−out sensitivity analysis for
'CPVL' on 'Bladder cancer'

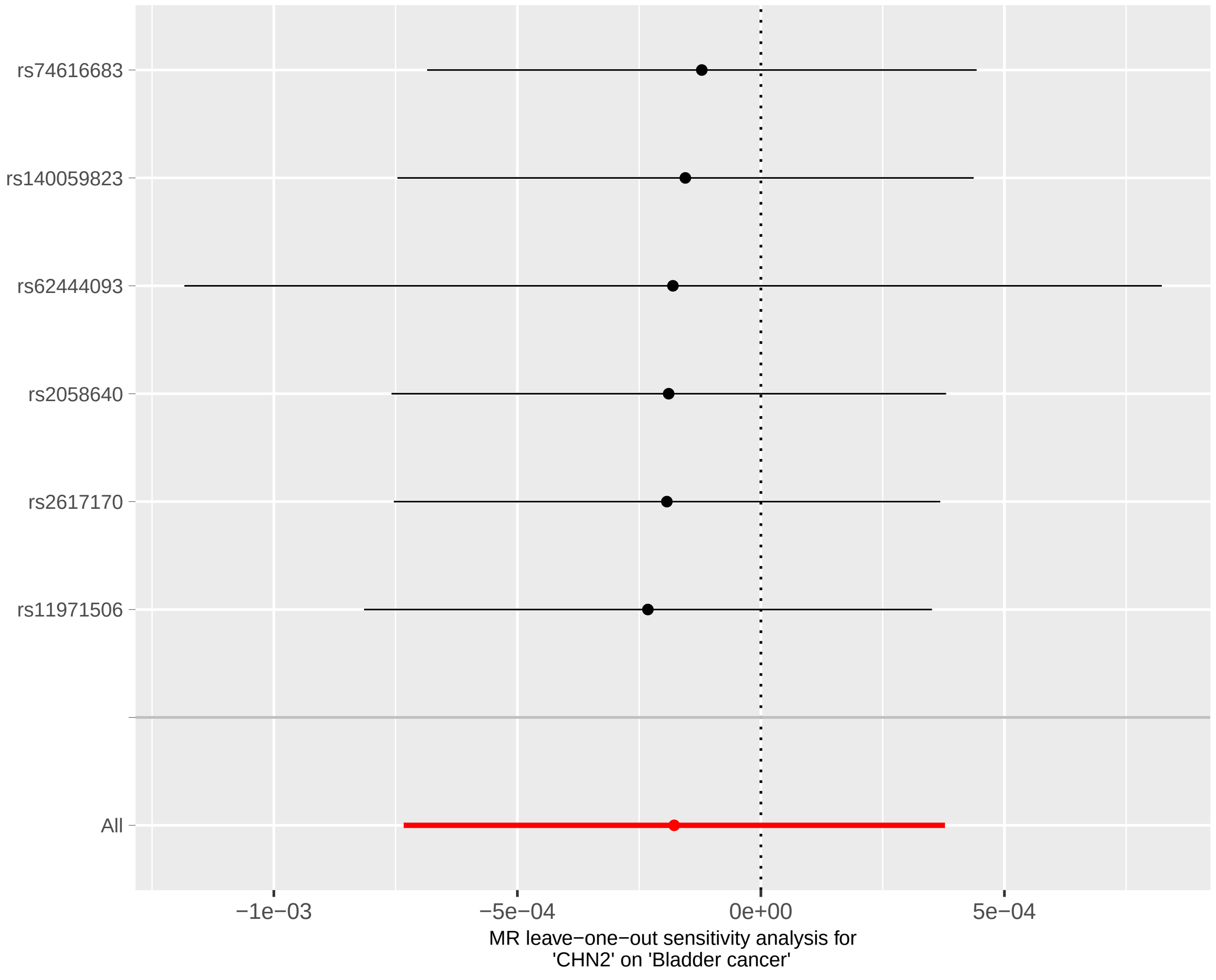

rs74616683
rs140059823
rs62444093
rs2058640
rs2617170
rs11971506
All
−1e−03
−5e−04
0e+00
5e−04
MR leave−one−out sensitivity analysis for
'CHN2' on 'Bladder cancer'
