# Supplementary figures and images for: Integrative multi-omics and single-cell transcriptomics reveal ARHGEF12 driving chemoresistance in bladder cancer
Source: Hereditas. 2025 Nov 27;162:234. doi: 10.1186/s41065-025-00606-1 (PMC12661753; doi:10.1186/s41065-025-00606-1)

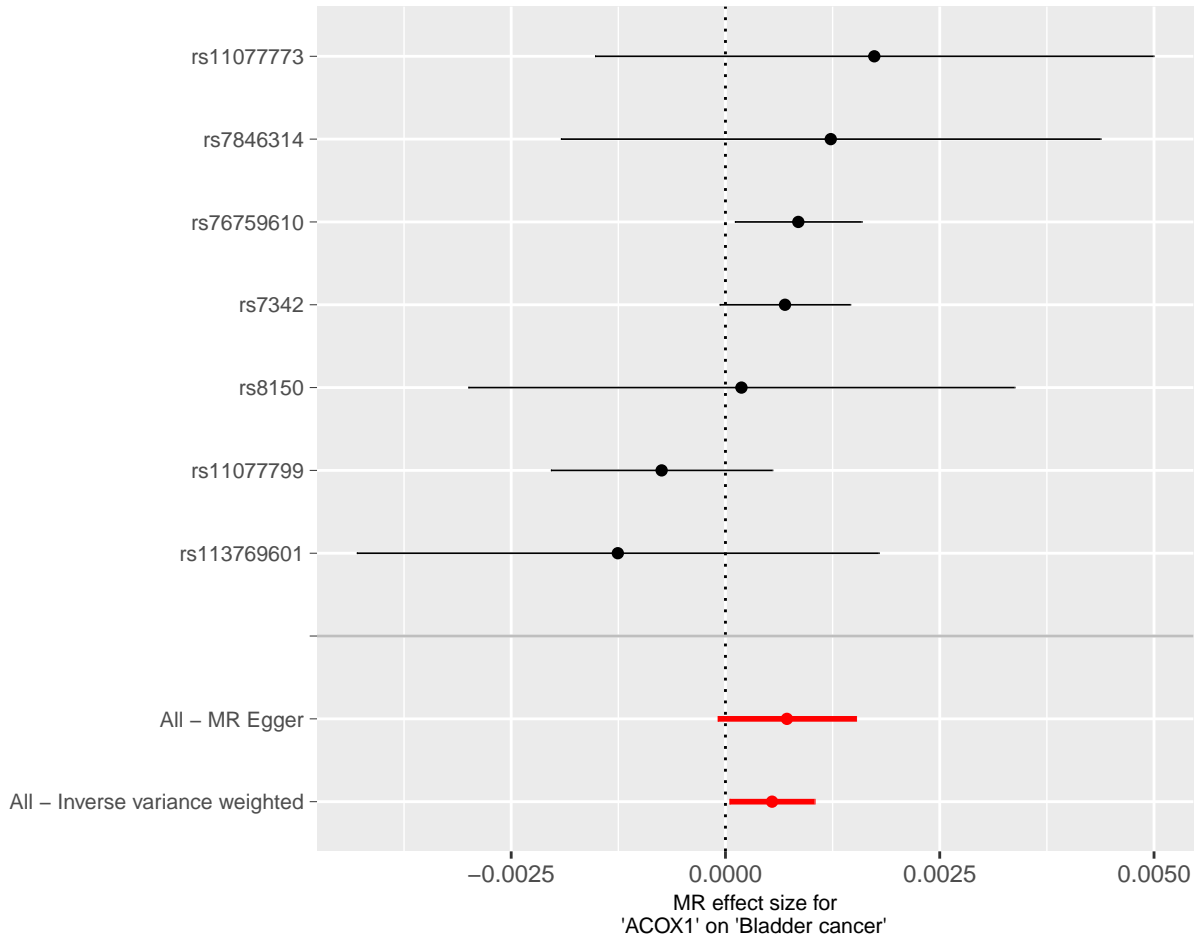

Supplement: Supplementary file 1 — Supplementary Material 1. [file 41065_2025_606_MOESM1_ESM.zip › Supplementary1/Supplementary - MR/eQTL-MR/MRpic/ACOX1.forest.pdf]

# MR Method

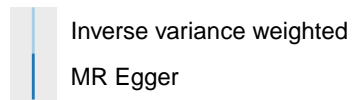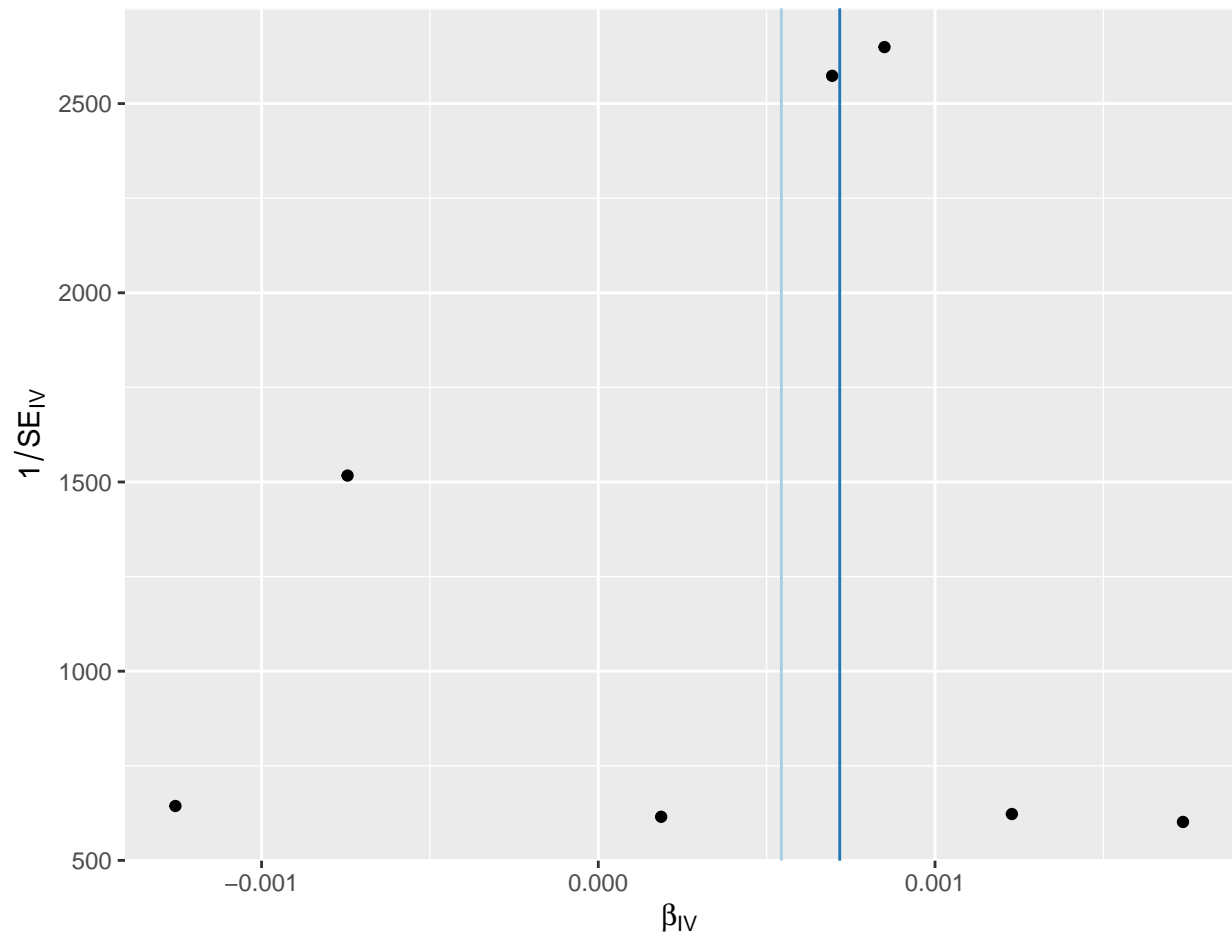

Supplement: Supplementary file 1 — Supplementary Material 1. [file 41065_2025_606_MOESM1_ESM.zip › Supplementary1/Supplementary - MR/eQTL-MR/MRpic/ACOX1.funnel_plot.pdf]

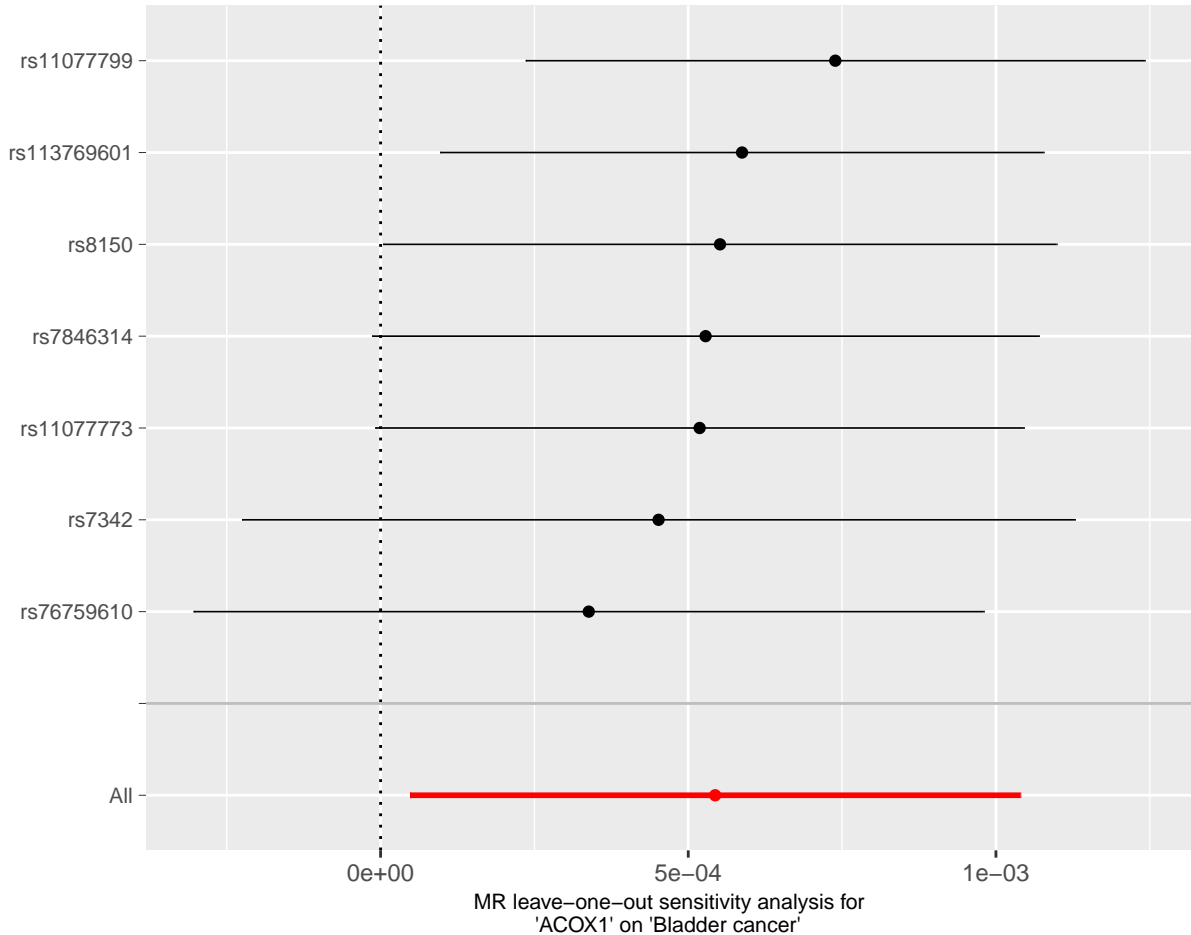

Supplement: Supplementary file 1 — Supplementary Material 1. [file 41065_2025_606_MOESM1_ESM.zip › Supplementary1/Supplementary - MR/eQTL-MR/MRpic/ACOX1.leaveoneout.pdf]

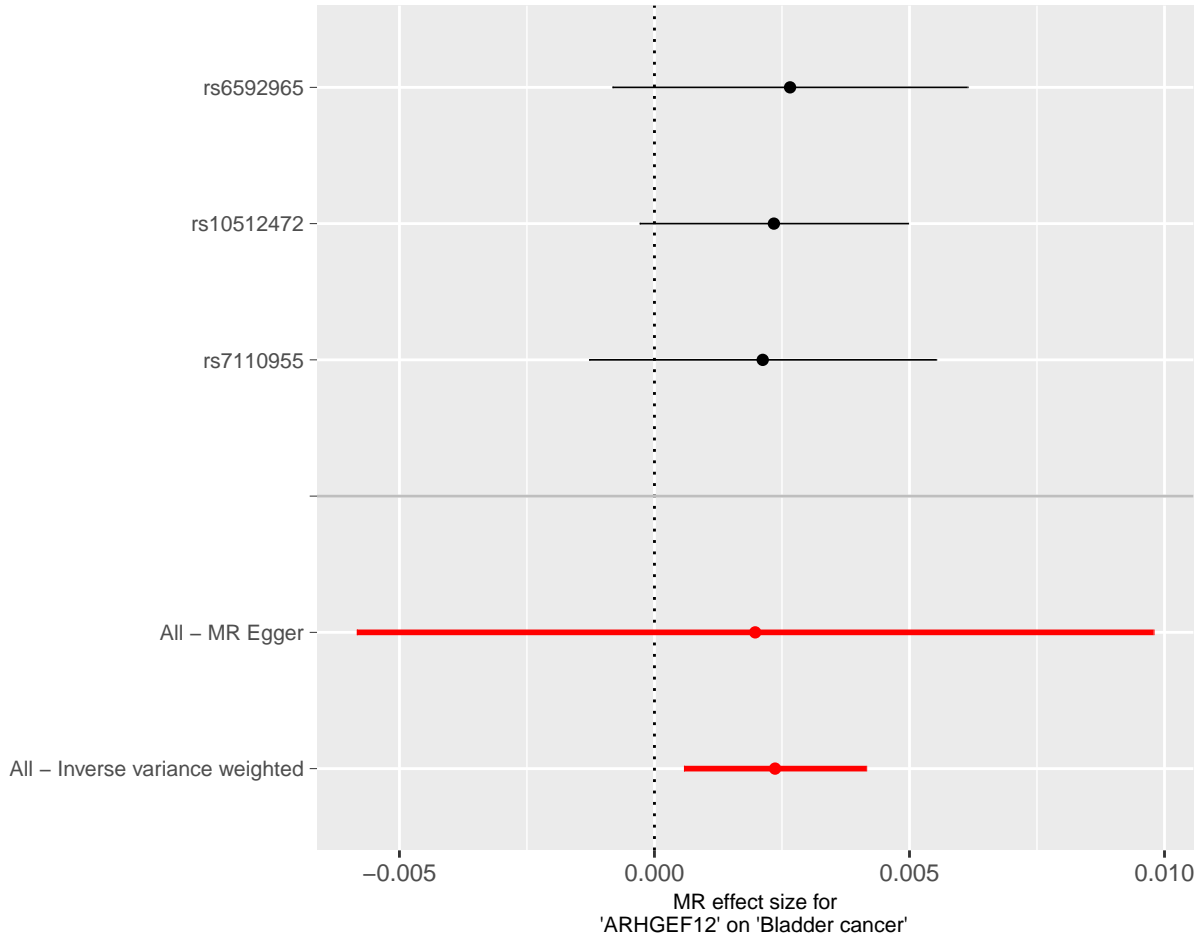

Supplement: Supplementary file 1 — Supplementary Material 1. [file 41065_2025_606_MOESM1_ESM.zip › Supplementary1/Supplementary - MR/eQTL-MR/MRpic/ARHGEF12.forest.pdf]

# MR Method

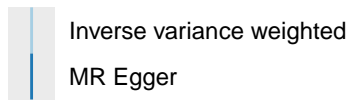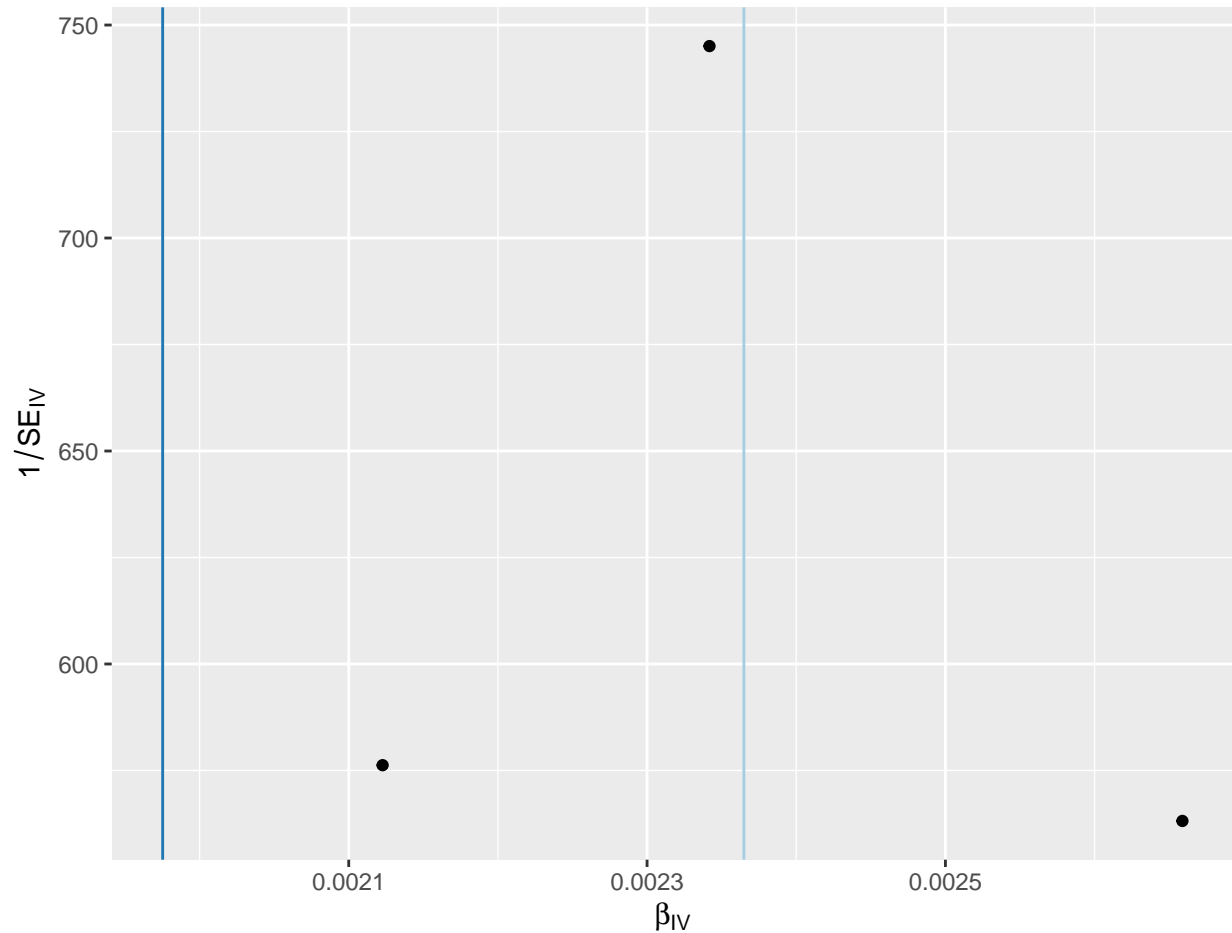

Supplement: Supplementary file 1 — Supplementary Material 1. [file 41065_2025_606_MOESM1_ESM.zip › Supplementary1/Supplementary - MR/eQTL-MR/MRpic/ARHGEF12.funnel_plot.pdf]

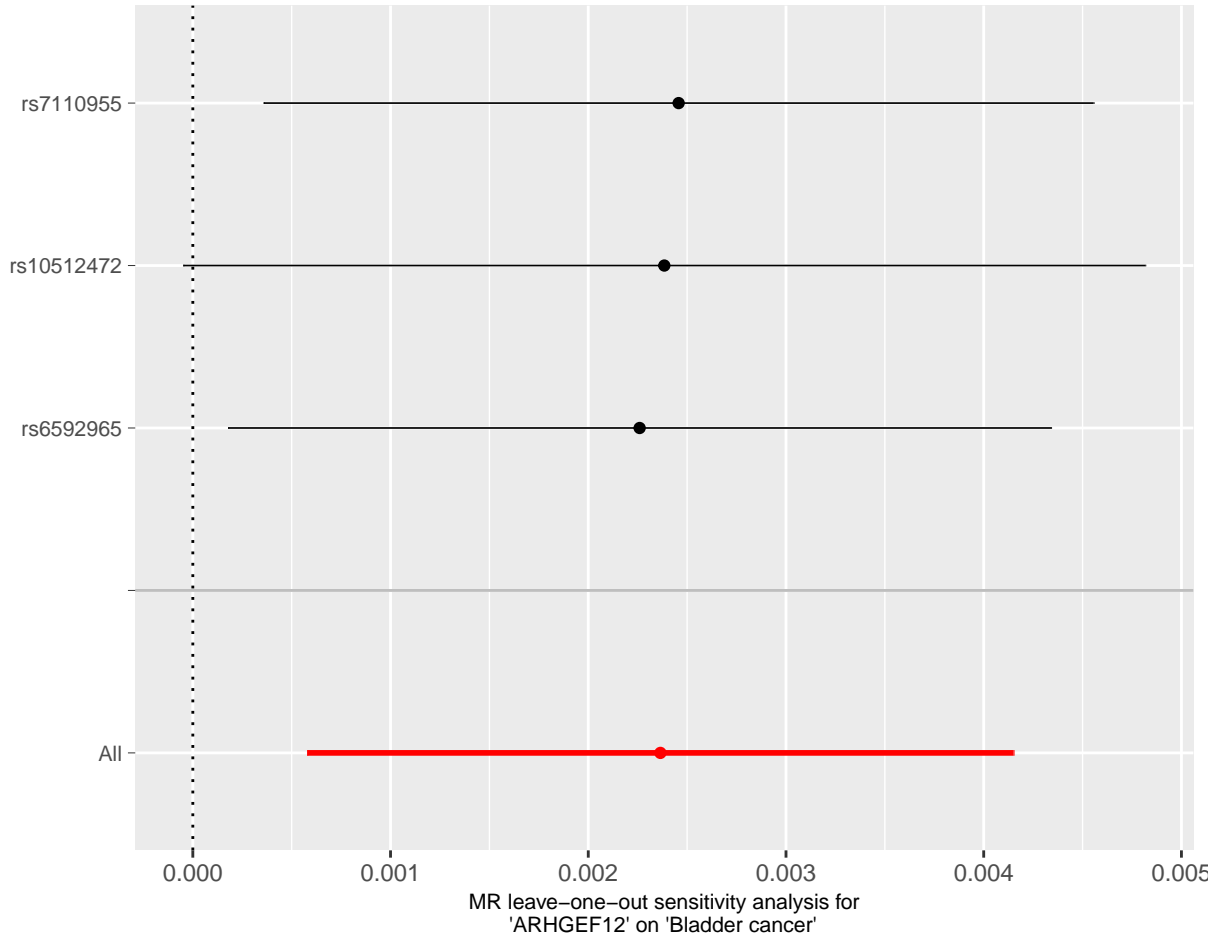

Supplement: Supplementary file 1 — Supplementary Material 1. [file 41065_2025_606_MOESM1_ESM.zip › Supplementary1/Supplementary - MR/eQTL-MR/MRpic/ARHGEF12.leaveoneout.pdf]

# MR Test

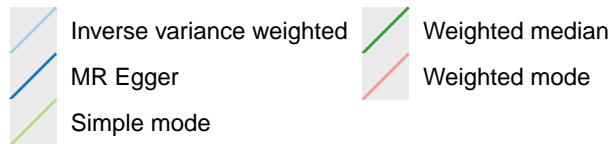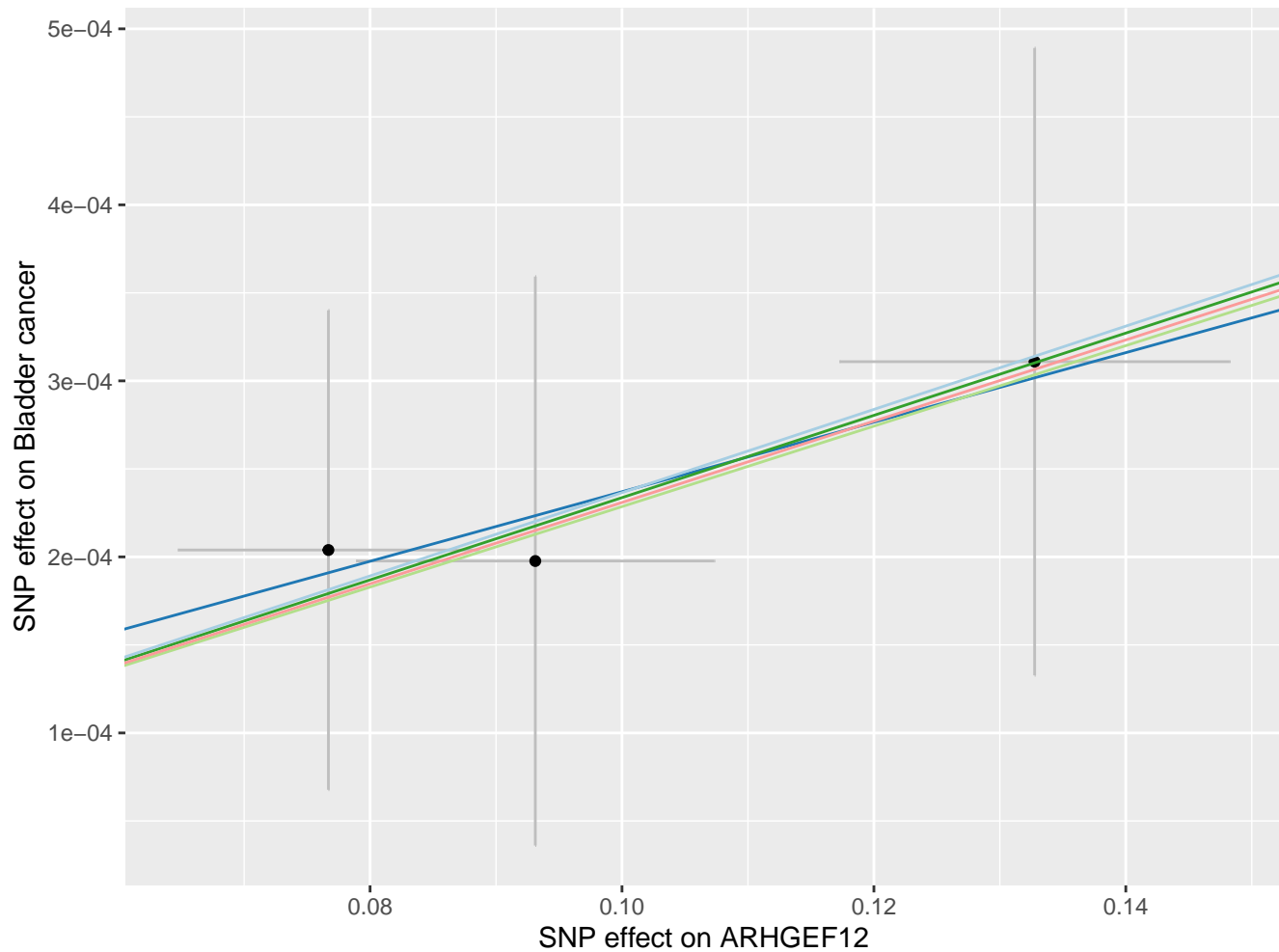

Supplement: Supplementary file 1 — Supplementary Material 1. [file 41065_2025_606_MOESM1_ESM.zip › Supplementary1/Supplementary - MR/eQTL-MR/MRpic/ARHGEF12.scatter_plot.pdf]

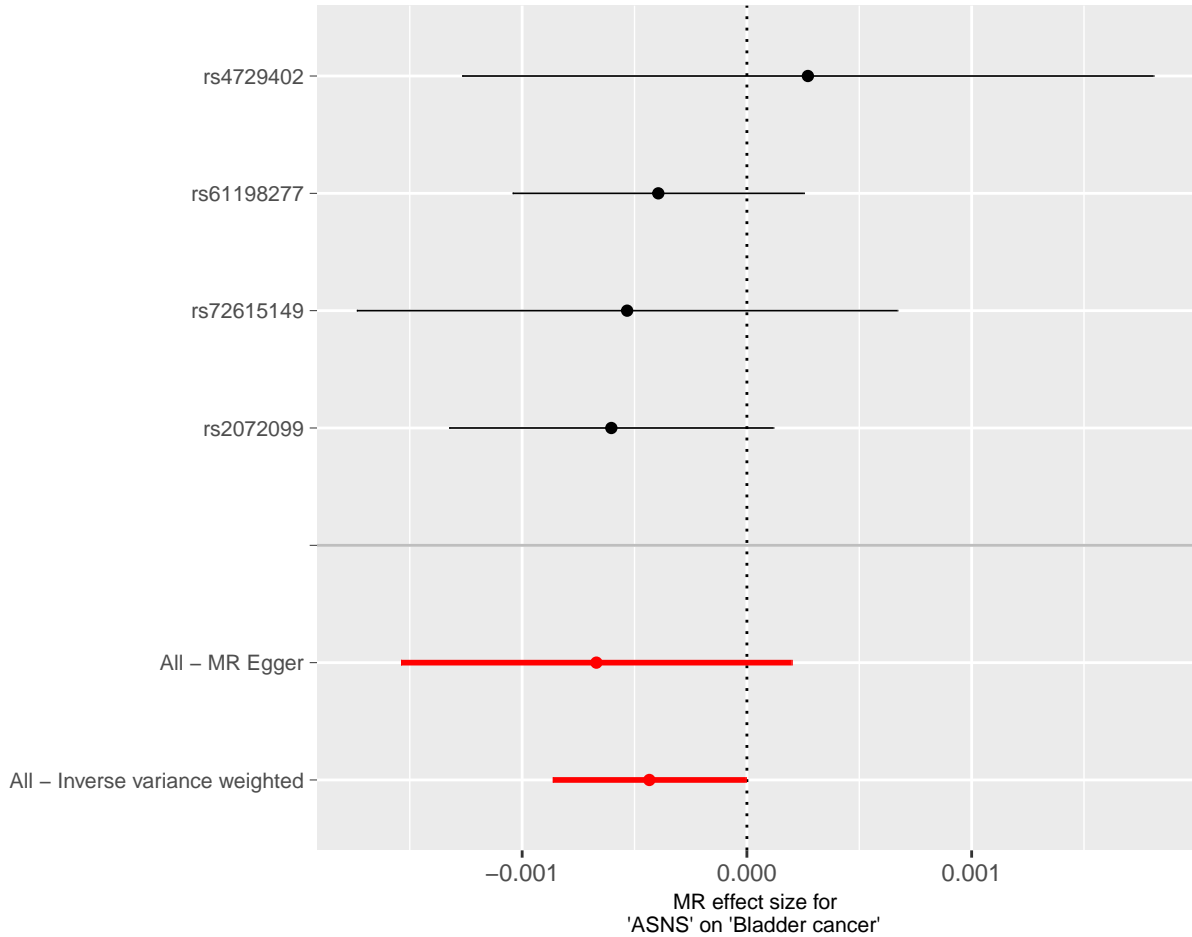

Supplement: Supplementary file 1 — Supplementary Material 1. [file 41065_2025_606_MOESM1_ESM.zip › Supplementary1/Supplementary - MR/eQTL-MR/MRpic/ASNS.forest.pdf]

# MR Method

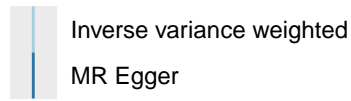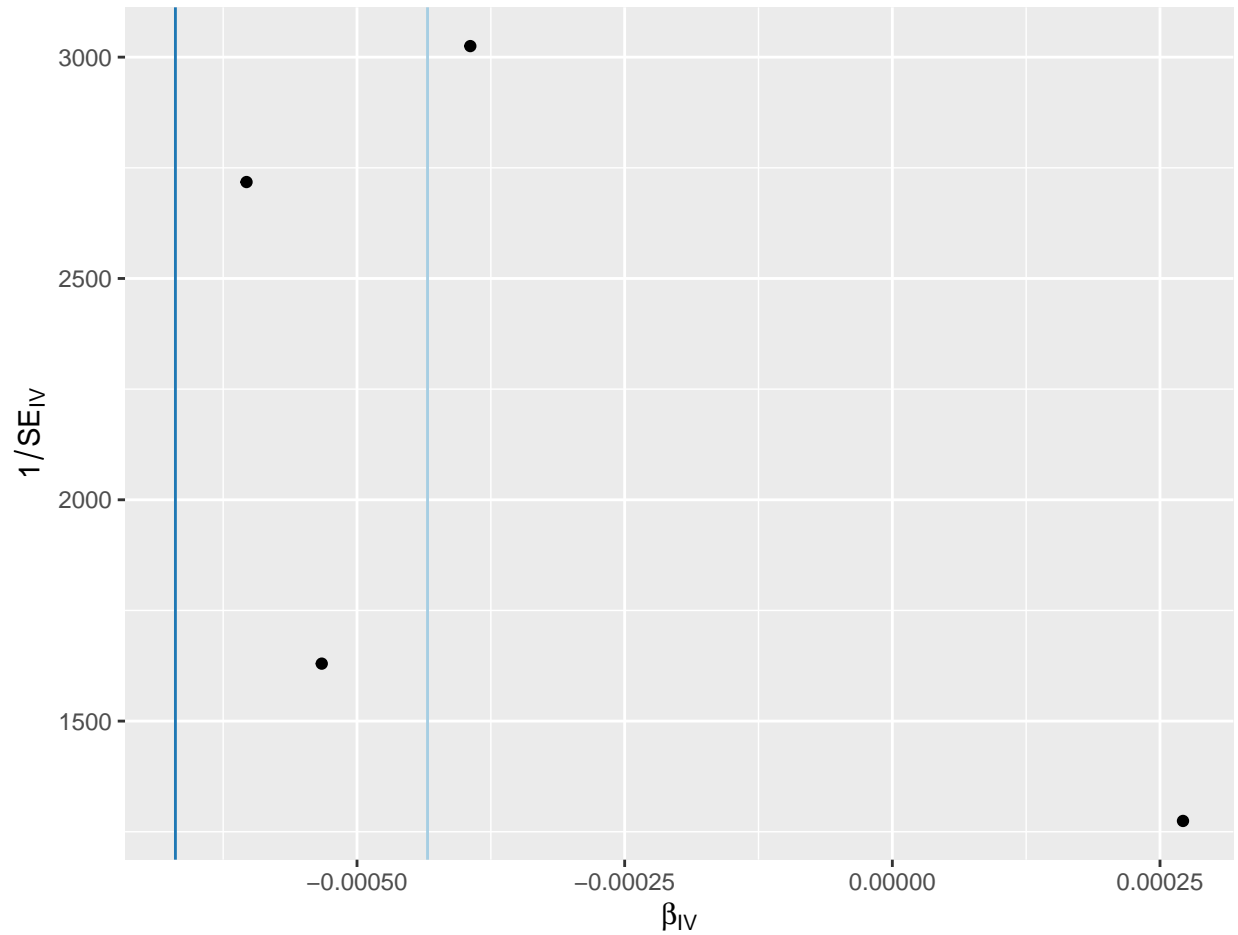

Supplement: Supplementary file 1 — Supplementary Material 1. [file 41065_2025_606_MOESM1_ESM.zip › Supplementary1/Supplementary - MR/eQTL-MR/MRpic/ASNS.funnel_plot.pdf]

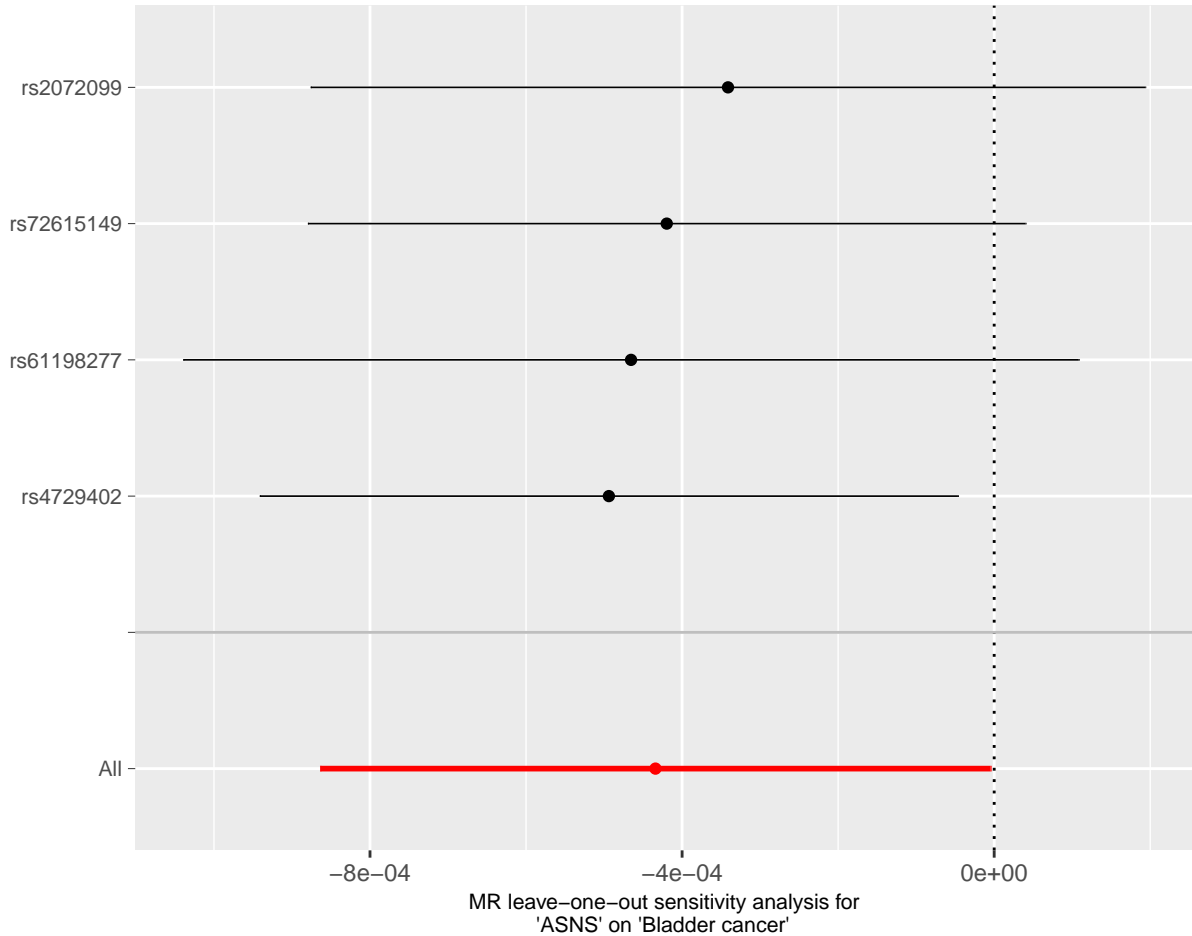

Supplement: Supplementary file 1 — Supplementary Material 1. [file 41065_2025_606_MOESM1_ESM.zip › Supplementary1/Supplementary - MR/eQTL-MR/MRpic/ASNS.leaveoneout.pdf]

# MR Test

- Inverse variance weighted
- MR Egger
- Simple mode
- Weighted median
- Weighted mode

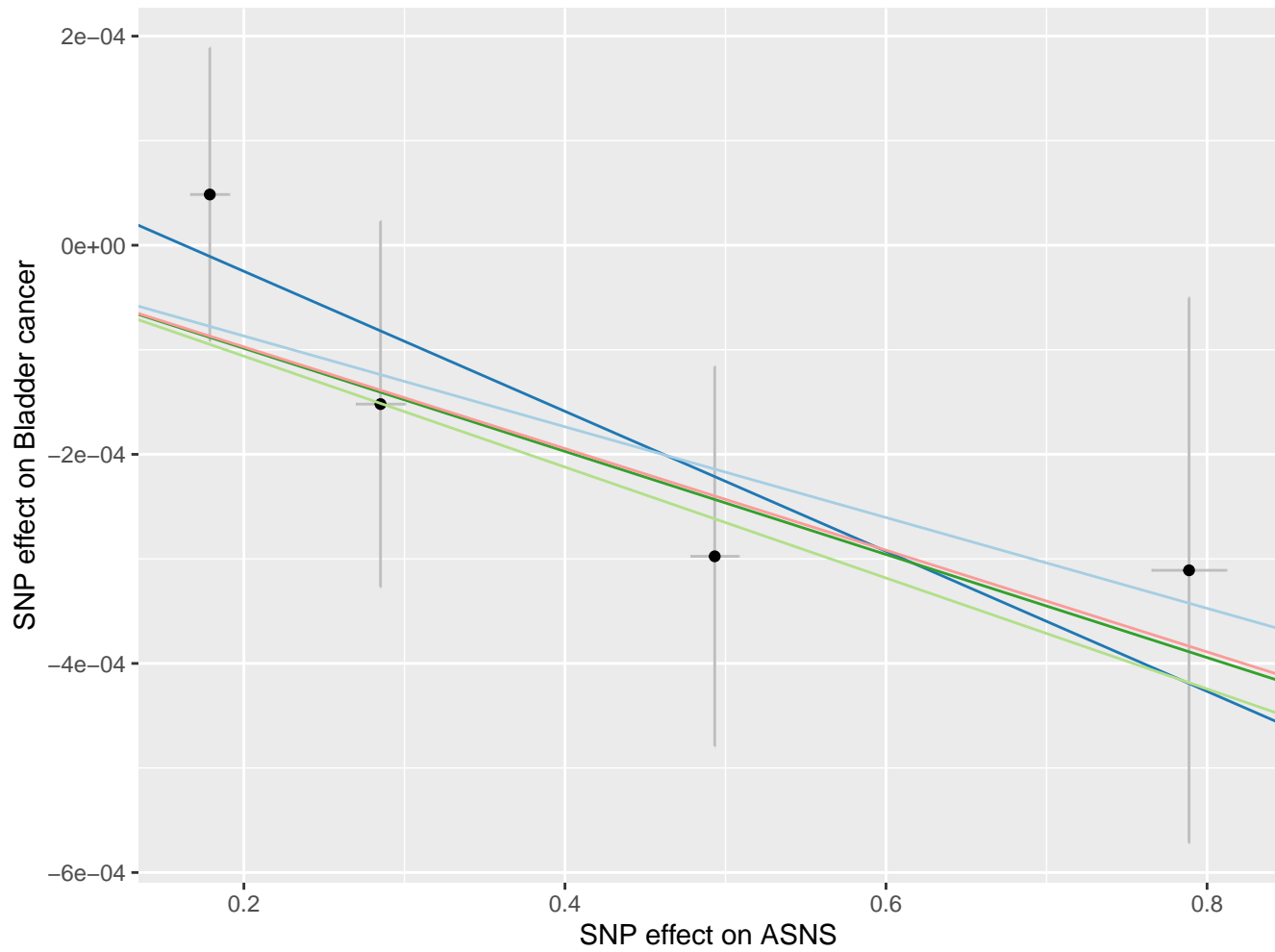

Supplement: Supplementary file 1 — Supplementary Material 1. [file 41065_2025_606_MOESM1_ESM.zip › Supplementary1/Supplementary - MR/eQTL-MR/MRpic/ASNS.scatter_plot.pdf]

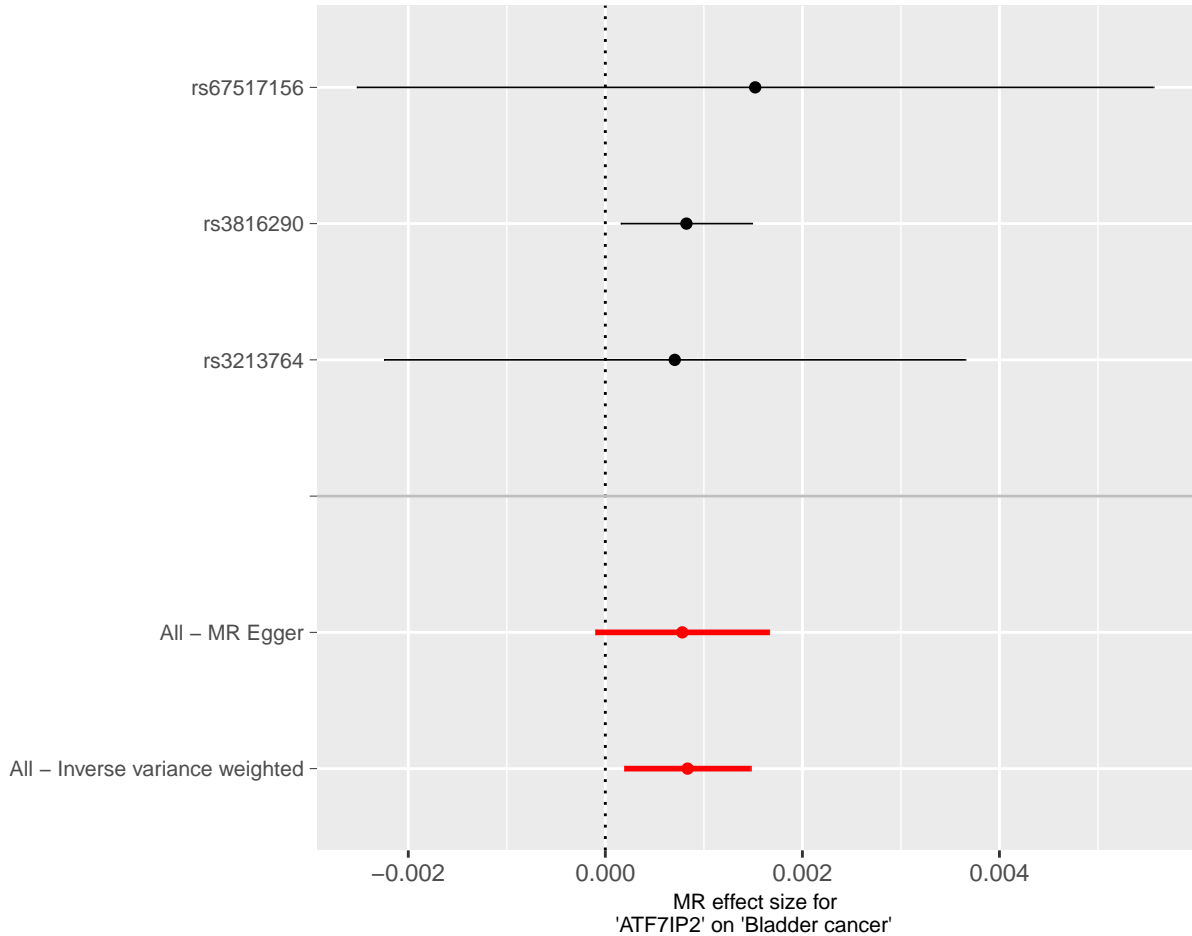

Supplement: Supplementary file 1 — Supplementary Material 1. [file 41065_2025_606_MOESM1_ESM.zip › Supplementary1/Supplementary - MR/eQTL-MR/MRpic/ATF7IP2.forest.pdf]

# MR Method

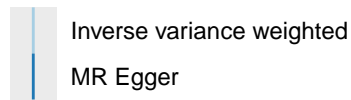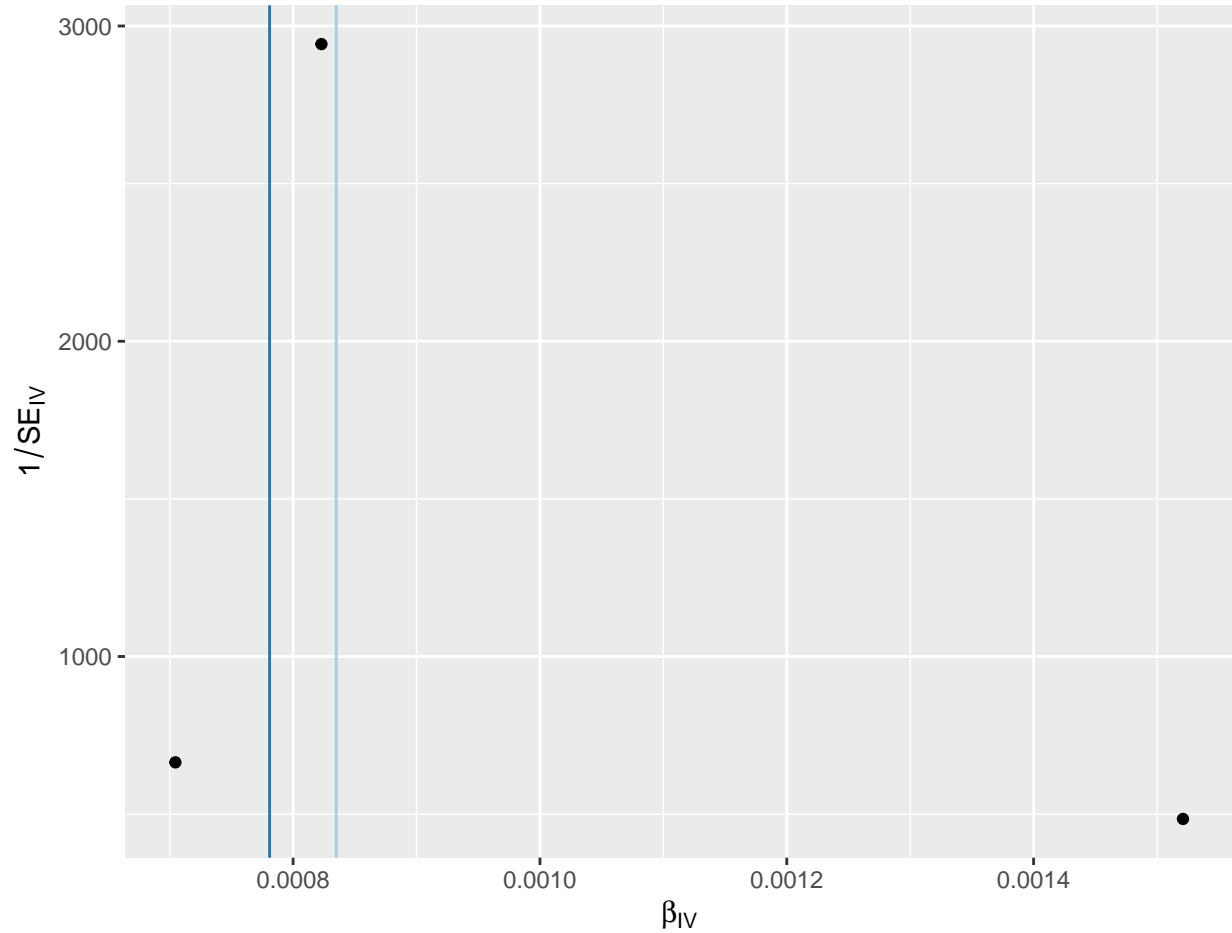

Supplement: Supplementary file 1 — Supplementary Material 1. [file 41065_2025_606_MOESM1_ESM.zip › Supplementary1/Supplementary - MR/eQTL-MR/MRpic/ATF7IP2.funnel_plot.pdf]

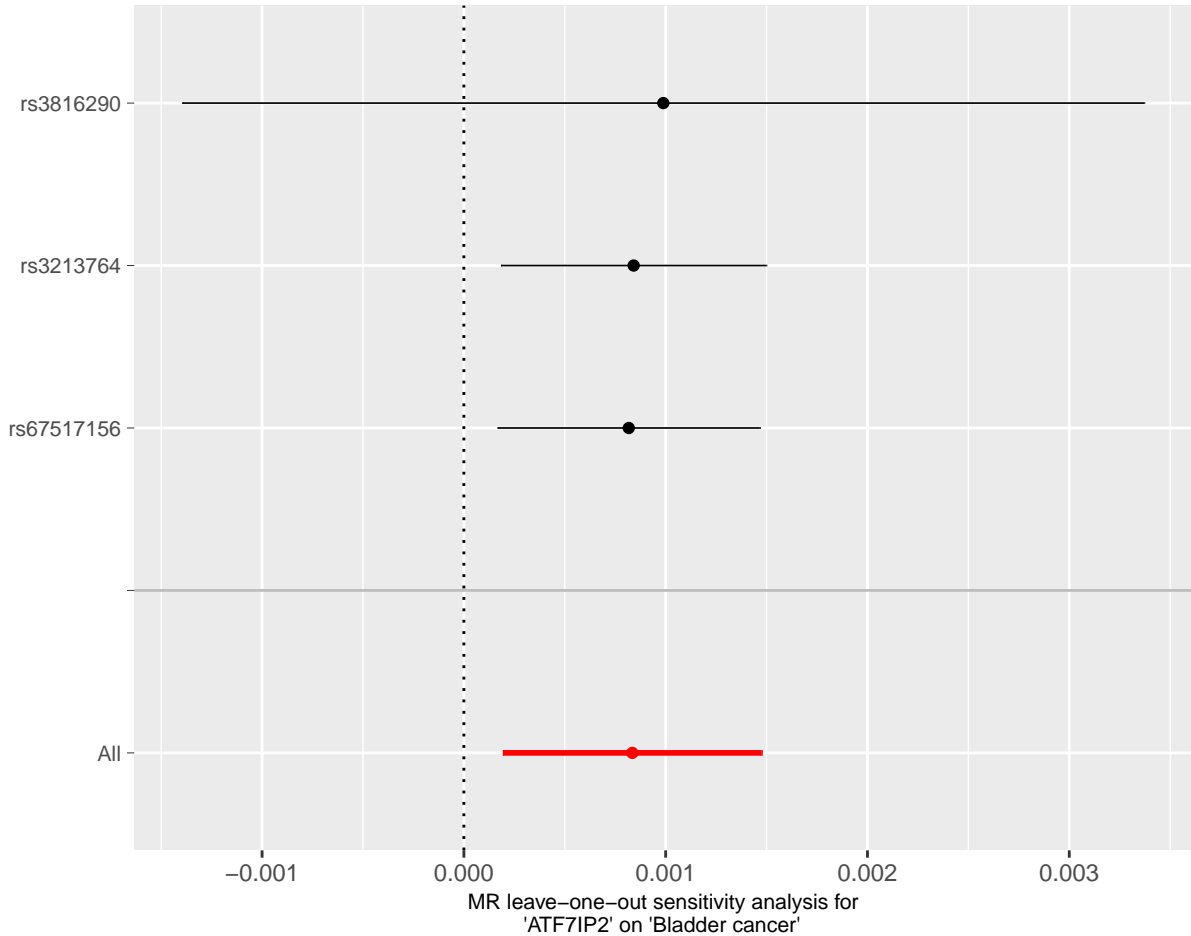

Supplement: Supplementary file 1 — Supplementary Material 1. [file 41065_2025_606_MOESM1_ESM.zip › Supplementary1/Supplementary - MR/eQTL-MR/MRpic/ATF7IP2.leaveoneout.pdf]

# MR Test

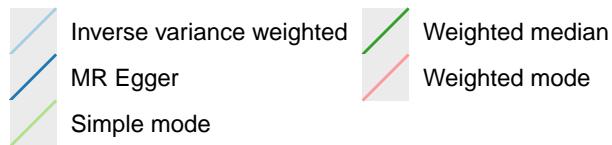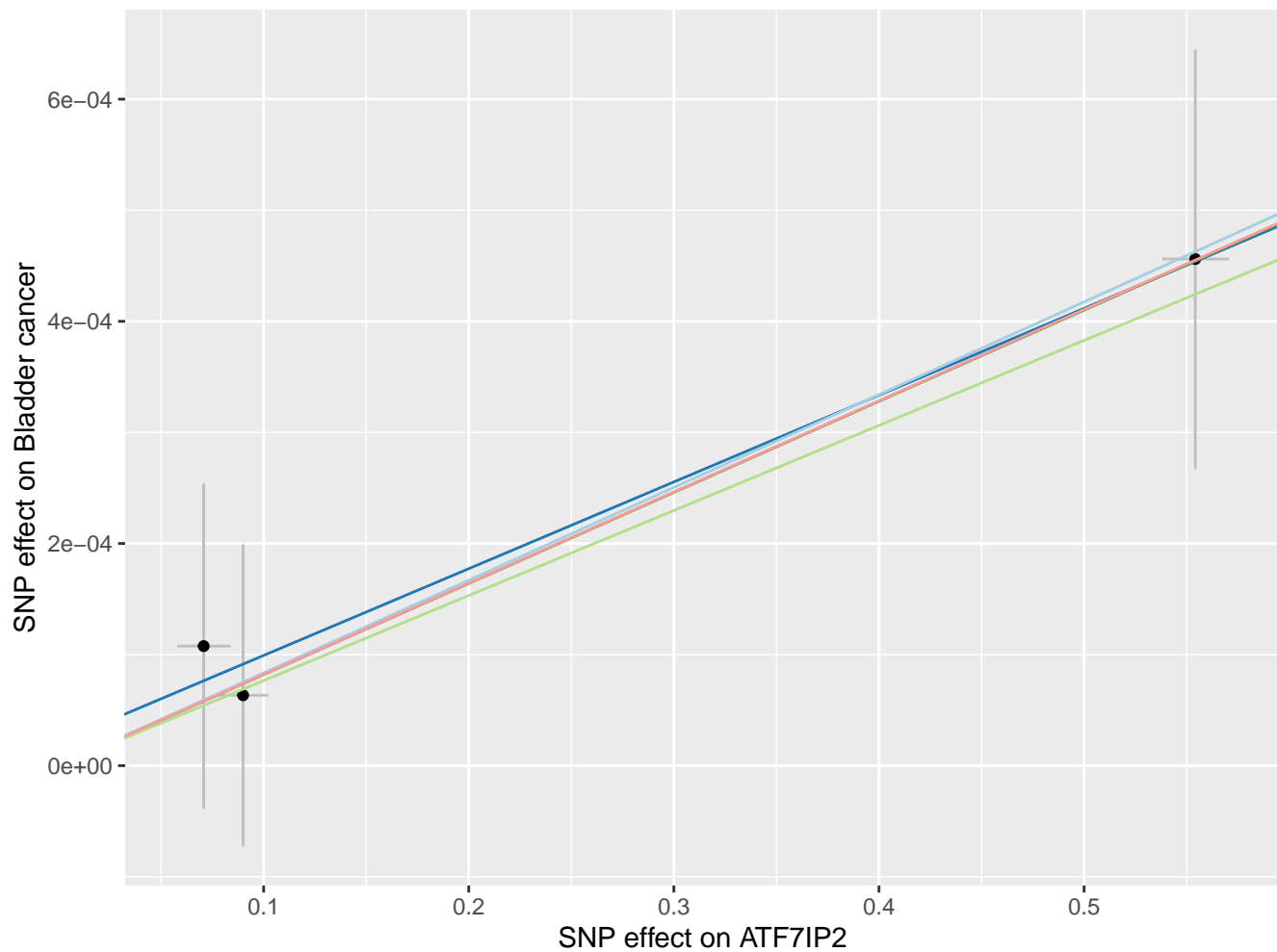

Supplement: Supplementary file 1 — Supplementary Material 1. [file 41065_2025_606_MOESM1_ESM.zip › Supplementary1/Supplementary - MR/eQTL-MR/MRpic/ATF7IP2.scatter_plot.pdf]

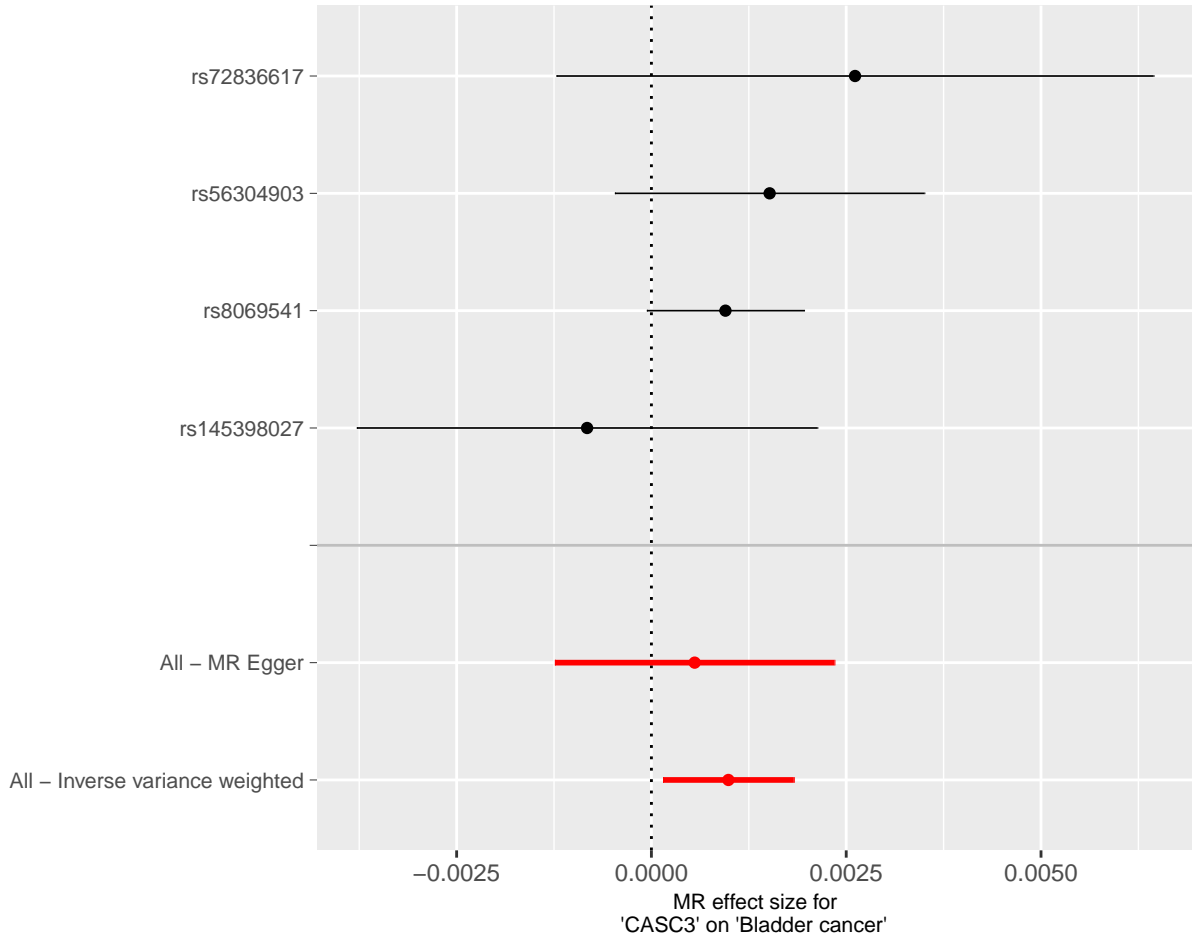

Supplement: Supplementary file 1 — Supplementary Material 1. [file 41065_2025_606_MOESM1_ESM.zip › Supplementary1/Supplementary - MR/eQTL-MR/MRpic/CASC3.forest.pdf]

# MR Method

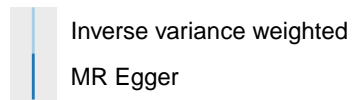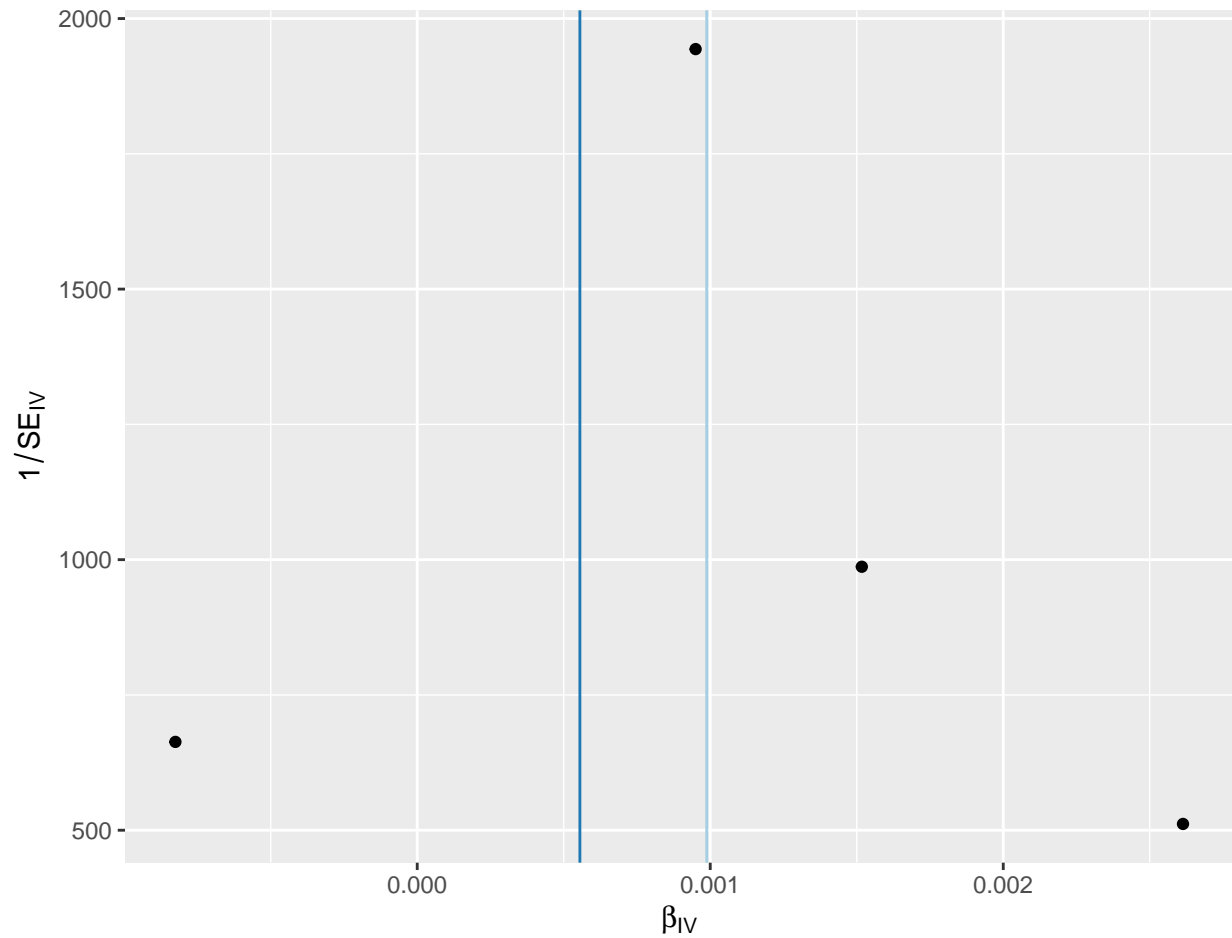

Supplement: Supplementary file 1 — Supplementary Material 1. [file 41065_2025_606_MOESM1_ESM.zip › Supplementary1/Supplementary - MR/eQTL-MR/MRpic/CASC3.funnel_plot.pdf]

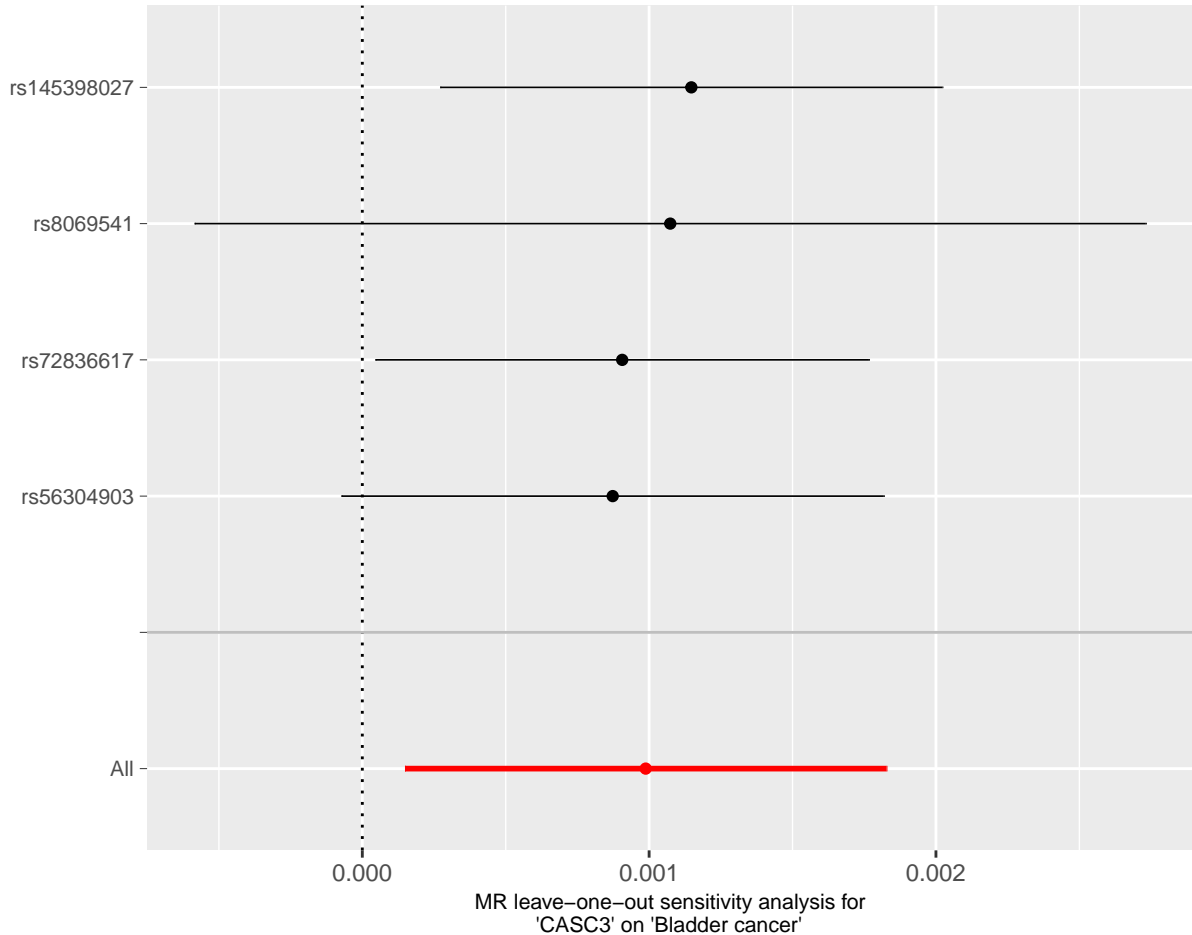

Supplement: Supplementary file 1 — Supplementary Material 1. [file 41065_2025_606_MOESM1_ESM.zip › Supplementary1/Supplementary - MR/eQTL-MR/MRpic/CASC3.leaveoneout.pdf]

# MR Test

- Inverse variance weighted
- MR Egger
- Simple mode
- Weighted median
- Weighted mode

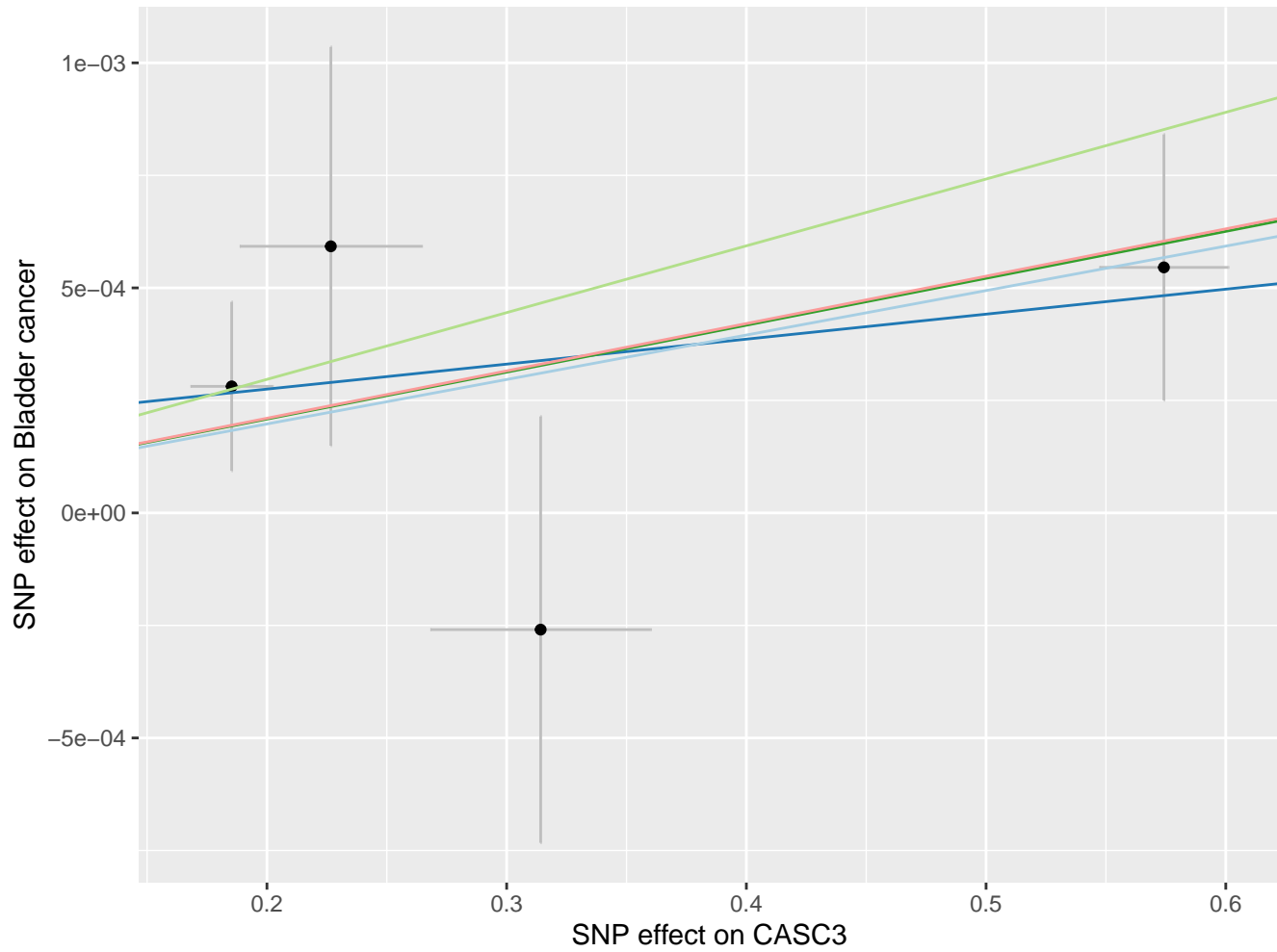

Supplement: Supplementary file 1 — Supplementary Material 1. [file 41065_2025_606_MOESM1_ESM.zip › Supplementary1/Supplementary - MR/eQTL-MR/MRpic/CASC3.scatter_plot.pdf]

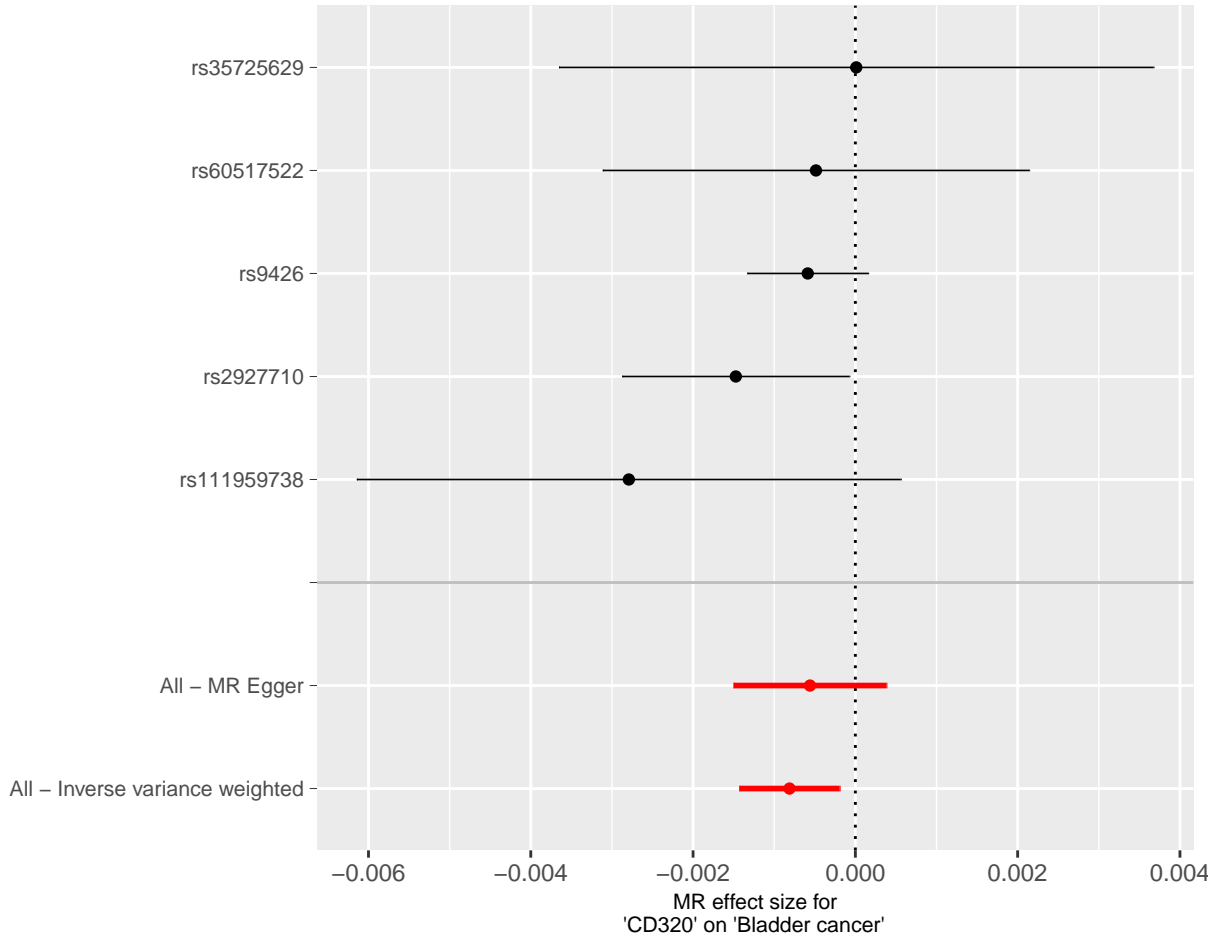

Supplement: Supplementary file 1 — Supplementary Material 1. [file 41065_2025_606_MOESM1_ESM.zip › Supplementary1/Supplementary - MR/eQTL-MR/MRpic/CD320.forest.pdf]

# MR Method

- Inverse variance weighted
- MR Egger

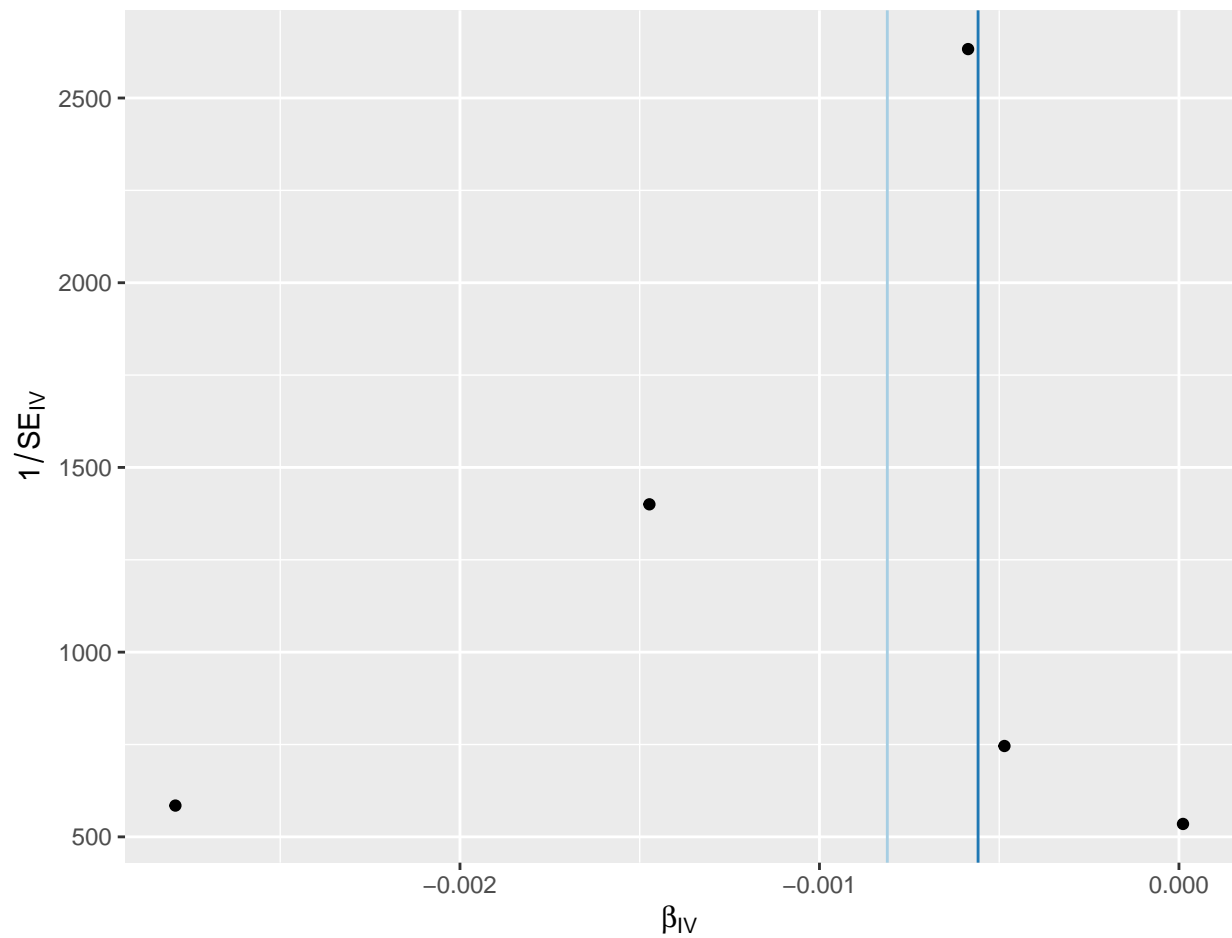

Supplement: Supplementary file 1 — Supplementary Material 1. [file 41065_2025_606_MOESM1_ESM.zip › Supplementary1/Supplementary - MR/eQTL-MR/MRpic/CD320.funnel_plot.pdf]

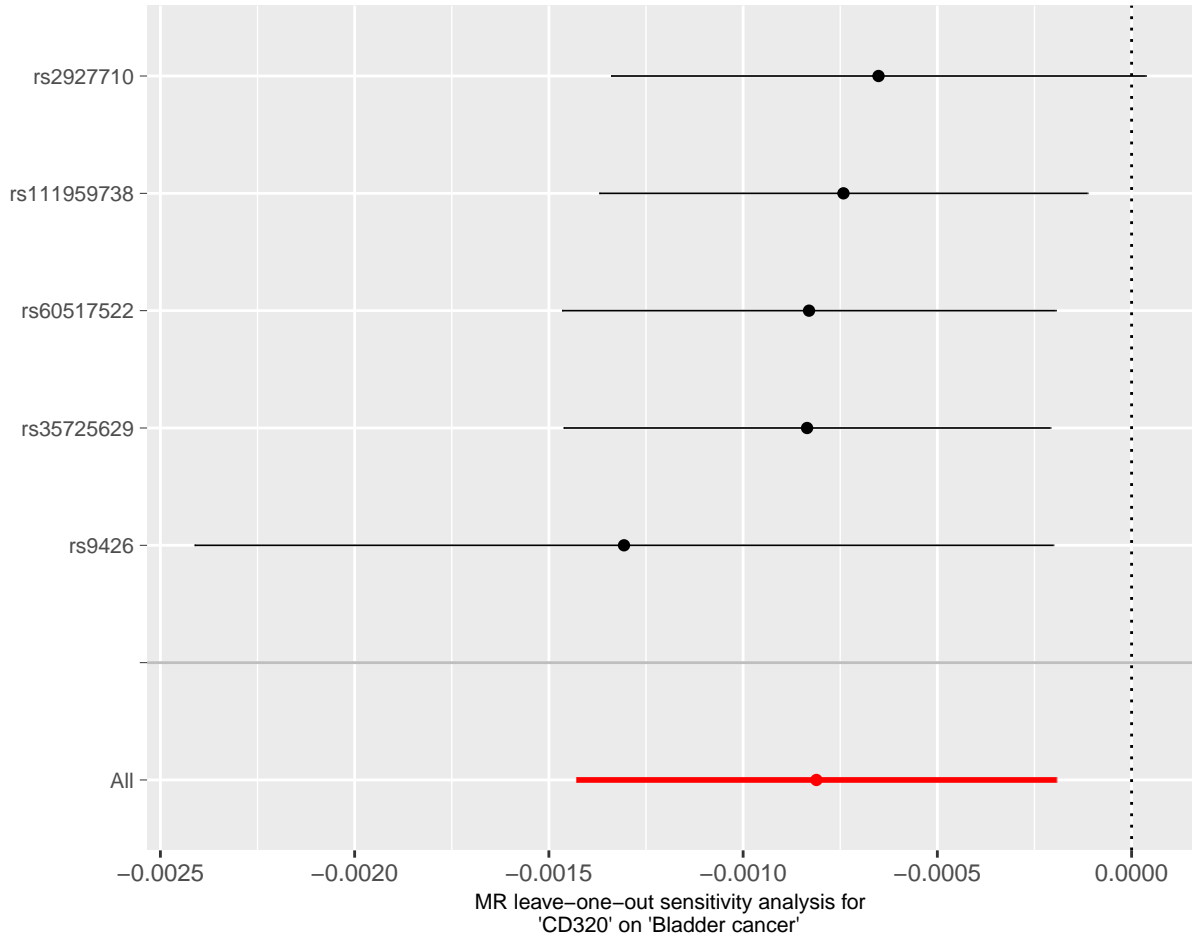

Supplement: Supplementary file 1 — Supplementary Material 1. [file 41065_2025_606_MOESM1_ESM.zip › Supplementary1/Supplementary - MR/eQTL-MR/MRpic/CD320.leaveoneout.pdf]

# MR Test

- Inverse variance weighted
- MR Egger
- Simple mode
- Weighted median
- Weighted mode

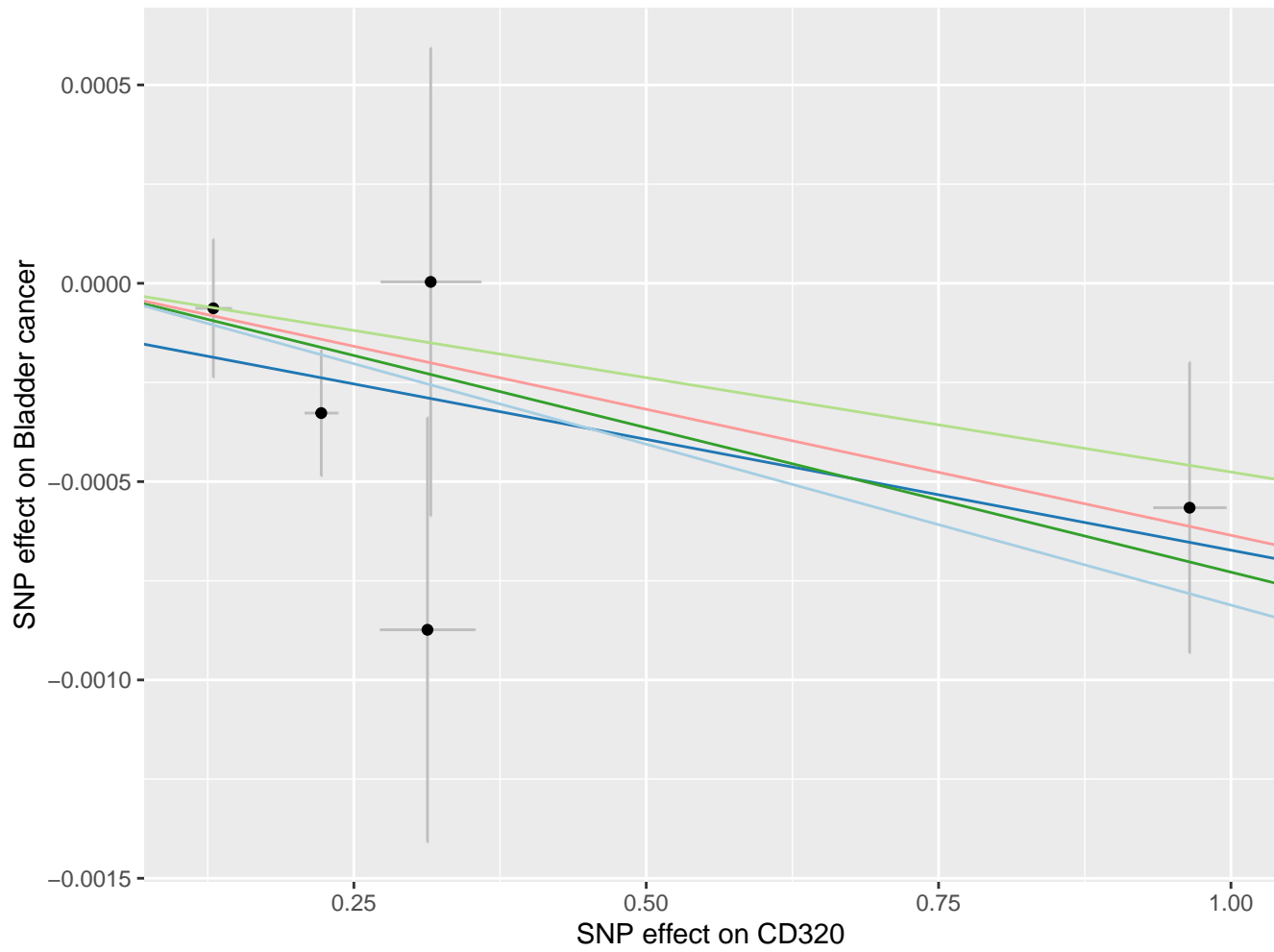

Supplement: Supplementary file 1 — Supplementary Material 1. [file 41065_2025_606_MOESM1_ESM.zip › Supplementary1/Supplementary - MR/eQTL-MR/MRpic/CD320.scatter_plot.pdf]

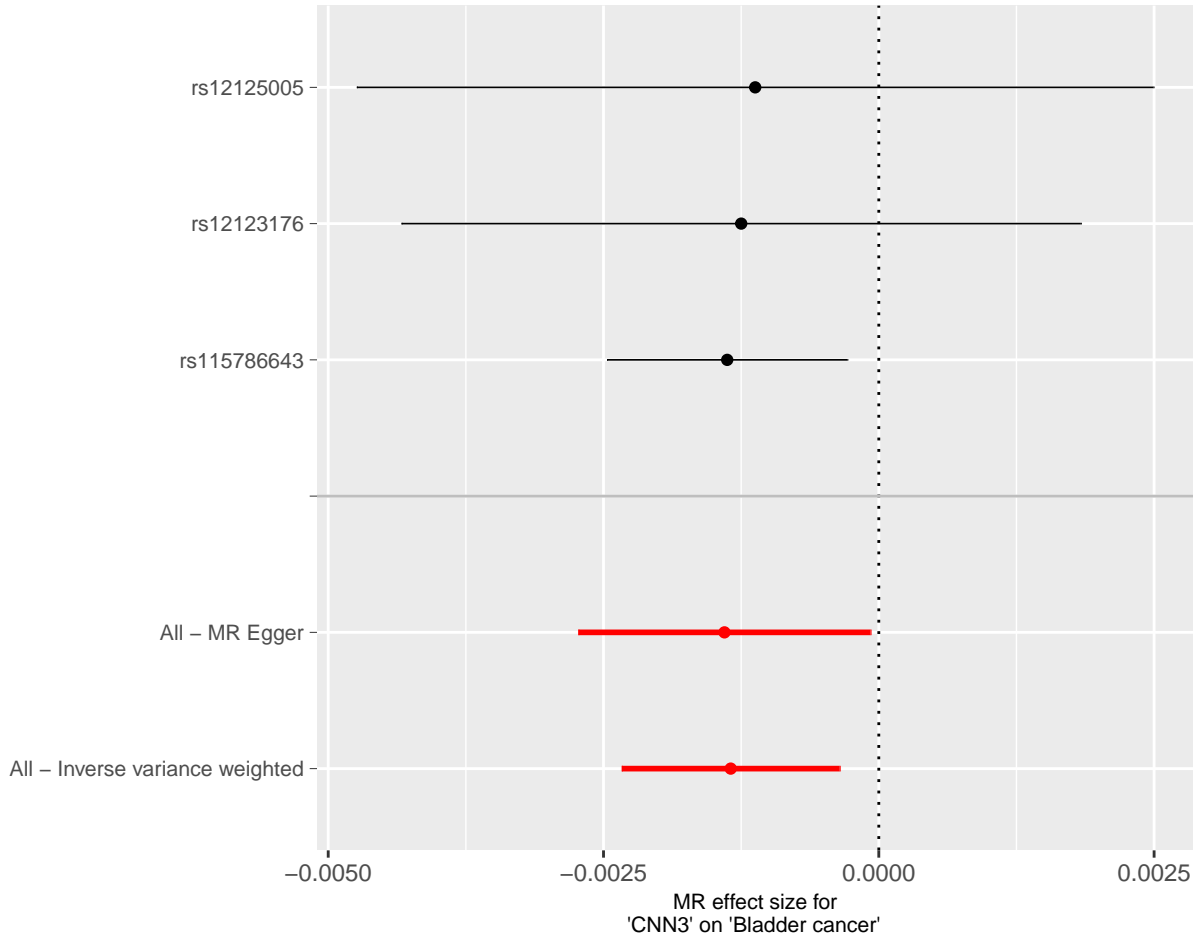

Supplement: Supplementary file 1 — Supplementary Material 1. [file 41065_2025_606_MOESM1_ESM.zip › Supplementary1/Supplementary - MR/eQTL-MR/MRpic/CNN3.forest.pdf]

# MR Method

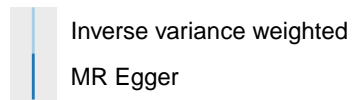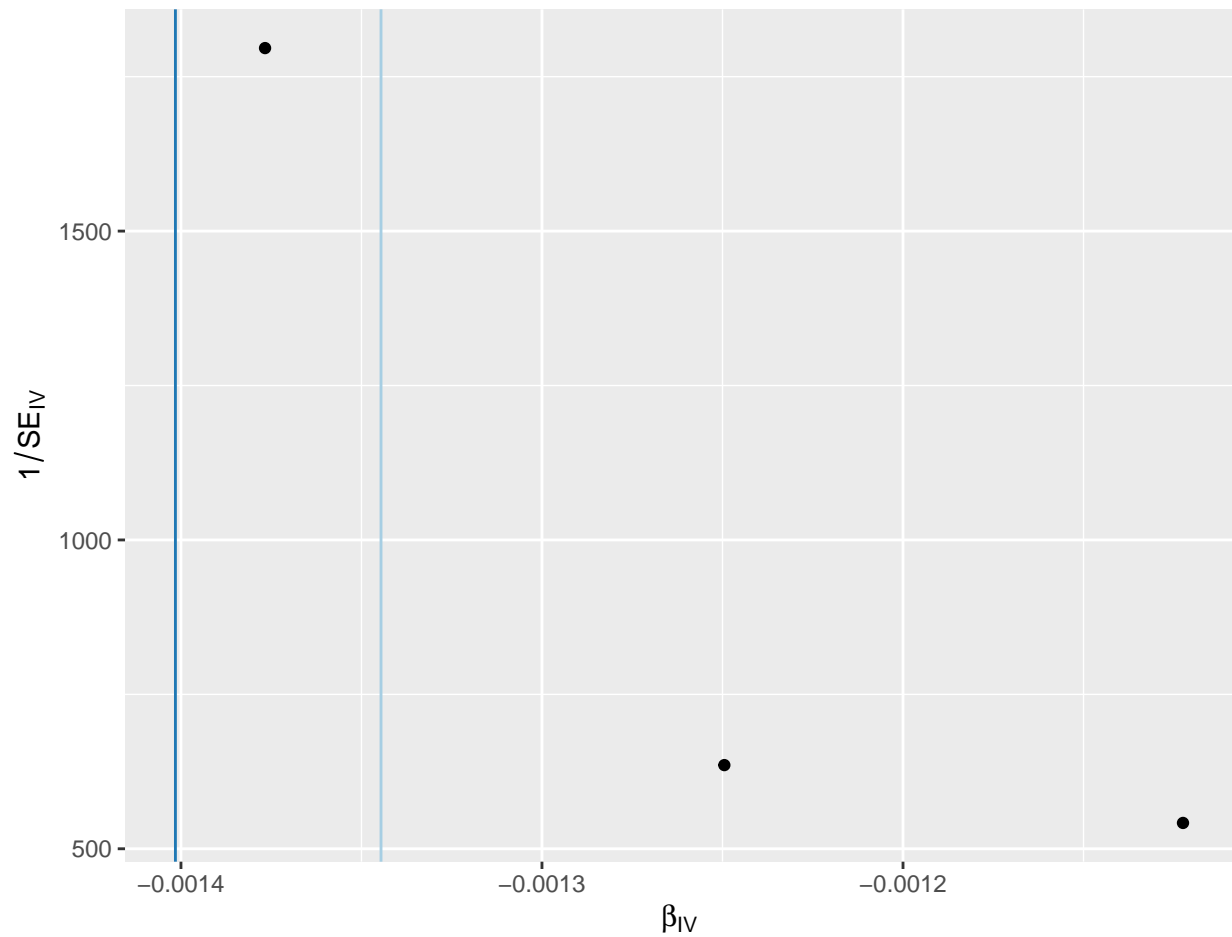

Supplement: Supplementary file 1 — Supplementary Material 1. [file 41065_2025_606_MOESM1_ESM.zip › Supplementary1/Supplementary - MR/eQTL-MR/MRpic/CNN3.funnel_plot.pdf]

rs115786643

rs12123176

rs12125005

All

-0.003

-0.002

-0.001

0.000

0.001

MR leave-one-out sensitivity analysis for  
'CNN3' on 'Bladder cancer'

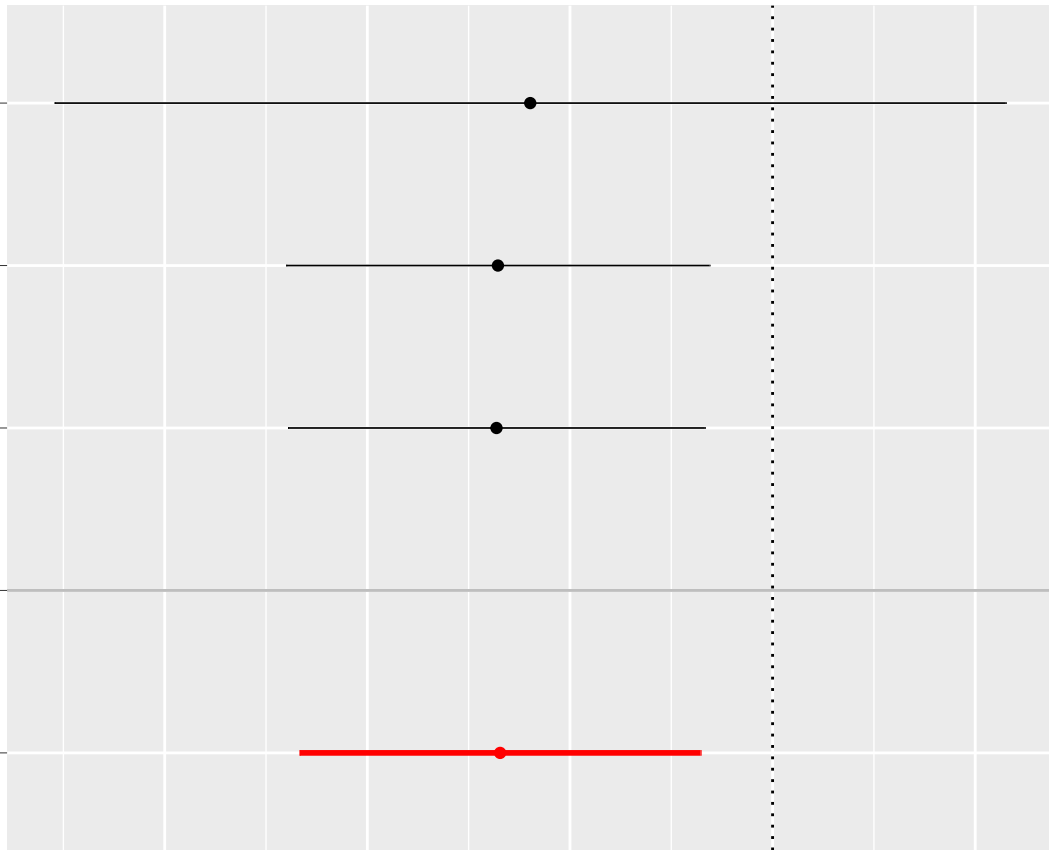

Supplement: Supplementary file 1 — Supplementary Material 1. [file 41065_2025_606_MOESM1_ESM.zip › Supplementary1/Supplementary - MR/eQTL-MR/MRpic/CNN3.leaveoneout.pdf]

# MR Test

- Inverse variance weighted
- MR Egger
- Simple mode
- Weighted median
- Weighted mode

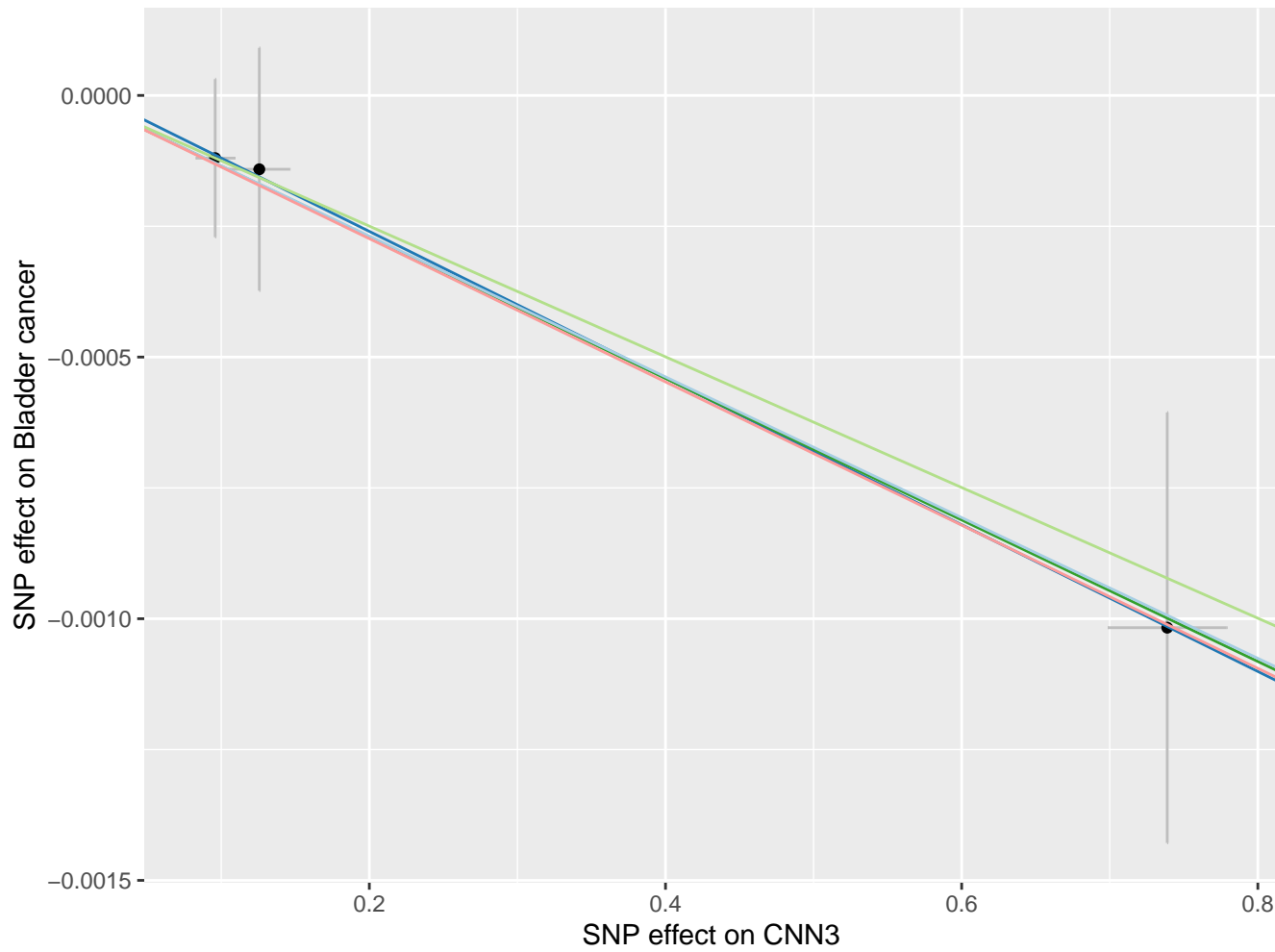

Supplement: Supplementary file 1 — Supplementary Material 1. [file 41065_2025_606_MOESM1_ESM.zip › Supplementary1/Supplementary - MR/eQTL-MR/MRpic/CNN3.scatter_plot.pdf]

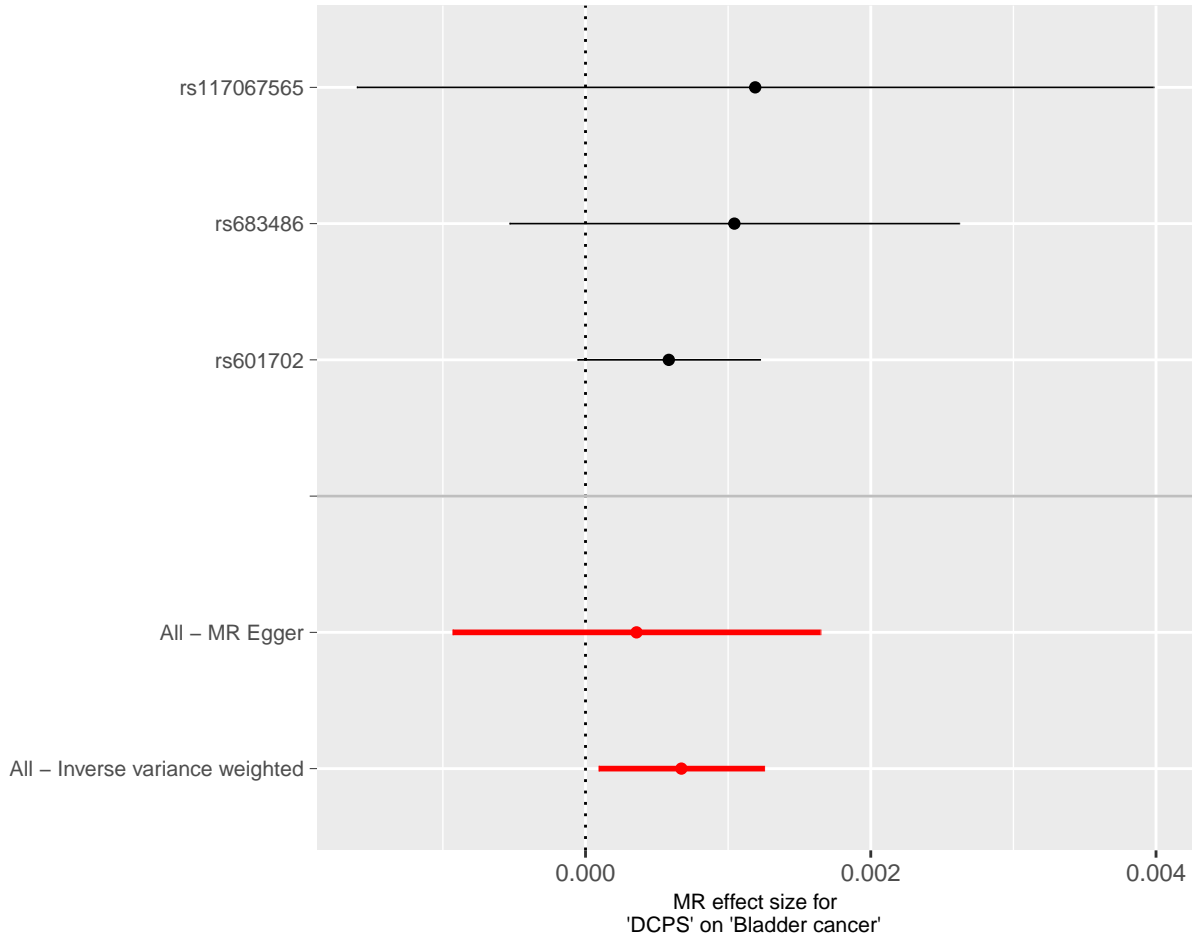

Supplement: Supplementary file 1 — Supplementary Material 1. [file 41065_2025_606_MOESM1_ESM.zip › Supplementary1/Supplementary - MR/eQTL-MR/MRpic/DCPS.forest.pdf]

# MR Method

- Inverse variance weighted
- MR Egger

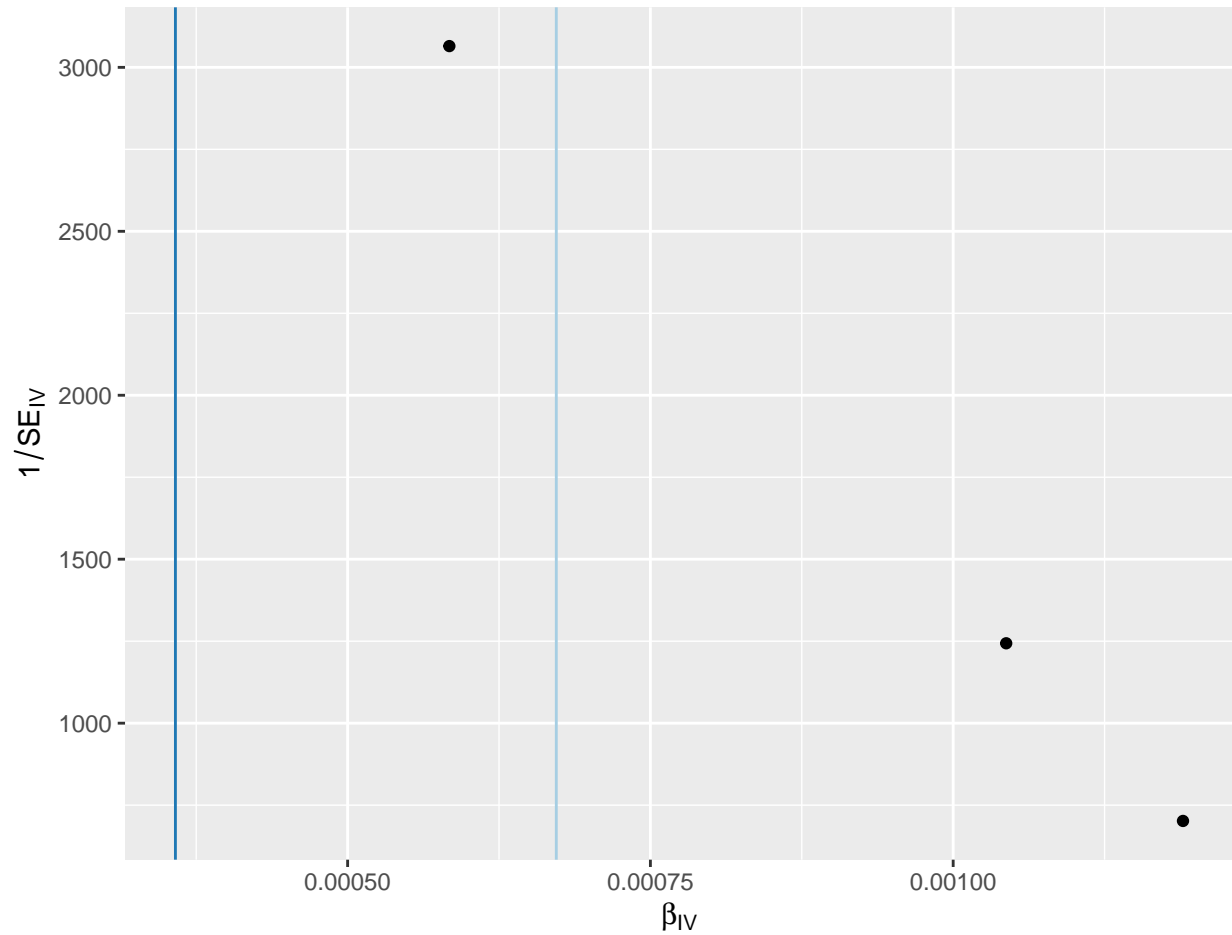

Supplement: Supplementary file 1 — Supplementary Material 1. [file 41065_2025_606_MOESM1_ESM.zip › Supplementary1/Supplementary - MR/eQTL-MR/MRpic/DCPS.funnel_plot.pdf]

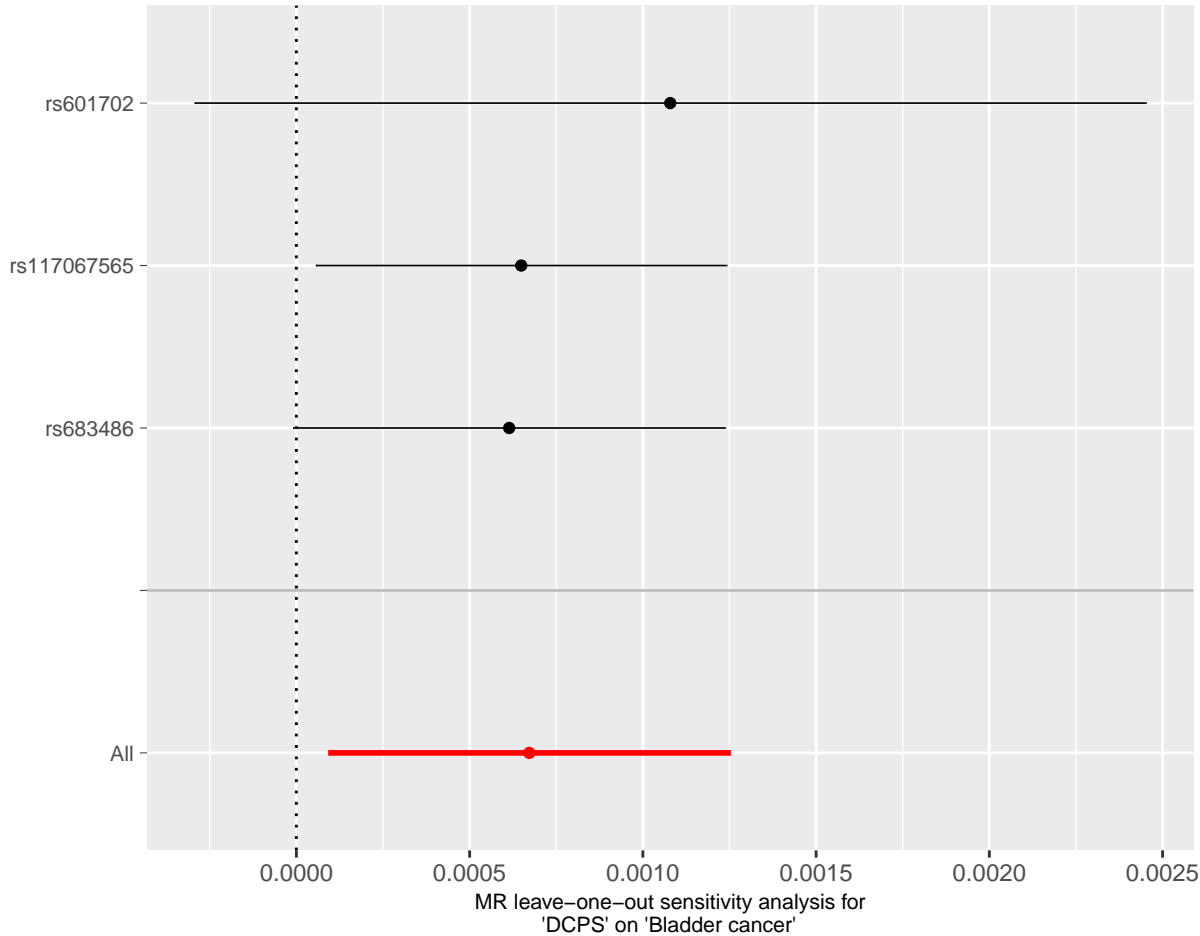

Supplement: Supplementary file 1 — Supplementary Material 1. [file 41065_2025_606_MOESM1_ESM.zip › Supplementary1/Supplementary - MR/eQTL-MR/MRpic/DCPS.leaveoneout.pdf]

# MR Test

- Inverse variance weighted
- MR Egger
- Simple mode
- Weighted median
- Weighted mode

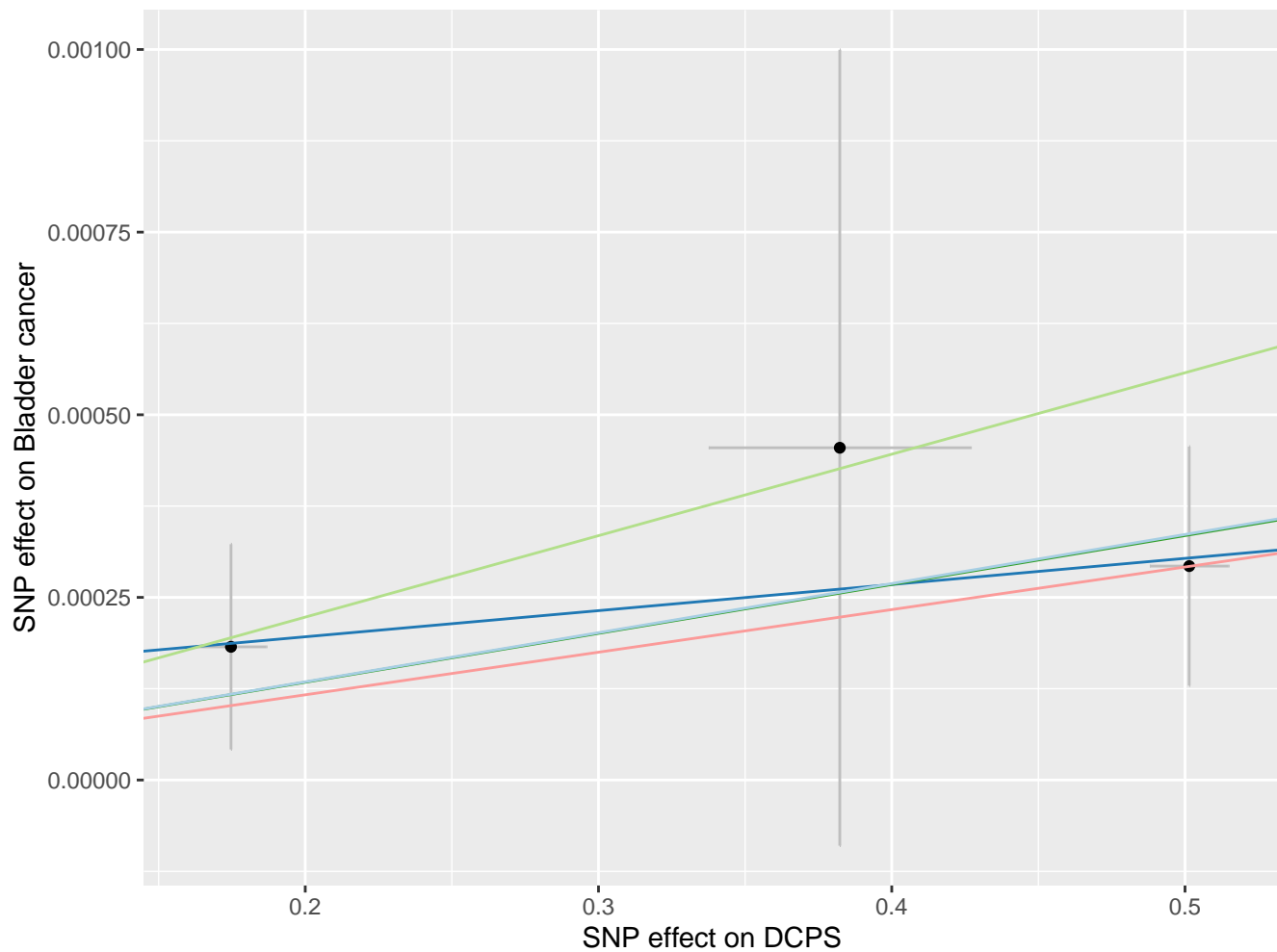

Supplement: Supplementary file 1 — Supplementary Material 1. [file 41065_2025_606_MOESM1_ESM.zip › Supplementary1/Supplementary - MR/eQTL-MR/MRpic/DCPS.scatter_plot.pdf]

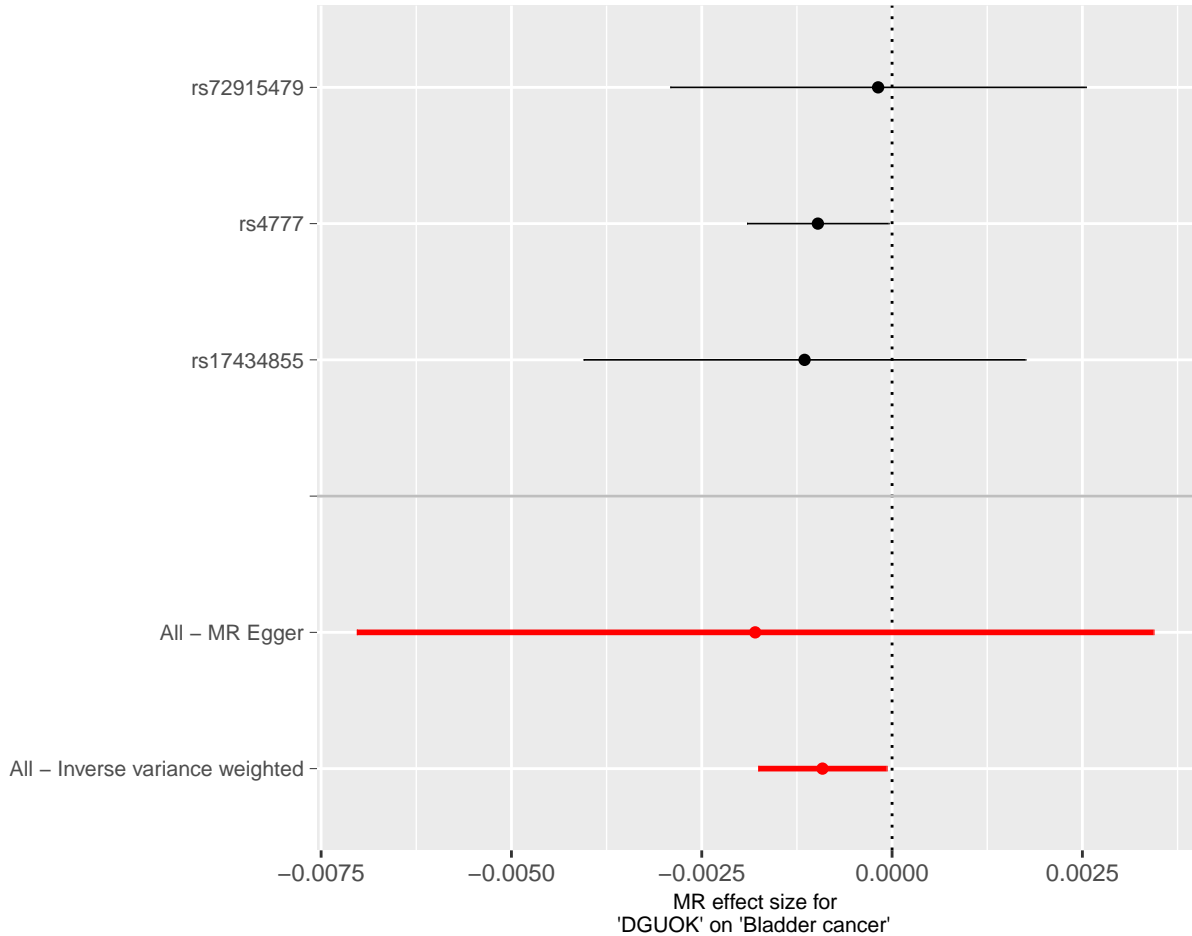

Supplement: Supplementary file 1 — Supplementary Material 1. [file 41065_2025_606_MOESM1_ESM.zip › Supplementary1/Supplementary - MR/eQTL-MR/MRpic/DGUOK.forest.pdf]

# MR Method

- Inverse variance weighted
- MR Egger

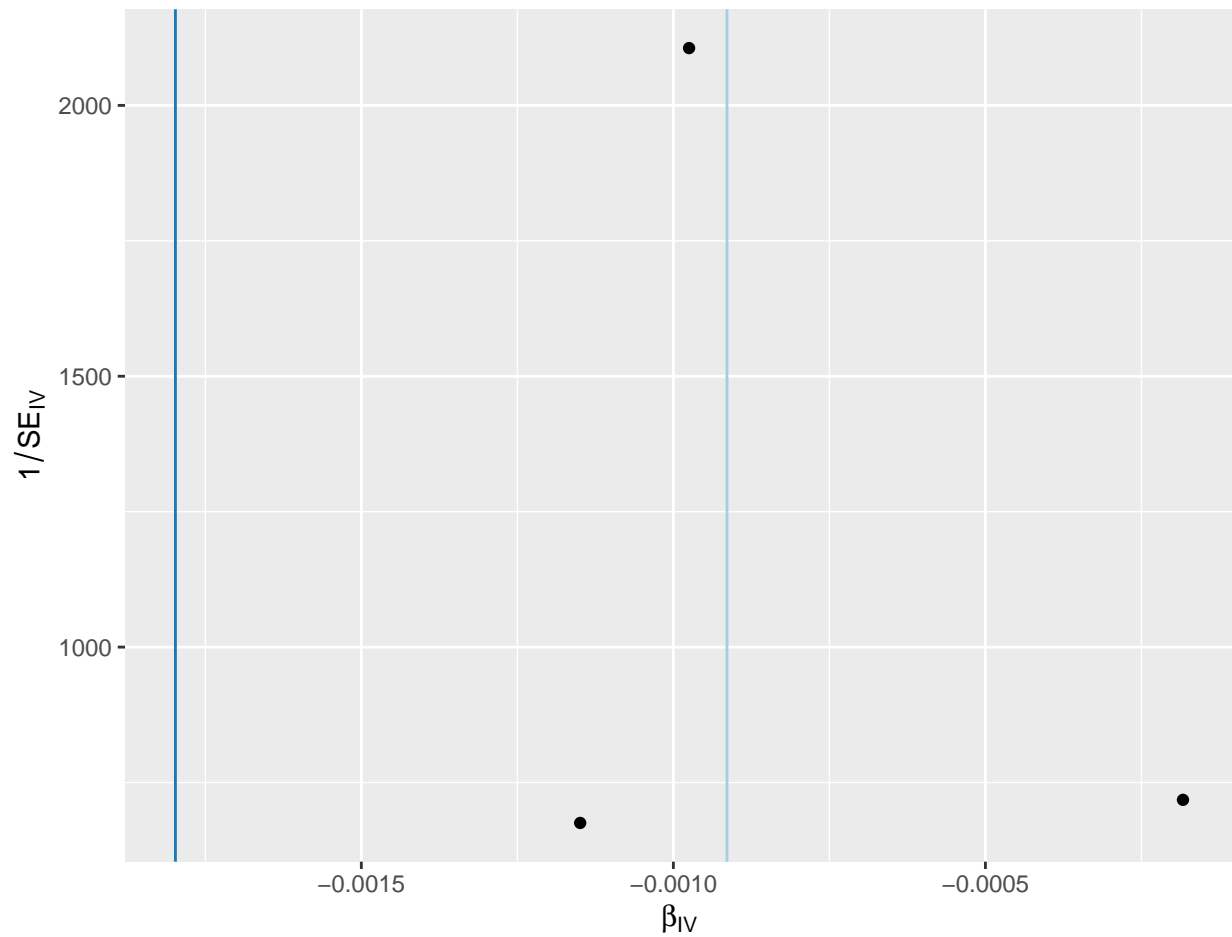

Supplement: Supplementary file 1 — Supplementary Material 1. [file 41065_2025_606_MOESM1_ESM.zip › Supplementary1/Supplementary - MR/eQTL-MR/MRpic/DGUOK.funnel_plot.pdf]

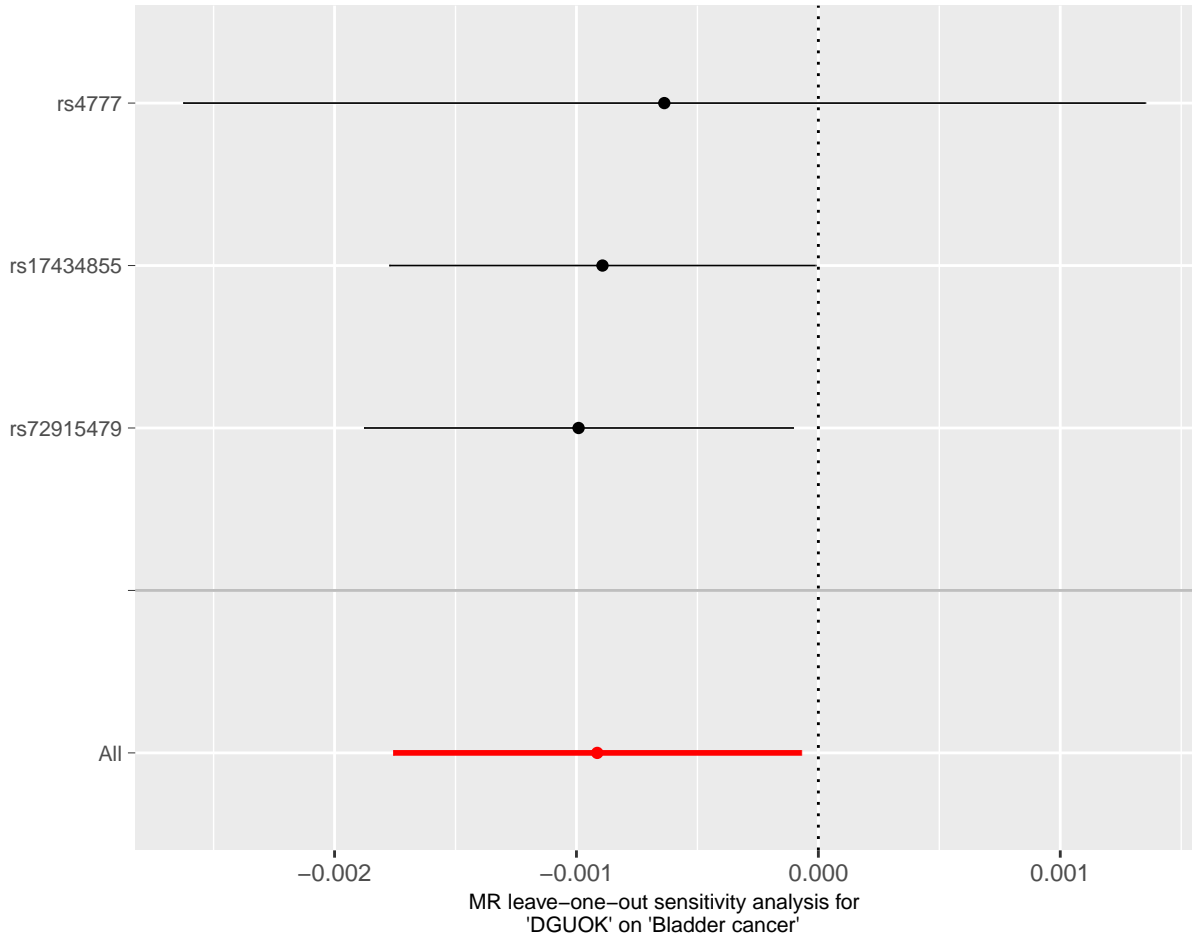

Supplement: Supplementary file 1 — Supplementary Material 1. [file 41065_2025_606_MOESM1_ESM.zip › Supplementary1/Supplementary - MR/eQTL-MR/MRpic/DGUOK.leaveoneout.pdf]

# MR Test

- Inverse variance weighted
- MR Egger
- Simple mode
- Weighted median
- Weighted mode

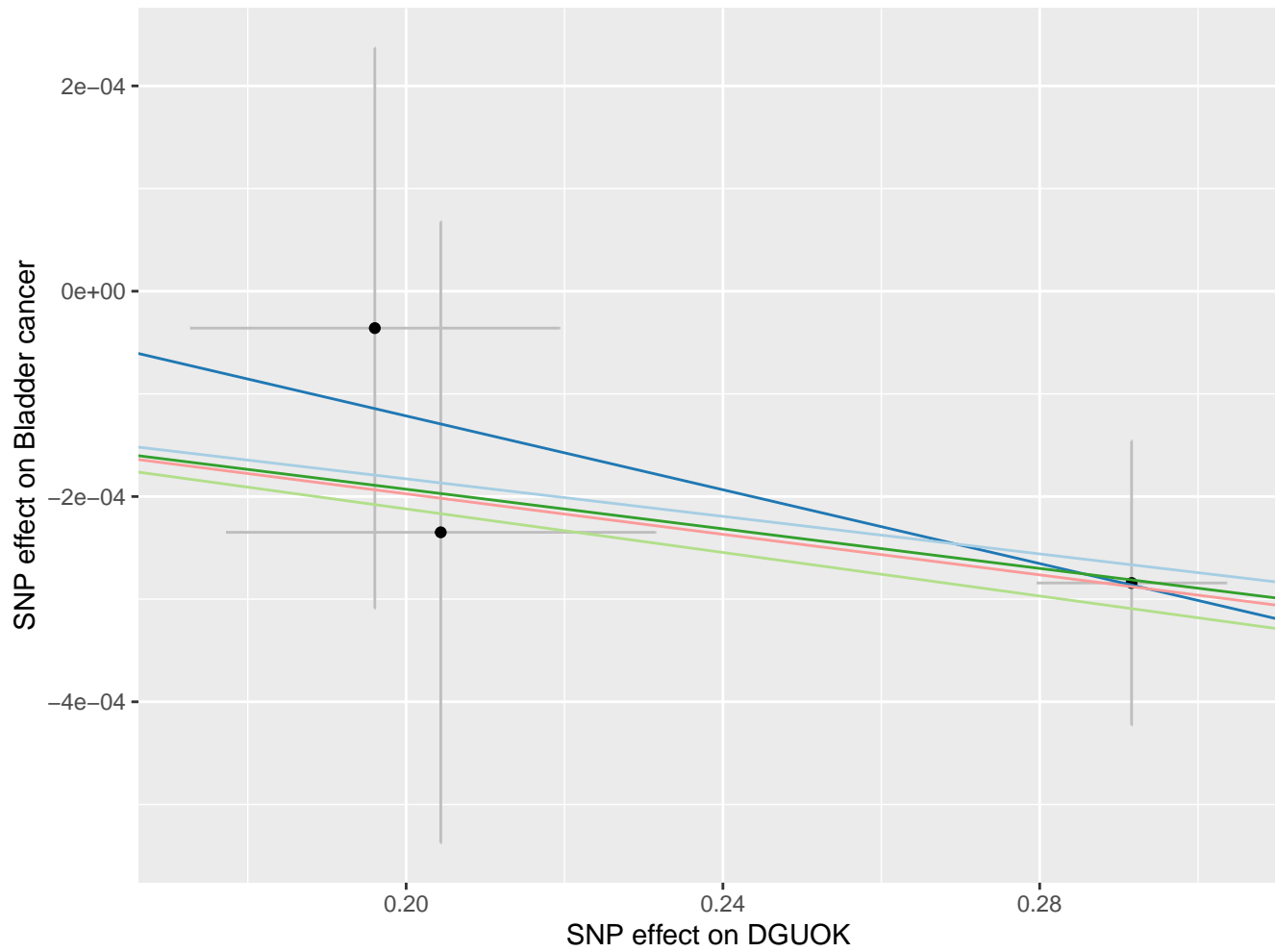

Supplement: Supplementary file 1 — Supplementary Material 1. [file 41065_2025_606_MOESM1_ESM.zip › Supplementary1/Supplementary - MR/eQTL-MR/MRpic/DGUOK.scatter_plot.pdf]

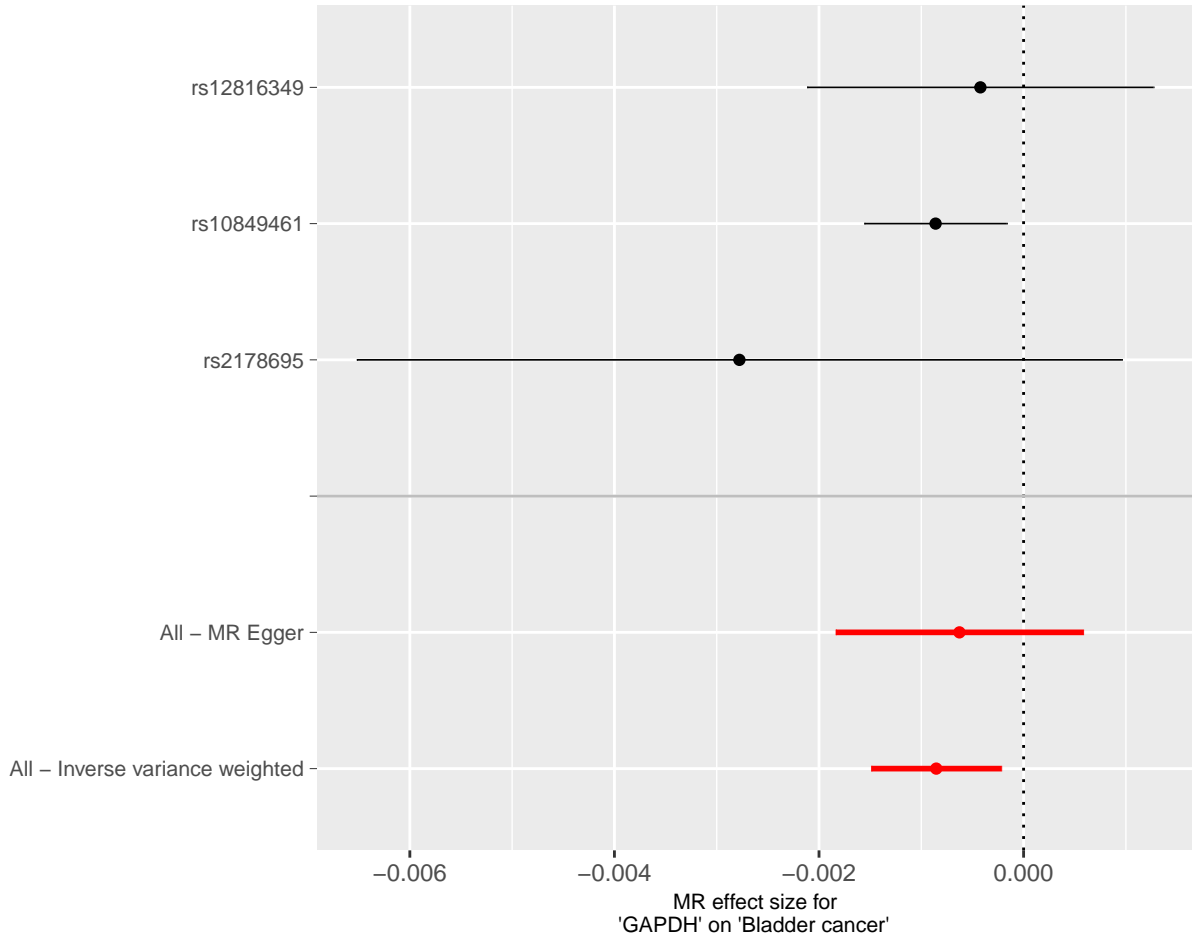

Supplement: Supplementary file 1 — Supplementary Material 1. [file 41065_2025_606_MOESM1_ESM.zip › Supplementary1/Supplementary - MR/eQTL-MR/MRpic/GAPDH.forest.pdf]

# MR Method

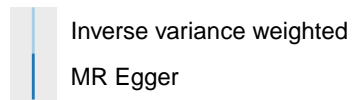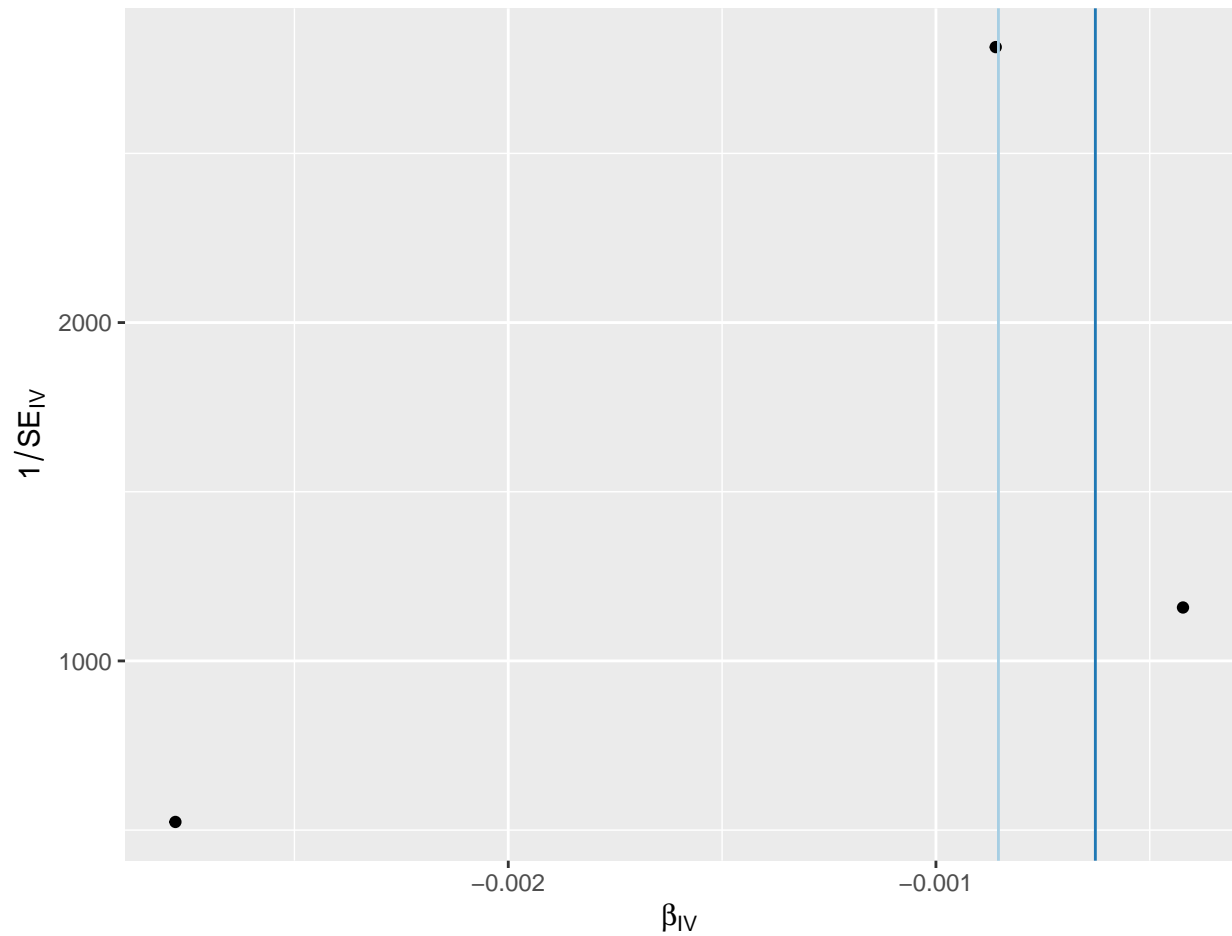

Supplement: Supplementary file 1 — Supplementary Material 1. [file 41065_2025_606_MOESM1_ESM.zip › Supplementary1/Supplementary - MR/eQTL-MR/MRpic/GAPDH.funnel_plot.pdf]

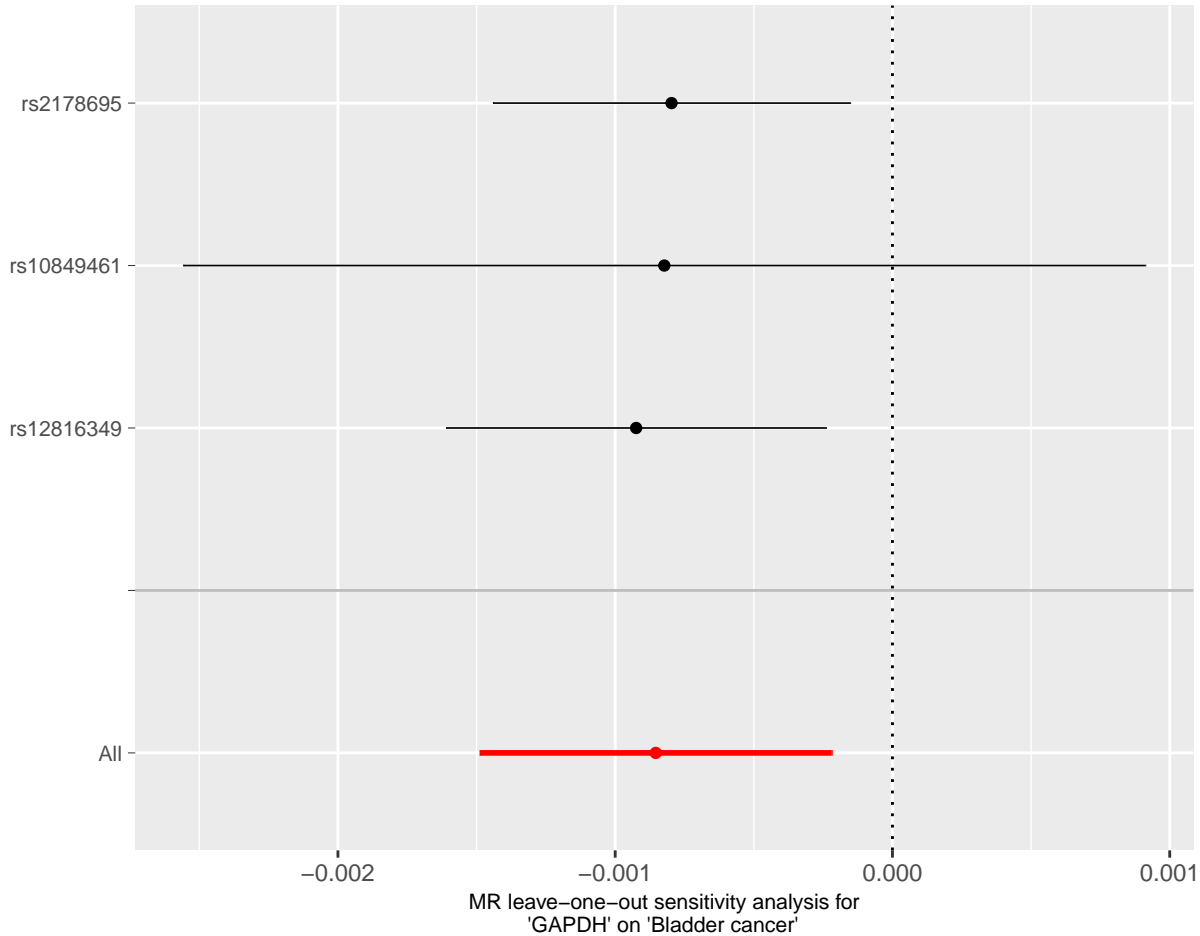

Supplement: Supplementary file 1 — Supplementary Material 1. [file 41065_2025_606_MOESM1_ESM.zip › Supplementary1/Supplementary - MR/eQTL-MR/MRpic/GAPDH.leaveoneout.pdf]

# MR Test

- Inverse variance weighted
- MR Egger
- Simple mode
- Weighted median
- Weighted mode

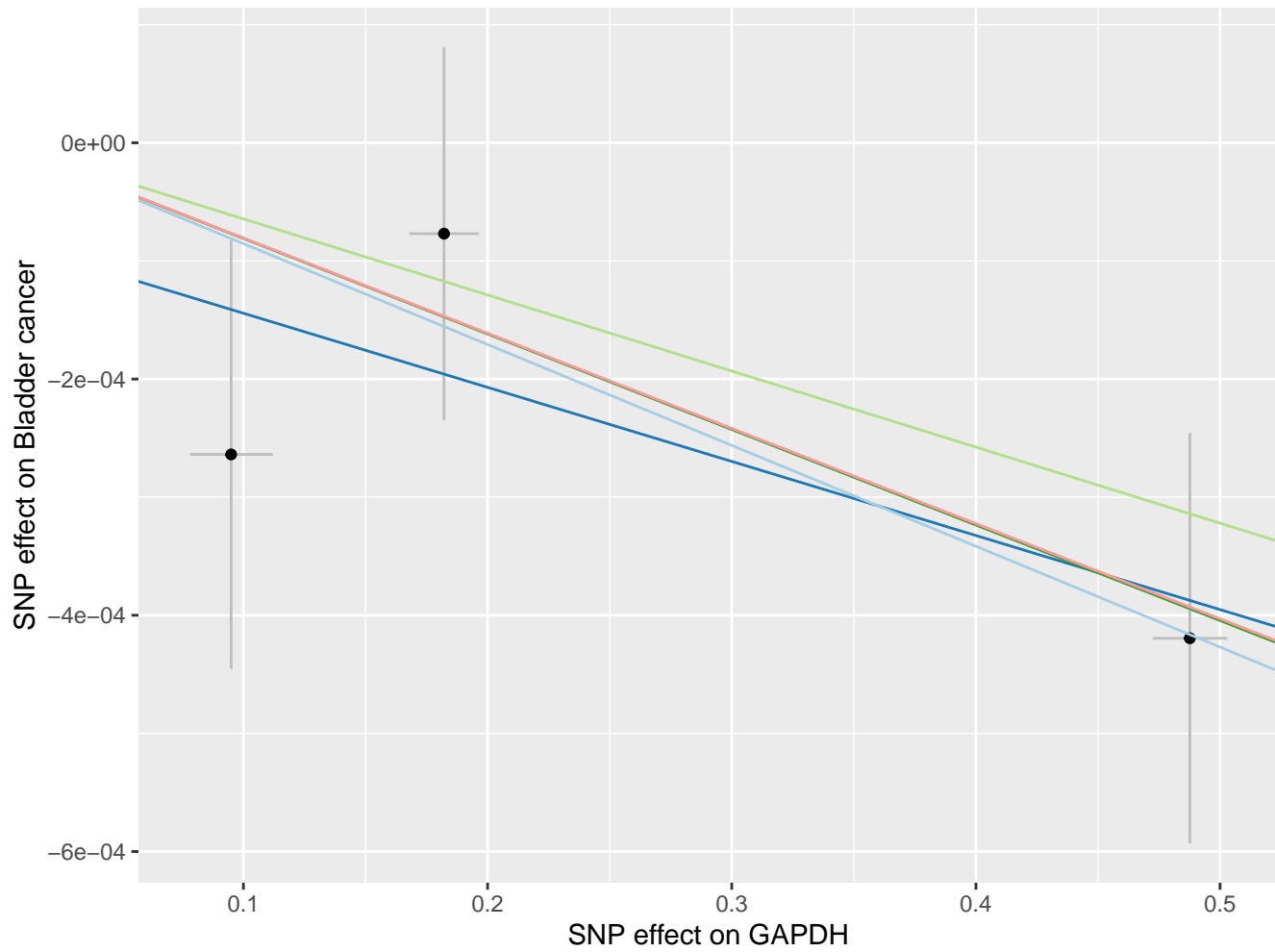

Supplement: Supplementary file 1 — Supplementary Material 1. [file 41065_2025_606_MOESM1_ESM.zip › Supplementary1/Supplementary - MR/eQTL-MR/MRpic/GAPDH.scatter_plot.pdf]

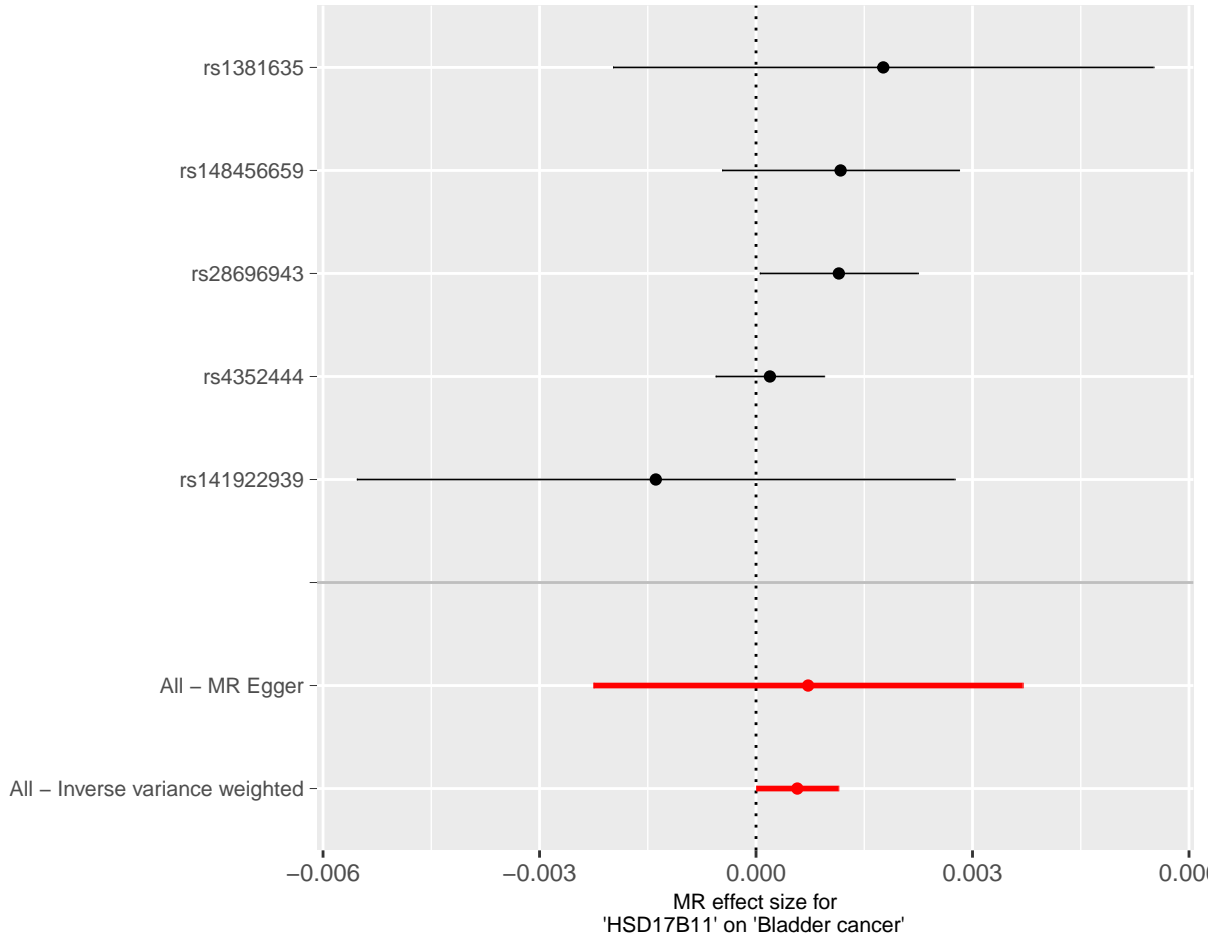

Supplement: Supplementary file 1 — Supplementary Material 1. [file 41065_2025_606_MOESM1_ESM.zip › Supplementary1/Supplementary - MR/eQTL-MR/MRpic/HSD17B11.forest.pdf]

# MR Method

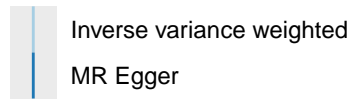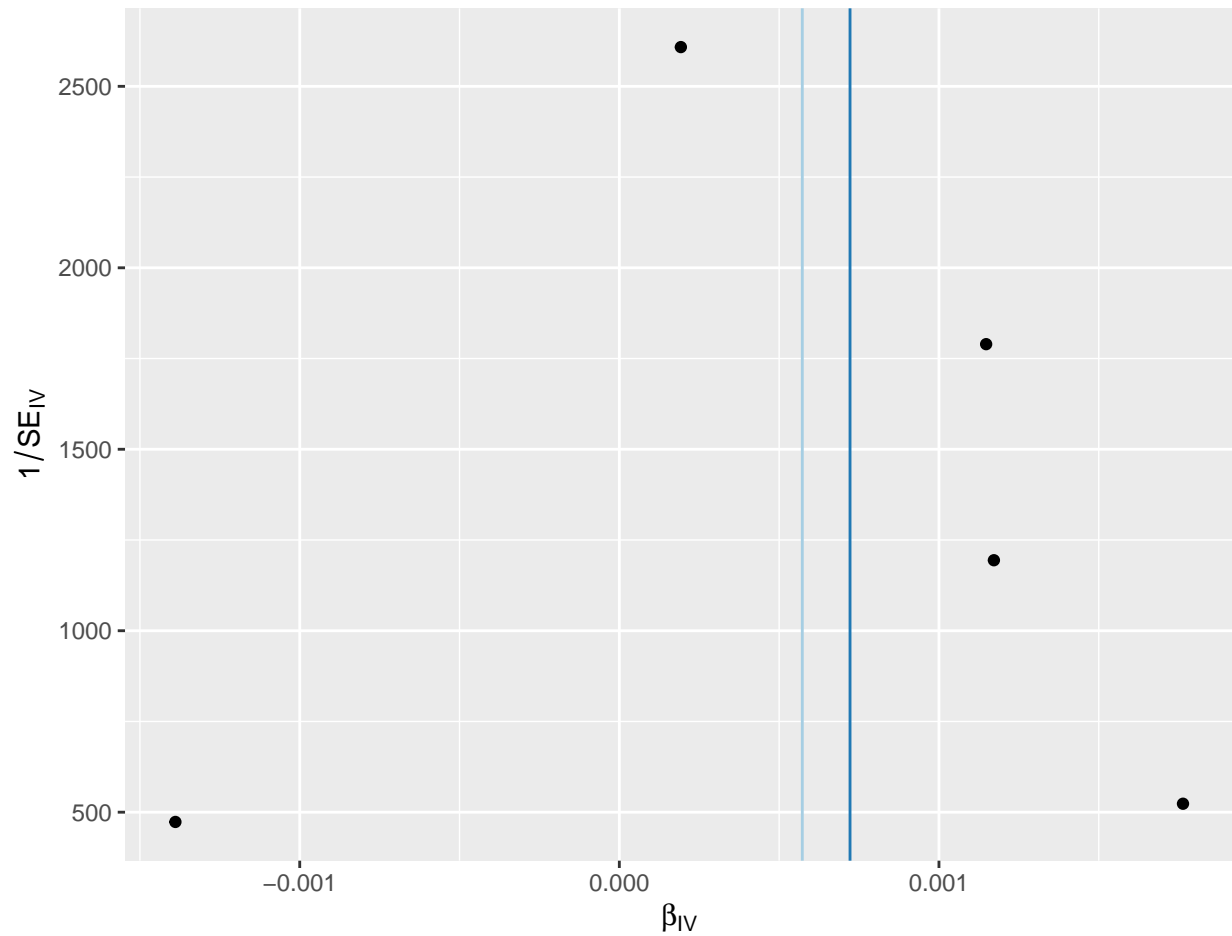

Supplement: Supplementary file 1 — Supplementary Material 1. [file 41065_2025_606_MOESM1_ESM.zip › Supplementary1/Supplementary - MR/eQTL-MR/MRpic/HSD17B11.funnel_plot.pdf]

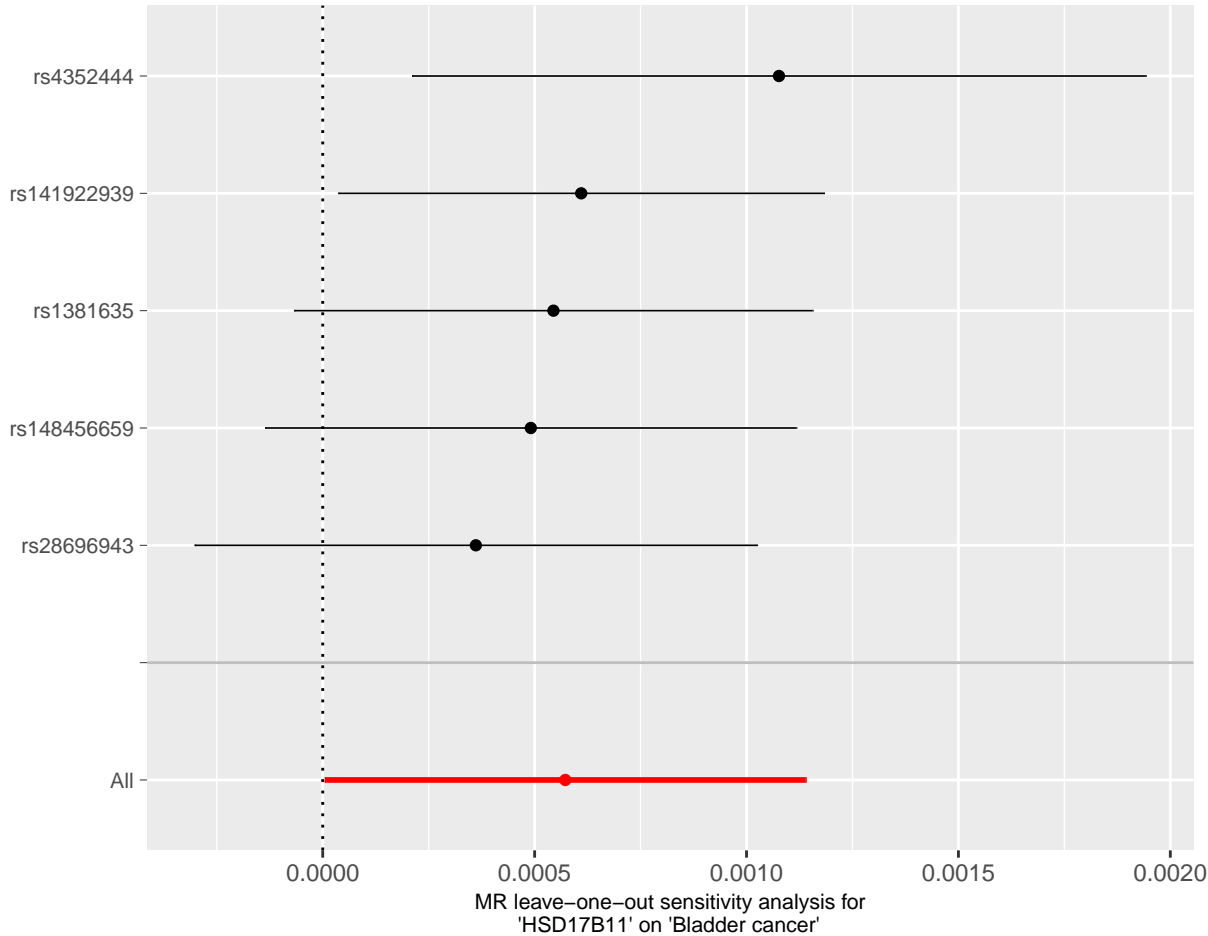

Supplement: Supplementary file 1 — Supplementary Material 1. [file 41065_2025_606_MOESM1_ESM.zip › Supplementary1/Supplementary - MR/eQTL-MR/MRpic/HSD17B11.leaveoneout.pdf]

# MR Test

- Inverse variance weighted
- MR Egger
- Simple mode
- Weighted median
- Weighted mode

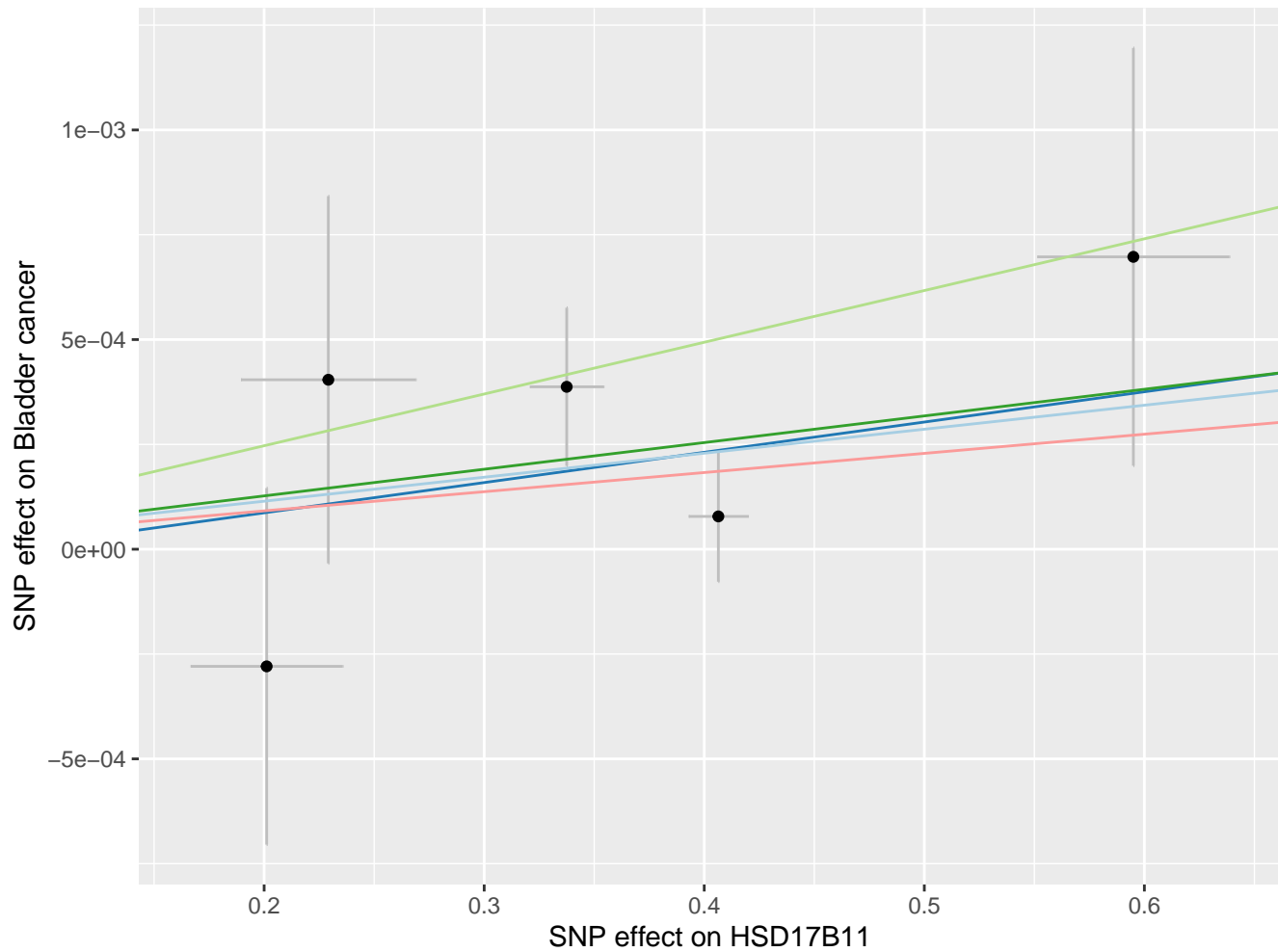

Supplement: Supplementary file 1 — Supplementary Material 1. [file 41065_2025_606_MOESM1_ESM.zip › Supplementary1/Supplementary - MR/eQTL-MR/MRpic/HSD17B11.scatter_plot.pdf]

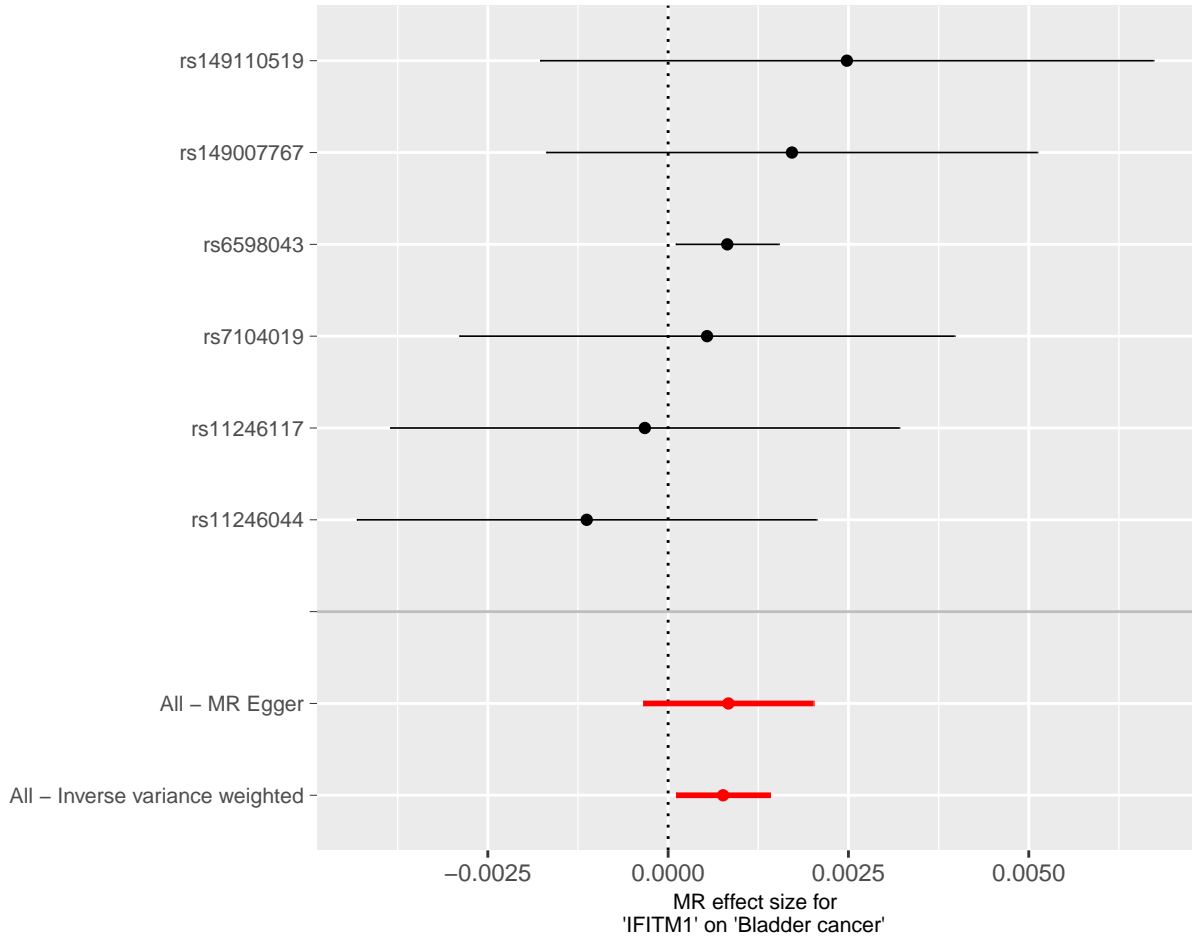

Supplement: Supplementary file 1 — Supplementary Material 1. [file 41065_2025_606_MOESM1_ESM.zip › Supplementary1/Supplementary - MR/eQTL-MR/MRpic/IFITM1.forest.pdf]

# MR Method

- Inverse variance weighted
- MR Egger

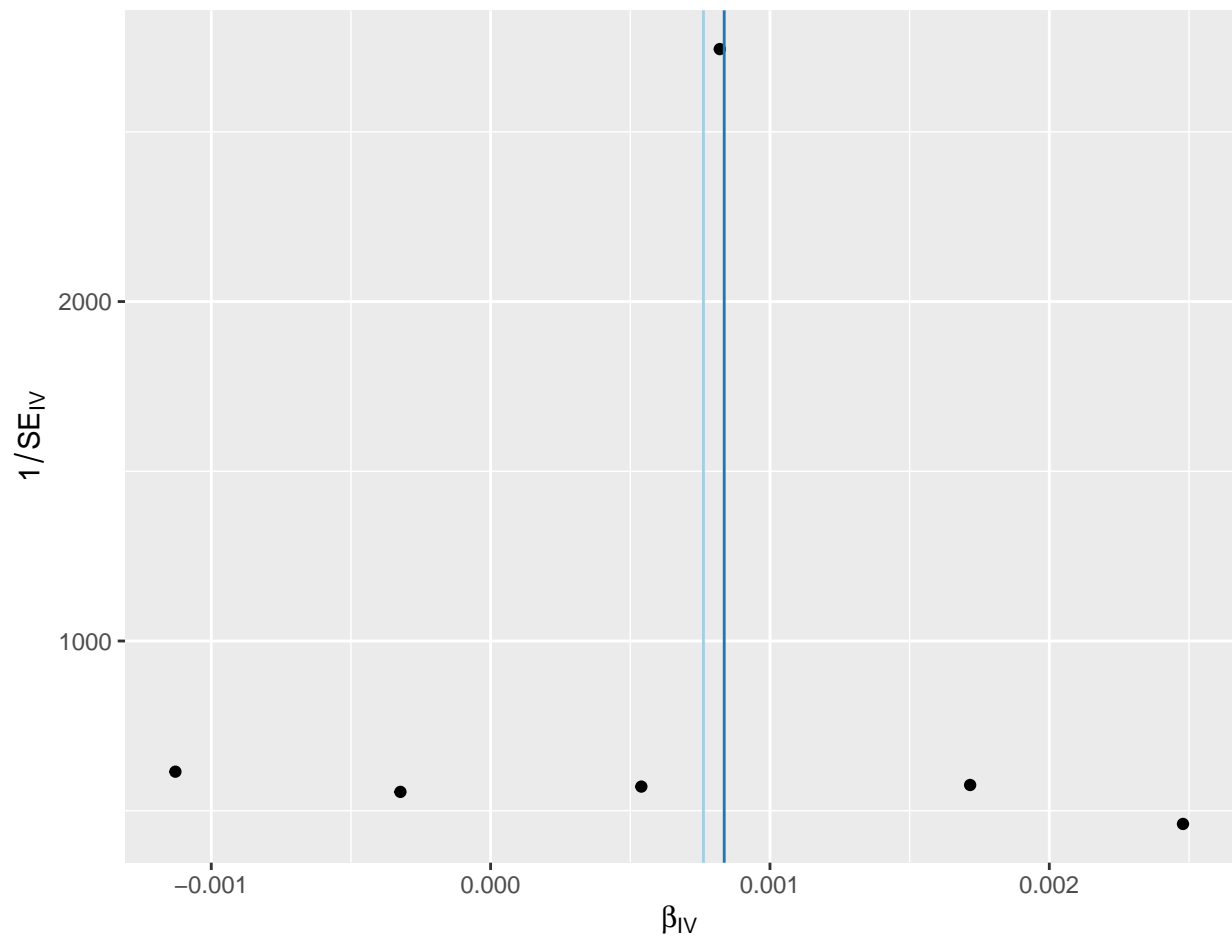

Supplement: Supplementary file 1 — Supplementary Material 1. [file 41065_2025_606_MOESM1_ESM.zip › Supplementary1/Supplementary - MR/eQTL-MR/MRpic/IFITM1.funnel_plot.pdf]

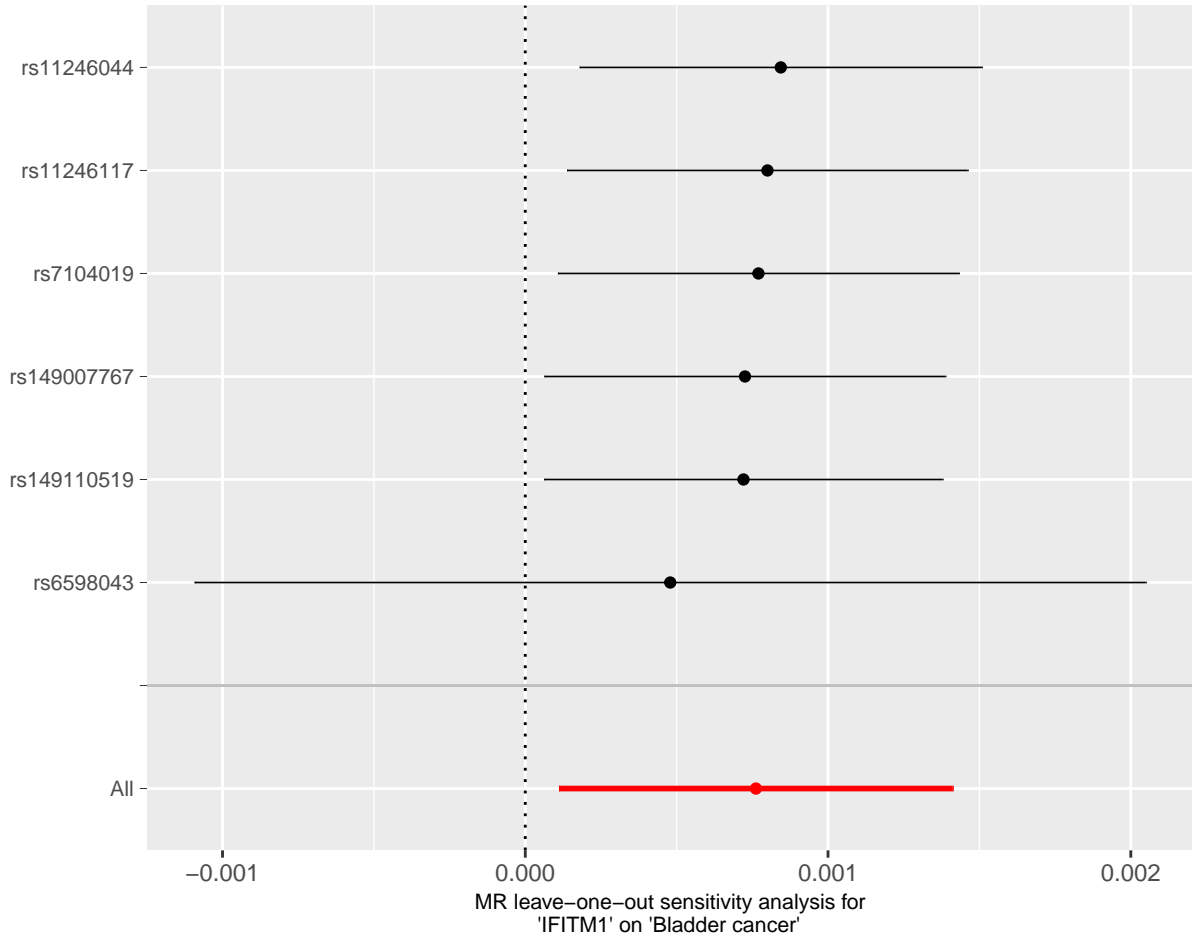

Supplement: Supplementary file 1 — Supplementary Material 1. [file 41065_2025_606_MOESM1_ESM.zip › Supplementary1/Supplementary - MR/eQTL-MR/MRpic/IFITM1.leaveoneout.pdf]

# MR Test

- Inverse variance weighted
- MR Egger
- Simple mode
- Weighted median
- Weighted mode

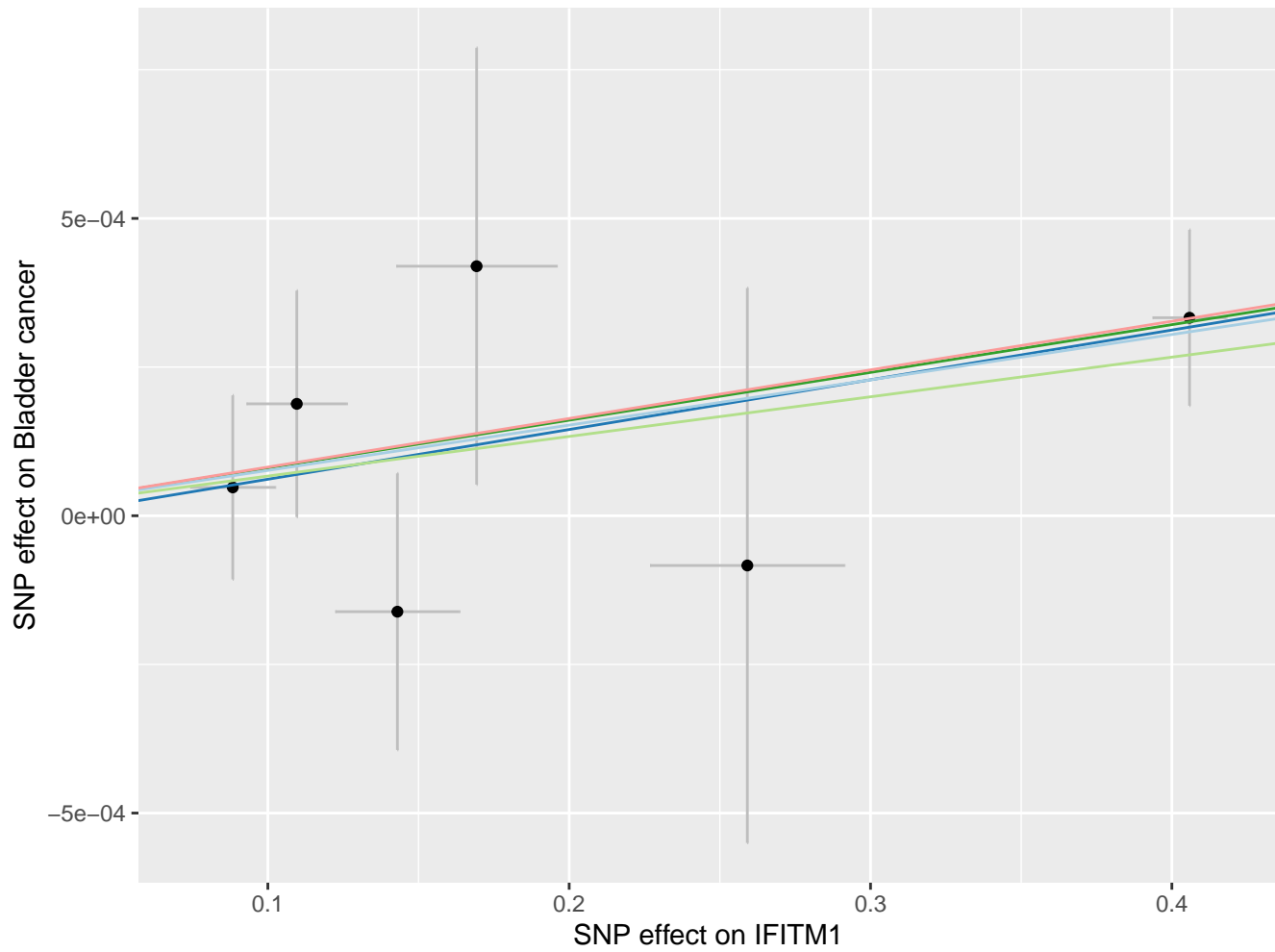

Supplement: Supplementary file 1 — Supplementary Material 1. [file 41065_2025_606_MOESM1_ESM.zip › Supplementary1/Supplementary - MR/eQTL-MR/MRpic/IFITM1.scatter_plot.pdf]

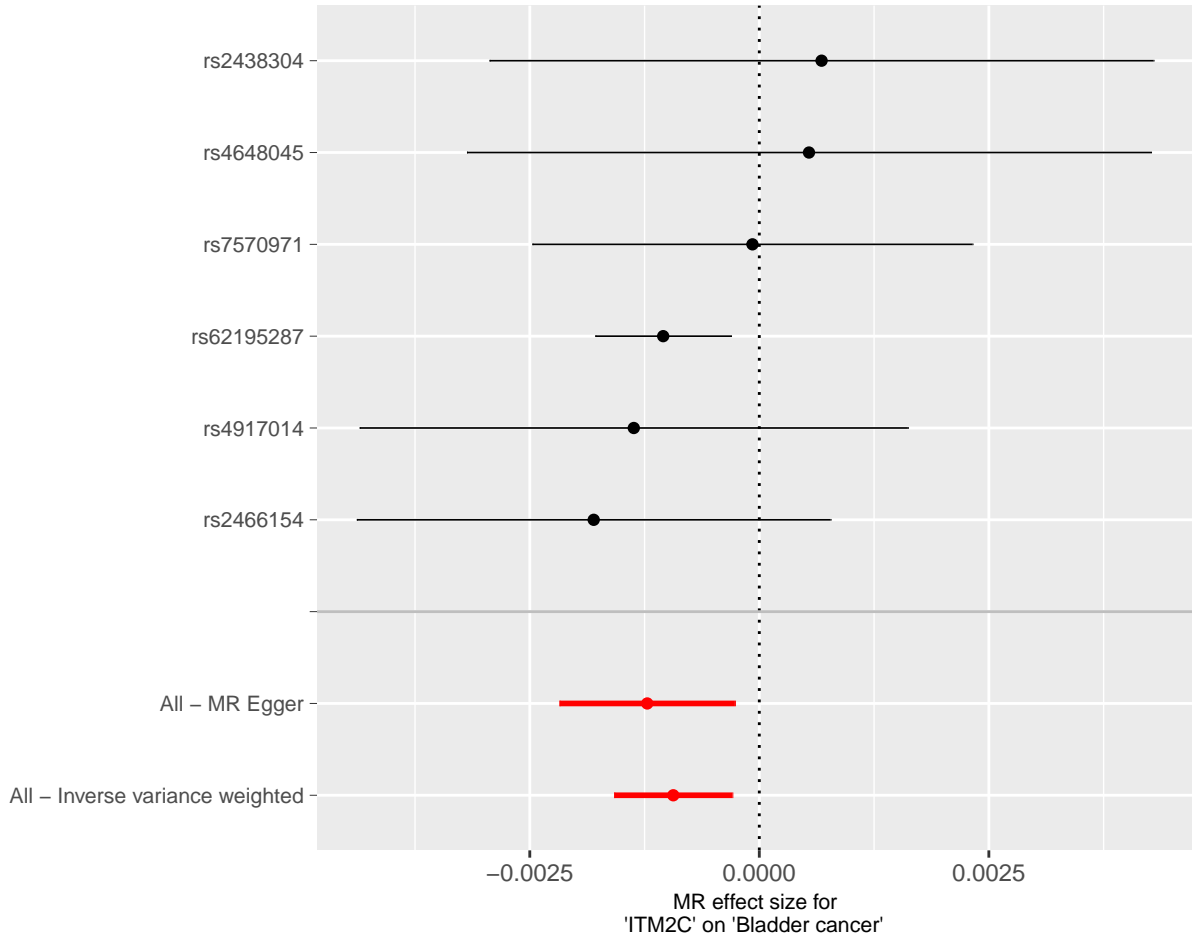

Supplement: Supplementary file 1 — Supplementary Material 1. [file 41065_2025_606_MOESM1_ESM.zip › Supplementary1/Supplementary - MR/eQTL-MR/MRpic/ITM2C.forest.pdf]

# MR Method

- Inverse variance weighted
- MR Egger

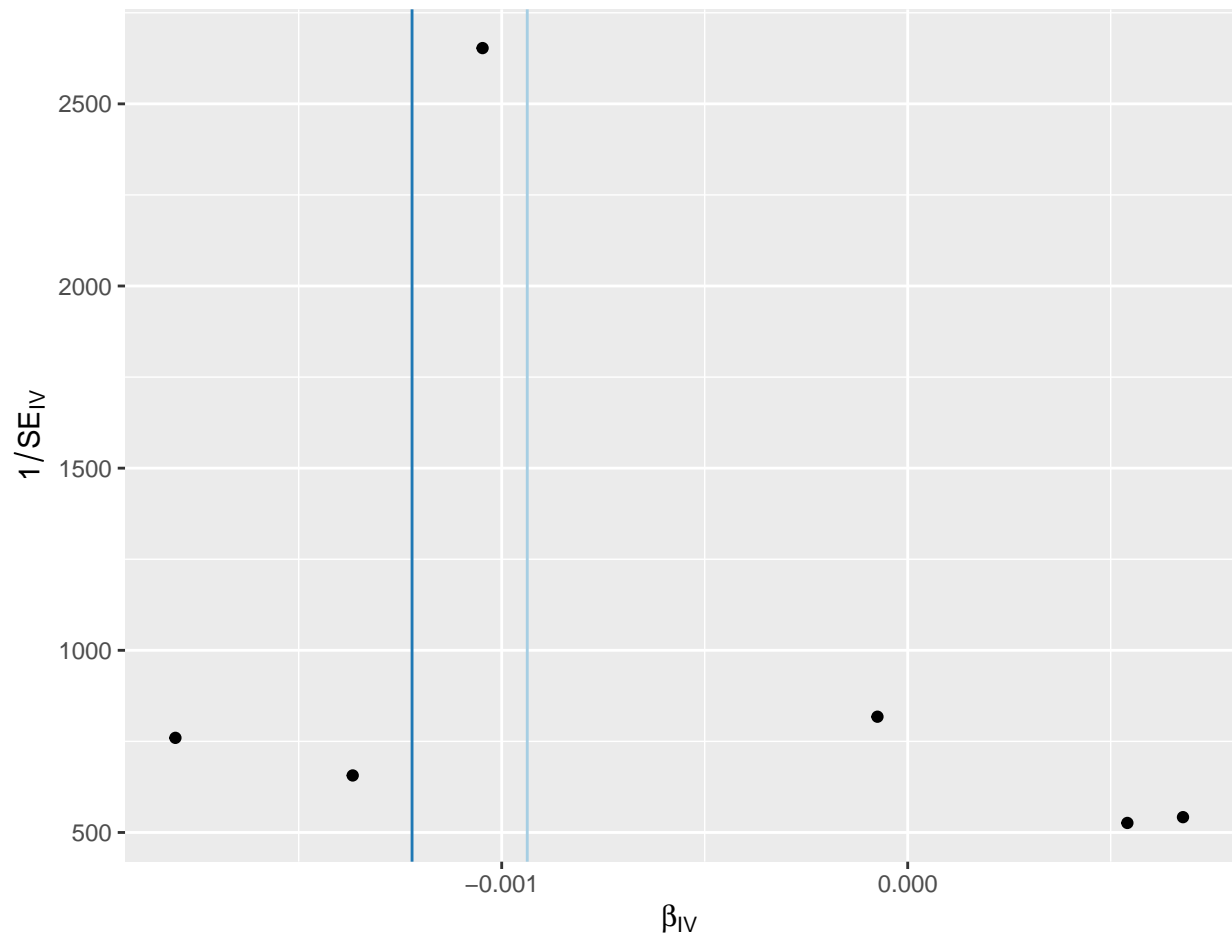

Supplement: Supplementary file 1 — Supplementary Material 1. [file 41065_2025_606_MOESM1_ESM.zip › Supplementary1/Supplementary - MR/eQTL-MR/MRpic/ITM2C.funnel_plot.pdf]

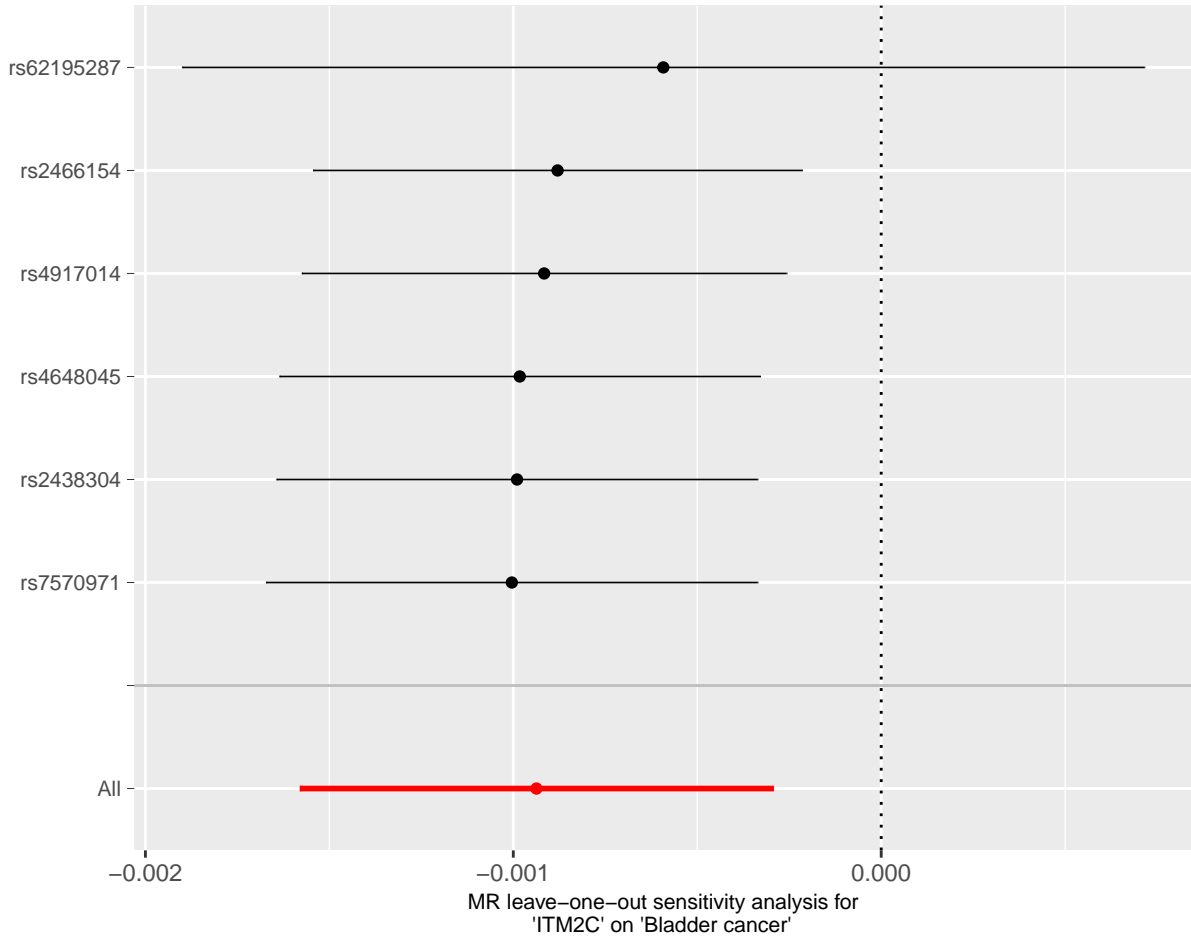

Supplement: Supplementary file 1 — Supplementary Material 1. [file 41065_2025_606_MOESM1_ESM.zip › Supplementary1/Supplementary - MR/eQTL-MR/MRpic/ITM2C.leaveoneout.pdf]

# MR Test

- Inverse variance weighted
- MR Egger
- Simple mode
- Weighted median
- Weighted mode

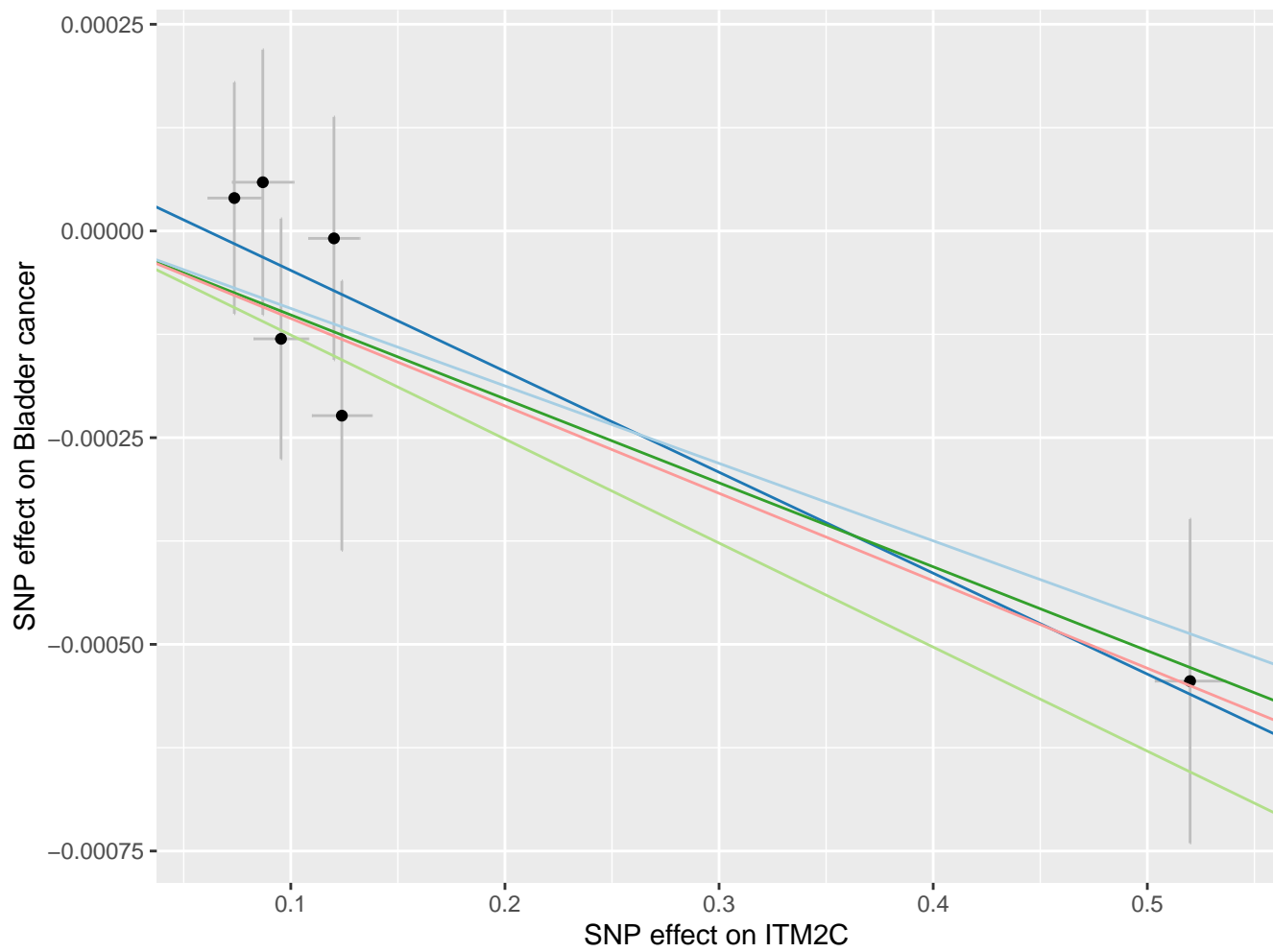

Supplement: Supplementary file 1 — Supplementary Material 1. [file 41065_2025_606_MOESM1_ESM.zip › Supplementary1/Supplementary - MR/eQTL-MR/MRpic/ITM2C.scatter_plot.pdf]

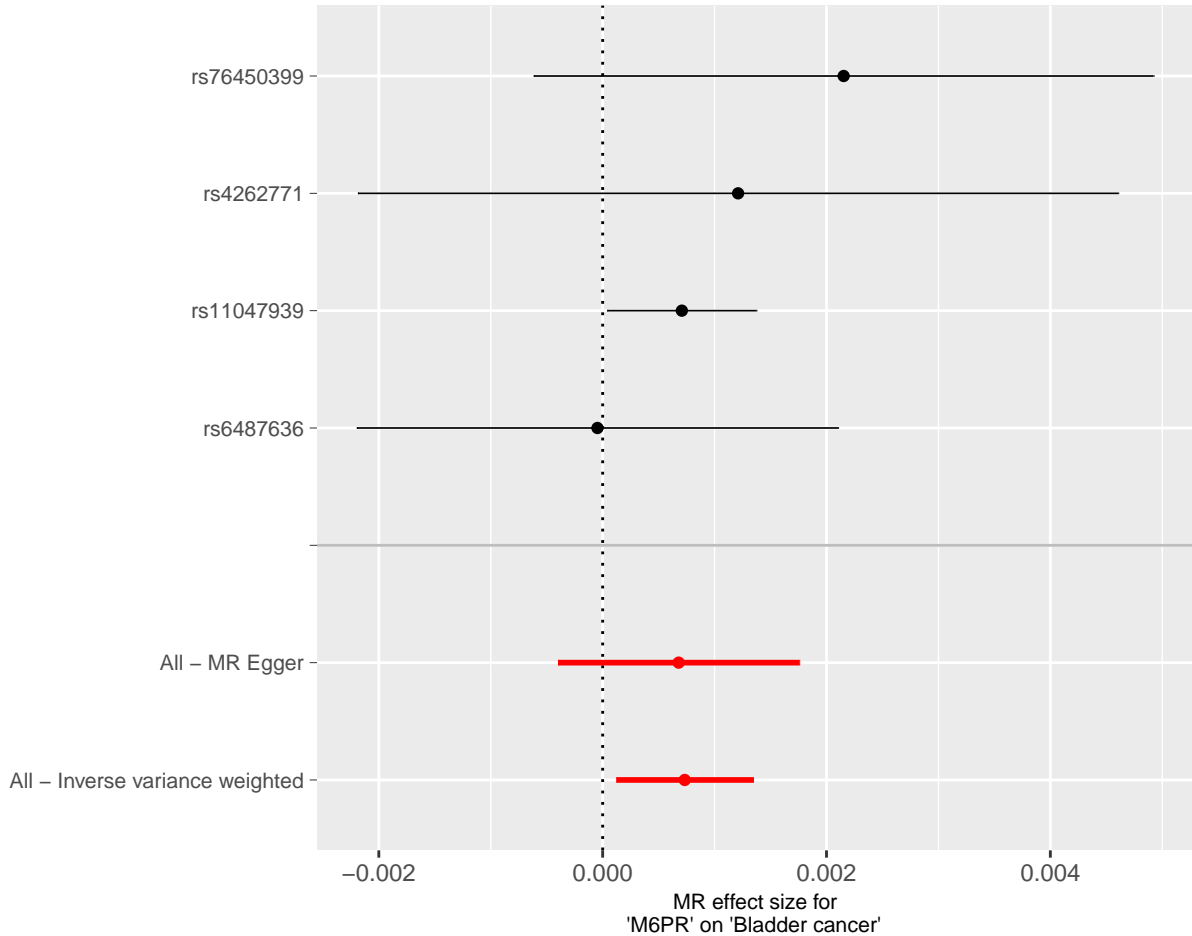

Supplement: Supplementary file 1 — Supplementary Material 1. [file 41065_2025_606_MOESM1_ESM.zip › Supplementary1/Supplementary - MR/eQTL-MR/MRpic/M6PR.forest.pdf]

# MR Method

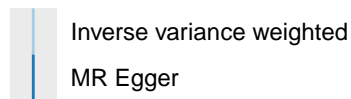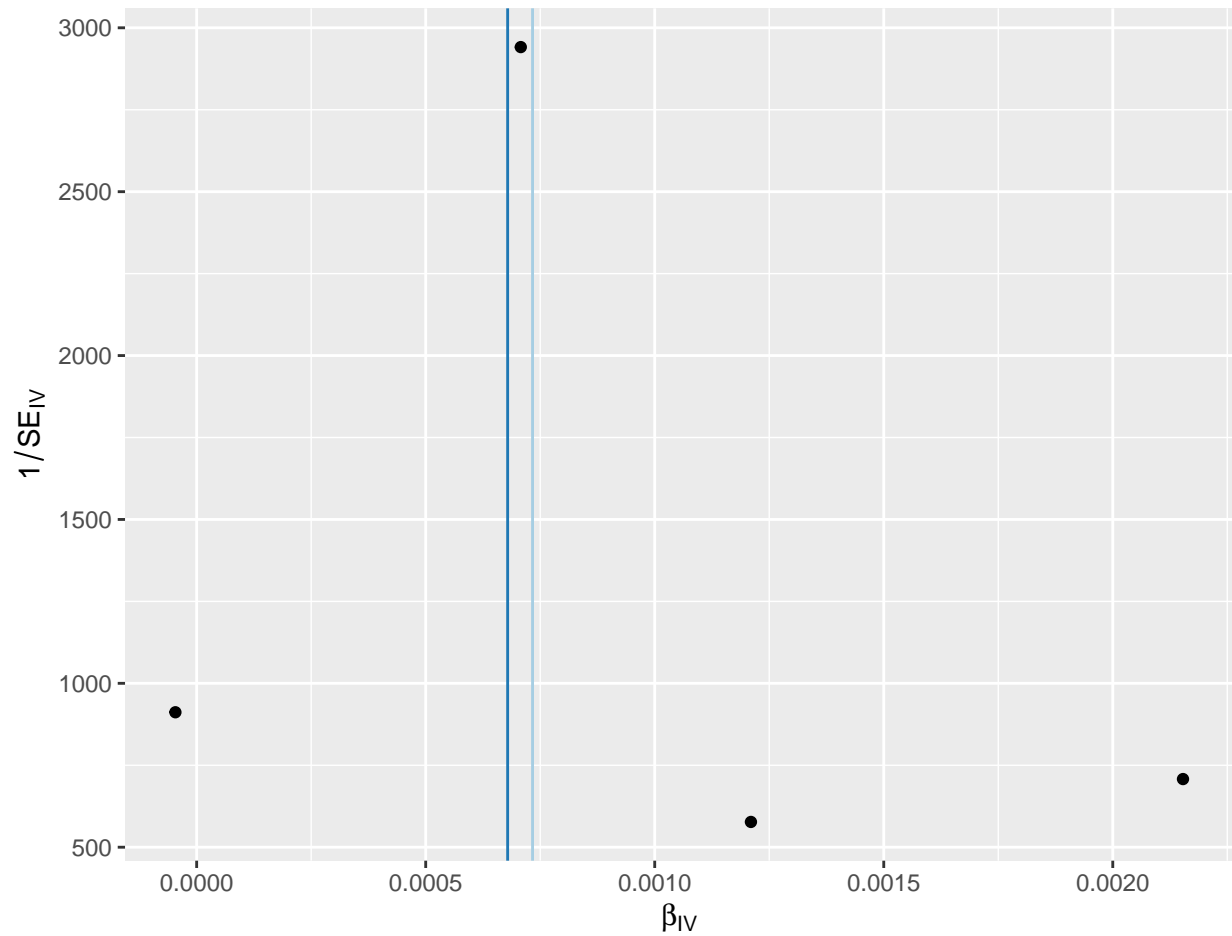

Supplement: Supplementary file 1 — Supplementary Material 1. [file 41065_2025_606_MOESM1_ESM.zip › Supplementary1/Supplementary - MR/eQTL-MR/MRpic/M6PR.funnel_plot.pdf]

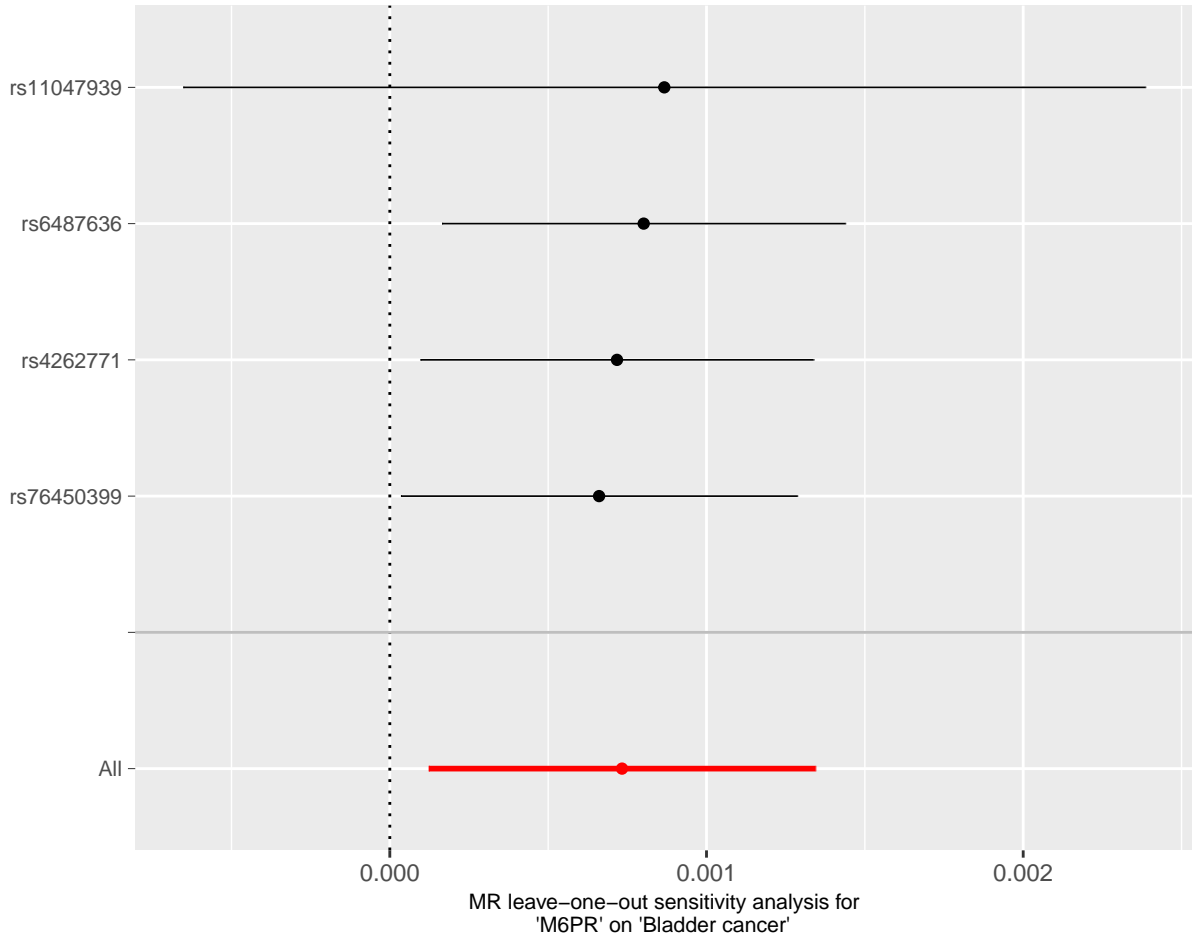

Supplement: Supplementary file 1 — Supplementary Material 1. [file 41065_2025_606_MOESM1_ESM.zip › Supplementary1/Supplementary - MR/eQTL-MR/MRpic/M6PR.leaveoneout.pdf]

# MR Test

- Inverse variance weighted
- MR Egger
- Simple mode
- Weighted median
- Weighted mode

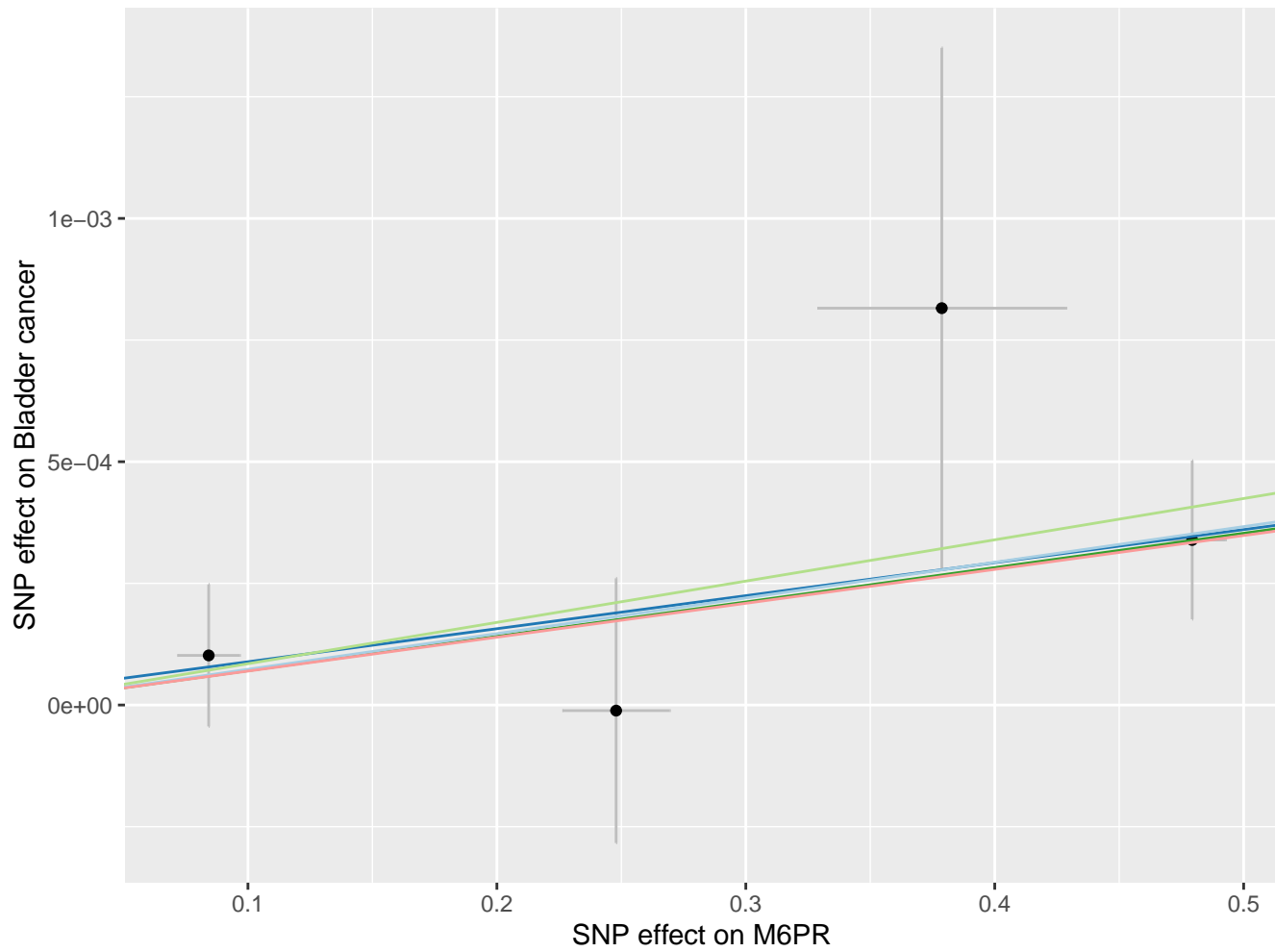

Supplement: Supplementary file 1 — Supplementary Material 1. [file 41065_2025_606_MOESM1_ESM.zip › Supplementary1/Supplementary - MR/eQTL-MR/MRpic/M6PR.scatter_plot.pdf]

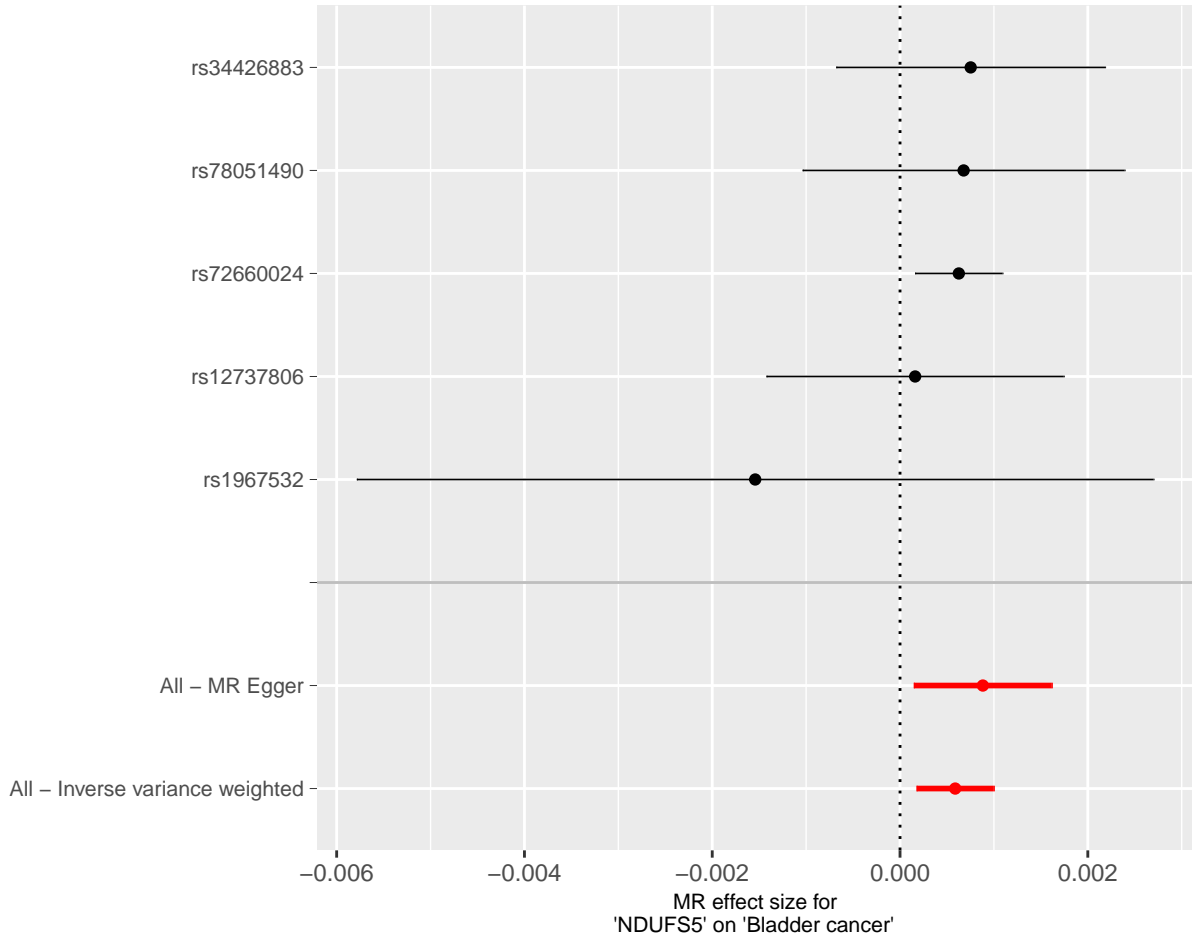

Supplement: Supplementary file 1 — Supplementary Material 1. [file 41065_2025_606_MOESM1_ESM.zip › Supplementary1/Supplementary - MR/eQTL-MR/MRpic/NDUFS5.forest.pdf]

# MR Method

- Inverse variance weighted
- MR Egger

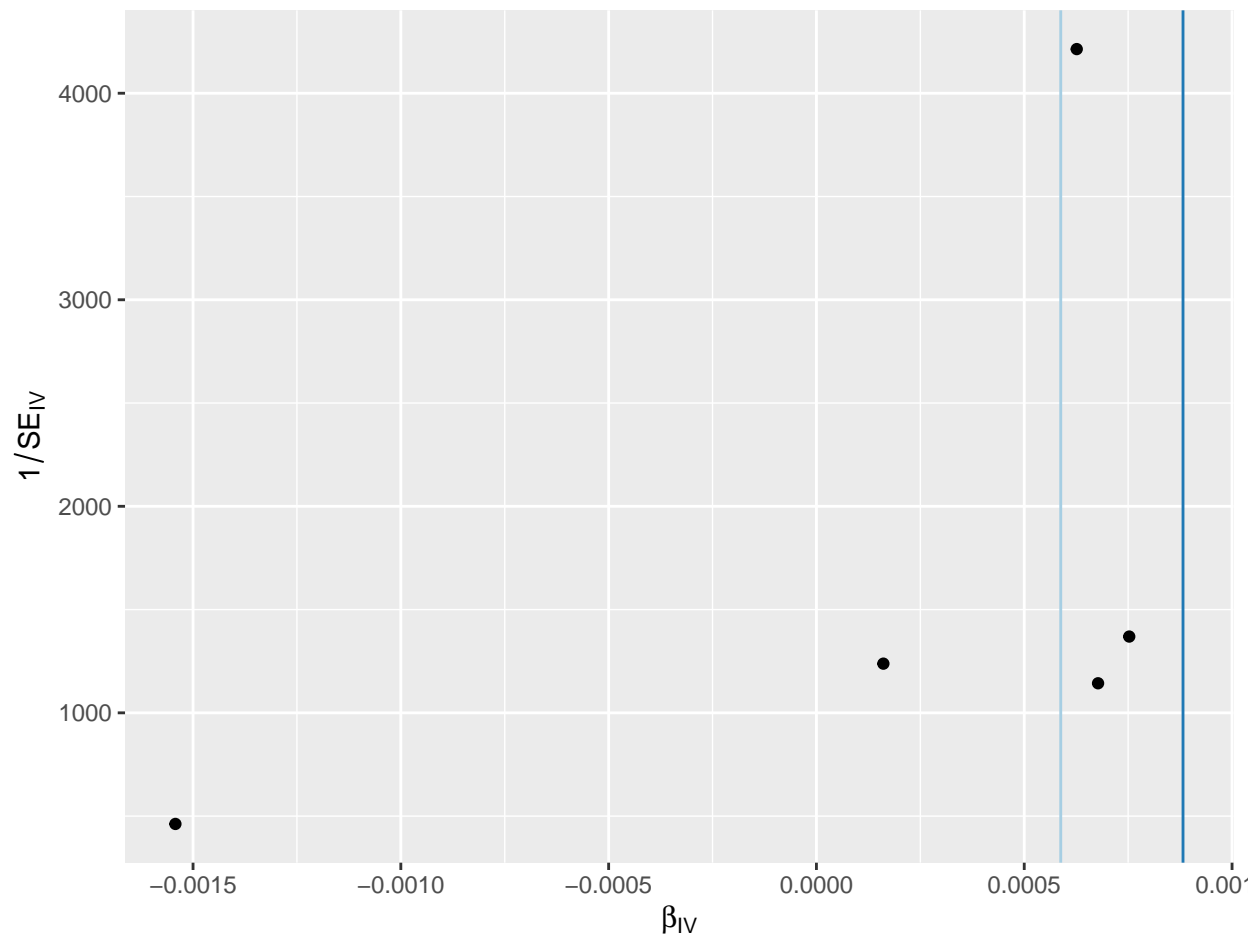

Supplement: Supplementary file 1 — Supplementary Material 1. [file 41065_2025_606_MOESM1_ESM.zip › Supplementary1/Supplementary - MR/eQTL-MR/MRpic/NDUFS5.funnel_plot.pdf]

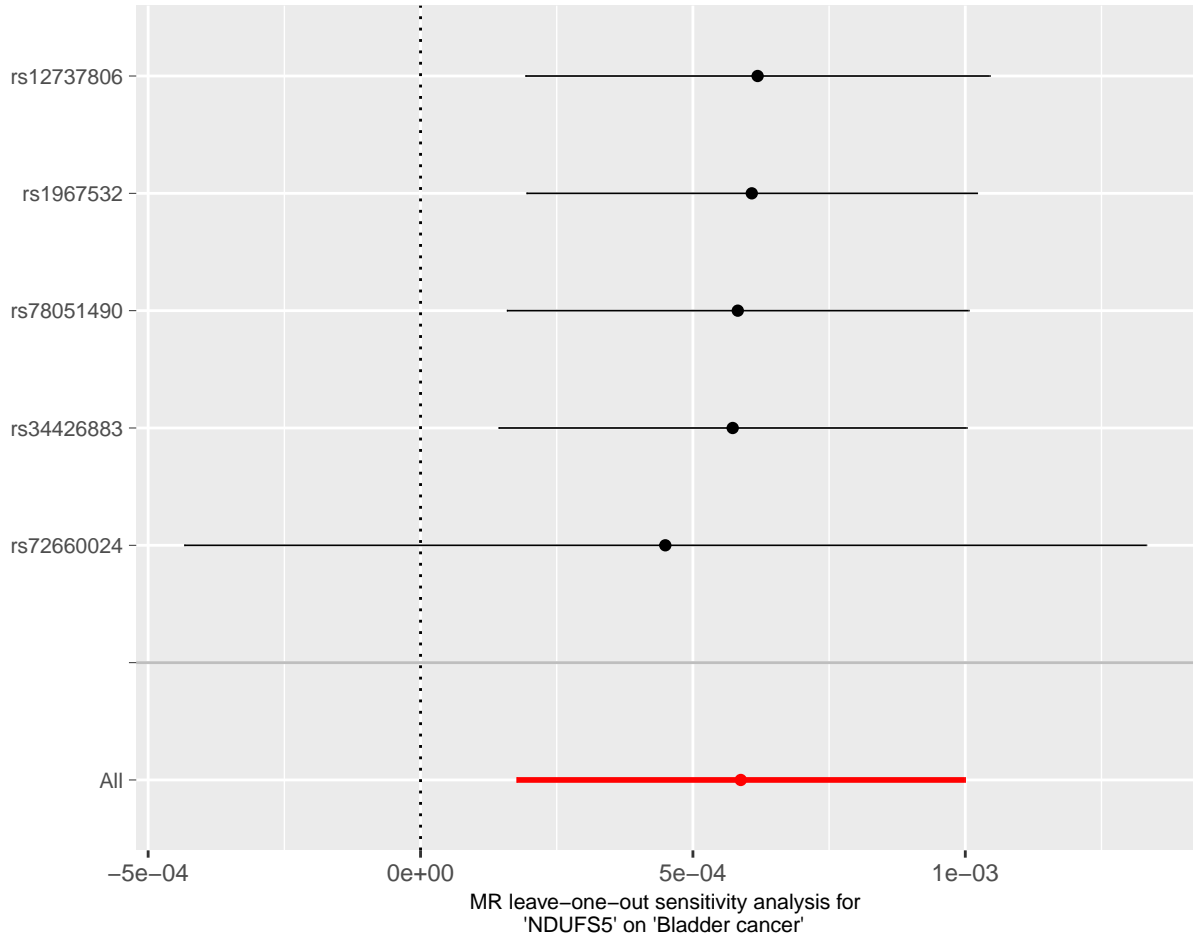

Supplement: Supplementary file 1 — Supplementary Material 1. [file 41065_2025_606_MOESM1_ESM.zip › Supplementary1/Supplementary - MR/eQTL-MR/MRpic/NDUFS5.leaveoneout.pdf]

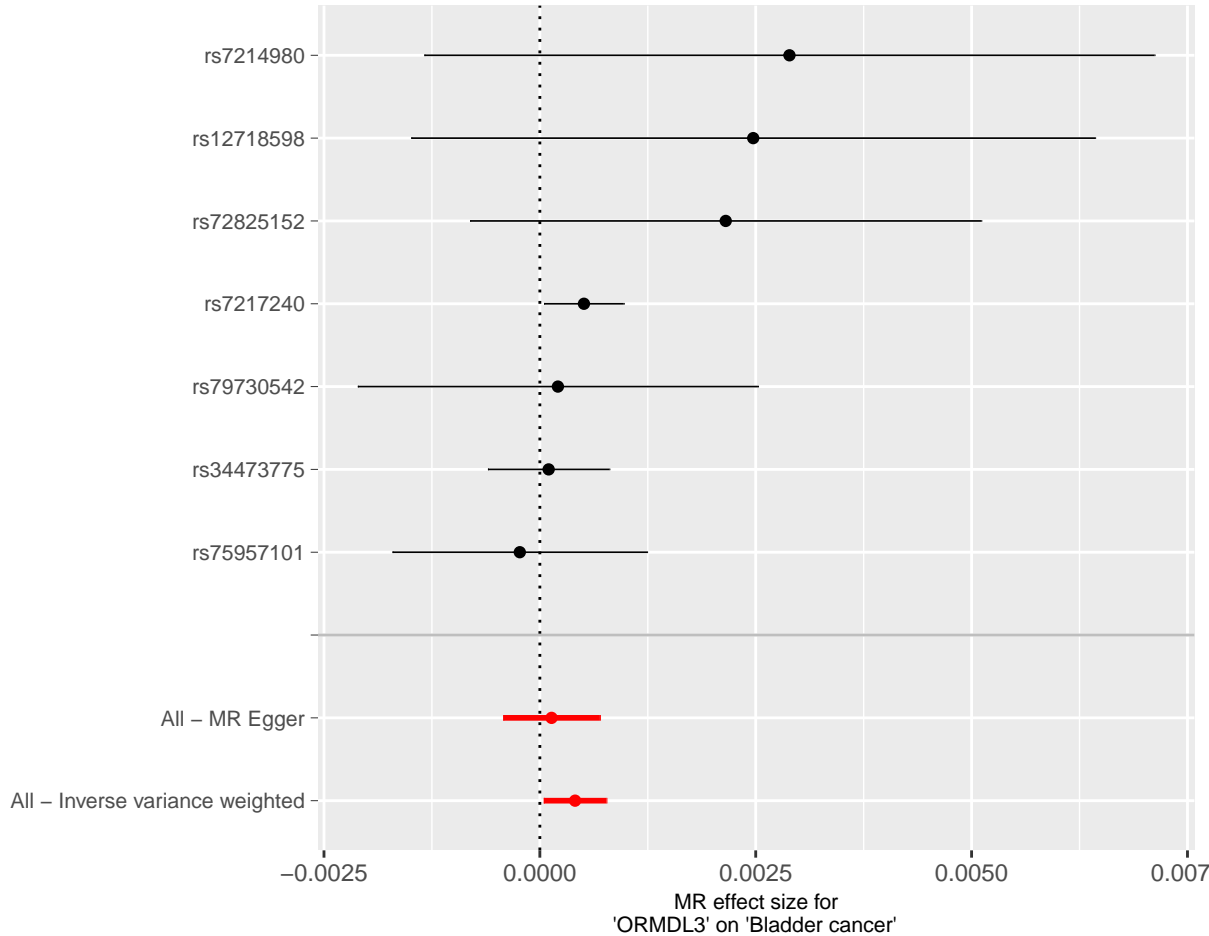

Supplement: Supplementary file 1 — Supplementary Material 1. [file 41065_2025_606_MOESM1_ESM.zip › Supplementary1/Supplementary - MR/eQTL-MR/MRpic/ORMDL3.forest.pdf]

# MR Method

- Inverse variance weighted
- MR Egger

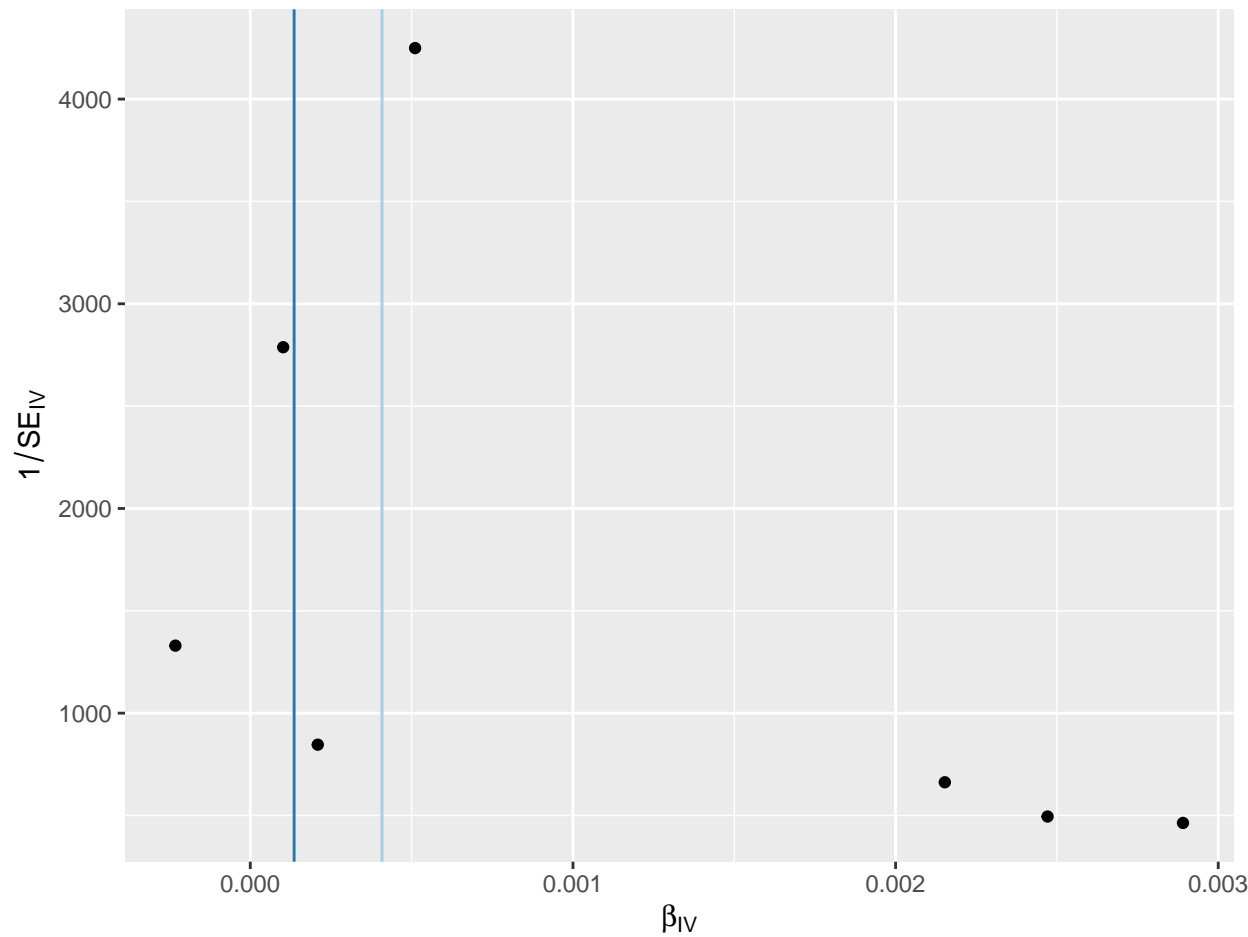

Supplement: Supplementary file 1 — Supplementary Material 1. [file 41065_2025_606_MOESM1_ESM.zip › Supplementary1/Supplementary - MR/eQTL-MR/MRpic/ORMDL3.funnel_plot.pdf]

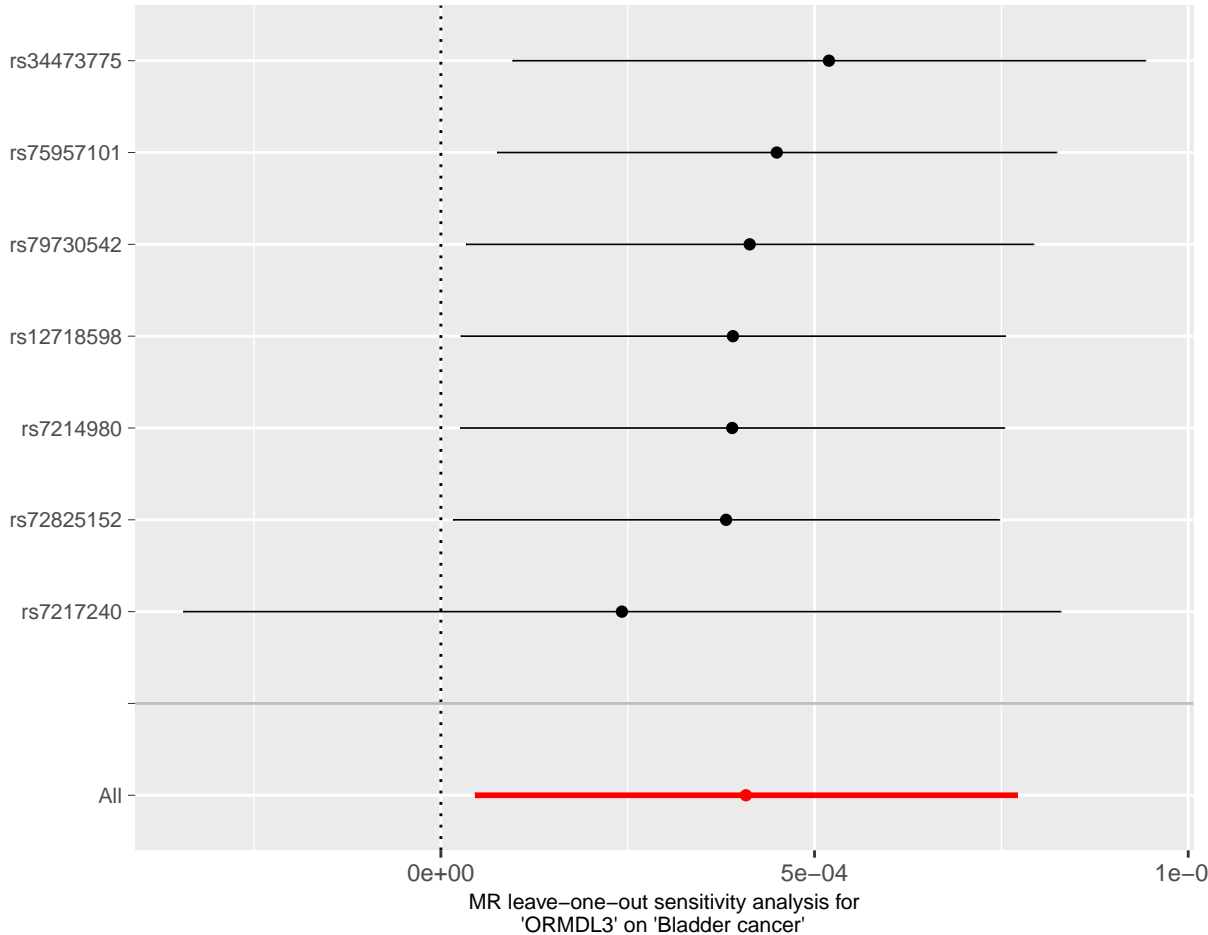

Supplement: Supplementary file 1 — Supplementary Material 1. [file 41065_2025_606_MOESM1_ESM.zip › Supplementary1/Supplementary - MR/eQTL-MR/MRpic/ORMDL3.leaveoneout.pdf]

# MR Test

- Inverse variance weighted
- MR Egger
- Simple mode
- Weighted median
- Weighted mode

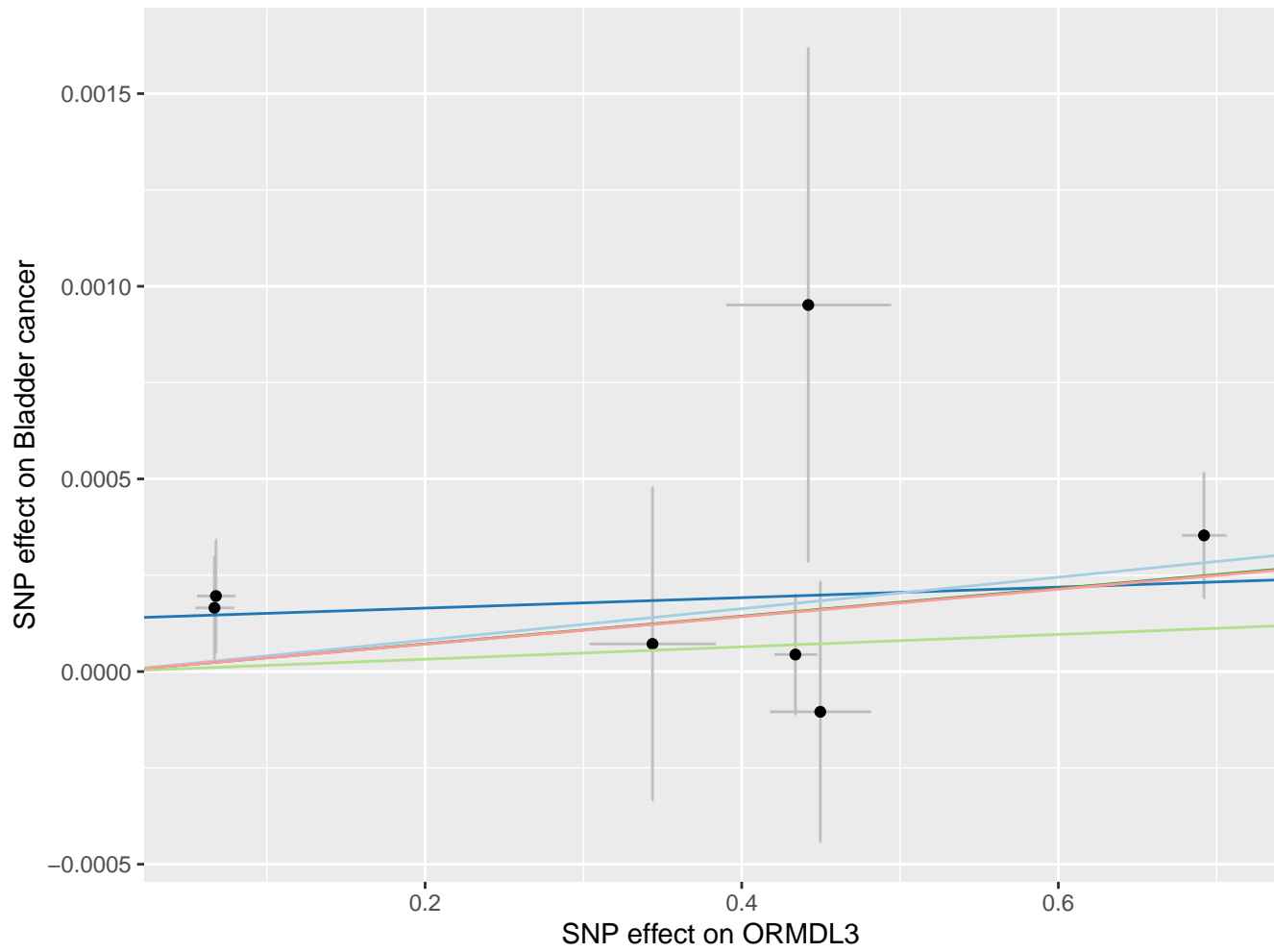

Supplement: Supplementary file 1 — Supplementary Material 1. [file 41065_2025_606_MOESM1_ESM.zip › Supplementary1/Supplementary - MR/eQTL-MR/MRpic/ORMDL3.scatter_plot.pdf]

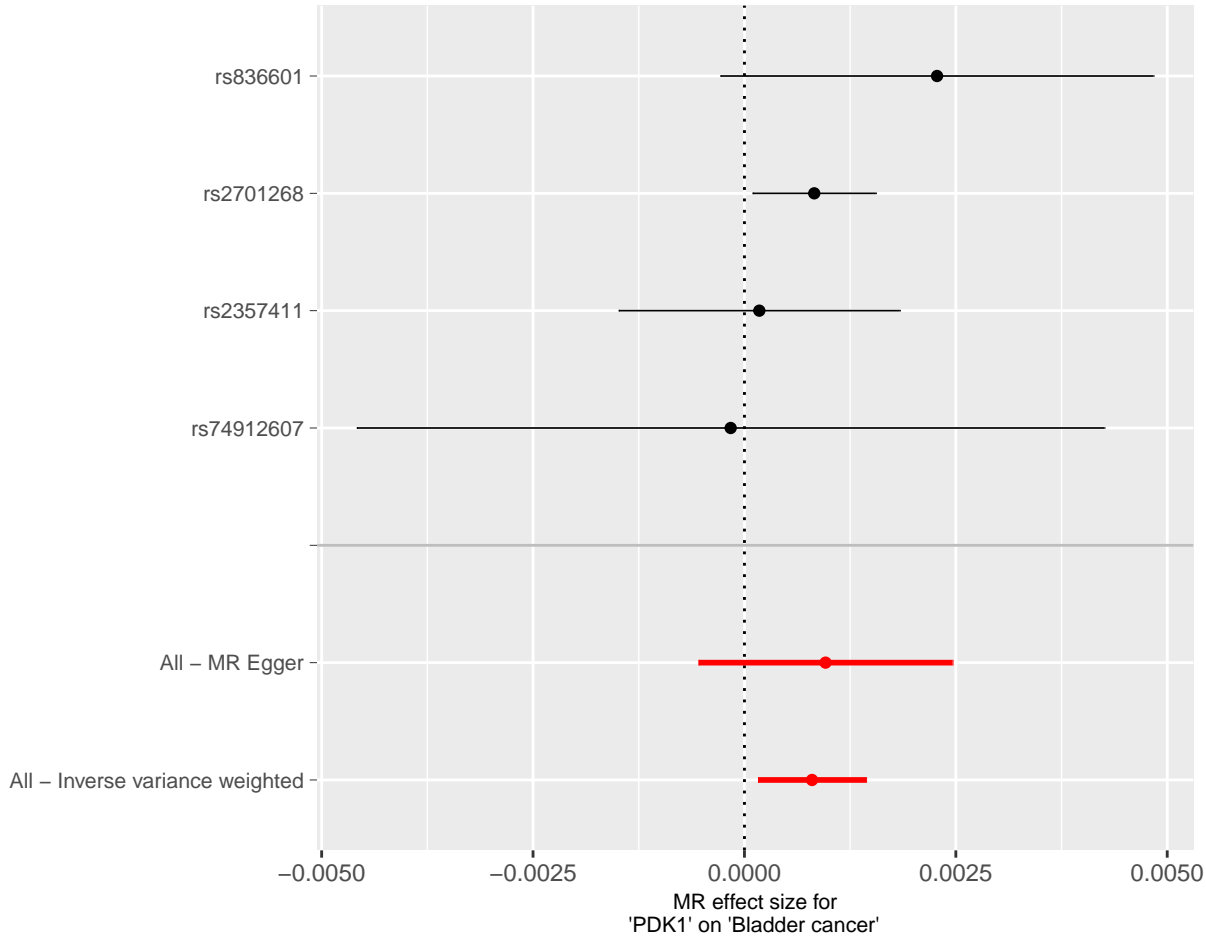

Supplement: Supplementary file 1 — Supplementary Material 1. [file 41065_2025_606_MOESM1_ESM.zip › Supplementary1/Supplementary - MR/eQTL-MR/MRpic/PDK1.forest.pdf]

# MR Method

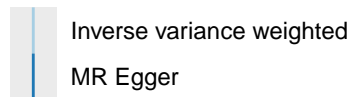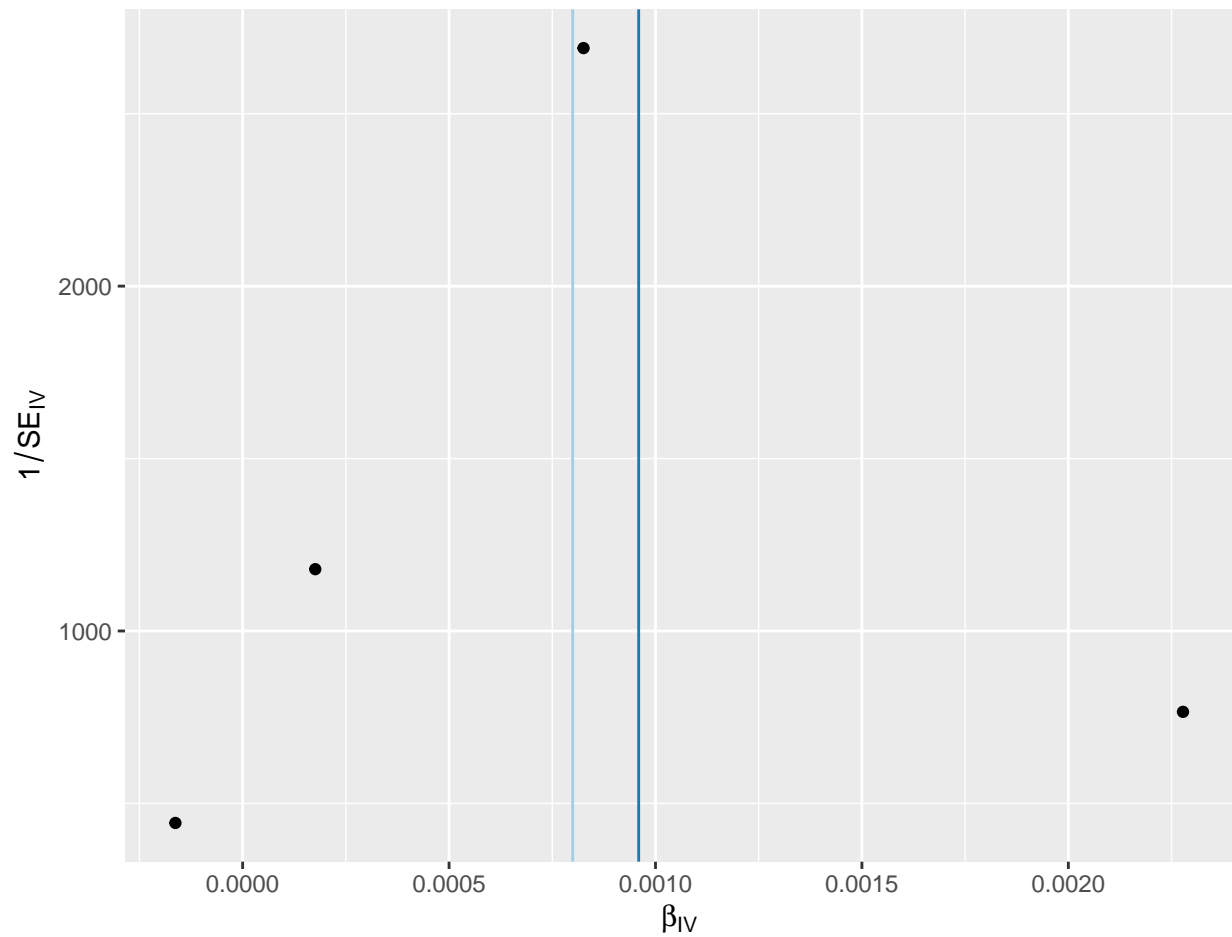

Supplement: Supplementary file 1 — Supplementary Material 1. [file 41065_2025_606_MOESM1_ESM.zip › Supplementary1/Supplementary - MR/eQTL-MR/MRpic/PDK1.funnel_plot.pdf]

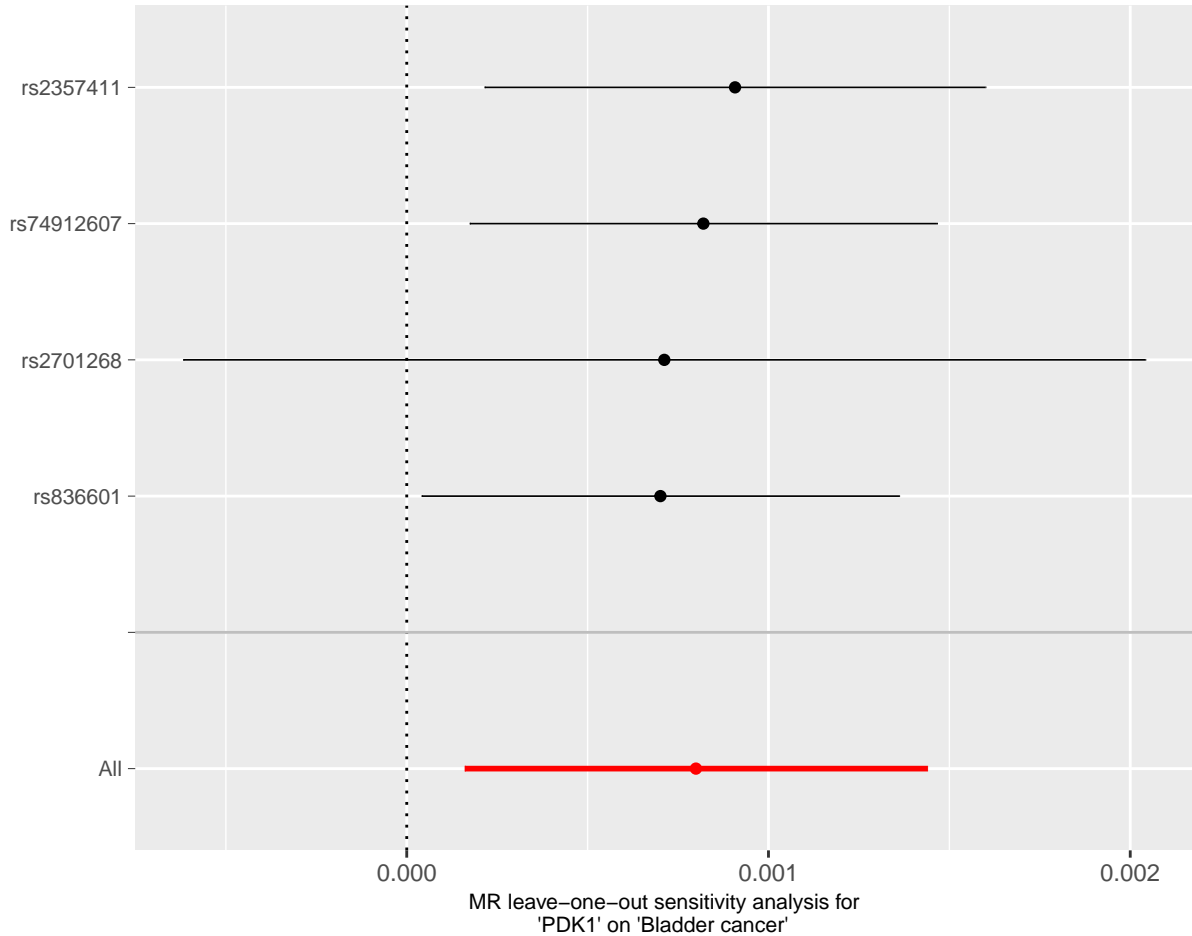

Supplement: Supplementary file 1 — Supplementary Material 1. [file 41065_2025_606_MOESM1_ESM.zip › Supplementary1/Supplementary - MR/eQTL-MR/MRpic/PDK1.leaveoneout.pdf]

# MR Test

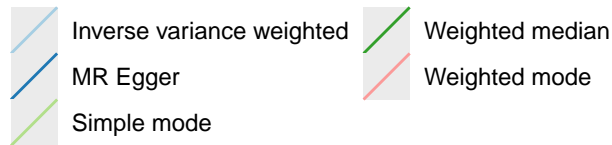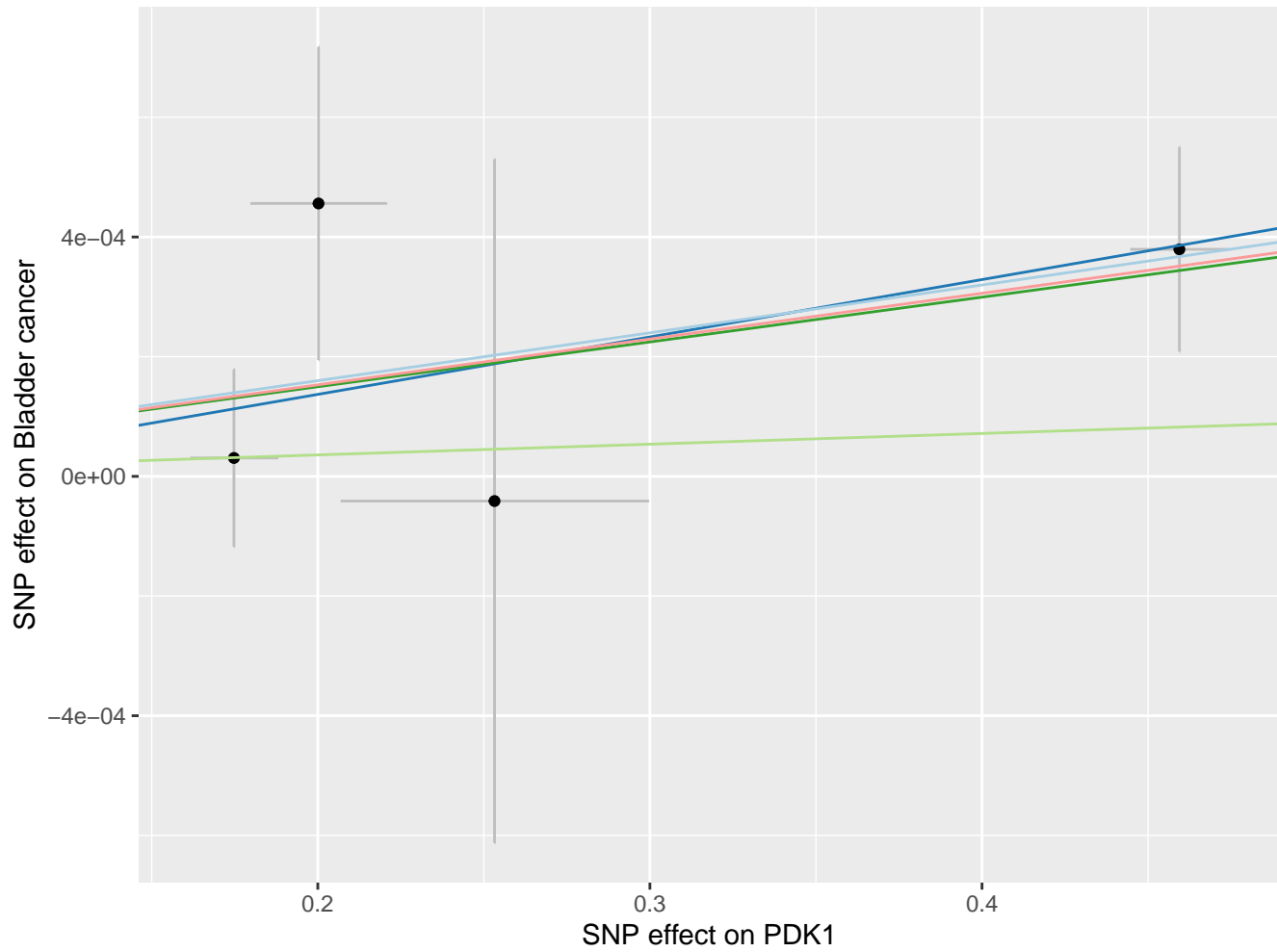

Supplement: Supplementary file 1 — Supplementary Material 1. [file 41065_2025_606_MOESM1_ESM.zip › Supplementary1/Supplementary - MR/eQTL-MR/MRpic/PDK1.scatter_plot.pdf]

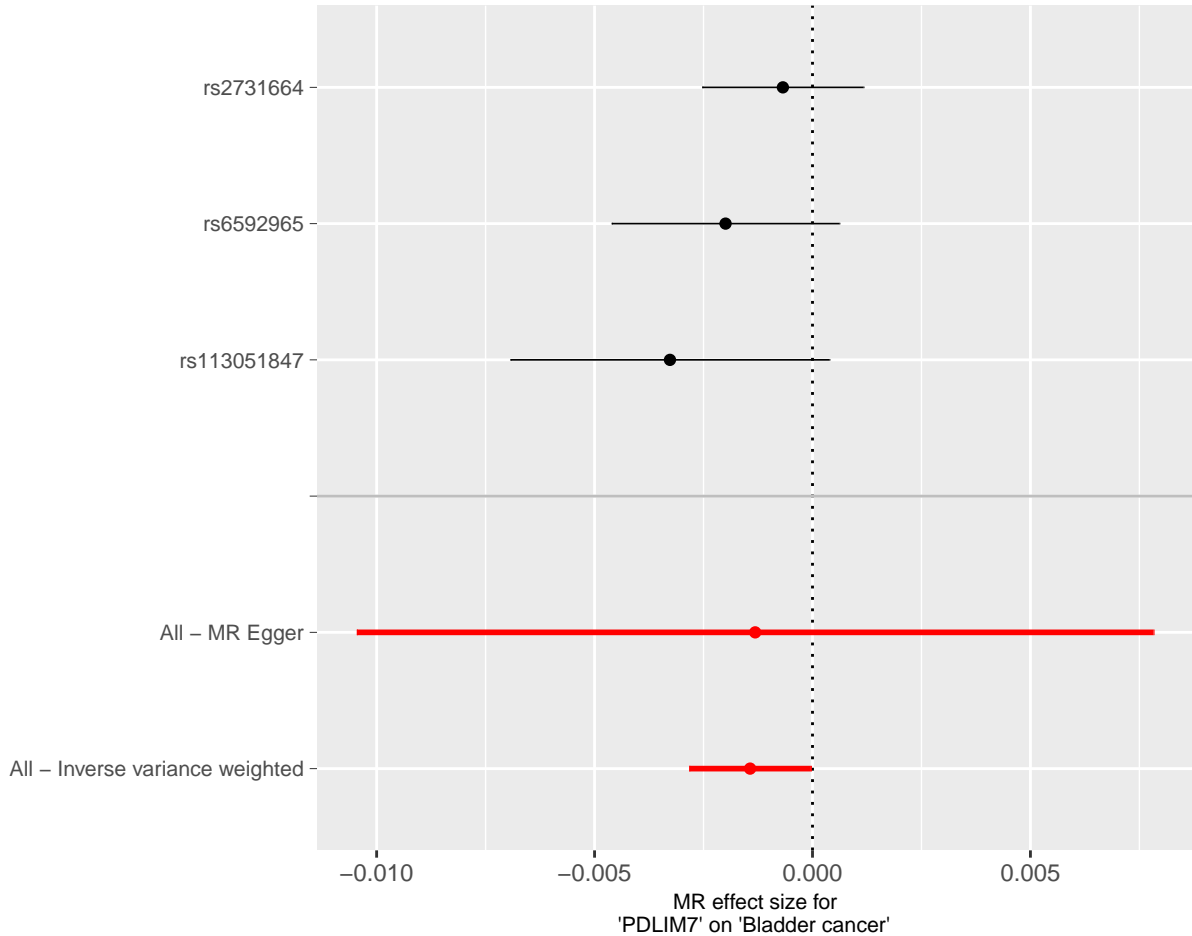

Supplement: Supplementary file 1 — Supplementary Material 1. [file 41065_2025_606_MOESM1_ESM.zip › Supplementary1/Supplementary - MR/eQTL-MR/MRpic/PDLIM7.forest.pdf]

# MR Method

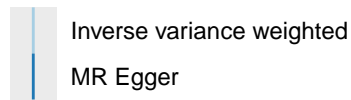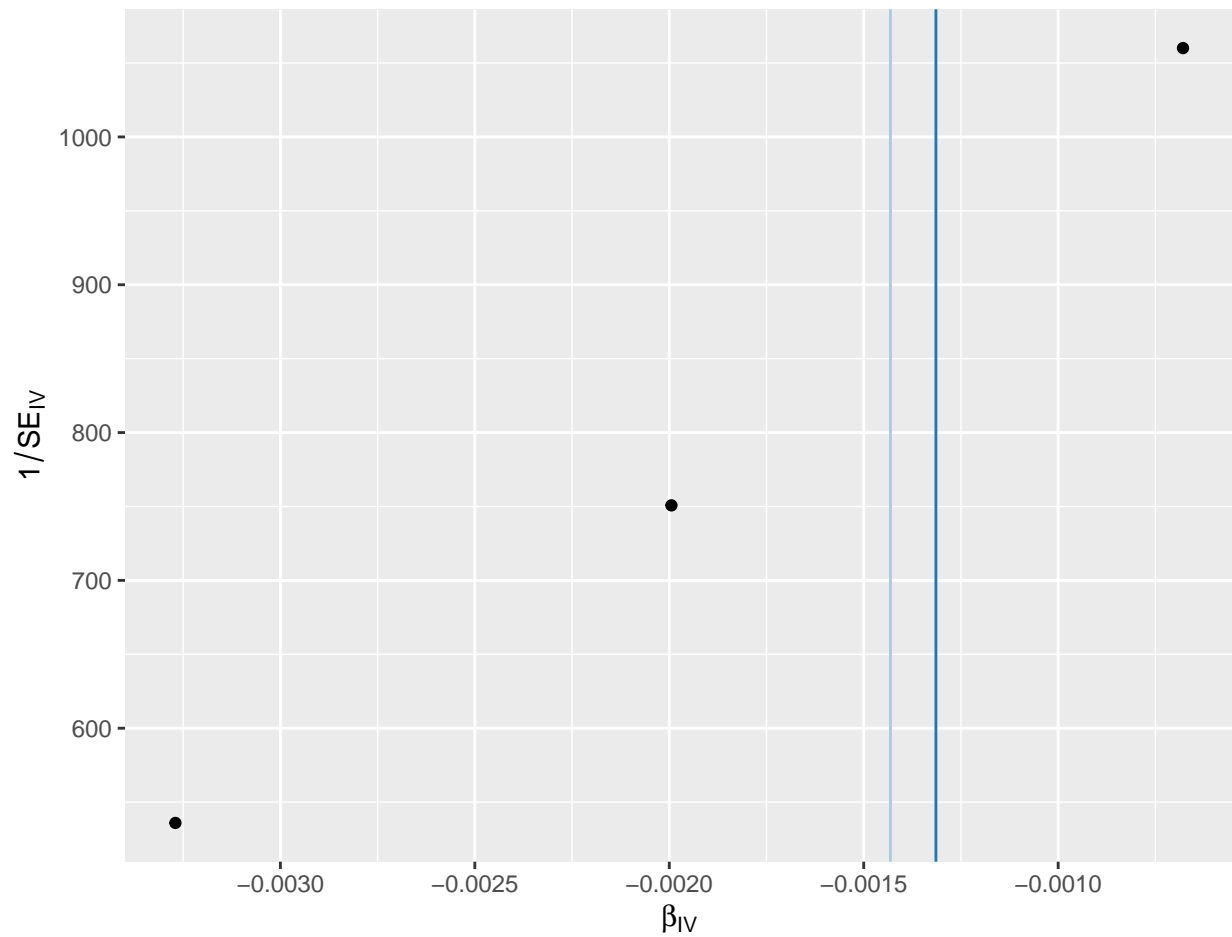

Supplement: Supplementary file 1 — Supplementary Material 1. [file 41065_2025_606_MOESM1_ESM.zip › Supplementary1/Supplementary - MR/eQTL-MR/MRpic/PDLIM7.funnel_plot.pdf]

rs113051847

rs6592965

rs2731664

All

-0.004

-0.003

-0.002

-0.001

0.000

0.001

MR leave-one-out sensitivity analysis for  
'PDLIM7' on 'Bladder cancer'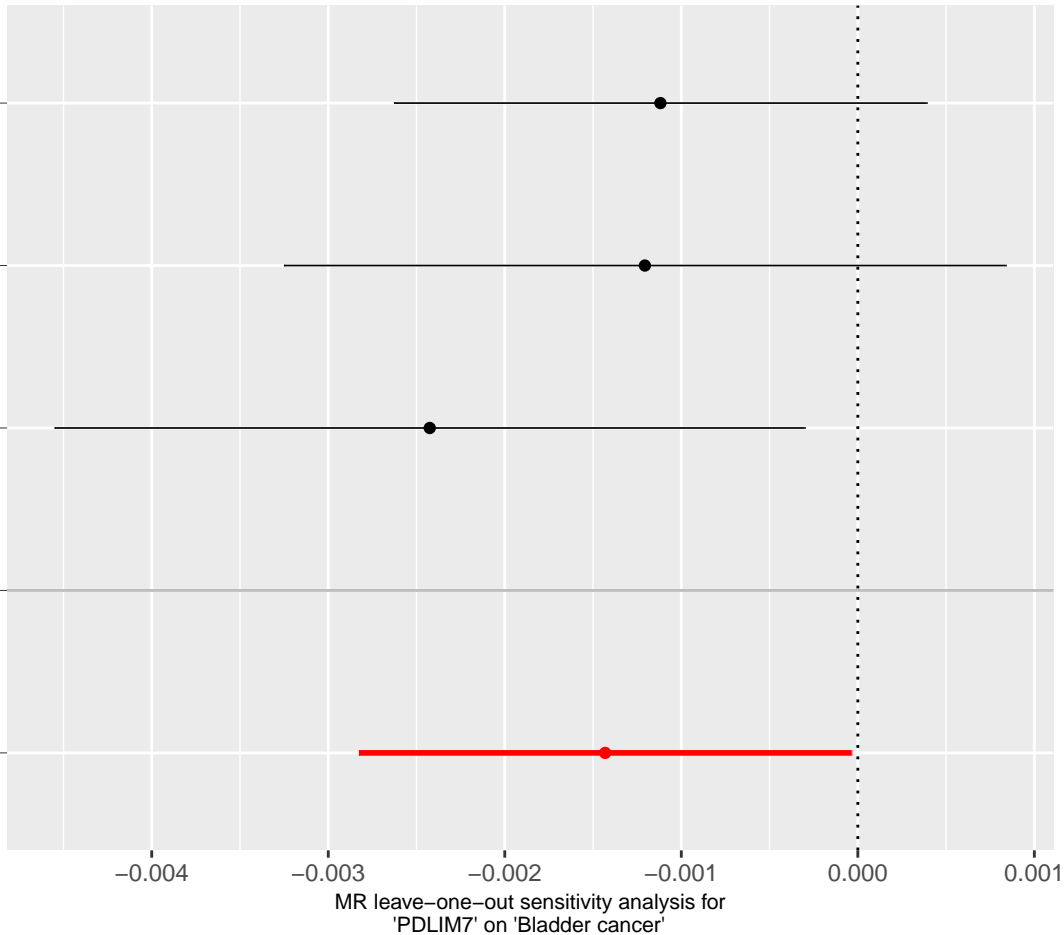

Supplement: Supplementary file 1 — Supplementary Material 1. [file 41065_2025_606_MOESM1_ESM.zip › Supplementary1/Supplementary - MR/eQTL-MR/MRpic/PDLIM7.leaveoneout.pdf]

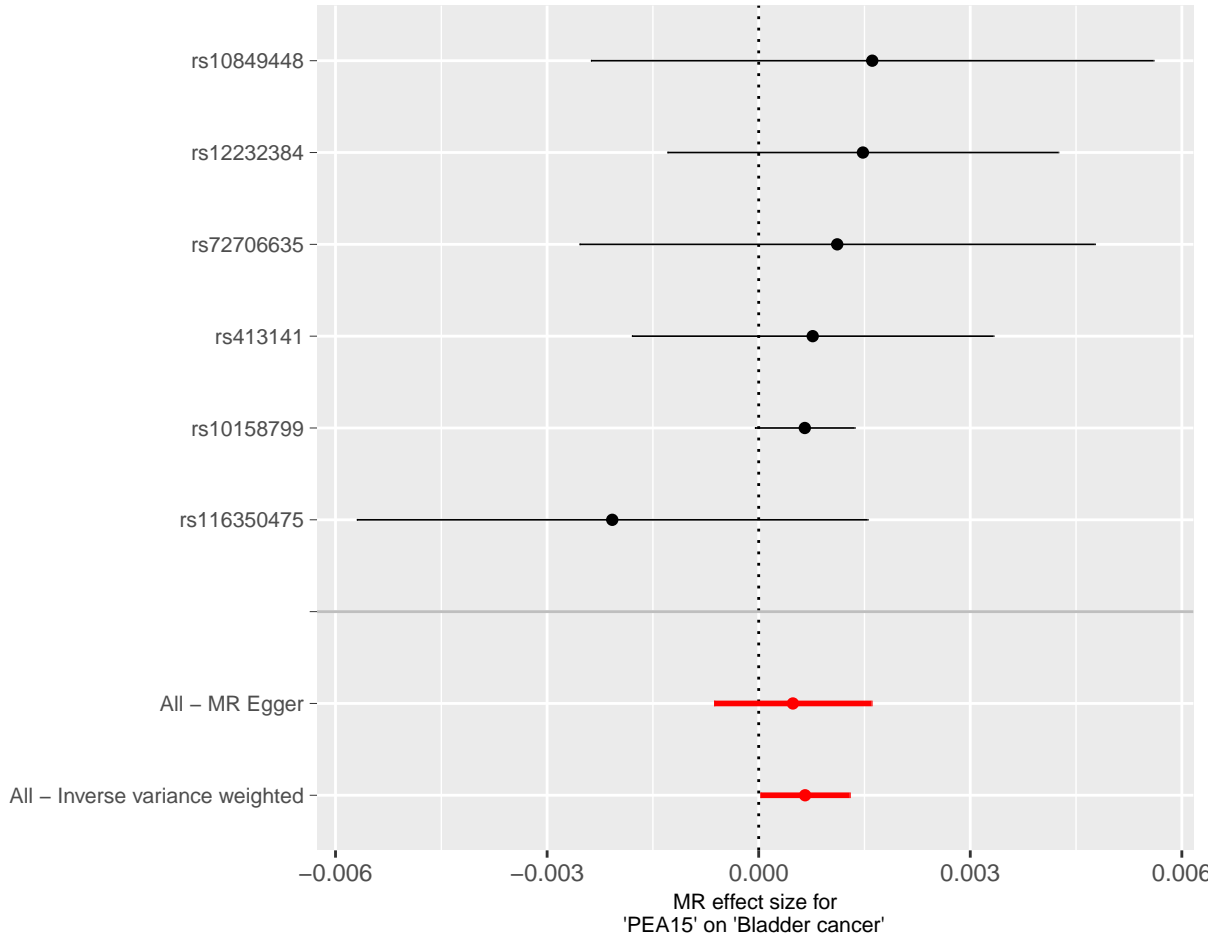

Supplement: Supplementary file 1 — Supplementary Material 1. [file 41065_2025_606_MOESM1_ESM.zip › Supplementary1/Supplementary - MR/eQTL-MR/MRpic/PEA15.forest.pdf]

# MR Method

- Inverse variance weighted
- MR Egger

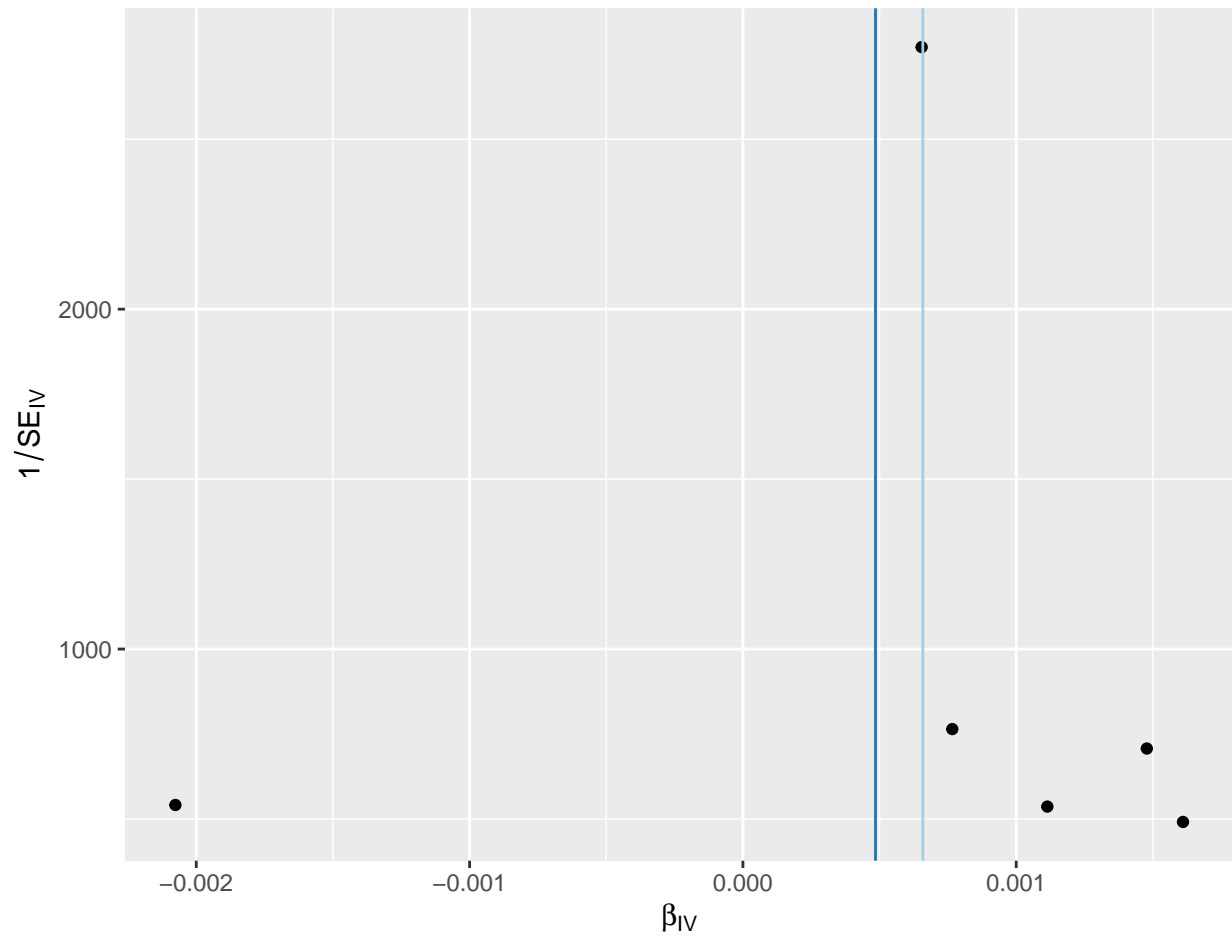

Supplement: Supplementary file 1 — Supplementary Material 1. [file 41065_2025_606_MOESM1_ESM.zip › Supplementary1/Supplementary - MR/eQTL-MR/MRpic/PEA15.funnel_plot.pdf]

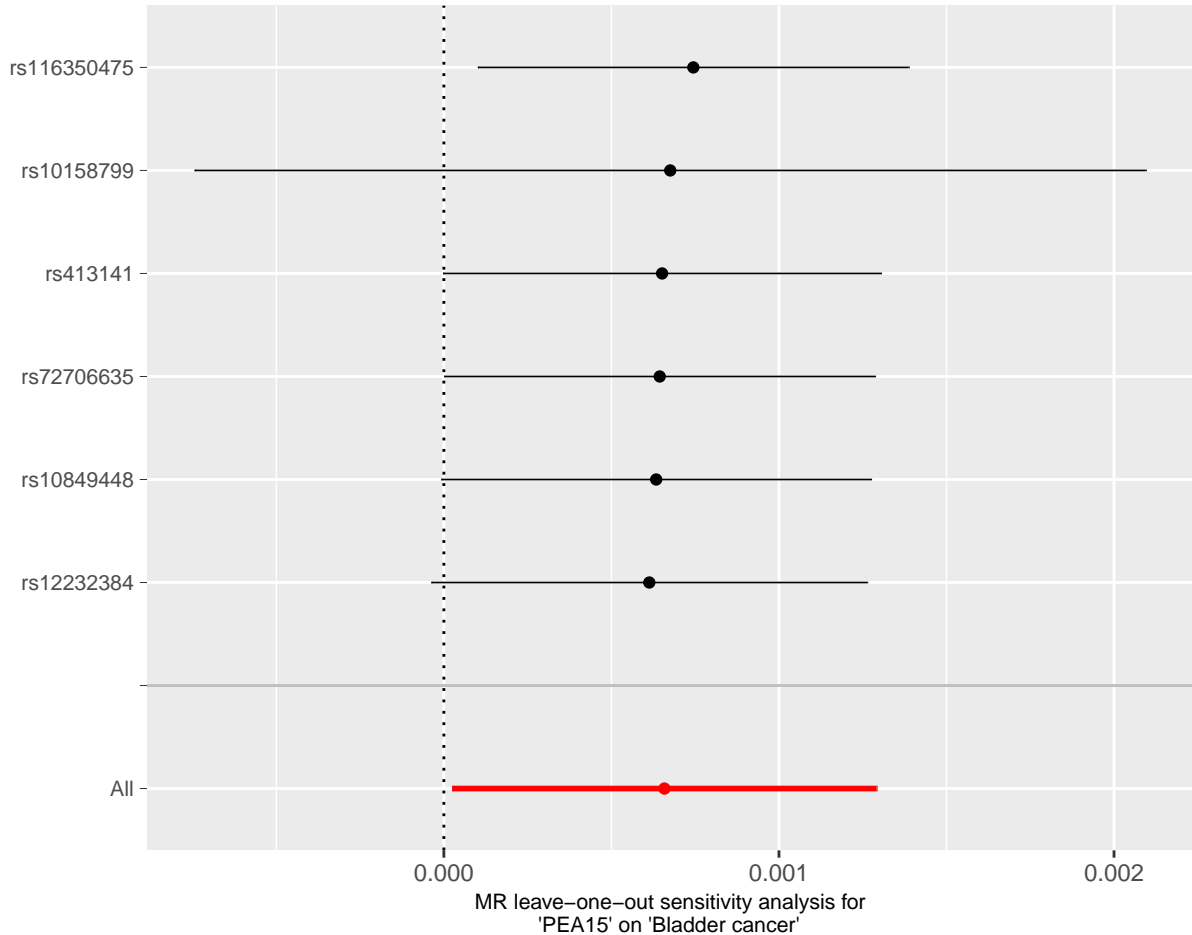

Supplement: Supplementary file 1 — Supplementary Material 1. [file 41065_2025_606_MOESM1_ESM.zip › Supplementary1/Supplementary - MR/eQTL-MR/MRpic/PEA15.leaveoneout.pdf]

# MR Test

- Inverse variance weighted
- MR Egger
- Simple mode
- Weighted median
- Weighted mode

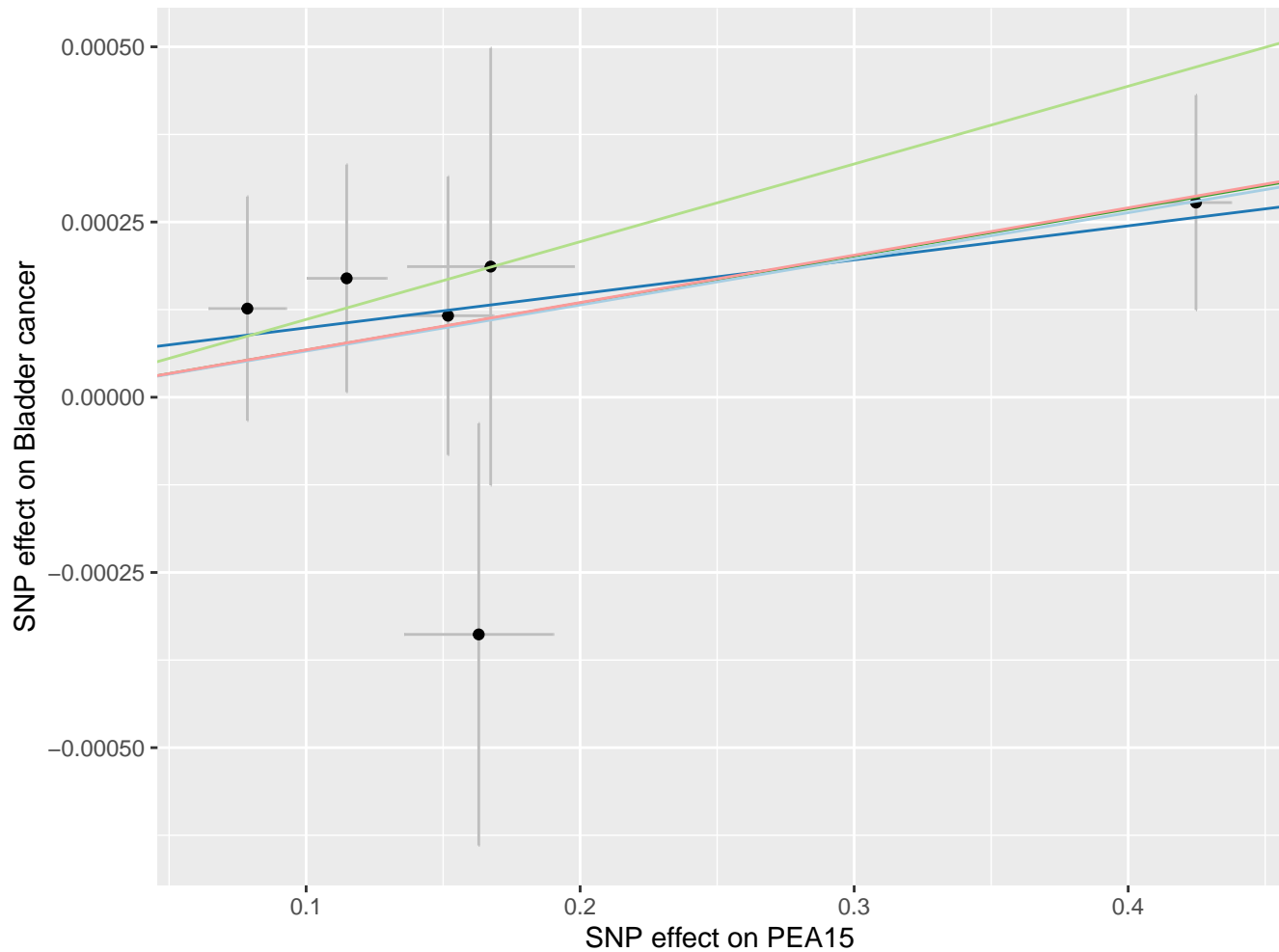

Supplement: Supplementary file 1 — Supplementary Material 1. [file 41065_2025_606_MOESM1_ESM.zip › Supplementary1/Supplementary - MR/eQTL-MR/MRpic/PEA15.scatter_plot.pdf]

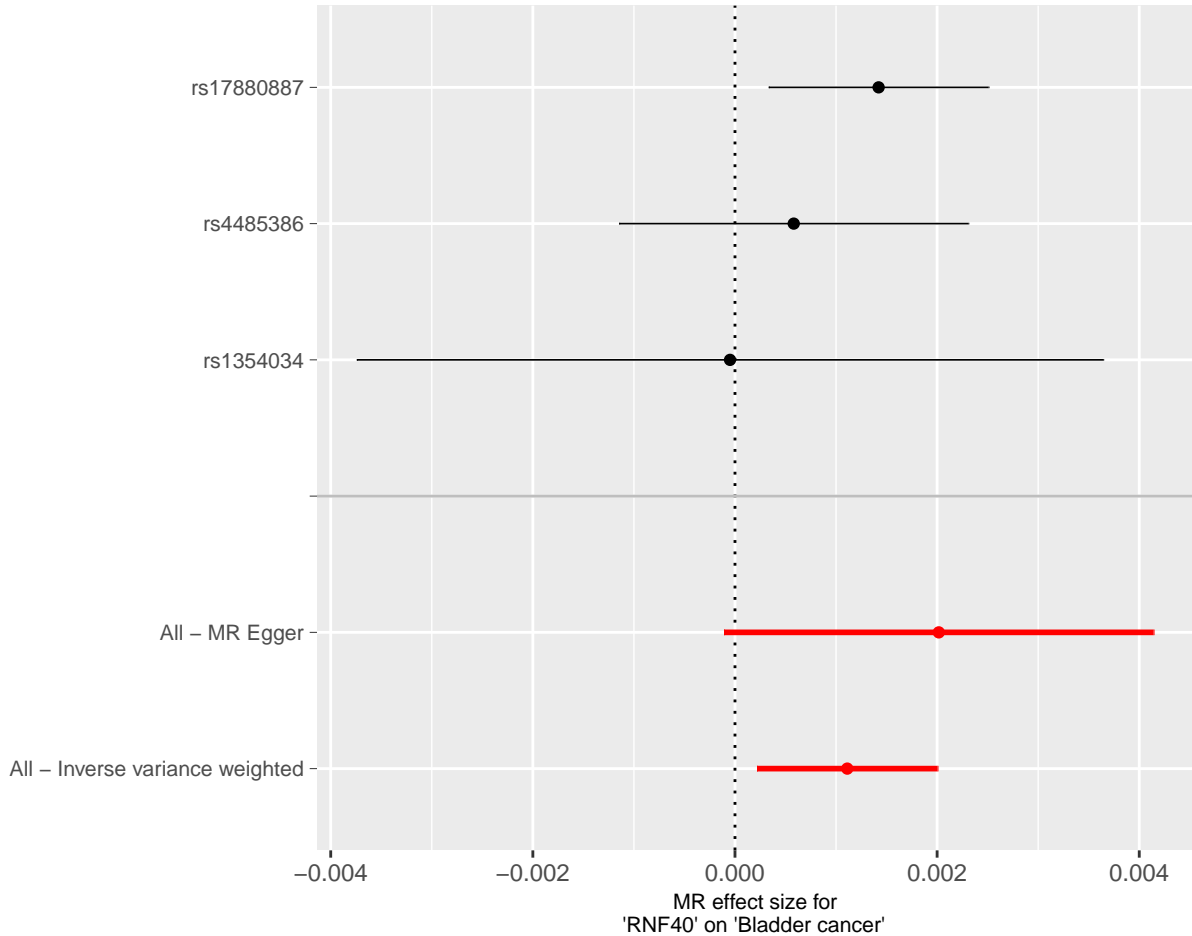

Supplement: Supplementary file 1 — Supplementary Material 1. [file 41065_2025_606_MOESM1_ESM.zip › Supplementary1/Supplementary - MR/eQTL-MR/MRpic/RNF40.forest.pdf]

# MR Method

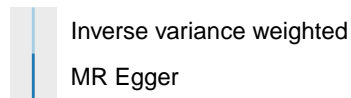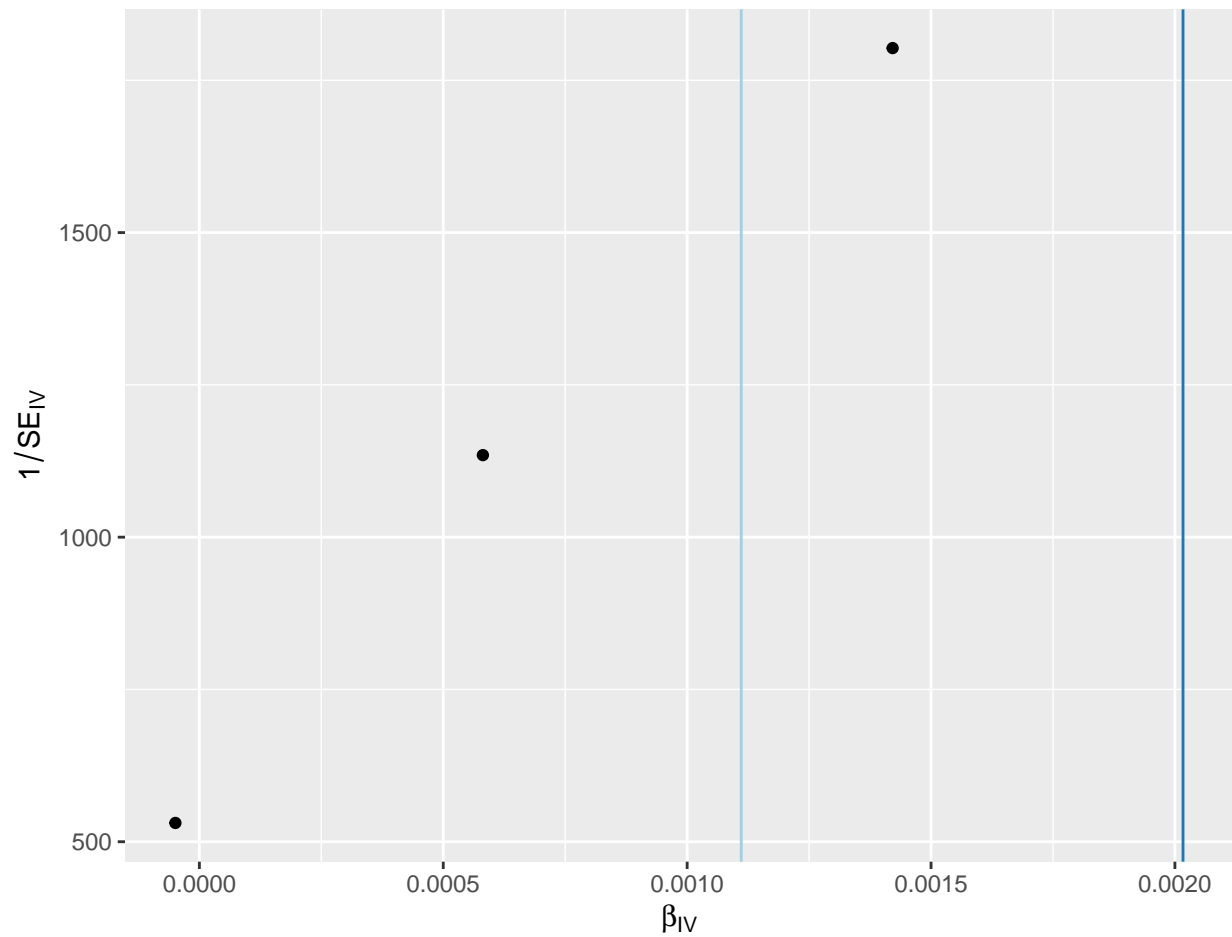

Supplement: Supplementary file 1 — Supplementary Material 1. [file 41065_2025_606_MOESM1_ESM.zip › Supplementary1/Supplementary - MR/eQTL-MR/MRpic/RNF40.funnel_plot.pdf]

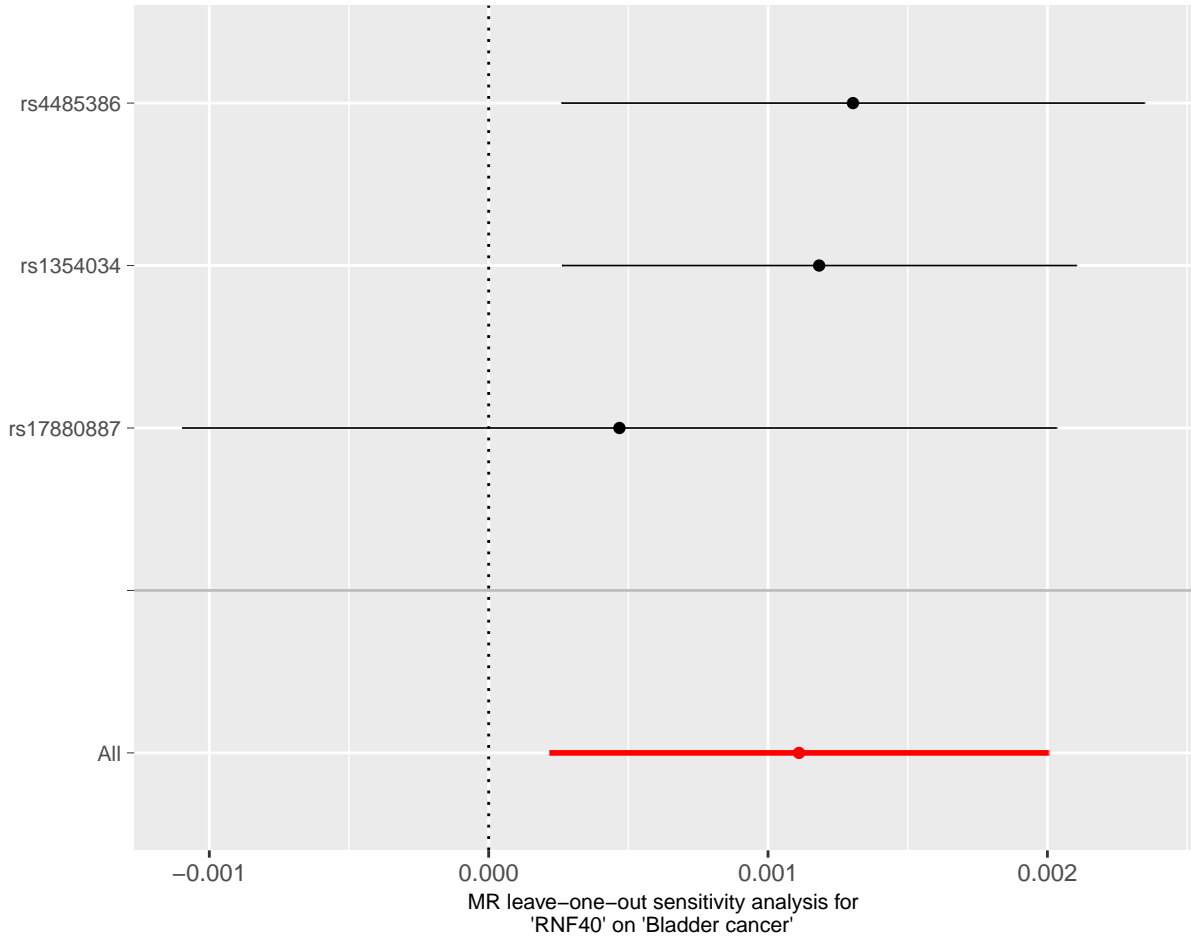

Supplement: Supplementary file 1 — Supplementary Material 1. [file 41065_2025_606_MOESM1_ESM.zip › Supplementary1/Supplementary - MR/eQTL-MR/MRpic/RNF40.leaveoneout.pdf]

# MR Test

- Inverse variance weighted
- MR Egger
- Simple mode
- Weighted median
- Weighted mode

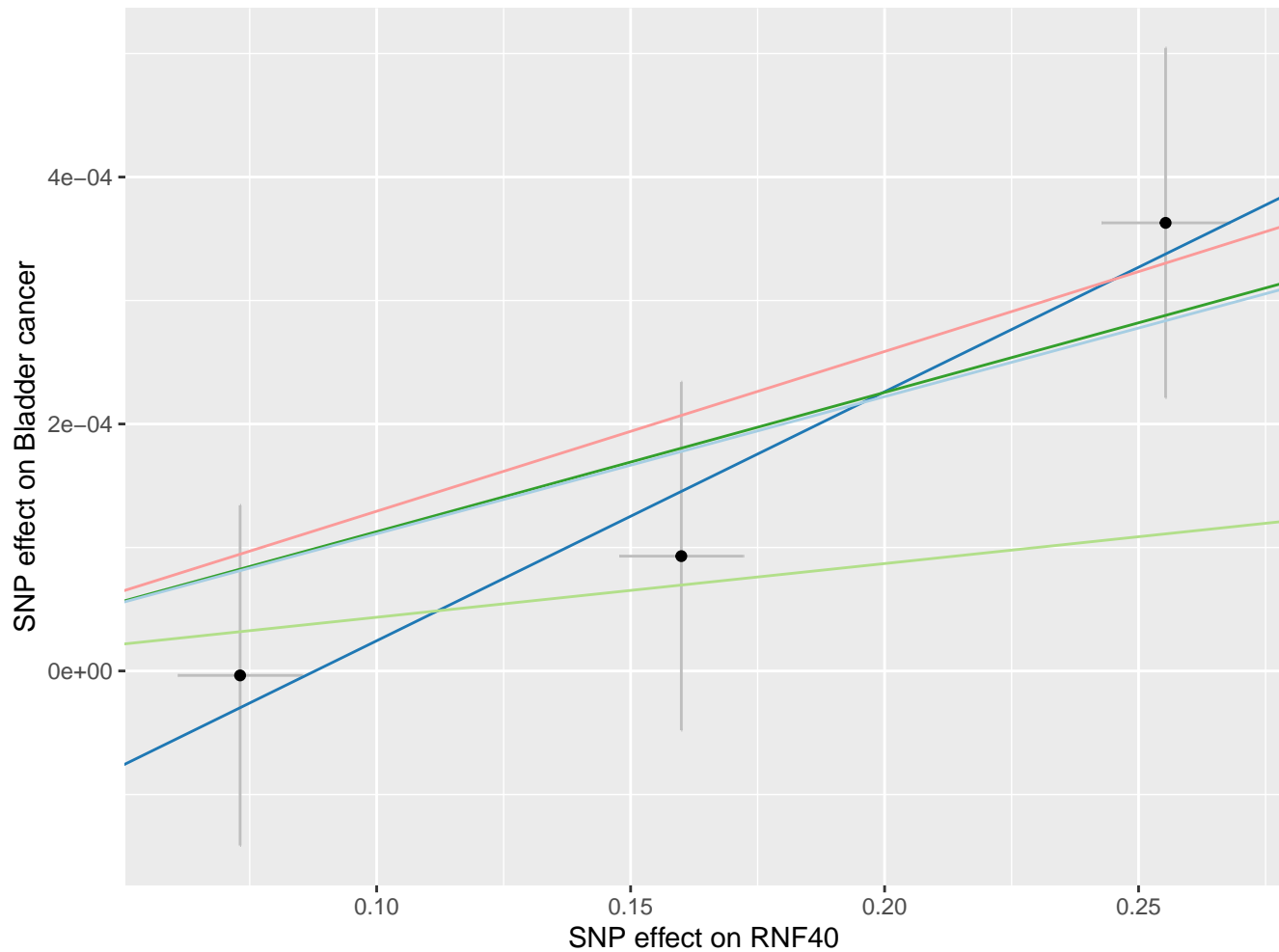

Supplement: Supplementary file 1 — Supplementary Material 1. [file 41065_2025_606_MOESM1_ESM.zip › Supplementary1/Supplementary - MR/eQTL-MR/MRpic/RNF40.scatter_plot.pdf]

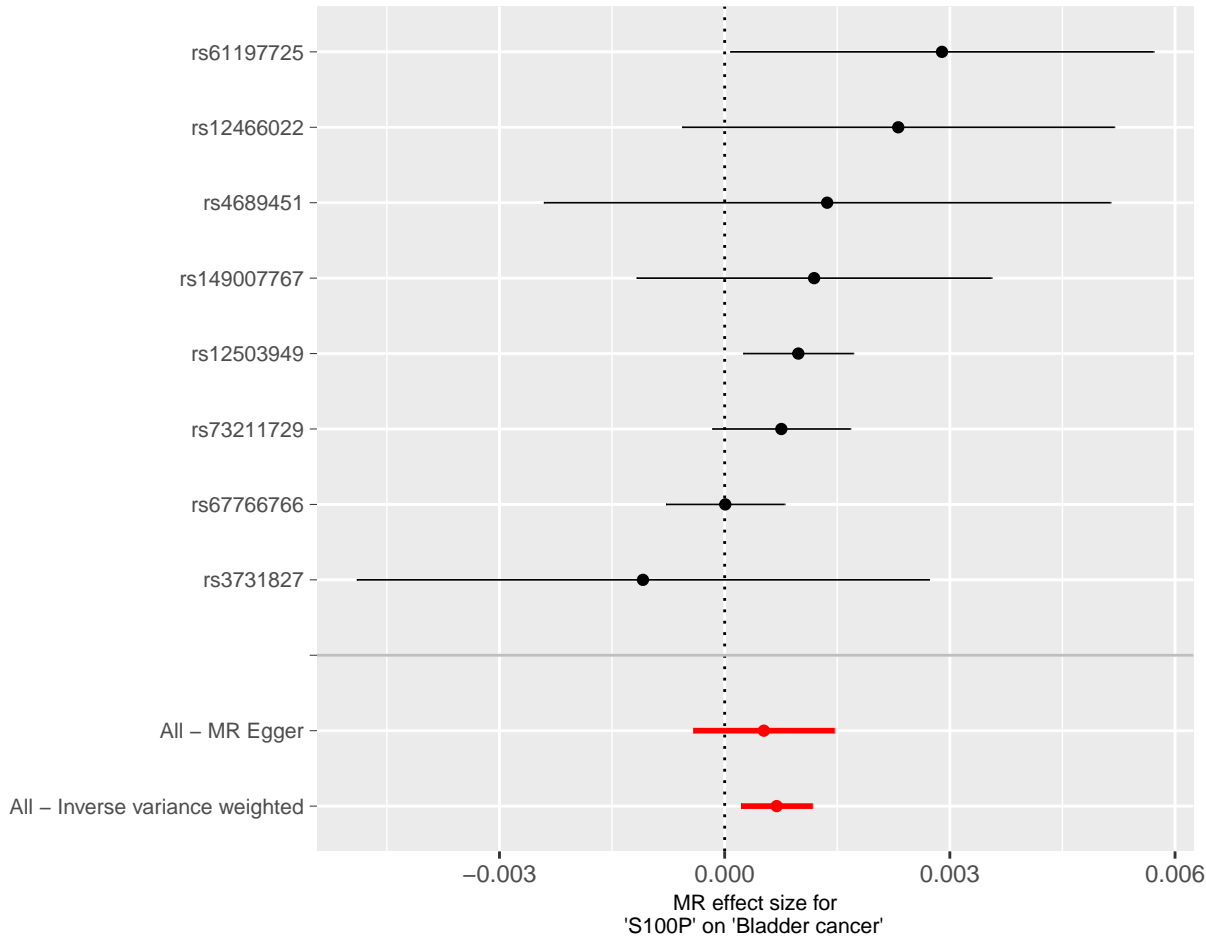

Supplement: Supplementary file 1 — Supplementary Material 1. [file 41065_2025_606_MOESM1_ESM.zip › Supplementary1/Supplementary - MR/eQTL-MR/MRpic/S100P.forest.pdf]

# MR Method

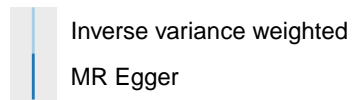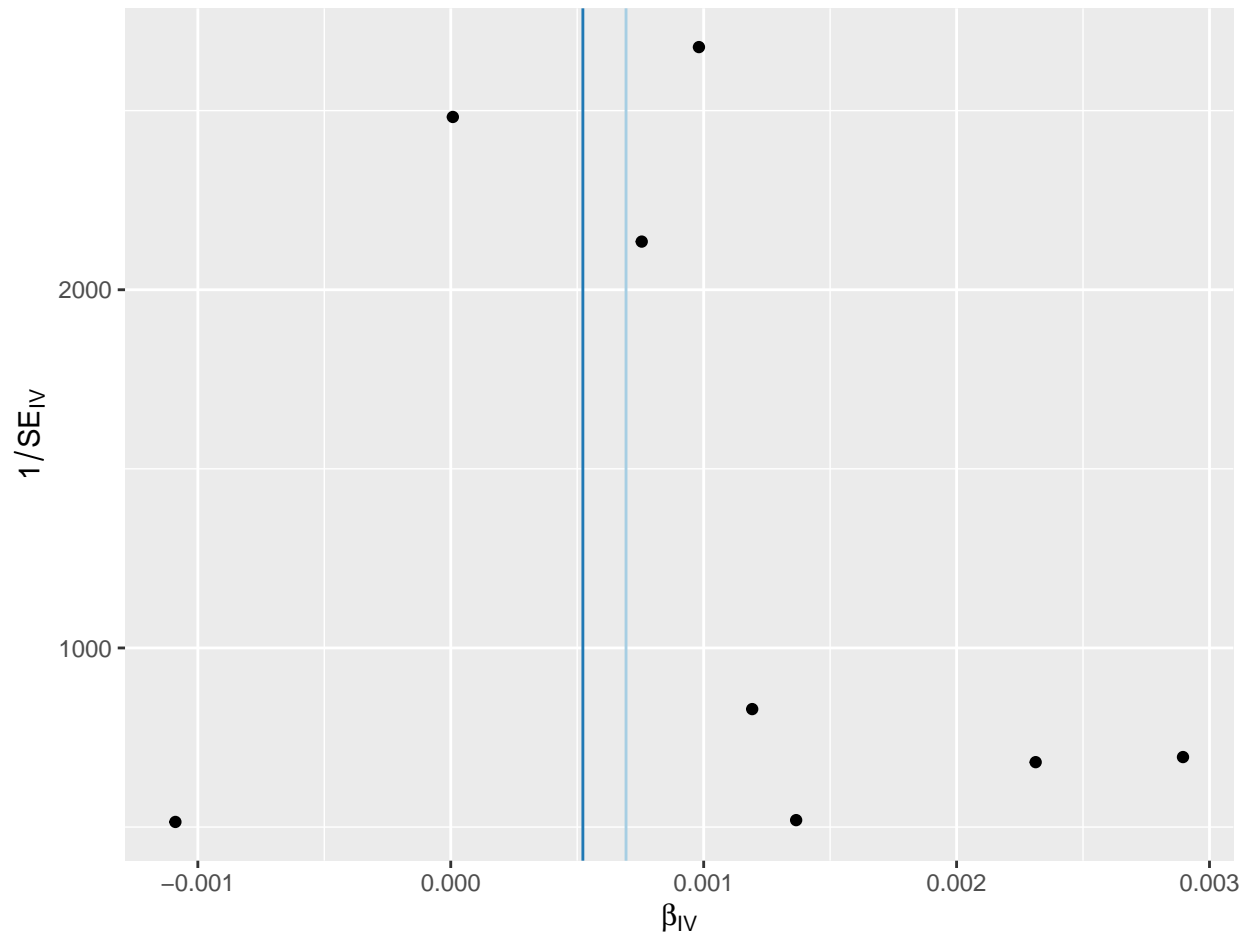

Supplement: Supplementary file 1 — Supplementary Material 1. [file 41065_2025_606_MOESM1_ESM.zip › Supplementary1/Supplementary - MR/eQTL-MR/MRpic/S100P.funnel_plot.pdf]

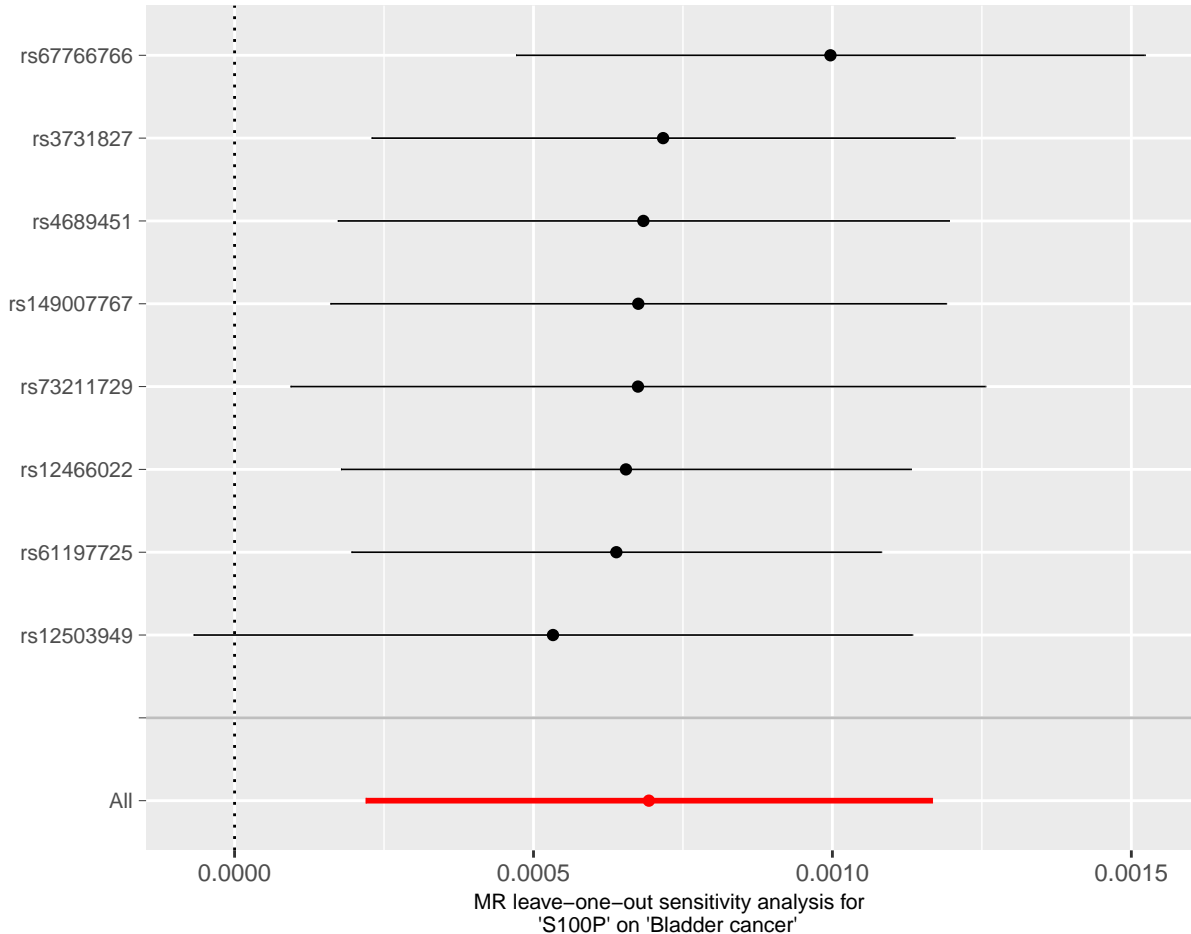

Supplement: Supplementary file 1 — Supplementary Material 1. [file 41065_2025_606_MOESM1_ESM.zip › Supplementary1/Supplementary - MR/eQTL-MR/MRpic/S100P.leaveoneout.pdf]

# MR Test

- Inverse variance weighted
- MR Egger
- Simple mode
- Weighted median
- Weighted mode

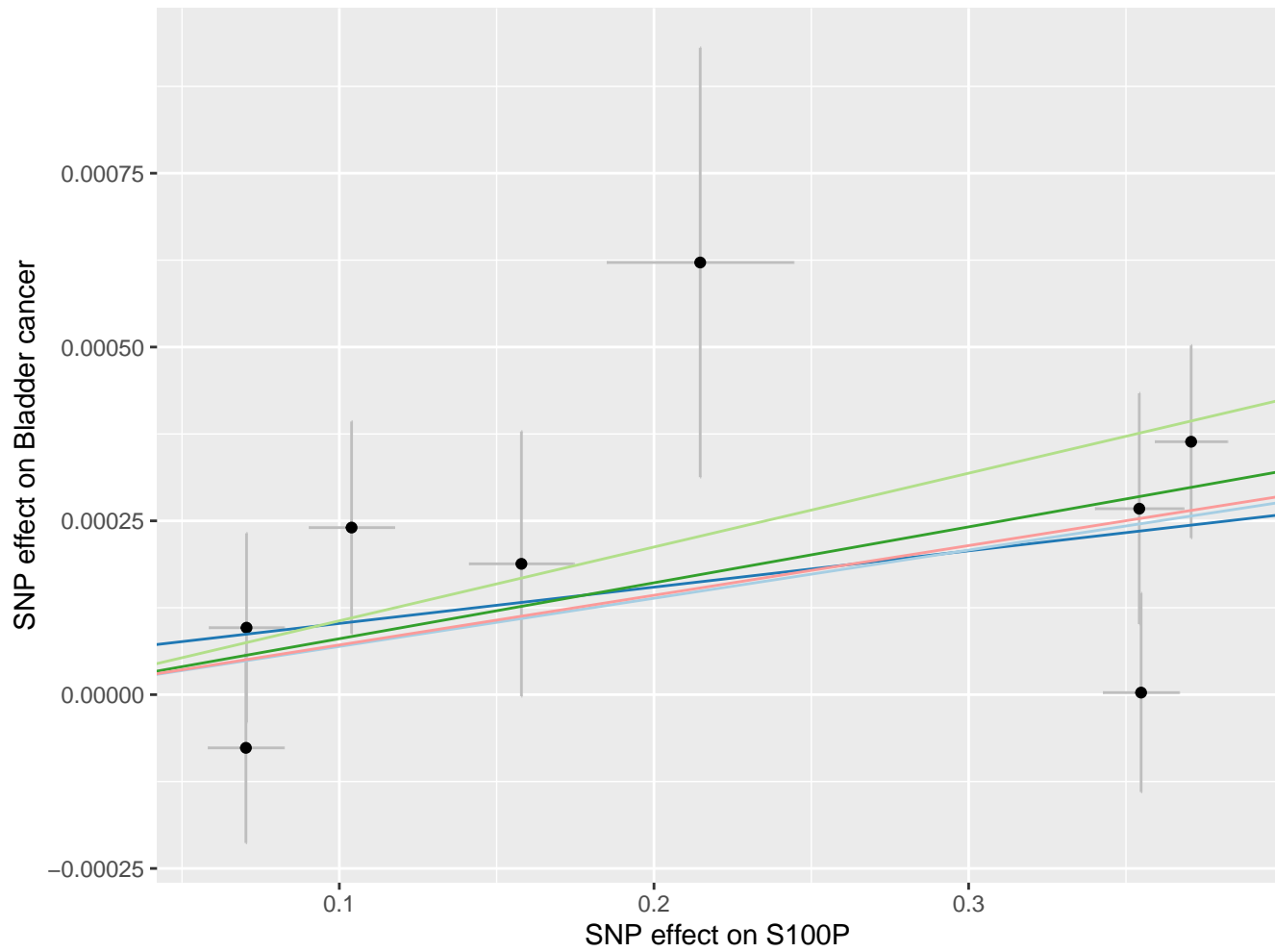

Supplement: Supplementary file 1 — Supplementary Material 1. [file 41065_2025_606_MOESM1_ESM.zip › Supplementary1/Supplementary - MR/eQTL-MR/MRpic/S100P.scatter_plot.pdf]
