# Supplementary material for: Integrative multi-omics and single-cell transcriptomics reveal ARHGEF12 driving chemoresistance in bladder cancer
Source: Hereditas. 2025 Nov 27;162:234. doi: 10.1186/s41065-025-00606-1 (PMC12661753; doi:10.1186/s41065-025-00606-1)
Supplement: Supplementary file 1 — Supplementary Material 1. [file pic.scatter_plot.pdf › Supplementary1/Supplementary - MR/eQTL-MR/MR/pic.scatter_plot.pdf]

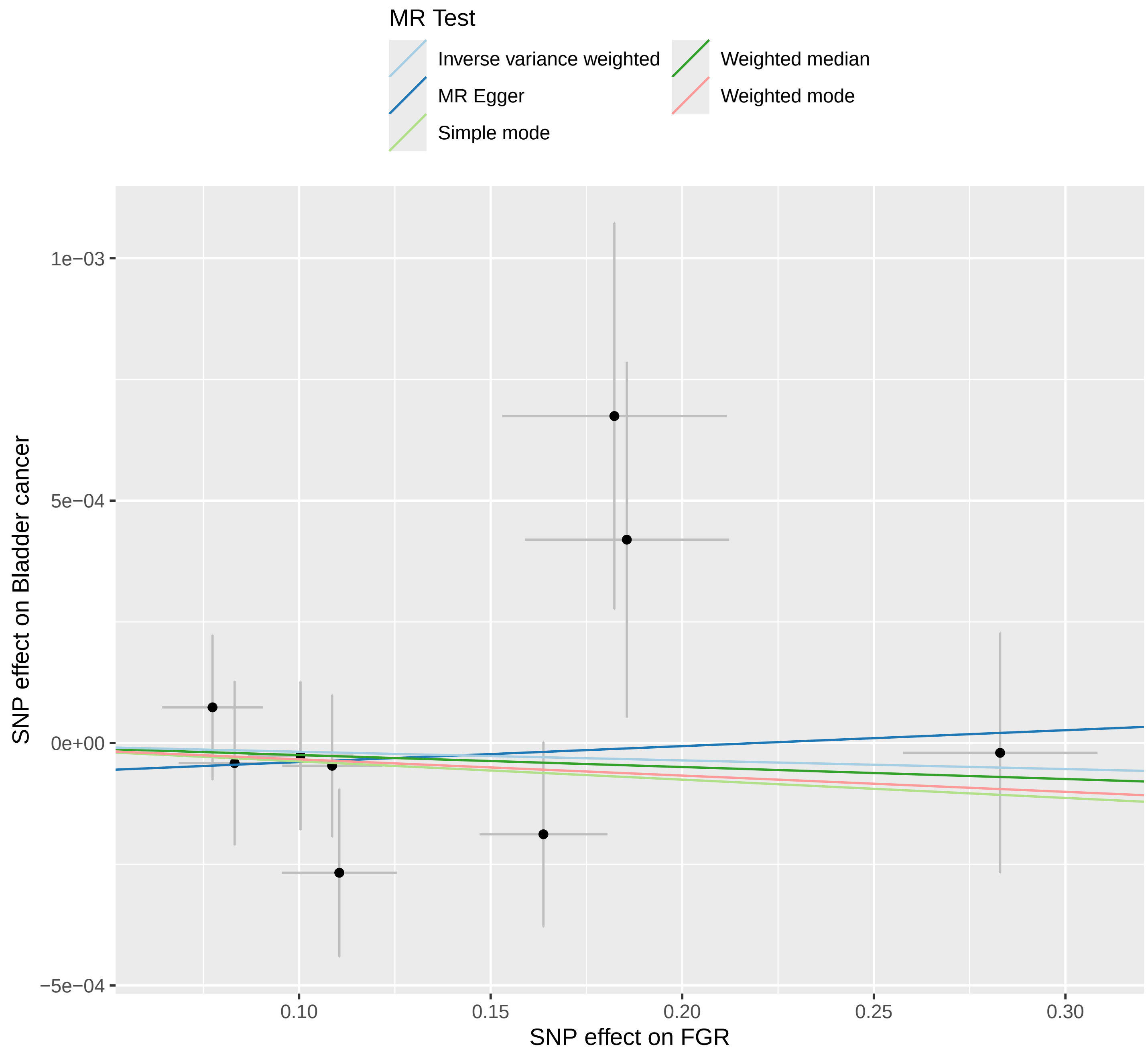
MR Test
Inverse variance weighted
MR Egger
Simple mode
Weighted median
Weighted mode
SNP effect on Bladder cancer
1e−03
5e−04
0e+00
−5e−04
0.10
0.15
0.20
0.25
0.30
SNP effect on FGR

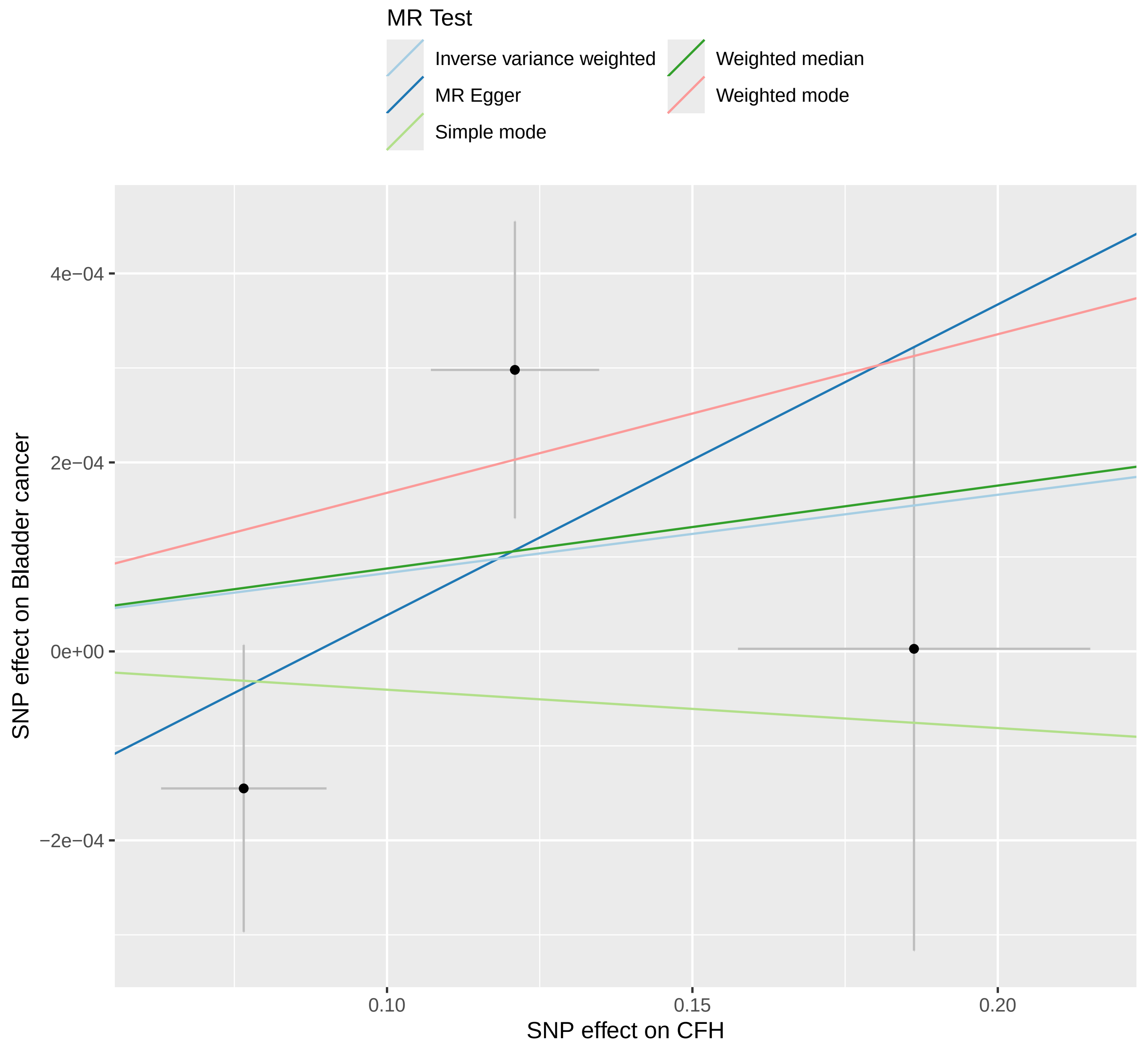

MR Test
Inverse variance weighted
MR Egger
Simple mode
Weighted median
Weighted mode
4e−04
2e−04
0e+00
−2e−04
0.10
0.15
0.20
SNP effect on Bladder cancer
SNP effect on CFH

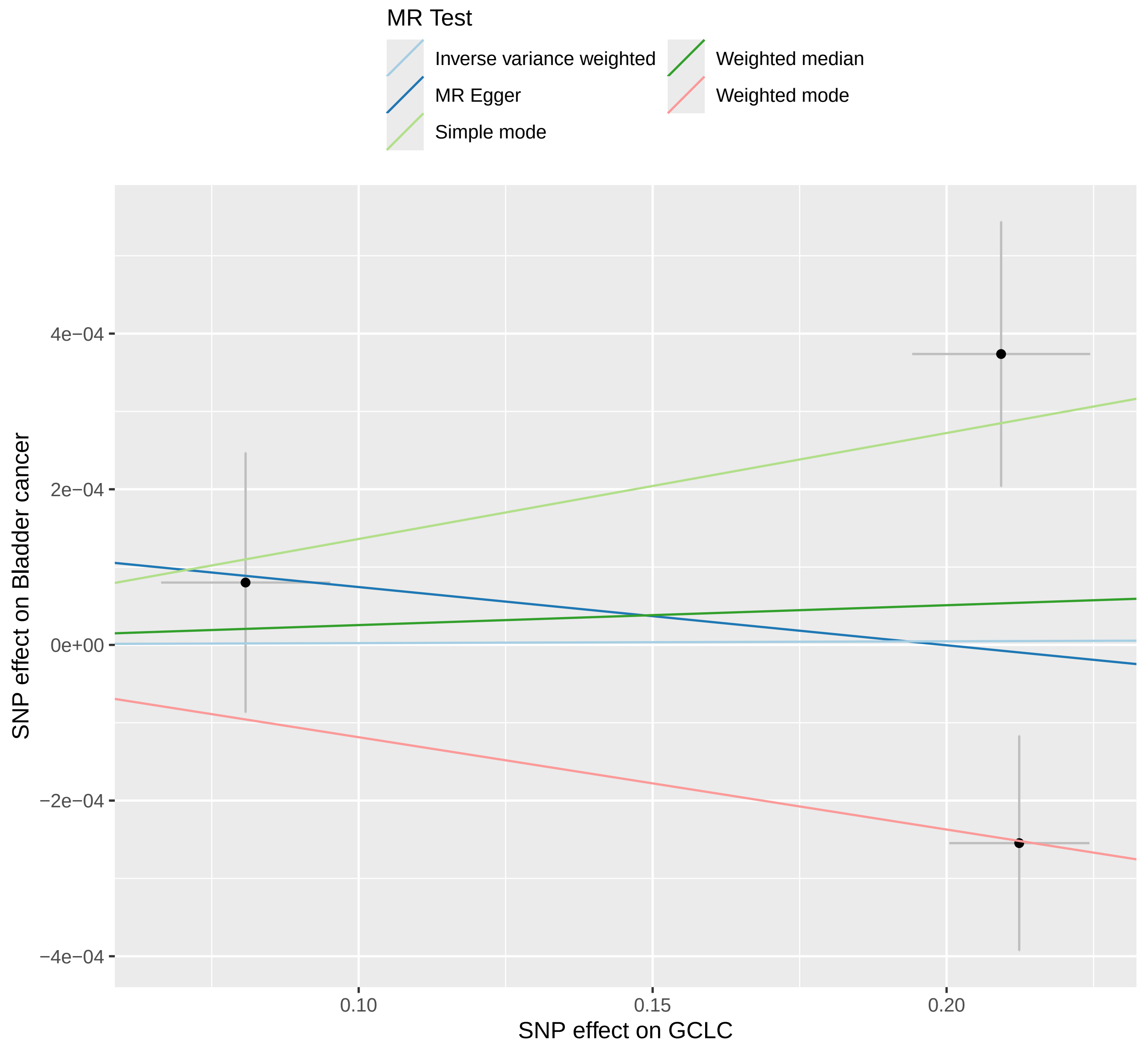

MR Test
Inverse variance weighted
MR Egger
Simple mode
Weighted median
Weighted mode
SNP effect on Bladder cancer
4e−04
2e−04
0e+00
−2e−04
−4e−04
0.10
0.15
0.20
SNP effect on GCLC

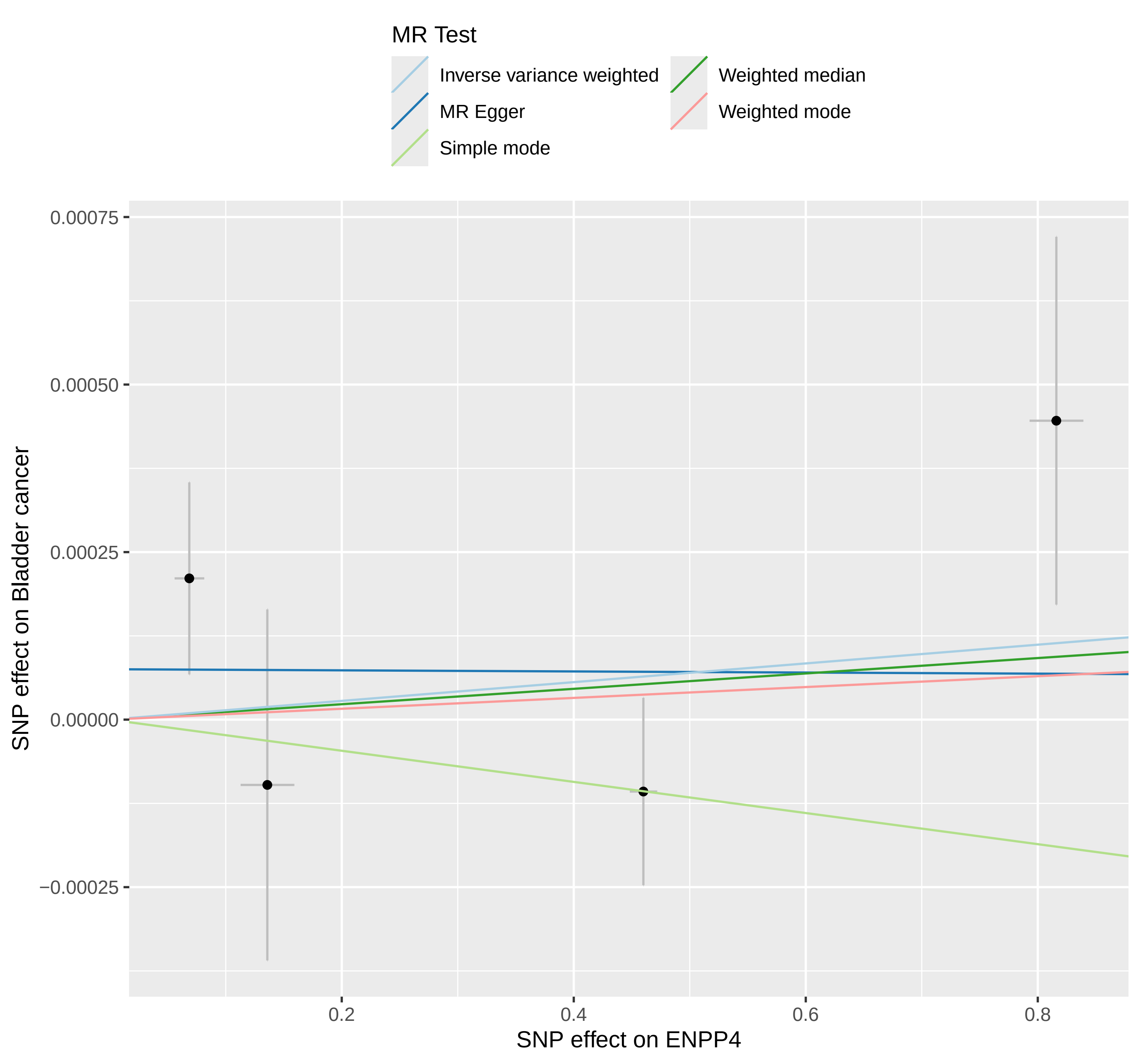
MR Test
Inverse variance weighted
MR Egger
Simple mode
Weighted median
Weighted mode
0.00075
0.00050
0.00025
0.00000
−0.00025
SNP effect on Bladder cancer
0.2
0.4
0.6
0.8
SNP effect on ENPP4

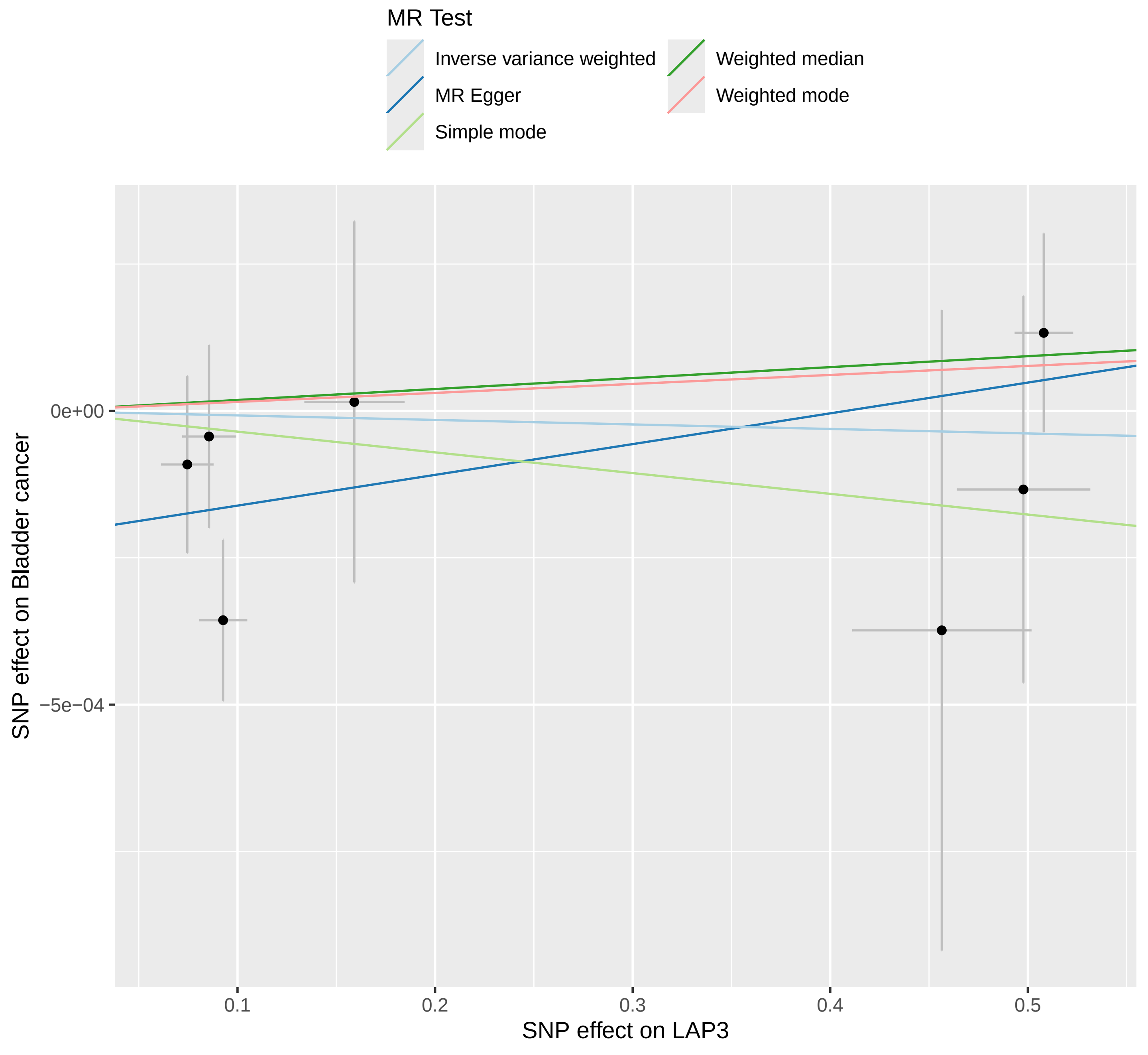

MR Test
Inverse variance weighted
MR Egger
Simple mode
Weighted median
Weighted mode
SNP effect on Bladder cancer
0e+00
−5e−04
0.1
0.2
0.3
0.4
0.5
SNP effect on LAP3

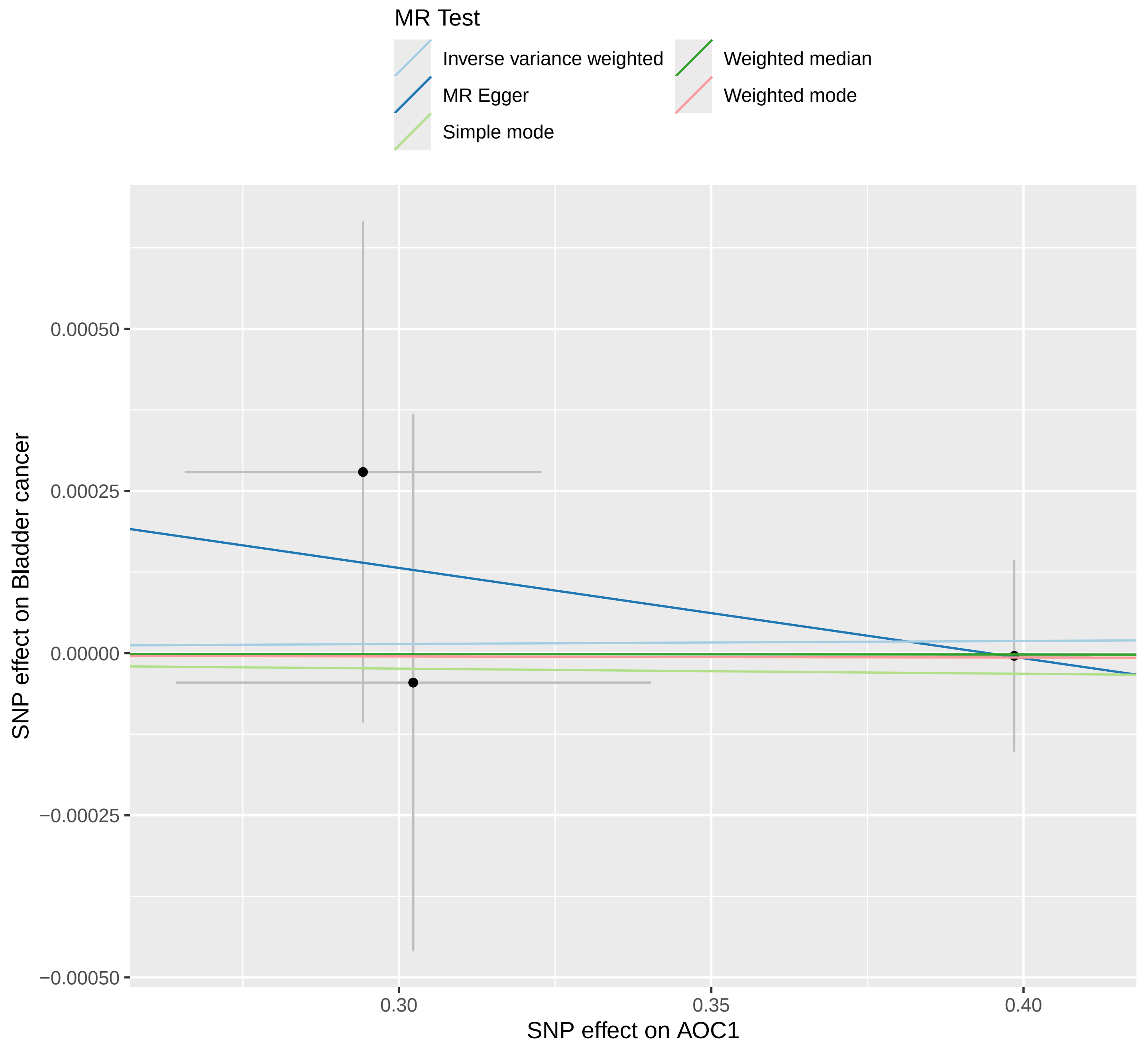
MR Test
Inverse variance weighted
MR Egger
Simple mode
Weighted median
Weighted mode
0.00050
0.00025
0.00000
−0.00025
−0.00050
SNP effect on Bladder cancer
0.30
0.35
0.40
SNP effect on AOC1

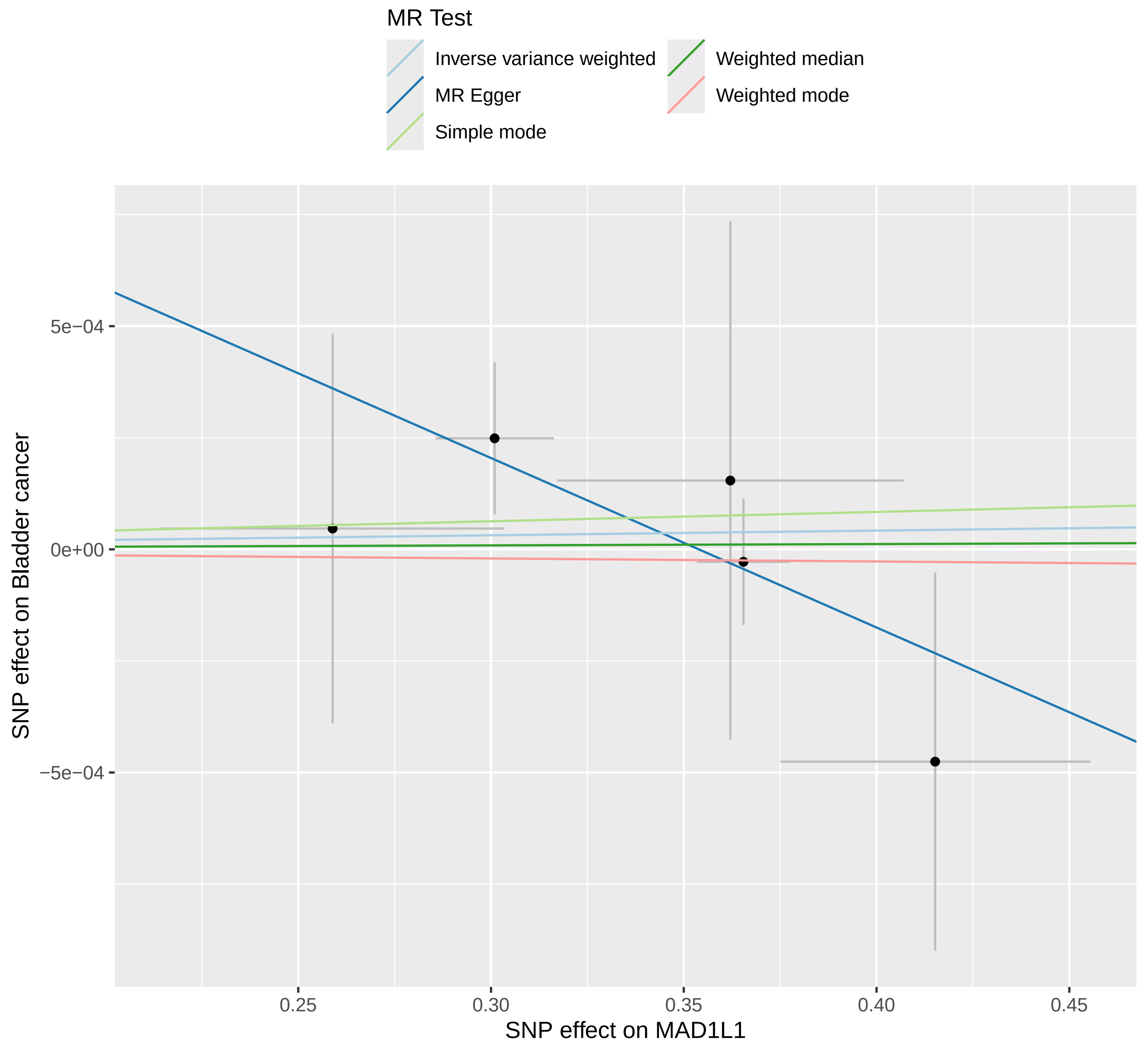

MR Test
Inverse variance weighted
MR Egger
Simple mode
Weighted median
Weighted mode
5e−04
0e+00
−5e−04
SNP effect on Bladder cancer
0.25
0.30
0.35
0.40
0.45
SNP effect on MAD1L1

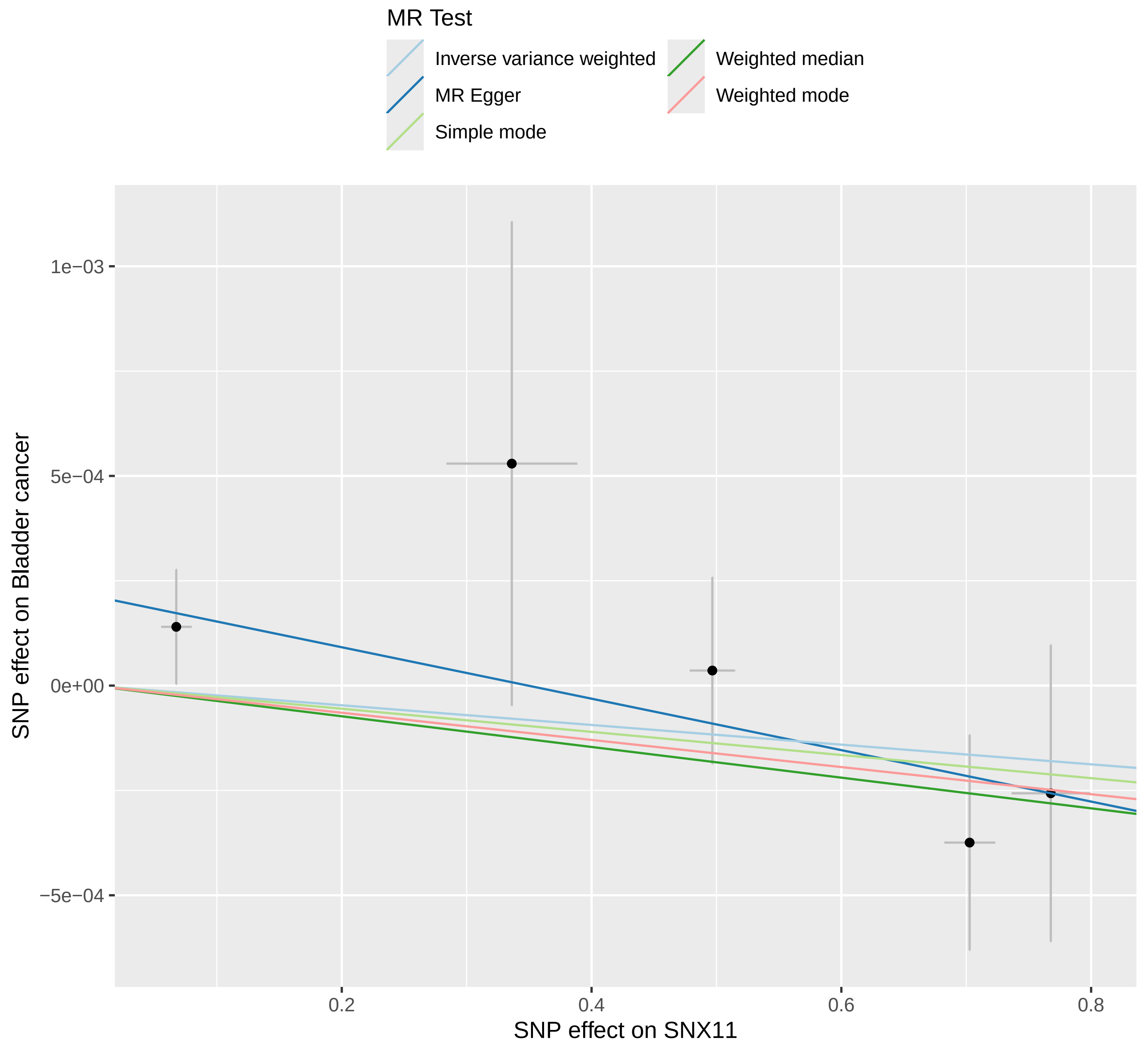

MR Test
Inverse variance weighted
MR Egger
Simple mode
Weighted median
Weighted mode
SNP effect on Bladder cancer
1e−03
5e−04
0e+00
−5e−04
SNP effect on SNX11
0.2
0.4
0.6
0.8

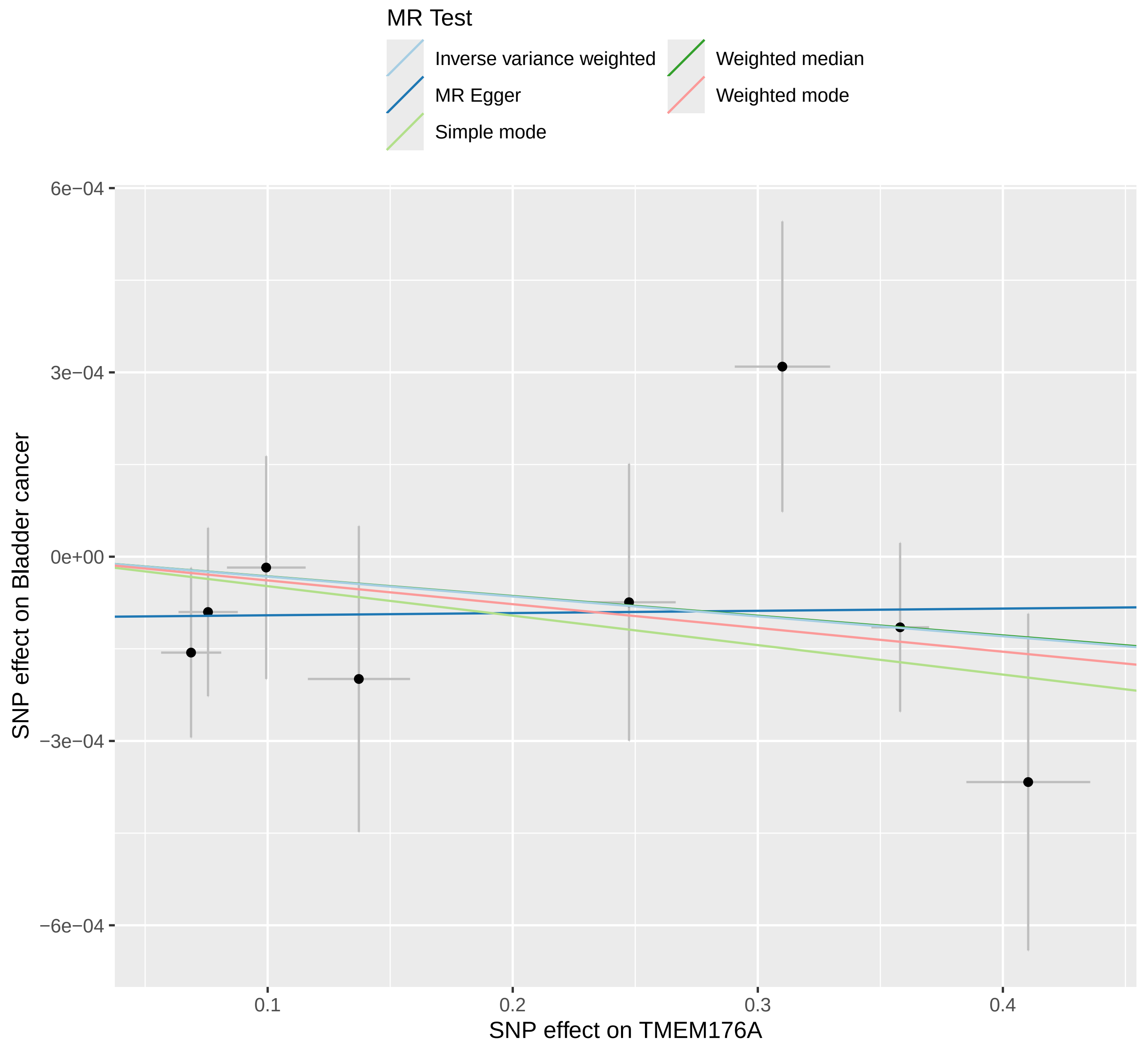
MR Test
Inverse variance weighted
MR Egger
Simple mode
Weighted median
Weighted mode
6e−04
3e−04
0e+00
−3e−04
−6e−04
0.1
0.2
0.3
0.4
SNP effect on TMEM176A
SNP effect on Bladder cancer

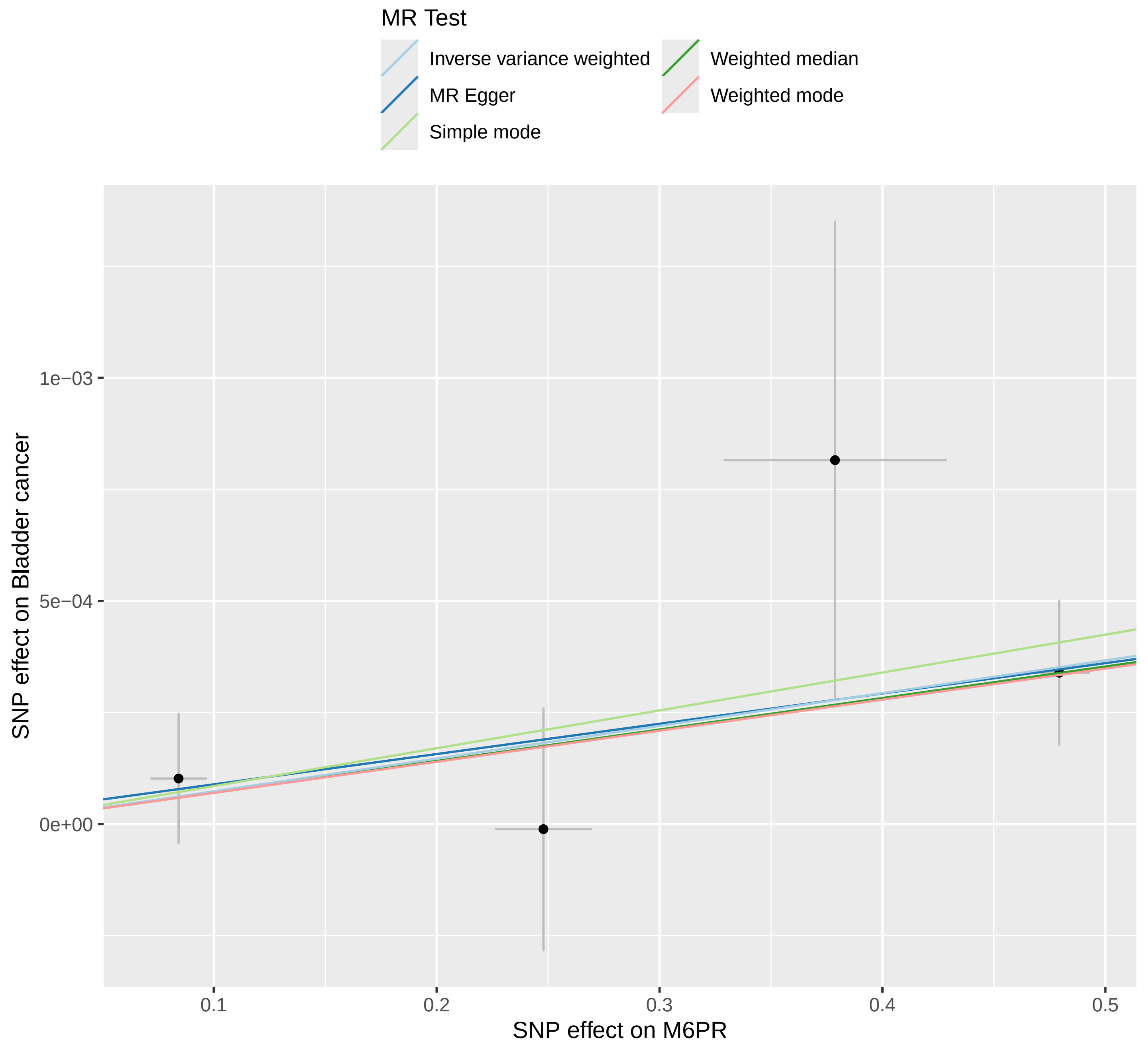
MR Test
Inverse variance weighted
MR Egger
Simple mode
Weighted median
Weighted mode
SNP effect on Bladder cancer
1e−03
5e−04
0e+00
0.1
0.2
0.3
0.4
0.5
SNP effect on M6PR

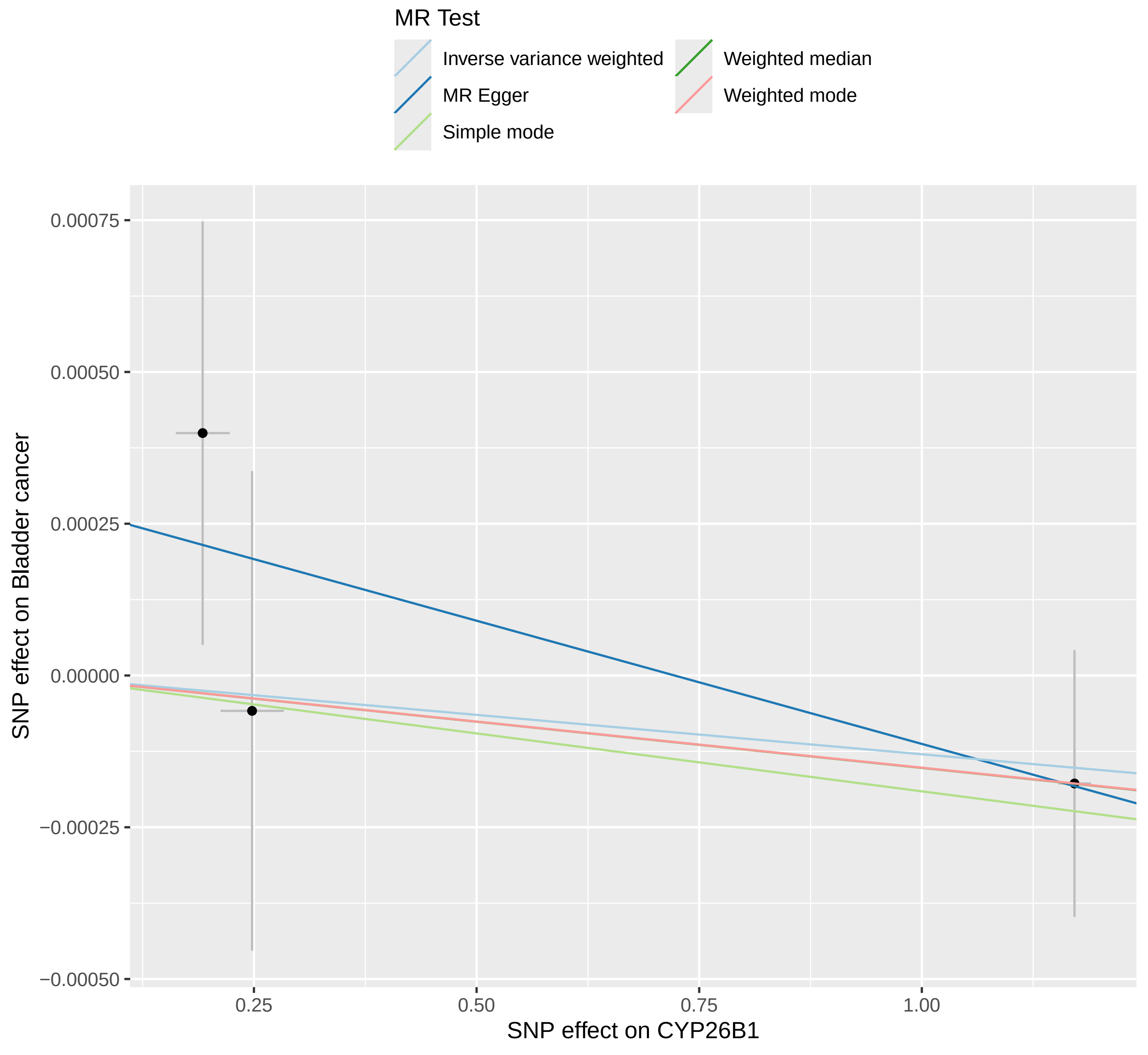

MR Test
Inverse variance weighted
MR Egger
Simple mode
Weighted median
Weighted mode
0.00075
0.00050
0.00025
0.00000
−0.00025
−0.00050
SNP effect on Bladder cancer
0.25
0.50
0.75
1.00
SNP effect on CYP26B1

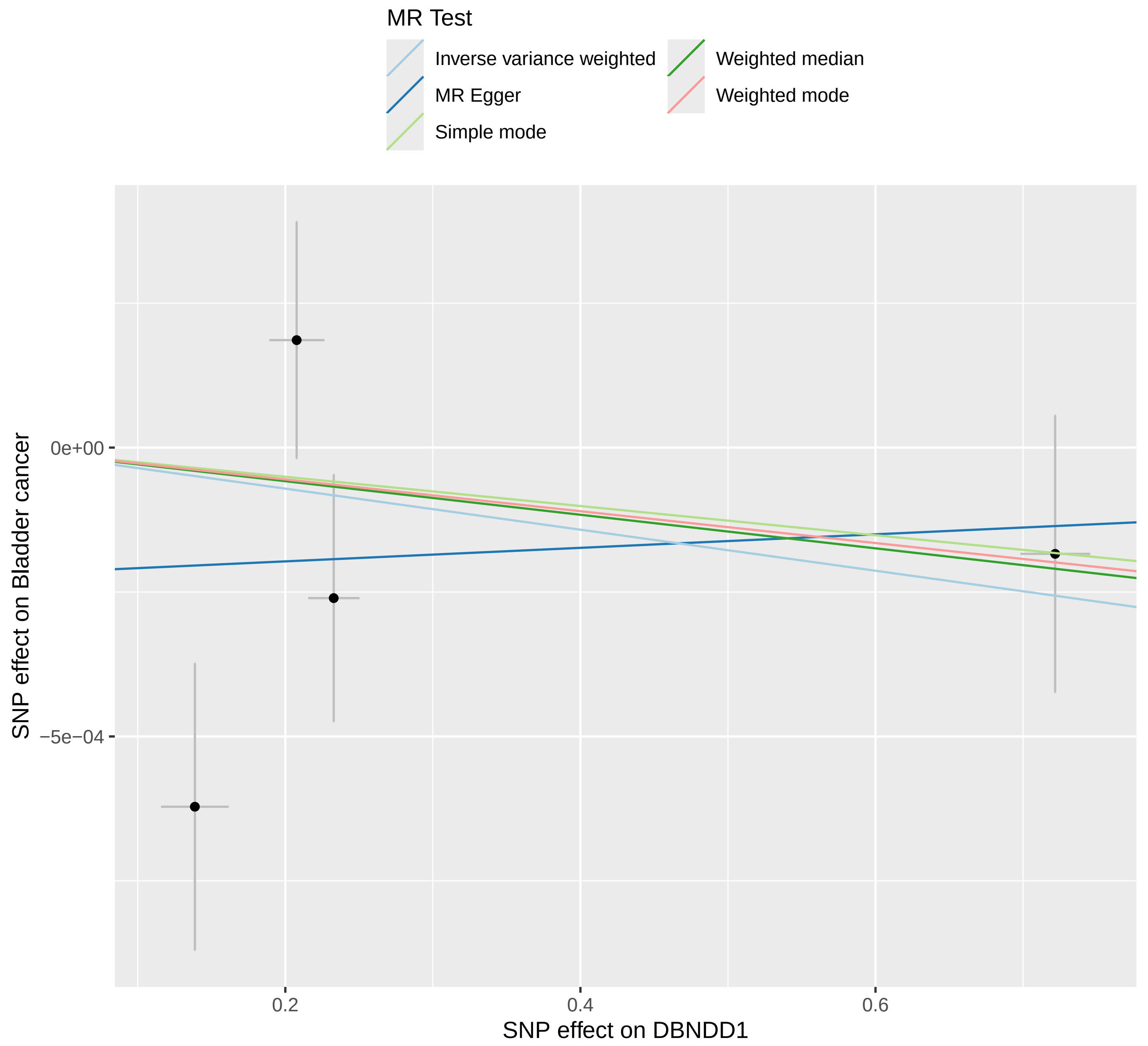

MR Test
Inverse variance weighted
MR Egger
Simple mode
Weighted median
Weighted mode
SNP effect on Bladder cancer
0e+00
−5e−04
0.2
0.4
0.6
SNP effect on DBNDD1

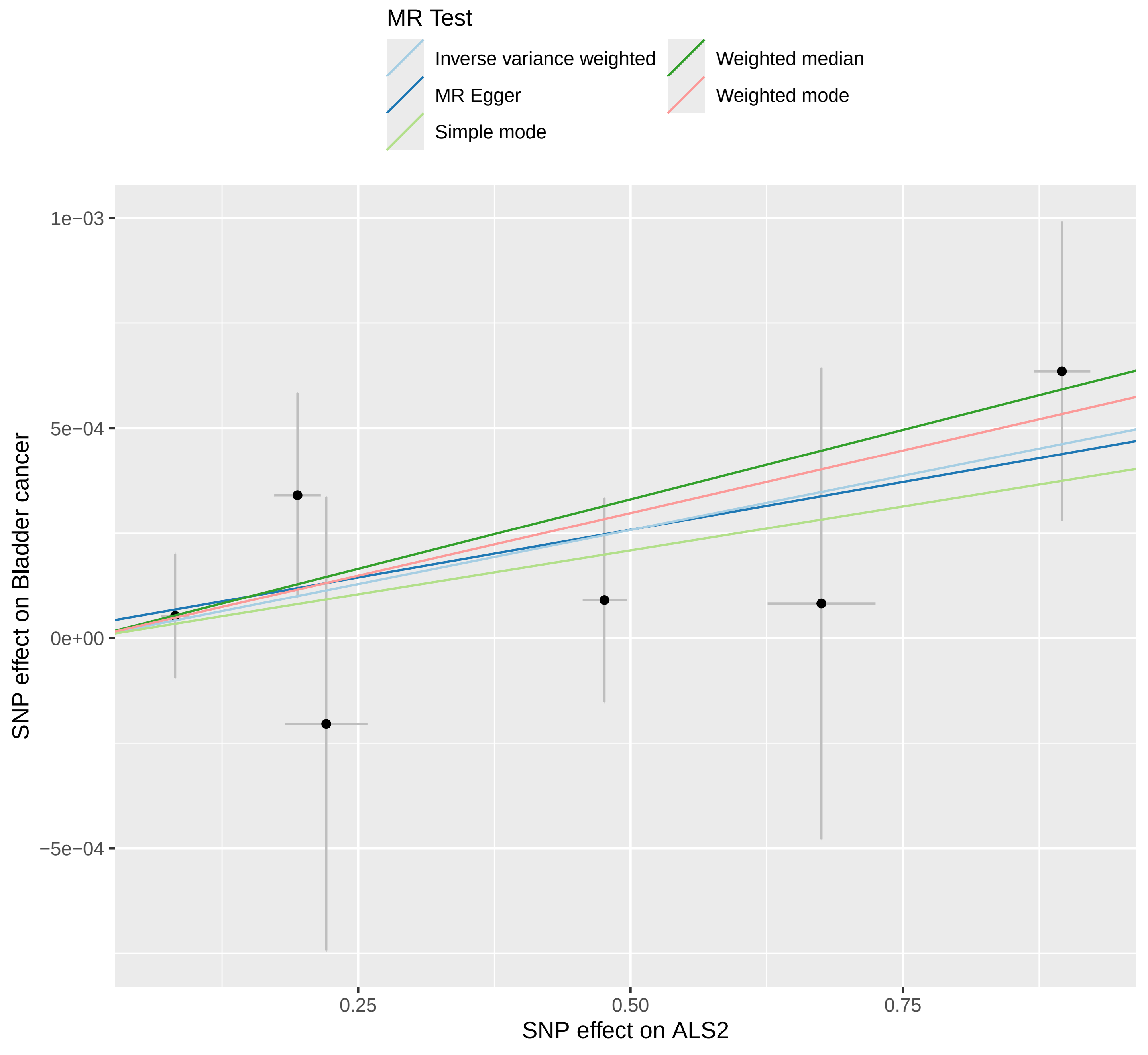
MR Test
Inverse variance weighted
MR Egger
Simple mode
Weighted median
Weighted mode
1e−03
5e−04
0e+00
−5e−04
SNP effect on Bladder cancer
0.25
0.50
0.75
SNP effect on ALS2

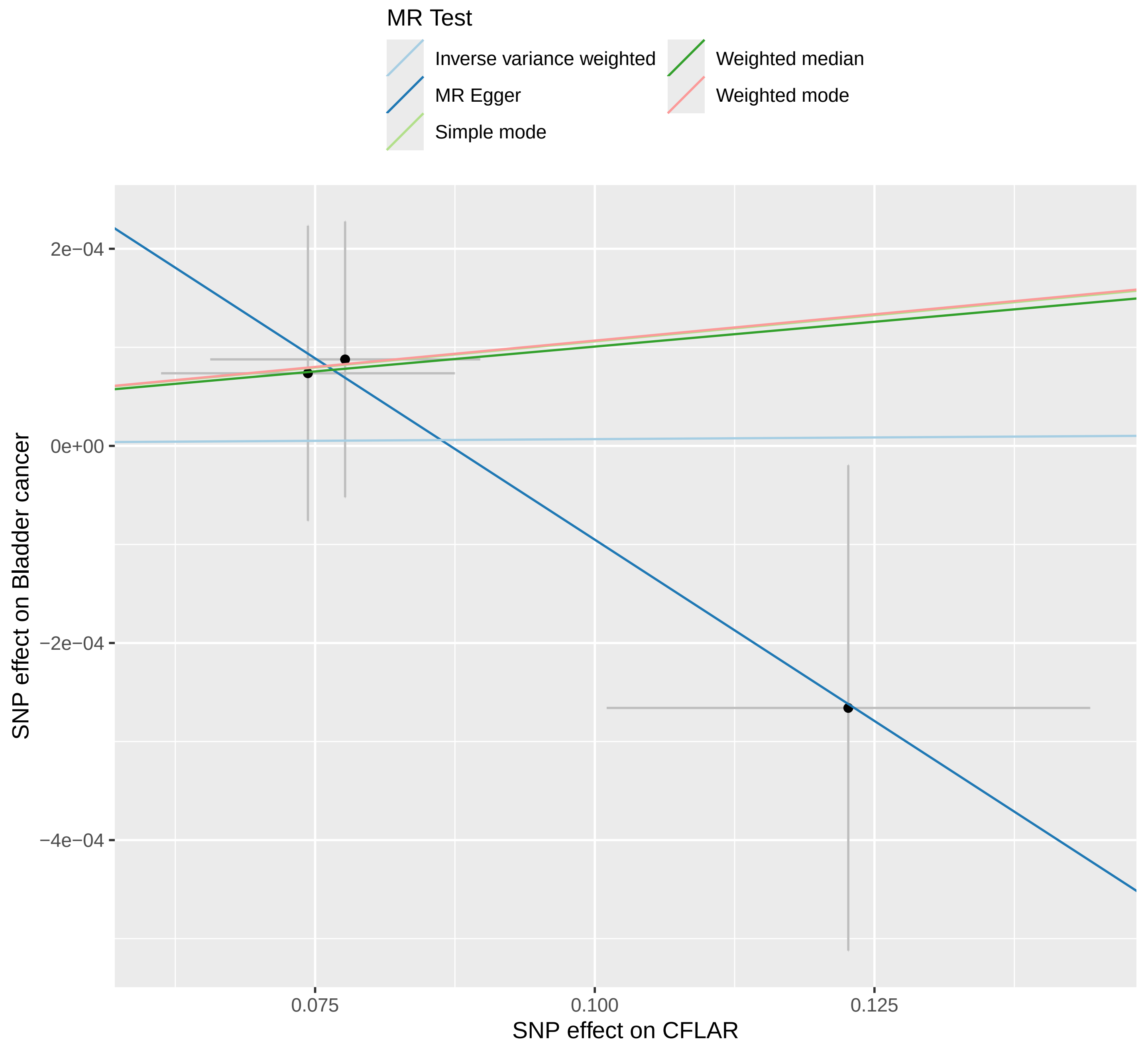
MR Test
Inverse variance weighted
MR Egger
Simple mode
Weighted median
Weighted mode
2e−04
0e+00
−2e−04
−4e−04
0.075
0.100
0.125
SNP effect on CFLAR
SNP effect on Bladder cancer

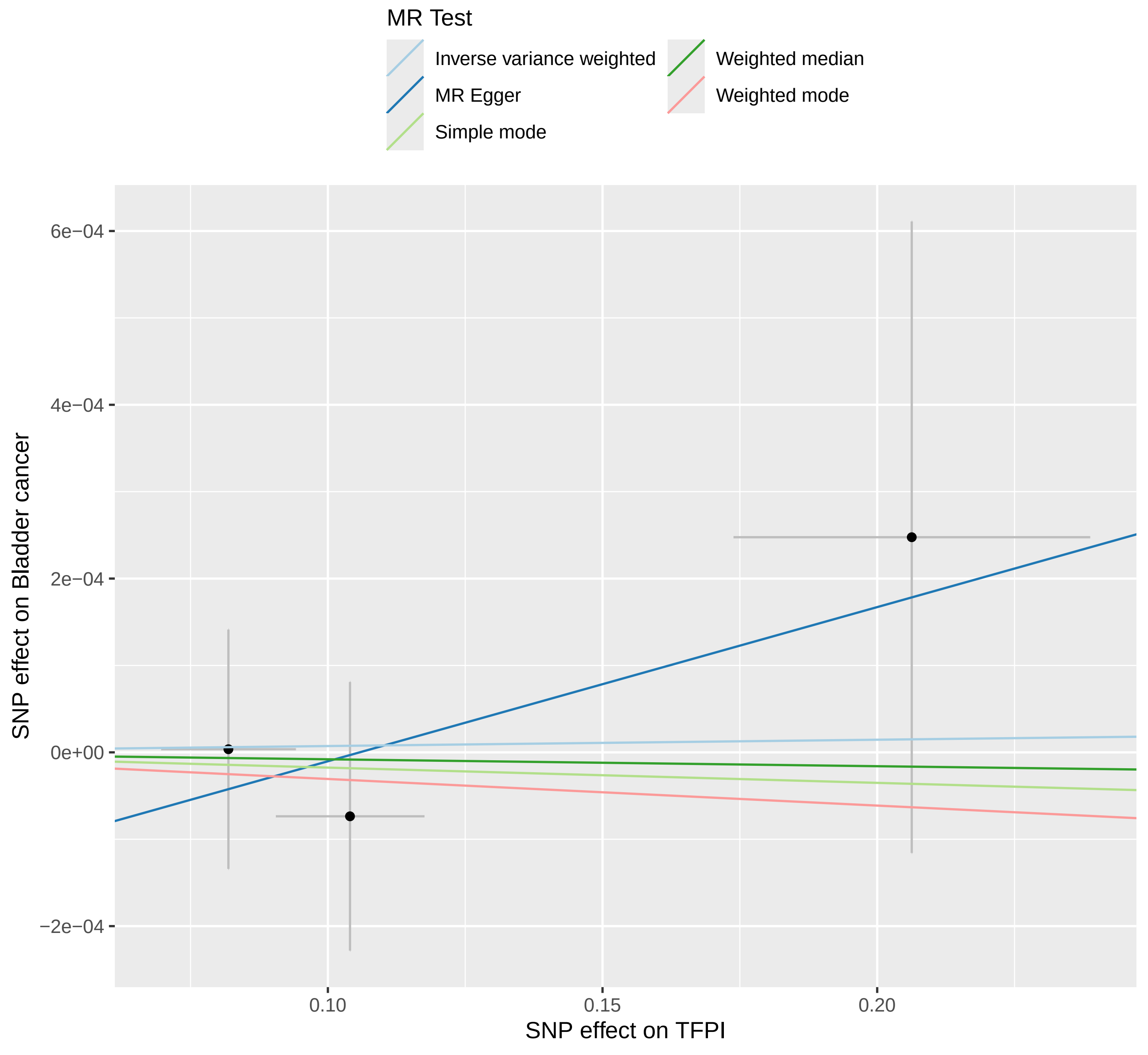

MR Test
Inverse variance weighted
MR Egger
Simple mode
Weighted median
Weighted mode
6e−04
4e−04
2e−04
0e+00
−2e−04
0.10
0.15
0.20
SNP effect on TFPI
SNP effect on Bladder cancer

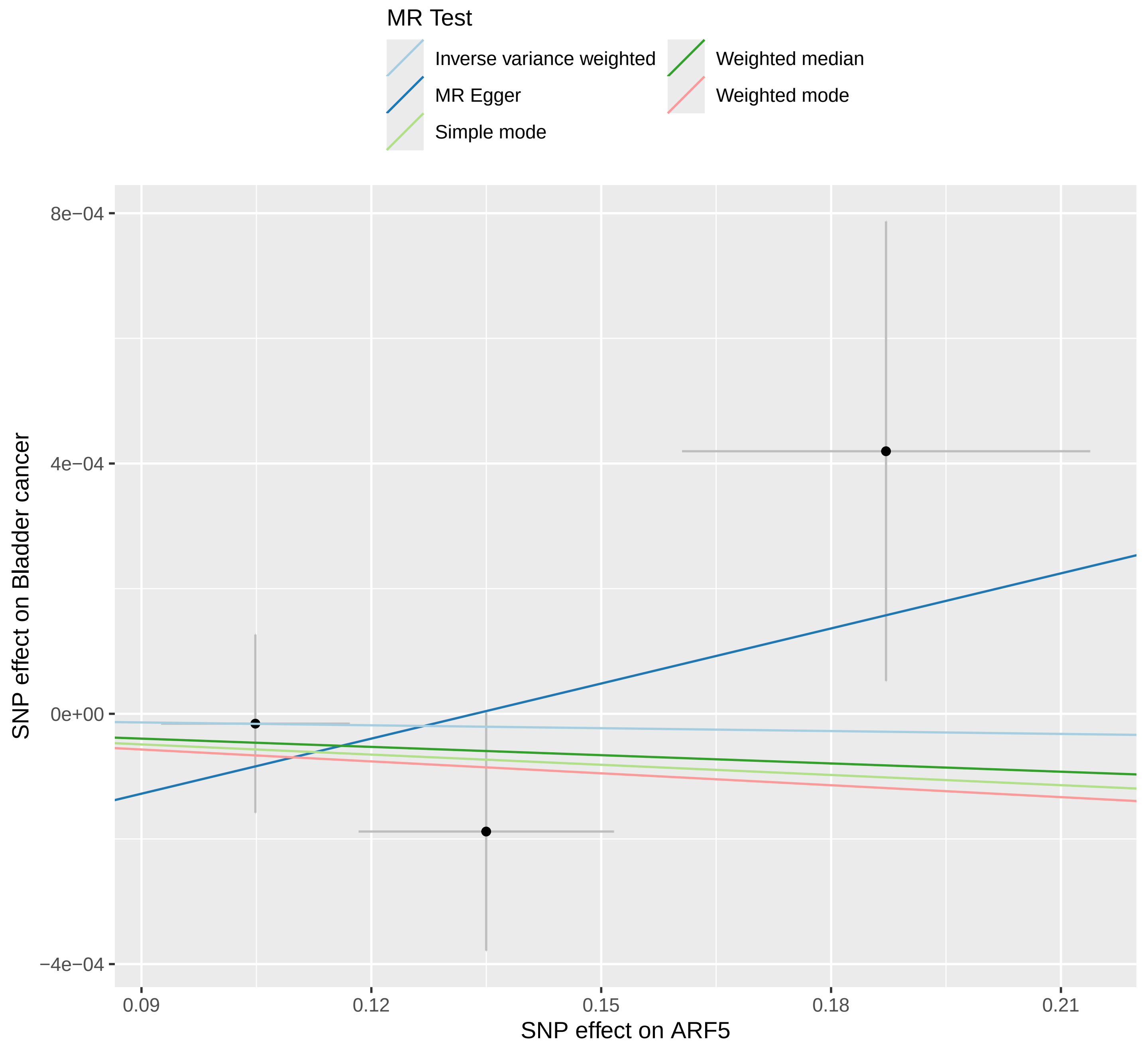

MR Test
Inverse variance weighted
MR Egger
Simple mode
Weighted median
Weighted mode
8e−04
4e−04
0e+00
−4e−04
SNP effect on Bladder cancer
0.09
0.12
0.15
0.18
0.21
SNP effect on ARF5

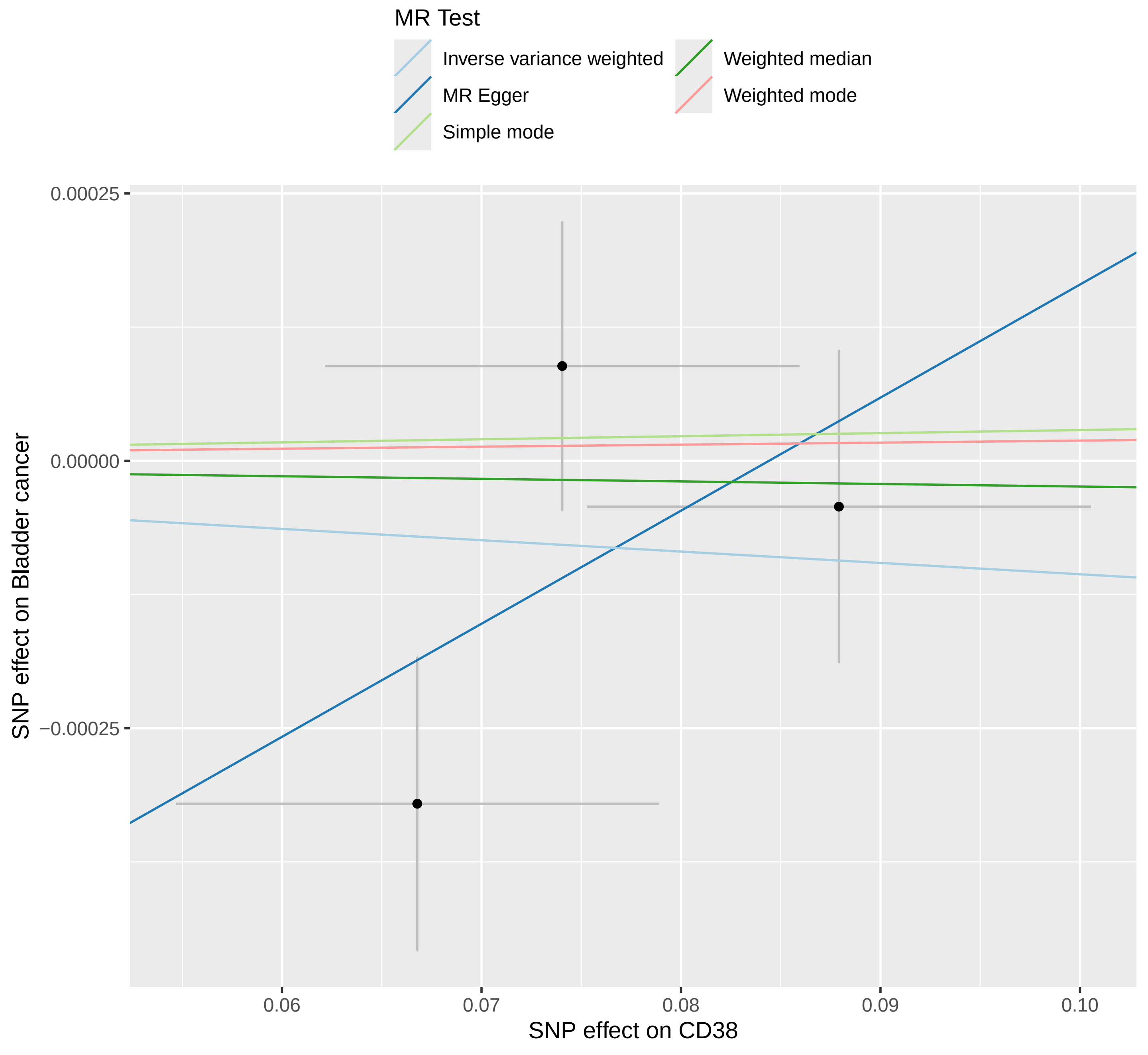

MR Test
Inverse variance weighted
MR Egger
Simple mode
Weighted median
Weighted mode
0.00025
0.00000
−0.00025
0.06
0.07
0.08
0.09
0.10
SNP effect on Bladder cancer
SNP effect on CD38

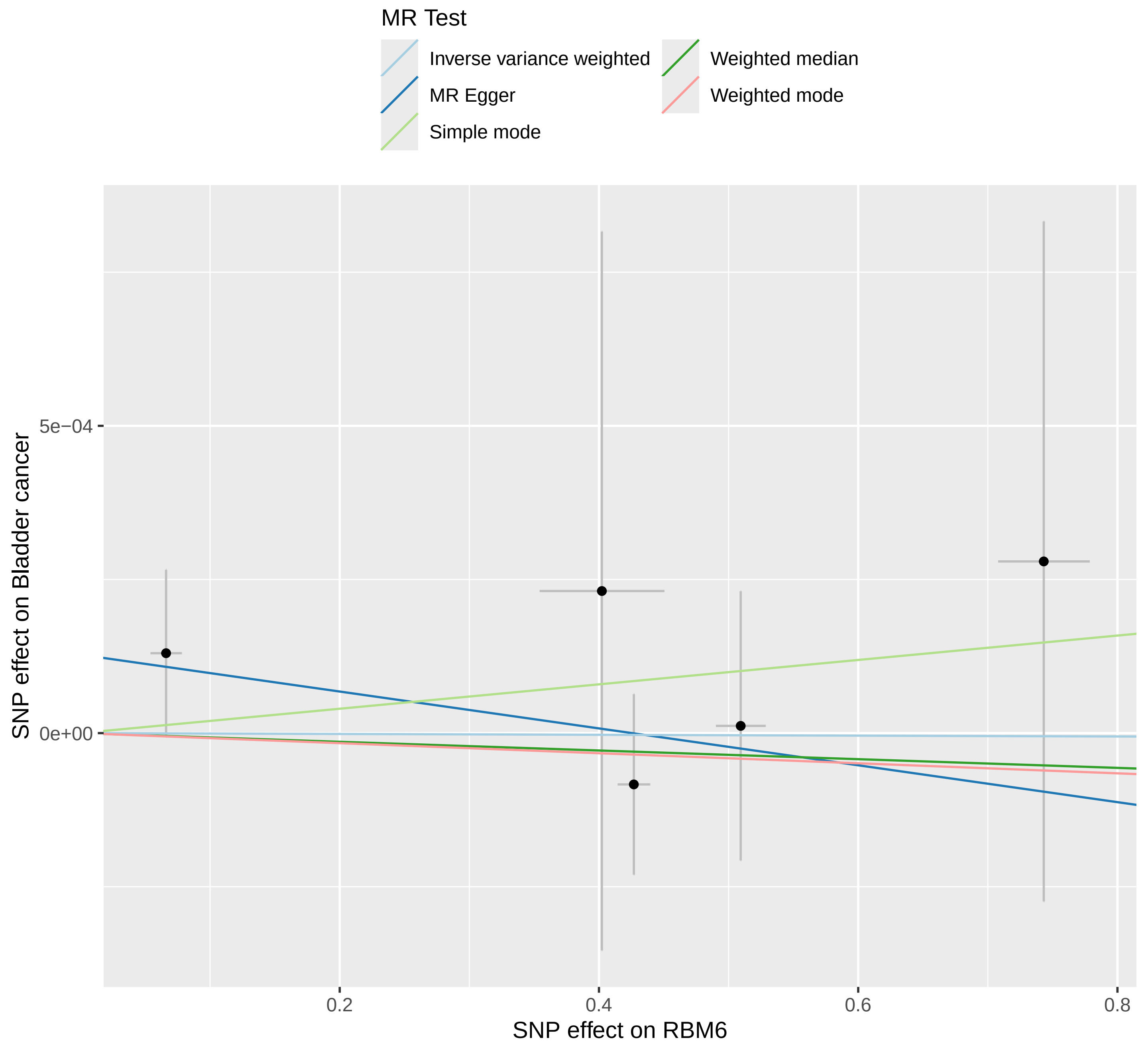

MR Test
Inverse variance weighted
MR Egger
Simple mode
Weighted median
Weighted mode
SNP effect on Bladder cancer
5e−04
0e+00
0.2
0.4
0.6
0.8
SNP effect on RBM6

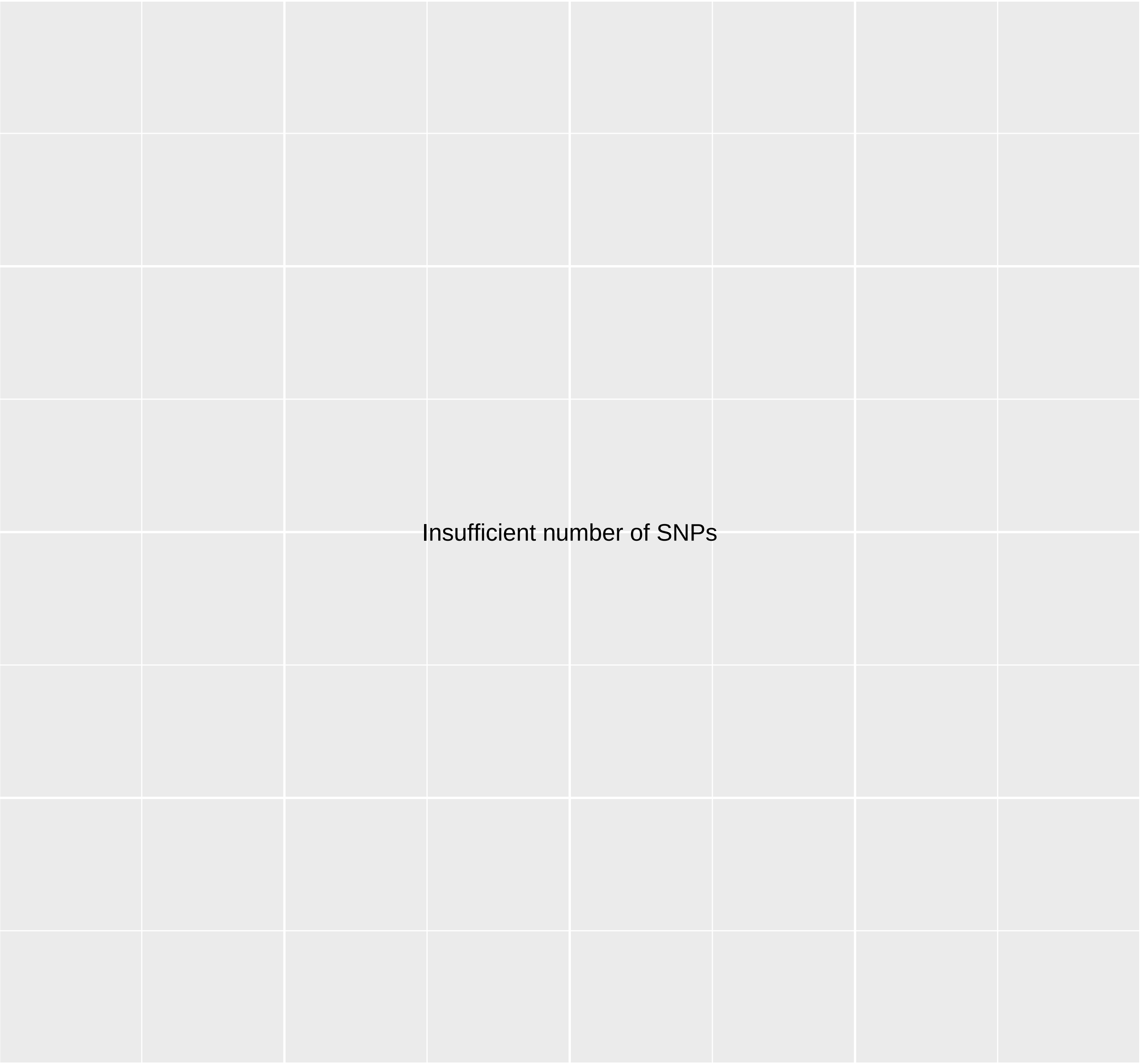
Insufficient number of SNPs

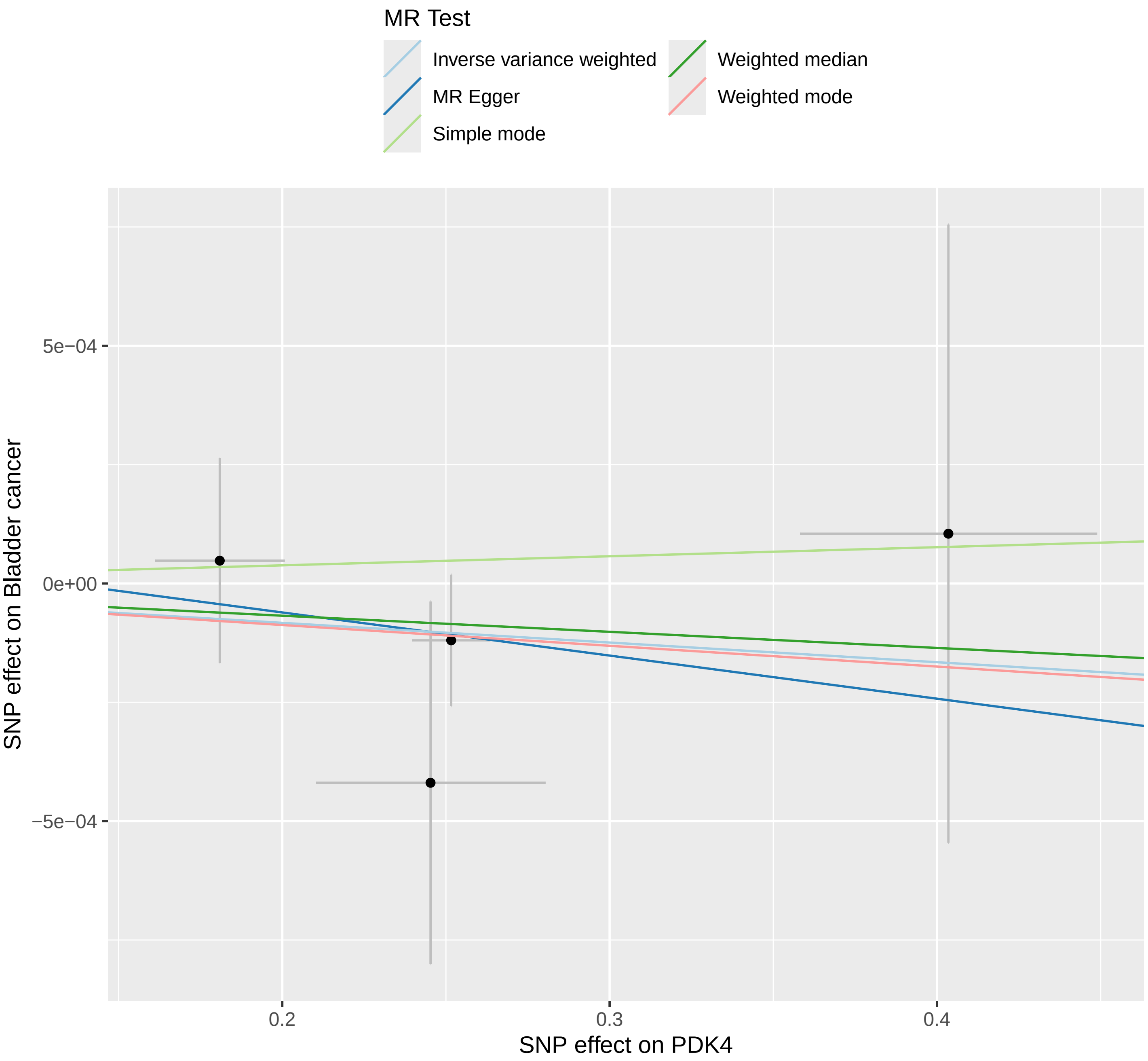
MR Test
Inverse variance weighted
MR Egger
Simple mode
Weighted median
Weighted mode
5e−04
0e+00
−5e−04
0.2
0.3
0.4
SNP effect on Bladder cancer
SNP effect on PDK4

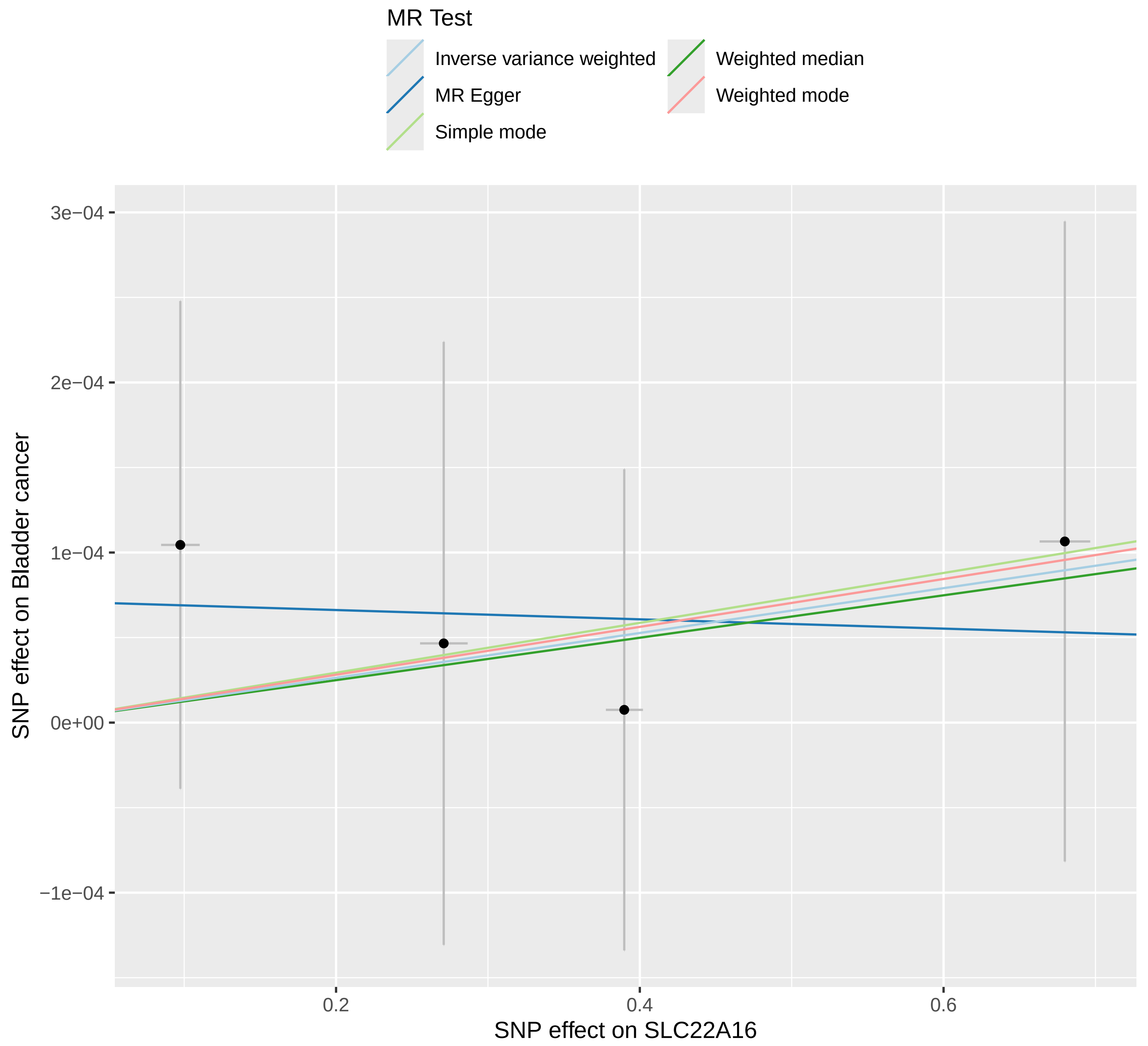
MR Test
Inverse variance weighted
MR Egger
Simple mode
Weighted median
Weighted mode
3e−04
2e−04
1e−04
0e+00
−1e−04
SNP effect on Bladder cancer
0.2
0.4
0.6
SNP effect on SLC22A16

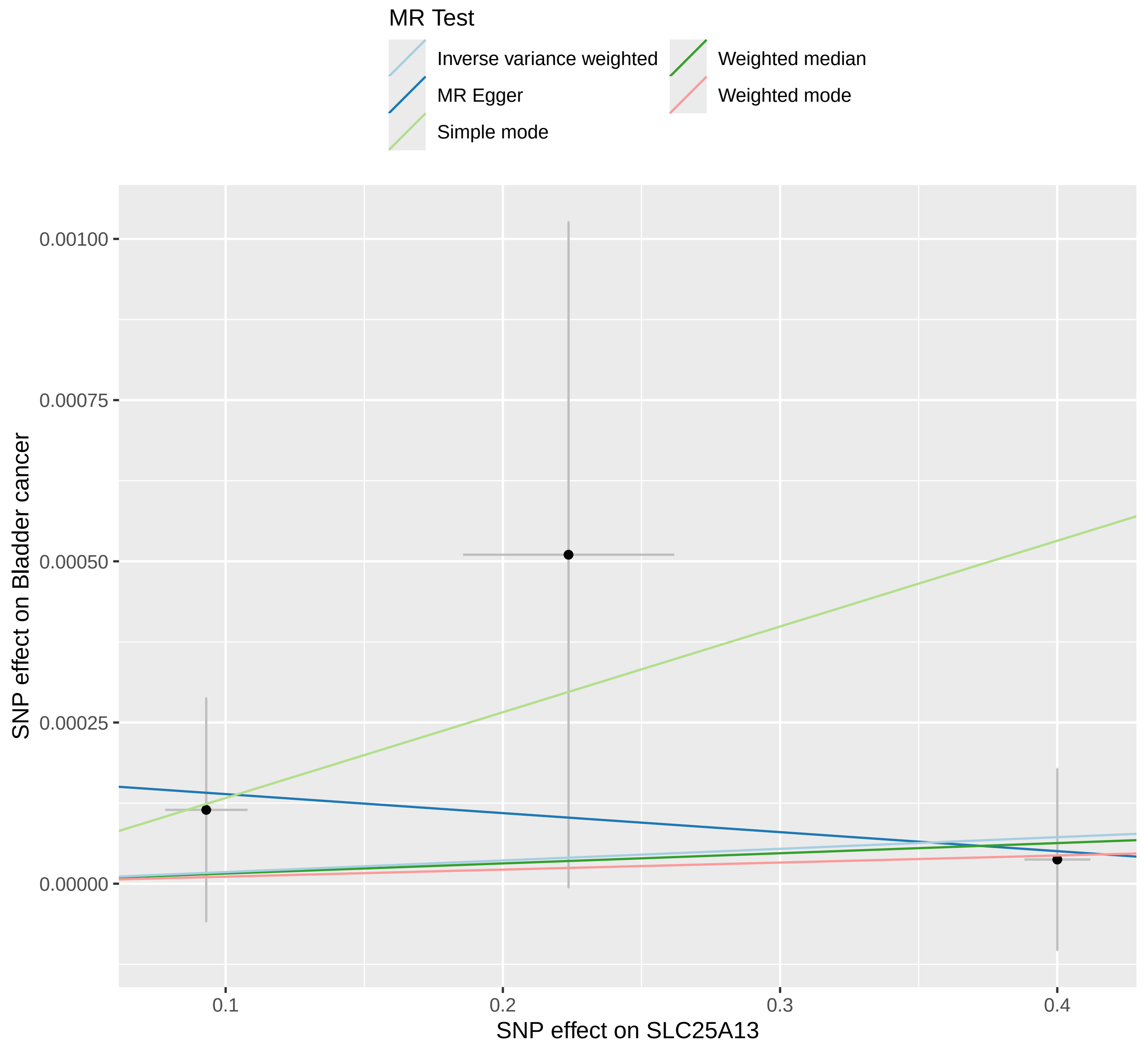
MR Test
Inverse variance weighted
MR Egger
Simple mode
Weighted median
Weighted mode
0.00100
0.00075
0.00050
0.00025
0.00000
SNP effect on Bladder cancer
0.1
0.2
0.3
0.4
SNP effect on SLC25A13

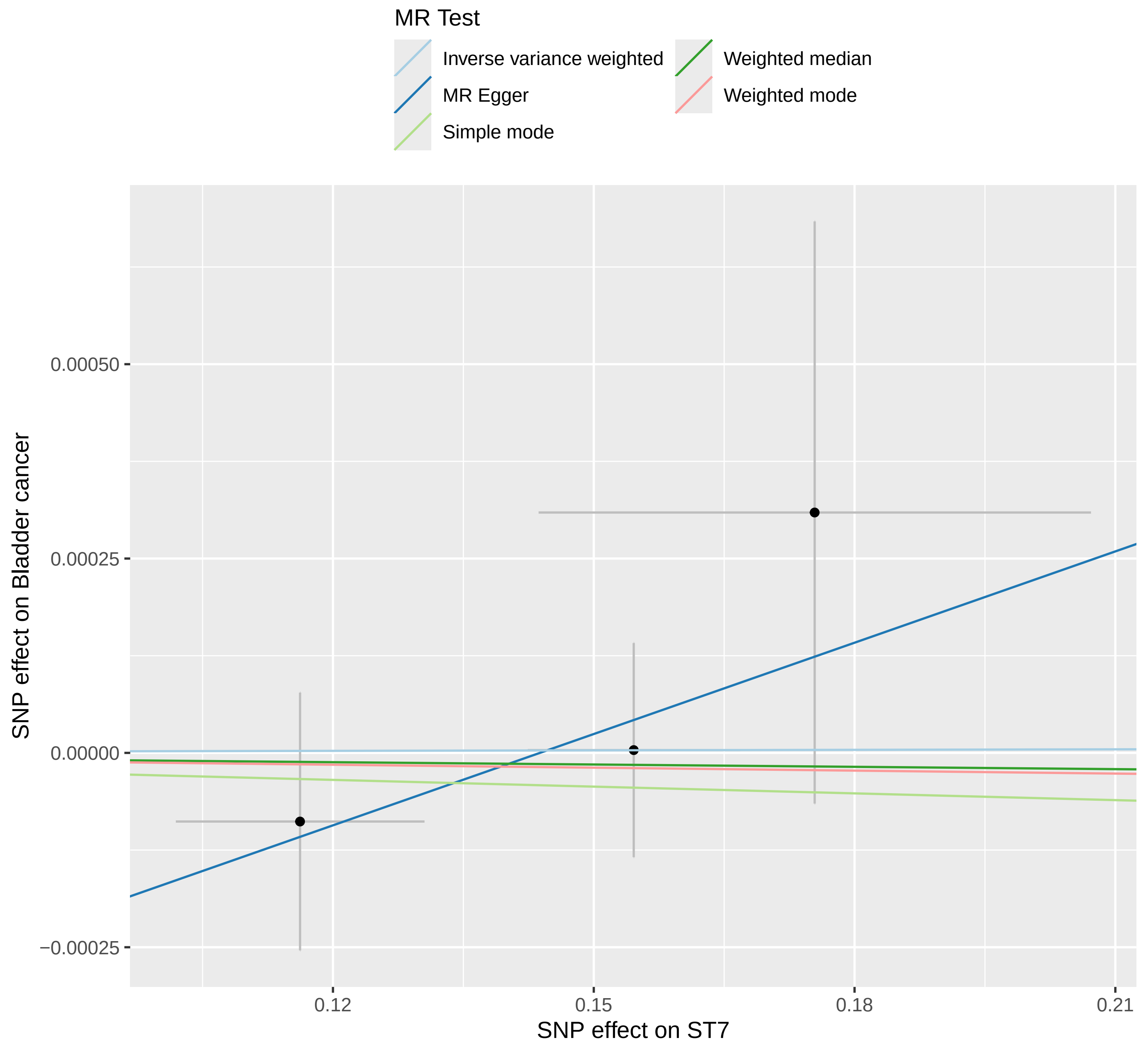
MR Test
Inverse variance weighted
MR Egger
Simple mode
Weighted median
Weighted mode
0.00050
0.00025
0.00000
−0.00025
SNP effect on Bladder cancer
0.12
0.15
0.18
0.21
SNP effect on ST7

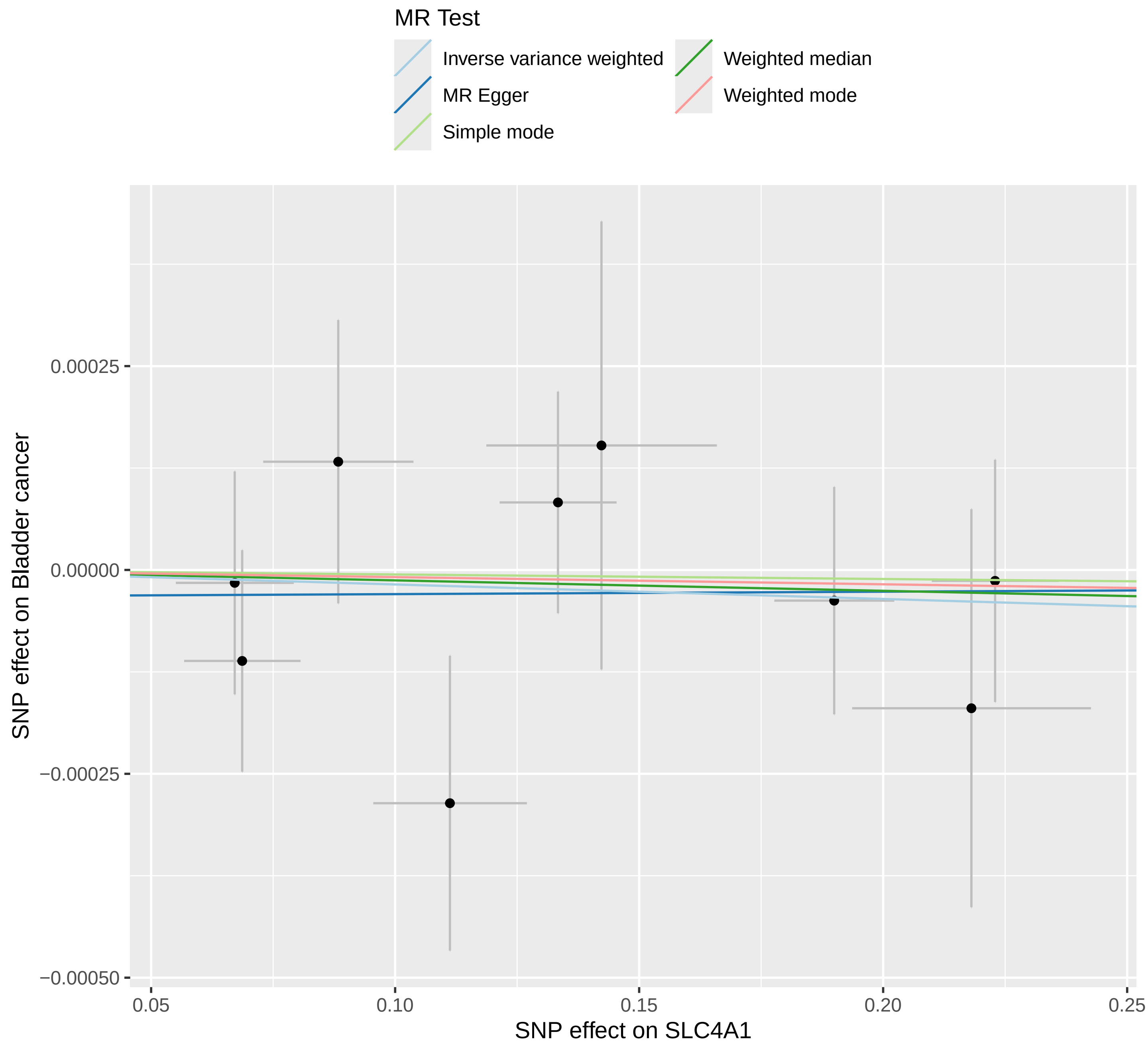
MR Test
Inverse variance weighted
MR Egger
Simple mode
Weighted median
Weighted mode
0.00025
0.00000
−0.00025
−0.00050
SNP effect on Bladder cancer
0.05
0.10
0.15
0.20
0.25
SNP effect on SLC4A1

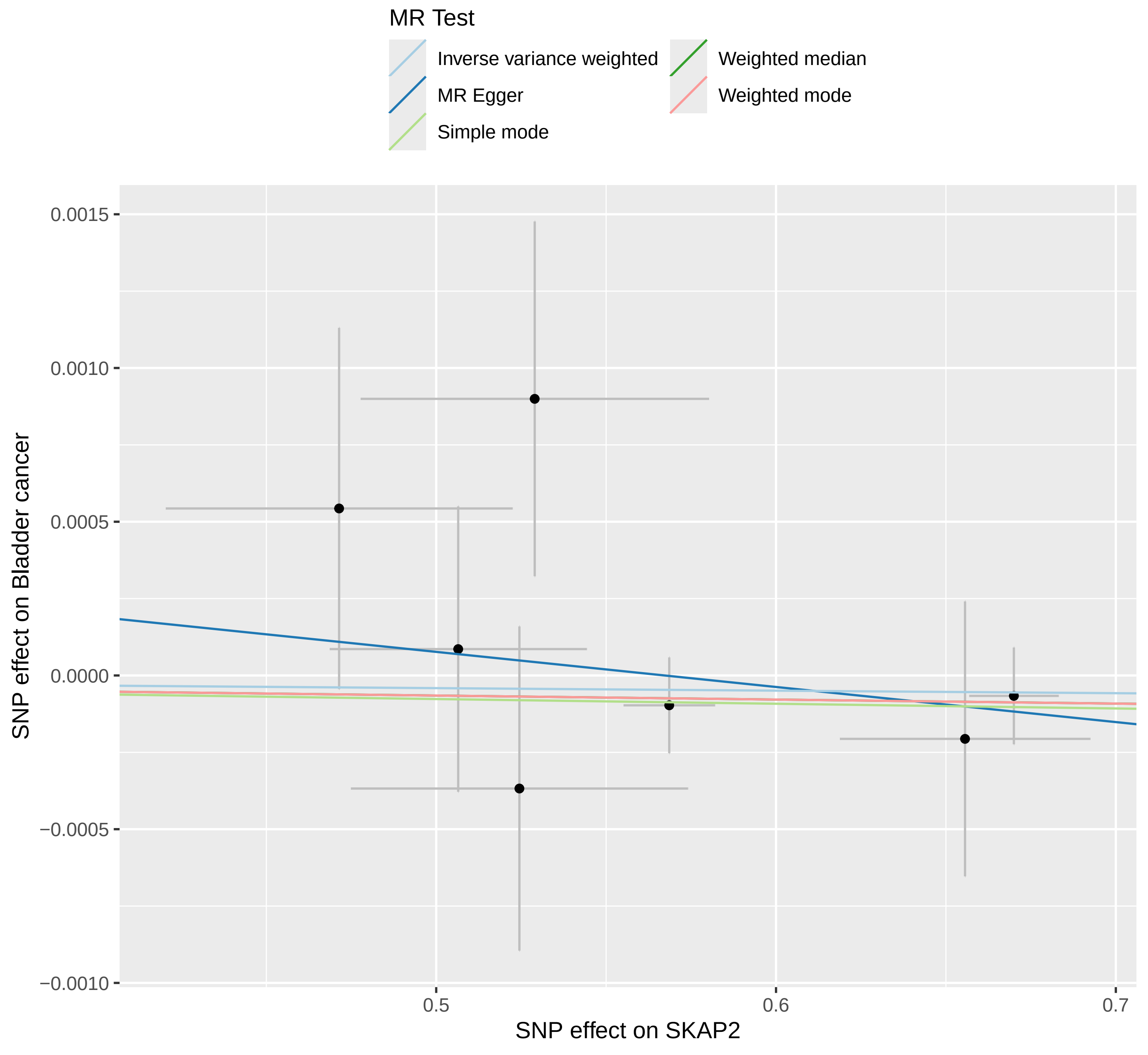

MR Test
Inverse variance weighted
MR Egger
Simple mode
Weighted median
Weighted mode
0.0015
0.0010
0.0005
0.0000
−0.0005
−0.0010
SNP effect on Bladder cancer
0.5
0.6
0.7
SNP effect on SKAP2

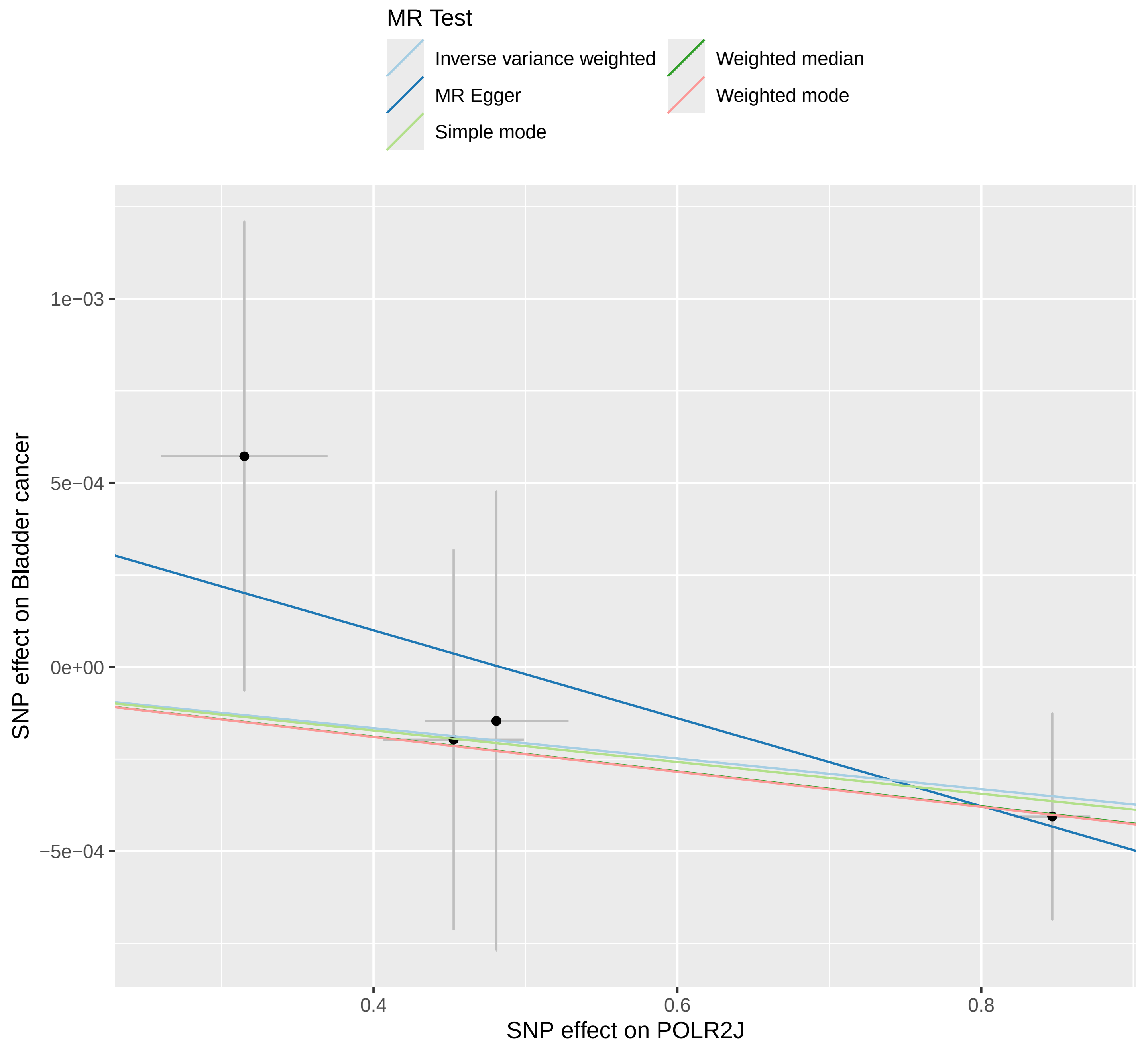

MR Test
Inverse variance weighted
MR Egger
Simple mode
Weighted median
Weighted mode
1e−03
5e−04
0e+00
−5e−04
SNP effect on Bladder cancer
0.4
0.6
0.8
SNP effect on POLR2J

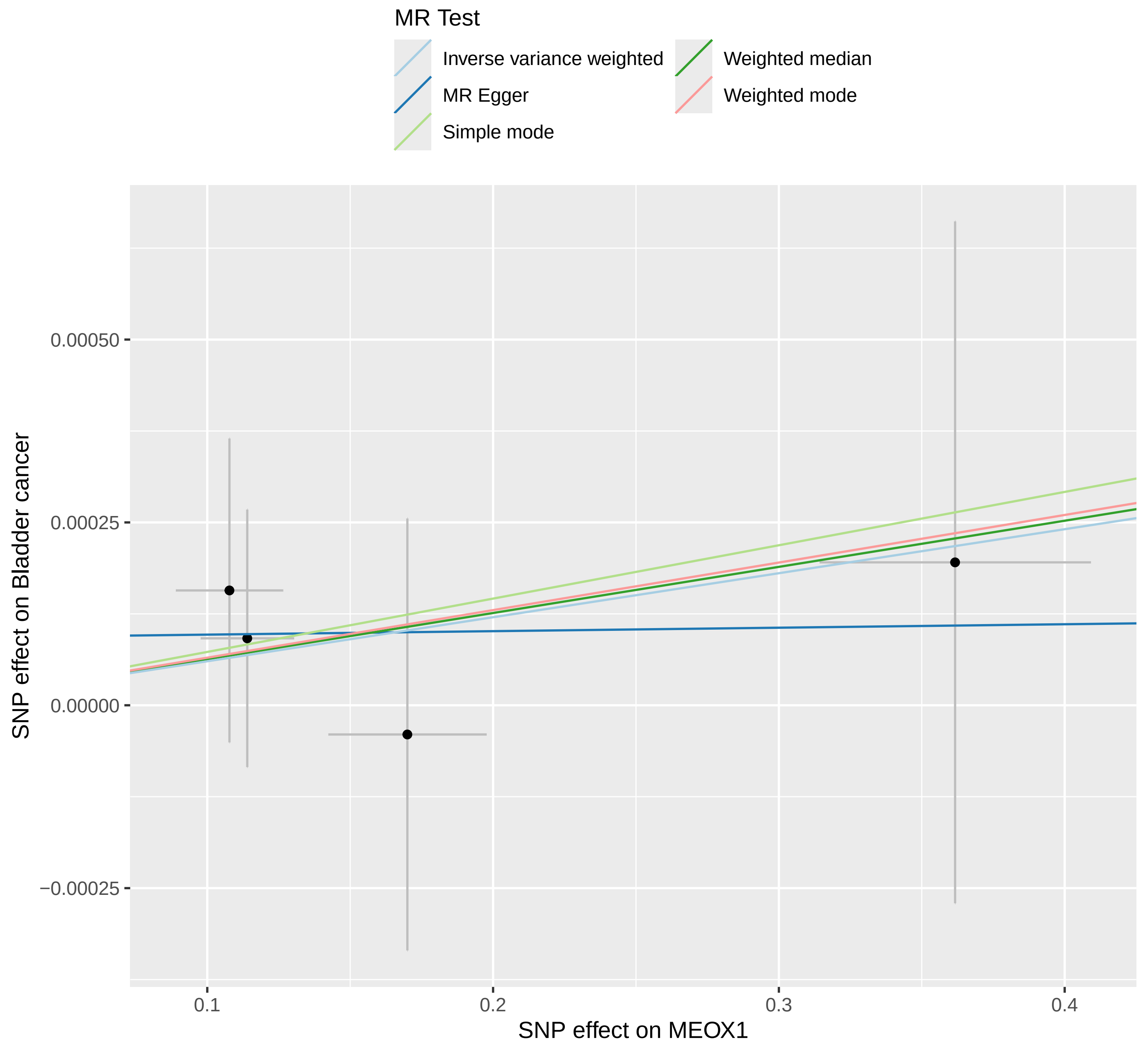

MR Test
Inverse variance weighted
MR Egger
Simple mode
Weighted median
Weighted mode
0.00050
0.00025
0.00000
−0.00025
SNP effect on Bladder cancer
0.1
0.2
0.3
0.4
SNP effect on MEOX1

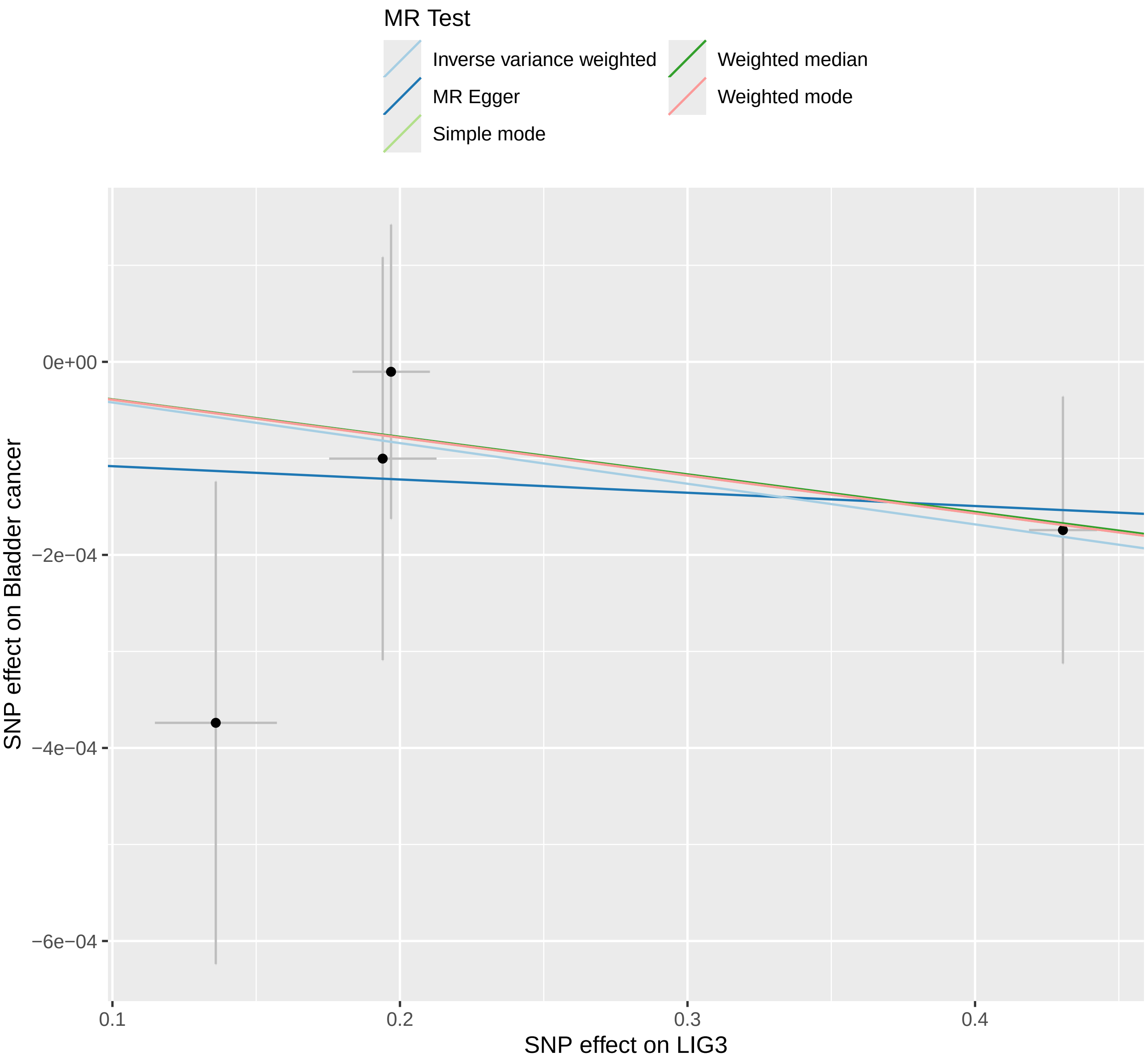
MR Test
Inverse variance weighted
MR Egger
Simple mode
Weighted median
Weighted mode
0e+00
−2e−04
−4e−04
−6e−04
0.1
0.2
0.3
0.4
SNP effect on Bladder cancer
SNP effect on LIG3

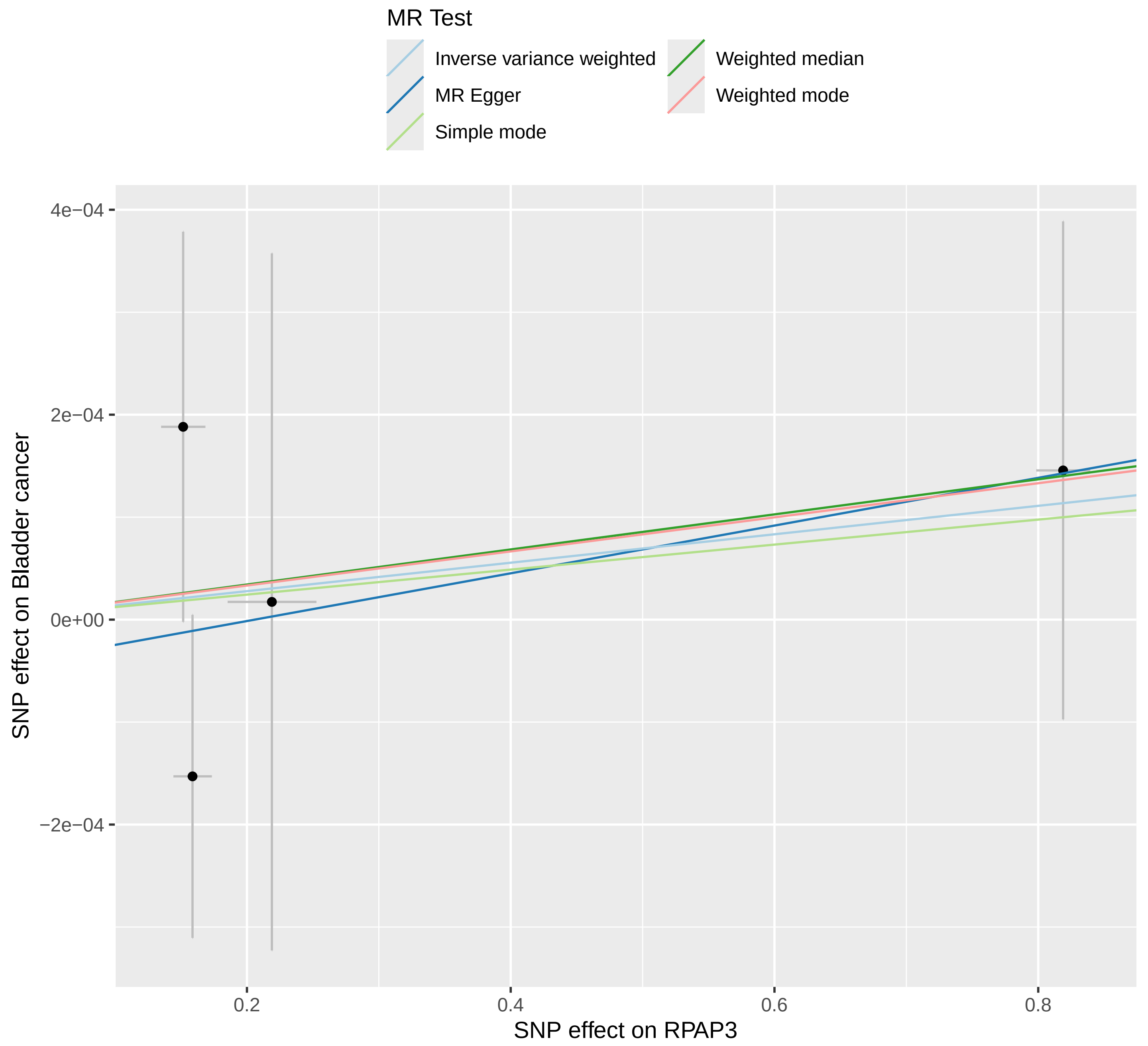
MR Test
Inverse variance weighted
MR Egger
Simple mode
Weighted median
Weighted mode
4e−04
2e−04
0e+00
−2e−04
0.2
0.4
0.6
0.8
SNP effect on Bladder cancer
SNP effect on RPAP3

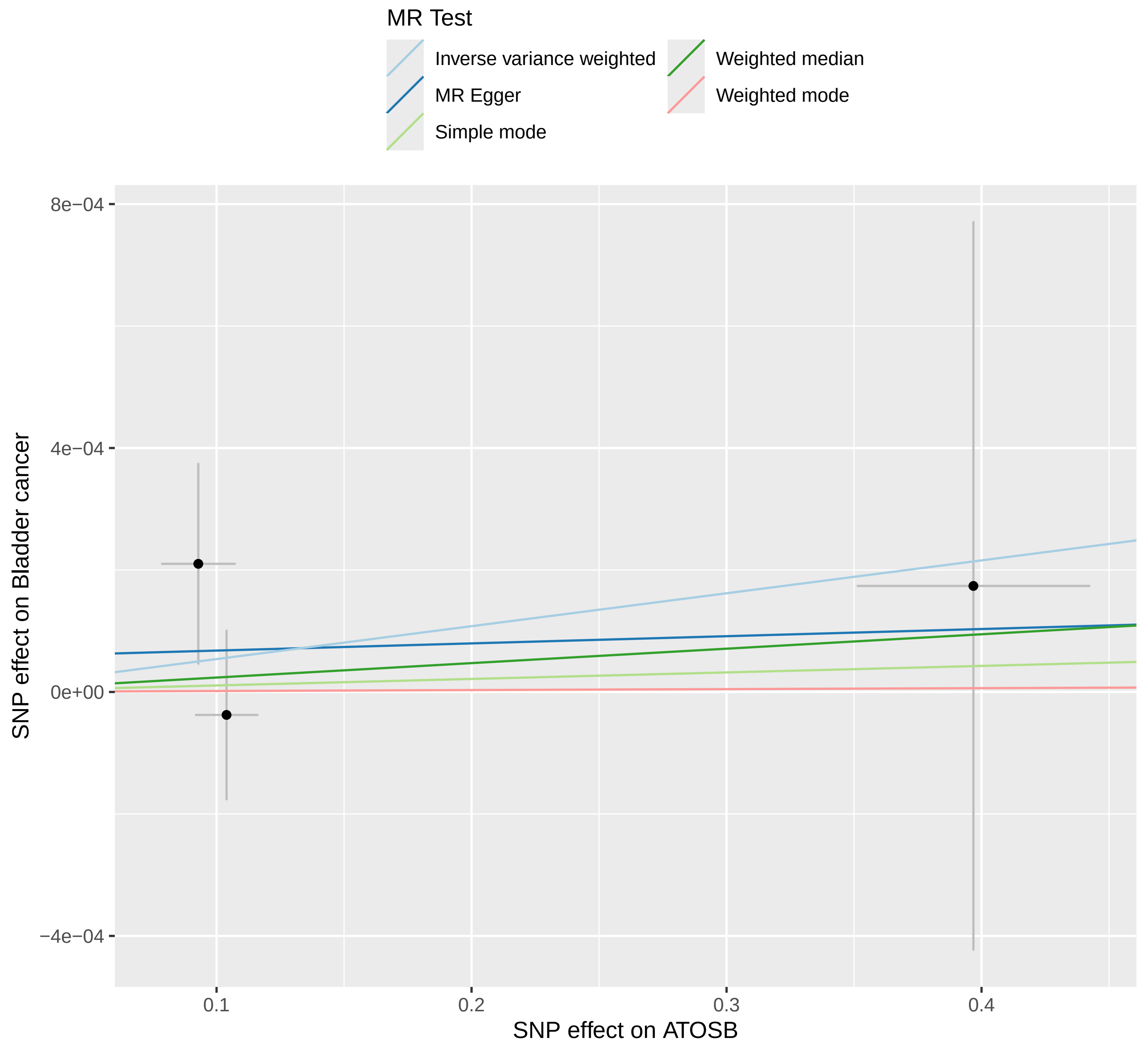
MR Test
Inverse variance weighted
MR Egger
Simple mode
Weighted median
Weighted mode
8e−04
4e−04
0e+00
−4e−04
0.1
0.2
0.3
0.4
SNP effect on Bladder cancer
SNP effect on ATOSB

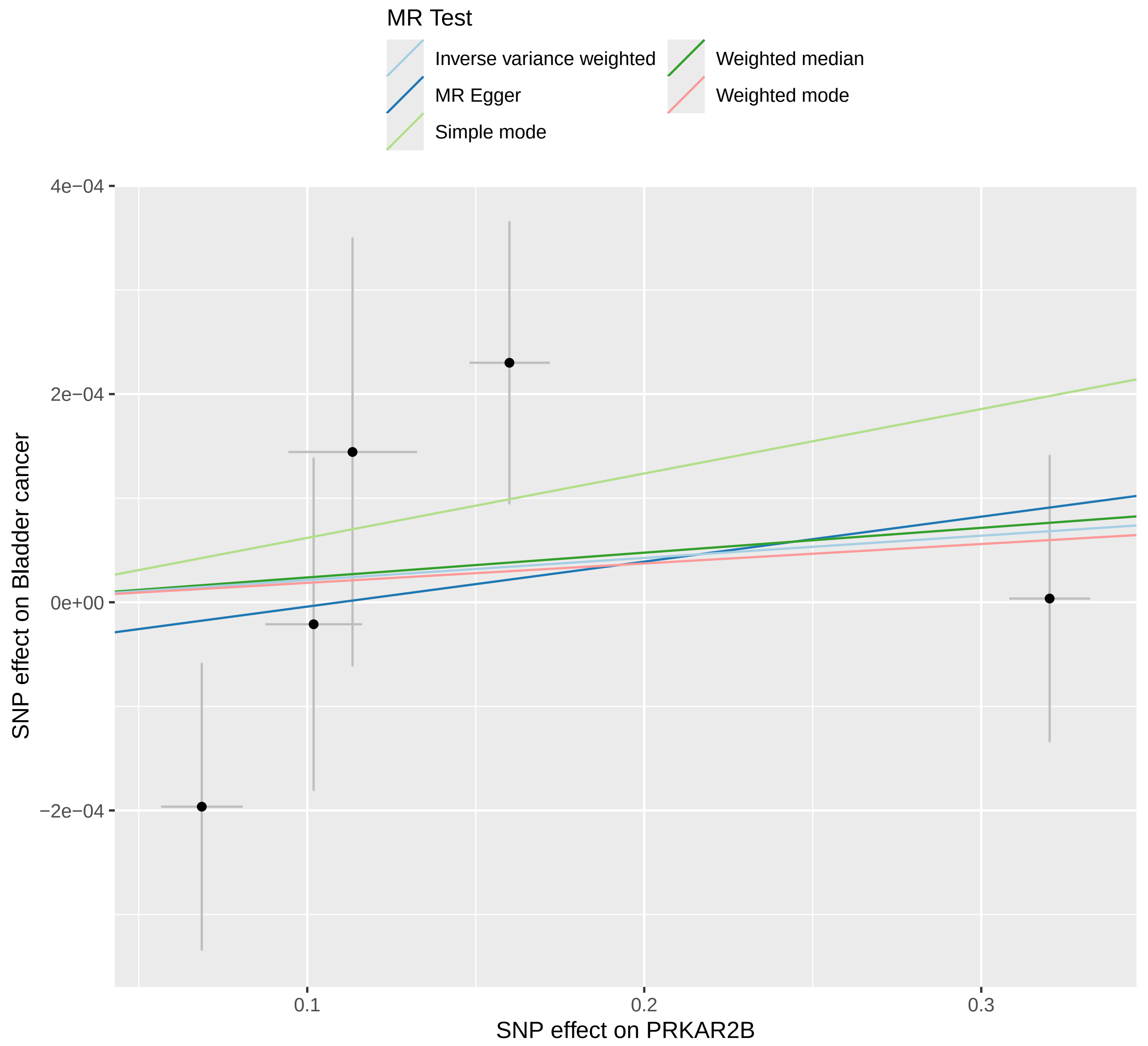
MR Test
Inverse variance weighted
MR Egger
Simple mode
Weighted median
Weighted mode
4e−04
2e−04
0e+00
−2e−04
SNP effect on Bladder cancer
0.1
0.2
0.3
SNP effect on PRKAR2B

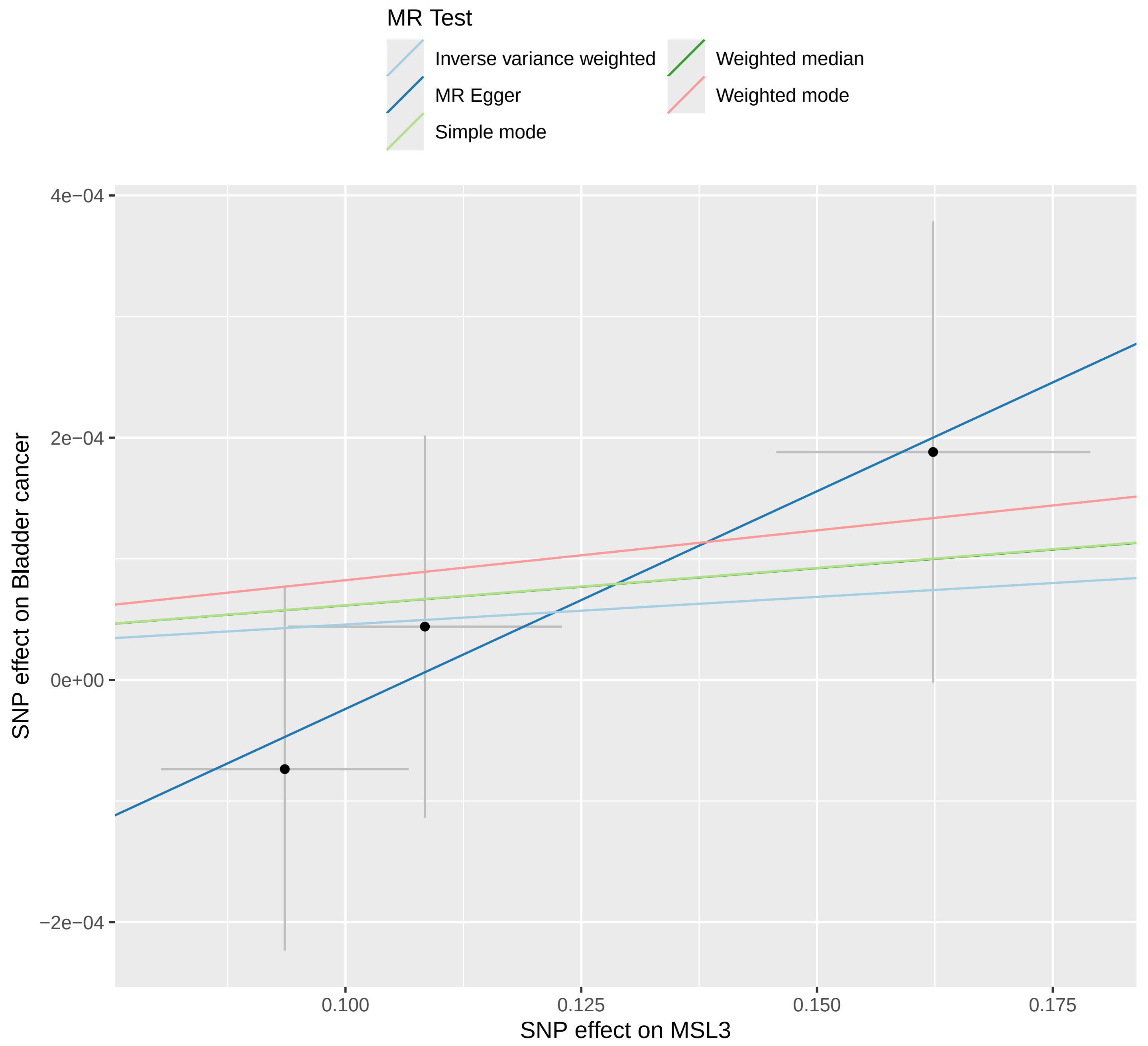

MR Test
Inverse variance weighted
MR Egger
Simple mode
Weighted median
Weighted mode
4e−04
2e−04
0e+00
−2e−04
0.100
0.125
0.150
0.175
SNP effect on MSL3
SNP effect on Bladder cancer

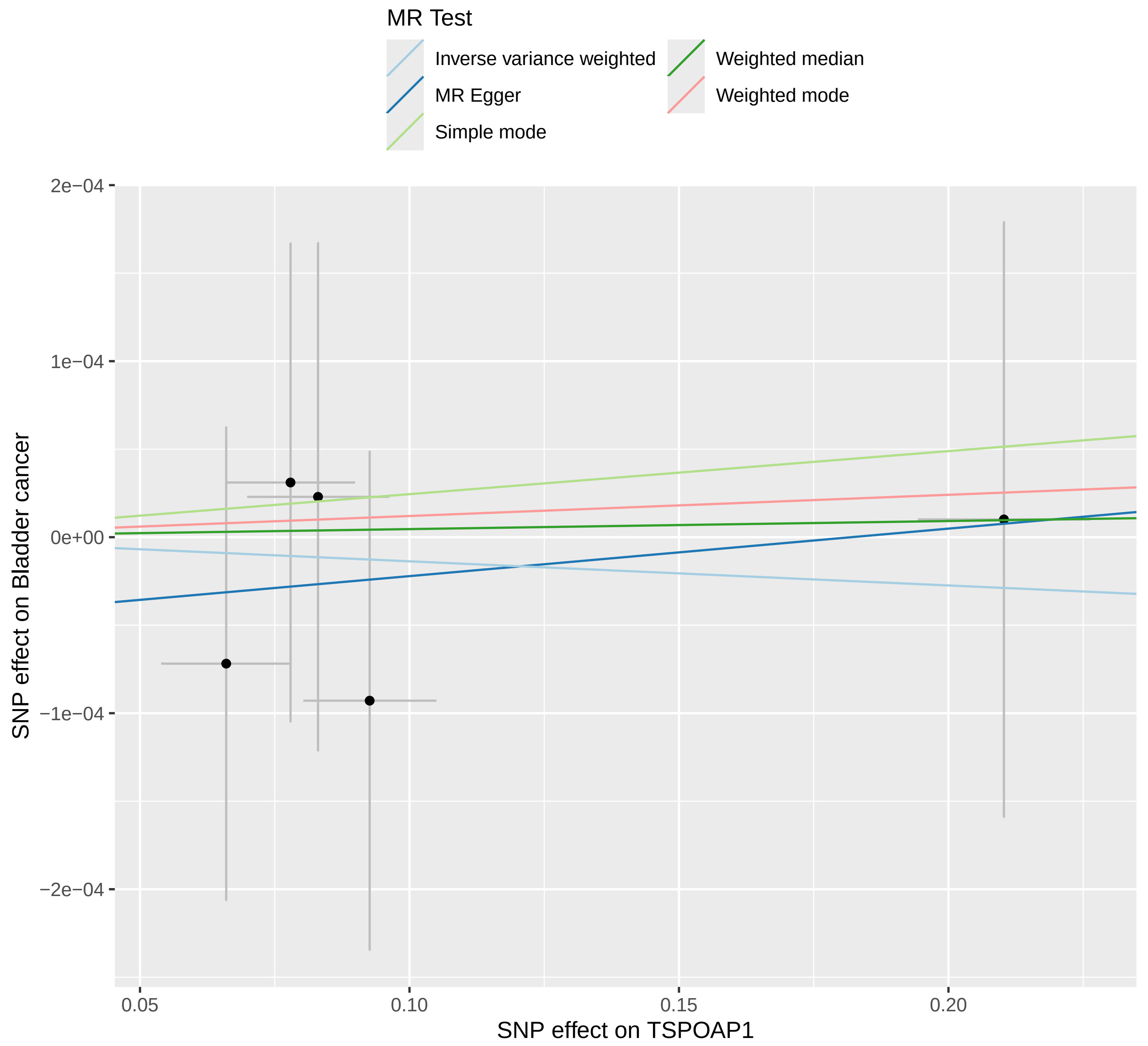
MR Test
Inverse variance weighted
MR Egger
Simple mode
Weighted median
Weighted mode
2e−04
1e−04
0e+00
−1e−04
−2e−04
SNP effect on Bladder cancer
0.05
0.10
0.15
0.20
SNP effect on TSPOAP1

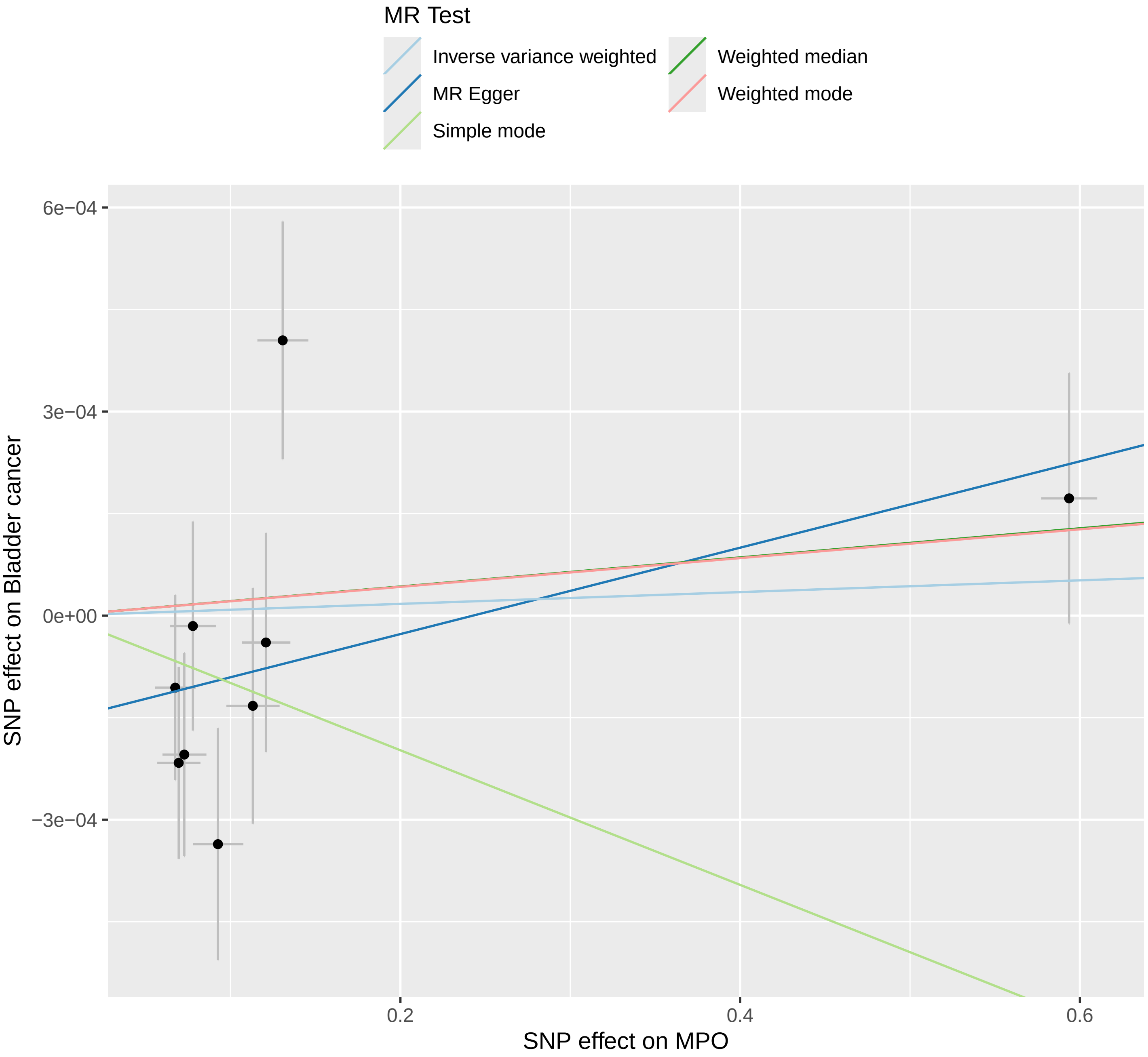

MR Test
Inverse variance weighted
MR Egger
Simple mode
Weighted median
Weighted mode
6e−04
3e−04
0e+00
−3e−04
0.2
0.4
0.6
SNP effect on Bladder cancer
SNP effect on MPO

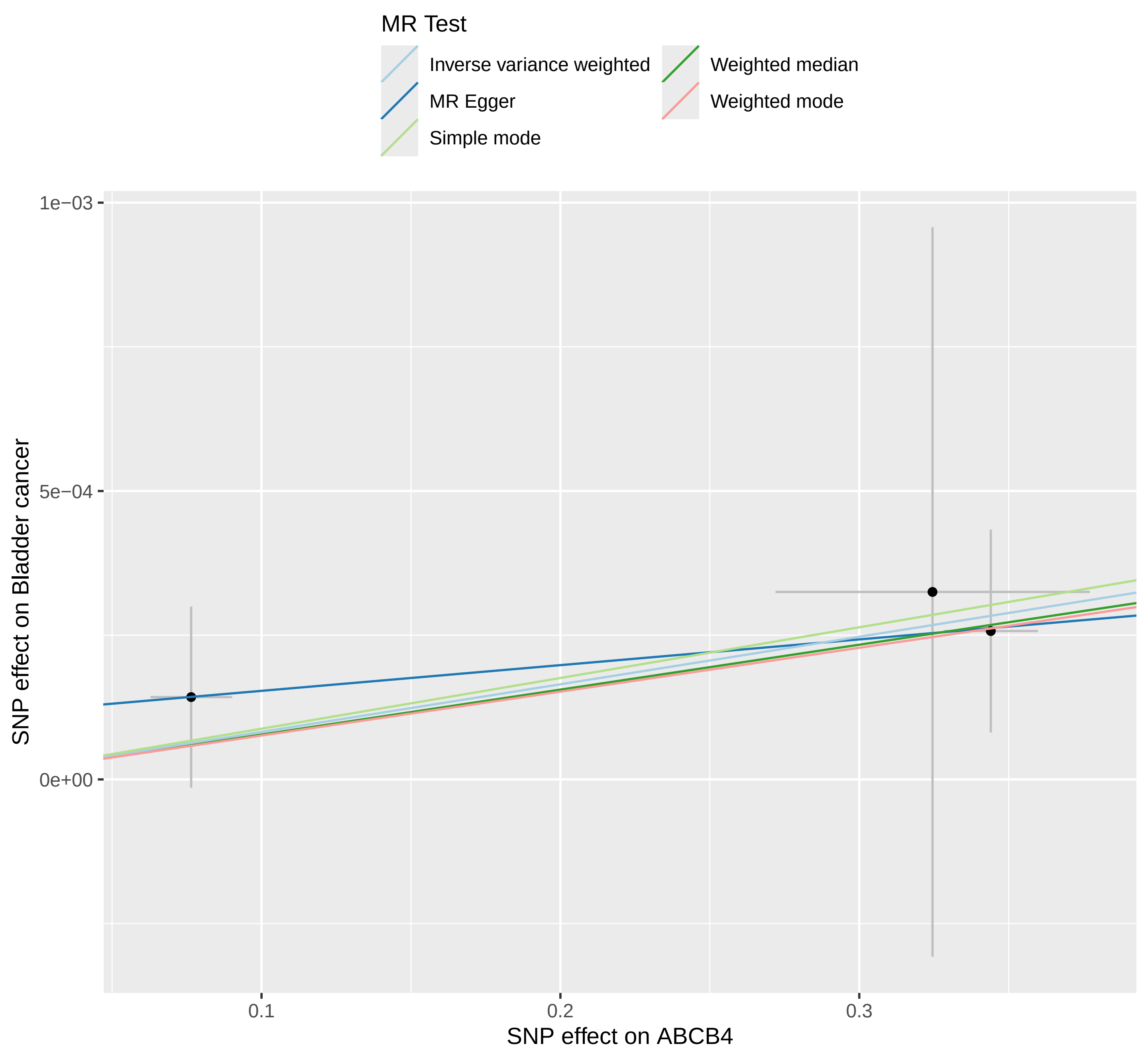
MR Test
Inverse variance weighted
MR Egger
Simple mode
Weighted median
Weighted mode
1e−03
5e−04
0e+00
SNP effect on Bladder cancer
0.1
0.2
0.3
SNP effect on ABCB4

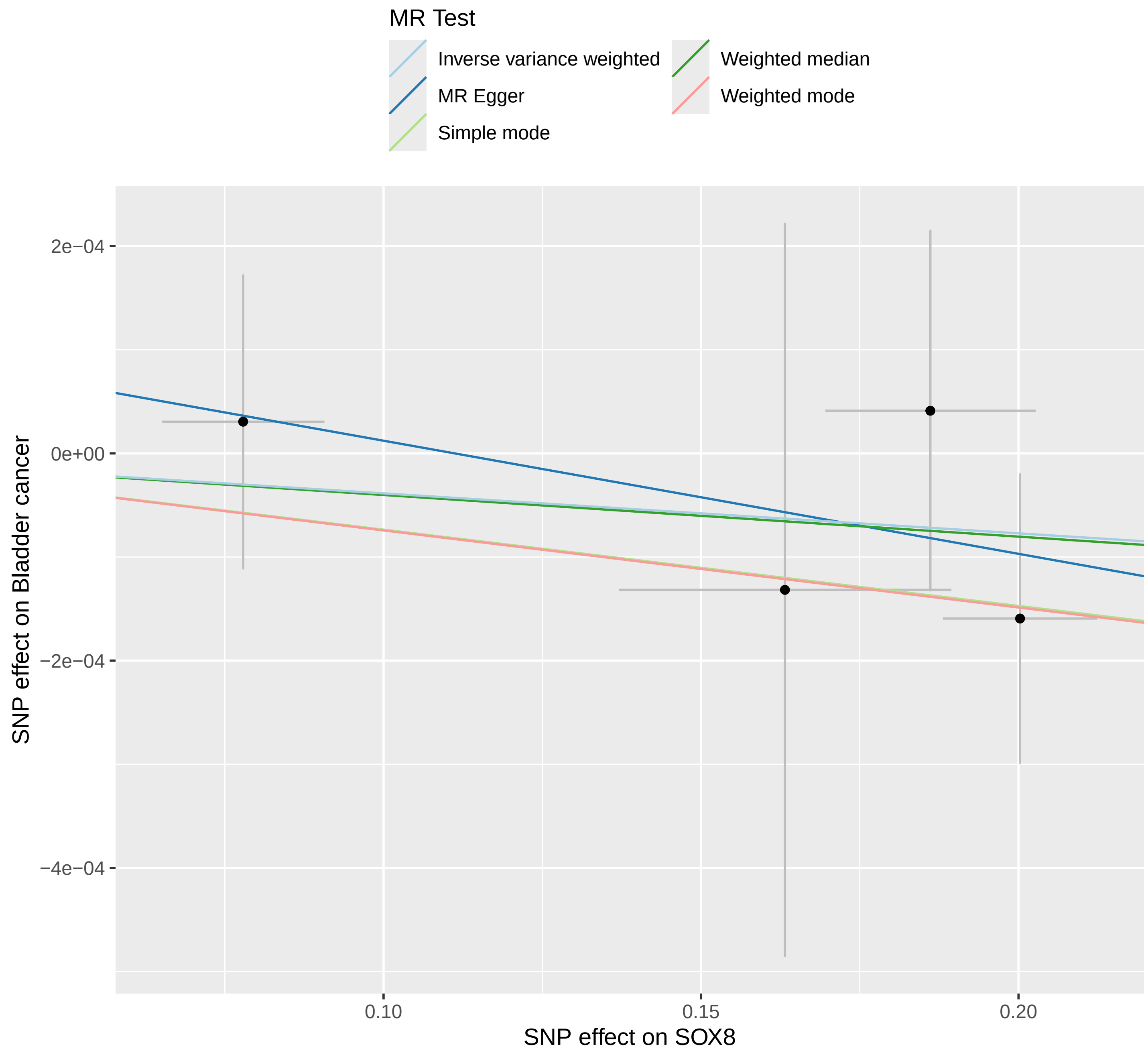
MR Test
Inverse variance weighted
MR Egger
Simple mode
Weighted median
Weighted mode
2e−04
0e+00
−2e−04
−4e−04
SNP effect on Bladder cancer
0.10
0.15
0.20
SNP effect on SOX8

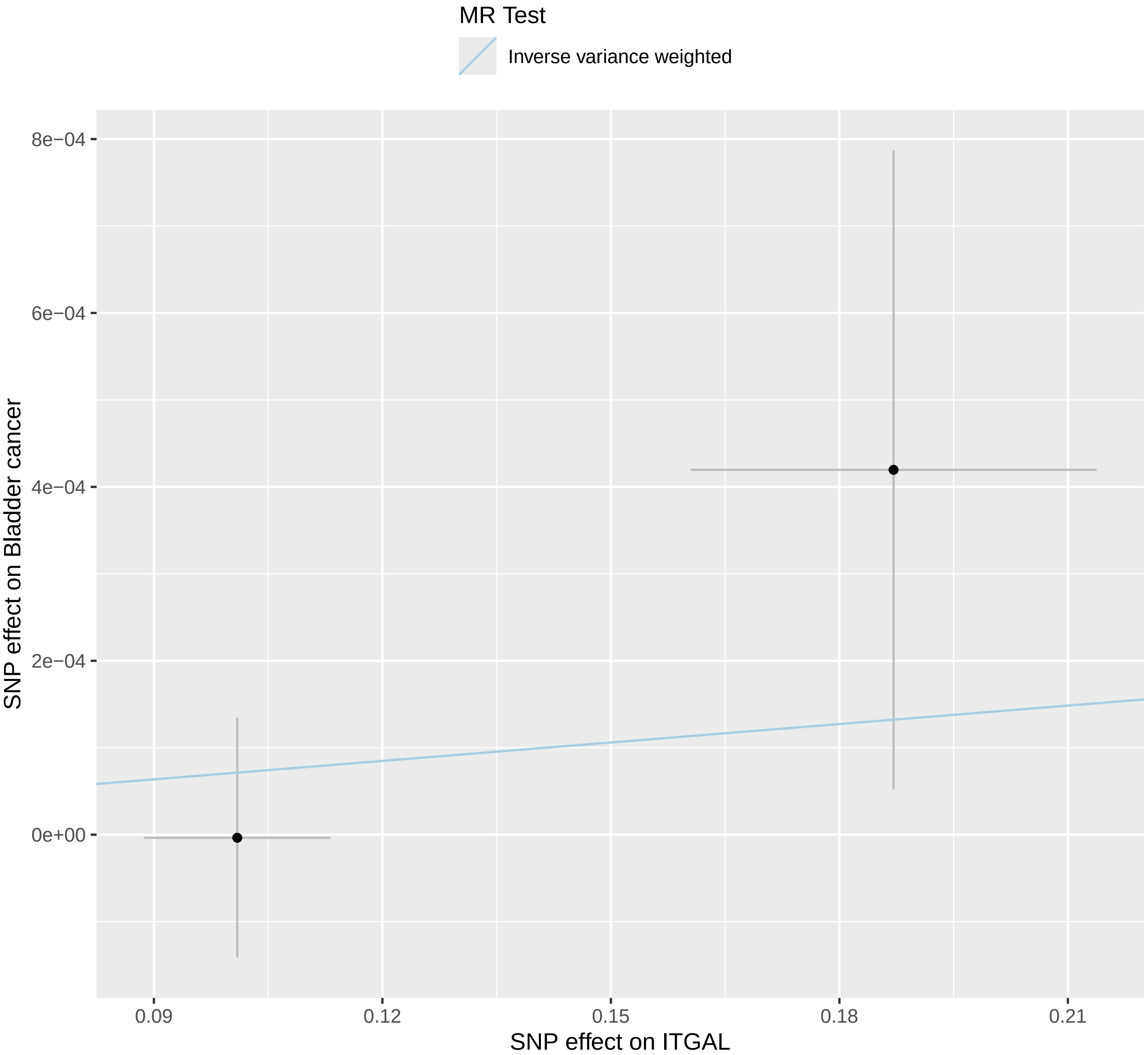

MR Test
Inverse variance weighted
8e−04
6e−04
4e−04
2e−04
0e+00
SNP effect on Bladder cancer
0.09
0.12
0.15
0.18
0.21
SNP effect on ITGAL

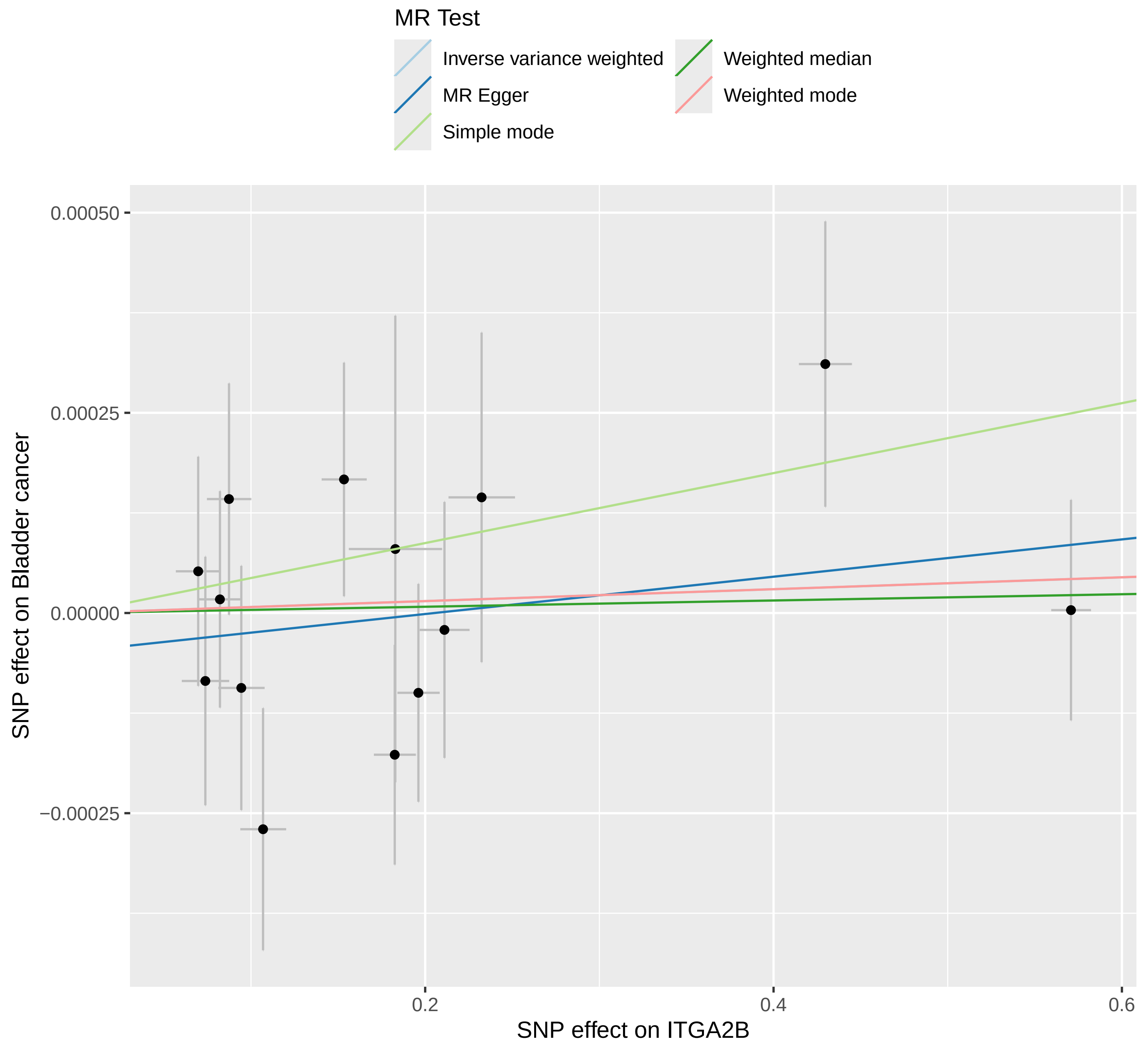
MR Test
Inverse variance weighted
MR Egger
Simple mode
Weighted median
Weighted mode
0.00050
0.00025
0.00000
−0.00025
SNP effect on Bladder cancer
0.2
0.4
0.6
SNP effect on ITGA2B

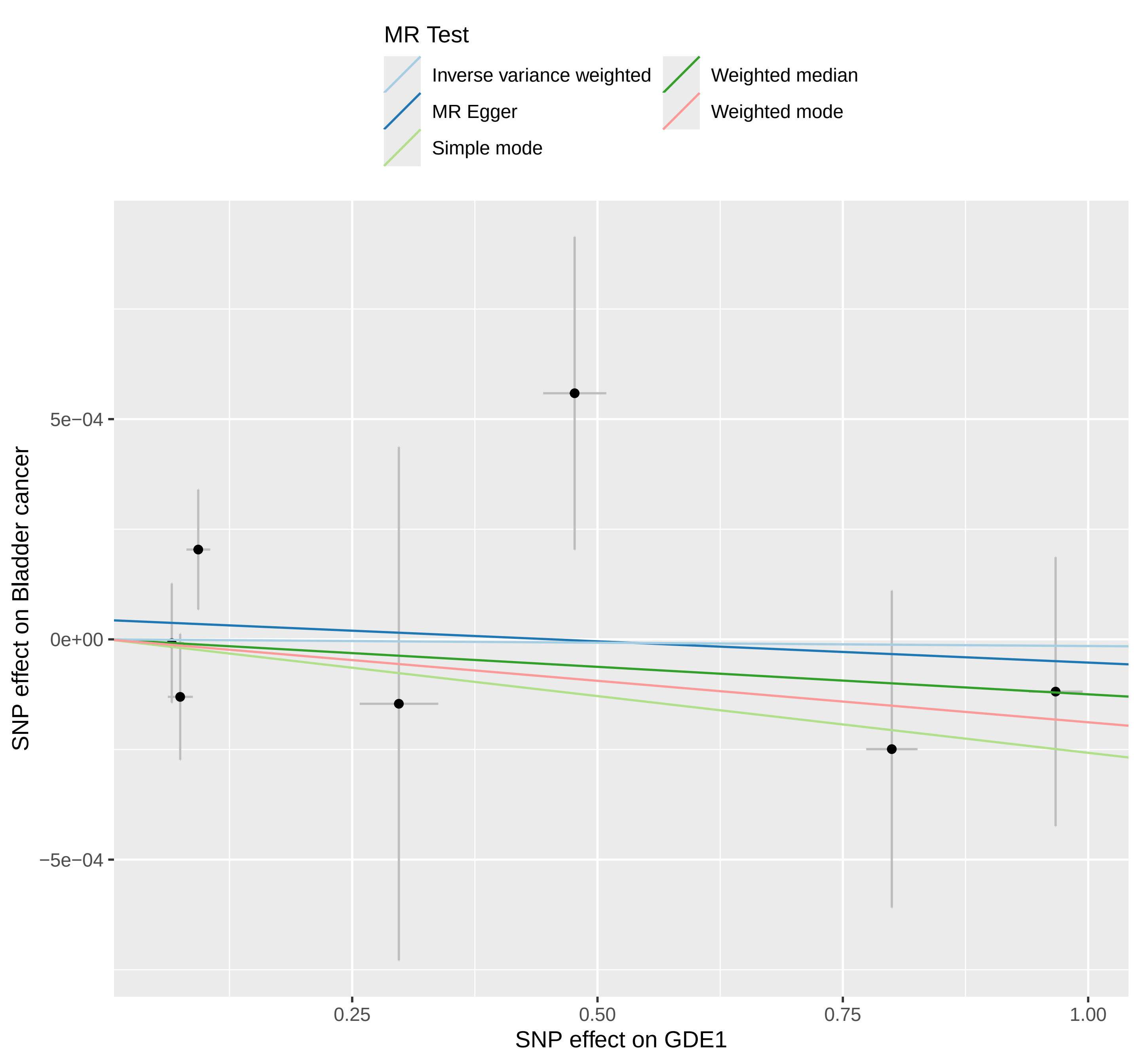

MR Test
Inverse variance weighted
MR Egger
Simple mode
Weighted median
Weighted mode
5e−04
0e+00
−5e−04
SNP effect on Bladder cancer
0.25
0.50
0.75
1.00
SNP effect on GDE1

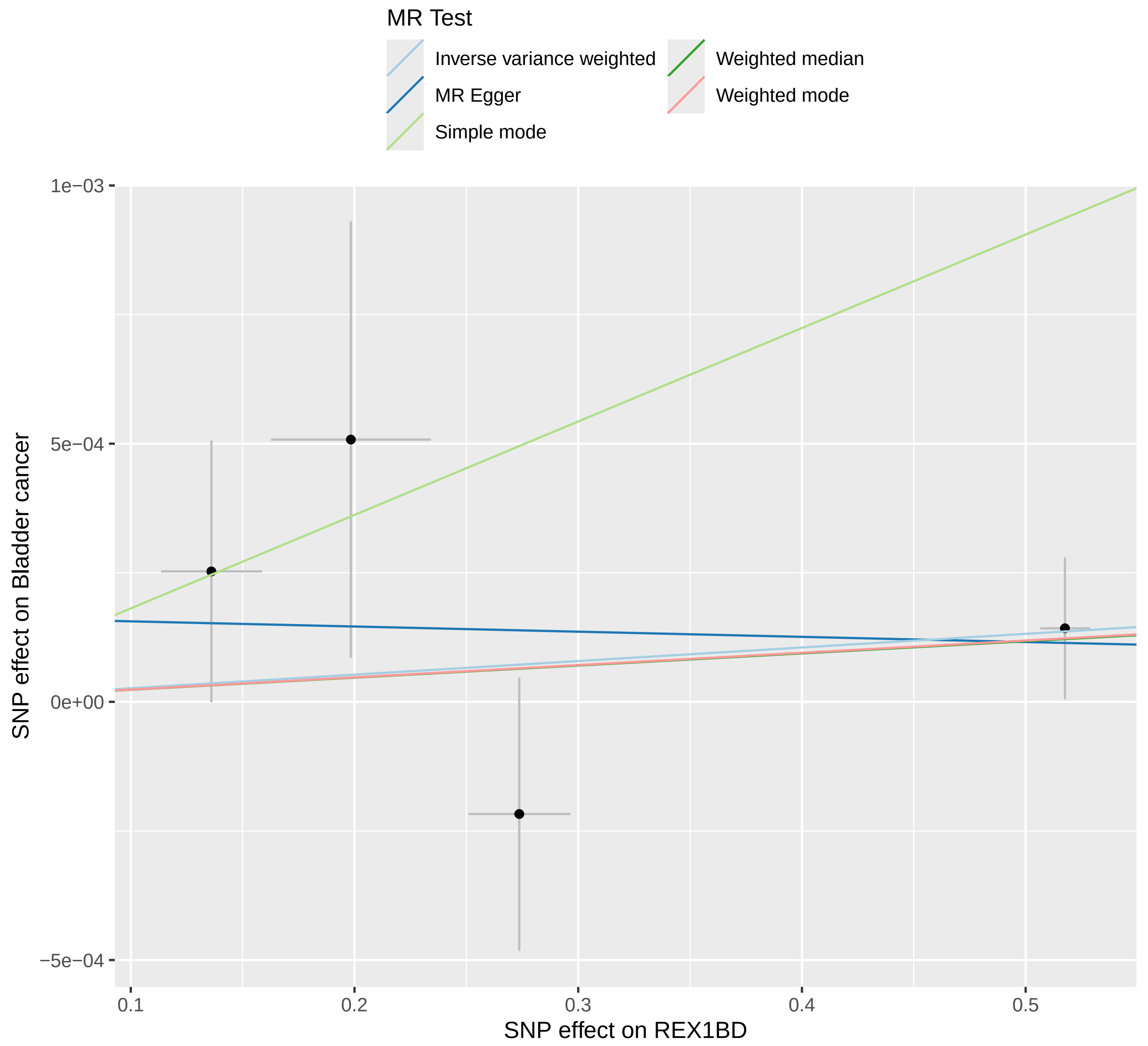
MR Test
Inverse variance weighted
MR Egger
Simple mode
Weighted median
Weighted mode
1e−03
5e−04
0e+00
−5e−04
0.1
0.2
0.3
0.4
0.5
SNP effect on REX1BD
SNP effect on Bladder cancer

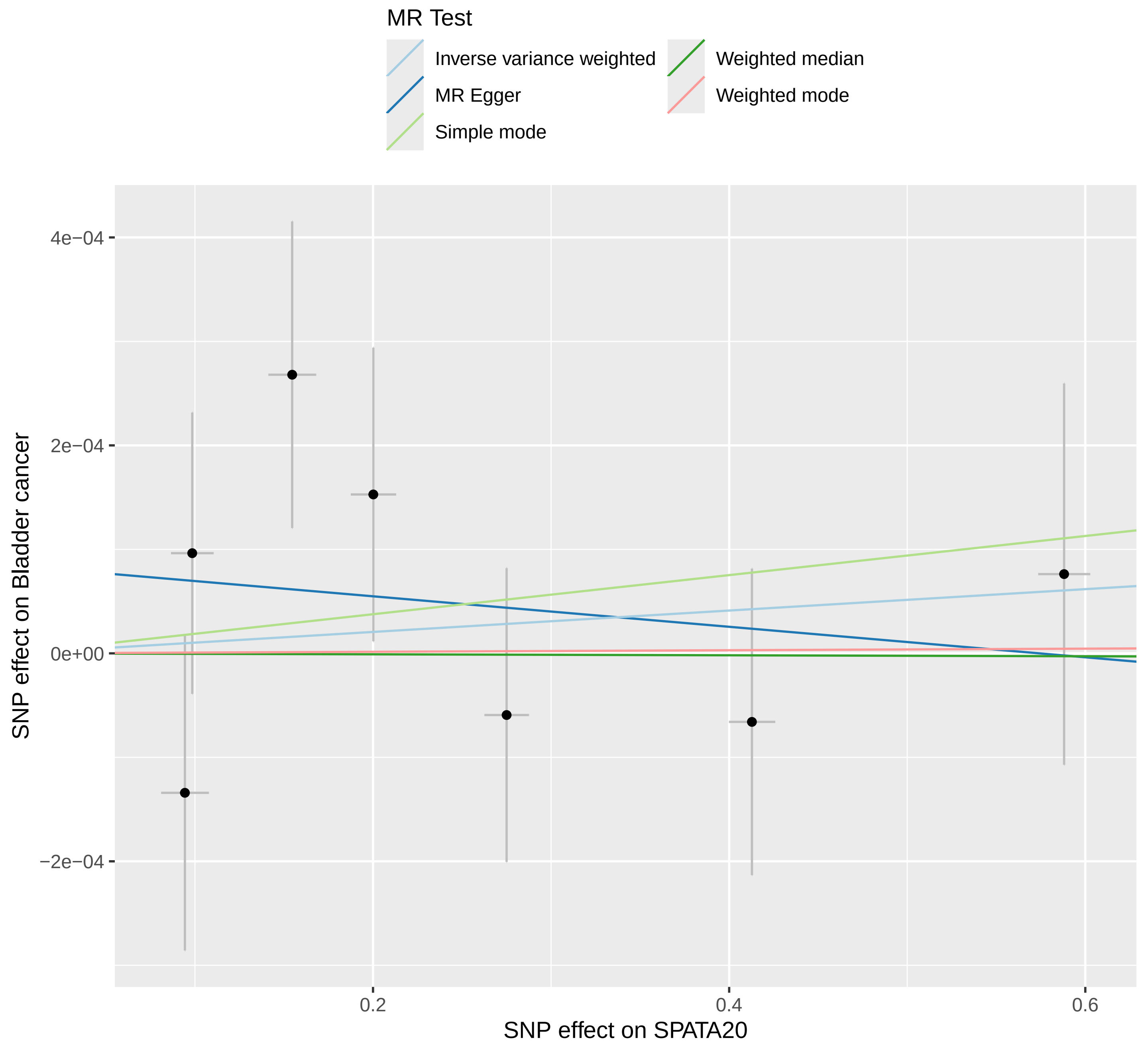

MR Test
Inverse variance weighted
MR Egger
Simple mode
Weighted median
Weighted mode
4e−04
2e−04
0e+00
−2e−04
0.2
0.4
0.6
SNP effect on Bladder cancer
SNP effect on SPATA20

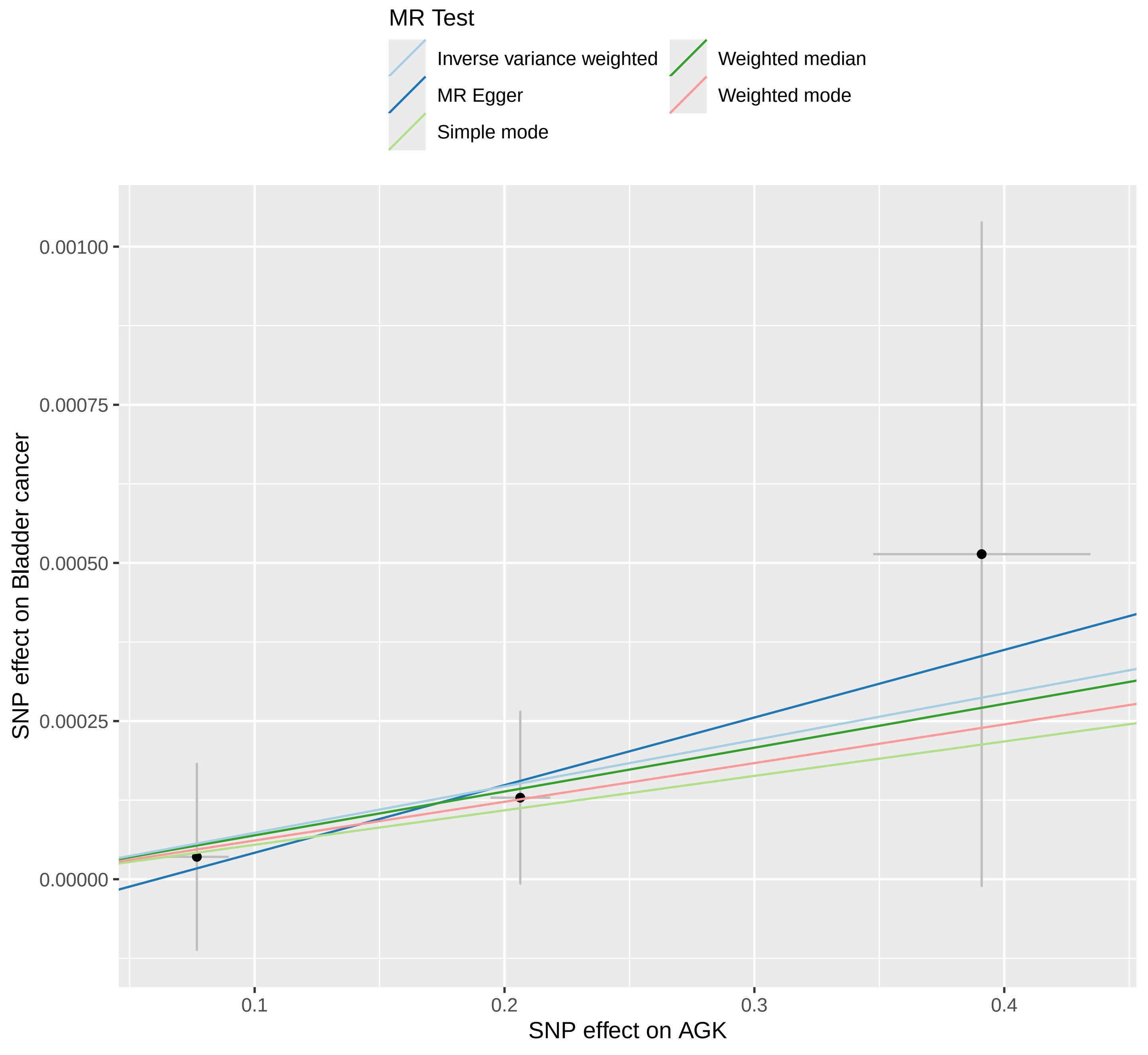

MR Test
Inverse variance weighted
MR Egger
Simple mode
Weighted median
Weighted mode
0.00100
0.00075
0.00050
0.00025
0.00000
SNP effect on Bladder cancer
0.1
0.2
0.3
0.4
SNP effect on AGK

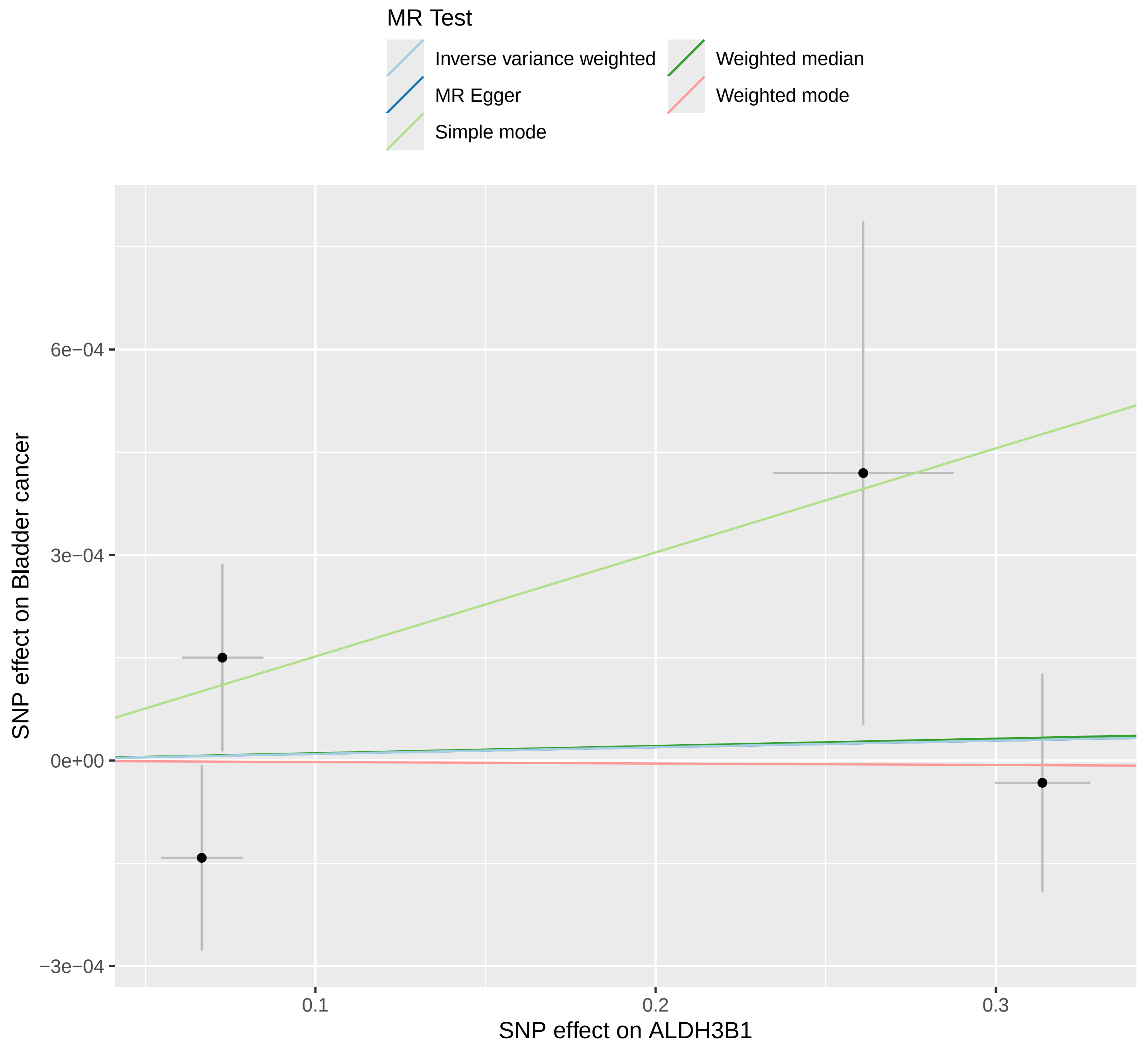

MR Test
Inverse variance weighted
MR Egger
Simple mode
Weighted median
Weighted mode
6e−04
3e−04
0e+00
−3e−04
0.1
0.2
0.3
SNP effect on Bladder cancer
SNP effect on ALDH3B1

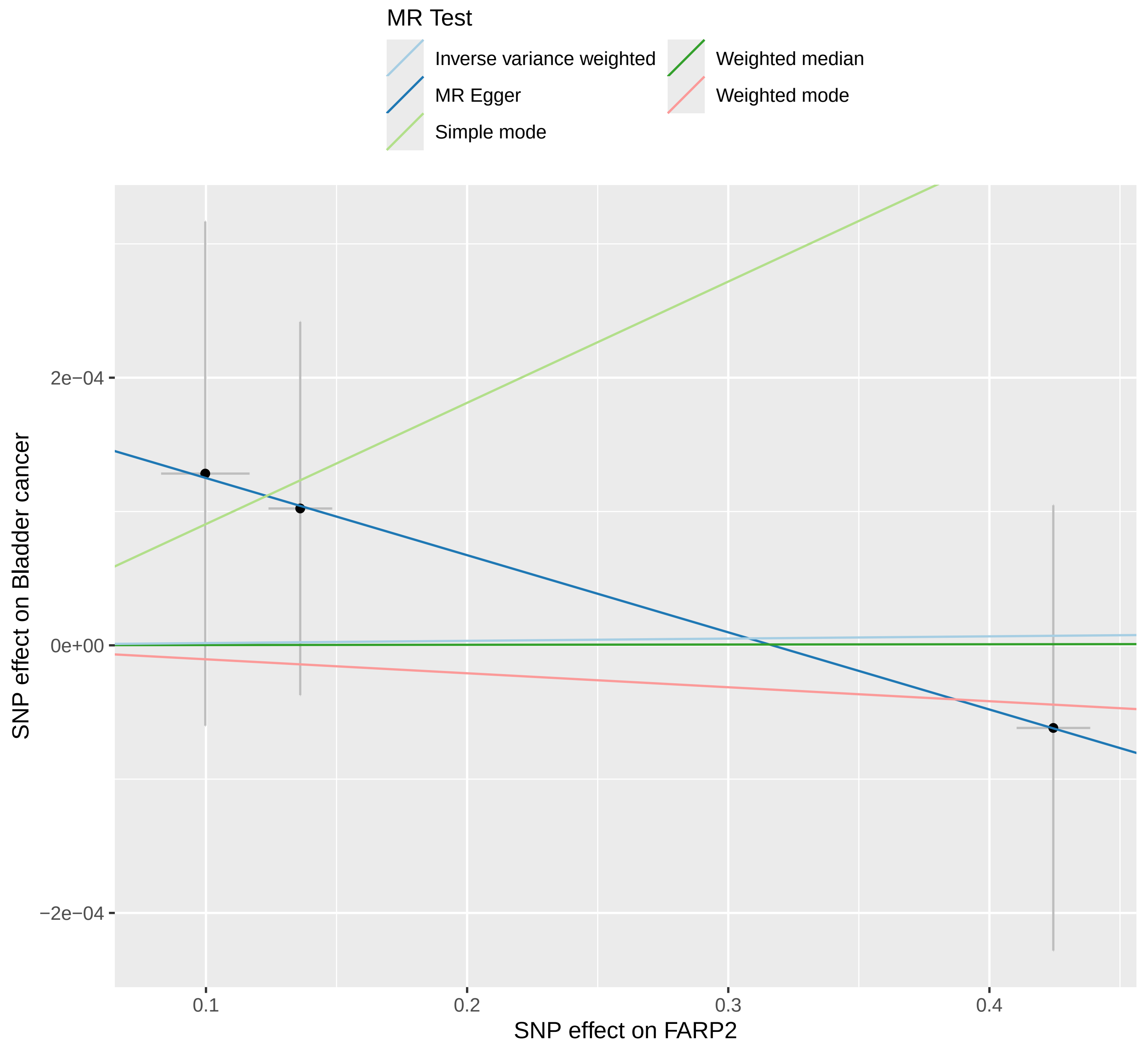
MR Test
Inverse variance weighted
MR Egger
Simple mode
Weighted median
Weighted mode
2e−04
0e+00
−2e−04
SNP effect on Bladder cancer
0.1
0.2
0.3
0.4
SNP effect on FARP2

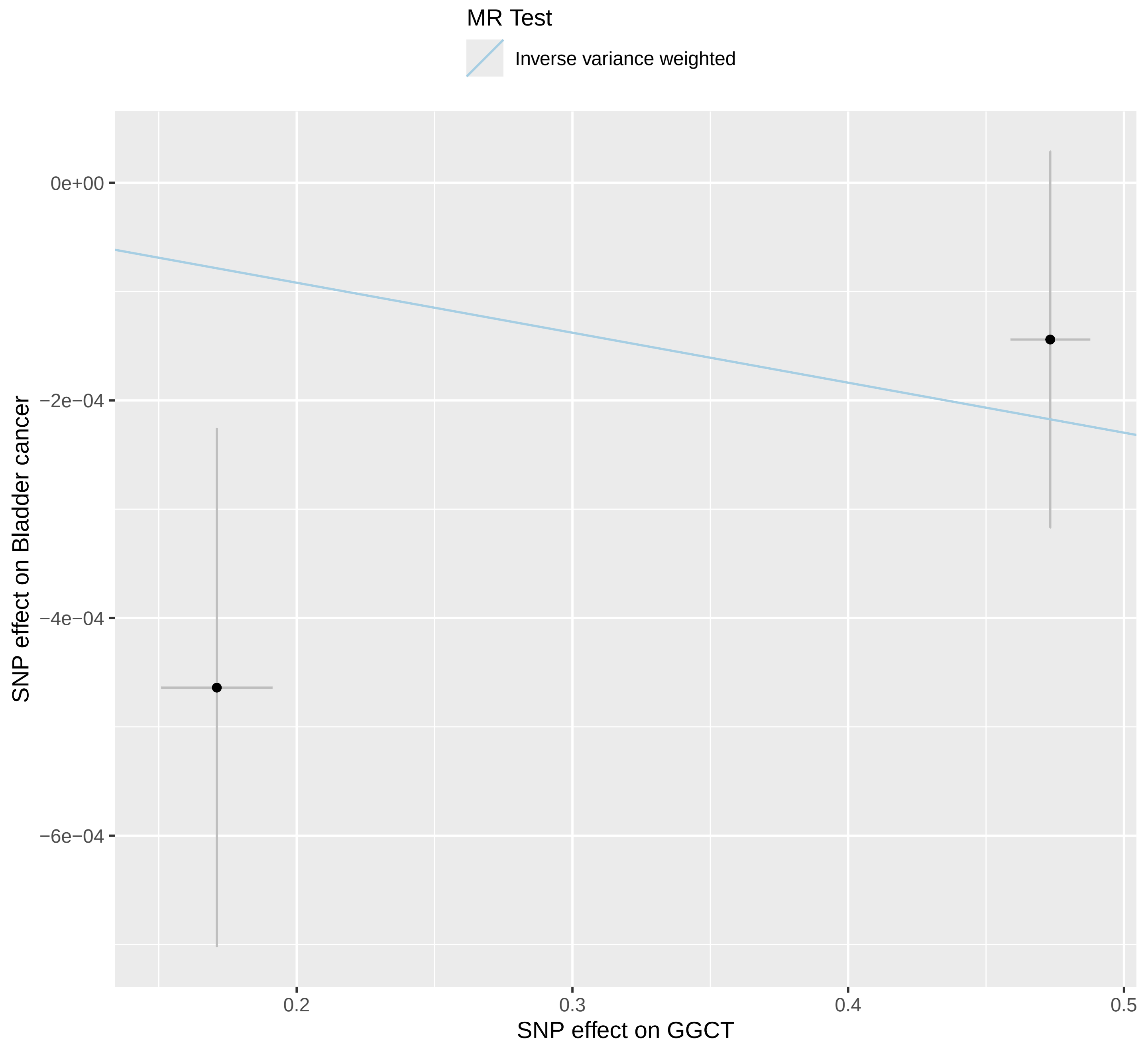

MR Test
Inverse variance weighted
0e+00
−2e−04
−4e−04
−6e−04
SNP effect on Bladder cancer
0.2
0.3
0.4
0.5
SNP effect on GGCT

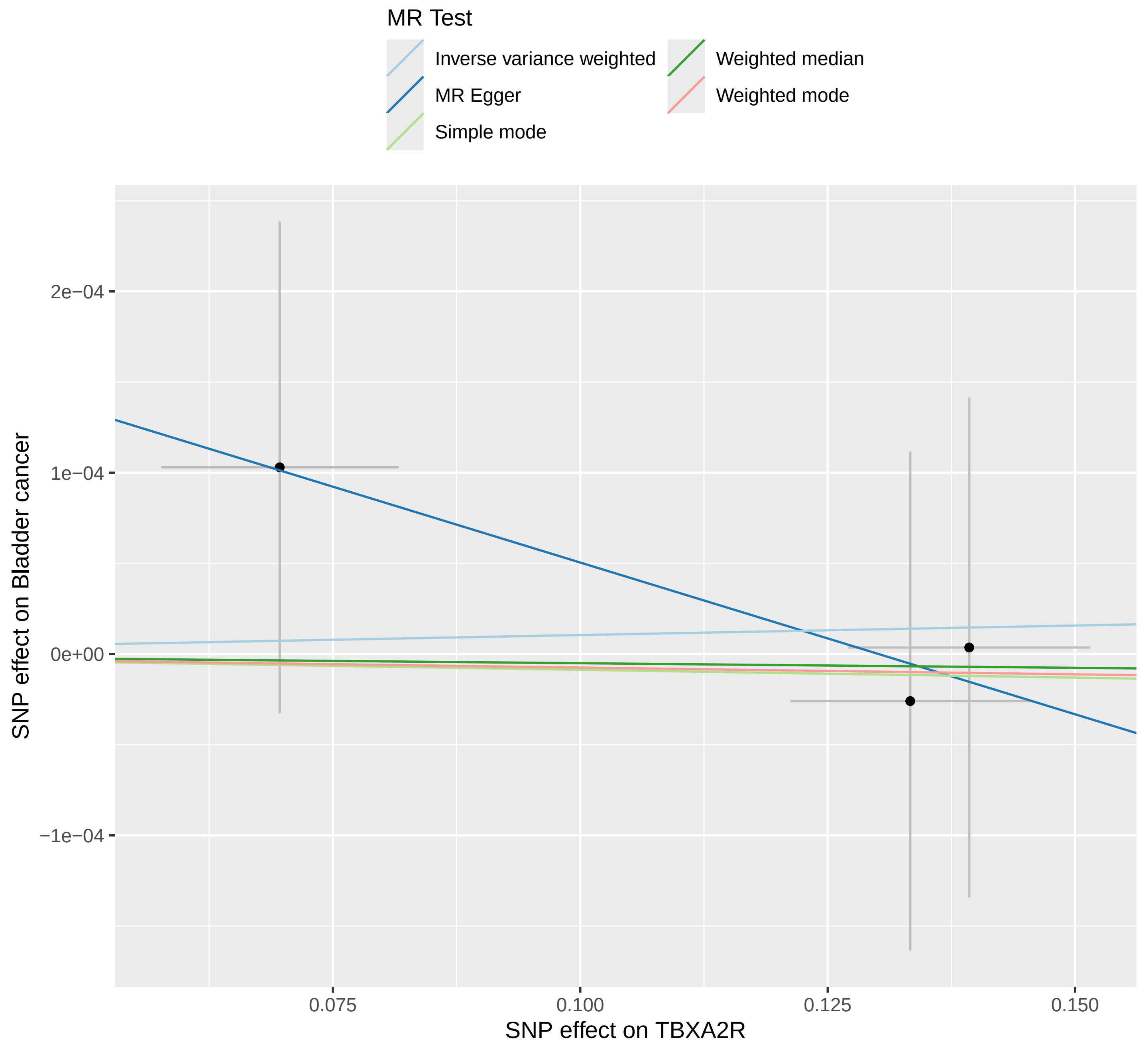
MR Test
Inverse variance weighted
MR Egger
Simple mode
Weighted median
Weighted mode
2e−04
1e−04
0e+00
−1e−04
SNP effect on Bladder cancer
0.075
0.100
0.125
0.150
SNP effect on TBXA2R

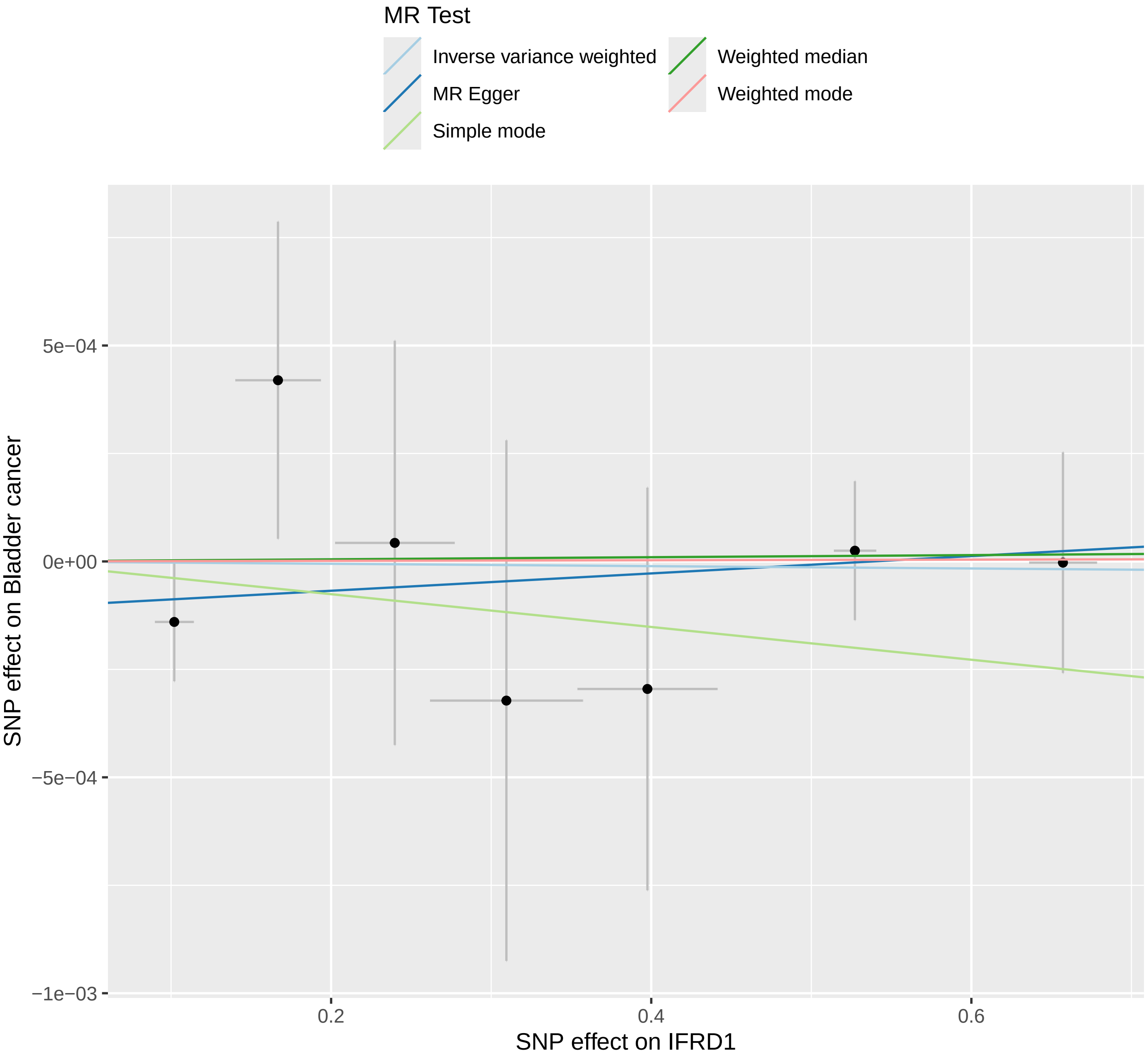

MR Test
Inverse variance weighted
MR Egger
Simple mode
Weighted median
Weighted mode
5e−04
0e+00
−5e−04
−1e−03
SNP effect on Bladder cancer
0.2
0.4
0.6
SNP effect on IFRD1

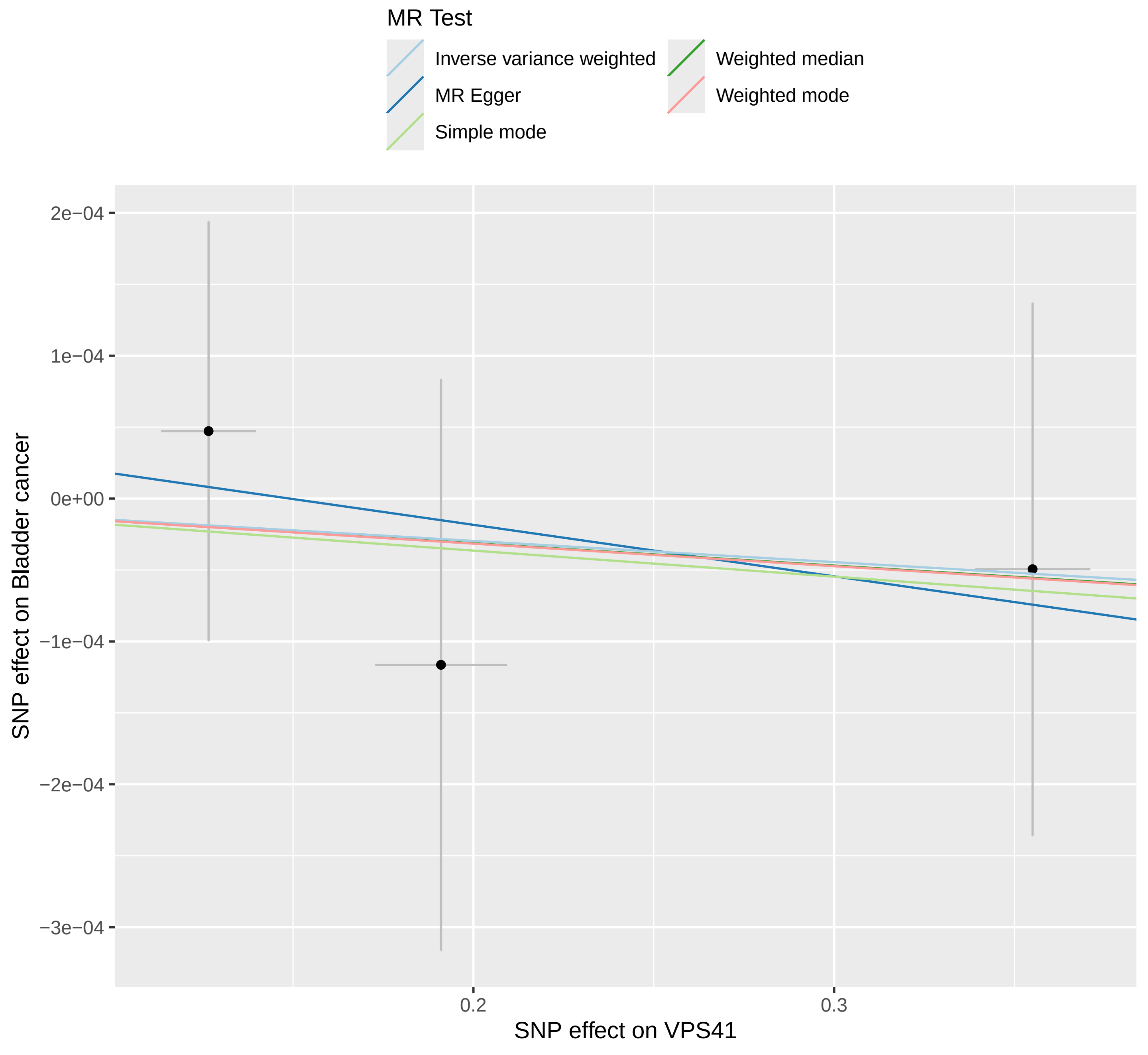
MR Test
Inverse variance weighted
MR Egger
Simple mode
Weighted median
Weighted mode
2e−04
1e−04
0e+00
−1e−04
−2e−04
−3e−04
0.2
0.3
SNP effect on VPS41
SNP effect on Bladder cancer

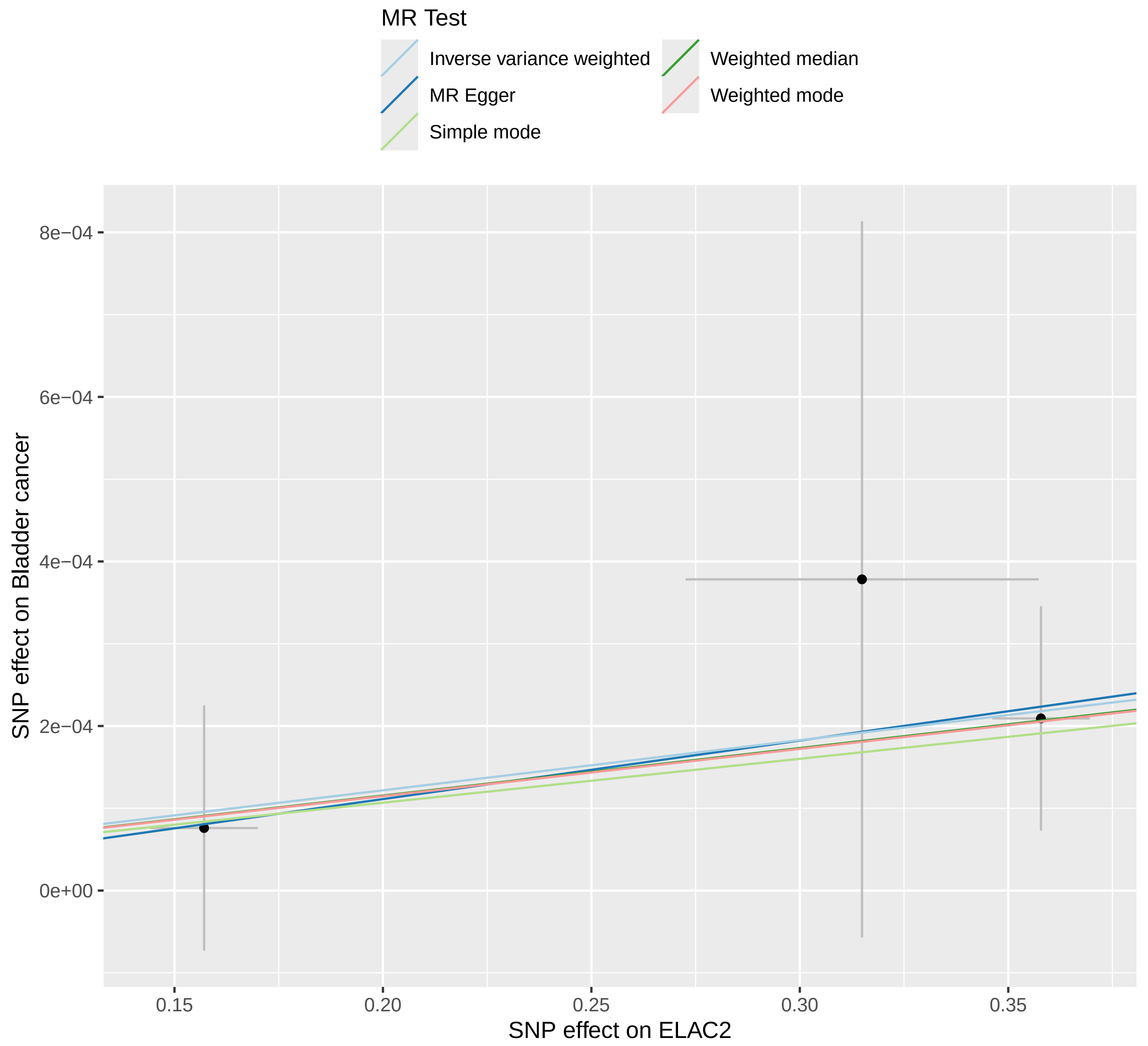
MR Test
Inverse variance weighted
MR Egger
Simple mode
Weighted median
Weighted mode
8e−04
6e−04
4e−04
2e−04
0e+00
SNP effect on Bladder cancer
0.15
0.20
0.25
0.30
0.35
SNP effect on ELAC2

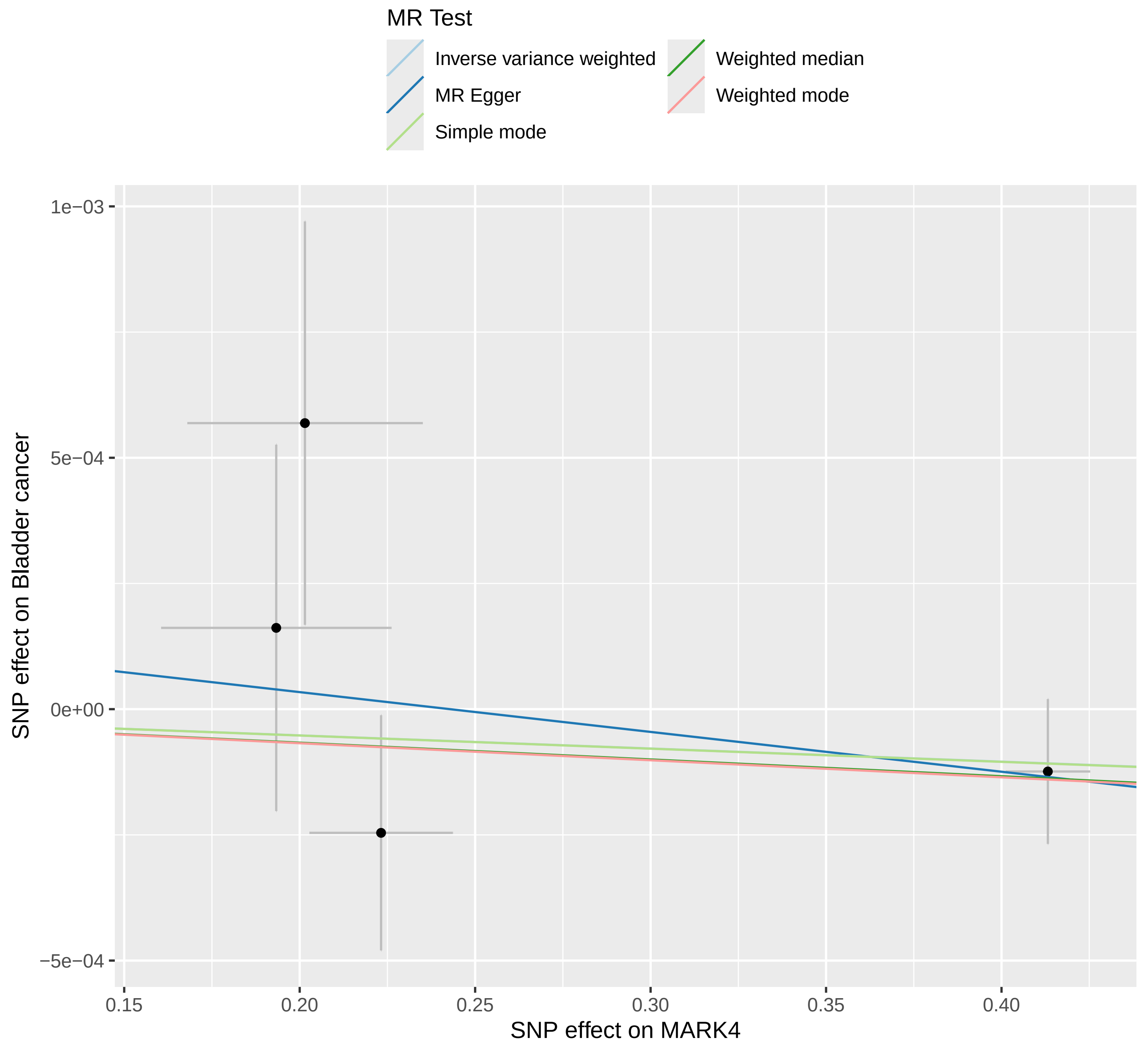
MR Test
Inverse variance weighted
MR Egger
Simple mode
Weighted median
Weighted mode
1e−03
5e−04
0e+00
−5e−04
0.15
0.20
0.25
0.30
0.35
0.40
SNP effect on Bladder cancer
SNP effect on MARK4

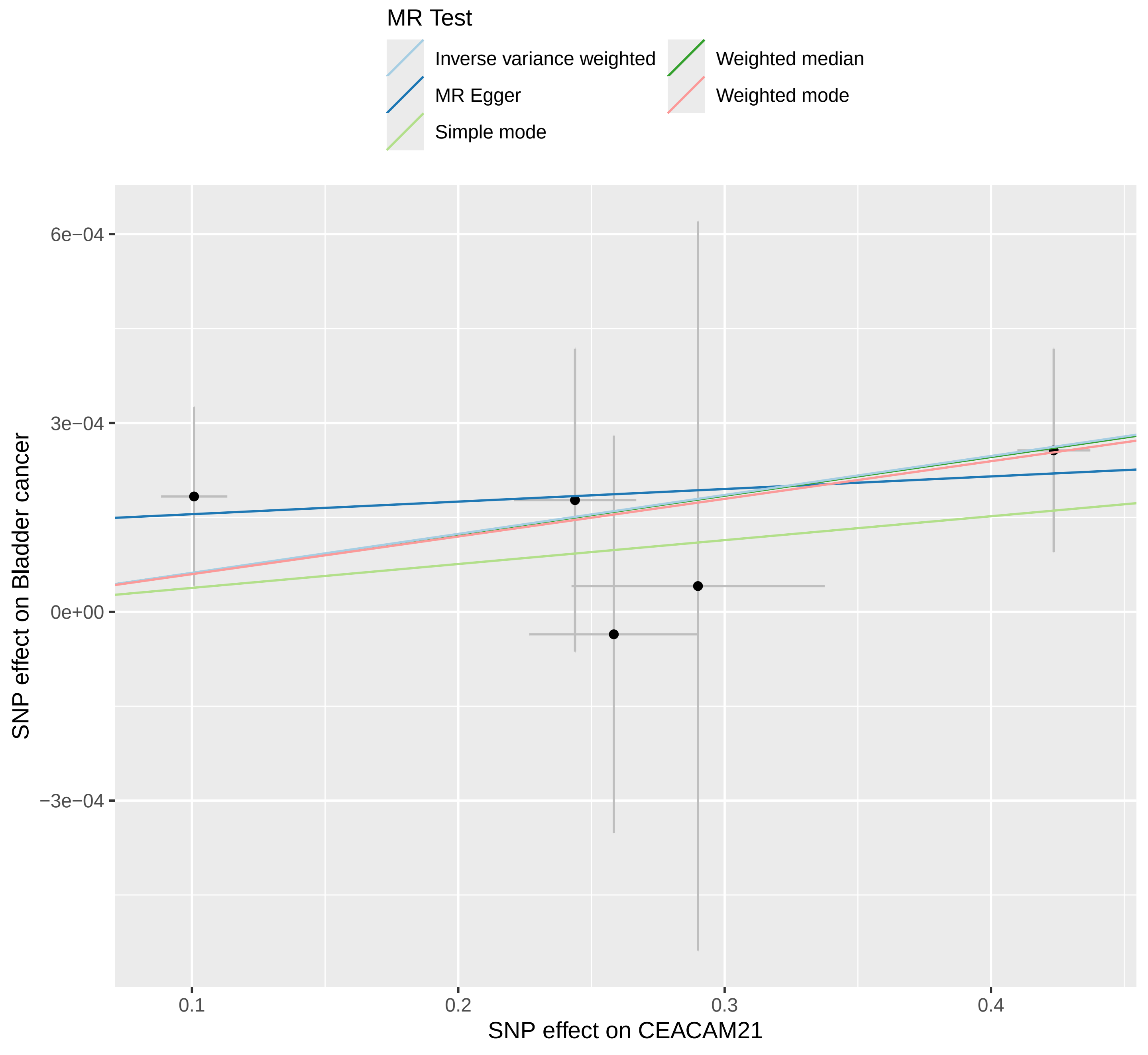
MR Test
Inverse variance weighted
MR Egger
Simple mode
Weighted median
Weighted mode
6e−04
3e−04
0e+00
−3e−04
0.1
0.2
0.3
0.4
SNP effect on Bladder cancer
SNP effect on CEACAM21

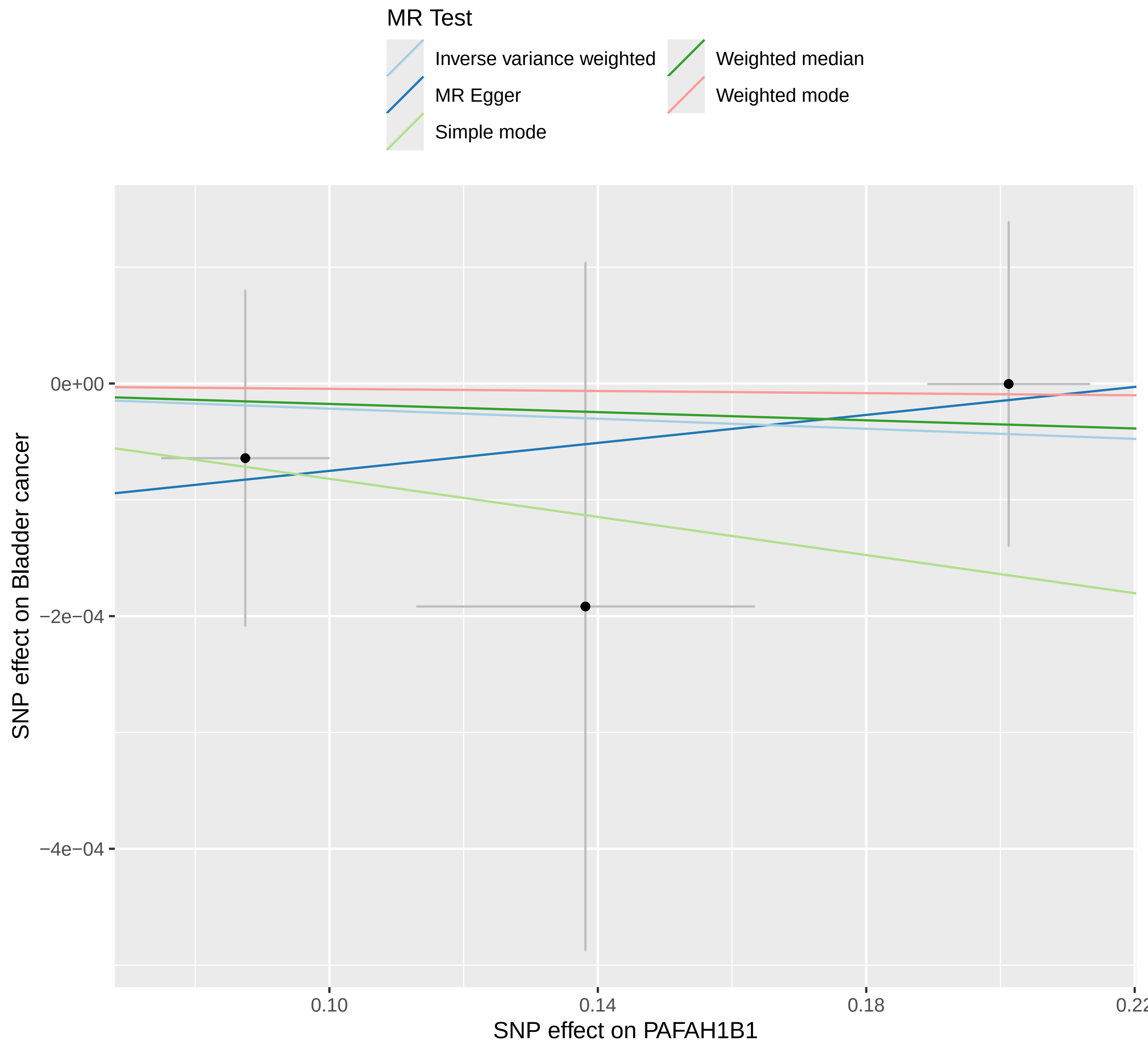

MR Test
Inverse variance weighted
MR Egger
Simple mode
Weighted median
Weighted mode
0e+00
−2e−04
−4e−04
SNP effect on Bladder cancer
0.10
0.14
0.18
0.22
SNP effect on PAFAH1B1

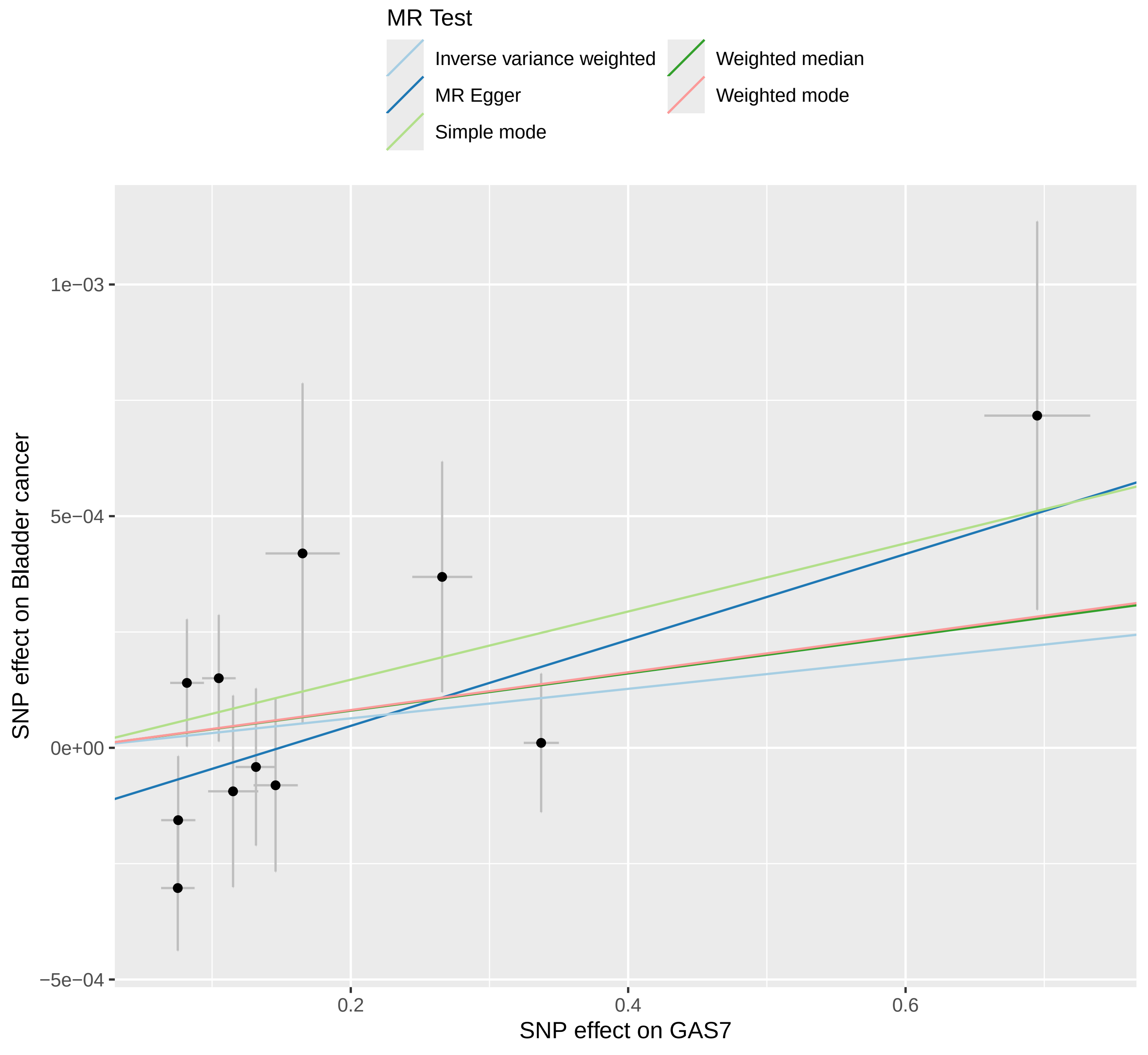

MR Test
Inverse variance weighted
MR Egger
Simple mode
Weighted median
Weighted mode
SNP effect on Bladder cancer
1e−03
5e−04
0e+00
−5e−04
0.2
0.4
0.6
SNP effect on GAS7

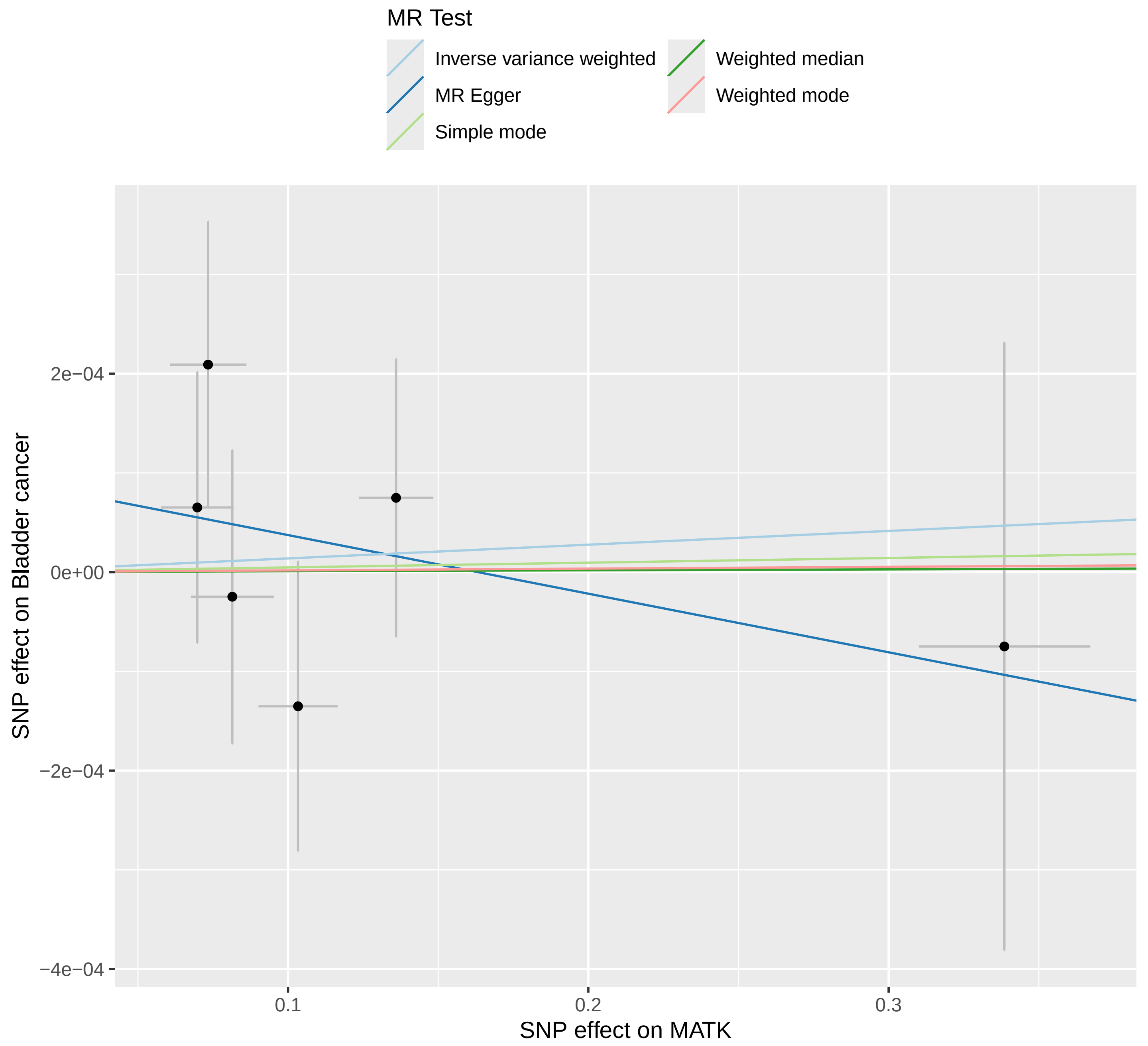

MR Test
Inverse variance weighted
MR Egger
Simple mode
Weighted median
Weighted mode
2e−04
0e+00
−2e−04
−4e−04
0.1
0.2
0.3
SNP effect on Bladder cancer
SNP effect on MATK

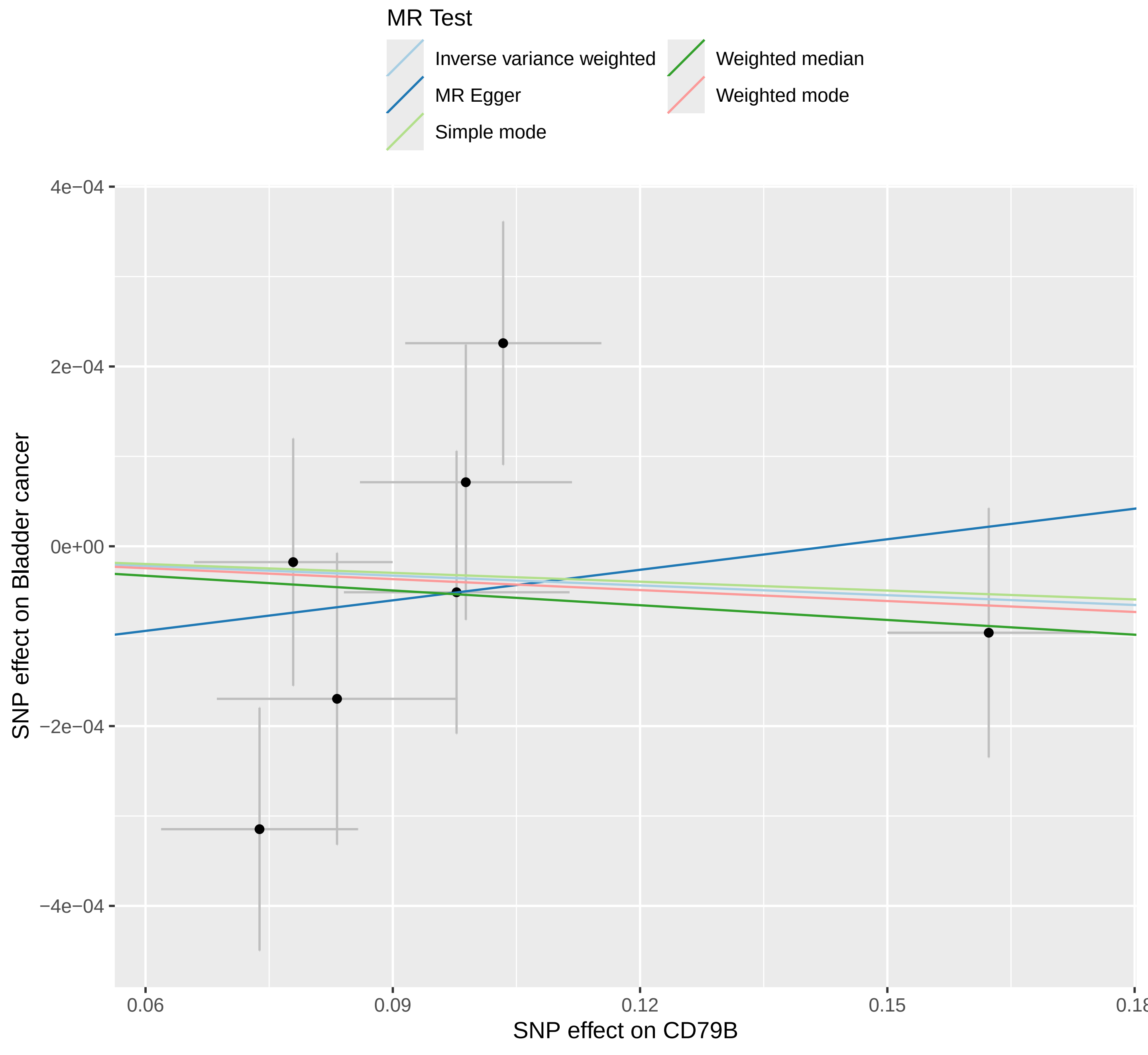

MR Test
Inverse variance weighted
MR Egger
Simple mode
Weighted median
Weighted mode
4e−04
2e−04
0e+00
−2e−04
−4e−04
SNP effect on Bladder cancer
0.06
0.09
0.12
0.15
0.18
SNP effect on CD79B

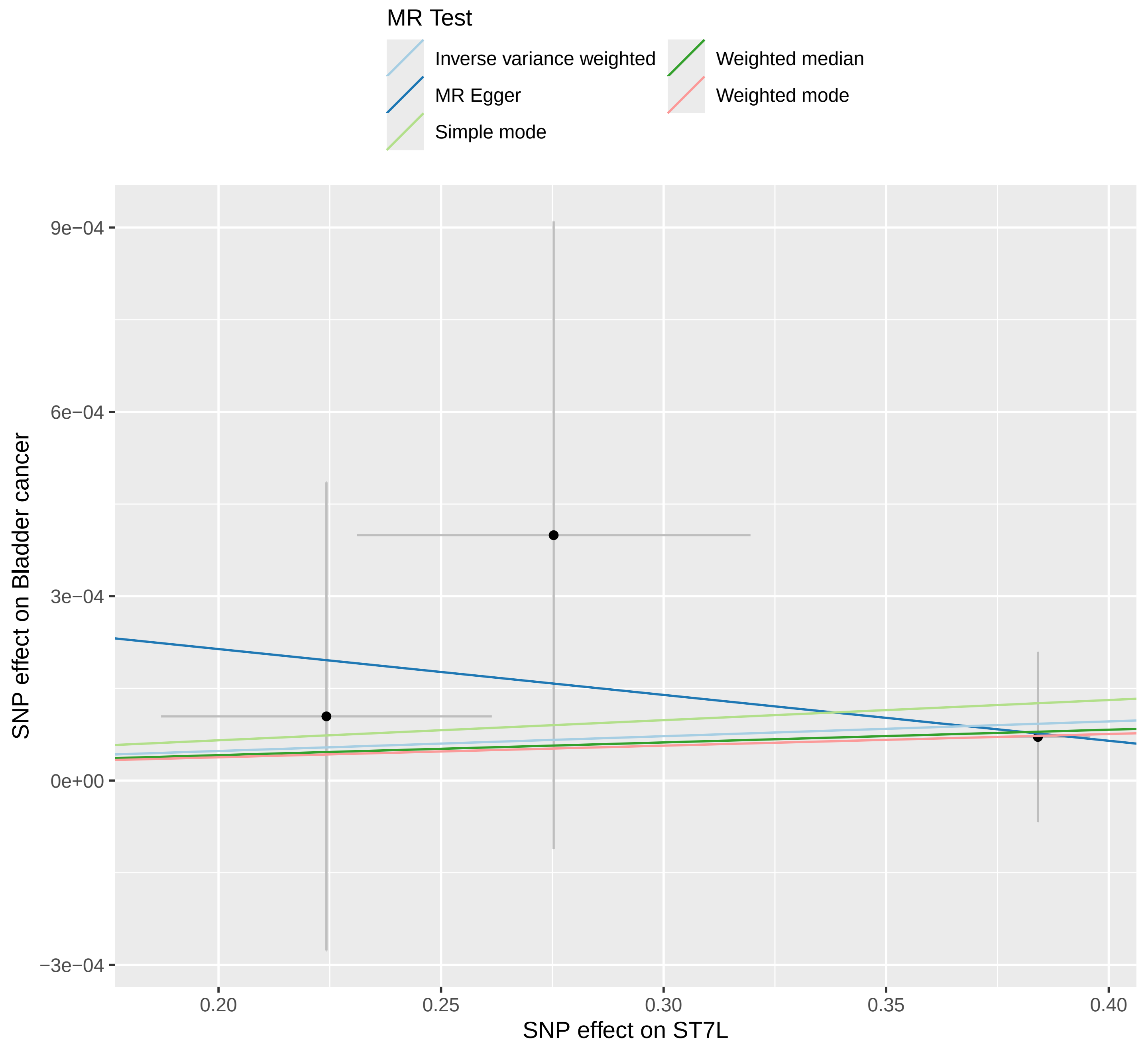

MR Test
Inverse variance weighted
MR Egger
Simple mode
Weighted median
Weighted mode
9e−04
6e−04
3e−04
0e+00
−3e−04
SNP effect on Bladder cancer
0.20
0.25
0.30
0.35
0.40
SNP effect on ST7L

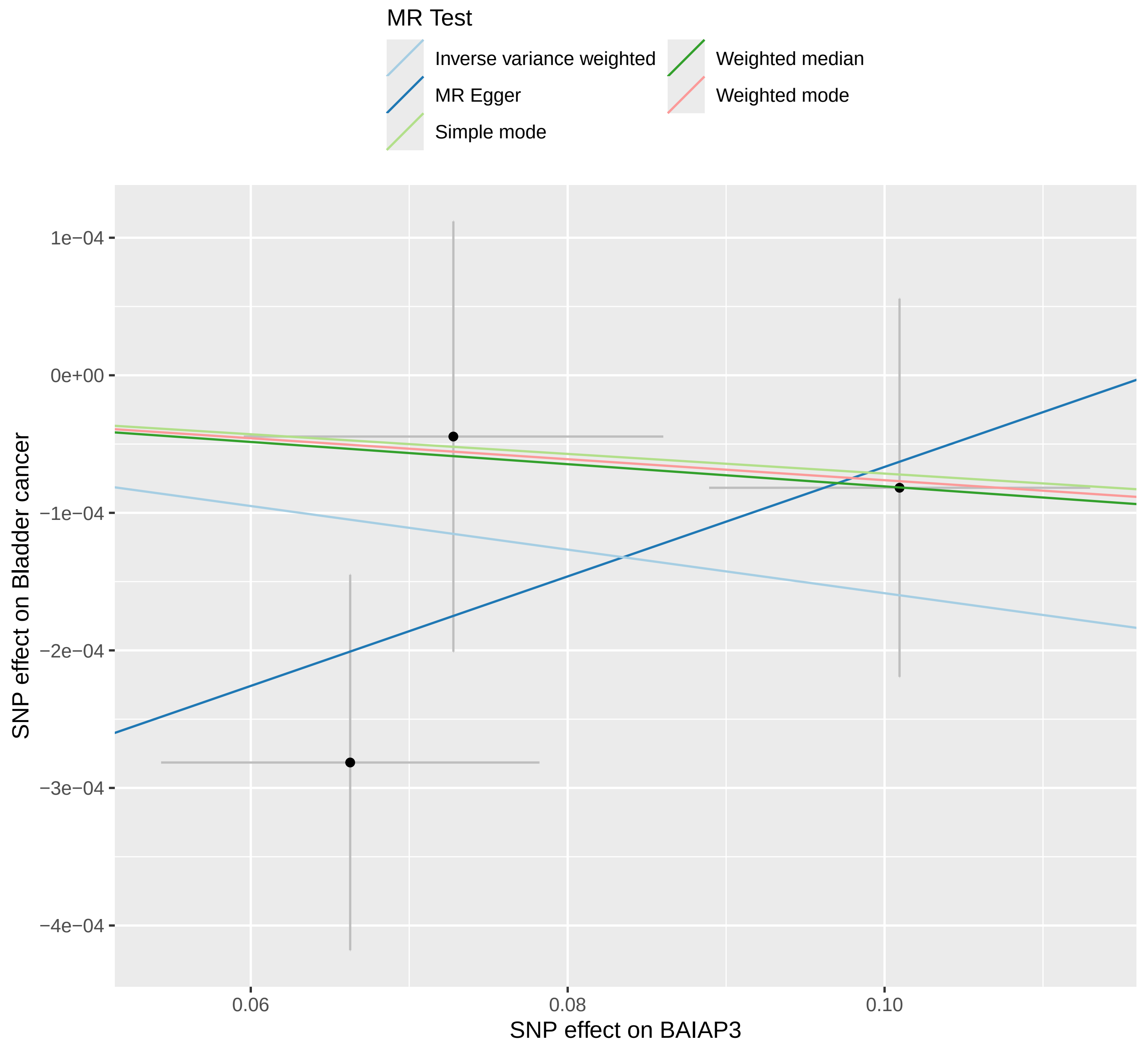
MR Test
Inverse variance weighted
MR Egger
Simple mode
Weighted median
Weighted mode
1e−04
0e+00
−1e−04
−2e−04
−3e−04
−4e−04
SNP effect on Bladder cancer
0.06
0.08
0.10
SNP effect on BAIAP3

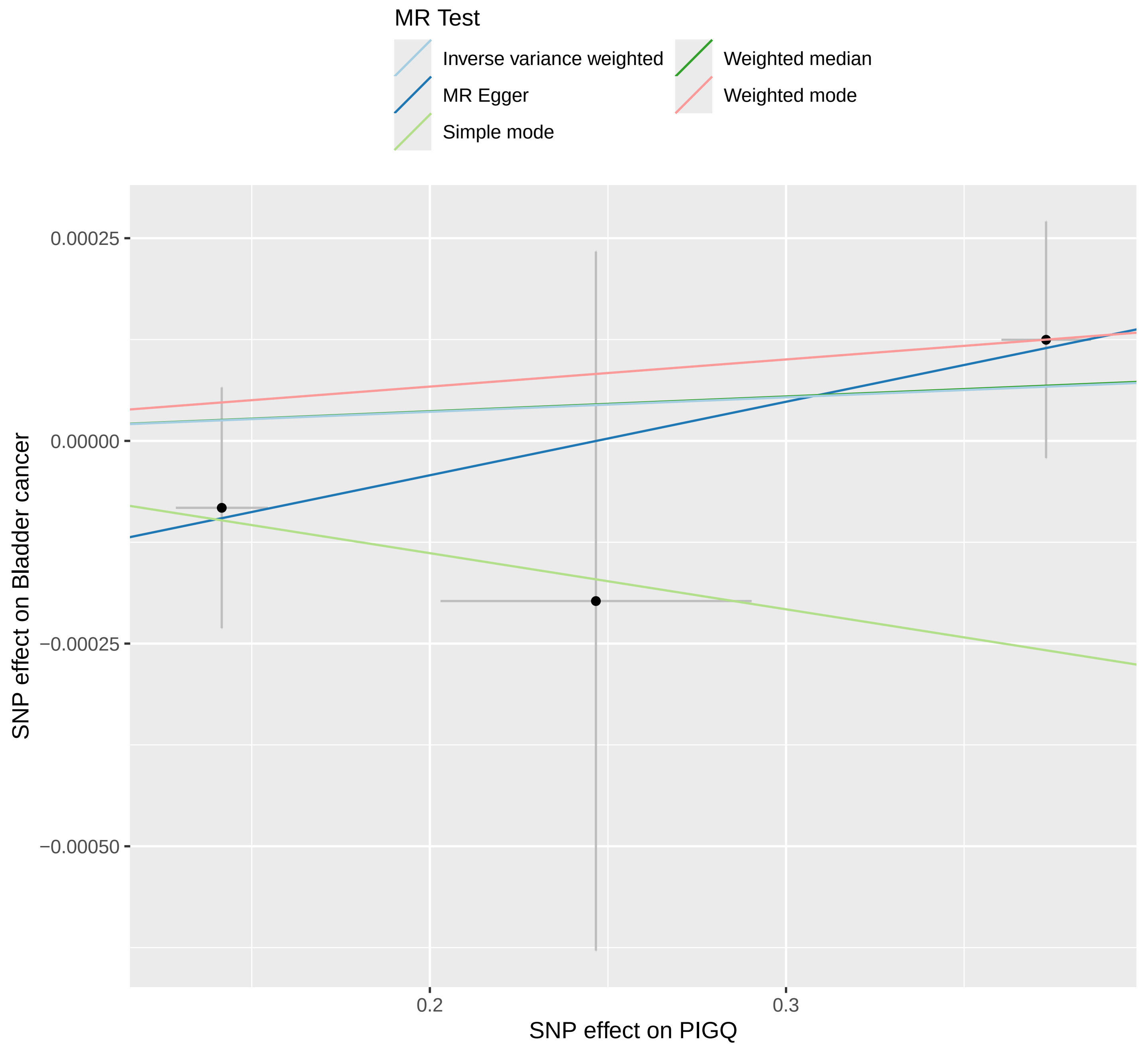

MR Test
Inverse variance weighted
MR Egger
Simple mode
Weighted median
Weighted mode
0.00025
0.00000
−0.00025
−0.00050
SNP effect on Bladder cancer
0.2
0.3
SNP effect on PIGQ

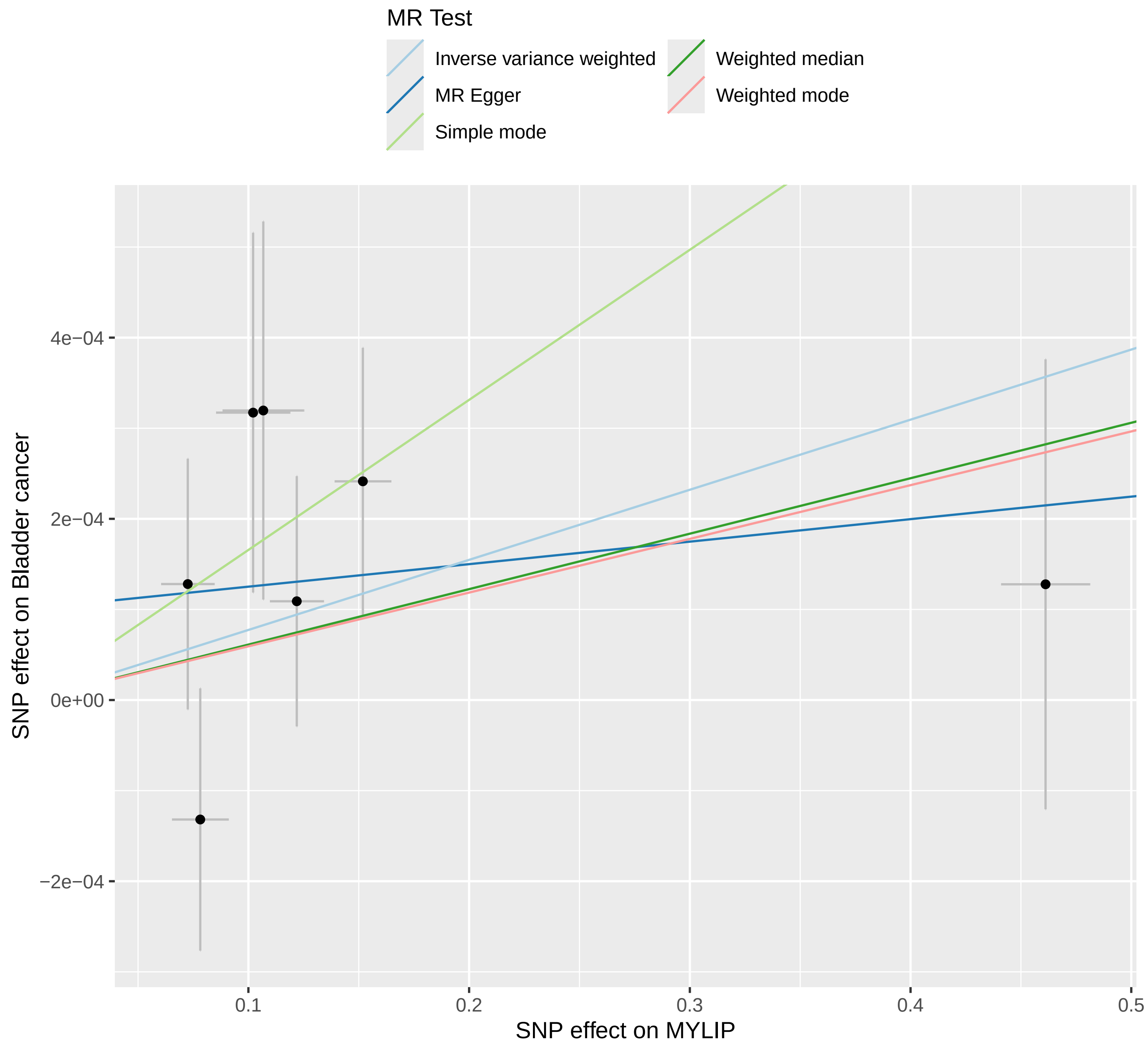

MR Test
Inverse variance weighted
MR Egger
Simple mode
Weighted median
Weighted mode
4e−04
2e−04
0e+00
−2e−04
0.1
0.2
0.3
0.4
0.5
SNP effect on MYLIP
SNP effect on Bladder cancer

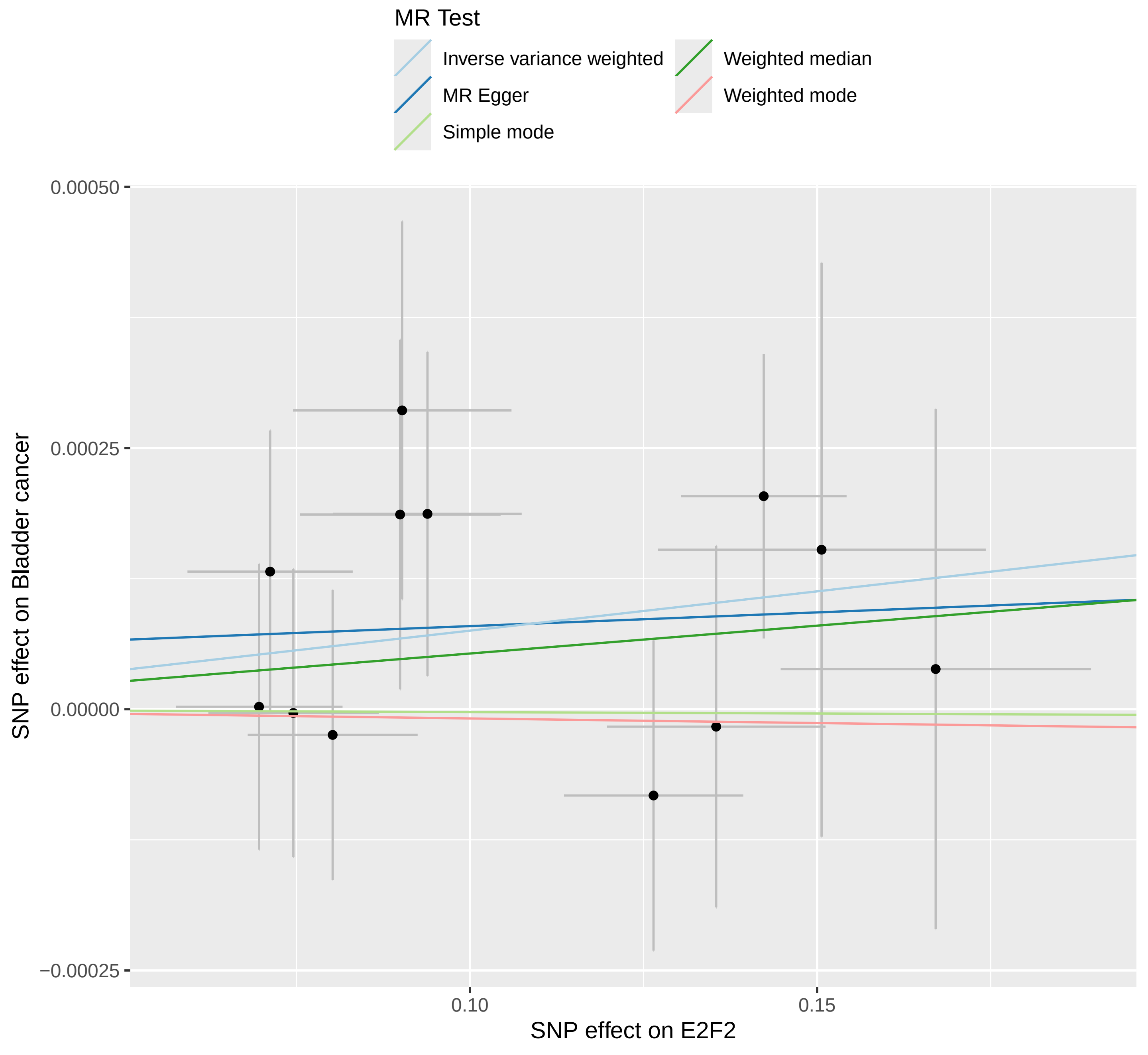
MR Test
Inverse variance weighted
MR Egger
Simple mode
Weighted median
Weighted mode
0.00050
0.00025
0.00000
−0.00025
0.10
0.15
SNP effect on Bladder cancer
SNP effect on E2F2

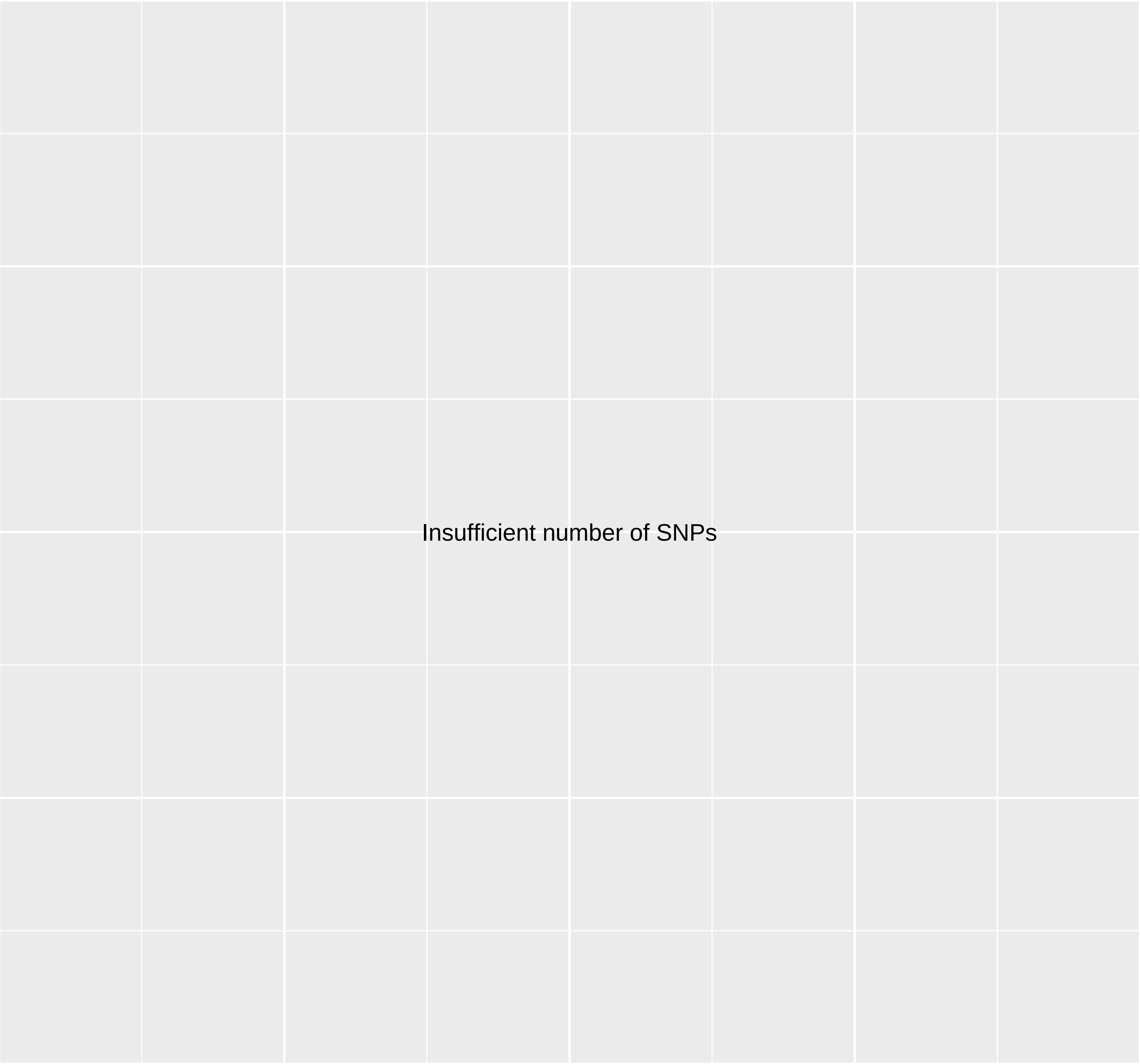
Insufficient number of SNPs

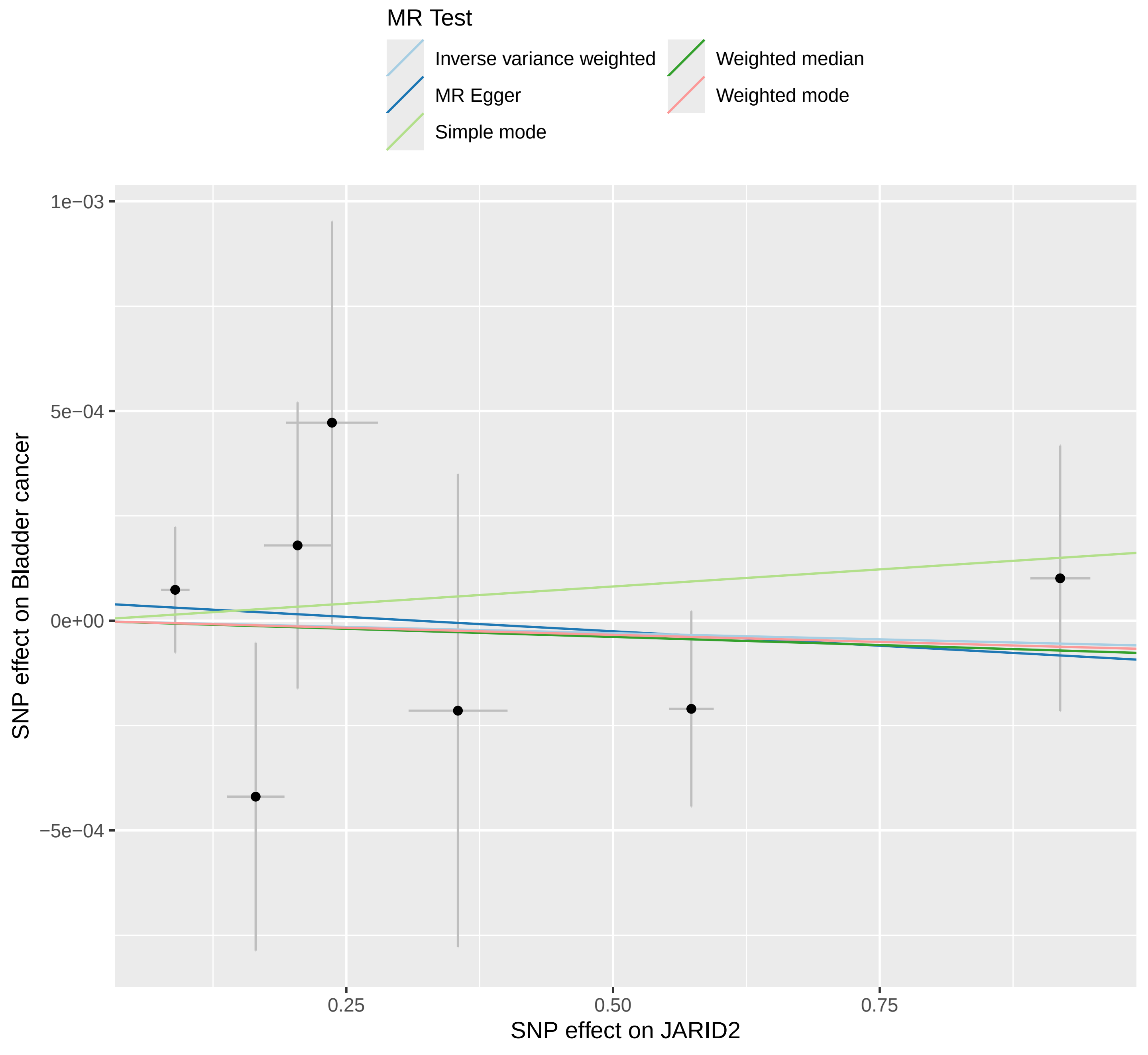
MR Test
Inverse variance weighted
MR Egger
Simple mode
Weighted median
Weighted mode
1e−03
5e−04
0e+00
−5e−04
0.25
0.50
0.75
SNP effect on Bladder cancer
SNP effect on JARID2

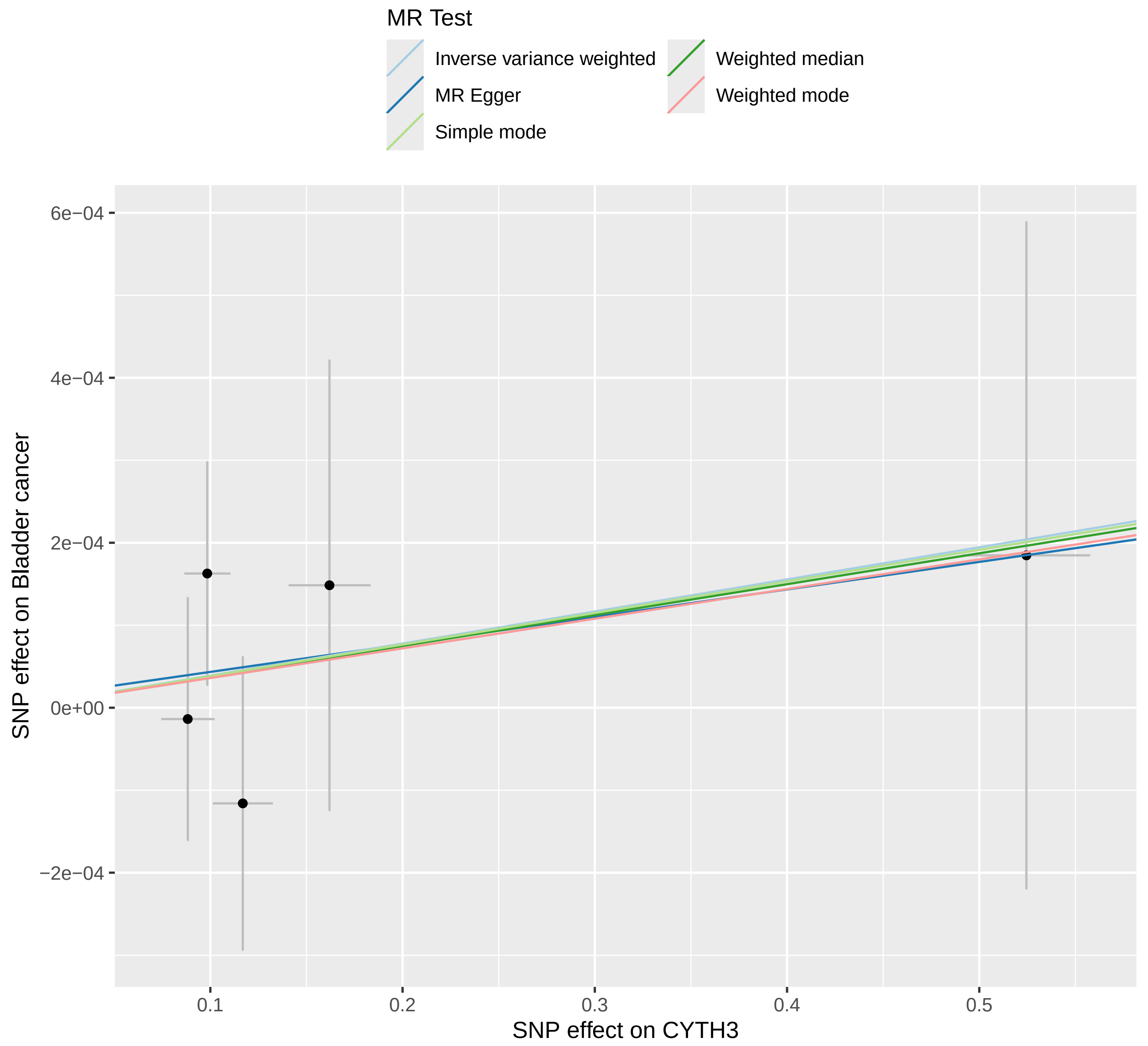
MR Test
Inverse variance weighted
MR Egger
Simple mode
Weighted median
Weighted mode
6e−04
4e−04
2e−04
0e+00
−2e−04
SNP effect on Bladder cancer
0.1
0.2
0.3
0.4
0.5
SNP effect on CYTH3

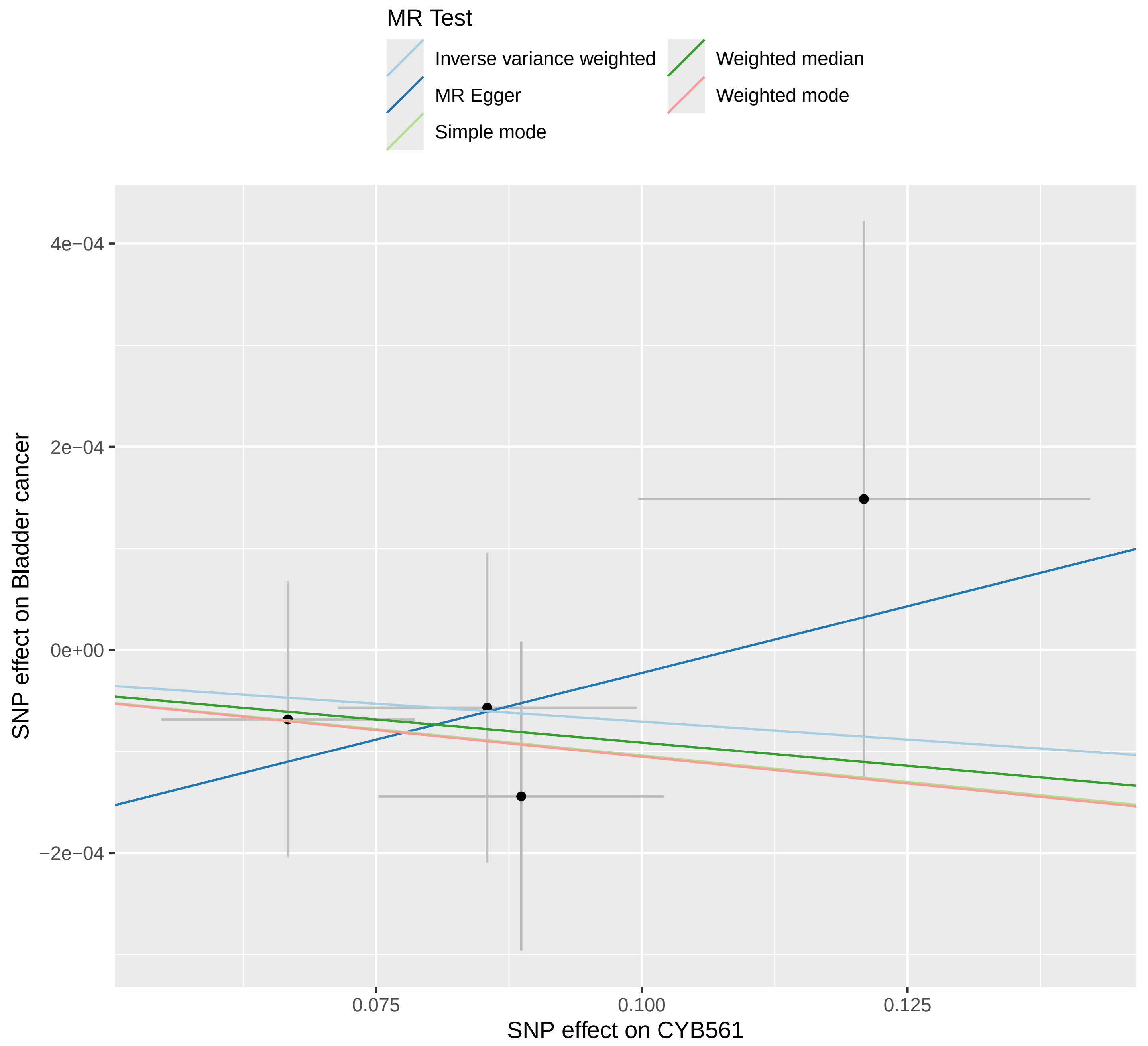
MR Test
Inverse variance weighted
MR Egger
Simple mode
Weighted median
Weighted mode
4e−04
2e−04
0e+00
−2e−04
SNP effect on Bladder cancer
0.075
0.100
0.125
SNP effect on CYB561

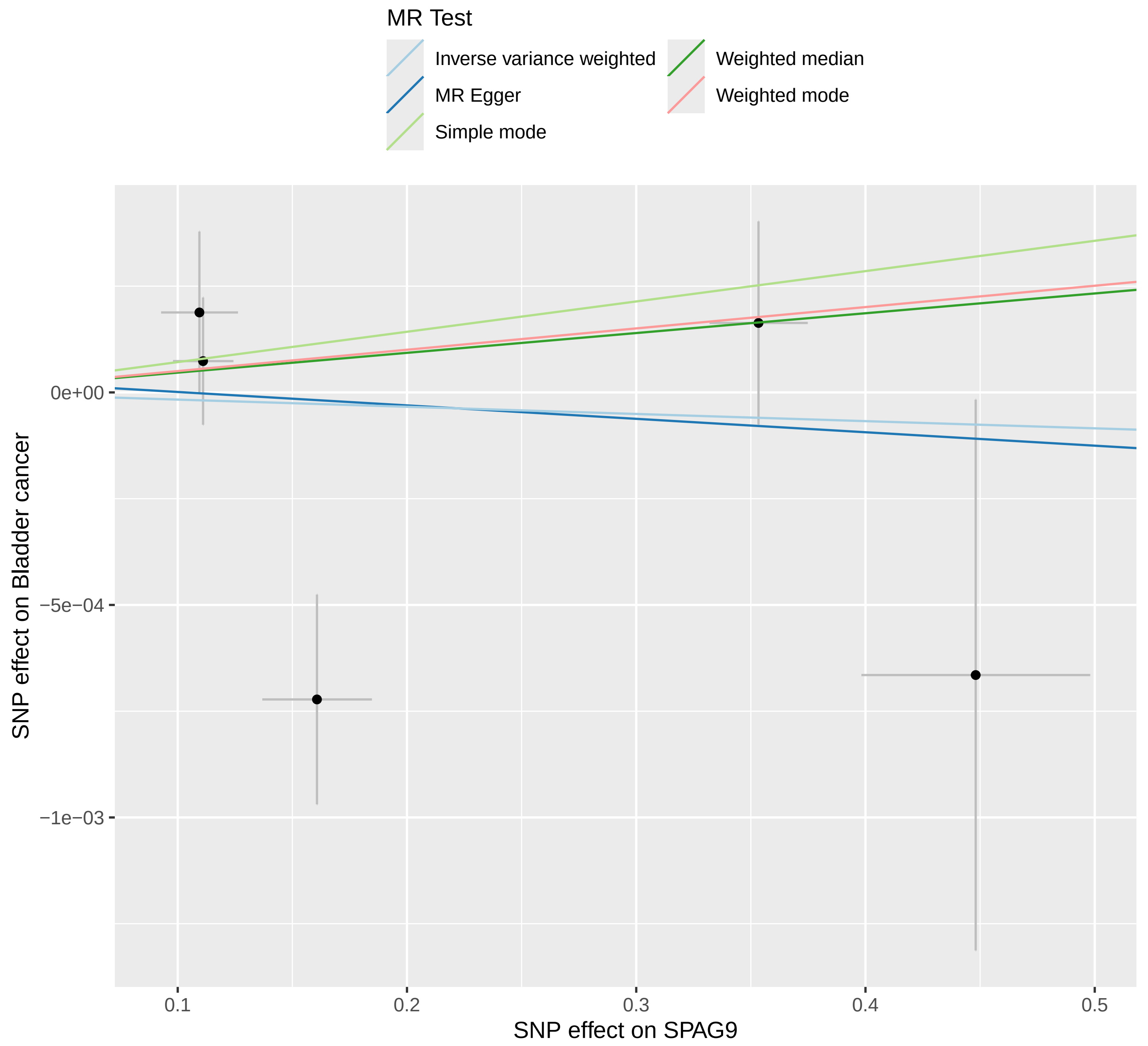

MR Test
Inverse variance weighted
MR Egger
Simple mode
Weighted median
Weighted mode
0e+00
−5e−04
−1e−03
0.1
0.2
0.3
0.4
0.5
SNP effect on Bladder cancer
SNP effect on SPAG9

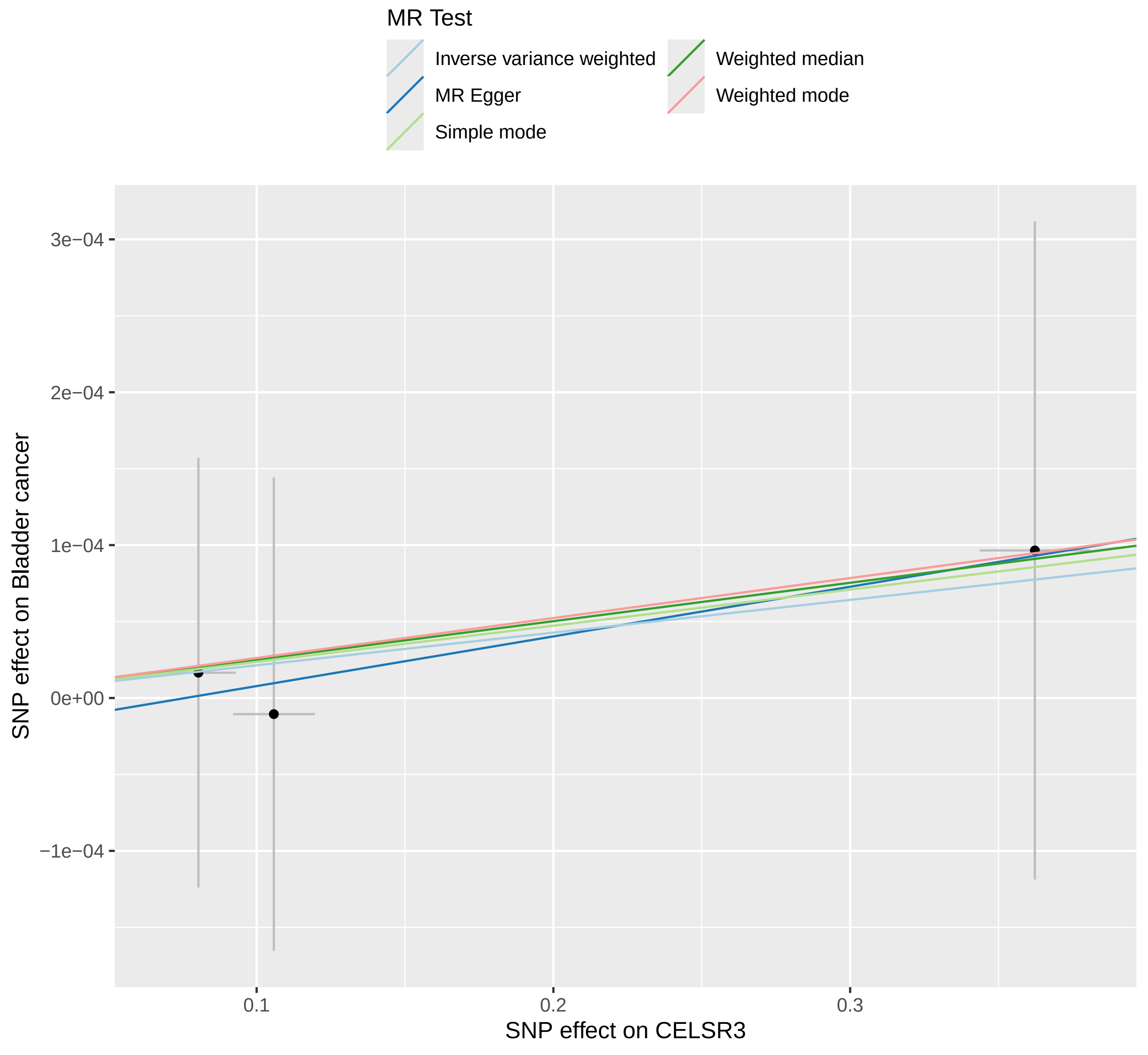

MR Test
Inverse variance weighted
MR Egger
Simple mode
Weighted median
Weighted mode
3e−04
2e−04
1e−04
0e+00
−1e−04
SNP effect on Bladder cancer
0.1
0.2
0.3
SNP effect on CELSR3

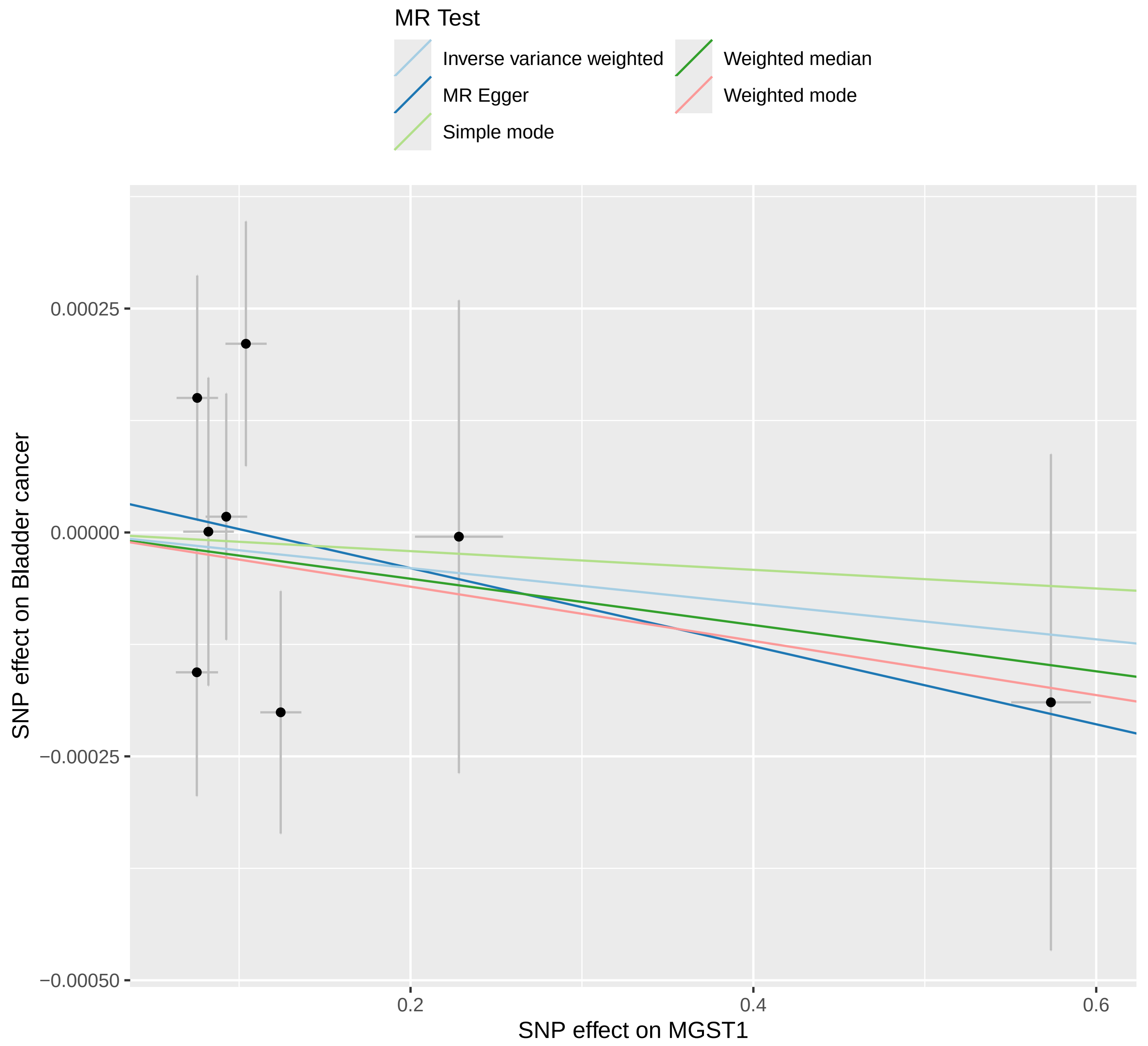

MR Test
Inverse variance weighted
MR Egger
Simple mode
Weighted median
Weighted mode
0.00025
0.00000
−0.00025
−0.00050
SNP effect on Bladder cancer
0.2
0.4
0.6
SNP effect on MGST1

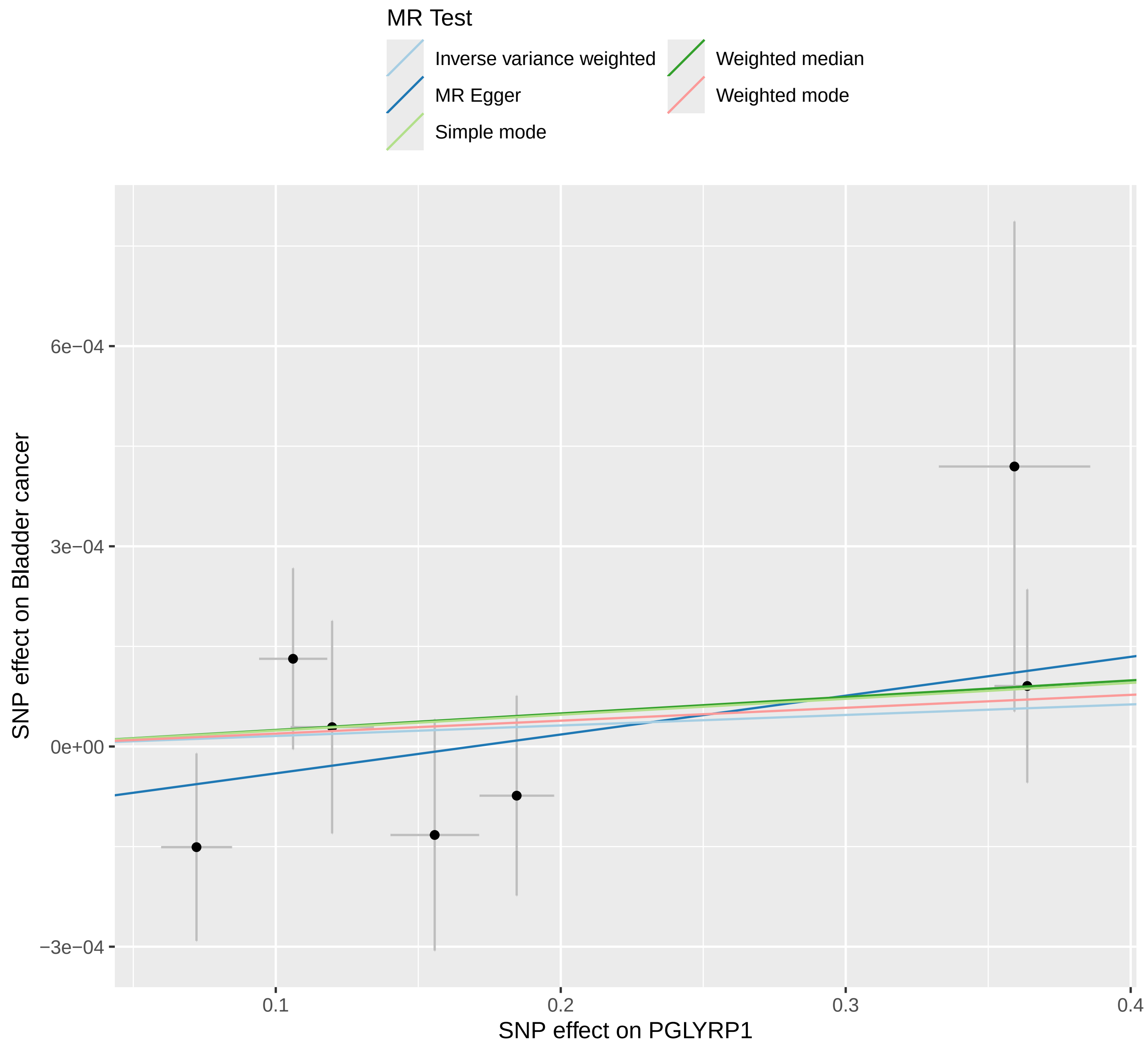
MR Test
Inverse variance weighted
MR Egger
Simple mode
Weighted median
Weighted mode
6e−04
3e−04
0e+00
−3e−04
SNP effect on Bladder cancer
0.1
0.2
0.3
0.4
SNP effect on PGLYRP1

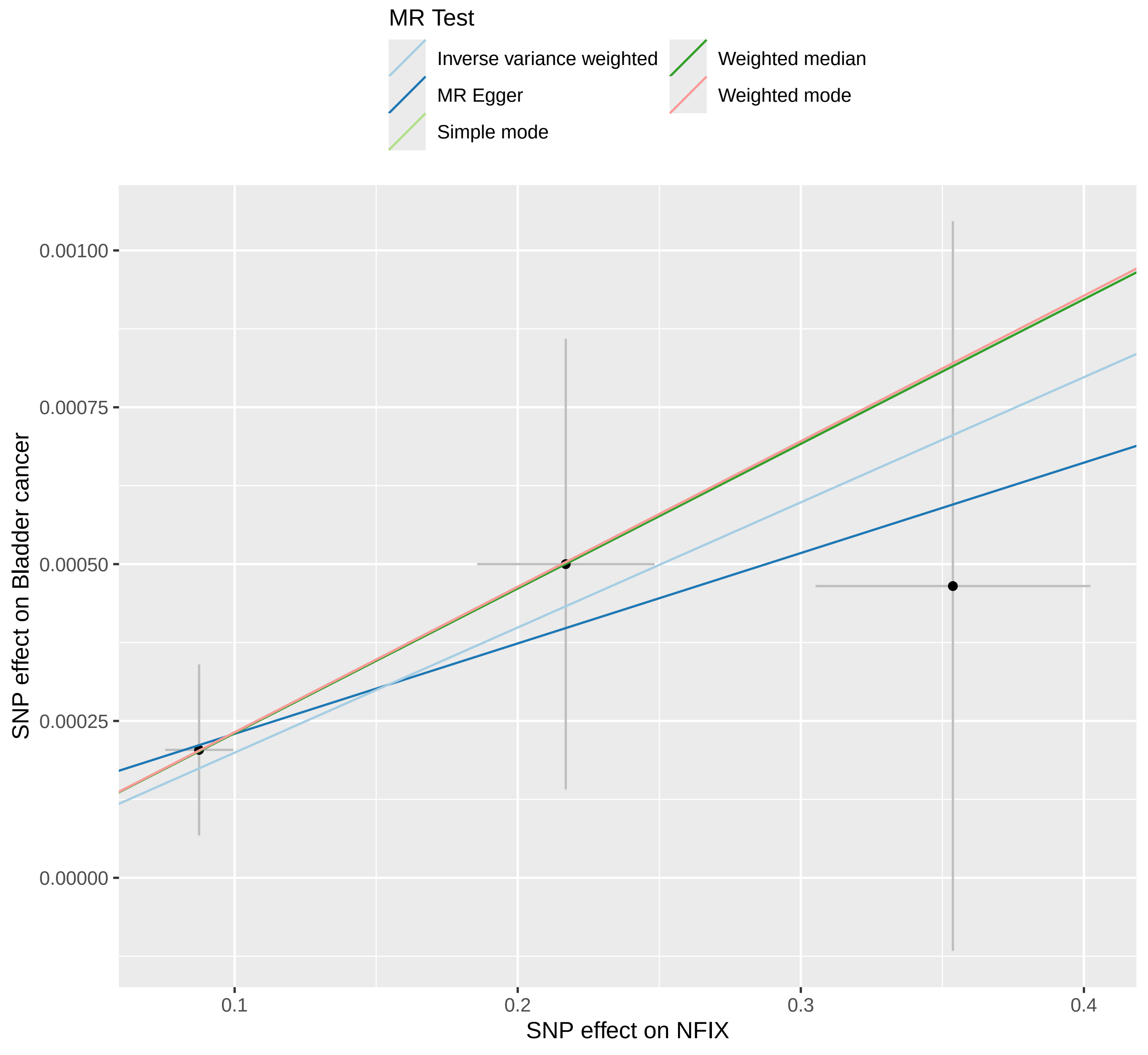
MR Test
Inverse variance weighted
MR Egger
Simple mode
Weighted median
Weighted mode
0.00100
0.00075
0.00050
0.00025
0.00000
SNP effect on Bladder cancer
0.1
0.2
0.3
0.4
SNP effect on NFIX

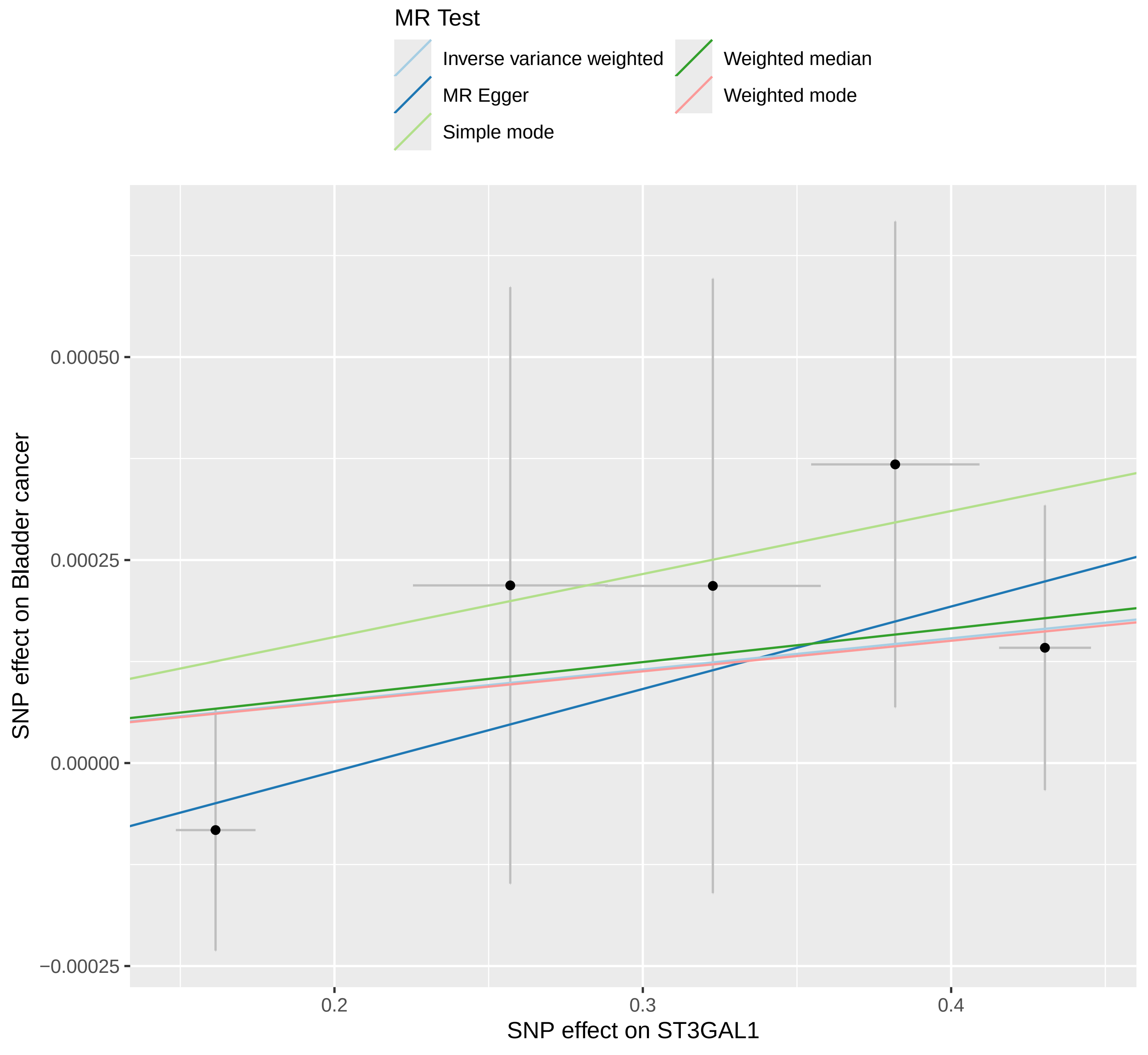
MR Test
Inverse variance weighted
MR Egger
Simple mode
Weighted median
Weighted mode
0.00050
0.00025
0.00000
−0.00025
SNP effect on Bladder cancer
0.2
0.3
0.4
SNP effect on ST3GAL1

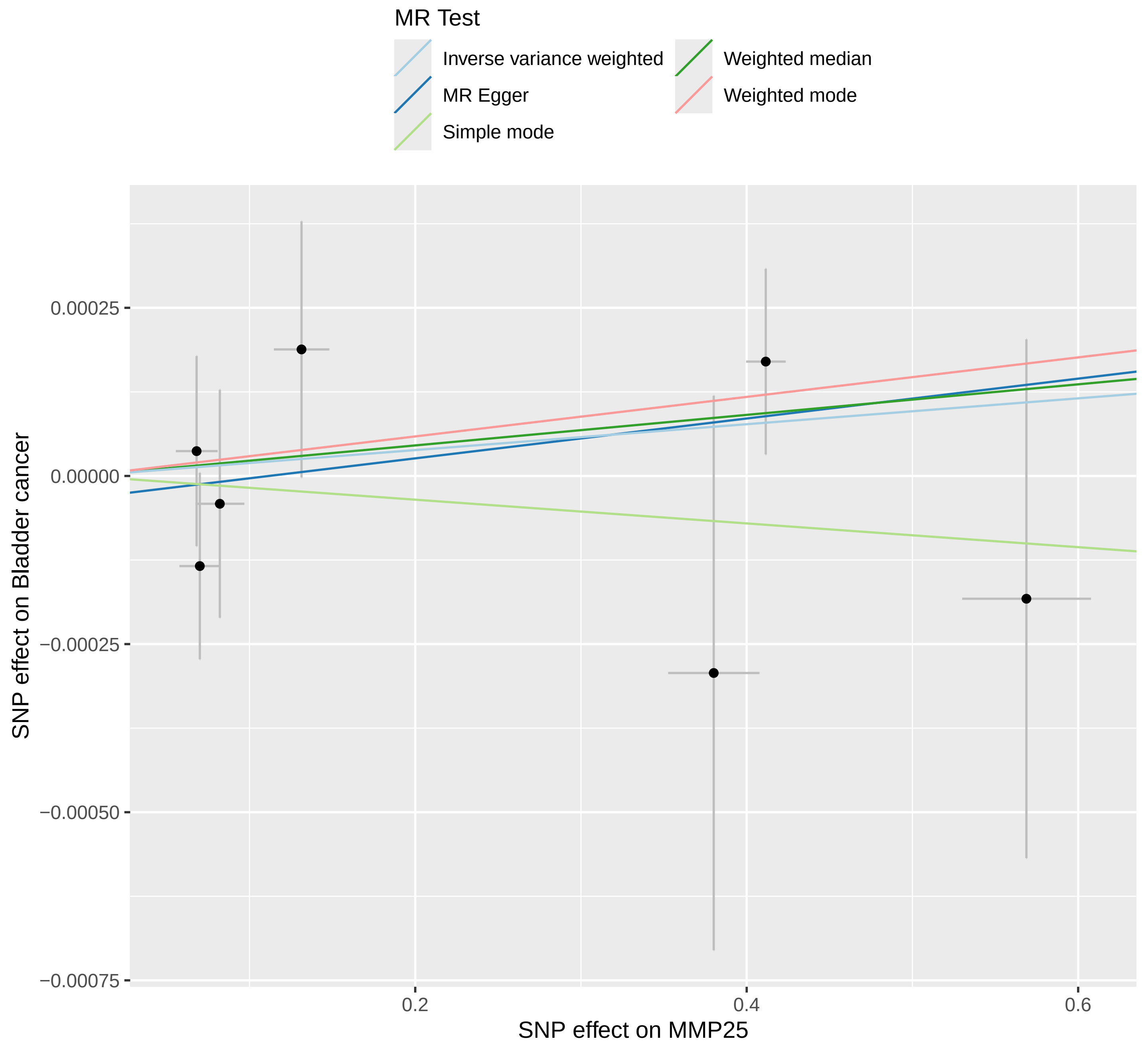

MR Test
Inverse variance weighted
MR Egger
Simple mode
Weighted median
Weighted mode
0.00025
0.00000
−0.00025
−0.00050
−0.00075
SNP effect on Bladder cancer
0.2
0.4
0.6
SNP effect on MMP25

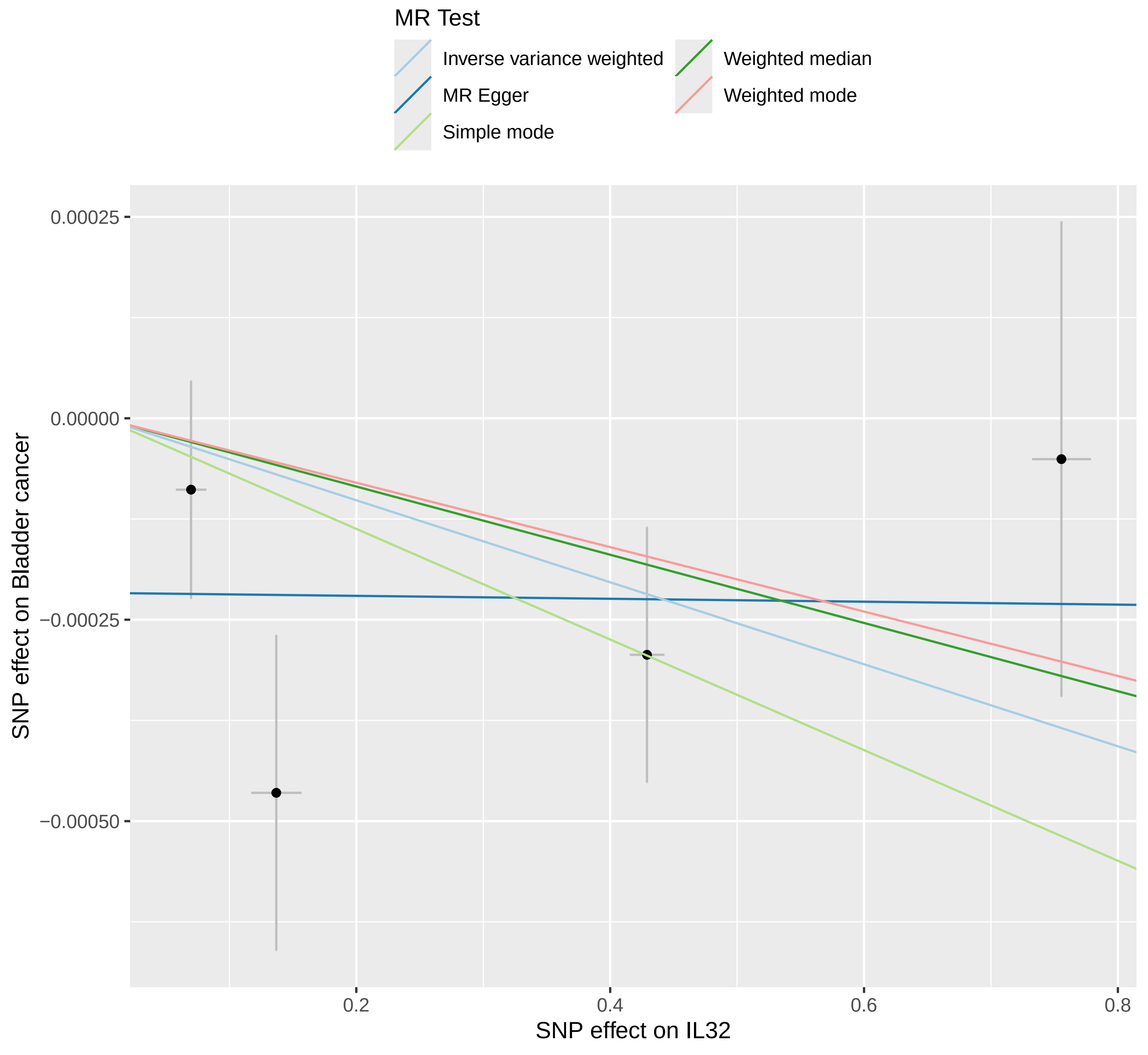

MR Test
Inverse variance weighted
MR Egger
Simple mode
Weighted median
Weighted mode
0.00025
0.00000
−0.00025
−0.00050
SNP effect on Bladder cancer
0.2
0.4
0.6
0.8
SNP effect on IL32

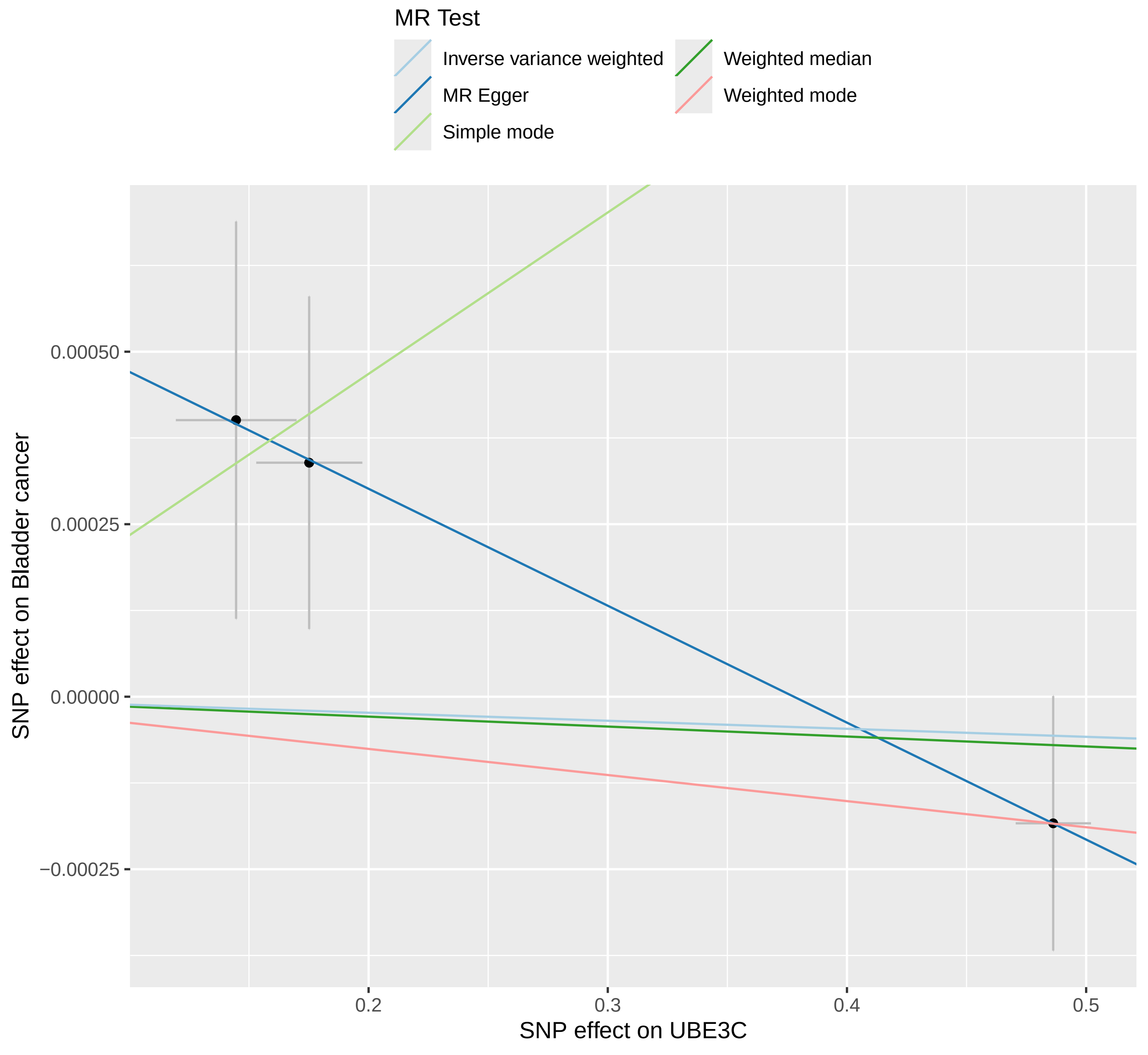

MR Test
Inverse variance weighted
MR Egger
Simple mode
Weighted median
Weighted mode
0.00050
0.00025
0.00000
−0.00025
0.2
0.3
0.4
0.5
SNP effect on UBE3C
SNP effect on Bladder cancer

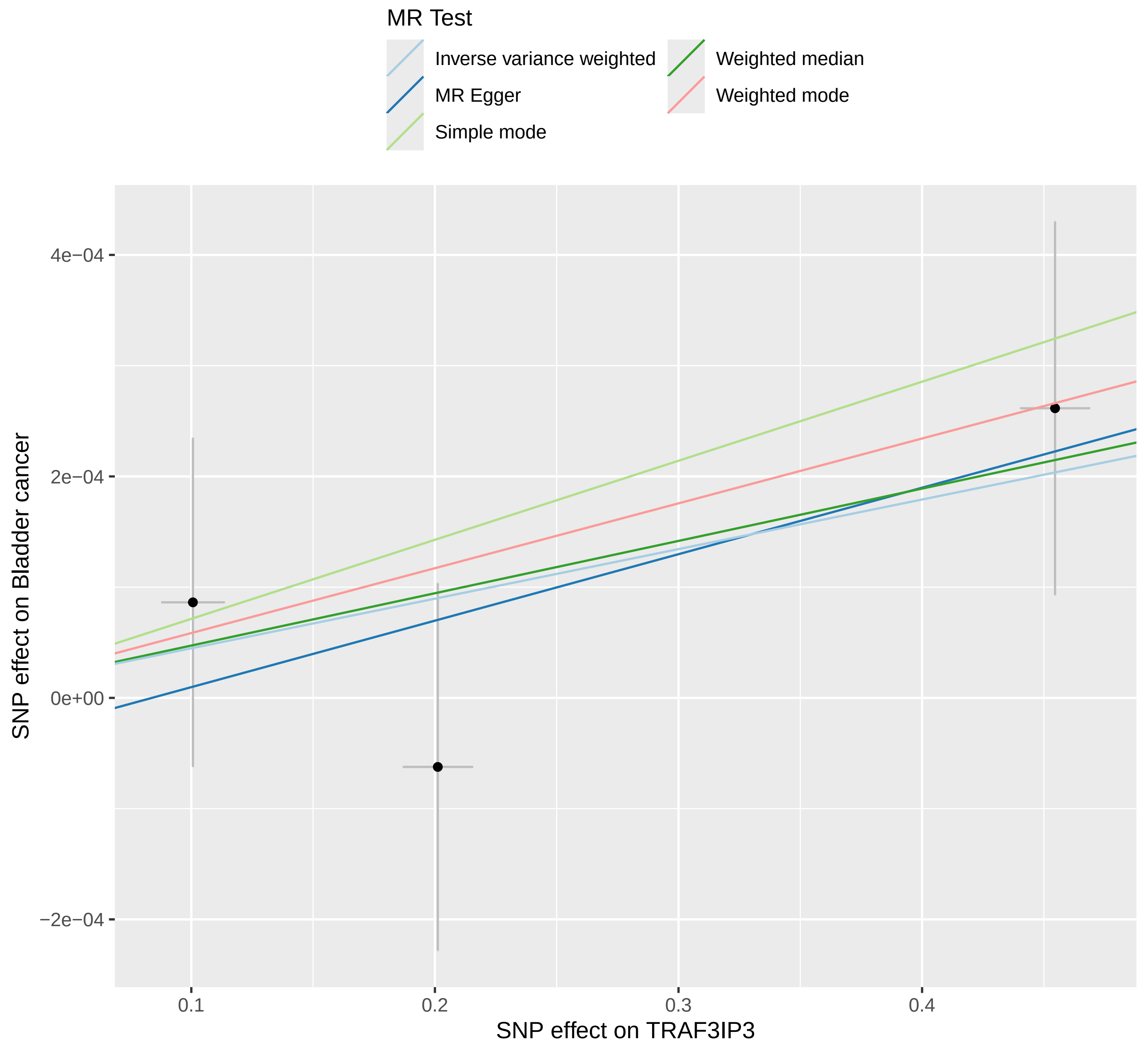
MR Test
Inverse variance weighted
MR Egger
Simple mode
Weighted median
Weighted mode
4e−04
2e−04
0e+00
−2e−04
0.1
0.2
0.3
0.4
SNP effect on Bladder cancer
SNP effect on TRAF3IP3

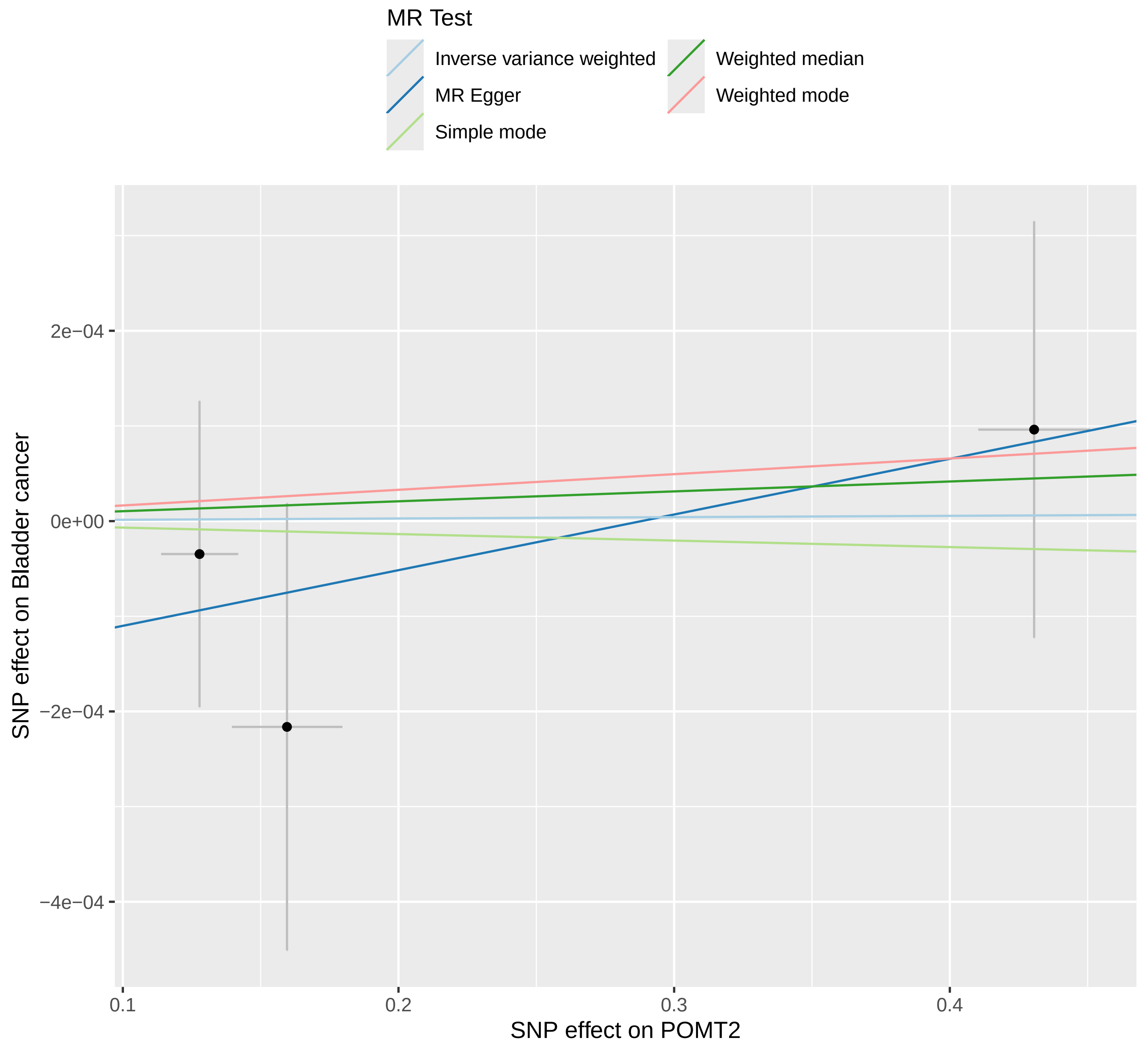
MR Test
Inverse variance weighted
MR Egger
Simple mode
Weighted median
Weighted mode
2e−04
0e+00
−2e−04
−4e−04
0.1
0.2
0.3
0.4
SNP effect on Bladder cancer
SNP effect on POMT2

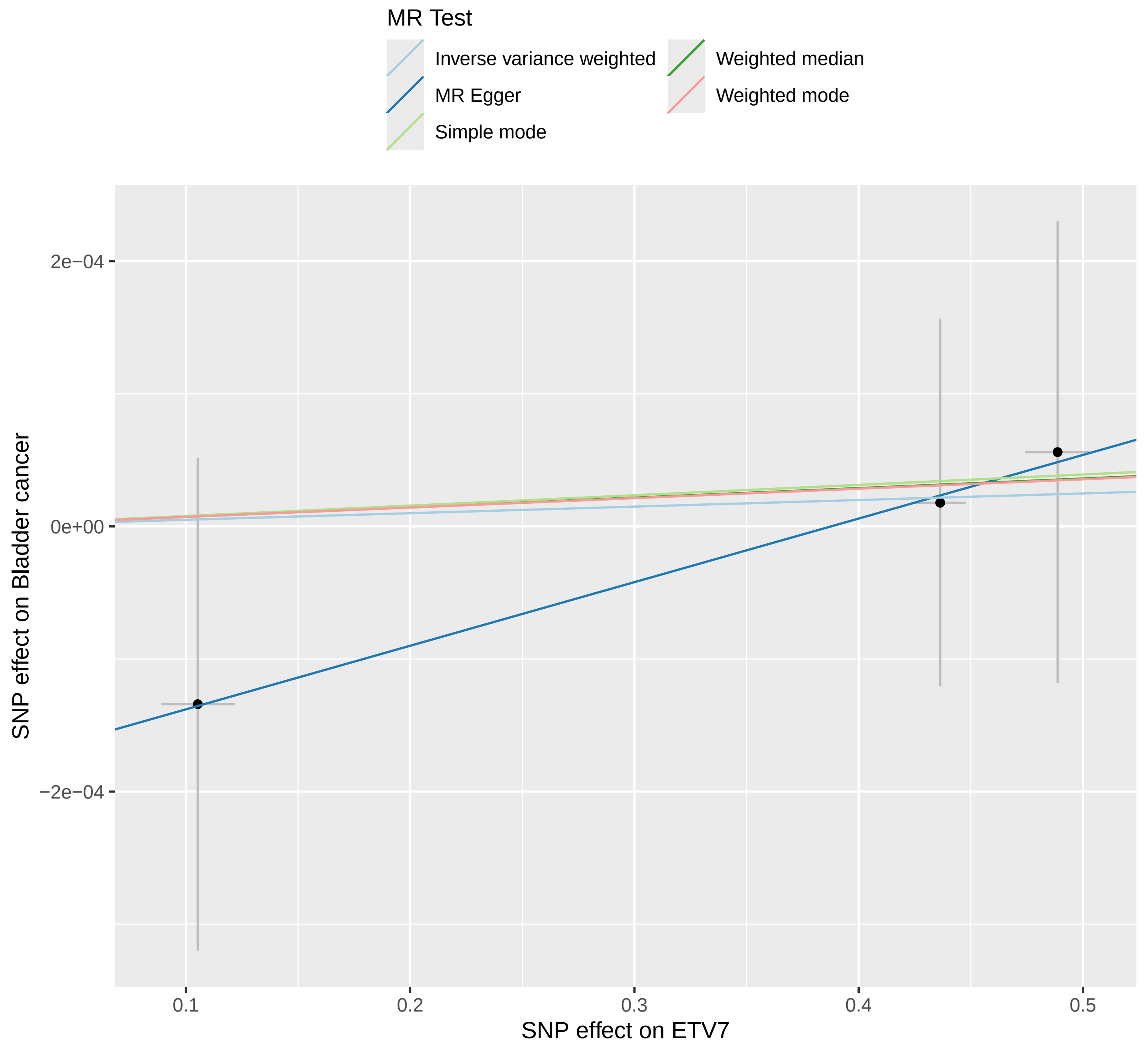
MR Test
Inverse variance weighted
MR Egger
Simple mode
Weighted median
Weighted mode
2e−04
0e+00
−2e−04
SNP effect on Bladder cancer
0.1
0.2
0.3
0.4
0.5
SNP effect on ETV7

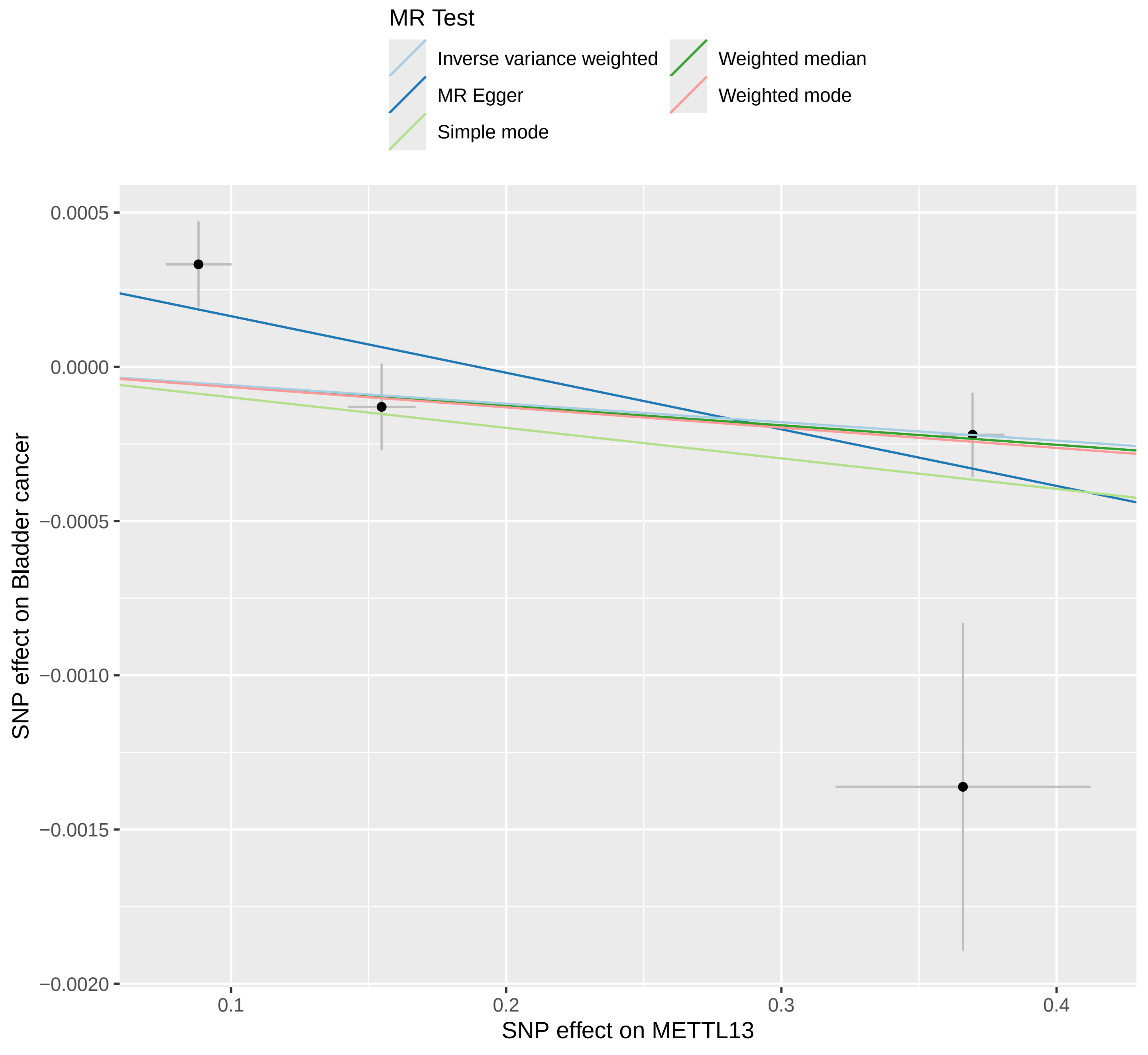
MR Test
Inverse variance weighted
MR Egger
Simple mode
Weighted median
Weighted mode
0.0005
0.0000
−0.0005
−0.0010
−0.0015
−0.0020
SNP effect on Bladder cancer
0.1
0.2
0.3
0.4
SNP effect on METTL13

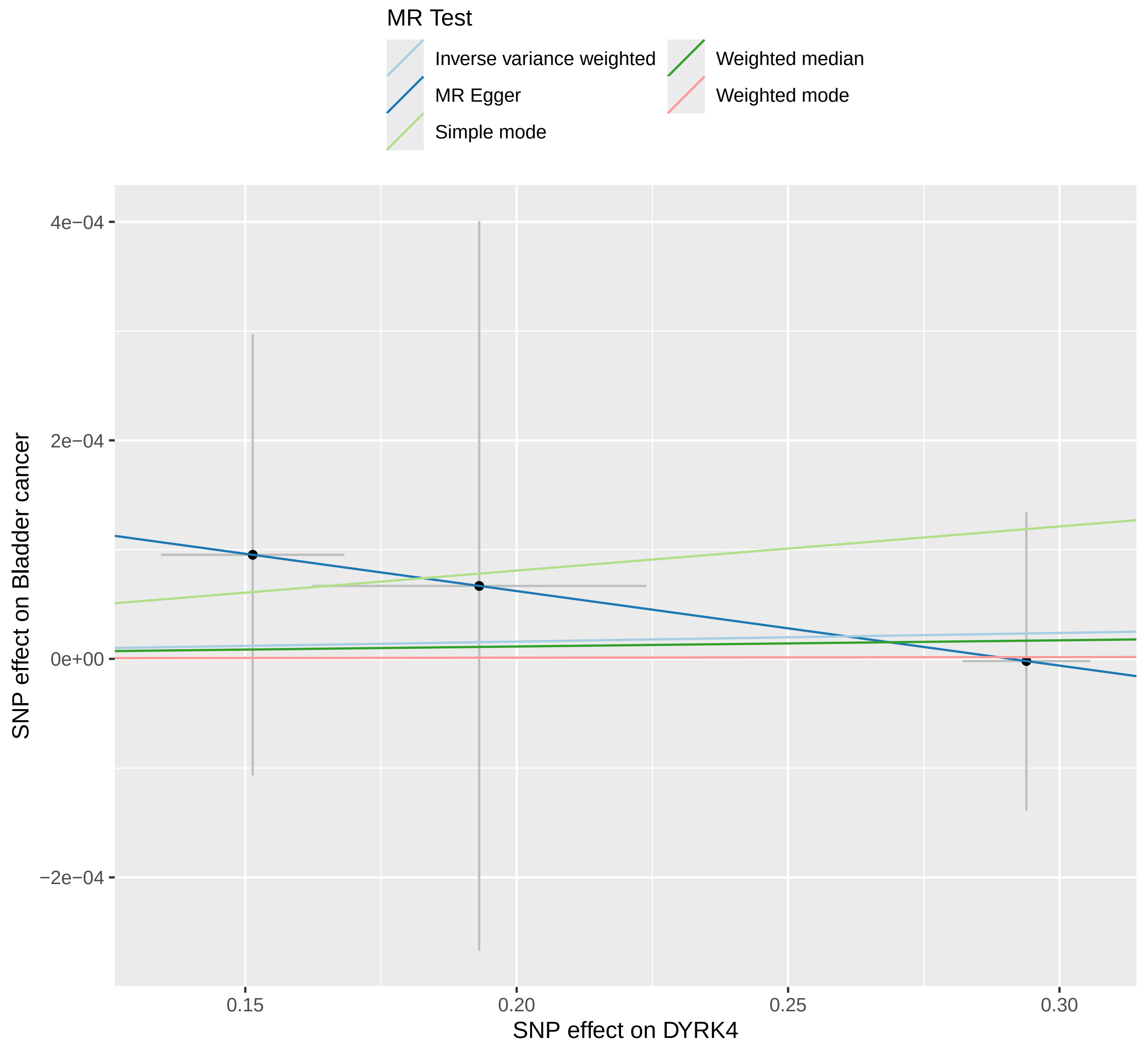

MR Test
Inverse variance weighted
MR Egger
Simple mode
Weighted median
Weighted mode
4e−04
2e−04
0e+00
−2e−04
SNP effect on Bladder cancer
0.15
0.20
0.25
0.30
SNP effect on DYRK4

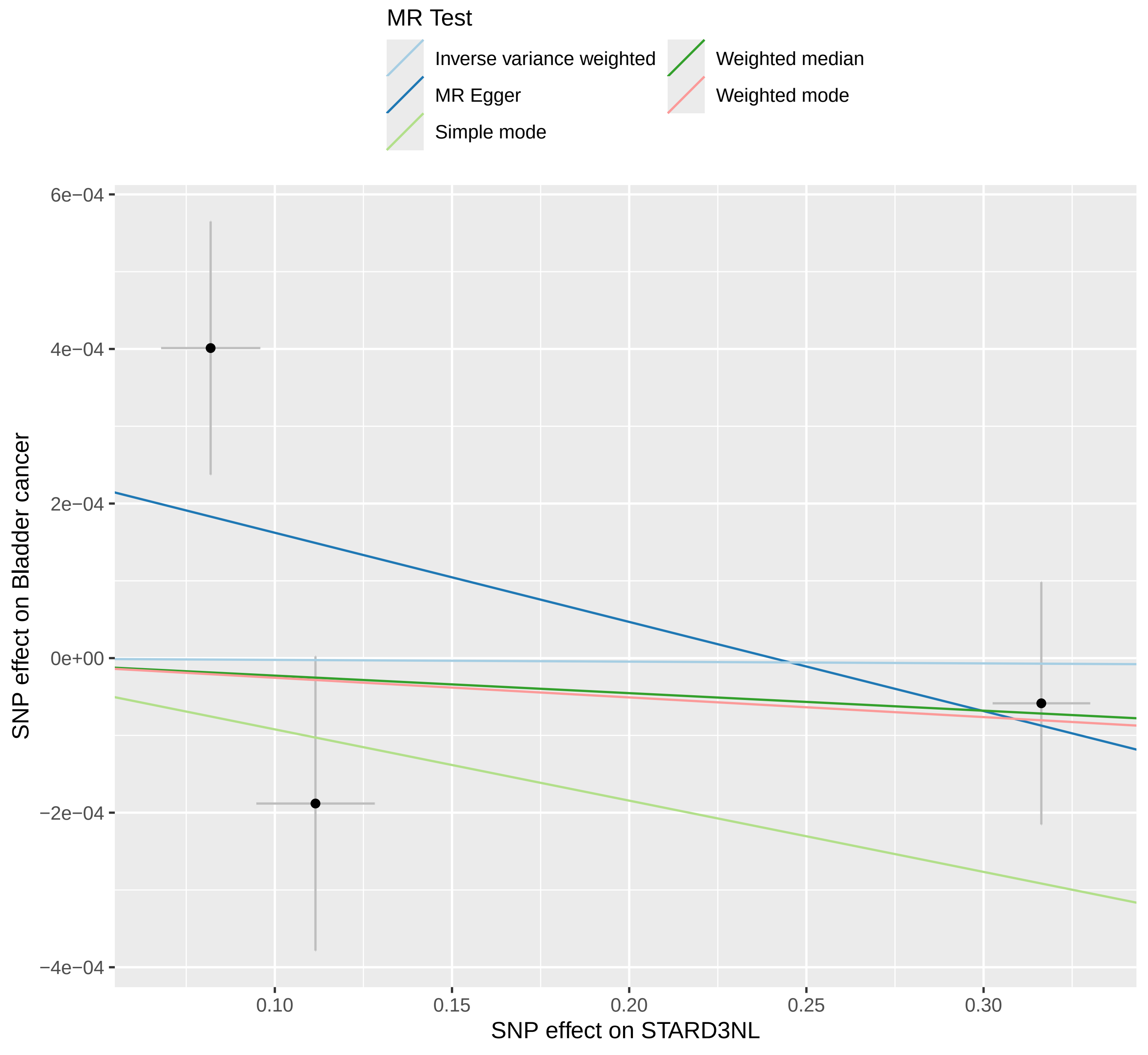

MR Test
Inverse variance weighted
MR Egger
Simple mode
Weighted median
Weighted mode
6e−04
4e−04
2e−04
0e+00
−2e−04
−4e−04
0.10
0.15
0.20
0.25
0.30
SNP effect on Bladder cancer
SNP effect on STARD3NL

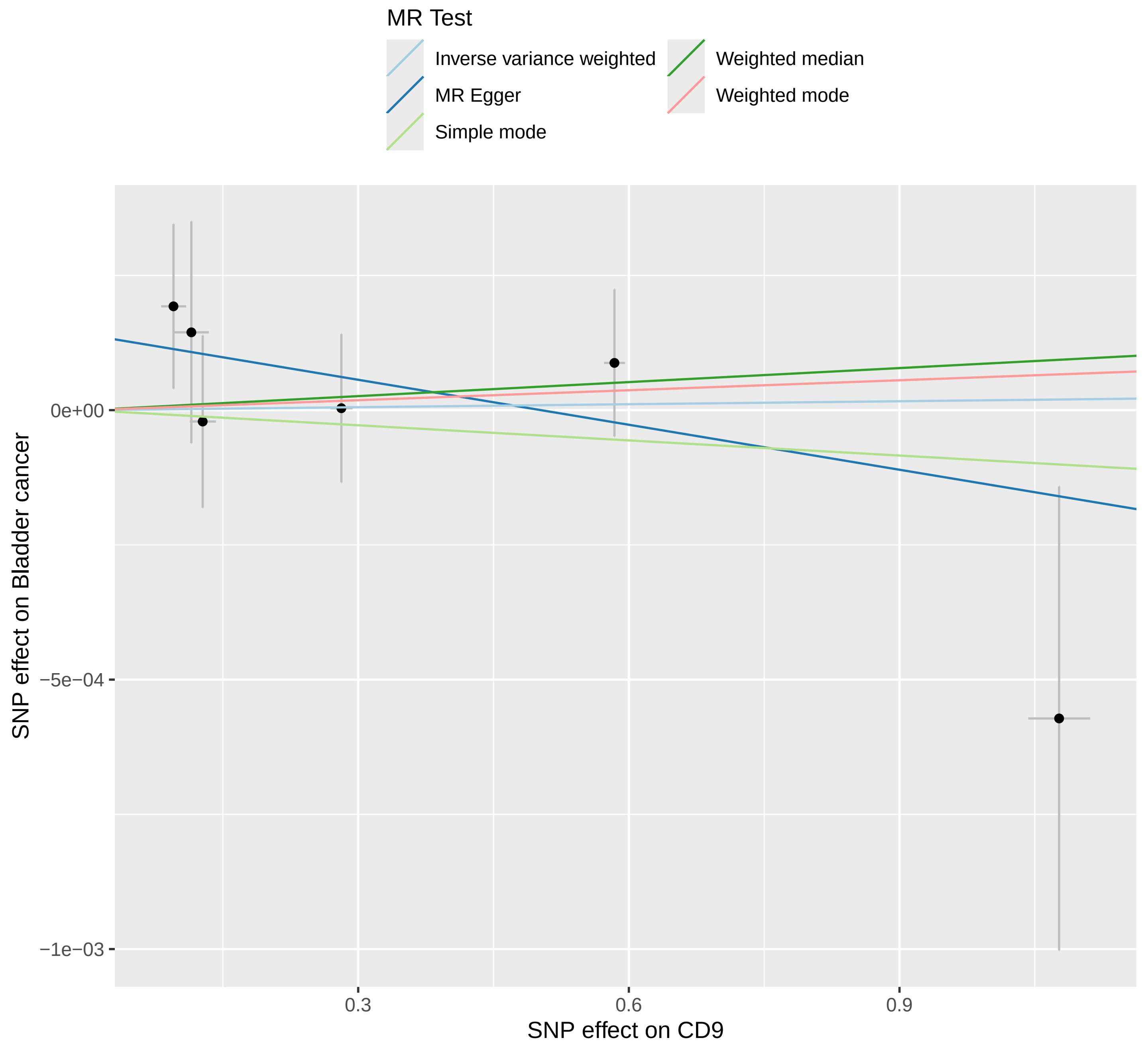
MR Test
Inverse variance weighted
MR Egger
Simple mode
Weighted median
Weighted mode
0e+00
−5e−04
−1e−03
0.3
0.6
0.9
SNP effect on Bladder cancer
SNP effect on CD9

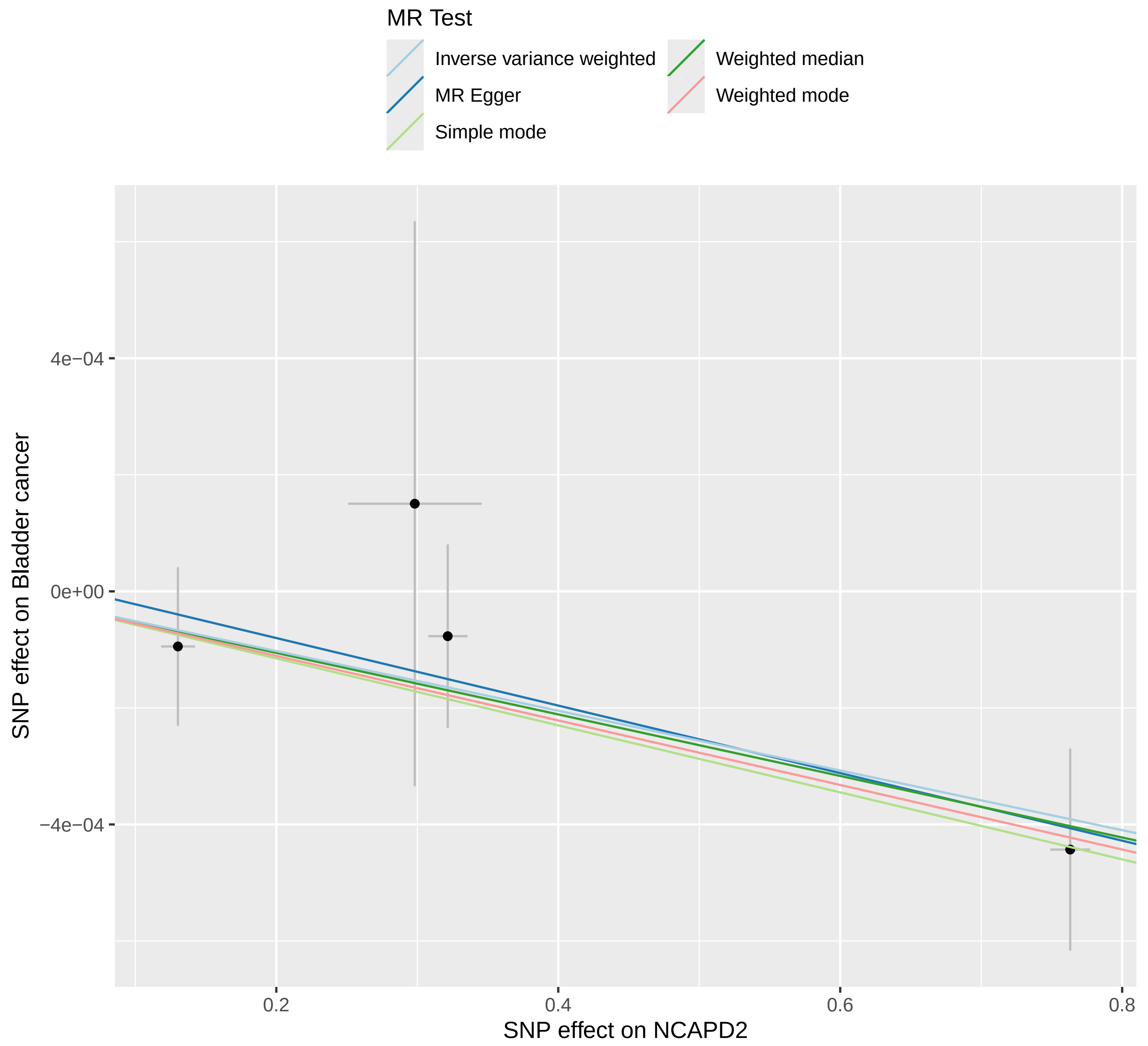

MR Test
Inverse variance weighted
MR Egger
Simple mode
Weighted median
Weighted mode
4e−04
0e+00
−4e−04
0.2
0.4
0.6
0.8
SNP effect on Bladder cancer
SNP effect on NCAPD2

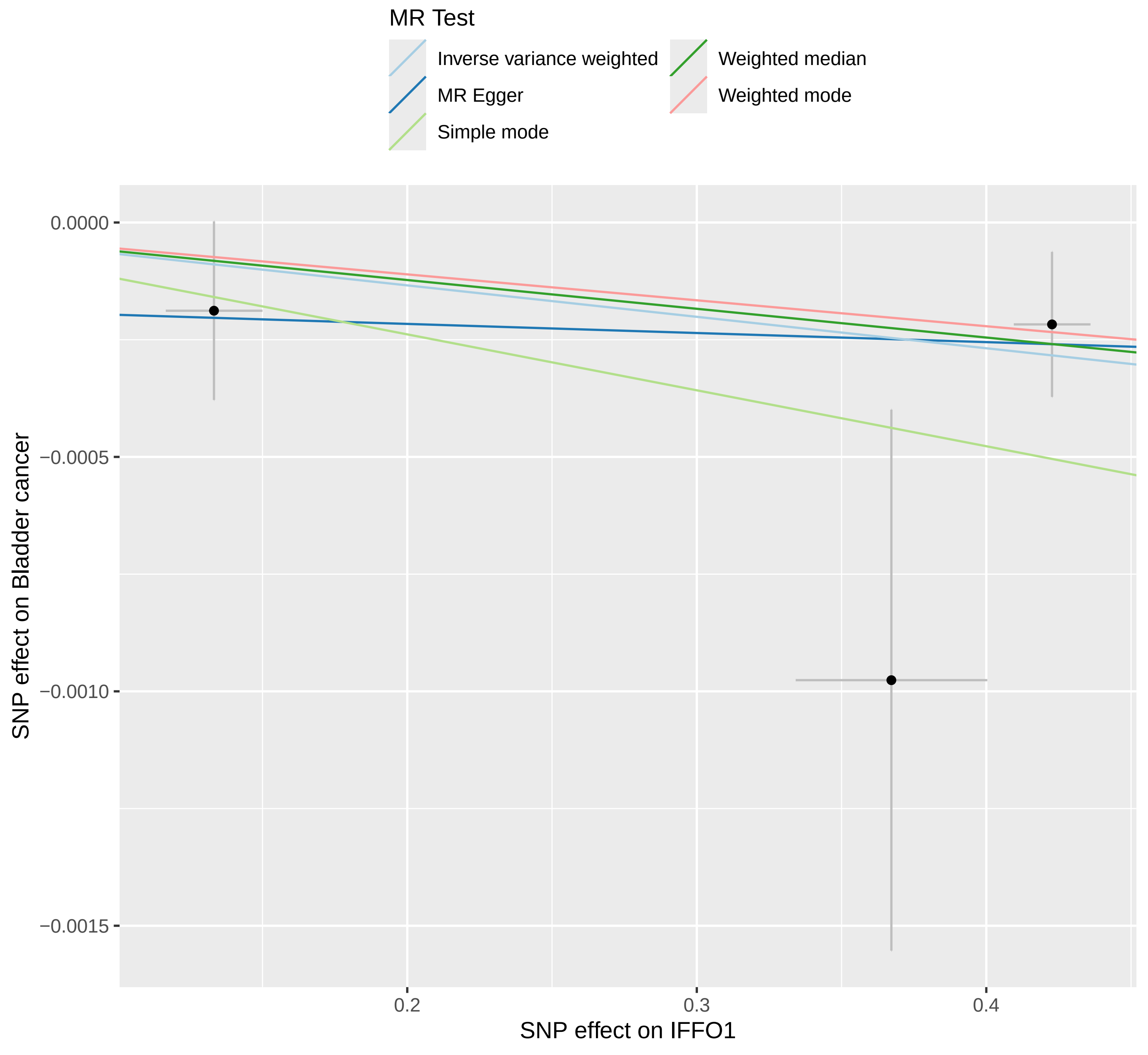

MR Test
Inverse variance weighted
MR Egger
Simple mode
Weighted median
Weighted mode
0.0000
−0.0005
−0.0010
−0.0015
SNP effect on Bladder cancer
0.2
0.3
0.4
SNP effect on IFFO1

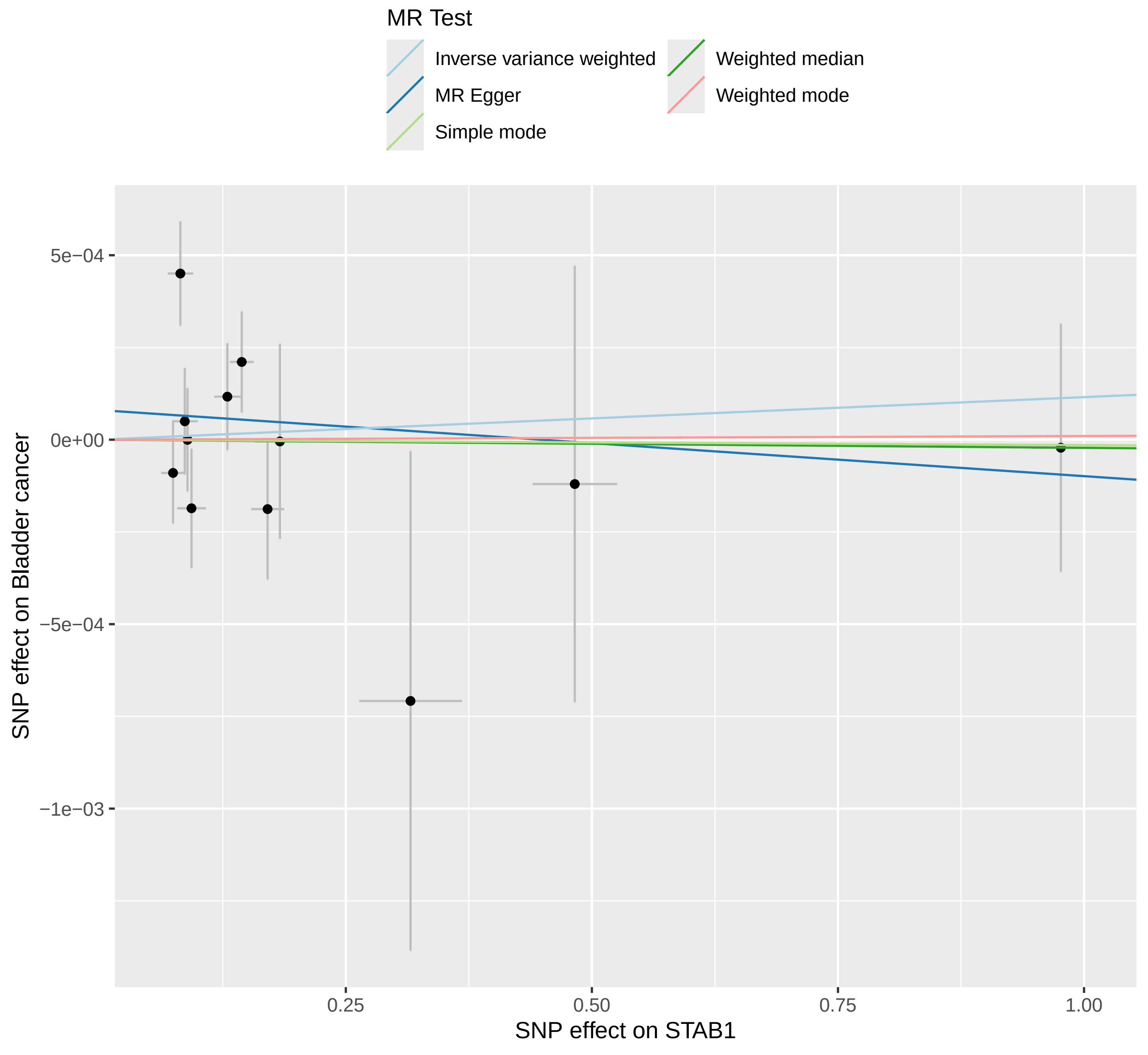
MR Test
Inverse variance weighted
MR Egger
Simple mode
Weighted median
Weighted mode
SNP effect on Bladder cancer
5e−04
0e+00
−5e−04
−1e−03
0.25
0.50
0.75
1.00
SNP effect on STAB1

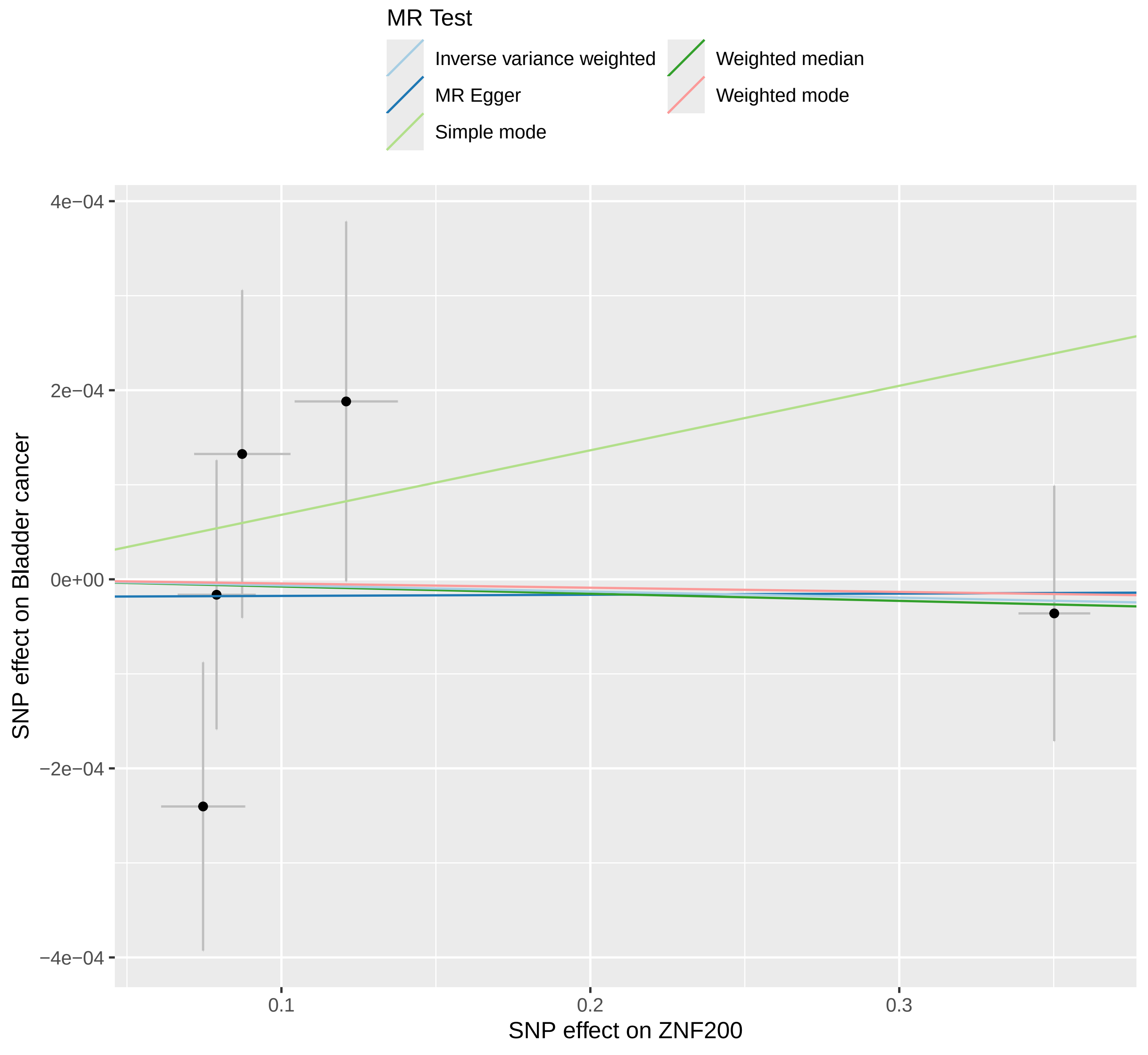

MR Test
Inverse variance weighted
MR Egger
Simple mode
Weighted median
Weighted mode
4e−04
2e−04
0e+00
−2e−04
−4e−04
SNP effect on Bladder cancer
0.1
0.2
0.3
SNP effect on ZNF200

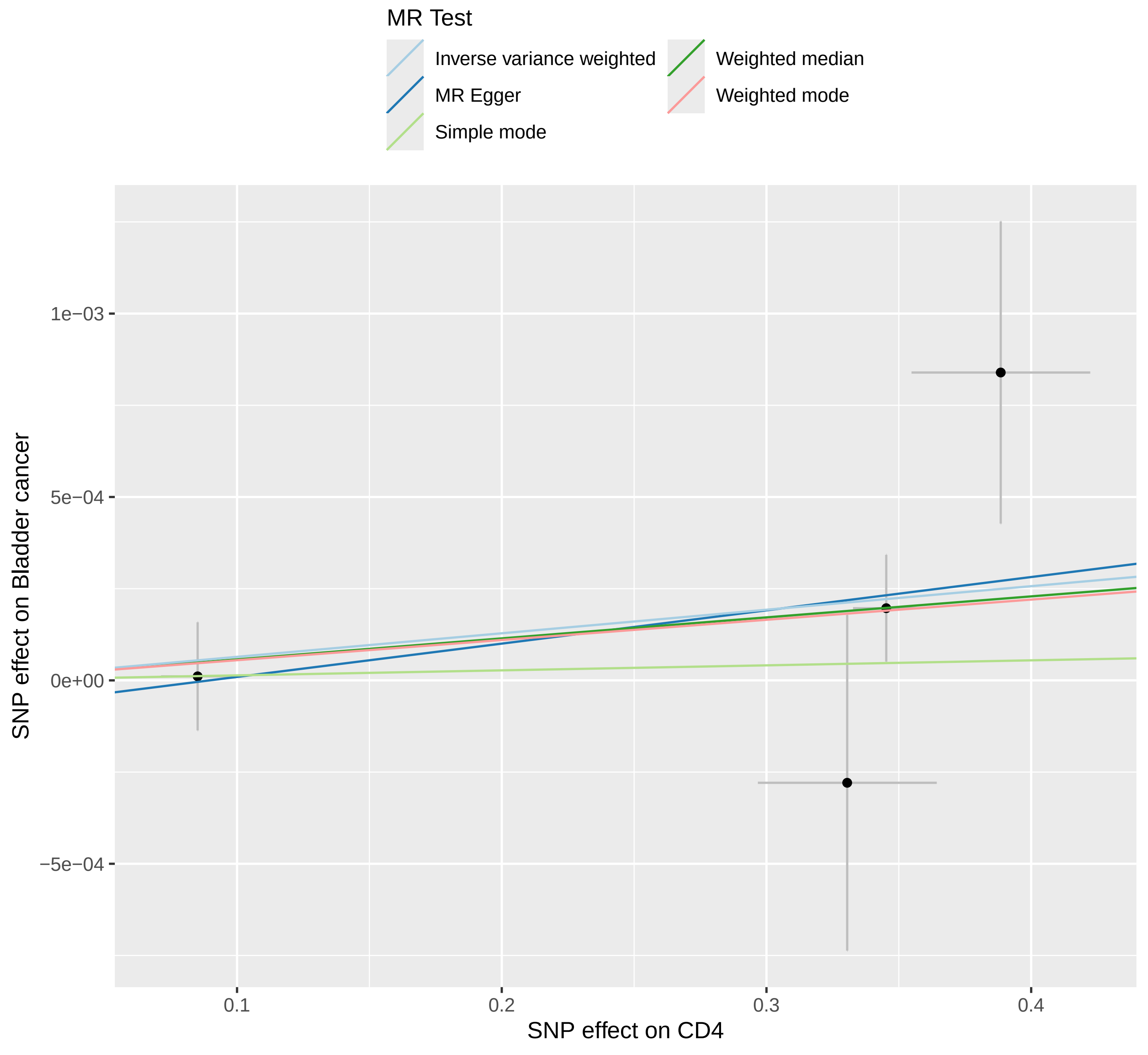
MR Test
Inverse variance weighted
MR Egger
Simple mode
Weighted median
Weighted mode
1e−03
5e−04
0e+00
−5e−04
0.1
0.2
0.3
0.4
SNP effect on Bladder cancer
SNP effect on CD4

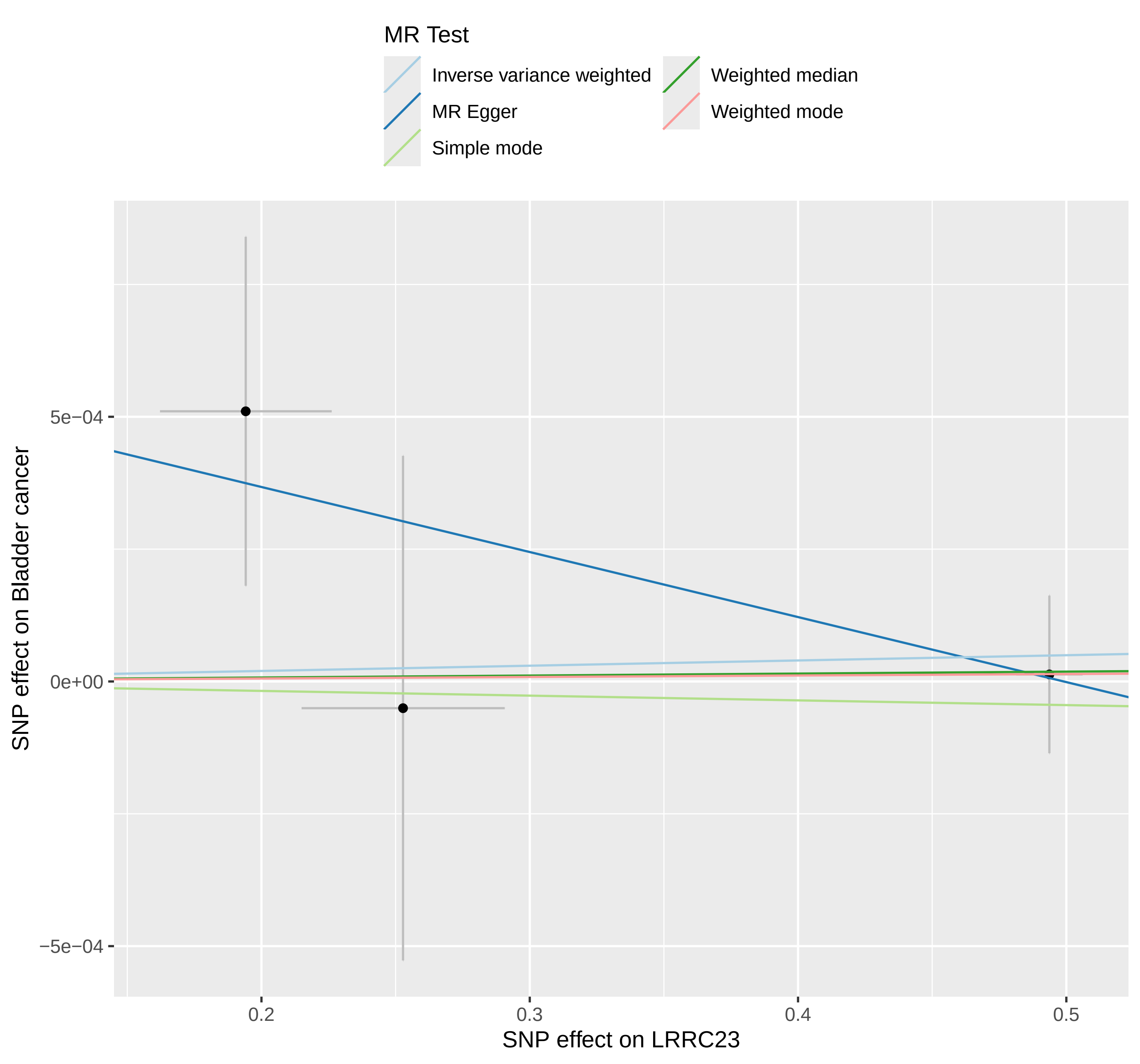

MR Test
Inverse variance weighted
MR Egger
Simple mode
Weighted median
Weighted mode
SNP effect on Bladder cancer
5e−04
0e+00
−5e−04
0.2
0.3
0.4
0.5
SNP effect on LRRC23

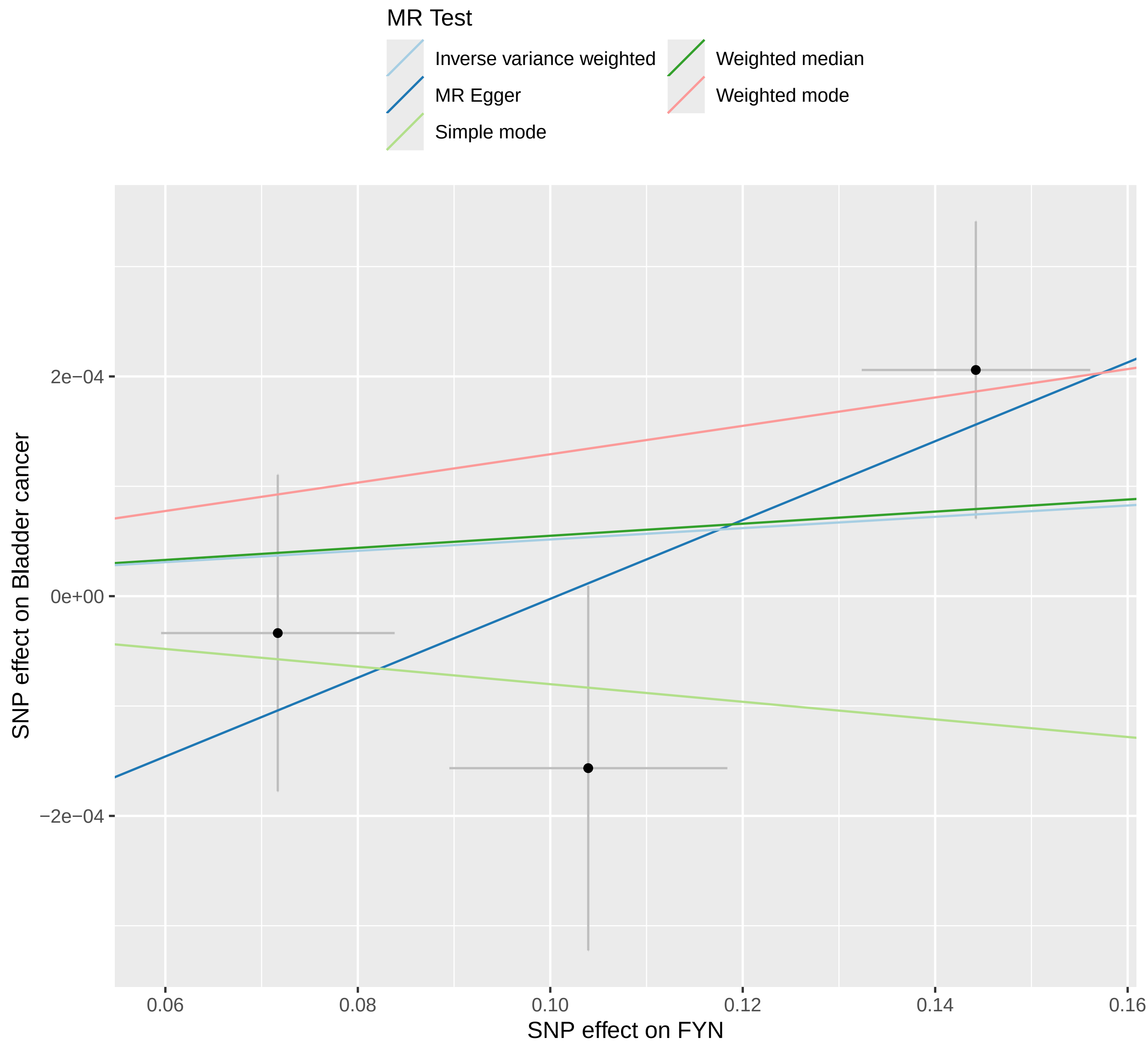

MR Test
Inverse variance weighted
MR Egger
Simple mode
Weighted median
Weighted mode
2e−04
0e+00
−2e−04
SNP effect on Bladder cancer
0.06
0.08
0.10
0.12
0.14
0.16
SNP effect on FYN

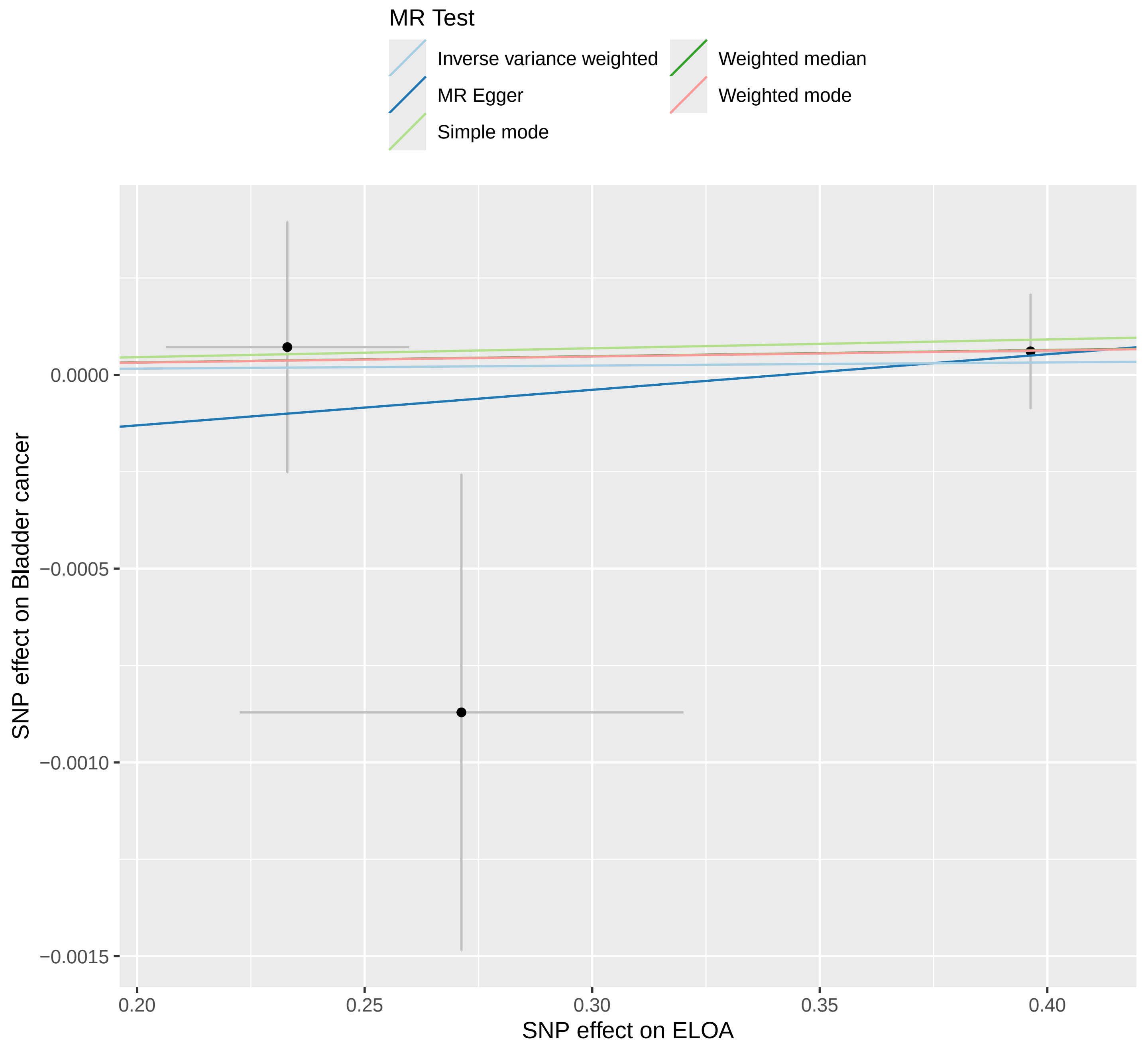
MR Test
Inverse variance weighted
MR Egger
Simple mode
Weighted median
Weighted mode
0.0000
−0.0005
−0.0010
−0.0015
SNP effect on Bladder cancer
0.20
0.25
0.30
0.35
0.40
SNP effect on ELOA

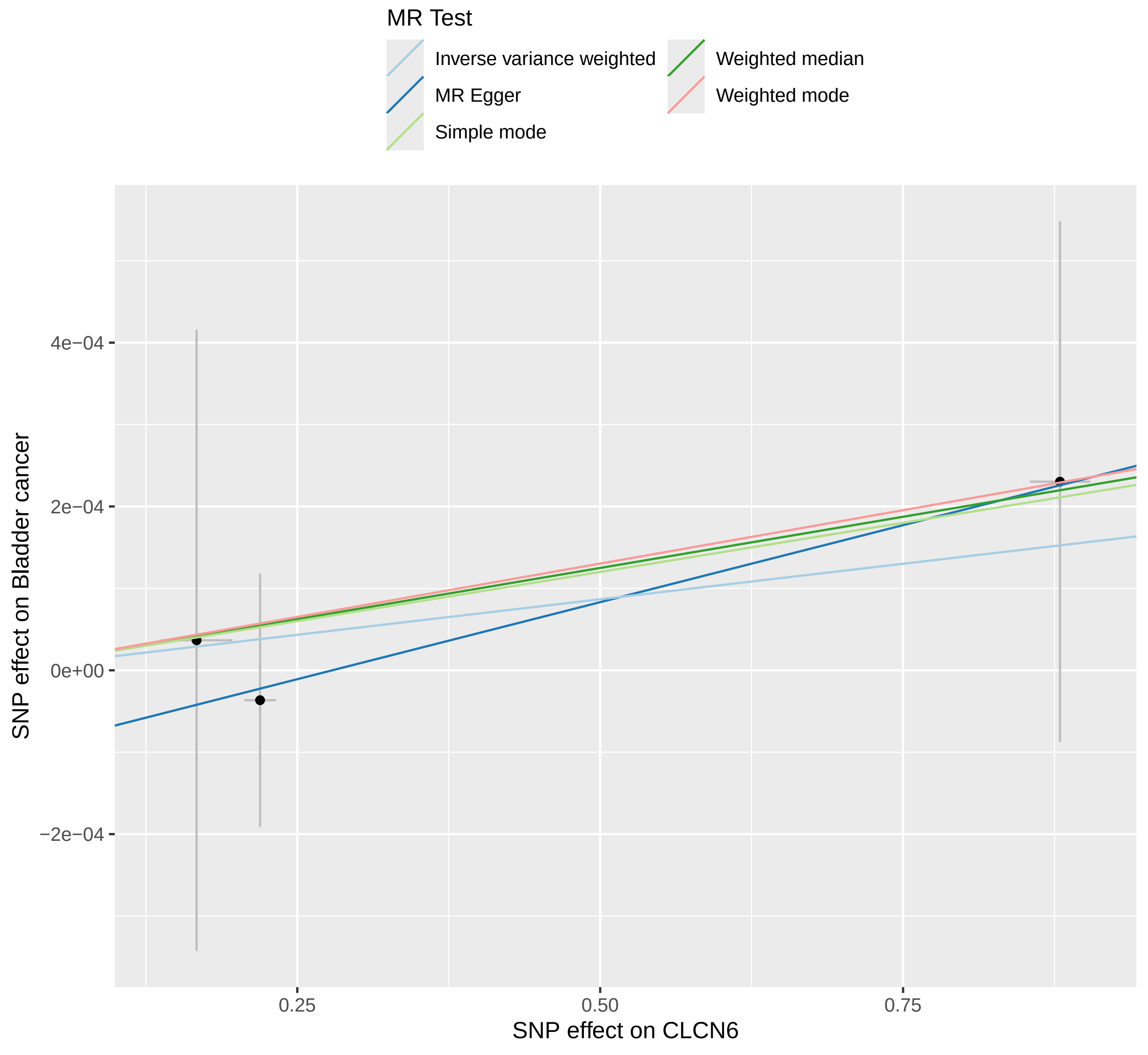
MR Test
Inverse variance weighted
MR Egger
Simple mode
Weighted median
Weighted mode
4e−04
2e−04
0e+00
−2e−04
SNP effect on Bladder cancer
0.25
0.50
0.75
SNP effect on CLCN6

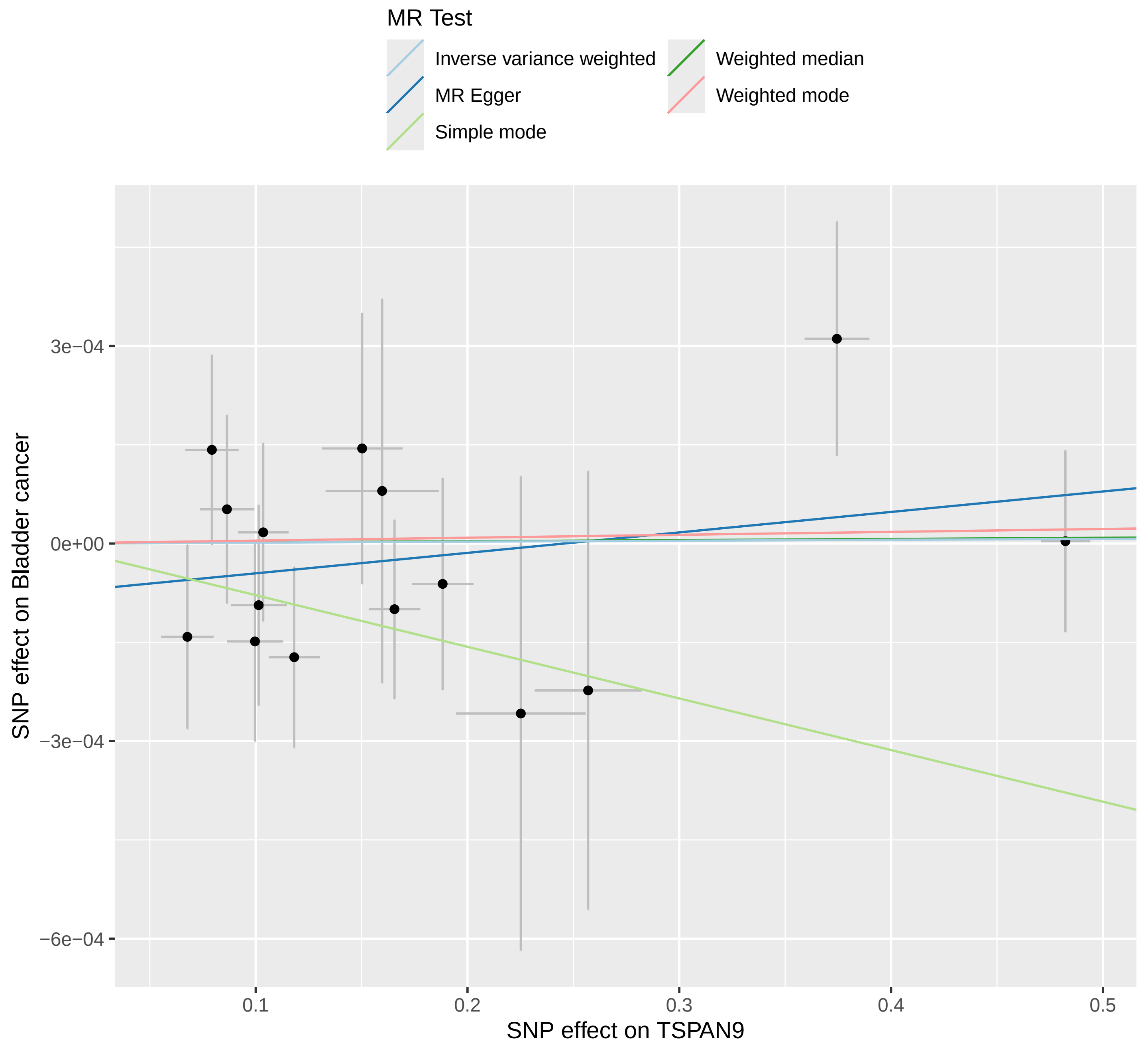
MR Test
Inverse variance weighted
MR Egger
Simple mode
Weighted median
Weighted mode
SNP effect on Bladder cancer
3e−04
0e+00
−3e−04
−6e−04
0.1
0.2
0.3
0.4
0.5
SNP effect on TSPAN9

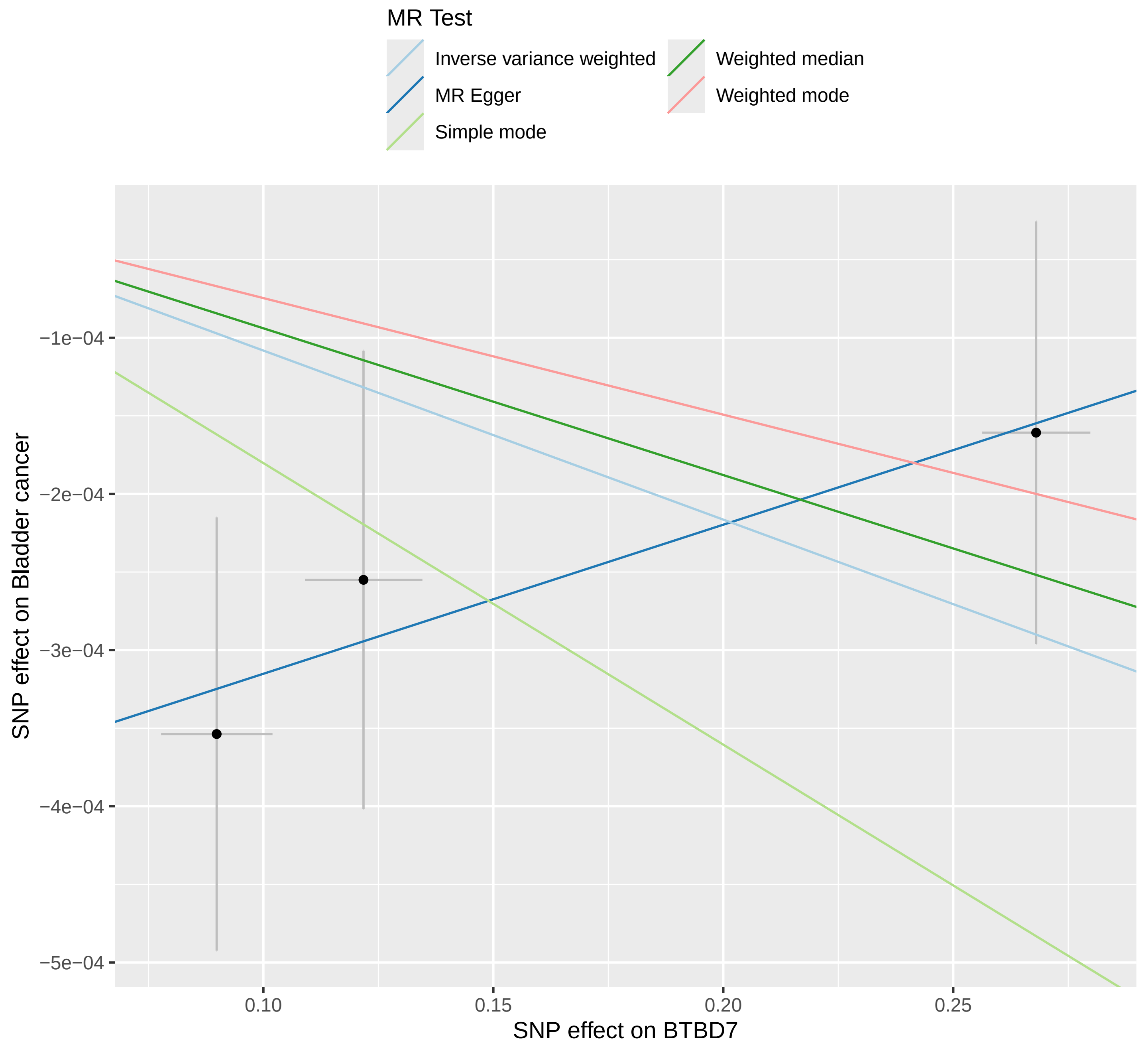

MR Test
Inverse variance weighted
MR Egger
Simple mode
Weighted median
Weighted mode
−1e−04
−2e−04
−3e−04
−4e−04
−5e−04
0.10
0.15
0.20
0.25
SNP effect on BTBD7
SNP effect on Bladder cancer

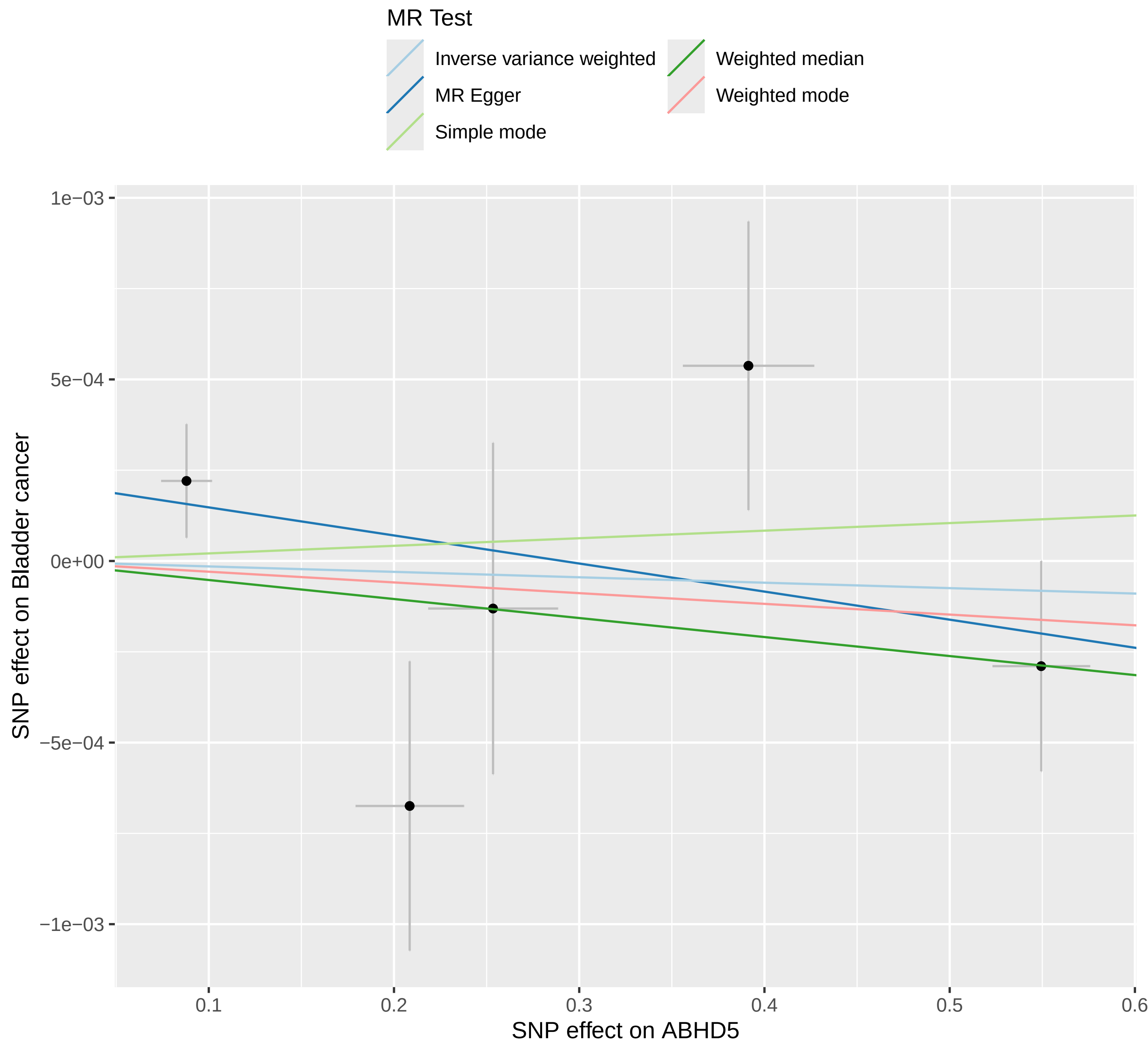
MR Test
Inverse variance weighted
MR Egger
Simple mode
Weighted median
Weighted mode
1e−03
5e−04
0e+00
−5e−04
−1e−03
SNP effect on Bladder cancer
0.1
0.2
0.3
0.4
0.5
0.6
SNP effect on ABHD5

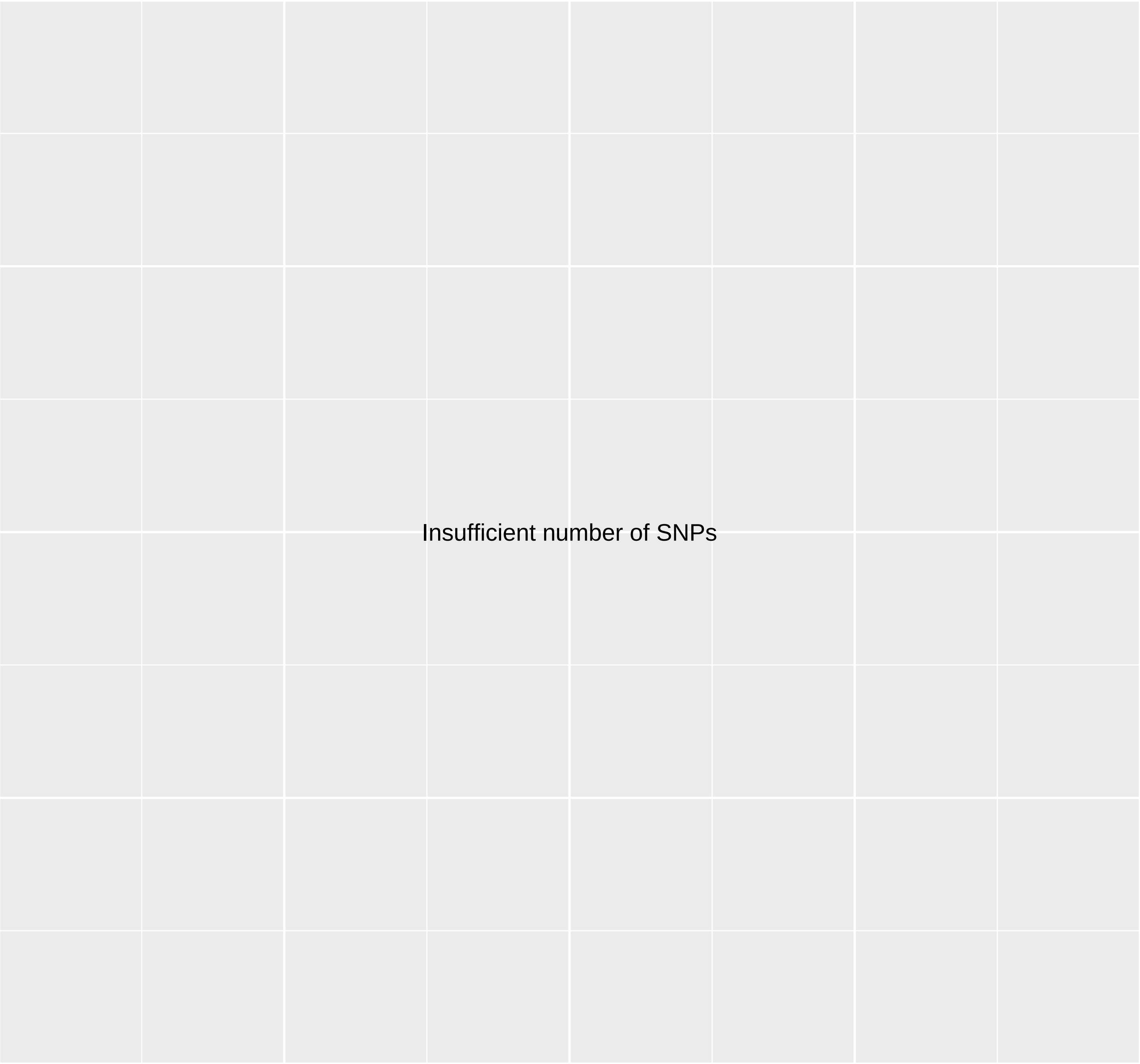
Insufficient number of SNPs

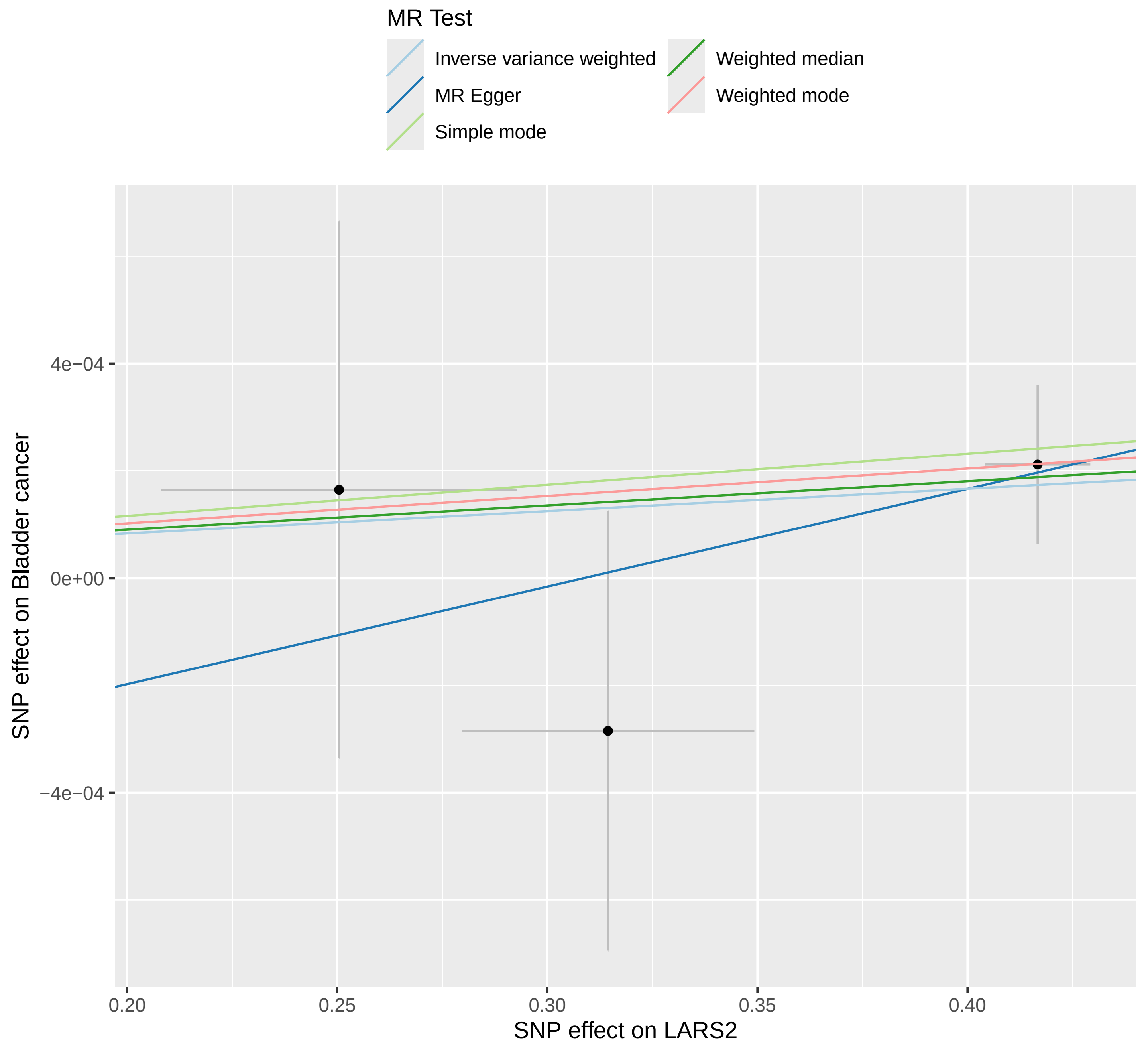
MR Test
Inverse variance weighted
MR Egger
Simple mode
Weighted median
Weighted mode
4e−04
0e+00
−4e−04
SNP effect on Bladder cancer
0.20
0.25
0.30
0.35
0.40
SNP effect on LARS2

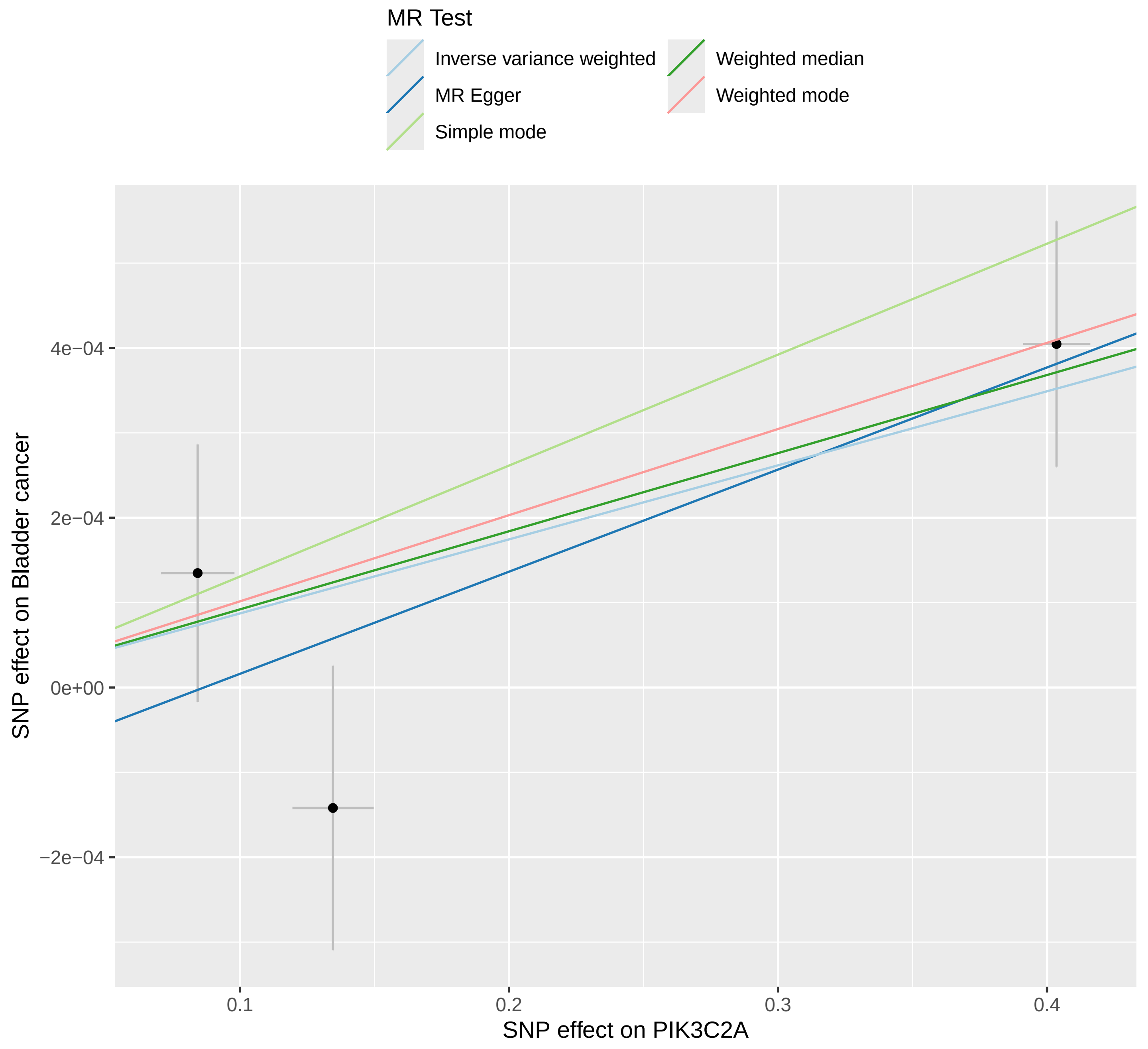

MR Test
Inverse variance weighted
MR Egger
Simple mode
Weighted median
Weighted mode
4e−04
2e−04
0e+00
−2e−04
SNP effect on Bladder cancer
0.1
0.2
0.3
0.4
SNP effect on PIK3C2A

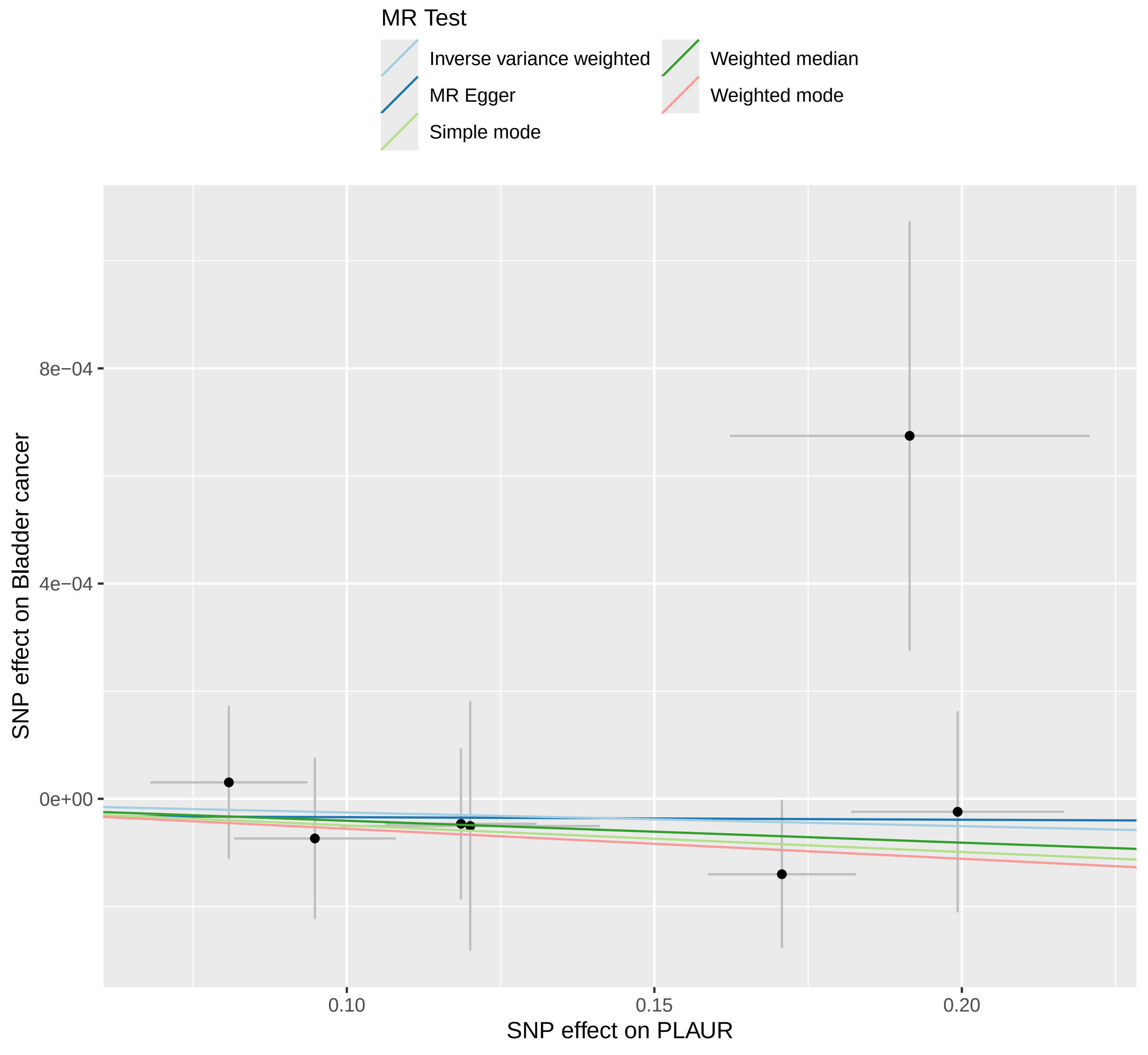
MR Test
Inverse variance weighted
MR Egger
Simple mode
Weighted median
Weighted mode
8e−04
4e−04
0e+00
SNP effect on Bladder cancer
0.10
0.15
0.20
SNP effect on PLAUR

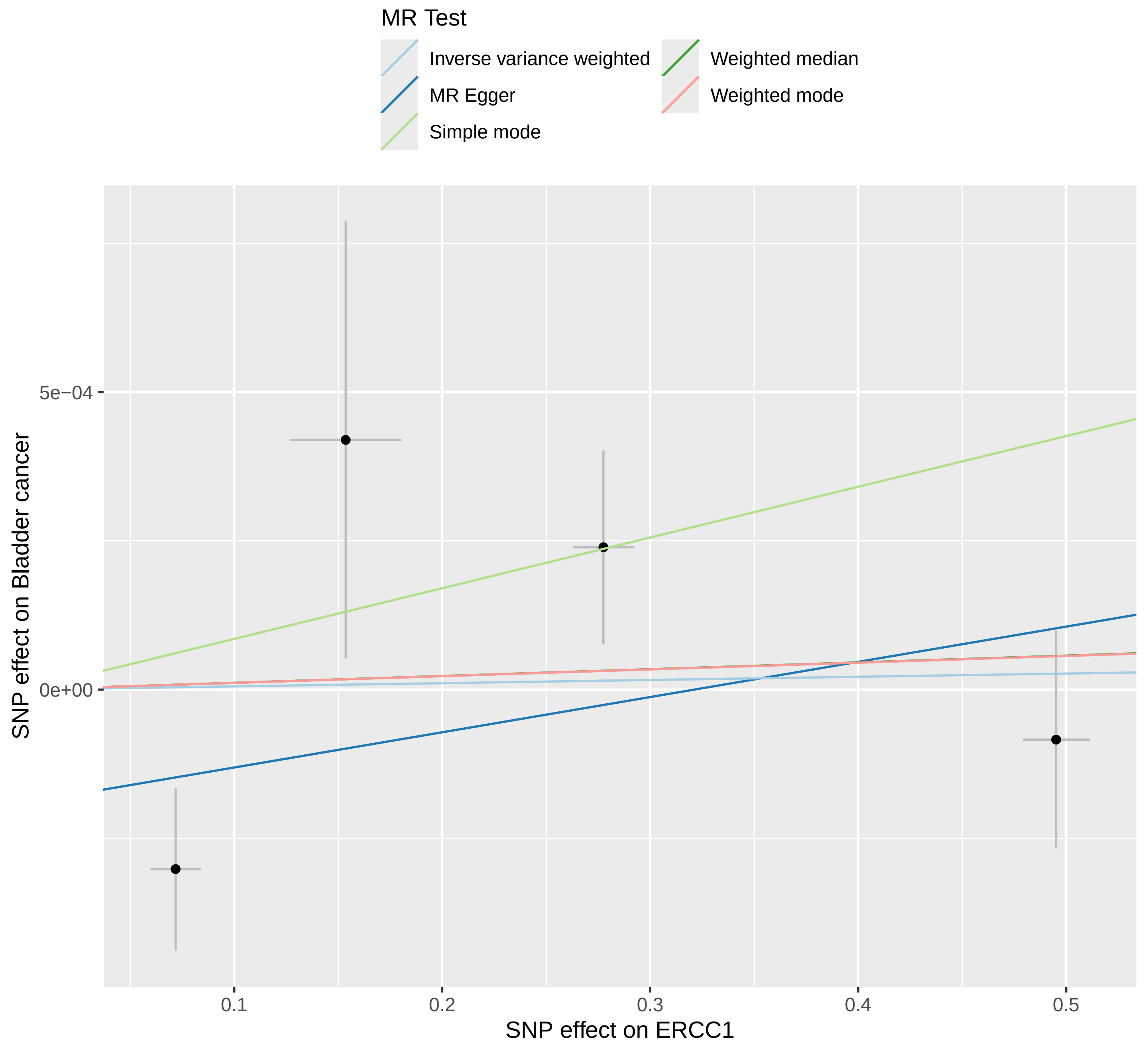

MR Test
Inverse variance weighted
MR Egger
Simple mode
Weighted median
Weighted mode
5e−04
0e+00
0.1
0.2
0.3
0.4
0.5
SNP effect on ERCC1
SNP effect on Bladder cancer

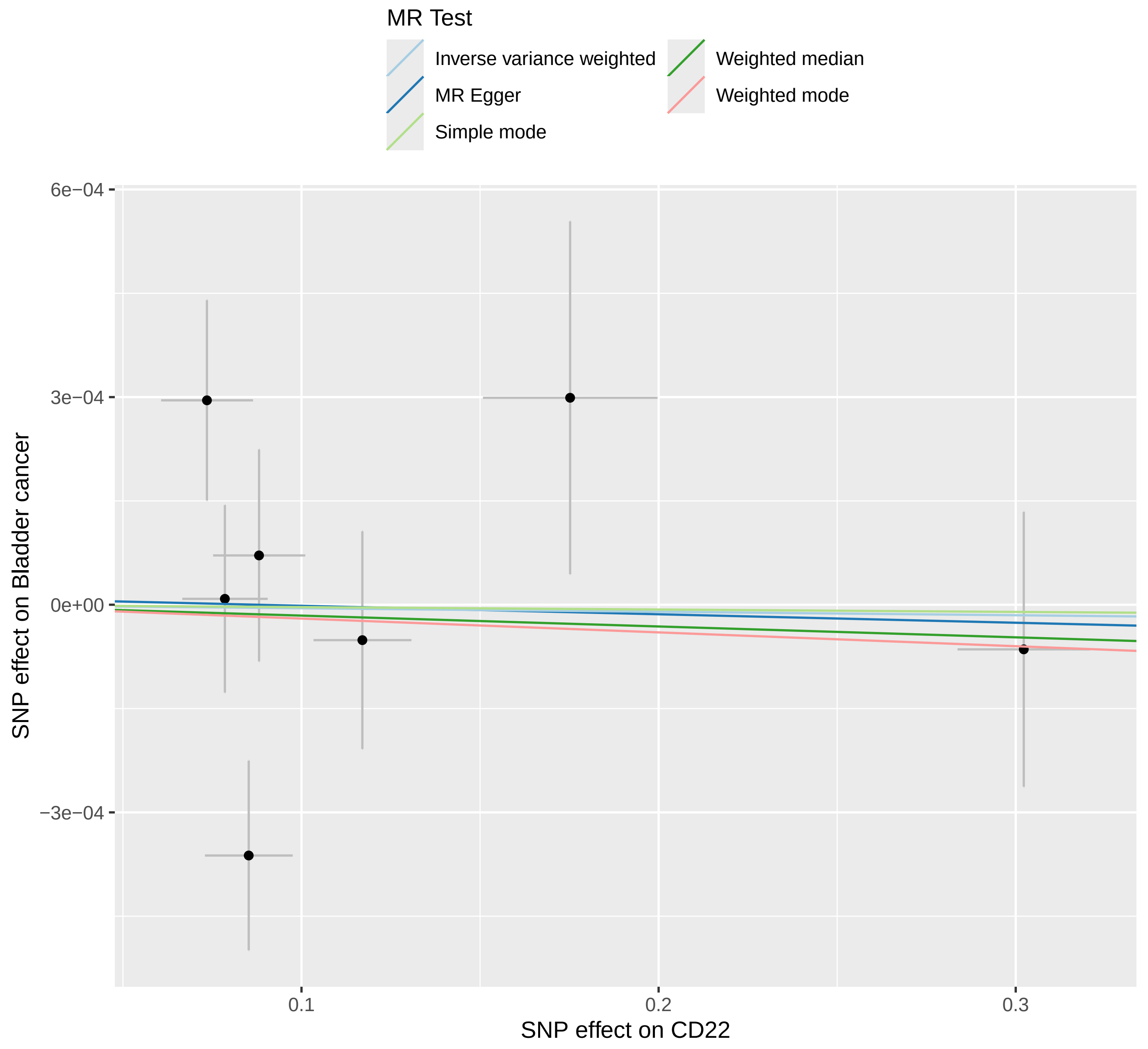

MR Test
Inverse variance weighted
MR Egger
Simple mode
Weighted median
Weighted mode
6e−04
3e−04
0e+00
−3e−04
SNP effect on Bladder cancer
0.1
0.2
0.3
SNP effect on CD22

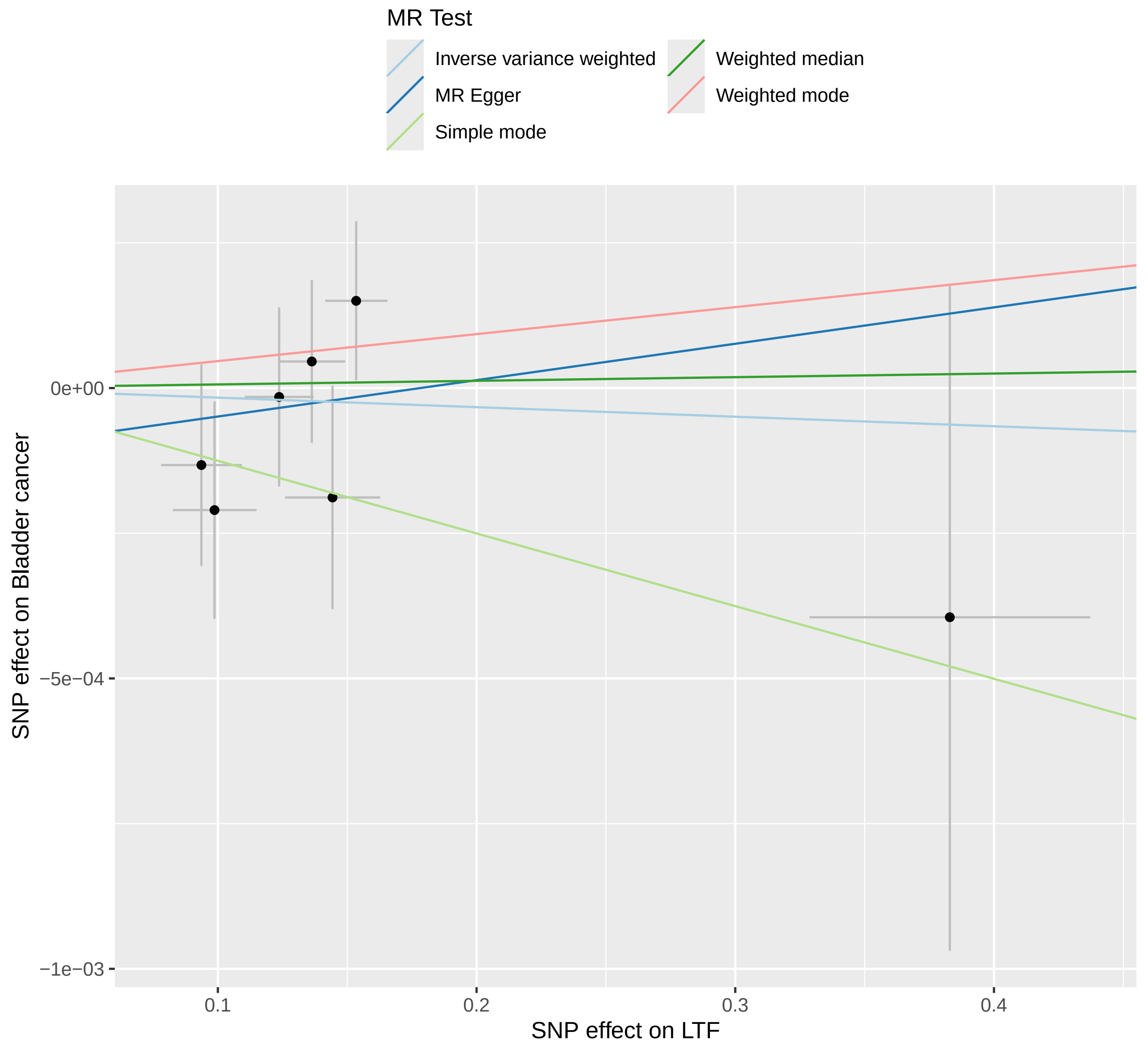
MR Test
Inverse variance weighted
MR Egger
Simple mode
Weighted median
Weighted mode
0e+00
−5e−04
−1e−03
SNP effect on Bladder cancer
0.1
0.2
0.3
0.4
SNP effect on LTF

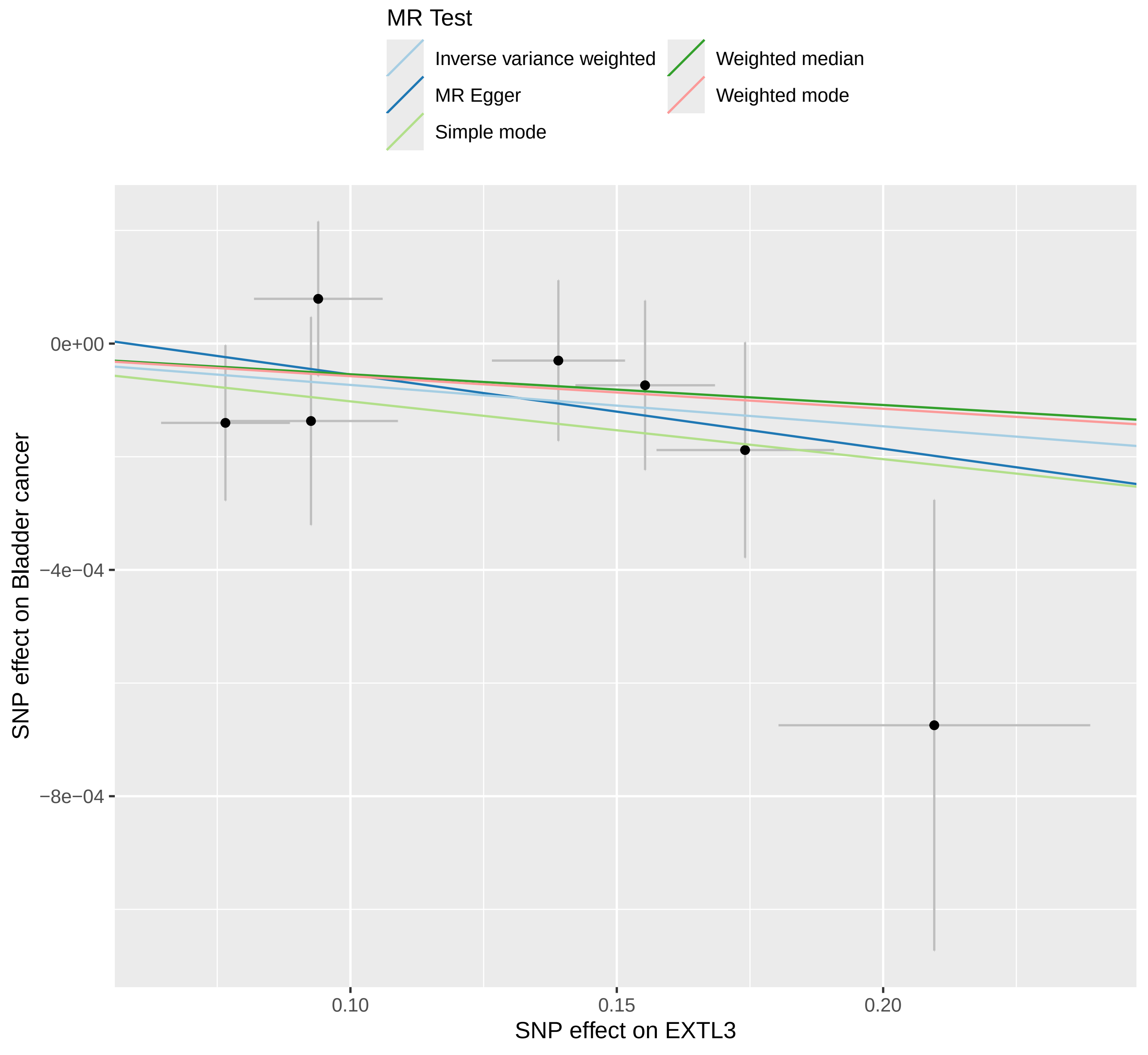
MR Test
Inverse variance weighted
MR Egger
Simple mode
Weighted median
Weighted mode
0e+00
−4e−04
−8e−04
SNP effect on Bladder cancer
0.10
0.15
0.20
SNP effect on EXTL3

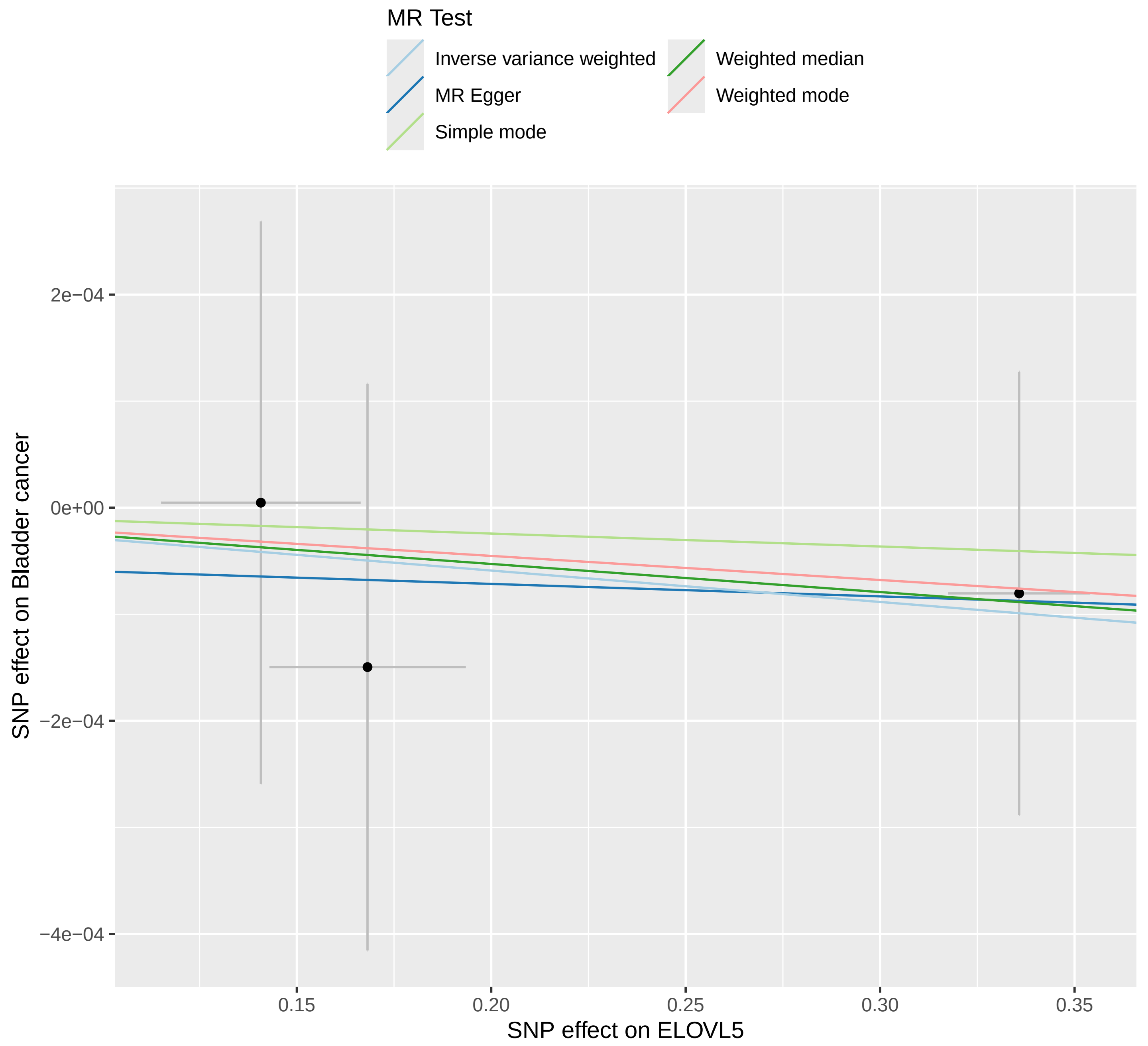
MR Test
Inverse variance weighted
MR Egger
Simple mode
Weighted median
Weighted mode
2e−04
0e+00
−2e−04
−4e−04
0.15
0.20
0.25
0.30
0.35
SNP effect on ELOVL5
SNP effect on Bladder cancer

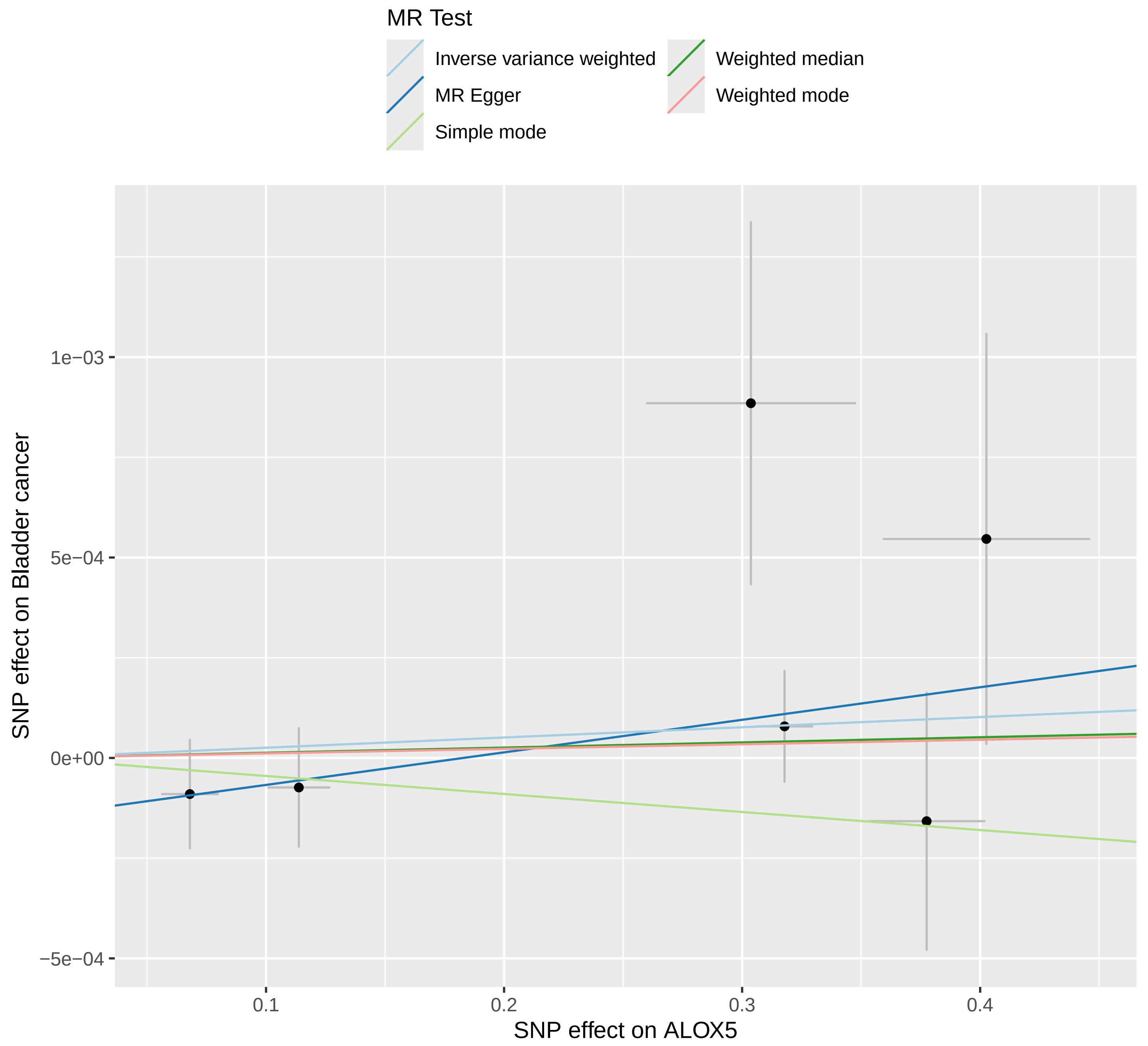

MR Test
Inverse variance weighted
MR Egger
Simple mode
Weighted median
Weighted mode
1e−03
5e−04
0e+00
−5e−04
0.1
0.2
0.3
0.4
SNP effect on ALOX5
SNP effect on Bladder cancer

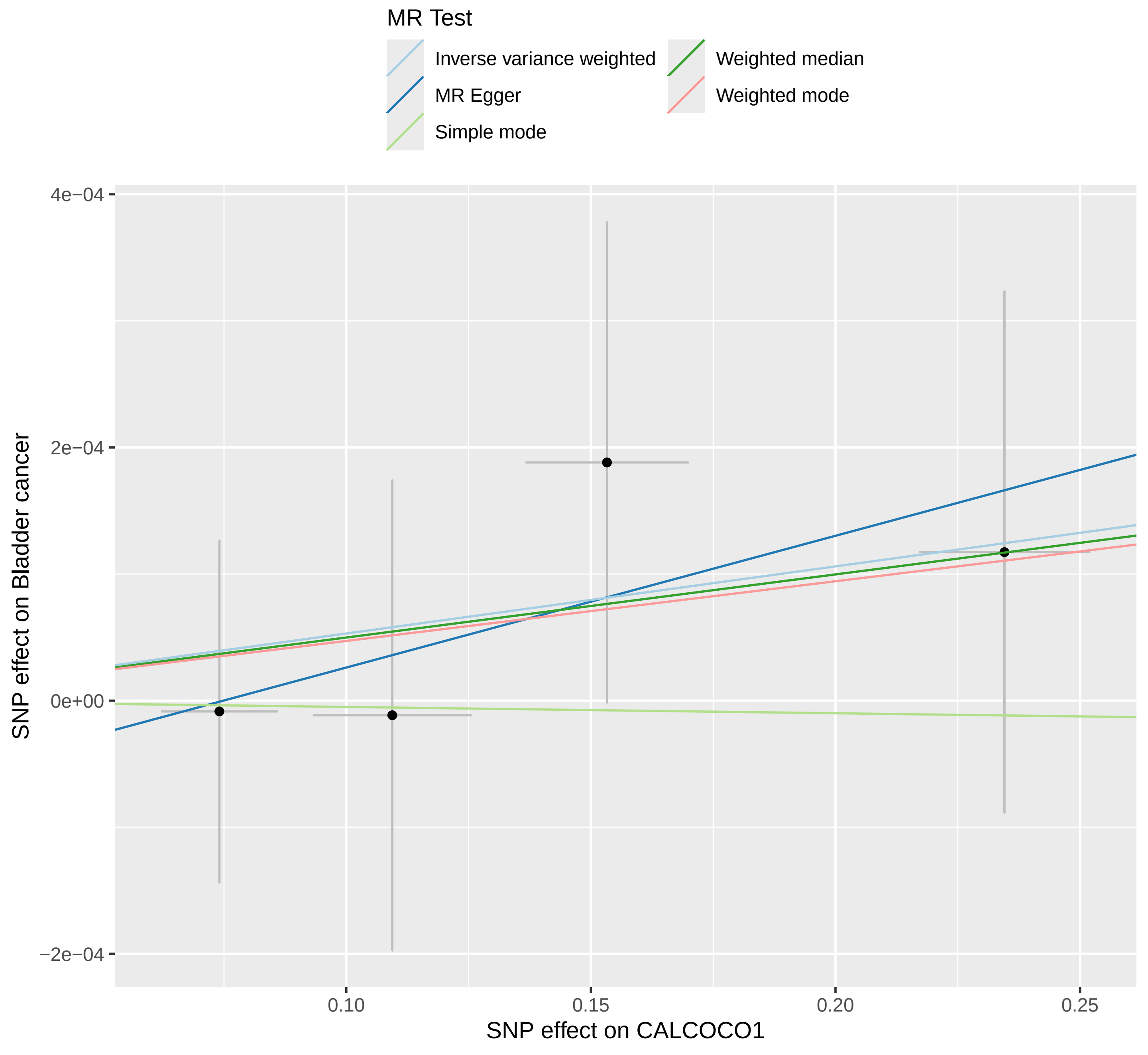
MR Test
Inverse variance weighted
MR Egger
Simple mode
Weighted median
Weighted mode
4e−04
2e−04
0e+00
−2e−04
0.10
0.15
0.20
0.25
SNP effect on Bladder cancer
SNP effect on CALCOCO1

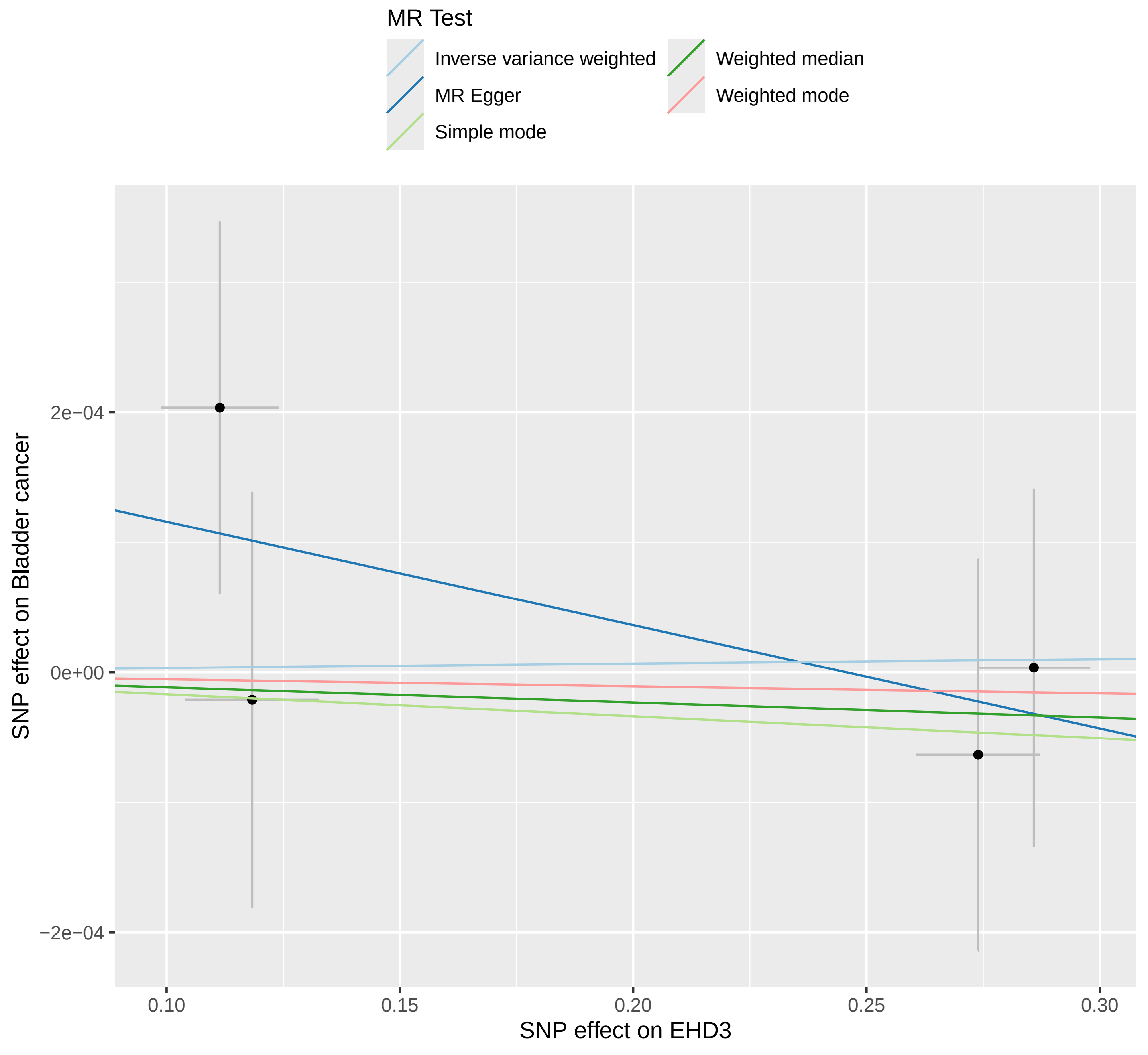
MR Test
Inverse variance weighted
MR Egger
Simple mode
Weighted median
Weighted mode
2e−04
0e+00
−2e−04
SNP effect on Bladder cancer
0.10
0.15
0.20
0.25
0.30
SNP effect on EHD3

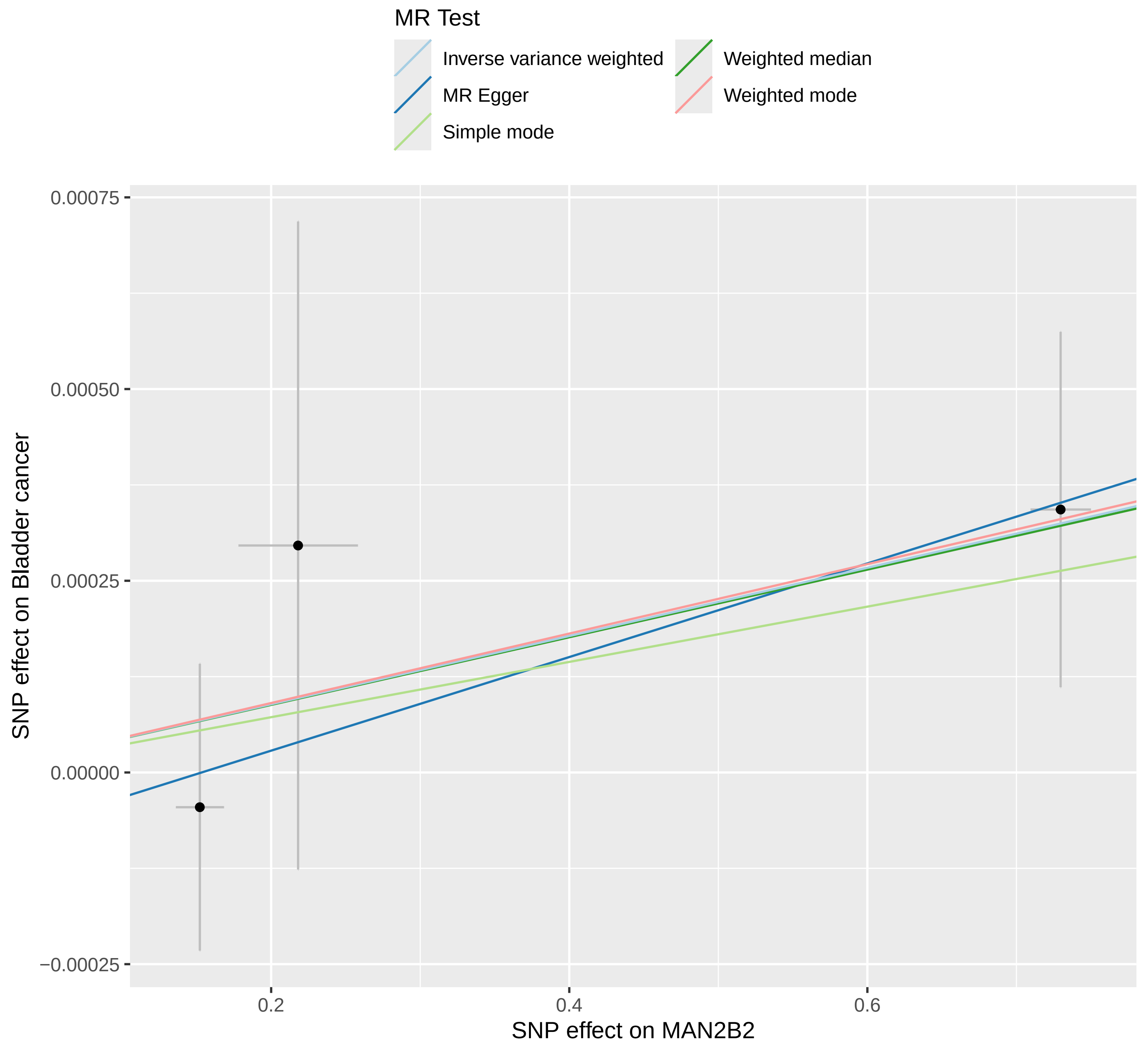

MR Test
Inverse variance weighted
MR Egger
Simple mode
Weighted median
Weighted mode
0.00075
0.00050
0.00025
0.00000
−0.00025
SNP effect on Bladder cancer
0.2
0.4
0.6
SNP effect on MAN2B2

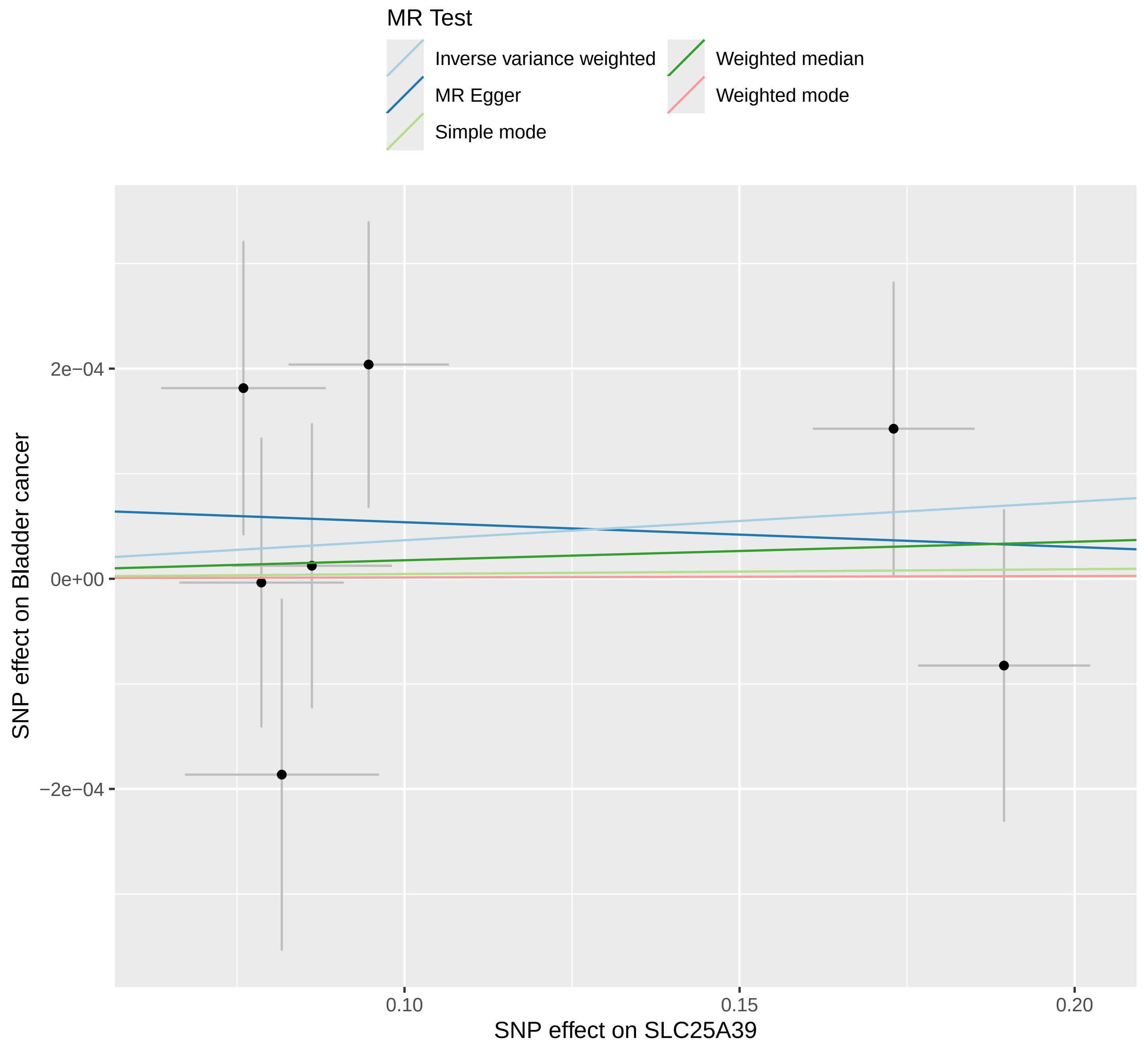
MR Test
Inverse variance weighted
MR Egger
Simple mode
Weighted median
Weighted mode
2e−04
0e+00
−2e−04
SNP effect on Bladder cancer
0.10
0.15
0.20
SNP effect on SLC25A39

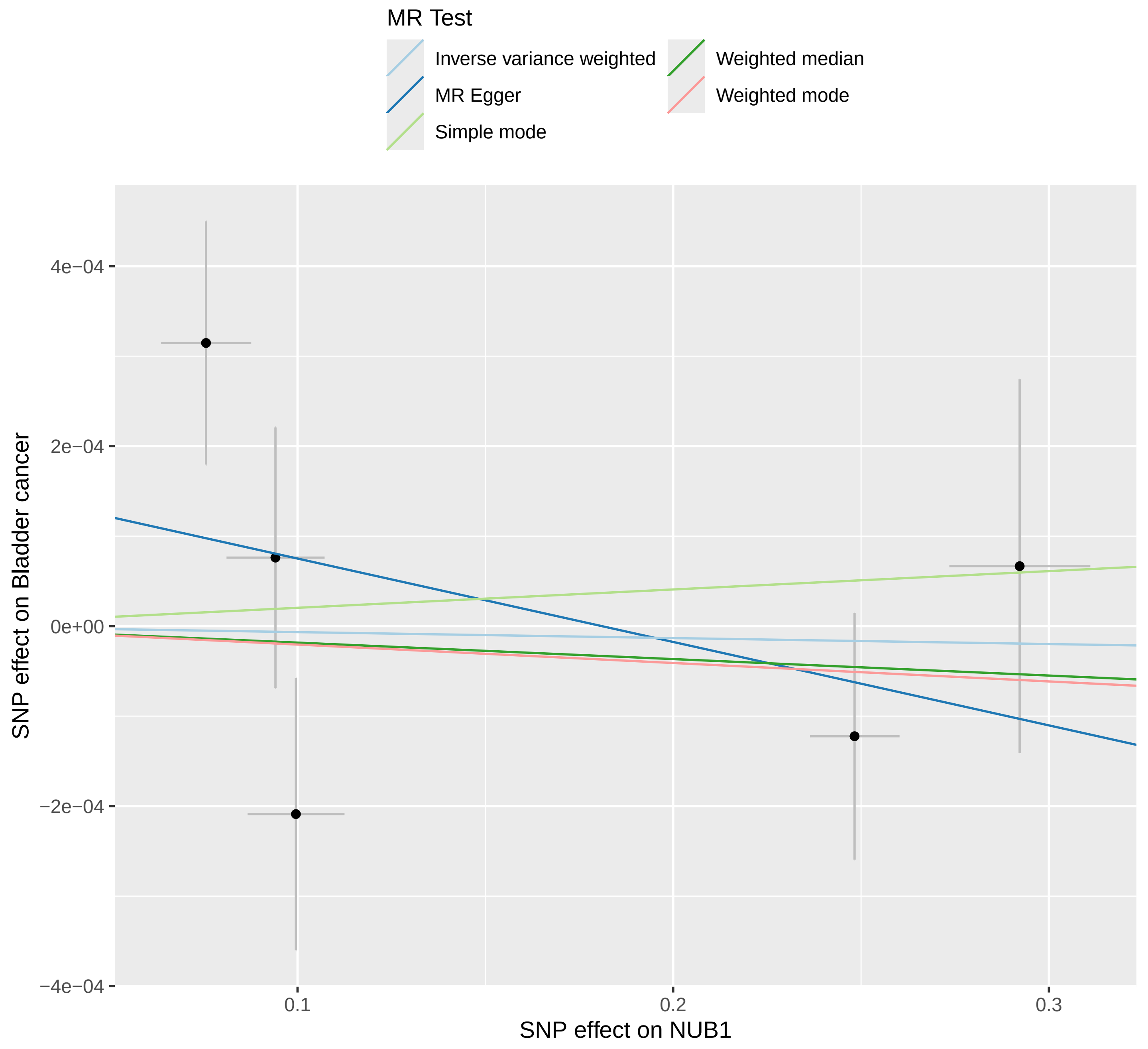

MR Test
Inverse variance weighted
MR Egger
Simple mode
Weighted median
Weighted mode
4e−04
2e−04
0e+00
−2e−04
−4e−04
0.1
0.2
0.3
SNP effect on Bladder cancer
SNP effect on NUB1

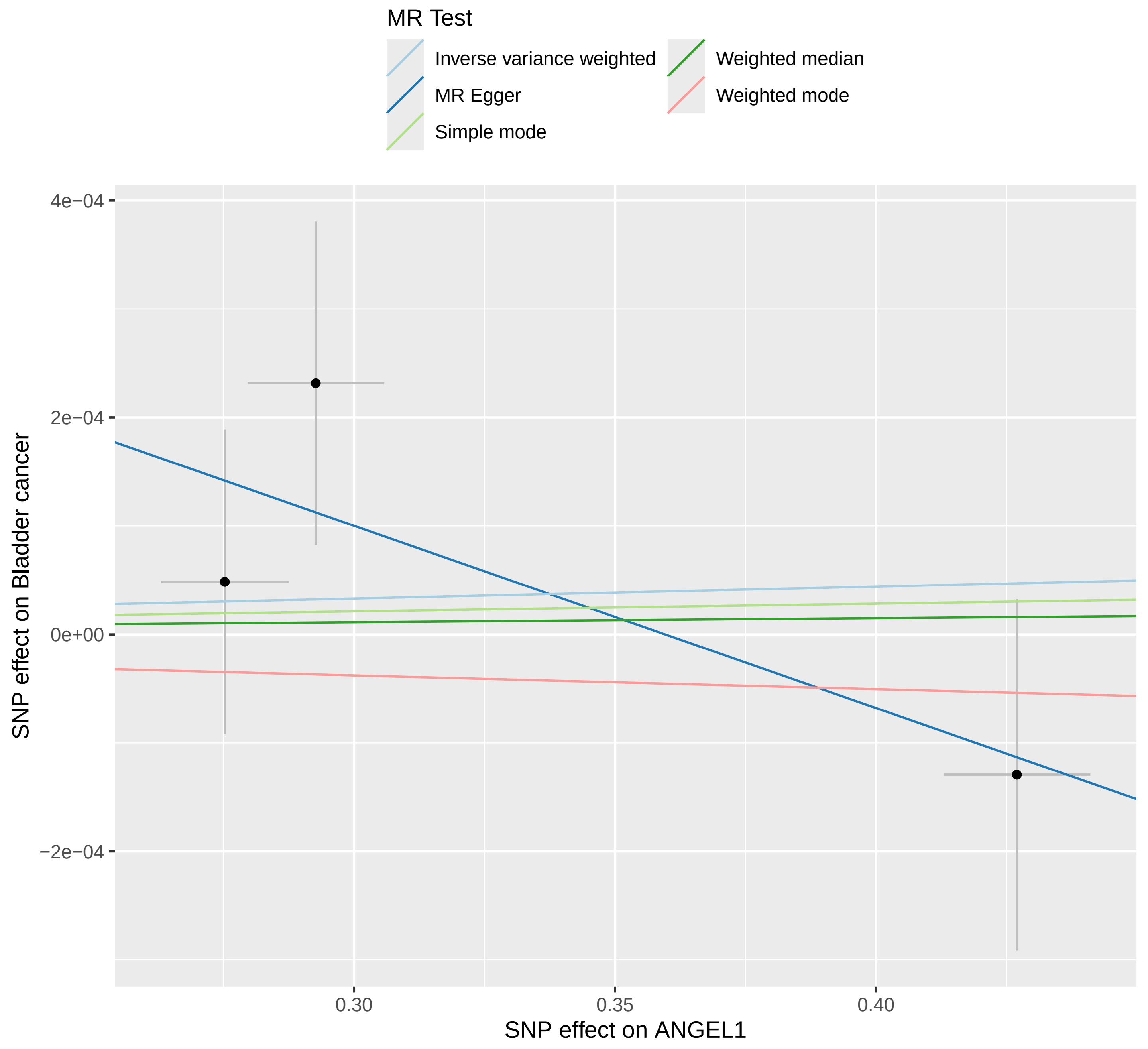

MR Test
Inverse variance weighted
MR Egger
Simple mode
Weighted median
Weighted mode
4e−04
2e−04
0e+00
−2e−04
0.30
0.35
0.40
SNP effect on Bladder cancer
SNP effect on ANGEL1

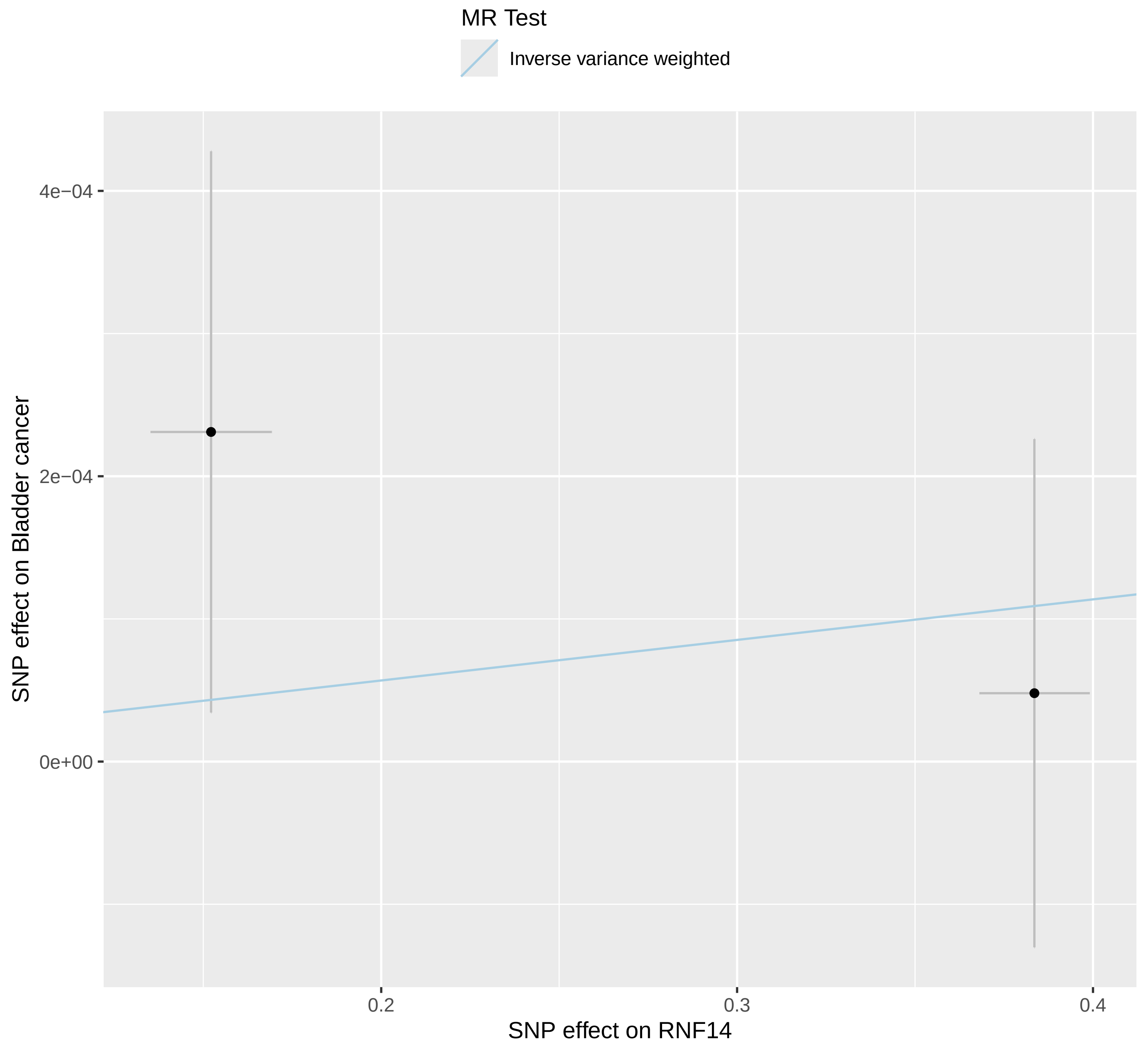
MR Test
Inverse variance weighted
4e−04
2e−04
0e+00
0.2
0.3
0.4
SNP effect on RNF14
SNP effect on Bladder cancer

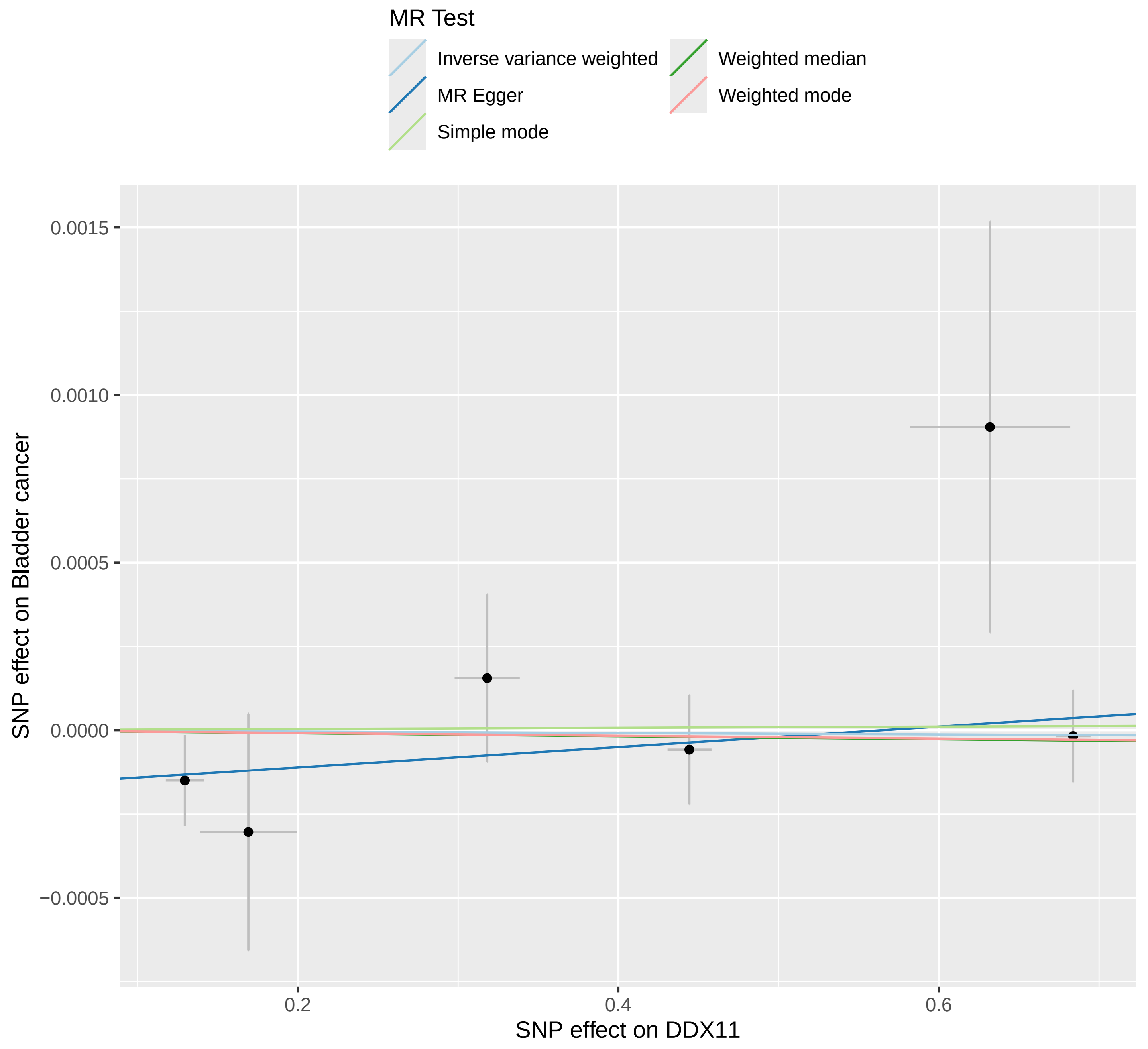
MR Test
Inverse variance weighted
MR Egger
Simple mode
Weighted median
Weighted mode
0.0015
0.0010
0.0005
0.0000
−0.0005
SNP effect on Bladder cancer
0.2
0.4
0.6
SNP effect on DDX11

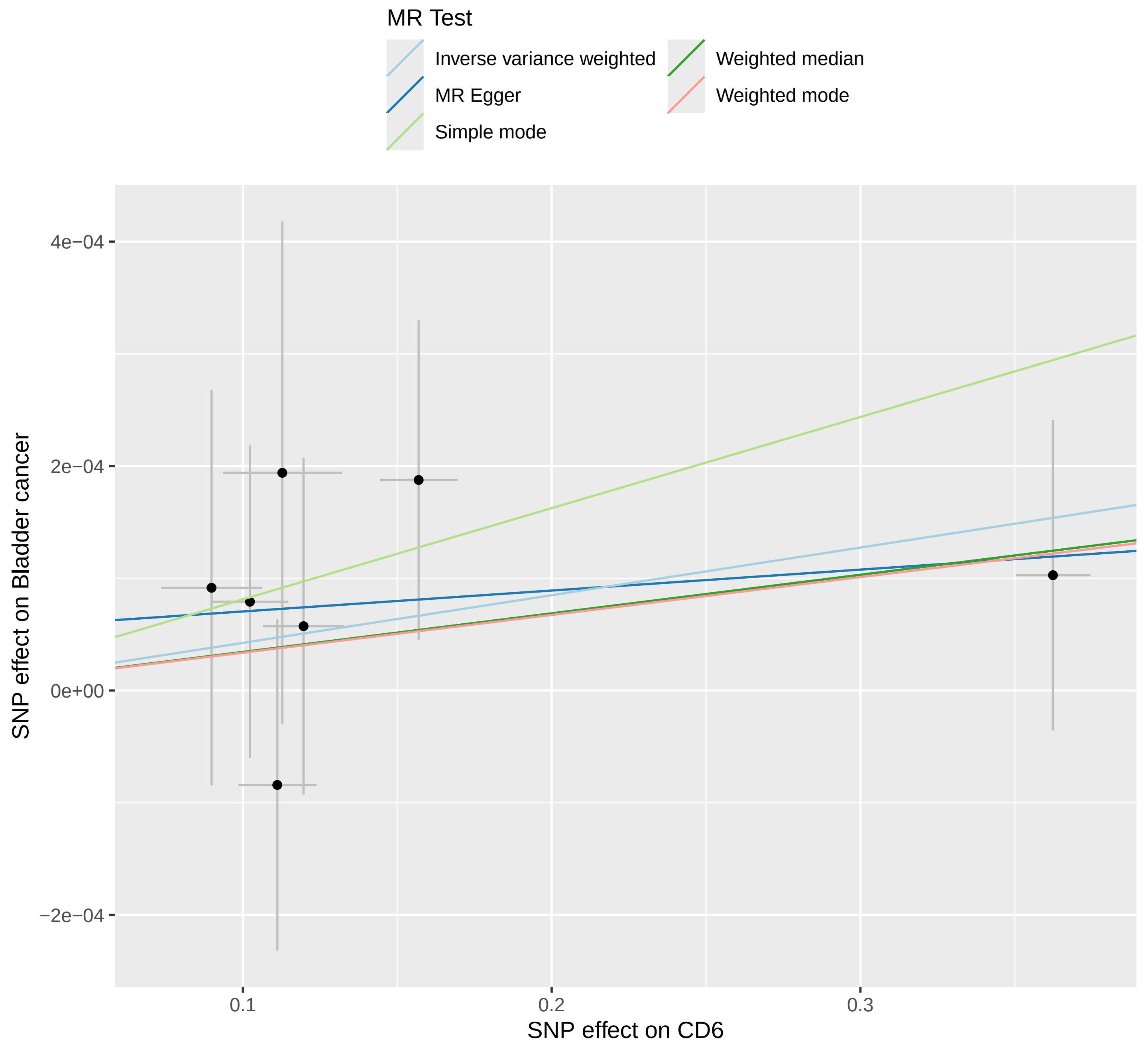

MR Test
Inverse variance weighted
MR Egger
Simple mode
Weighted median
Weighted mode
4e−04
2e−04
0e+00
−2e−04
0.1
0.2
0.3
SNP effect on CD6
SNP effect on Bladder cancer

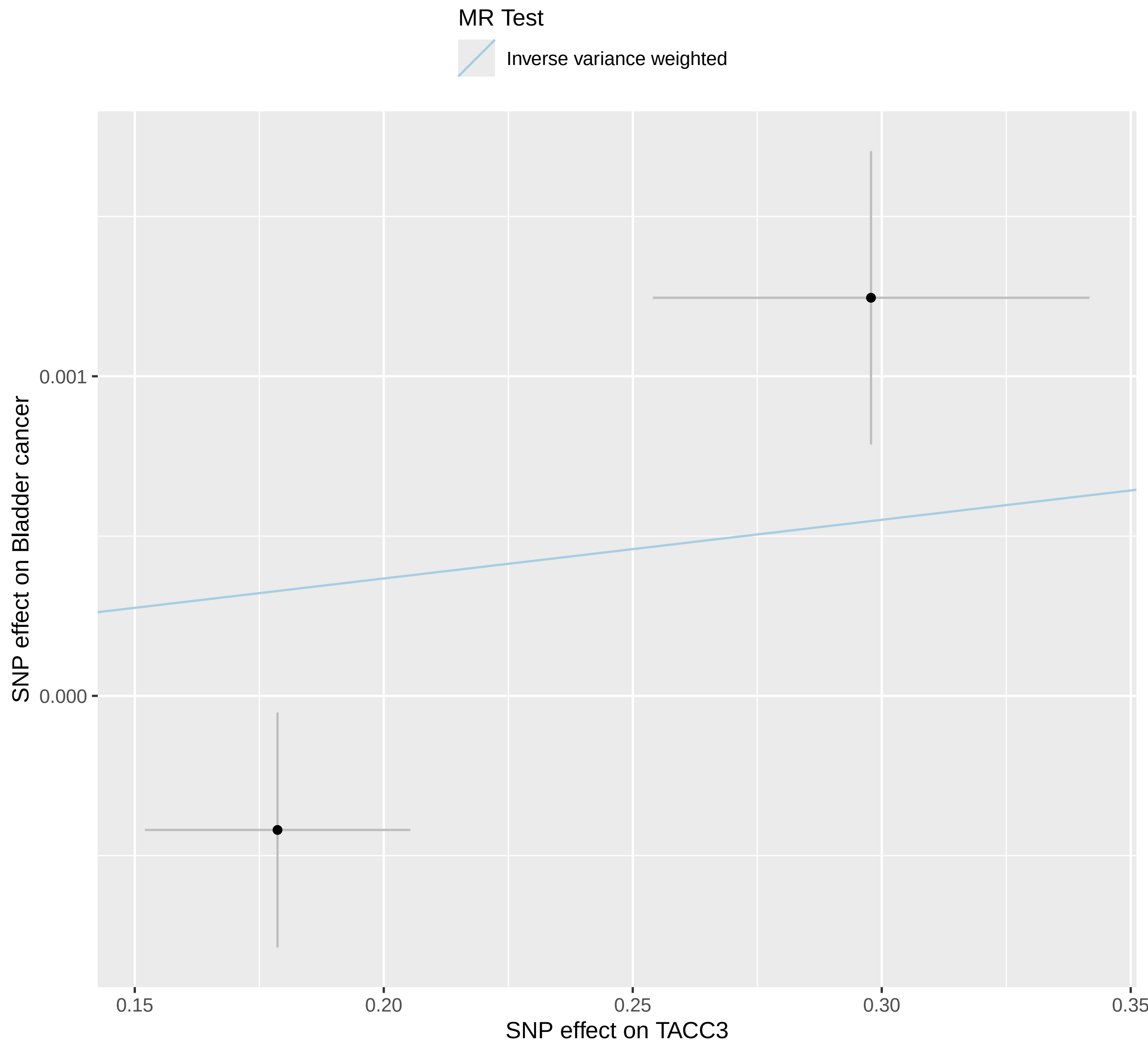

MR Test
Inverse variance weighted
0.001
0.000
SNP effect on Bladder cancer
0.15
0.20
0.25
0.30
0.35
SNP effect on TACC3

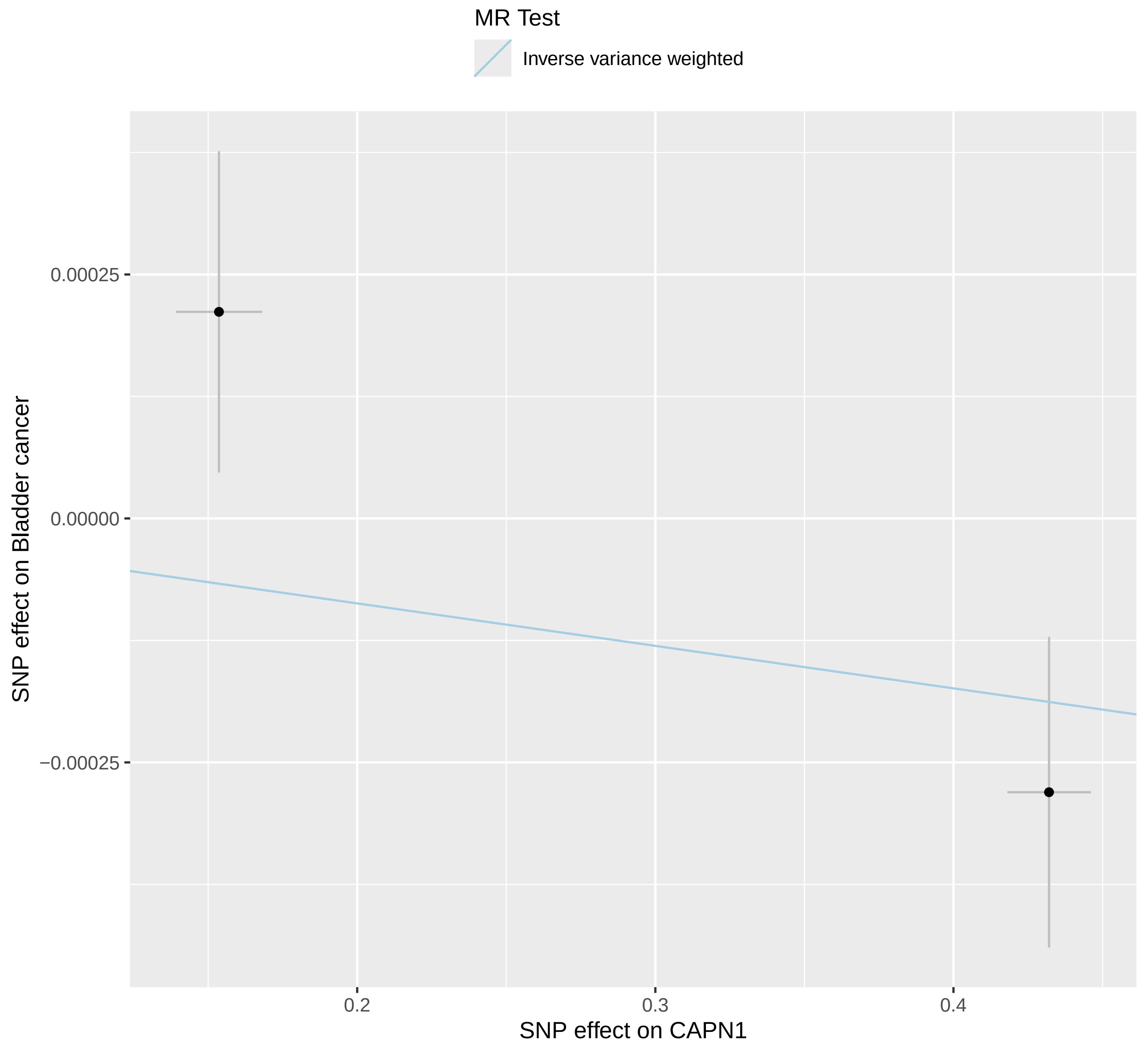

MR Test
Inverse variance weighted
0.00025
0.00000
−0.00025
0.2
0.3
0.4
SNP effect on Bladder cancer
SNP effect on CAPN1

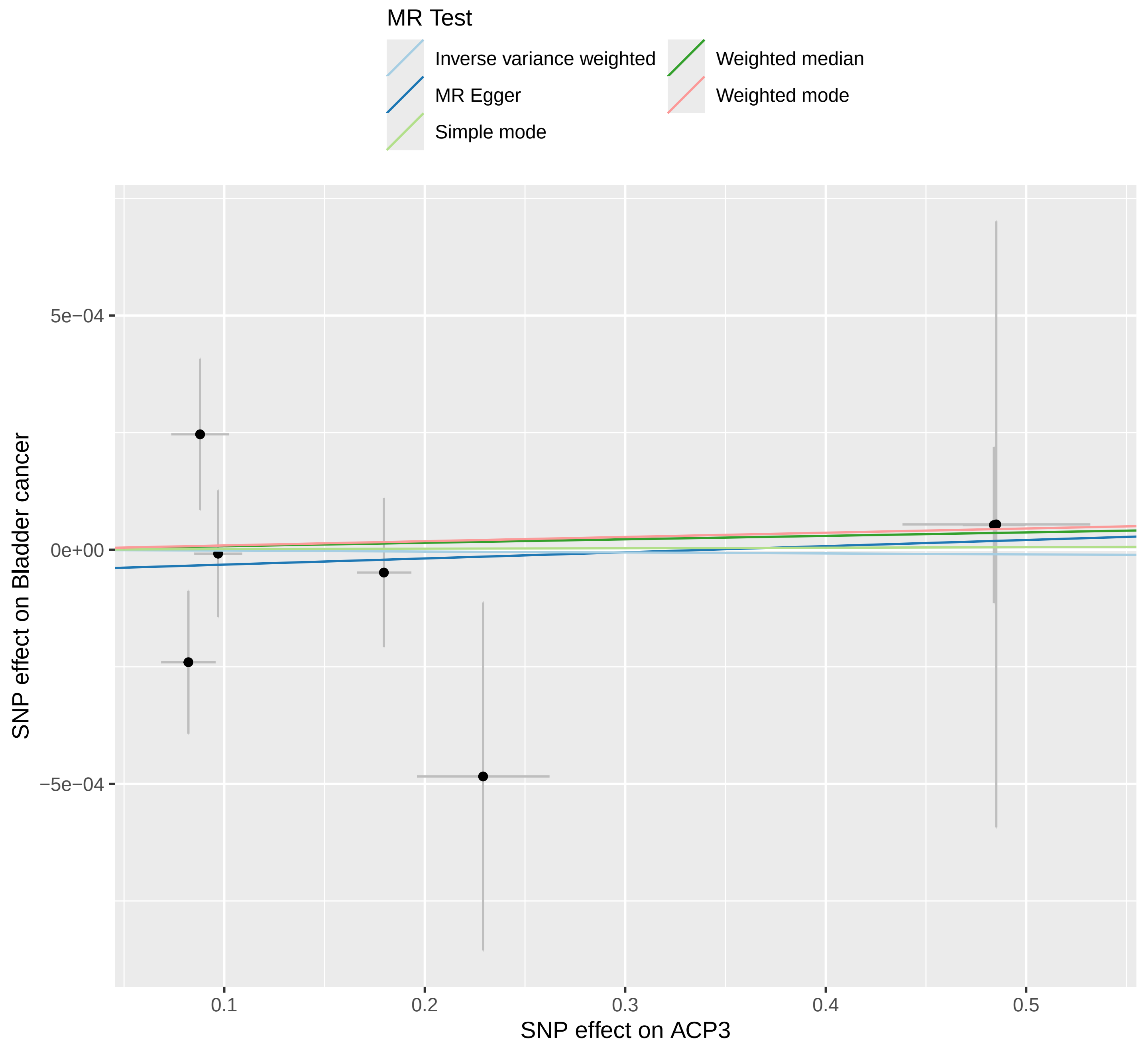

MR Test
Inverse variance weighted
MR Egger
Simple mode
Weighted median
Weighted mode
5e−04
0e+00
−5e−04
SNP effect on Bladder cancer
0.1
0.2
0.3
0.4
0.5
SNP effect on ACP3

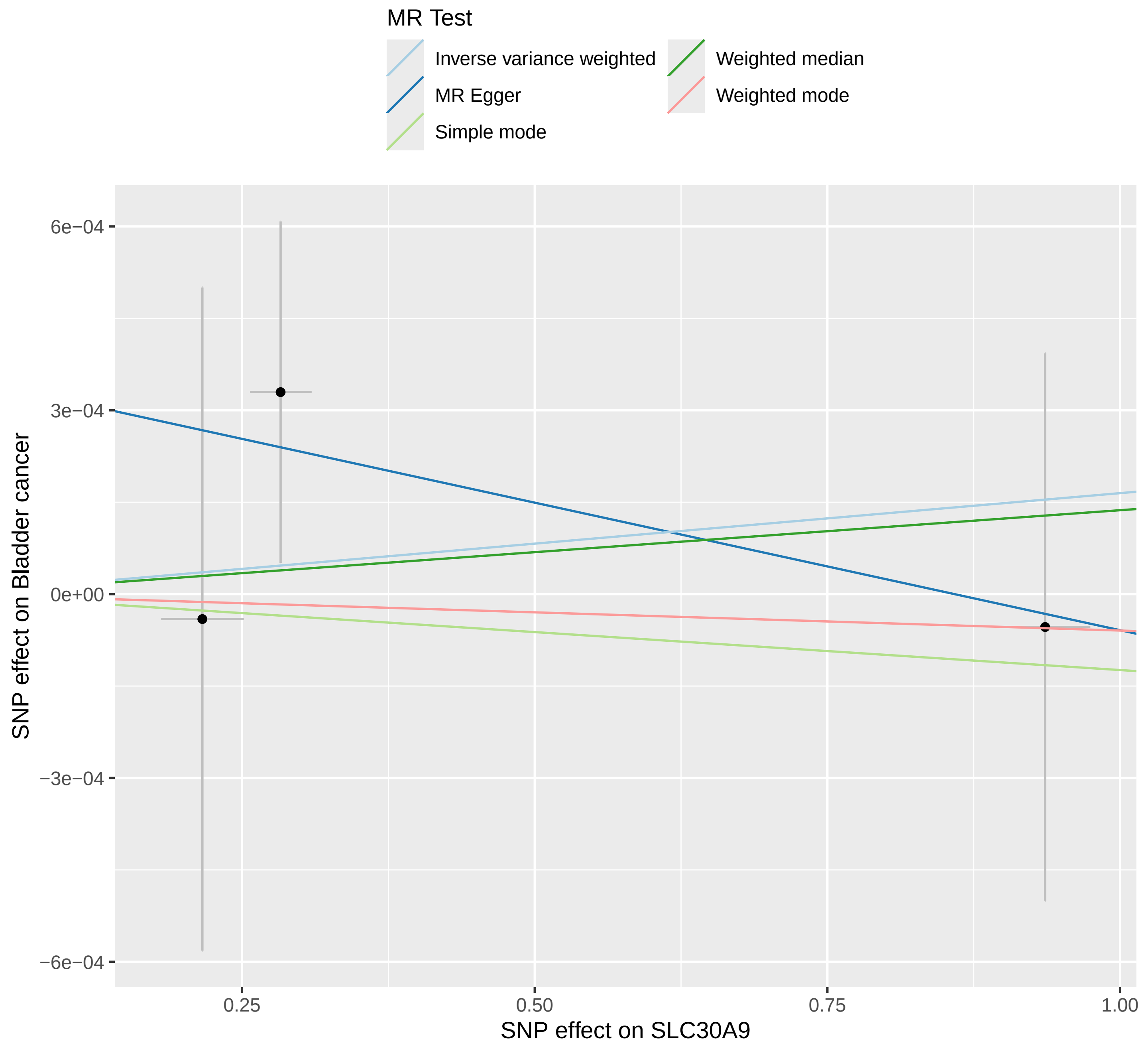
MR Test
Inverse variance weighted
MR Egger
Simple mode
Weighted median
Weighted mode
6e−04
3e−04
0e+00
−3e−04
−6e−04
0.25
0.50
0.75
1.00
SNP effect on Bladder cancer
SNP effect on SLC30A9

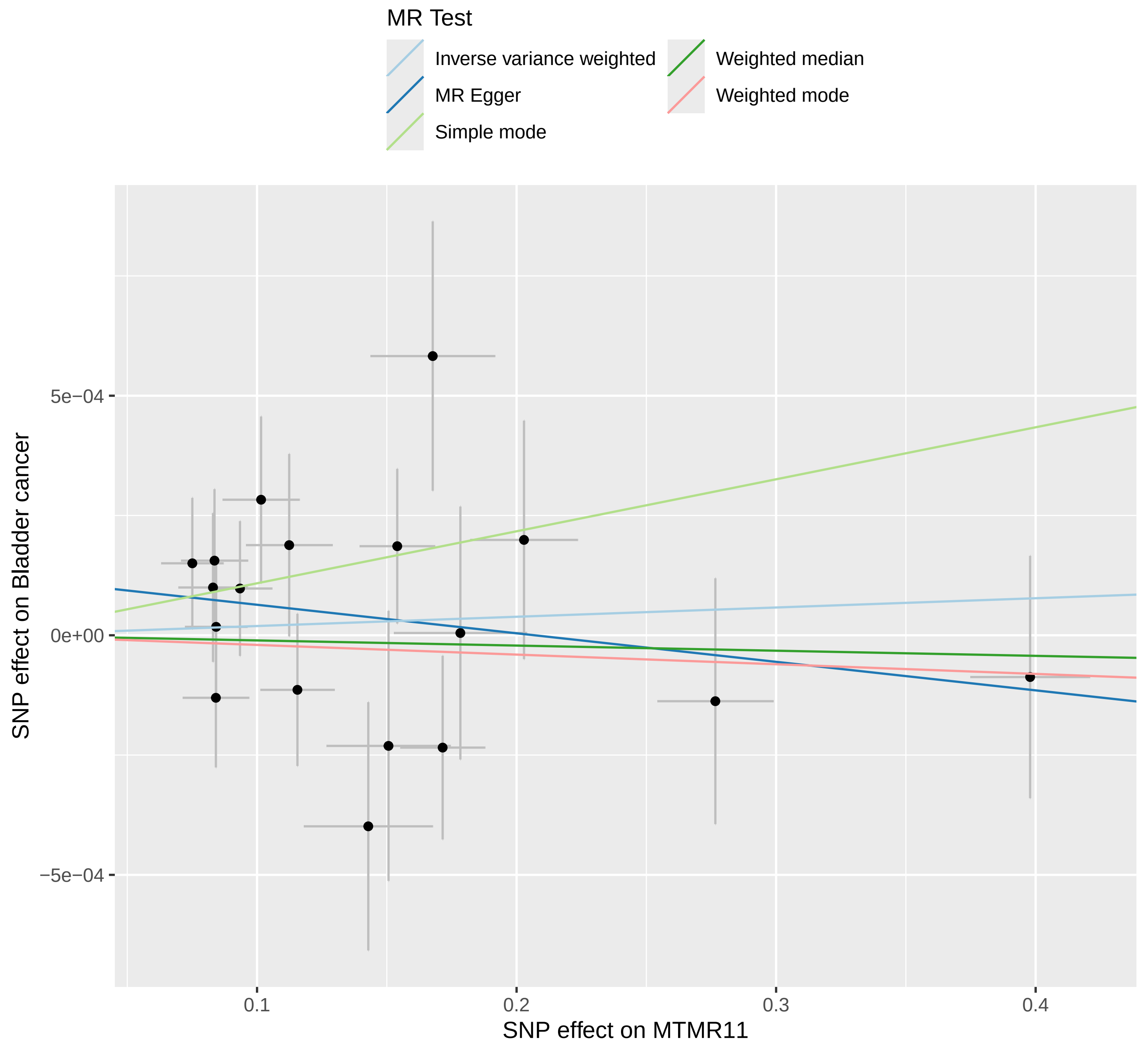
MR Test
Inverse variance weighted
MR Egger
Simple mode
Weighted median
Weighted mode
5e−04
0e+00
−5e−04
SNP effect on Bladder cancer
0.1
0.2
0.3
0.4
SNP effect on MTMR11

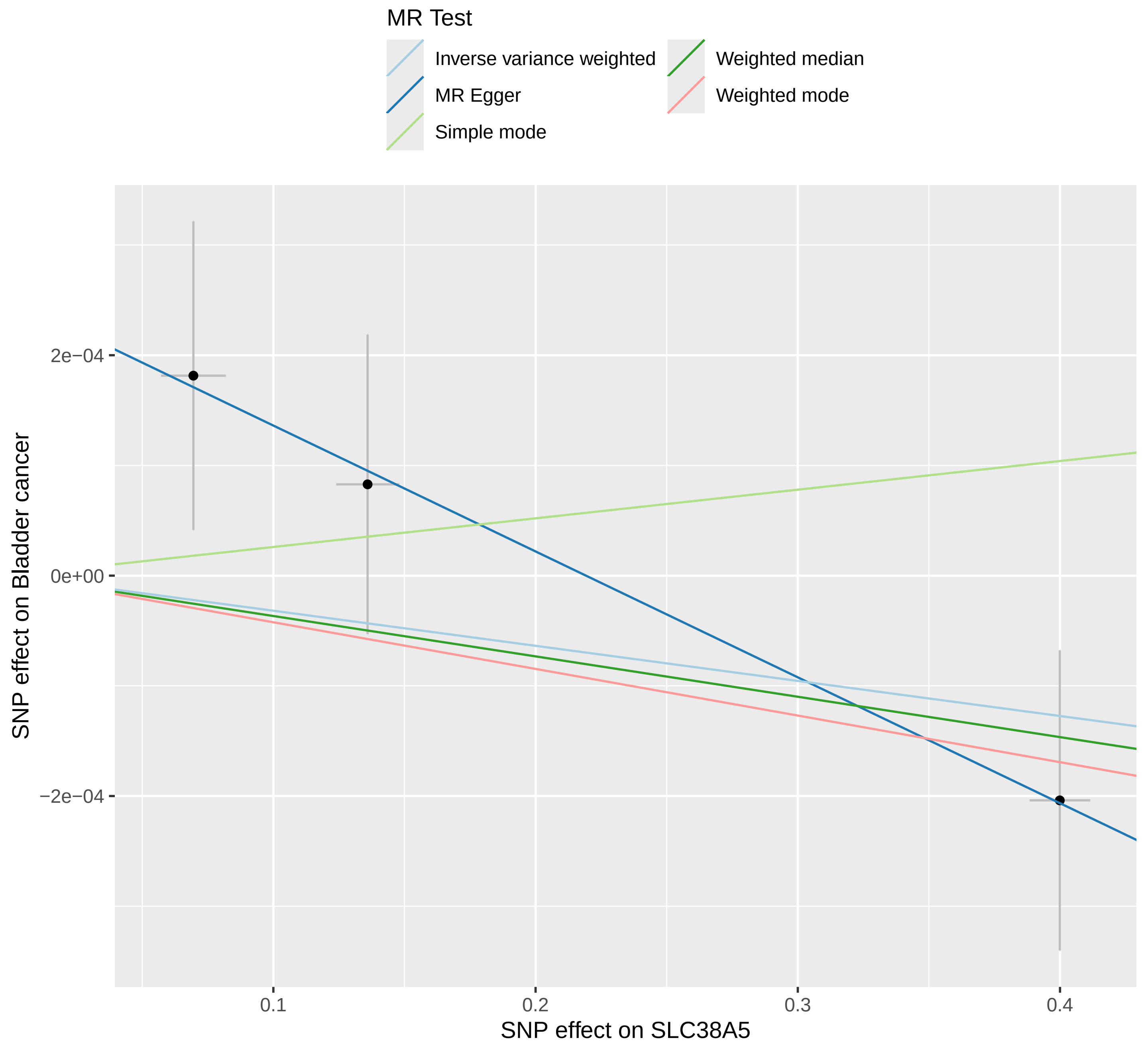

MR Test
Inverse variance weighted
MR Egger
Simple mode
Weighted median
Weighted mode
2e−04
0e+00
−2e−04
0.1
0.2
0.3
0.4
SNP effect on SLC38A5
SNP effect on Bladder cancer

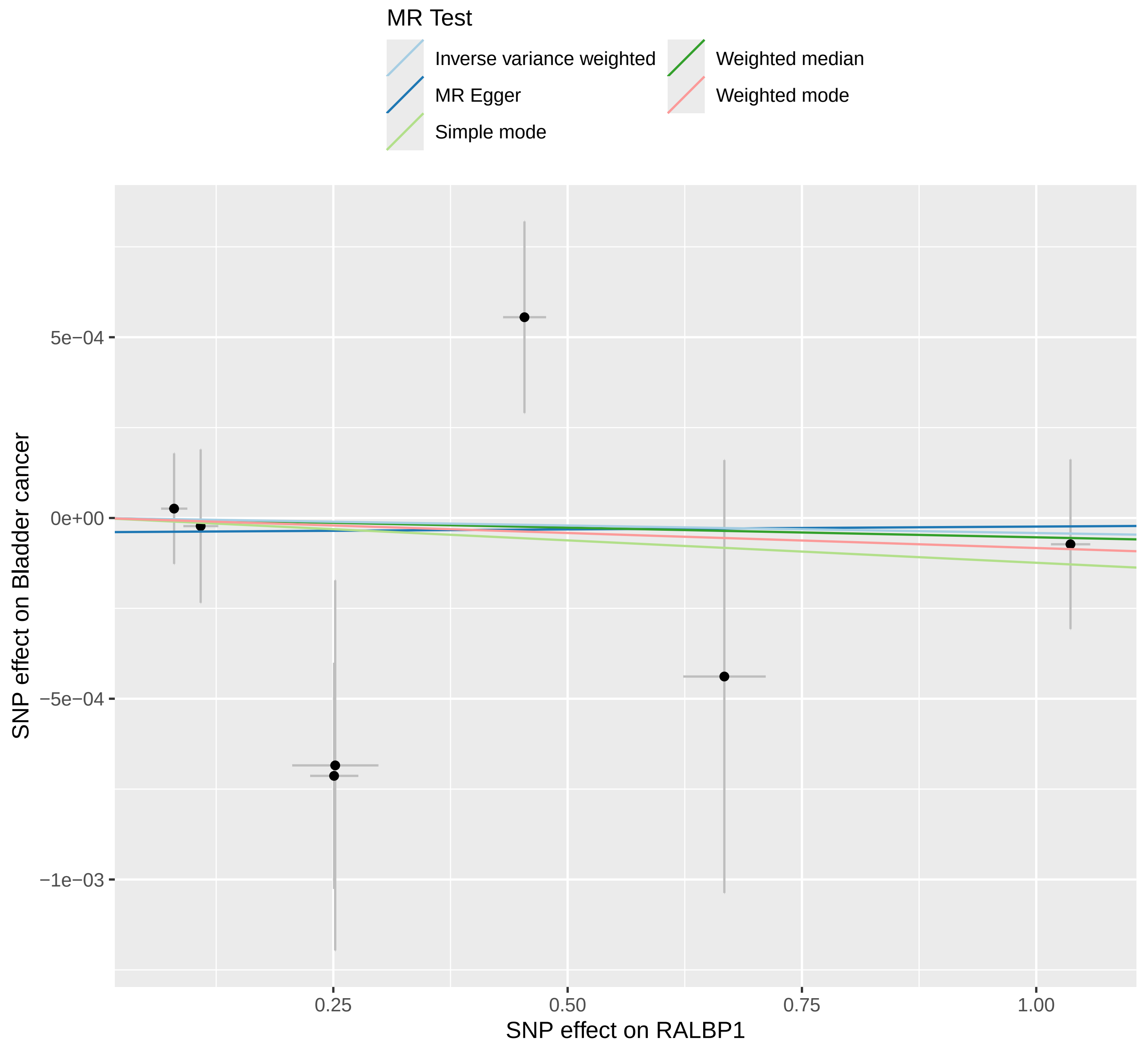
MR Test
Inverse variance weighted
MR Egger
Simple mode
Weighted median
Weighted mode
5e−04
0e+00
−5e−04
−1e−03
0.25
0.50
0.75
1.00
SNP effect on RALBP1
SNP effect on Bladder cancer

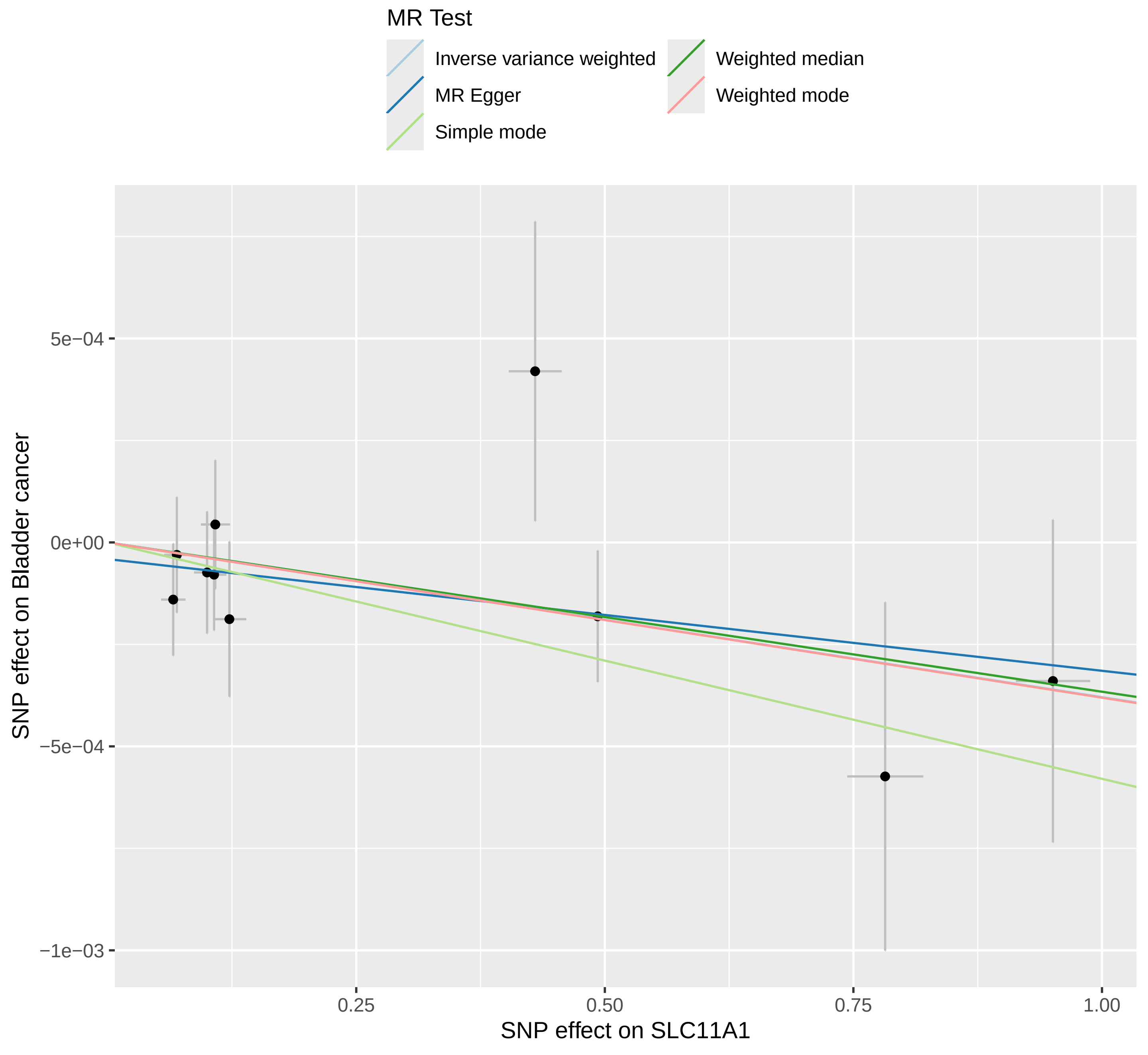
MR Test
Inverse variance weighted
MR Egger
Simple mode
Weighted median
Weighted mode
5e−04
0e+00
−5e−04
−1e−03
0.25
0.50
0.75
1.00
SNP effect on Bladder cancer
SNP effect on SLC11A1

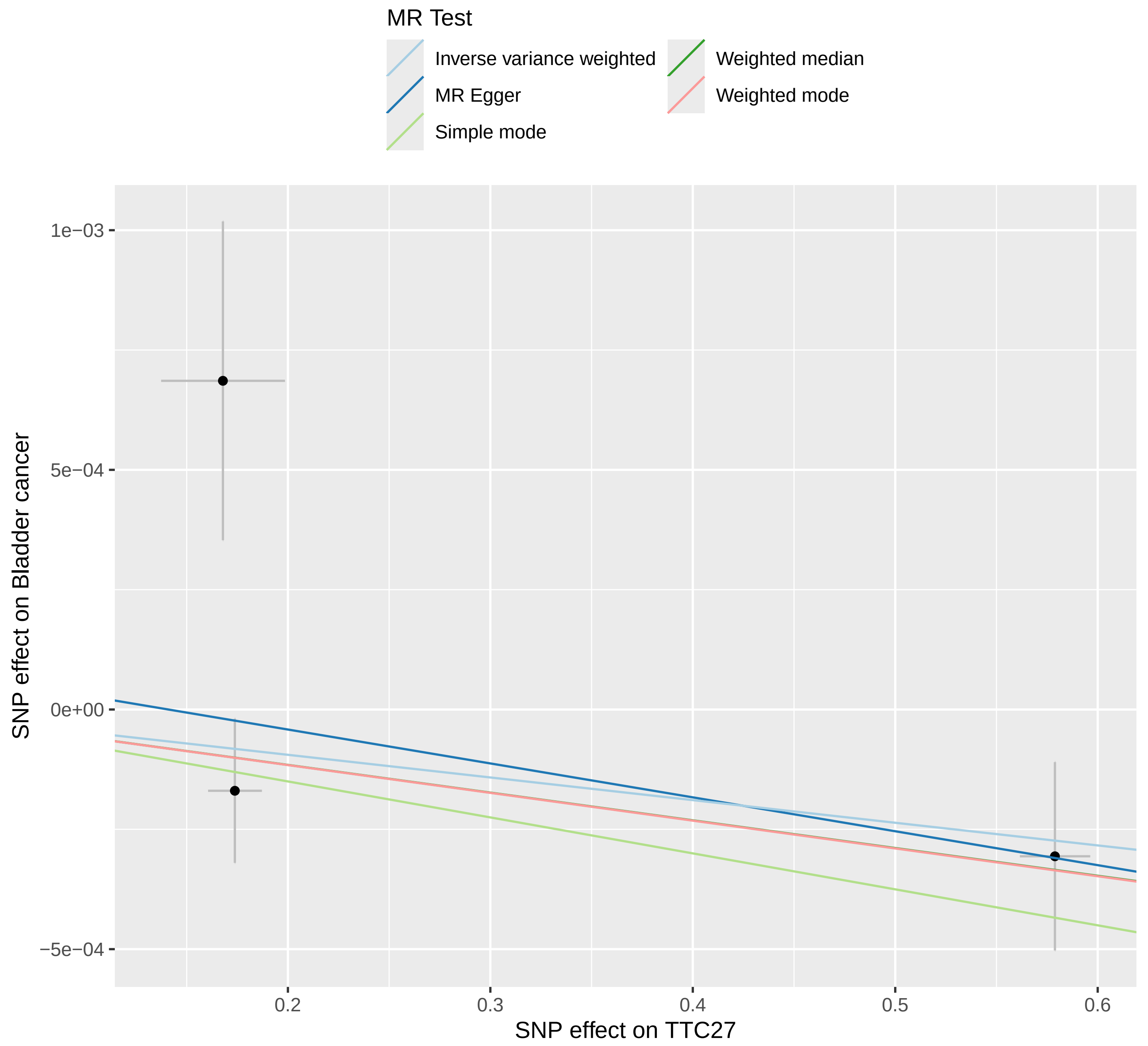

MR Test
Inverse variance weighted
MR Egger
Simple mode
Weighted median
Weighted mode
1e−03
5e−04
0e+00
−5e−04
0.2
0.3
0.4
0.5
0.6
SNP effect on TTC27
SNP effect on Bladder cancer

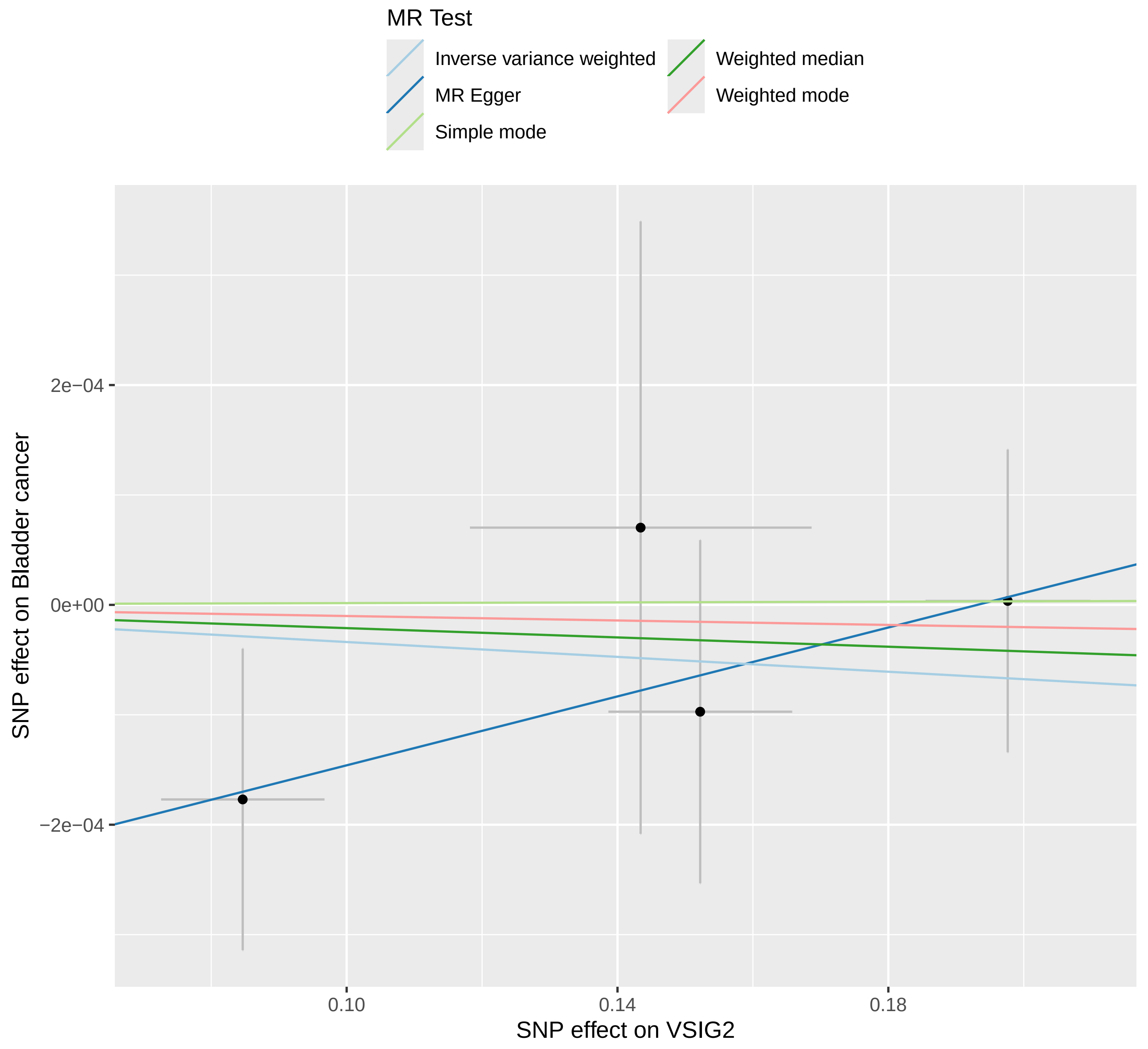
MR Test
Inverse variance weighted
MR Egger
Simple mode
Weighted median
Weighted mode
2e−04
0e+00
−2e−04
SNP effect on Bladder cancer
0.10
0.14
0.18
SNP effect on VSIG2

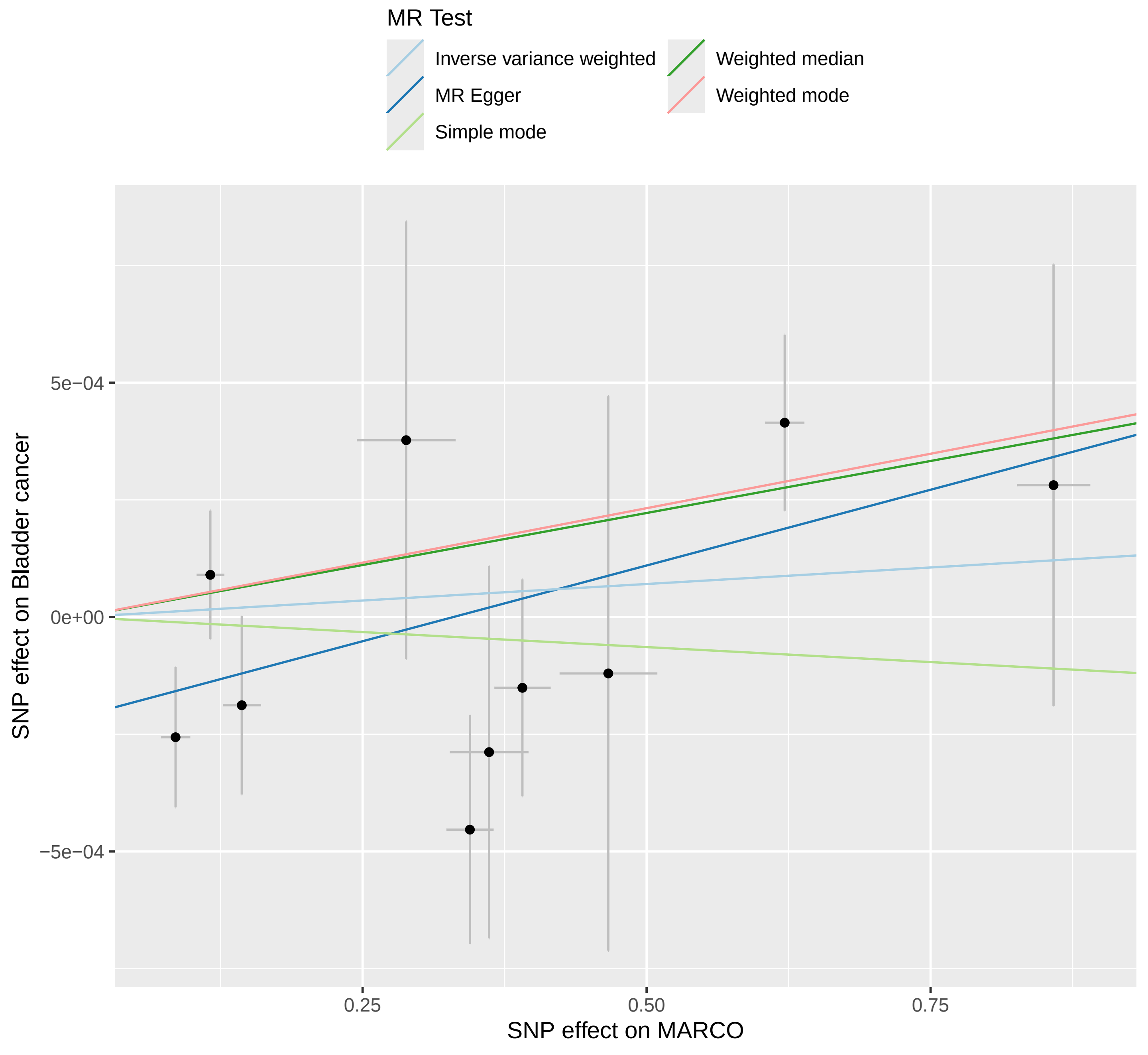

MR Test
Inverse variance weighted
MR Egger
Simple mode
Weighted median
Weighted mode
5e−04
0e+00
−5e−04
0.25
0.50
0.75
SNP effect on Bladder cancer
SNP effect on MARCO

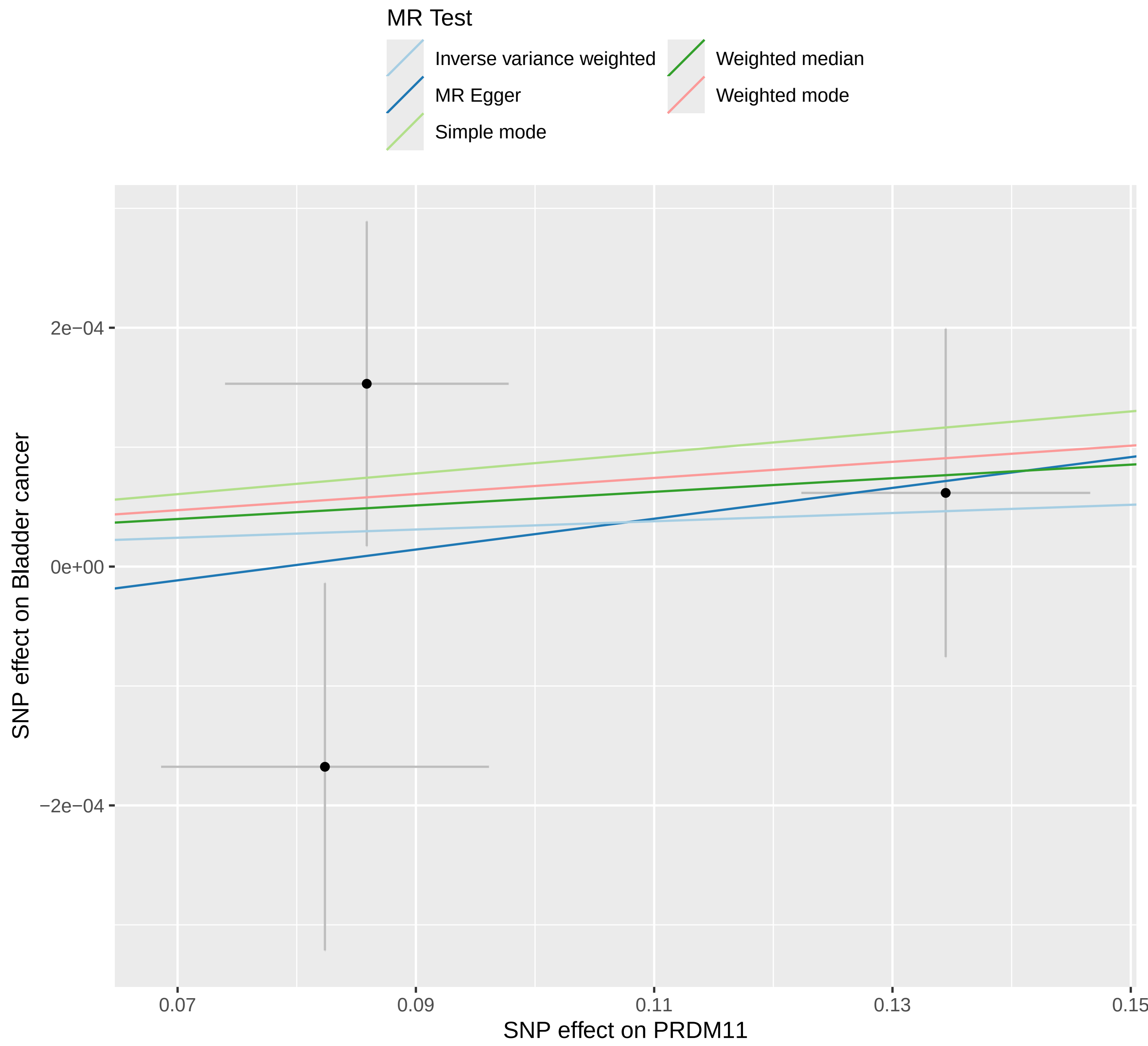
MR Test
Inverse variance weighted
MR Egger
Simple mode
Weighted median
Weighted mode
2e−04
0e+00
−2e−04
SNP effect on Bladder cancer
0.07
0.09
0.11
0.13
0.15
SNP effect on PRDM11

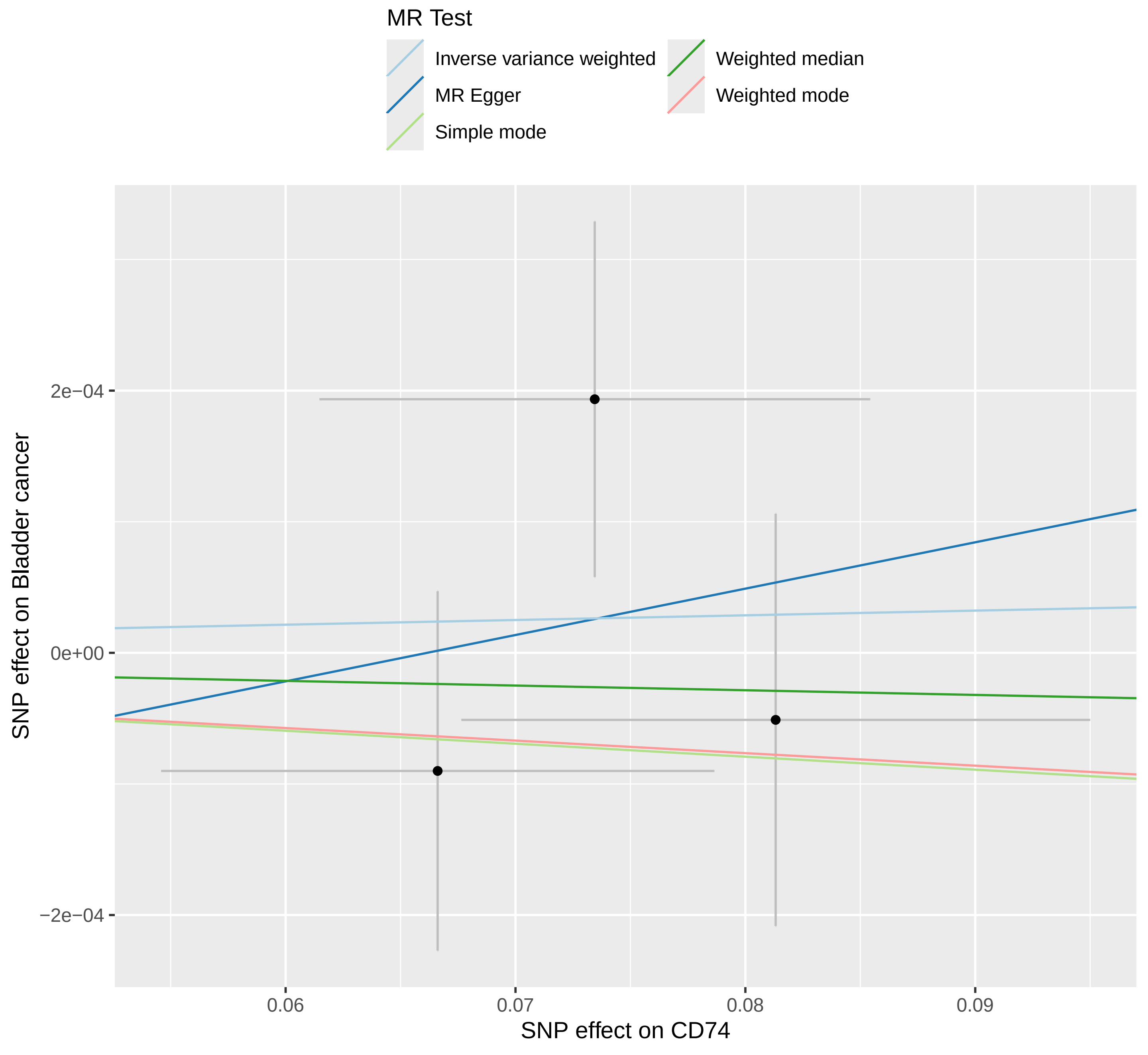
MR Test
Inverse variance weighted
MR Egger
Simple mode
Weighted median
Weighted mode
2e−04
0e+00
−2e−04
0.06
0.07
0.08
0.09
SNP effect on Bladder cancer
SNP effect on CD74

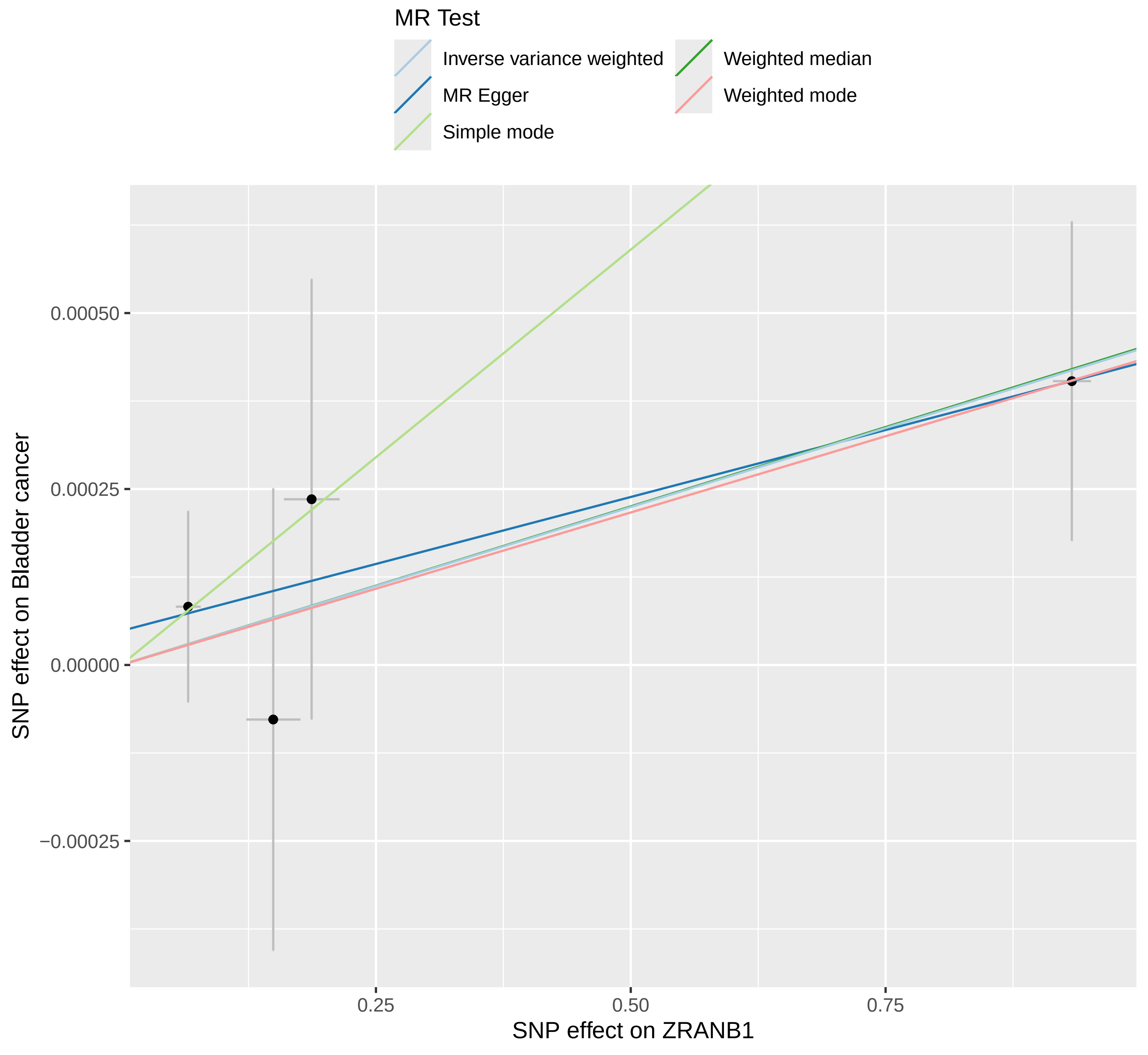

MR Test
Inverse variance weighted
MR Egger
Simple mode
Weighted median
Weighted mode
0.00050
0.00025
0.00000
−0.00025
SNP effect on Bladder cancer
0.25
0.50
0.75
SNP effect on ZRANB1

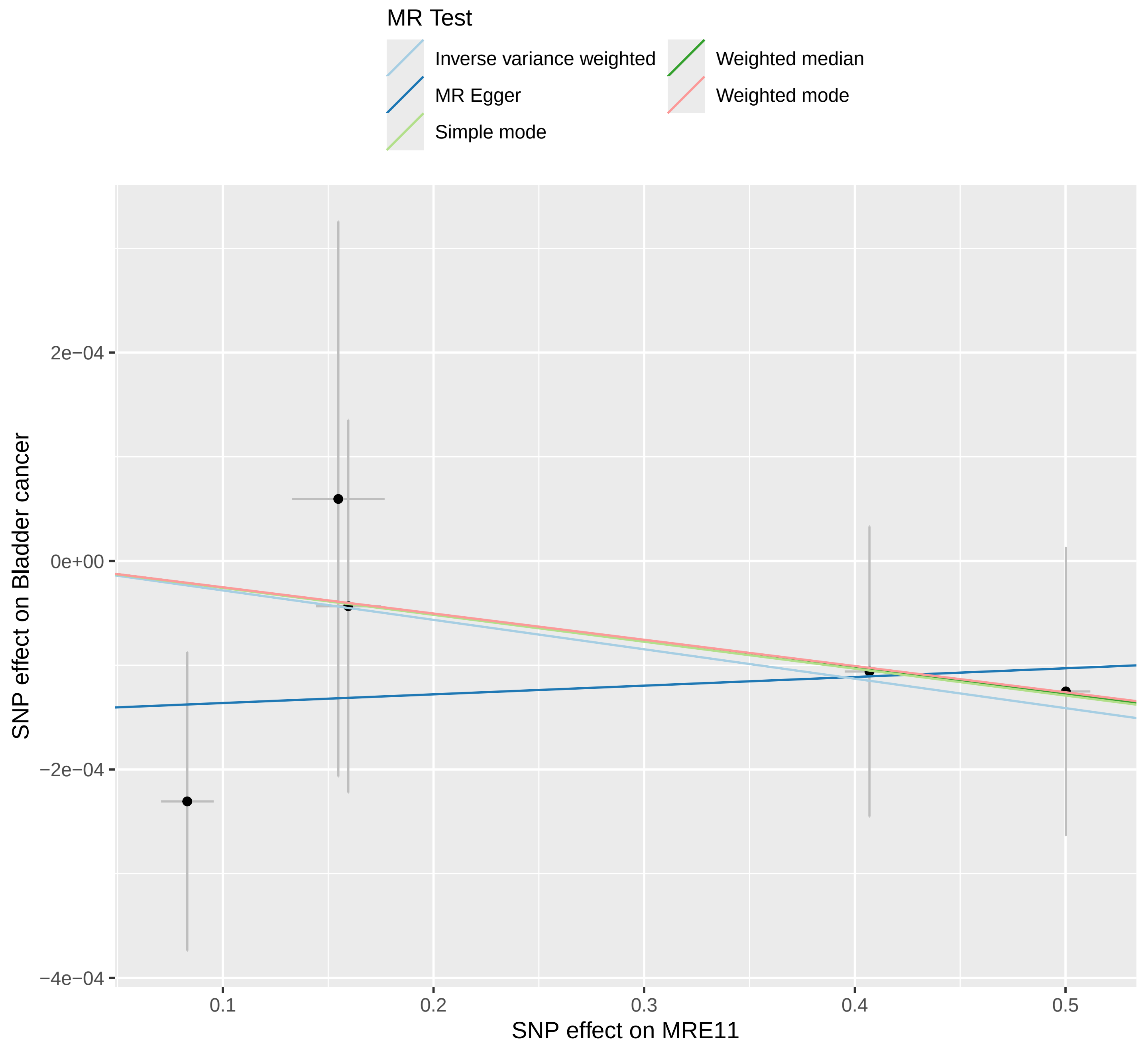
MR Test
Inverse variance weighted
MR Egger
Simple mode
Weighted median
Weighted mode
2e−04
0e+00
−2e−04
−4e−04
SNP effect on Bladder cancer
0.1
0.2
0.3
0.4
0.5
SNP effect on MRE11

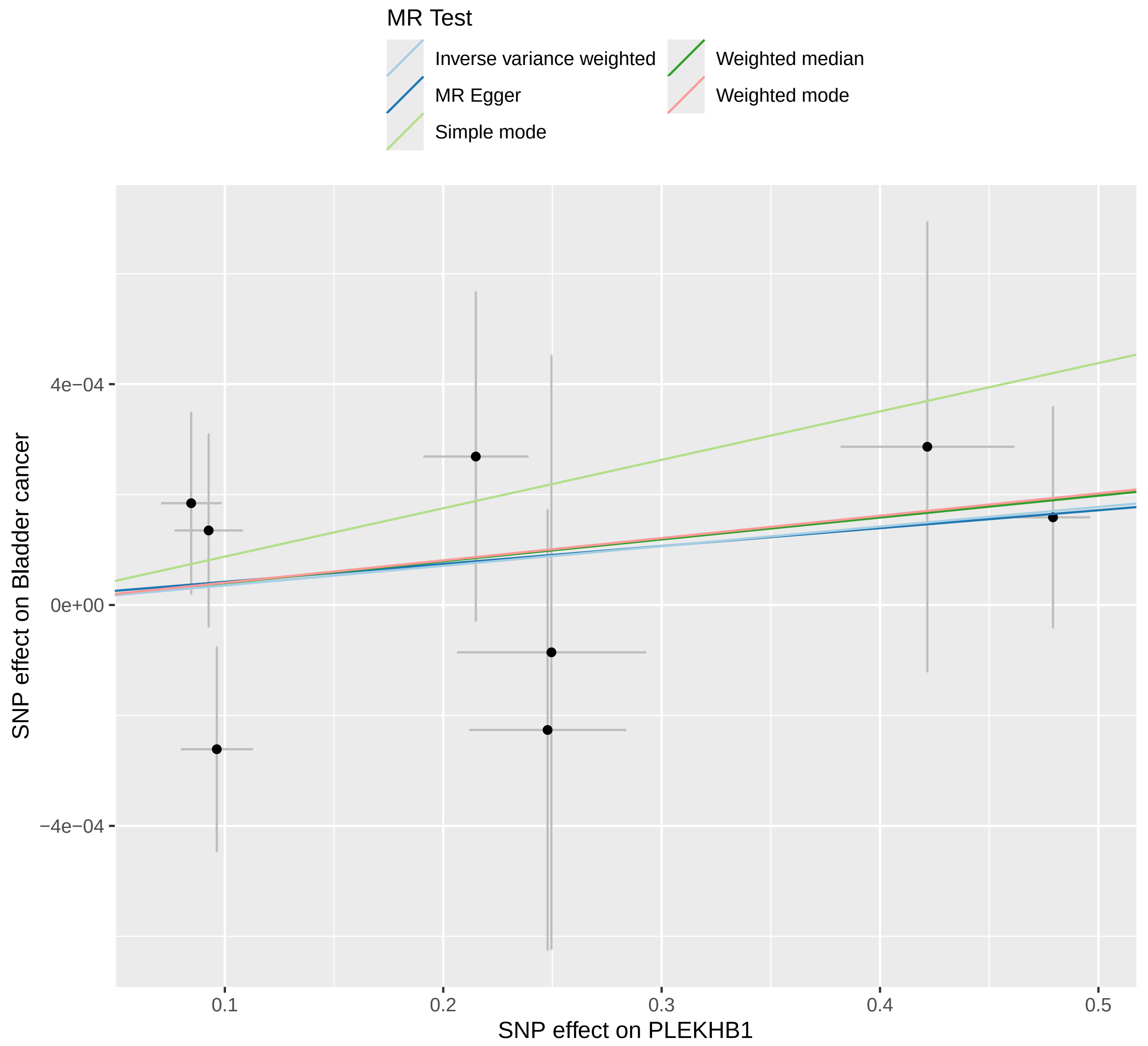
MR Test
Inverse variance weighted
MR Egger
Simple mode
Weighted median
Weighted mode
4e−04
0e+00
−4e−04
SNP effect on Bladder cancer
0.1
0.2
0.3
0.4
0.5
SNP effect on PLEKHB1

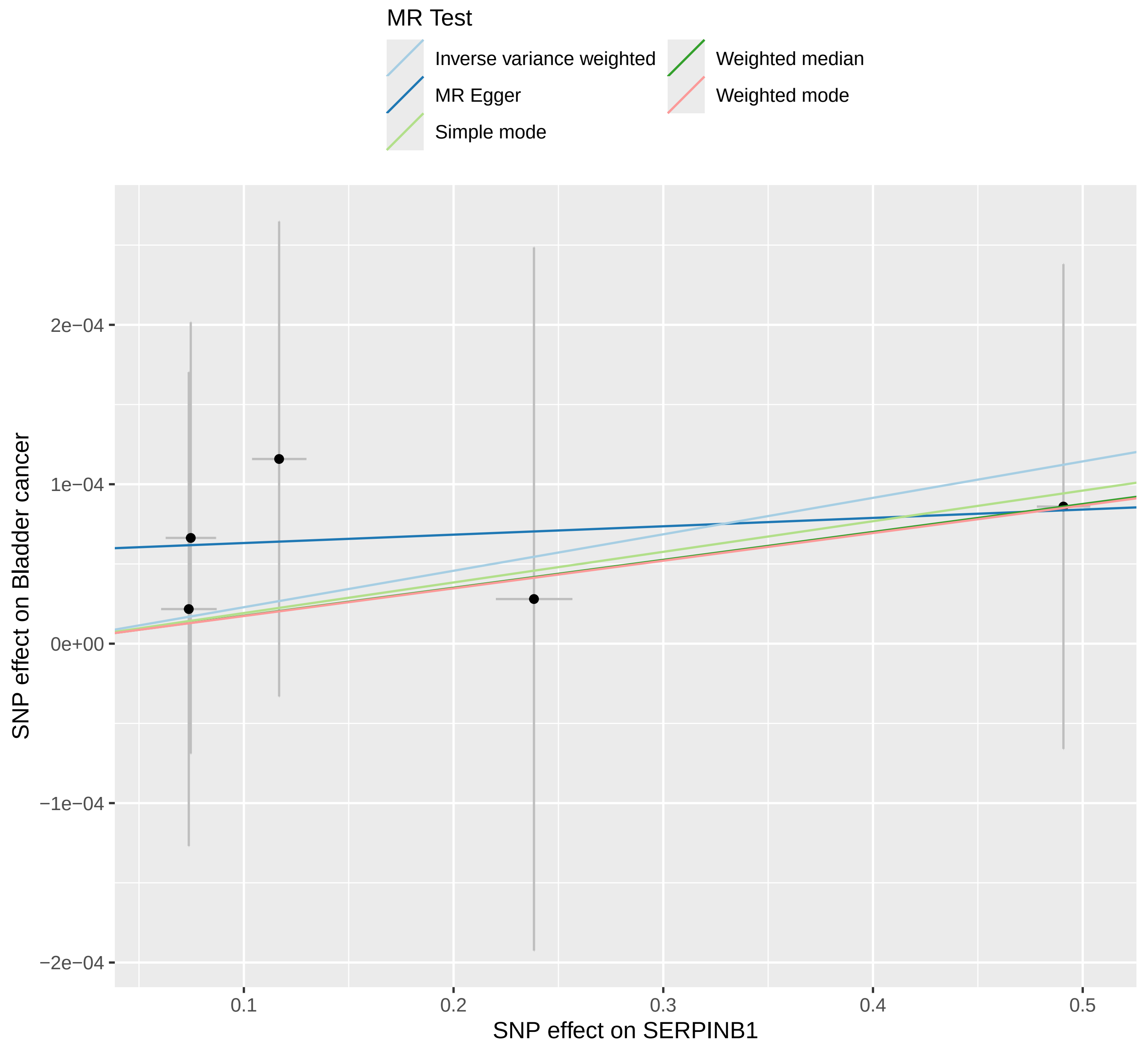

MR Test
Inverse variance weighted
MR Egger
Simple mode
Weighted median
Weighted mode
SNP effect on Bladder cancer
2e−04
1e−04
0e+00
−1e−04
−2e−04
0.1
0.2
0.3
0.4
0.5
SNP effect on SERPINB1

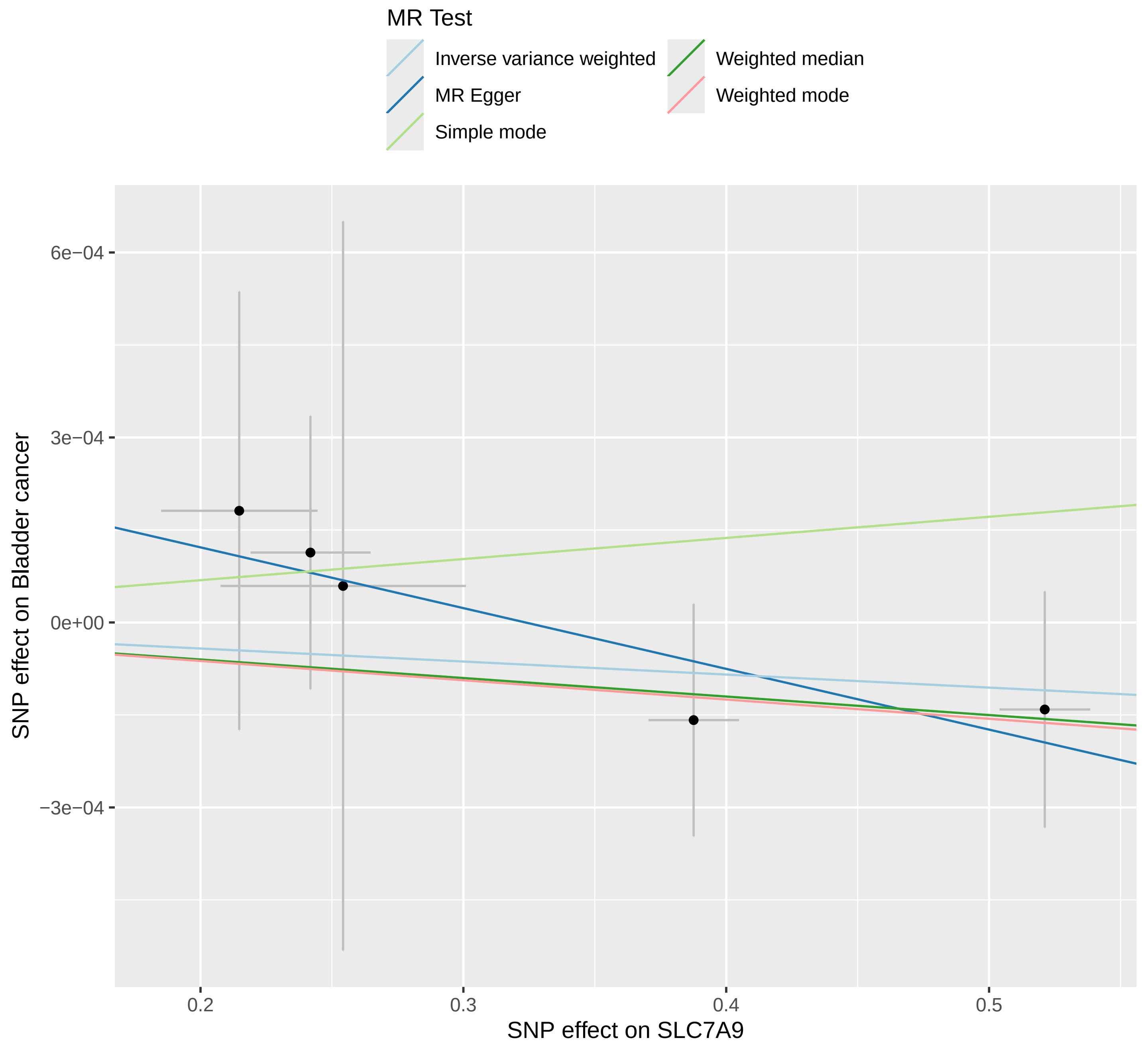
MR Test
Inverse variance weighted
MR Egger
Simple mode
Weighted median
Weighted mode
6e−04
3e−04
0e+00
−3e−04
0.2
0.3
0.4
0.5
SNP effect on Bladder cancer
SNP effect on SLC7A9

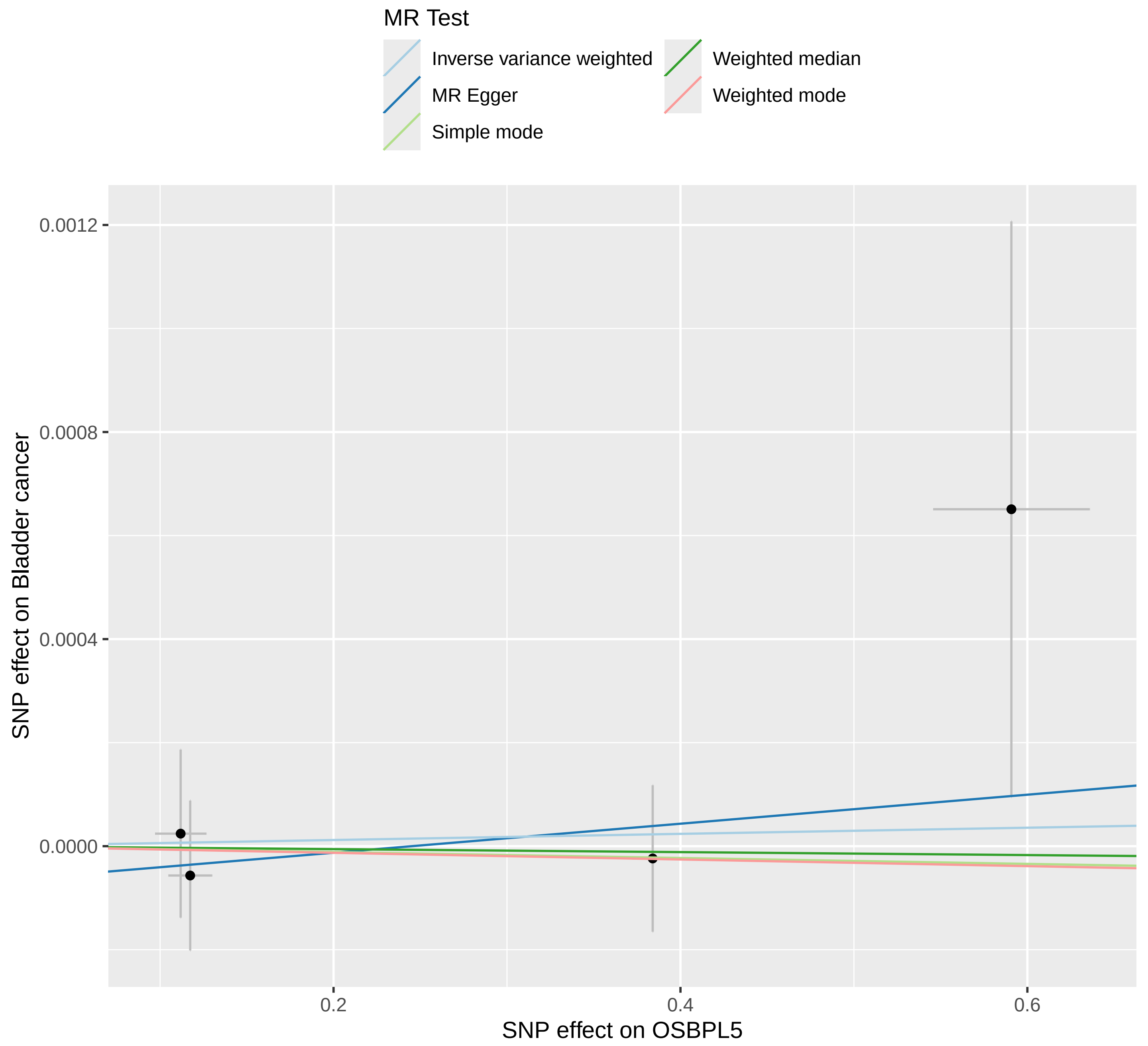
MR Test
Inverse variance weighted
MR Egger
Simple mode
Weighted median
Weighted mode
0.0012
0.0008
0.0004
0.0000
0.2
0.4
0.6
SNP effect on Bladder cancer
SNP effect on OSBPL5

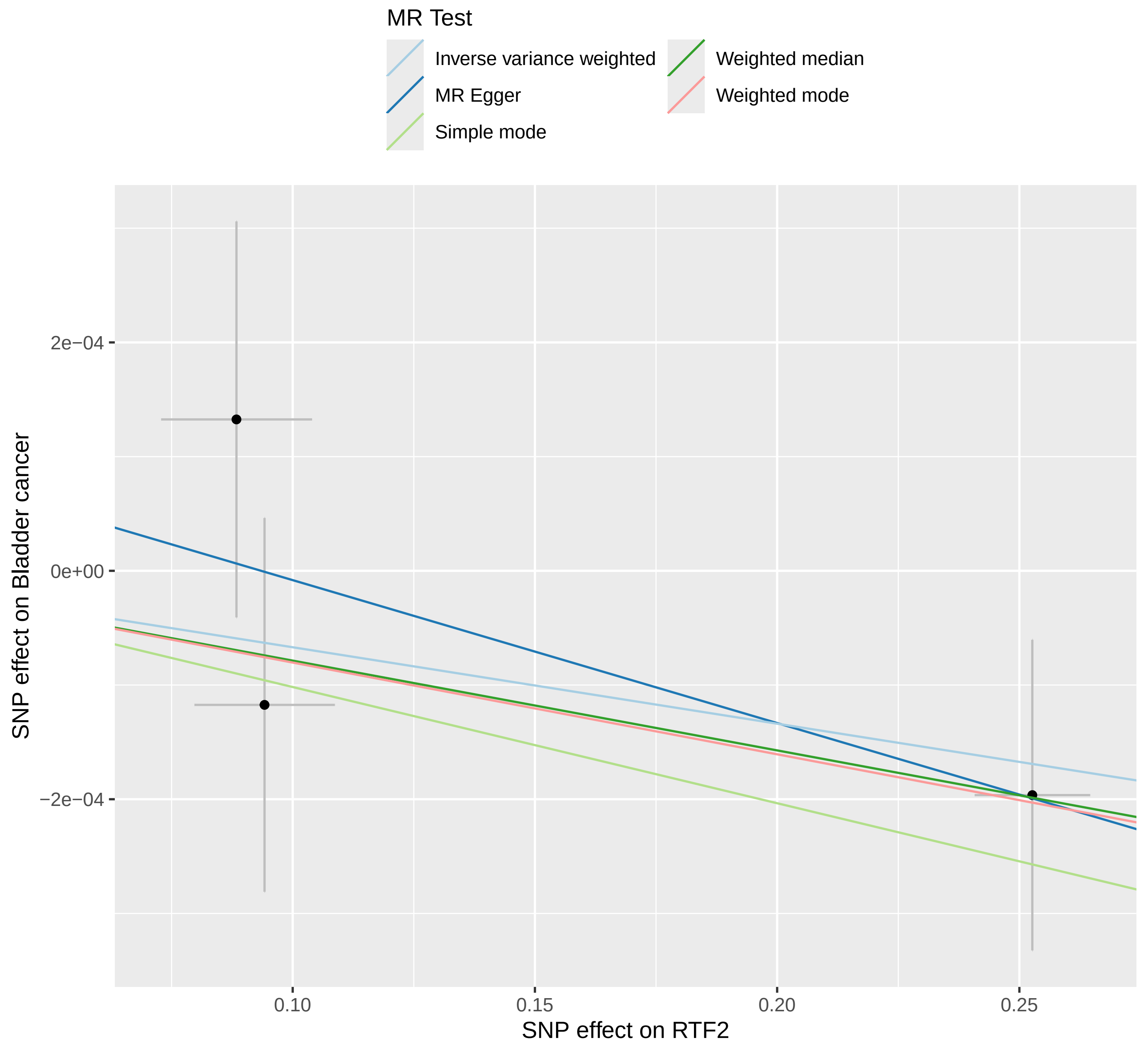

MR Test
Inverse variance weighted
MR Egger
Simple mode
Weighted median
Weighted mode
2e−04
0e+00
−2e−04
SNP effect on Bladder cancer
0.10
0.15
0.20
0.25
SNP effect on RTF2

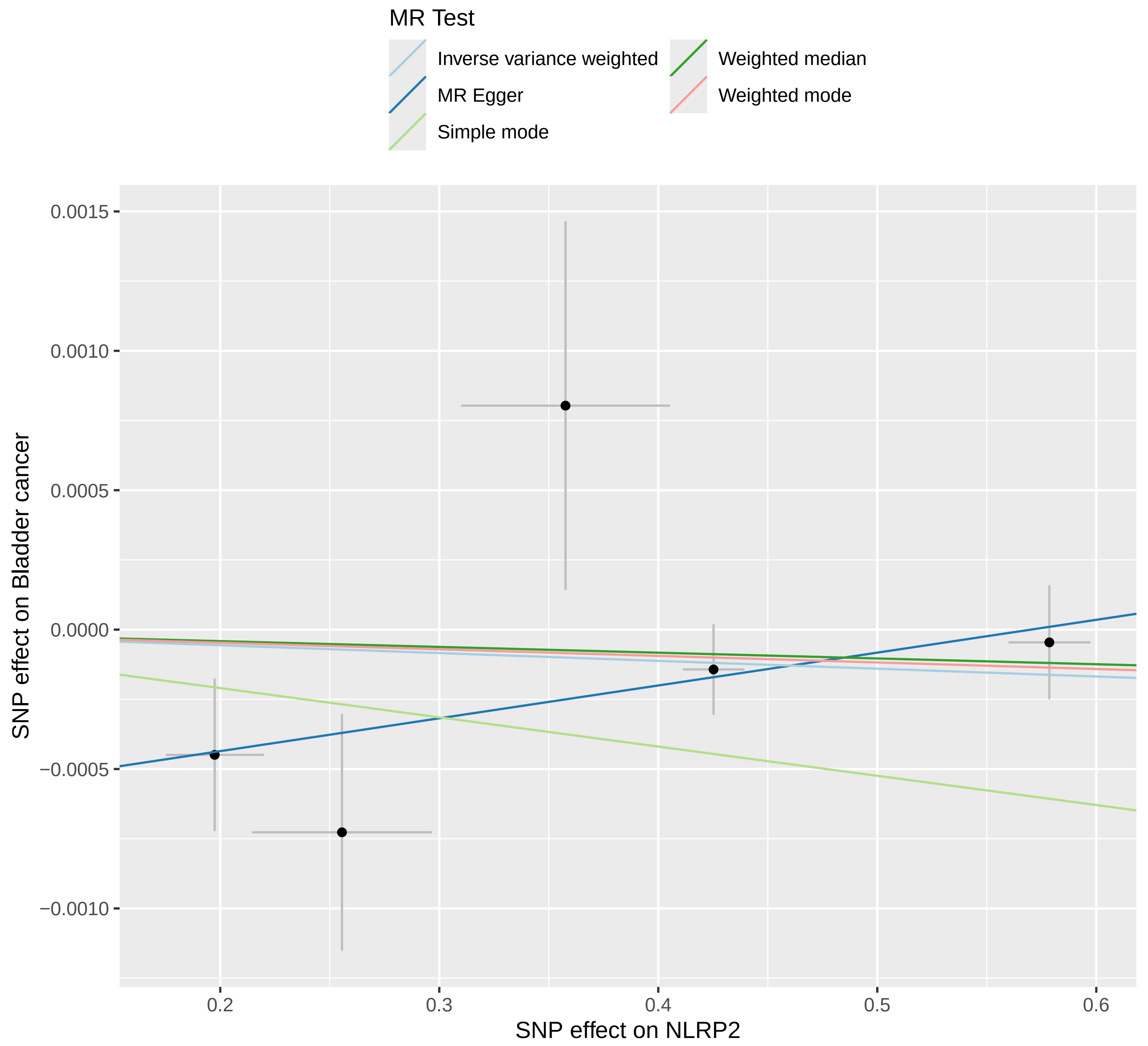

MR Test
Inverse variance weighted
MR Egger
Simple mode
Weighted median
Weighted mode
0.0015
0.0010
0.0005
0.0000
−0.0005
−0.0010
SNP effect on Bladder cancer
0.2
0.3
0.4
0.5
0.6
SNP effect on NLRP2

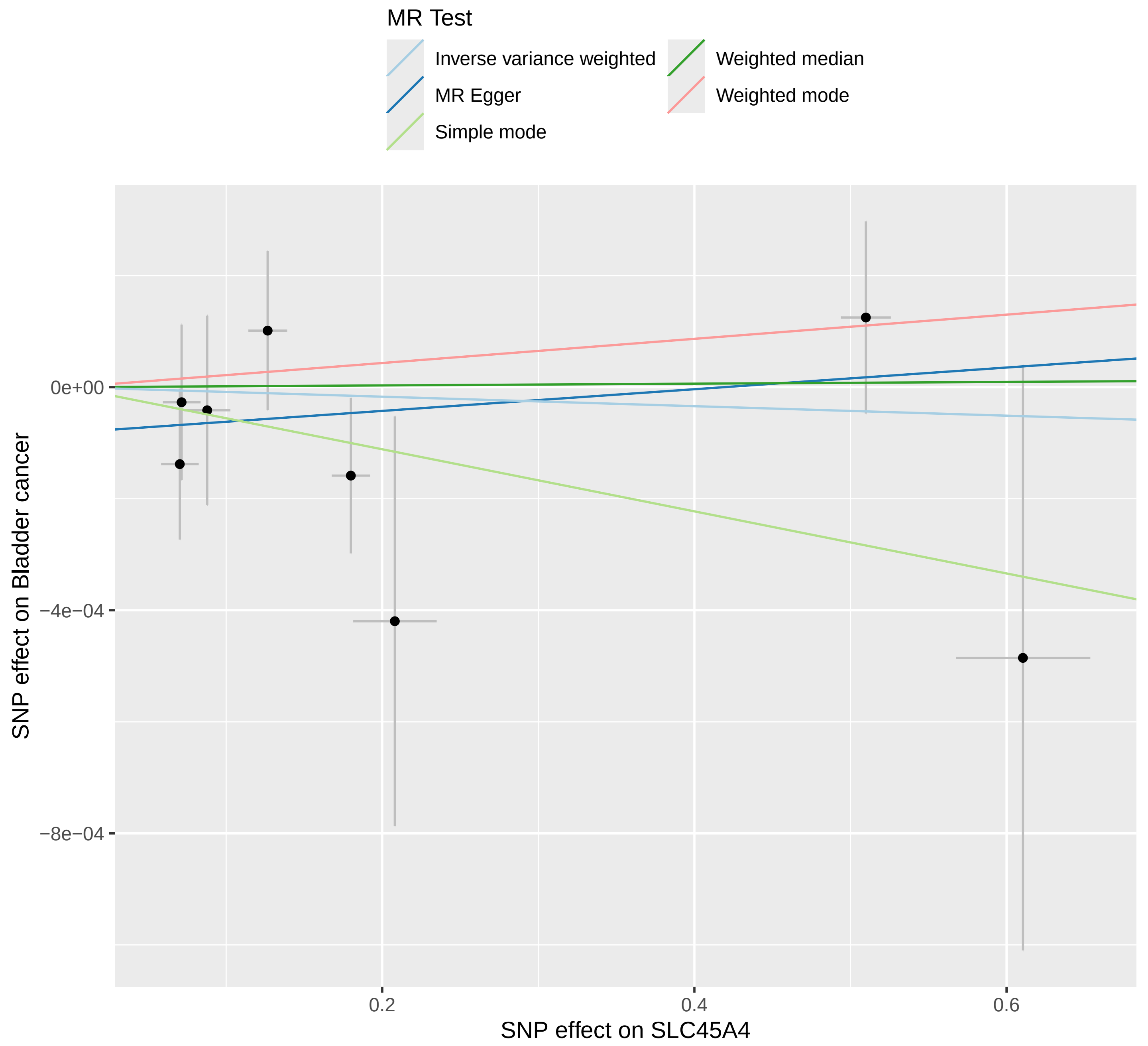

MR Test
Inverse variance weighted
MR Egger
Simple mode
Weighted median
Weighted mode
0e+00
−4e−04
−8e−04
SNP effect on Bladder cancer
0.2
0.4
0.6
SNP effect on SLC45A4

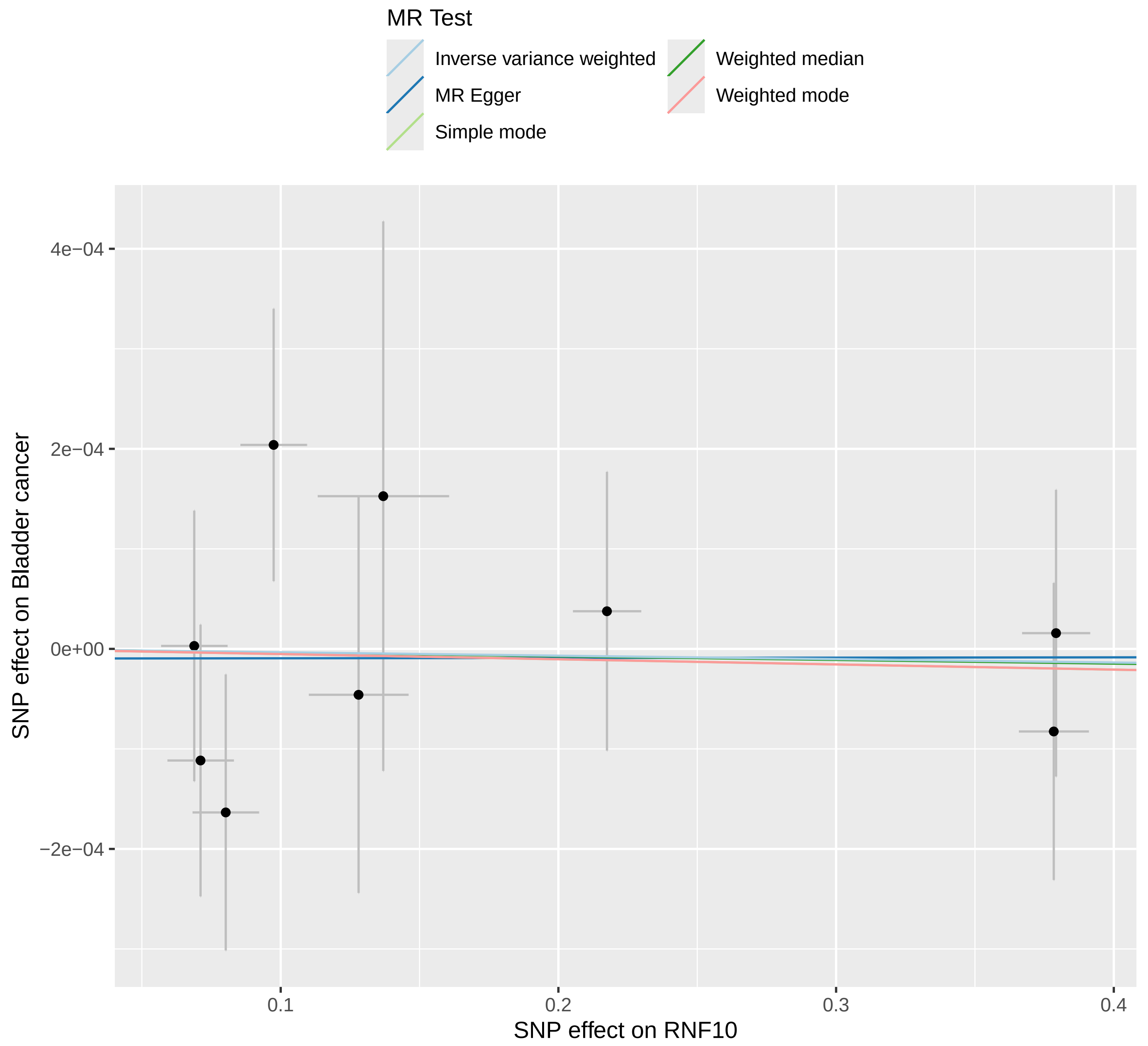

MR Test
Inverse variance weighted
MR Egger
Simple mode
Weighted median
Weighted mode
4e−04
2e−04
0e+00
−2e−04
0.1
0.2
0.3
0.4
SNP effect on Bladder cancer
SNP effect on RNF10

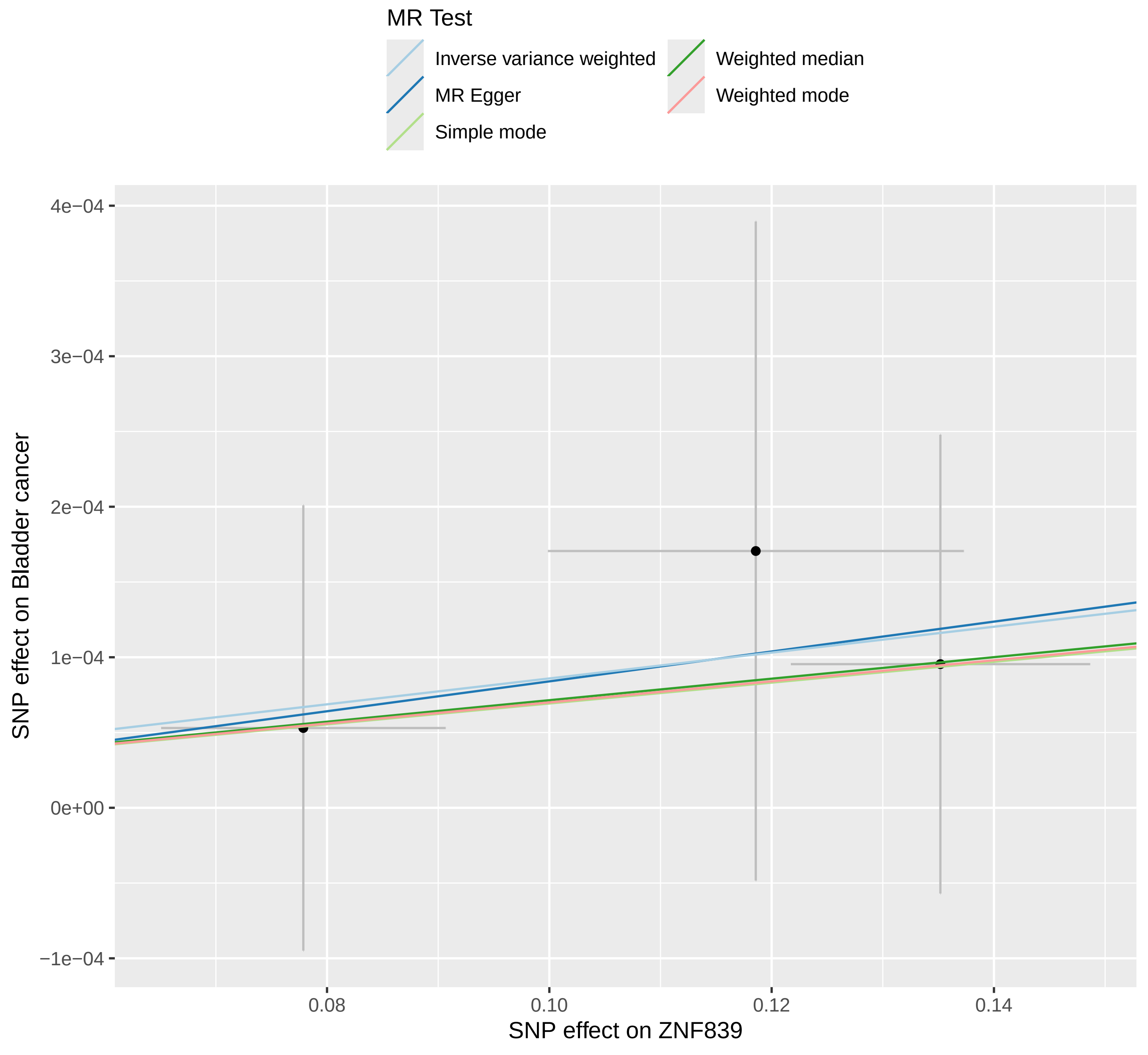
MR Test
Inverse variance weighted
MR Egger
Simple mode
Weighted median
Weighted mode
4e−04
3e−04
2e−04
1e−04
0e+00
−1e−04
0.08
0.10
0.12
0.14
SNP effect on Bladder cancer
SNP effect on ZNF839

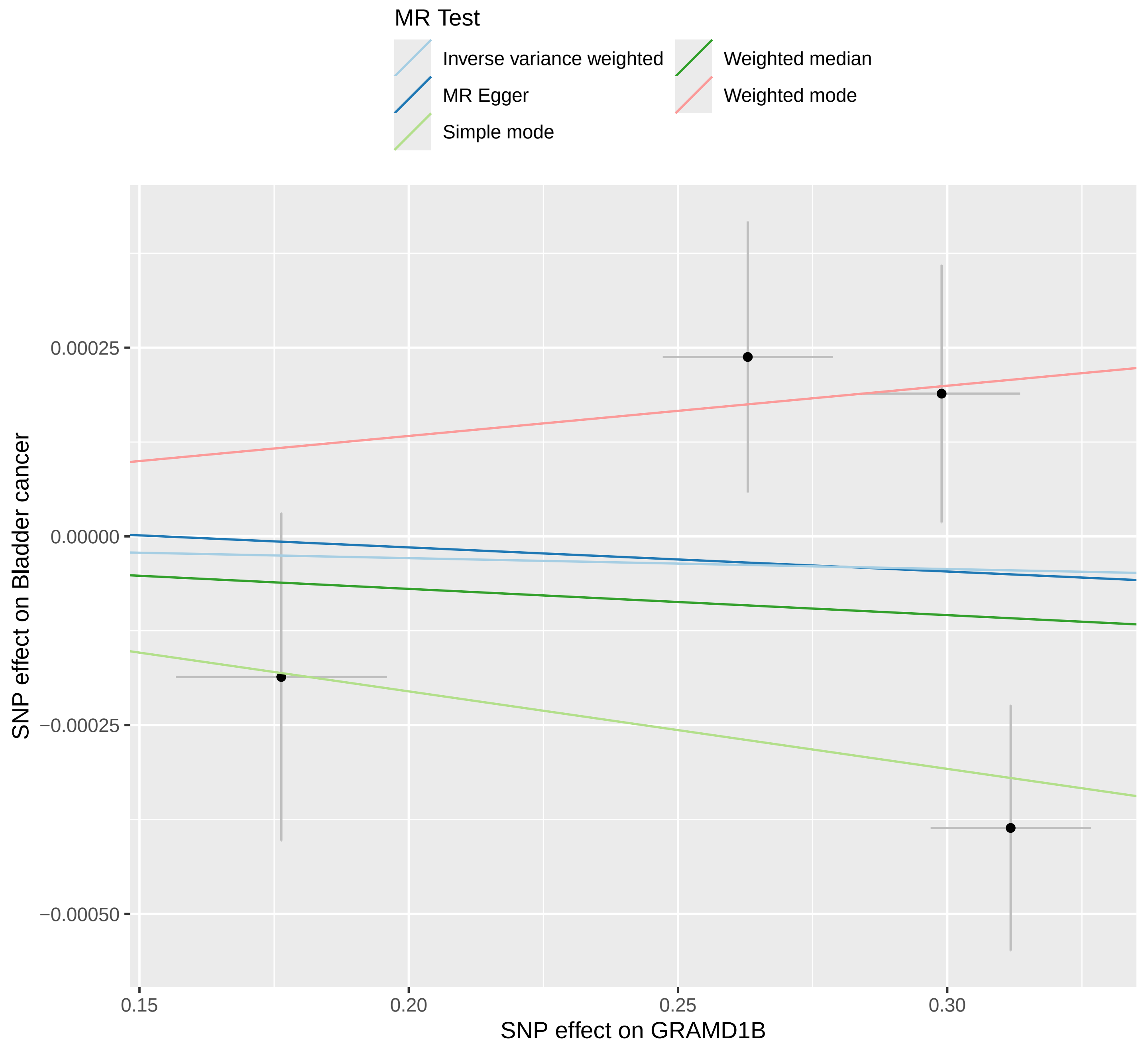

MR Test
Inverse variance weighted
MR Egger
Simple mode
Weighted median
Weighted mode
0.00025
0.00000
−0.00025
−0.00050
SNP effect on Bladder cancer
0.15
0.20
0.25
0.30
SNP effect on GRAMD1B

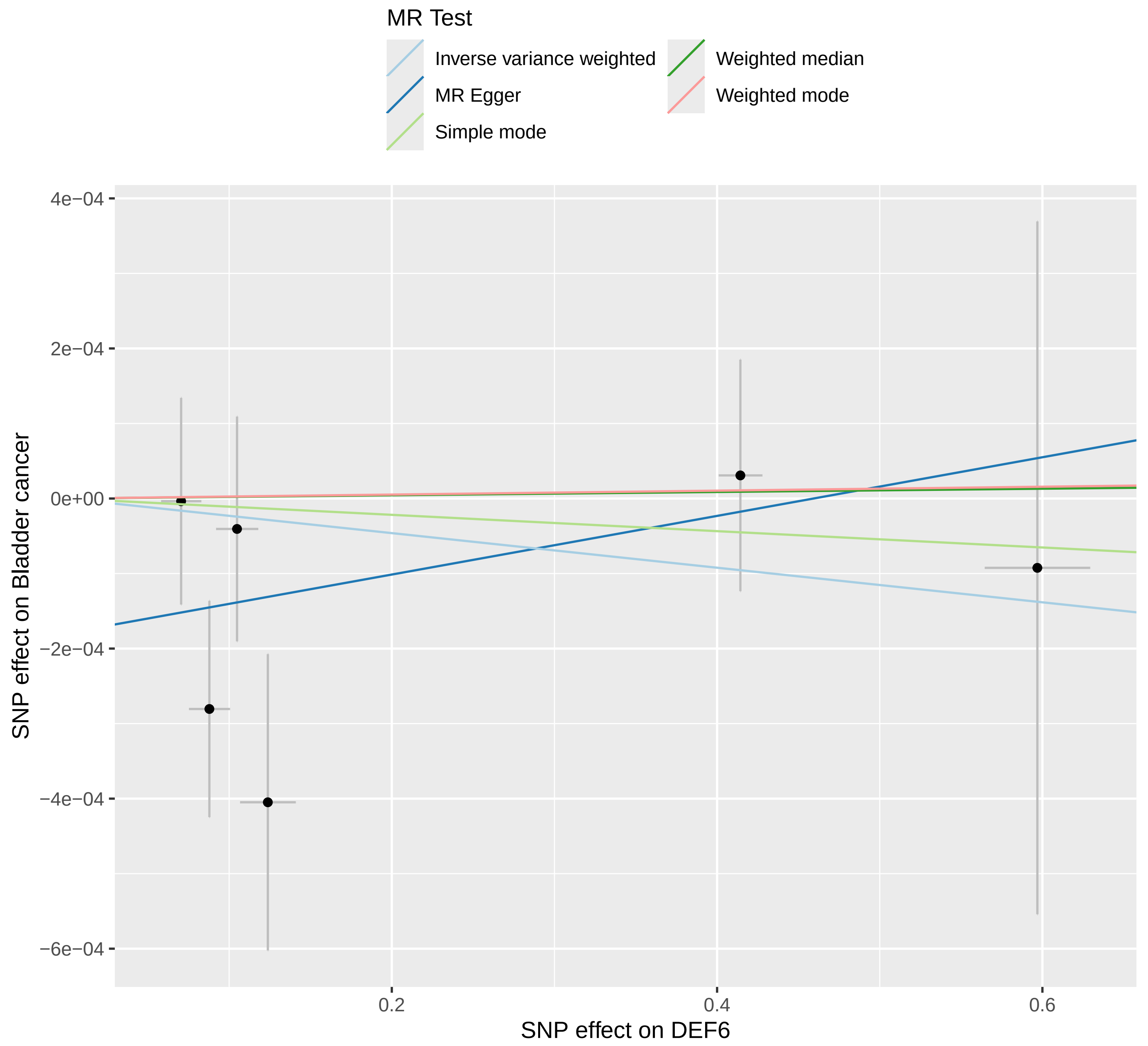

MR Test
Inverse variance weighted
MR Egger
Simple mode
Weighted median
Weighted mode
4e−04
2e−04
0e+00
−2e−04
−4e−04
−6e−04
0.2
0.4
0.6
SNP effect on Bladder cancer
SNP effect on DEF6

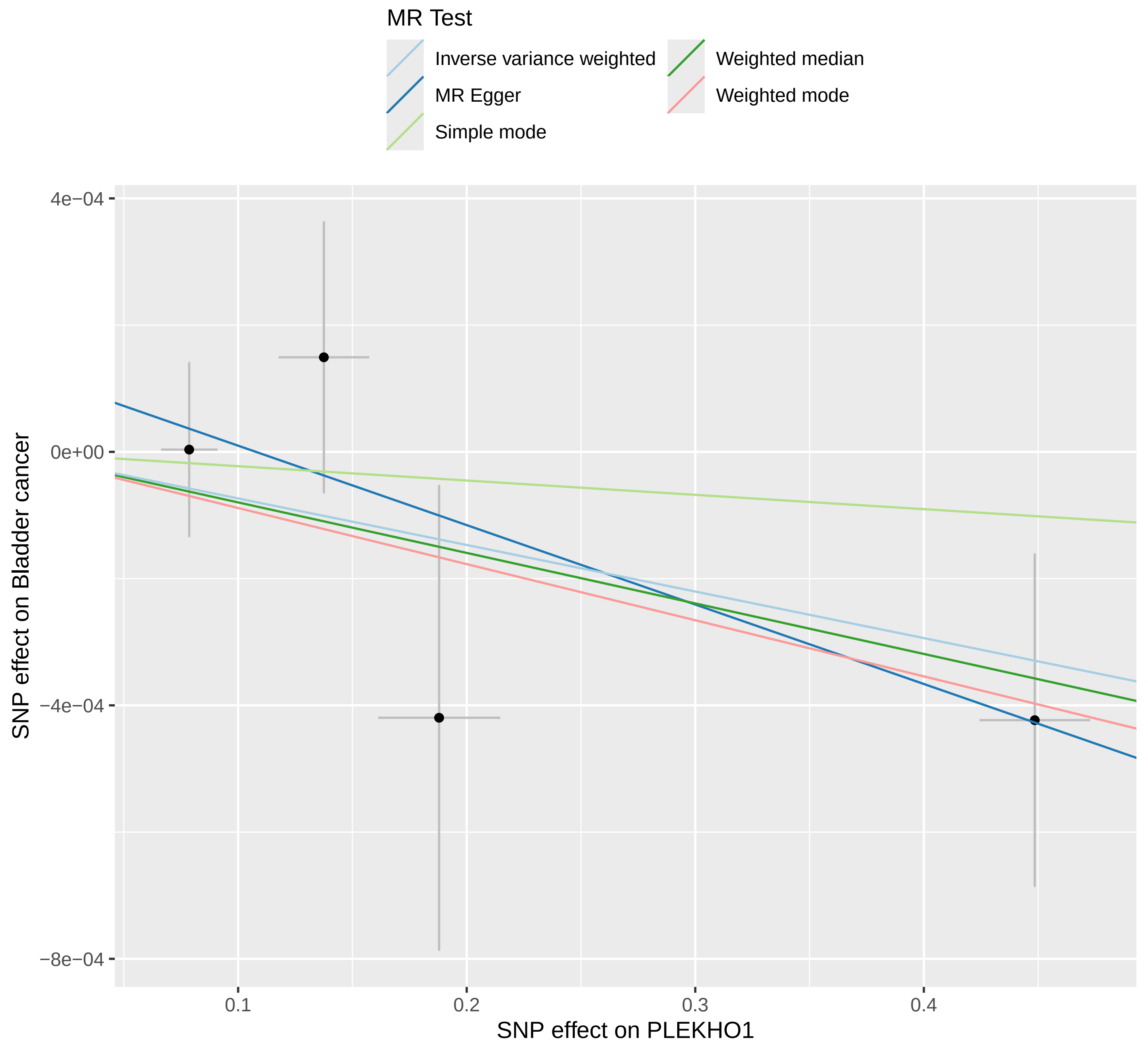
MR Test
Inverse variance weighted
MR Egger
Simple mode
Weighted median
Weighted mode
4e−04
0e+00
−4e−04
−8e−04
SNP effect on Bladder cancer
0.1
0.2
0.3
0.4
SNP effect on PLEKHO1

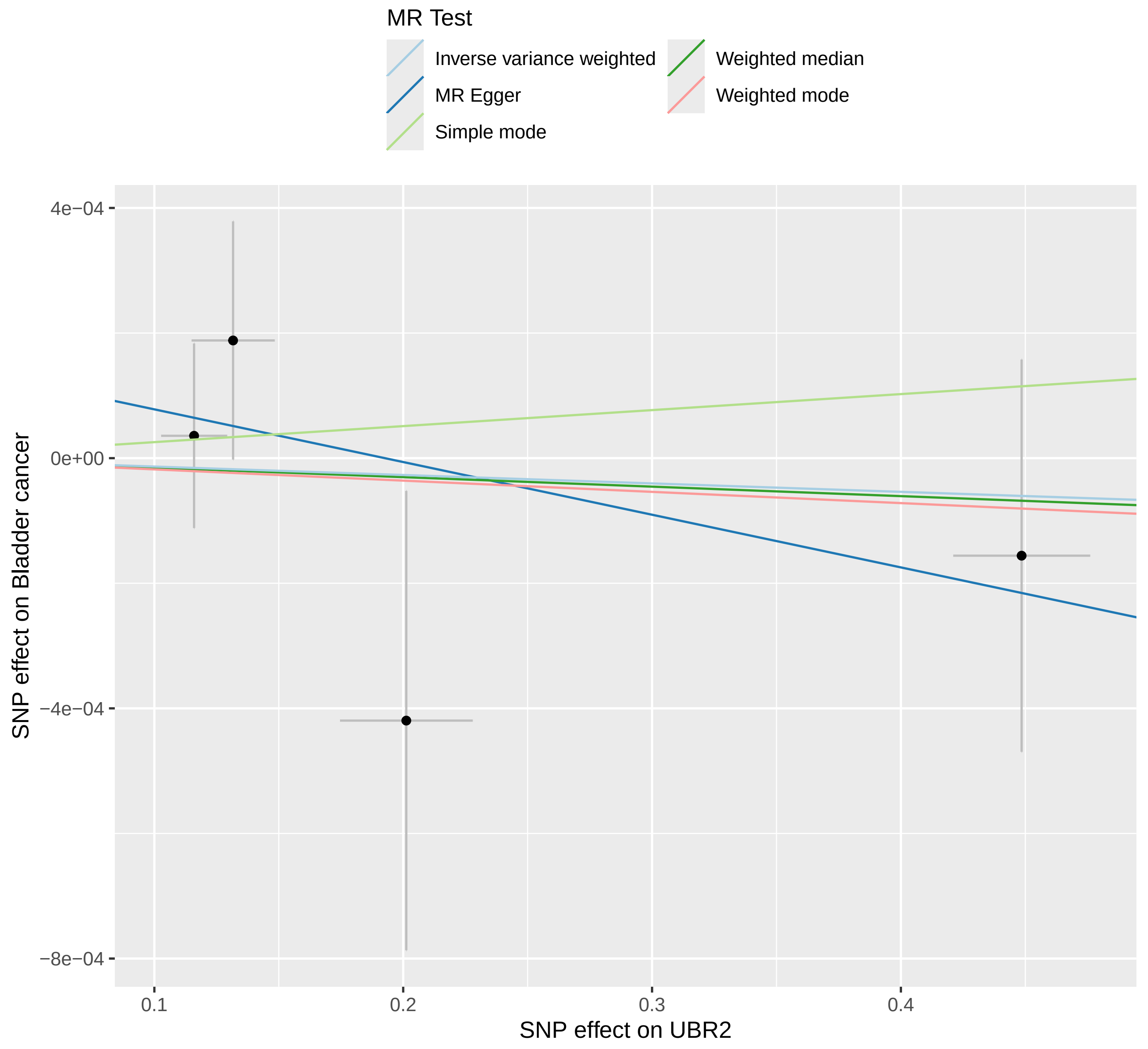

MR Test
Inverse variance weighted
MR Egger
Simple mode
Weighted median
Weighted mode
4e−04
0e+00
−4e−04
−8e−04
SNP effect on Bladder cancer
0.1
0.2
0.3
0.4
SNP effect on UBR2

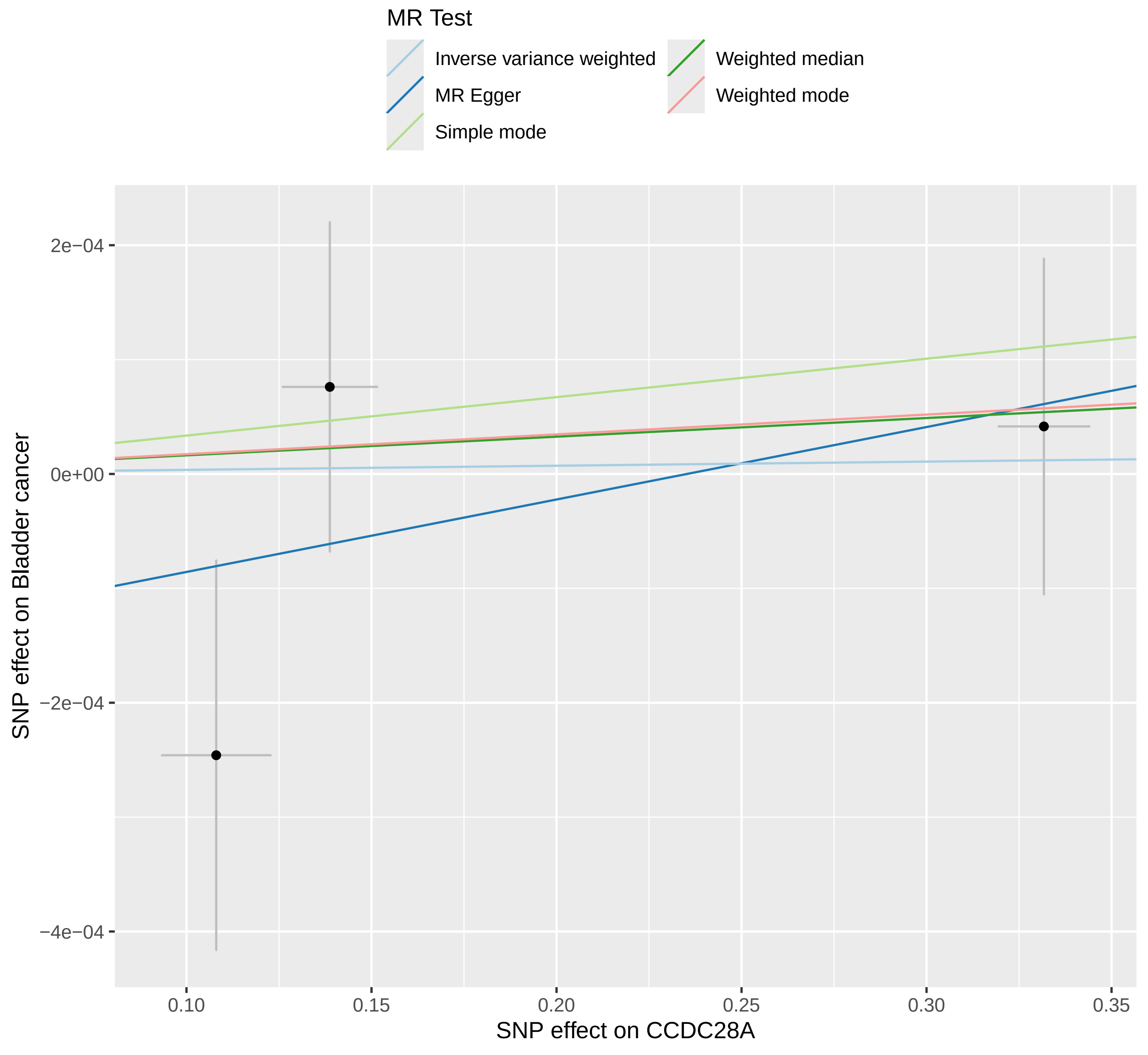
MR Test
Inverse variance weighted
MR Egger
Simple mode
Weighted median
Weighted mode
2e−04
0e+00
−2e−04
−4e−04
0.10
0.15
0.20
0.25
0.30
0.35
SNP effect on Bladder cancer
SNP effect on CCDC28A

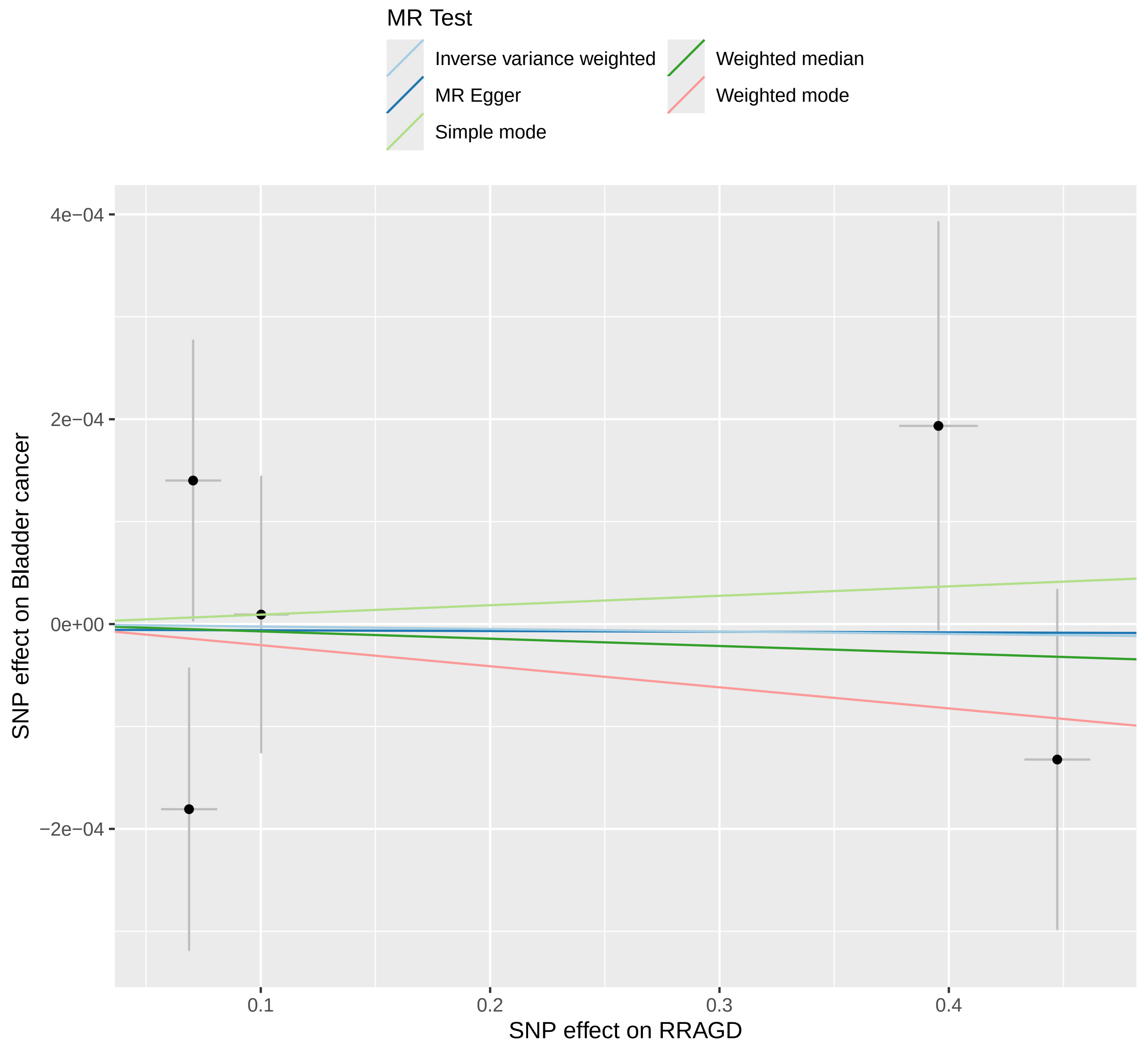
MR Test
Inverse variance weighted
MR Egger
Simple mode
Weighted median
Weighted mode
4e−04
2e−04
0e+00
−2e−04
0.1
0.2
0.3
0.4
SNP effect on Bladder cancer
SNP effect on RRAGD

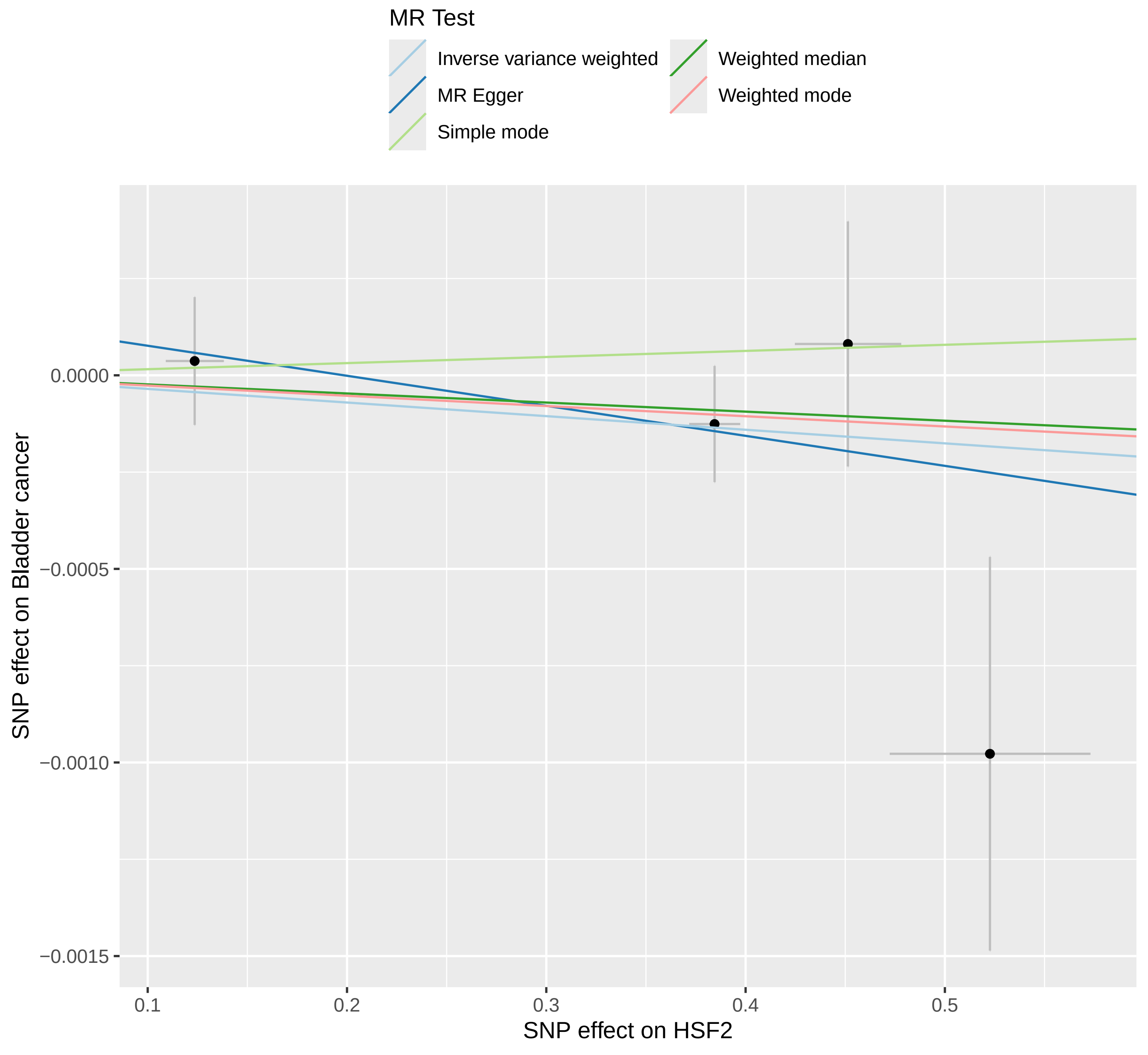

MR Test
Inverse variance weighted
MR Egger
Simple mode
Weighted median
Weighted mode
0.0000
−0.0005
−0.0010
−0.0015
SNP effect on Bladder cancer
0.1
0.2
0.3
0.4
0.5
SNP effect on HSF2

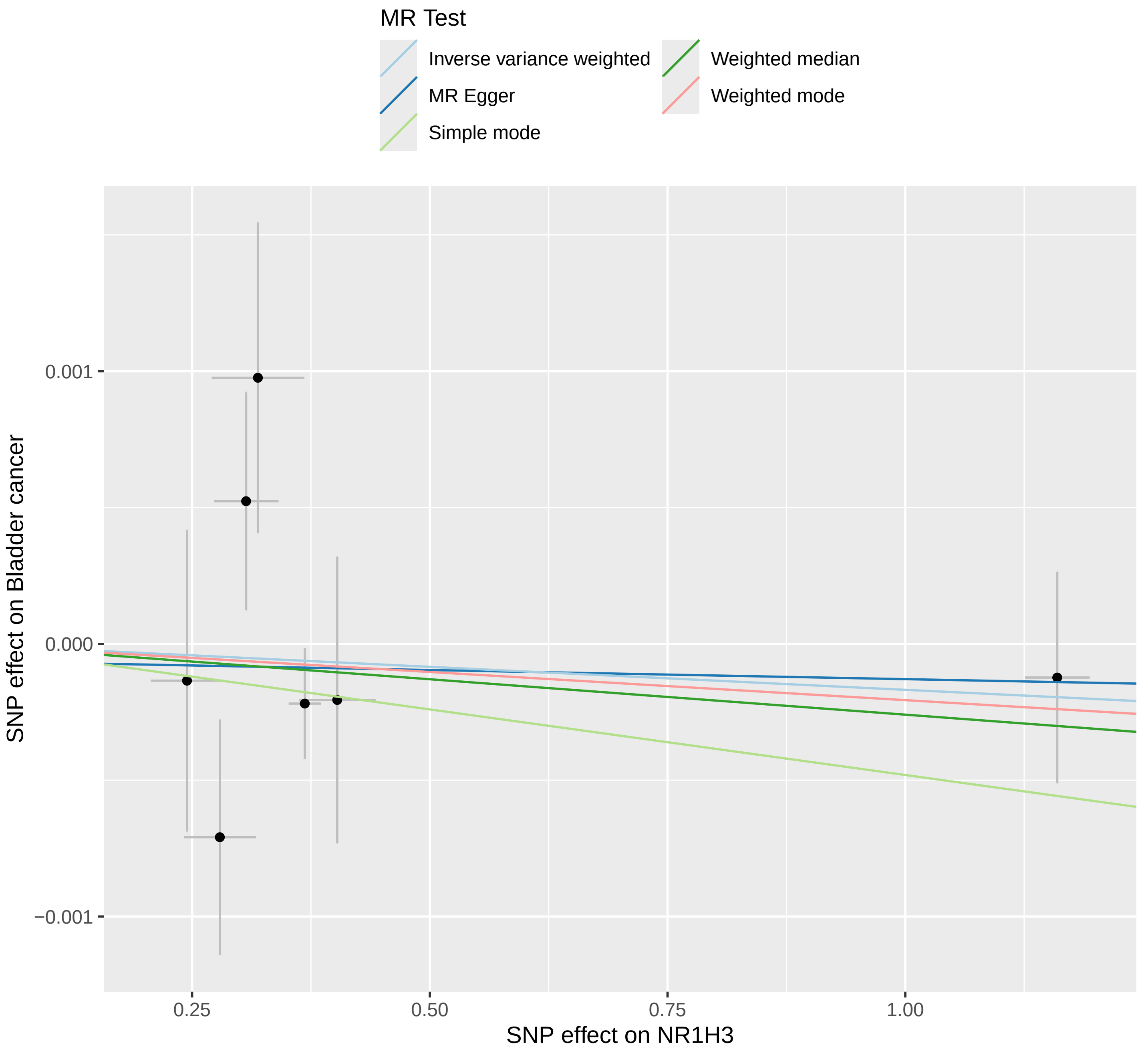

MR Test
Inverse variance weighted
MR Egger
Simple mode
Weighted median
Weighted mode
0.001
0.000
−0.001
SNP effect on Bladder cancer
0.25
0.50
0.75
1.00
SNP effect on NR1H3

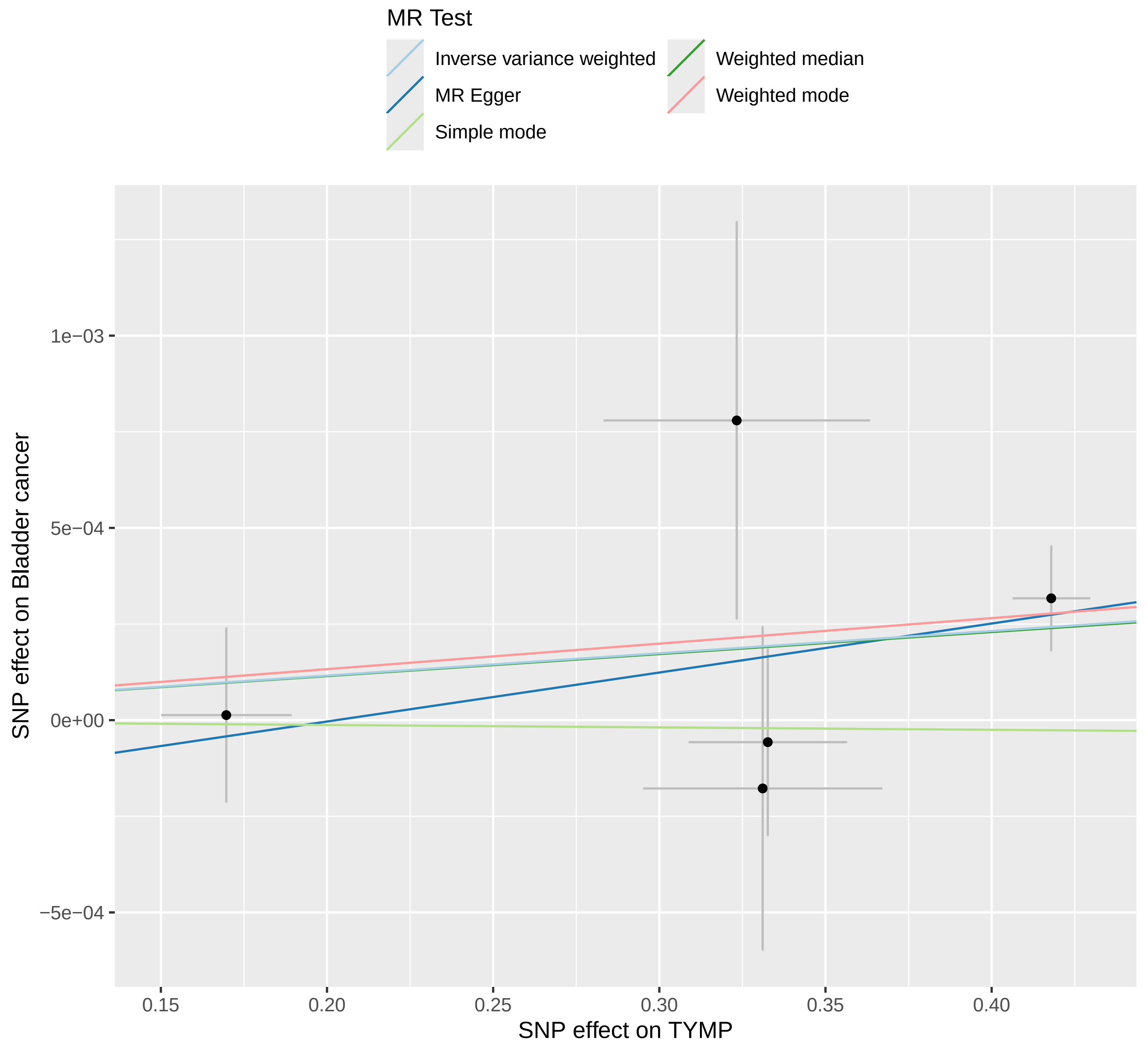

MR Test
Inverse variance weighted
MR Egger
Simple mode
Weighted median
Weighted mode
1e−03
5e−04
0e+00
−5e−04
0.15
0.20
0.25
0.30
0.35
0.40
SNP effect on TYMP
SNP effect on Bladder cancer

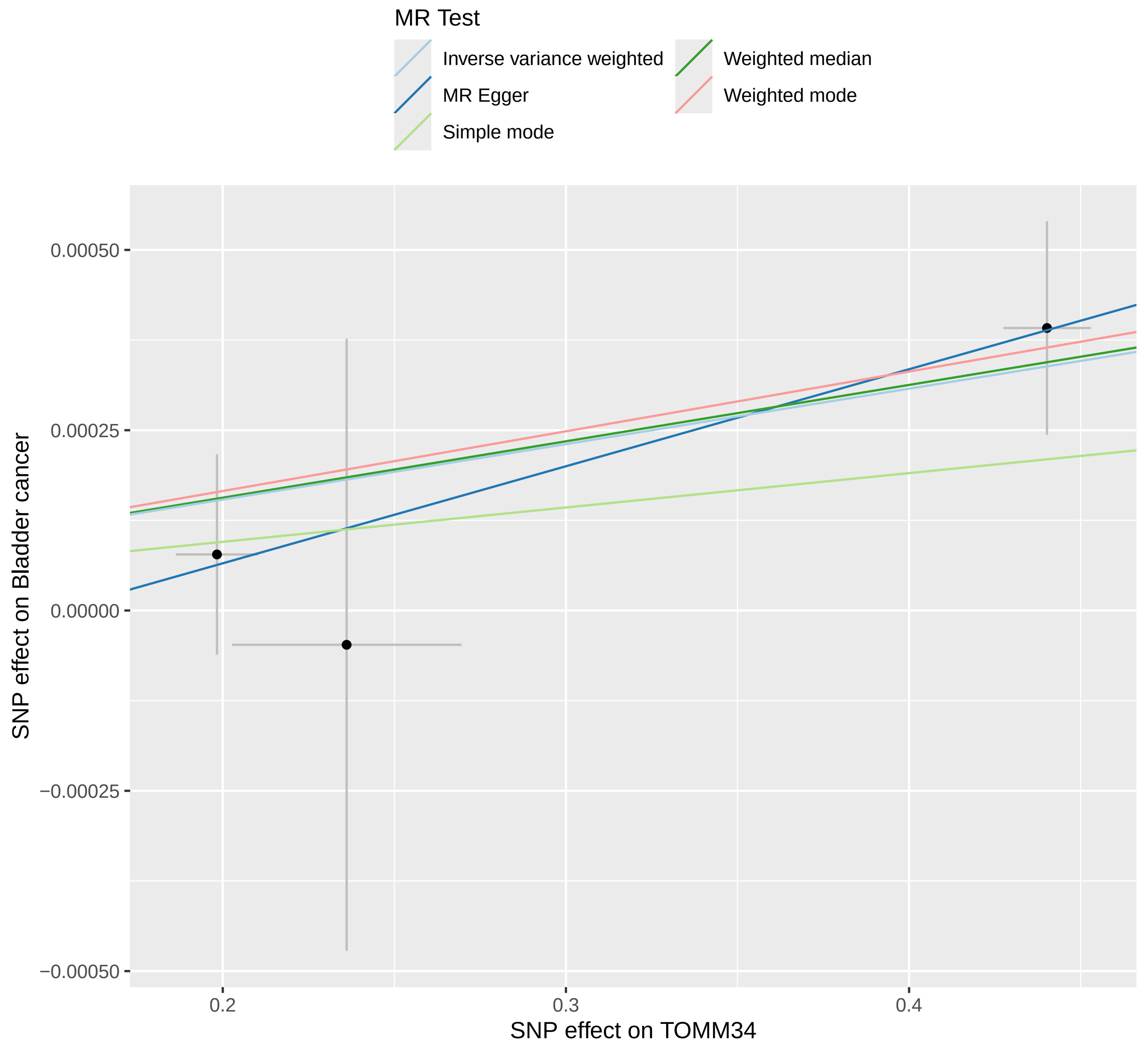

MR Test
Inverse variance weighted
MR Egger
Simple mode
Weighted median
Weighted mode
0.00050
0.00025
0.00000
−0.00025
−0.00050
SNP effect on Bladder cancer
0.2
0.3
0.4
SNP effect on TOMM34

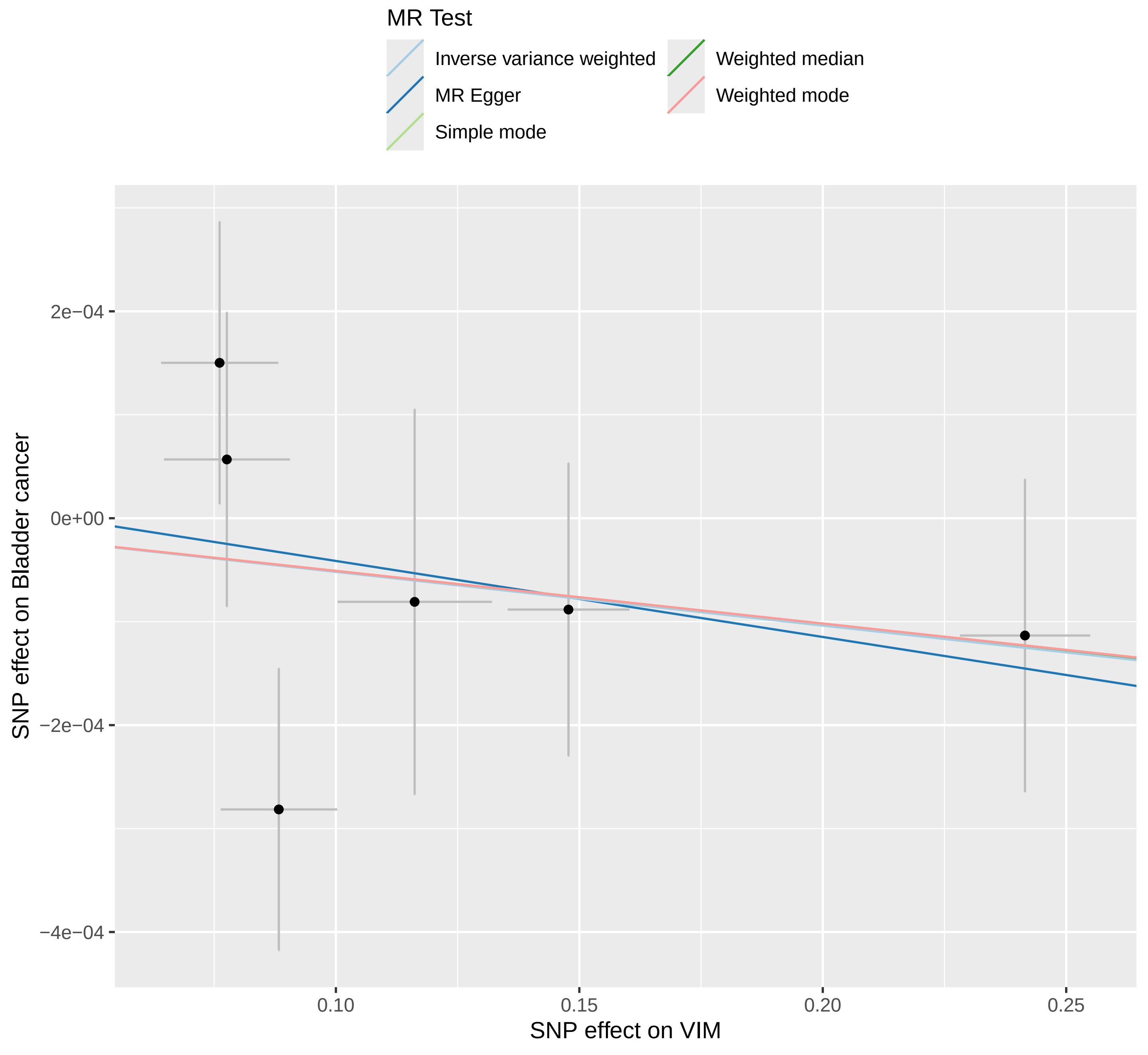
MR Test
Inverse variance weighted
MR Egger
Simple mode
Weighted median
Weighted mode
2e−04
0e+00
−2e−04
−4e−04
0.10
0.15
0.20
0.25
SNP effect on Bladder cancer
SNP effect on VIM

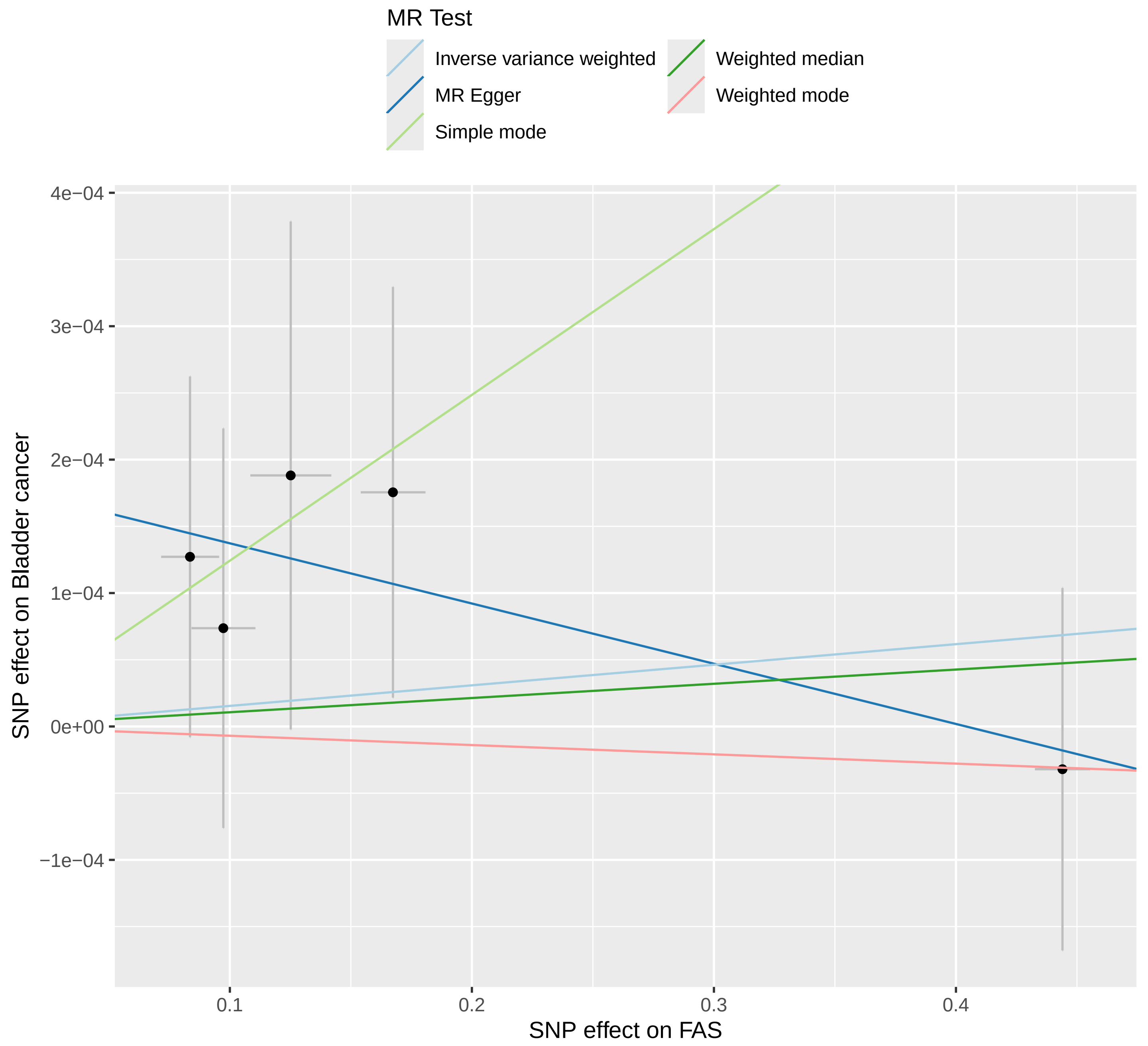
MR Test
Inverse variance weighted
MR Egger
Simple mode
Weighted median
Weighted mode
4e−04
3e−04
2e−04
1e−04
0e+00
−1e−04
SNP effect on Bladder cancer
0.1
0.2
0.3
0.4
SNP effect on FAS

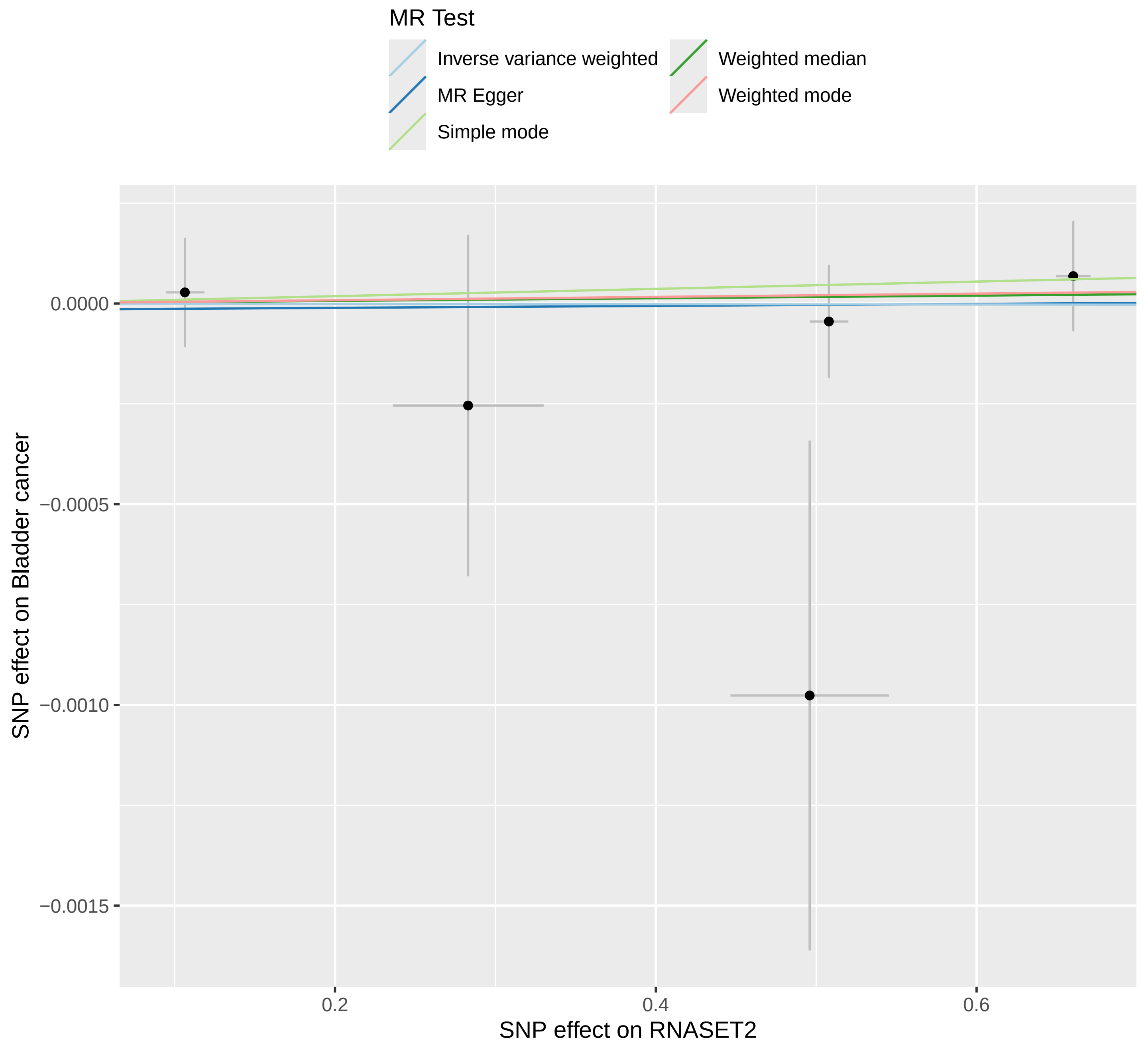
MR Test
Inverse variance weighted
MR Egger
Simple mode
Weighted median
Weighted mode
0.0000
−0.0005
−0.0010
−0.0015
SNP effect on Bladder cancer
0.2
0.4
0.6
SNP effect on RNASET2

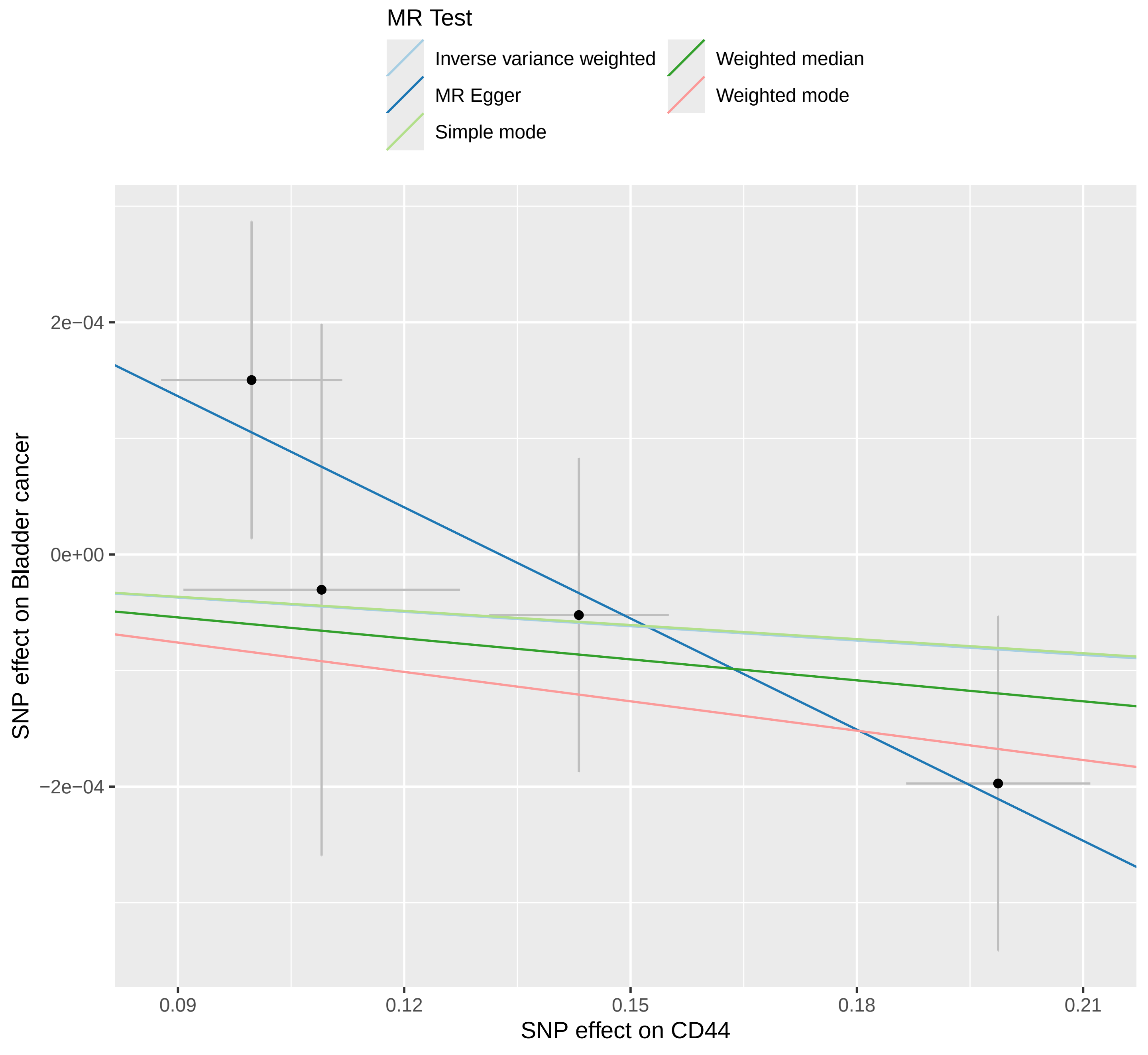
MR Test
Inverse variance weighted
MR Egger
Simple mode
Weighted median
Weighted mode
SNP effect on Bladder cancer
2e−04
0e+00
−2e−04
0.09
0.12
0.15
0.18
0.21
SNP effect on CD44

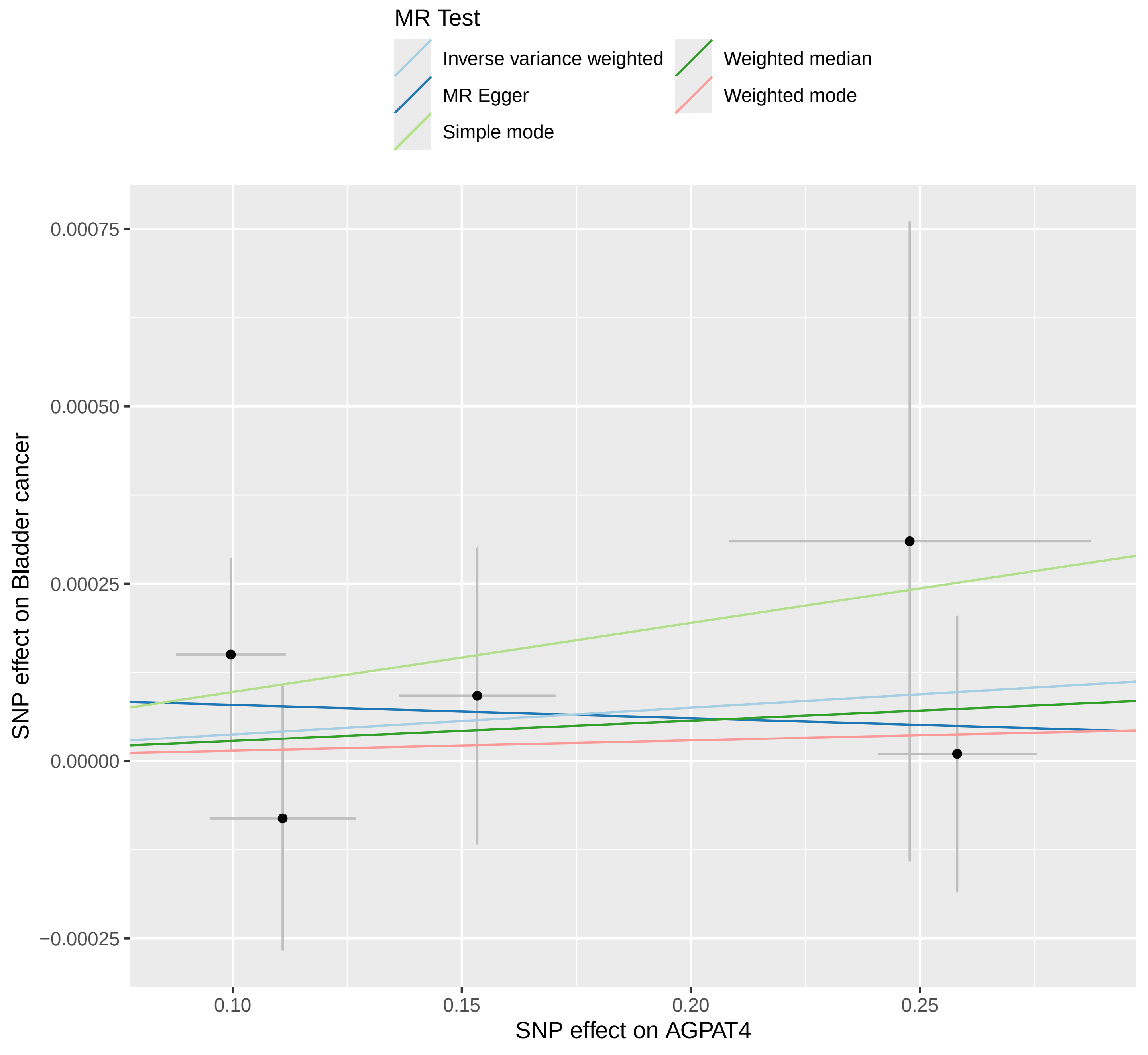

MR Test
Inverse variance weighted
MR Egger
Simple mode
Weighted median
Weighted mode
0.00075
0.00050
0.00025
0.00000
−0.00025
SNP effect on Bladder cancer
0.10
0.15
0.20
0.25
SNP effect on AGPAT4

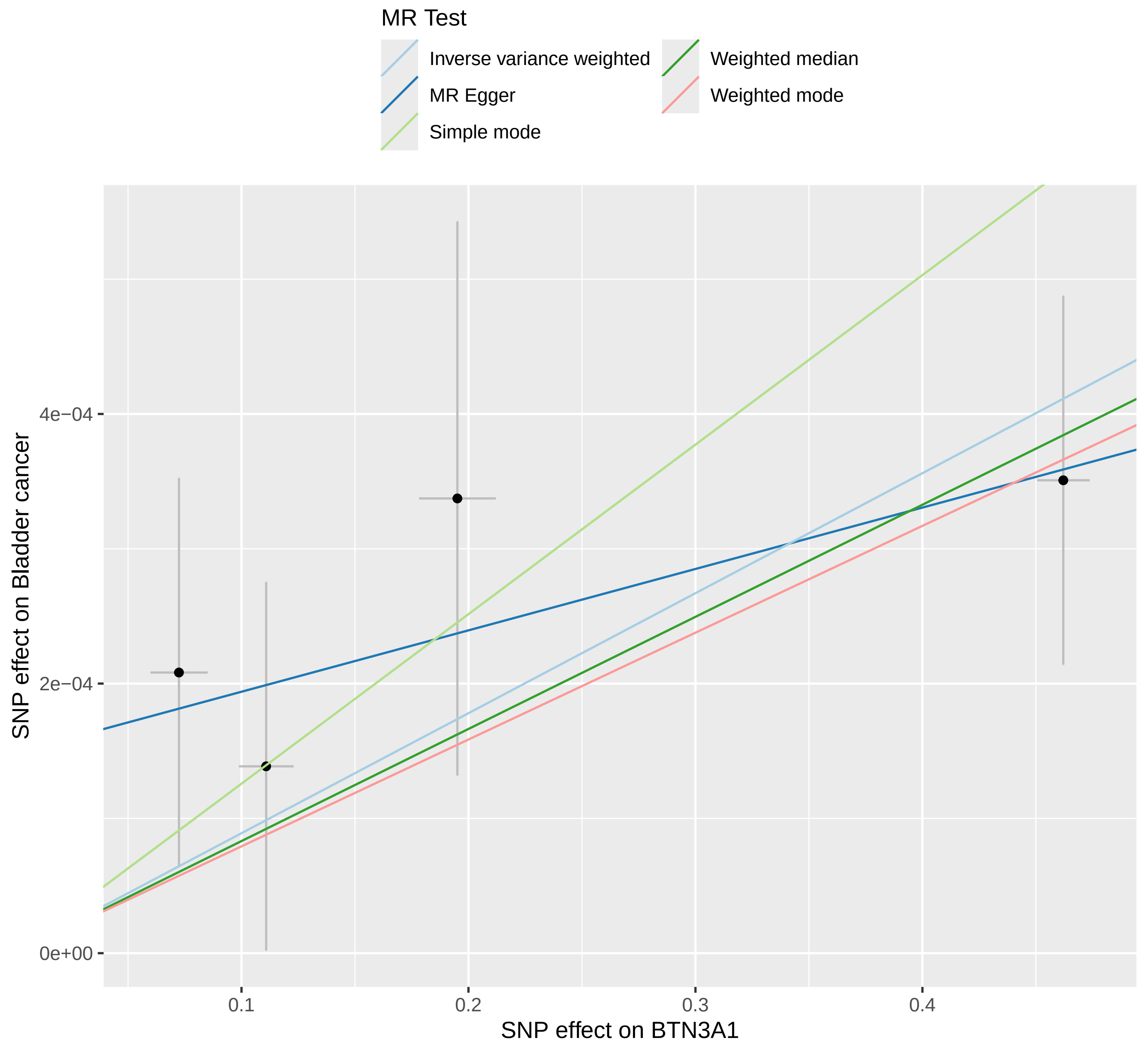

MR Test
Inverse variance weighted
MR Egger
Simple mode
Weighted median
Weighted mode
SNP effect on Bladder cancer
4e−04
2e−04
0e+00
0.1
0.2
0.3
0.4
SNP effect on BTN3A1

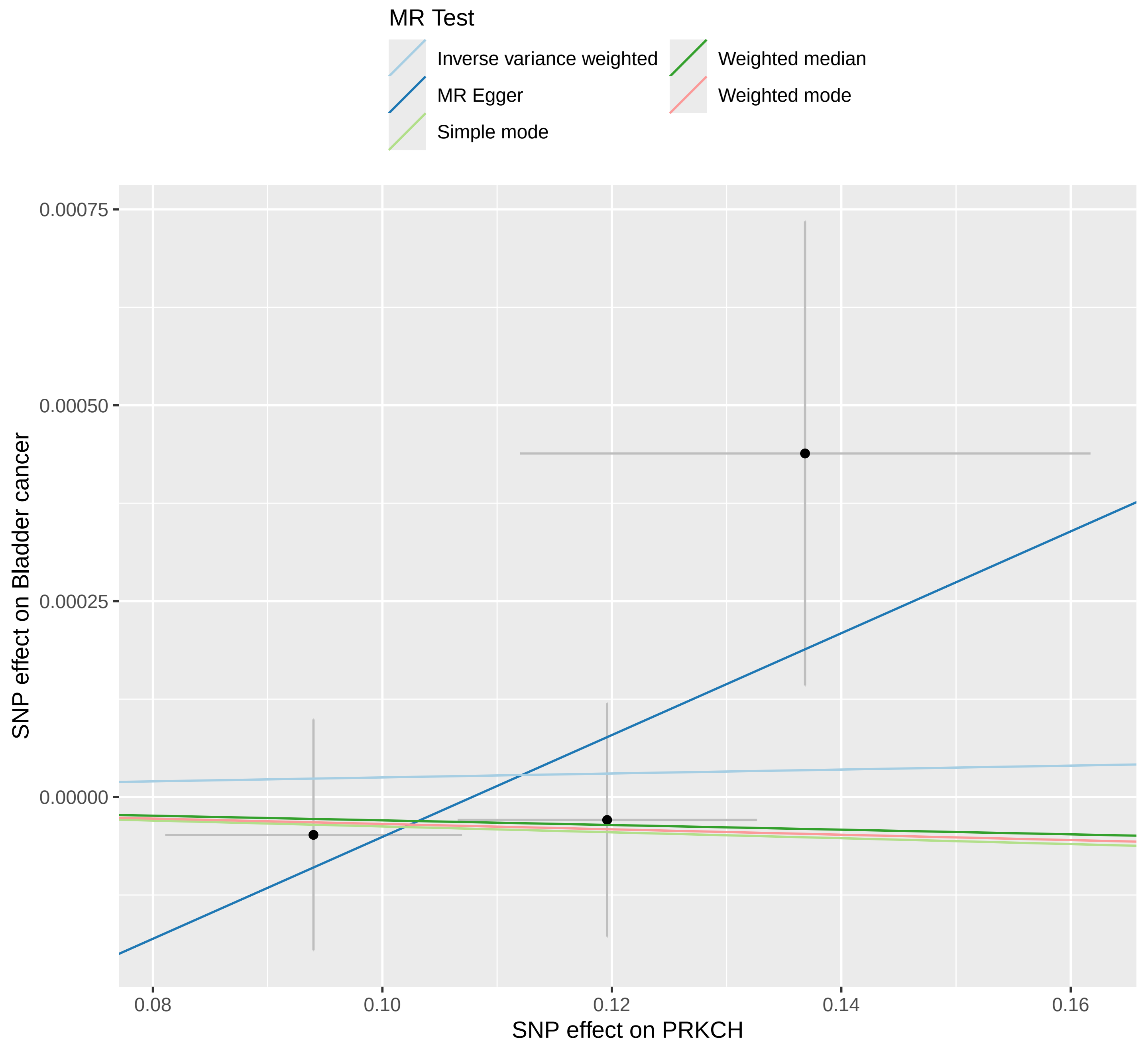

MR Test
Inverse variance weighted
MR Egger
Simple mode
Weighted median
Weighted mode
0.00075
0.00050
0.00025
0.00000
SNP effect on Bladder cancer
0.08
0.10
0.12
0.14
0.16
SNP effect on PRKCH

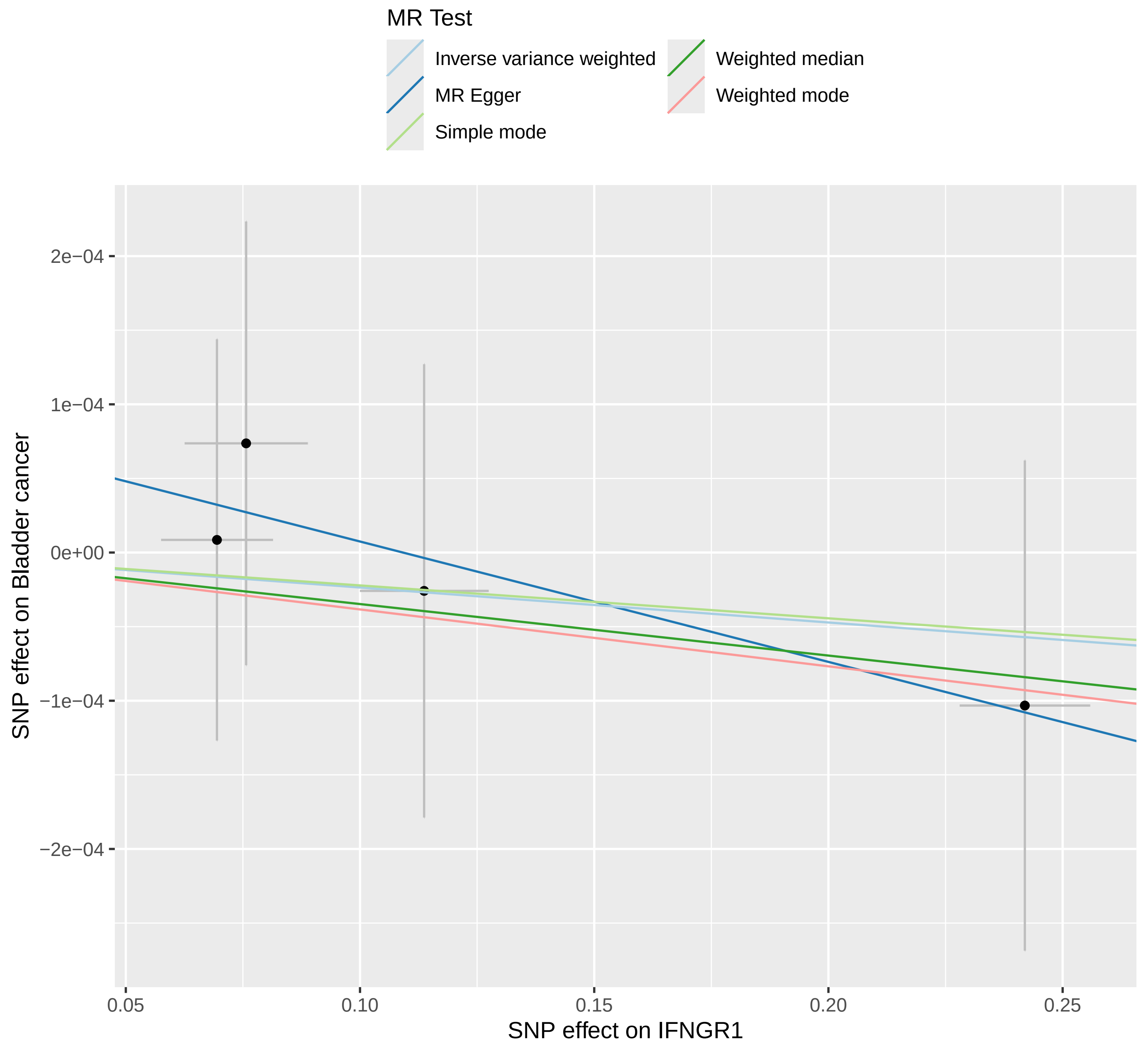
MR Test
Inverse variance weighted
Weighted median
MR Egger
Weighted mode
Simple mode
2e−04
1e−04
0e+00
−1e−04
−2e−04
SNP effect on Bladder cancer
0.05
0.10
0.15
0.20
0.25
SNP effect on IFNGR1

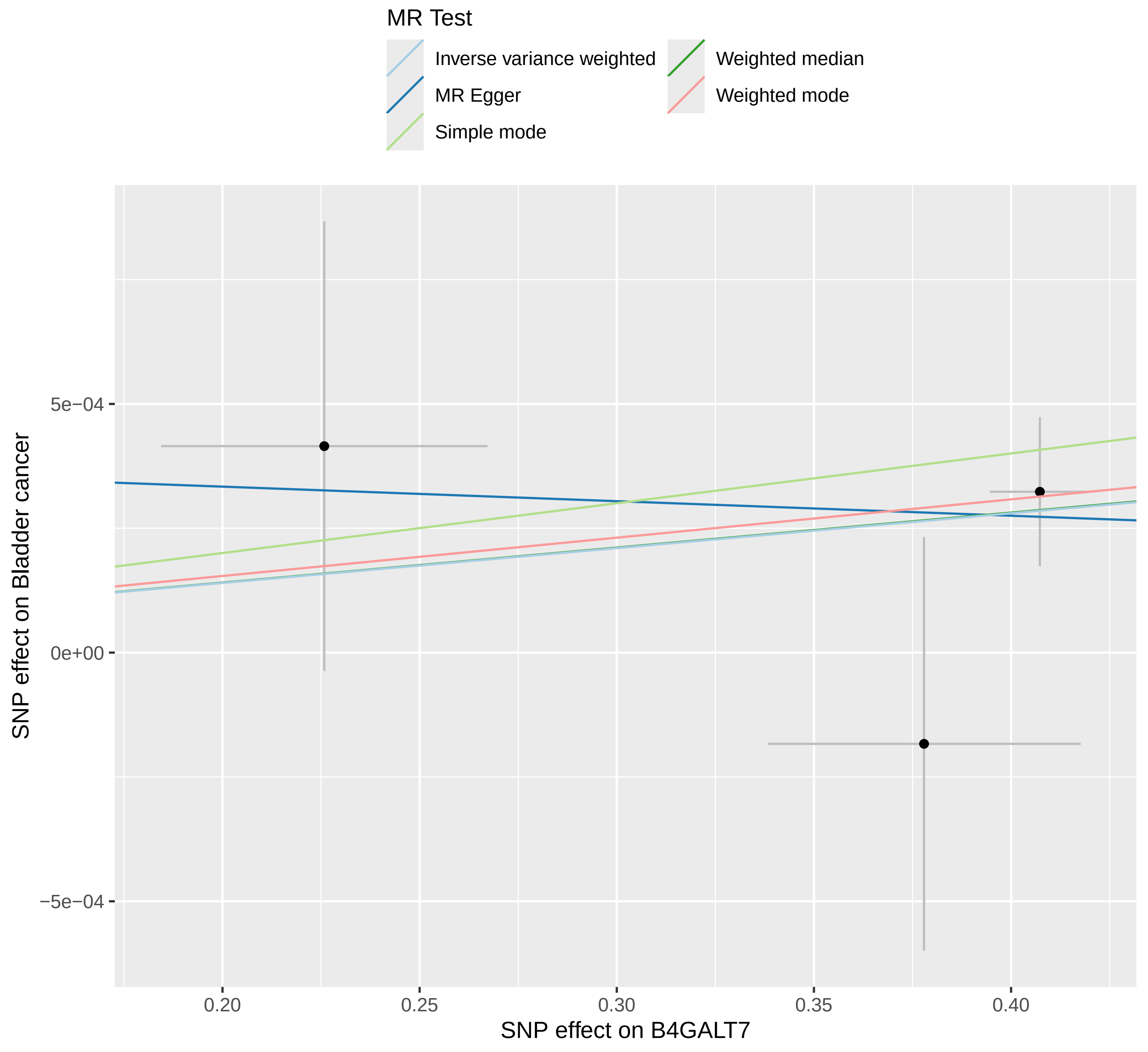
MR Test
Inverse variance weighted
MR Egger
Simple mode
Weighted median
Weighted mode
5e−04
0e+00
−5e−04
SNP effect on Bladder cancer
0.20
0.25
0.30
0.35
0.40
SNP effect on B4GALT7

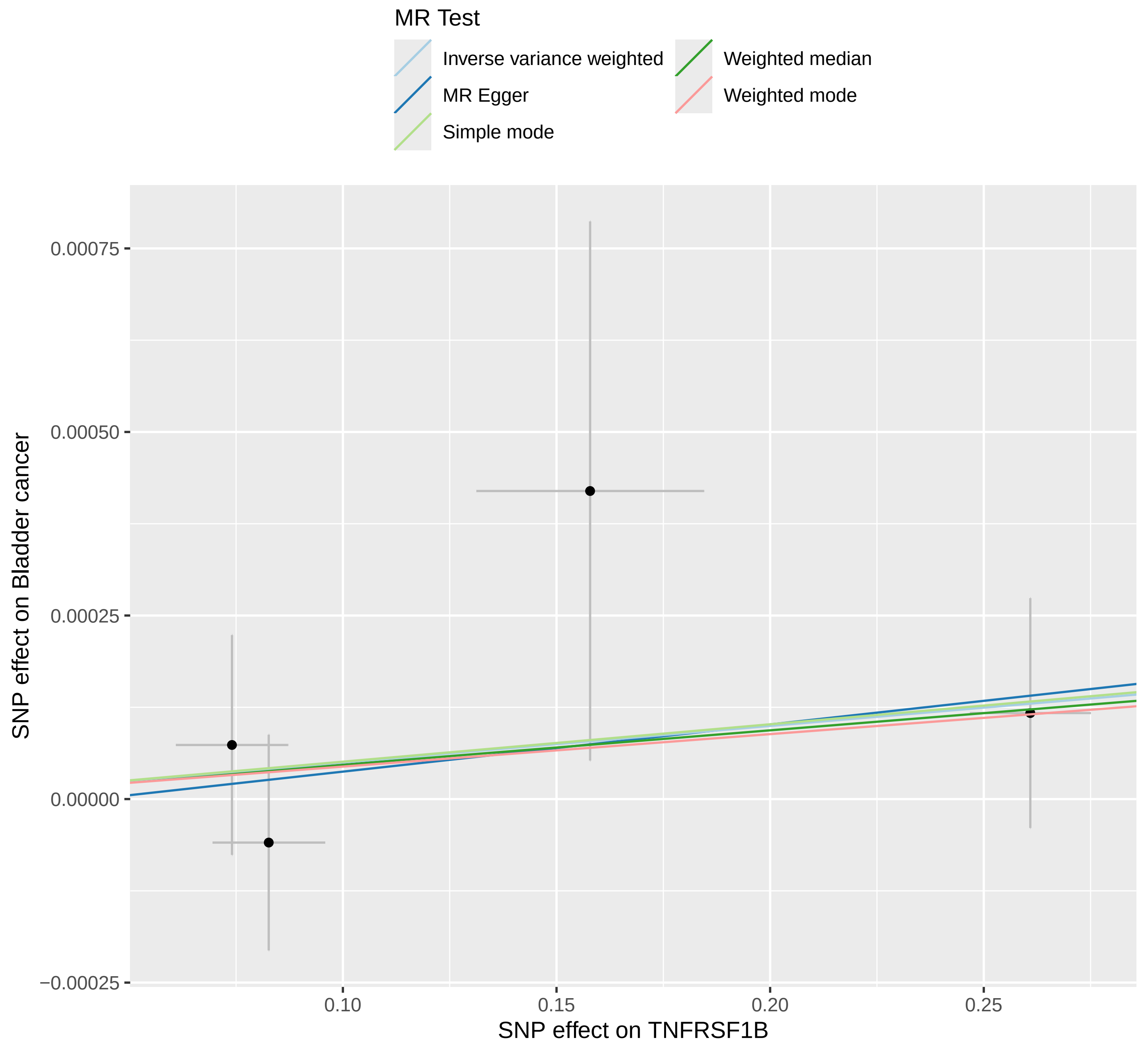
MR Test
Inverse variance weighted
MR Egger
Simple mode
Weighted median
Weighted mode
0.00075
0.00050
0.00025
0.00000
−0.00025
SNP effect on Bladder cancer
0.10
0.15
0.20
0.25
SNP effect on TNFRSF1B

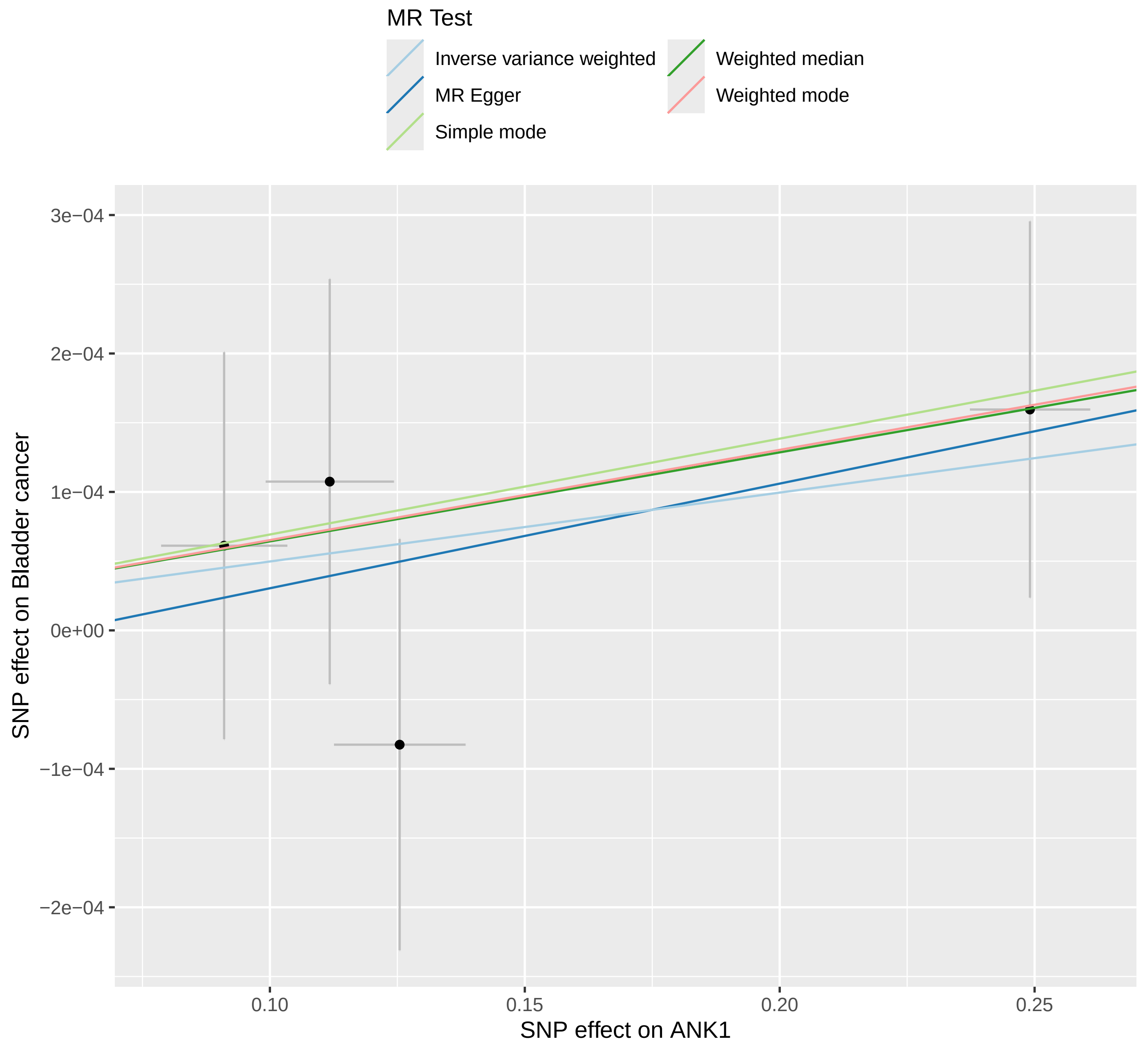

MR Test
Inverse variance weighted
MR Egger
Simple mode
Weighted median
Weighted mode
3e−04
2e−04
1e−04
0e+00
−1e−04
−2e−04
SNP effect on Bladder cancer
0.10
0.15
0.20
0.25
SNP effect on ANK1

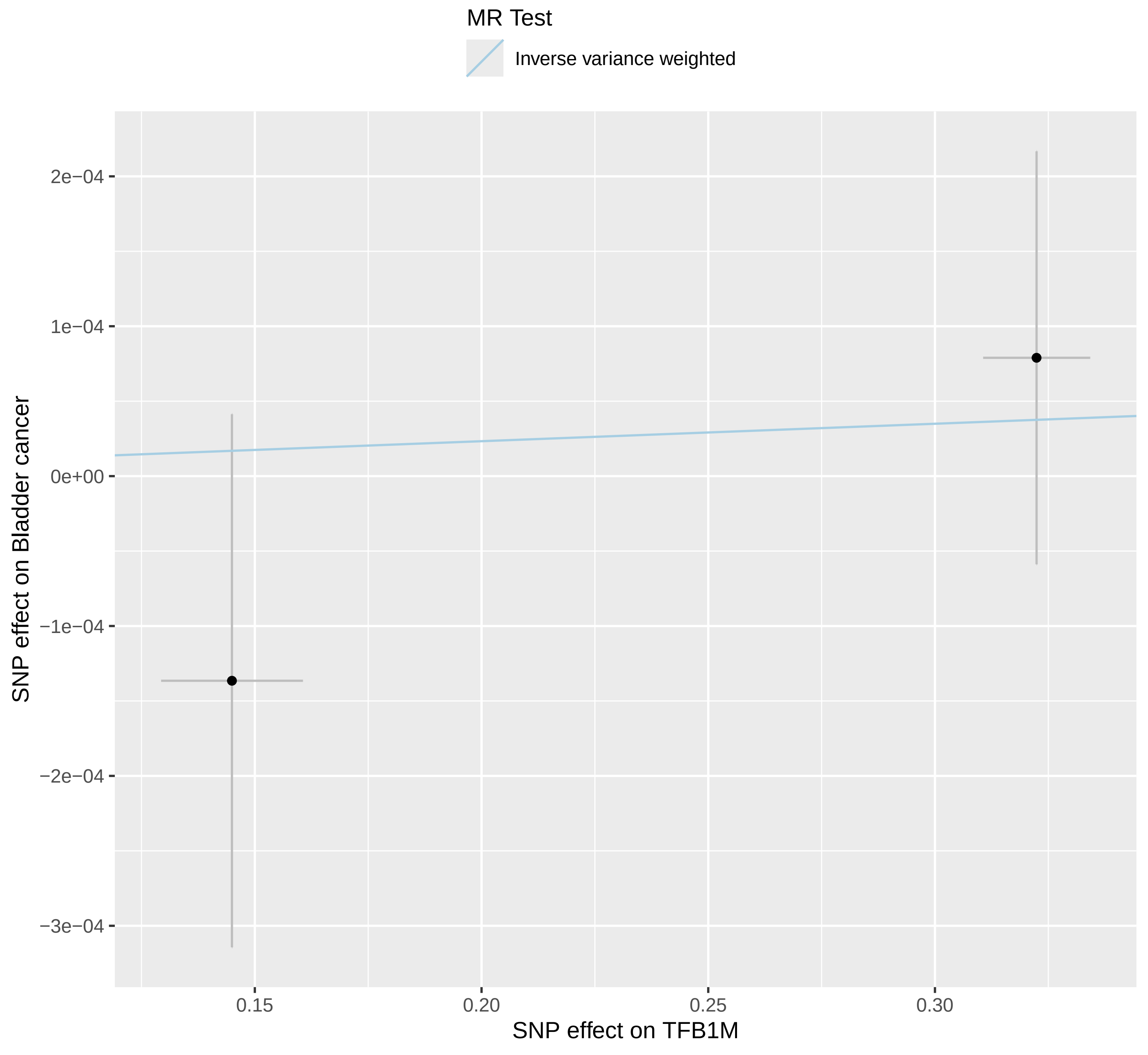

MR Test
Inverse variance weighted
2e−04
1e−04
0e+00
−1e−04
−2e−04
−3e−04
SNP effect on Bladder cancer
0.15
0.20
0.25
0.30
SNP effect on TFB1M

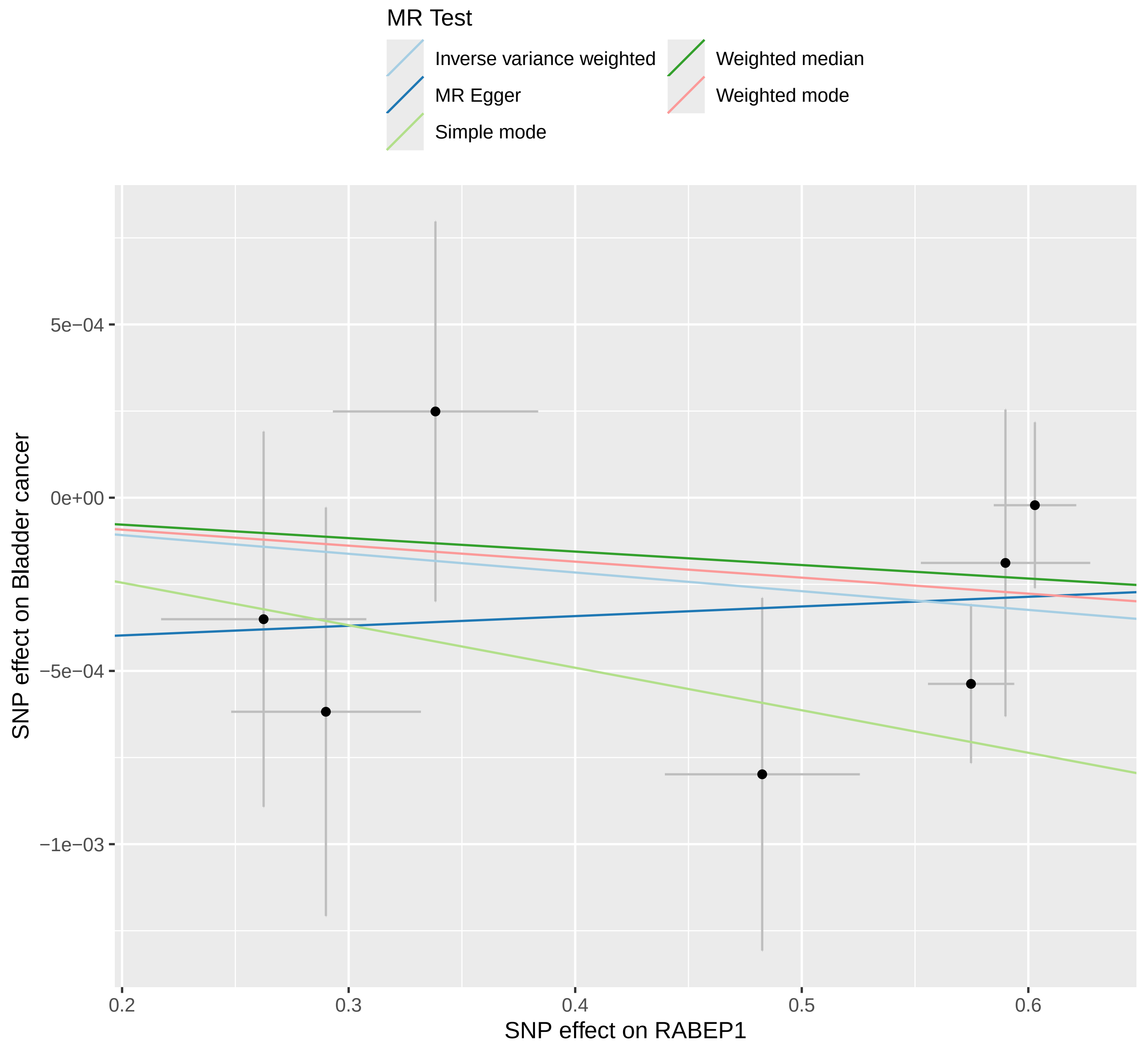

MR Test
Inverse variance weighted
MR Egger
Simple mode
Weighted median
Weighted mode
5e−04
0e+00
−5e−04
−1e−03
SNP effect on Bladder cancer
0.2
0.3
0.4
0.5
0.6
SNP effect on RABEP1

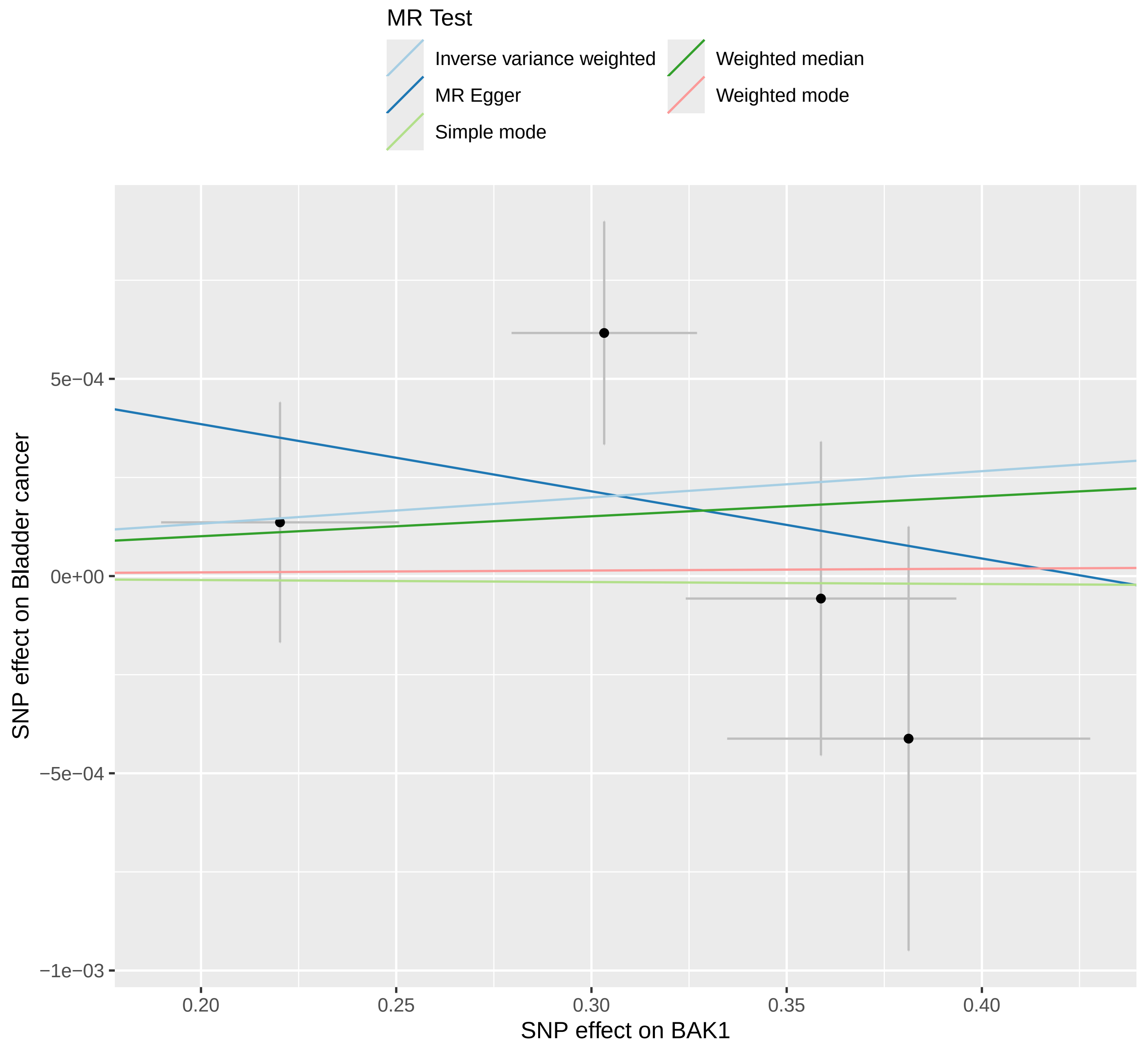
MR Test
Inverse variance weighted
MR Egger
Simple mode
Weighted median
Weighted mode
5e−04
0e+00
−5e−04
−1e−03
0.20
0.25
0.30
0.35
0.40
SNP effect on Bladder cancer
SNP effect on BAK1

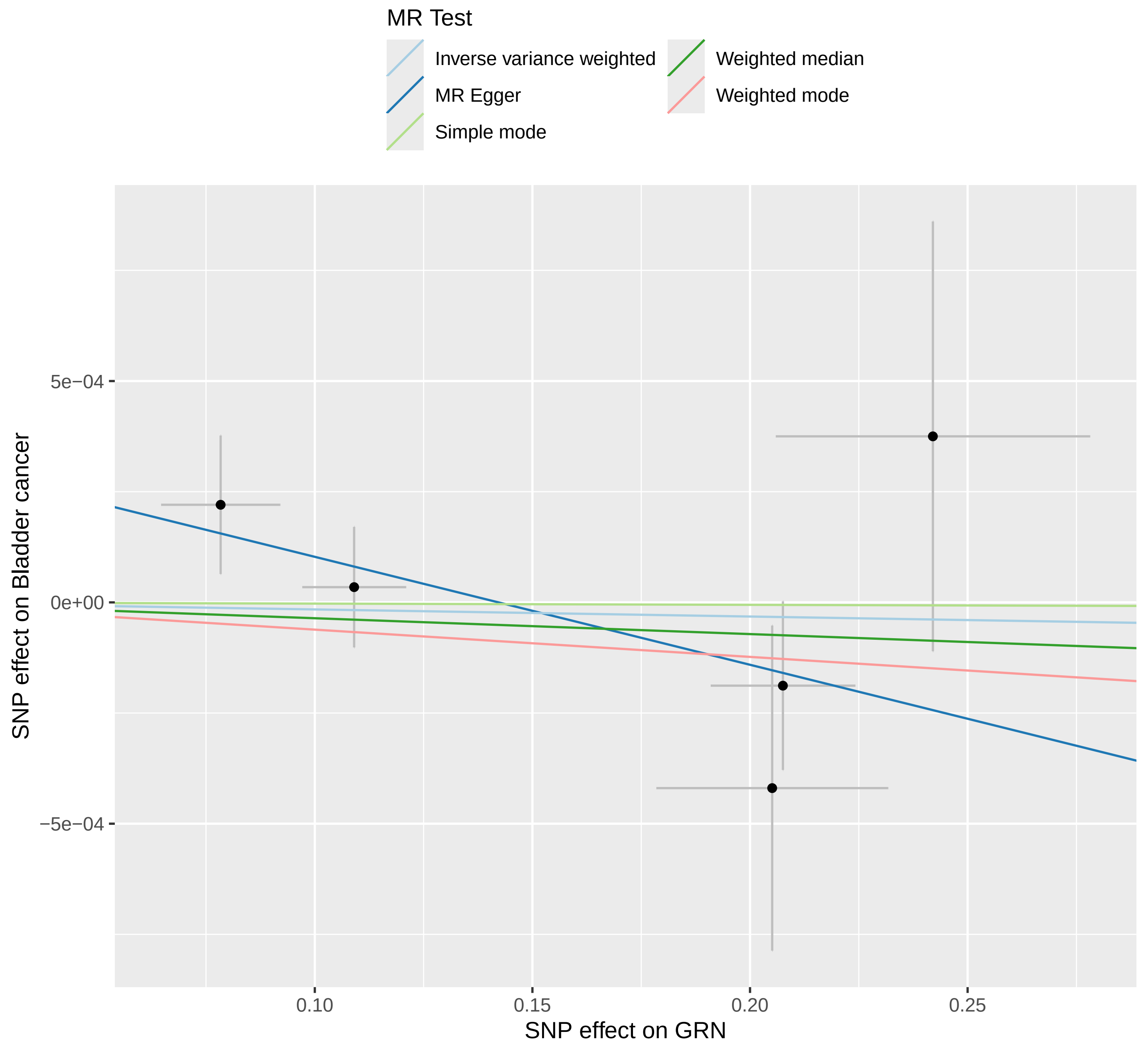

MR Test
Inverse variance weighted
MR Egger
Simple mode
Weighted median
Weighted mode
5e−04
0e+00
−5e−04
SNP effect on Bladder cancer
0.10
0.15
0.20
0.25
SNP effect on GRN

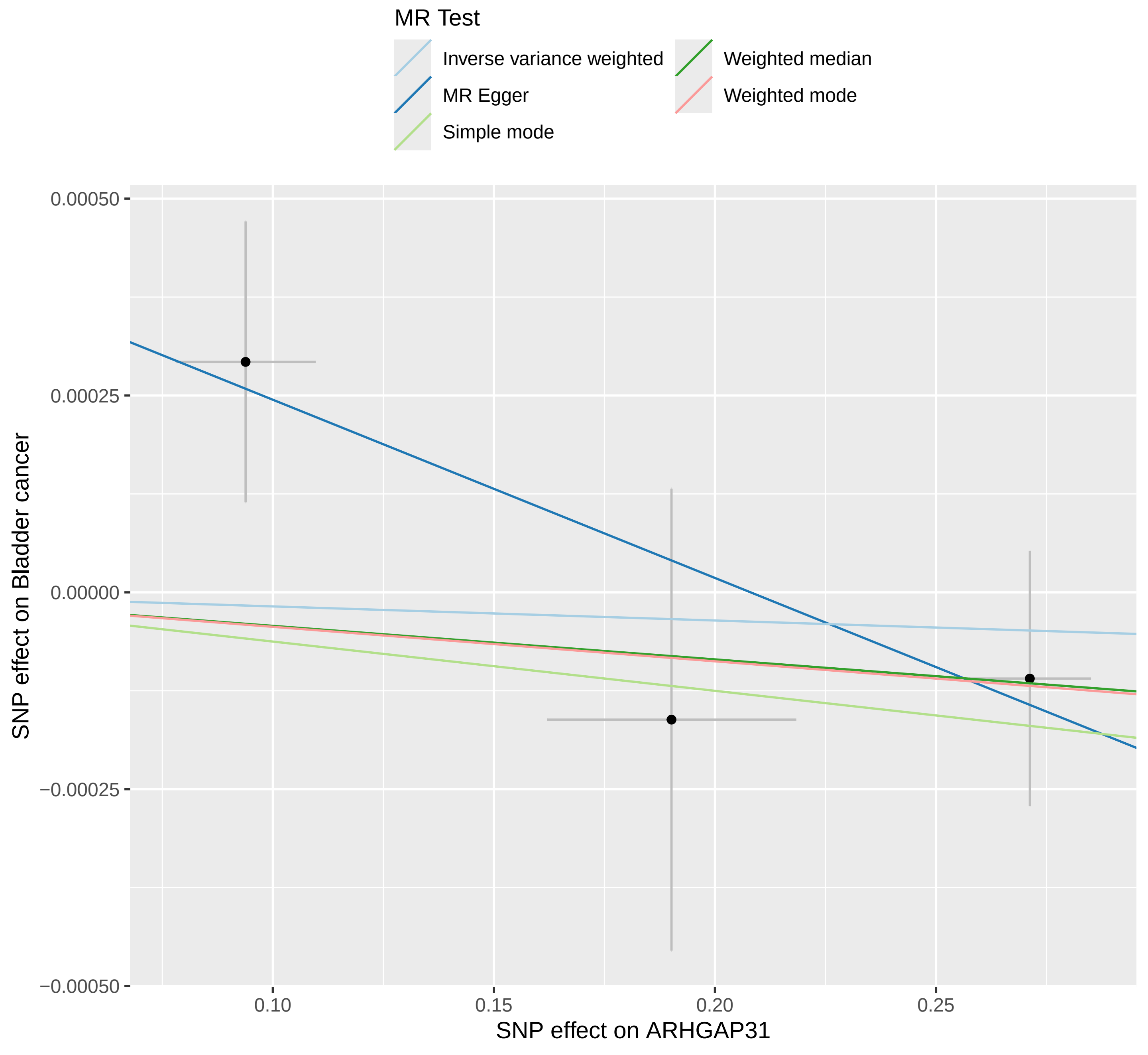
MR Test
Inverse variance weighted
MR Egger
Simple mode
Weighted median
Weighted mode
0.00050
0.00025
0.00000
−0.00025
−0.00050
SNP effect on Bladder cancer
0.10
0.15
0.20
0.25
SNP effect on ARHGAP31

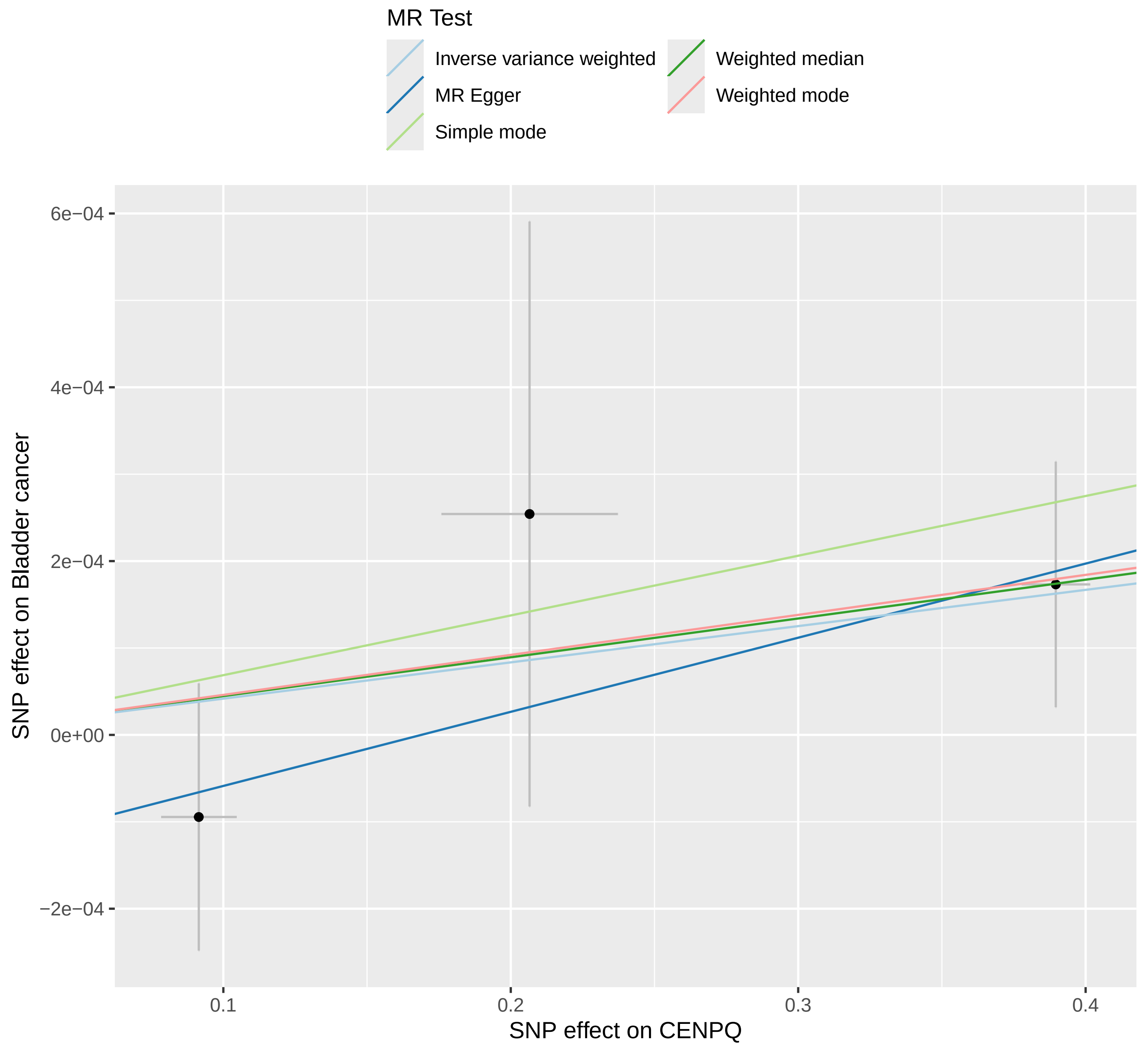

MR Test
Inverse variance weighted
MR Egger
Simple mode
Weighted median
Weighted mode
6e−04
4e−04
2e−04
0e+00
−2e−04
SNP effect on Bladder cancer
0.1
0.2
0.3
0.4
SNP effect on CENPQ

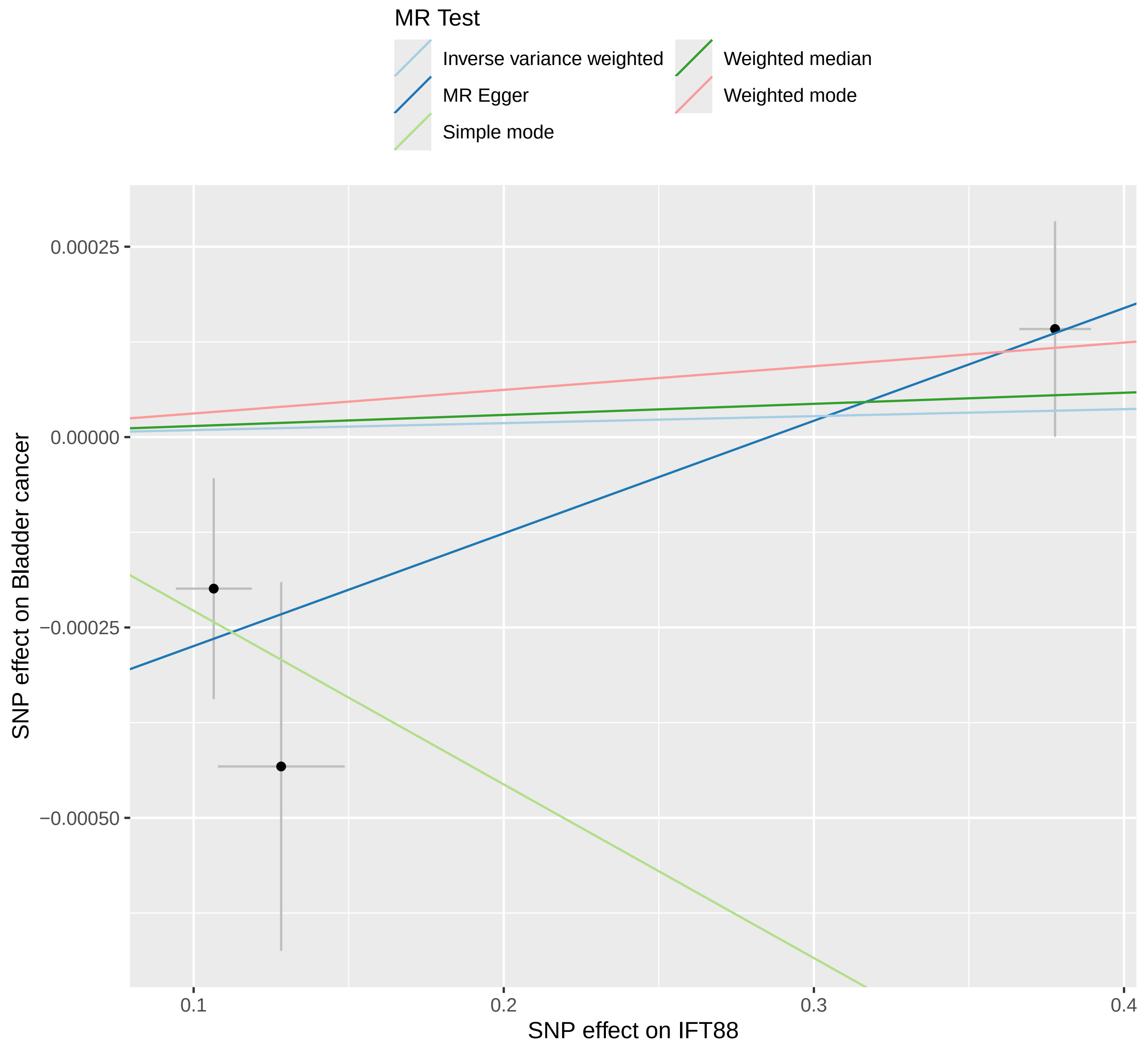

MR Test
Inverse variance weighted
MR Egger
Simple mode
Weighted median
Weighted mode
0.00025
0.00000
−0.00025
−0.00050
SNP effect on Bladder cancer
0.1
0.2
0.3
0.4
SNP effect on IFT88

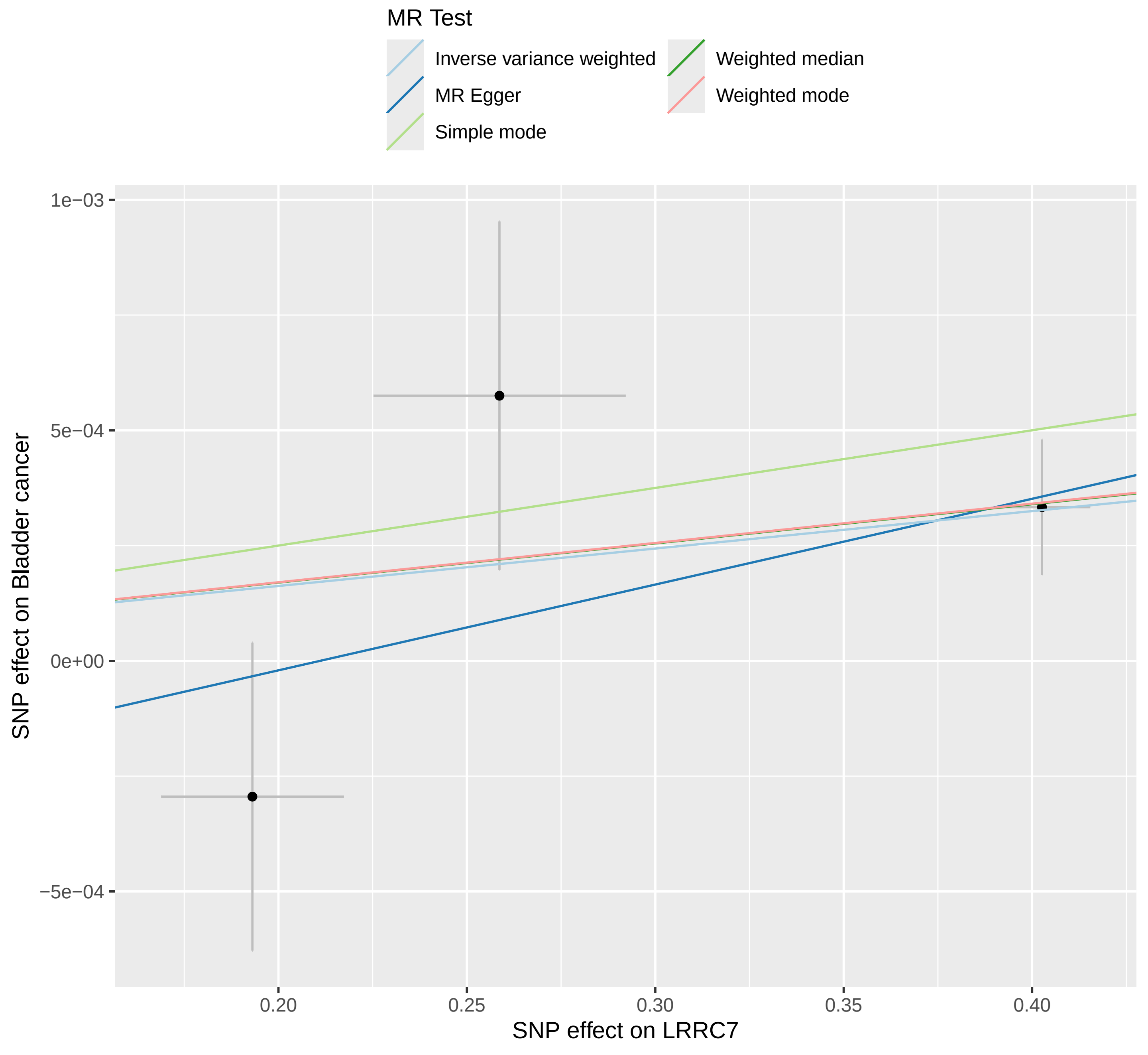
MR Test
Inverse variance weighted
MR Egger
Simple mode
Weighted median
Weighted mode
1e−03
5e−04
0e+00
−5e−04
0.20
0.25
0.30
0.35
0.40
SNP effect on Bladder cancer
SNP effect on LRRC7

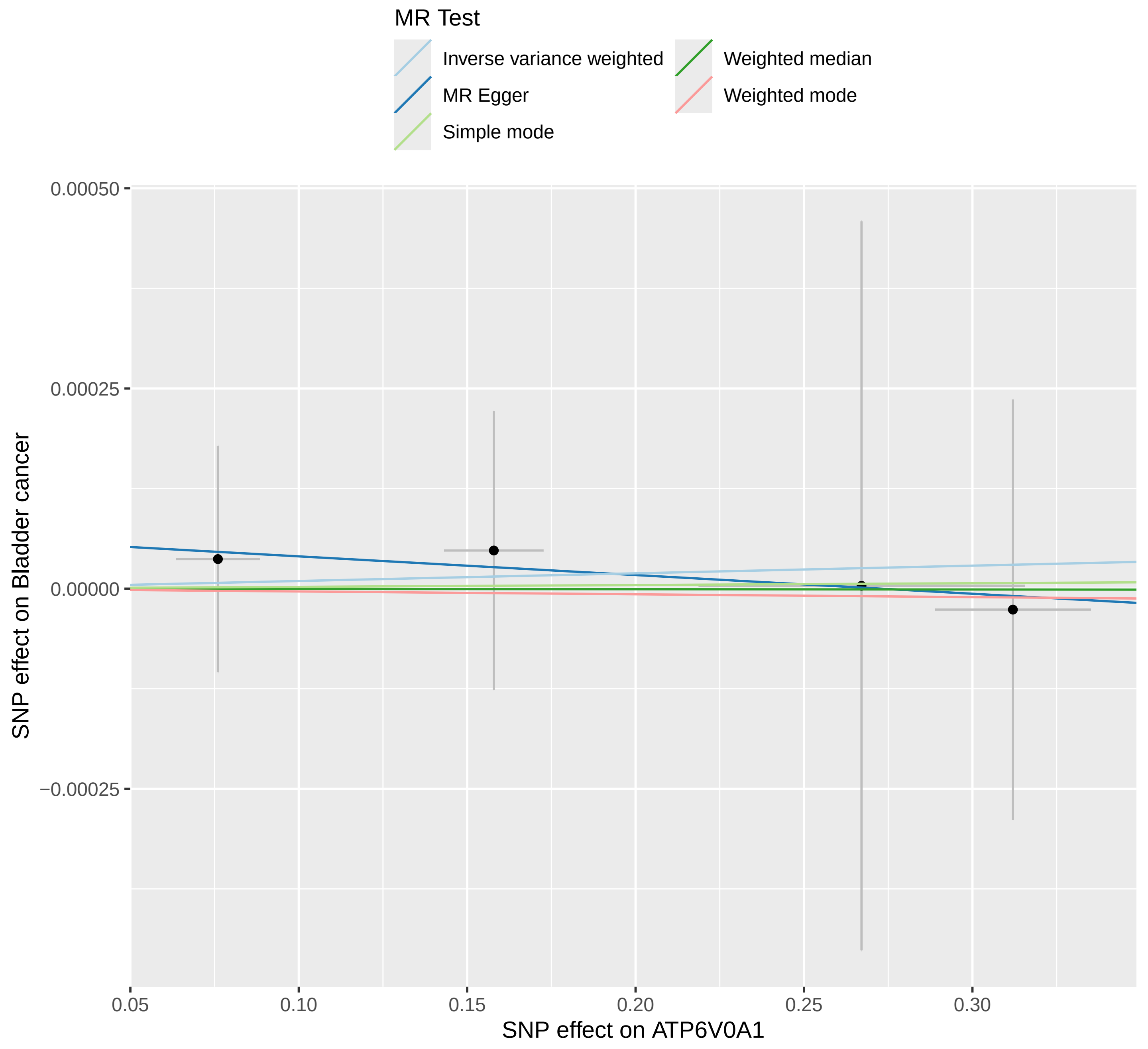
MR Test
Inverse variance weighted
MR Egger
Simple mode
Weighted median
Weighted mode
0.00050
0.00025
0.00000
−0.00025
SNP effect on Bladder cancer
0.05
0.10
0.15
0.20
0.25
0.30
SNP effect on ATP6V0A1

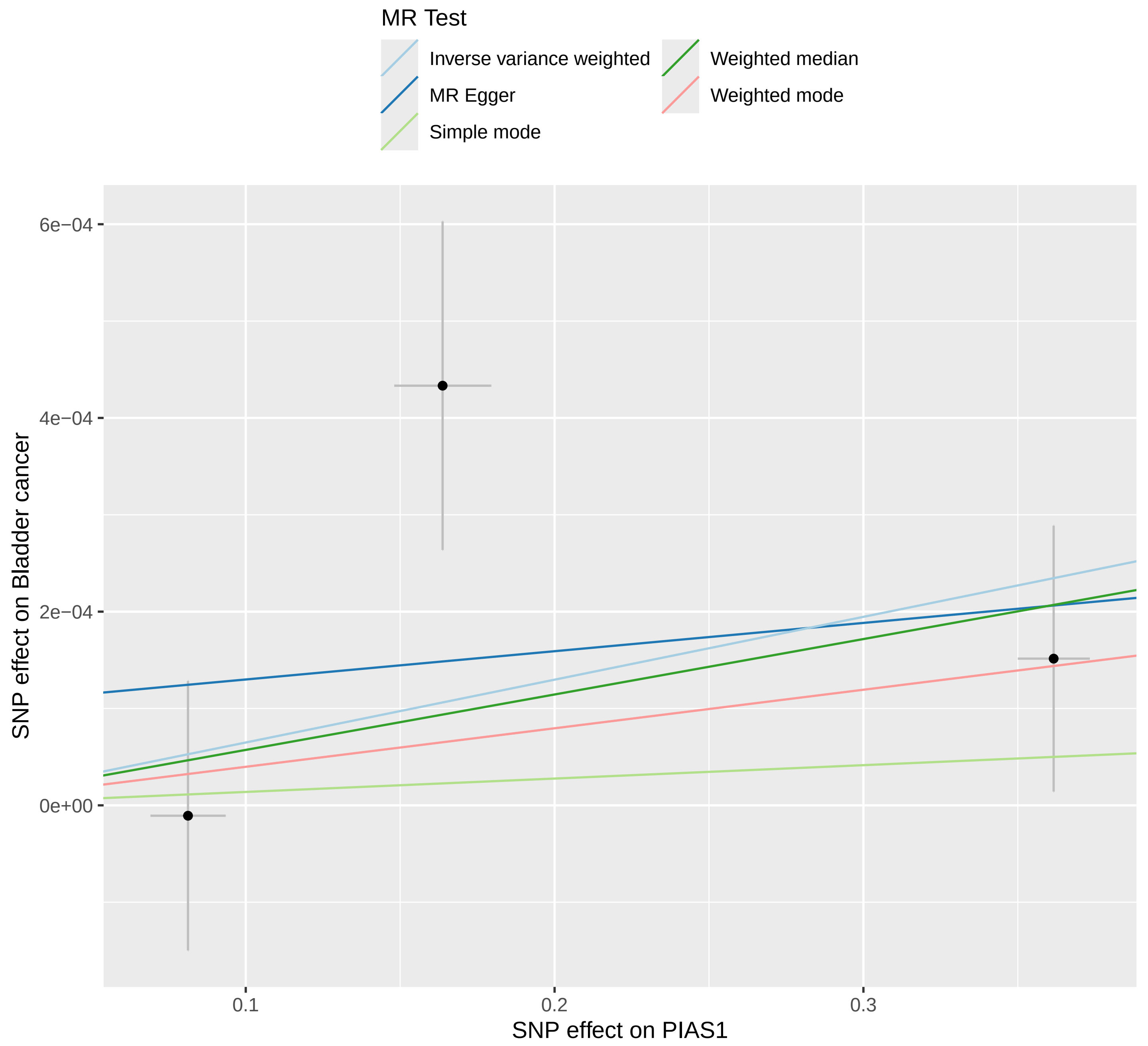

MR Test
Inverse variance weighted
MR Egger
Simple mode
Weighted median
Weighted mode
6e−04
4e−04
2e−04
0e+00
SNP effect on Bladder cancer
0.1
0.2
0.3
SNP effect on PIAS1

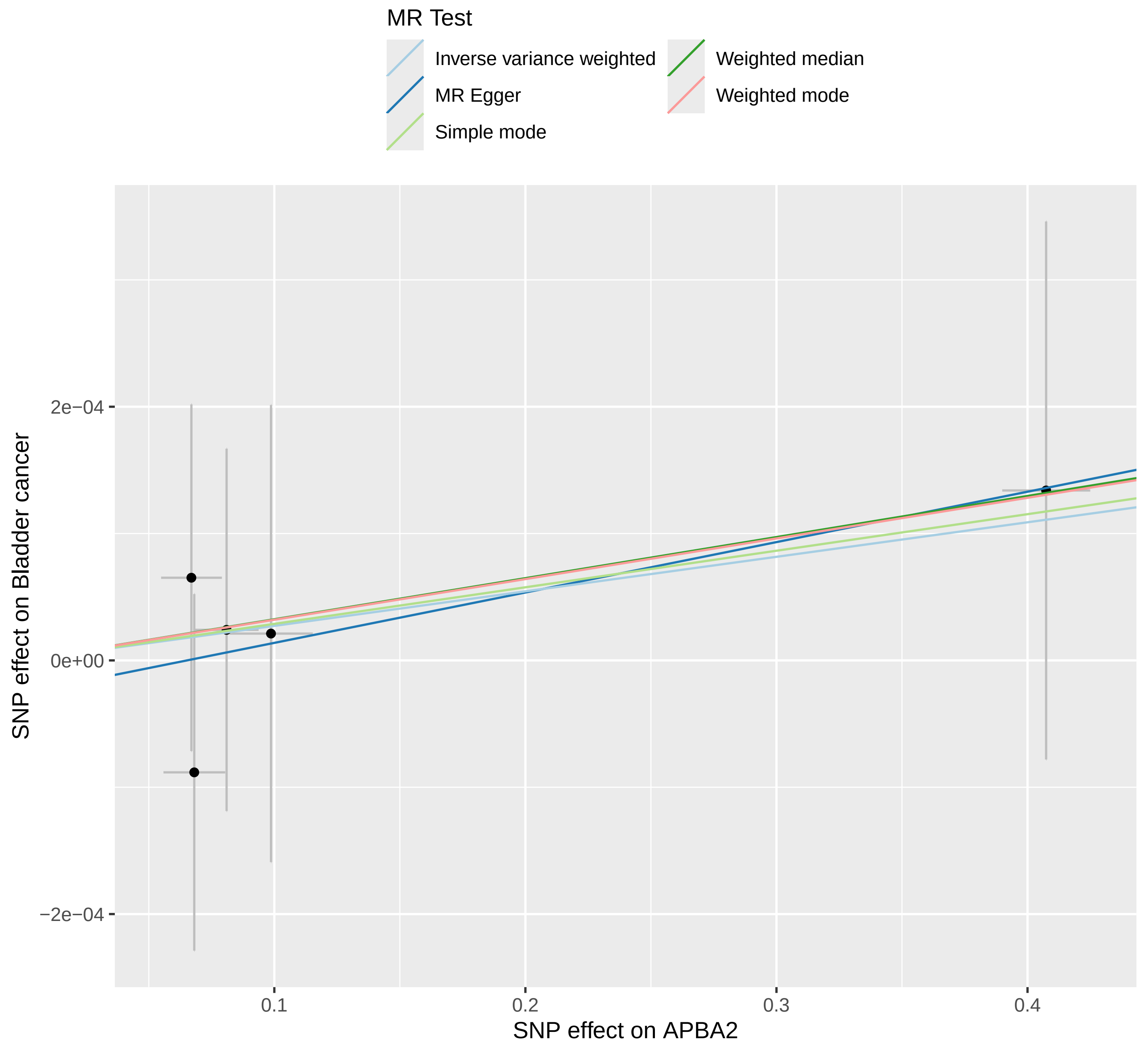

MR Test
Inverse variance weighted
MR Egger
Simple mode
Weighted median
Weighted mode
2e−04
0e+00
−2e−04
SNP effect on Bladder cancer
0.1
0.2
0.3
0.4
SNP effect on APBA2

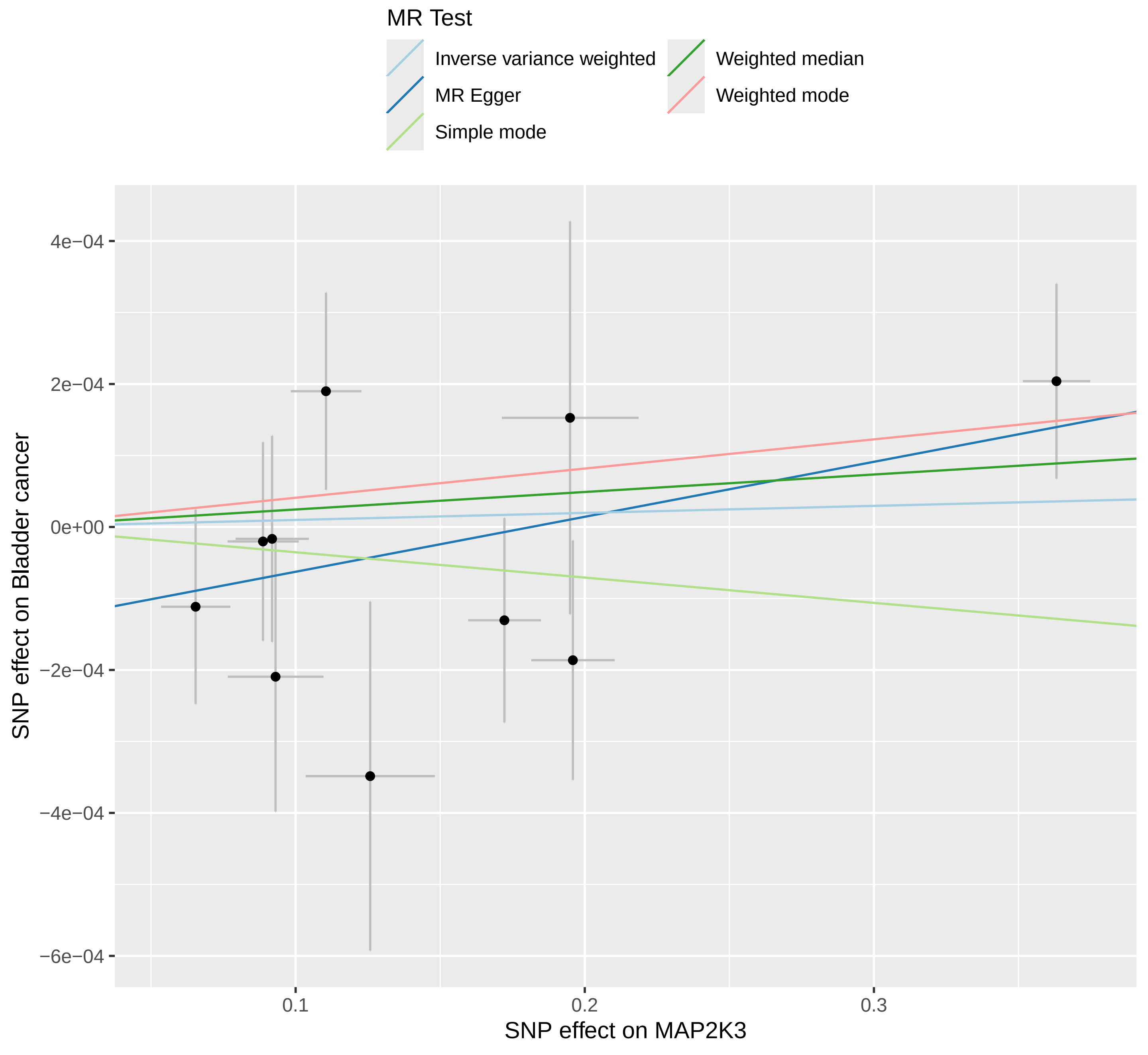
MR Test
Inverse variance weighted
MR Egger
Simple mode
Weighted median
Weighted mode
4e−04
2e−04
0e+00
−2e−04
−4e−04
−6e−04
SNP effect on Bladder cancer
0.1
0.2
0.3
SNP effect on MAP2K3

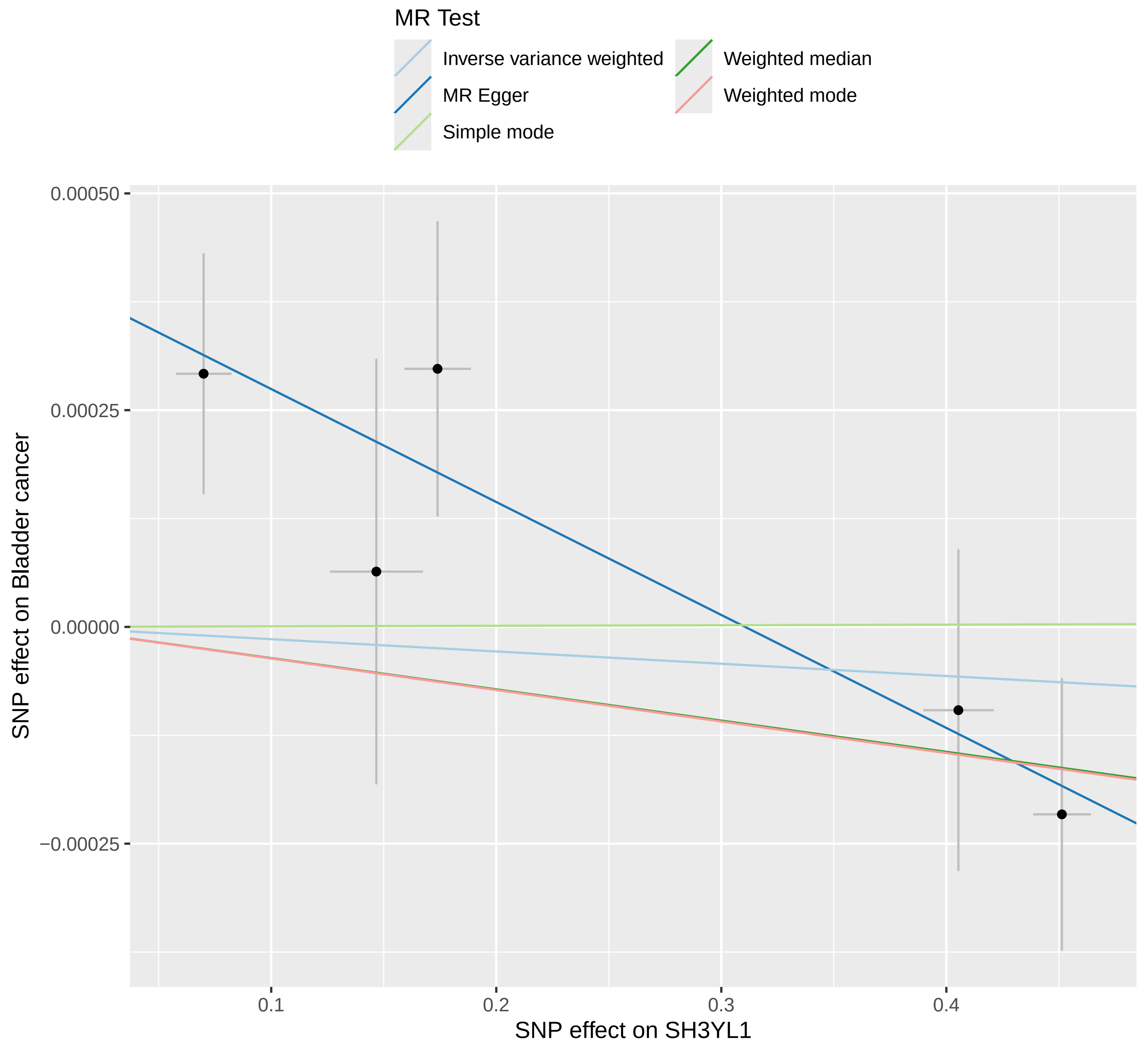

MR Test
Inverse variance weighted
MR Egger
Simple mode
Weighted median
Weighted mode
0.00050
0.00025
0.00000
−0.00025
SNP effect on Bladder cancer
0.1
0.2
0.3
0.4
SNP effect on SH3YL1

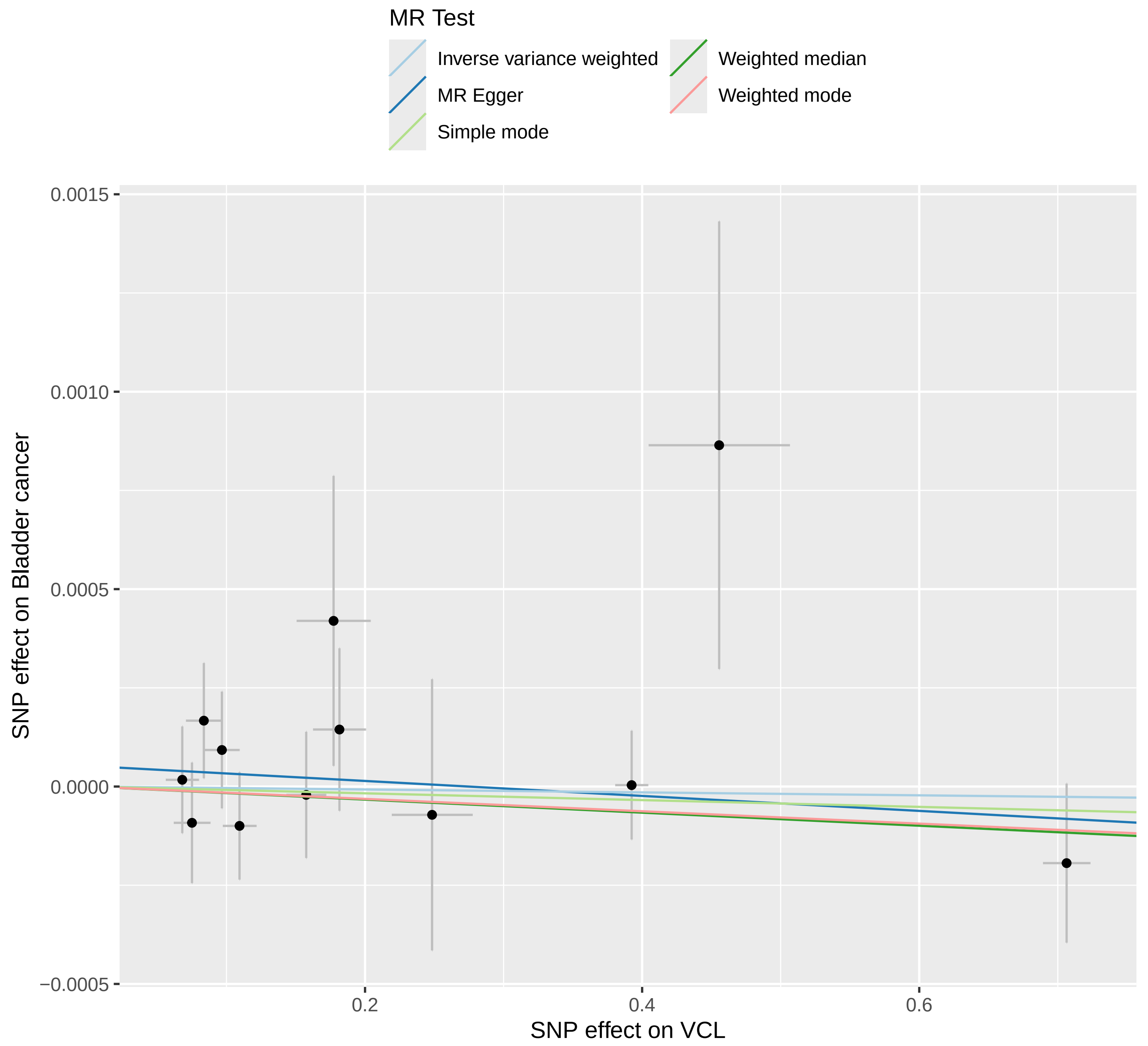

MR Test
Inverse variance weighted
MR Egger
Simple mode
Weighted median
Weighted mode
0.0015
0.0010
0.0005
0.0000
−0.0005
0.2
0.4
0.6
SNP effect on Bladder cancer
SNP effect on VCL

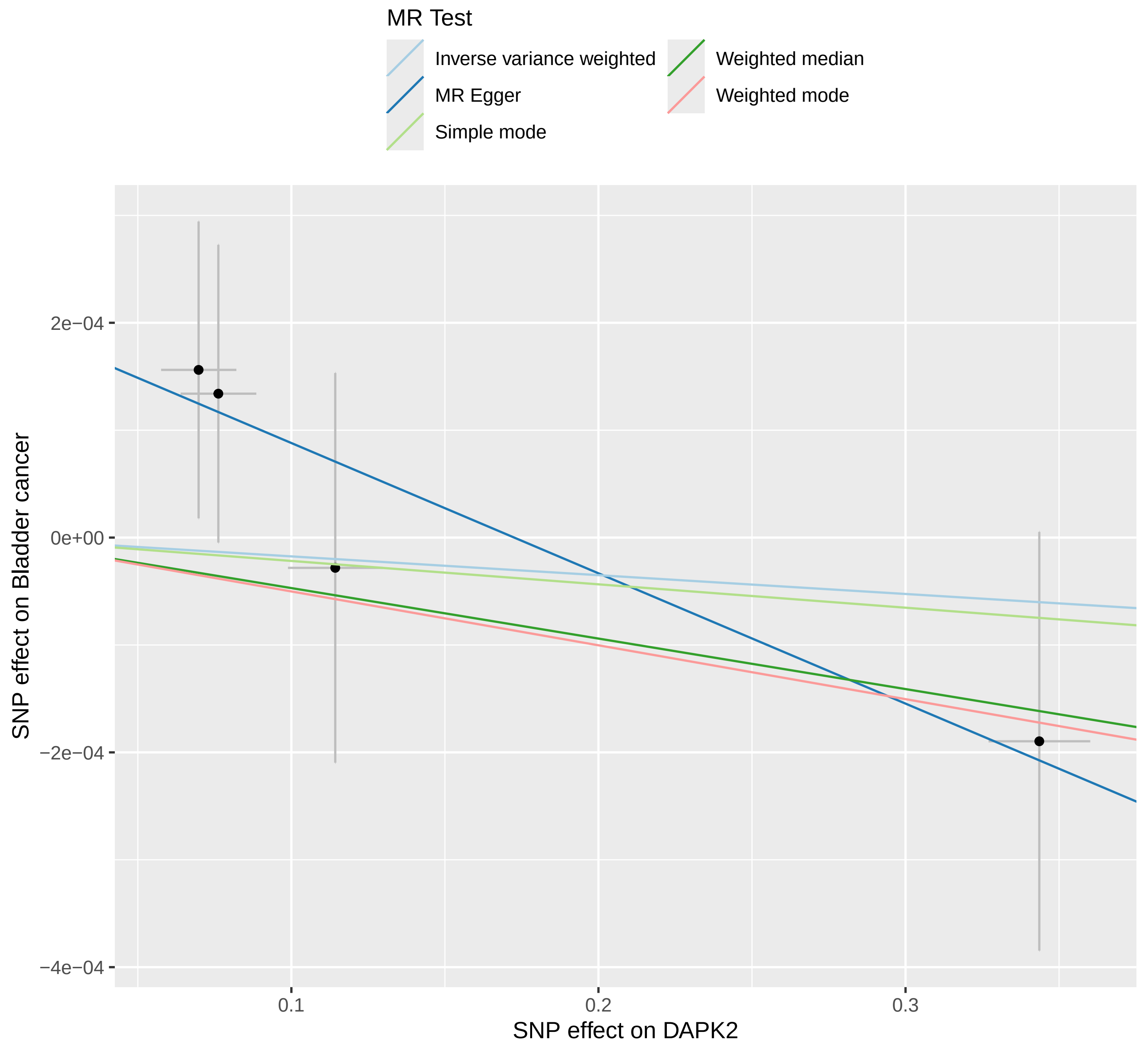
MR Test
Inverse variance weighted
MR Egger
Simple mode
Weighted median
Weighted mode
2e−04
0e+00
−2e−04
−4e−04
0.1
0.2
0.3
SNP effect on Bladder cancer
SNP effect on DAPK2

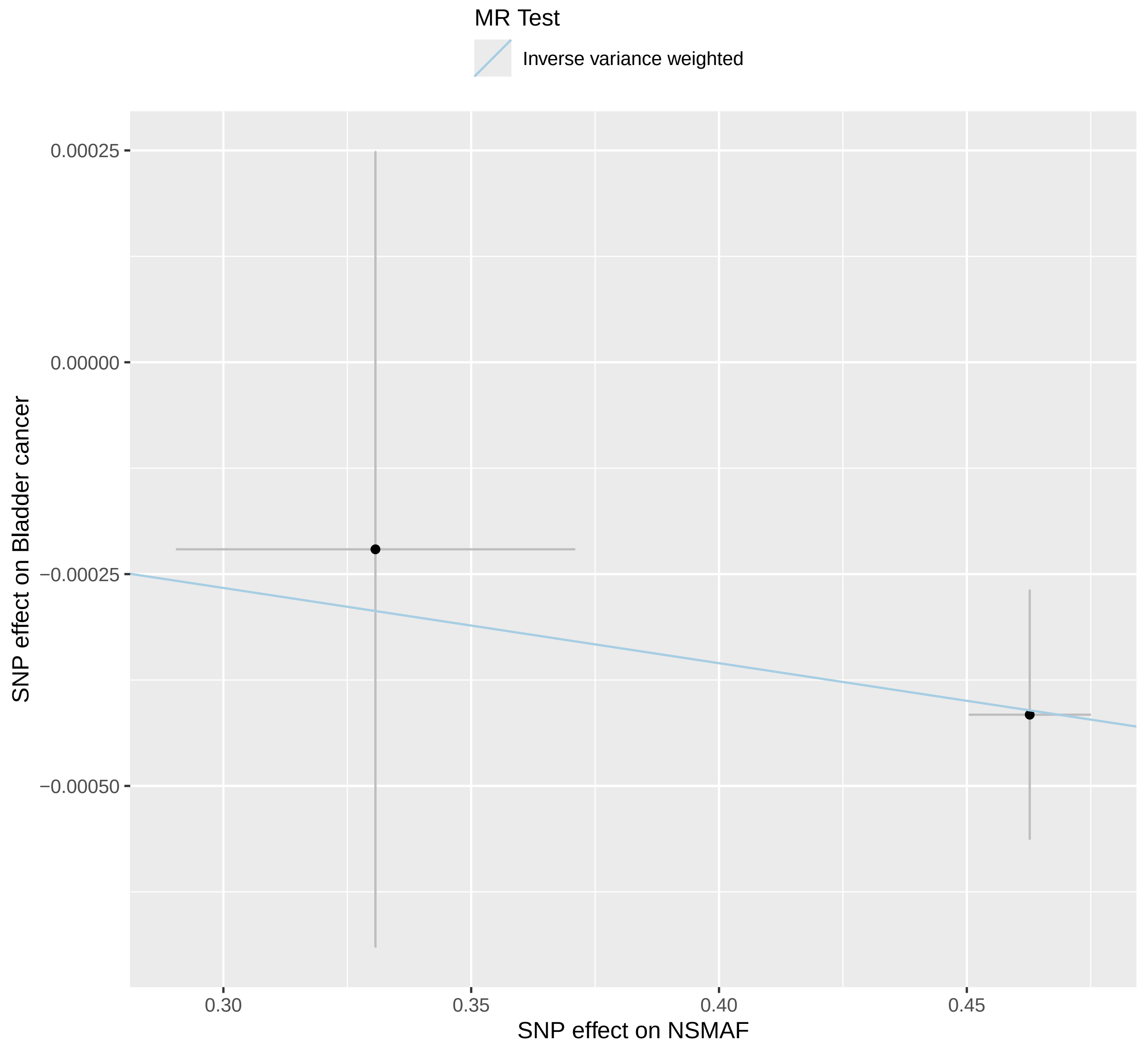

MR Test
Inverse variance weighted
0.00025
0.00000
−0.00025
−0.00050
0.30
0.35
0.40
0.45
SNP effect on NSMAF
SNP effect on Bladder cancer

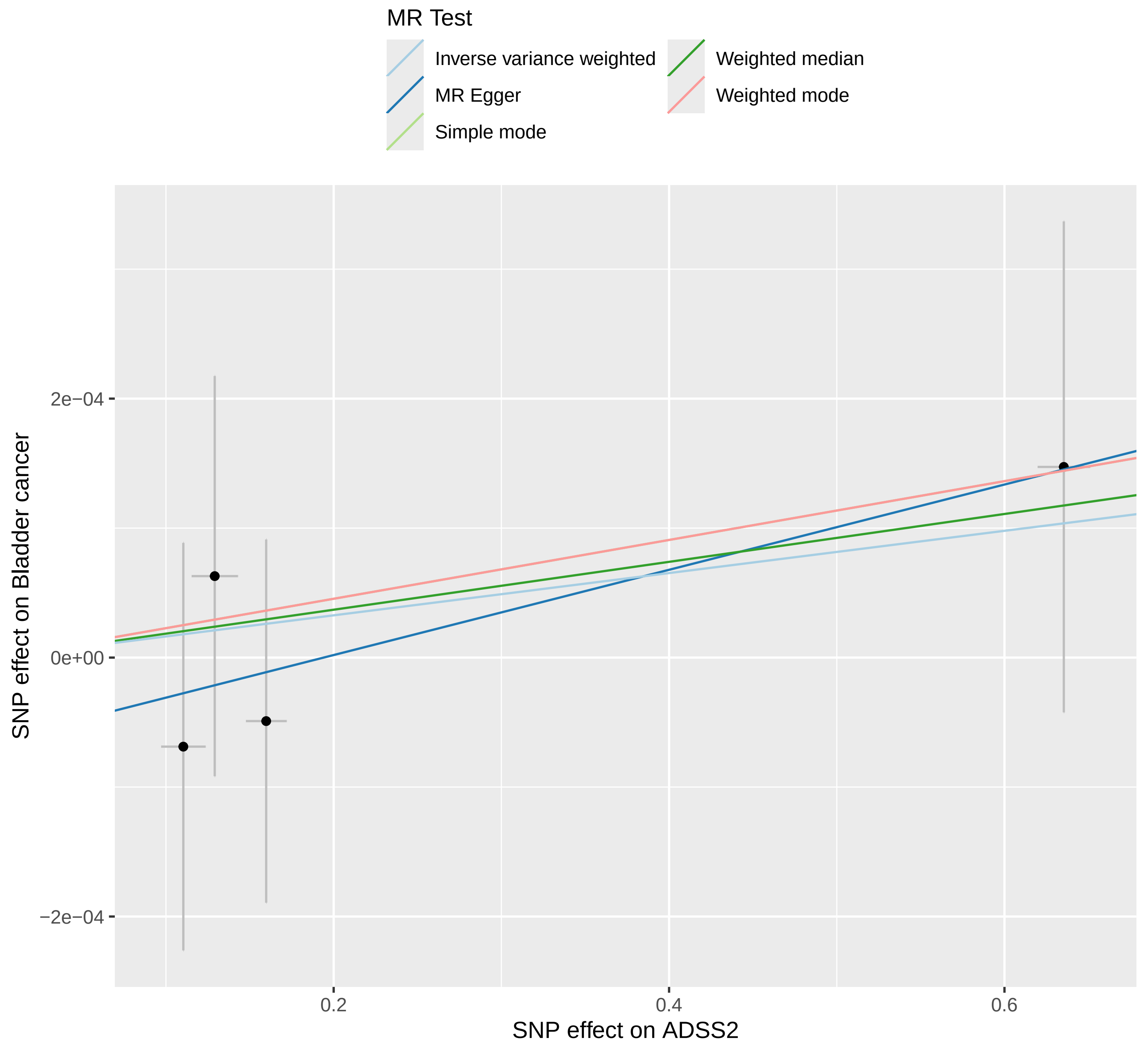

MR Test
Inverse variance weighted
MR Egger
Simple mode
Weighted median
Weighted mode
2e−04
0e+00
−2e−04
0.2
0.4
0.6
SNP effect on Bladder cancer
SNP effect on ADSS2

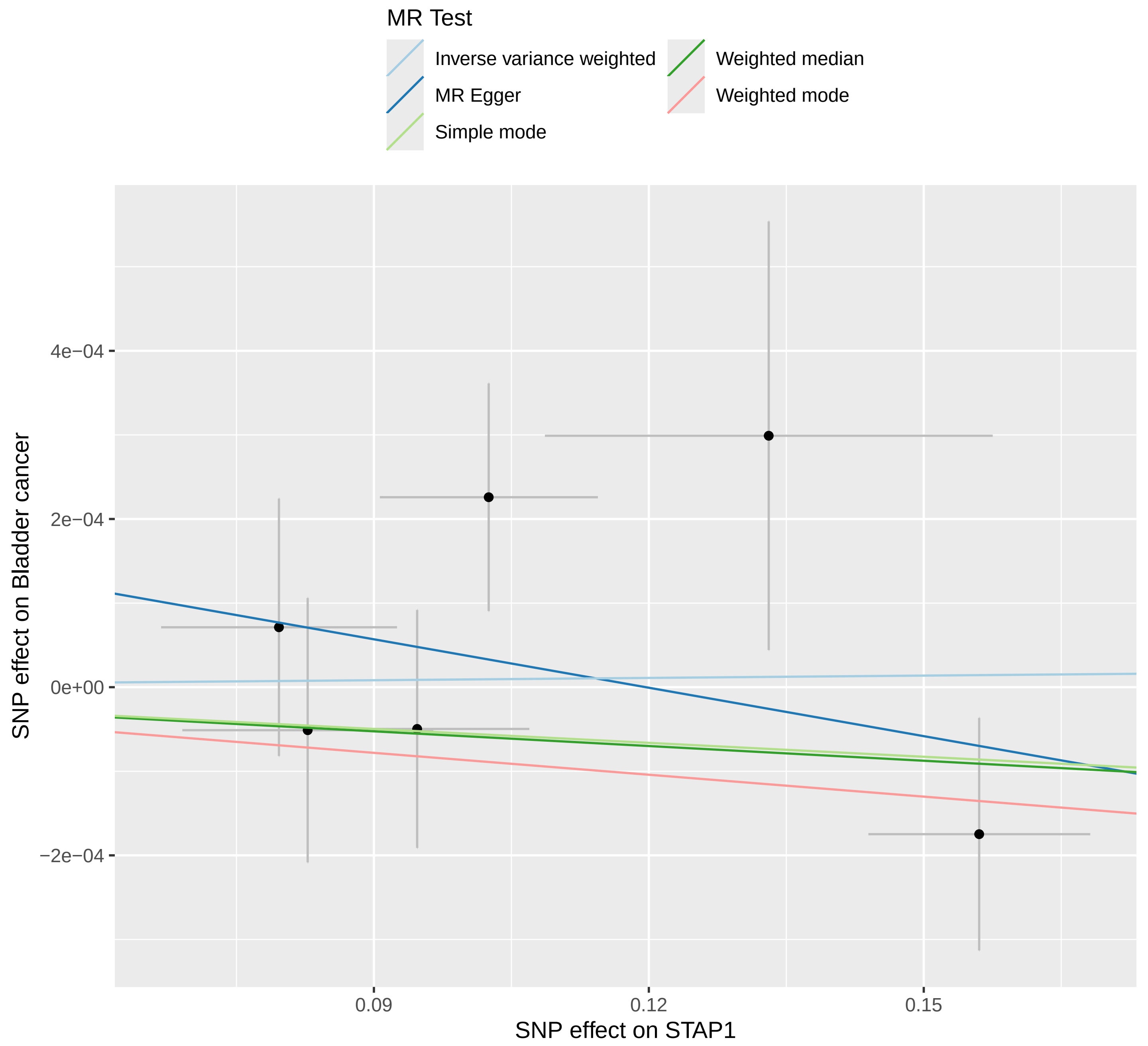
MR Test
Inverse variance weighted
MR Egger
Simple mode
Weighted median
Weighted mode
4e−04
2e−04
0e+00
−2e−04
0.09
0.12
0.15
SNP effect on Bladder cancer
SNP effect on STAP1

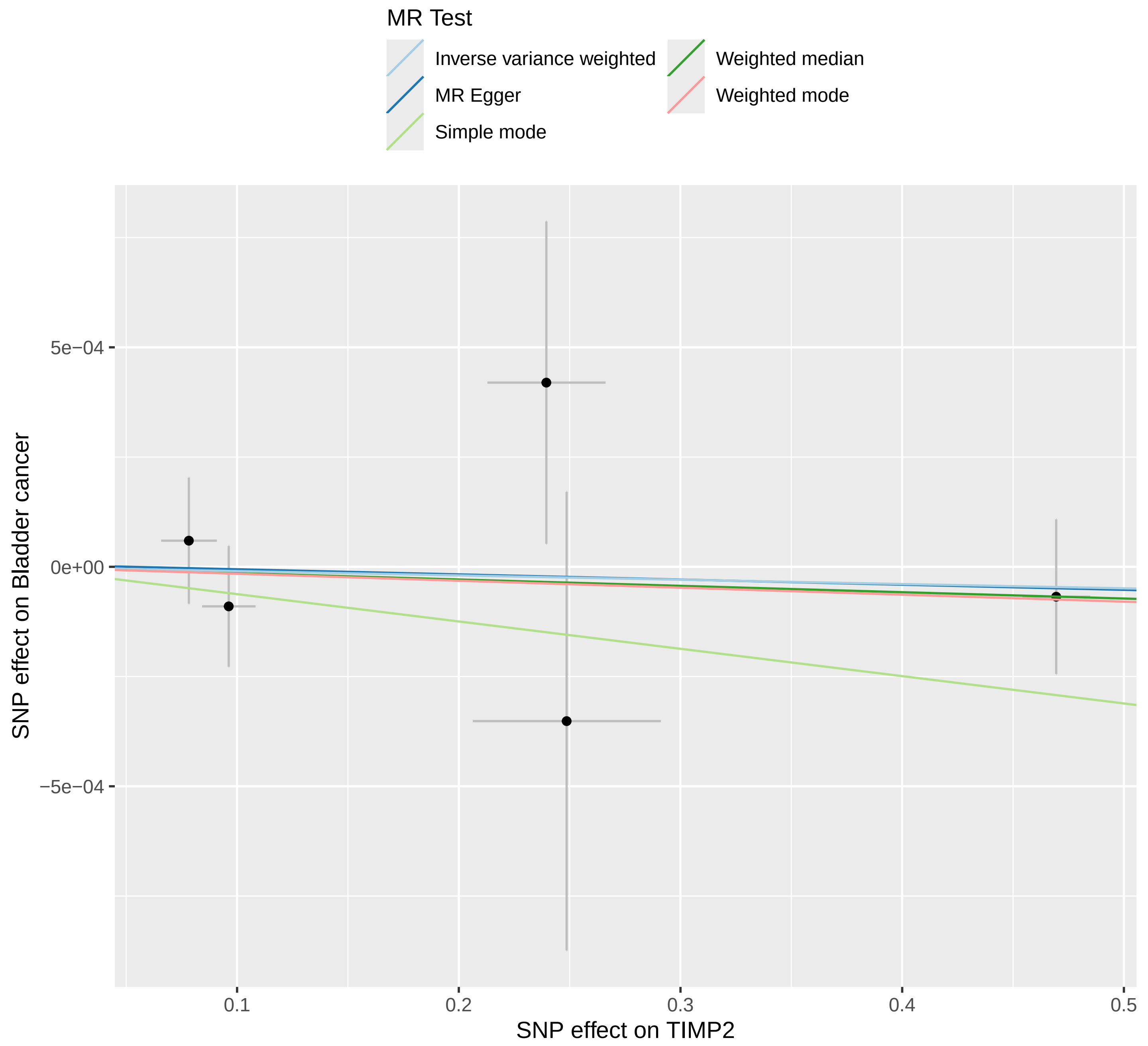

MR Test
Inverse variance weighted
MR Egger
Simple mode
Weighted median
Weighted mode
5e−04
0e+00
−5e−04
SNP effect on Bladder cancer
0.1
0.2
0.3
0.4
0.5
SNP effect on TIMP2

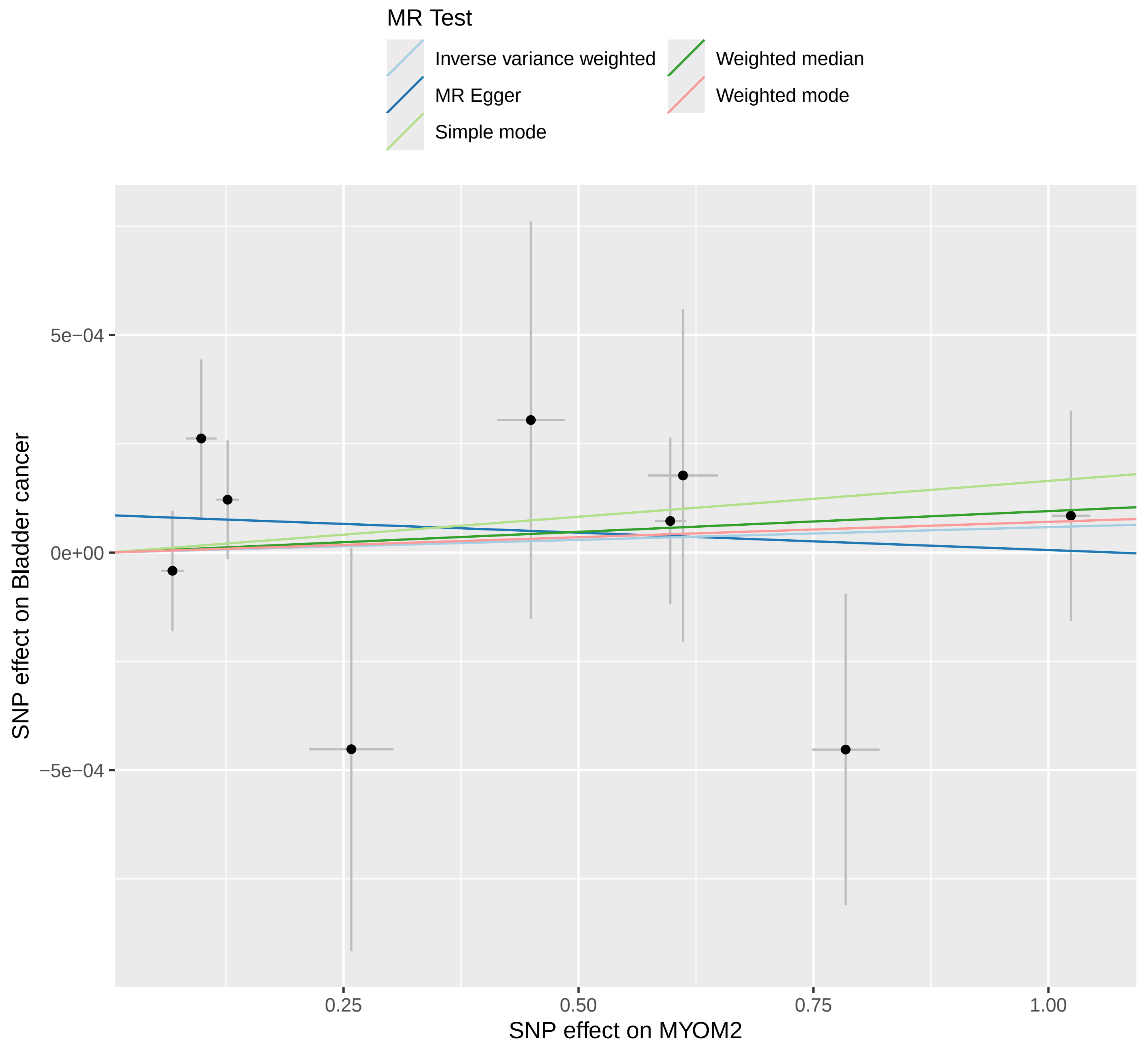
MR Test
Inverse variance weighted
MR Egger
Simple mode
Weighted median
Weighted mode
5e−04
0e+00
−5e−04
0.25
0.50
0.75
1.00
SNP effect on MYOM2
SNP effect on Bladder cancer

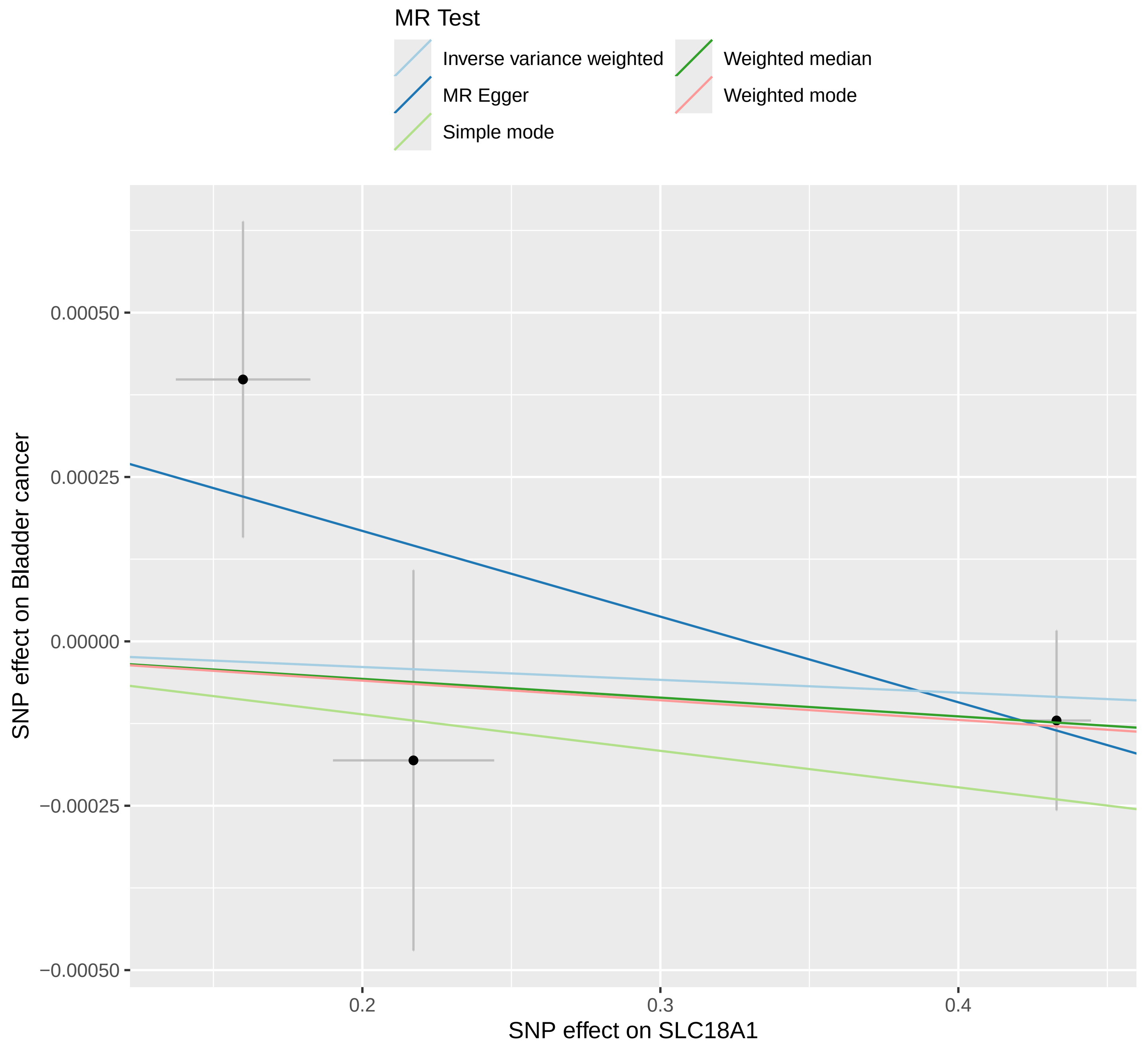
MR Test
Inverse variance weighted
MR Egger
Simple mode
Weighted median
Weighted mode
SNP effect on Bladder cancer
0.00050
0.00025
0.00000
−0.00025
−0.00050
0.2
0.3
0.4
SNP effect on SLC18A1

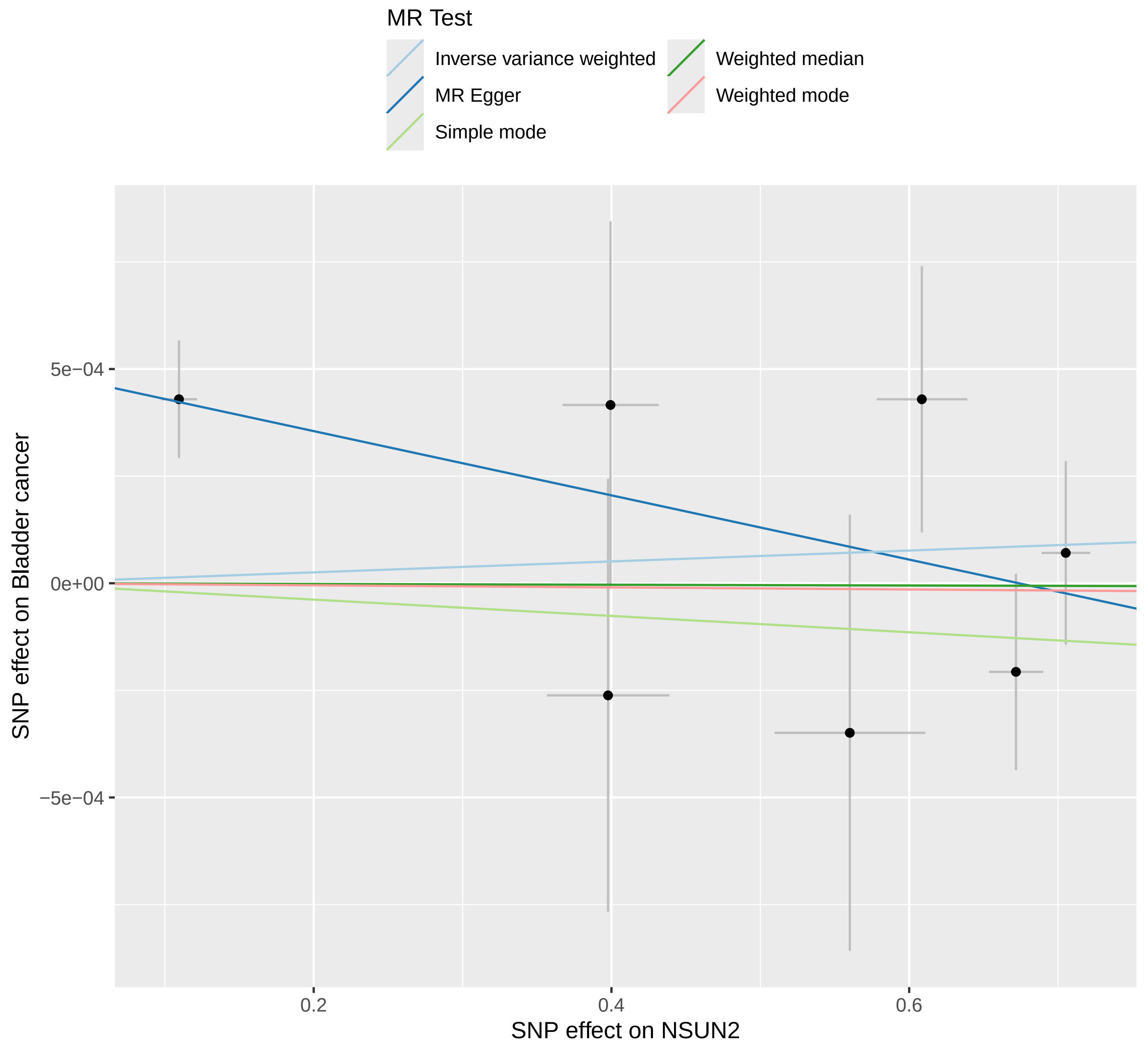

MR Test
Inverse variance weighted
MR Egger
Simple mode
Weighted median
Weighted mode
5e−04
0e+00
−5e−04
SNP effect on Bladder cancer
0.2
0.4
0.6
SNP effect on NSUN2

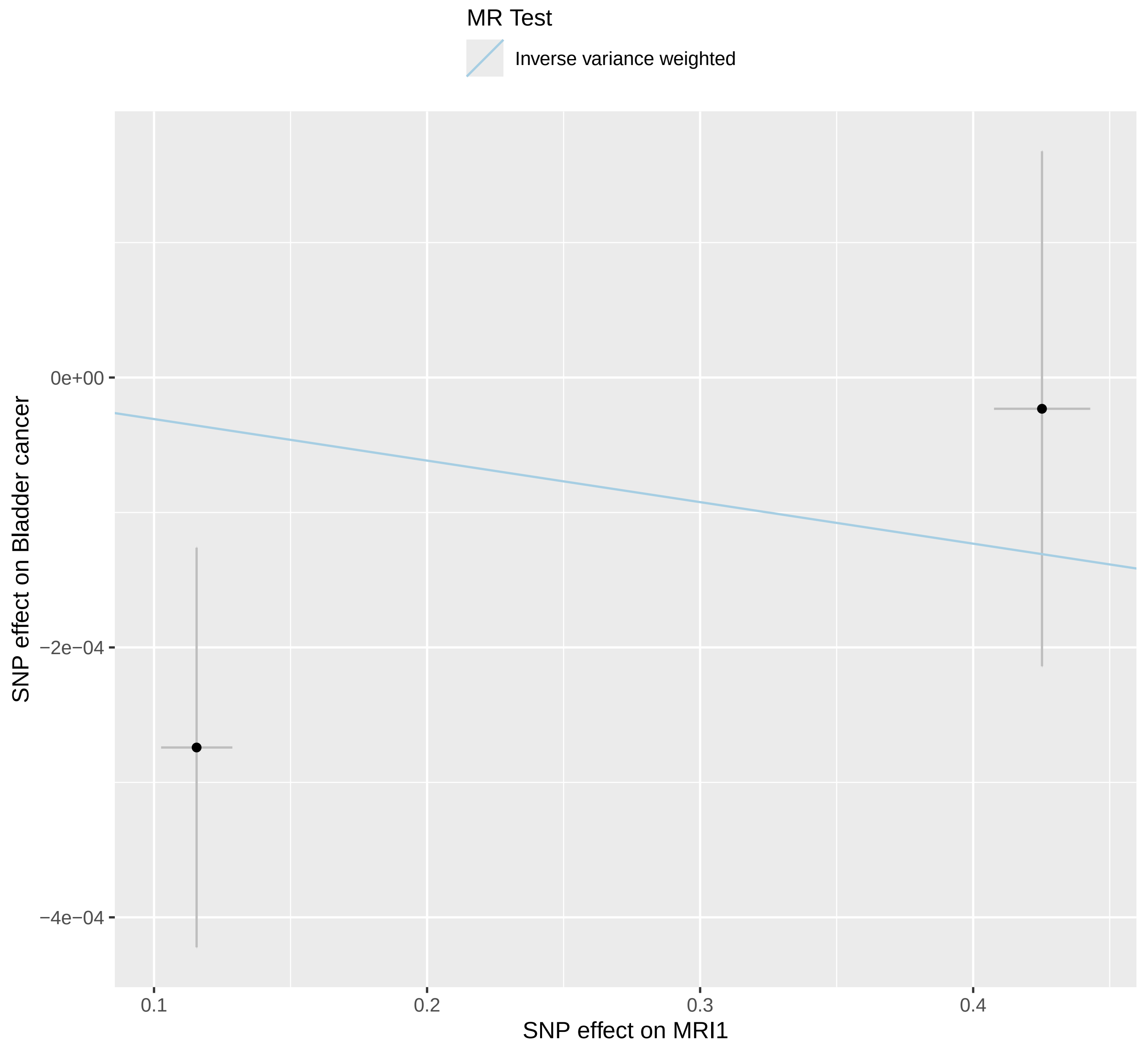
MR Test
Inverse variance weighted
0e+00
−2e−04
−4e−04
SNP effect on Bladder cancer
0.1
0.2
0.3
0.4
SNP effect on MRI1

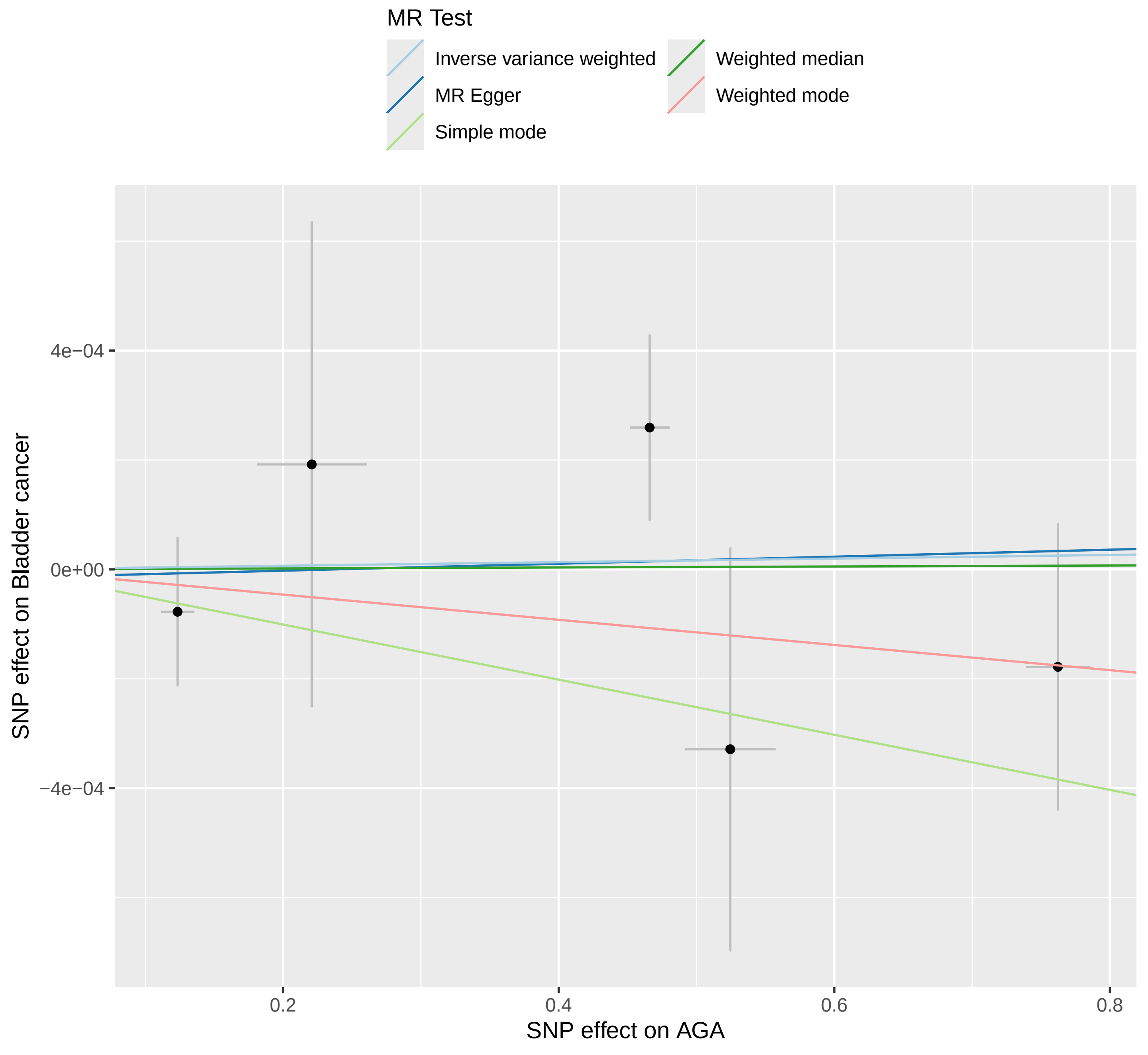
MR Test
Inverse variance weighted
MR Egger
Simple mode
Weighted median
Weighted mode
4e−04
0e+00
−4e−04
SNP effect on Bladder cancer
0.2
0.4
0.6
0.8
SNP effect on AGA

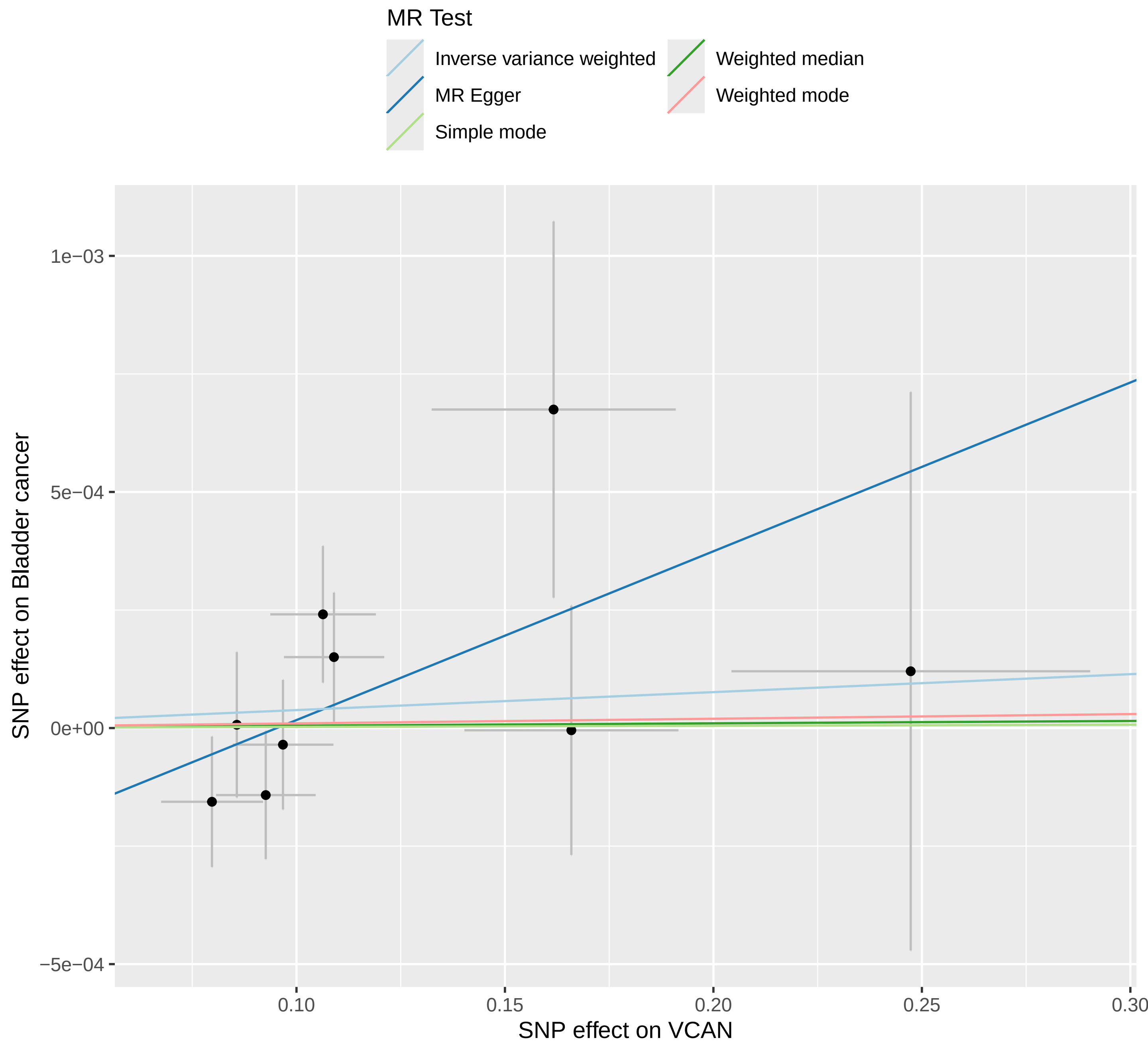

MR Test
Inverse variance weighted
MR Egger
Simple mode
Weighted median
Weighted mode
1e−03
5e−04
0e+00
−5e−04
0.10
0.15
0.20
0.25
0.30
SNP effect on VCAN
SNP effect on Bladder cancer

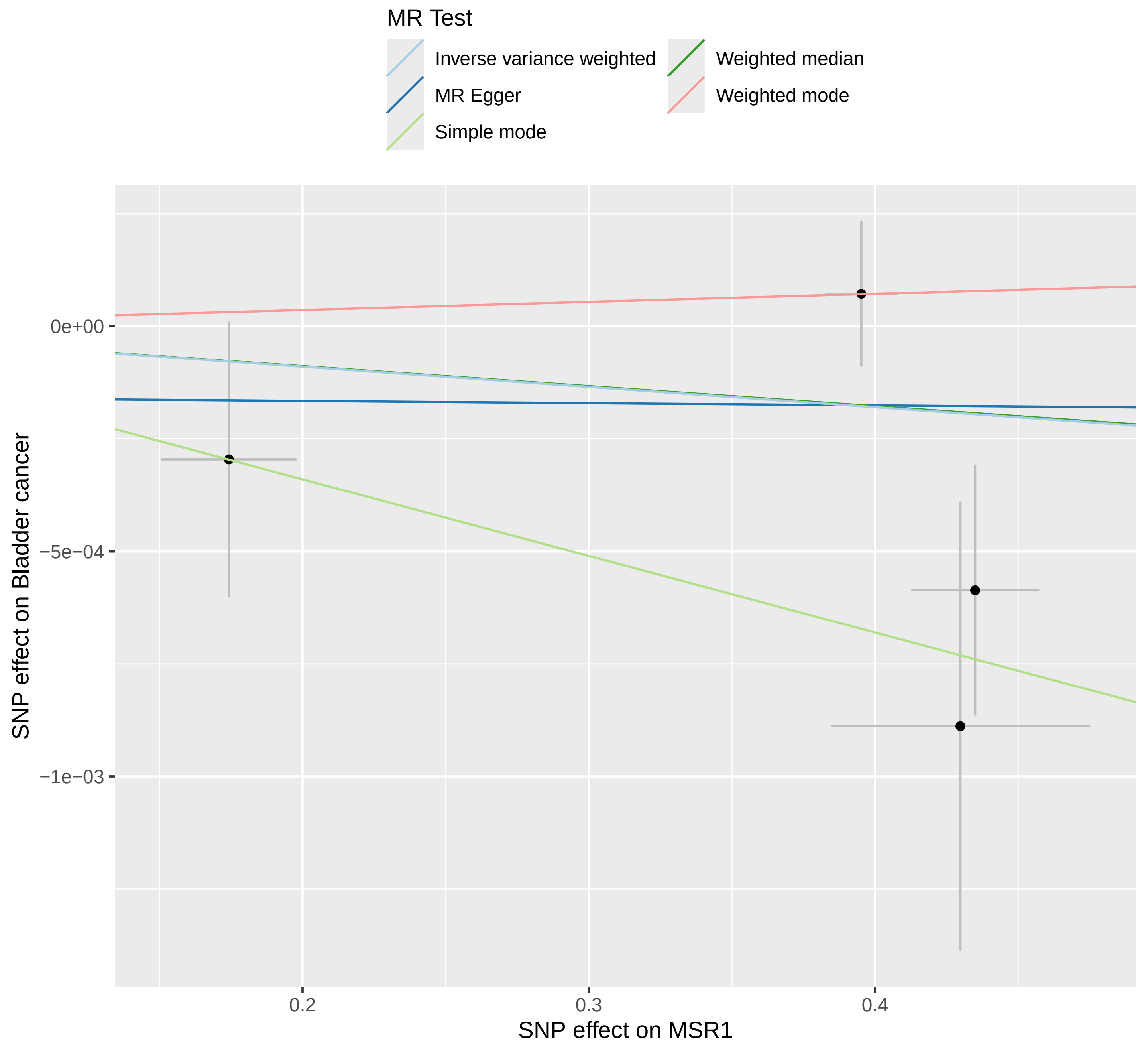
MR Test
Inverse variance weighted
MR Egger
Simple mode
Weighted median
Weighted mode
0e+00
−5e−04
−1e−03
SNP effect on Bladder cancer
0.2
0.3
0.4
SNP effect on MSR1

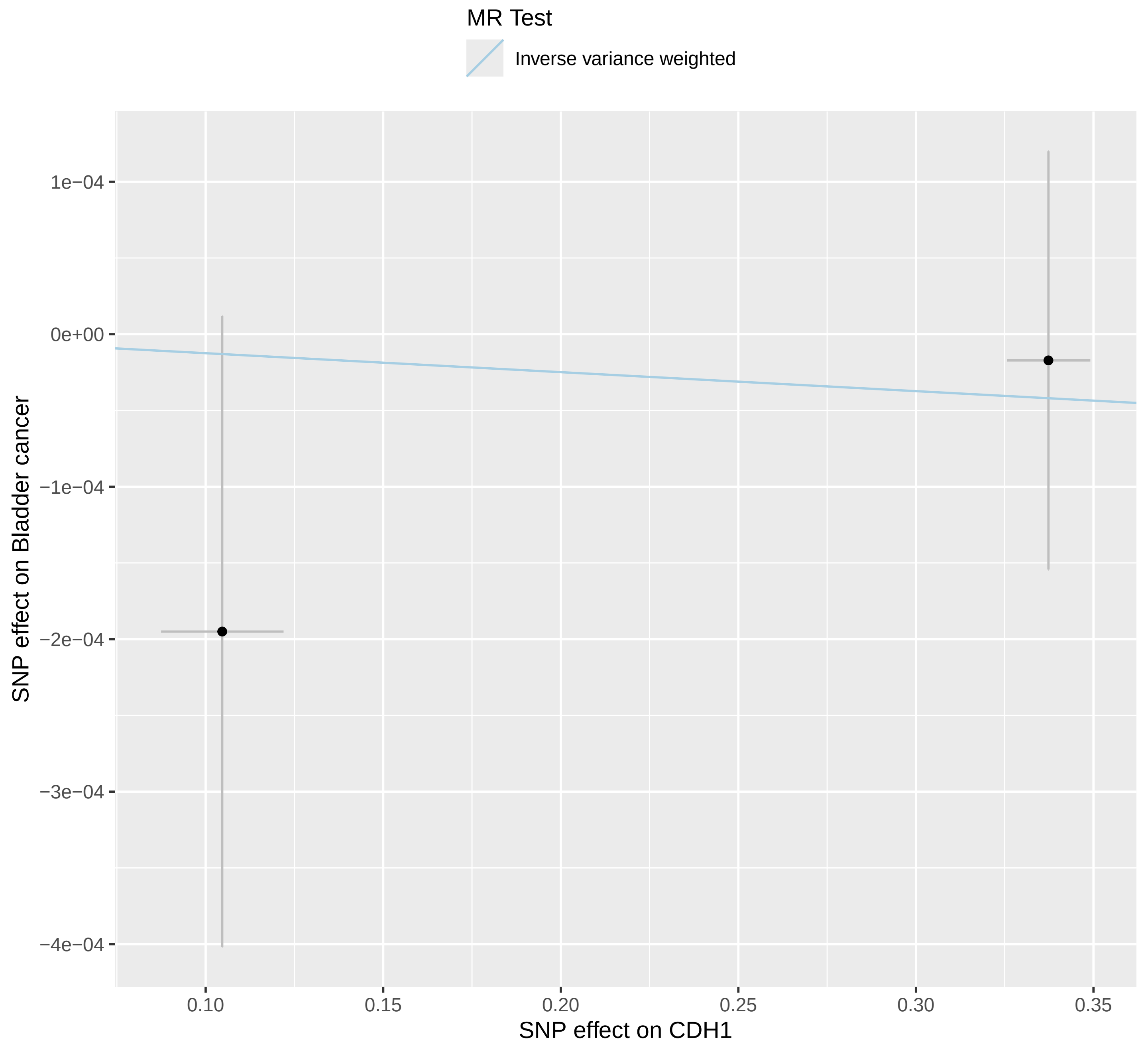

MR Test
Inverse variance weighted
1e−04
0e+00
−1e−04
−2e−04
−3e−04
−4e−04
SNP effect on Bladder cancer
0.10
0.15
0.20
0.25
0.30
0.35
SNP effect on CDH1

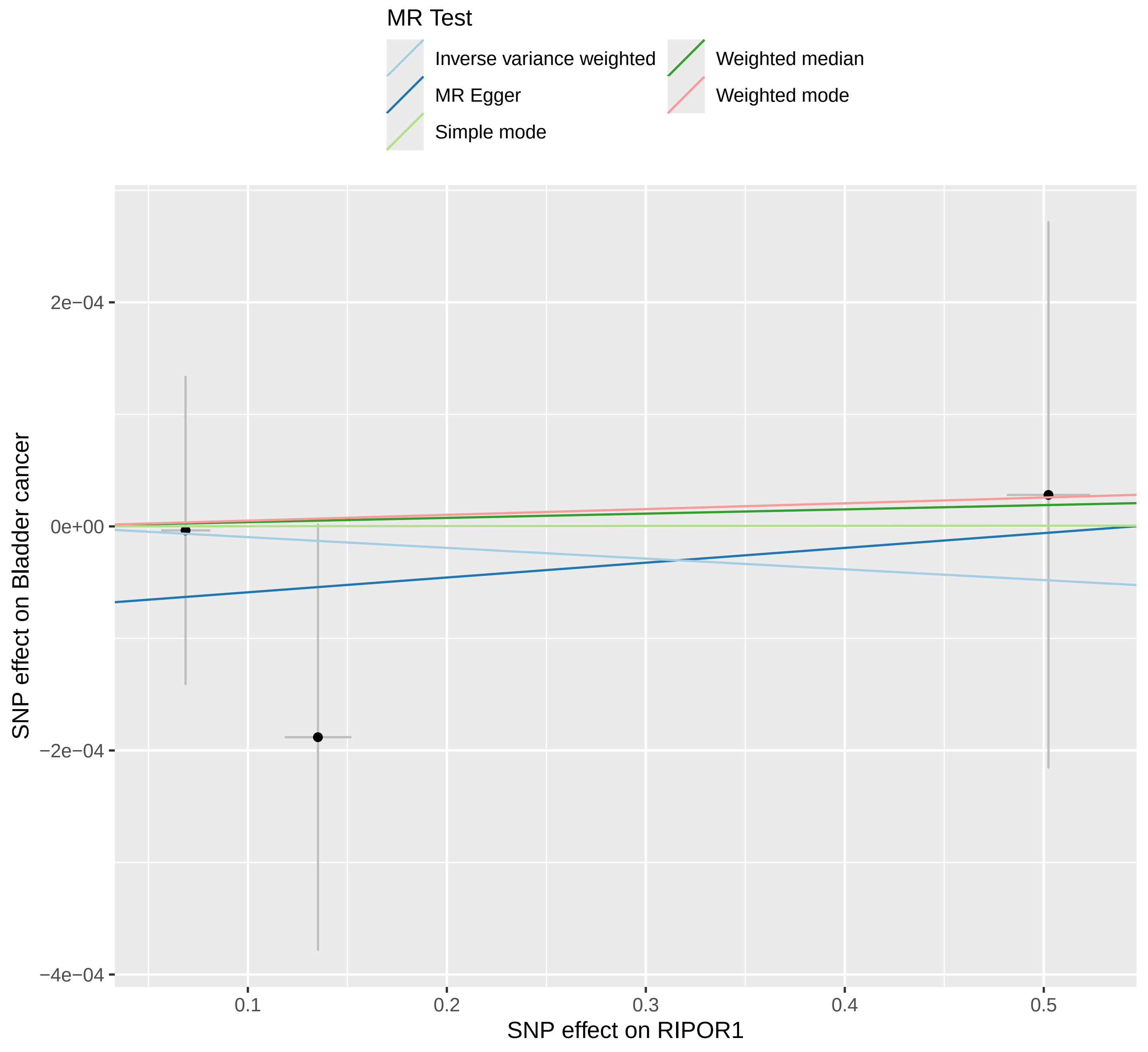
MR Test
Inverse variance weighted
MR Egger
Simple mode
Weighted median
Weighted mode
2e−04
0e+00
−2e−04
−4e−04
0.1
0.2
0.3
0.4
0.5
SNP effect on RIPOR1
SNP effect on Bladder cancer

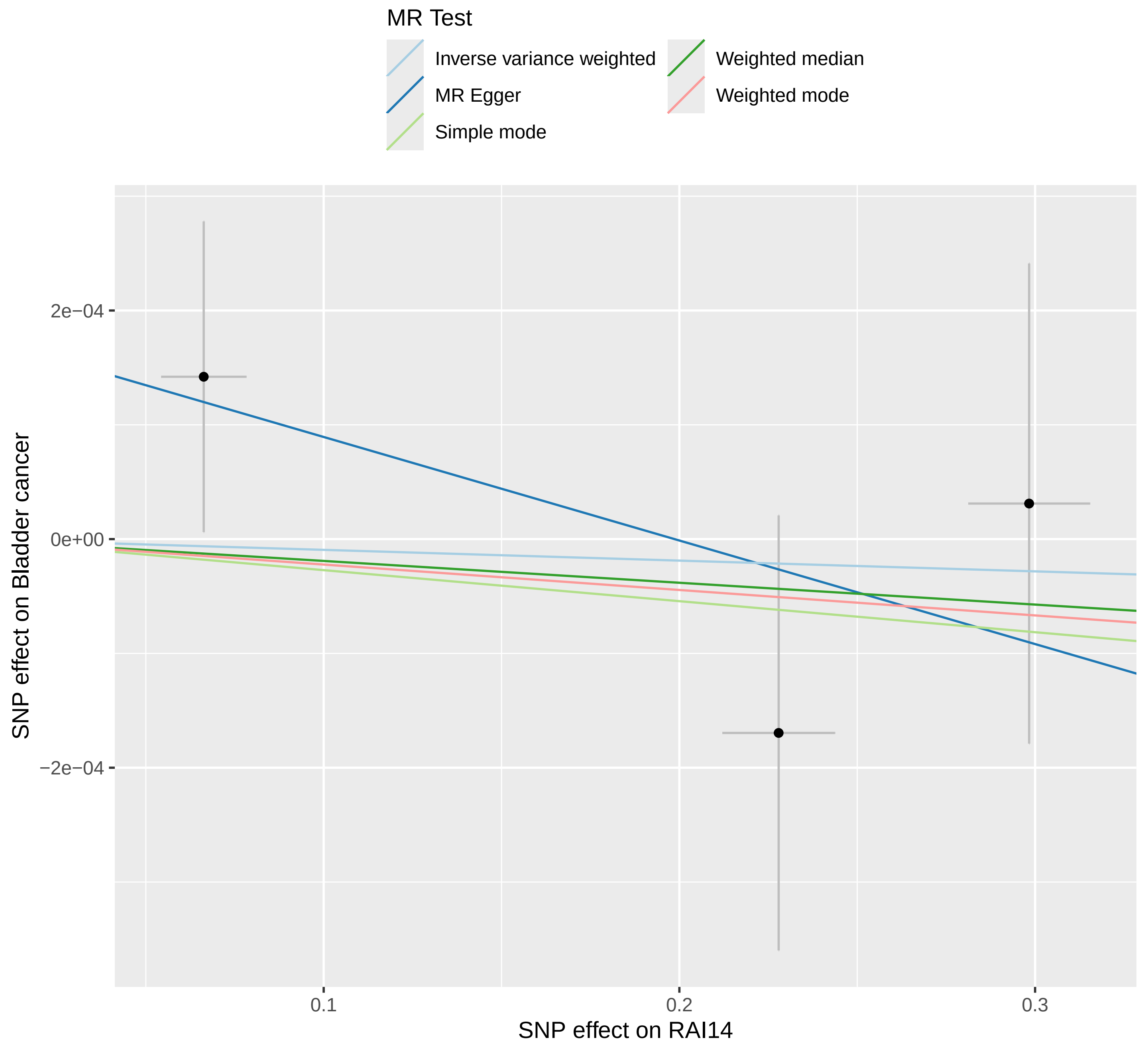
MR Test
Inverse variance weighted
MR Egger
Simple mode
Weighted median
Weighted mode
2e−04
0e+00
−2e−04
SNP effect on Bladder cancer
0.1
0.2
0.3
SNP effect on RAI14

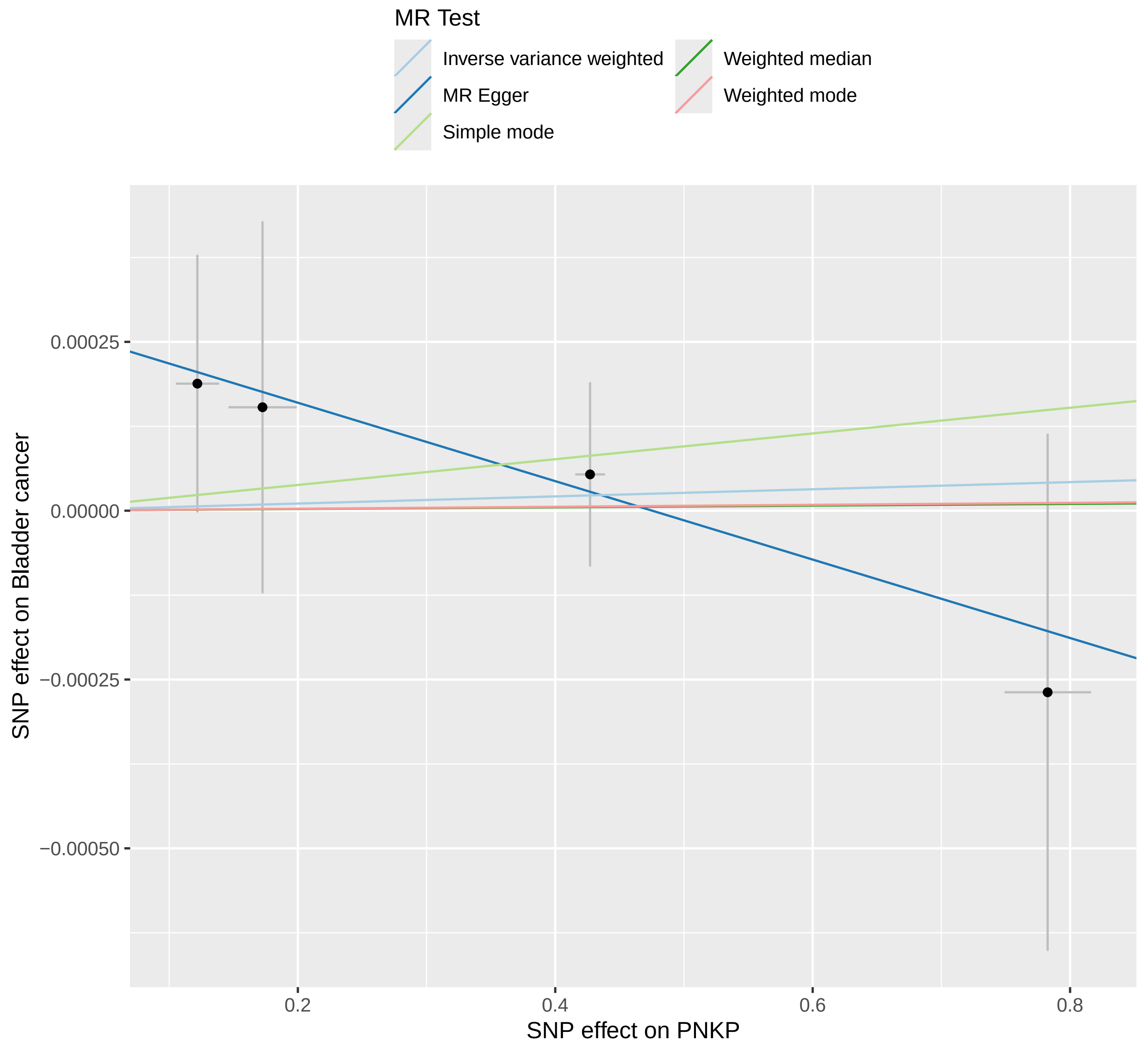
MR Test
Inverse variance weighted
MR Egger
Simple mode
Weighted median
Weighted mode
0.00025
0.00000
−0.00025
−0.00050
SNP effect on Bladder cancer
0.2
0.4
0.6
0.8
SNP effect on PNKP

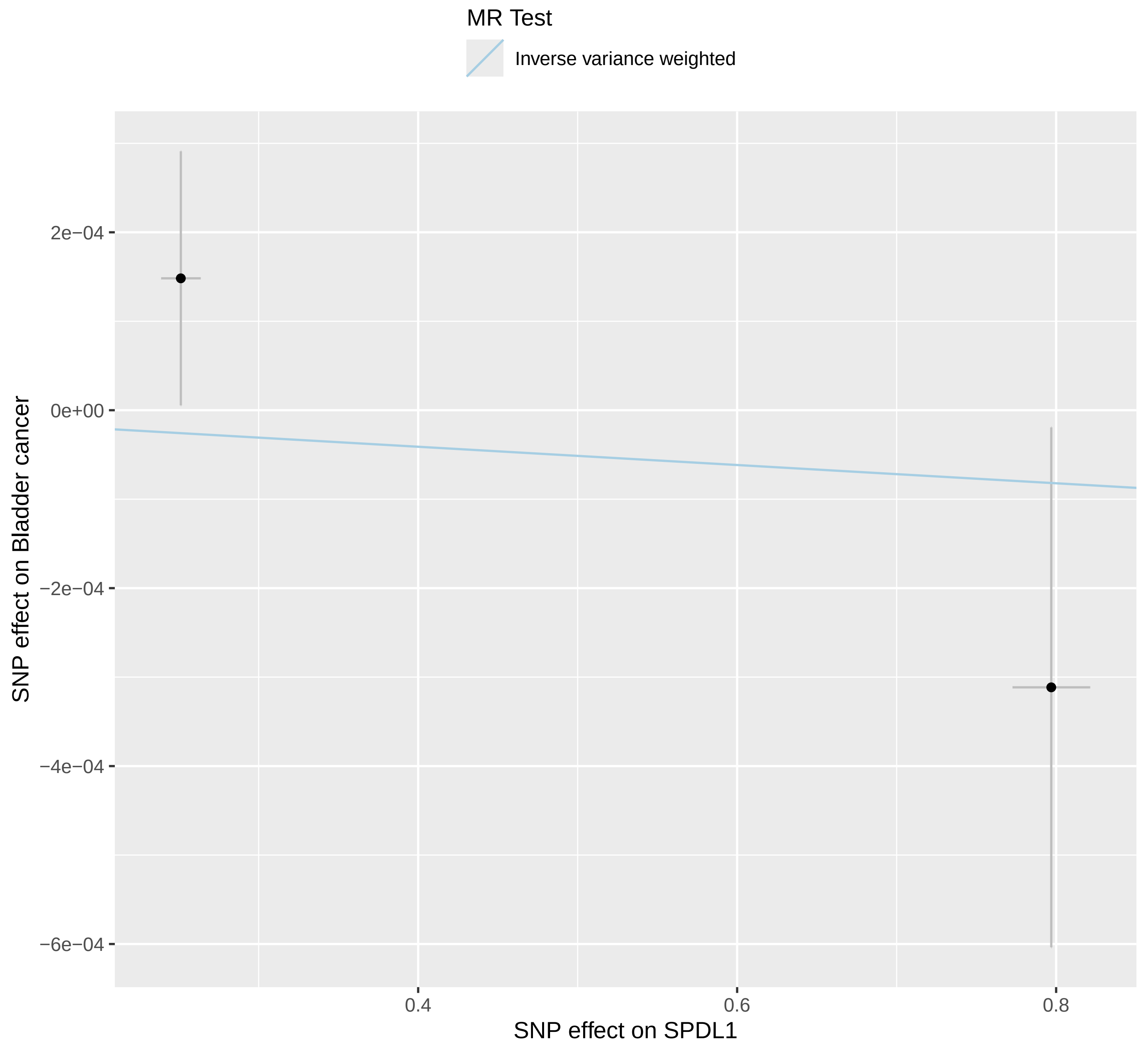

MR Test
Inverse variance weighted
2e−04
0e+00
−2e−04
−4e−04
−6e−04
SNP effect on Bladder cancer
0.4
0.6
0.8
SNP effect on SPDL1

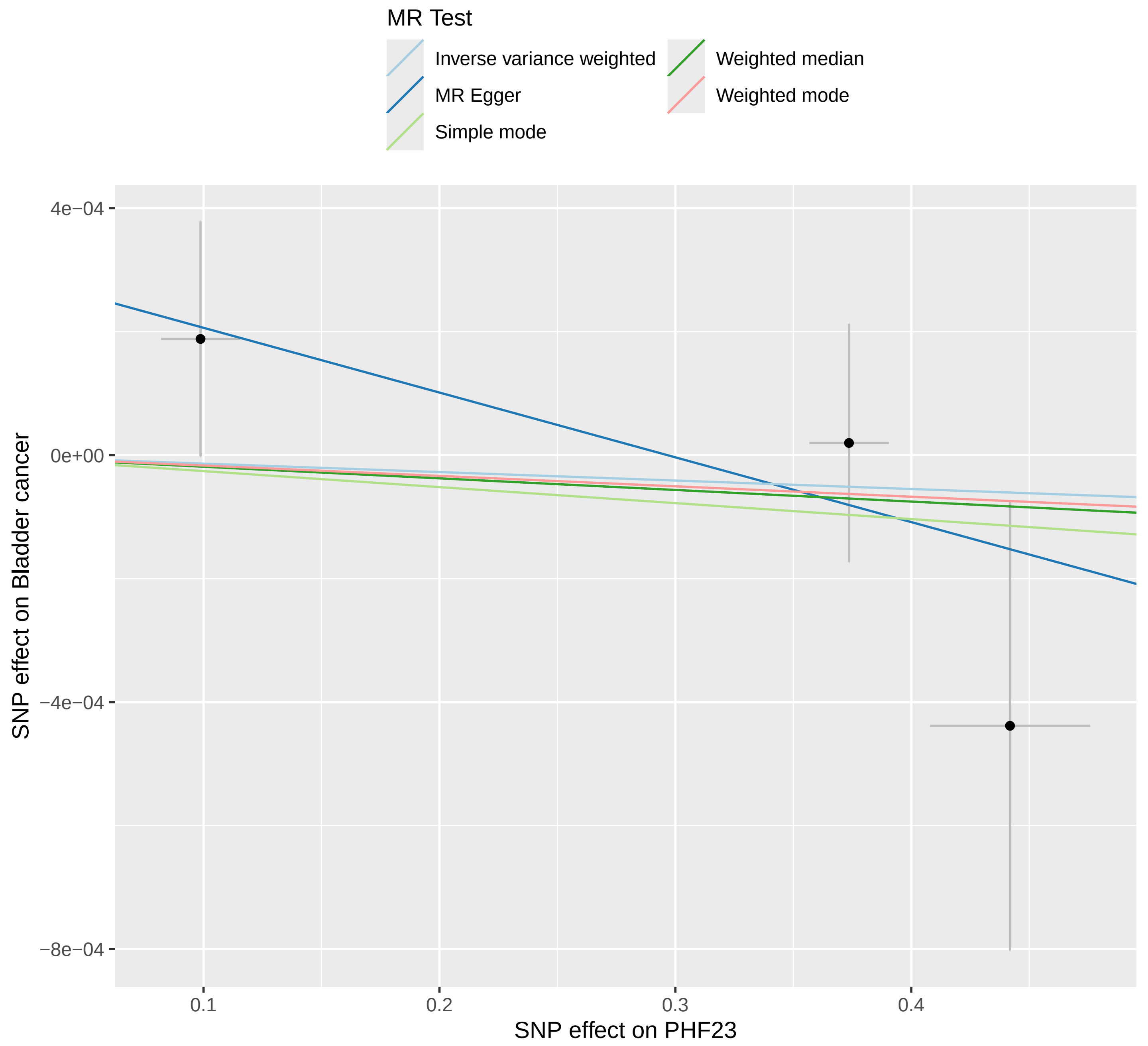
MR Test
Inverse variance weighted
MR Egger
Simple mode
Weighted median
Weighted mode
4e−04
0e+00
−4e−04
−8e−04
SNP effect on Bladder cancer
0.1
0.2
0.3
0.4
SNP effect on PHF23

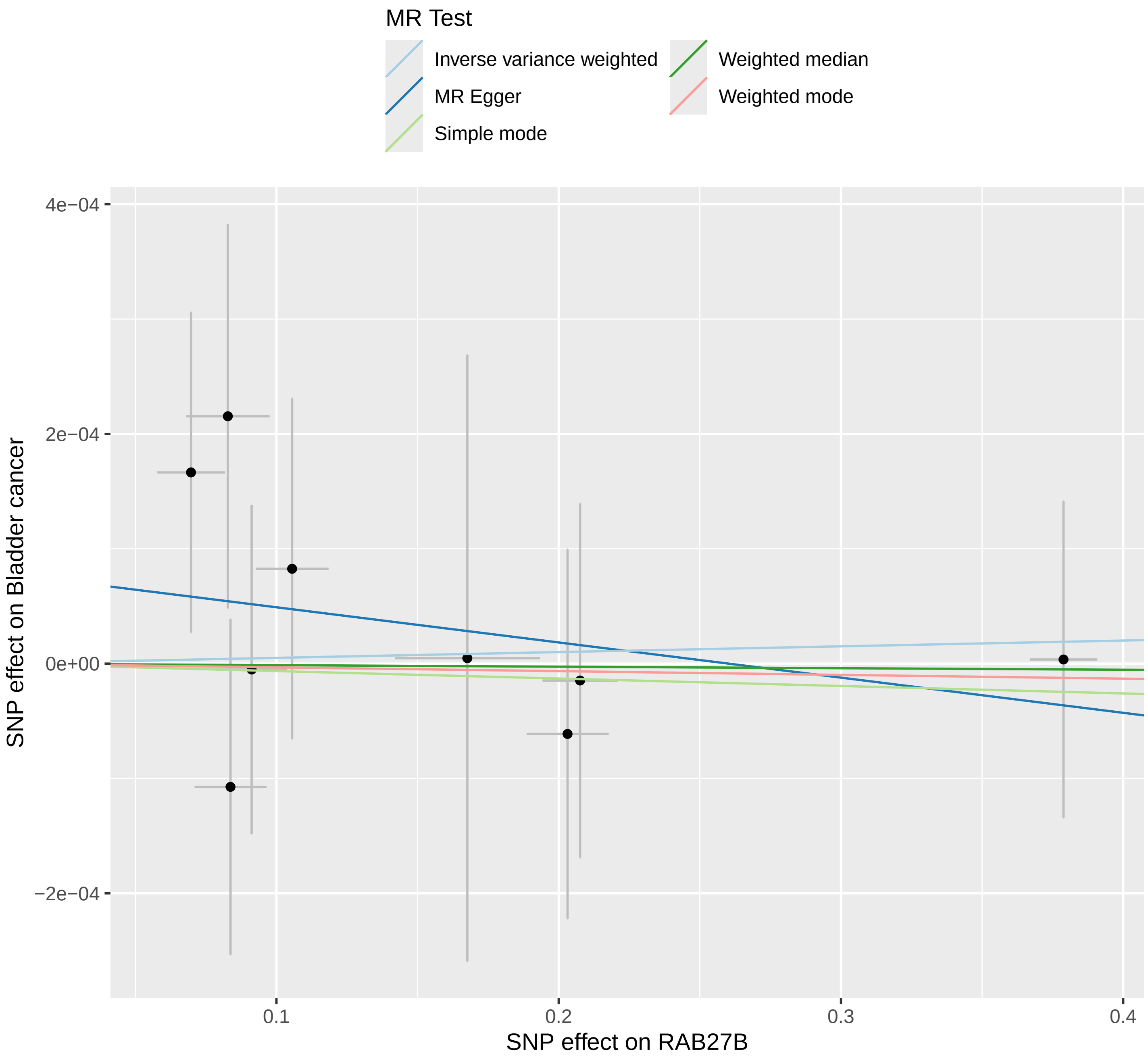
MR Test
Inverse variance weighted
MR Egger
Simple mode
Weighted median
Weighted mode
4e−04
2e−04
0e+00
−2e−04
SNP effect on Bladder cancer
0.1
0.2
0.3
0.4
SNP effect on RAB27B

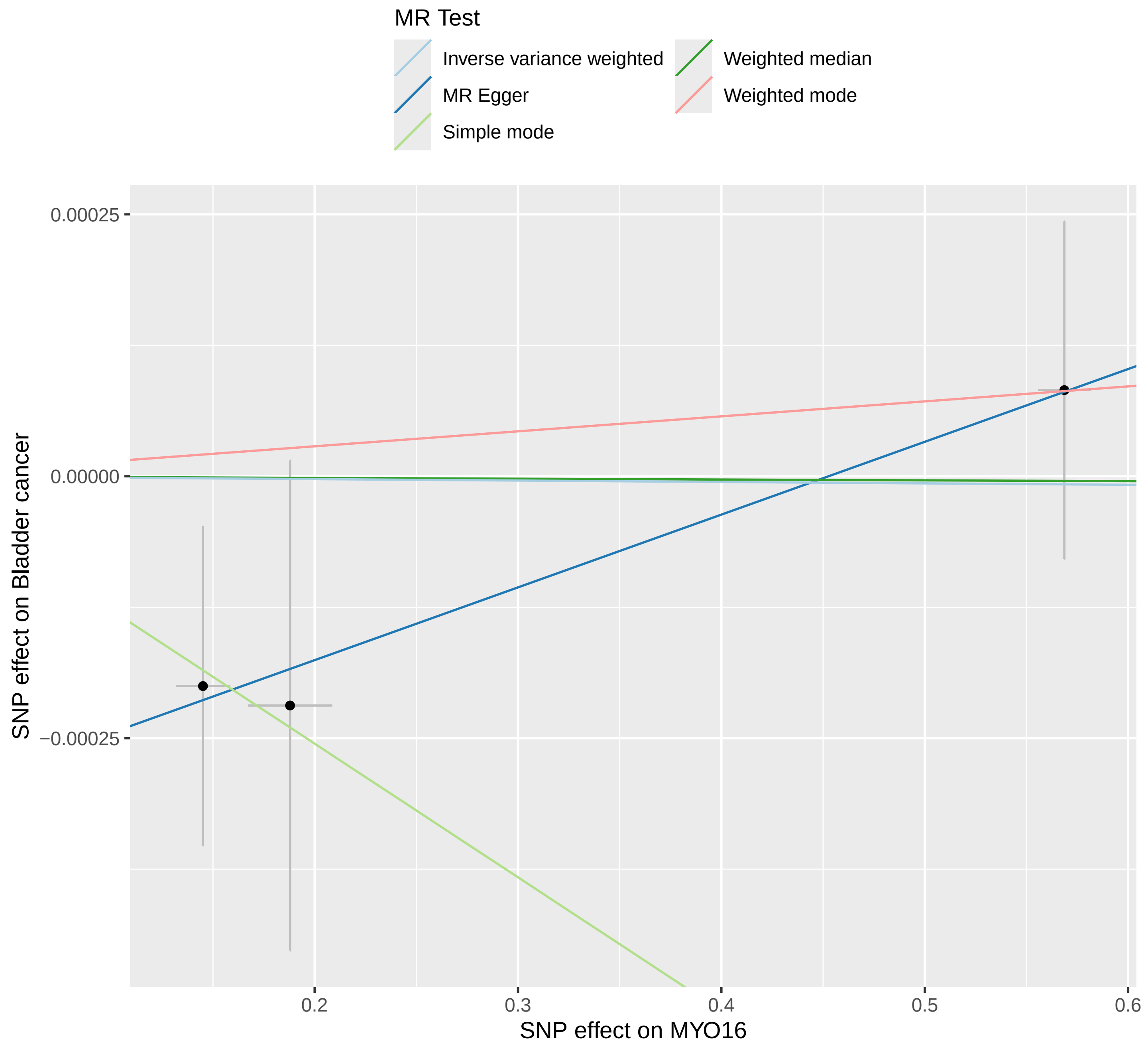

MR Test
Inverse variance weighted
MR Egger
Simple mode
Weighted median
Weighted mode
0.00025
0.00000
−0.00025
SNP effect on Bladder cancer
0.2
0.3
0.4
0.5
0.6
SNP effect on MYO16

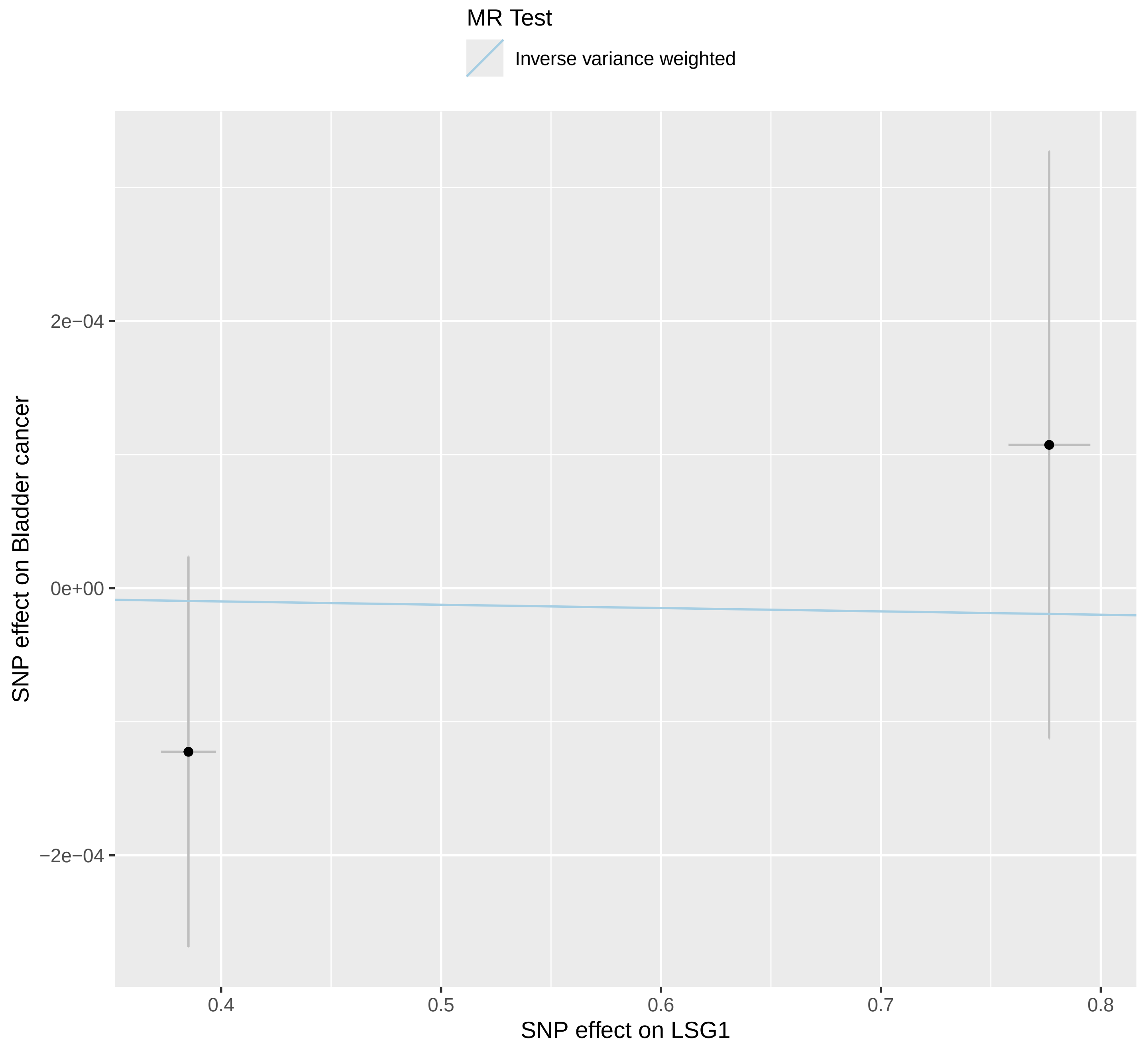

MR Test
Inverse variance weighted
2e−04
0e+00
−2e−04
SNP effect on Bladder cancer
0.4
0.5
0.6
0.7
0.8
SNP effect on LSG1

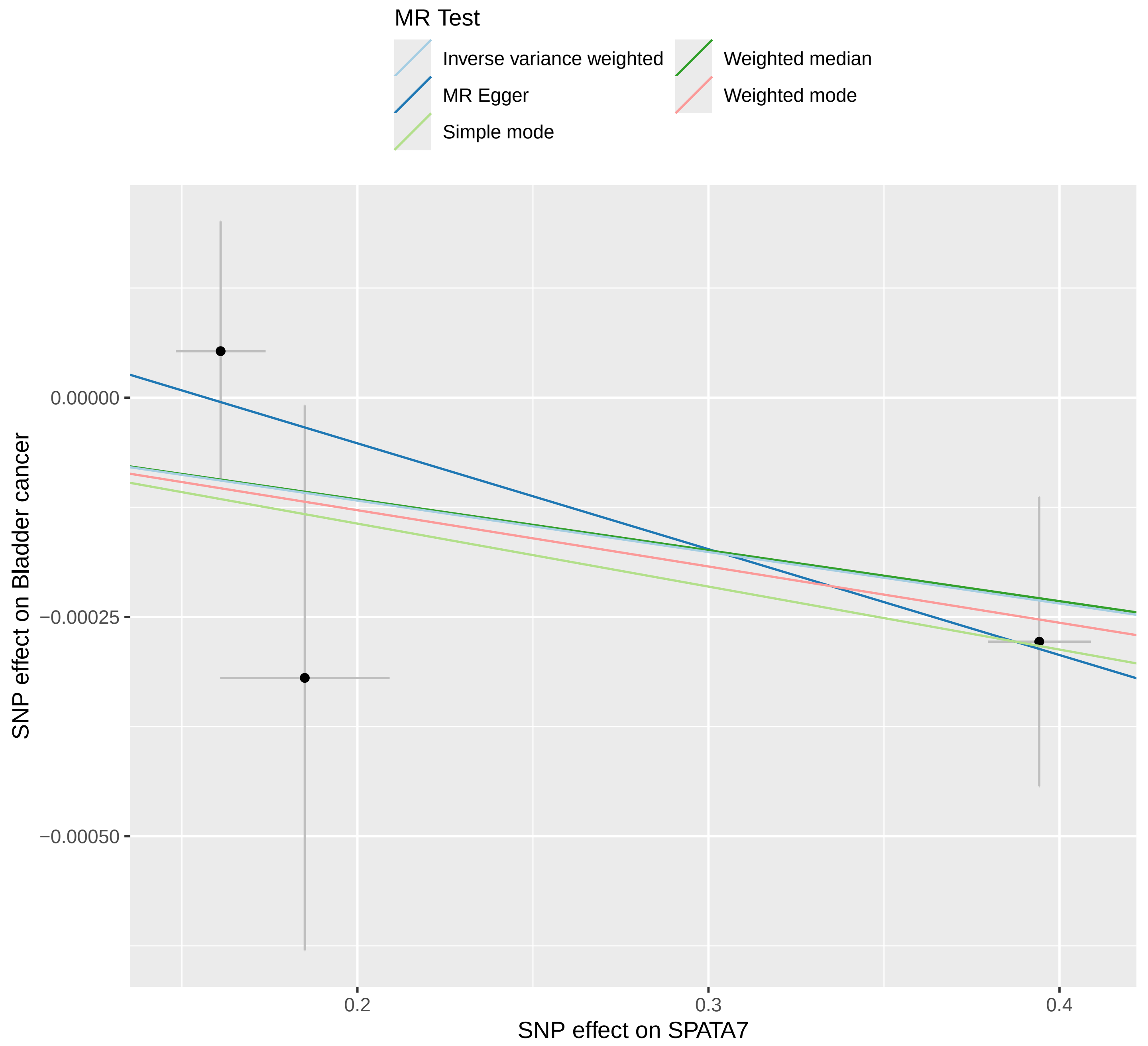
MR Test
Inverse variance weighted
MR Egger
Simple mode
Weighted median
Weighted mode
0.00000
−0.00025
−0.00050
SNP effect on Bladder cancer
0.2
0.3
0.4
SNP effect on SPATA7

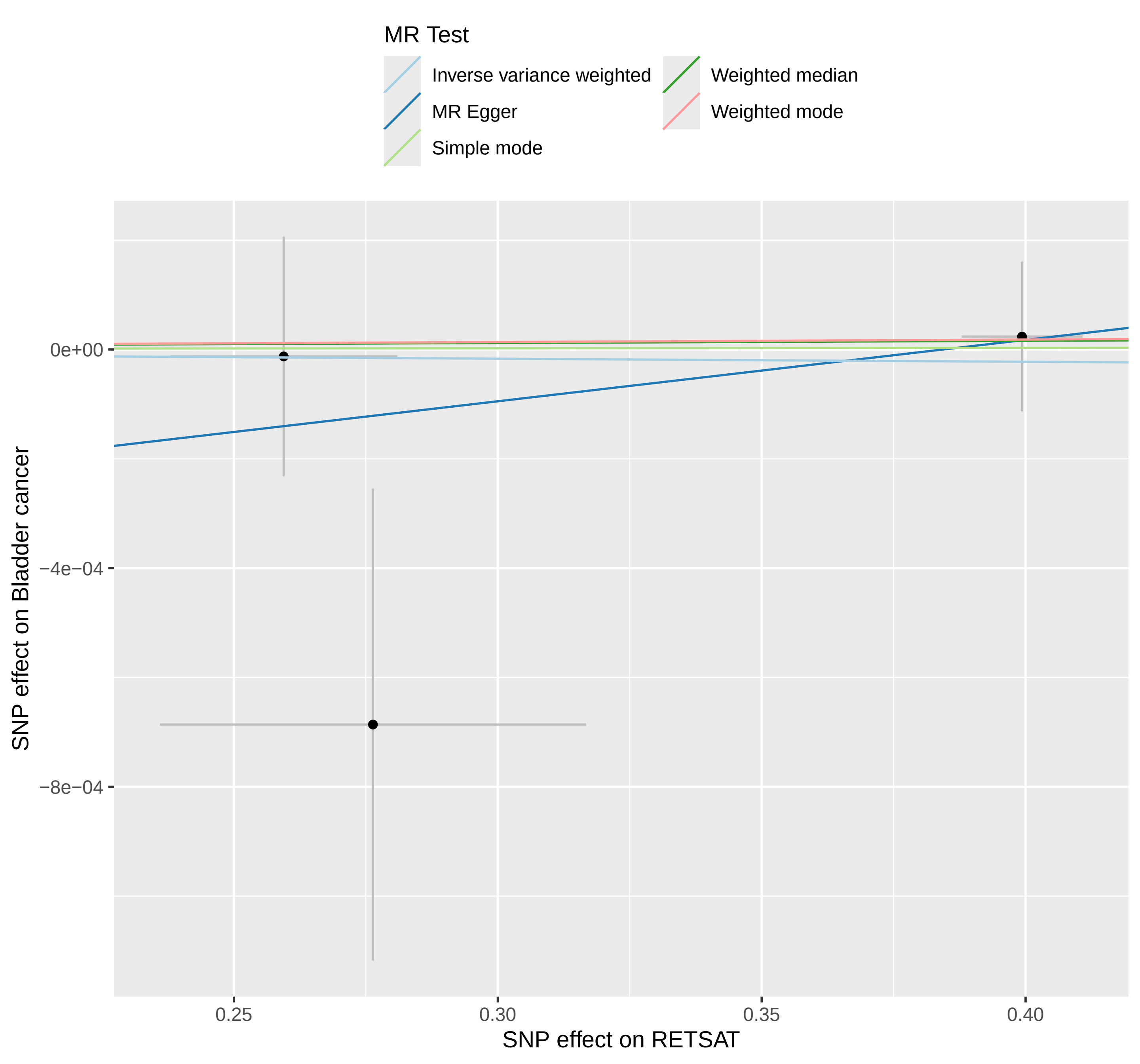
MR Test
Inverse variance weighted
MR Egger
Simple mode
Weighted median
Weighted mode
0e+00
−4e−04
−8e−04
SNP effect on Bladder cancer
0.25
0.30
0.35
0.40
SNP effect on RETSAT

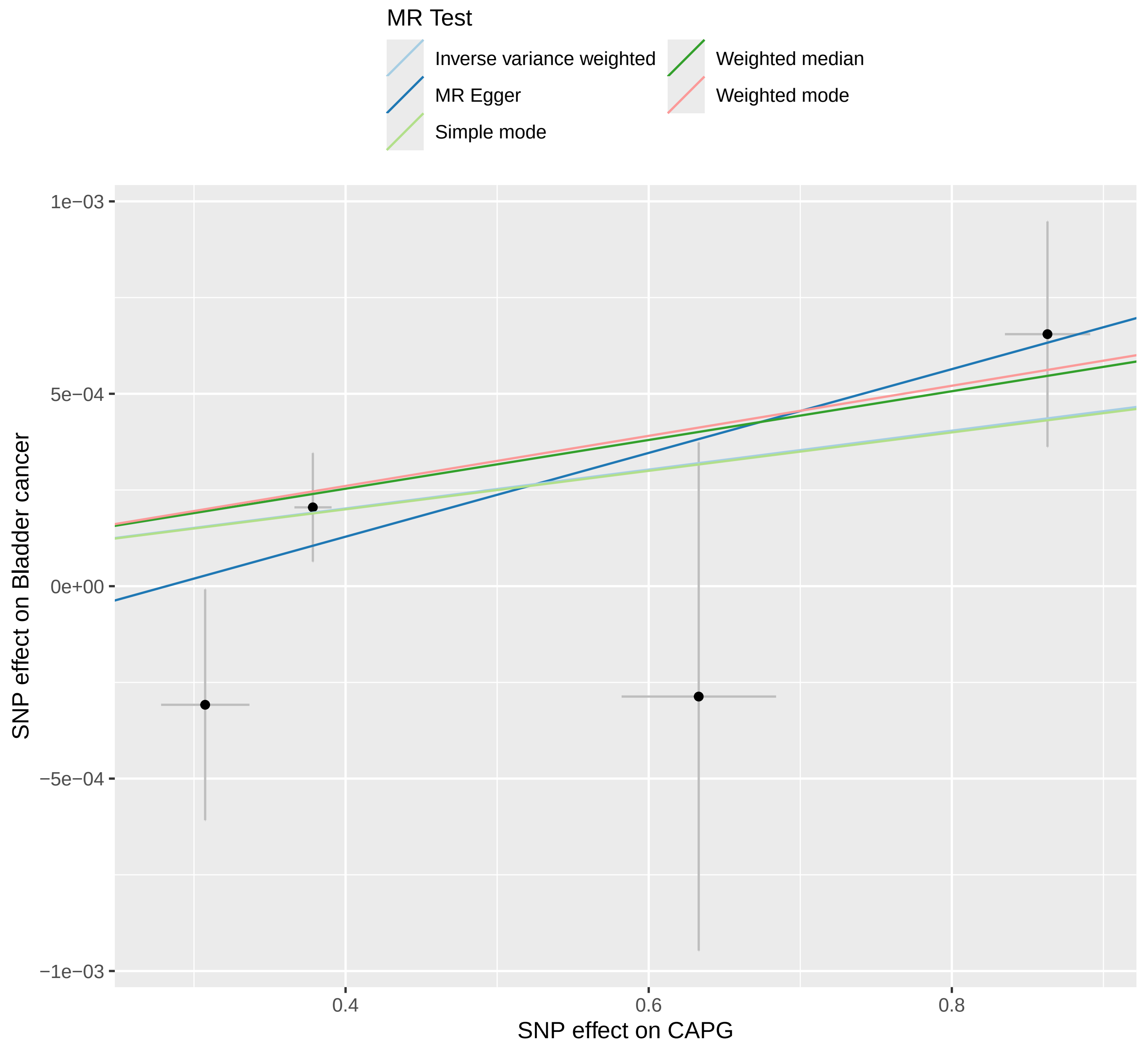
MR Test
Inverse variance weighted
MR Egger
Simple mode
Weighted median
Weighted mode
1e−03
5e−04
0e+00
−5e−04
−1e−03
SNP effect on Bladder cancer
0.4
0.6
0.8
SNP effect on CAPG

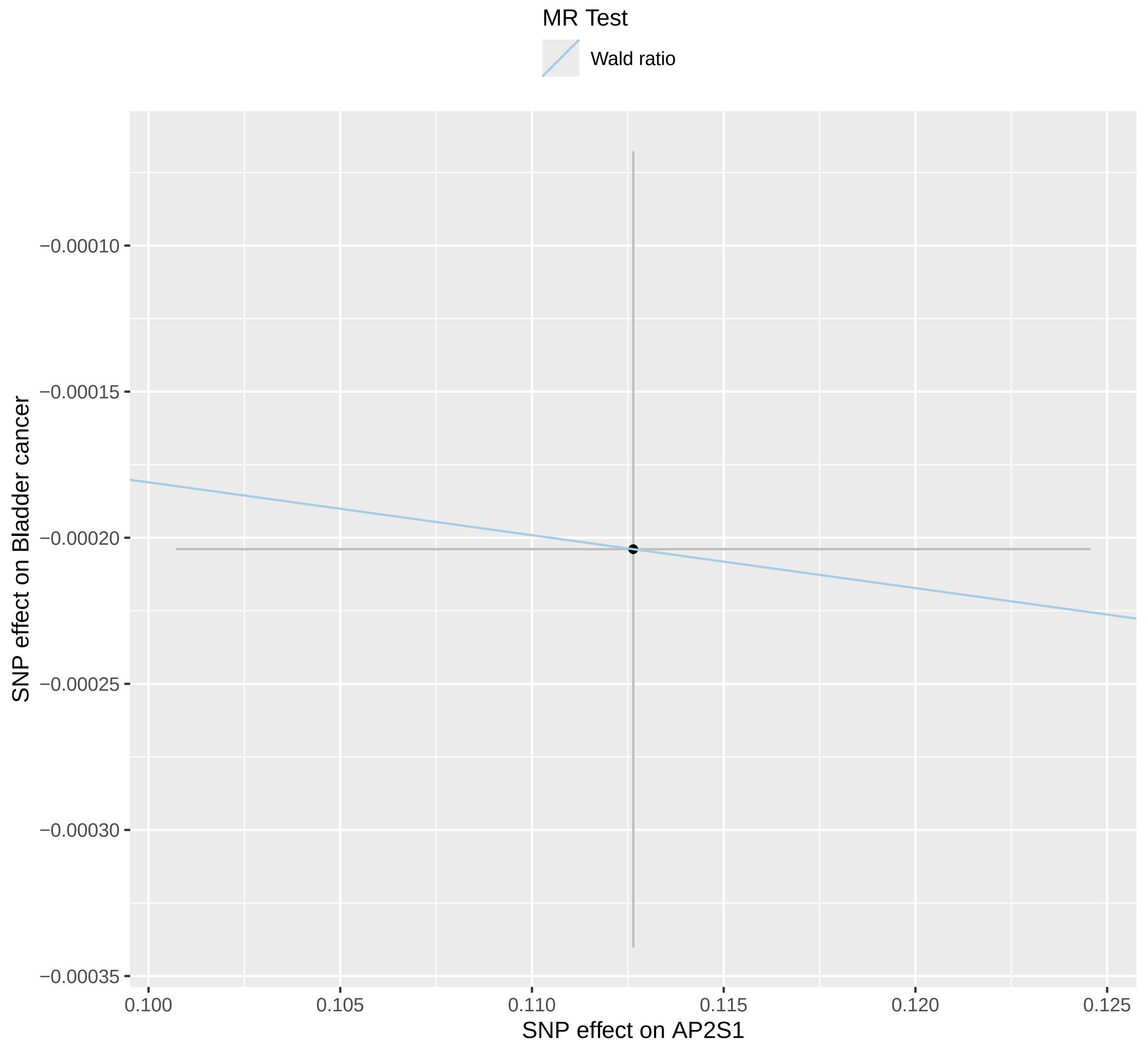

MR Test
Wald ratio
−0.00010
−0.00015
−0.00020
−0.00025
−0.00030
−0.00035
SNP effect on Bladder cancer
0.100
0.105
0.110
0.115
0.120
0.125
SNP effect on AP2S1

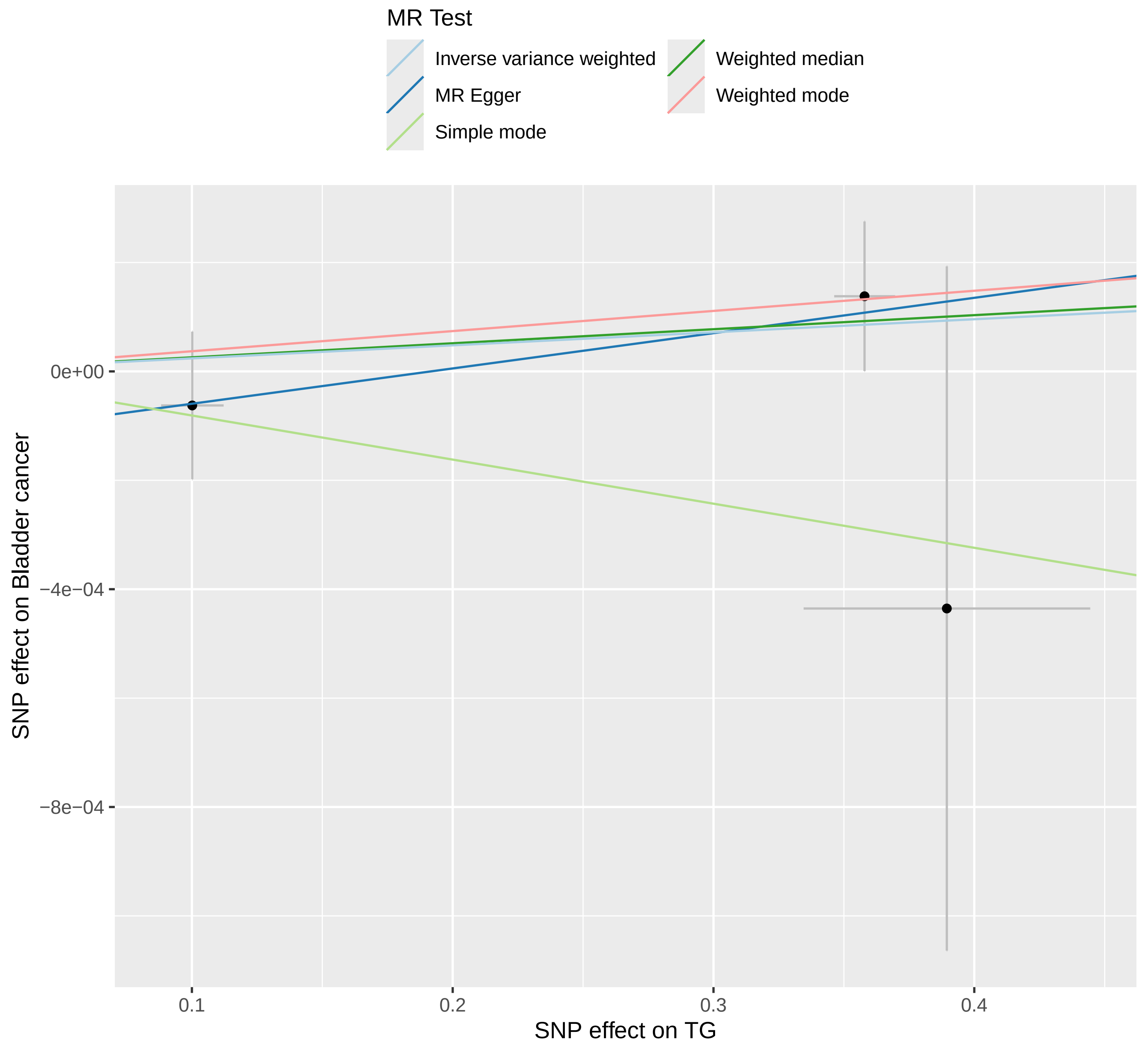

MR Test
Inverse variance weighted
MR Egger
Simple mode
Weighted median
Weighted mode
0e+00
−4e−04
−8e−04
SNP effect on Bladder cancer
0.1
0.2
0.3
0.4
SNP effect on TG

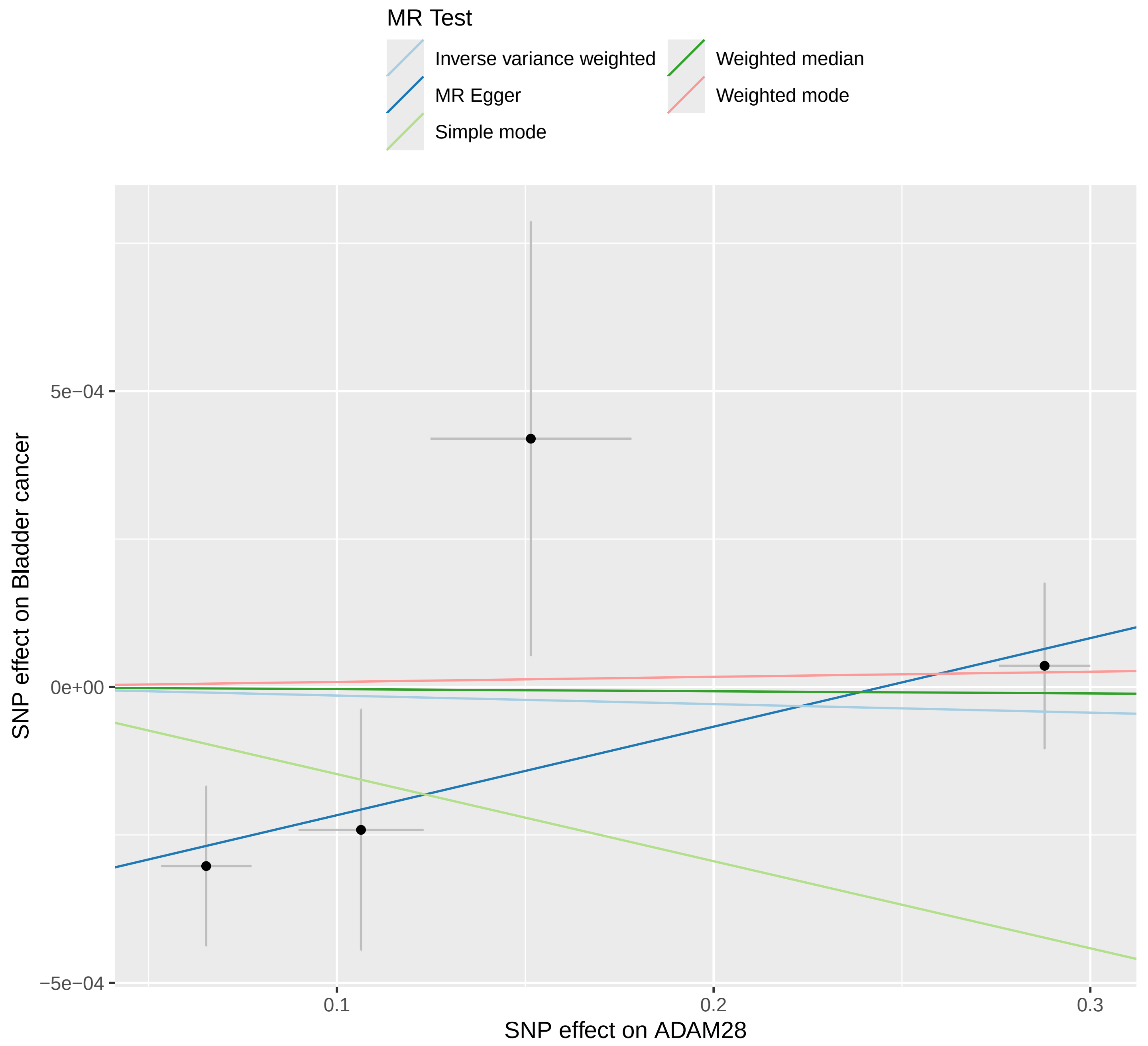
MR Test
Inverse variance weighted
MR Egger
Simple mode
Weighted median
Weighted mode
5e−04
0e+00
−5e−04
0.1
0.2
0.3
SNP effect on ADAM28
SNP effect on Bladder cancer

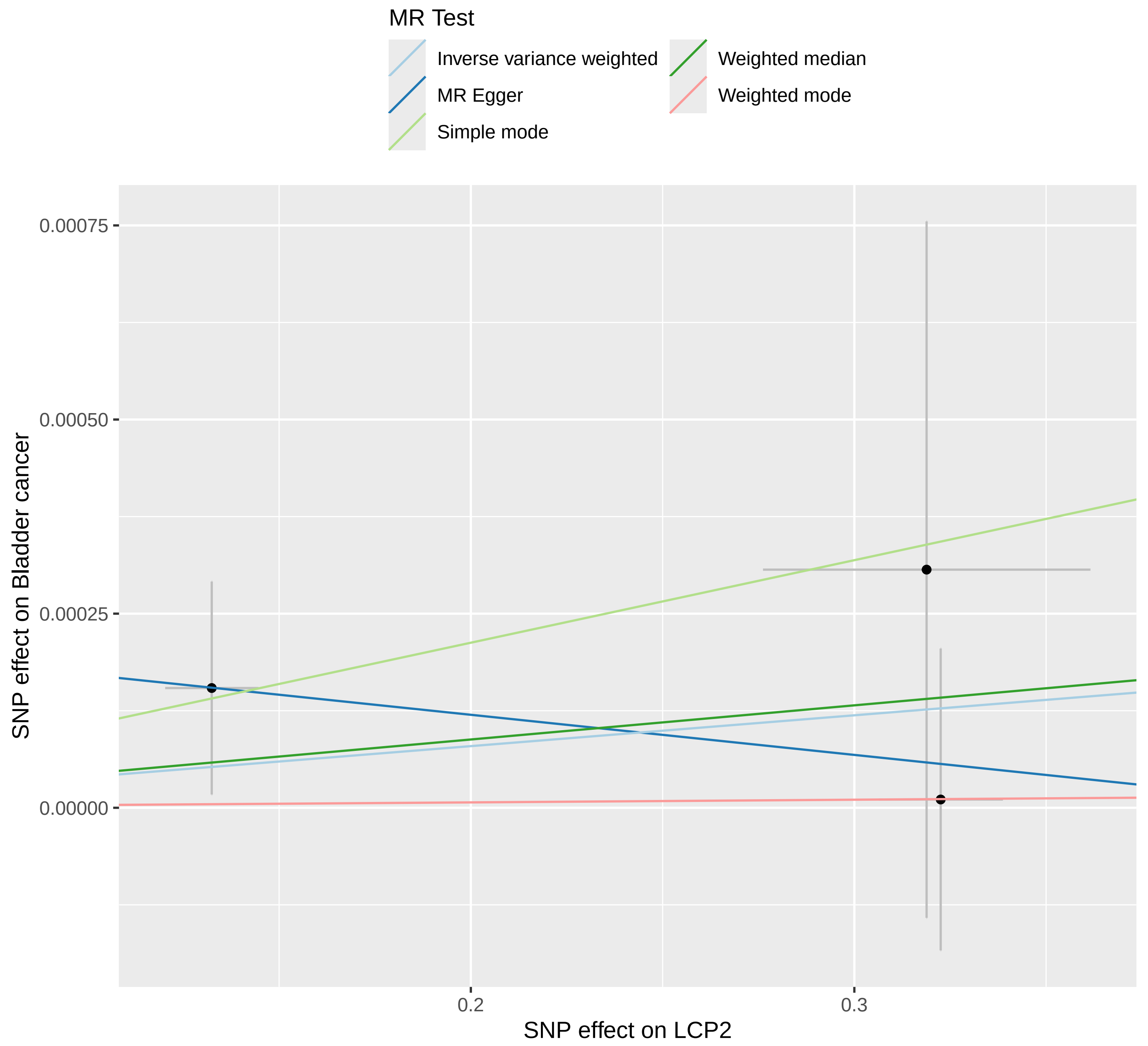

MR Test
Inverse variance weighted
MR Egger
Simple mode
Weighted median
Weighted mode
0.00075
0.00050
0.00025
0.00000
SNP effect on Bladder cancer
0.2
0.3
SNP effect on LCP2

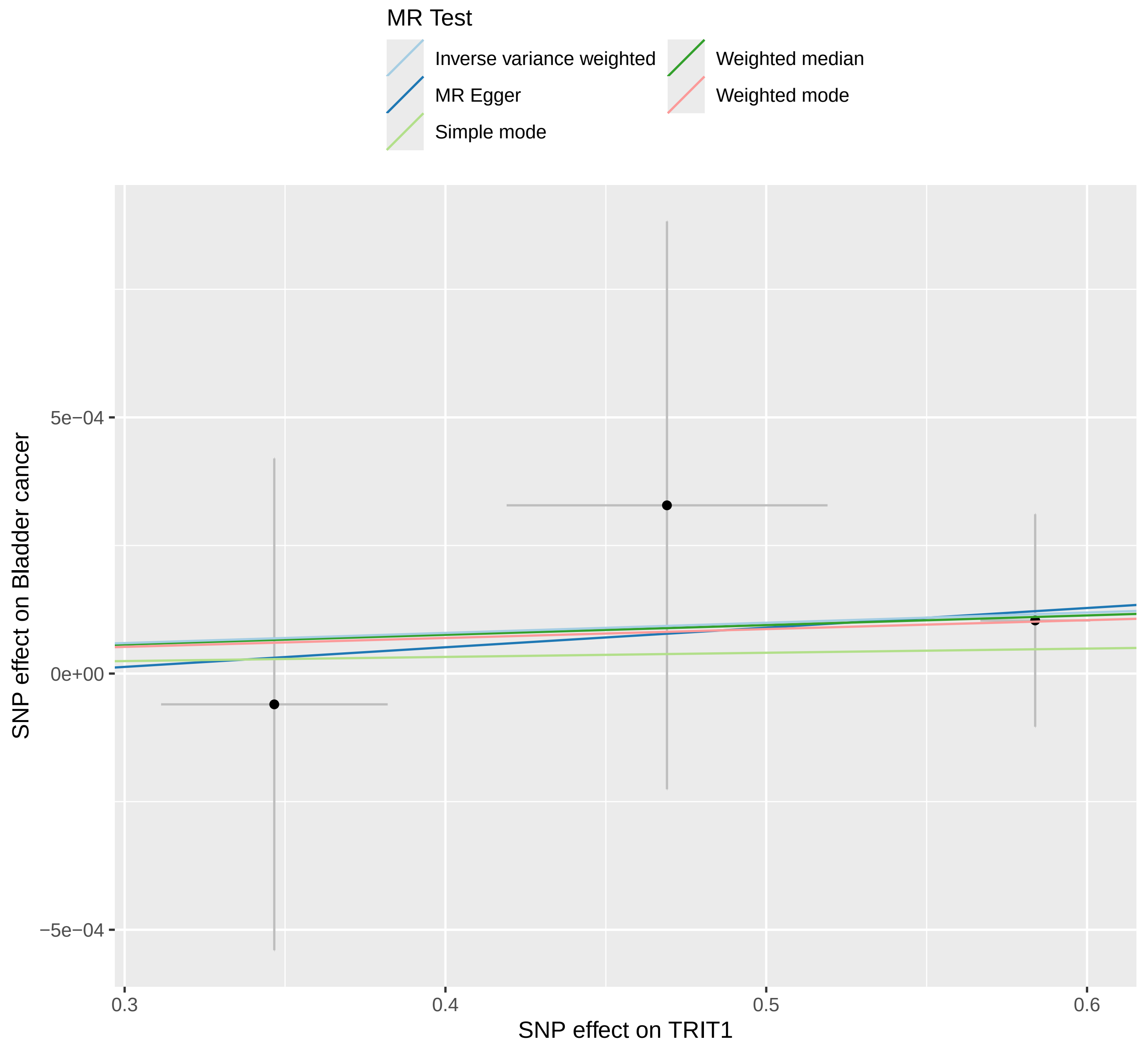

MR Test
Inverse variance weighted
MR Egger
Simple mode
Weighted median
Weighted mode
5e−04
0e+00
−5e−04
SNP effect on Bladder cancer
0.3
0.4
0.5
0.6
SNP effect on TRIT1

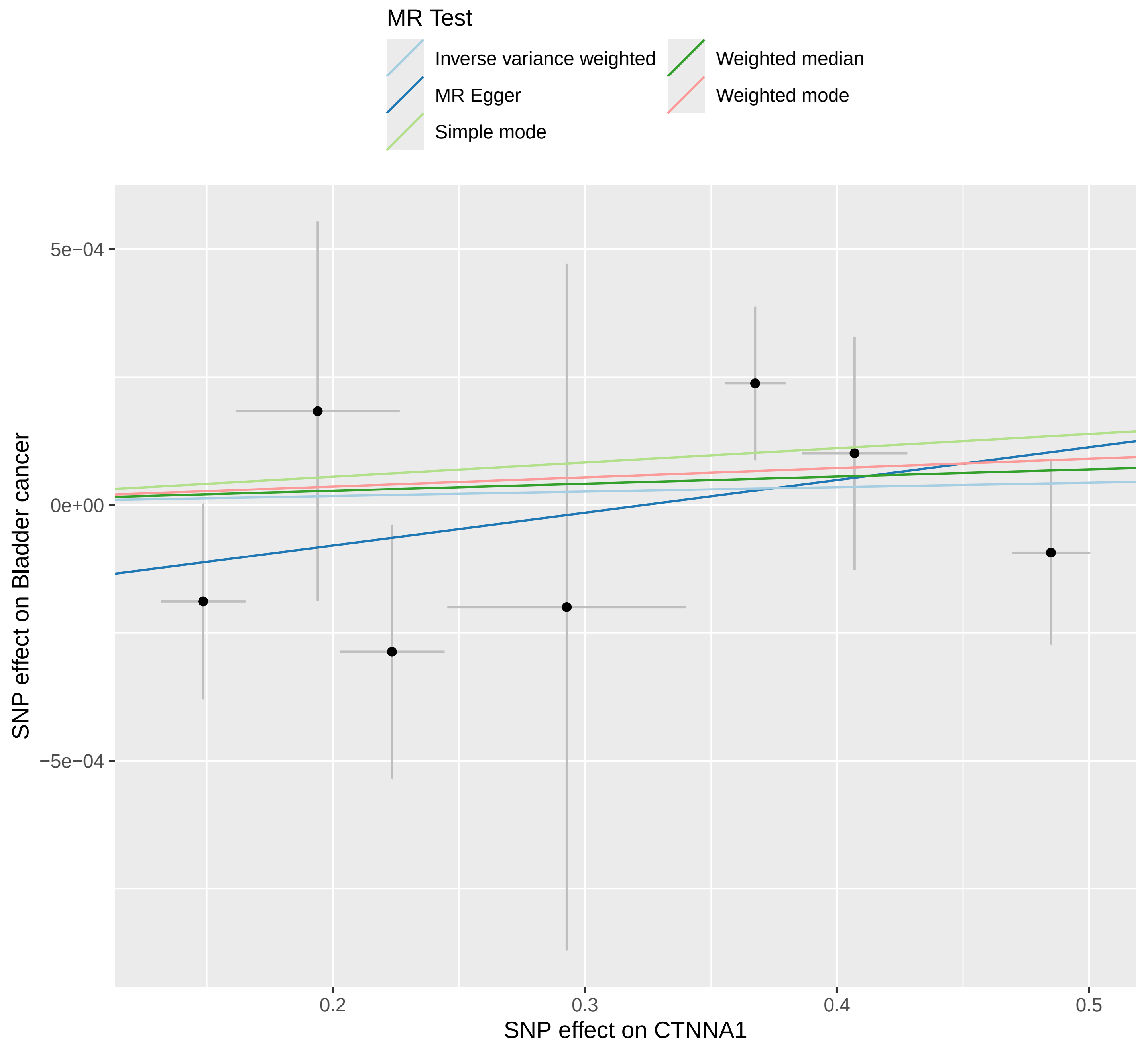
MR Test
Inverse variance weighted
MR Egger
Simple mode
Weighted median
Weighted mode
5e−04
0e+00
−5e−04
SNP effect on Bladder cancer
0.2
0.3
0.4
0.5
SNP effect on CTNNA1

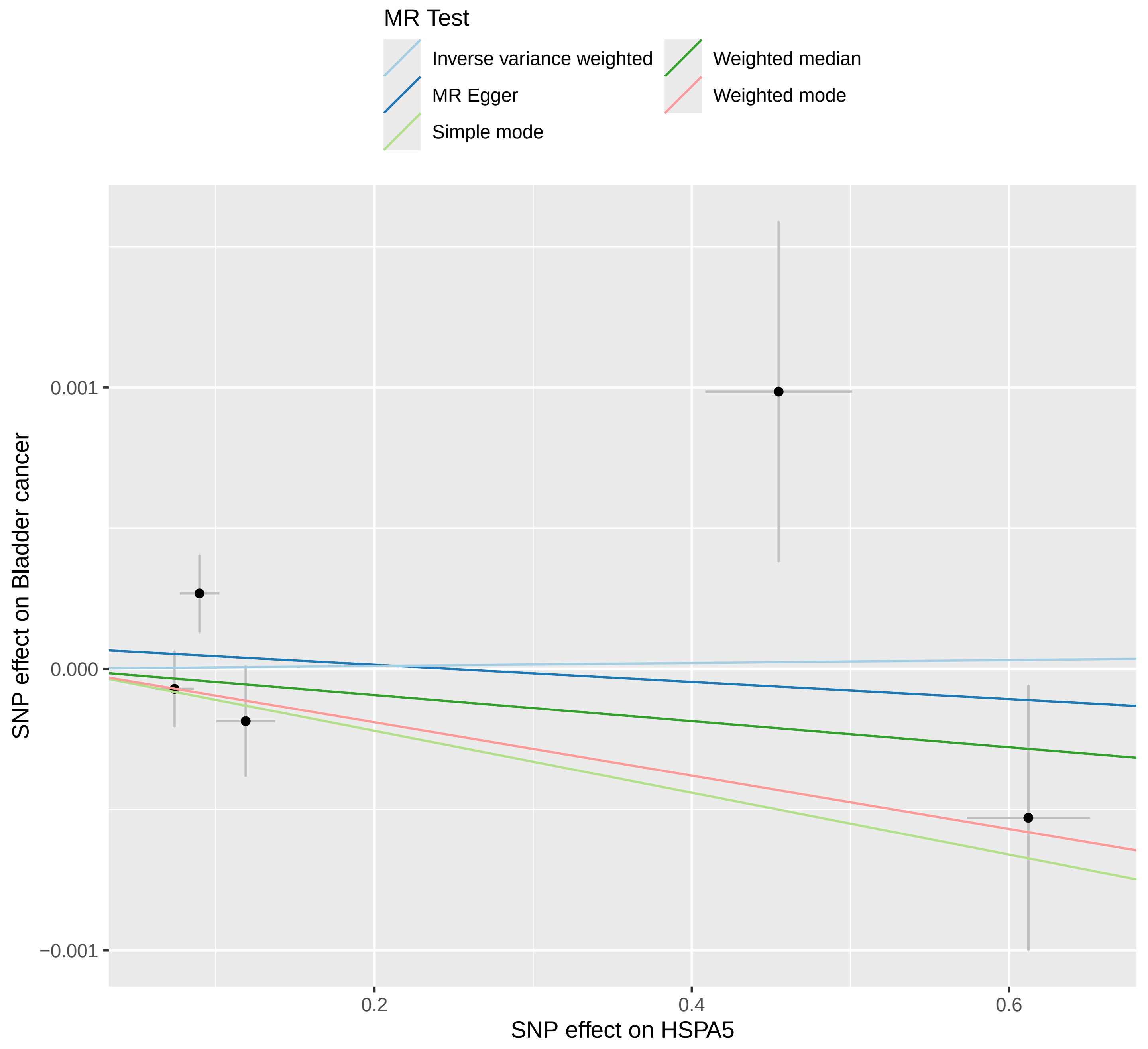
MR Test
Inverse variance weighted
MR Egger
Simple mode
Weighted median
Weighted mode
0.001
0.000
−0.001
0.2
0.4
0.6
SNP effect on HSPA5
SNP effect on Bladder cancer

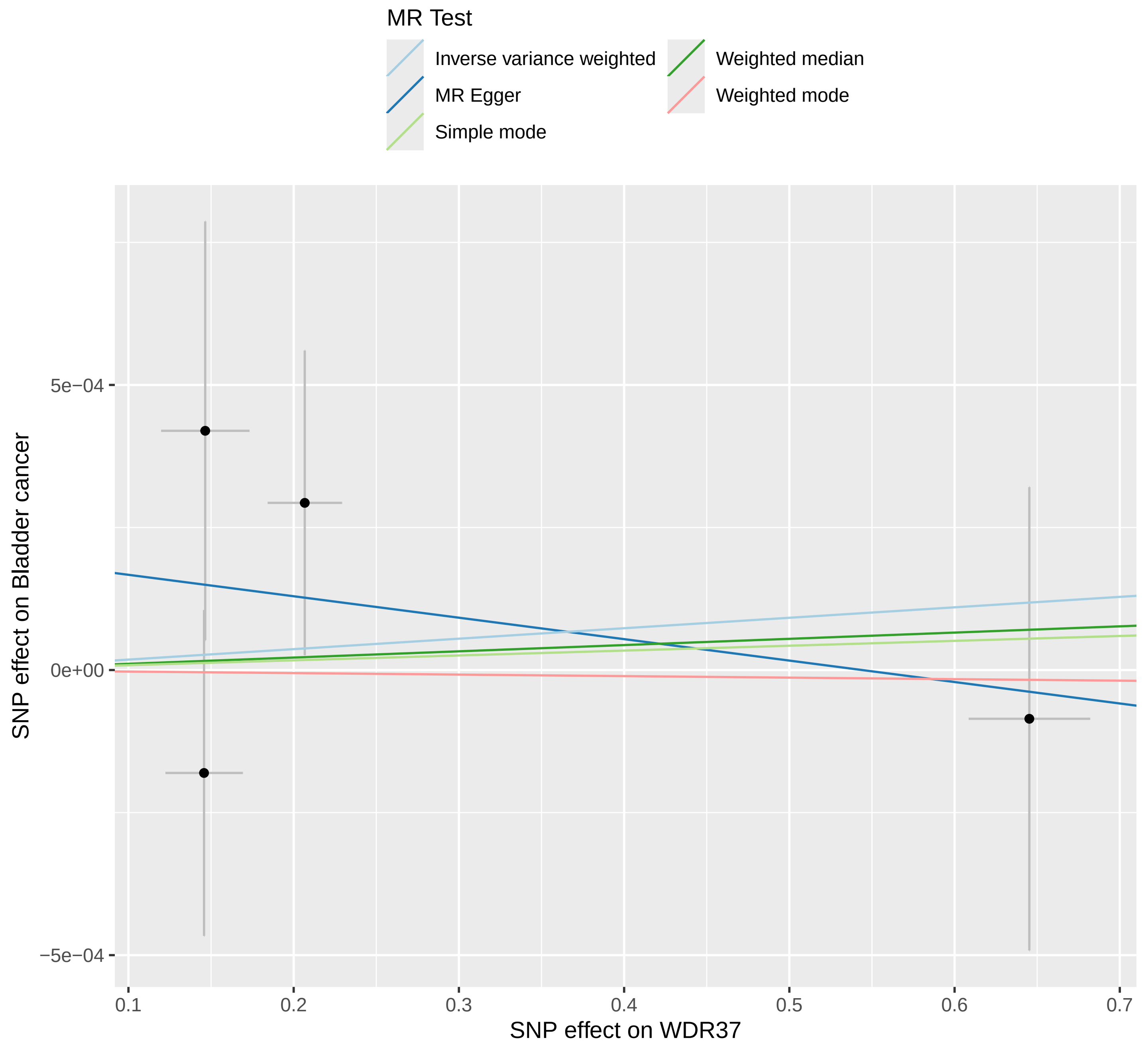
MR Test
Inverse variance weighted
MR Egger
Simple mode
Weighted median
Weighted mode
5e−04
0e+00
−5e−04
SNP effect on Bladder cancer
0.1
0.2
0.3
0.4
0.5
0.6
0.7
SNP effect on WDR37

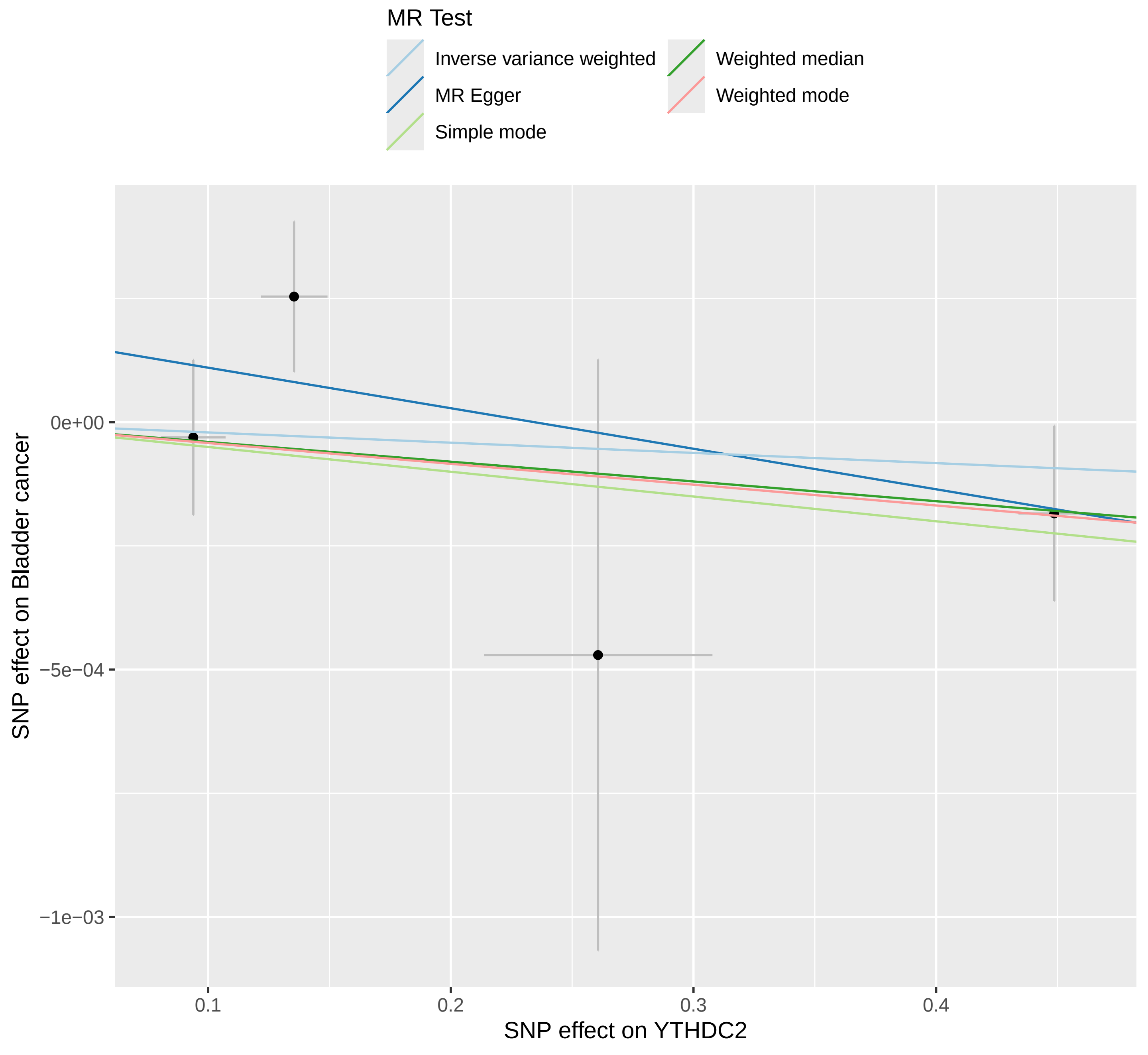

MR Test
Inverse variance weighted
MR Egger
Simple mode
Weighted median
Weighted mode
0e+00
−5e−04
−1e−03
0.1
0.2
0.3
0.4
SNP effect on Bladder cancer
SNP effect on YTHDC2

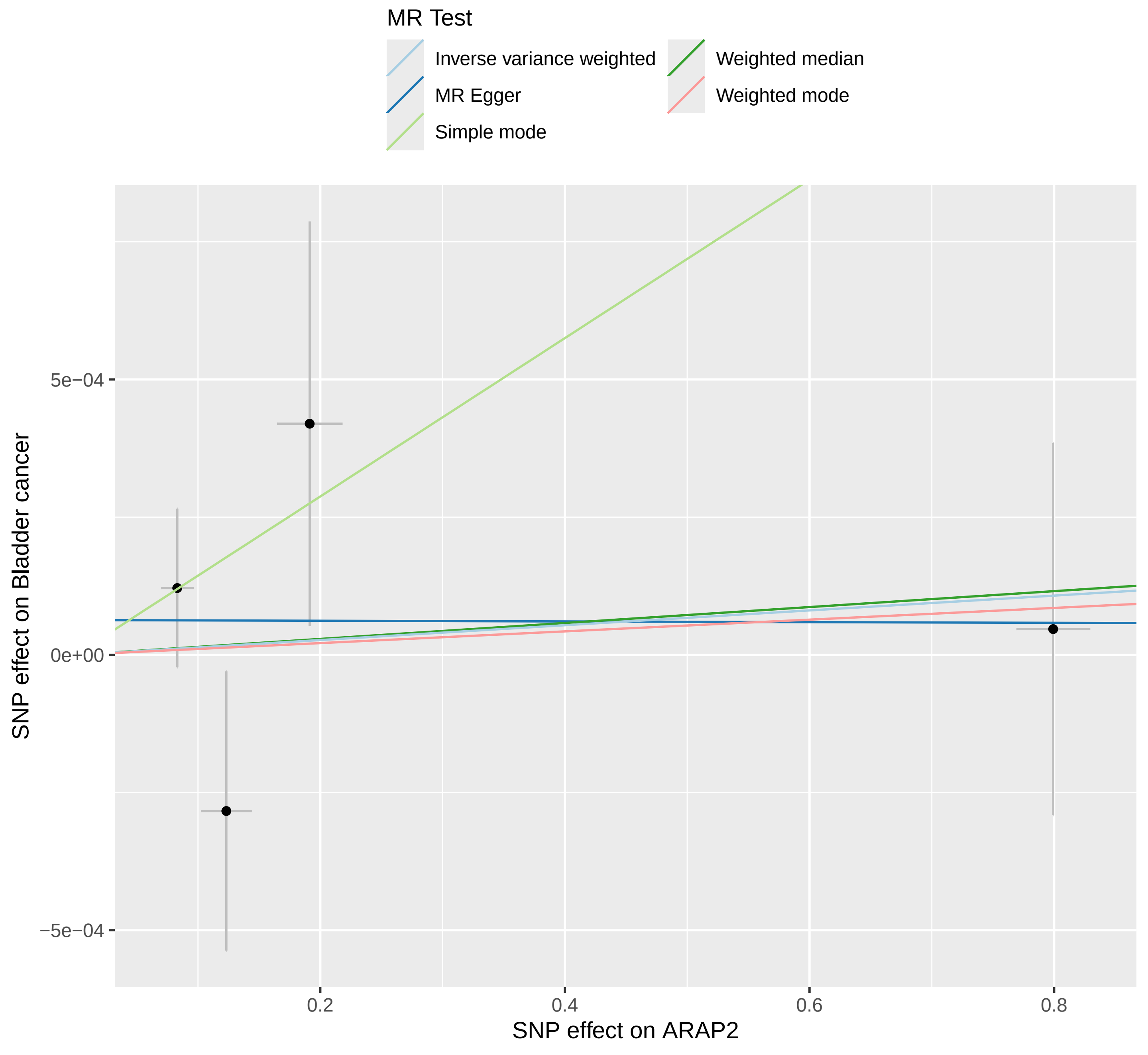

MR Test
Inverse variance weighted
MR Egger
Simple mode
Weighted median
Weighted mode
5e−04
0e+00
−5e−04
SNP effect on Bladder cancer
0.2
0.4
0.6
0.8
SNP effect on ARAP2

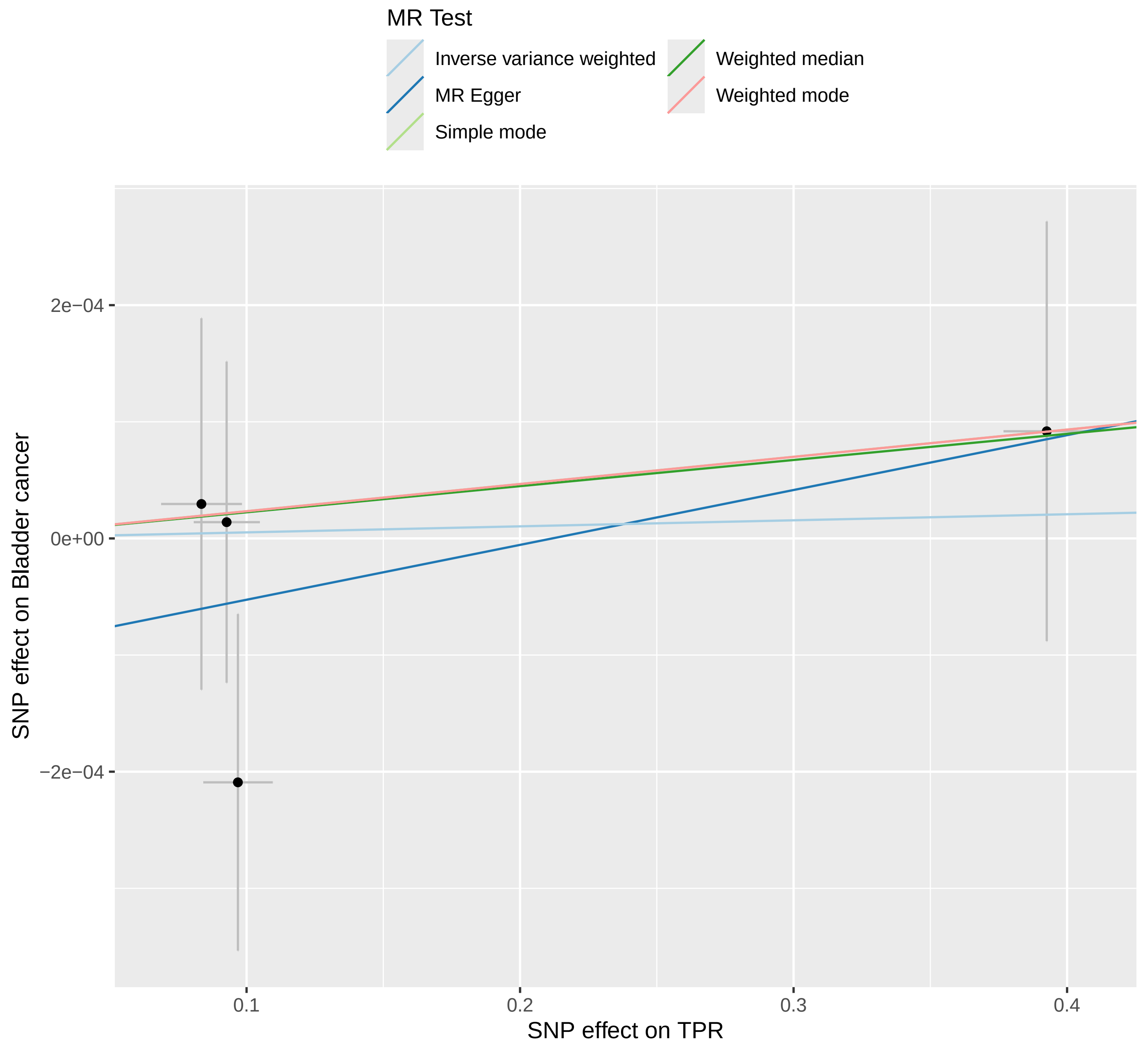

MR Test
Inverse variance weighted
MR Egger
Simple mode
Weighted median
Weighted mode
2e−04
0e+00
−2e−04
0.1
0.2
0.3
0.4
SNP effect on Bladder cancer
SNP effect on TPR

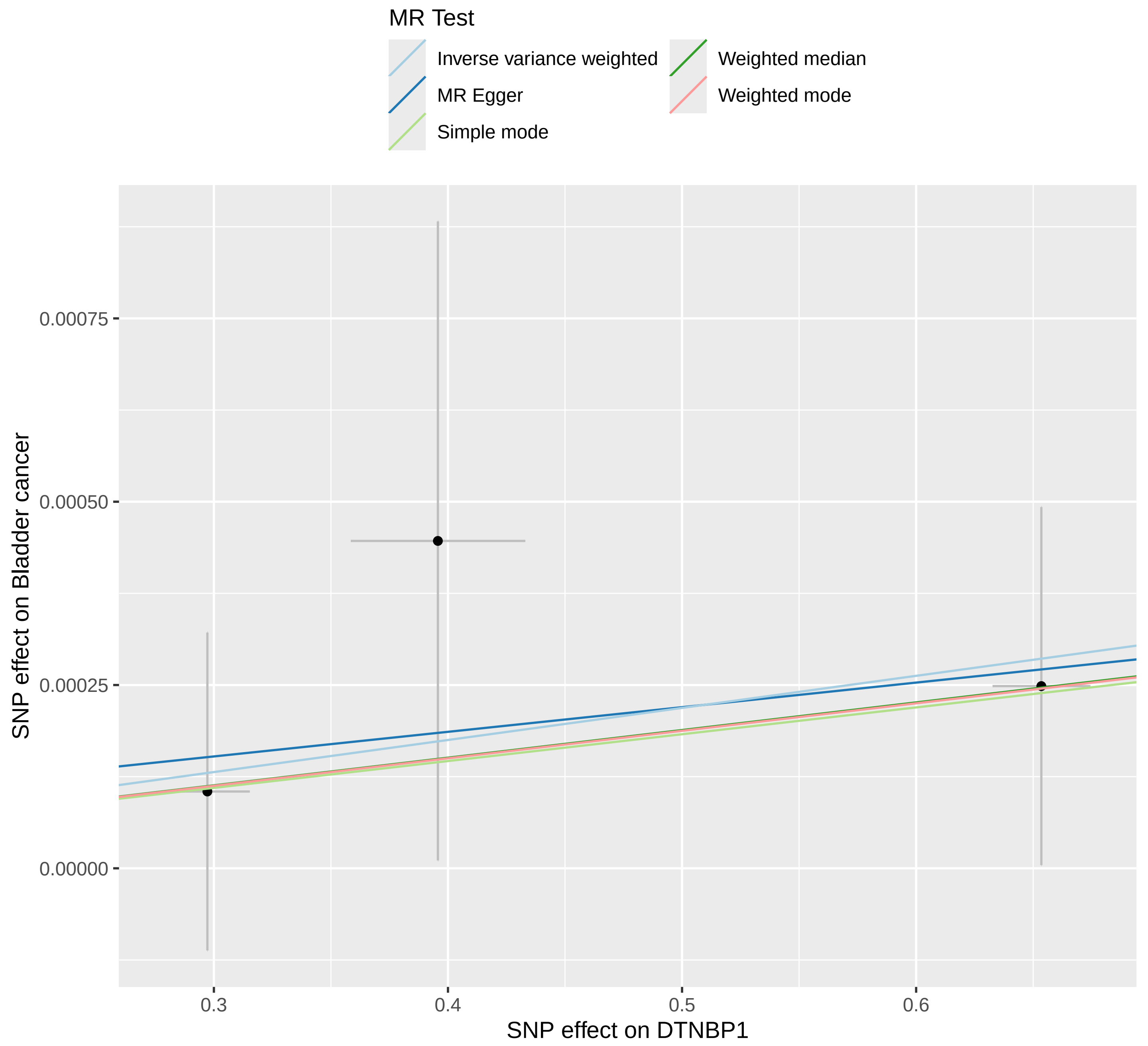
MR Test
Inverse variance weighted
MR Egger
Simple mode
Weighted median
Weighted mode
0.00075
0.00050
0.00025
0.00000
0.3
0.4
0.5
0.6
SNP effect on DTNBP1
SNP effect on Bladder cancer

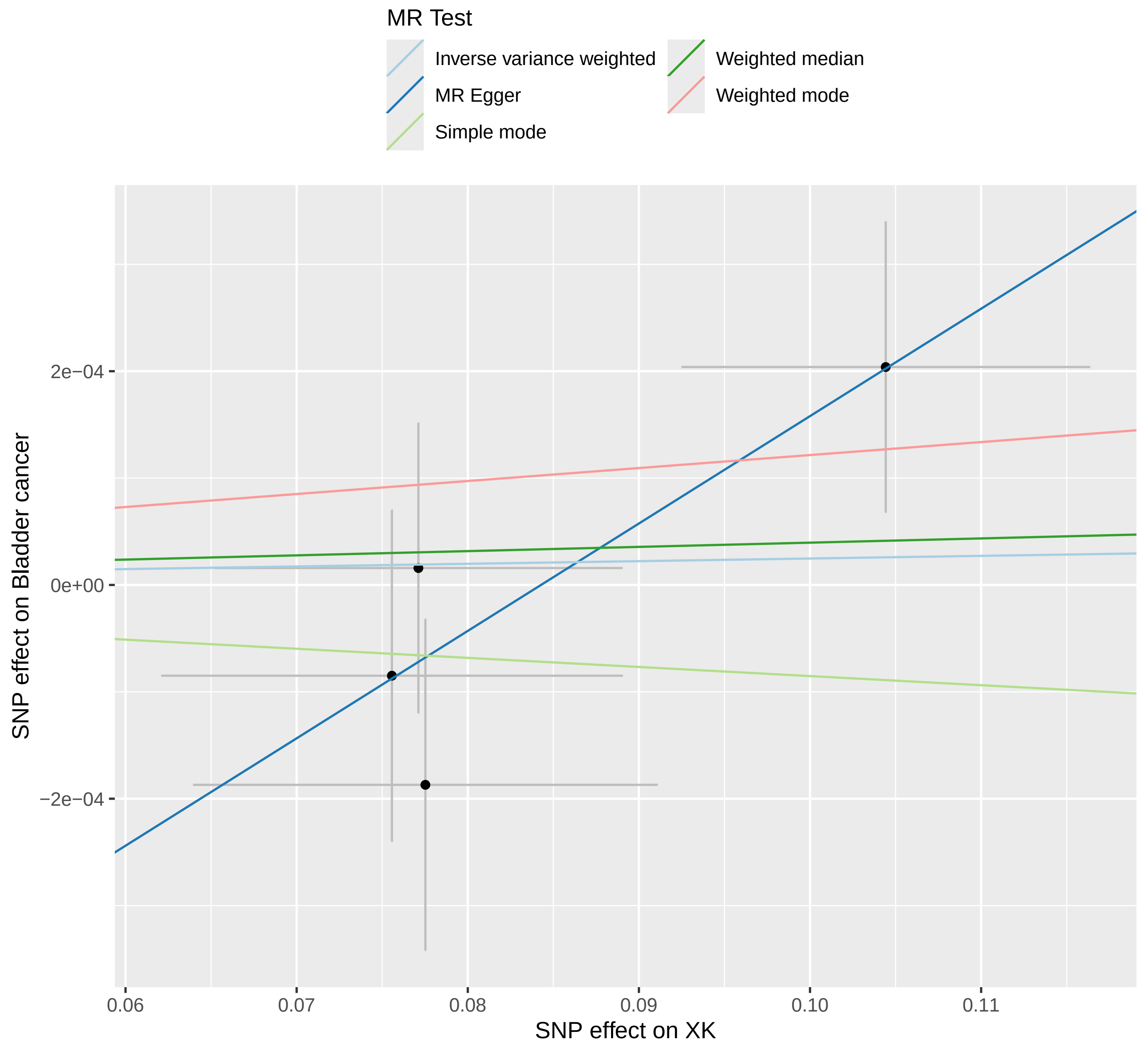
MR Test
Inverse variance weighted
MR Egger
Simple mode
Weighted median
Weighted mode
2e−04
0e+00
−2e−04
0.06
0.07
0.08
0.09
0.10
0.11
SNP effect on XK
SNP effect on Bladder cancer

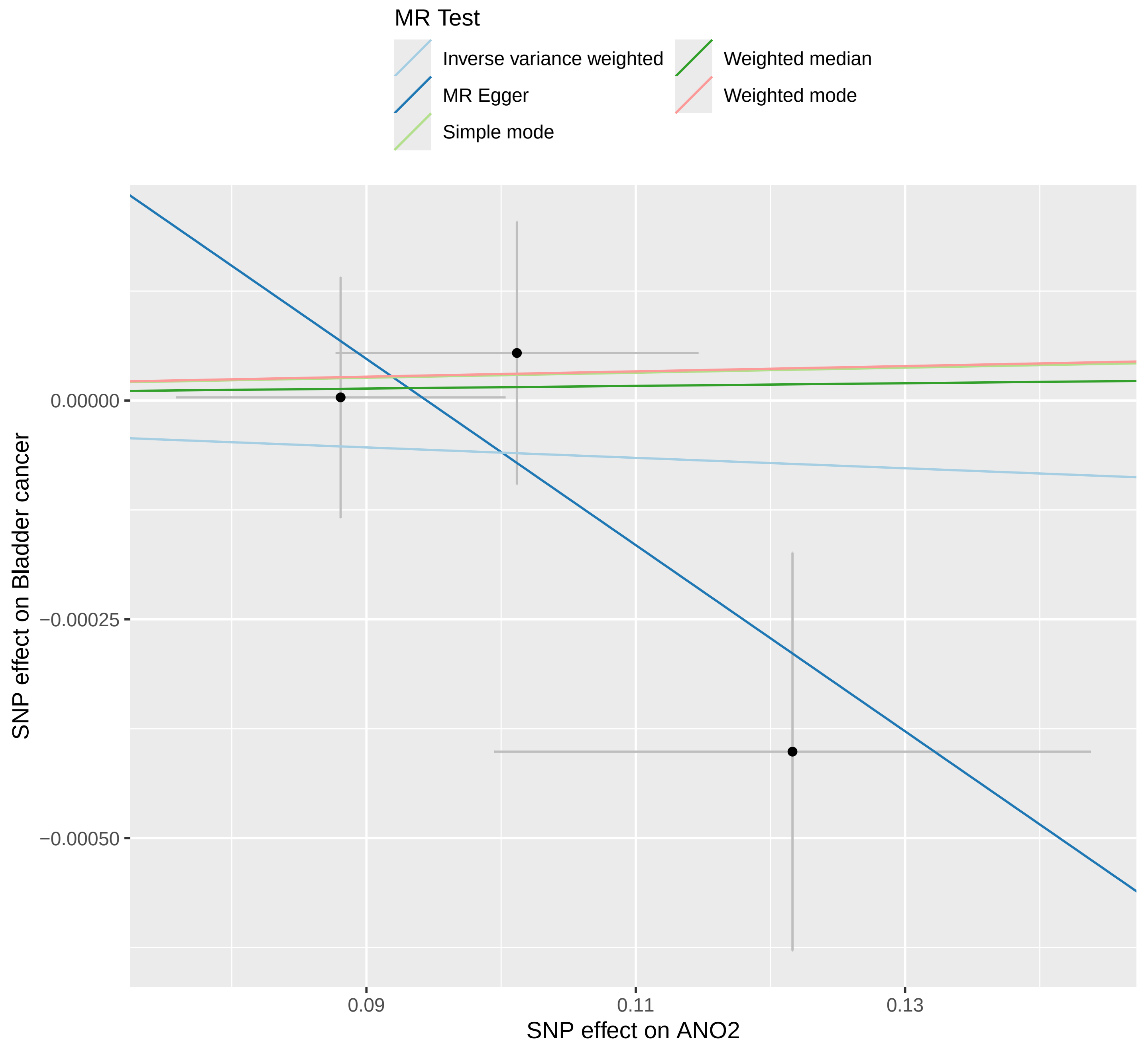

MR Test
Inverse variance weighted
MR Egger
Simple mode
Weighted median
Weighted mode
0.00000
−0.00025
−0.00050
SNP effect on Bladder cancer
0.09
0.11
0.13
SNP effect on ANO2

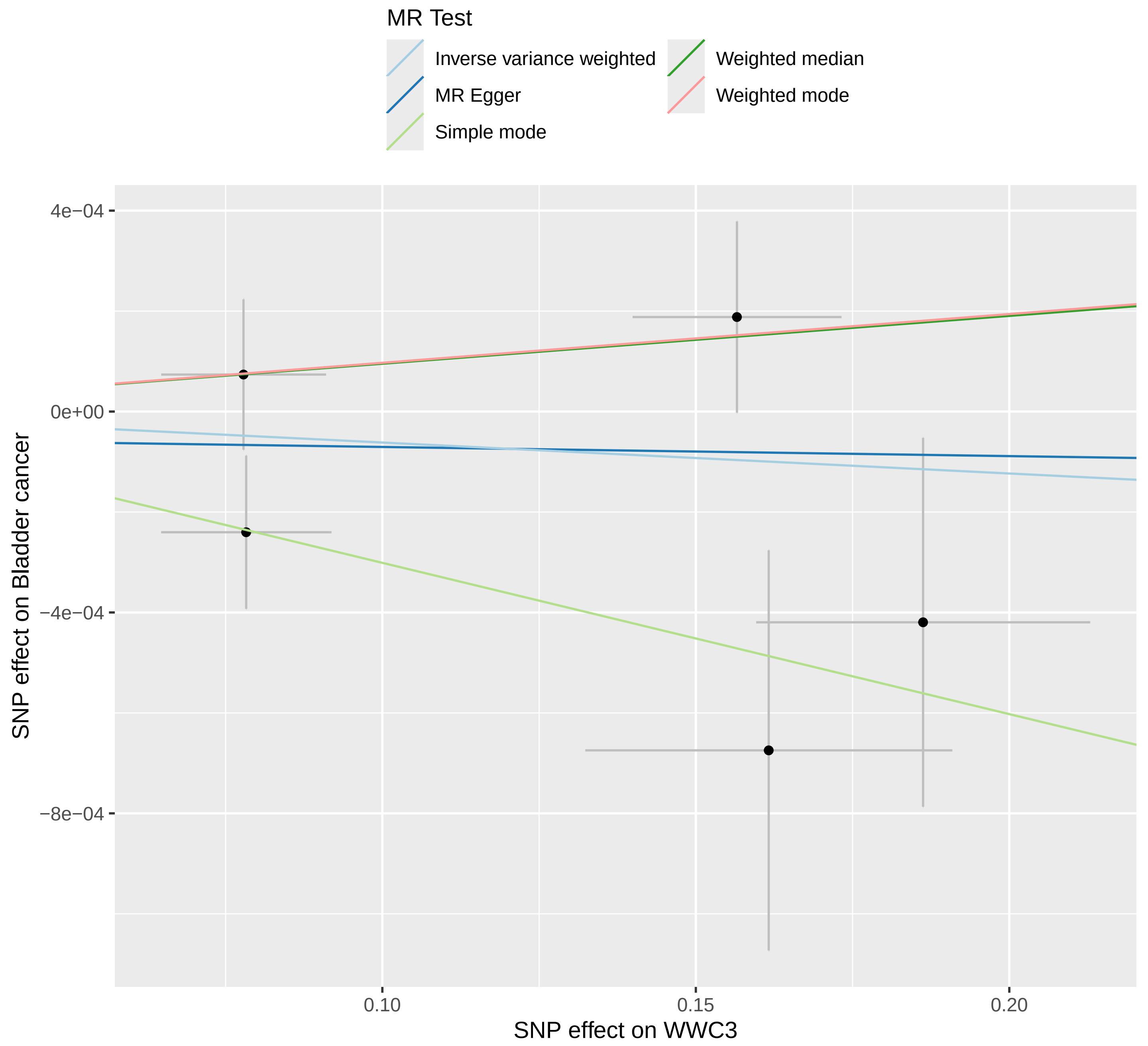
MR Test
Inverse variance weighted
MR Egger
Simple mode
Weighted median
Weighted mode
4e−04
0e+00
−4e−04
−8e−04
0.10
0.15
0.20
SNP effect on Bladder cancer
SNP effect on WWC3

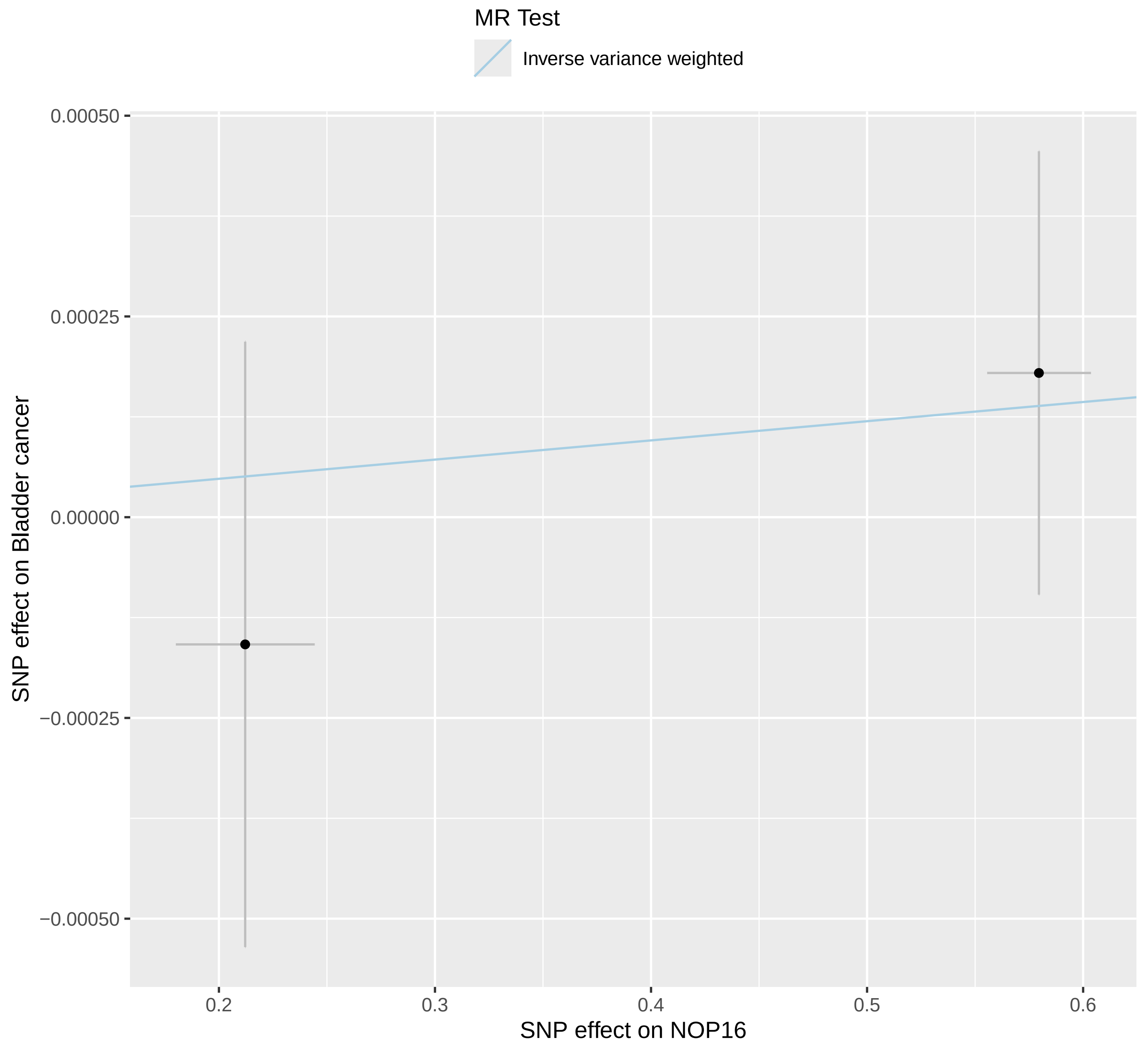

MR Test
Inverse variance weighted
0.00050
0.00025
0.00000
−0.00025
−0.00050
0.2
0.3
0.4
0.5
0.6
SNP effect on NOP16
SNP effect on Bladder cancer

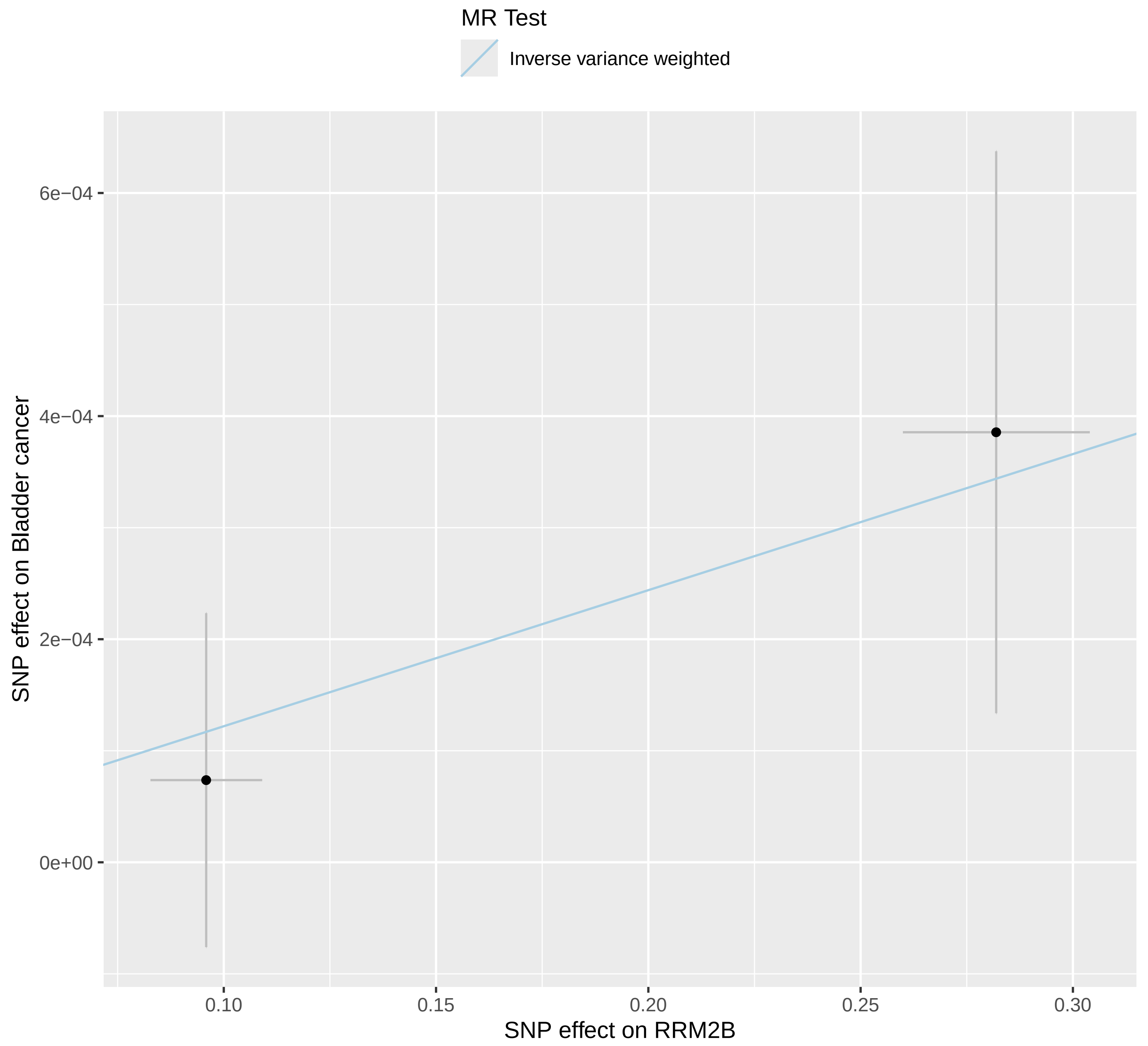

MR Test
Inverse variance weighted
6e−04
4e−04
2e−04
0e+00
0.10
0.15
0.20
0.25
0.30
SNP effect on RRM2B
SNP effect on Bladder cancer

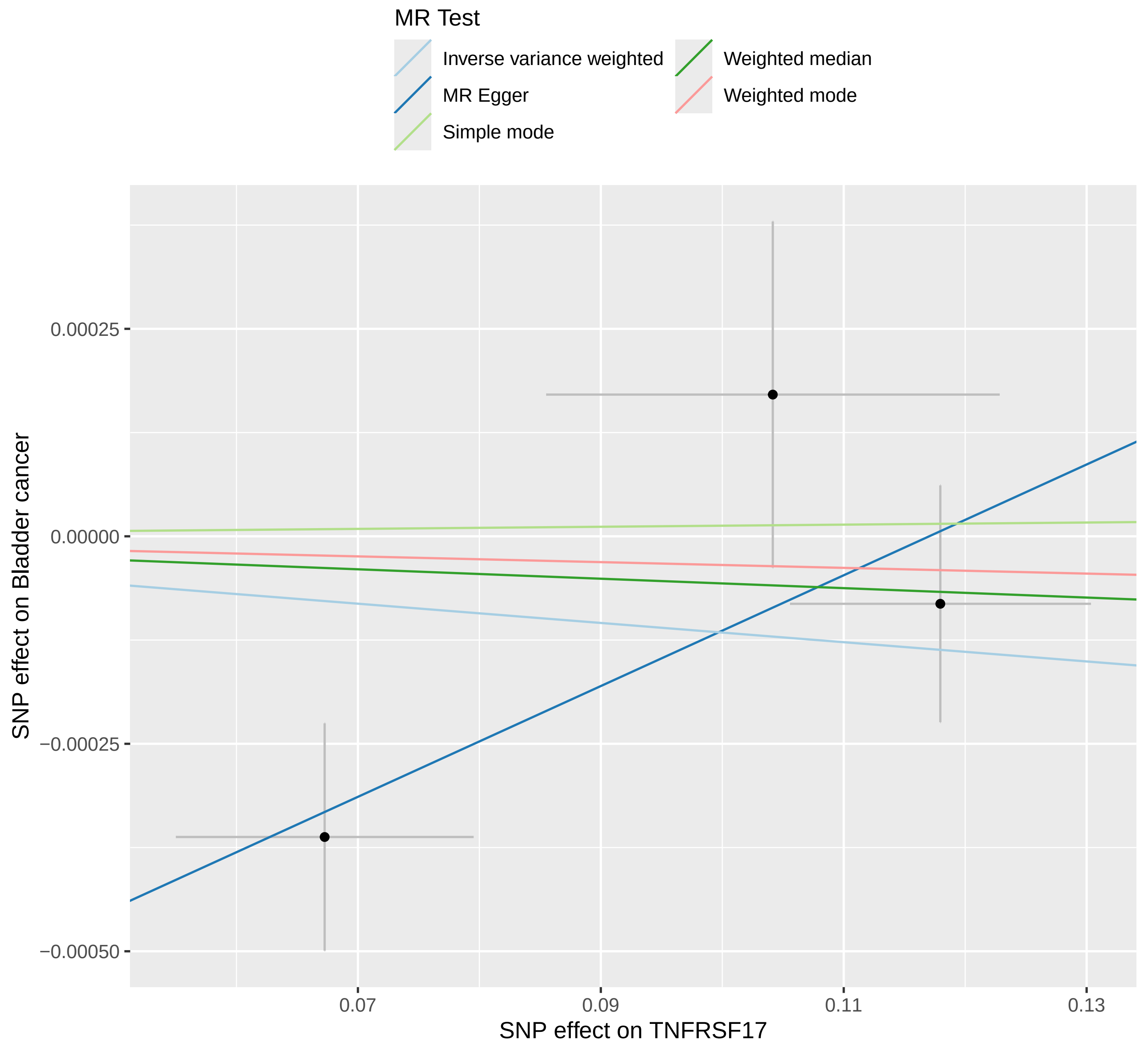

MR Test
Inverse variance weighted
MR Egger
Simple mode
Weighted median
Weighted mode
0.00025
0.00000
−0.00025
−0.00050
SNP effect on Bladder cancer
0.07
0.09
0.11
0.13
SNP effect on TNFRSF17

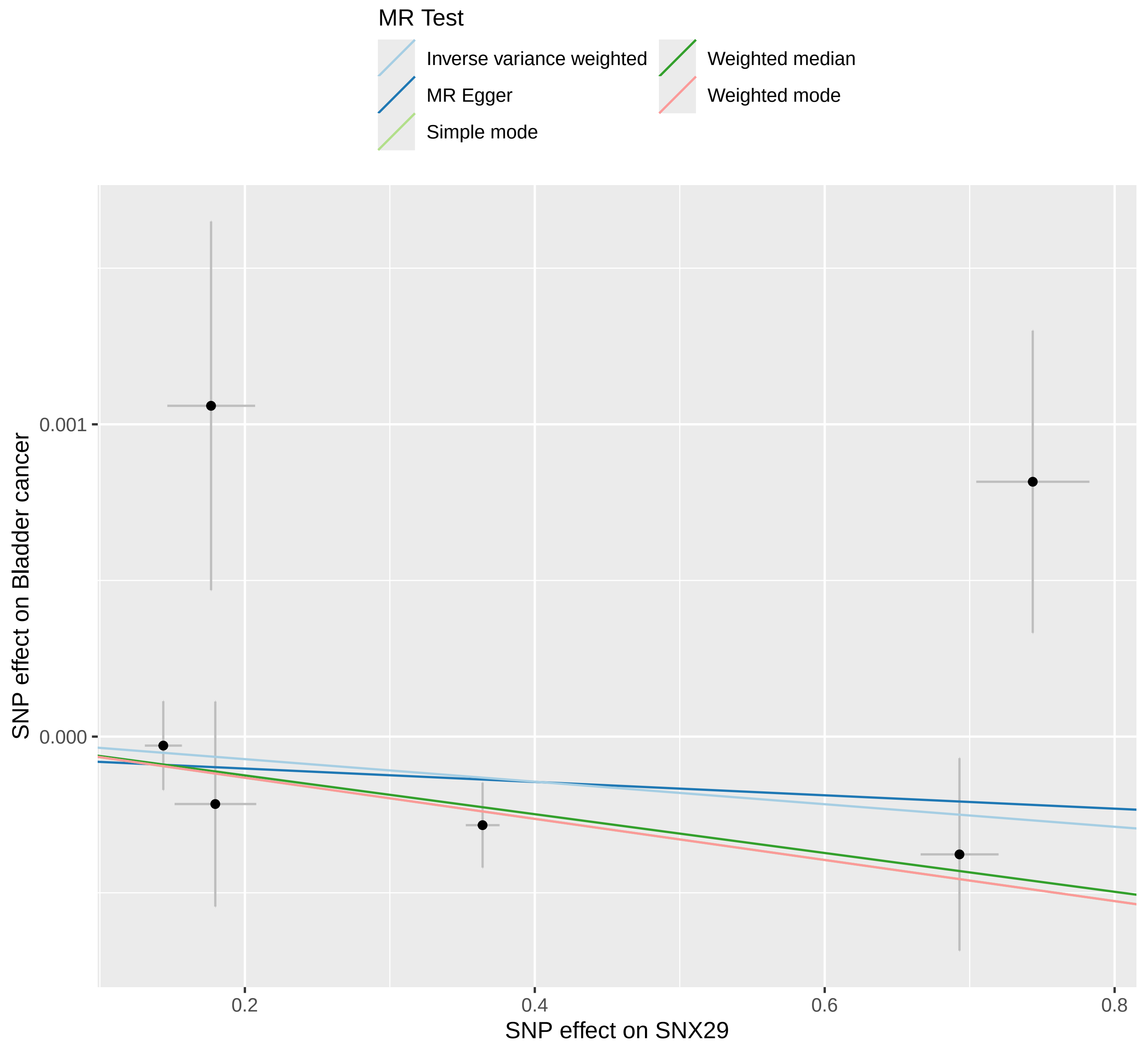
MR Test
Inverse variance weighted
MR Egger
Simple mode
Weighted median
Weighted mode
SNP effect on Bladder cancer
0.001
0.000
0.2
0.4
0.6
0.8
SNP effect on SNX29

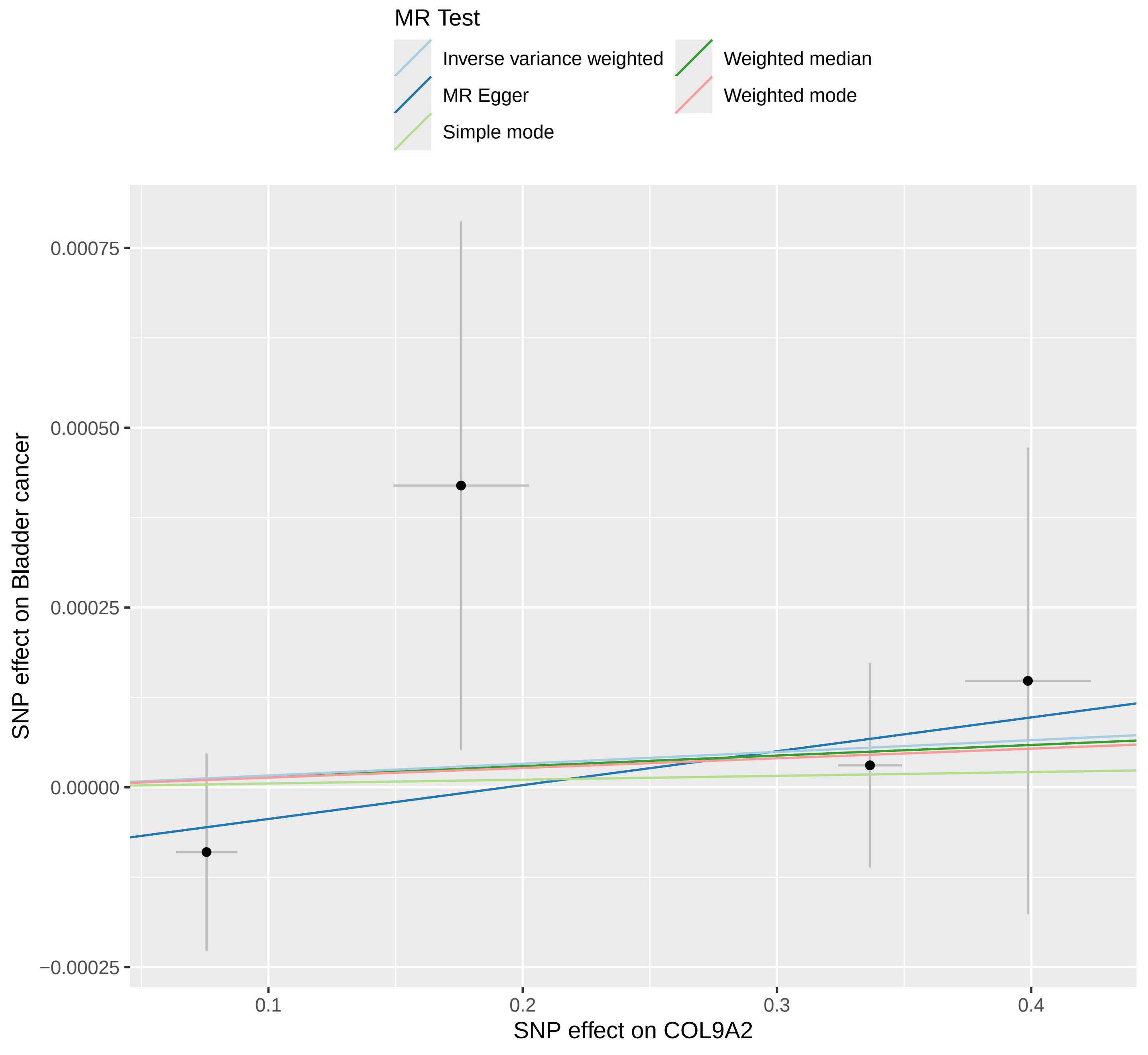

MR Test
Inverse variance weighted
MR Egger
Simple mode
Weighted median
Weighted mode
0.00075
0.00050
0.00025
0.00000
−0.00025
SNP effect on Bladder cancer
0.1
0.2
0.3
0.4
SNP effect on COL9A2

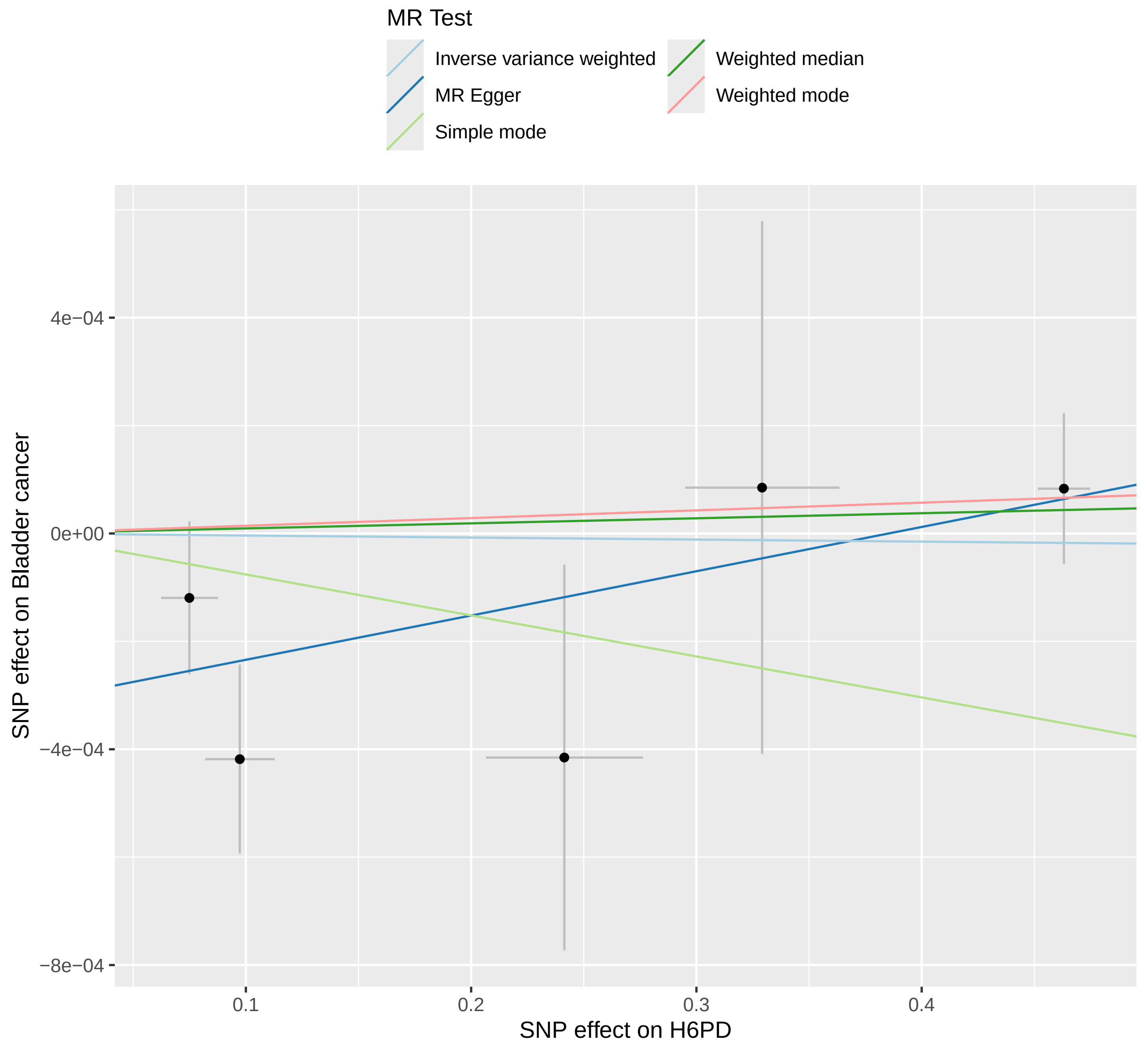
MR Test
Inverse variance weighted
MR Egger
Simple mode
Weighted median
Weighted mode
4e−04
0e+00
−4e−04
−8e−04
0.1
0.2
0.3
0.4
SNP effect on H6PD
SNP effect on Bladder cancer

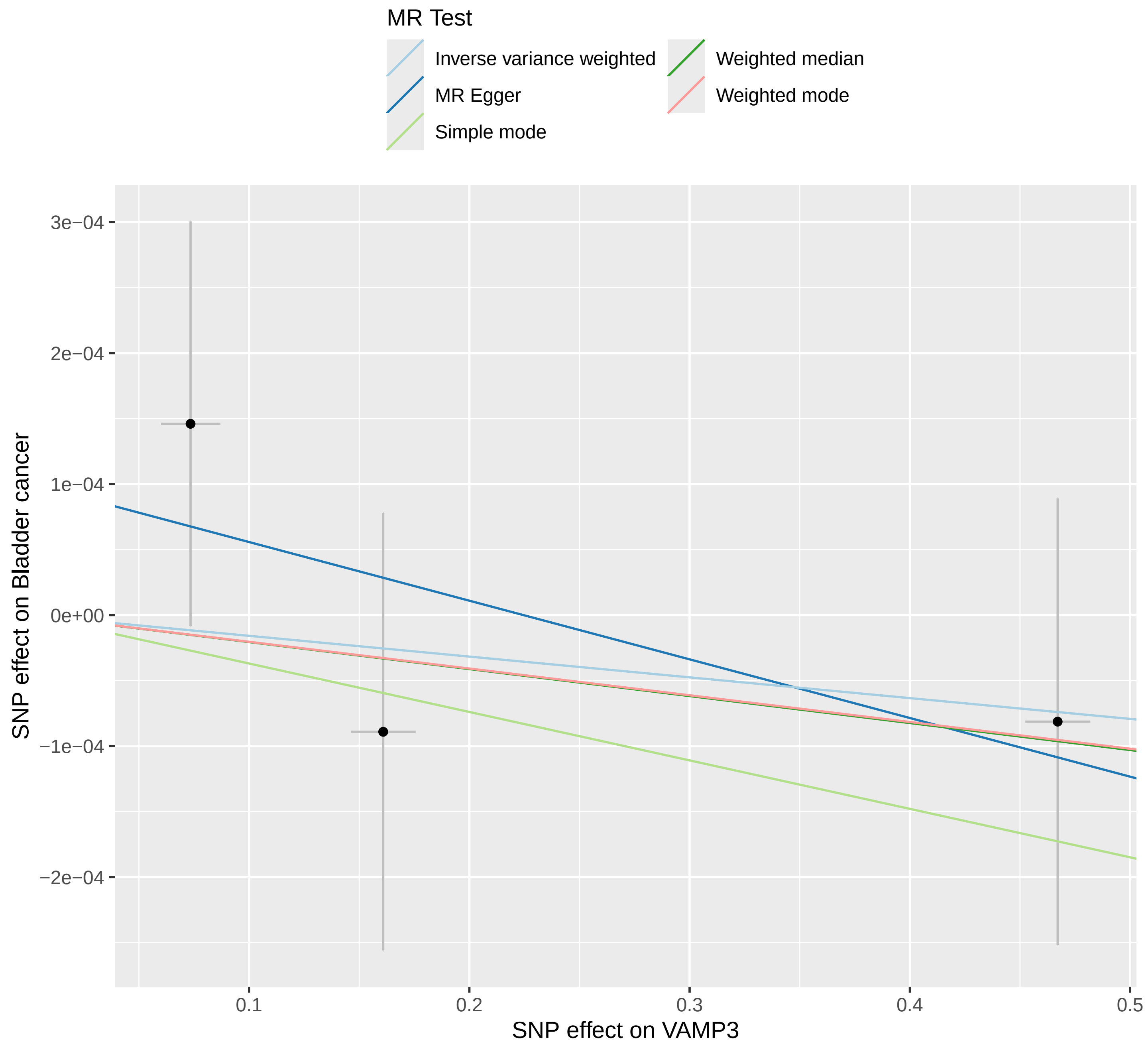

MR Test
Inverse variance weighted
MR Egger
Simple mode
Weighted median
Weighted mode
3e−04
2e−04
1e−04
0e+00
−1e−04
−2e−04
SNP effect on Bladder cancer
0.1
0.2
0.3
0.4
0.5
SNP effect on VAMP3

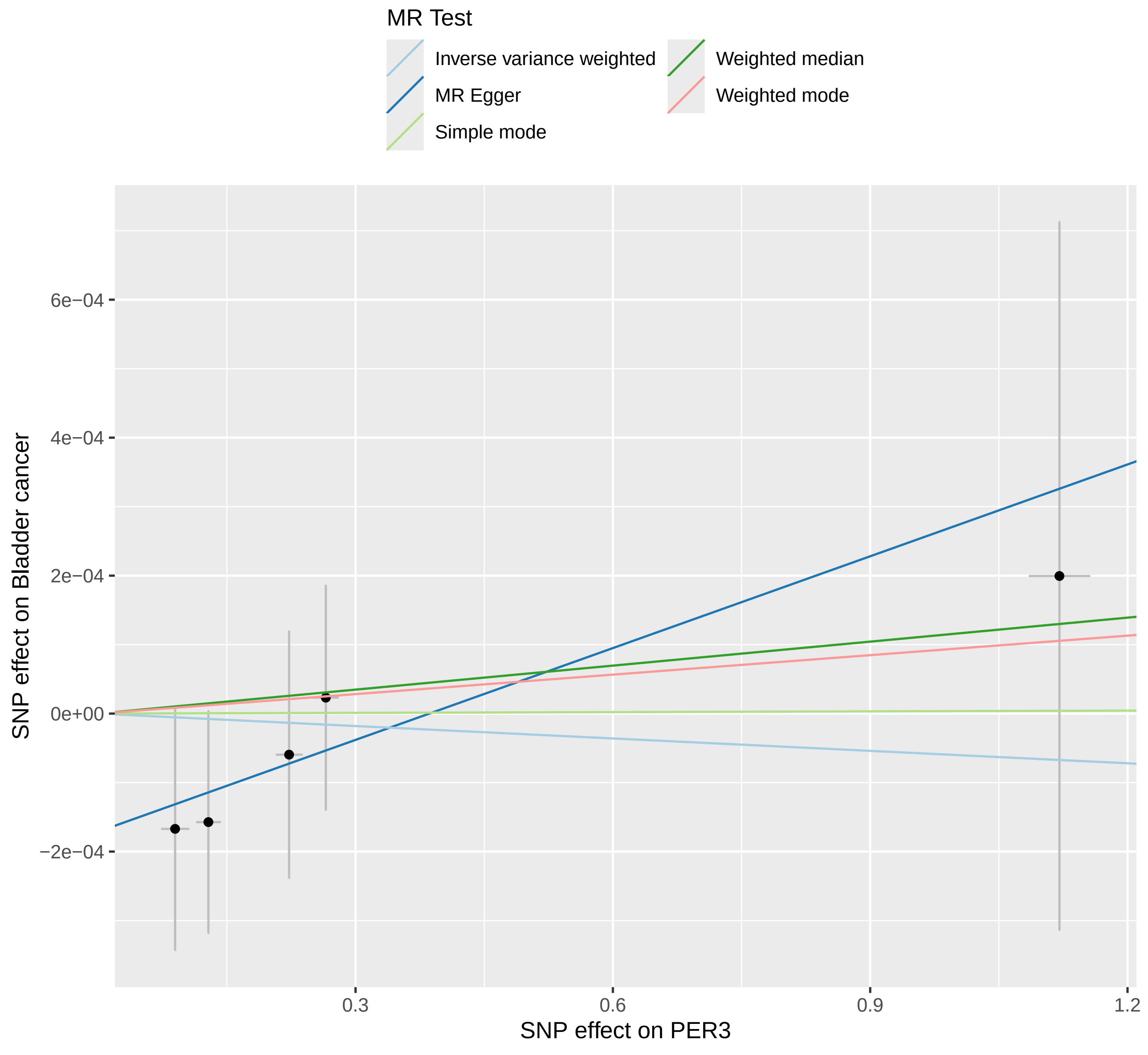
MR Test
Inverse variance weighted
MR Egger
Simple mode
Weighted median
Weighted mode
6e−04
4e−04
2e−04
0e+00
−2e−04
SNP effect on Bladder cancer
0.3
0.6
0.9
1.2
SNP effect on PER3

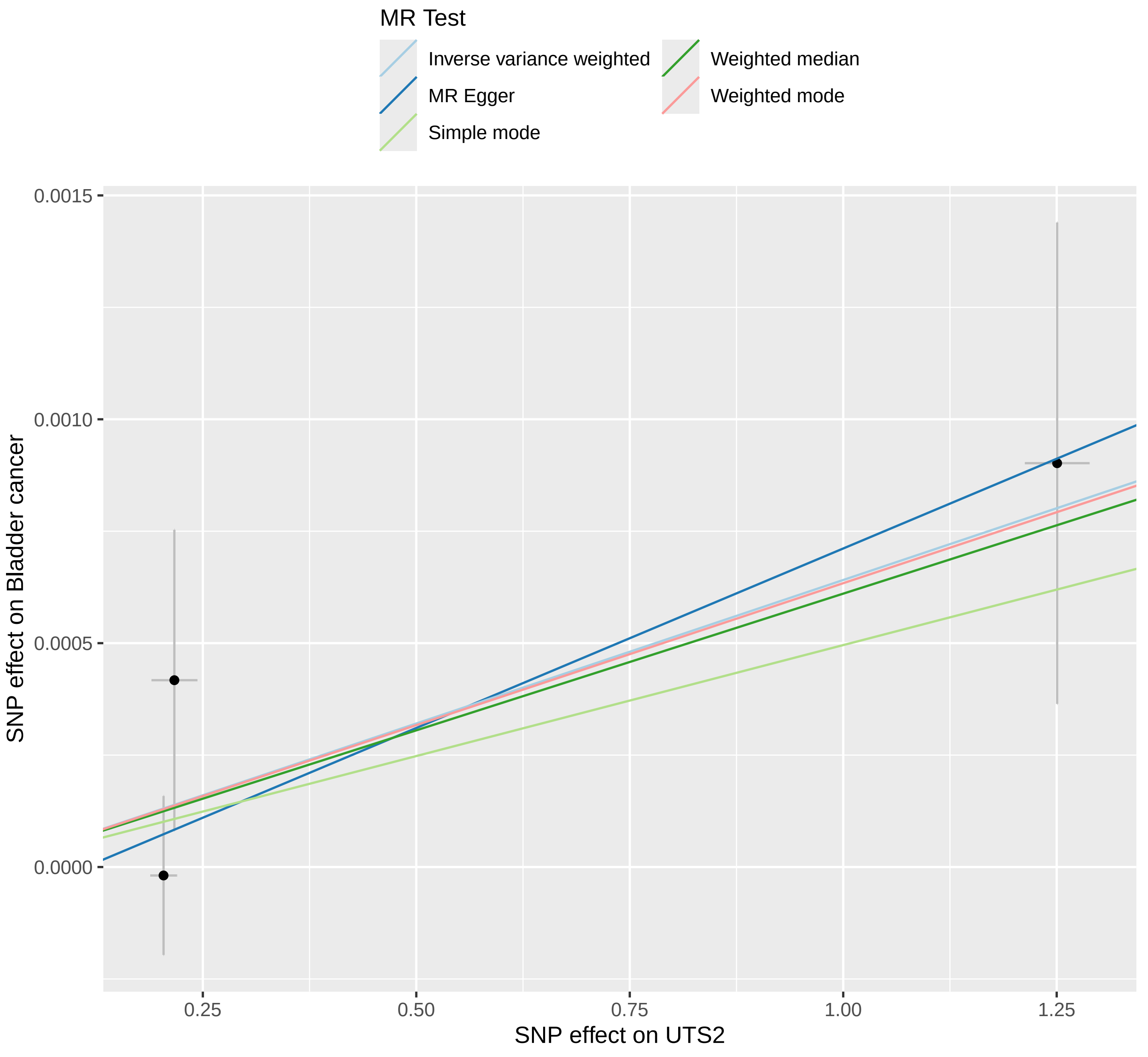
MR Test
Inverse variance weighted
MR Egger
Simple mode
Weighted median
Weighted mode
0.0015
0.0010
0.0005
0.0000
SNP effect on Bladder cancer
0.25
0.50
0.75
1.00
1.25
SNP effect on UTS2

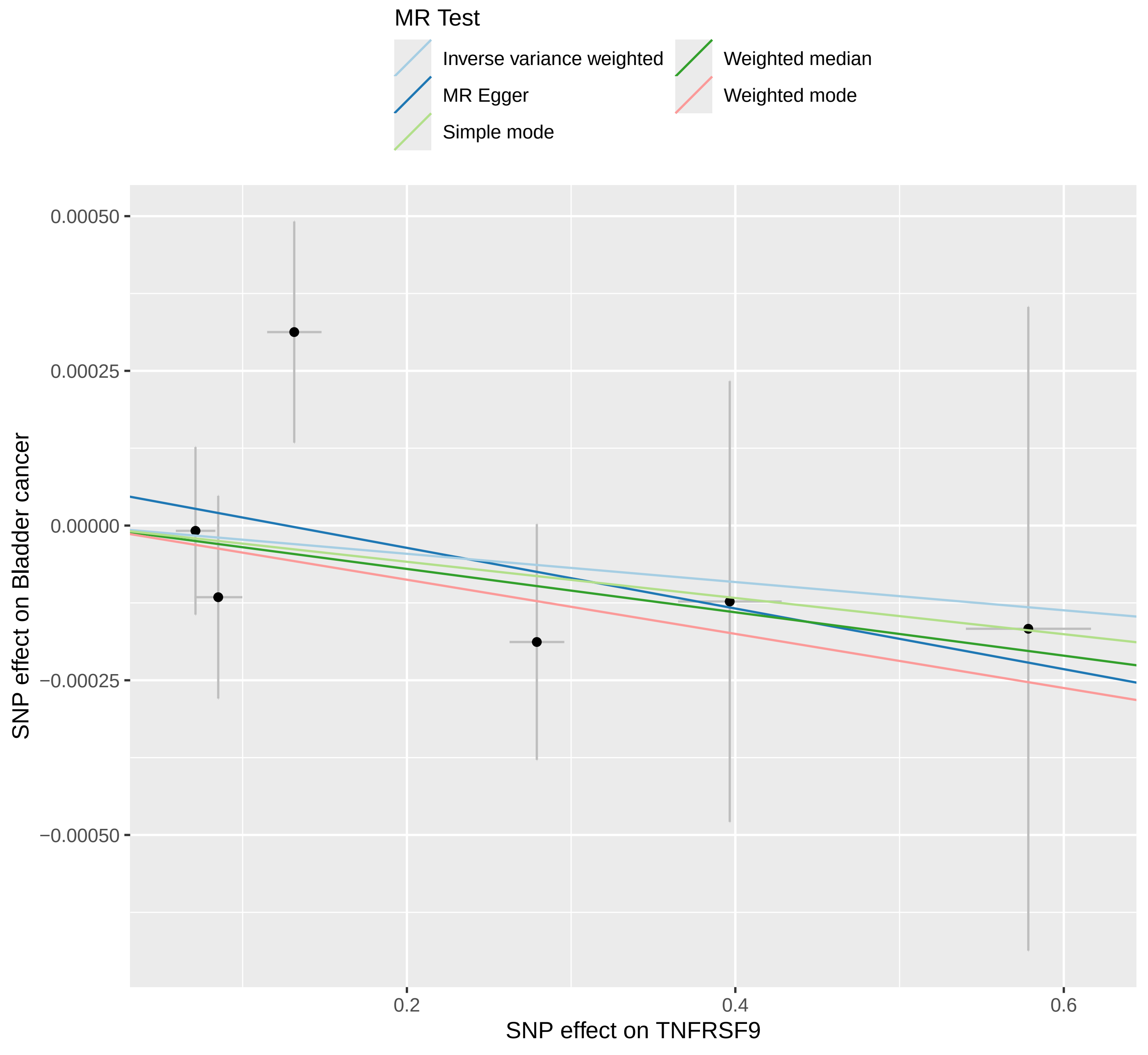
MR Test
Inverse variance weighted
MR Egger
Simple mode
Weighted median
Weighted mode
0.00050
0.00025
0.00000
−0.00025
−0.00050
SNP effect on Bladder cancer
0.2
0.4
0.6
SNP effect on TNFRSF9

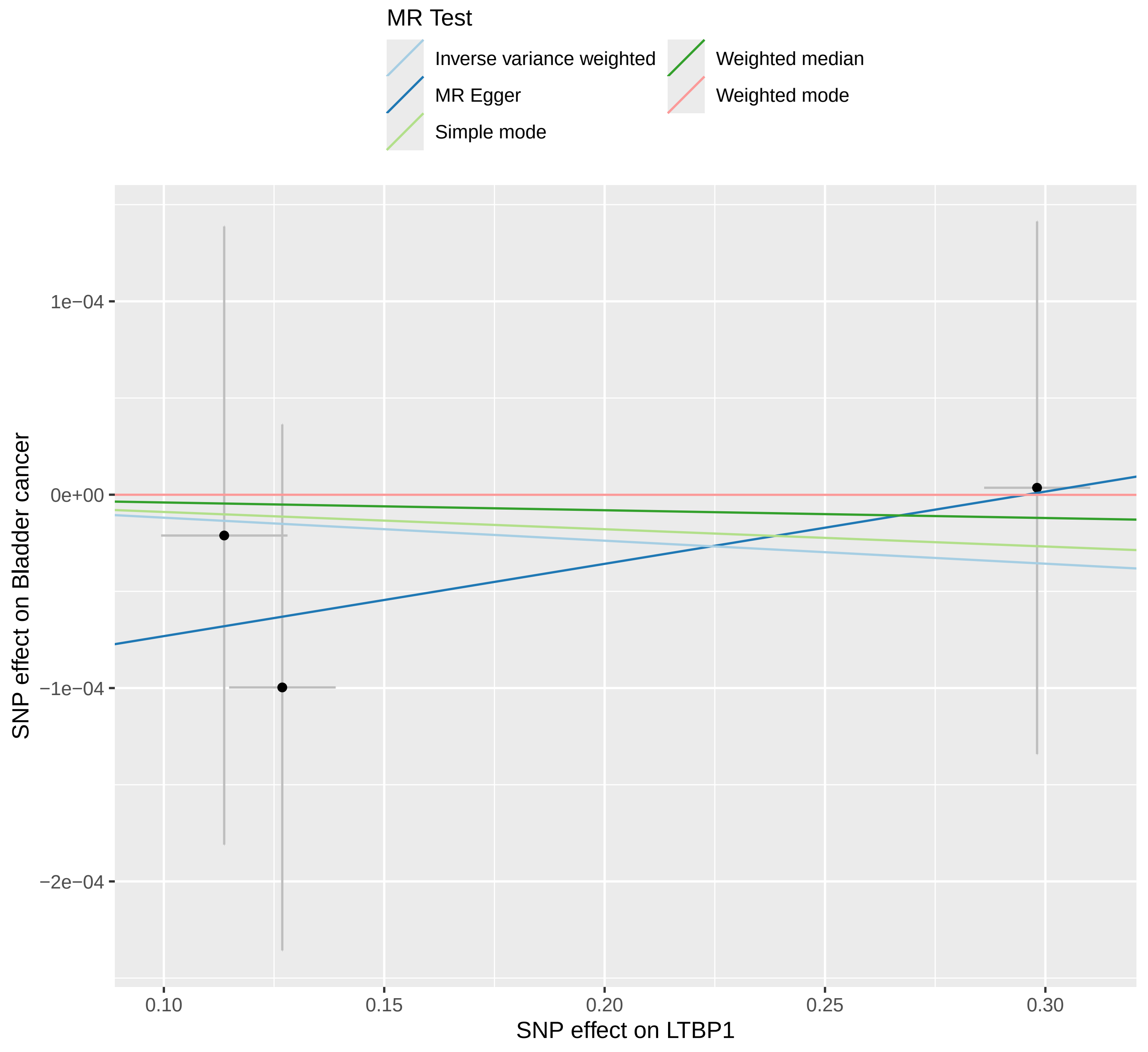
MR Test
Inverse variance weighted
MR Egger
Simple mode
Weighted median
Weighted mode
1e−04
0e+00
−1e−04
−2e−04
SNP effect on Bladder cancer
0.10
0.15
0.20
0.25
0.30
SNP effect on LTBP1

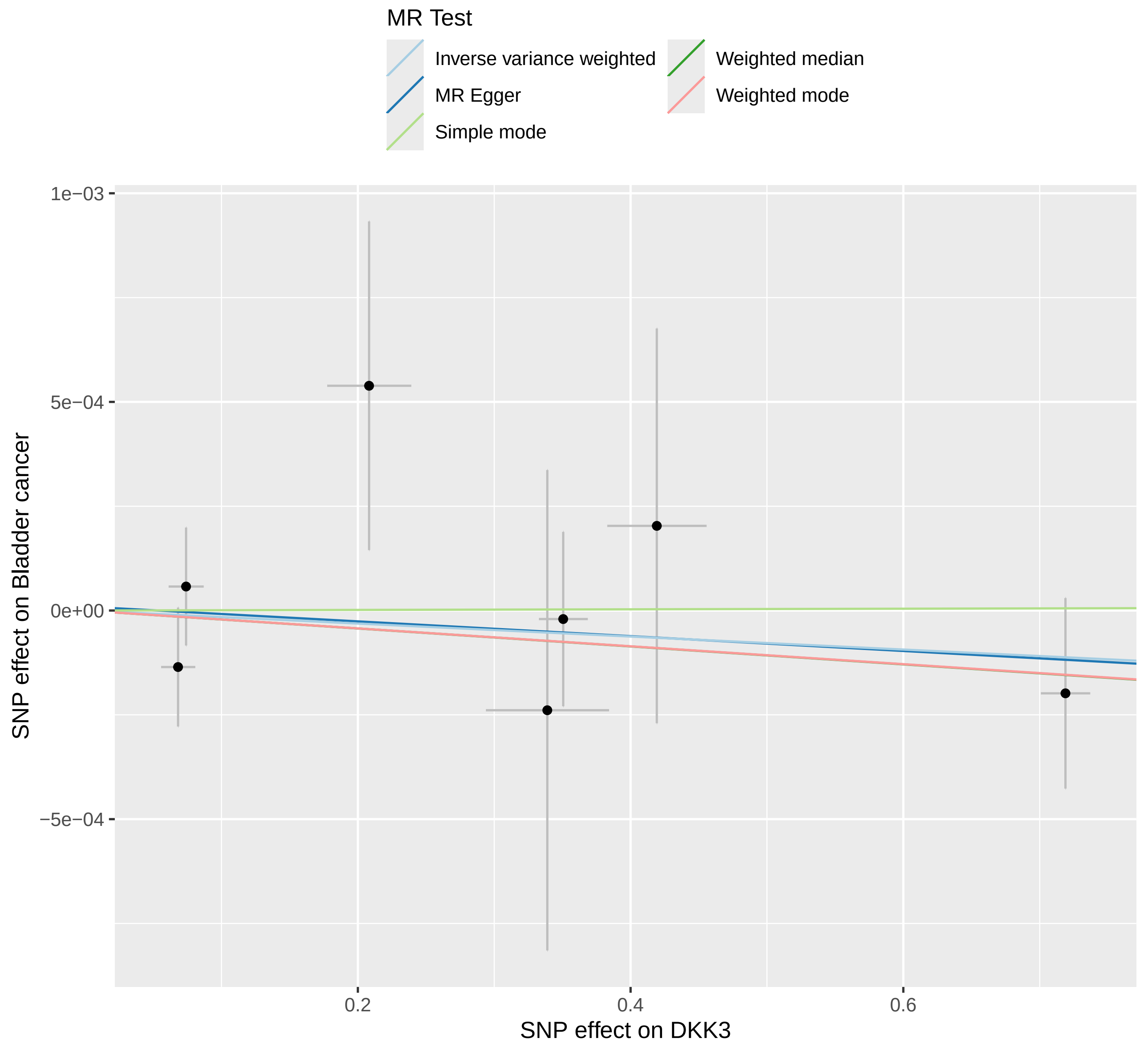

MR Test
Inverse variance weighted
MR Egger
Simple mode
Weighted median
Weighted mode
1e−03
5e−04
0e+00
−5e−04
0.2
0.4
0.6
SNP effect on Bladder cancer
SNP effect on DKK3

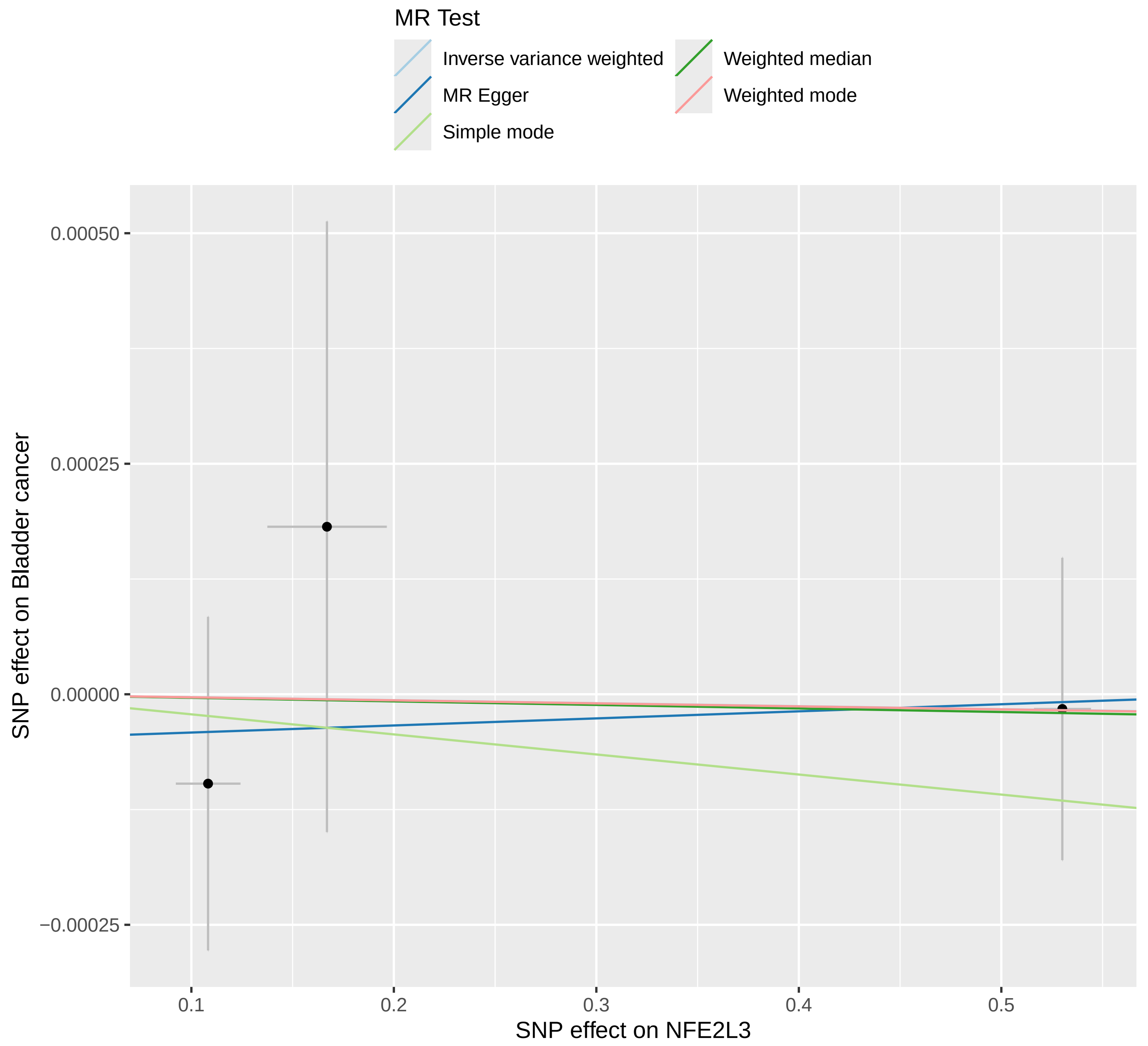

MR Test
Inverse variance weighted
MR Egger
Simple mode
Weighted median
Weighted mode
0.00050
0.00025
0.00000
−0.00025
SNP effect on Bladder cancer
0.1
0.2
0.3
0.4
0.5
SNP effect on NFE2L3

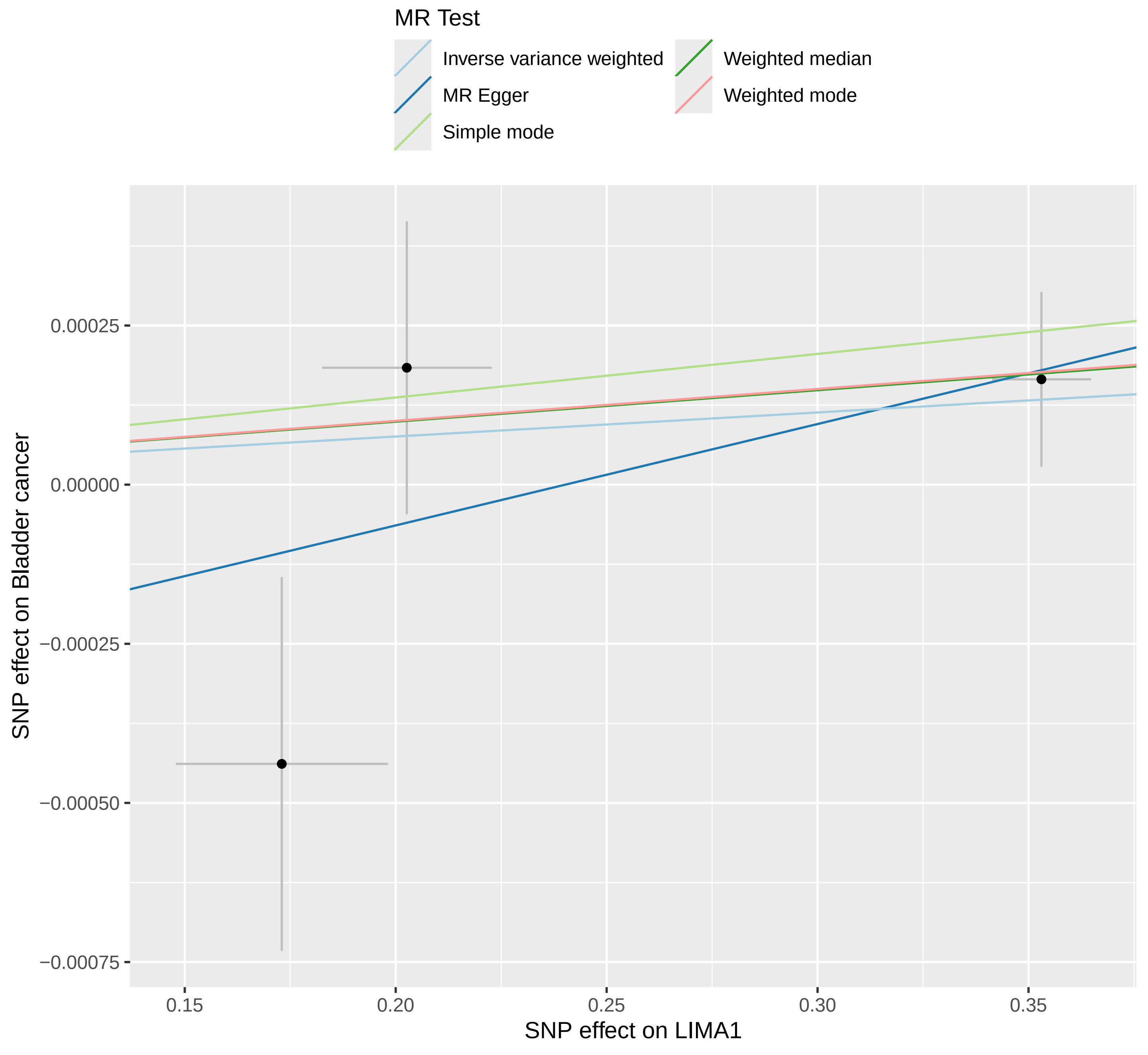

MR Test
Inverse variance weighted
MR Egger
Simple mode
Weighted median
Weighted mode
0.00025
0.00000
−0.00025
−0.00050
−0.00075
SNP effect on Bladder cancer
0.15
0.20
0.25
0.30
0.35
SNP effect on LIMA1

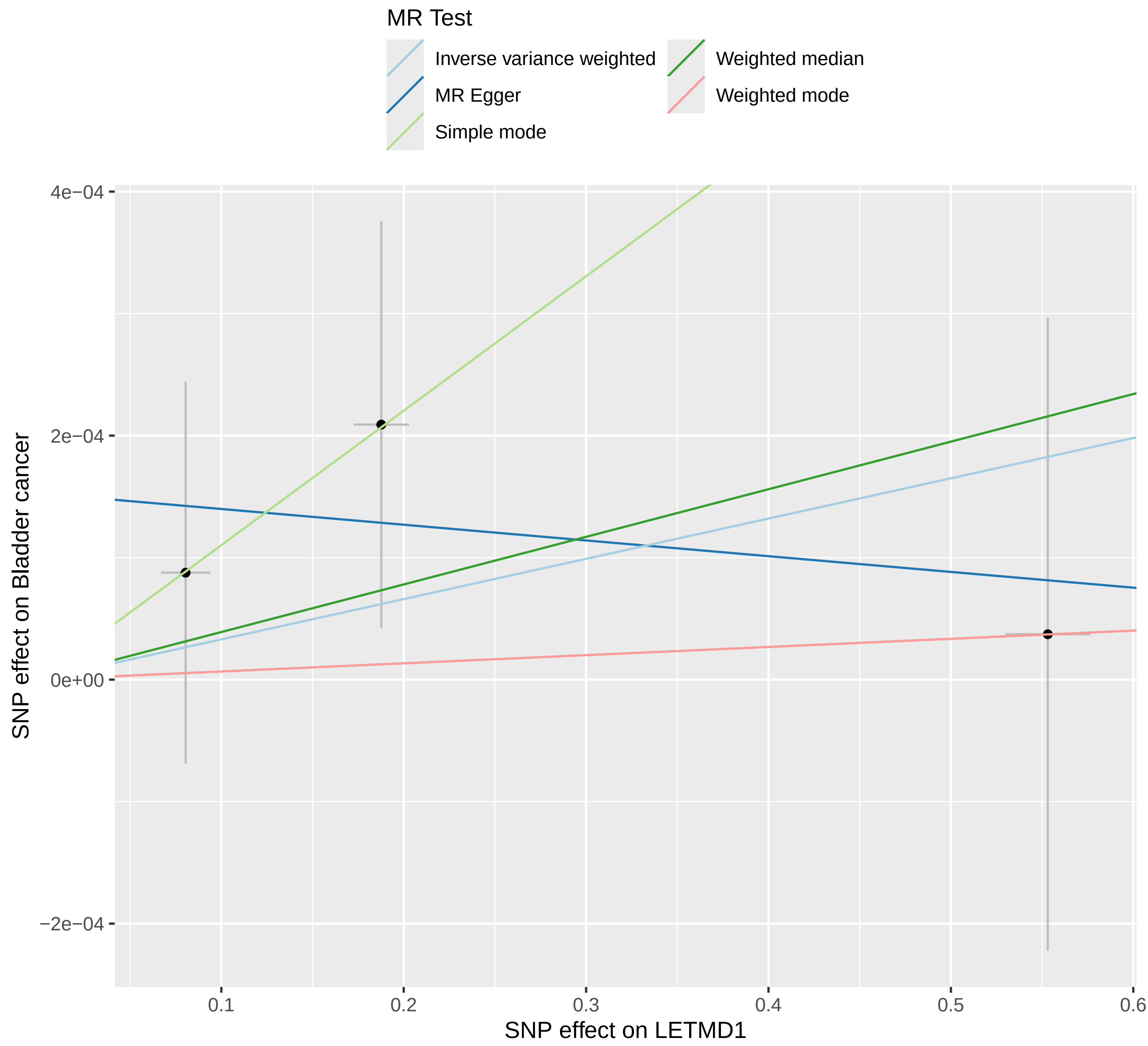

MR Test
Inverse variance weighted
MR Egger
Simple mode
Weighted median
Weighted mode
4e−04
2e−04
0e+00
−2e−04
0.1
0.2
0.3
0.4
0.5
0.6
SNP effect on Bladder cancer
SNP effect on LETMD1

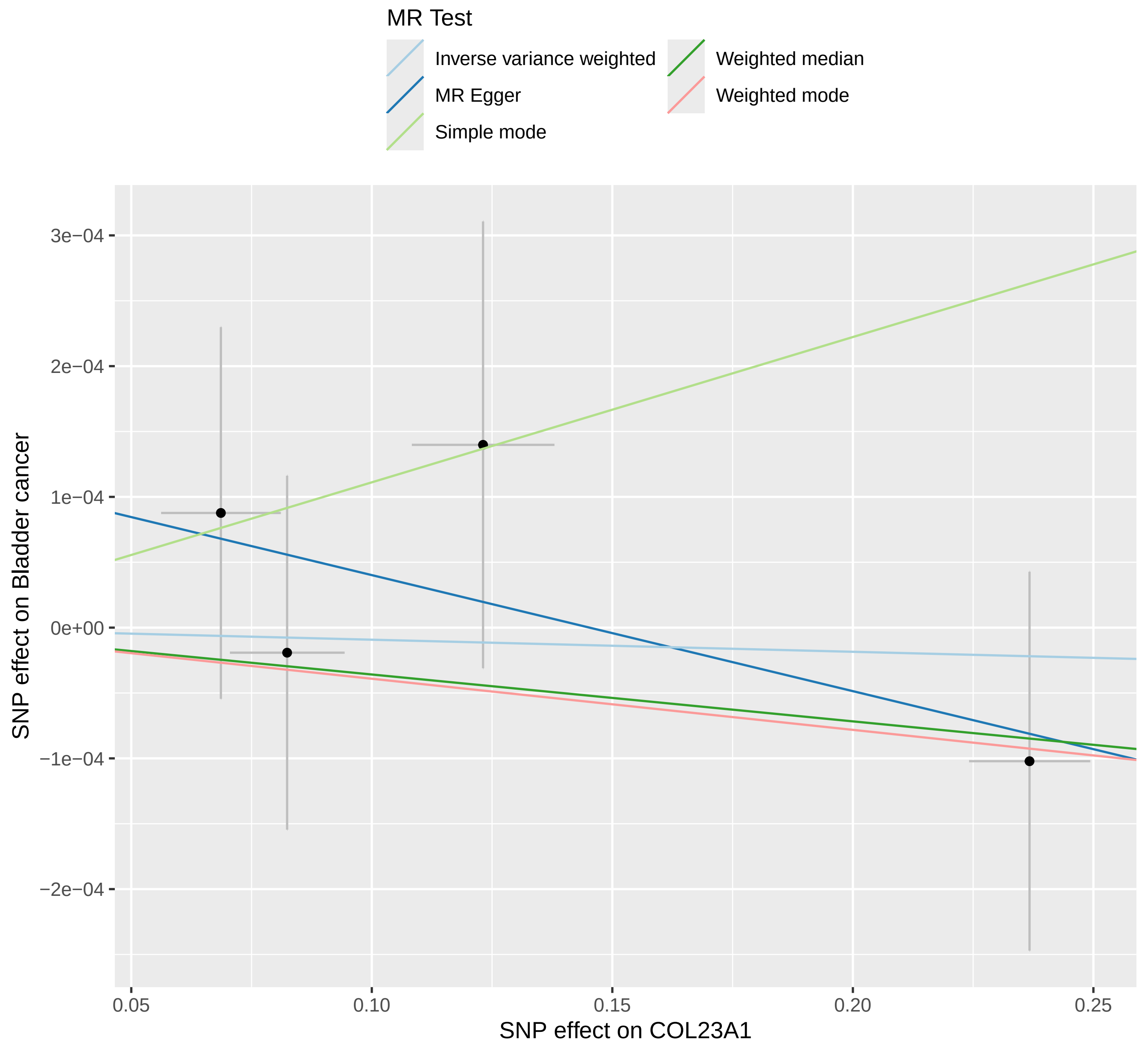
MR Test
Inverse variance weighted
MR Egger
Simple mode
Weighted median
Weighted mode
3e−04
2e−04
1e−04
0e+00
−1e−04
−2e−04
SNP effect on Bladder cancer
0.05
0.10
0.15
0.20
0.25
SNP effect on COL23A1

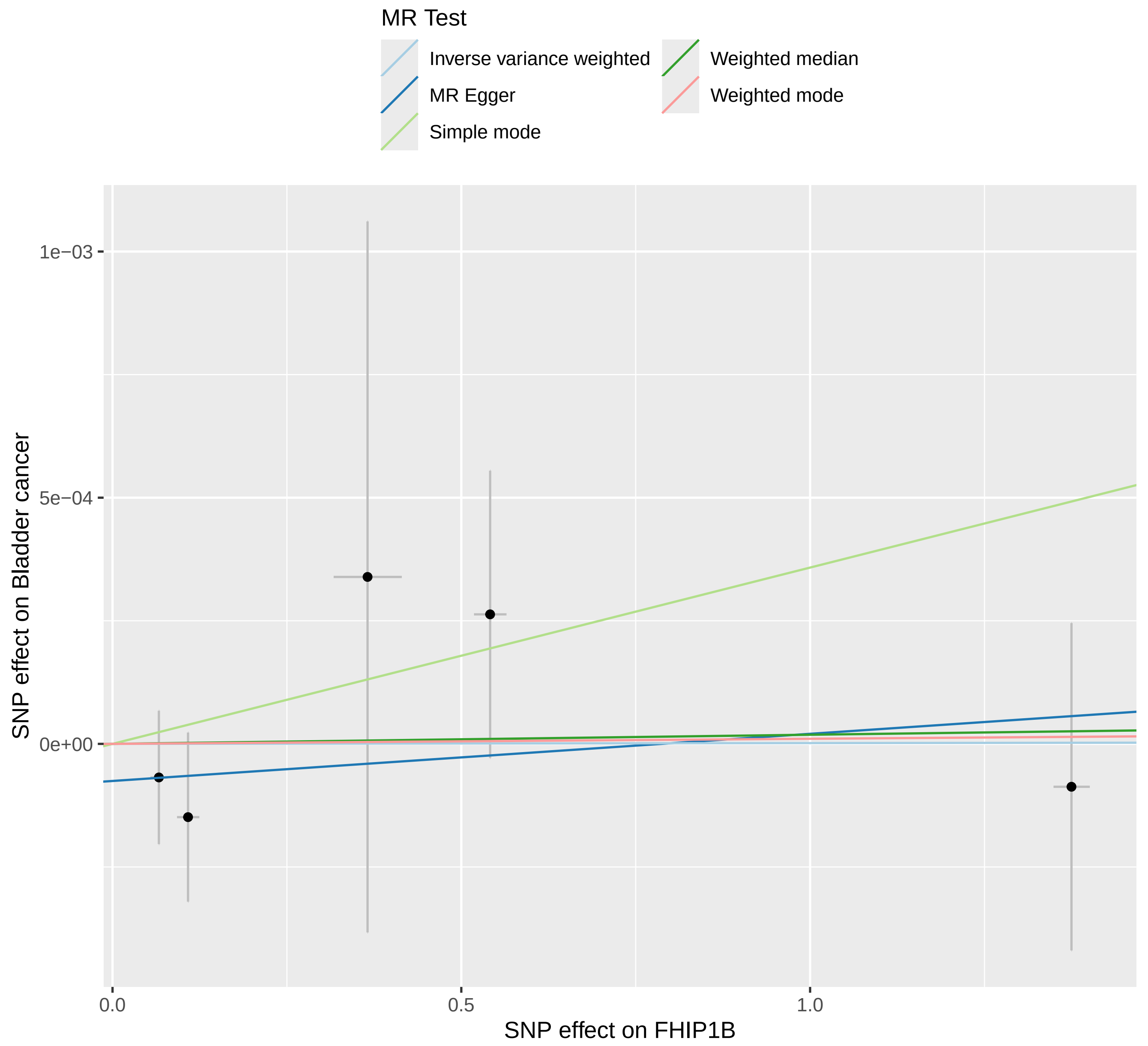
MR Test
Inverse variance weighted
MR Egger
Simple mode
Weighted median
Weighted mode
1e−03
5e−04
0e+00
SNP effect on Bladder cancer
0.0
0.5
1.0
SNP effect on FHIP1B

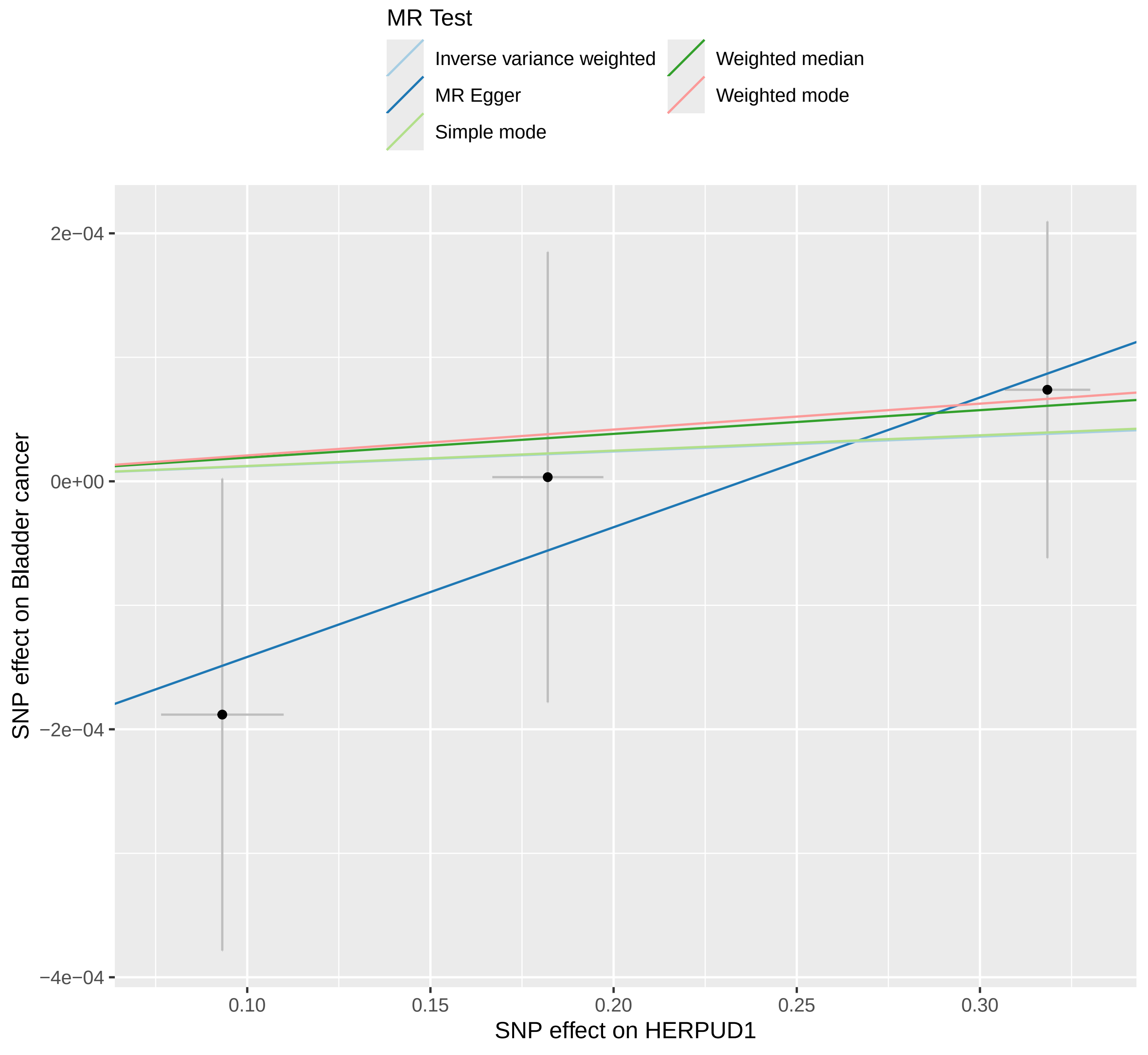
MR Test
Inverse variance weighted
MR Egger
Simple mode
Weighted median
Weighted mode
SNP effect on Bladder cancer
2e−04
0e+00
−2e−04
−4e−04
0.10
0.15
0.20
0.25
0.30
SNP effect on HERPUD1

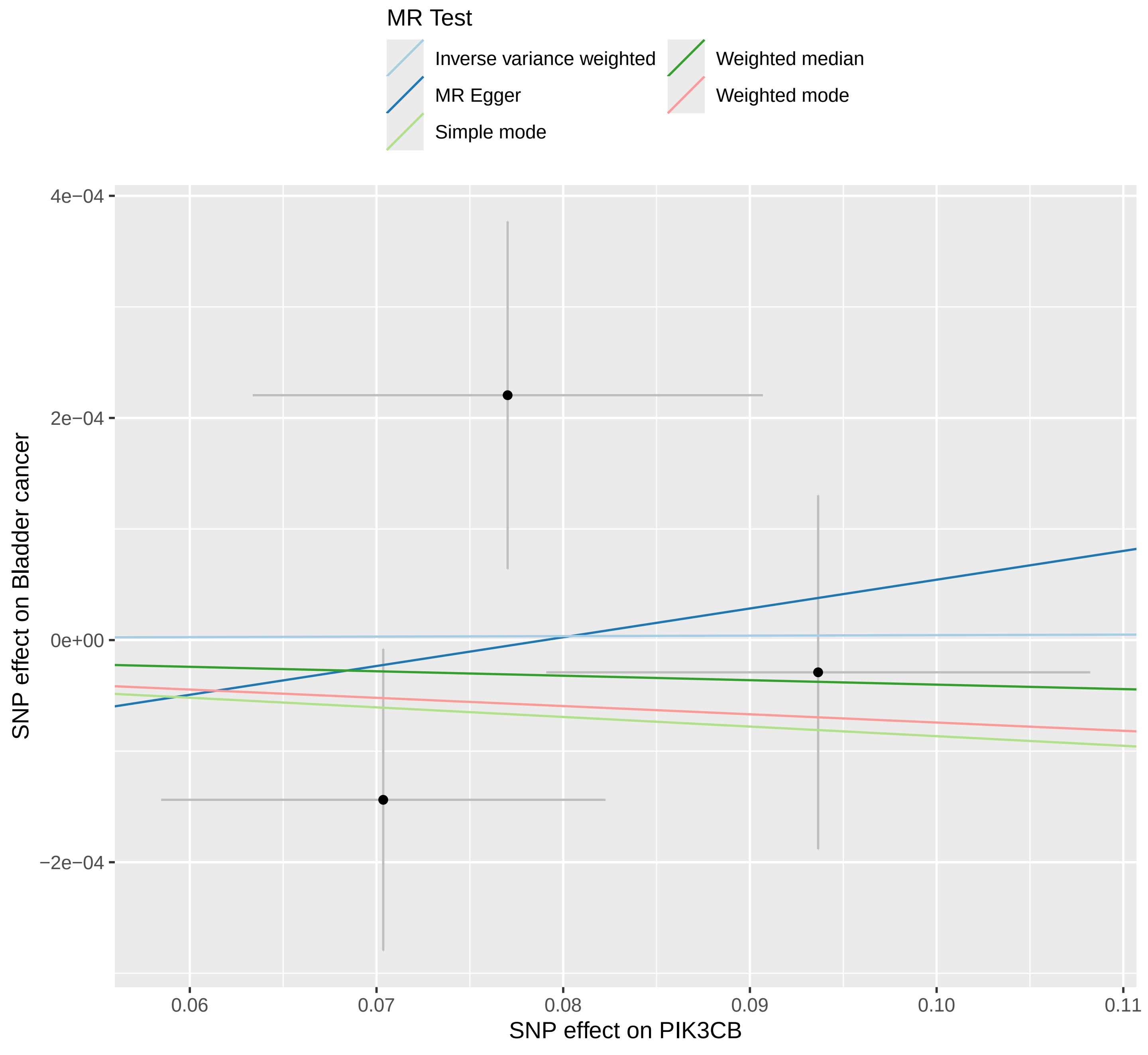

MR Test
Inverse variance weighted
MR Egger
Simple mode
Weighted median
Weighted mode
4e−04
2e−04
0e+00
−2e−04
SNP effect on Bladder cancer
0.06
0.07
0.08
0.09
0.10
0.11
SNP effect on PIK3CB

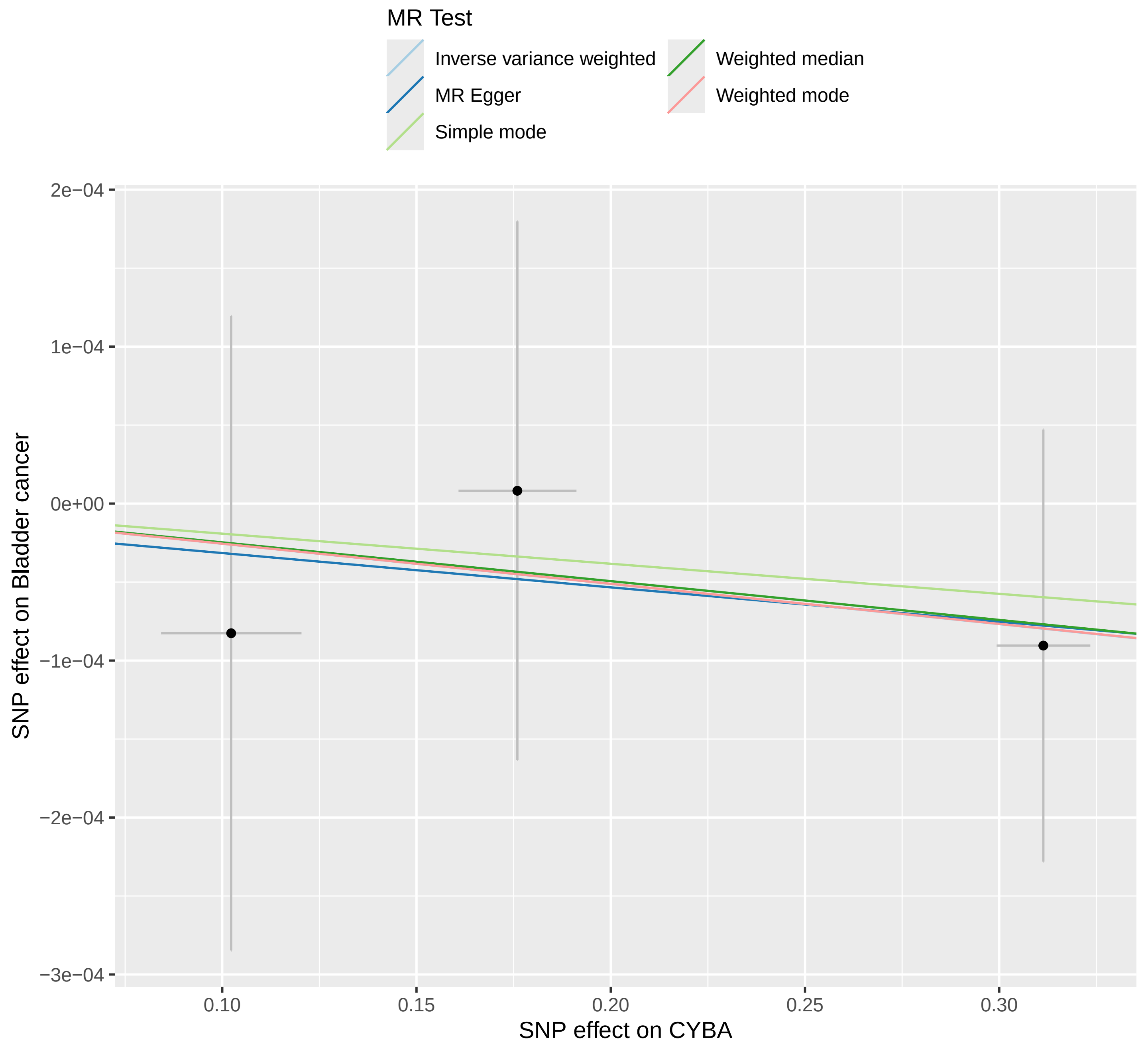

MR Test
Inverse variance weighted
MR Egger
Simple mode
Weighted median
Weighted mode
2e−04
1e−04
0e+00
−1e−04
−2e−04
−3e−04
SNP effect on Bladder cancer
0.10
0.15
0.20
0.25
0.30
SNP effect on CYBA

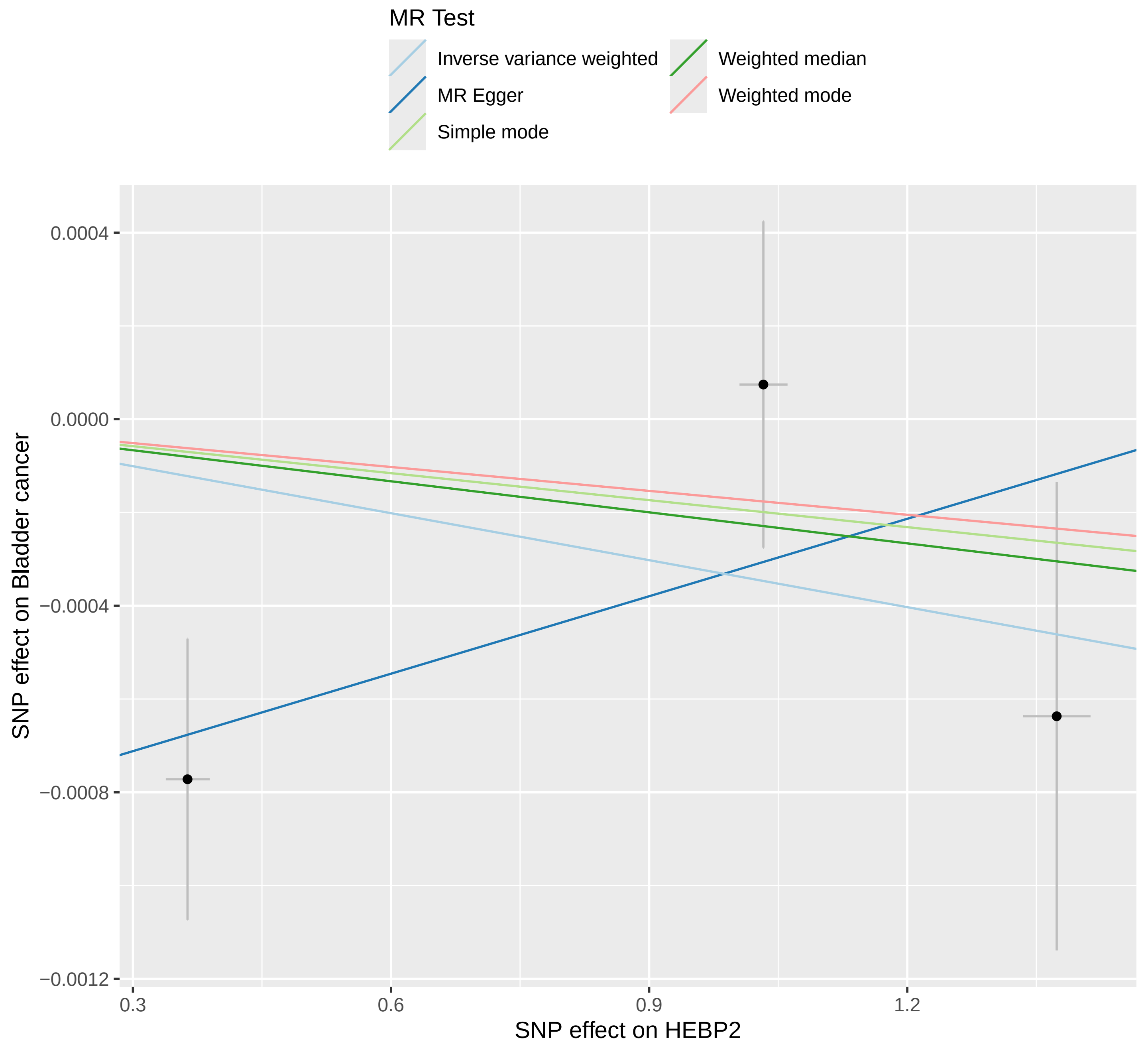

MR Test
Inverse variance weighted
MR Egger
Simple mode
Weighted median
Weighted mode
0.0004
0.0000
−0.0004
−0.0008
−0.0012
SNP effect on Bladder cancer
0.3
0.6
0.9
1.2
SNP effect on HEBP2

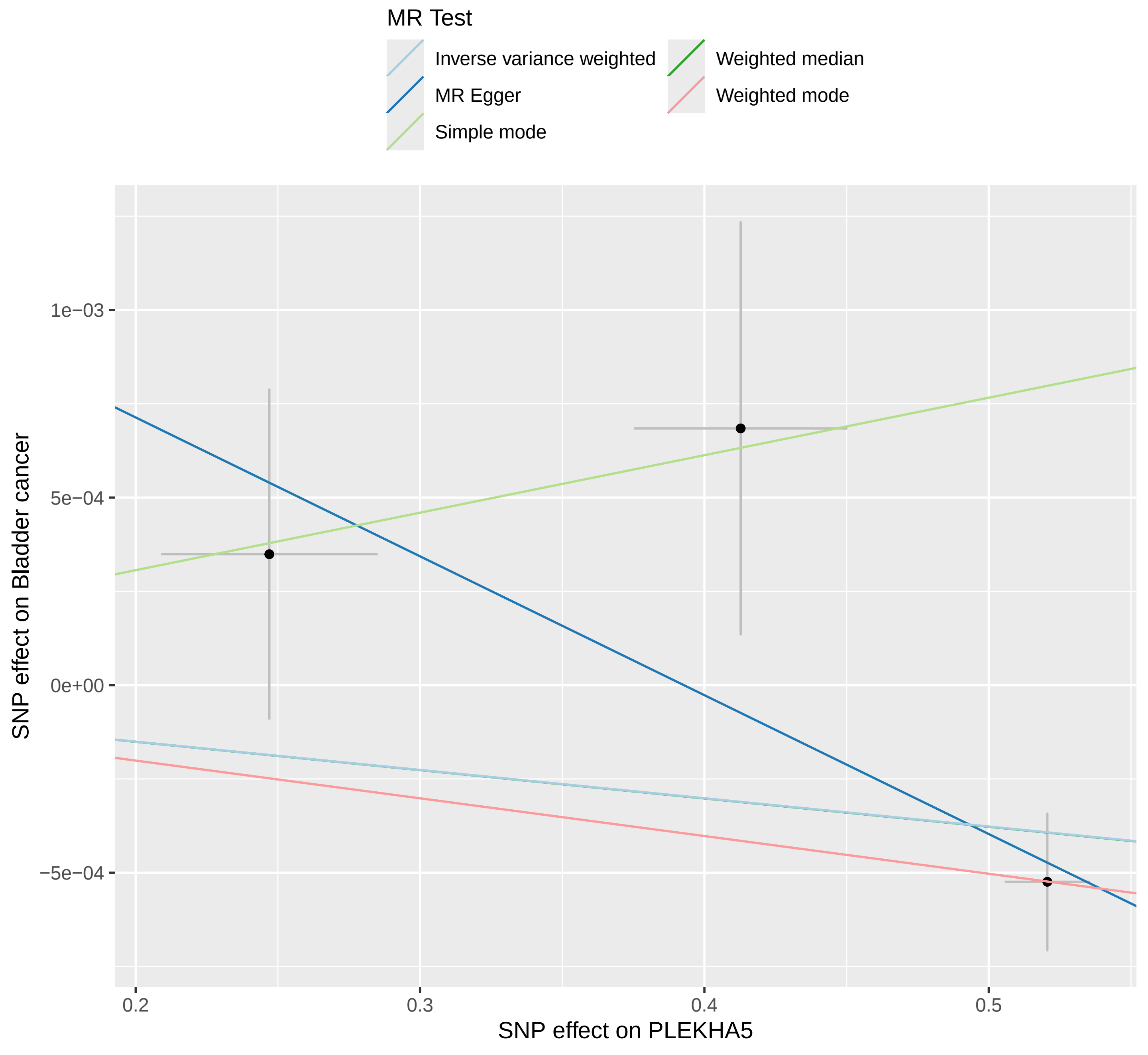
MR Test
Inverse variance weighted
MR Egger
Simple mode
Weighted median
Weighted mode
1e−03
5e−04
0e+00
−5e−04
0.2
0.3
0.4
0.5
SNP effect on Bladder cancer
SNP effect on PLEKHA5

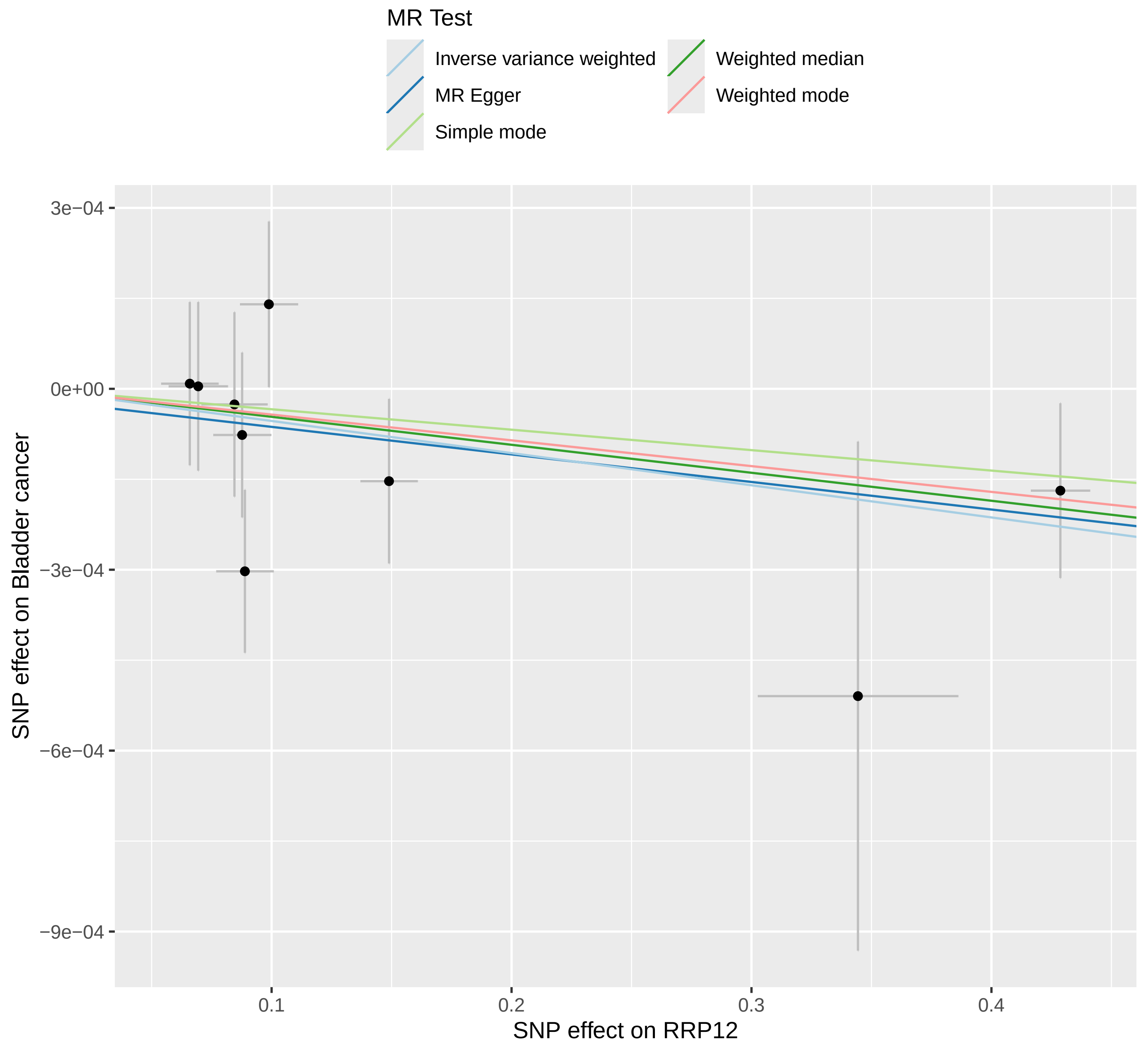
MR Test
Inverse variance weighted
MR Egger
Simple mode
Weighted median
Weighted mode
3e−04
0e+00
−3e−04
−6e−04
−9e−04
SNP effect on Bladder cancer
0.1
0.2
0.3
0.4
SNP effect on RRP12

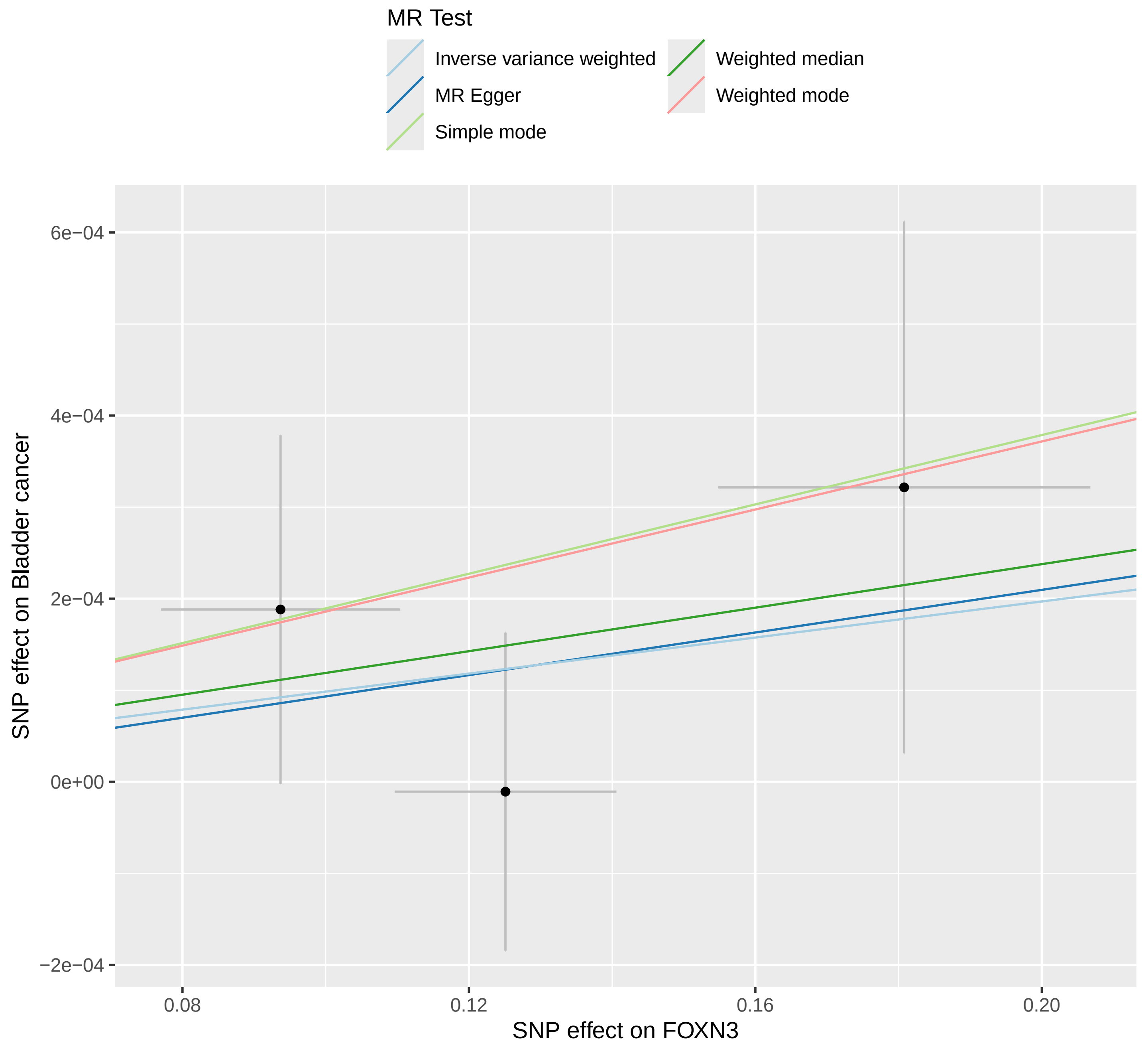
MR Test
Inverse variance weighted
MR Egger
Simple mode
Weighted median
Weighted mode
SNP effect on Bladder cancer
6e−04
4e−04
2e−04
0e+00
−2e−04
0.08
0.12
0.16
0.20
SNP effect on FOXN3

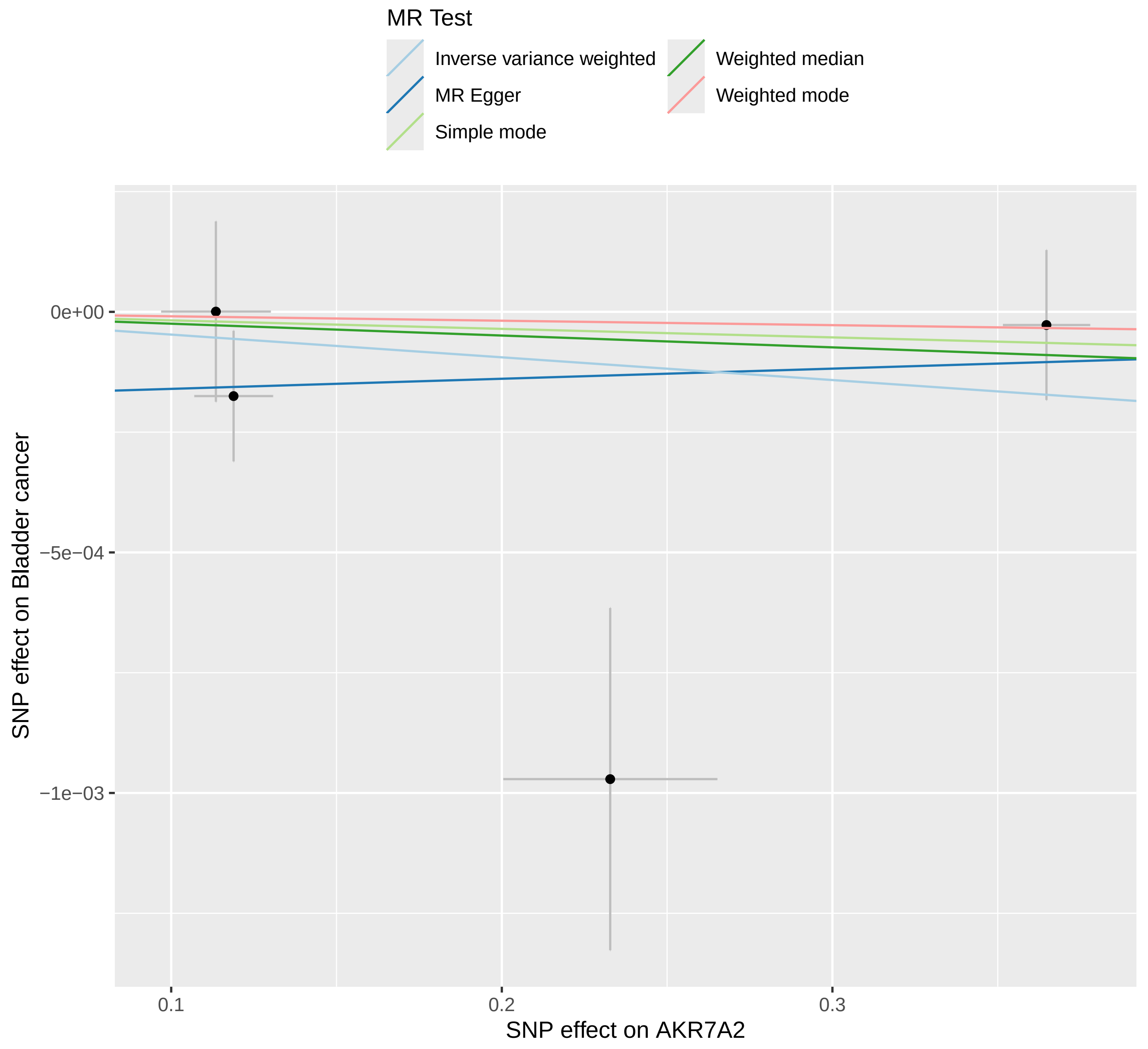

MR Test
Inverse variance weighted
MR Egger
Simple mode
Weighted median
Weighted mode
0e+00
−5e−04
−1e−03
0.1
0.2
0.3
SNP effect on Bladder cancer
SNP effect on AKR7A2

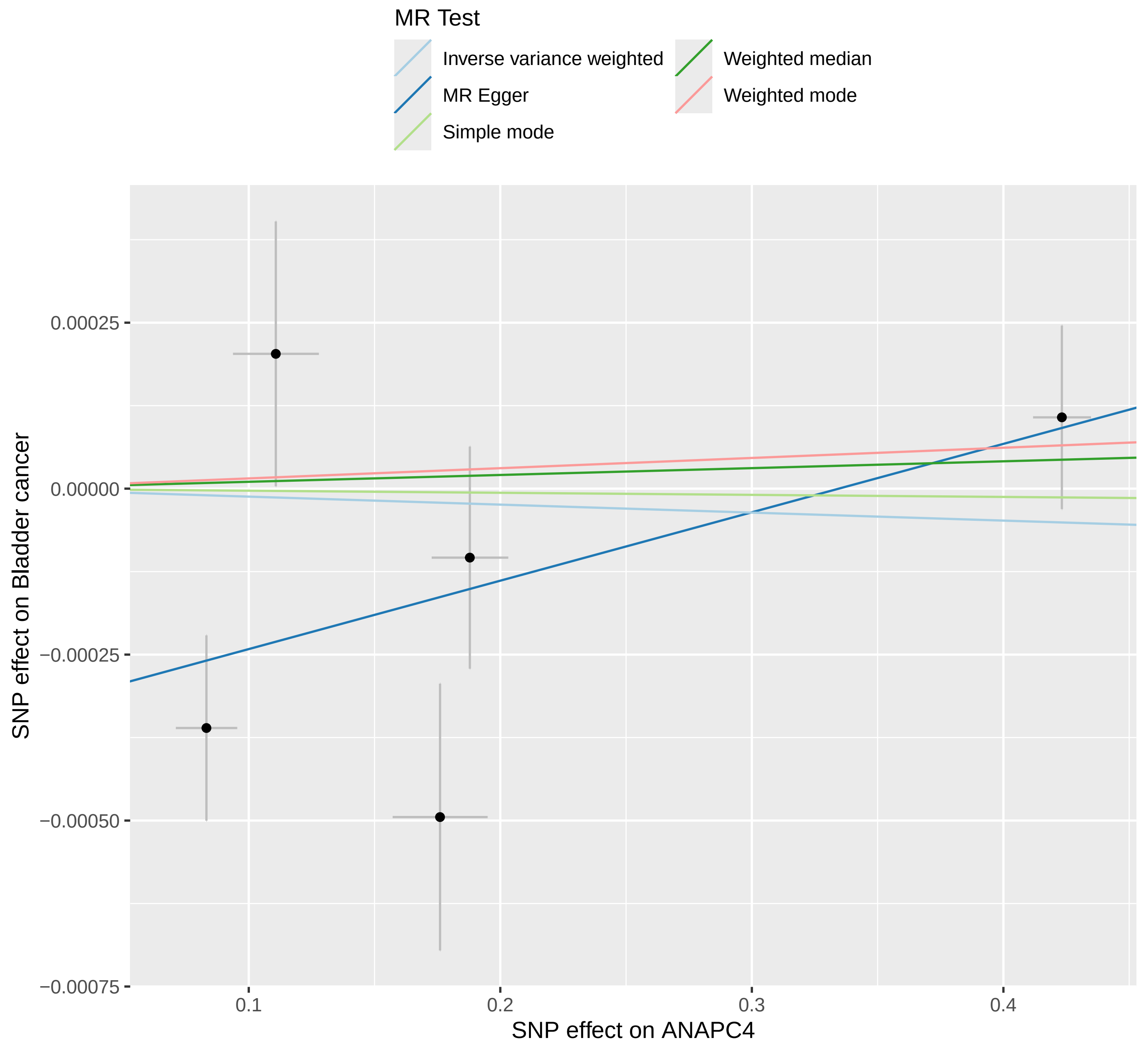

MR Test
Inverse variance weighted
MR Egger
Simple mode
Weighted median
Weighted mode
SNP effect on Bladder cancer
0.00025
0.00000
−0.00025
−0.00050
−0.00075
0.1
0.2
0.3
0.4
SNP effect on ANAPC4

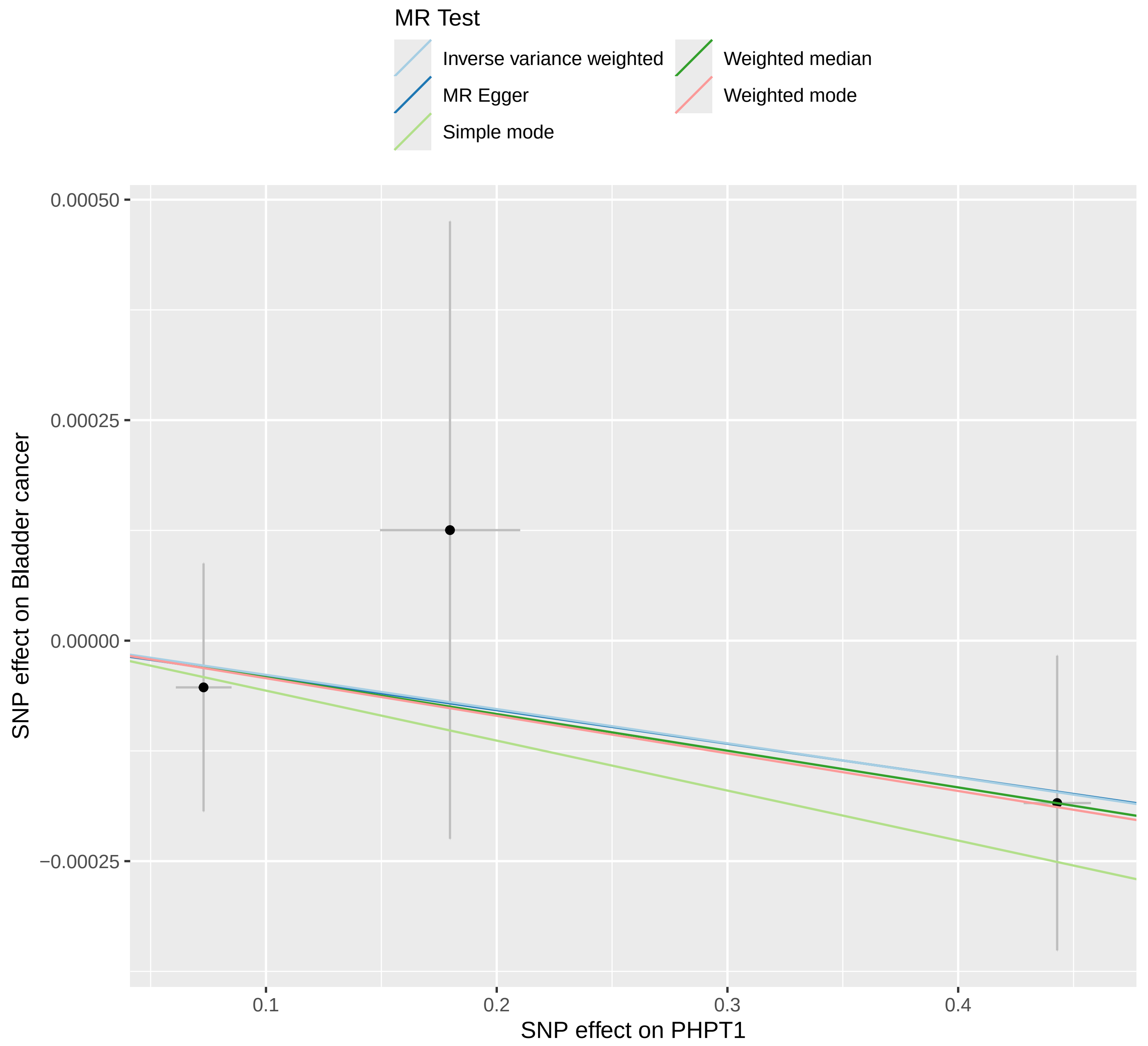

MR Test
Inverse variance weighted
MR Egger
Simple mode
Weighted median
Weighted mode
0.00050
0.00025
0.00000
−0.00025
SNP effect on Bladder cancer
0.1
0.2
0.3
0.4
SNP effect on PHPT1

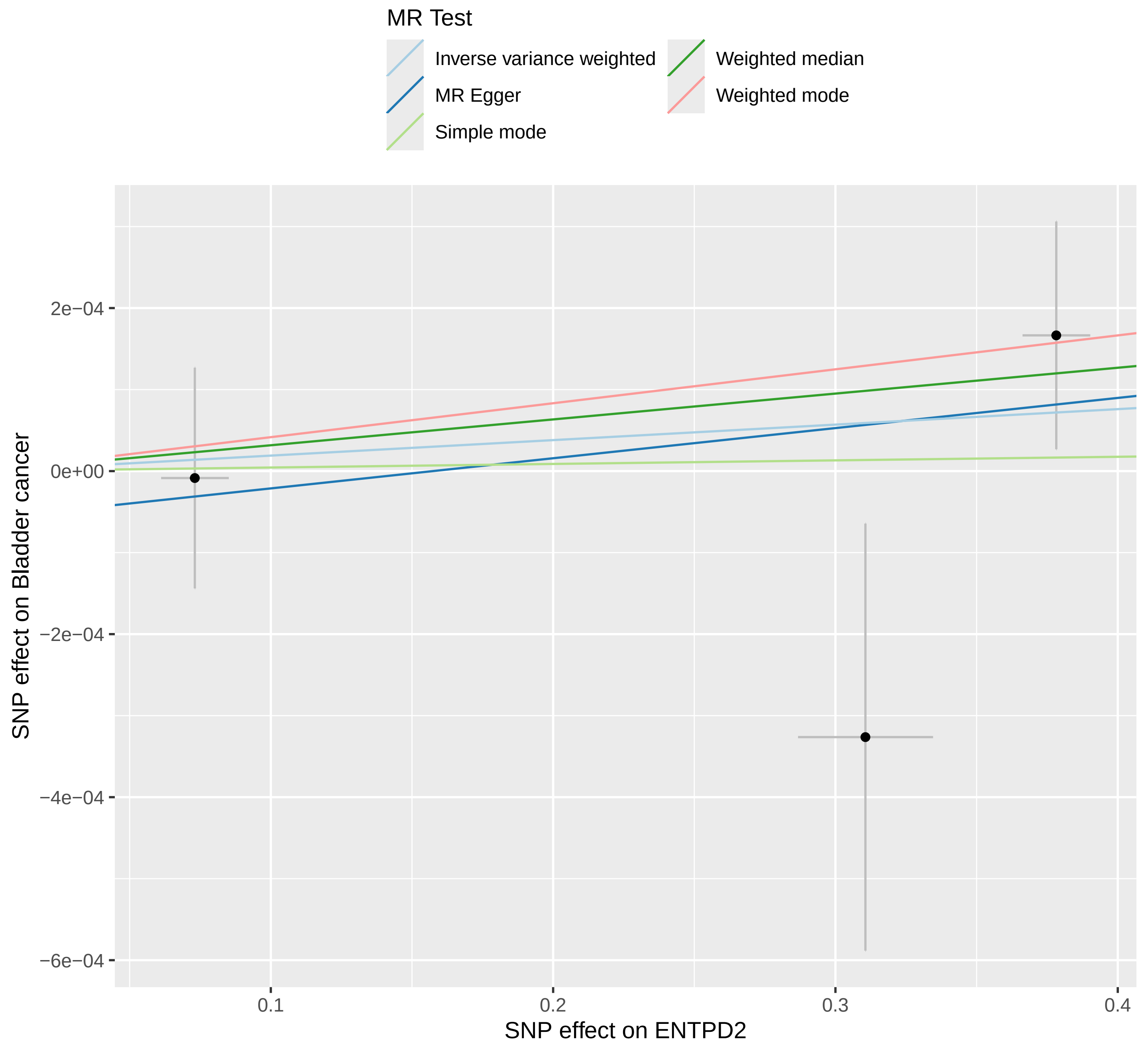

MR Test
Inverse variance weighted
MR Egger
Simple mode
Weighted median
Weighted mode
2e−04
0e+00
−2e−04
−4e−04
−6e−04
SNP effect on Bladder cancer
0.1
0.2
0.3
0.4
SNP effect on ENTPD2

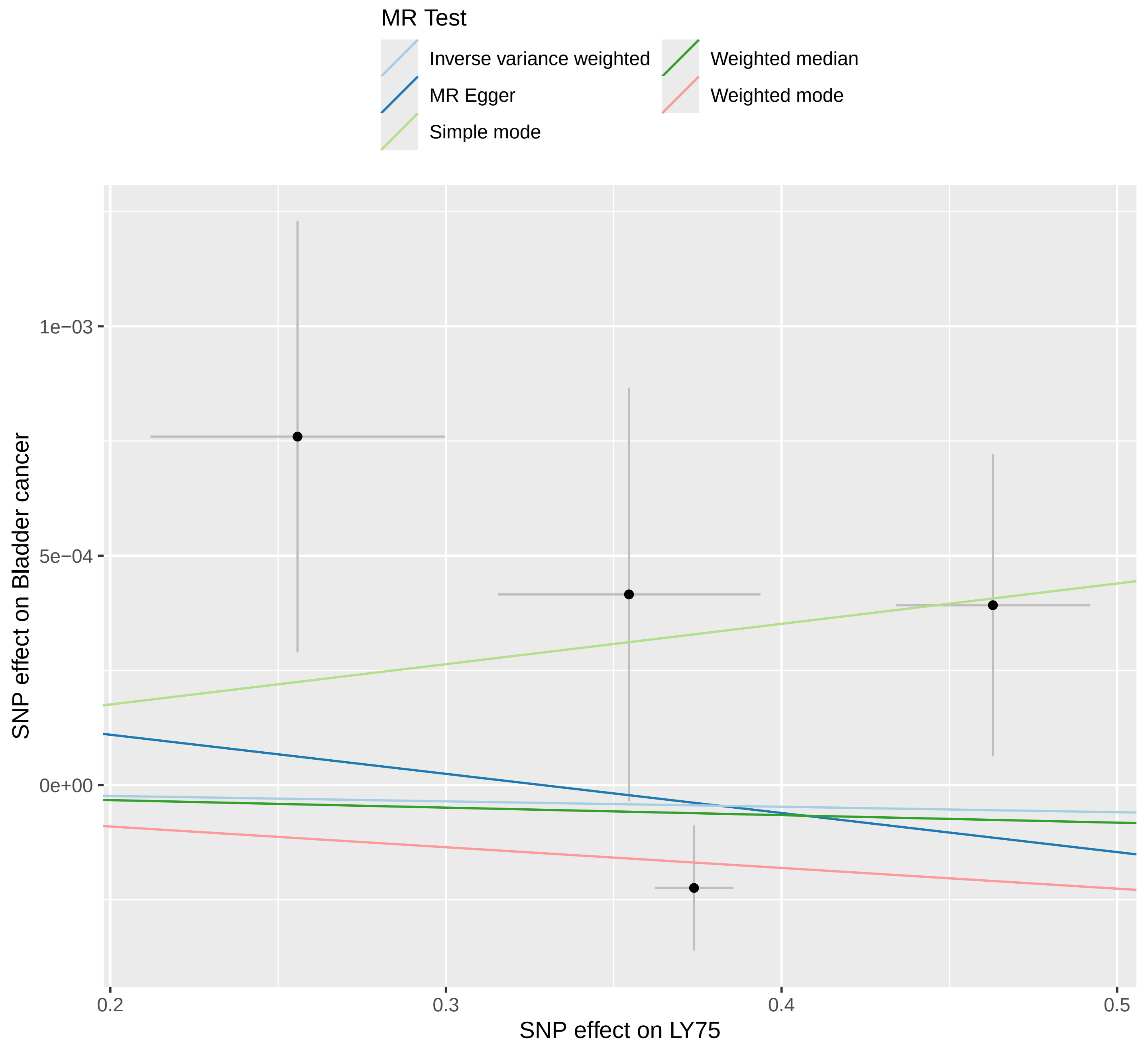

MR Test
Inverse variance weighted
MR Egger
Simple mode
Weighted median
Weighted mode
1e−03
5e−04
0e+00
0.2
0.3
0.4
0.5
SNP effect on LY75
SNP effect on Bladder cancer

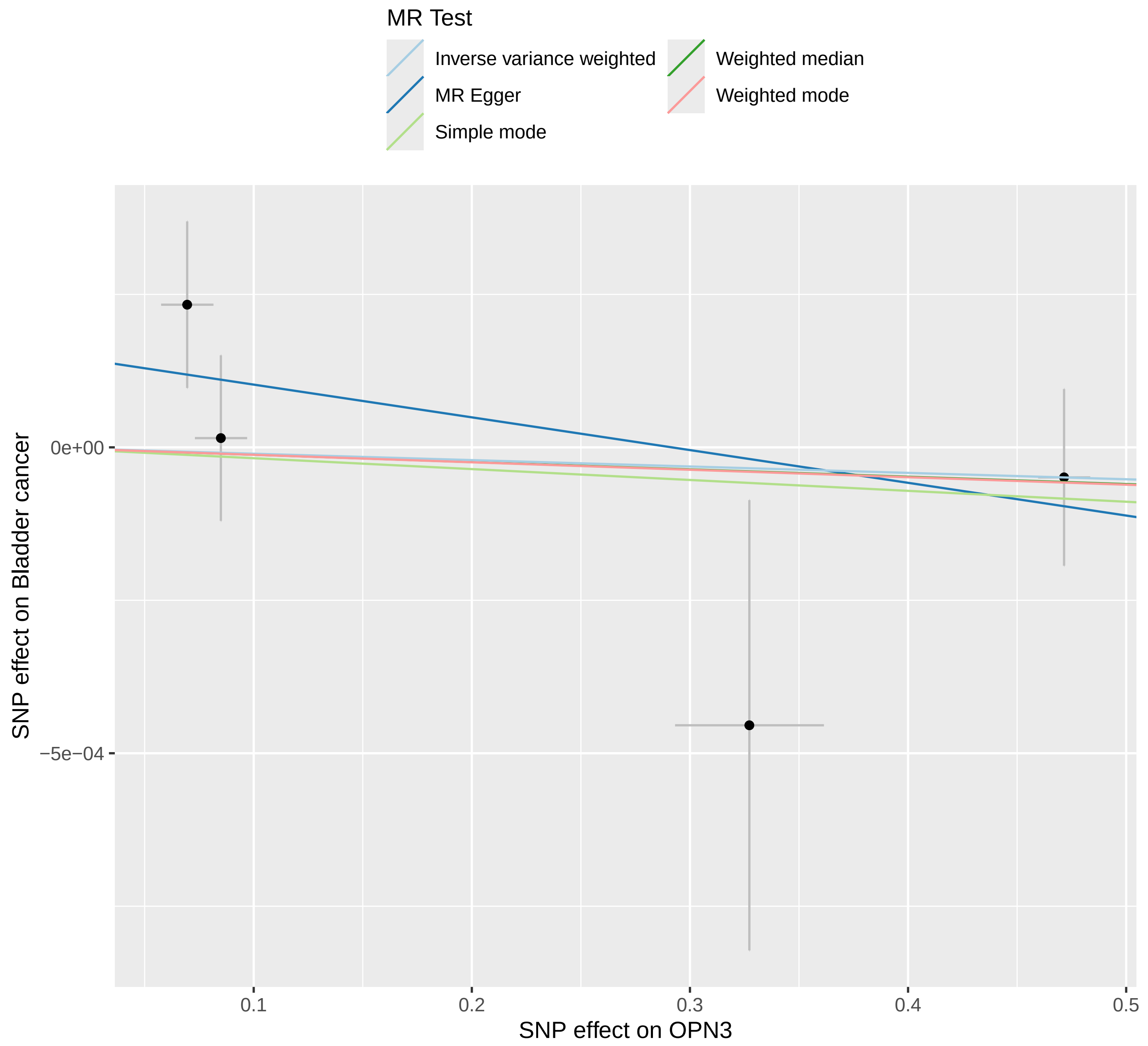
MR Test
Inverse variance weighted
MR Egger
Simple mode
Weighted median
Weighted mode
0e+00
−5e−04
0.1
0.2
0.3
0.4
0.5
SNP effect on OPN3
SNP effect on Bladder cancer

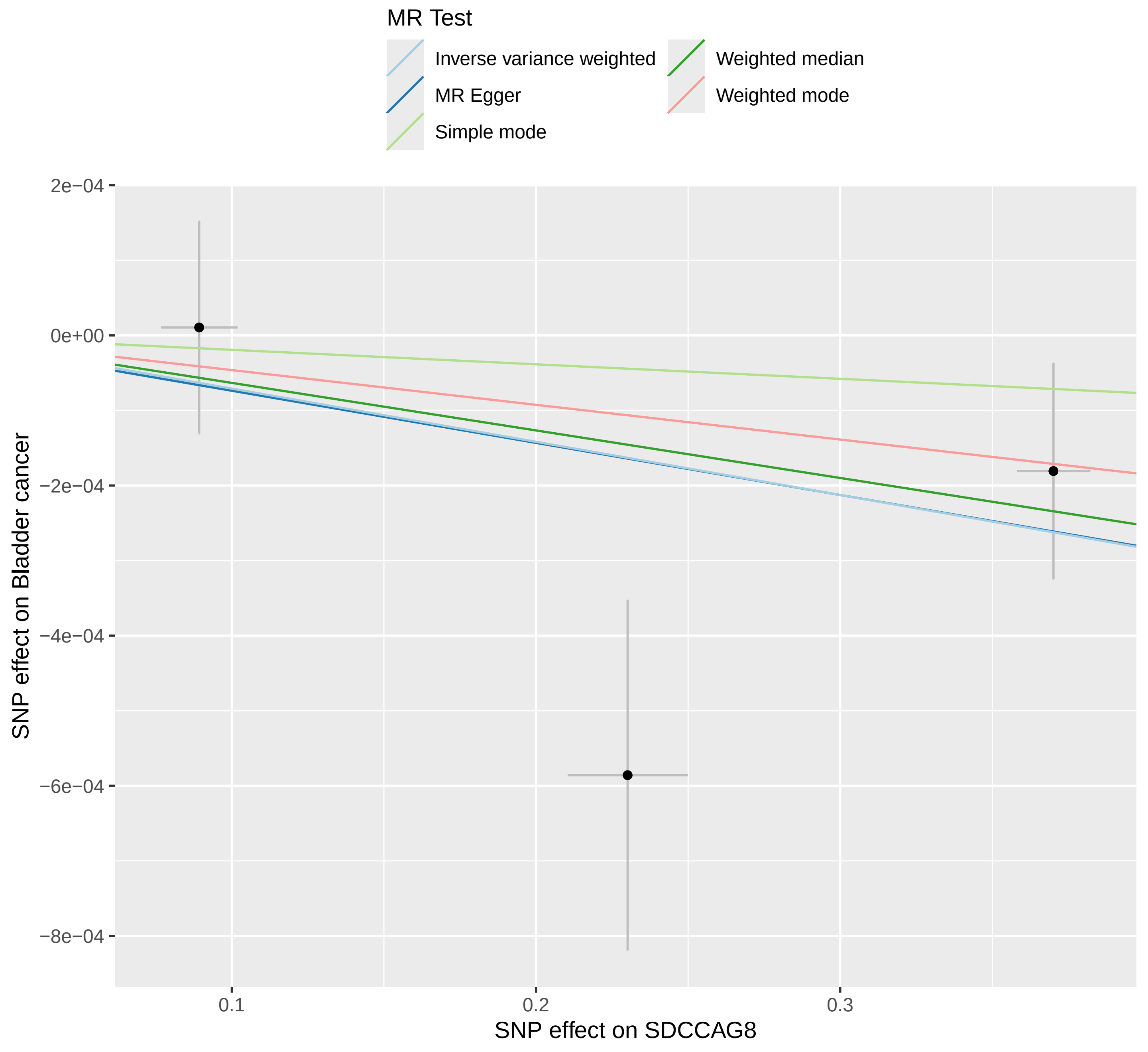

MR Test
Inverse variance weighted
MR Egger
Simple mode
Weighted median
Weighted mode
2e−04
0e+00
−2e−04
−4e−04
−6e−04
−8e−04
SNP effect on Bladder cancer
0.1
0.2
0.3
SNP effect on SDCCAG8

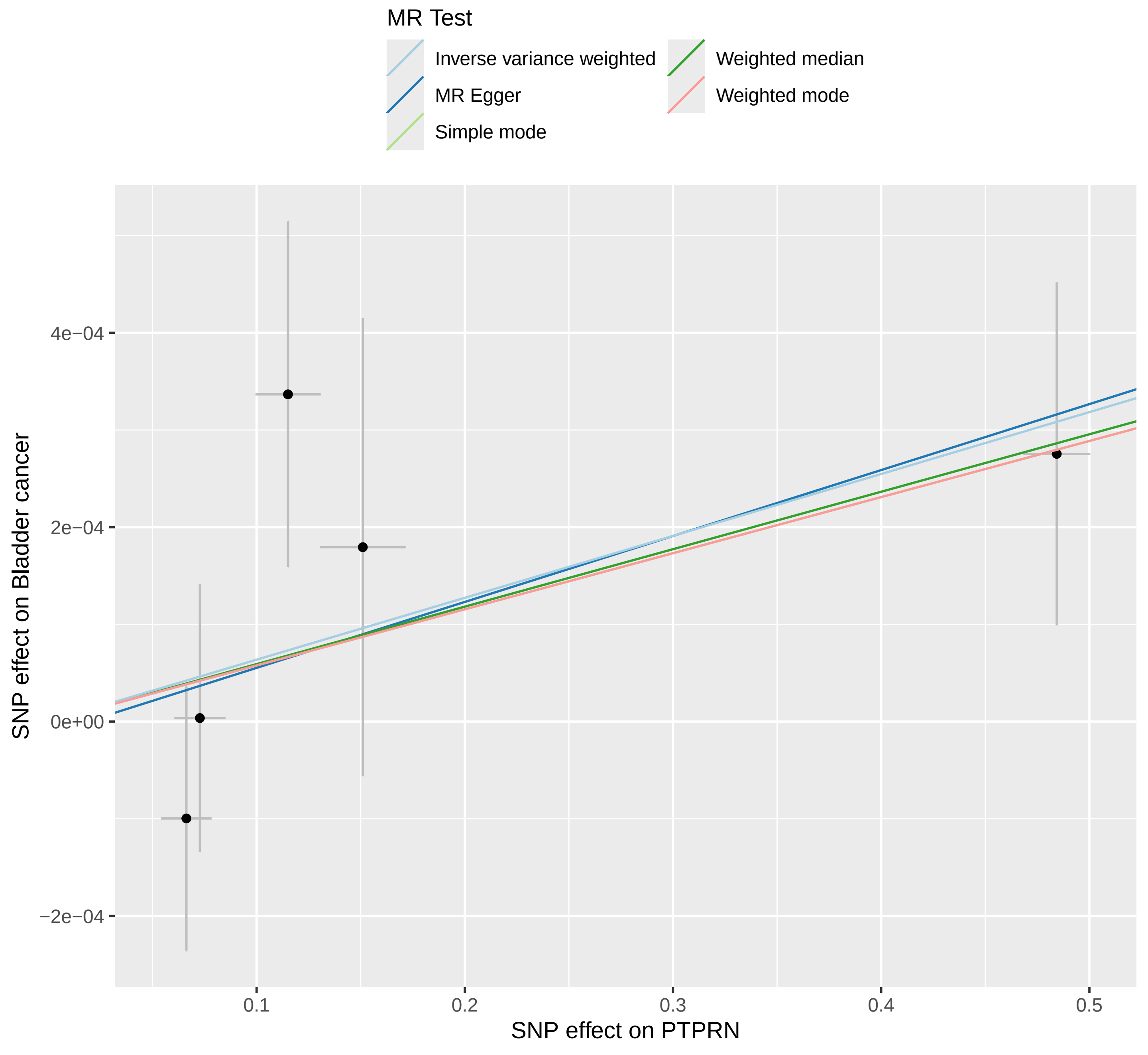

MR Test
Inverse variance weighted
MR Egger
Simple mode
Weighted median
Weighted mode
4e−04
2e−04
0e+00
−2e−04
0.1
0.2
0.3
0.4
0.5
SNP effect on Bladder cancer
SNP effect on PTPRN

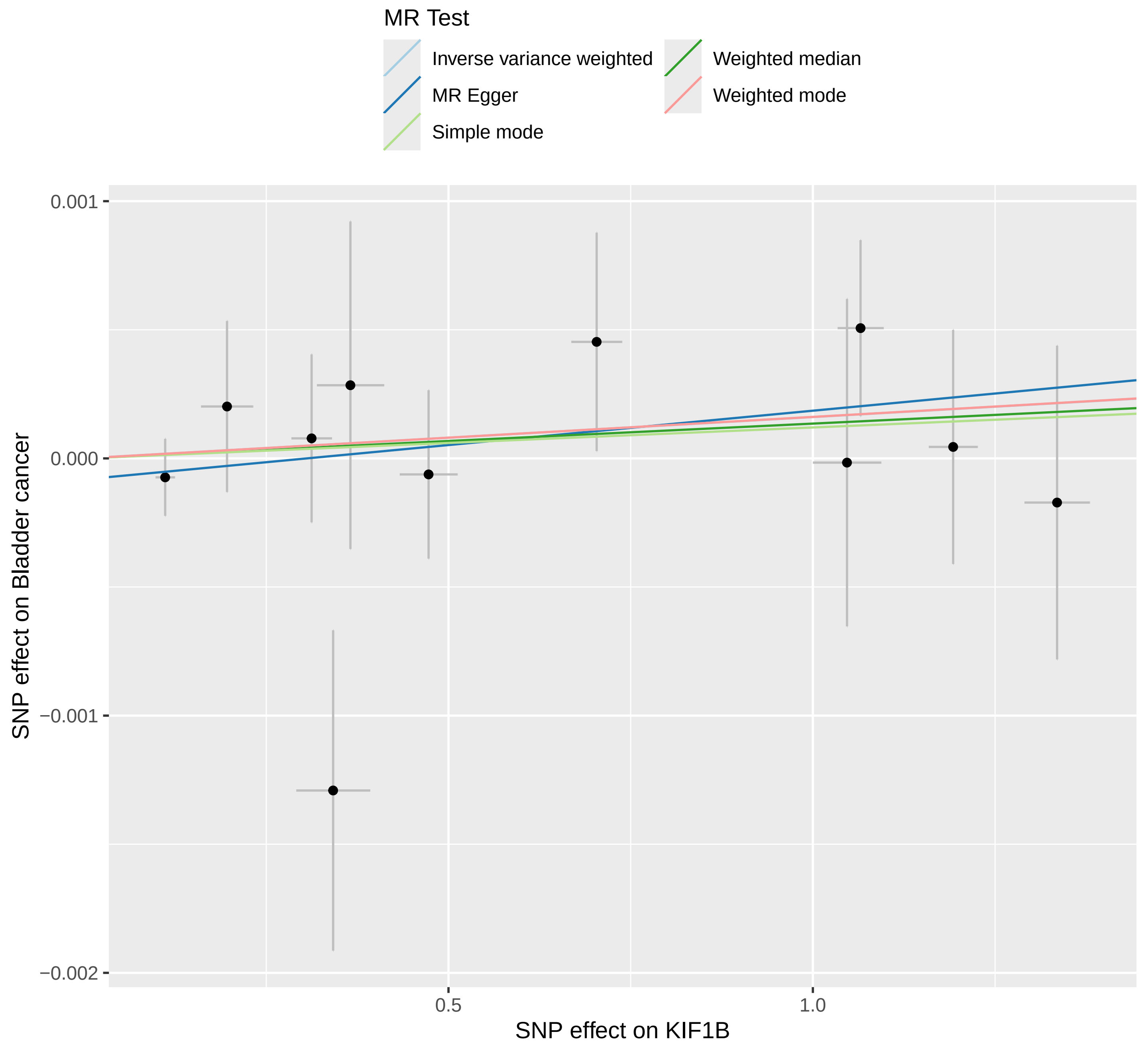

MR Test
Inverse variance weighted
MR Egger
Simple mode
Weighted median
Weighted mode
0.001
0.000
−0.001
−0.002
0.5
1.0
SNP effect on Bladder cancer
SNP effect on KIF1B

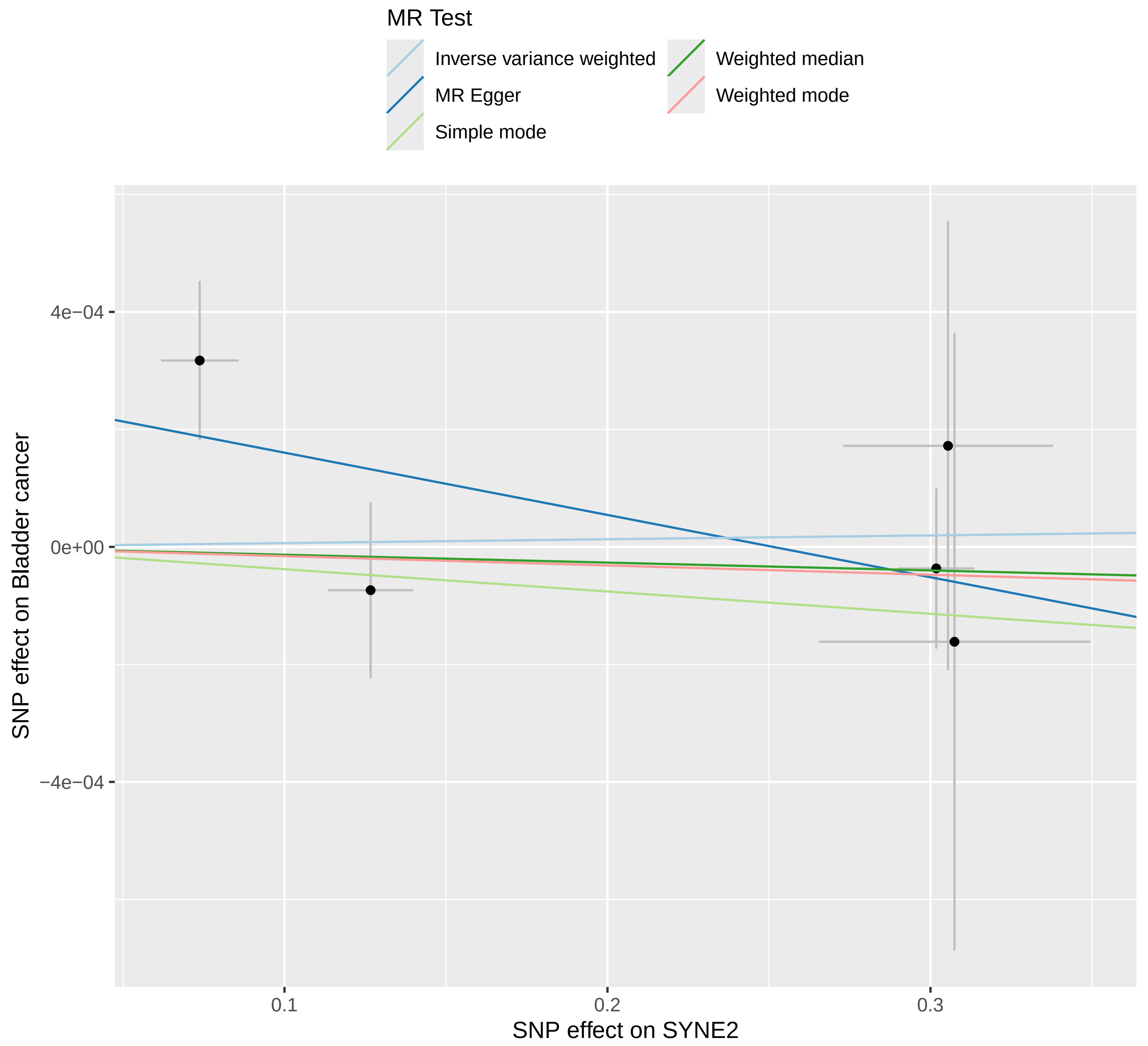

MR Test
Inverse variance weighted
MR Egger
Simple mode
Weighted median
Weighted mode
4e−04
0e+00
−4e−04
SNP effect on Bladder cancer
0.1
0.2
0.3
SNP effect on SYNE2

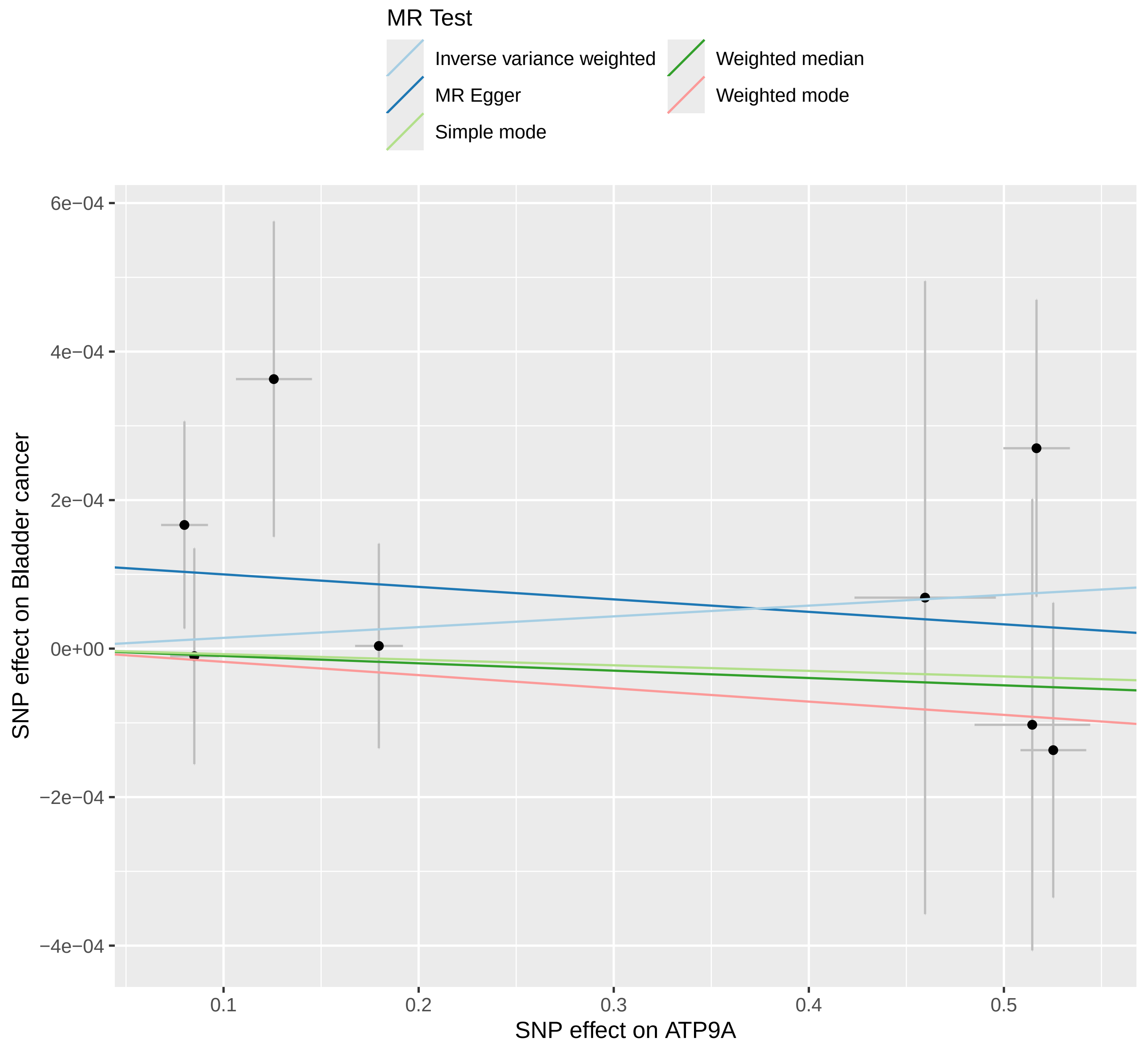
MR Test
Inverse variance weighted
MR Egger
Simple mode
Weighted median
Weighted mode
6e−04
4e−04
2e−04
0e+00
−2e−04
−4e−04
SNP effect on Bladder cancer
0.1
0.2
0.3
0.4
0.5
SNP effect on ATP9A

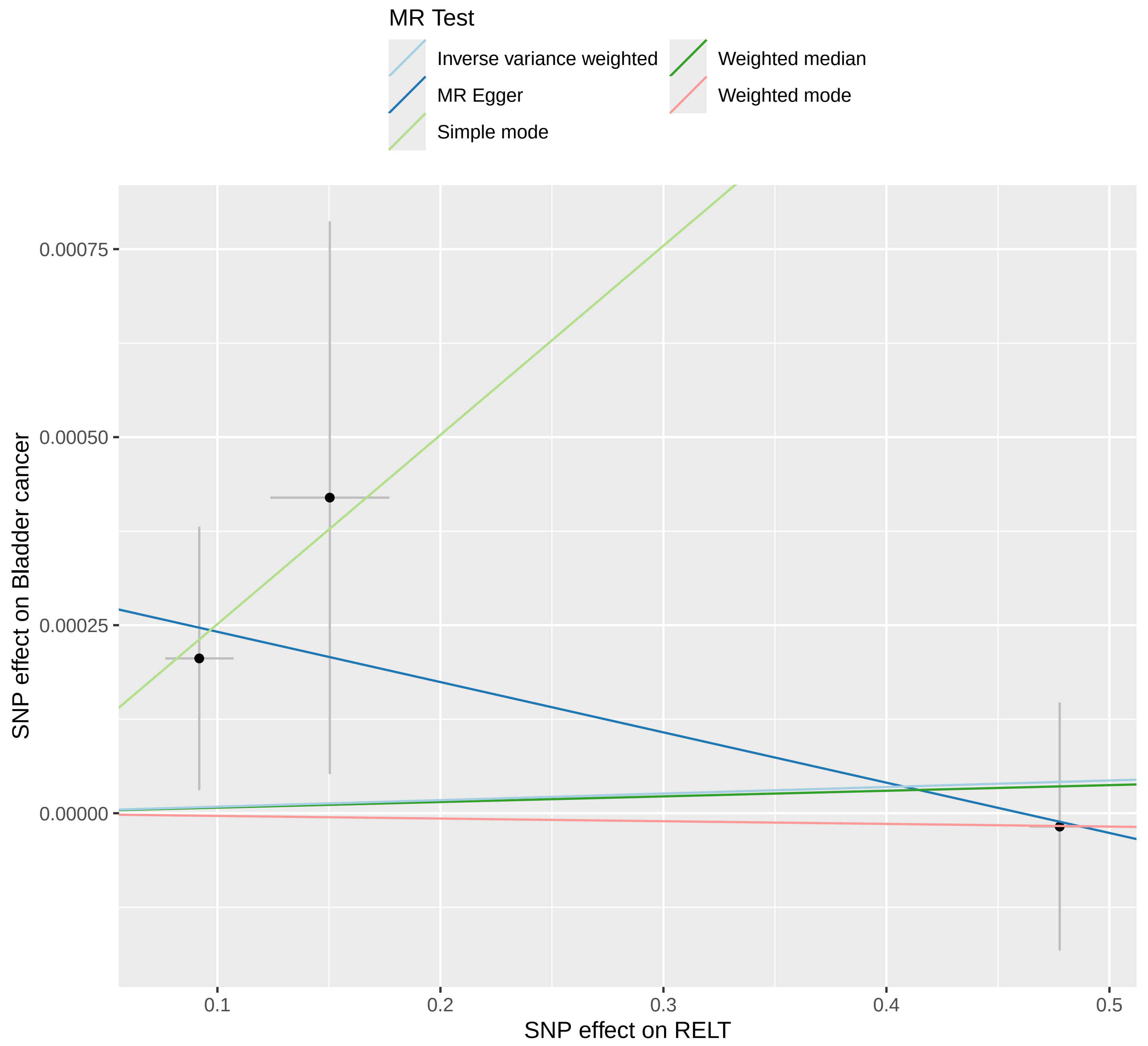

MR Test
Inverse variance weighted
MR Egger
Simple mode
Weighted median
Weighted mode
0.00075
0.00050
0.00025
0.00000
SNP effect on Bladder cancer
0.1
0.2
0.3
0.4
0.5
SNP effect on RELT

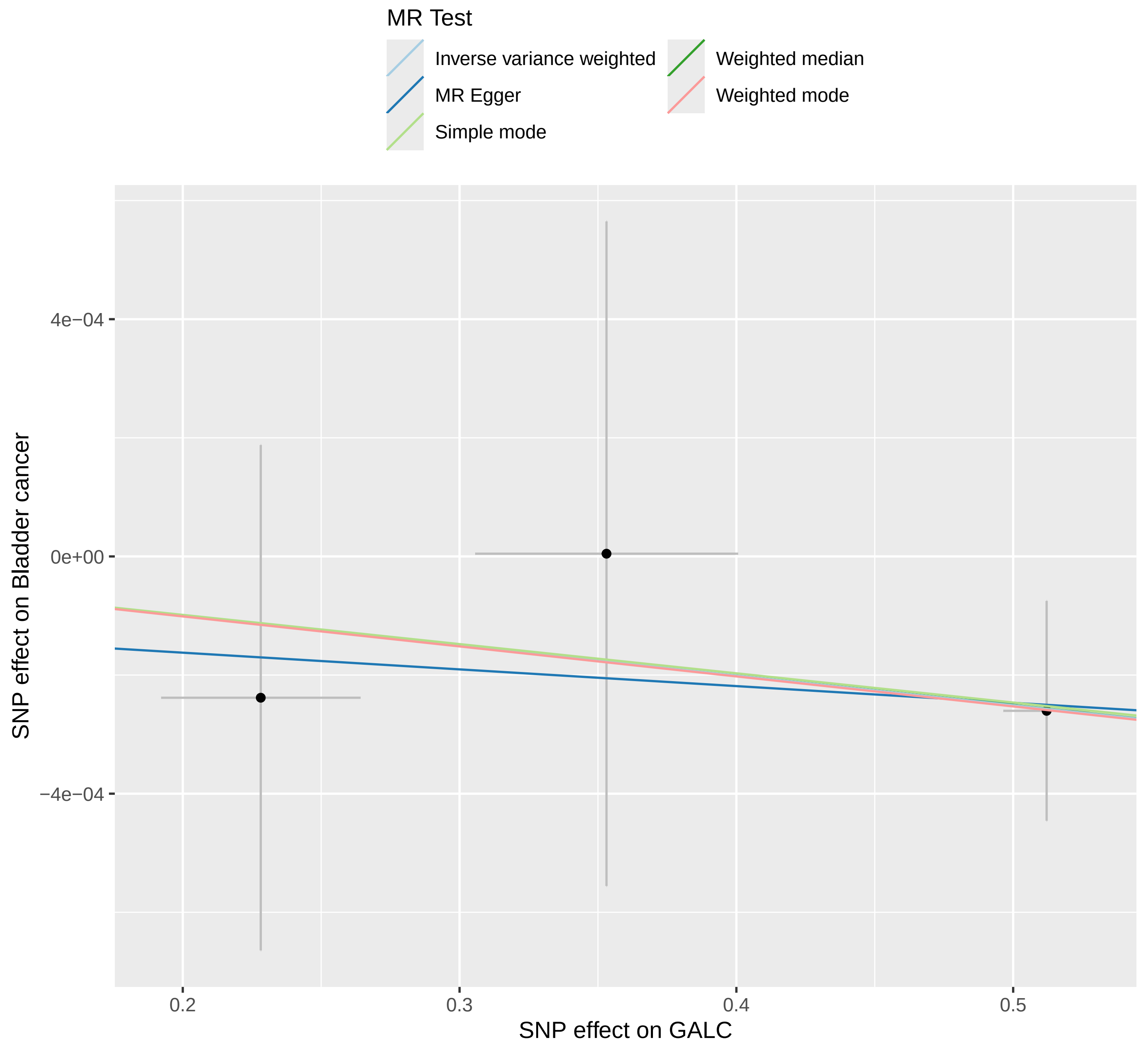
MR Test
Inverse variance weighted
MR Egger
Simple mode
Weighted median
Weighted mode
4e−04
0e+00
−4e−04
0.2
0.3
0.4
0.5
SNP effect on Bladder cancer
SNP effect on GALC

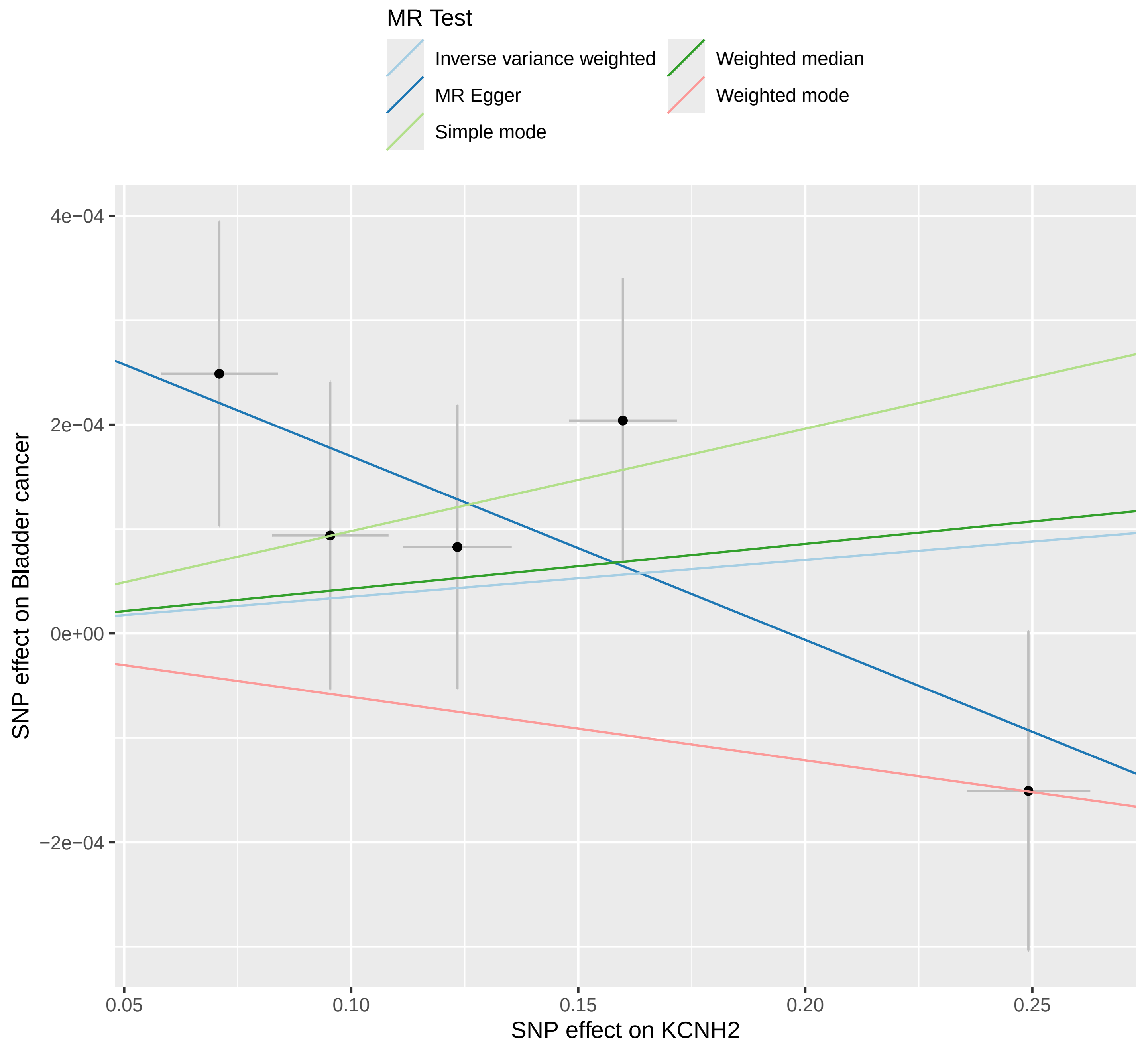
MR Test
Inverse variance weighted
MR Egger
Simple mode
Weighted median
Weighted mode
4e−04
2e−04
0e+00
−2e−04
SNP effect on Bladder cancer
0.05
0.10
0.15
0.20
0.25
SNP effect on KCNH2

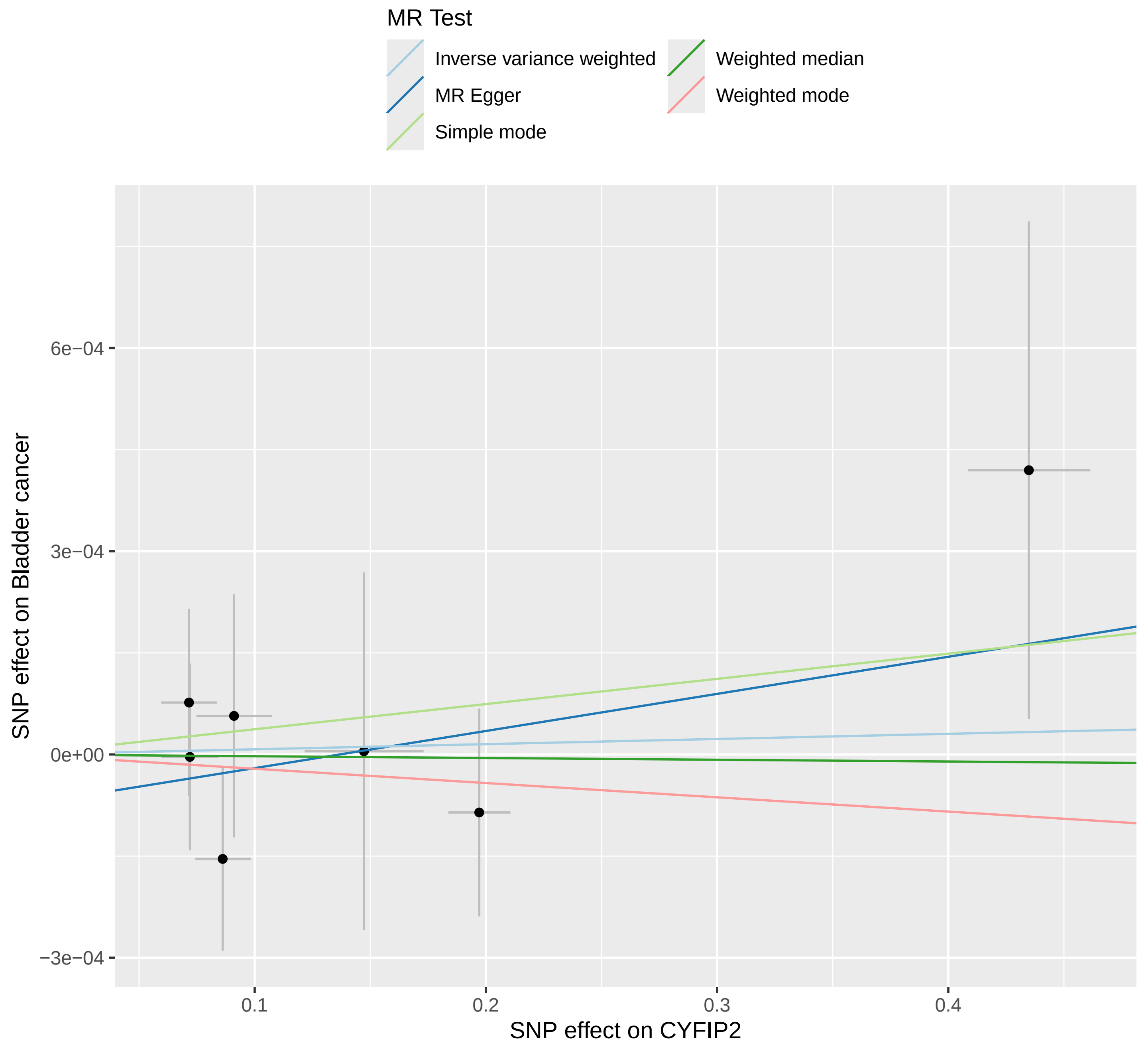
MR Test
Inverse variance weighted
MR Egger
Simple mode
Weighted median
Weighted mode
6e−04
3e−04
0e+00
−3e−04
0.1
0.2
0.3
0.4
SNP effect on Bladder cancer
SNP effect on CYFIP2

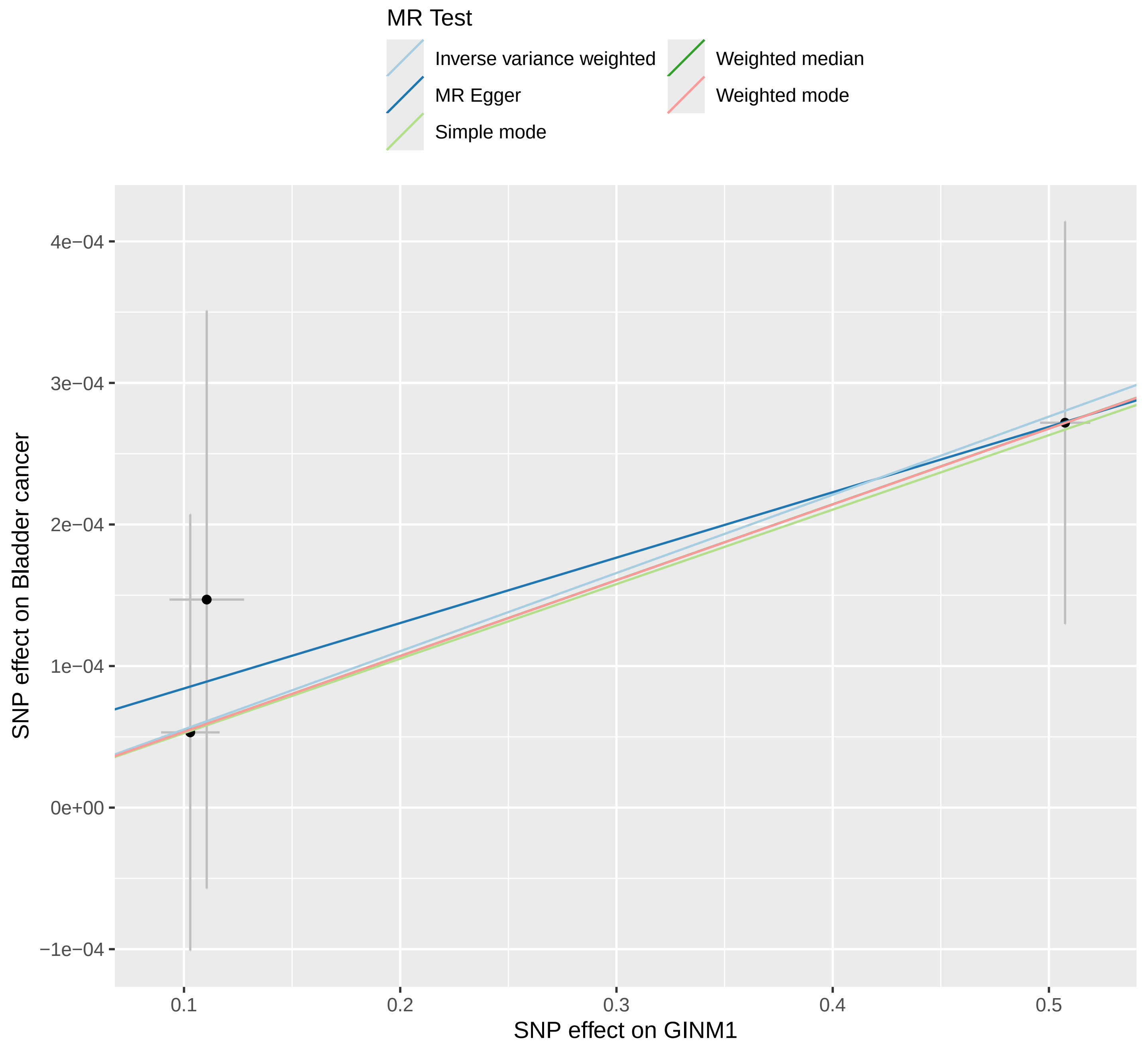

MR Test
Inverse variance weighted
MR Egger
Simple mode
Weighted median
Weighted mode
4e−04
3e−04
2e−04
1e−04
0e+00
−1e−04
0.1
0.2
0.3
0.4
0.5
SNP effect on Bladder cancer
SNP effect on GINM1

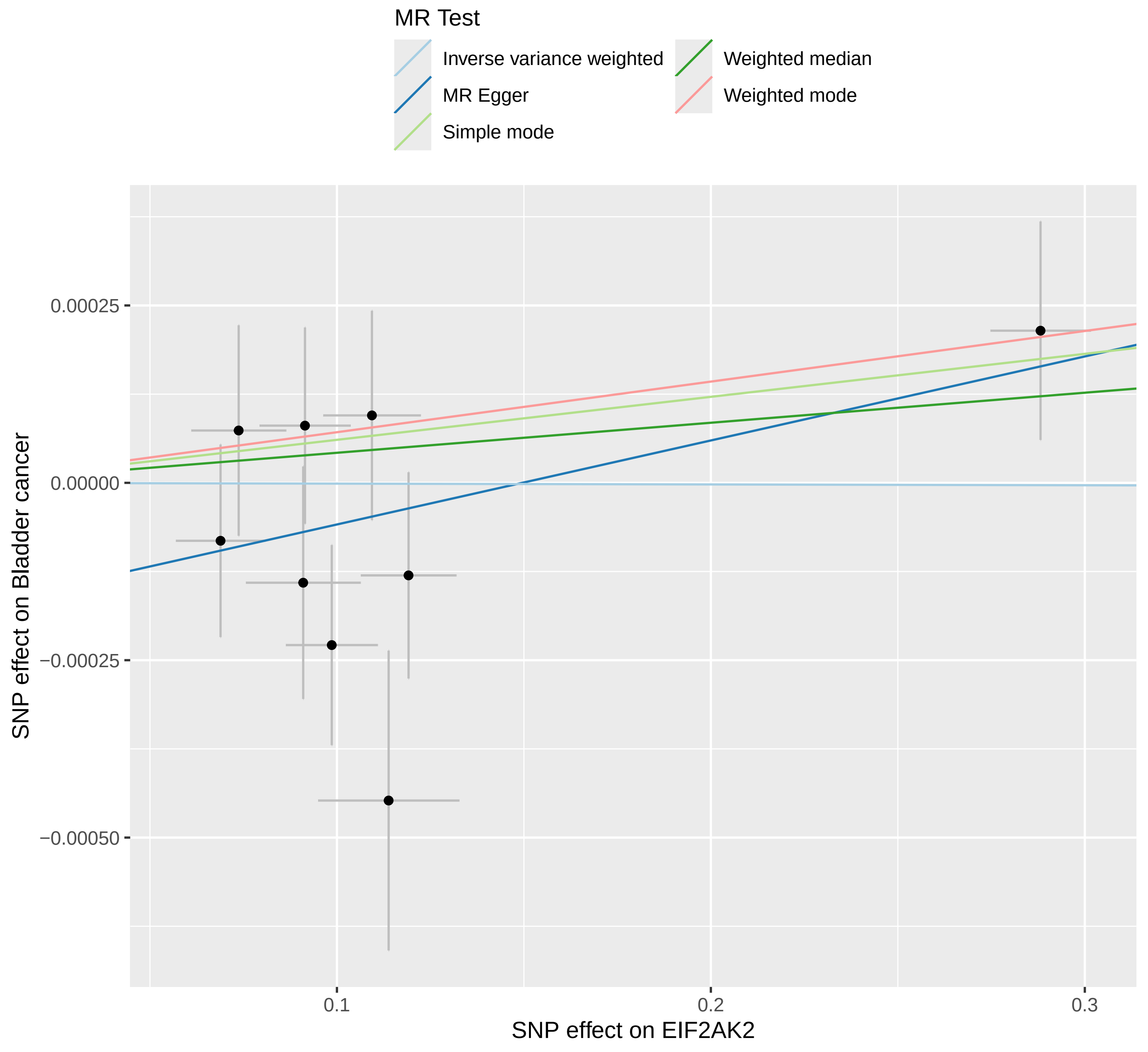

MR Test
Inverse variance weighted
MR Egger
Simple mode
Weighted median
Weighted mode
0.00025
0.00000
−0.00025
−0.00050
0.1
0.2
0.3
SNP effect on Bladder cancer
SNP effect on EIF2AK2

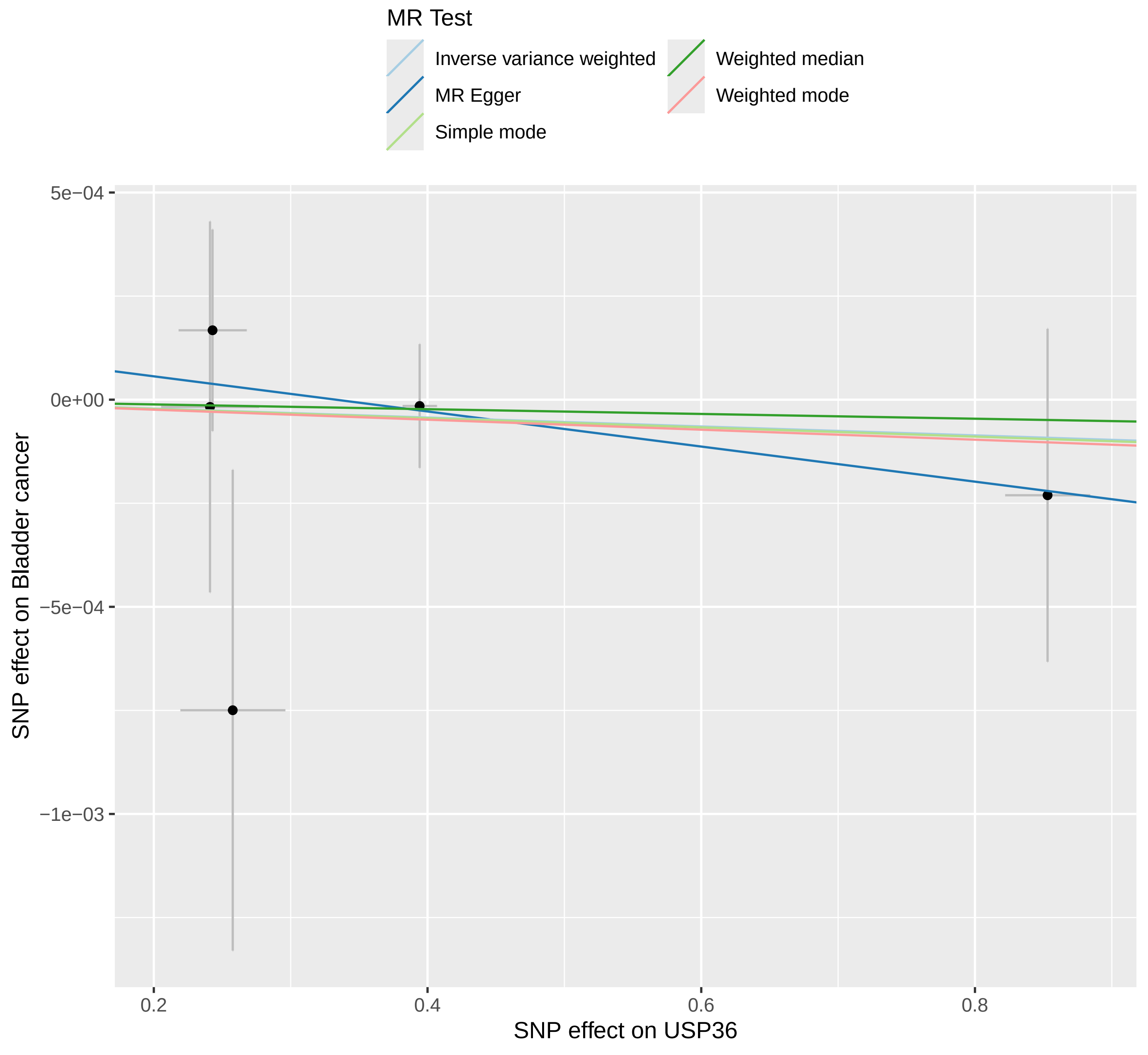
MR Test
Inverse variance weighted
MR Egger
Simple mode
Weighted median
Weighted mode
5e−04
0e+00
−5e−04
−1e−03
SNP effect on Bladder cancer
0.2
0.4
0.6
0.8
SNP effect on USP36

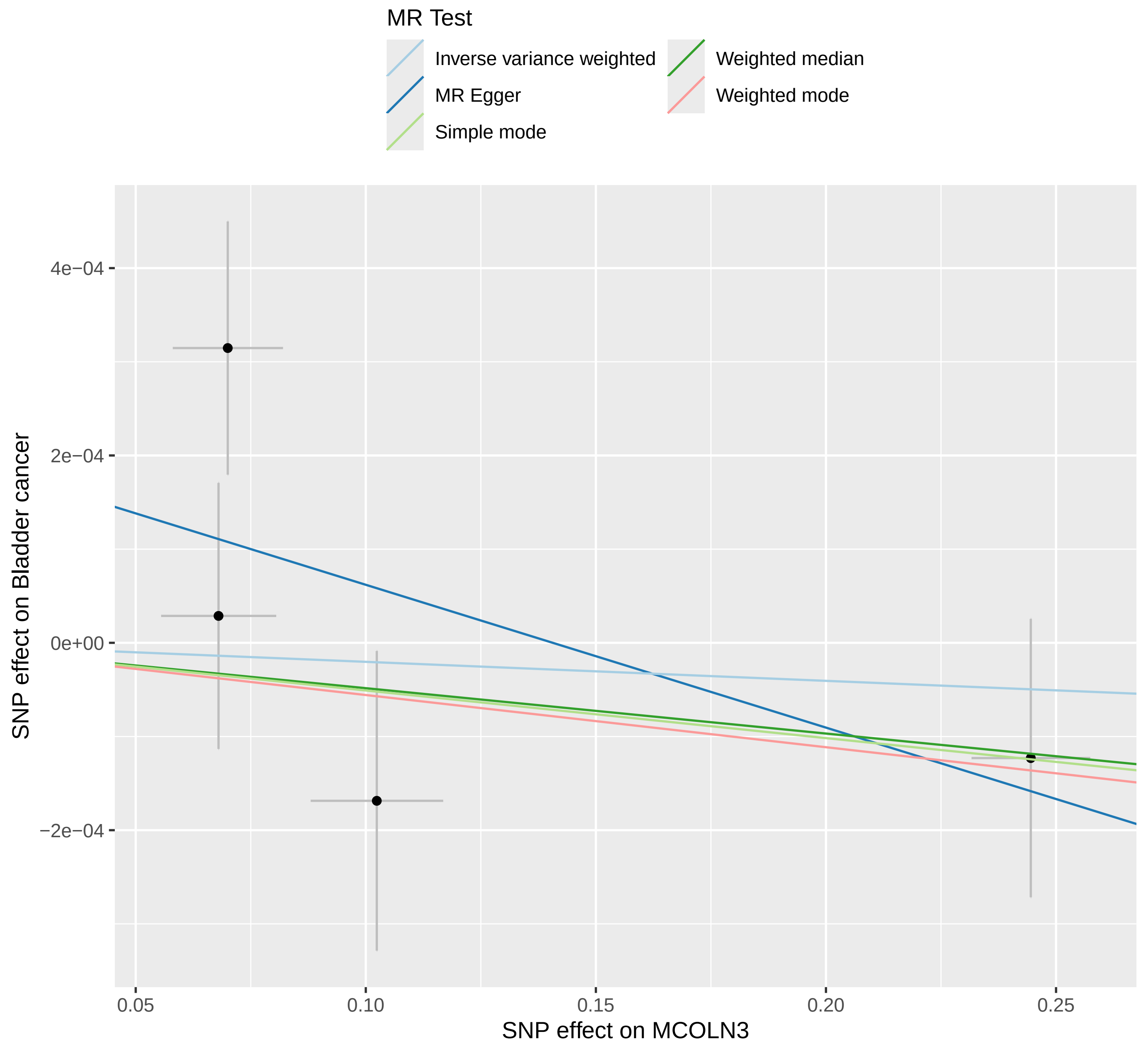
MR Test
Inverse variance weighted
MR Egger
Simple mode
Weighted median
Weighted mode
4e−04
2e−04
0e+00
−2e−04
SNP effect on Bladder cancer
0.05
0.10
0.15
0.20
0.25
SNP effect on MCOLN3

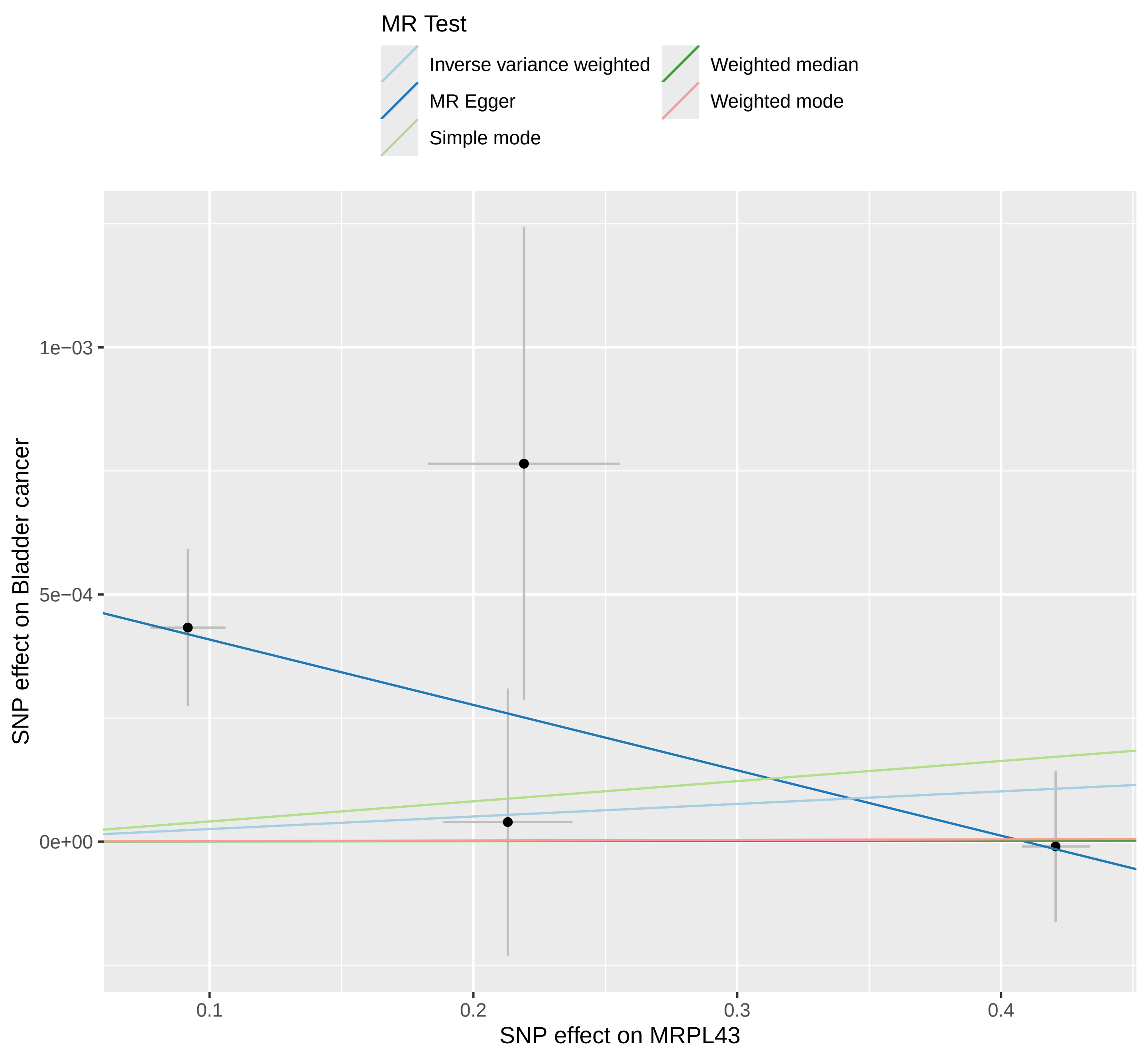

MR Test
Inverse variance weighted
MR Egger
Simple mode
Weighted median
Weighted mode
1e−03
5e−04
0e+00
SNP effect on Bladder cancer
0.1
0.2
0.3
0.4
SNP effect on MRPL43

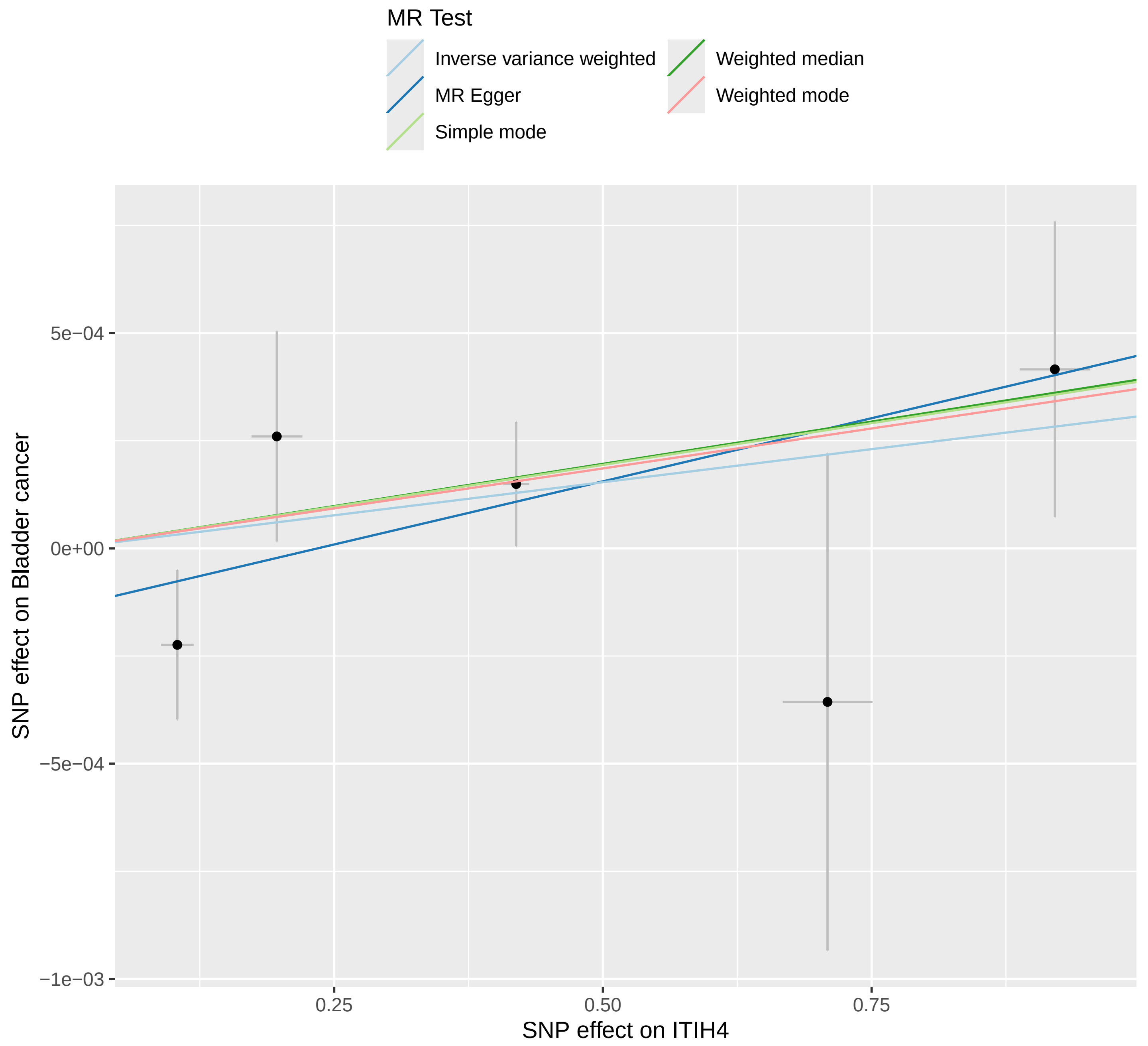

MR Test
Inverse variance weighted
MR Egger
Simple mode
Weighted median
Weighted mode
5e−04
0e+00
−5e−04
−1e−03
0.25
0.50
0.75
SNP effect on Bladder cancer
SNP effect on ITIH4

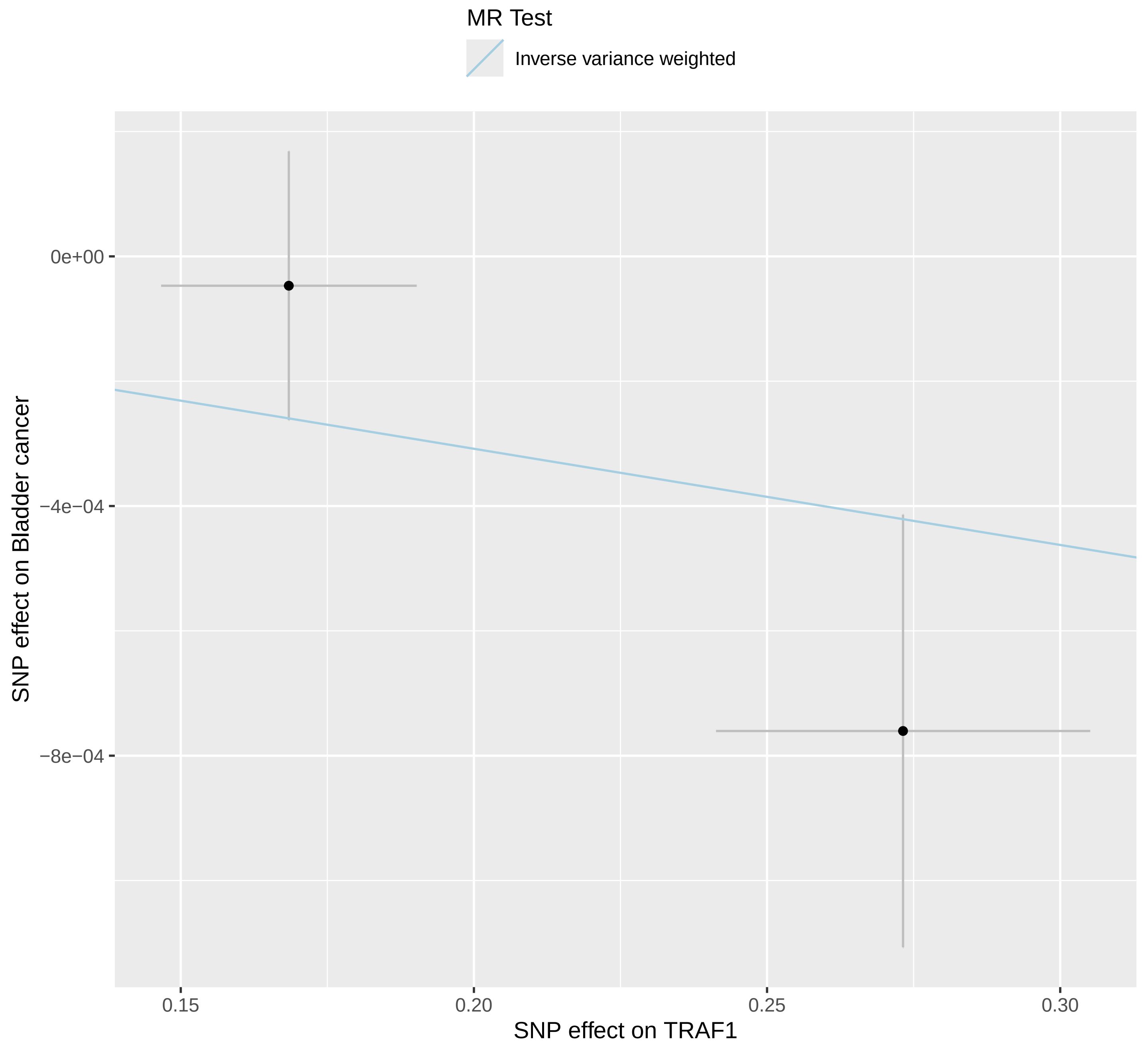

MR Test
Inverse variance weighted
0e+00
−4e−04
−8e−04
0.15
0.20
0.25
0.30
SNP effect on Bladder cancer
SNP effect on TRAF1

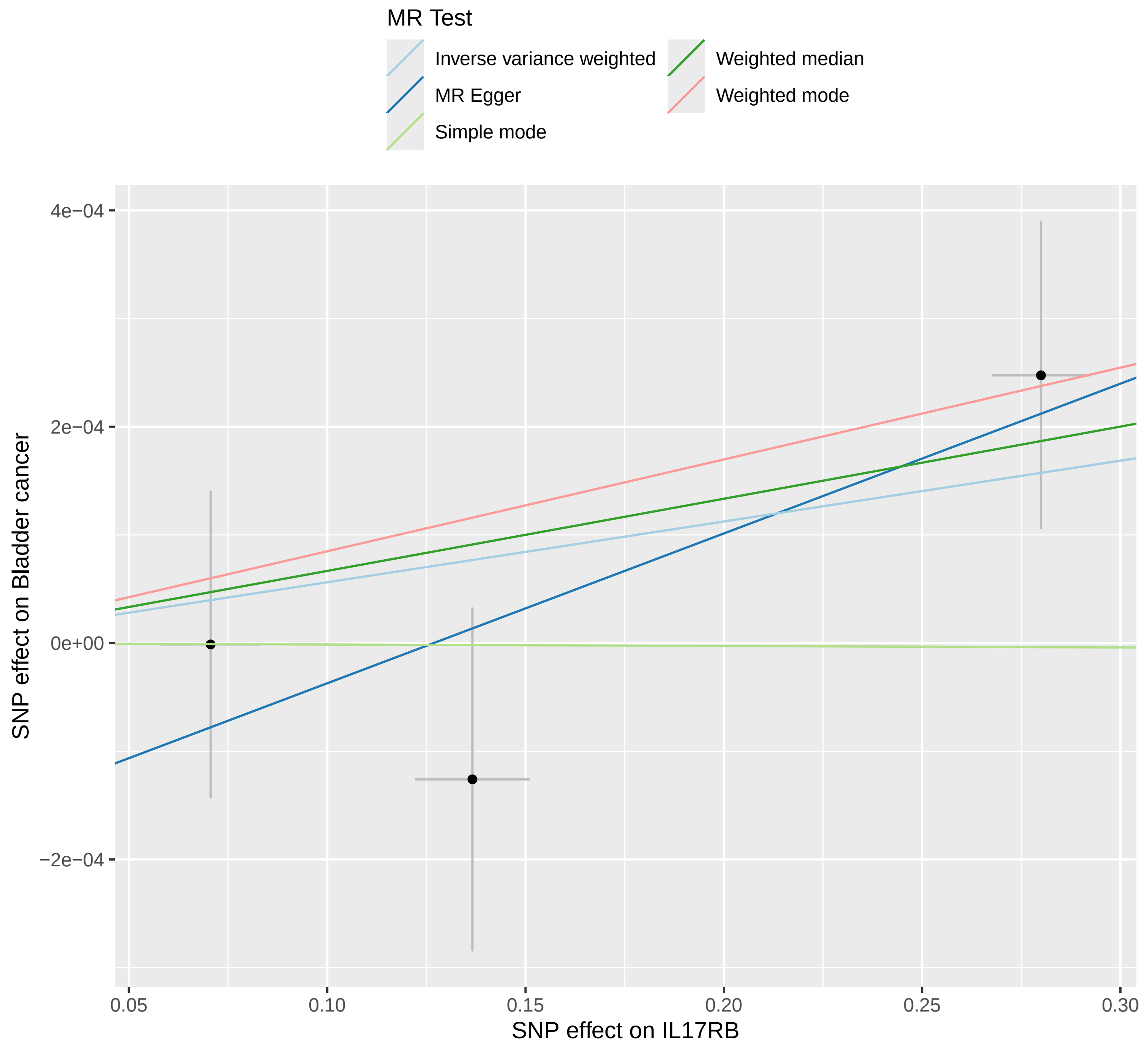
MR Test
Inverse variance weighted
MR Egger
Simple mode
Weighted median
Weighted mode
4e−04
2e−04
0e+00
−2e−04
SNP effect on Bladder cancer
0.05
0.10
0.15
0.20
0.25
0.30
SNP effect on IL17RB

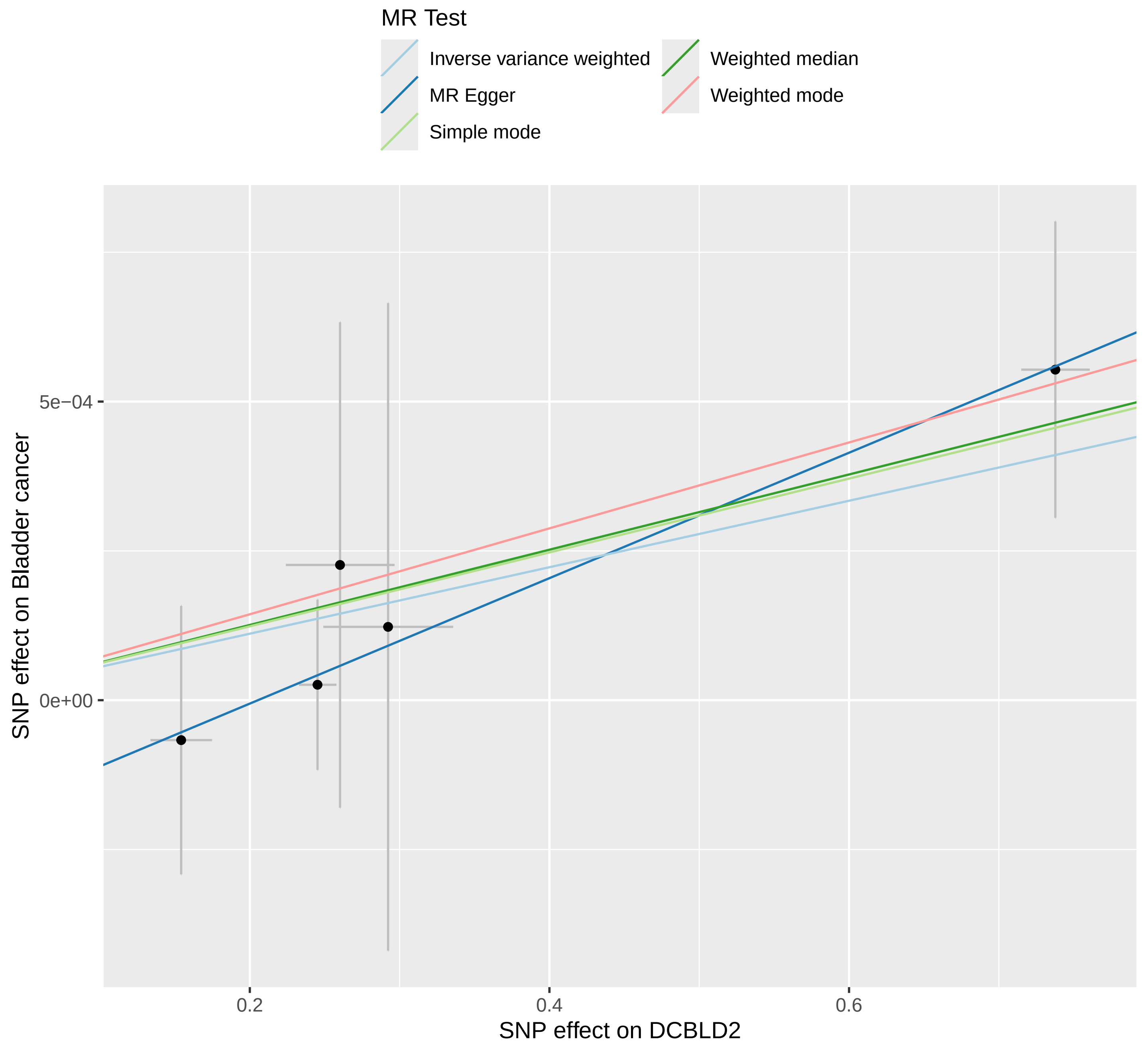

MR Test
Inverse variance weighted
MR Egger
Simple mode
Weighted median
Weighted mode
SNP effect on Bladder cancer
5e−04
0e+00
0.2
0.4
0.6
SNP effect on DCBLD2

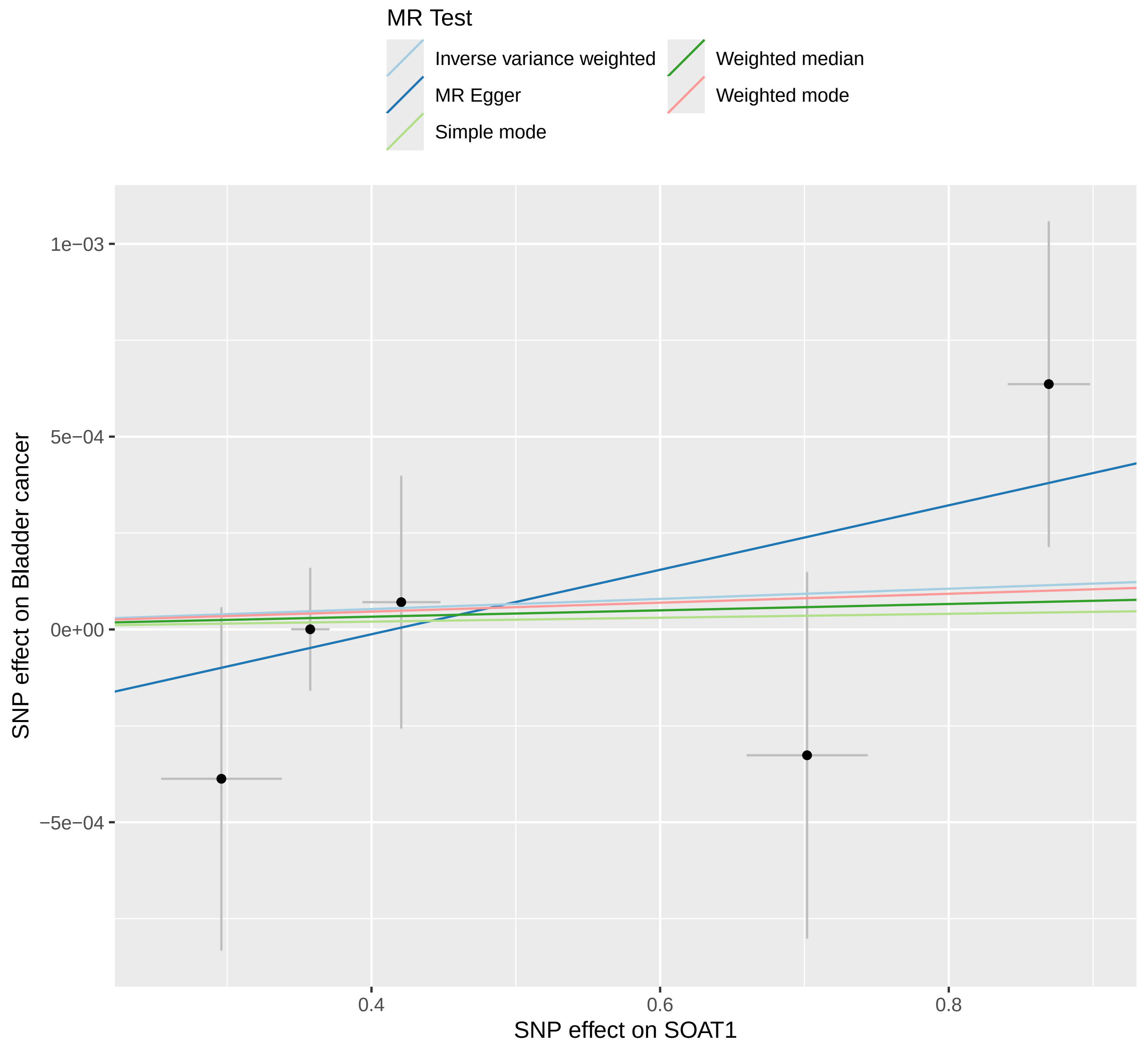
MR Test
Inverse variance weighted
MR Egger
Simple mode
Weighted median
Weighted mode
1e−03
5e−04
0e+00
−5e−04
SNP effect on Bladder cancer
0.4
0.6
0.8
SNP effect on SOAT1

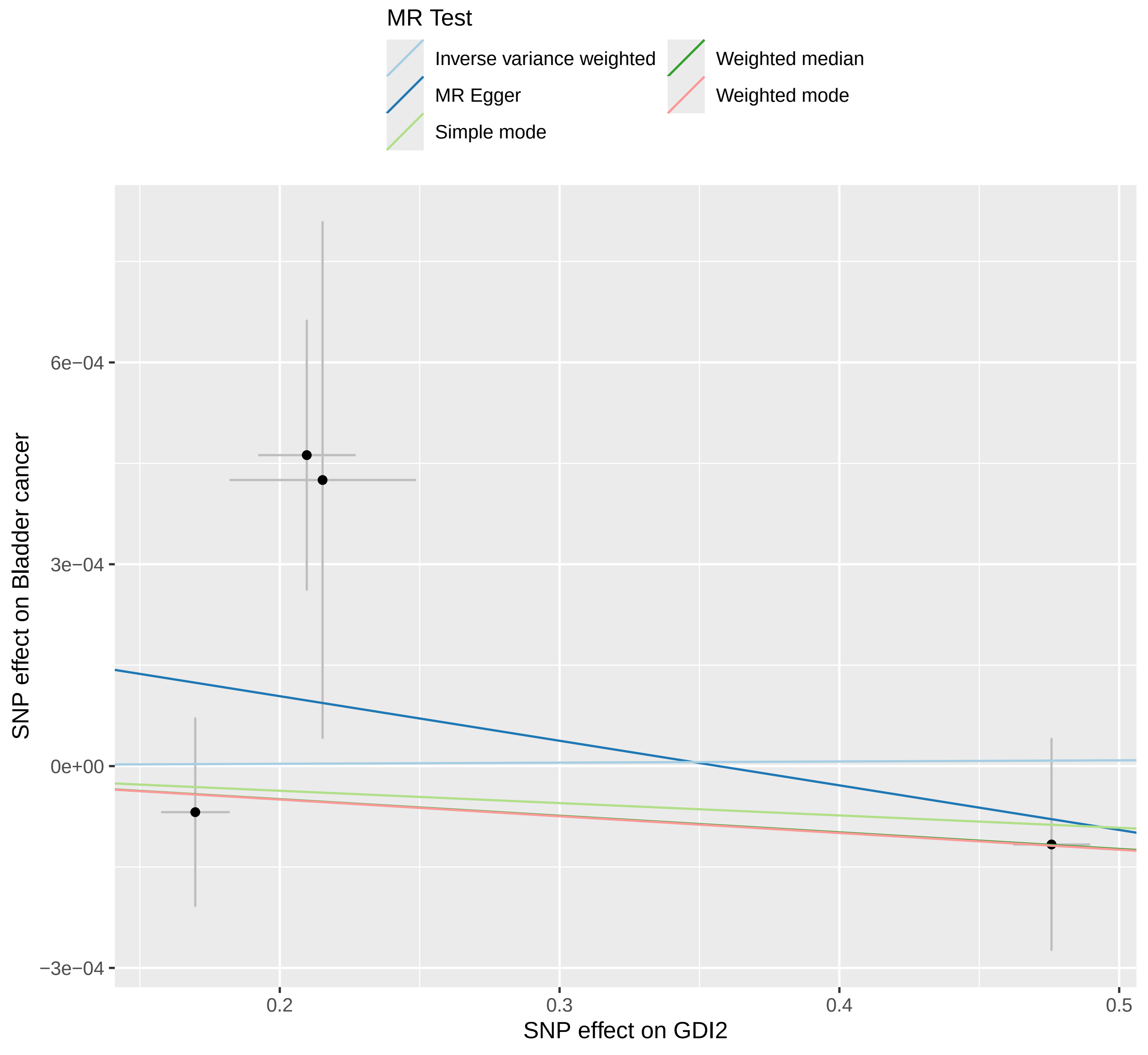
MR Test
Inverse variance weighted
MR Egger
Simple mode
Weighted median
Weighted mode
SNP effect on Bladder cancer
6e−04
3e−04
0e+00
−3e−04
0.2
0.3
0.4
0.5
SNP effect on GDI2

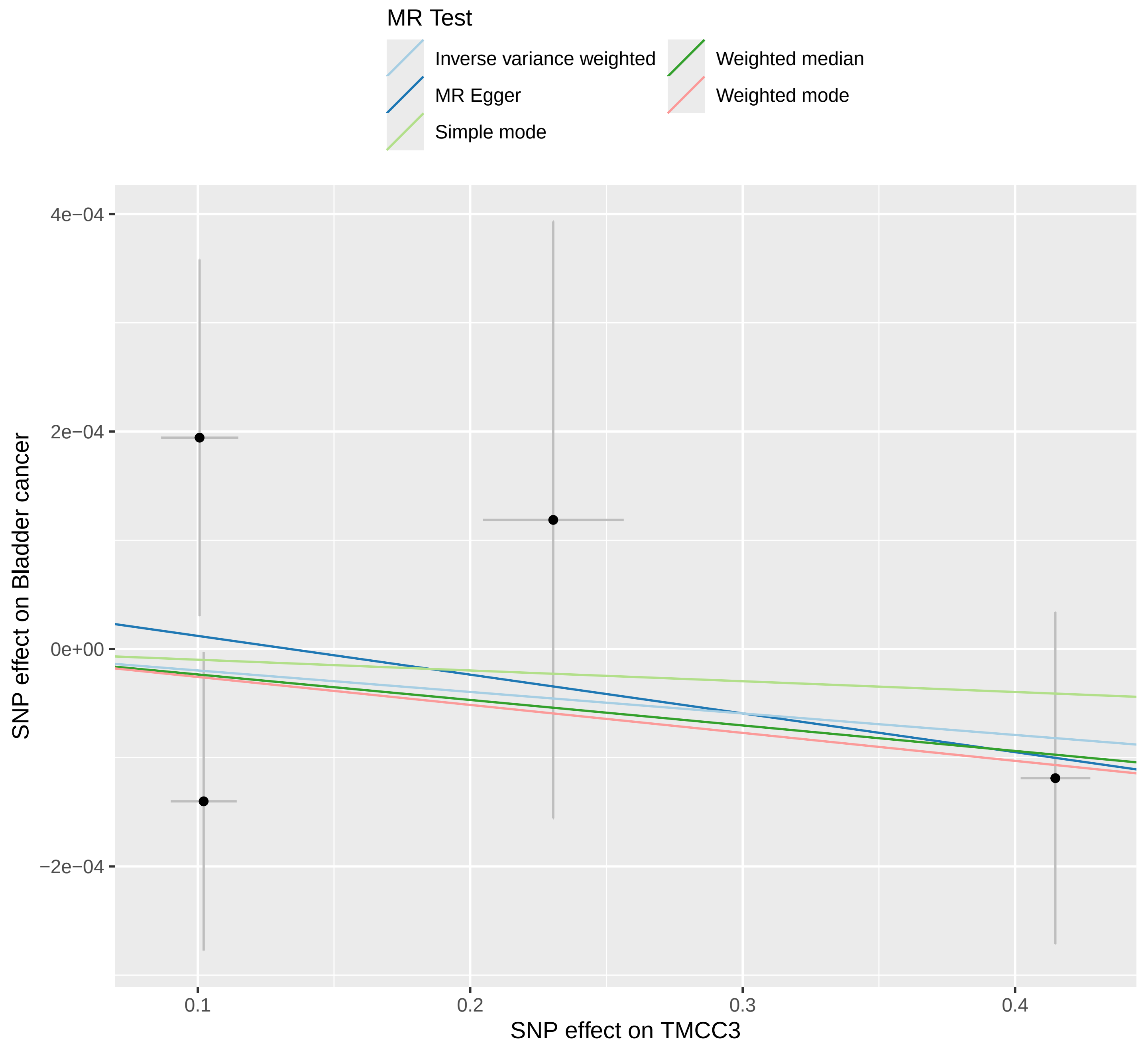

MR Test
Inverse variance weighted
MR Egger
Simple mode
Weighted median
Weighted mode
4e−04
2e−04
0e+00
−2e−04
0.1
0.2
0.3
0.4
SNP effect on TMCC3
SNP effect on Bladder cancer

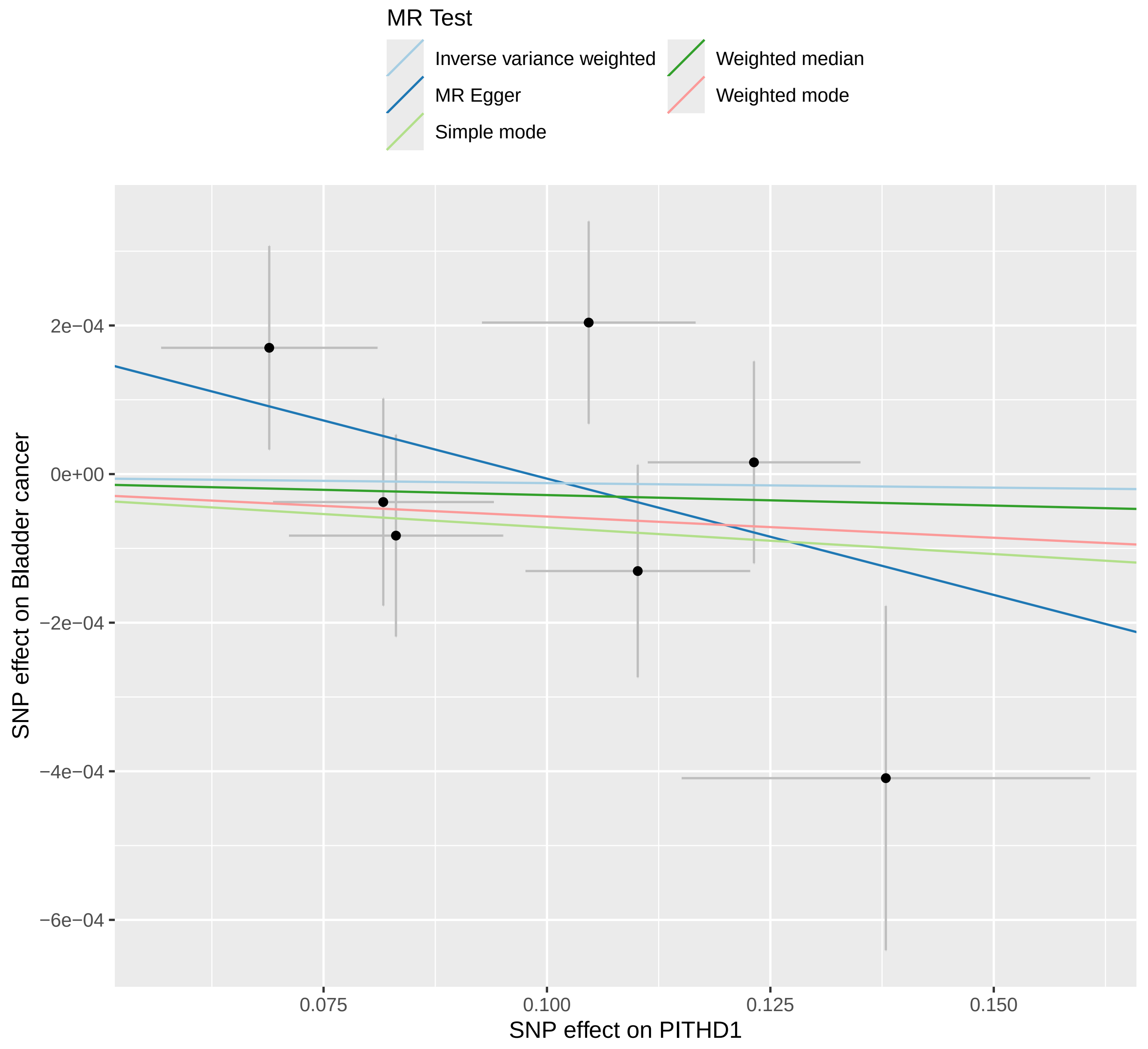
MR Test
Inverse variance weighted
MR Egger
Simple mode
Weighted median
Weighted mode
2e−04
0e+00
−2e−04
−4e−04
−6e−04
SNP effect on Bladder cancer
0.075
0.100
0.125
0.150
SNP effect on PITHD1

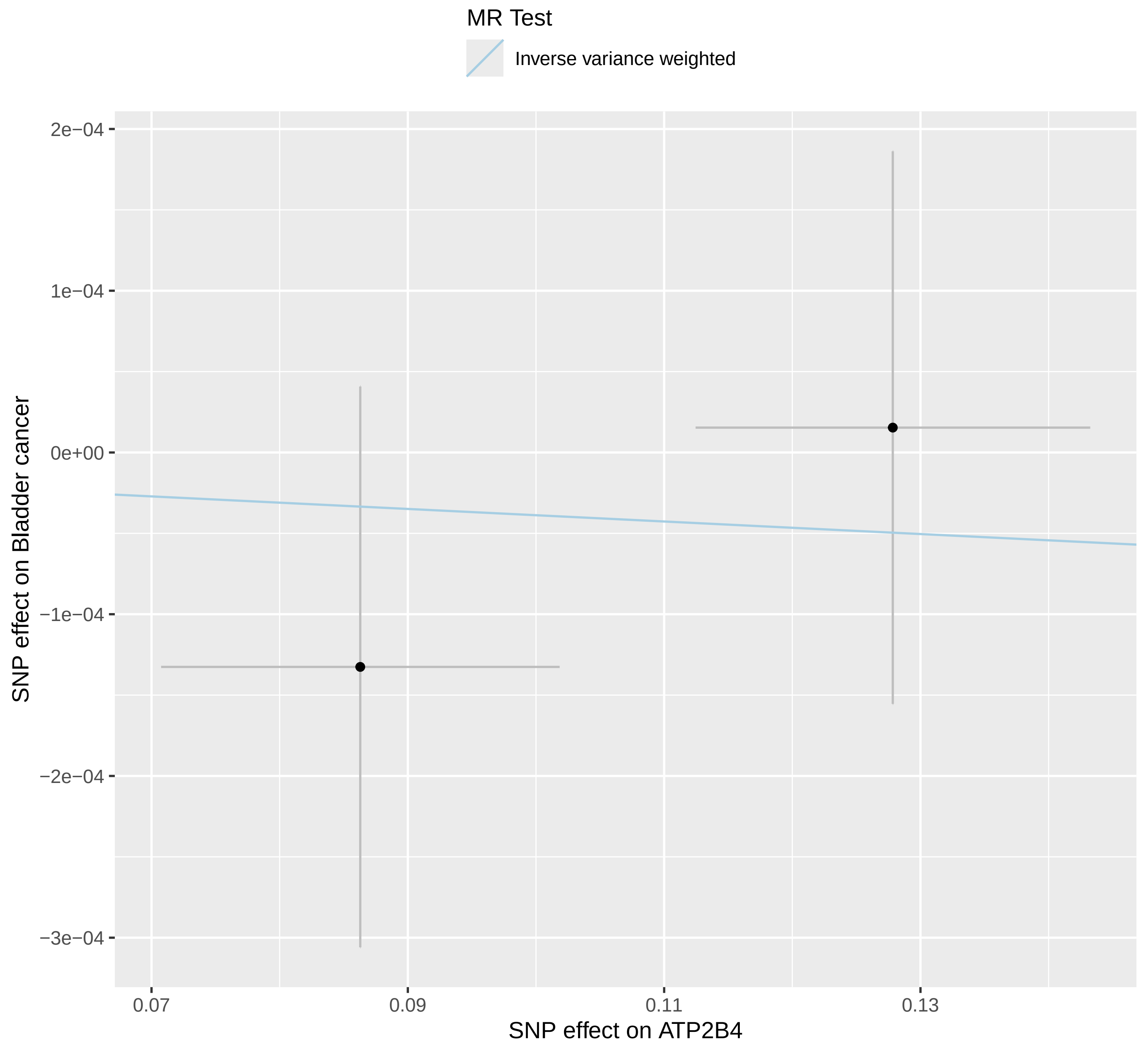
MR Test
Inverse variance weighted
2e−04
1e−04
0e+00
−1e−04
−2e−04
−3e−04
0.07
0.09
0.11
0.13
SNP effect on ATP2B4
SNP effect on Bladder cancer

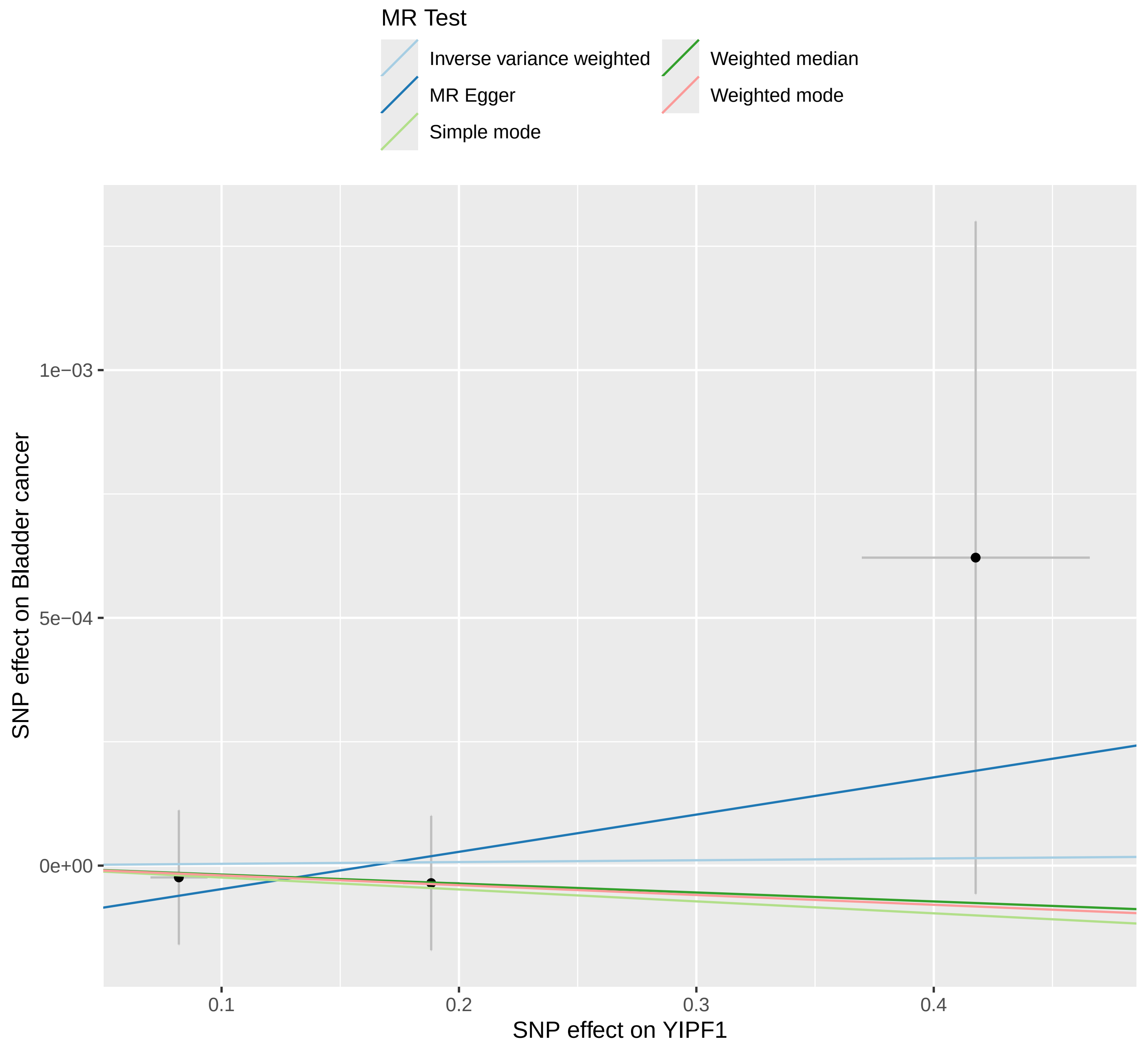

MR Test
Inverse variance weighted
MR Egger
Simple mode
Weighted median
Weighted mode
1e−03
5e−04
0e+00
SNP effect on Bladder cancer
0.1
0.2
0.3
0.4
SNP effect on YIPF1

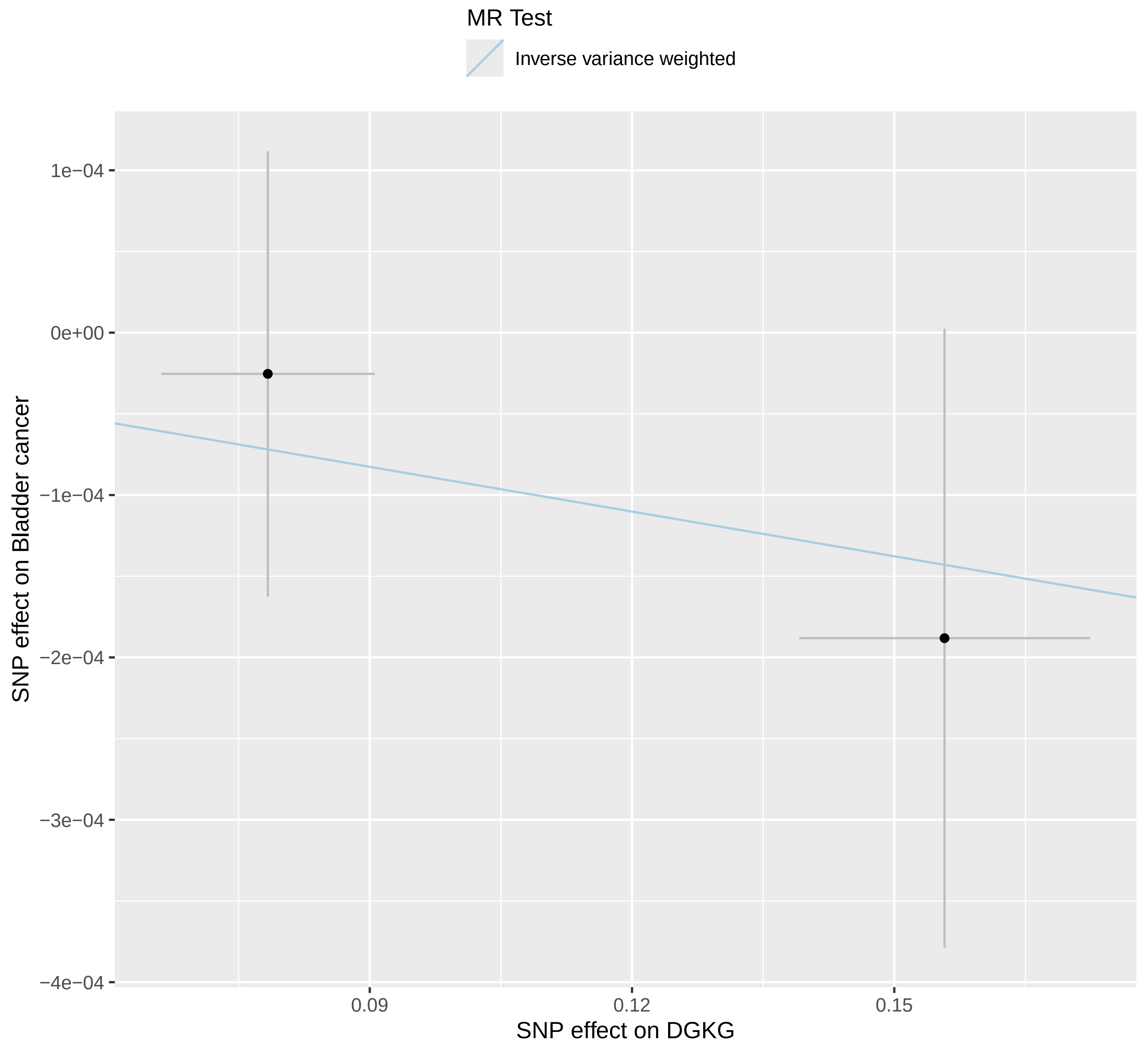
MR Test
Inverse variance weighted
1e−04
0e+00
−1e−04
−2e−04
−3e−04
−4e−04
SNP effect on Bladder cancer
0.09
0.12
0.15
SNP effect on DGKG

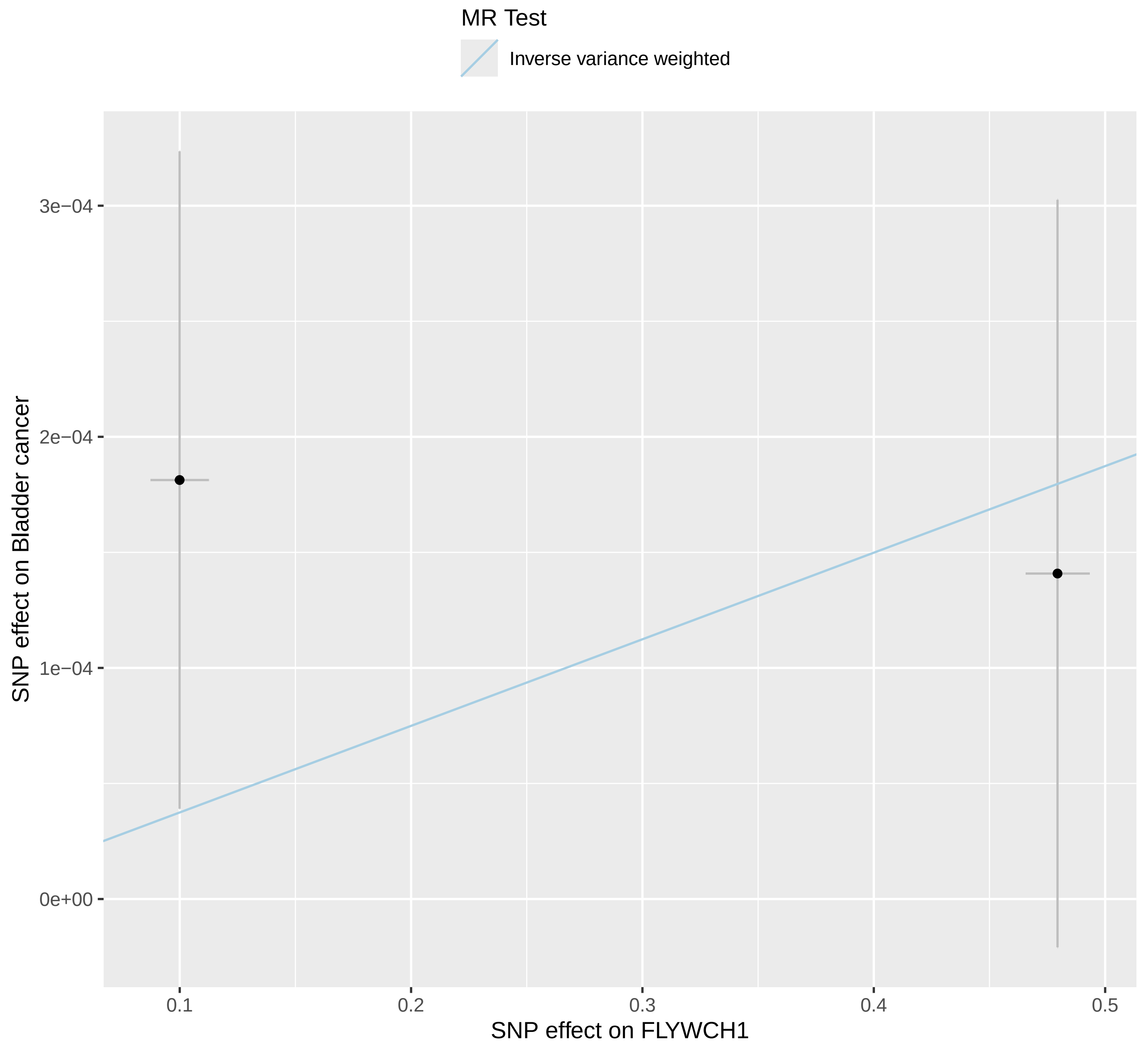
MR Test
Inverse variance weighted
SNP effect on Bladder cancer
3e−04
2e−04
1e−04
0e+00
0.1
0.2
0.3
0.4
0.5
SNP effect on FLYWCH1

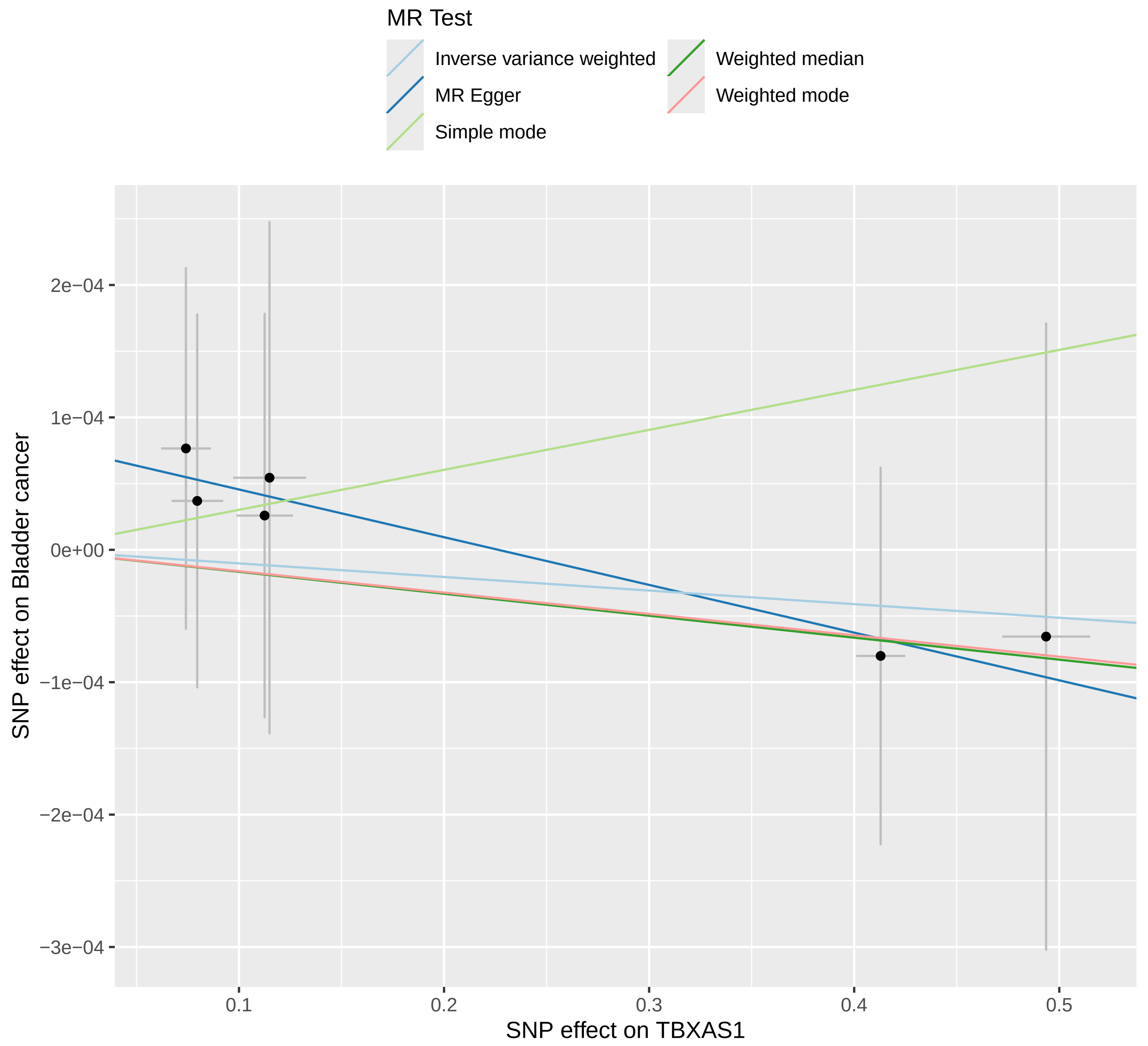

MR Test
Inverse variance weighted
MR Egger
Simple mode
Weighted median
Weighted mode
2e−04
1e−04
0e+00
−1e−04
−2e−04
−3e−04
0.1
0.2
0.3
0.4
0.5
SNP effect on TBXAS1
SNP effect on Bladder cancer

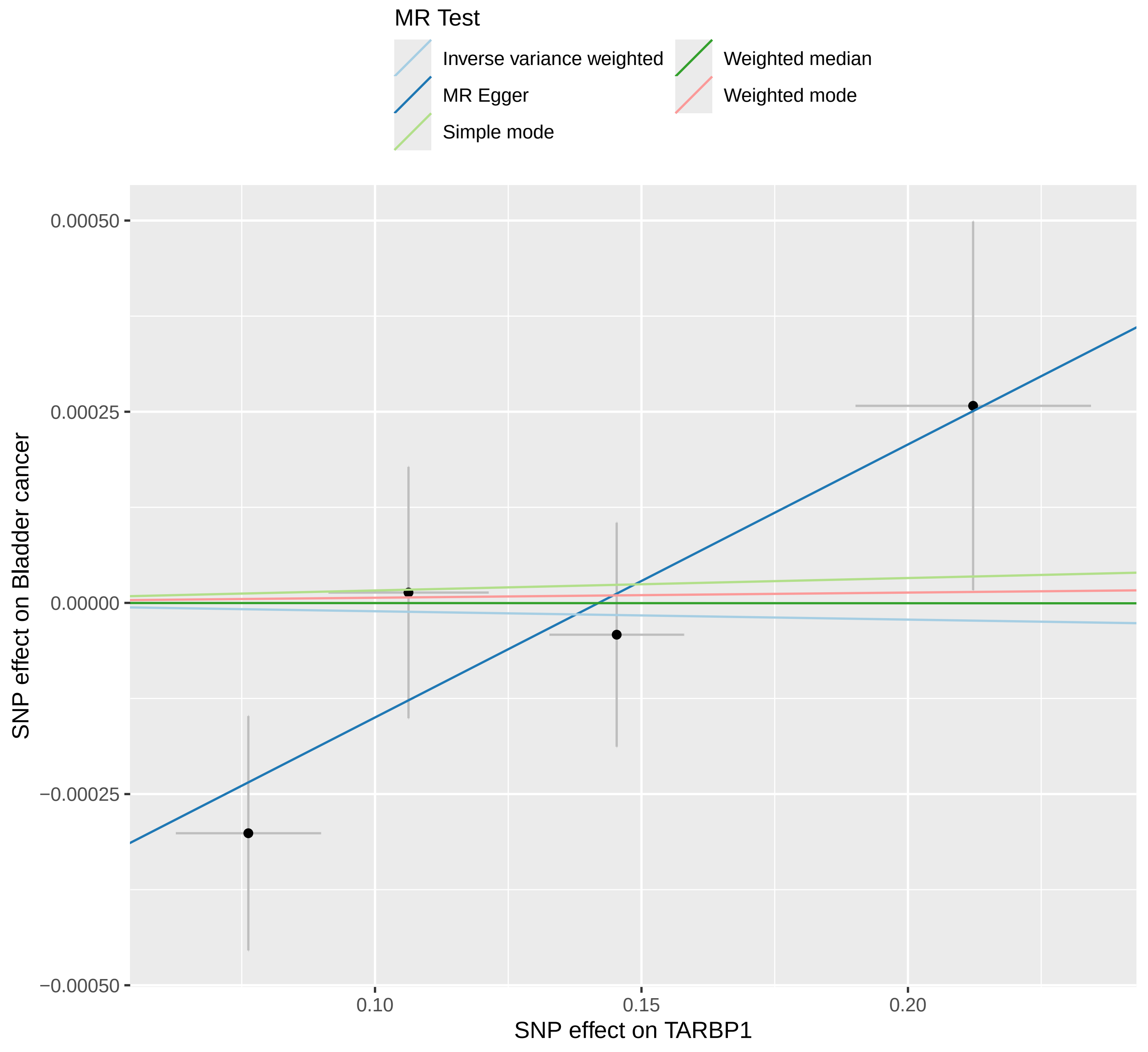

MR Test
Inverse variance weighted
MR Egger
Simple mode
Weighted median
Weighted mode
0.00050
0.00025
0.00000
−0.00025
−0.00050
SNP effect on Bladder cancer
0.10
0.15
0.20
SNP effect on TARBP1

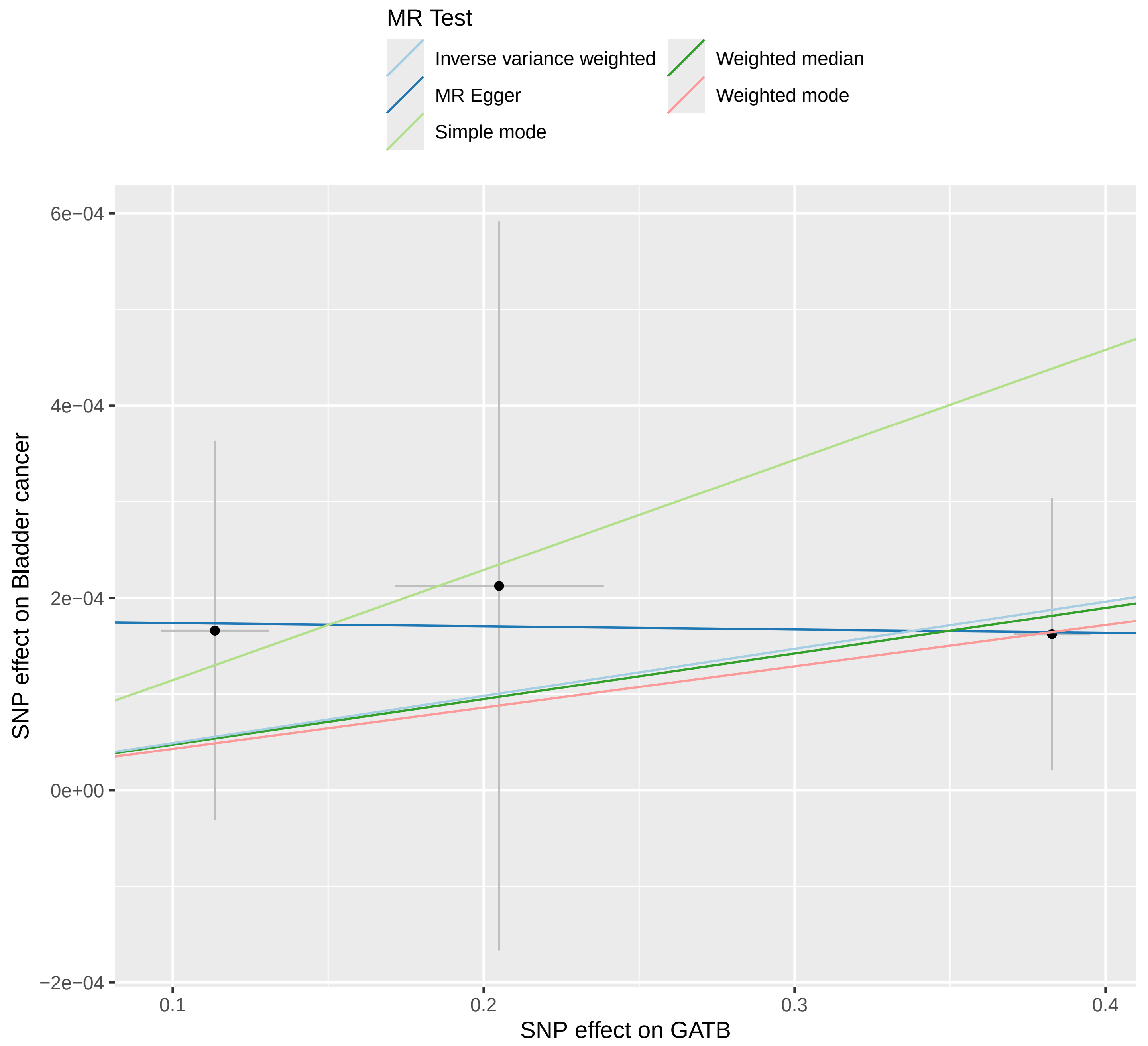
MR Test
Inverse variance weighted
MR Egger
Simple mode
Weighted median
Weighted mode
6e−04
4e−04
2e−04
0e+00
−2e−04
SNP effect on Bladder cancer
0.1
0.2
0.3
0.4
SNP effect on GATB

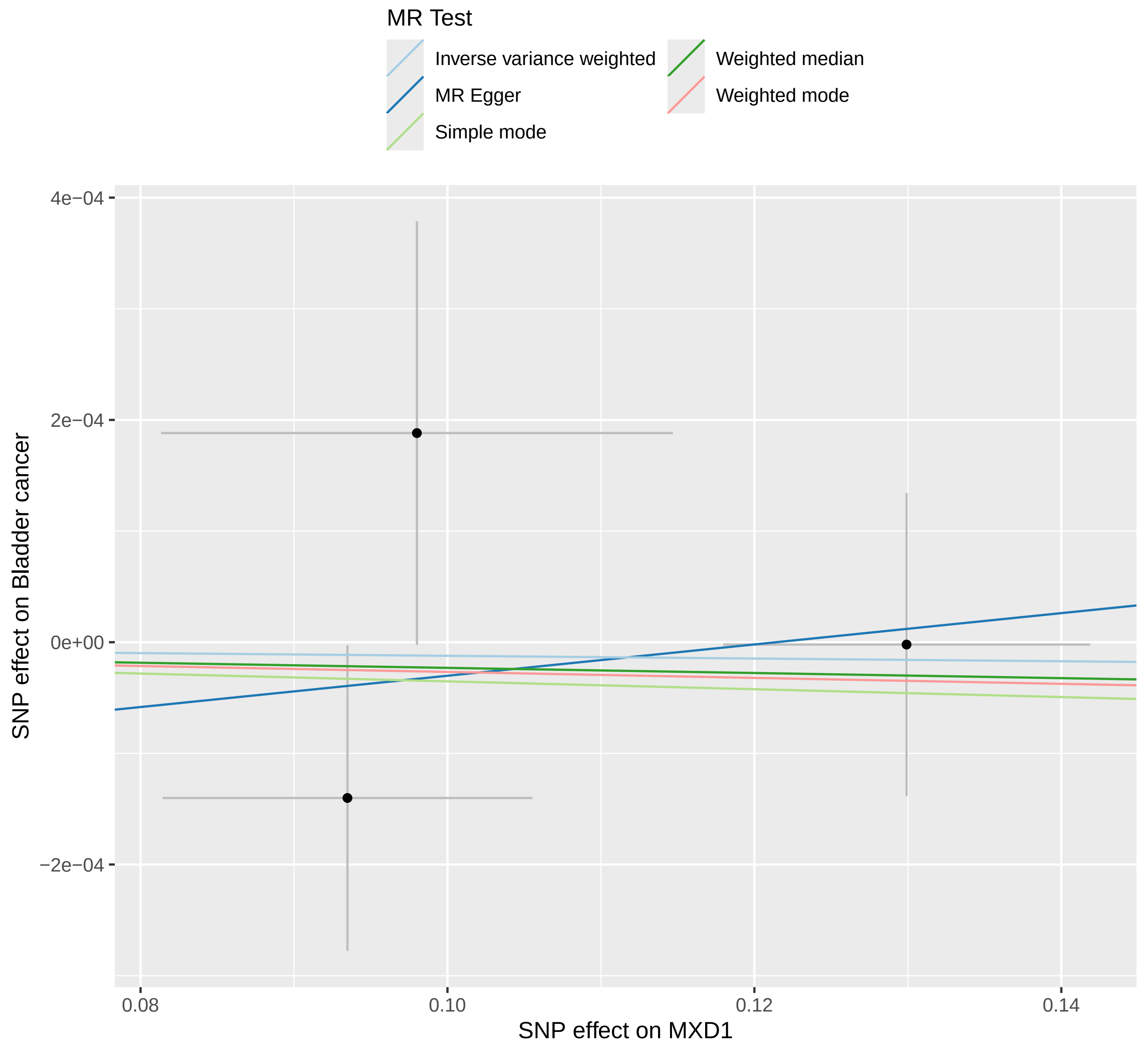

MR Test
Inverse variance weighted
MR Egger
Simple mode
Weighted median
Weighted mode
4e−04
2e−04
0e+00
−2e−04
SNP effect on Bladder cancer
0.08
0.10
0.12
0.14
SNP effect on MXD1

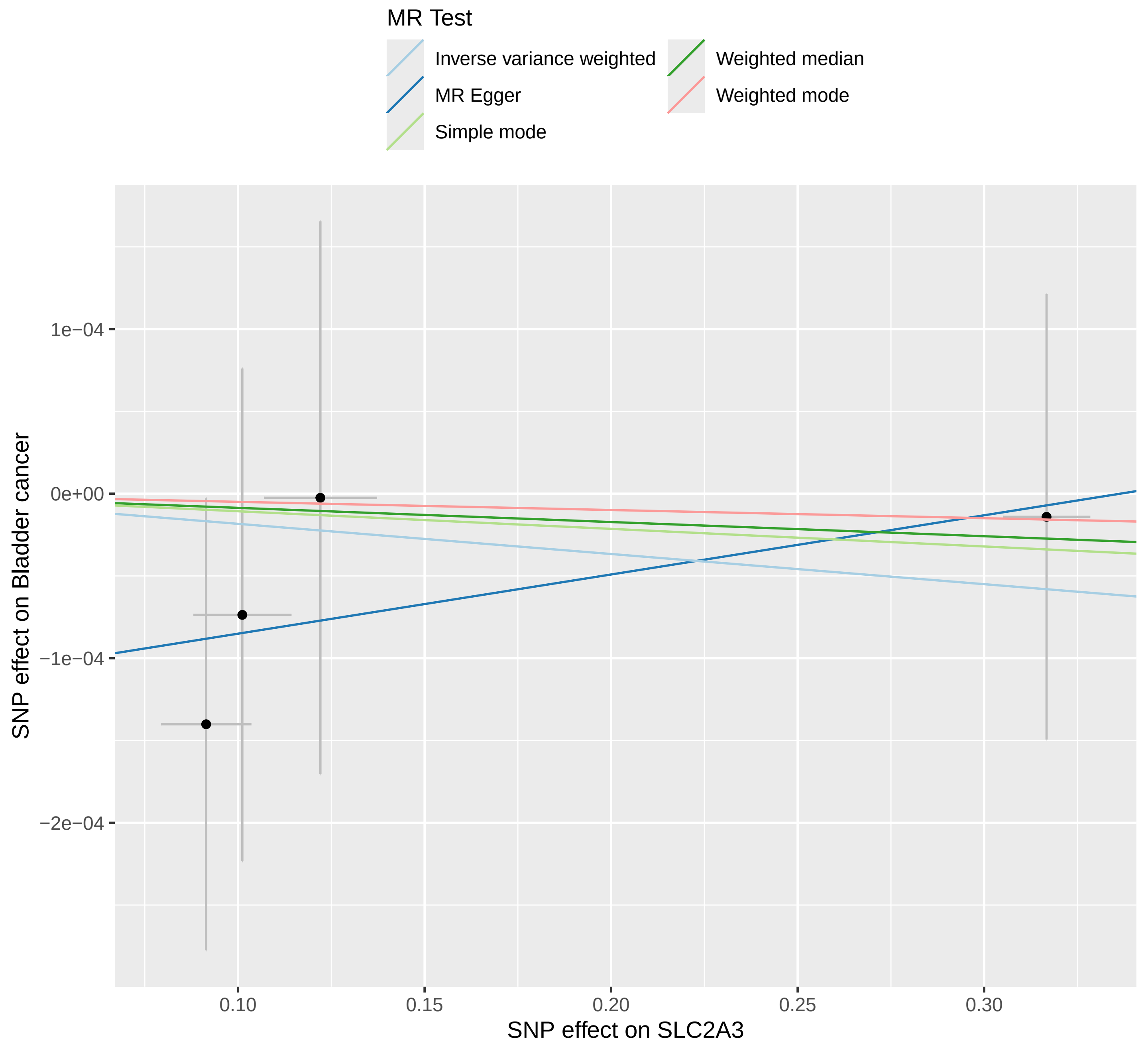

MR Test
Inverse variance weighted
MR Egger
Simple mode
Weighted median
Weighted mode
1e−04
0e+00
−1e−04
−2e−04
0.10
0.15
0.20
0.25
0.30
SNP effect on SLC2A3
SNP effect on Bladder cancer

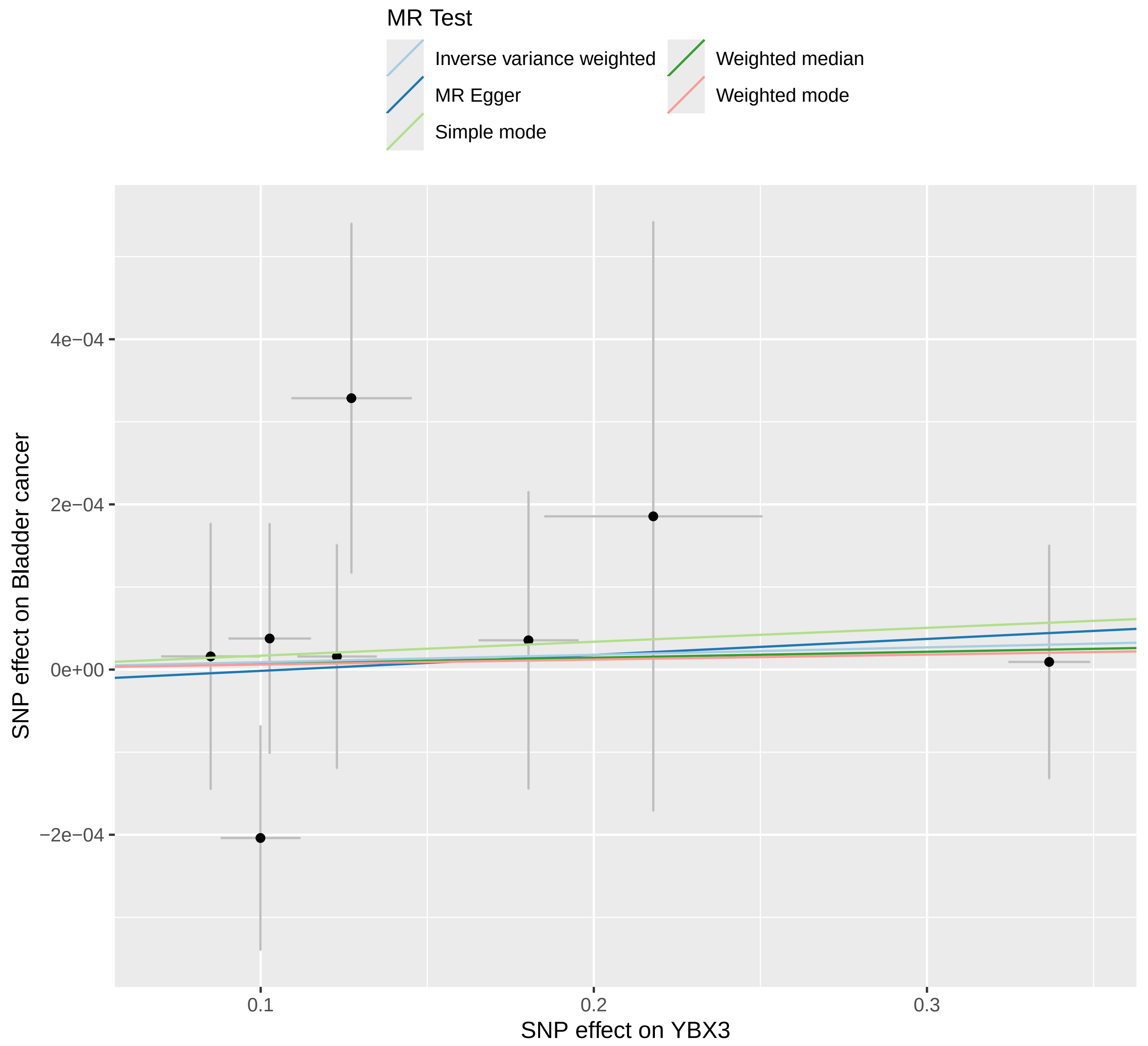

MR Test
Inverse variance weighted
MR Egger
Simple mode
Weighted median
Weighted mode
4e−04
2e−04
0e+00
−2e−04
SNP effect on Bladder cancer
0.1
0.2
0.3
SNP effect on YBX3

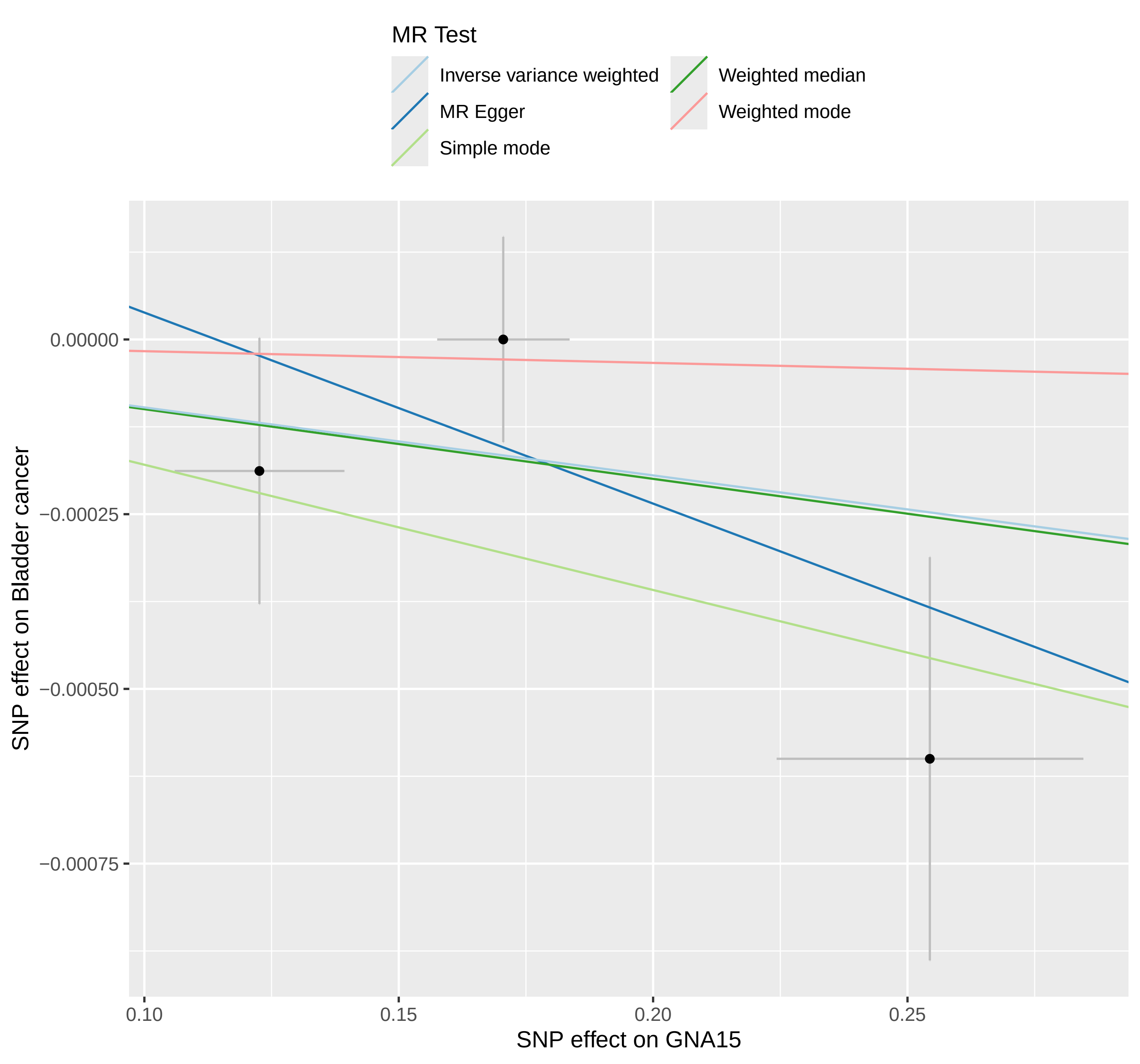

MR Test
Inverse variance weighted
MR Egger
Simple mode
Weighted median
Weighted mode
0.00000
−0.00025
−0.00050
−0.00075
SNP effect on Bladder cancer
0.10
0.15
0.20
0.25
SNP effect on GNA15

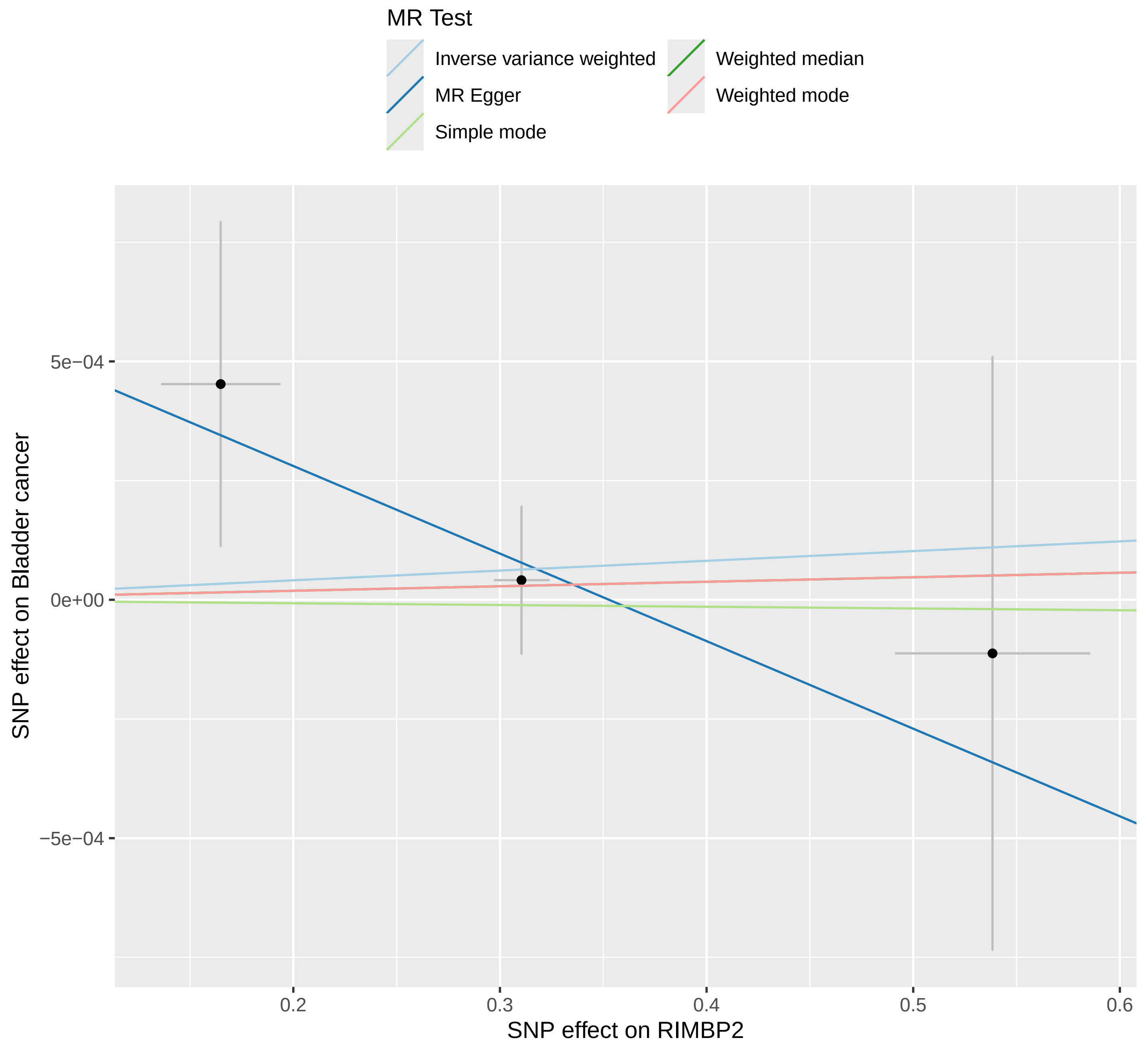

MR Test
Inverse variance weighted
MR Egger
Simple mode
Weighted median
Weighted mode
5e−04
0e+00
−5e−04
SNP effect on Bladder cancer
0.2
0.3
0.4
0.5
0.6
SNP effect on RIMBP2

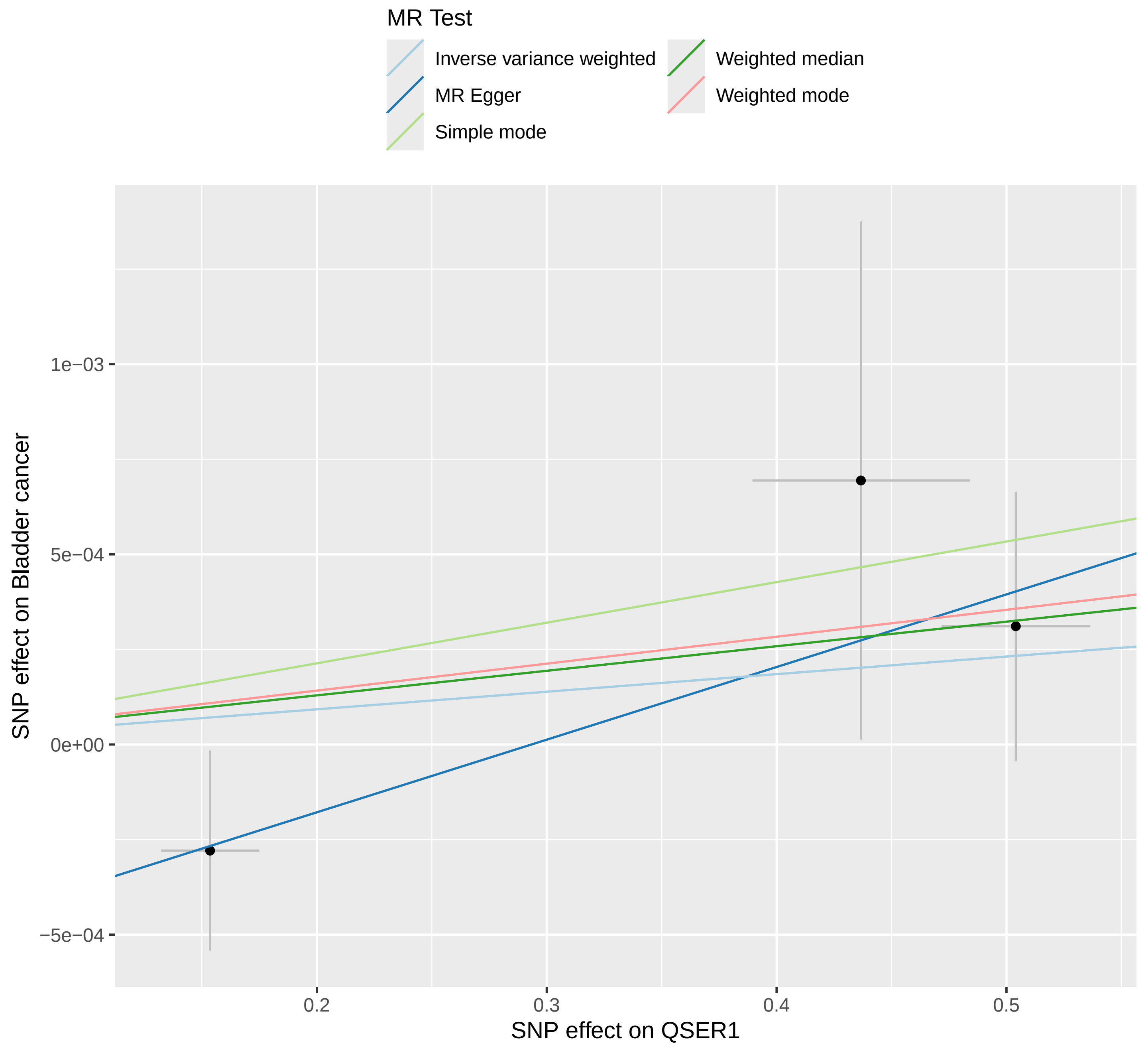
MR Test
Inverse variance weighted
MR Egger
Simple mode
Weighted median
Weighted mode
1e−03
5e−04
0e+00
−5e−04
0.2
0.3
0.4
0.5
SNP effect on QSER1
SNP effect on Bladder cancer

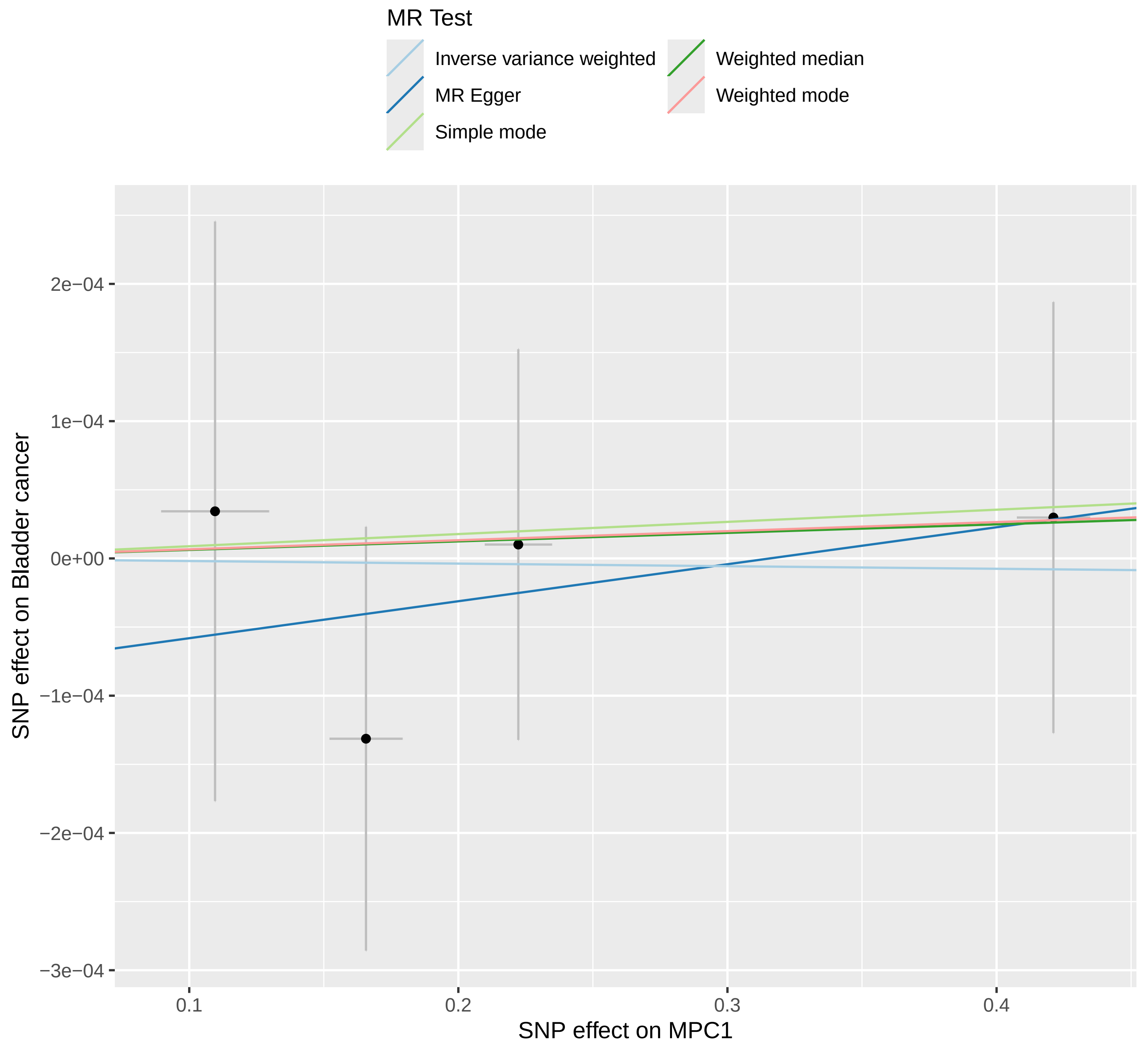

MR Test
Inverse variance weighted
MR Egger
Simple mode
Weighted median
Weighted mode
SNP effect on Bladder cancer
2e−04
1e−04
0e+00
−1e−04
−2e−04
−3e−04
0.1
0.2
0.3
0.4
SNP effect on MPC1

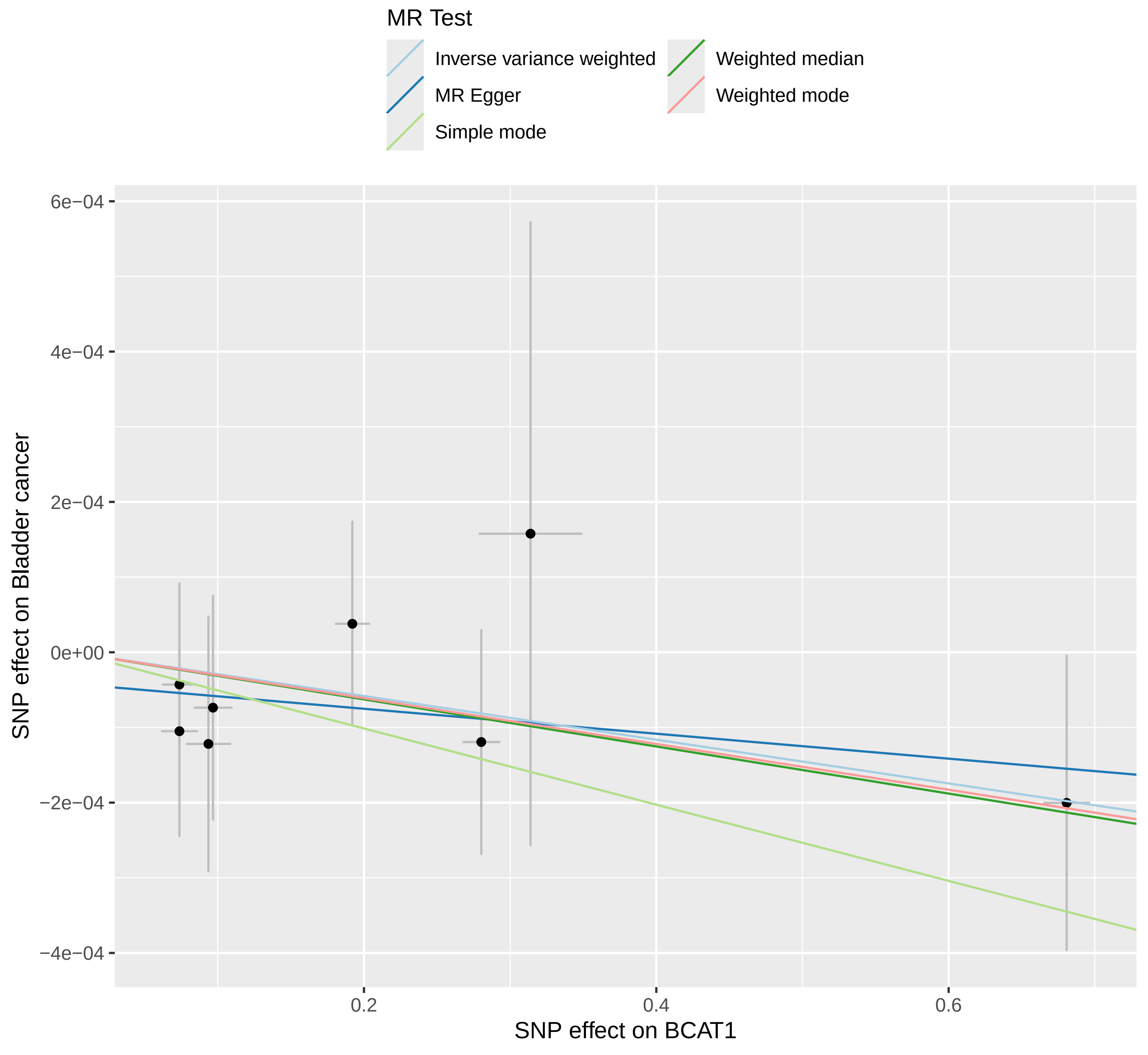

MR Test
Inverse variance weighted
MR Egger
Simple mode
Weighted median
Weighted mode
6e−04
4e−04
2e−04
0e+00
−2e−04
−4e−04
SNP effect on Bladder cancer
0.2
0.4
0.6
SNP effect on BCAT1

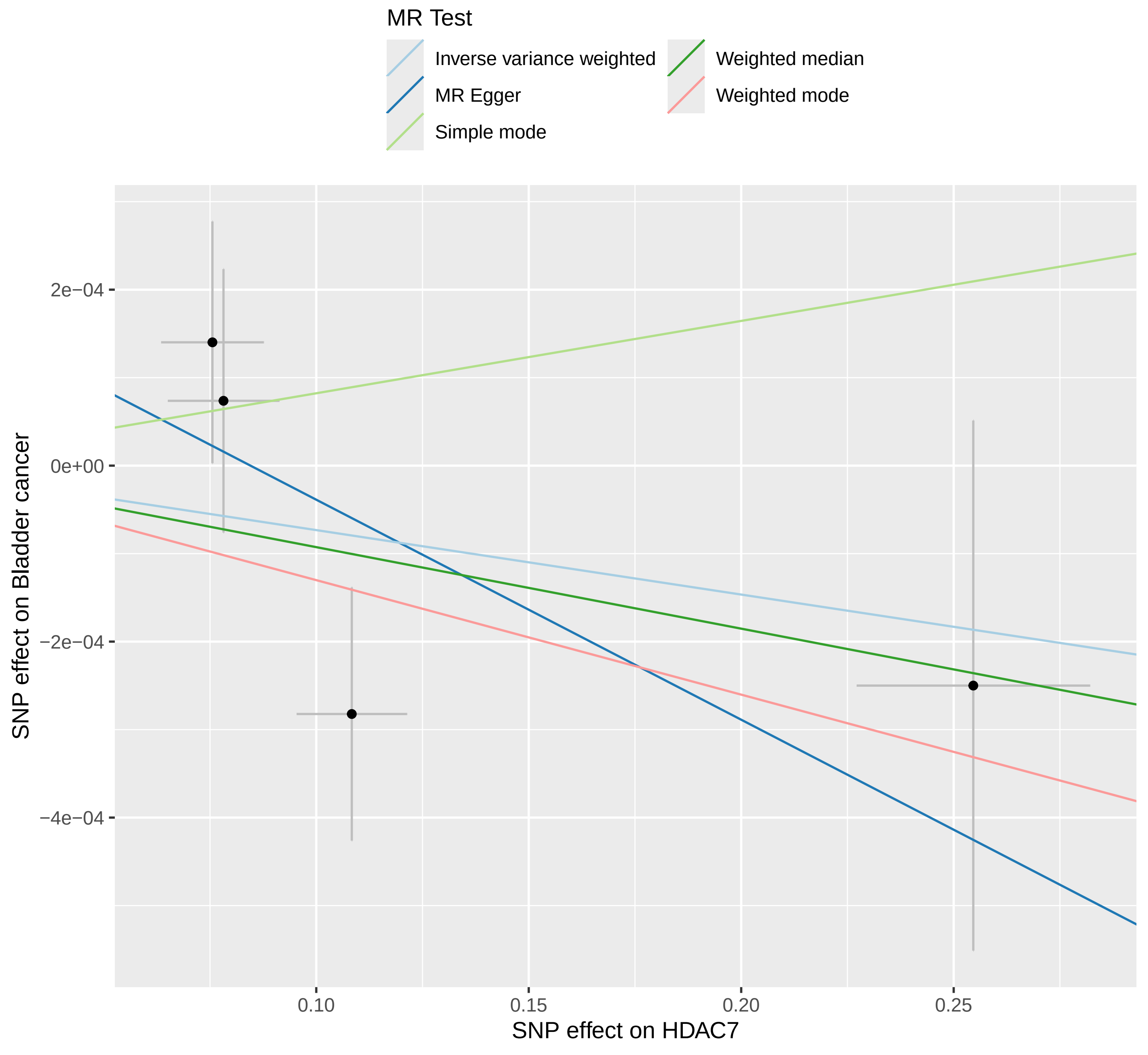
MR Test
Inverse variance weighted
MR Egger
Simple mode
Weighted median
Weighted mode
2e−04
0e+00
−2e−04
−4e−04
SNP effect on Bladder cancer
0.10
0.15
0.20
0.25
SNP effect on HDAC7

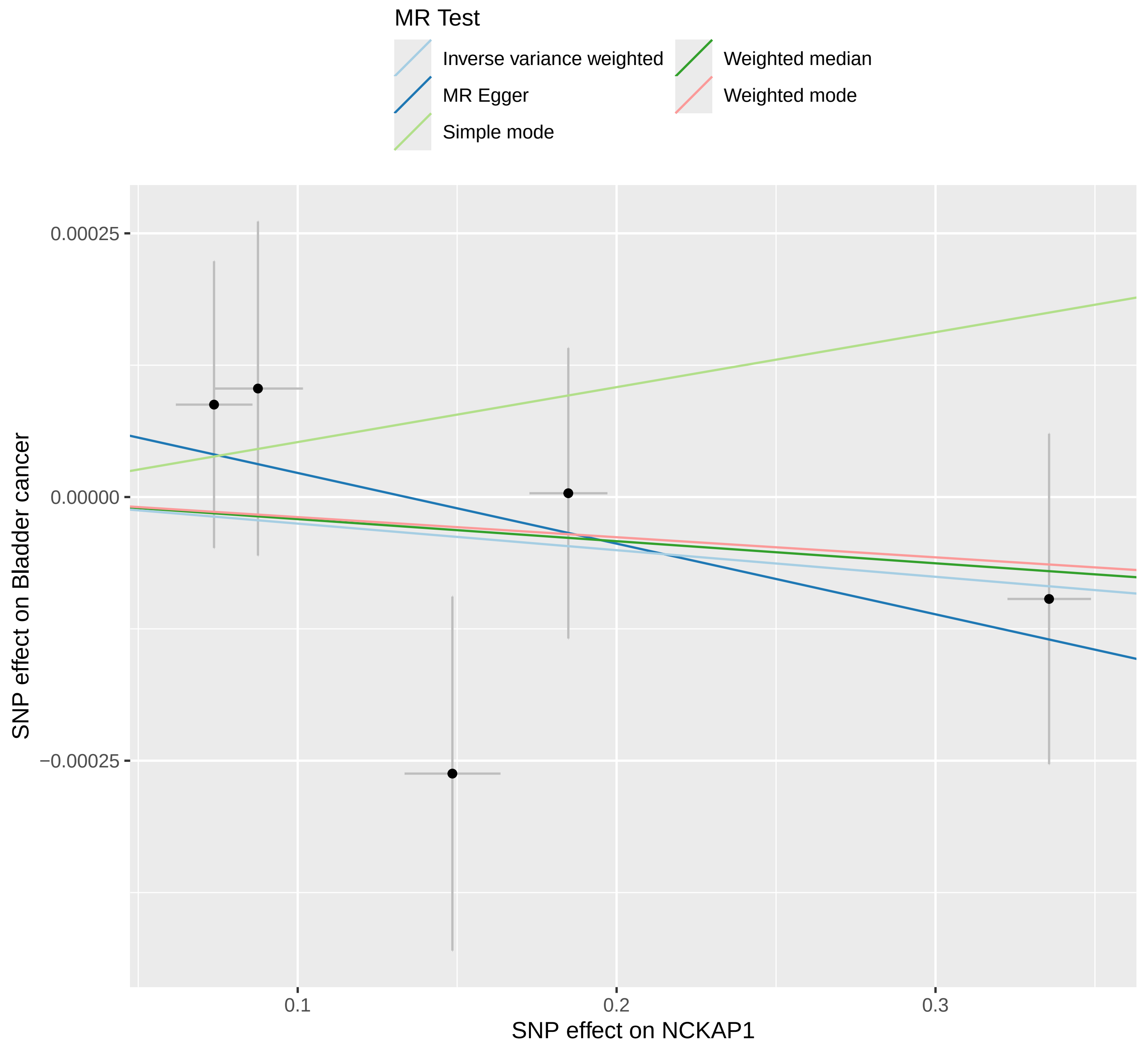
MR Test
Inverse variance weighted
MR Egger
Simple mode
Weighted median
Weighted mode
0.00025
0.00000
−0.00025
0.1
0.2
0.3
SNP effect on Bladder cancer
SNP effect on NCKAP1

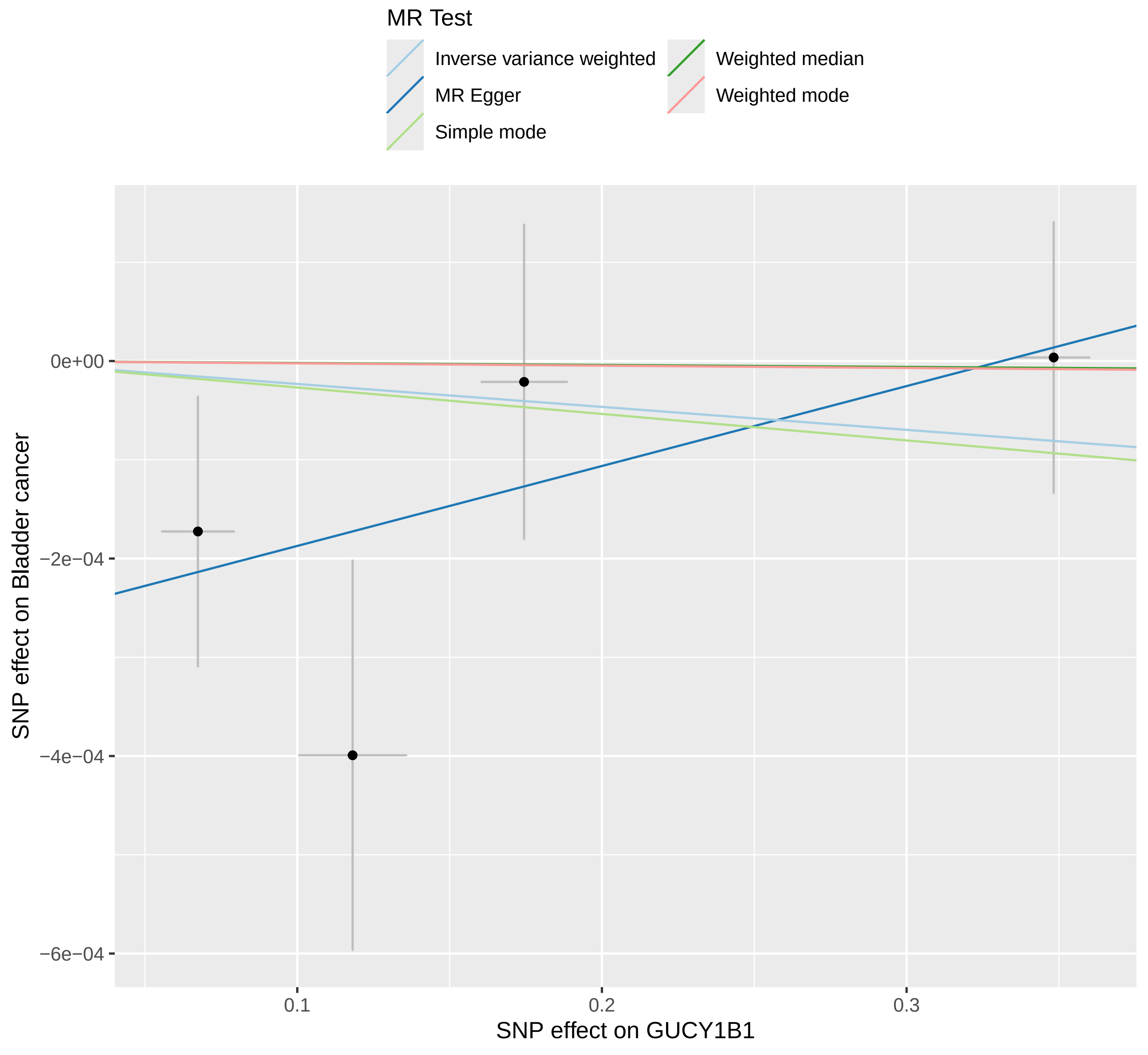
MR Test
Inverse variance weighted
MR Egger
Simple mode
Weighted median
Weighted mode
0e+00
−2e−04
−4e−04
−6e−04
SNP effect on Bladder cancer
0.1
0.2
0.3
SNP effect on GUCY1B1

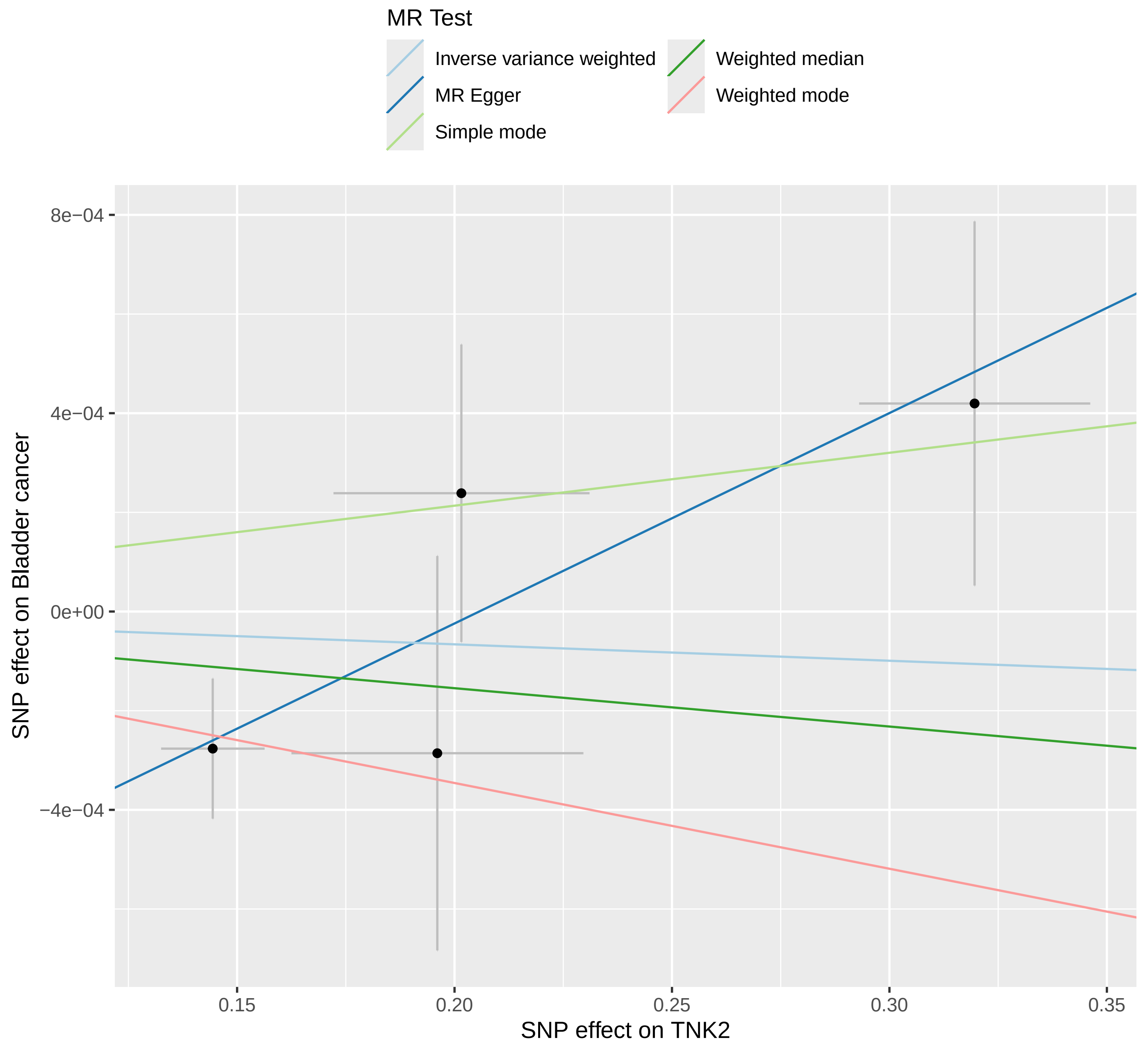
MR Test
Inverse variance weighted
MR Egger
Simple mode
Weighted median
Weighted mode
8e−04
4e−04
0e+00
−4e−04
SNP effect on Bladder cancer
0.15
0.20
0.25
0.30
0.35
SNP effect on TNK2

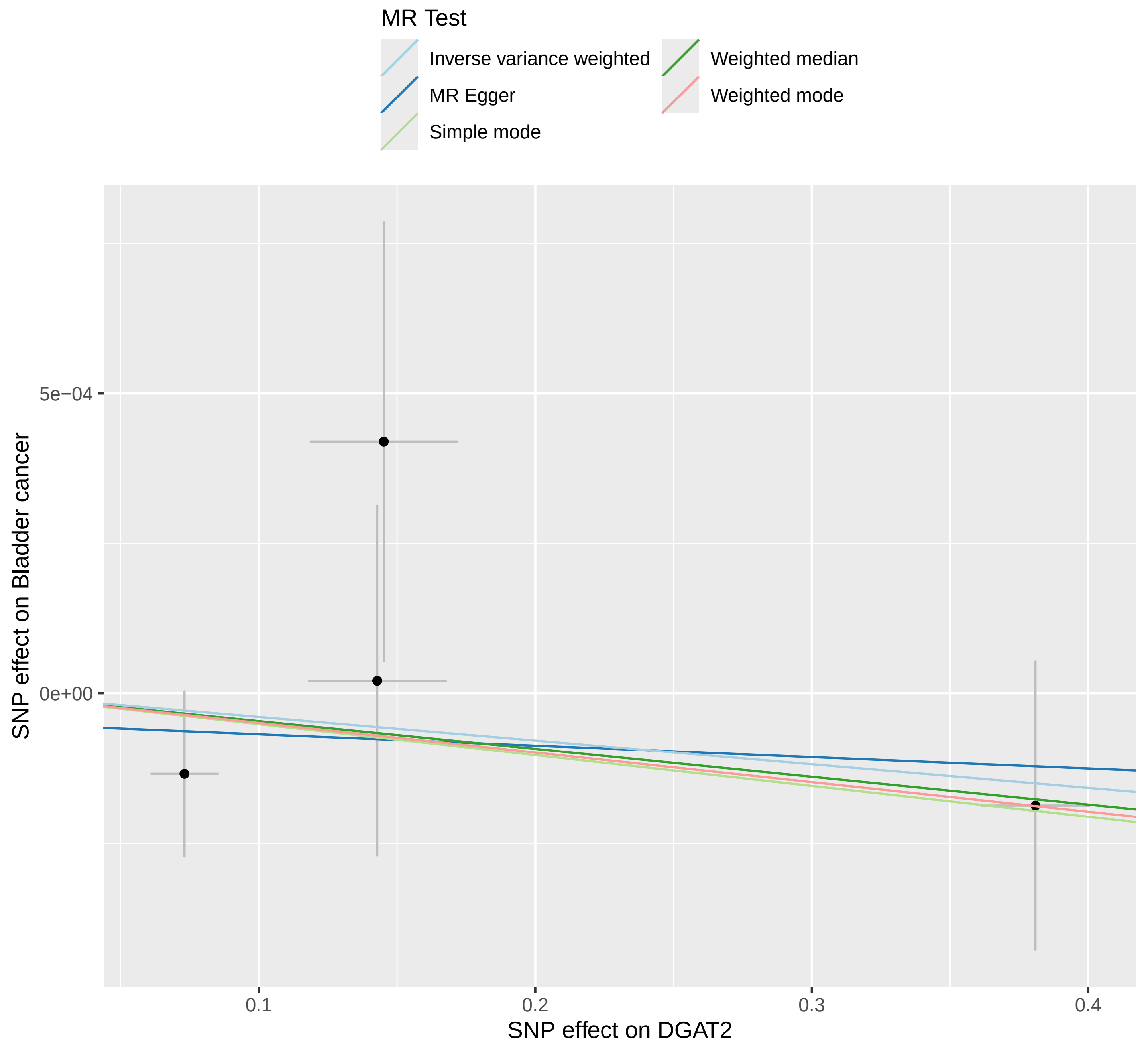

MR Test
Inverse variance weighted
MR Egger
Simple mode
Weighted median
Weighted mode
5e−04
0e+00
SNP effect on Bladder cancer
0.1
0.2
0.3
0.4
SNP effect on DGAT2

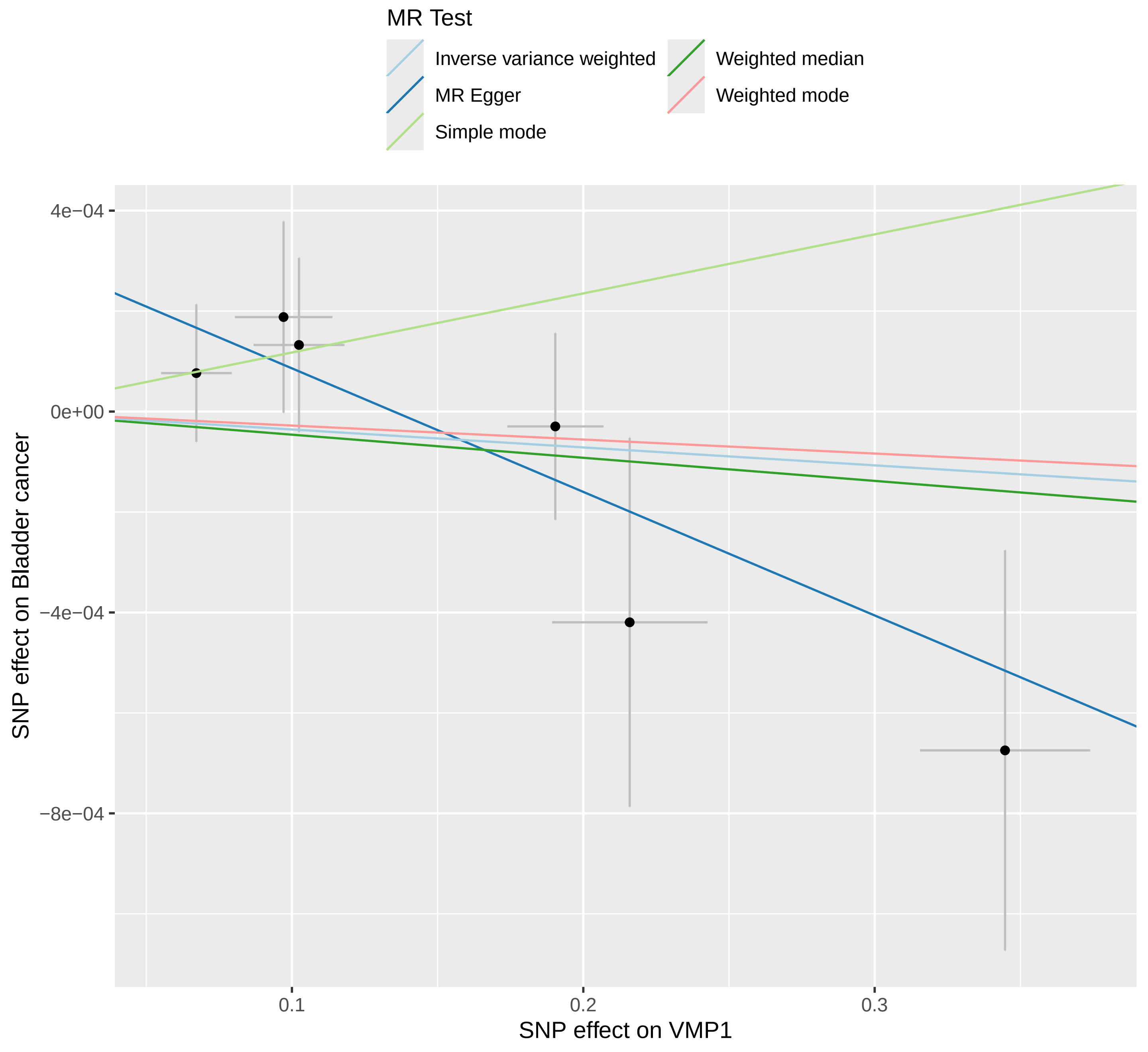

MR Test
Inverse variance weighted
MR Egger
Simple mode
Weighted median
Weighted mode
4e−04
0e+00
−4e−04
−8e−04
0.1
0.2
0.3
SNP effect on VMP1
SNP effect on Bladder cancer

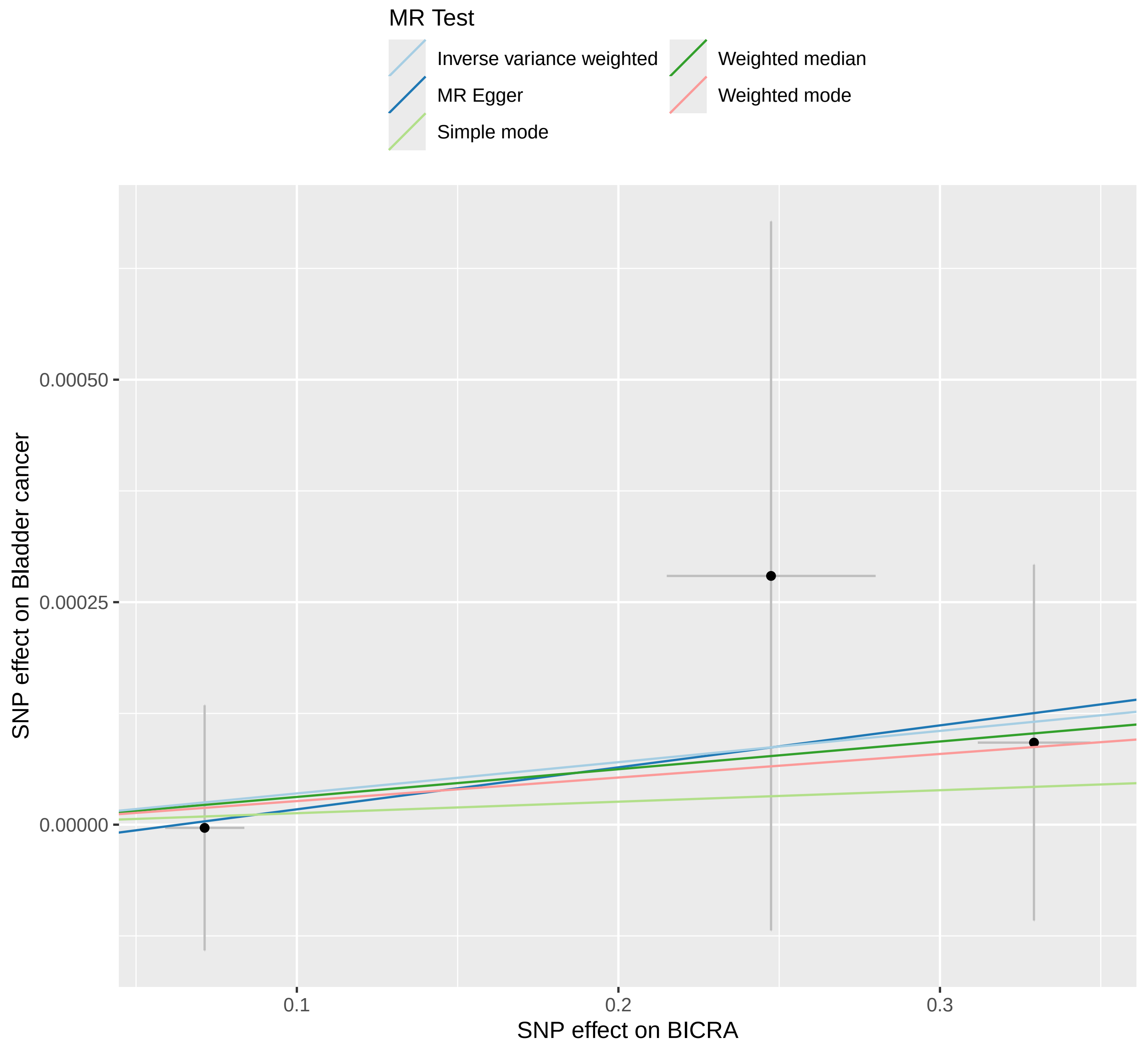

MR Test
Inverse variance weighted
MR Egger
Simple mode
Weighted median
Weighted mode
0.00050
0.00025
0.00000
SNP effect on Bladder cancer
0.1
0.2
0.3
SNP effect on BICRA

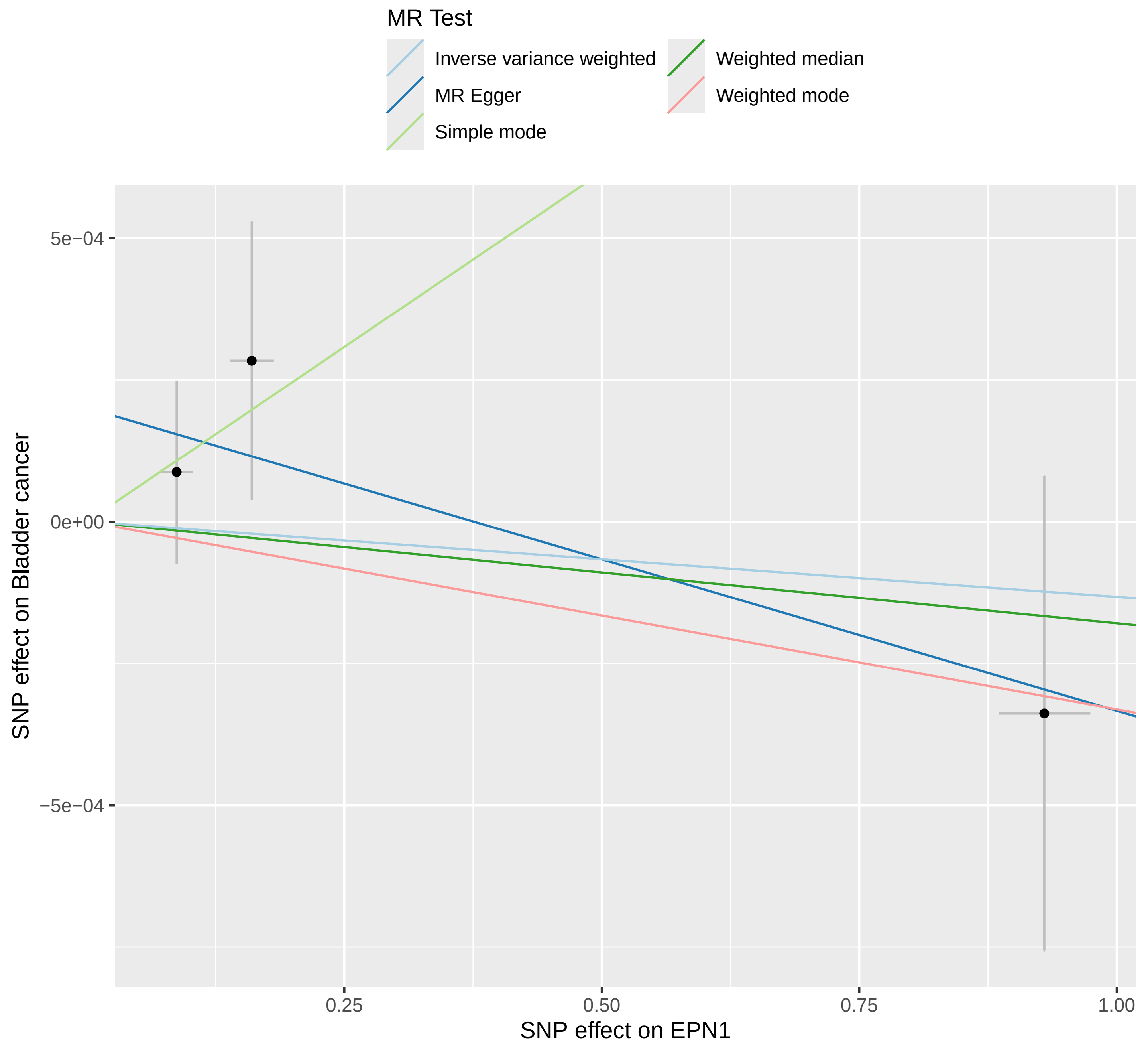
MR Test
Inverse variance weighted
MR Egger
Simple mode
Weighted median
Weighted mode
5e−04
0e+00
−5e−04
0.25
0.50
0.75
1.00
SNP effect on Bladder cancer
SNP effect on EPN1

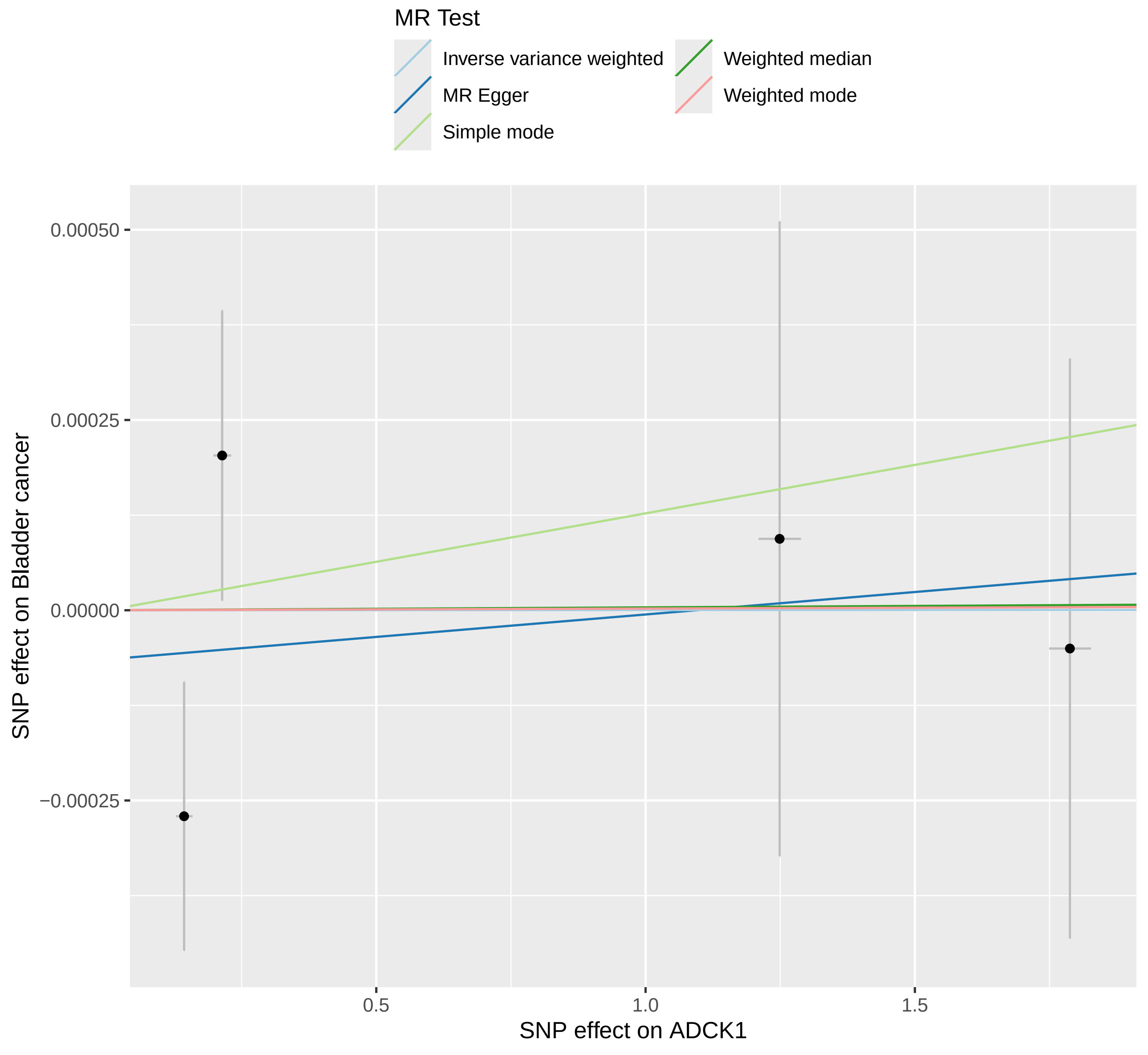

MR Test
Inverse variance weighted
MR Egger
Simple mode
Weighted median
Weighted mode
0.00050
0.00025
0.00000
−0.00025
SNP effect on Bladder cancer
0.5
1.0
1.5
SNP effect on ADCK1

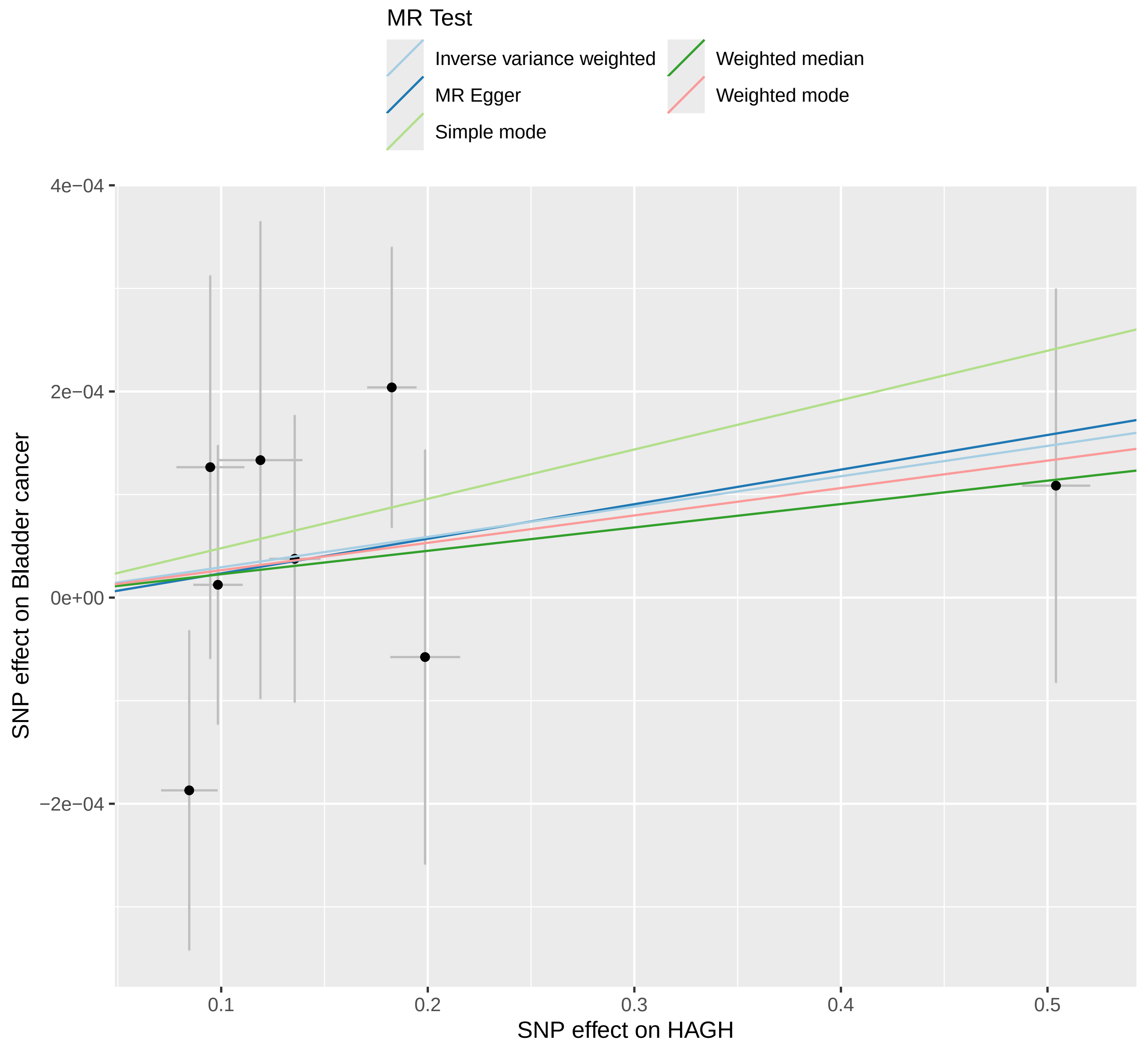

MR Test
Inverse variance weighted
MR Egger
Simple mode
Weighted median
Weighted mode
4e−04
2e−04
0e+00
−2e−04
SNP effect on Bladder cancer
0.1
0.2
0.3
0.4
0.5
SNP effect on HAGH

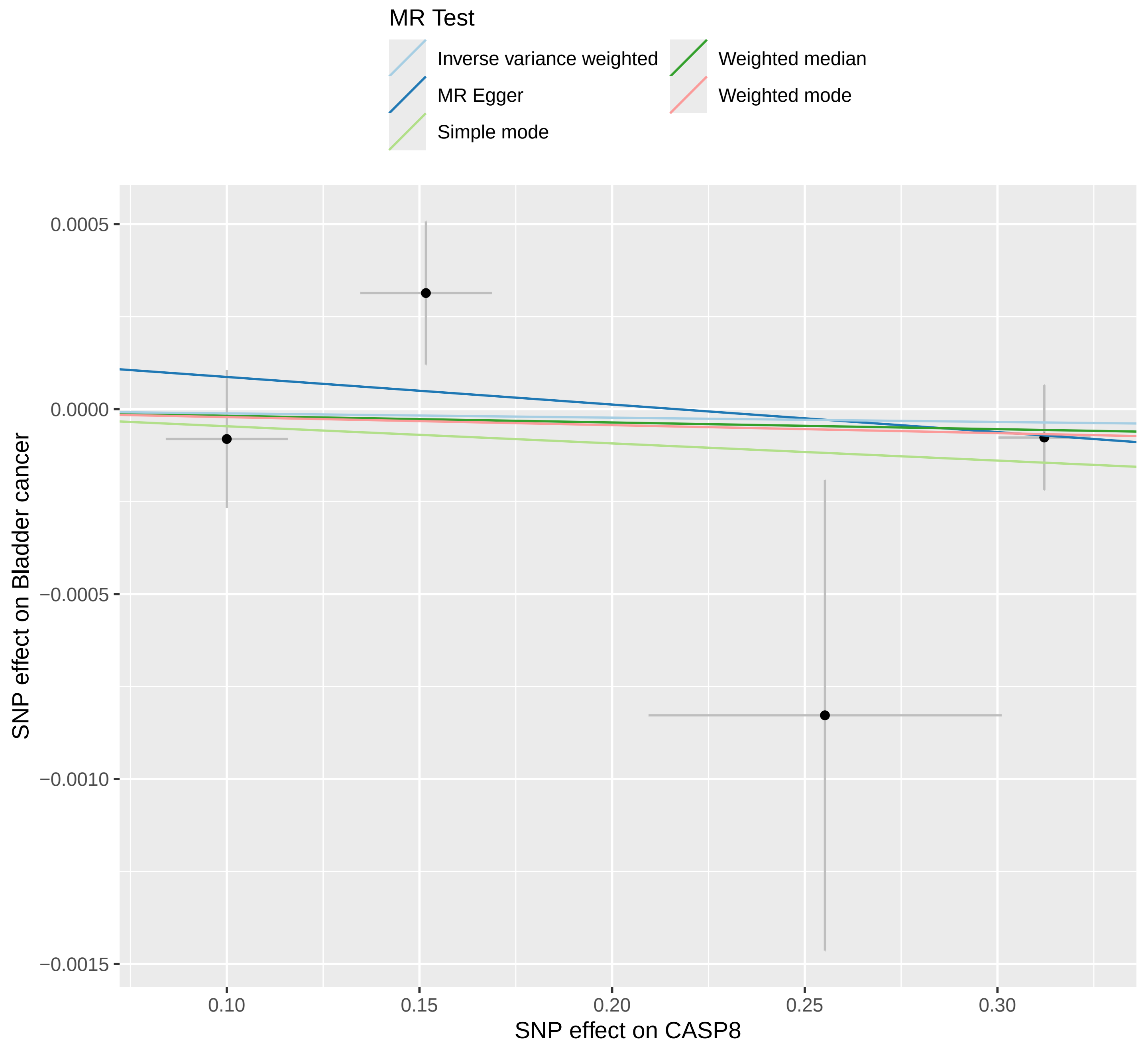

MR Test
Inverse variance weighted
MR Egger
Simple mode
Weighted median
Weighted mode
0.0005
0.0000
−0.0005
−0.0010
−0.0015
SNP effect on Bladder cancer
0.10
0.15
0.20
0.25
0.30
SNP effect on CASP8

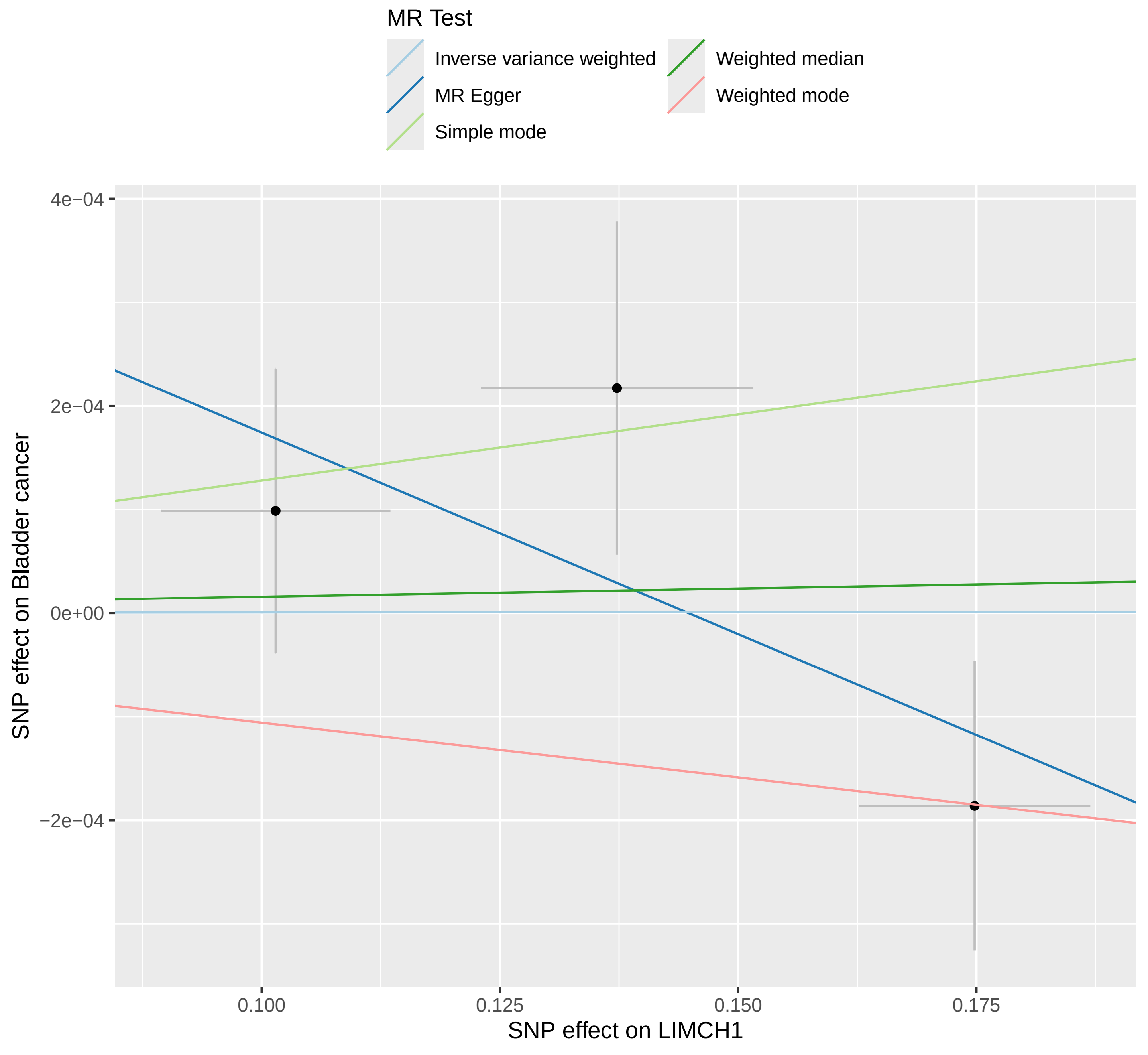
MR Test
Inverse variance weighted
MR Egger
Simple mode
Weighted median
Weighted mode
4e−04
2e−04
0e+00
−2e−04
0.100
0.125
0.150
0.175
SNP effect on LIMCH1
SNP effect on Bladder cancer

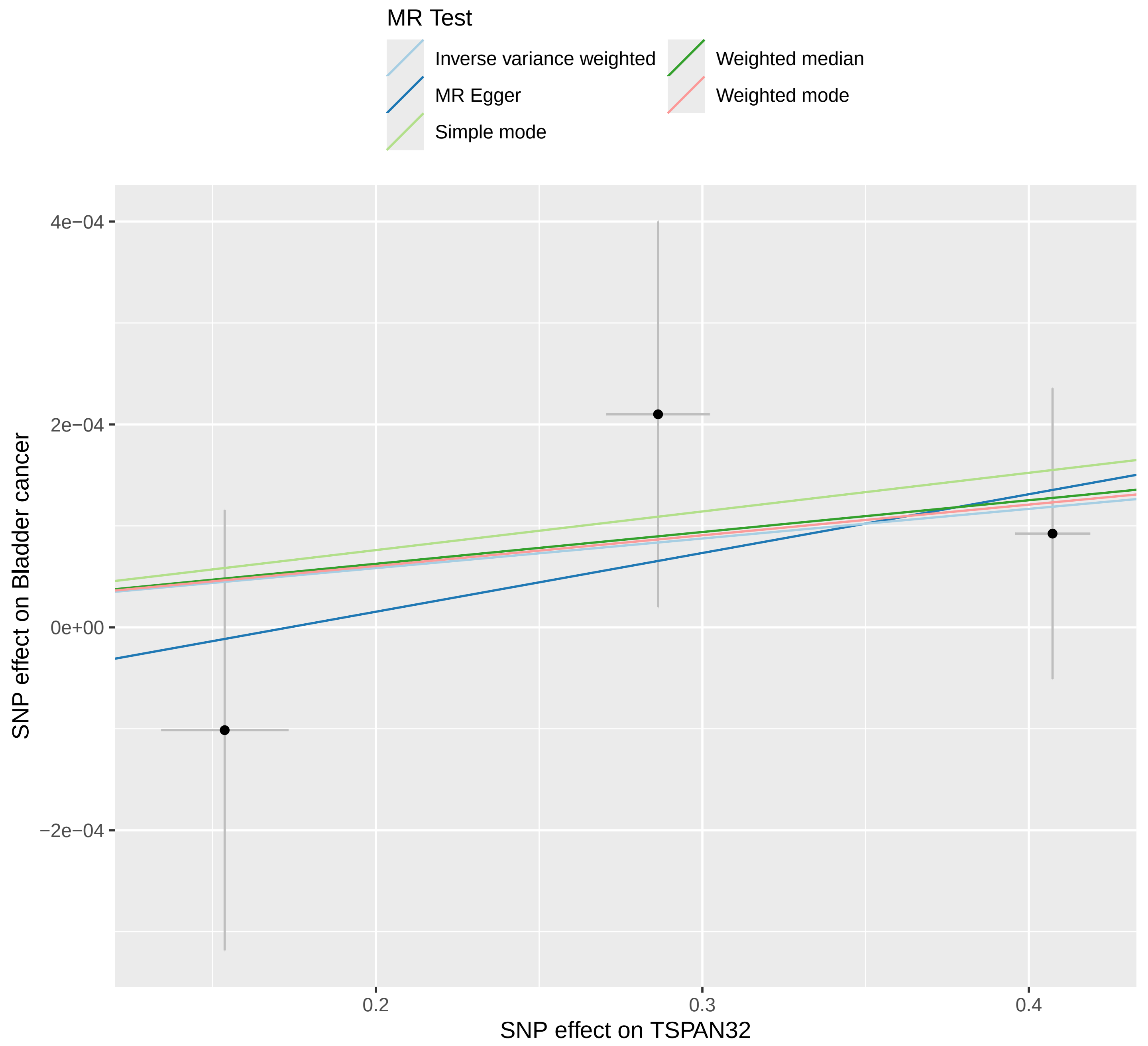
MR Test
Inverse variance weighted
MR Egger
Simple mode
Weighted median
Weighted mode
4e−04
2e−04
0e+00
−2e−04
0.2
0.3
0.4
SNP effect on Bladder cancer
SNP effect on TSPAN32

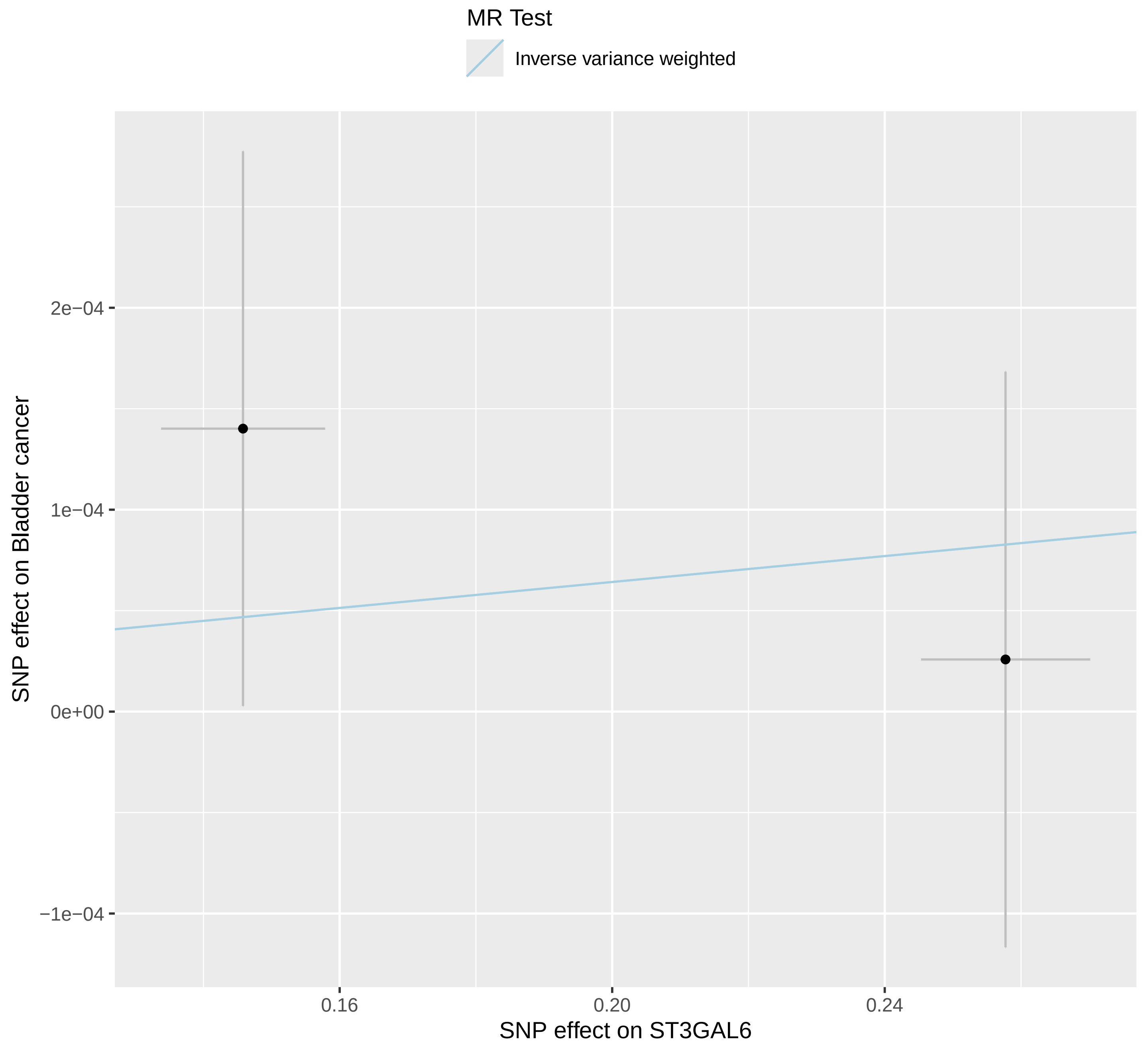

MR Test
Inverse variance weighted
2e−04
1e−04
0e+00
−1e−04
0.16
0.20
0.24
SNP effect on Bladder cancer
SNP effect on ST3GAL6

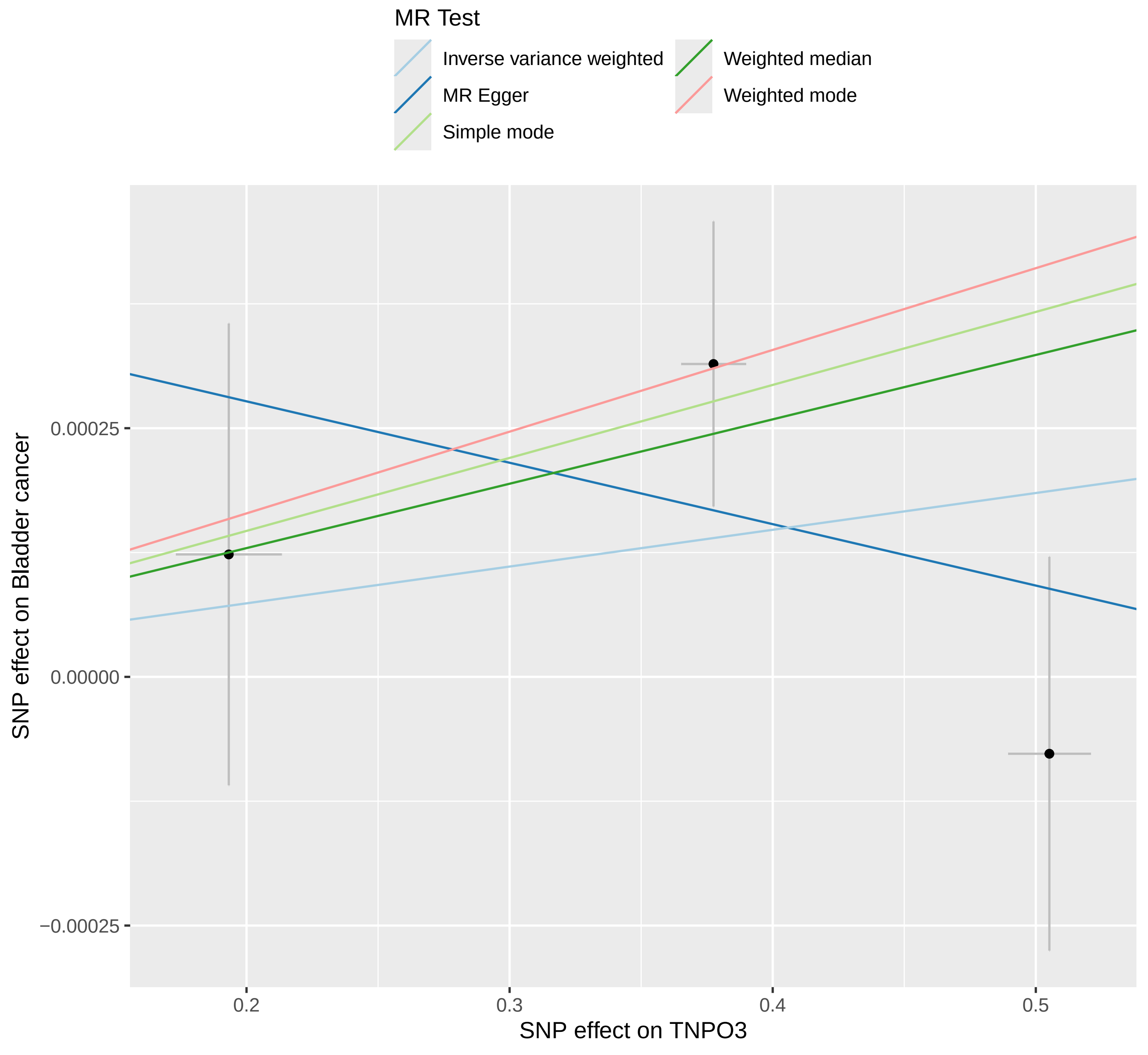
MR Test
Inverse variance weighted
MR Egger
Simple mode
Weighted median
Weighted mode
SNP effect on Bladder cancer
0.00025
0.00000
−0.00025
0.2
0.3
0.4
0.5
SNP effect on TNPO3

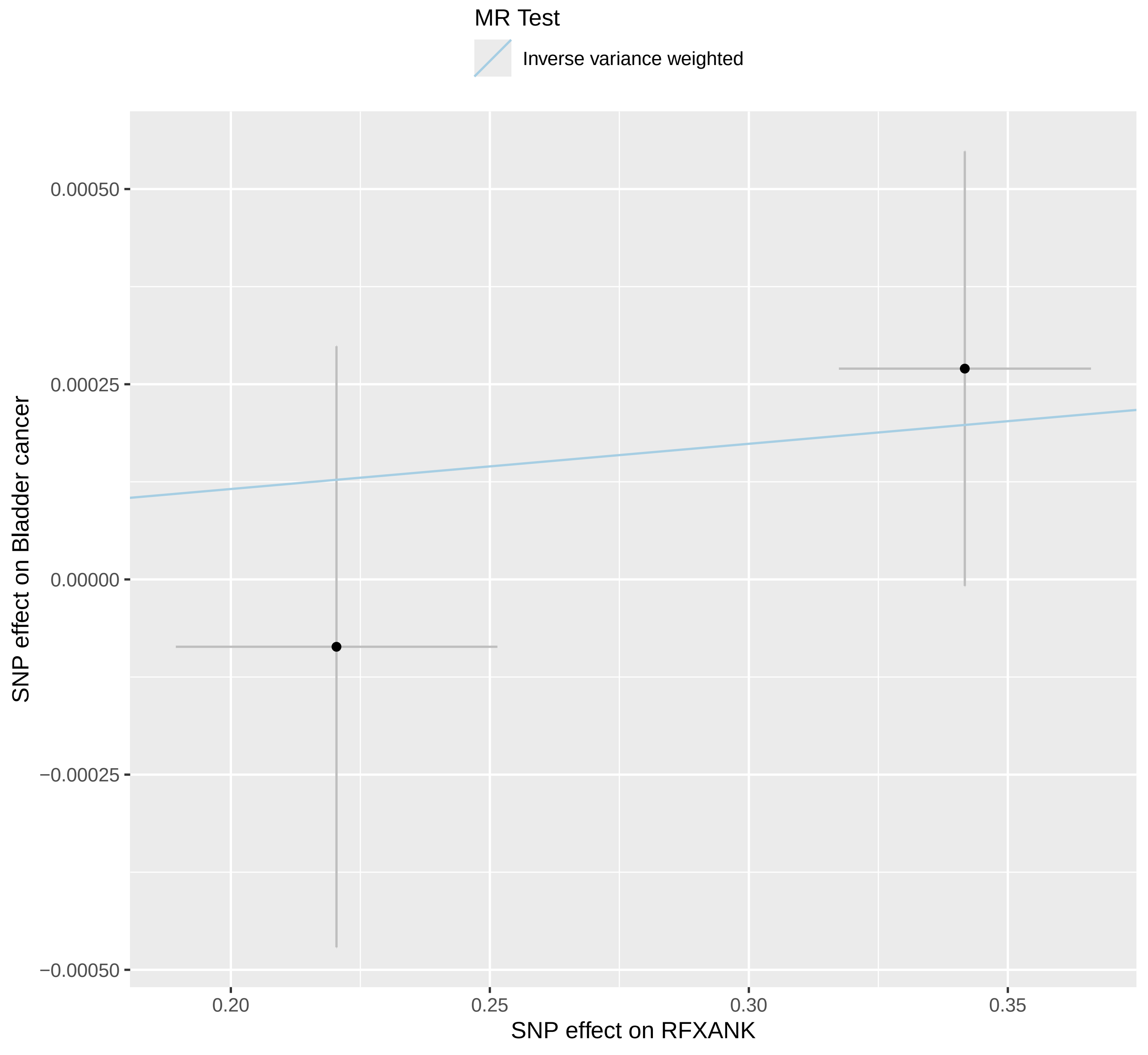

MR Test
Inverse variance weighted
0.00050
0.00025
0.00000
−0.00025
−0.00050
0.20
0.25
0.30
0.35
SNP effect on RFXANK
SNP effect on Bladder cancer

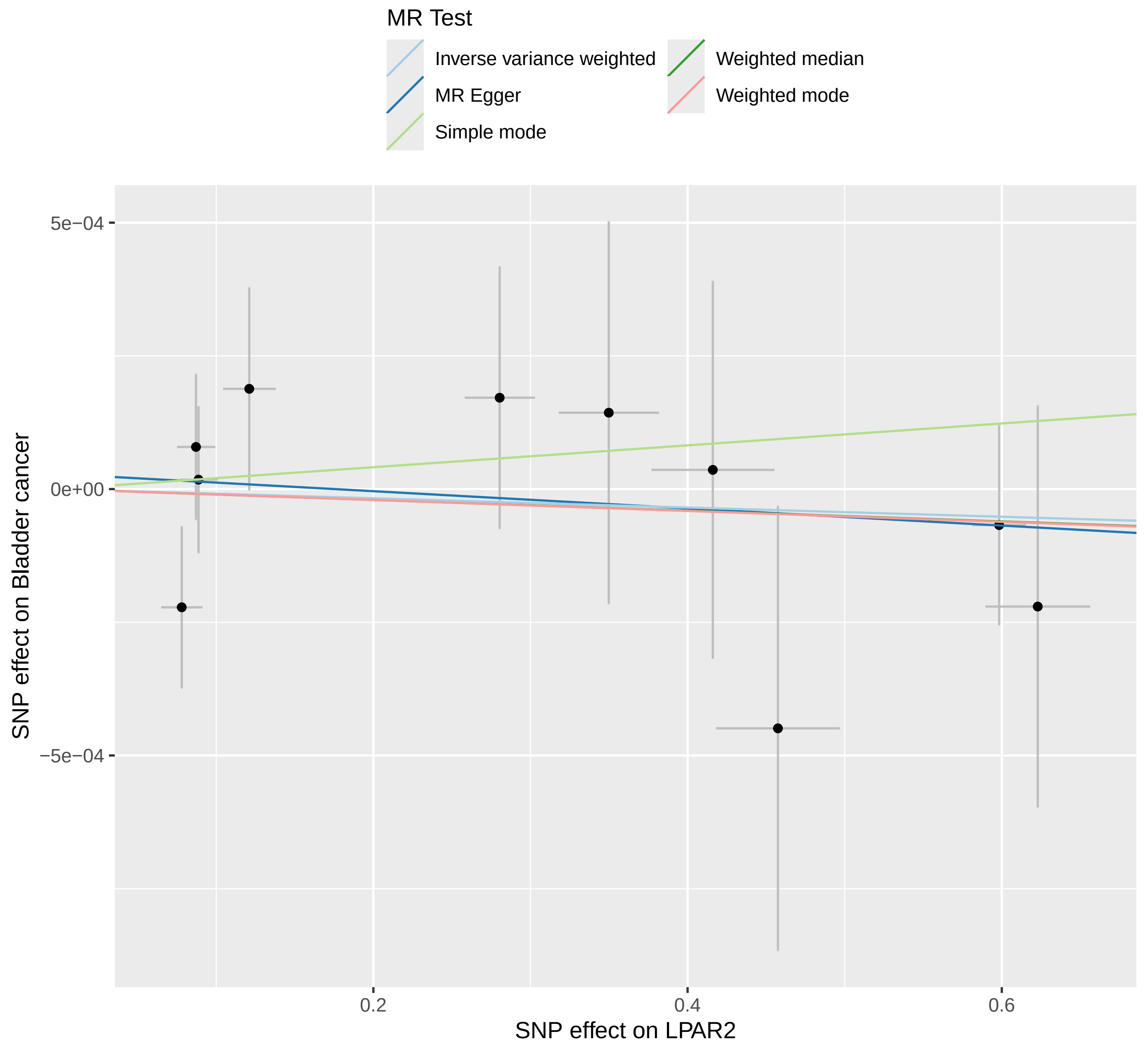
MR Test
Inverse variance weighted
MR Egger
Simple mode
Weighted median
Weighted mode
5e−04
0e+00
−5e−04
0.2
0.4
0.6
SNP effect on Bladder cancer
SNP effect on LPAR2

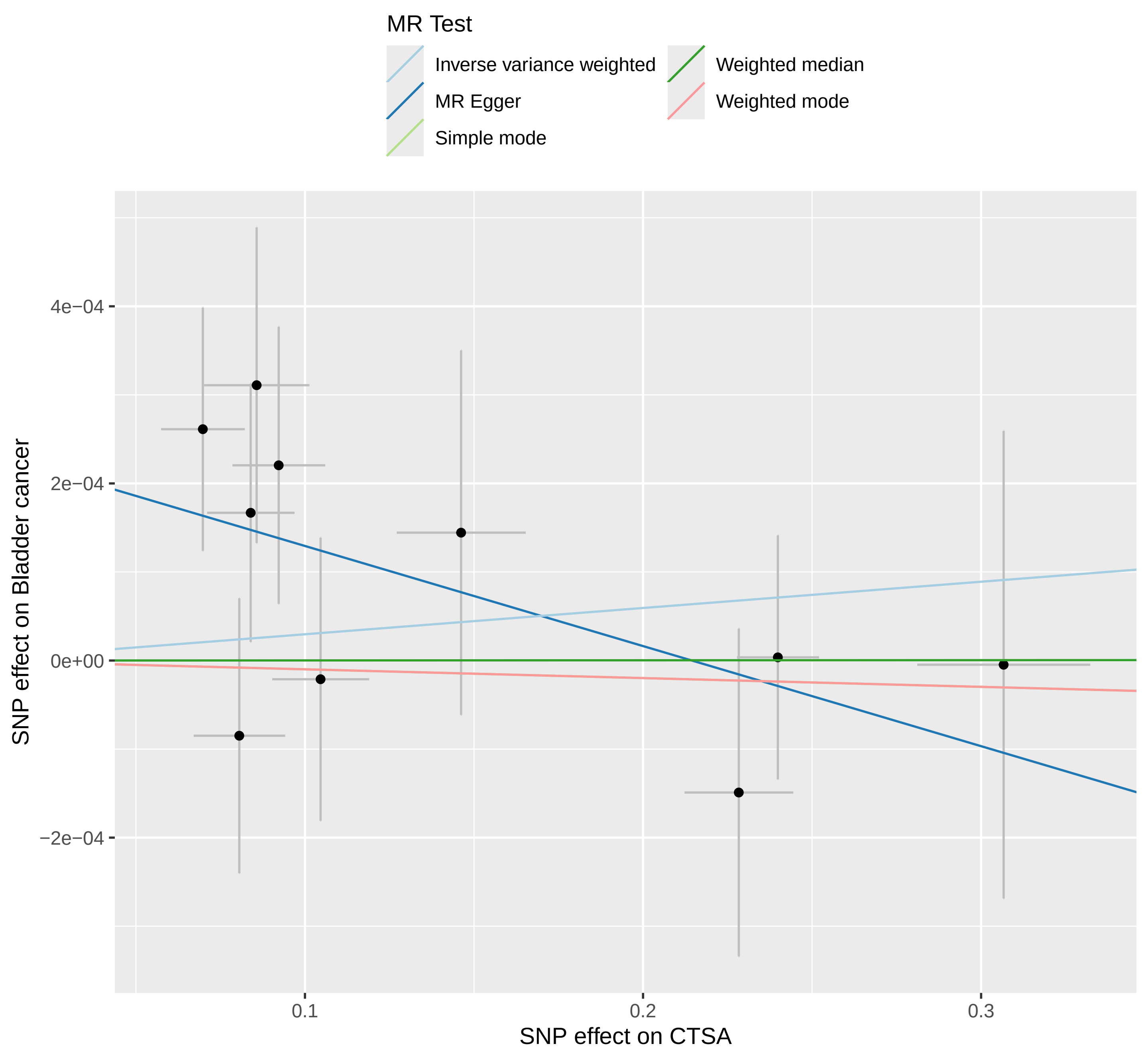
MR Test
Inverse variance weighted
MR Egger
Simple mode
Weighted median
Weighted mode
4e−04
2e−04
0e+00
−2e−04
SNP effect on Bladder cancer
0.1
0.2
0.3
SNP effect on CTSA

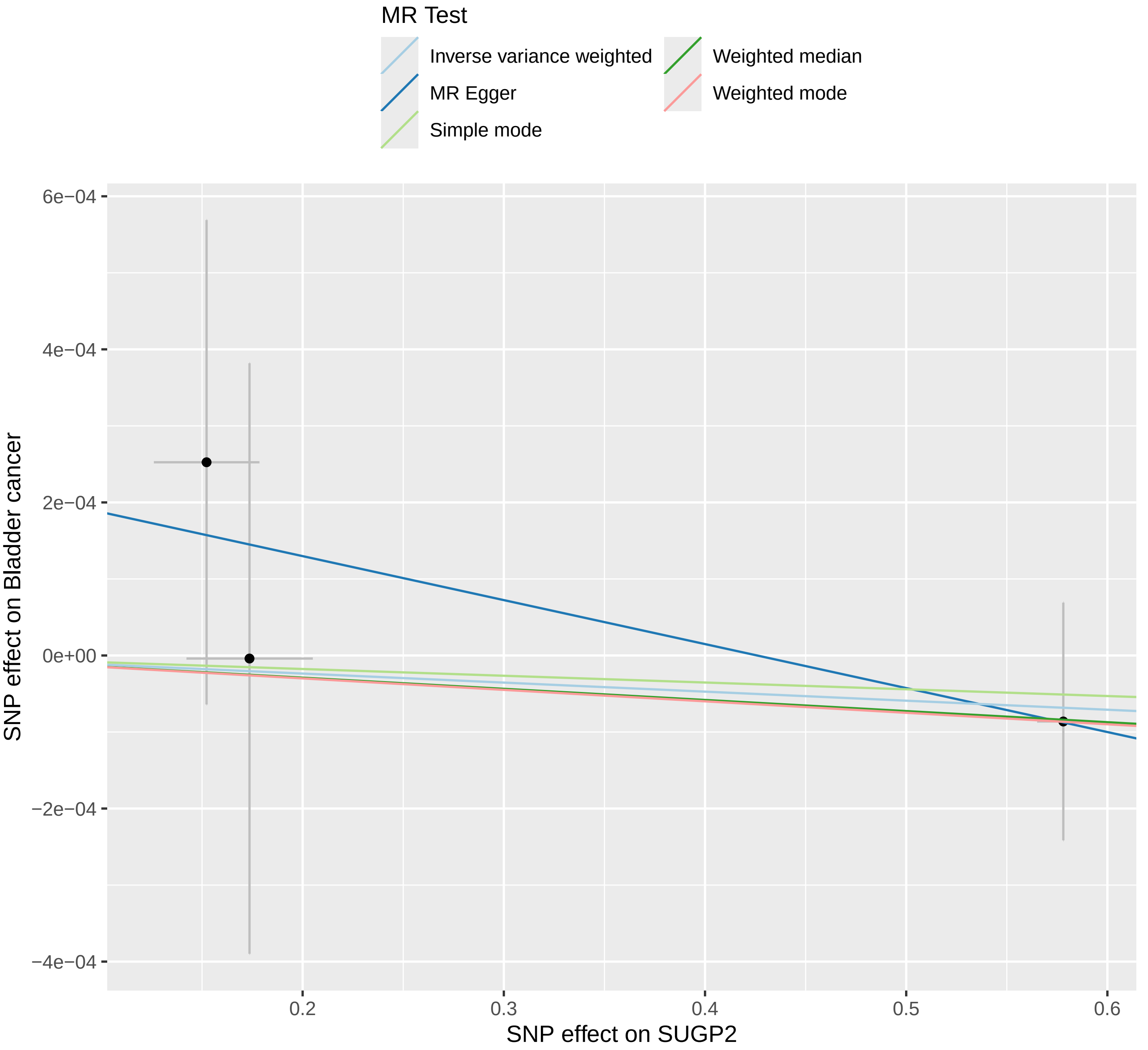
MR Test
Inverse variance weighted
MR Egger
Simple mode
Weighted median
Weighted mode
6e−04
4e−04
2e−04
0e+00
−2e−04
−4e−04
SNP effect on Bladder cancer
0.2
0.3
0.4
0.5
0.6
SNP effect on SUGP2

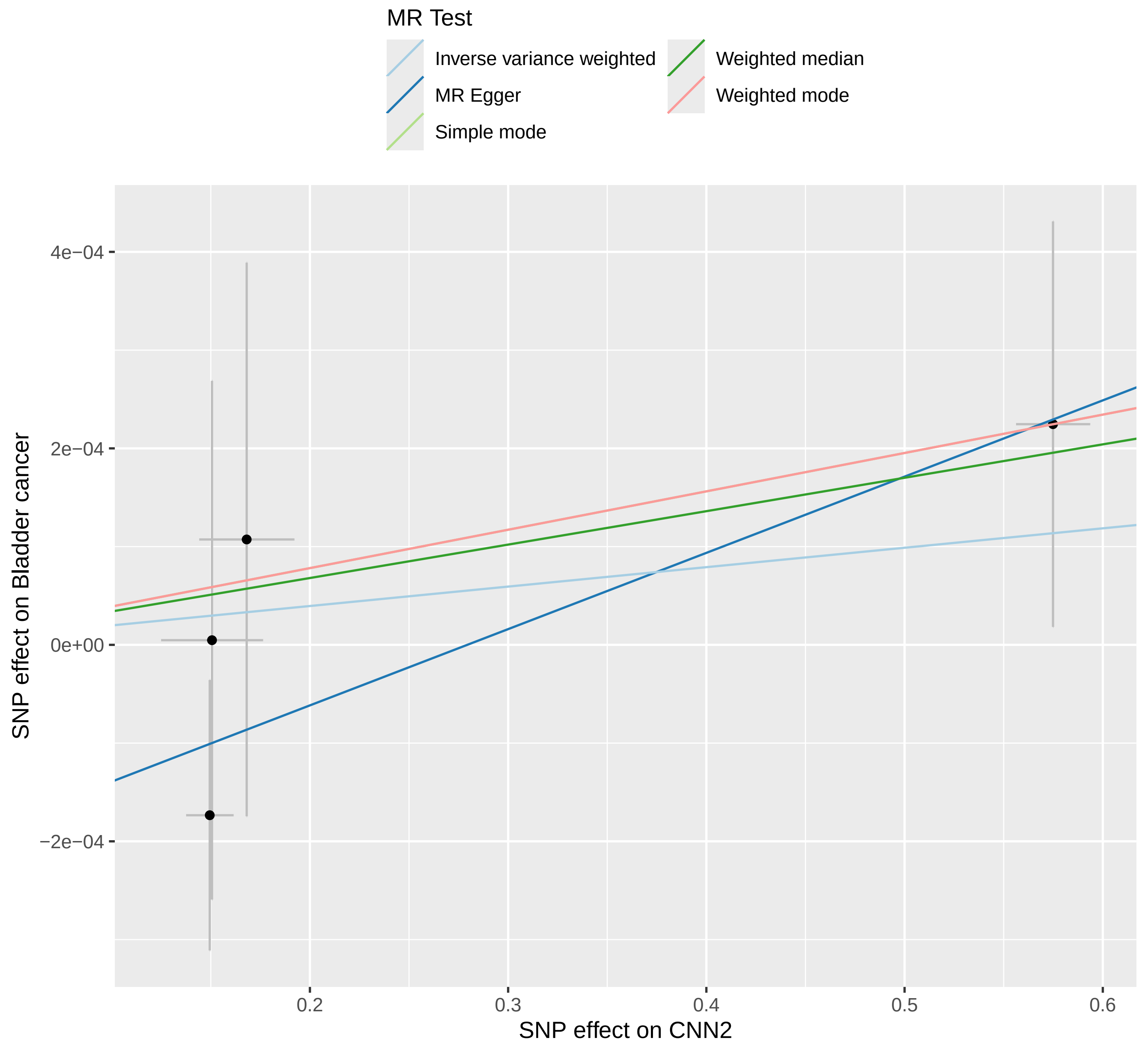
MR Test
Inverse variance weighted
MR Egger
Simple mode
Weighted median
Weighted mode
4e−04
2e−04
0e+00
−2e−04
SNP effect on Bladder cancer
0.2
0.3
0.4
0.5
0.6
SNP effect on CNN2

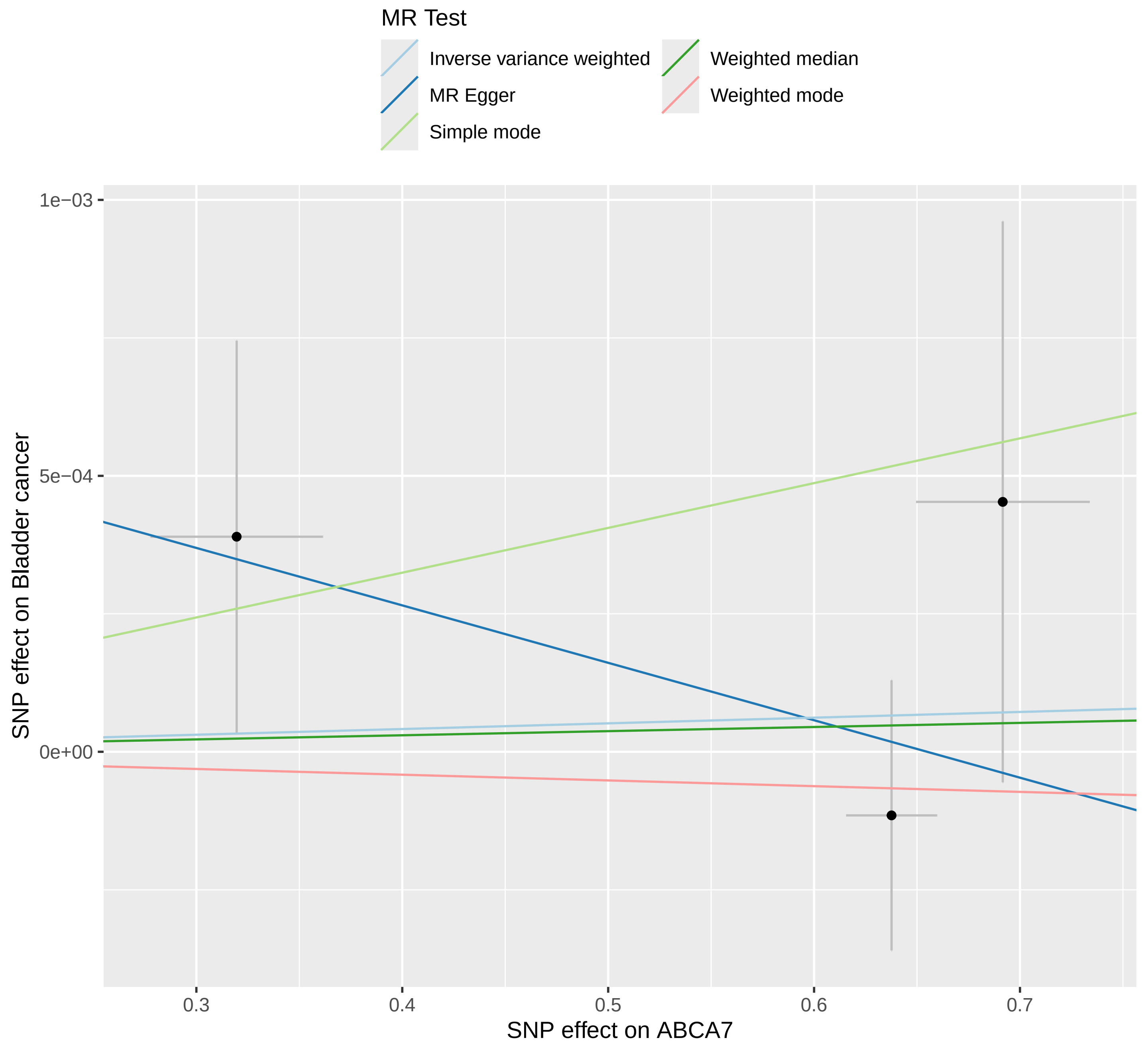

MR Test
Inverse variance weighted
MR Egger
Simple mode
Weighted median
Weighted mode
1e−03
5e−04
0e+00
SNP effect on Bladder cancer
0.3
0.4
0.5
0.6
0.7
SNP effect on ABCA7

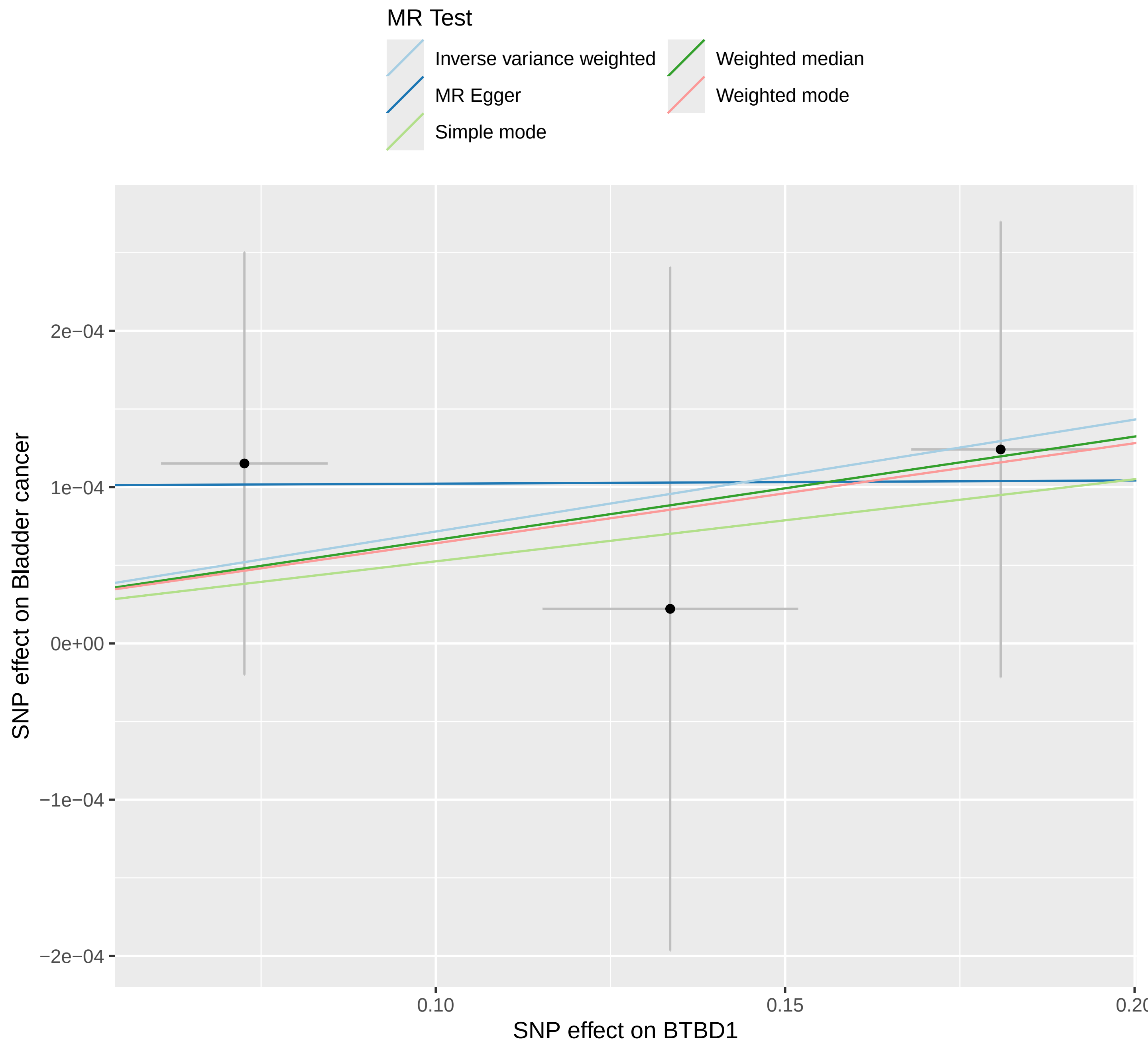

MR Test
Inverse variance weighted
MR Egger
Simple mode
Weighted median
Weighted mode
2e−04
1e−04
0e+00
−1e−04
−2e−04
0.10
0.15
0.20
SNP effect on BTBD1
SNP effect on Bladder cancer

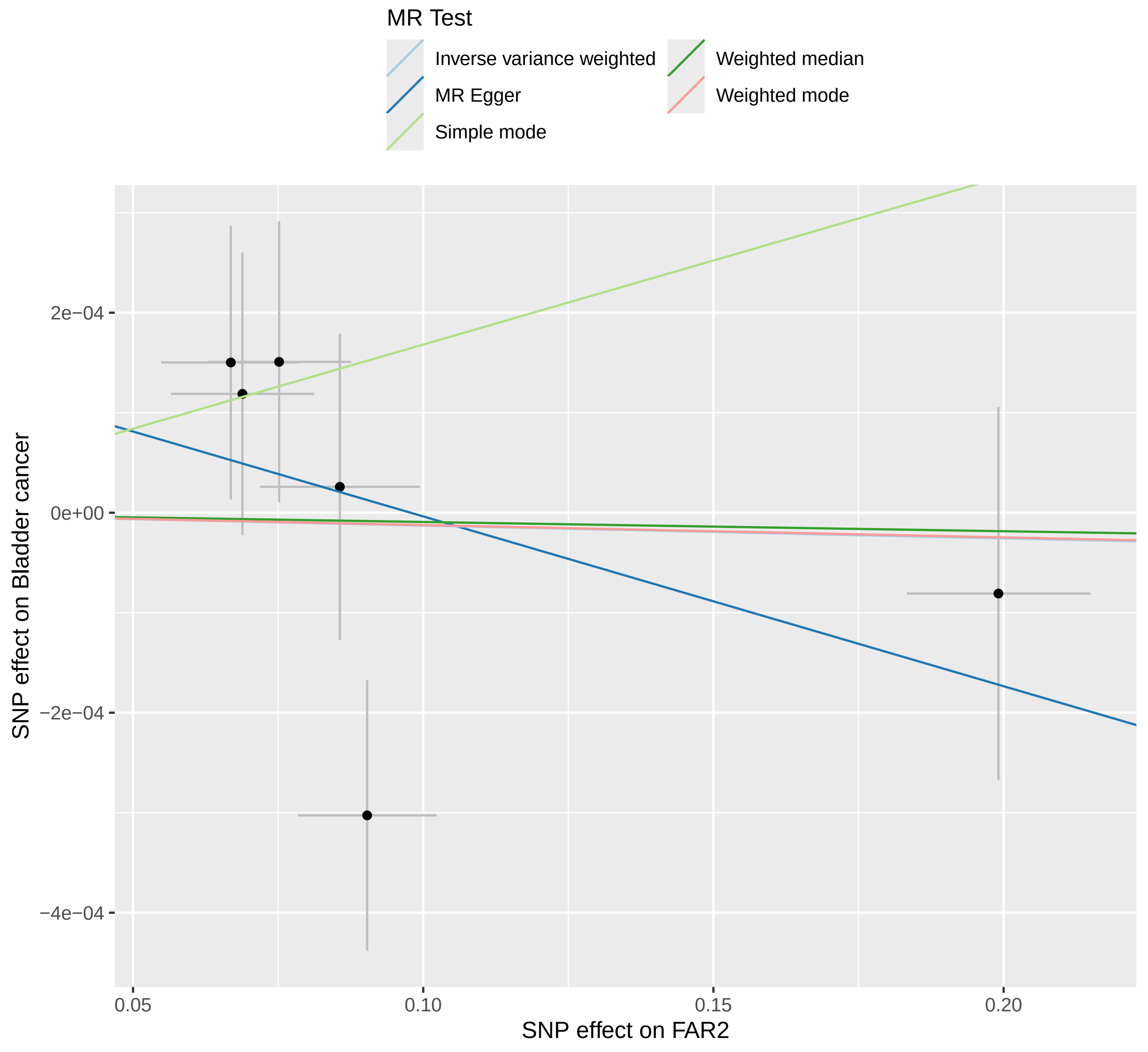
MR Test
Inverse variance weighted
MR Egger
Simple mode
Weighted median
Weighted mode
2e−04
0e+00
−2e−04
−4e−04
SNP effect on Bladder cancer
0.05
0.10
0.15
0.20
SNP effect on FAR2

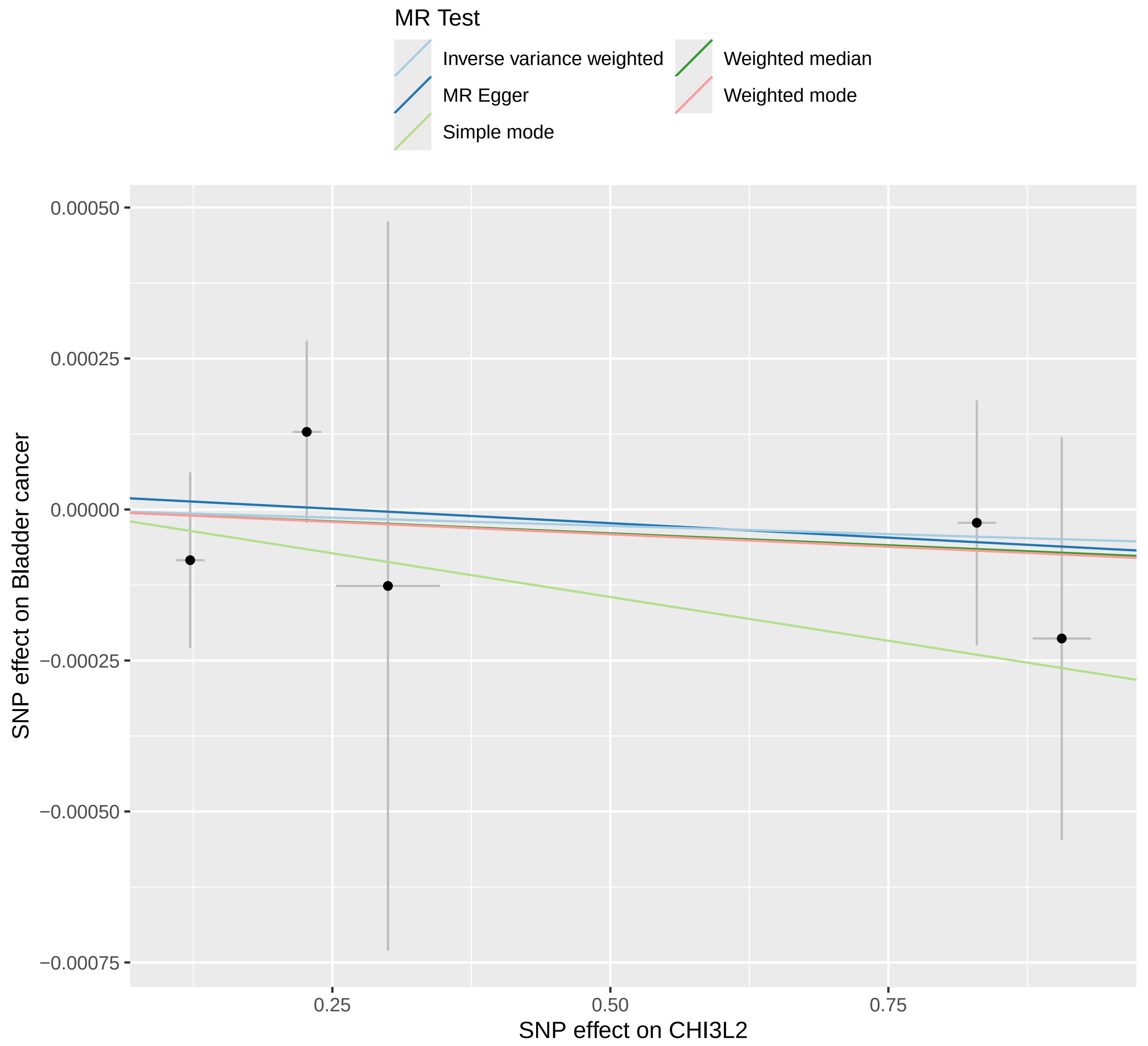

MR Test
Inverse variance weighted
MR Egger
Simple mode
Weighted median
Weighted mode
0.00050
0.00025
0.00000
−0.00025
−0.00050
−0.00075
SNP effect on Bladder cancer
0.25
0.50
0.75
SNP effect on CHI3L2

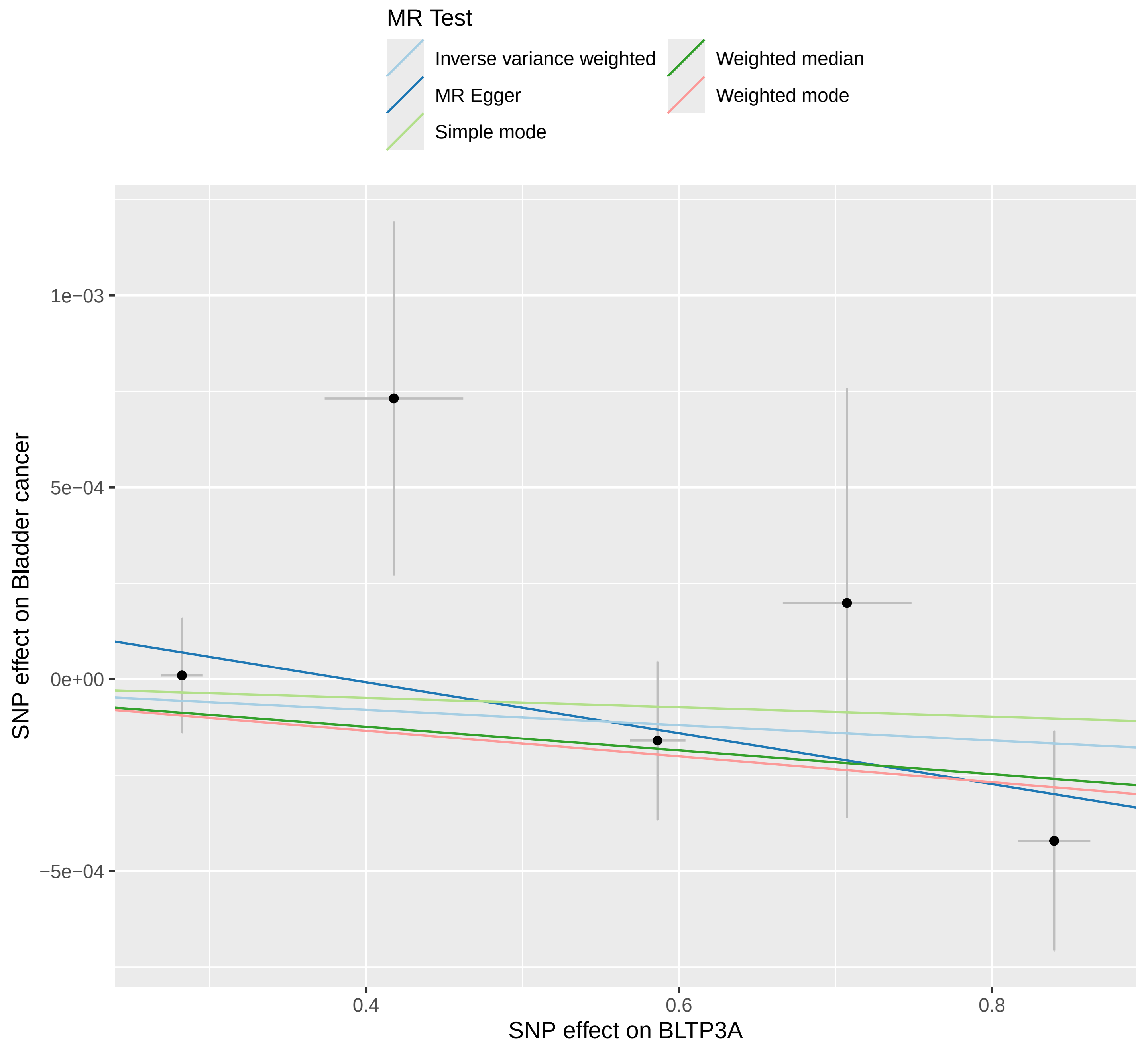

MR Test
Inverse variance weighted
MR Egger
Simple mode
Weighted median
Weighted mode
1e−03
5e−04
0e+00
−5e−04
SNP effect on Bladder cancer
0.4
0.6
0.8
SNP effect on BLTP3A

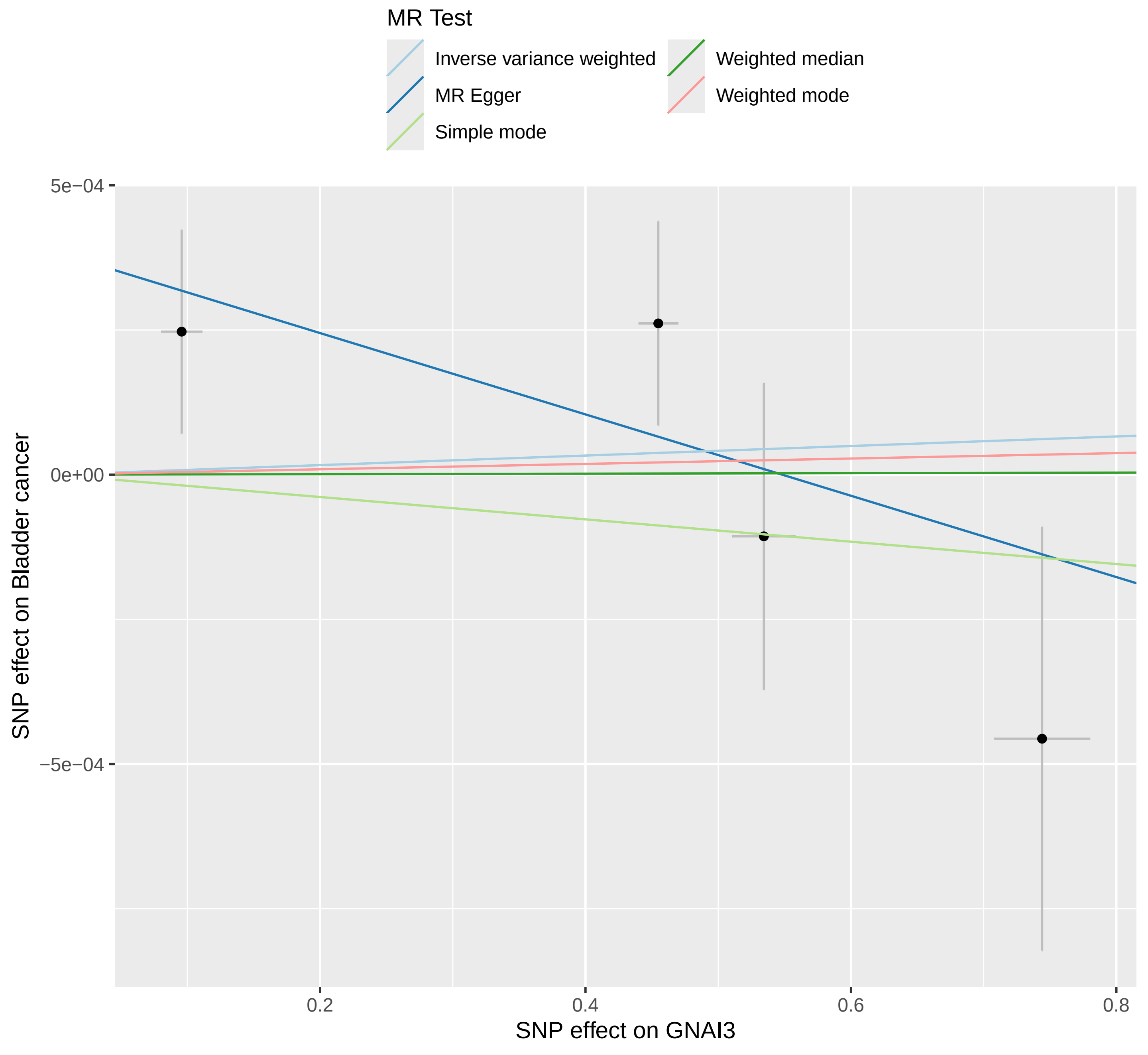
MR Test
Inverse variance weighted
MR Egger
Simple mode
Weighted median
Weighted mode
5e−04
0e+00
−5e−04
SNP effect on Bladder cancer
0.2
0.4
0.6
0.8
SNP effect on GNAI3

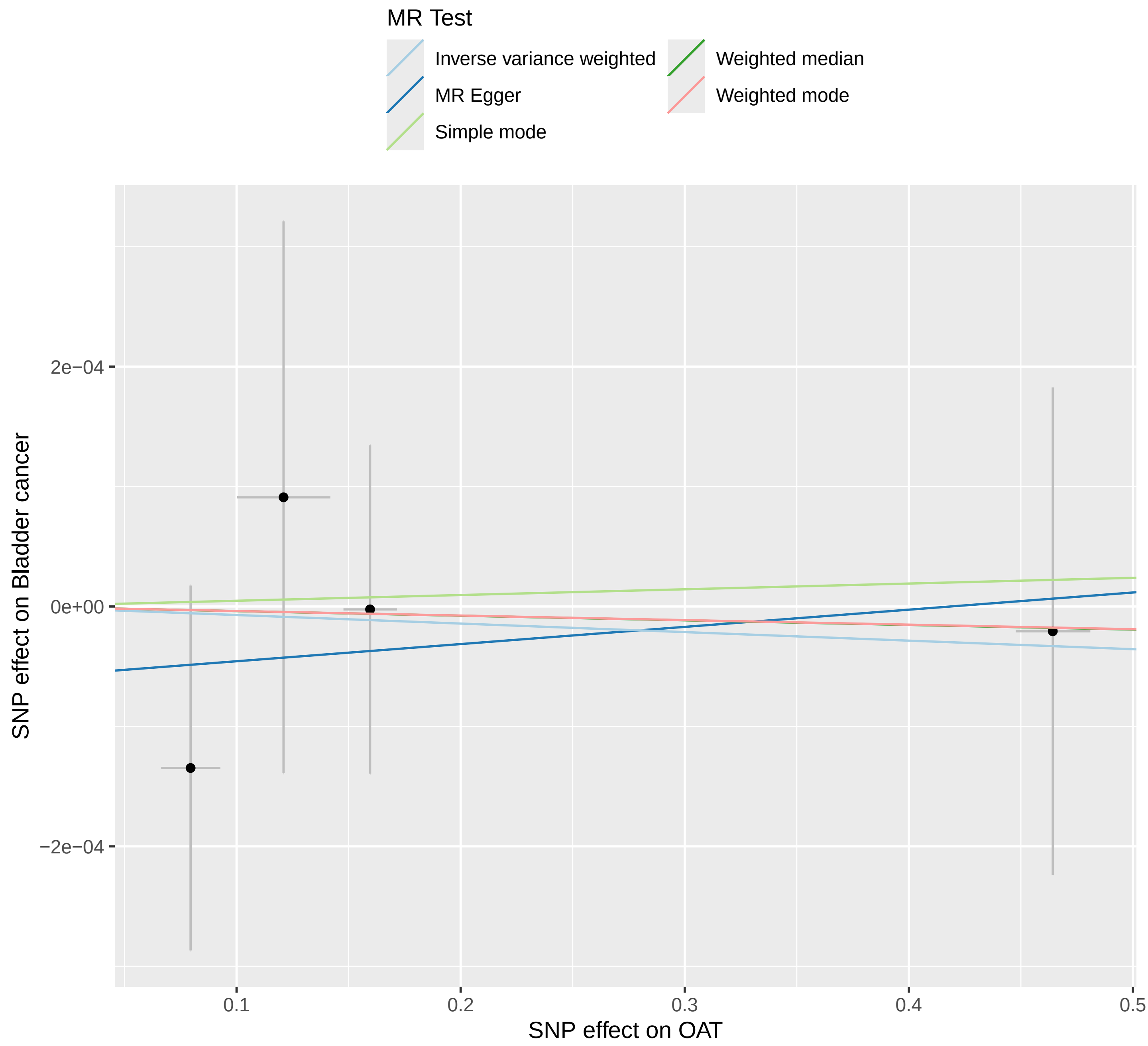
MR Test
Inverse variance weighted
MR Egger
Simple mode
Weighted median
Weighted mode
2e−04
0e+00
−2e−04
0.1
0.2
0.3
0.4
0.5
SNP effect on OAT
SNP effect on Bladder cancer

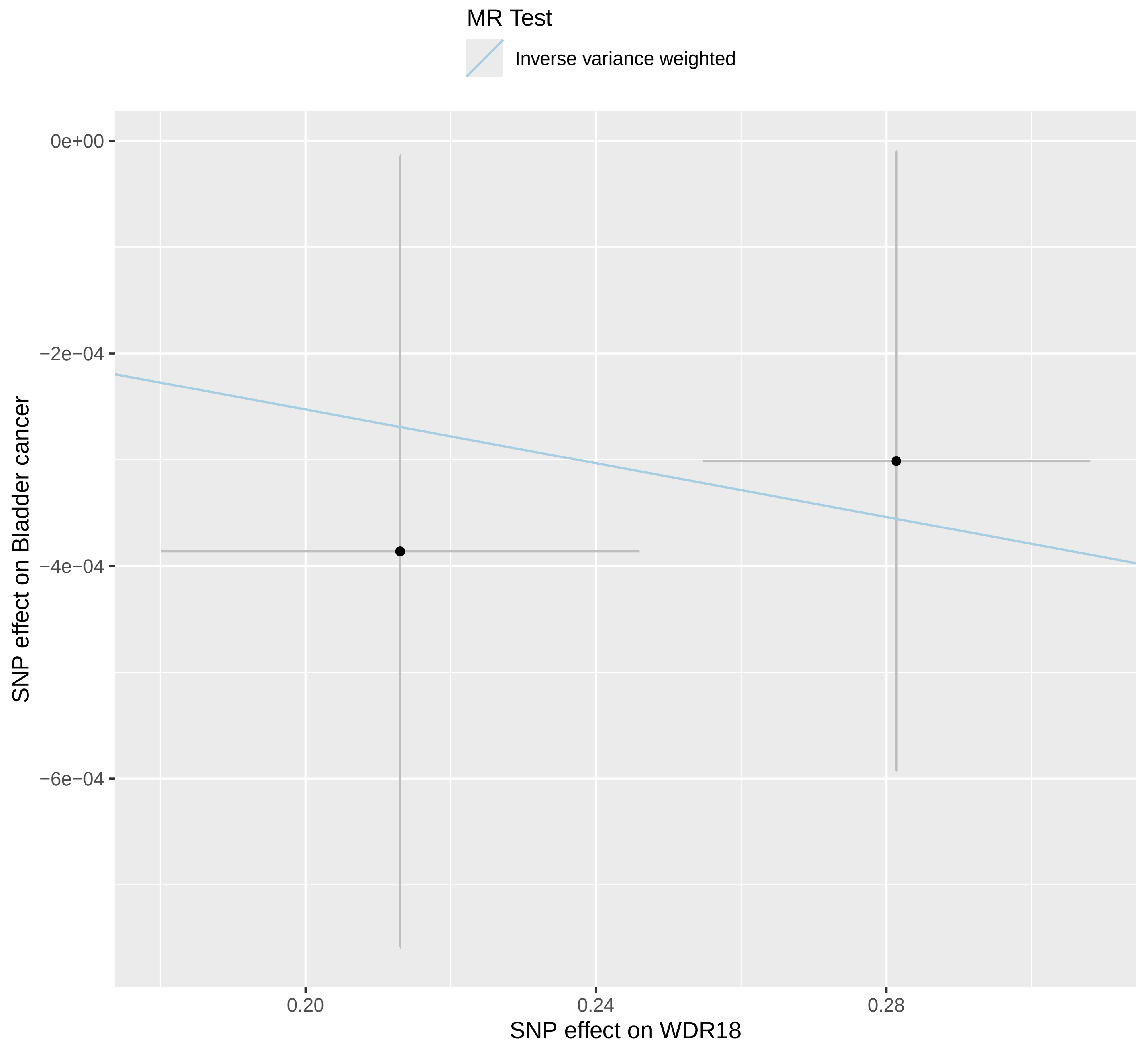
MR Test
Inverse variance weighted
0e+00
−2e−04
−4e−04
−6e−04
SNP effect on Bladder cancer
0.20
0.24
0.28
SNP effect on WDR18

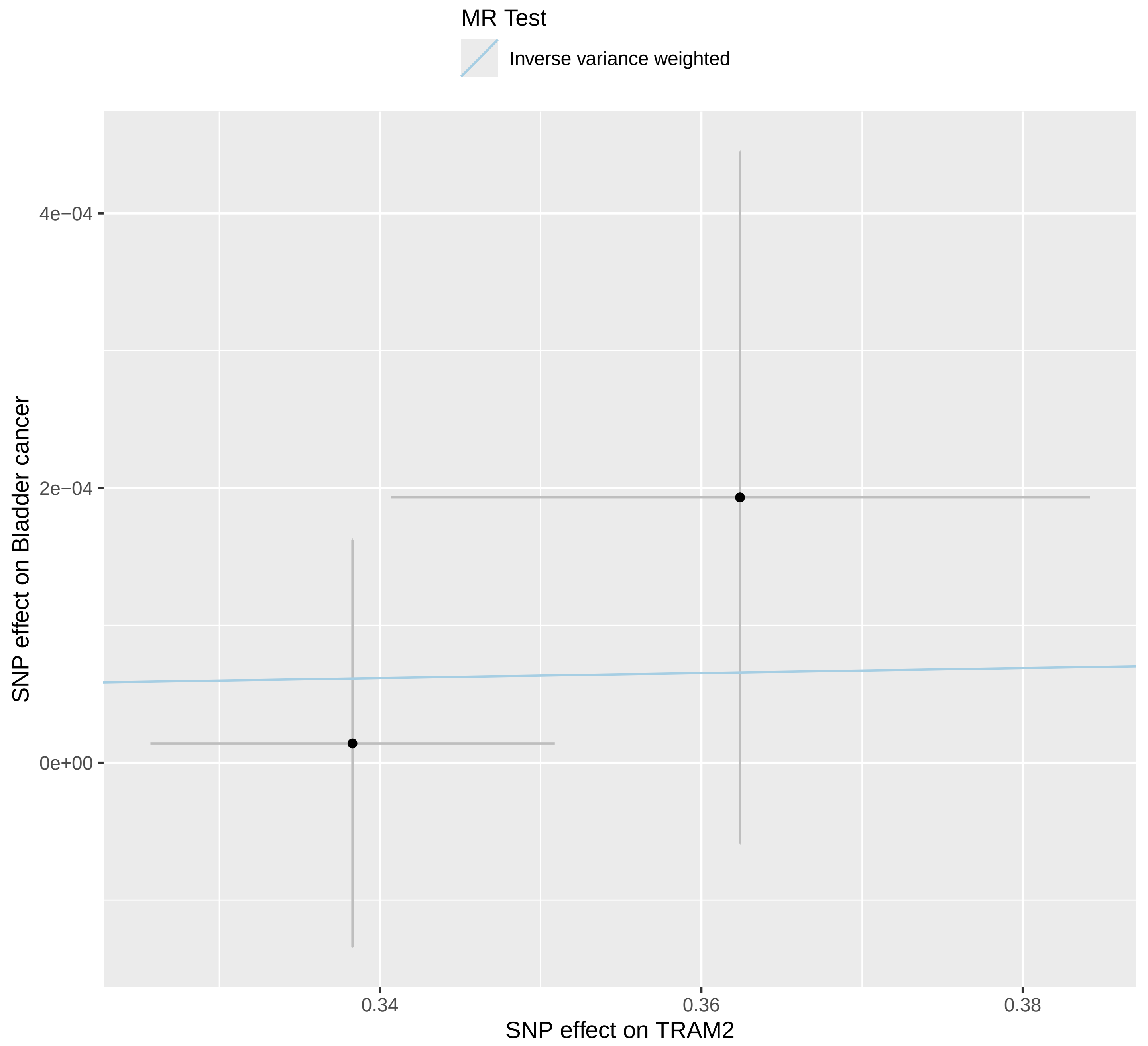
MR Test
Inverse variance weighted
4e−04
2e−04
0e+00
0.34
0.36
0.38
SNP effect on Bladder cancer
SNP effect on TRAM2

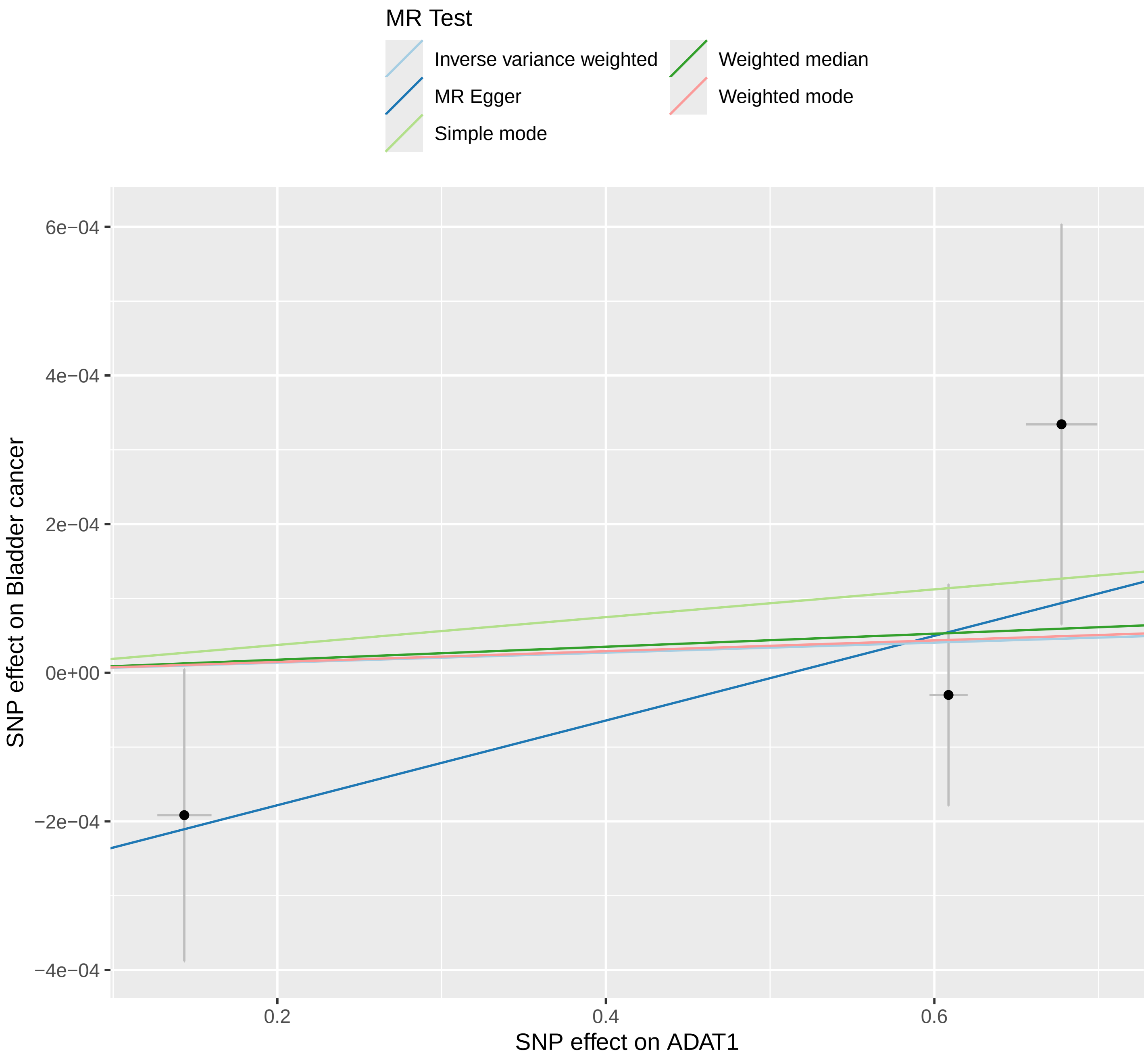

MR Test
Inverse variance weighted
MR Egger
Simple mode
Weighted median
Weighted mode
6e−04
4e−04
2e−04
0e+00
−2e−04
−4e−04
0.2
0.4
0.6
SNP effect on ADAT1
SNP effect on Bladder cancer

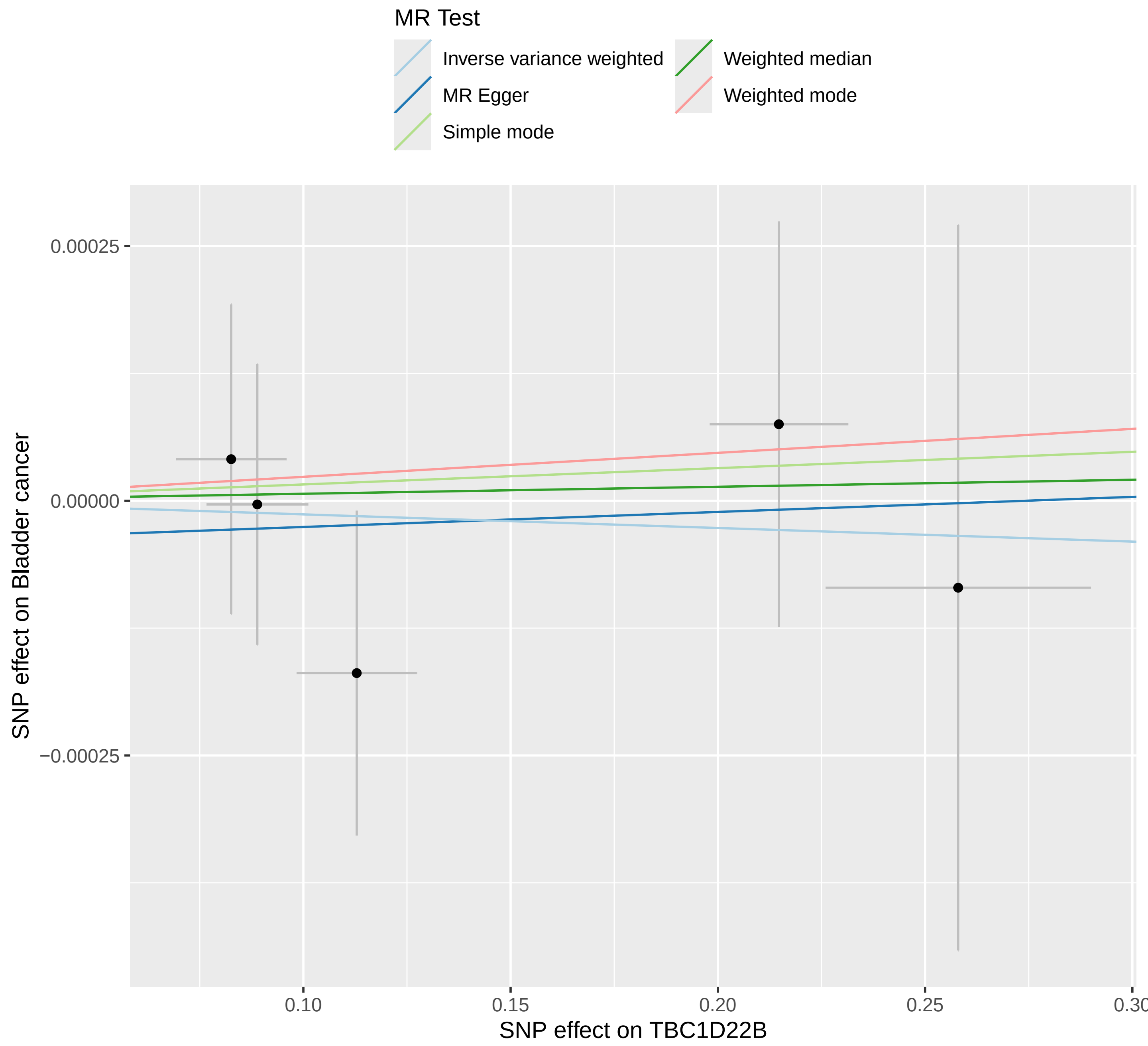
MR Test
Inverse variance weighted
MR Egger
Simple mode
Weighted median
Weighted mode
0.00025
0.00000
−0.00025
0.10
0.15
0.20
0.25
0.30
SNP effect on TBC1D22B
SNP effect on Bladder cancer

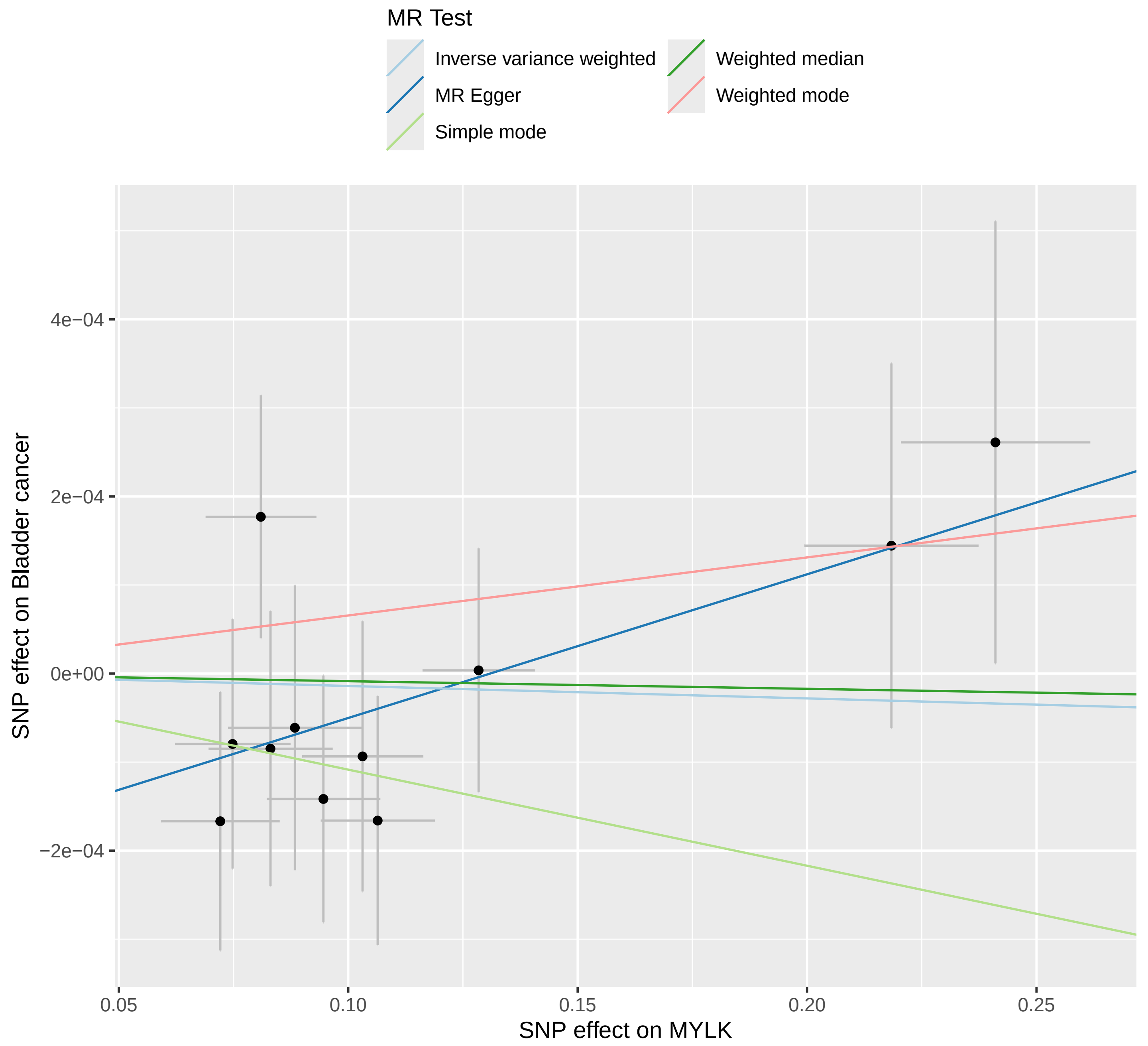

MR Test
Inverse variance weighted
MR Egger
Simple mode
Weighted median
Weighted mode
4e−04
2e−04
0e+00
−2e−04
SNP effect on Bladder cancer
0.05
0.10
0.15
0.20
0.25
SNP effect on MYLK

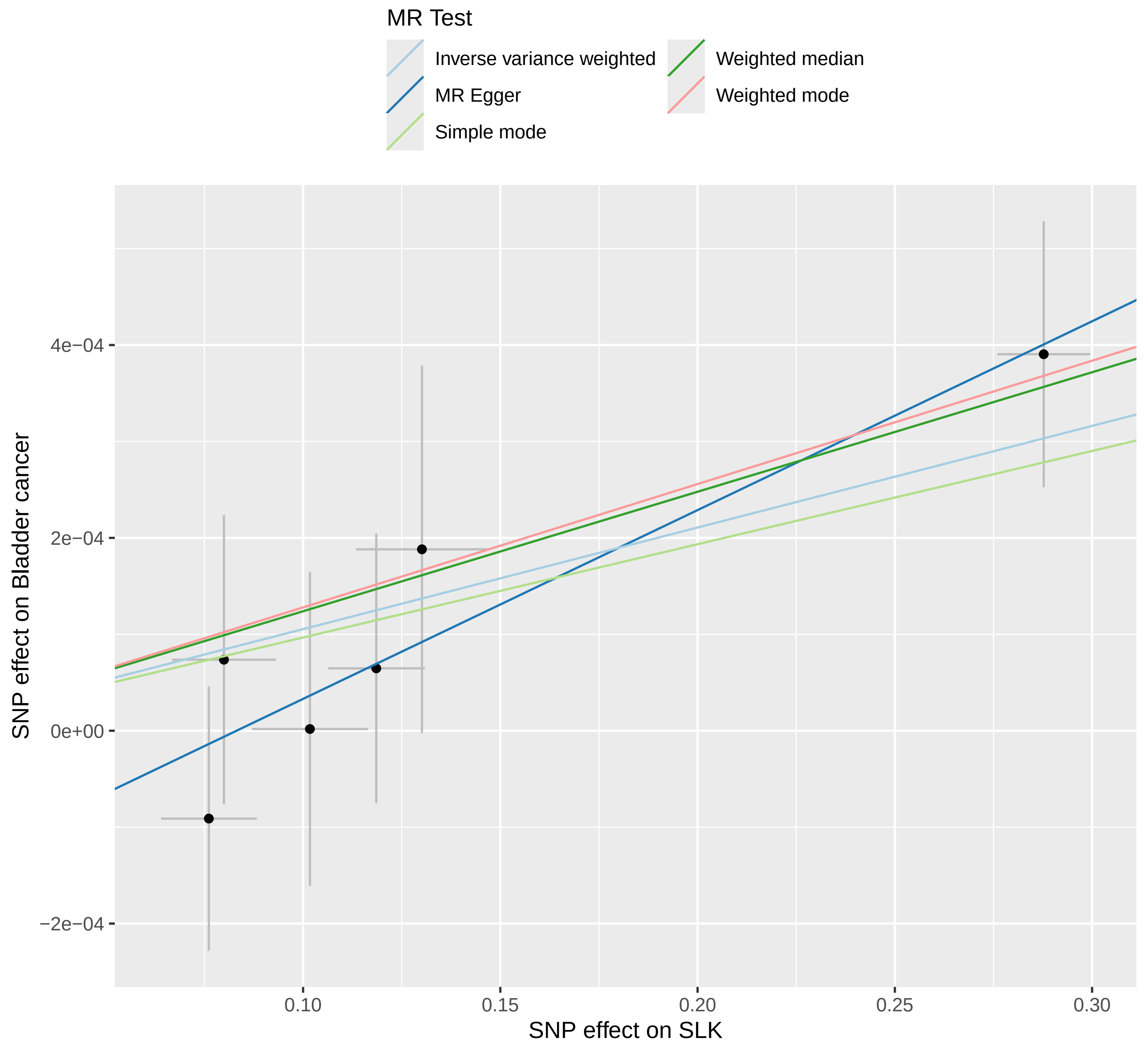

MR Test
Inverse variance weighted
MR Egger
Simple mode
Weighted median
Weighted mode
4e−04
2e−04
0e+00
−2e−04
0.10
0.15
0.20
0.25
0.30
SNP effect on SLK
SNP effect on Bladder cancer

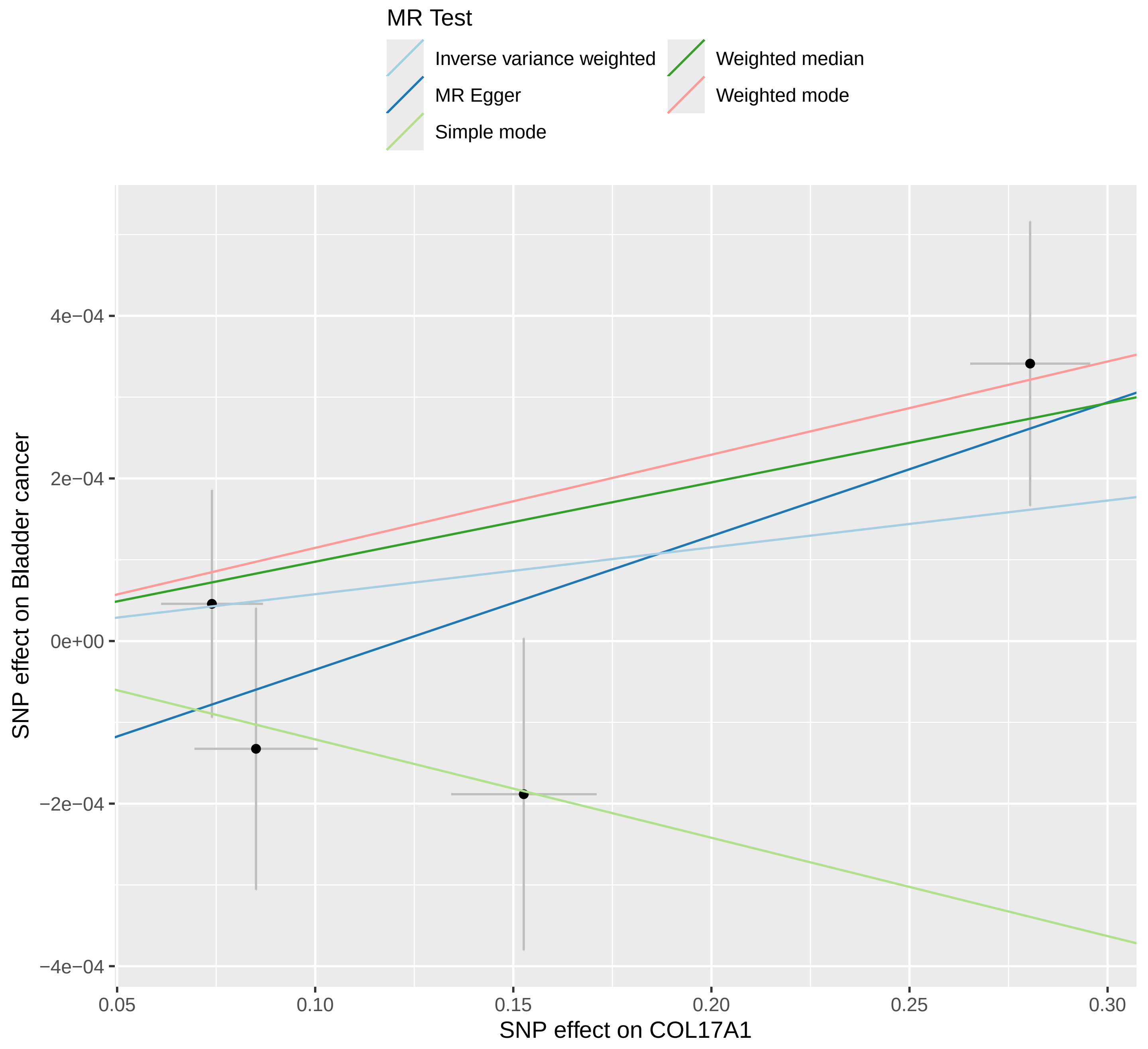
MR Test
Inverse variance weighted
MR Egger
Simple mode
Weighted median
Weighted mode
4e−04
2e−04
0e+00
−2e−04
−4e−04
SNP effect on Bladder cancer
0.05
0.10
0.15
0.20
0.25
0.30
SNP effect on COL17A1

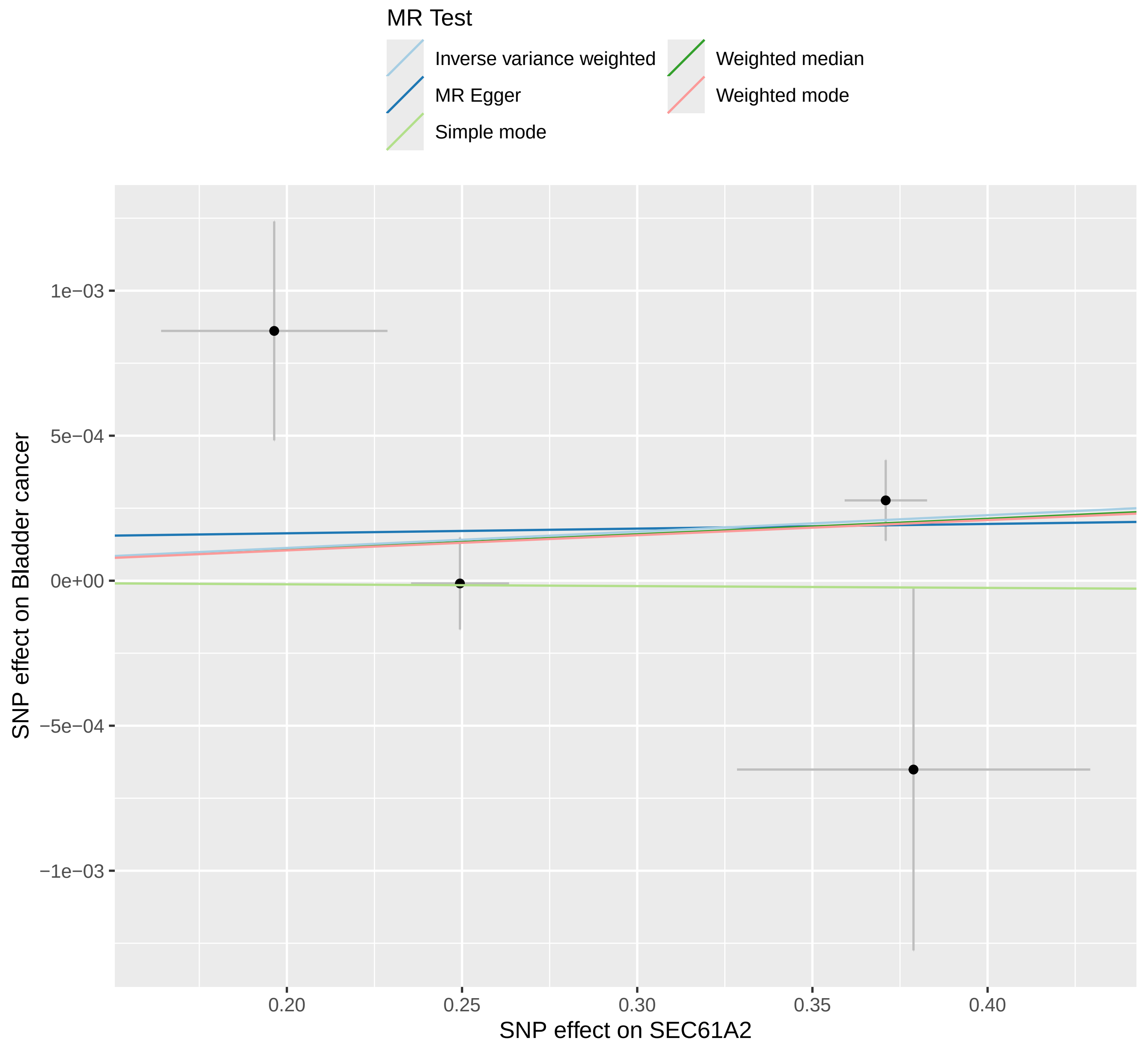
MR Test
Inverse variance weighted
MR Egger
Simple mode
Weighted median
Weighted mode
1e−03
5e−04
0e+00
−5e−04
−1e−03
0.20
0.25
0.30
0.35
0.40
SNP effect on Bladder cancer
SNP effect on SEC61A2

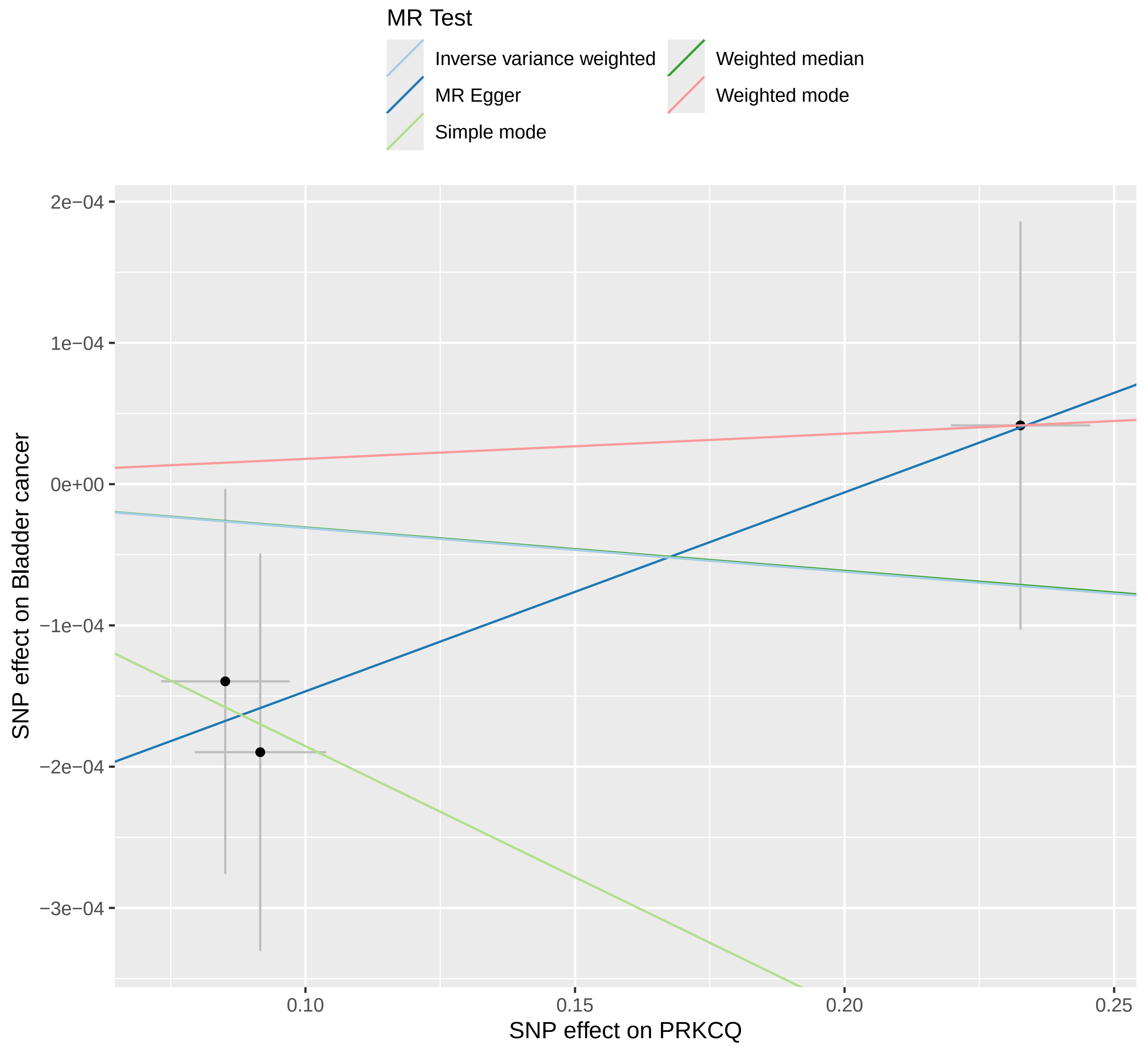

MR Test
Inverse variance weighted
MR Egger
Simple mode
Weighted median
Weighted mode
2e−04
1e−04
0e+00
−1e−04
−2e−04
−3e−04
0.10
0.15
0.20
0.25
SNP effect on Bladder cancer
SNP effect on PRKCQ

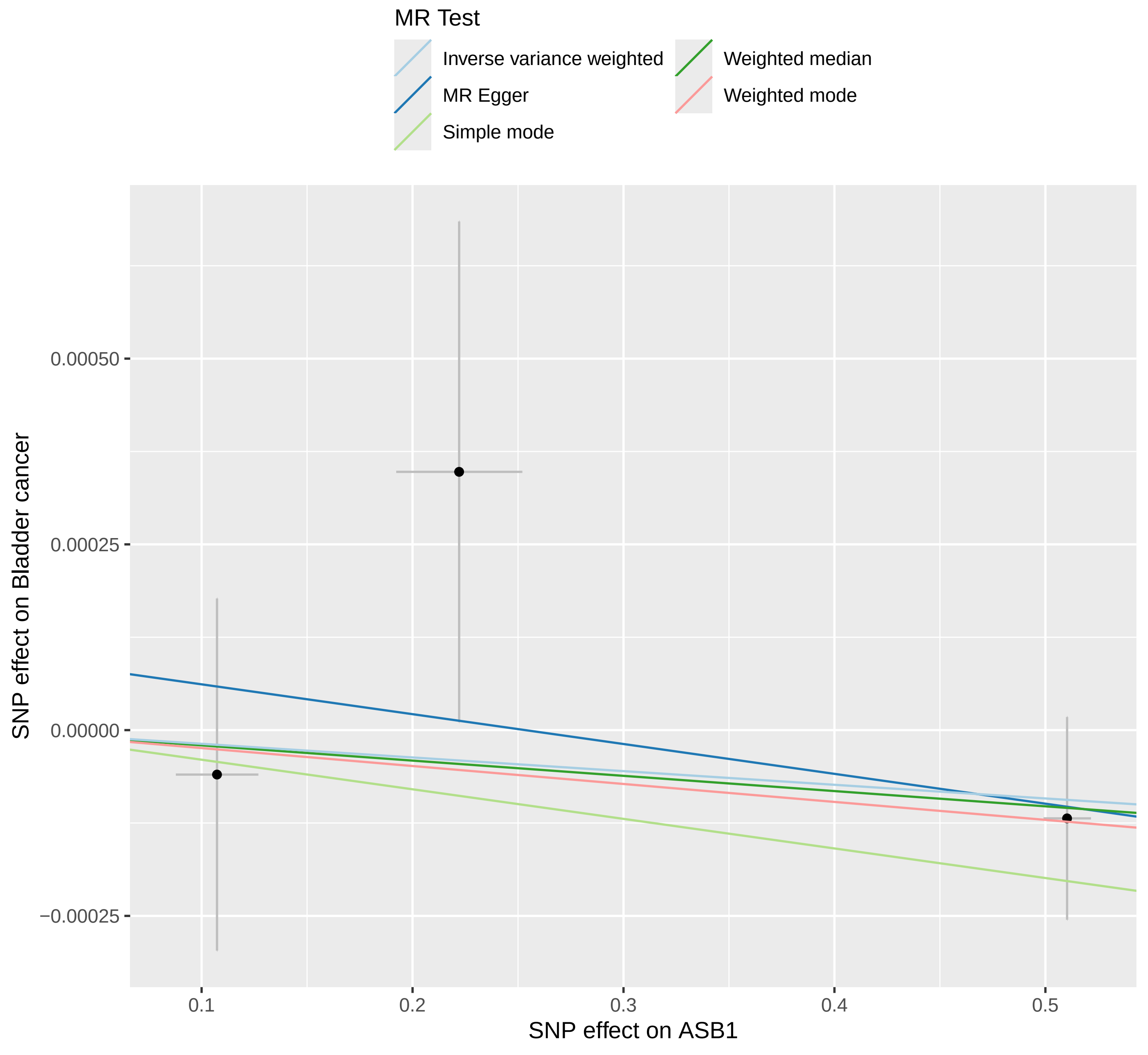
MR Test
Inverse variance weighted
MR Egger
Simple mode
Weighted median
Weighted mode
0.00050
0.00025
0.00000
−0.00025
SNP effect on Bladder cancer
0.1
0.2
0.3
0.4
0.5
SNP effect on ASB1

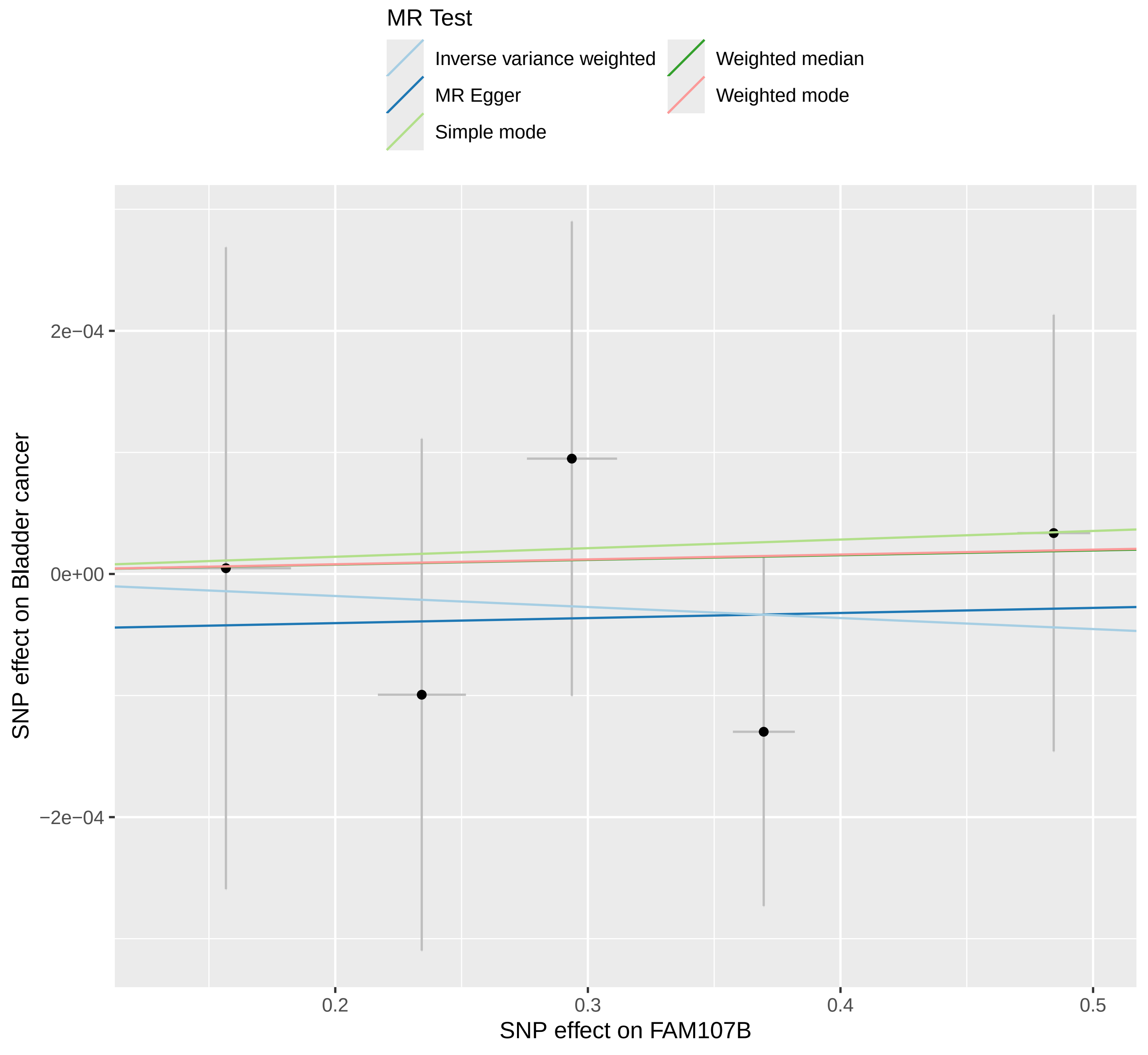
MR Test
Inverse variance weighted
MR Egger
Simple mode
Weighted median
Weighted mode
SNP effect on Bladder cancer
2e−04
0e+00
−2e−04
0.2
0.3
0.4
0.5
SNP effect on FAM107B

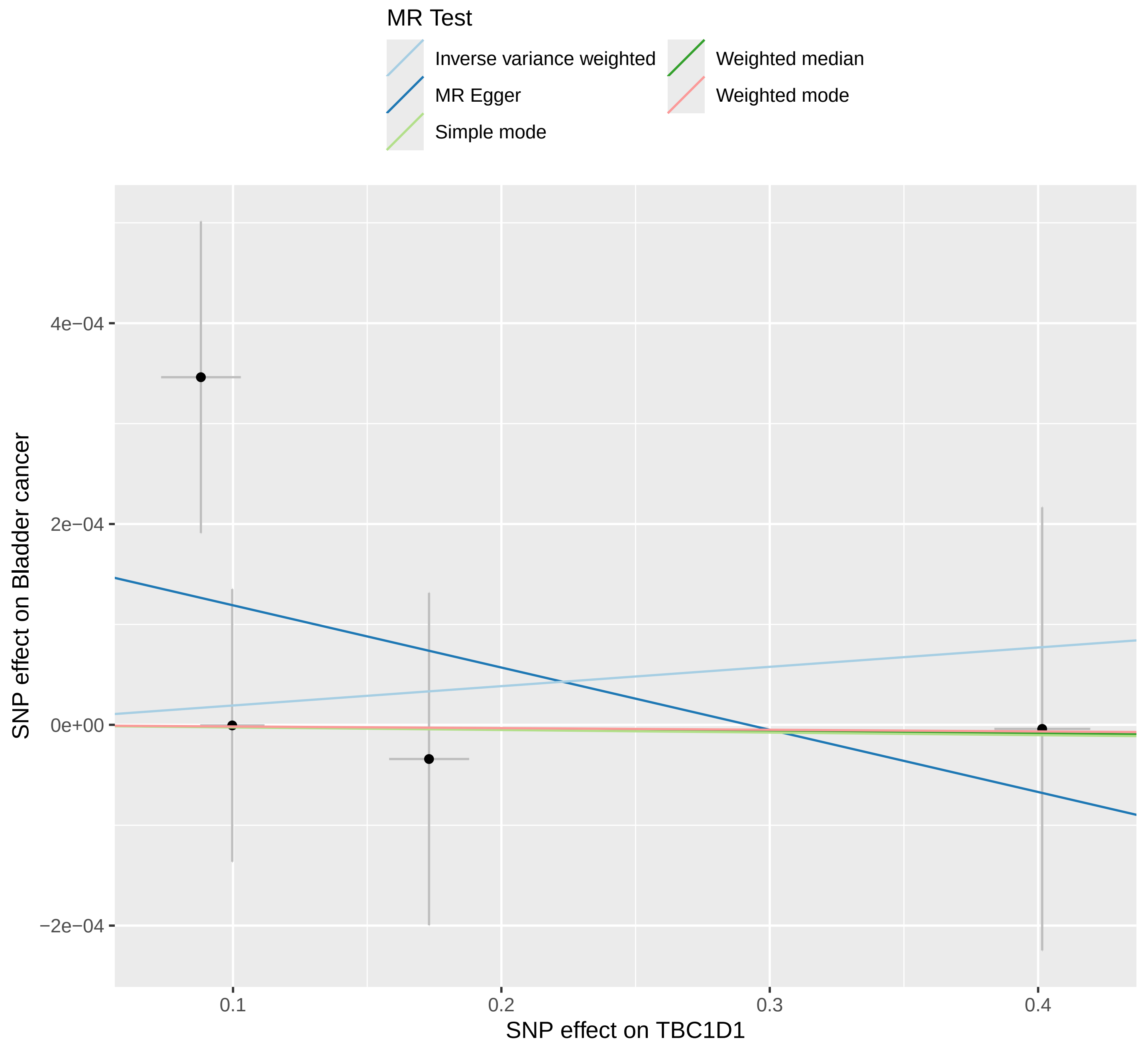
MR Test
Inverse variance weighted
MR Egger
Simple mode
Weighted median
Weighted mode
4e−04
2e−04
0e+00
−2e−04
SNP effect on Bladder cancer
0.1
0.2
0.3
0.4
SNP effect on TBC1D1

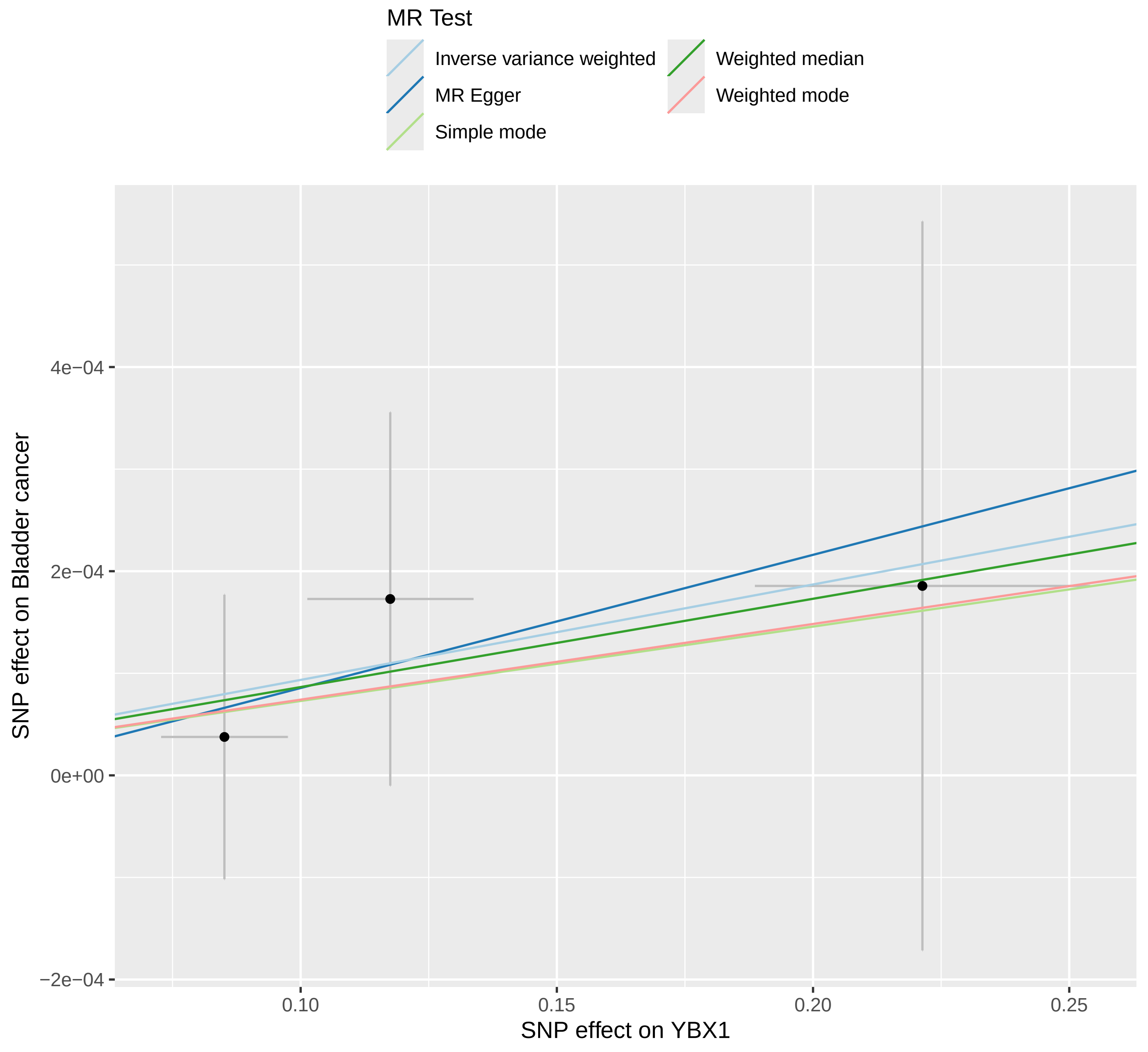

MR Test
Inverse variance weighted
MR Egger
Simple mode
Weighted median
Weighted mode
4e−04
2e−04
0e+00
−2e−04
0.10
0.15
0.20
0.25
SNP effect on Bladder cancer
SNP effect on YBX1

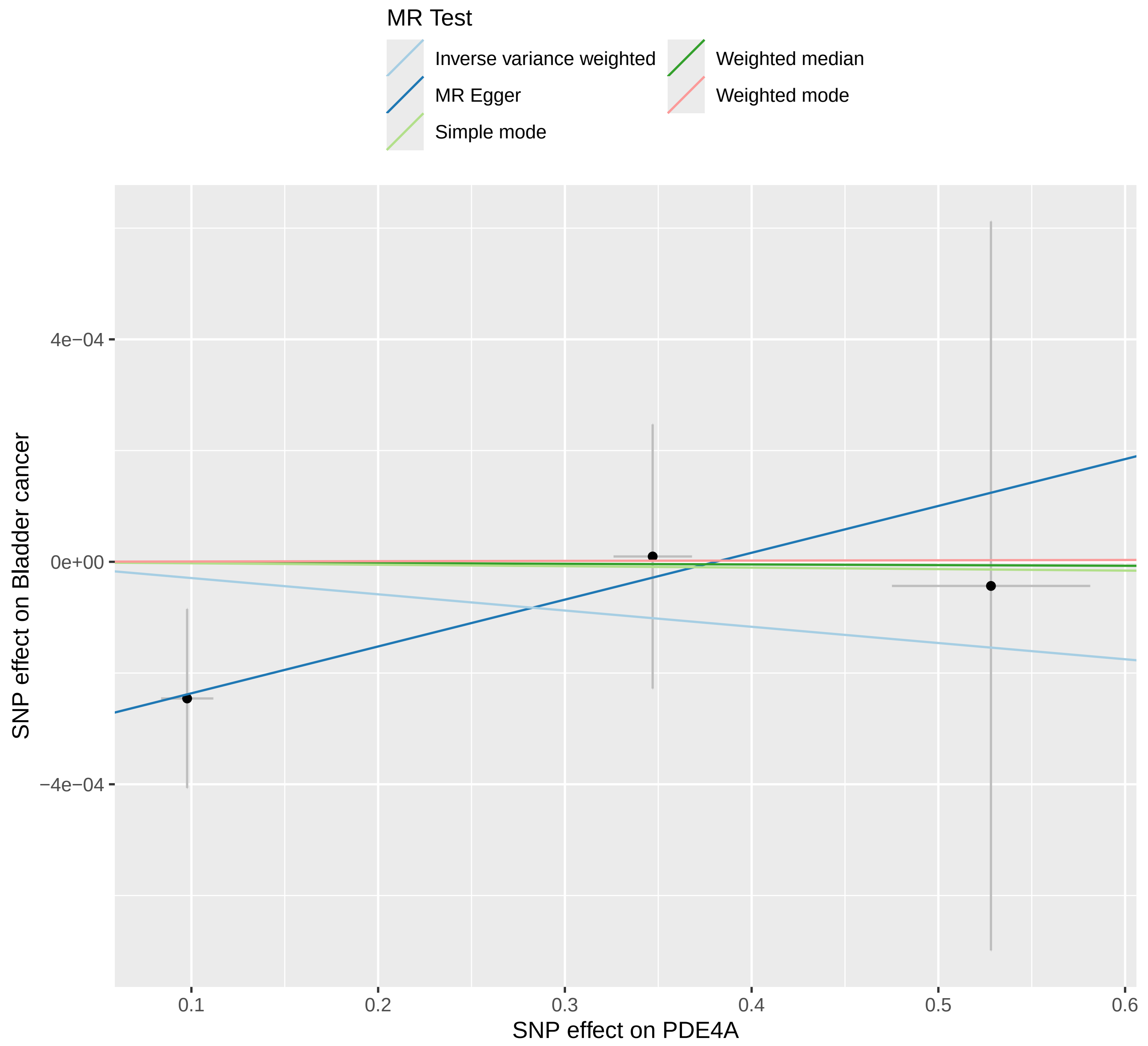
MR Test
Inverse variance weighted
MR Egger
Simple mode
Weighted median
Weighted mode
4e−04
0e+00
−4e−04
SNP effect on Bladder cancer
0.1
0.2
0.3
0.4
0.5
0.6
SNP effect on PDE4A

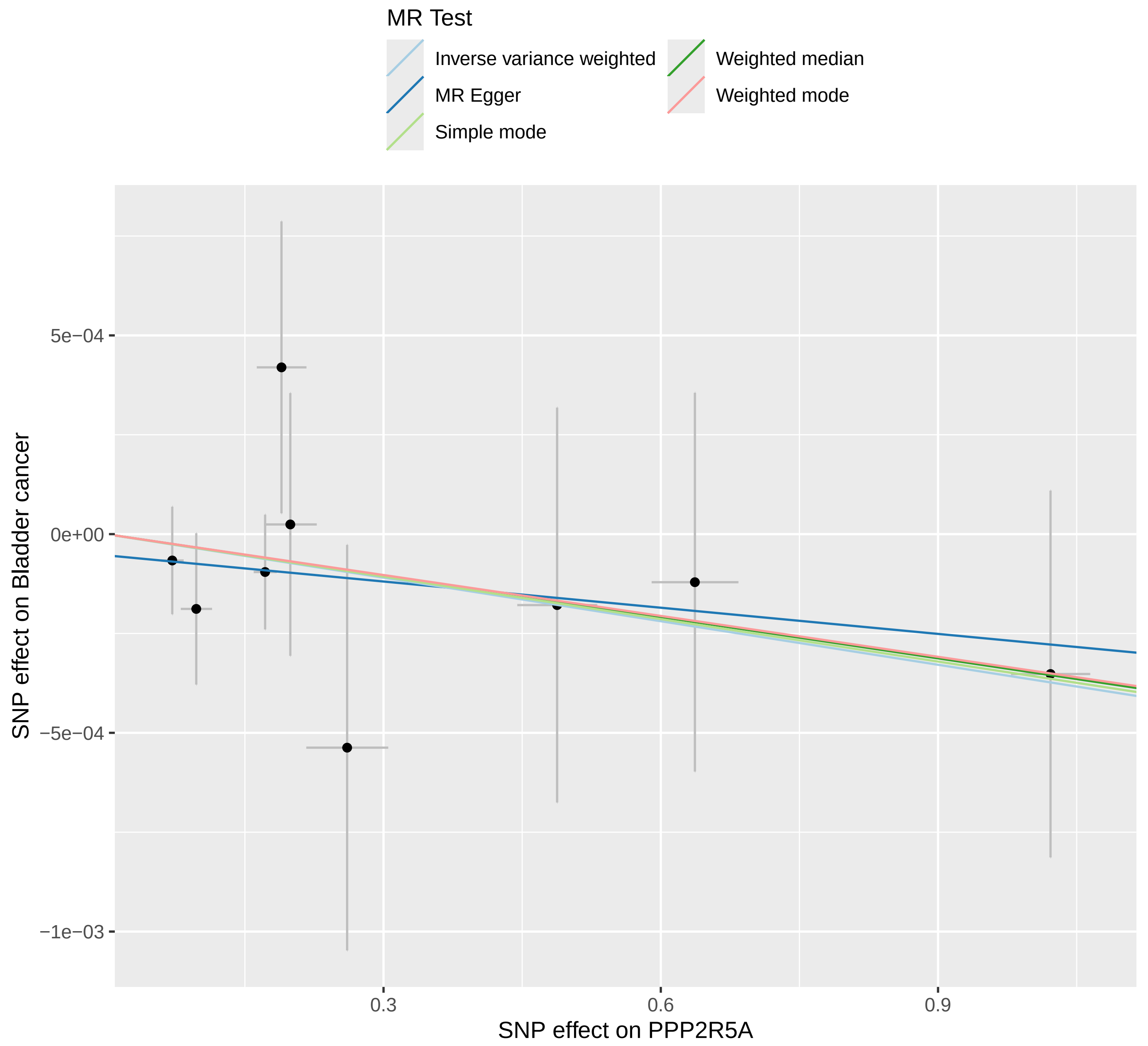

MR Test
Inverse variance weighted
MR Egger
Simple mode
Weighted median
Weighted mode
5e−04
0e+00
−5e−04
−1e−03
SNP effect on Bladder cancer
0.3
0.6
0.9
SNP effect on PPP2R5A

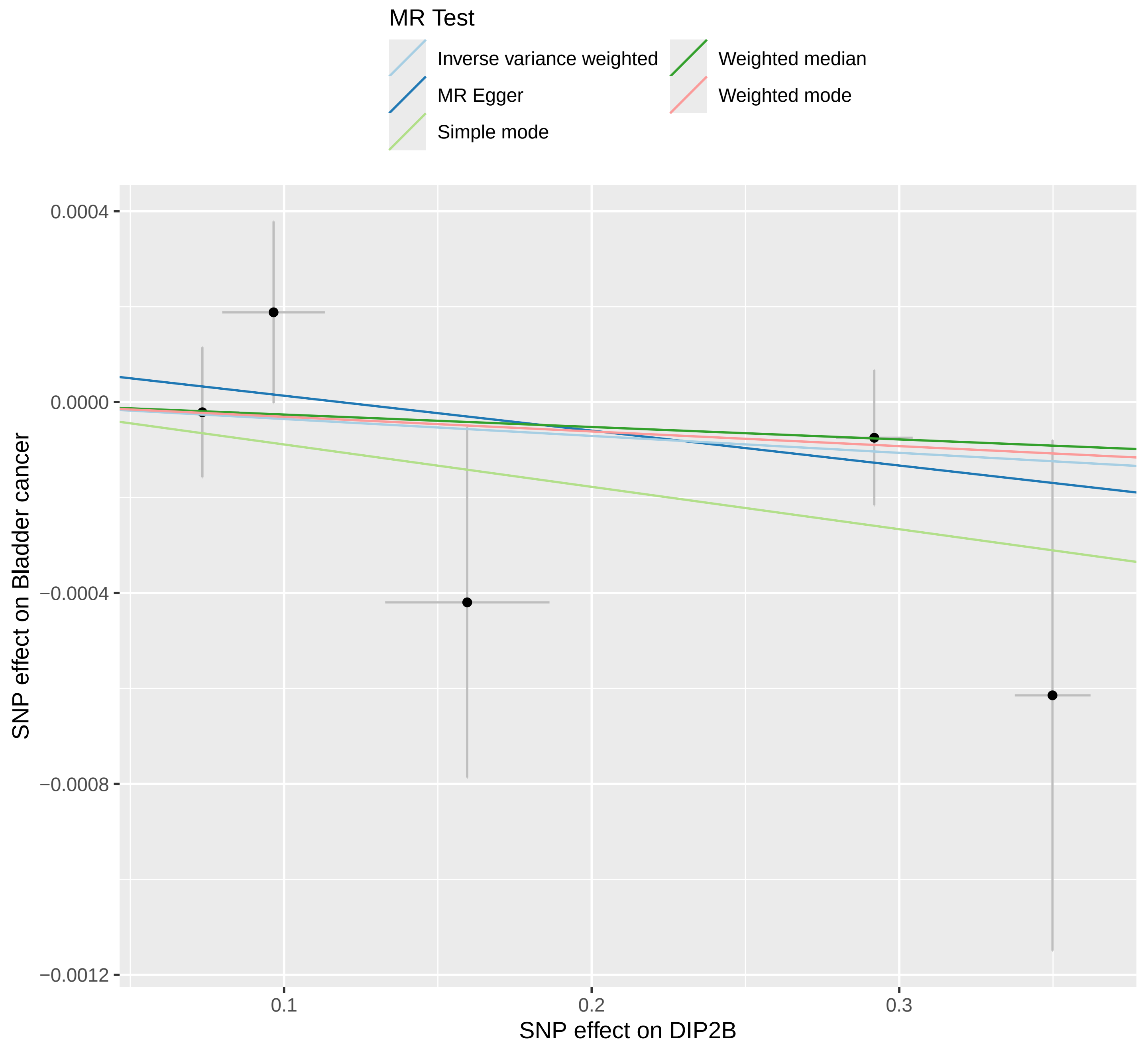

MR Test
Inverse variance weighted
MR Egger
Simple mode
Weighted median
Weighted mode
0.0004
0.0000
−0.0004
−0.0008
−0.0012
0.1
0.2
0.3
SNP effect on Bladder cancer
SNP effect on DIP2B

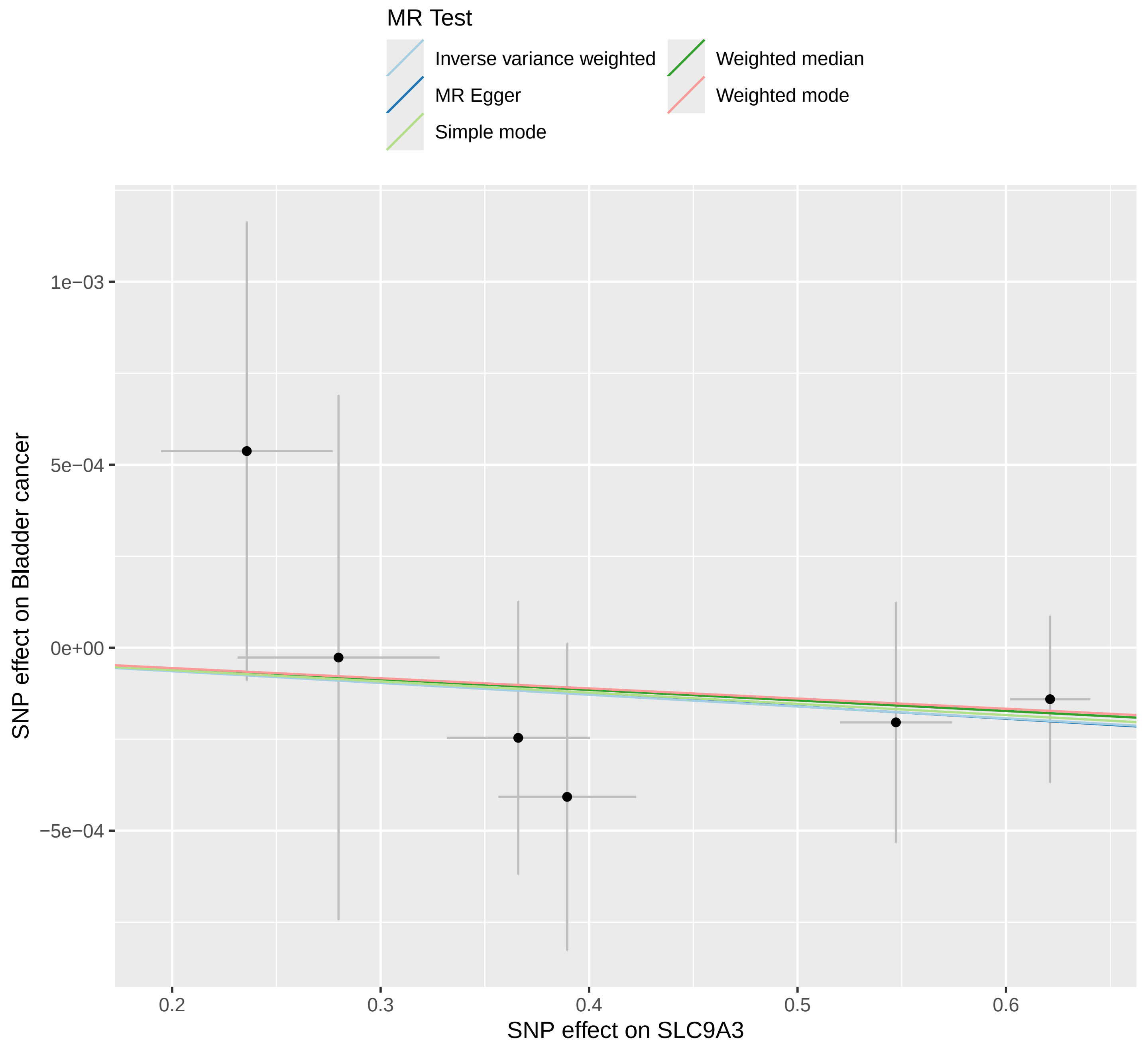
MR Test
Inverse variance weighted
MR Egger
Simple mode
Weighted median
Weighted mode
1e−03
5e−04
0e+00
−5e−04
SNP effect on Bladder cancer
0.2
0.3
0.4
0.5
0.6
SNP effect on SLC9A3

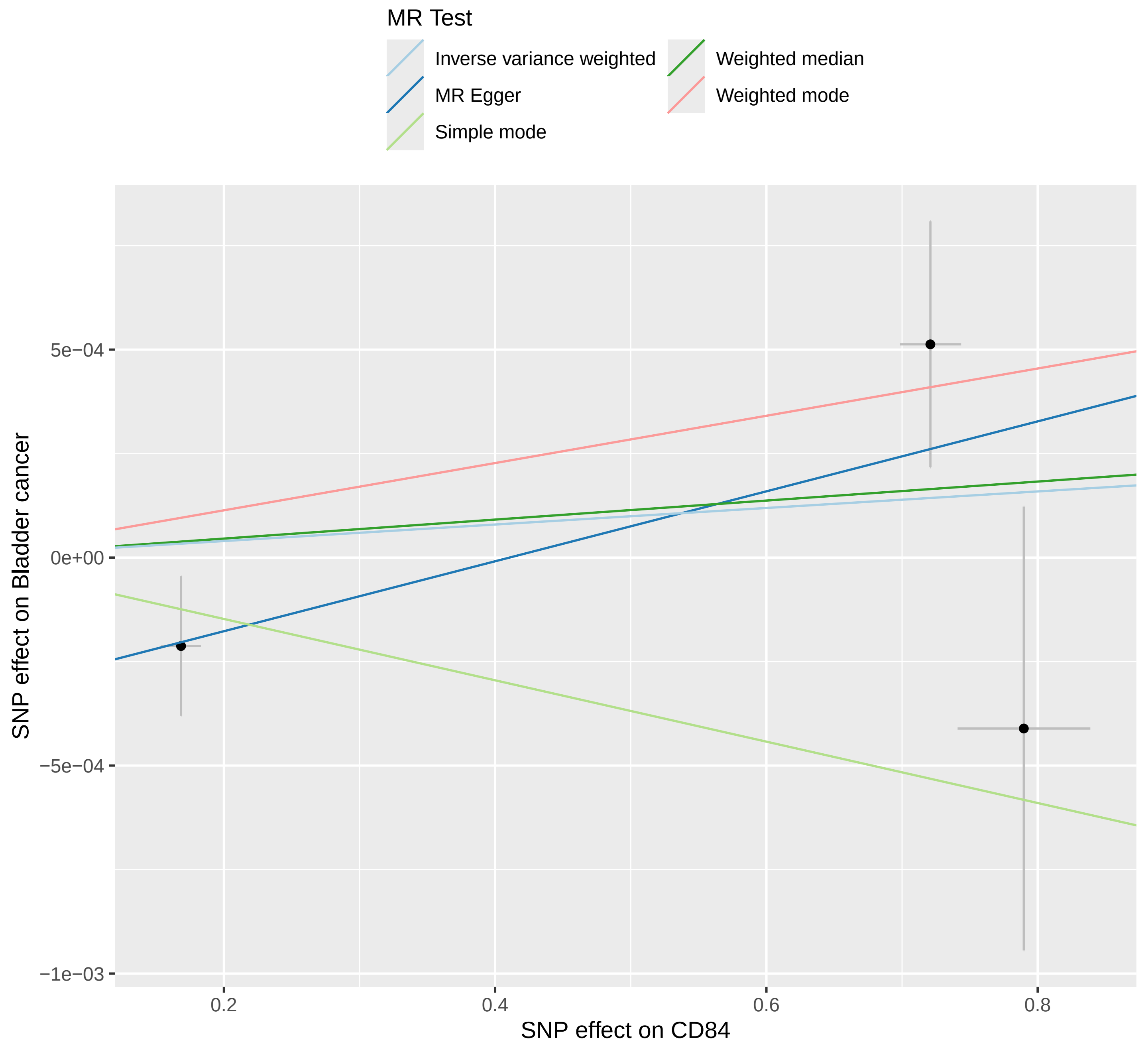
MR Test
Inverse variance weighted
MR Egger
Simple mode
Weighted median
Weighted mode
5e−04
0e+00
−5e−04
−1e−03
0.2
0.4
0.6
0.8
SNP effect on Bladder cancer
SNP effect on CD84

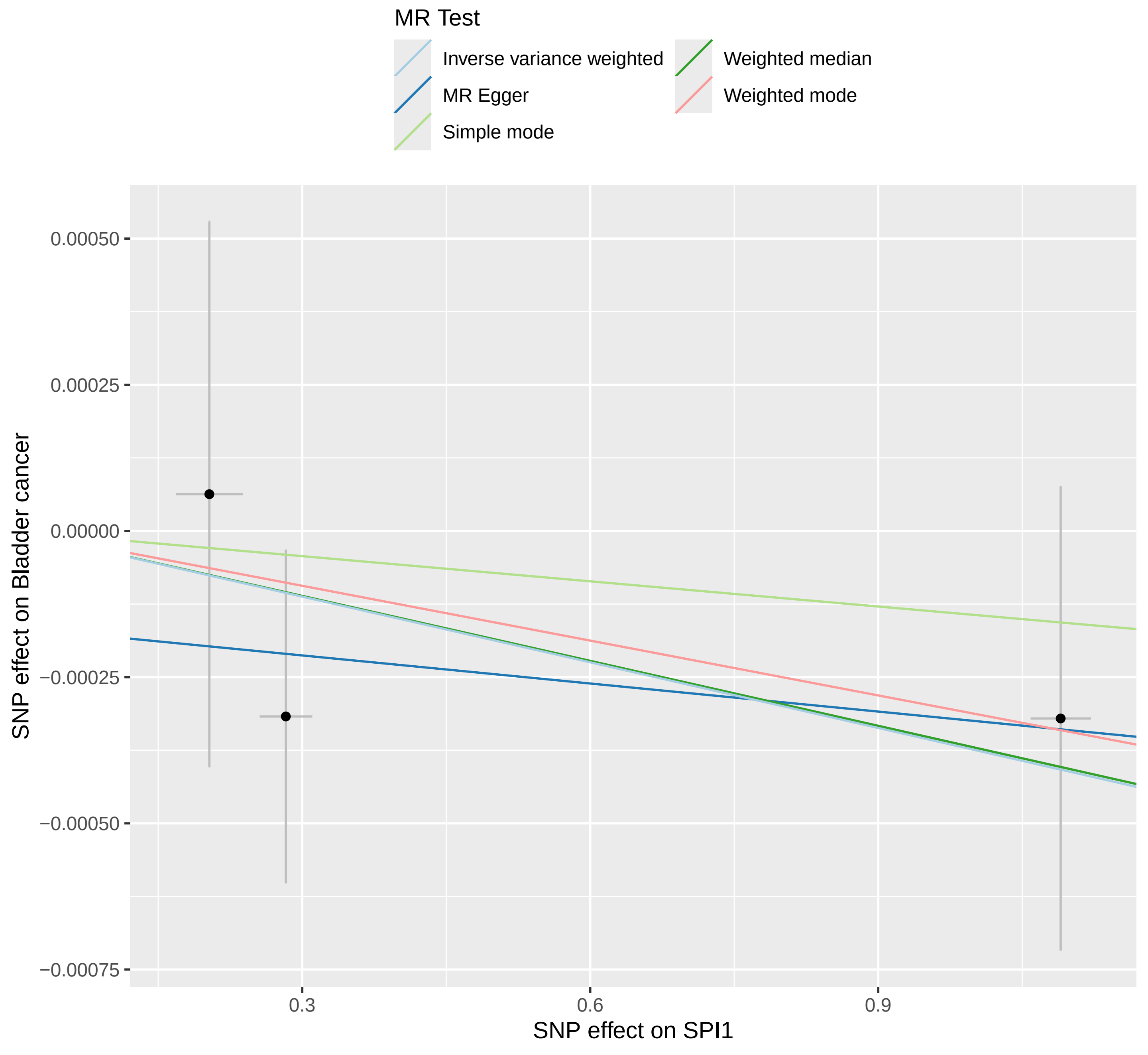
MR Test
Inverse variance weighted
MR Egger
Simple mode
Weighted median
Weighted mode
0.00050
0.00025
0.00000
−0.00025
−0.00050
−0.00075
SNP effect on Bladder cancer
0.3
0.6
0.9
SNP effect on SPI1

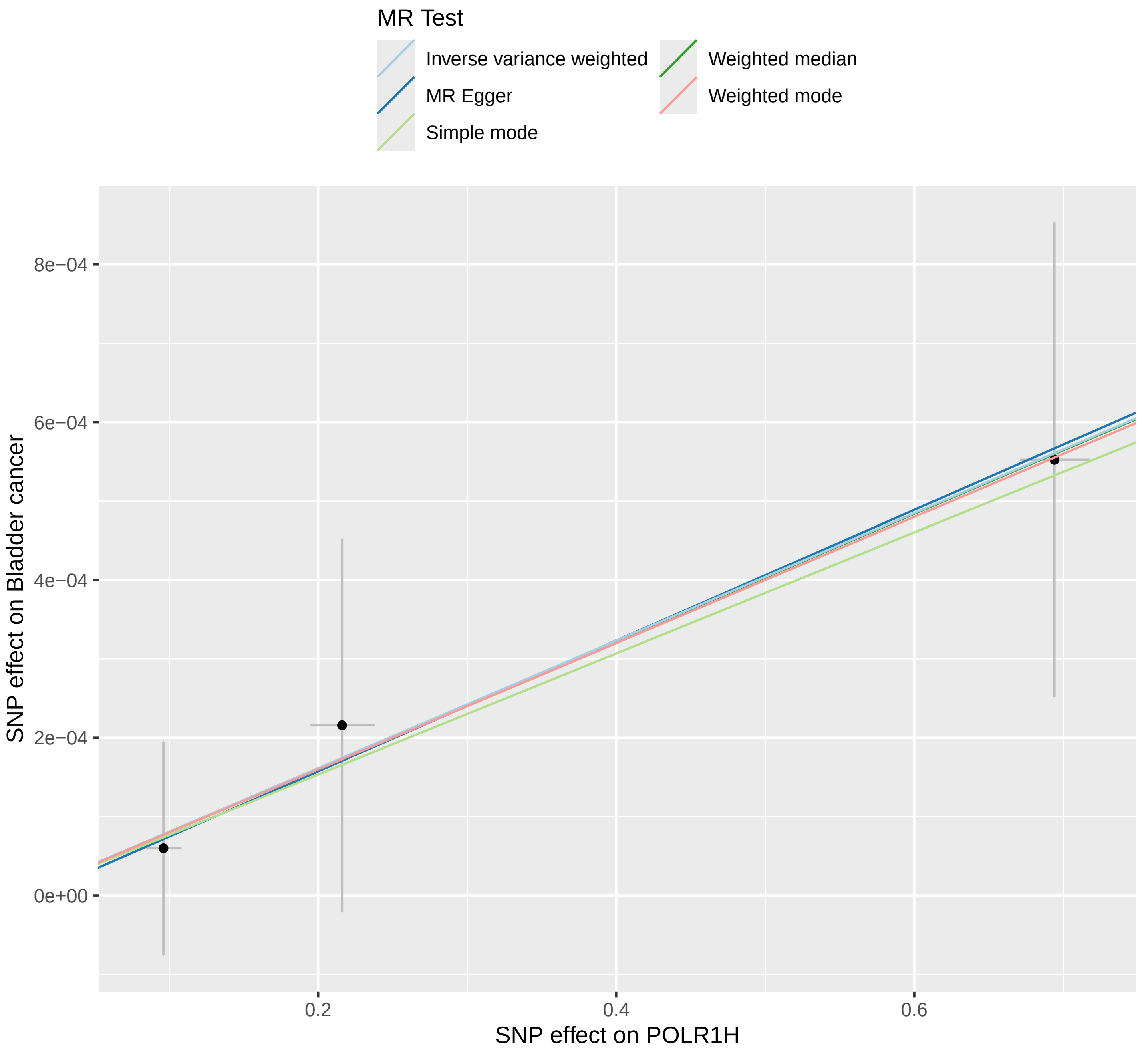
MR Test
Inverse variance weighted
MR Egger
Simple mode
Weighted median
Weighted mode
SNP effect on Bladder cancer
8e−04
6e−04
4e−04
2e−04
0e+00
0.2
0.4
0.6
SNP effect on POLR1H

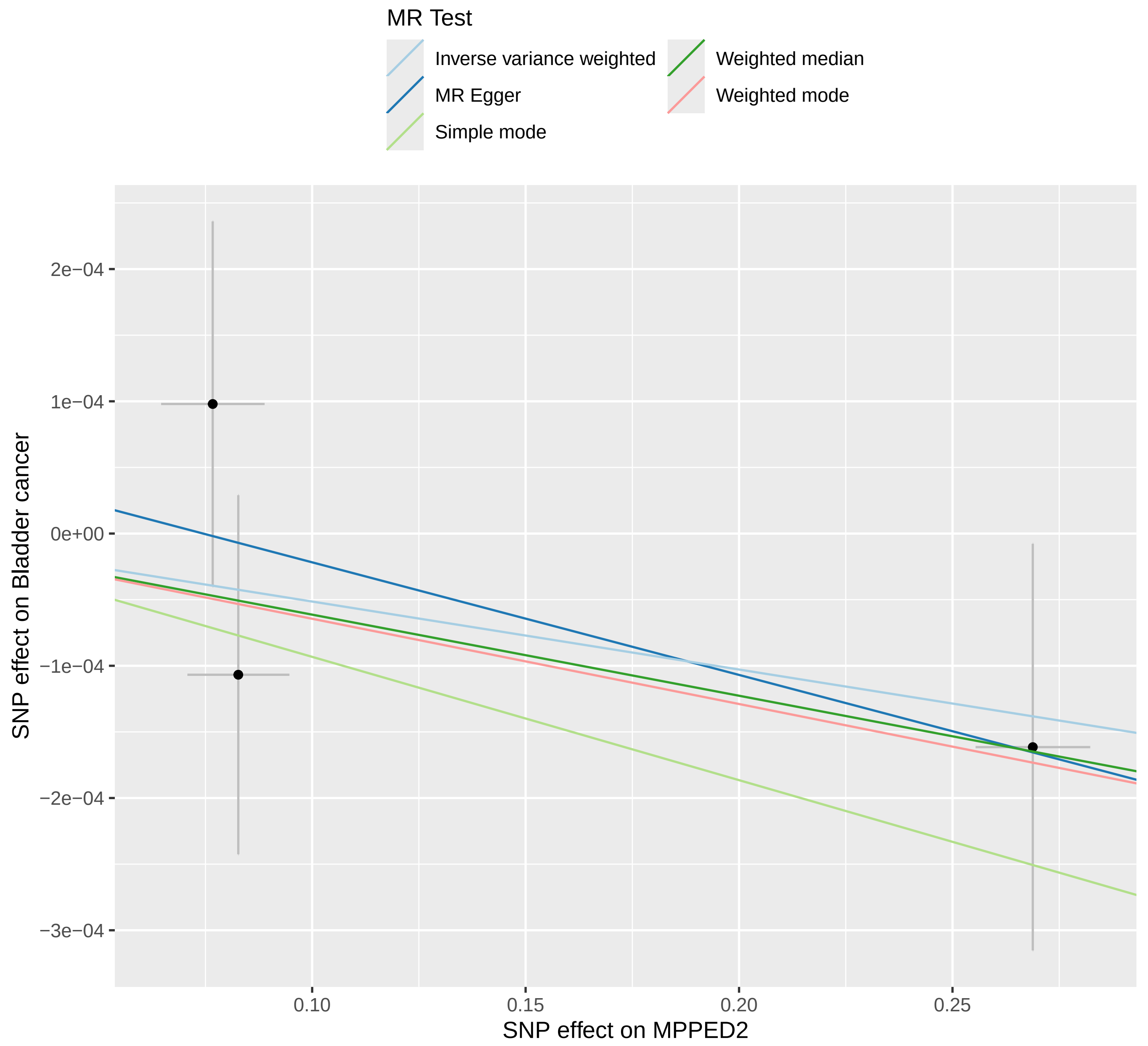

MR Test
Inverse variance weighted
MR Egger
Simple mode
Weighted median
Weighted mode
2e−04
1e−04
0e+00
−1e−04
−2e−04
−3e−04
0.10
0.15
0.20
0.25
SNP effect on MPPED2
SNP effect on Bladder cancer

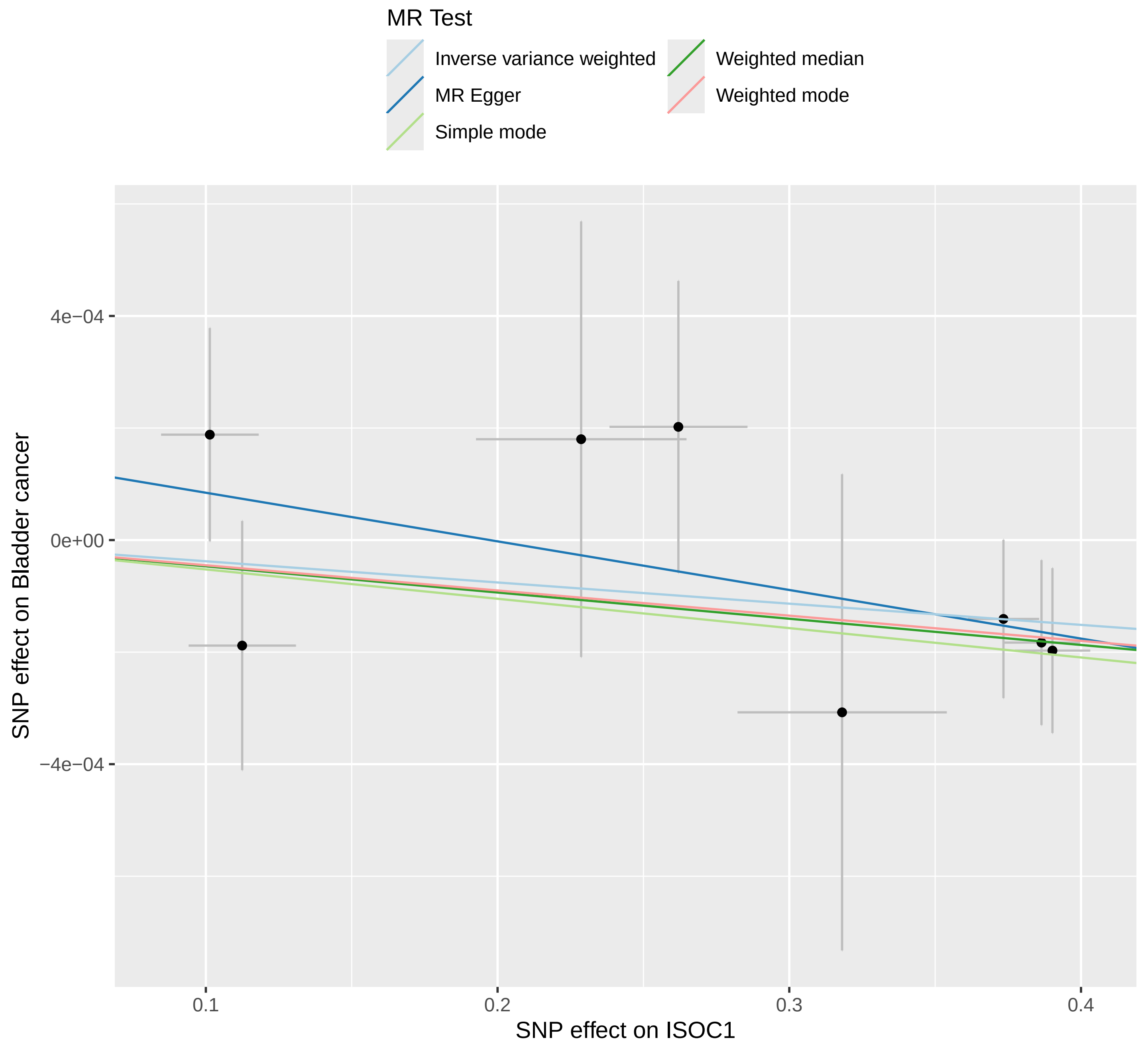

MR Test
Inverse variance weighted
MR Egger
Simple mode
Weighted median
Weighted mode
4e−04
0e+00
−4e−04
0.1
0.2
0.3
0.4
SNP effect on ISOC1
SNP effect on Bladder cancer

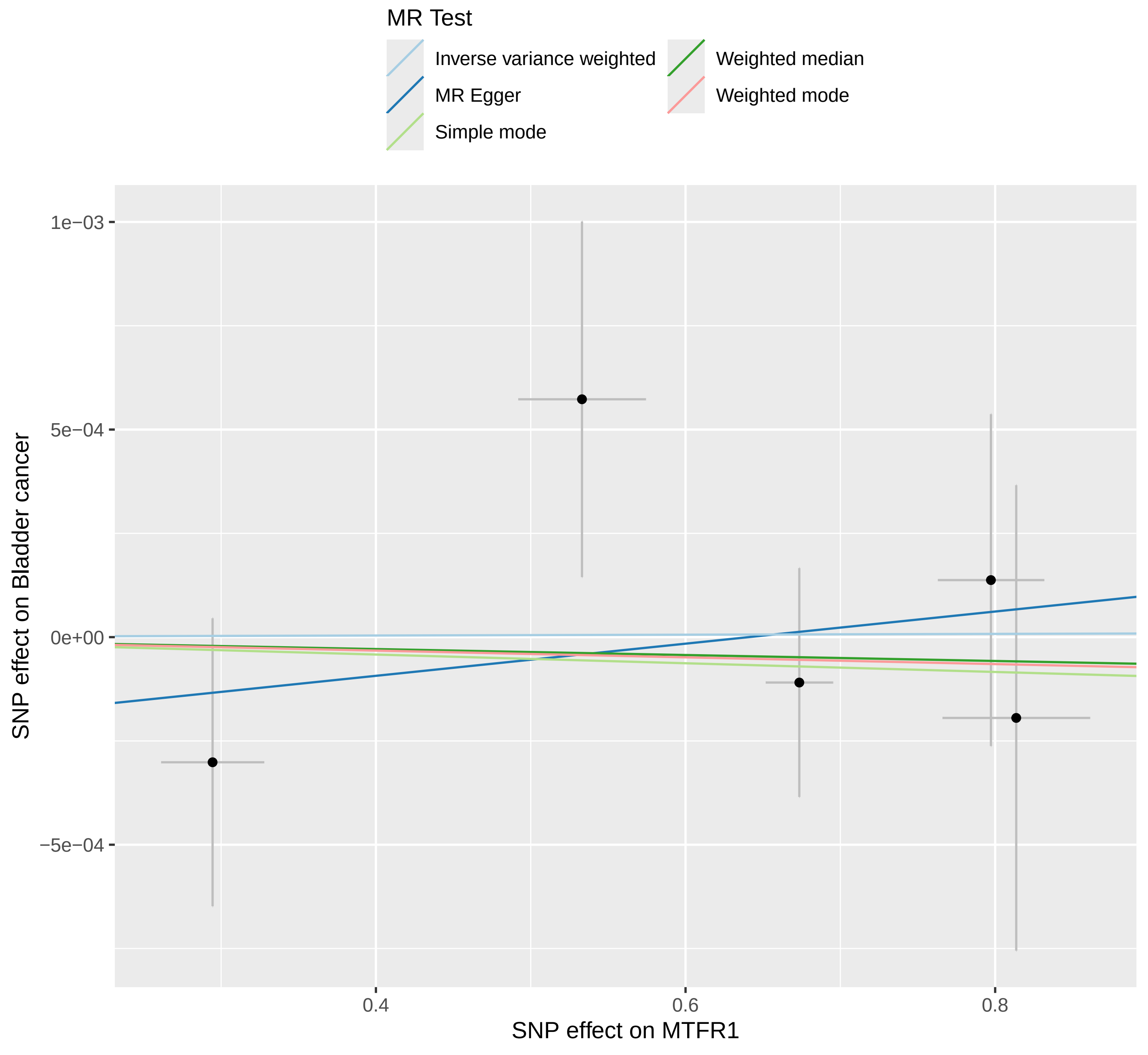
MR Test
Inverse variance weighted
MR Egger
Simple mode
Weighted median
Weighted mode
1e−03
5e−04
0e+00
−5e−04
0.4
0.6
0.8
SNP effect on MTFR1
SNP effect on Bladder cancer

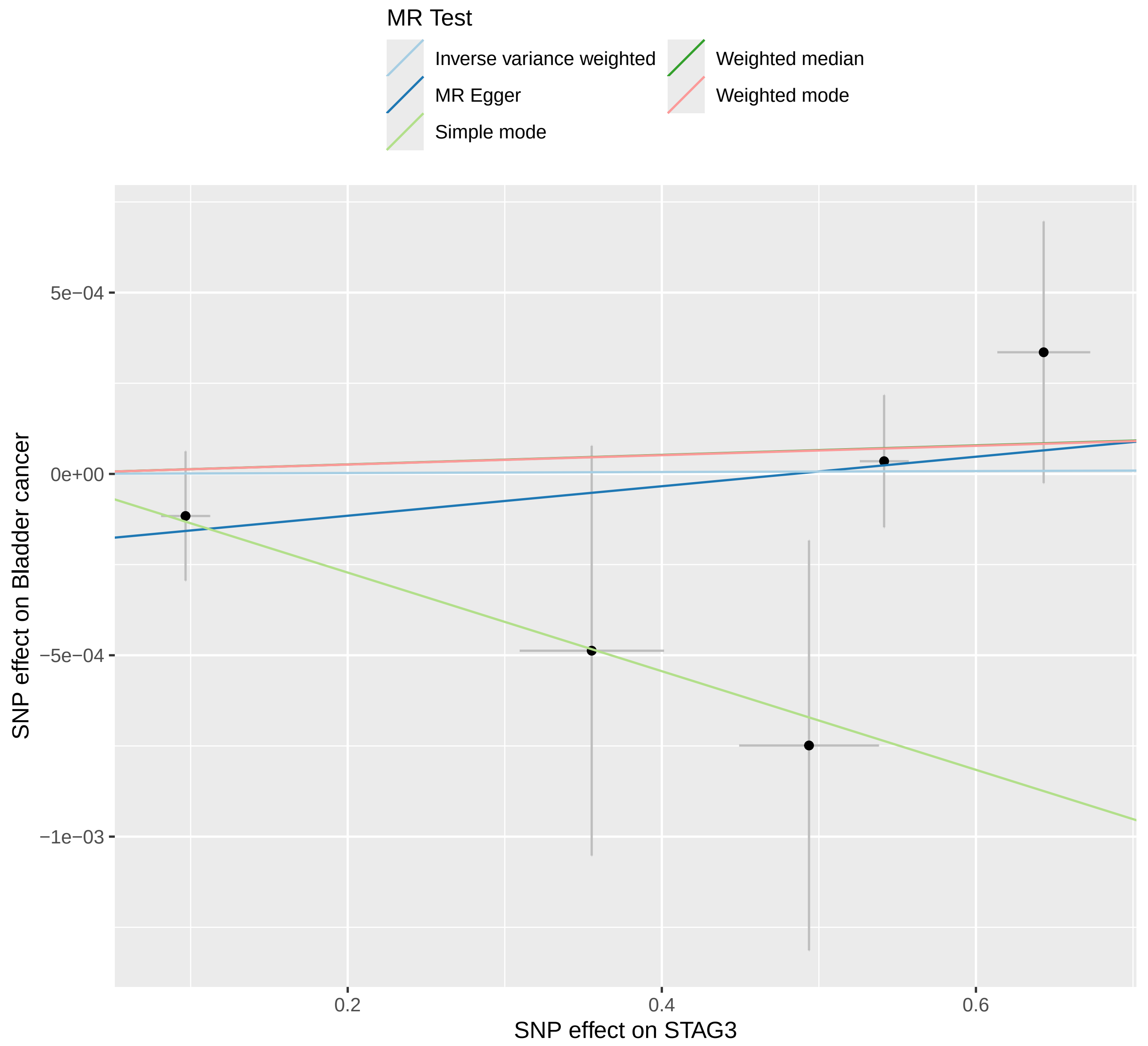
MR Test
Inverse variance weighted
MR Egger
Simple mode
Weighted median
Weighted mode
5e−04
0e+00
−5e−04
−1e−03
SNP effect on Bladder cancer
0.2
0.4
0.6
SNP effect on STAG3

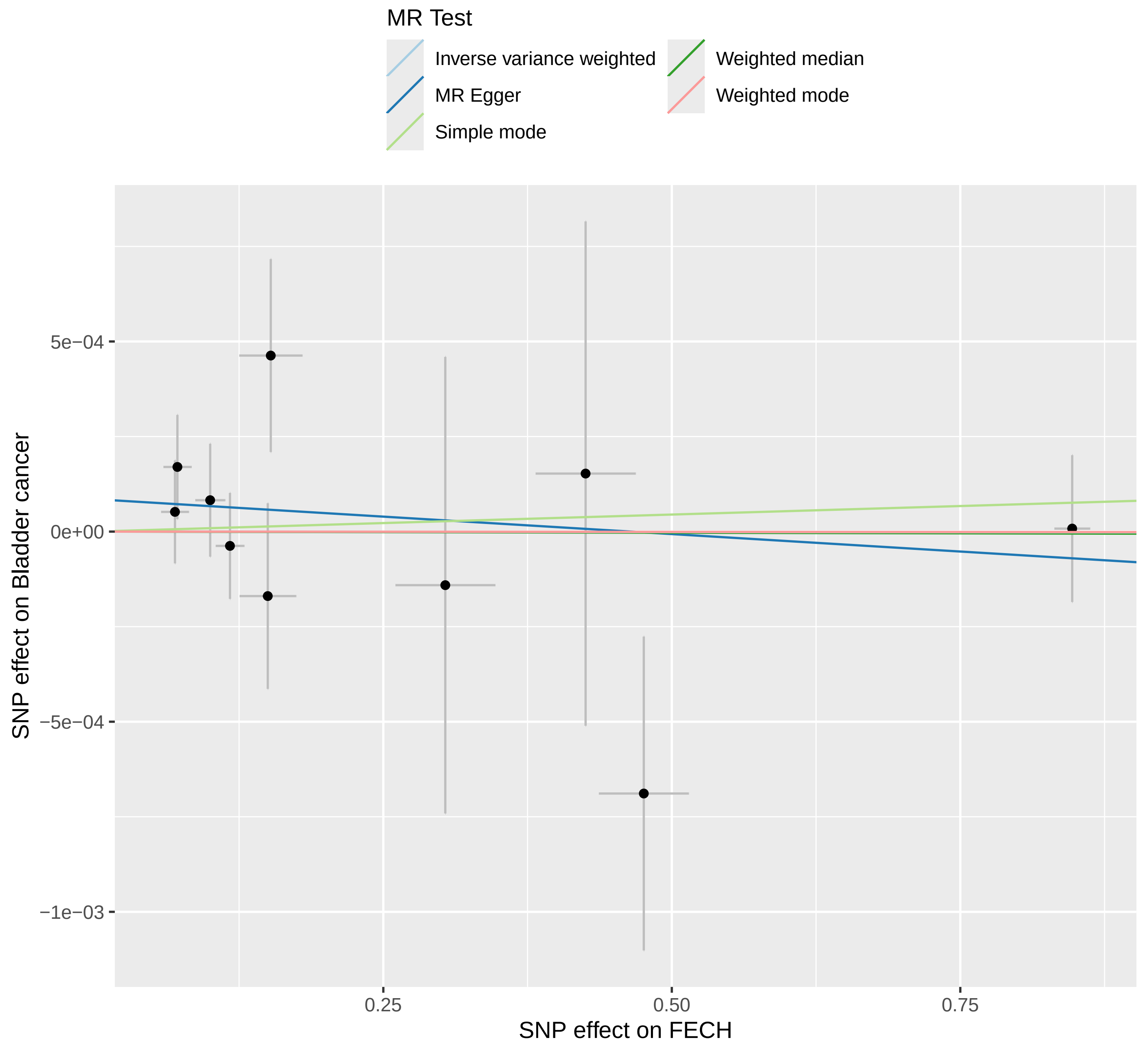

MR Test
Inverse variance weighted
MR Egger
Simple mode
Weighted median
Weighted mode
5e−04
0e+00
−5e−04
−1e−03
SNP effect on Bladder cancer
0.25
0.50
0.75
SNP effect on FECH

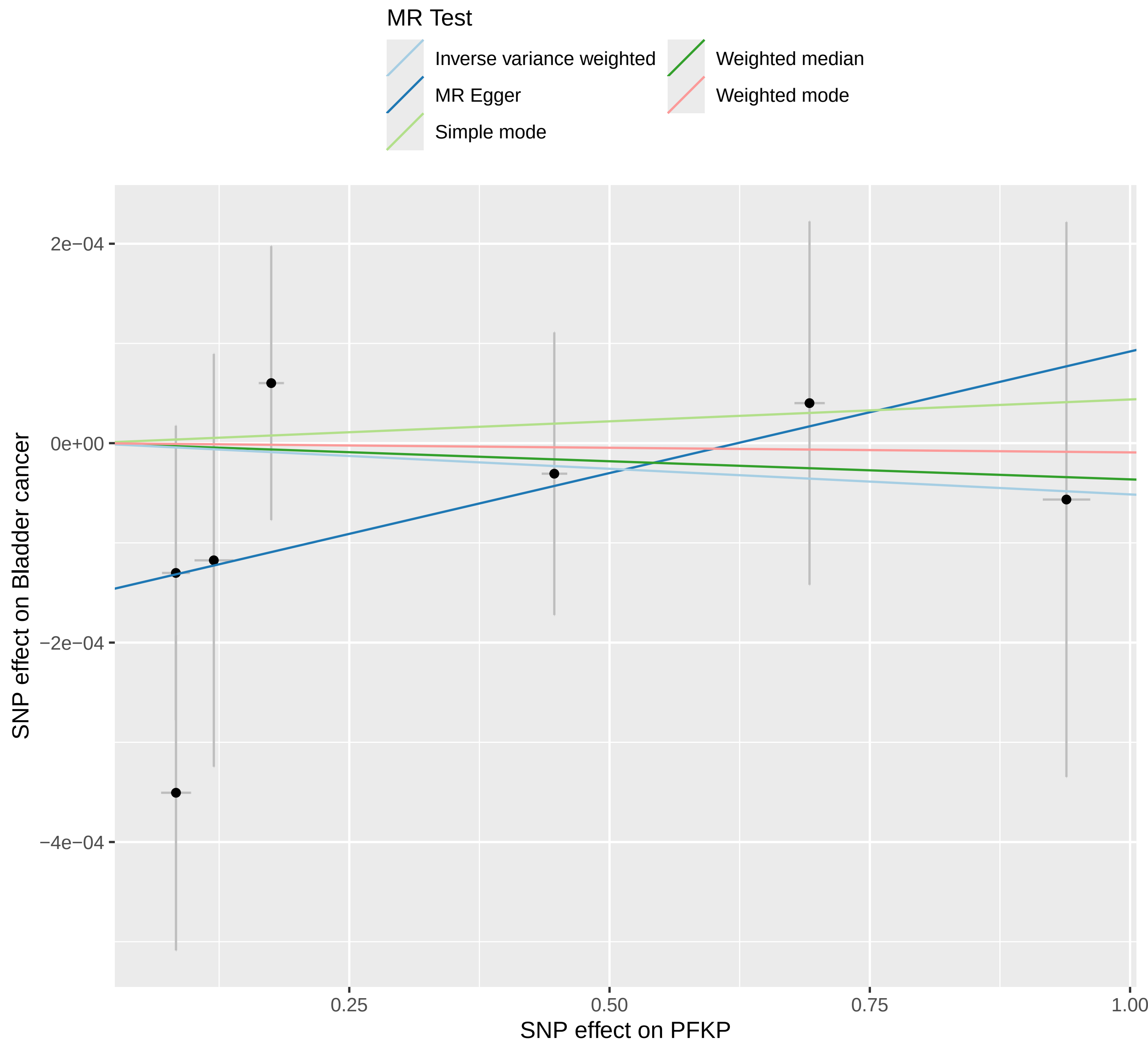

MR Test
Inverse variance weighted
MR Egger
Simple mode
Weighted median
Weighted mode
2e−04
0e+00
−2e−04
−4e−04
SNP effect on Bladder cancer
0.25
0.50
0.75
1.00
SNP effect on PFKP

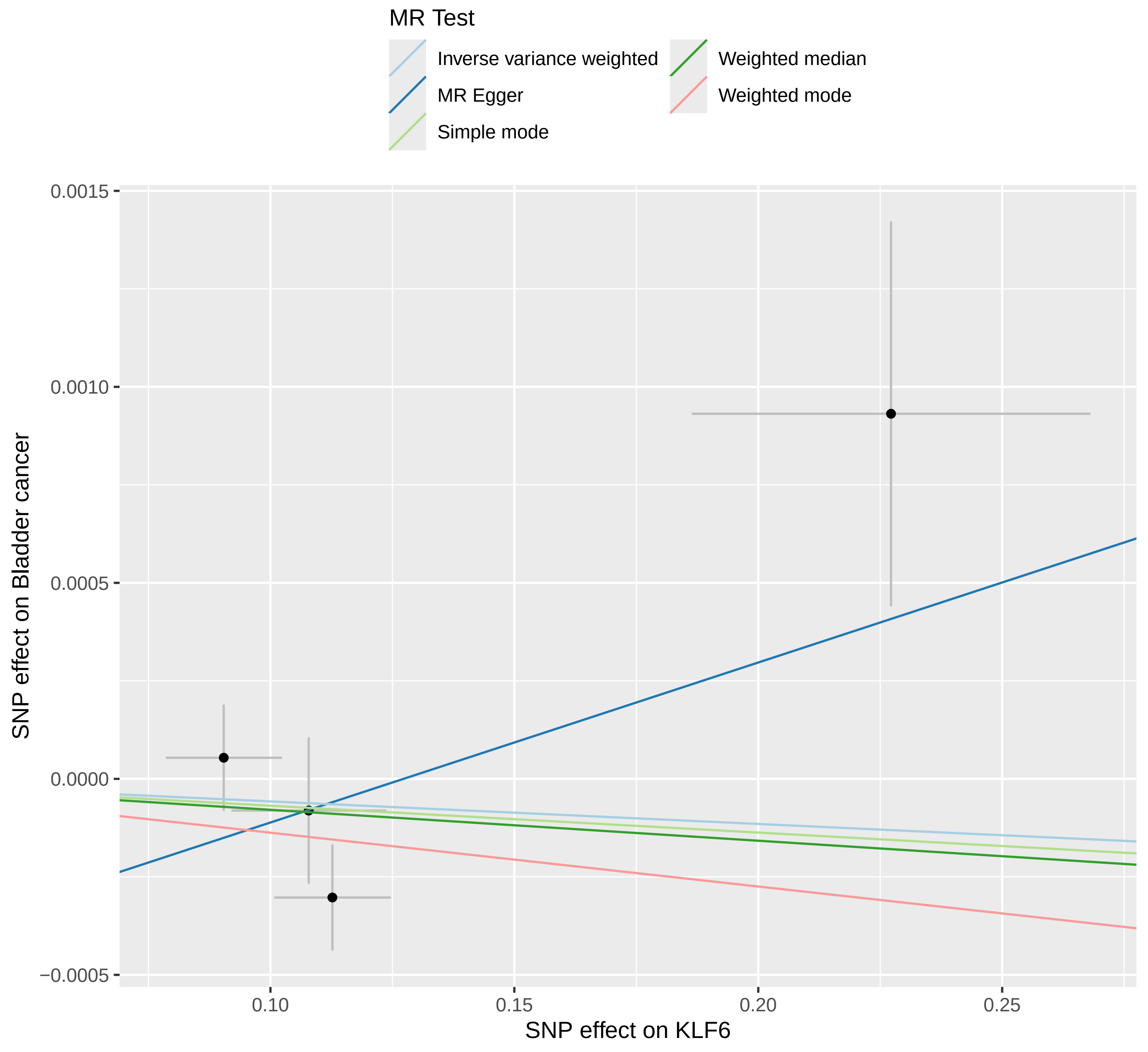

MR Test
Inverse variance weighted
MR Egger
Simple mode
Weighted median
Weighted mode
0.0015
0.0010
0.0005
0.0000
−0.0005
0.10
0.15
0.20
0.25
SNP effect on KLF6
SNP effect on Bladder cancer

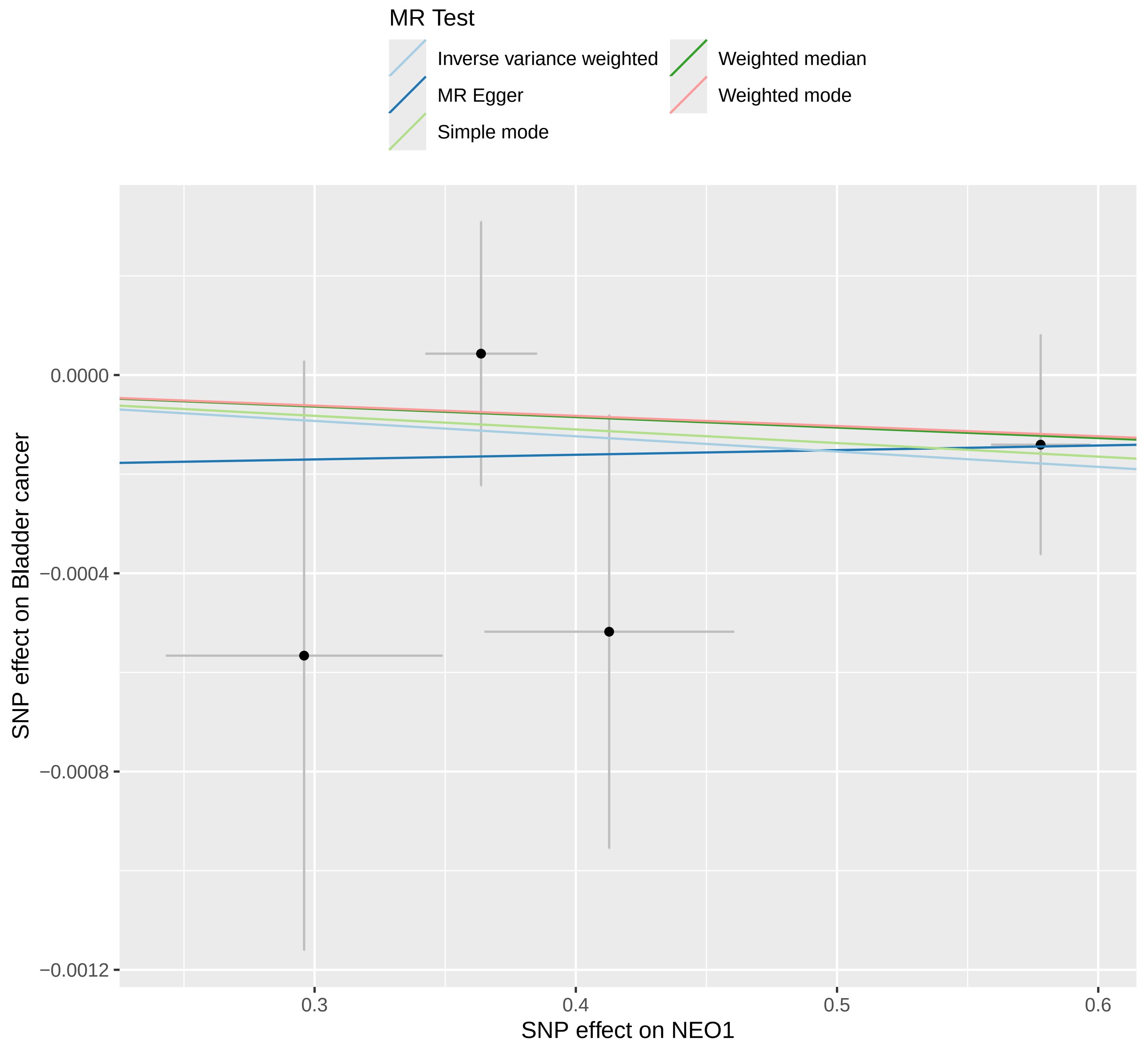

MR Test
Inverse variance weighted
MR Egger
Simple mode
Weighted median
Weighted mode
0.0000
−0.0004
−0.0008
−0.0012
SNP effect on Bladder cancer
0.3
0.4
0.5
0.6
SNP effect on NEO1

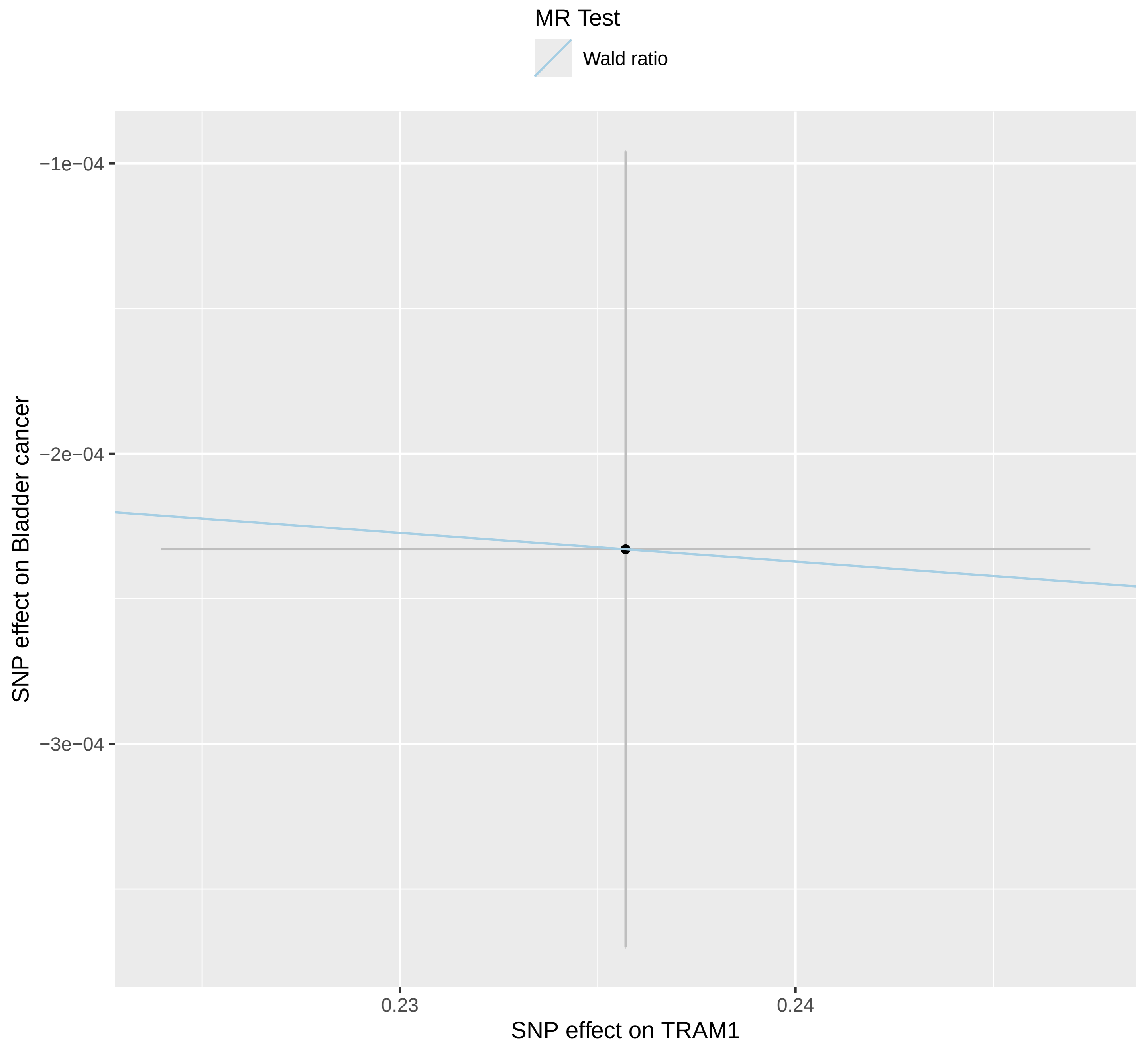
MR Test
Wald ratio
−1e−04
−2e−04
−3e−04
SNP effect on Bladder cancer
0.23
0.24
SNP effect on TRAM1

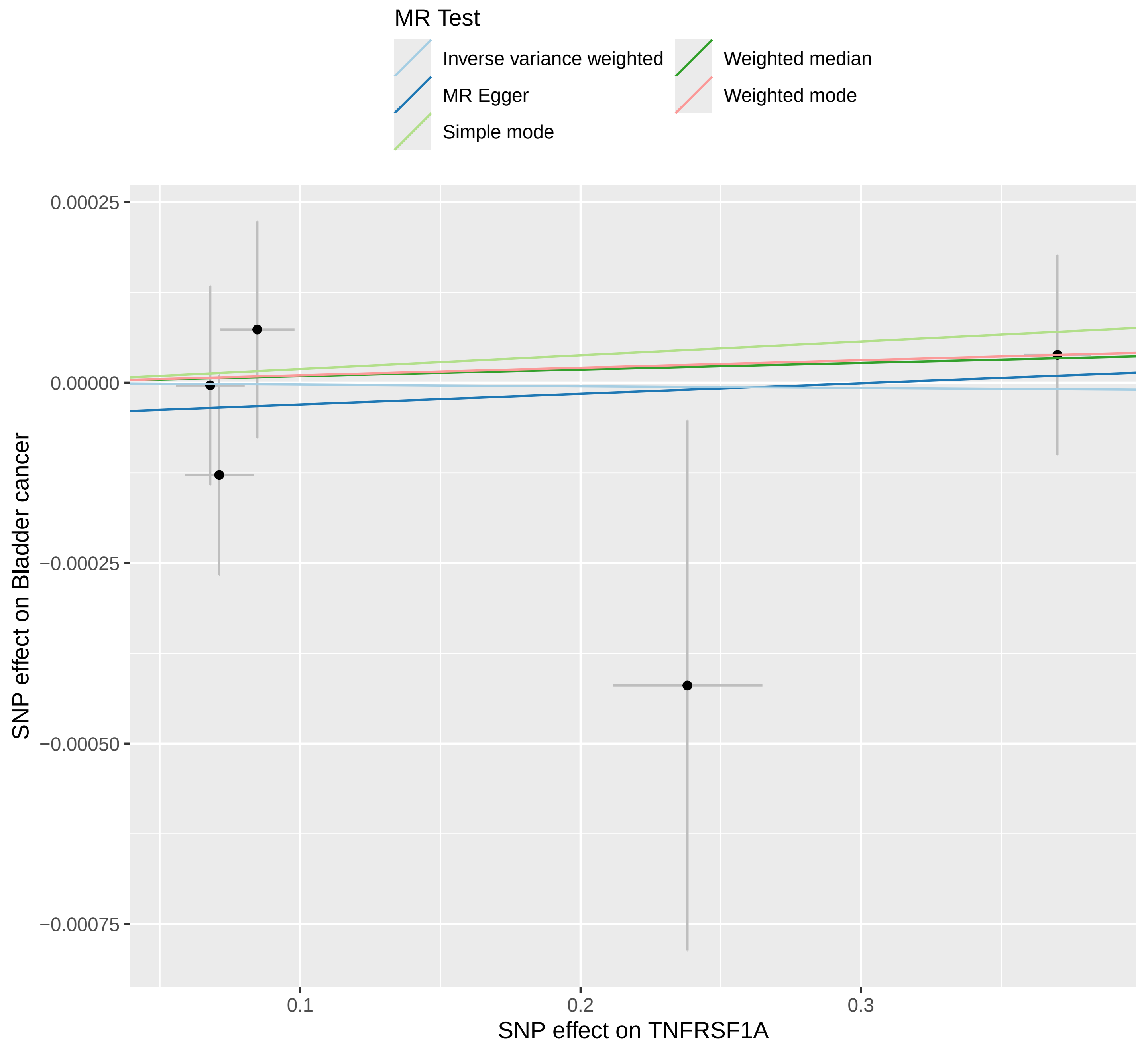
MR Test
Inverse variance weighted
MR Egger
Simple mode
Weighted median
Weighted mode
0.00025
0.00000
−0.00025
−0.00050
−0.00075
0.1
0.2
0.3
SNP effect on Bladder cancer
SNP effect on TNFRSF1A

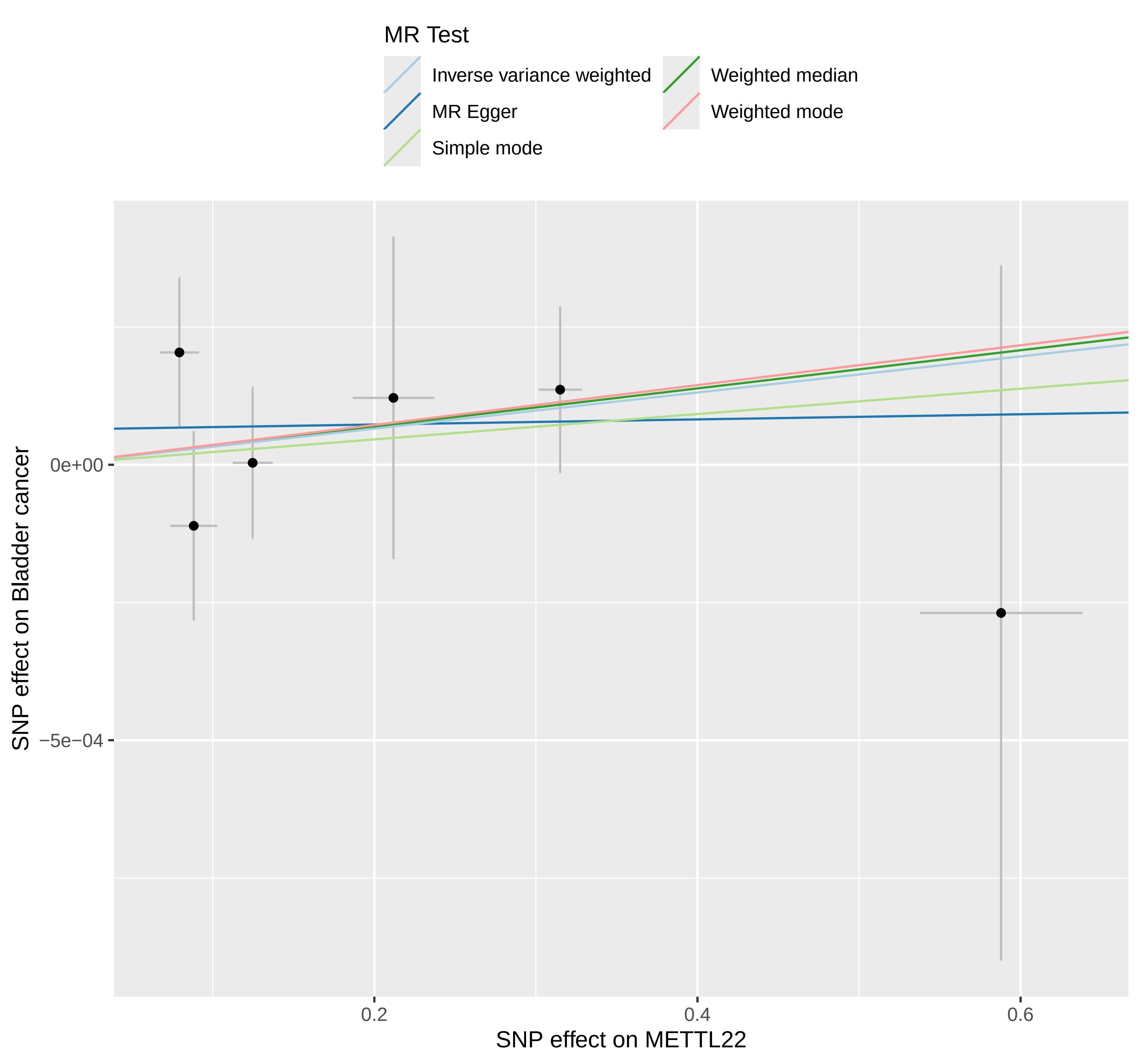
MR Test
Inverse variance weighted
MR Egger
Simple mode
Weighted median
Weighted mode
0e+00
−5e−04
SNP effect on Bladder cancer
0.2
0.4
0.6
SNP effect on METTL22

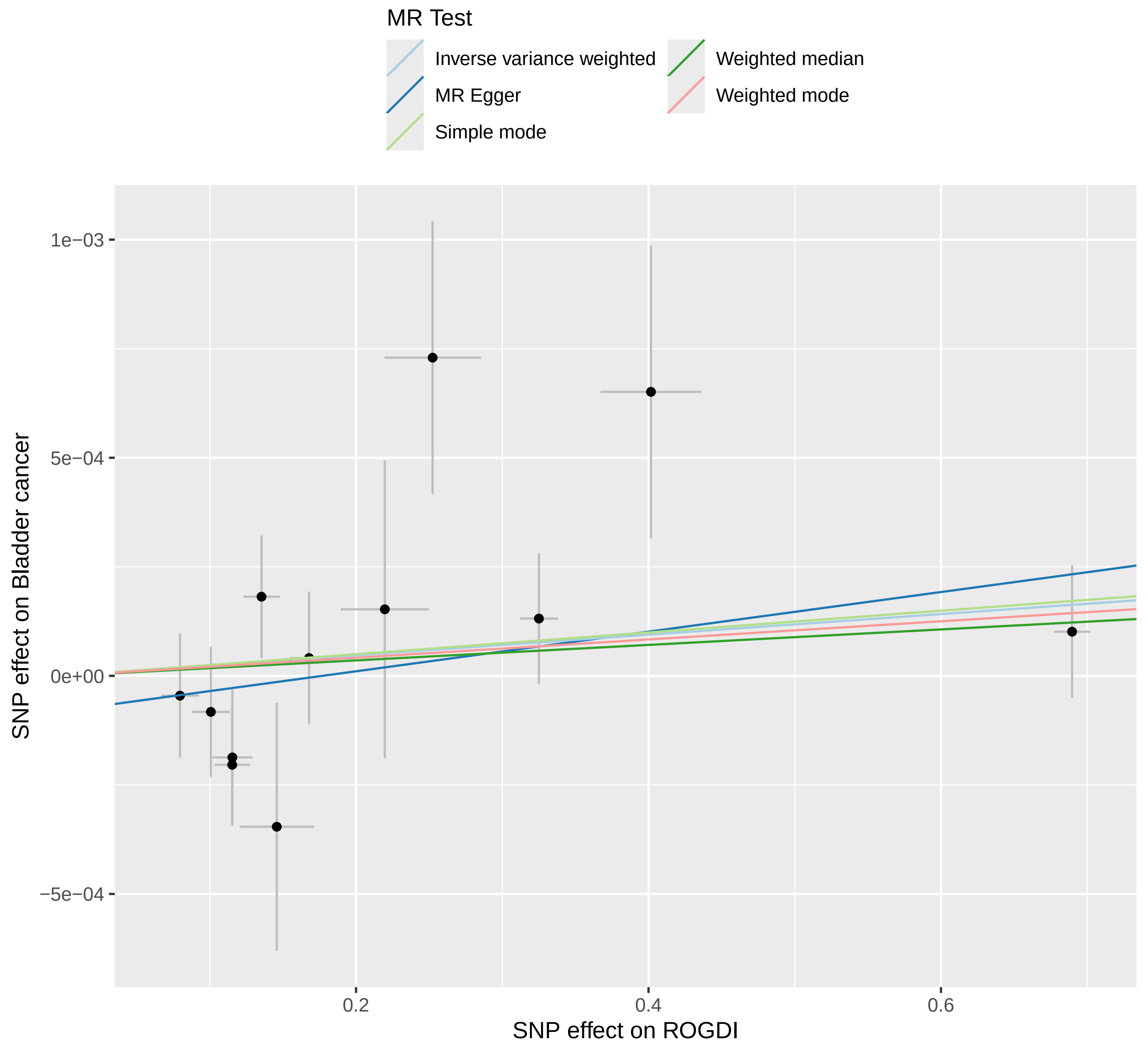

MR Test
Inverse variance weighted
MR Egger
Simple mode
Weighted median
Weighted mode
1e−03
5e−04
0e+00
−5e−04
SNP effect on Bladder cancer
0.2
0.4
0.6
SNP effect on ROGDI

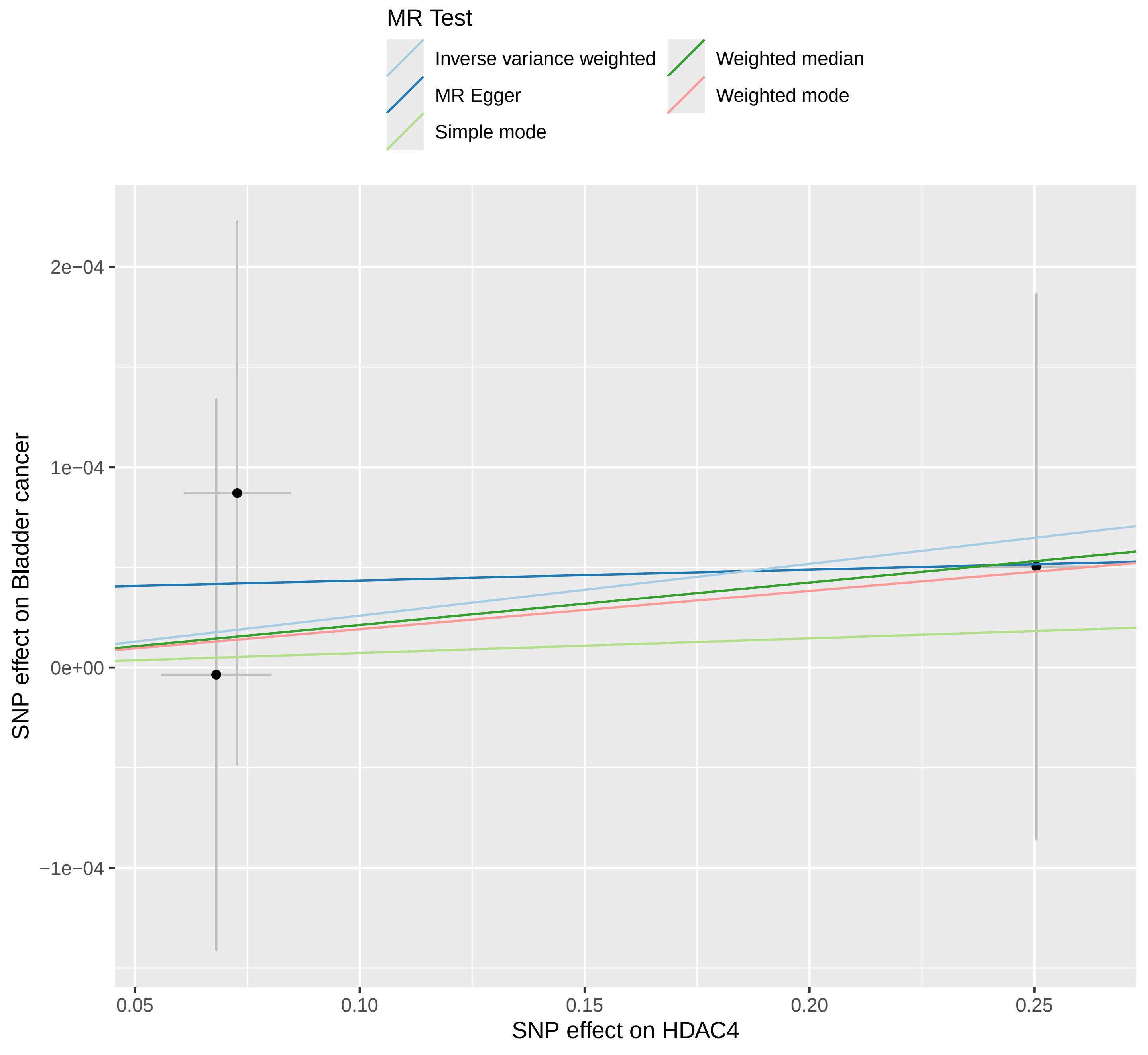

MR Test
Inverse variance weighted
MR Egger
Simple mode
Weighted median
Weighted mode
2e−04
1e−04
0e+00
−1e−04
SNP effect on Bladder cancer
0.05
0.10
0.15
0.20
0.25
SNP effect on HDAC4

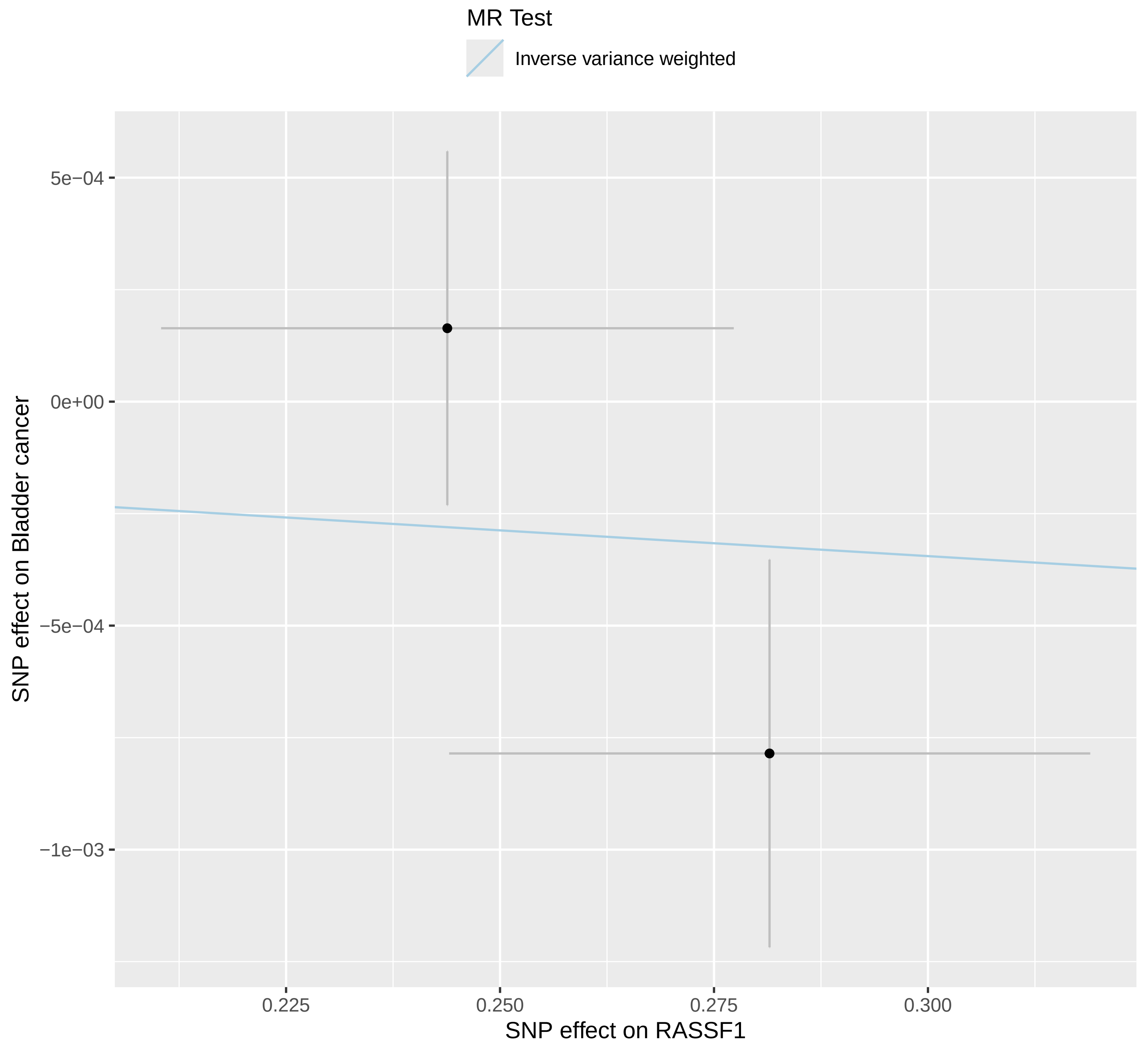

MR Test
Inverse variance weighted
5e−04
0e+00
−5e−04
−1e−03
SNP effect on Bladder cancer
0.225
0.250
0.275
0.300
SNP effect on RASSF1

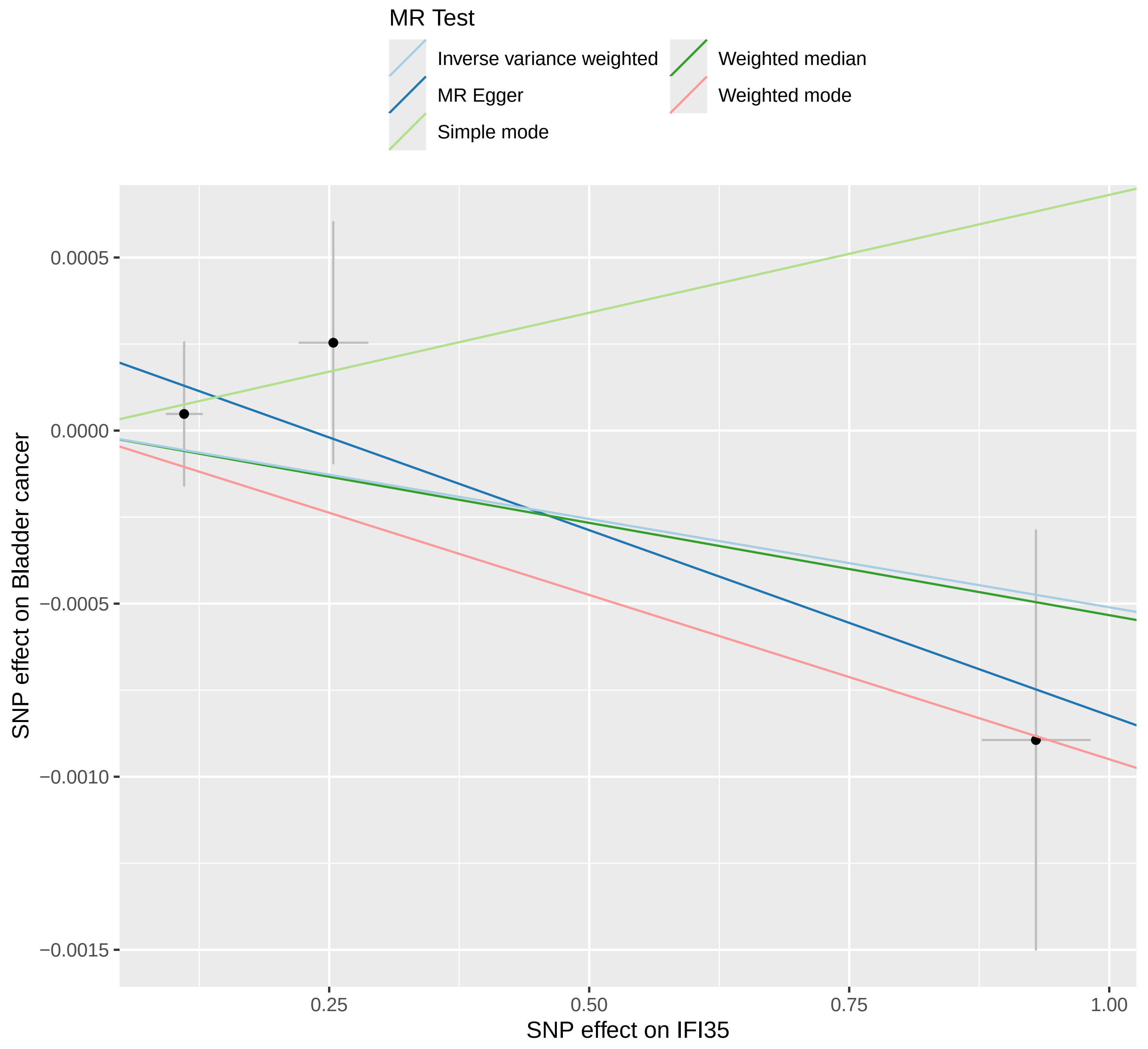
MR Test
Inverse variance weighted
MR Egger
Simple mode
Weighted median
Weighted mode
0.0005
0.0000
−0.0005
−0.0010
−0.0015
SNP effect on Bladder cancer
0.25
0.50
0.75
1.00
SNP effect on IFI35

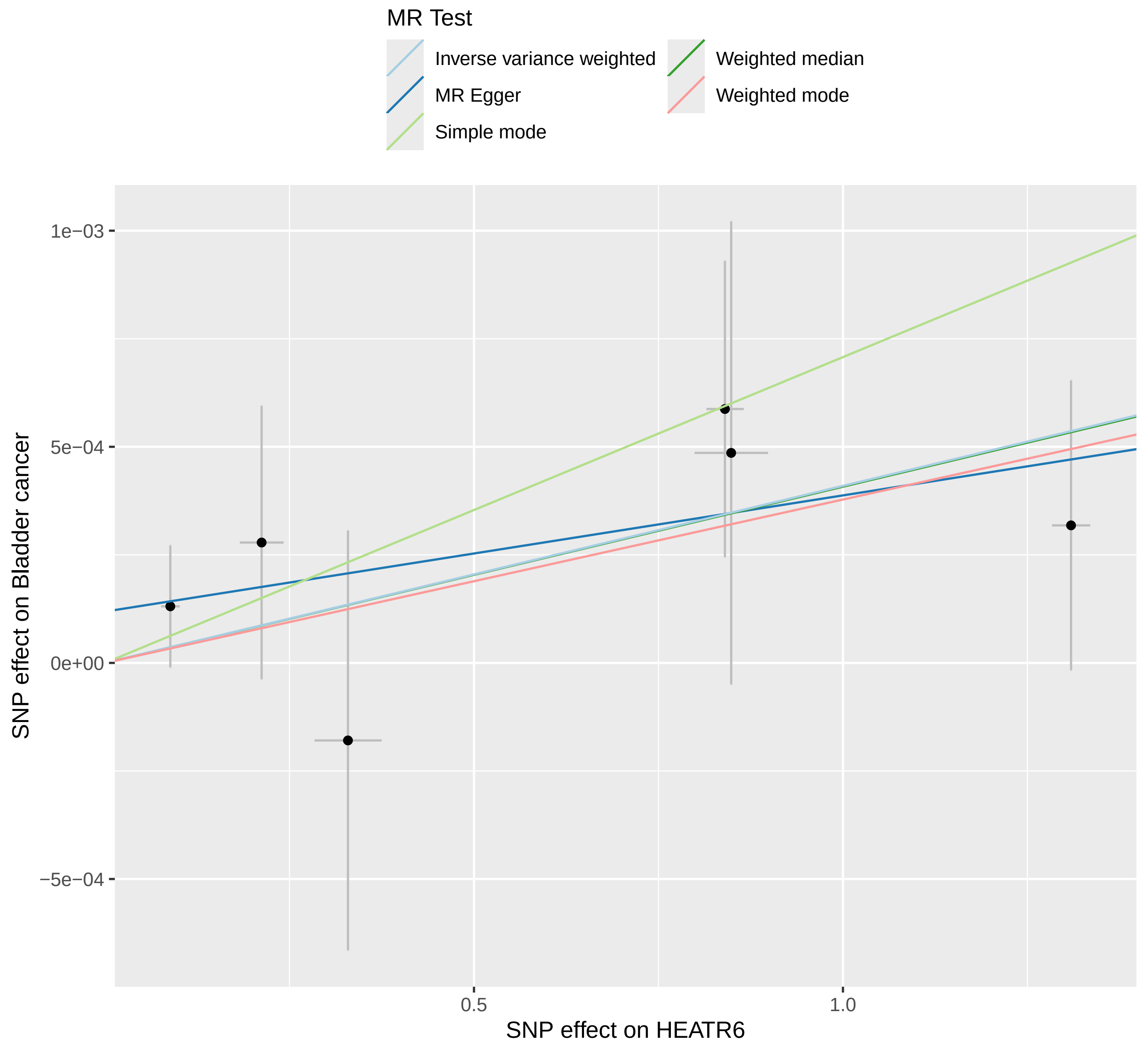

MR Test
Inverse variance weighted
MR Egger
Simple mode
Weighted median
Weighted mode
1e−03
5e−04
0e+00
−5e−04
0.5
1.0
SNP effect on Bladder cancer
SNP effect on HEATR6

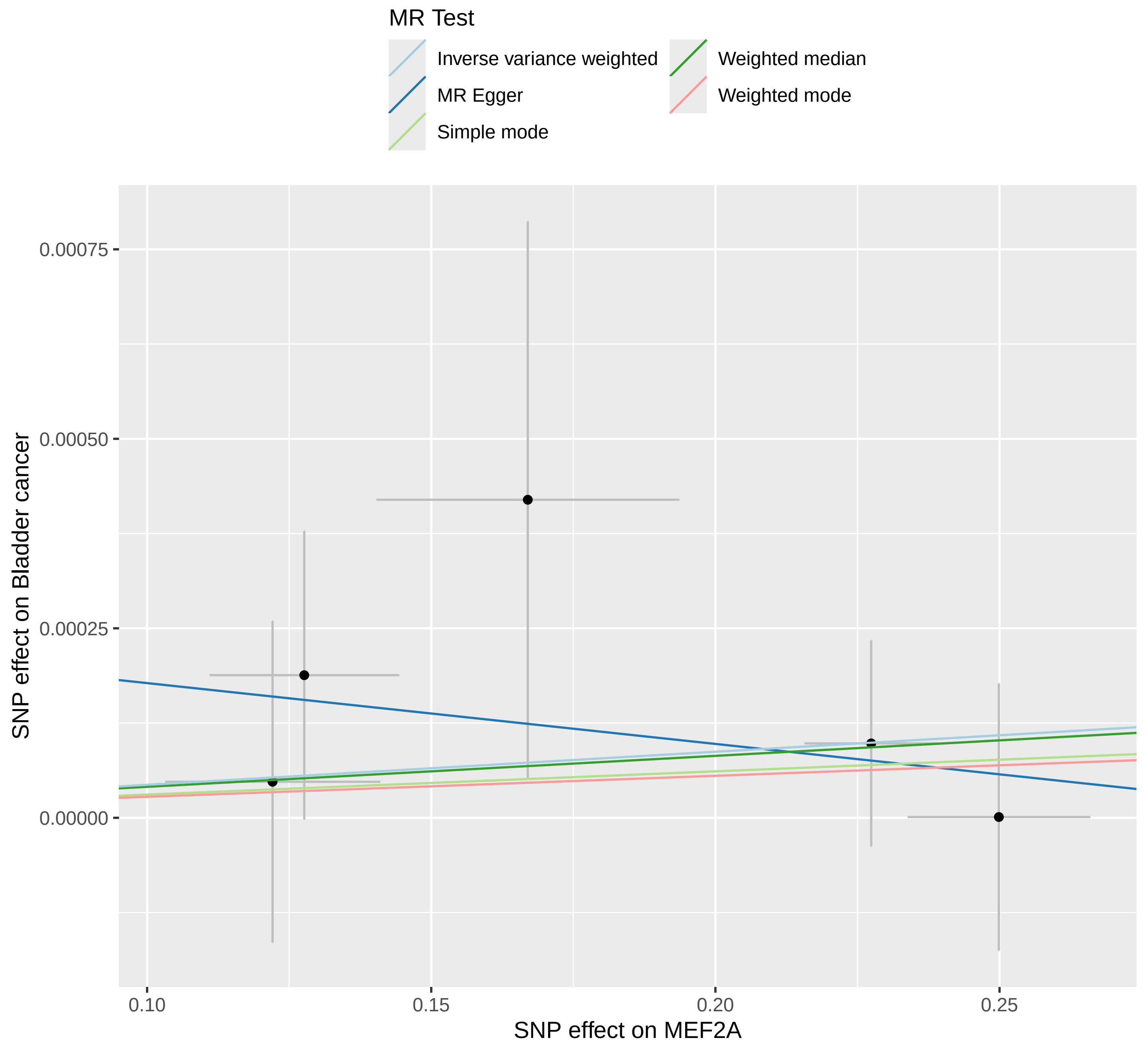
MR Test
Inverse variance weighted
MR Egger
Simple mode
Weighted median
Weighted mode
0.00075
0.00050
0.00025
0.00000
SNP effect on Bladder cancer
0.10
0.15
0.20
0.25
SNP effect on MEF2A

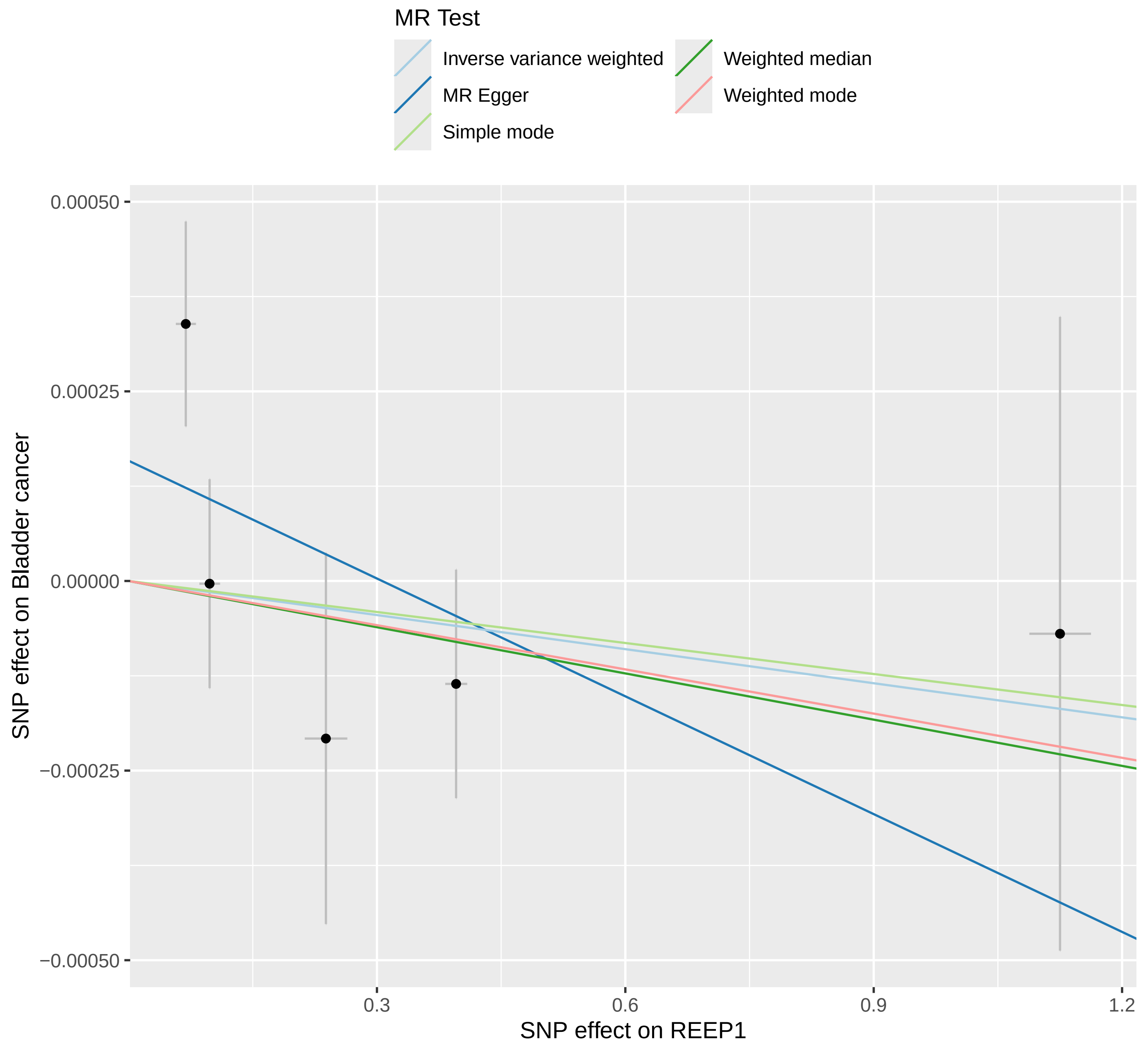

MR Test
Inverse variance weighted
MR Egger
Simple mode
Weighted median
Weighted mode
0.00050
0.00025
0.00000
−0.00025
−0.00050
SNP effect on Bladder cancer
0.3
0.6
0.9
1.2
SNP effect on REEP1

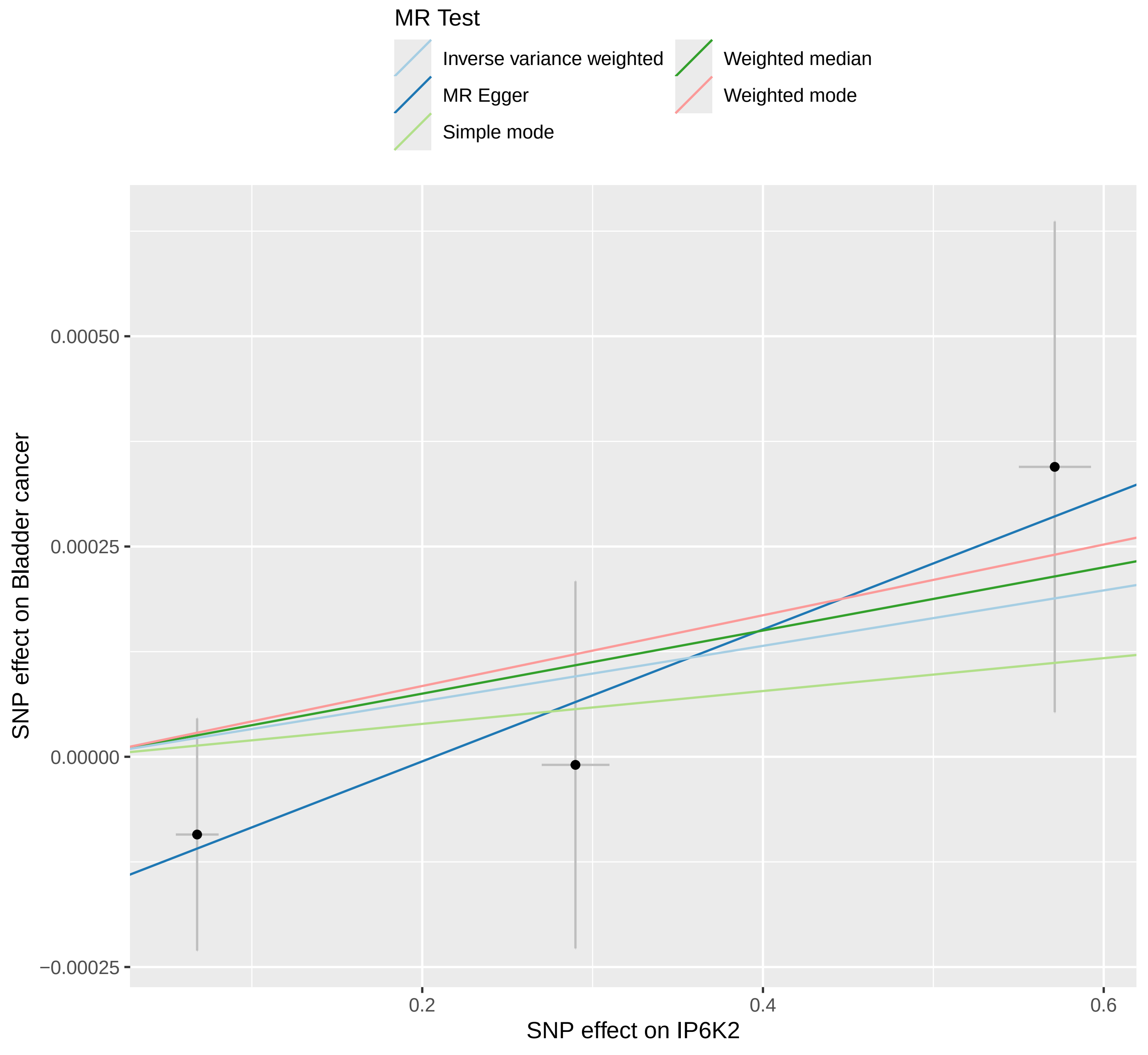
MR Test
Inverse variance weighted
MR Egger
Simple mode
Weighted median
Weighted mode
0.00050
0.00025
0.00000
−0.00025
SNP effect on Bladder cancer
0.2
0.4
0.6
SNP effect on IP6K2

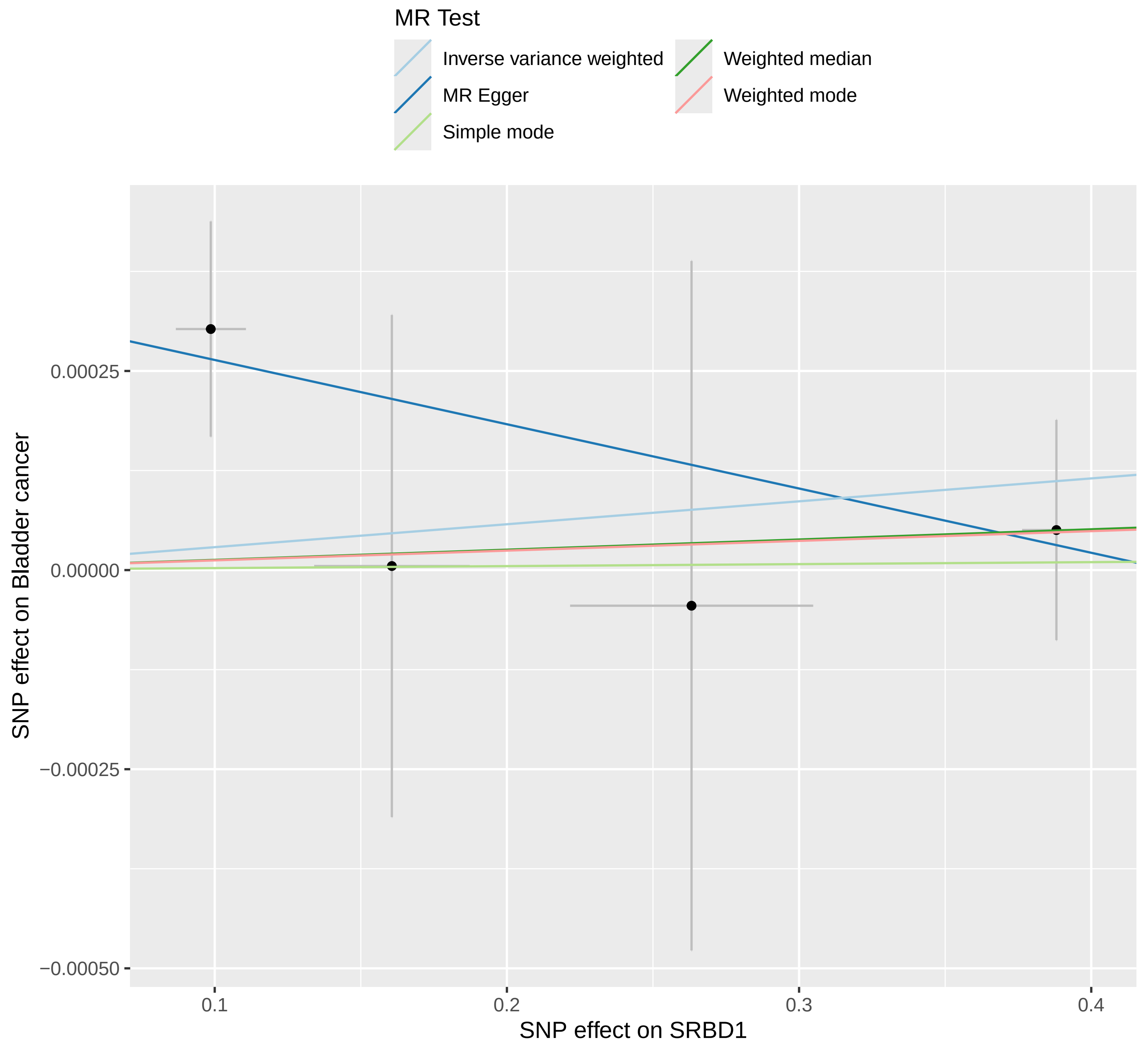

MR Test
Inverse variance weighted
MR Egger
Simple mode
Weighted median
Weighted mode
SNP effect on Bladder cancer
0.00025
0.00000
−0.00025
−0.00050
0.1
0.2
0.3
0.4
SNP effect on SRBD1

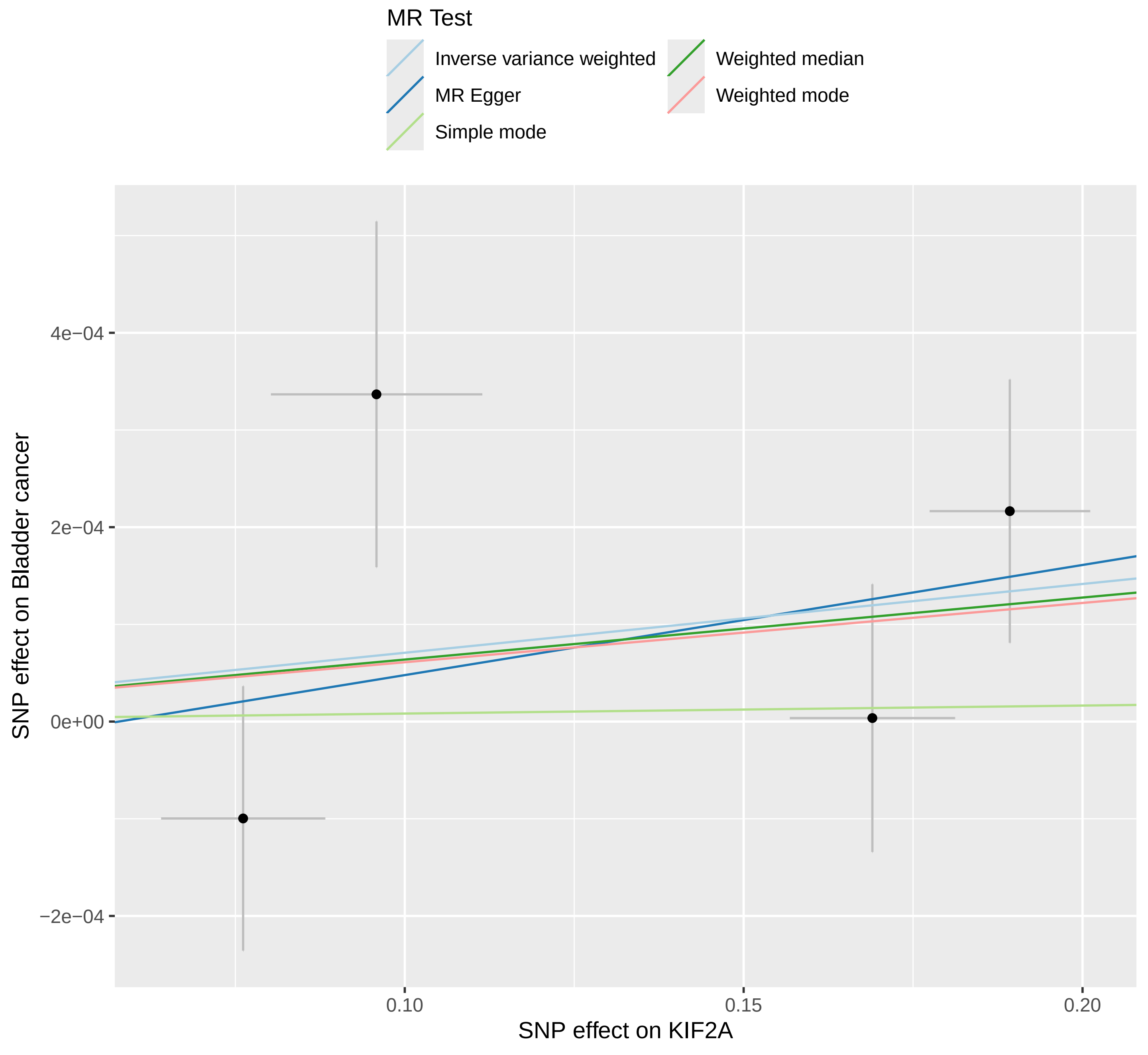

MR Test
Inverse variance weighted
MR Egger
Simple mode
Weighted median
Weighted mode
4e−04
2e−04
0e+00
−2e−04
SNP effect on Bladder cancer
0.10
0.15
0.20
SNP effect on KIF2A

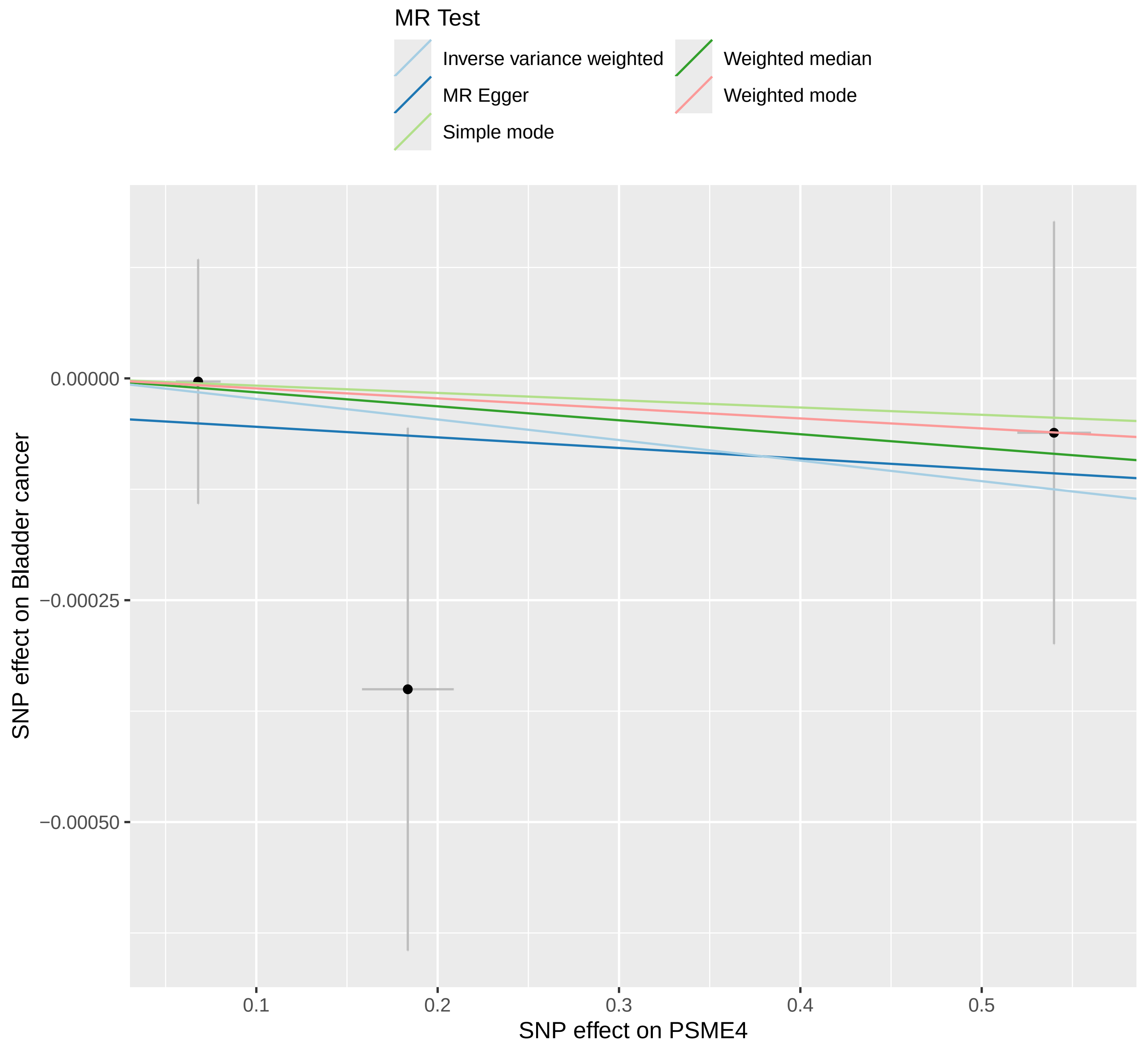

MR Test
Inverse variance weighted
MR Egger
Simple mode
Weighted median
Weighted mode
0.00000
−0.00025
−0.00050
SNP effect on Bladder cancer
0.1
0.2
0.3
0.4
0.5
SNP effect on PSME4

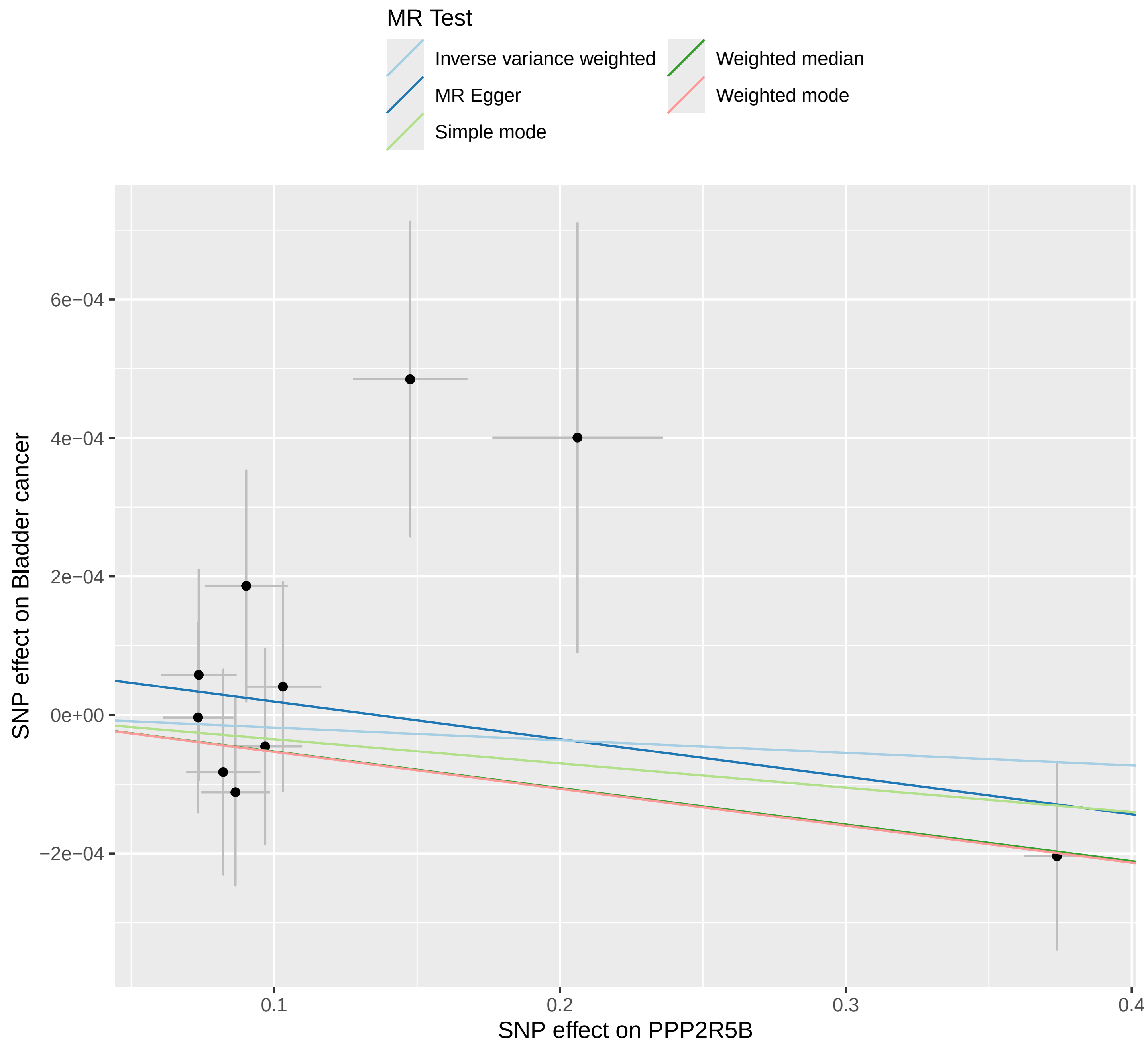
MR Test
Inverse variance weighted
MR Egger
Simple mode
Weighted median
Weighted mode
6e−04
4e−04
2e−04
0e+00
−2e−04
SNP effect on Bladder cancer
0.1
0.2
0.3
0.4
SNP effect on PPP2R5B

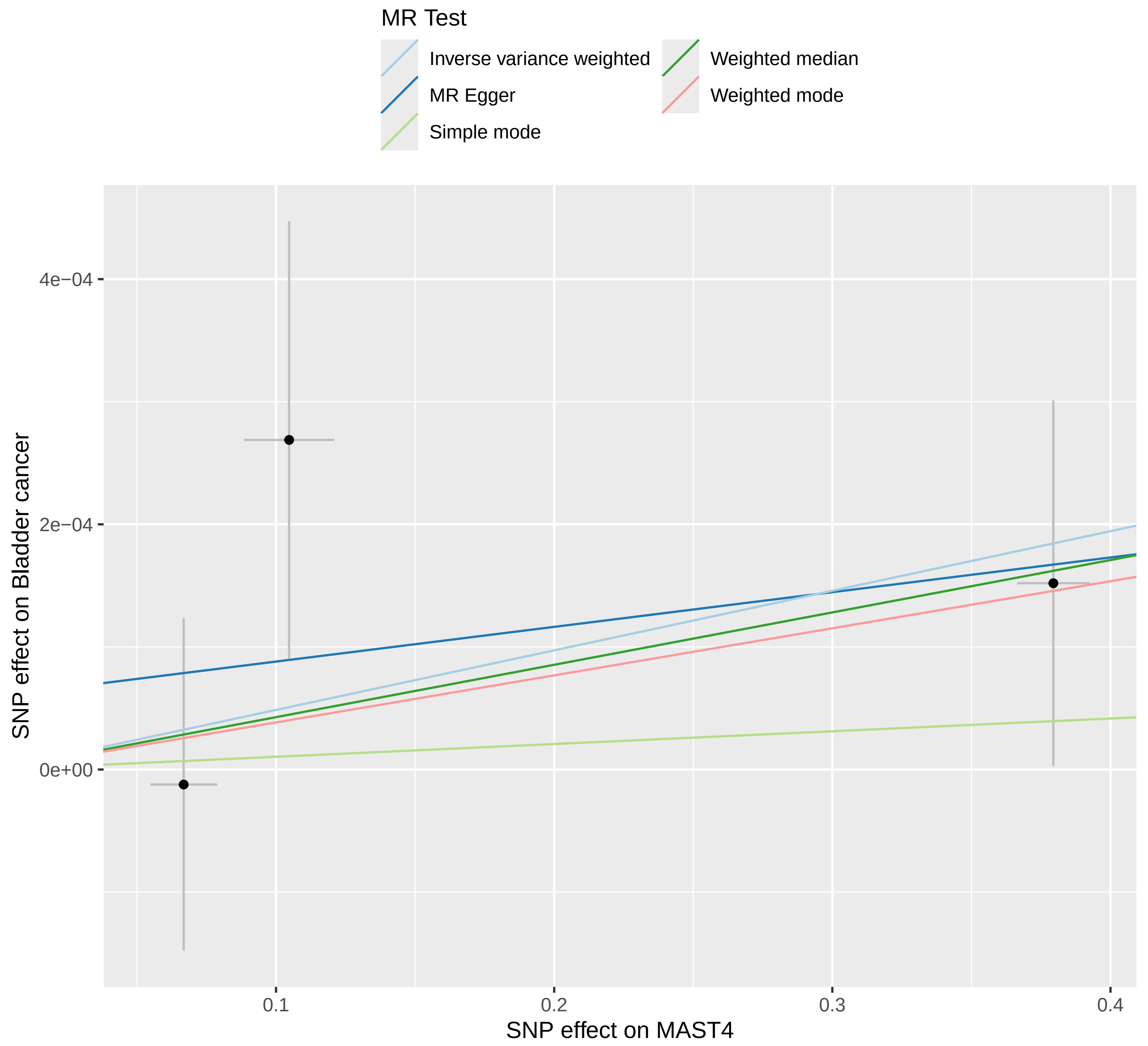

MR Test
Inverse variance weighted
MR Egger
Simple mode
Weighted median
Weighted mode
4e−04
2e−04
0e+00
SNP effect on Bladder cancer
0.1
0.2
0.3
0.4
SNP effect on MAST4

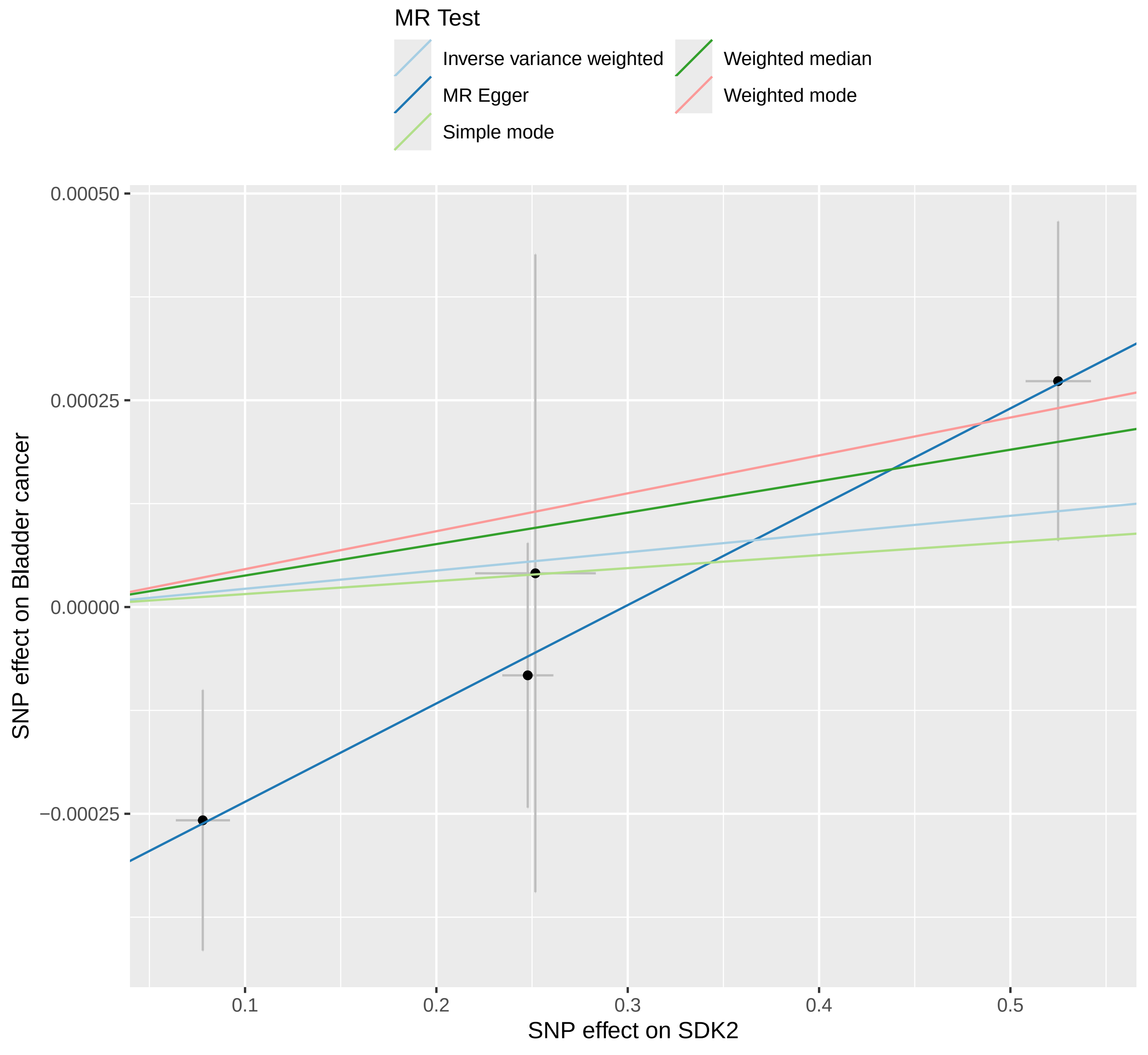
MR Test
Inverse variance weighted
MR Egger
Simple mode
Weighted median
Weighted mode
0.00050
0.00025
0.00000
−0.00025
SNP effect on Bladder cancer
0.1
0.2
0.3
0.4
0.5
SNP effect on SDK2

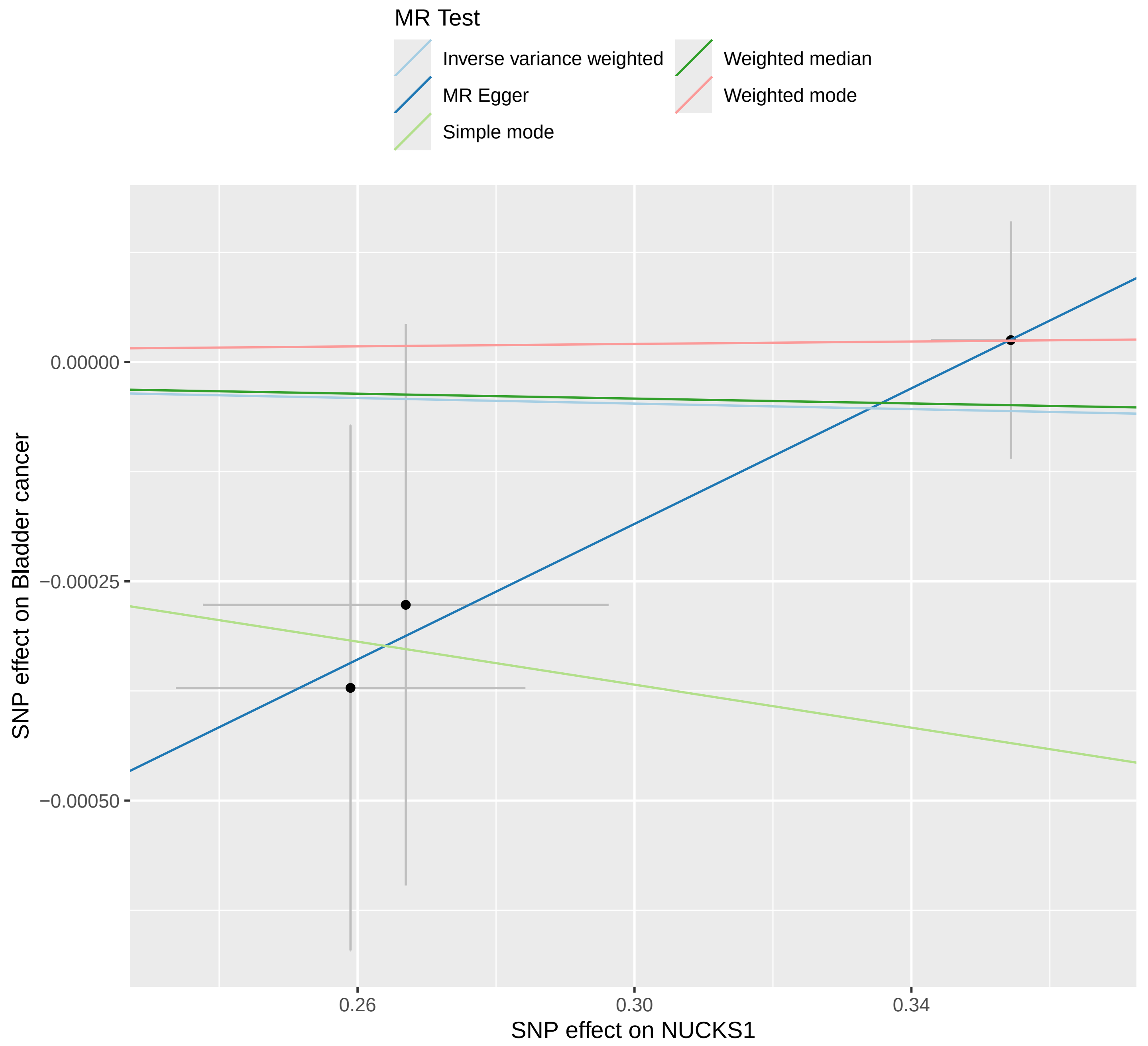

MR Test
Inverse variance weighted
MR Egger
Simple mode
Weighted median
Weighted mode
0.00000
−0.00025
−0.00050
SNP effect on Bladder cancer
0.26
0.30
0.34
SNP effect on NUCKS1

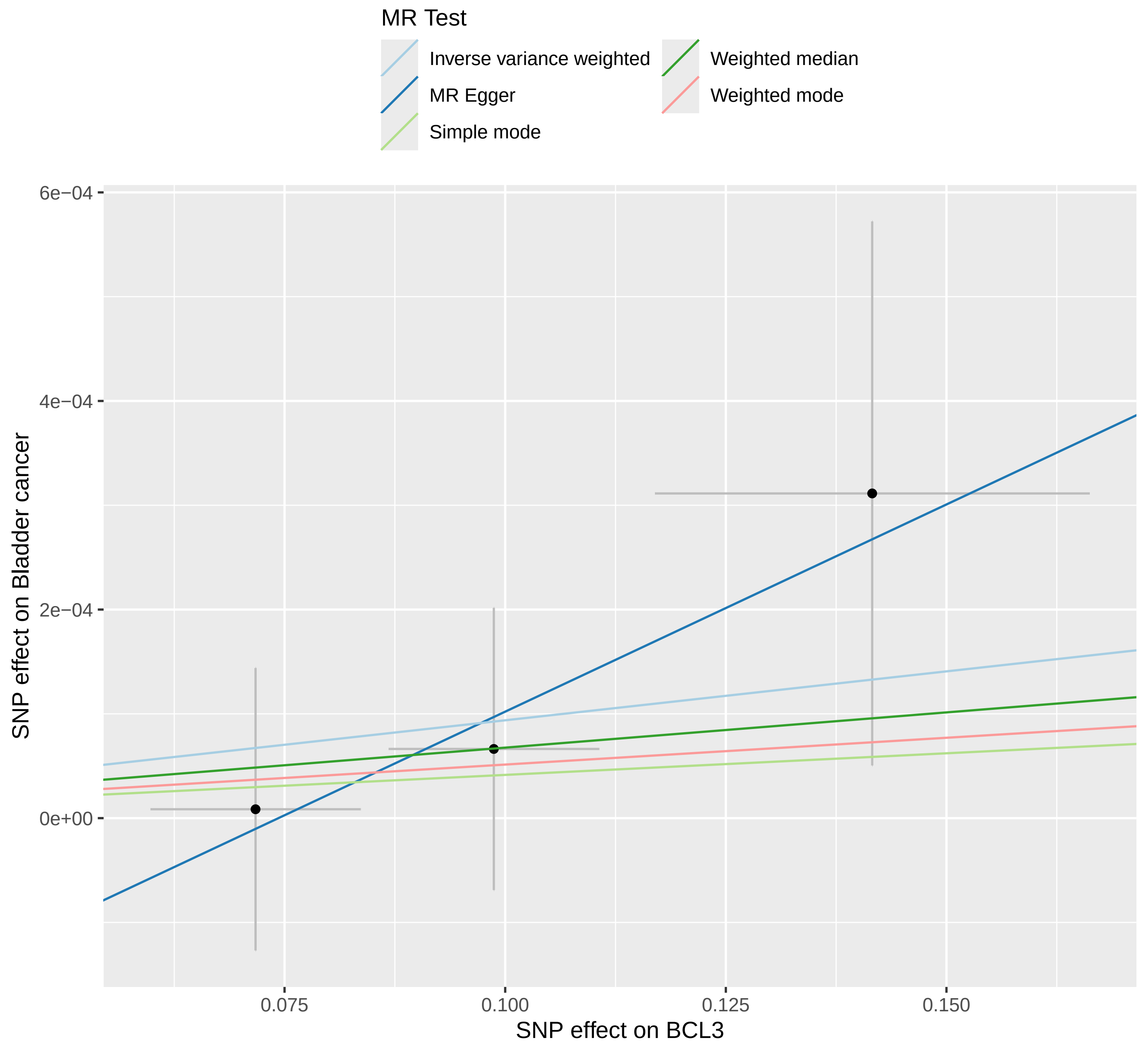
MR Test
Inverse variance weighted
MR Egger
Simple mode
Weighted median
Weighted mode
6e−04
4e−04
2e−04
0e+00
SNP effect on Bladder cancer
0.075
0.100
0.125
0.150
SNP effect on BCL3

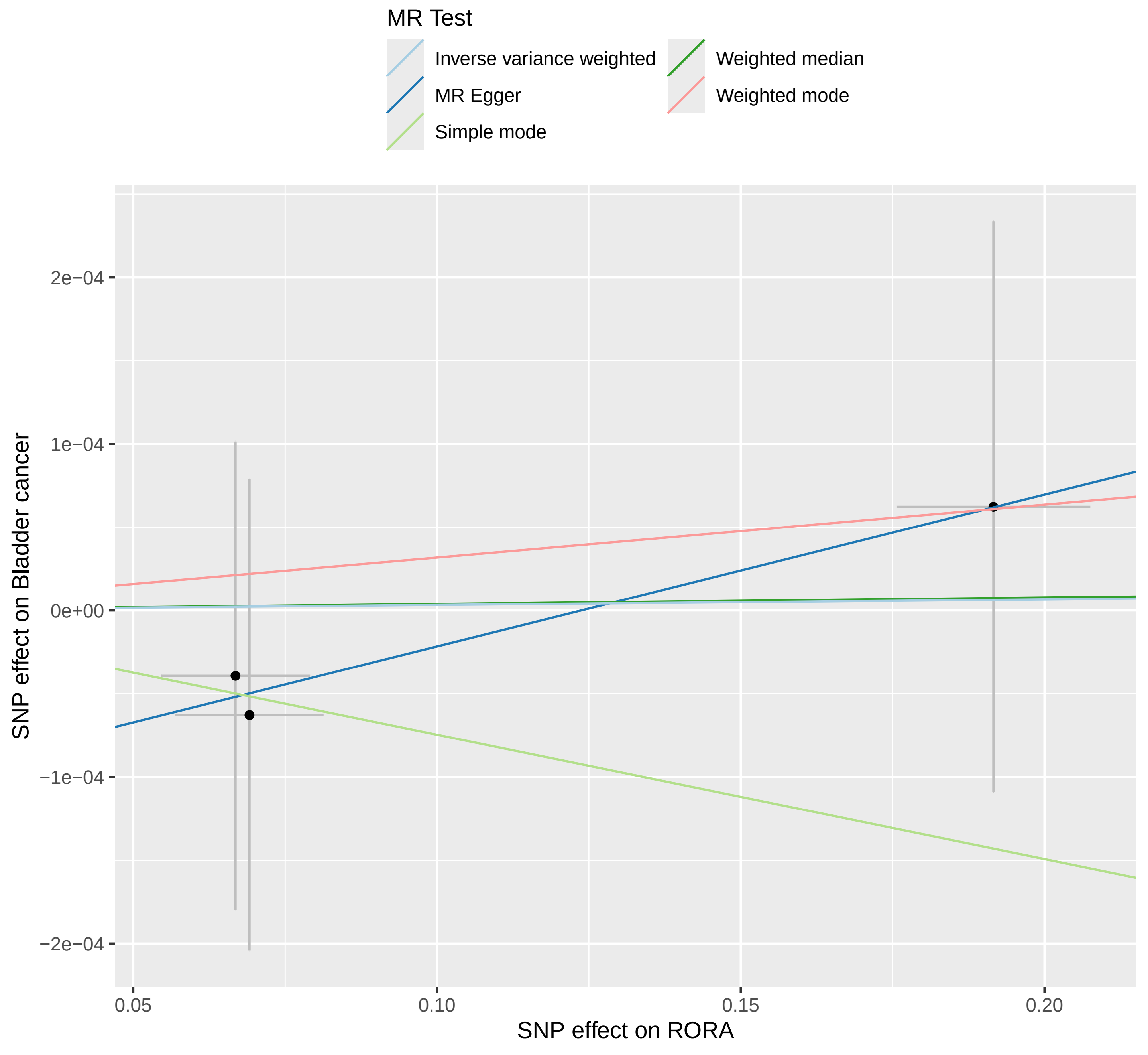

MR Test
Inverse variance weighted
MR Egger
Simple mode
Weighted median
Weighted mode
2e−04
1e−04
0e+00
−1e−04
−2e−04
SNP effect on Bladder cancer
0.05
0.10
0.15
0.20
SNP effect on RORA

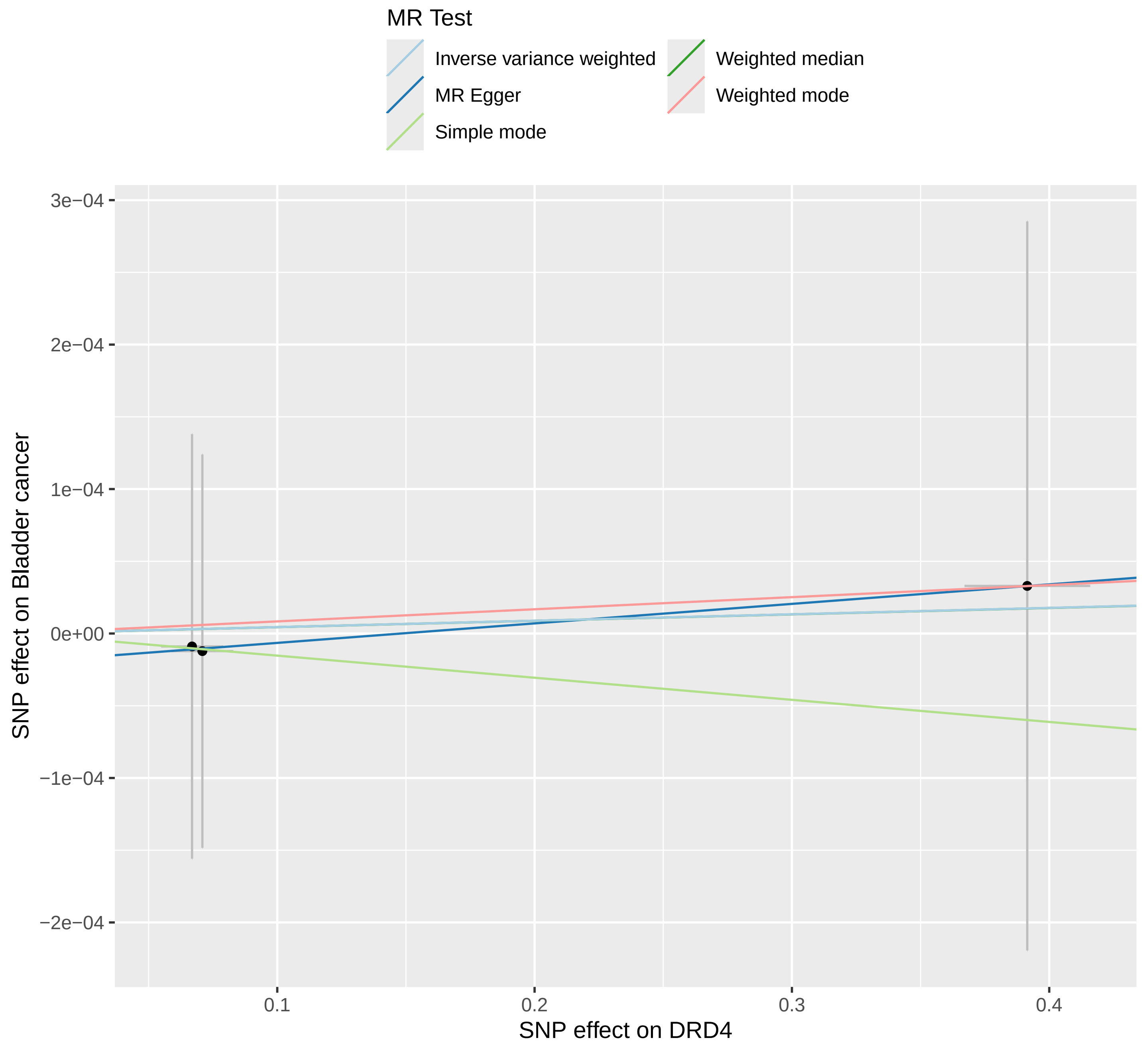
MR Test
Inverse variance weighted
MR Egger
Simple mode
Weighted median
Weighted mode
3e−04
2e−04
1e−04
0e+00
−1e−04
−2e−04
SNP effect on Bladder cancer
0.1
0.2
0.3
0.4
SNP effect on DRD4

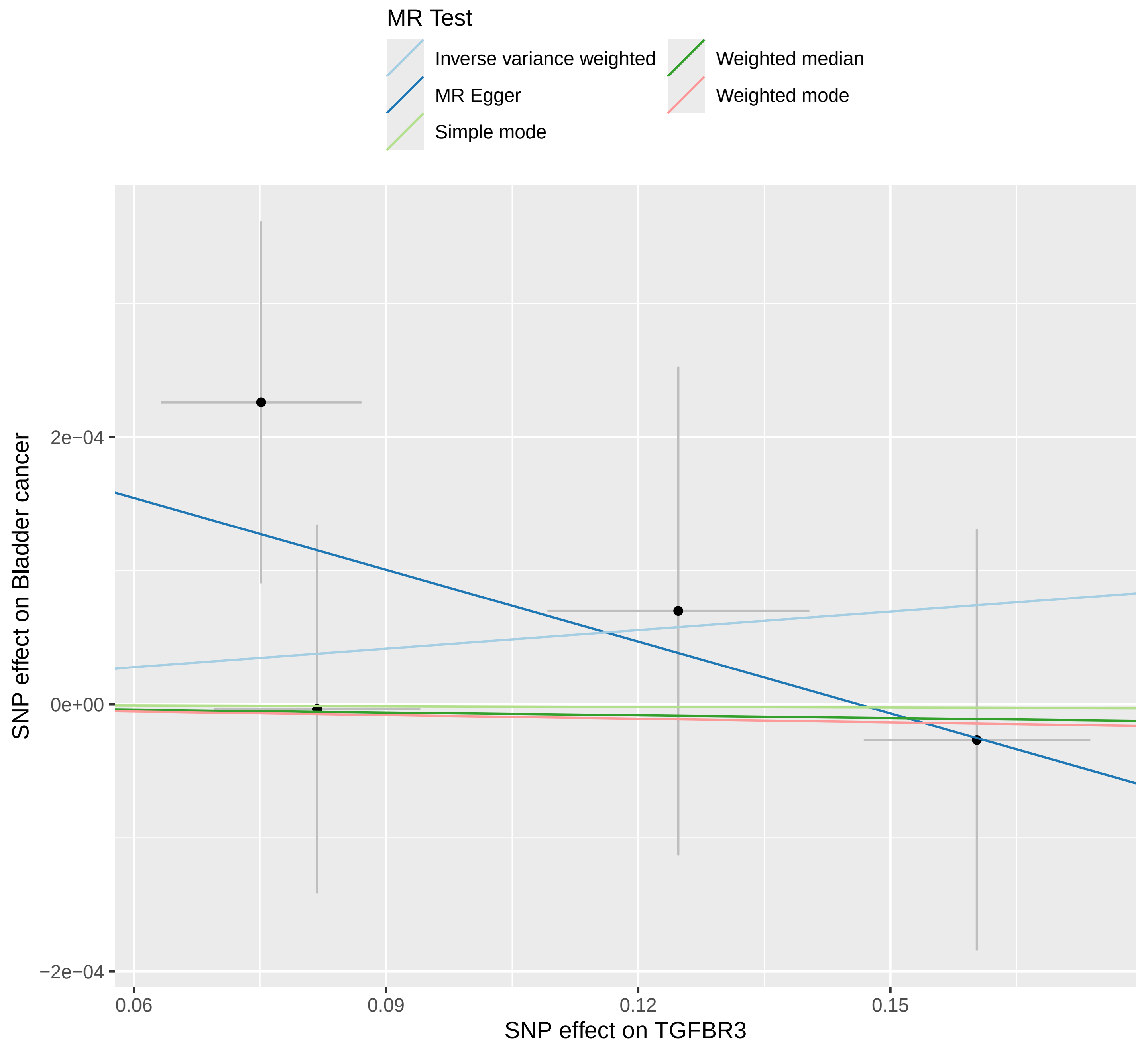

MR Test
Inverse variance weighted
MR Egger
Simple mode
Weighted median
Weighted mode
SNP effect on Bladder cancer
2e−04
0e+00
−2e−04
0.06
0.09
0.12
0.15
SNP effect on TGFBR3

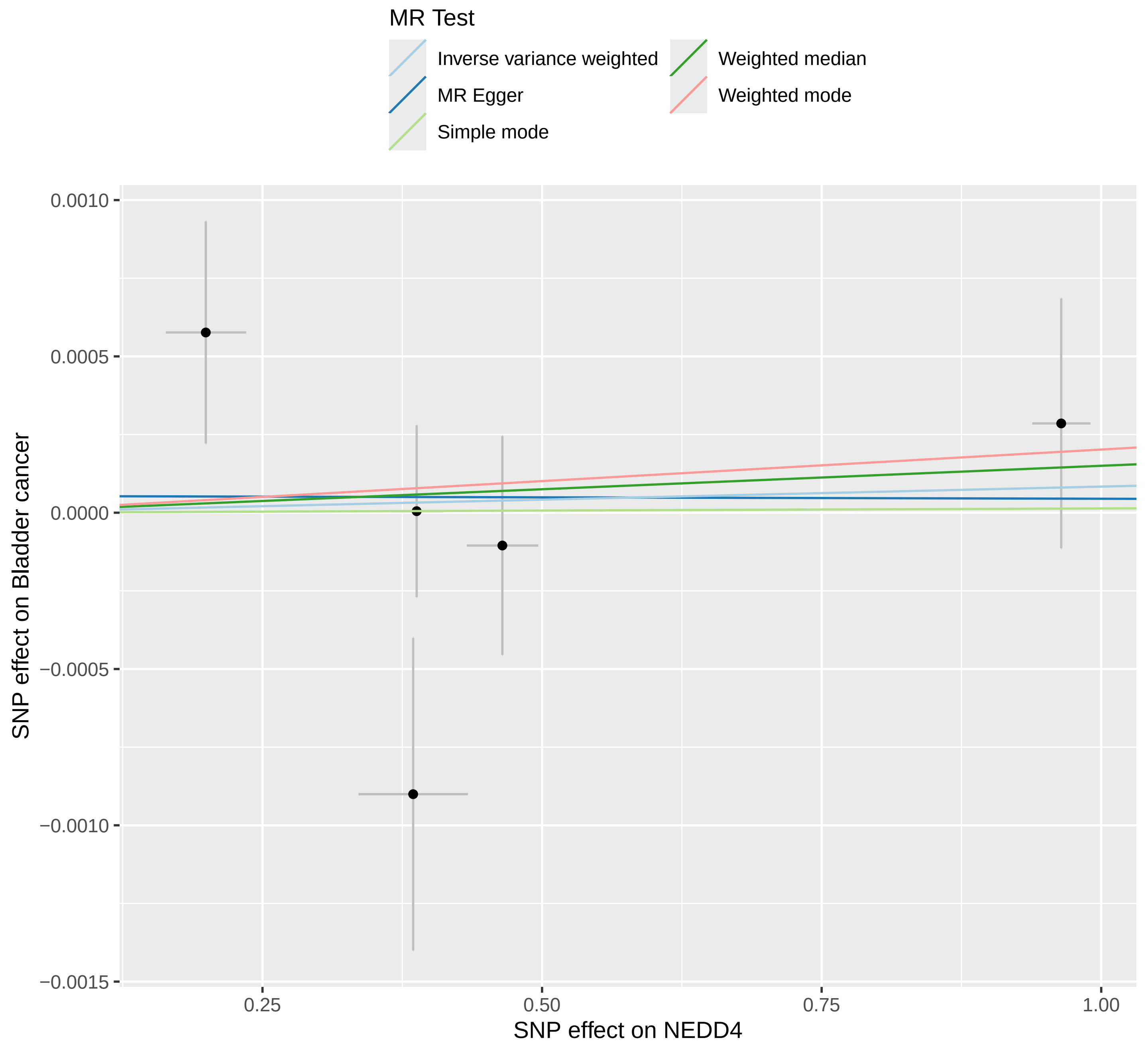

MR Test
Inverse variance weighted
MR Egger
Simple mode
Weighted median
Weighted mode
0.0010
0.0005
0.0000
−0.0005
−0.0010
−0.0015
SNP effect on Bladder cancer
0.25
0.50
0.75
1.00
SNP effect on NEDD4

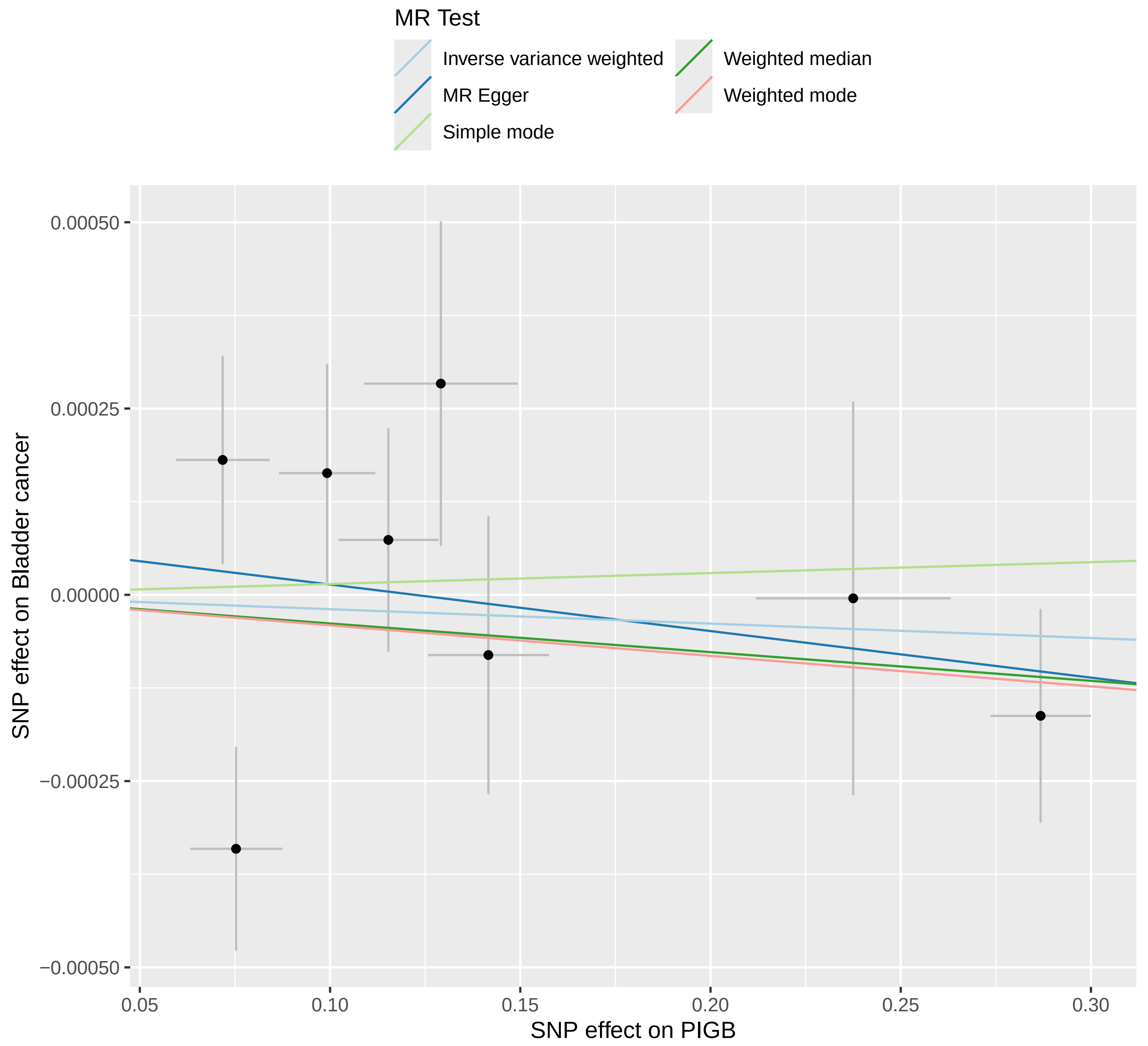

MR Test
Inverse variance weighted
MR Egger
Simple mode
Weighted median
Weighted mode
0.00050
0.00025
0.00000
−0.00025
−0.00050
SNP effect on Bladder cancer
0.05
0.10
0.15
0.20
0.25
0.30
SNP effect on PIGB

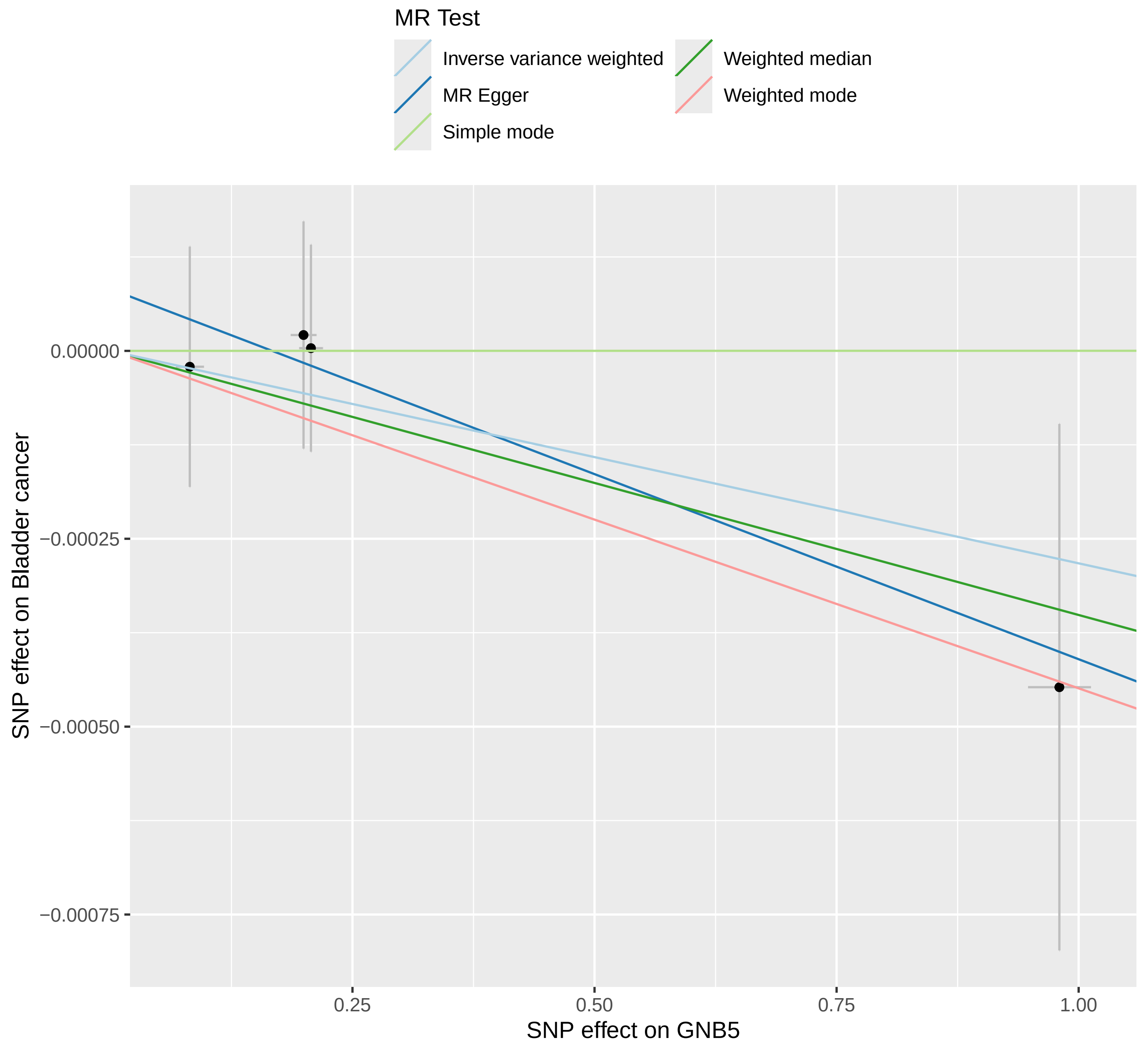

MR Test
Inverse variance weighted
MR Egger
Simple mode
Weighted median
Weighted mode
0.00000
−0.00025
−0.00050
−0.00075
SNP effect on Bladder cancer
0.25
0.50
0.75
1.00
SNP effect on GNB5

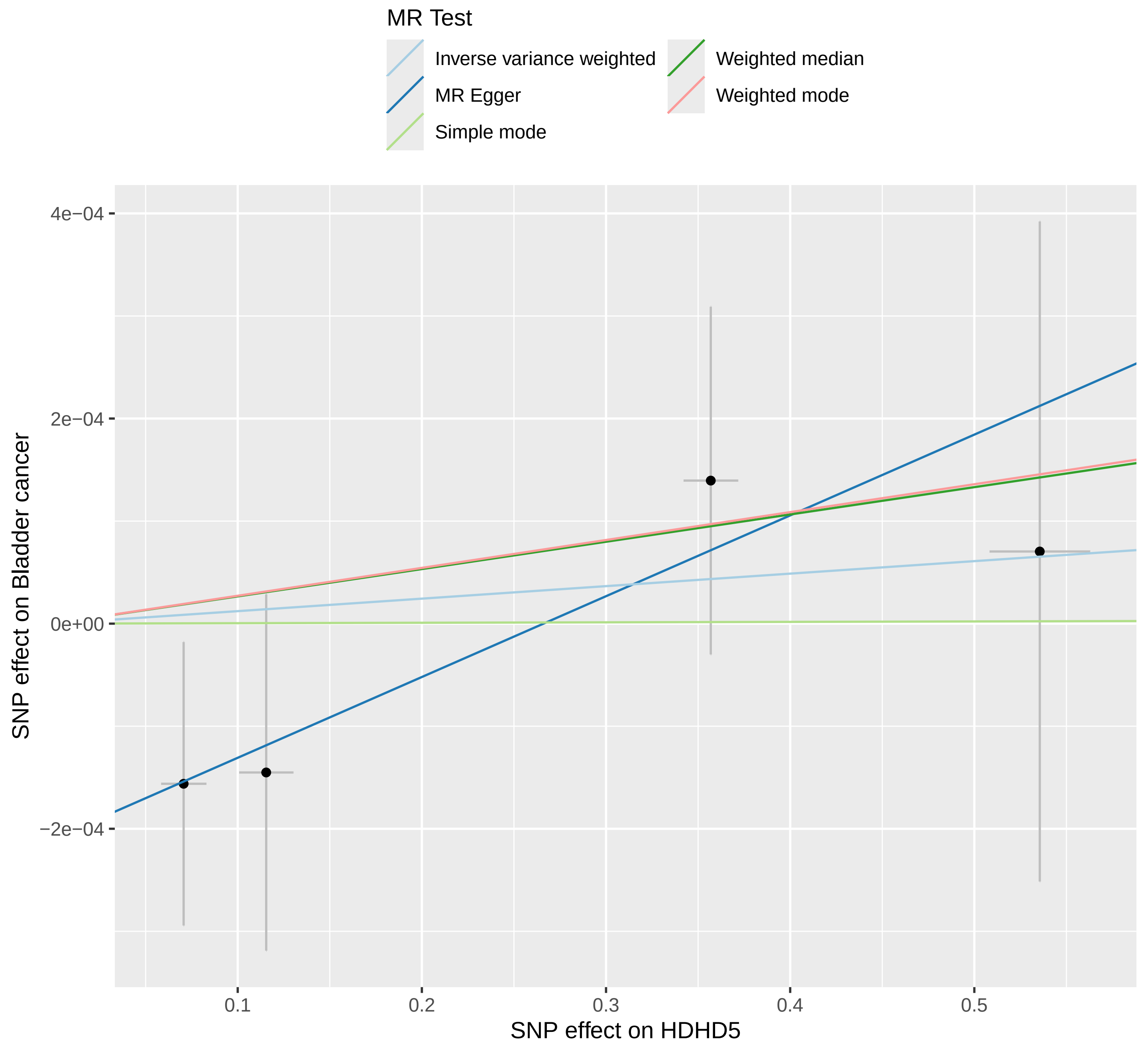

MR Test
Inverse variance weighted
MR Egger
Simple mode
Weighted median
Weighted mode
4e−04
2e−04
0e+00
−2e−04
SNP effect on Bladder cancer
0.1
0.2
0.3
0.4
0.5
SNP effect on HDHD5

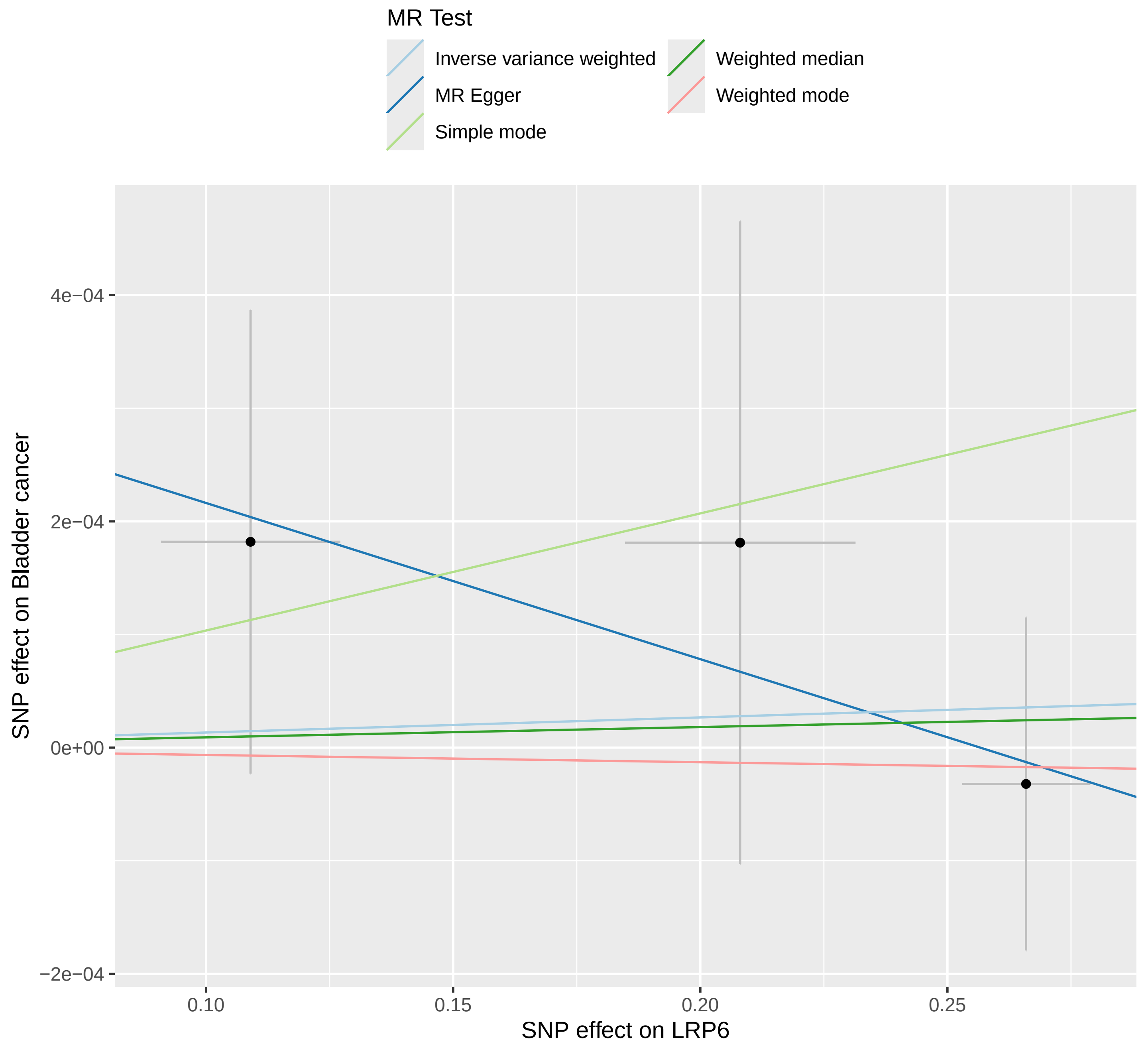
MR Test
Inverse variance weighted
MR Egger
Simple mode
Weighted median
Weighted mode
4e−04
2e−04
0e+00
−2e−04
0.10
0.15
0.20
0.25
SNP effect on Bladder cancer
SNP effect on LRP6

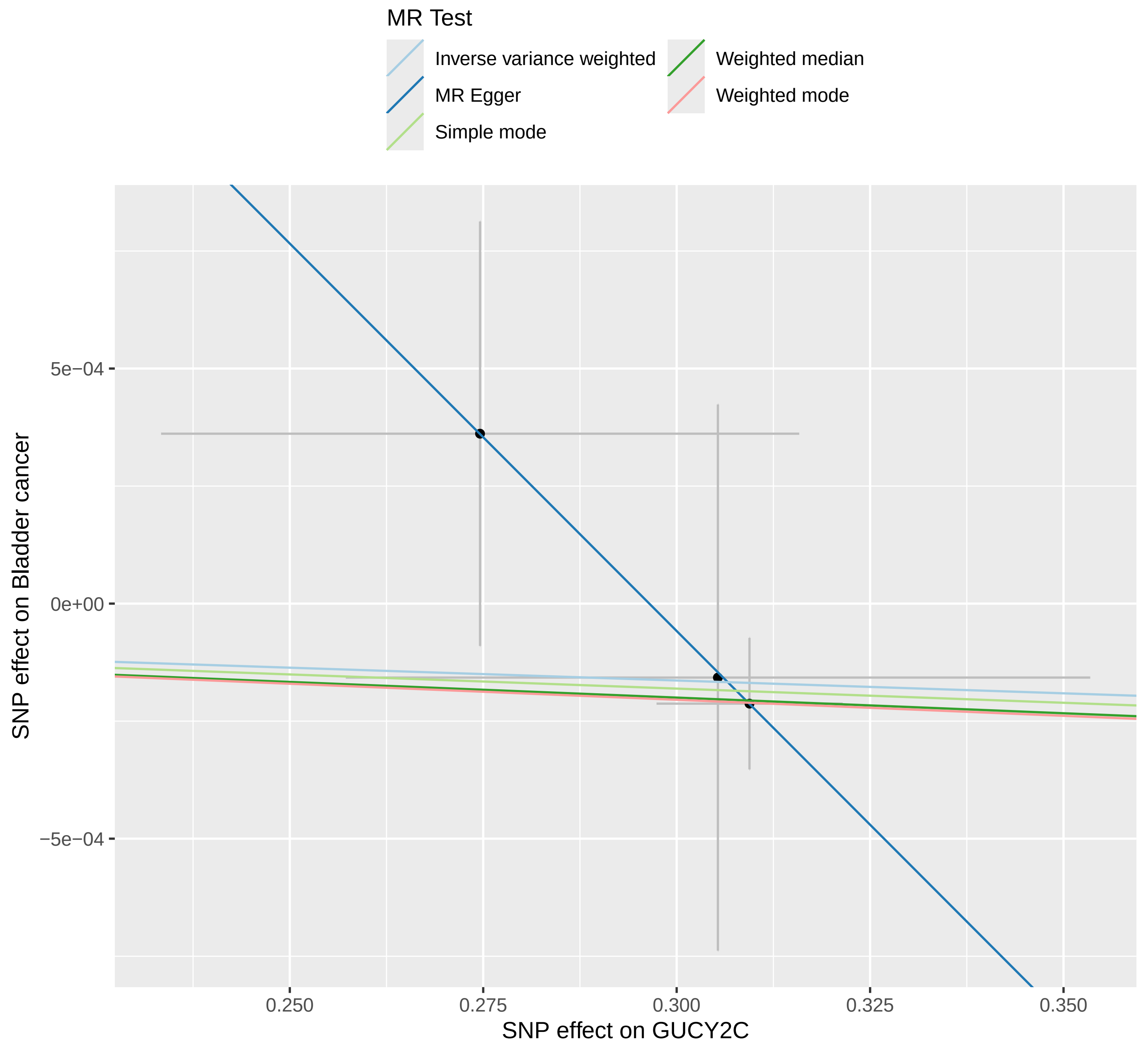

MR Test
Inverse variance weighted
MR Egger
Simple mode
Weighted median
Weighted mode
5e−04
0e+00
−5e−04
SNP effect on Bladder cancer
0.250
0.275
0.300
0.325
0.350
SNP effect on GUCY2C

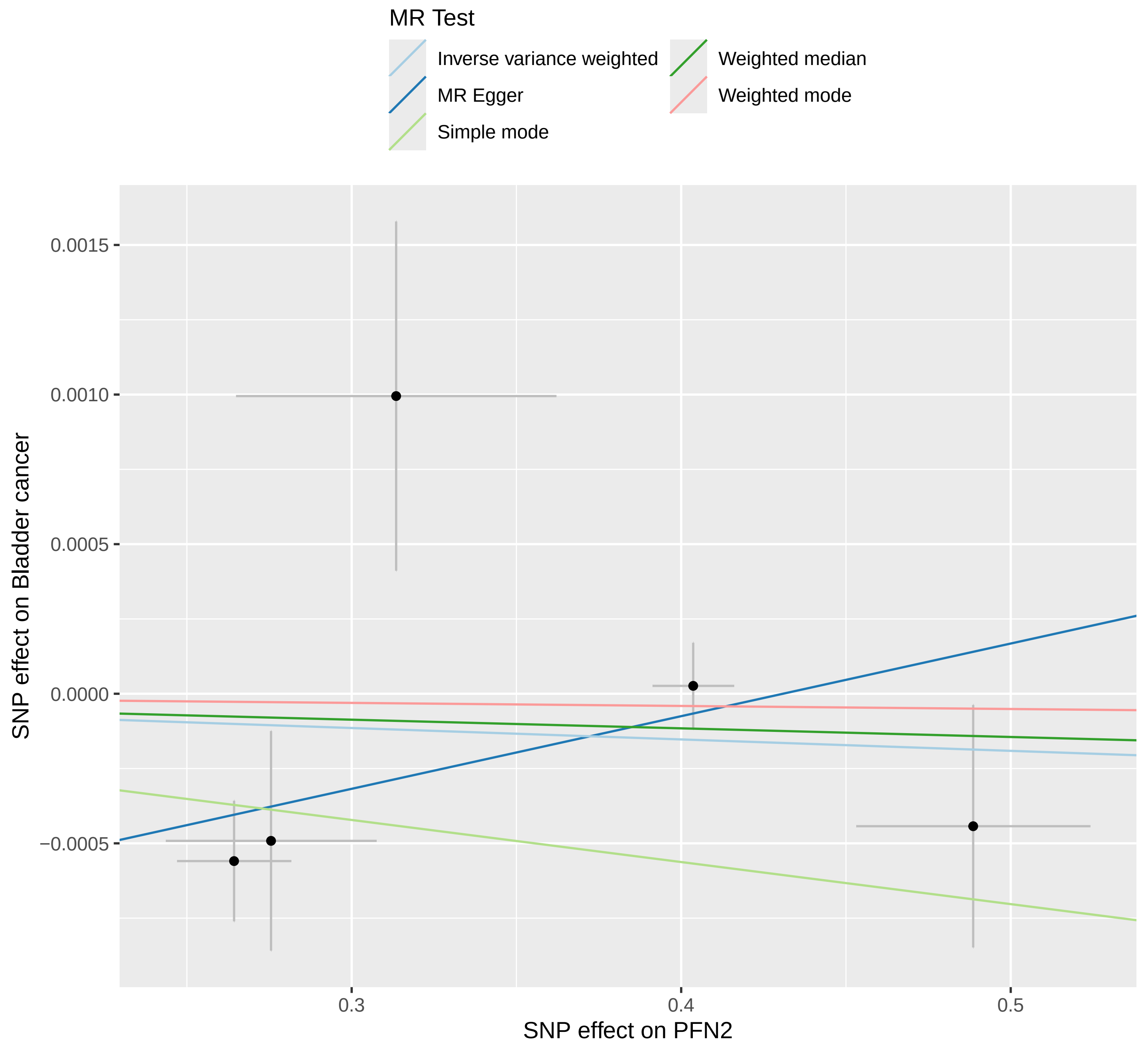

MR Test
Inverse variance weighted
MR Egger
Simple mode
Weighted median
Weighted mode
0.0015
0.0010
0.0005
0.0000
−0.0005
SNP effect on Bladder cancer
0.3
0.4
0.5
SNP effect on PFN2

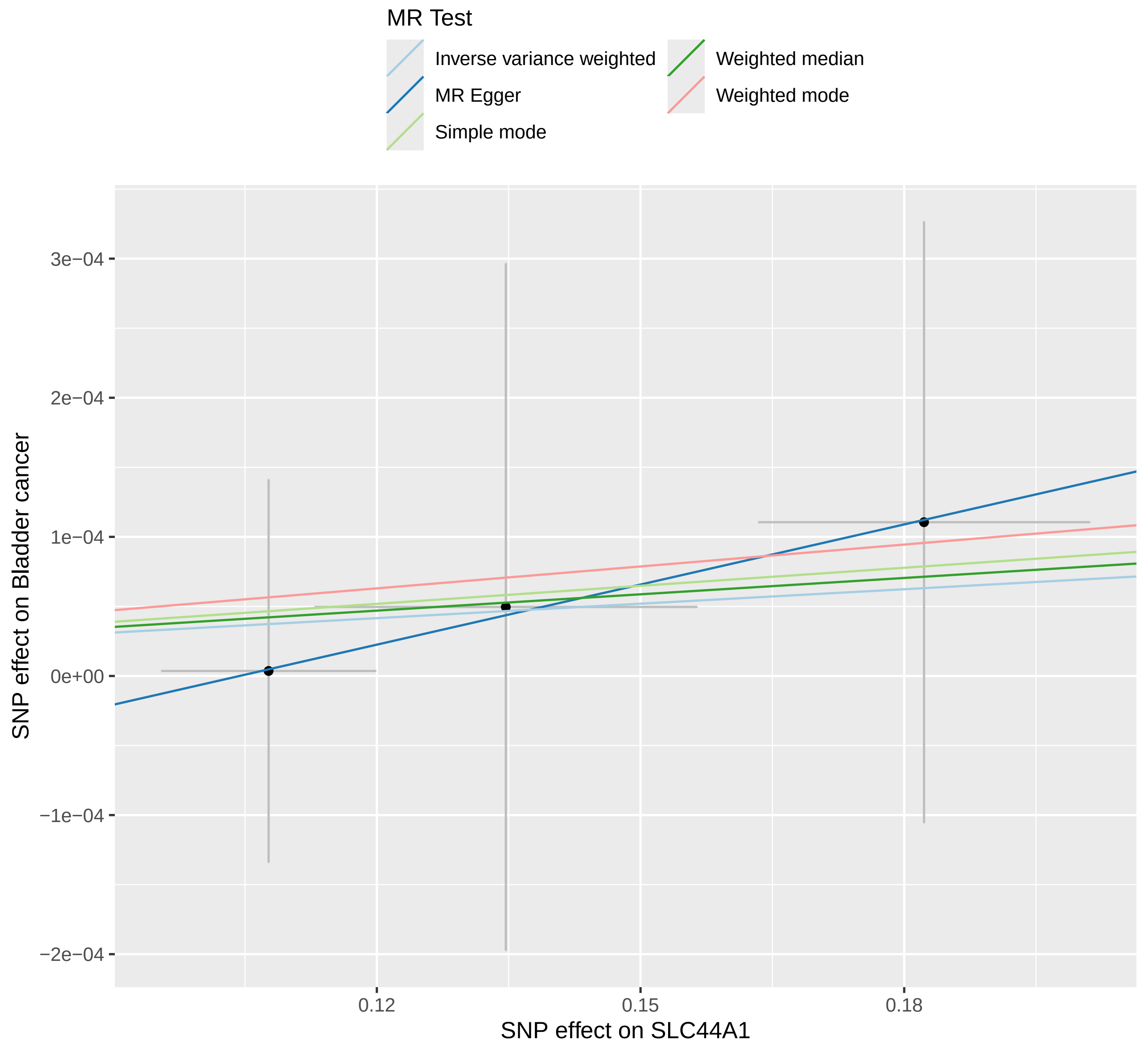

MR Test
Inverse variance weighted
MR Egger
Simple mode
Weighted median
Weighted mode
3e−04
2e−04
1e−04
0e+00
−1e−04
−2e−04
SNP effect on Bladder cancer
0.12
0.15
0.18
SNP effect on SLC44A1

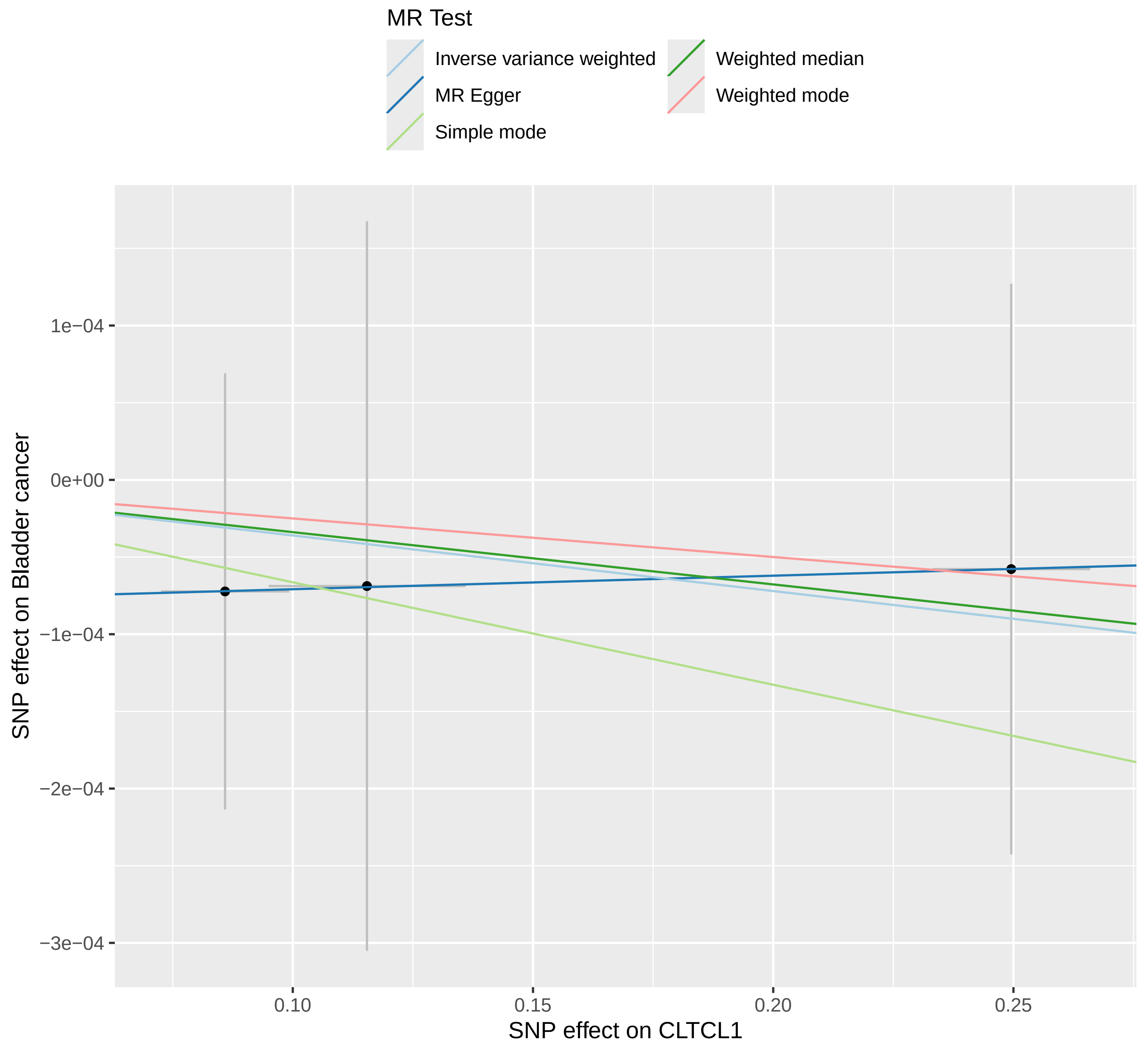
MR Test
Inverse variance weighted
MR Egger
Simple mode
Weighted median
Weighted mode
1e−04
0e+00
−1e−04
−2e−04
−3e−04
SNP effect on Bladder cancer
0.10
0.15
0.20
0.25
SNP effect on CLTCL1

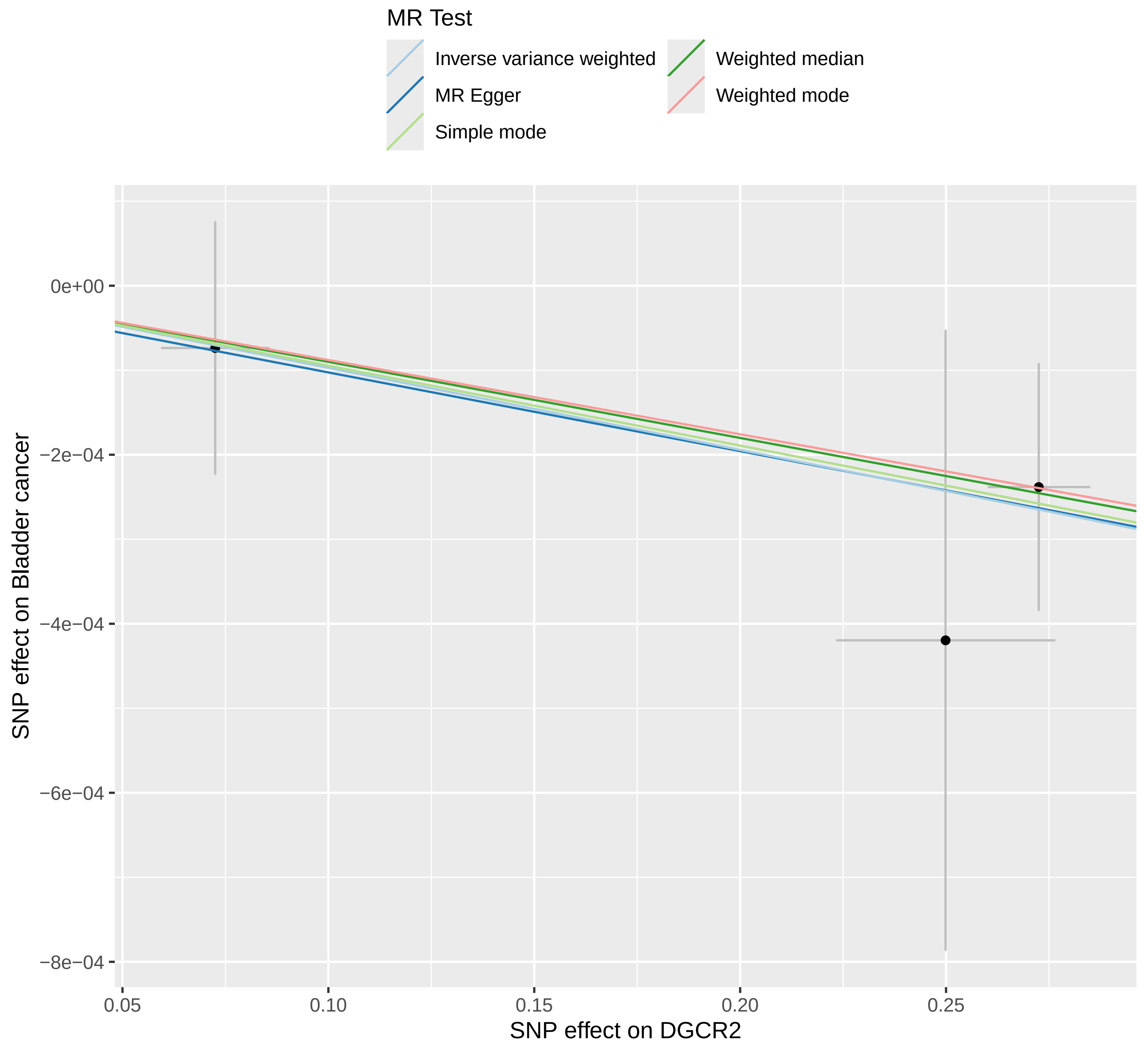
MR Test
Inverse variance weighted
MR Egger
Simple mode
Weighted median
Weighted mode
0e+00
−2e−04
−4e−04
−6e−04
−8e−04
SNP effect on Bladder cancer
0.05
0.10
0.15
0.20
0.25
SNP effect on DGCR2

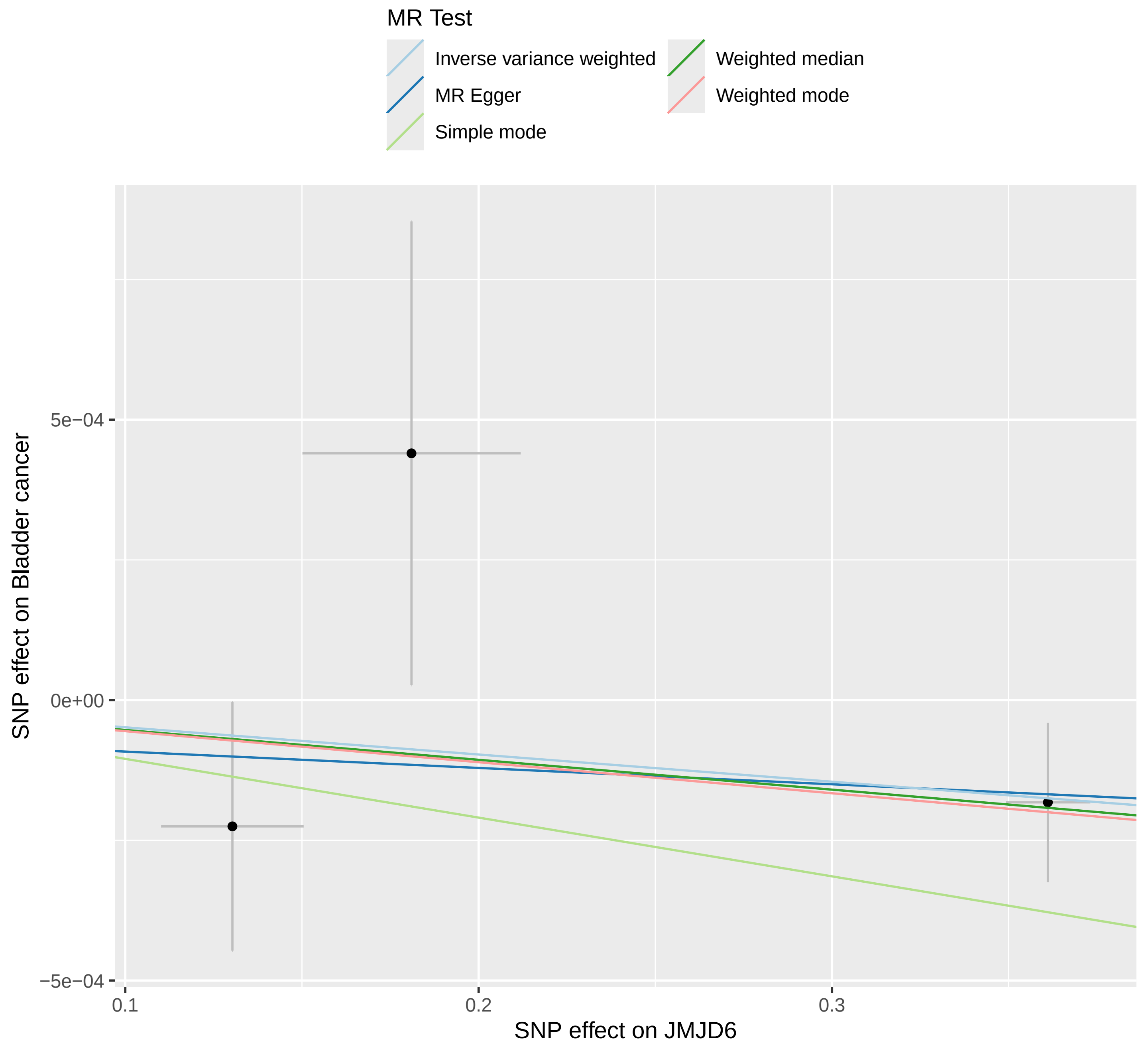

MR Test
Inverse variance weighted
MR Egger
Simple mode
Weighted median
Weighted mode
5e−04
0e+00
−5e−04
0.1
0.2
0.3
SNP effect on Bladder cancer
SNP effect on JMJD6

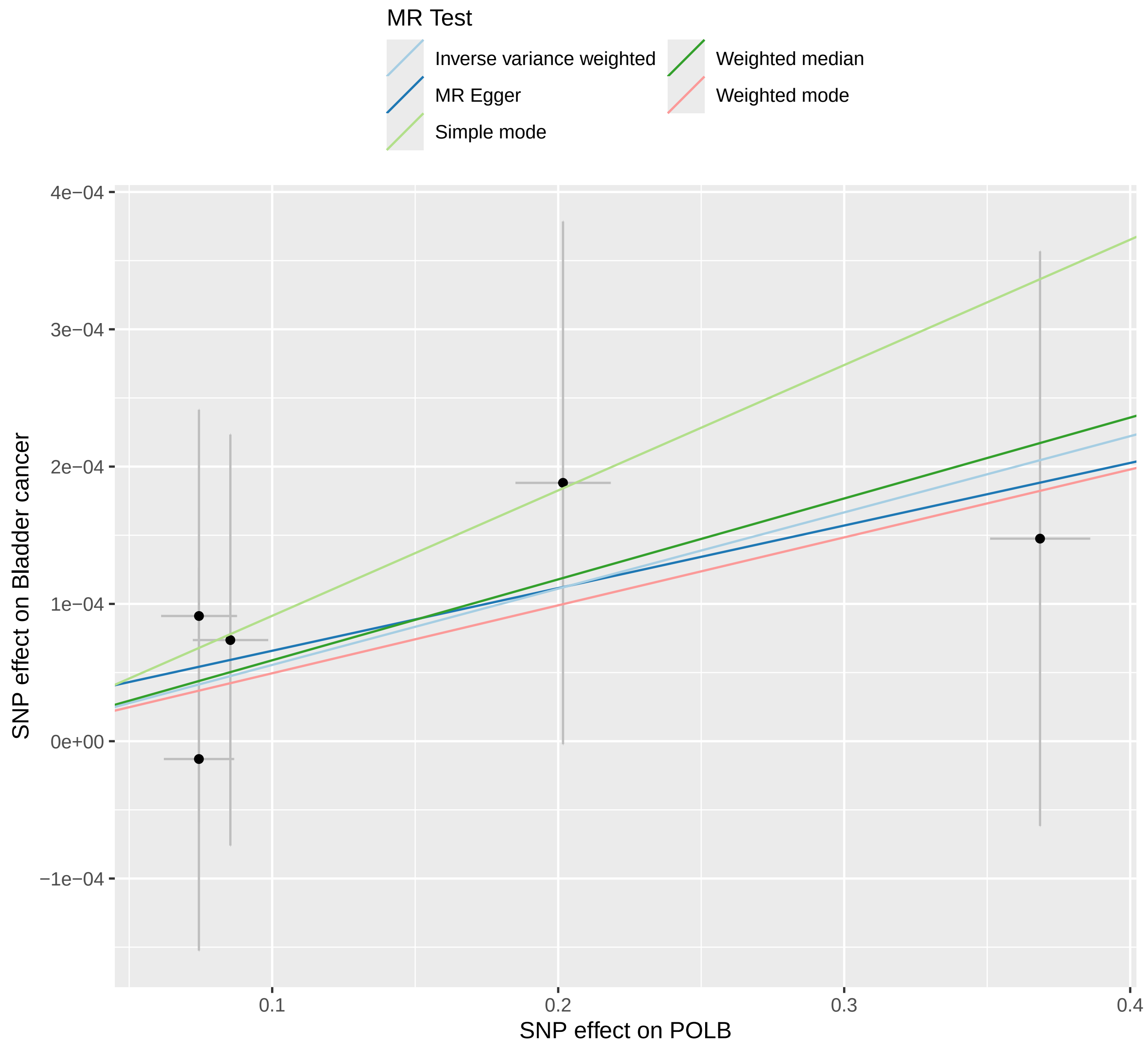
MR Test
Inverse variance weighted
MR Egger
Simple mode
Weighted median
Weighted mode
4e−04
3e−04
2e−04
1e−04
0e+00
−1e−04
SNP effect on Bladder cancer
0.1
0.2
0.3
0.4
SNP effect on POLB

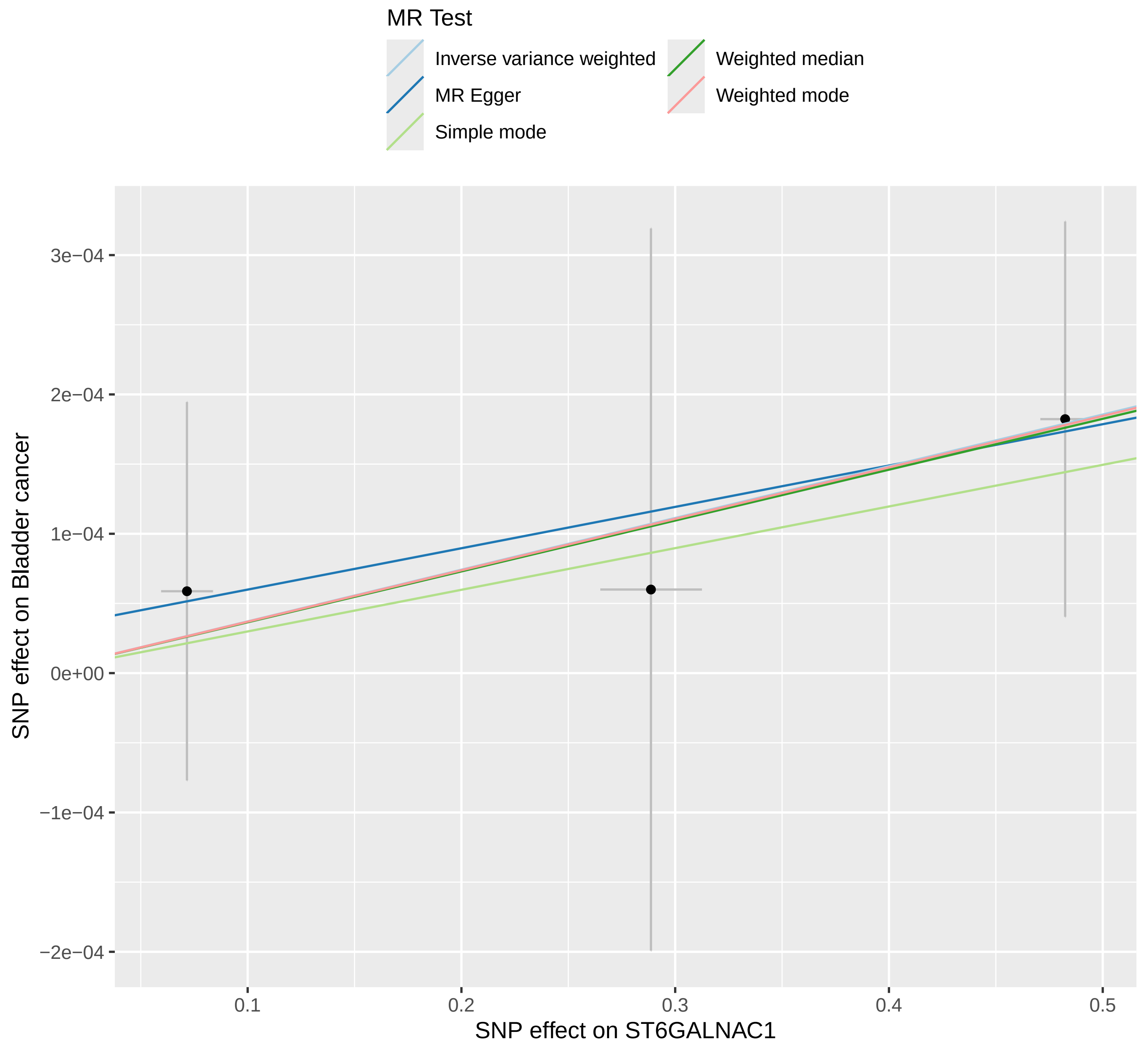

MR Test
Inverse variance weighted
MR Egger
Simple mode
Weighted median
Weighted mode
3e−04
2e−04
1e−04
0e+00
−1e−04
−2e−04
SNP effect on Bladder cancer
0.1
0.2
0.3
0.4
0.5
SNP effect on ST6GALNAC1

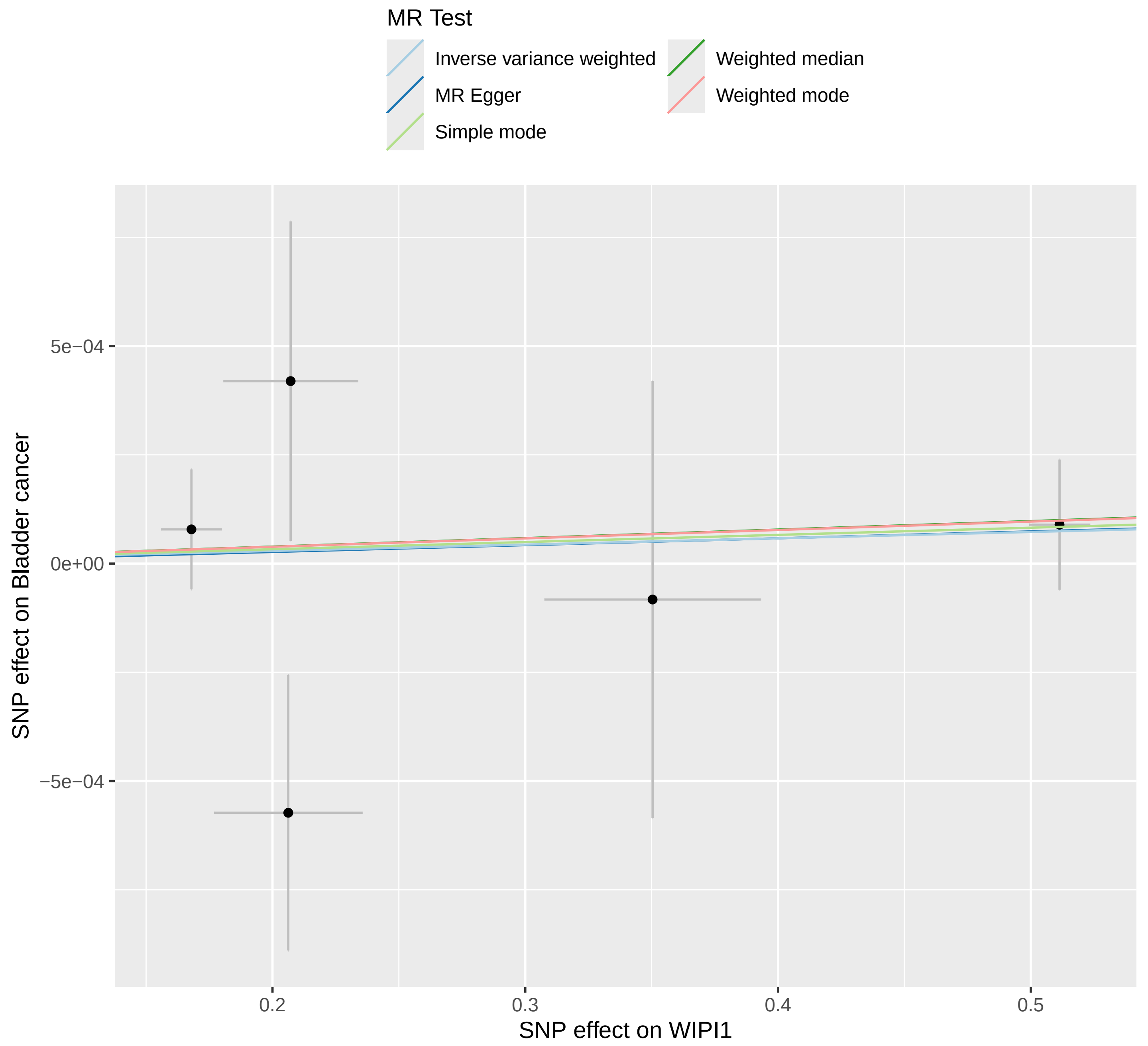

MR Test
Inverse variance weighted
MR Egger
Simple mode
Weighted median
Weighted mode
5e−04
0e+00
−5e−04
0.2
0.3
0.4
0.5
SNP effect on WIPI1
SNP effect on Bladder cancer

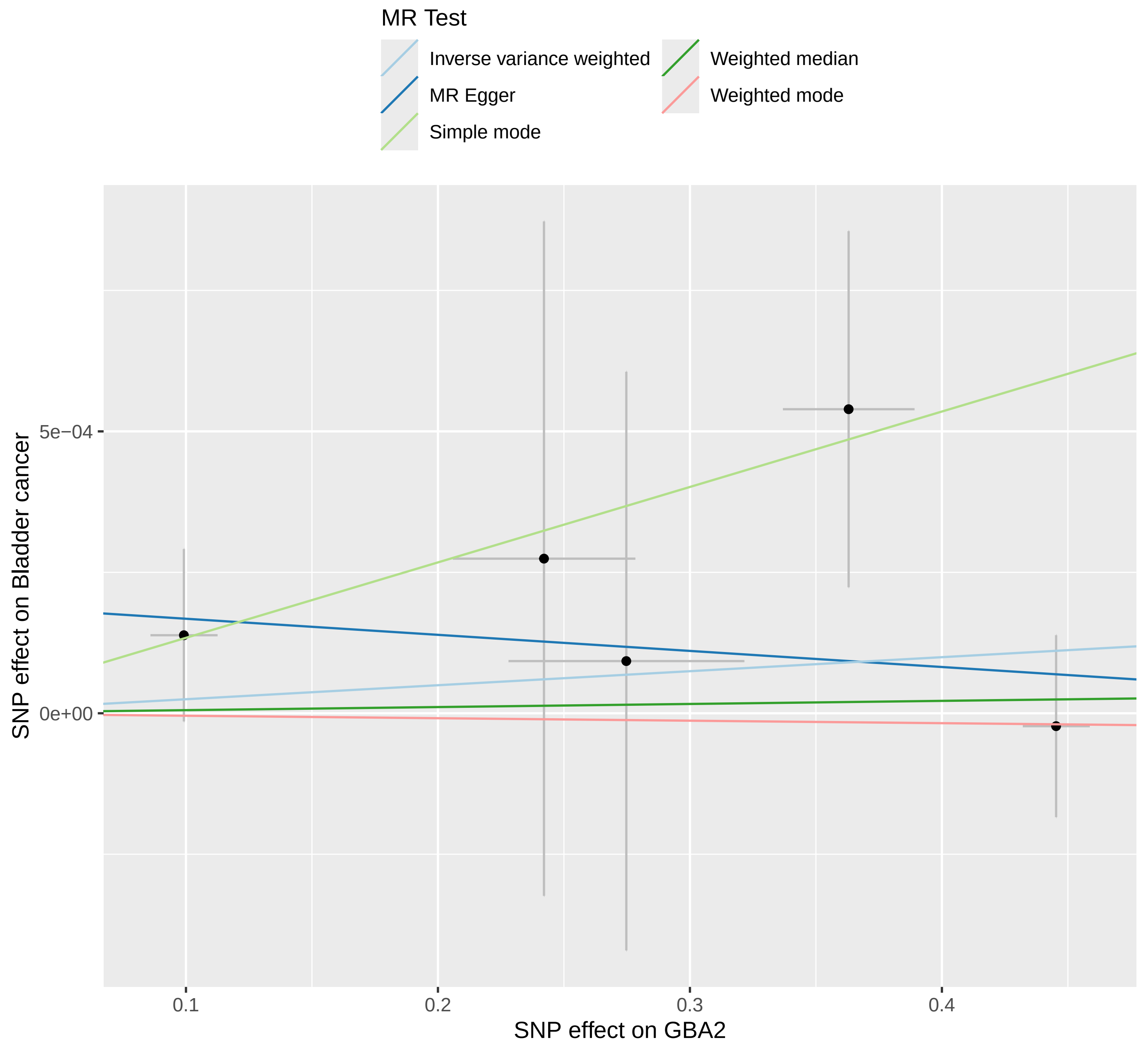

MR Test
Inverse variance weighted
MR Egger
Simple mode
Weighted median
Weighted mode
5e−04
0e+00
0.1
0.2
0.3
0.4
SNP effect on GBA2
SNP effect on Bladder cancer

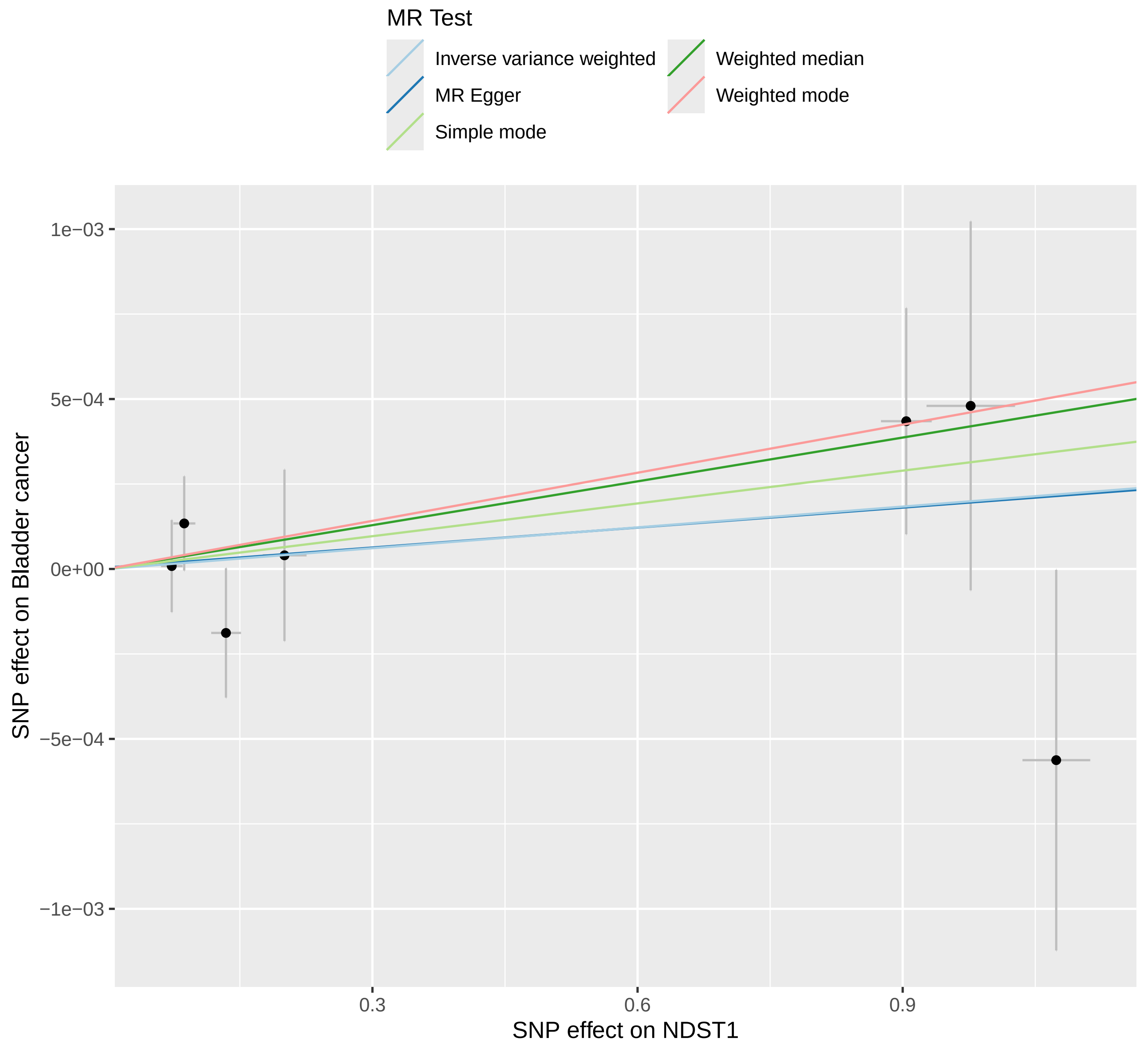
MR Test
Inverse variance weighted
MR Egger
Simple mode
Weighted median
Weighted mode
1e−03
5e−04
0e+00
−5e−04
−1e−03
SNP effect on Bladder cancer
0.3
0.6
0.9
SNP effect on NDST1

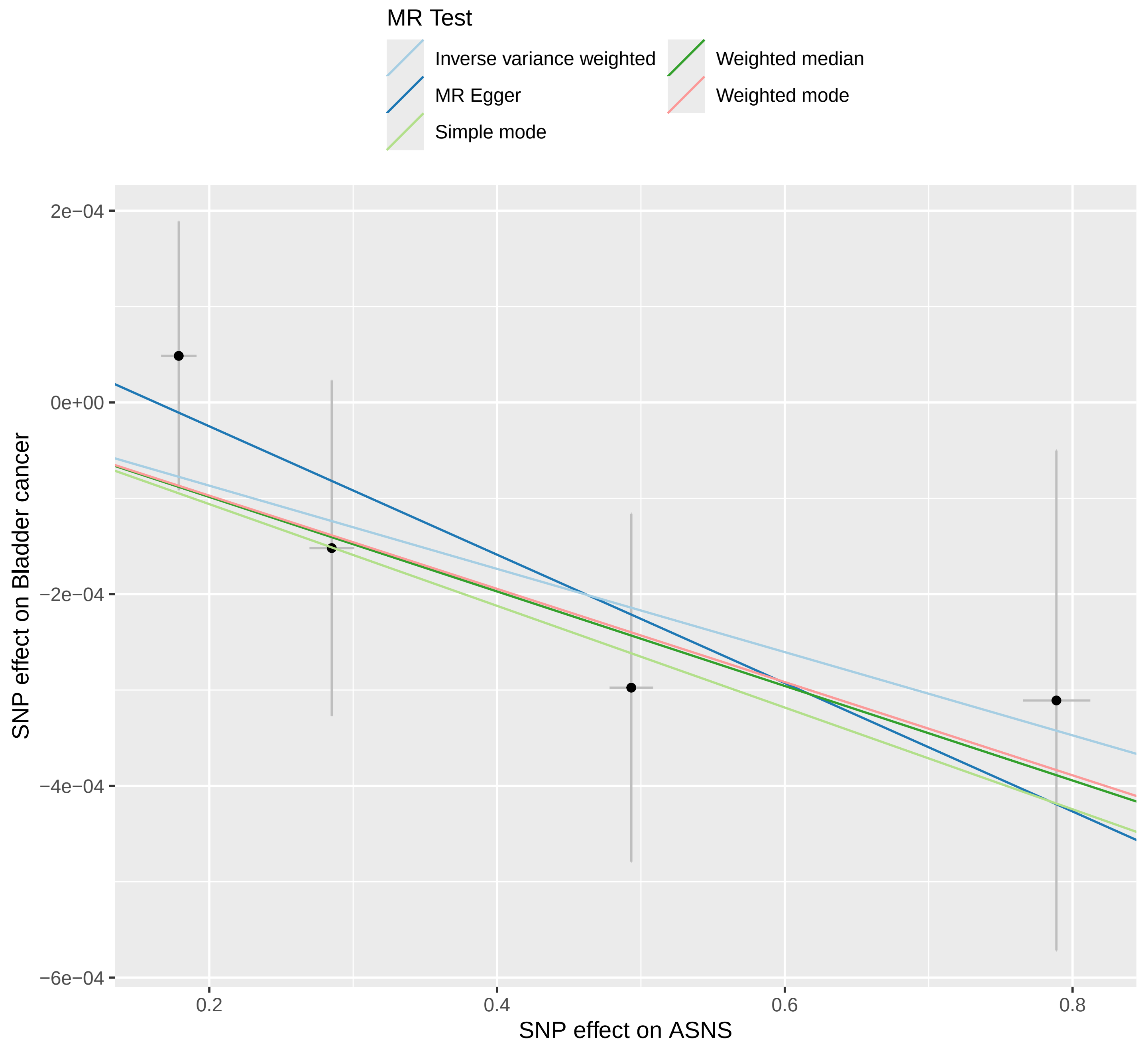

MR Test
Inverse variance weighted
MR Egger
Simple mode
Weighted median
Weighted mode
2e−04
0e+00
−2e−04
−4e−04
−6e−04
0.2
0.4
0.6
0.8
SNP effect on ASNS
SNP effect on Bladder cancer

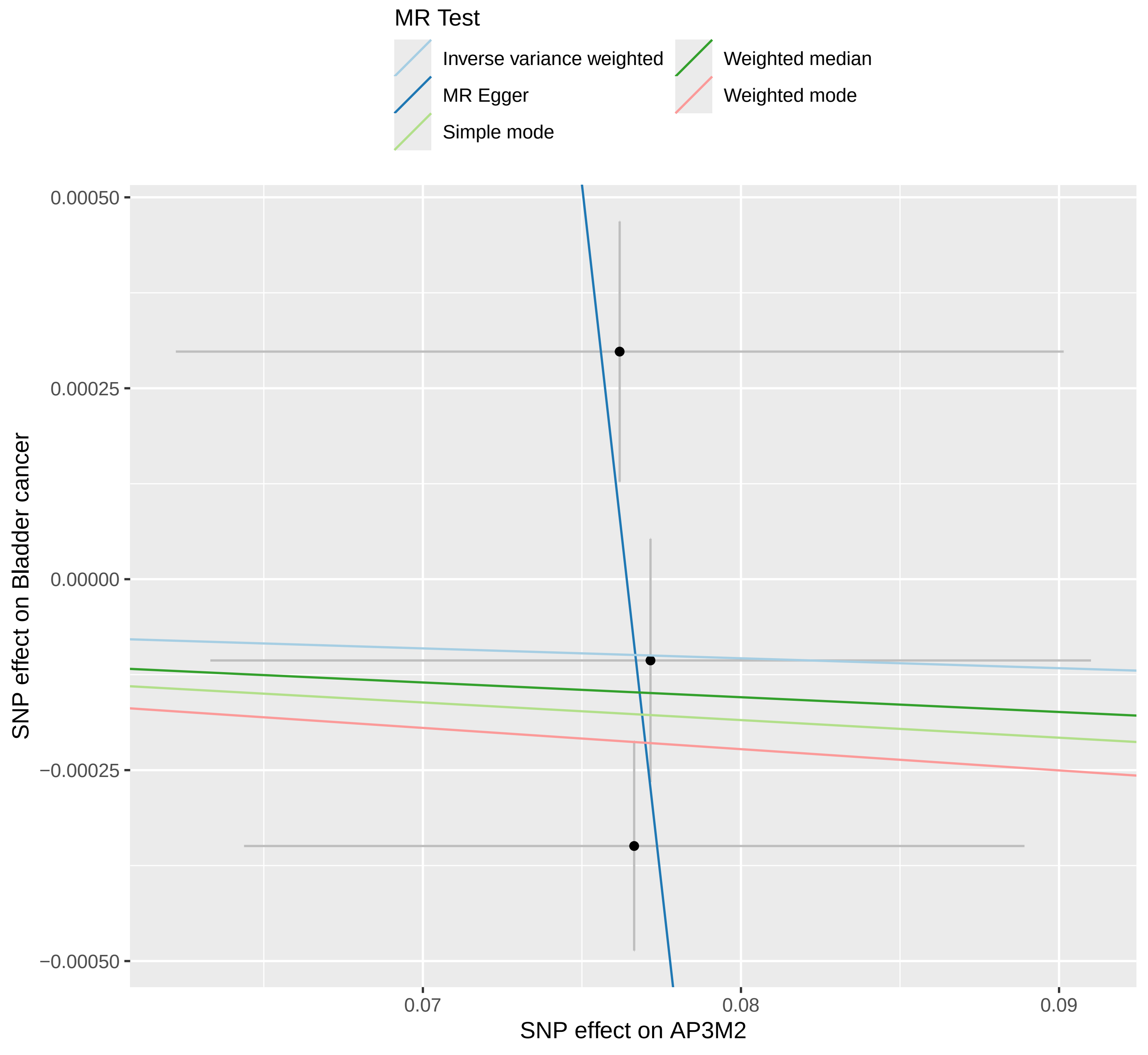
MR Test
Inverse variance weighted
MR Egger
Simple mode
Weighted median
Weighted mode
0.00050
0.00025
0.00000
−0.00025
−0.00050
SNP effect on Bladder cancer
0.07
0.08
0.09
SNP effect on AP3M2

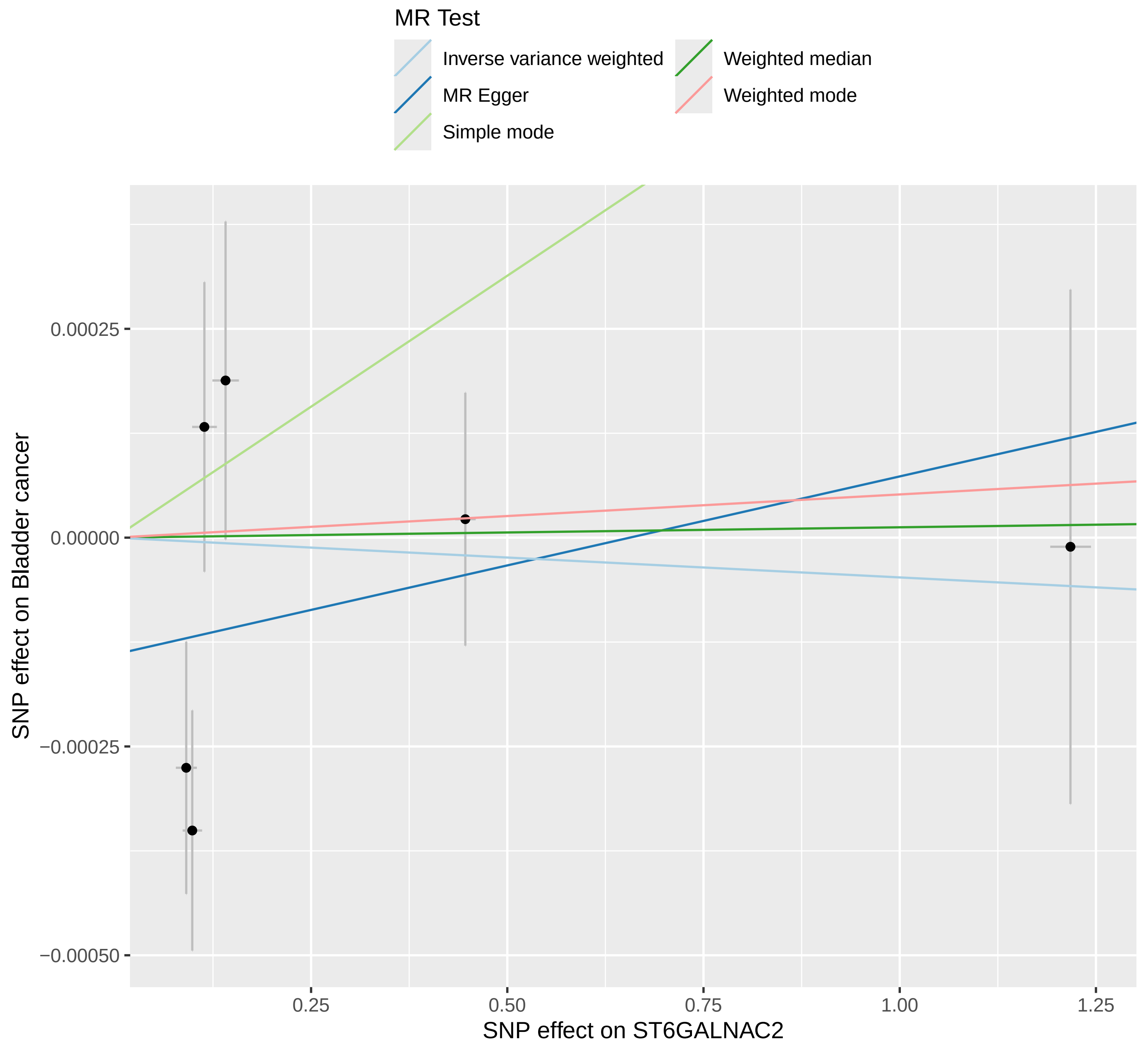

MR Test
Inverse variance weighted
MR Egger
Simple mode
Weighted median
Weighted mode
0.00025
0.00000
−0.00025
−0.00050
SNP effect on Bladder cancer
0.25
0.50
0.75
1.00
1.25
SNP effect on ST6GALNAC2

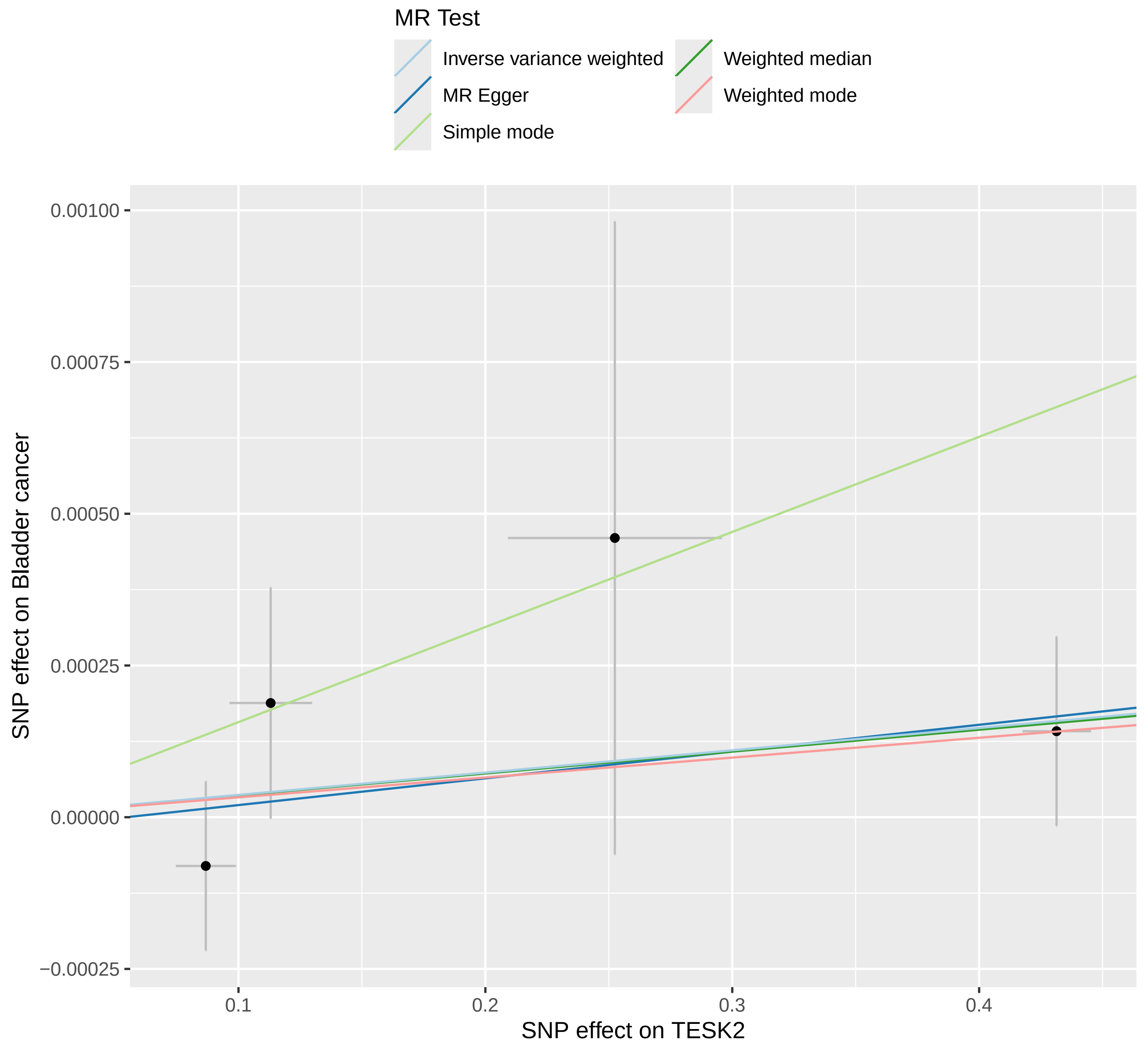
MR Test
Inverse variance weighted
MR Egger
Simple mode
Weighted median
Weighted mode
0.00100
0.00075
0.00050
0.00025
0.00000
−0.00025
SNP effect on Bladder cancer
0.1
0.2
0.3
0.4
SNP effect on TESK2

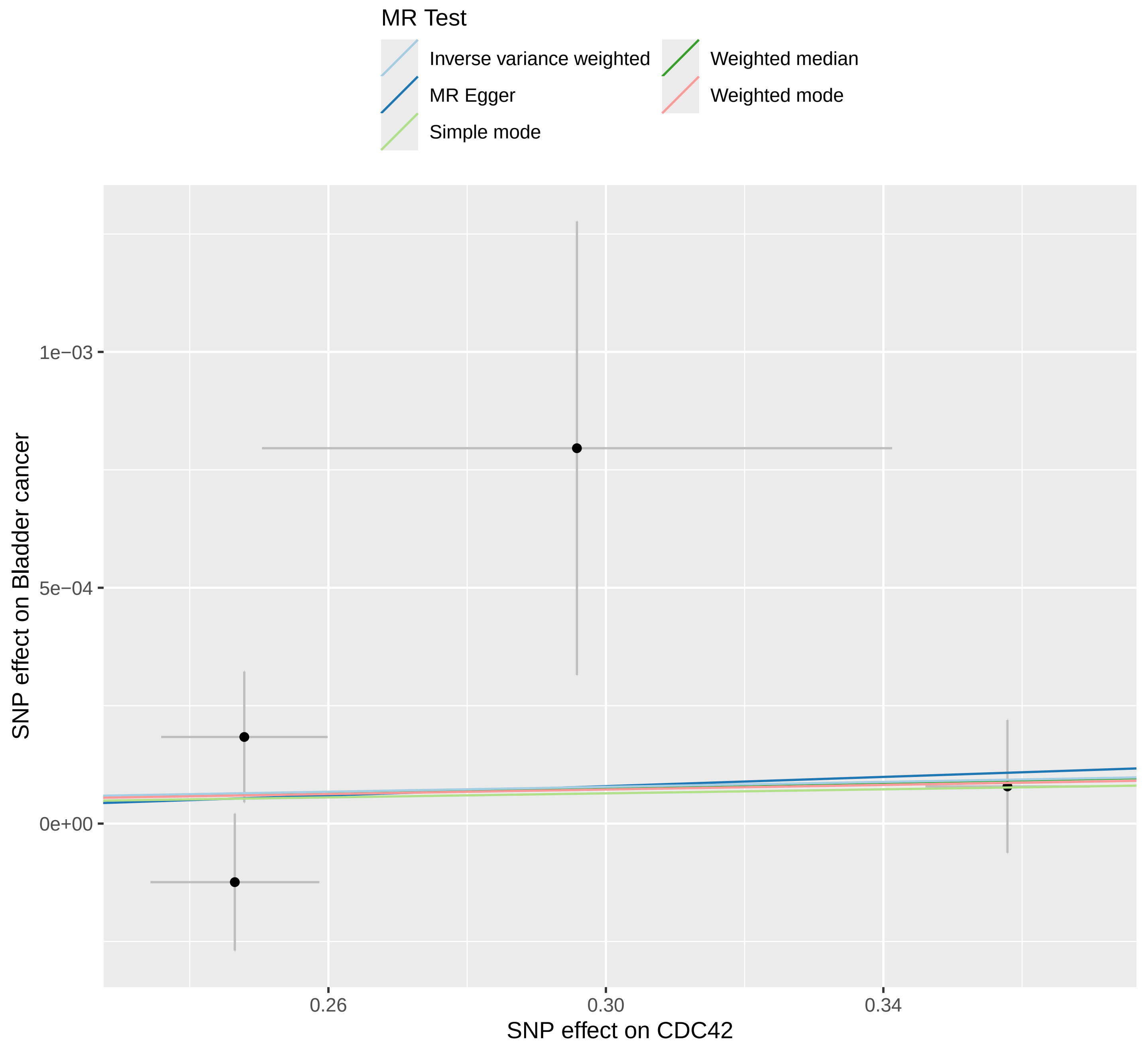
MR Test
Inverse variance weighted
MR Egger
Simple mode
Weighted median
Weighted mode
SNP effect on Bladder cancer
1e−03
5e−04
0e+00
0.26
0.30
0.34
SNP effect on CDC42

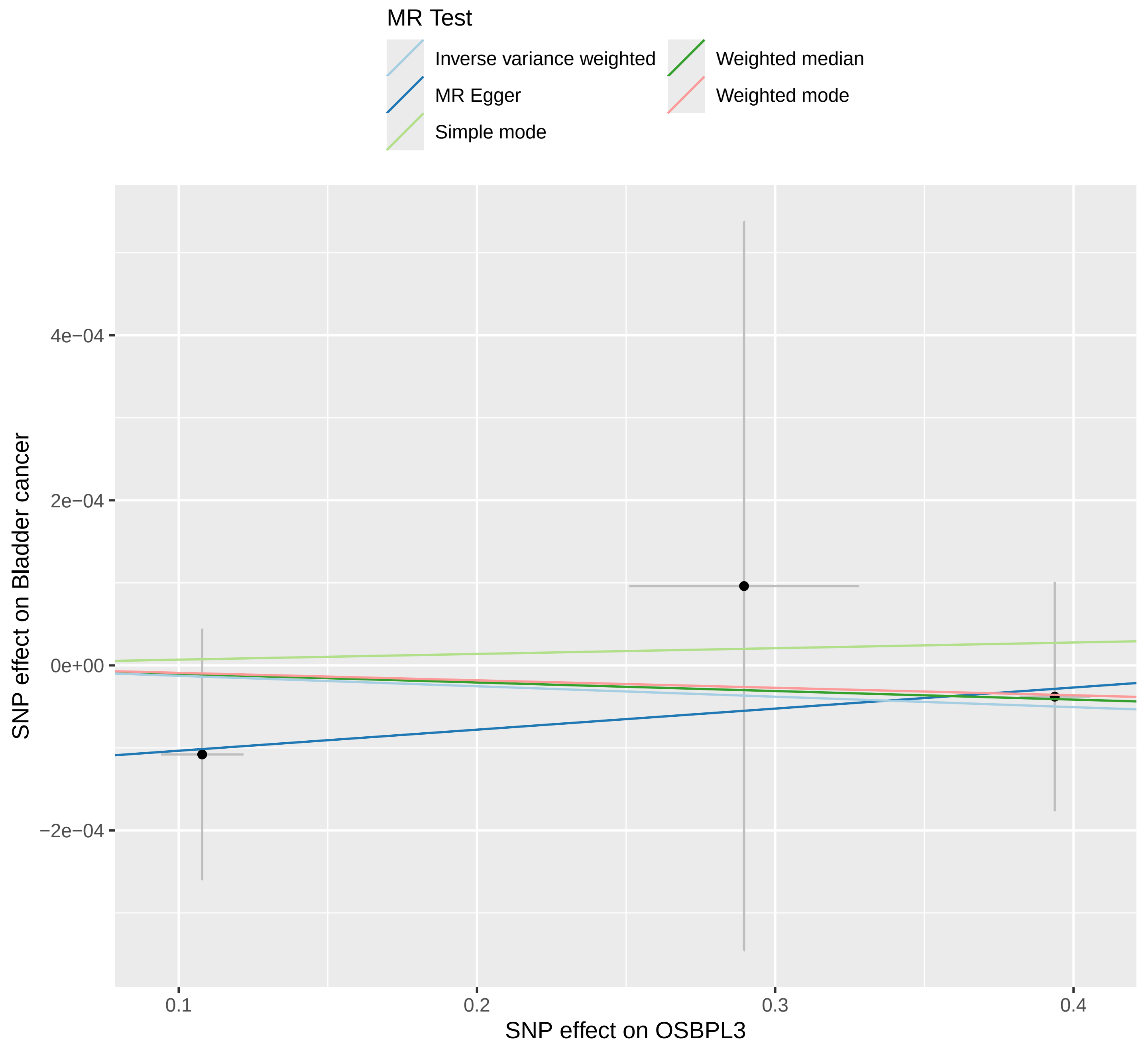

MR Test
Inverse variance weighted
MR Egger
Simple mode
Weighted median
Weighted mode
4e−04
2e−04
0e+00
−2e−04
SNP effect on Bladder cancer
0.1
0.2
0.3
0.4
SNP effect on OSBPL3

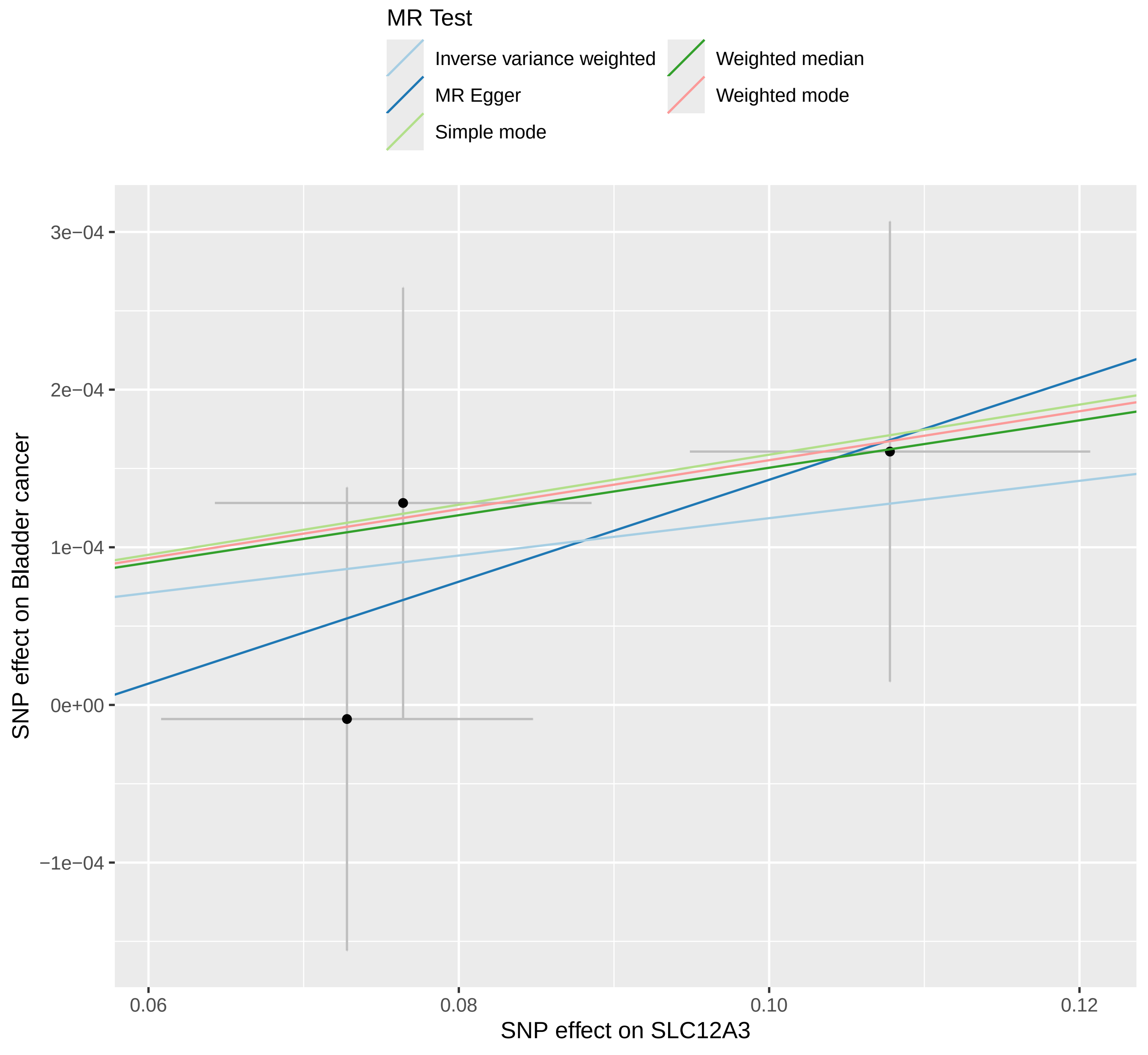
MR Test
Inverse variance weighted
MR Egger
Simple mode
Weighted median
Weighted mode
3e−04
2e−04
1e−04
0e+00
−1e−04
SNP effect on Bladder cancer
0.06
0.08
0.10
0.12
SNP effect on SLC12A3

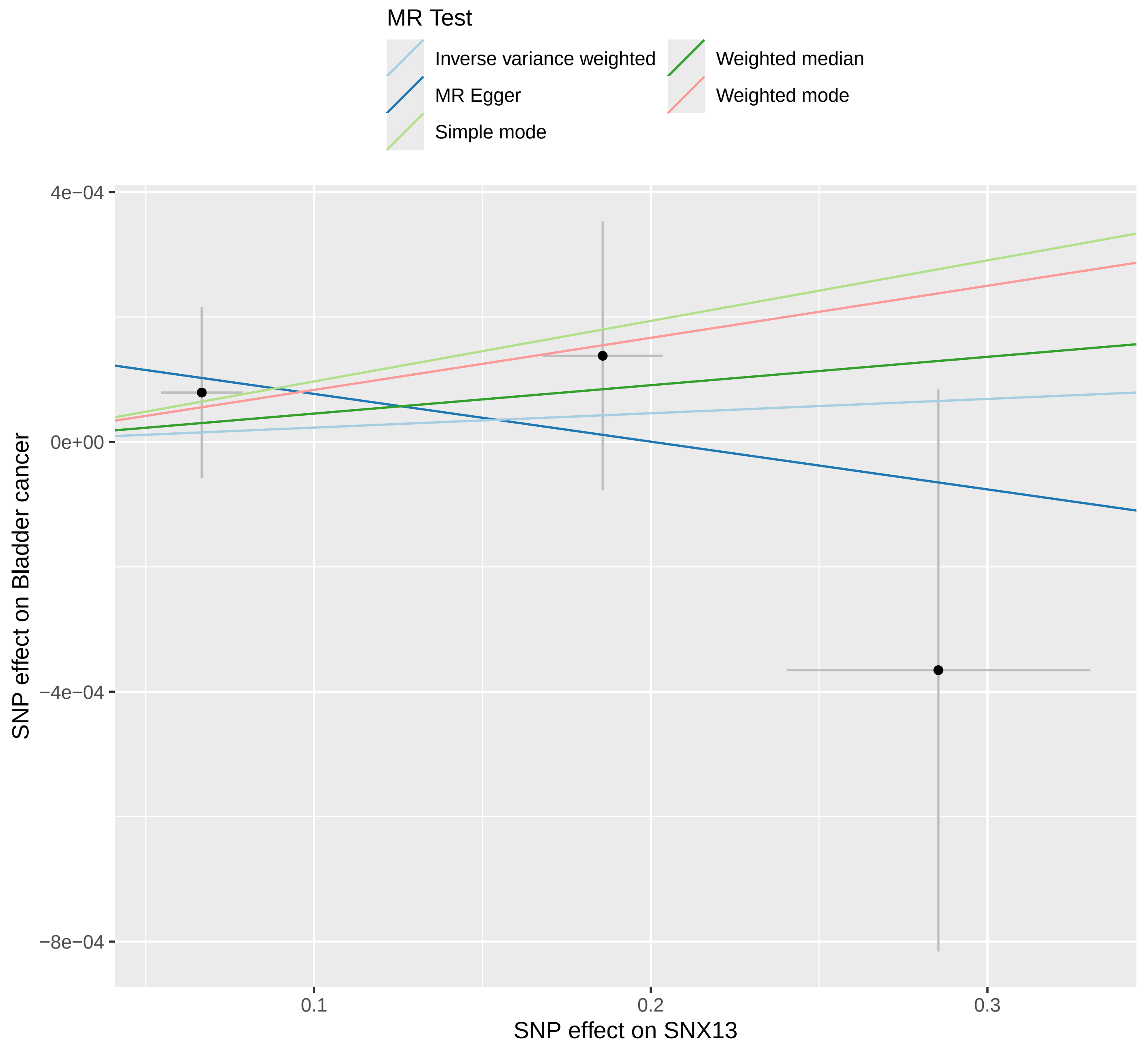
MR Test
Inverse variance weighted
MR Egger
Simple mode
Weighted median
Weighted mode
4e−04
0e+00
−4e−04
−8e−04
SNP effect on Bladder cancer
0.1
0.2
0.3
SNP effect on SNX13

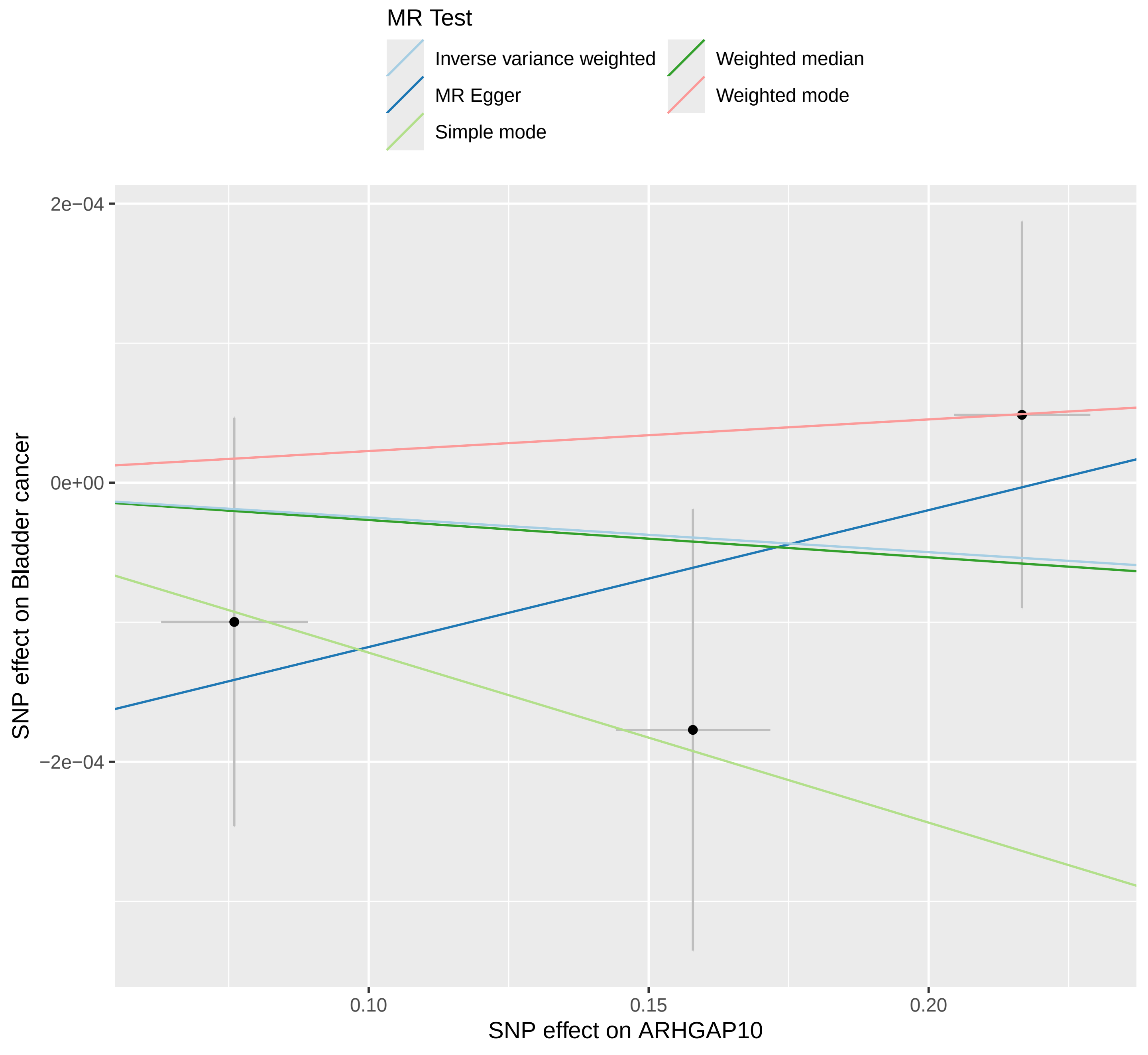
MR Test
Inverse variance weighted
MR Egger
Simple mode
Weighted median
Weighted mode
2e−04
0e+00
−2e−04
SNP effect on Bladder cancer
0.10
0.15
0.20
SNP effect on ARHGAP10

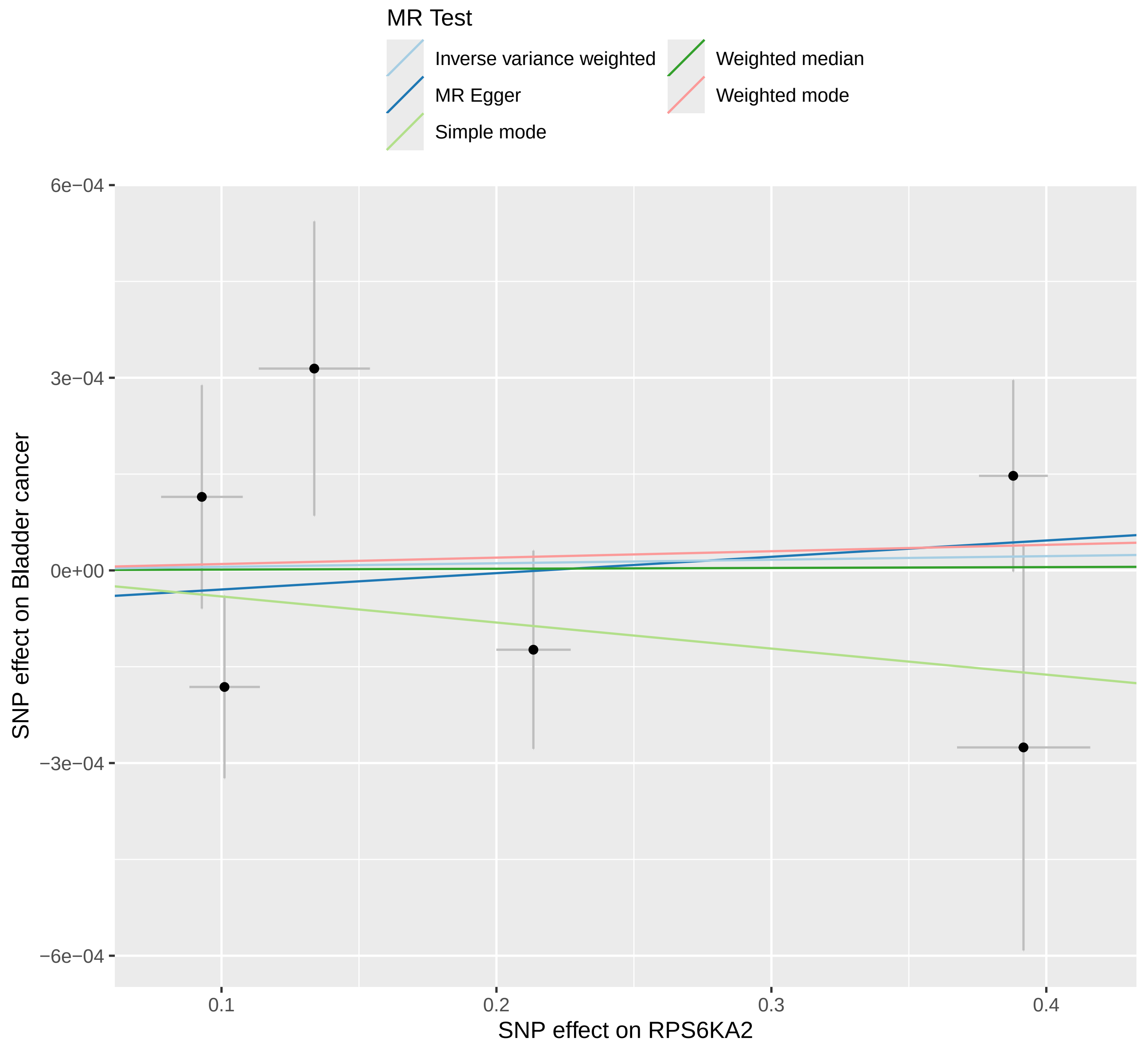
MR Test
Inverse variance weighted
MR Egger
Simple mode
Weighted median
Weighted mode
6e−04
3e−04
0e+00
−3e−04
−6e−04
SNP effect on Bladder cancer
0.1
0.2
0.3
0.4
SNP effect on RPS6KA2

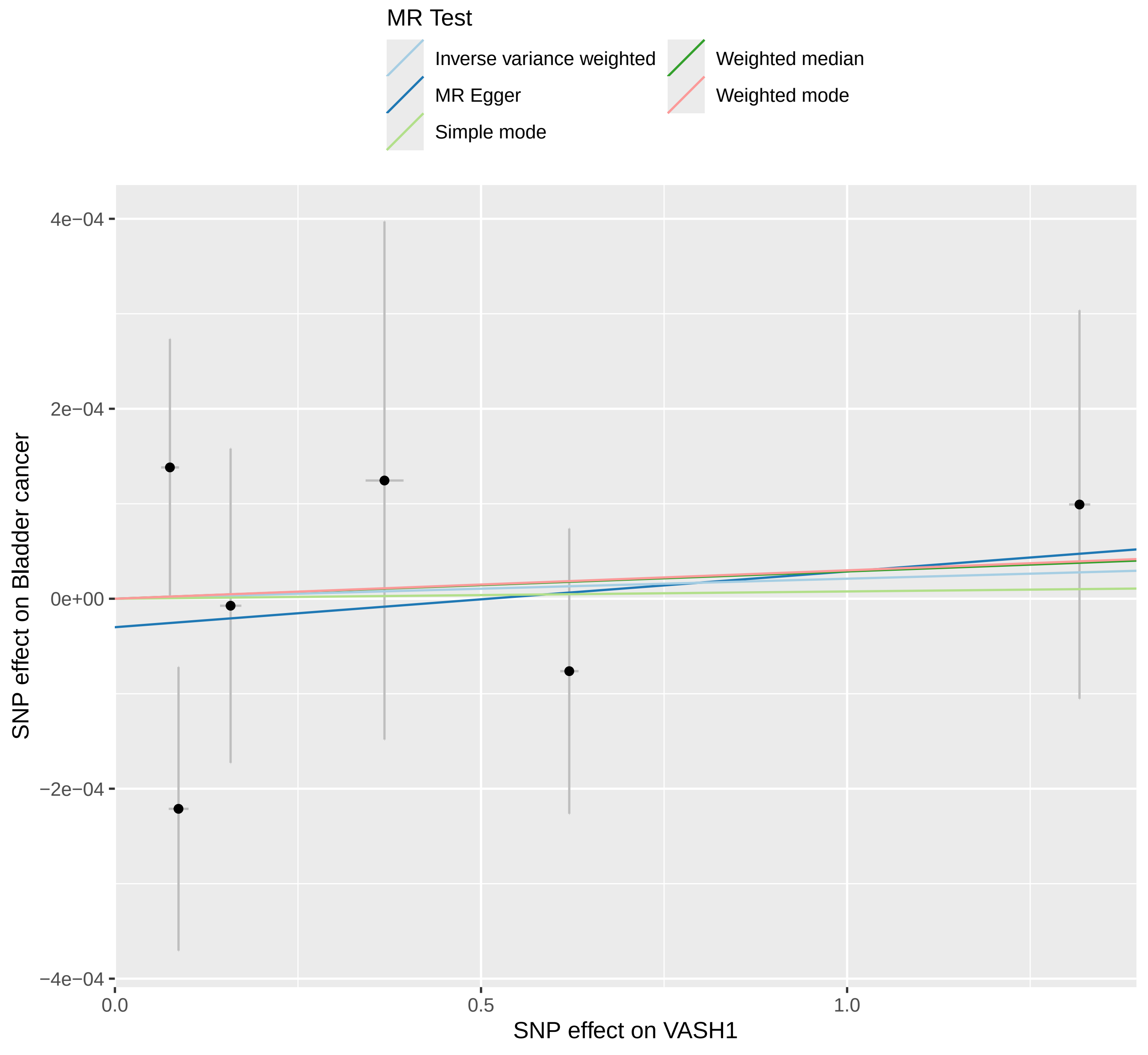
MR Test
Inverse variance weighted
MR Egger
Simple mode
Weighted median
Weighted mode
4e−04
2e−04
0e+00
−2e−04
−4e−04
SNP effect on Bladder cancer
0.0
0.5
1.0
SNP effect on VASH1

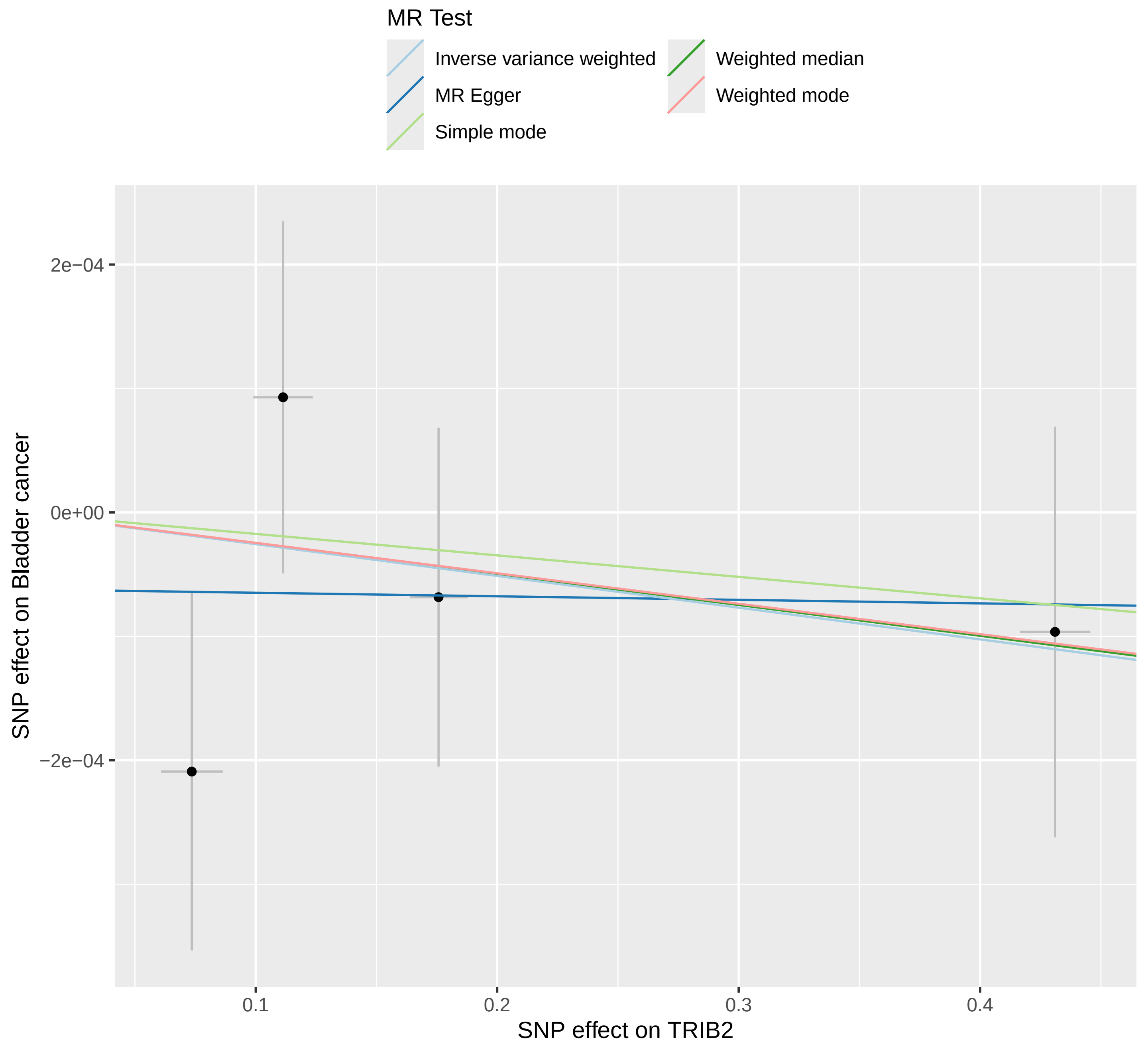
MR Test
Inverse variance weighted
MR Egger
Simple mode
Weighted median
Weighted mode
2e−04
0e+00
−2e−04
SNP effect on Bladder cancer
0.1
0.2
0.3
0.4
SNP effect on TRIB2

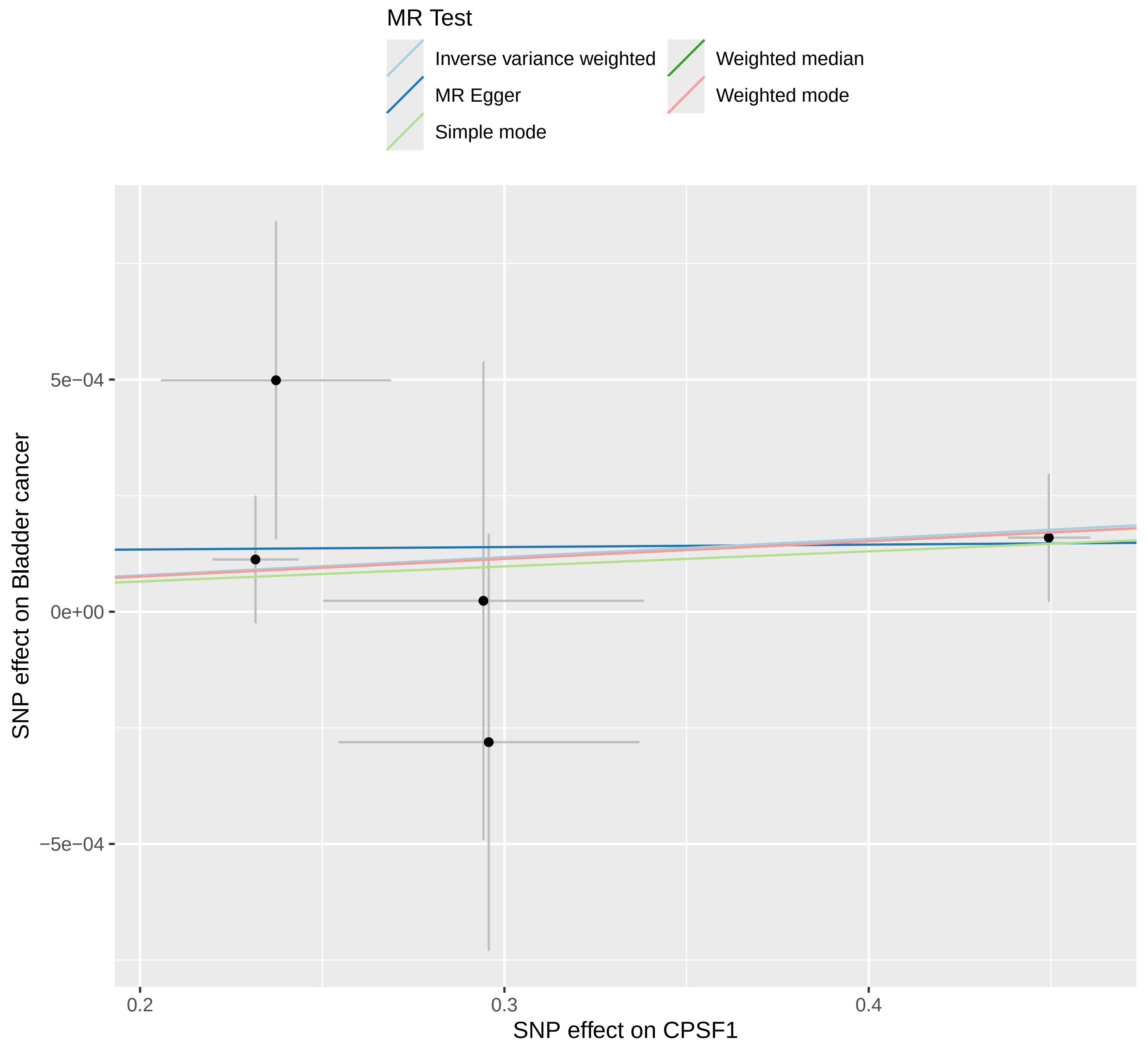

MR Test
Inverse variance weighted
MR Egger
Simple mode
Weighted median
Weighted mode
5e−04
0e+00
−5e−04
SNP effect on Bladder cancer
0.2
0.3
0.4
SNP effect on CPSF1

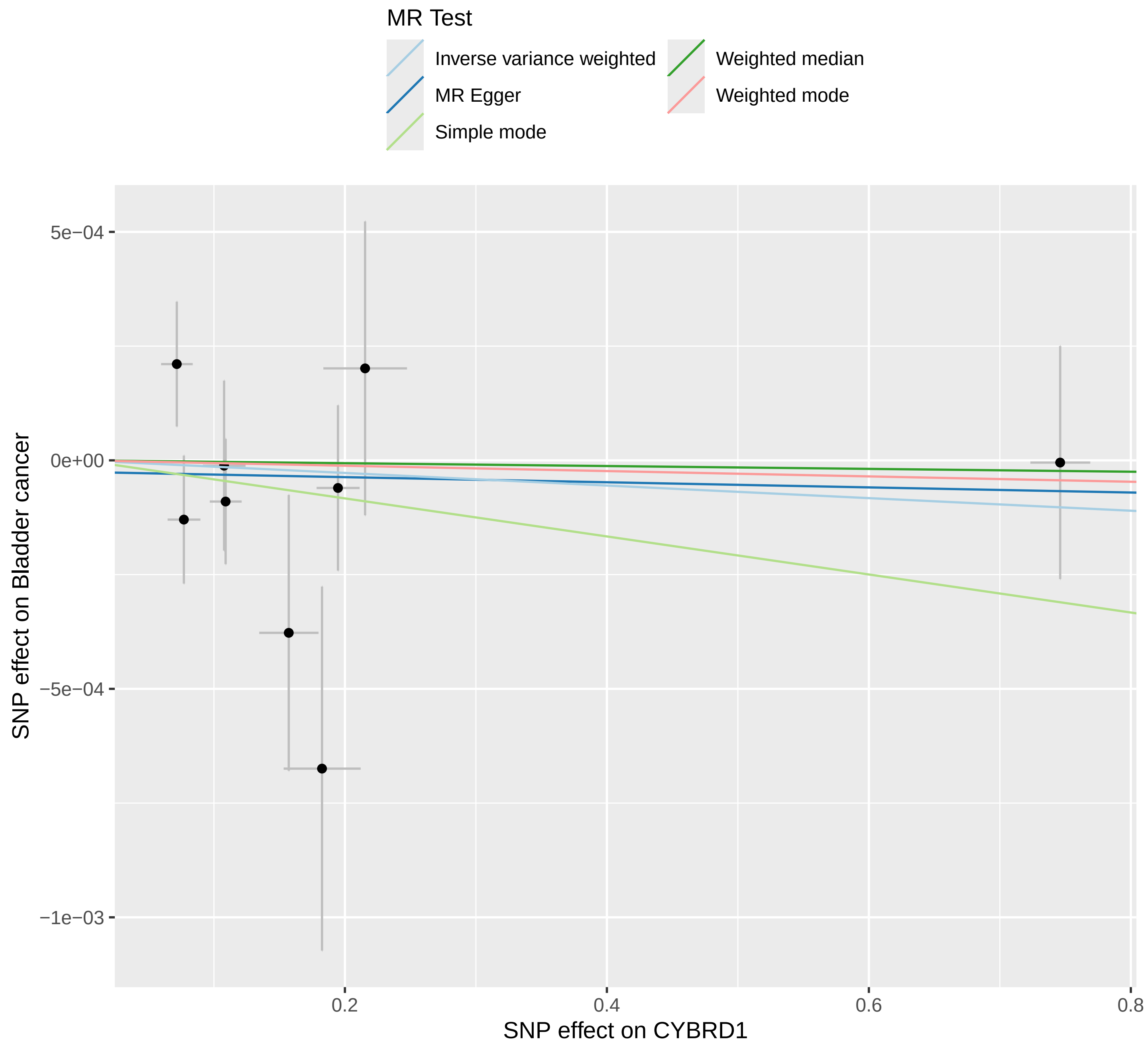

MR Test
Inverse variance weighted
MR Egger
Simple mode
Weighted median
Weighted mode
5e−04
0e+00
−5e−04
−1e−03
SNP effect on Bladder cancer
0.2
0.4
0.6
0.8
SNP effect on CYBRD1

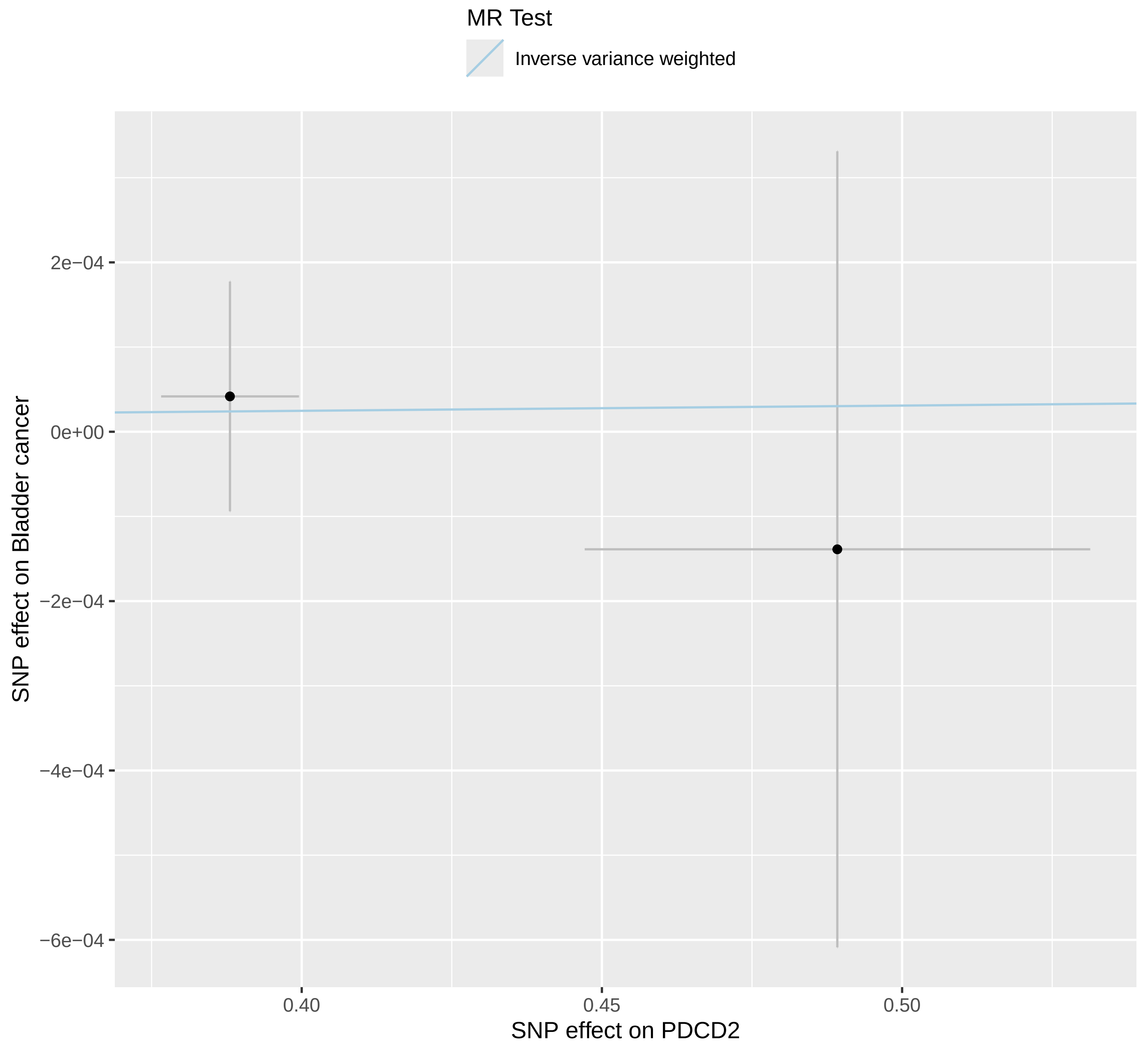

MR Test
Inverse variance weighted
SNP effect on Bladder cancer
2e−04
0e+00
−2e−04
−4e−04
−6e−04
0.40
0.45
0.50
SNP effect on PDCD2

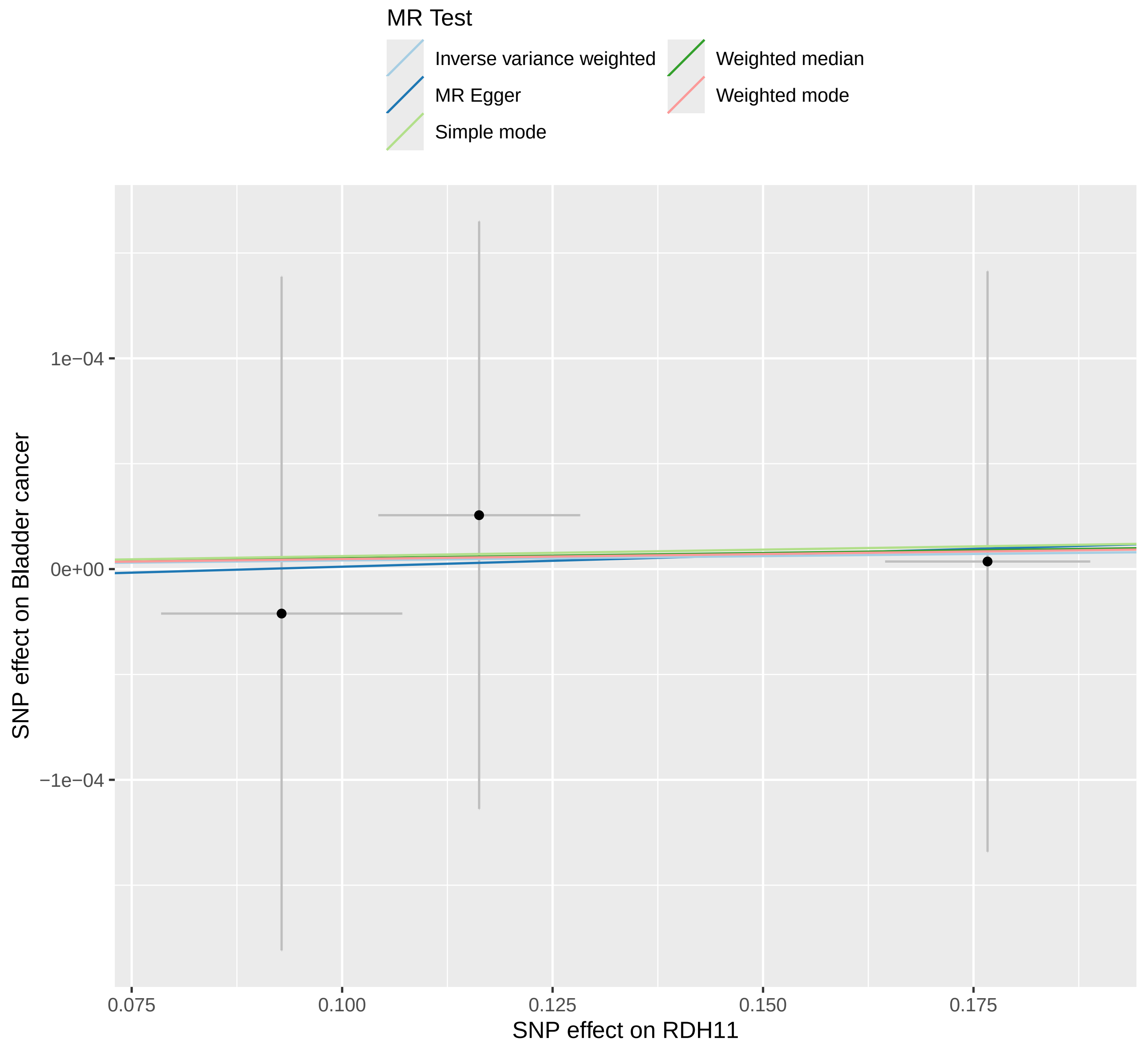
MR Test
Inverse variance weighted
MR Egger
Simple mode
Weighted median
Weighted mode
1e−04
0e+00
−1e−04
SNP effect on Bladder cancer
0.075
0.100
0.125
0.150
0.175
SNP effect on RDH11

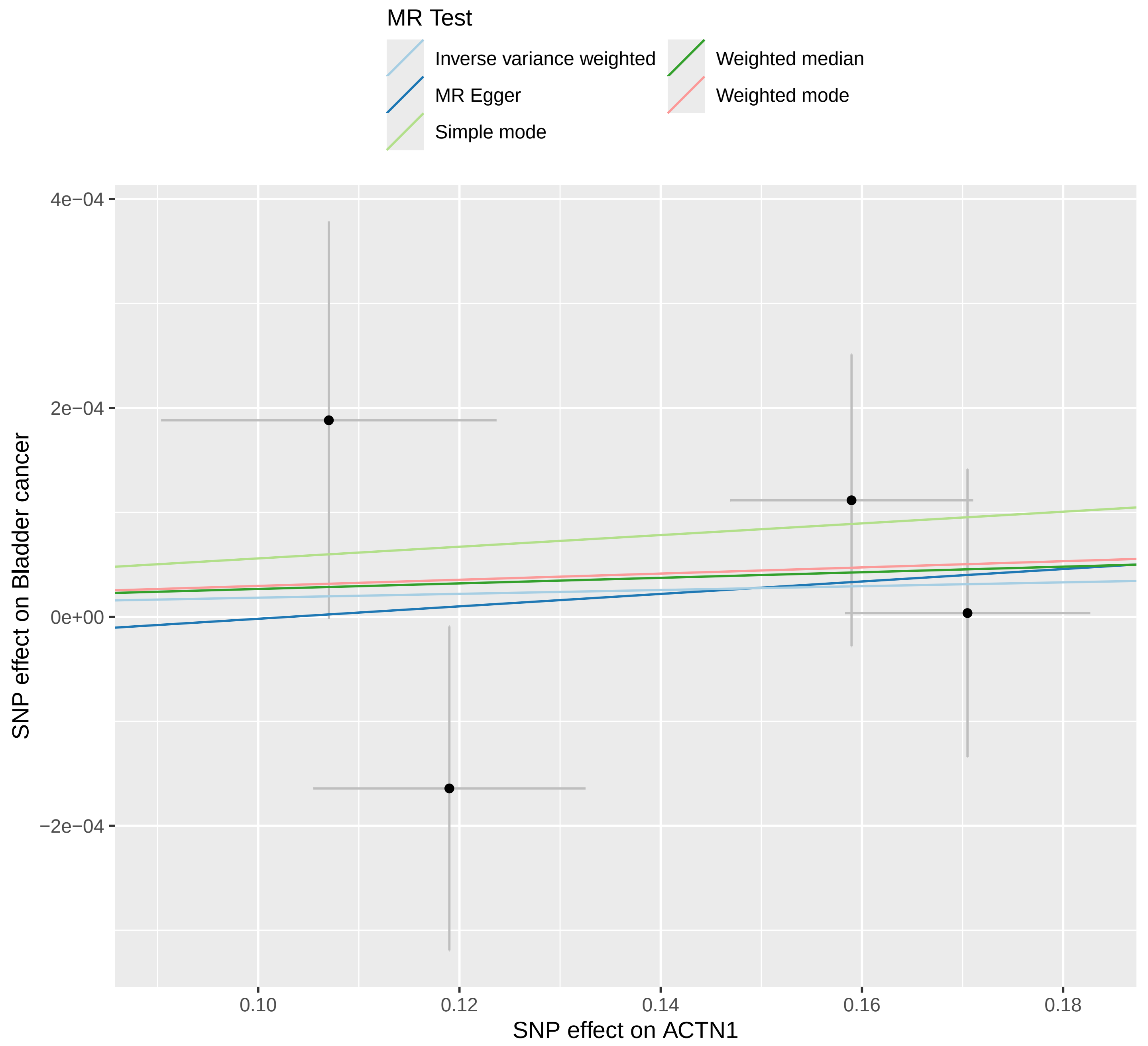

MR Test
Inverse variance weighted
MR Egger
Simple mode
Weighted median
Weighted mode
4e−04
2e−04
0e+00
−2e−04
SNP effect on Bladder cancer
0.10
0.12
0.14
0.16
0.18
SNP effect on ACTN1

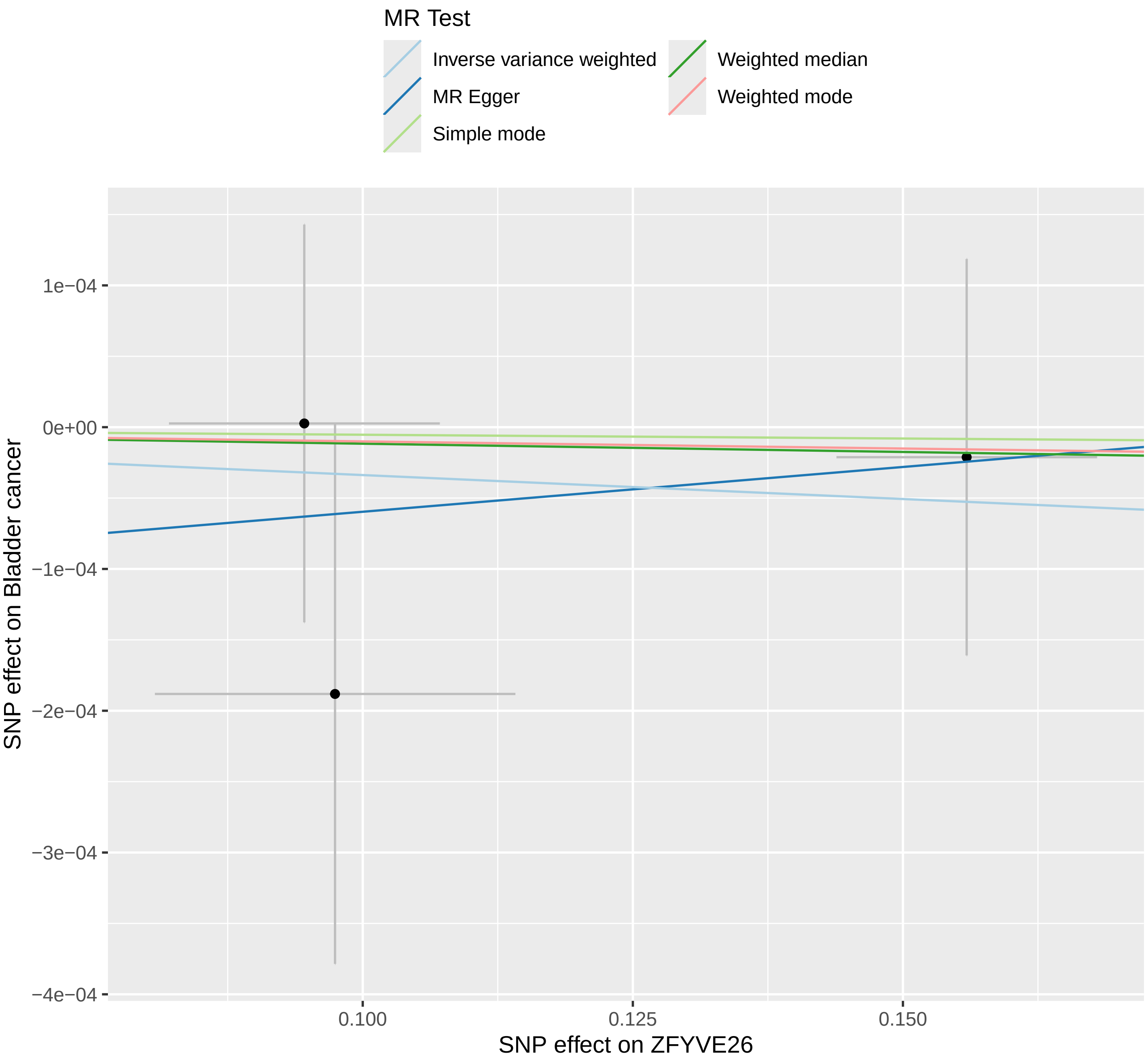

MR Test
Inverse variance weighted
MR Egger
Simple mode
Weighted median
Weighted mode
1e−04
0e+00
−1e−04
−2e−04
−3e−04
−4e−04
SNP effect on Bladder cancer
0.100
0.125
0.150
SNP effect on ZFYVE26

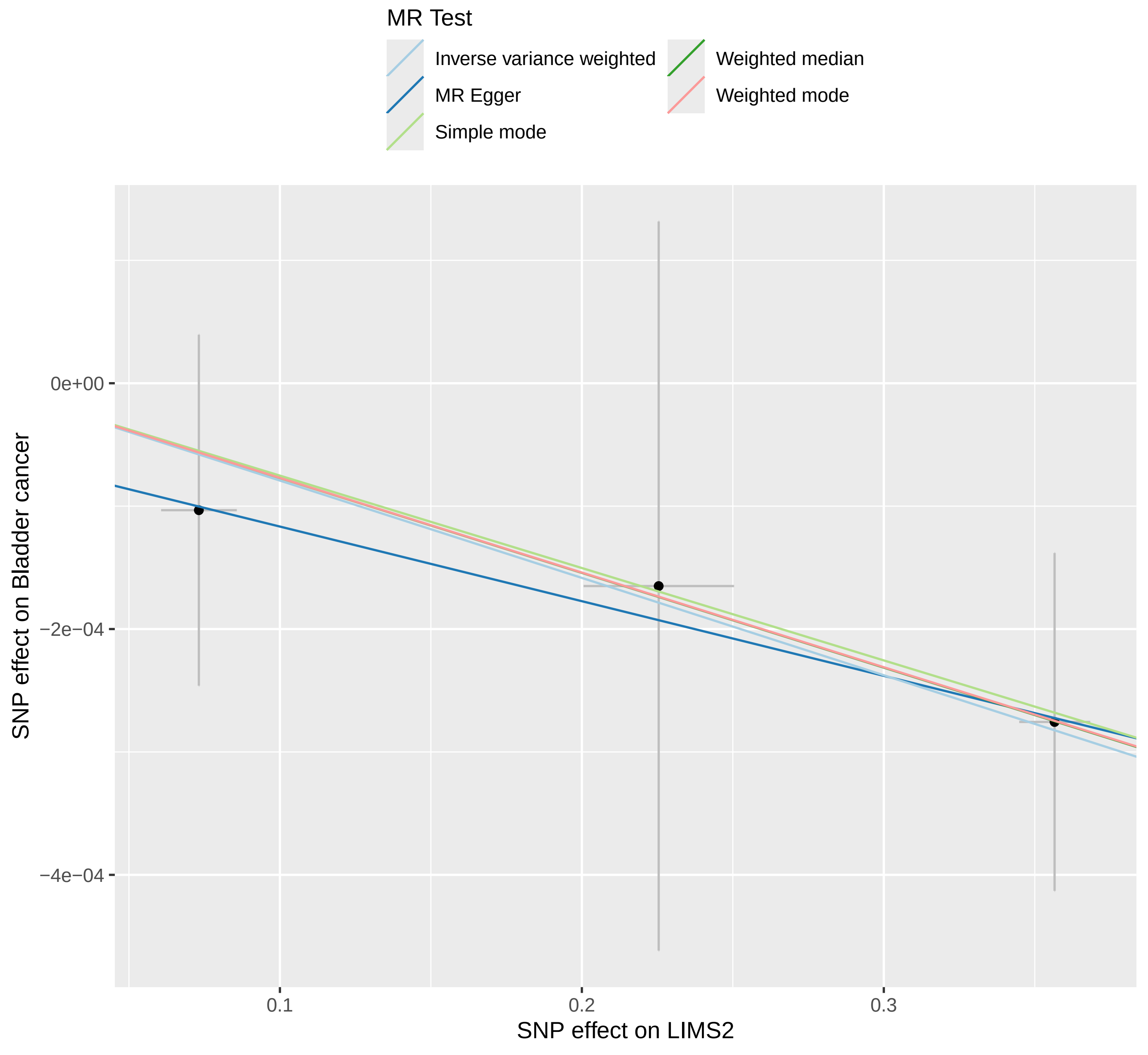

MR Test
Inverse variance weighted
MR Egger
Simple mode
Weighted median
Weighted mode
0e+00
−2e−04
−4e−04
SNP effect on Bladder cancer
0.1
0.2
0.3
SNP effect on LIMS2

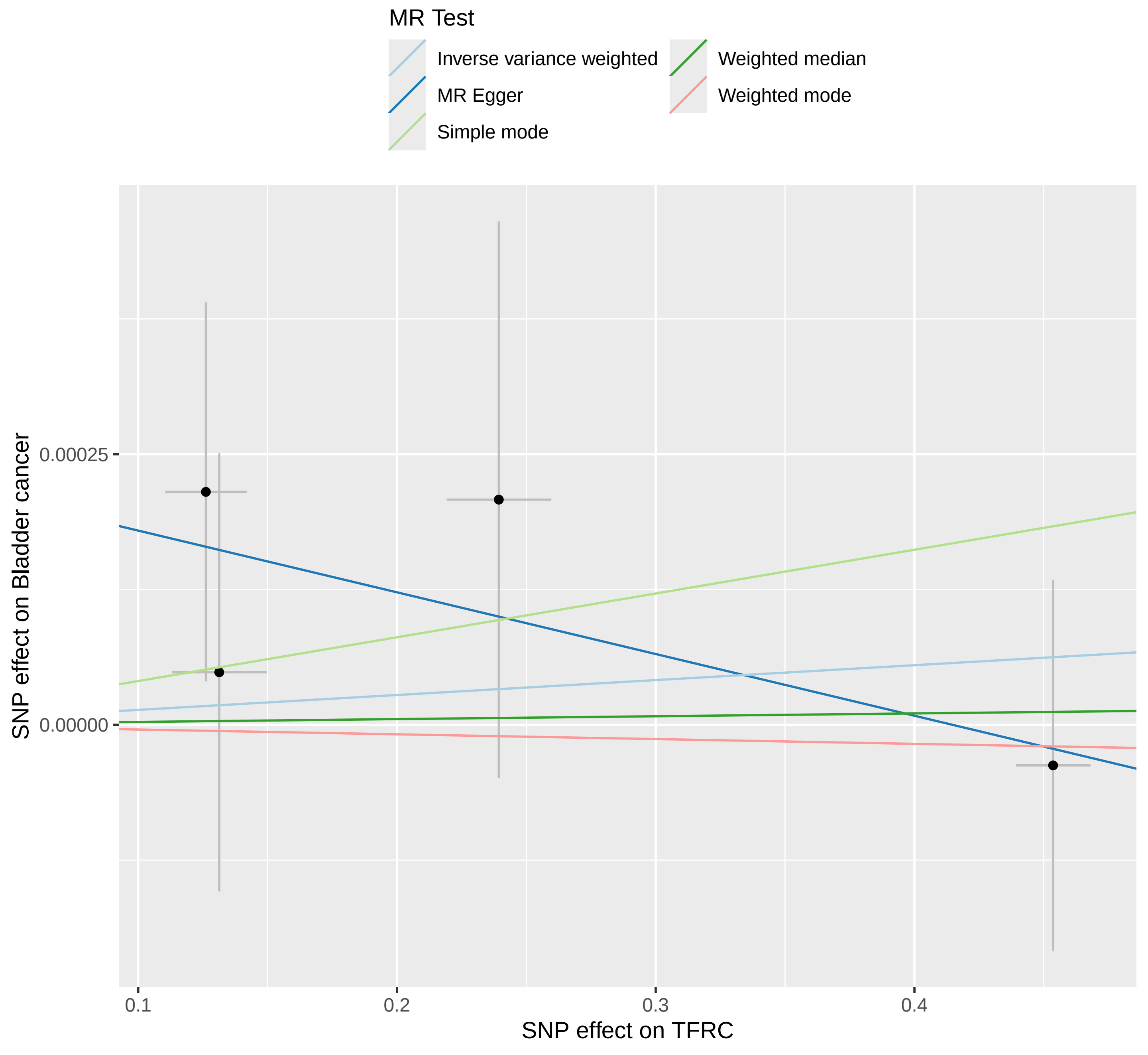
MR Test
Inverse variance weighted
MR Egger
Simple mode
Weighted median
Weighted mode
0.00025
0.00000
SNP effect on Bladder cancer
0.1
0.2
0.3
0.4
SNP effect on TFRC

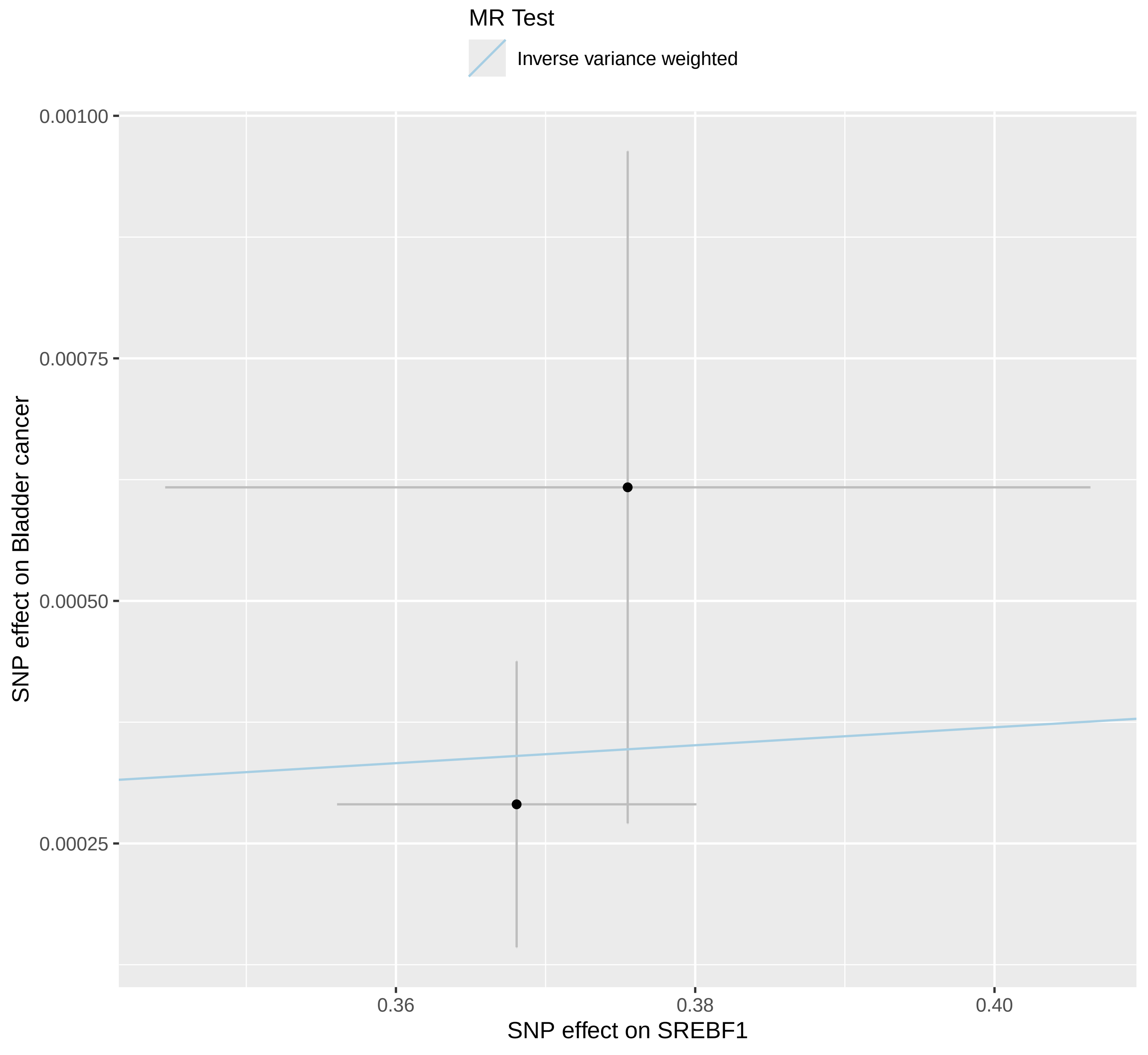
MR Test
Inverse variance weighted
0.00100
0.00075
0.00050
0.00025
SNP effect on Bladder cancer
0.36
0.38
0.40
SNP effect on SREBF1

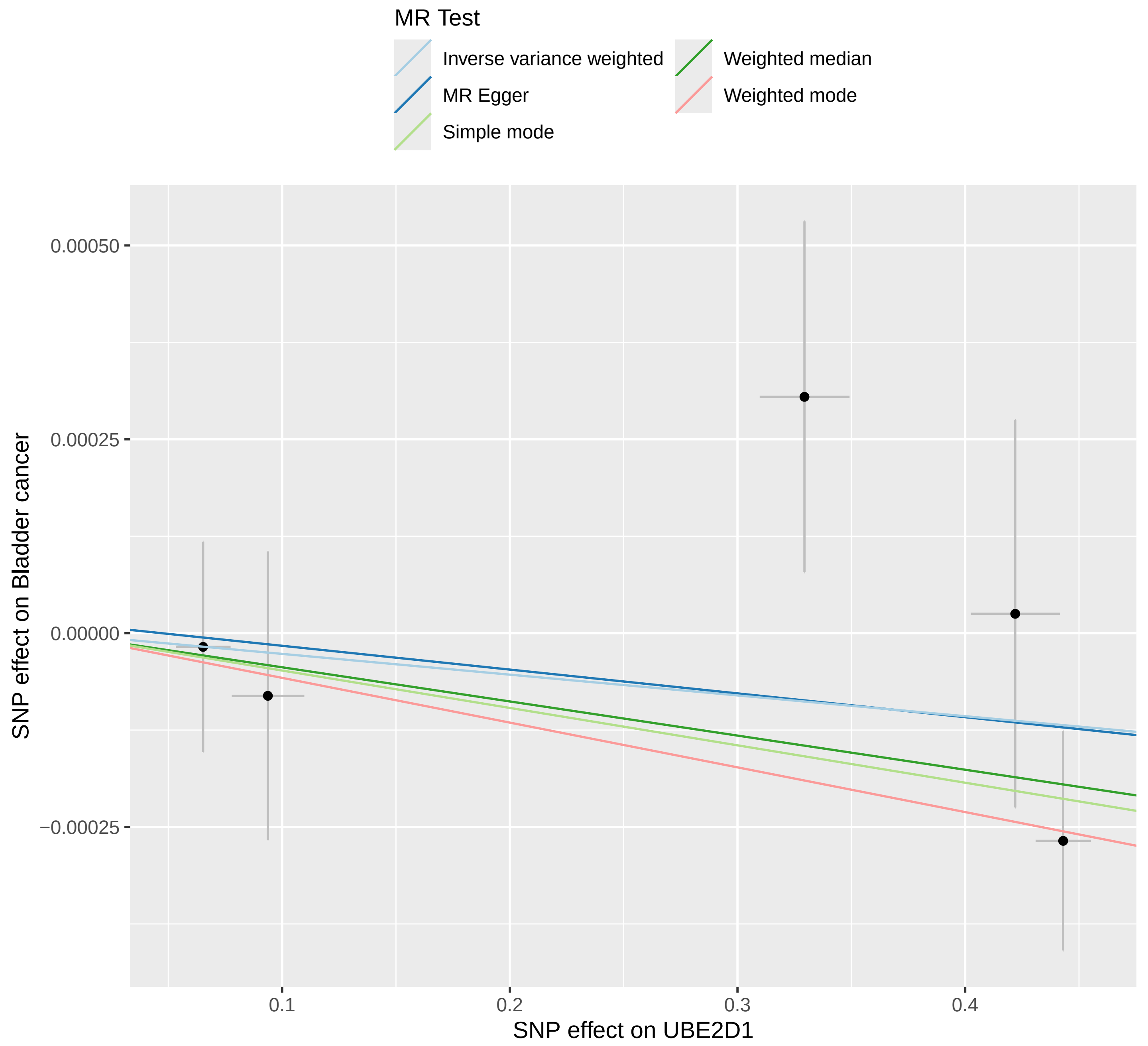
MR Test
Inverse variance weighted
MR Egger
Simple mode
Weighted median
Weighted mode
0.00050
0.00025
0.00000
−0.00025
SNP effect on Bladder cancer
0.1
0.2
0.3
0.4
SNP effect on UBE2D1

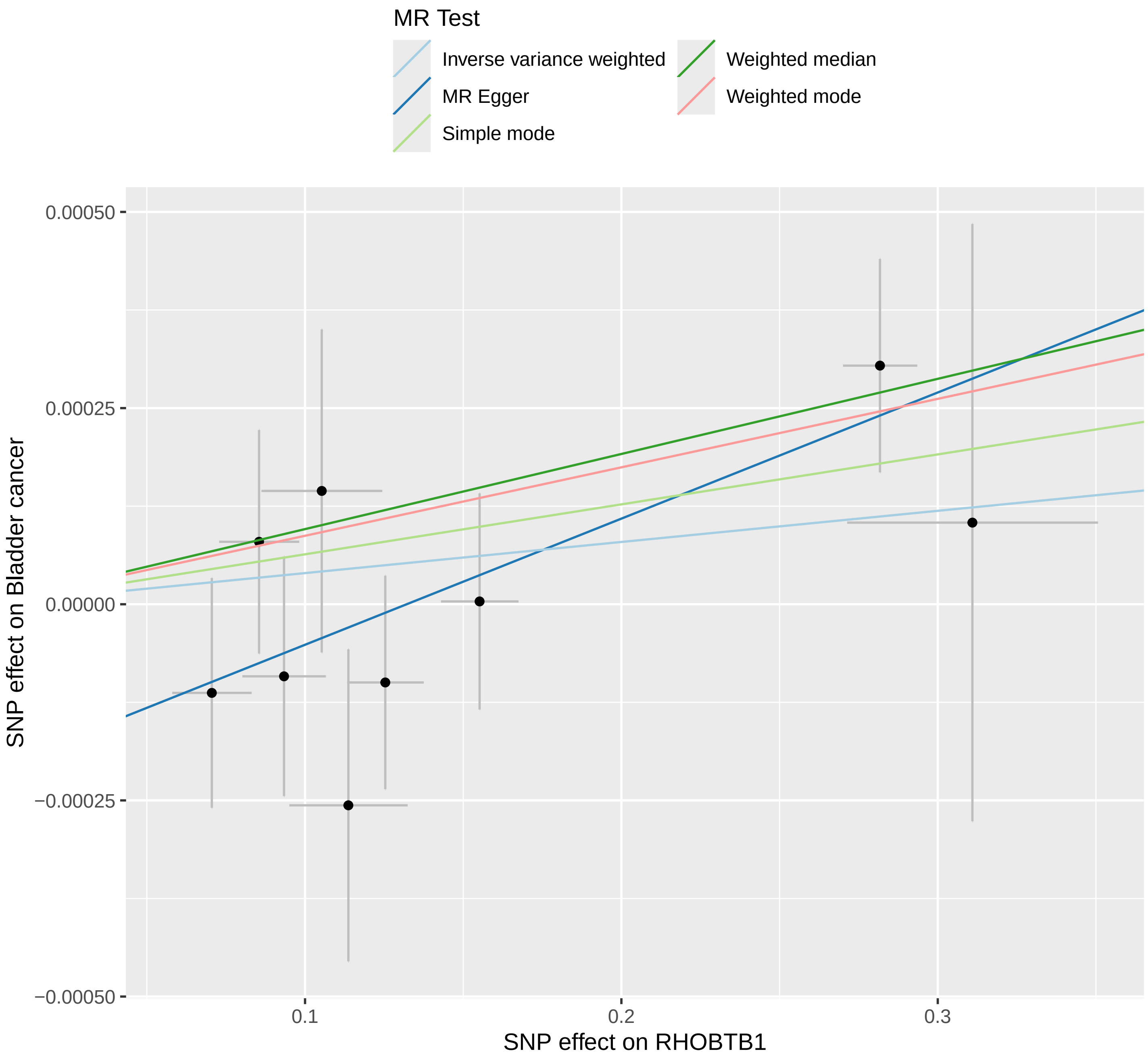

MR Test
Inverse variance weighted
MR Egger
Simple mode
Weighted median
Weighted mode
0.00050
0.00025
0.00000
−0.00025
−0.00050
SNP effect on Bladder cancer
0.1
0.2
0.3
SNP effect on RHOBTB1

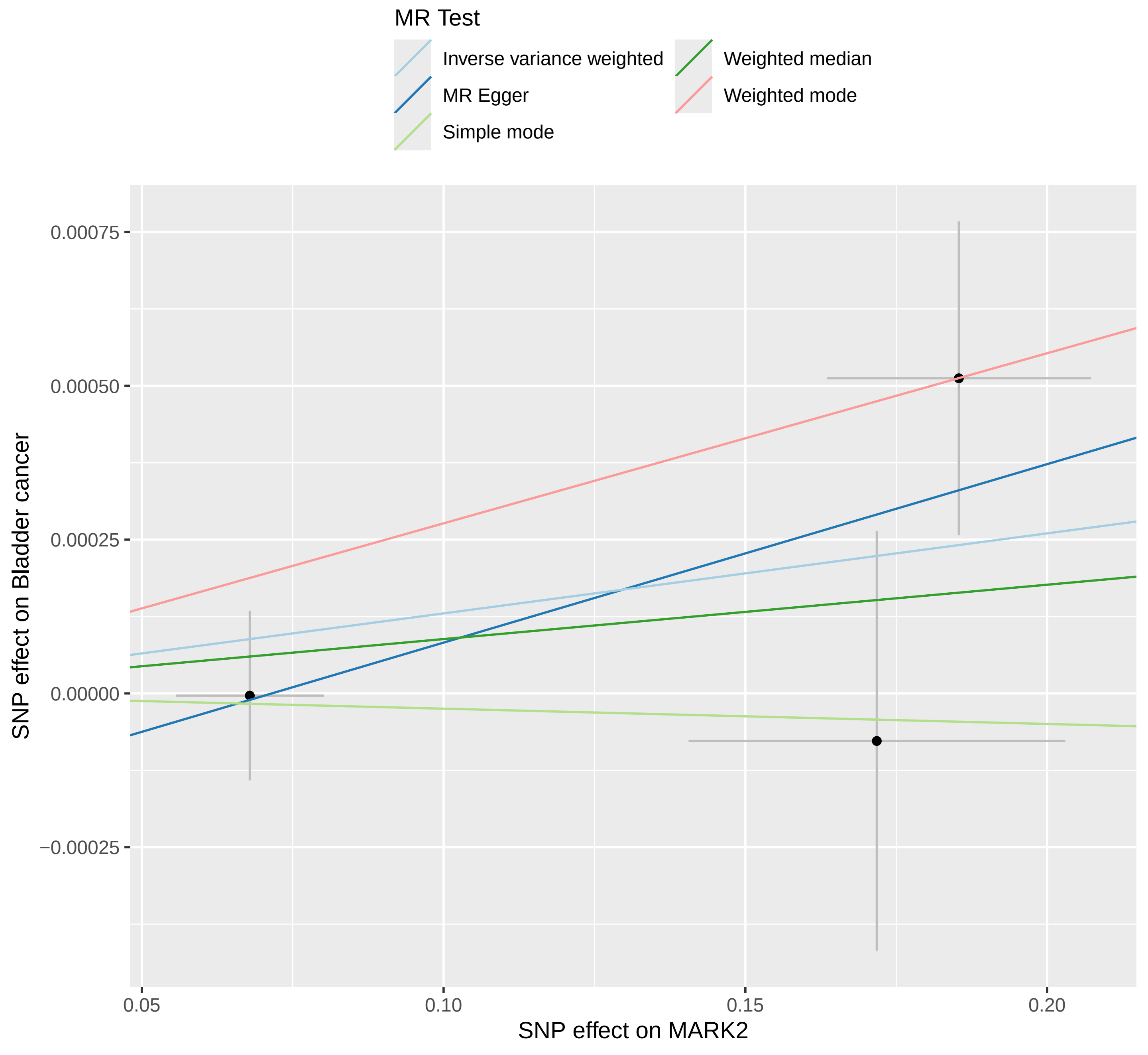

MR Test
Inverse variance weighted
MR Egger
Simple mode
Weighted median
Weighted mode
0.00075
0.00050
0.00025
0.00000
−0.00025
SNP effect on Bladder cancer
0.05
0.10
0.15
0.20
SNP effect on MARK2

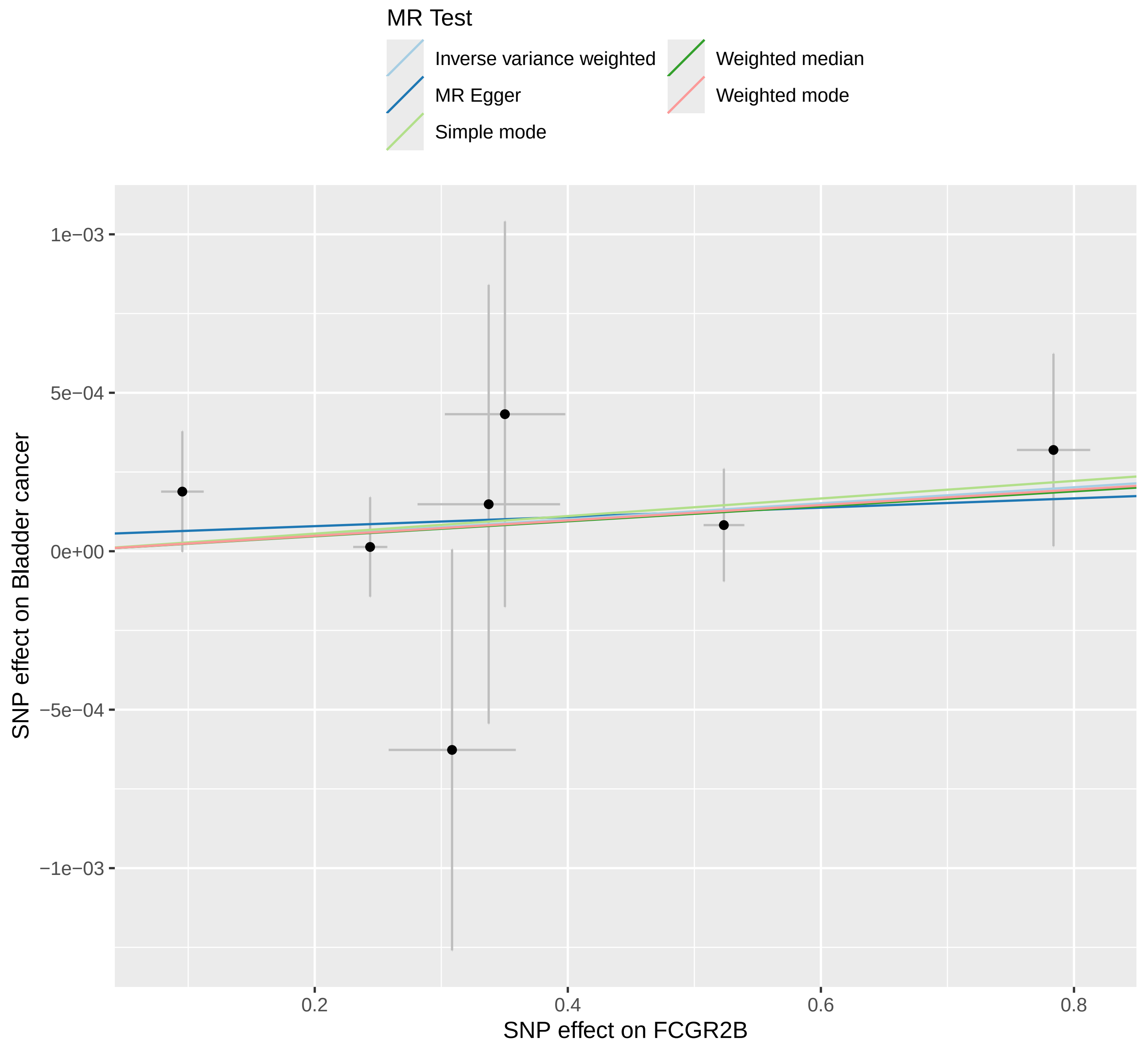

MR Test
Inverse variance weighted
MR Egger
Simple mode
Weighted median
Weighted mode
1e−03
5e−04
0e+00
−5e−04
−1e−03
0.2
0.4
0.6
0.8
SNP effect on FCGR2B
SNP effect on Bladder cancer

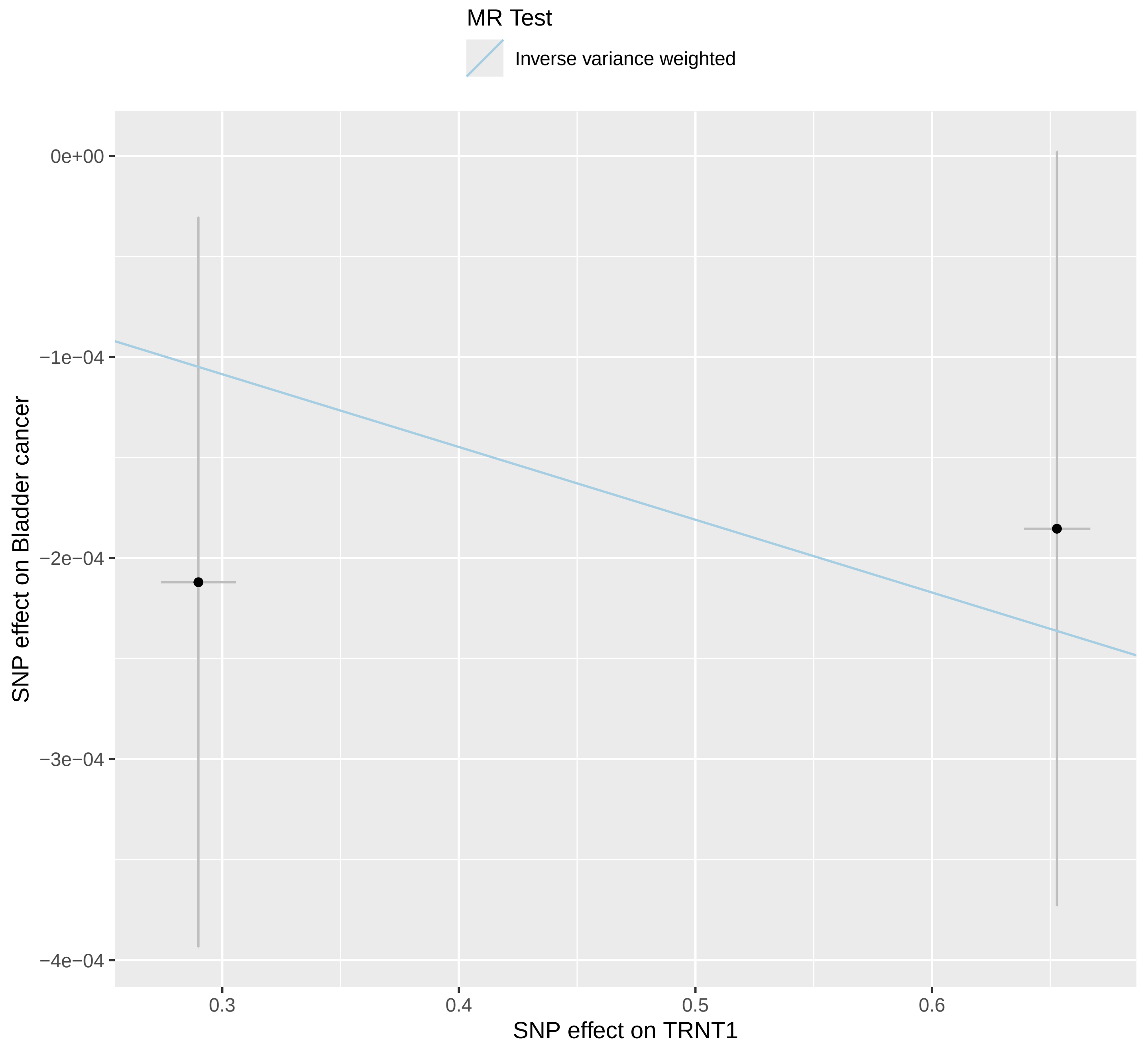

MR Test
Inverse variance weighted
0e+00
−1e−04
−2e−04
−3e−04
−4e−04
0.3
0.4
0.5
0.6
SNP effect on Bladder cancer
SNP effect on TRNT1

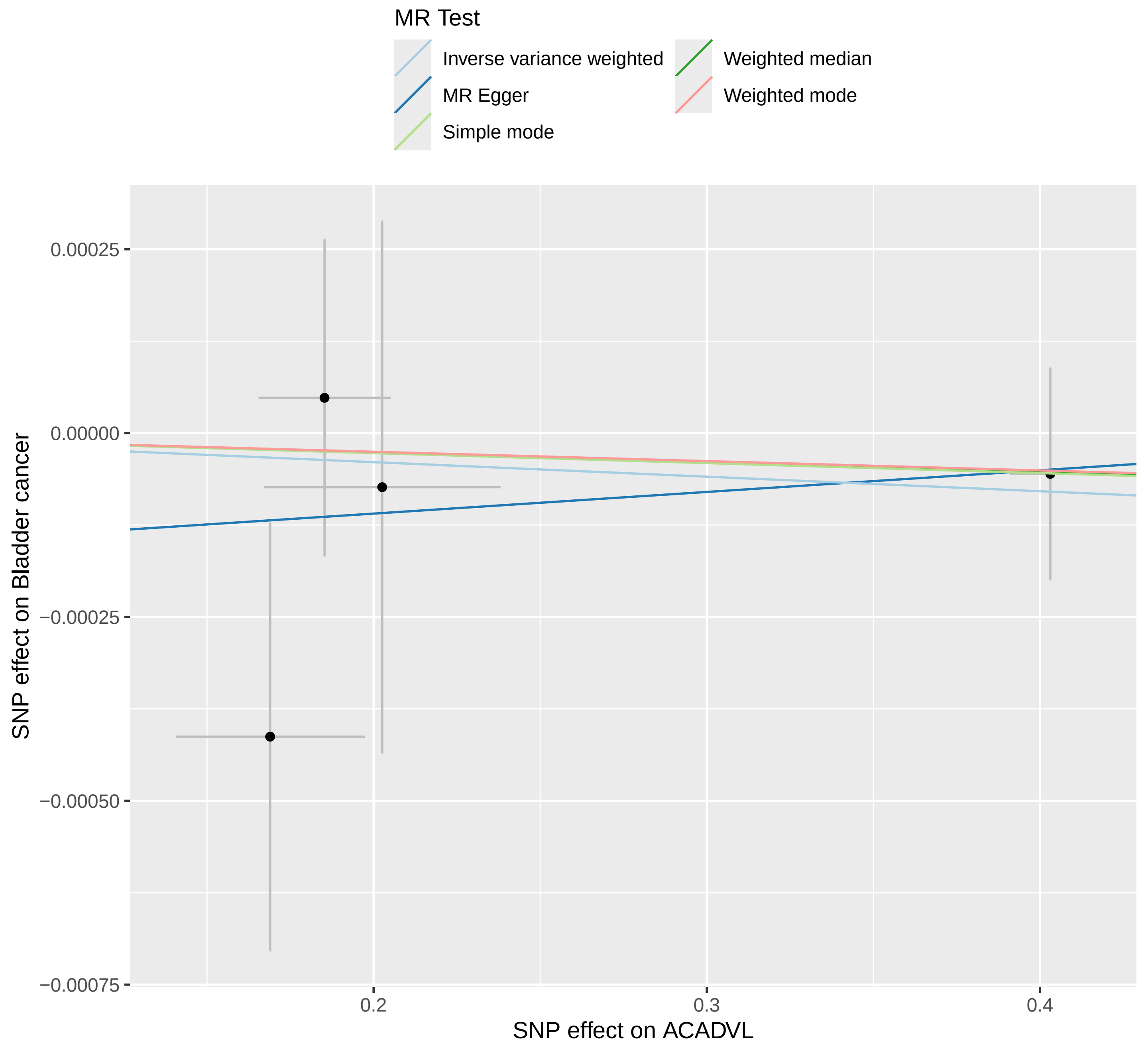

MR Test
Inverse variance weighted
MR Egger
Simple mode
Weighted median
Weighted mode
0.00025
0.00000
−0.00025
−0.00050
−0.00075
SNP effect on Bladder cancer
0.2
0.3
0.4
SNP effect on ACADVL

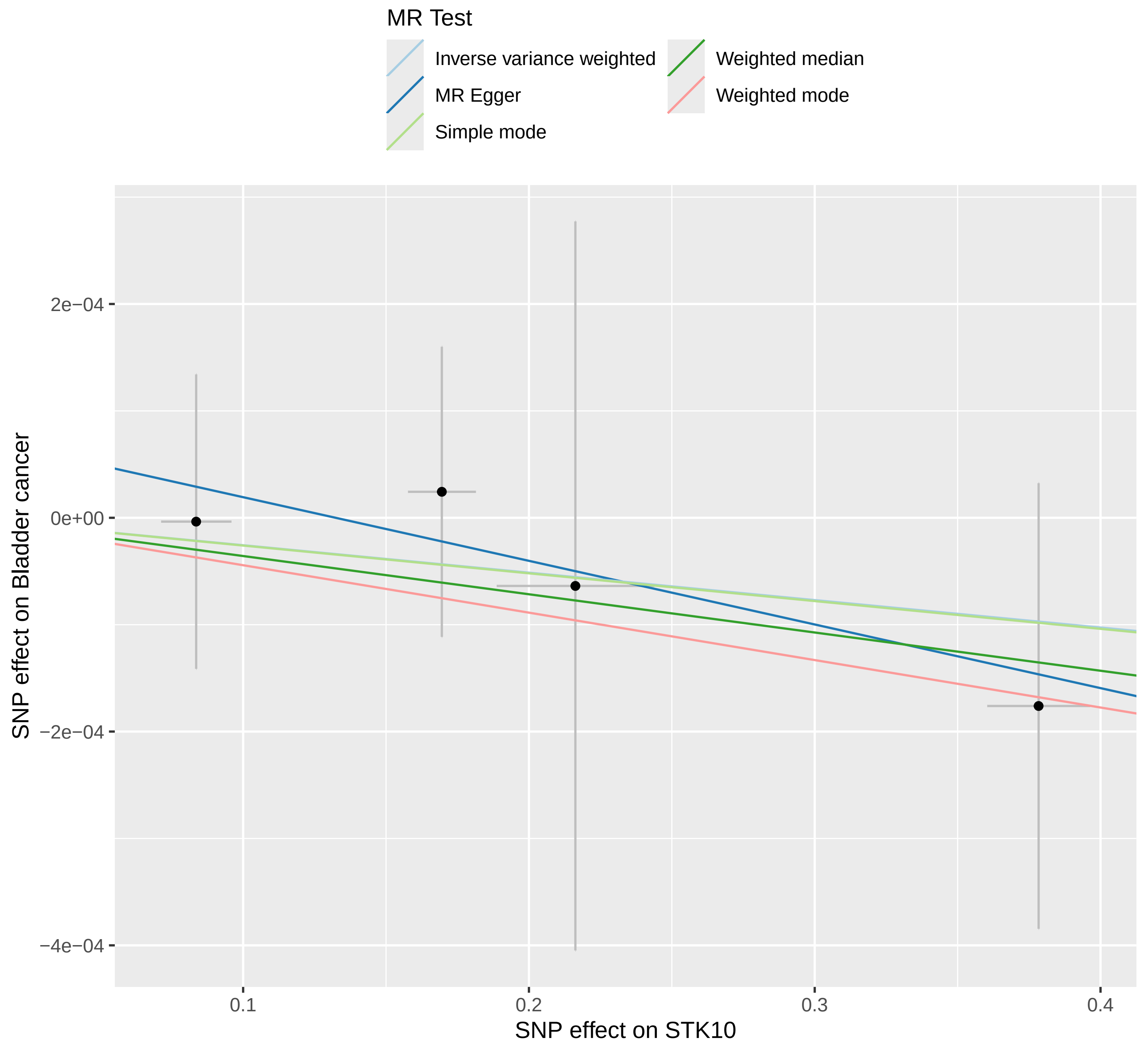

MR Test
Inverse variance weighted
MR Egger
Simple mode
Weighted median
Weighted mode
2e−04
0e+00
−2e−04
−4e−04
0.1
0.2
0.3
0.4
SNP effect on STK10
SNP effect on Bladder cancer

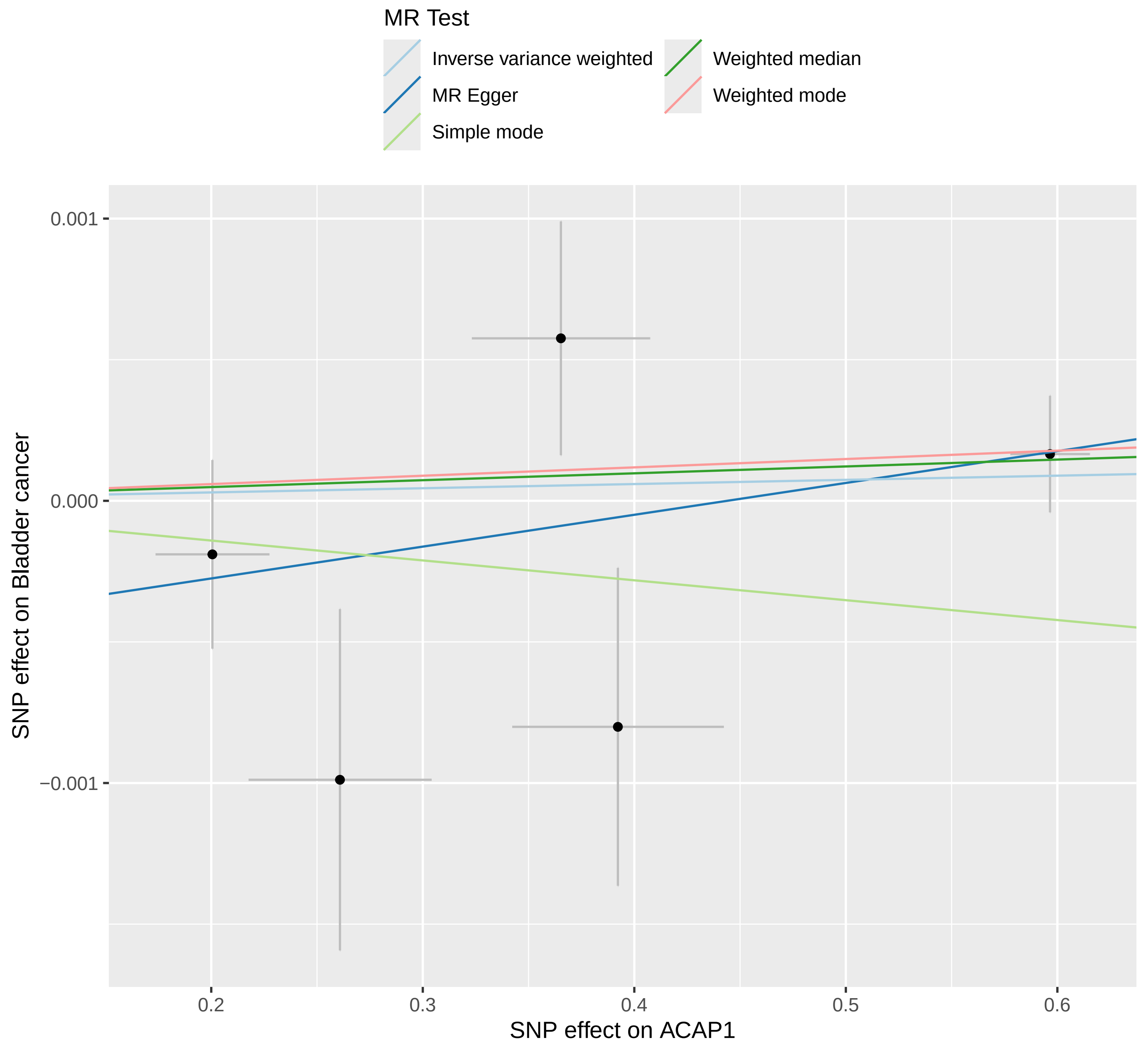

MR Test
Inverse variance weighted
MR Egger
Simple mode
Weighted median
Weighted mode
0.001
0.000
−0.001
0.2
0.3
0.4
0.5
0.6
SNP effect on ACAP1
SNP effect on Bladder cancer

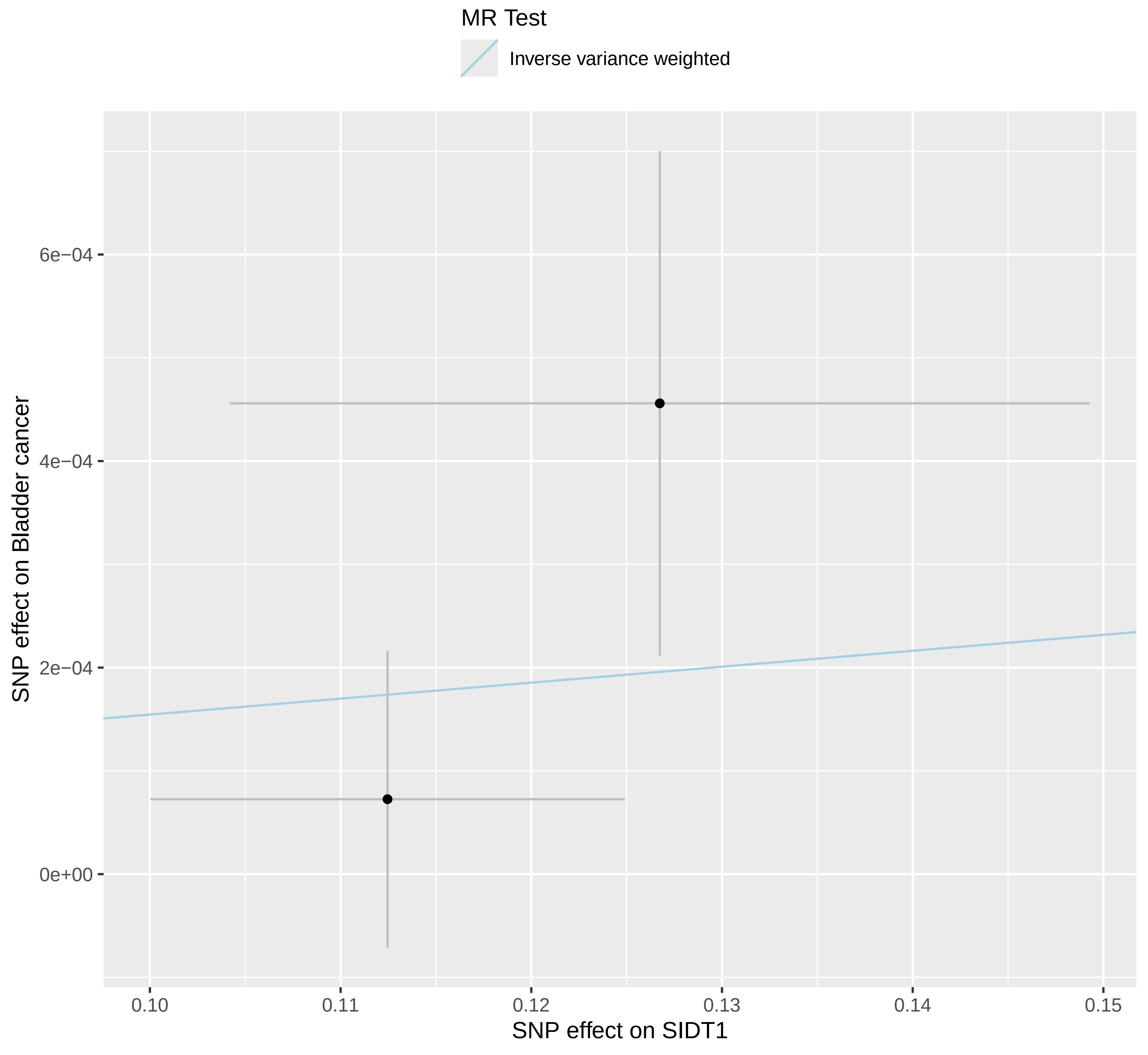
MR Test
Inverse variance weighted
SNP effect on Bladder cancer
6e−04
4e−04
2e−04
0e+00
0.10
0.11
0.12
0.13
0.14
0.15
SNP effect on SIDT1

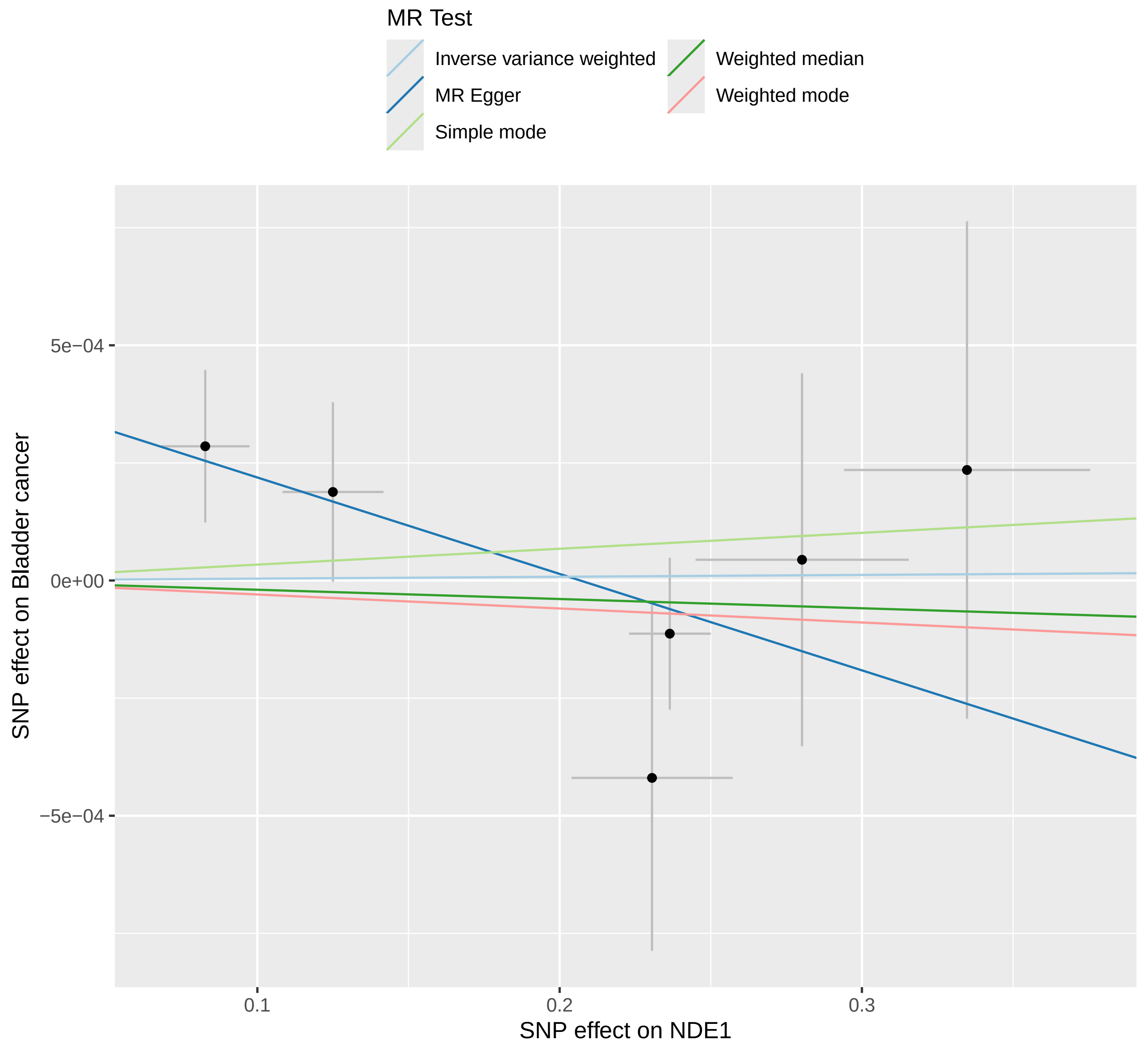

MR Test
Inverse variance weighted
MR Egger
Simple mode
Weighted median
Weighted mode
5e−04
0e+00
−5e−04
SNP effect on Bladder cancer
0.1
0.2
0.3
SNP effect on NDE1

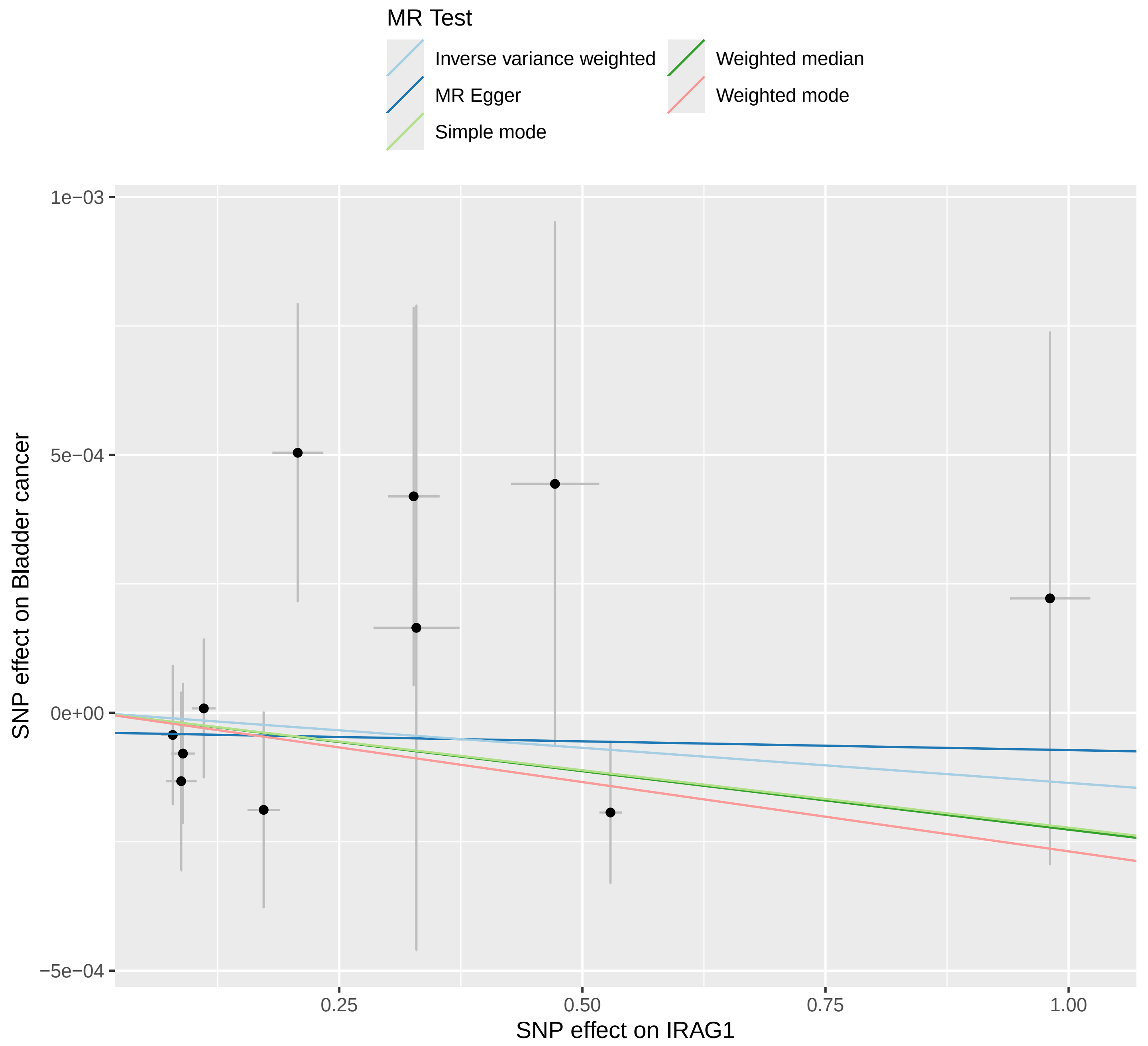
MR Test
Inverse variance weighted
MR Egger
Simple mode
Weighted median
Weighted mode
1e−03
5e−04
0e+00
−5e−04
0.25
0.50
0.75
1.00
SNP effect on Bladder cancer
SNP effect on IRAG1

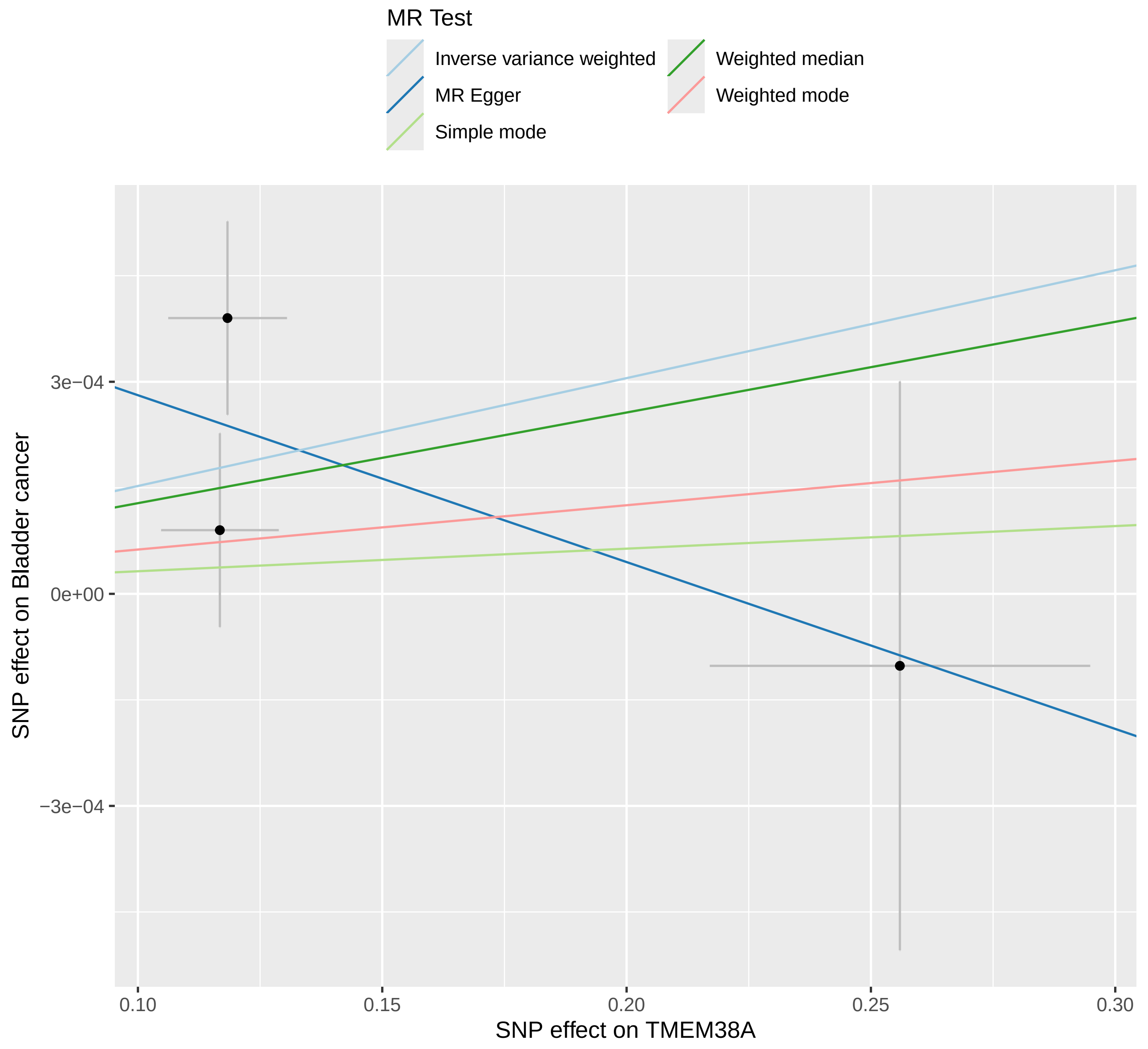
MR Test
Inverse variance weighted
MR Egger
Simple mode
Weighted median
Weighted mode
3e−04
0e+00
−3e−04
SNP effect on Bladder cancer
0.10
0.15
0.20
0.25
0.30
SNP effect on TMEM38A

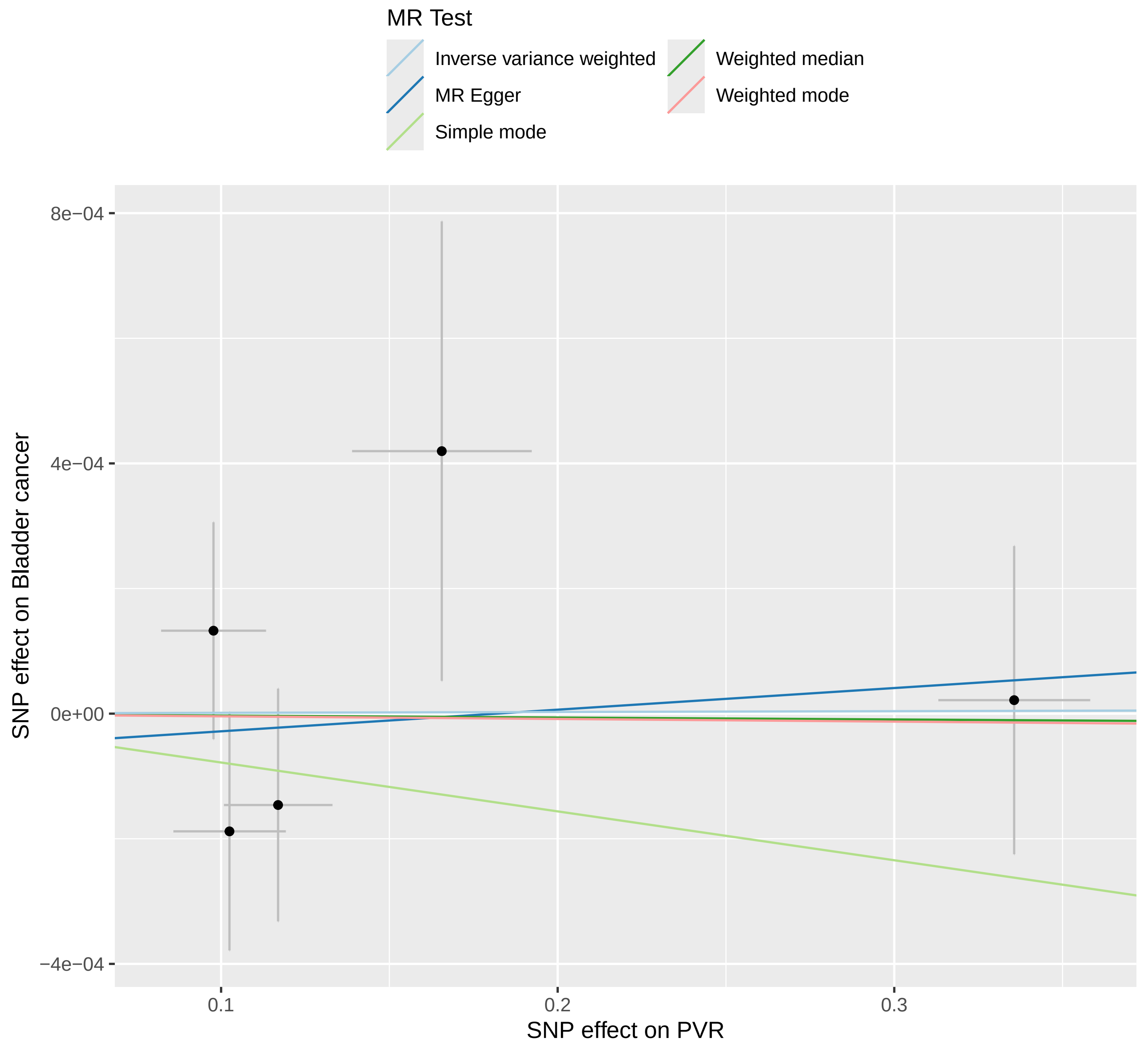
MR Test
Inverse variance weighted
MR Egger
Simple mode
Weighted median
Weighted mode
8e−04
4e−04
0e+00
−4e−04
SNP effect on Bladder cancer
0.1
0.2
0.3
SNP effect on PVR

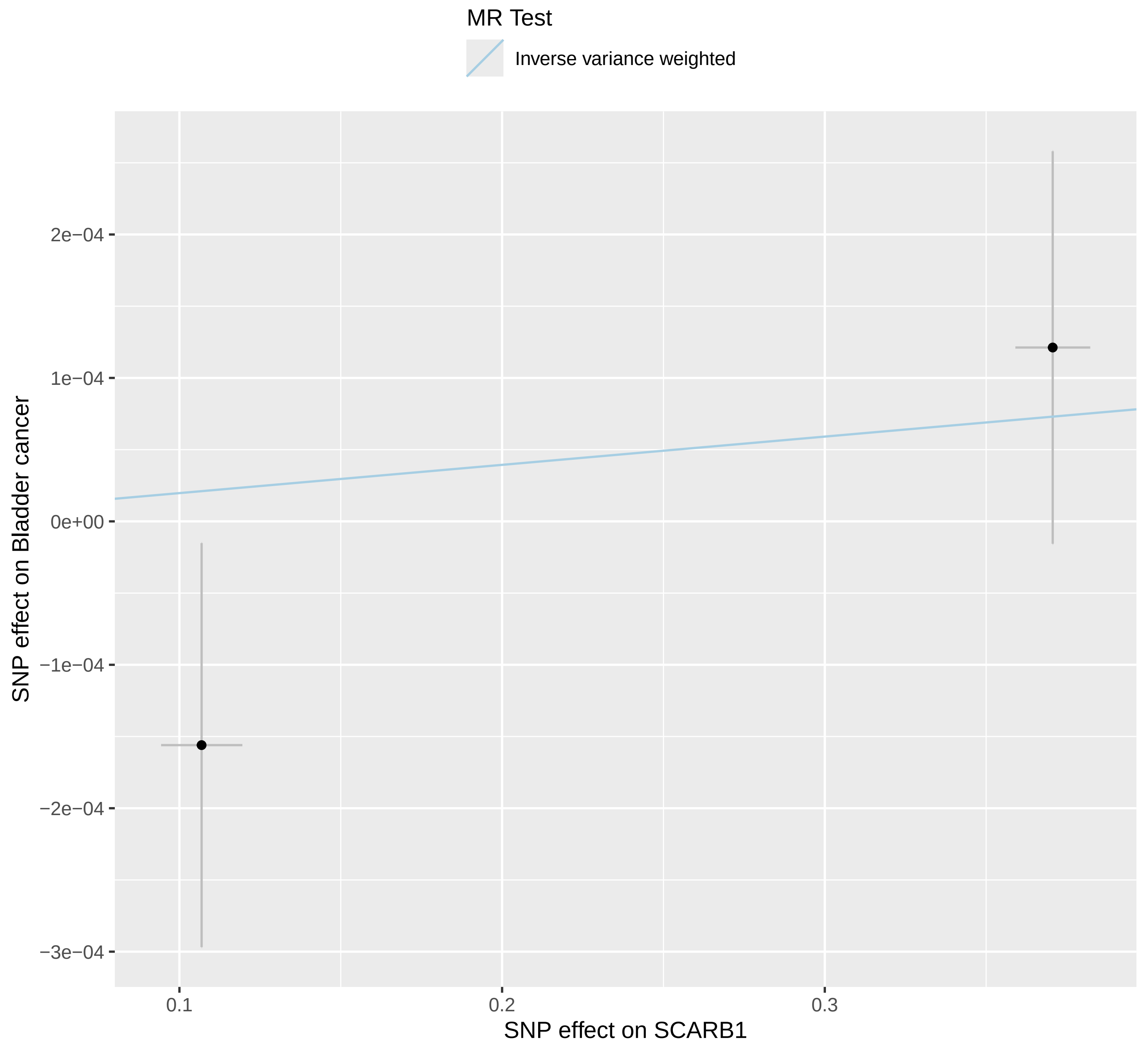

MR Test
Inverse variance weighted
2e−04
1e−04
0e+00
−1e−04
−2e−04
−3e−04
0.1
0.2
0.3
SNP effect on SCARB1
SNP effect on Bladder cancer

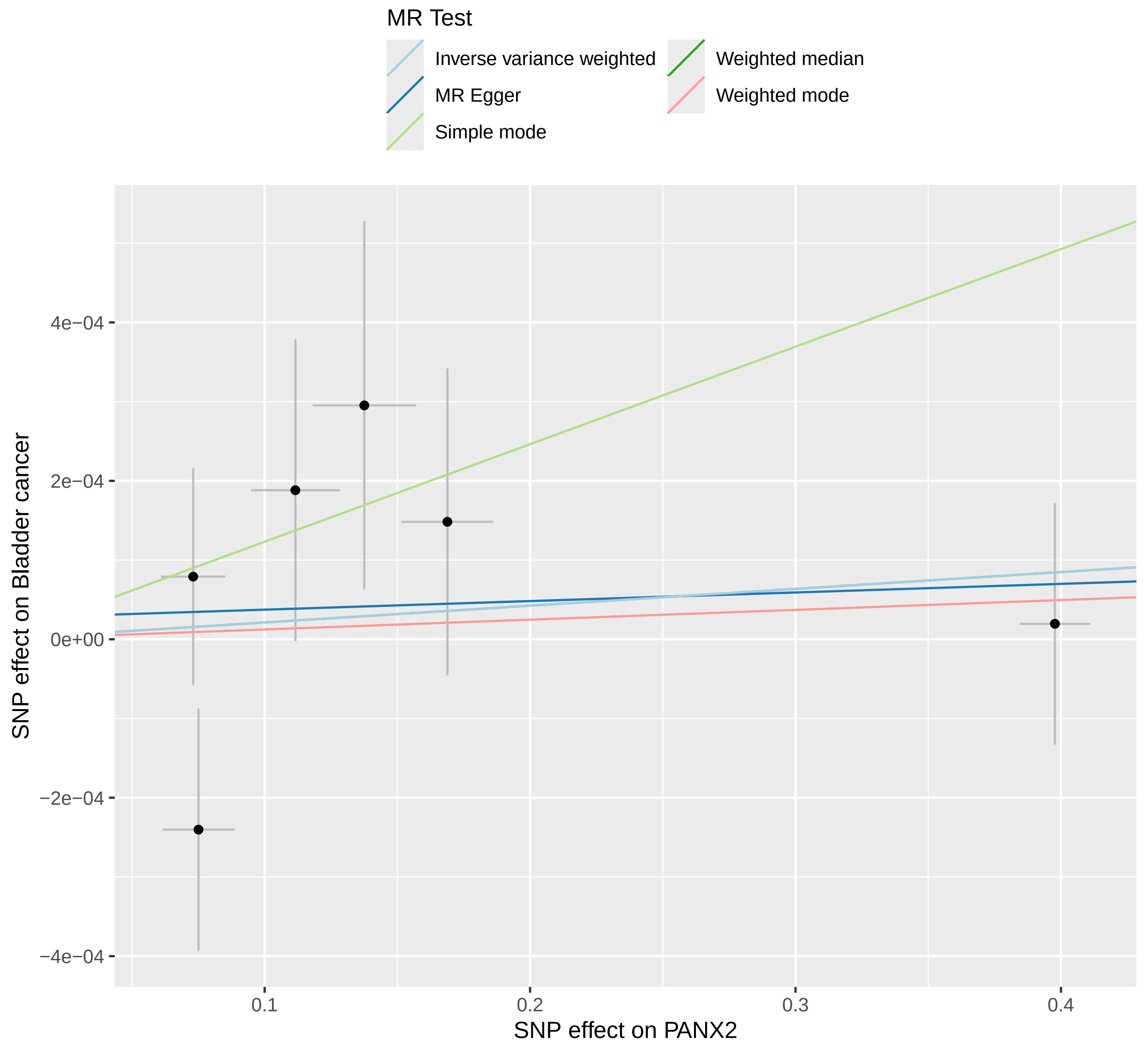
MR Test
Inverse variance weighted
MR Egger
Simple mode
Weighted median
Weighted mode
4e−04
2e−04
0e+00
−2e−04
−4e−04
SNP effect on Bladder cancer
0.1
0.2
0.3
0.4
SNP effect on PANX2

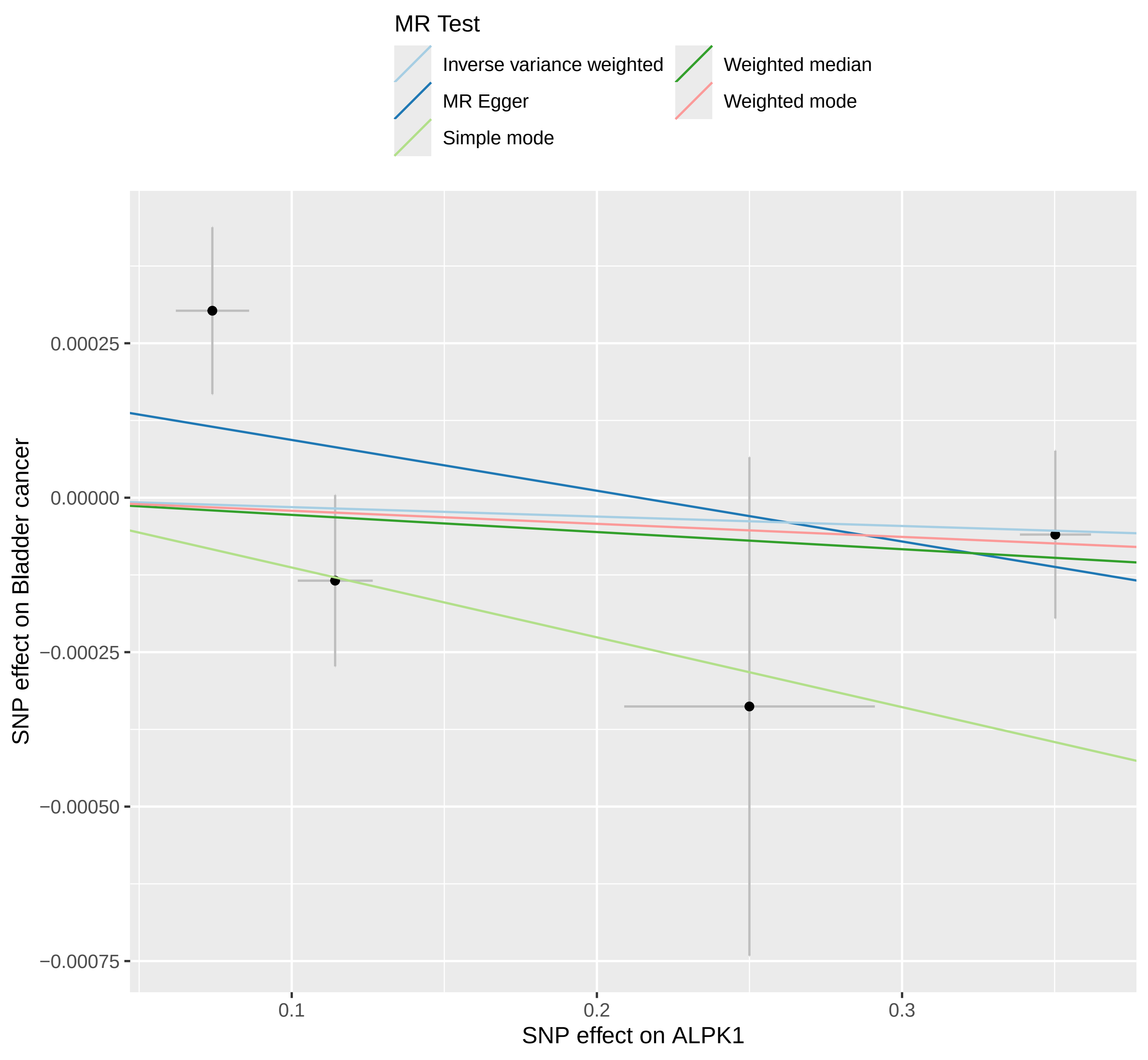
MR Test
Inverse variance weighted
MR Egger
Simple mode
Weighted median
Weighted mode
SNP effect on Bladder cancer
0.00025
0.00000
−0.00025
−0.00050
−0.00075
0.1
0.2
0.3
SNP effect on ALPK1

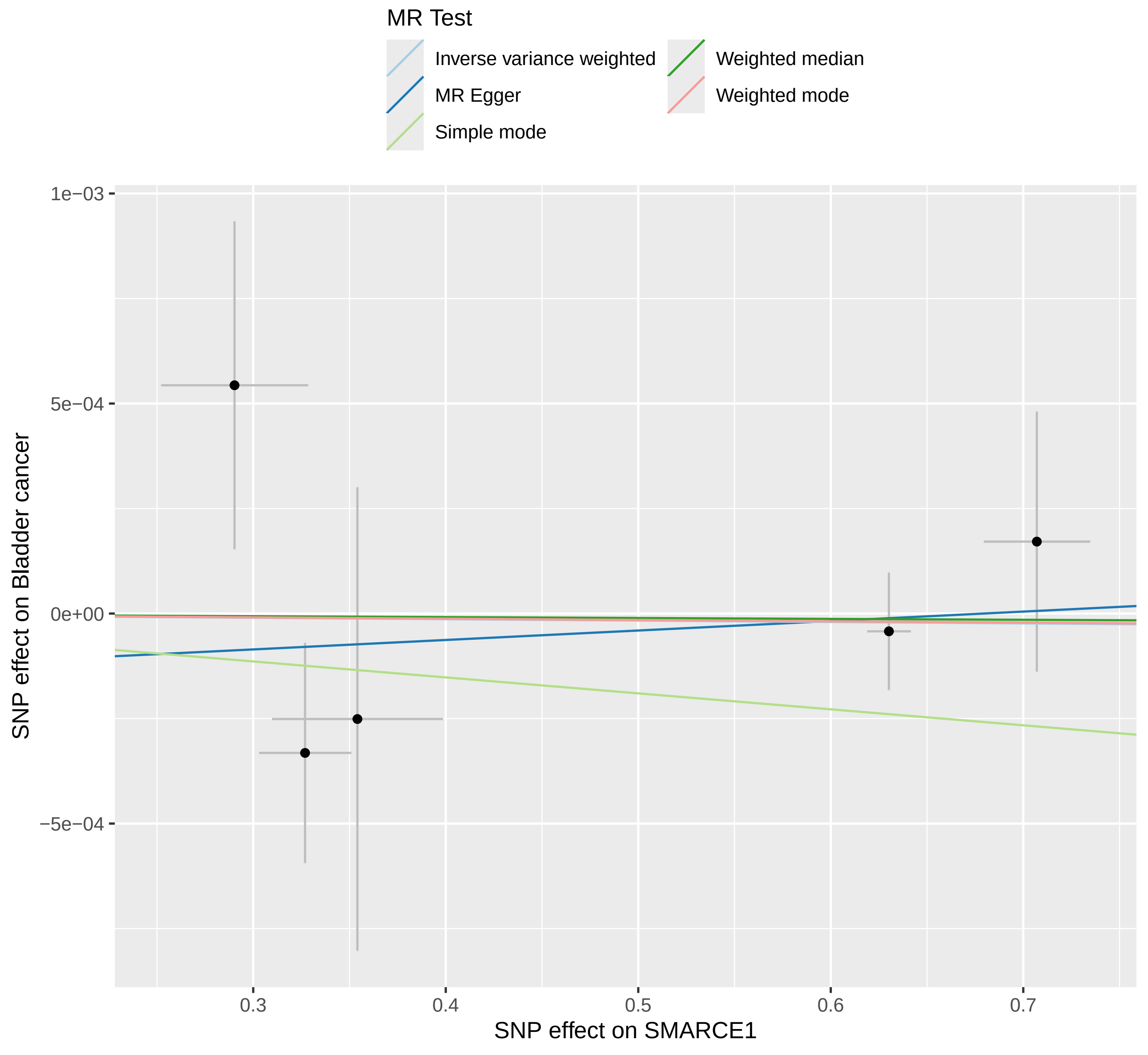
MR Test
Inverse variance weighted
MR Egger
Simple mode
Weighted median
Weighted mode
1e−03
5e−04
0e+00
−5e−04
0.3
0.4
0.5
0.6
0.7
SNP effect on Bladder cancer
SNP effect on SMARCE1

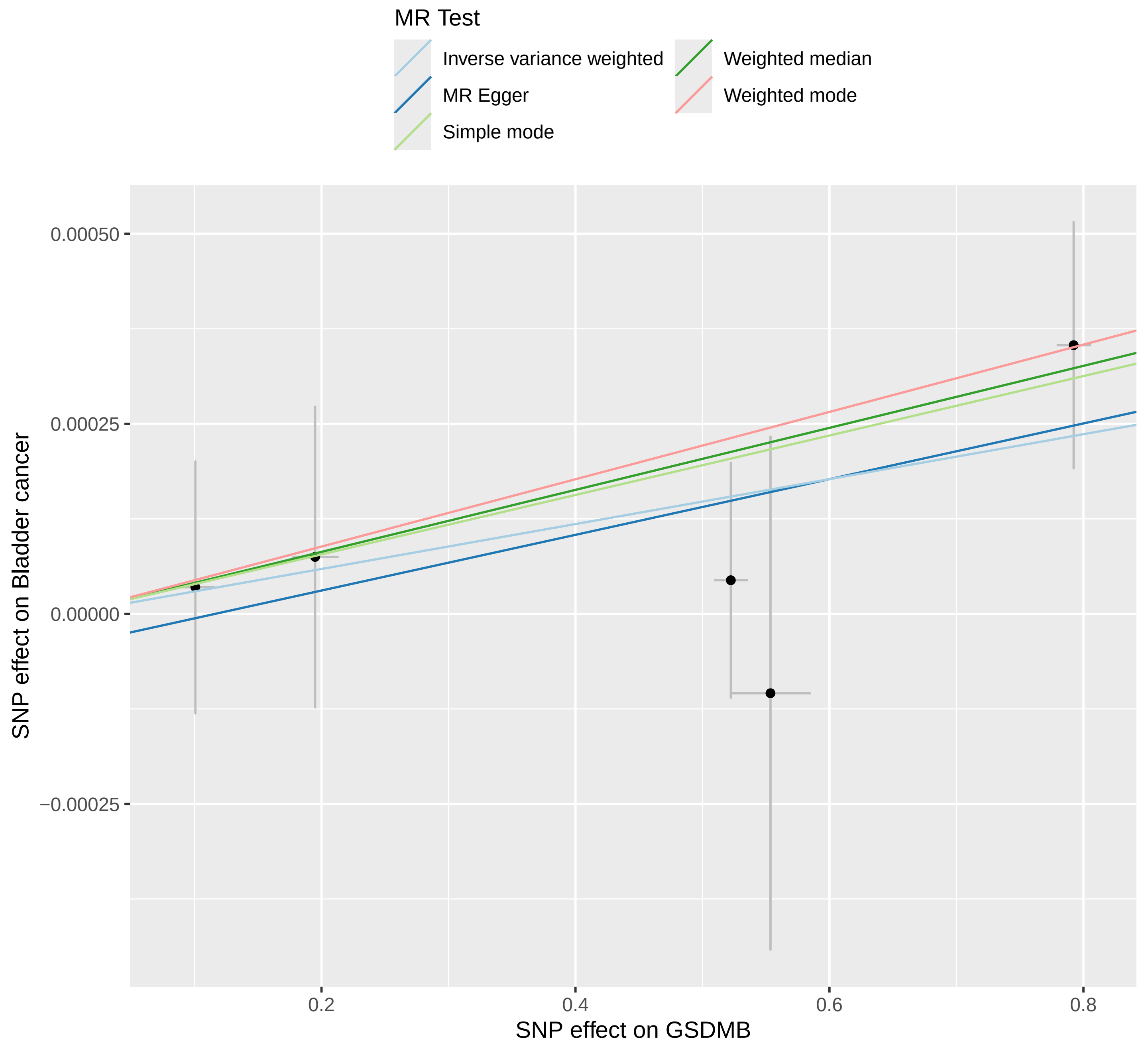
MR Test
Inverse variance weighted
MR Egger
Simple mode
Weighted median
Weighted mode
0.00050
0.00025
0.00000
−0.00025
0.2
0.4
0.6
0.8
SNP effect on Bladder cancer
SNP effect on GSDMB

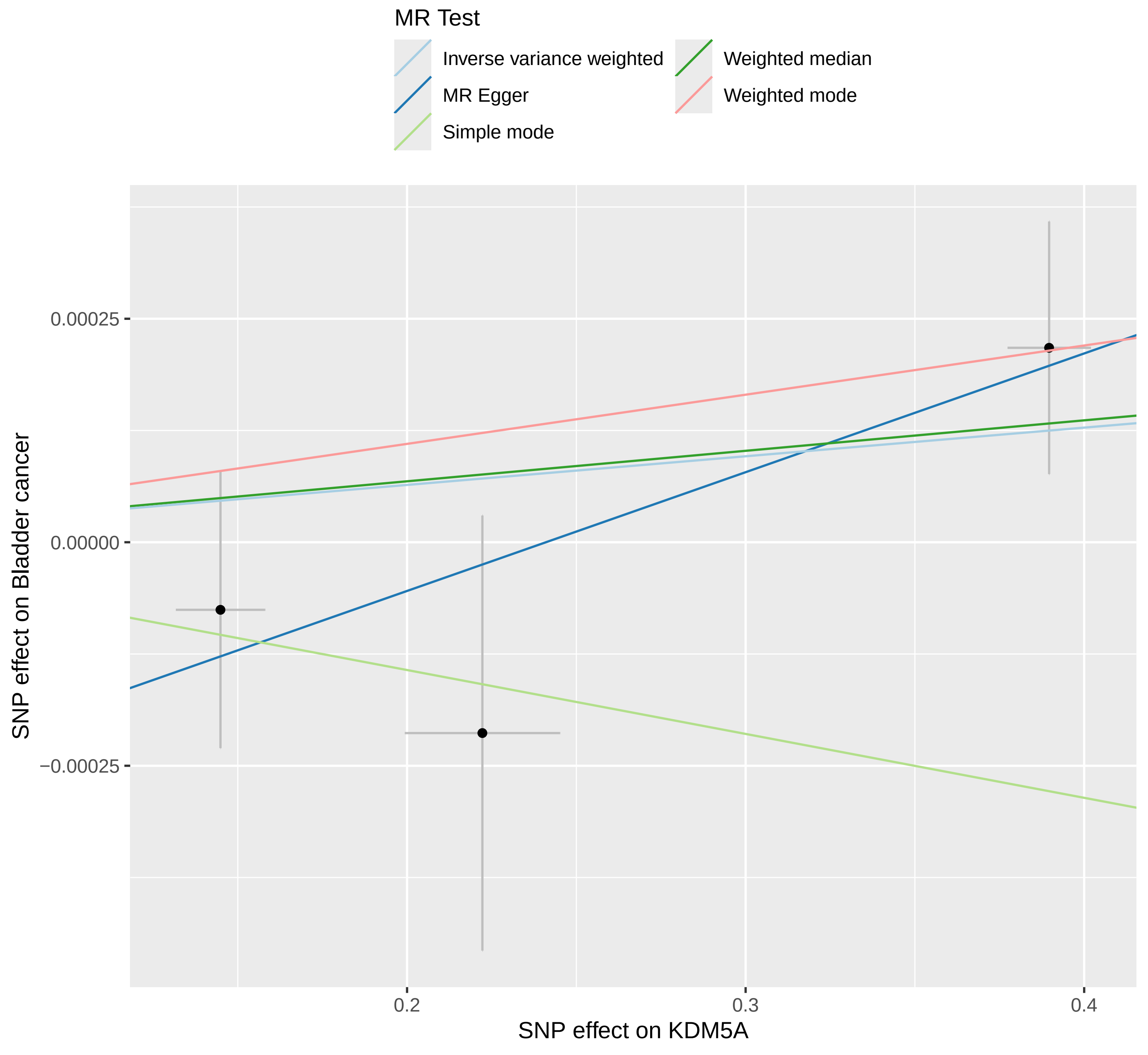
MR Test
Inverse variance weighted
MR Egger
Simple mode
Weighted median
Weighted mode
0.00025
0.00000
−0.00025
0.2
0.3
0.4
SNP effect on KDM5A
SNP effect on Bladder cancer

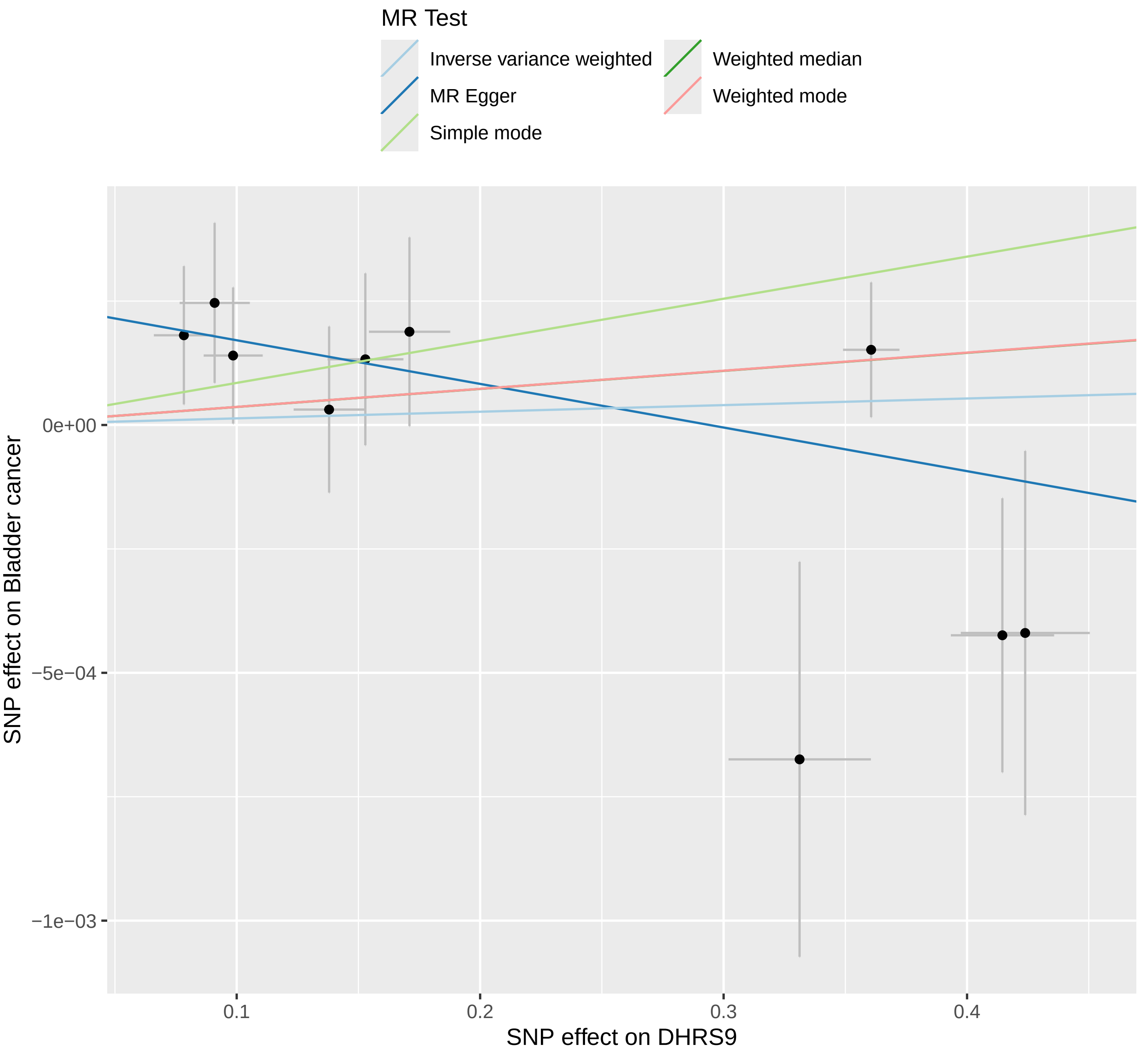
MR Test
Inverse variance weighted
MR Egger
Simple mode
Weighted median
Weighted mode
0e+00
−5e−04
−1e−03
SNP effect on Bladder cancer
0.1
0.2
0.3
0.4
SNP effect on DHRS9

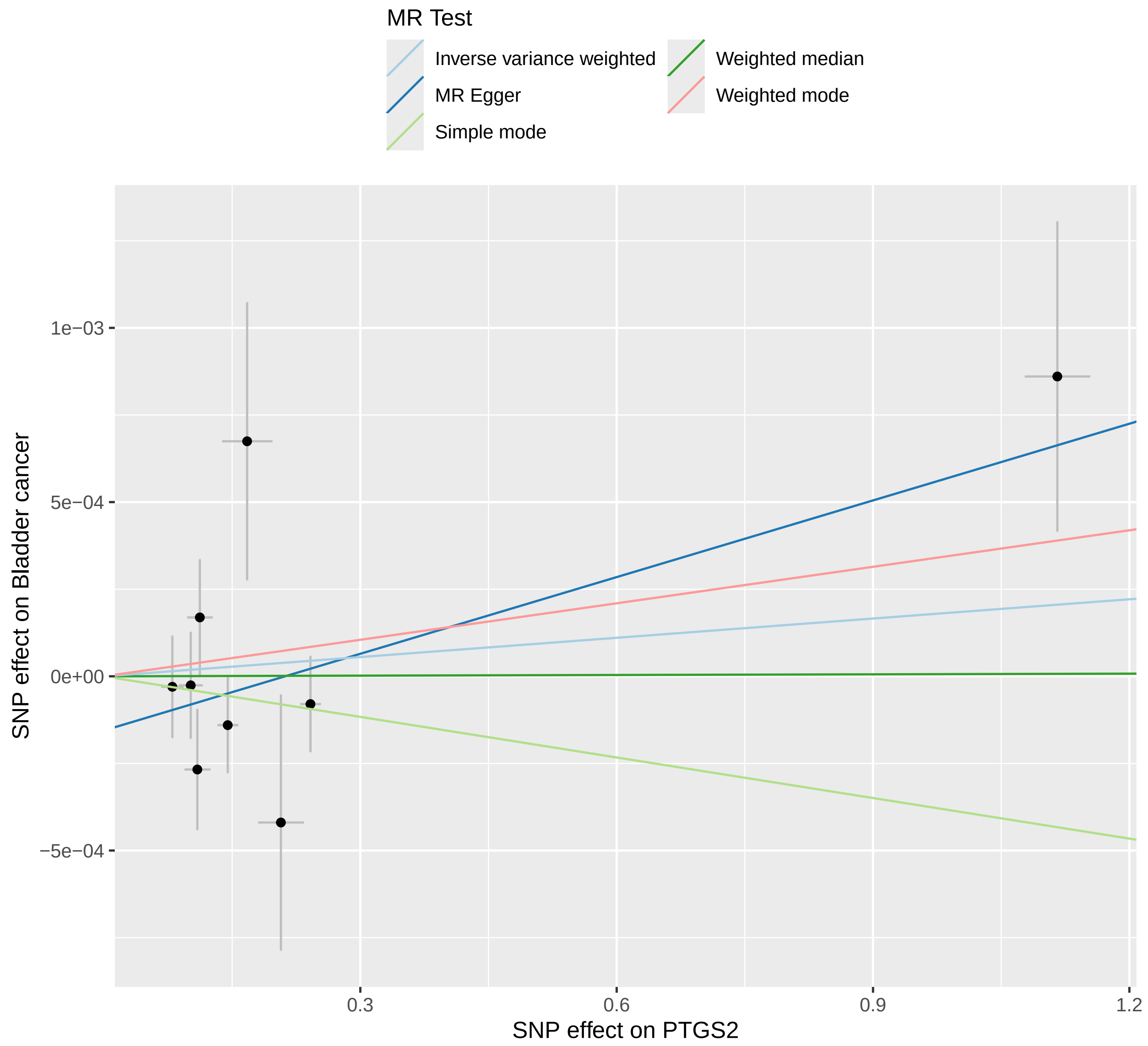
MR Test
Inverse variance weighted
MR Egger
Simple mode
Weighted median
Weighted mode
SNP effect on Bladder cancer
1e−03
5e−04
0e+00
−5e−04
0.3
0.6
0.9
1.2
SNP effect on PTGS2

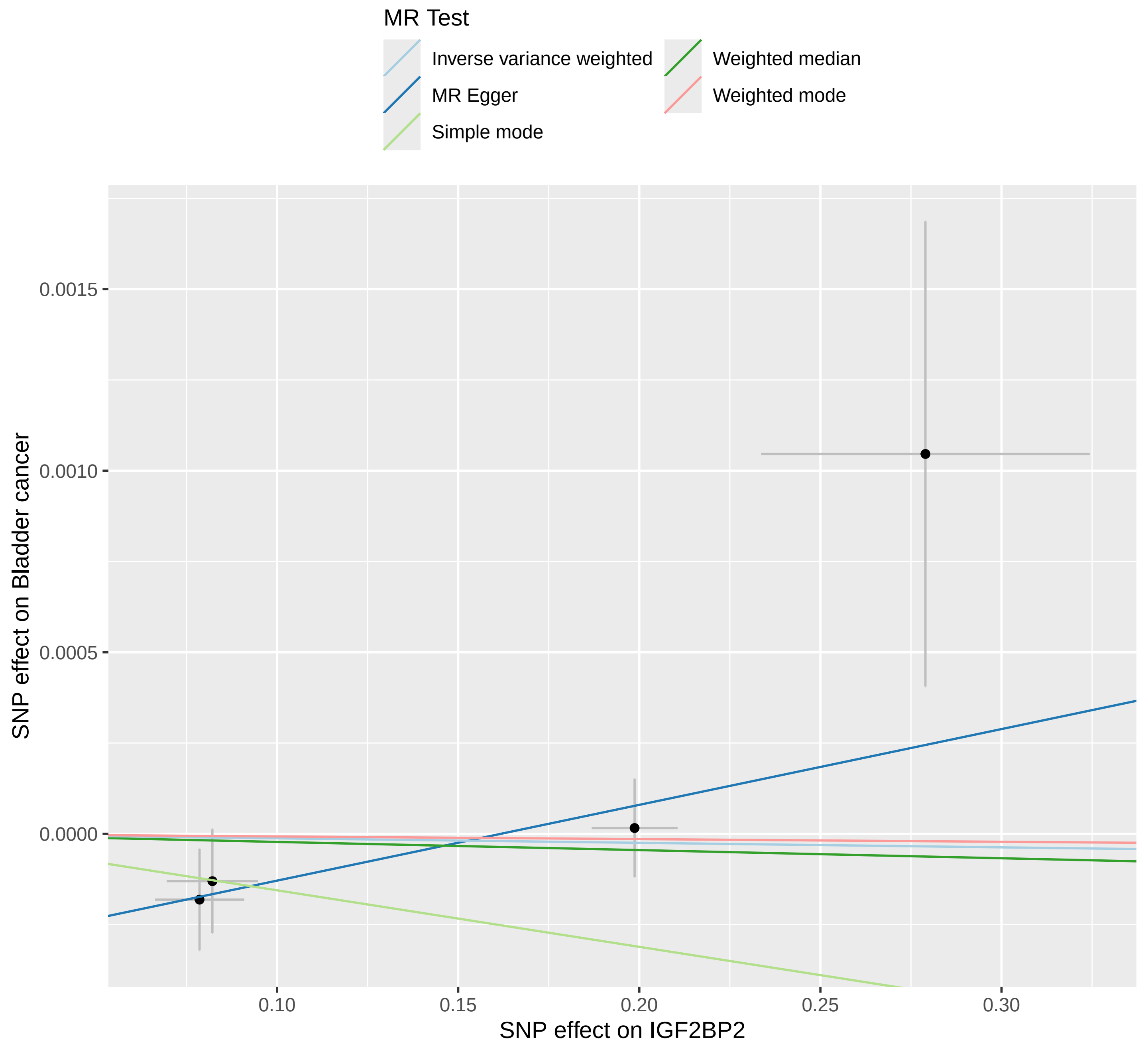
MR Test
Inverse variance weighted
MR Egger
Simple mode
Weighted median
Weighted mode
0.0015
0.0010
0.0005
0.0000
SNP effect on Bladder cancer
0.10
0.15
0.20
0.25
0.30
SNP effect on IGF2BP2

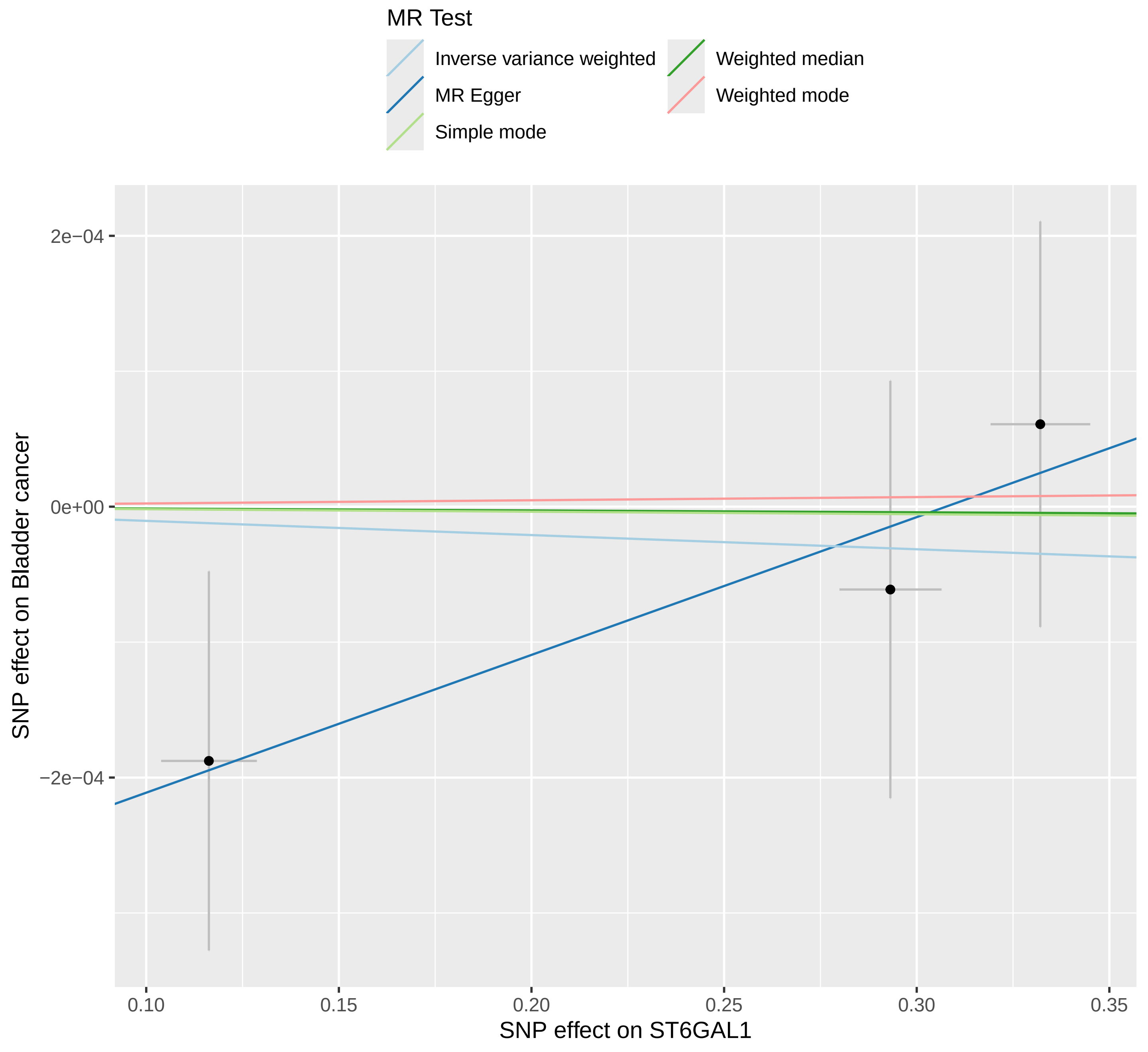
MR Test
Inverse variance weighted
MR Egger
Simple mode
Weighted median
Weighted mode
2e−04
0e+00
−2e−04
0.10
0.15
0.20
0.25
0.30
0.35
SNP effect on ST6GAL1
SNP effect on Bladder cancer

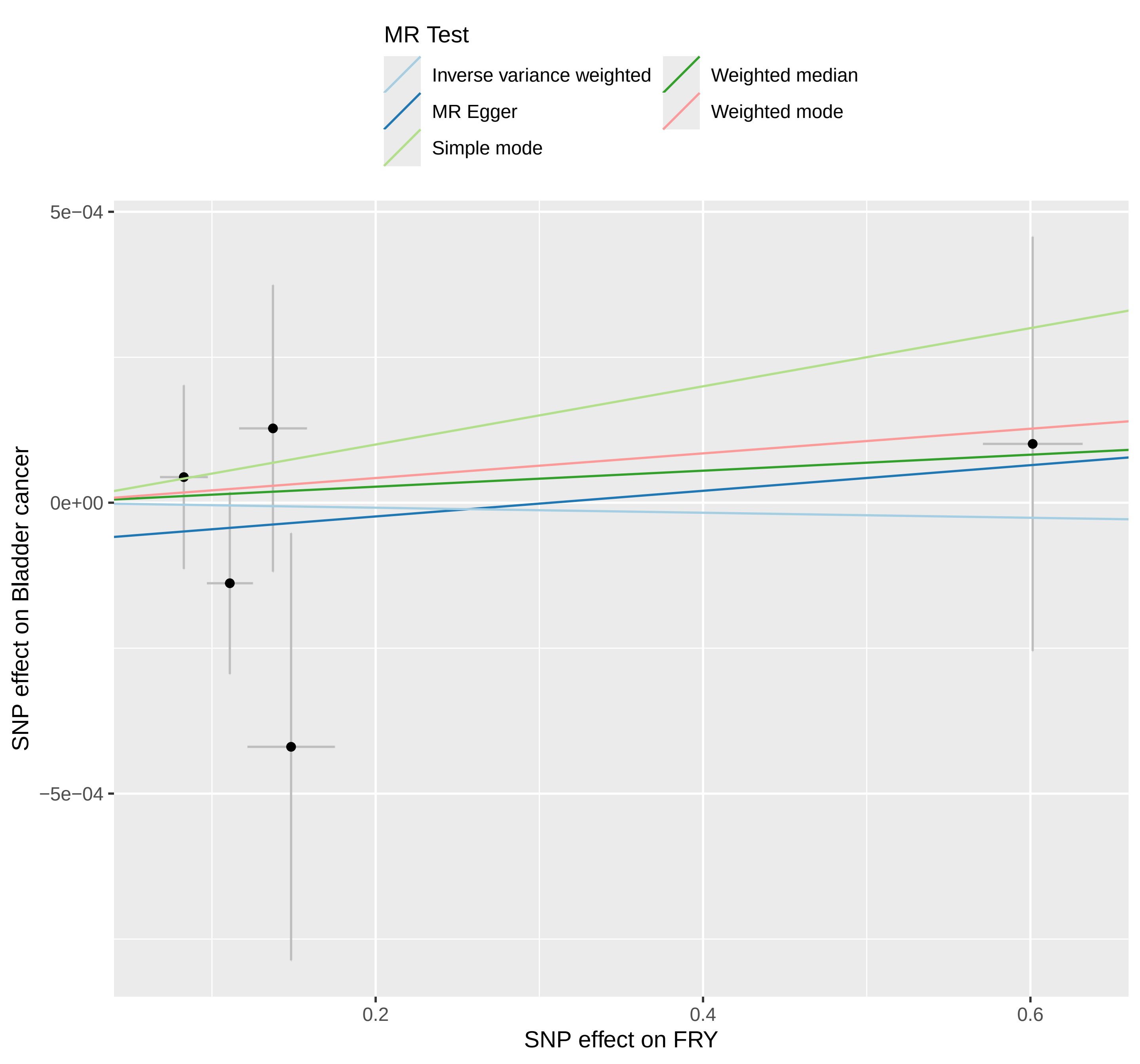
MR Test
Inverse variance weighted
MR Egger
Simple mode
Weighted median
Weighted mode
5e−04
0e+00
−5e−04
0.2
0.4
0.6
SNP effect on FRY
SNP effect on Bladder cancer

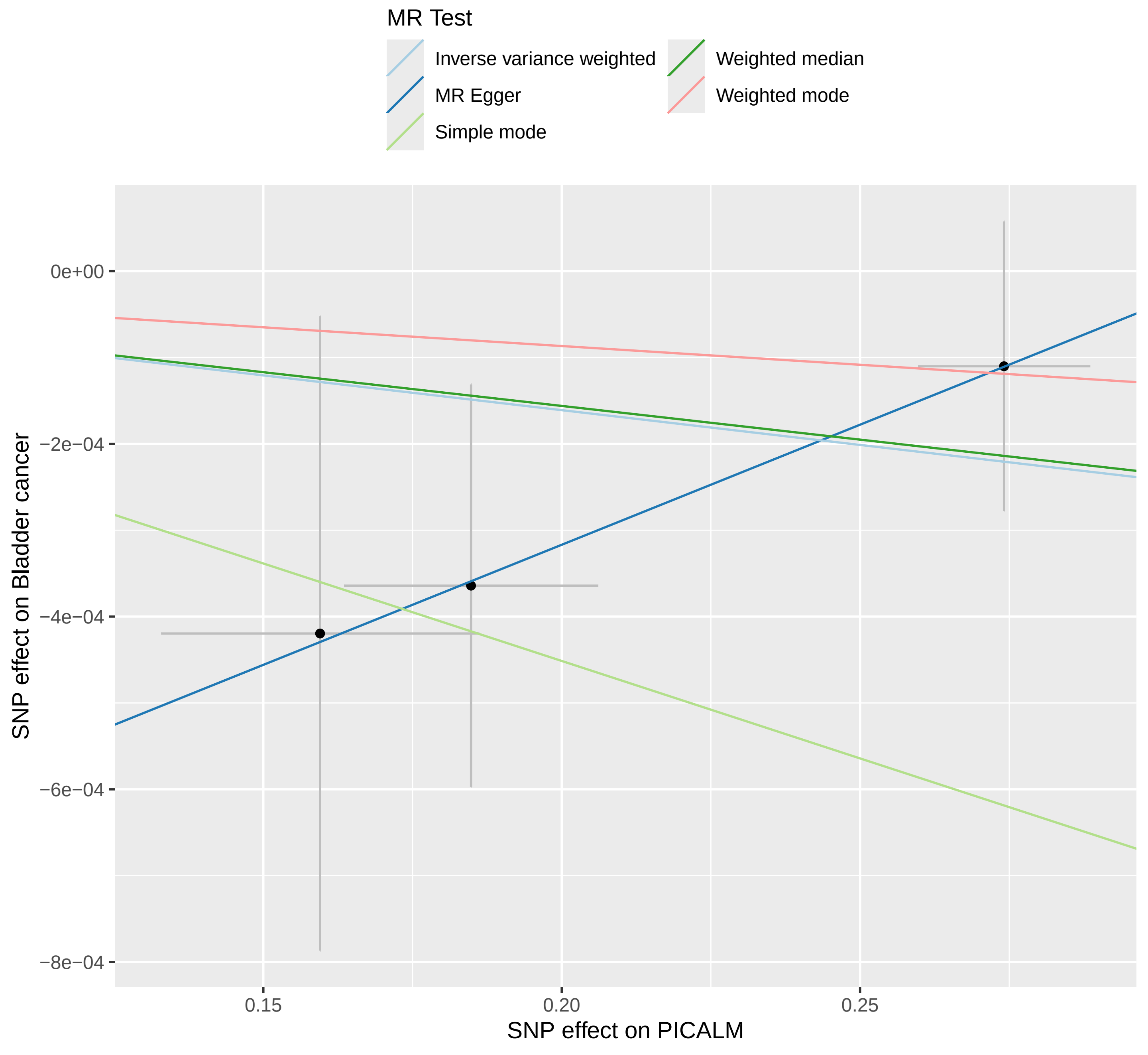

MR Test
Inverse variance weighted
MR Egger
Simple mode
Weighted median
Weighted mode
0e+00
−2e−04
−4e−04
−6e−04
−8e−04
0.15
0.20
0.25
SNP effect on Bladder cancer
SNP effect on PICALM

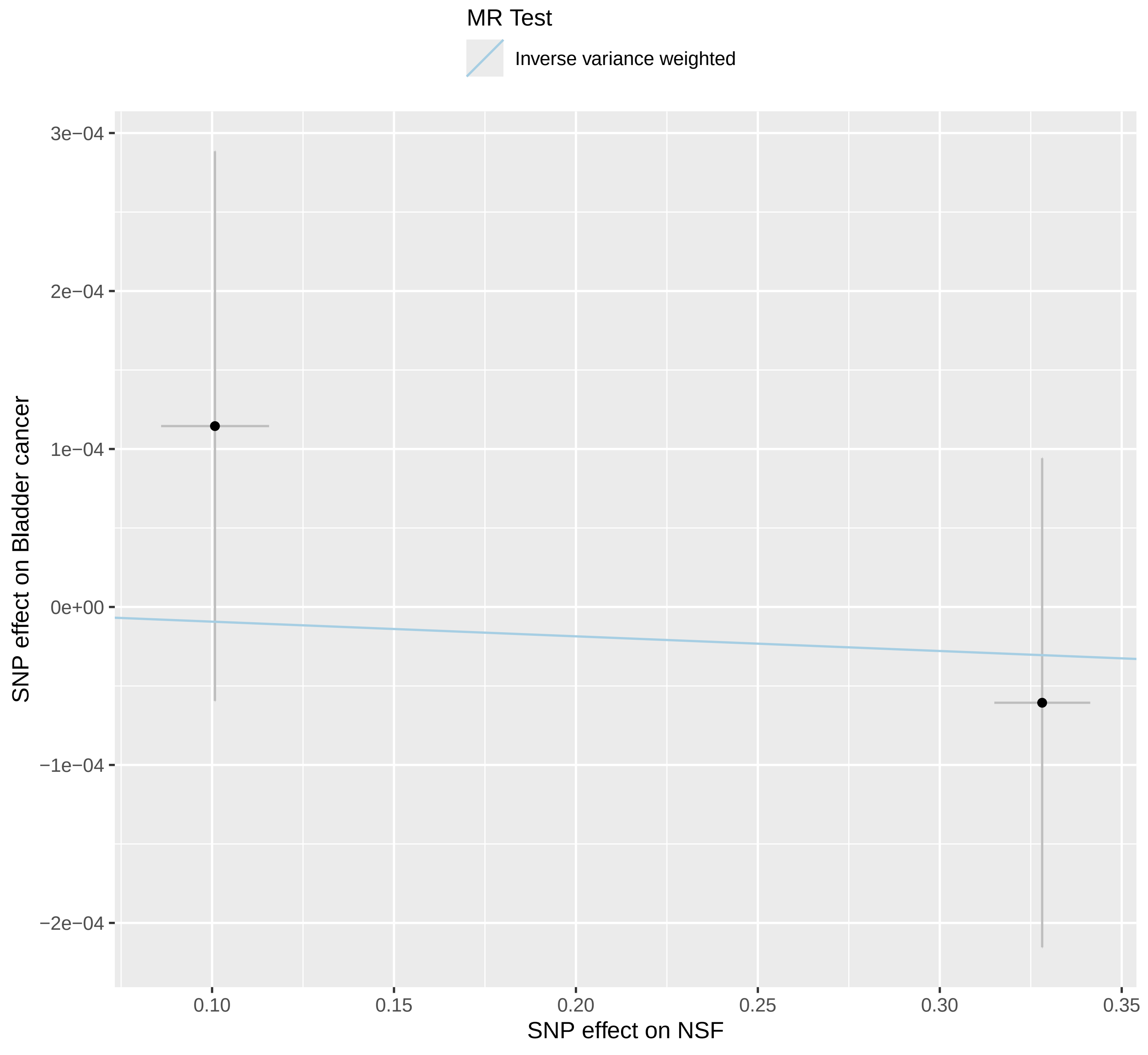

MR Test
Inverse variance weighted
3e−04
2e−04
1e−04
0e+00
−1e−04
−2e−04
0.10
0.15
0.20
0.25
0.30
0.35
SNP effect on NSF
SNP effect on Bladder cancer

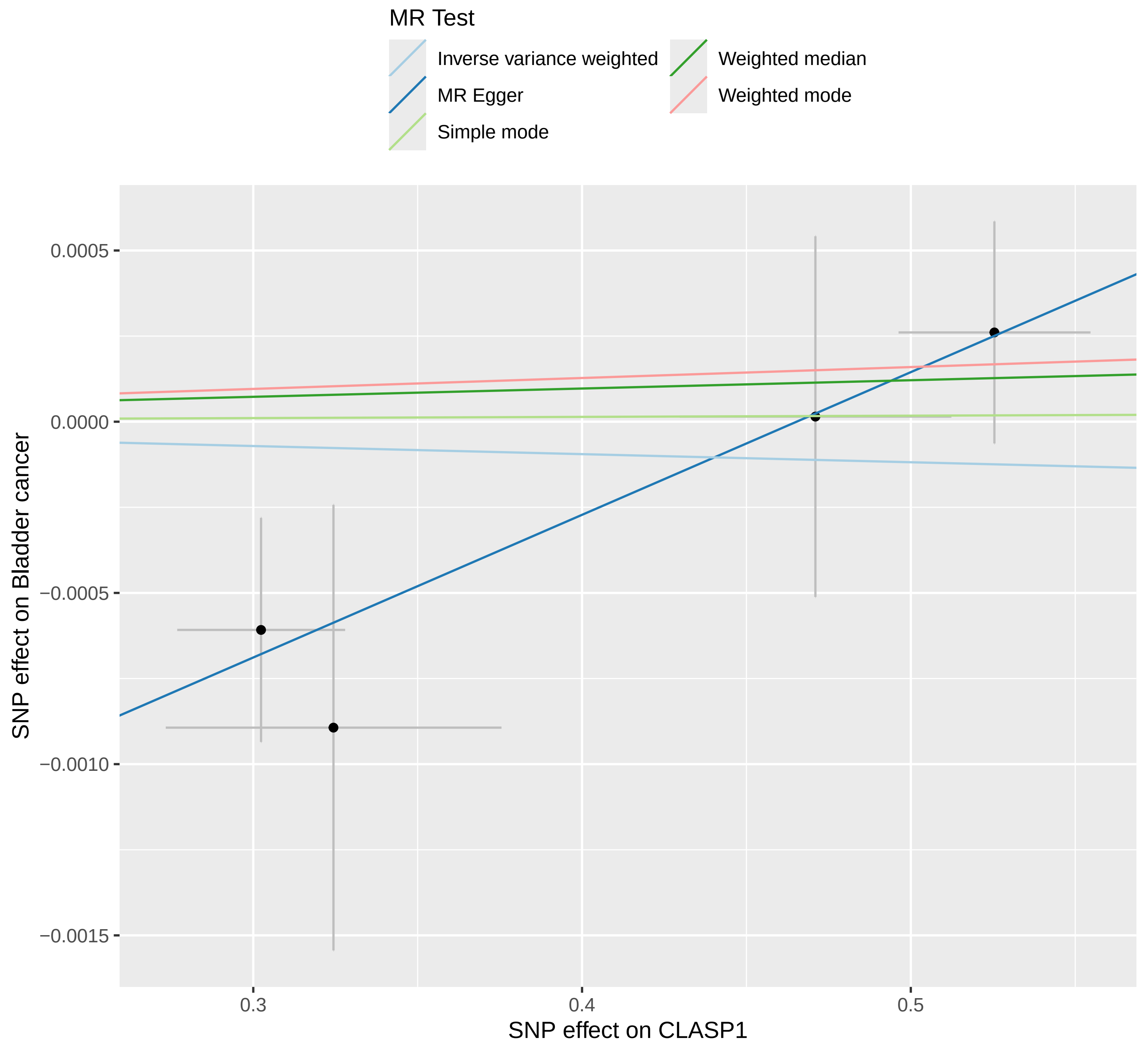

MR Test
Inverse variance weighted
MR Egger
Simple mode
Weighted median
Weighted mode
0.0005
0.0000
−0.0005
−0.0010
−0.0015
0.3
0.4
0.5
SNP effect on CLASP1
SNP effect on Bladder cancer

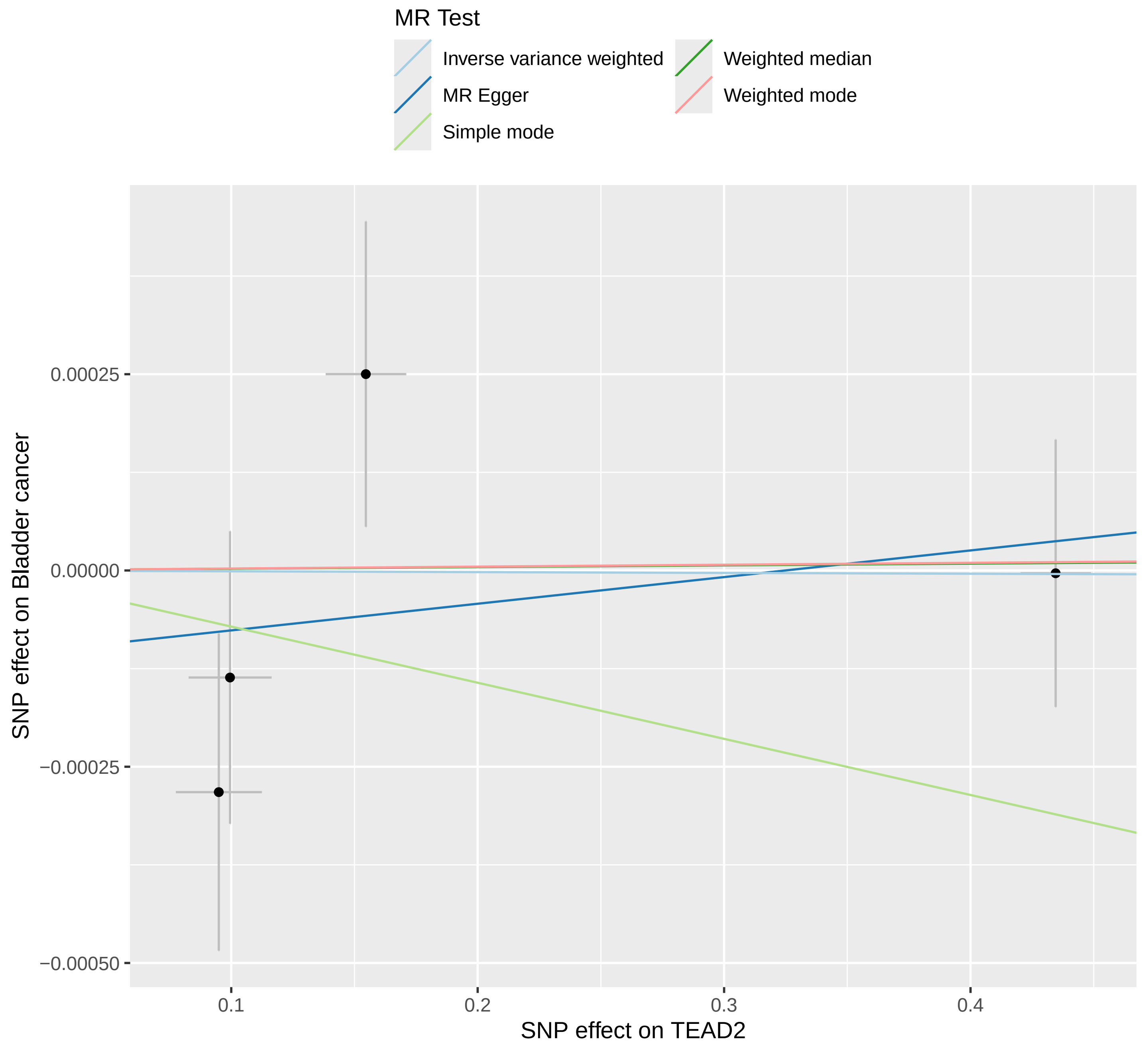
MR Test
Inverse variance weighted
MR Egger
Simple mode
Weighted median
Weighted mode
0.00025
0.00000
−0.00025
−0.00050
0.1
0.2
0.3
0.4
SNP effect on Bladder cancer
SNP effect on TEAD2

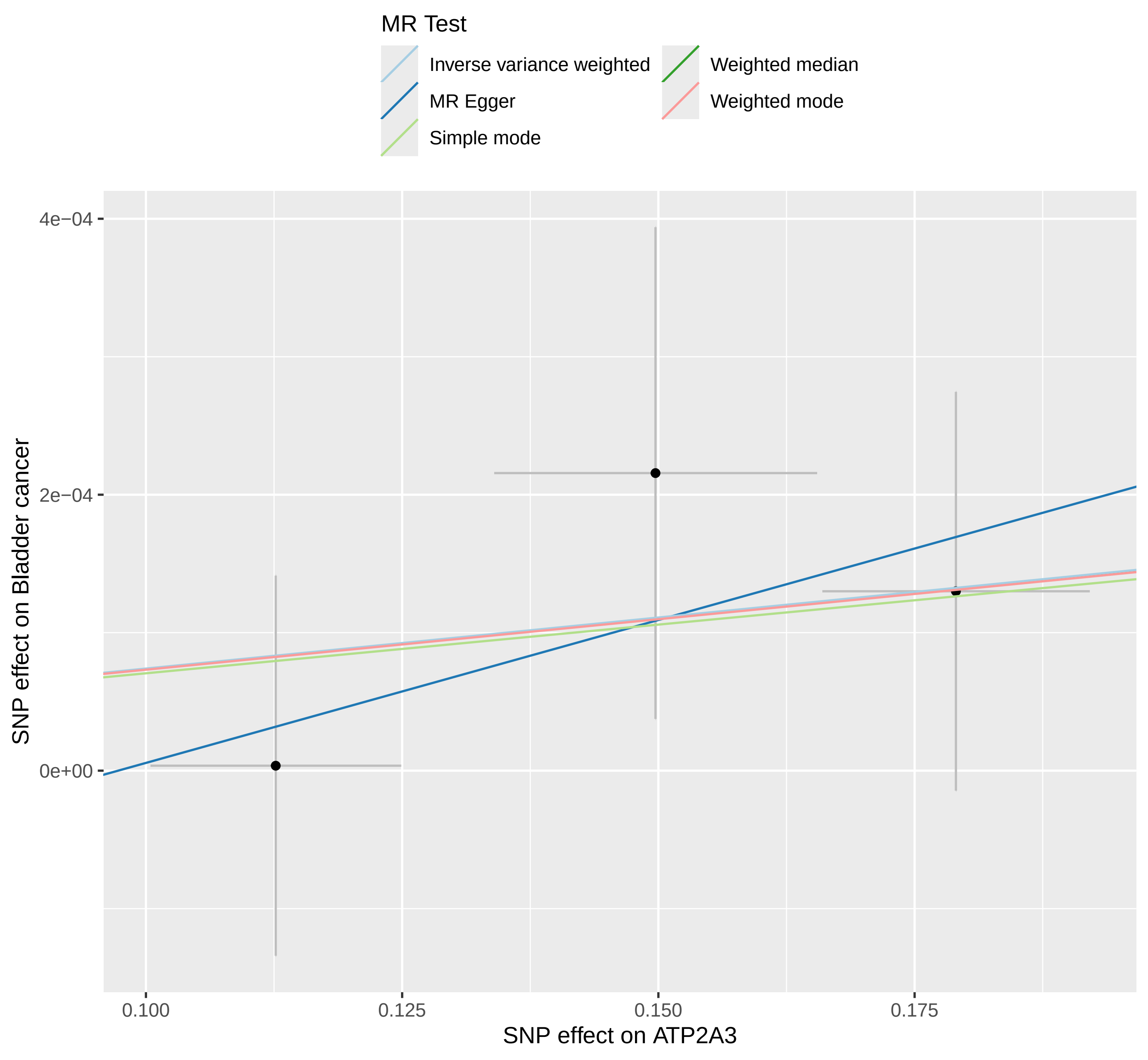

MR Test
Inverse variance weighted
MR Egger
Simple mode
Weighted median
Weighted mode
4e−04
2e−04
0e+00
SNP effect on Bladder cancer
0.100
0.125
0.150
0.175
SNP effect on ATP2A3

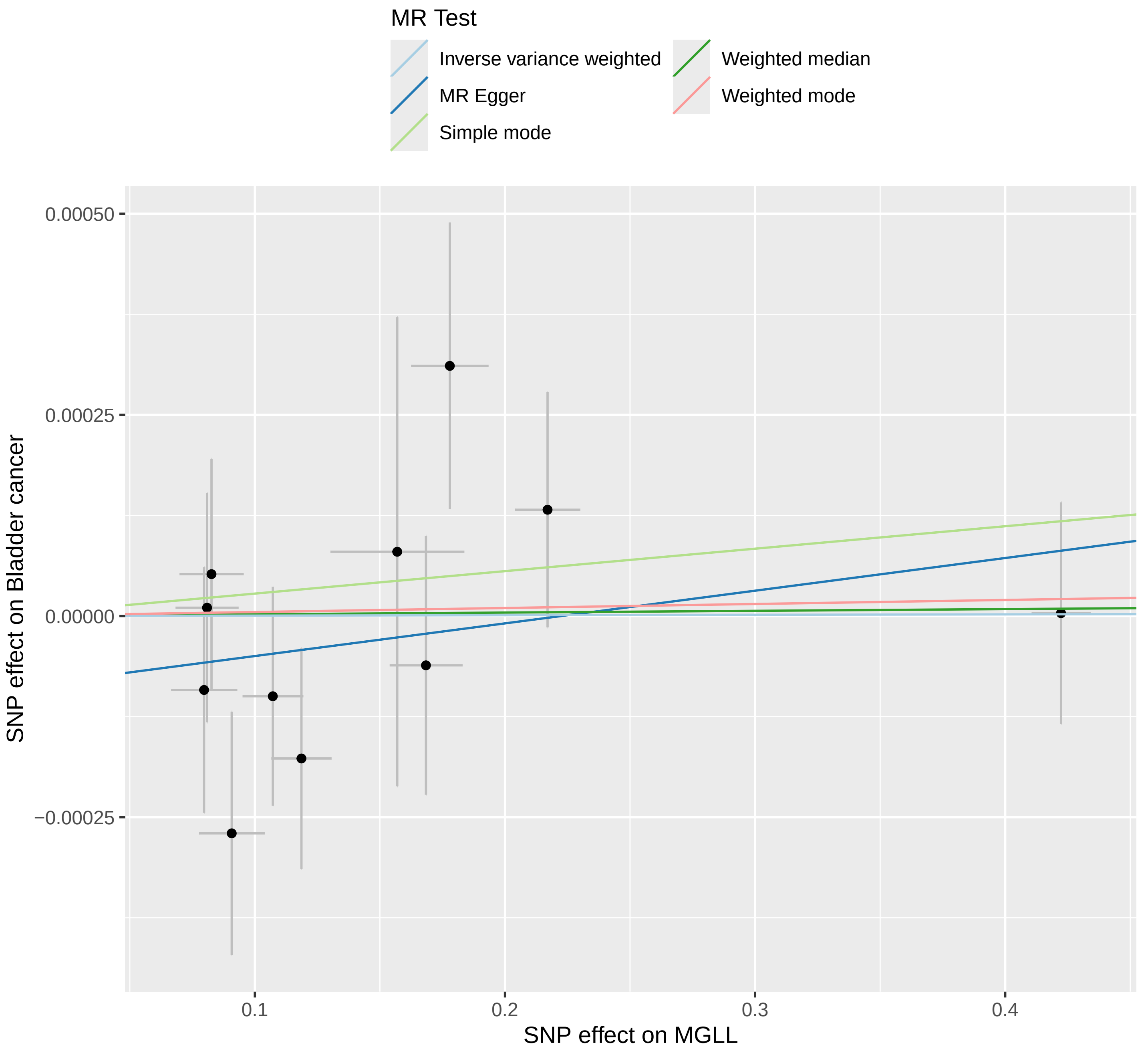

MR Test
Inverse variance weighted
MR Egger
Simple mode
Weighted median
Weighted mode
0.00050
0.00025
0.00000
−0.00025
SNP effect on Bladder cancer
0.1
0.2
0.3
0.4
SNP effect on MGLL

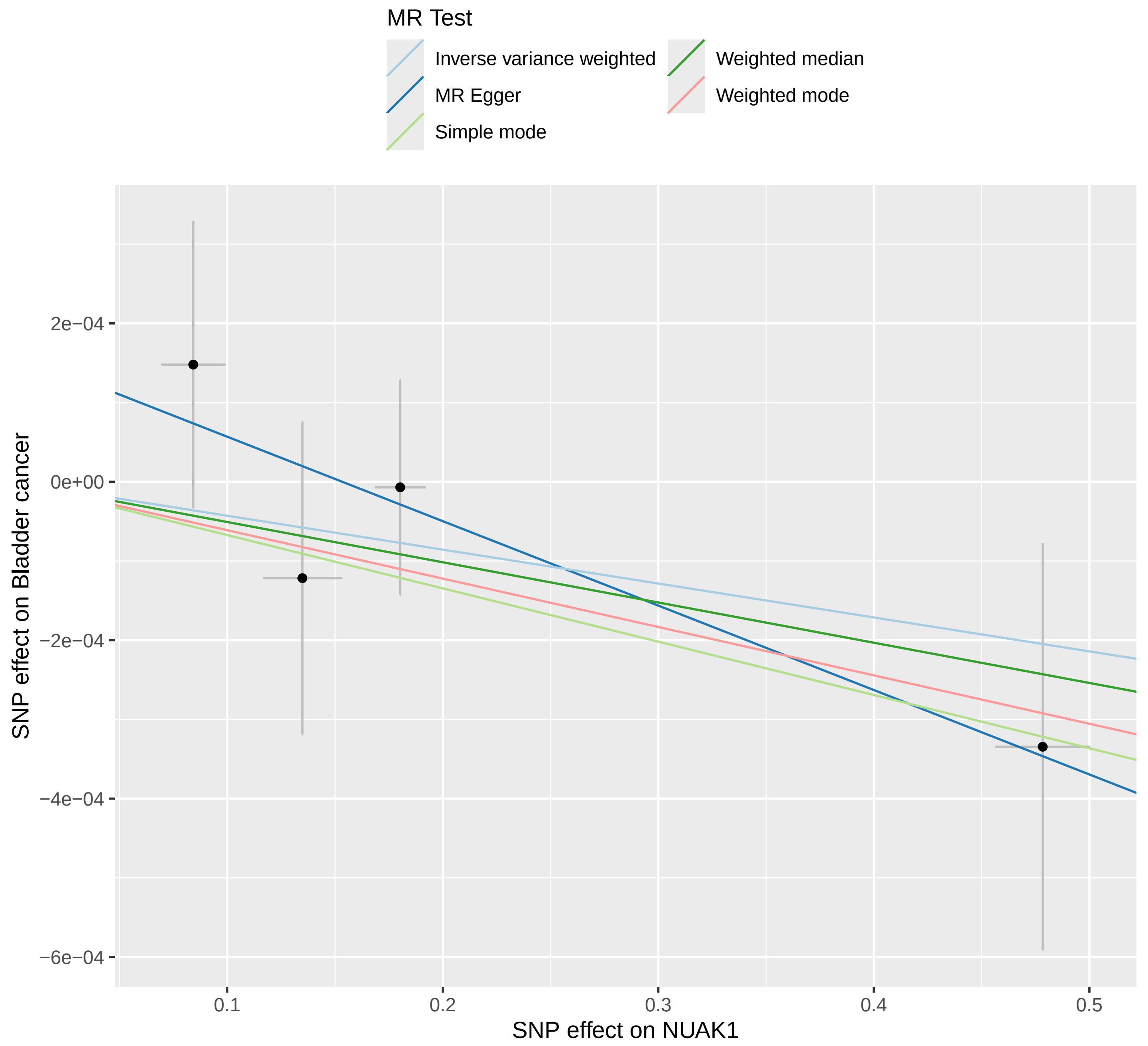
MR Test
Inverse variance weighted
MR Egger
Simple mode
Weighted median
Weighted mode
2e−04
0e+00
−2e−04
−4e−04
−6e−04
0.1
0.2
0.3
0.4
0.5
SNP effect on Bladder cancer
SNP effect on NUAK1

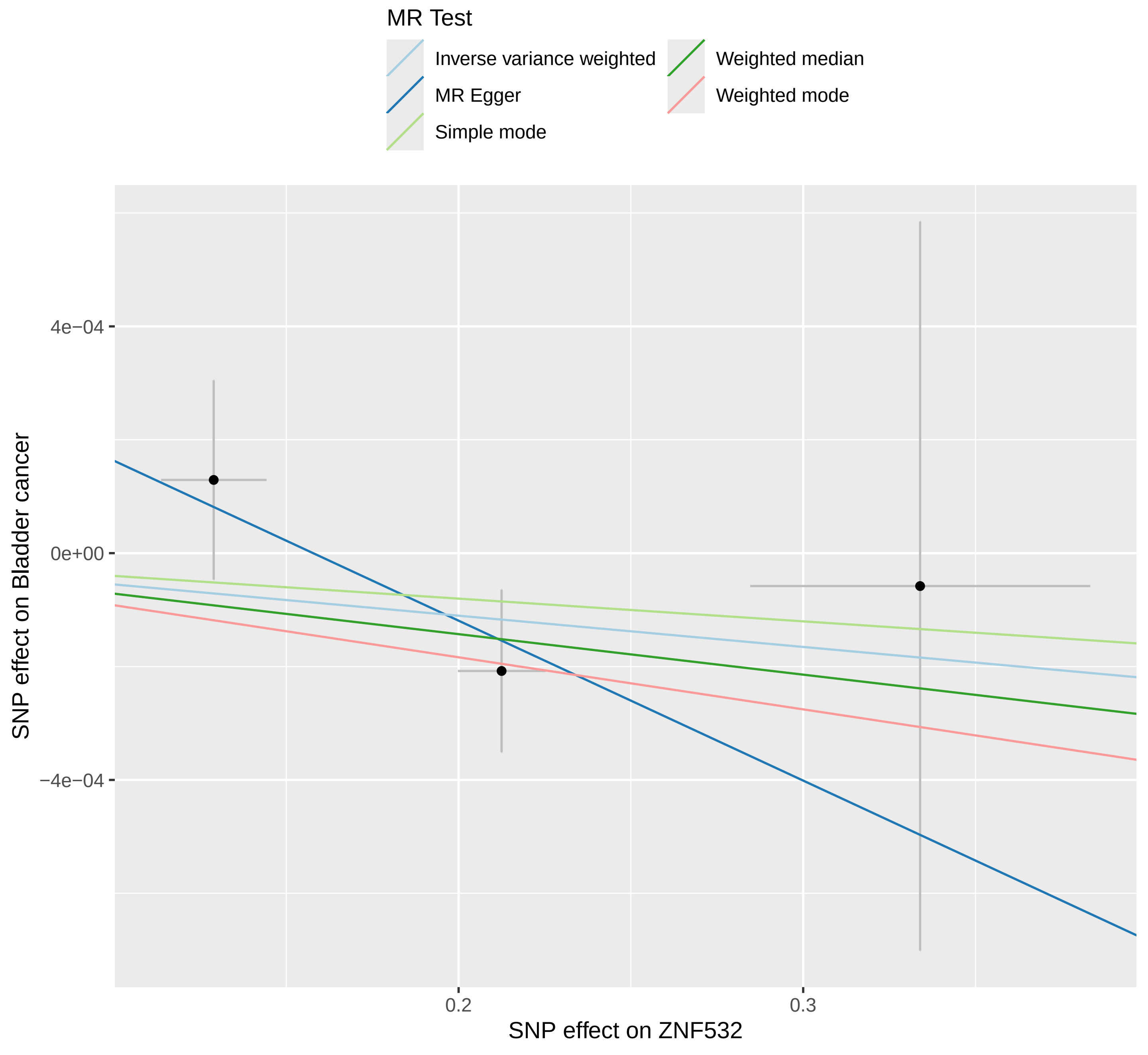
MR Test
Inverse variance weighted
MR Egger
Simple mode
Weighted median
Weighted mode
4e−04
0e+00
−4e−04
SNP effect on Bladder cancer
0.2
0.3
SNP effect on ZNF532

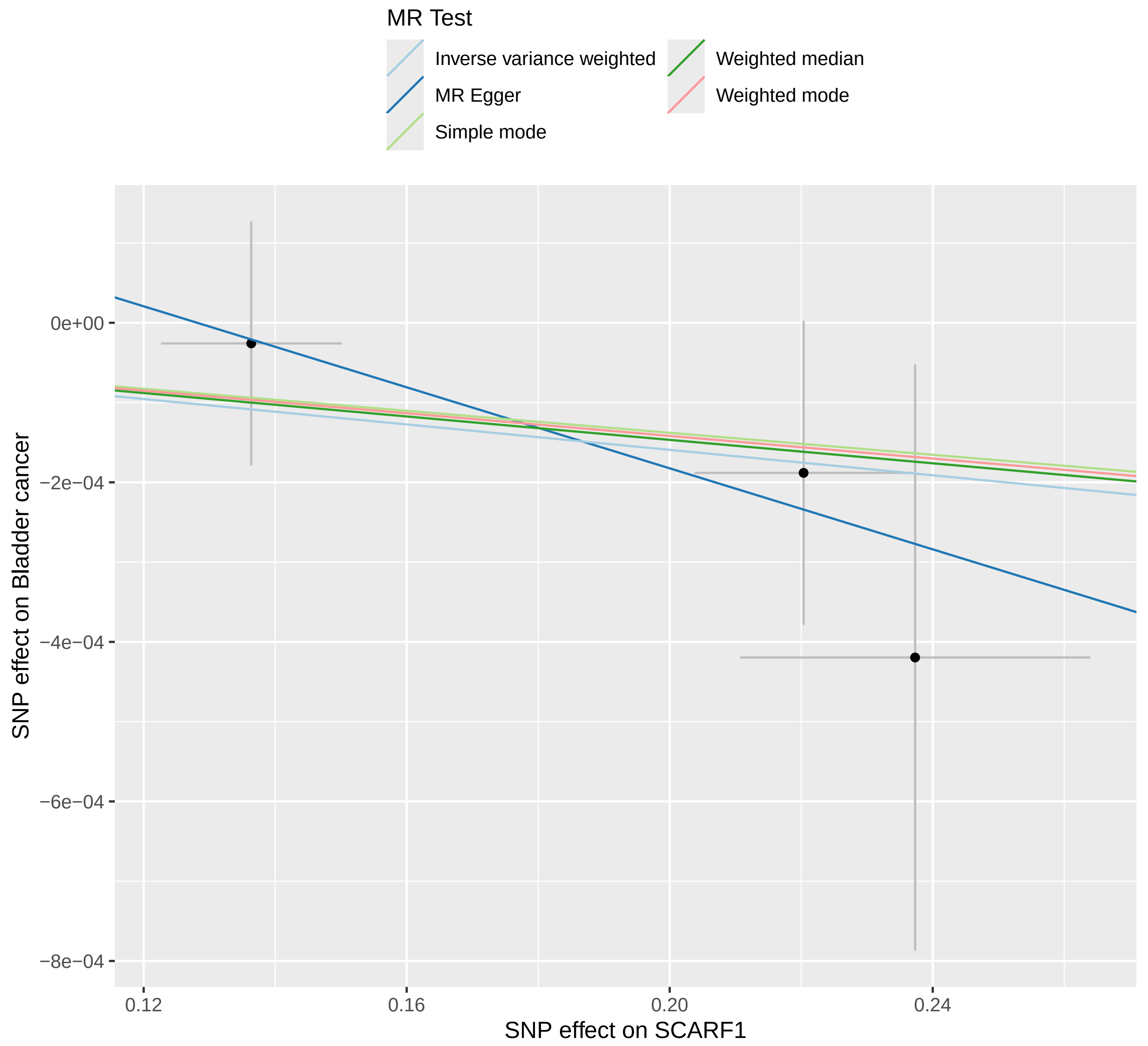

MR Test
Inverse variance weighted
MR Egger
Simple mode
Weighted median
Weighted mode
0e+00
−2e−04
−4e−04
−6e−04
−8e−04
0.12
0.16
0.20
0.24
SNP effect on Bladder cancer
SNP effect on SCARF1

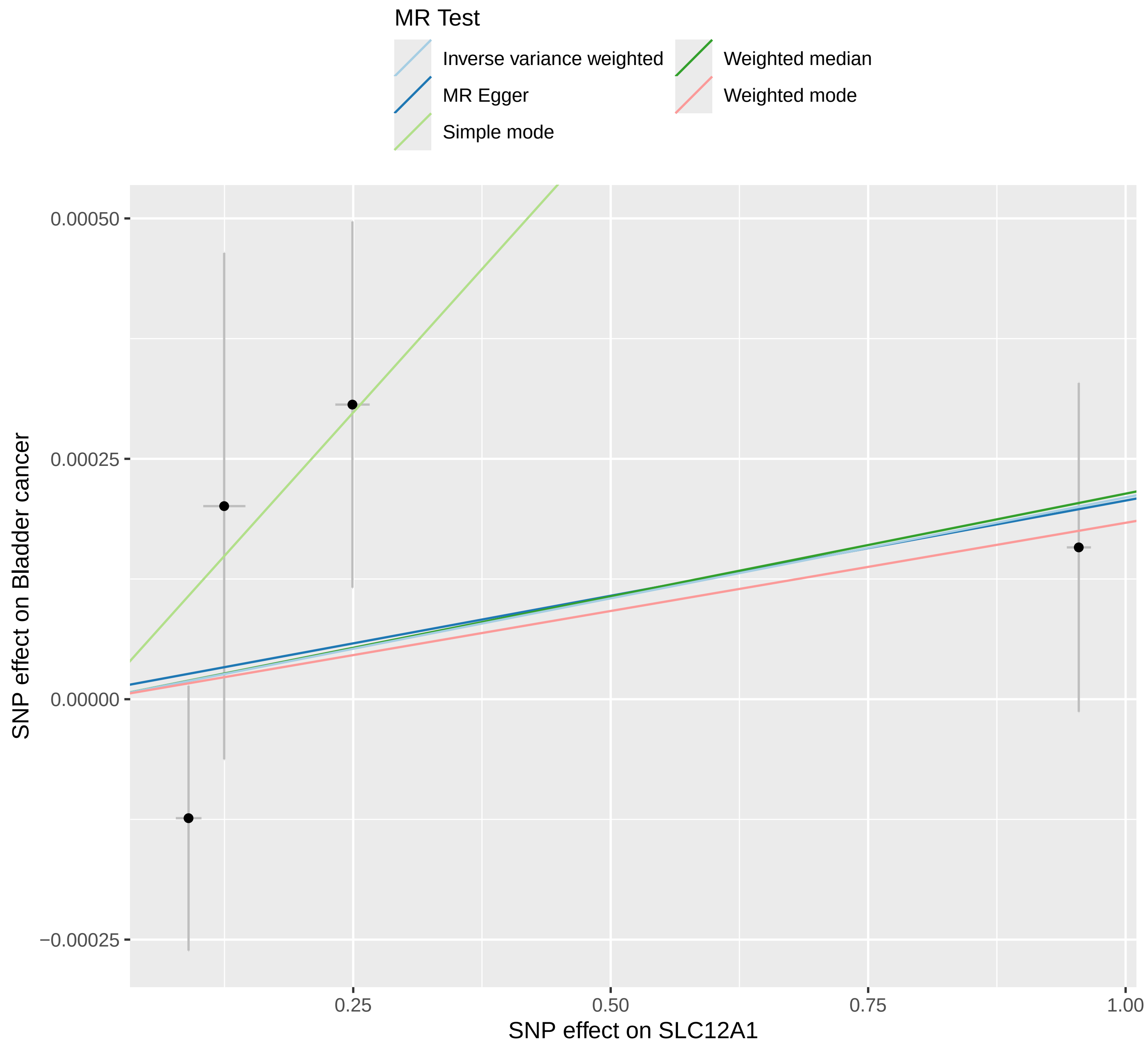
MR Test
Inverse variance weighted
MR Egger
Simple mode
Weighted median
Weighted mode
0.00050
0.00025
0.00000
−0.00025
SNP effect on Bladder cancer
0.25
0.50
0.75
1.00
SNP effect on SLC12A1

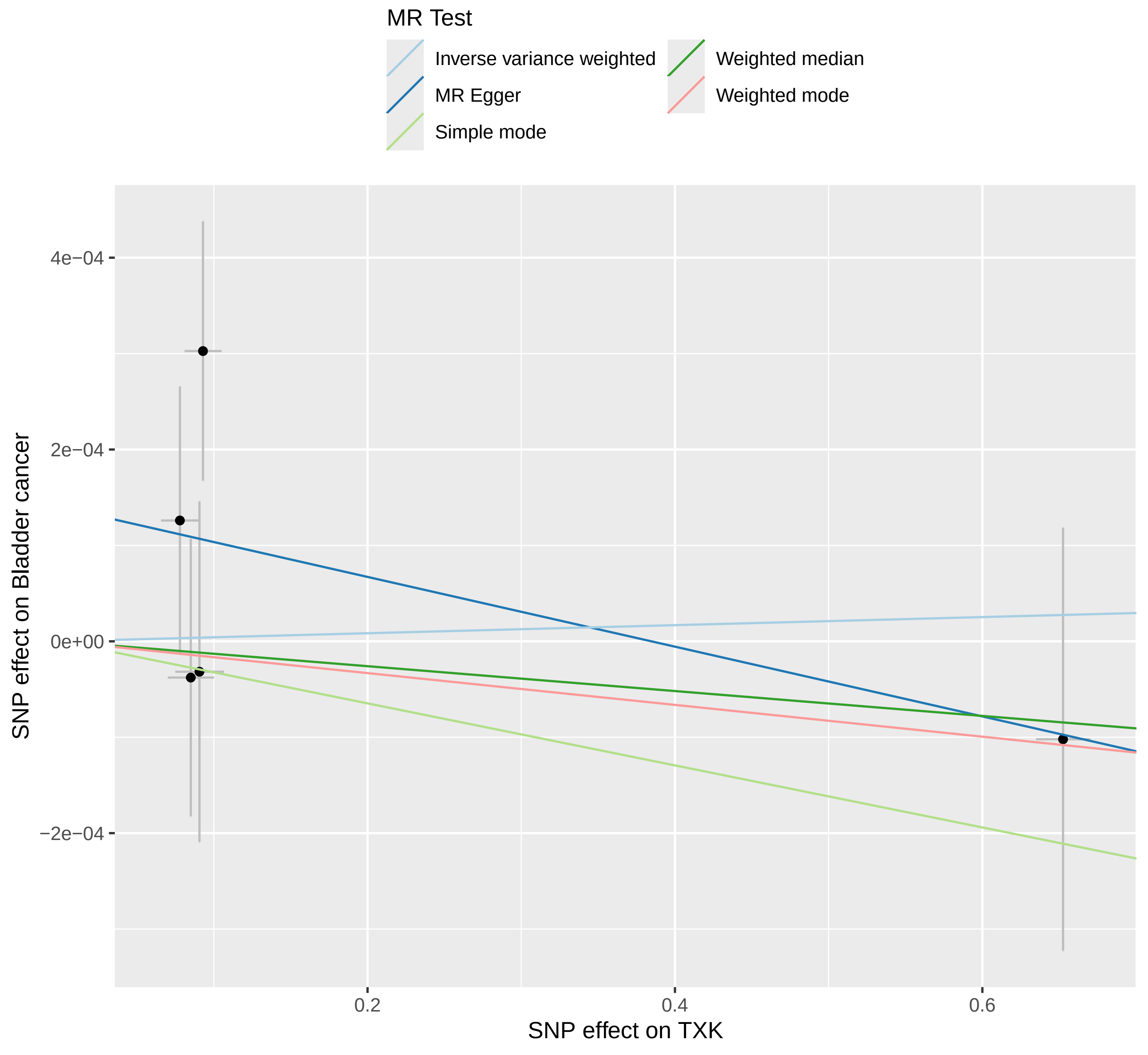
MR Test
Inverse variance weighted
MR Egger
Simple mode
Weighted median
Weighted mode
4e−04
2e−04
0e+00
−2e−04
SNP effect on Bladder cancer
0.2
0.4
0.6
SNP effect on TXK

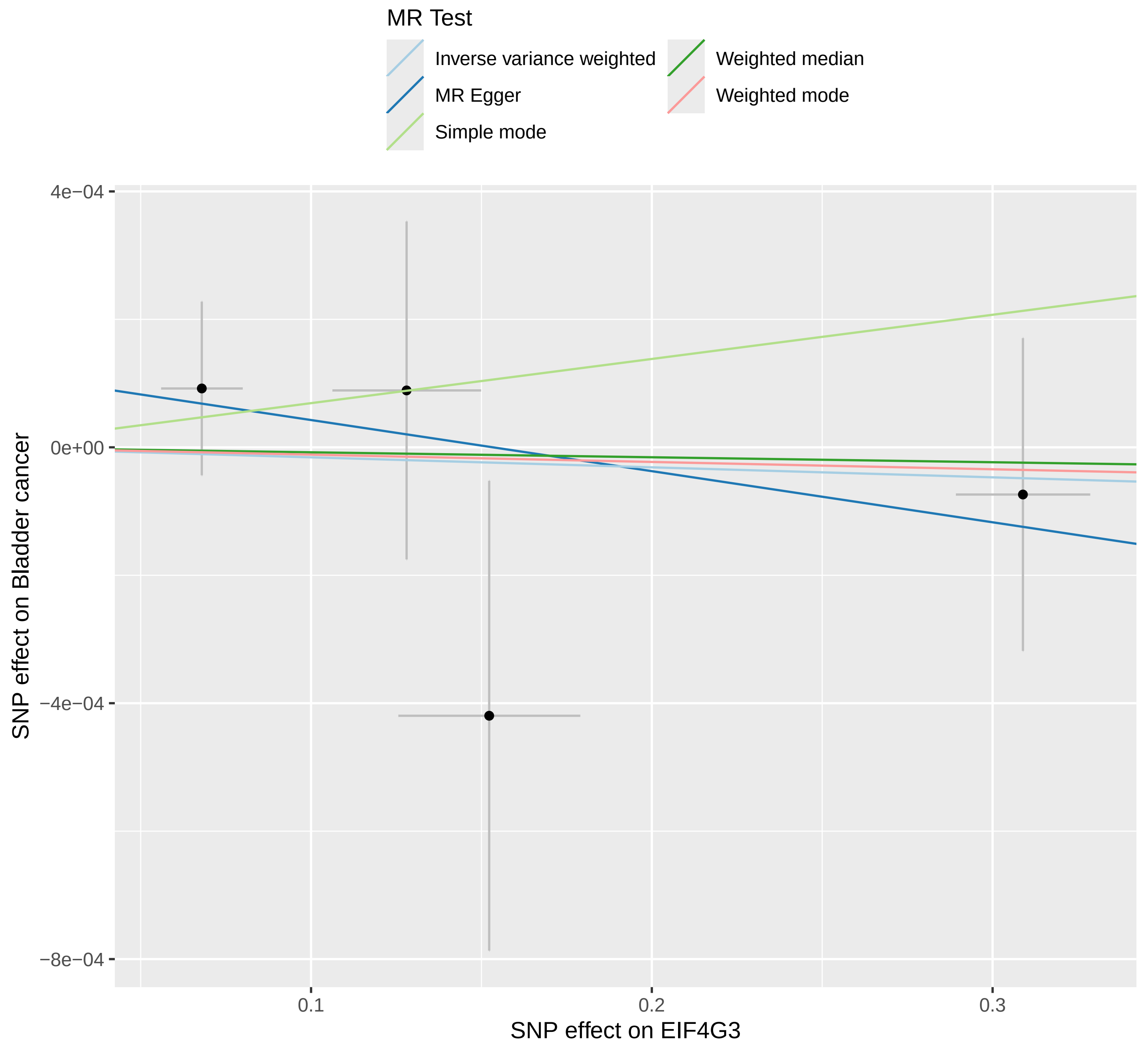

MR Test
Inverse variance weighted
MR Egger
Simple mode
Weighted median
Weighted mode
4e−04
0e+00
−4e−04
−8e−04
0.1
0.2
0.3
SNP effect on EIF4G3
SNP effect on Bladder cancer

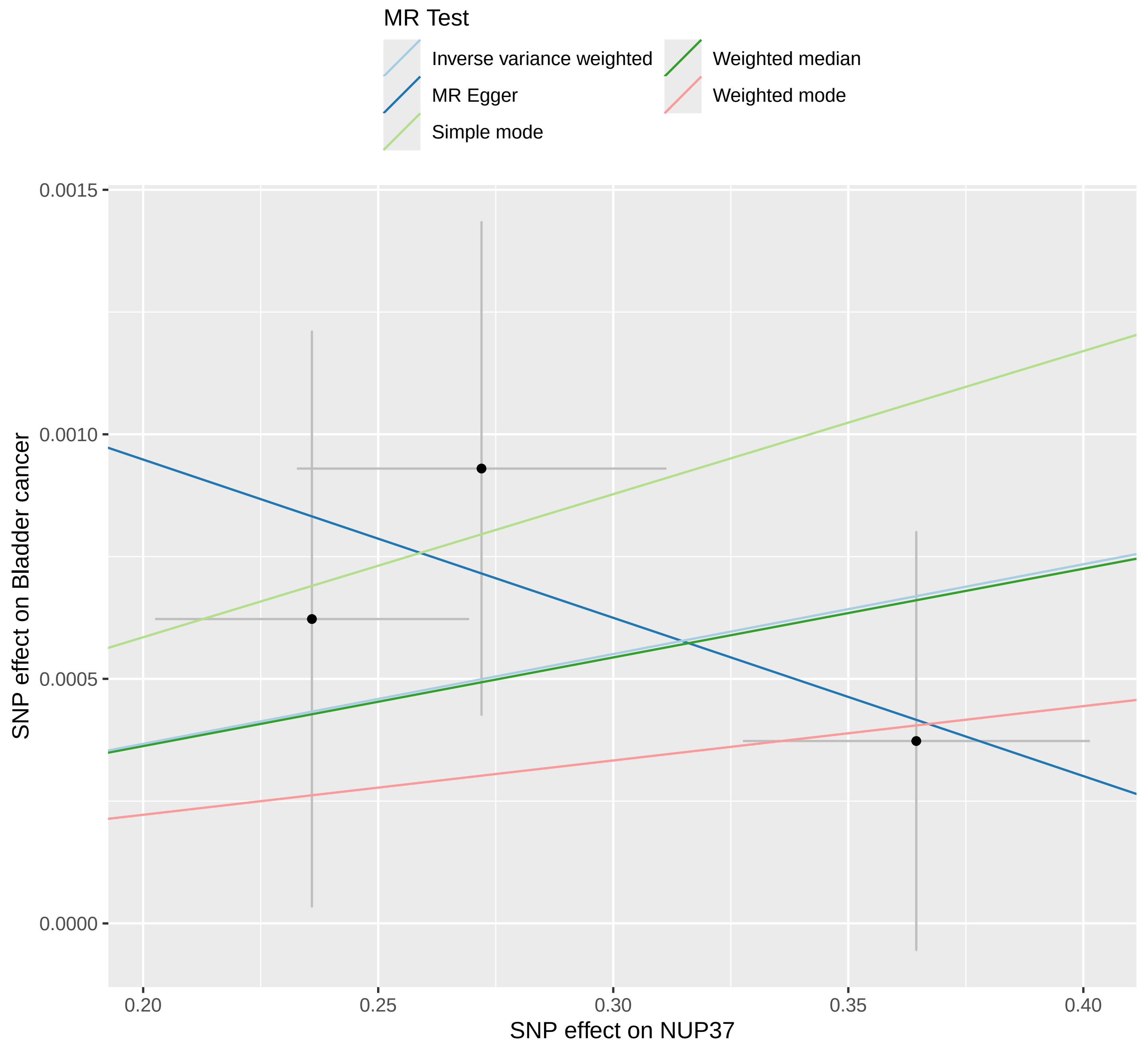

MR Test
Inverse variance weighted
MR Egger
Simple mode
Weighted median
Weighted mode
0.0015
0.0010
0.0005
0.0000
SNP effect on Bladder cancer
0.20
0.25
0.30
0.35
0.40
SNP effect on NUP37

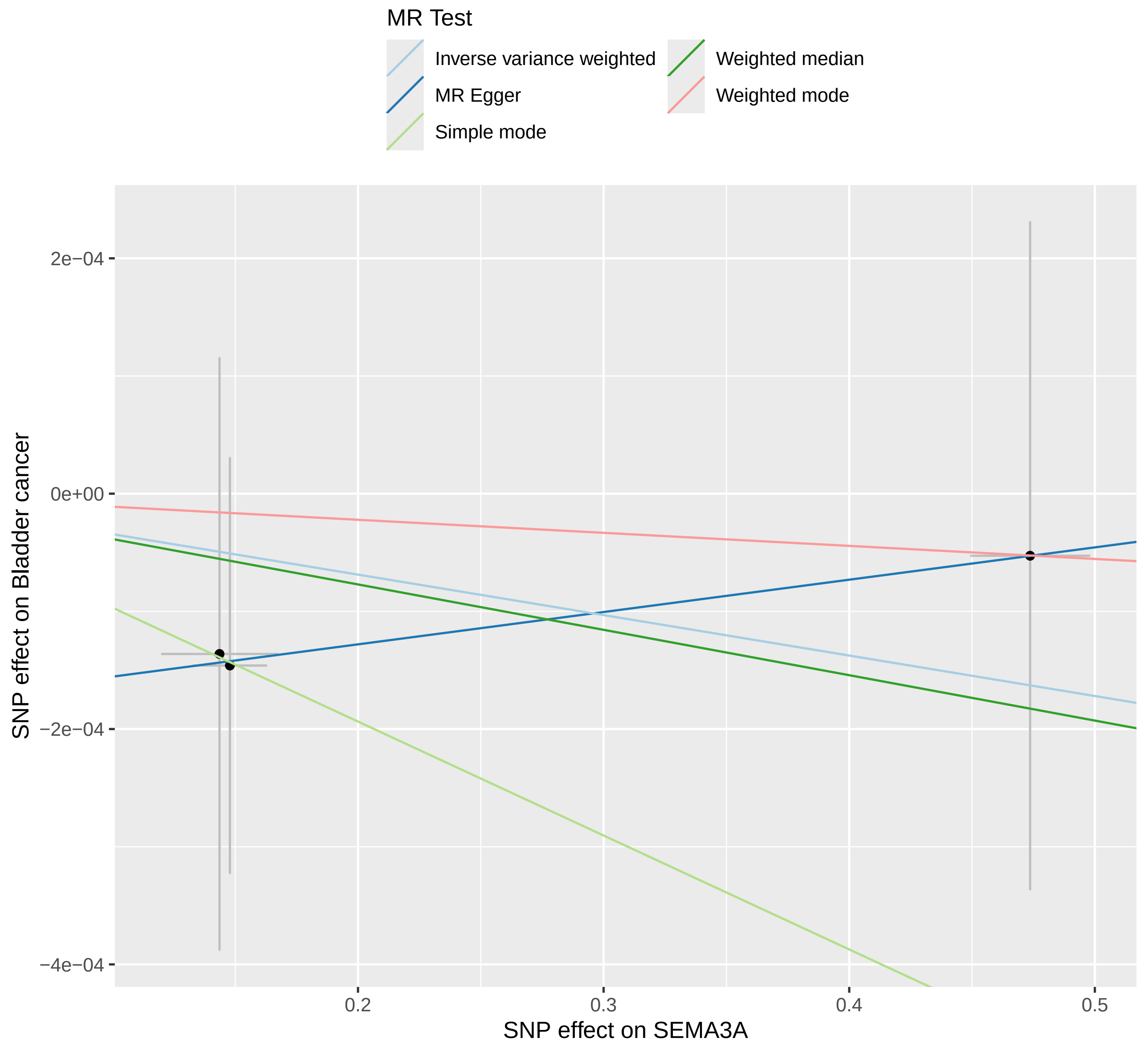
MR Test
Inverse variance weighted
MR Egger
Simple mode
Weighted median
Weighted mode
2e−04
0e+00
−2e−04
−4e−04
SNP effect on Bladder cancer
0.2
0.3
0.4
0.5
SNP effect on SEMA3A

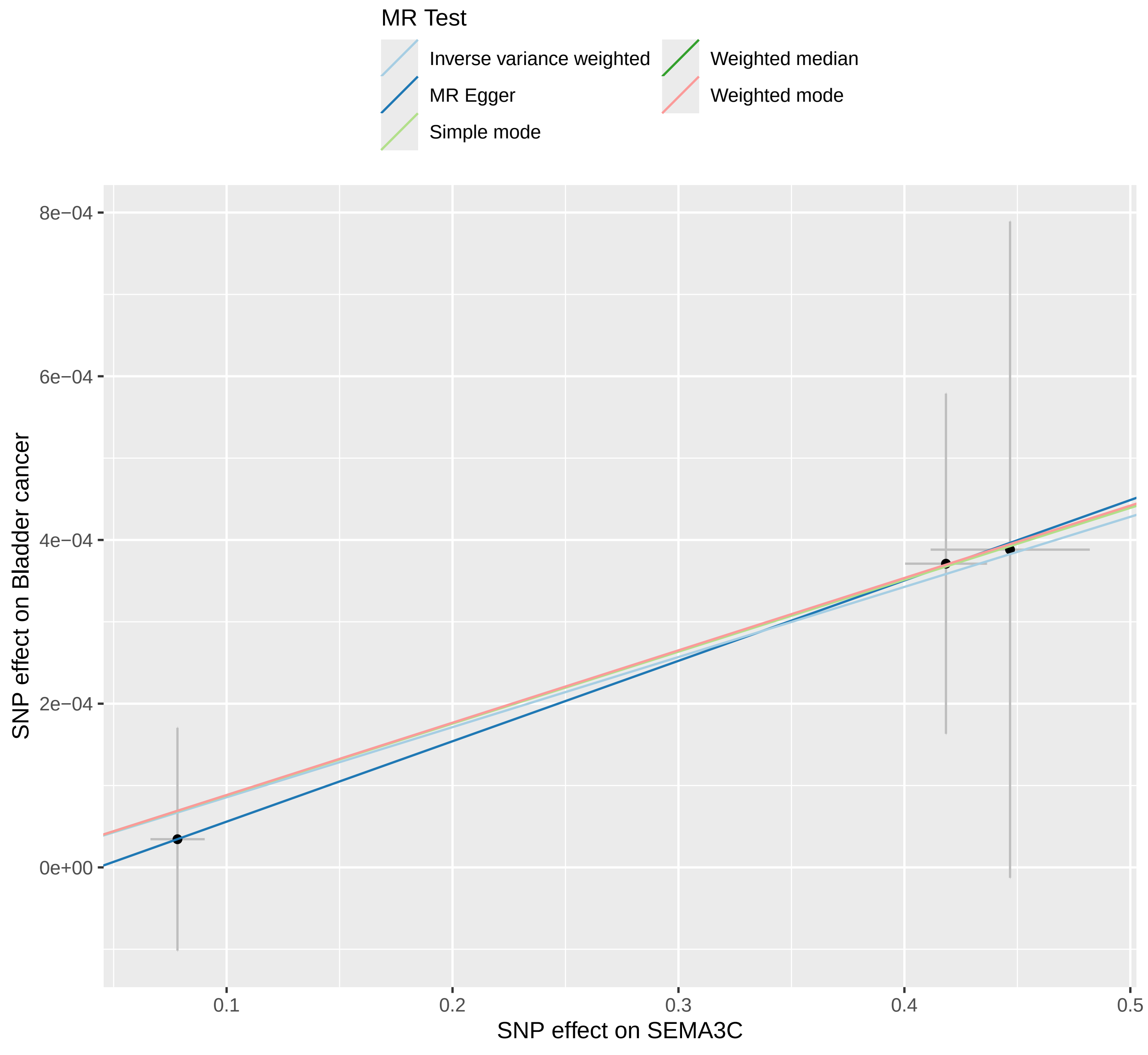
MR Test
Inverse variance weighted
MR Egger
Simple mode
Weighted median
Weighted mode
8e−04
6e−04
4e−04
2e−04
0e+00
SNP effect on Bladder cancer
0.1
0.2
0.3
0.4
0.5
SNP effect on SEMA3C

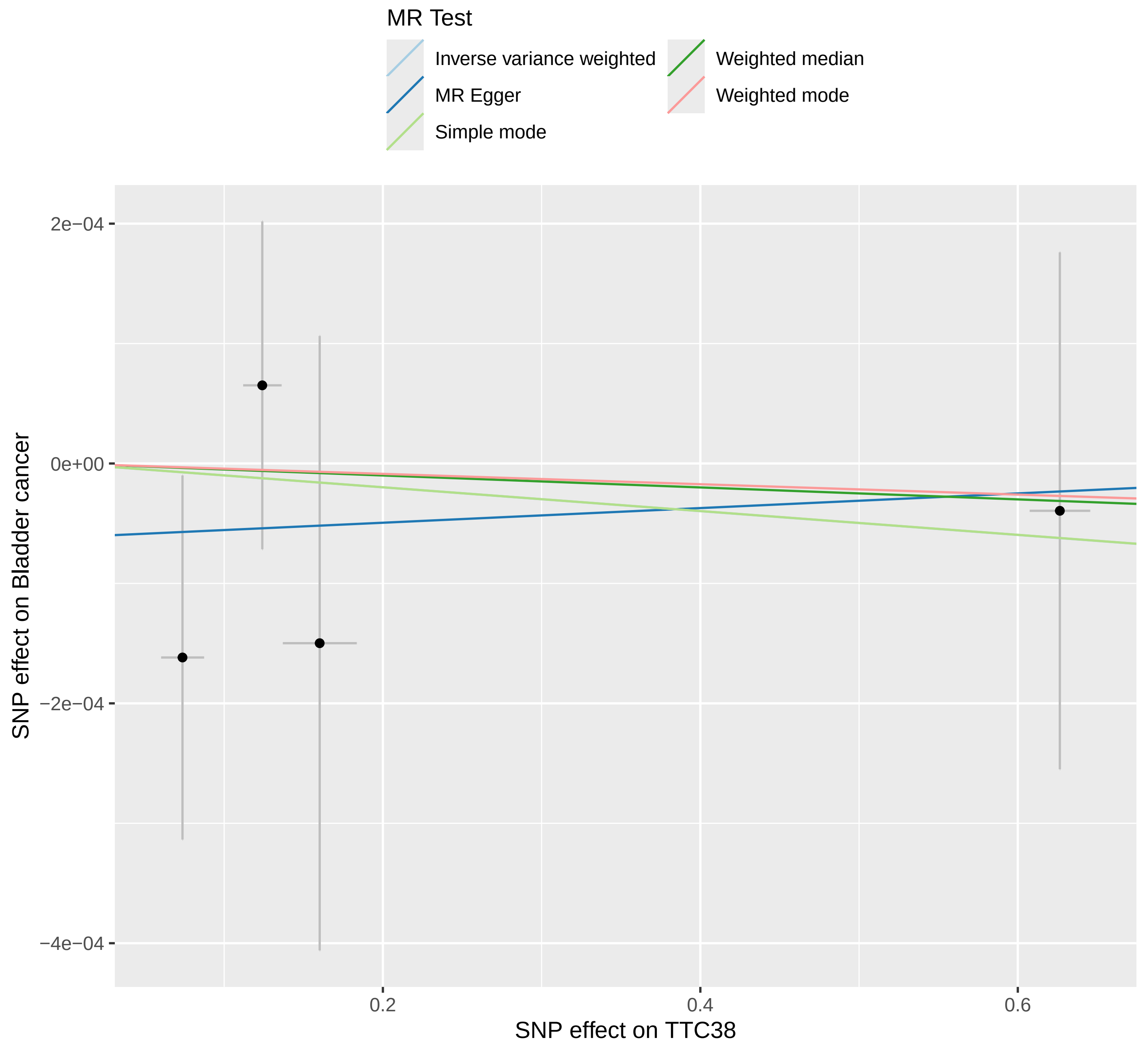

MR Test
Inverse variance weighted
MR Egger
Simple mode
Weighted median
Weighted mode
2e−04
0e+00
−2e−04
−4e−04
0.2
0.4
0.6
SNP effect on Bladder cancer
SNP effect on TTC38

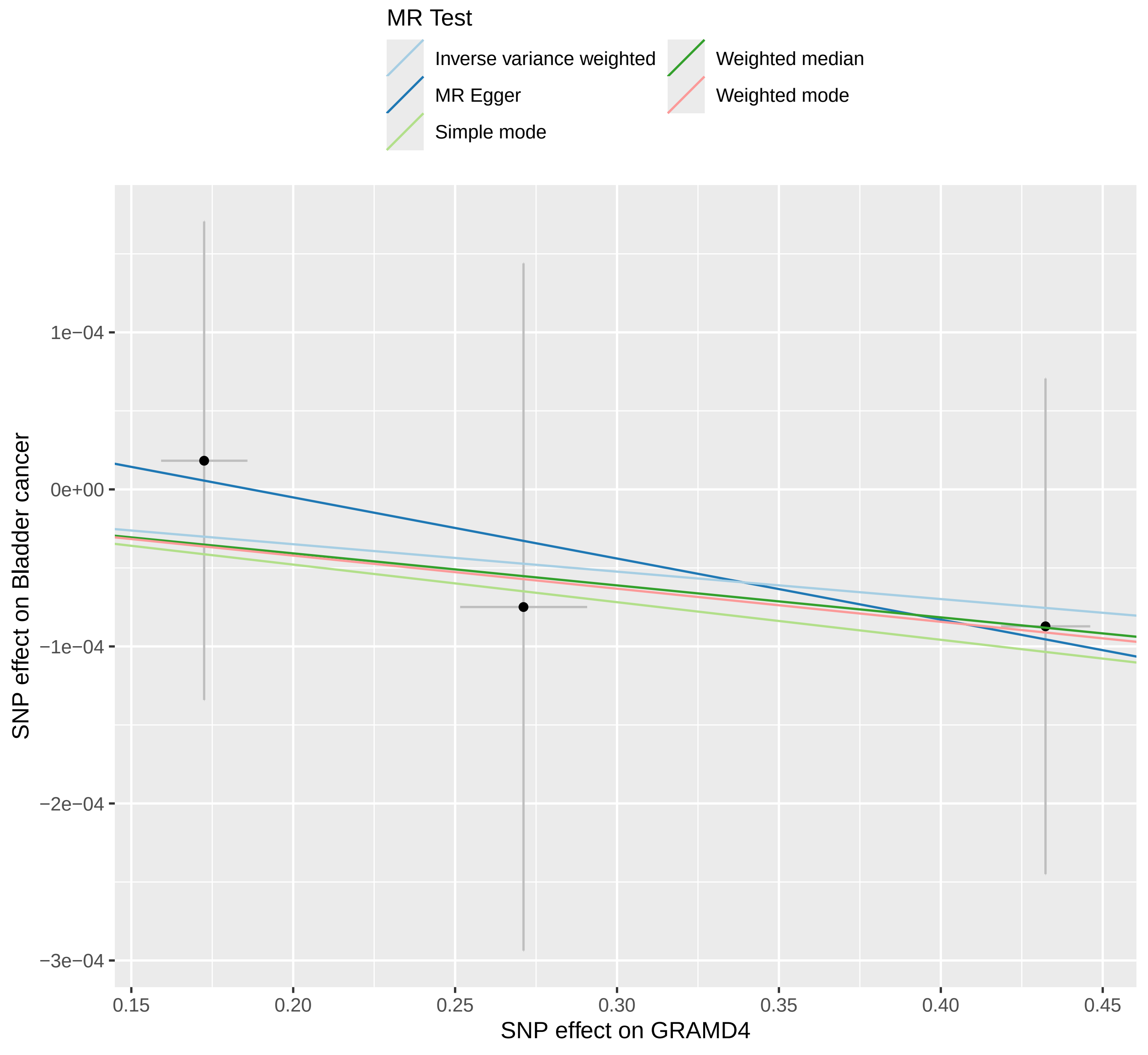

MR Test
Inverse variance weighted
MR Egger
Simple mode
Weighted median
Weighted mode
1e−04
0e+00
−1e−04
−2e−04
−3e−04
SNP effect on Bladder cancer
0.15
0.20
0.25
0.30
0.35
0.40
0.45
SNP effect on GRAMD4

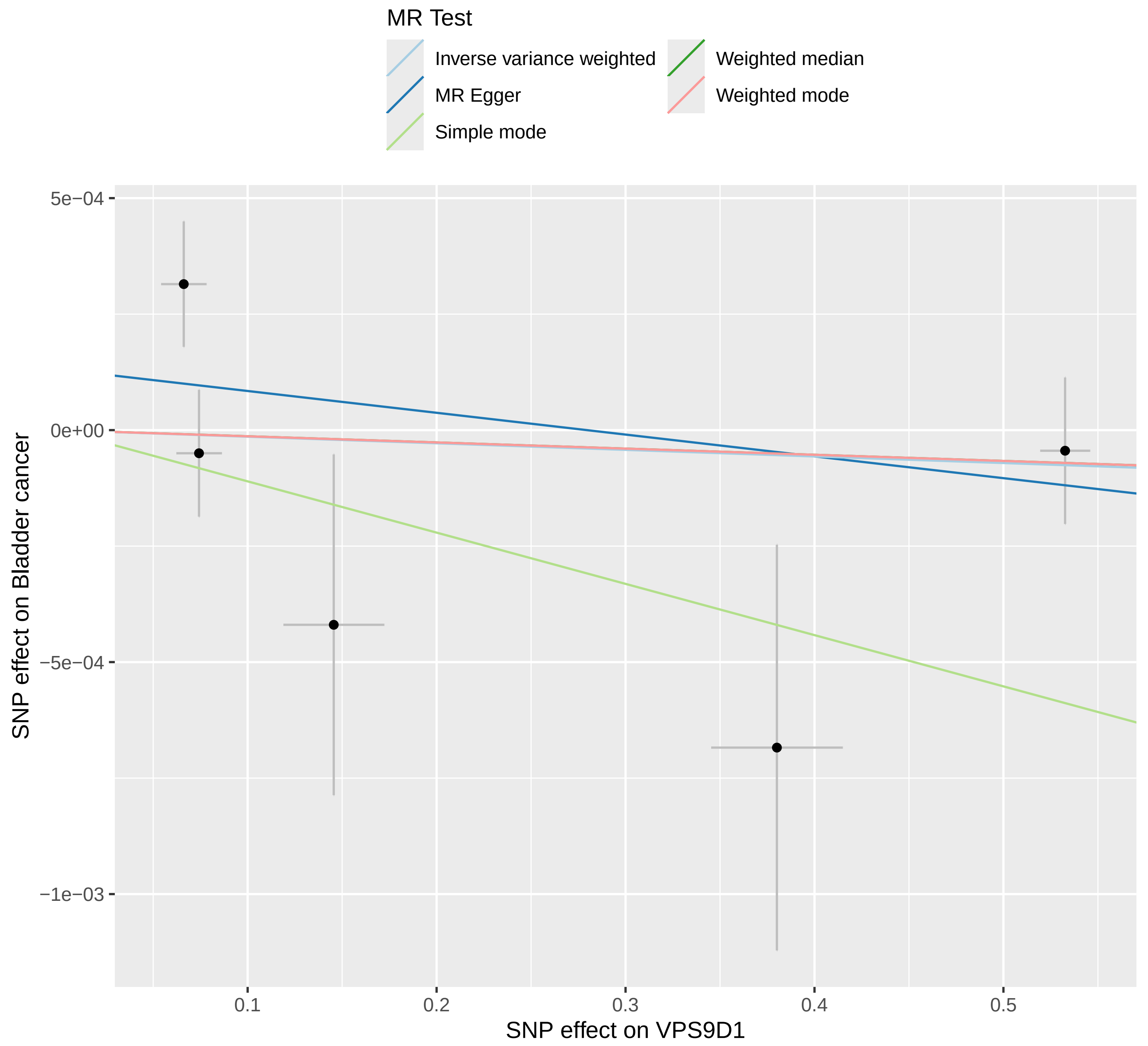

MR Test
Inverse variance weighted
MR Egger
Simple mode
Weighted median
Weighted mode
5e−04
0e+00
−5e−04
−1e−03
SNP effect on Bladder cancer
0.1
0.2
0.3
0.4
0.5
SNP effect on VPS9D1

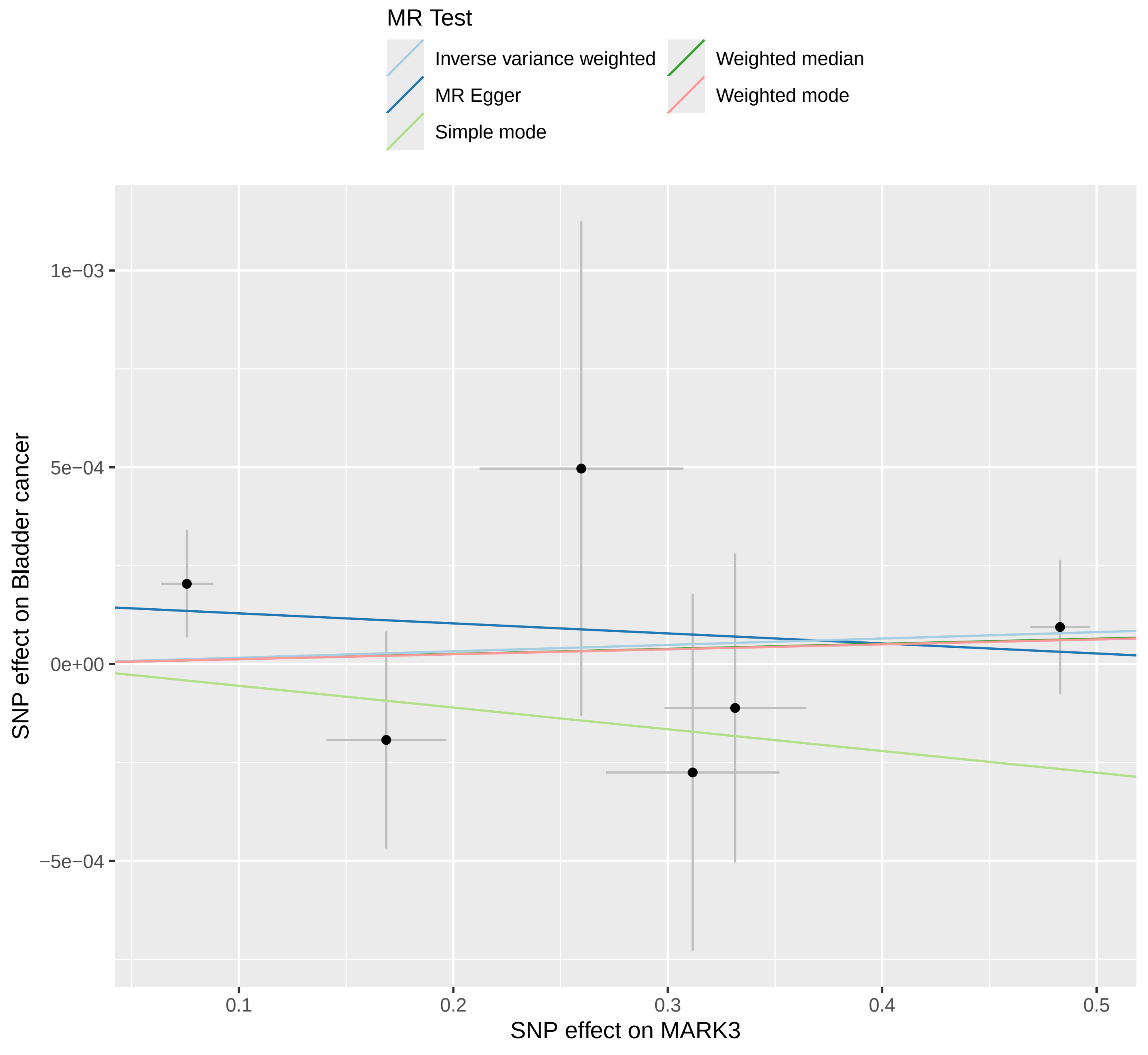

MR Test
Inverse variance weighted
MR Egger
Simple mode
Weighted median
Weighted mode
1e−03
5e−04
0e+00
−5e−04
SNP effect on Bladder cancer
0.1
0.2
0.3
0.4
0.5
SNP effect on MARK3

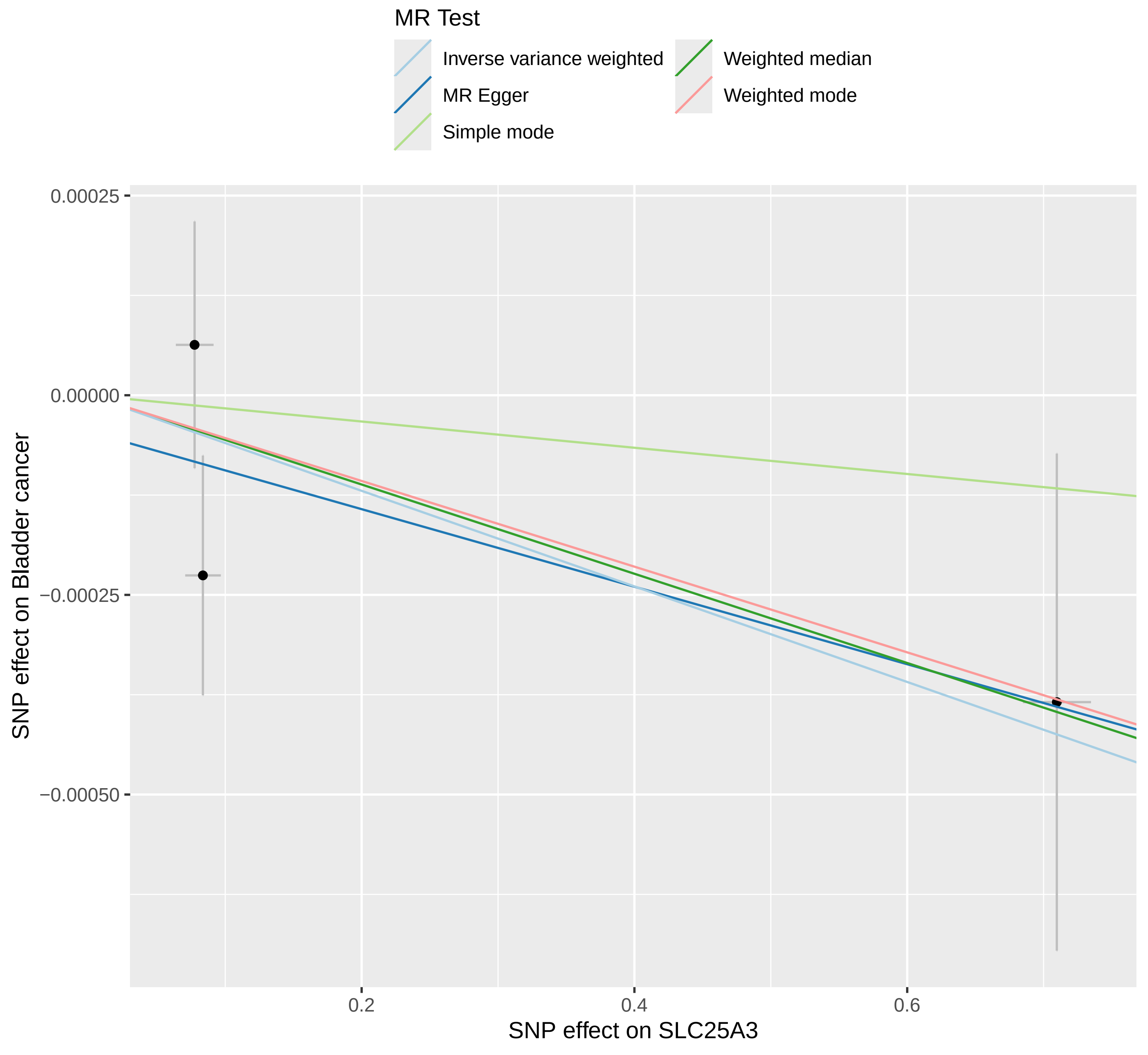

MR Test
Inverse variance weighted
MR Egger
Simple mode
Weighted median
Weighted mode
0.00025
0.00000
−0.00025
−0.00050
SNP effect on Bladder cancer
0.2
0.4
0.6
SNP effect on SLC25A3

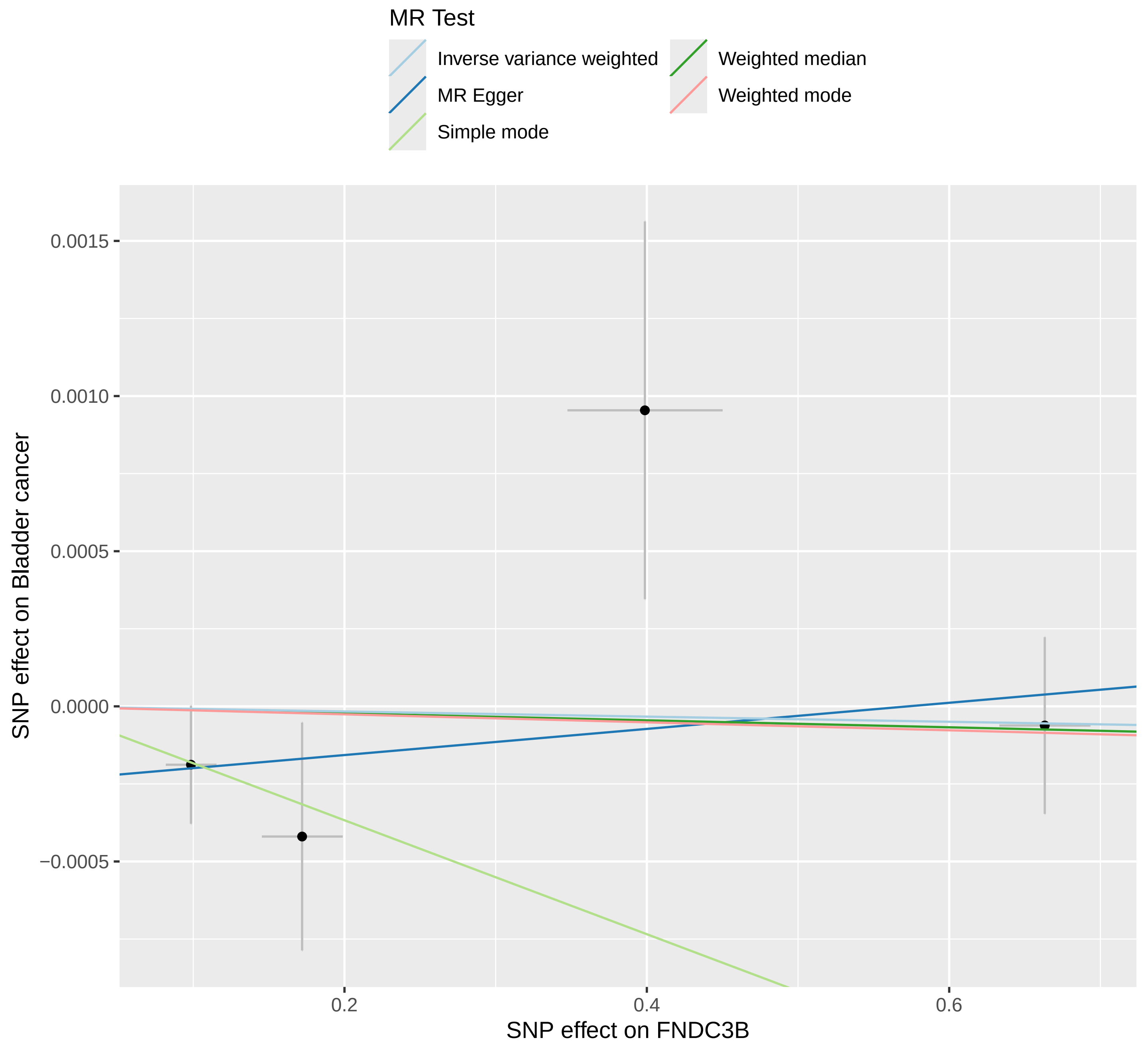
MR Test
Inverse variance weighted
MR Egger
Simple mode
Weighted median
Weighted mode
0.0015
0.0010
0.0005
0.0000
−0.0005
SNP effect on Bladder cancer
0.2
0.4
0.6
SNP effect on FNDC3B

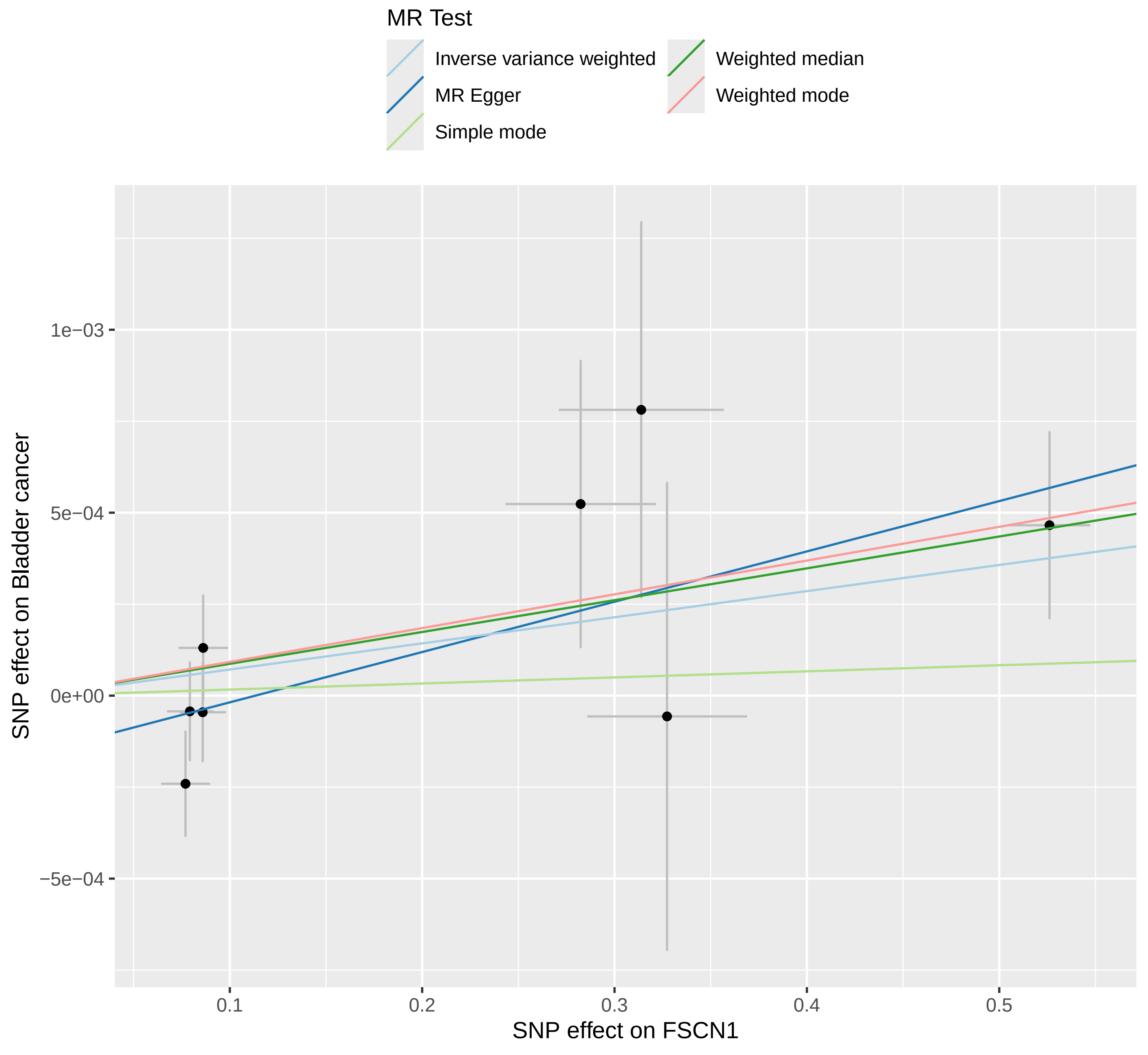

MR Test
Inverse variance weighted
MR Egger
Simple mode
Weighted median
Weighted mode
SNP effect on Bladder cancer
1e−03
5e−04
0e+00
−5e−04
0.1
0.2
0.3
0.4
0.5
SNP effect on FSCN1

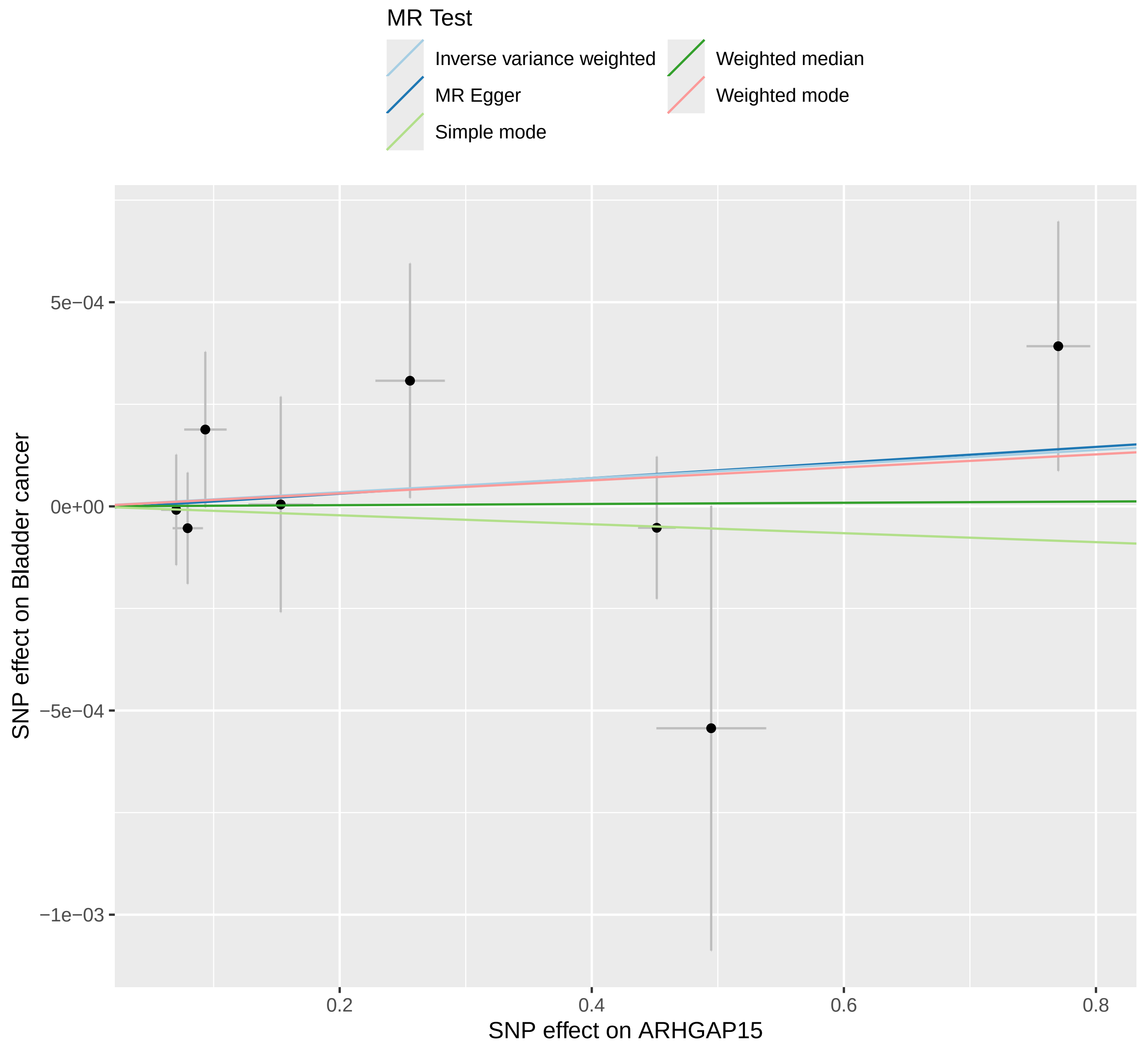
MR Test
Inverse variance weighted
MR Egger
Simple mode
Weighted median
Weighted mode
5e−04
0e+00
−5e−04
−1e−03
0.2
0.4
0.6
0.8
SNP effect on Bladder cancer
SNP effect on ARHGAP15

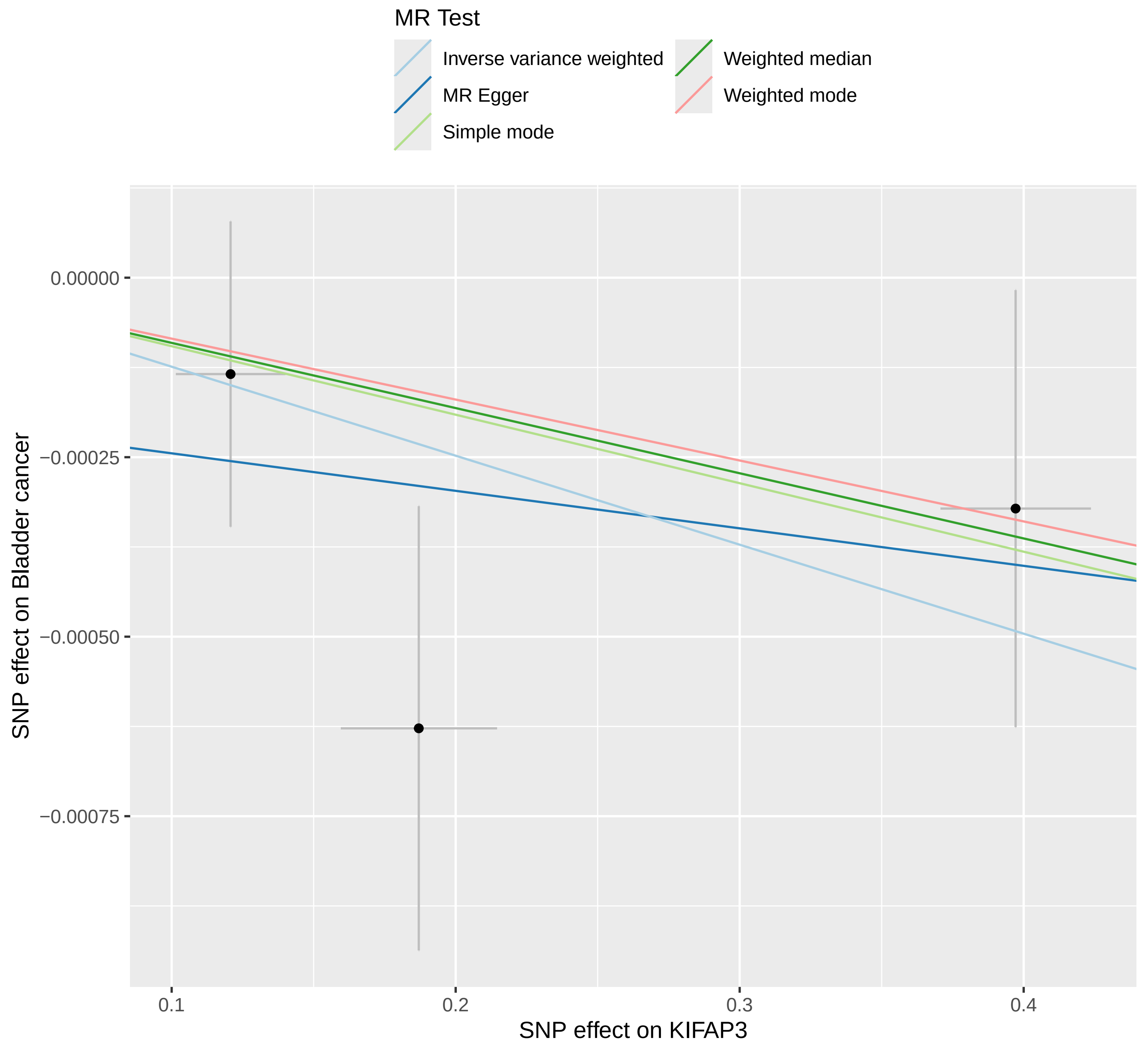

MR Test
Inverse variance weighted
MR Egger
Simple mode
Weighted median
Weighted mode
0.00000
−0.00025
−0.00050
−0.00075
SNP effect on Bladder cancer
0.1
0.2
0.3
0.4
SNP effect on KIFAP3

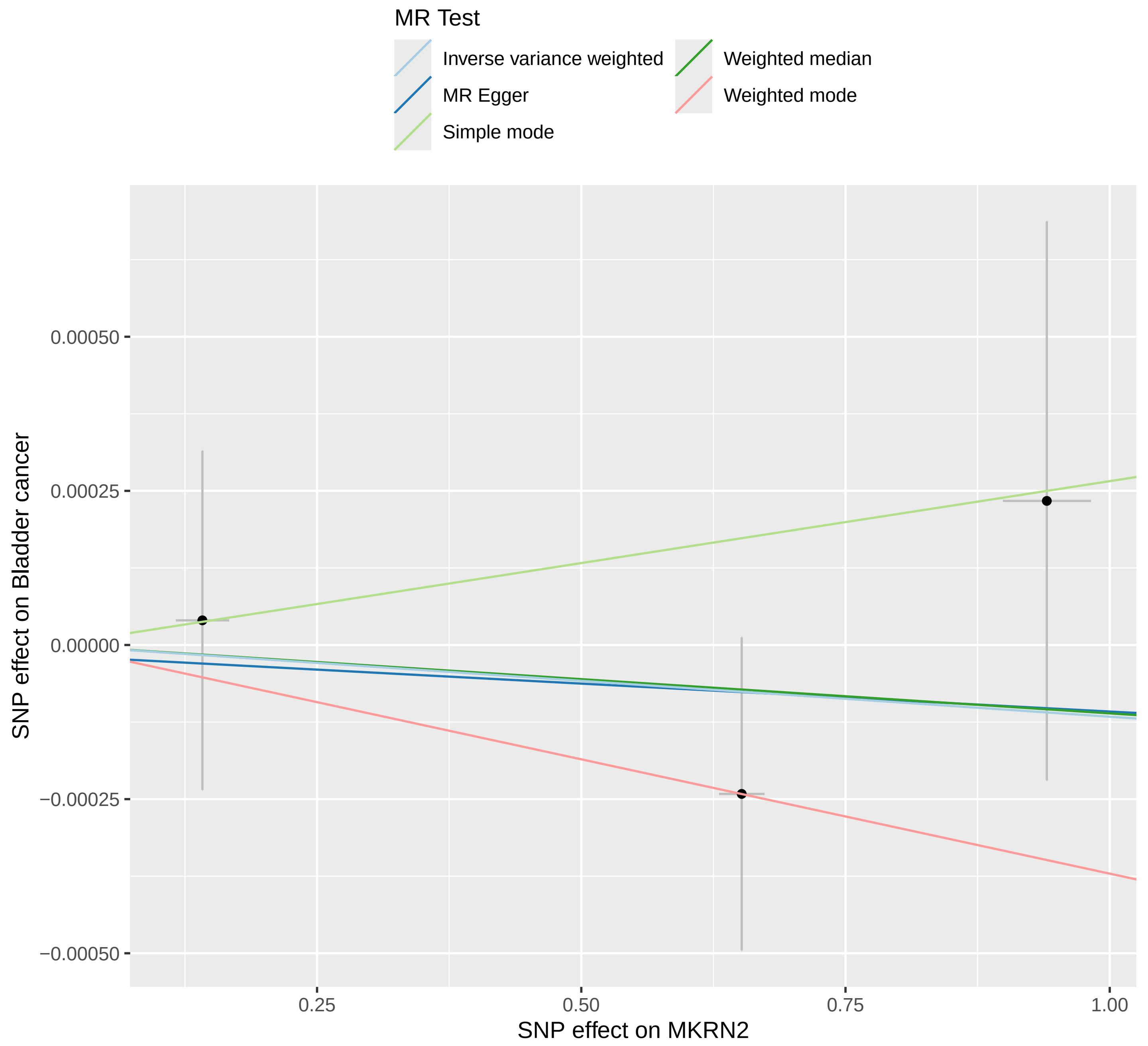

MR Test
Inverse variance weighted
MR Egger
Simple mode
Weighted median
Weighted mode
SNP effect on Bladder cancer
0.00050
0.00025
0.00000
−0.00025
−0.00050
0.25
0.50
0.75
1.00
SNP effect on MKRN2

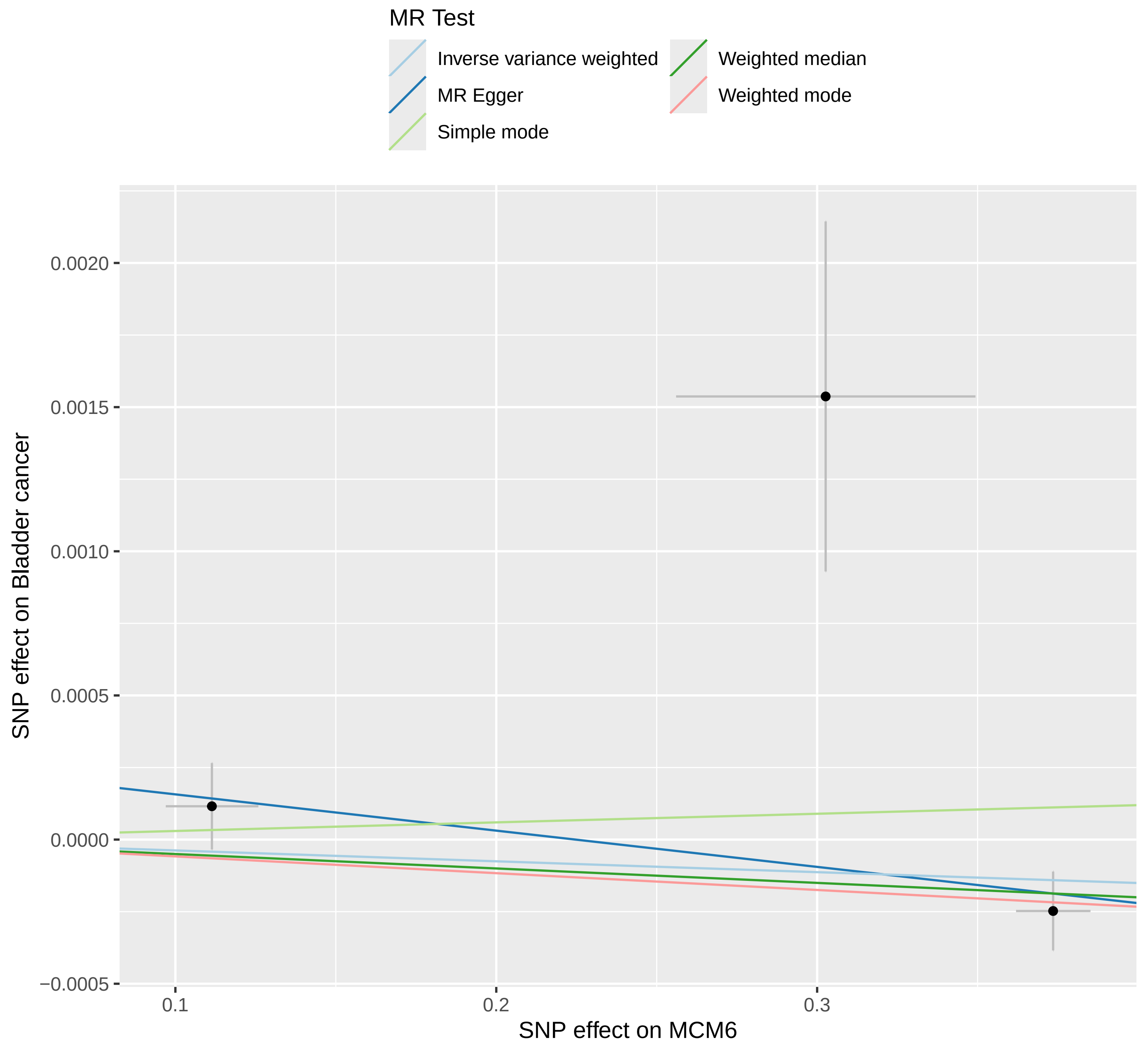
MR Test
Inverse variance weighted
MR Egger
Simple mode
Weighted median
Weighted mode
0.0020
0.0015
0.0010
0.0005
0.0000
−0.0005
SNP effect on Bladder cancer
0.1
0.2
0.3
SNP effect on MCM6

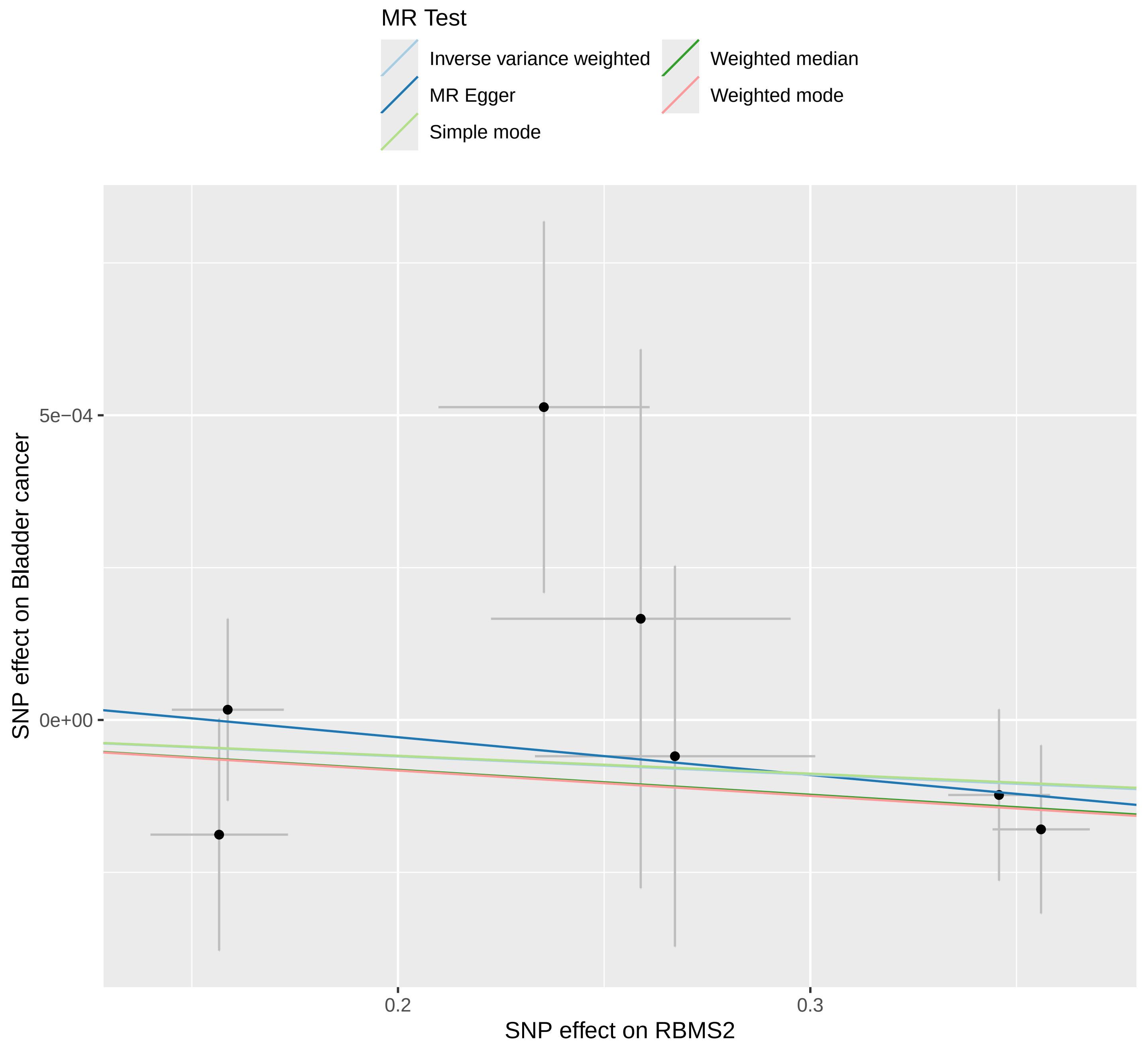

MR Test
Inverse variance weighted
MR Egger
Simple mode
Weighted median
Weighted mode
5e−04
0e+00
SNP effect on Bladder cancer
0.2
0.3
SNP effect on RBMS2

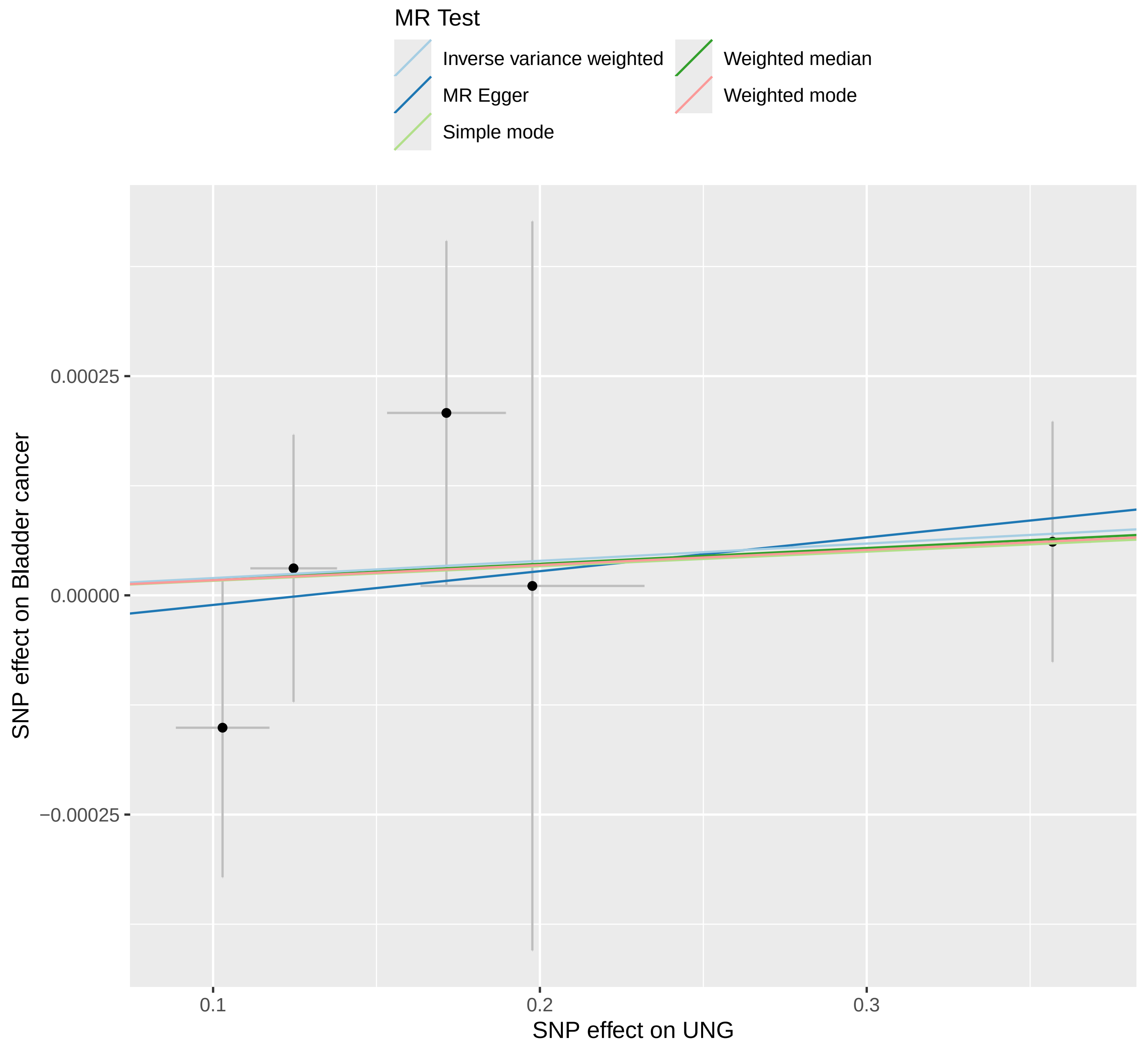

MR Test
Inverse variance weighted
MR Egger
Simple mode
Weighted median
Weighted mode
0.00025
0.00000
−0.00025
SNP effect on Bladder cancer
0.1
0.2
0.3
SNP effect on UNG

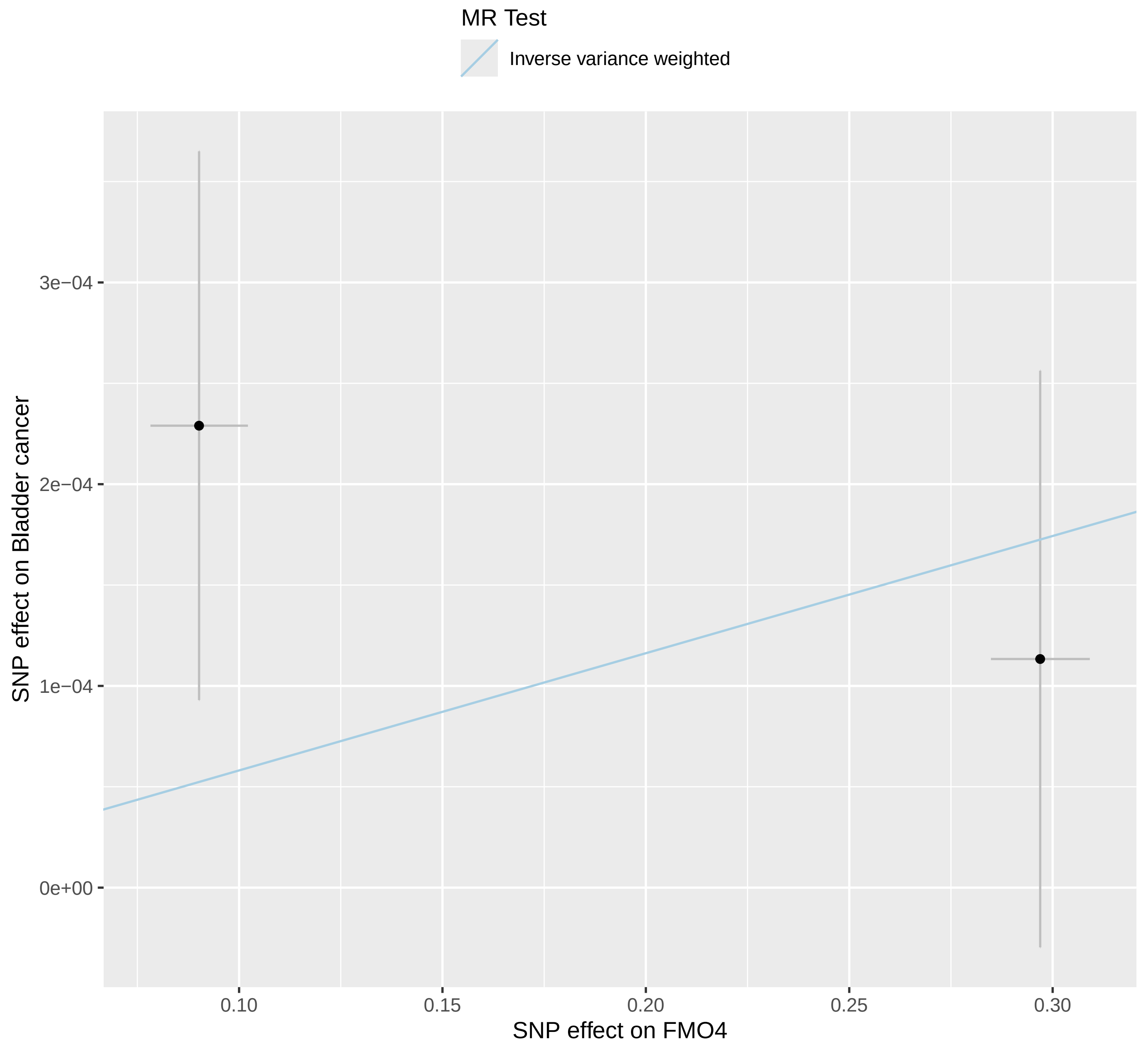

MR Test
Inverse variance weighted
SNP effect on Bladder cancer
3e−04
2e−04
1e−04
0e+00
0.10
0.15
0.20
0.25
0.30
SNP effect on FMO4

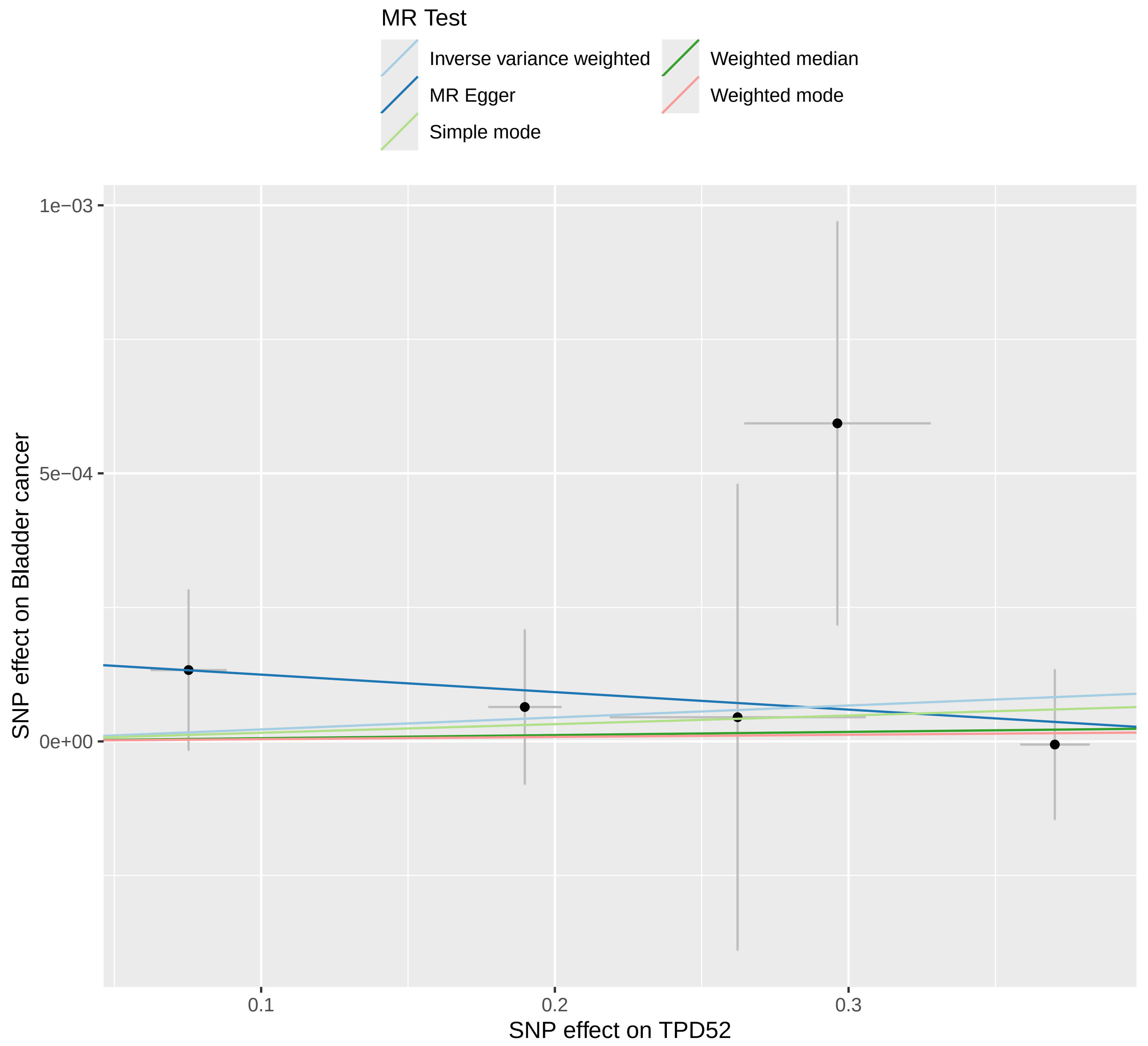
MR Test
Inverse variance weighted
MR Egger
Simple mode
Weighted median
Weighted mode
1e−03
5e−04
0e+00
0.1
0.2
0.3
SNP effect on TPD52
SNP effect on Bladder cancer

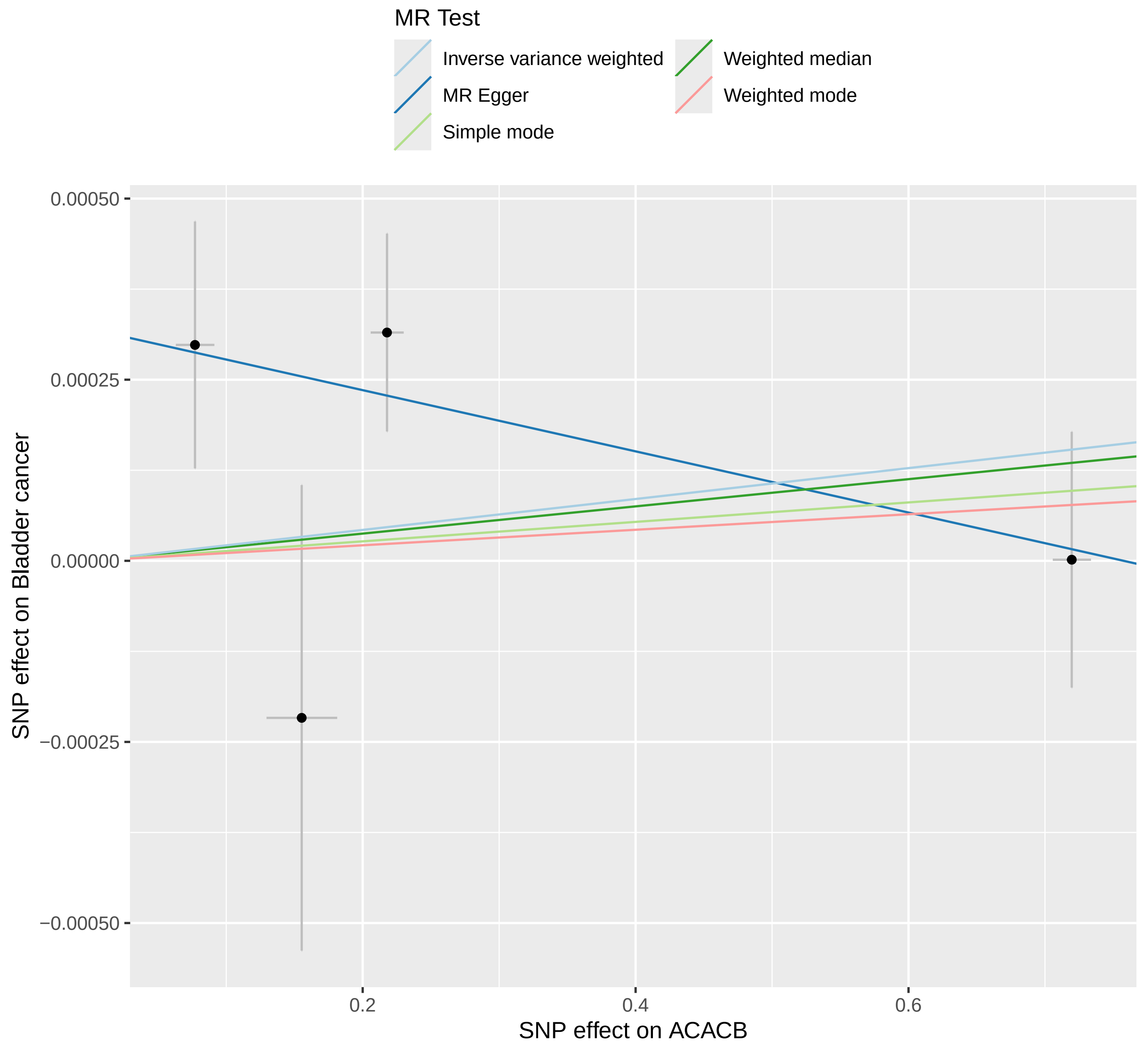

MR Test
Inverse variance weighted
MR Egger
Simple mode
Weighted median
Weighted mode
0.00050
0.00025
0.00000
−0.00025
−0.00050
SNP effect on Bladder cancer
0.2
0.4
0.6
SNP effect on ACACB

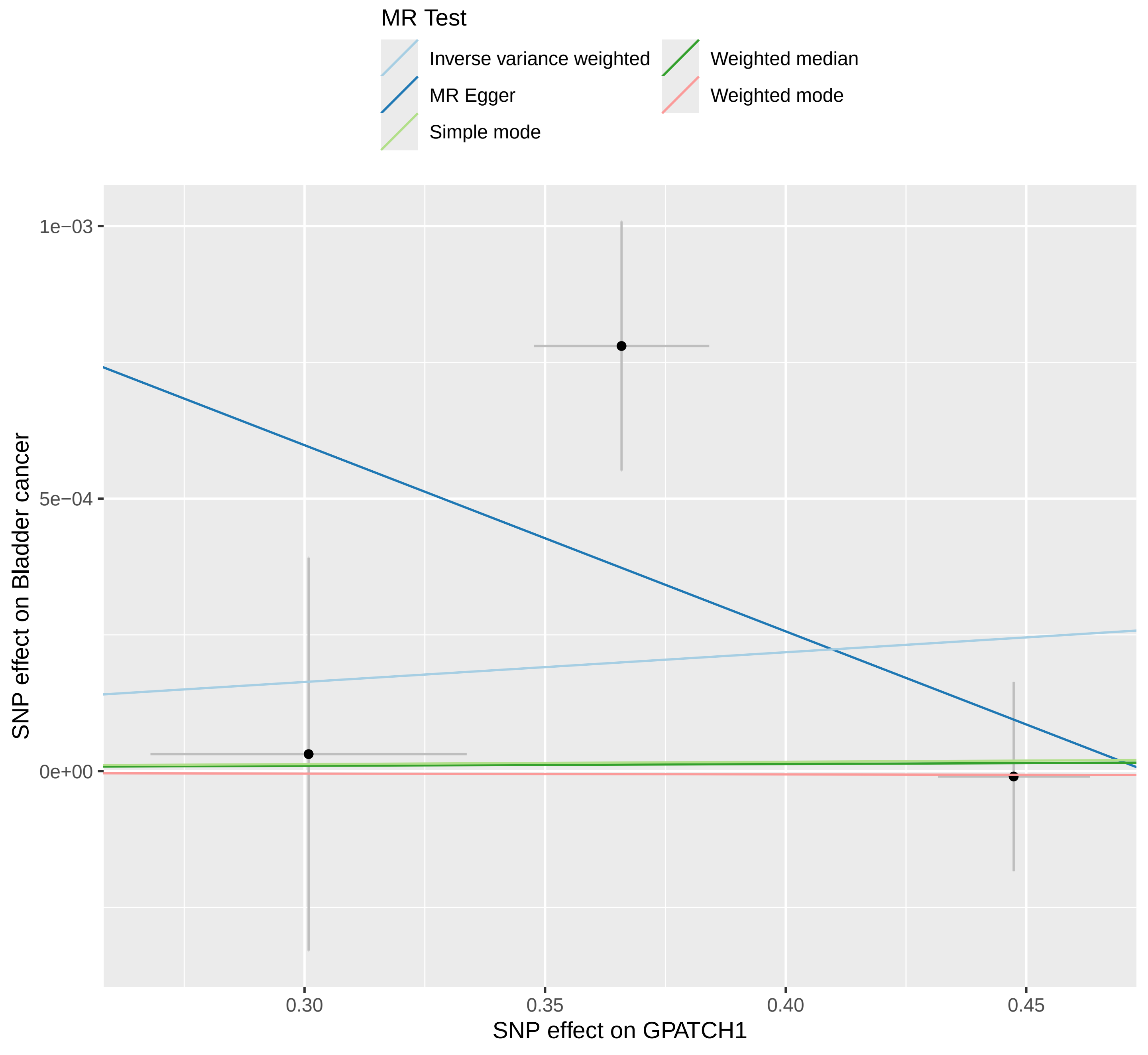
MR Test
Inverse variance weighted
MR Egger
Simple mode
Weighted median
Weighted mode
1e−03
5e−04
0e+00
0.30
0.35
0.40
0.45
SNP effect on GPATCH1
SNP effect on Bladder cancer

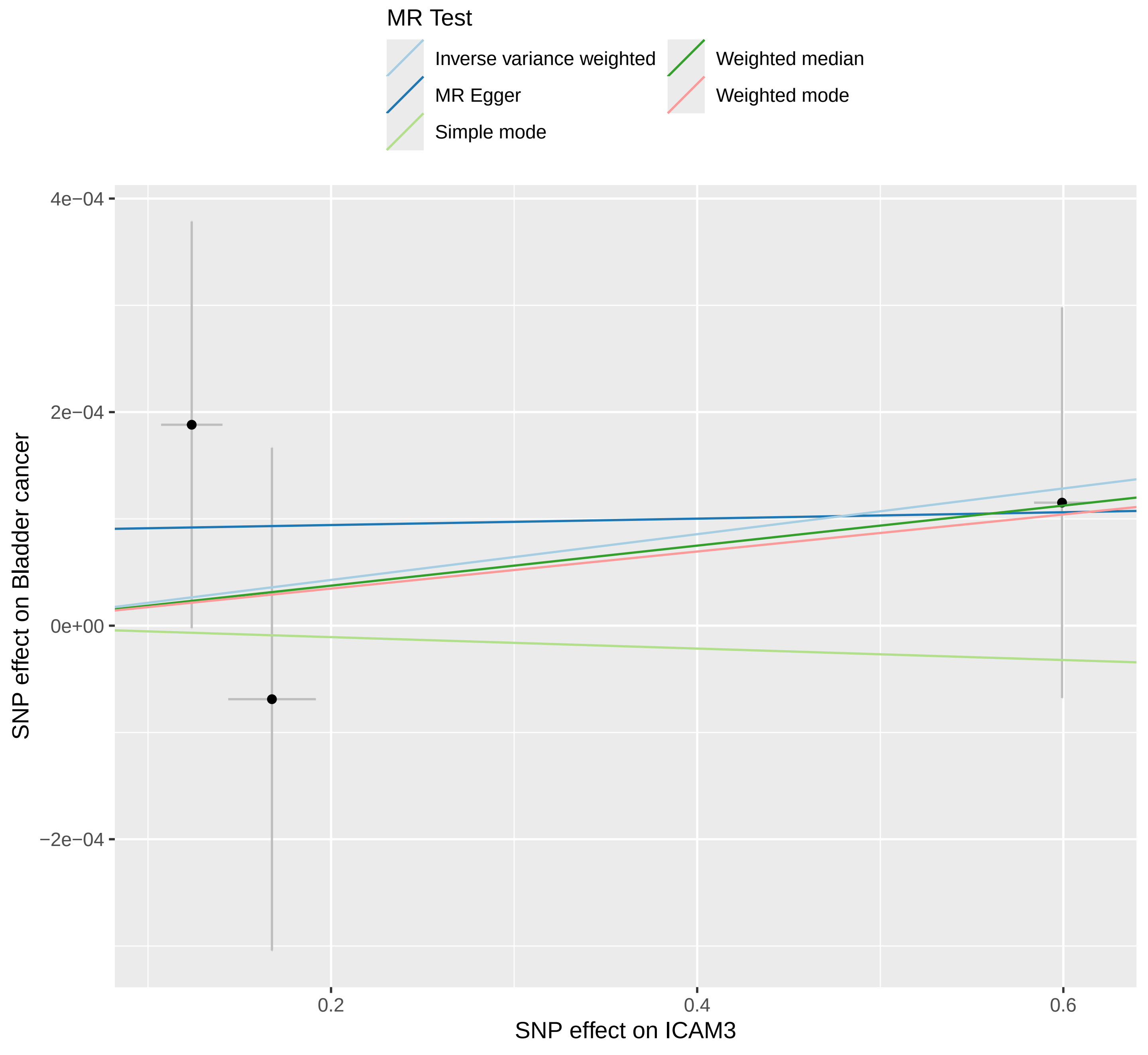

MR Test
Inverse variance weighted
MR Egger
Simple mode
Weighted median
Weighted mode
4e−04
2e−04
0e+00
−2e−04
SNP effect on Bladder cancer
0.2
0.4
0.6
SNP effect on ICAM3

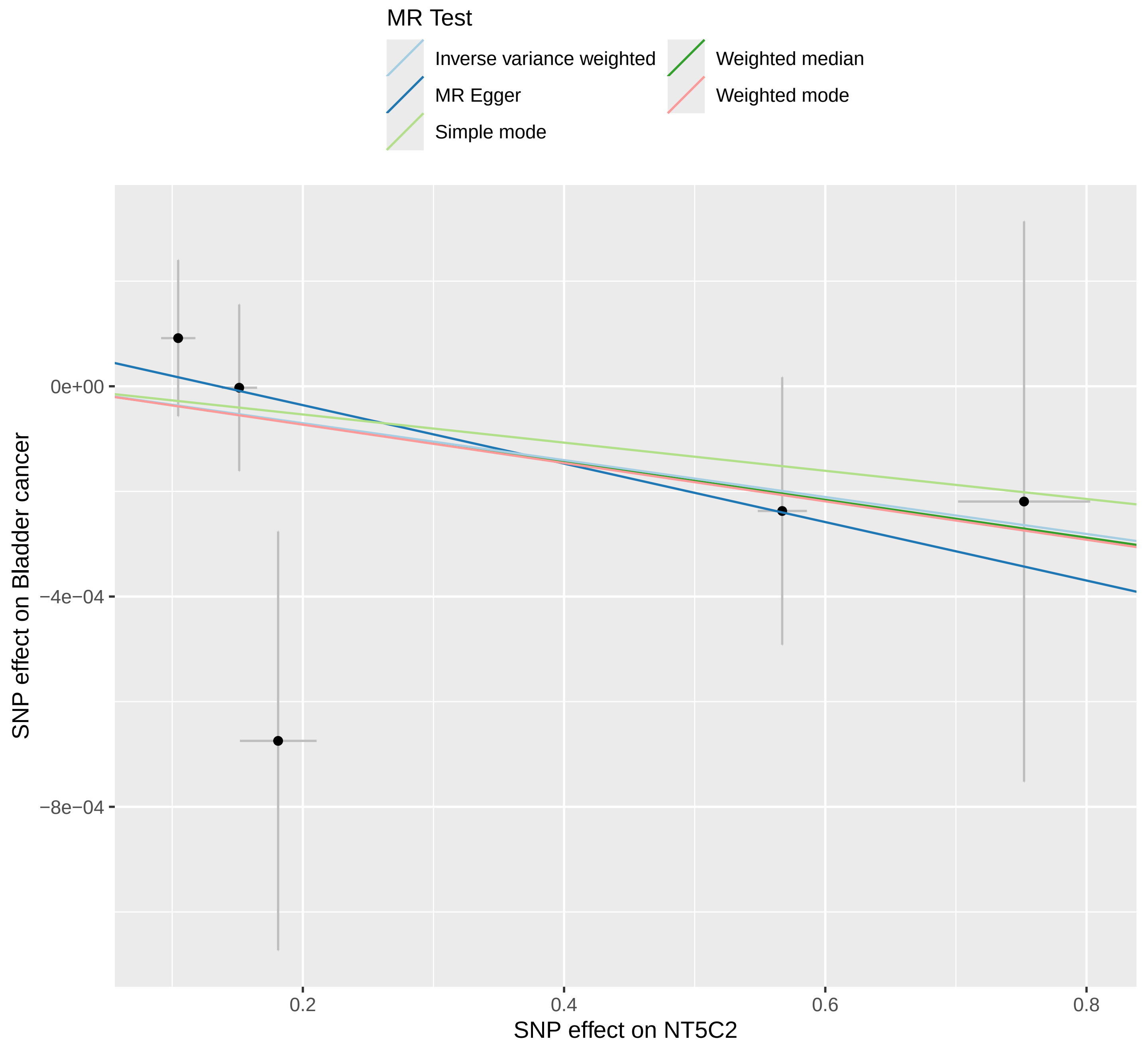
MR Test
Inverse variance weighted
MR Egger
Simple mode
Weighted median
Weighted mode
0e+00
−4e−04
−8e−04
0.2
0.4
0.6
0.8
SNP effect on NT5C2
SNP effect on Bladder cancer

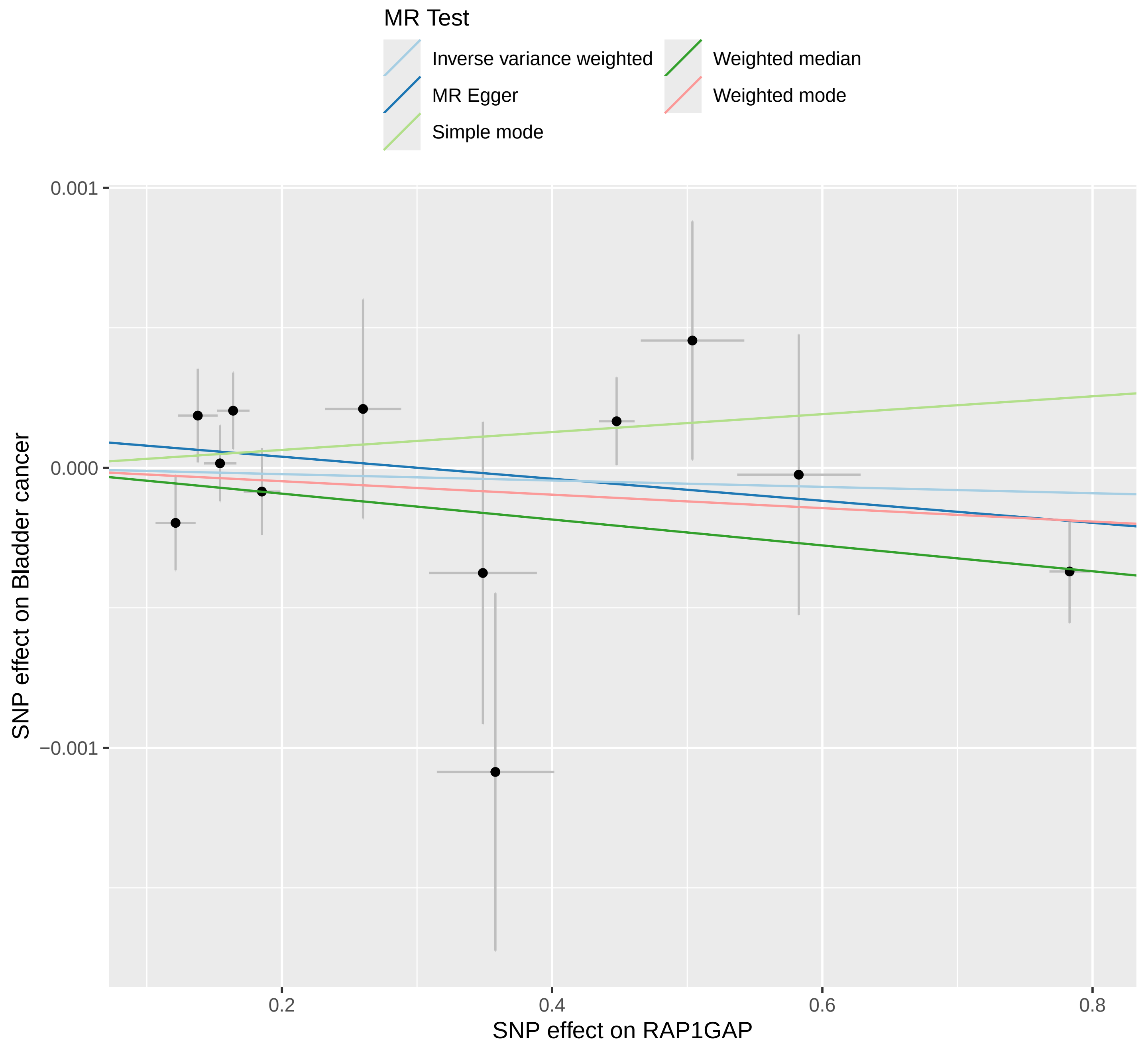
MR Test
Inverse variance weighted
MR Egger
Simple mode
Weighted median
Weighted mode
0.001
0.000
−0.001
SNP effect on Bladder cancer
0.2
0.4
0.6
0.8
SNP effect on RAP1GAP

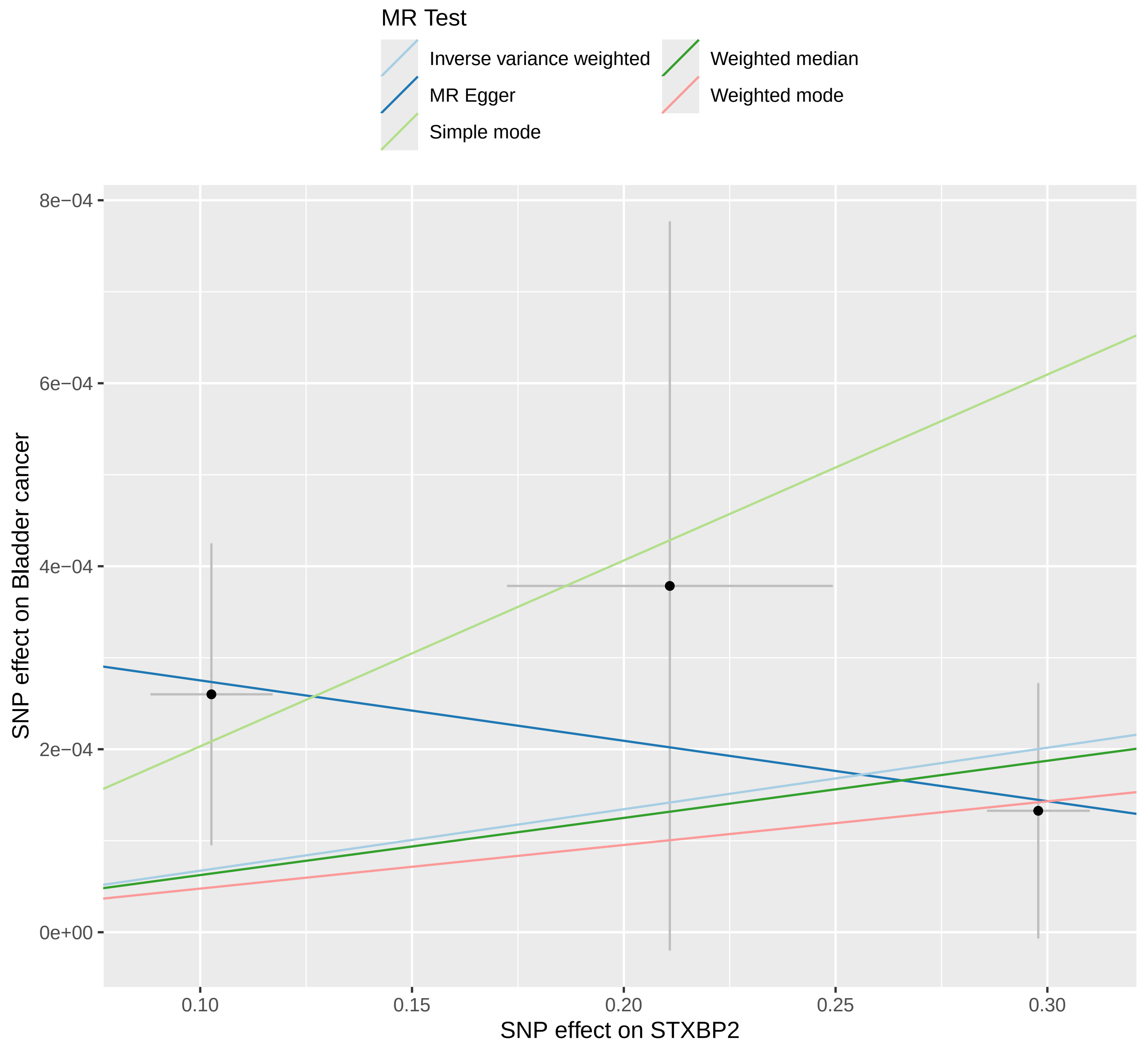

MR Test
Inverse variance weighted
MR Egger
Simple mode
Weighted median
Weighted mode
8e−04
6e−04
4e−04
2e−04
0e+00
SNP effect on Bladder cancer
0.10
0.15
0.20
0.25
0.30
SNP effect on STXBP2

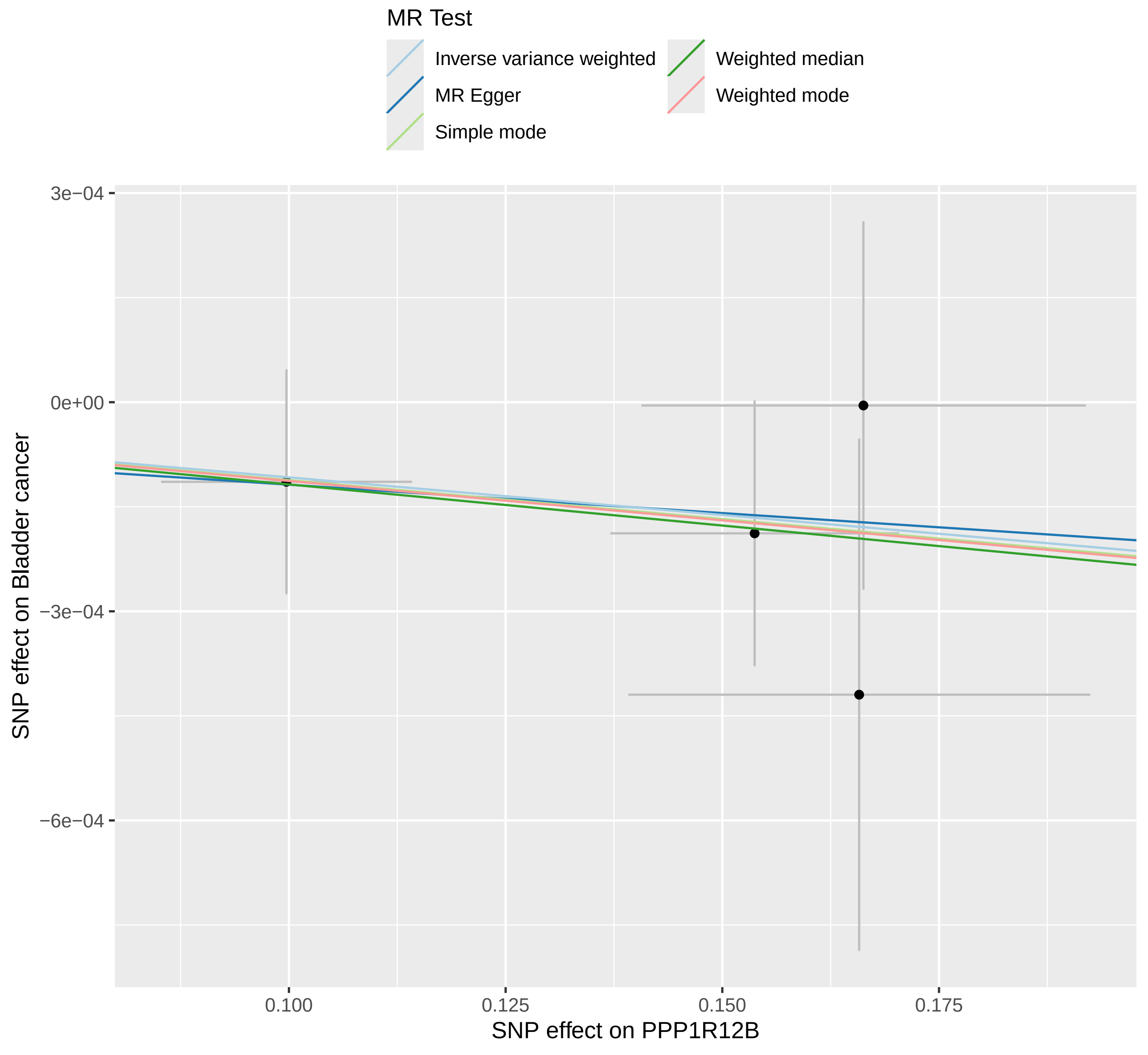

MR Test
Inverse variance weighted
MR Egger
Simple mode
Weighted median
Weighted mode
3e−04
0e+00
−3e−04
−6e−04
SNP effect on Bladder cancer
0.100
0.125
0.150
0.175
SNP effect on PPP1R12B

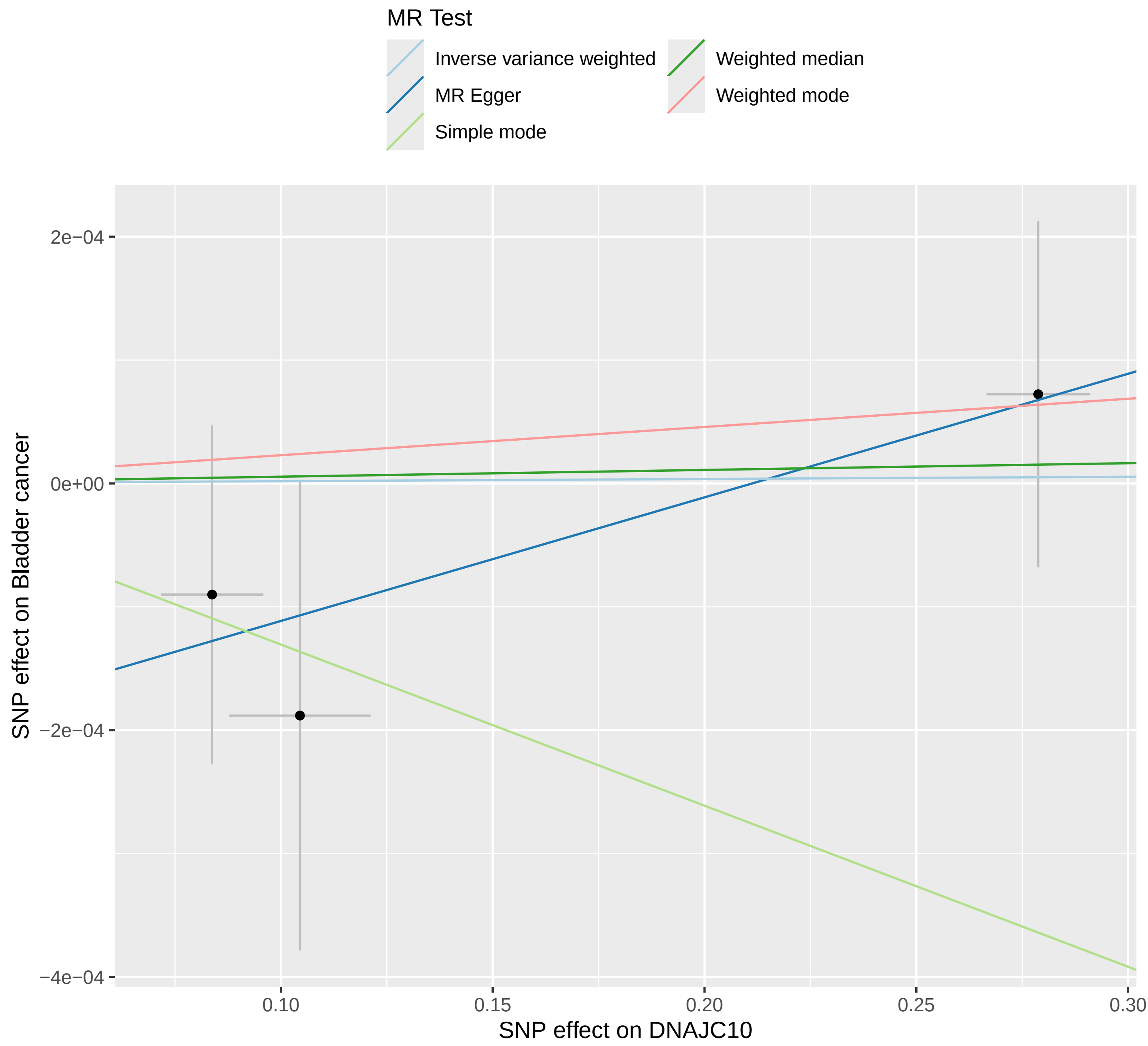
MR Test
Inverse variance weighted
MR Egger
Simple mode
Weighted median
Weighted mode
2e−04
0e+00
−2e−04
−4e−04
SNP effect on Bladder cancer
0.10
0.15
0.20
0.25
0.30
SNP effect on DNAJC10

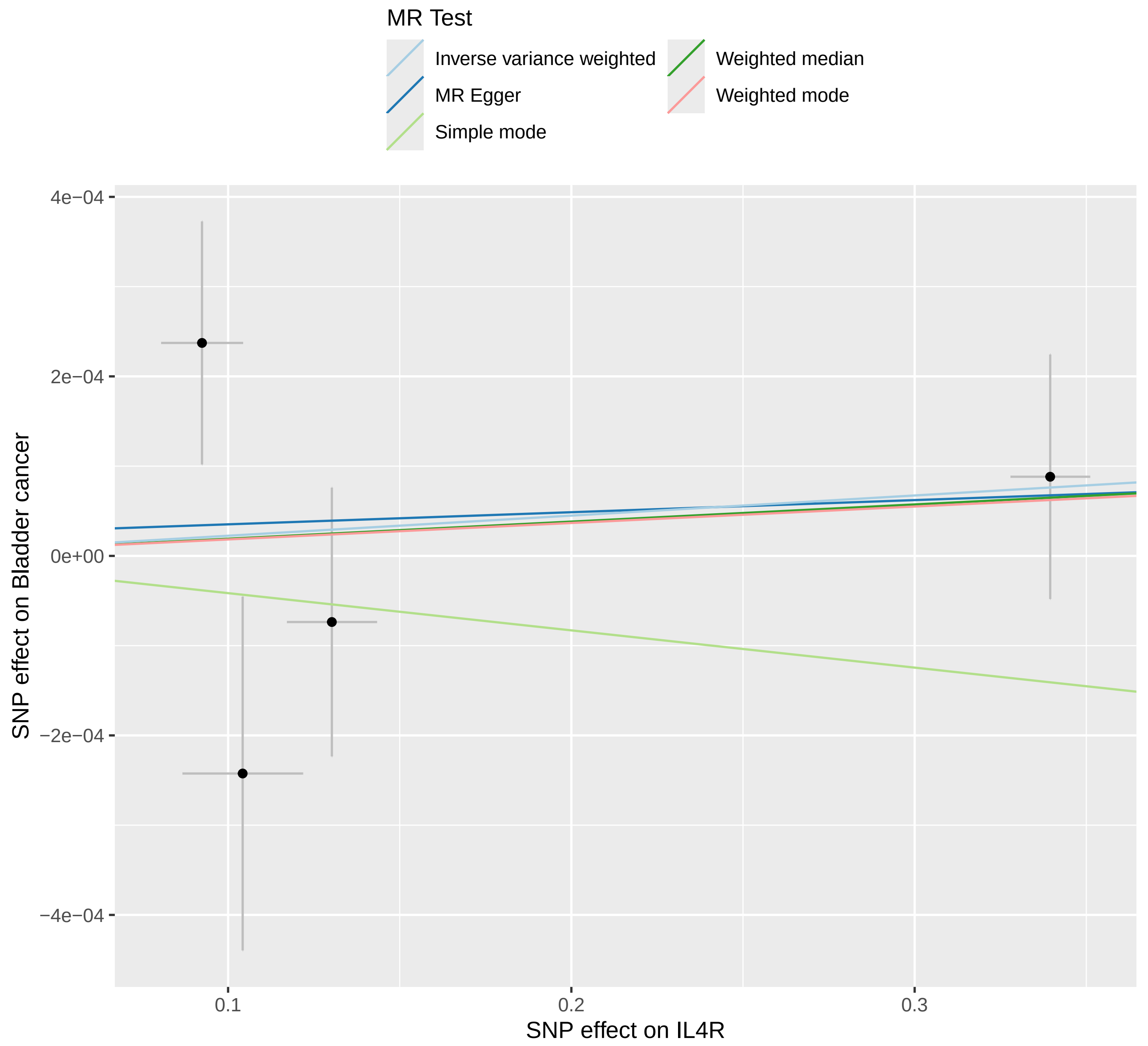

MR Test
Inverse variance weighted
MR Egger
Simple mode
Weighted median
Weighted mode
4e−04
2e−04
0e+00
−2e−04
−4e−04
SNP effect on Bladder cancer
0.1
0.2
0.3
SNP effect on IL4R

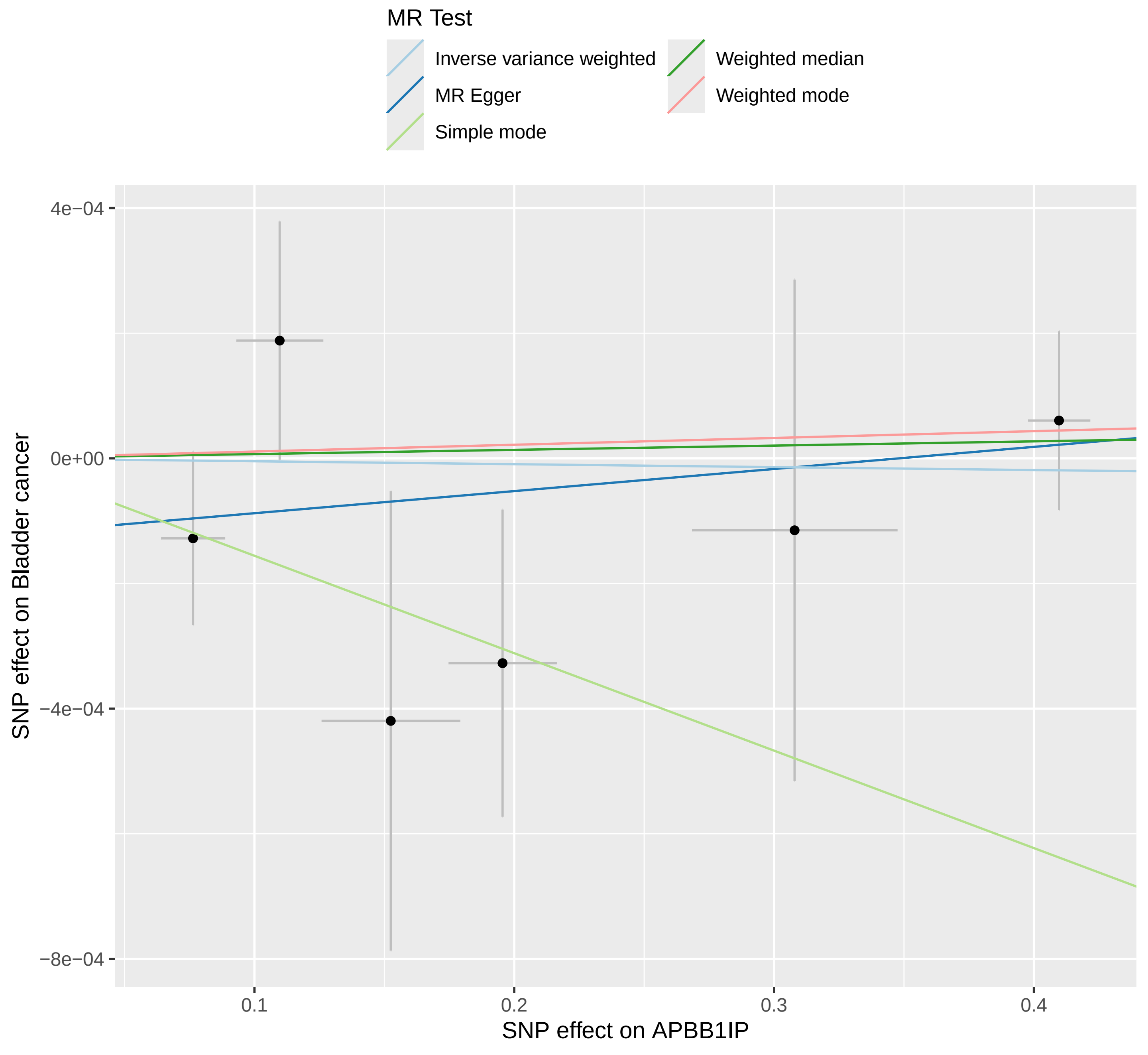

MR Test
Inverse variance weighted
MR Egger
Simple mode
Weighted median
Weighted mode
4e−04
0e+00
−4e−04
−8e−04
0.1
0.2
0.3
0.4
SNP effect on APBB1IP
SNP effect on Bladder cancer

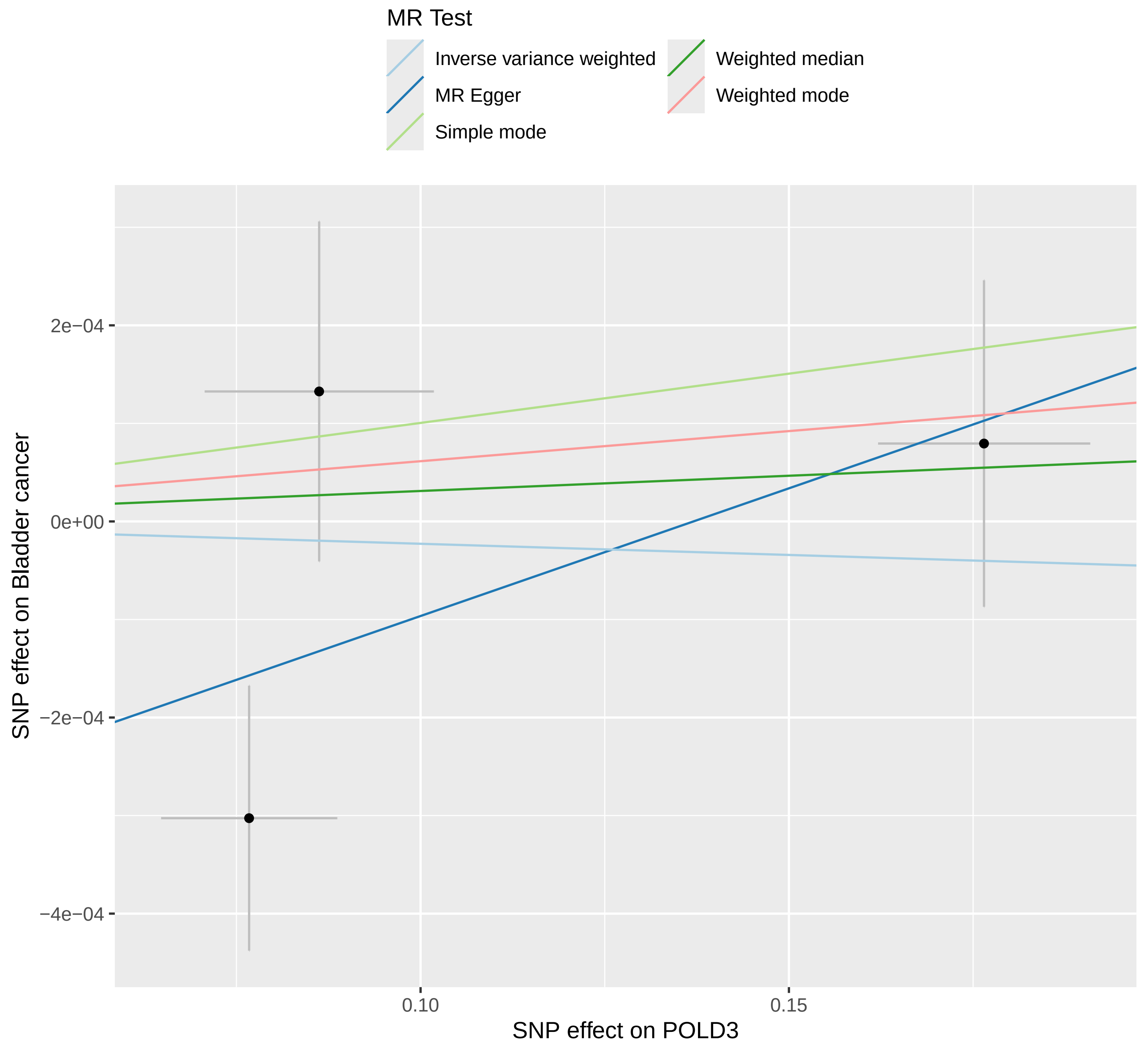

MR Test
Inverse variance weighted
MR Egger
Simple mode
Weighted median
Weighted mode
2e−04
0e+00
−2e−04
−4e−04
0.10
0.15
SNP effect on Bladder cancer
SNP effect on POLD3

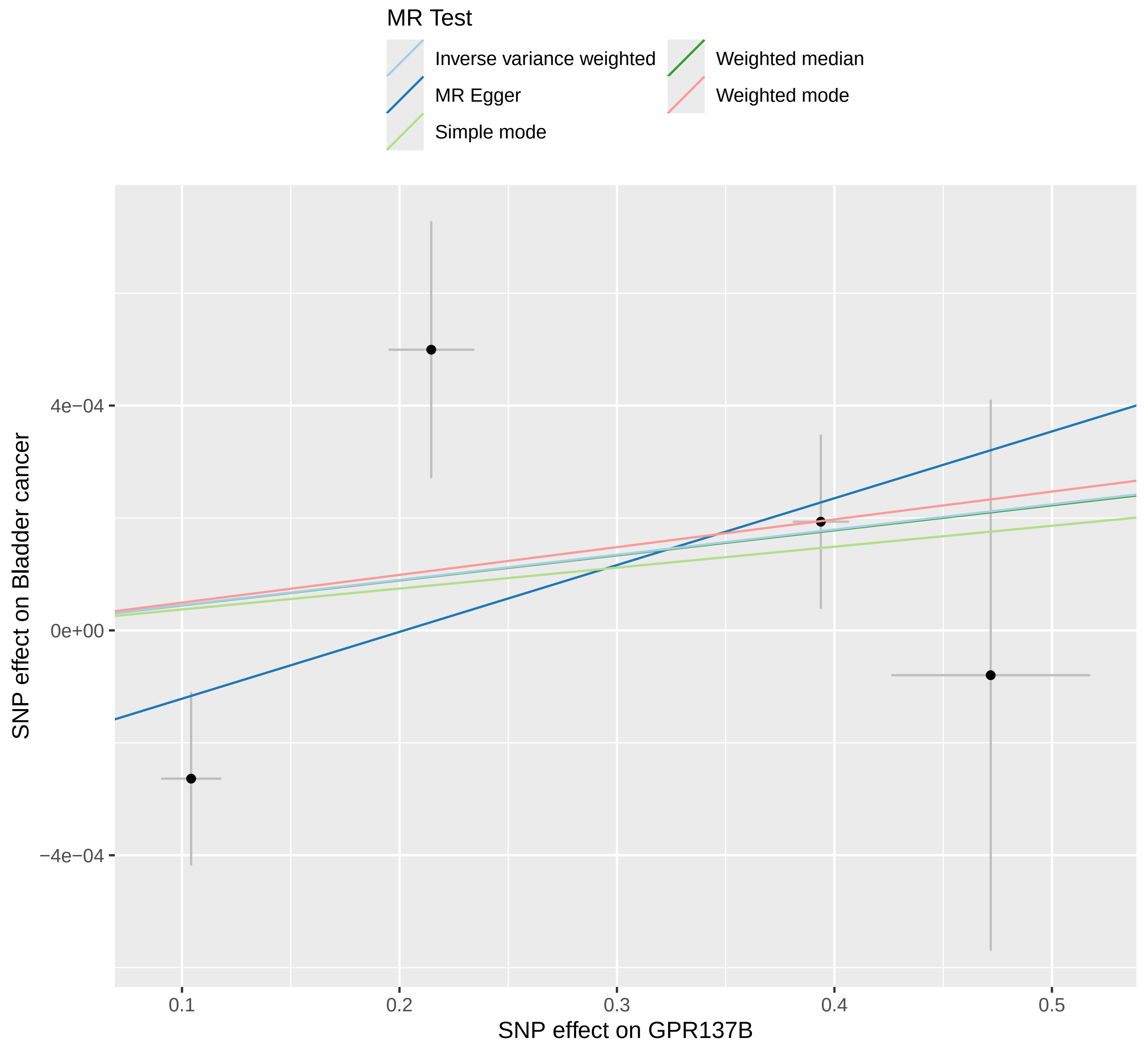
MR Test
Inverse variance weighted
MR Egger
Simple mode
Weighted median
Weighted mode
4e−04
0e+00
−4e−04
SNP effect on Bladder cancer
0.1
0.2
0.3
0.4
0.5
SNP effect on GPR137B

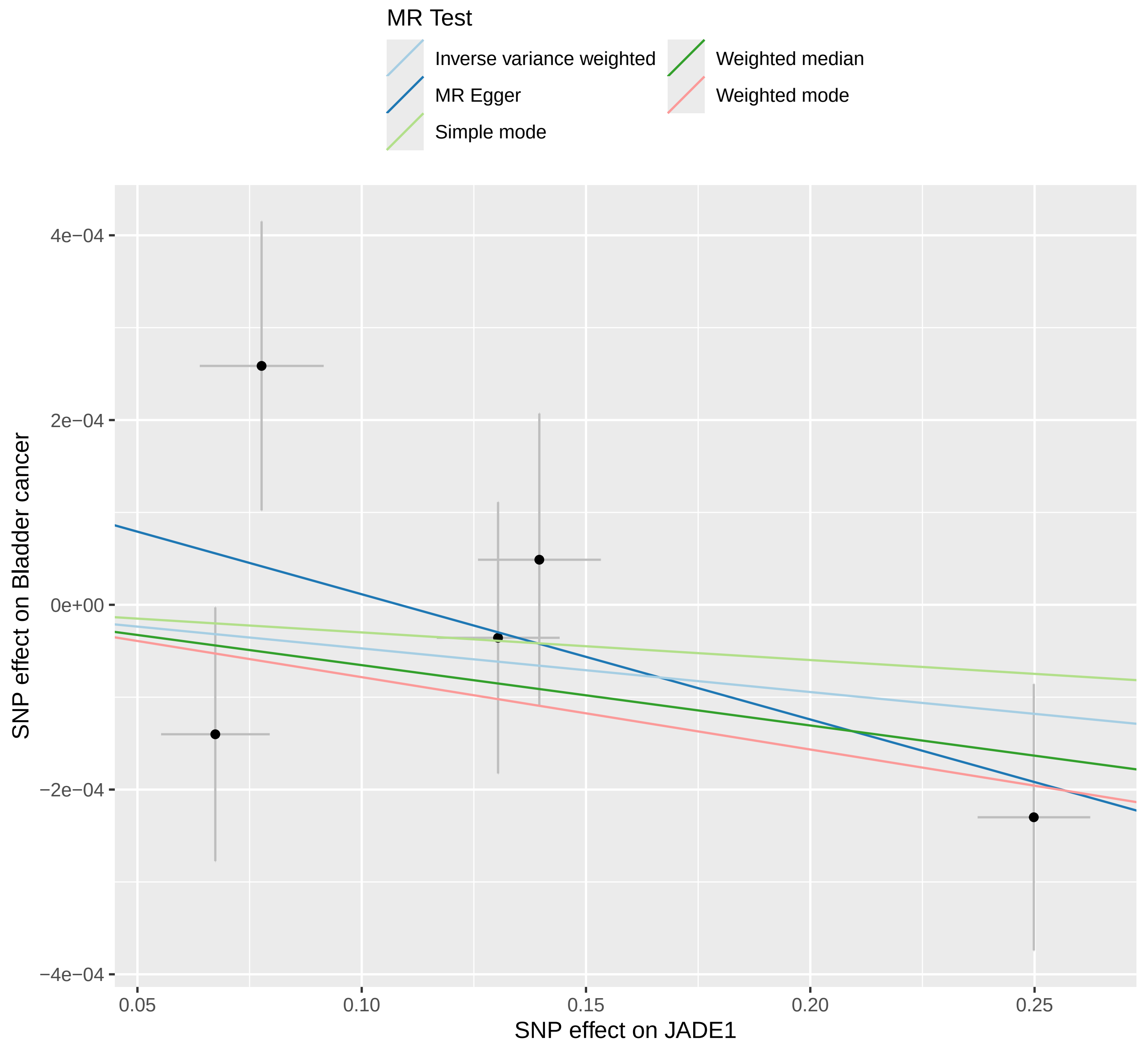
MR Test
Inverse variance weighted
MR Egger
Simple mode
Weighted median
Weighted mode
SNP effect on Bladder cancer
4e−04
2e−04
0e+00
−2e−04
−4e−04
0.05
0.10
0.15
0.20
0.25
SNP effect on JADE1

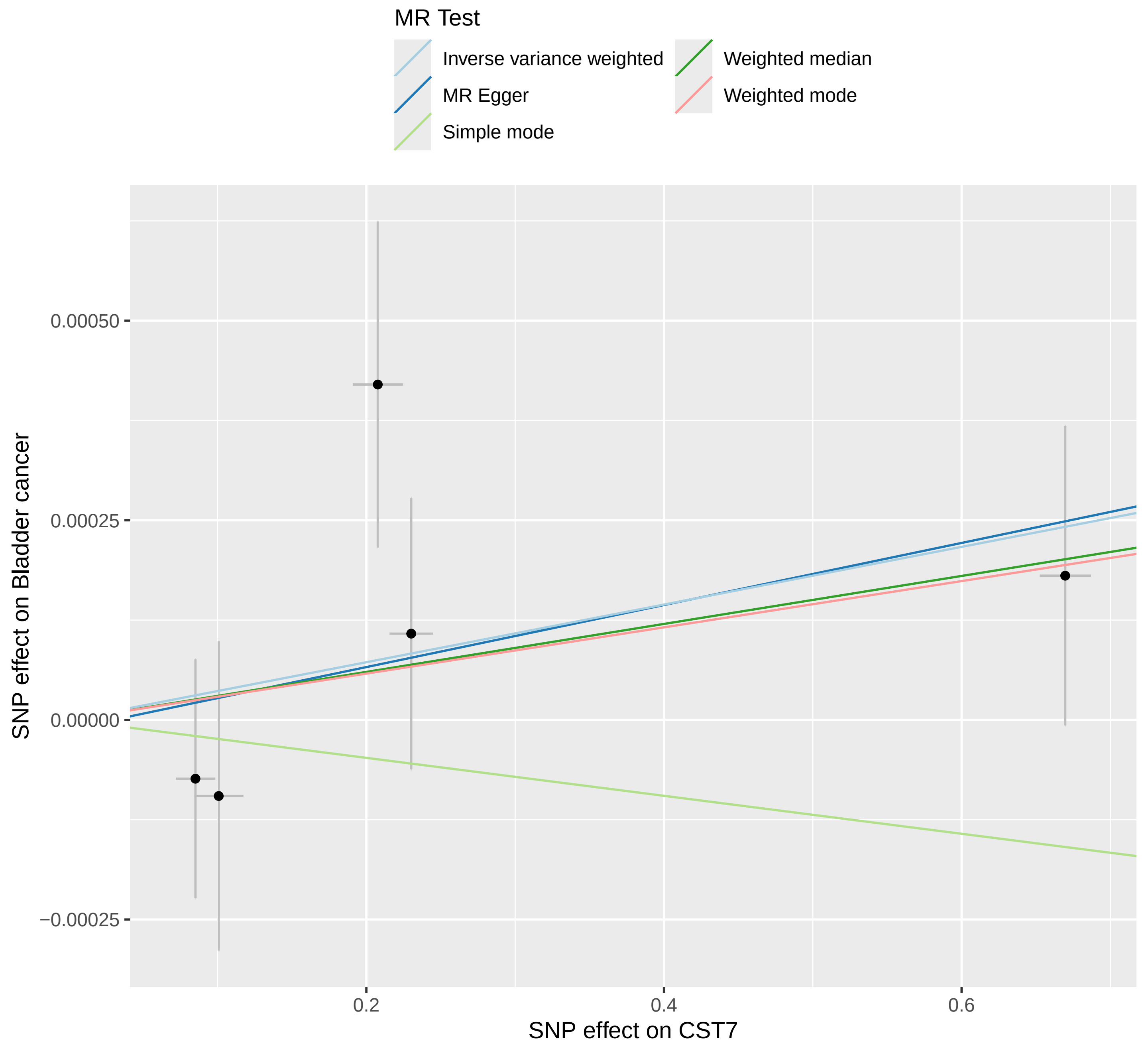

MR Test
Inverse variance weighted
MR Egger
Simple mode
Weighted median
Weighted mode
SNP effect on Bladder cancer
0.00050
0.00025
0.00000
−0.00025
0.2
0.4
0.6
SNP effect on CST7

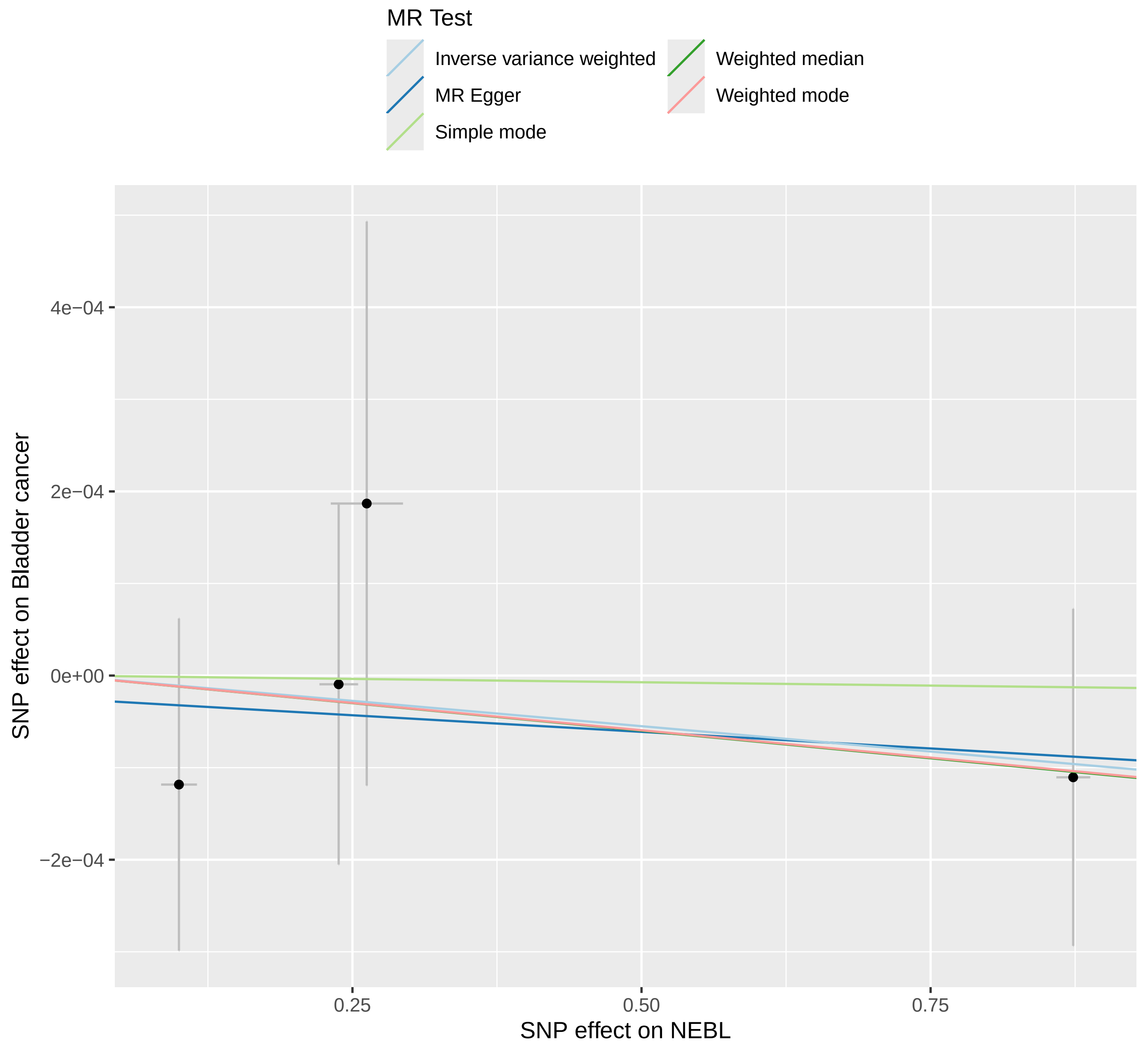
MR Test
Inverse variance weighted
MR Egger
Simple mode
Weighted median
Weighted mode
SNP effect on Bladder cancer
4e−04
2e−04
0e+00
−2e−04
0.25
0.50
0.75
SNP effect on NEBL

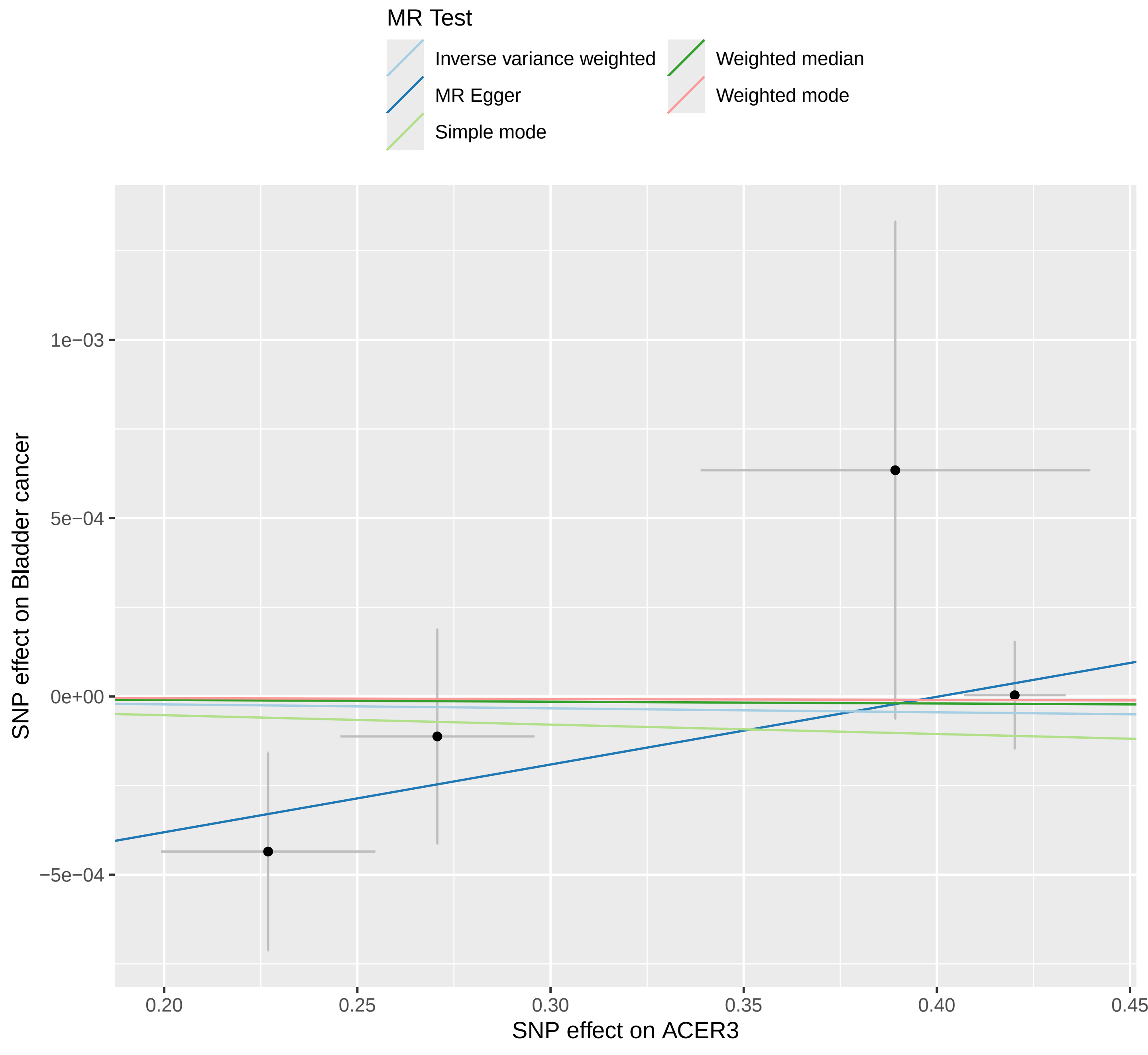
MR Test
Inverse variance weighted
MR Egger
Simple mode
Weighted median
Weighted mode
1e−03
5e−04
0e+00
−5e−04
0.20
0.25
0.30
0.35
0.40
0.45
SNP effect on ACER3
SNP effect on Bladder cancer

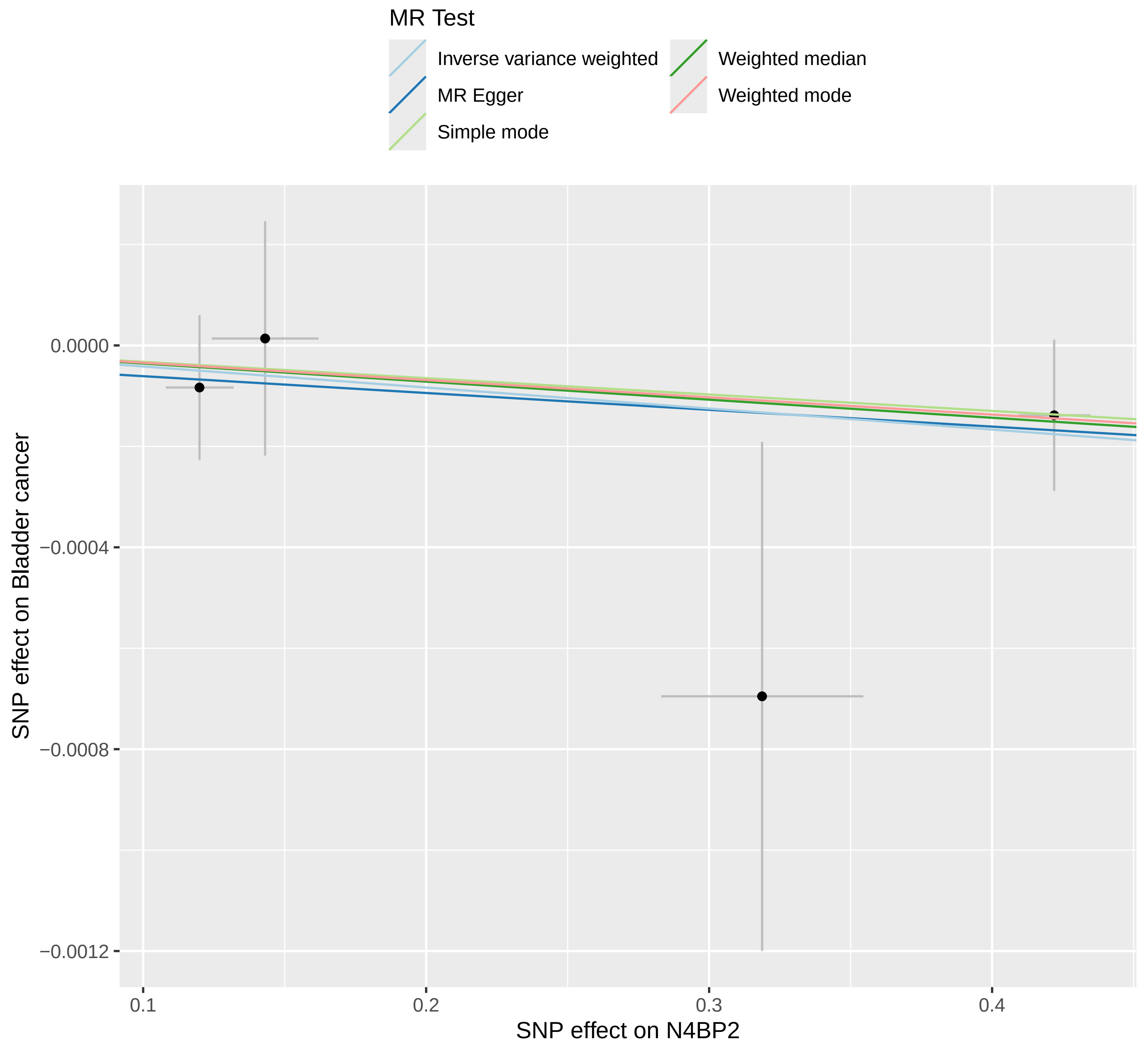

MR Test
Inverse variance weighted
MR Egger
Simple mode
Weighted median
Weighted mode
0.0000
−0.0004
−0.0008
−0.0012
SNP effect on Bladder cancer
0.1
0.2
0.3
0.4
SNP effect on N4BP2

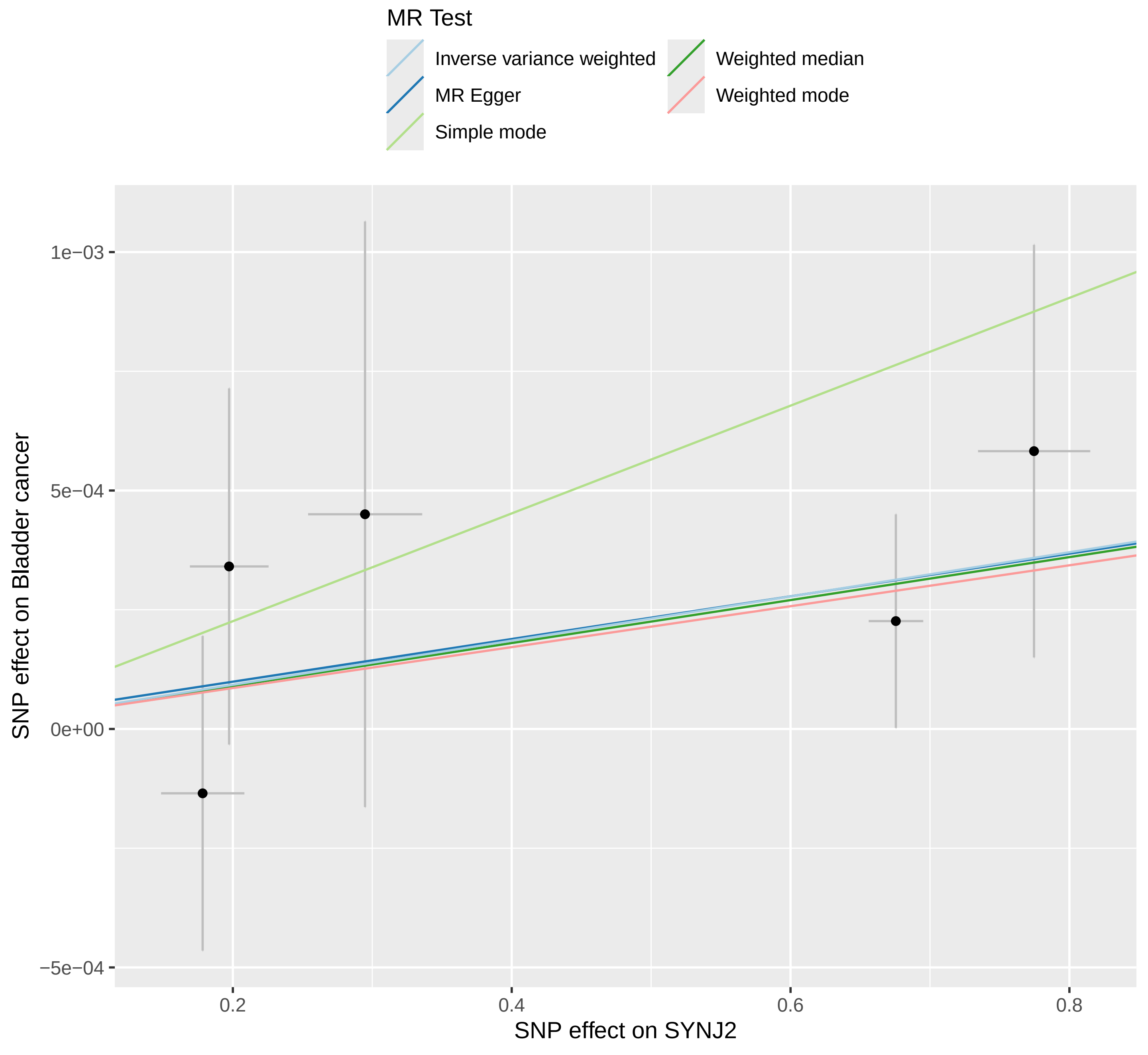

MR Test
Inverse variance weighted
MR Egger
Simple mode
Weighted median
Weighted mode
1e−03
5e−04
0e+00
−5e−04
SNP effect on Bladder cancer
0.2
0.4
0.6
0.8
SNP effect on SYNJ2

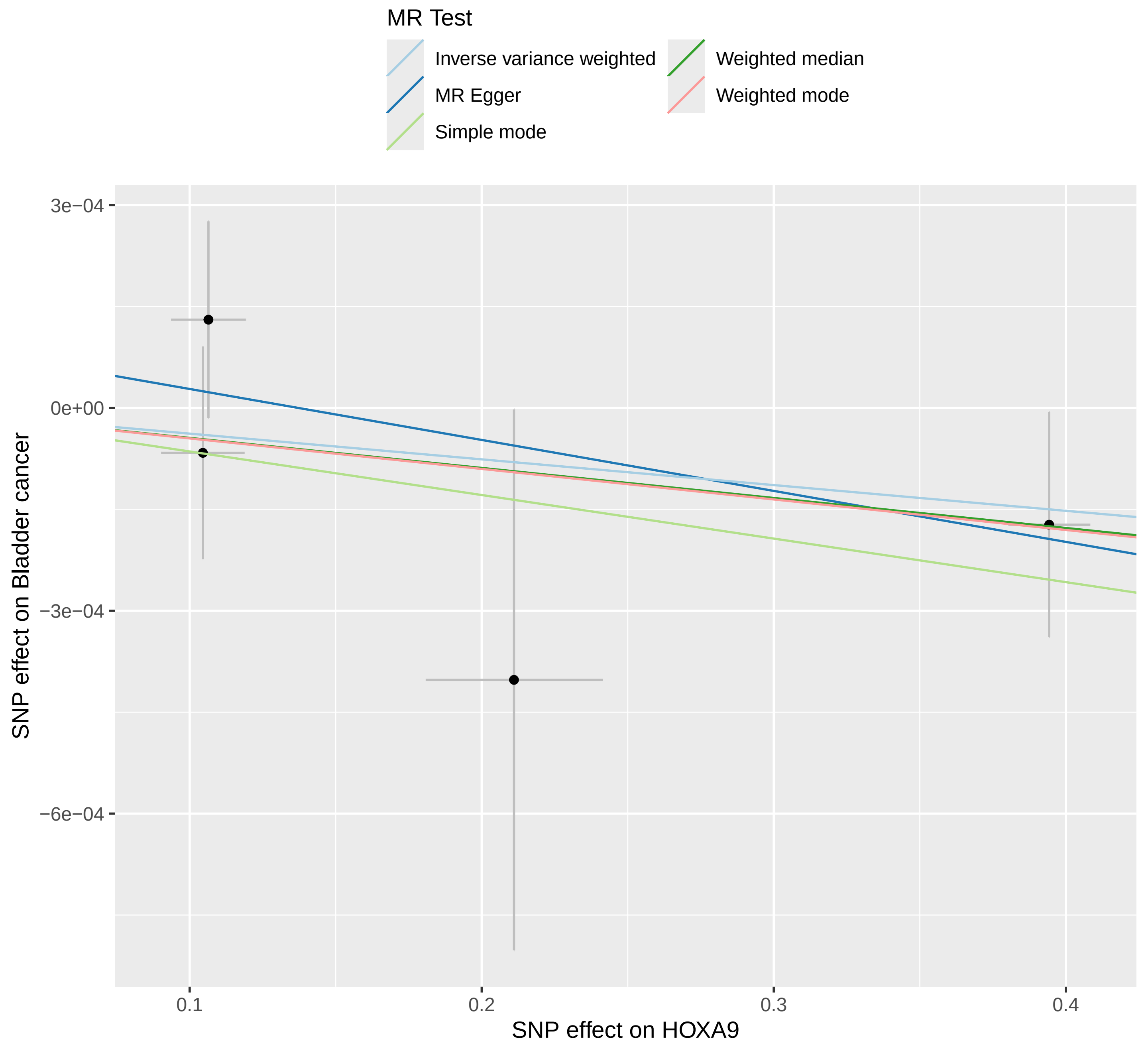
MR Test
Inverse variance weighted
MR Egger
Simple mode
Weighted median
Weighted mode
3e−04
0e+00
−3e−04
−6e−04
0.1
0.2
0.3
0.4
SNP effect on HOXA9
SNP effect on Bladder cancer

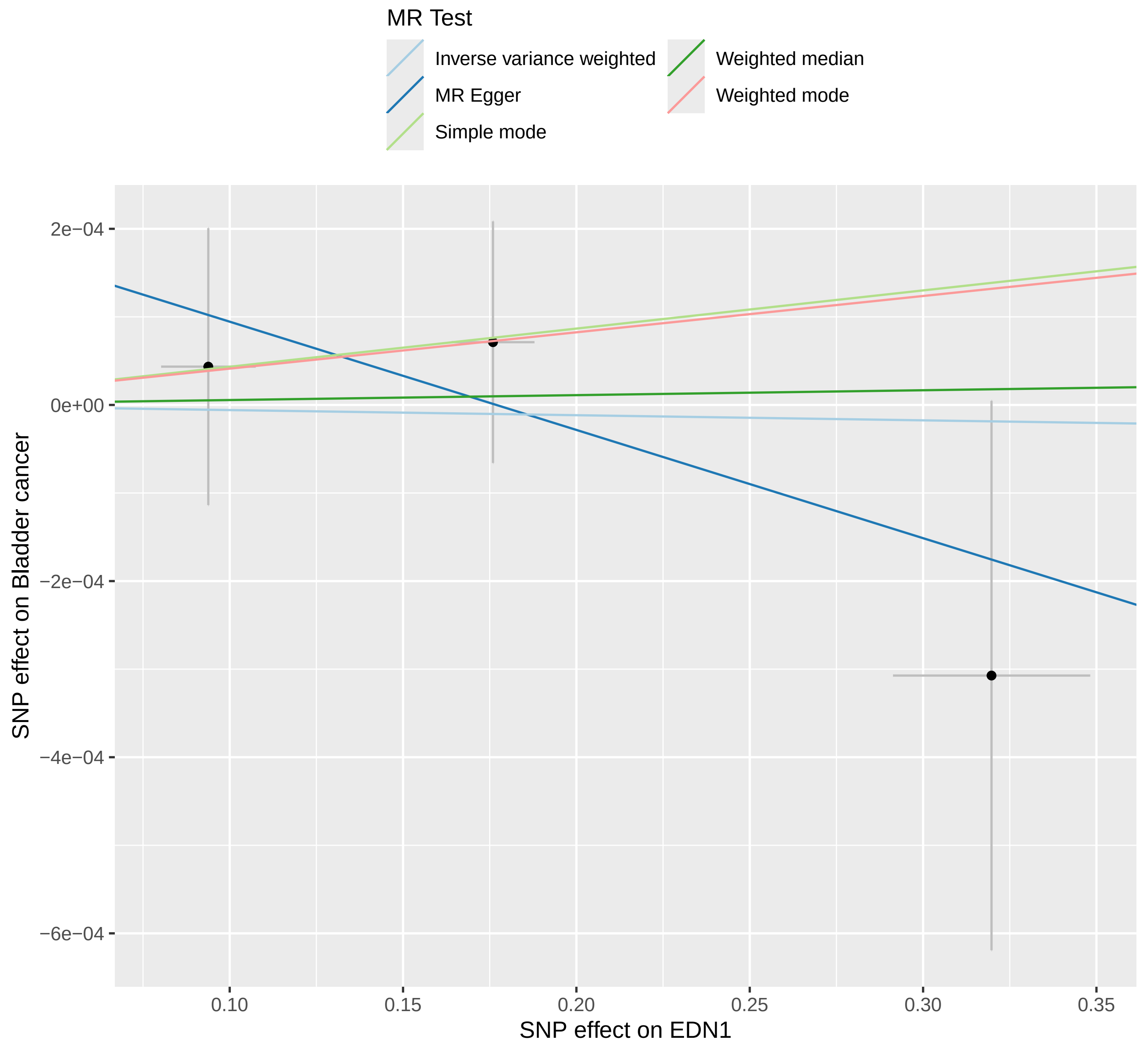

MR Test
Inverse variance weighted
MR Egger
Simple mode
Weighted median
Weighted mode
2e−04
0e+00
−2e−04
−4e−04
−6e−04
SNP effect on Bladder cancer
0.10
0.15
0.20
0.25
0.30
0.35
SNP effect on EDN1

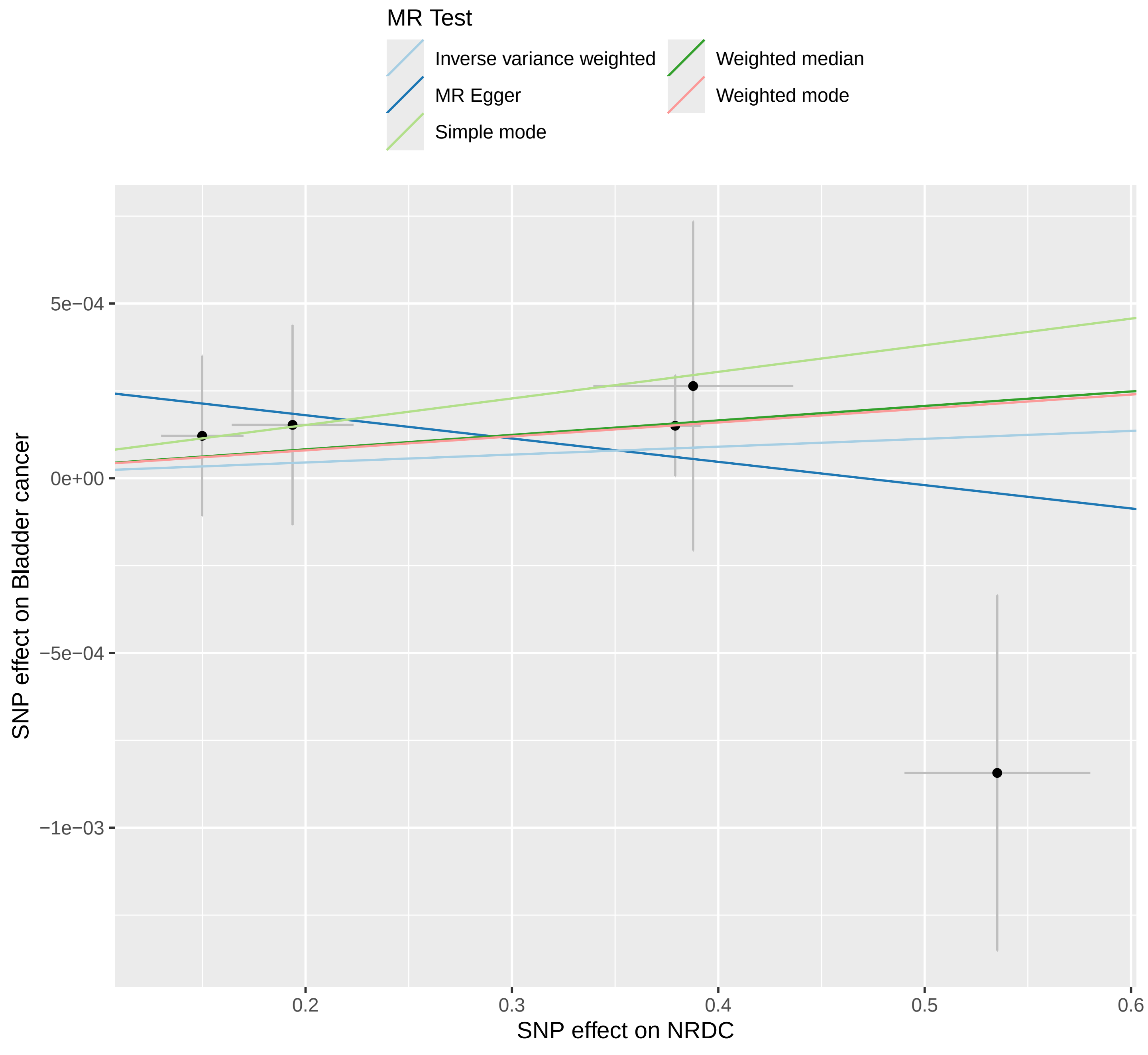

MR Test
Inverse variance weighted
MR Egger
Simple mode
Weighted median
Weighted mode
5e−04
0e+00
−5e−04
−1e−03
SNP effect on Bladder cancer
0.2
0.3
0.4
0.5
0.6
SNP effect on NRDC

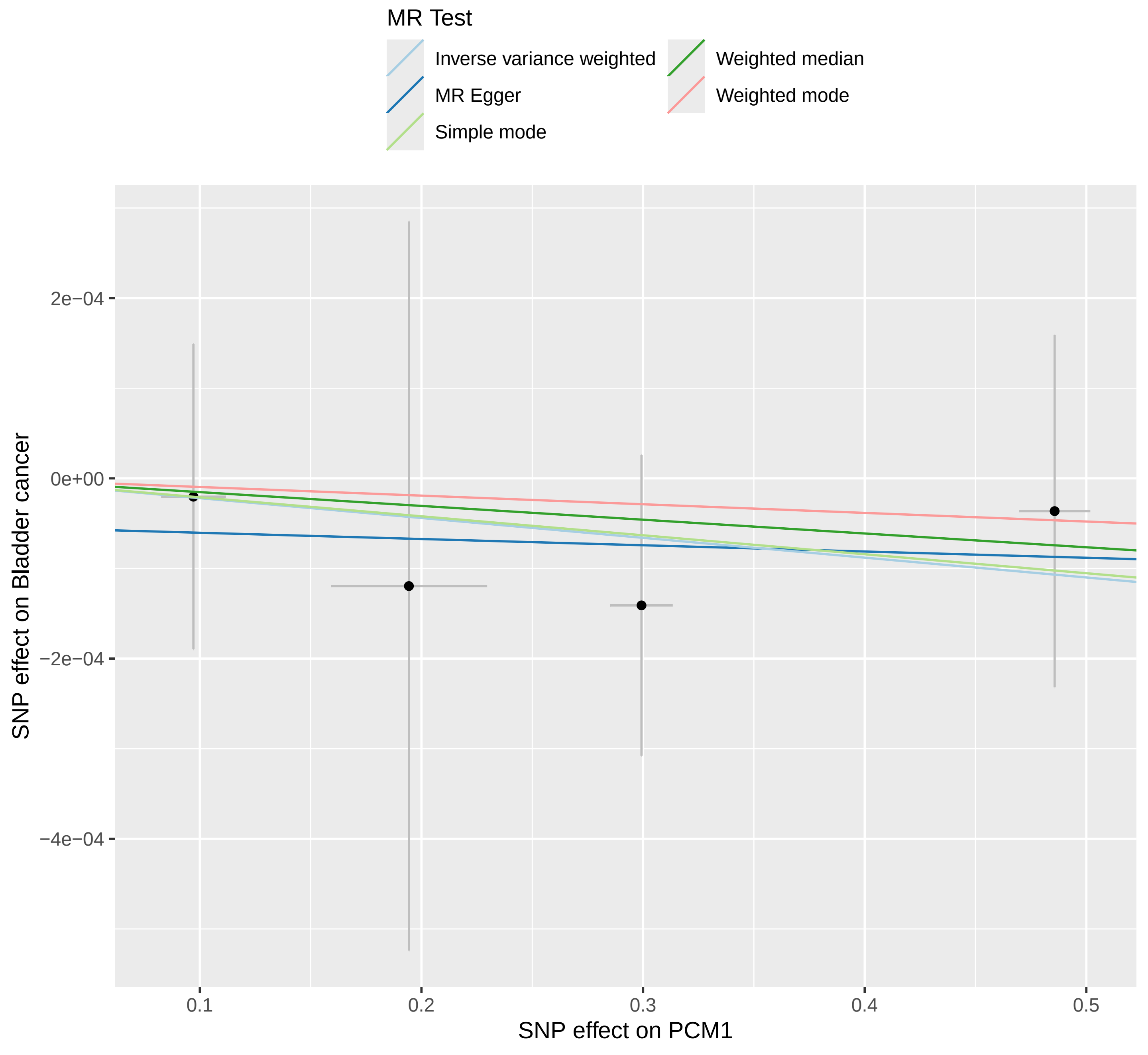
MR Test
Inverse variance weighted
MR Egger
Simple mode
Weighted median
Weighted mode
SNP effect on Bladder cancer
2e−04
0e+00
−2e−04
−4e−04
0.1
0.2
0.3
0.4
0.5
SNP effect on PCM1

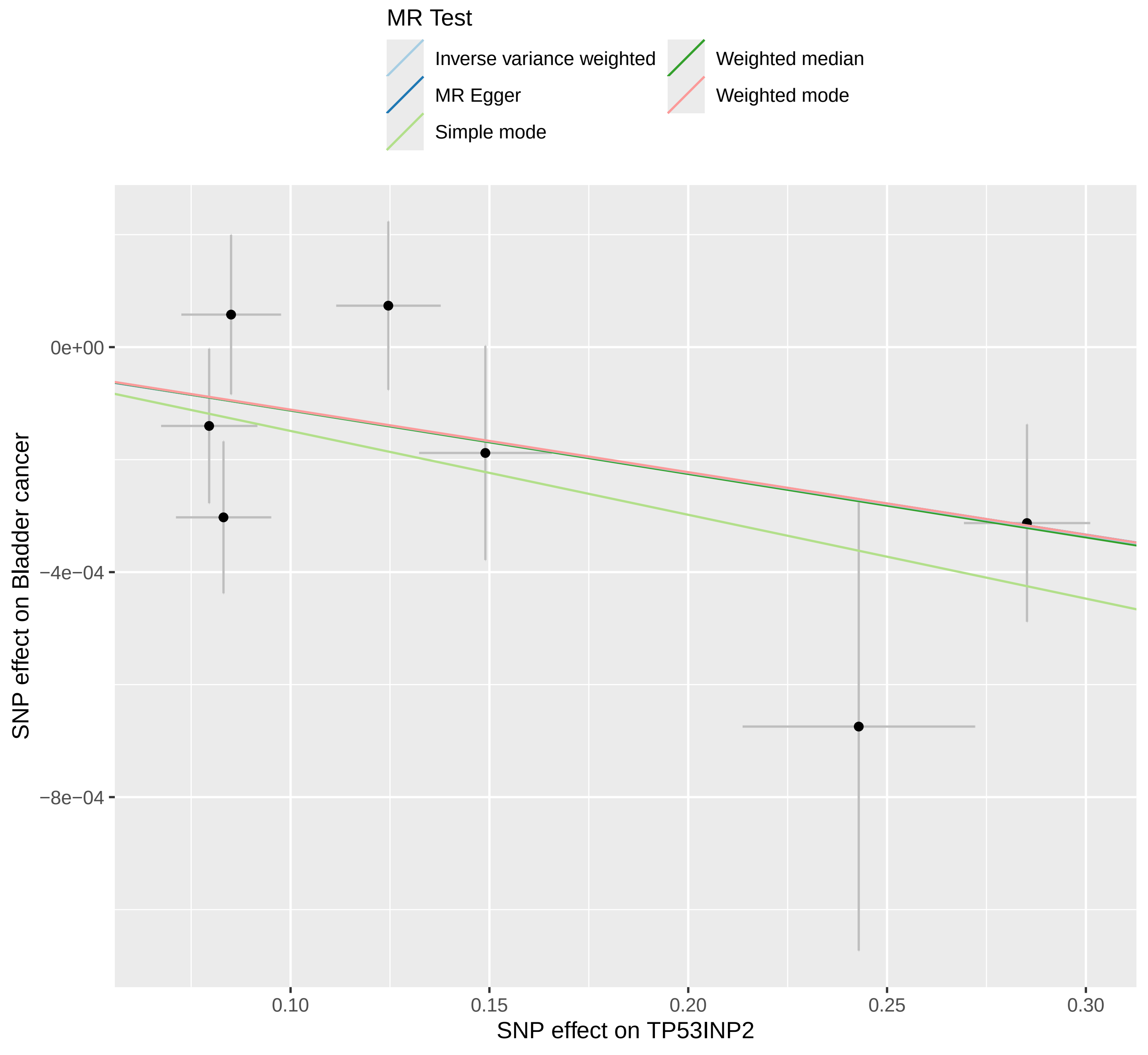
MR Test
Inverse variance weighted
MR Egger
Simple mode
Weighted median
Weighted mode
0e+00
−4e−04
−8e−04
SNP effect on Bladder cancer
0.10
0.15
0.20
0.25
0.30
SNP effect on TP53INP2

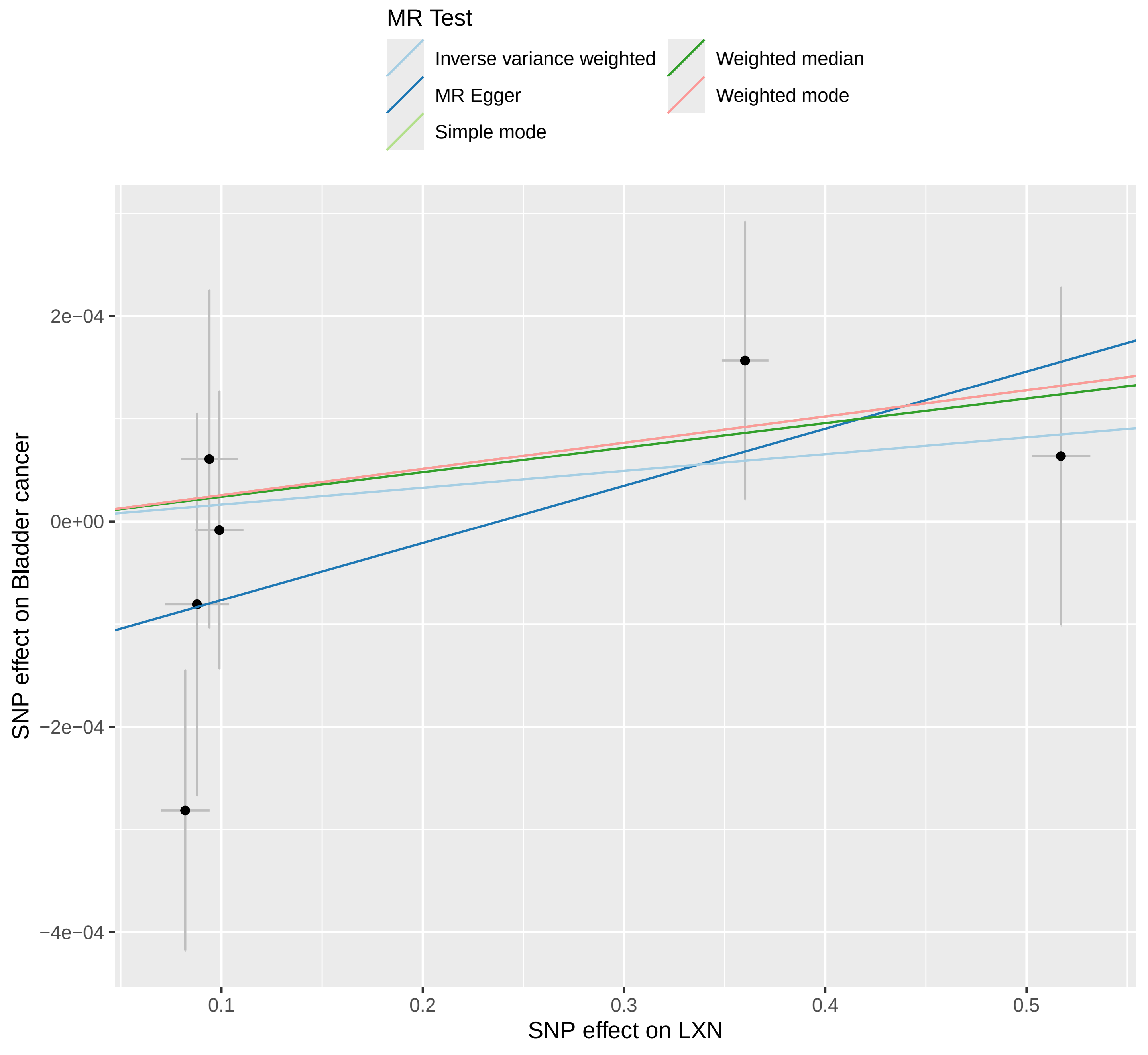
MR Test
Inverse variance weighted
MR Egger
Simple mode
Weighted median
Weighted mode
2e−04
0e+00
−2e−04
−4e−04
0.1
0.2
0.3
0.4
0.5
SNP effect on LXN
SNP effect on Bladder cancer

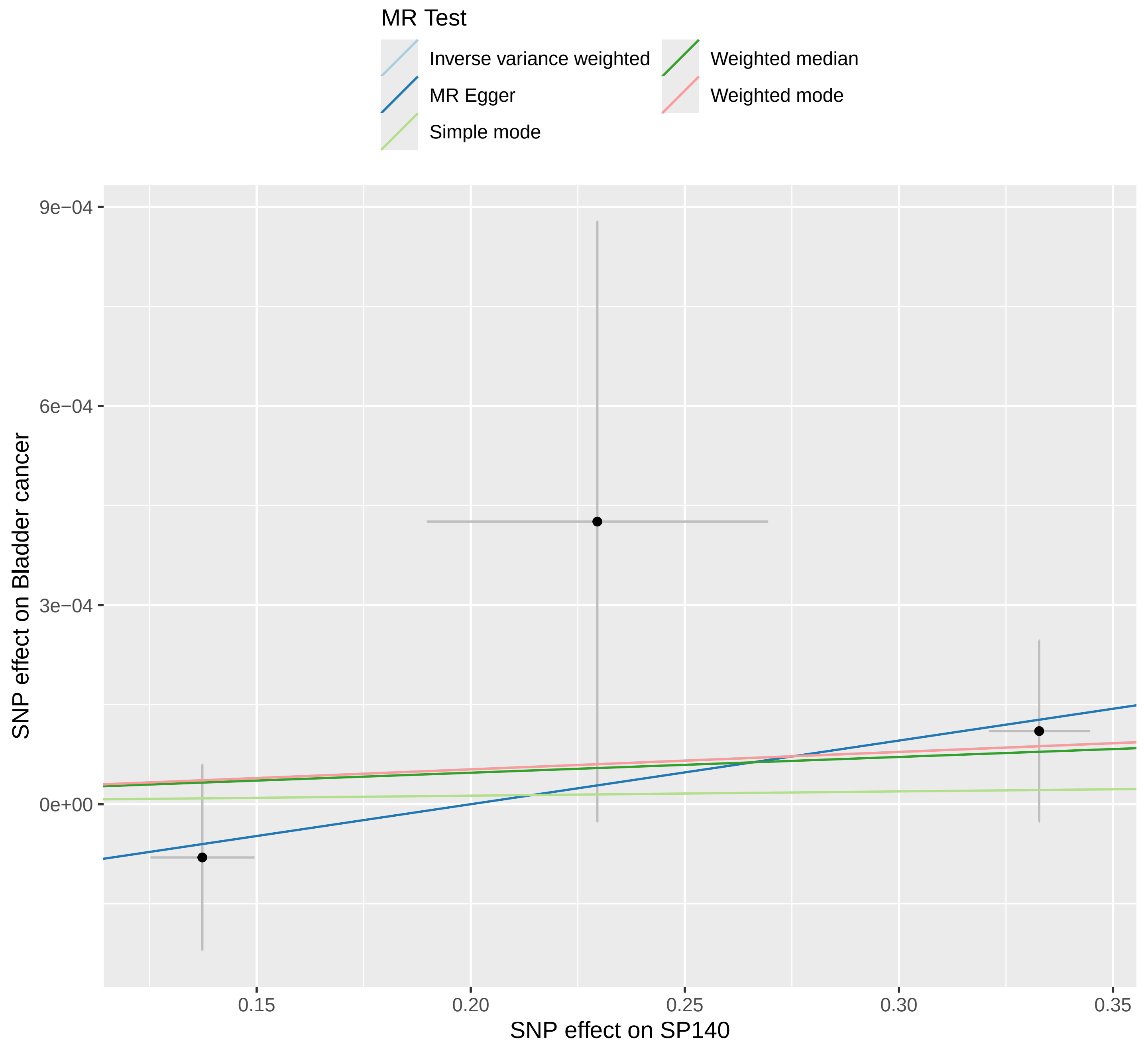

MR Test
Inverse variance weighted
MR Egger
Simple mode
Weighted median
Weighted mode
9e−04
6e−04
3e−04
0e+00
SNP effect on Bladder cancer
0.15
0.20
0.25
0.30
0.35
SNP effect on SP140

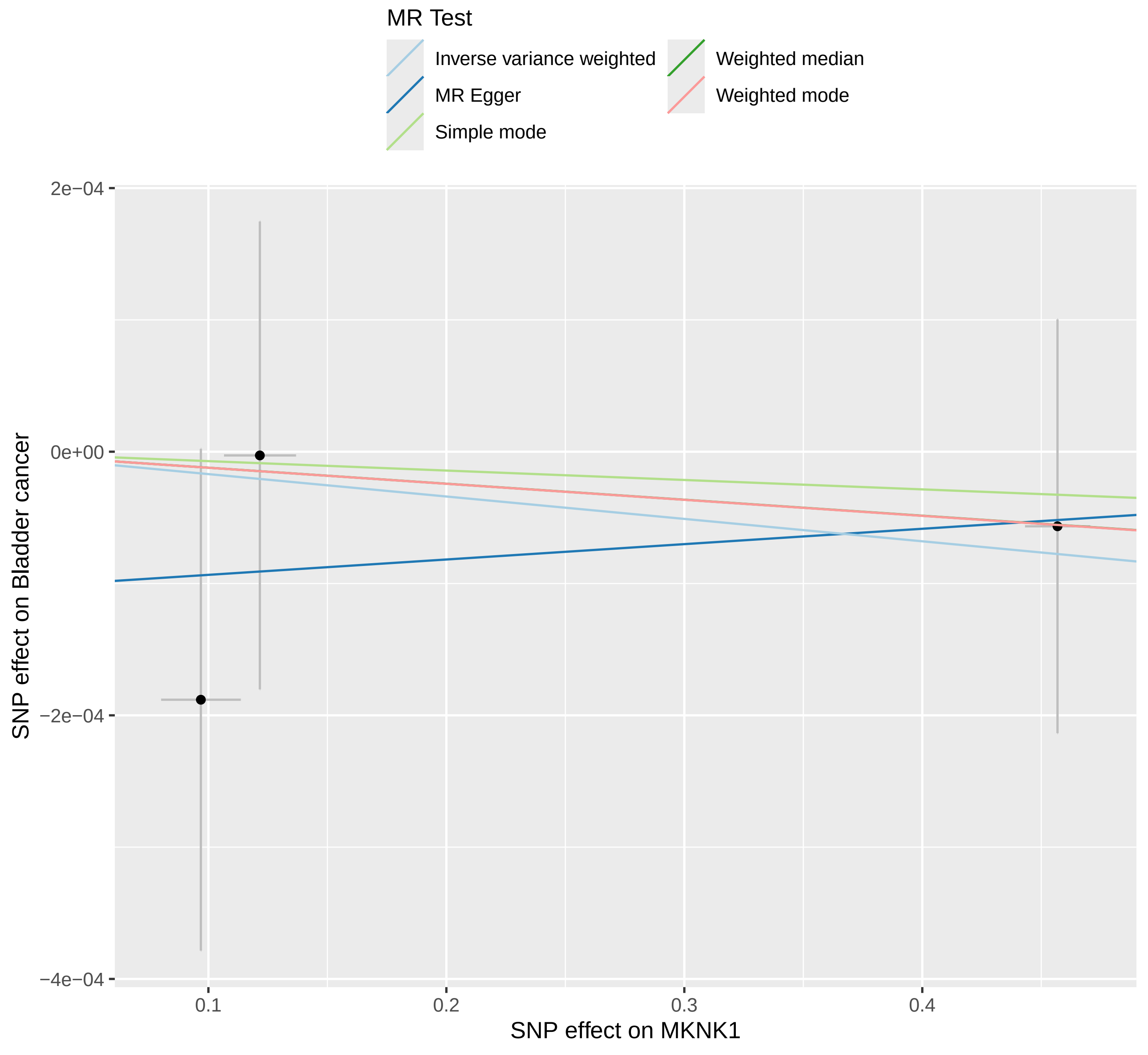
MR Test
Inverse variance weighted
MR Egger
Simple mode
Weighted median
Weighted mode
2e−04
0e+00
−2e−04
−4e−04
0.1
0.2
0.3
0.4
SNP effect on Bladder cancer
SNP effect on MKNK1

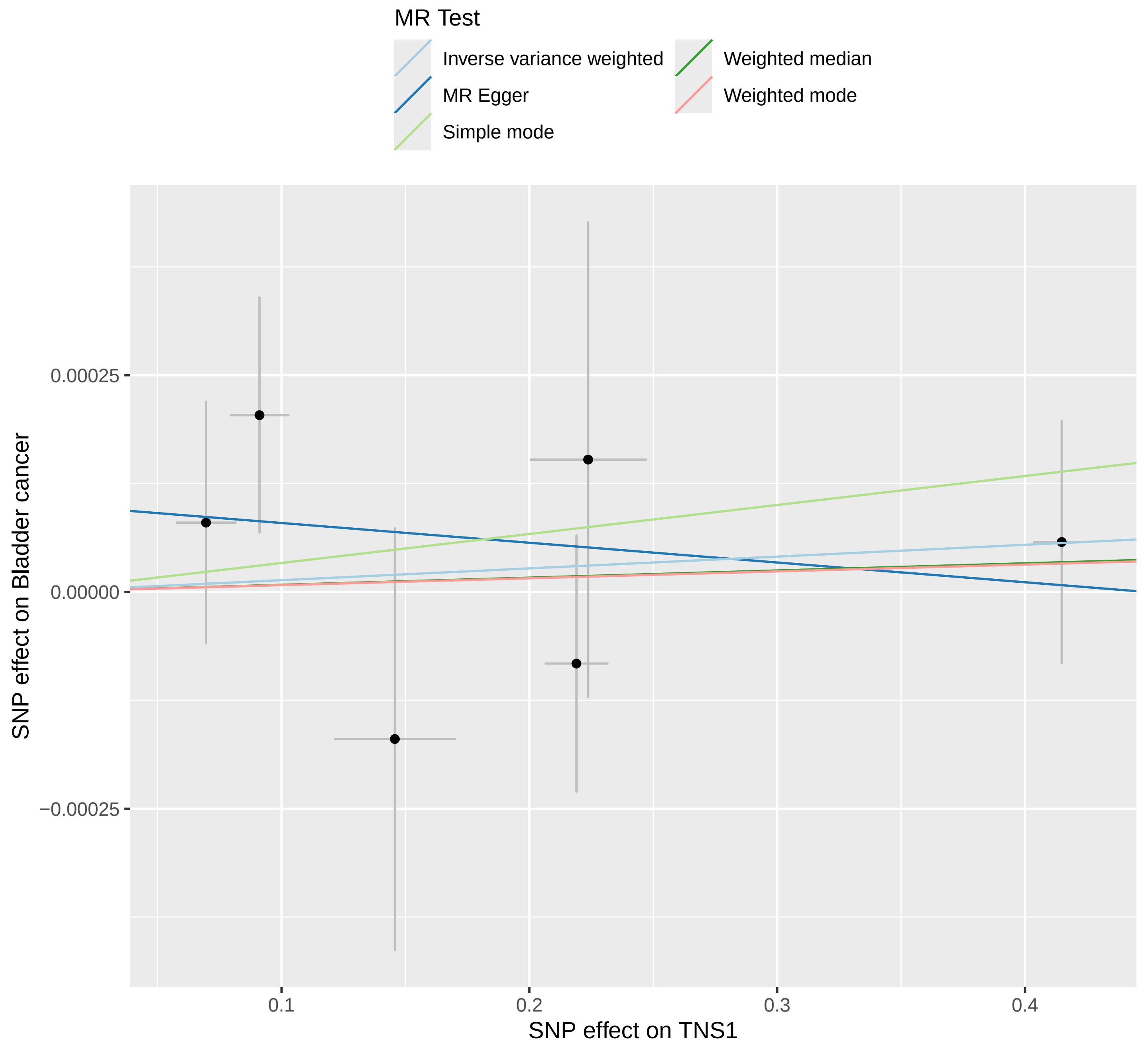
MR Test
Inverse variance weighted
MR Egger
Simple mode
Weighted median
Weighted mode
0.00025
0.00000
−0.00025
0.1
0.2
0.3
0.4
SNP effect on TNS1
SNP effect on Bladder cancer

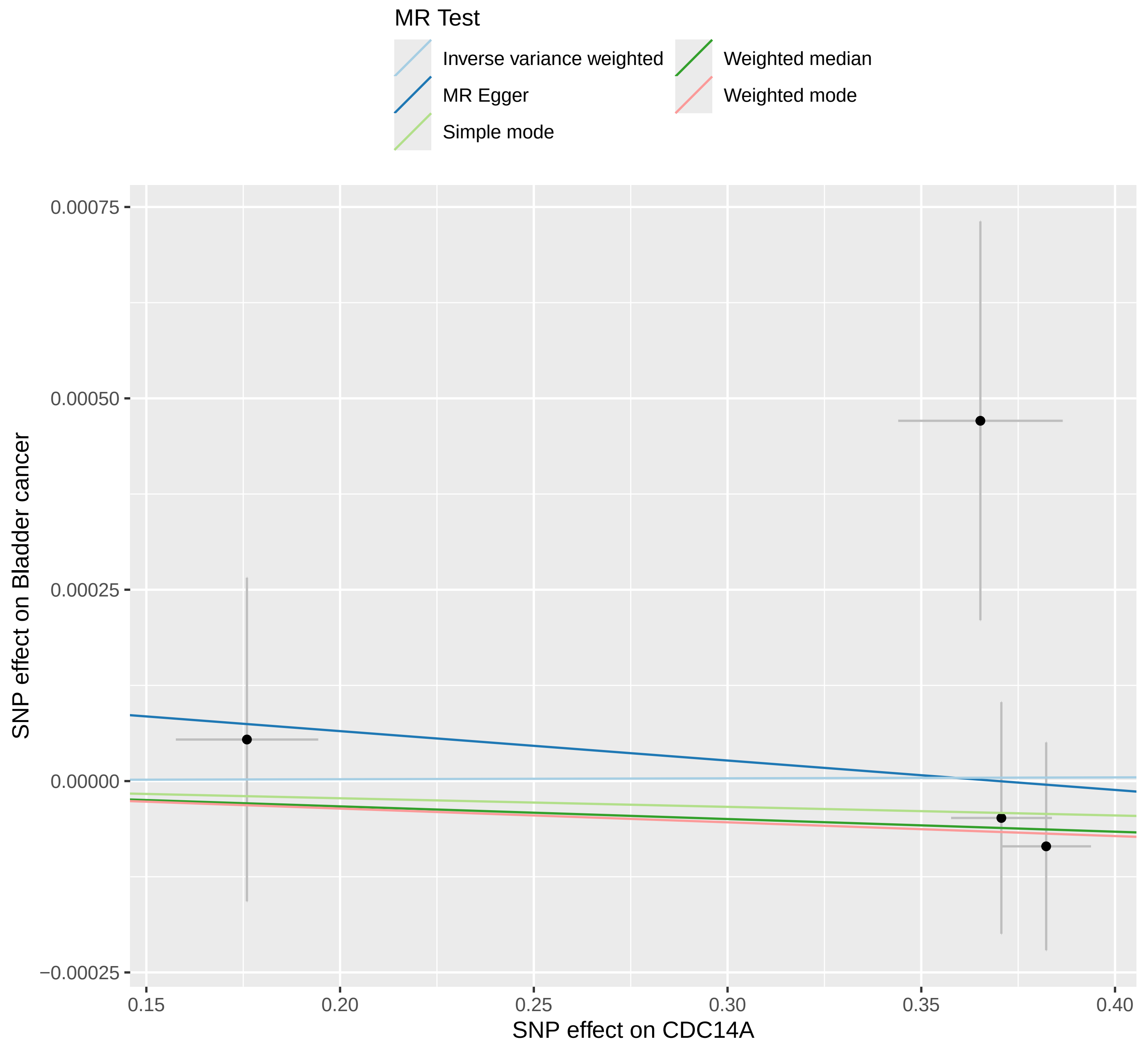
MR Test
Inverse variance weighted
MR Egger
Simple mode
Weighted median
Weighted mode
0.00075
0.00050
0.00025
0.00000
−0.00025
SNP effect on Bladder cancer
0.15
0.20
0.25
0.30
0.35
0.40
SNP effect on CDC14A

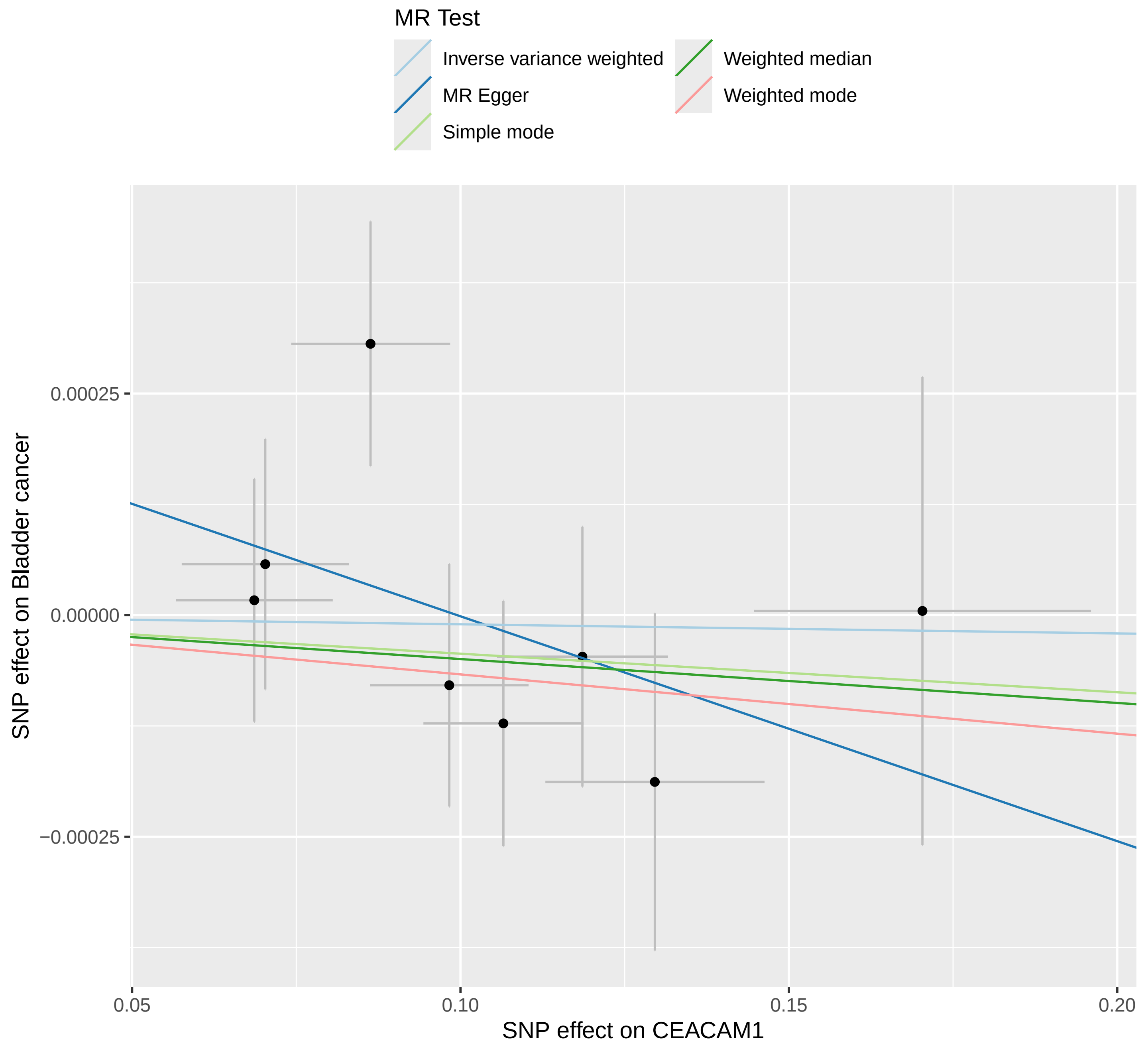

MR Test
Inverse variance weighted
MR Egger
Simple mode
Weighted median
Weighted mode
0.00025
0.00000
−0.00025
SNP effect on Bladder cancer
0.05
0.10
0.15
0.20
SNP effect on CEACAM1

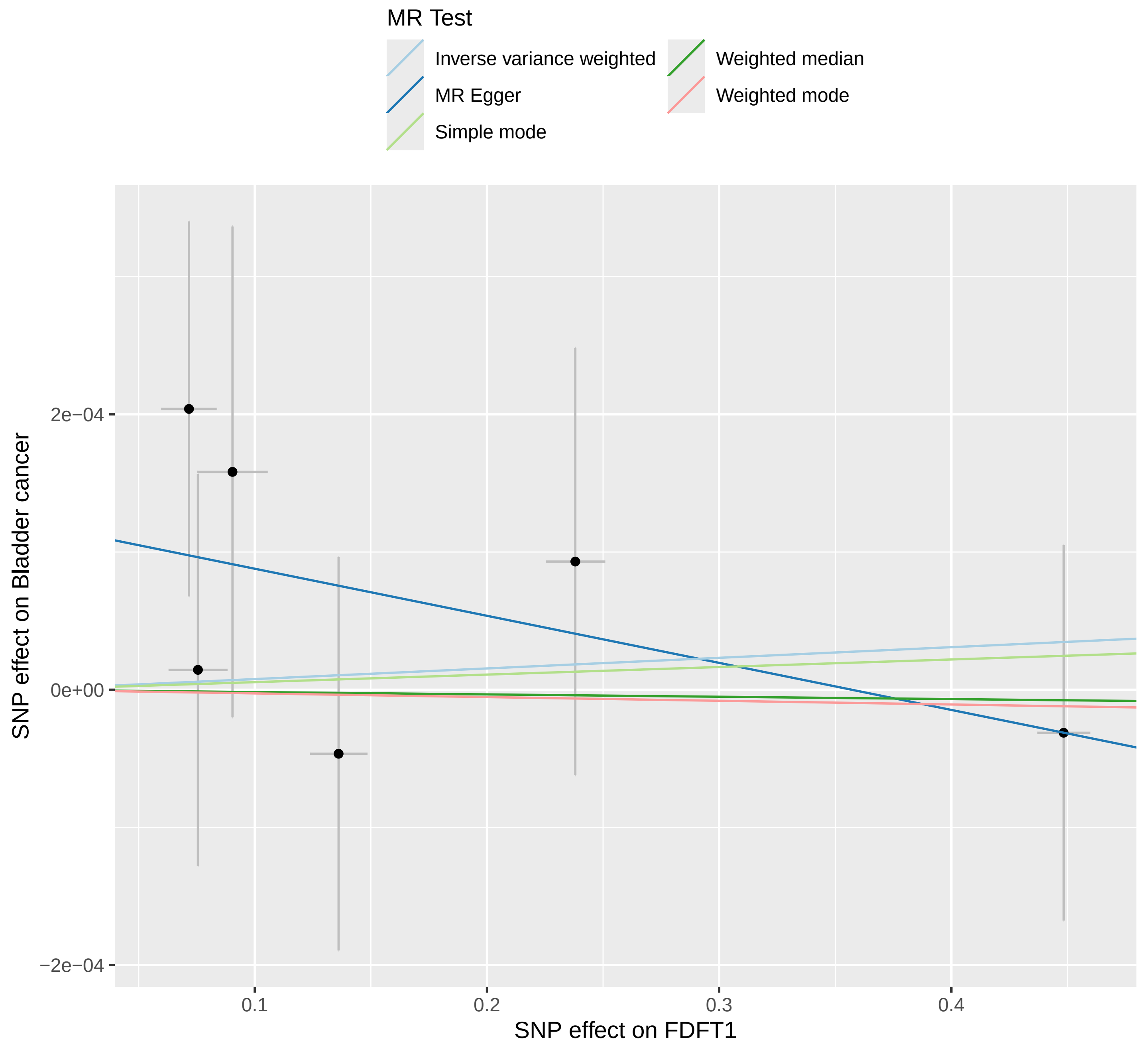

MR Test
Inverse variance weighted
MR Egger
Simple mode
Weighted median
Weighted mode
2e−04
0e+00
−2e−04
SNP effect on Bladder cancer
0.1
0.2
0.3
0.4
SNP effect on FDFT1

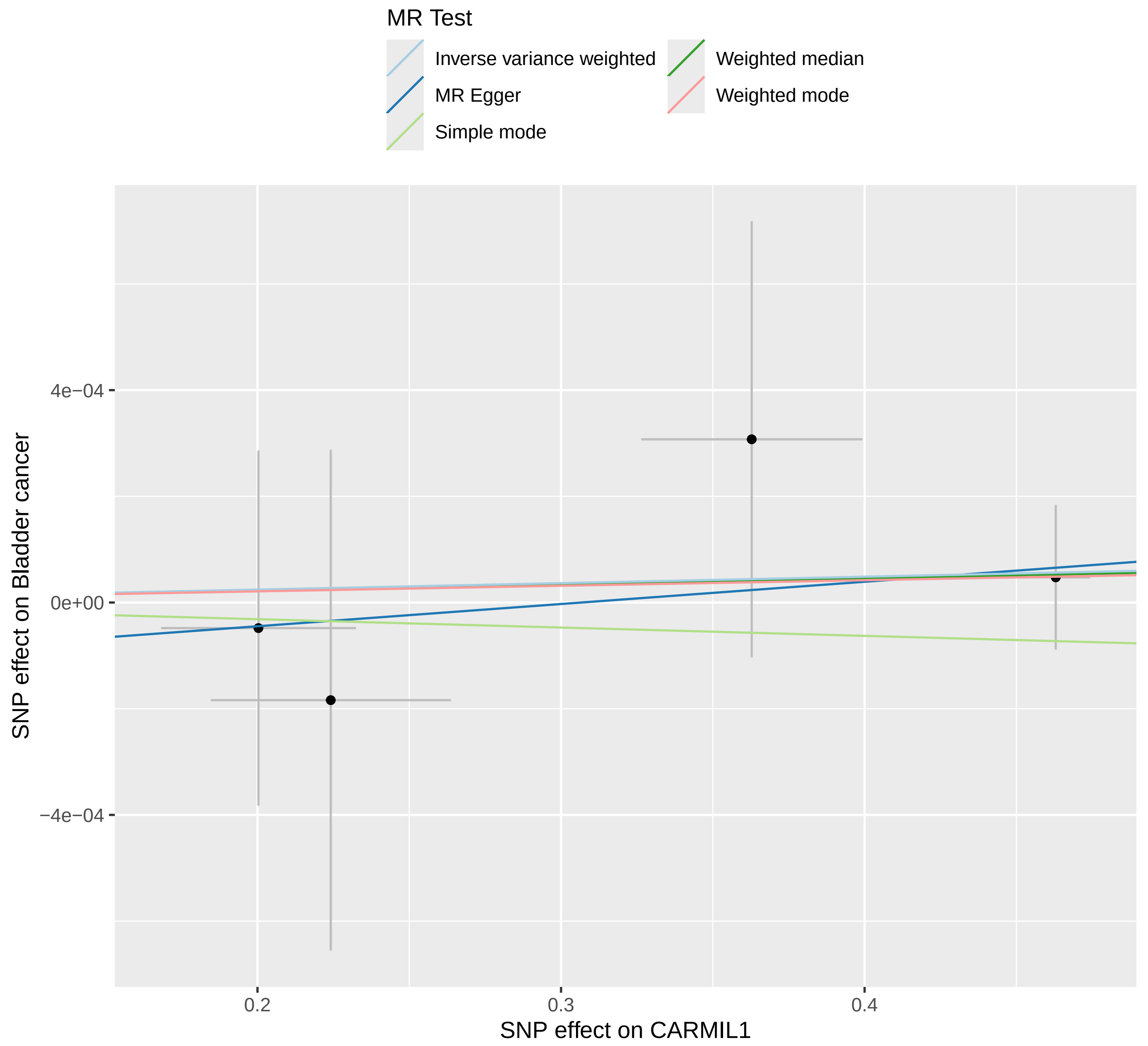
MR Test
Inverse variance weighted
MR Egger
Simple mode
Weighted median
Weighted mode
4e−04
0e+00
−4e−04
SNP effect on Bladder cancer
0.2
0.3
0.4
SNP effect on CARMIL1

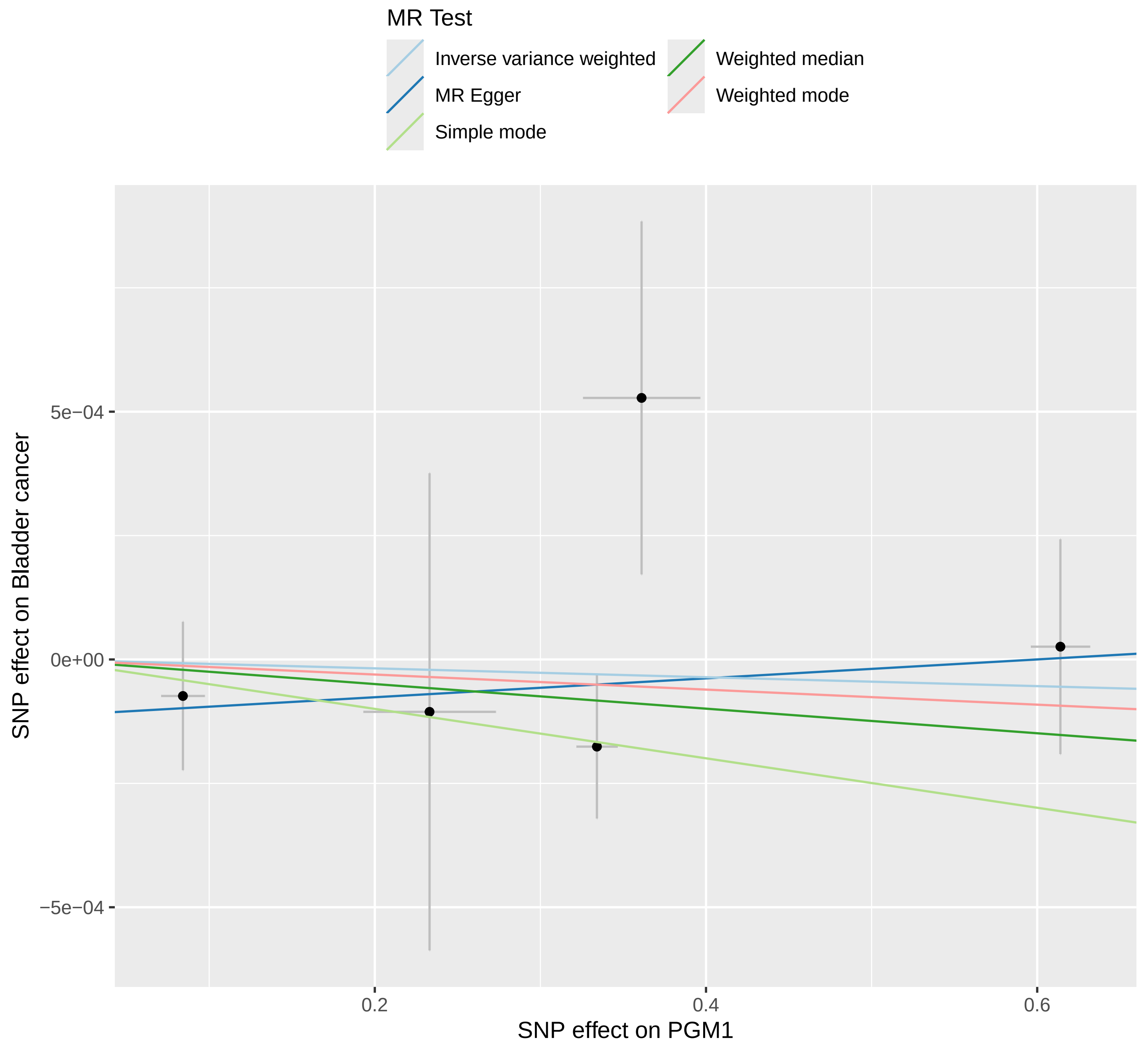
MR Test
Inverse variance weighted
MR Egger
Simple mode
Weighted median
Weighted mode
5e−04
0e+00
−5e−04
SNP effect on Bladder cancer
0.2
0.4
0.6
SNP effect on PGM1

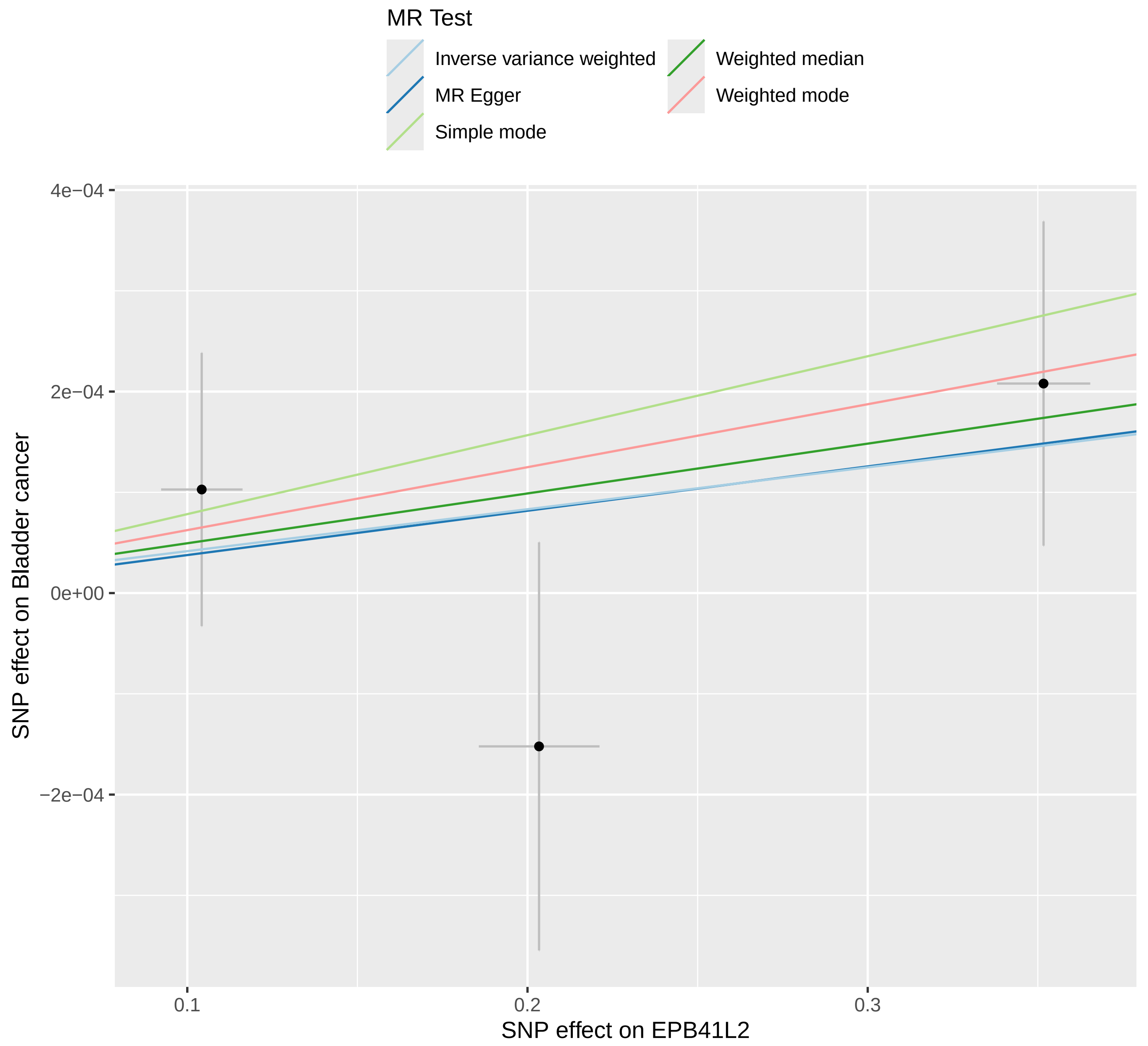
MR Test
Inverse variance weighted
MR Egger
Simple mode
Weighted median
Weighted mode
4e−04
2e−04
0e+00
−2e−04
0.1
0.2
0.3
SNP effect on Bladder cancer
SNP effect on EPB41L2

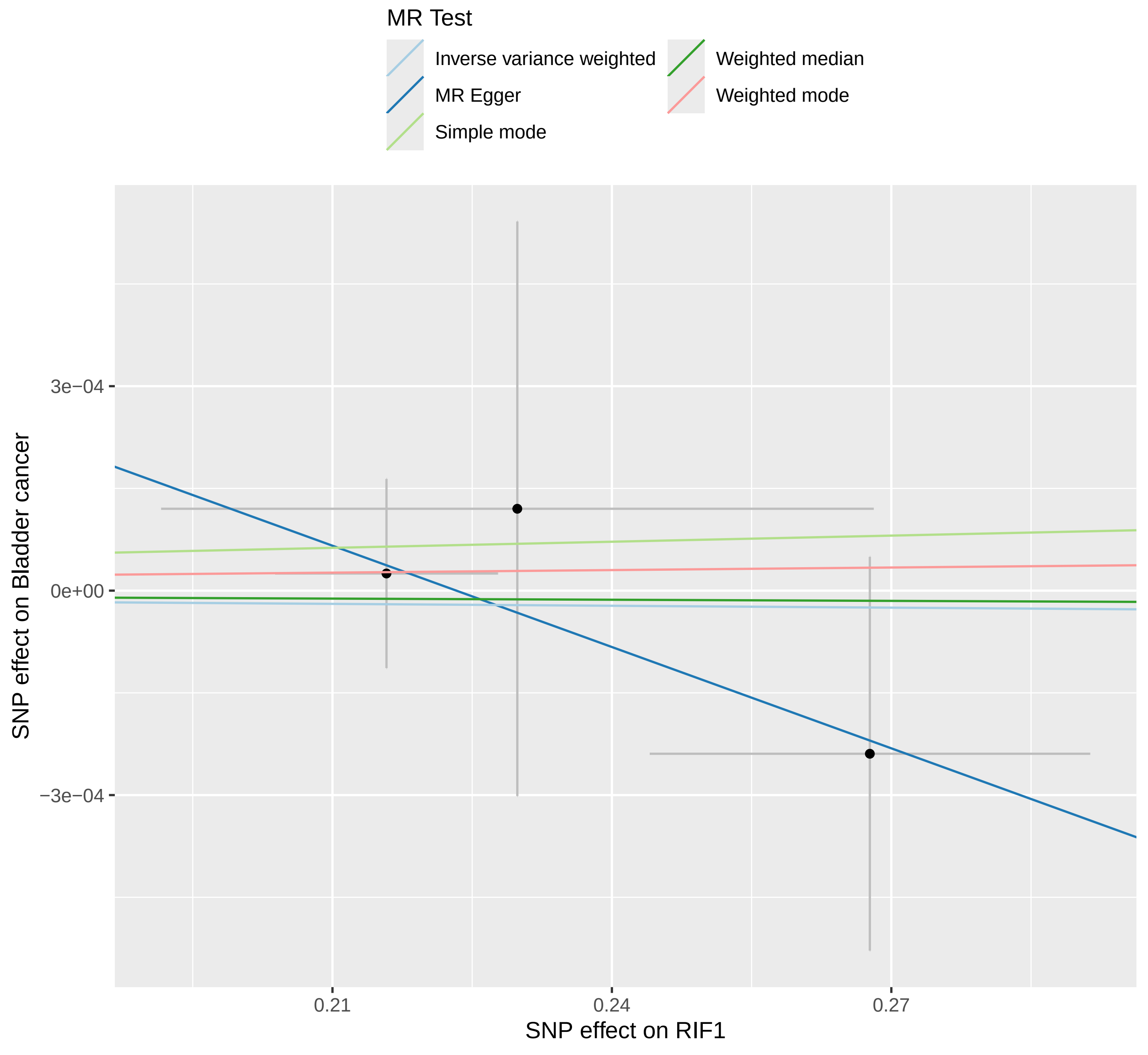
MR Test
Inverse variance weighted
MR Egger
Simple mode
Weighted median
Weighted mode
3e−04
0e+00
−3e−04
SNP effect on Bladder cancer
0.21
0.24
0.27
SNP effect on RIF1

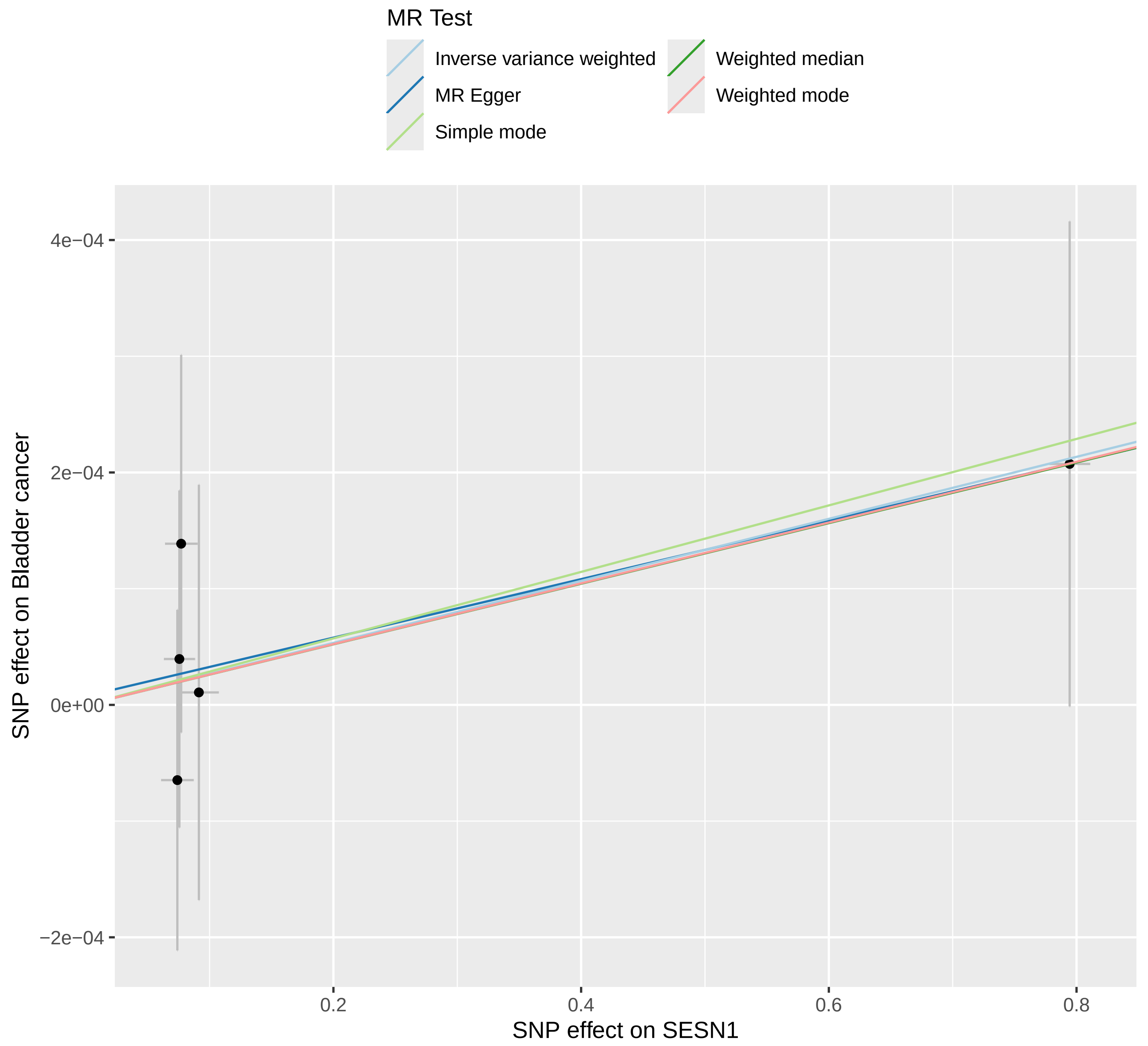
MR Test
Inverse variance weighted
MR Egger
Simple mode
Weighted median
Weighted mode
4e−04
2e−04
0e+00
−2e−04
SNP effect on Bladder cancer
0.2
0.4
0.6
0.8
SNP effect on SESN1

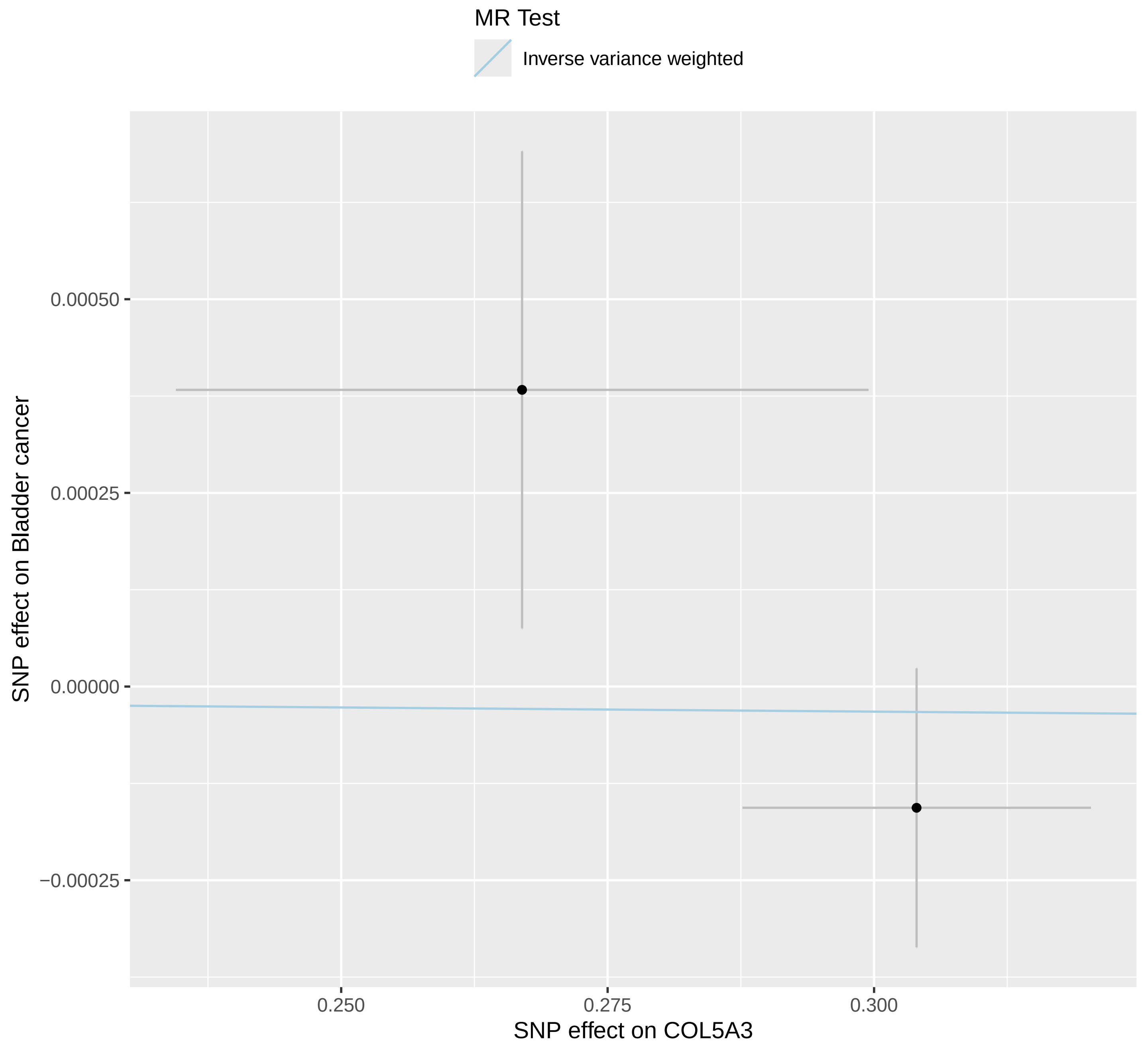

MR Test
Inverse variance weighted
0.00050
0.00025
0.00000
−0.00025
0.250
0.275
0.300
SNP effect on COL5A3
SNP effect on Bladder cancer

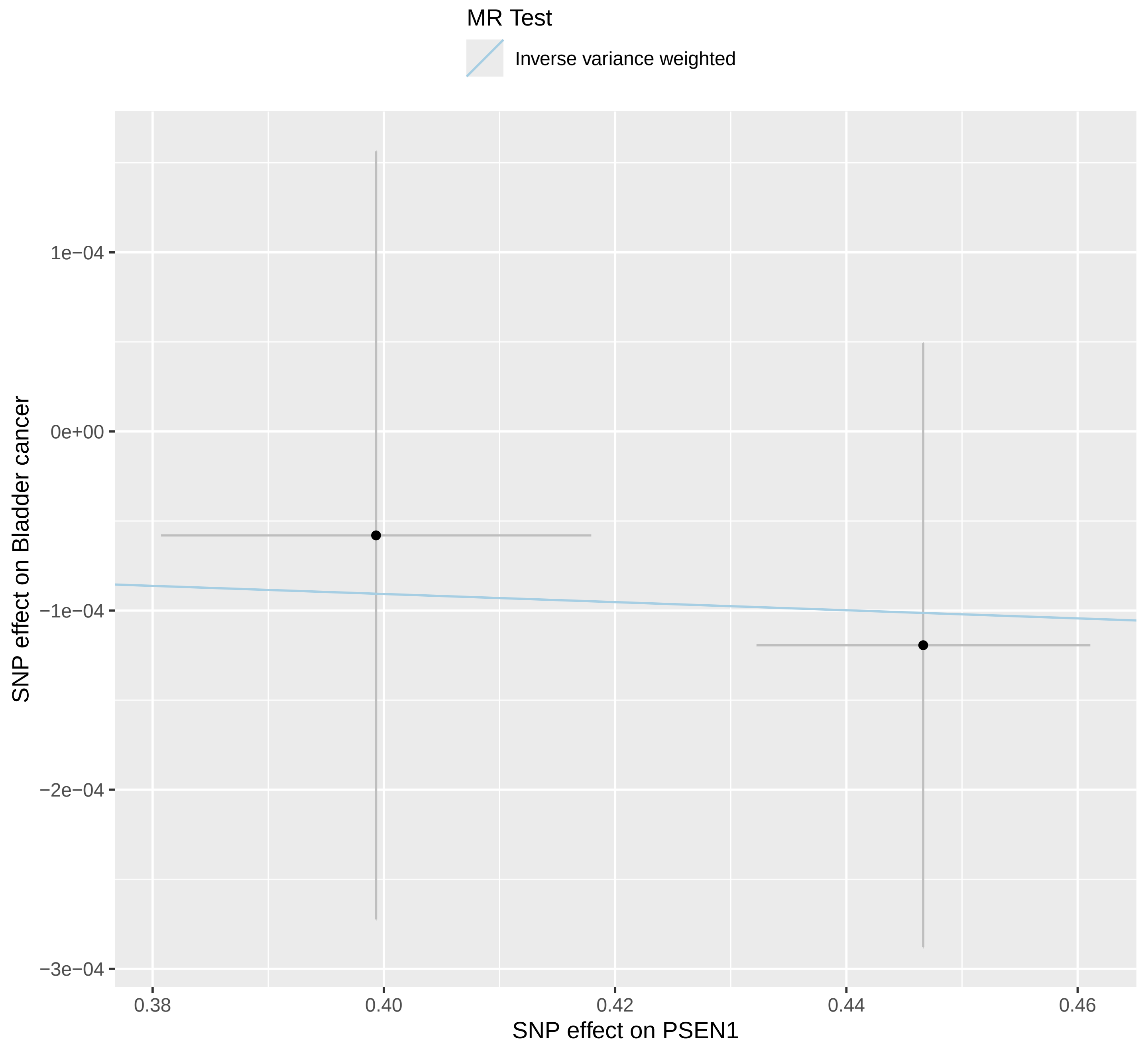
MR Test
Inverse variance weighted
1e−04
0e+00
−1e−04
−2e−04
−3e−04
0.38
0.40
0.42
0.44
0.46
SNP effect on PSEN1
SNP effect on Bladder cancer

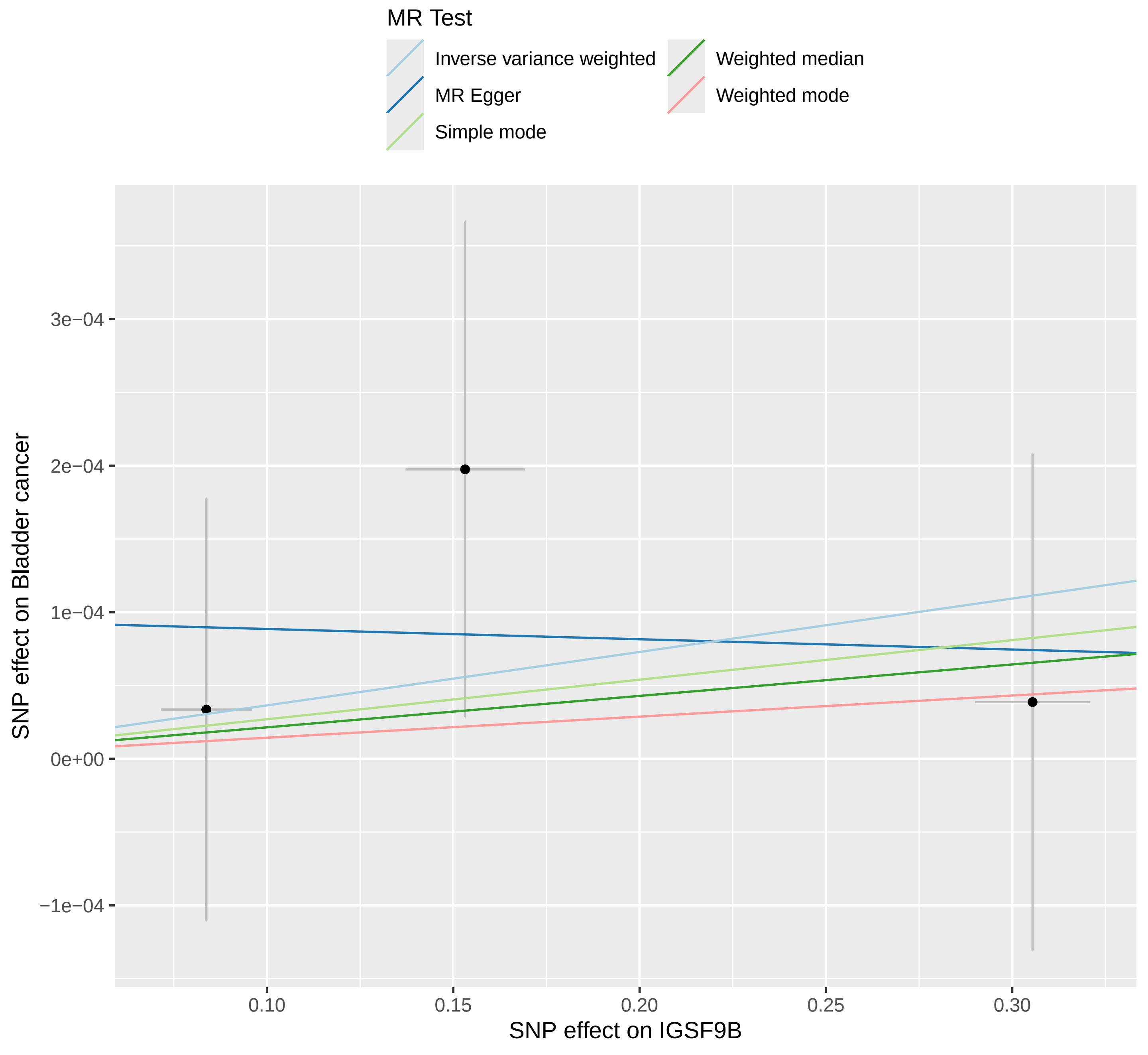
MR Test
Inverse variance weighted
MR Egger
Simple mode
Weighted median
Weighted mode
SNP effect on Bladder cancer
3e−04
2e−04
1e−04
0e+00
−1e−04
0.10
0.15
0.20
0.25
0.30
SNP effect on IGSF9B

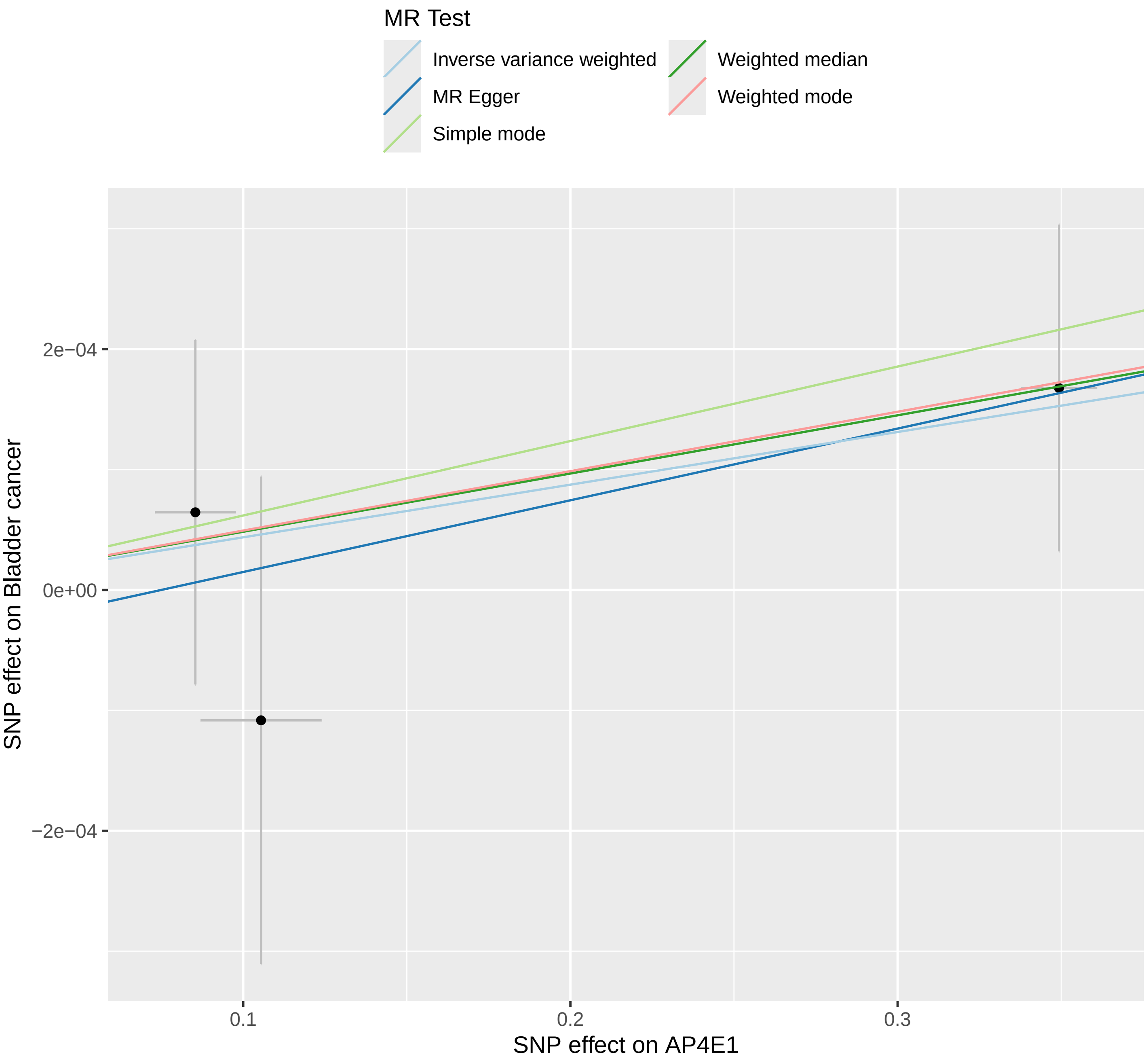
MR Test
Inverse variance weighted
MR Egger
Simple mode
Weighted median
Weighted mode
2e−04
0e+00
−2e−04
0.1
0.2
0.3
SNP effect on Bladder cancer
SNP effect on AP4E1

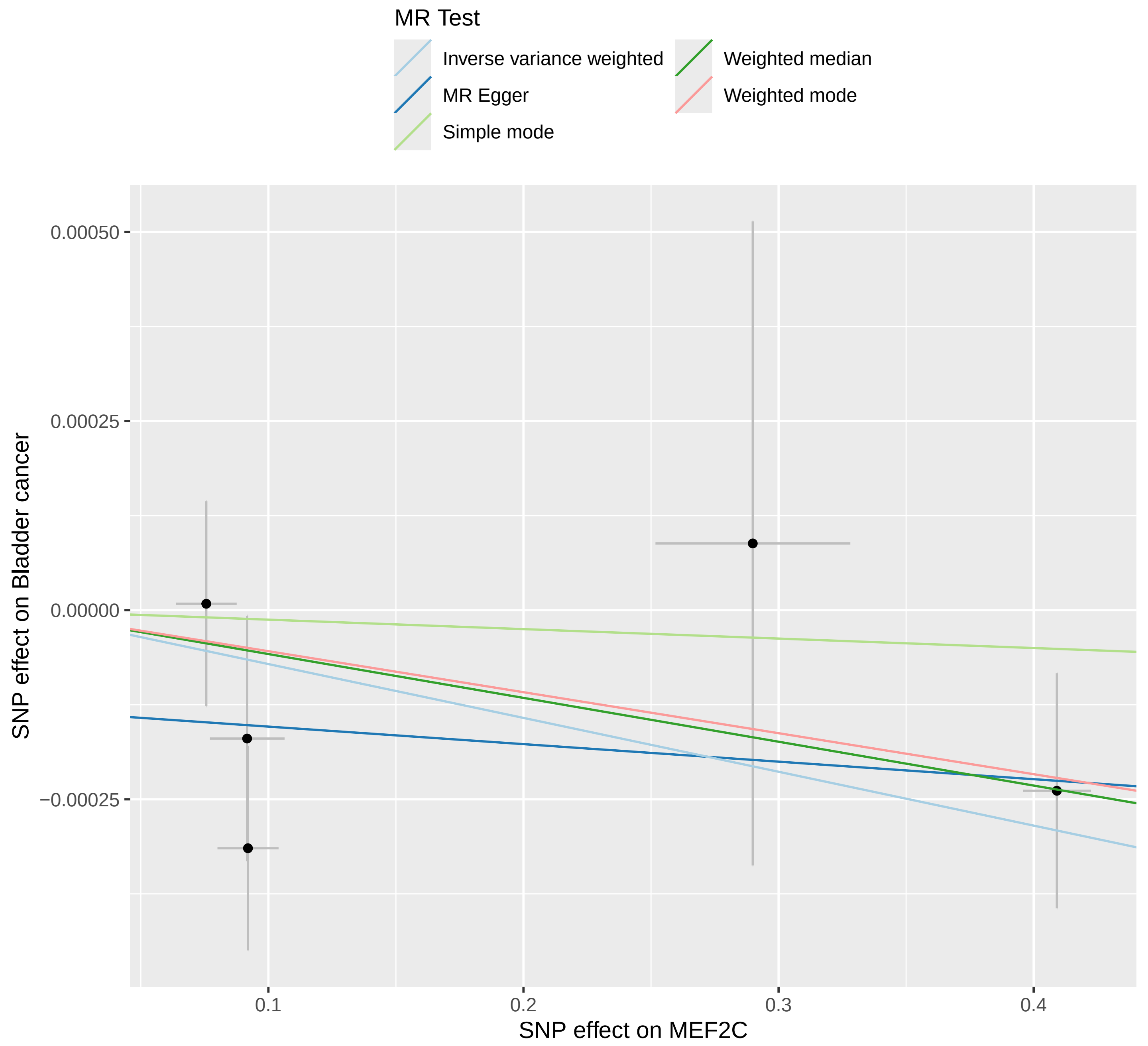
MR Test
Inverse variance weighted
MR Egger
Simple mode
Weighted median
Weighted mode
0.00050
0.00025
0.00000
−0.00025
SNP effect on Bladder cancer
0.1
0.2
0.3
0.4
SNP effect on MEF2C

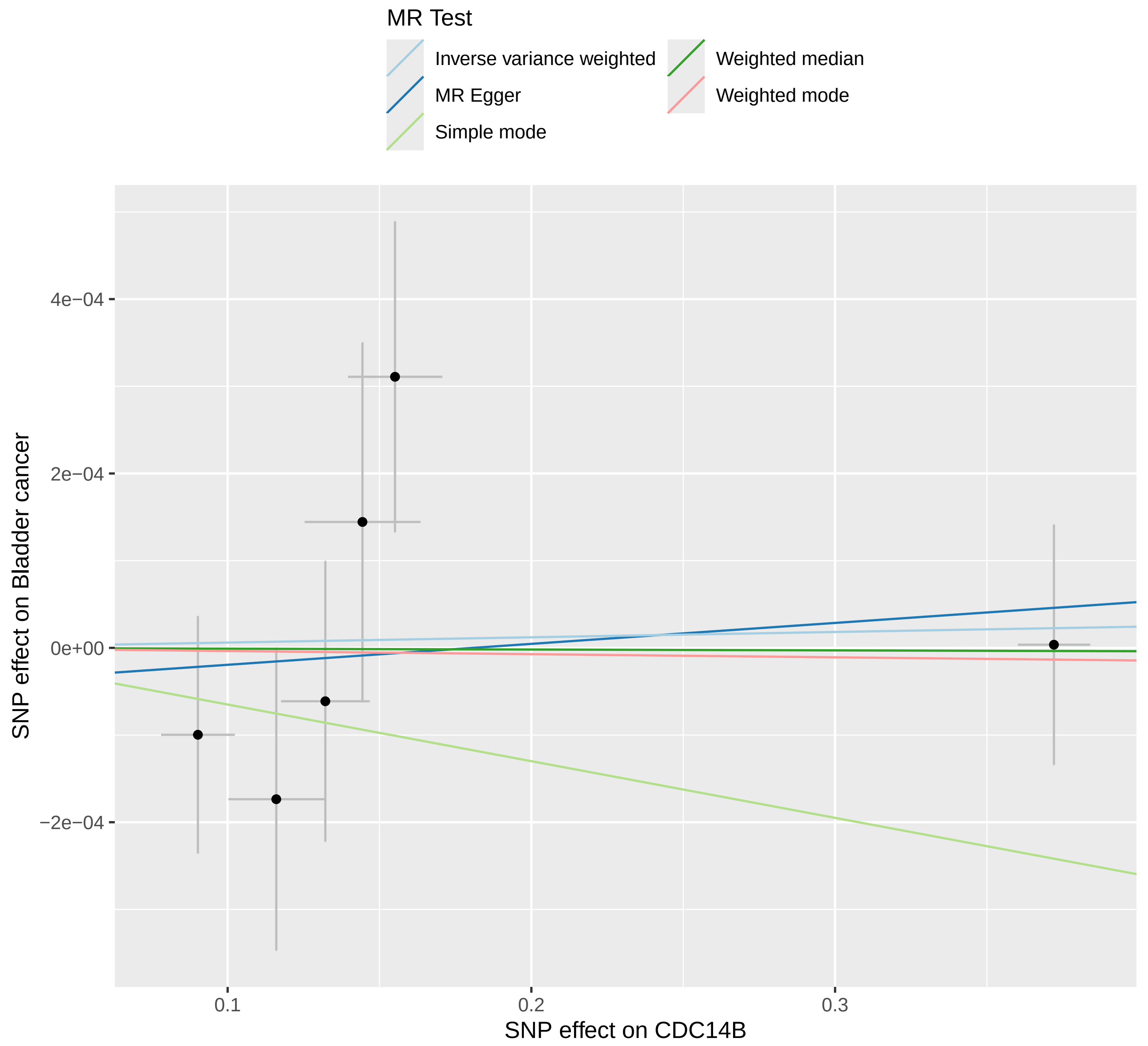

MR Test
Inverse variance weighted
Weighted median
MR Egger
Weighted mode
Simple mode
4e−04
2e−04
0e+00
−2e−04
0.1
0.2
0.3
SNP effect on Bladder cancer
SNP effect on CDC14B

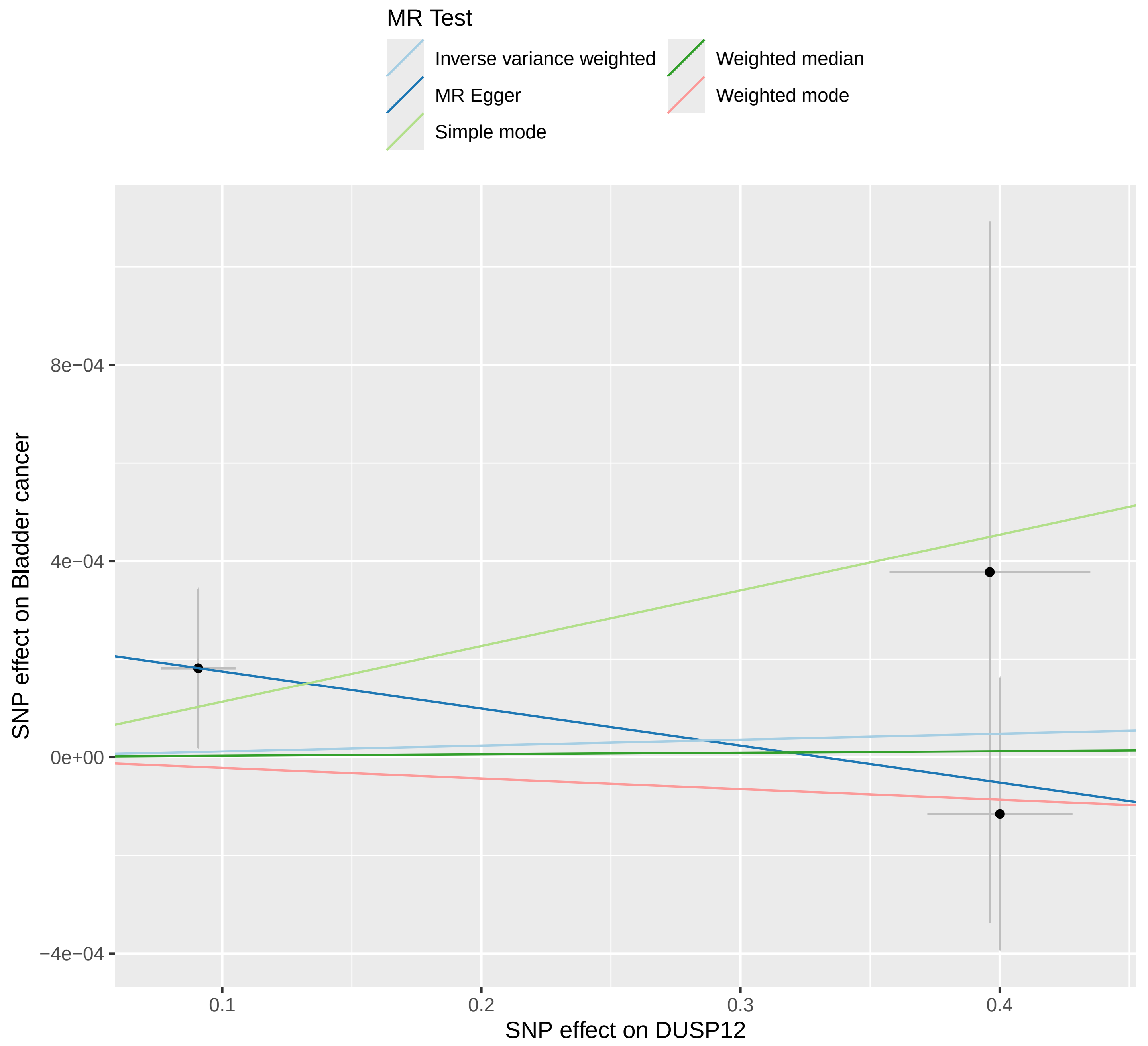
MR Test
Inverse variance weighted
MR Egger
Simple mode
Weighted median
Weighted mode
8e−04
4e−04
0e+00
−4e−04
0.1
0.2
0.3
0.4
SNP effect on DUSP12
SNP effect on Bladder cancer

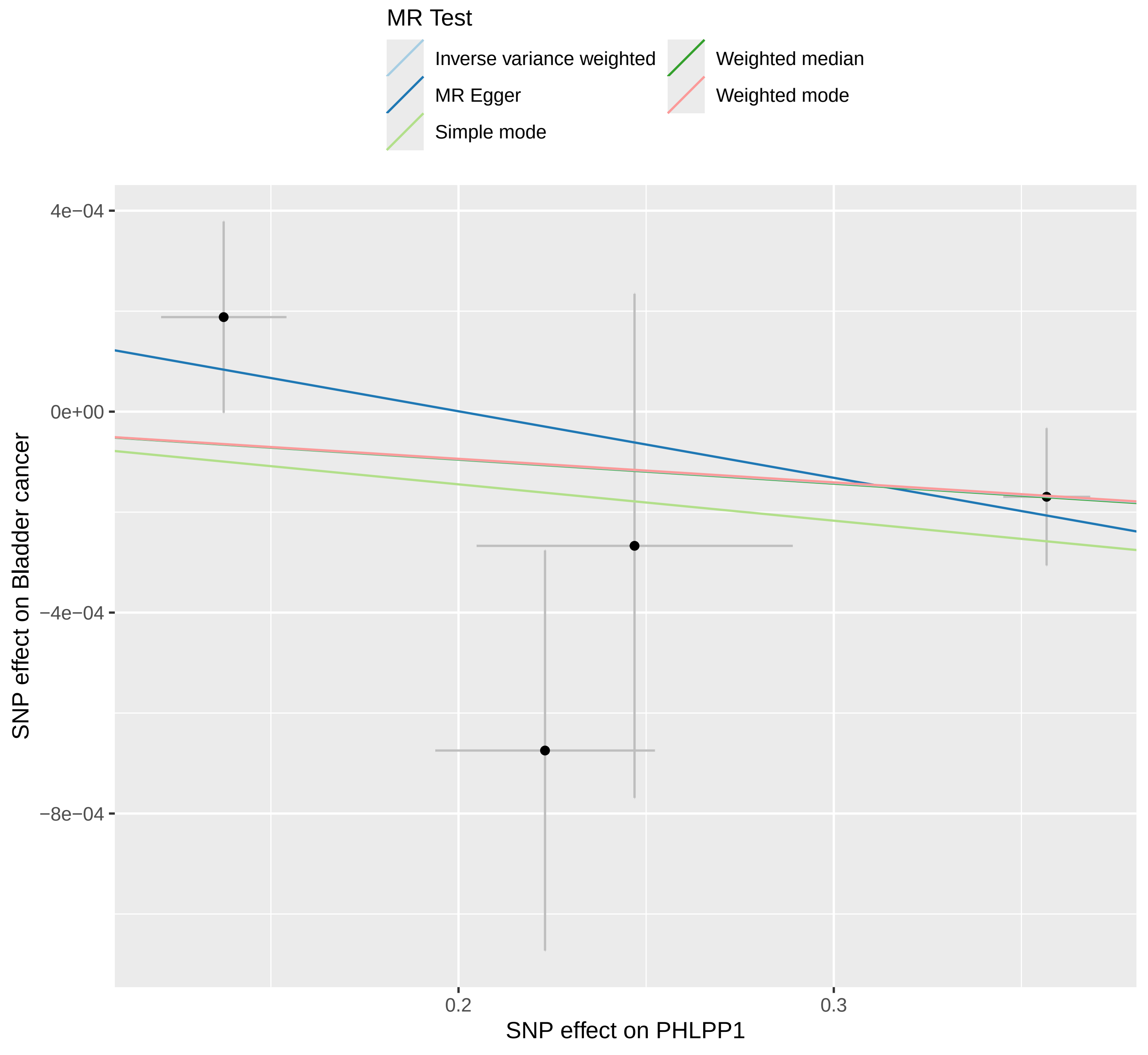

MR Test
Inverse variance weighted
MR Egger
Simple mode
Weighted median
Weighted mode
4e−04
0e+00
−4e−04
−8e−04
0.2
0.3
SNP effect on Bladder cancer
SNP effect on PHLPP1

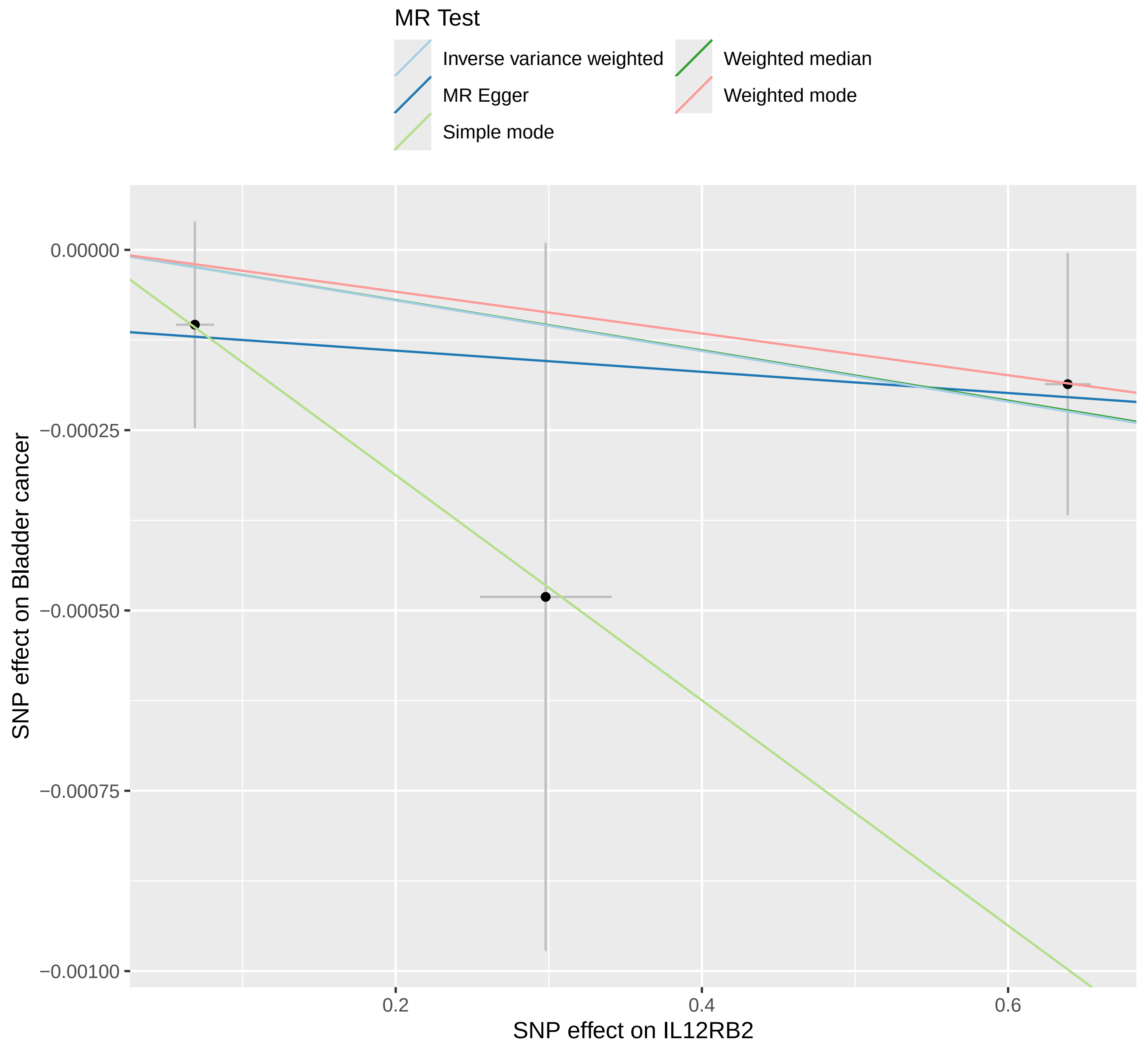
MR Test
Inverse variance weighted
MR Egger
Simple mode
Weighted median
Weighted mode
0.00000
−0.00025
−0.00050
−0.00075
−0.00100
0.2
0.4
0.6
SNP effect on Bladder cancer
SNP effect on IL12RB2

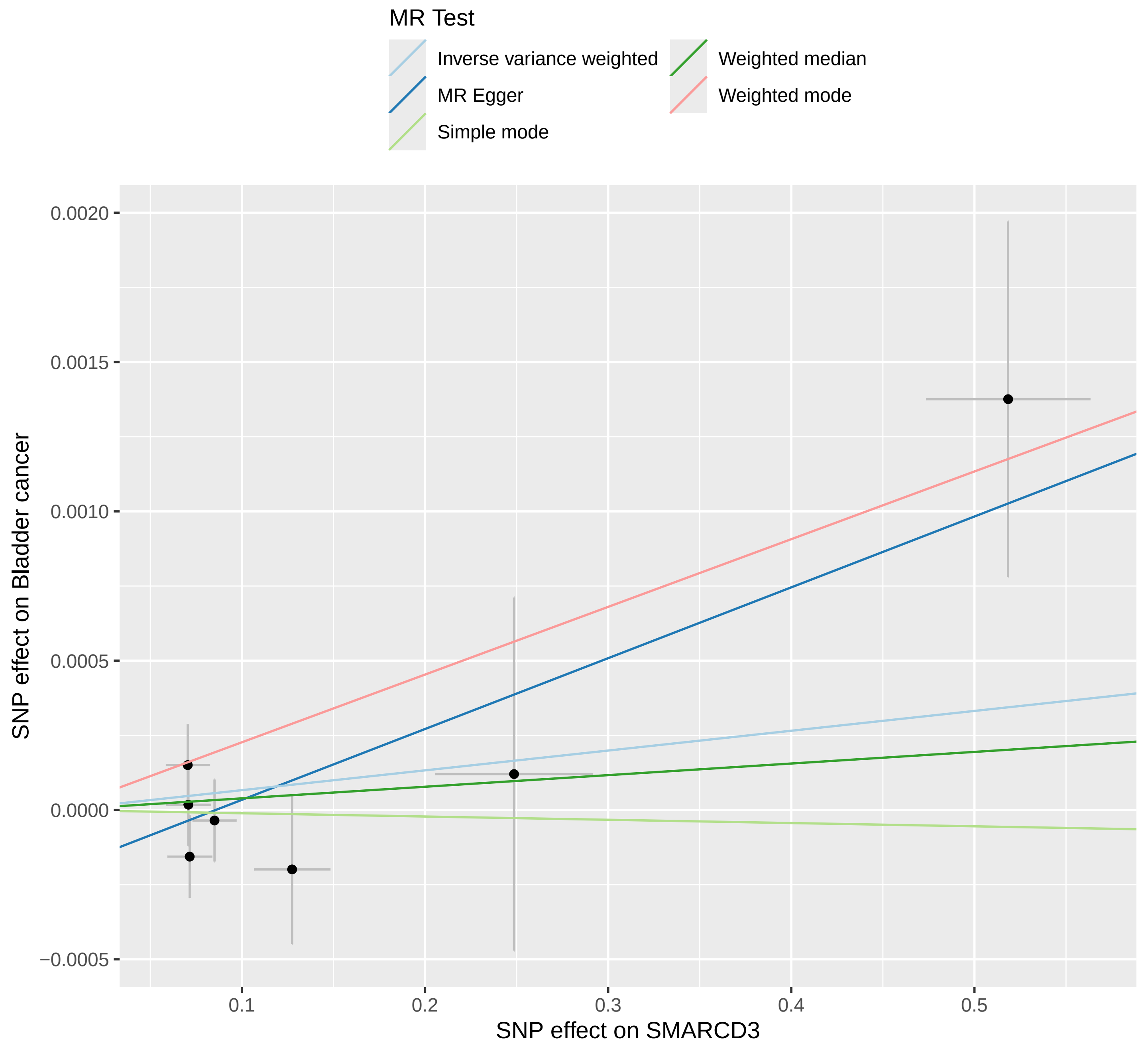

MR Test
Inverse variance weighted
MR Egger
Simple mode
Weighted median
Weighted mode
0.0020
0.0015
0.0010
0.0005
0.0000
−0.0005
SNP effect on Bladder cancer
0.1
0.2
0.3
0.4
0.5
SNP effect on SMARCD3

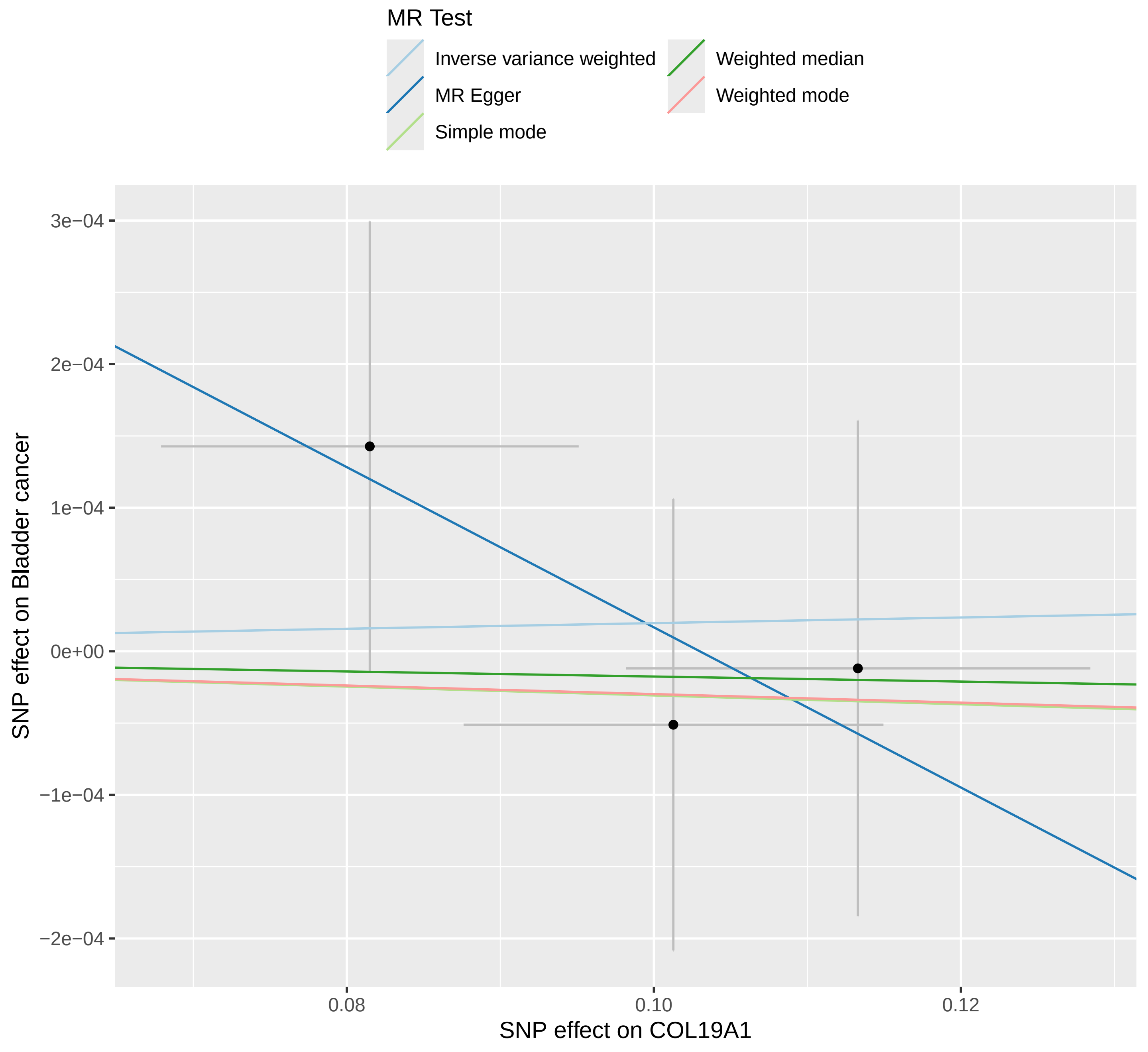

MR Test
Inverse variance weighted
MR Egger
Simple mode
Weighted median
Weighted mode
3e−04
2e−04
1e−04
0e+00
−1e−04
−2e−04
SNP effect on Bladder cancer
0.08
0.10
0.12
SNP effect on COL19A1

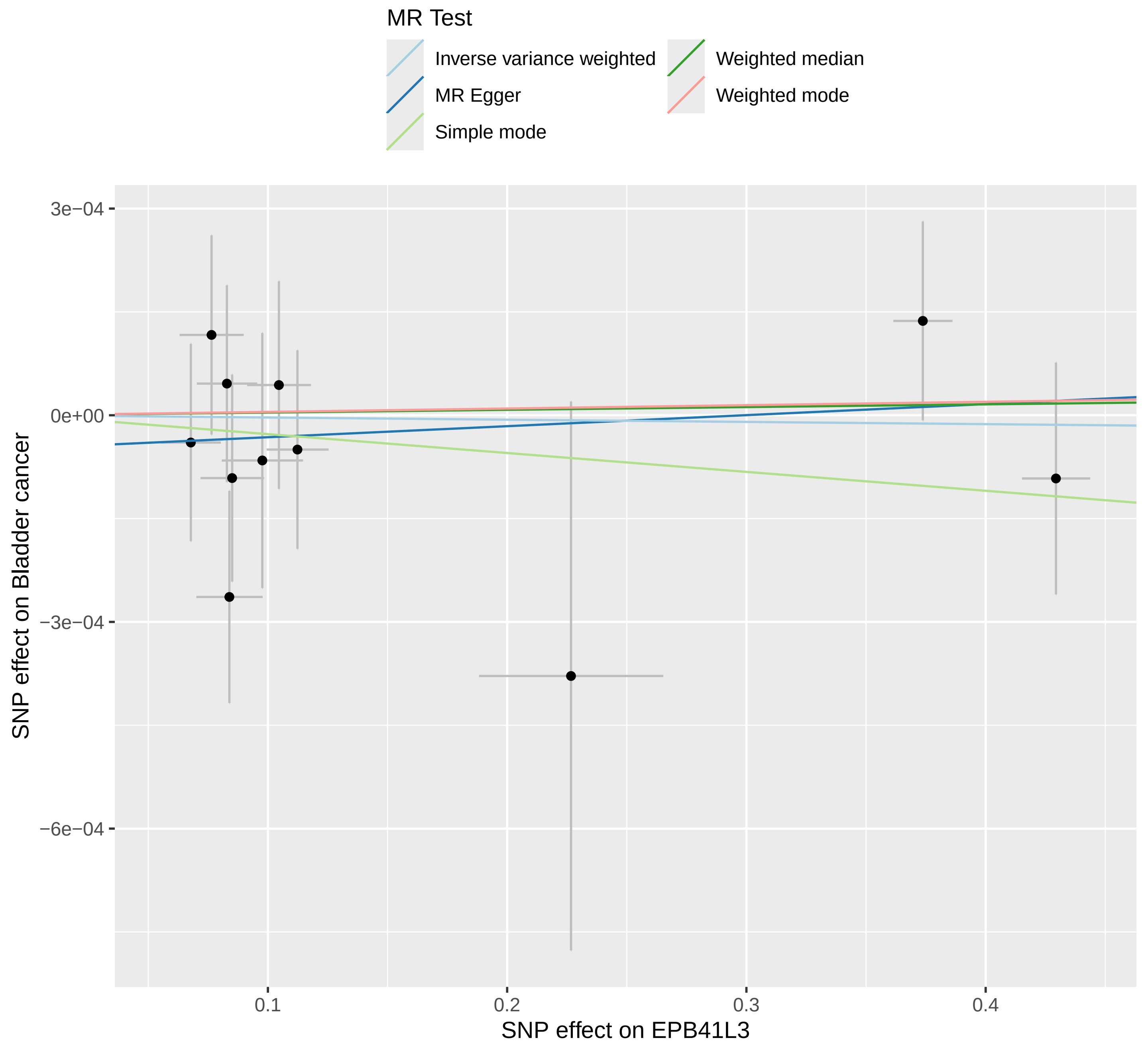

MR Test
Inverse variance weighted
MR Egger
Simple mode
Weighted median
Weighted mode
3e−04
0e+00
−3e−04
−6e−04
0.1
0.2
0.3
0.4
SNP effect on EPB41L3
SNP effect on Bladder cancer

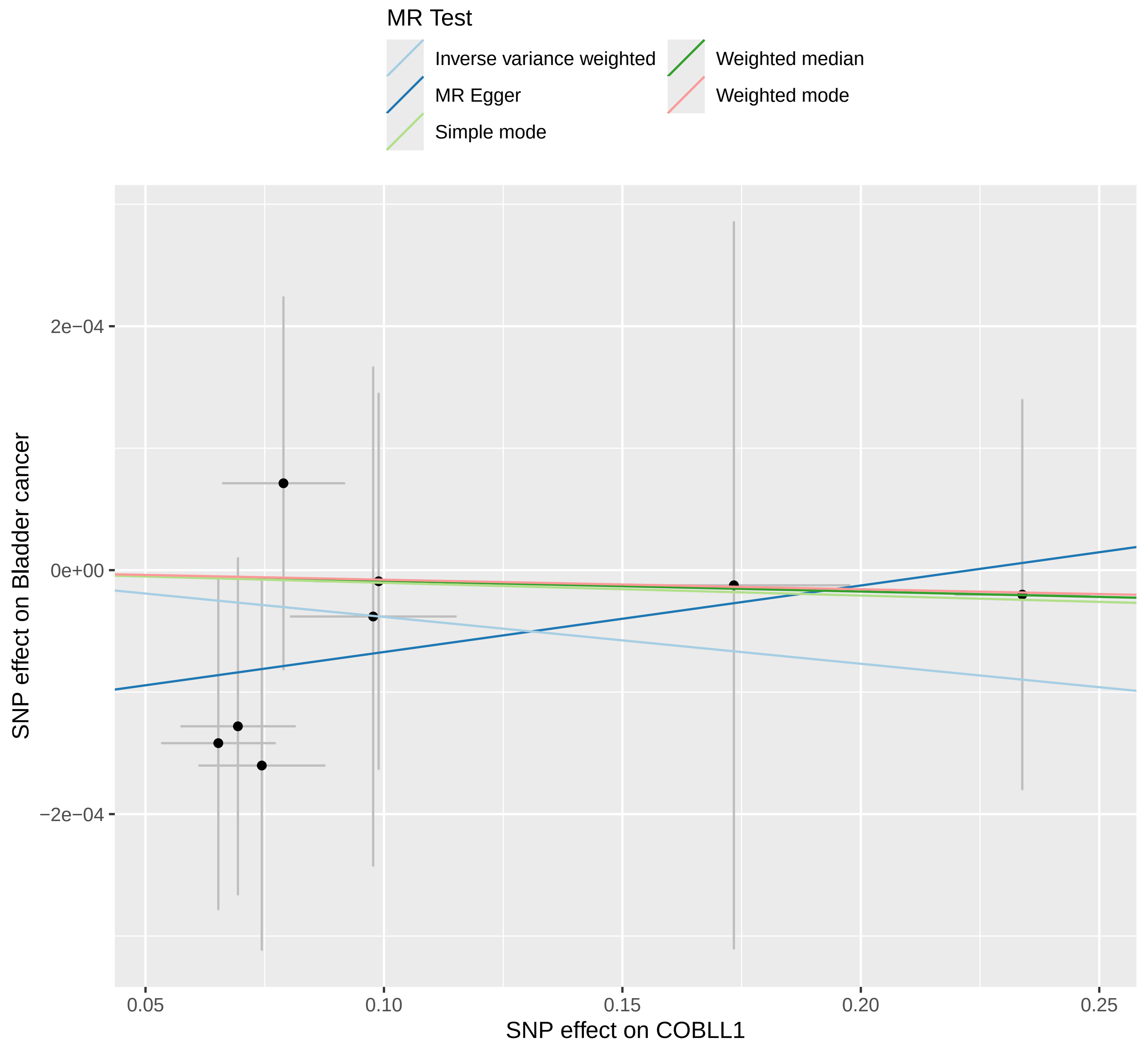
MR Test
Inverse variance weighted
MR Egger
Simple mode
Weighted median
Weighted mode
2e−04
0e+00
−2e−04
SNP effect on Bladder cancer
0.05
0.10
0.15
0.20
0.25
SNP effect on COBLL1

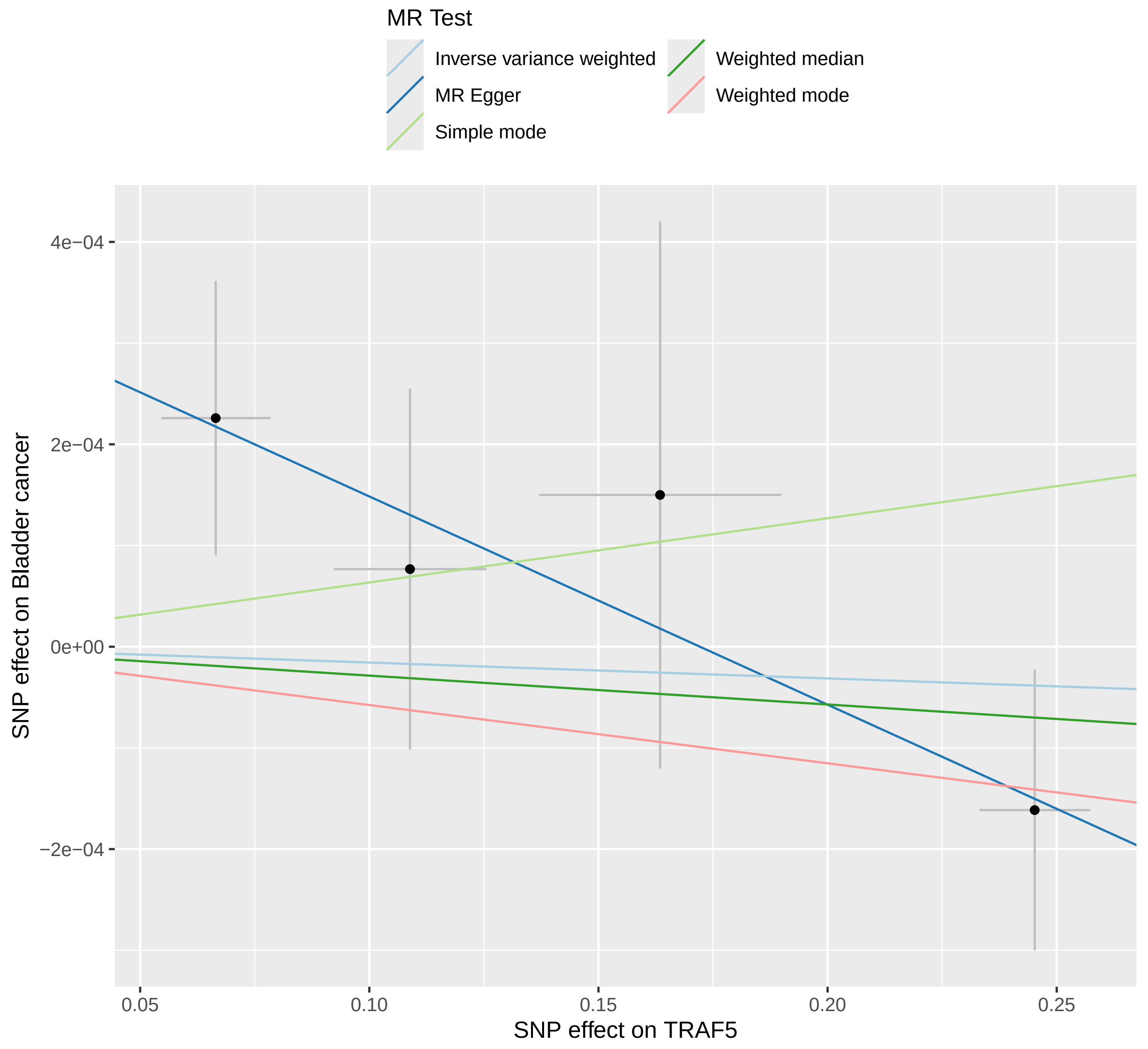
MR Test
Inverse variance weighted
MR Egger
Simple mode
Weighted median
Weighted mode
4e−04
2e−04
0e+00
−2e−04
SNP effect on Bladder cancer
0.05
0.10
0.15
0.20
0.25
SNP effect on TRAF5

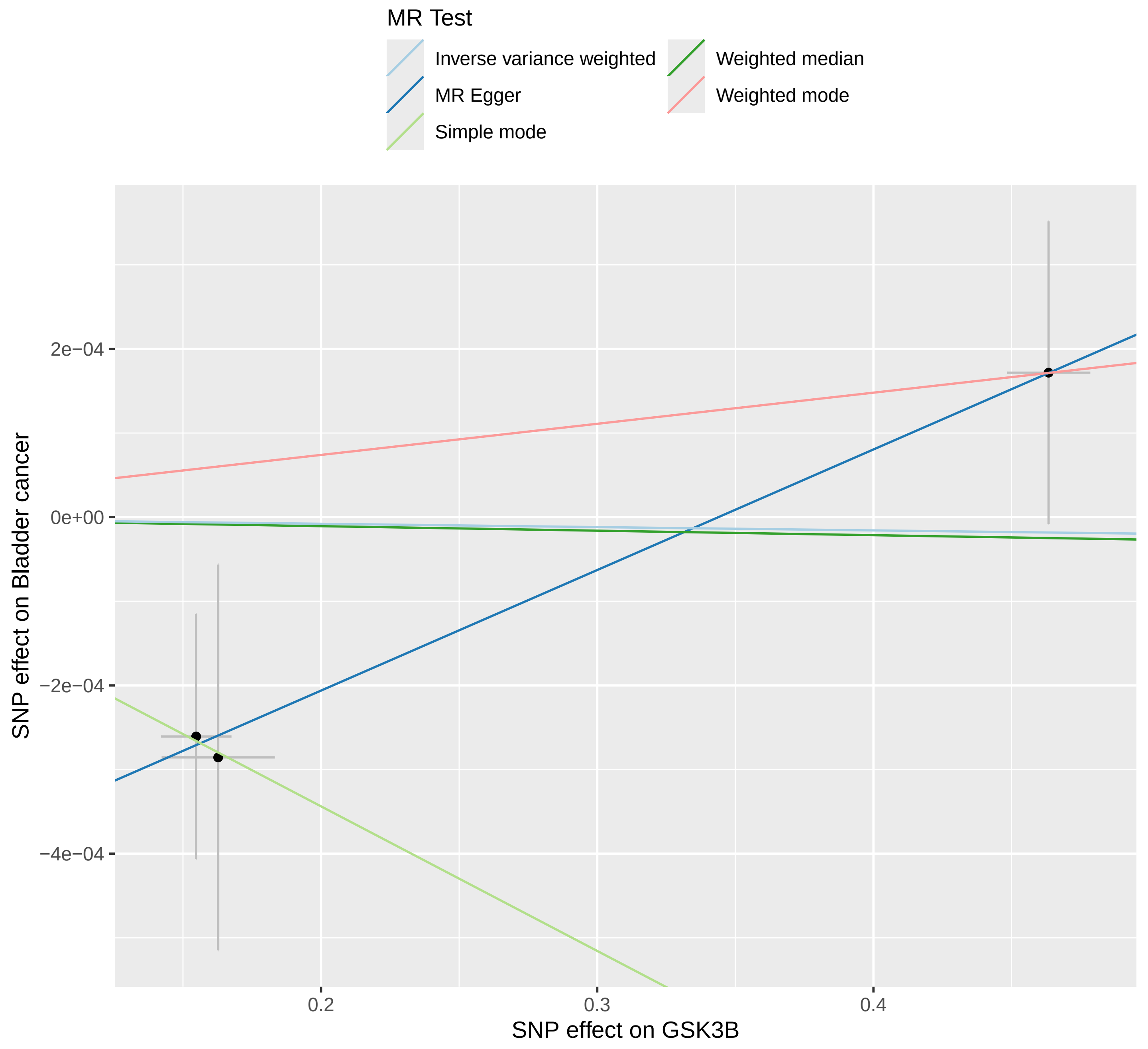
MR Test
Inverse variance weighted
MR Egger
Simple mode
Weighted median
Weighted mode
SNP effect on Bladder cancer
2e−04
0e+00
−2e−04
−4e−04
0.2
0.3
0.4
SNP effect on GSK3B

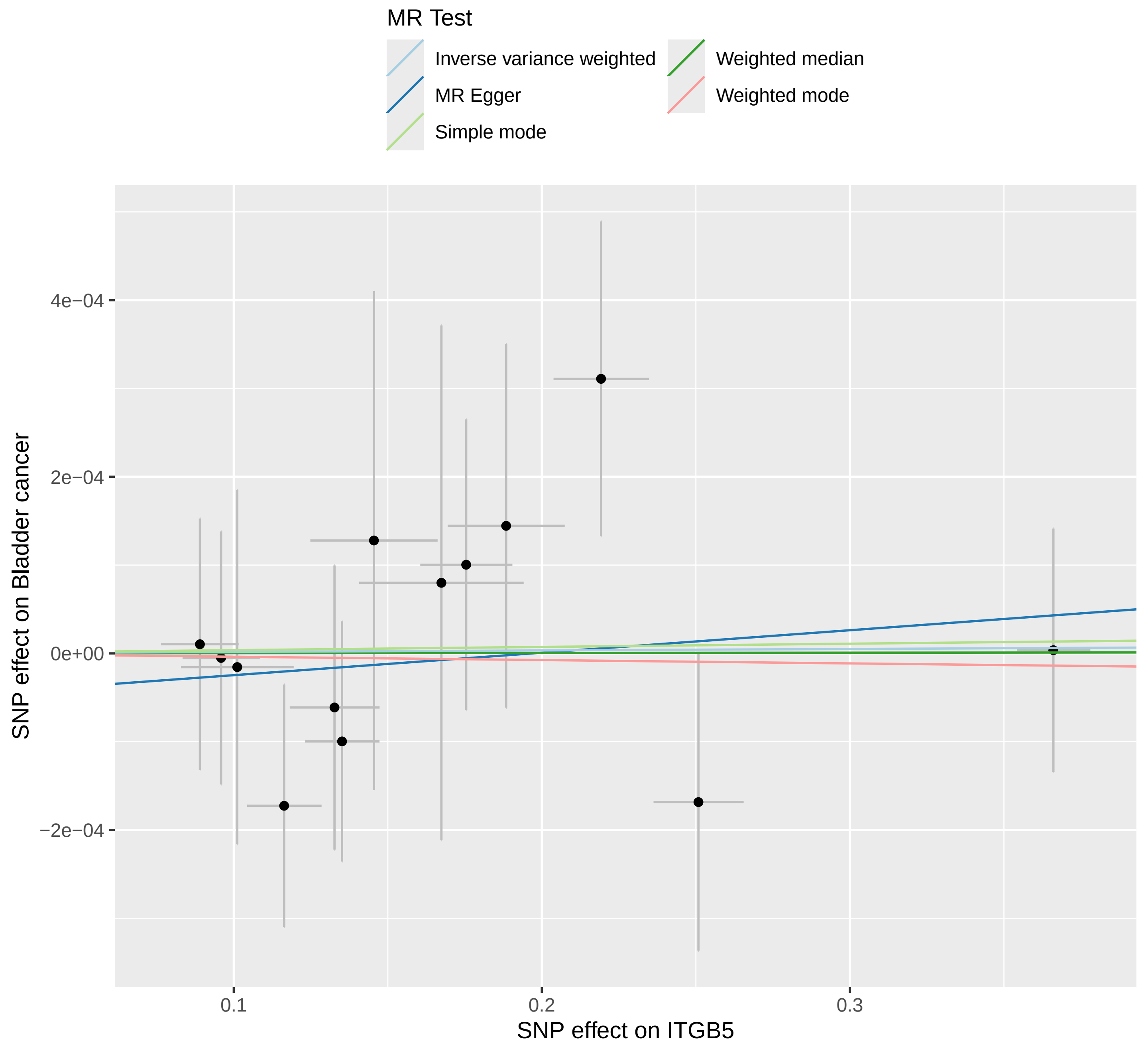

MR Test
Inverse variance weighted
MR Egger
Simple mode
Weighted median
Weighted mode
4e−04
2e−04
0e+00
−2e−04
0.1
0.2
0.3
SNP effect on Bladder cancer
SNP effect on ITGB5

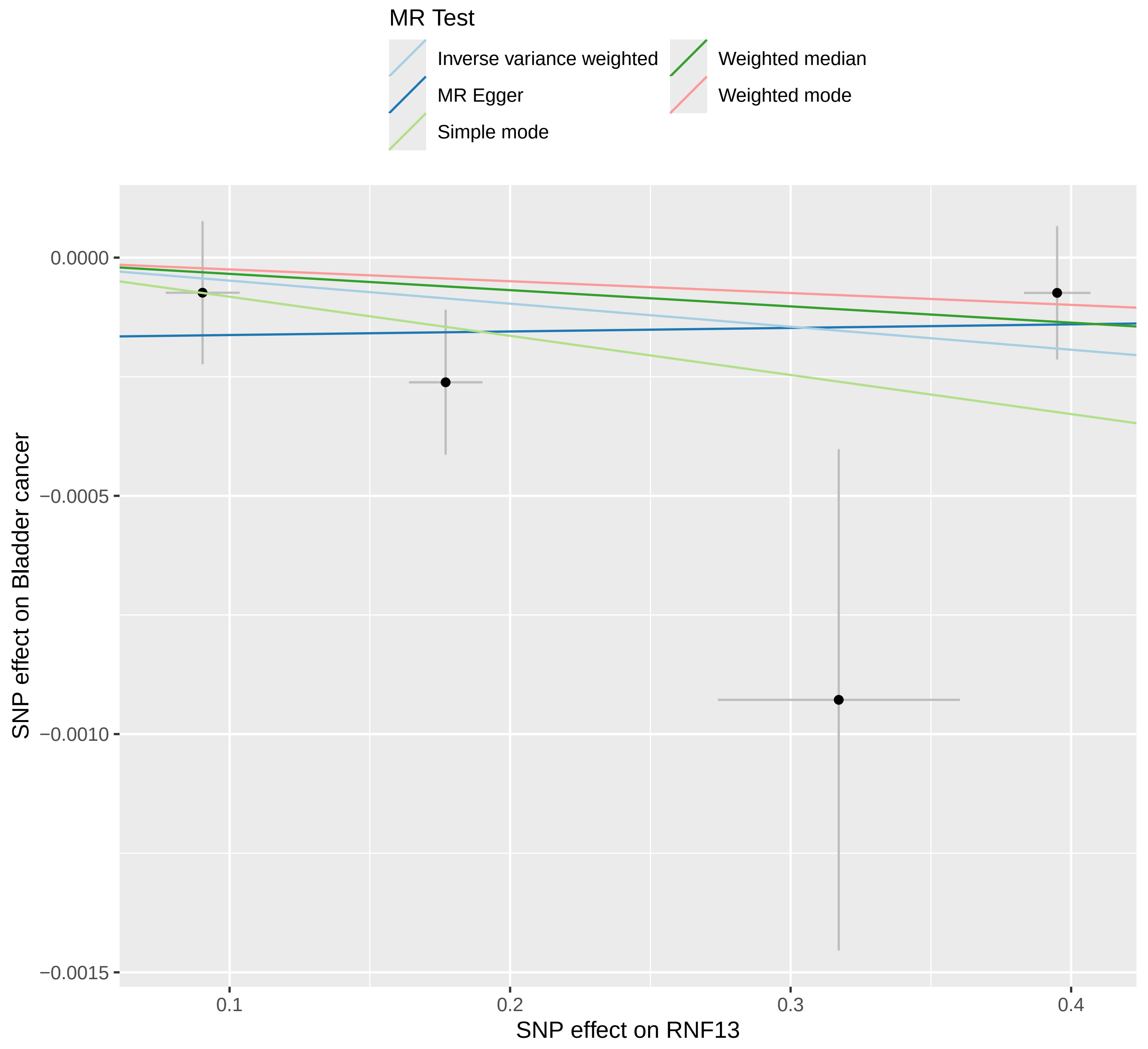

MR Test
Inverse variance weighted
MR Egger
Simple mode
Weighted median
Weighted mode
0.0000
−0.0005
−0.0010
−0.0015
SNP effect on Bladder cancer
0.1
0.2
0.3
0.4
SNP effect on RNF13

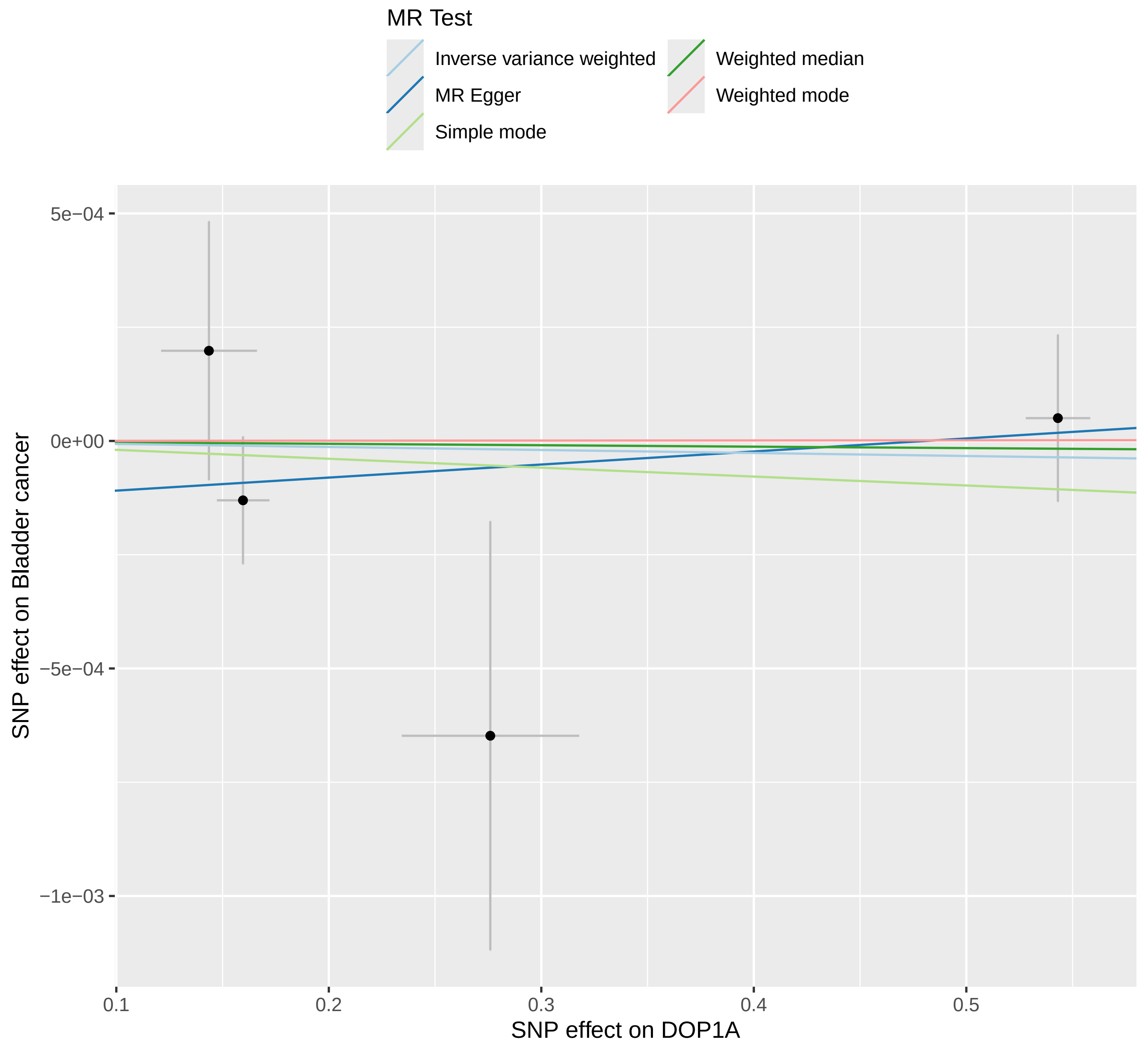

MR Test
Inverse variance weighted
MR Egger
Simple mode
Weighted median
Weighted mode
5e−04
0e+00
−5e−04
−1e−03
SNP effect on Bladder cancer
0.1
0.2
0.3
0.4
0.5
SNP effect on DOP1A

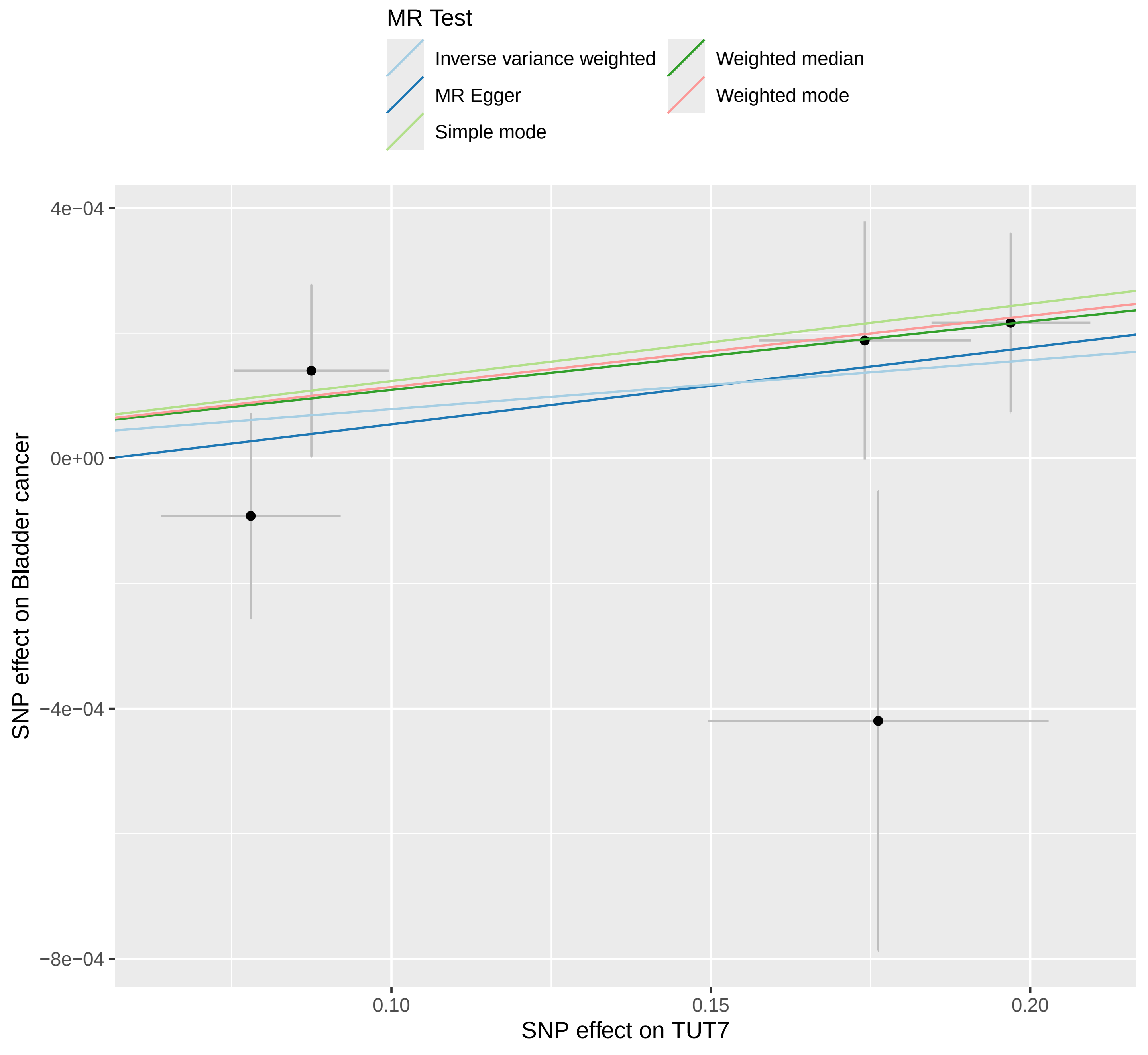
MR Test
Inverse variance weighted
MR Egger
Simple mode
Weighted median
Weighted mode
4e−04
0e+00
−4e−04
−8e−04
0.10
0.15
0.20
SNP effect on TUT7
SNP effect on Bladder cancer

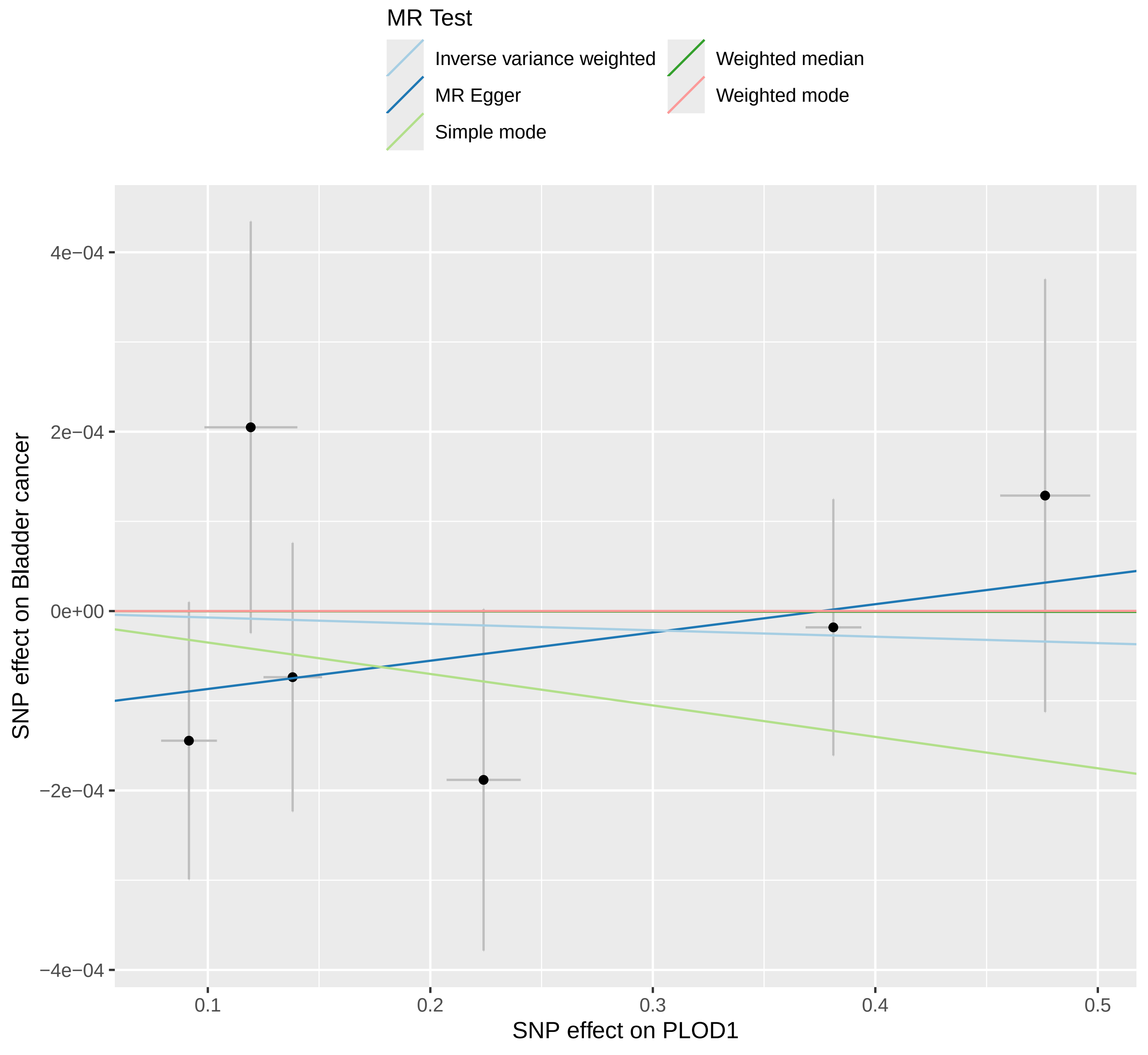

MR Test
Inverse variance weighted
MR Egger
Simple mode
Weighted median
Weighted mode
4e−04
2e−04
0e+00
−2e−04
−4e−04
SNP effect on Bladder cancer
0.1
0.2
0.3
0.4
0.5
SNP effect on PLOD1

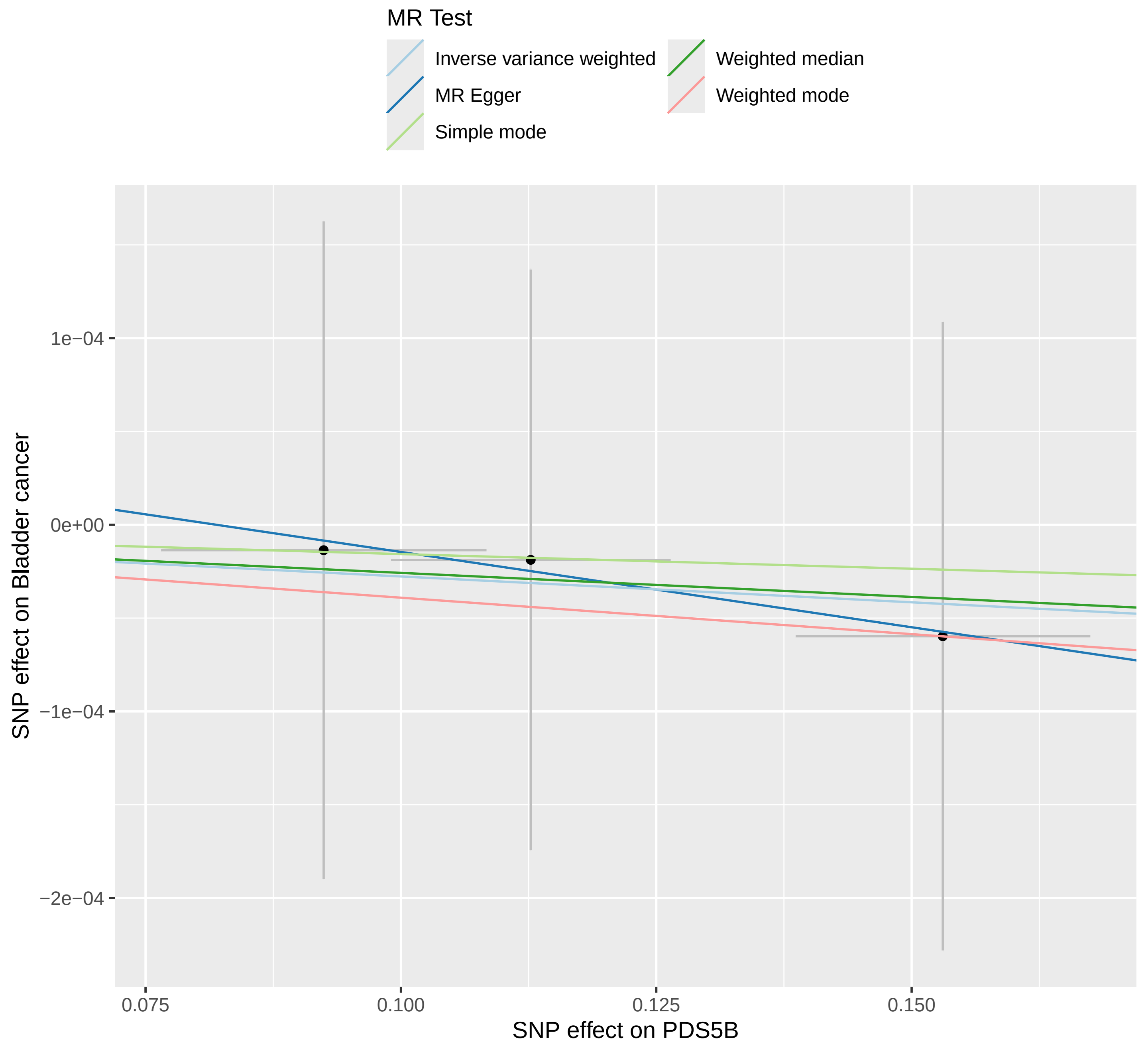
MR Test
Inverse variance weighted
MR Egger
Simple mode
Weighted median
Weighted mode
1e−04
0e+00
−1e−04
−2e−04
0.075
0.100
0.125
0.150
SNP effect on Bladder cancer
SNP effect on PDS5B

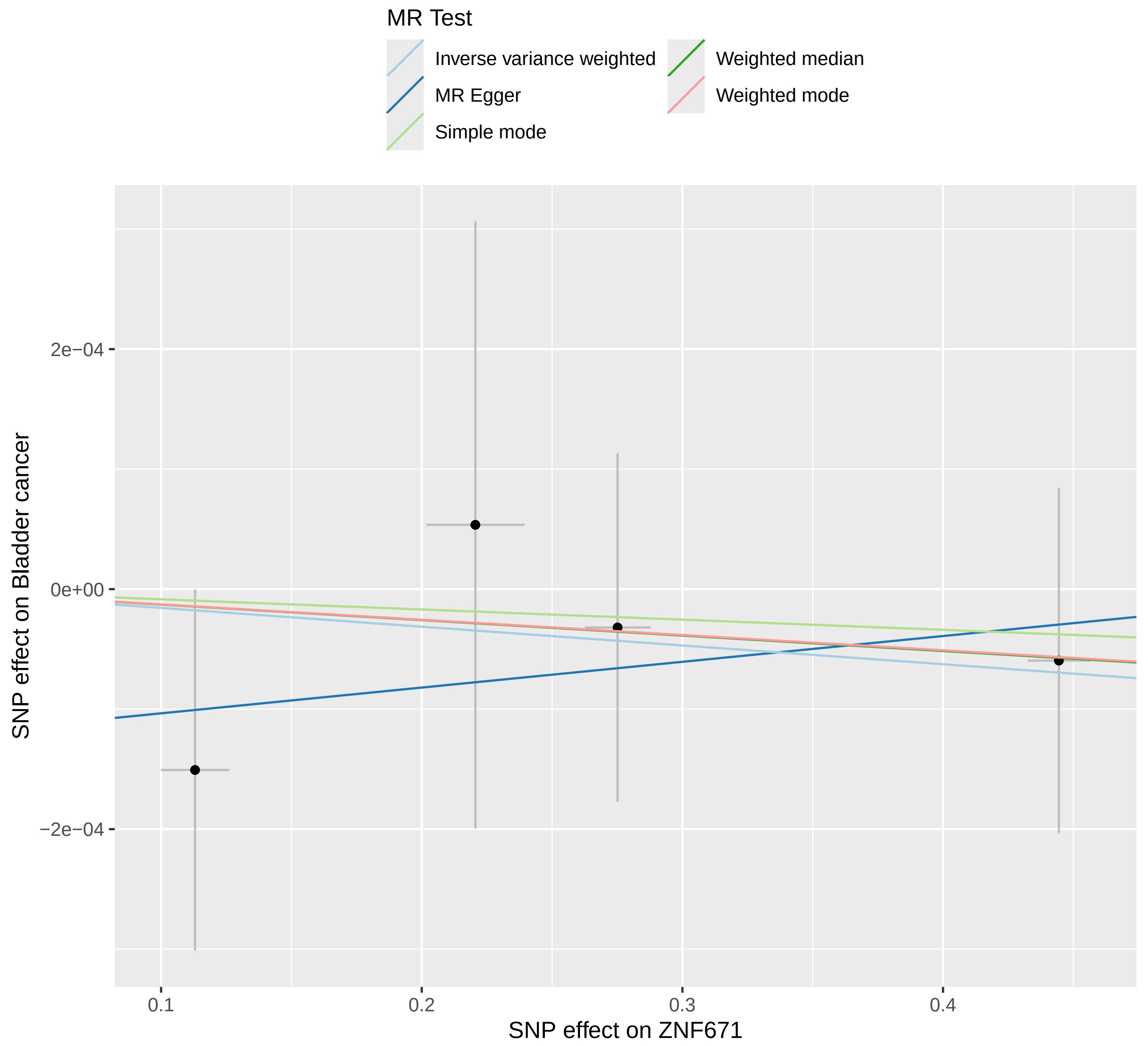
MR Test
Inverse variance weighted
MR Egger
Simple mode
Weighted median
Weighted mode
2e−04
0e+00
−2e−04
0.1
0.2
0.3
0.4
SNP effect on ZNF671
SNP effect on Bladder cancer

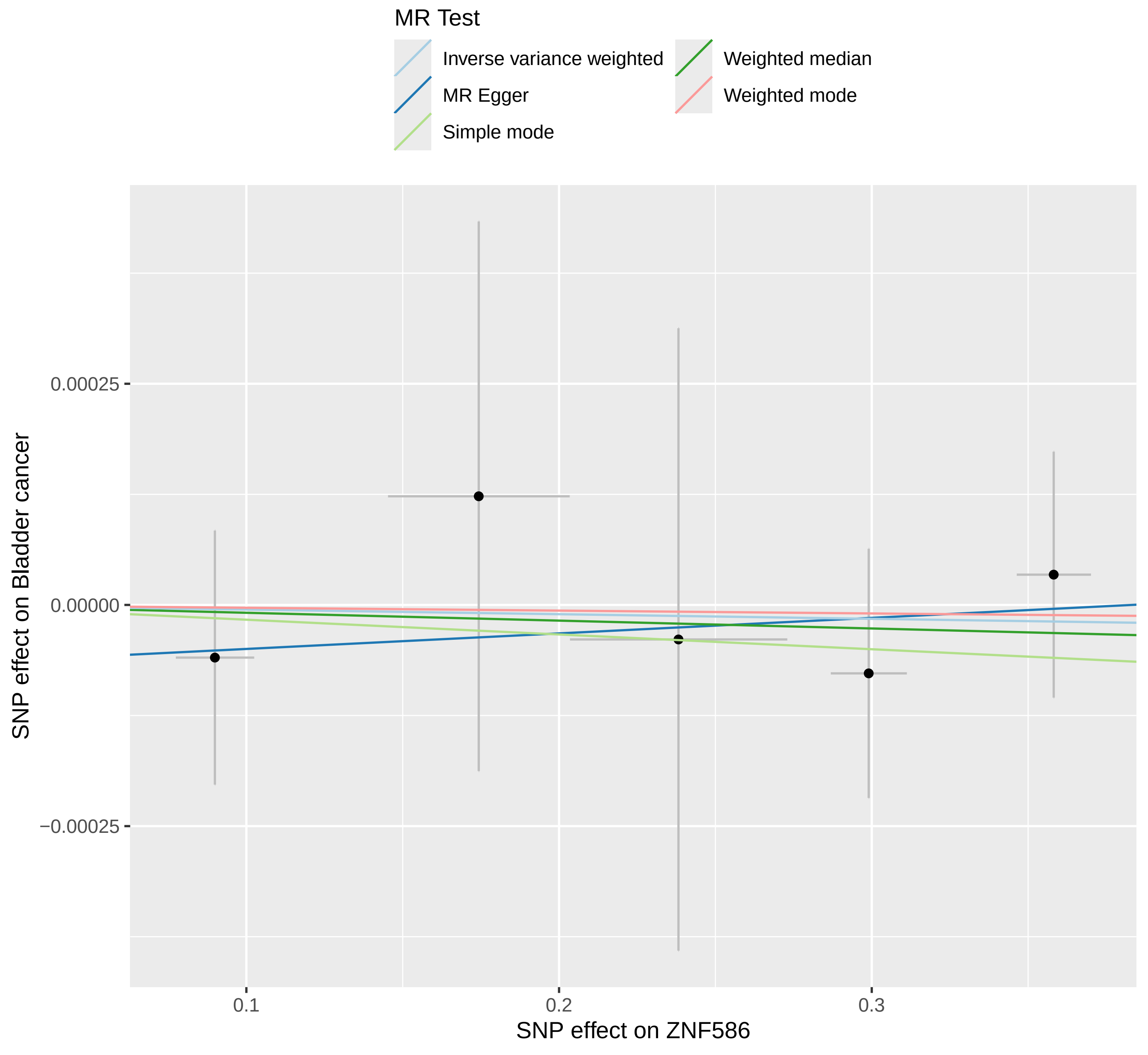
MR Test
Inverse variance weighted
MR Egger
Simple mode
Weighted median
Weighted mode
0.00025
0.00000
−0.00025
SNP effect on Bladder cancer
0.1
0.2
0.3
SNP effect on ZNF586

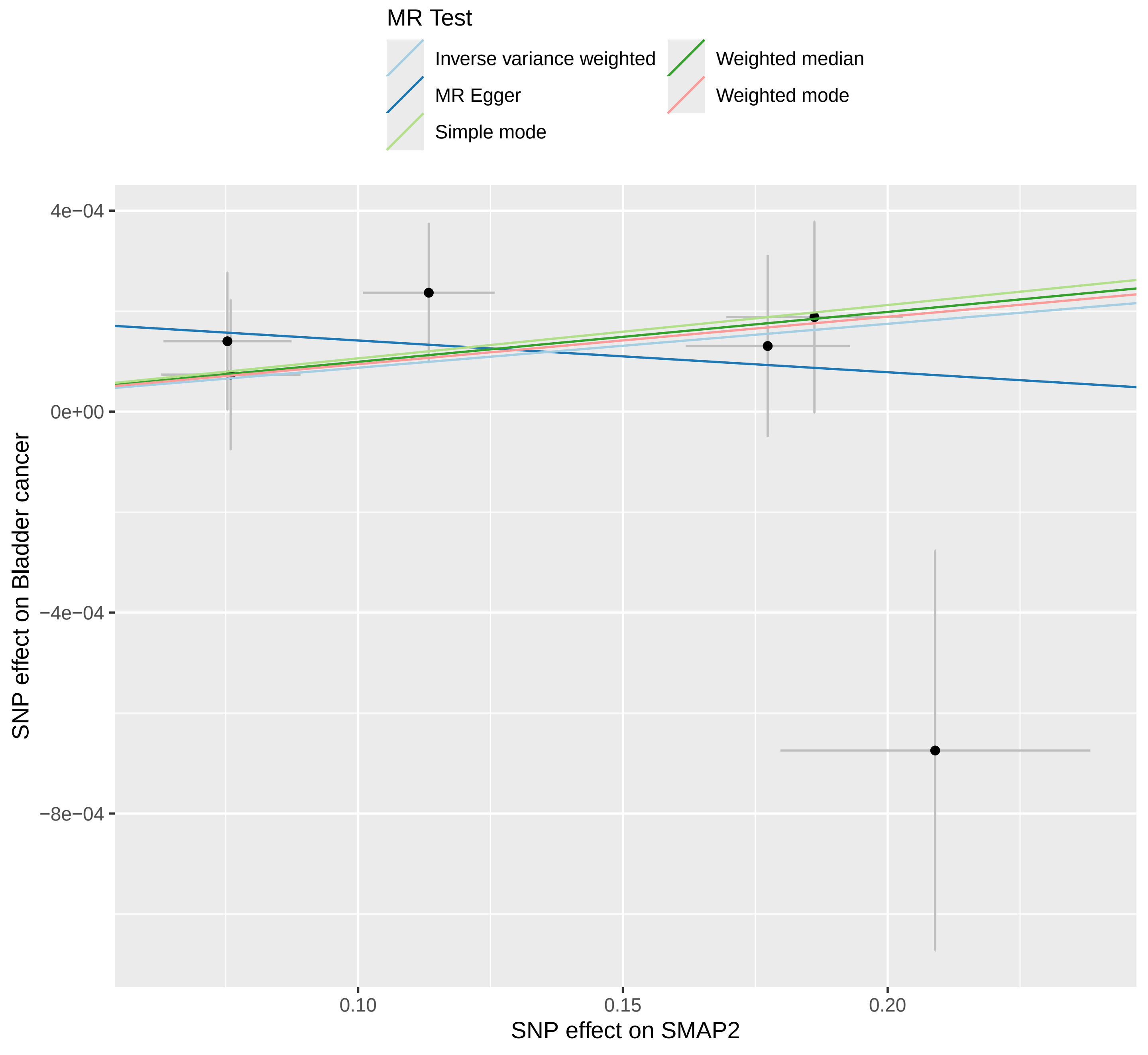

MR Test
Inverse variance weighted
MR Egger
Simple mode
Weighted median
Weighted mode
4e−04
0e+00
−4e−04
−8e−04
SNP effect on Bladder cancer
0.10
0.15
0.20
SNP effect on SMAP2

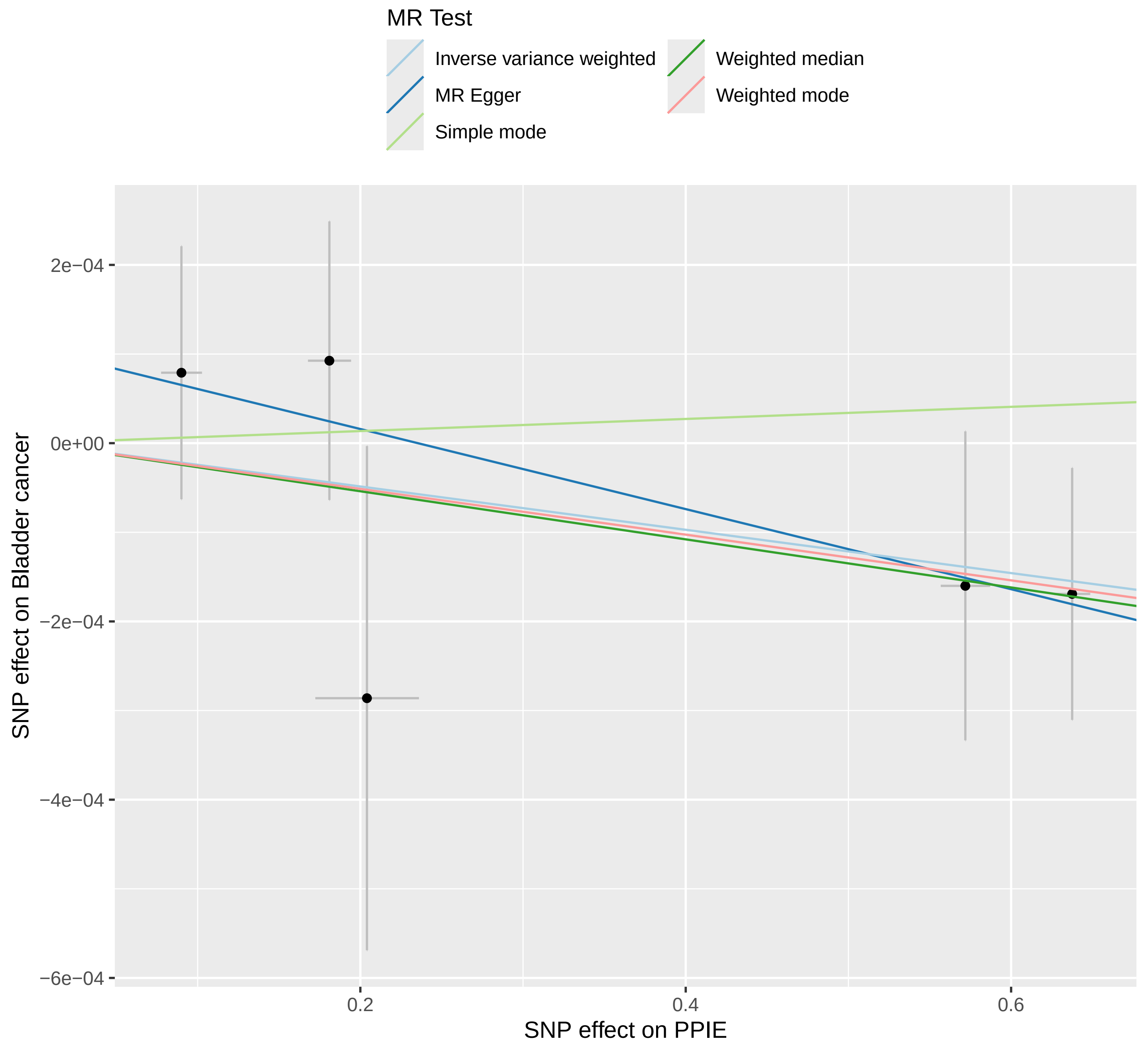
MR Test
Inverse variance weighted
MR Egger
Simple mode
Weighted median
Weighted mode
SNP effect on Bladder cancer
2e−04
0e+00
−2e−04
−4e−04
−6e−04
0.2
0.4
0.6
SNP effect on PPIE

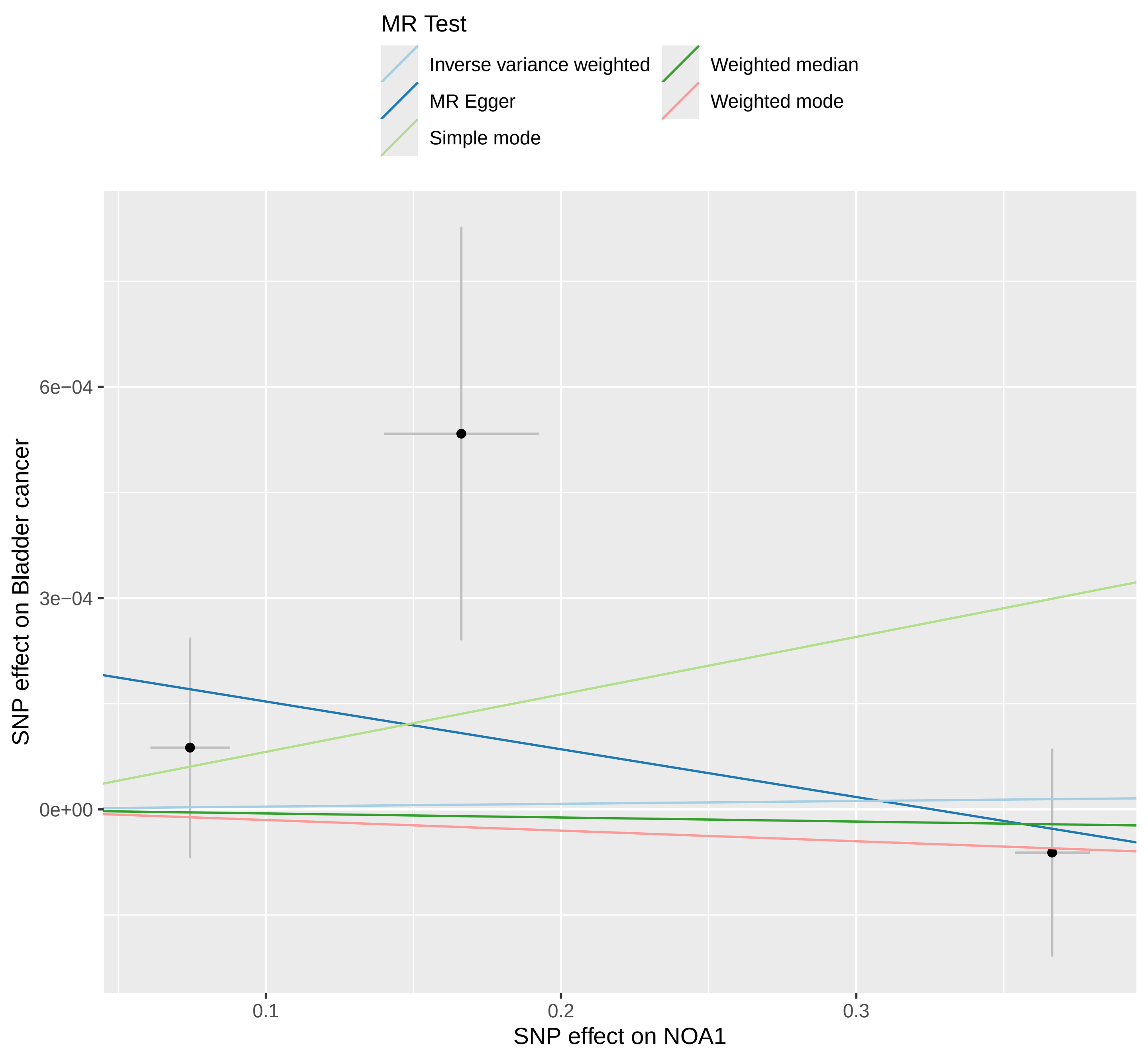
MR Test
Inverse variance weighted
MR Egger
Simple mode
Weighted median
Weighted mode
6e−04
3e−04
0e+00
SNP effect on Bladder cancer
0.1
0.2
0.3
SNP effect on NOA1

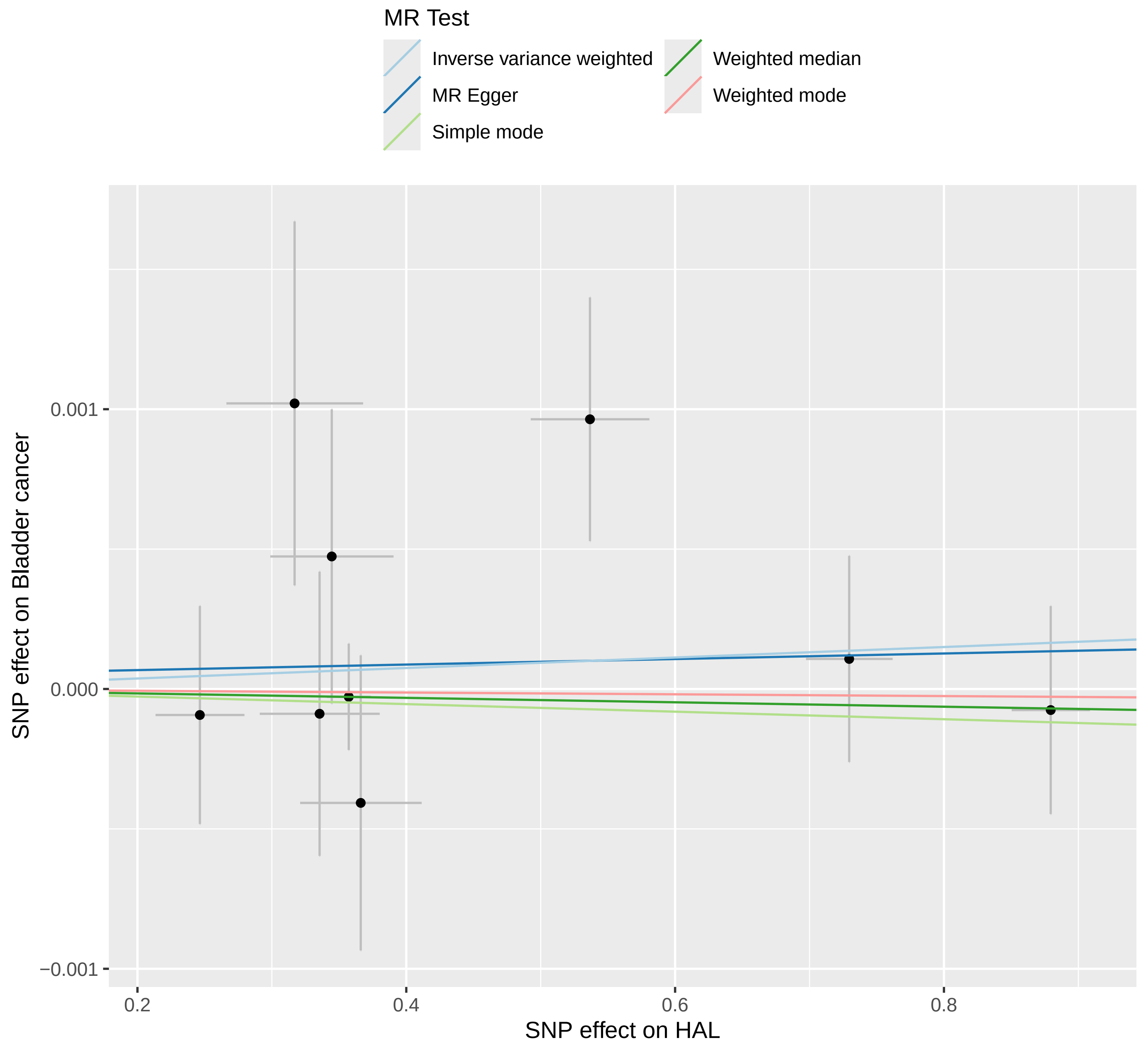

MR Test
Inverse variance weighted
MR Egger
Simple mode
Weighted median
Weighted mode
SNP effect on Bladder cancer
0.001
0.000
−0.001
0.2
0.4
0.6
0.8
SNP effect on HAL

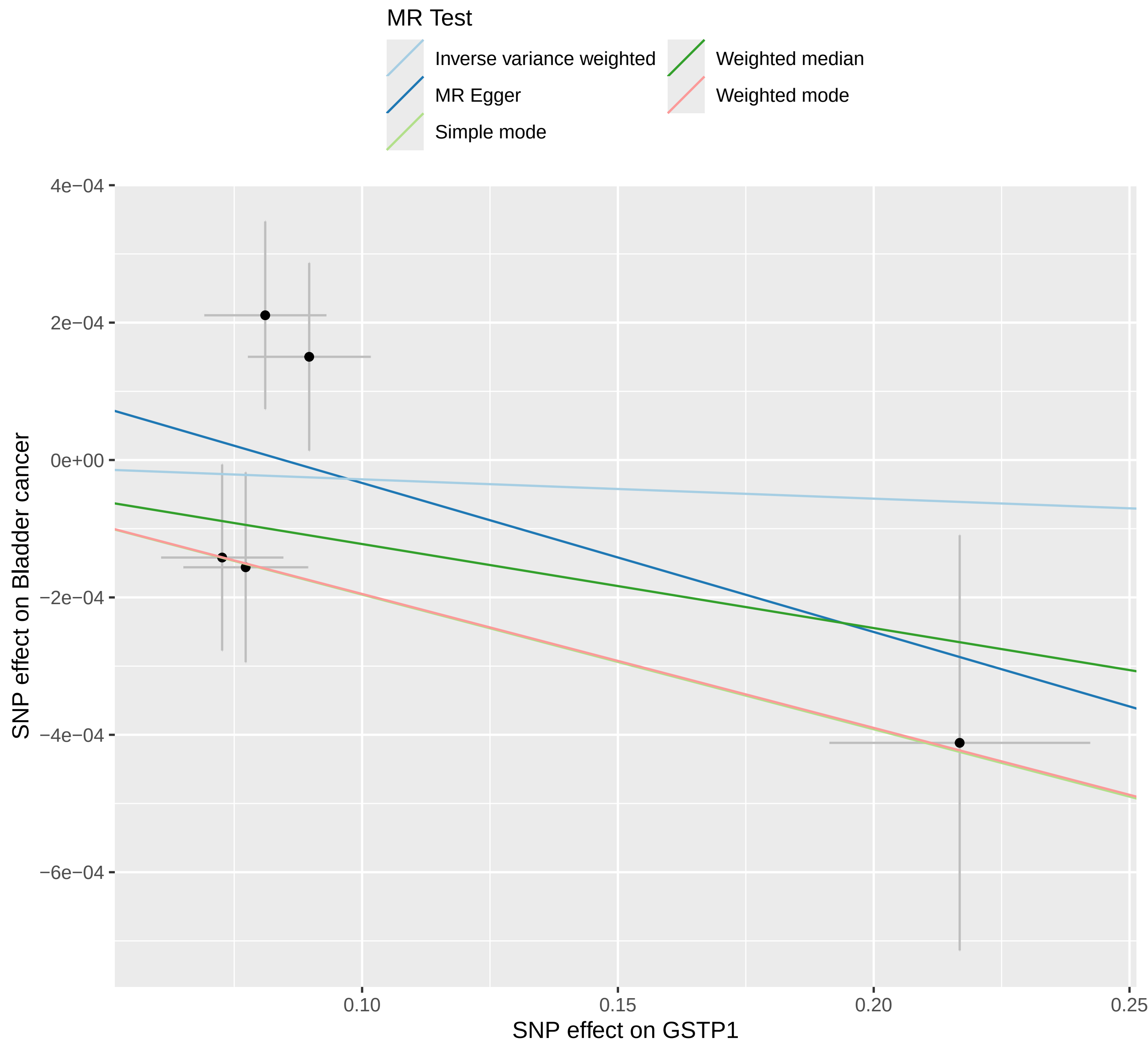

MR Test
Inverse variance weighted
MR Egger
Simple mode
Weighted median
Weighted mode
4e−04
2e−04
0e+00
−2e−04
−4e−04
−6e−04
0.10
0.15
0.20
0.25
SNP effect on GSTP1
SNP effect on Bladder cancer

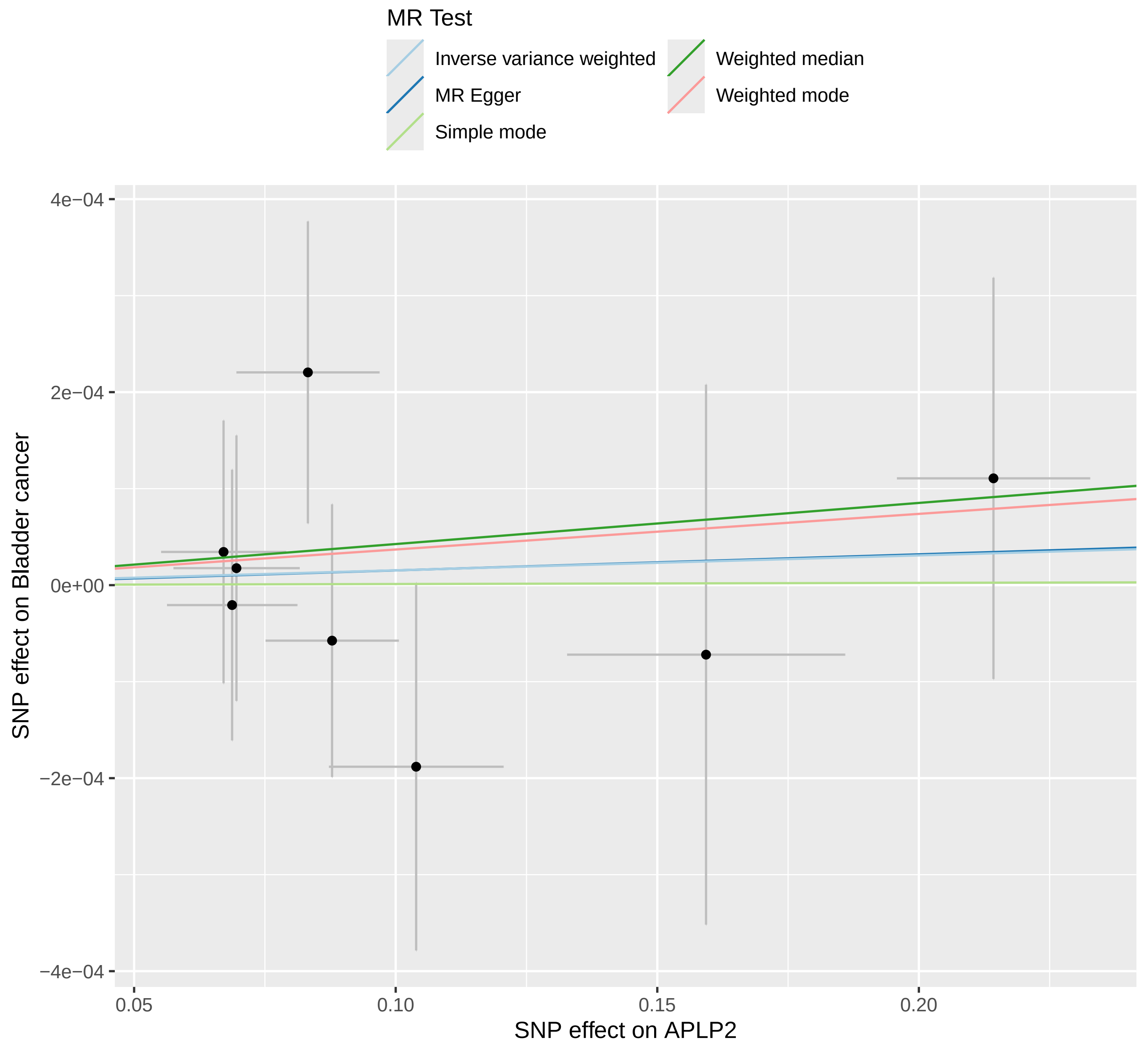
MR Test
Inverse variance weighted
MR Egger
Simple mode
Weighted median
Weighted mode
4e−04
2e−04
0e+00
−2e−04
−4e−04
SNP effect on Bladder cancer
0.05
0.10
0.15
0.20
SNP effect on APLP2

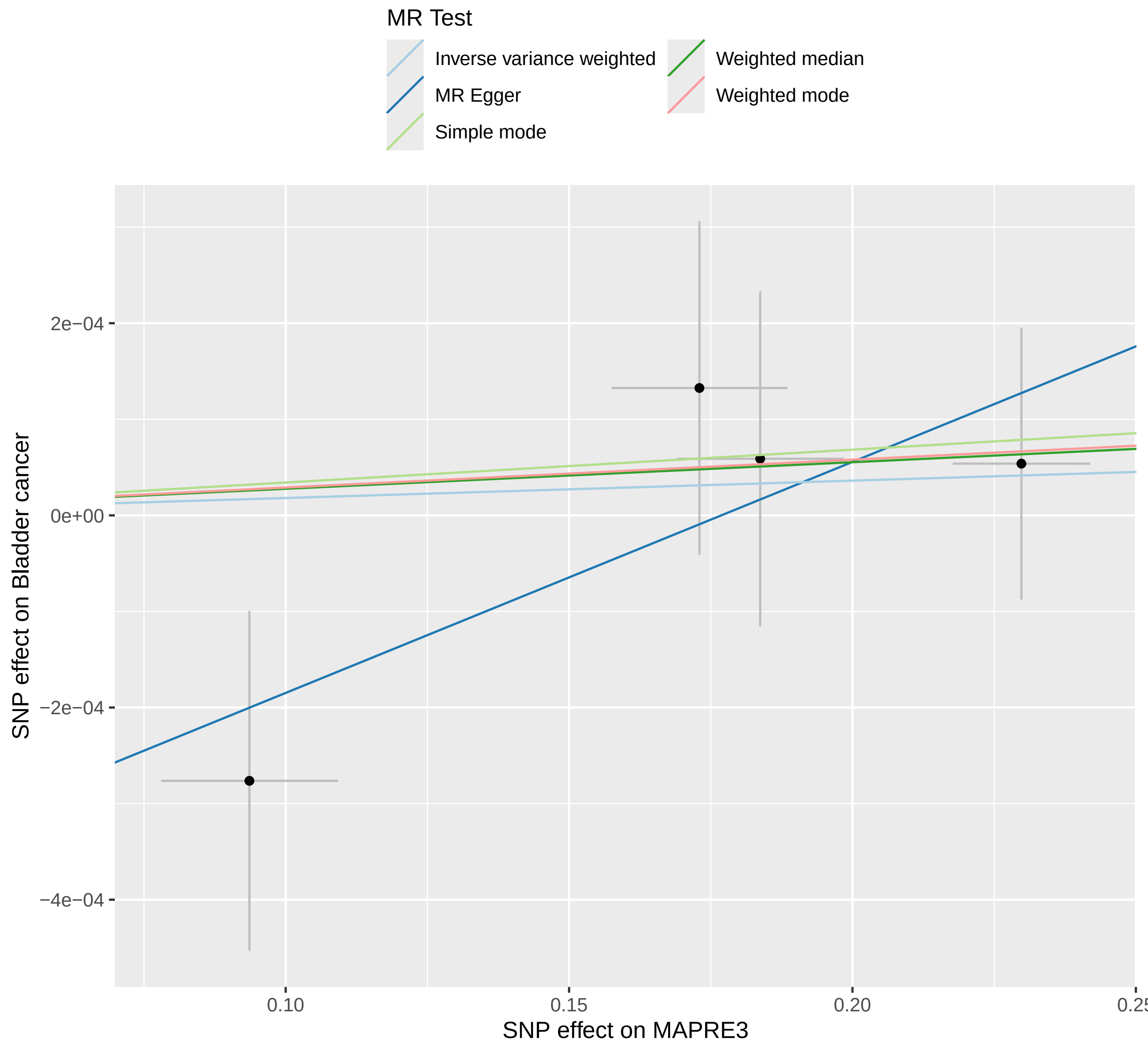

MR Test
Inverse variance weighted
MR Egger
Simple mode
Weighted median
Weighted mode
2e−04
0e+00
−2e−04
−4e−04
0.10
0.15
0.20
0.25
SNP effect on Bladder cancer
SNP effect on MAPRE3

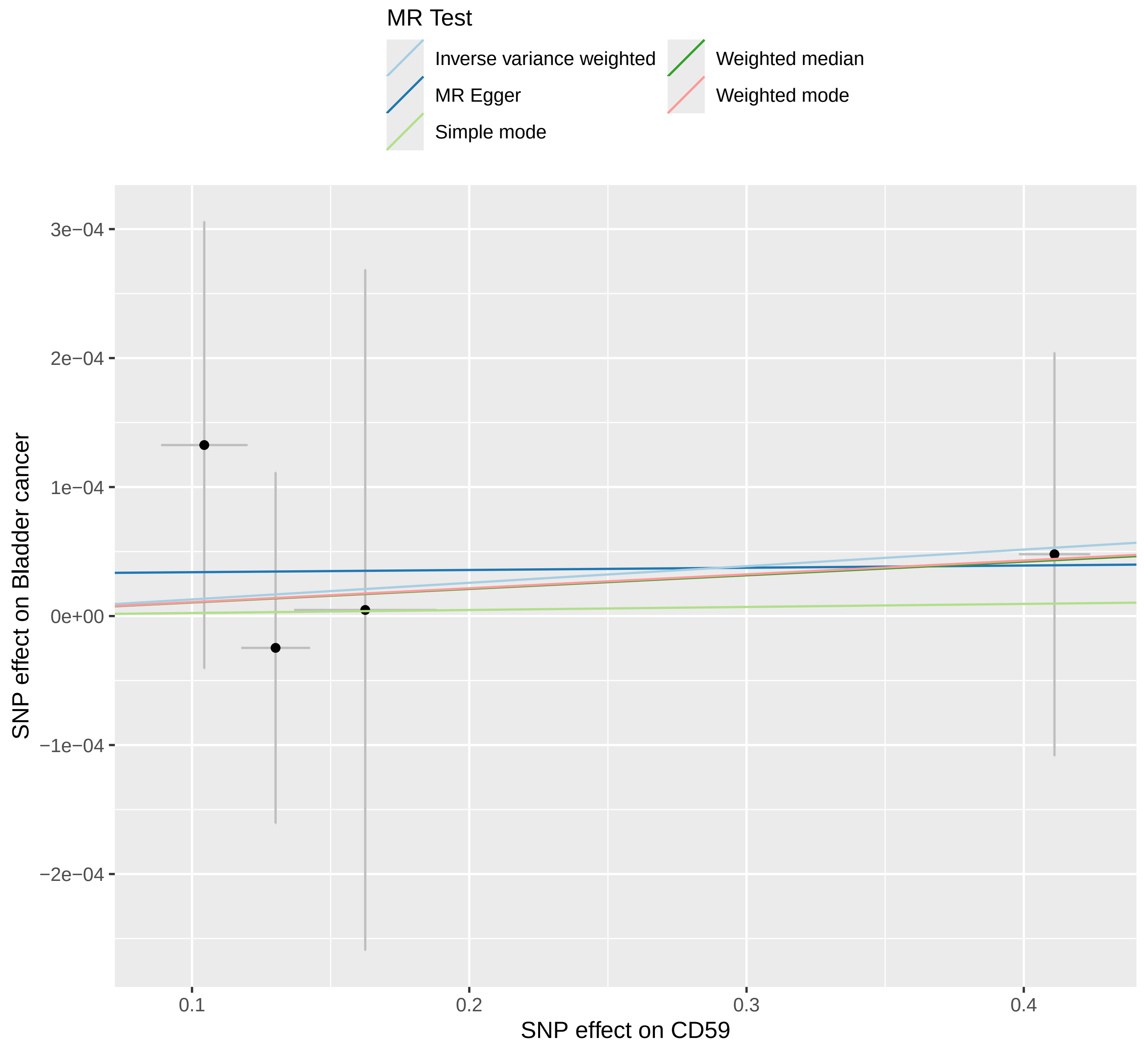
MR Test
Inverse variance weighted
MR Egger
Simple mode
Weighted median
Weighted mode
3e−04
2e−04
1e−04
0e+00
−1e−04
−2e−04
SNP effect on Bladder cancer
0.1
0.2
0.3
0.4
SNP effect on CD59

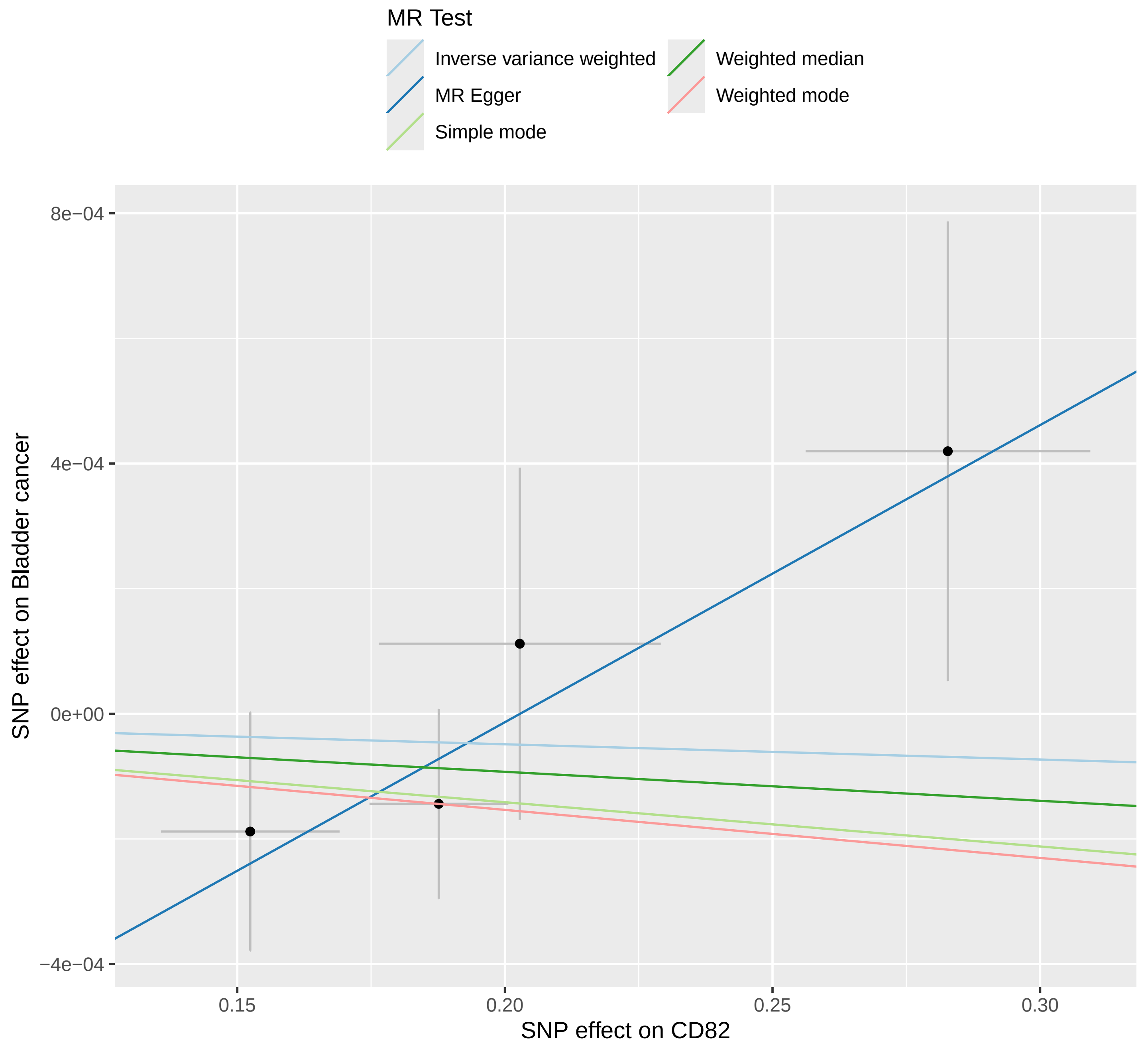

MR Test
Inverse variance weighted
MR Egger
Simple mode
Weighted median
Weighted mode
8e−04
4e−04
0e+00
−4e−04
SNP effect on Bladder cancer
0.15
0.20
0.25
0.30
SNP effect on CD82

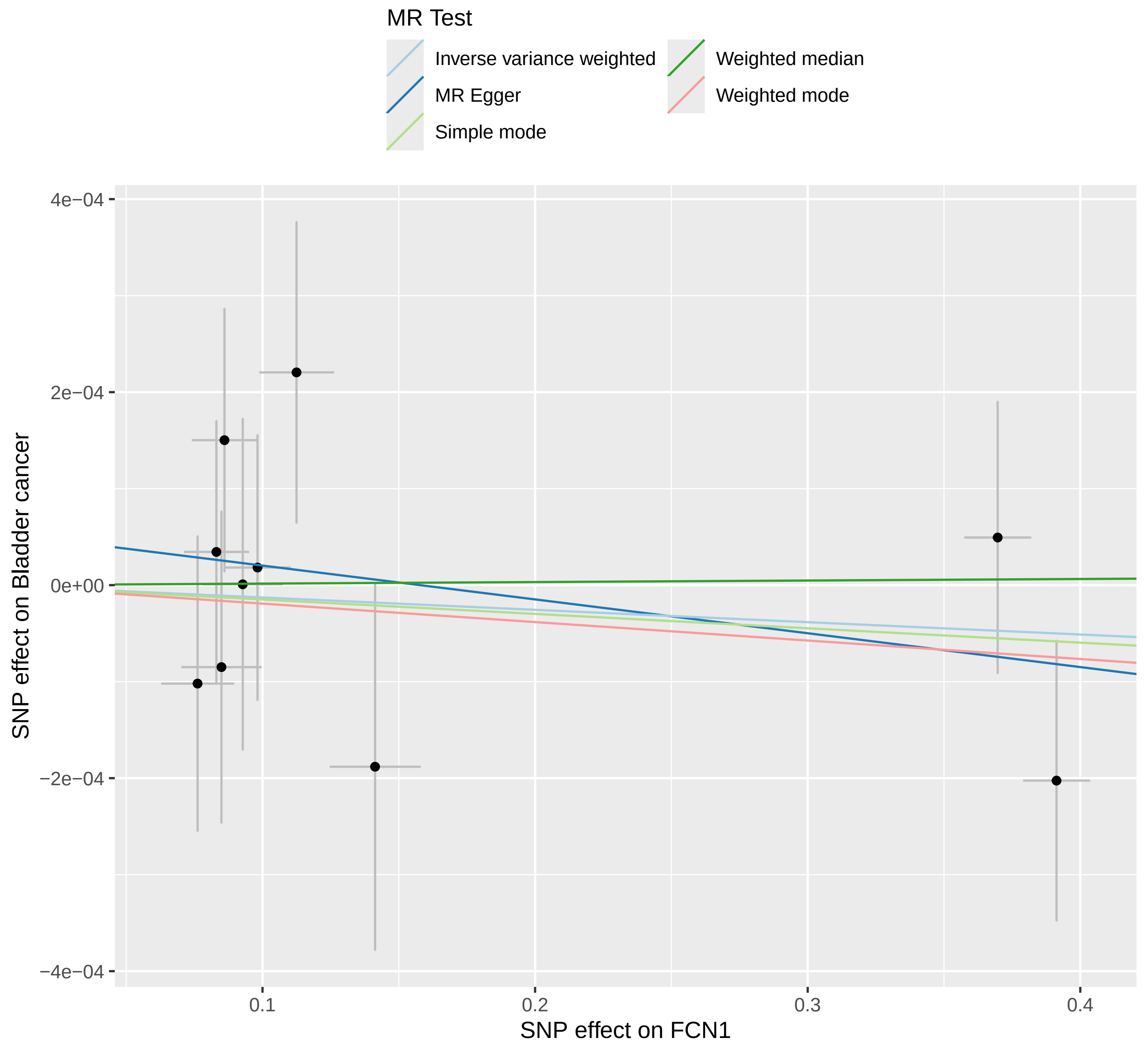
MR Test
Inverse variance weighted
MR Egger
Simple mode
Weighted median
Weighted mode
4e−04
2e−04
0e+00
−2e−04
−4e−04
SNP effect on Bladder cancer
0.1
0.2
0.3
0.4
SNP effect on FCN1

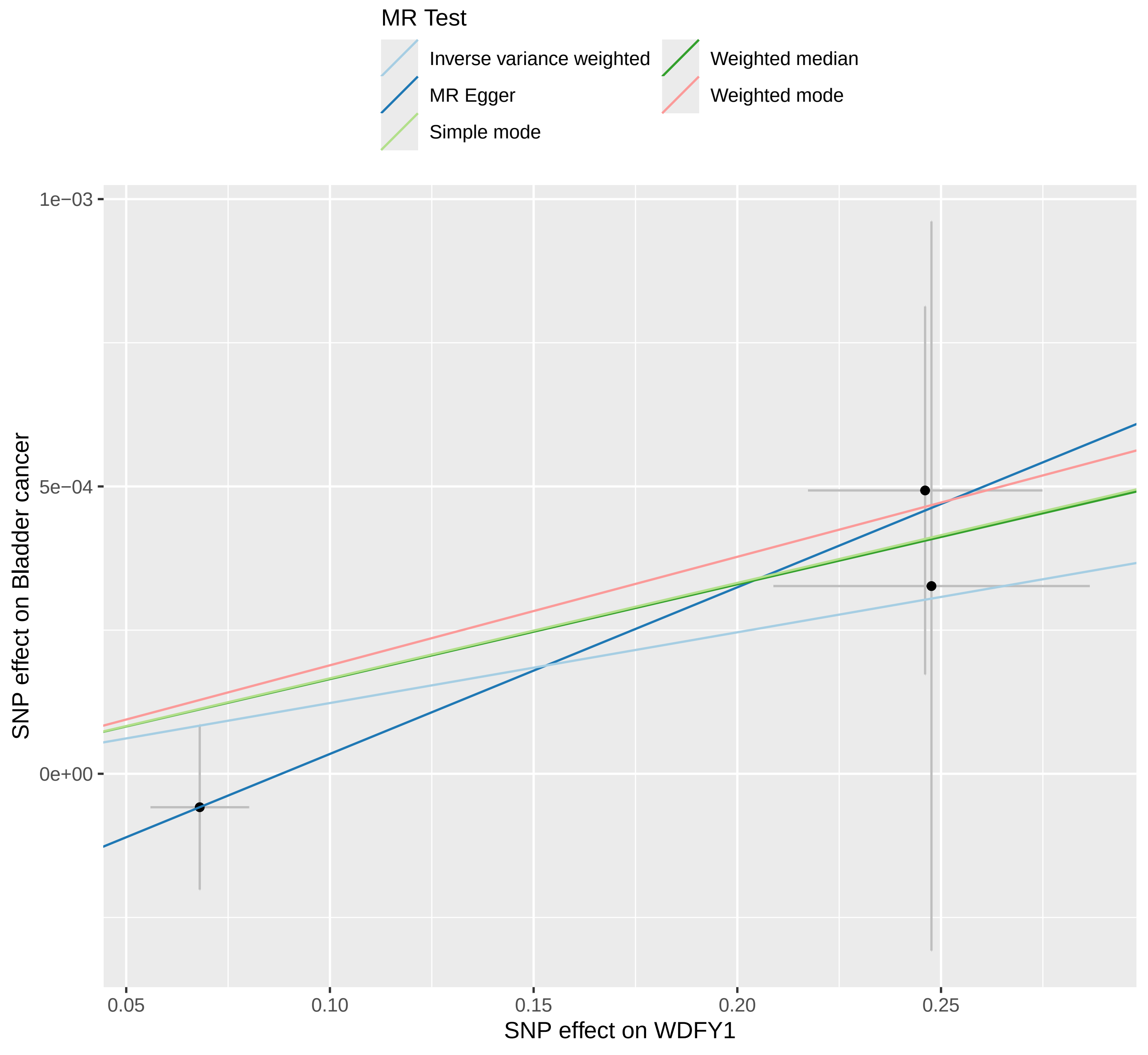
MR Test
Inverse variance weighted
MR Egger
Simple mode
Weighted median
Weighted mode
1e−03
5e−04
0e+00
0.05
0.10
0.15
0.20
0.25
SNP effect on Bladder cancer
SNP effect on WDFY1

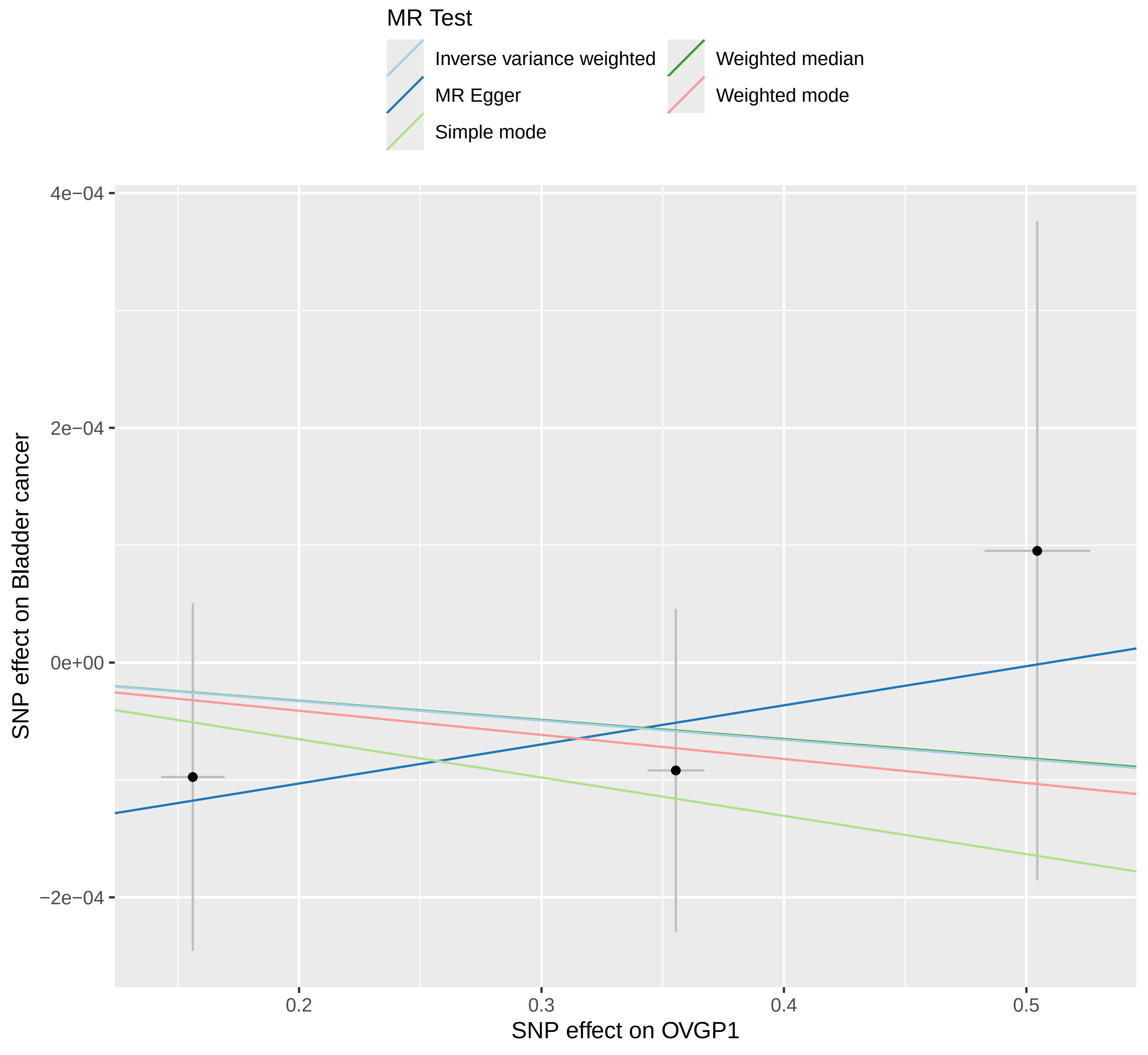
MR Test
Inverse variance weighted
MR Egger
Simple mode
Weighted median
Weighted mode
4e−04
2e−04
0e+00
−2e−04
SNP effect on Bladder cancer
0.2
0.3
0.4
0.5
SNP effect on OVGP1

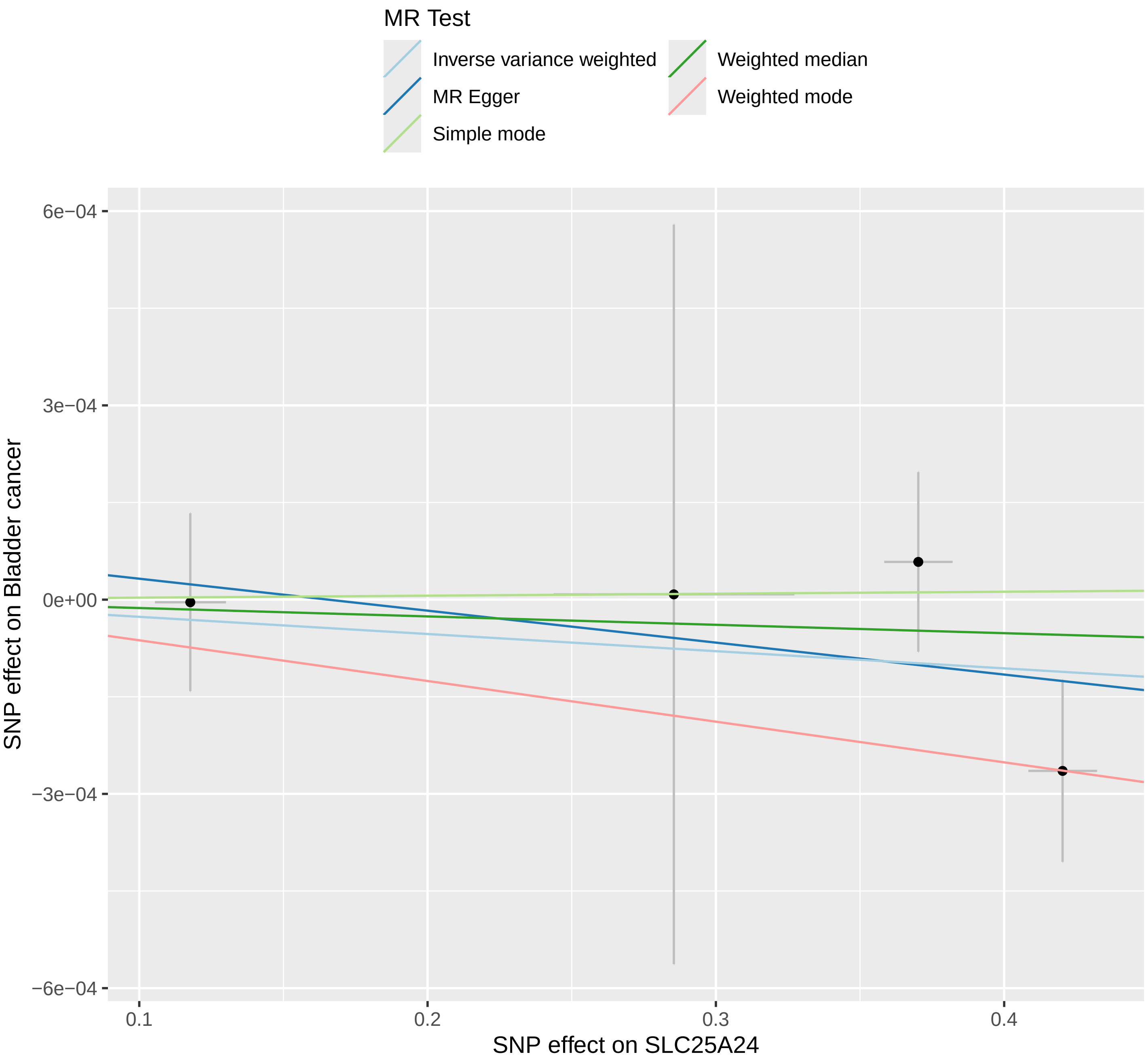
MR Test
Inverse variance weighted
MR Egger
Simple mode
Weighted median
Weighted mode
6e−04
3e−04
0e+00
−3e−04
−6e−04
SNP effect on Bladder cancer
0.1
0.2
0.3
0.4
SNP effect on SLC25A24

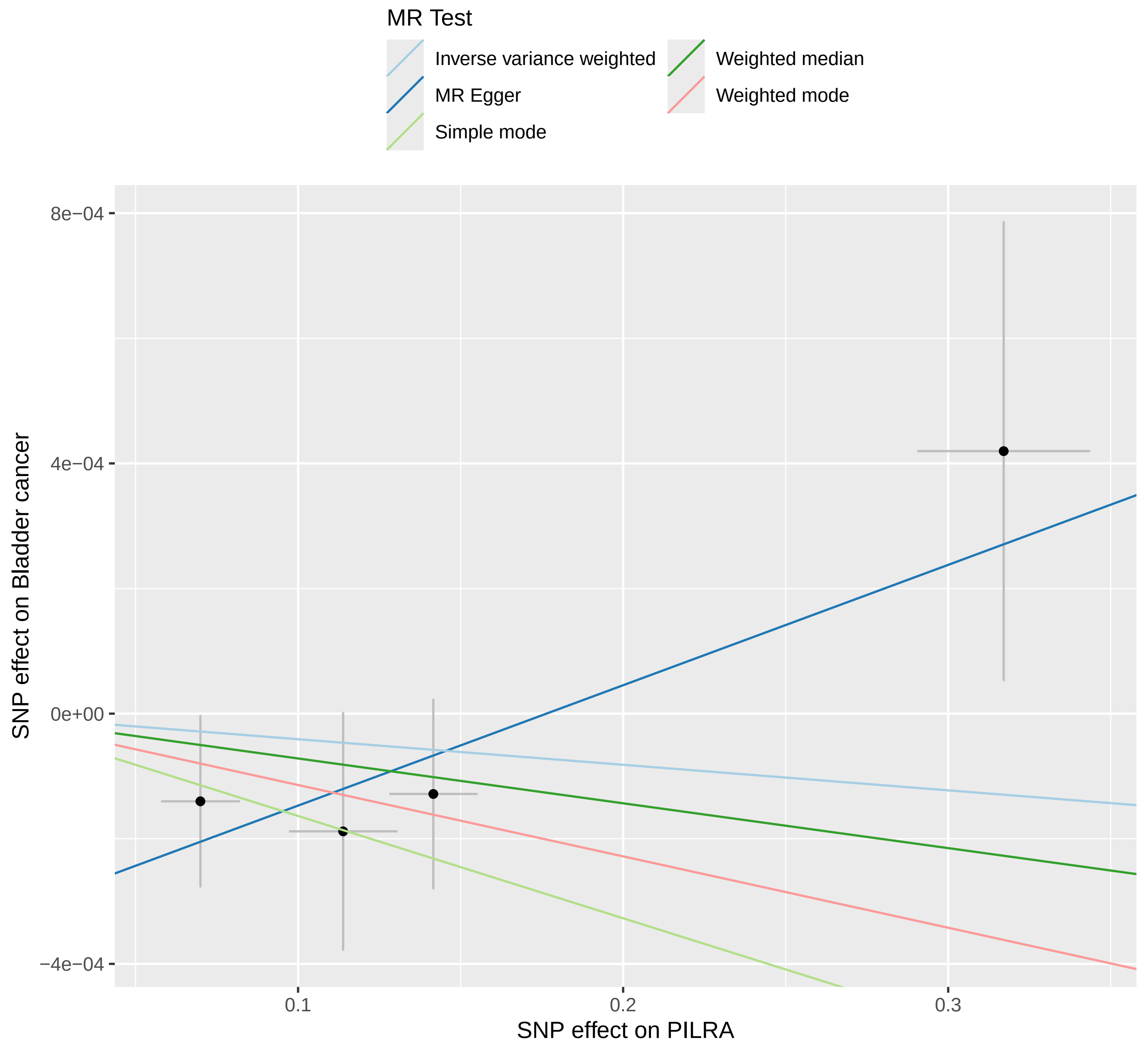

MR Test
Inverse variance weighted
MR Egger
Simple mode
Weighted median
Weighted mode
8e−04
4e−04
0e+00
−4e−04
0.1
0.2
0.3
SNP effect on Bladder cancer
SNP effect on PILRA

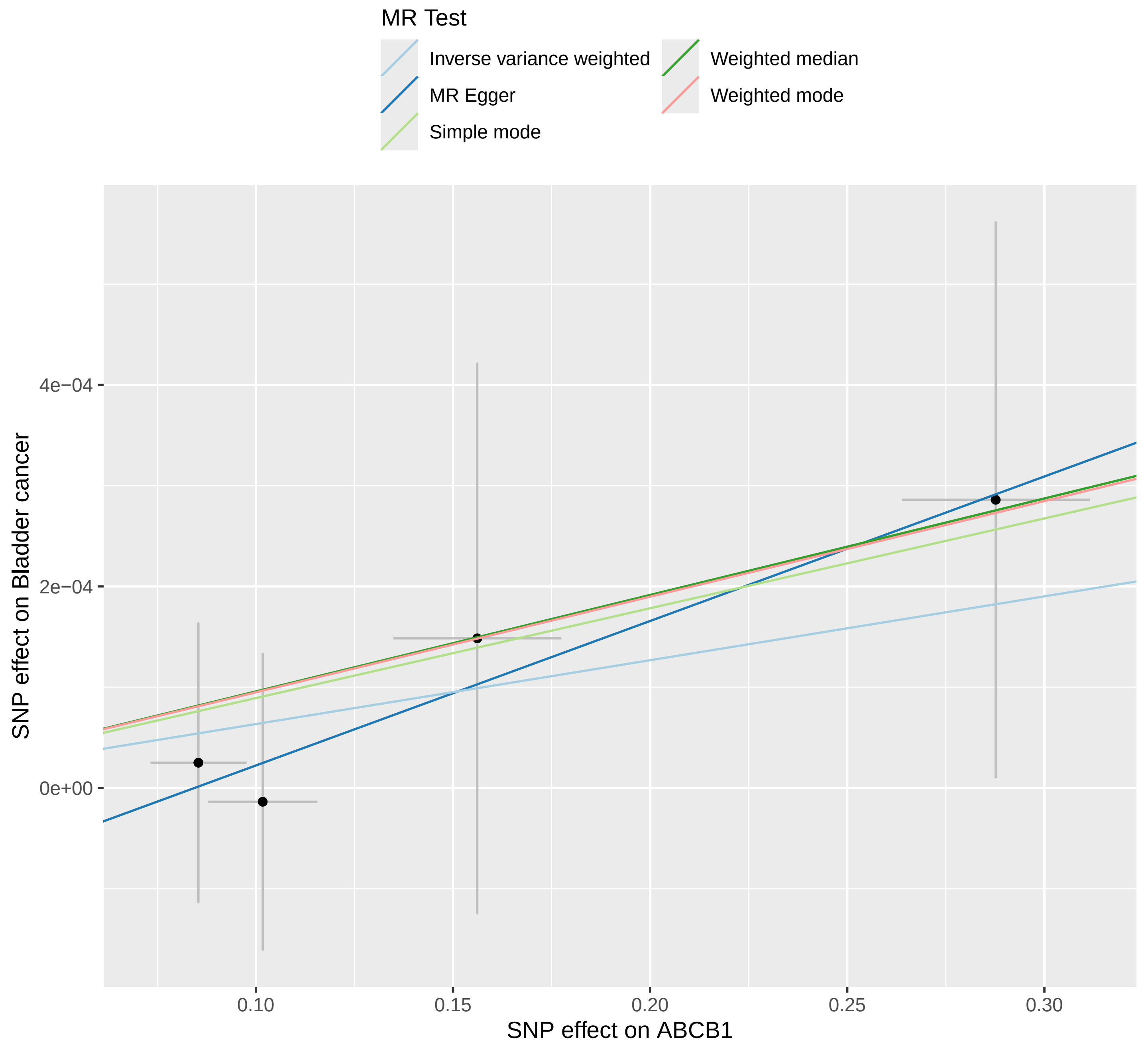
MR Test
Inverse variance weighted
MR Egger
Simple mode
Weighted median
Weighted mode
4e−04
2e−04
0e+00
SNP effect on Bladder cancer
0.10
0.15
0.20
0.25
0.30
SNP effect on ABCB1

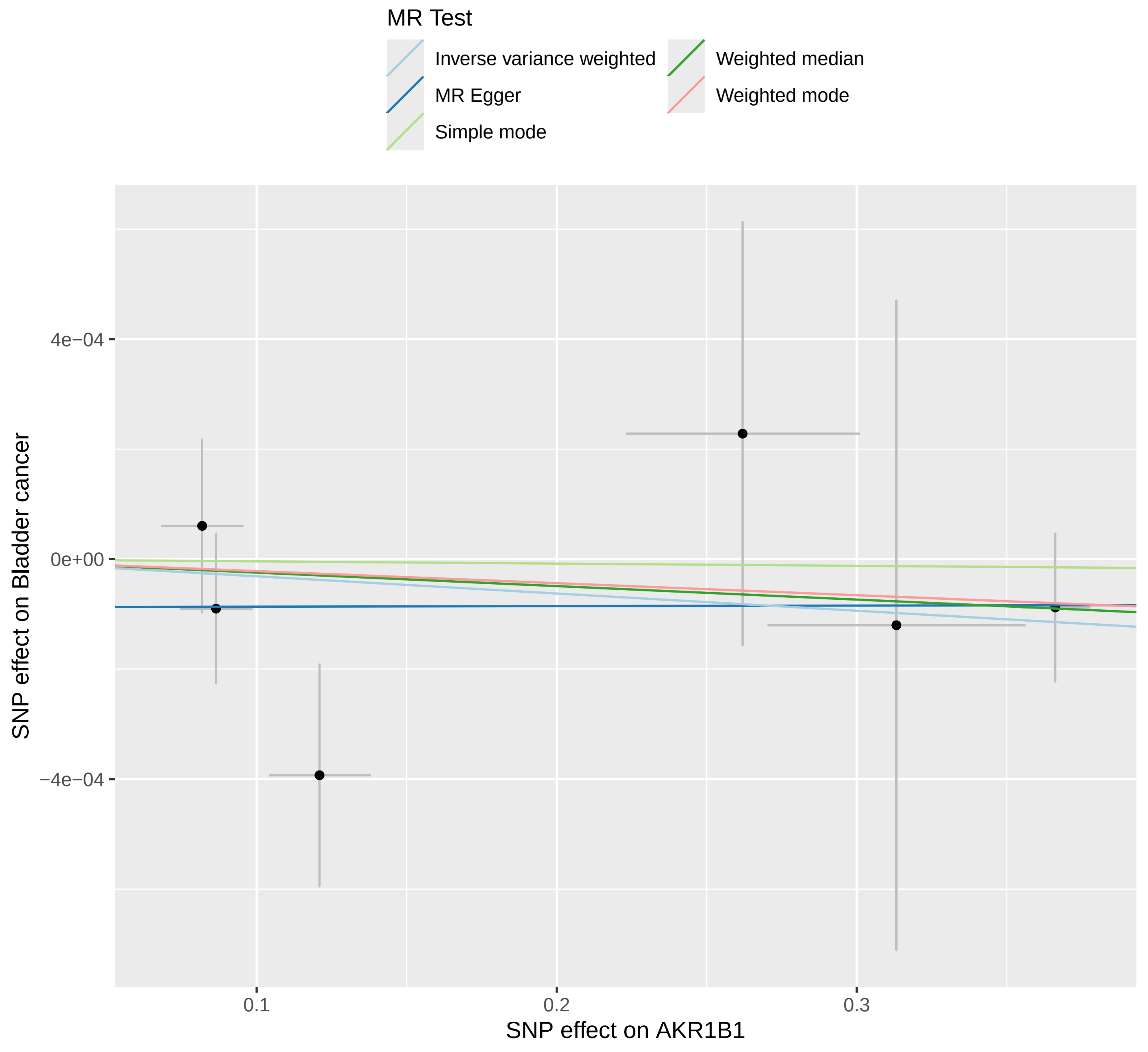

MR Test
Inverse variance weighted
MR Egger
Simple mode
Weighted median
Weighted mode
4e−04
0e+00
−4e−04
0.1
0.2
0.3
SNP effect on AKR1B1
SNP effect on Bladder cancer

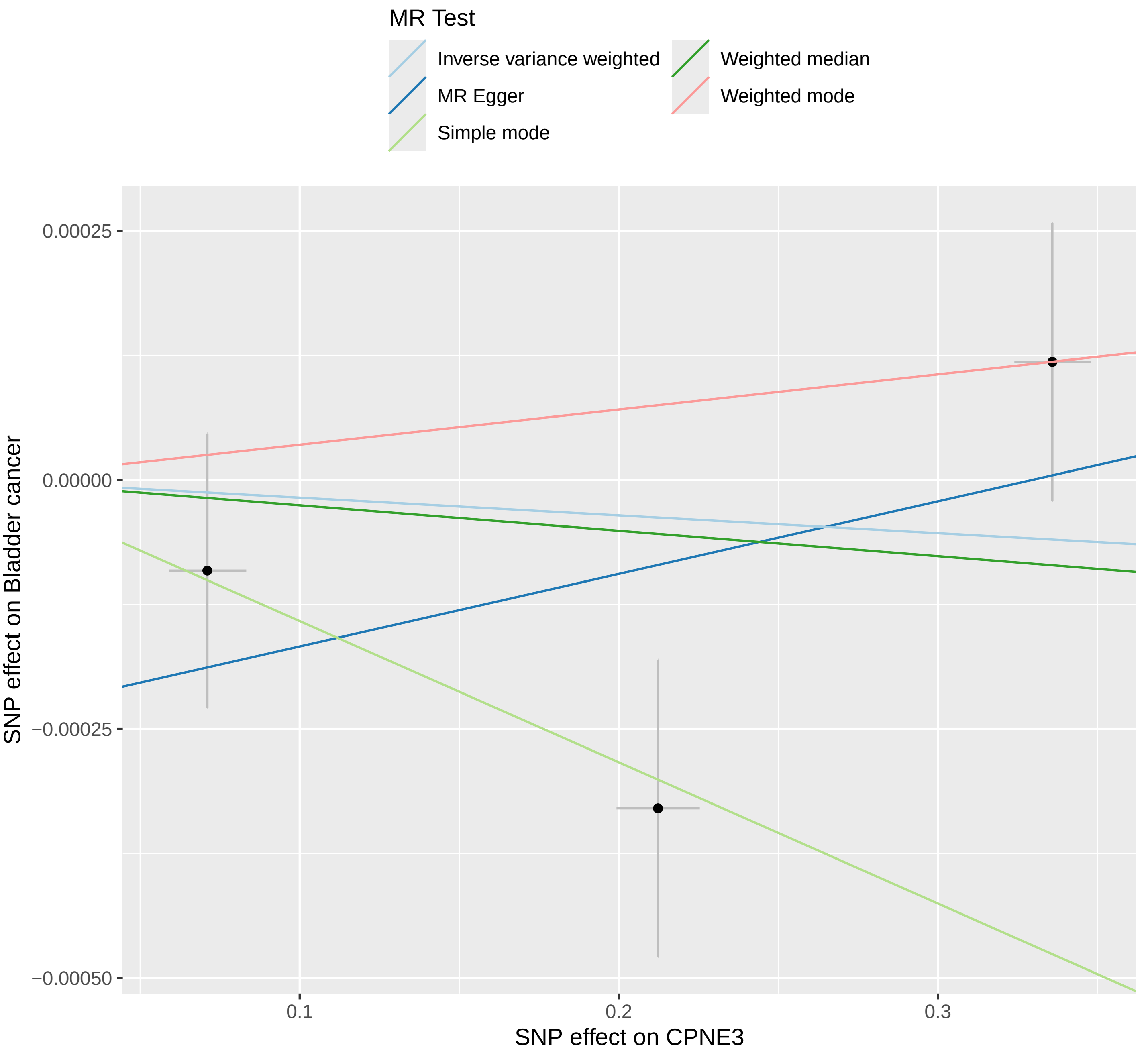
MR Test
Inverse variance weighted
MR Egger
Simple mode
Weighted median
Weighted mode
0.00025
0.00000
−0.00025
−0.00050
0.1
0.2
0.3
SNP effect on Bladder cancer
SNP effect on CPNE3

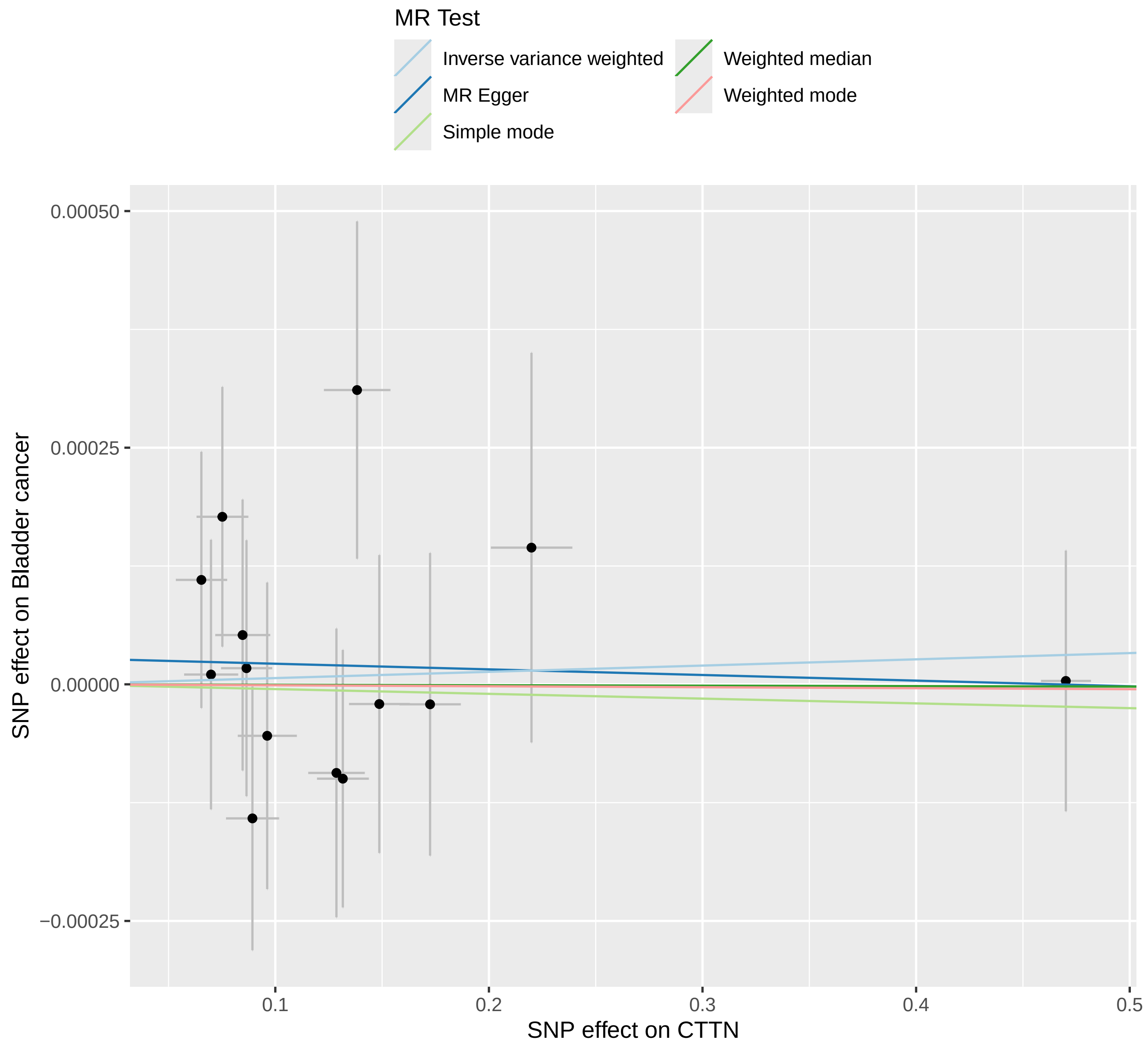

MR Test
Inverse variance weighted
MR Egger
Simple mode
Weighted median
Weighted mode
0.00050
0.00025
0.00000
−0.00025
0.1
0.2
0.3
0.4
0.5
SNP effect on CTTN
SNP effect on Bladder cancer

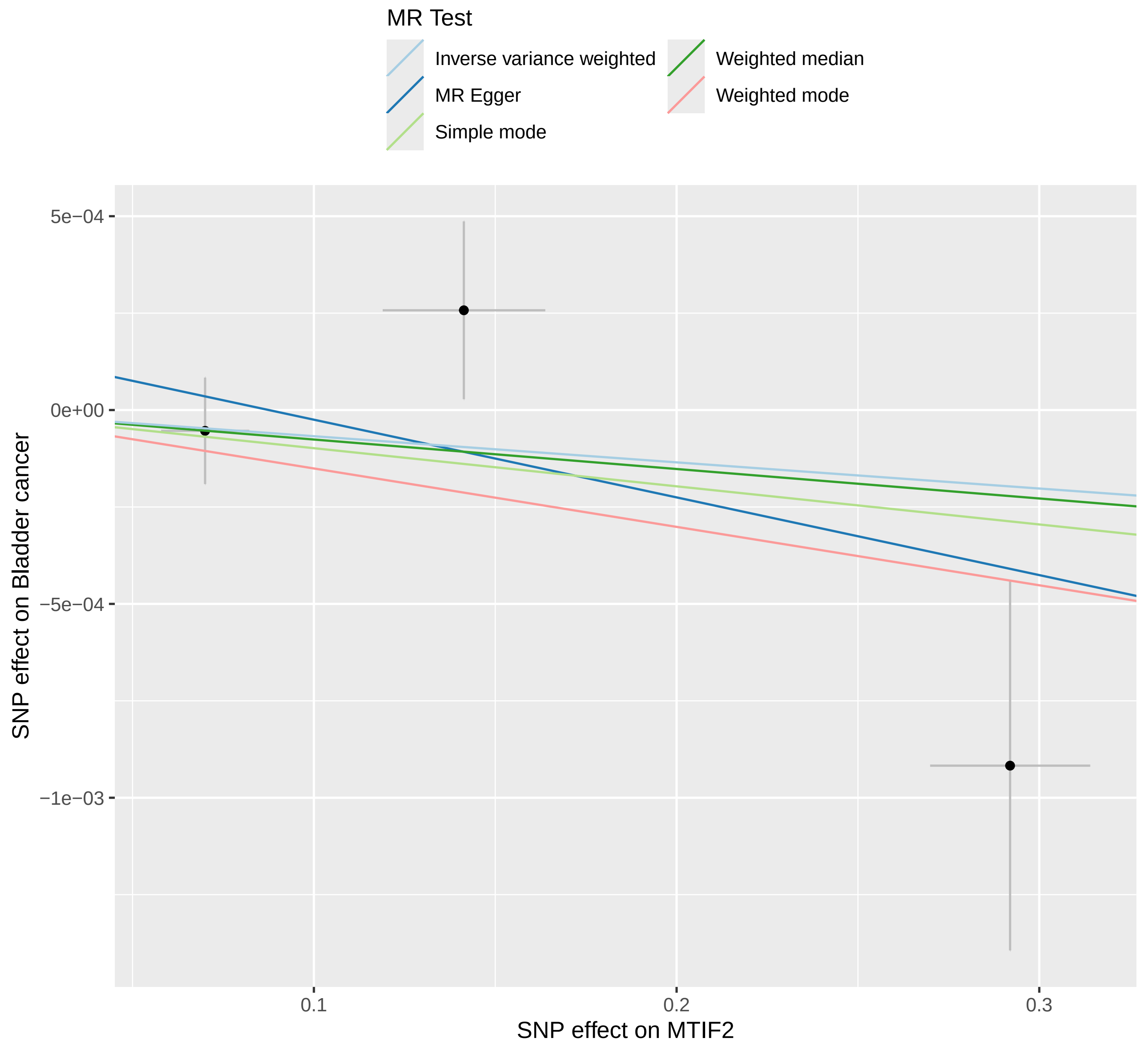

MR Test
Inverse variance weighted
MR Egger
Simple mode
Weighted median
Weighted mode
5e−04
0e+00
−5e−04
−1e−03
0.1
0.2
0.3
SNP effect on Bladder cancer
SNP effect on MTIF2

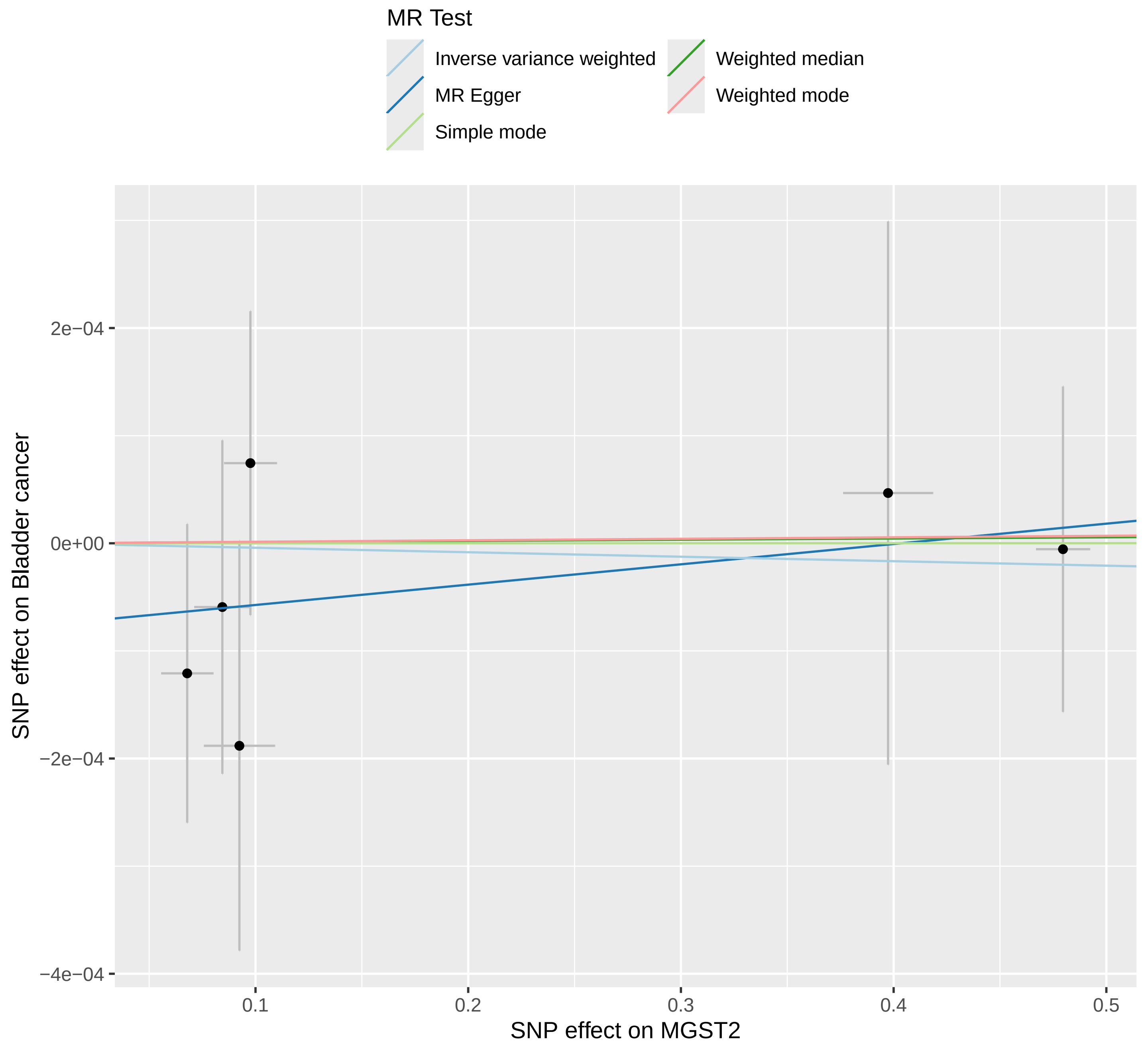
MR Test
Inverse variance weighted
MR Egger
Simple mode
Weighted median
Weighted mode
2e−04
0e+00
−2e−04
−4e−04
SNP effect on Bladder cancer
0.1
0.2
0.3
0.4
0.5
SNP effect on MGST2

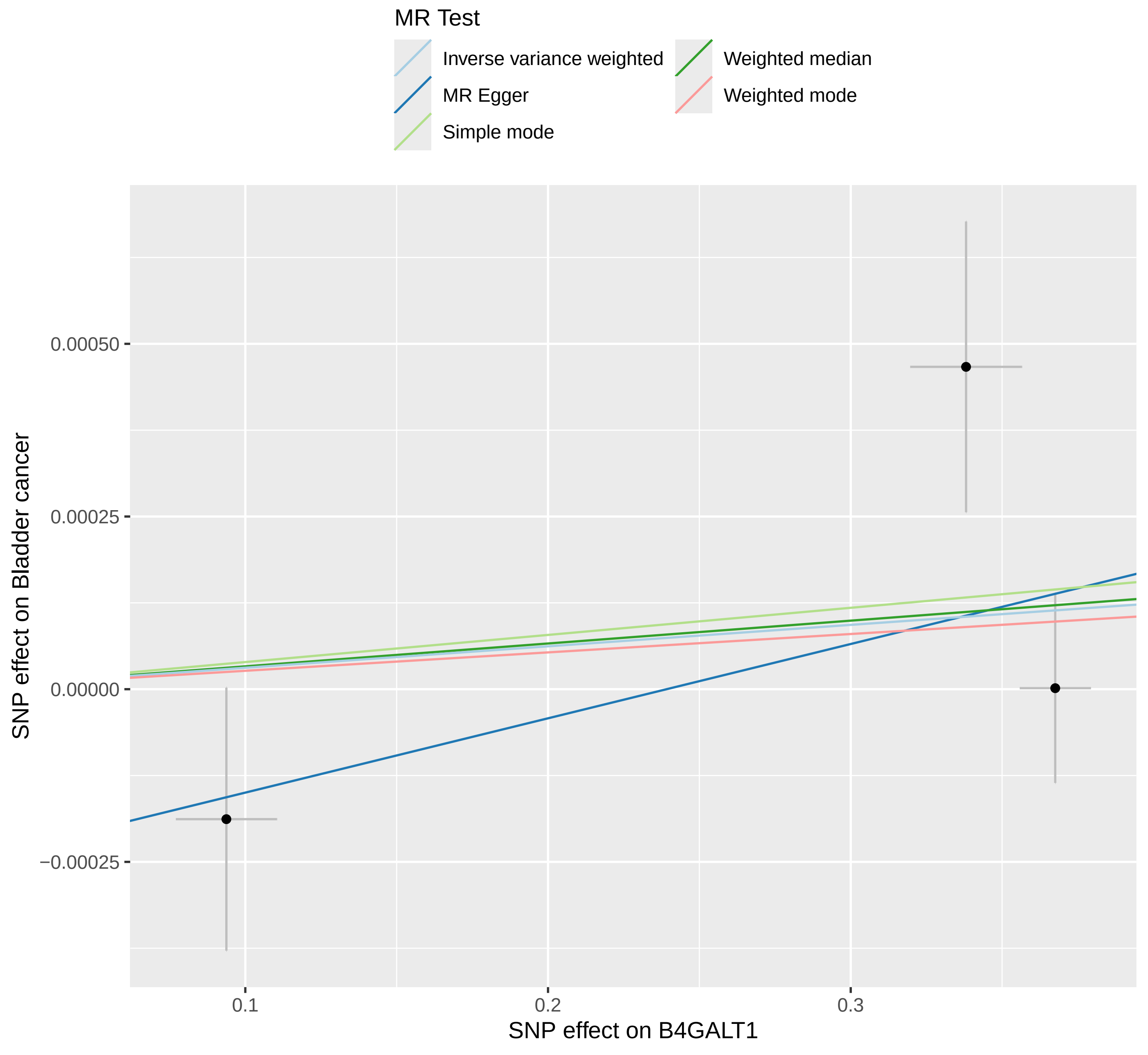

MR Test
Inverse variance weighted
MR Egger
Simple mode
Weighted median
Weighted mode
0.00050
0.00025
0.00000
−0.00025
SNP effect on Bladder cancer
0.1
0.2
0.3
SNP effect on B4GALT1

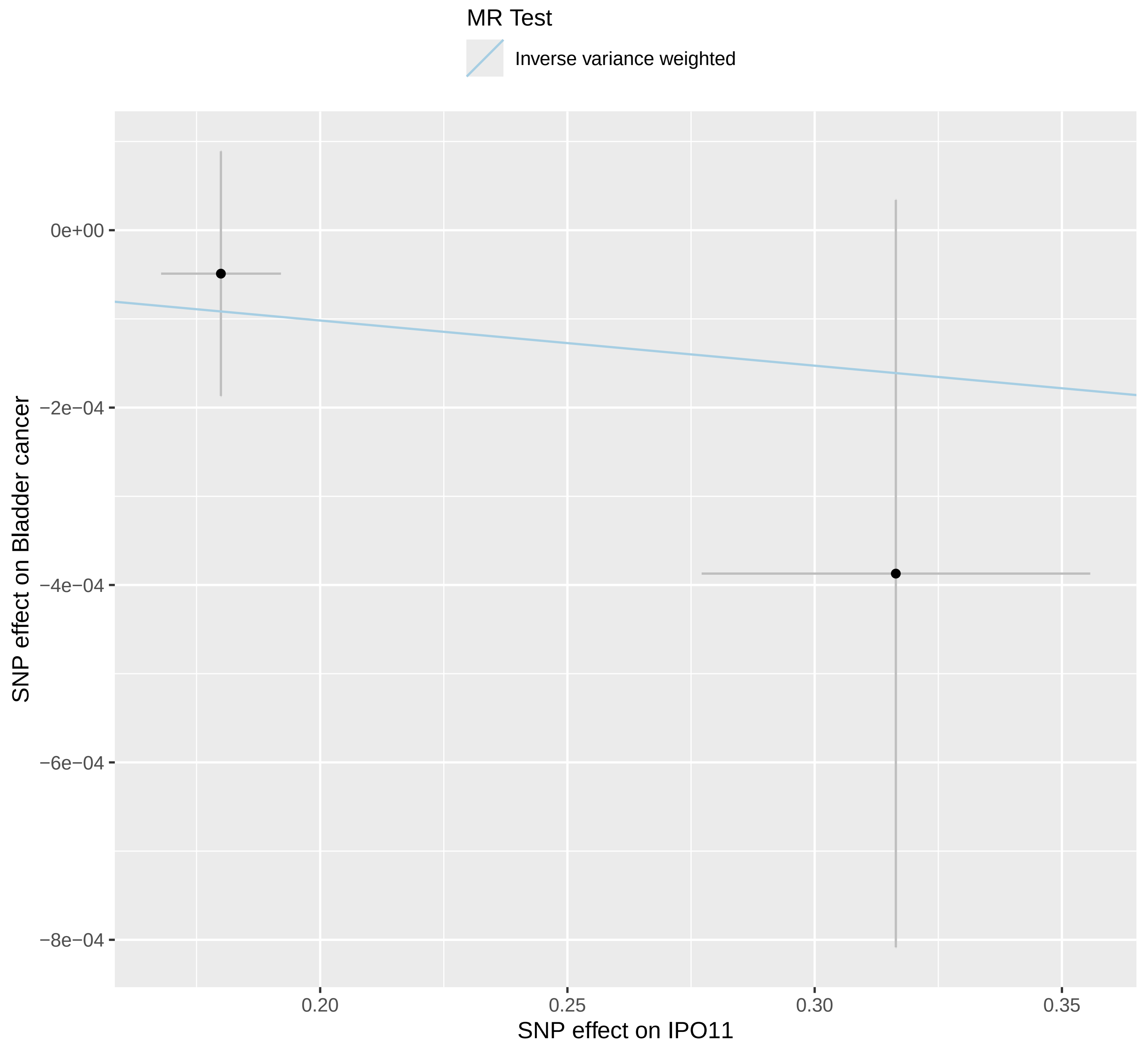

MR Test
Inverse variance weighted
0e+00
−2e−04
−4e−04
−6e−04
−8e−04
0.20
0.25
0.30
0.35
SNP effect on IPO11
SNP effect on Bladder cancer

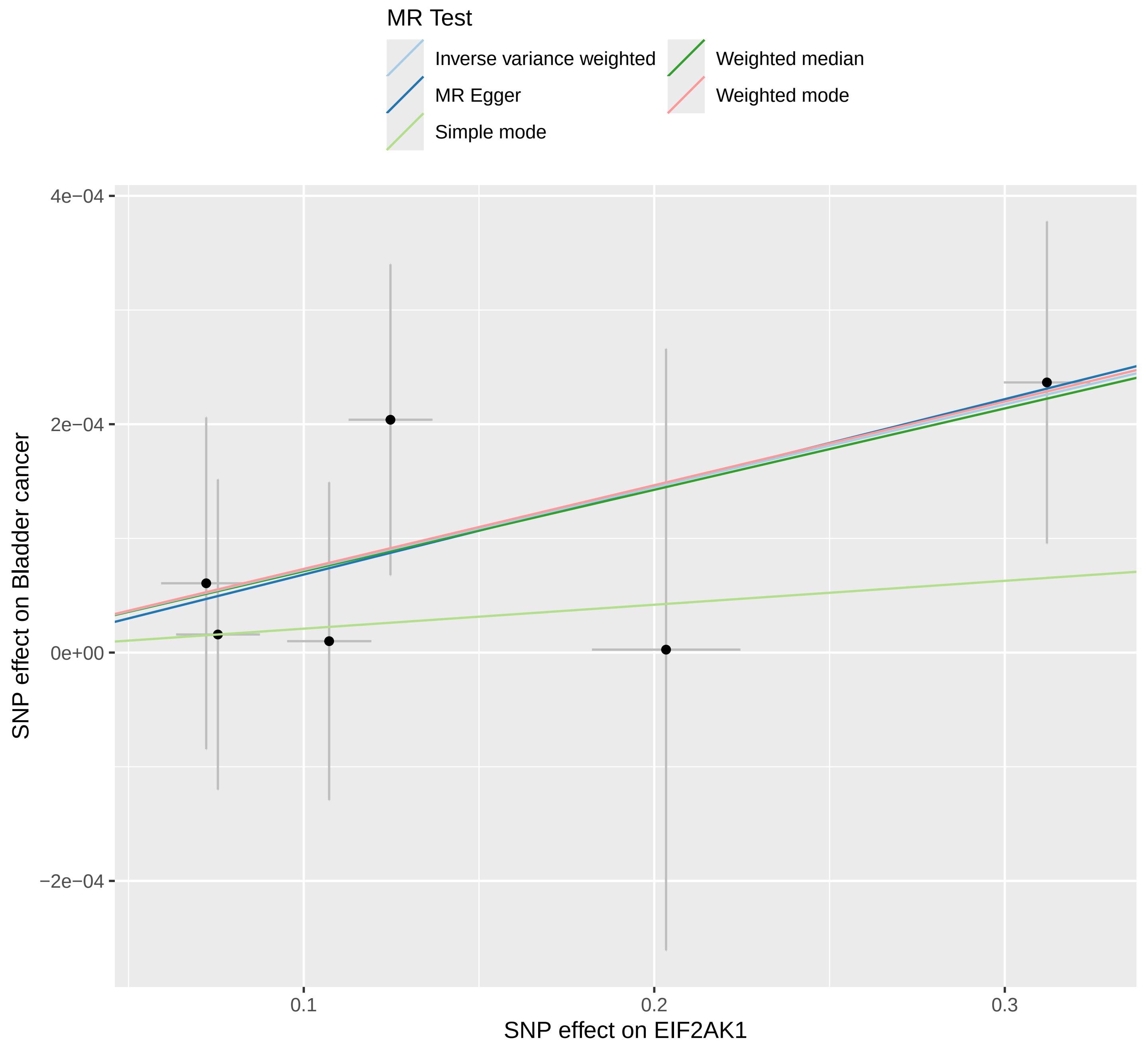
MR Test
Inverse variance weighted
MR Egger
Simple mode
Weighted median
Weighted mode
4e−04
2e−04
0e+00
−2e−04
0.1
0.2
0.3
SNP effect on Bladder cancer
SNP effect on EIF2AK1

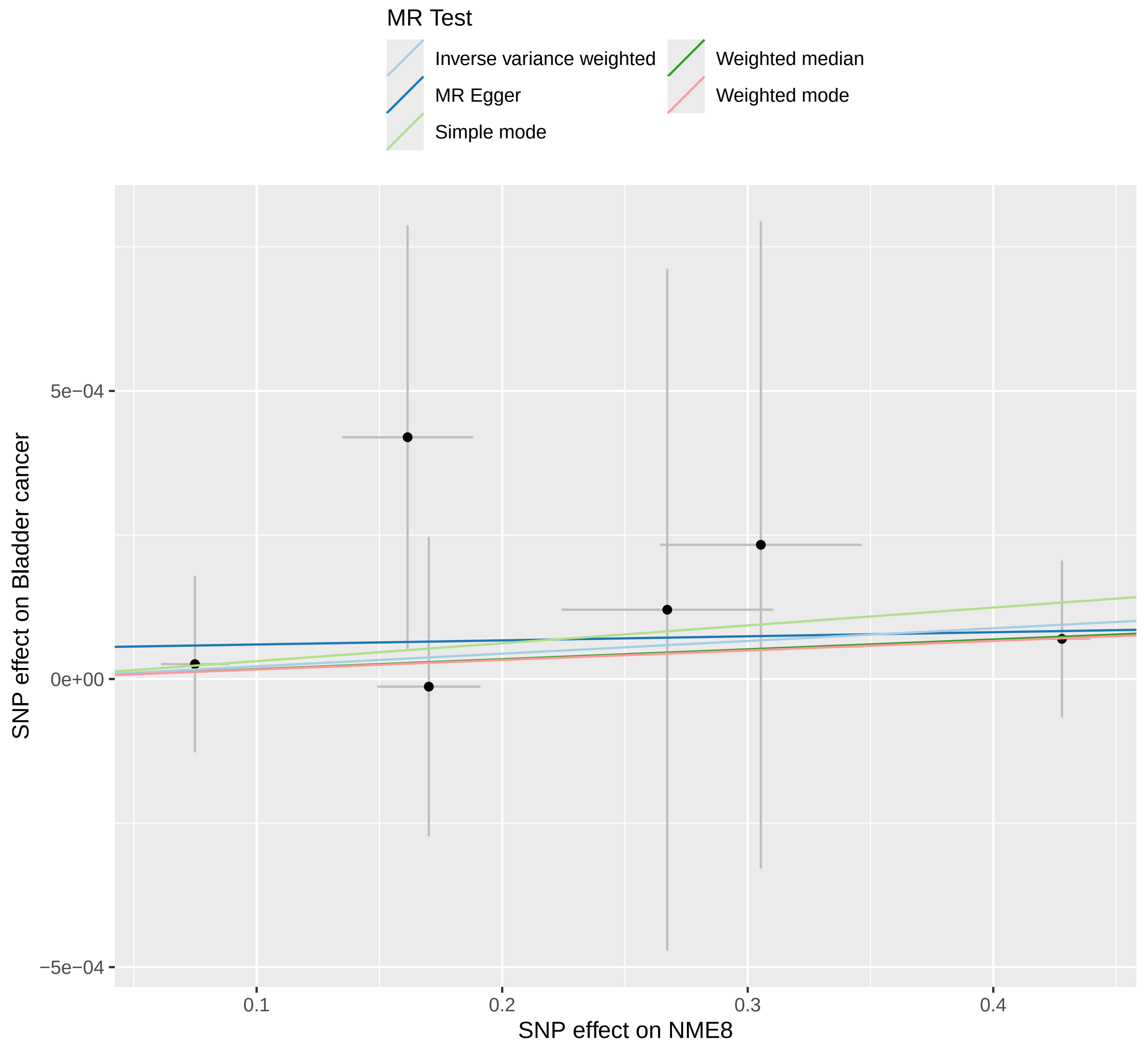
MR Test
Inverse variance weighted
MR Egger
Simple mode
Weighted median
Weighted mode
5e−04
0e+00
−5e−04
0.1
0.2
0.3
0.4
SNP effect on Bladder cancer
SNP effect on NME8

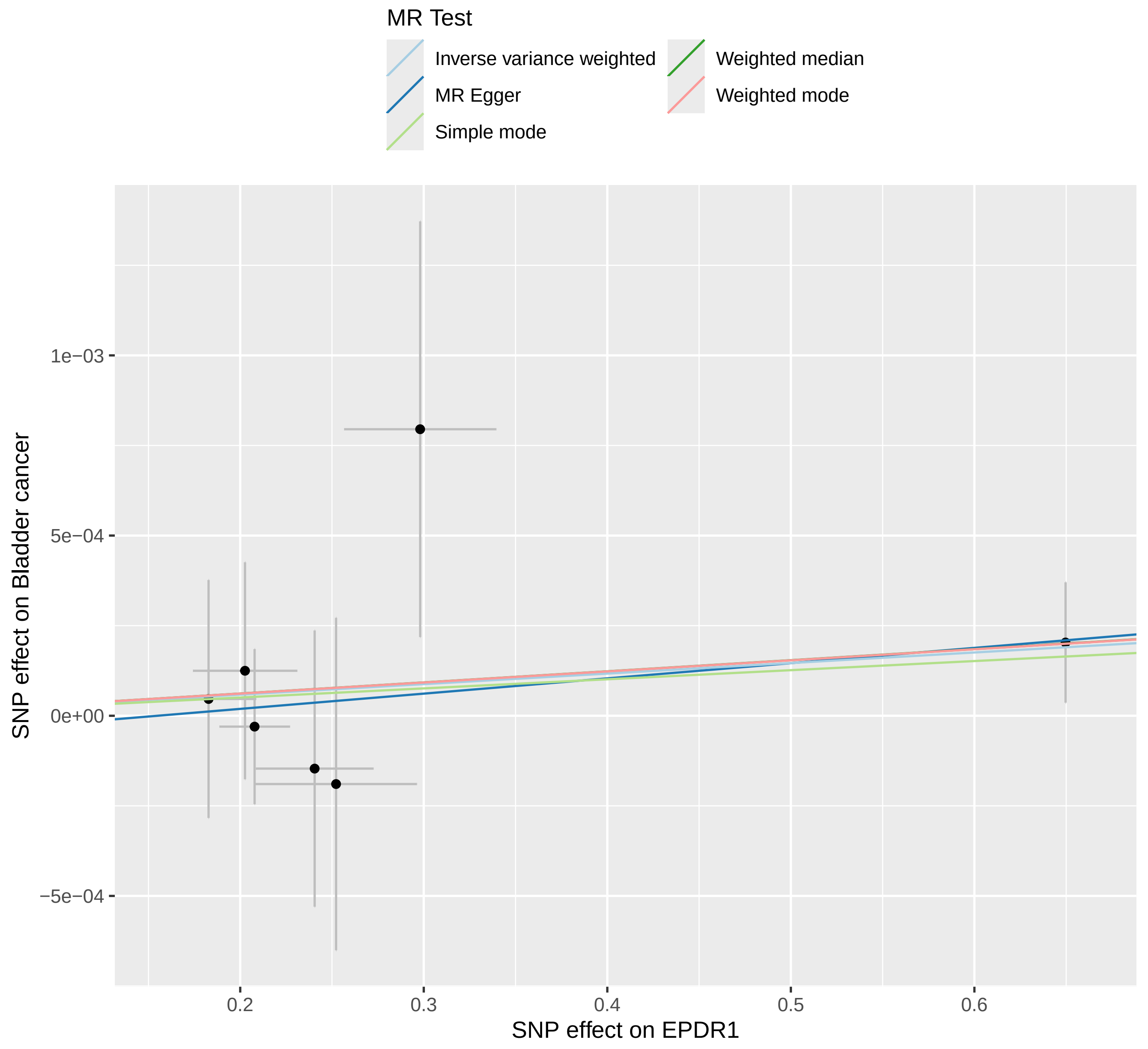

MR Test
Inverse variance weighted
MR Egger
Simple mode
Weighted median
Weighted mode
SNP effect on Bladder cancer
1e−03
5e−04
0e+00
−5e−04
0.2
0.3
0.4
0.5
0.6
SNP effect on EPDR1

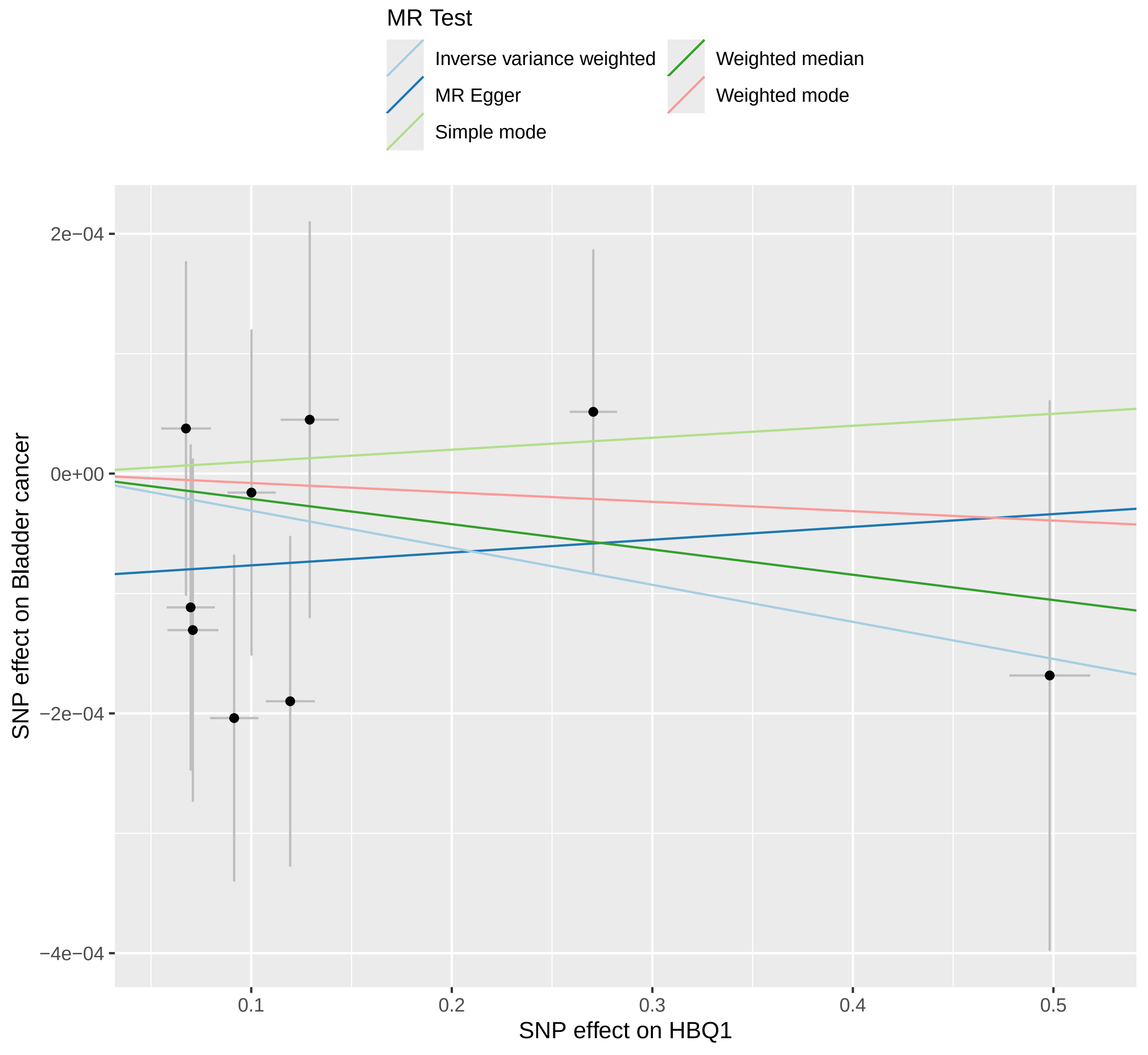

MR Test
Inverse variance weighted
MR Egger
Simple mode
Weighted median
Weighted mode
2e−04
0e+00
−2e−04
−4e−04
SNP effect on Bladder cancer
0.1
0.2
0.3
0.4
0.5
SNP effect on HBQ1

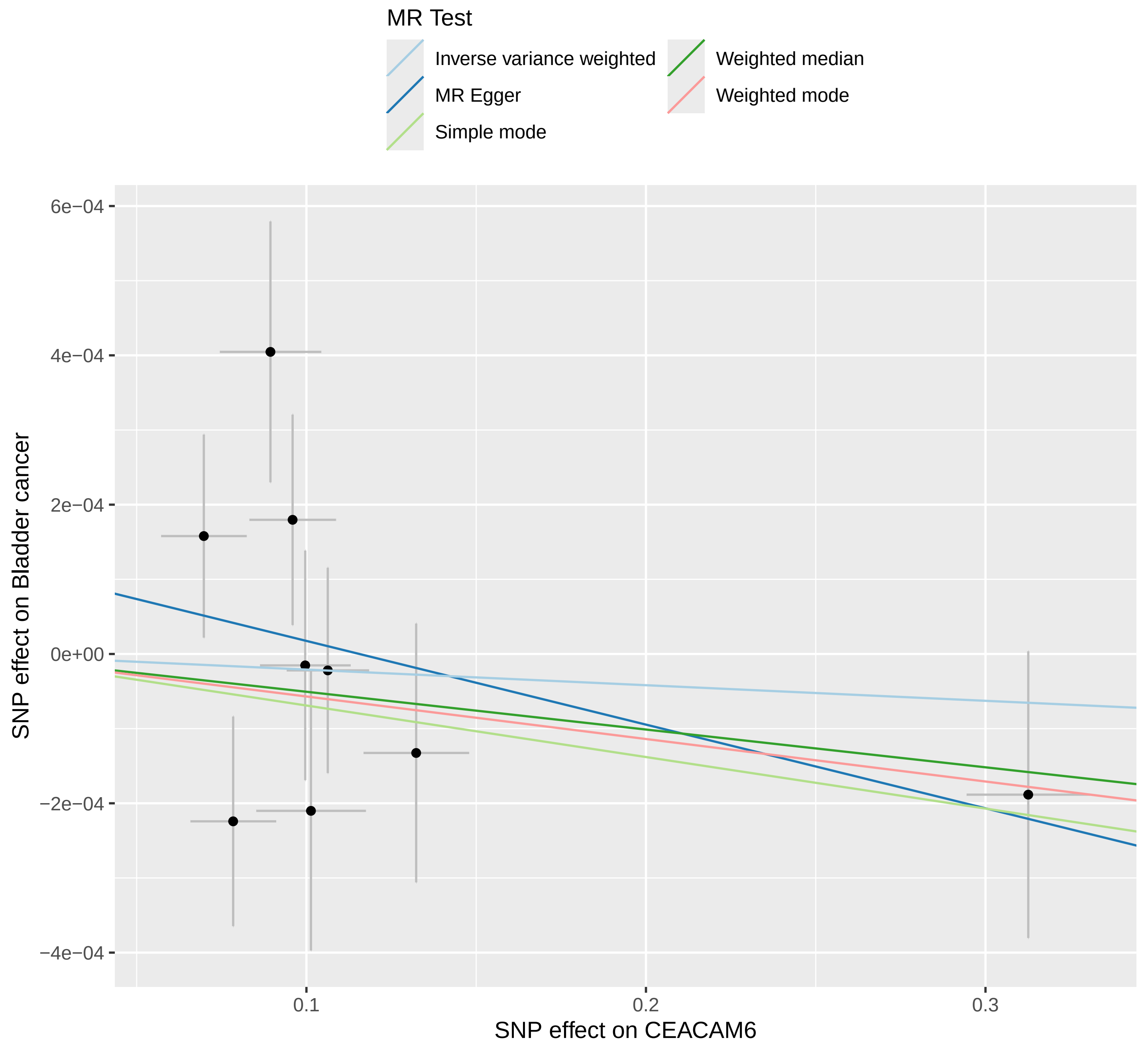
MR Test
Inverse variance weighted
MR Egger
Simple mode
Weighted median
Weighted mode
6e−04
4e−04
2e−04
0e+00
−2e−04
−4e−04
SNP effect on Bladder cancer
0.1
0.2
0.3
SNP effect on CEACAM6

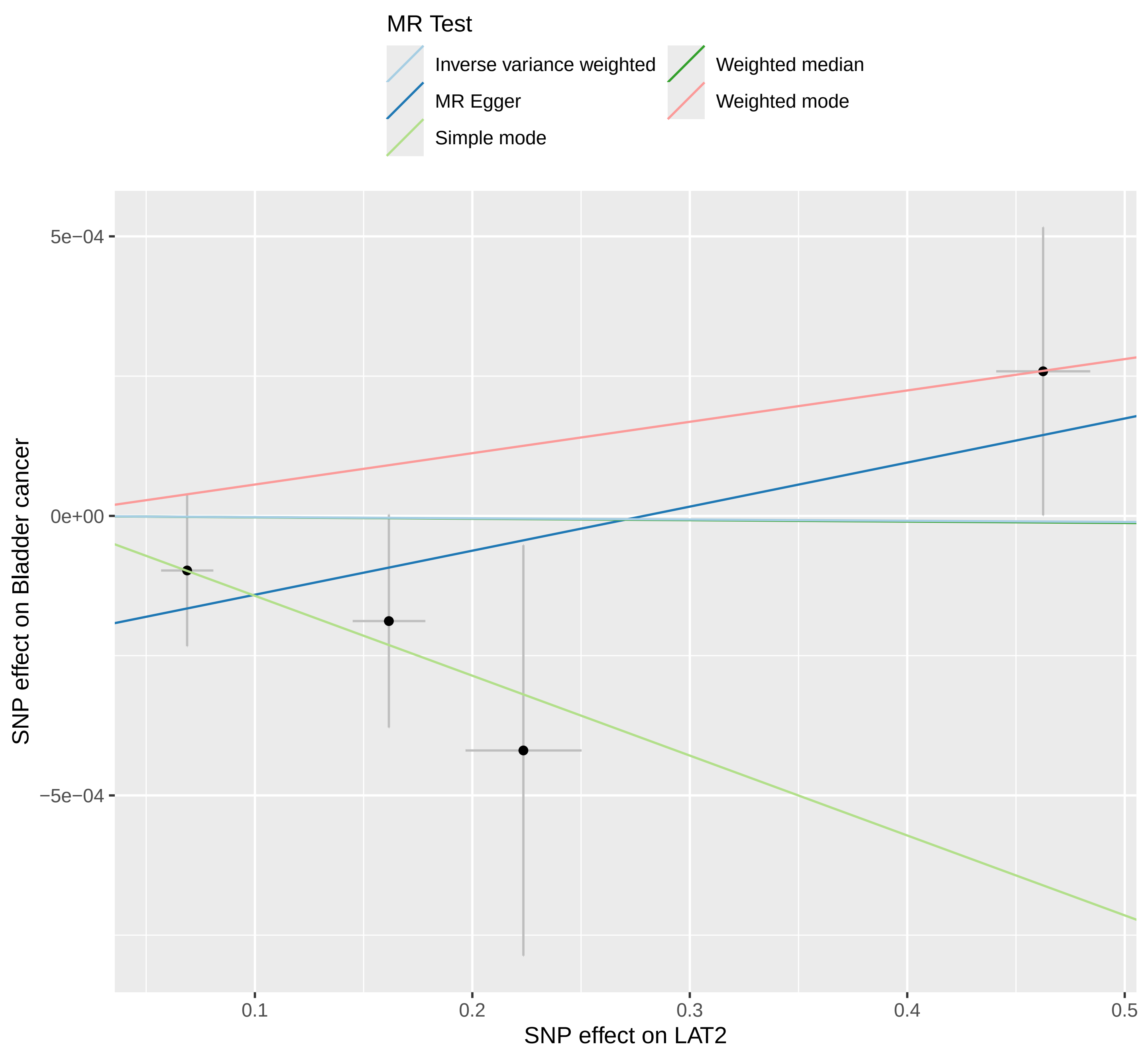
MR Test
Inverse variance weighted
Weighted median
MR Egger
Weighted mode
Simple mode
5e−04
0e+00
−5e−04
SNP effect on Bladder cancer
0.1
0.2
0.3
0.4
0.5
SNP effect on LAT2

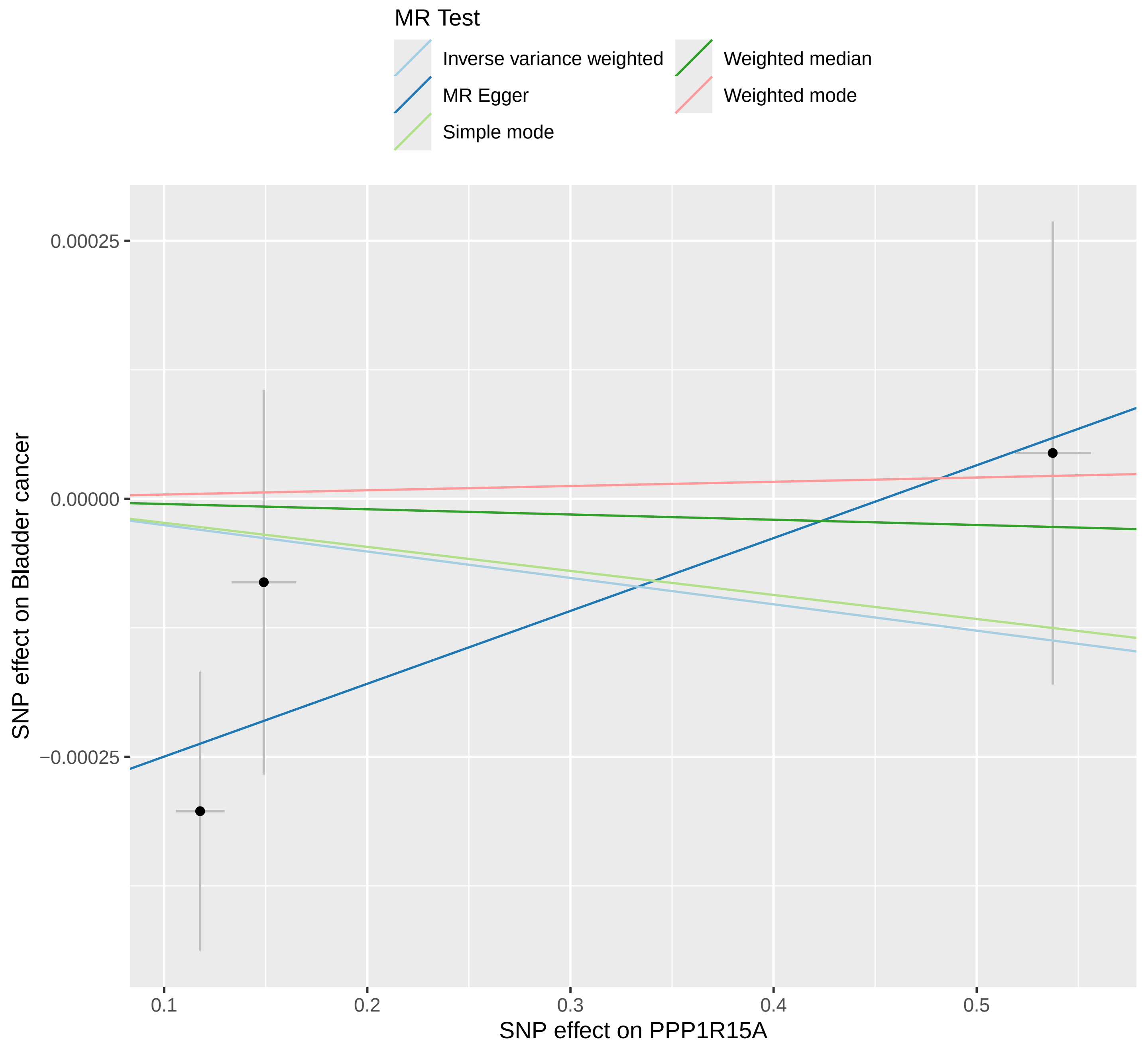

MR Test
Inverse variance weighted
MR Egger
Simple mode
Weighted median
Weighted mode
0.00025
0.00000
−0.00025
0.1
0.2
0.3
0.4
0.5
SNP effect on PPP1R15A
SNP effect on Bladder cancer

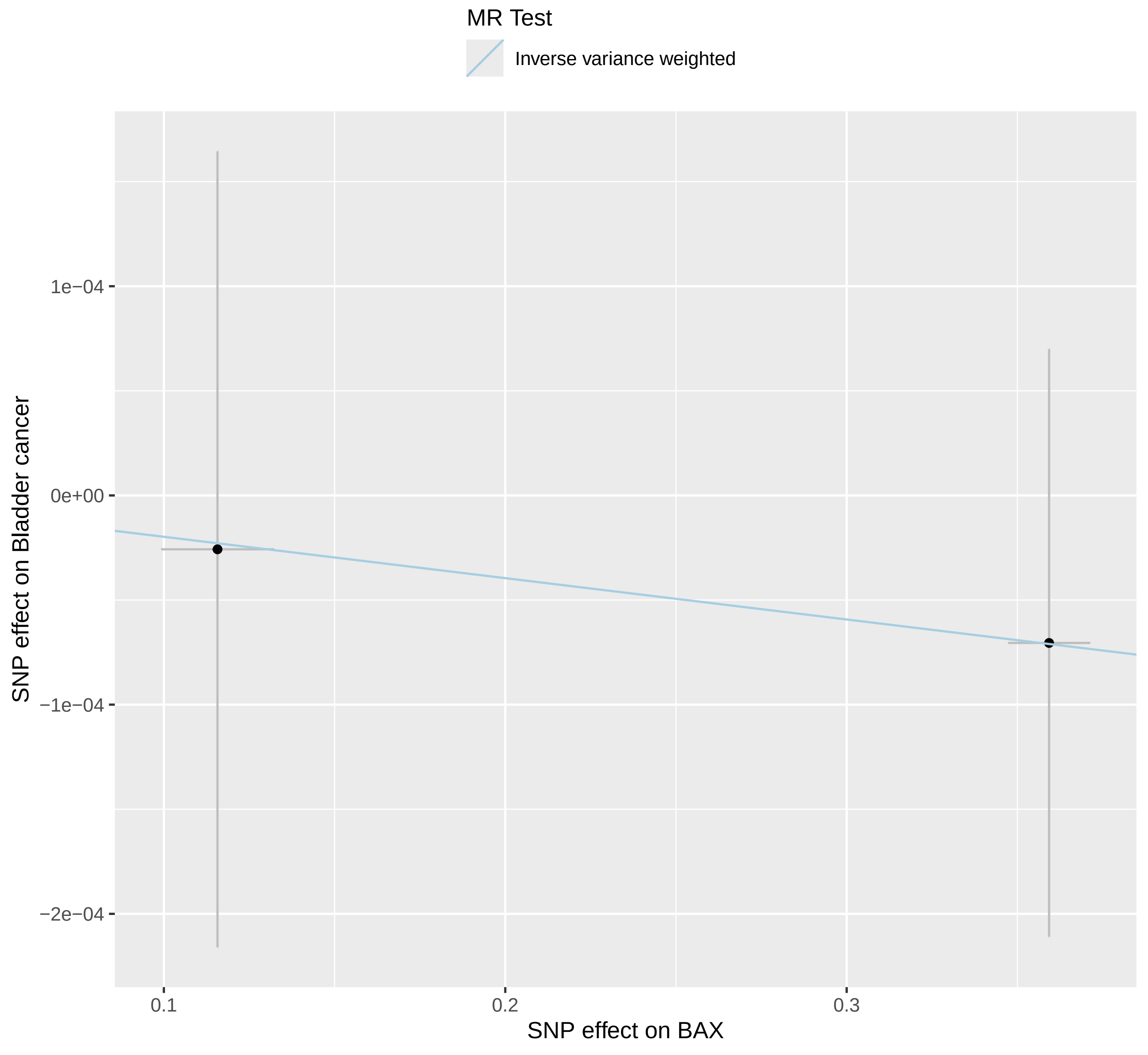

MR Test
Inverse variance weighted
1e−04
0e+00
−1e−04
−2e−04
0.1
0.2
0.3
SNP effect on BAX
SNP effect on Bladder cancer

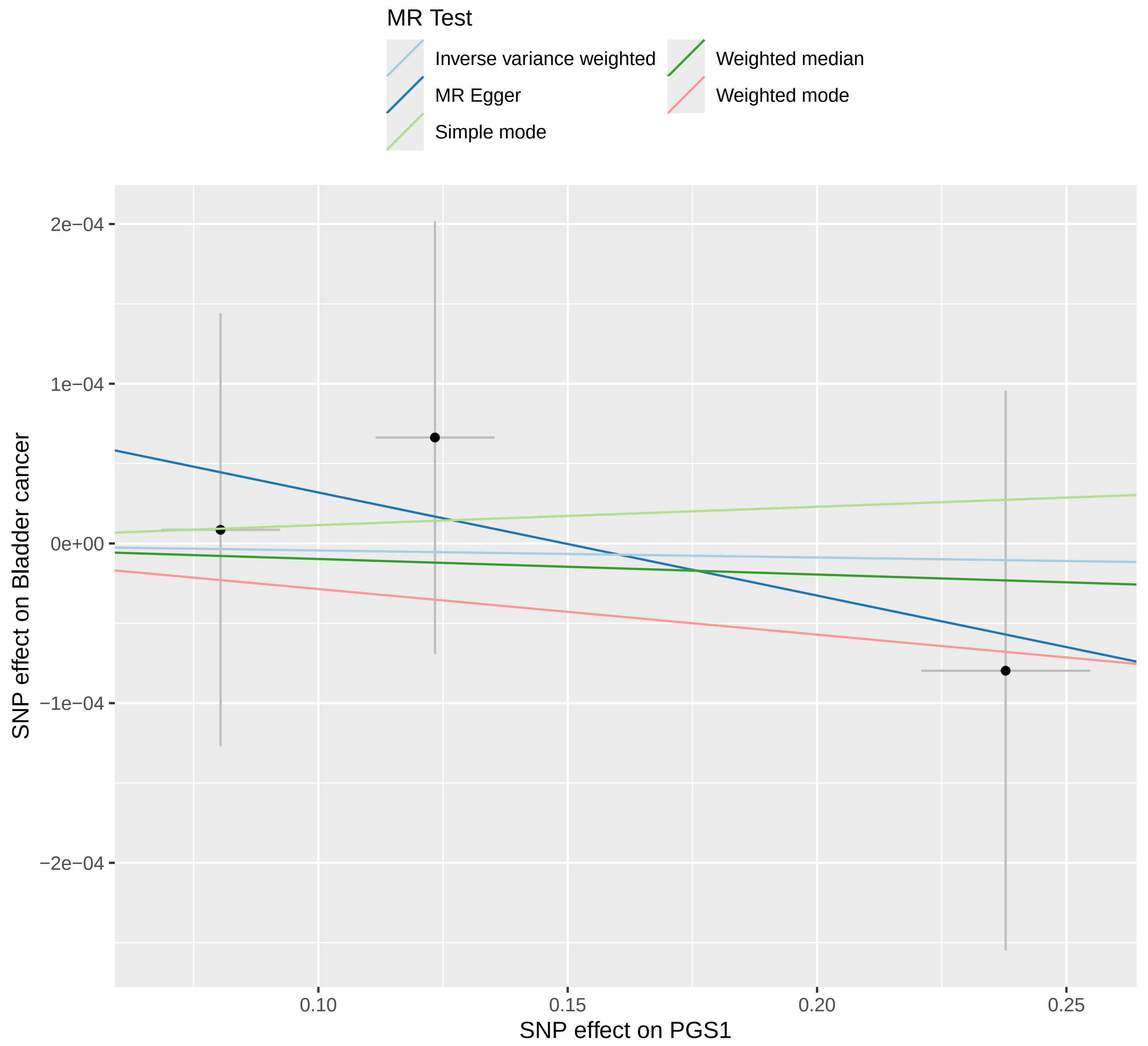

MR Test
Inverse variance weighted
MR Egger
Simple mode
Weighted median
Weighted mode
2e−04
1e−04
0e+00
−1e−04
−2e−04
SNP effect on Bladder cancer
0.10
0.15
0.20
0.25
SNP effect on PGS1

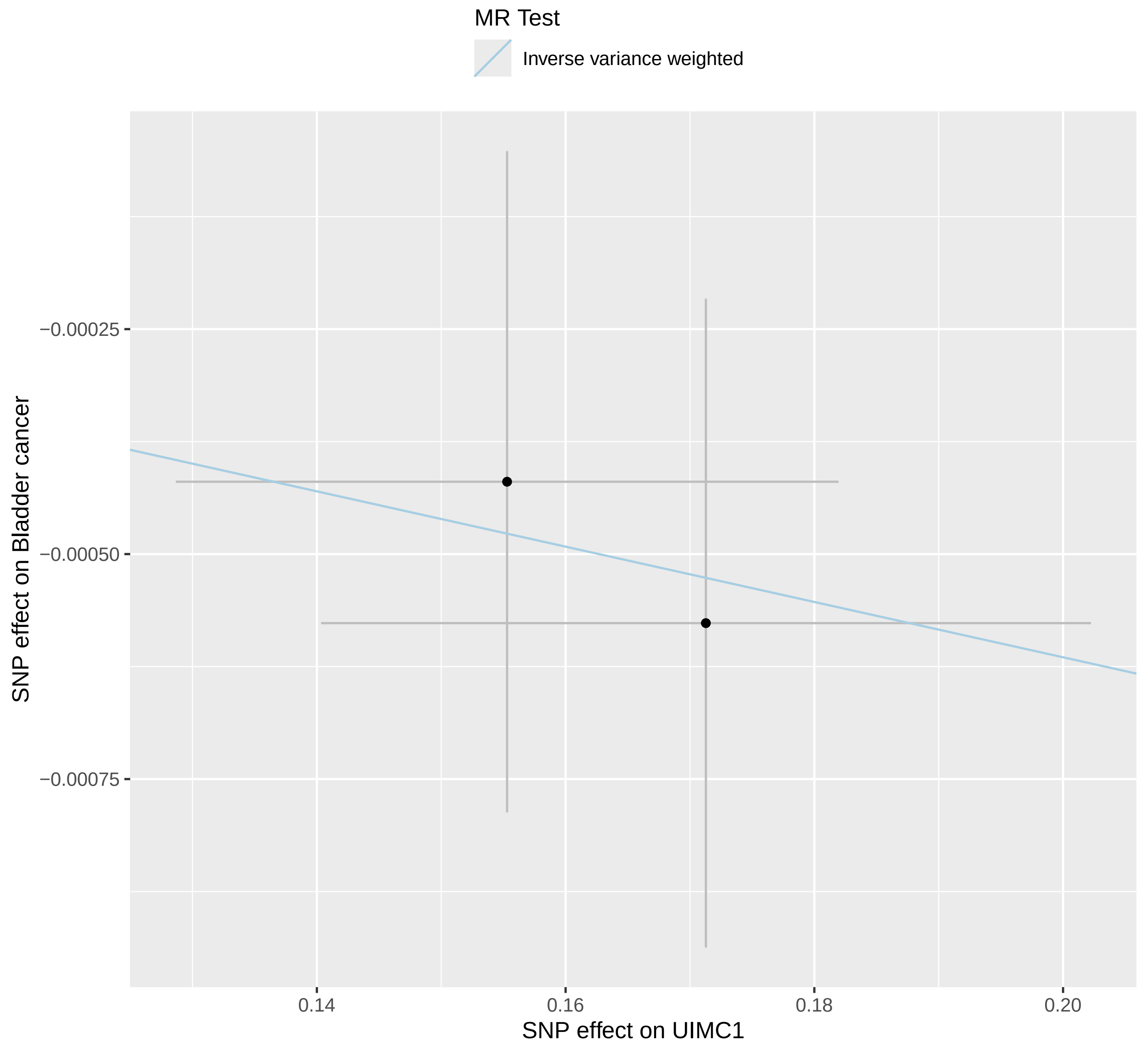
MR Test
Inverse variance weighted
−0.00025
−0.00050
−0.00075
SNP effect on Bladder cancer
0.14
0.16
0.18
0.20
SNP effect on UIMC1

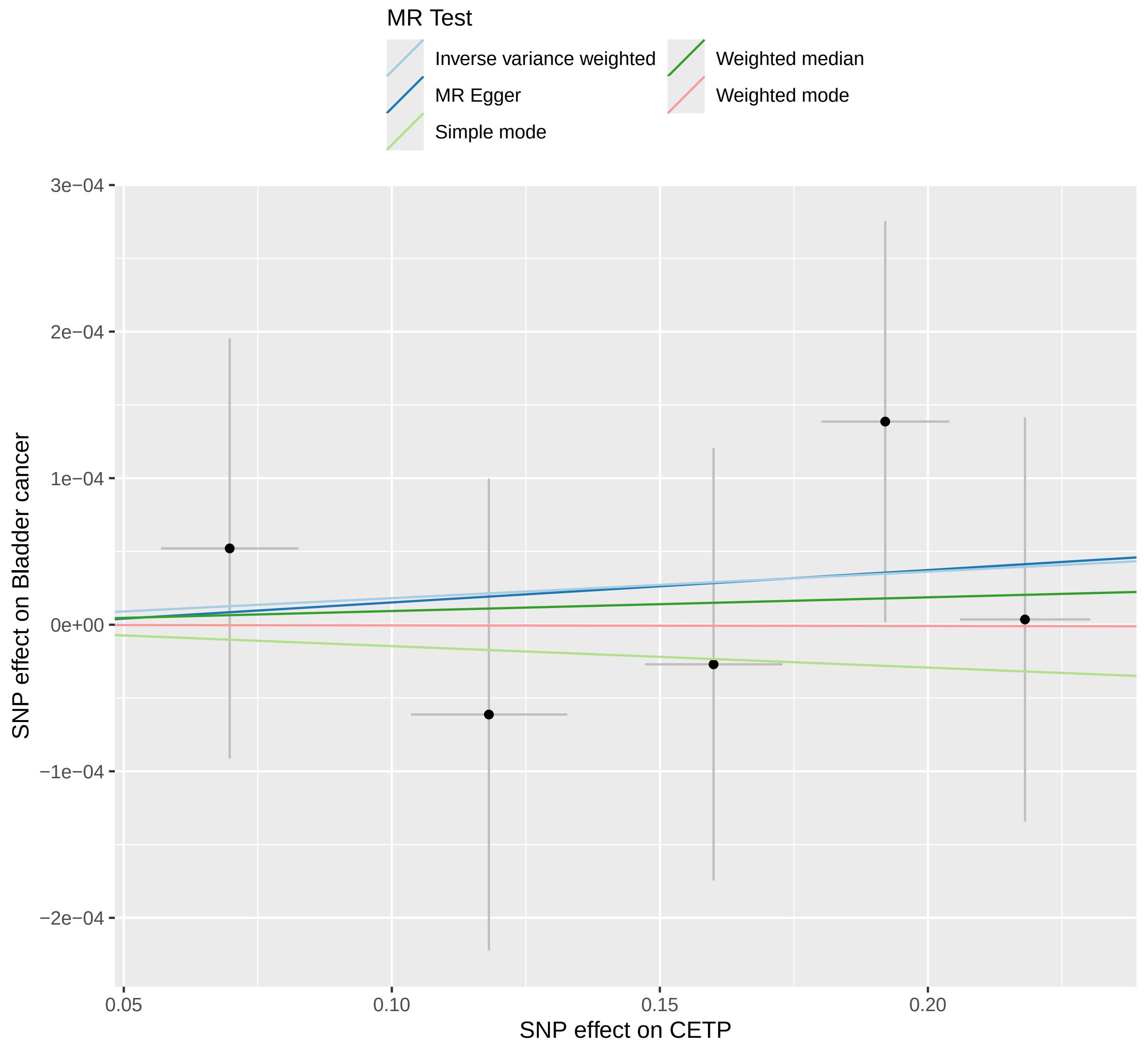
MR Test
Inverse variance weighted
MR Egger
Simple mode
Weighted median
Weighted mode
3e−04
2e−04
1e−04
0e+00
−1e−04
−2e−04
SNP effect on Bladder cancer
0.05
0.10
0.15
0.20
SNP effect on CETP

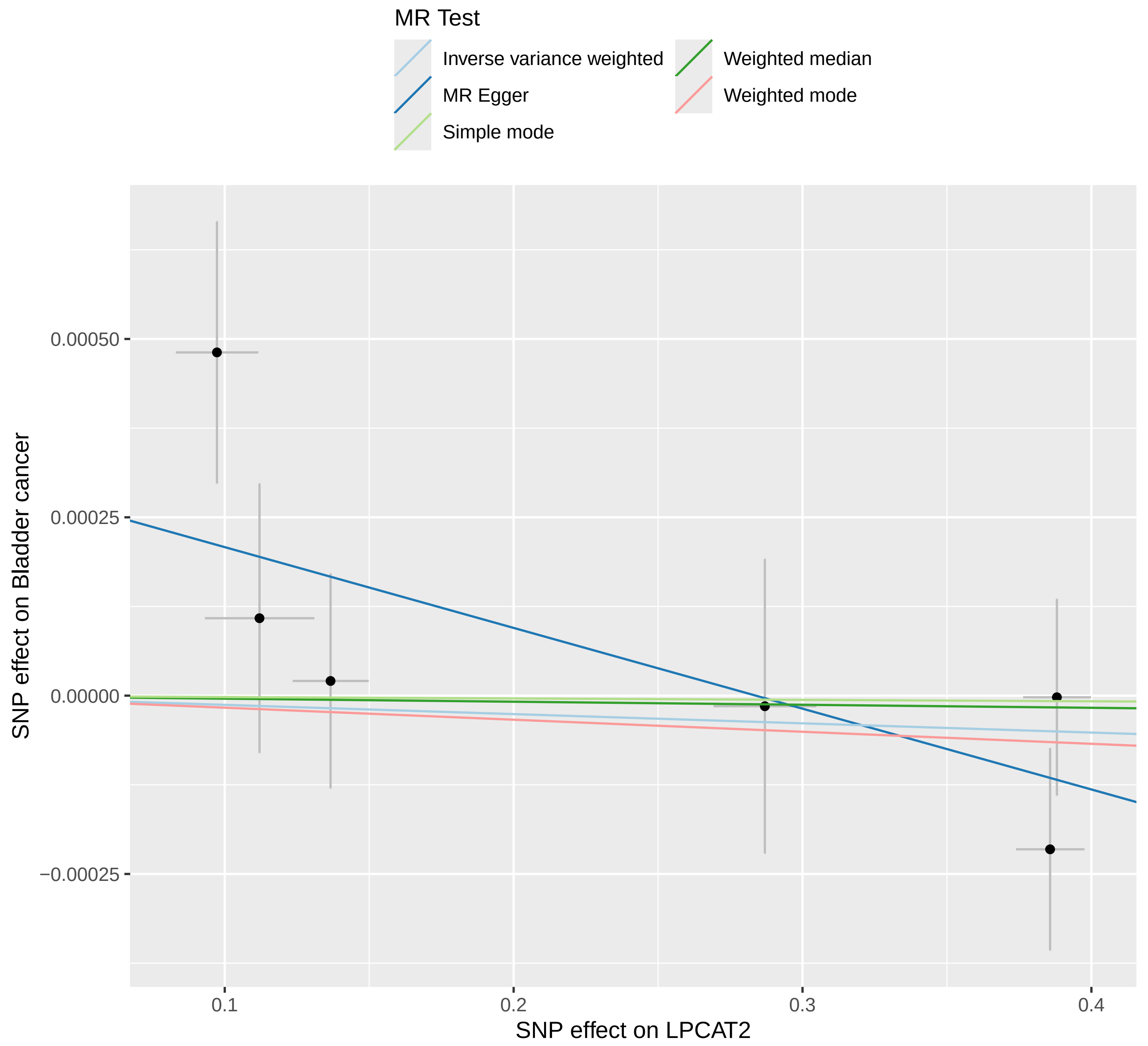

MR Test
Inverse variance weighted
MR Egger
Simple mode
Weighted median
Weighted mode
0.00050
0.00025
0.00000
−0.00025
SNP effect on Bladder cancer
0.1
0.2
0.3
0.4
SNP effect on LPCAT2

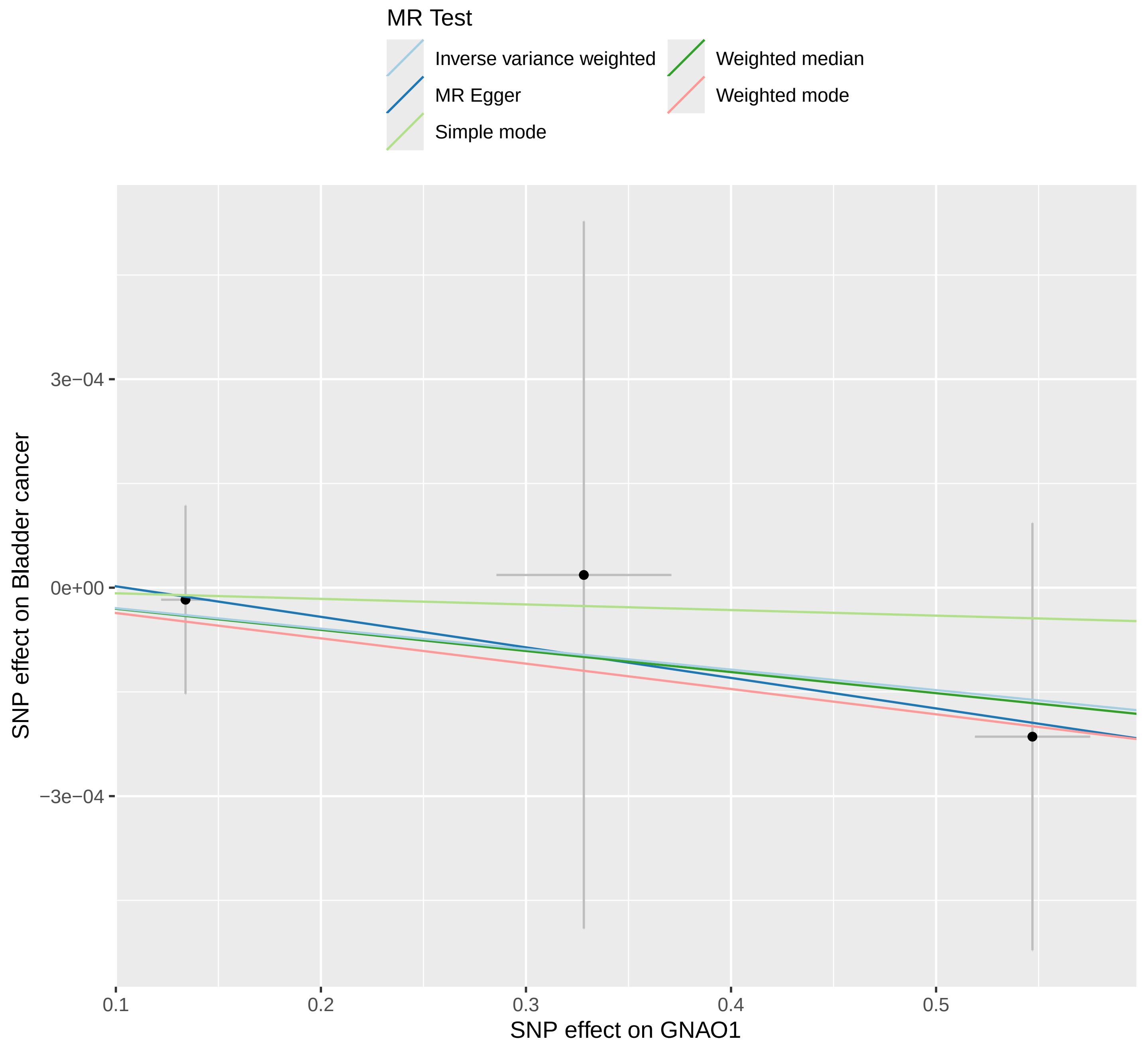

MR Test
Inverse variance weighted
MR Egger
Simple mode
Weighted median
Weighted mode
3e−04
0e+00
−3e−04
SNP effect on Bladder cancer
0.1
0.2
0.3
0.4
0.5
SNP effect on GNAO1

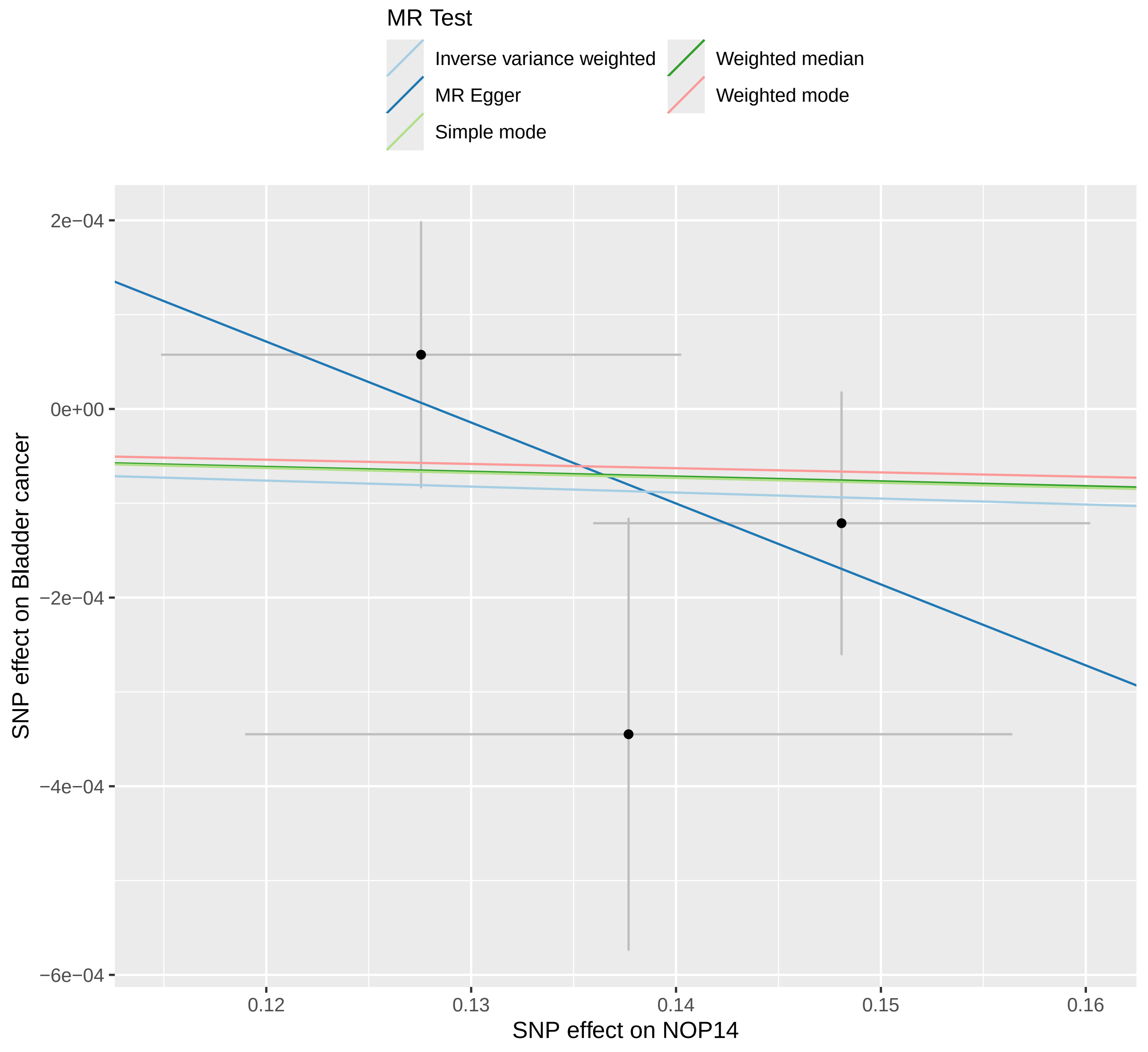
MR Test
Inverse variance weighted
MR Egger
Simple mode
Weighted median
Weighted mode
2e−04
0e+00
−2e−04
−4e−04
−6e−04
0.12
0.13
0.14
0.15
0.16
SNP effect on NOP14
SNP effect on Bladder cancer

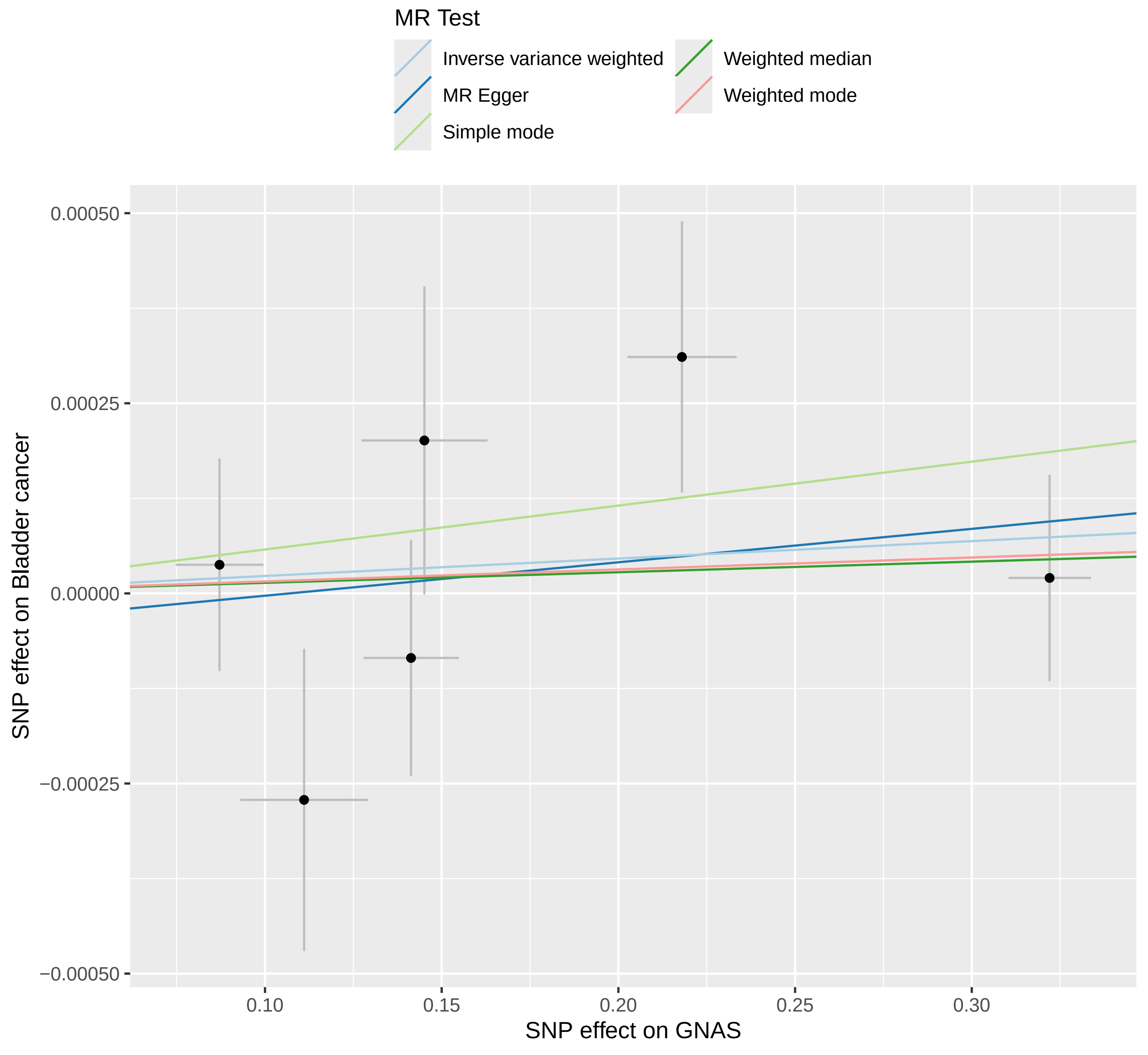

MR Test
Inverse variance weighted
MR Egger
Simple mode
Weighted median
Weighted mode
0.00050
0.00025
0.00000
−0.00025
−0.00050
SNP effect on Bladder cancer
0.10
0.15
0.20
0.25
0.30
SNP effect on GNAS

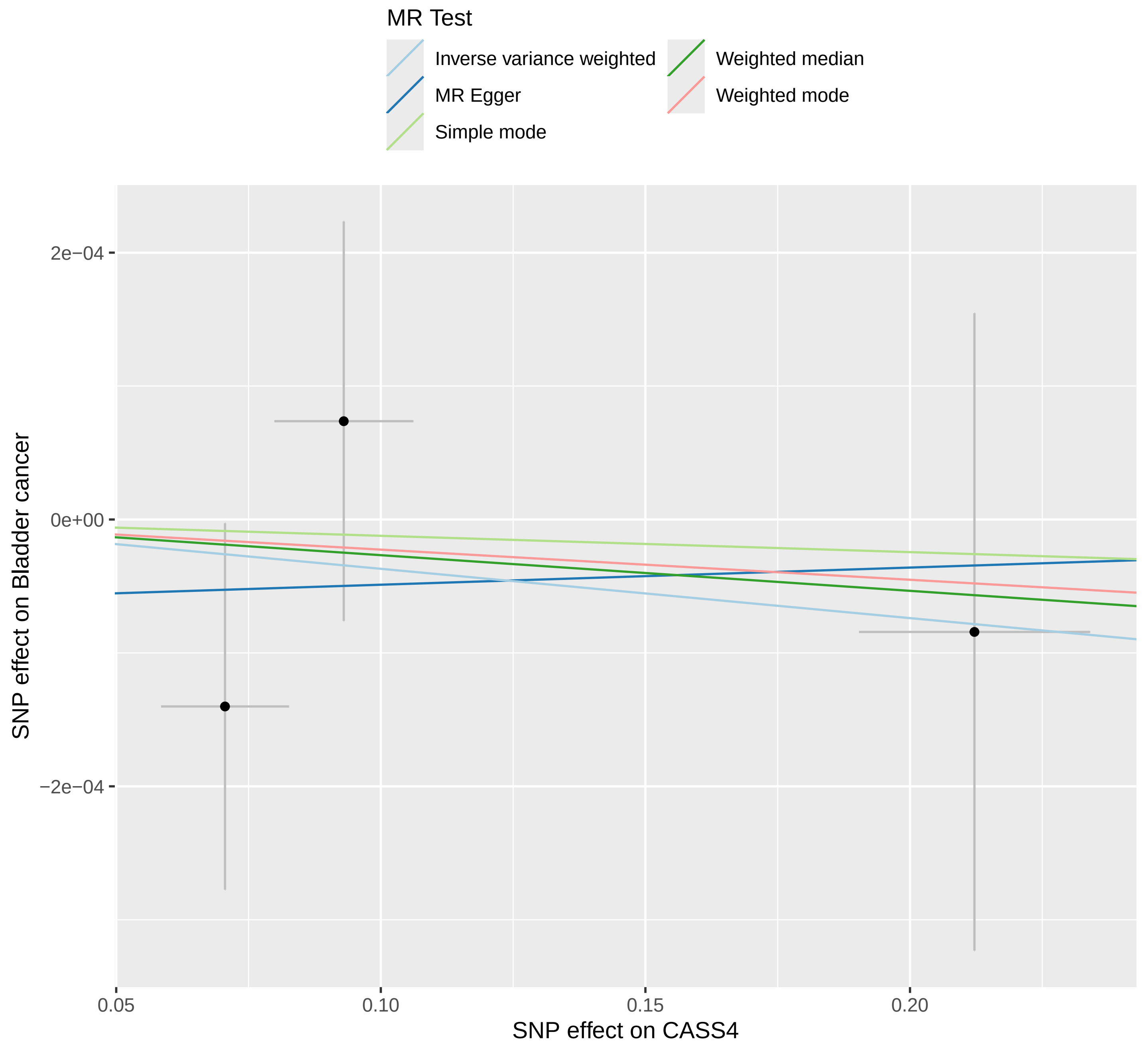
MR Test
Inverse variance weighted
MR Egger
Simple mode
Weighted median
Weighted mode
2e−04
0e+00
−2e−04
SNP effect on Bladder cancer
0.05
0.10
0.15
0.20
SNP effect on CASS4

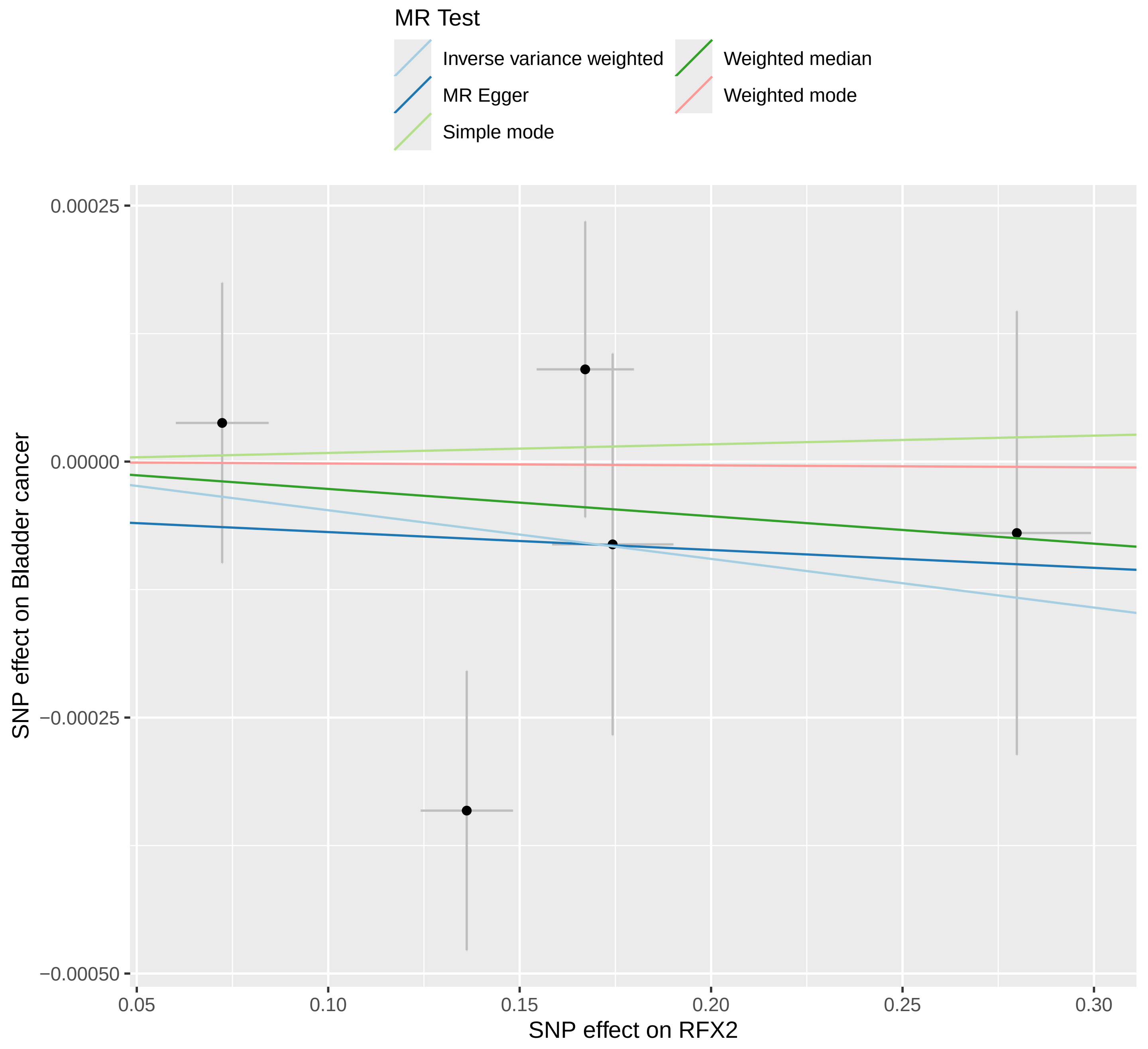

MR Test
Inverse variance weighted
MR Egger
Simple mode
Weighted median
Weighted mode
0.00025
0.00000
−0.00025
−0.00050
SNP effect on Bladder cancer
0.05
0.10
0.15
0.20
0.25
0.30
SNP effect on RFX2

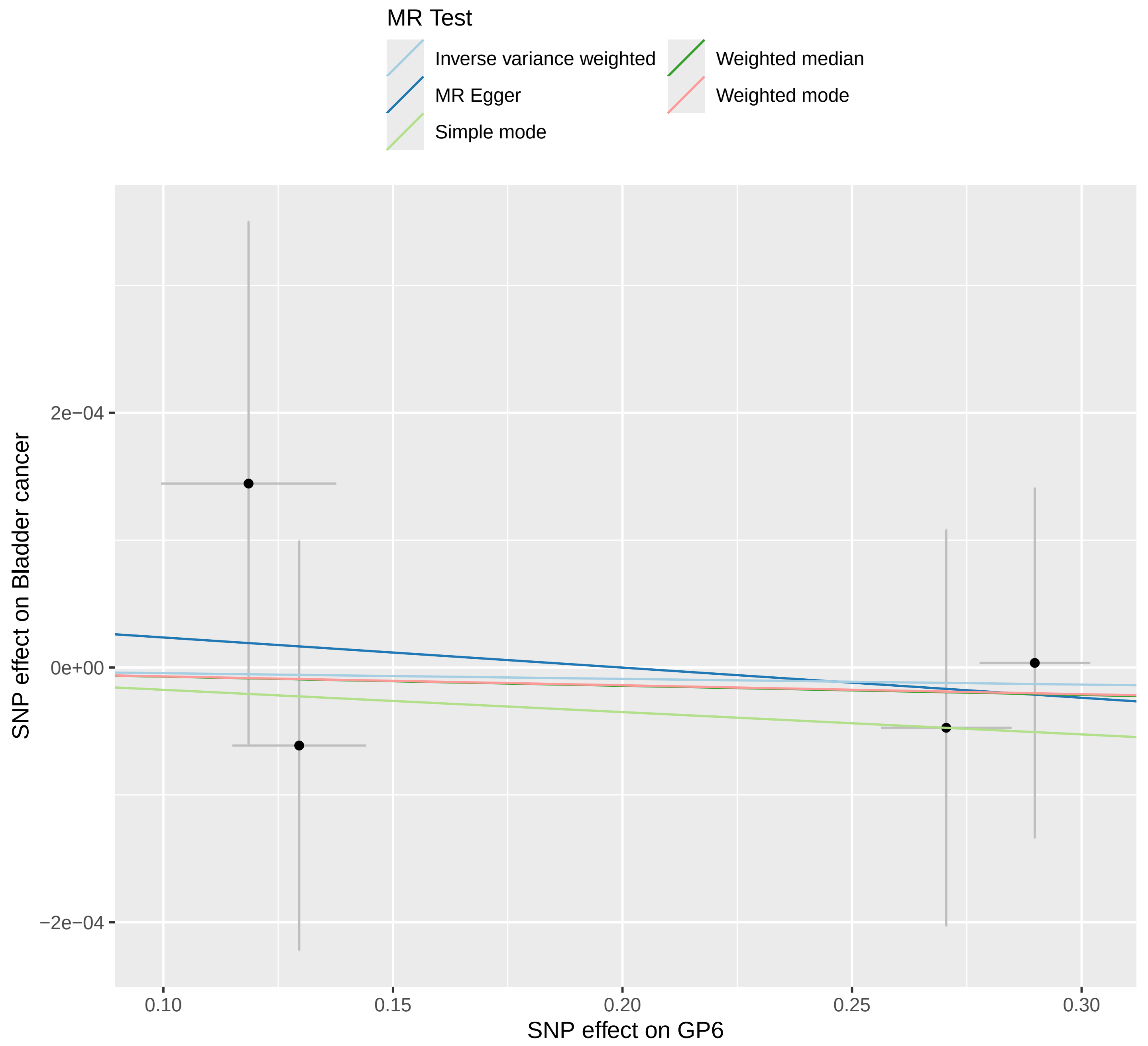

MR Test
Inverse variance weighted
MR Egger
Simple mode
Weighted median
Weighted mode
2e−04
0e+00
−2e−04
SNP effect on Bladder cancer
0.10
0.15
0.20
0.25
0.30
SNP effect on GP6

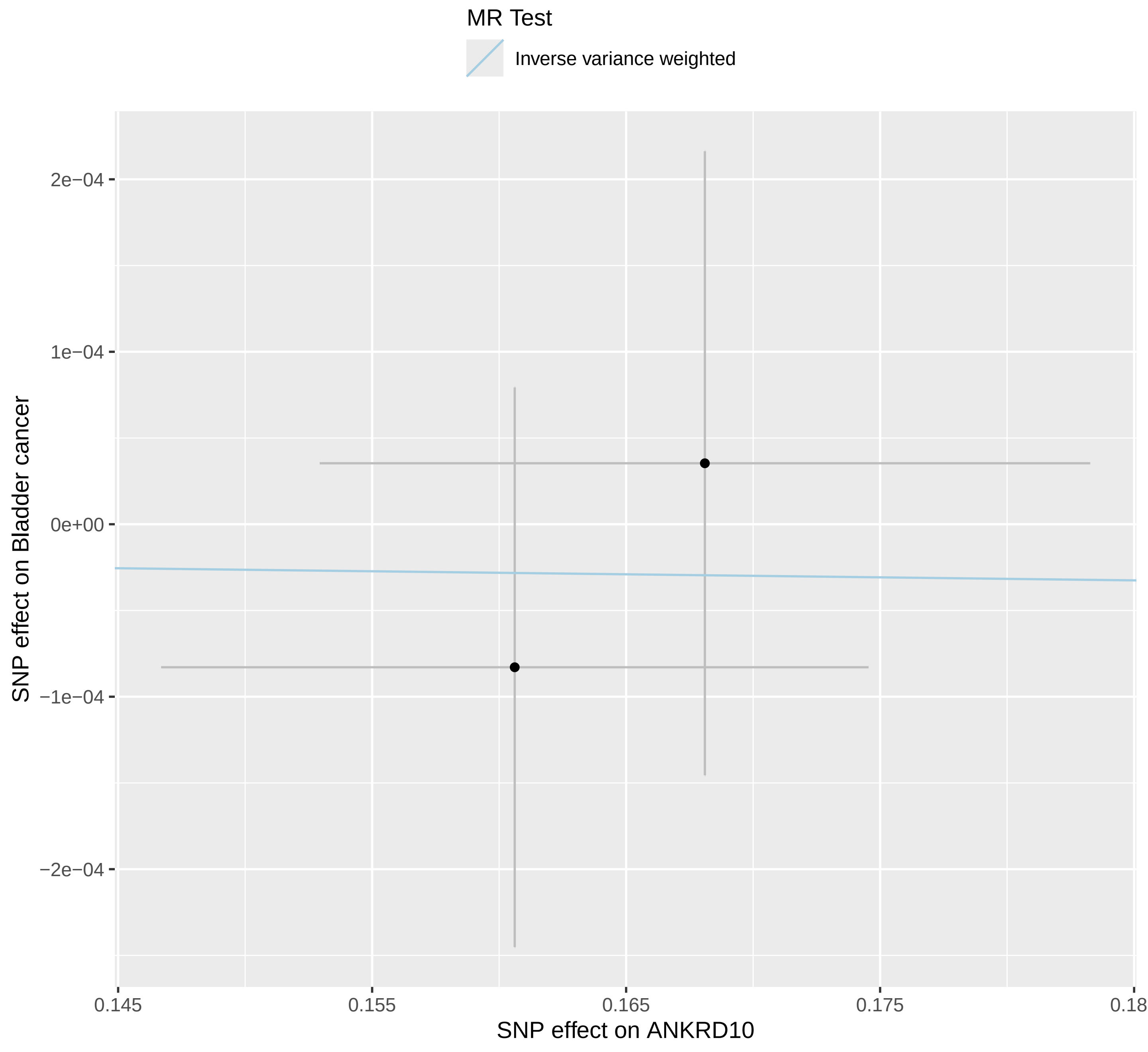

MR Test
Inverse variance weighted
SNP effect on Bladder cancer
2e−04
1e−04
0e+00
−1e−04
−2e−04
0.145
0.155
0.165
0.175
0.18
SNP effect on ANKRD10

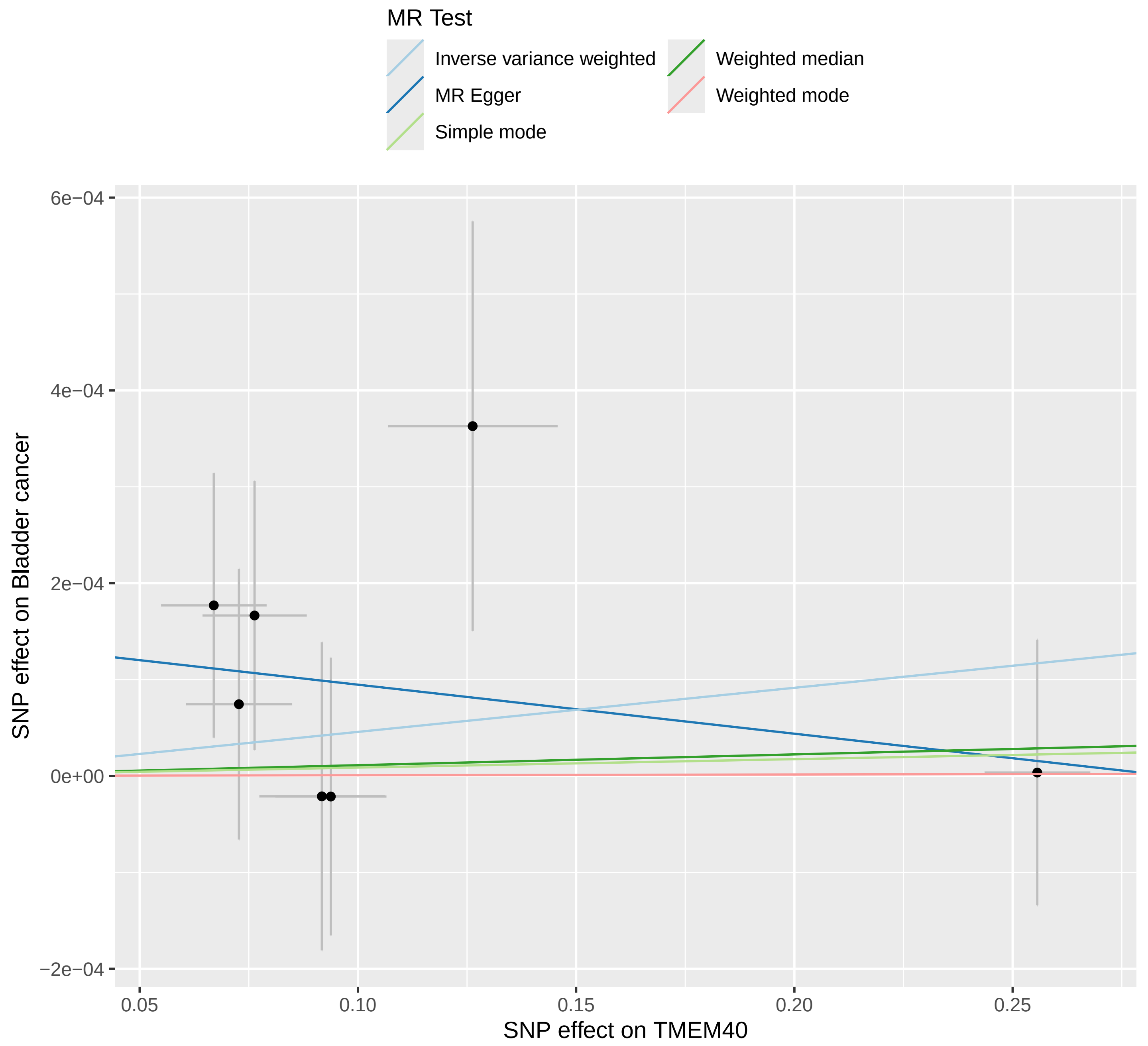

MR Test
Inverse variance weighted
MR Egger
Simple mode
Weighted median
Weighted mode
6e−04
4e−04
2e−04
0e+00
−2e−04
SNP effect on Bladder cancer
0.05
0.10
0.15
0.20
0.25
SNP effect on TMEM40

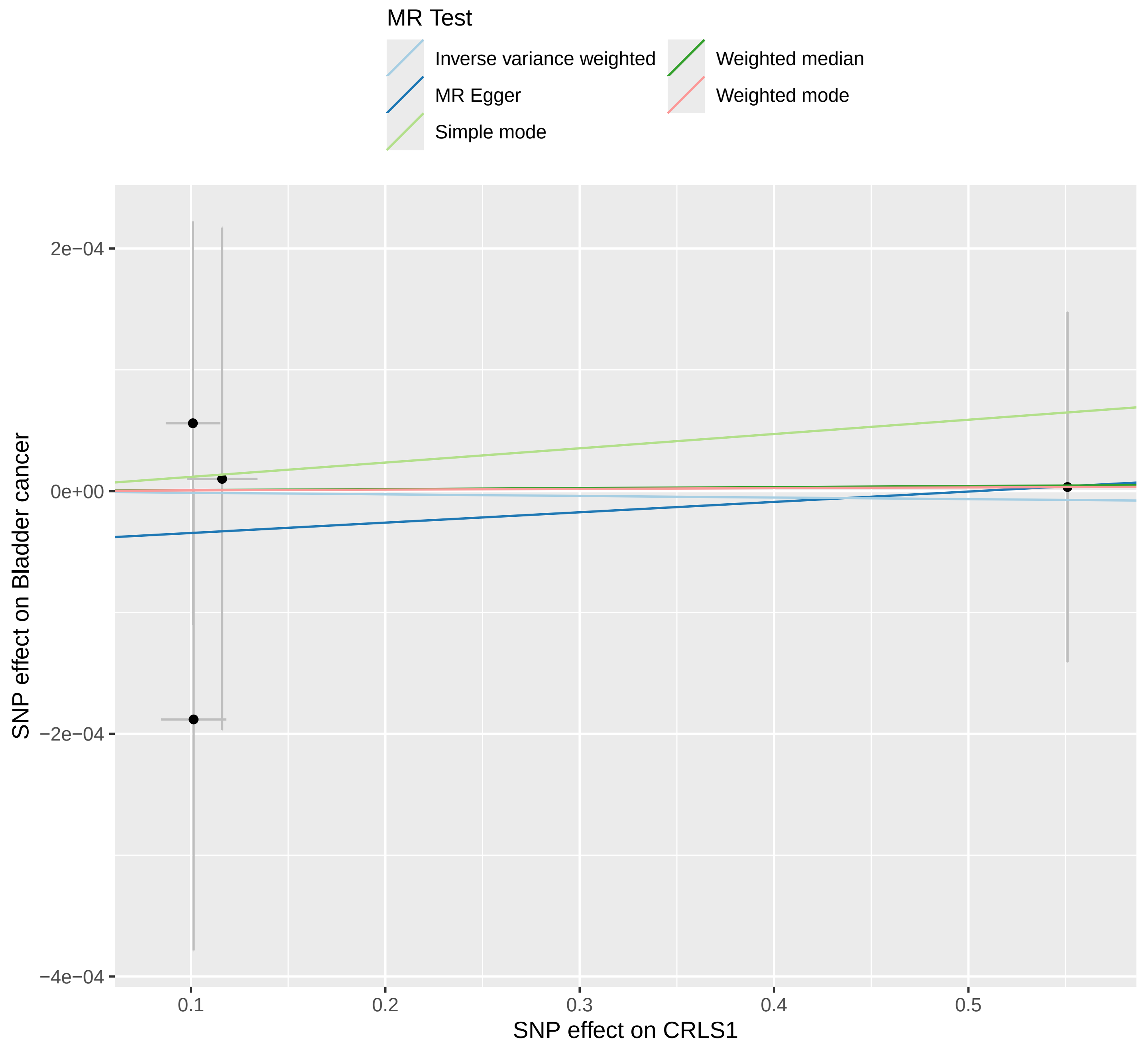
MR Test
Inverse variance weighted
MR Egger
Simple mode
Weighted median
Weighted mode
2e−04
0e+00
−2e−04
−4e−04
SNP effect on Bladder cancer
0.1
0.2
0.3
0.4
0.5
SNP effect on CRLS1

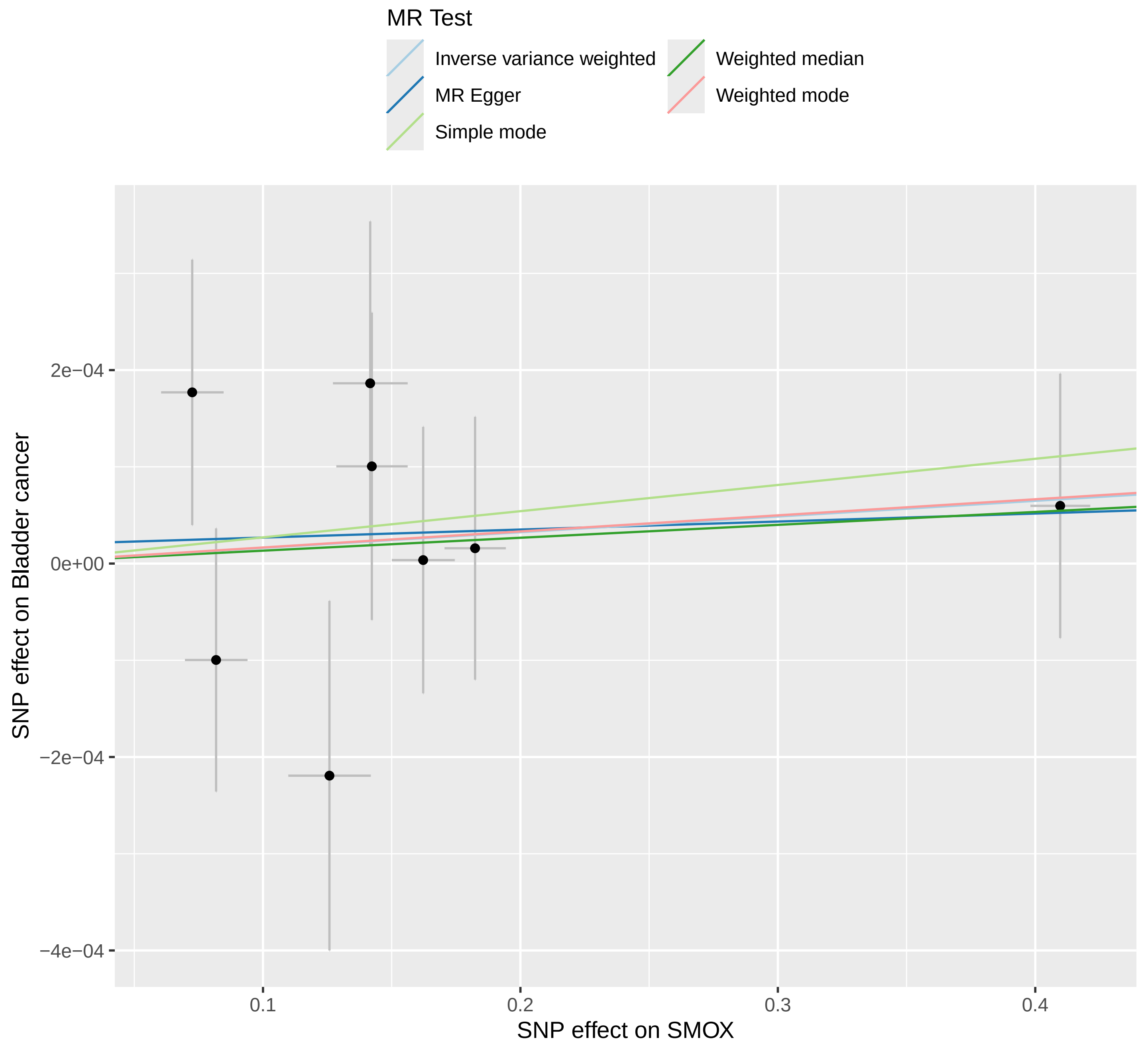

MR Test
Inverse variance weighted
MR Egger
Simple mode
Weighted median
Weighted mode
2e−04
0e+00
−2e−04
−4e−04
0.1
0.2
0.3
0.4
SNP effect on SMOX
SNP effect on Bladder cancer

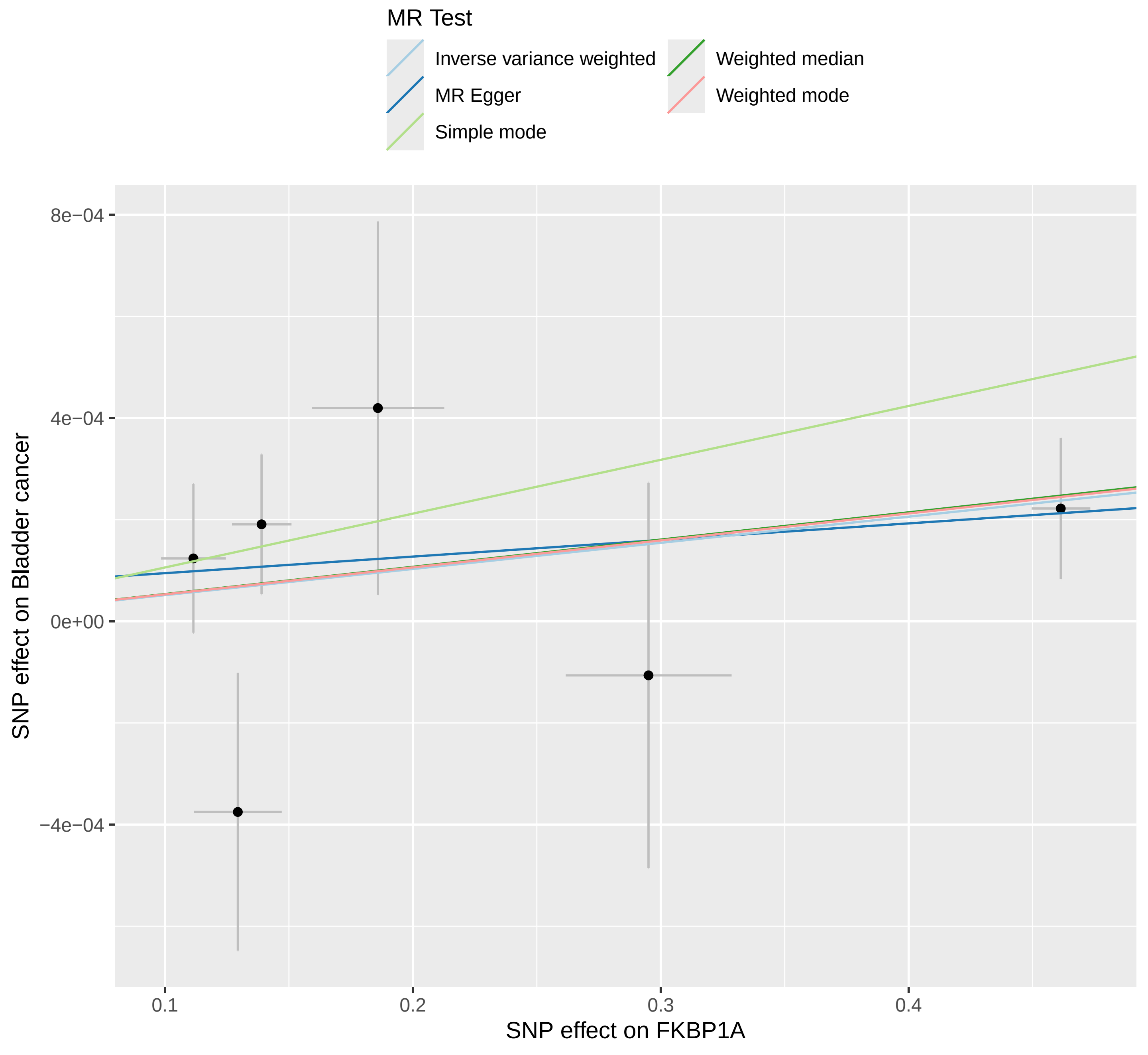
MR Test
Inverse variance weighted
MR Egger
Simple mode
Weighted median
Weighted mode
8e−04
4e−04
0e+00
−4e−04
SNP effect on Bladder cancer
0.1
0.2
0.3
0.4
SNP effect on FKBP1A

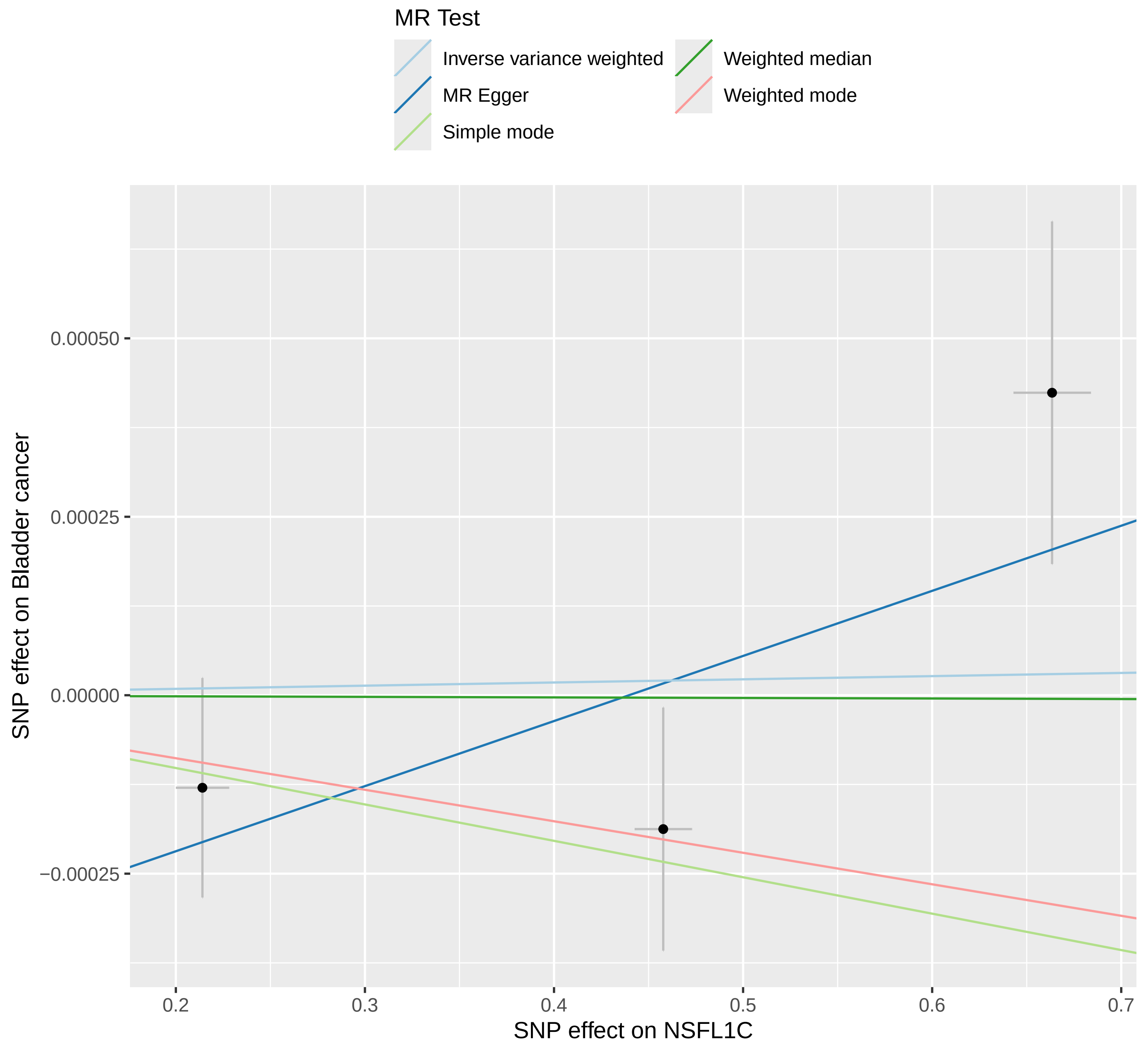
MR Test
Inverse variance weighted
MR Egger
Simple mode
Weighted median
Weighted mode
SNP effect on Bladder cancer
0.00050
0.00025
0.00000
−0.00025
0.2
0.3
0.4
0.5
0.6
0.7
SNP effect on NSFL1C

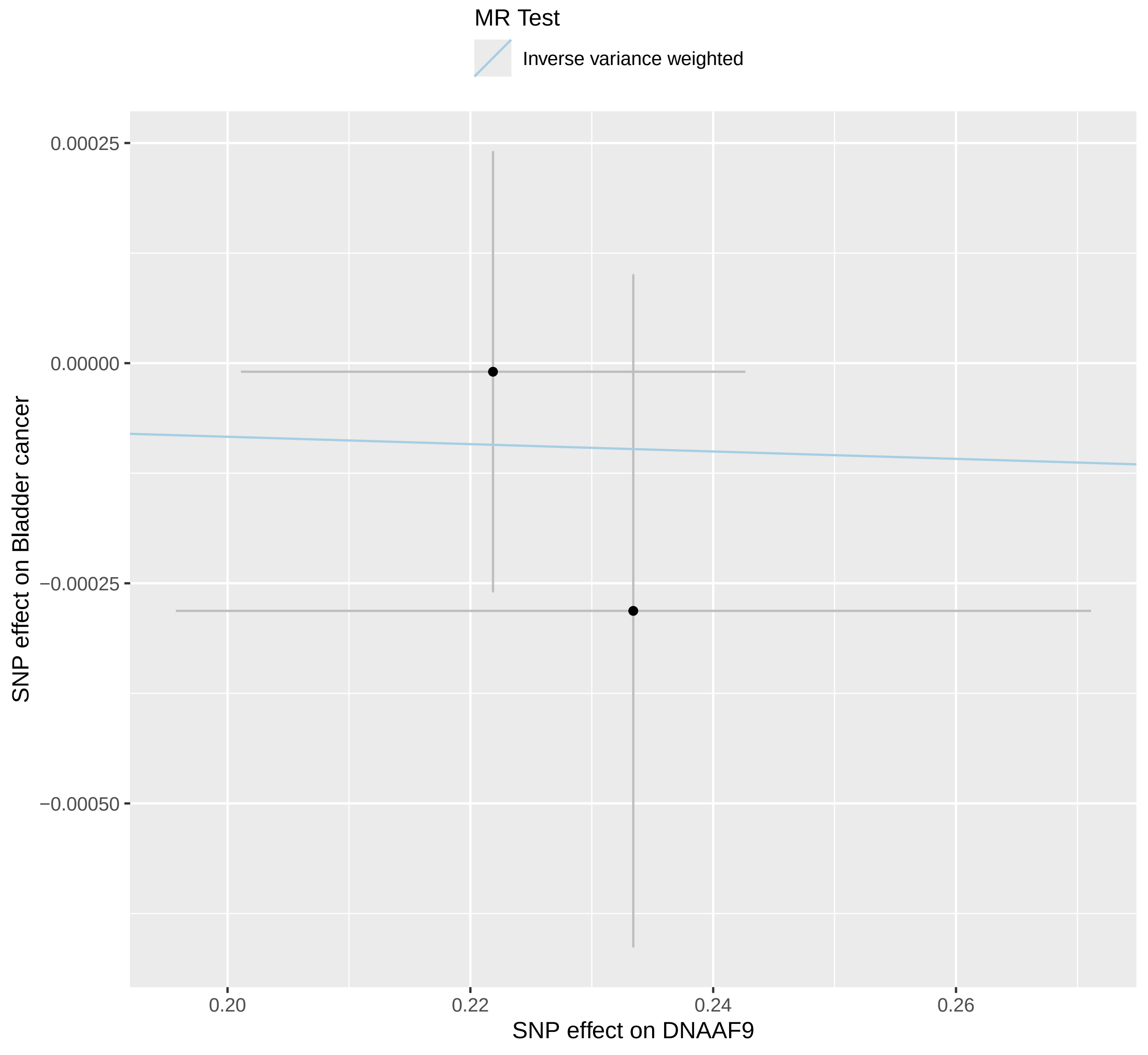

MR Test
Inverse variance weighted
0.00025
0.00000
−0.00025
−0.00050
SNP effect on Bladder cancer
0.20
0.22
0.24
0.26
SNP effect on DNAAF9

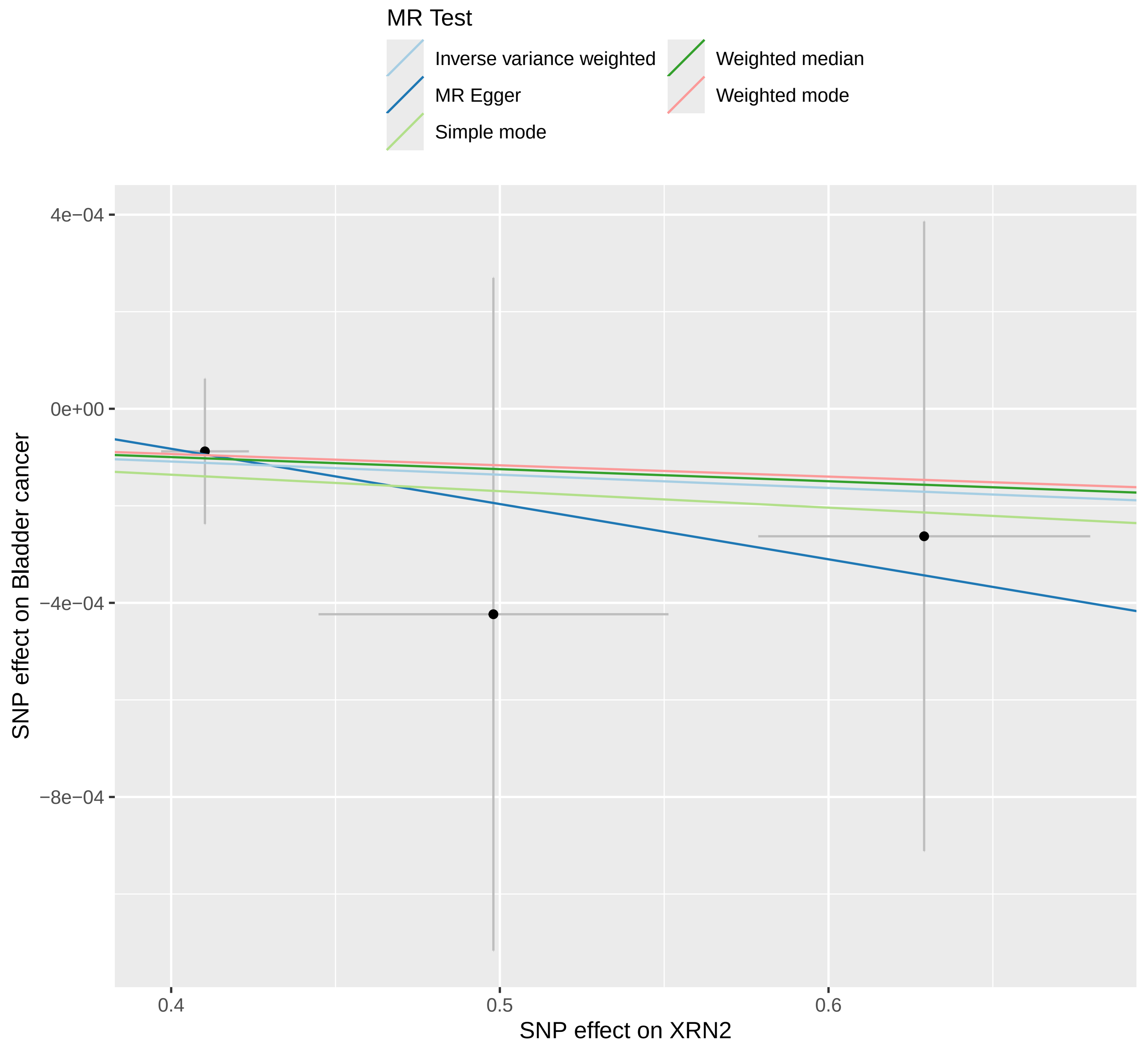
MR Test
Inverse variance weighted
MR Egger
Simple mode
Weighted median
Weighted mode
4e−04
0e+00
−4e−04
−8e−04
SNP effect on Bladder cancer
0.4
0.5
0.6
SNP effect on XRN2

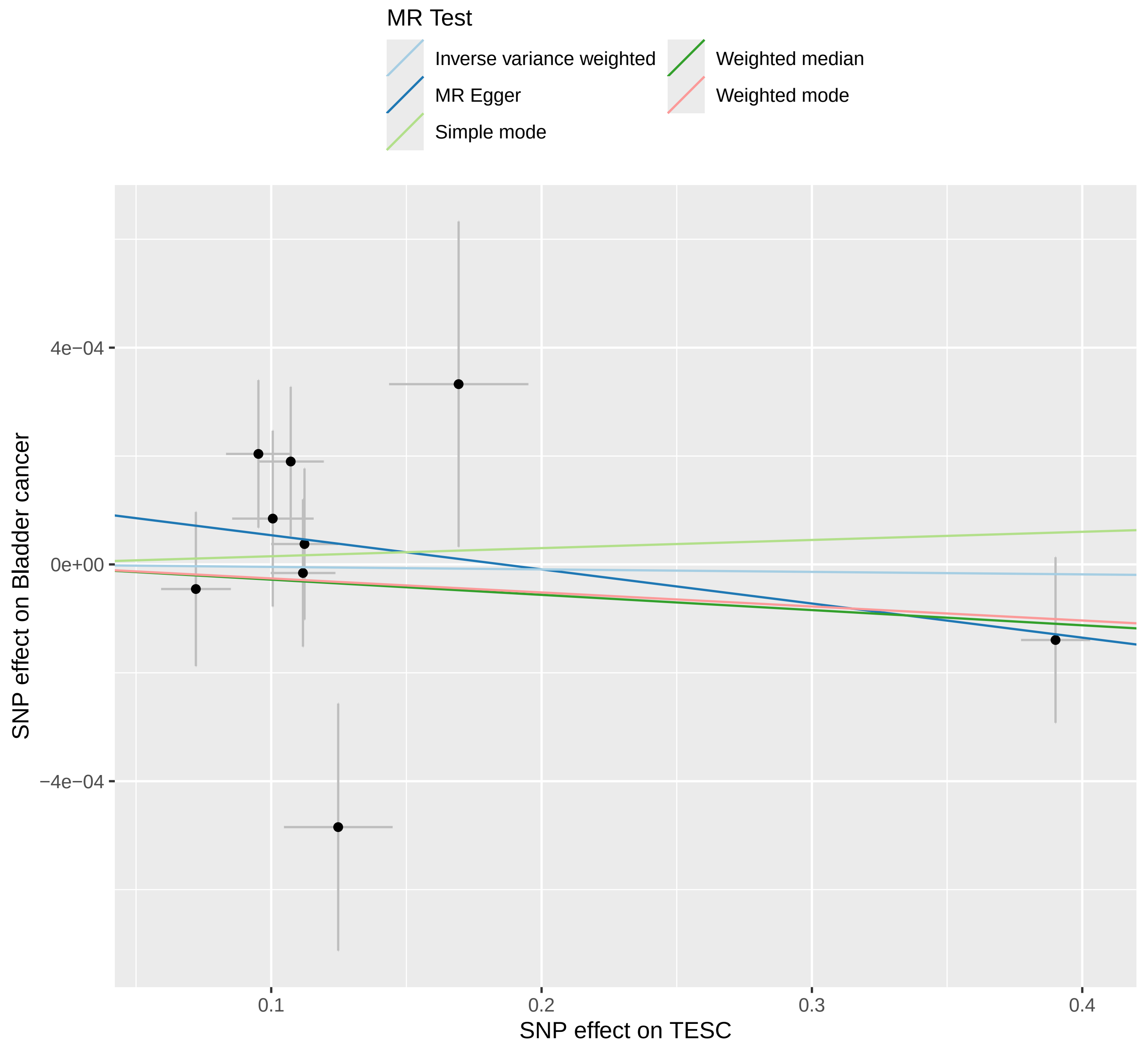
MR Test
Inverse variance weighted
MR Egger
Simple mode
Weighted median
Weighted mode
4e−04
0e+00
−4e−04
SNP effect on Bladder cancer
0.1
0.2
0.3
0.4
SNP effect on TESC

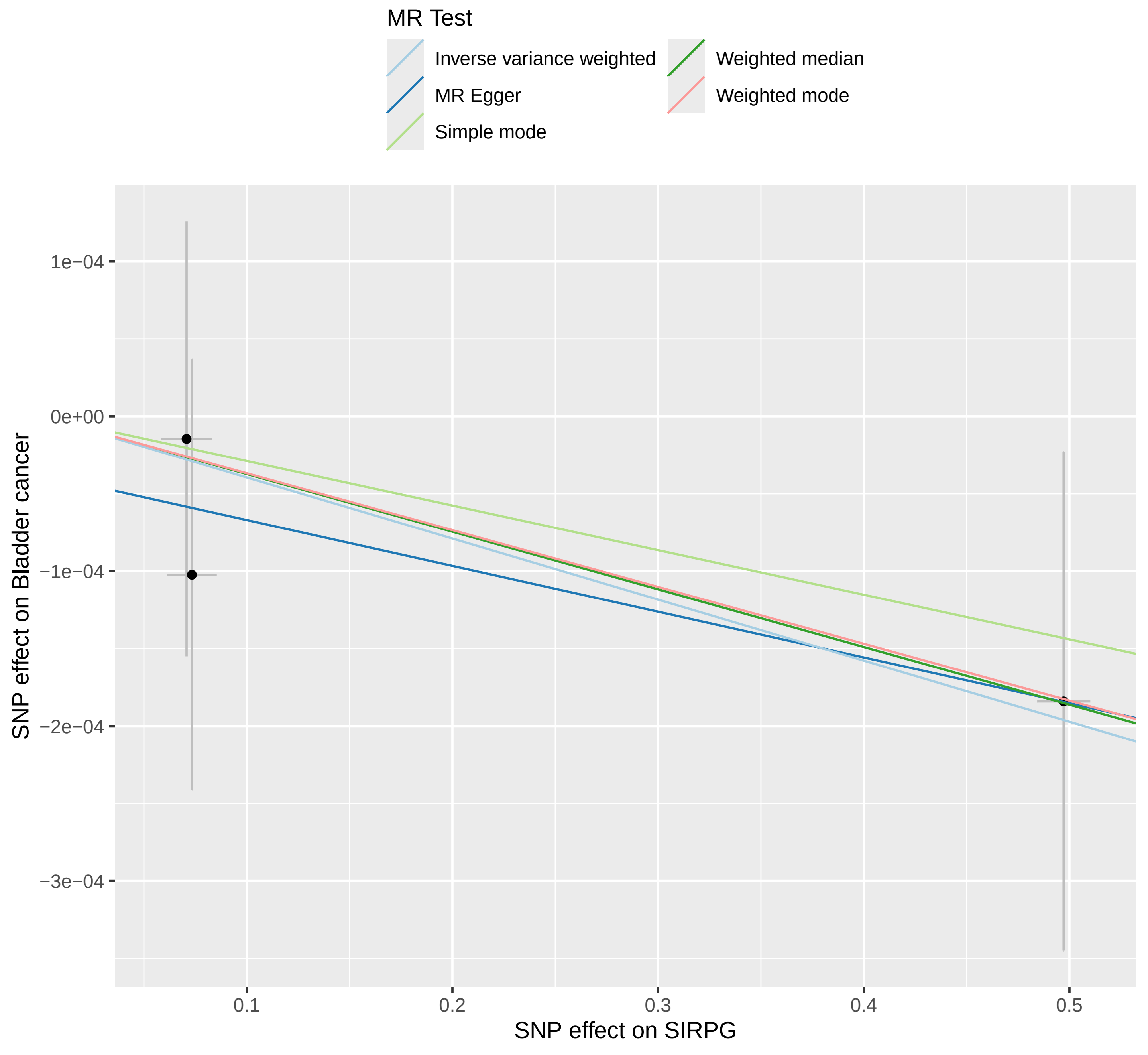
MR Test
Inverse variance weighted
MR Egger
Simple mode
Weighted median
Weighted mode
1e−04
0e+00
−1e−04
−2e−04
−3e−04
SNP effect on Bladder cancer
0.1
0.2
0.3
0.4
0.5
SNP effect on SIRPG

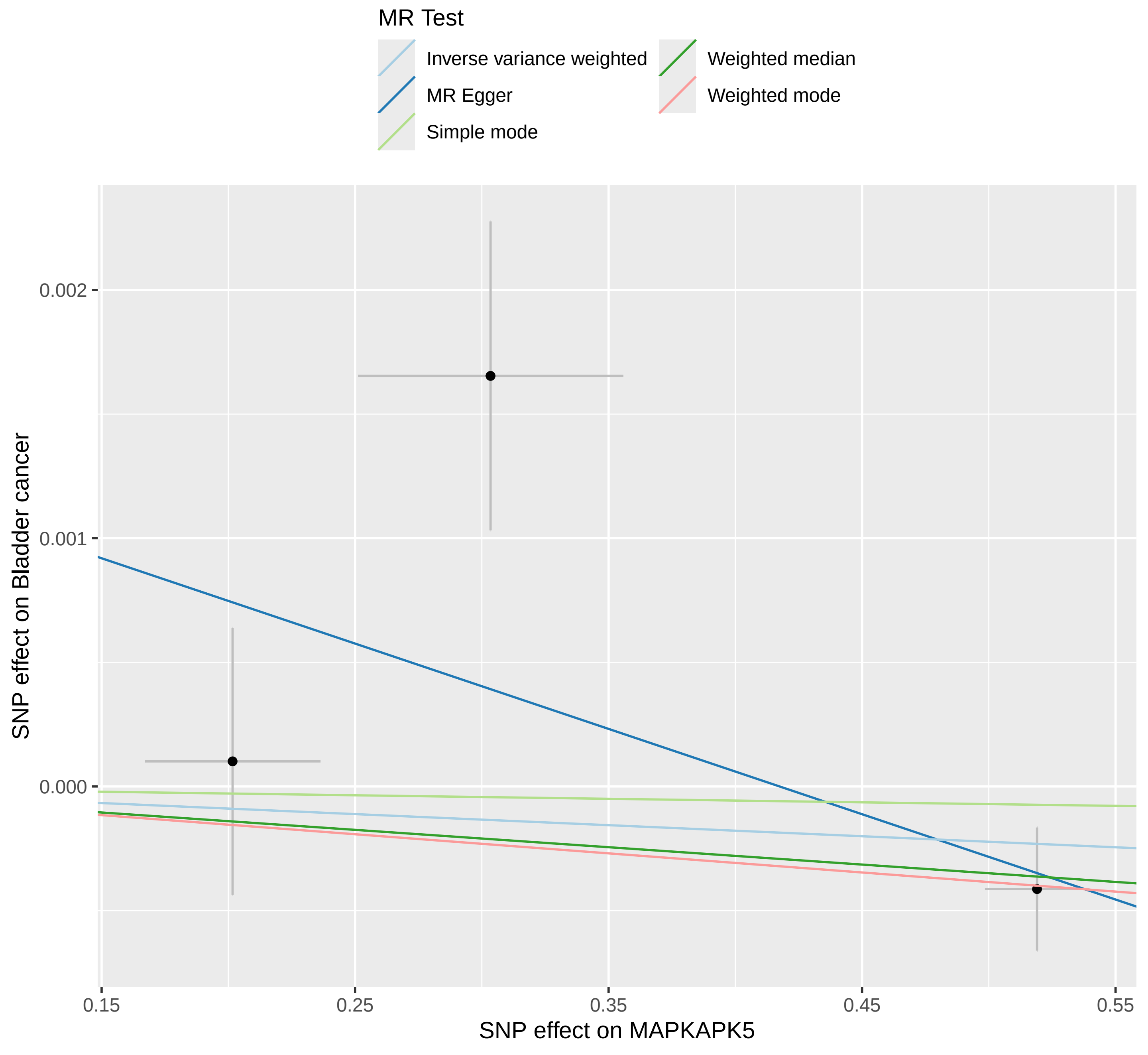
MR Test
Inverse variance weighted
MR Egger
Simple mode
Weighted median
Weighted mode
0.002
0.001
0.000
0.15
0.25
0.35
0.45
0.55
SNP effect on MAPKAPK5
SNP effect on Bladder cancer

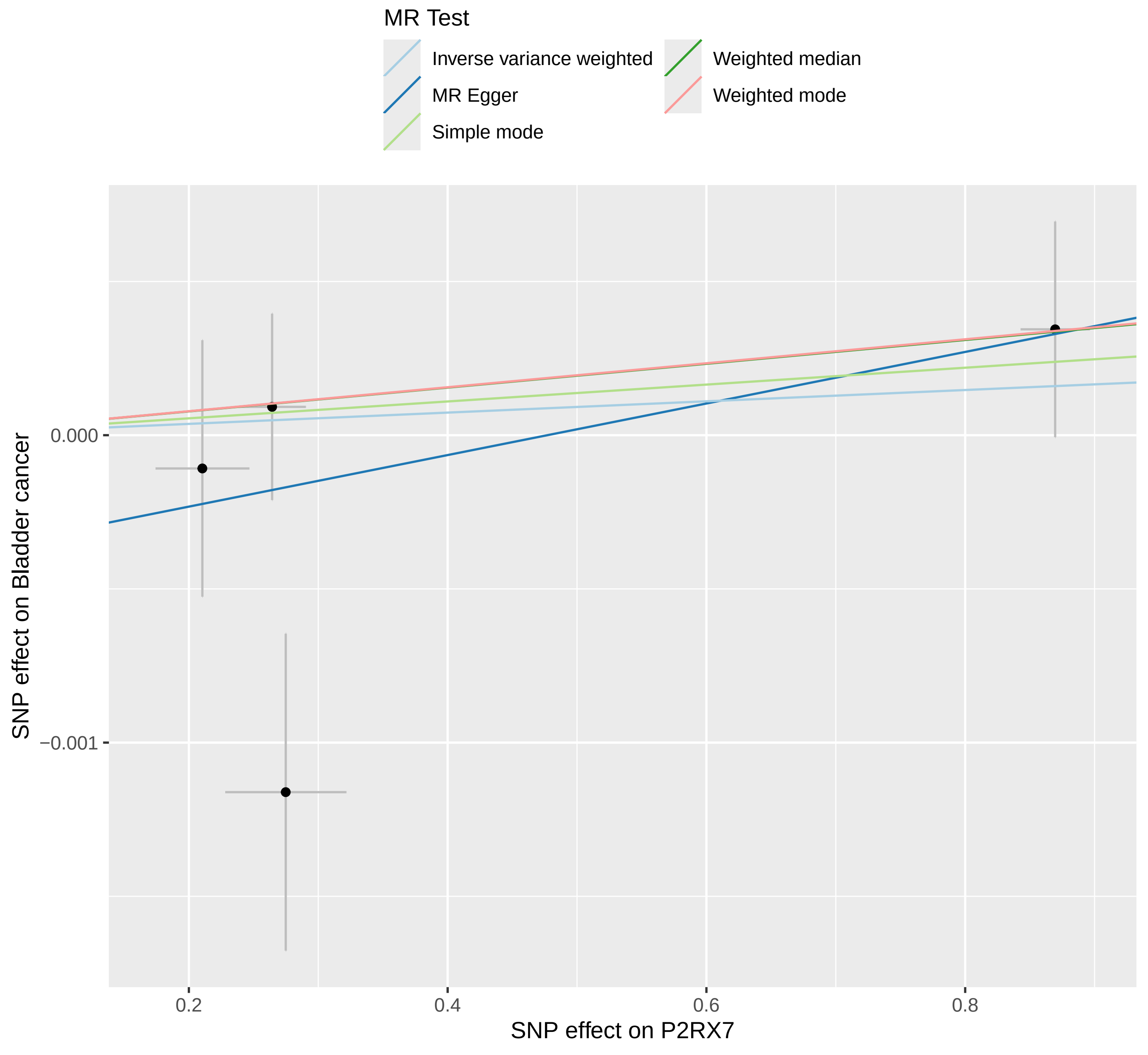

MR Test
Inverse variance weighted
MR Egger
Simple mode
Weighted median
Weighted mode
0.000
−0.001
SNP effect on Bladder cancer
0.2
0.4
0.6
0.8
SNP effect on P2RX7

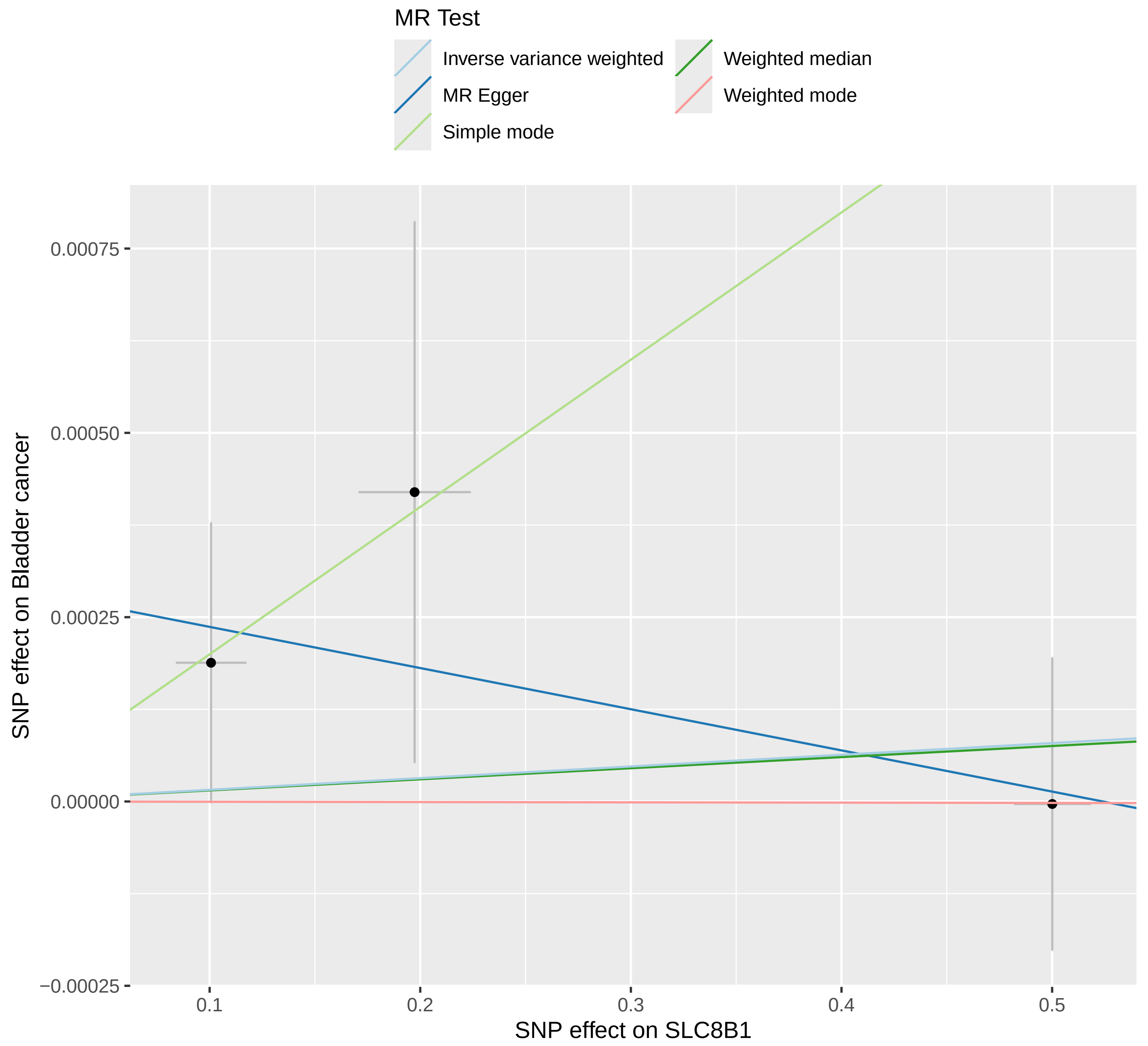

MR Test
Inverse variance weighted
MR Egger
Simple mode
Weighted median
Weighted mode
0.00075
0.00050
0.00025
0.00000
−0.00025
SNP effect on Bladder cancer
0.1
0.2
0.3
0.4
0.5
SNP effect on SLC8B1

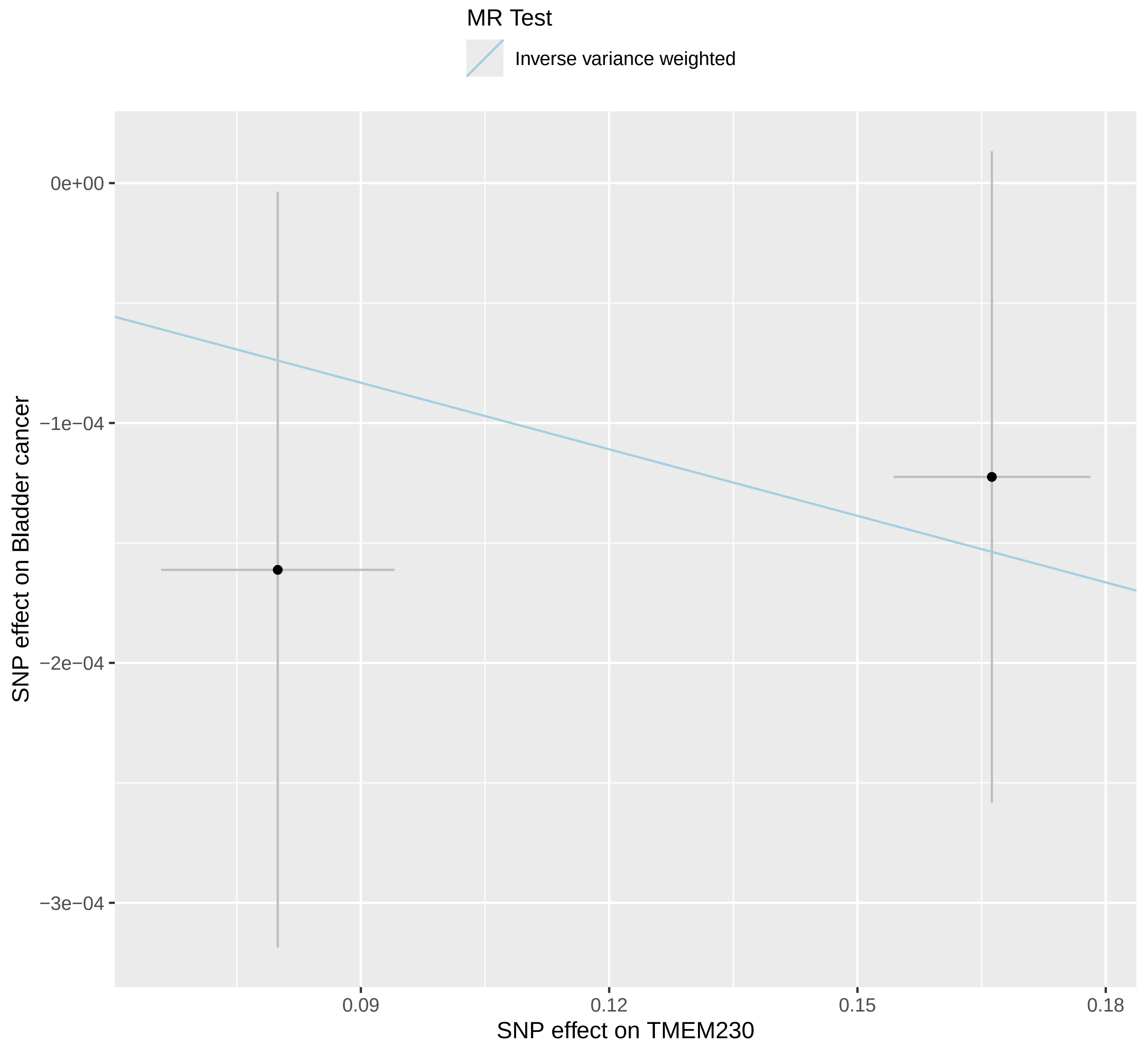

MR Test
Inverse variance weighted
0e+00
−1e−04
−2e−04
−3e−04
0.09
0.12
0.15
0.18
SNP effect on TMEM230
SNP effect on Bladder cancer

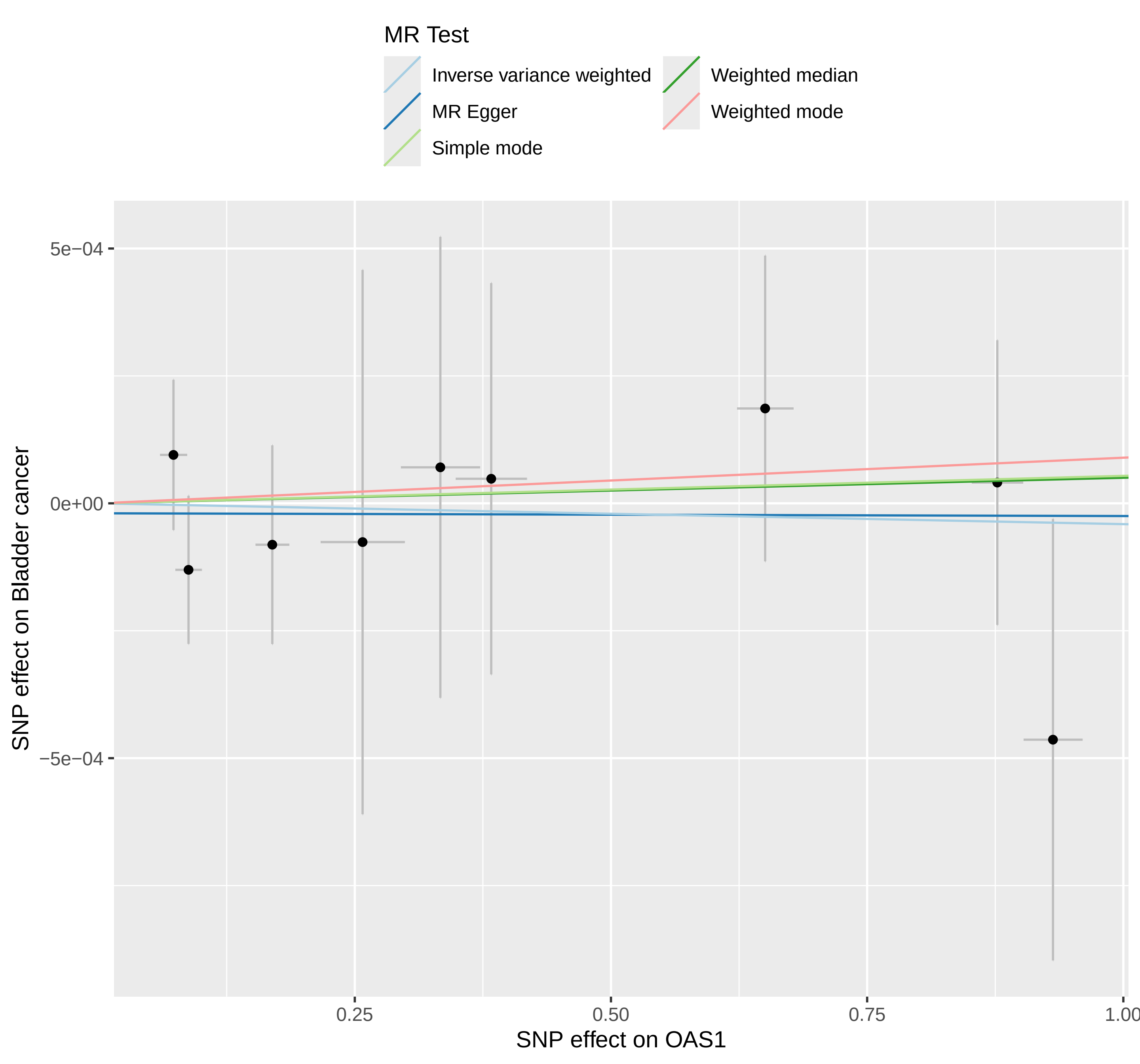

MR Test
Inverse variance weighted
MR Egger
Simple mode
Weighted median
Weighted mode
5e−04
0e+00
−5e−04
0.25
0.50
0.75
1.00
SNP effect on OAS1
SNP effect on Bladder cancer

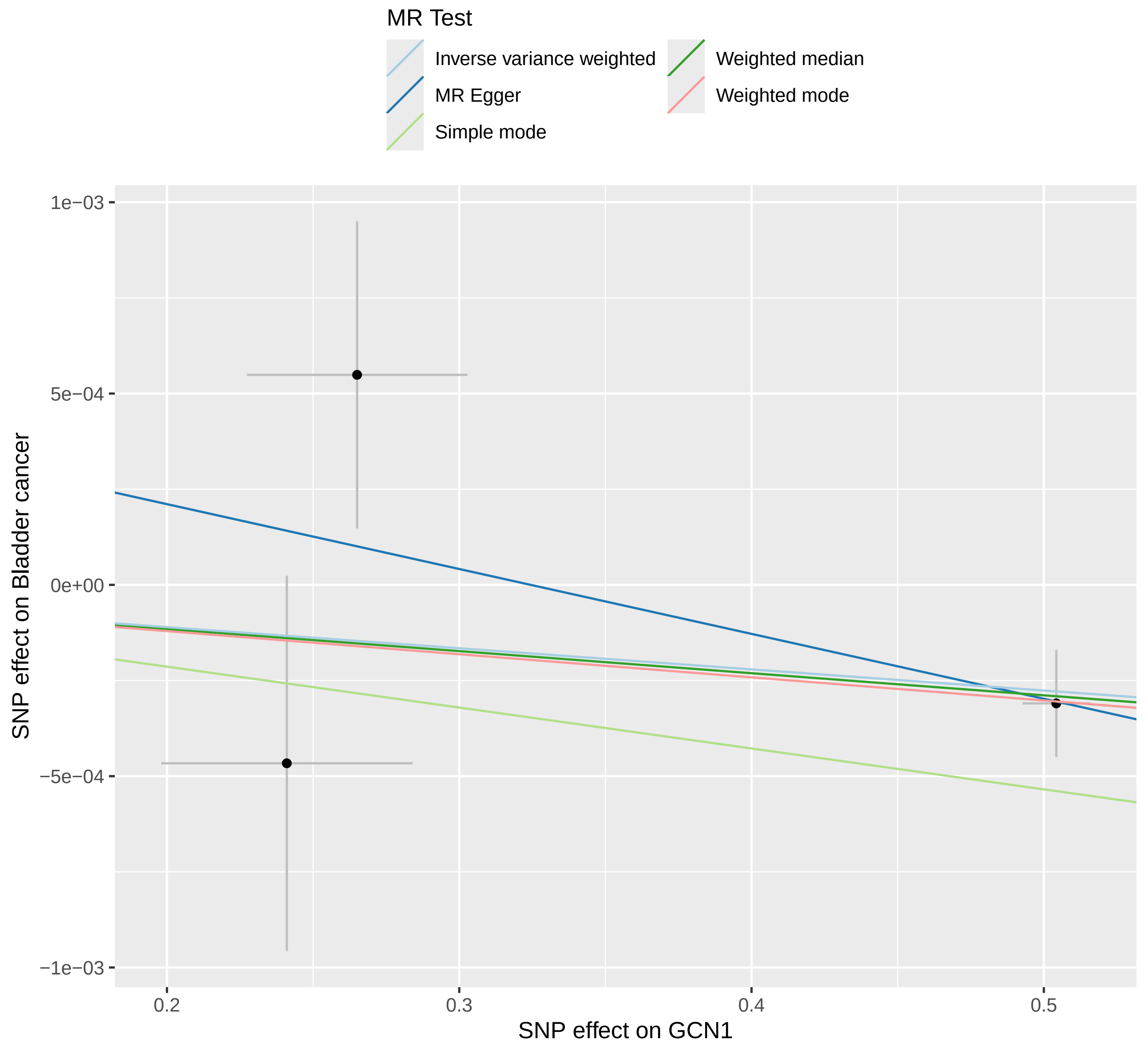
MR Test
Inverse variance weighted
MR Egger
Simple mode
Weighted median
Weighted mode
1e−03
5e−04
0e+00
−5e−04
−1e−03
SNP effect on Bladder cancer
0.2
0.3
0.4
0.5
SNP effect on GCN1

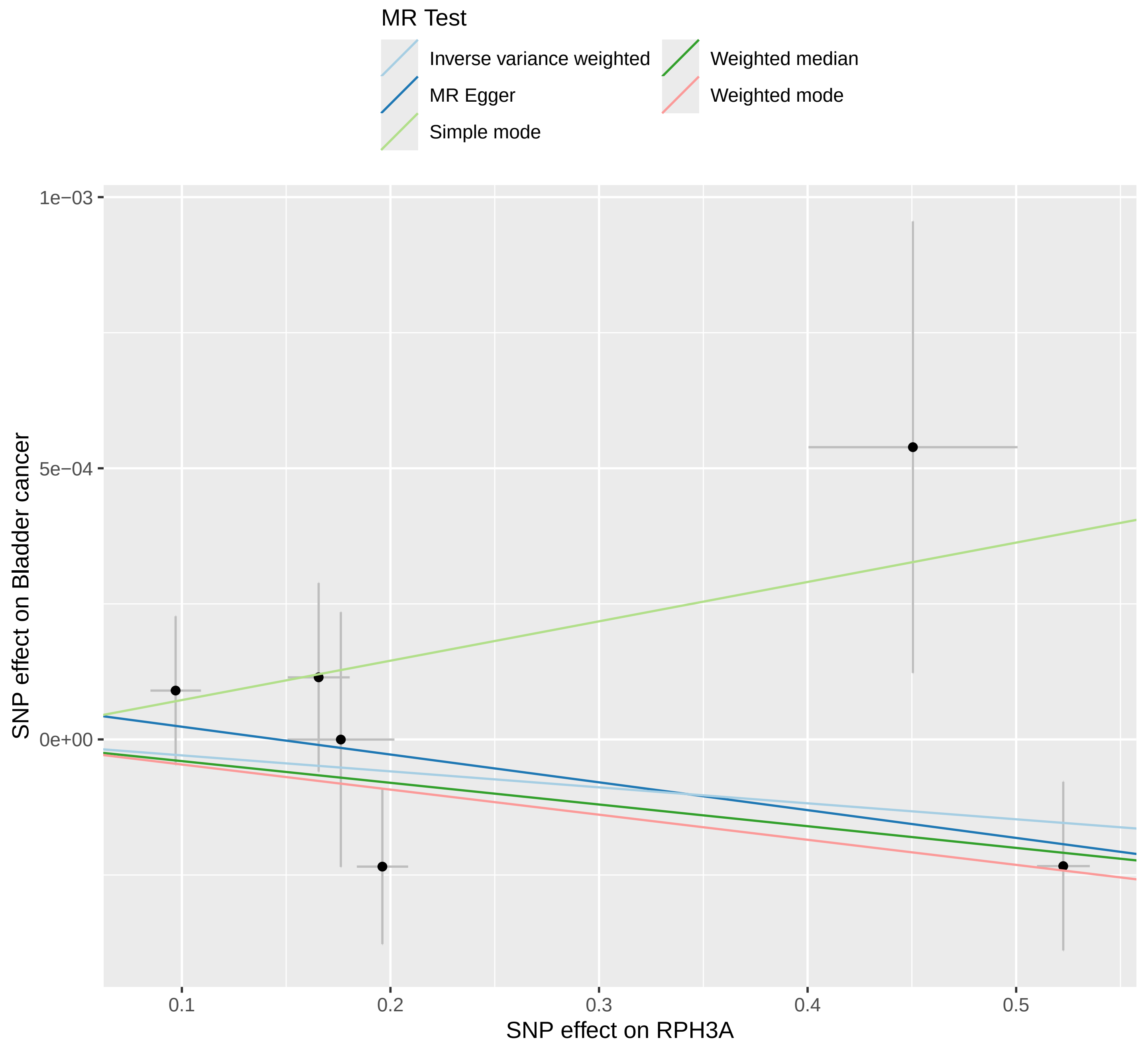

MR Test
Inverse variance weighted
MR Egger
Simple mode
Weighted median
Weighted mode
1e−03
5e−04
0e+00
0.1
0.2
0.3
0.4
0.5
SNP effect on RPH3A
SNP effect on Bladder cancer

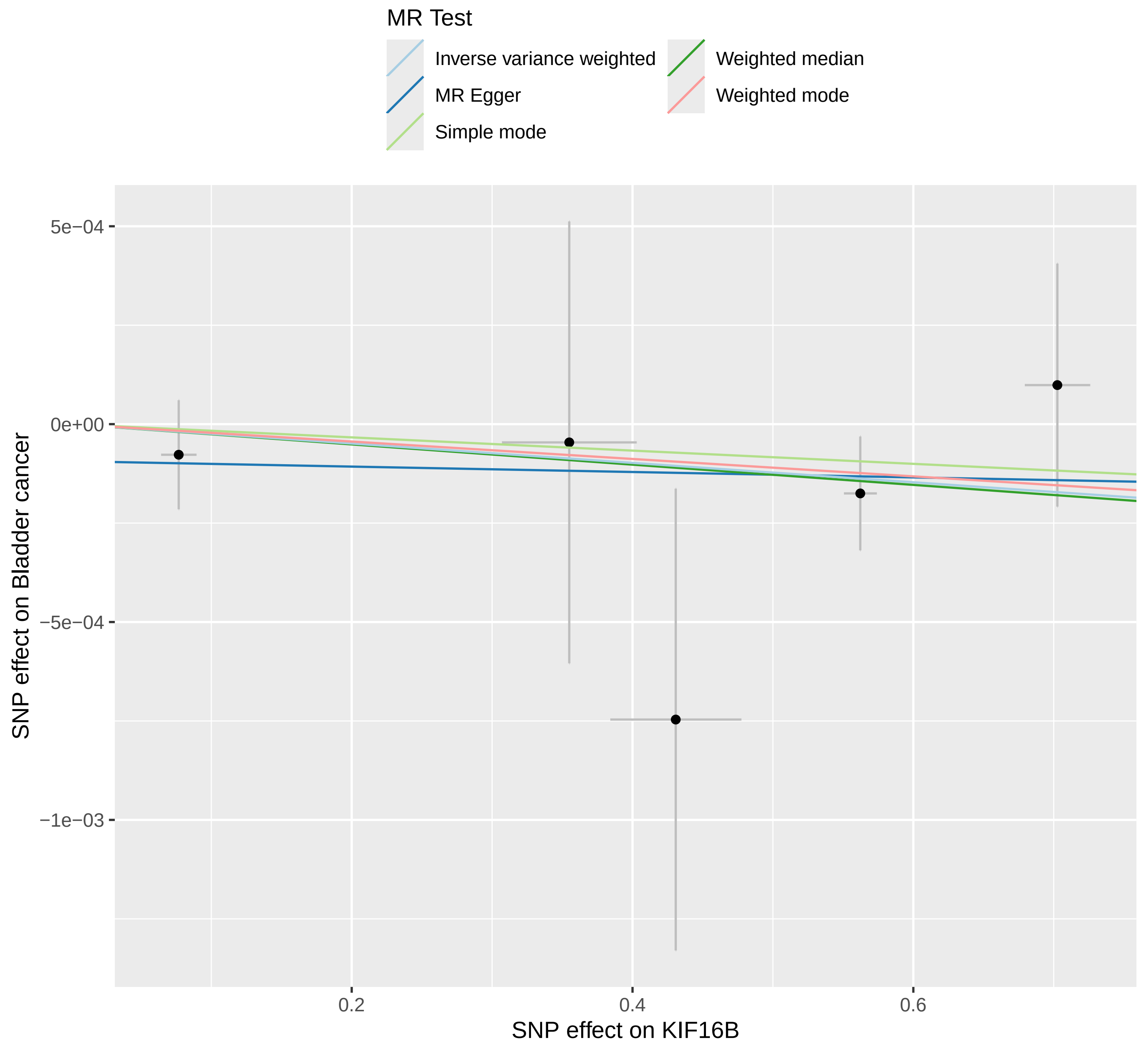

MR Test
Inverse variance weighted
MR Egger
Simple mode
Weighted median
Weighted mode
5e−04
0e+00
−5e−04
−1e−03
SNP effect on Bladder cancer
0.2
0.4
0.6
SNP effect on KIF16B

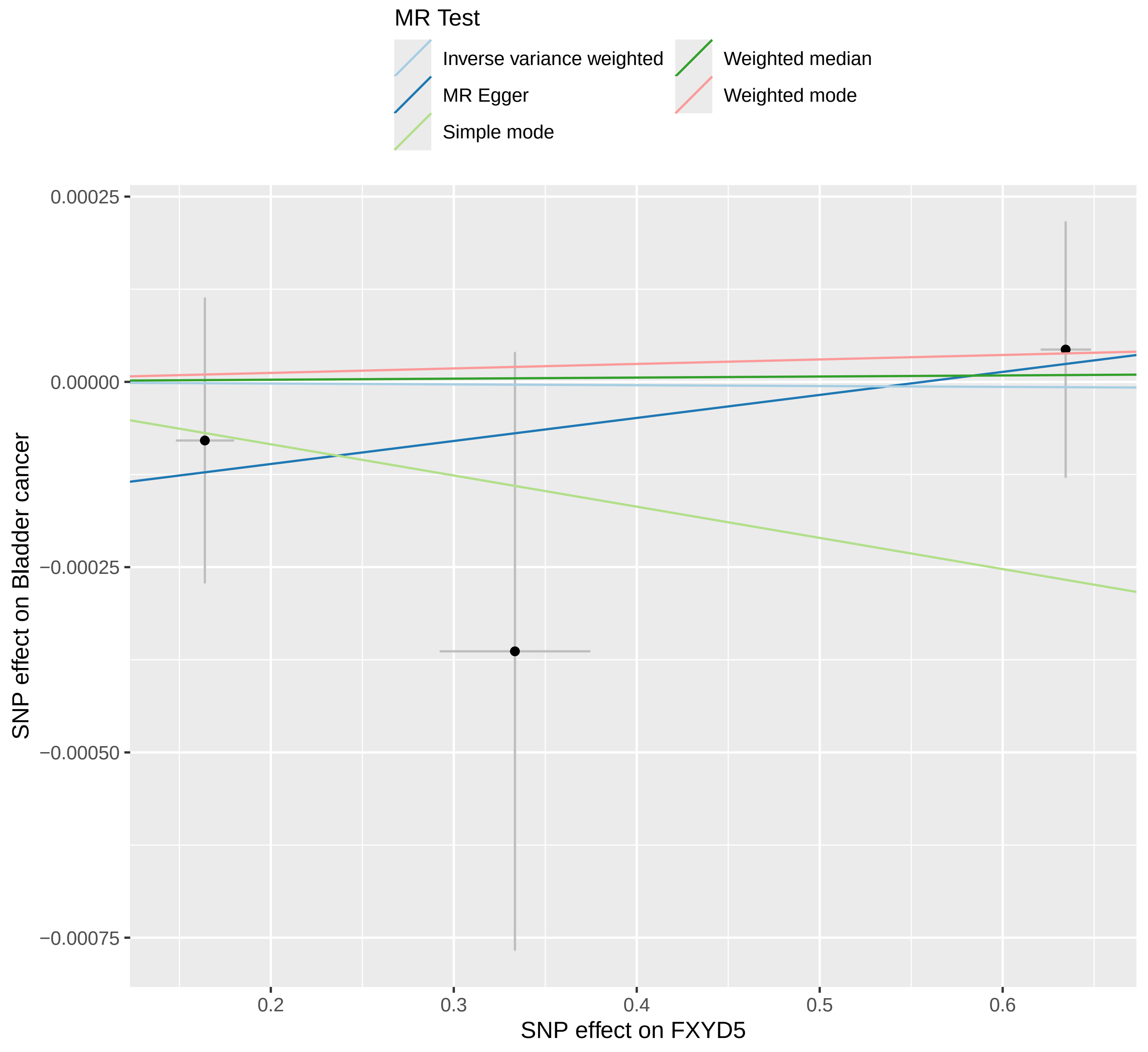

MR Test
Inverse variance weighted
MR Egger
Simple mode
Weighted median
Weighted mode
0.00025
0.00000
−0.00025
−0.00050
−0.00075
SNP effect on Bladder cancer
0.2
0.3
0.4
0.5
0.6
SNP effect on FXYD5

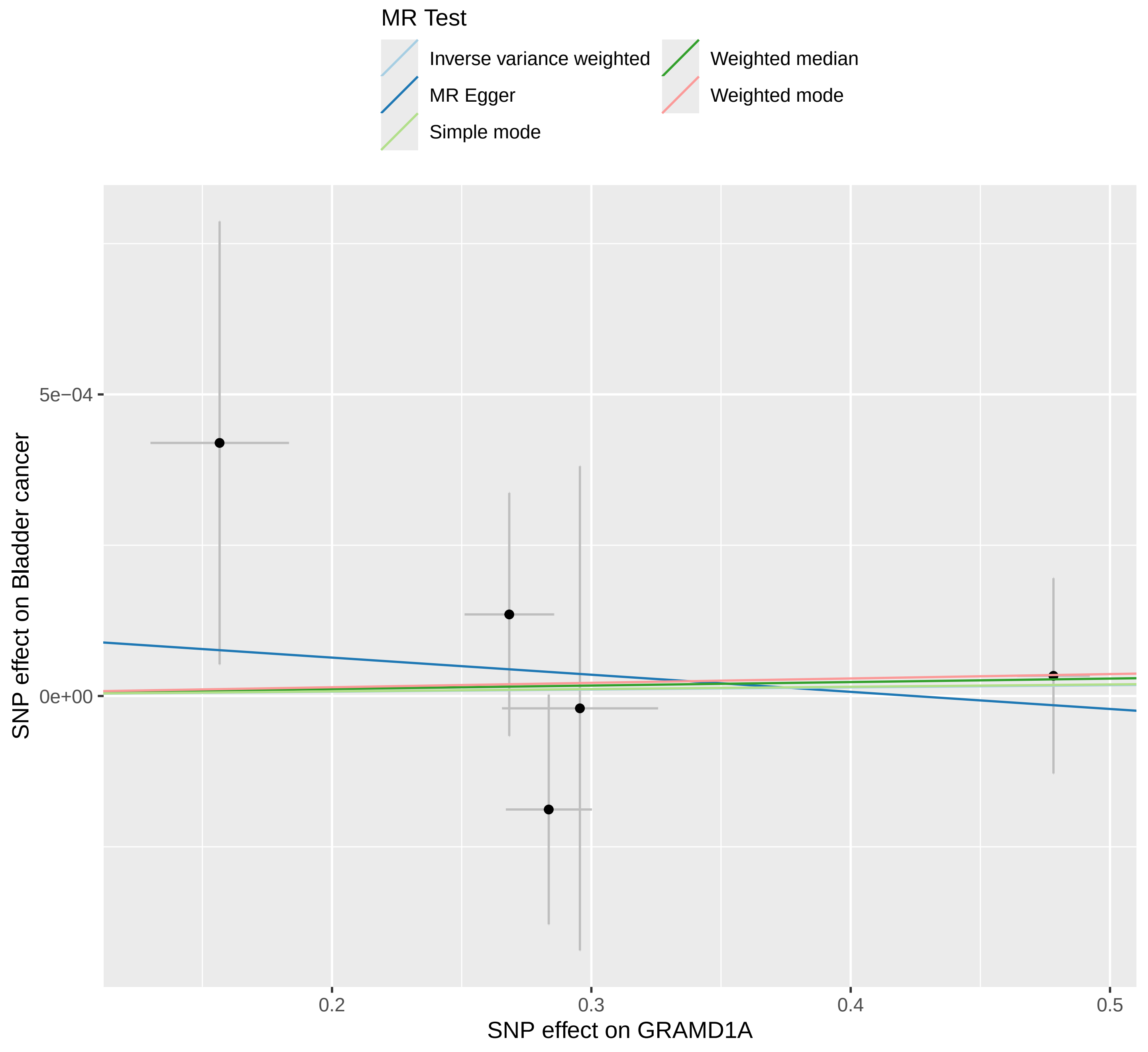

MR Test
Inverse variance weighted
MR Egger
Simple mode
Weighted median
Weighted mode
5e−04
0e+00
SNP effect on Bladder cancer
0.2
0.3
0.4
0.5
SNP effect on GRAMD1A

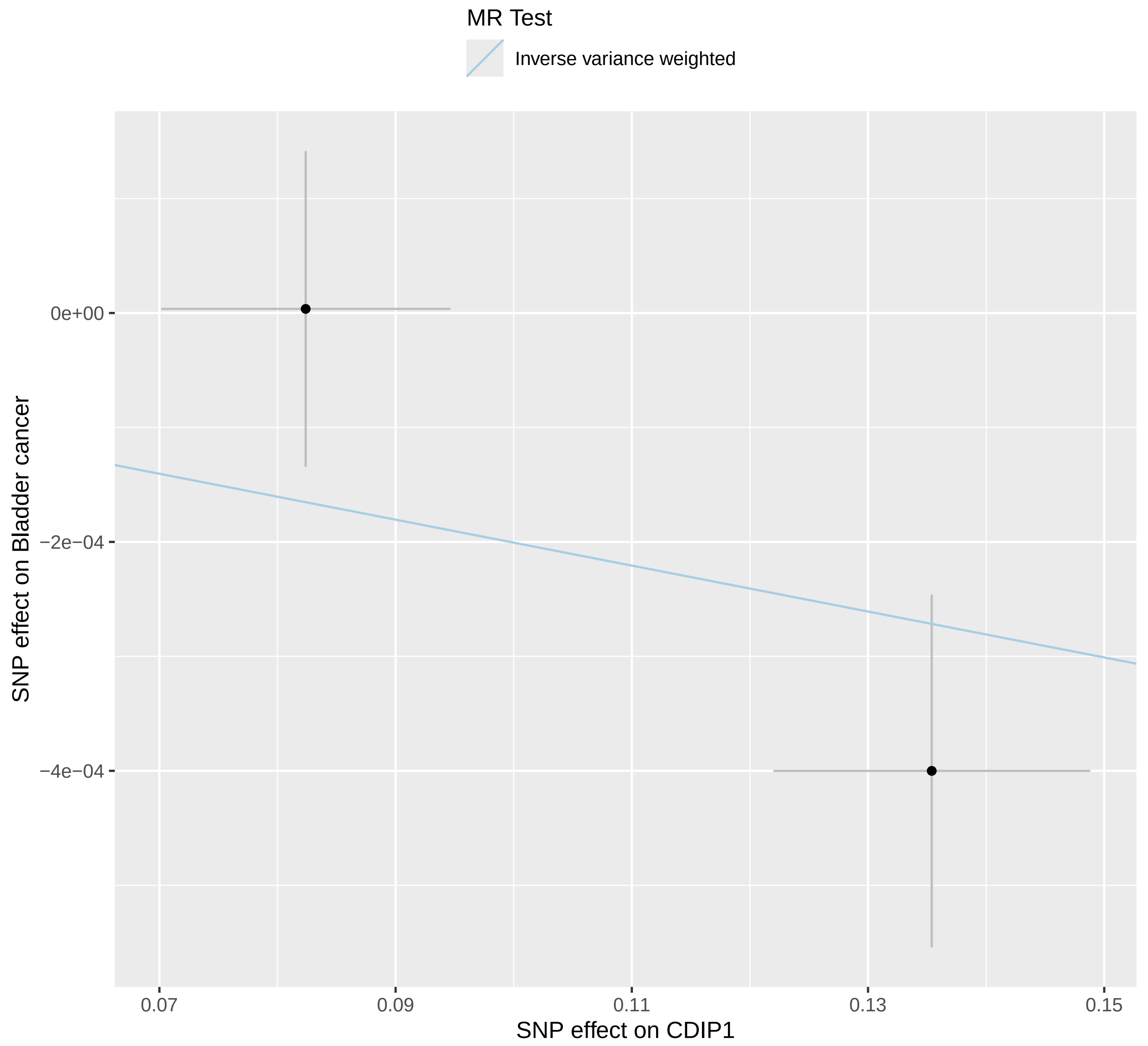

MR Test
Inverse variance weighted
0e+00
−2e−04
−4e−04
0.07
0.09
0.11
0.13
0.15
SNP effect on CDIP1
SNP effect on Bladder cancer

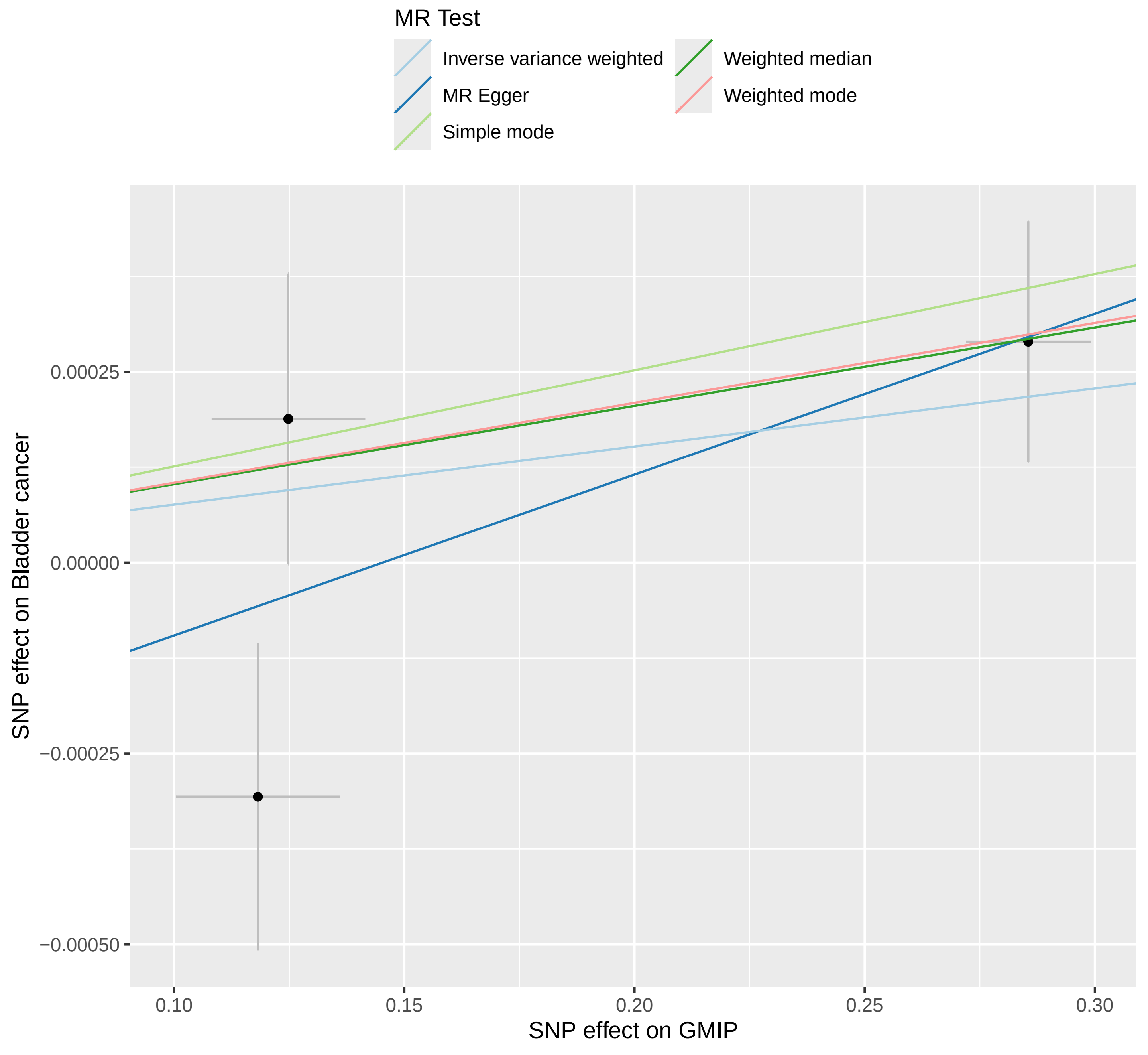
MR Test
Inverse variance weighted
MR Egger
Simple mode
Weighted median
Weighted mode
SNP effect on Bladder cancer
0.00025
0.00000
−0.00025
−0.00050
0.10
0.15
0.20
0.25
0.30
SNP effect on GMIP

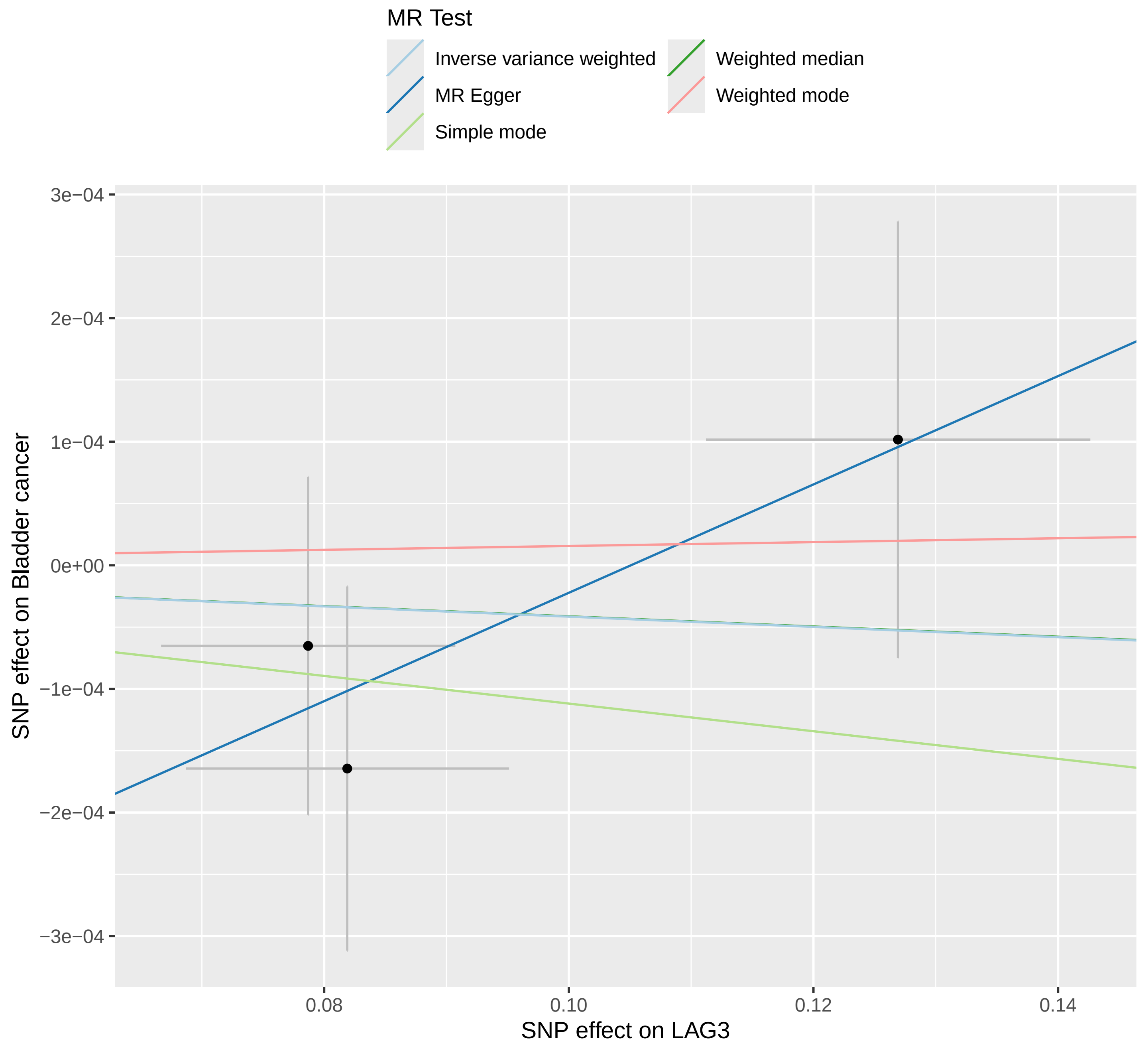
MR Test
Inverse variance weighted
MR Egger
Simple mode
Weighted median
Weighted mode
3e−04
2e−04
1e−04
0e+00
−1e−04
−2e−04
−3e−04
SNP effect on Bladder cancer
0.08
0.10
0.12
0.14
SNP effect on LAG3

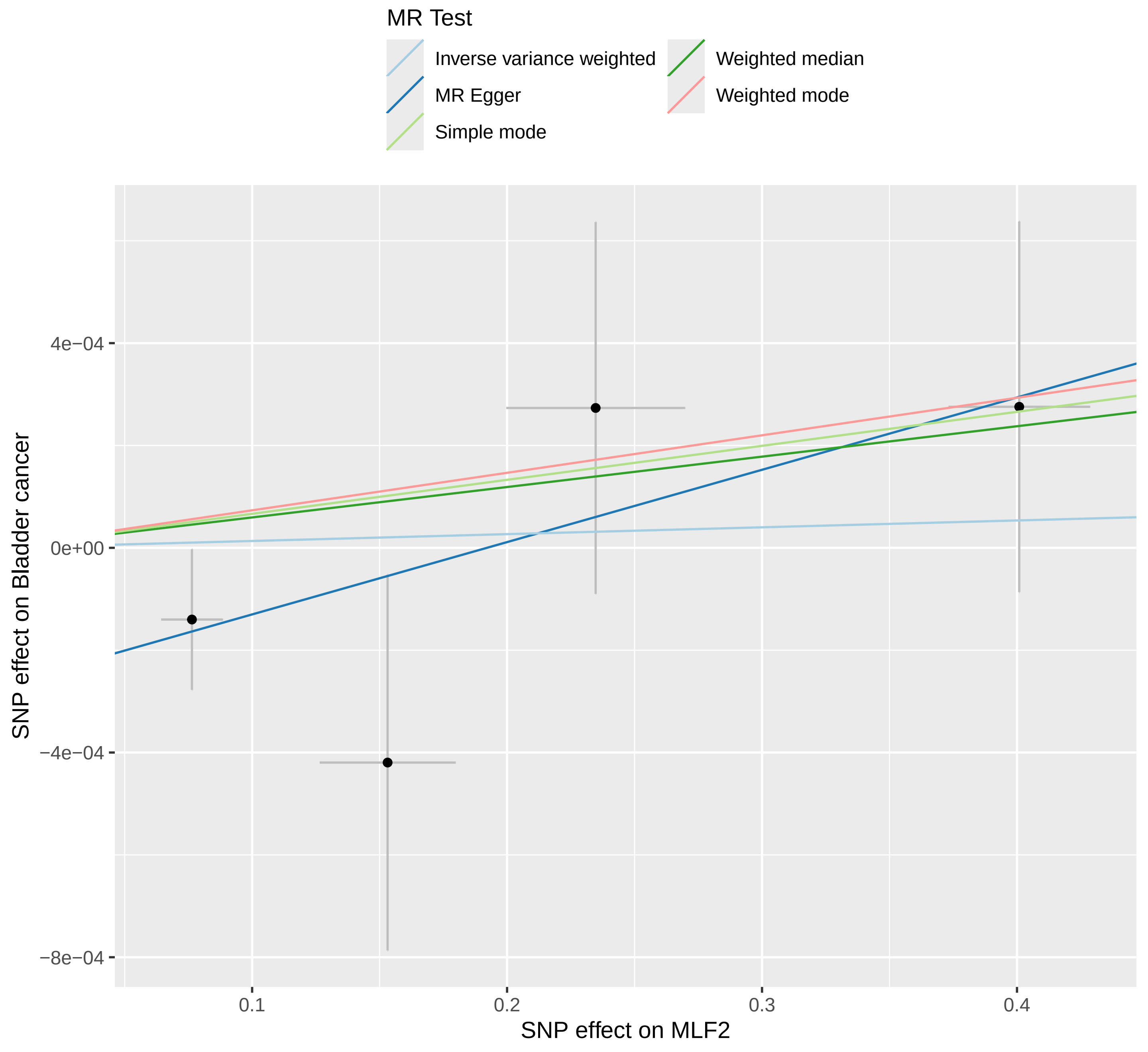
MR Test
Inverse variance weighted
MR Egger
Simple mode
Weighted median
Weighted mode
4e−04
0e+00
−4e−04
−8e−04
SNP effect on Bladder cancer
0.1
0.2
0.3
0.4
SNP effect on MLF2

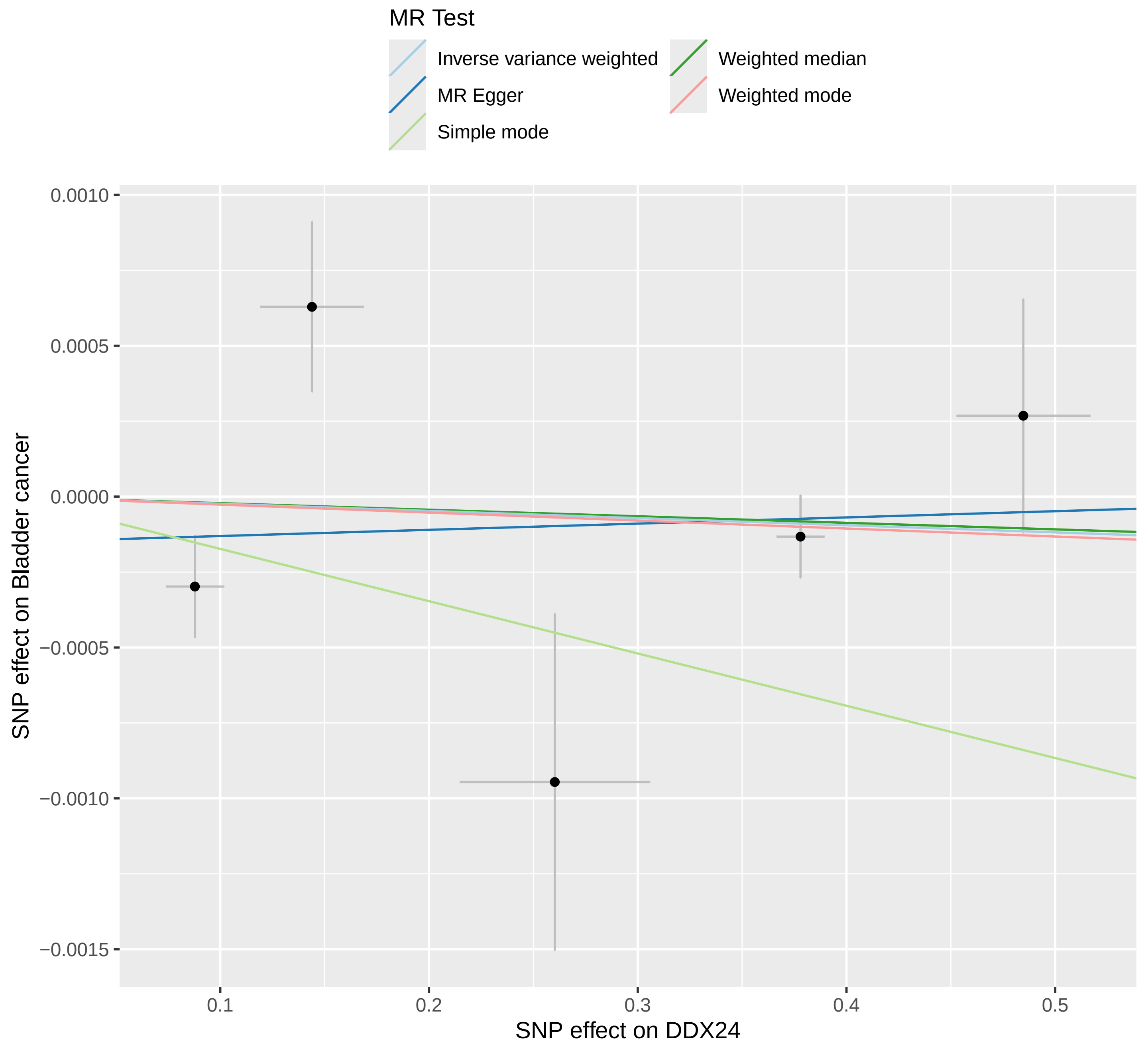
MR Test
Inverse variance weighted
MR Egger
Simple mode
Weighted median
Weighted mode
0.0010
0.0005
0.0000
−0.0005
−0.0010
−0.0015
SNP effect on Bladder cancer
0.1
0.2
0.3
0.4
0.5
SNP effect on DDX24

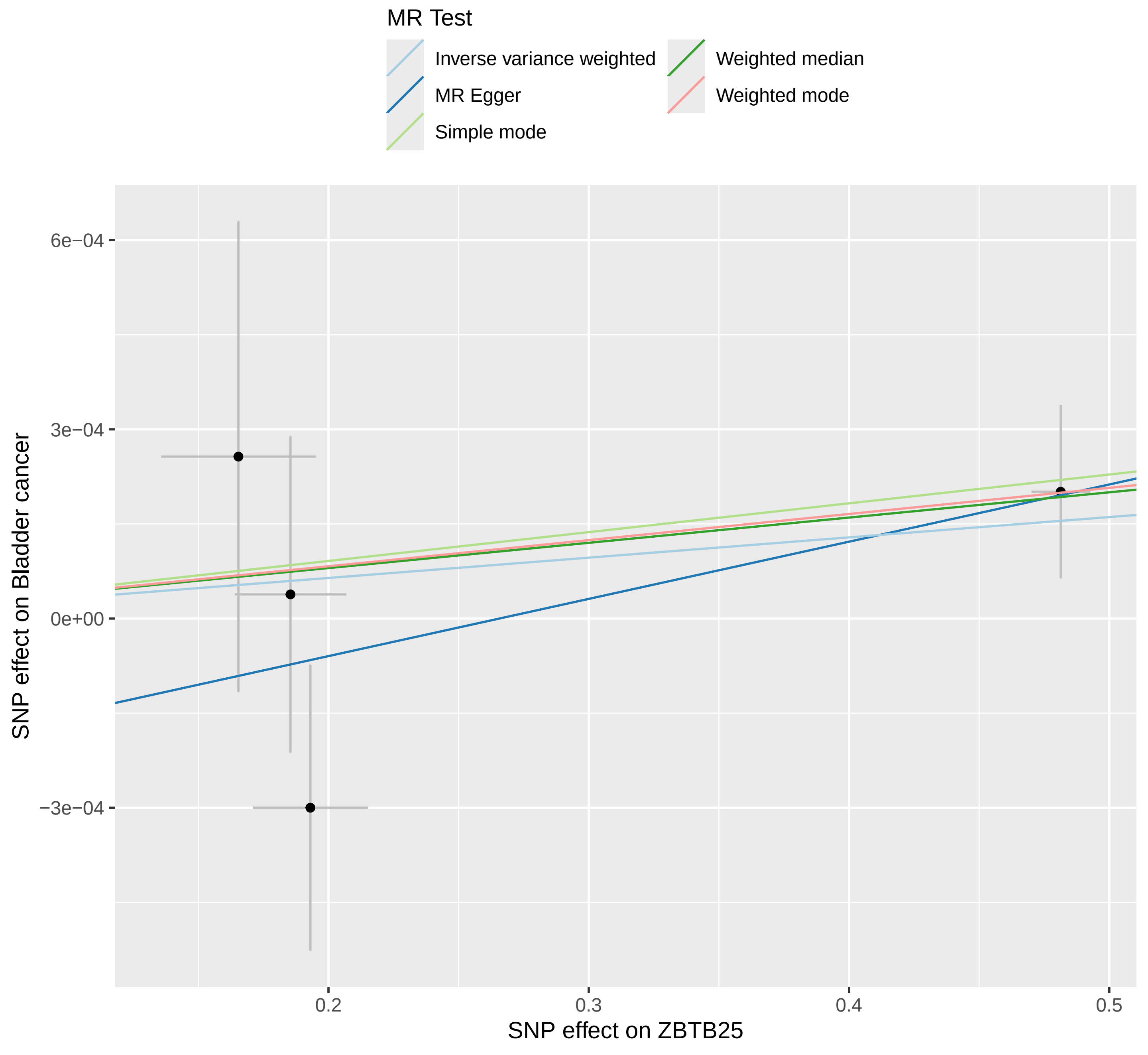
MR Test
Inverse variance weighted
MR Egger
Simple mode
Weighted median
Weighted mode
6e−04
3e−04
0e+00
−3e−04
SNP effect on Bladder cancer
0.2
0.3
0.4
0.5
SNP effect on ZBTB25

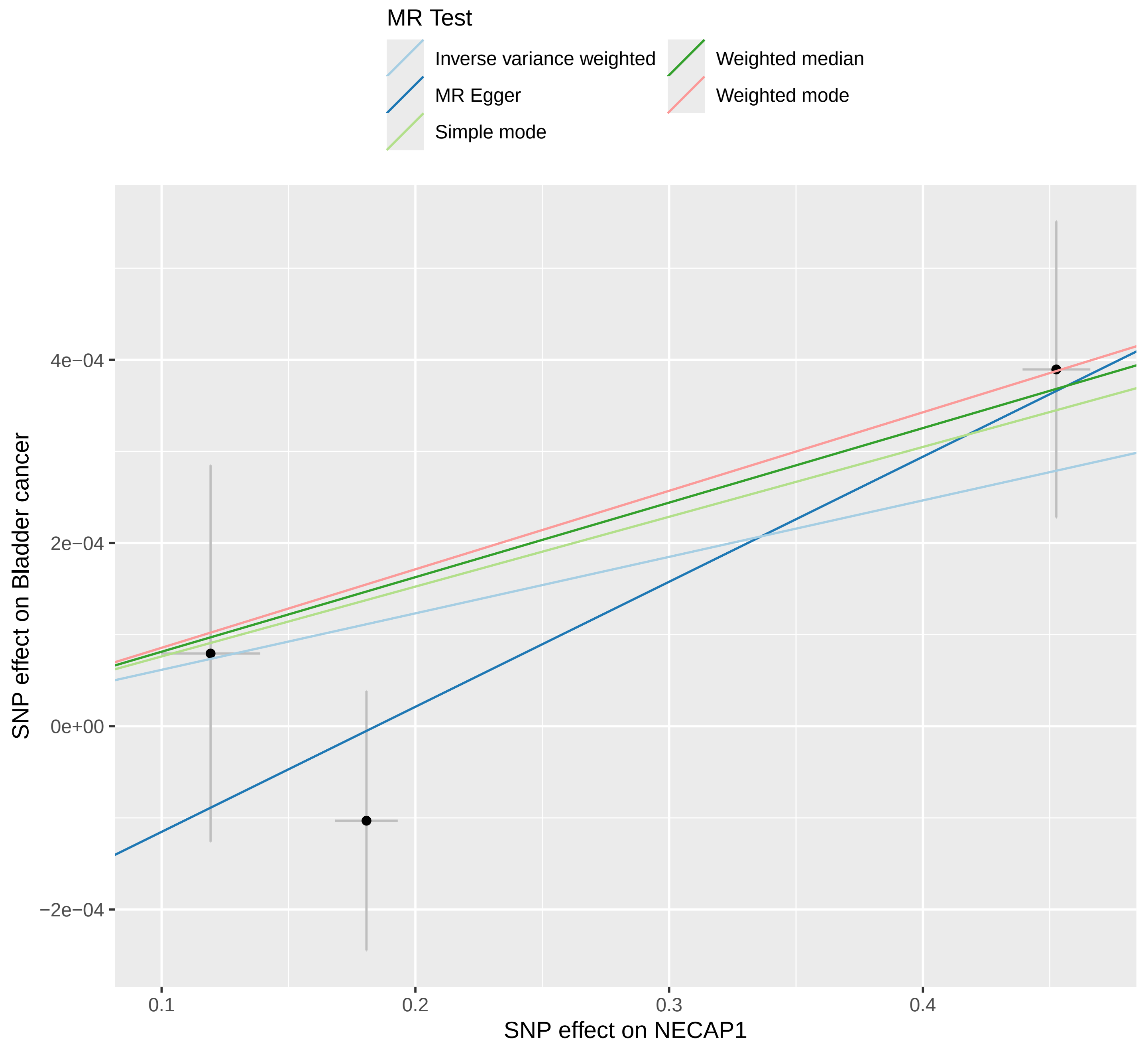

MR Test
Inverse variance weighted
MR Egger
Simple mode
Weighted median
Weighted mode
4e−04
2e−04
0e+00
−2e−04
0.1
0.2
0.3
0.4
SNP effect on NECAP1
SNP effect on Bladder cancer

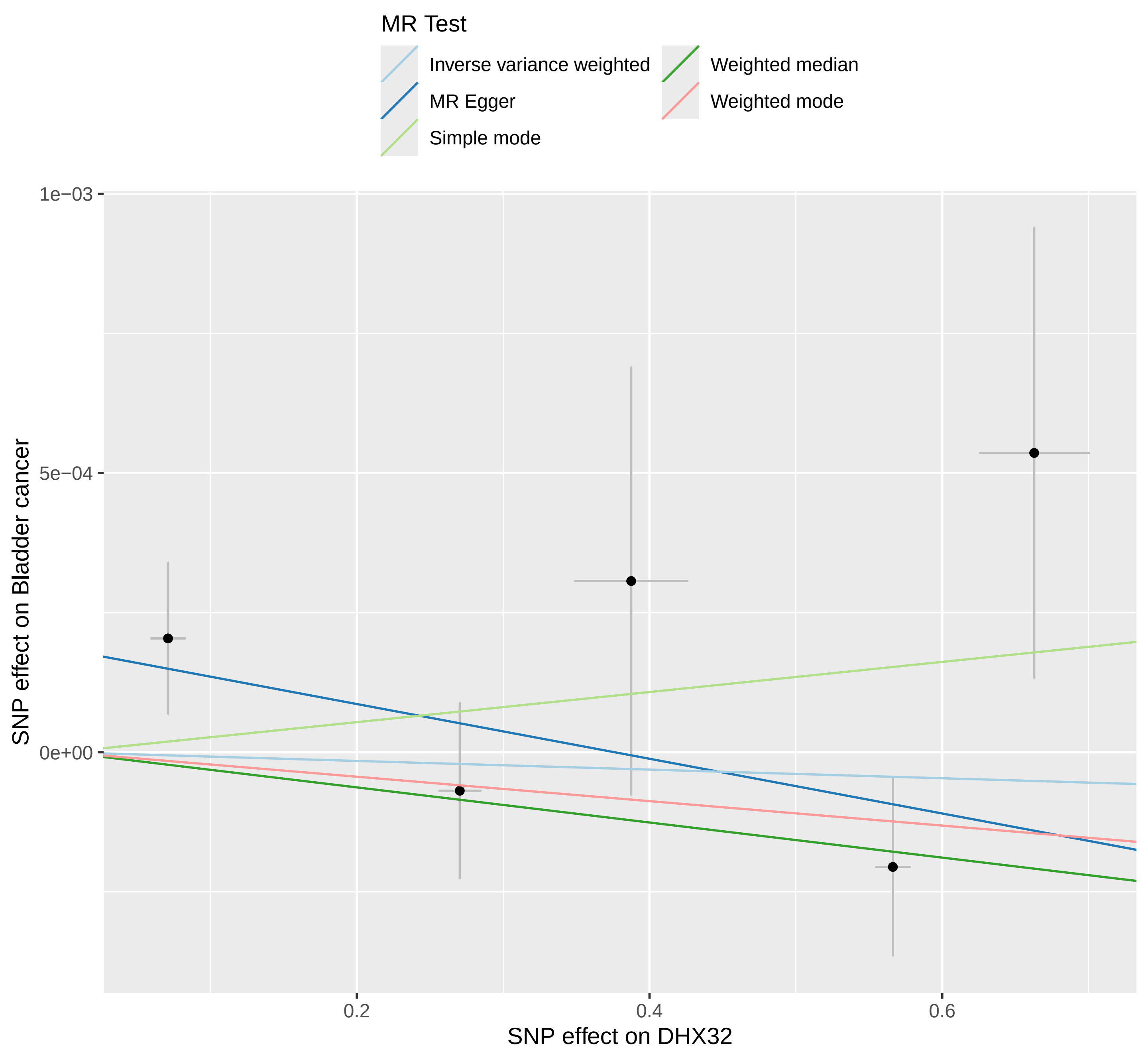

MR Test
Inverse variance weighted
MR Egger
Simple mode
Weighted median
Weighted mode
1e−03
5e−04
0e+00
0.2
0.4
0.6
SNP effect on DHX32
SNP effect on Bladder cancer

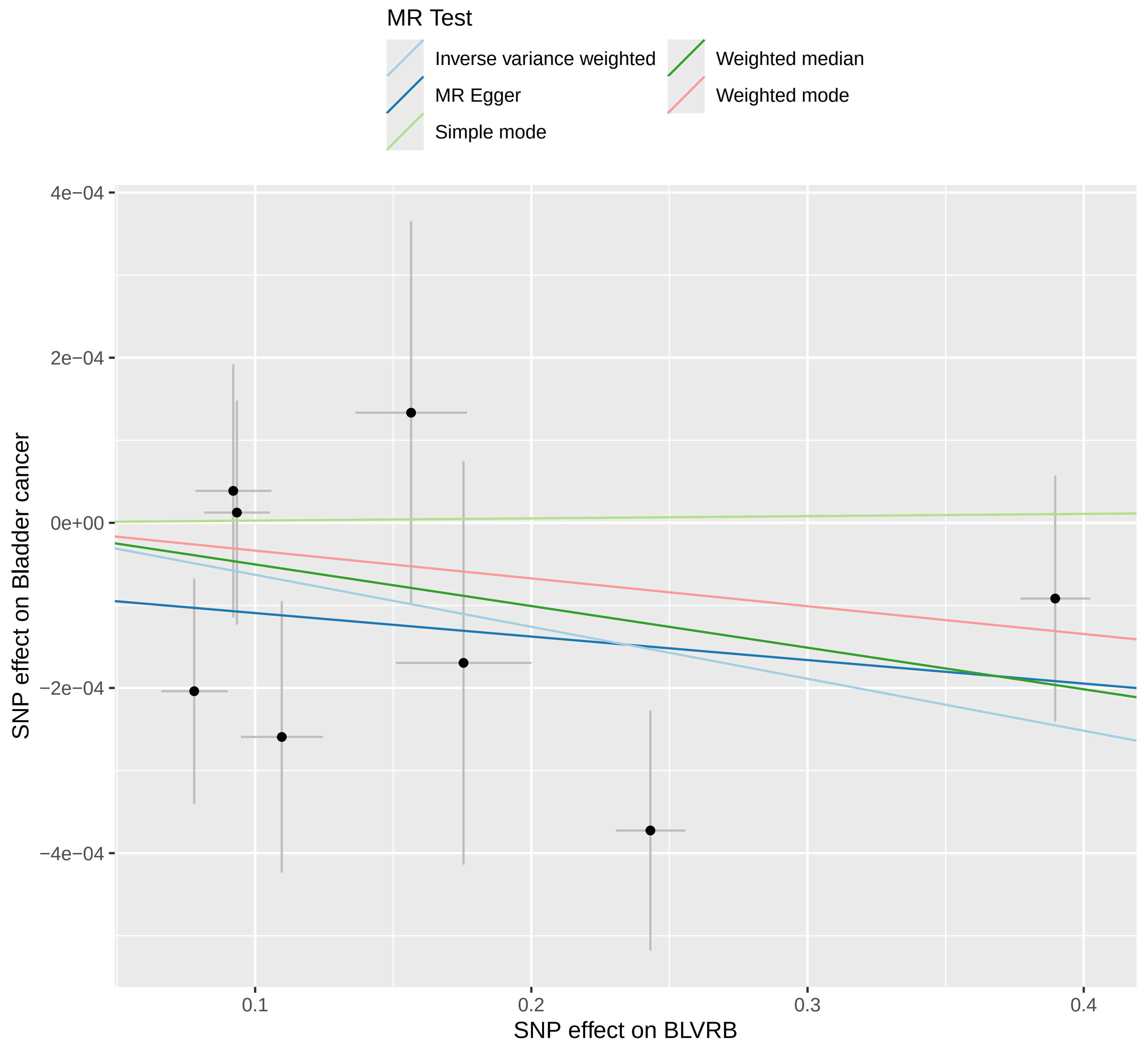

MR Test
Inverse variance weighted
MR Egger
Simple mode
Weighted median
Weighted mode
4e−04
2e−04
0e+00
−2e−04
−4e−04
SNP effect on Bladder cancer
0.1
0.2
0.3
0.4
SNP effect on BLVRB

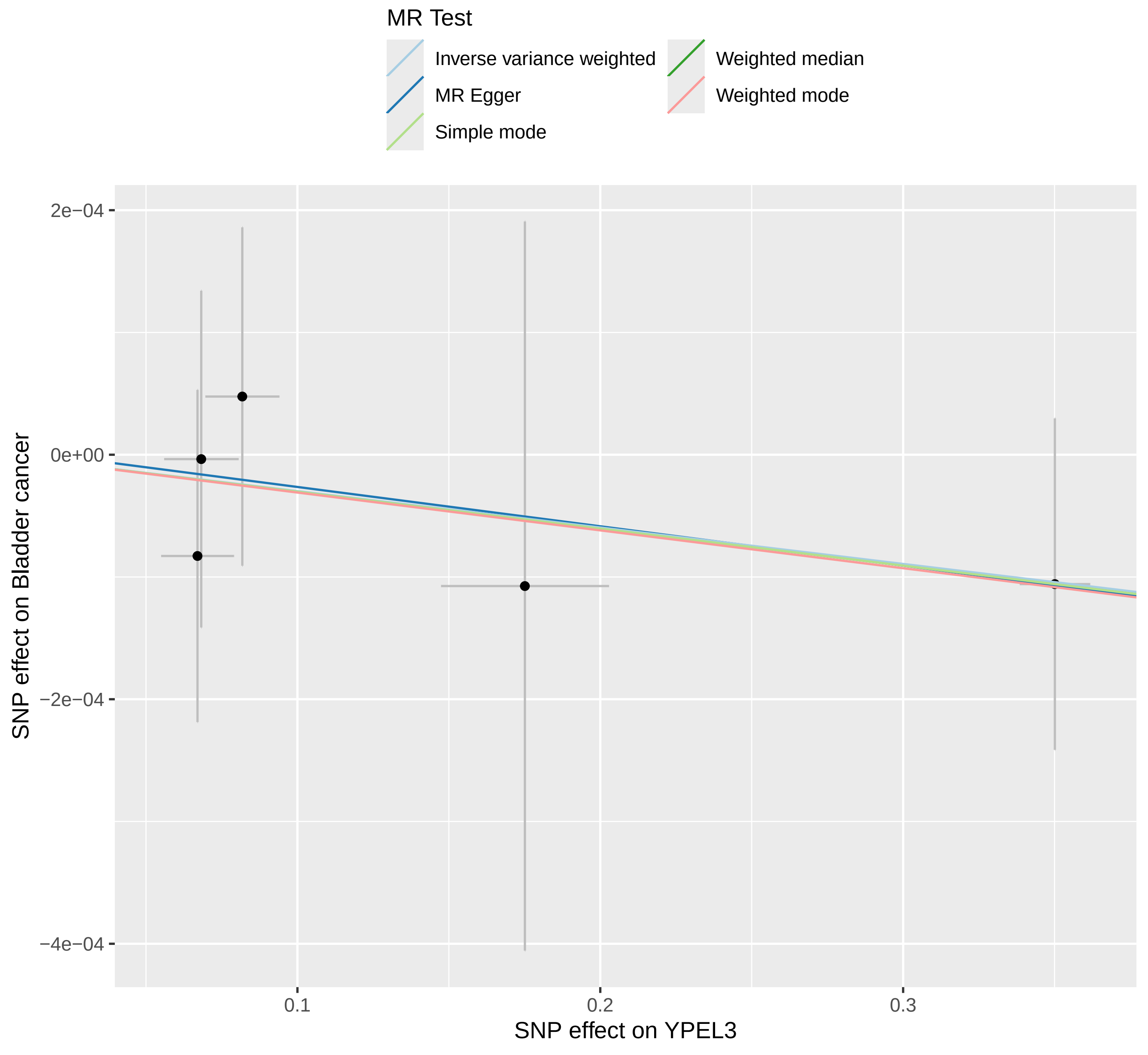
MR Test
Inverse variance weighted
MR Egger
Simple mode
Weighted median
Weighted mode
2e−04
0e+00
−2e−04
−4e−04
SNP effect on Bladder cancer
0.1
0.2
0.3
SNP effect on YPEL3

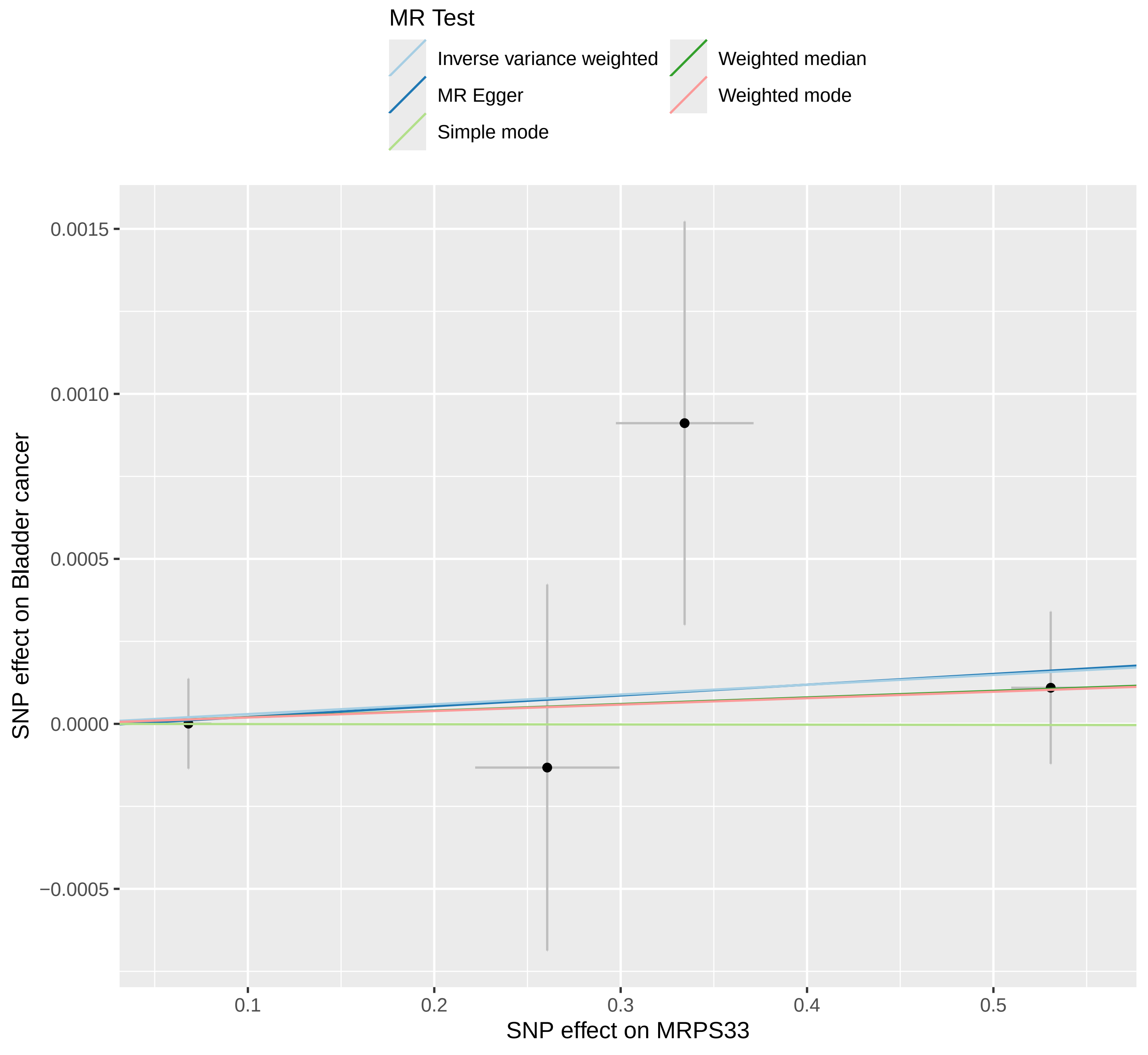
MR Test
Inverse variance weighted
MR Egger
Simple mode
Weighted median
Weighted mode
0.0015
0.0010
0.0005
0.0000
−0.0005
0.1
0.2
0.3
0.4
0.5
SNP effect on MRPS33
SNP effect on Bladder cancer

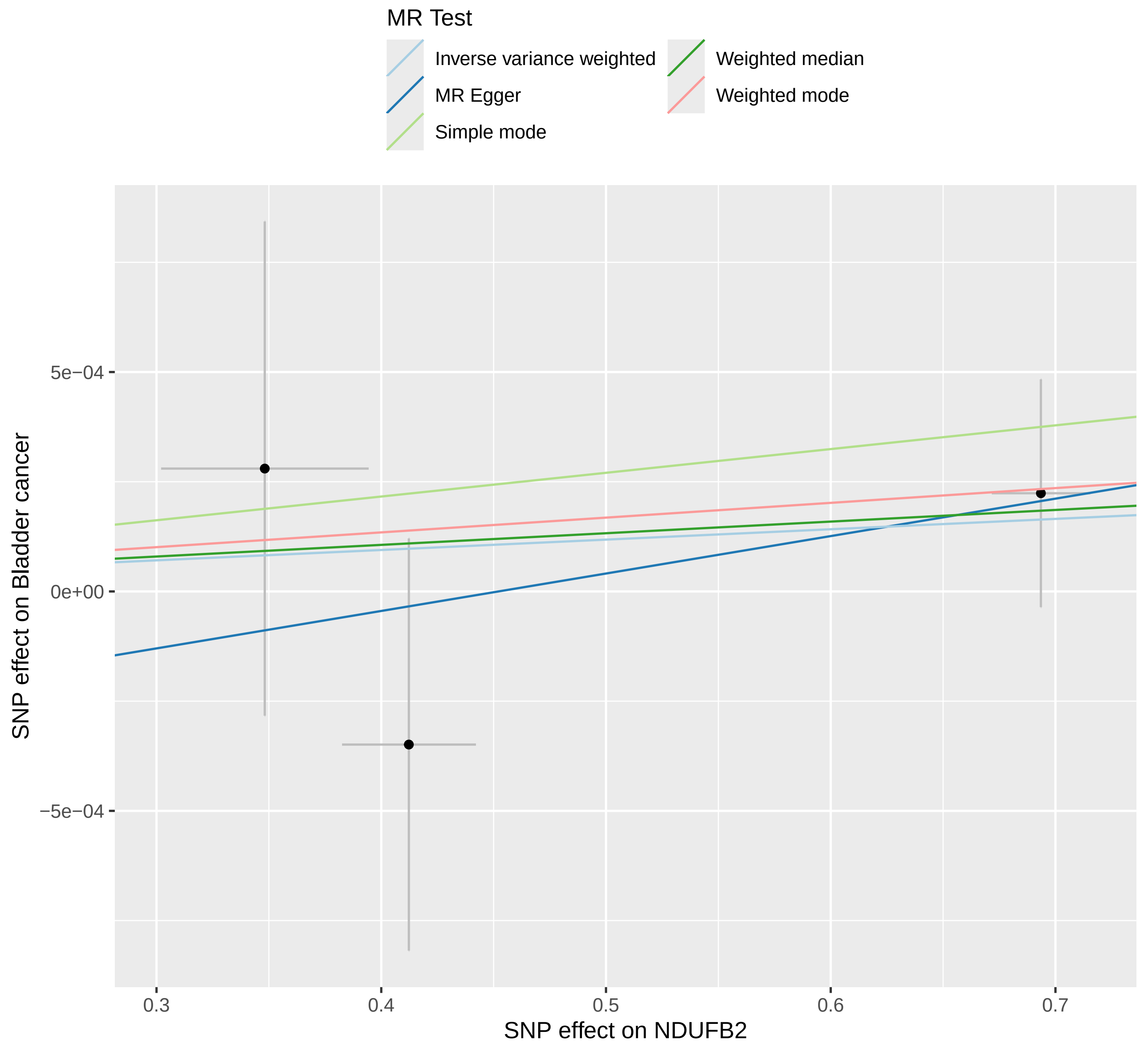

MR Test
Inverse variance weighted
MR Egger
Simple mode
Weighted median
Weighted mode
5e−04
0e+00
−5e−04
0.3
0.4
0.5
0.6
0.7
SNP effect on NDUFB2
SNP effect on Bladder cancer

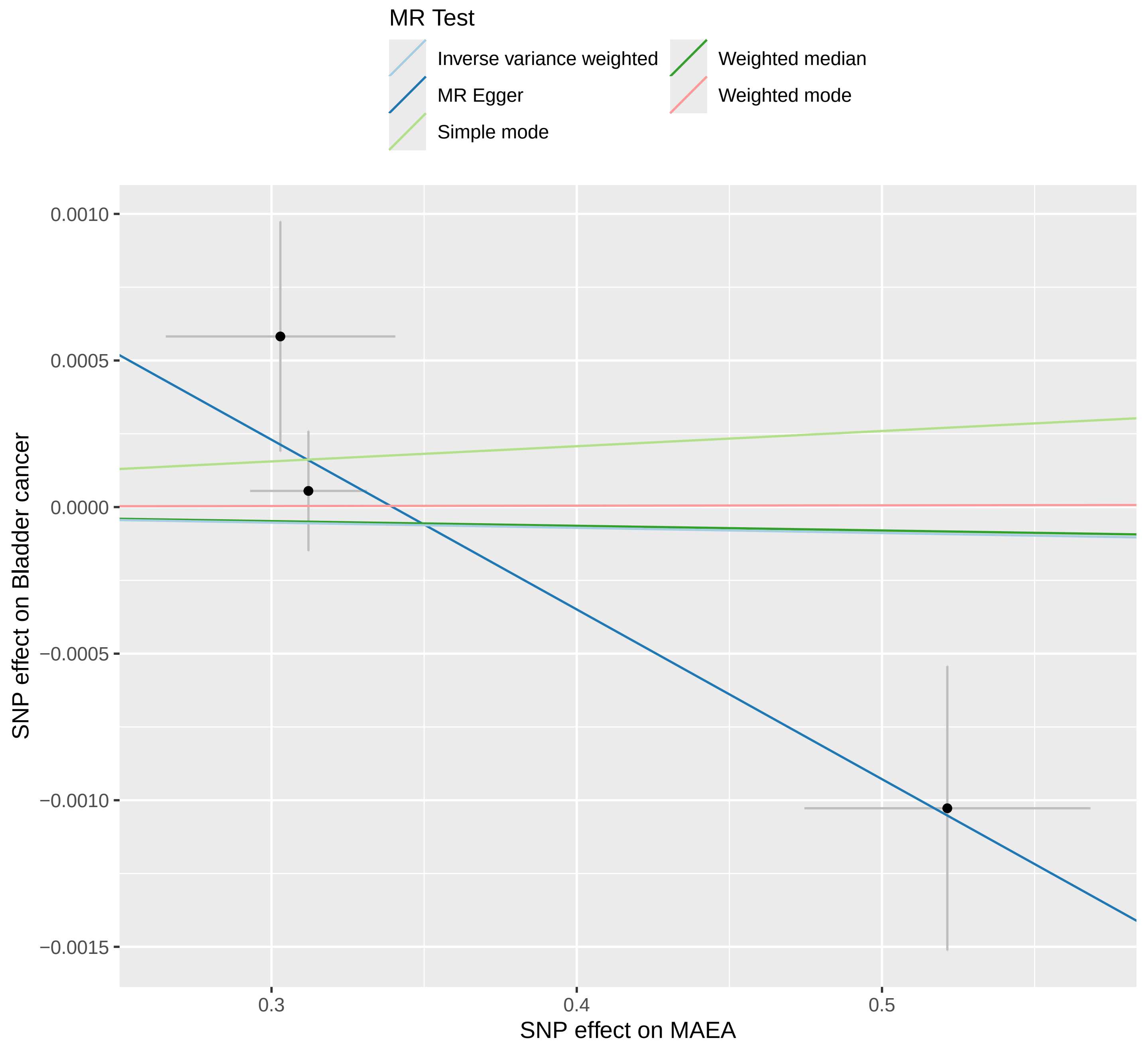
MR Test
Inverse variance weighted
MR Egger
Simple mode
Weighted median
Weighted mode
0.0010
0.0005
0.0000
−0.0005
−0.0010
−0.0015
SNP effect on Bladder cancer
0.3
0.4
0.5
SNP effect on MAEA

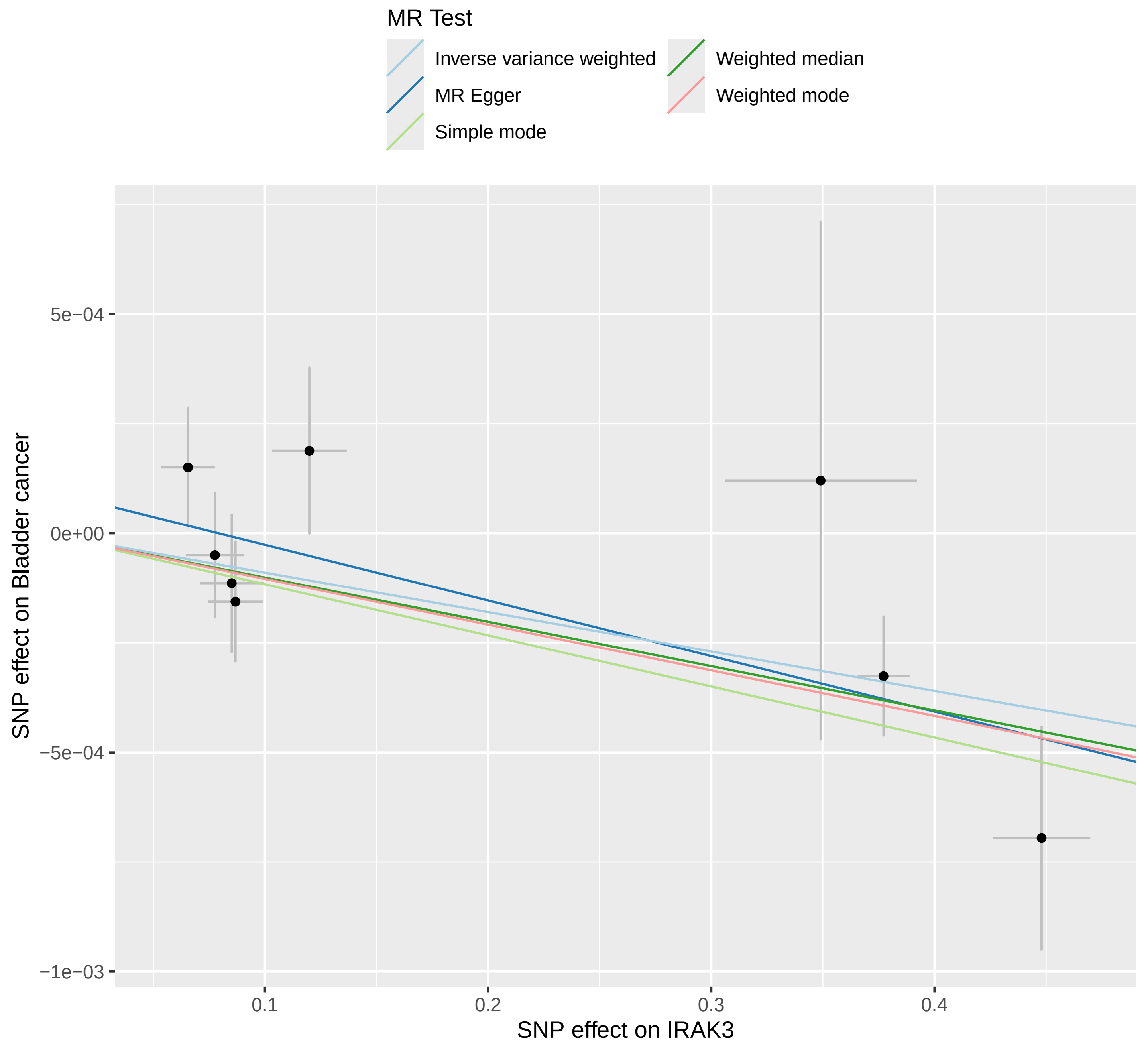
MR Test
Inverse variance weighted
MR Egger
Simple mode
Weighted median
Weighted mode
5e−04
0e+00
−5e−04
−1e−03
SNP effect on Bladder cancer
0.1
0.2
0.3
0.4
SNP effect on IRAK3

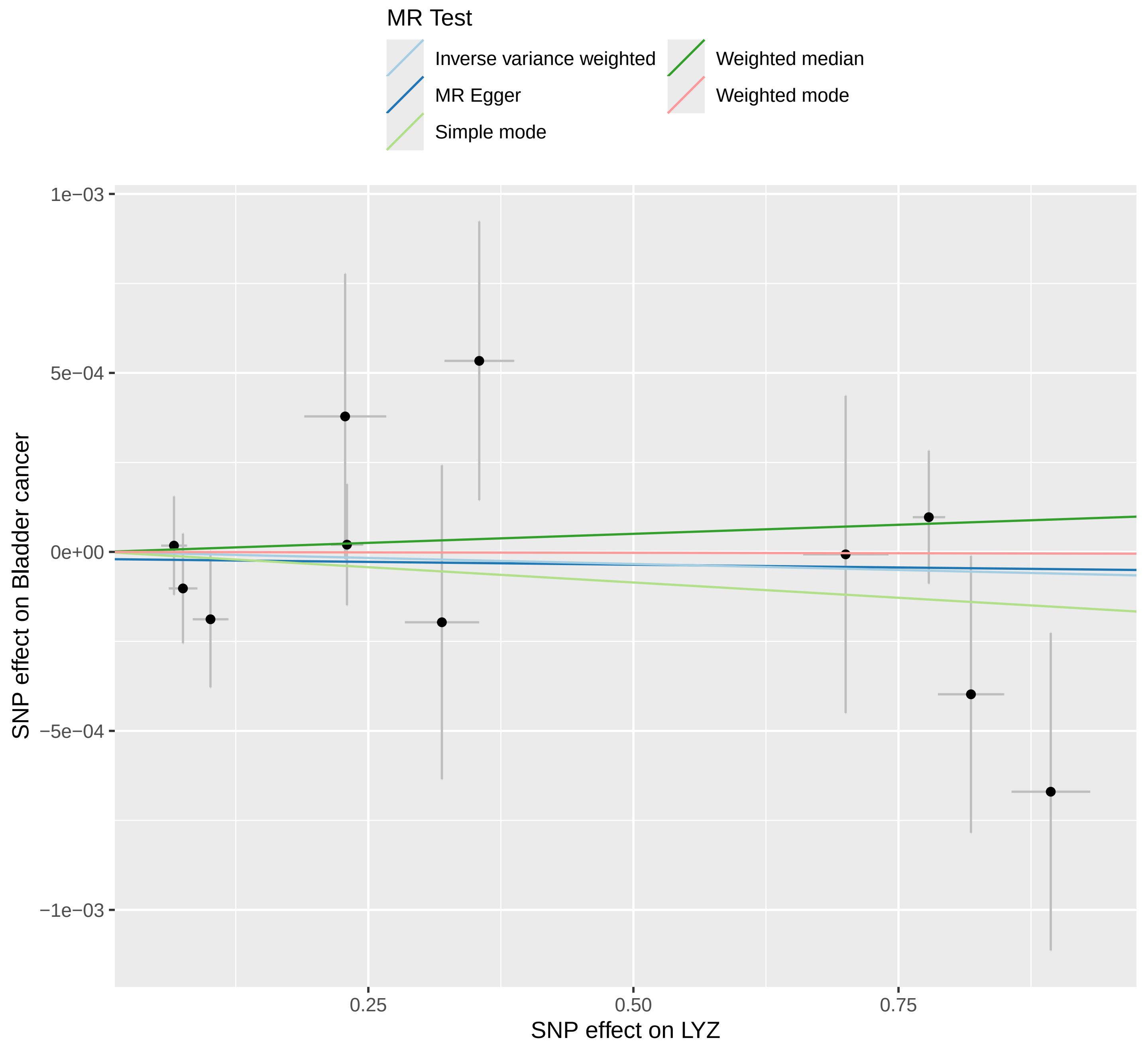

MR Test
Inverse variance weighted
MR Egger
Simple mode
Weighted median
Weighted mode
1e−03
5e−04
0e+00
−5e−04
−1e−03
SNP effect on Bladder cancer
0.25
0.50
0.75
SNP effect on LYZ

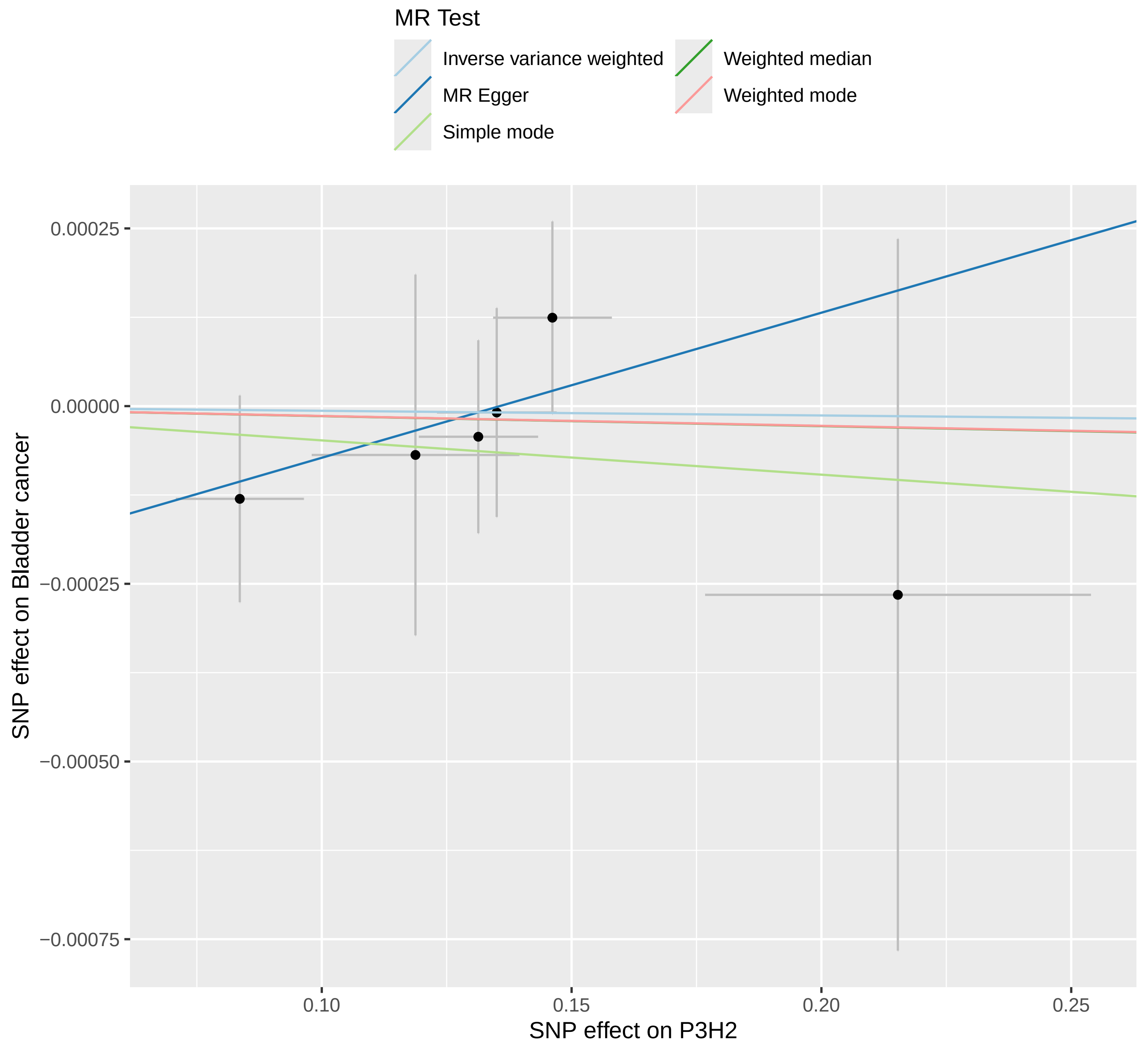

MR Test
Inverse variance weighted
MR Egger
Simple mode
Weighted median
Weighted mode
0.00025
0.00000
−0.00025
−0.00050
−0.00075
SNP effect on Bladder cancer
0.10
0.15
0.20
0.25
SNP effect on P3H2

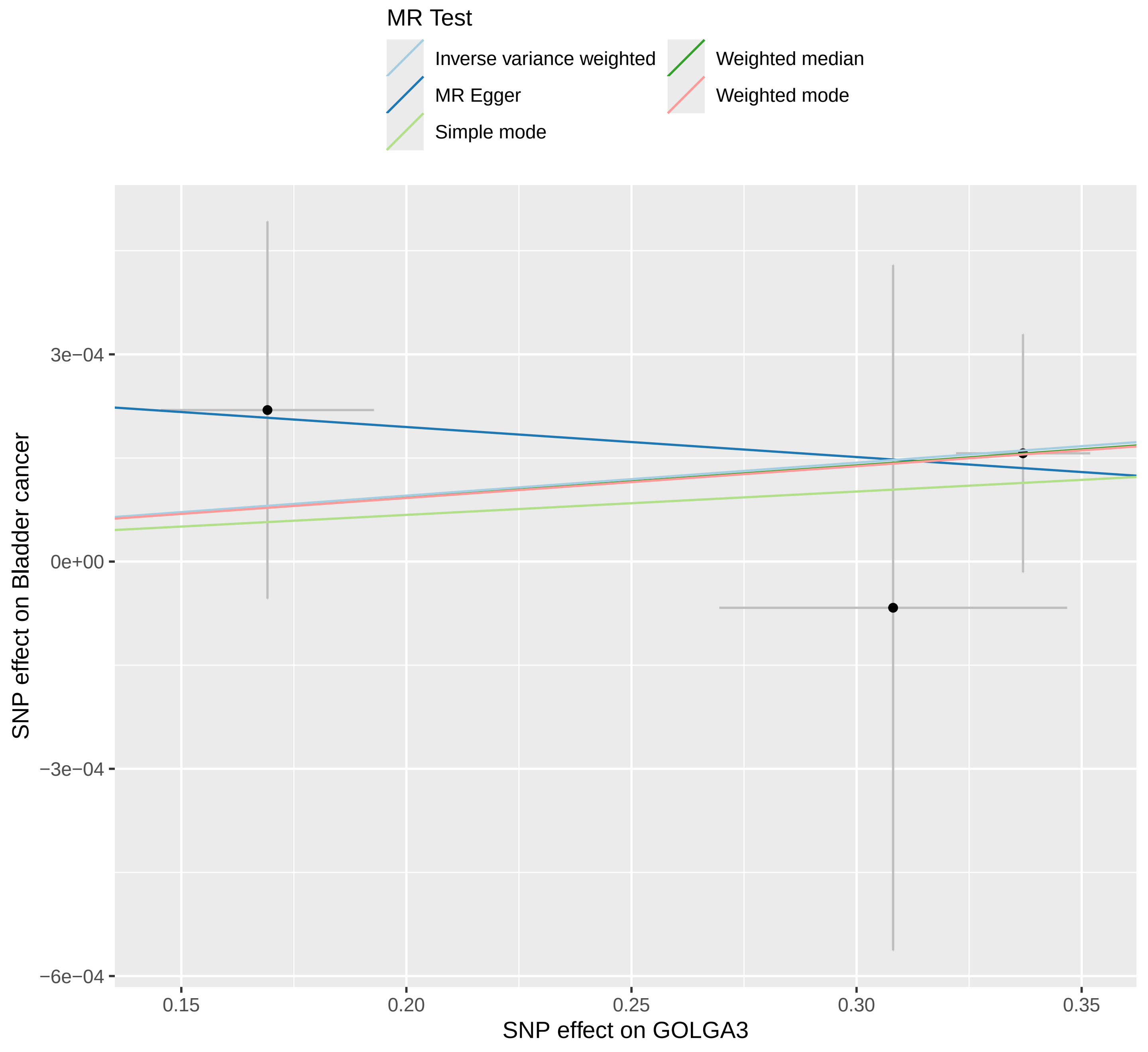
MR Test
Inverse variance weighted
MR Egger
Simple mode
Weighted median
Weighted mode
3e−04
0e+00
−3e−04
−6e−04
SNP effect on Bladder cancer
0.15
0.20
0.25
0.30
0.35
SNP effect on GOLGA3

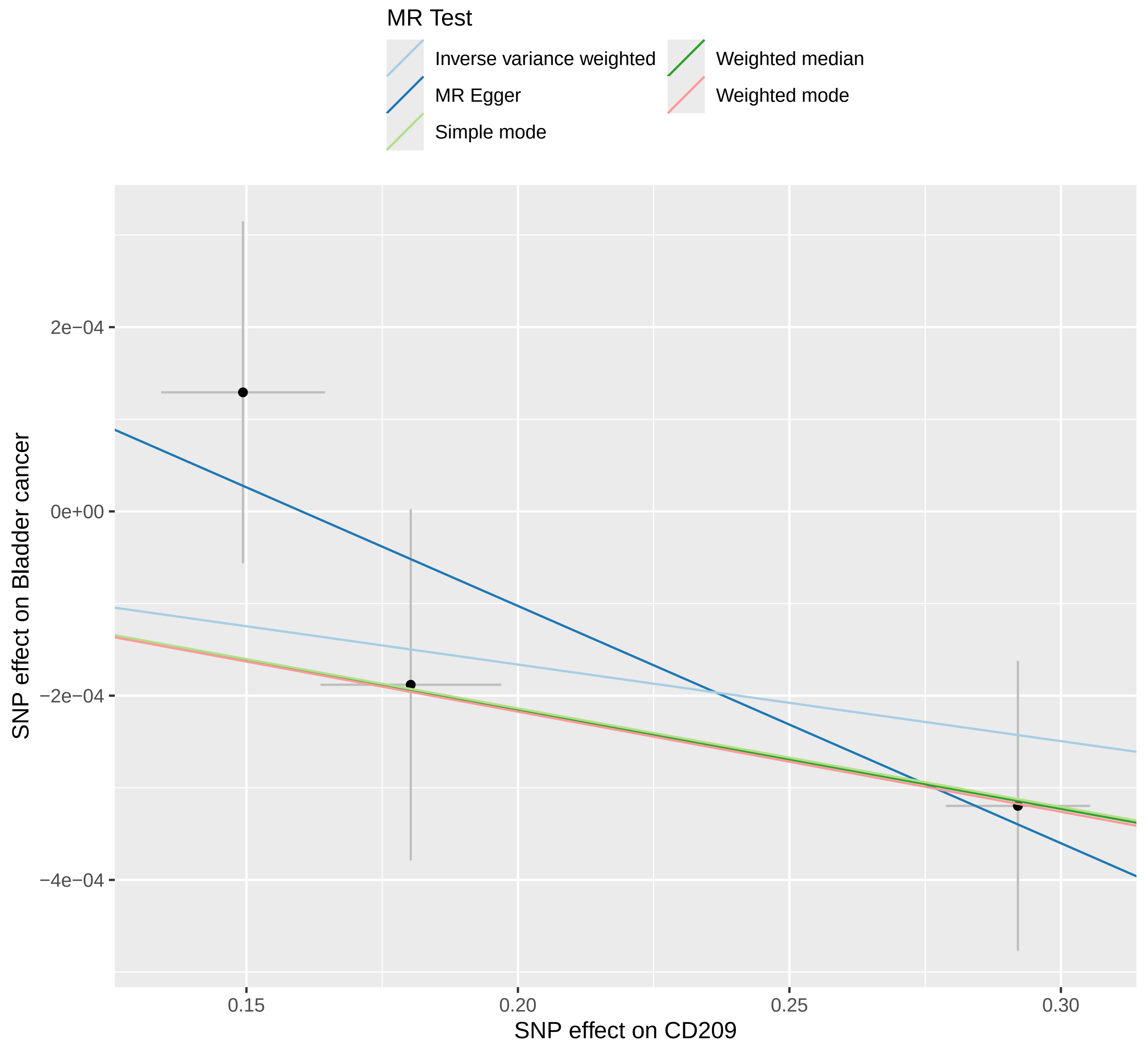
MR Test
Inverse variance weighted
MR Egger
Simple mode
Weighted median
Weighted mode
2e−04
0e+00
−2e−04
−4e−04
SNP effect on Bladder cancer
0.15
0.20
0.25
0.30
SNP effect on CD209

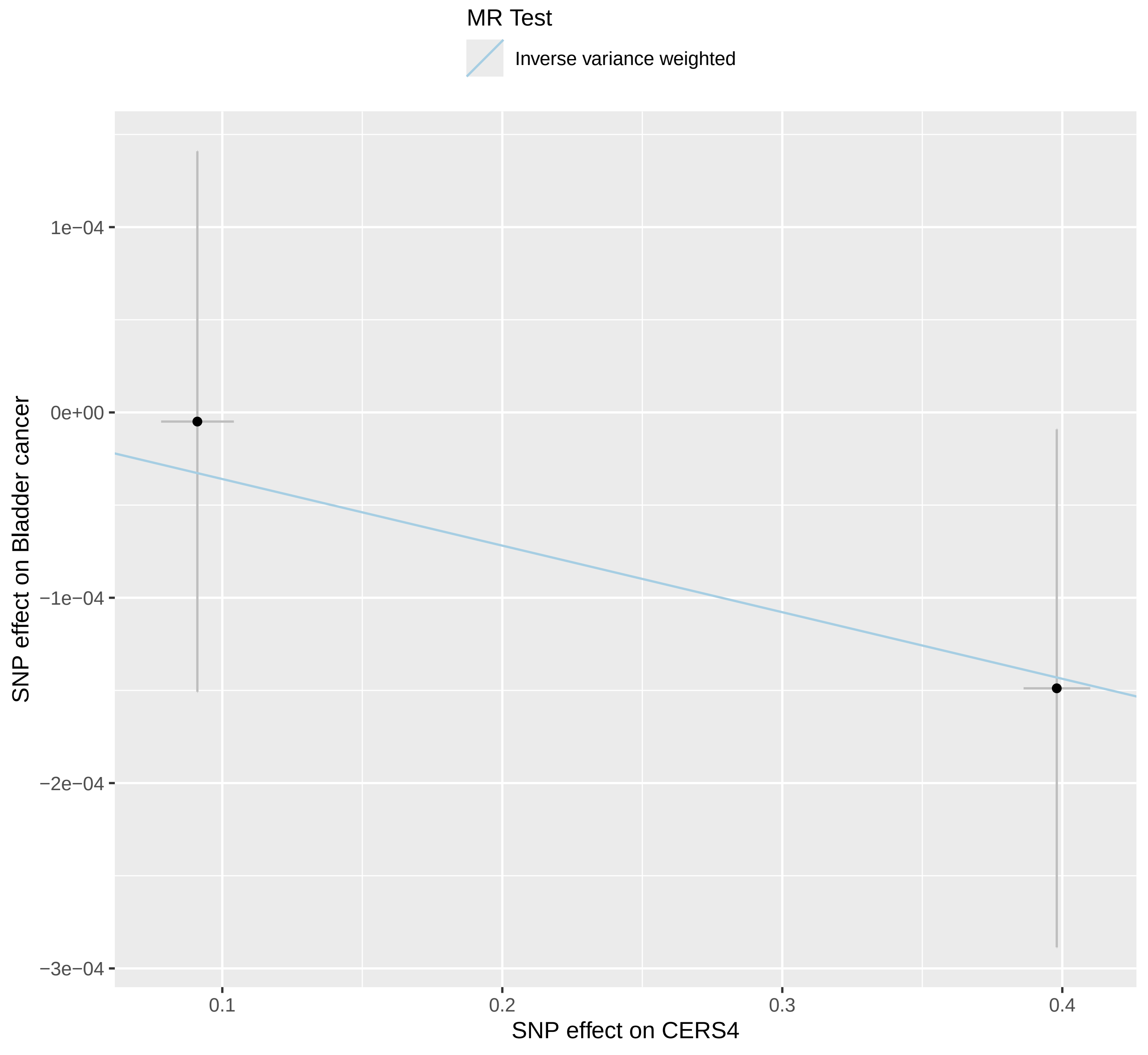

MR Test
Inverse variance weighted
1e−04
0e+00
−1e−04
−2e−04
−3e−04
0.1
0.2
0.3
0.4
SNP effect on CERS4
SNP effect on Bladder cancer

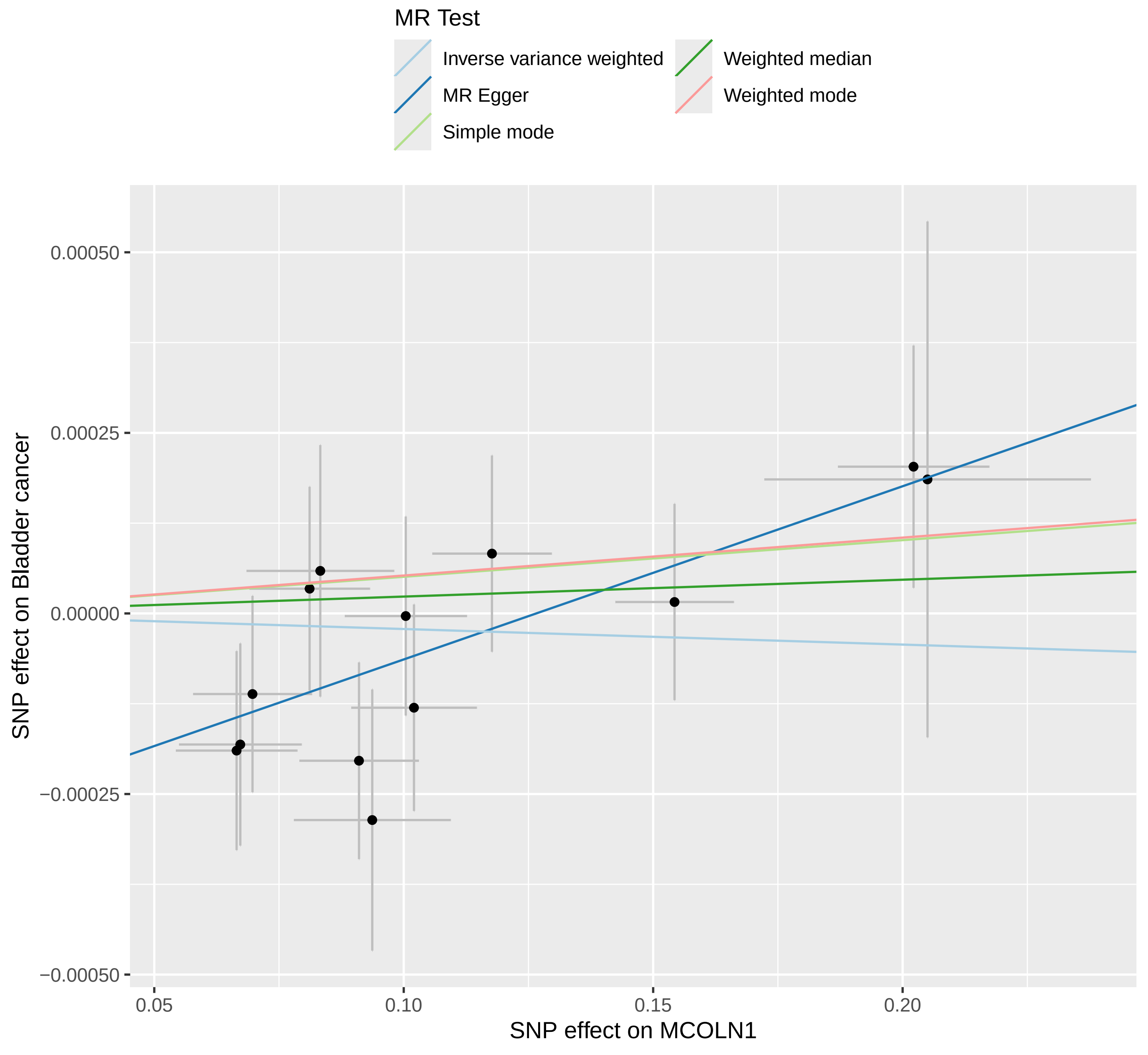

MR Test
Inverse variance weighted
MR Egger
Simple mode
Weighted median
Weighted mode
0.00050
0.00025
0.00000
−0.00025
−0.00050
0.05
0.10
0.15
0.20
SNP effect on Bladder cancer
SNP effect on MCOLN1

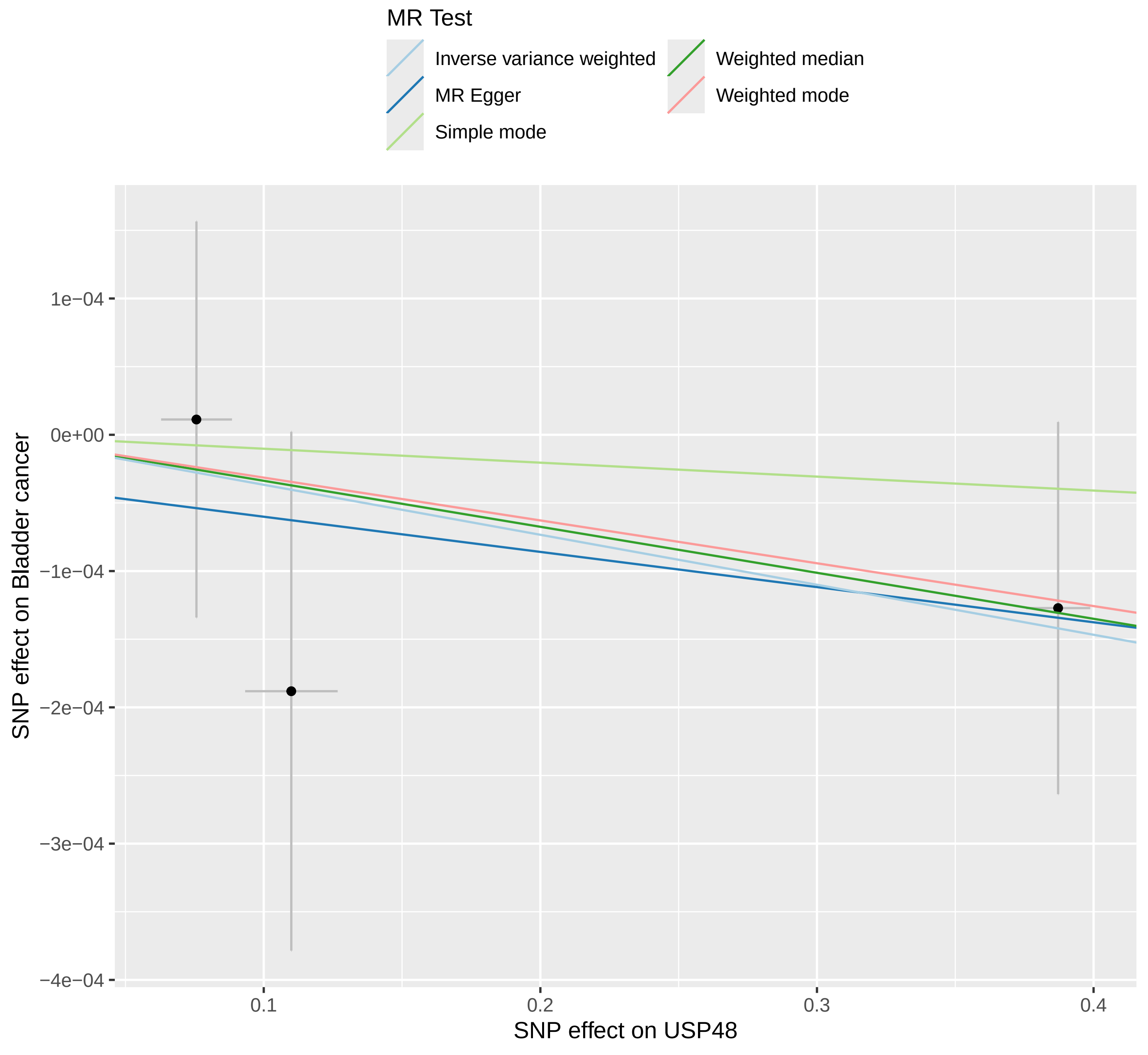

MR Test
Inverse variance weighted
MR Egger
Simple mode
Weighted median
Weighted mode
1e−04
0e+00
−1e−04
−2e−04
−3e−04
−4e−04
SNP effect on Bladder cancer
0.1
0.2
0.3
0.4
SNP effect on USP48

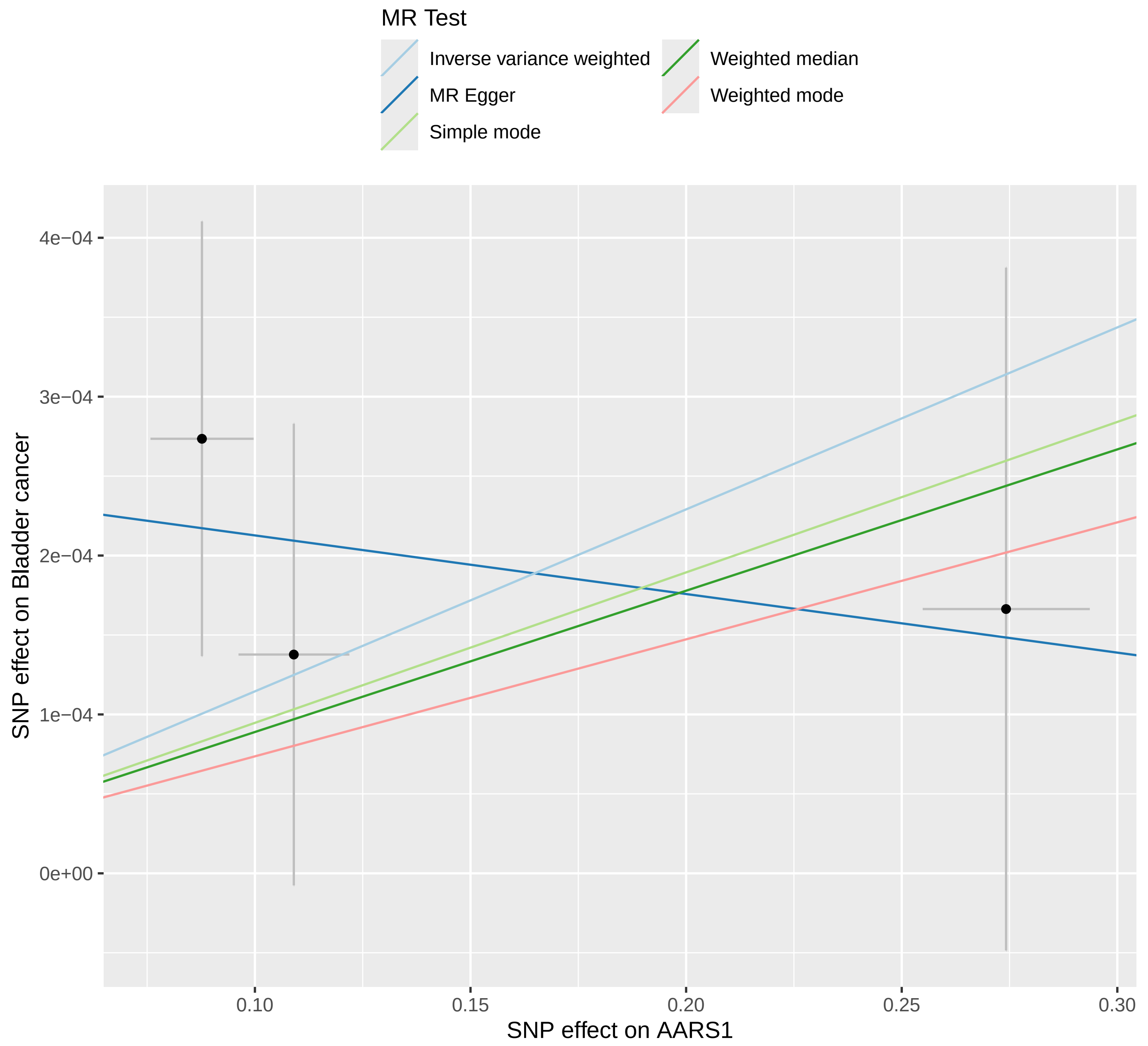

MR Test
Inverse variance weighted
MR Egger
Simple mode
Weighted median
Weighted mode
4e−04
3e−04
2e−04
1e−04
0e+00
0.10
0.15
0.20
0.25
0.30
SNP effect on AARS1
SNP effect on Bladder cancer

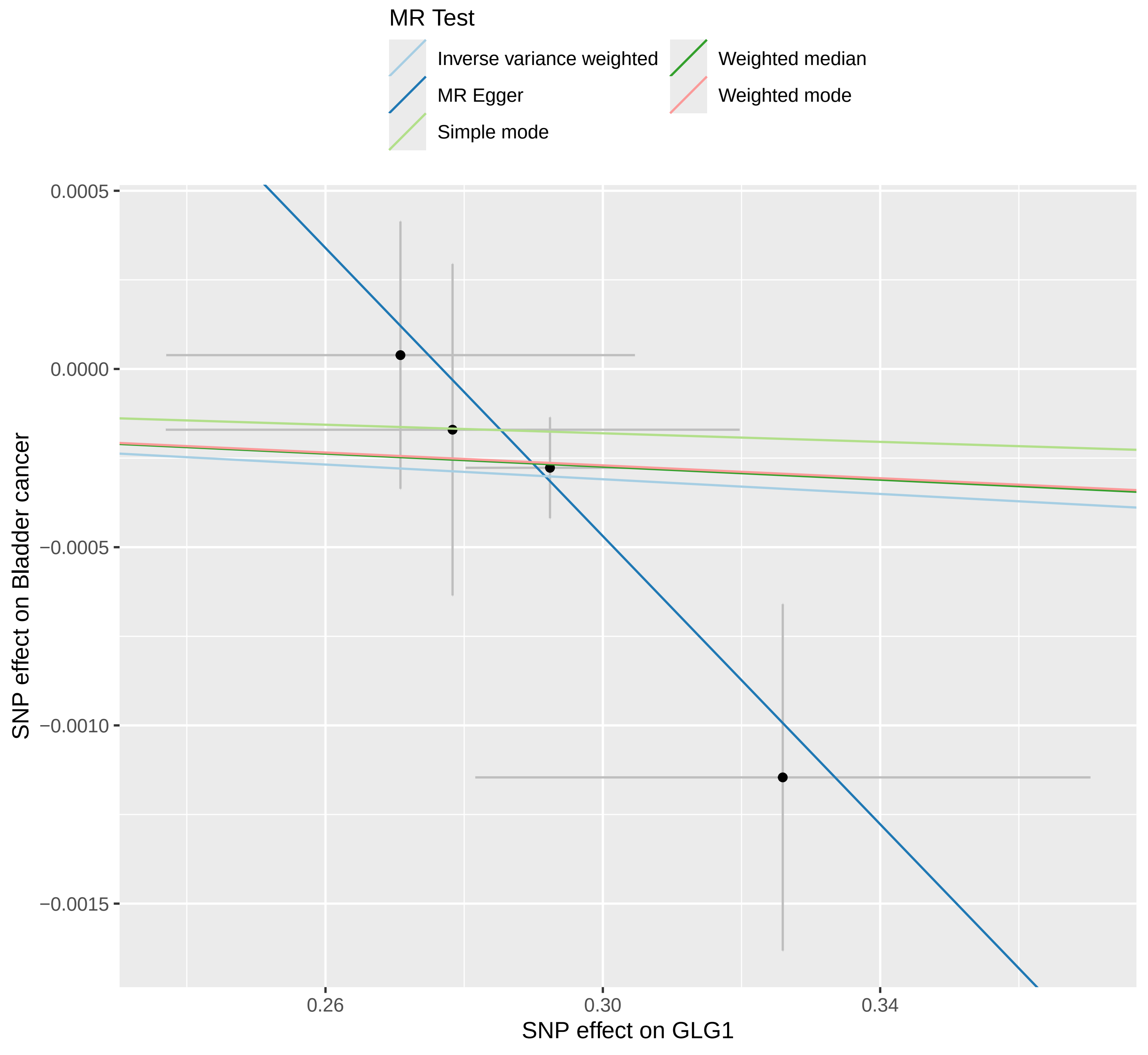
MR Test
Inverse variance weighted
MR Egger
Simple mode
Weighted median
Weighted mode
0.0005
0.0000
−0.0005
−0.0010
−0.0015
SNP effect on Bladder cancer
0.26
0.30
0.34
SNP effect on GLG1

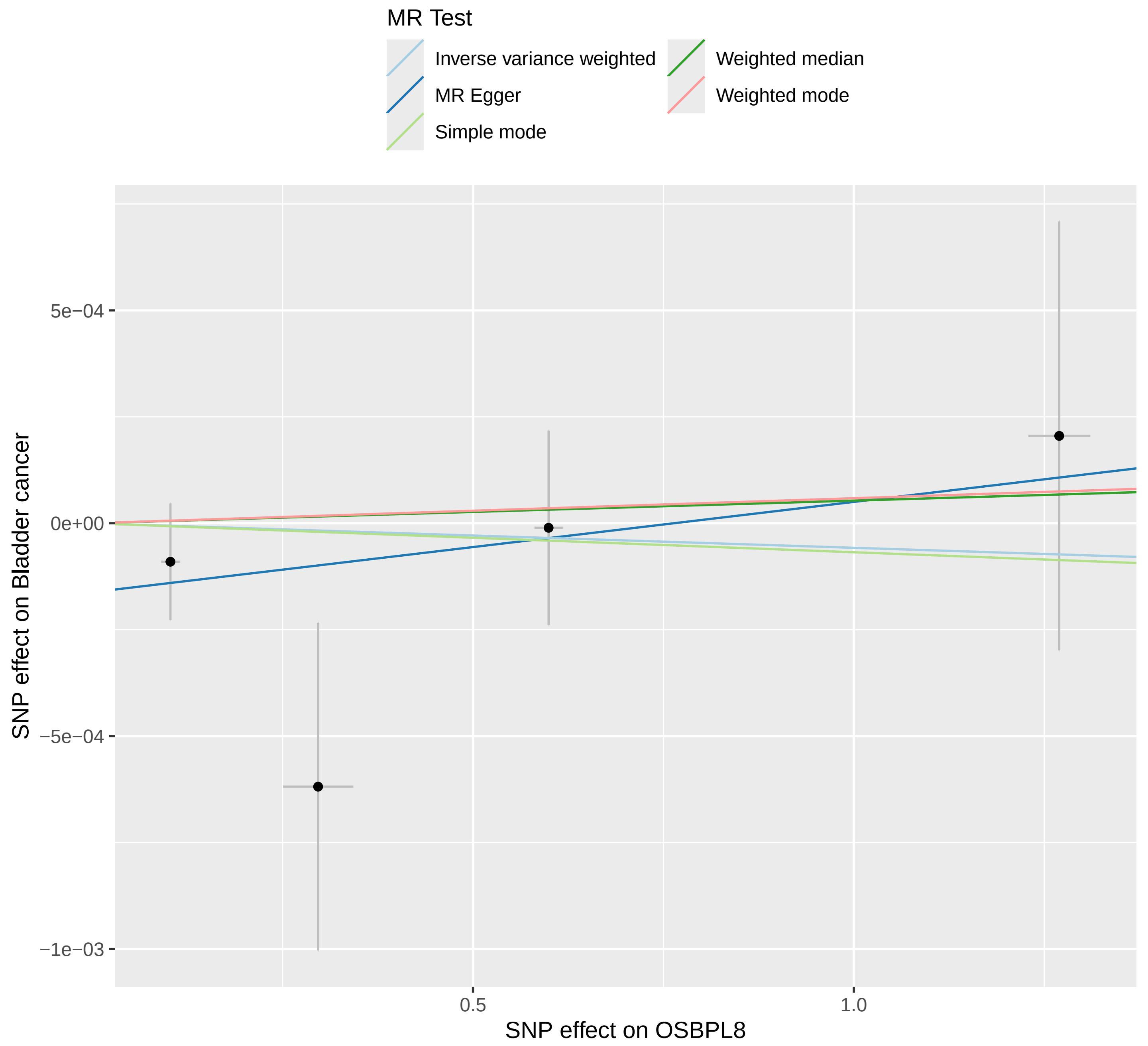
MR Test
Inverse variance weighted
MR Egger
Simple mode
Weighted median
Weighted mode
5e−04
0e+00
−5e−04
−1e−03
SNP effect on Bladder cancer
0.5
1.0
SNP effect on OSBPL8

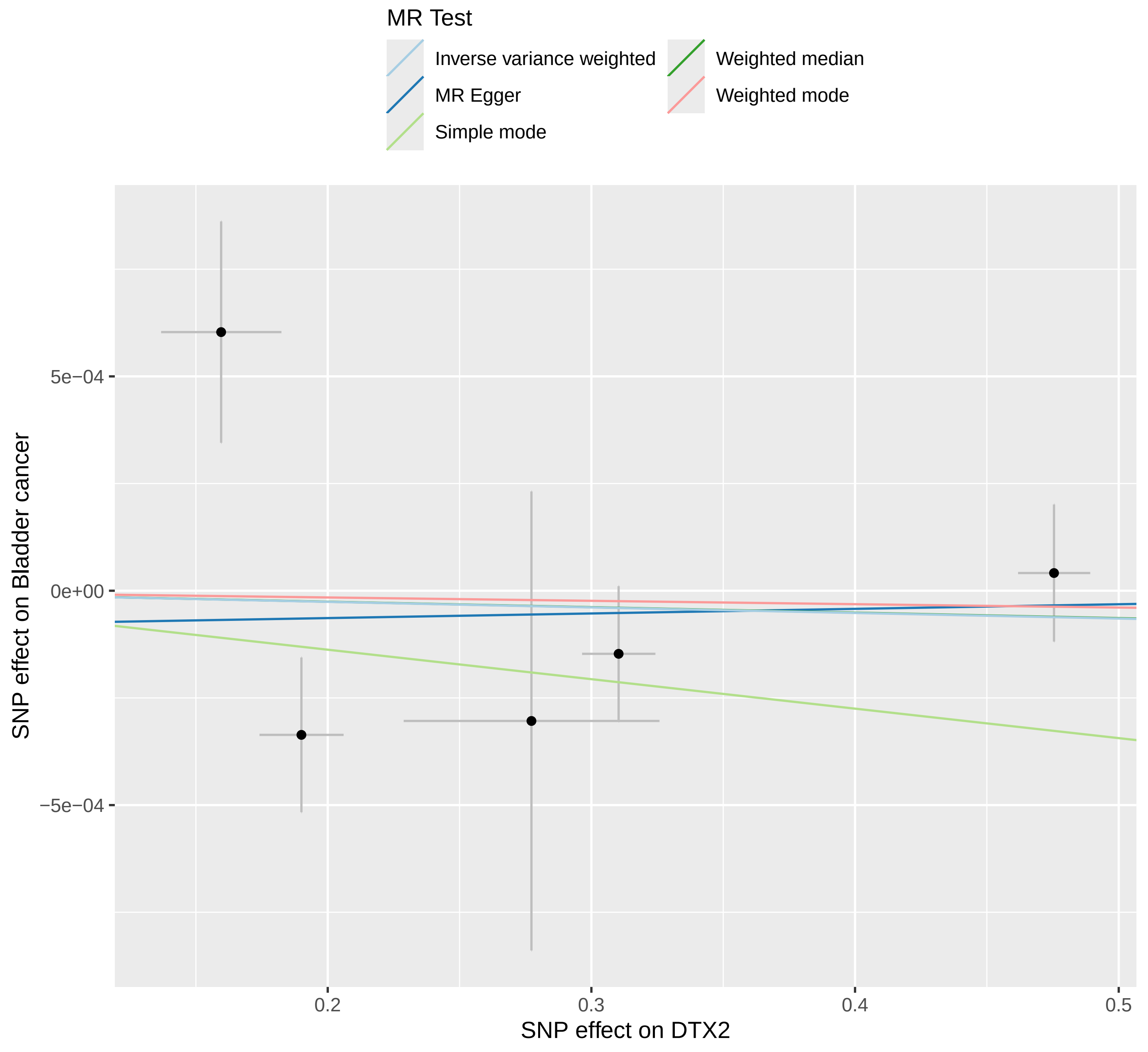
MR Test
Inverse variance weighted
MR Egger
Simple mode
Weighted median
Weighted mode
5e−04
0e+00
−5e−04
0.2
0.3
0.4
0.5
SNP effect on Bladder cancer
SNP effect on DTX2

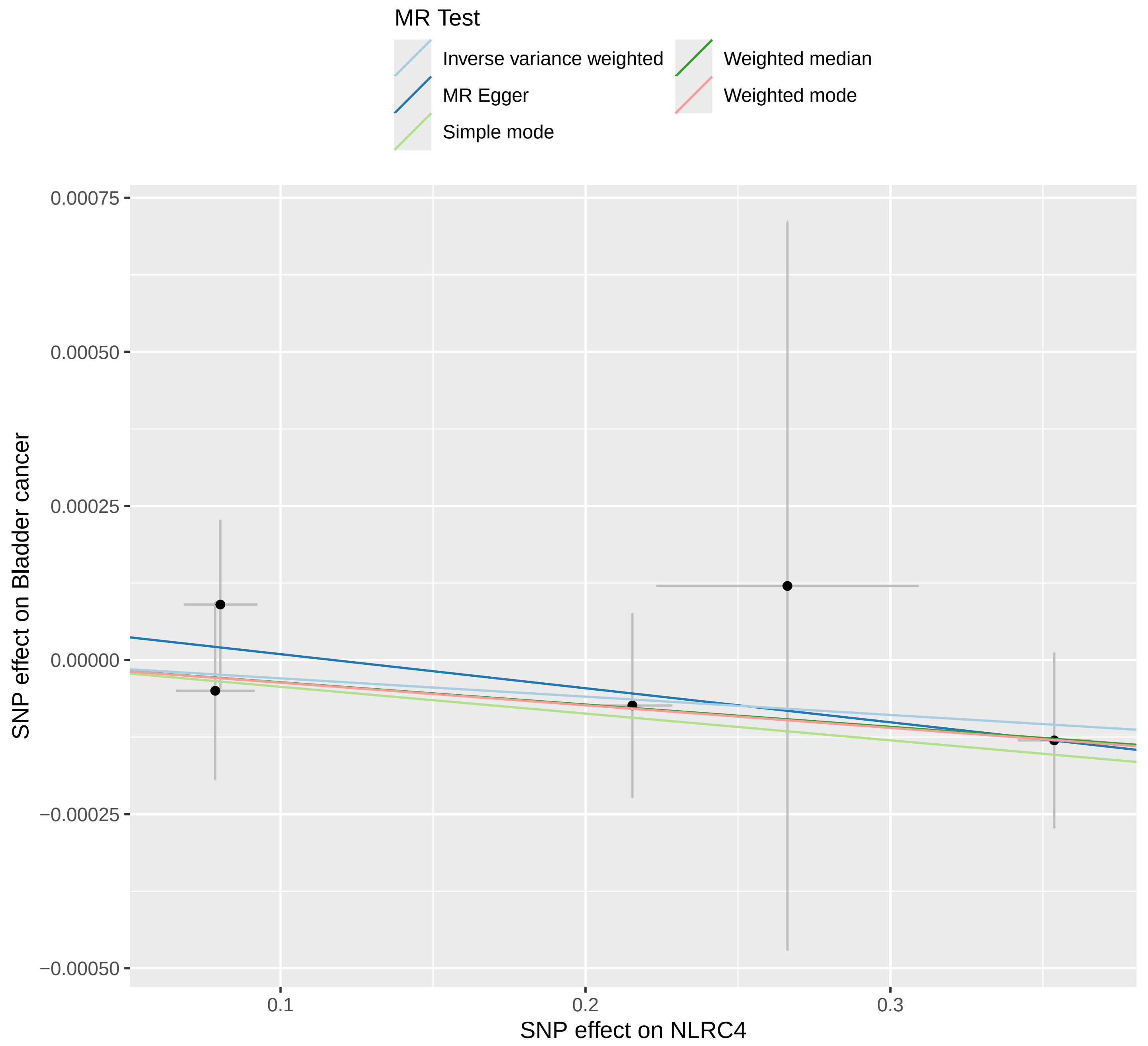
MR Test
Inverse variance weighted
MR Egger
Simple mode
Weighted median
Weighted mode
0.00075
0.00050
0.00025
0.00000
−0.00025
−0.00050
SNP effect on Bladder cancer
0.1
0.2
0.3
SNP effect on NLRC4

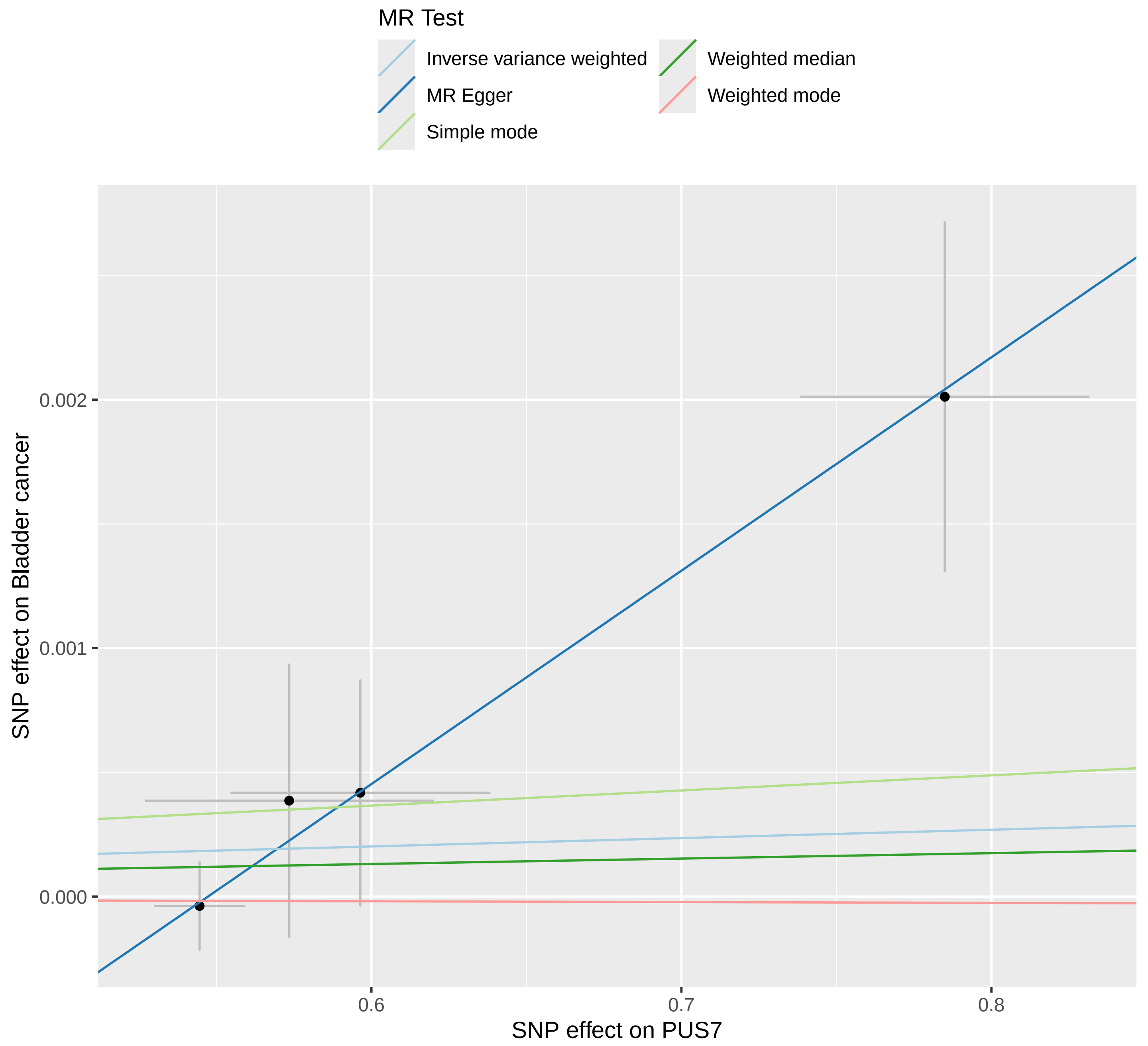
MR Test
Inverse variance weighted
MR Egger
Simple mode
Weighted median
Weighted mode
0.002
0.001
0.000
SNP effect on Bladder cancer
0.6
0.7
0.8
SNP effect on PUS7

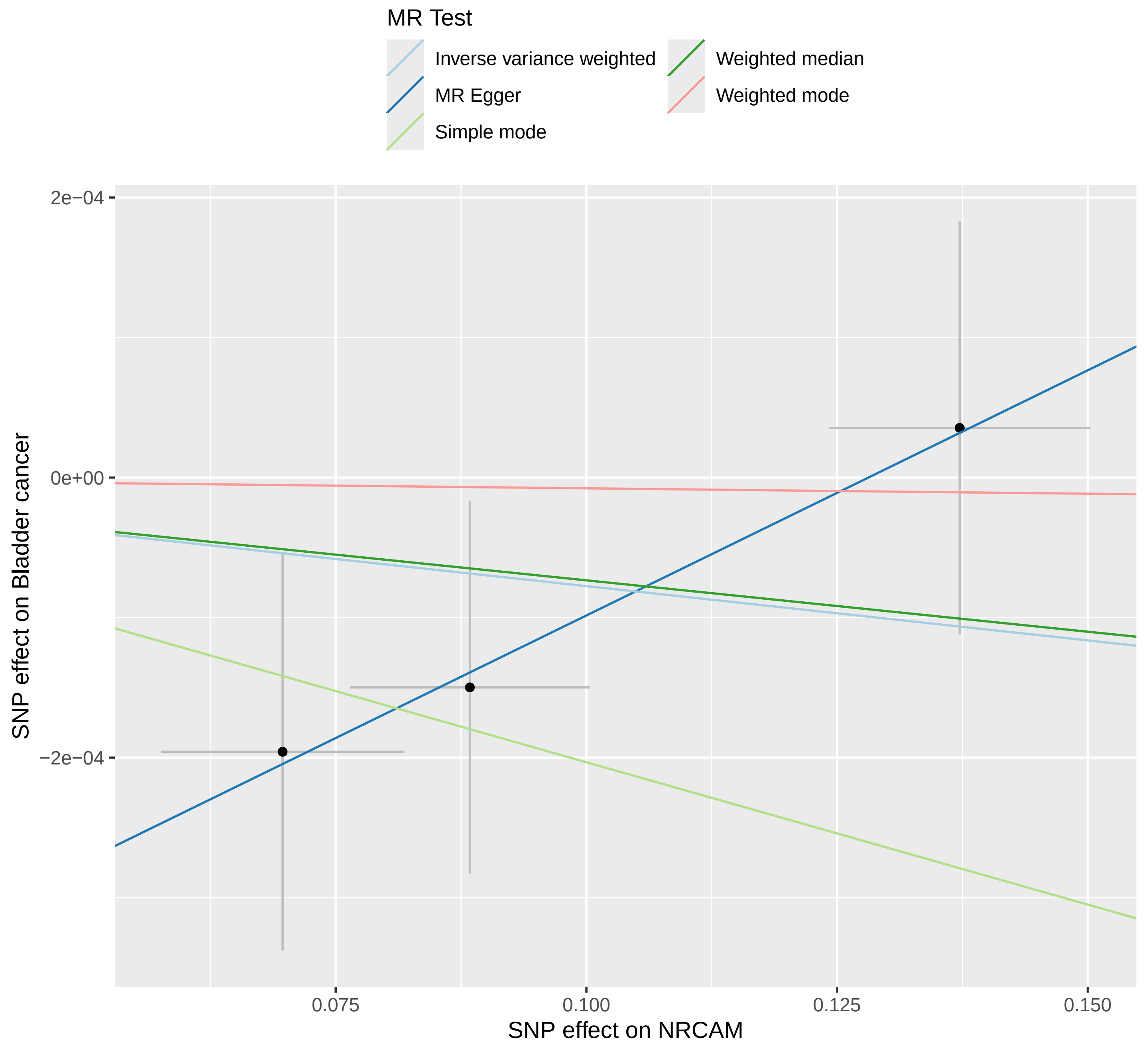
MR Test
Inverse variance weighted
MR Egger
Simple mode
Weighted median
Weighted mode
2e−04
0e+00
−2e−04
0.075
0.100
0.125
0.150
SNP effect on Bladder cancer
SNP effect on NRCAM

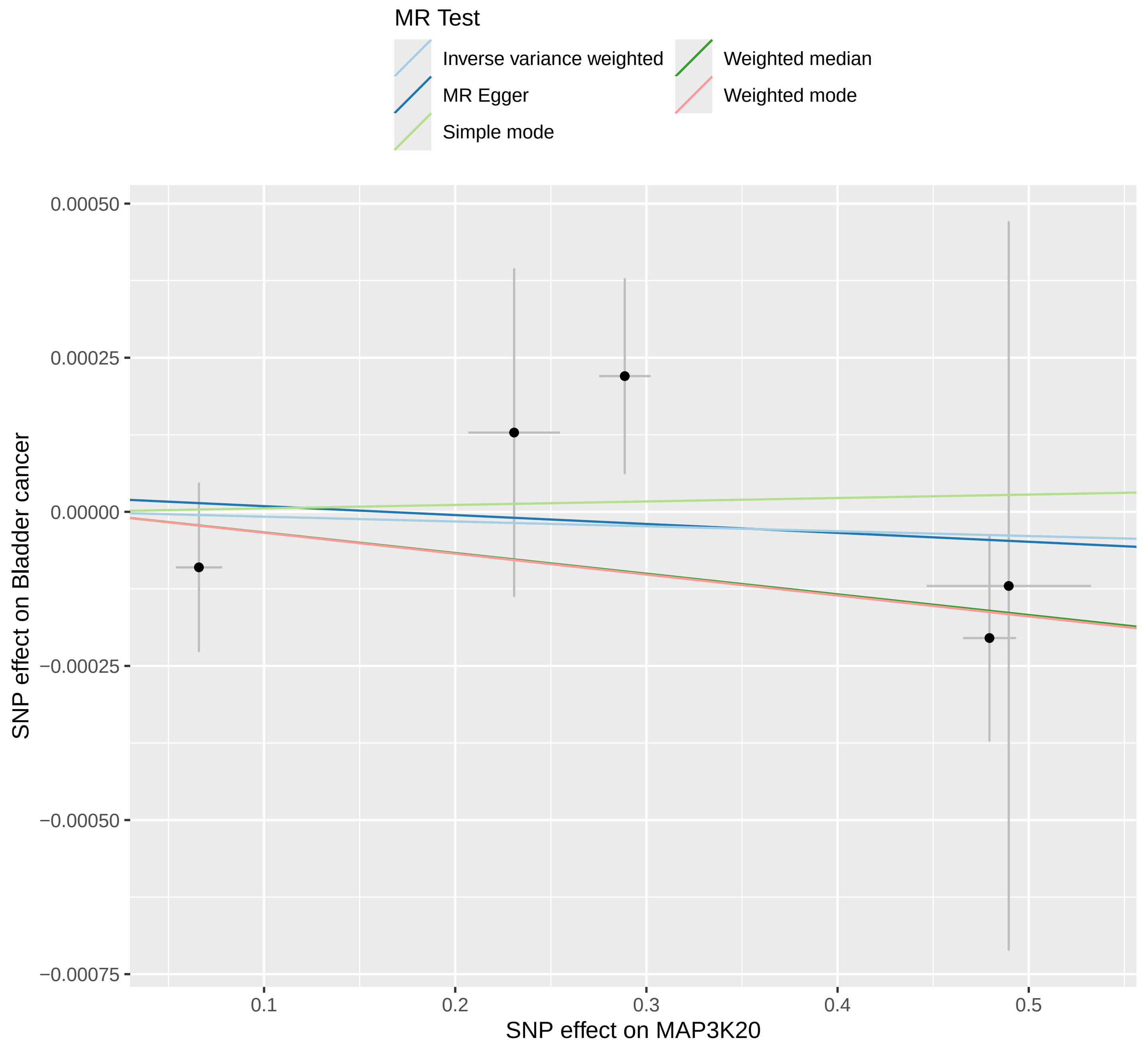

MR Test
Inverse variance weighted
MR Egger
Simple mode
Weighted median
Weighted mode
0.00050
0.00025
0.00000
−0.00025
−0.00050
−0.00075
SNP effect on Bladder cancer
0.1
0.2
0.3
0.4
0.5
SNP effect on MAP3K20

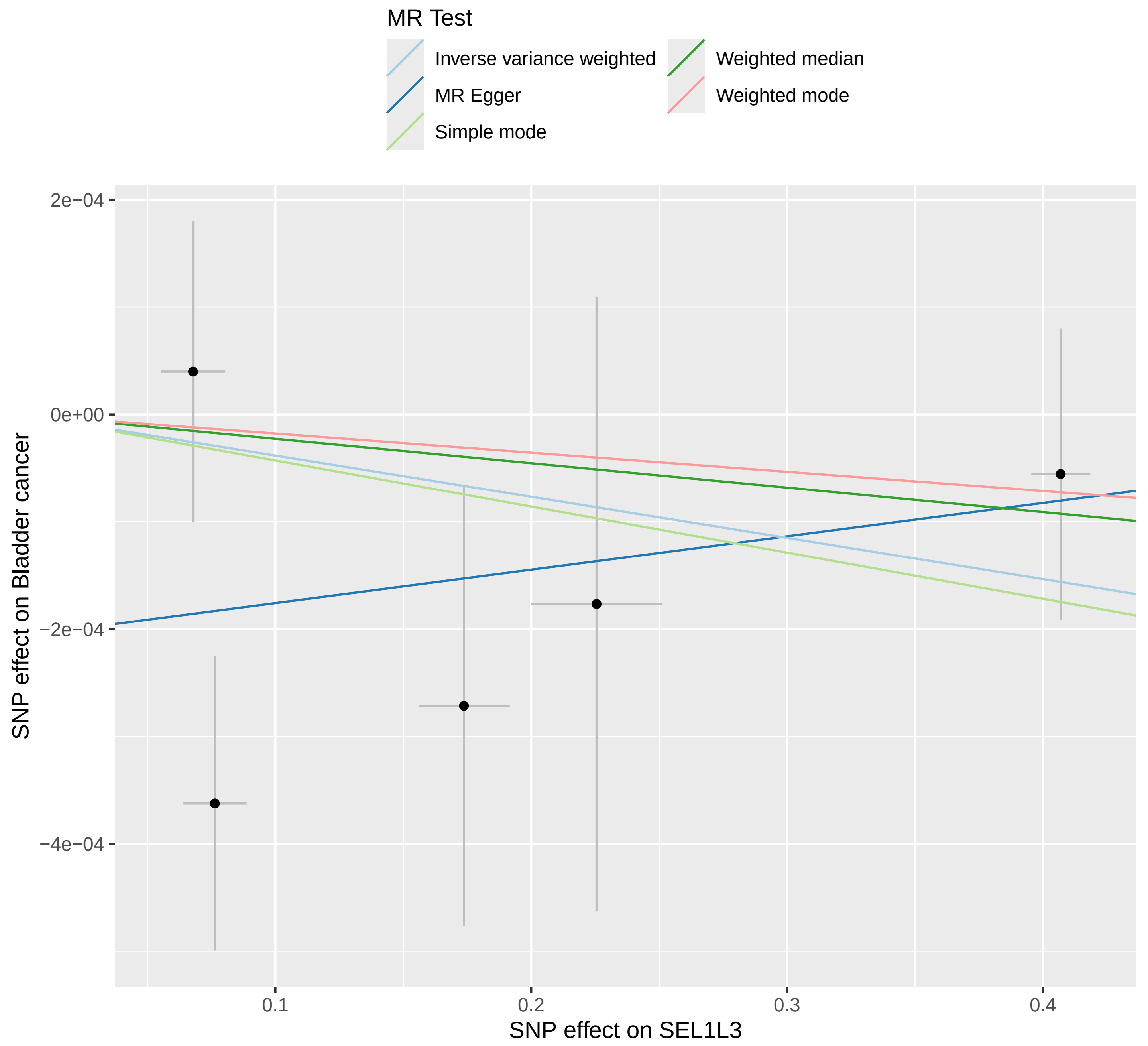

MR Test
Inverse variance weighted
MR Egger
Simple mode
Weighted median
Weighted mode
2e−04
0e+00
−2e−04
−4e−04
SNP effect on Bladder cancer
0.1
0.2
0.3
0.4
SNP effect on SEL1L3

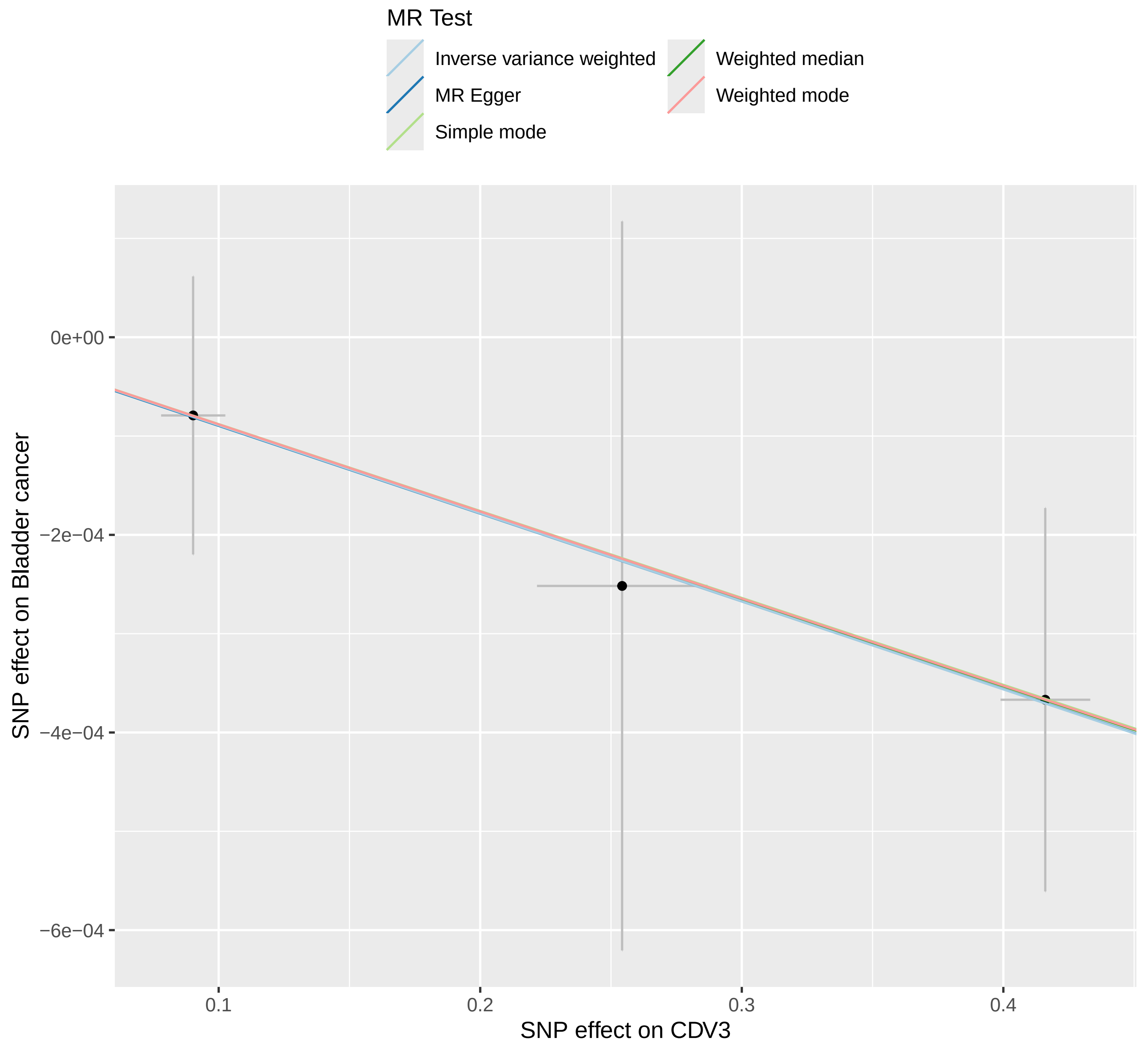
MR Test
Inverse variance weighted
MR Egger
Simple mode
Weighted median
Weighted mode
0e+00
−2e−04
−4e−04
−6e−04
0.1
0.2
0.3
0.4
SNP effect on Bladder cancer
SNP effect on CDV3

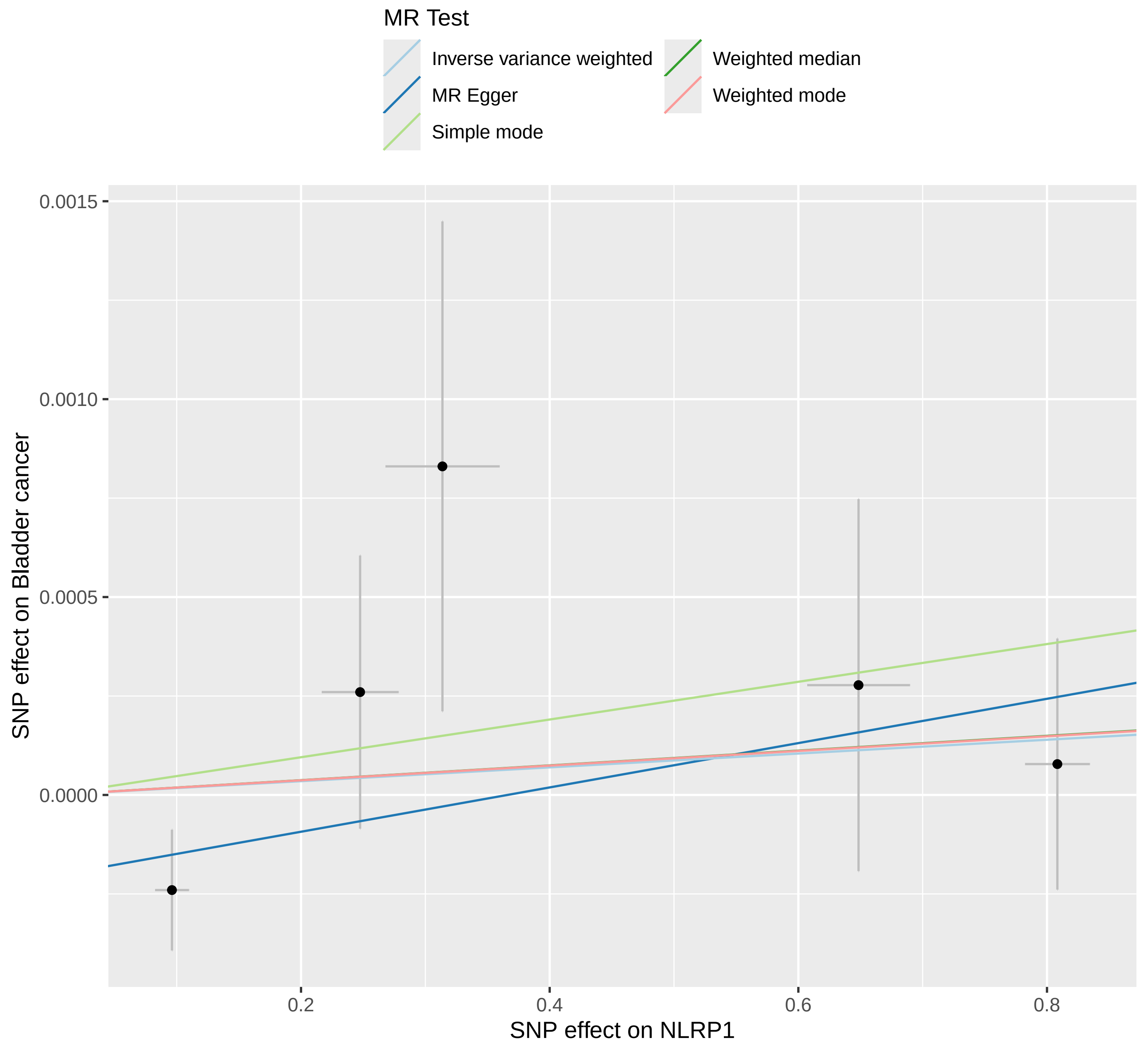

MR Test
Inverse variance weighted
MR Egger
Simple mode
Weighted median
Weighted mode
0.0015
0.0010
0.0005
0.0000
SNP effect on Bladder cancer
0.2
0.4
0.6
0.8
SNP effect on NLRP1

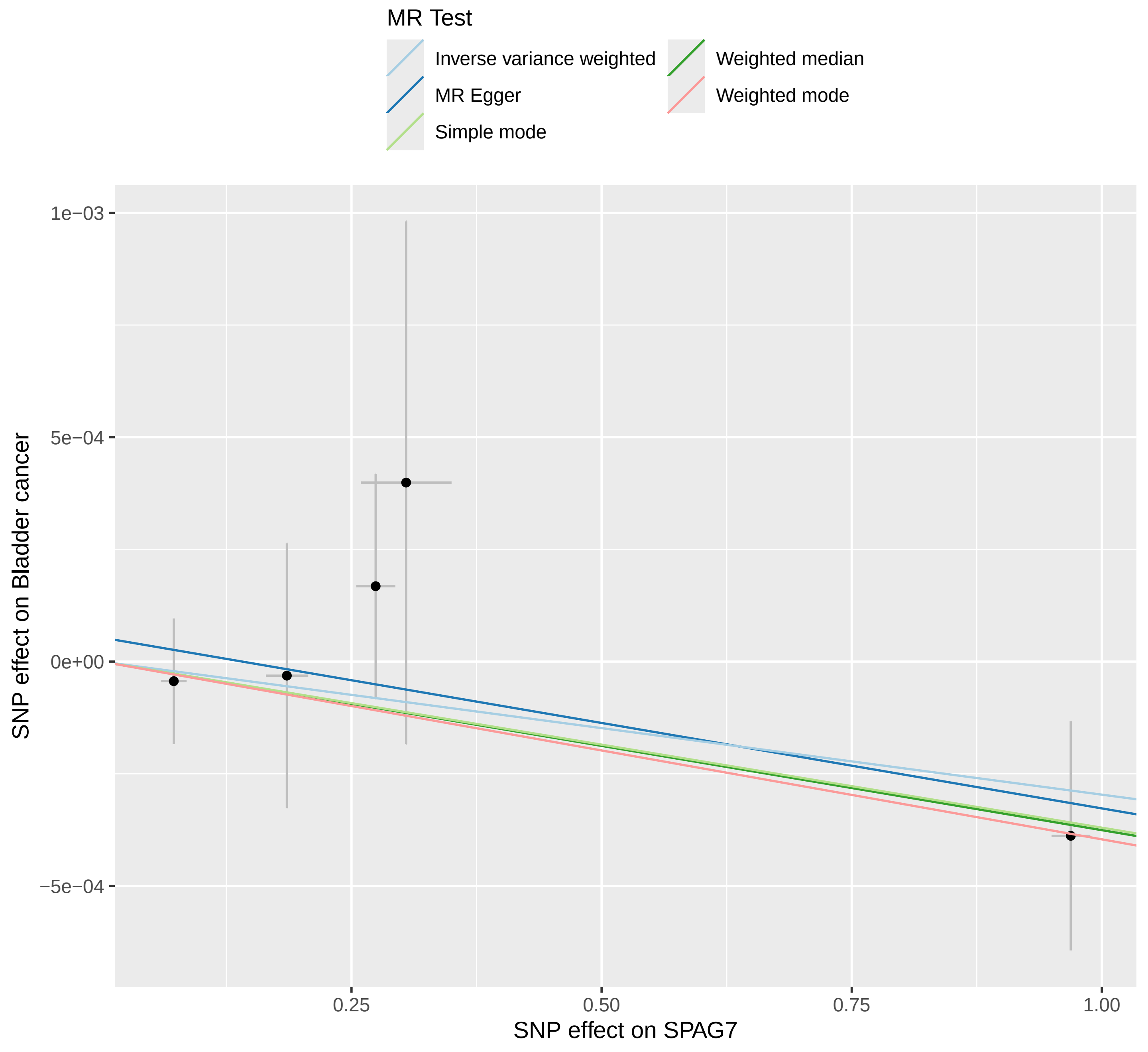
MR Test
Inverse variance weighted
MR Egger
Simple mode
Weighted median
Weighted mode
1e−03
5e−04
0e+00
−5e−04
SNP effect on Bladder cancer
0.25
0.50
0.75
1.00
SNP effect on SPAG7

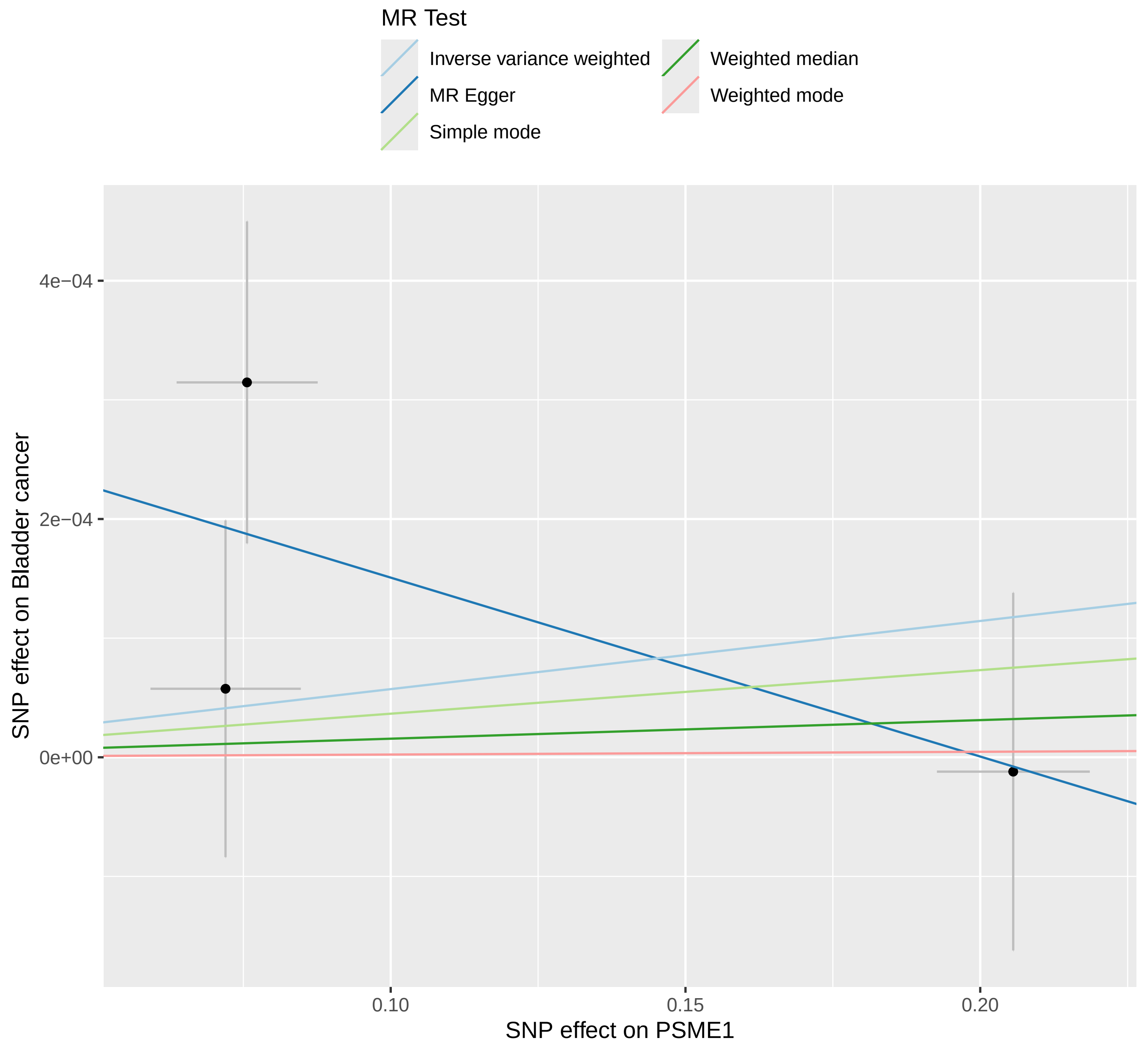

MR Test
Inverse variance weighted
MR Egger
Simple mode
Weighted median
Weighted mode
4e−04
2e−04
0e+00
SNP effect on Bladder cancer
0.10
0.15
0.20
SNP effect on PSME1

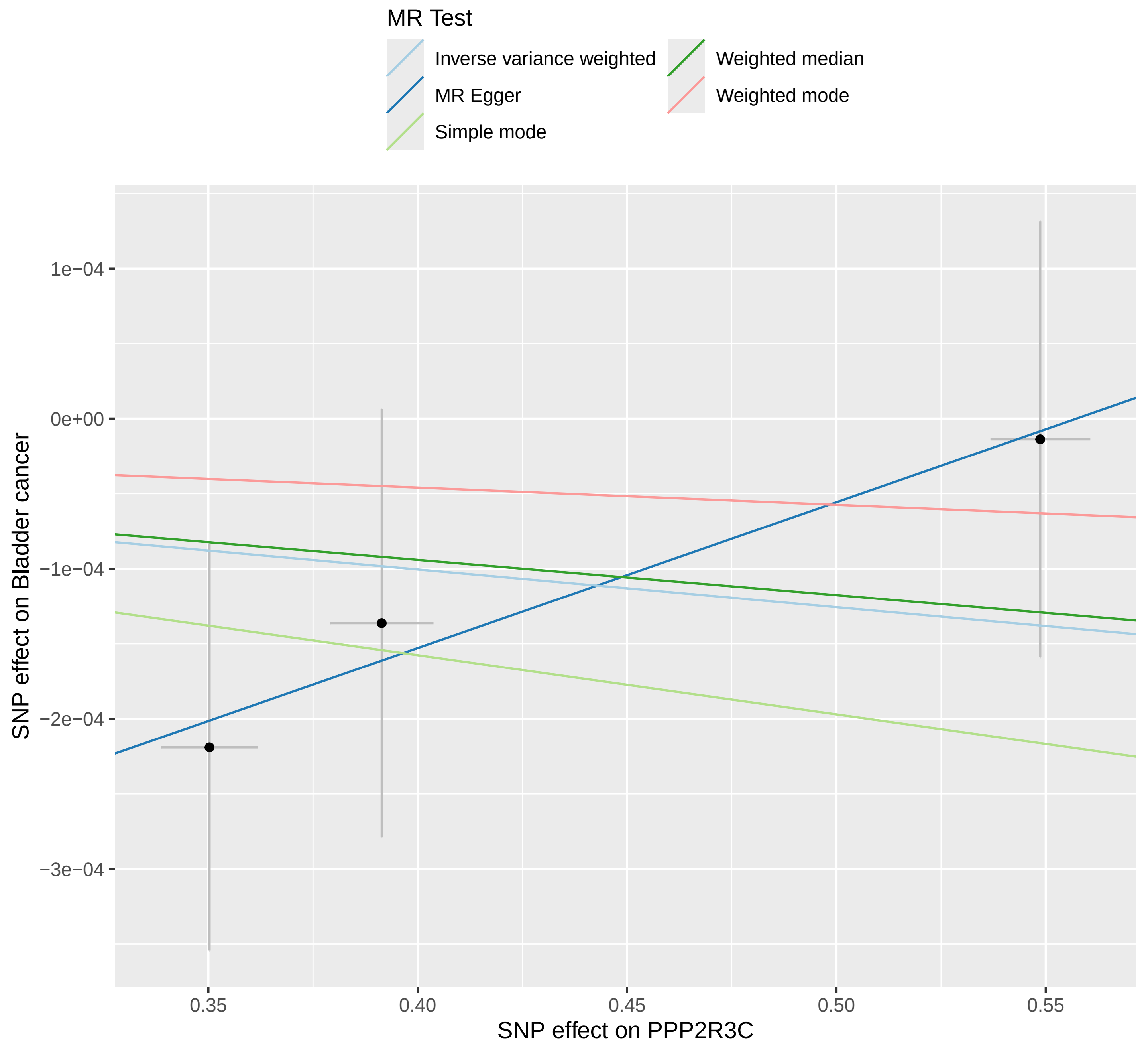

MR Test
Inverse variance weighted
MR Egger
Simple mode
Weighted median
Weighted mode
1e−04
0e+00
−1e−04
−2e−04
−3e−04
SNP effect on Bladder cancer
0.35
0.40
0.45
0.50
0.55
SNP effect on PPP2R3C

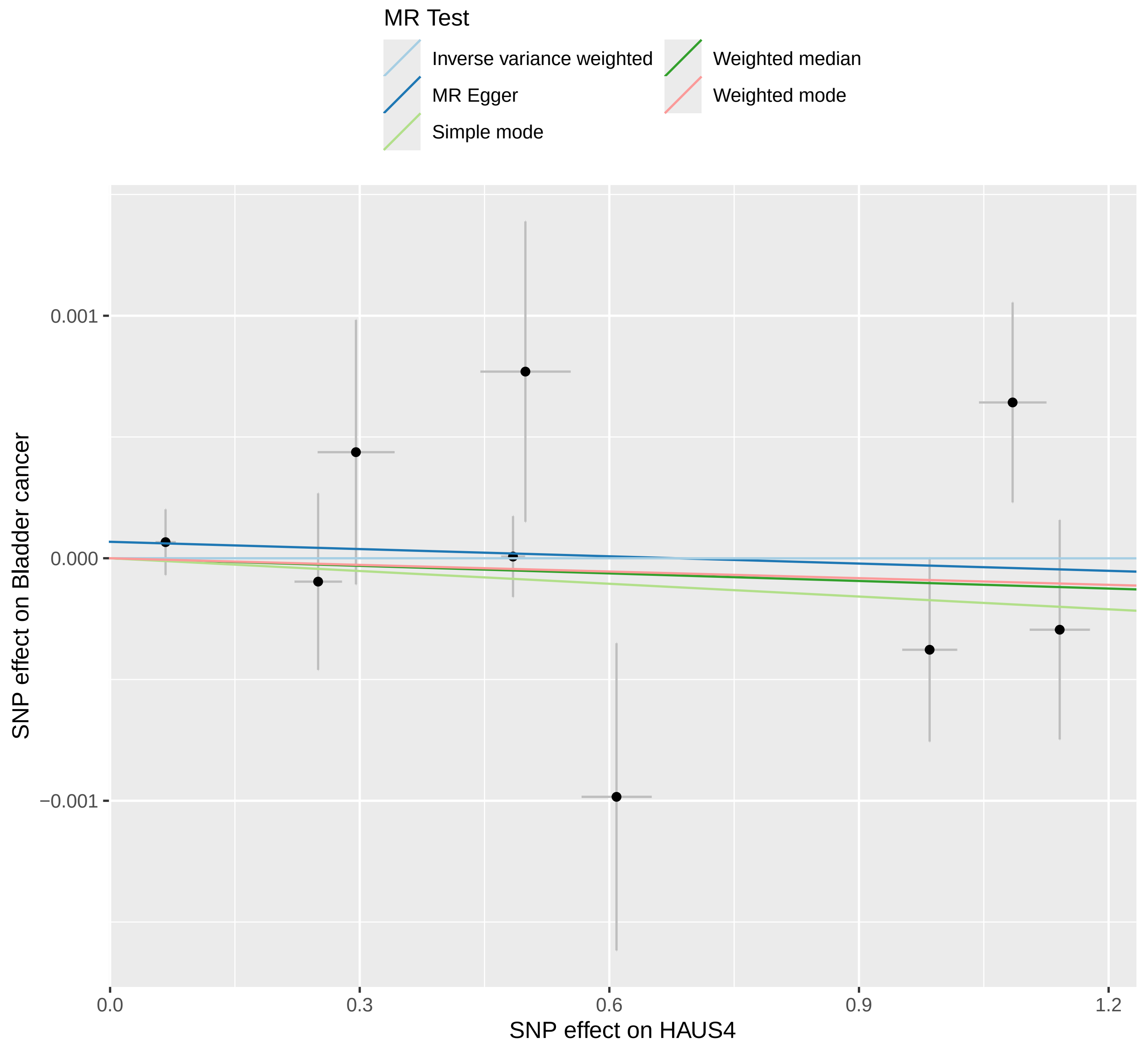

MR Test
Inverse variance weighted
MR Egger
Simple mode
Weighted median
Weighted mode
0.001
0.000
−0.001
0.0
0.3
0.6
0.9
1.2
SNP effect on Bladder cancer
SNP effect on HAUS4

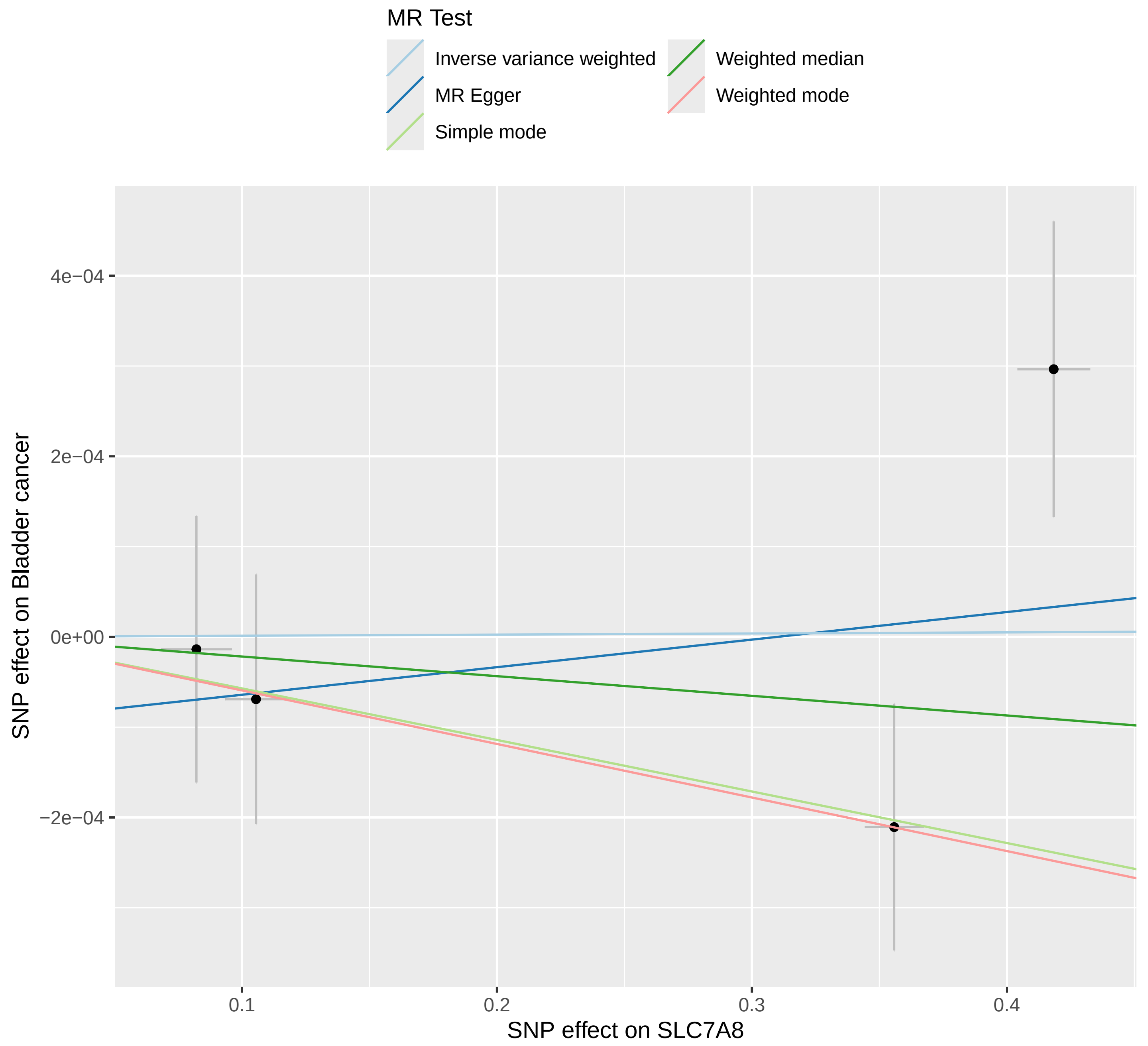
MR Test
Inverse variance weighted
MR Egger
Simple mode
Weighted median
Weighted mode
4e−04
2e−04
0e+00
−2e−04
SNP effect on Bladder cancer
0.1
0.2
0.3
0.4
SNP effect on SLC7A8

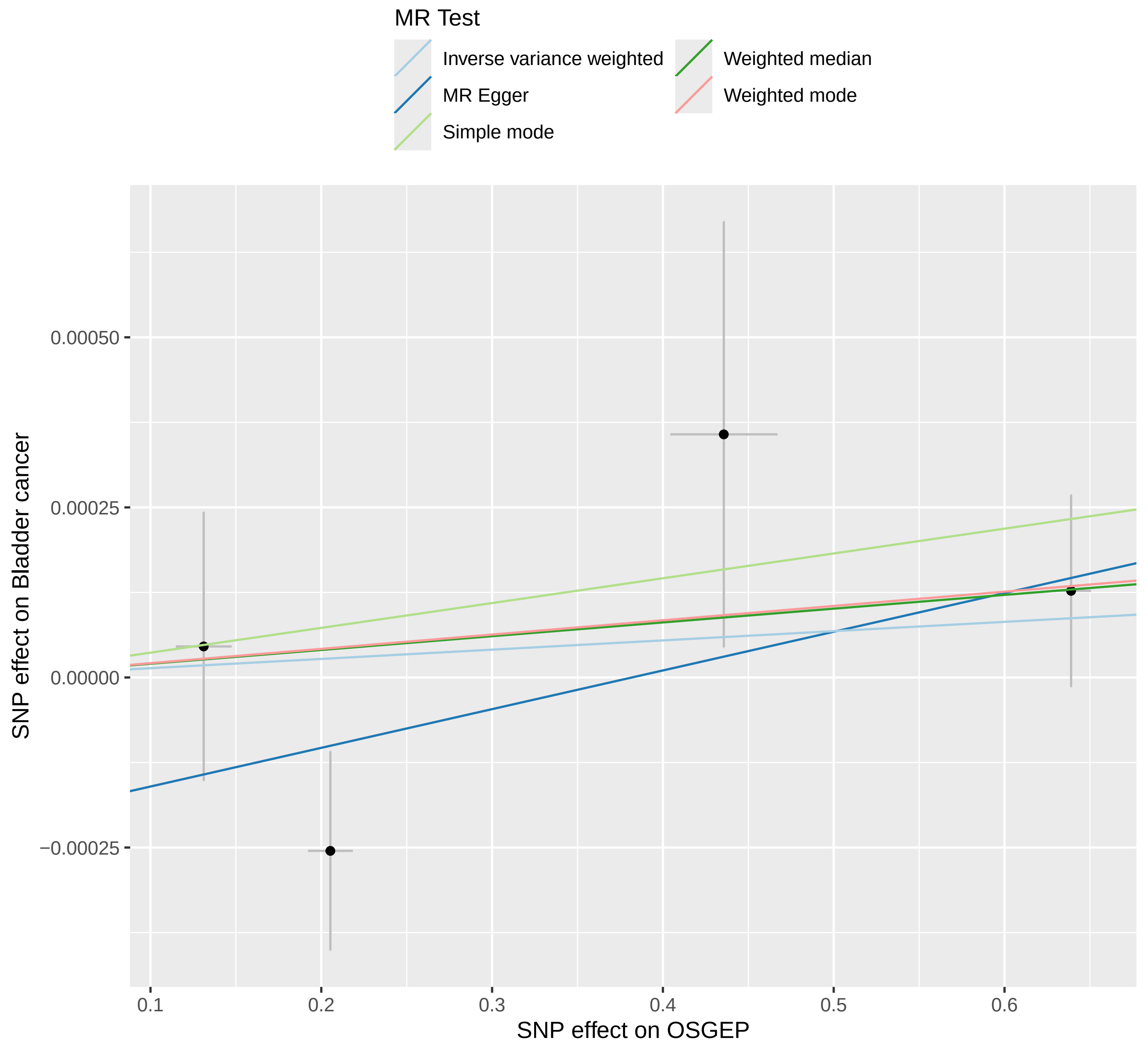
MR Test
Inverse variance weighted
MR Egger
Simple mode
Weighted median
Weighted mode
0.00050
0.00025
0.00000
−0.00025
SNP effect on Bladder cancer
0.1
0.2
0.3
0.4
0.5
0.6
SNP effect on OSGEP

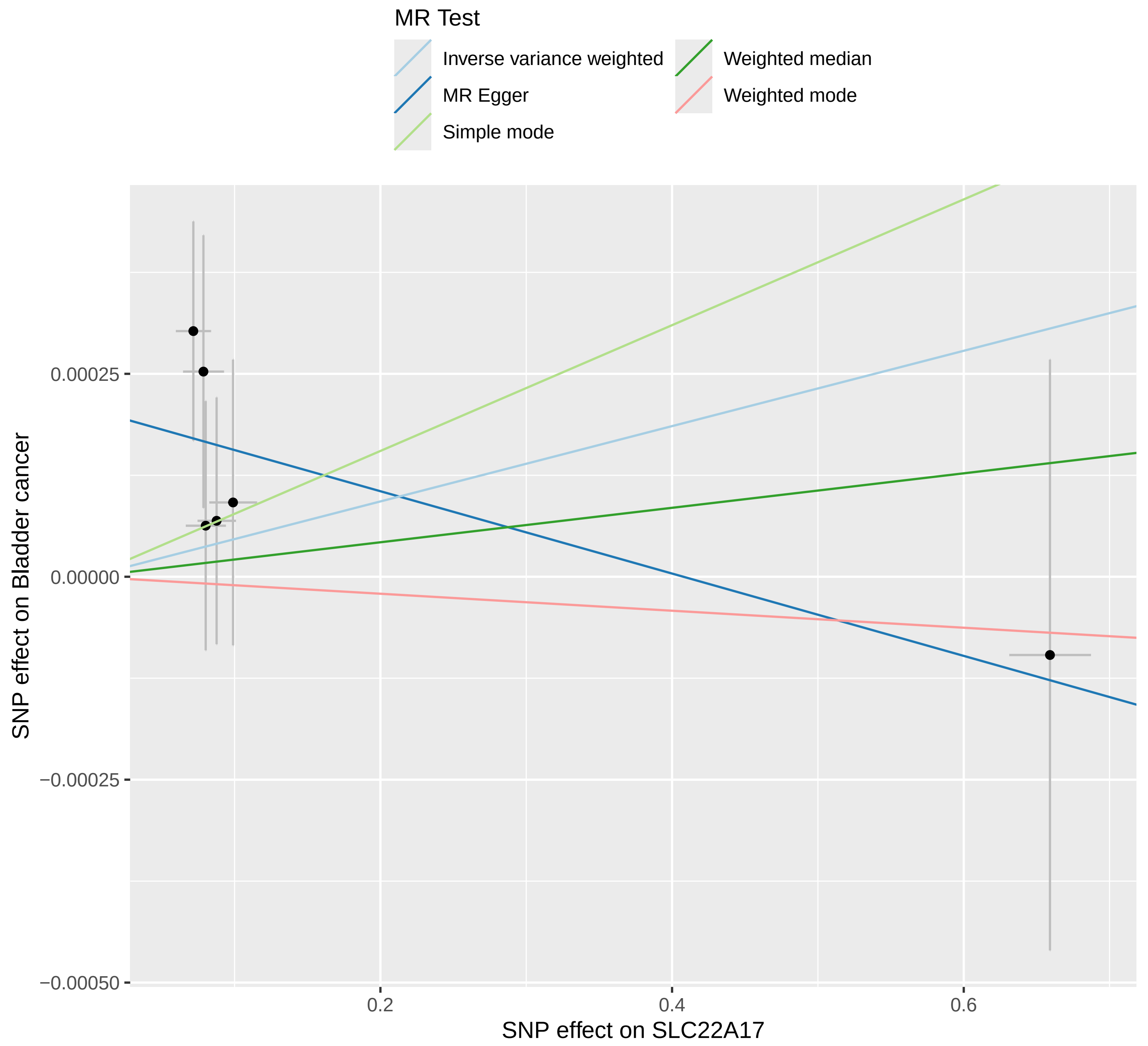

MR Test
Inverse variance weighted
MR Egger
Simple mode
Weighted median
Weighted mode
0.00025
0.00000
−0.00025
−0.00050
0.2
0.4
0.6
SNP effect on Bladder cancer
SNP effect on SLC22A17

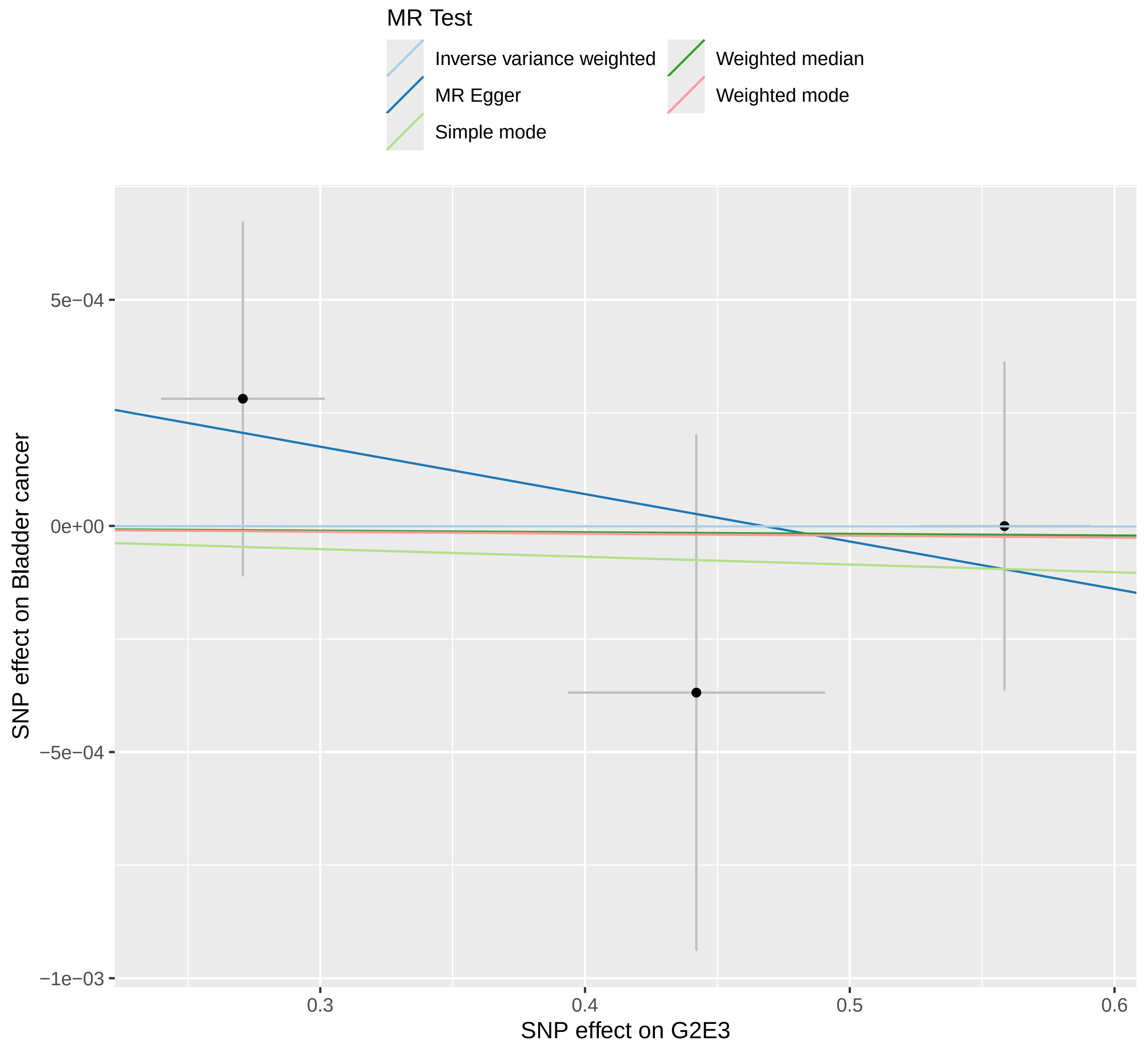

MR Test
Inverse variance weighted
MR Egger
Simple mode
Weighted median
Weighted mode
5e−04
0e+00
−5e−04
−1e−03
SNP effect on Bladder cancer
0.3
0.4
0.5
0.6
SNP effect on G2E3

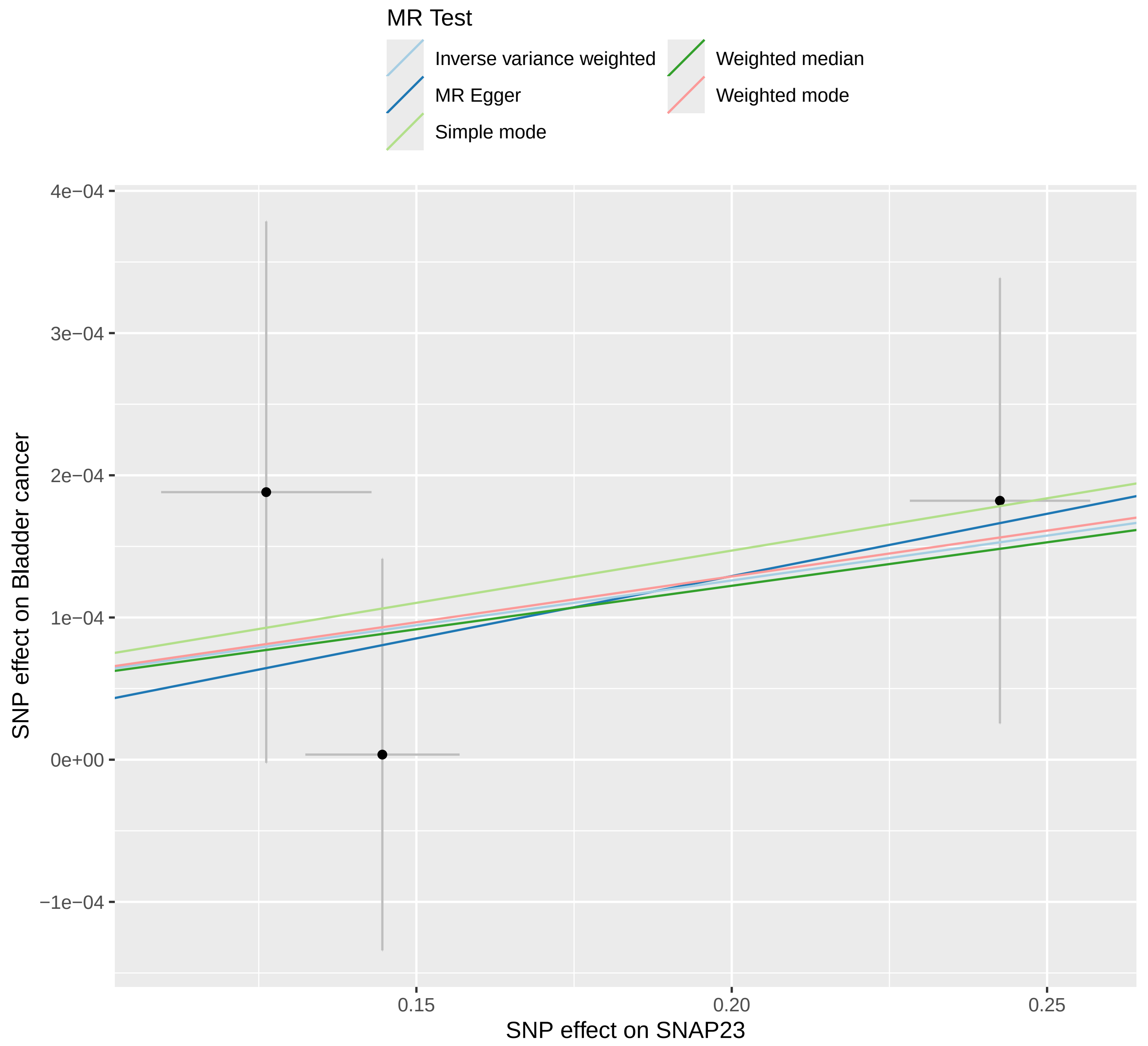
MR Test
Inverse variance weighted
MR Egger
Simple mode
Weighted median
Weighted mode
4e−04
3e−04
2e−04
1e−04
0e+00
−1e−04
SNP effect on Bladder cancer
0.15
0.20
0.25
SNP effect on SNAP23

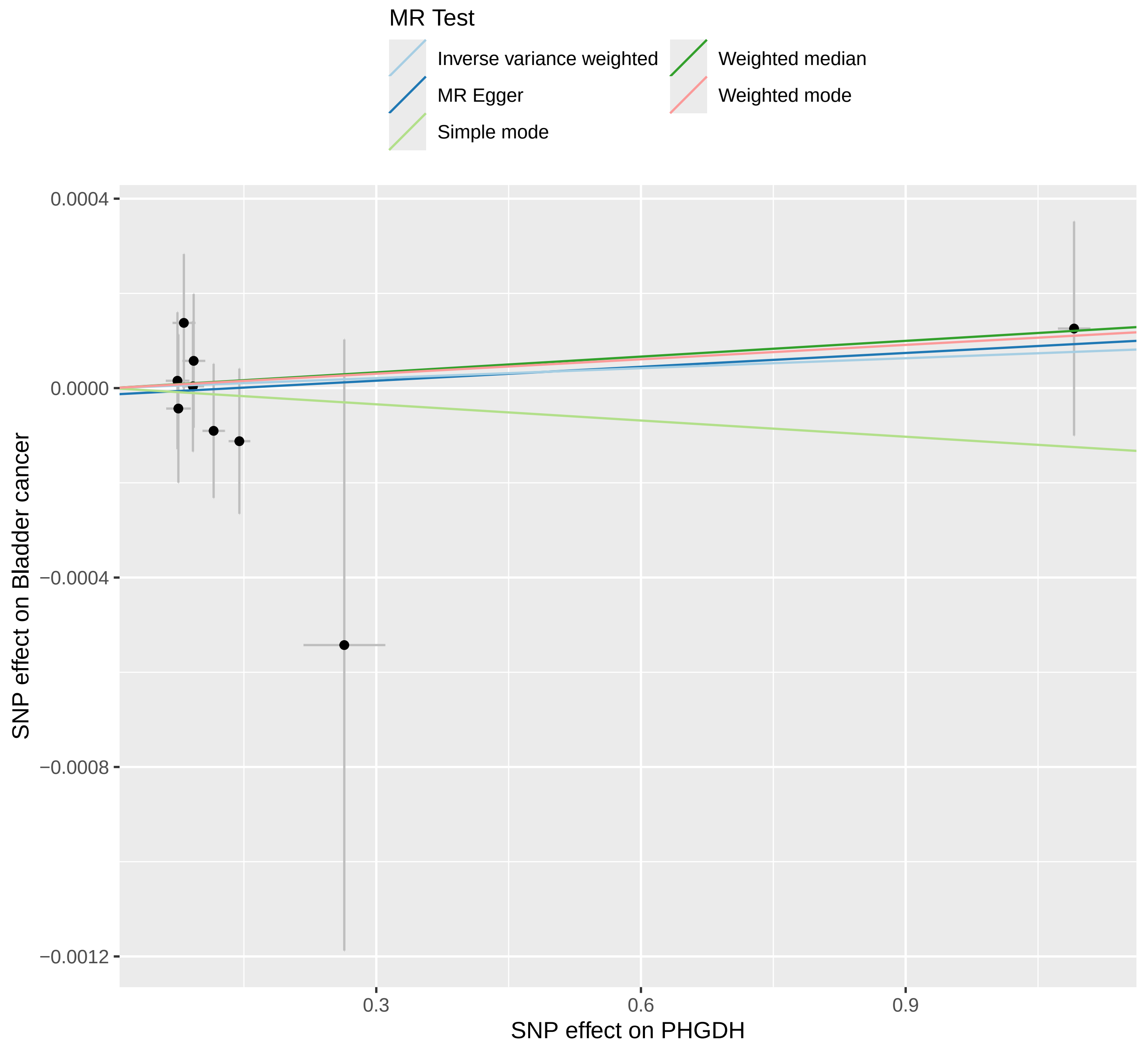

MR Test
Inverse variance weighted
MR Egger
Simple mode
Weighted median
Weighted mode
0.0004
0.0000
−0.0004
−0.0008
−0.0012
SNP effect on Bladder cancer
0.3
0.6
0.9
SNP effect on PHGDH

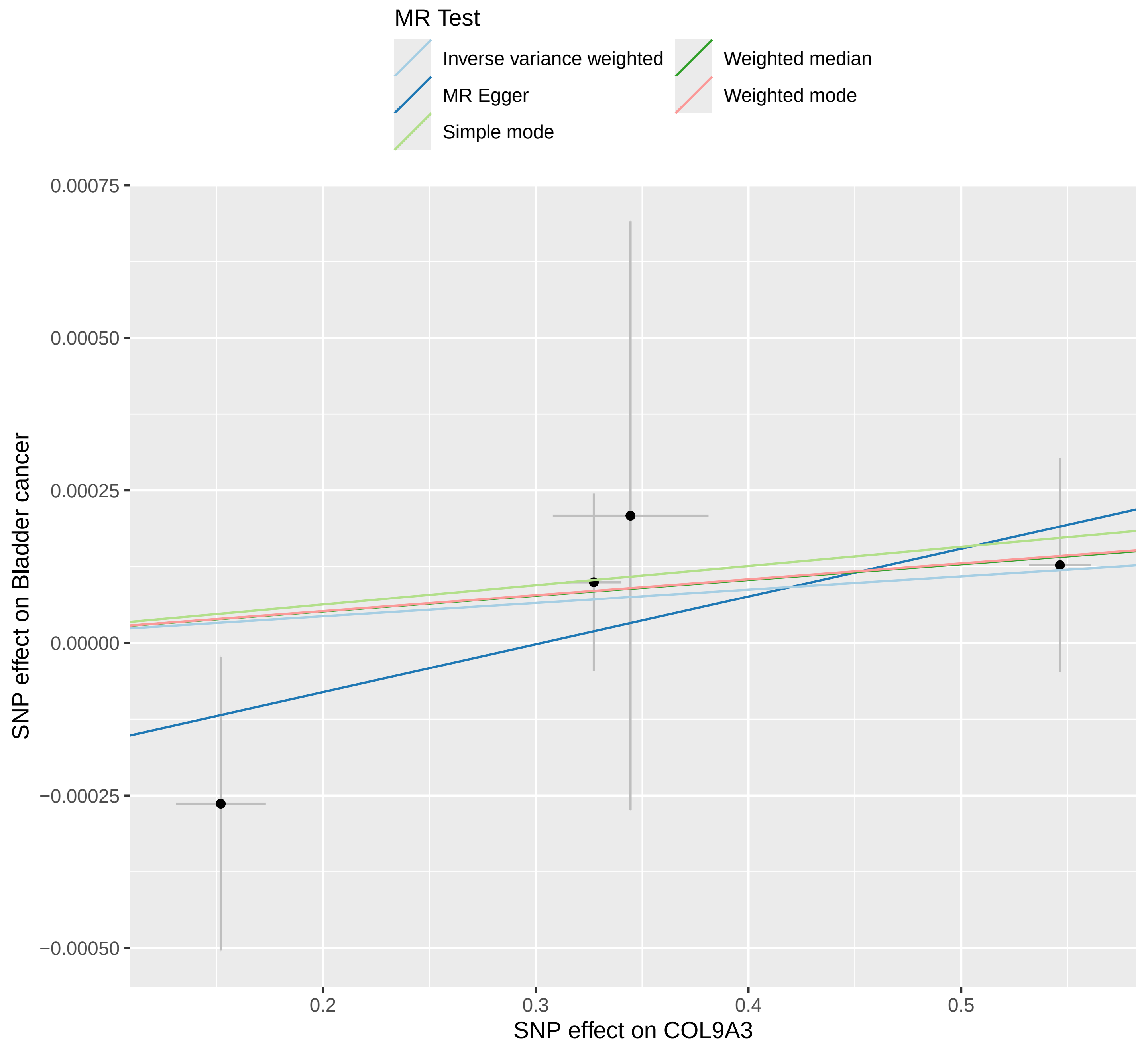

MR Test
Inverse variance weighted
MR Egger
Simple mode
Weighted median
Weighted mode
0.00075
0.00050
0.00025
0.00000
−0.00025
−0.00050
SNP effect on Bladder cancer
0.2
0.3
0.4
0.5
SNP effect on COL9A3

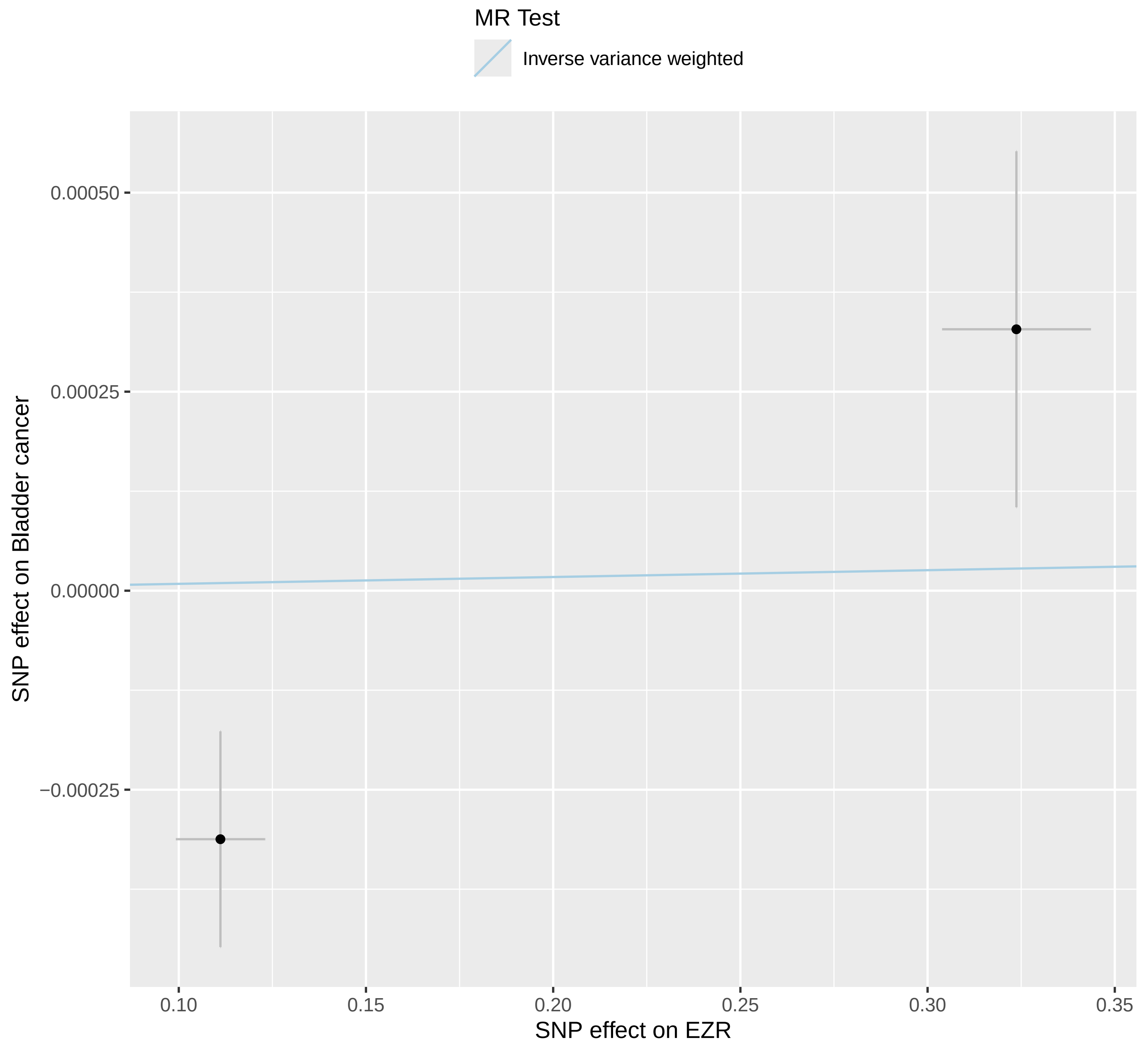
MR Test
Inverse variance weighted
SNP effect on Bladder cancer
0.00050
0.00025
0.00000
−0.00025
0.10
0.15
0.20
0.25
0.30
0.35
SNP effect on EZR

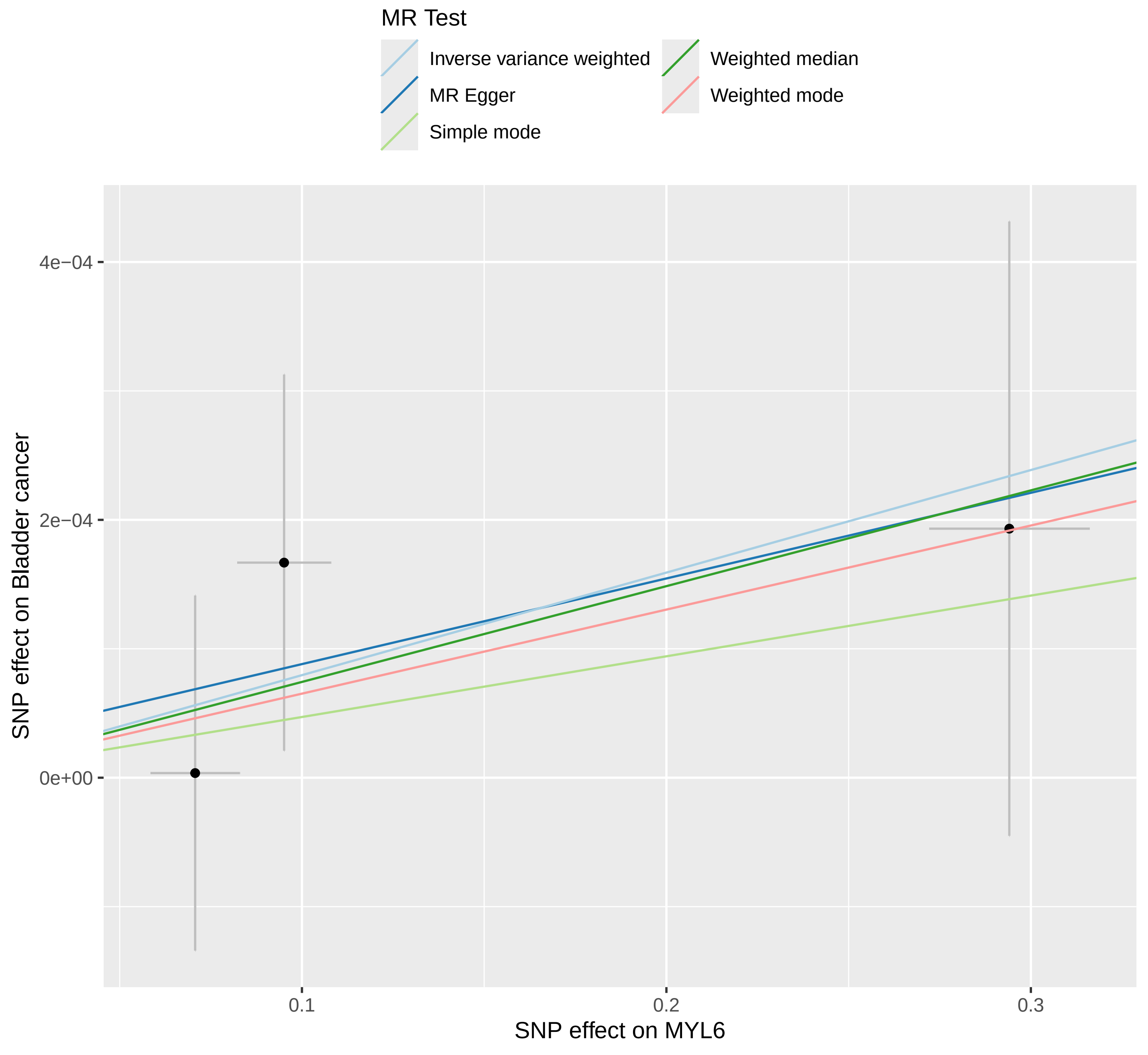
MR Test
Inverse variance weighted
MR Egger
Simple mode
Weighted median
Weighted mode
4e−04
2e−04
0e+00
SNP effect on Bladder cancer
0.1
0.2
0.3
SNP effect on MYL6

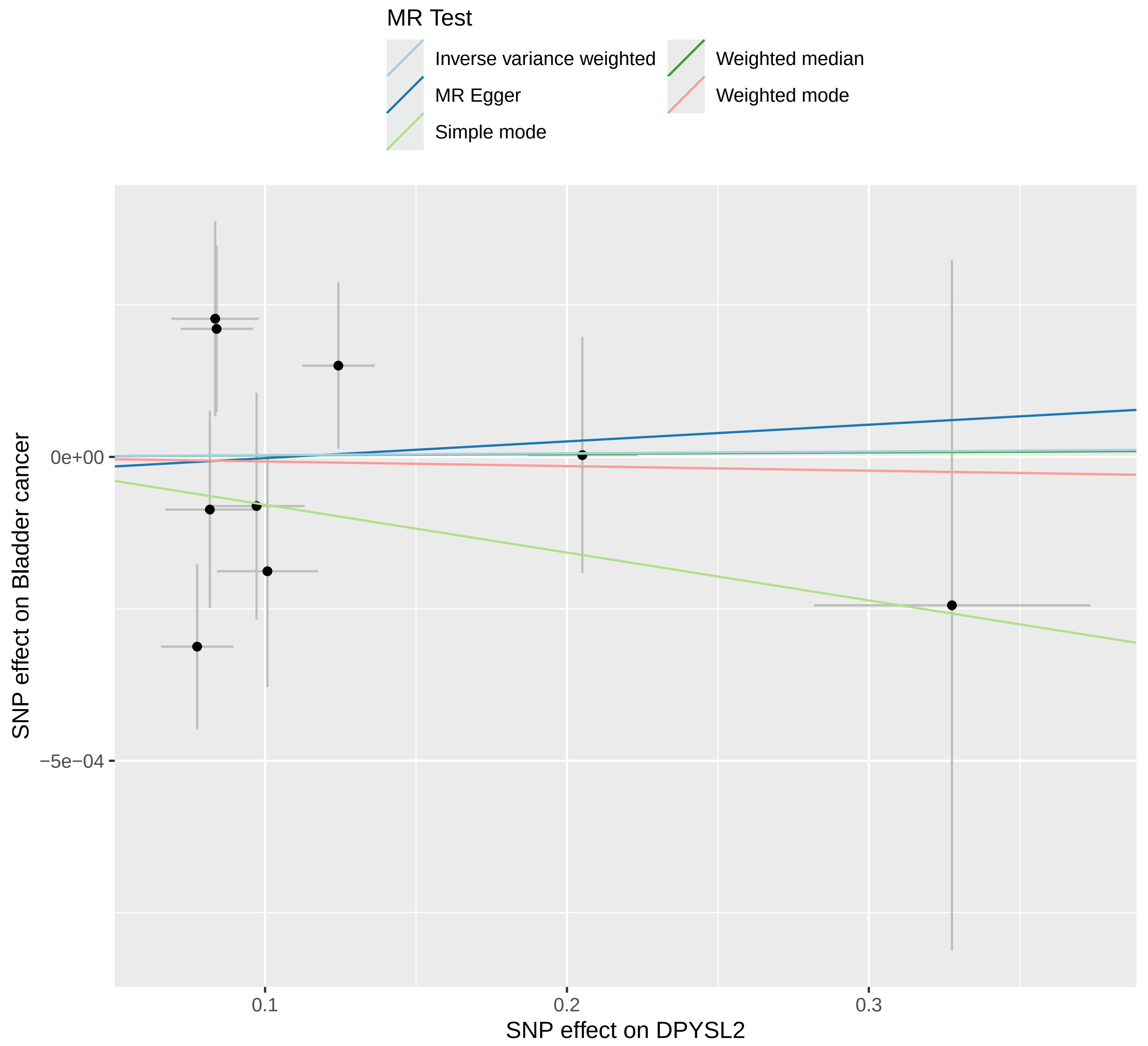
MR Test
Inverse variance weighted
MR Egger
Simple mode
Weighted median
Weighted mode
0e+00
−5e−04
0.1
0.2
0.3
SNP effect on DPYSL2
SNP effect on Bladder cancer

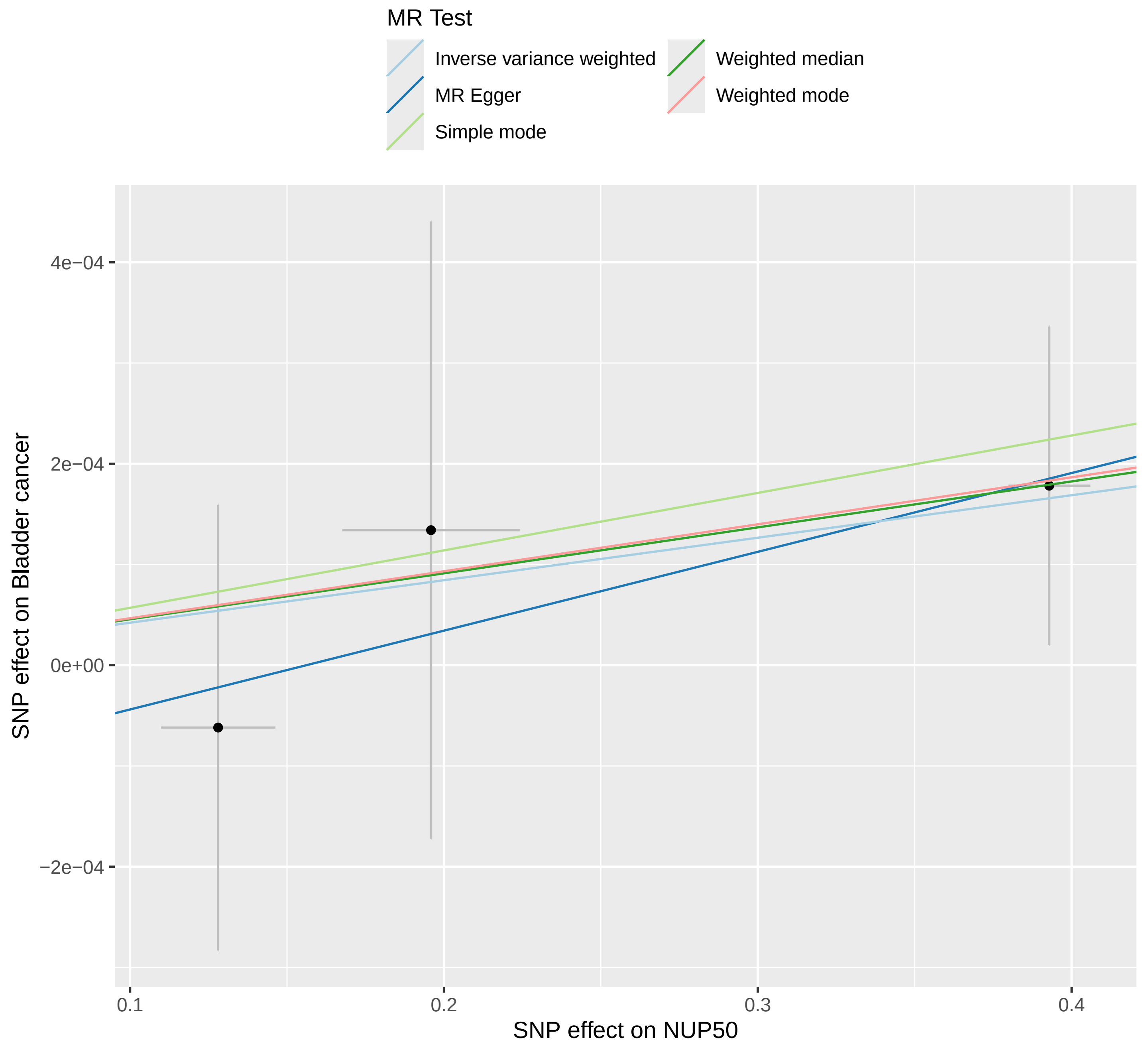

MR Test
Inverse variance weighted
MR Egger
Simple mode
Weighted median
Weighted mode
4e−04
2e−04
0e+00
−2e−04
SNP effect on Bladder cancer
0.1
0.2
0.3
0.4
SNP effect on NUP50

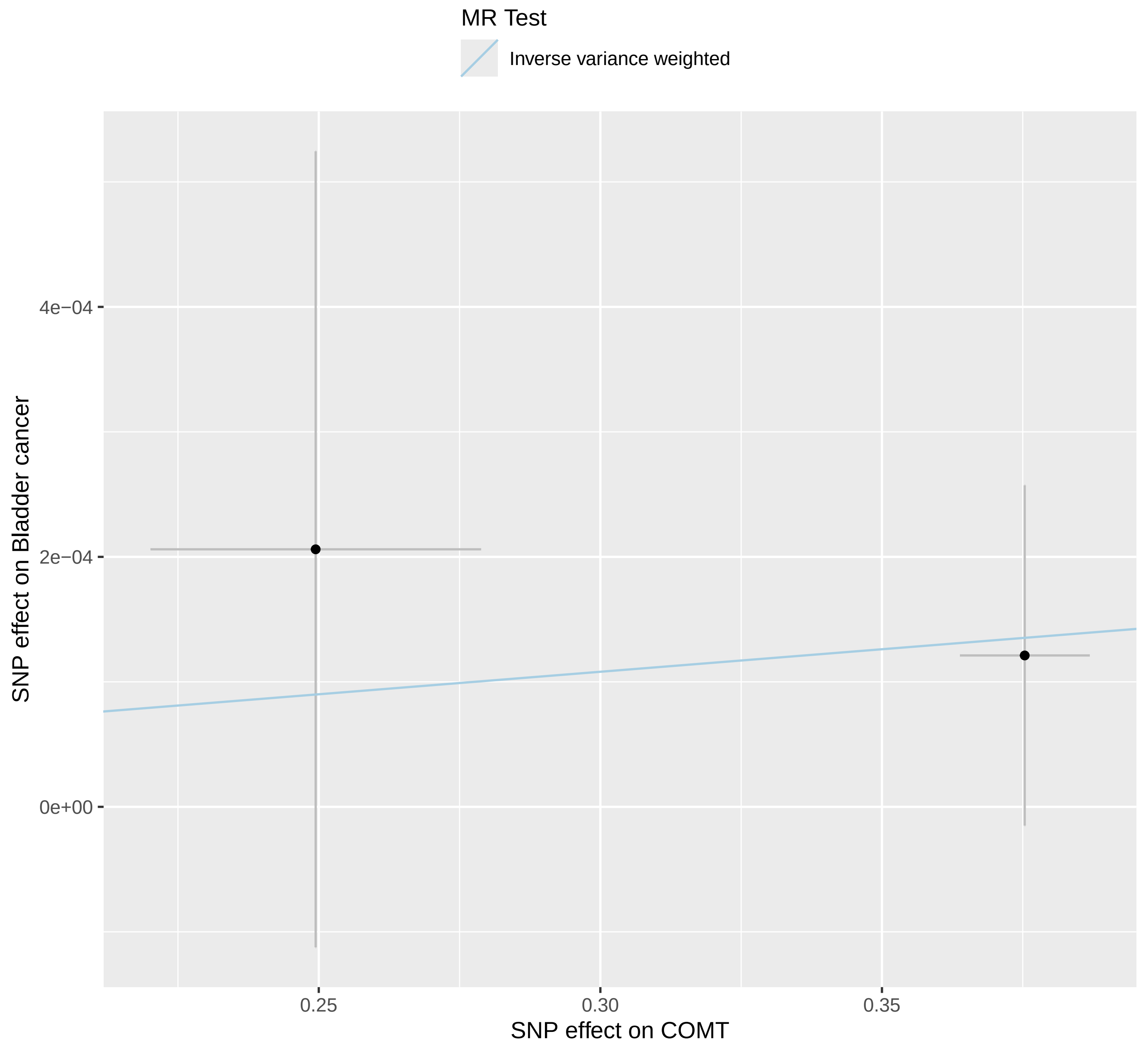
MR Test
Inverse variance weighted
4e−04
2e−04
0e+00
SNP effect on Bladder cancer
0.25
0.30
0.35
SNP effect on COMT

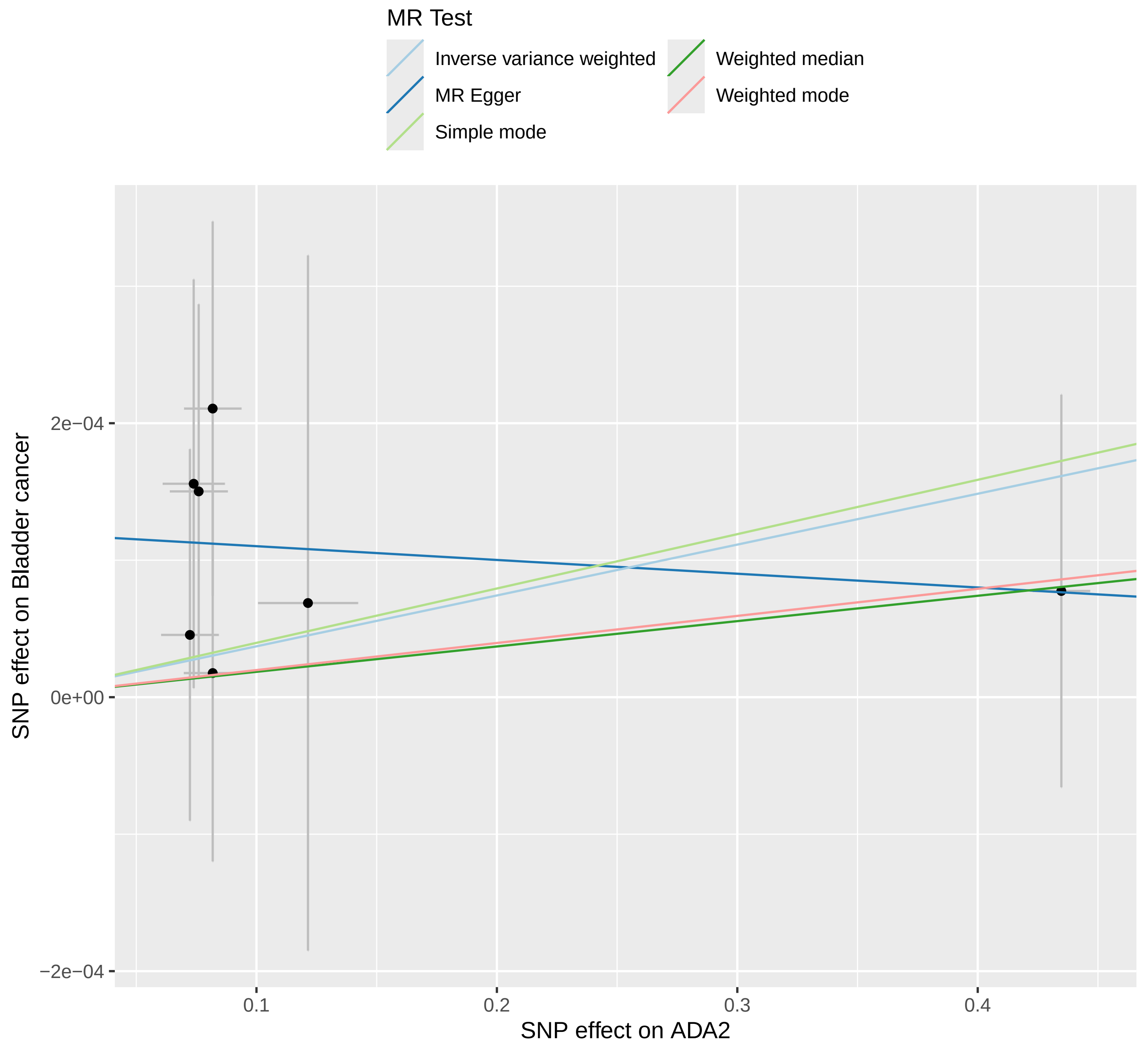
MR Test
Inverse variance weighted
MR Egger
Simple mode
Weighted median
Weighted mode
2e−04
0e+00
−2e−04
SNP effect on Bladder cancer
0.1
0.2
0.3
0.4
SNP effect on ADA2

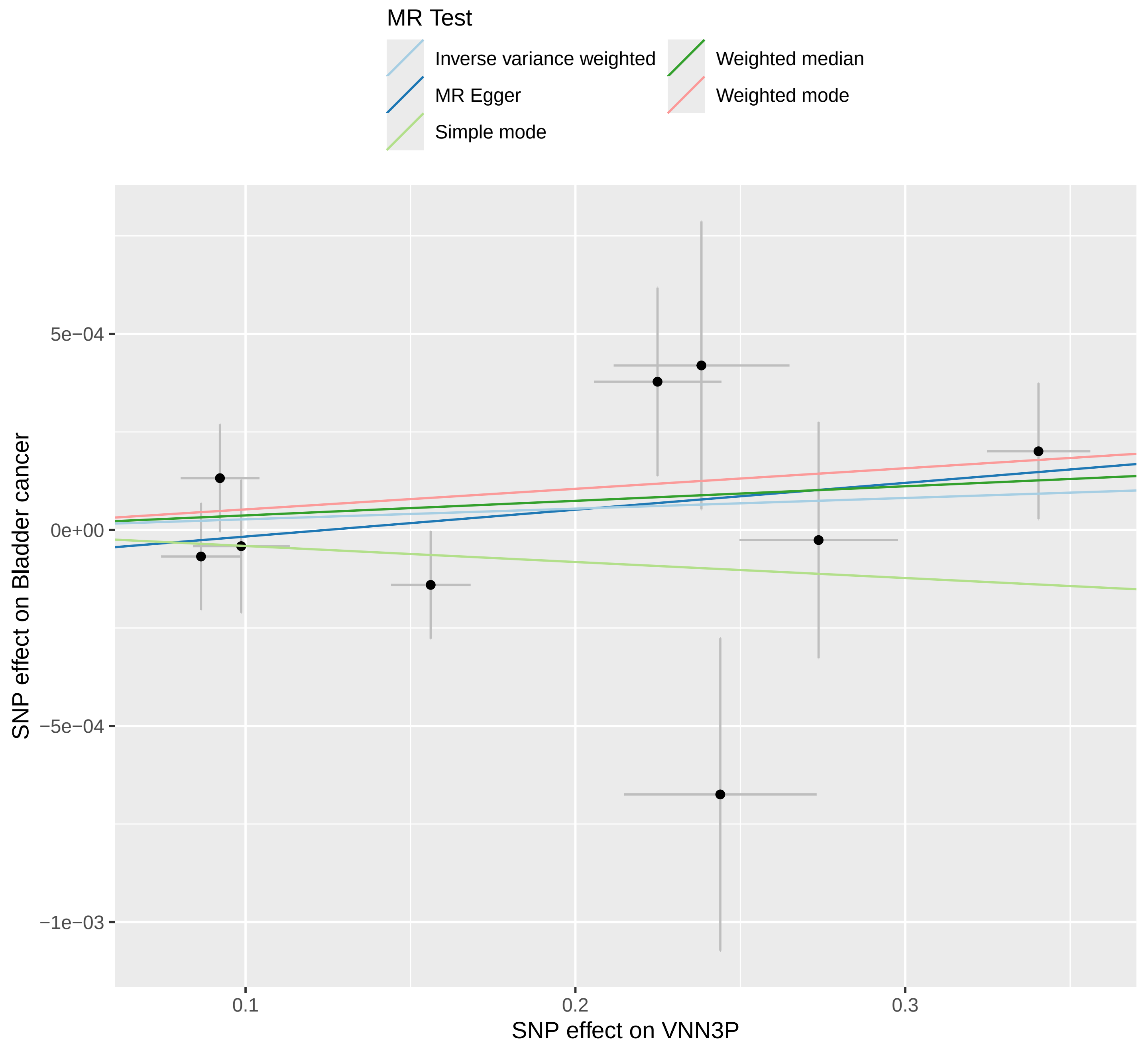

MR Test
Inverse variance weighted
MR Egger
Simple mode
Weighted median
Weighted mode
5e−04
0e+00
−5e−04
−1e−03
0.1
0.2
0.3
SNP effect on Bladder cancer
SNP effect on VNN3P

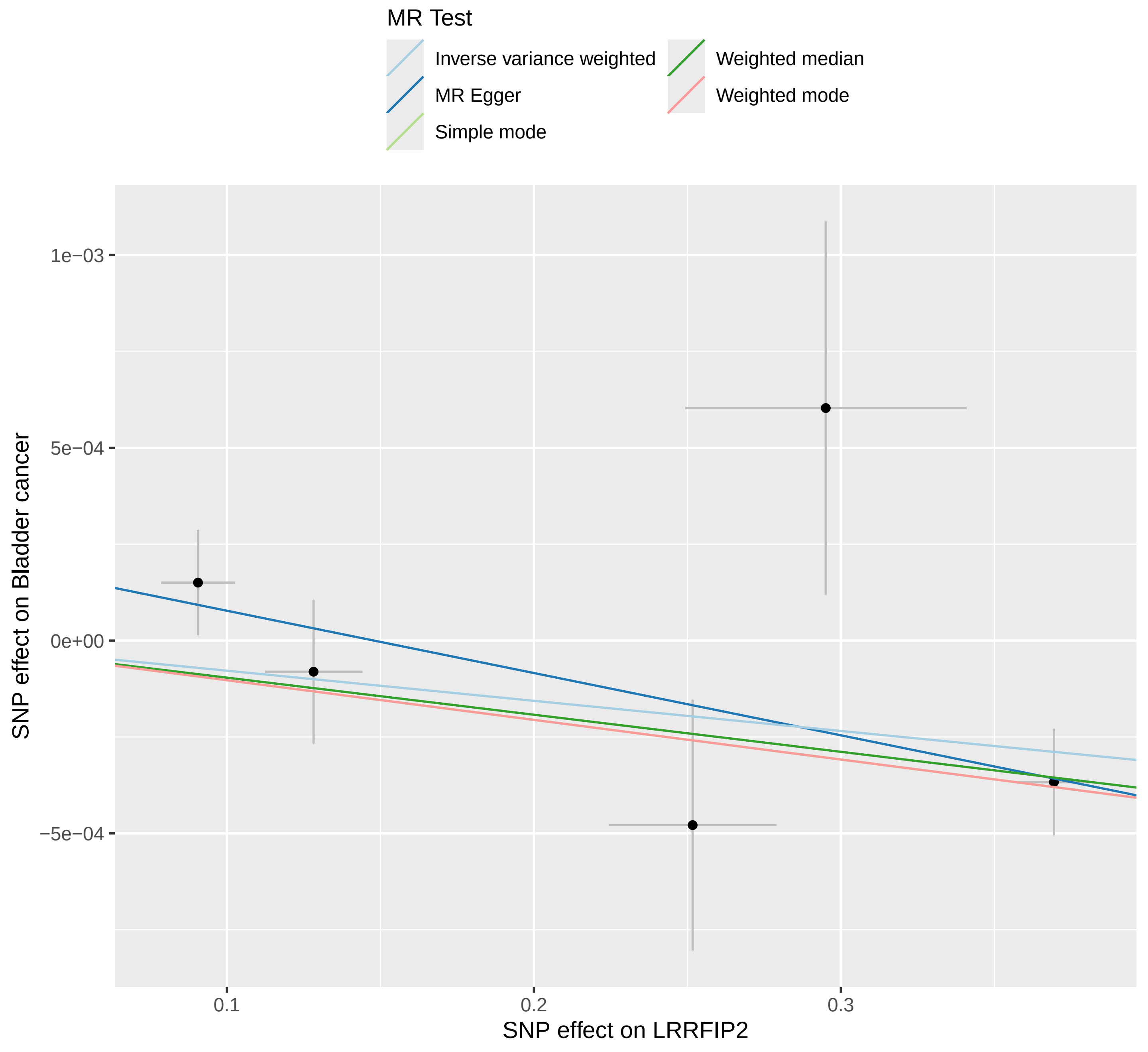
MR Test
Inverse variance weighted
MR Egger
Simple mode
Weighted median
Weighted mode
1e−03
5e−04
0e+00
−5e−04
0.1
0.2
0.3
SNP effect on LRRFIP2
SNP effect on Bladder cancer

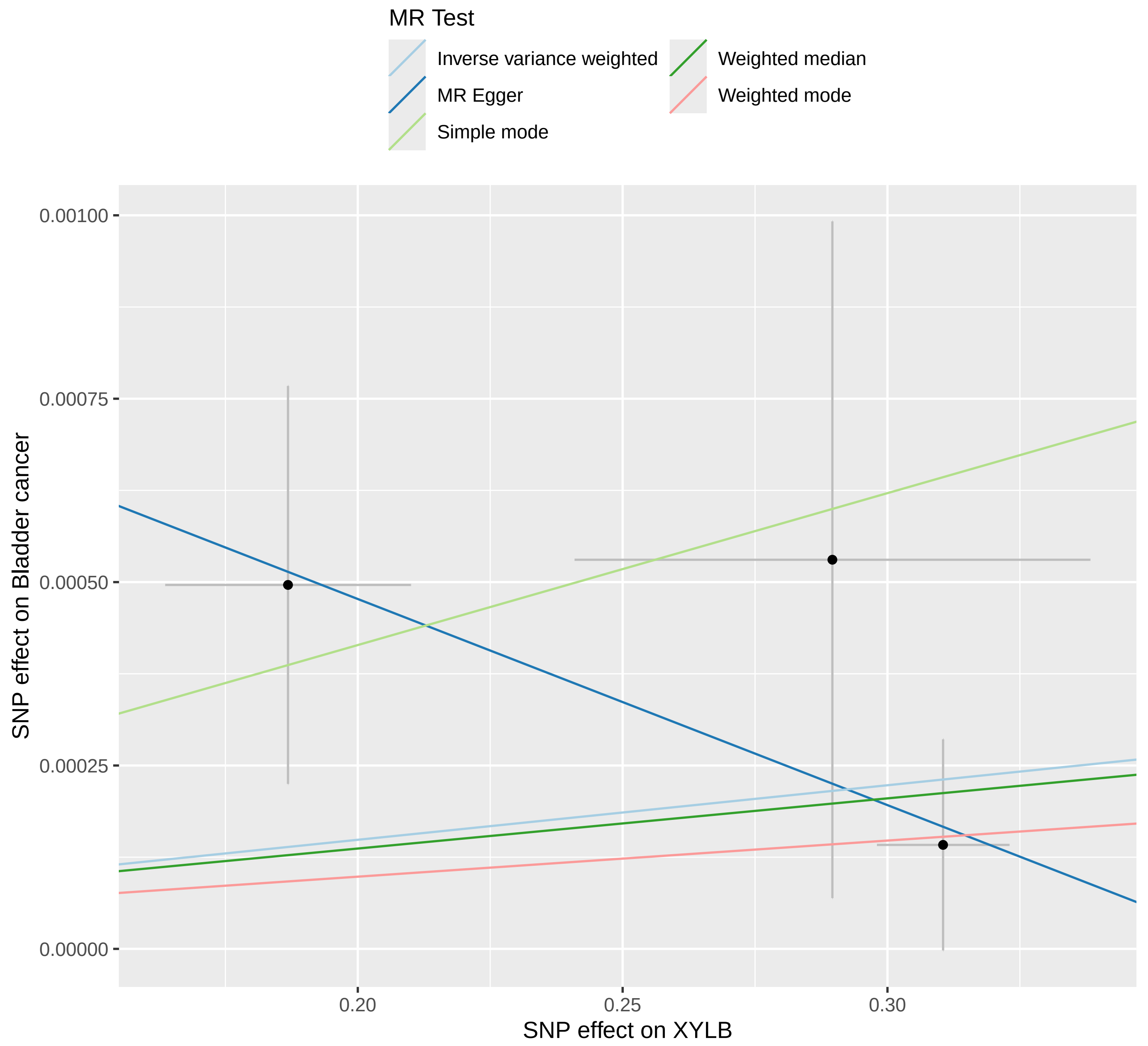

MR Test
Inverse variance weighted
MR Egger
Simple mode
Weighted median
Weighted mode
0.00100
0.00075
0.00050
0.00025
0.00000
SNP effect on Bladder cancer
0.20
0.25
0.30
SNP effect on XYLB

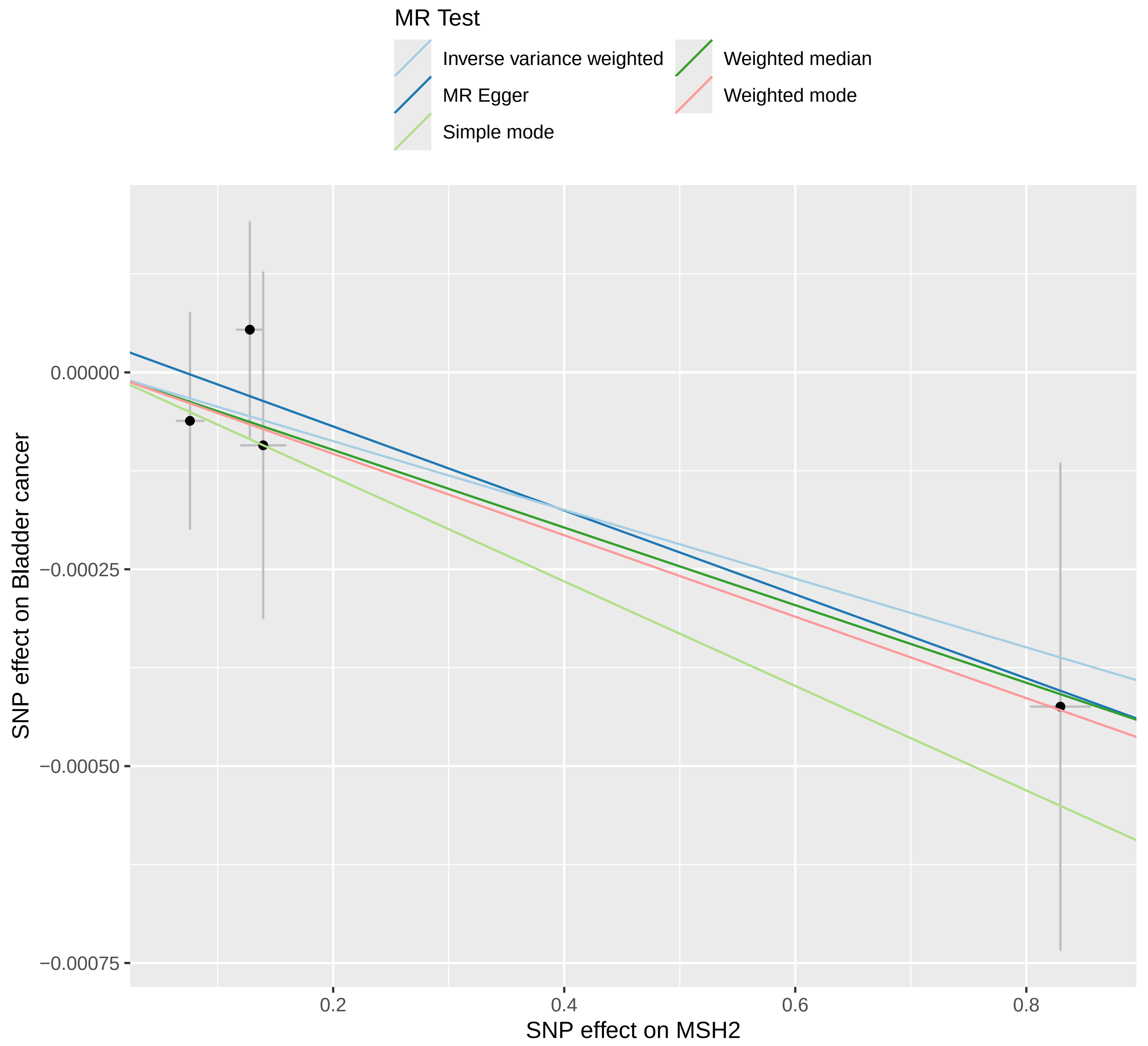

MR Test
Inverse variance weighted
MR Egger
Simple mode
Weighted median
Weighted mode
0.00000
−0.00025
−0.00050
−0.00075
SNP effect on Bladder cancer
0.2
0.4
0.6
0.8
SNP effect on MSH2

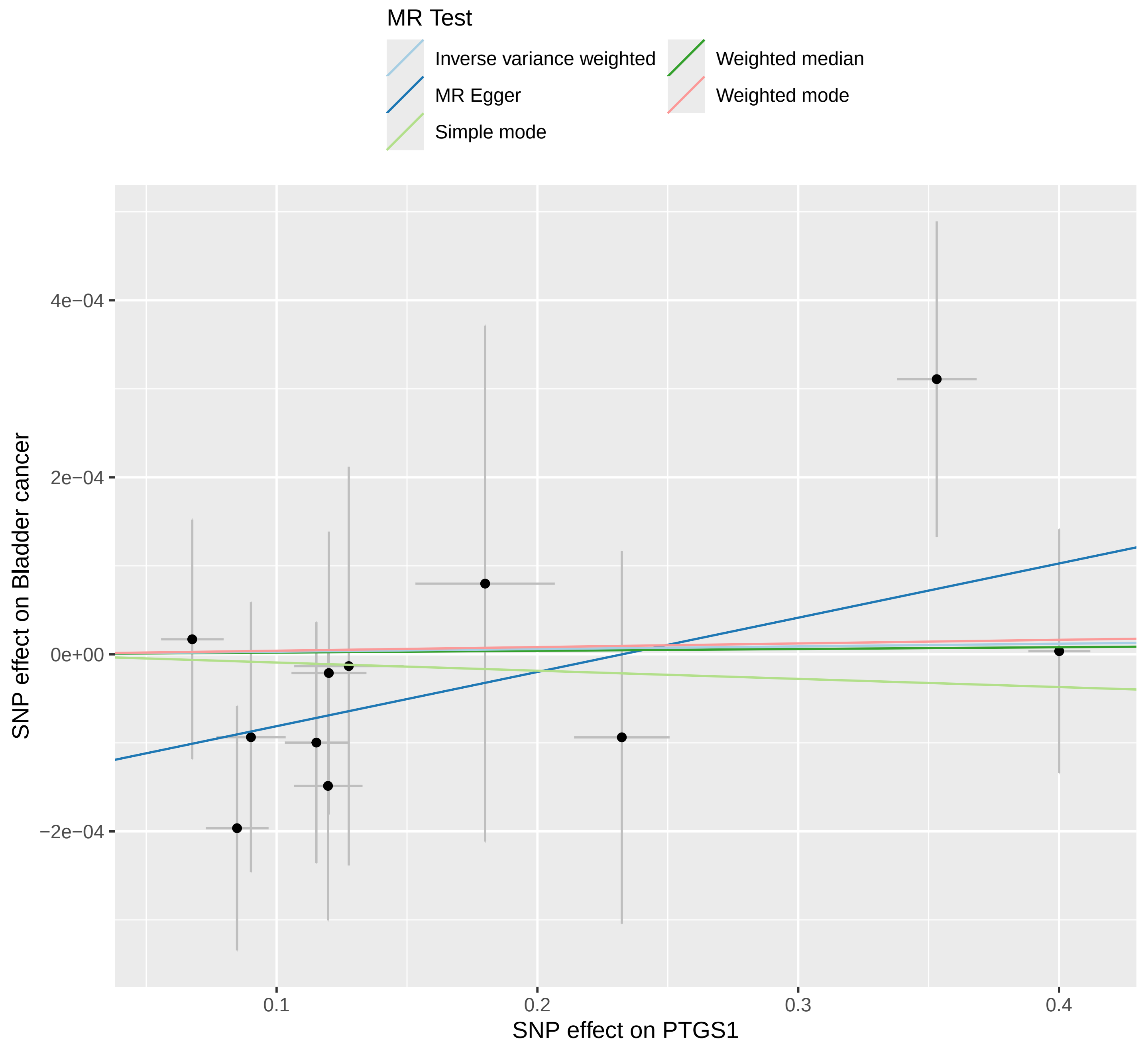
MR Test
Inverse variance weighted
MR Egger
Simple mode
Weighted median
Weighted mode
4e−04
2e−04
0e+00
−2e−04
SNP effect on Bladder cancer
0.1
0.2
0.3
0.4
SNP effect on PTGS1

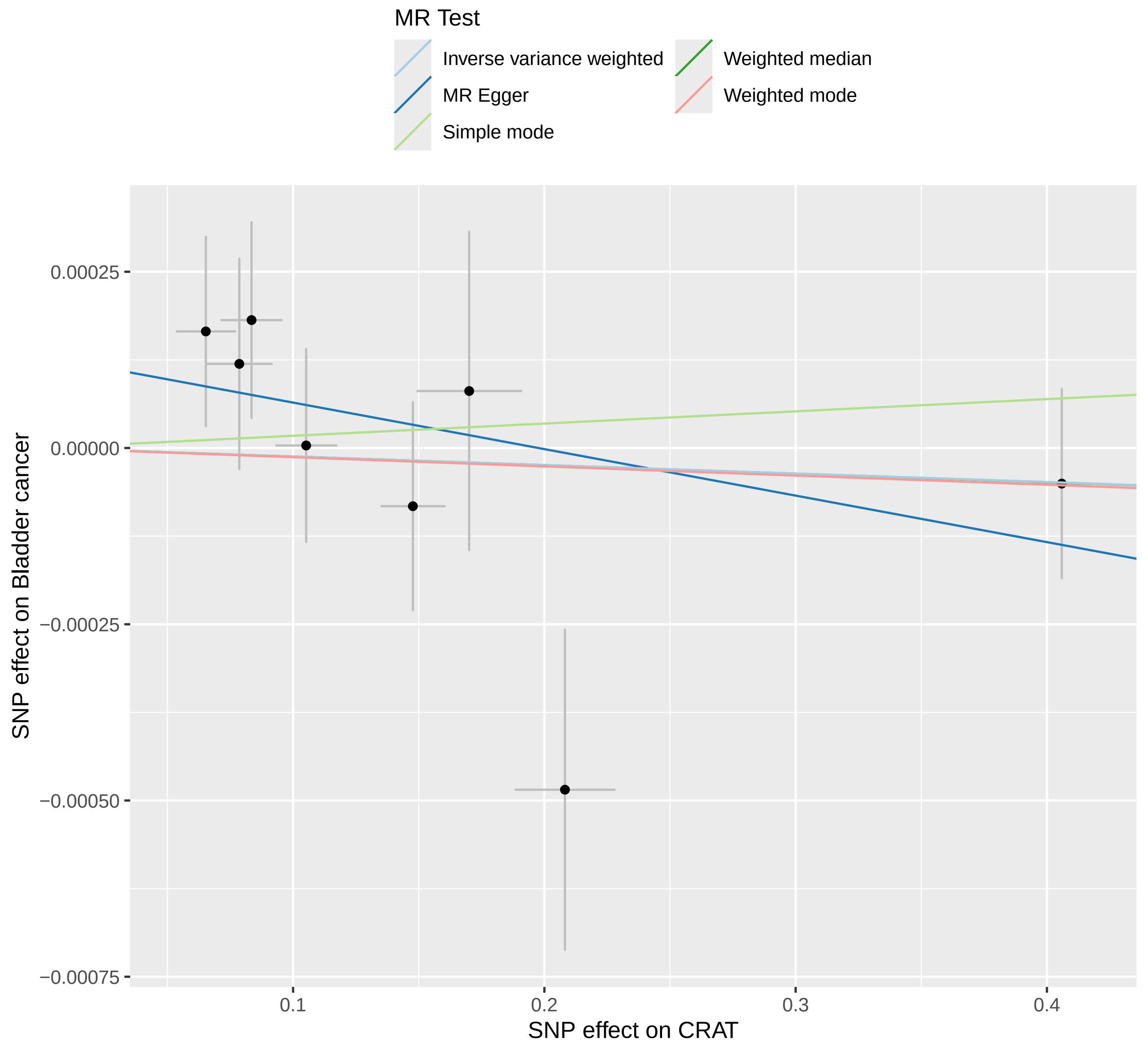

MR Test
Inverse variance weighted
MR Egger
Simple mode
Weighted median
Weighted mode
0.00025
0.00000
−0.00025
−0.00050
−0.00075
SNP effect on Bladder cancer
0.1
0.2
0.3
0.4
SNP effect on CRAT

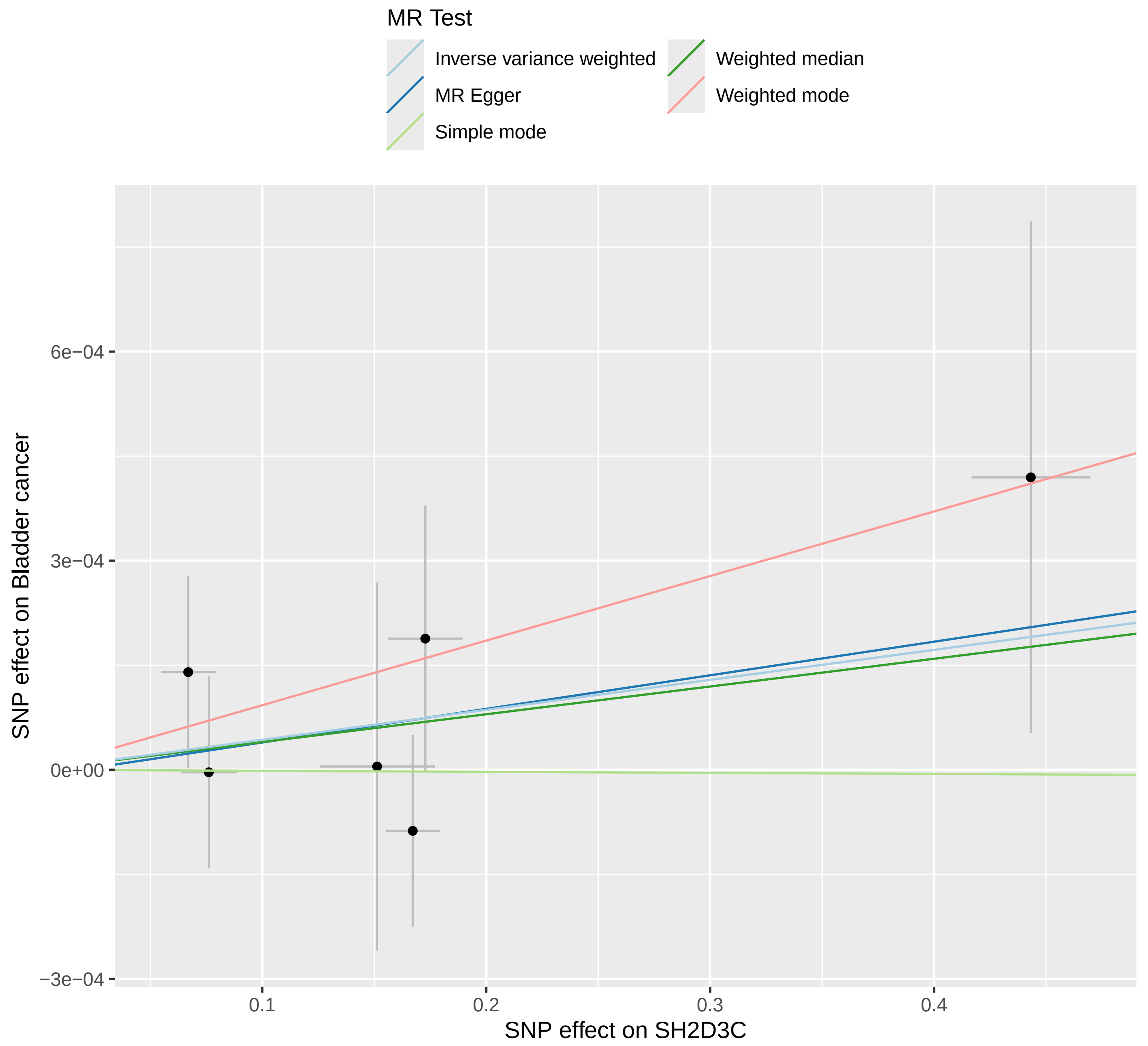
MR Test
Inverse variance weighted
MR Egger
Simple mode
Weighted median
Weighted mode
6e−04
3e−04
0e+00
−3e−04
0.1
0.2
0.3
0.4
SNP effect on Bladder cancer
SNP effect on SH2D3C

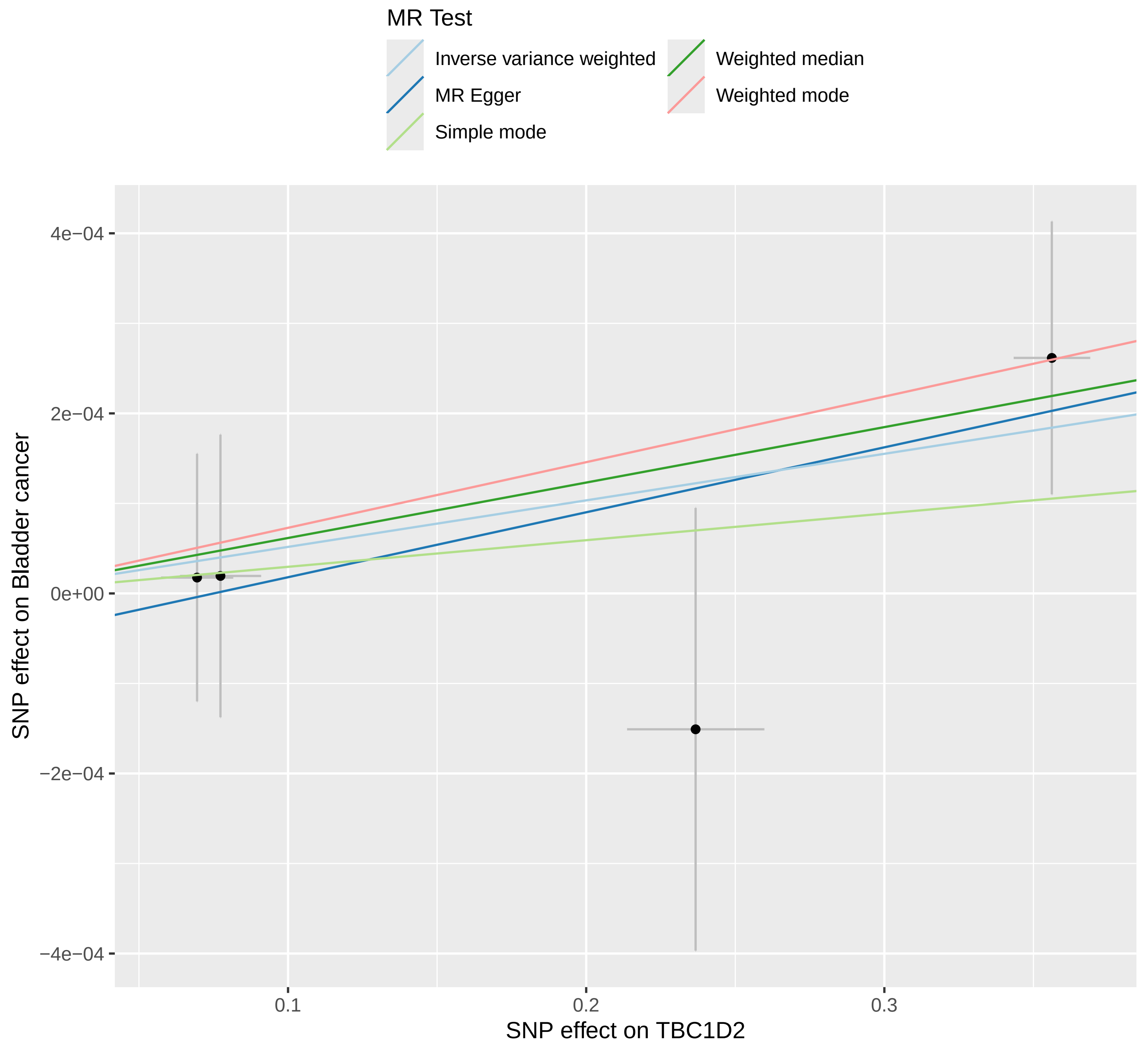

MR Test
Inverse variance weighted
MR Egger
Simple mode
Weighted median
Weighted mode
4e−04
2e−04
0e+00
−2e−04
−4e−04
SNP effect on Bladder cancer
0.1
0.2
0.3
SNP effect on TBC1D2

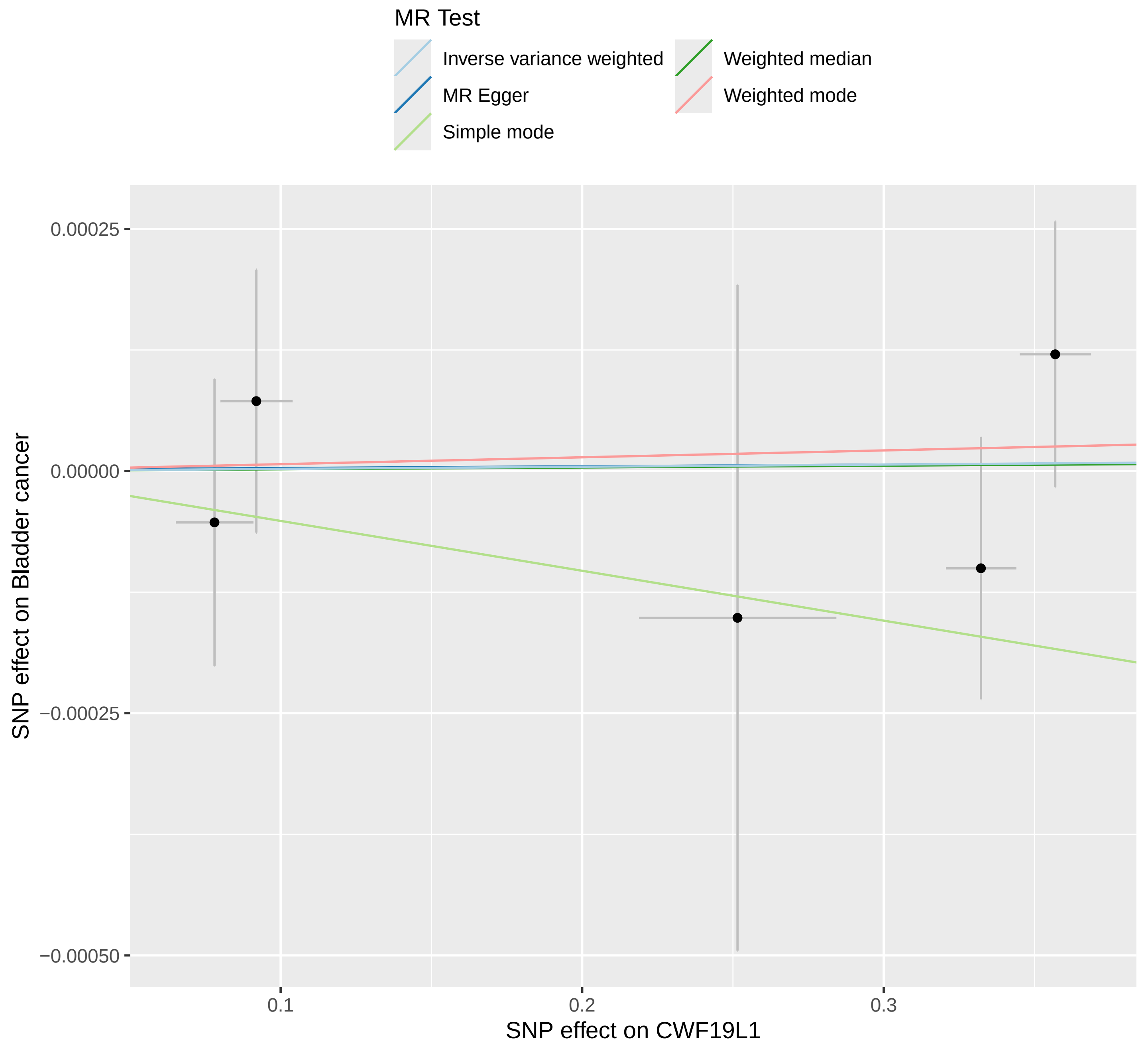

MR Test
Inverse variance weighted
MR Egger
Simple mode
Weighted median
Weighted mode
0.00025
0.00000
−0.00025
−0.00050
0.1
0.2
0.3
SNP effect on Bladder cancer
SNP effect on CWF19L1

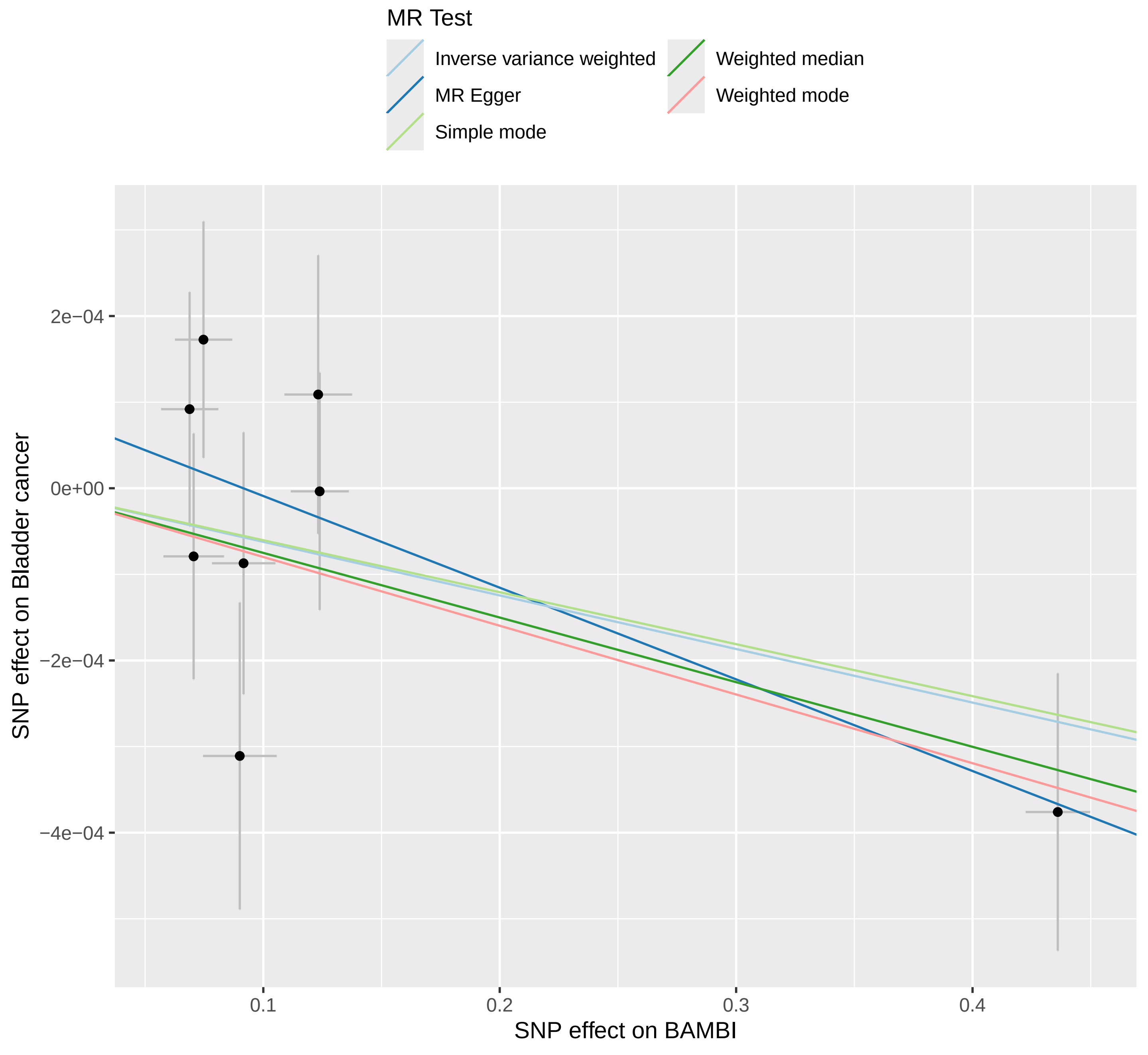

MR Test
Inverse variance weighted
MR Egger
Simple mode
Weighted median
Weighted mode
SNP effect on Bladder cancer
2e−04
0e+00
−2e−04
−4e−04
0.1
0.2
0.3
0.4
SNP effect on BAMBI

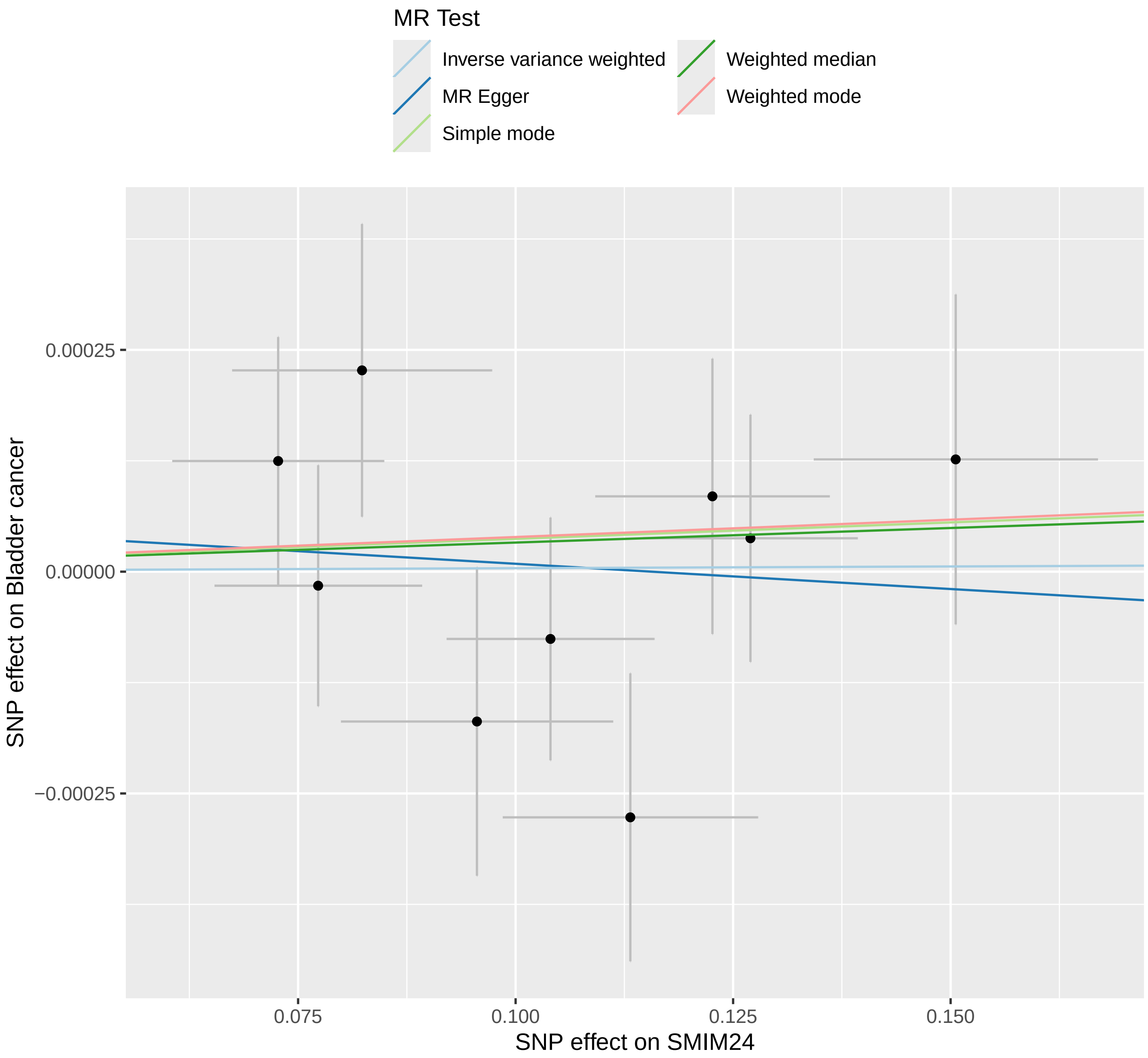
MR Test
Inverse variance weighted
MR Egger
Simple mode
Weighted median
Weighted mode
0.00025
0.00000
−0.00025
0.075
0.100
0.125
0.150
SNP effect on SMIM24
SNP effect on Bladder cancer

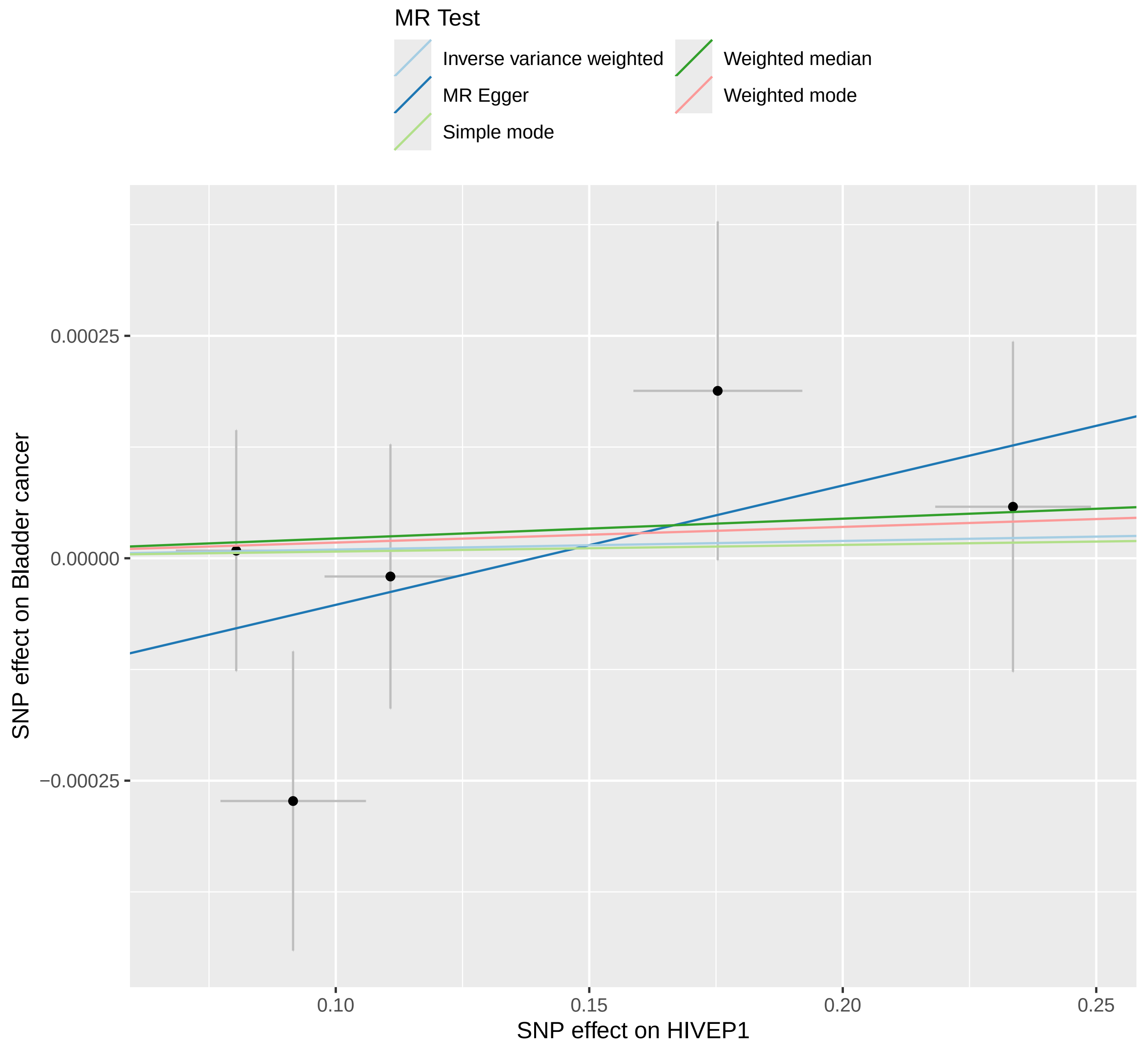
MR Test
Inverse variance weighted
MR Egger
Simple mode
Weighted median
Weighted mode
0.00025
0.00000
−0.00025
SNP effect on Bladder cancer
0.10
0.15
0.20
0.25
SNP effect on HIVEP1

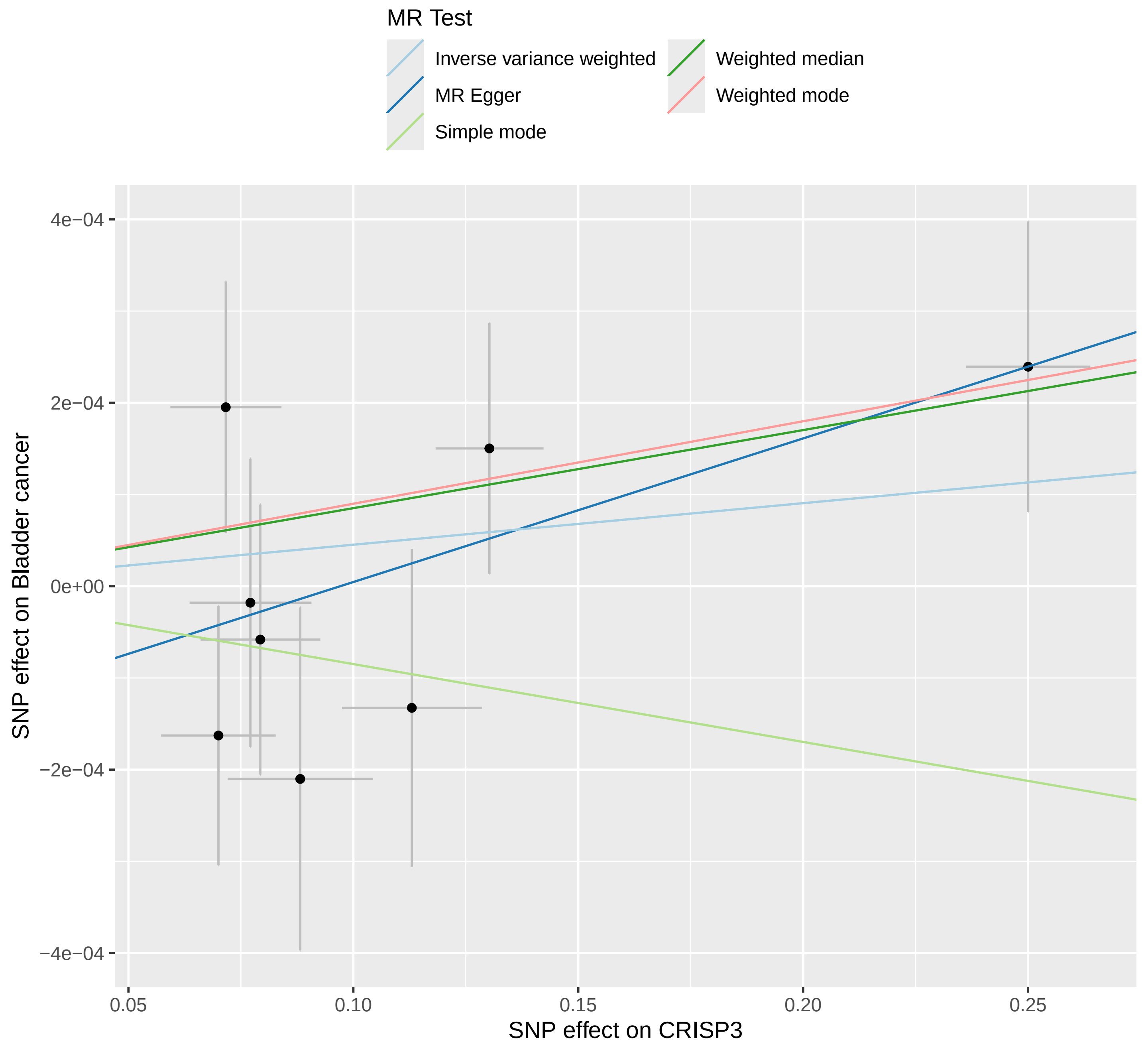
MR Test
Inverse variance weighted
MR Egger
Simple mode
Weighted median
Weighted mode
4e−04
2e−04
0e+00
−2e−04
−4e−04
SNP effect on Bladder cancer
0.05
0.10
0.15
0.20
0.25
SNP effect on CRISP3

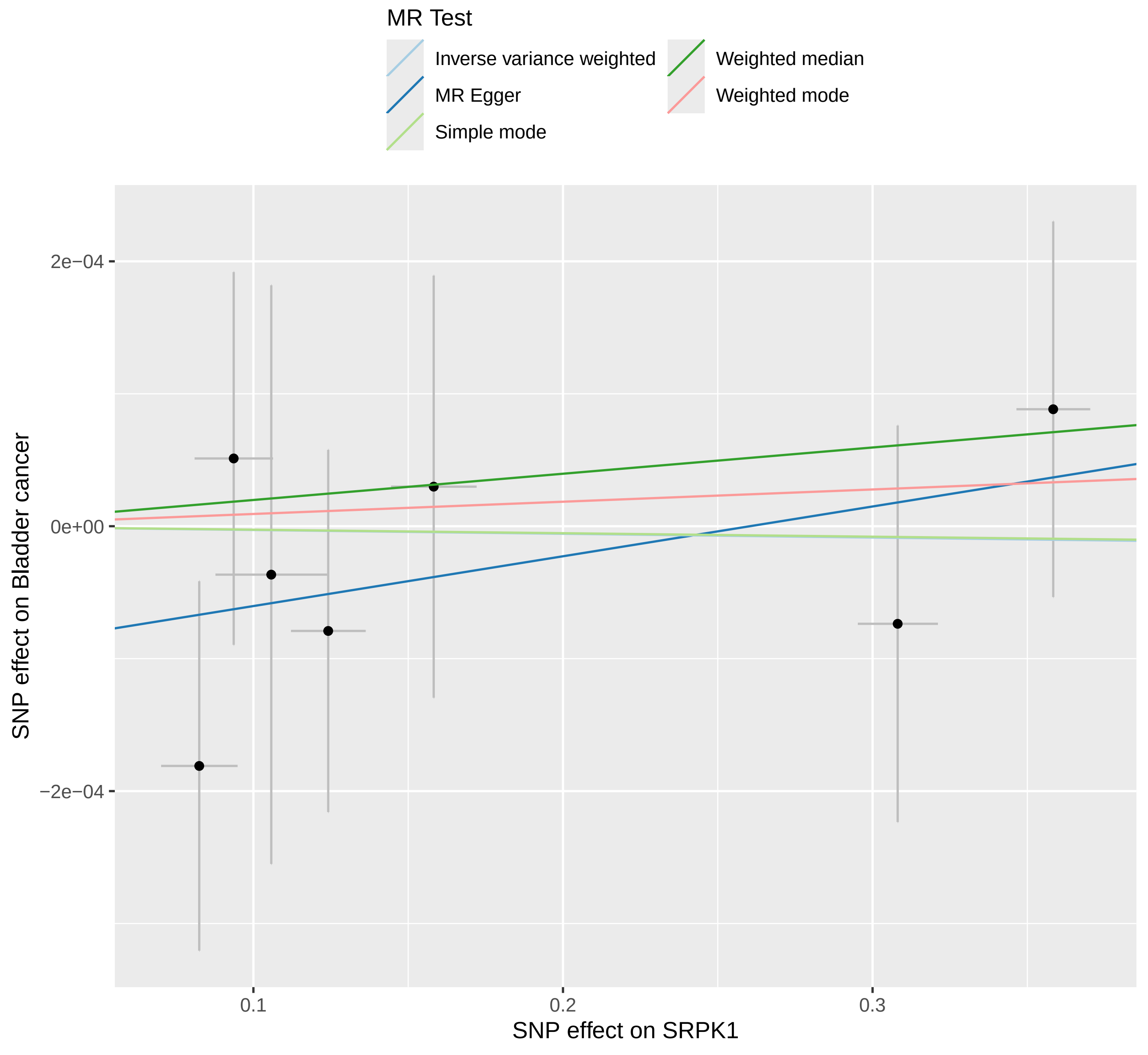

MR Test
Inverse variance weighted
MR Egger
Simple mode
Weighted median
Weighted mode
2e−04
0e+00
−2e−04
0.1
0.2
0.3
SNP effect on SRPK1
SNP effect on Bladder cancer

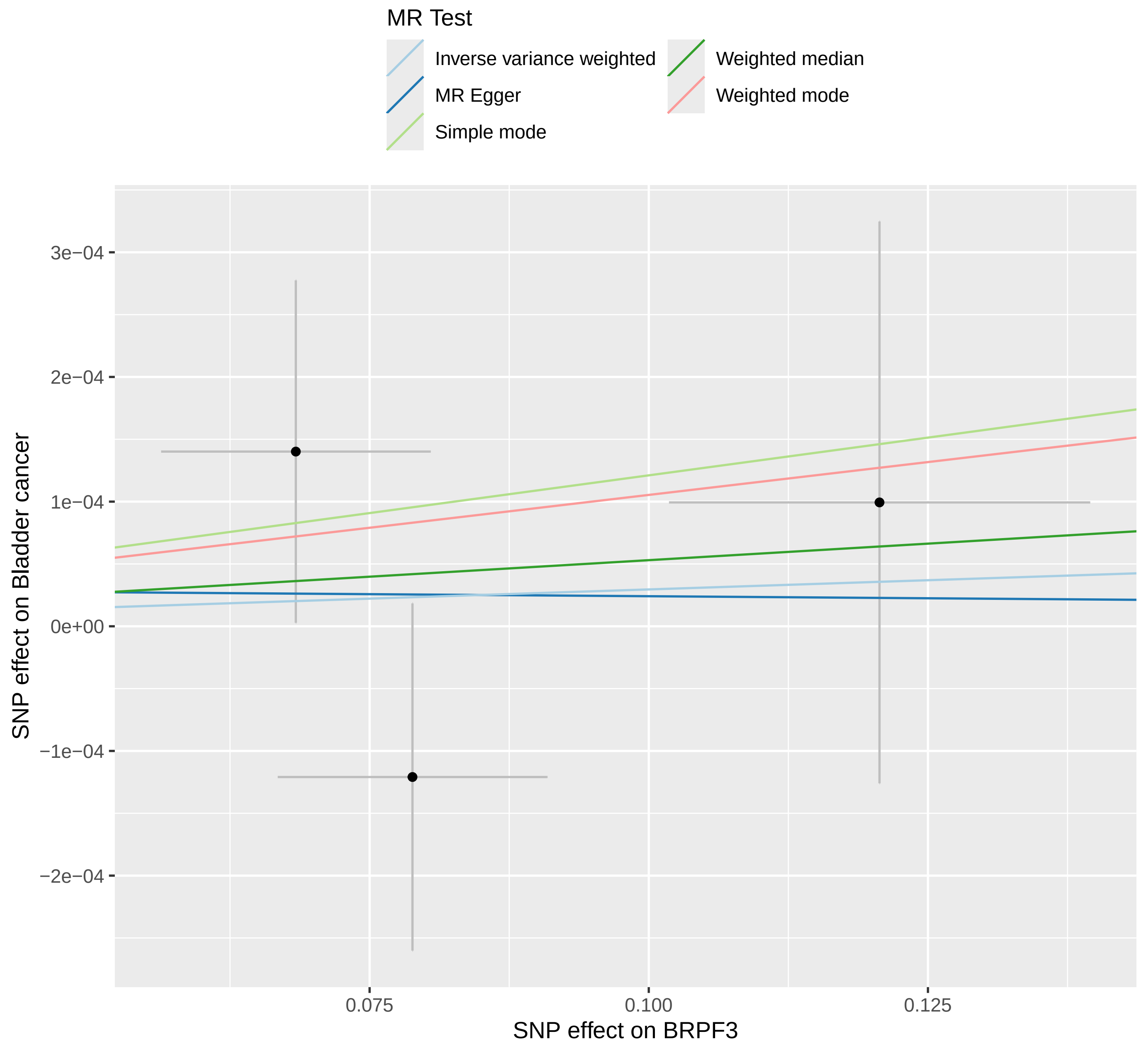
MR Test
Inverse variance weighted
MR Egger
Simple mode
Weighted median
Weighted mode
SNP effect on Bladder cancer
3e−04
2e−04
1e−04
0e+00
−1e−04
−2e−04
0.075
0.100
0.125
SNP effect on BRPF3

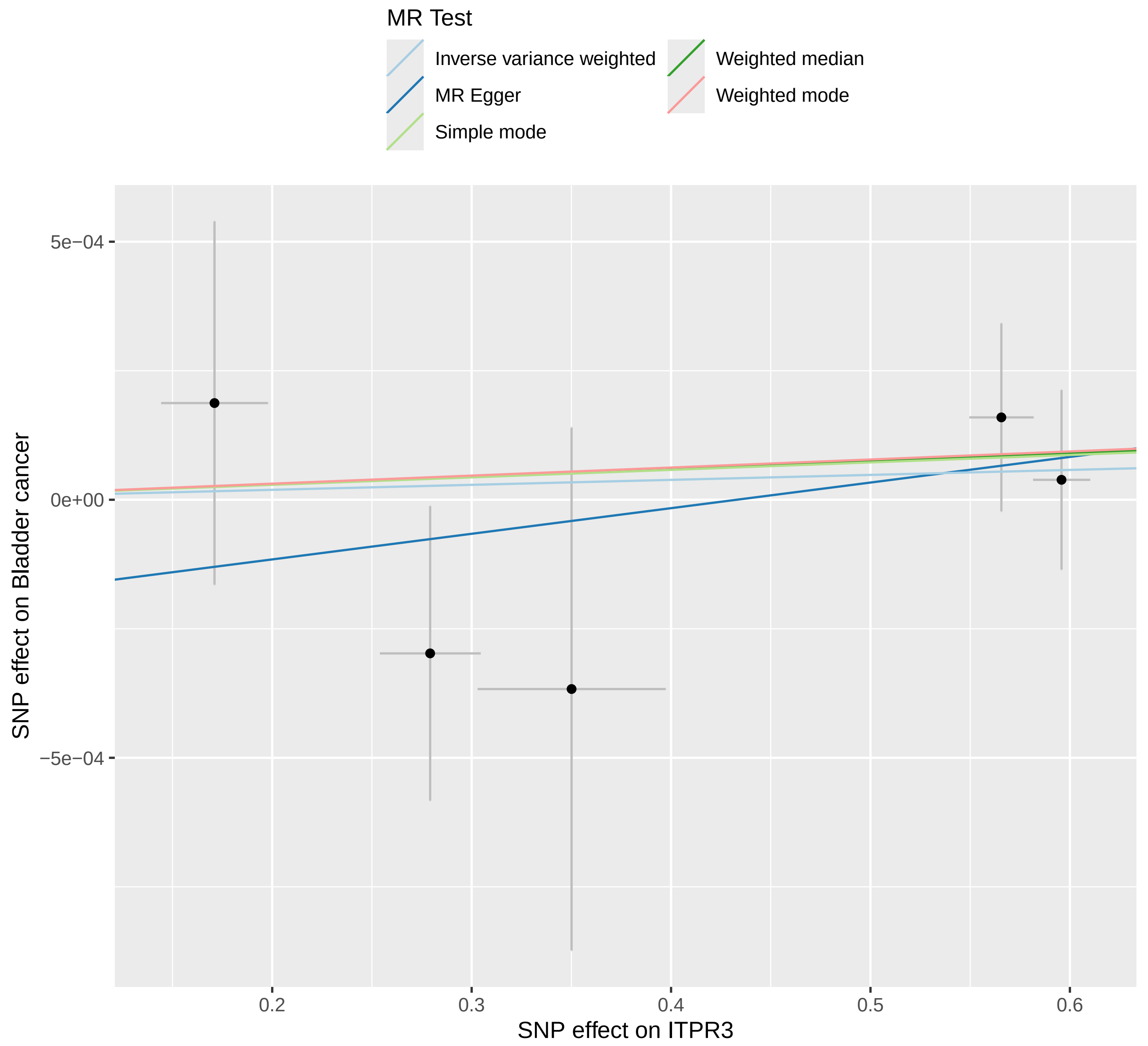
MR Test
Inverse variance weighted
MR Egger
Simple mode
Weighted median
Weighted mode
5e−04
0e+00
−5e−04
SNP effect on Bladder cancer
0.2
0.3
0.4
0.5
0.6
SNP effect on ITPR3

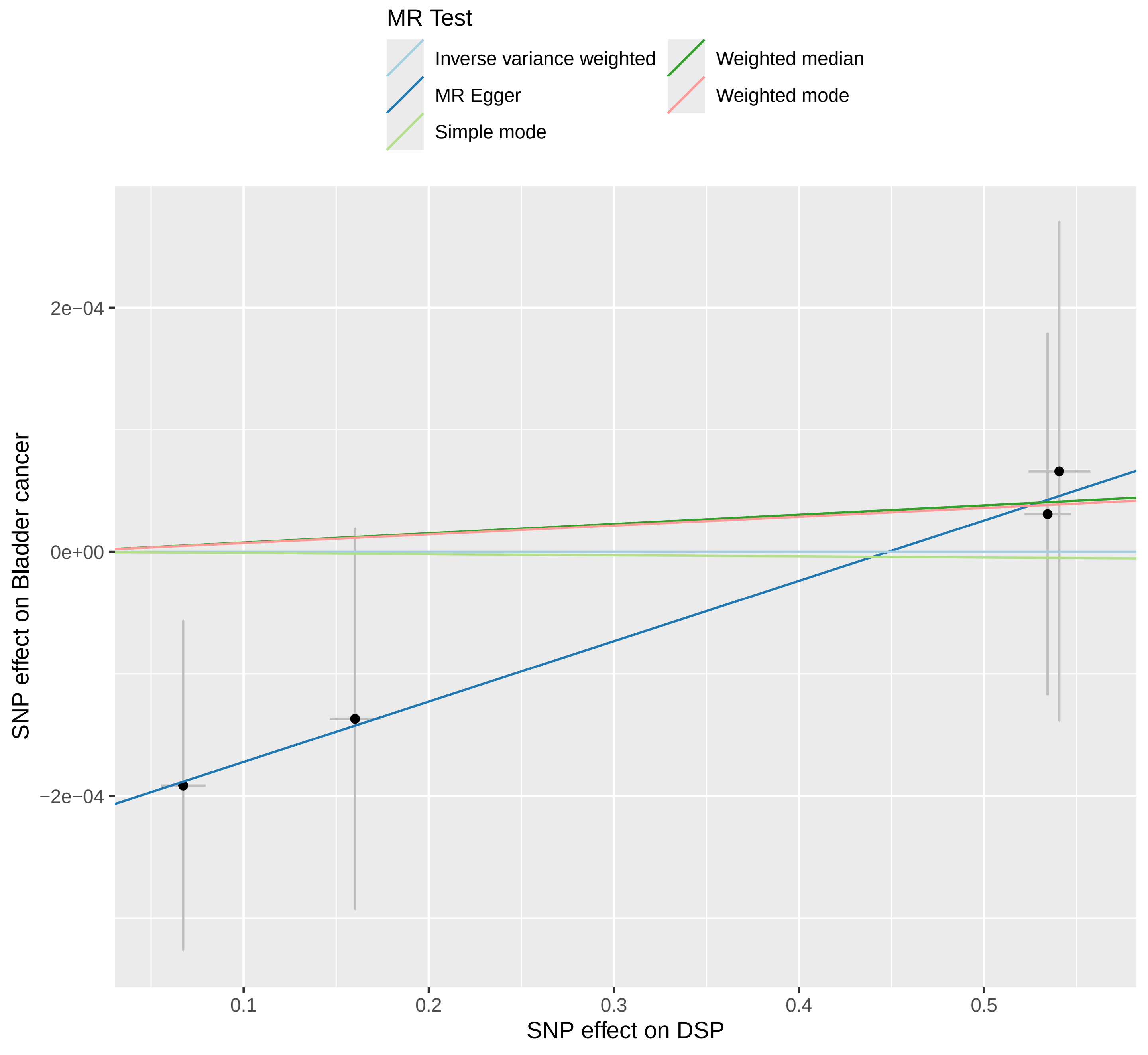
MR Test
Inverse variance weighted
MR Egger
Simple mode
Weighted median
Weighted mode
2e−04
0e+00
−2e−04
0.1
0.2
0.3
0.4
0.5
SNP effect on Bladder cancer
SNP effect on DSP

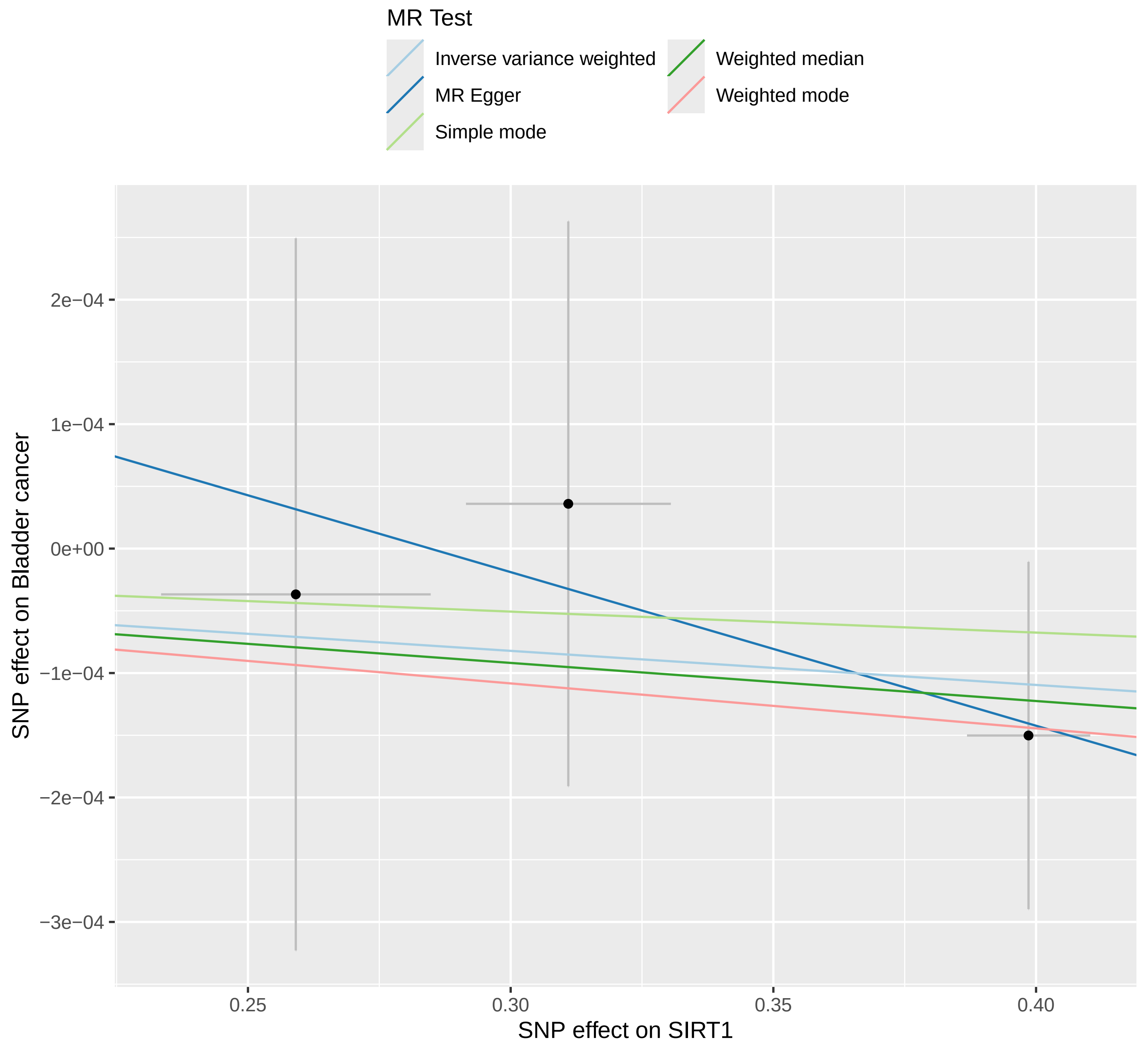

MR Test
Inverse variance weighted
MR Egger
Simple mode
Weighted median
Weighted mode
2e−04
1e−04
0e+00
−1e−04
−2e−04
−3e−04
SNP effect on Bladder cancer
0.25
0.30
0.35
0.40
SNP effect on SIRT1

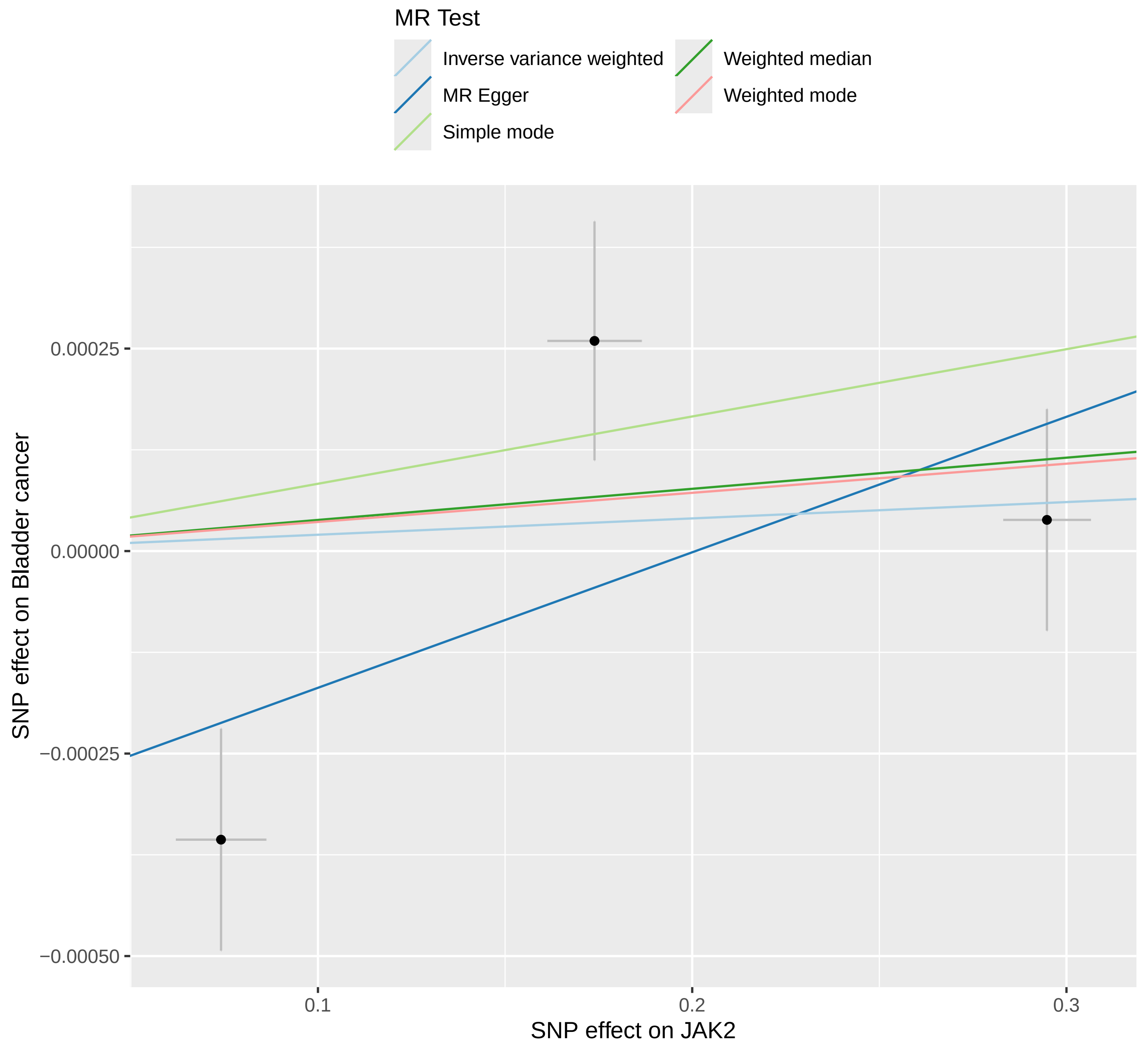

MR Test
Inverse variance weighted
MR Egger
Simple mode
Weighted median
Weighted mode
0.00025
0.00000
−0.00025
−0.00050
0.1
0.2
0.3
SNP effect on Bladder cancer
SNP effect on JAK2

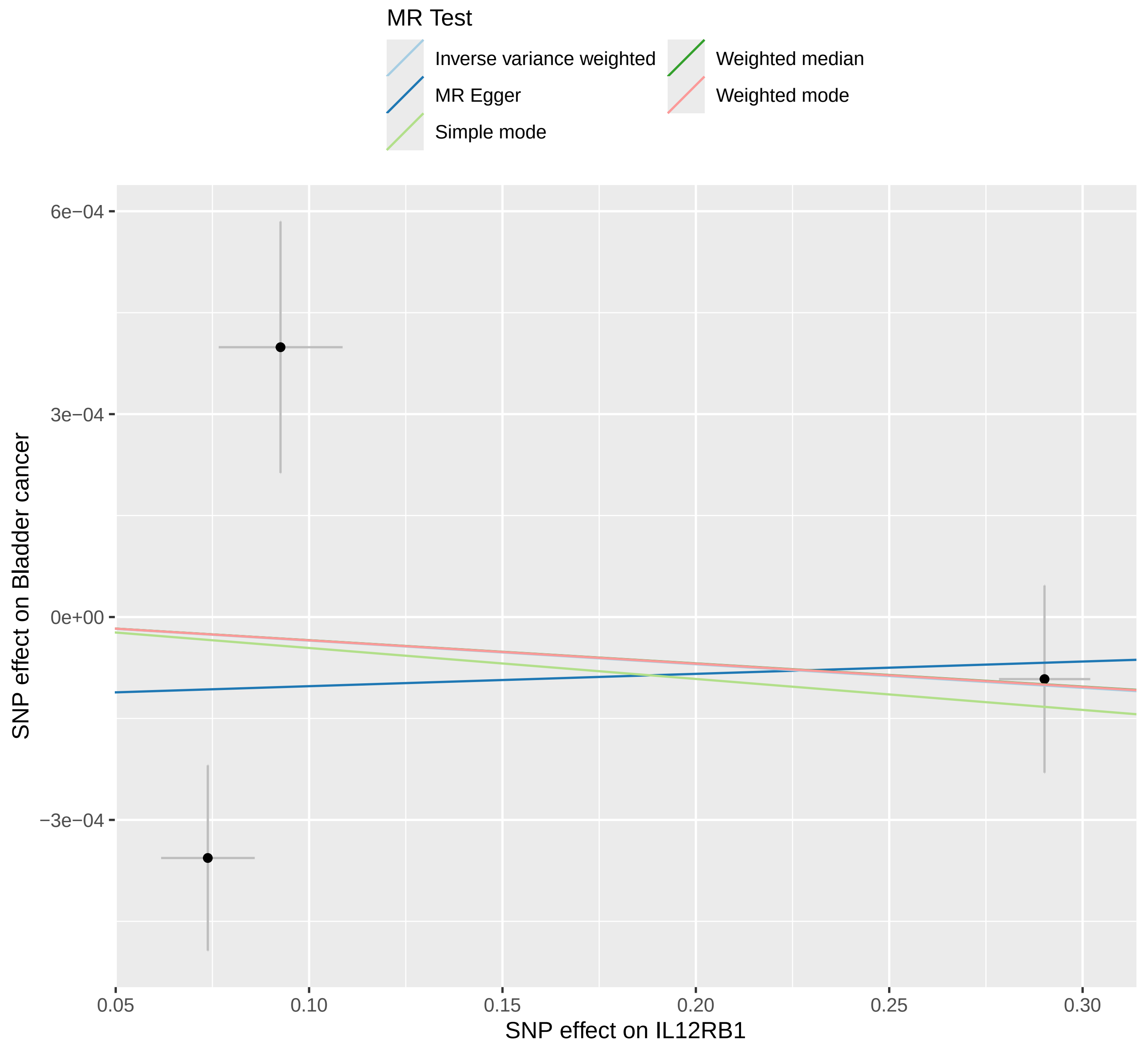

MR Test
Inverse variance weighted
MR Egger
Simple mode
Weighted median
Weighted mode
6e−04
3e−04
0e+00
−3e−04
SNP effect on Bladder cancer
0.05
0.10
0.15
0.20
0.25
0.30
SNP effect on IL12RB1

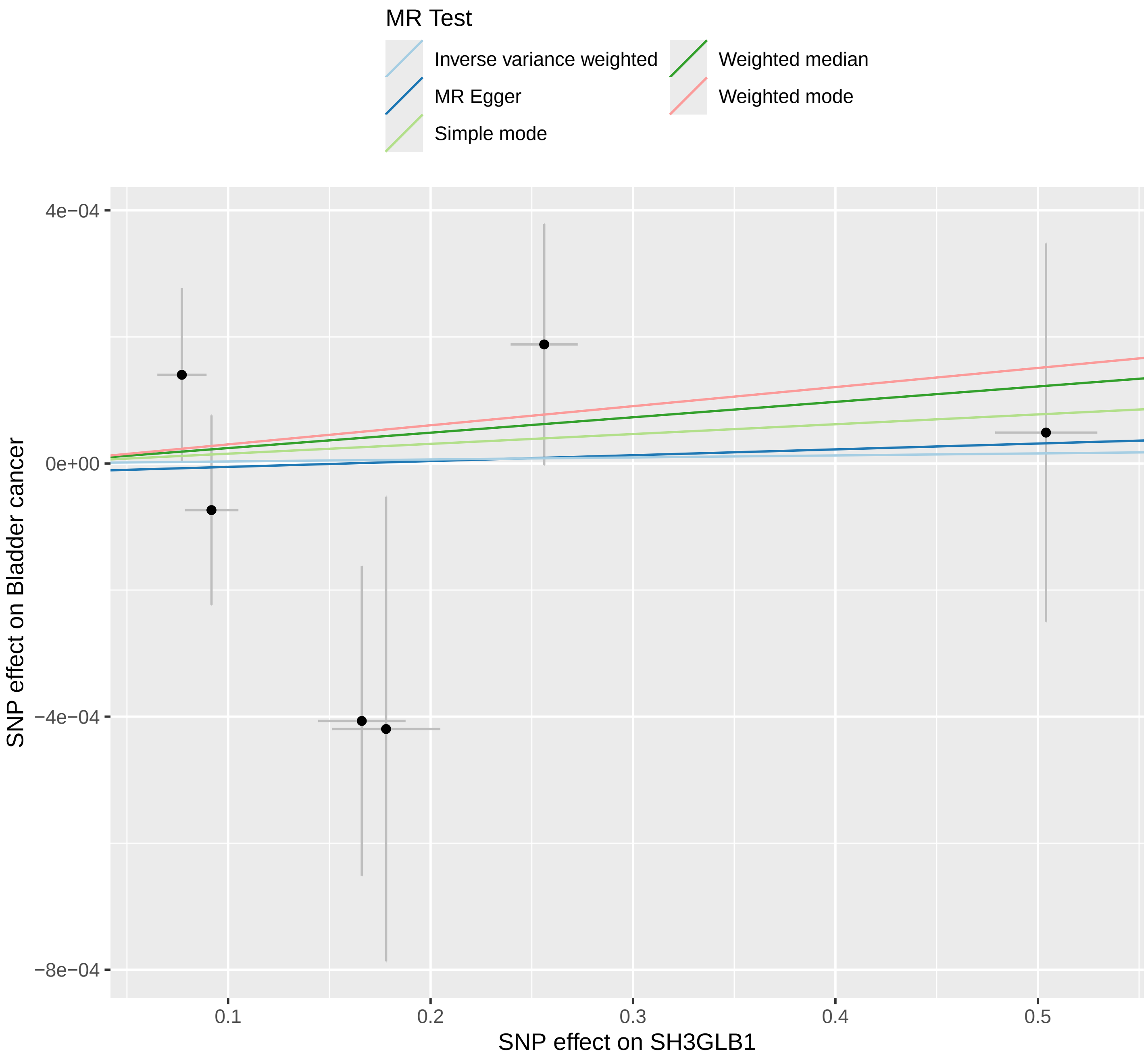

MR Test
Inverse variance weighted
MR Egger
Simple mode
Weighted median
Weighted mode
4e−04
0e+00
−4e−04
−8e−04
SNP effect on Bladder cancer
0.1
0.2
0.3
0.4
0.5
SNP effect on SH3GLB1

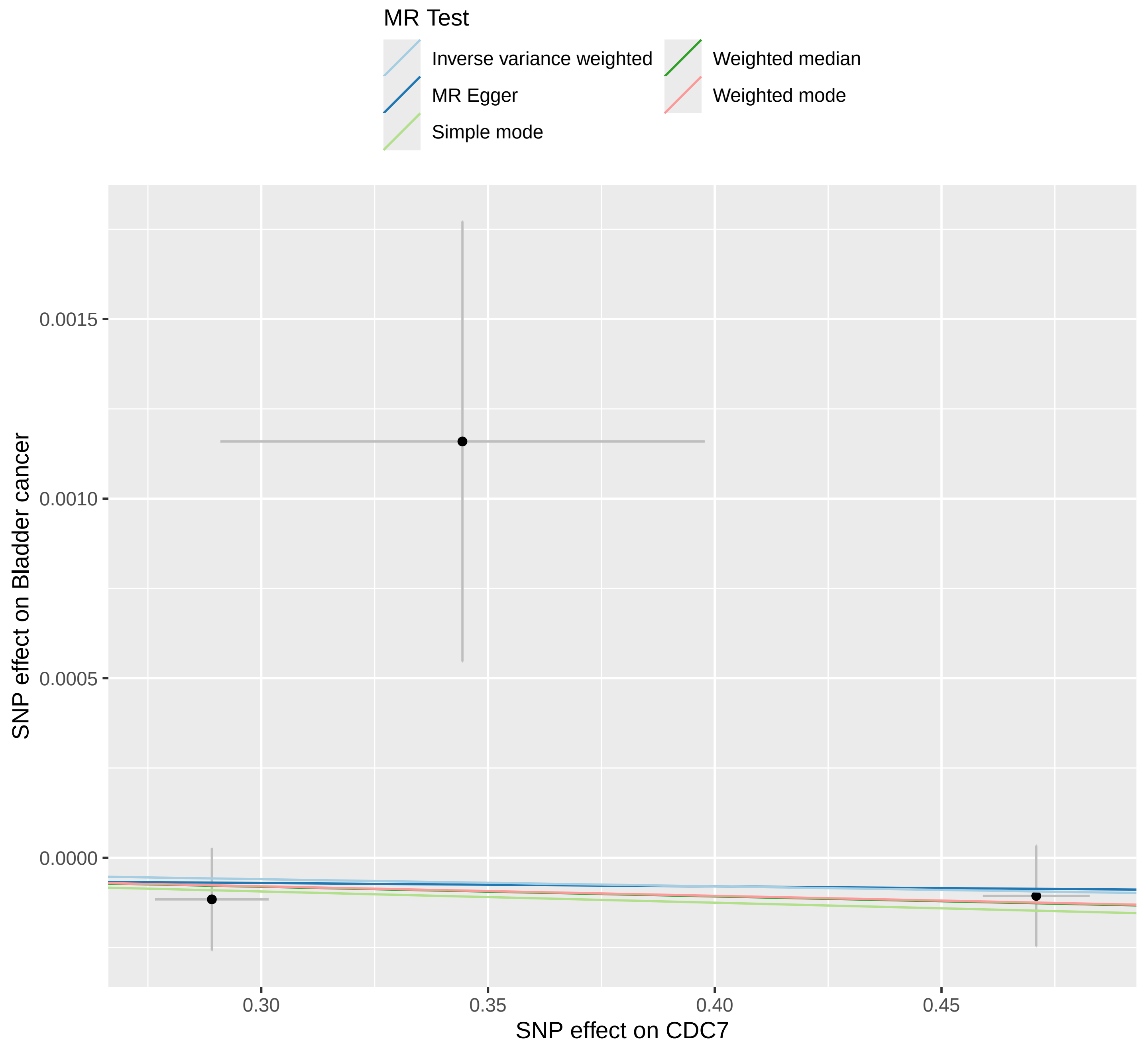
MR Test
Inverse variance weighted
MR Egger
Simple mode
Weighted median
Weighted mode
0.0015
0.0010
0.0005
0.0000
SNP effect on Bladder cancer
0.30
0.35
0.40
0.45
SNP effect on CDC7

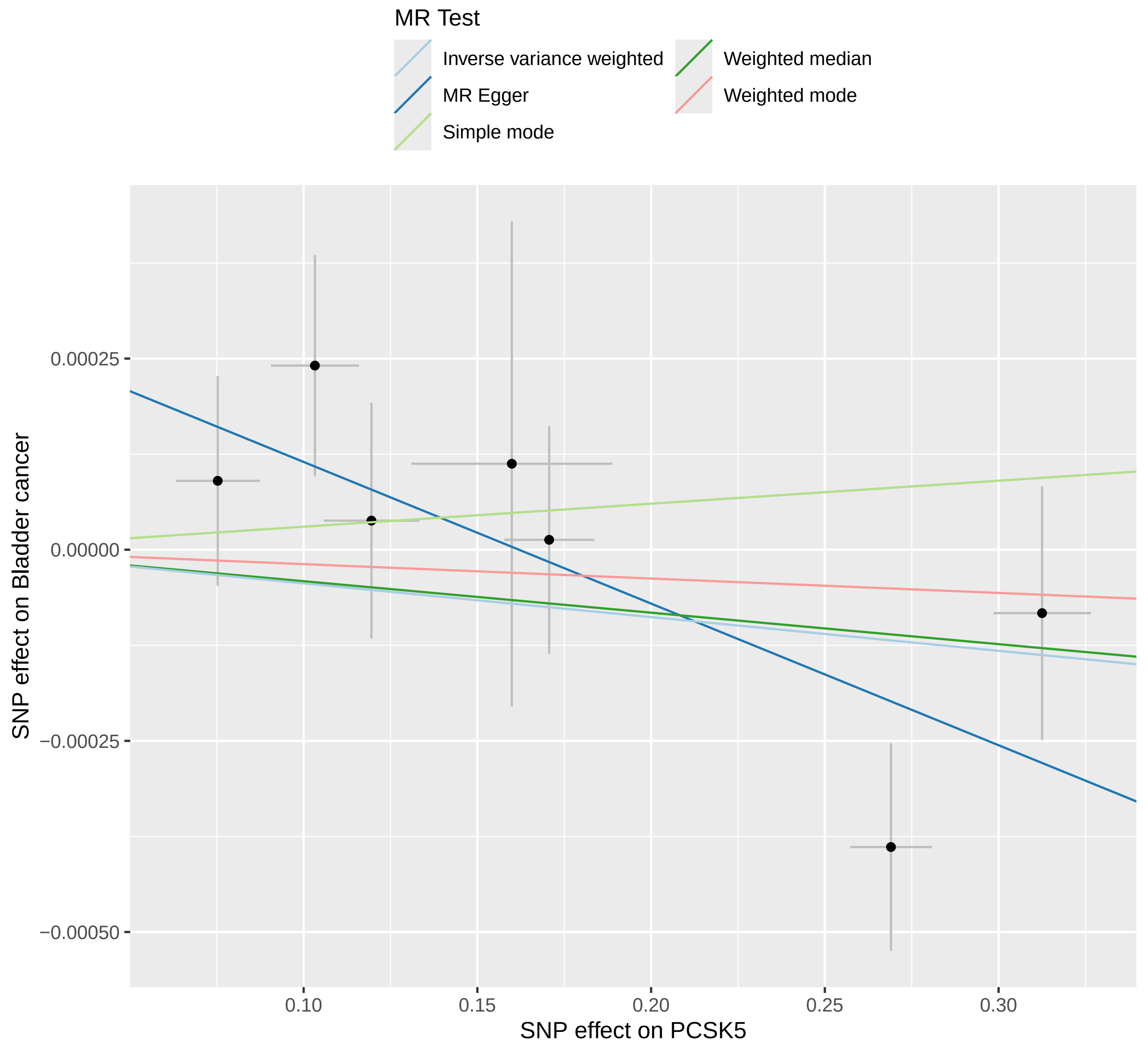

MR Test
Inverse variance weighted
MR Egger
Simple mode
Weighted median
Weighted mode
0.00025
0.00000
−0.00025
−0.00050
SNP effect on Bladder cancer
0.10
0.15
0.20
0.25
0.30
SNP effect on PCSK5

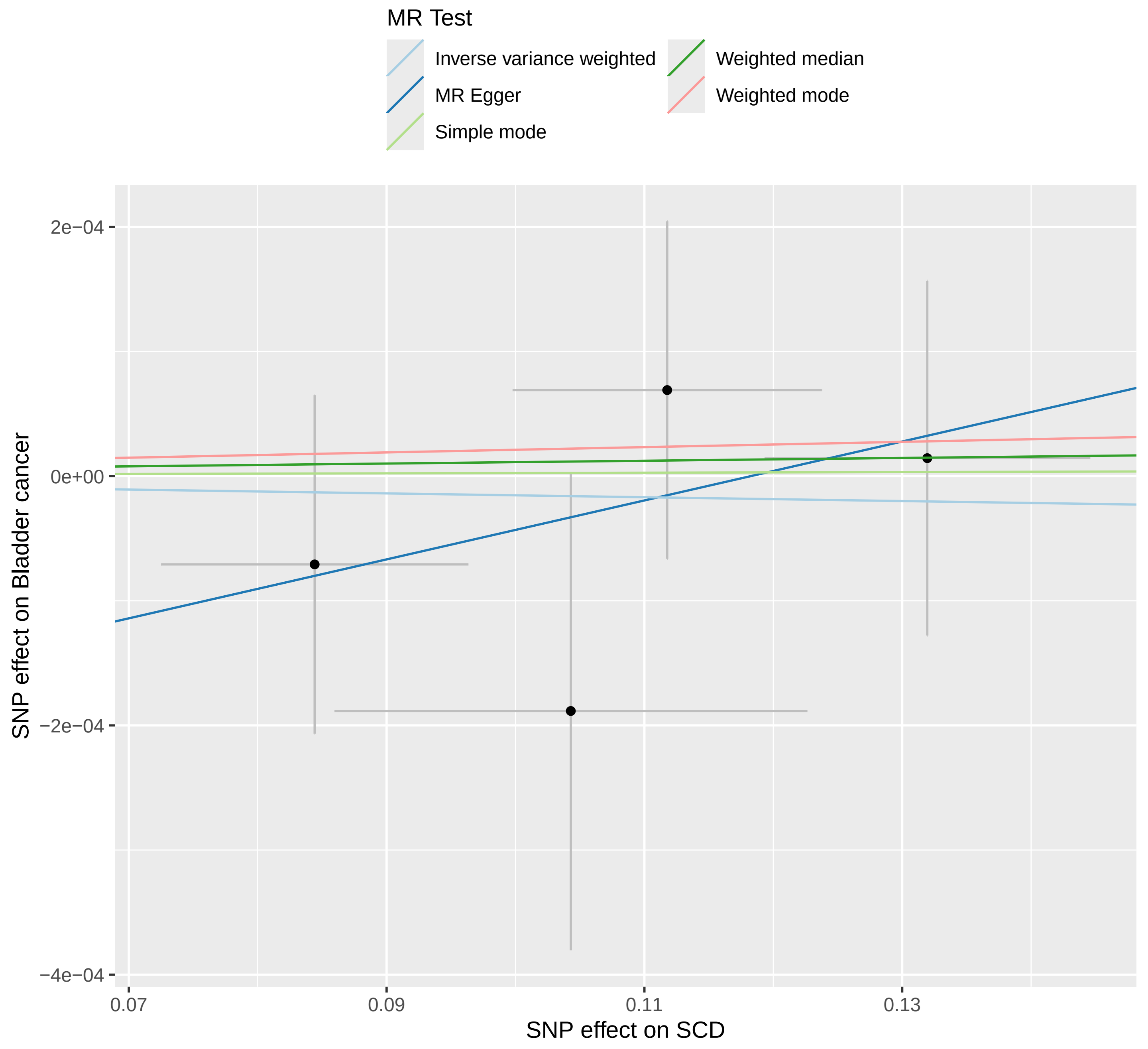

MR Test
Inverse variance weighted
MR Egger
Simple mode
Weighted median
Weighted mode
2e−04
0e+00
−2e−04
−4e−04
0.07
0.09
0.11
0.13
SNP effect on SCD
SNP effect on Bladder cancer

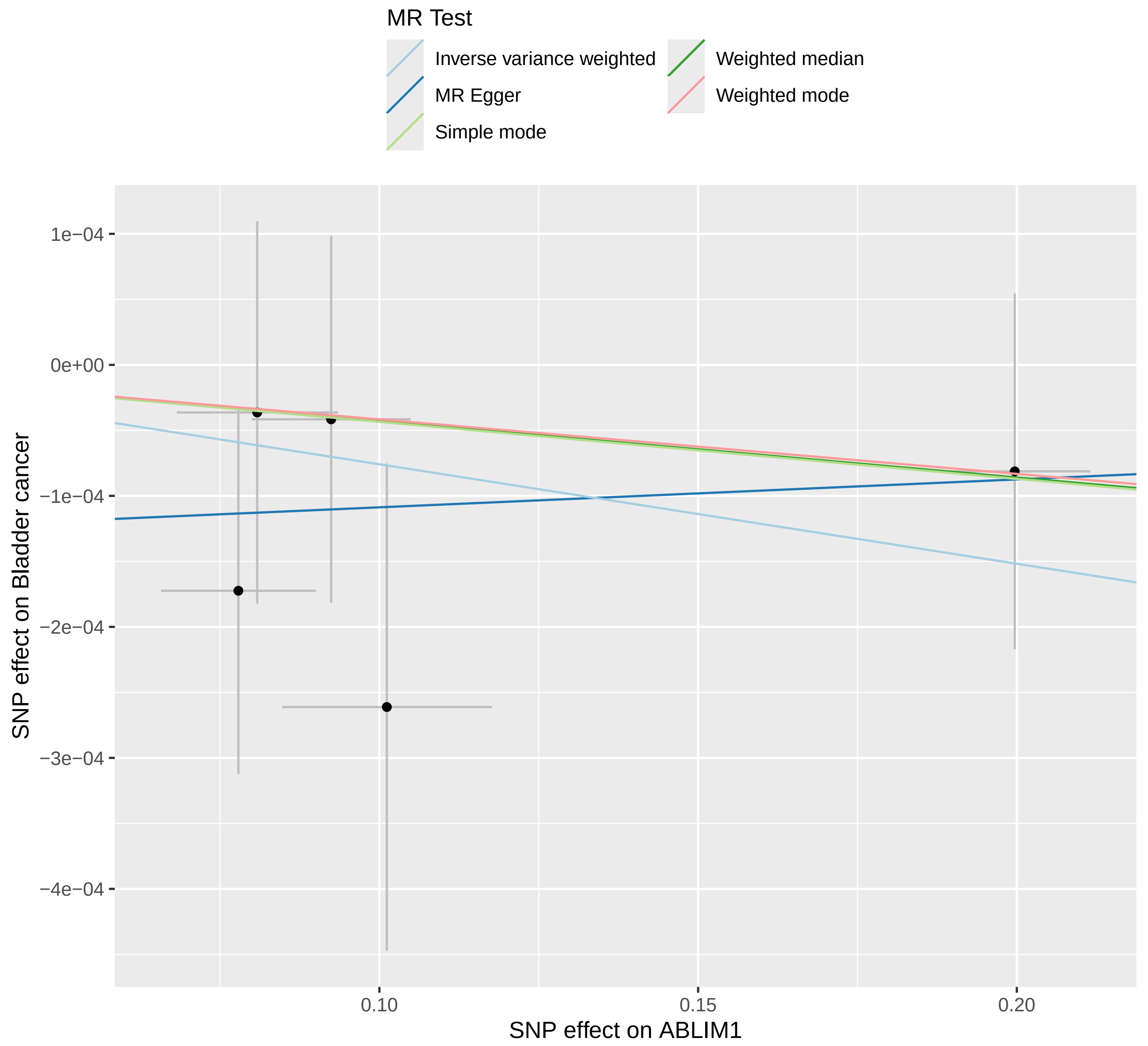

MR Test
Inverse variance weighted
MR Egger
Simple mode
Weighted median
Weighted mode
1e−04
0e+00
−1e−04
−2e−04
−3e−04
−4e−04
SNP effect on Bladder cancer
0.10
0.15
0.20
SNP effect on ABLIM1

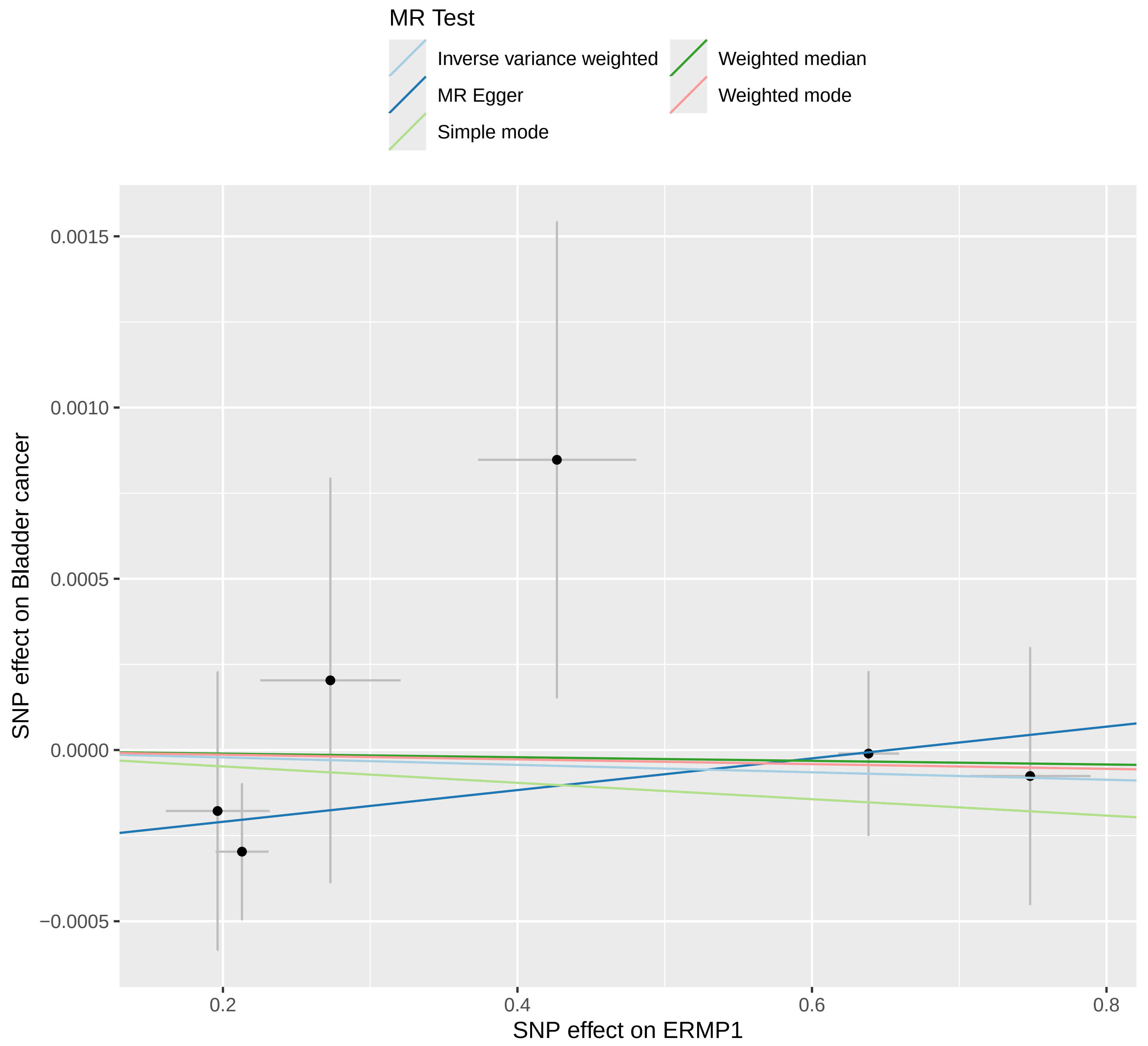

MR Test
Inverse variance weighted
MR Egger
Simple mode
Weighted median
Weighted mode
0.0015
0.0010
0.0005
0.0000
−0.0005
SNP effect on Bladder cancer
0.2
0.4
0.6
0.8
SNP effect on ERMP1

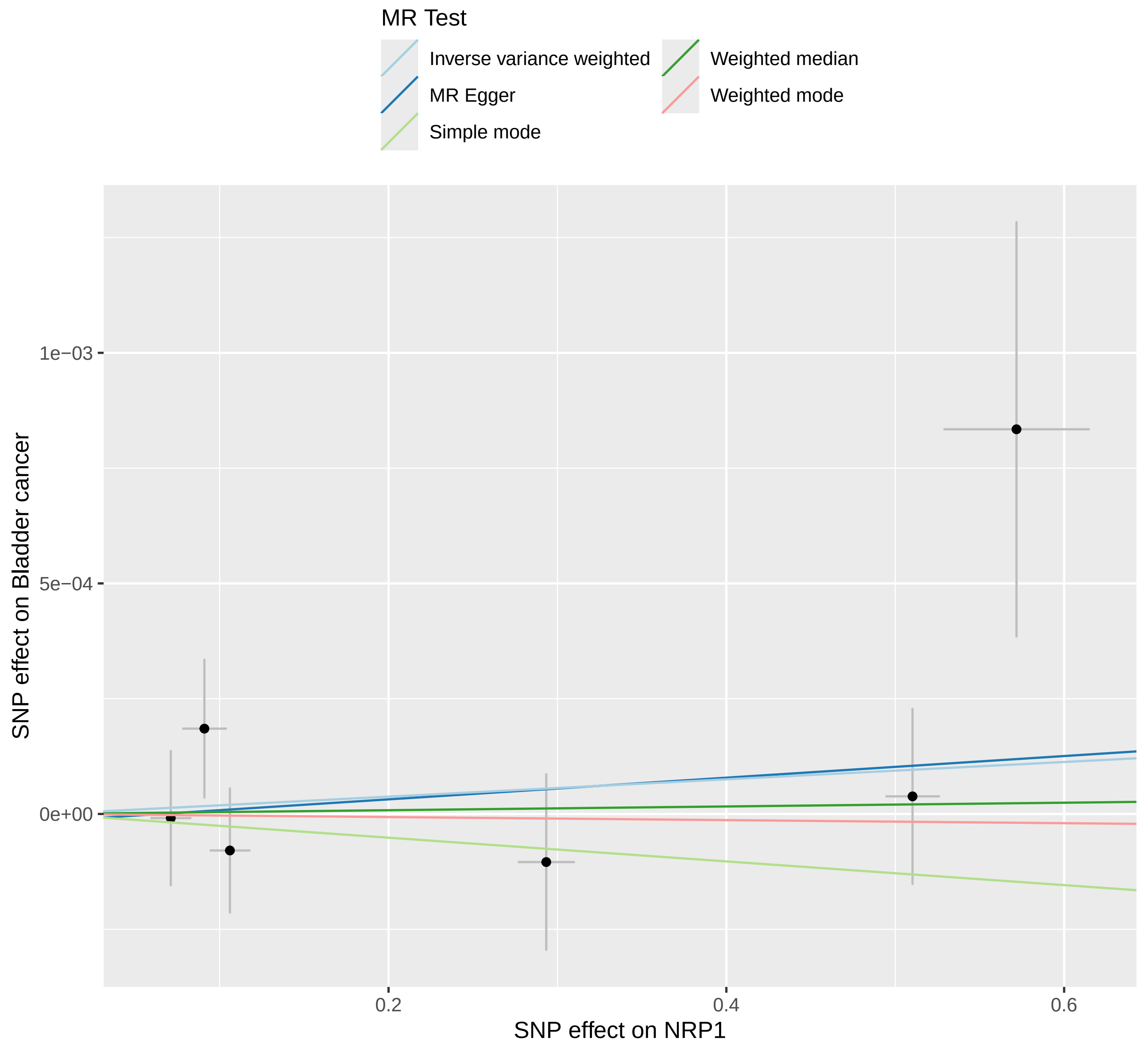

MR Test
Inverse variance weighted
MR Egger
Simple mode
Weighted median
Weighted mode
1e−03
5e−04
0e+00
SNP effect on Bladder cancer
0.2
0.4
0.6
SNP effect on NRP1

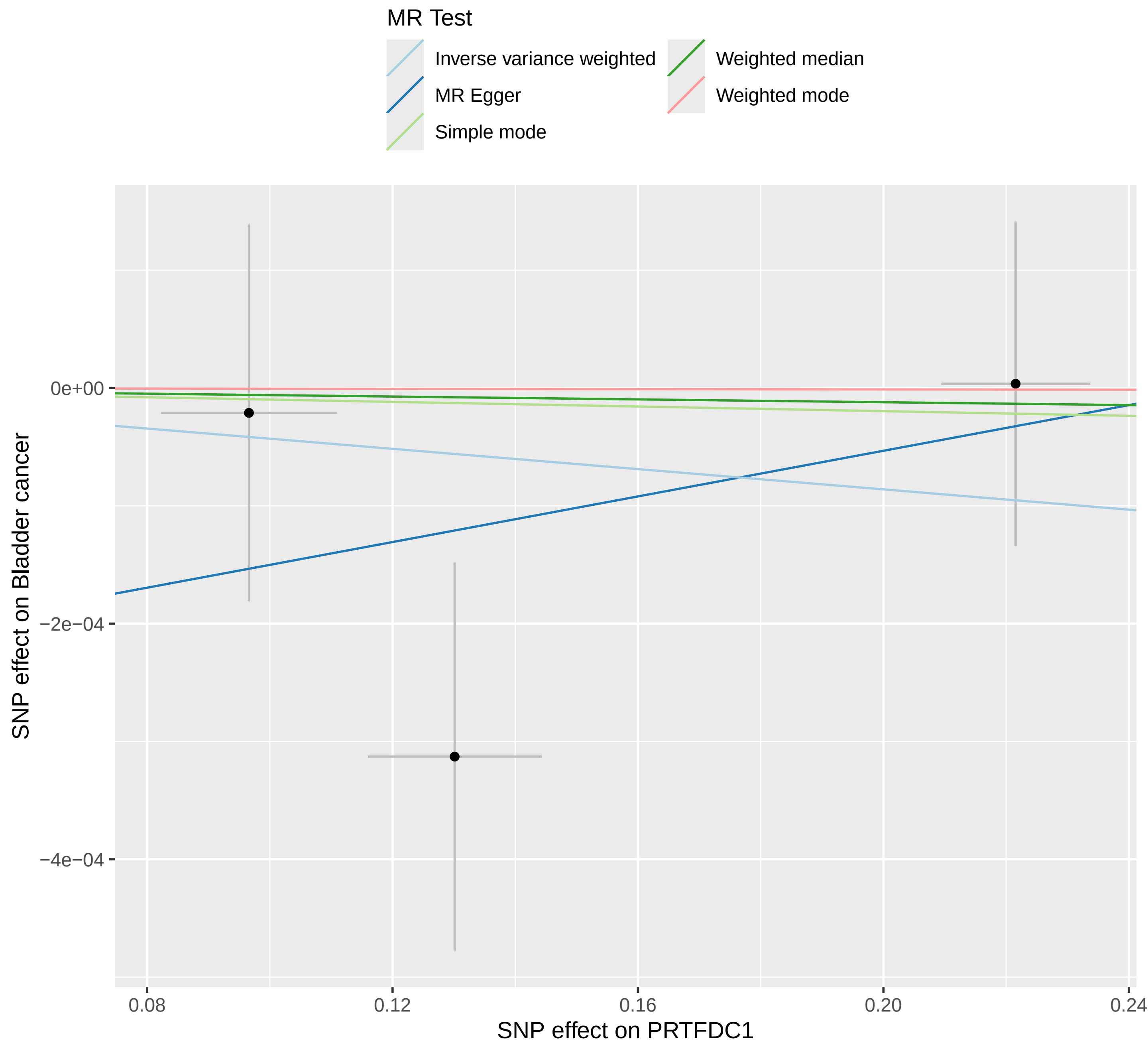

MR Test
Inverse variance weighted
MR Egger
Simple mode
Weighted median
Weighted mode
0e+00
−2e−04
−4e−04
SNP effect on Bladder cancer
0.08
0.12
0.16
0.20
0.24
SNP effect on PRTFDC1

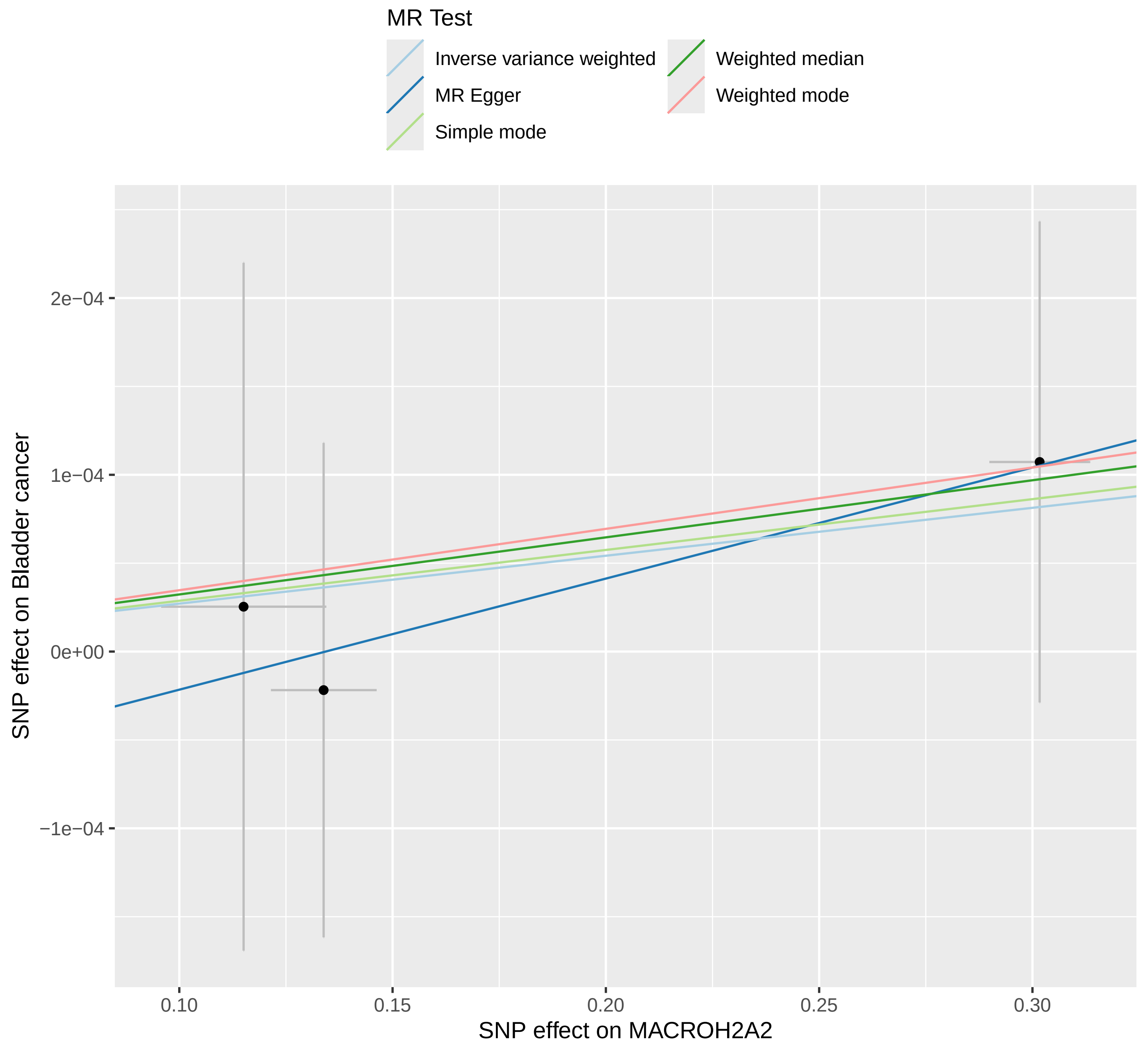

MR Test
Inverse variance weighted
MR Egger
Simple mode
Weighted median
Weighted mode
2e−04
1e−04
0e+00
−1e−04
SNP effect on Bladder cancer
0.10
0.15
0.20
0.25
0.30
SNP effect on MACROH2A2

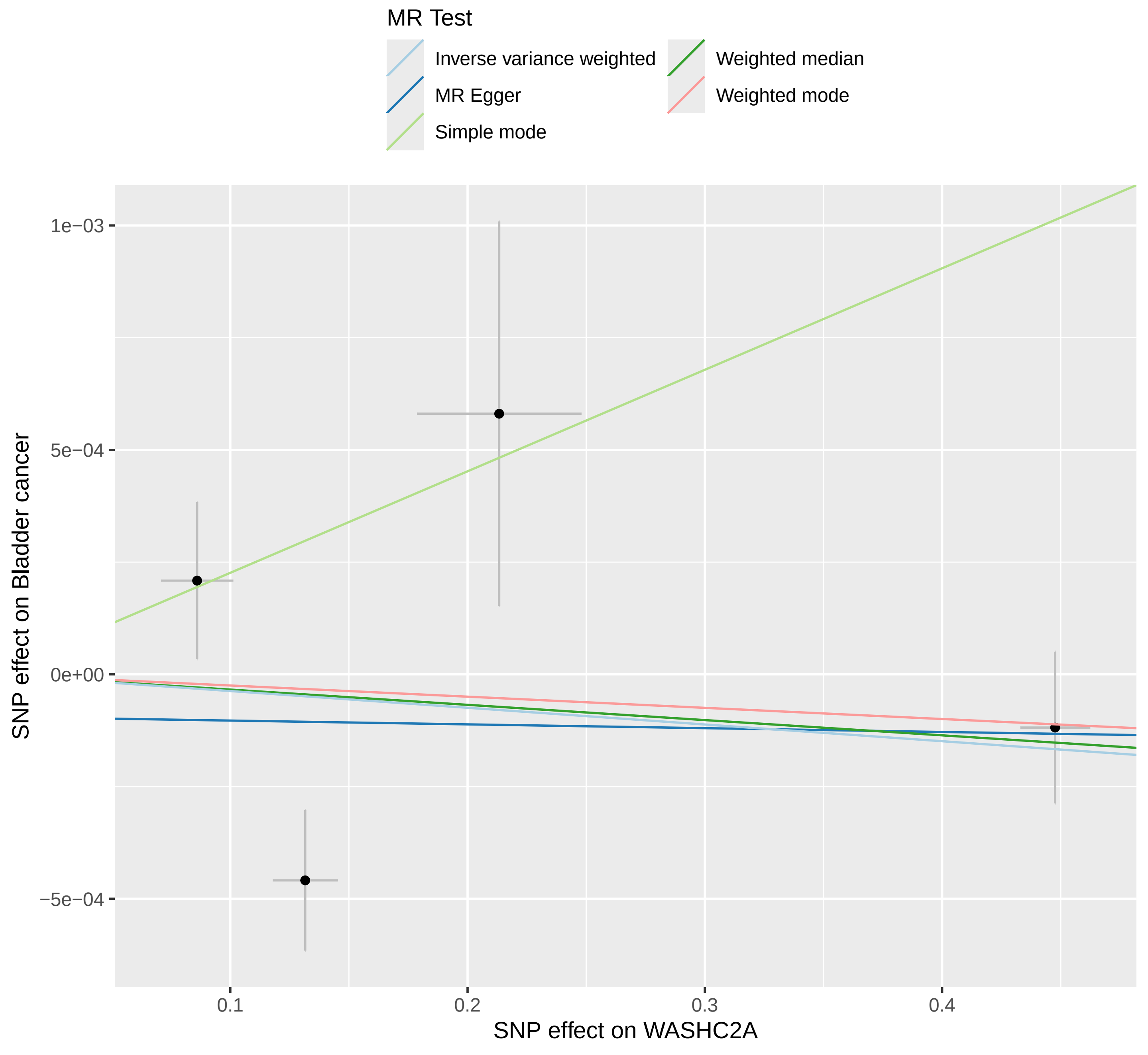
MR Test
Inverse variance weighted
MR Egger
Simple mode
Weighted median
Weighted mode
1e−03
5e−04
0e+00
−5e−04
0.1
0.2
0.3
0.4
SNP effect on WASHC2A
SNP effect on Bladder cancer

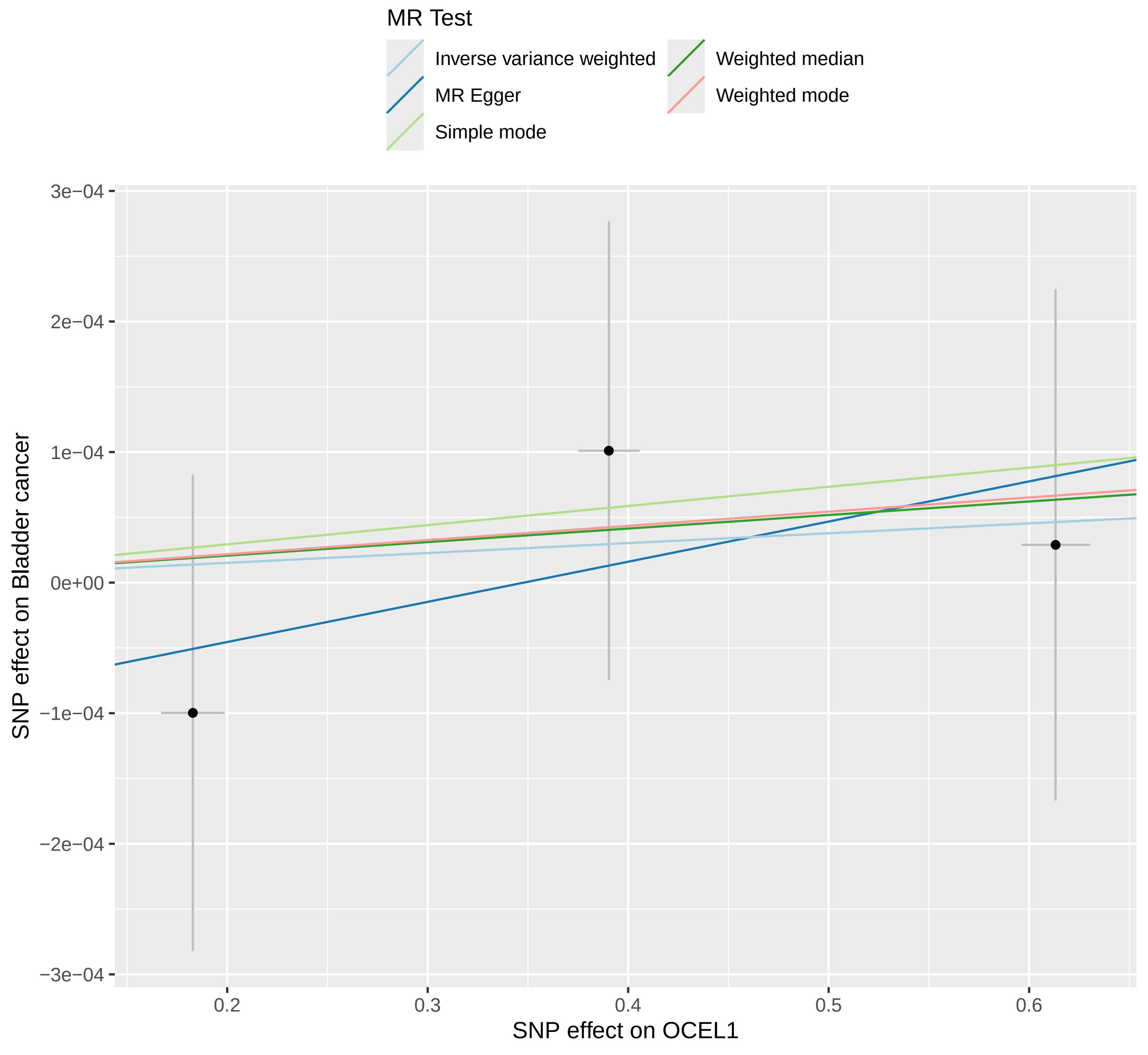
MR Test
Inverse variance weighted
MR Egger
Simple mode
Weighted median
Weighted mode
3e−04
2e−04
1e−04
0e+00
−1e−04
−2e−04
−3e−04
0.2
0.3
0.4
0.5
0.6
SNP effect on Bladder cancer
SNP effect on OCEL1

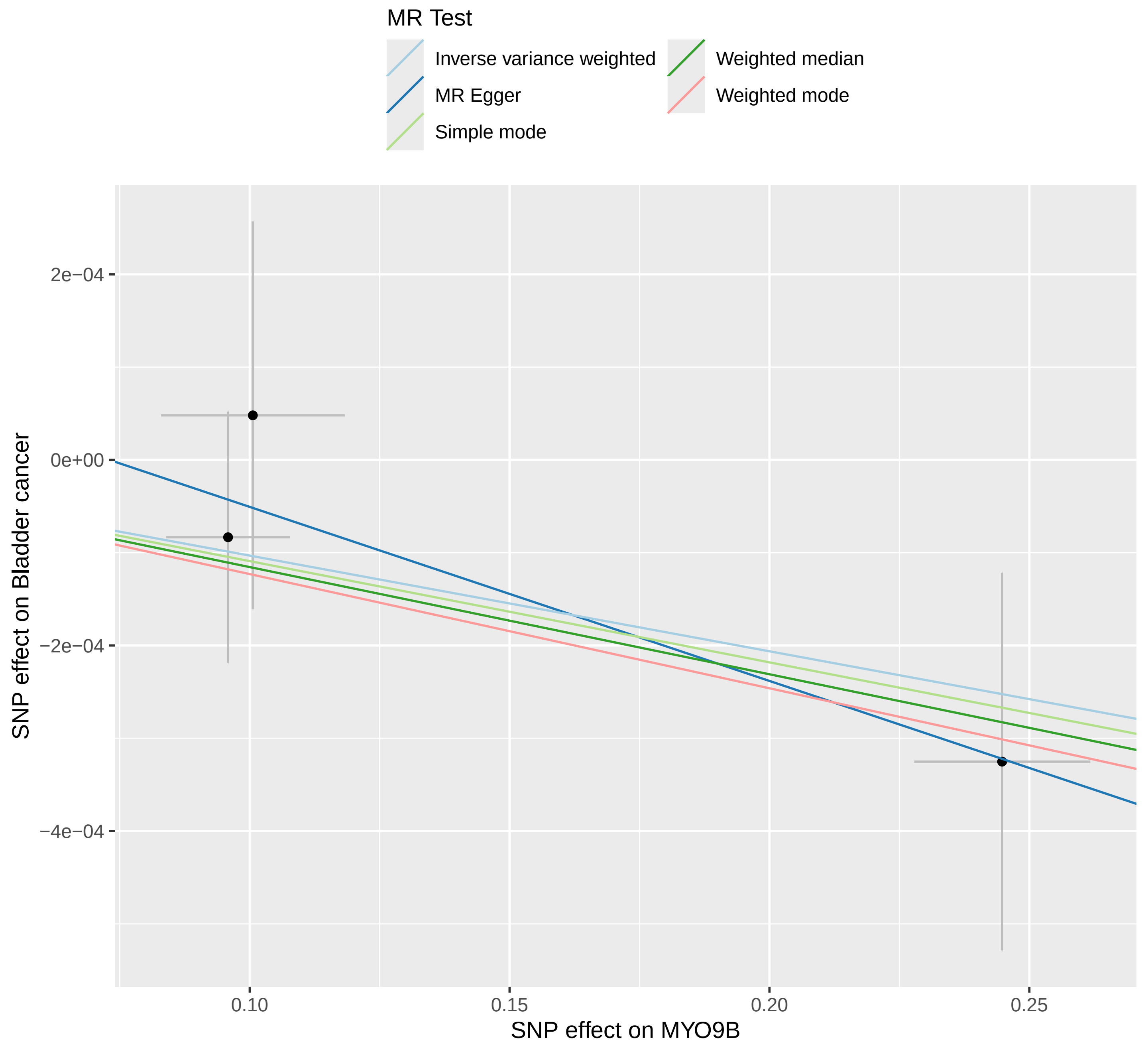
MR Test
Inverse variance weighted
MR Egger
Simple mode
Weighted median
Weighted mode
2e−04
0e+00
−2e−04
−4e−04
0.10
0.15
0.20
0.25
SNP effect on MYO9B
SNP effect on Bladder cancer

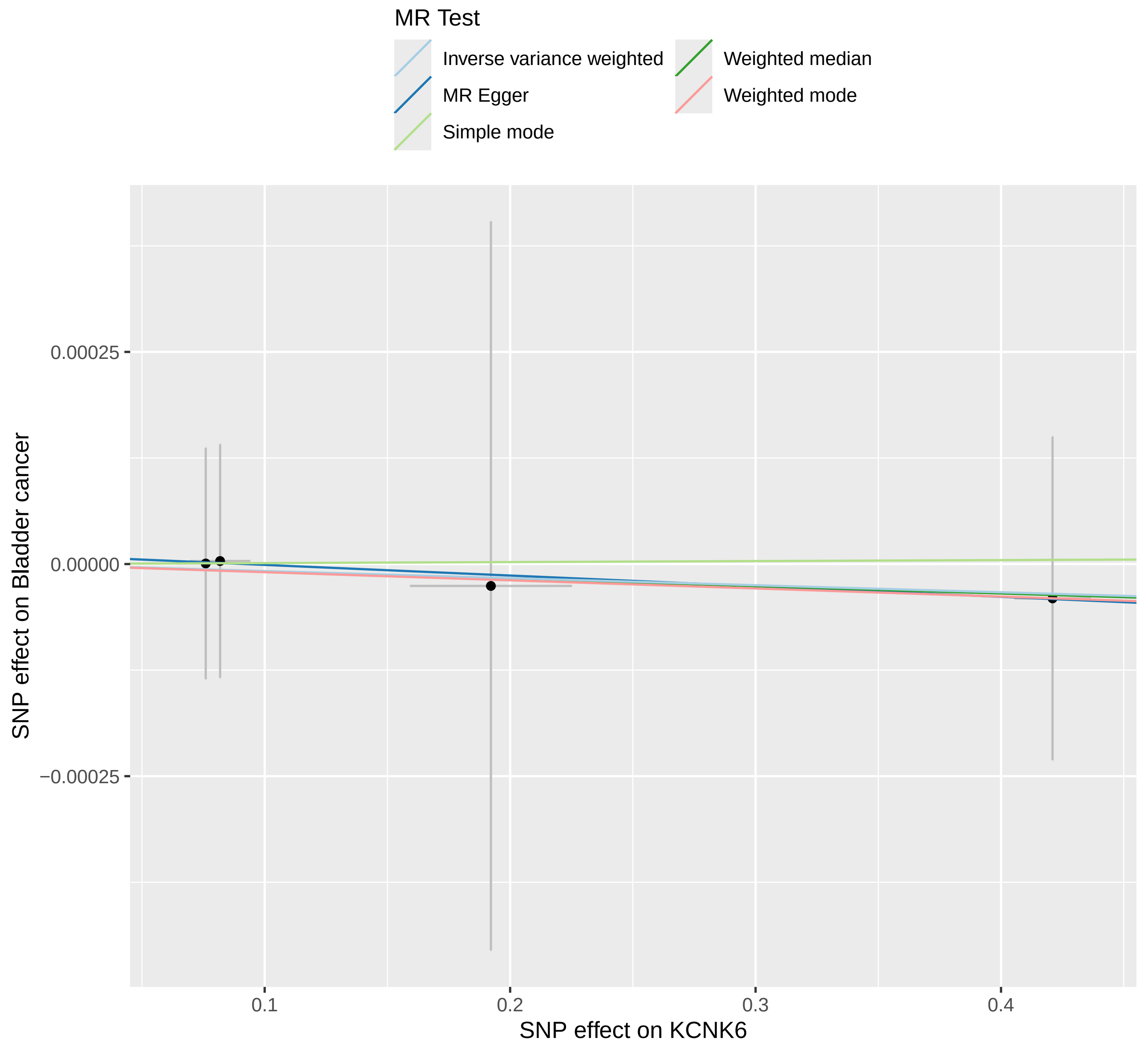

MR Test
Inverse variance weighted
MR Egger
Simple mode
Weighted median
Weighted mode
0.00025
0.00000
−0.00025
0.1
0.2
0.3
0.4
SNP effect on KCNK6
SNP effect on Bladder cancer

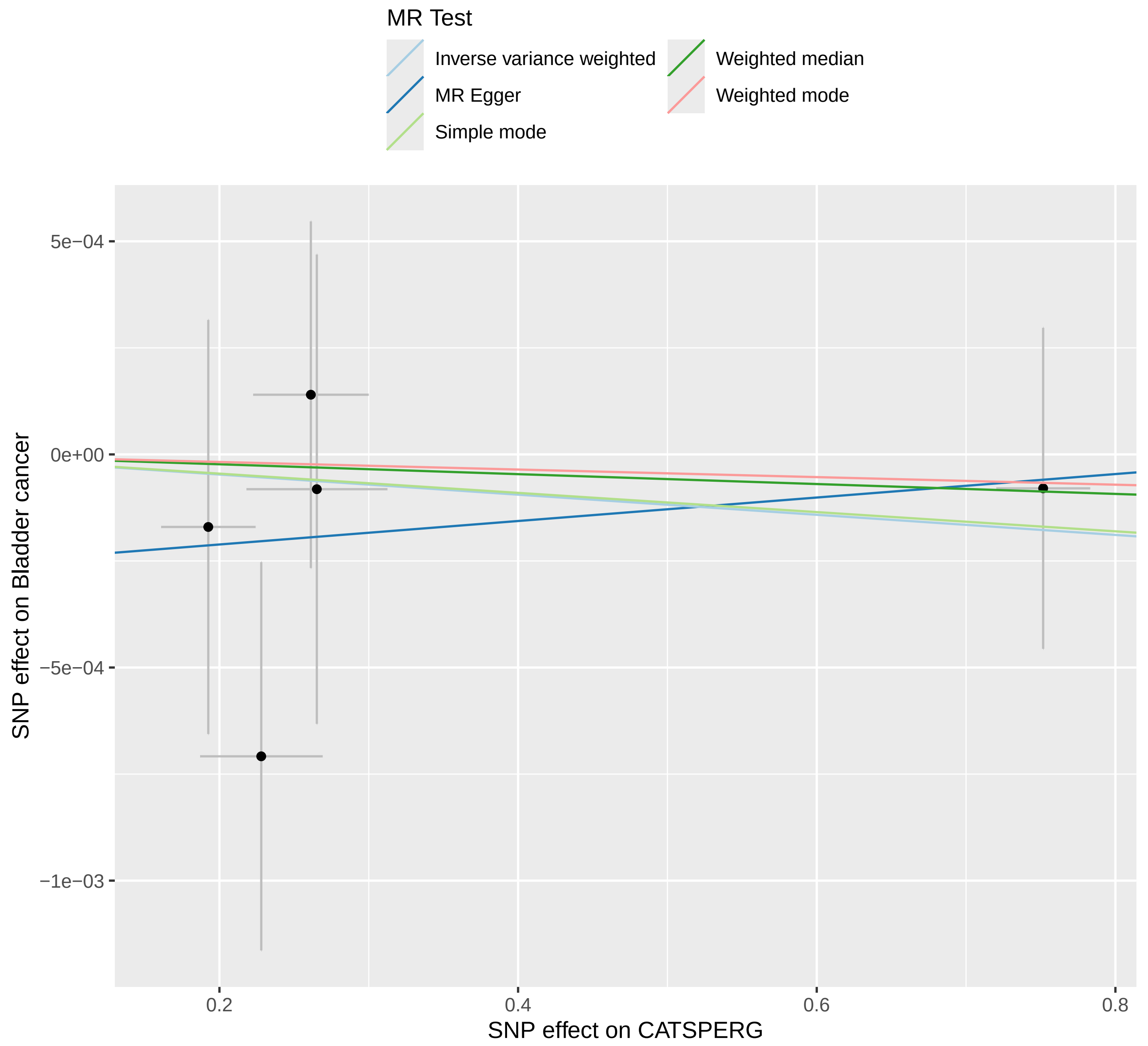
MR Test
Inverse variance weighted
MR Egger
Simple mode
Weighted median
Weighted mode
5e−04
0e+00
−5e−04
−1e−03
0.2
0.4
0.6
0.8
SNP effect on CATSPERG
SNP effect on Bladder cancer

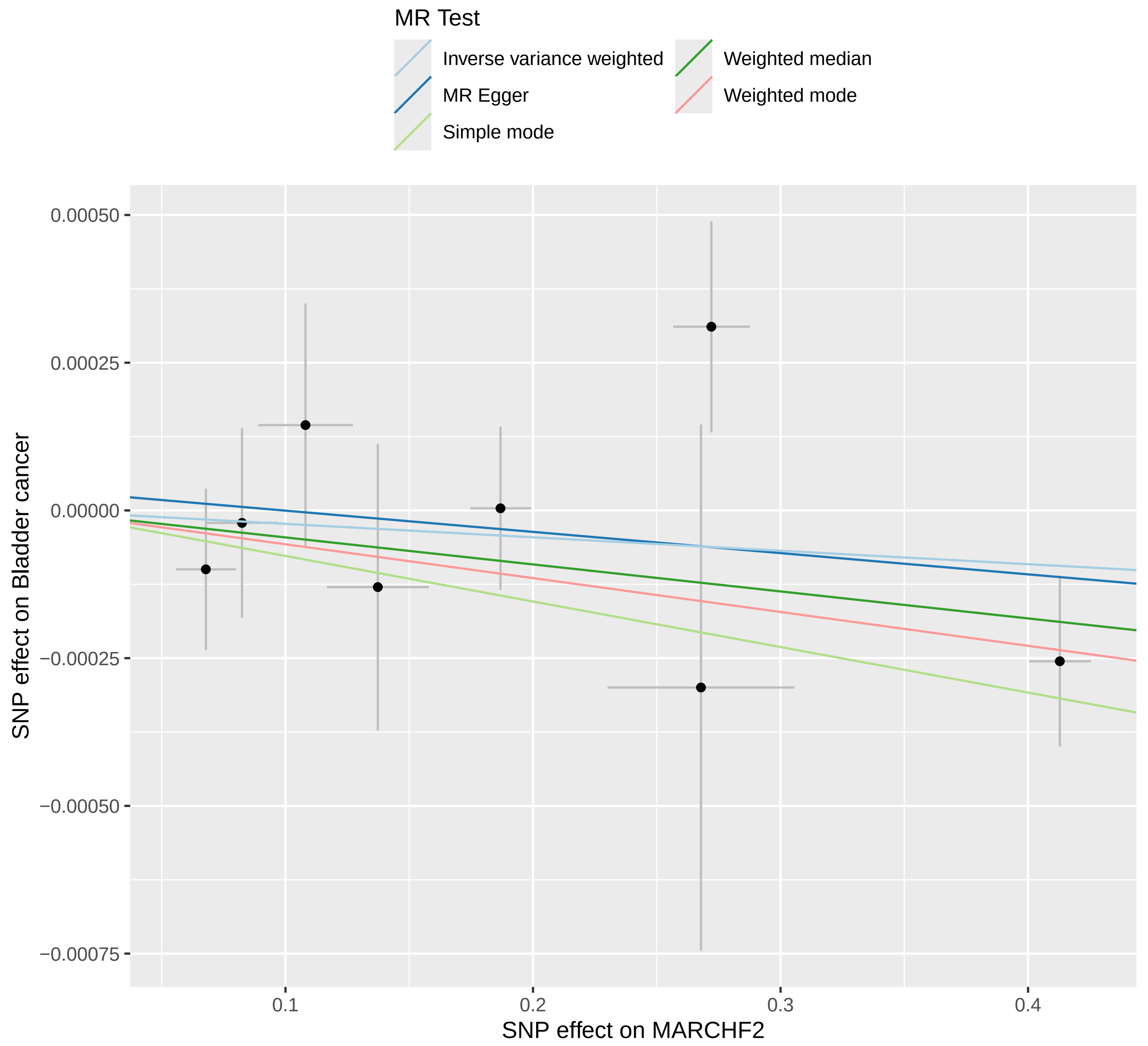

MR Test
Inverse variance weighted
MR Egger
Simple mode
Weighted median
Weighted mode
0.00050
0.00025
0.00000
−0.00025
−0.00050
−0.00075
SNP effect on Bladder cancer
0.1
0.2
0.3
0.4
SNP effect on MARCHF2

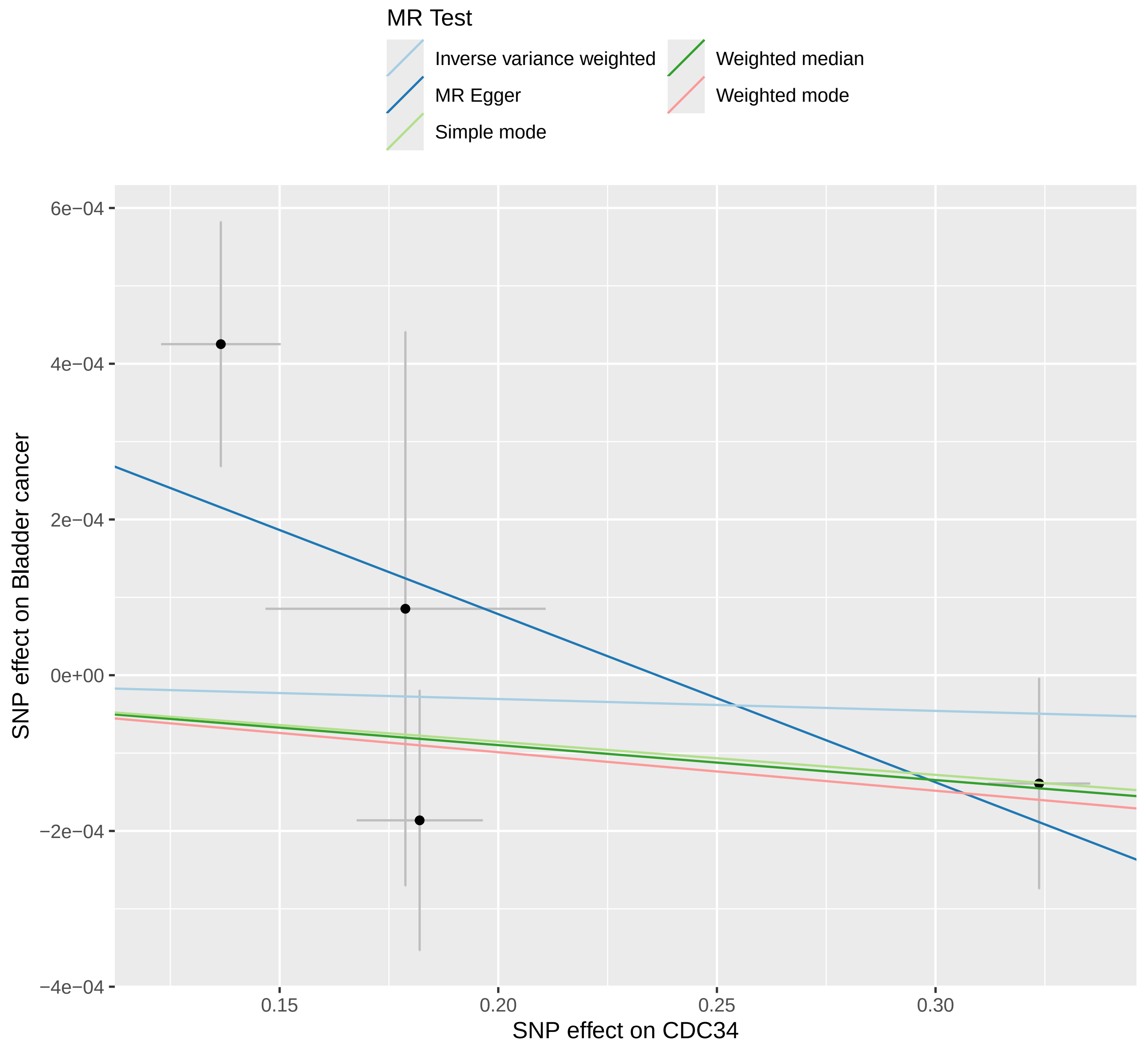

MR Test
Inverse variance weighted
MR Egger
Simple mode
Weighted median
Weighted mode
6e−04
4e−04
2e−04
0e+00
−2e−04
−4e−04
SNP effect on Bladder cancer
0.15
0.20
0.25
0.30
SNP effect on CDC34

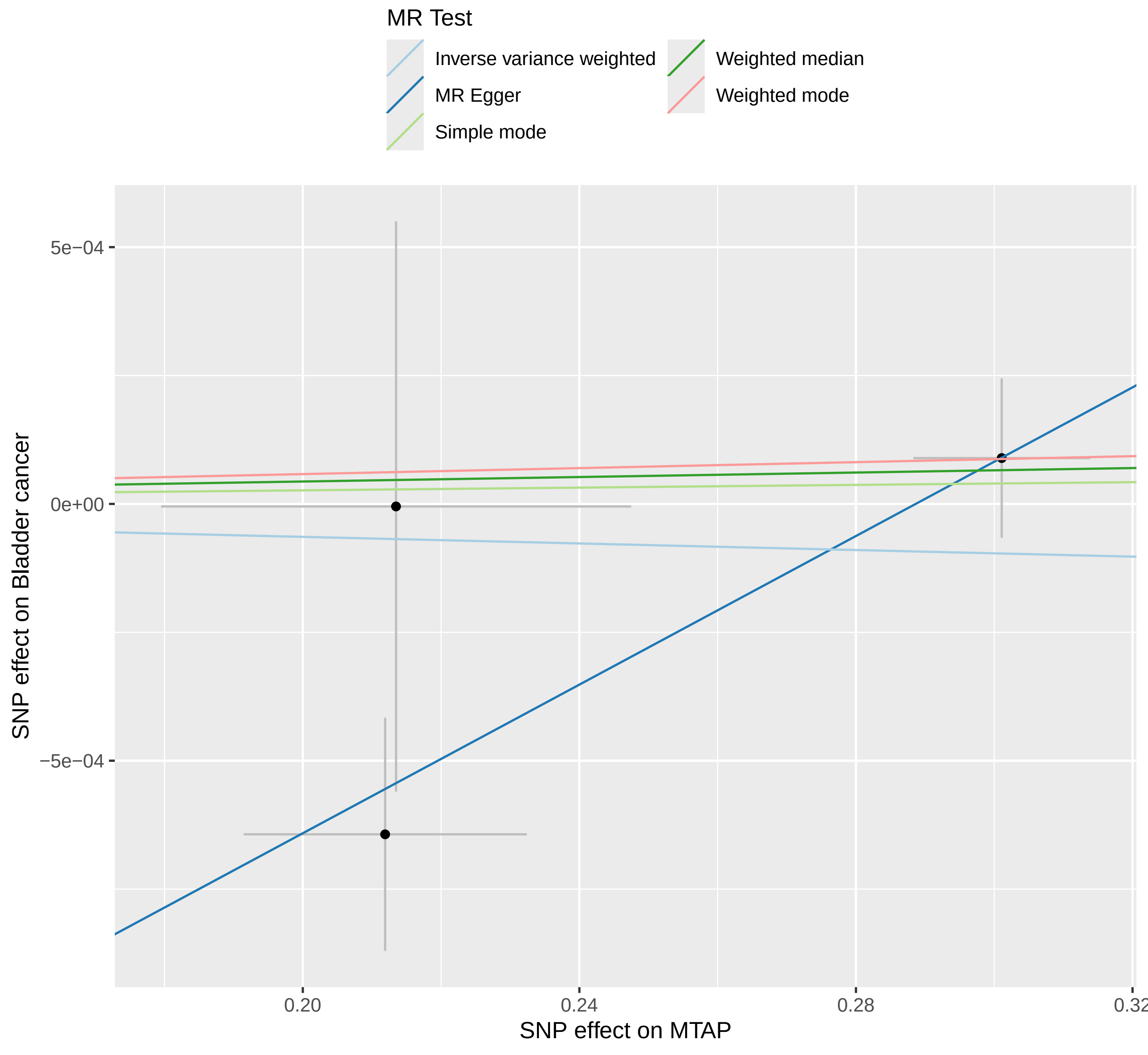

MR Test
Inverse variance weighted
MR Egger
Simple mode
Weighted median
Weighted mode
5e−04
0e+00
−5e−04
SNP effect on Bladder cancer
0.20
0.24
0.28
0.32
SNP effect on MTAP

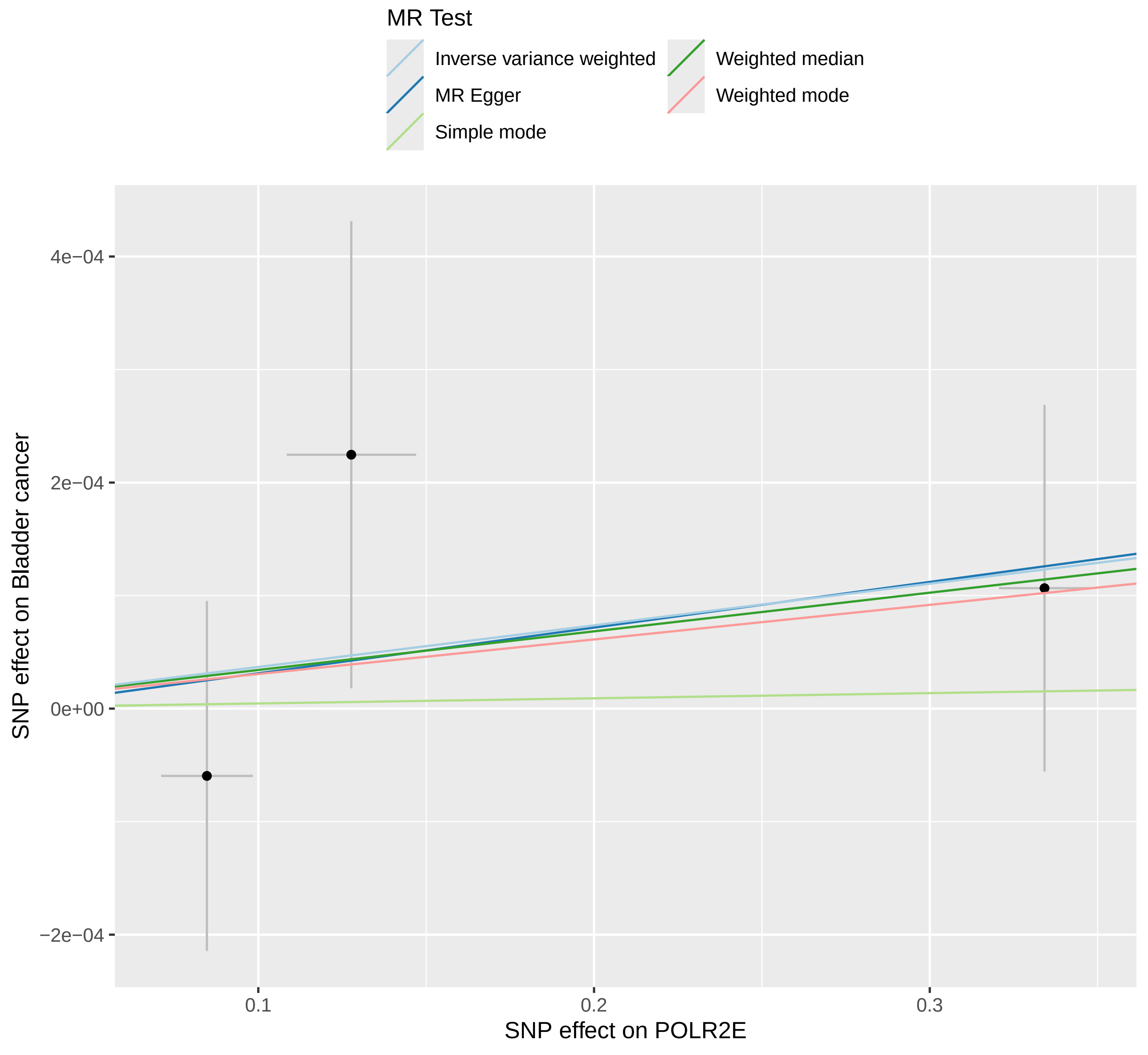

MR Test
Inverse variance weighted
MR Egger
Simple mode
Weighted median
Weighted mode
4e−04
2e−04
0e+00
−2e−04
0.1
0.2
0.3
SNP effect on Bladder cancer
SNP effect on POLR2E

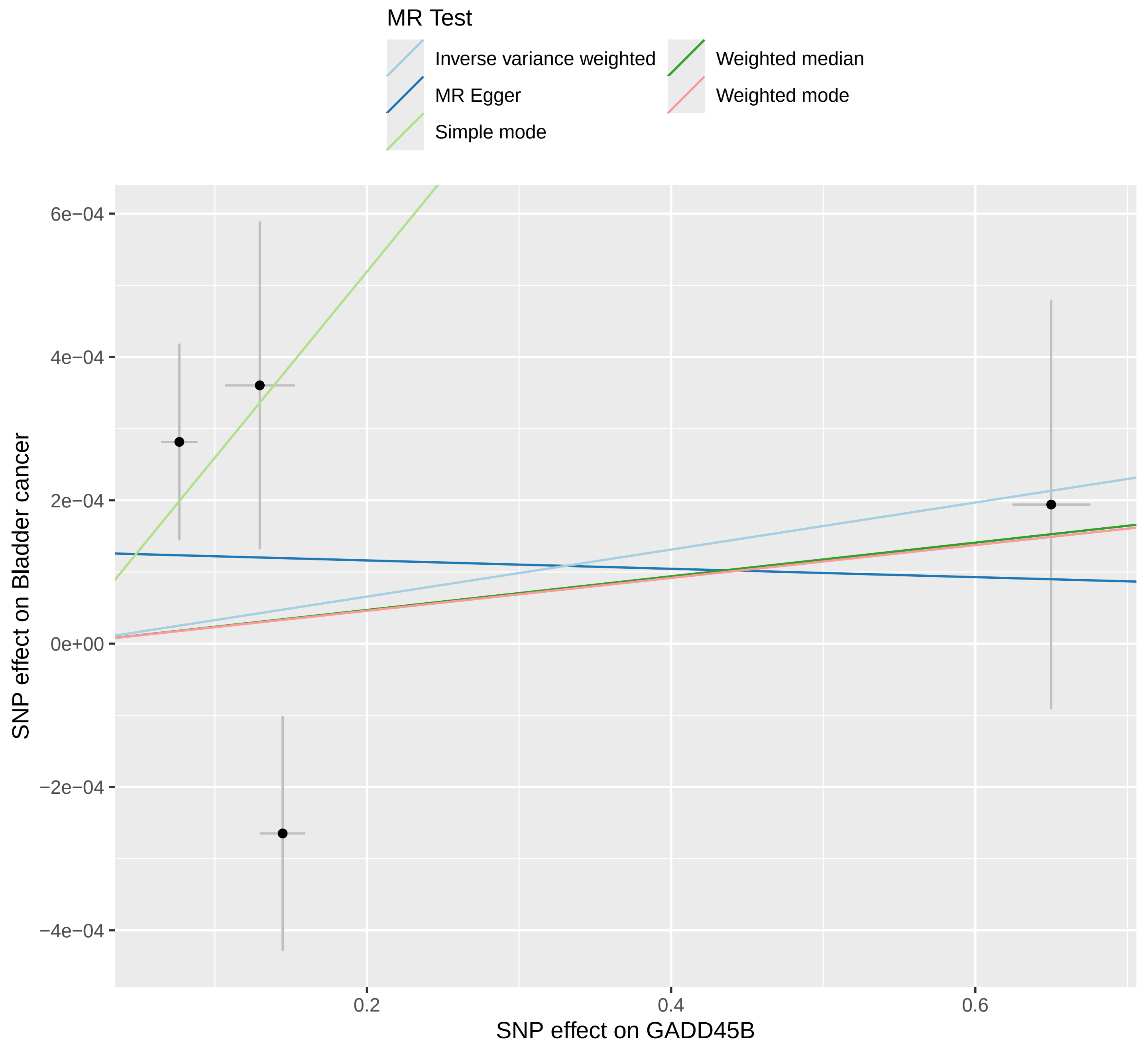

MR Test
Inverse variance weighted
MR Egger
Simple mode
Weighted median
Weighted mode
6e−04
4e−04
2e−04
0e+00
−2e−04
−4e−04
SNP effect on Bladder cancer
0.2
0.4
0.6
SNP effect on GADD45B

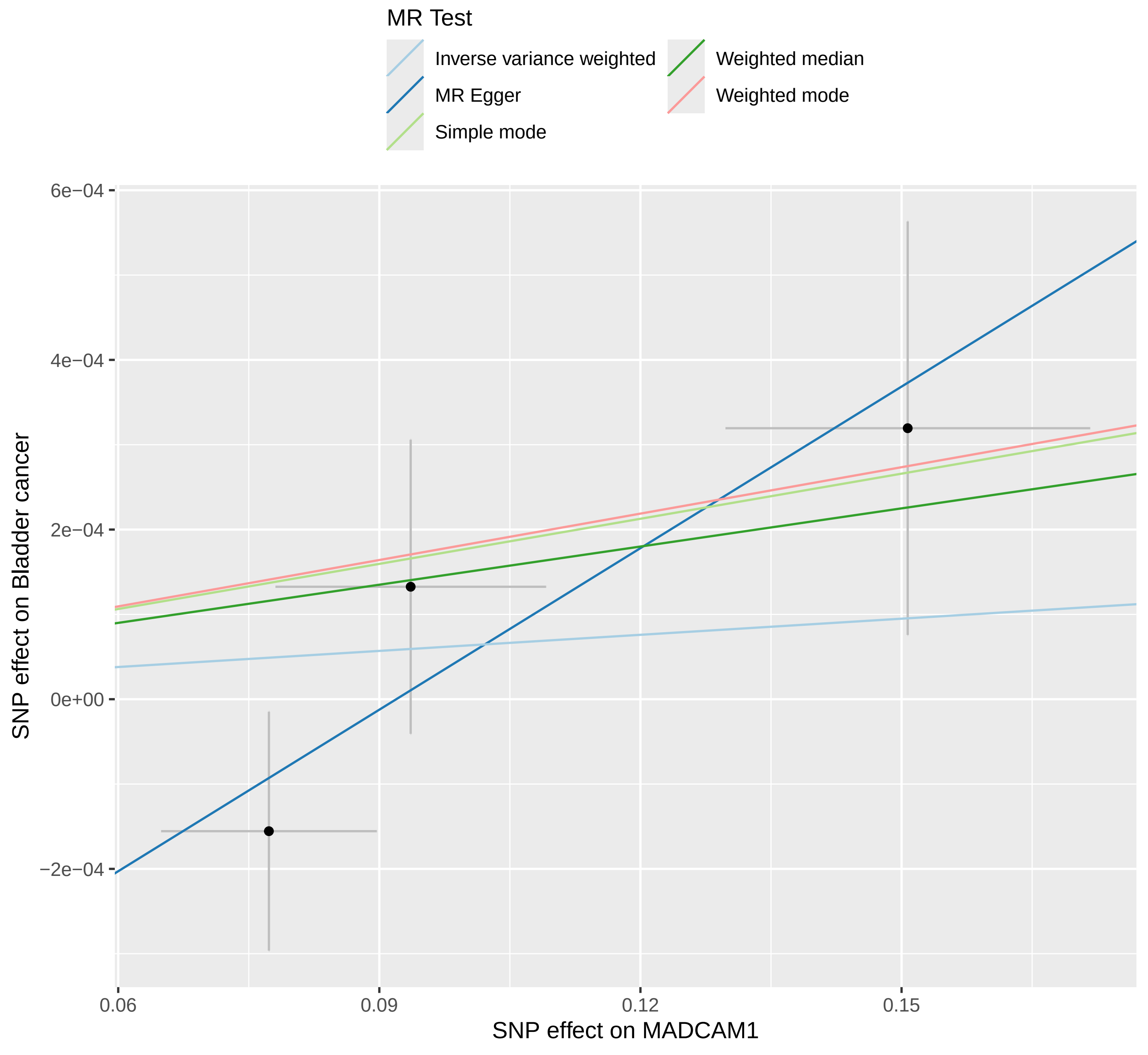

MR Test
Inverse variance weighted
MR Egger
Simple mode
Weighted median
Weighted mode
6e−04
4e−04
2e−04
0e+00
−2e−04
SNP effect on Bladder cancer
0.06
0.09
0.12
0.15
SNP effect on MADCAM1

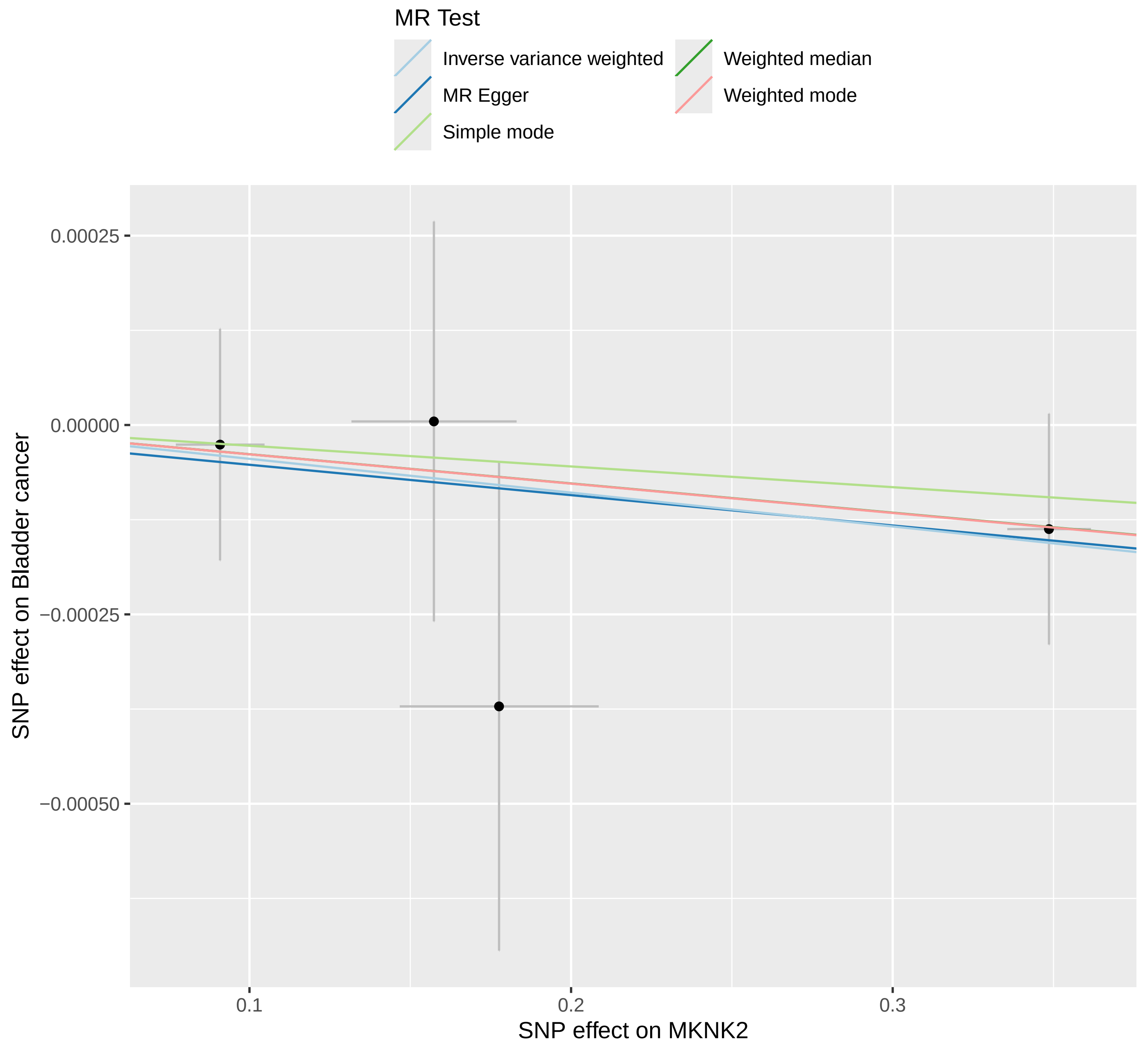
MR Test
Inverse variance weighted
MR Egger
Simple mode
Weighted median
Weighted mode
0.00025
0.00000
−0.00025
−0.00050
SNP effect on Bladder cancer
0.1
0.2
0.3
SNP effect on MKNK2

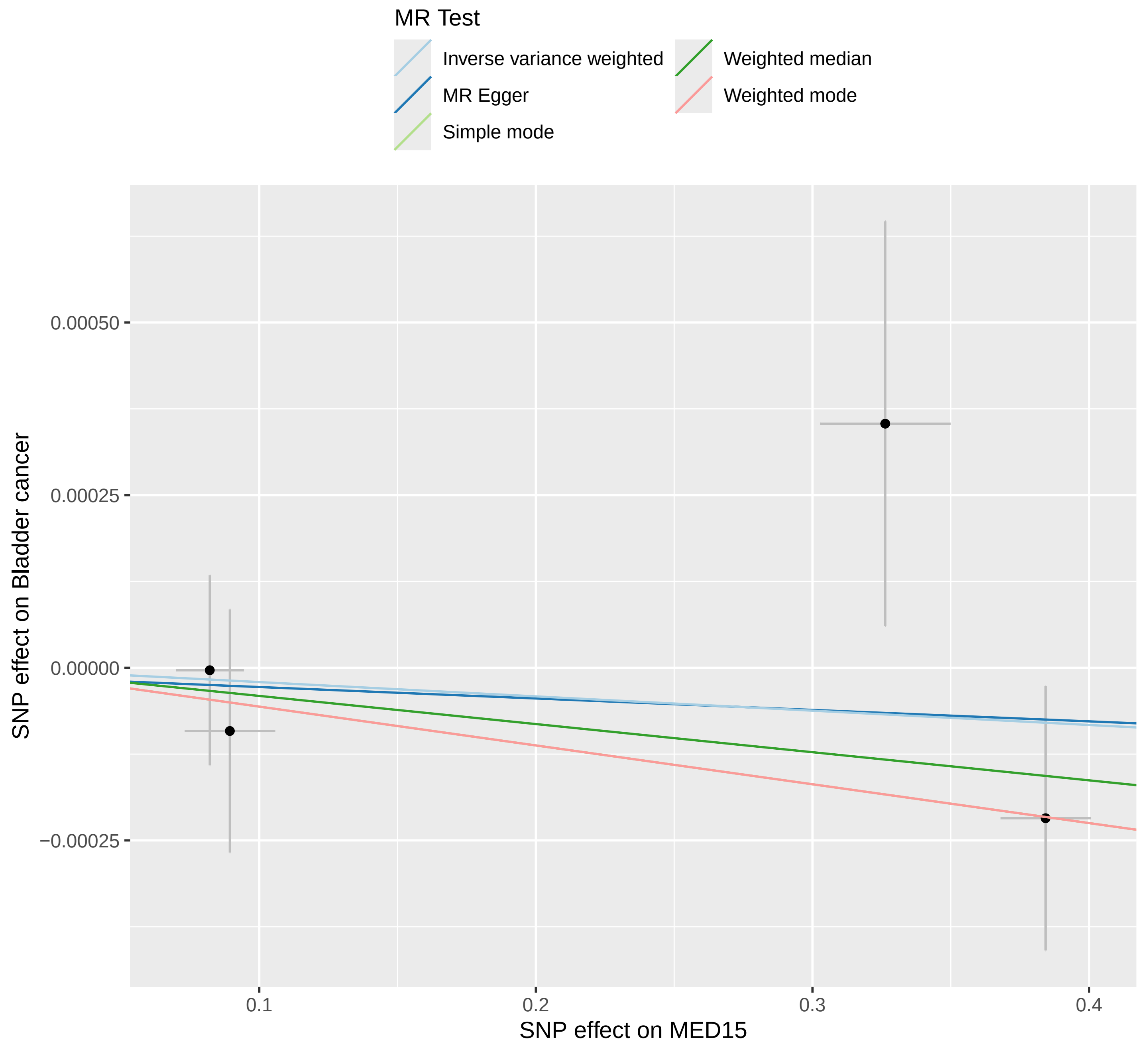
MR Test
Inverse variance weighted
MR Egger
Simple mode
Weighted median
Weighted mode
0.00050
0.00025
0.00000
−0.00025
0.1
0.2
0.3
0.4
SNP effect on Bladder cancer
SNP effect on MED15

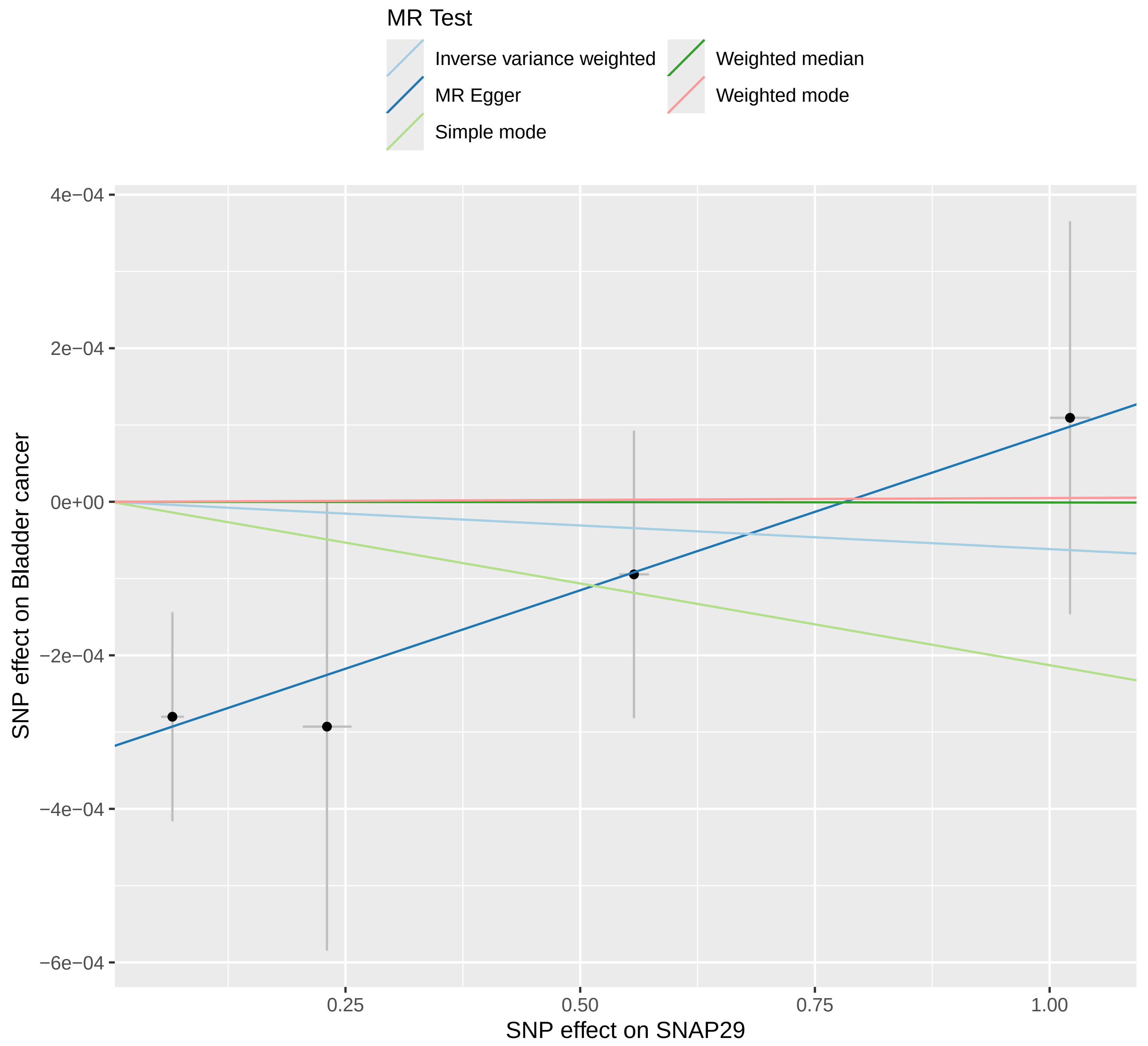

MR Test
Inverse variance weighted
MR Egger
Simple mode
Weighted median
Weighted mode
4e−04
2e−04
0e+00
−2e−04
−4e−04
−6e−04
0.25
0.50
0.75
1.00
SNP effect on Bladder cancer
SNP effect on SNAP29

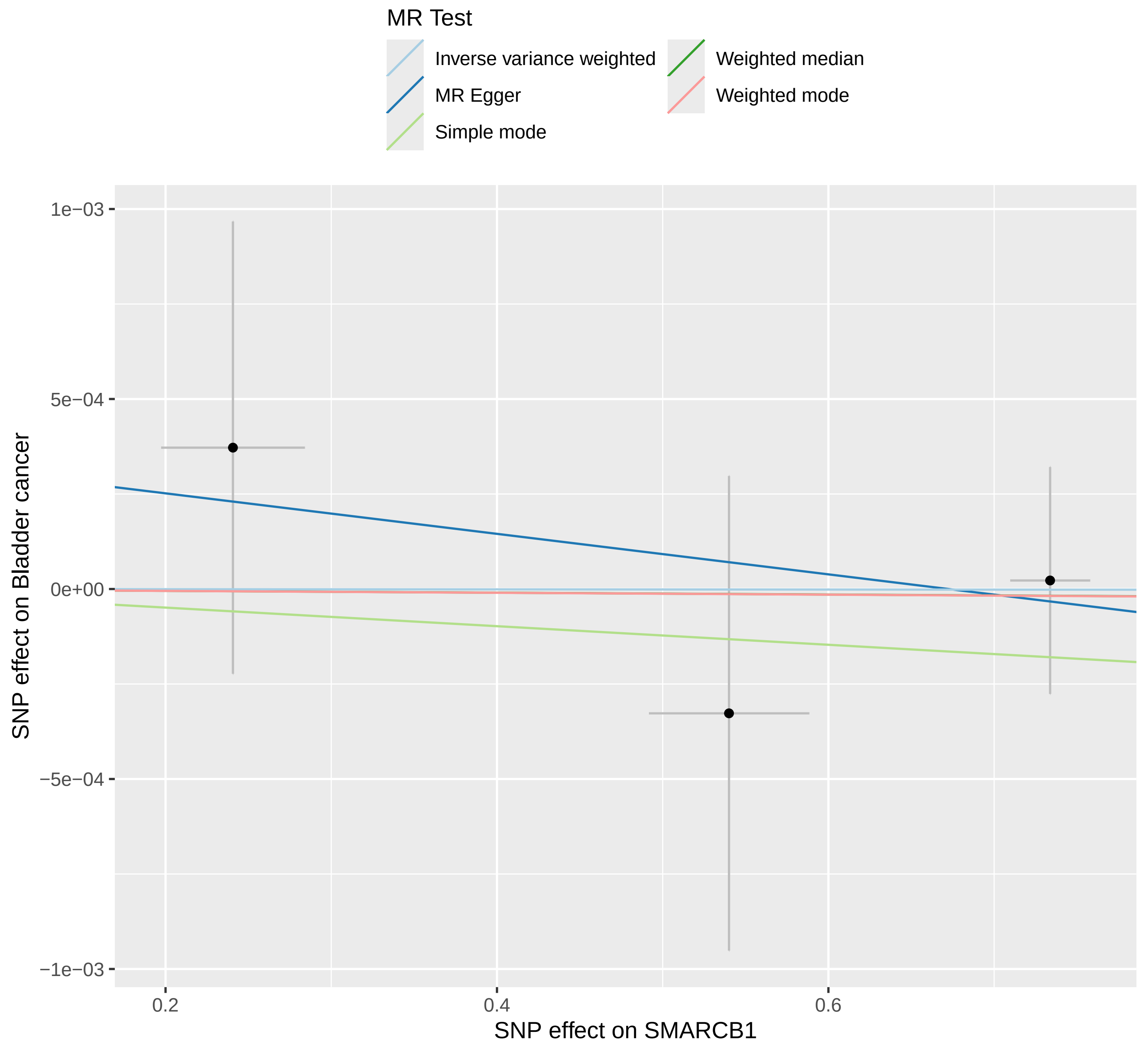
MR Test
Inverse variance weighted
MR Egger
Simple mode
Weighted median
Weighted mode
1e−03
5e−04
0e+00
−5e−04
−1e−03
0.2
0.4
0.6
SNP effect on Bladder cancer
SNP effect on SMARCB1

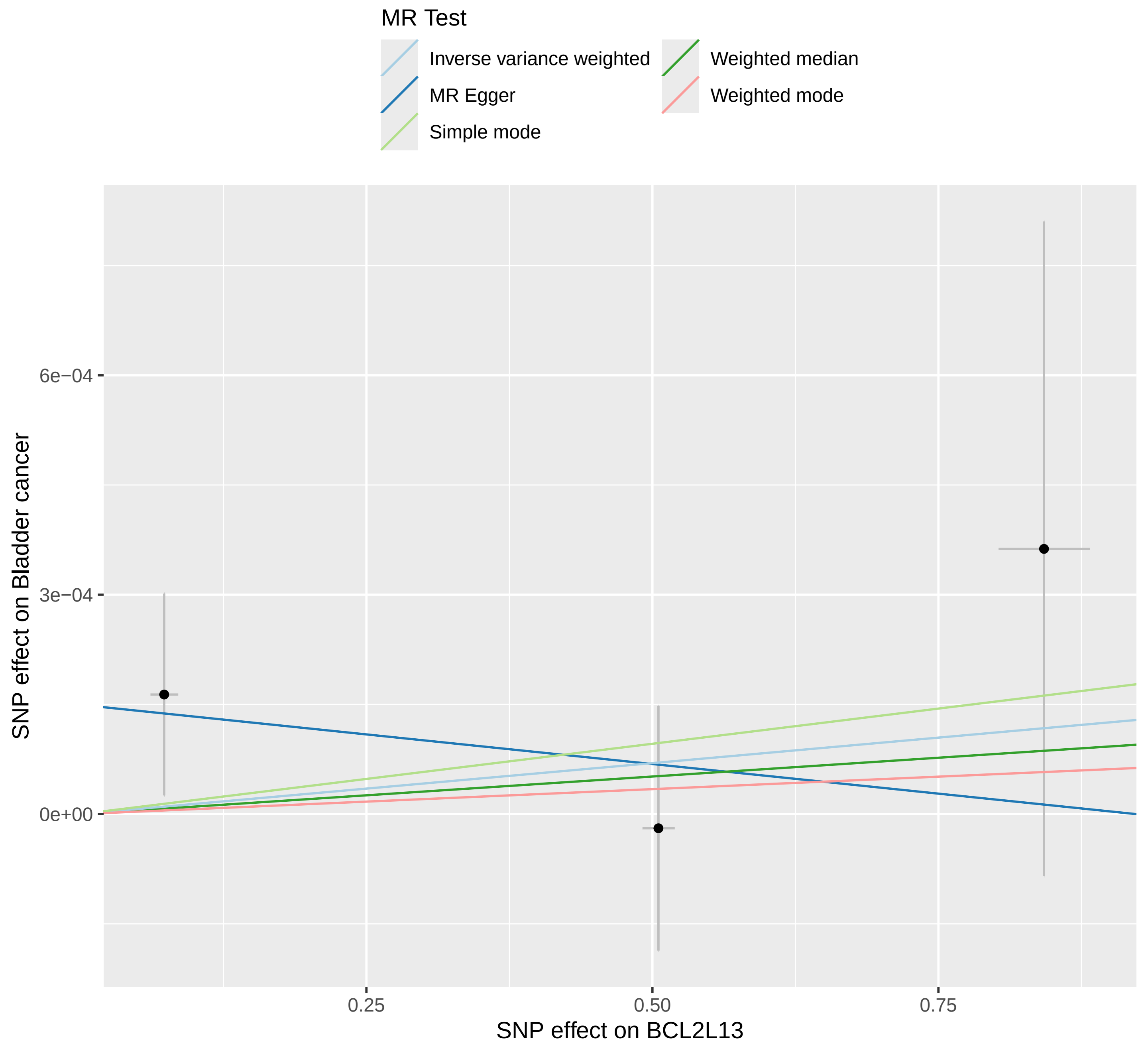

MR Test
Inverse variance weighted
MR Egger
Simple mode
Weighted median
Weighted mode
6e−04
3e−04
0e+00
0.25
0.50
0.75
SNP effect on BCL2L13
SNP effect on Bladder cancer

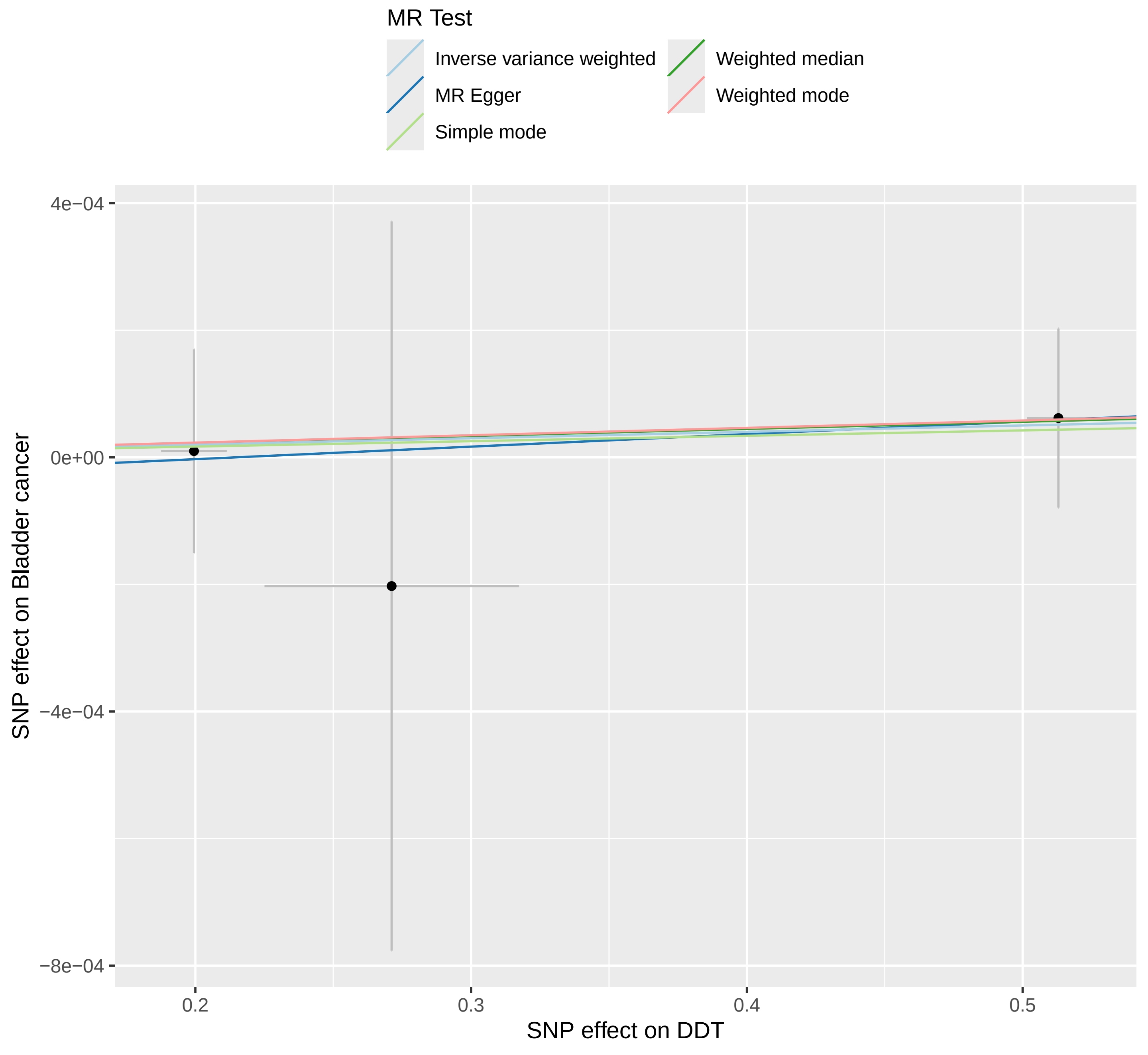
MR Test
Inverse variance weighted
MR Egger
Simple mode
Weighted median
Weighted mode
4e−04
0e+00
−4e−04
−8e−04
SNP effect on Bladder cancer
0.2
0.3
0.4
0.5
SNP effect on DDT

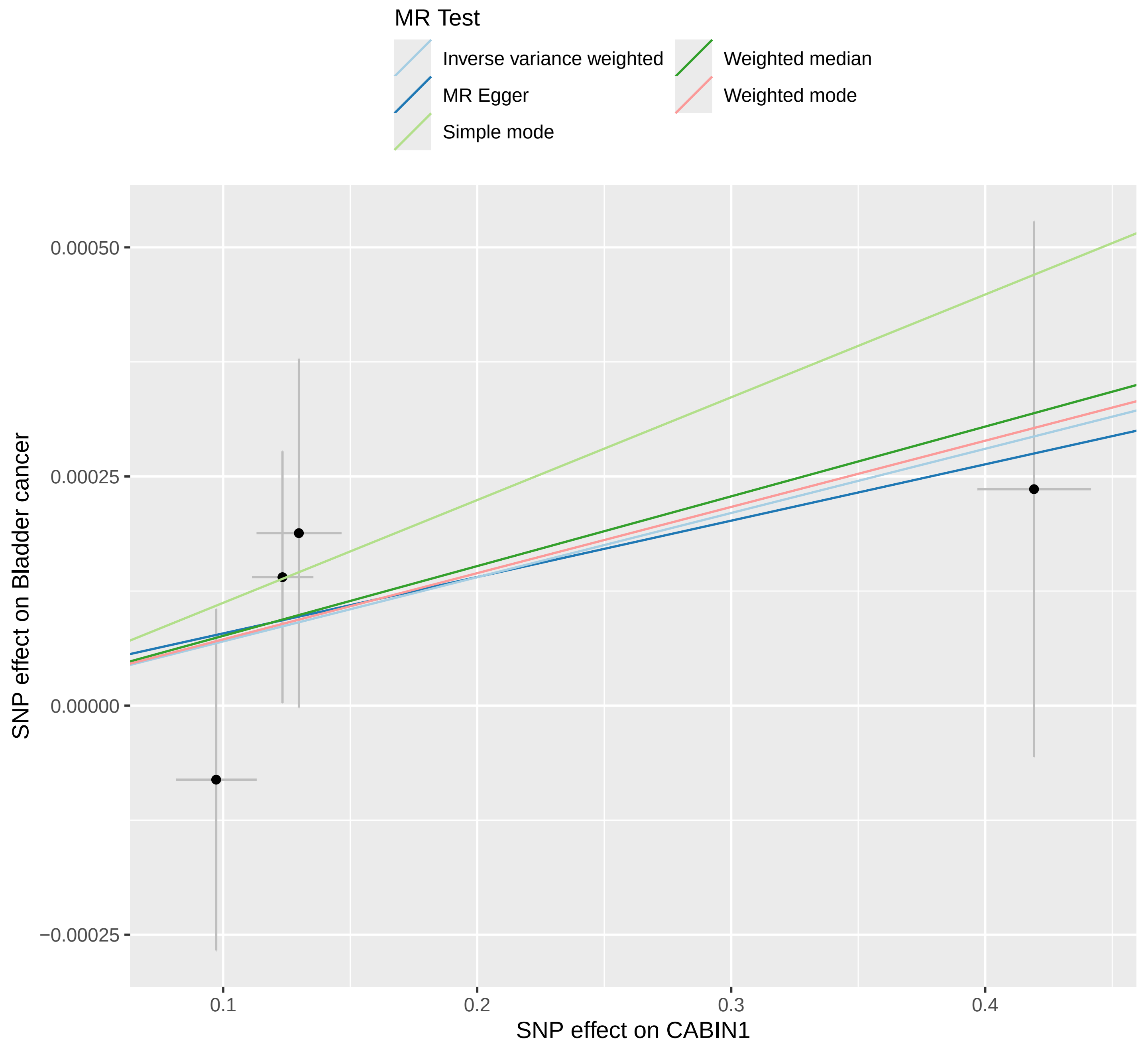

MR Test
Inverse variance weighted
MR Egger
Simple mode
Weighted median
Weighted mode
0.00050
0.00025
0.00000
−0.00025
SNP effect on Bladder cancer
0.1
0.2
0.3
0.4
SNP effect on CABIN1

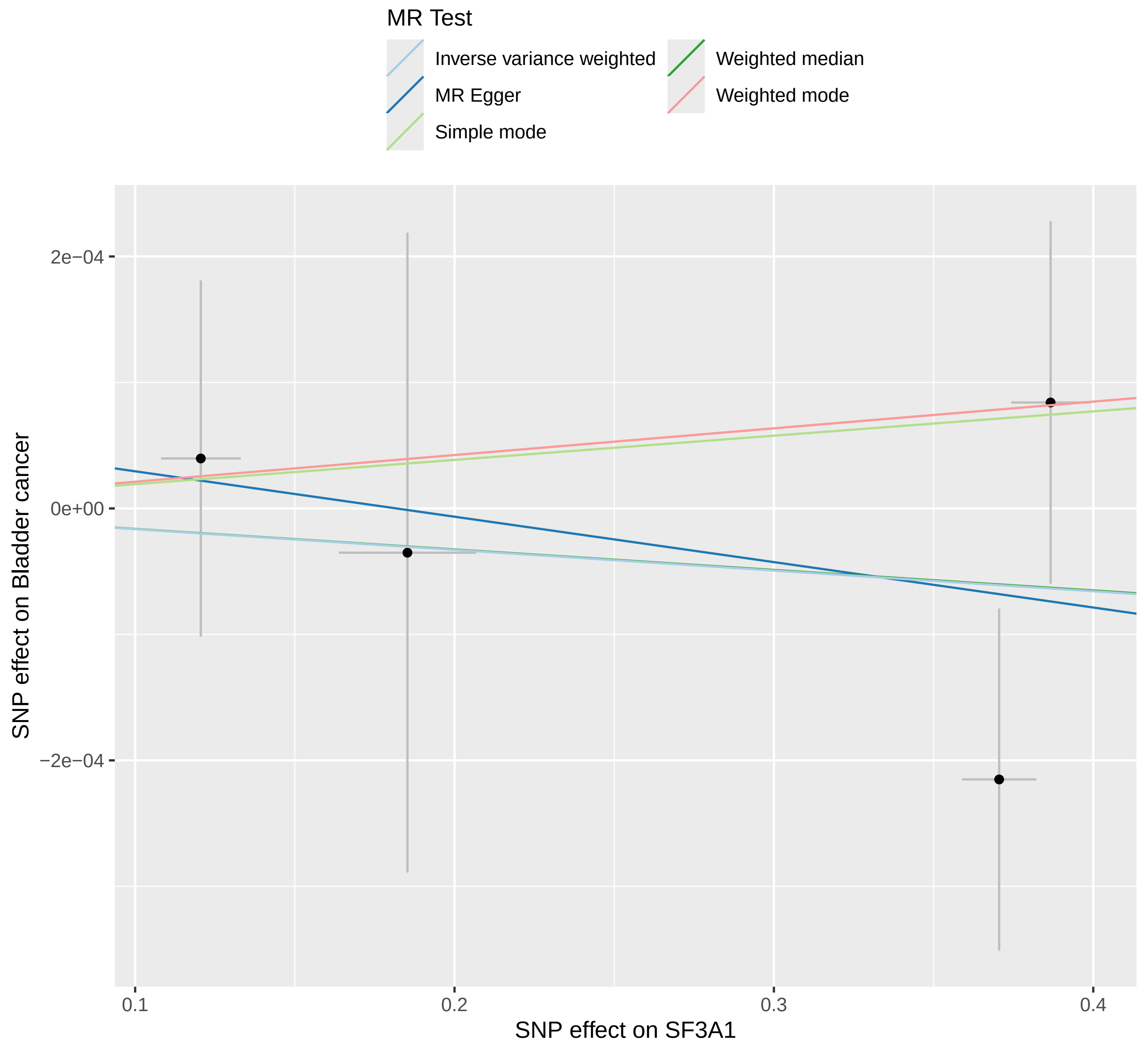
MR Test
Inverse variance weighted
MR Egger
Simple mode
Weighted median
Weighted mode
2e−04
0e+00
−2e−04
0.1
0.2
0.3
0.4
SNP effect on SF3A1
SNP effect on Bladder cancer

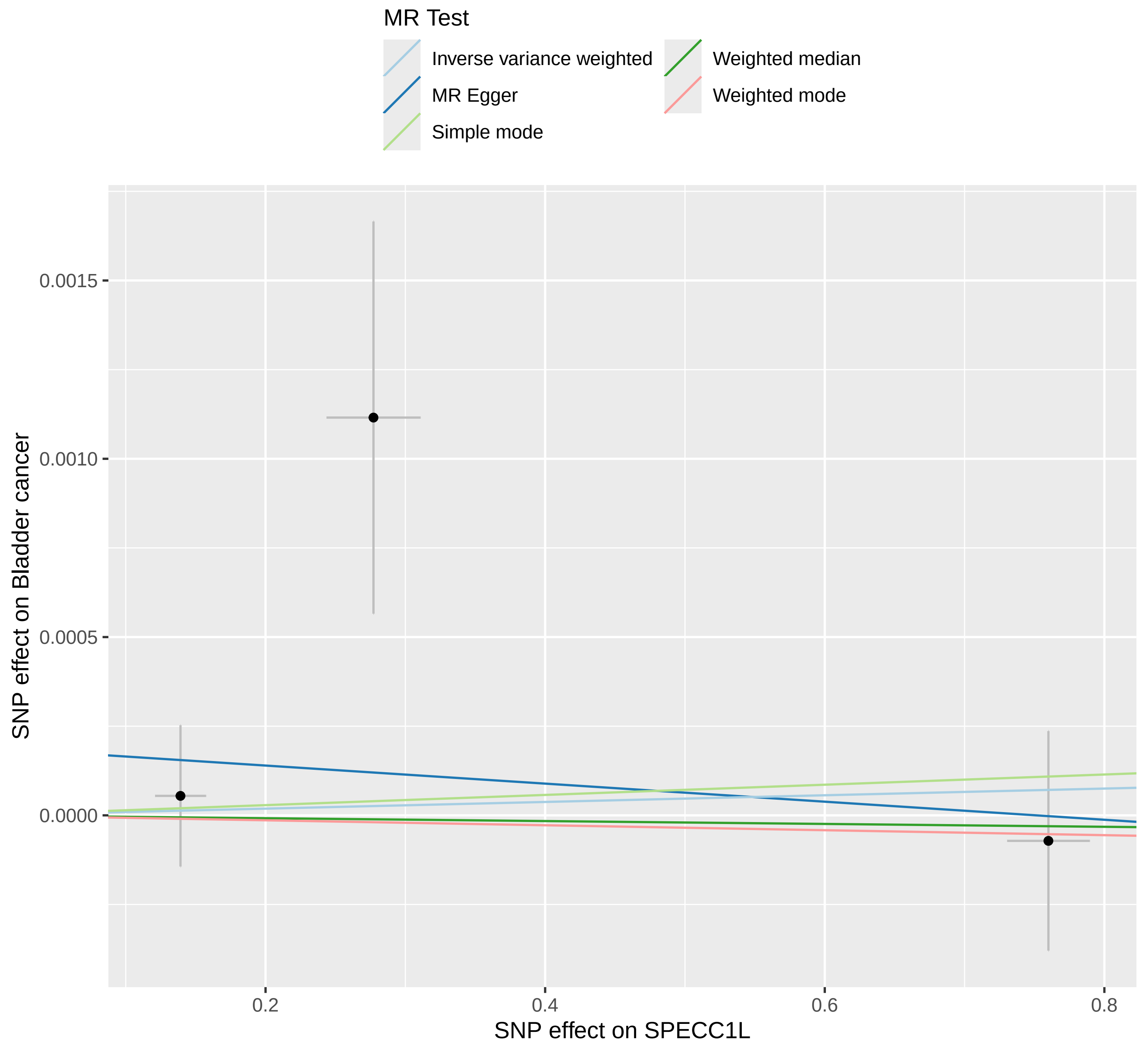
MR Test
Inverse variance weighted
MR Egger
Simple mode
Weighted median
Weighted mode
0.0015
0.0010
0.0005
0.0000
SNP effect on Bladder cancer
0.2
0.4
0.6
0.8
SNP effect on SPECC1L

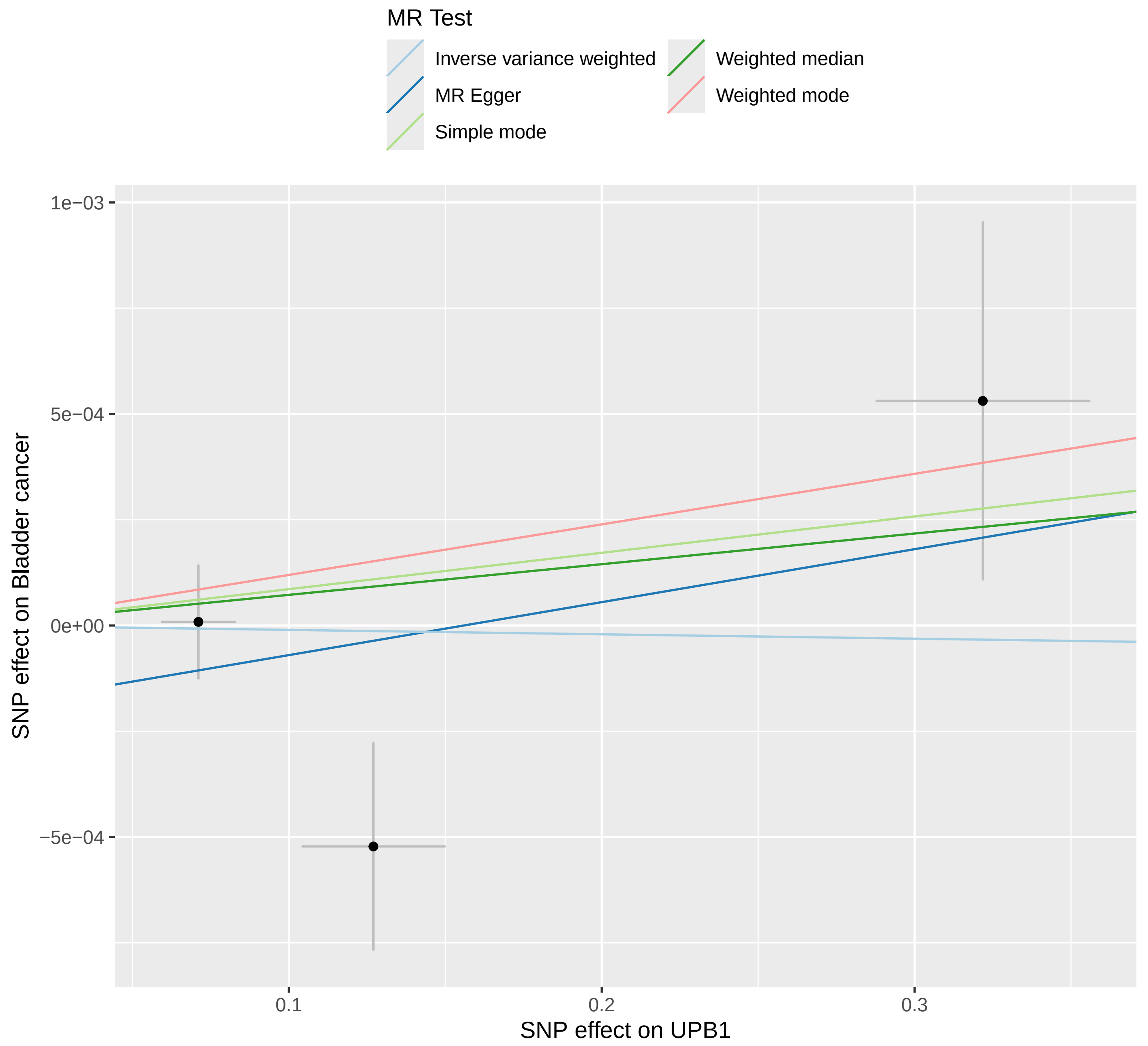

MR Test
Inverse variance weighted
MR Egger
Simple mode
Weighted median
Weighted mode
1e−03
5e−04
0e+00
−5e−04
0.1
0.2
0.3
SNP effect on UPB1
SNP effect on Bladder cancer

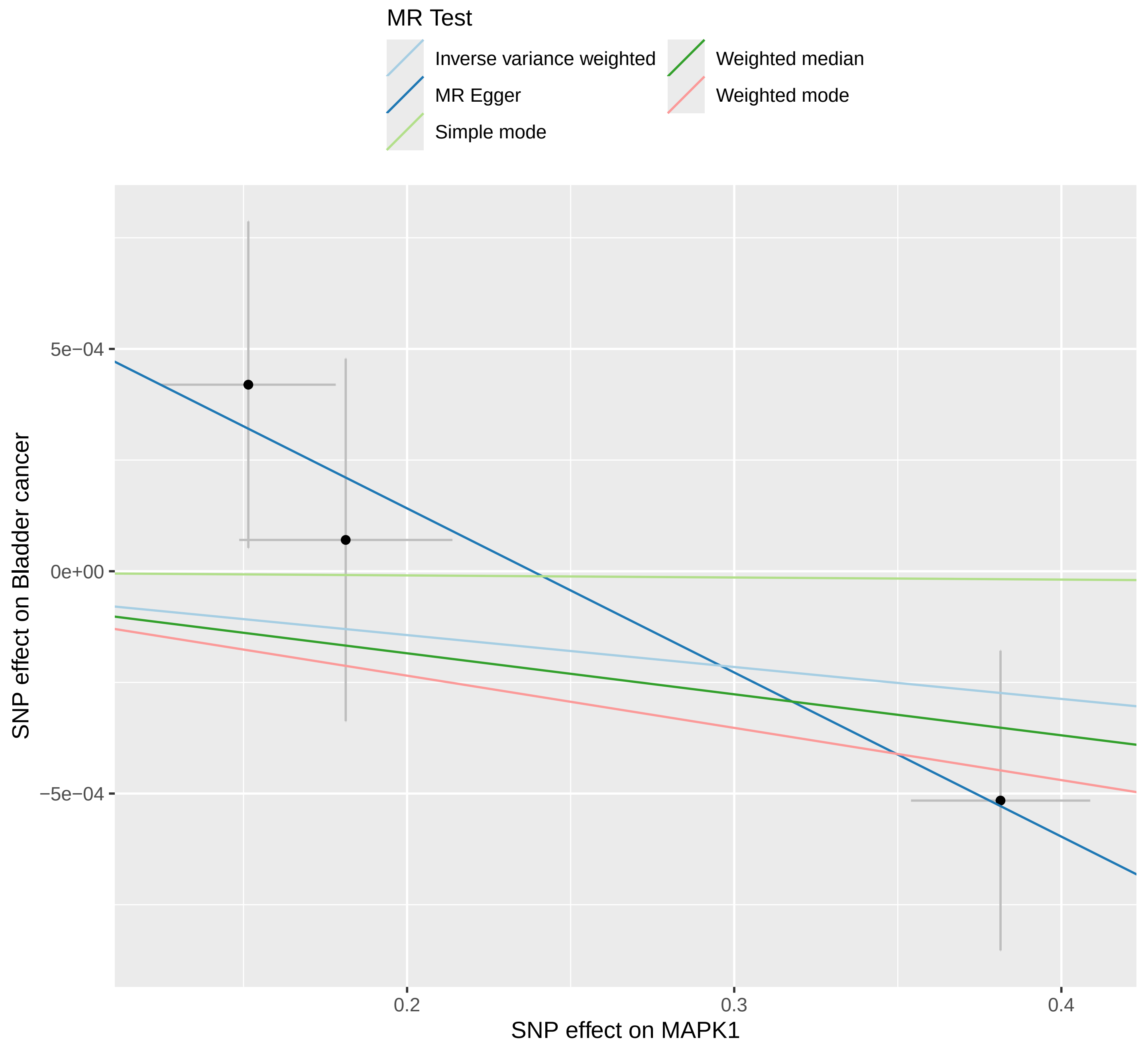

MR Test
Inverse variance weighted
MR Egger
Simple mode
Weighted median
Weighted mode
5e−04
0e+00
−5e−04
0.2
0.3
0.4
SNP effect on Bladder cancer
SNP effect on MAPK1

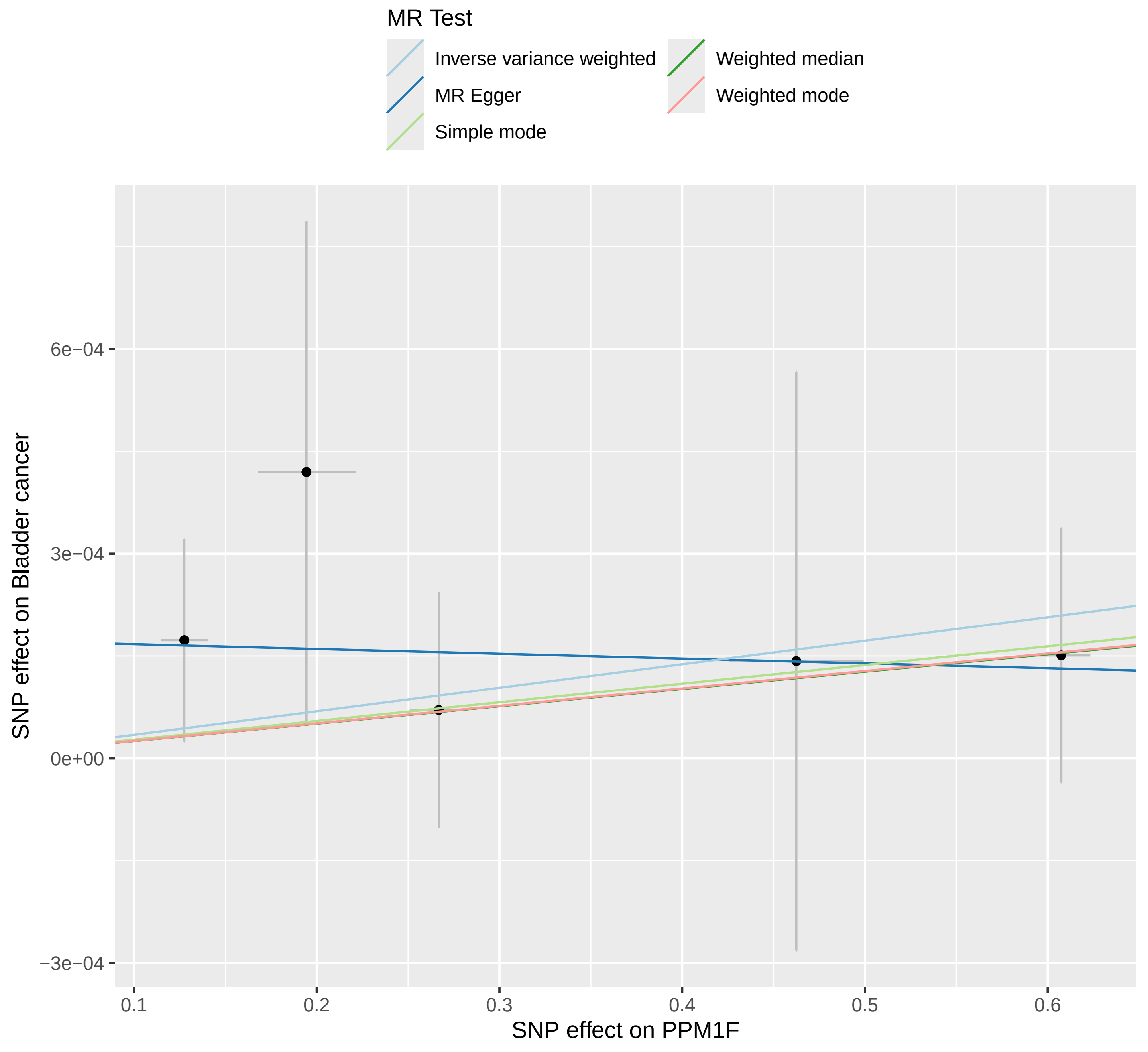

MR Test
Inverse variance weighted
MR Egger
Simple mode
Weighted median
Weighted mode
SNP effect on Bladder cancer
6e−04
3e−04
0e+00
−3e−04
0.1
0.2
0.3
0.4
0.5
0.6
SNP effect on PPM1F

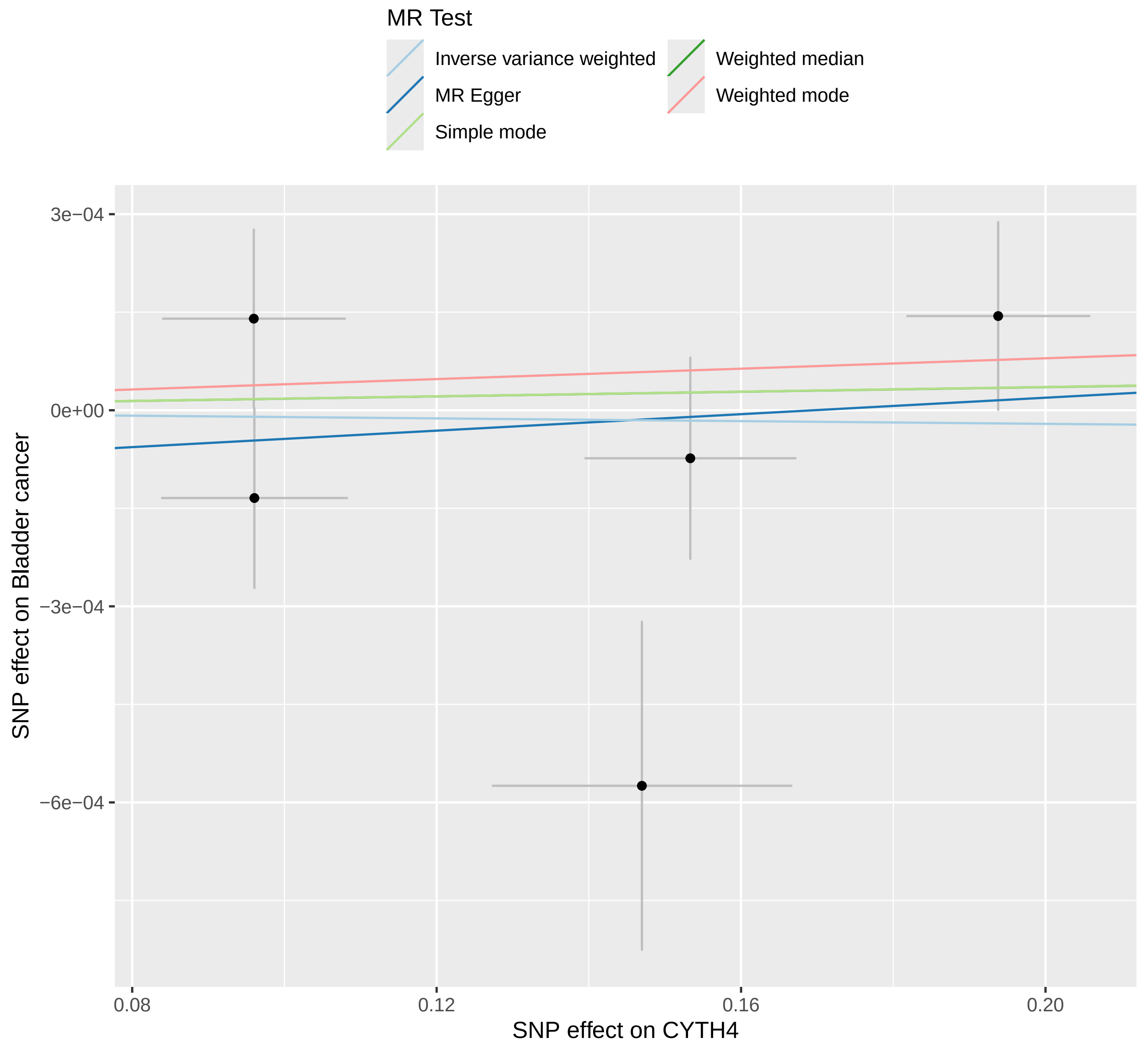

MR Test
Inverse variance weighted
MR Egger
Simple mode
Weighted median
Weighted mode
3e−04
0e+00
−3e−04
−6e−04
SNP effect on Bladder cancer
0.08
0.12
0.16
0.20
SNP effect on CYTH4

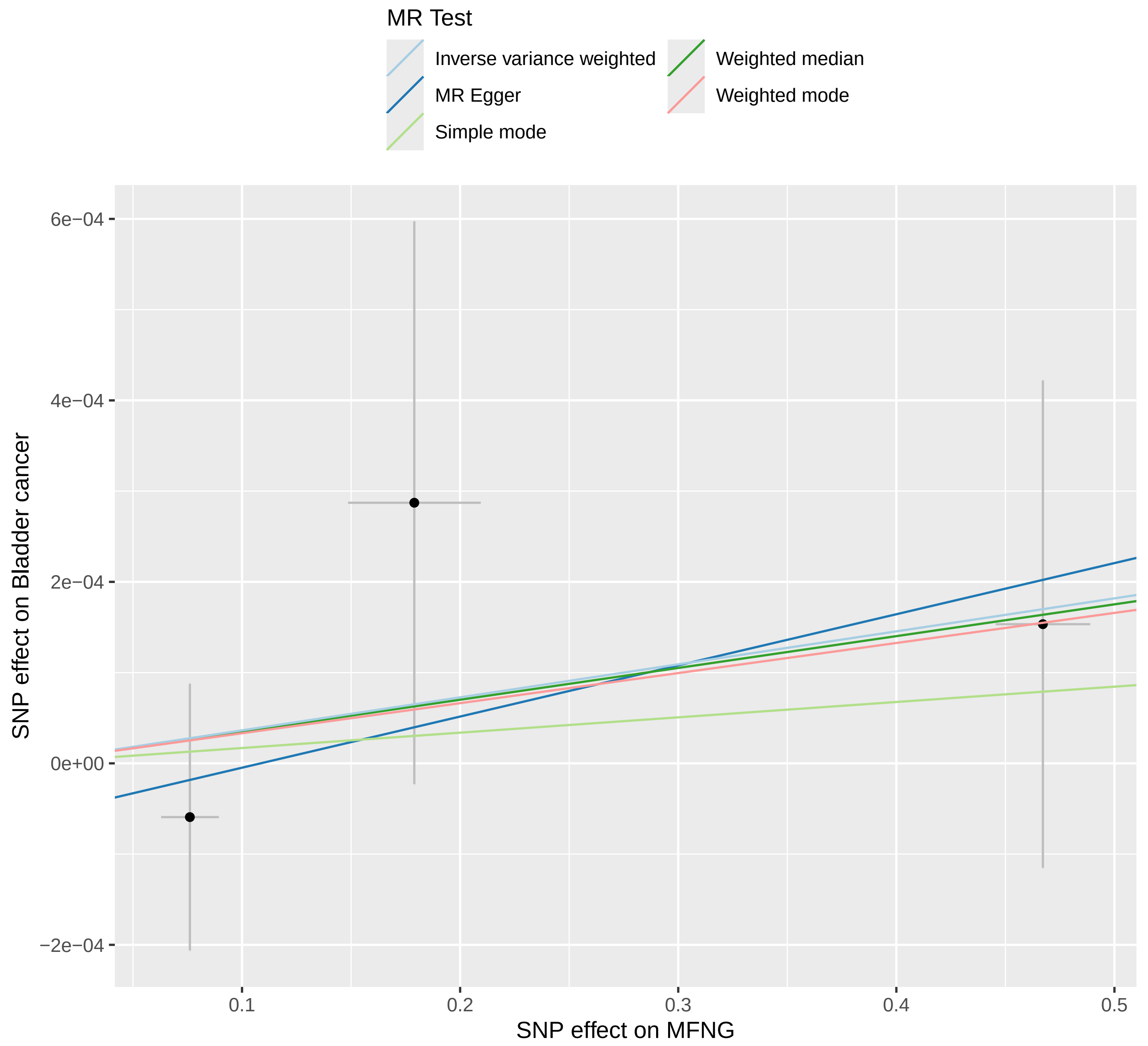
MR Test
Inverse variance weighted
MR Egger
Simple mode
Weighted median
Weighted mode
6e−04
4e−04
2e−04
0e+00
−2e−04
0.1
0.2
0.3
0.4
0.5
SNP effect on Bladder cancer
SNP effect on MFNG

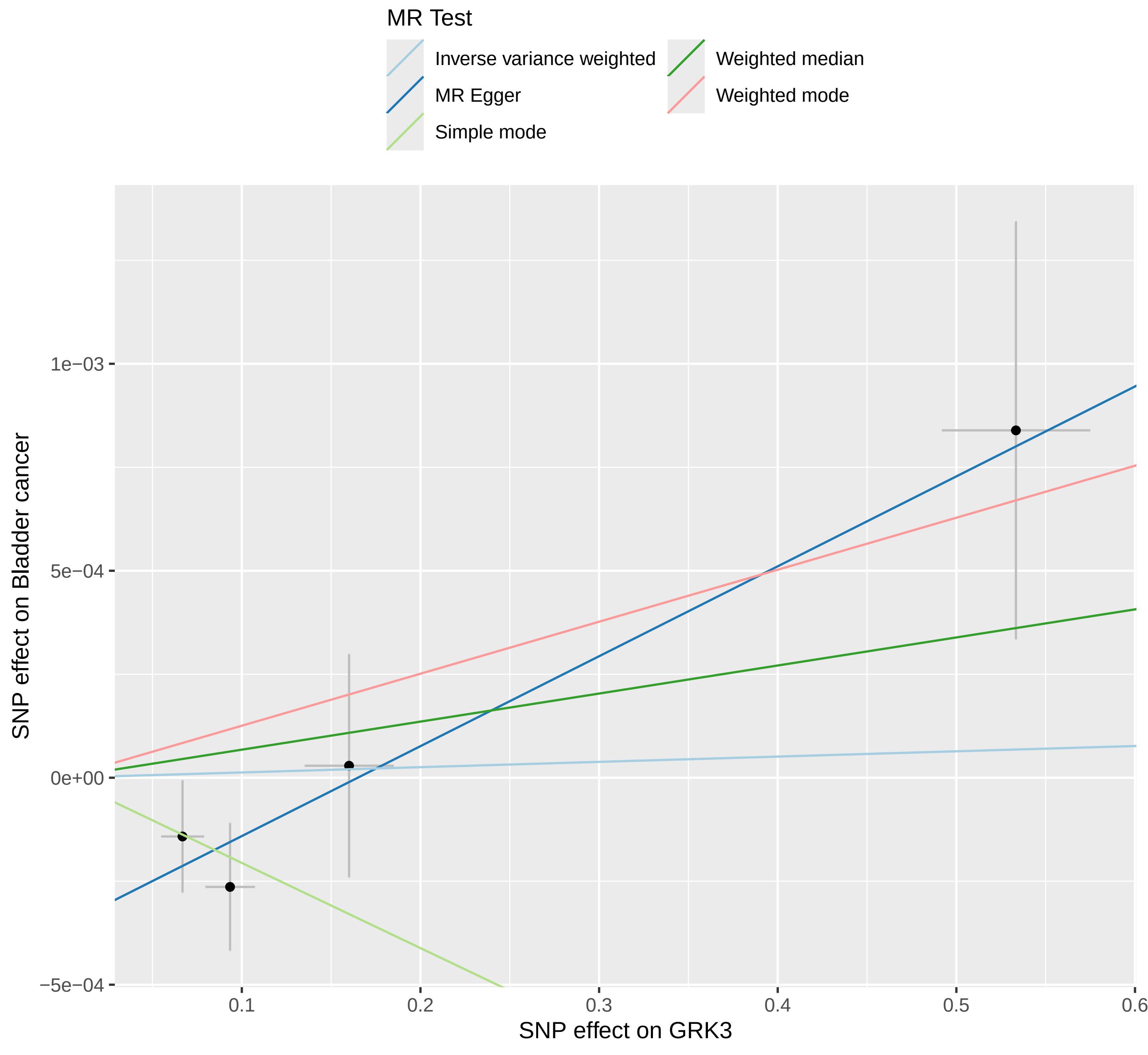
MR Test
Inverse variance weighted
MR Egger
Simple mode
Weighted median
Weighted mode
1e−03
5e−04
0e+00
−5e−04
0.1
0.2
0.3
0.4
0.5
0.6
SNP effect on Bladder cancer
SNP effect on GRK3

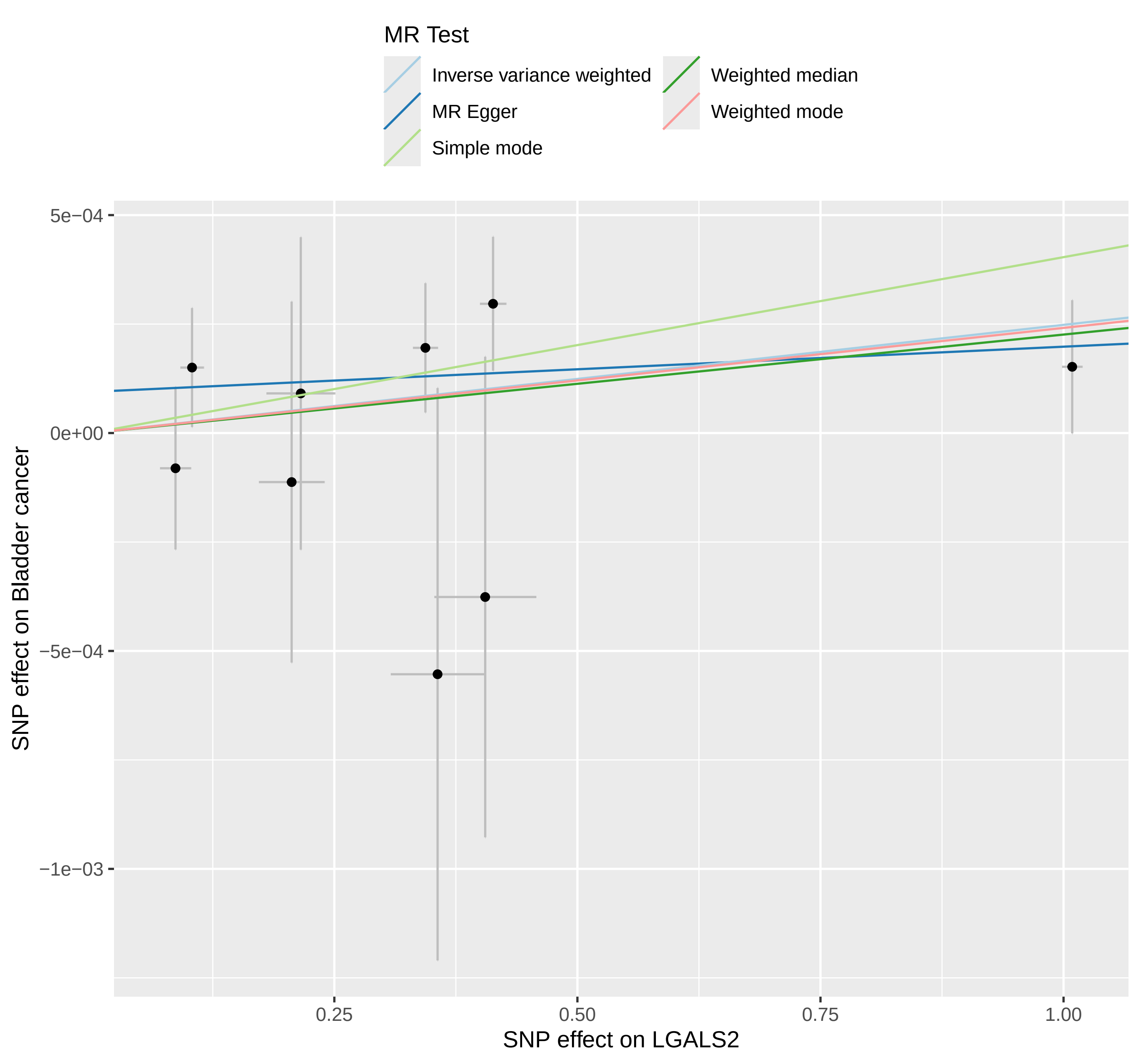
MR Test
Inverse variance weighted
MR Egger
Simple mode
Weighted median
Weighted mode
5e−04
0e+00
−5e−04
−1e−03
SNP effect on Bladder cancer
0.25
0.50
0.75
1.00
SNP effect on LGALS2

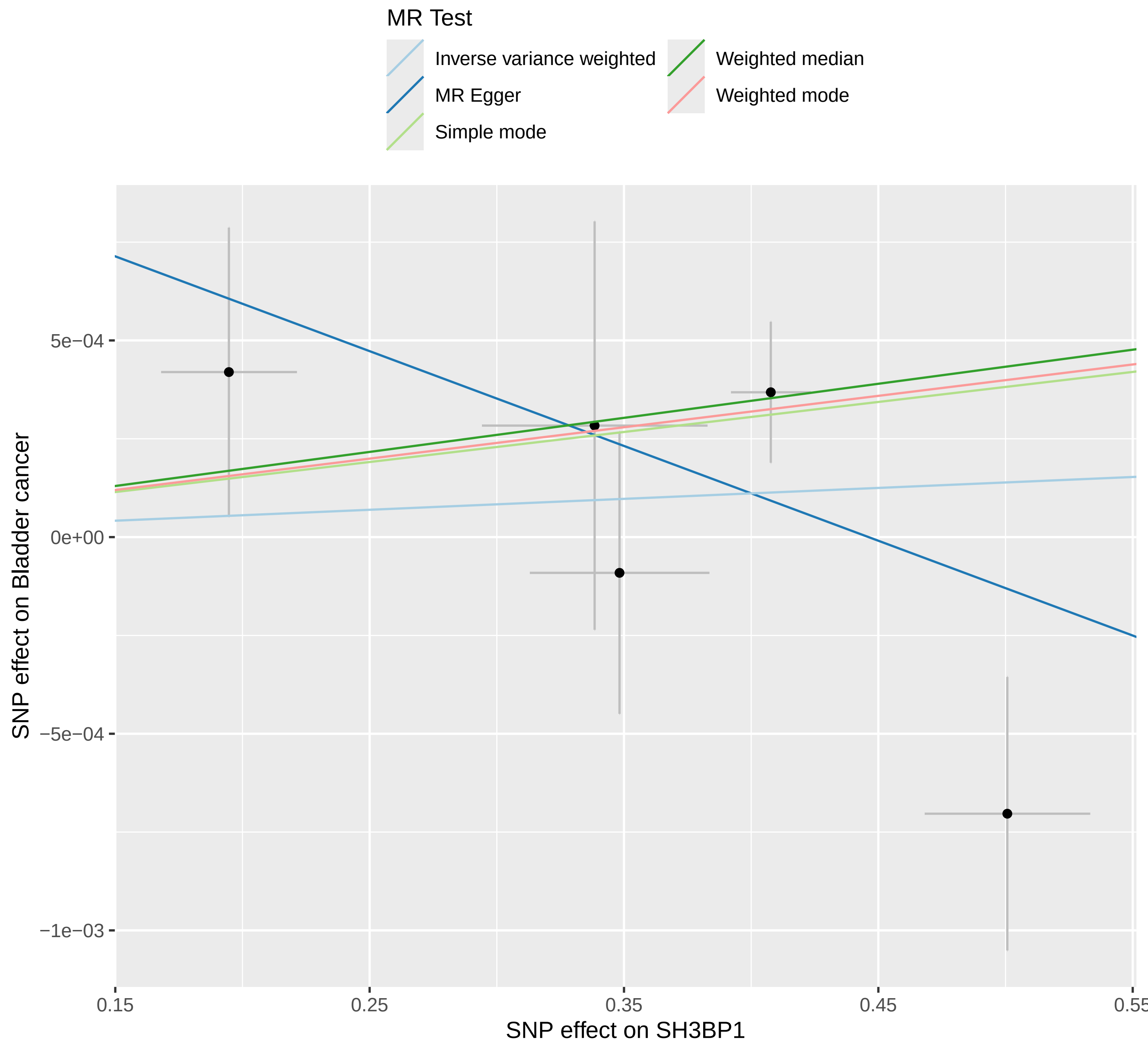

MR Test
Inverse variance weighted
MR Egger
Simple mode
Weighted median
Weighted mode
5e−04
0e+00
−5e−04
−1e−03
SNP effect on Bladder cancer
0.15
0.25
0.35
0.45
0.55
SNP effect on SH3BP1

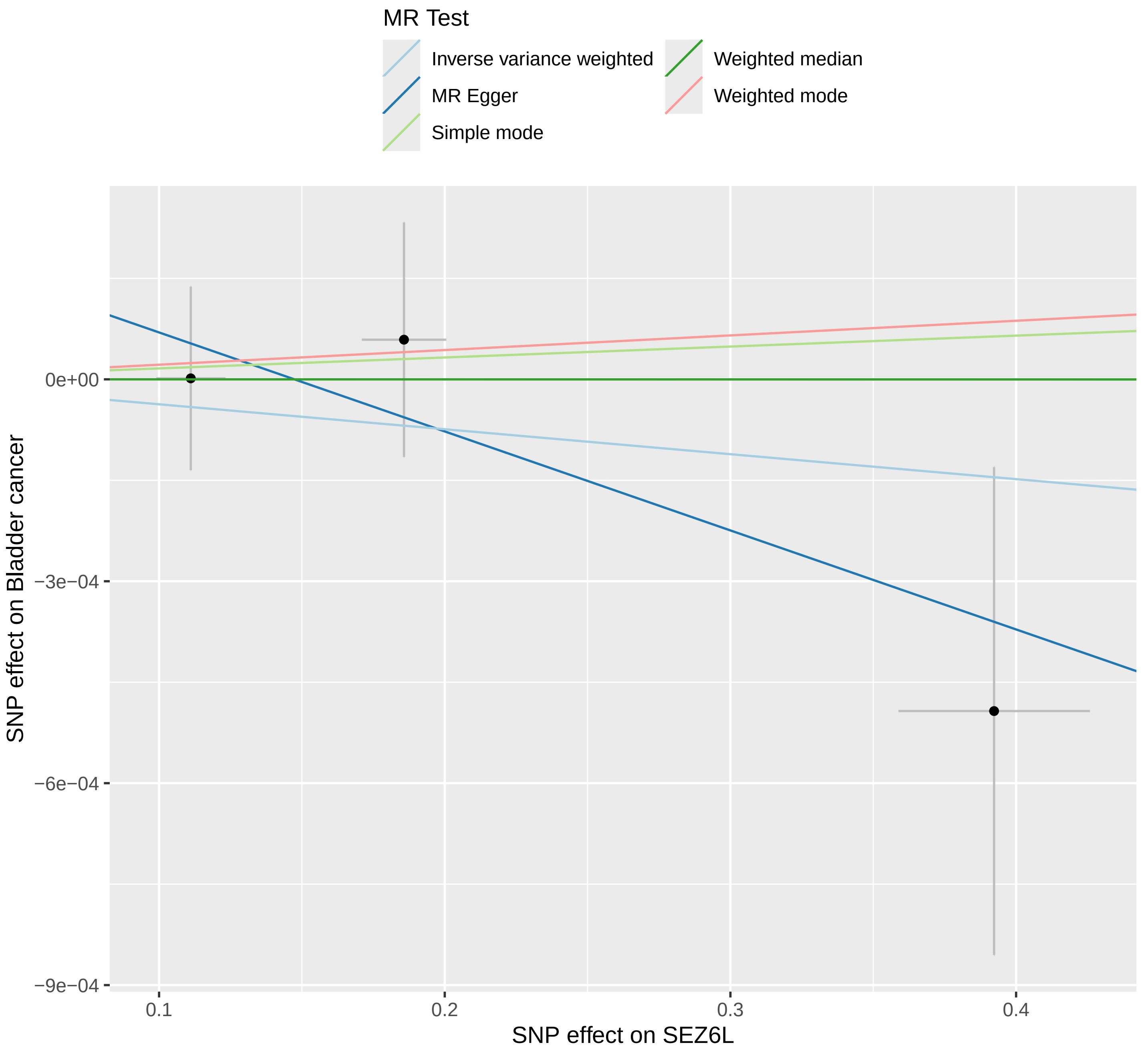
MR Test
Inverse variance weighted
MR Egger
Simple mode
Weighted median
Weighted mode
SNP effect on Bladder cancer
0e+00
−3e−04
−6e−04
−9e−04
0.1
0.2
0.3
0.4
SNP effect on SEZ6L

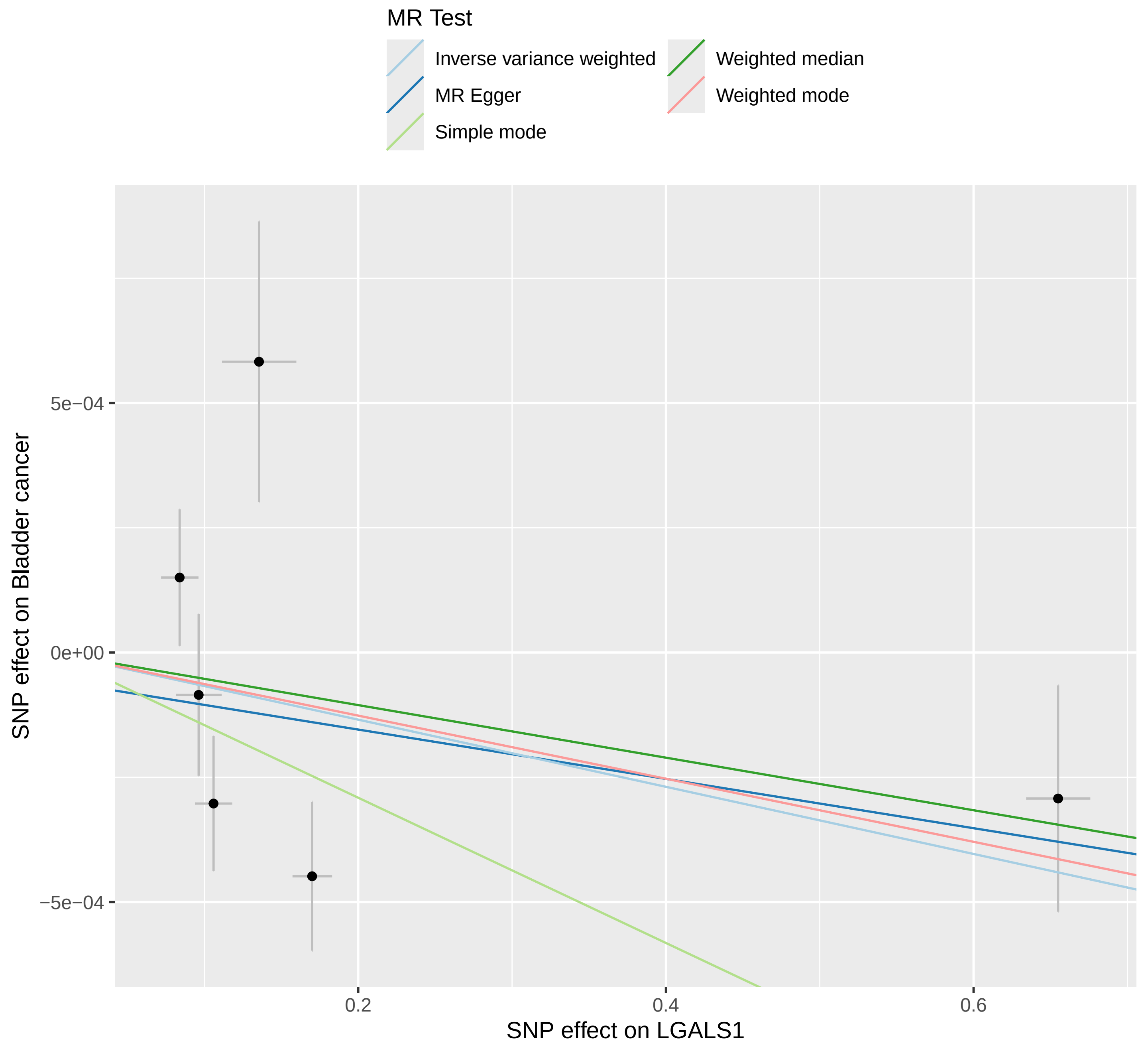
MR Test
Inverse variance weighted
MR Egger
Simple mode
Weighted median
Weighted mode
5e−04
0e+00
−5e−04
SNP effect on Bladder cancer
0.2
0.4
0.6
SNP effect on LGALS1

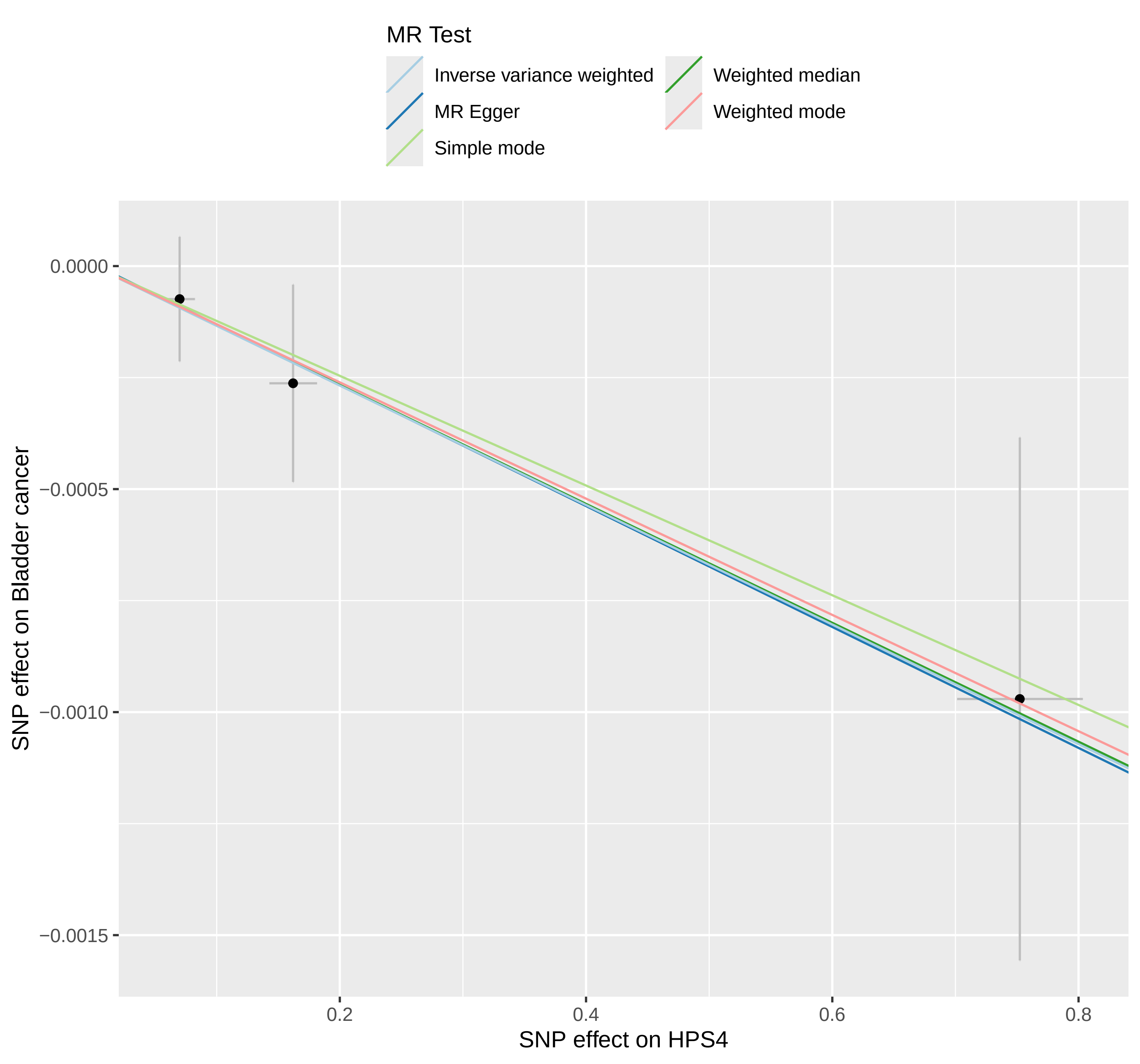
MR Test
Inverse variance weighted
MR Egger
Simple mode
Weighted median
Weighted mode
0.0000
−0.0005
−0.0010
−0.0015
SNP effect on Bladder cancer
0.2
0.4
0.6
0.8
SNP effect on HPS4

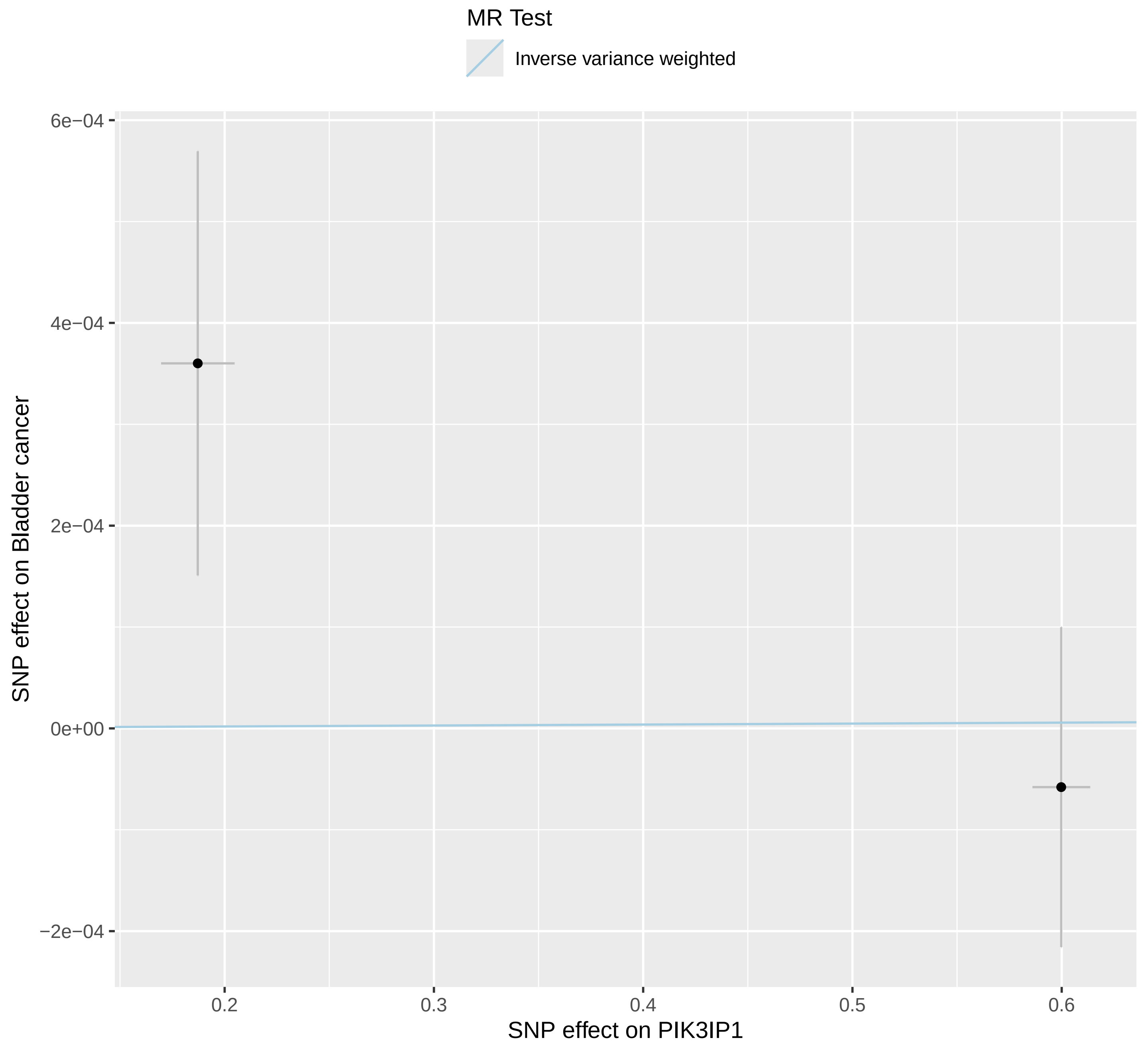
MR Test
Inverse variance weighted
6e−04
4e−04
2e−04
0e+00
−2e−04
SNP effect on Bladder cancer
0.2
0.3
0.4
0.5
0.6
SNP effect on PIK3IP1

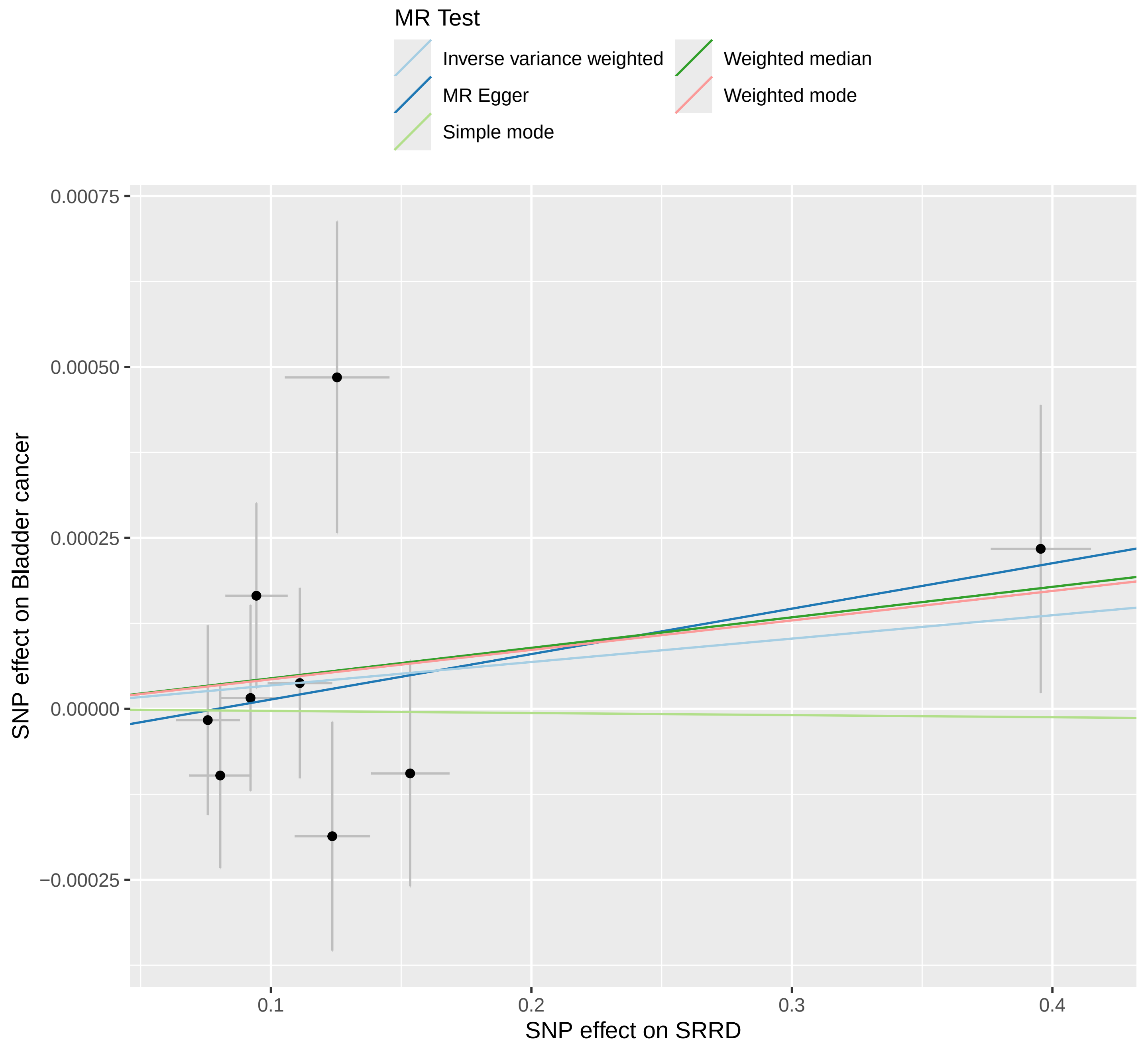
MR Test
Inverse variance weighted
MR Egger
Simple mode
Weighted median
Weighted mode
0.00075
0.00050
0.00025
0.00000
−0.00025
0.1
0.2
0.3
0.4
SNP effect on Bladder cancer
SNP effect on SRRD

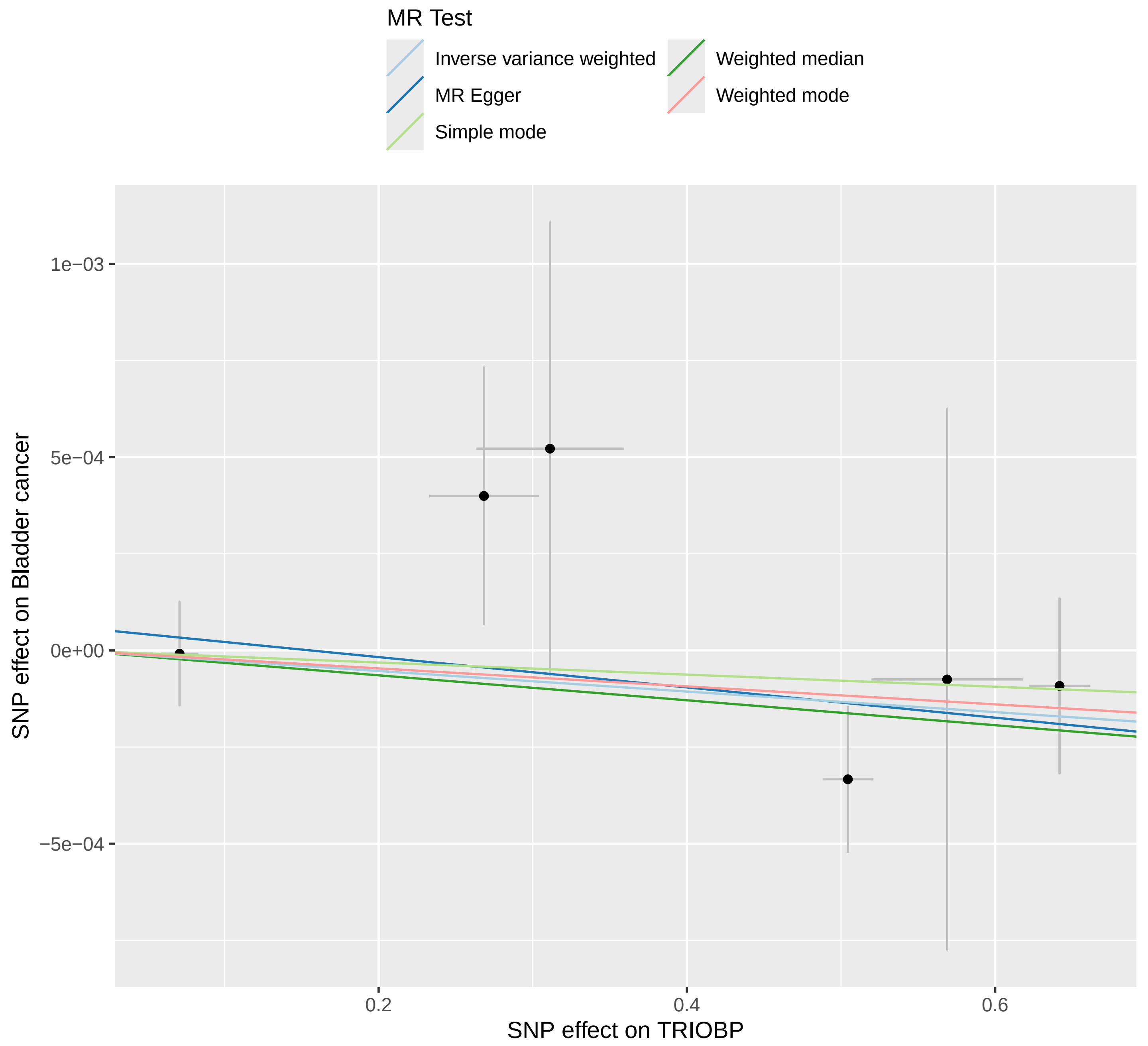
MR Test
Inverse variance weighted
MR Egger
Simple mode
Weighted median
Weighted mode
1e−03
5e−04
0e+00
−5e−04
0.2
0.4
0.6
SNP effect on Bladder cancer
SNP effect on TRIOBP

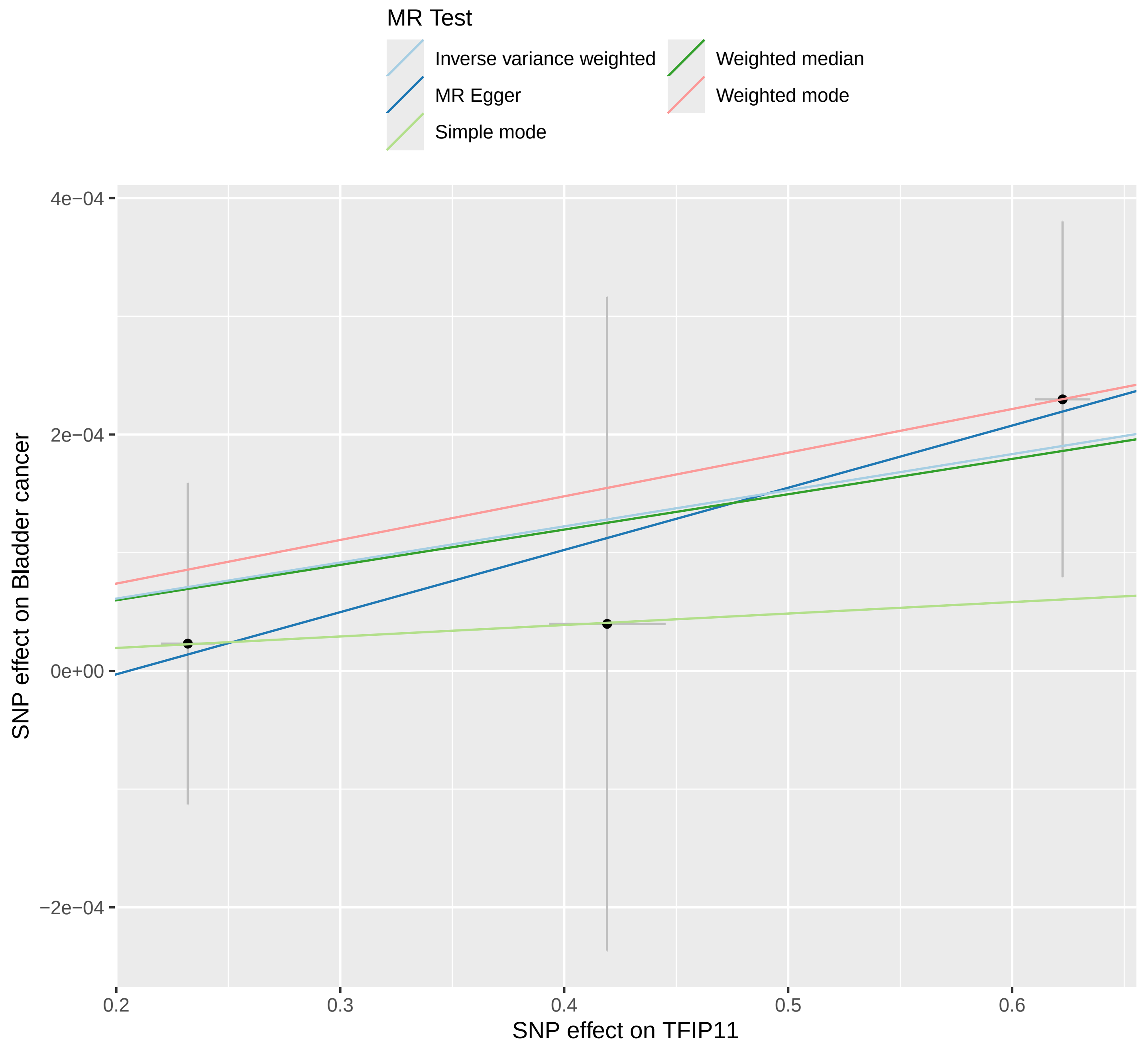

MR Test
Inverse variance weighted
MR Egger
Simple mode
Weighted median
Weighted mode
4e−04
2e−04
0e+00
−2e−04
0.2
0.3
0.4
0.5
0.6
SNP effect on TFIP11
SNP effect on Bladder cancer

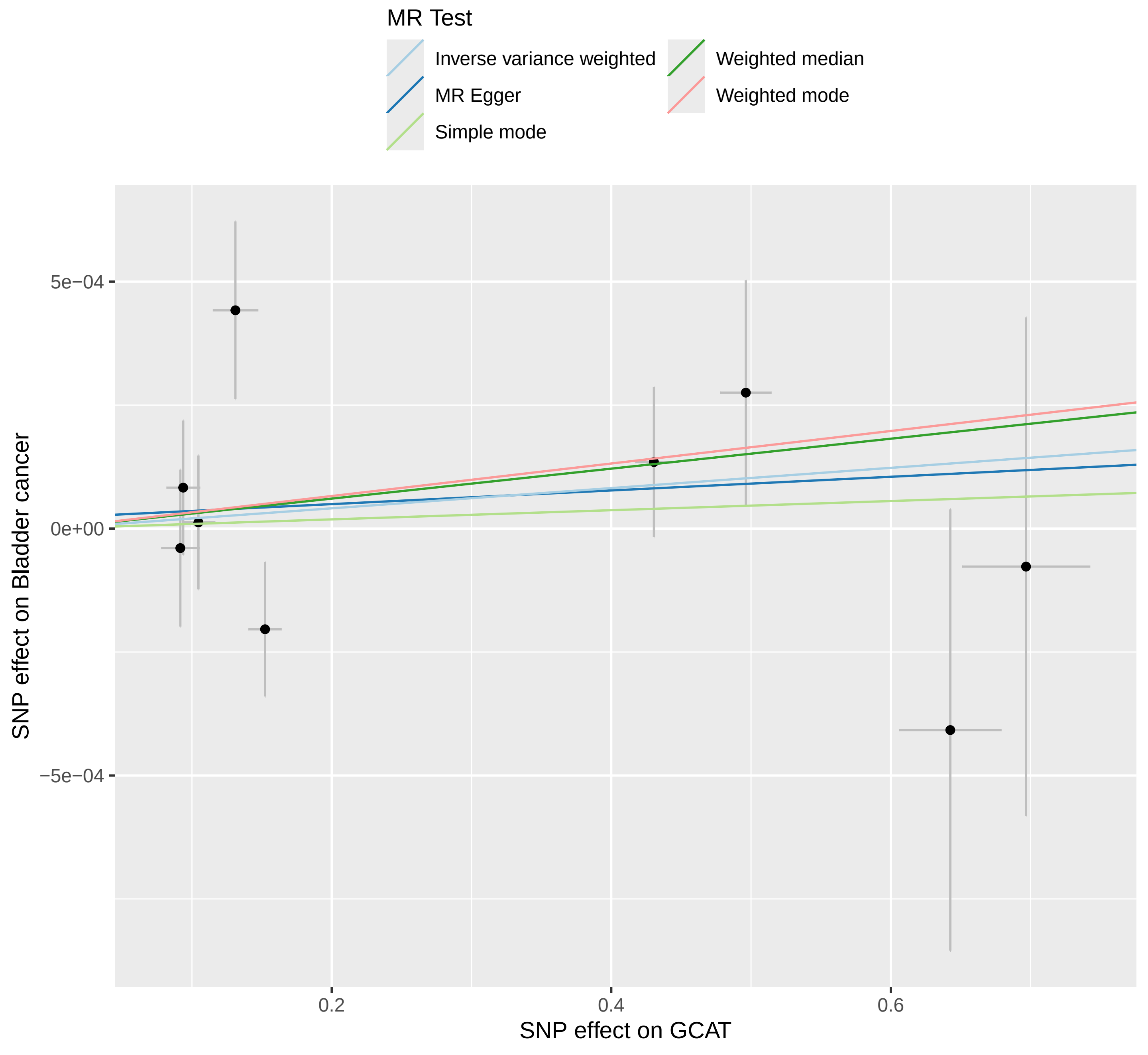
MR Test
Inverse variance weighted
MR Egger
Simple mode
Weighted median
Weighted mode
5e−04
0e+00
−5e−04
0.2
0.4
0.6
SNP effect on Bladder cancer
SNP effect on GCAT

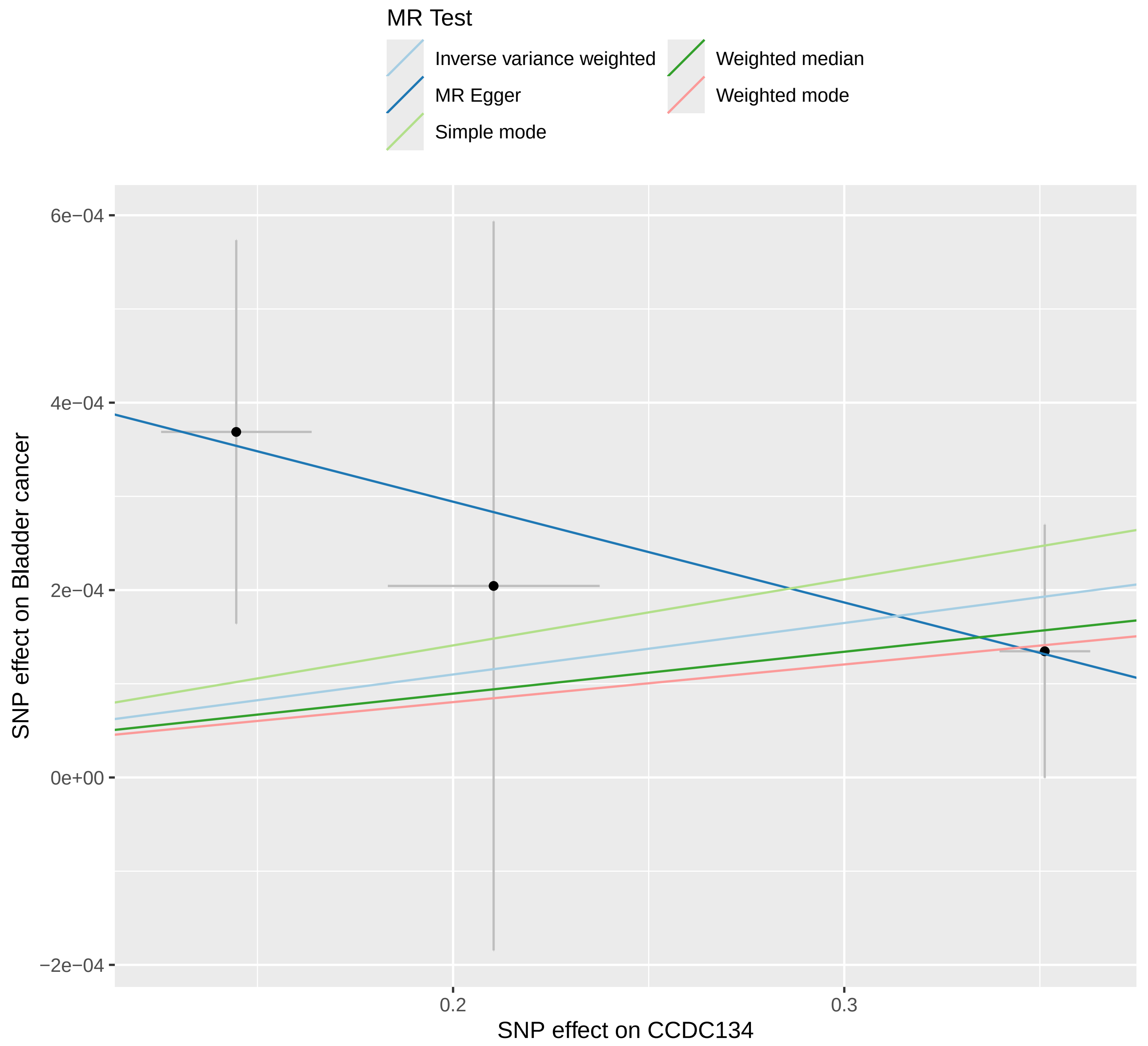

MR Test
Inverse variance weighted
MR Egger
Simple mode
Weighted median
Weighted mode
6e−04
4e−04
2e−04
0e+00
−2e−04
SNP effect on Bladder cancer
0.2
0.3
SNP effect on CCDC134

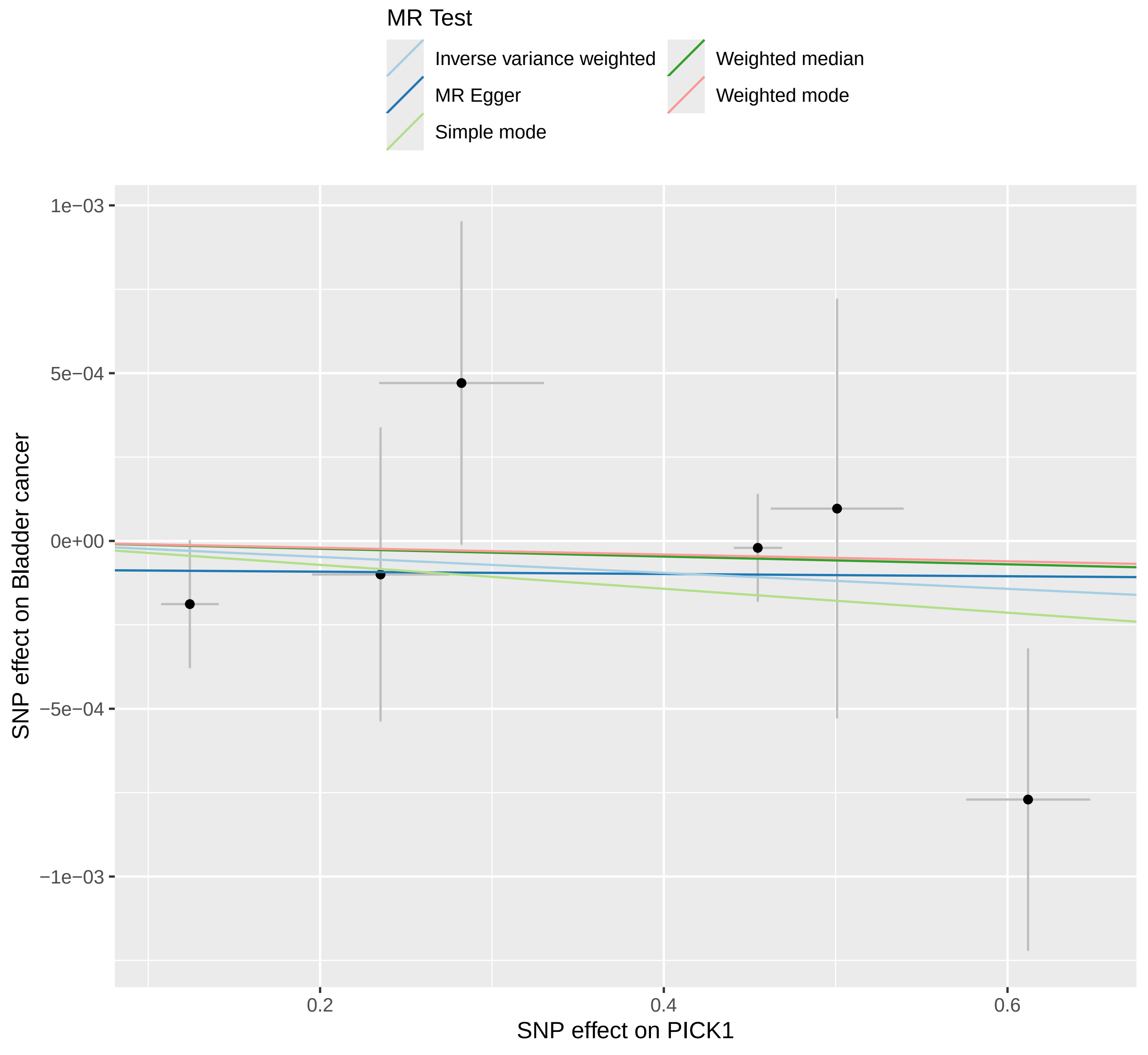
MR Test
Inverse variance weighted
MR Egger
Simple mode
Weighted median
Weighted mode
1e−03
5e−04
0e+00
−5e−04
−1e−03
SNP effect on Bladder cancer
0.2
0.4
0.6
SNP effect on PICK1

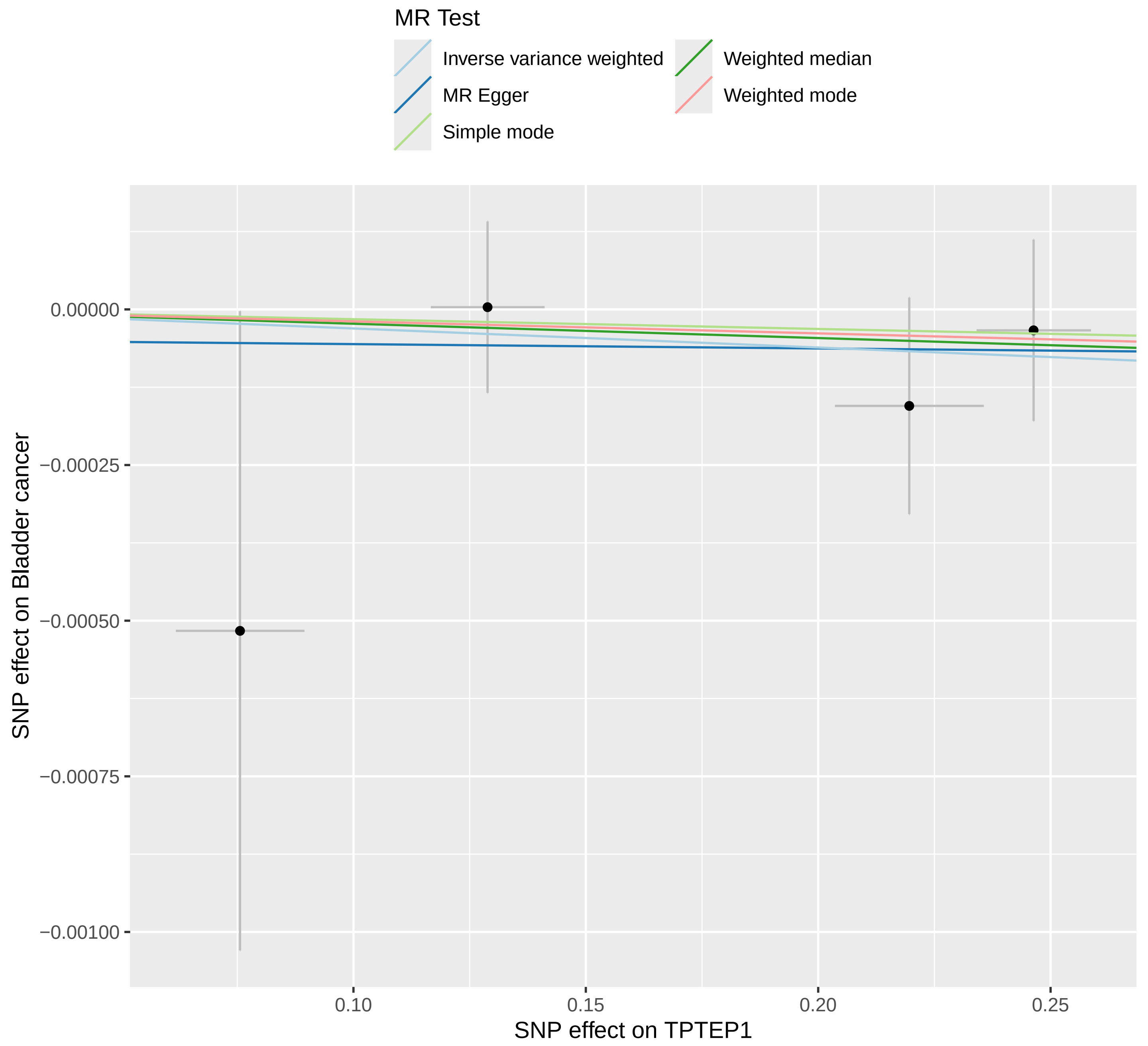
MR Test
Inverse variance weighted
MR Egger
Simple mode
Weighted median
Weighted mode
0.00000
−0.00025
−0.00050
−0.00075
−0.00100
SNP effect on Bladder cancer
0.10
0.15
0.20
0.25
SNP effect on TPTEP1

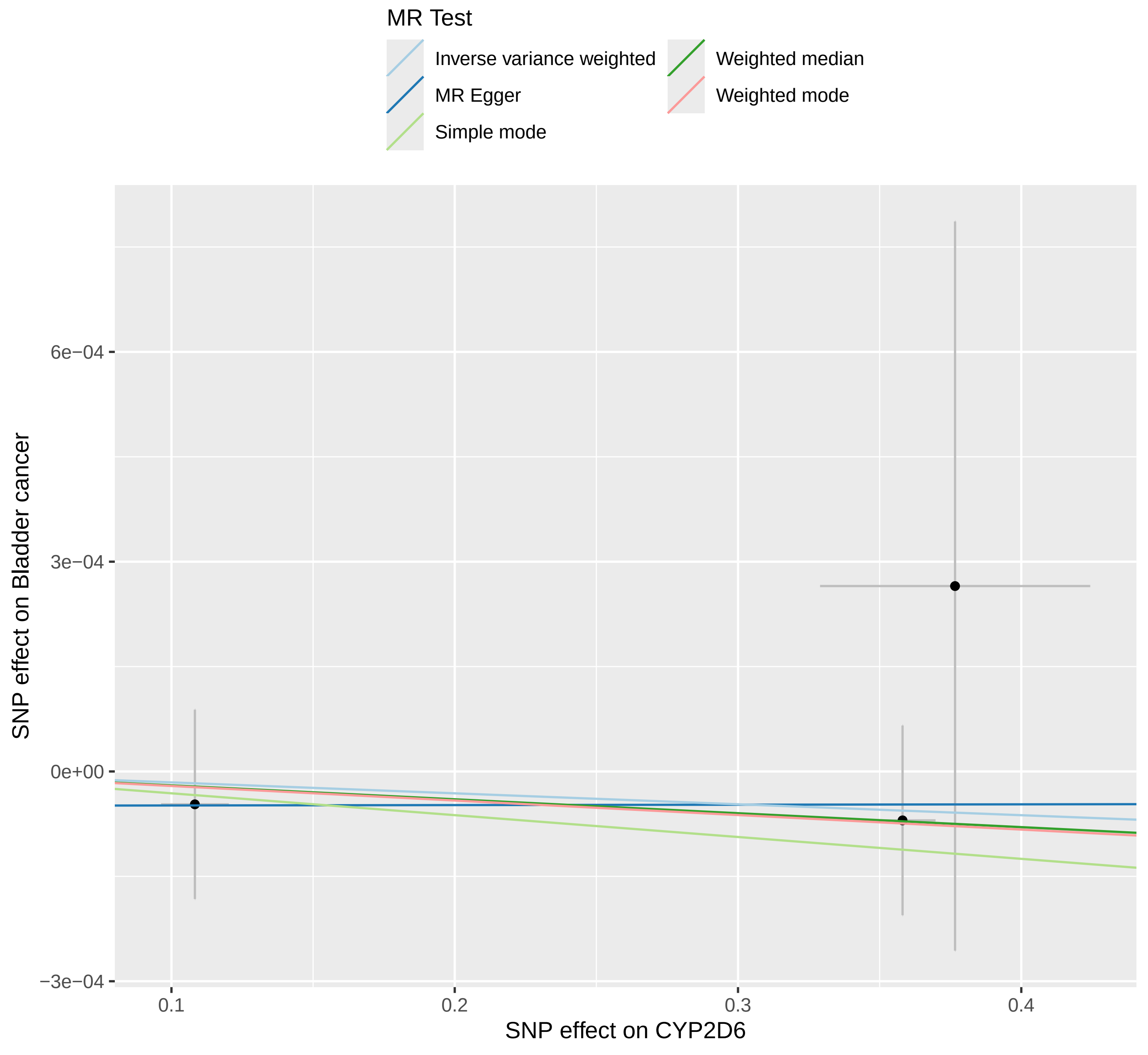
MR Test
Inverse variance weighted
MR Egger
Simple mode
Weighted median
Weighted mode
6e−04
3e−04
0e+00
−3e−04
SNP effect on Bladder cancer
0.1
0.2
0.3
0.4
SNP effect on CYP2D6

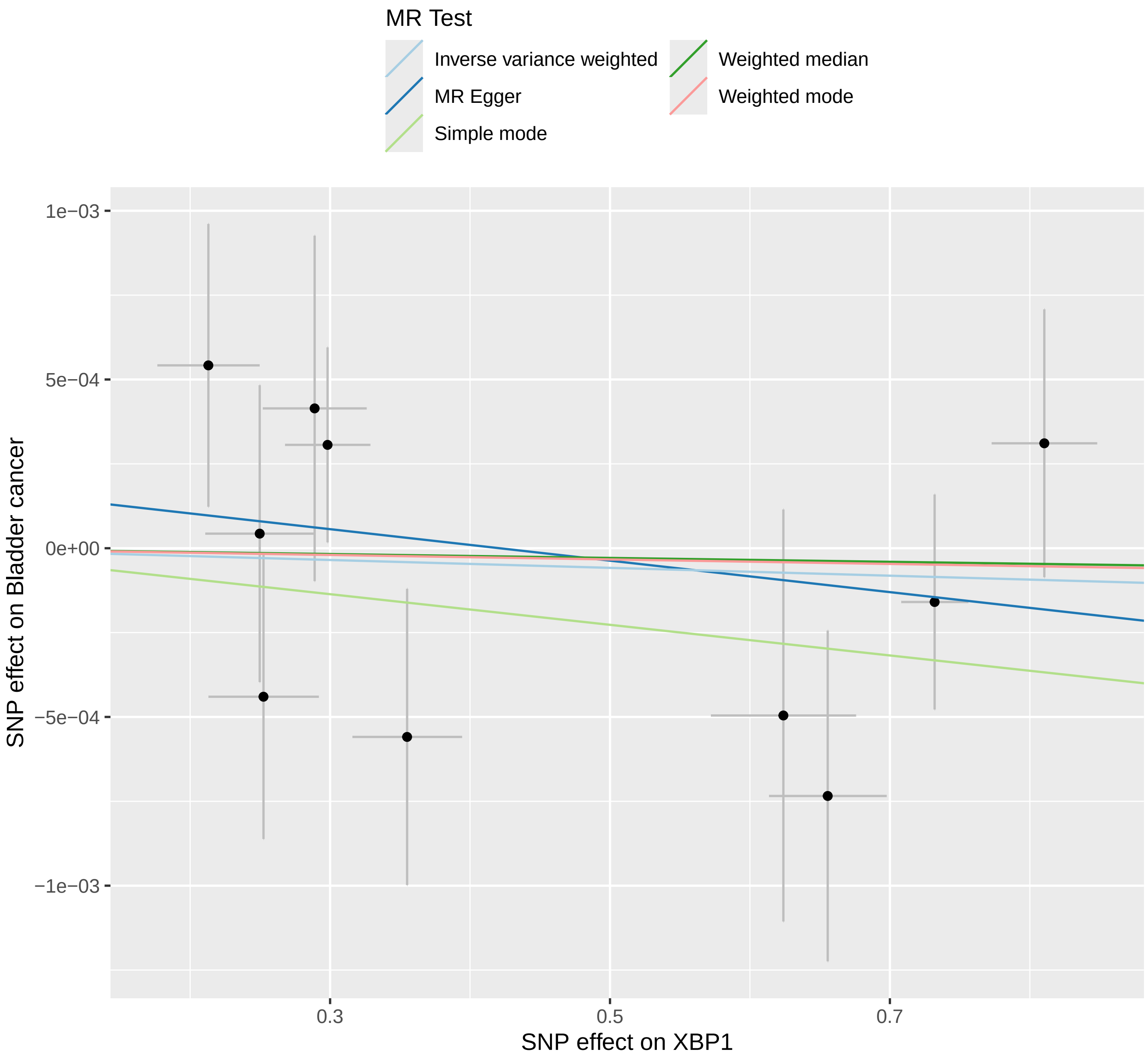

MR Test
Inverse variance weighted
MR Egger
Simple mode
Weighted median
Weighted mode
1e−03
5e−04
0e+00
−5e−04
−1e−03
SNP effect on Bladder cancer
0.3
0.5
0.7
SNP effect on XBP1

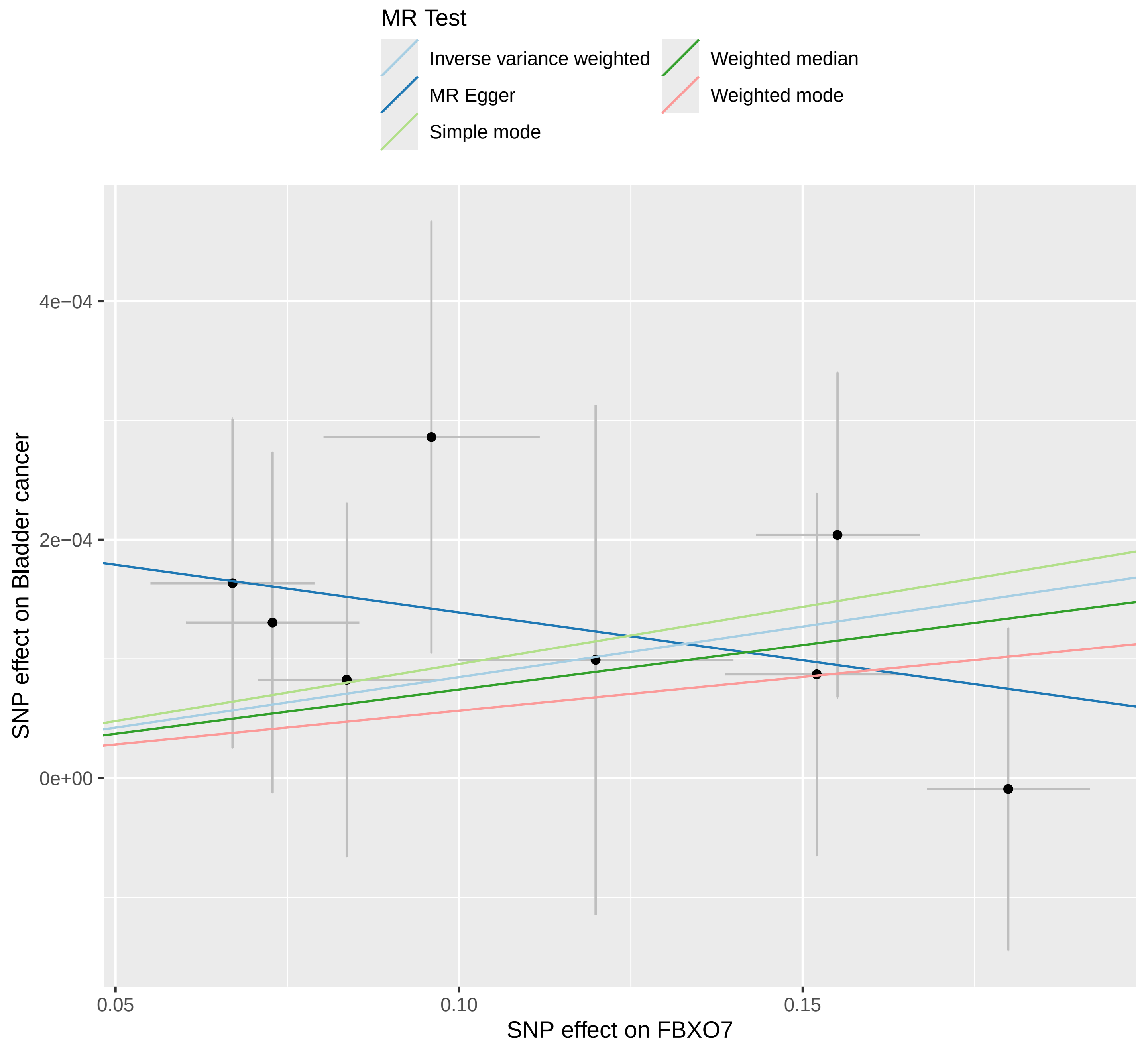

MR Test
Inverse variance weighted
MR Egger
Simple mode
Weighted median
Weighted mode
4e−04
2e−04
0e+00
0.05
0.10
0.15
SNP effect on Bladder cancer
SNP effect on FBXO7

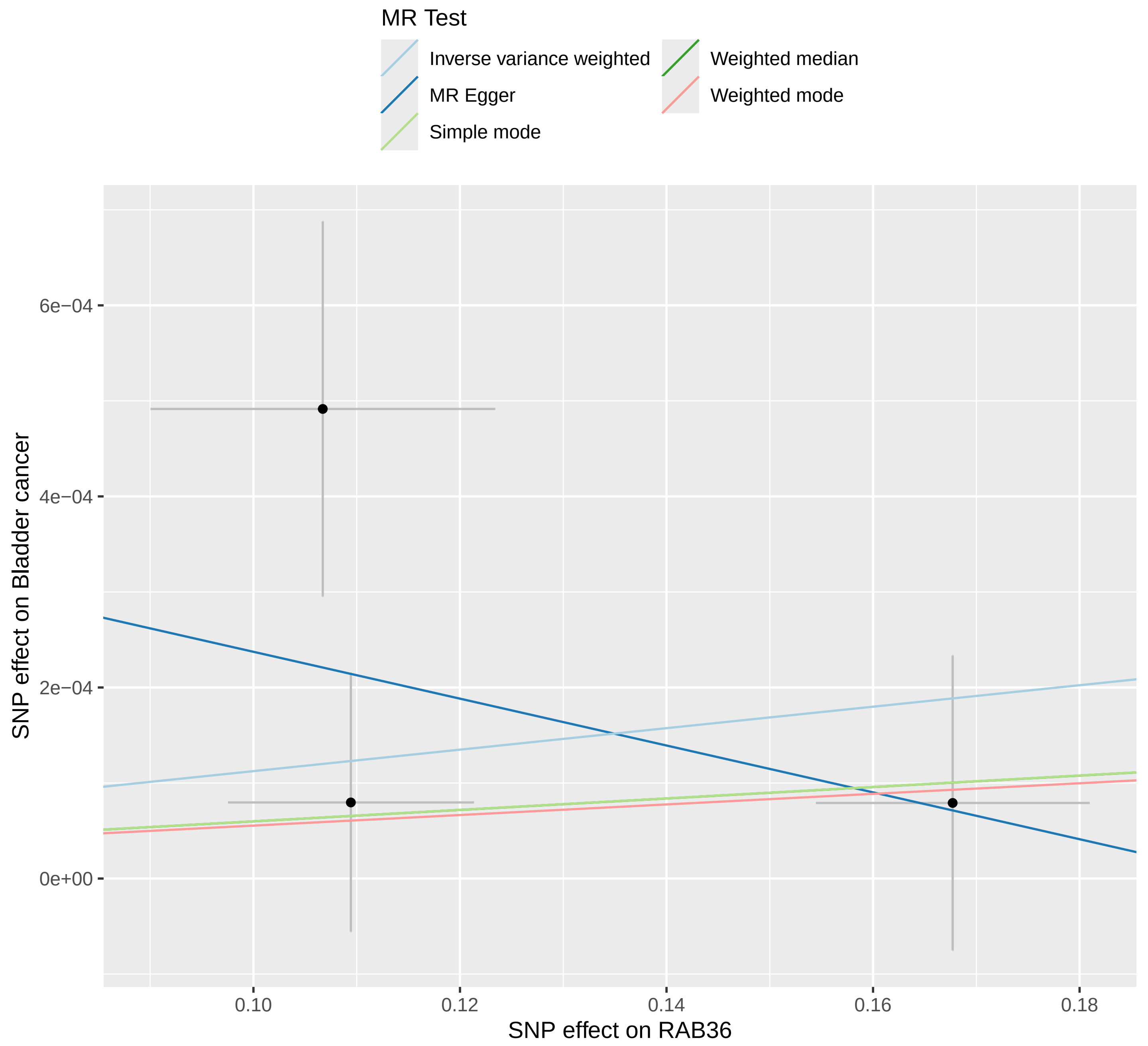

MR Test
Inverse variance weighted
MR Egger
Simple mode
Weighted median
Weighted mode
6e−04
4e−04
2e−04
0e+00
SNP effect on Bladder cancer
0.10
0.12
0.14
0.16
0.18
SNP effect on RAB36

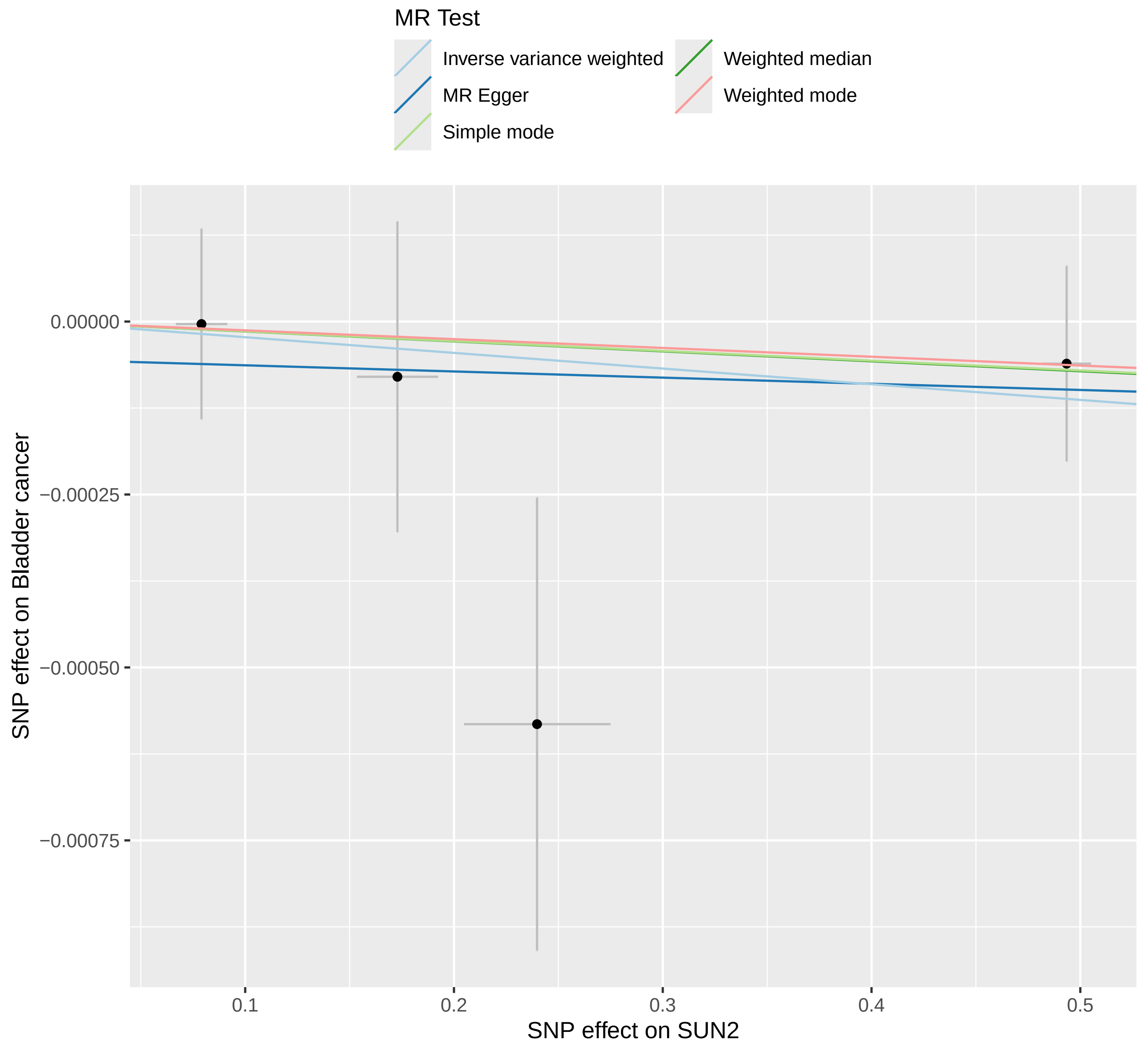
MR Test
Inverse variance weighted
MR Egger
Simple mode
Weighted median
Weighted mode
0.00000
−0.00025
−0.00050
−0.00075
SNP effect on Bladder cancer
0.1
0.2
0.3
0.4
0.5
SNP effect on SUN2

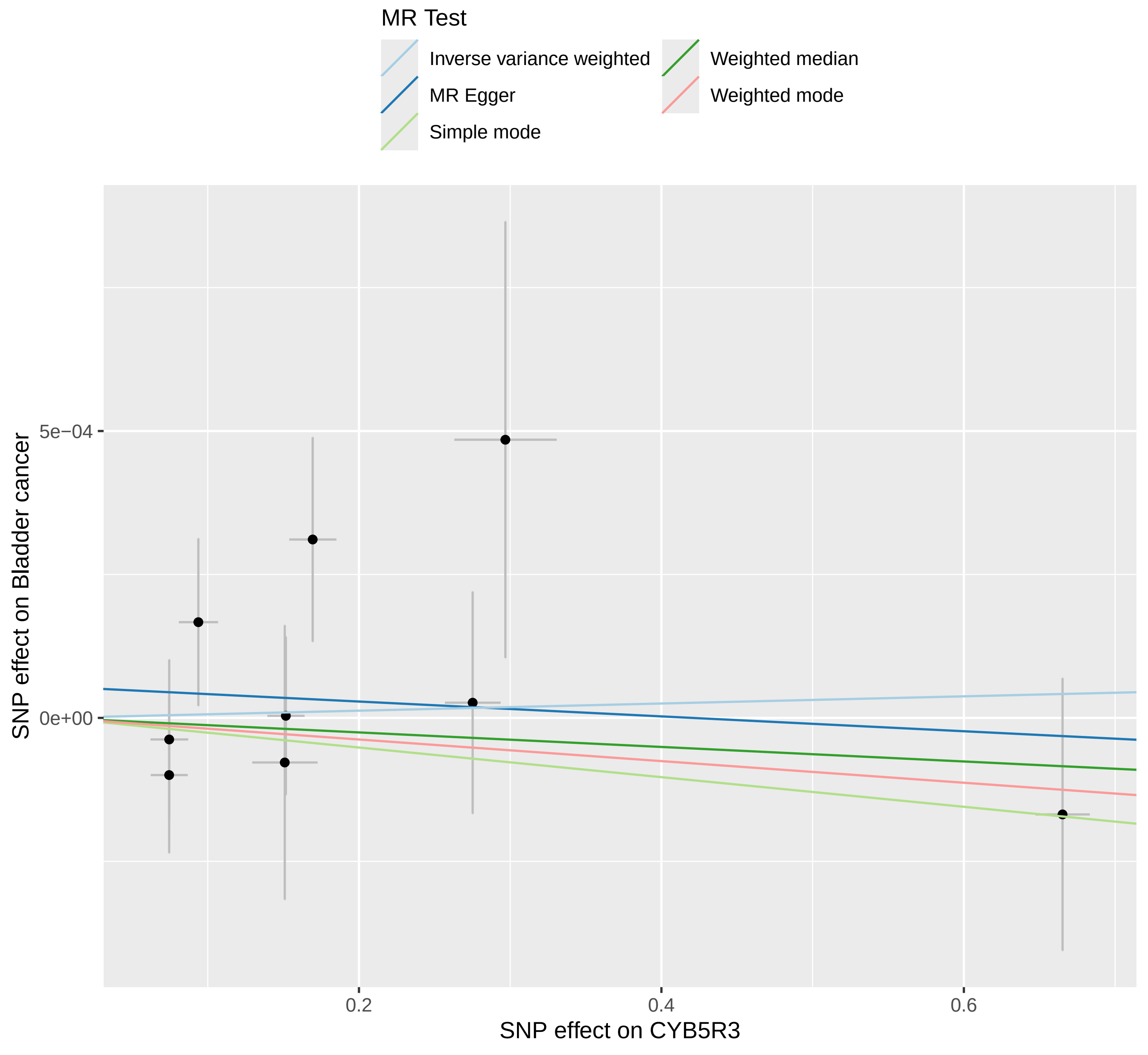

MR Test
Inverse variance weighted
MR Egger
Simple mode
Weighted median
Weighted mode
5e−04
0e+00
0.2
0.4
0.6
SNP effect on CYB5R3
SNP effect on Bladder cancer

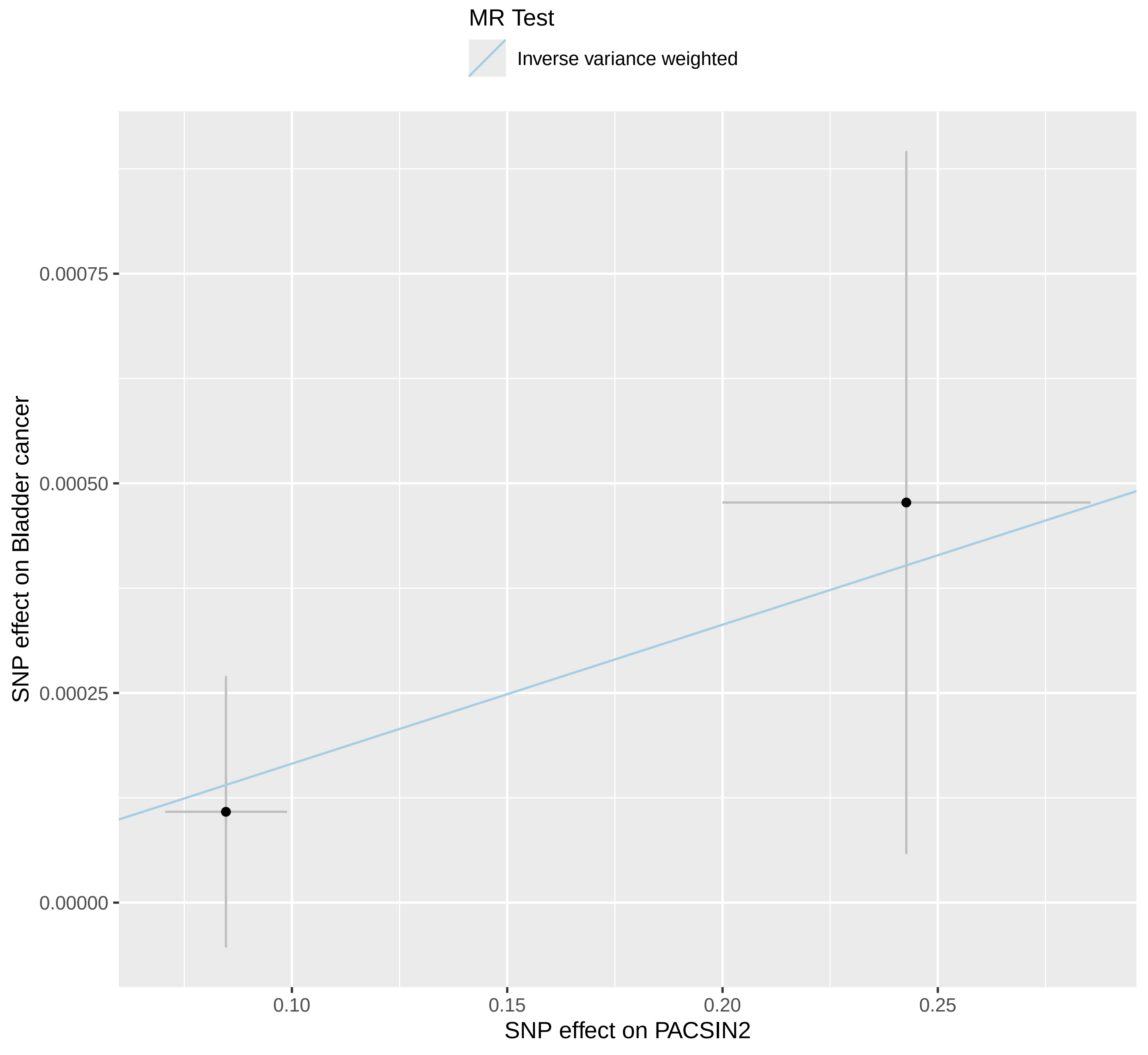

MR Test
Inverse variance weighted
0.00075
0.00050
0.00025
0.00000
SNP effect on Bladder cancer
0.10
0.15
0.20
0.25
SNP effect on PACSIN2

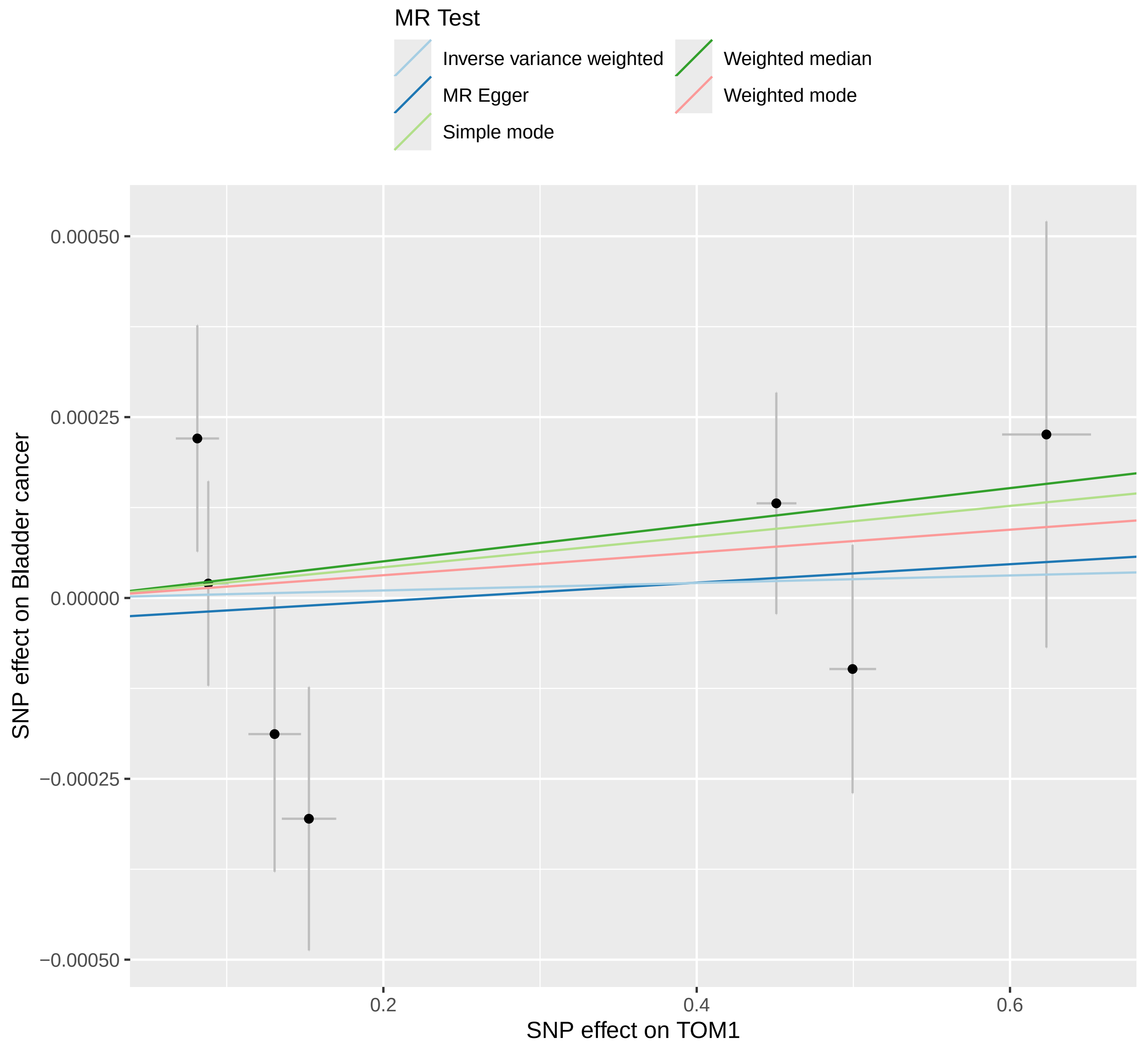
MR Test
Inverse variance weighted
MR Egger
Simple mode
Weighted median
Weighted mode
0.00050
0.00025
0.00000
−0.00025
−0.00050
SNP effect on Bladder cancer
0.2
0.4
0.6
SNP effect on TOM1

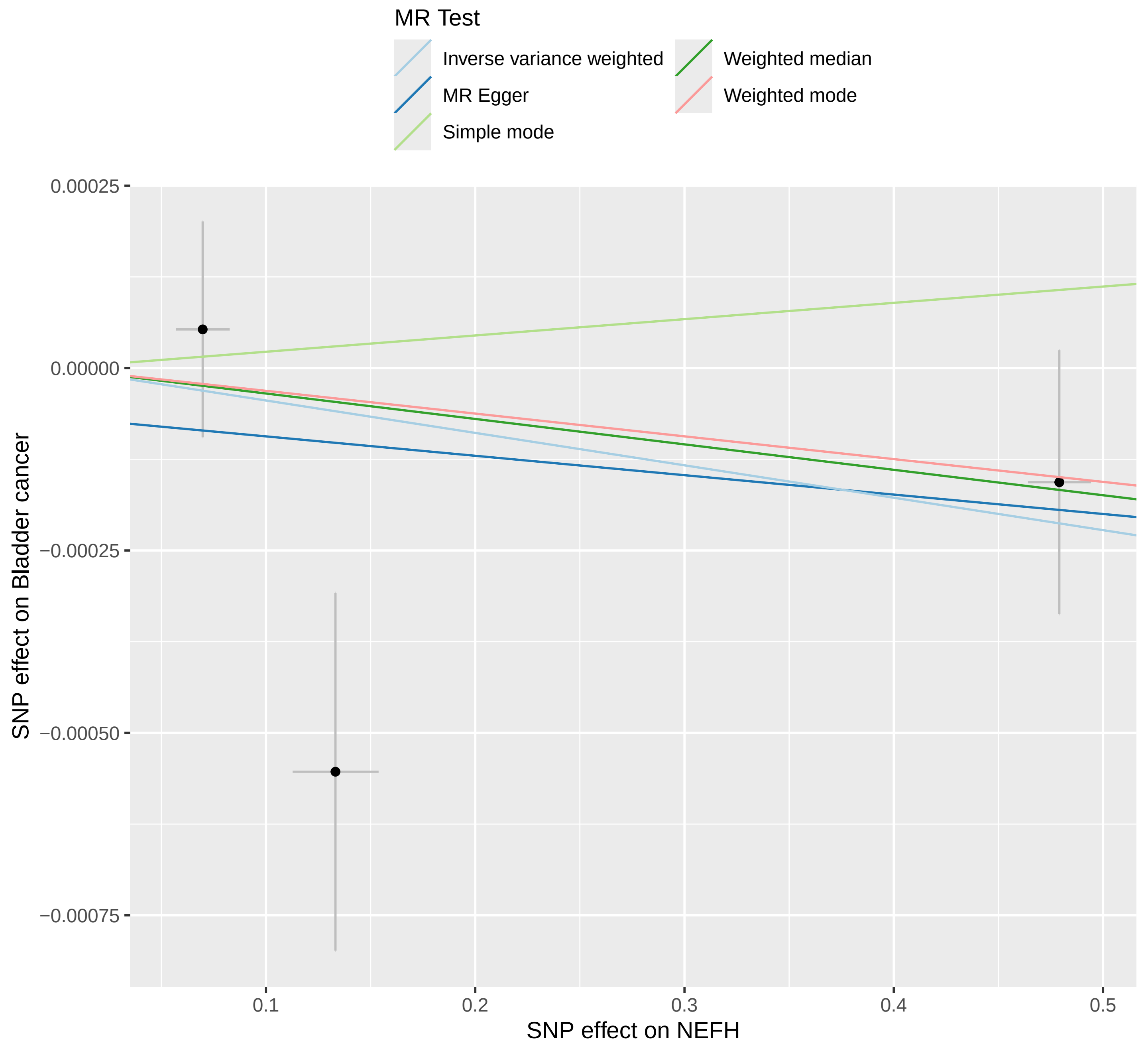
MR Test
Inverse variance weighted
MR Egger
Simple mode
Weighted median
Weighted mode
0.00025
0.00000
−0.00025
−0.00050
−0.00075
SNP effect on Bladder cancer
0.1
0.2
0.3
0.4
0.5
SNP effect on NEFH

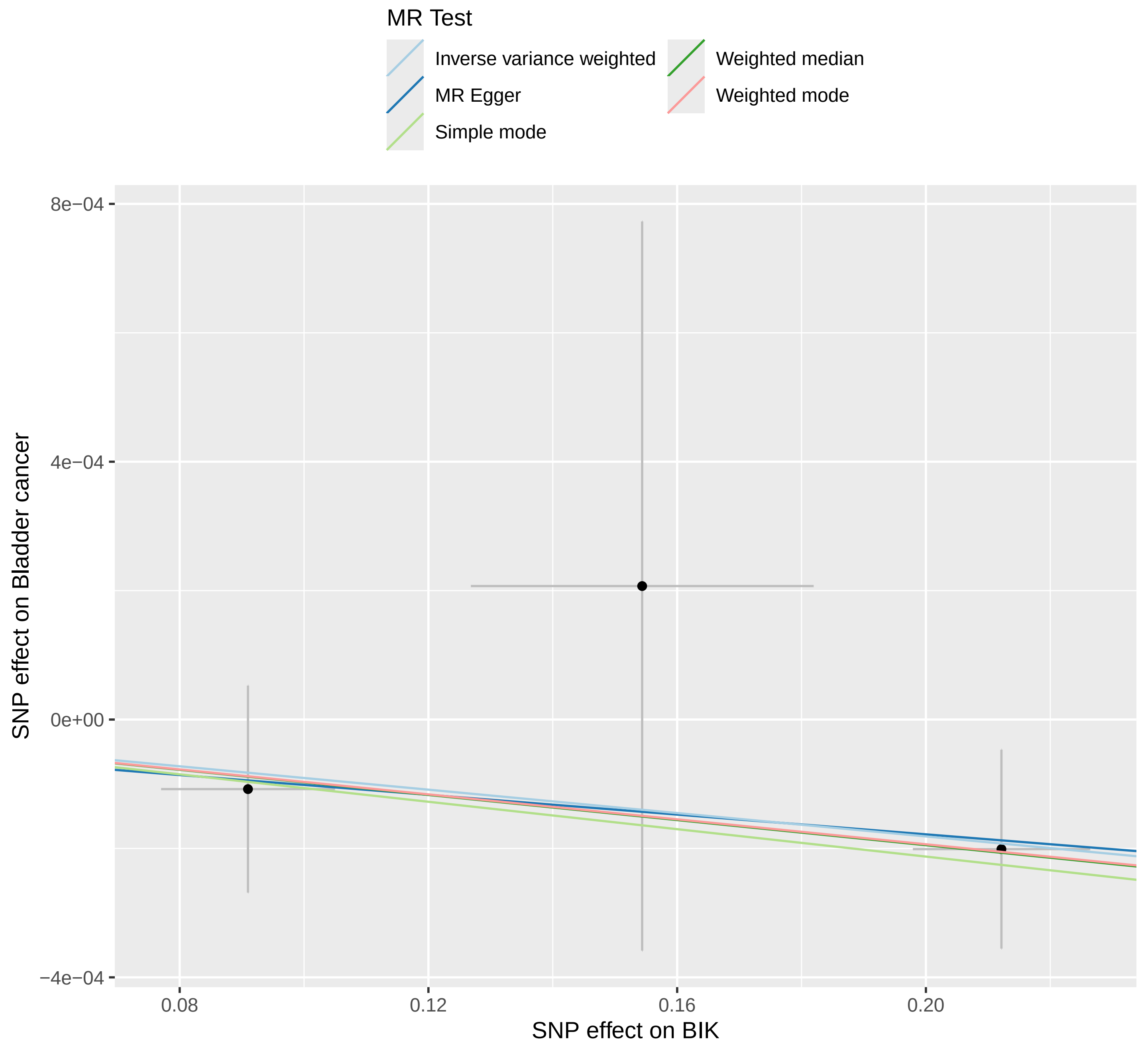

MR Test
Inverse variance weighted
MR Egger
Simple mode
Weighted median
Weighted mode
8e−04
4e−04
0e+00
−4e−04
0.08
0.12
0.16
0.20
SNP effect on Bladder cancer
SNP effect on BIK

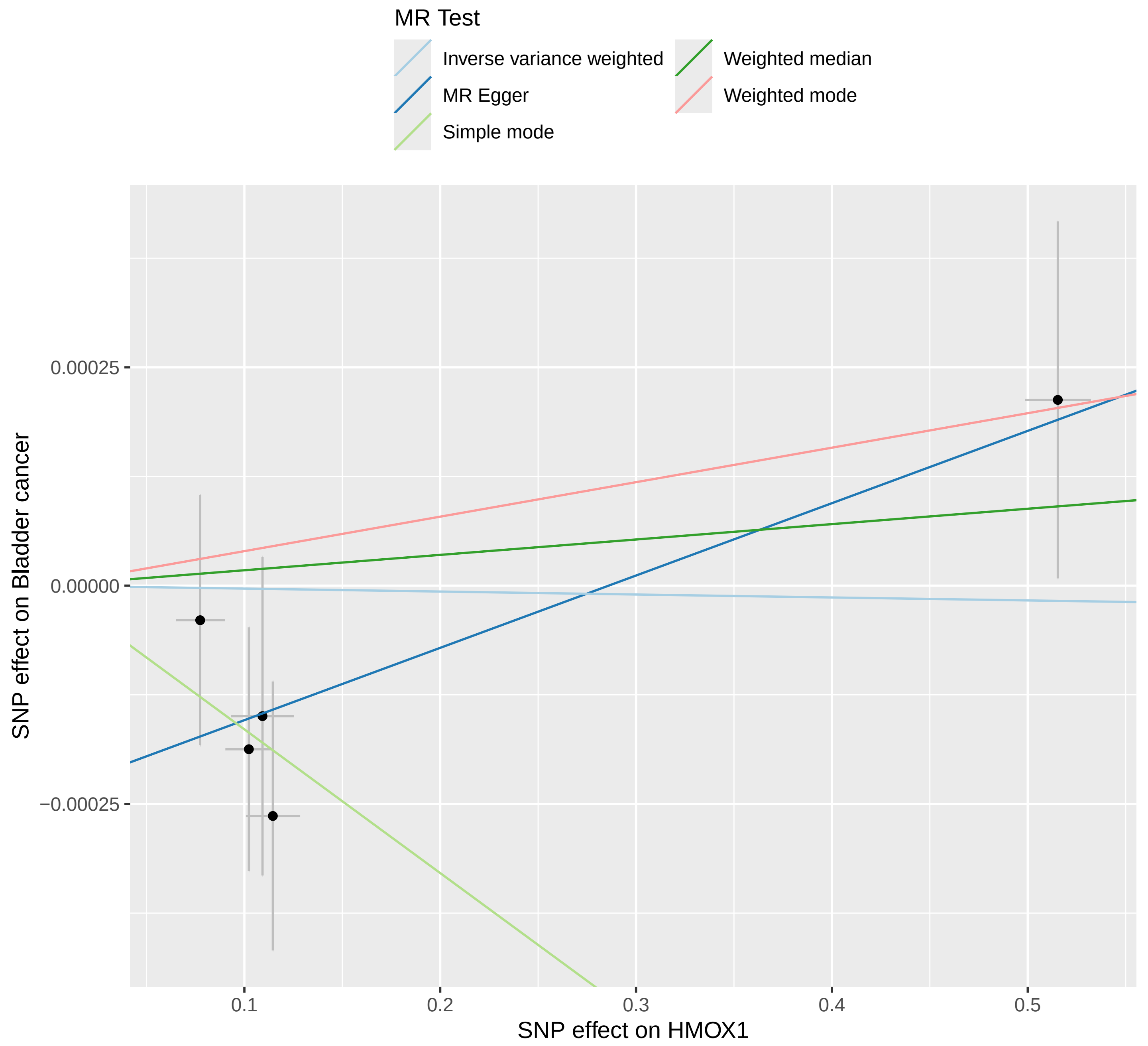
MR Test
Inverse variance weighted
MR Egger
Simple mode
Weighted median
Weighted mode
0.00025
0.00000
−0.00025
SNP effect on Bladder cancer
0.1
0.2
0.3
0.4
0.5
SNP effect on HMOX1

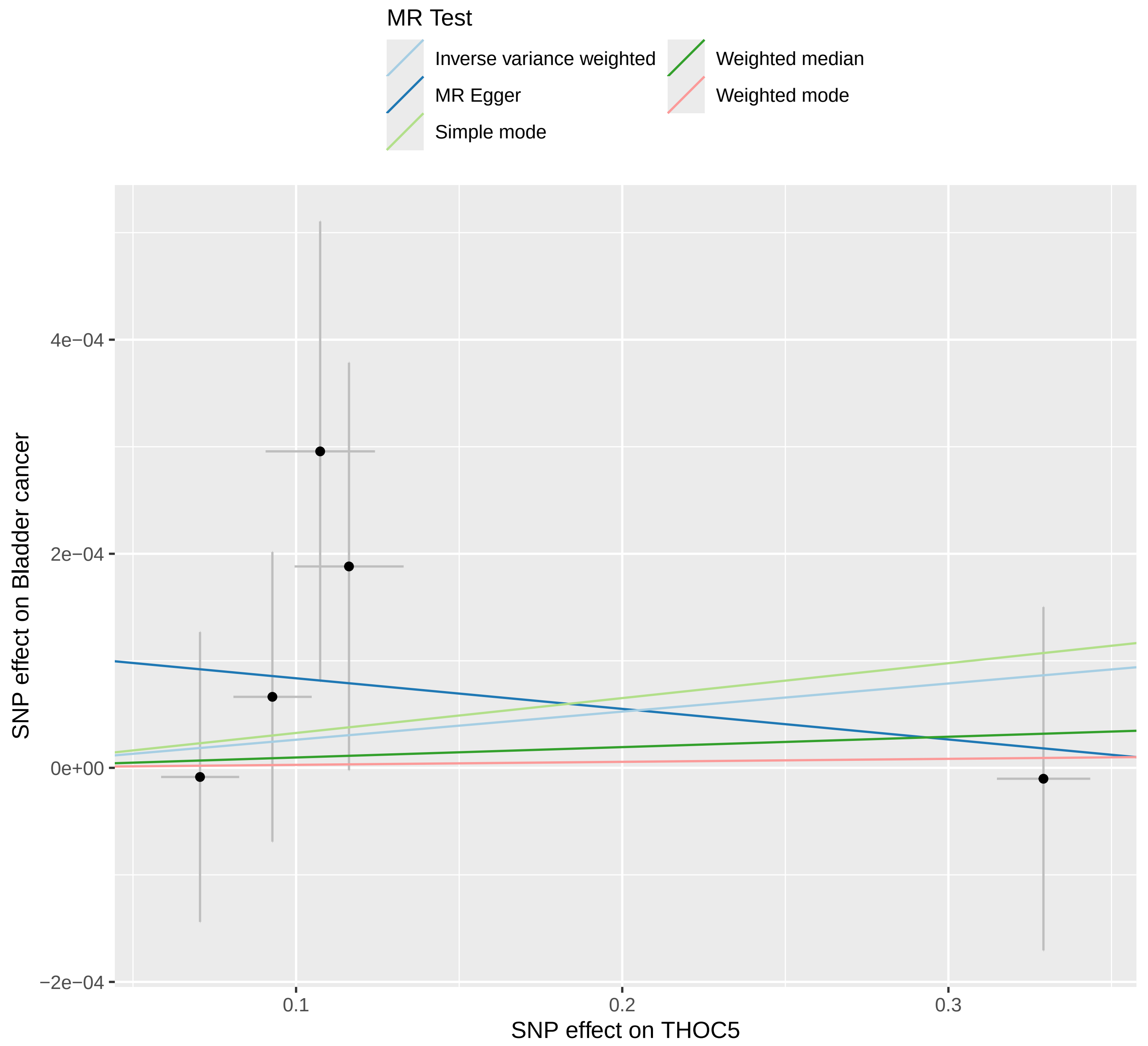

MR Test
Inverse variance weighted
MR Egger
Simple mode
Weighted median
Weighted mode
4e−04
2e−04
0e+00
−2e−04
0.1
0.2
0.3
SNP effect on Bladder cancer
SNP effect on THOC5

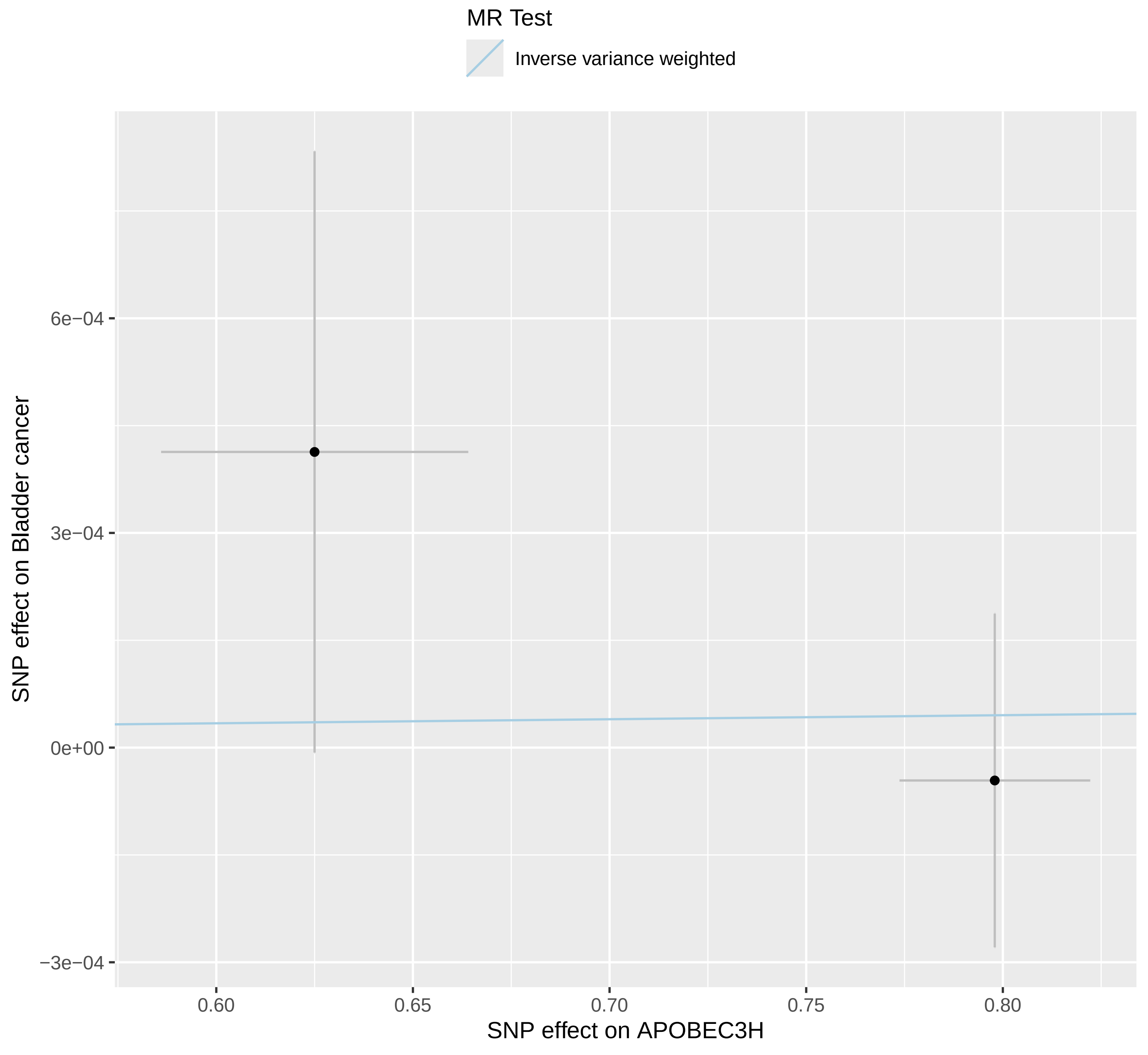

MR Test
Inverse variance weighted
6e−04
3e−04
0e+00
−3e−04
SNP effect on Bladder cancer
0.60
0.65
0.70
0.75
0.80
SNP effect on APOBEC3H

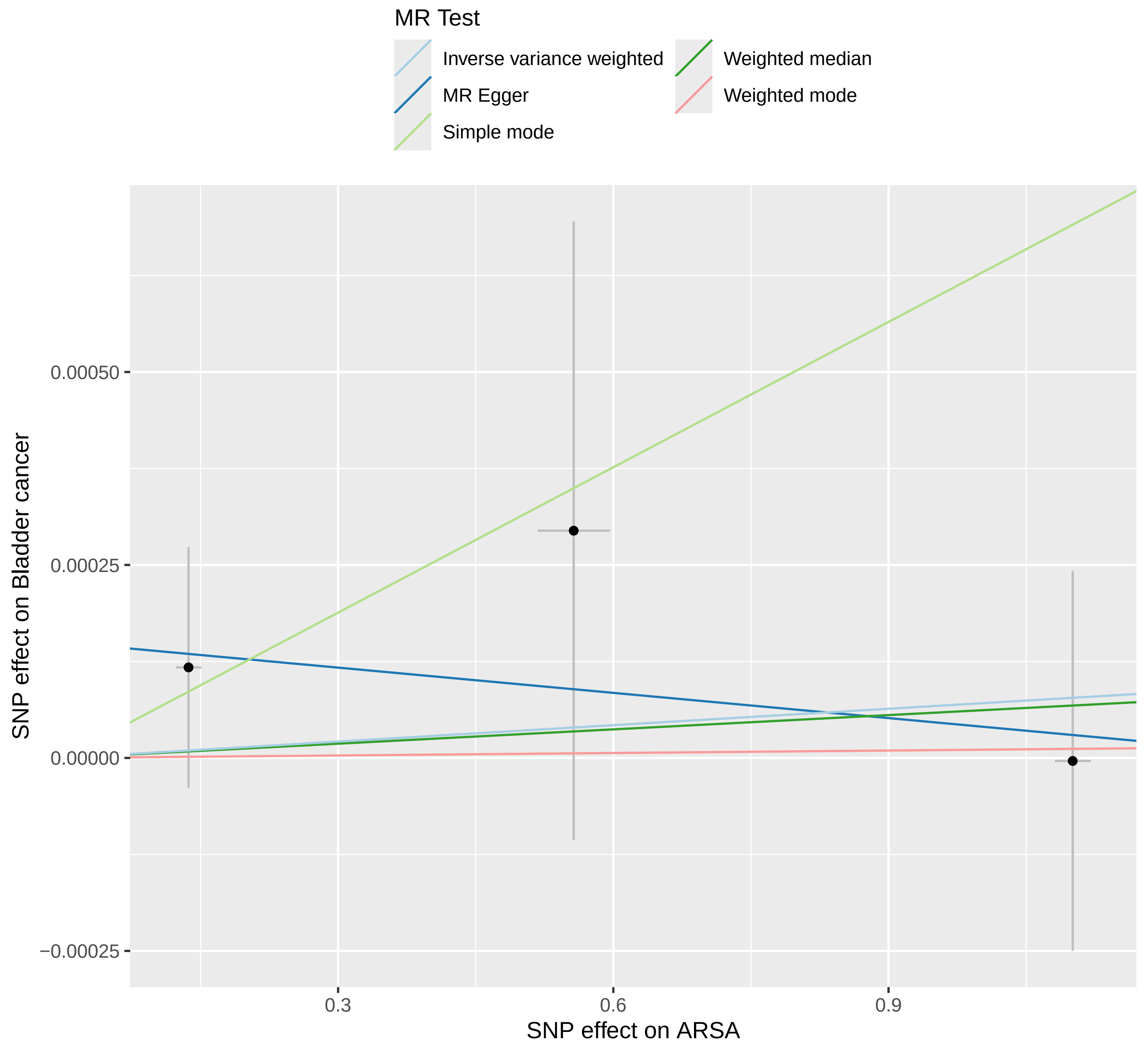
MR Test
Inverse variance weighted
MR Egger
Simple mode
Weighted median
Weighted mode
0.00050
0.00025
0.00000
−0.00025
SNP effect on Bladder cancer
0.3
0.6
0.9
SNP effect on ARSA

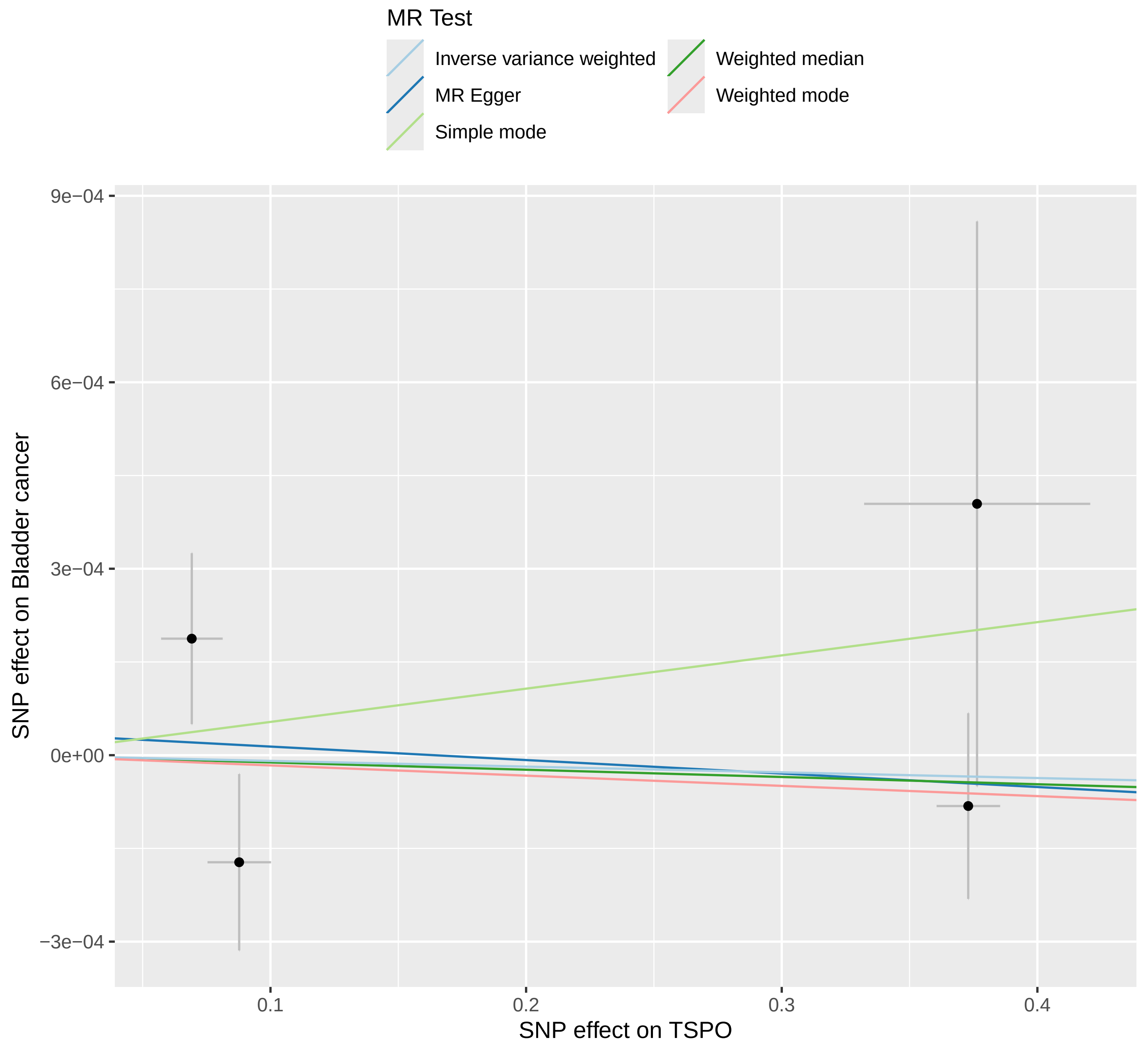

MR Test
Inverse variance weighted
MR Egger
Simple mode
Weighted median
Weighted mode
9e−04
6e−04
3e−04
0e+00
−3e−04
0.1
0.2
0.3
0.4
SNP effect on Bladder cancer
SNP effect on TSPO

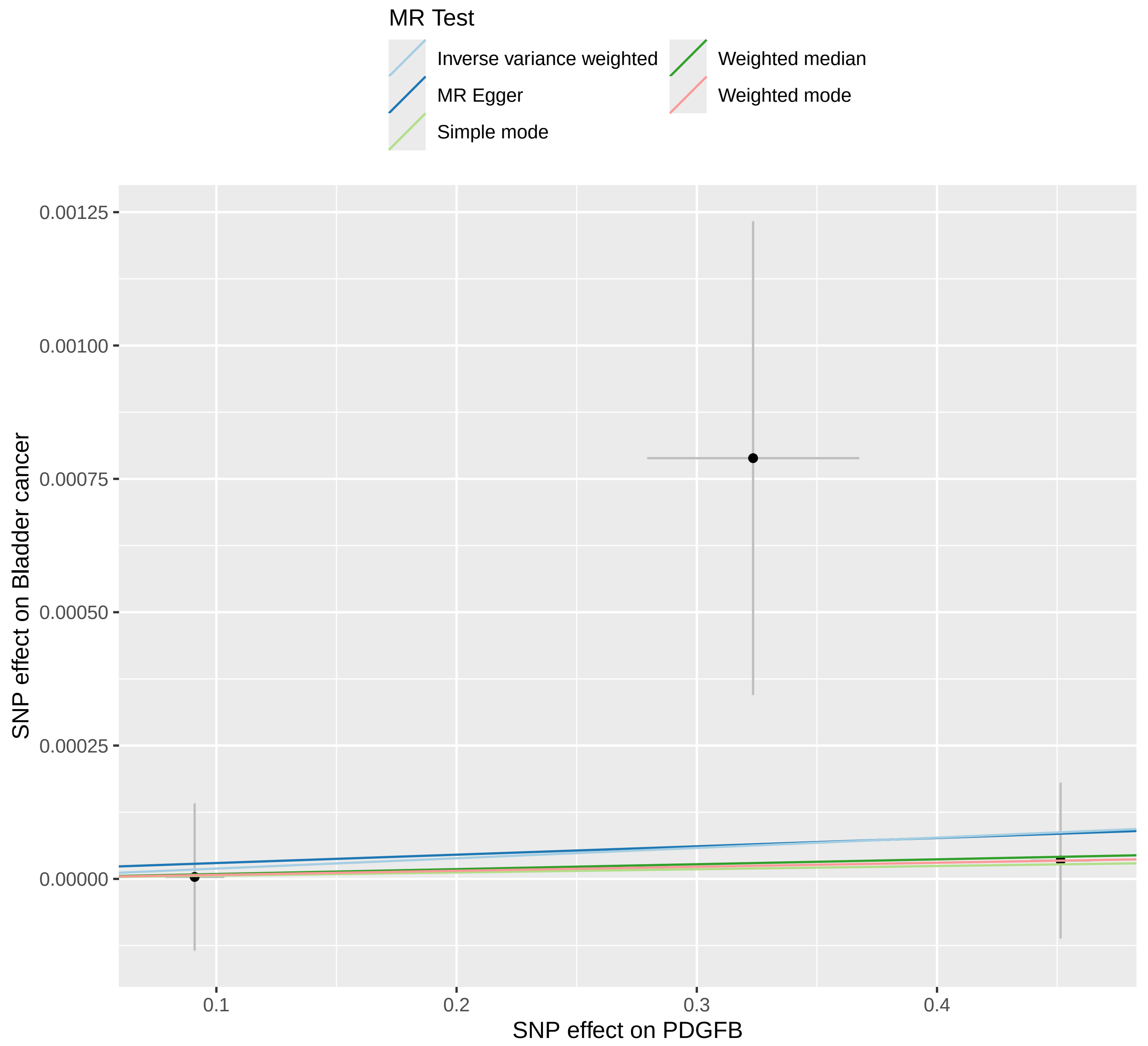
MR Test
Inverse variance weighted
MR Egger
Simple mode
Weighted median
Weighted mode
SNP effect on Bladder cancer
0.00125
0.00100
0.00075
0.00050
0.00025
0.00000
0.1
0.2
0.3
0.4
SNP effect on PDGFB

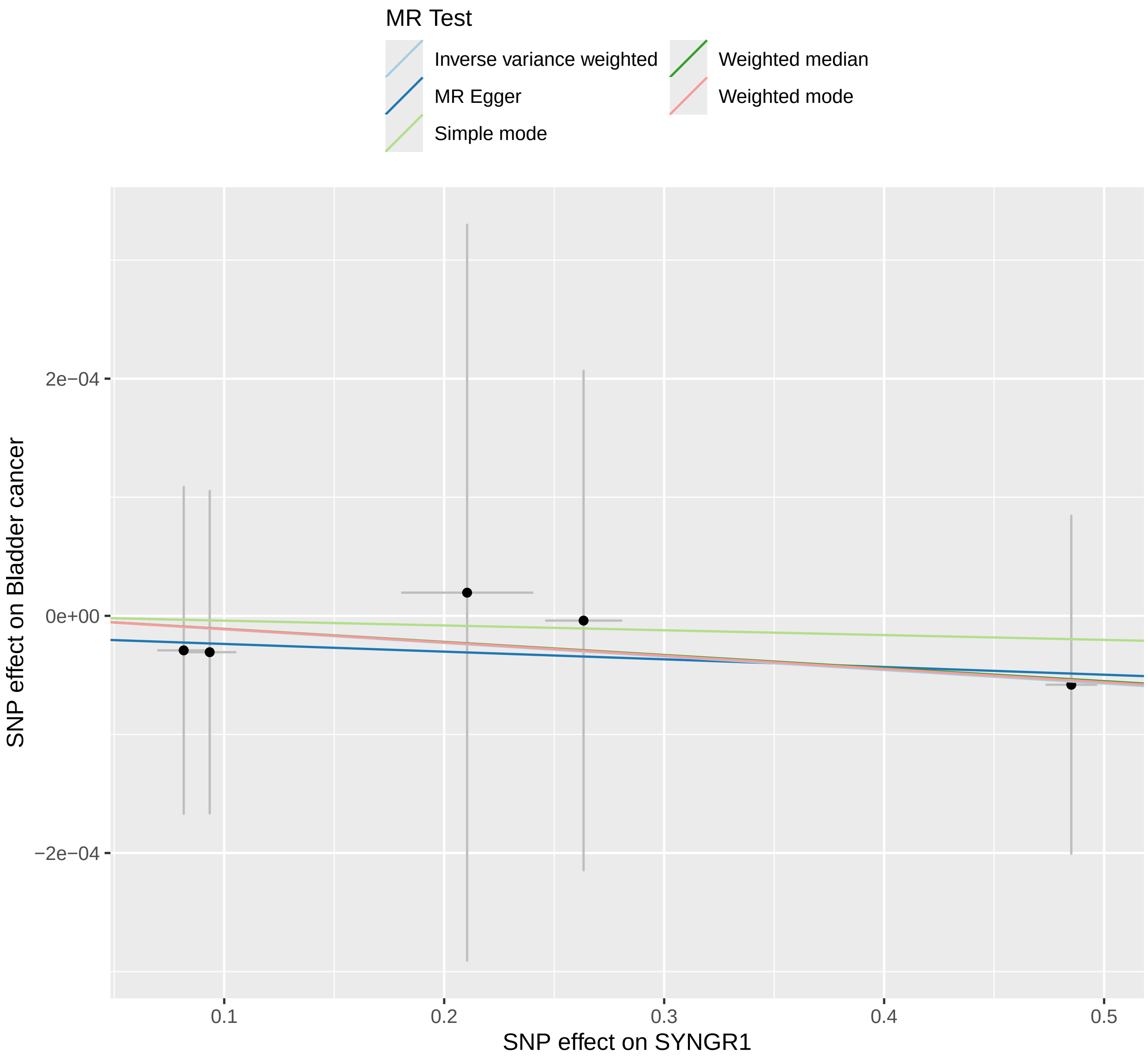

MR Test
Inverse variance weighted
MR Egger
Simple mode
Weighted median
Weighted mode
2e−04
0e+00
−2e−04
SNP effect on Bladder cancer
0.1
0.2
0.3
0.4
0.5
SNP effect on SYNGR1

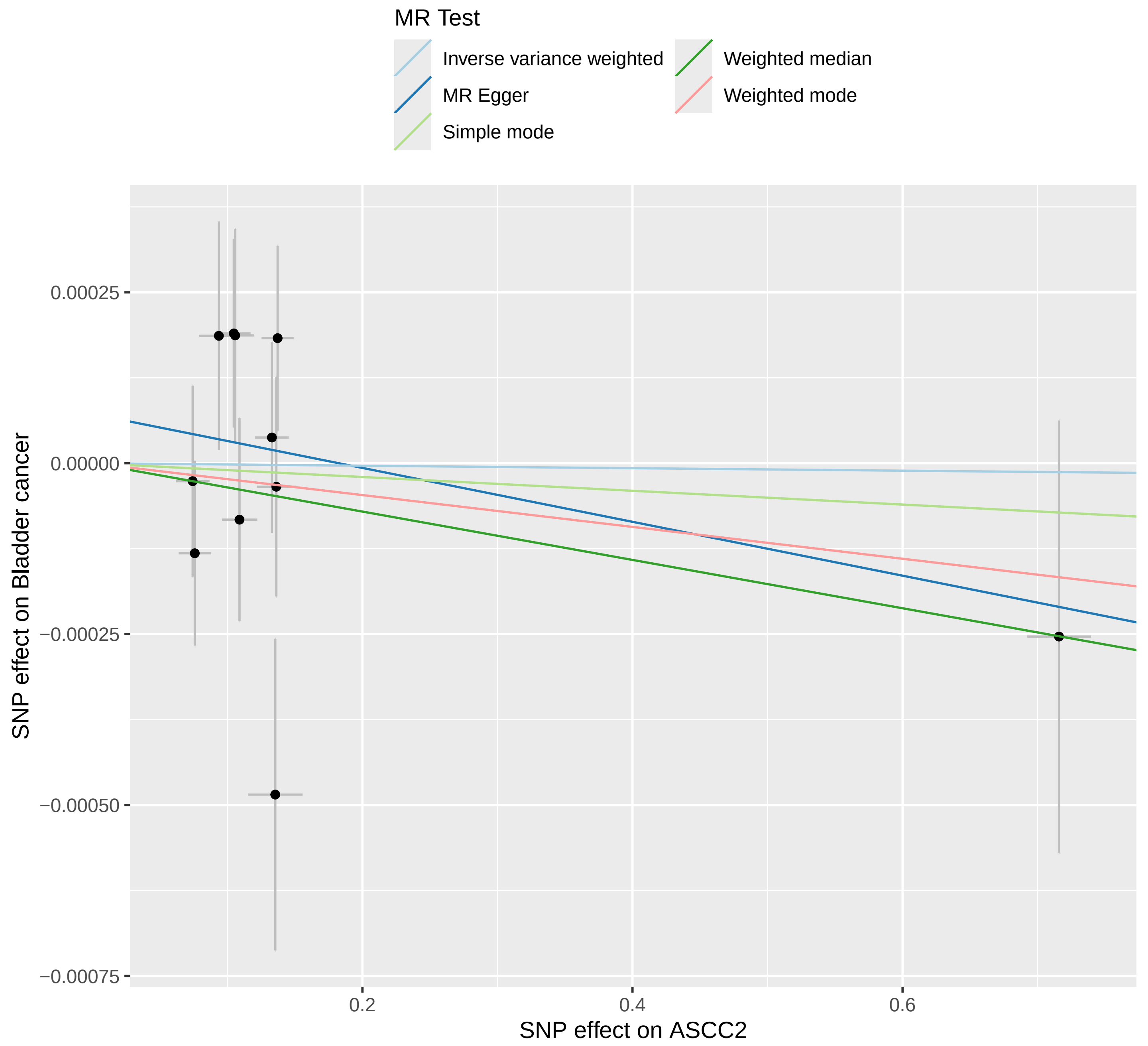
MR Test
Inverse variance weighted
MR Egger
Simple mode
Weighted median
Weighted mode
0.00025
0.00000
−0.00025
−0.00050
−0.00075
0.2
0.4
0.6
SNP effect on Bladder cancer
SNP effect on ASCC2

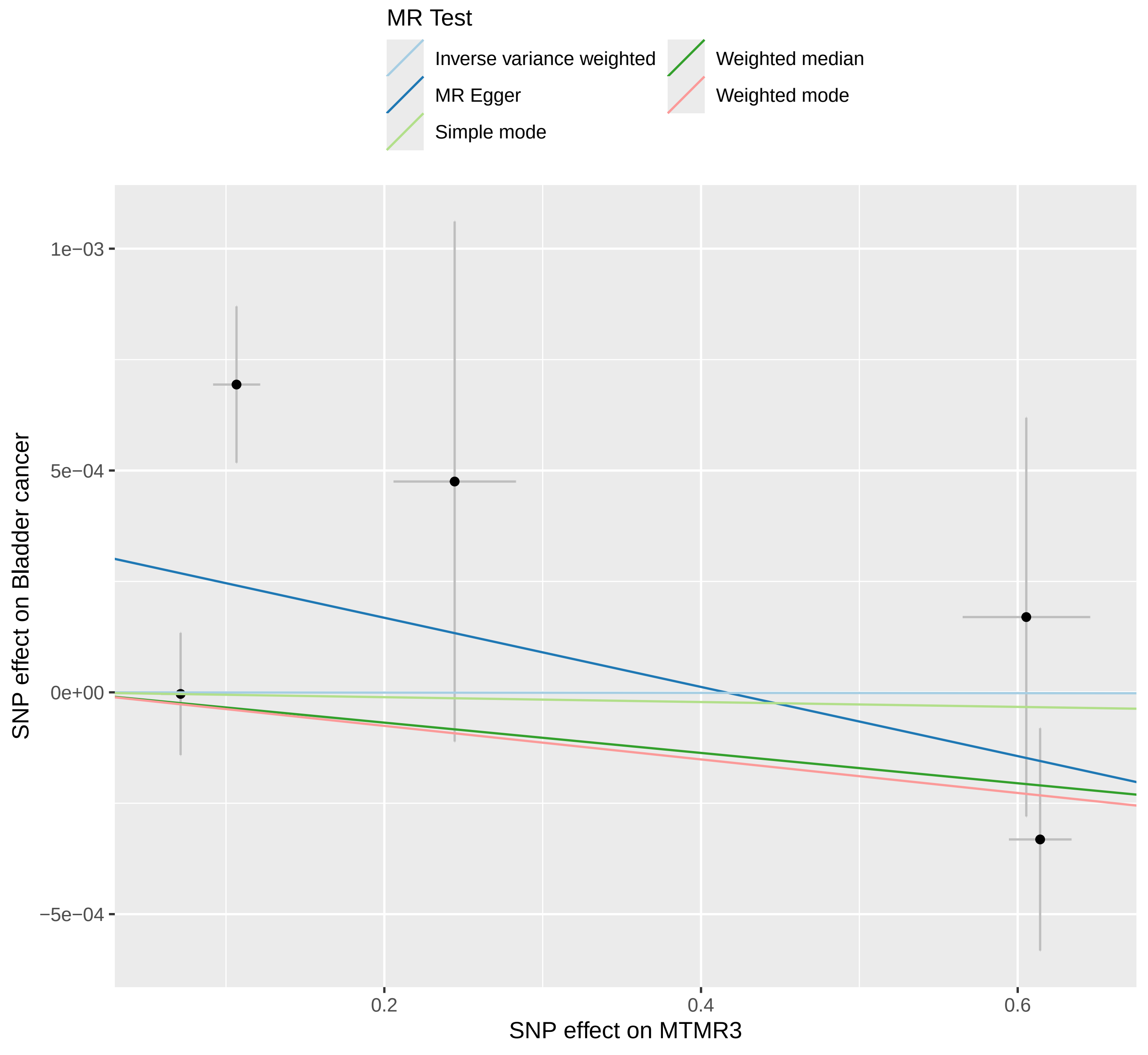

MR Test
Inverse variance weighted
MR Egger
Simple mode
Weighted median
Weighted mode
1e−03
5e−04
0e+00
−5e−04
0.2
0.4
0.6
SNP effect on Bladder cancer
SNP effect on MTMR3

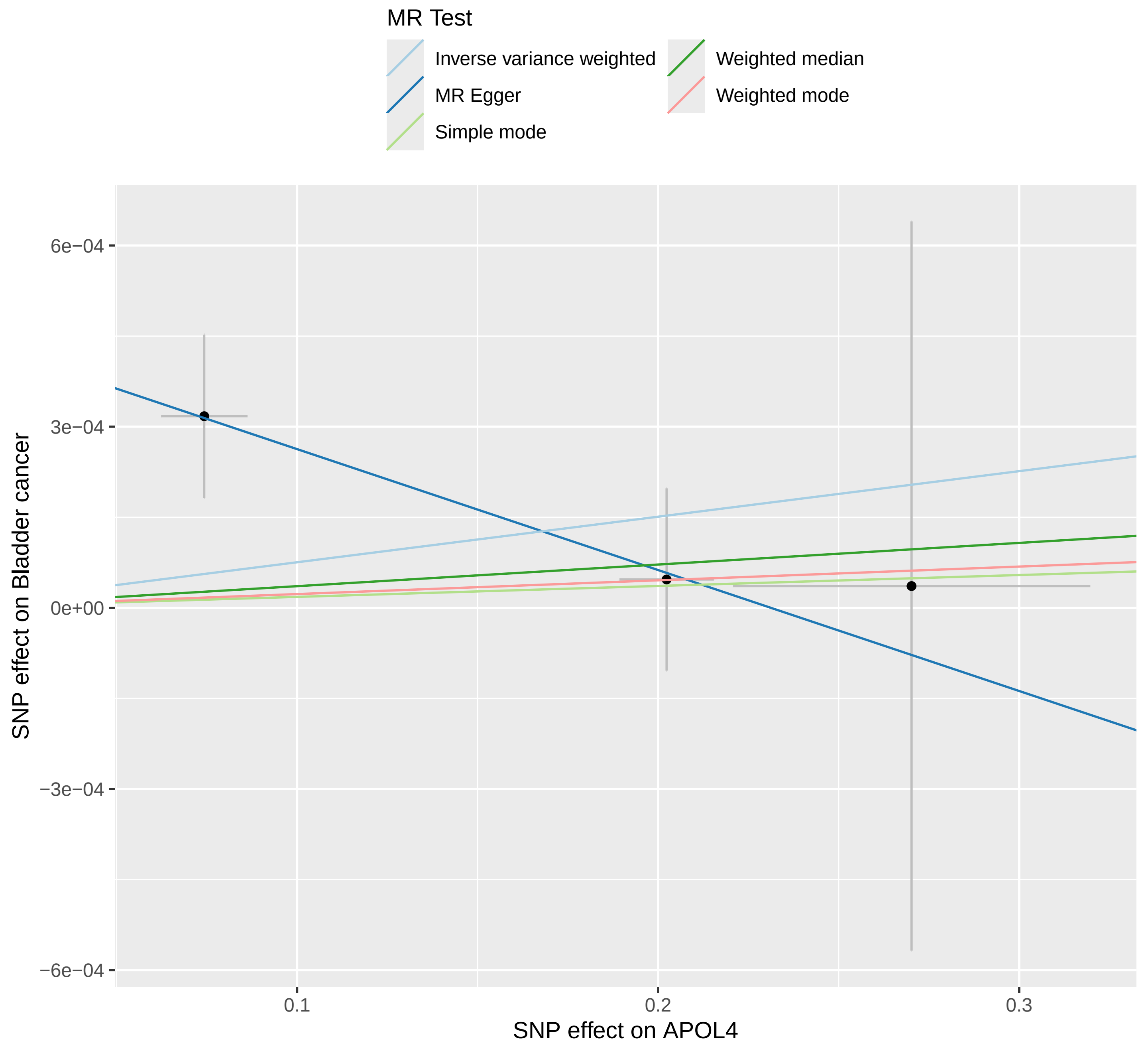
MR Test
Inverse variance weighted
MR Egger
Simple mode
Weighted median
Weighted mode
6e−04
3e−04
0e+00
−3e−04
−6e−04
0.1
0.2
0.3
SNP effect on Bladder cancer
SNP effect on APOL4

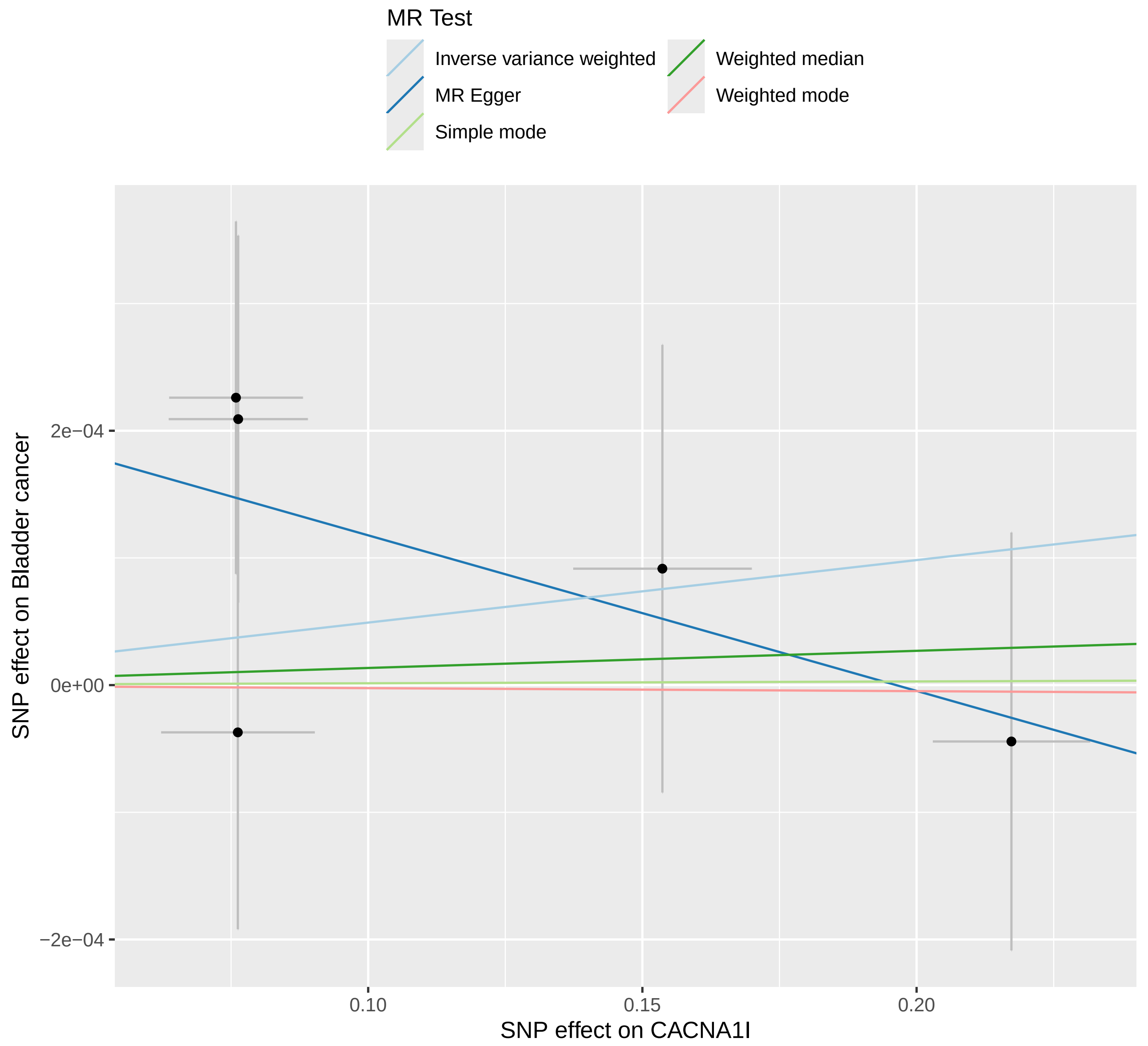
MR Test
Inverse variance weighted
MR Egger
Simple mode
Weighted median
Weighted mode
2e−04
0e+00
−2e−04
SNP effect on Bladder cancer
0.10
0.15
0.20
SNP effect on CACNA1I

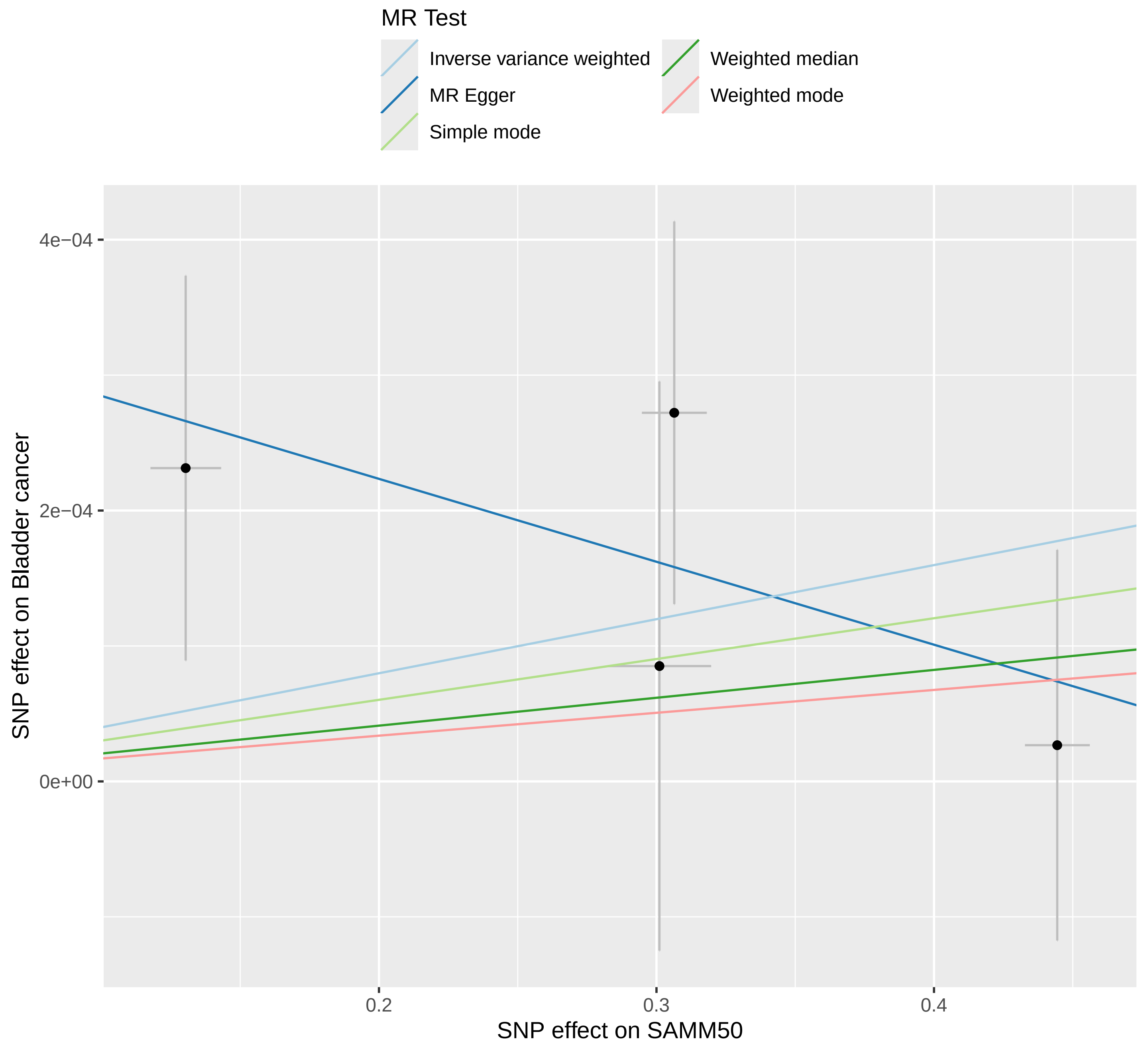

MR Test
Inverse variance weighted
MR Egger
Simple mode
Weighted median
Weighted mode
4e−04
2e−04
0e+00
0.2
0.3
0.4
SNP effect on Bladder cancer
SNP effect on SAMM50

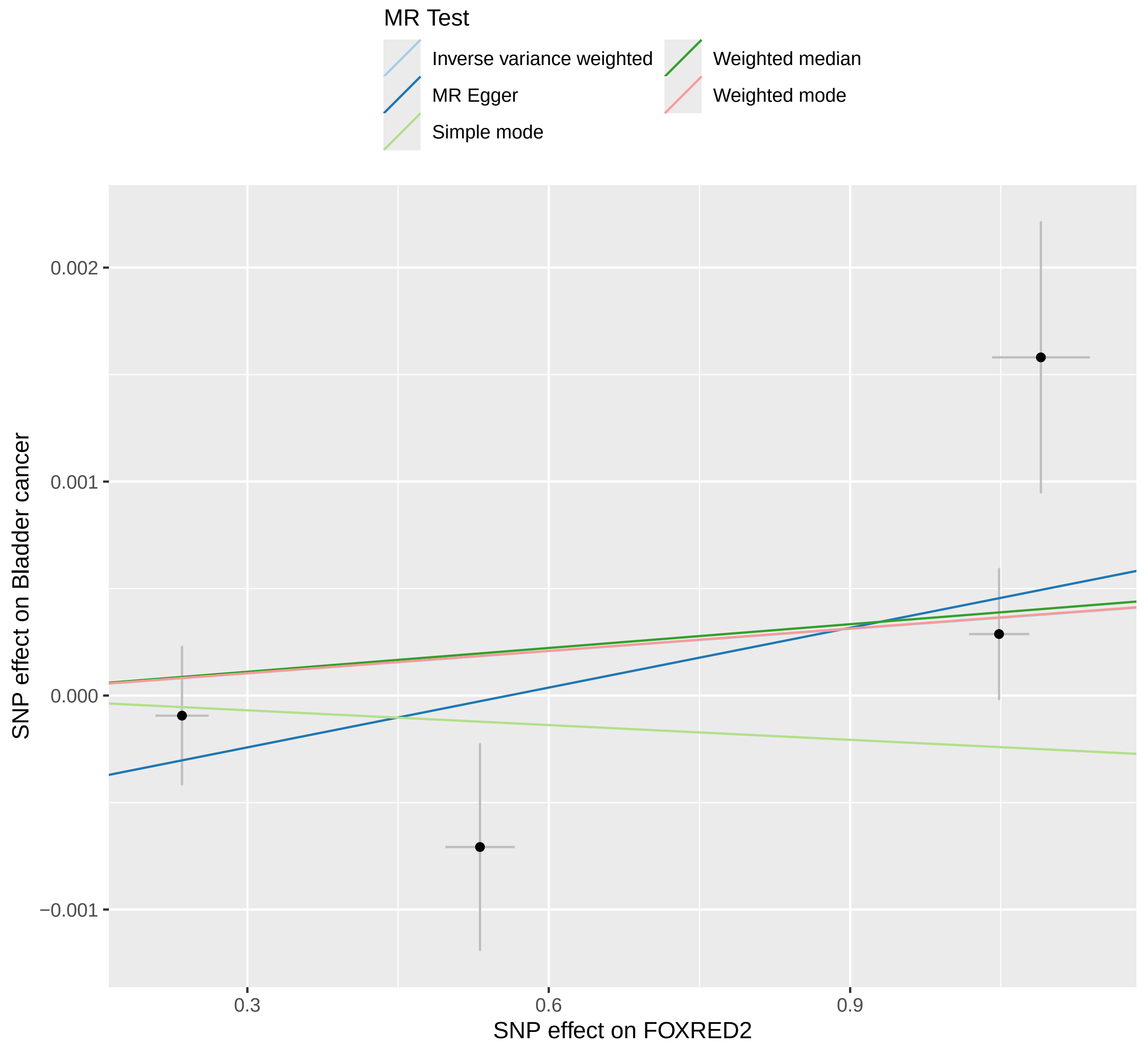

MR Test
Inverse variance weighted
MR Egger
Simple mode
Weighted median
Weighted mode
0.002
0.001
0.000
−0.001
0.3
0.6
0.9
SNP effect on Bladder cancer
SNP effect on FOXRED2

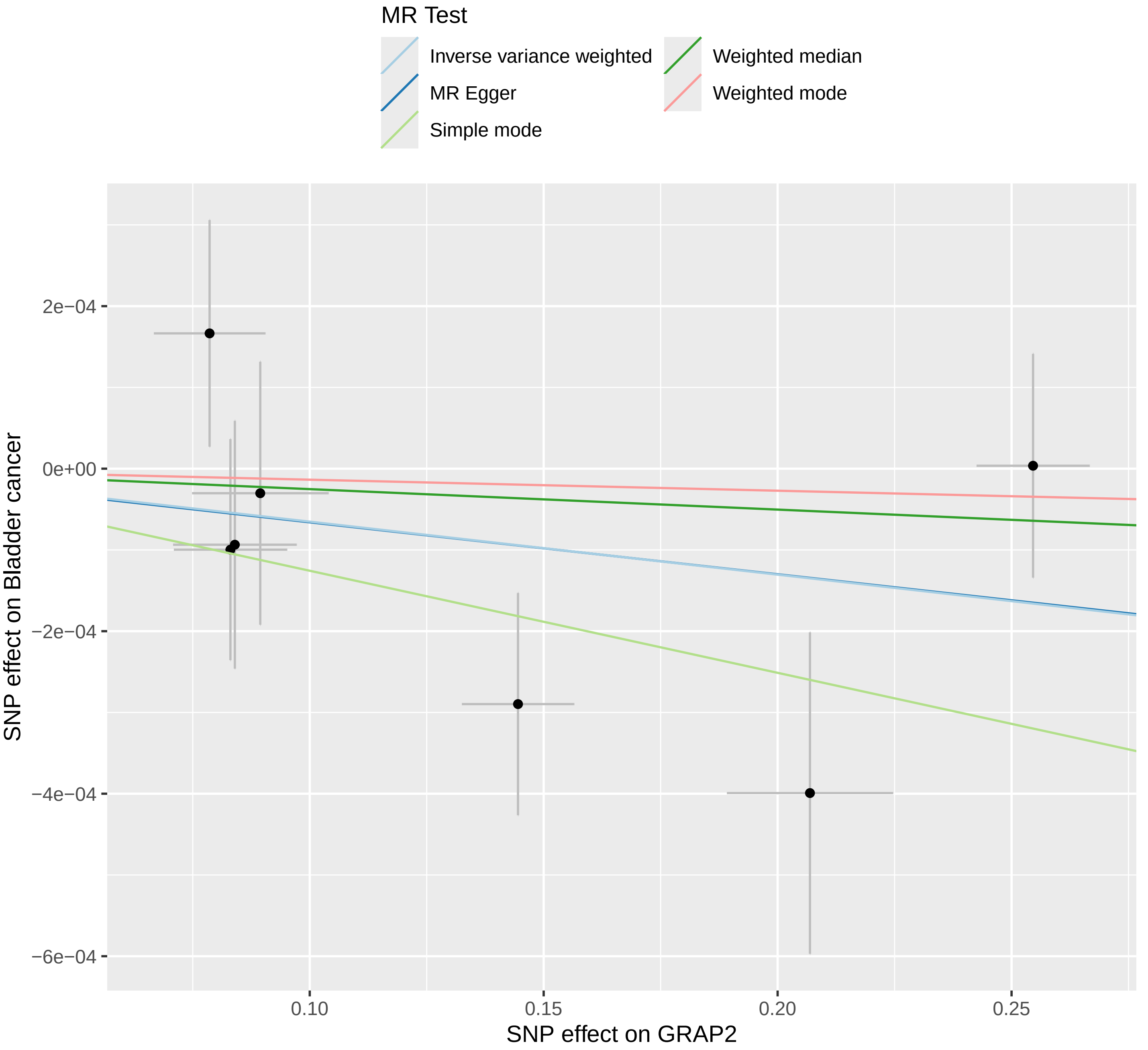
MR Test
Inverse variance weighted
MR Egger
Simple mode
Weighted median
Weighted mode
2e−04
0e+00
−2e−04
−4e−04
−6e−04
0.10
0.15
0.20
0.25
SNP effect on Bladder cancer
SNP effect on GRAP2

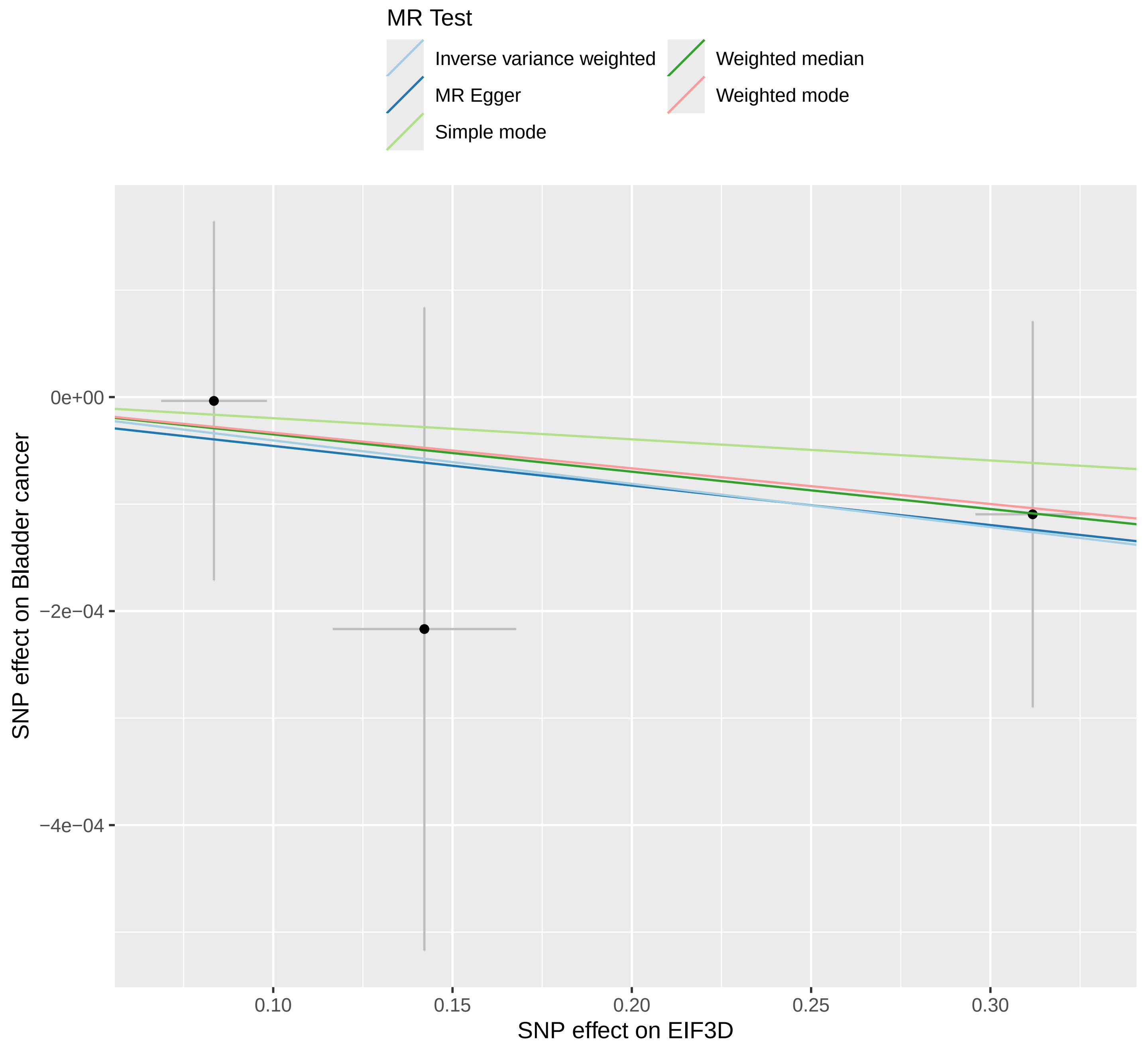
MR Test
Inverse variance weighted
MR Egger
Simple mode
Weighted median
Weighted mode
0e+00
−2e−04
−4e−04
SNP effect on Bladder cancer
0.10
0.15
0.20
0.25
0.30
SNP effect on EIF3D

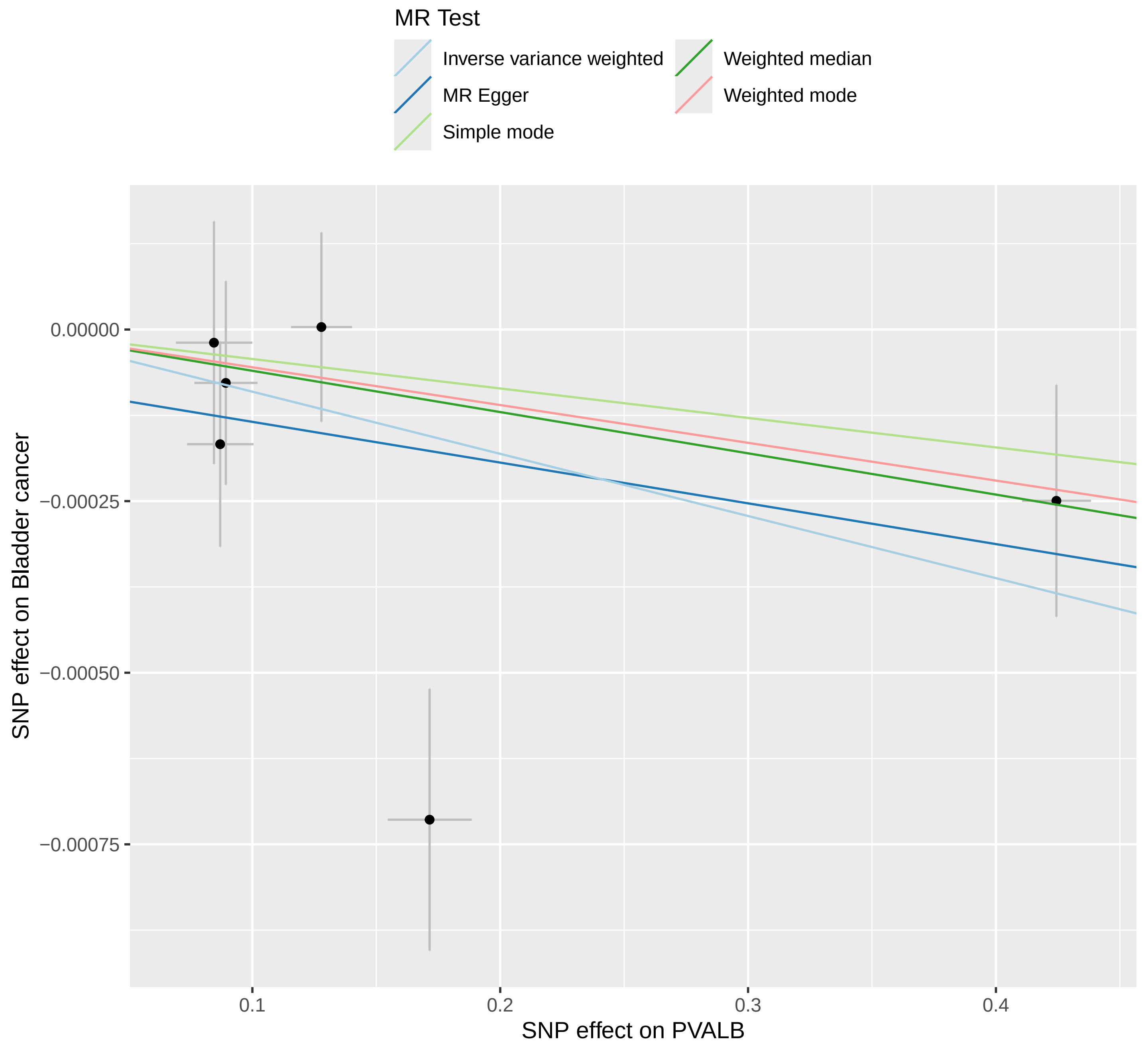
MR Test
Inverse variance weighted
MR Egger
Simple mode
Weighted median
Weighted mode
0.00000
−0.00025
−0.00050
−0.00075
SNP effect on Bladder cancer
0.1
0.2
0.3
0.4
SNP effect on PVALB

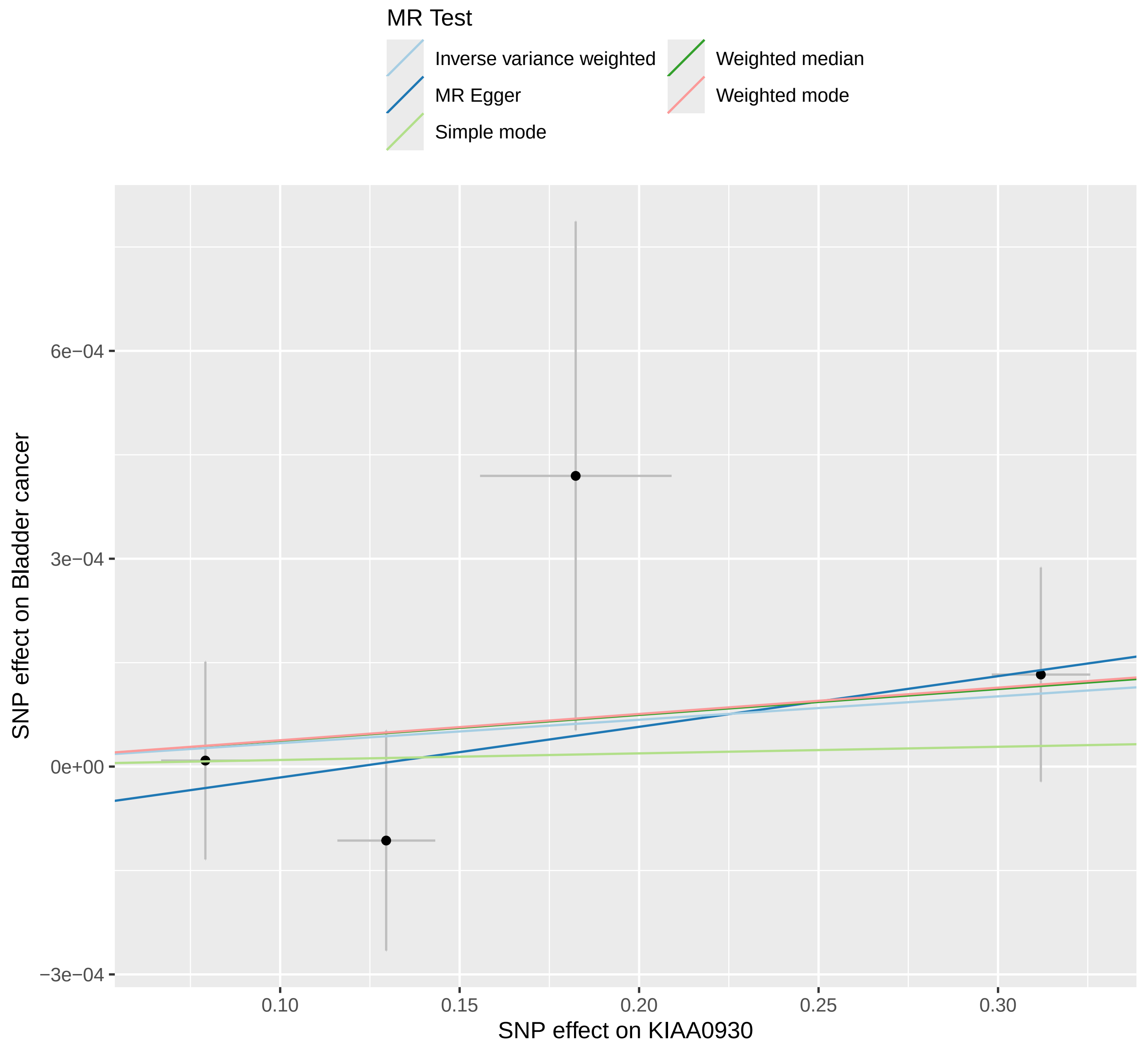
MR Test
Inverse variance weighted
MR Egger
Simple mode
Weighted median
Weighted mode
6e−04
3e−04
0e+00
−3e−04
0.10
0.15
0.20
0.25
0.30
SNP effect on KIAA0930
SNP effect on Bladder cancer

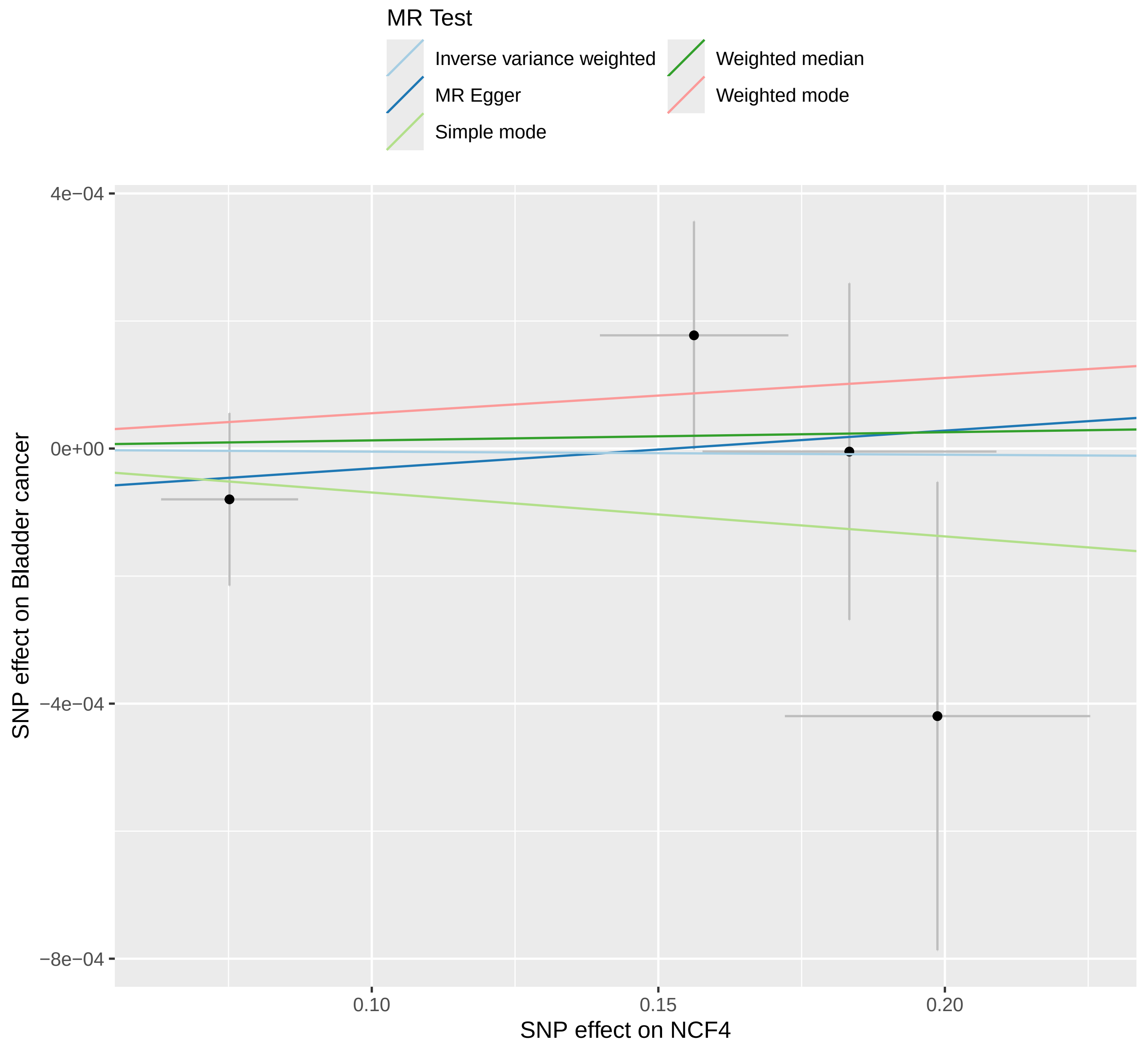

MR Test
Inverse variance weighted
MR Egger
Simple mode
Weighted median
Weighted mode
4e−04
0e+00
−4e−04
−8e−04
SNP effect on Bladder cancer
0.10
0.15
0.20
SNP effect on NCF4

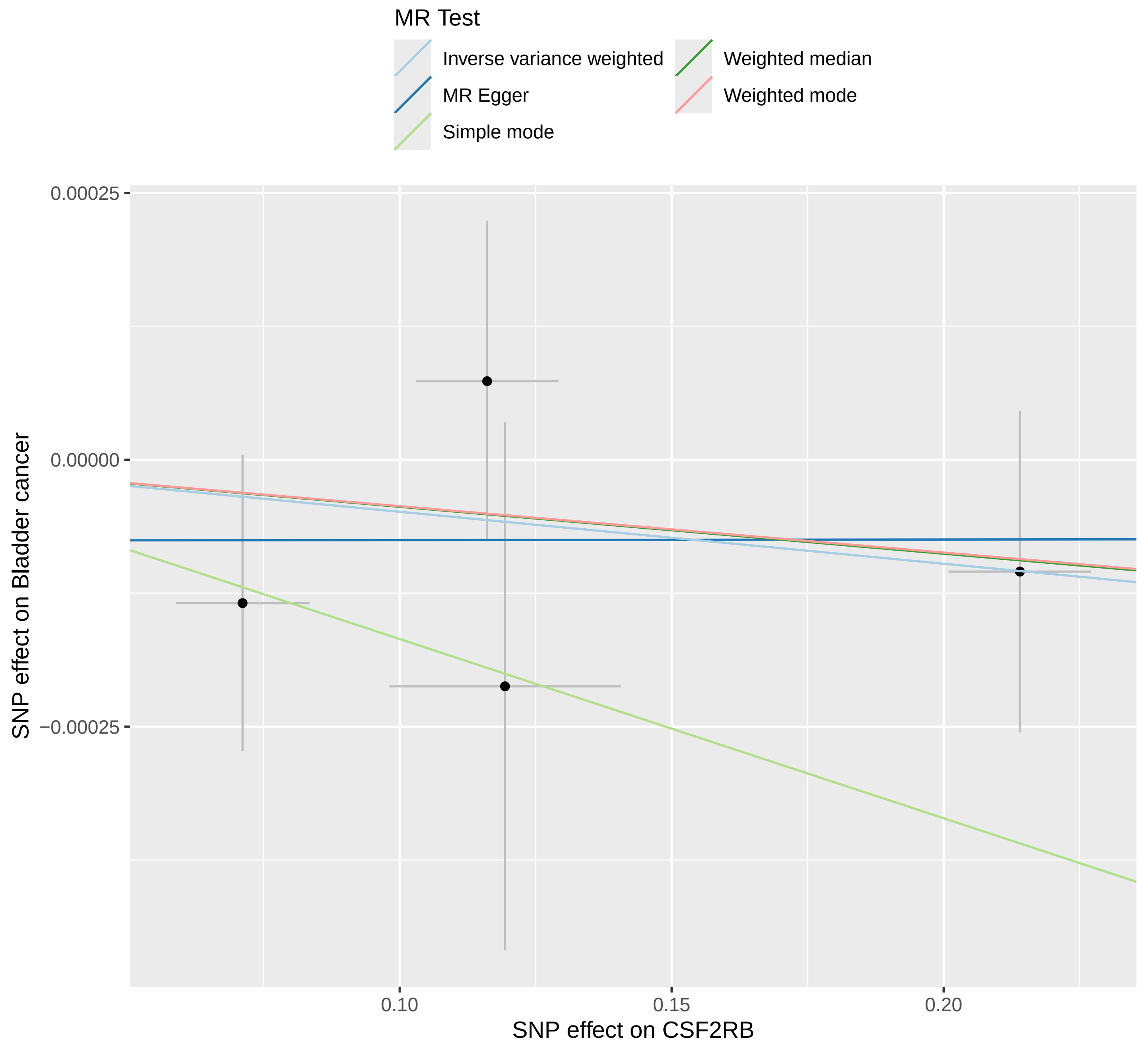

MR Test
Inverse variance weighted
MR Egger
Simple mode
Weighted median
Weighted mode
0.00025
0.00000
−0.00025
0.10
0.15
0.20
SNP effect on Bladder cancer
SNP effect on CSF2RB

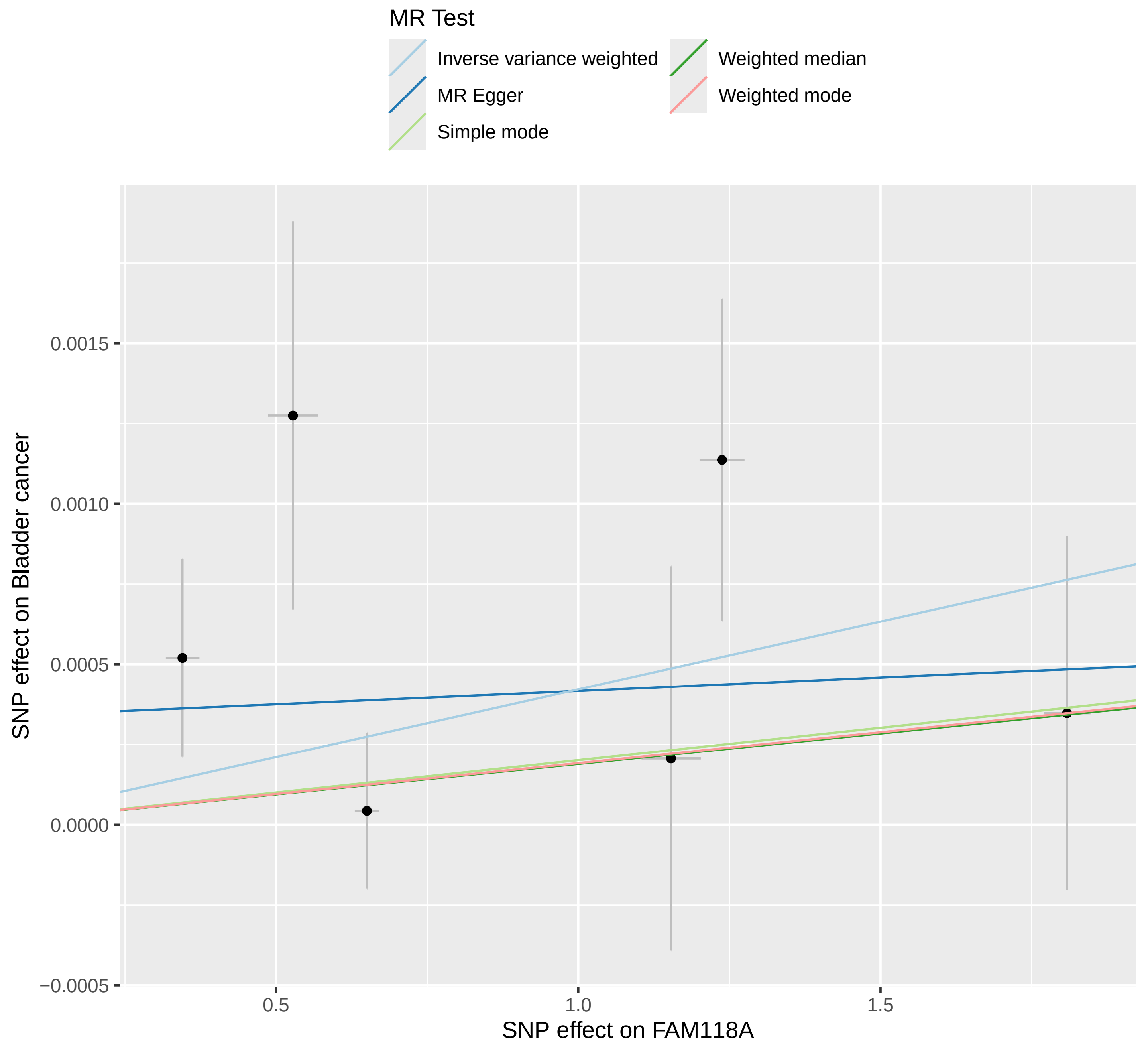

MR Test
Inverse variance weighted
MR Egger
Simple mode
Weighted median
Weighted mode
0.0015
0.0010
0.0005
0.0000
−0.0005
0.5
1.0
1.5
SNP effect on Bladder cancer
SNP effect on FAM118A

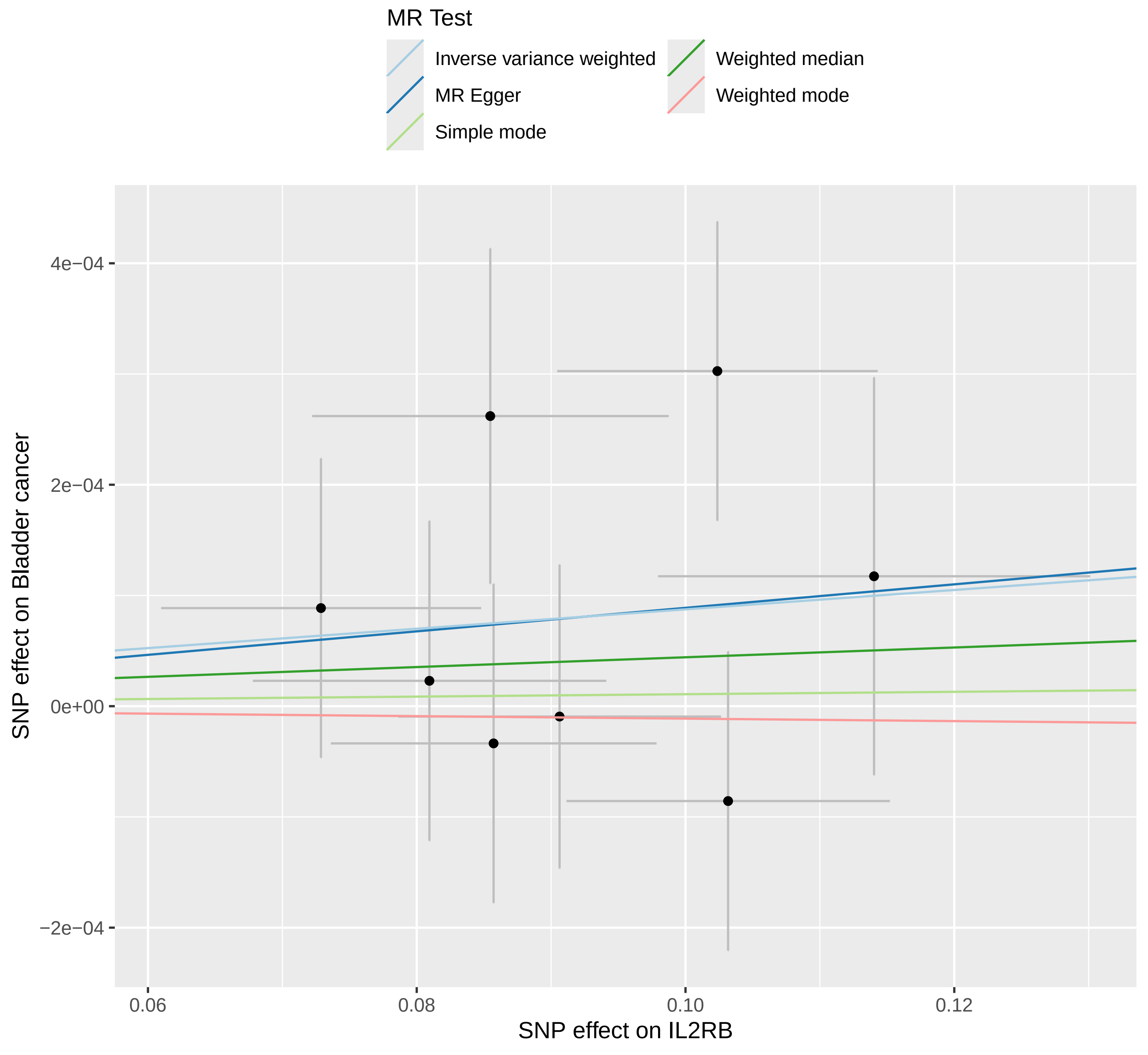

MR Test
Inverse variance weighted
MR Egger
Simple mode
Weighted median
Weighted mode
4e−04
2e−04
0e+00
−2e−04
0.06
0.08
0.10
0.12
SNP effect on IL2RB
SNP effect on Bladder cancer

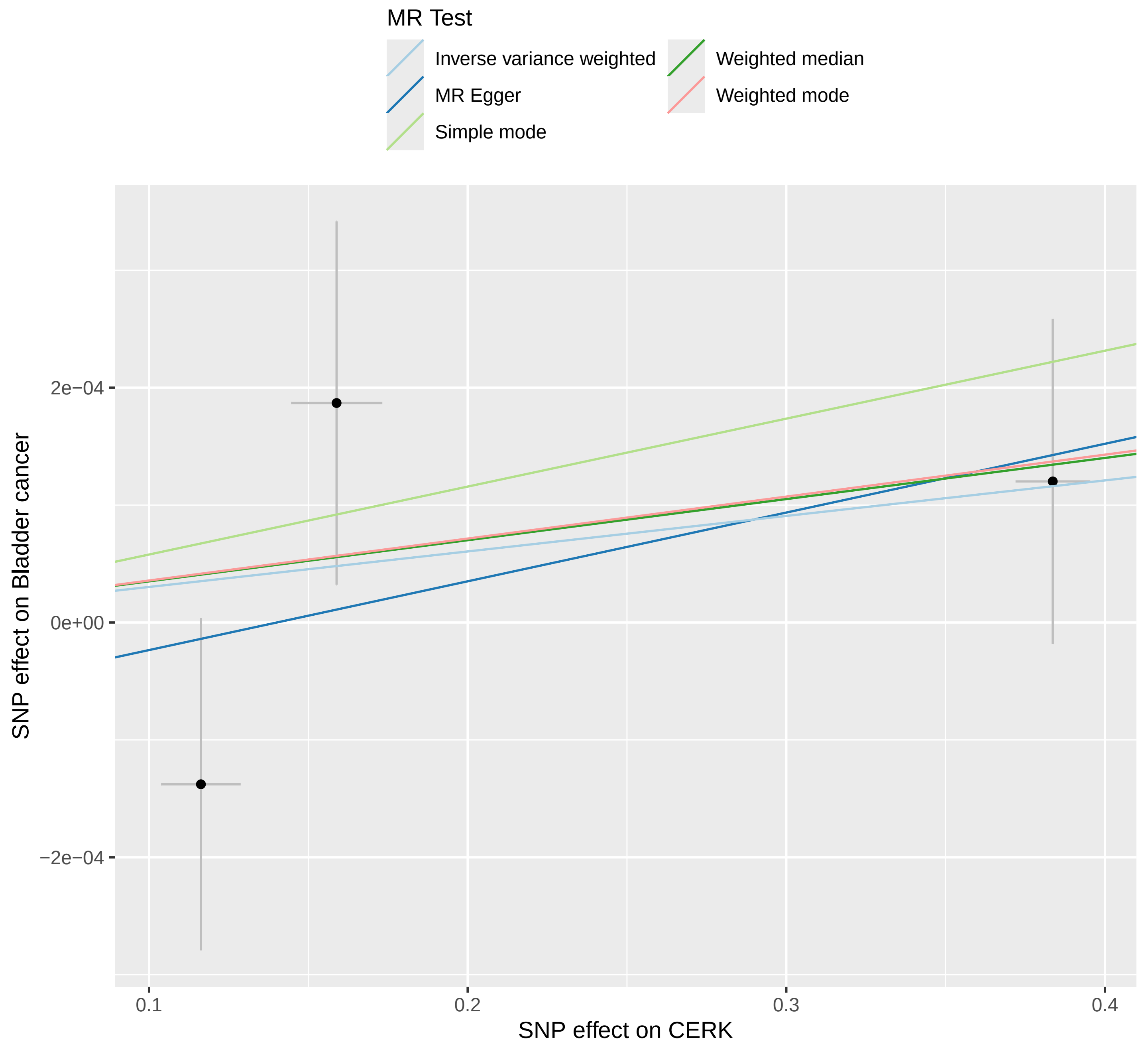

MR Test
Inverse variance weighted
MR Egger
Simple mode
Weighted median
Weighted mode
2e−04
0e+00
−2e−04
SNP effect on Bladder cancer
0.1
0.2
0.3
0.4
SNP effect on CERK

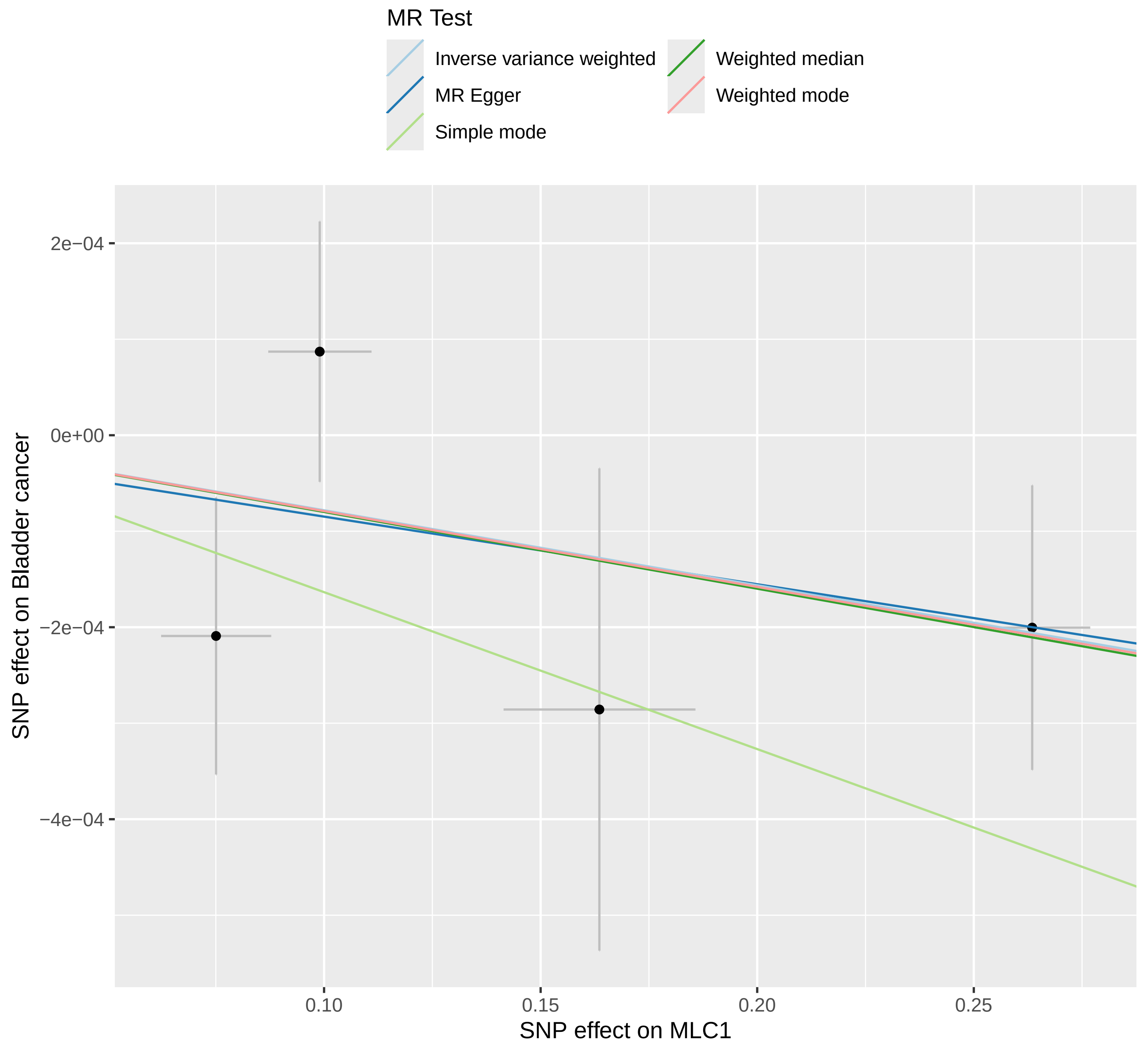
MR Test
Inverse variance weighted
MR Egger
Simple mode
Weighted median
Weighted mode
2e−04
0e+00
−2e−04
−4e−04
SNP effect on Bladder cancer
0.10
0.15
0.20
0.25
SNP effect on MLC1

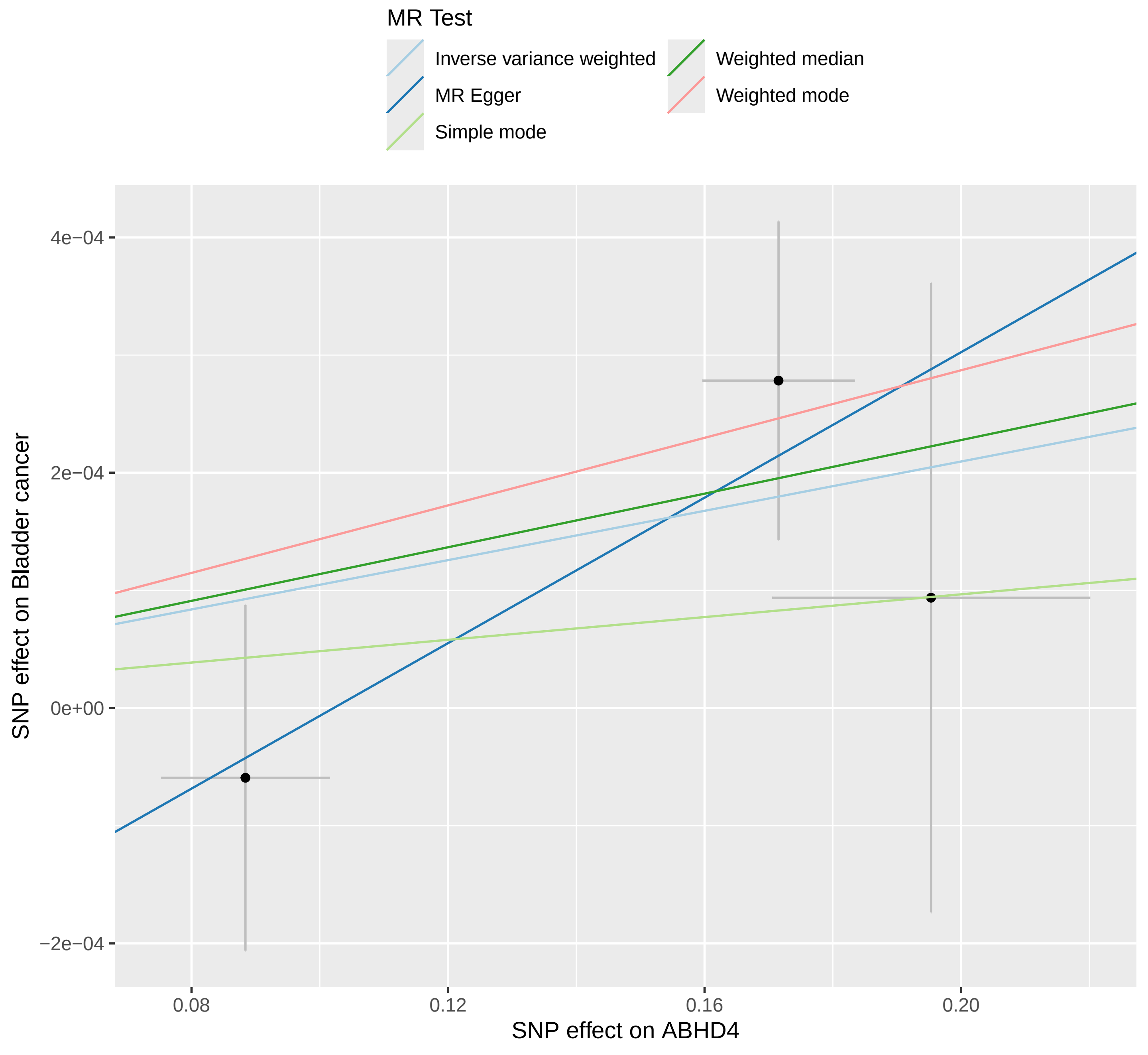

MR Test
Inverse variance weighted
MR Egger
Simple mode
Weighted median
Weighted mode
4e−04
2e−04
0e+00
−2e−04
SNP effect on Bladder cancer
0.08
0.12
0.16
0.20
SNP effect on ABHD4

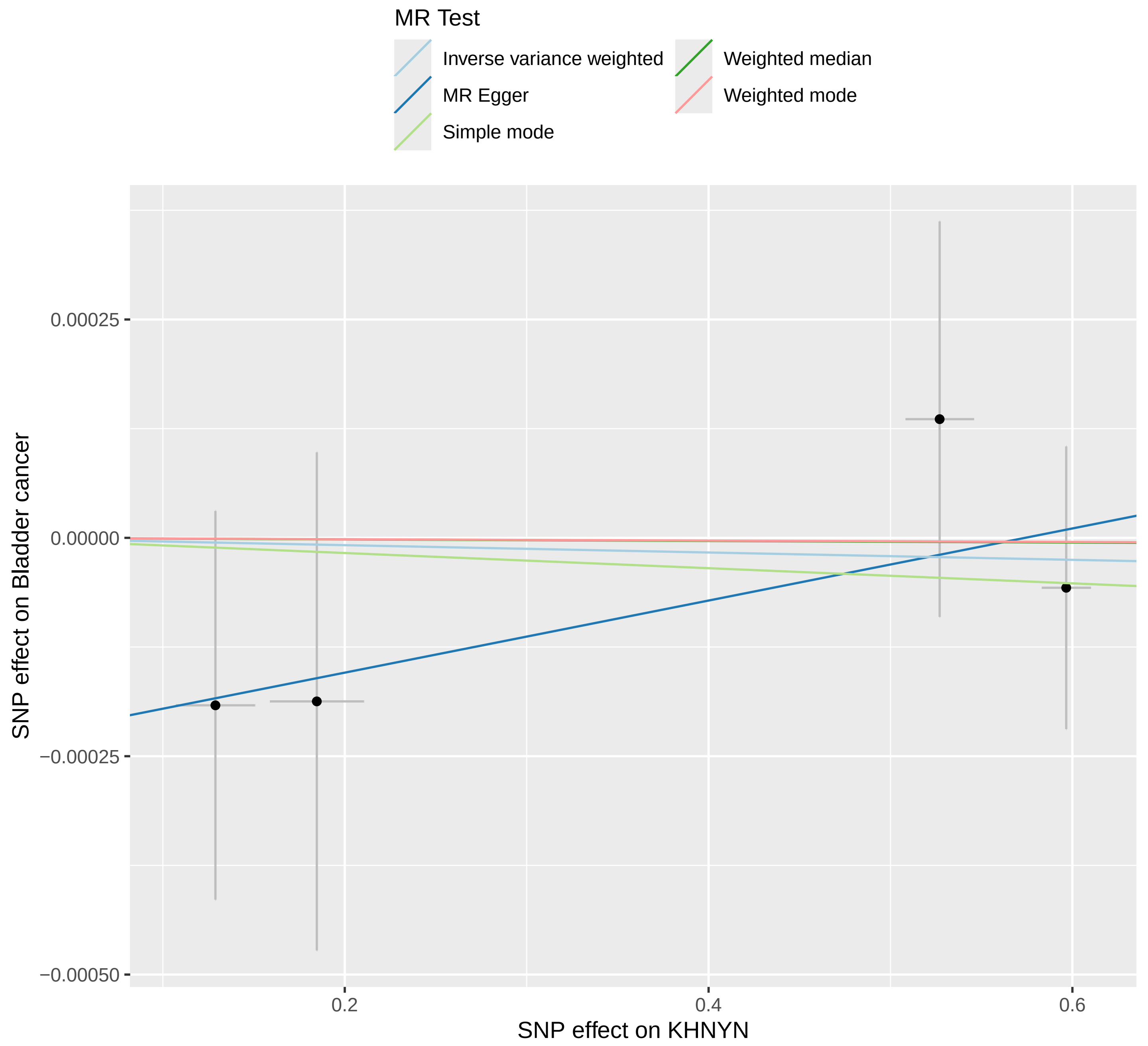
MR Test
Inverse variance weighted
MR Egger
Simple mode
Weighted median
Weighted mode
0.00025
0.00000
−0.00025
−0.00050
SNP effect on Bladder cancer
0.2
0.4
0.6
SNP effect on KHNYN

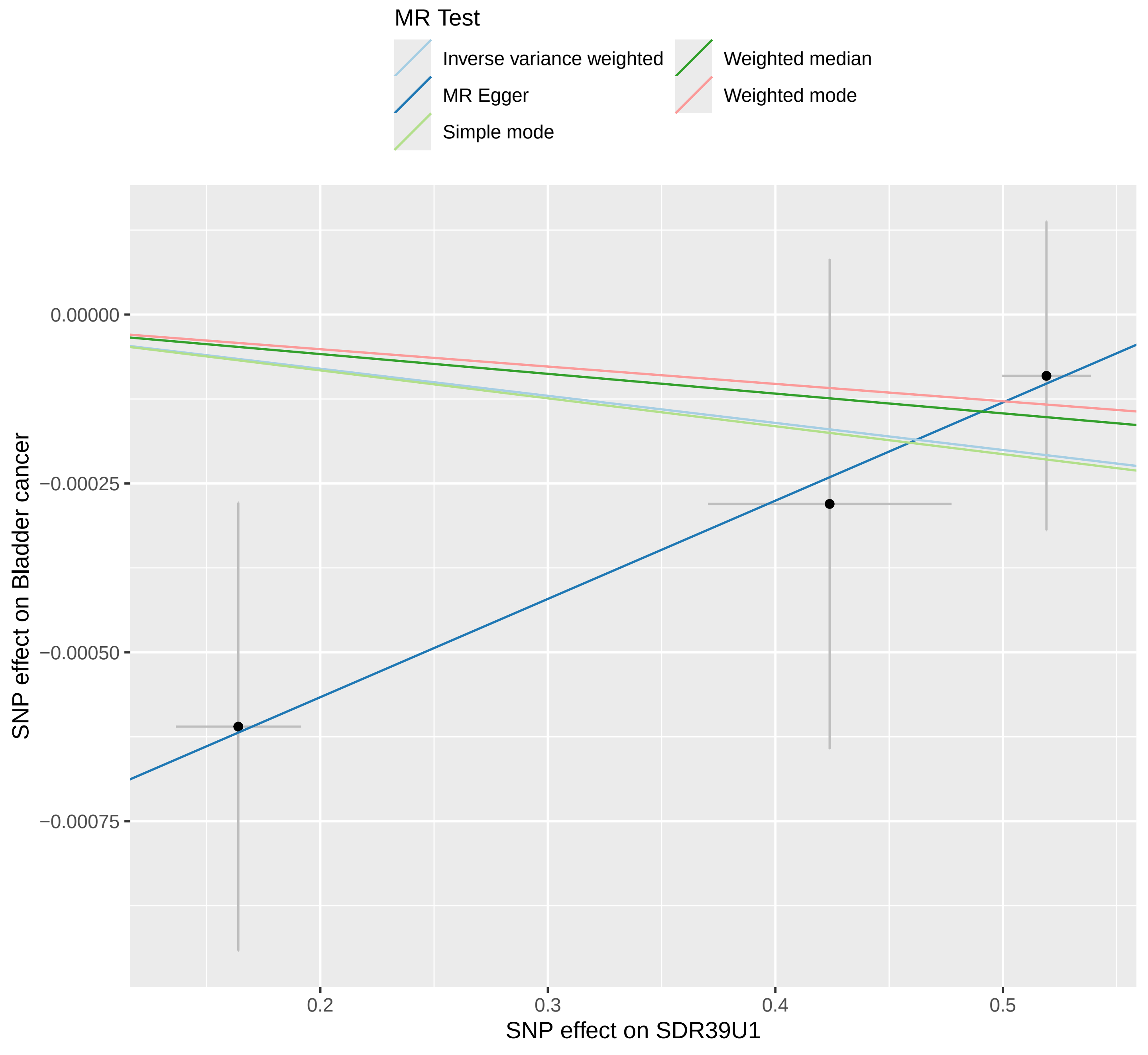
MR Test
Inverse variance weighted
MR Egger
Simple mode
Weighted median
Weighted mode
0.00000
−0.00025
−0.00050
−0.00075
0.2
0.3
0.4
0.5
SNP effect on SDR39U1
SNP effect on Bladder cancer

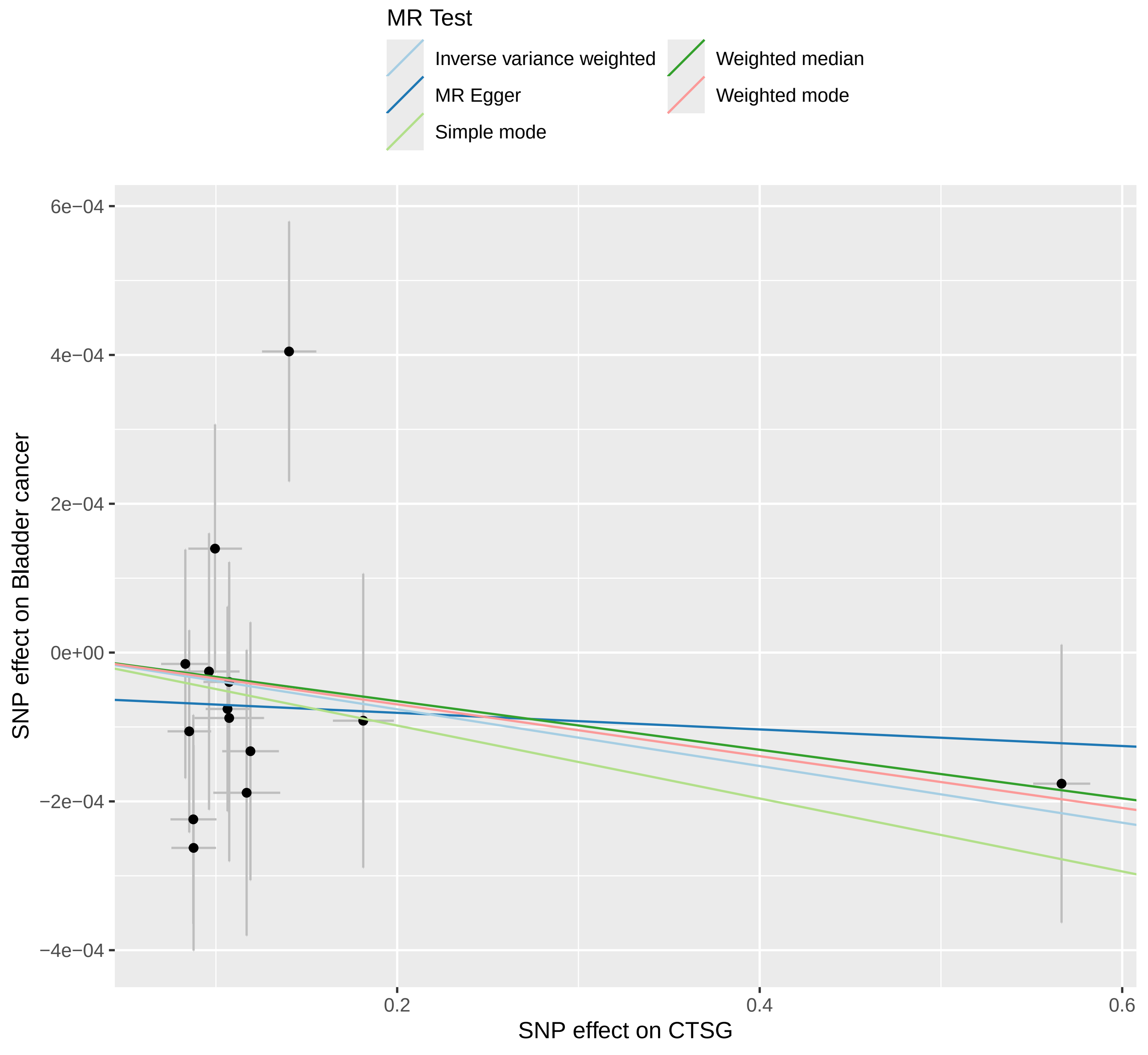

MR Test
Inverse variance weighted
MR Egger
Simple mode
Weighted median
Weighted mode
6e−04
4e−04
2e−04
0e+00
−2e−04
−4e−04
0.2
0.4
0.6
SNP effect on Bladder cancer
SNP effect on CTSG

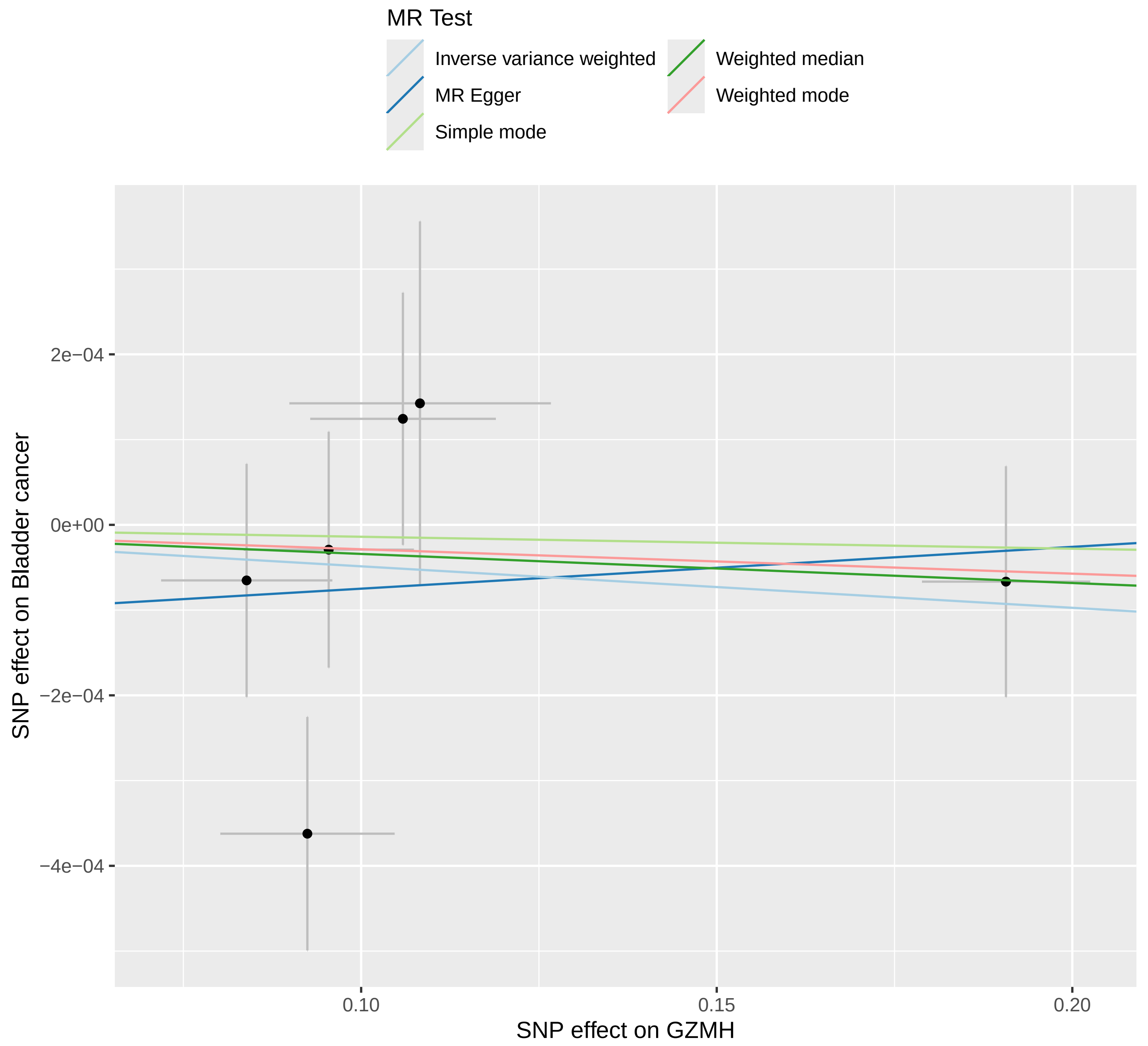
MR Test
Inverse variance weighted
MR Egger
Simple mode
Weighted median
Weighted mode
SNP effect on Bladder cancer
2e−04
0e+00
−2e−04
−4e−04
0.10
0.15
0.20
SNP effect on GZMH

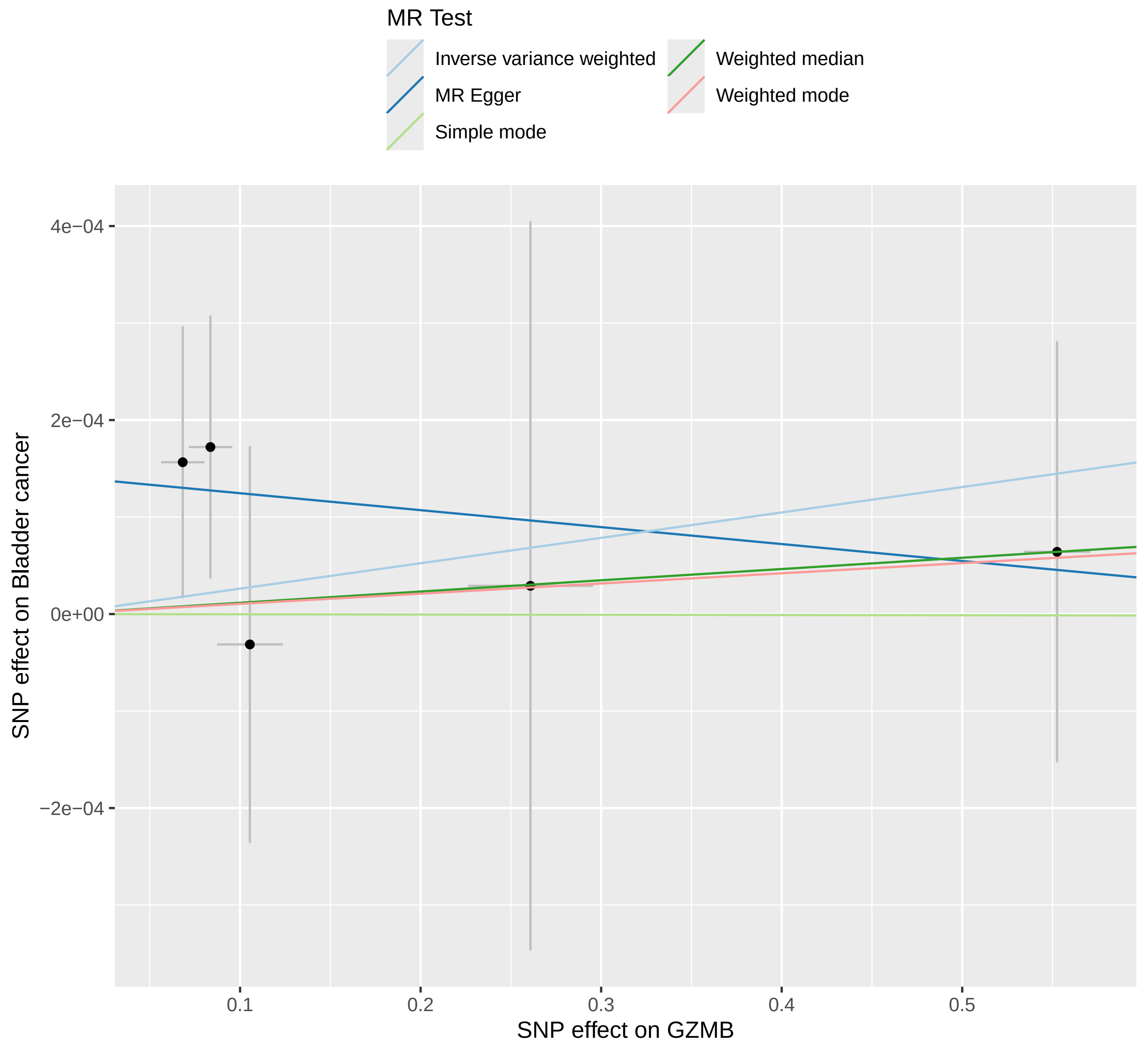
MR Test
Inverse variance weighted
MR Egger
Simple mode
Weighted median
Weighted mode
4e−04
2e−04
0e+00
−2e−04
SNP effect on Bladder cancer
0.1
0.2
0.3
0.4
0.5
SNP effect on GZMB

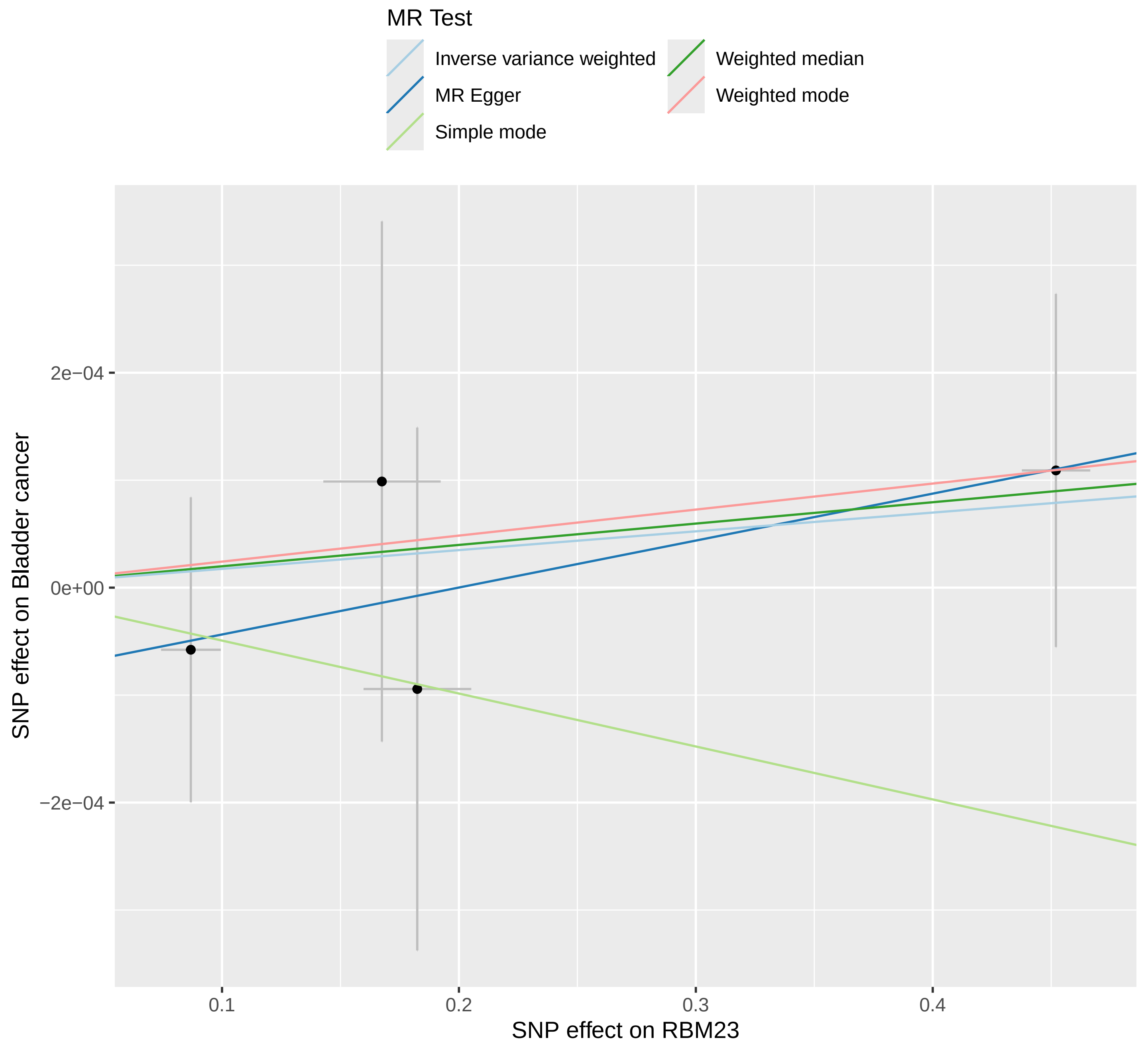
MR Test
Inverse variance weighted
MR Egger
Simple mode
Weighted median
Weighted mode
2e−04
0e+00
−2e−04
0.1
0.2
0.3
0.4
SNP effect on Bladder cancer
SNP effect on RBM23

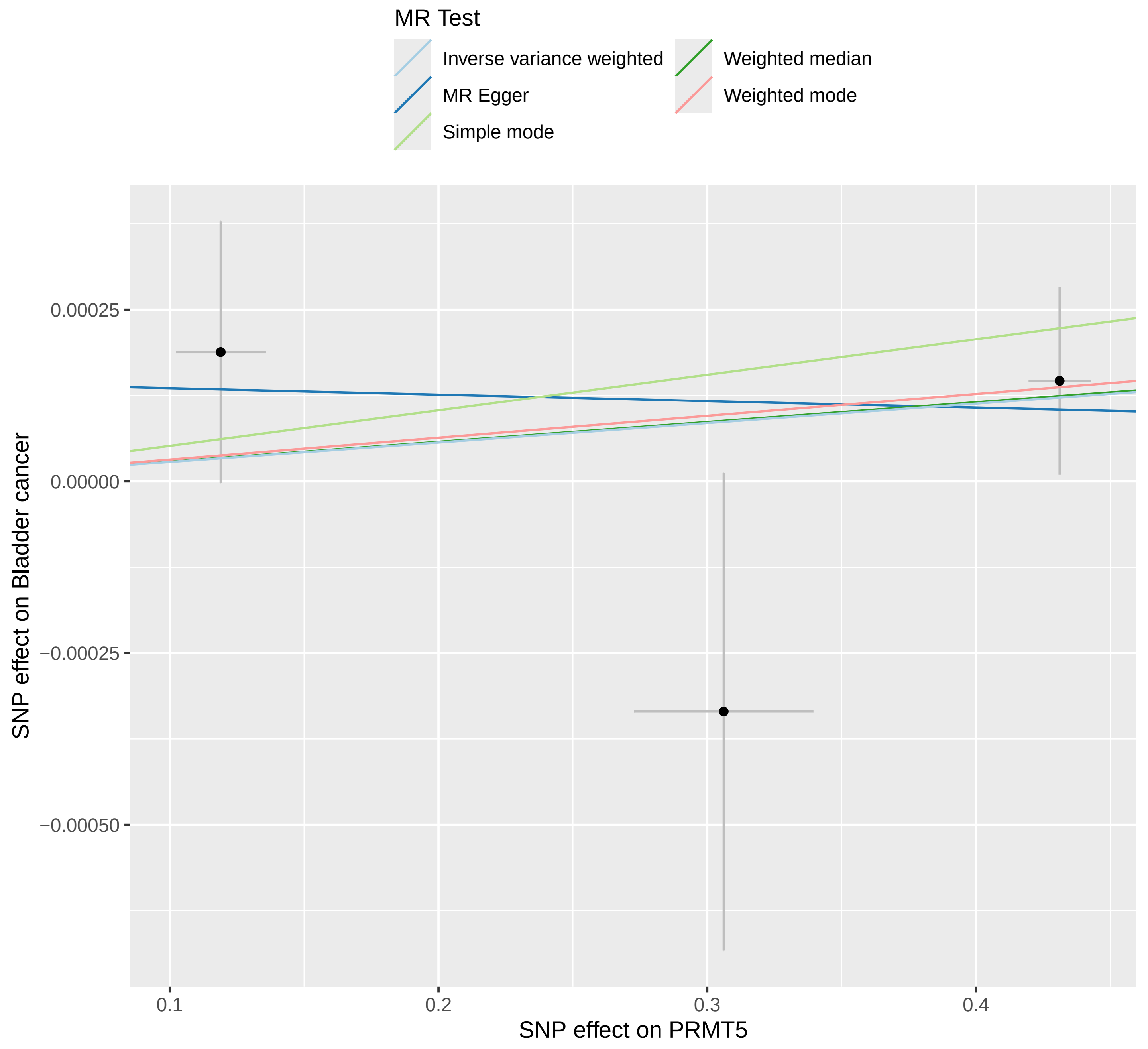

MR Test
Inverse variance weighted
MR Egger
Simple mode
Weighted median
Weighted mode
0.00025
0.00000
−0.00025
−0.00050
0.1
0.2
0.3
0.4
SNP effect on PRMT5
SNP effect on Bladder cancer

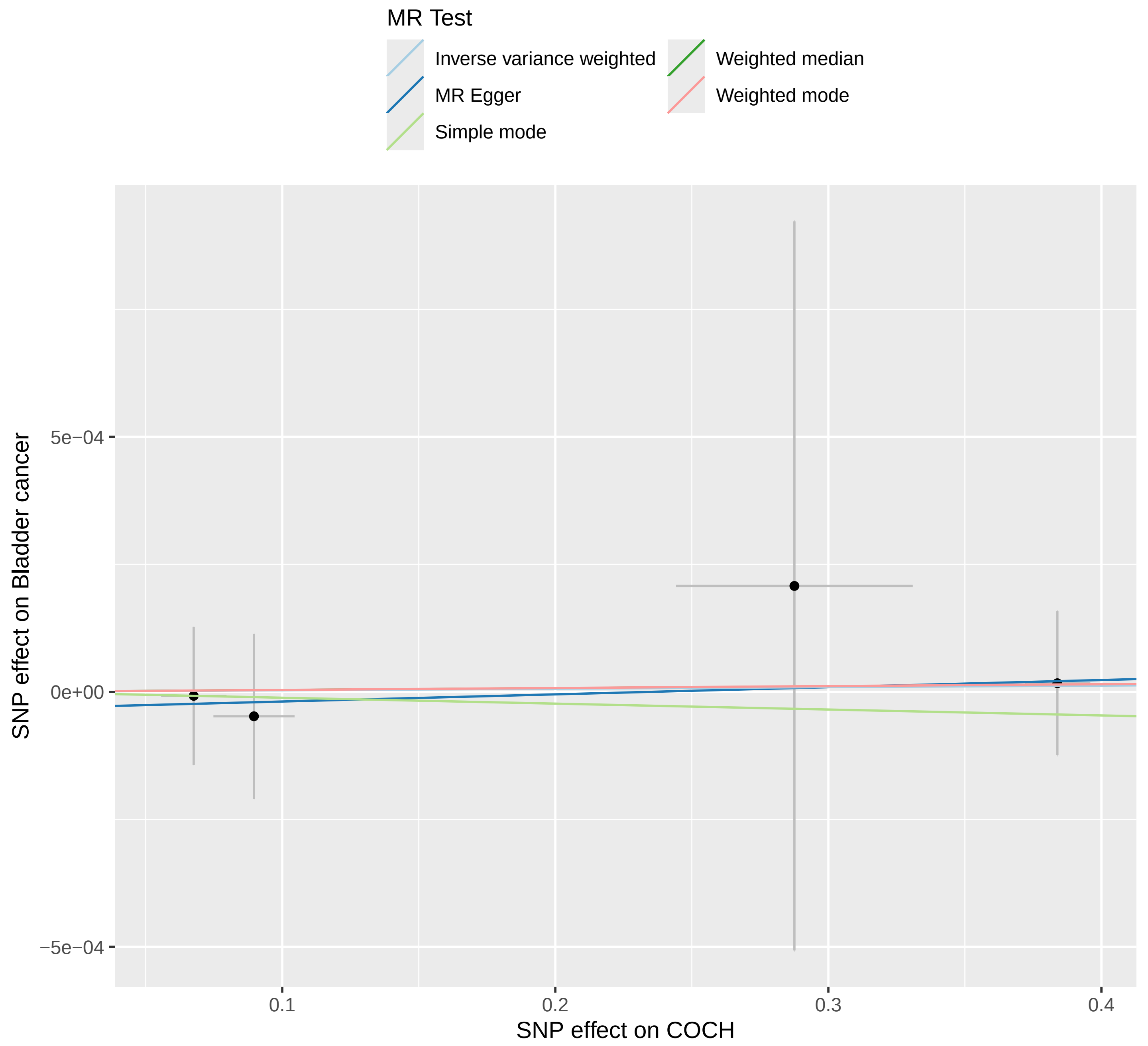

MR Test
Inverse variance weighted
MR Egger
Simple mode
Weighted median
Weighted mode
5e−04
0e+00
−5e−04
SNP effect on Bladder cancer
0.1
0.2
0.3
0.4
SNP effect on COCH

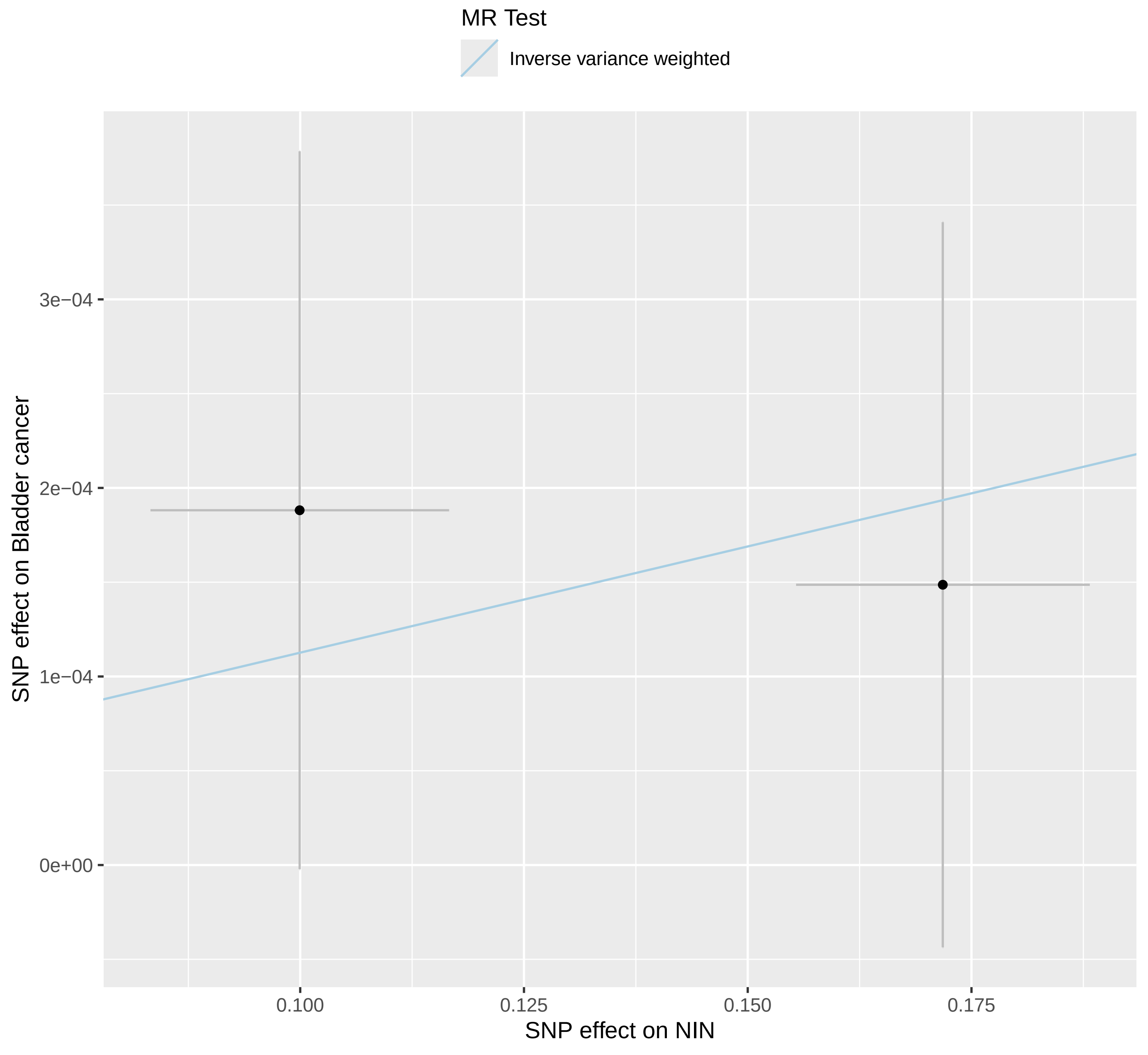
MR Test
Inverse variance weighted
3e−04
2e−04
1e−04
0e+00
SNP effect on Bladder cancer
0.100
0.125
0.150
0.175
SNP effect on NIN

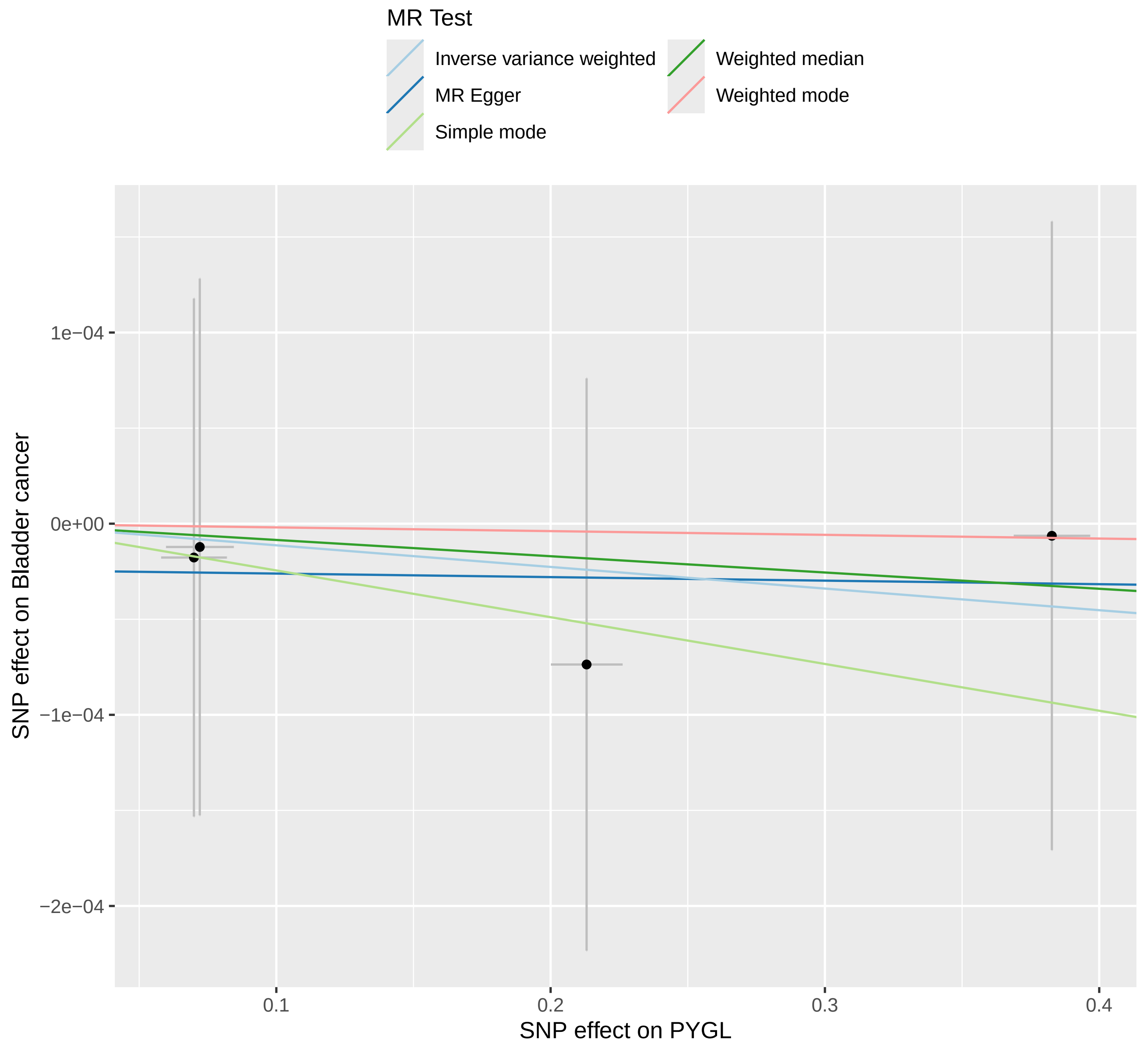

MR Test
Inverse variance weighted
MR Egger
Simple mode
Weighted median
Weighted mode
SNP effect on Bladder cancer
1e−04
0e+00
−1e−04
−2e−04
0.1
0.2
0.3
0.4
SNP effect on PYGL

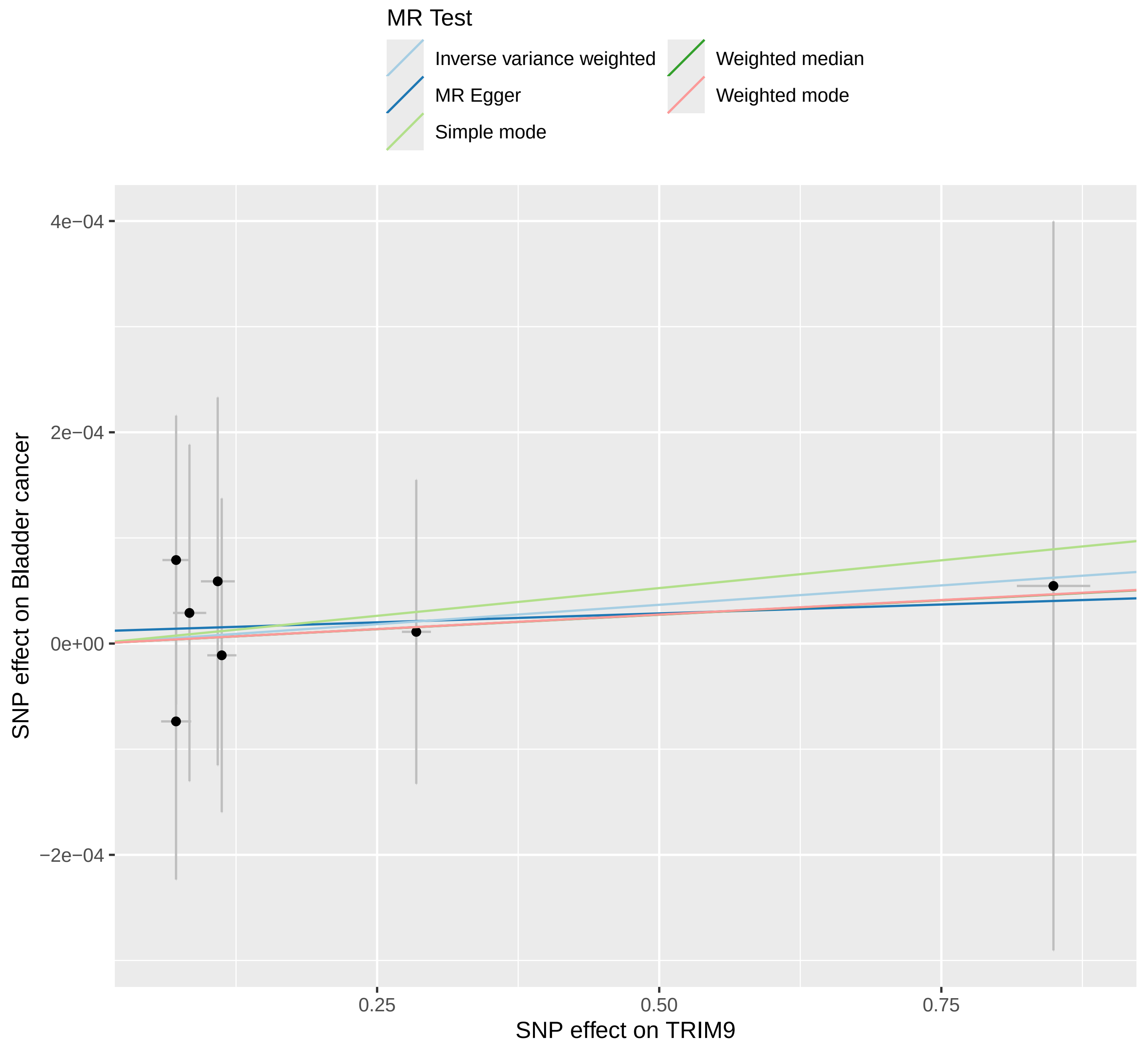
MR Test
Inverse variance weighted
MR Egger
Simple mode
Weighted median
Weighted mode
4e−04
2e−04
0e+00
−2e−04
SNP effect on Bladder cancer
0.25
0.50
0.75
SNP effect on TRIM9

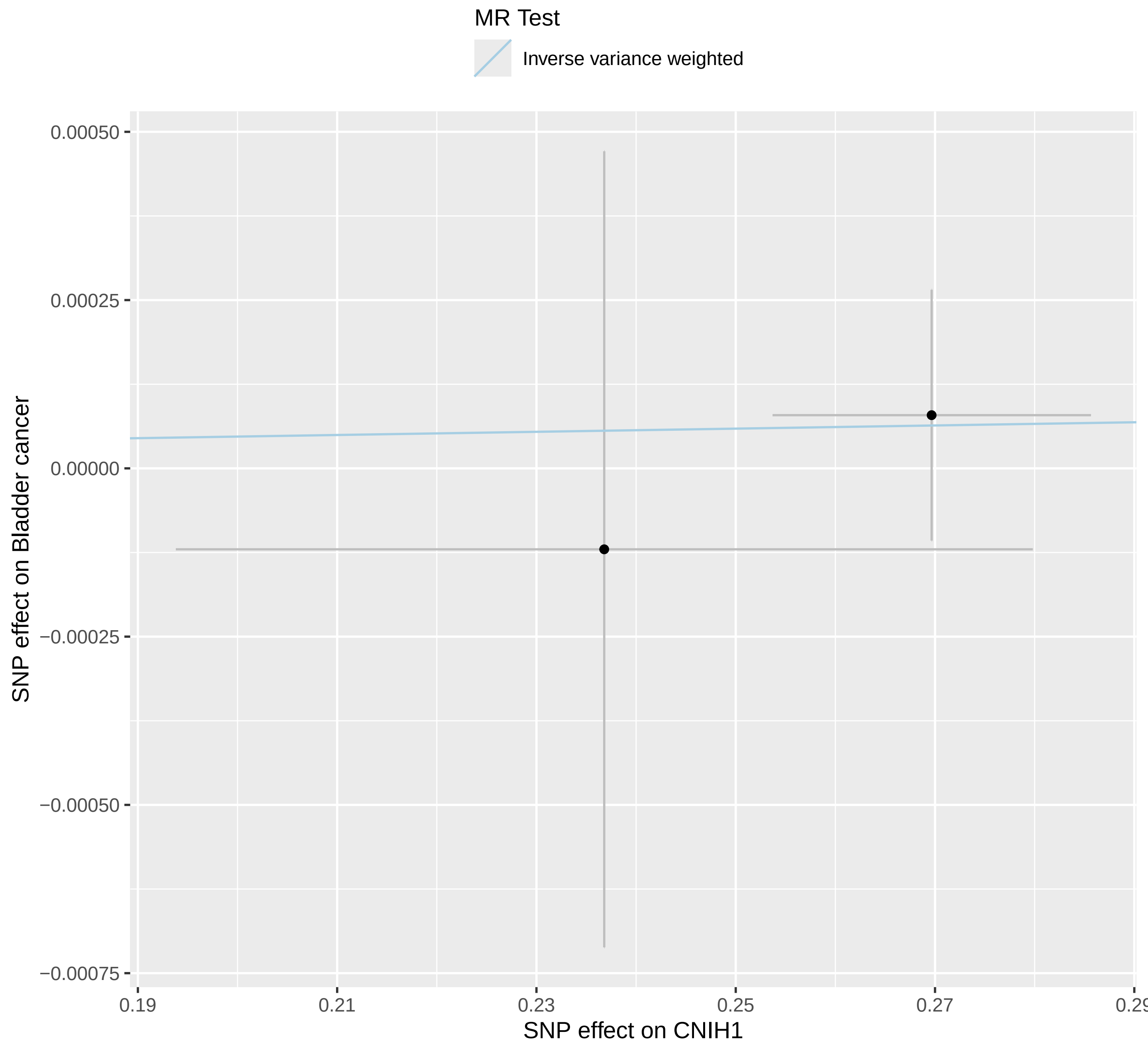

MR Test
Inverse variance weighted
0.00050
0.00025
0.00000
−0.00025
−0.00050
−0.00075
SNP effect on Bladder cancer
0.19
0.21
0.23
0.25
0.27
SNP effect on CNIH1

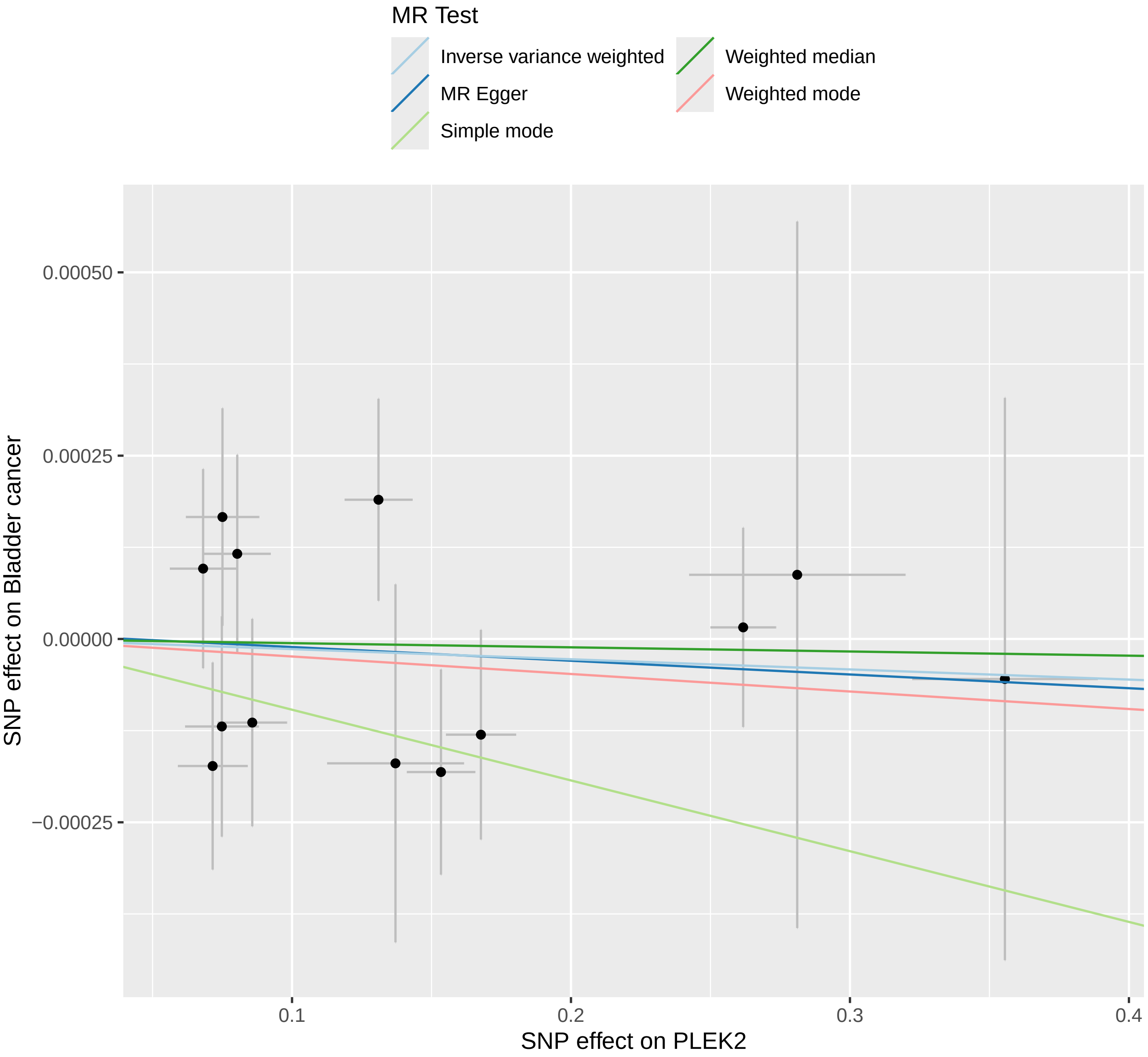

MR Test
Inverse variance weighted
MR Egger
Simple mode
Weighted median
Weighted mode
0.00050
0.00025
0.00000
−0.00025
SNP effect on Bladder cancer
0.1
0.2
0.3
0.4
SNP effect on PLEK2

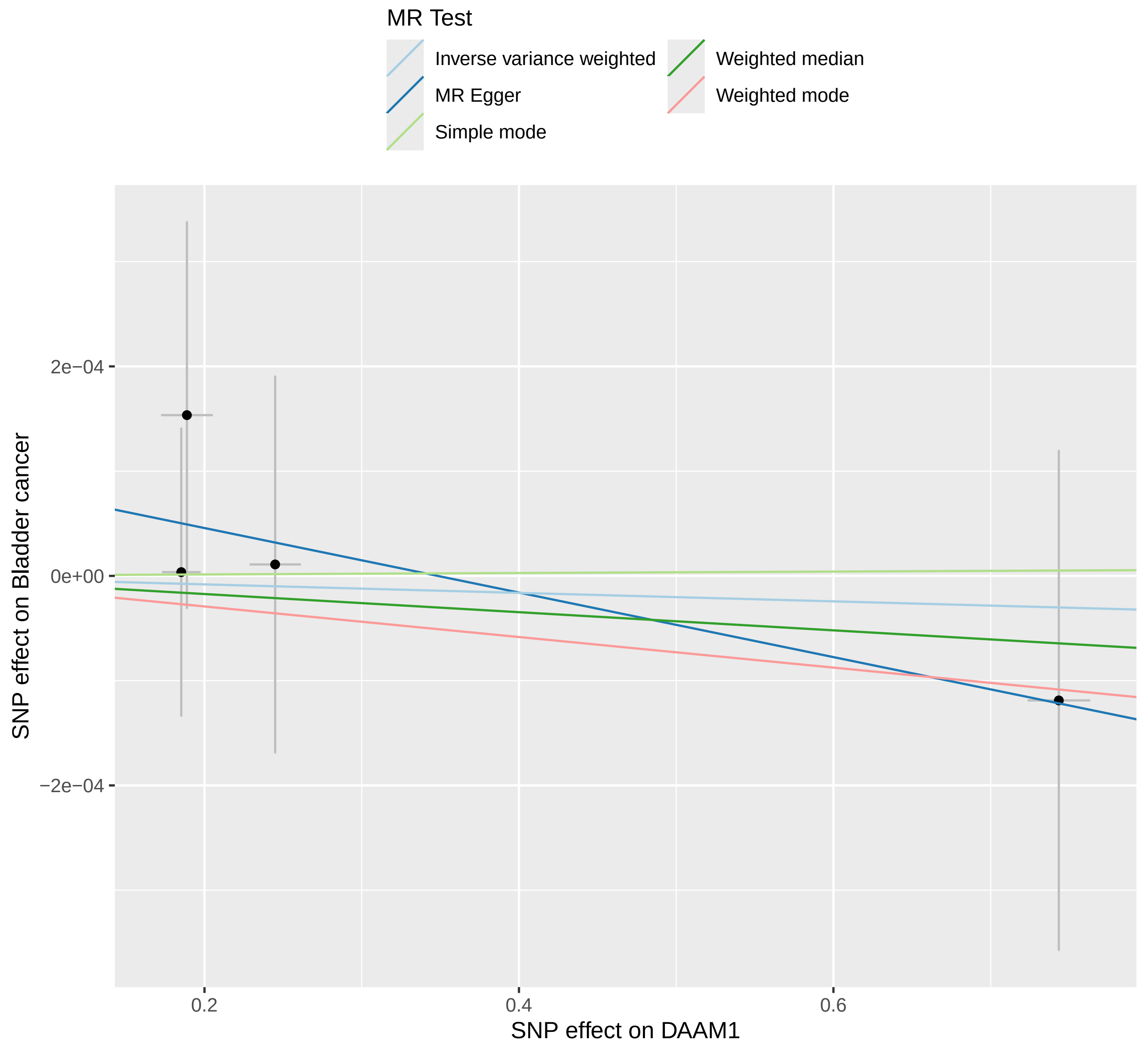
MR Test
Inverse variance weighted
MR Egger
Simple mode
Weighted median
Weighted mode
2e−04
0e+00
−2e−04
SNP effect on Bladder cancer
0.2
0.4
0.6
SNP effect on DAAM1

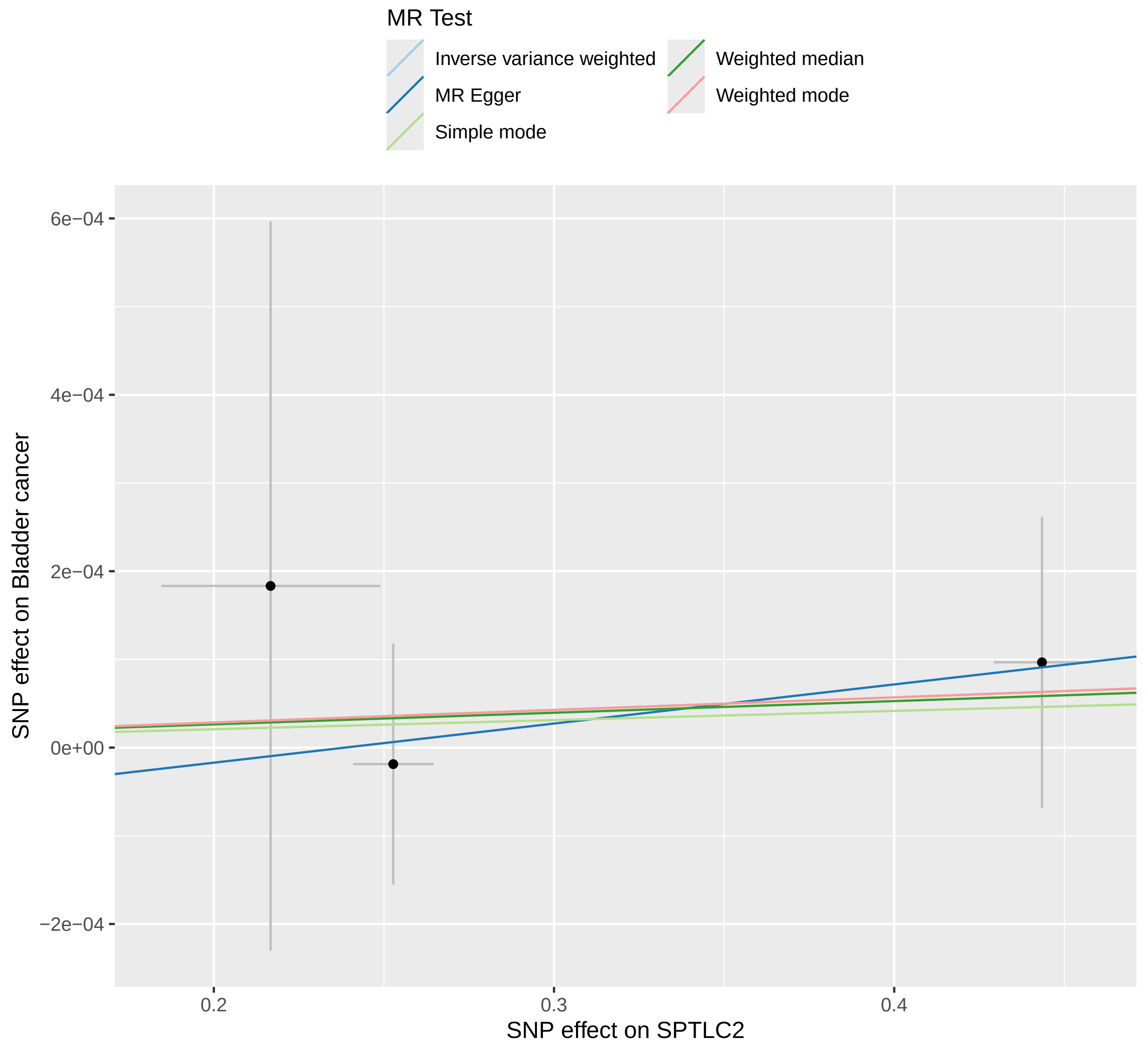
MR Test
Inverse variance weighted
MR Egger
Simple mode
Weighted median
Weighted mode
6e−04
4e−04
2e−04
0e+00
−2e−04
SNP effect on Bladder cancer
0.2
0.3
0.4
SNP effect on SPTLC2

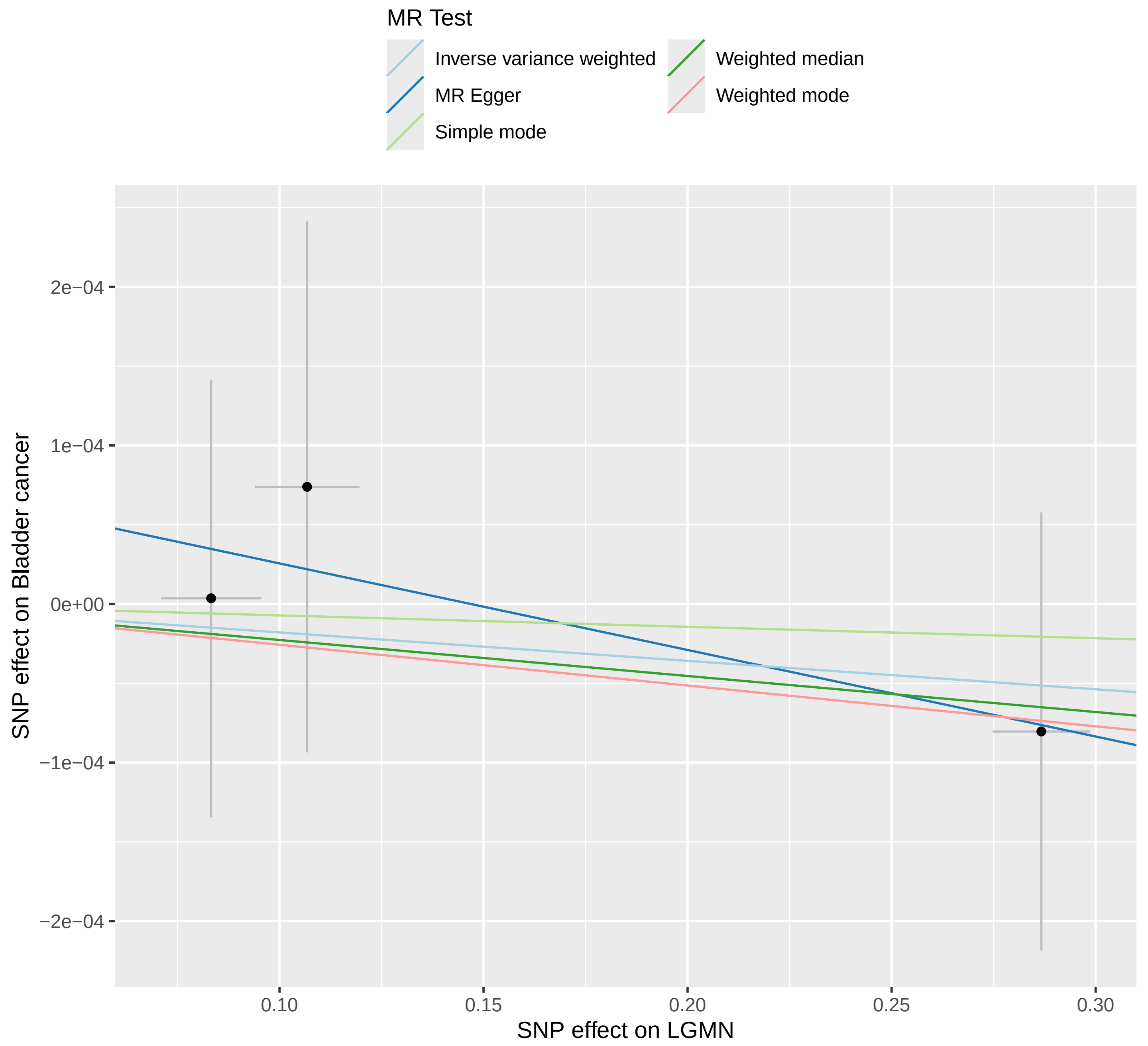

MR Test
Inverse variance weighted
MR Egger
Simple mode
Weighted median
Weighted mode
2e−04
1e−04
0e+00
−1e−04
−2e−04
SNP effect on Bladder cancer
0.10
0.15
0.20
0.25
0.30
SNP effect on LGMN

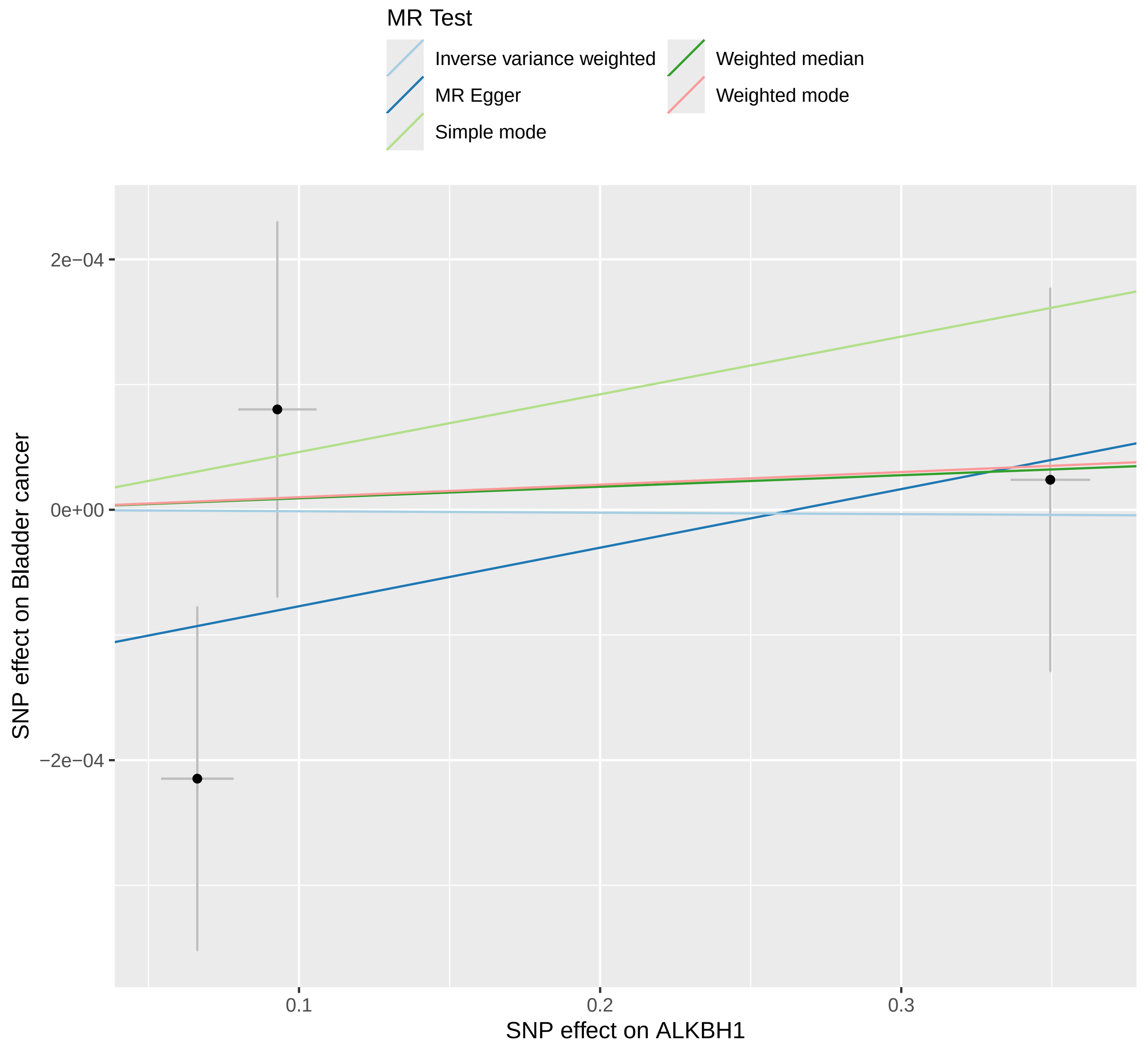
MR Test
Inverse variance weighted
MR Egger
Simple mode
Weighted median
Weighted mode
2e−04
0e+00
−2e−04
SNP effect on Bladder cancer
0.1
0.2
0.3
SNP effect on ALKBH1

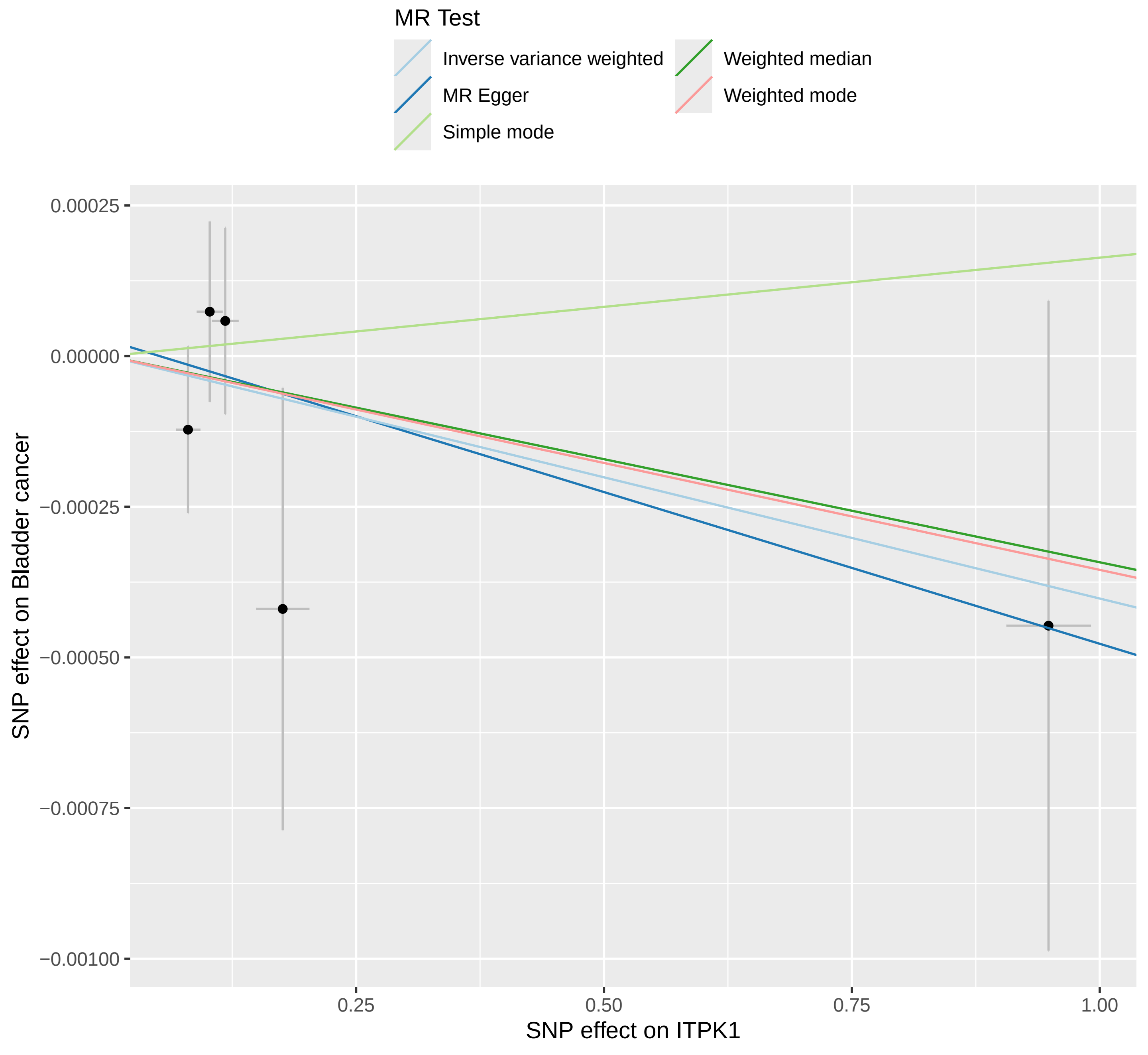

MR Test
Inverse variance weighted
MR Egger
Simple mode
Weighted median
Weighted mode
0.00025
0.00000
−0.00025
−0.00050
−0.00075
−0.00100
SNP effect on Bladder cancer
0.25
0.50
0.75
1.00
SNP effect on ITPK1

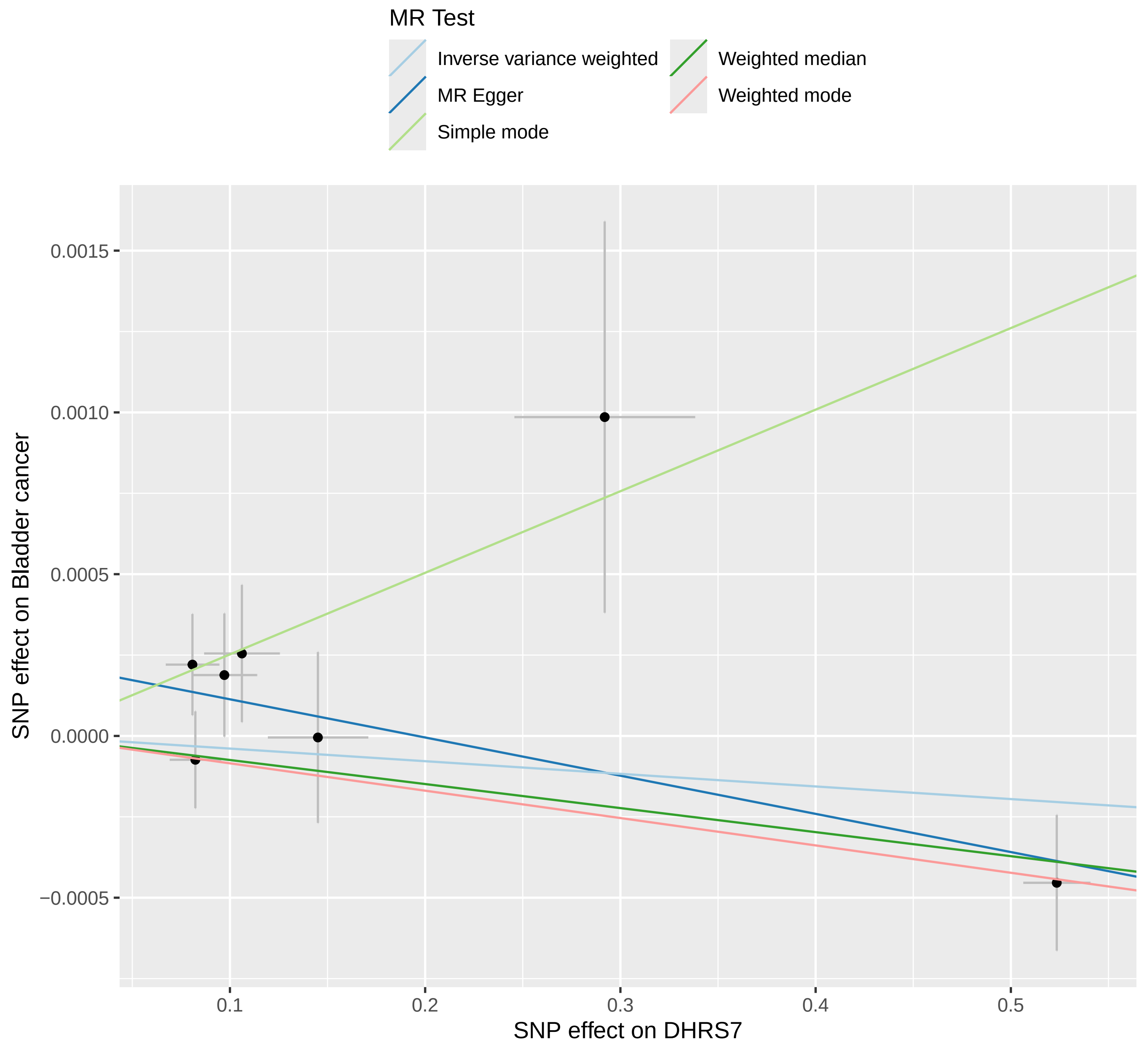

MR Test
Inverse variance weighted
MR Egger
Simple mode
Weighted median
Weighted mode
0.0015
0.0010
0.0005
0.0000
−0.0005
SNP effect on Bladder cancer
0.1
0.2
0.3
0.4
0.5
SNP effect on DHRS7

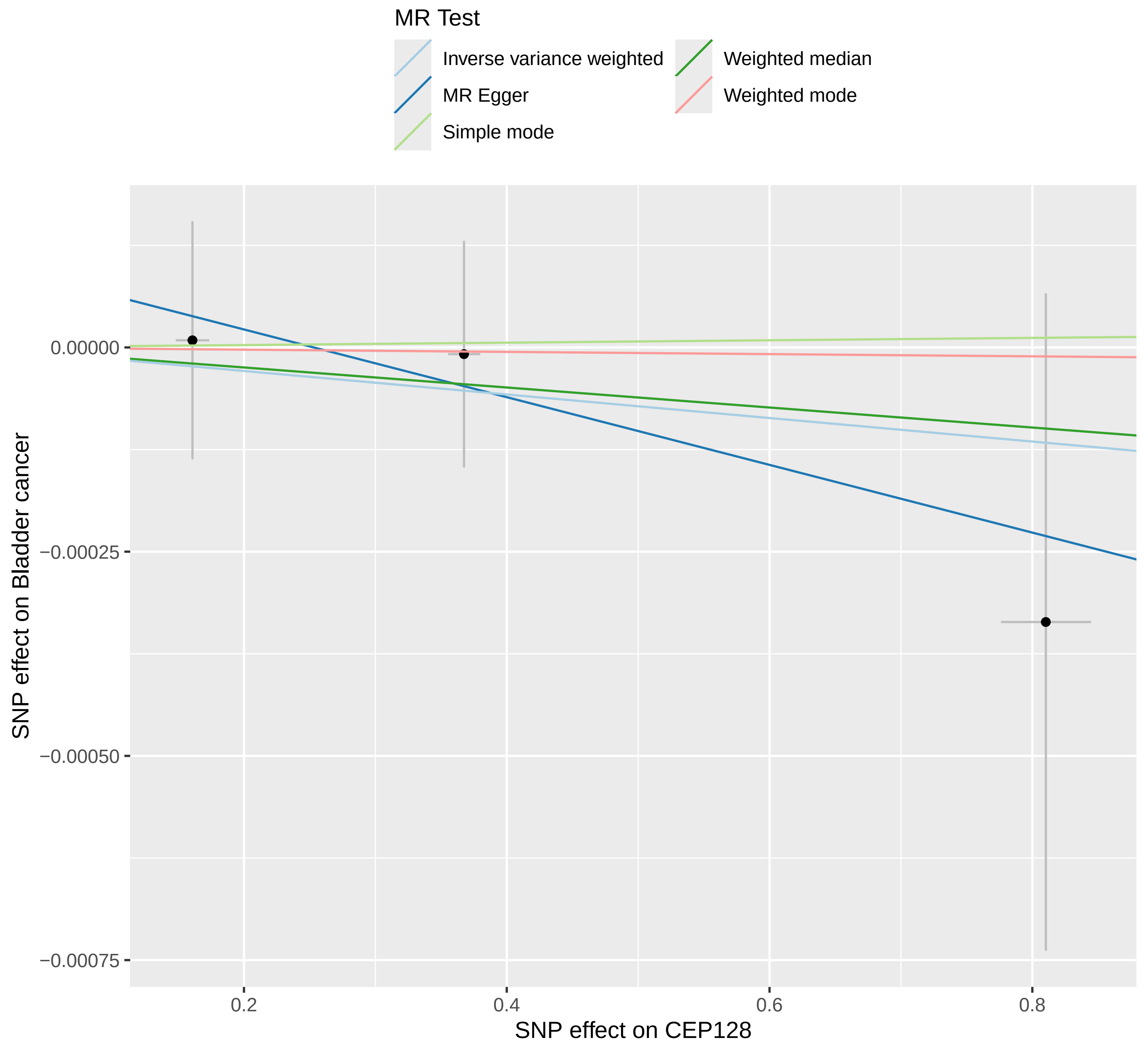

MR Test
Inverse variance weighted
MR Egger
Simple mode
Weighted median
Weighted mode
0.00000
−0.00025
−0.00050
−0.00075
SNP effect on Bladder cancer
0.2
0.4
0.6
0.8
SNP effect on CEP128

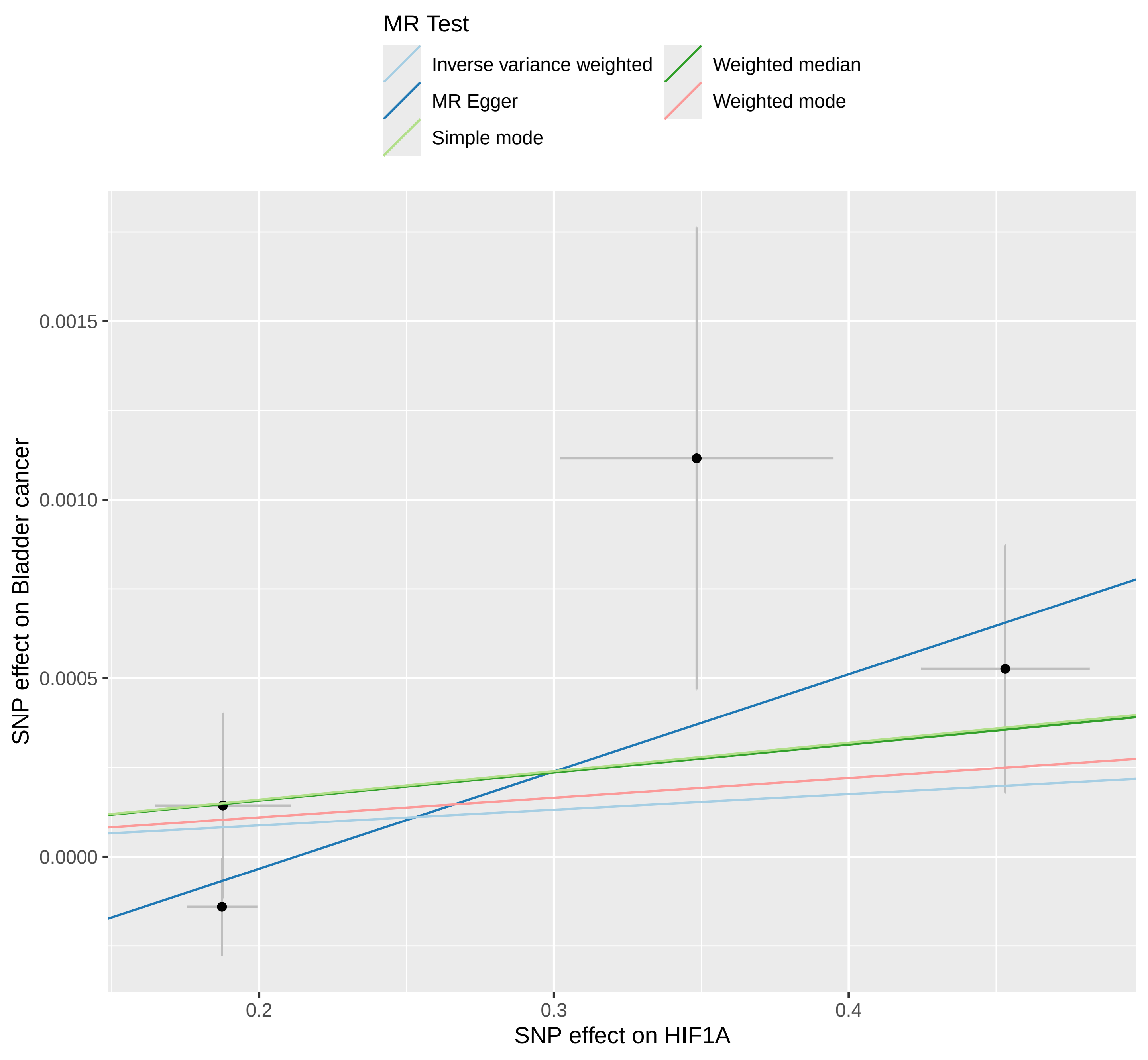
MR Test
Inverse variance weighted
MR Egger
Simple mode
Weighted median
Weighted mode
0.0015
0.0010
0.0005
0.0000
SNP effect on Bladder cancer
0.2
0.3
0.4
SNP effect on HIF1A

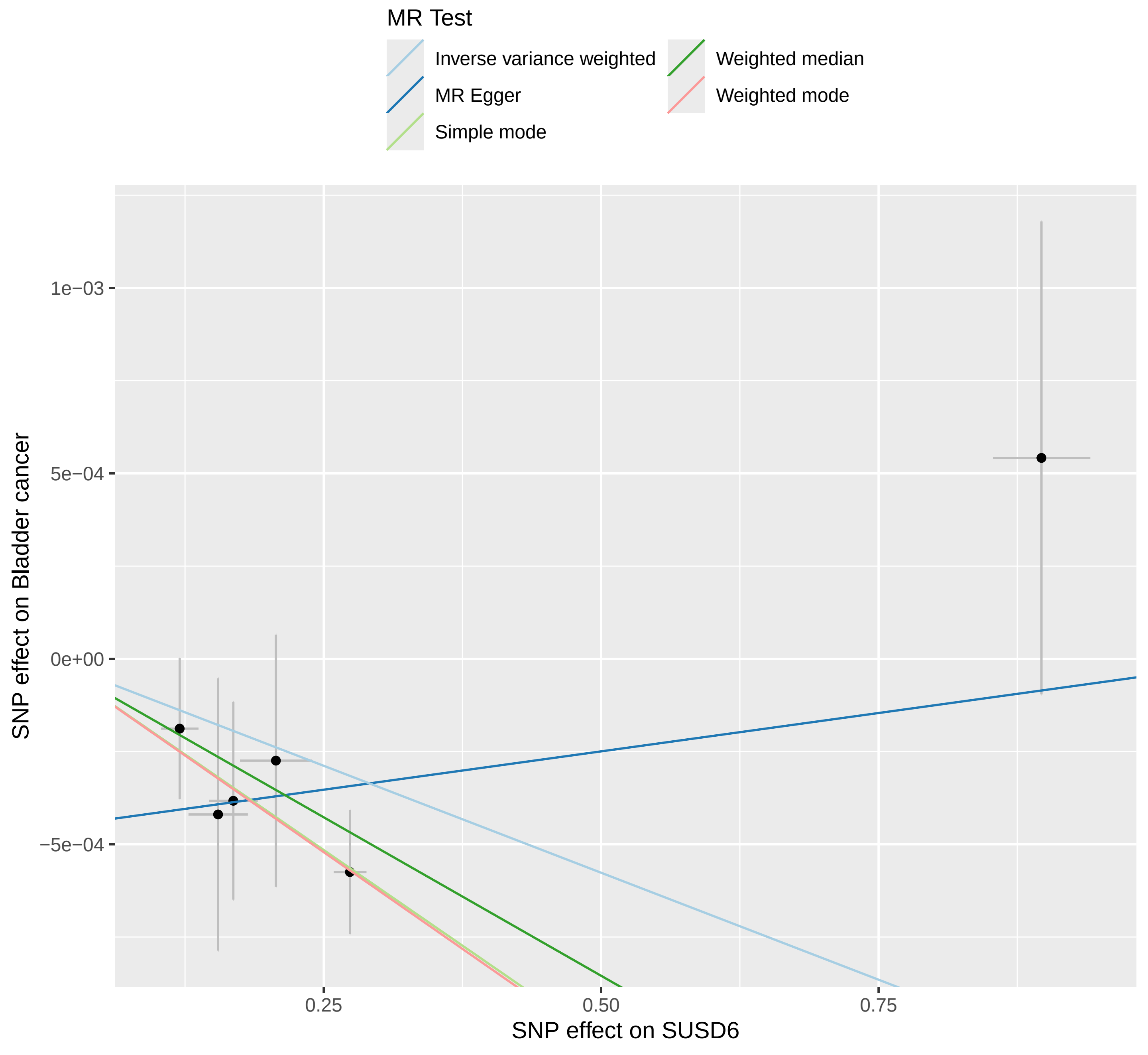
MR Test
Inverse variance weighted
MR Egger
Simple mode
Weighted median
Weighted mode
1e−03
5e−04
0e+00
−5e−04
SNP effect on Bladder cancer
0.25
0.50
0.75
SNP effect on SUSD6

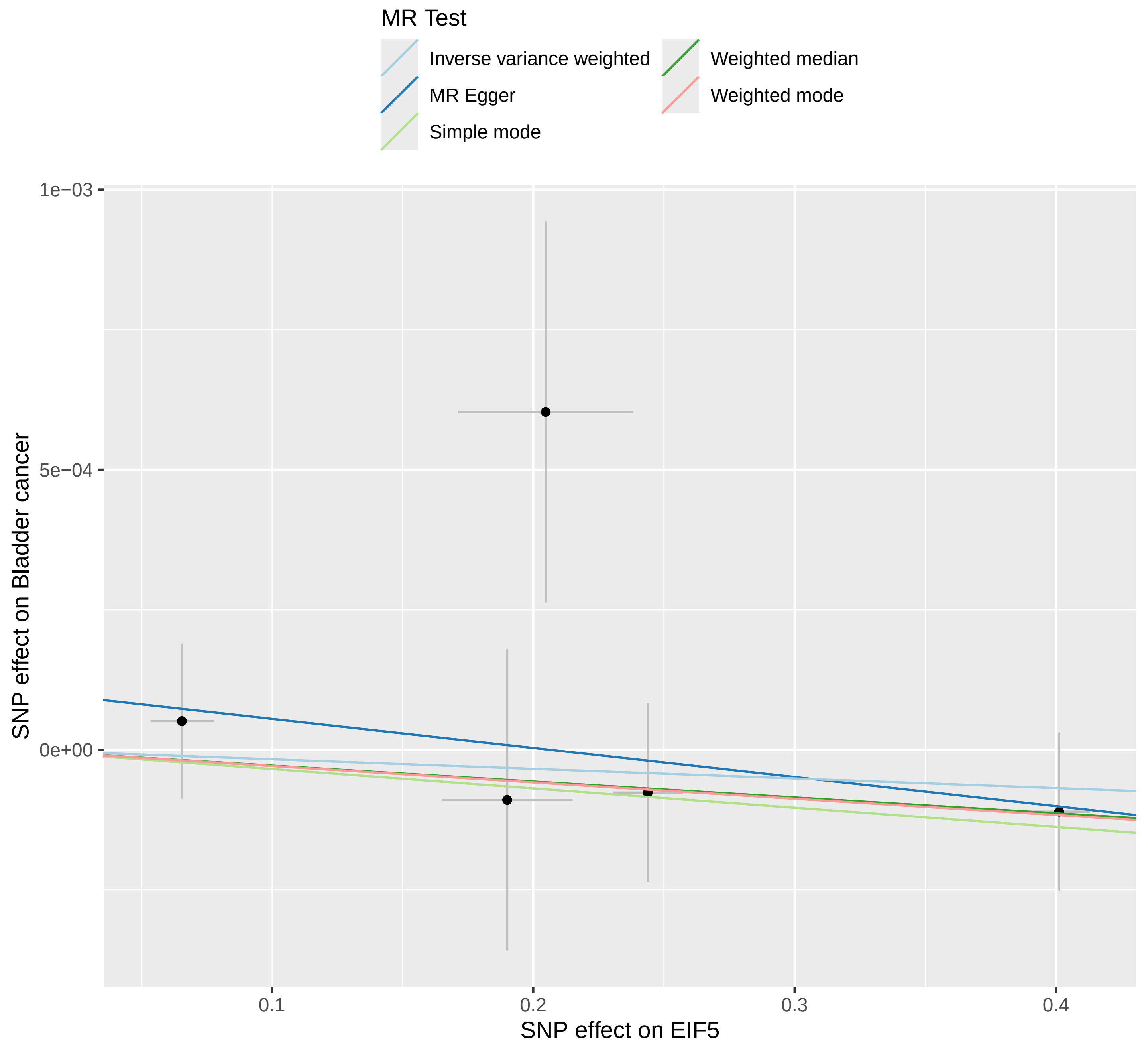
MR Test
Inverse variance weighted
MR Egger
Simple mode
Weighted median
Weighted mode
1e−03
5e−04
0e+00
0.1
0.2
0.3
0.4
SNP effect on EIF5
SNP effect on Bladder cancer

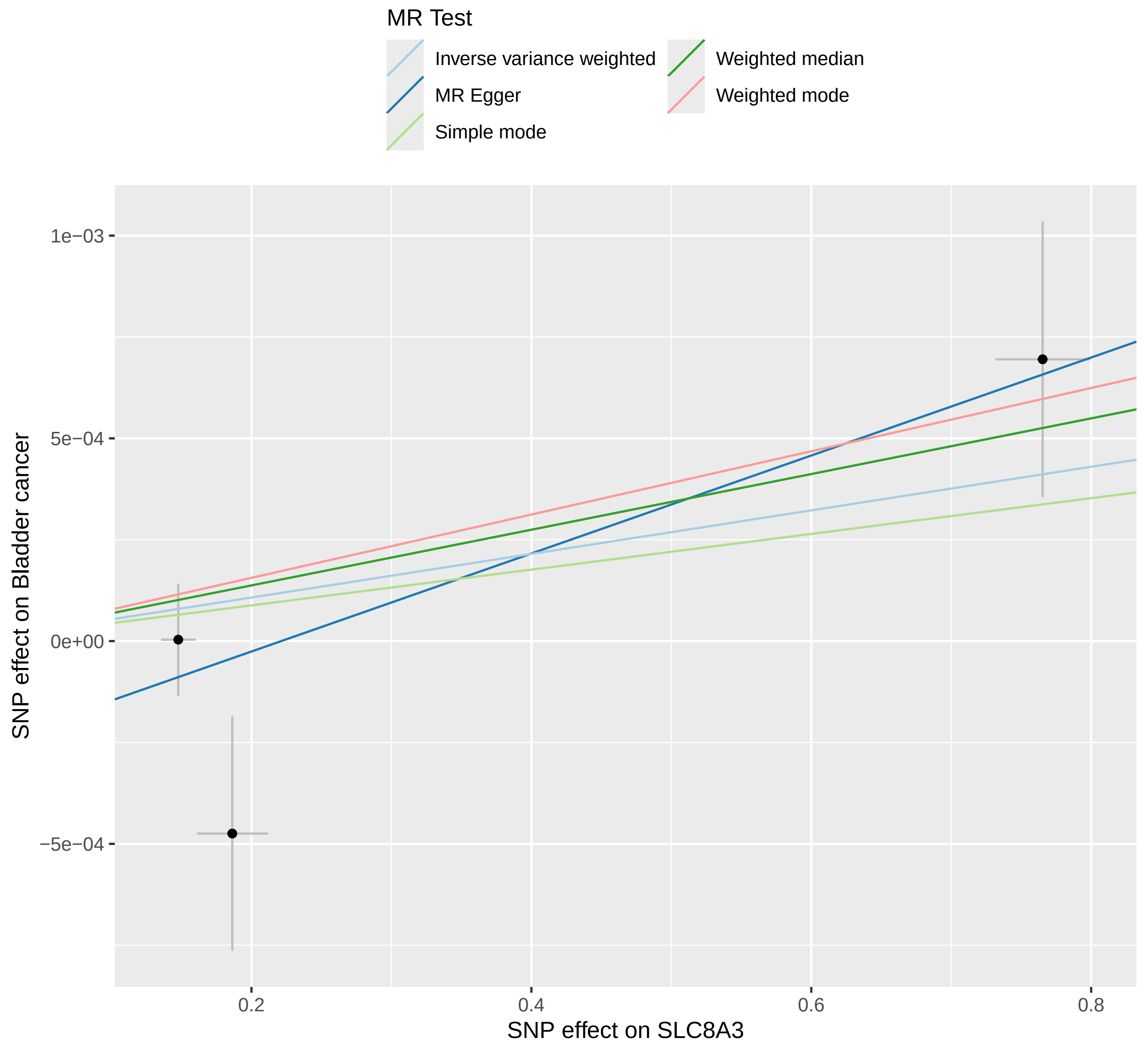
MR Test
Inverse variance weighted
MR Egger
Simple mode
Weighted median
Weighted mode
1e−03
5e−04
0e+00
−5e−04
SNP effect on Bladder cancer
0.2
0.4
0.6
0.8
SNP effect on SLC8A3

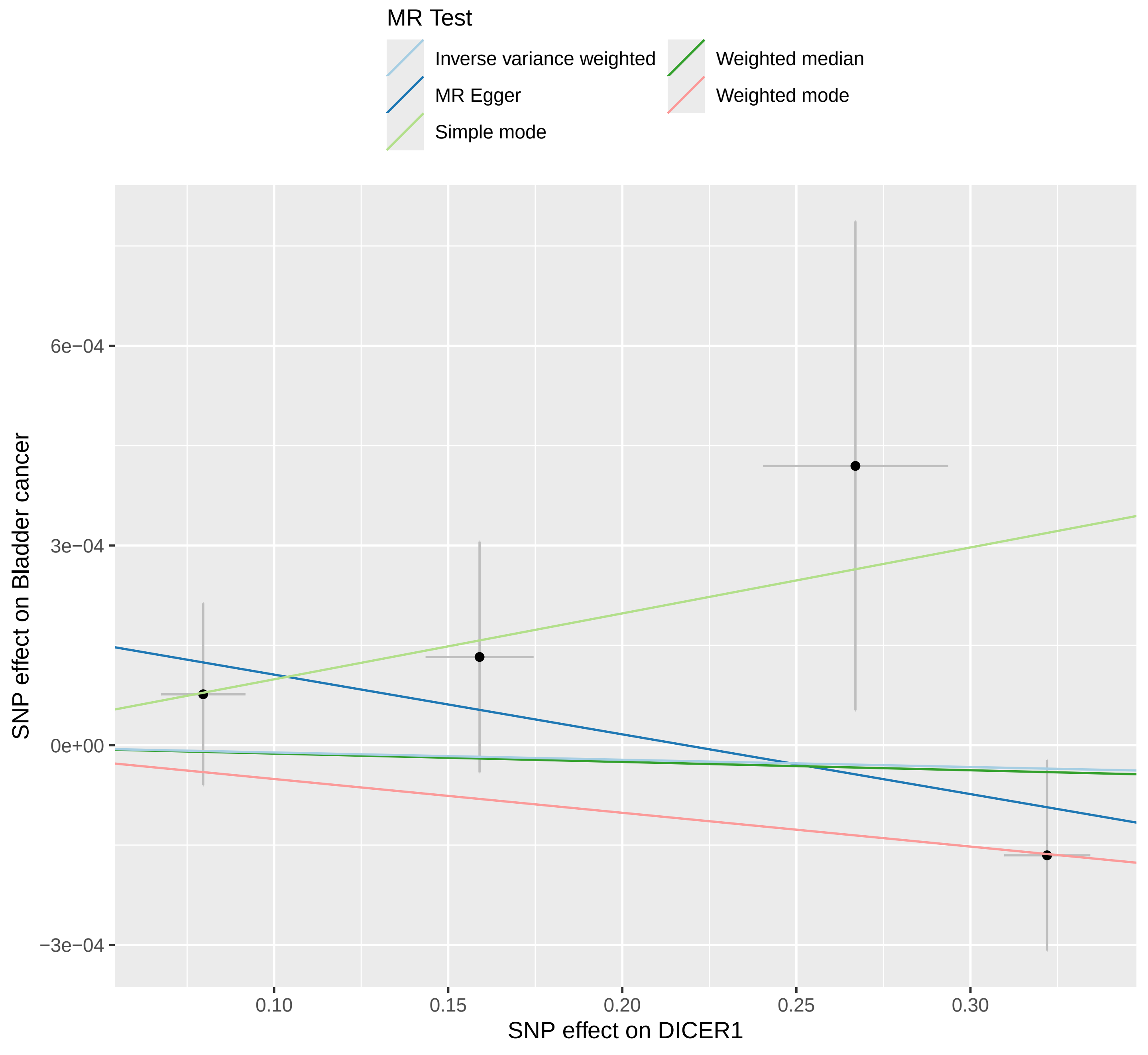

MR Test
Inverse variance weighted
MR Egger
Simple mode
Weighted median
Weighted mode
SNP effect on Bladder cancer
6e−04
3e−04
0e+00
−3e−04
0.10
0.15
0.20
0.25
0.30
SNP effect on DICER1

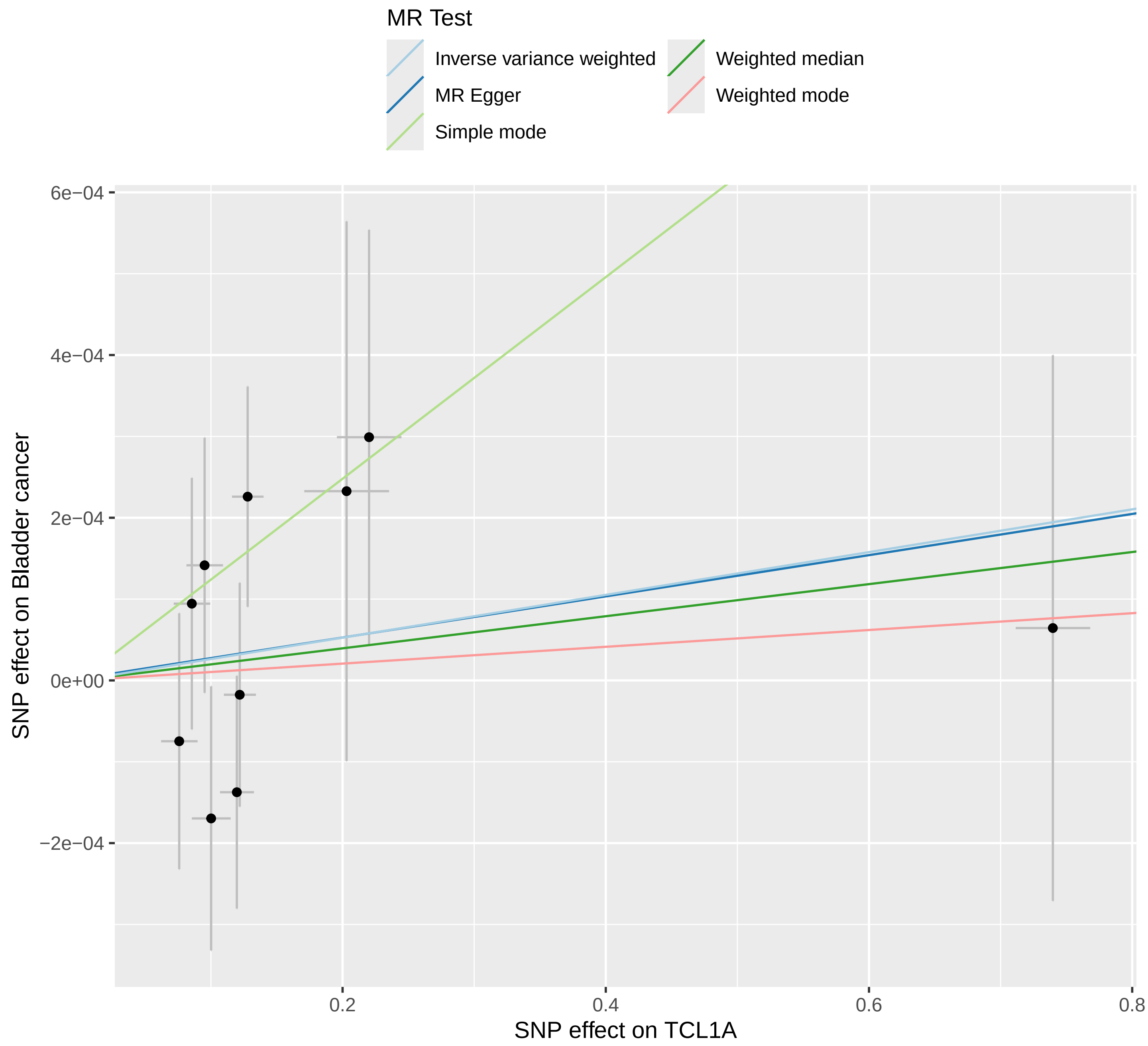

MR Test
Inverse variance weighted
MR Egger
Simple mode
Weighted median
Weighted mode
6e−04
4e−04
2e−04
0e+00
−2e−04
SNP effect on Bladder cancer
0.2
0.4
0.6
0.8
SNP effect on TCL1A

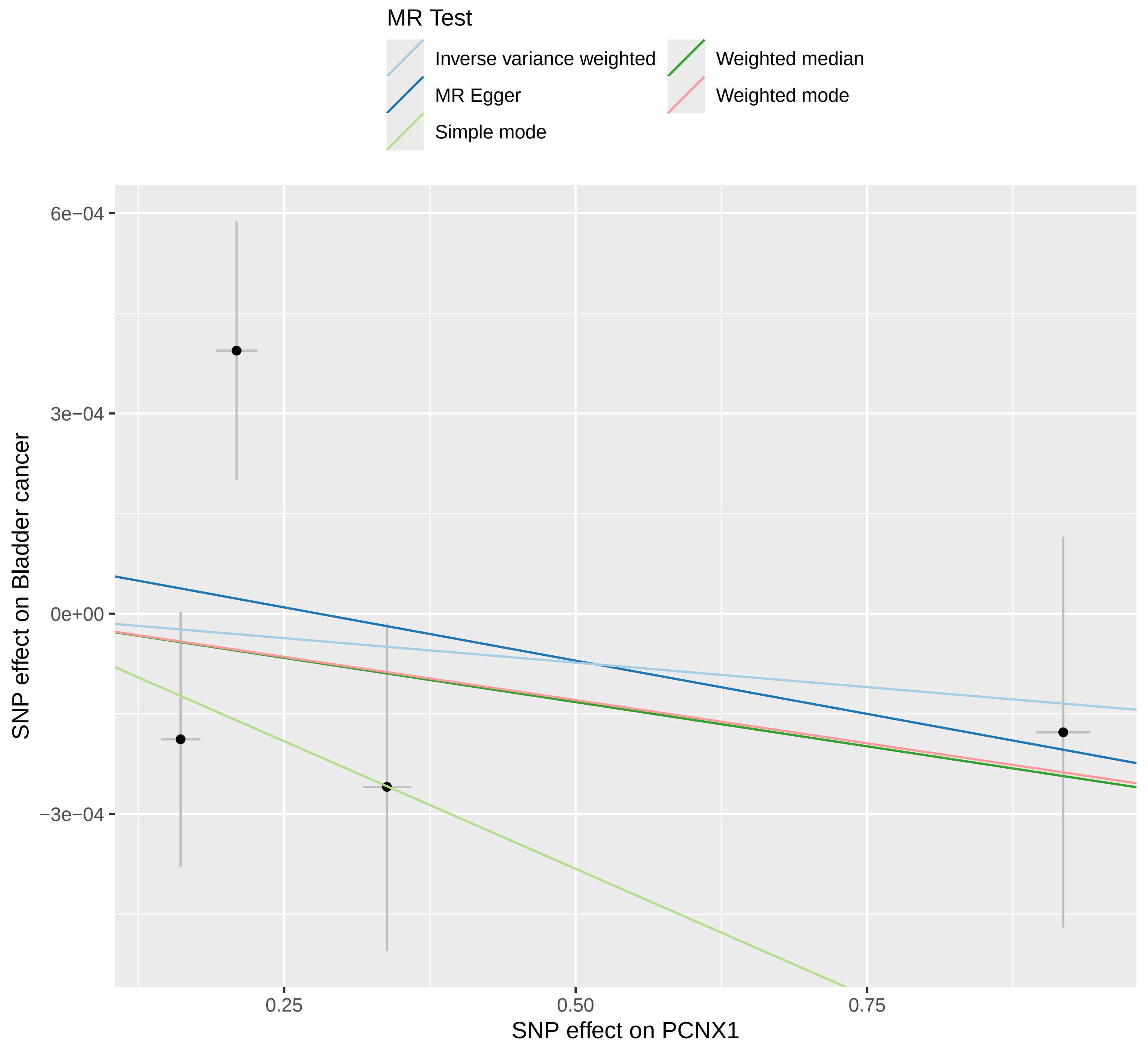
MR Test
Inverse variance weighted
MR Egger
Simple mode
Weighted median
Weighted mode
6e−04
3e−04
0e+00
−3e−04
SNP effect on Bladder cancer
0.25
0.50
0.75
SNP effect on PCNX1

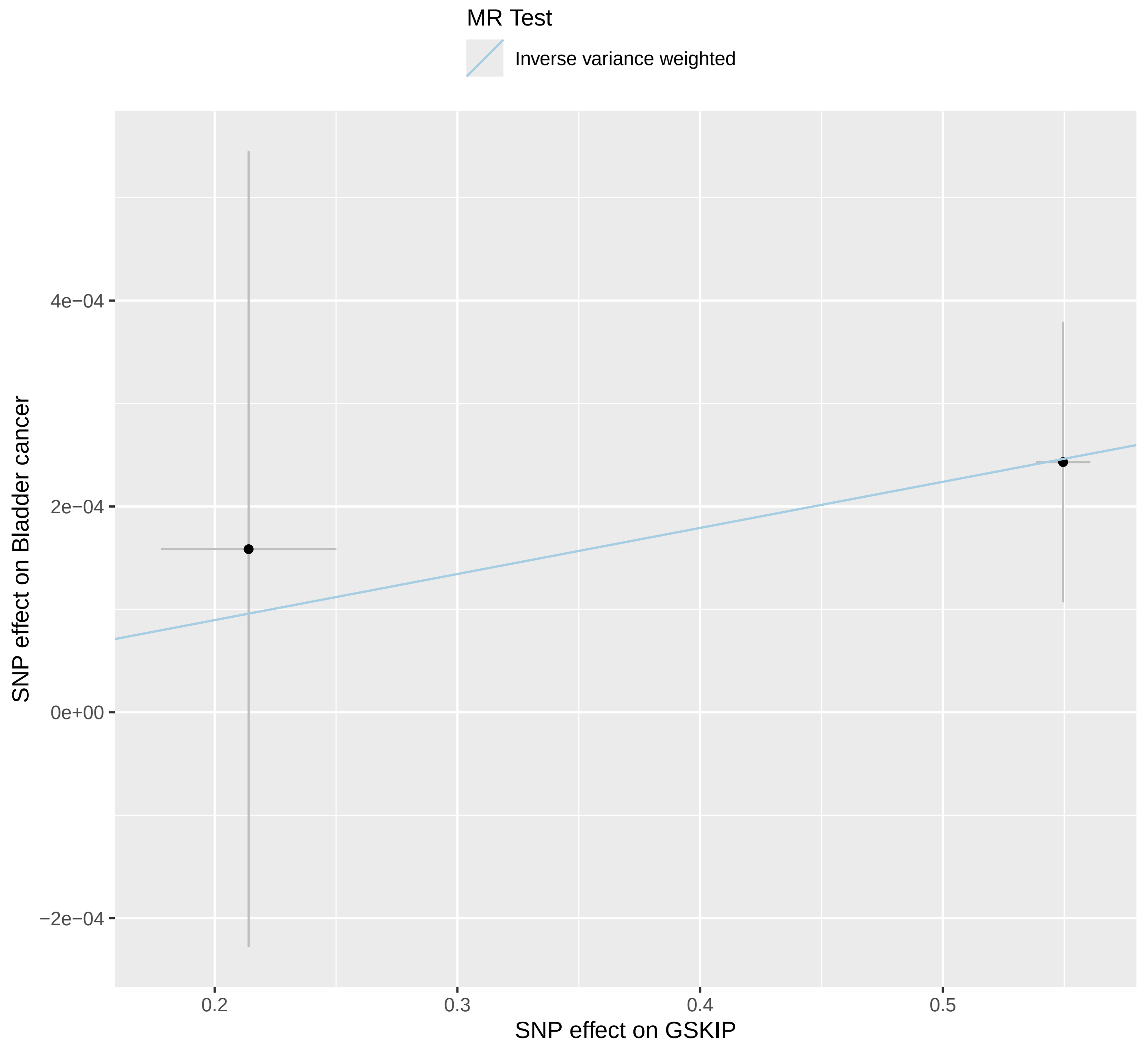

MR Test
Inverse variance weighted
SNP effect on Bladder cancer
4e−04
2e−04
0e+00
−2e−04
0.2
0.3
0.4
0.5
SNP effect on GSKIP

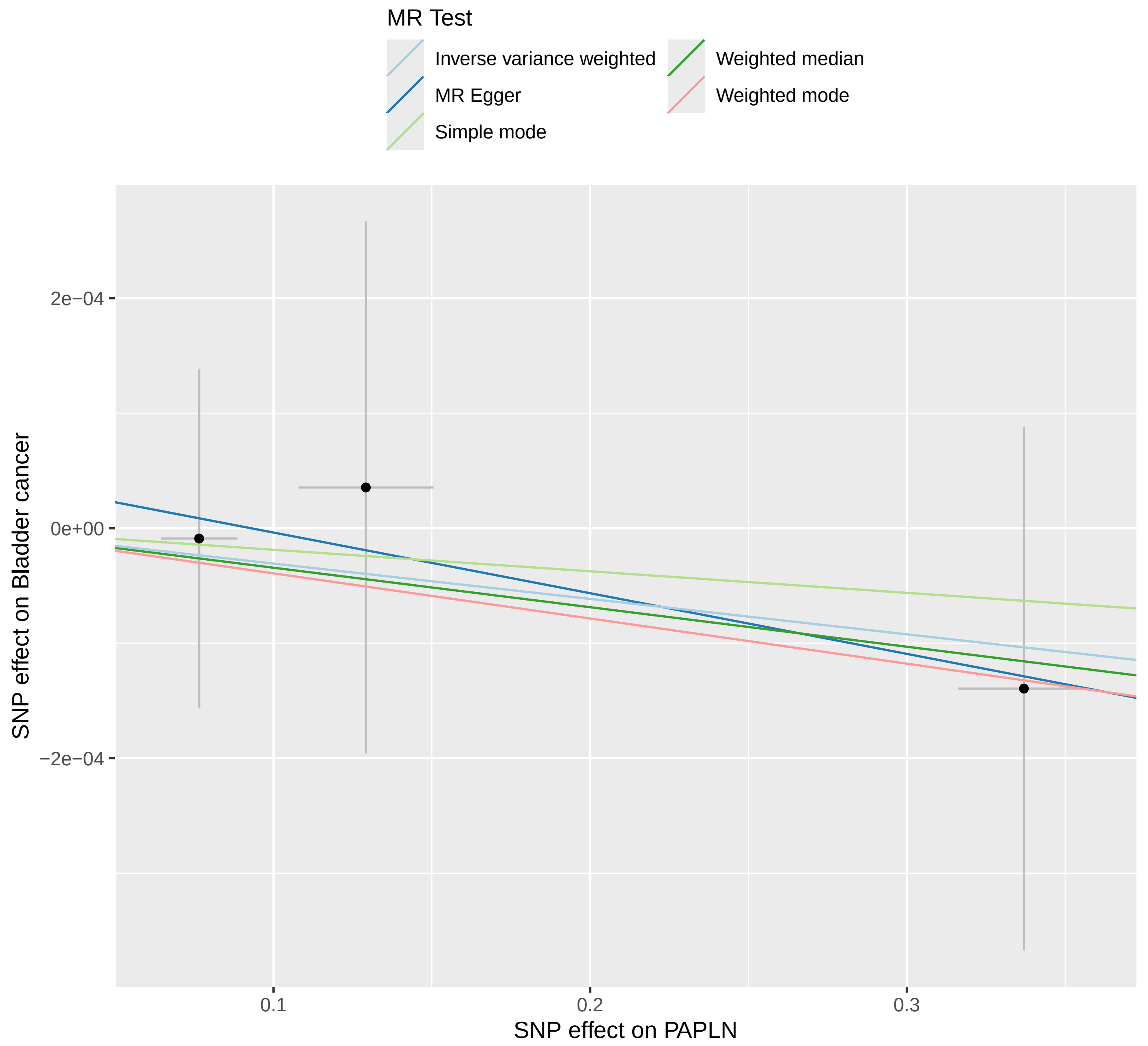

MR Test
Inverse variance weighted
MR Egger
Simple mode
Weighted median
Weighted mode
2e−04
0e+00
−2e−04
SNP effect on Bladder cancer
0.1
0.2
0.3
SNP effect on PAPLN

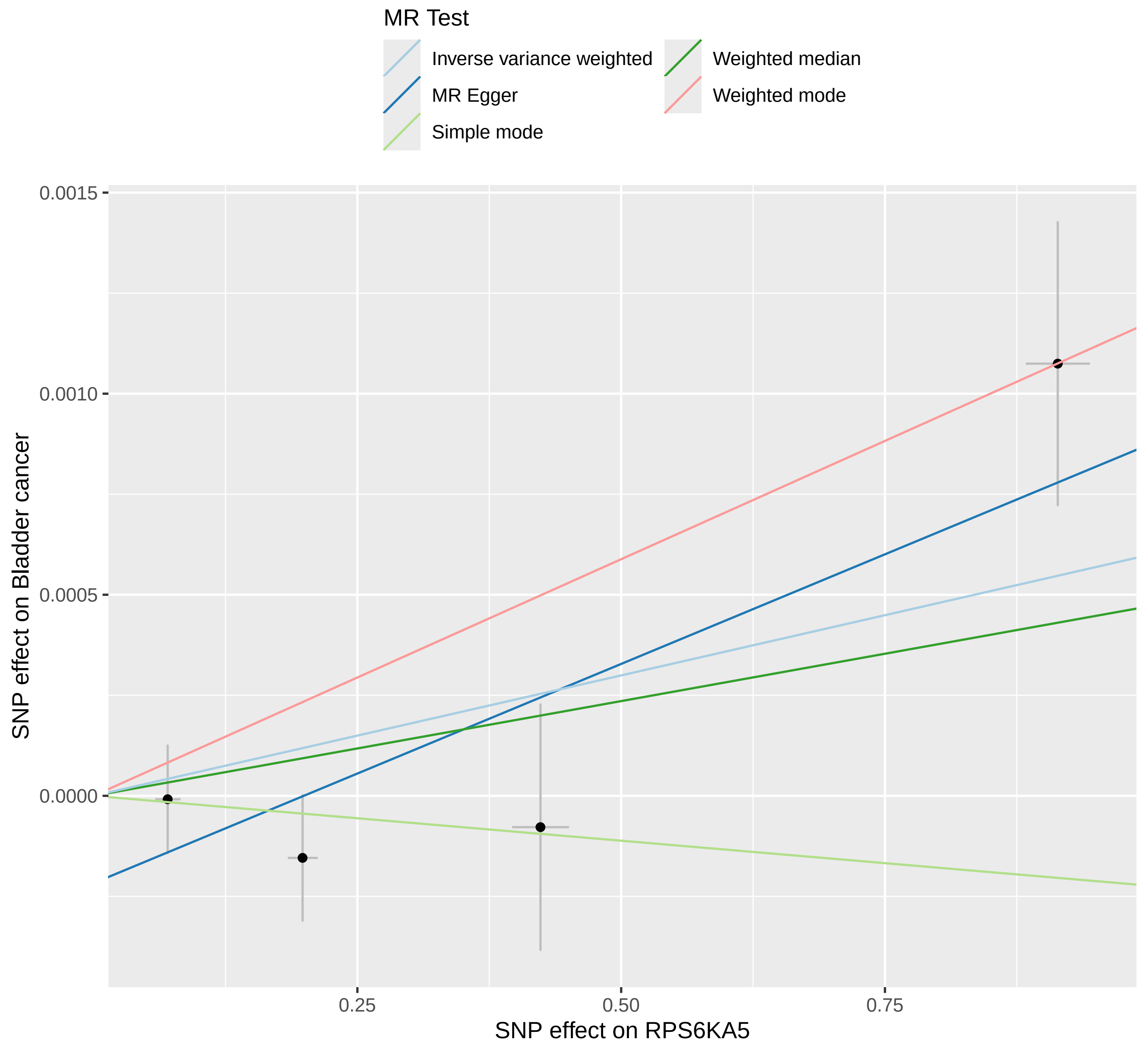

MR Test
Inverse variance weighted
MR Egger
Simple mode
Weighted median
Weighted mode
0.0015
0.0010
0.0005
0.0000
SNP effect on Bladder cancer
0.25
0.50
0.75
SNP effect on RPS6KA5

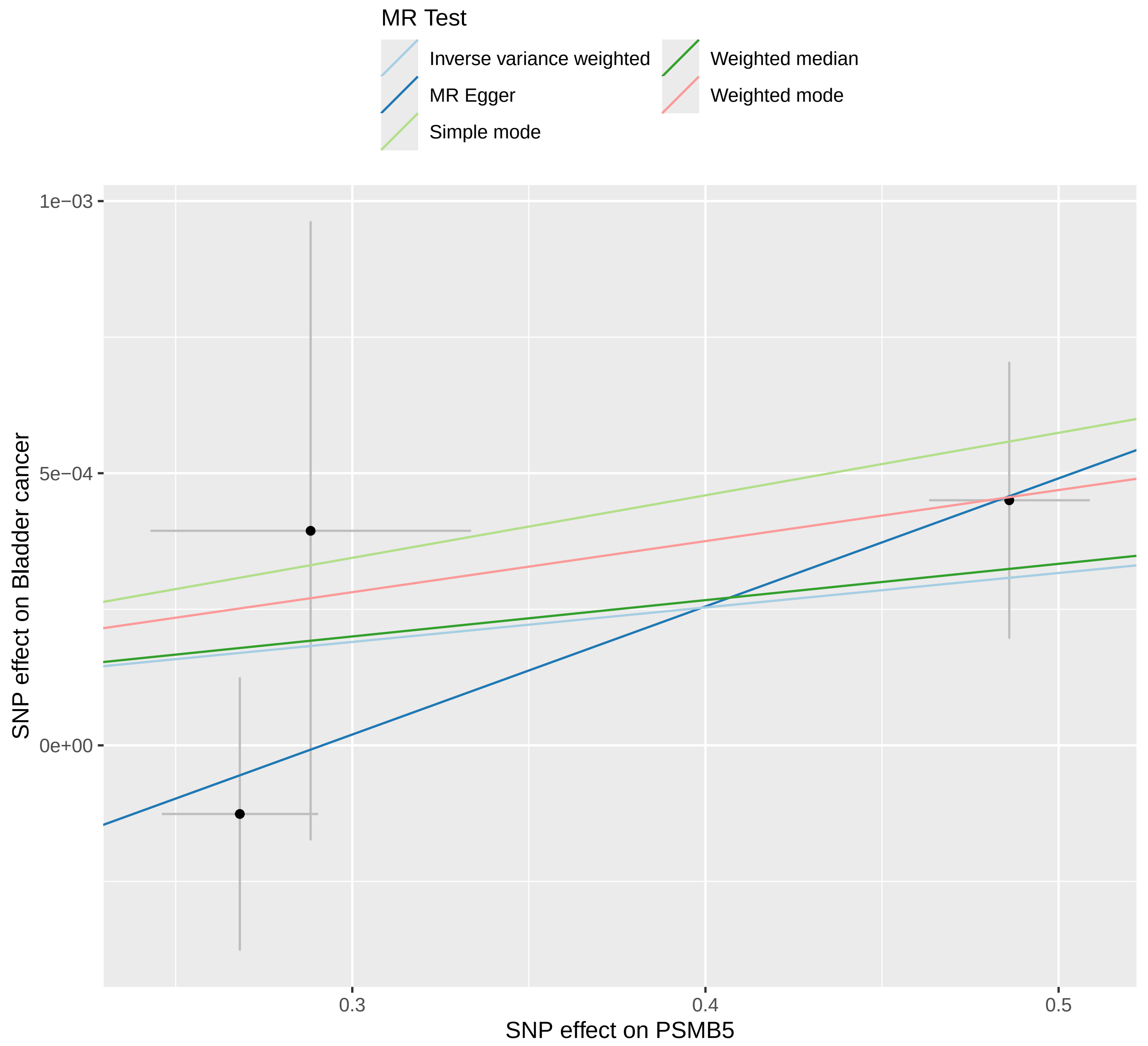
MR Test
Inverse variance weighted
MR Egger
Simple mode
Weighted median
Weighted mode
1e−03
5e−04
0e+00
0.3
0.4
0.5
SNP effect on PSMB5
SNP effect on Bladder cancer

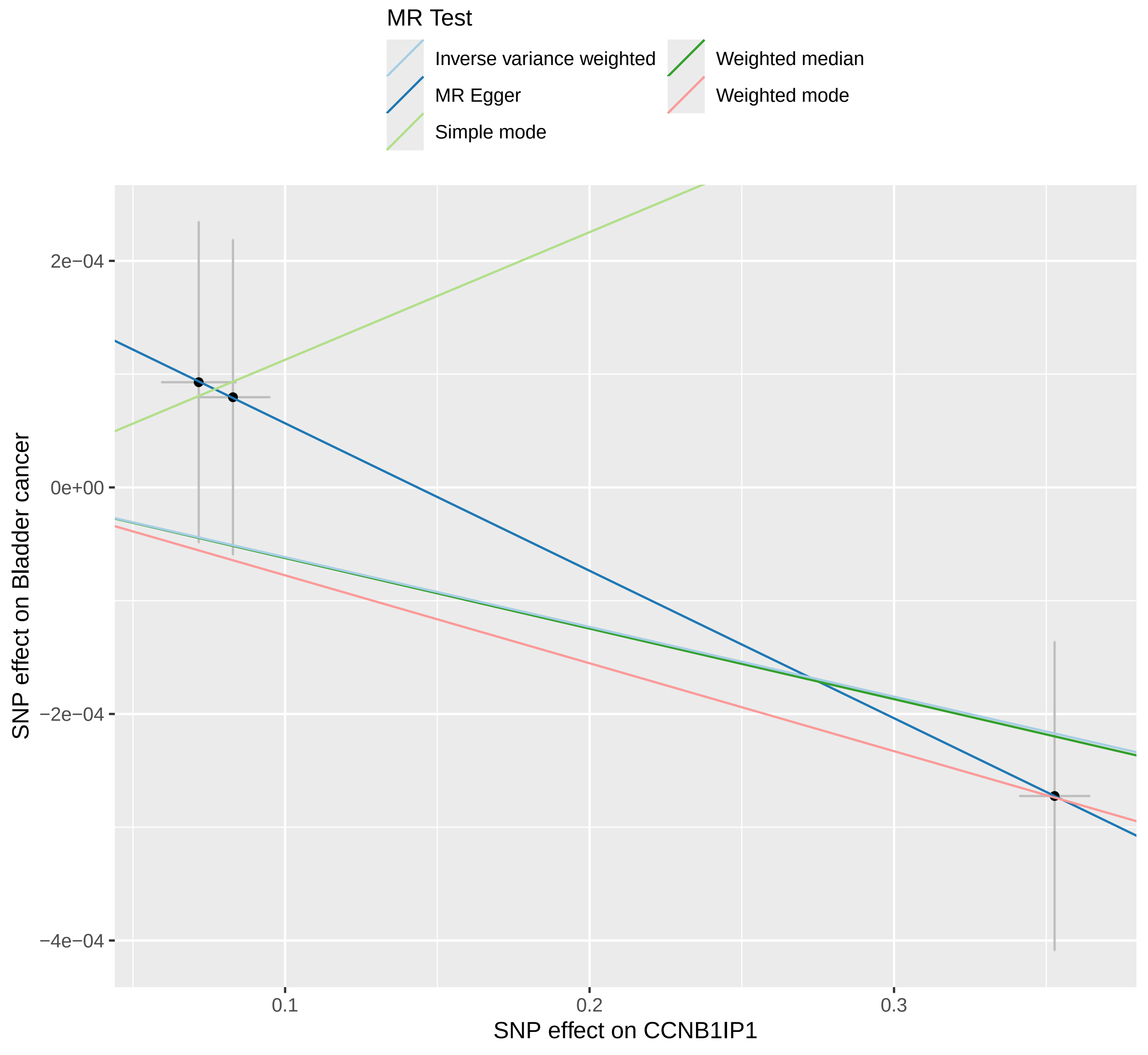
MR Test
Inverse variance weighted
MR Egger
Simple mode
Weighted median
Weighted mode
2e−04
0e+00
−2e−04
−4e−04
0.1
0.2
0.3
SNP effect on Bladder cancer
SNP effect on CCNB1IP1

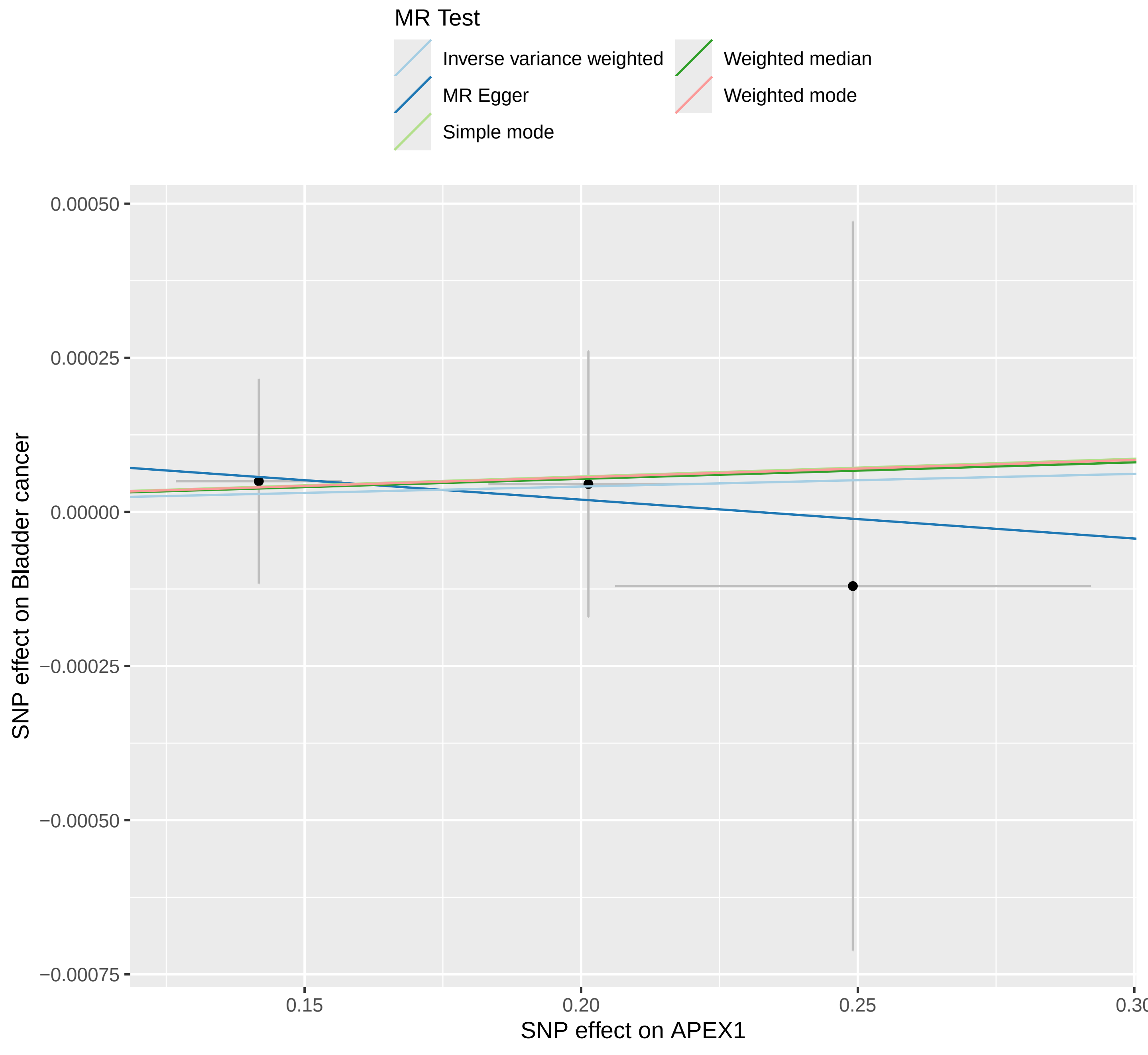

MR Test
Inverse variance weighted
MR Egger
Simple mode
Weighted median
Weighted mode
0.00050
0.00025
0.00000
−0.00025
−0.00050
−0.00075
0.15
0.20
0.25
0.30
SNP effect on Bladder cancer
SNP effect on APEX1

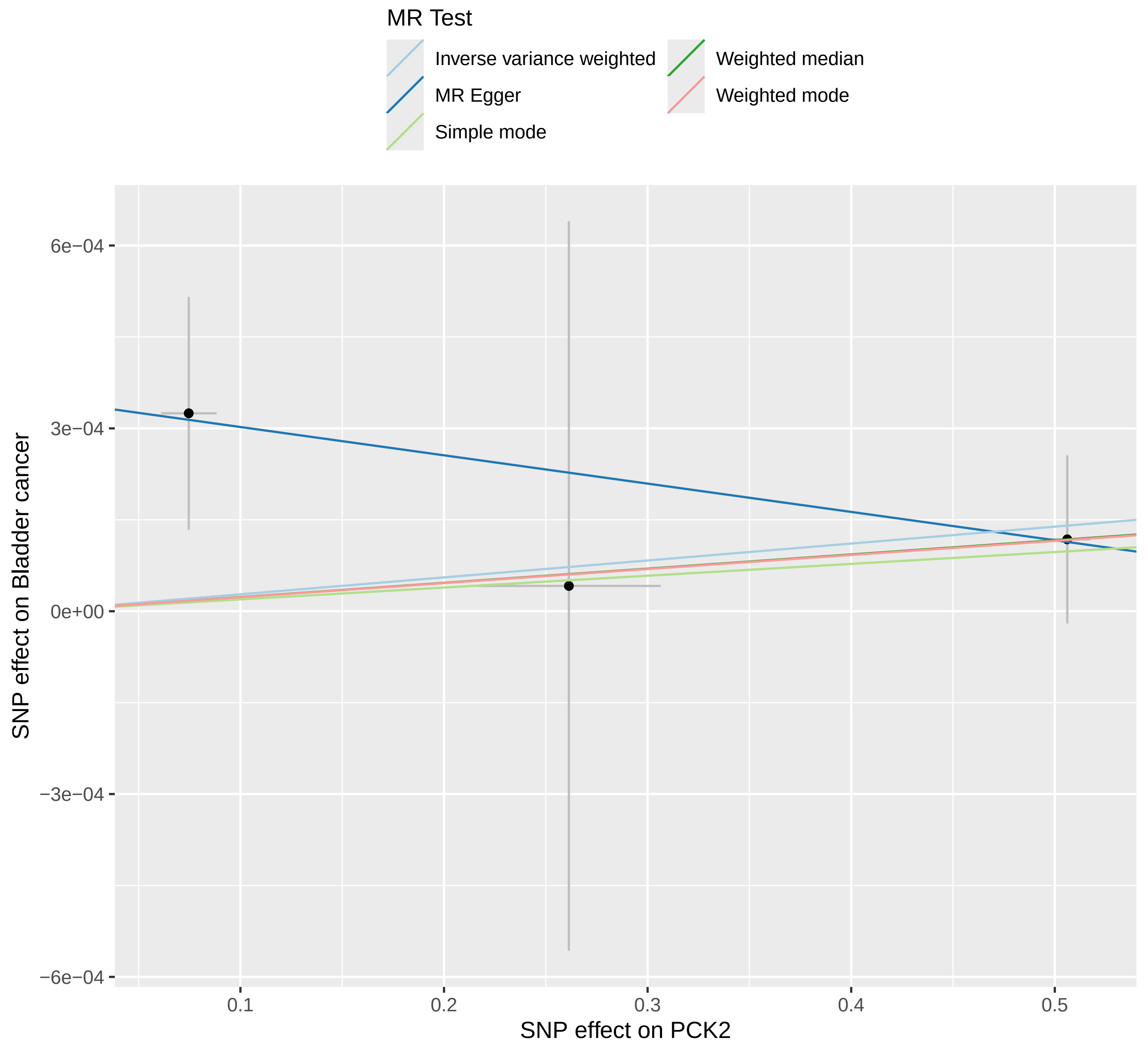

MR Test
Inverse variance weighted
MR Egger
Simple mode
Weighted median
Weighted mode
6e−04
3e−04
0e+00
−3e−04
−6e−04
0.1
0.2
0.3
0.4
0.5
SNP effect on Bladder cancer
SNP effect on PCK2

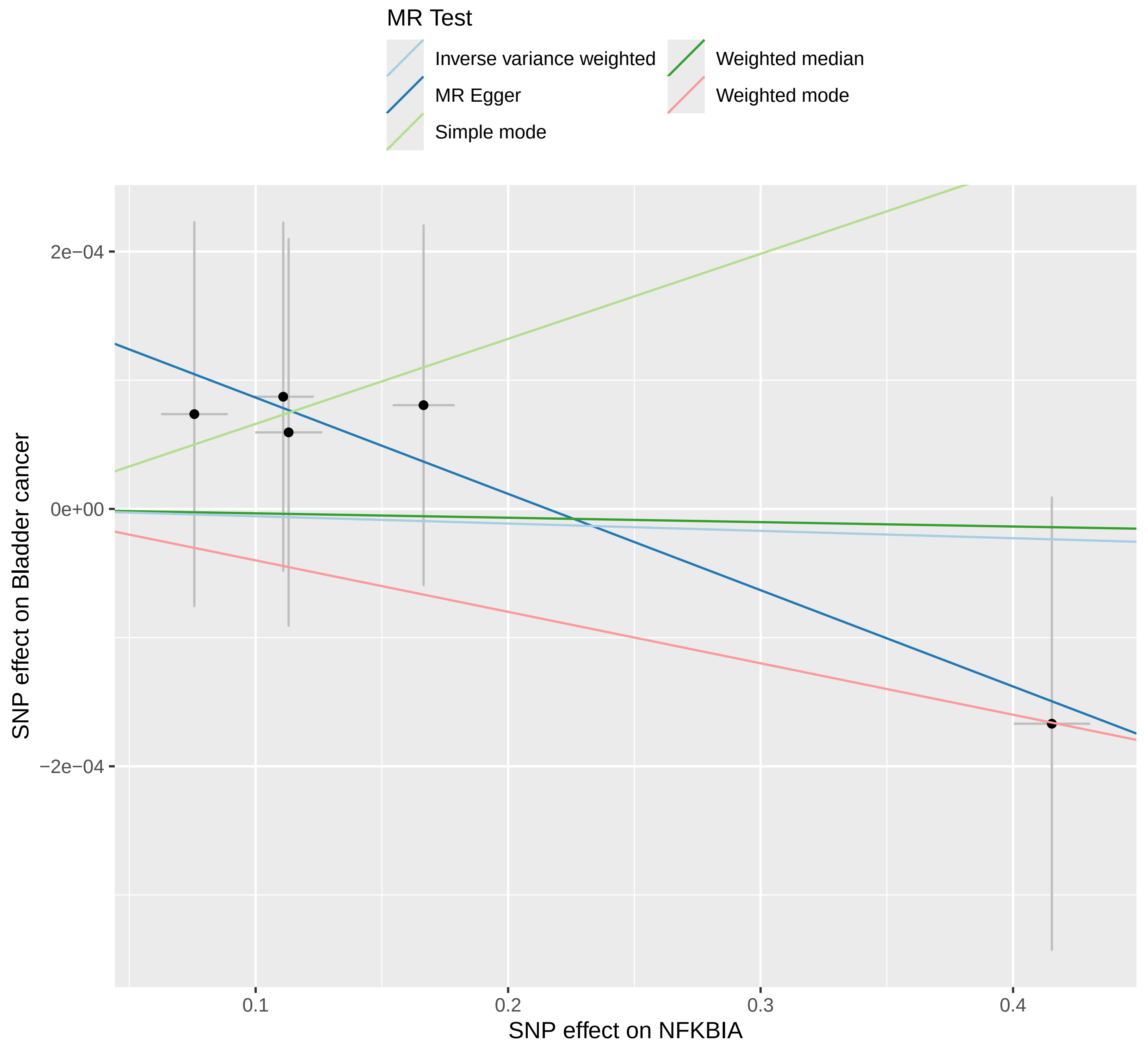

MR Test
Inverse variance weighted
MR Egger
Simple mode
Weighted median
Weighted mode
2e−04
0e+00
−2e−04
SNP effect on Bladder cancer
0.1
0.2
0.3
0.4
SNP effect on NFKBIA

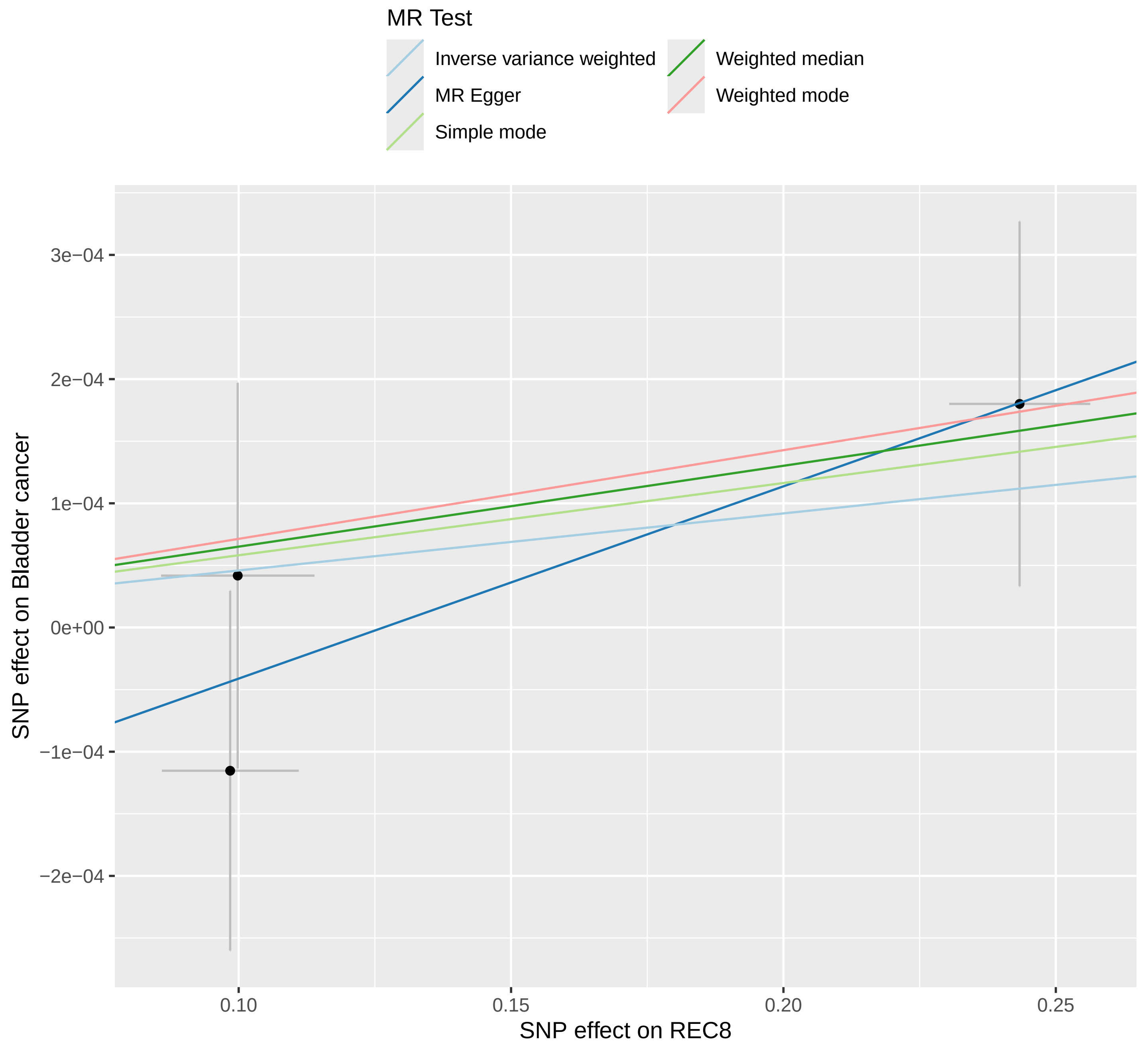
MR Test
Inverse variance weighted
MR Egger
Simple mode
Weighted median
Weighted mode
3e−04
2e−04
1e−04
0e+00
−1e−04
−2e−04
SNP effect on Bladder cancer
0.10
0.15
0.20
0.25
SNP effect on REC8

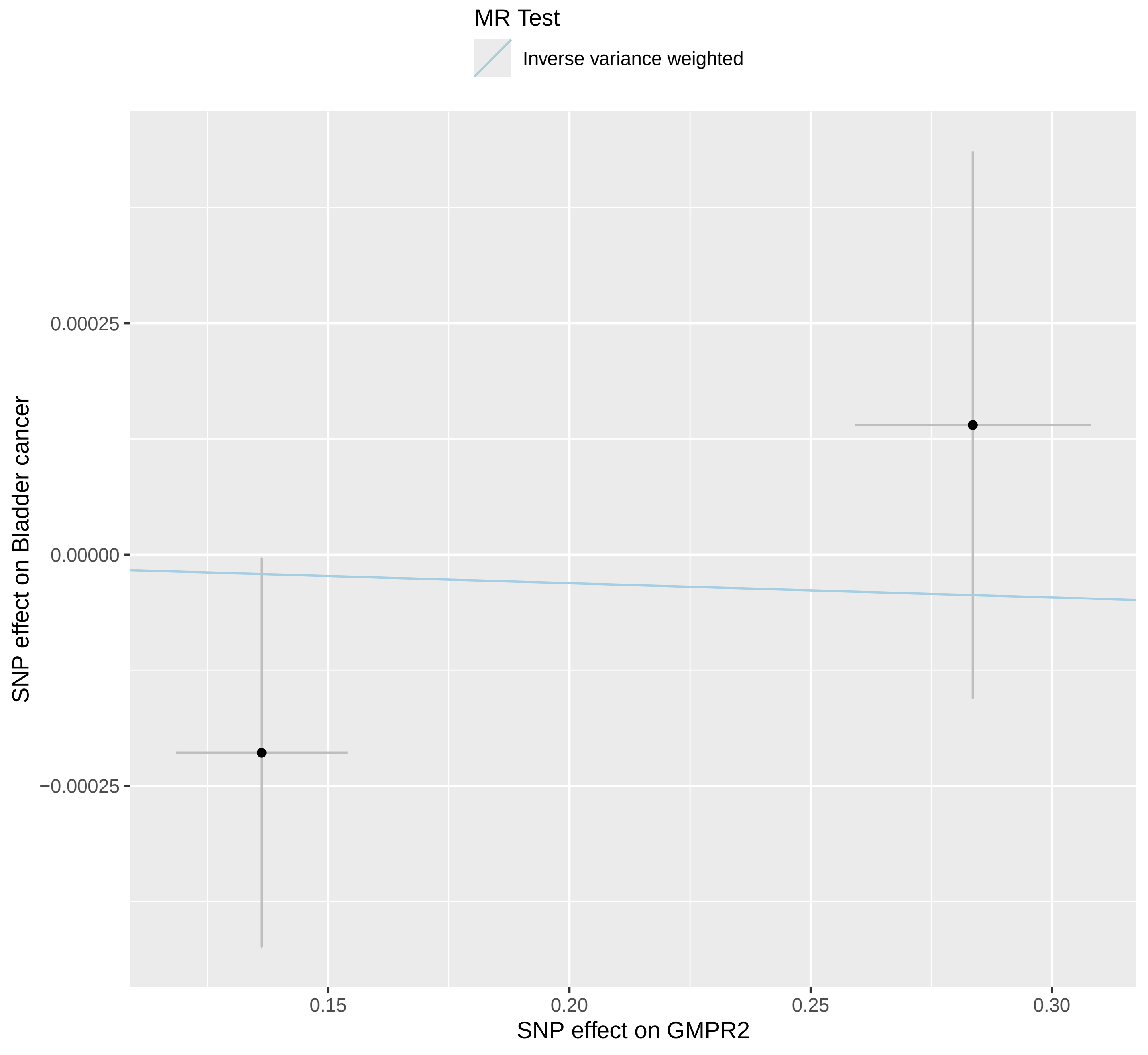

MR Test
Inverse variance weighted
0.00025
0.00000
−0.00025
0.15
0.20
0.25
0.30
SNP effect on GMPR2
SNP effect on Bladder cancer

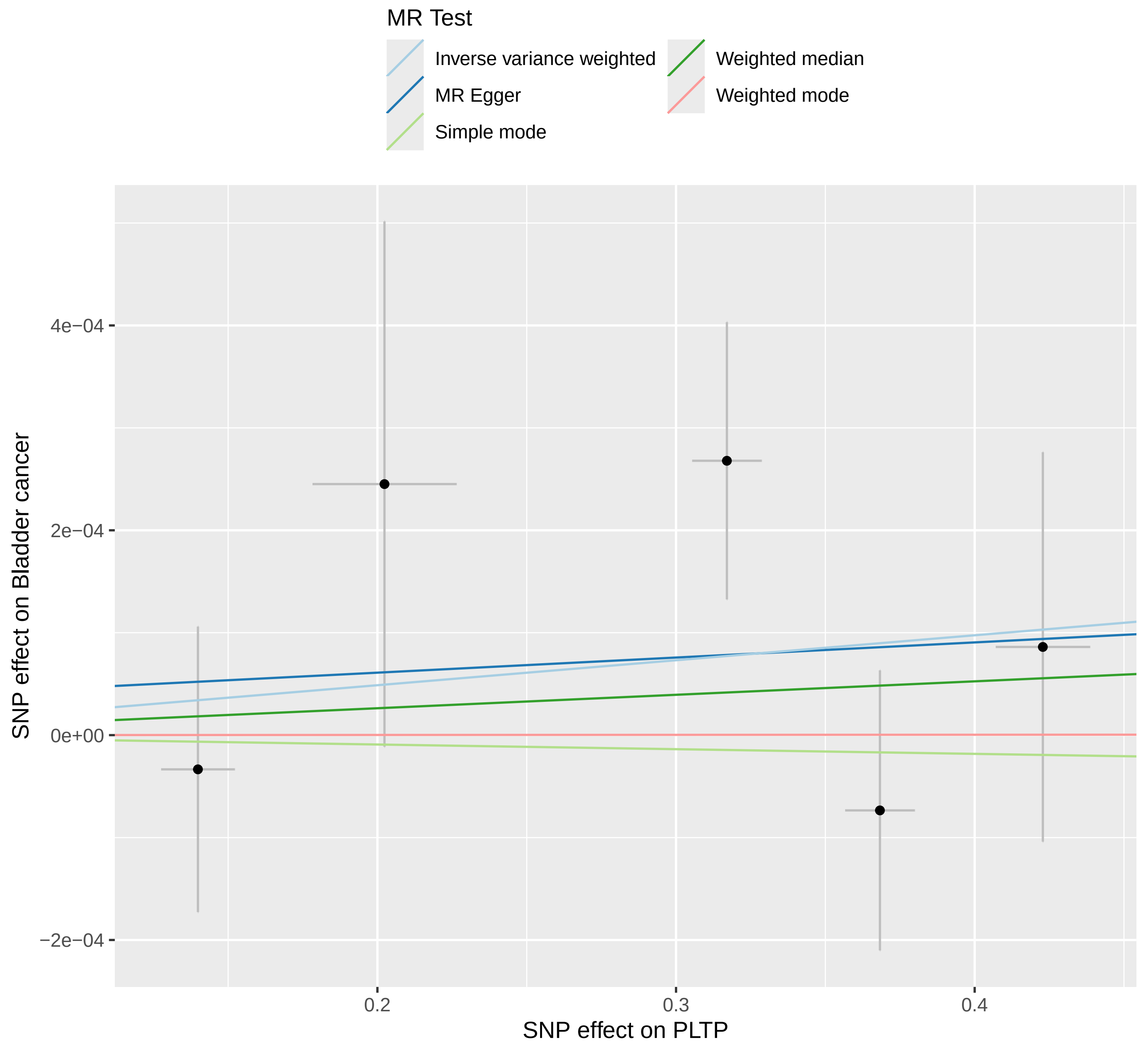
MR Test
Inverse variance weighted
MR Egger
Simple mode
Weighted median
Weighted mode
SNP effect on Bladder cancer
4e−04
2e−04
0e+00
−2e−04
0.2
0.3
0.4
SNP effect on PLTP

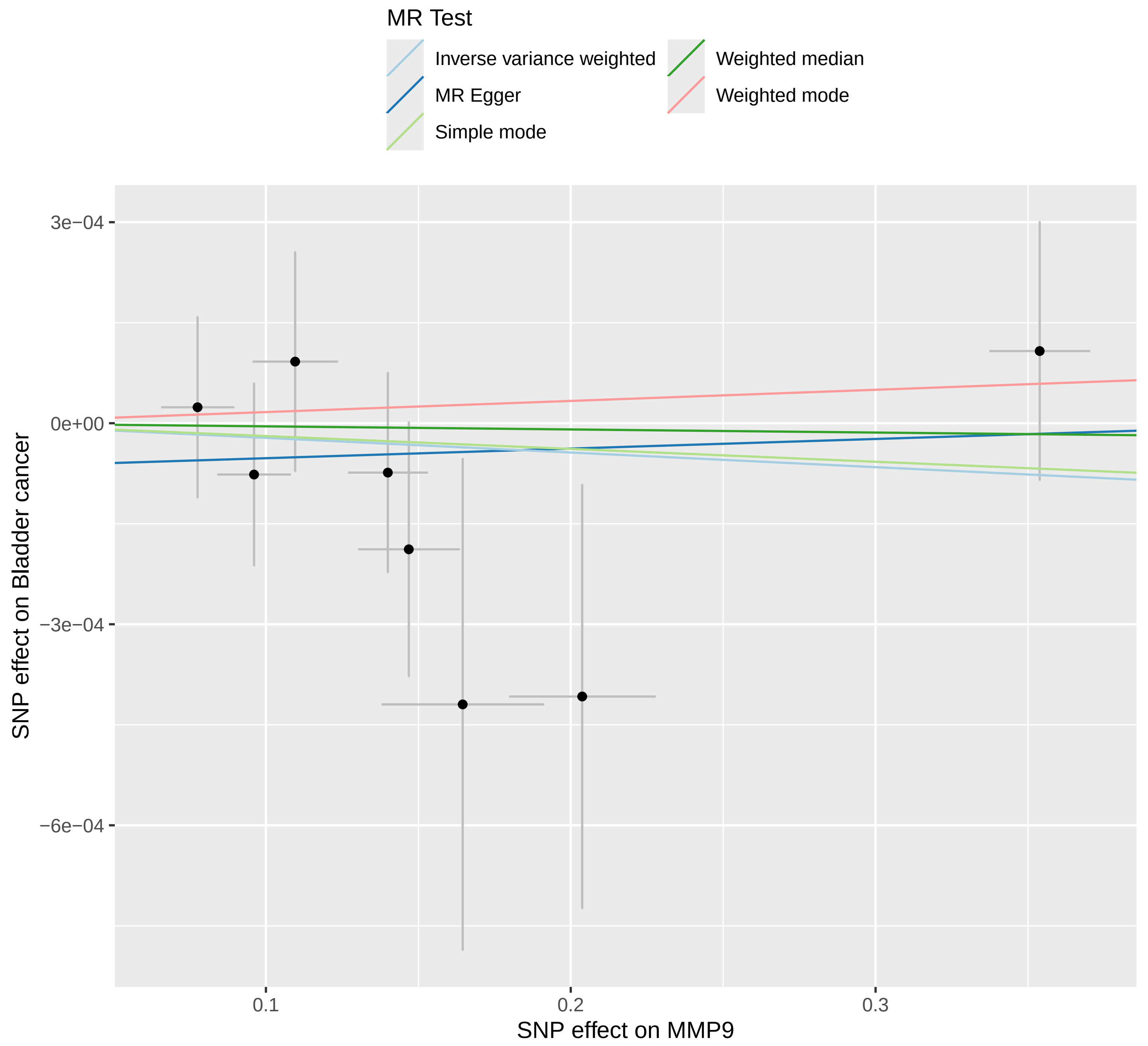
MR Test
Inverse variance weighted
MR Egger
Simple mode
Weighted median
Weighted mode
3e−04
0e+00
−3e−04
−6e−04
0.1
0.2
0.3
SNP effect on MMP9
SNP effect on Bladder cancer

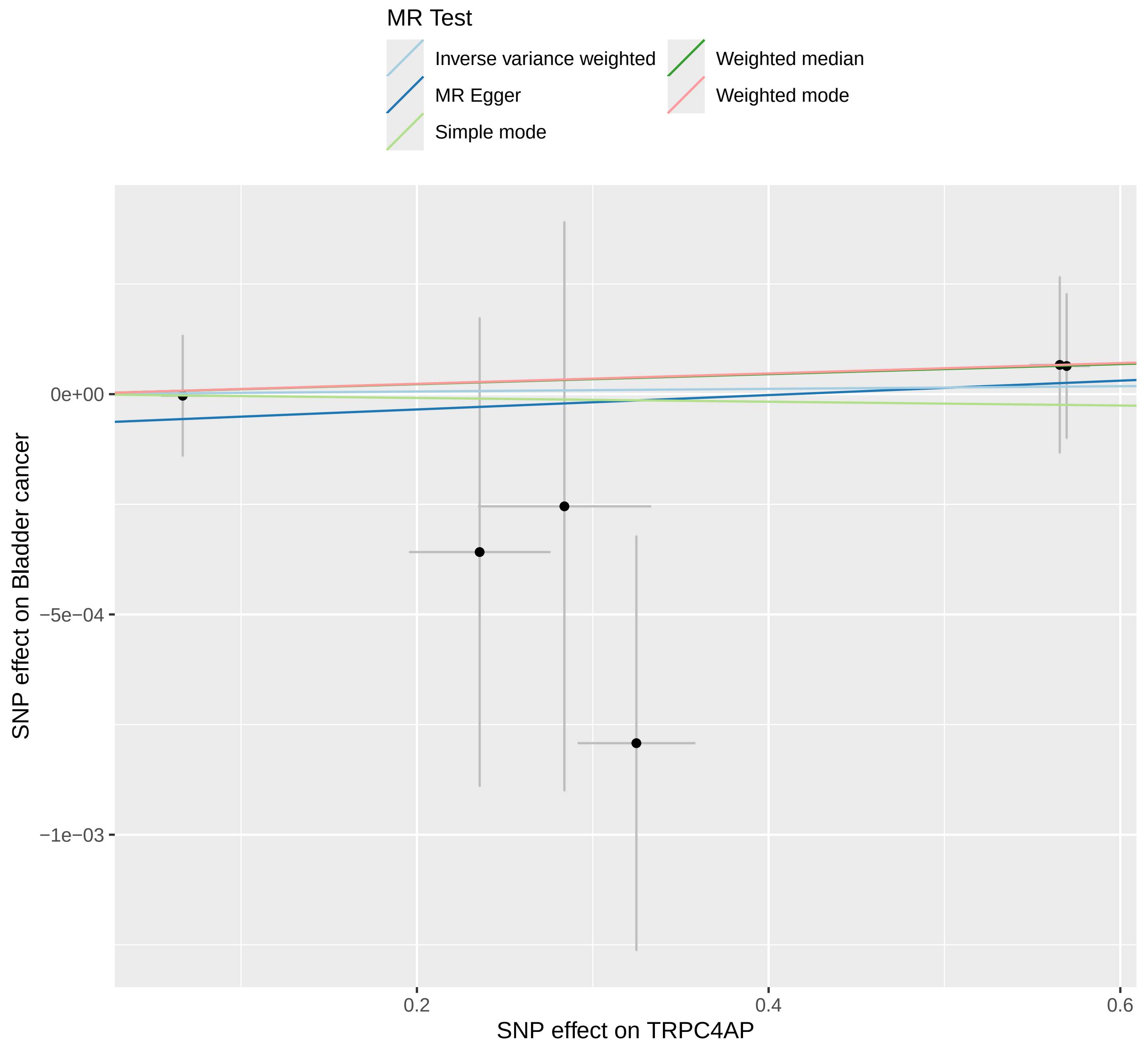

MR Test
Inverse variance weighted
MR Egger
Simple mode
Weighted median
Weighted mode
0e+00
−5e−04
−1e−03
0.2
0.4
0.6
SNP effect on TRPC4AP
SNP effect on Bladder cancer

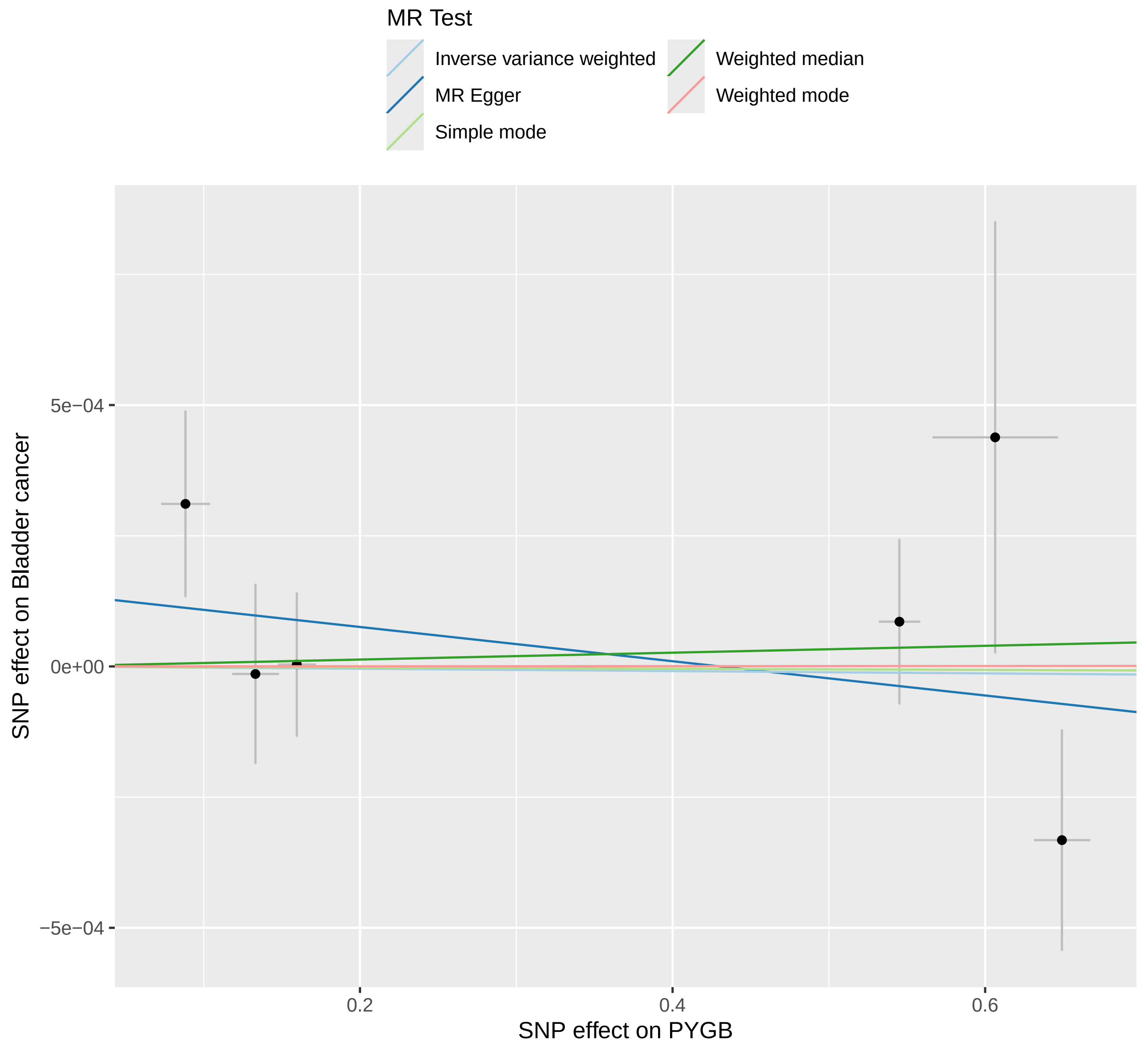

MR Test
Inverse variance weighted
MR Egger
Simple mode
Weighted median
Weighted mode
5e−04
0e+00
−5e−04
SNP effect on Bladder cancer
0.2
0.4
0.6
SNP effect on PYGB

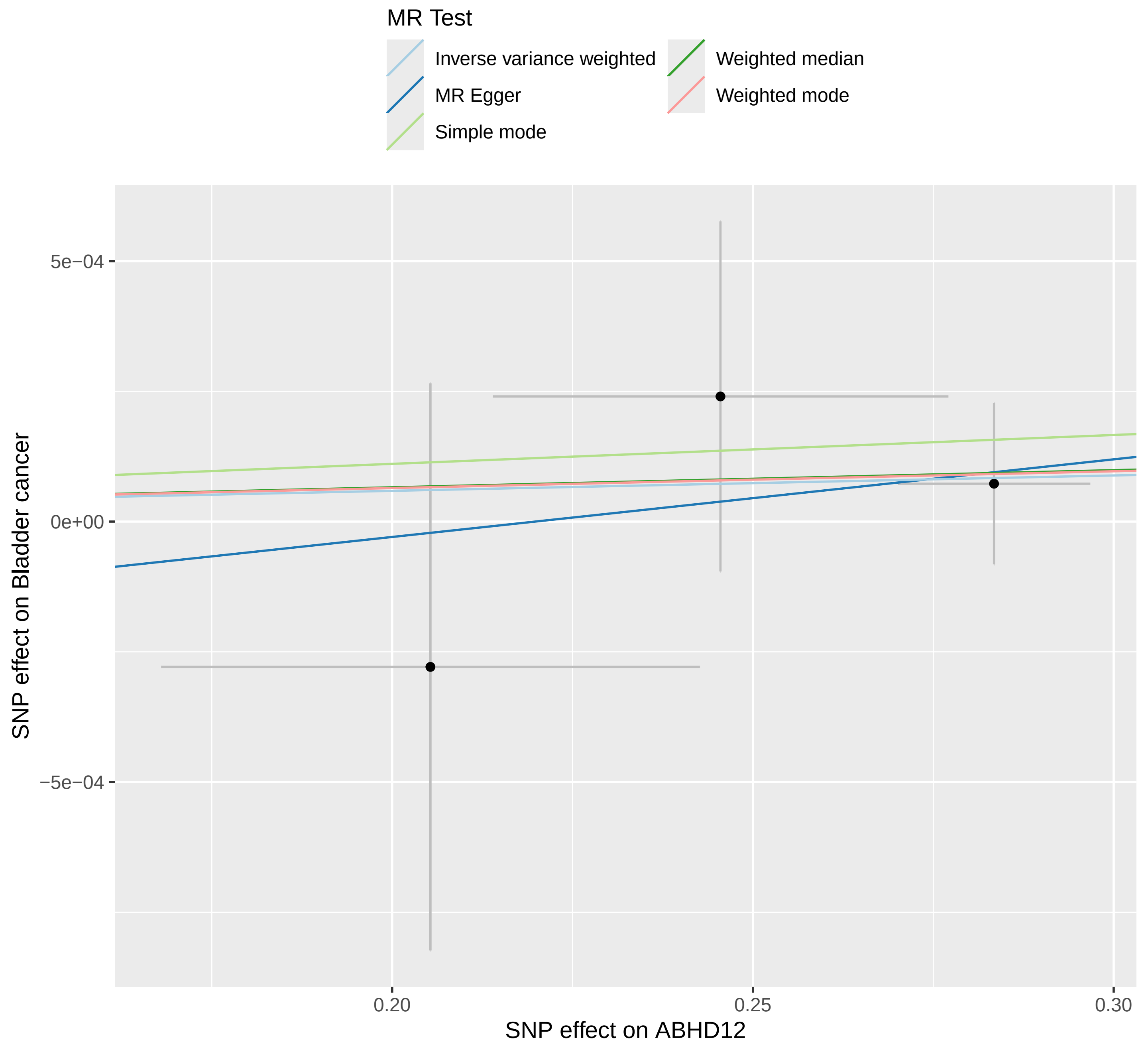
MR Test
Inverse variance weighted
MR Egger
Simple mode
Weighted median
Weighted mode
5e−04
0e+00
−5e−04
SNP effect on Bladder cancer
0.20
0.25
0.30
SNP effect on ABHD12

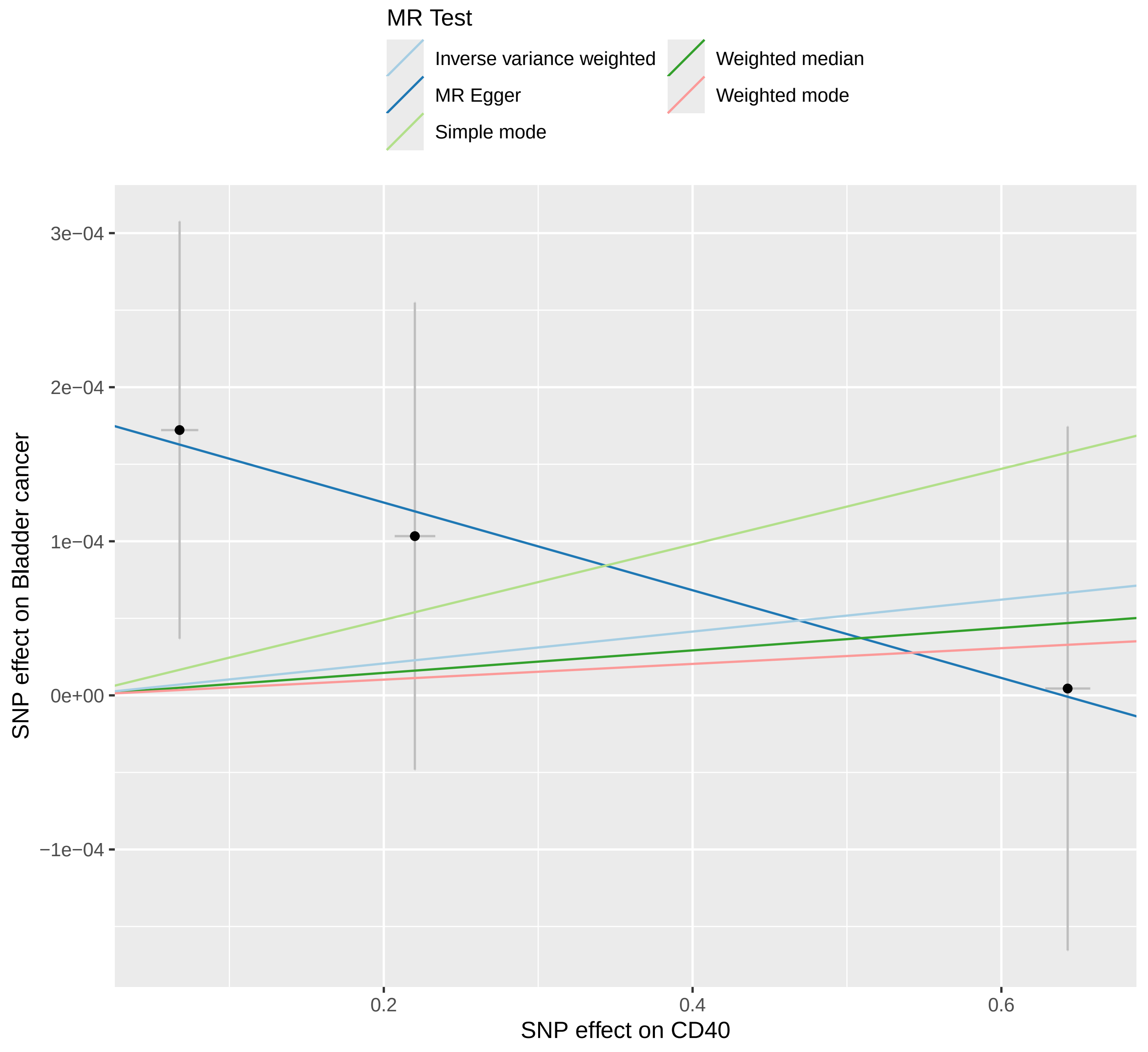
MR Test
Inverse variance weighted
MR Egger
Simple mode
Weighted median
Weighted mode
3e−04
2e−04
1e−04
0e+00
−1e−04
SNP effect on Bladder cancer
0.2
0.4
0.6
SNP effect on CD40

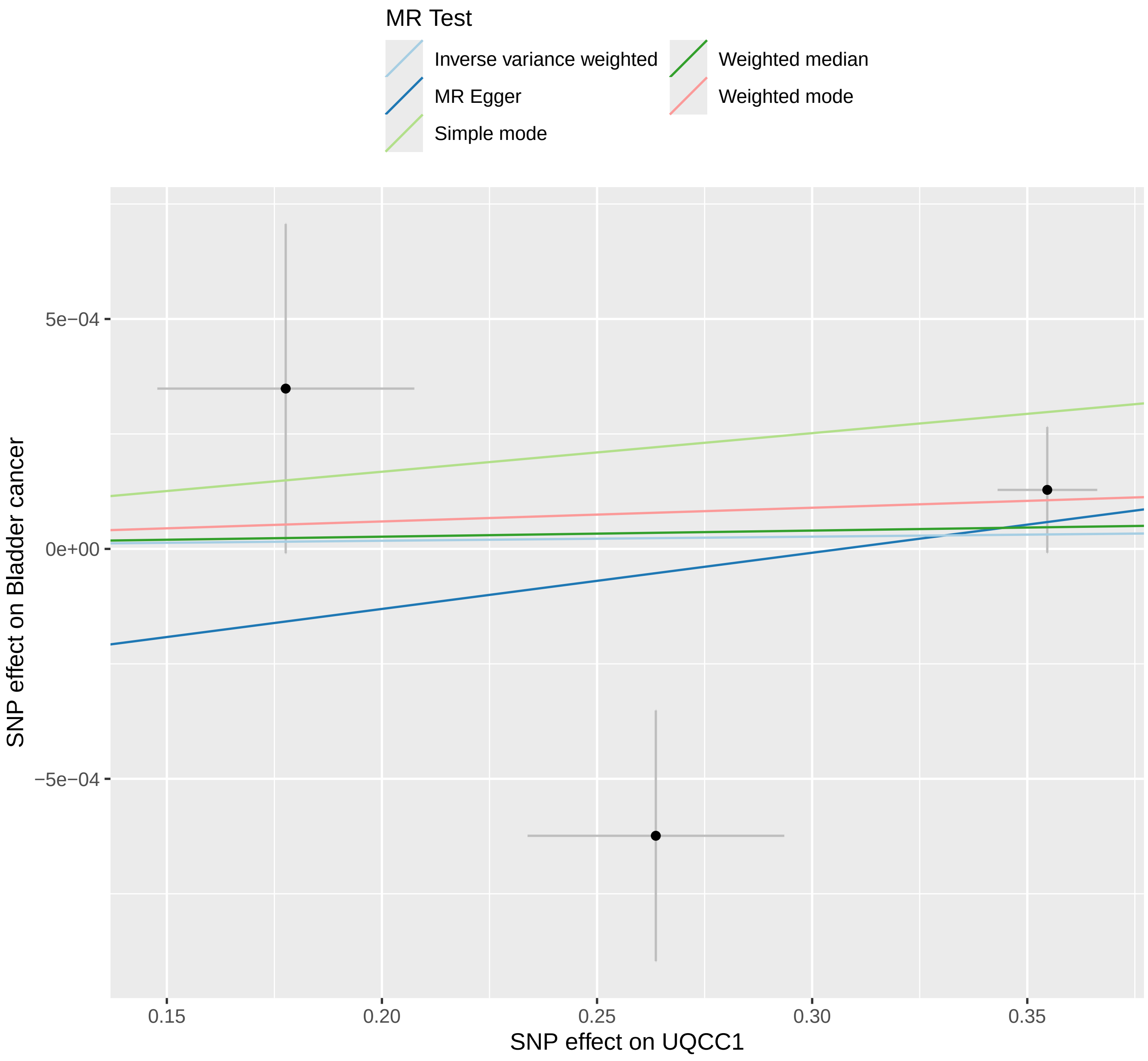
MR Test
Inverse variance weighted
MR Egger
Simple mode
Weighted median
Weighted mode
SNP effect on Bladder cancer
5e−04
0e+00
−5e−04
0.15
0.20
0.25
0.30
0.35
SNP effect on UQCC1

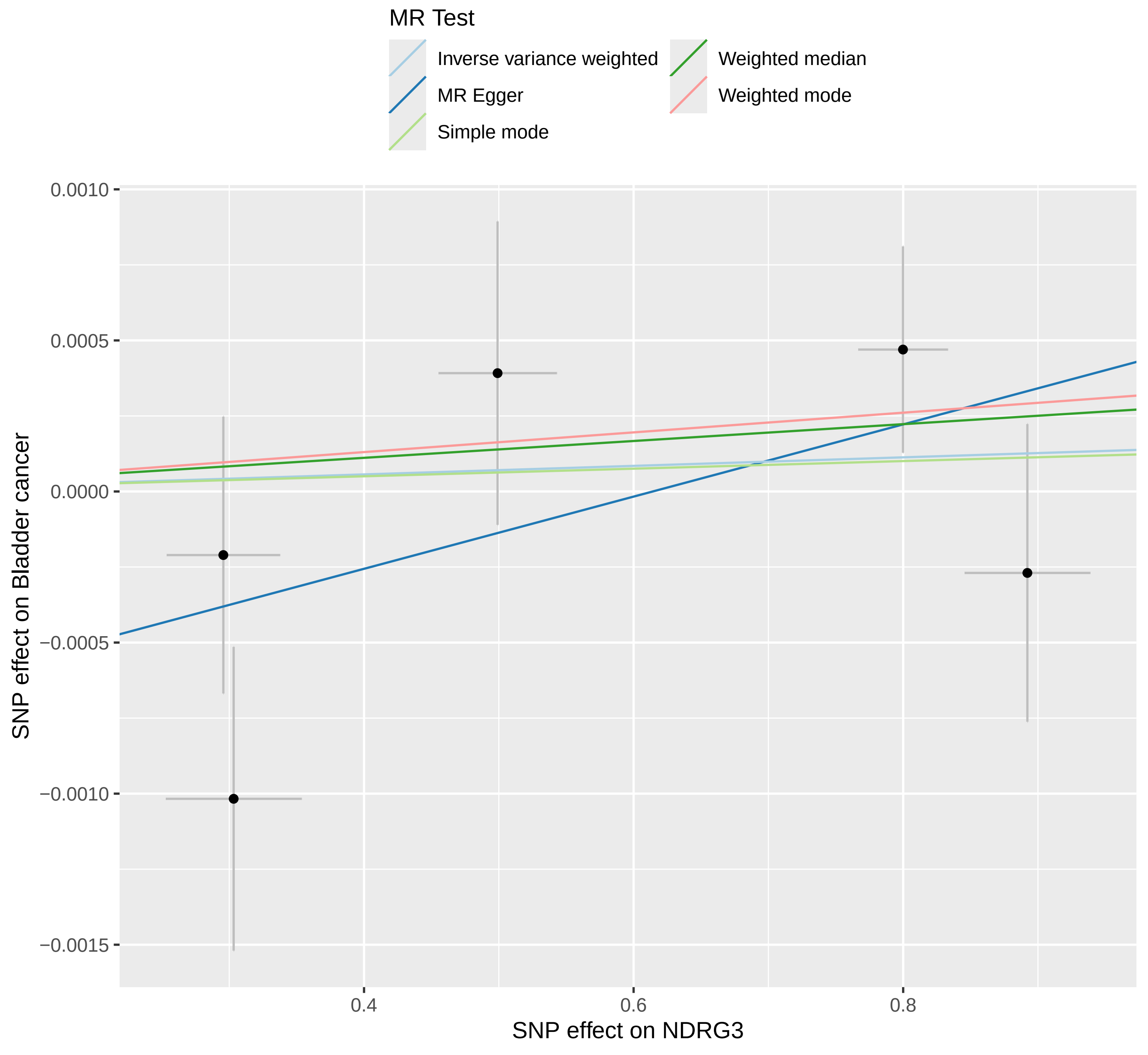
MR Test
Inverse variance weighted
MR Egger
Simple mode
Weighted median
Weighted mode
0.0010
0.0005
0.0000
−0.0005
−0.0010
−0.0015
SNP effect on Bladder cancer
0.4
0.6
0.8
SNP effect on NDRG3

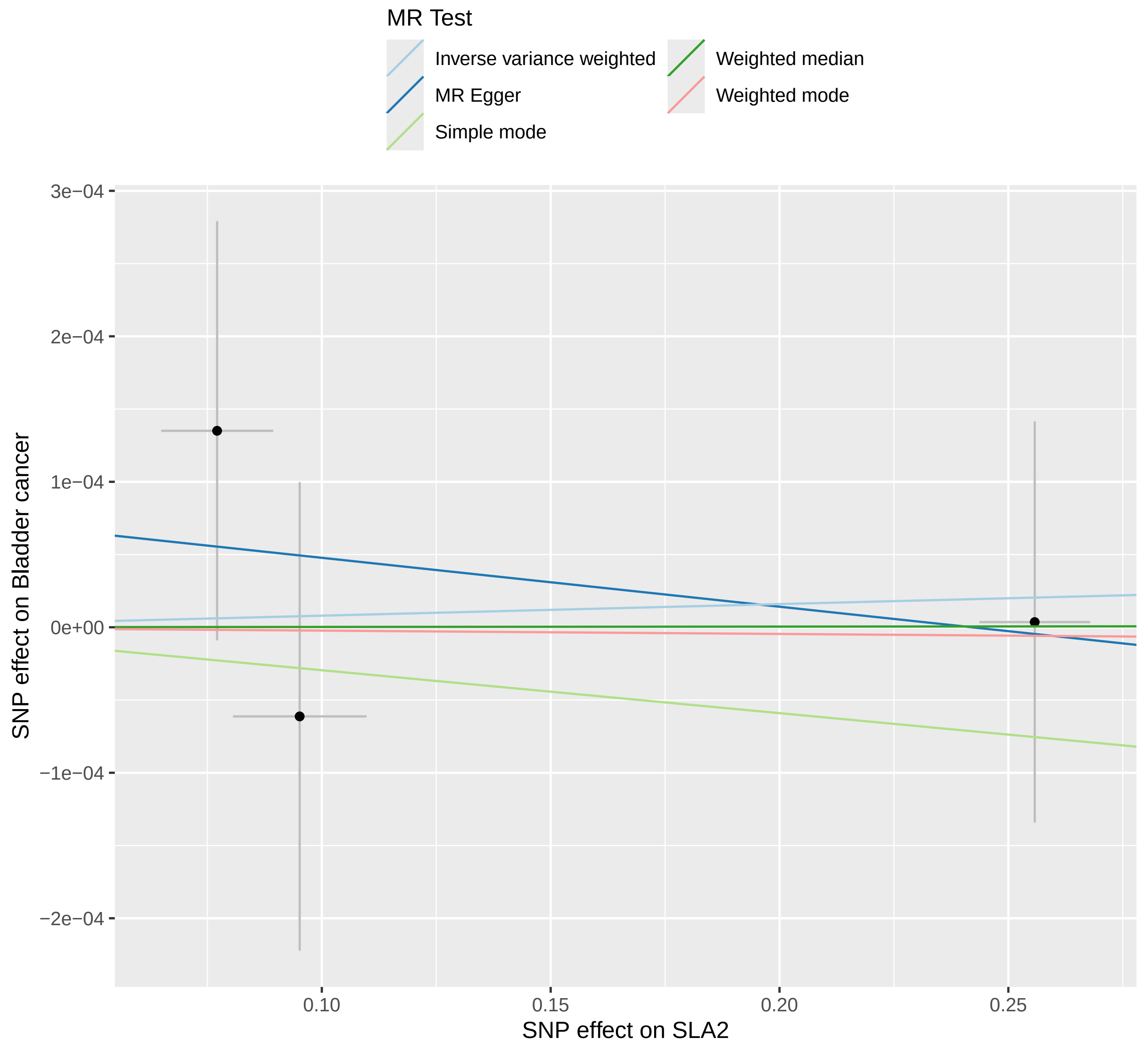
MR Test
Inverse variance weighted
MR Egger
Simple mode
Weighted median
Weighted mode
3e−04
2e−04
1e−04
0e+00
−1e−04
−2e−04
SNP effect on Bladder cancer
0.10
0.15
0.20
0.25
SNP effect on SLA2

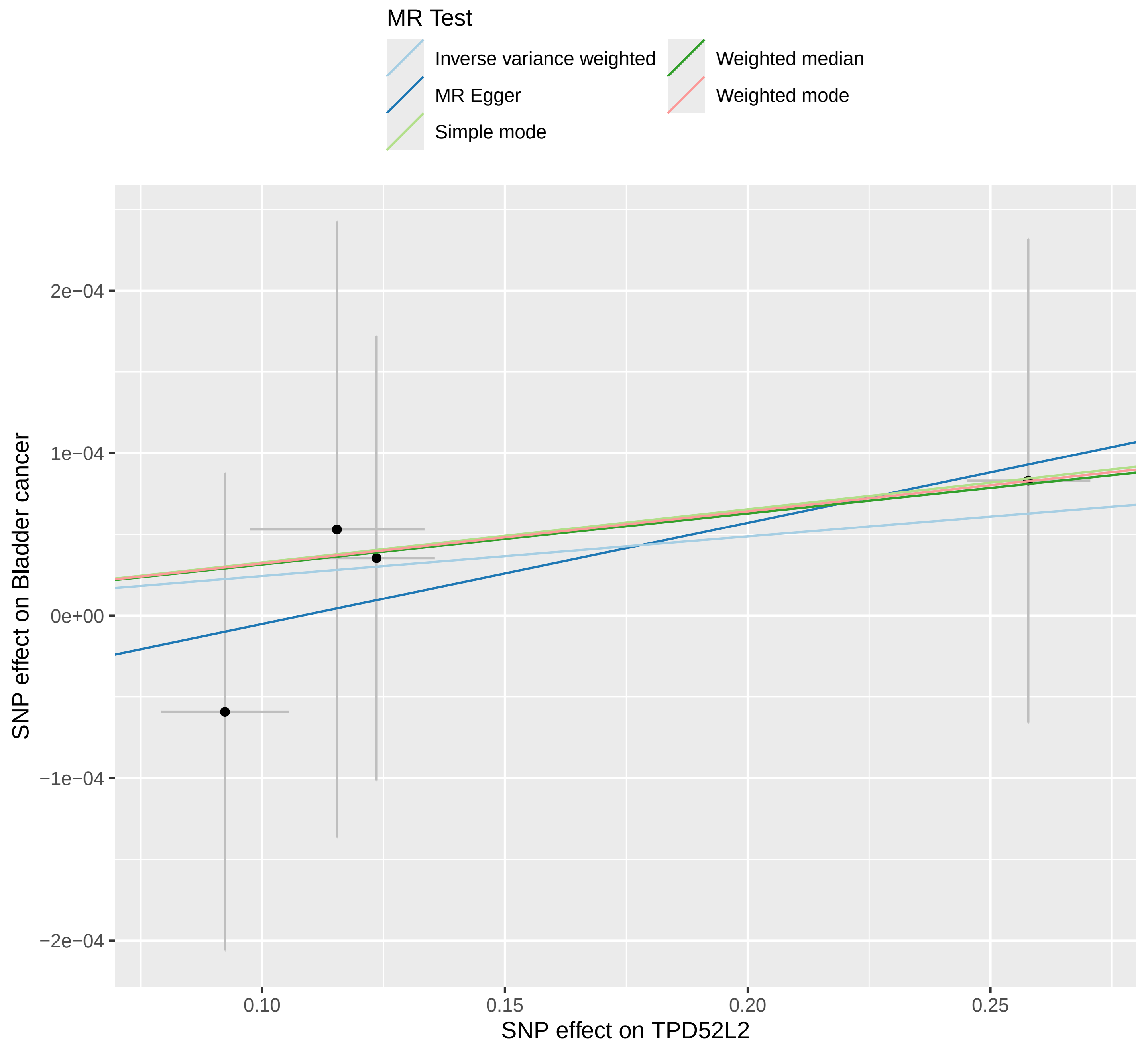

MR Test
Inverse variance weighted
MR Egger
Simple mode
Weighted median
Weighted mode
2e−04
1e−04
0e+00
−1e−04
−2e−04
SNP effect on Bladder cancer
0.10
0.15
0.20
0.25
SNP effect on TPD52L2

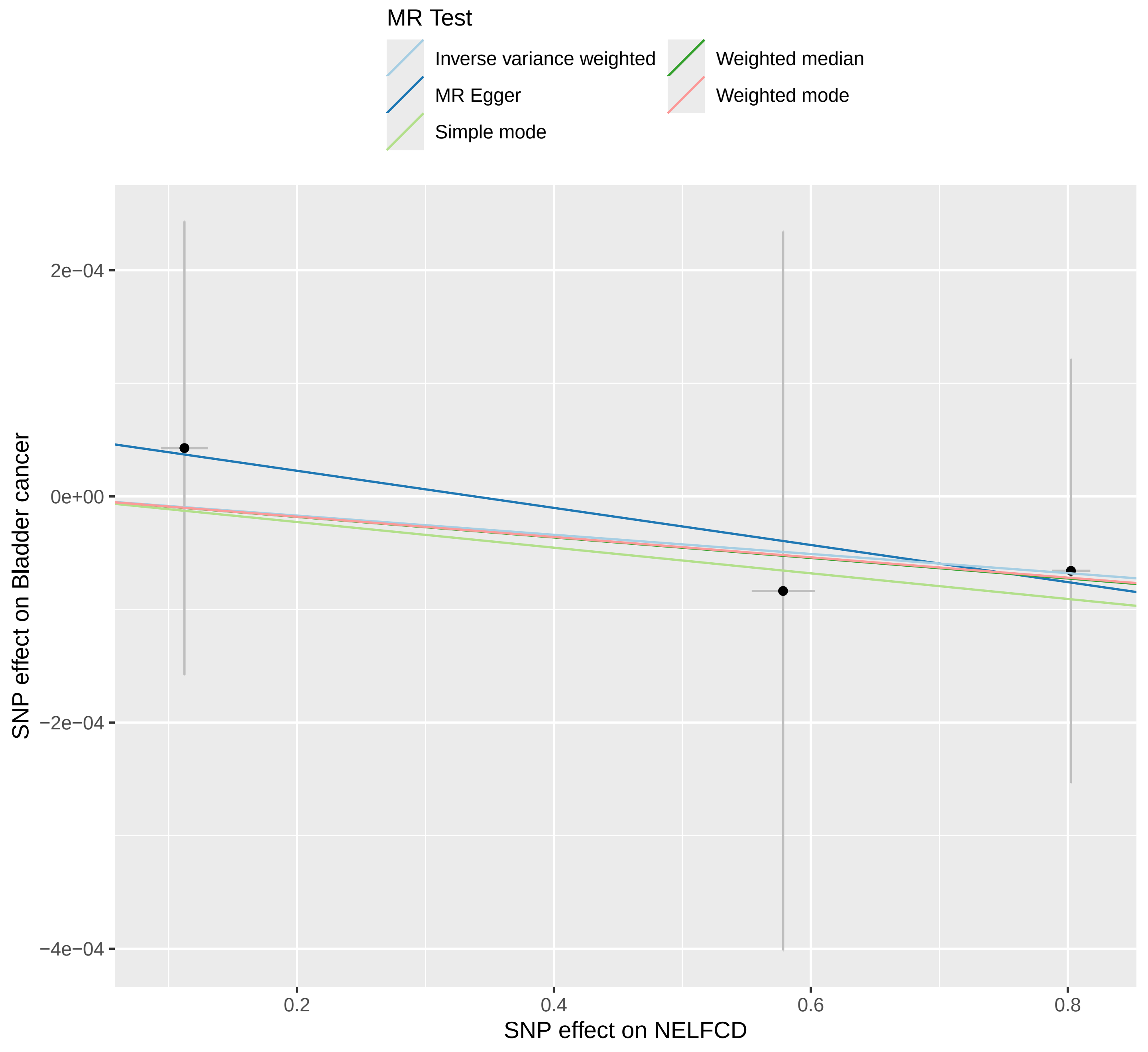
MR Test
Inverse variance weighted
MR Egger
Simple mode
Weighted median
Weighted mode
2e−04
0e+00
−2e−04
−4e−04
0.2
0.4
0.6
0.8
SNP effect on NELFCD
SNP effect on Bladder cancer

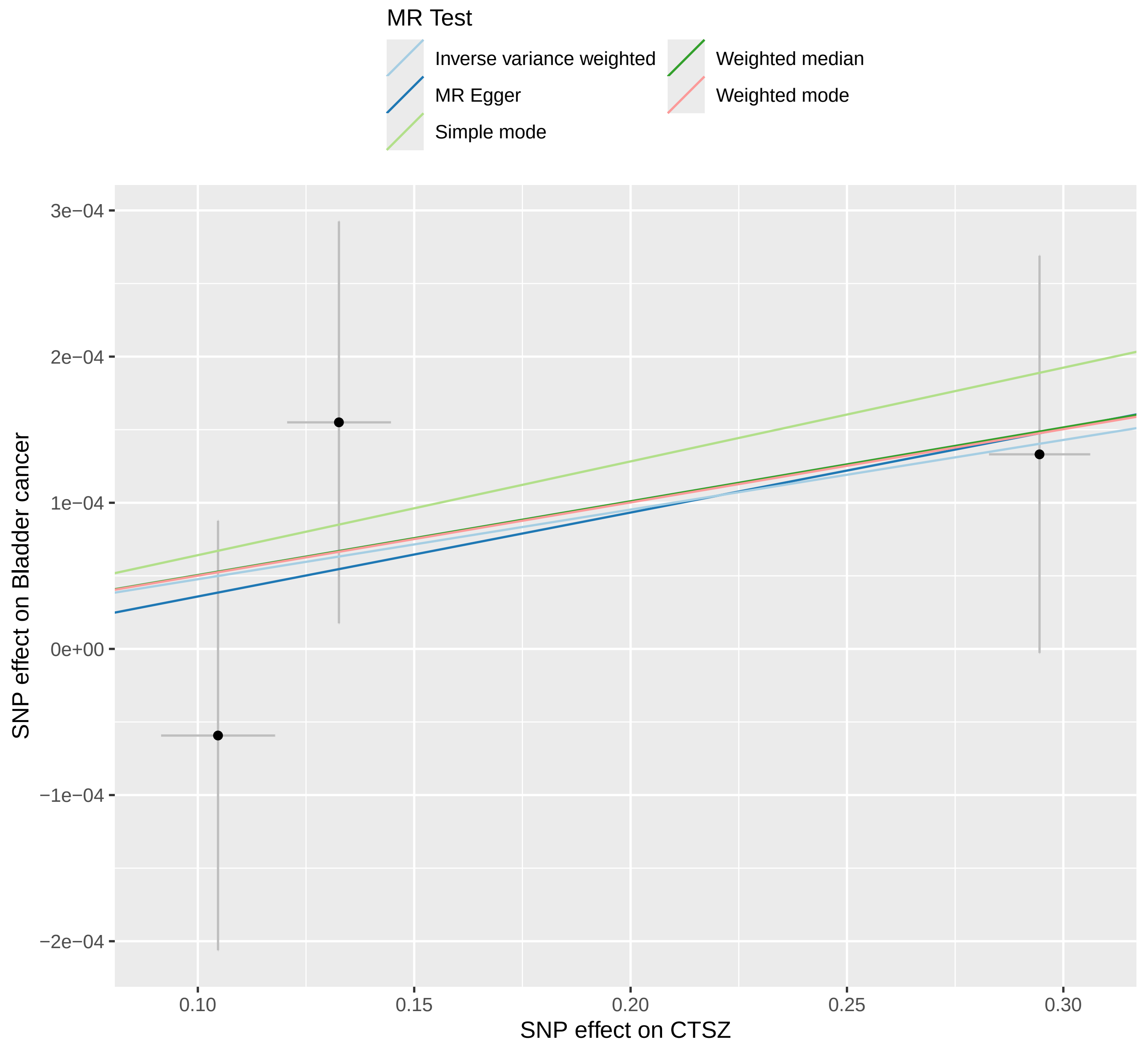
MR Test
Inverse variance weighted
MR Egger
Simple mode
Weighted median
Weighted mode
3e−04
2e−04
1e−04
0e+00
−1e−04
−2e−04
SNP effect on Bladder cancer
0.10
0.15
0.20
0.25
0.30
SNP effect on CTSZ

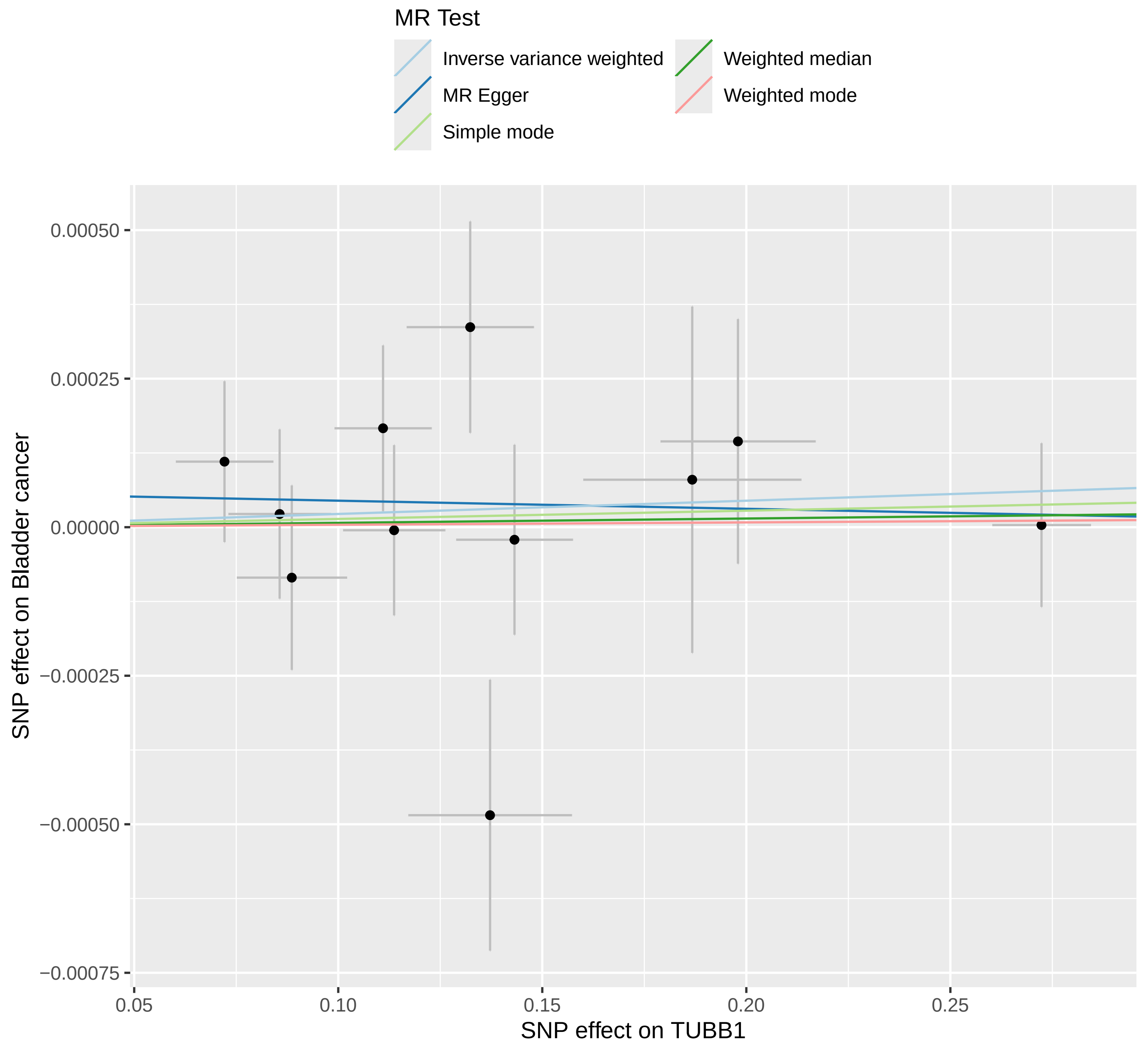

MR Test
Inverse variance weighted
MR Egger
Simple mode
Weighted median
Weighted mode
0.00050
0.00025
0.00000
−0.00025
−0.00050
−0.00075
0.05
0.10
0.15
0.20
0.25
SNP effect on Bladder cancer
SNP effect on TUBB1

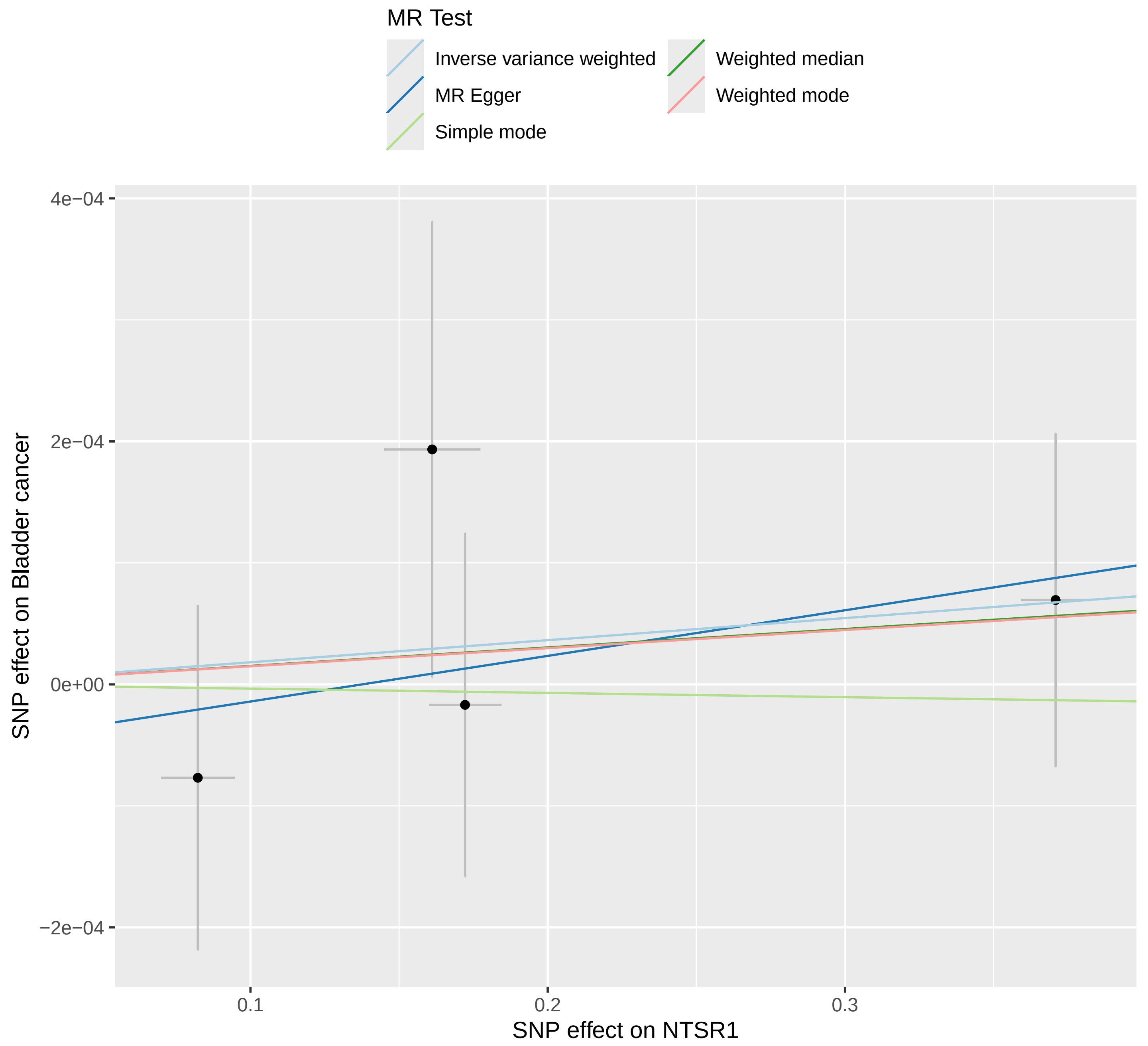

MR Test
Inverse variance weighted
MR Egger
Simple mode
Weighted median
Weighted mode
4e−04
2e−04
0e+00
−2e−04
SNP effect on Bladder cancer
0.1
0.2
0.3
SNP effect on NTSR1

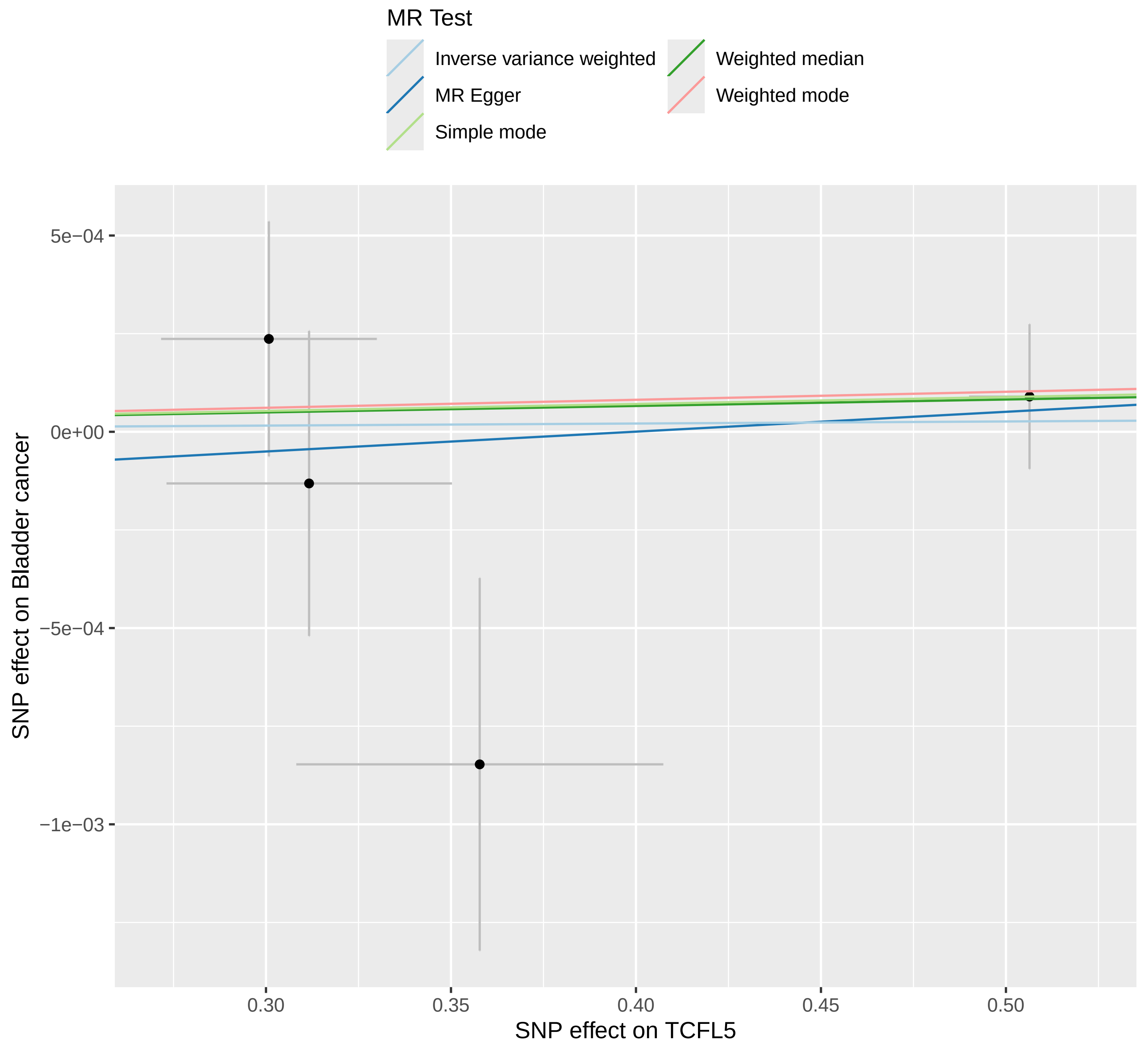

MR Test
Inverse variance weighted
MR Egger
Simple mode
Weighted median
Weighted mode
5e−04
0e+00
−5e−04
−1e−03
0.30
0.35
0.40
0.45
0.50
SNP effect on TCFL5
SNP effect on Bladder cancer

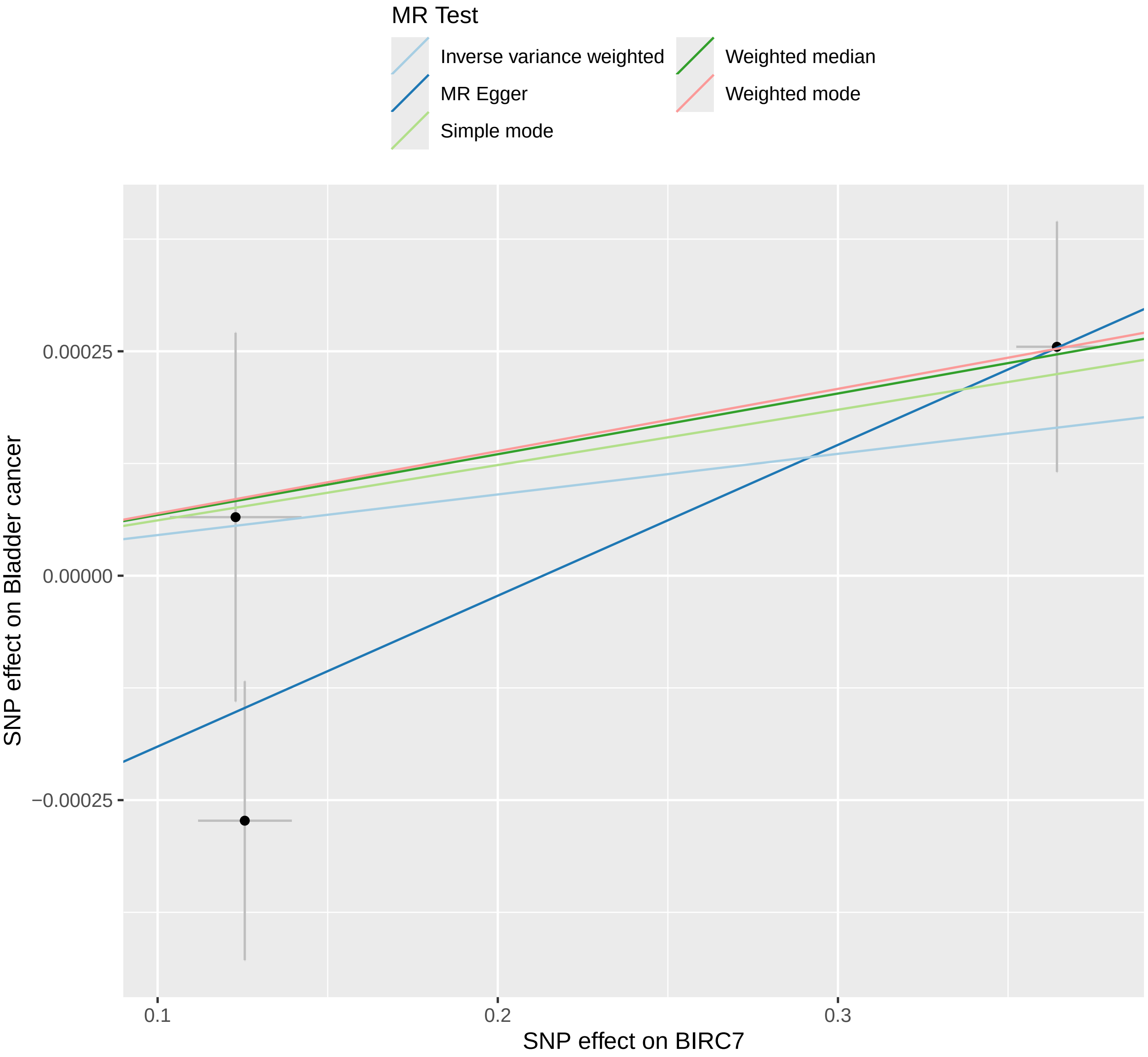
MR Test
Inverse variance weighted
MR Egger
Simple mode
Weighted median
Weighted mode
0.00025
0.00000
−0.00025
0.1
0.2
0.3
SNP effect on Bladder cancer
SNP effect on BIRC7

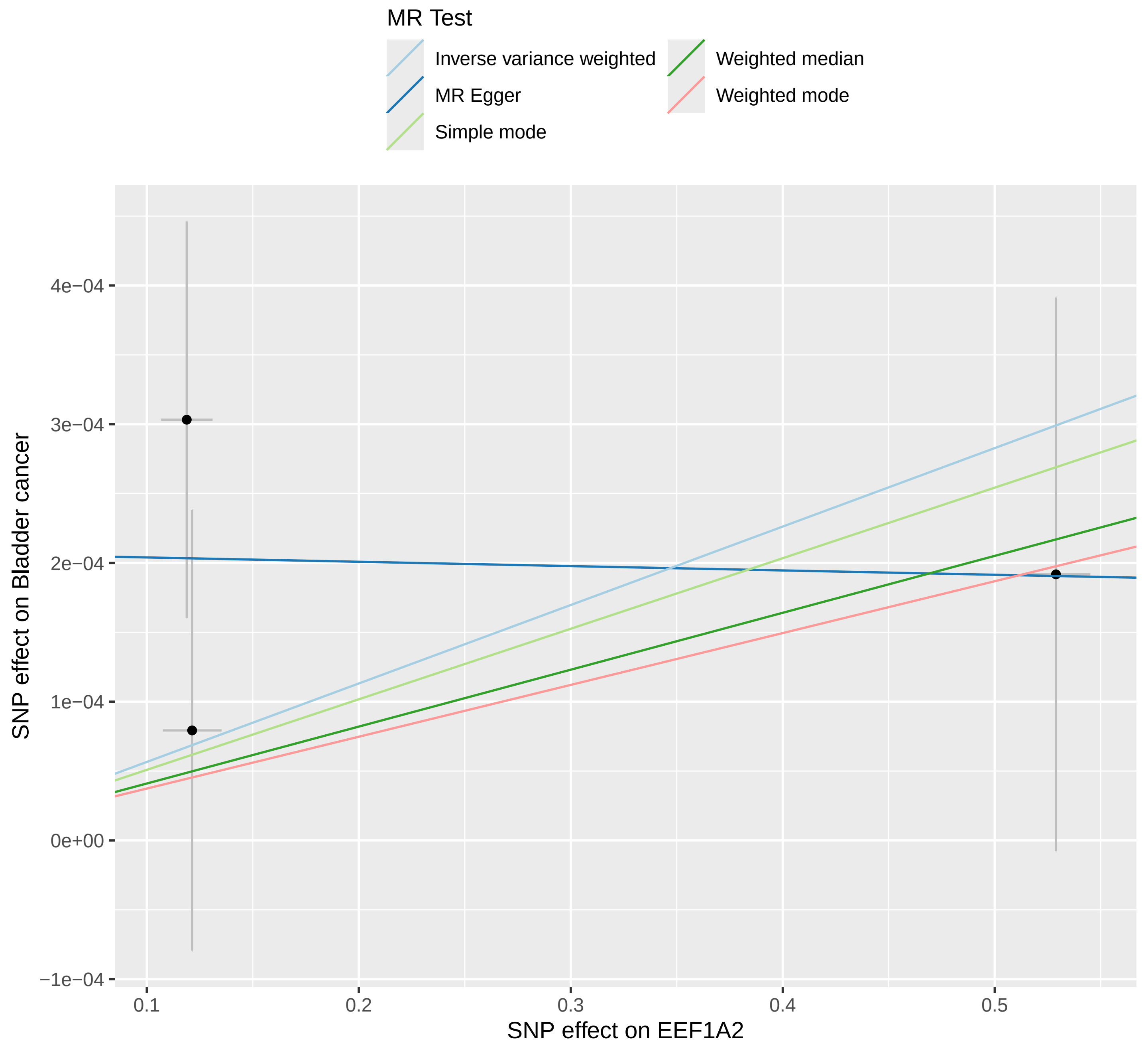

MR Test
Inverse variance weighted
MR Egger
Simple mode
Weighted median
Weighted mode
4e−04
3e−04
2e−04
1e−04
0e+00
−1e−04
0.1
0.2
0.3
0.4
0.5
SNP effect on Bladder cancer
SNP effect on EEF1A2

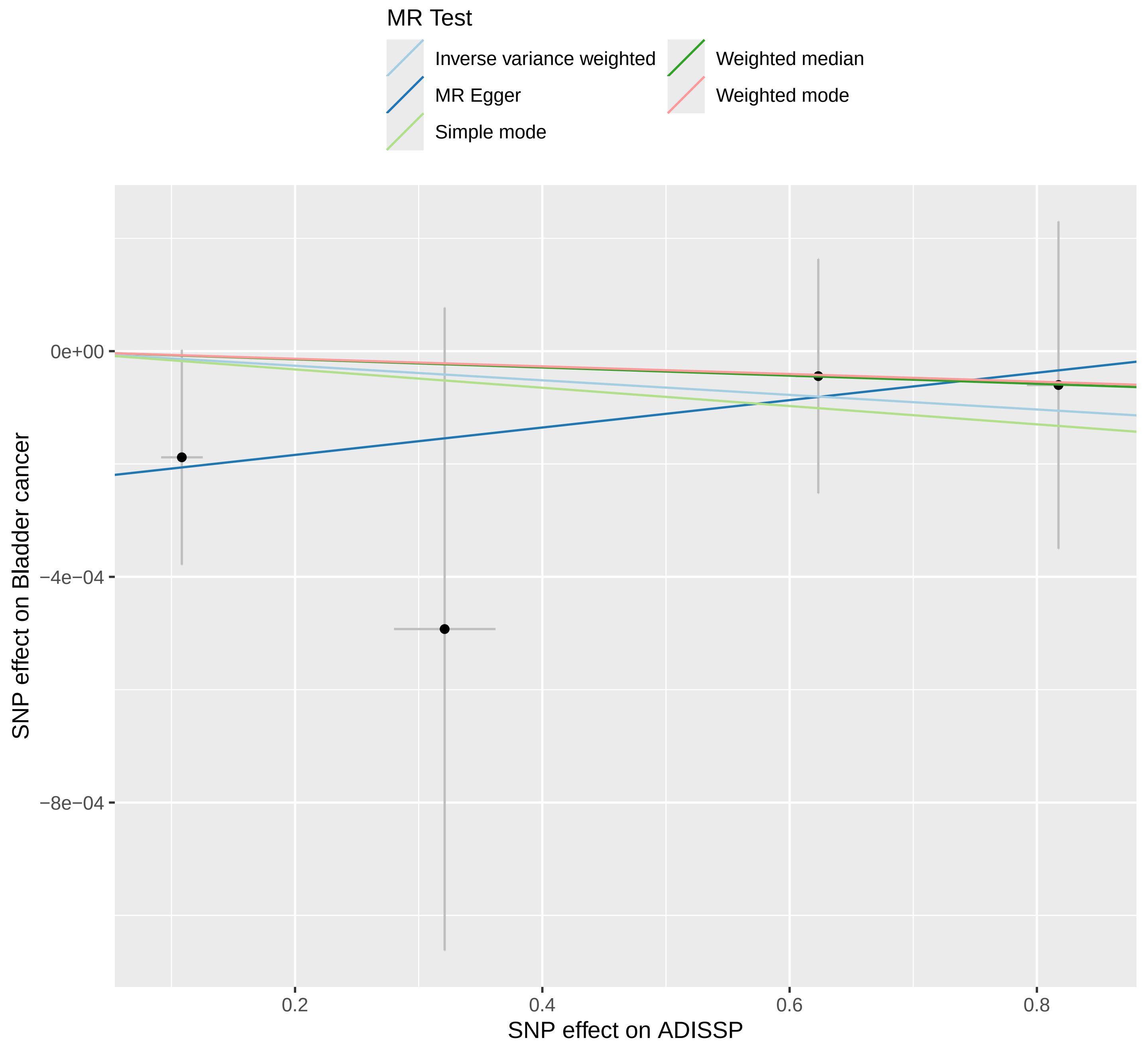

MR Test
Inverse variance weighted
MR Egger
Simple mode
Weighted median
Weighted mode
0e+00
−4e−04
−8e−04
SNP effect on Bladder cancer
0.2
0.4
0.6
0.8
SNP effect on ADISSP

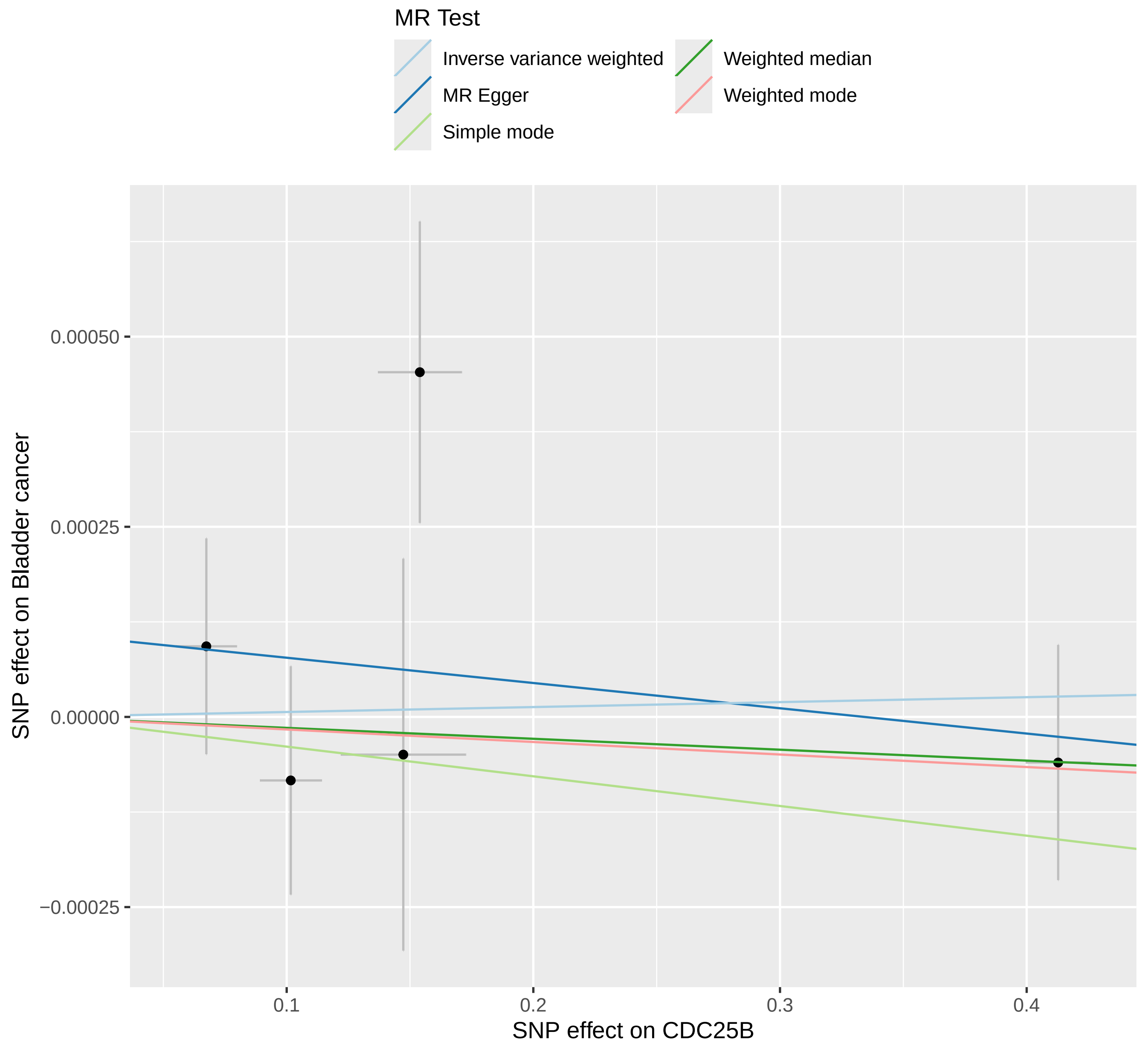

MR Test
Inverse variance weighted
MR Egger
Simple mode
Weighted median
Weighted mode
0.00050
0.00025
0.00000
−0.00025
SNP effect on Bladder cancer
0.1
0.2
0.3
0.4
SNP effect on CDC25B

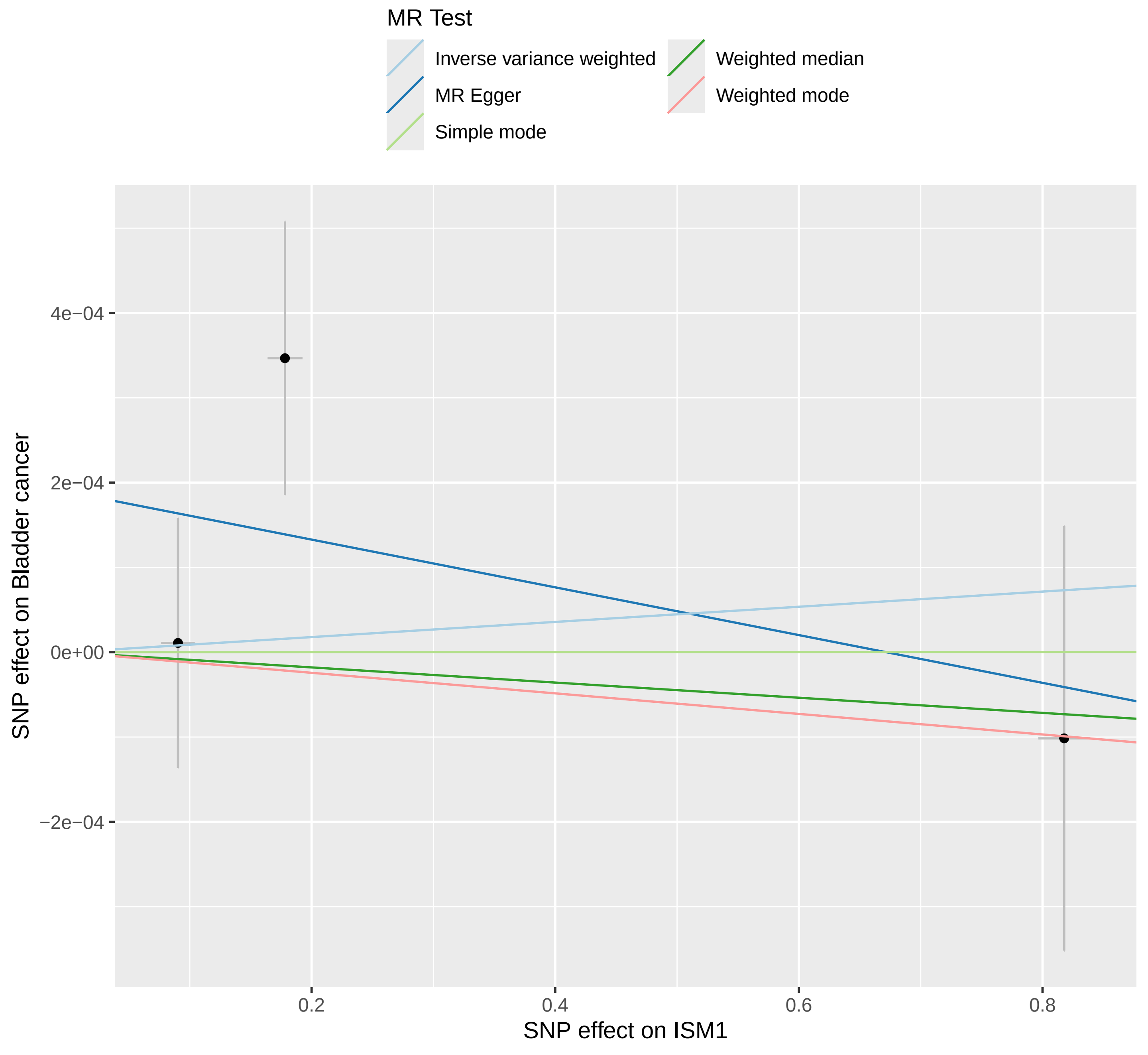
MR Test
Inverse variance weighted
MR Egger
Simple mode
Weighted median
Weighted mode
4e−04
2e−04
0e+00
−2e−04
SNP effect on Bladder cancer
0.2
0.4
0.6
0.8
SNP effect on ISM1

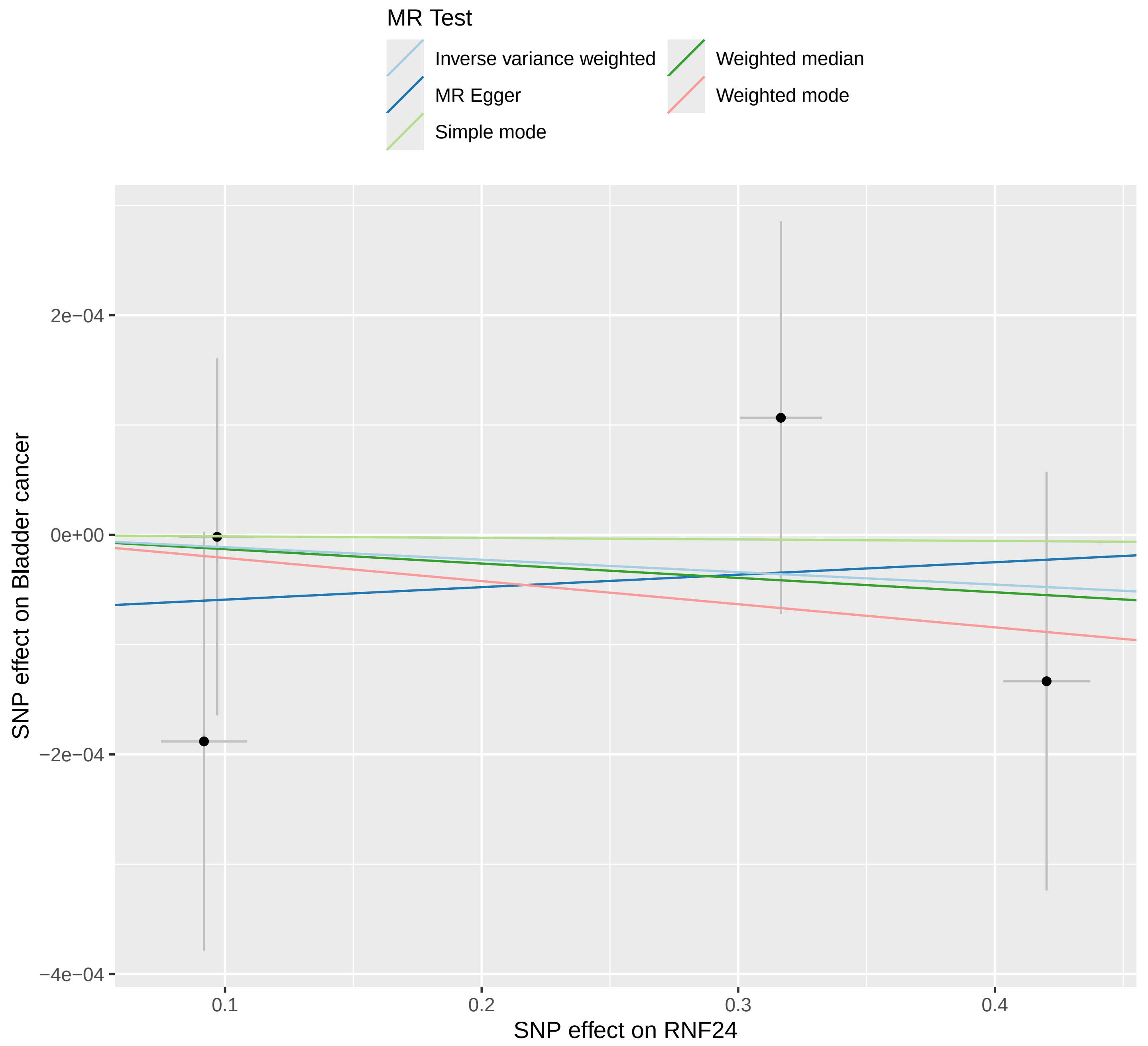

MR Test
Inverse variance weighted
MR Egger
Simple mode
Weighted median
Weighted mode
2e−04
0e+00
−2e−04
−4e−04
SNP effect on Bladder cancer
0.1
0.2
0.3
0.4
SNP effect on RNF24

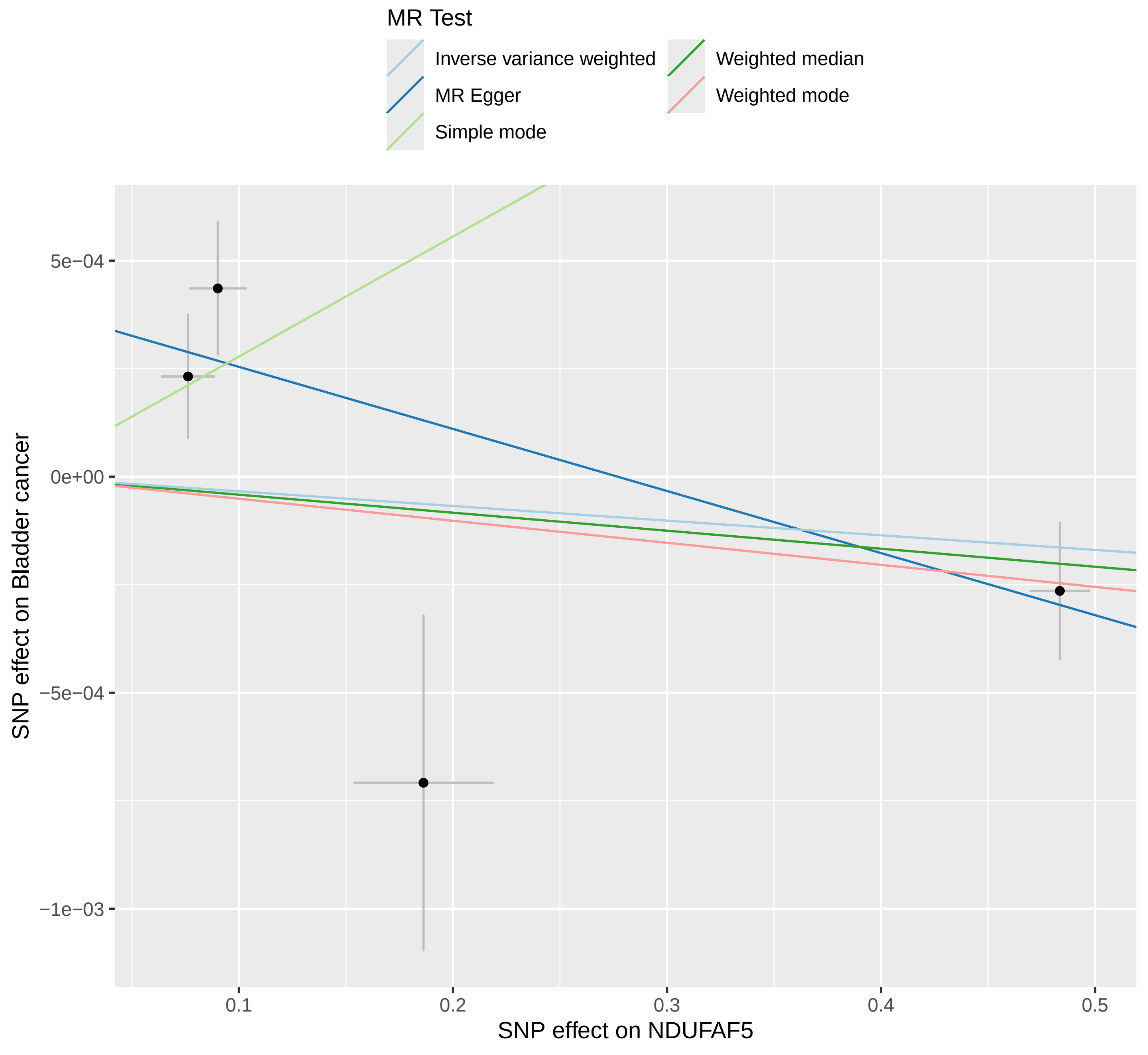

MR Test
Inverse variance weighted
MR Egger
Simple mode
Weighted median
Weighted mode
5e−04
0e+00
−5e−04
−1e−03
0.1
0.2
0.3
0.4
0.5
SNP effect on Bladder cancer
SNP effect on NDUFAF5

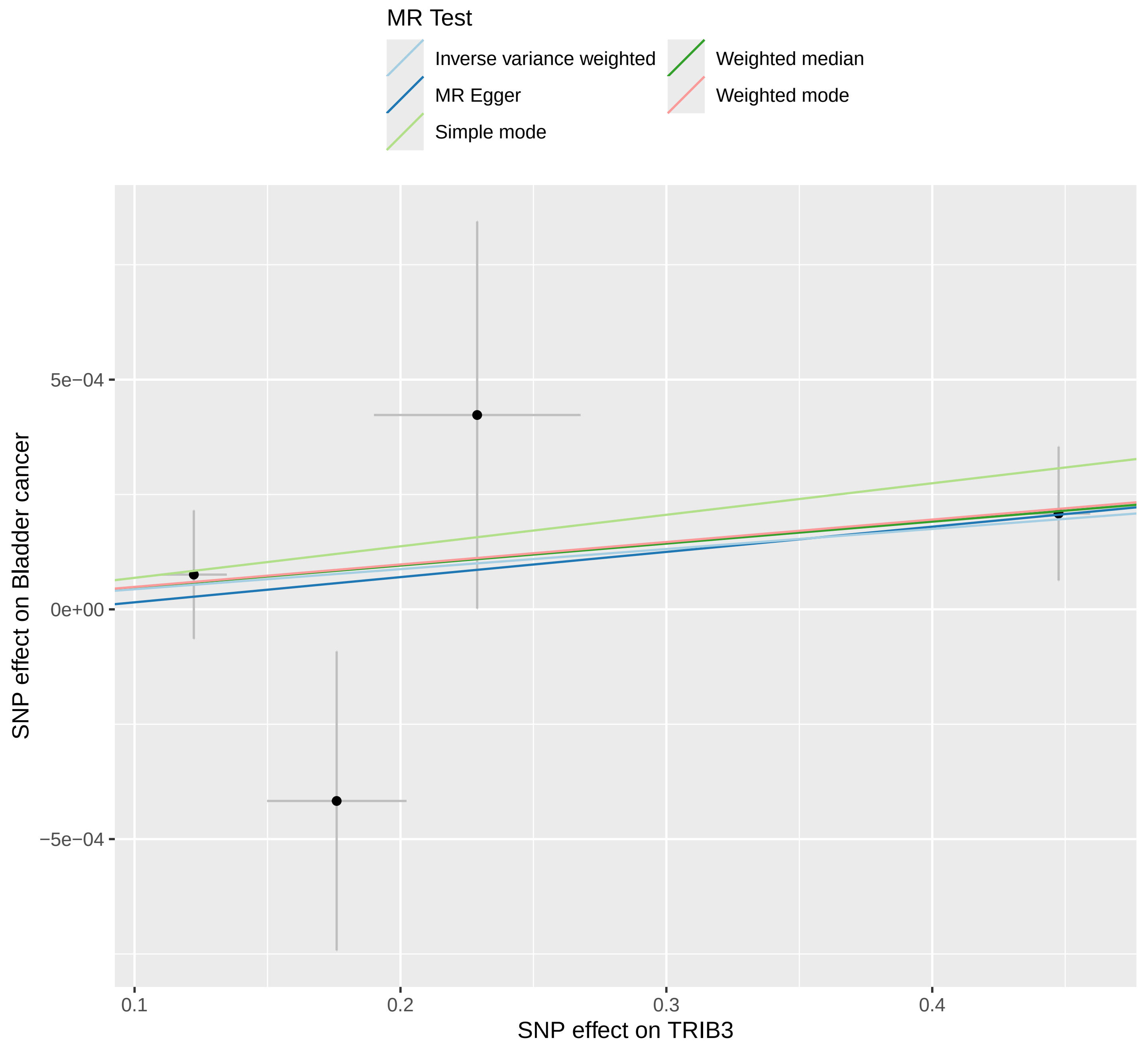

MR Test
Inverse variance weighted
MR Egger
Simple mode
Weighted median
Weighted mode
5e−04
0e+00
−5e−04
SNP effect on Bladder cancer
0.1
0.2
0.3
0.4
SNP effect on TRIB3

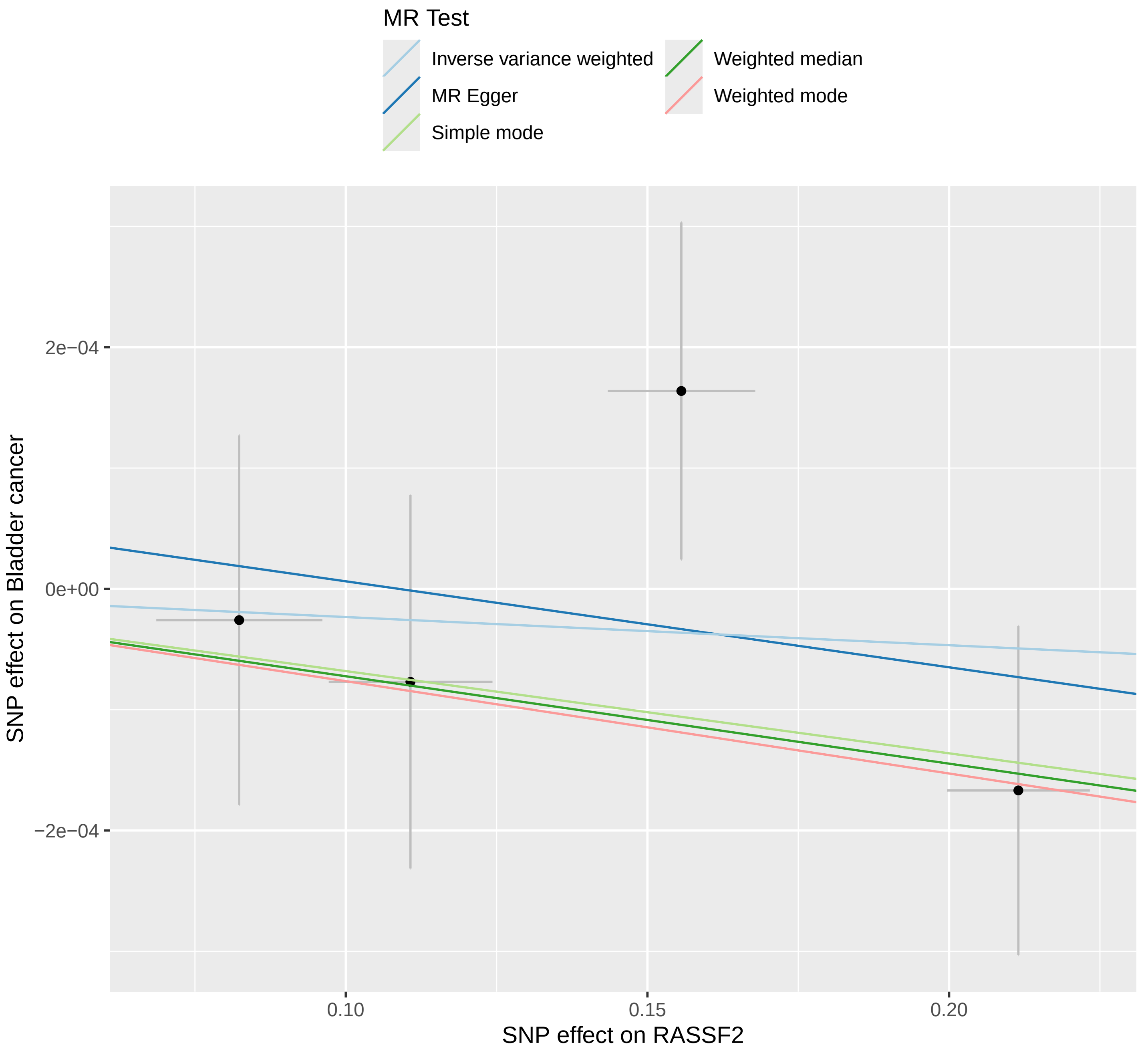

MR Test
Inverse variance weighted
MR Egger
Simple mode
Weighted median
Weighted mode
2e−04
0e+00
−2e−04
SNP effect on Bladder cancer
0.10
0.15
0.20
SNP effect on RASSF2

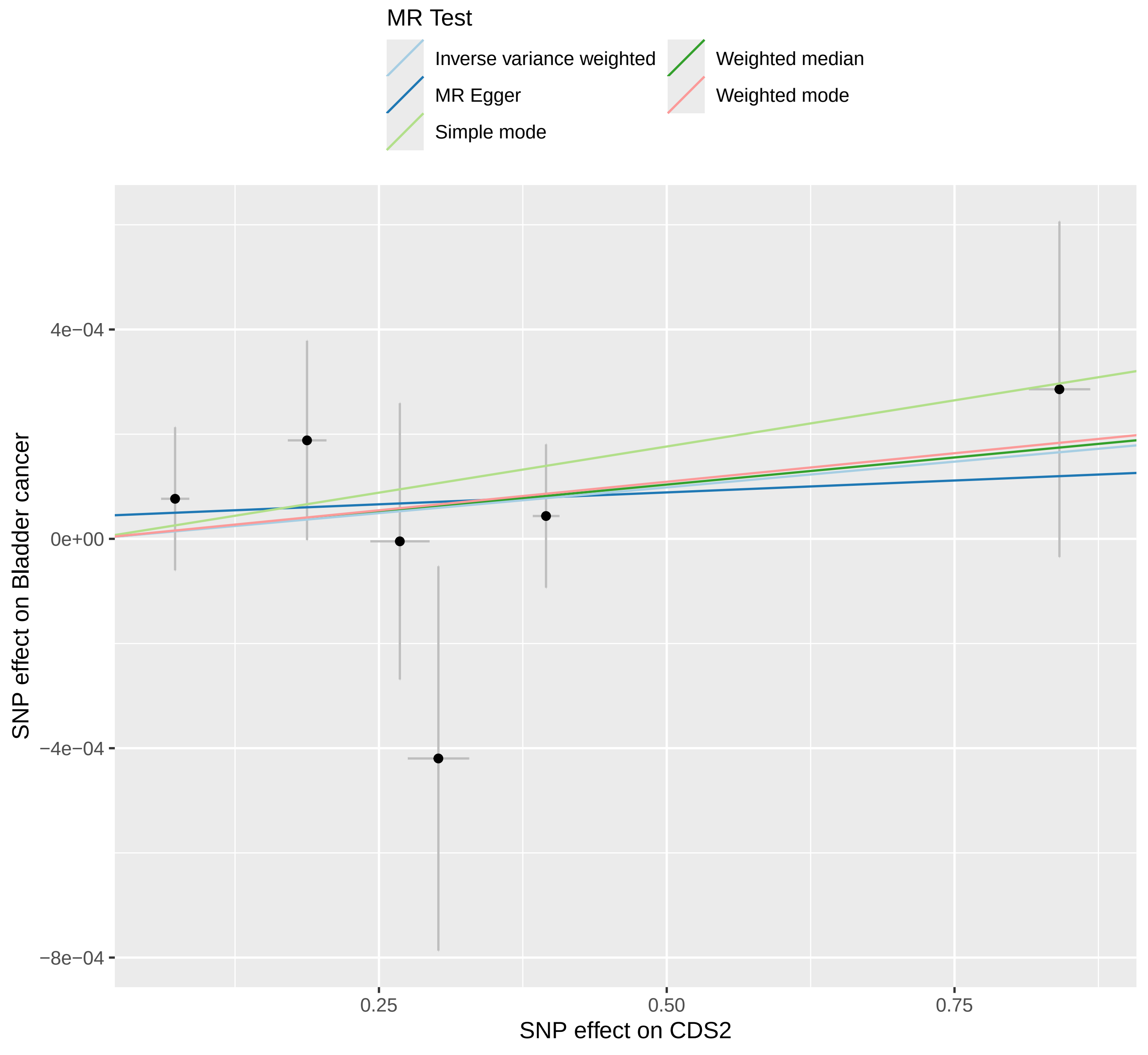

MR Test
Inverse variance weighted
MR Egger
Simple mode
Weighted median
Weighted mode
4e−04
0e+00
−4e−04
−8e−04
SNP effect on Bladder cancer
0.25
0.50
0.75
SNP effect on CDS2

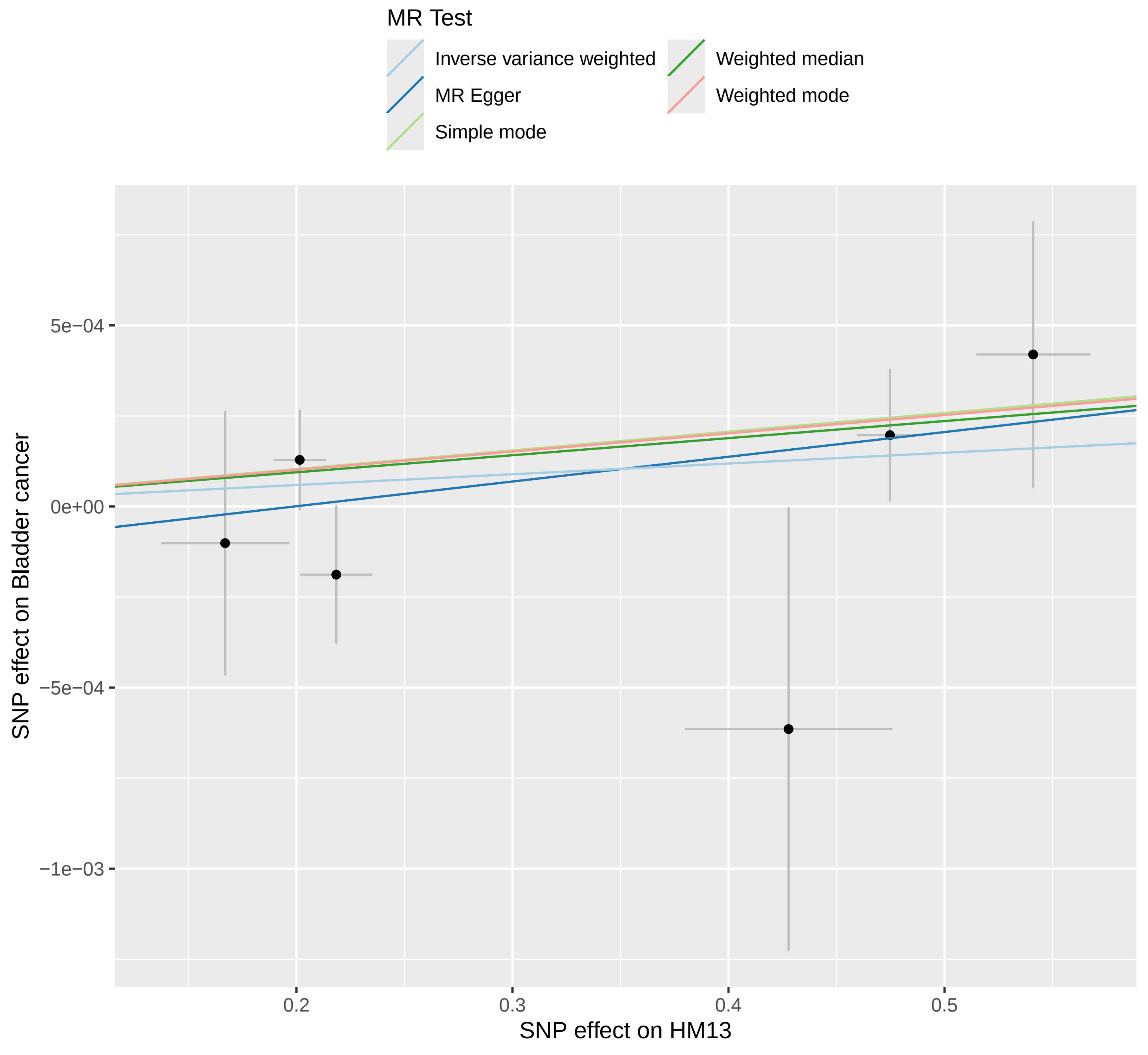

MR Test
Inverse variance weighted
MR Egger
Simple mode
Weighted median
Weighted mode
5e−04
0e+00
−5e−04
−1e−03
0.2
0.3
0.4
0.5
SNP effect on HM13
SNP effect on Bladder cancer

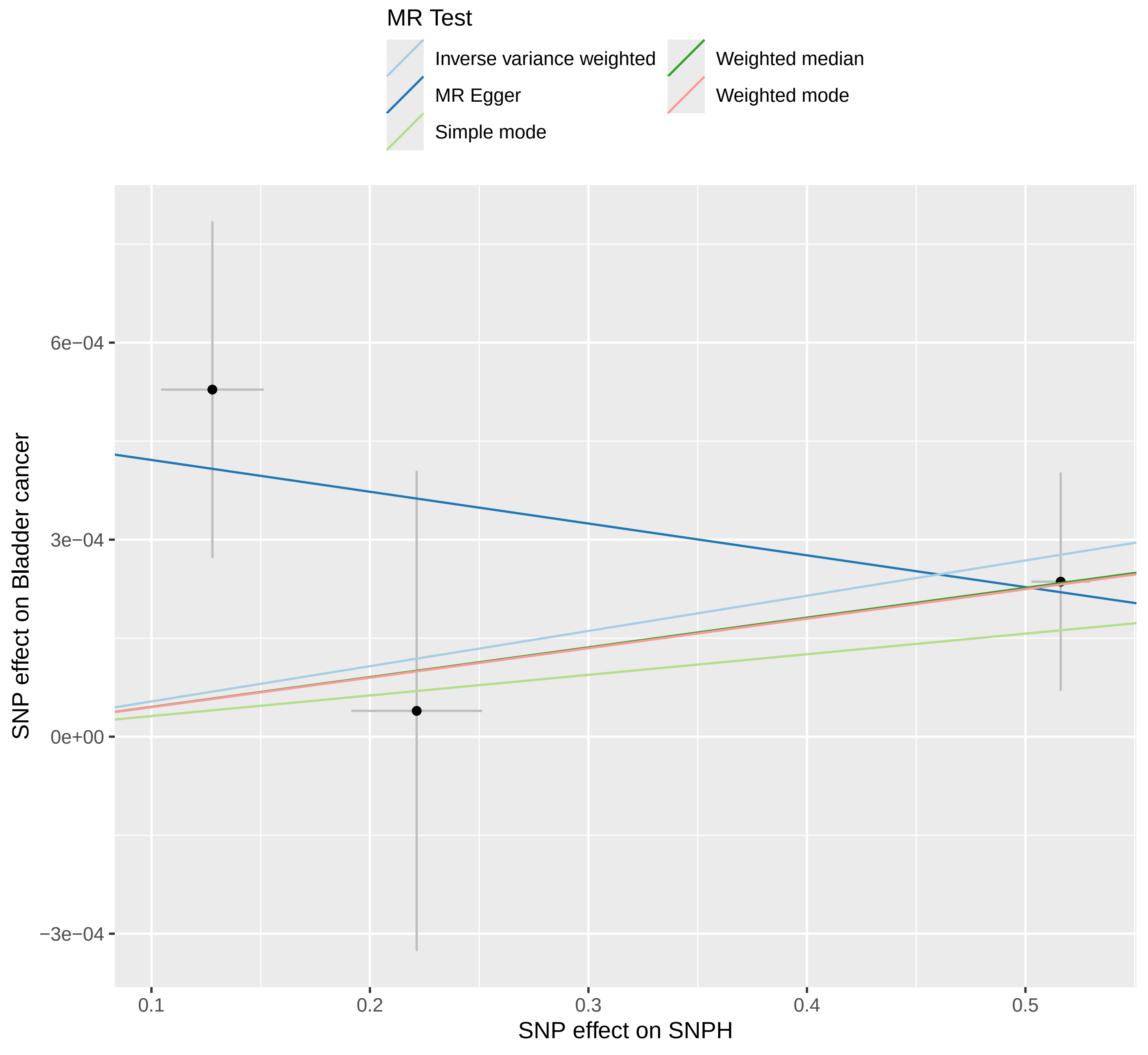

MR Test
Inverse variance weighted
MR Egger
Simple mode
Weighted median
Weighted mode
6e−04
3e−04
0e+00
−3e−04
SNP effect on Bladder cancer
0.1
0.2
0.3
0.4
0.5
SNP effect on SNPH

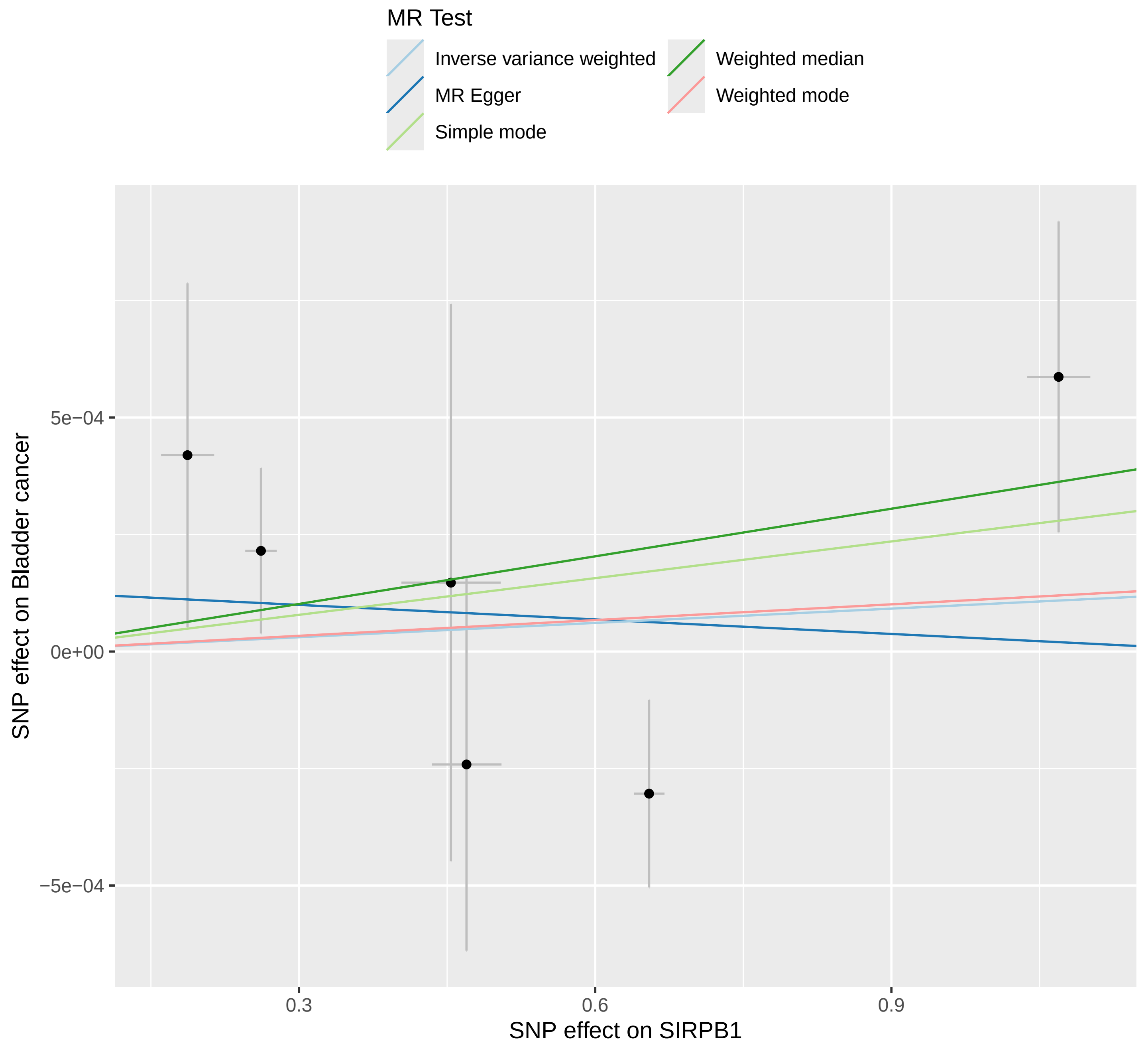

MR Test
Inverse variance weighted
MR Egger
Simple mode
Weighted median
Weighted mode
5e−04
0e+00
−5e−04
SNP effect on Bladder cancer
0.3
0.6
0.9
SNP effect on SIRPB1

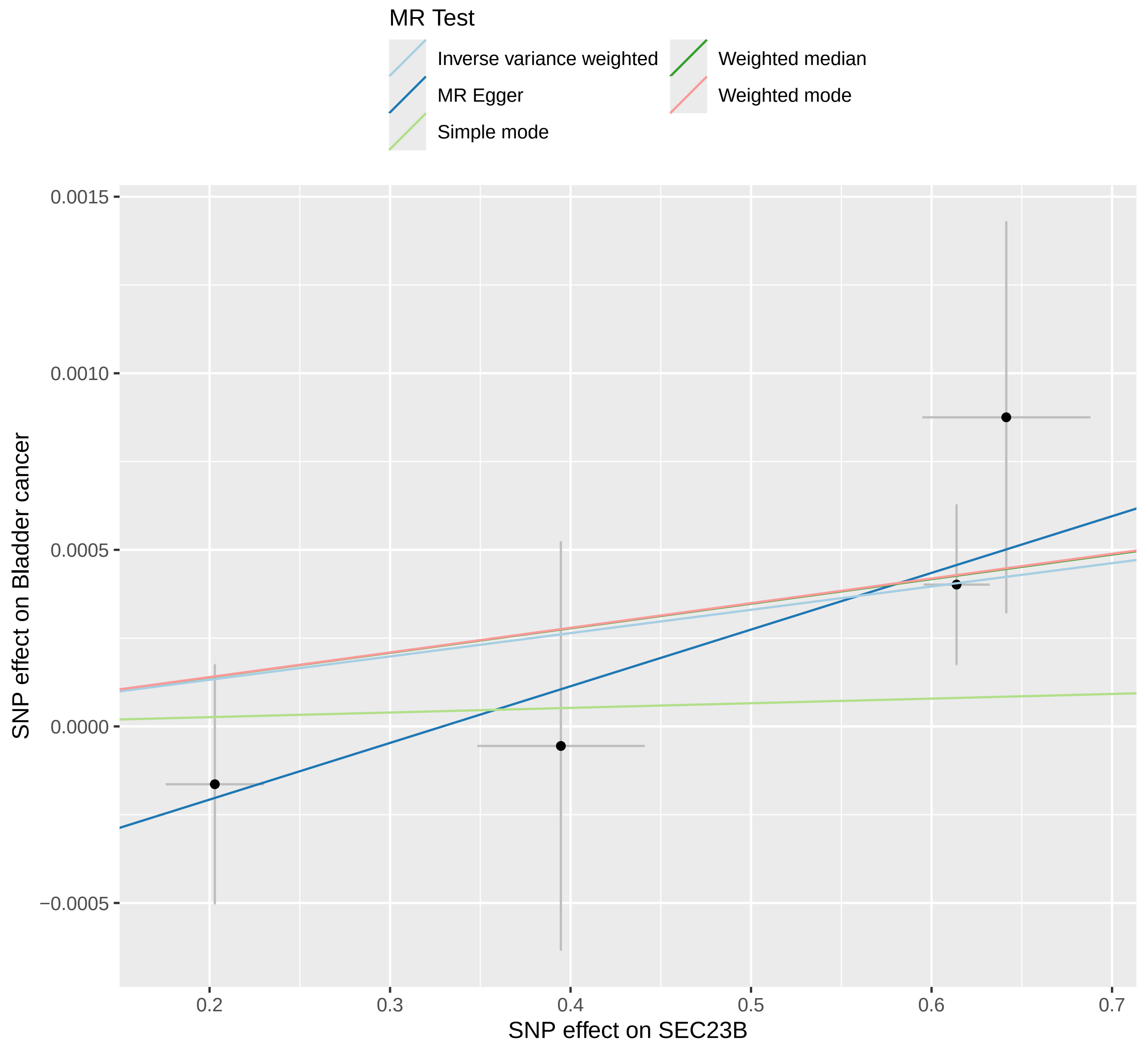
MR Test
Inverse variance weighted
MR Egger
Simple mode
Weighted median
Weighted mode
0.0015
0.0010
0.0005
0.0000
−0.0005
SNP effect on Bladder cancer
0.2
0.3
0.4
0.5
0.6
0.7
SNP effect on SEC23B

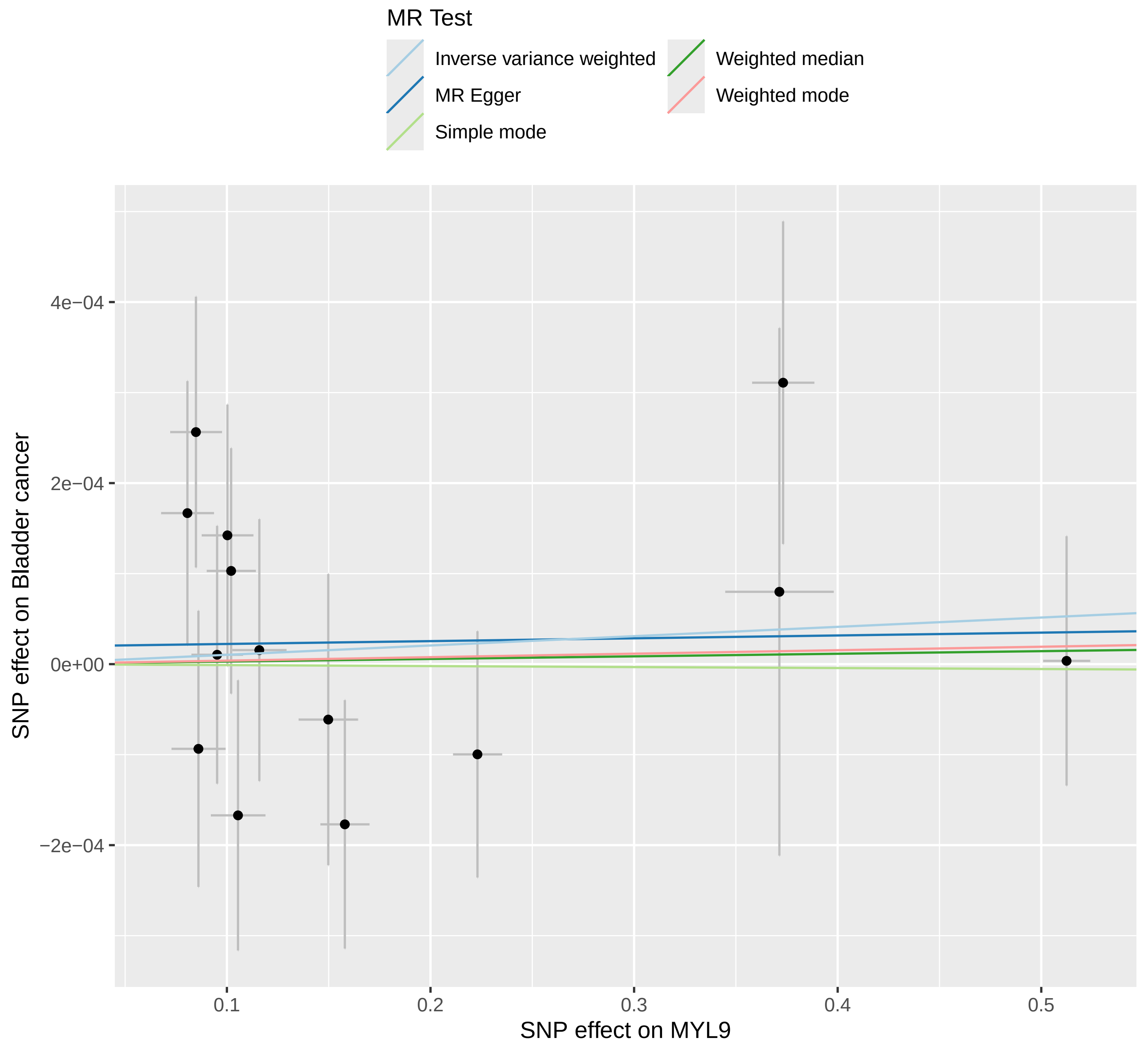
MR Test
Inverse variance weighted
MR Egger
Simple mode
Weighted median
Weighted mode
4e−04
2e−04
0e+00
−2e−04
0.1
0.2
0.3
0.4
0.5
SNP effect on MYL9
SNP effect on Bladder cancer

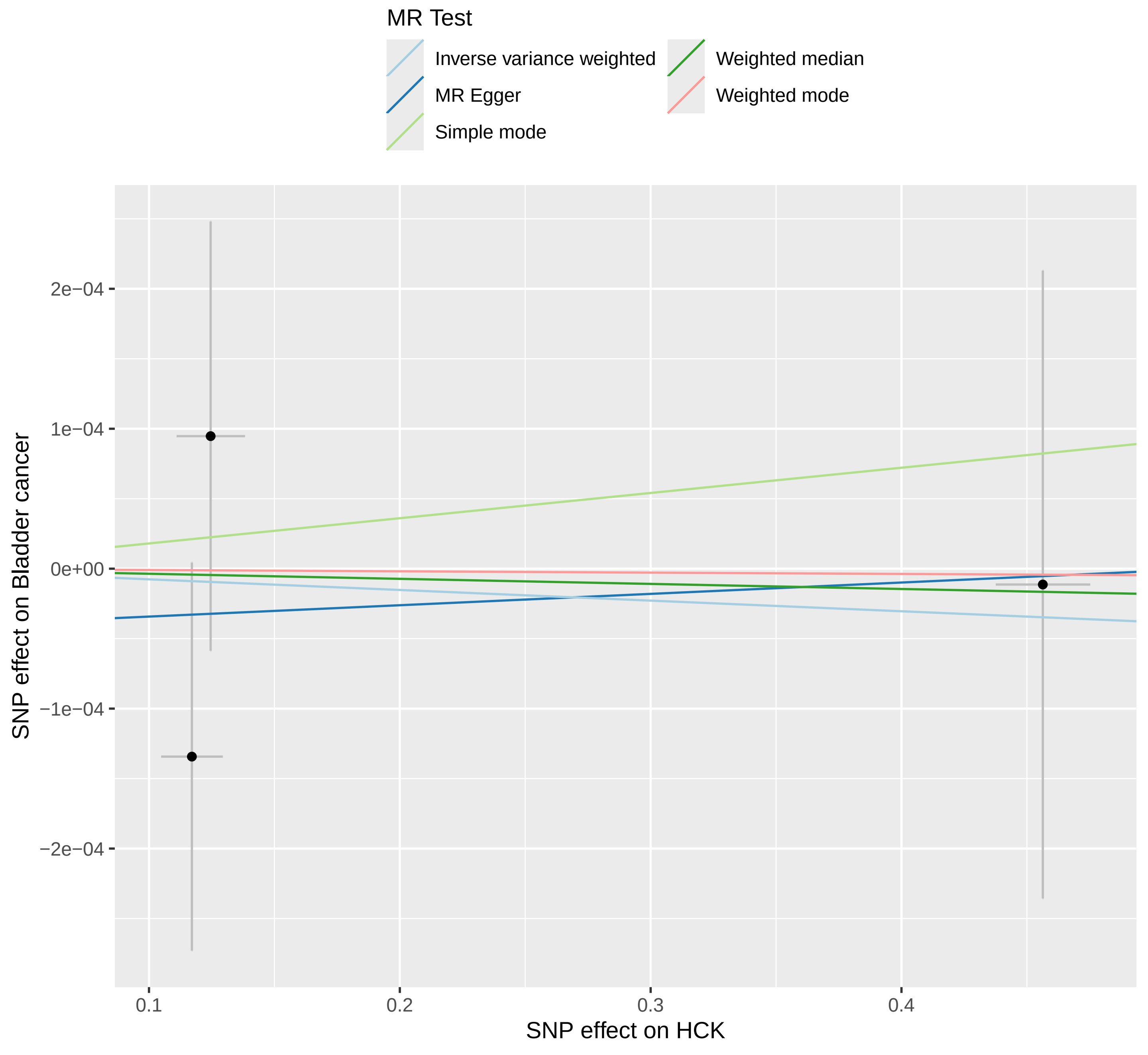
MR Test
Inverse variance weighted
MR Egger
Simple mode
Weighted median
Weighted mode
2e−04
1e−04
0e+00
−1e−04
−2e−04
SNP effect on Bladder cancer
0.1
0.2
0.3
0.4
SNP effect on HCK

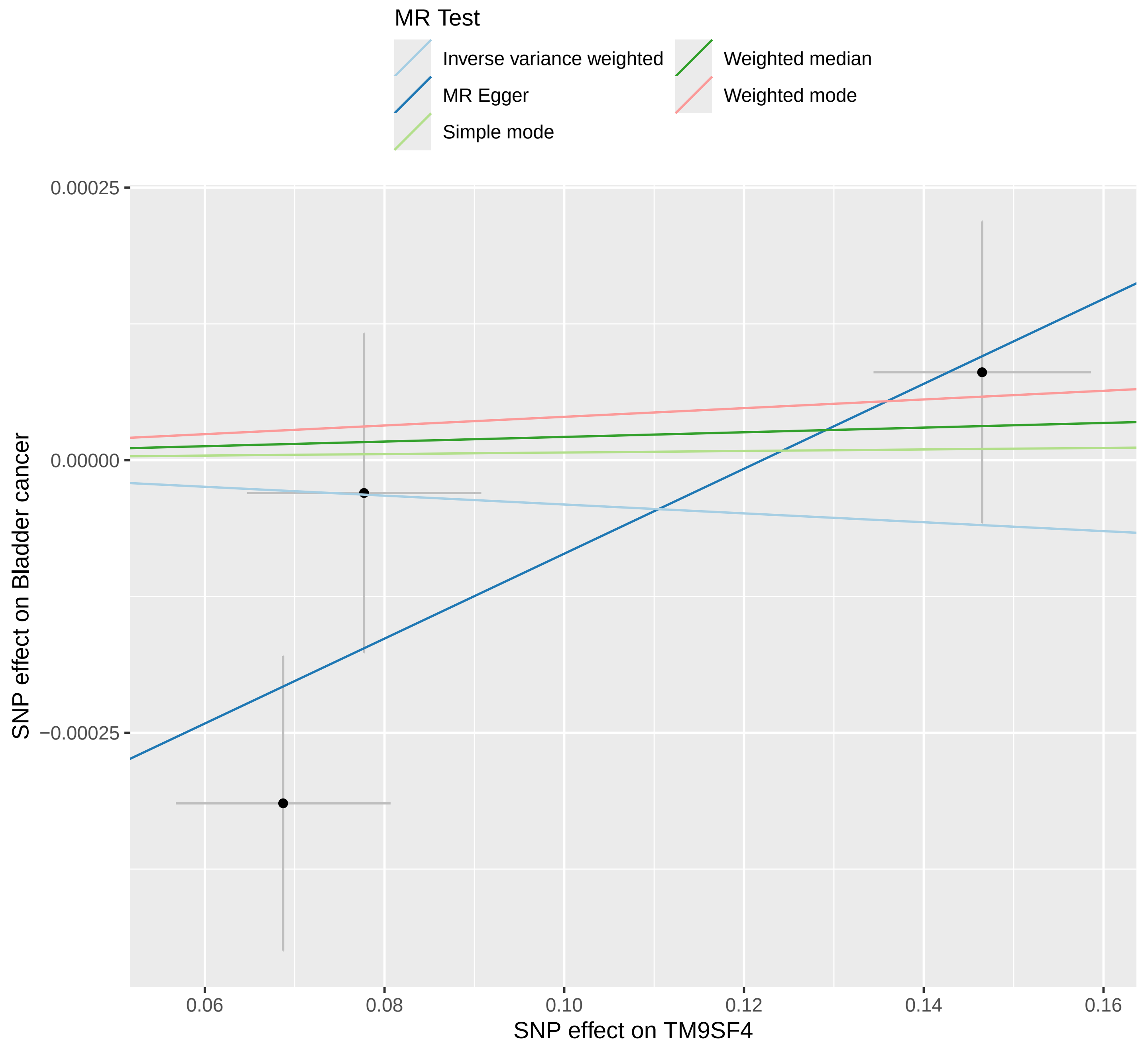

MR Test
Inverse variance weighted
MR Egger
Simple mode
Weighted median
Weighted mode
0.00025
0.00000
−0.00025
SNP effect on Bladder cancer
0.06
0.08
0.10
0.12
0.14
0.16
SNP effect on TM9SF4

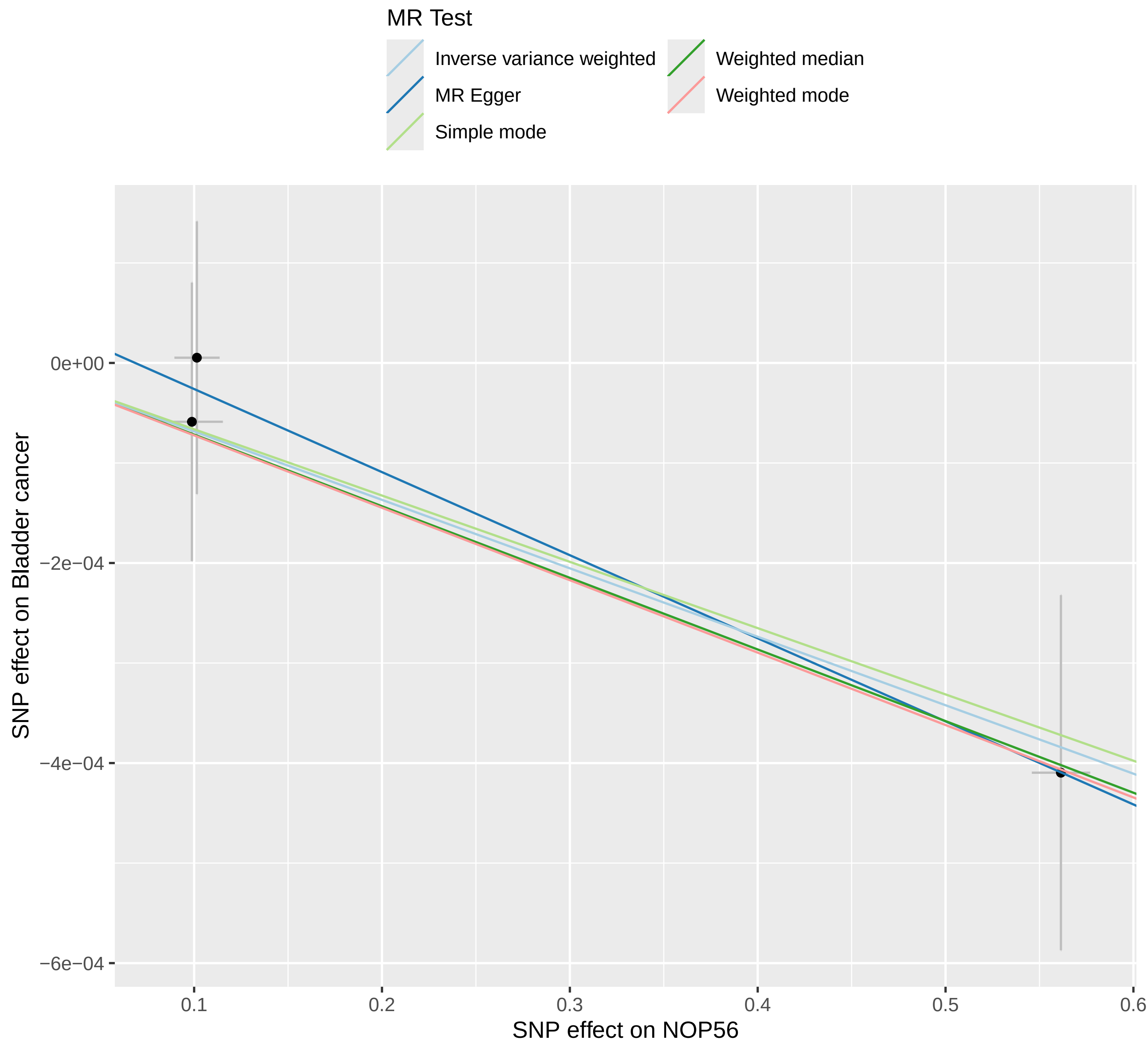

MR Test
Inverse variance weighted
MR Egger
Simple mode
Weighted median
Weighted mode
0e+00
−2e−04
−4e−04
−6e−04
SNP effect on Bladder cancer
0.1
0.2
0.3
0.4
0.5
0.6
SNP effect on NOP56

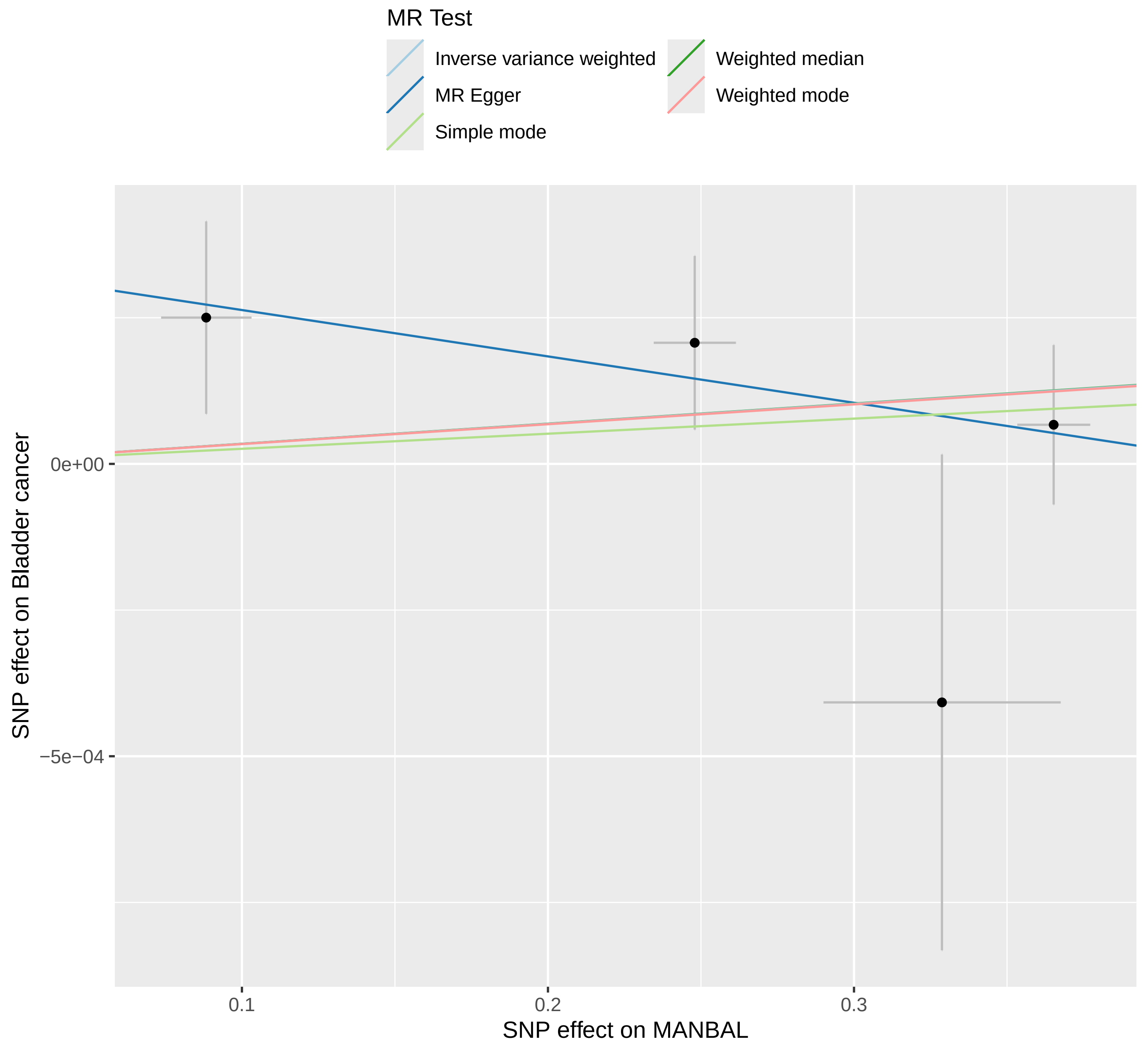

MR Test
Inverse variance weighted
MR Egger
Simple mode
Weighted median
Weighted mode
0e+00
−5e−04
0.1
0.2
0.3
SNP effect on Bladder cancer
SNP effect on MANBAL

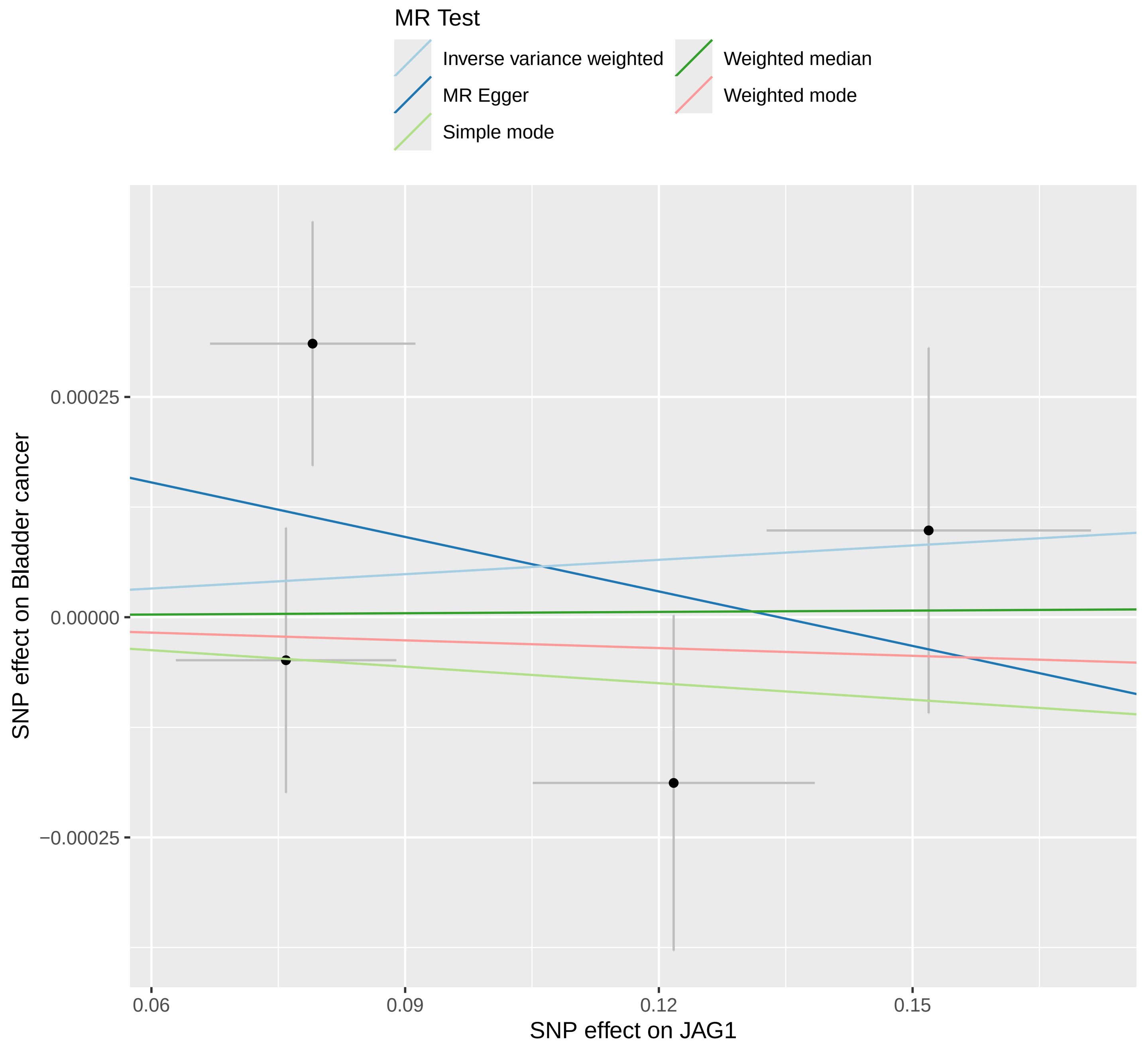
MR Test
Inverse variance weighted
MR Egger
Simple mode
Weighted median
Weighted mode
SNP effect on Bladder cancer
0.00025
0.00000
−0.00025
0.06
0.09
0.12
0.15
SNP effect on JAG1

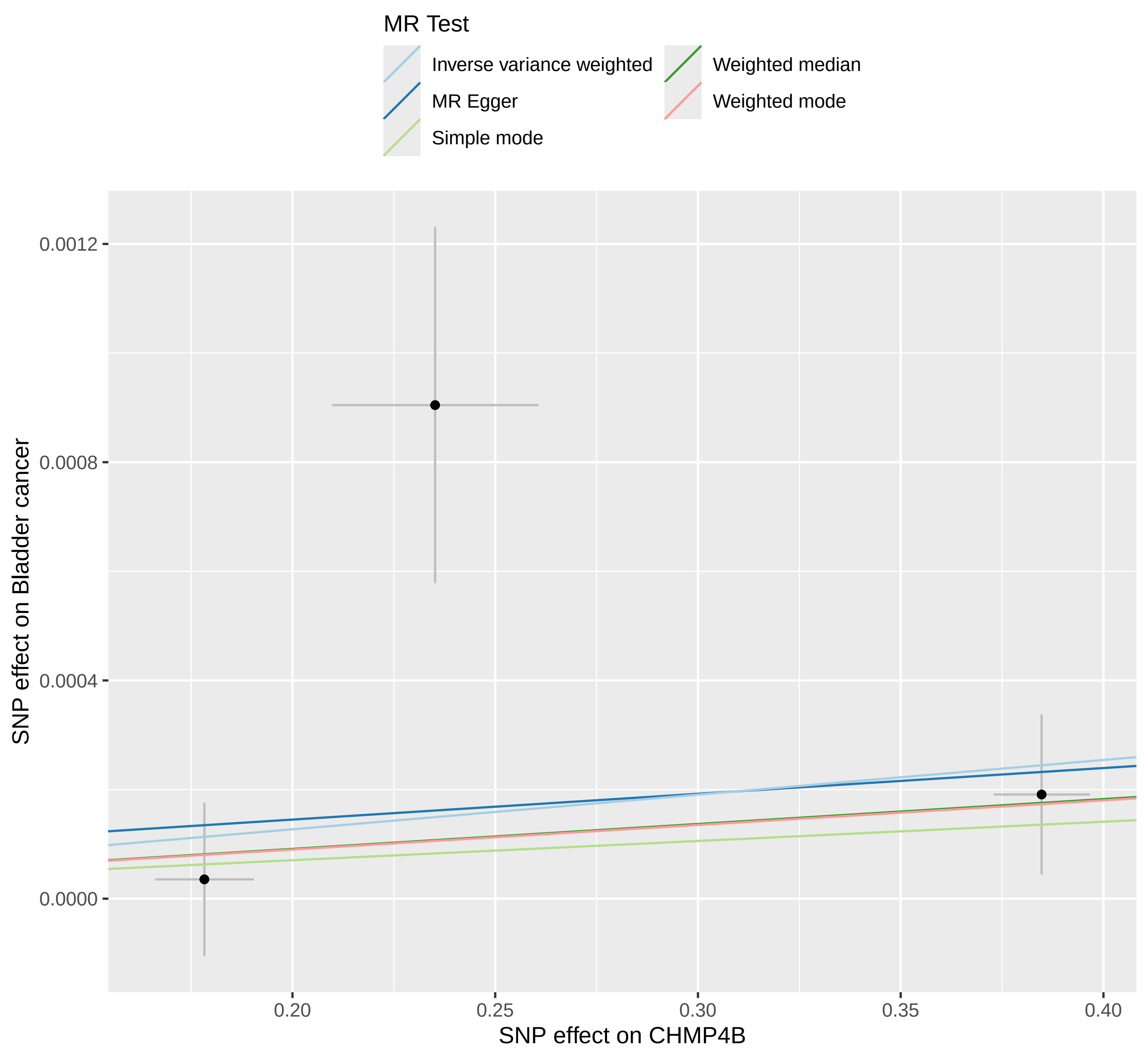
MR Test
Inverse variance weighted
MR Egger
Simple mode
Weighted median
Weighted mode
0.0012
0.0008
0.0004
0.0000
0.20
0.25
0.30
0.35
0.40
SNP effect on Bladder cancer
SNP effect on CHMP4B

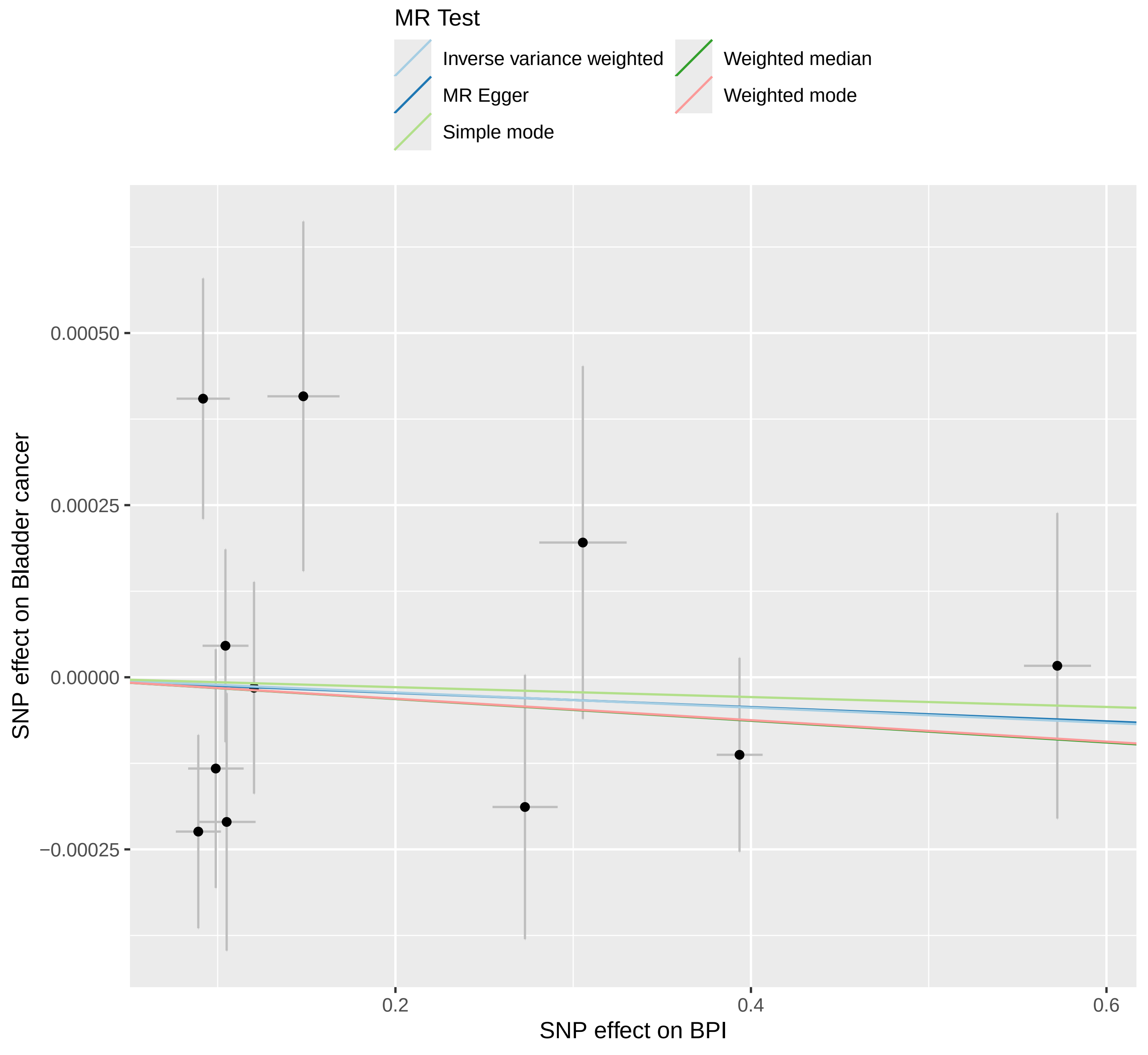

MR Test
Inverse variance weighted
MR Egger
Simple mode
Weighted median
Weighted mode
0.00050
0.00025
0.00000
−0.00025
SNP effect on Bladder cancer
0.2
0.4
0.6
SNP effect on BPI

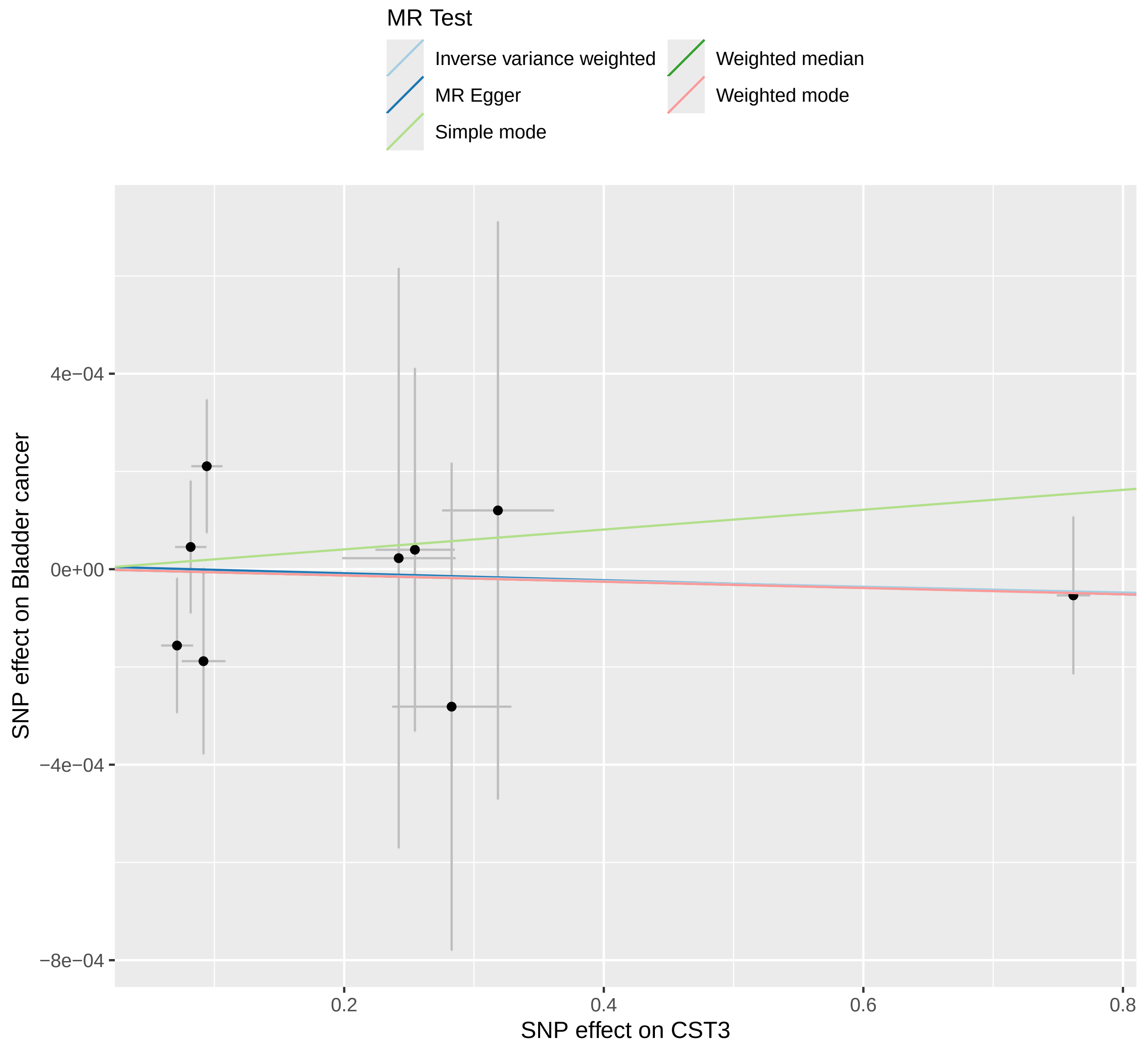

MR Test
Inverse variance weighted
MR Egger
Simple mode
Weighted median
Weighted mode
4e−04
0e+00
−4e−04
−8e−04
SNP effect on Bladder cancer
0.2
0.4
0.6
0.8
SNP effect on CST3

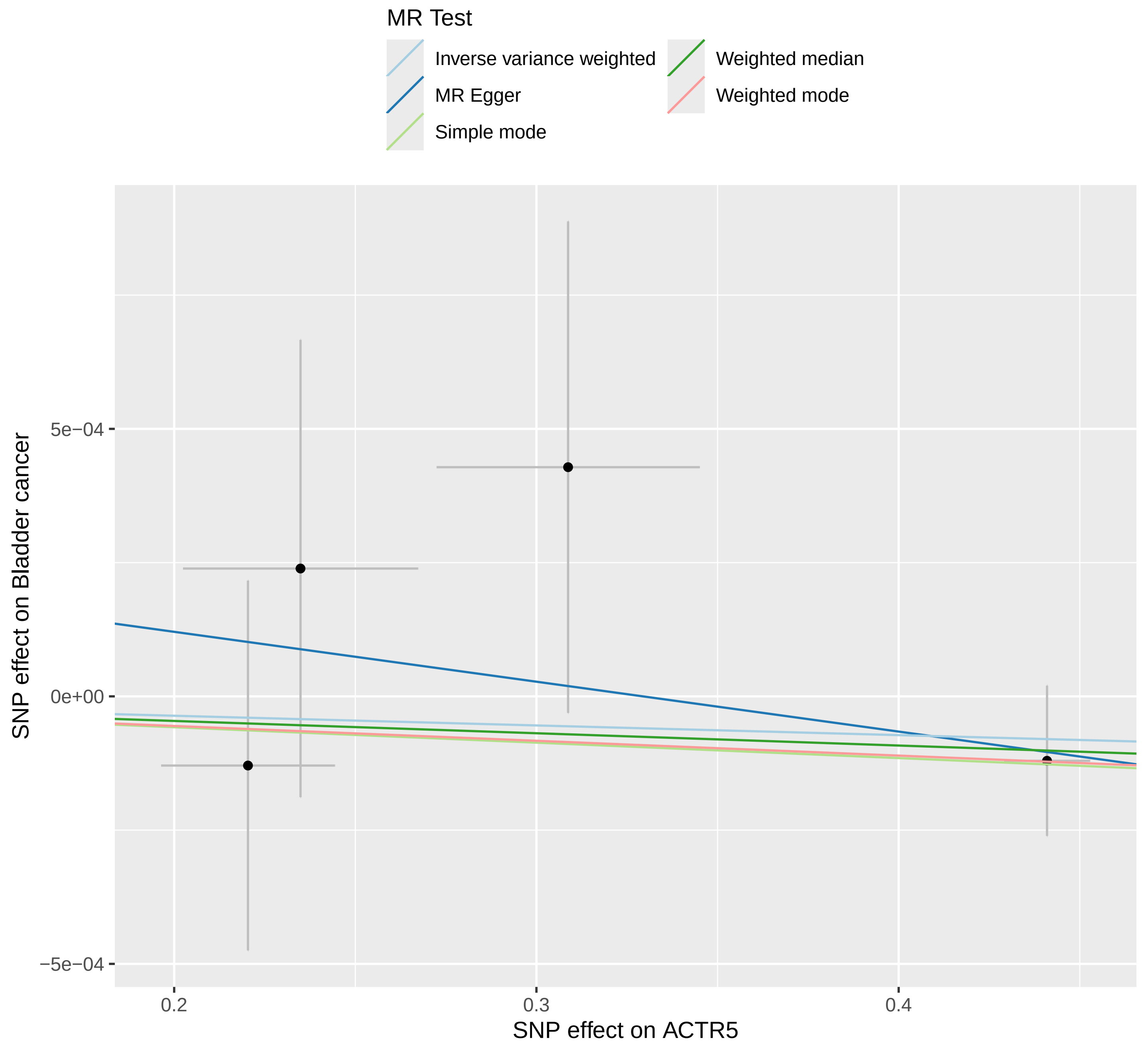

MR Test
Inverse variance weighted
MR Egger
Simple mode
Weighted median
Weighted mode
SNP effect on Bladder cancer
5e−04
0e+00
−5e−04
0.2
0.3
0.4
SNP effect on ACTR5

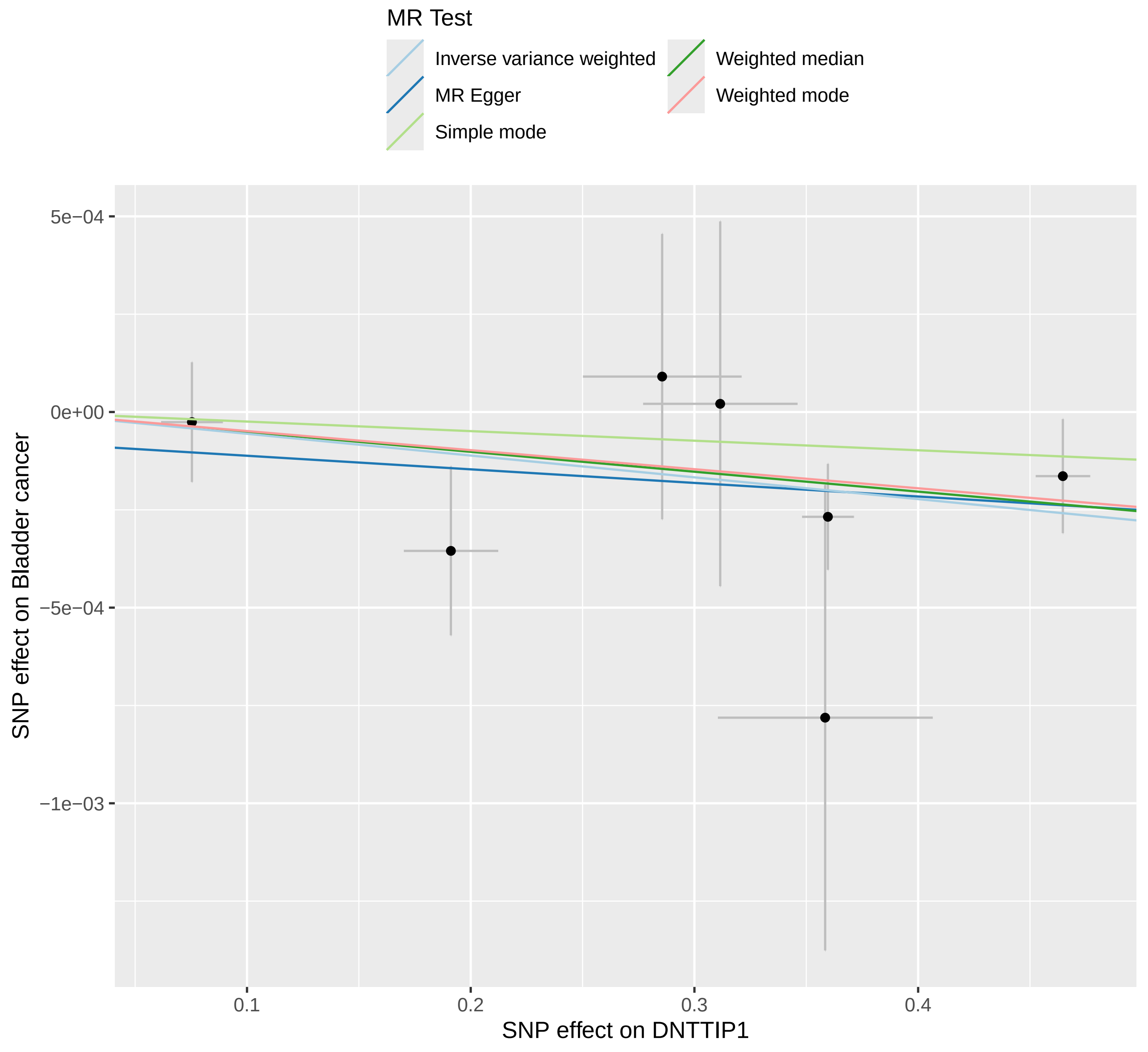

MR Test
Inverse variance weighted
MR Egger
Simple mode
Weighted median
Weighted mode
5e−04
0e+00
−5e−04
−1e−03
0.1
0.2
0.3
0.4
SNP effect on DNTTIP1
SNP effect on Bladder cancer

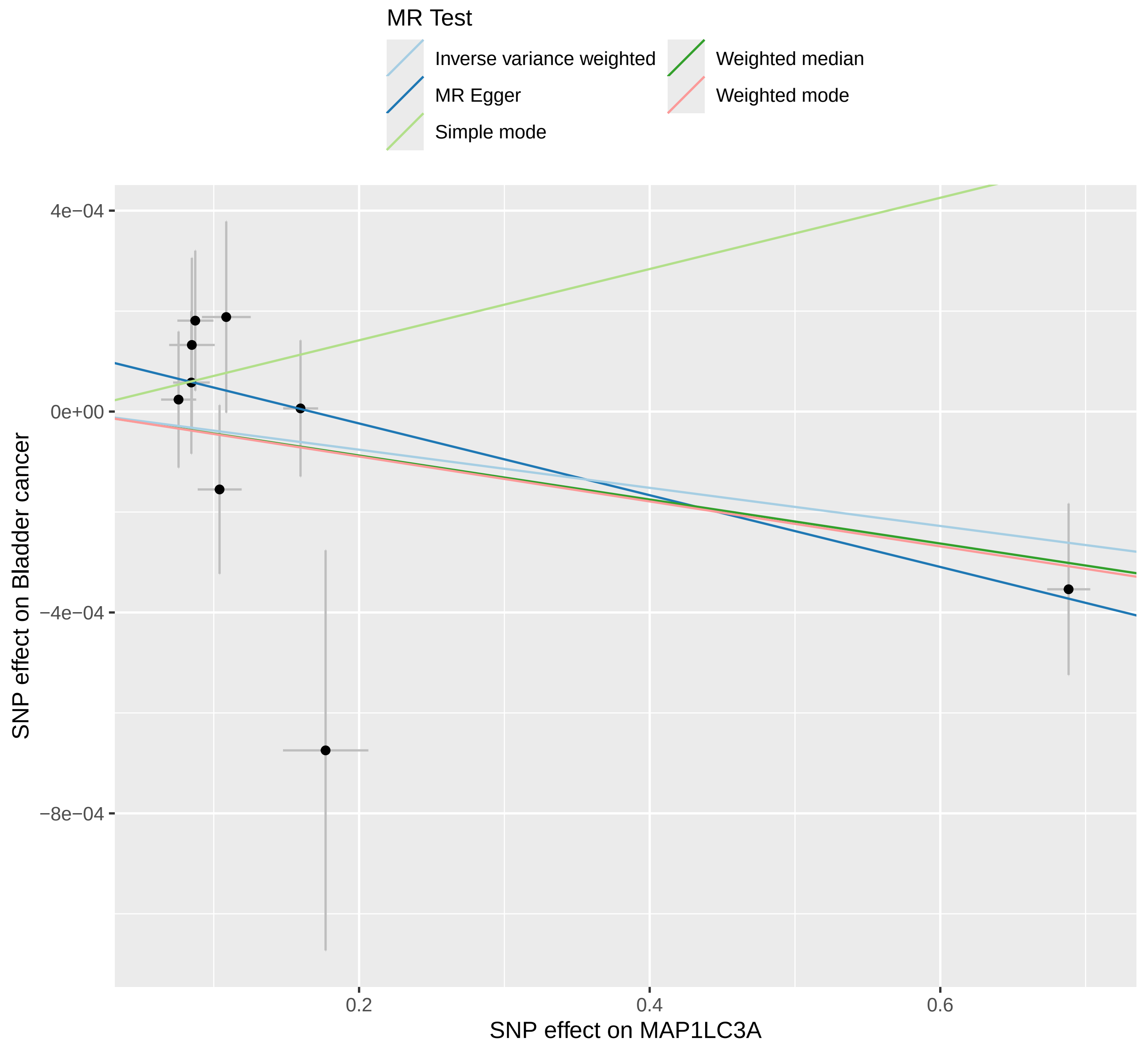
MR Test
Inverse variance weighted
MR Egger
Simple mode
Weighted median
Weighted mode
4e−04
0e+00
−4e−04
−8e−04
SNP effect on Bladder cancer
0.2
0.4
0.6
SNP effect on MAP1LC3A

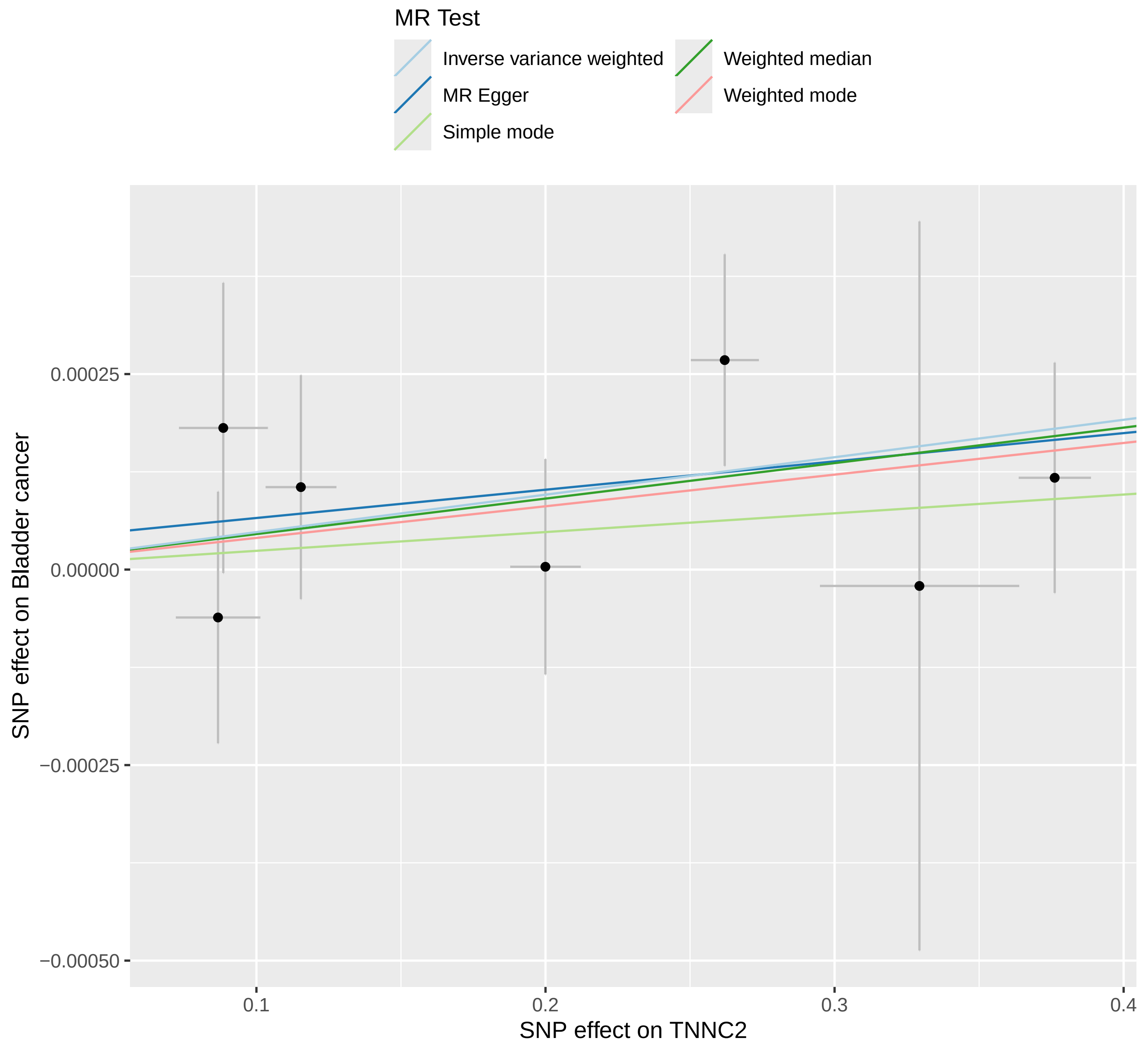

MR Test
Inverse variance weighted
MR Egger
Simple mode
Weighted median
Weighted mode
0.00025
0.00000
−0.00025
−0.00050
SNP effect on Bladder cancer
0.1
0.2
0.3
0.4
SNP effect on TNNC2

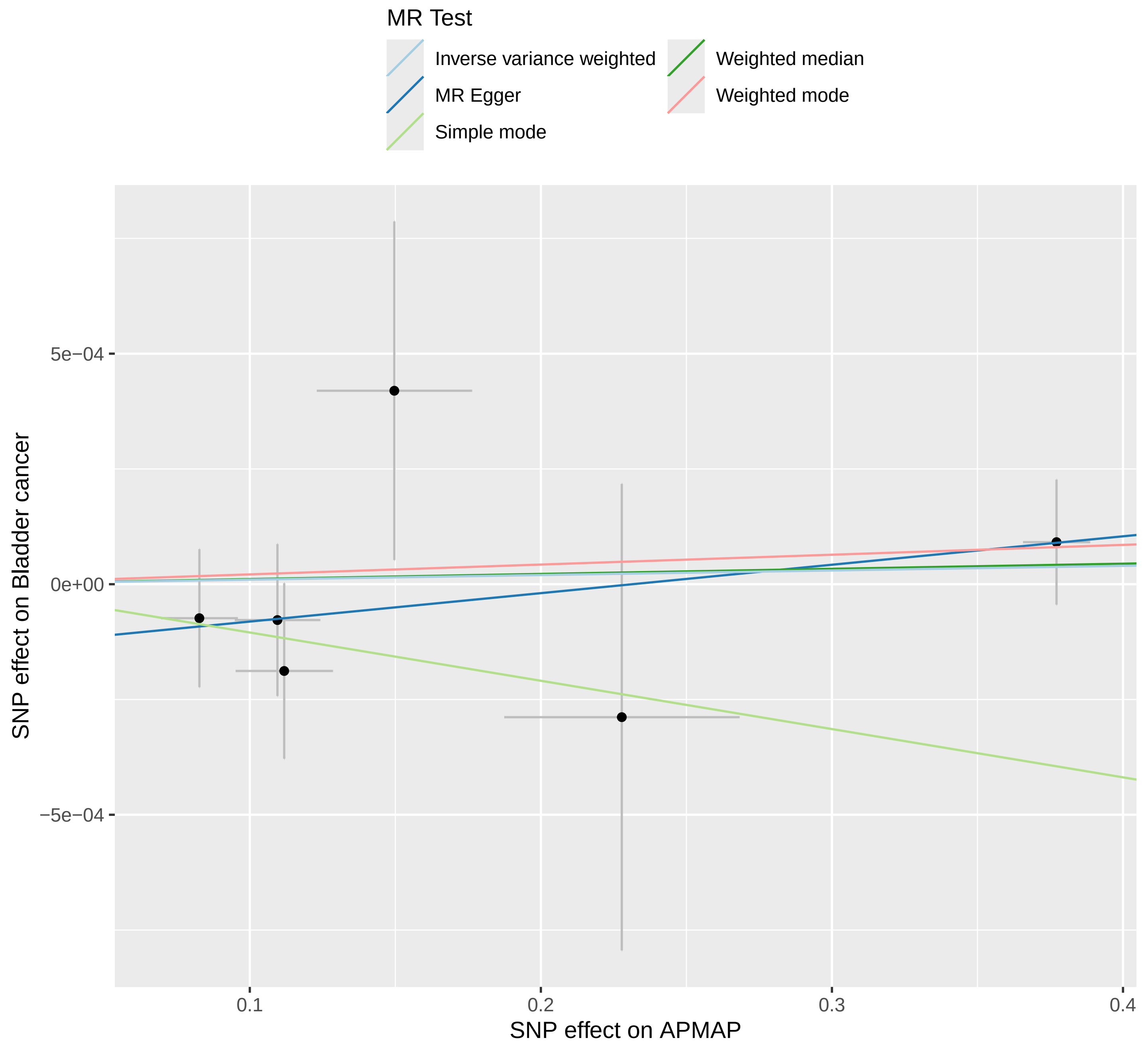

MR Test
Inverse variance weighted
MR Egger
Simple mode
Weighted median
Weighted mode
5e−04
0e+00
−5e−04
SNP effect on Bladder cancer
0.1
0.2
0.3
0.4
SNP effect on APMAP

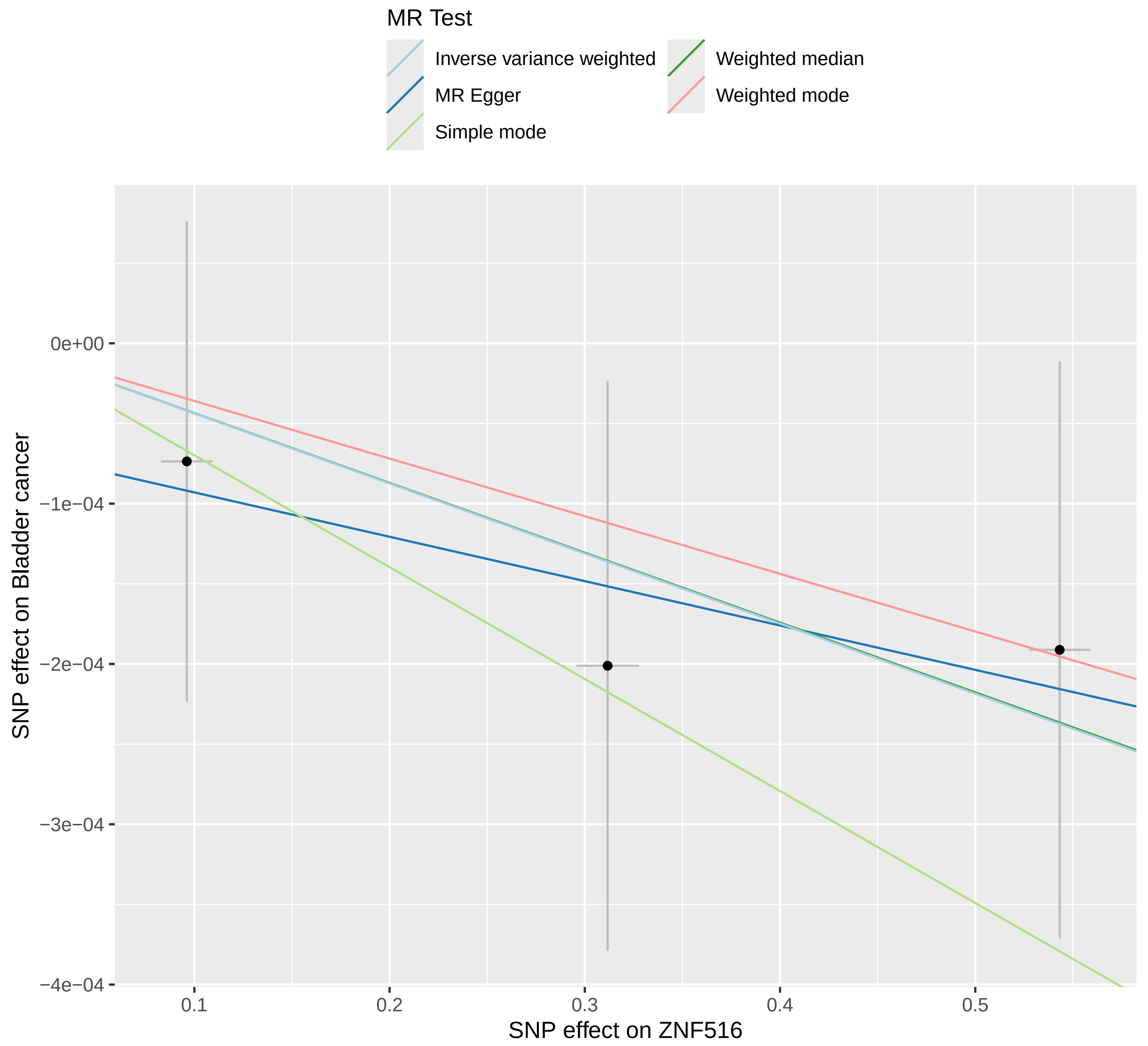

MR Test
Inverse variance weighted
MR Egger
Simple mode
Weighted median
Weighted mode
0e+00
−1e−04
−2e−04
−3e−04
−4e−04
0.1
0.2
0.3
0.4
0.5
SNP effect on Bladder cancer
SNP effect on ZNF516

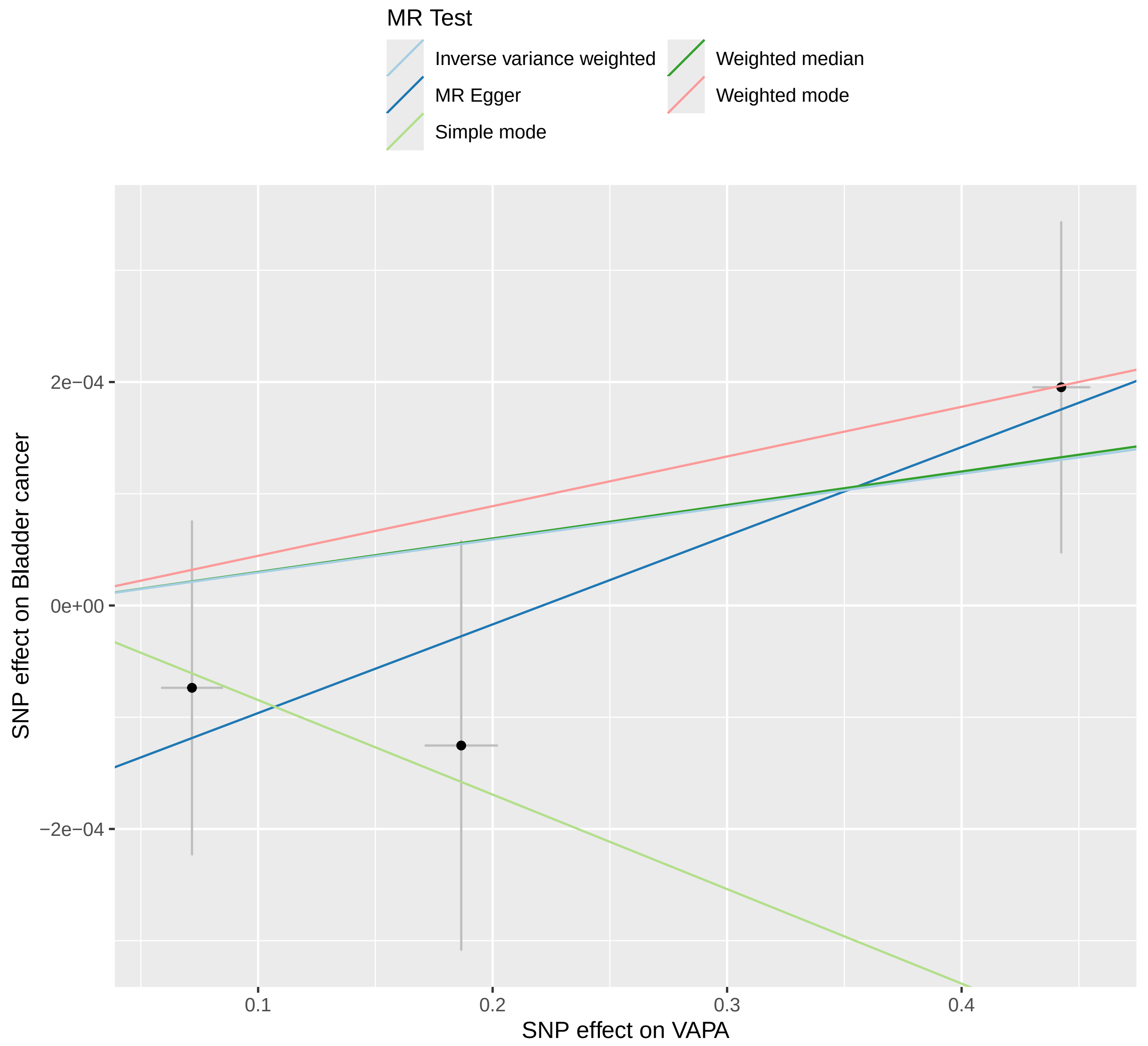
MR Test
Inverse variance weighted
MR Egger
Simple mode
Weighted median
Weighted mode
2e−04
0e+00
−2e−04
0.1
0.2
0.3
0.4
SNP effect on Bladder cancer
SNP effect on VAPA

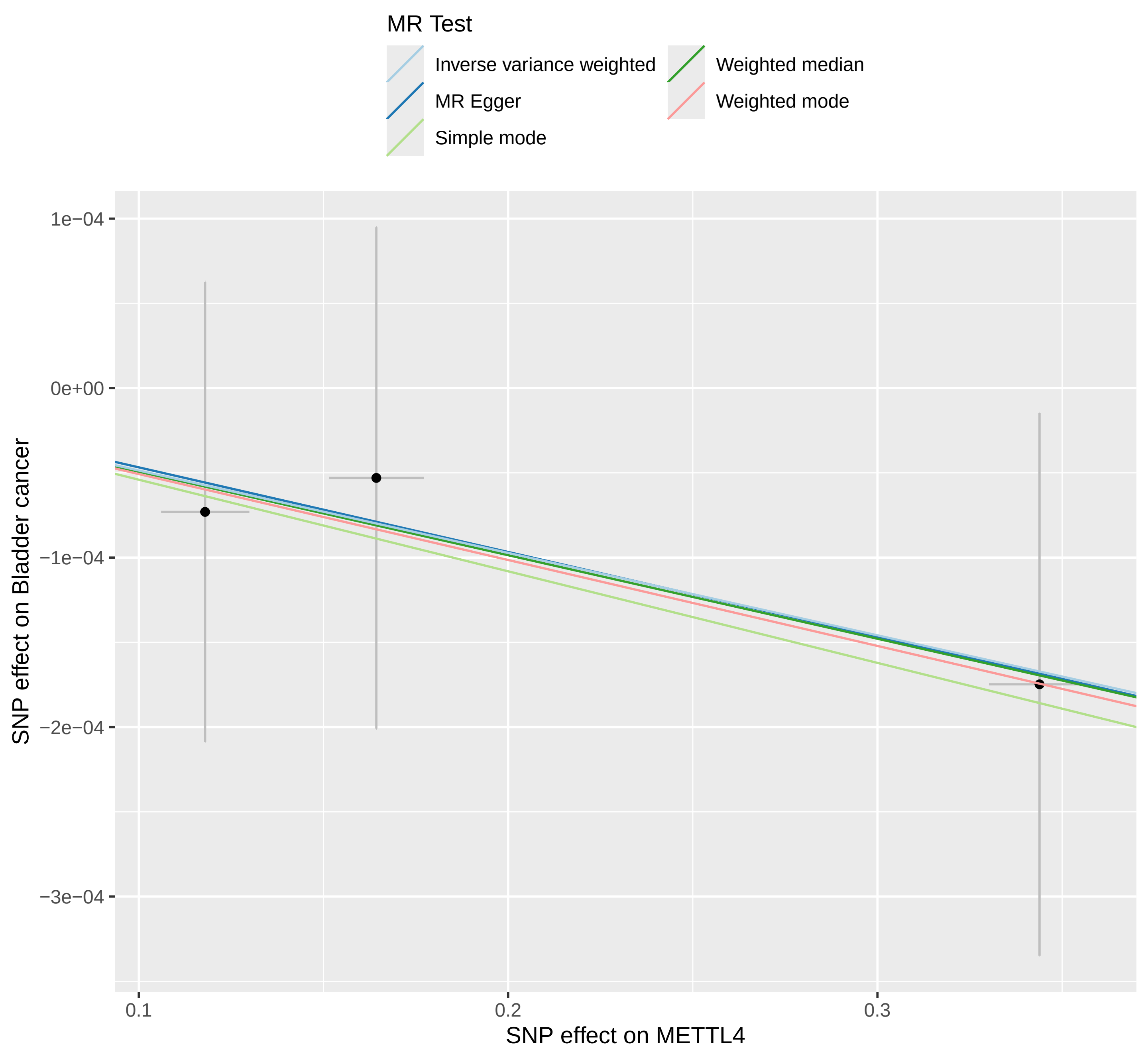

MR Test
Inverse variance weighted
MR Egger
Simple mode
Weighted median
Weighted mode
1e−04
0e+00
−1e−04
−2e−04
−3e−04
SNP effect on Bladder cancer
0.1
0.2
0.3
SNP effect on METTL4

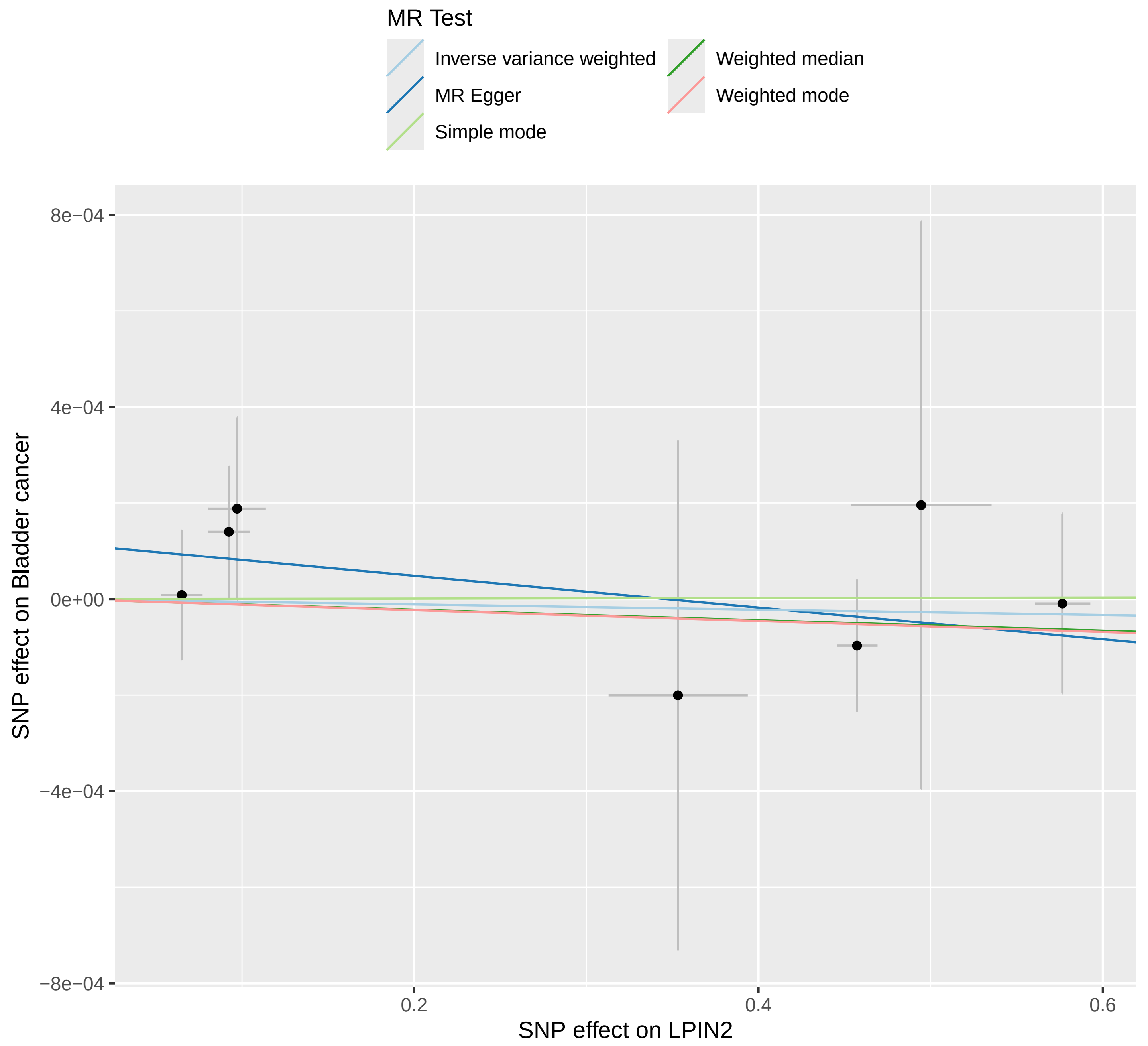
MR Test
Inverse variance weighted
MR Egger
Simple mode
Weighted median
Weighted mode
8e−04
4e−04
0e+00
−4e−04
−8e−04
SNP effect on Bladder cancer
0.2
0.4
0.6
SNP effect on LPIN2

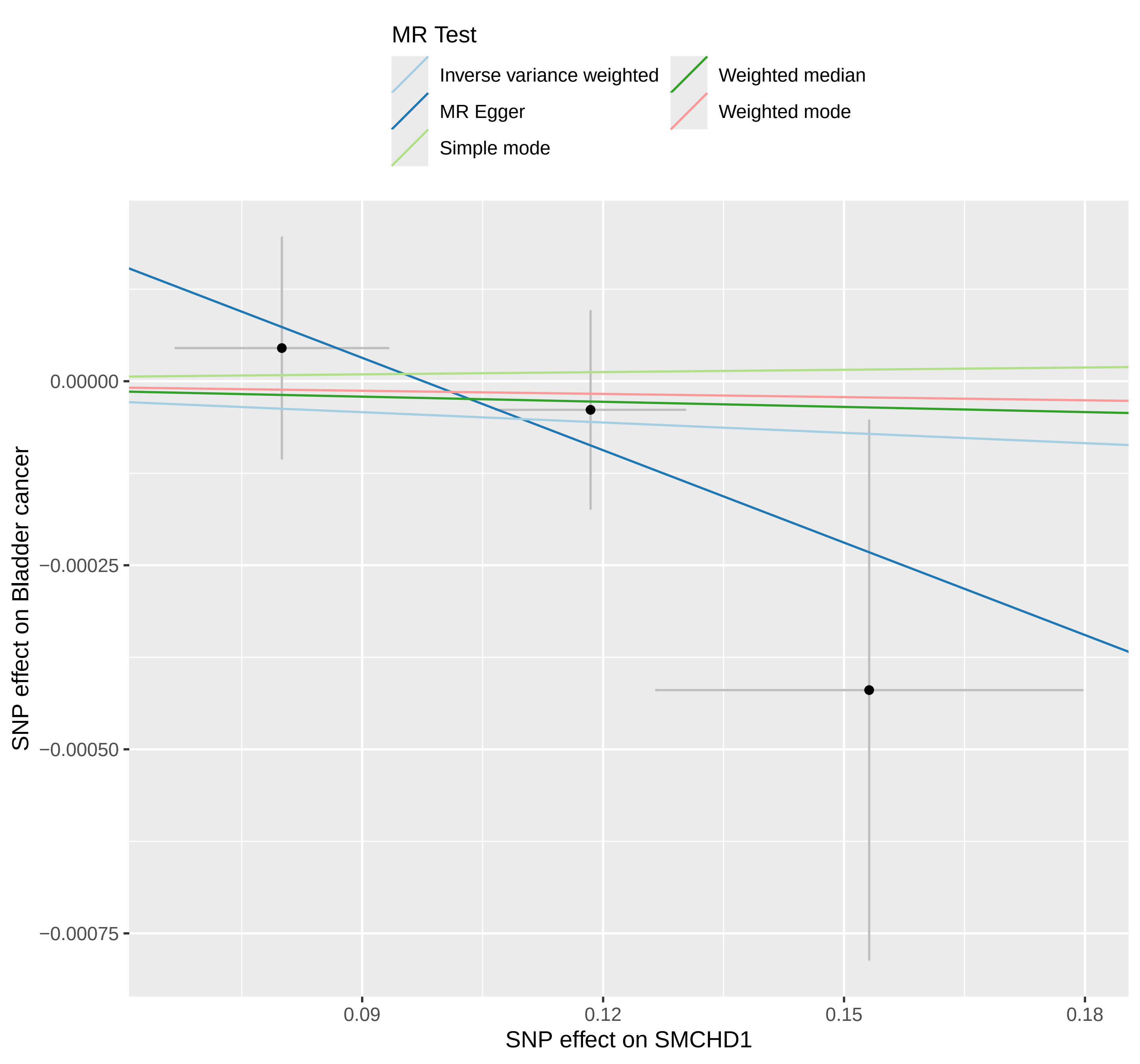
MR Test
Inverse variance weighted
MR Egger
Simple mode
Weighted median
Weighted mode
0.00000
−0.00025
−0.00050
−0.00075
0.09
0.12
0.15
0.18
SNP effect on Bladder cancer
SNP effect on SMCHD1

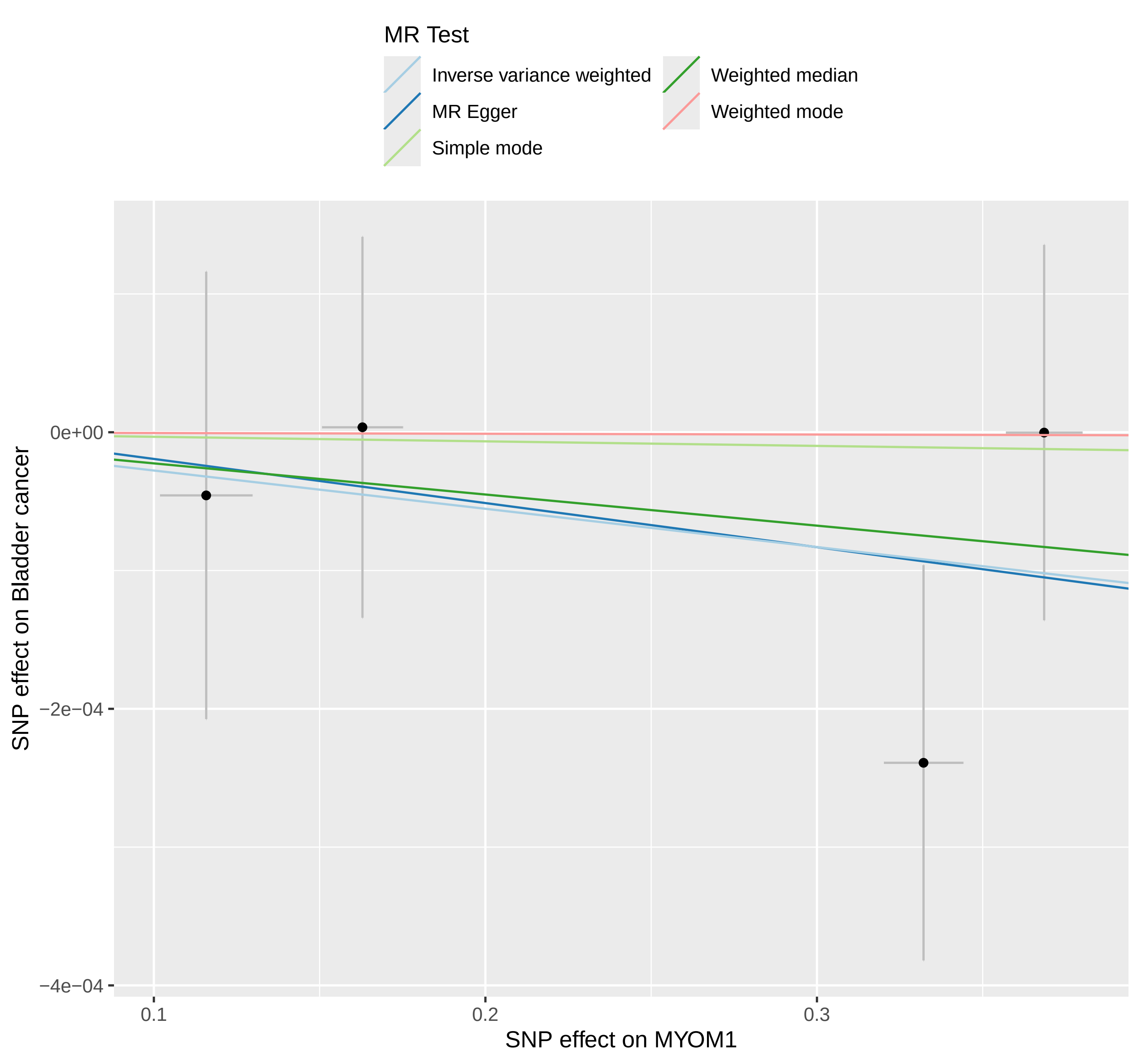

MR Test
Inverse variance weighted
MR Egger
Simple mode
Weighted median
Weighted mode
0e+00
−2e−04
−4e−04
SNP effect on Bladder cancer
0.1
0.2
0.3
SNP effect on MYOM1

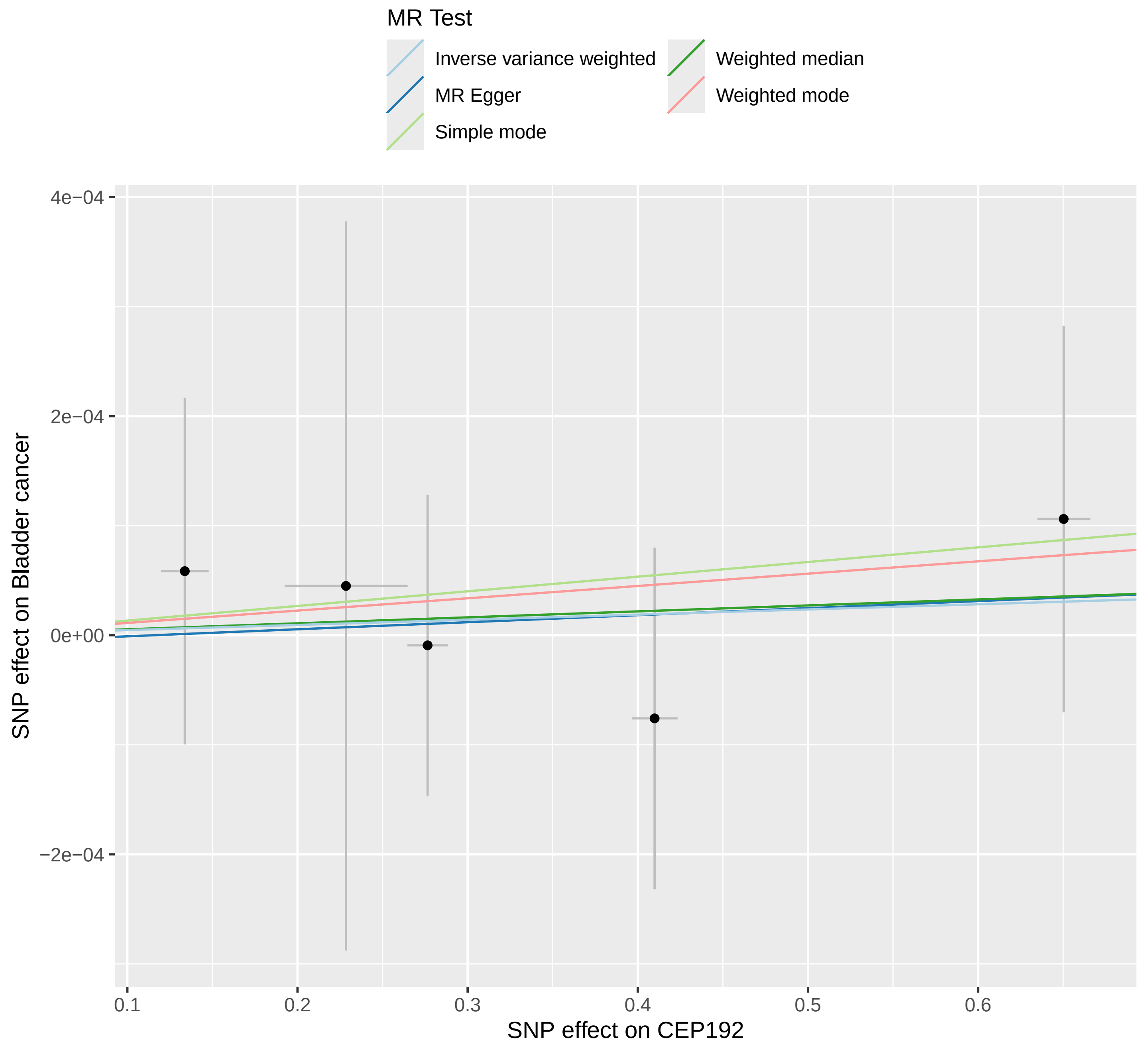
MR Test
Inverse variance weighted
MR Egger
Simple mode
Weighted median
Weighted mode
4e−04
2e−04
0e+00
−2e−04
0.1
0.2
0.3
0.4
0.5
0.6
SNP effect on Bladder cancer
SNP effect on CEP192

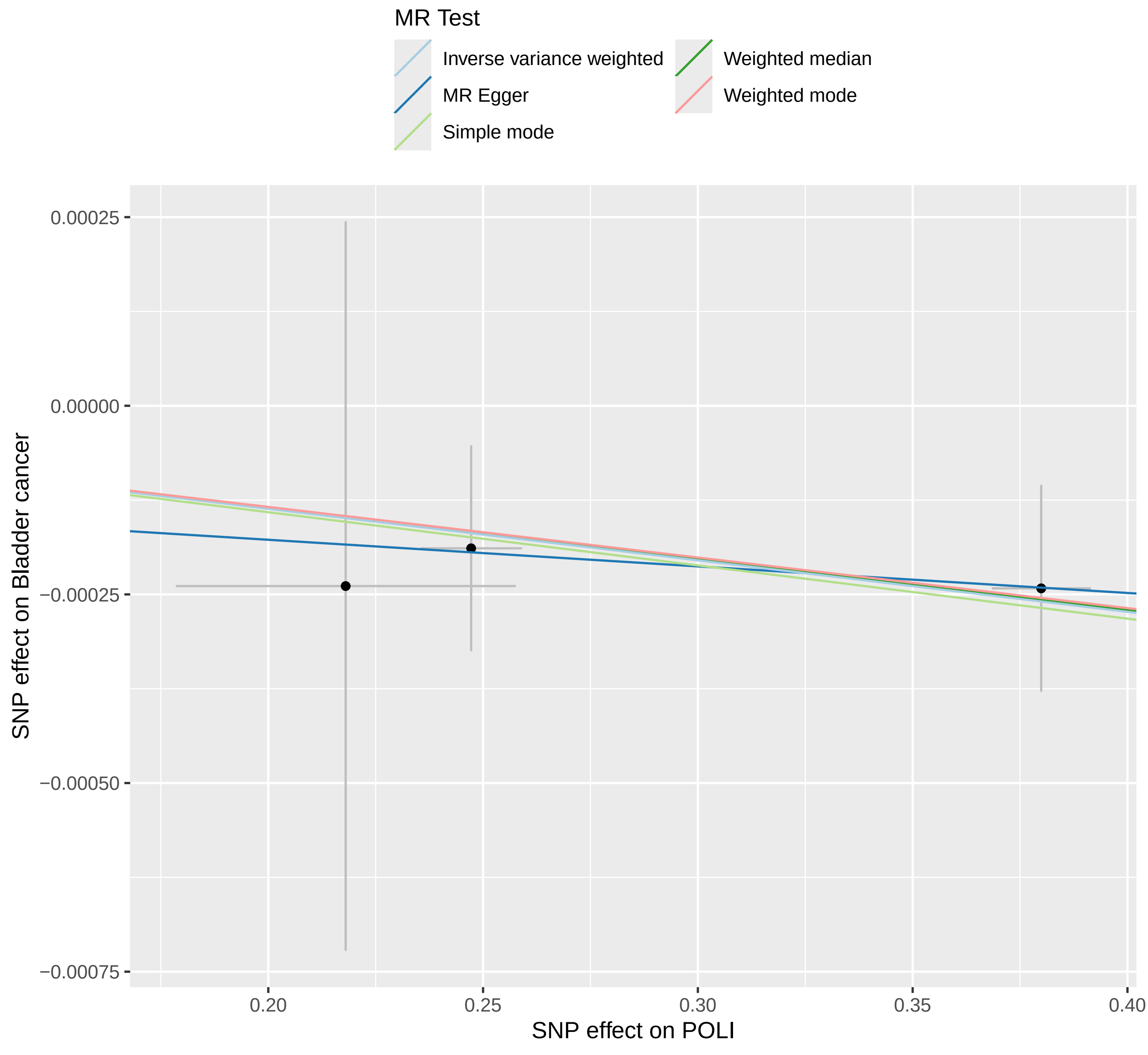
MR Test
Inverse variance weighted
MR Egger
Simple mode
Weighted median
Weighted mode
0.00025
0.00000
−0.00025
−0.00050
−0.00075
SNP effect on Bladder cancer
0.20
0.25
0.30
0.35
0.40
SNP effect on POLI

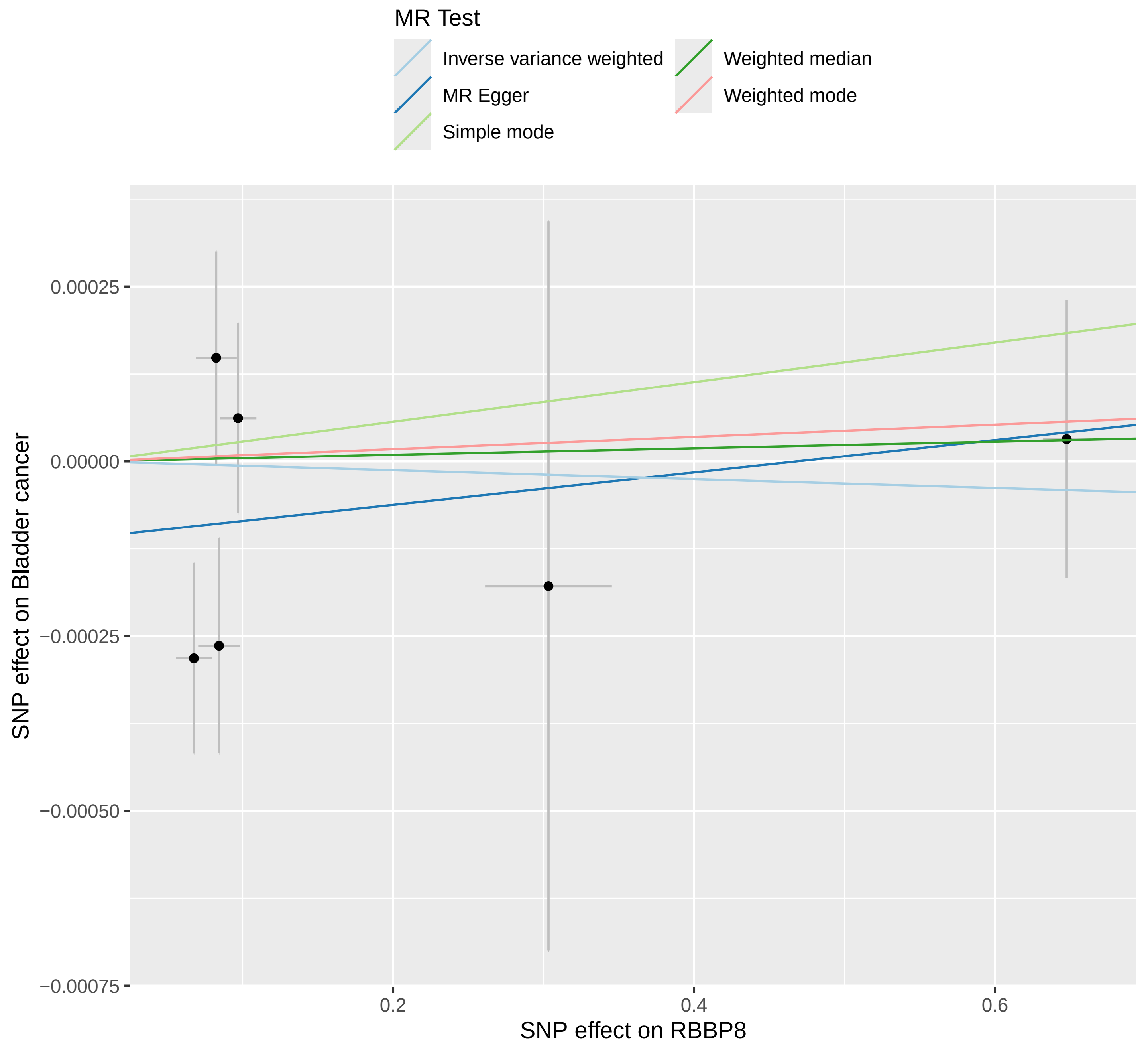
MR Test
Inverse variance weighted
MR Egger
Simple mode
Weighted median
Weighted mode
0.00025
0.00000
−0.00025
−0.00050
−0.00075
0.2
0.4
0.6
SNP effect on RBBP8
SNP effect on Bladder cancer

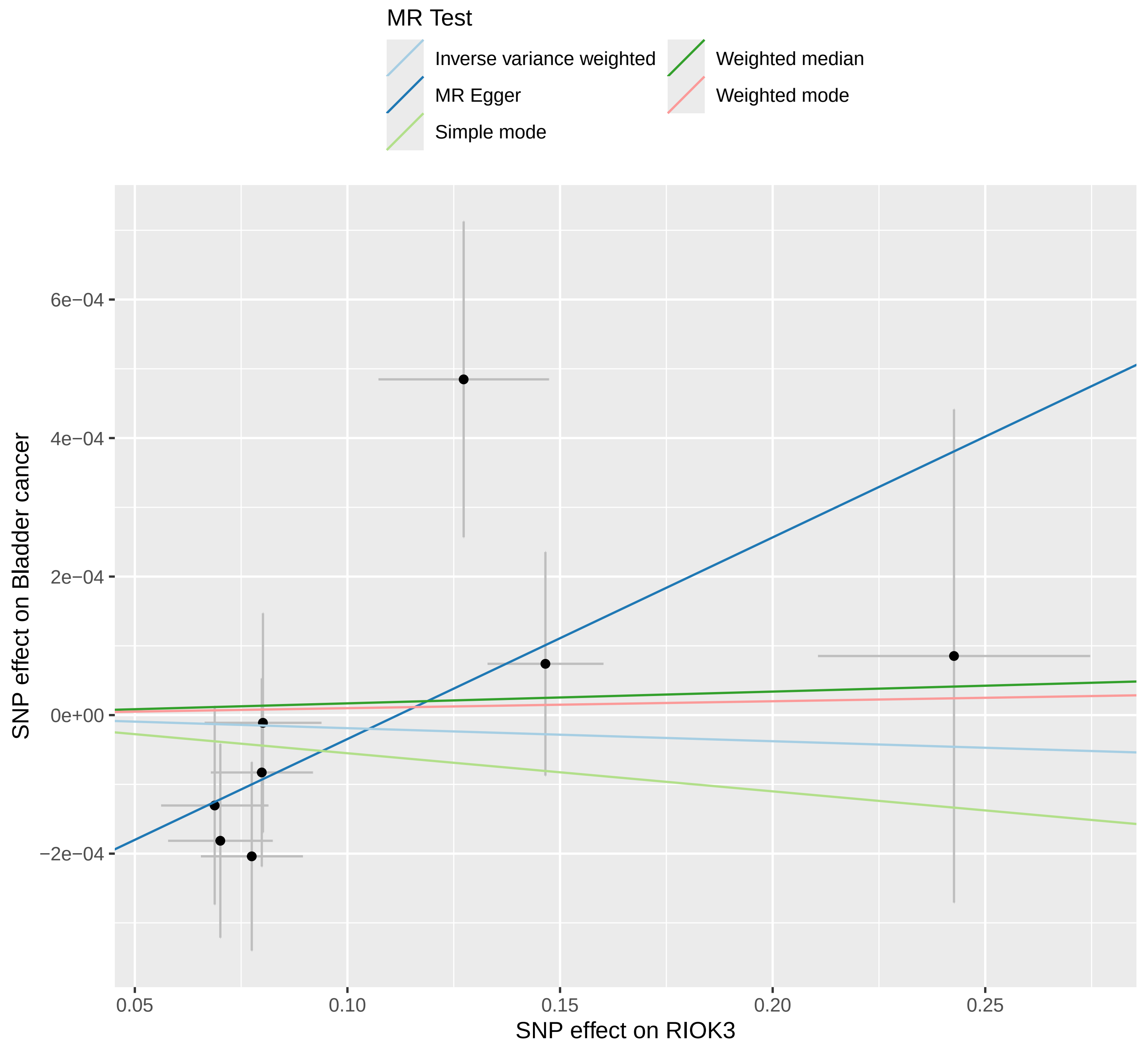
MR Test
Inverse variance weighted
MR Egger
Simple mode
Weighted median
Weighted mode
6e−04
4e−04
2e−04
0e+00
−2e−04
SNP effect on Bladder cancer
0.05
0.10
0.15
0.20
0.25
SNP effect on RIOK3

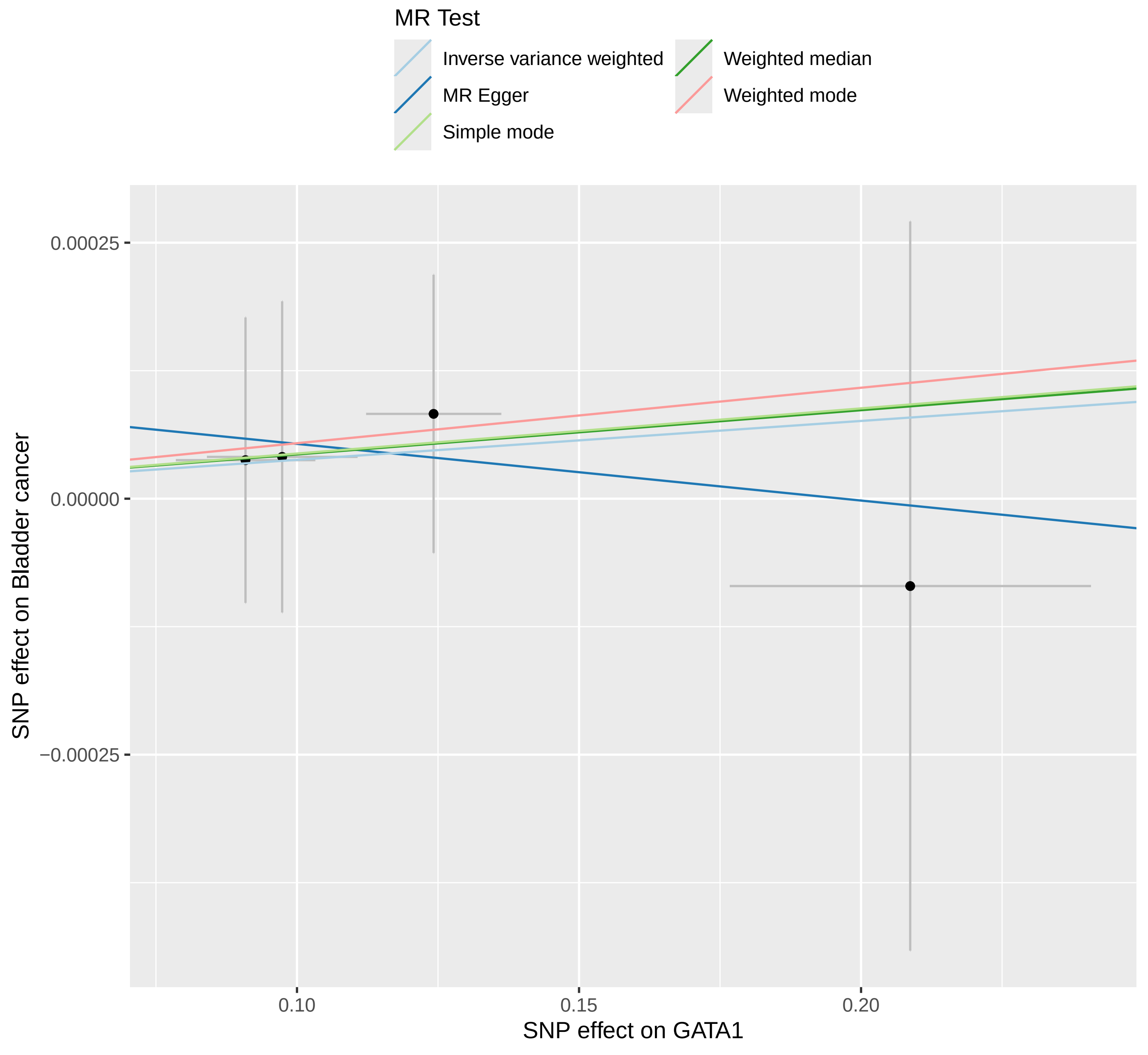

MR Test
Inverse variance weighted
MR Egger
Simple mode
Weighted median
Weighted mode
0.00025
0.00000
−0.00025
SNP effect on Bladder cancer
0.10
0.15
0.20
SNP effect on GATA1

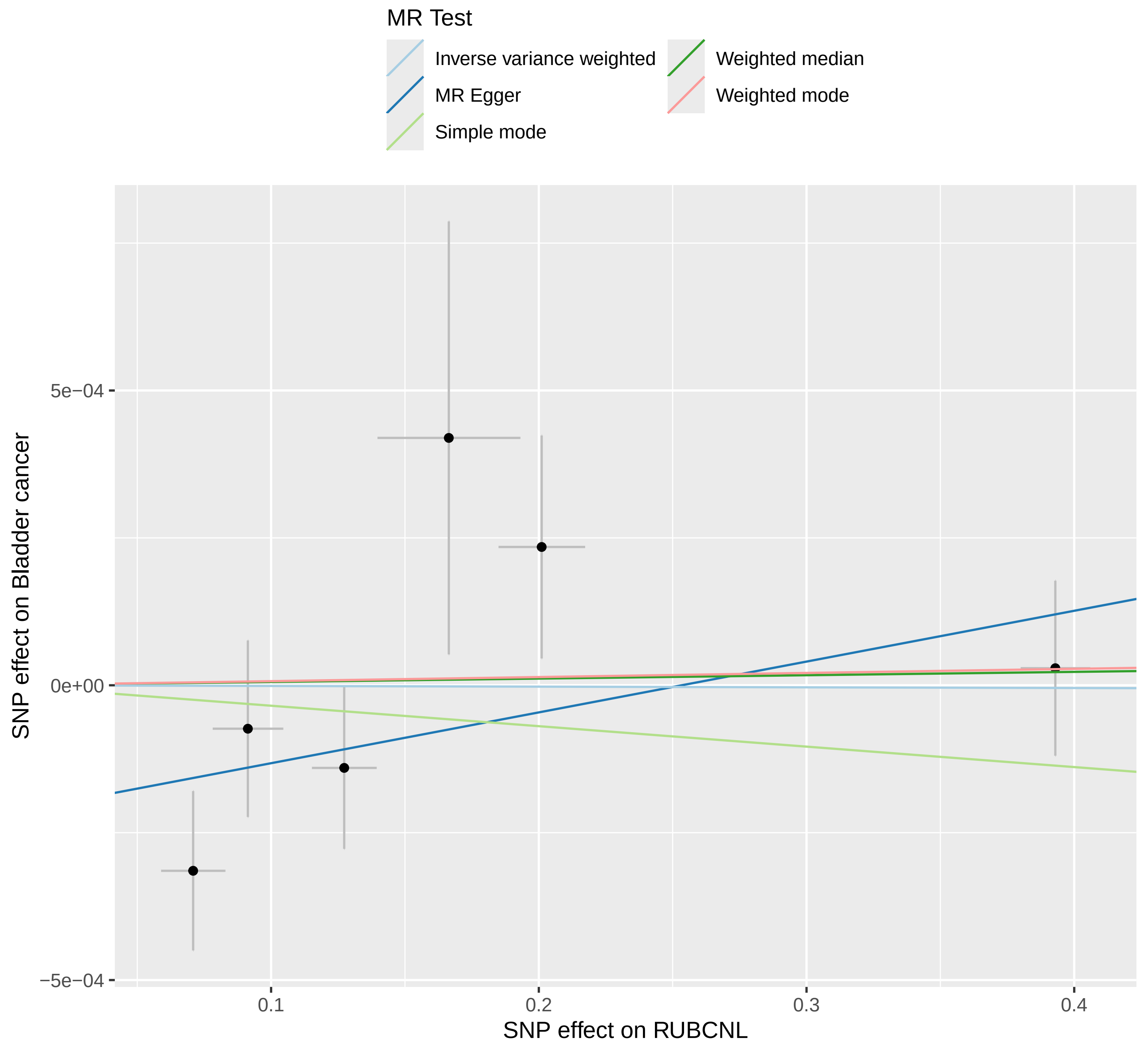

MR Test
Inverse variance weighted
MR Egger
Simple mode
Weighted median
Weighted mode
5e−04
0e+00
−5e−04
0.1
0.2
0.3
0.4
SNP effect on Bladder cancer
SNP effect on RUBCNL

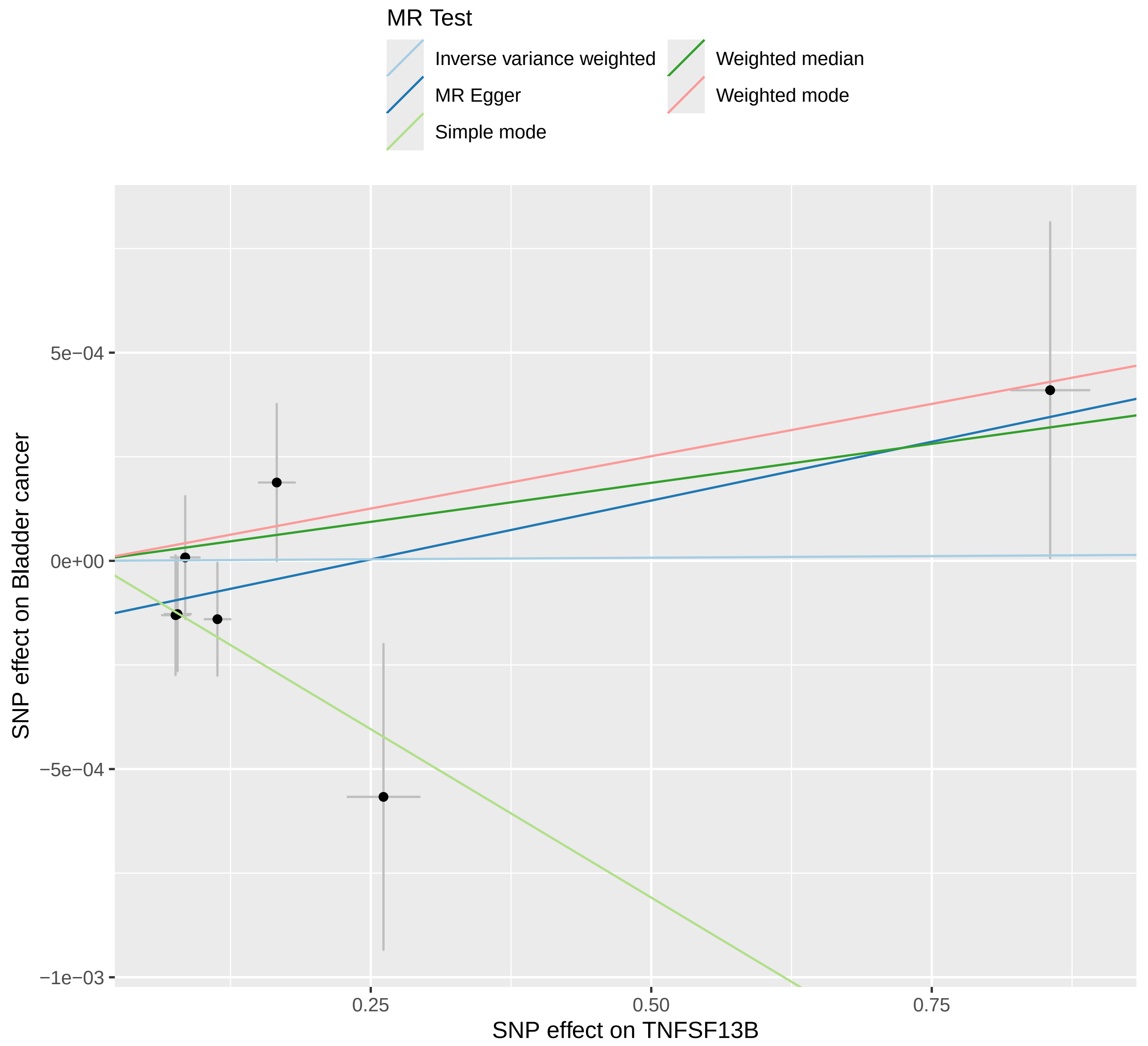
MR Test
Inverse variance weighted
MR Egger
Simple mode
Weighted median
Weighted mode
SNP effect on Bladder cancer
5e−04
0e+00
−5e−04
−1e−03
0.25
0.50
0.75
SNP effect on TNFSF13B

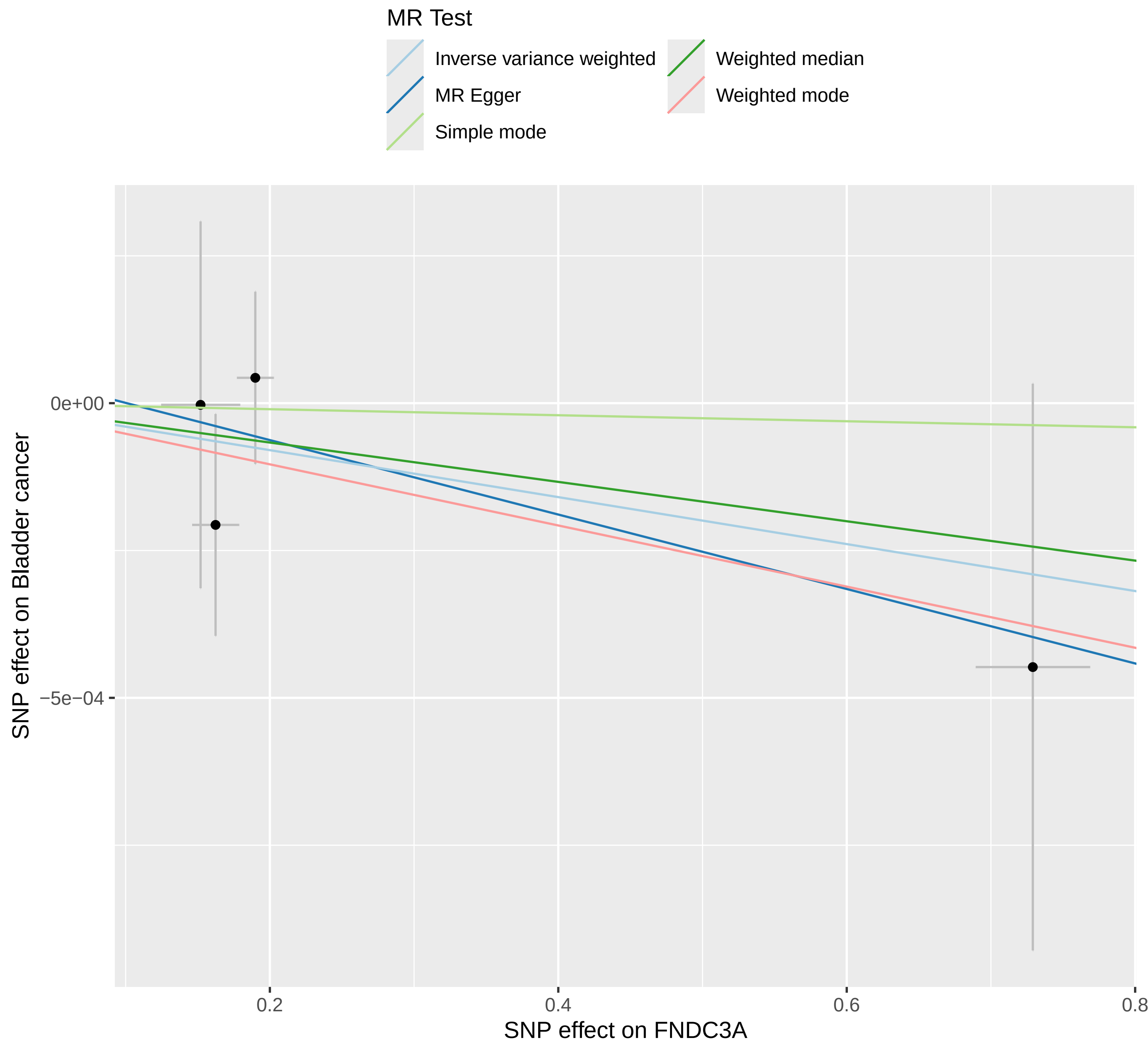
MR Test
Inverse variance weighted
MR Egger
Simple mode
Weighted median
Weighted mode
0e+00
−5e−04
SNP effect on Bladder cancer
0.2
0.4
0.6
0.8
SNP effect on FNDC3A

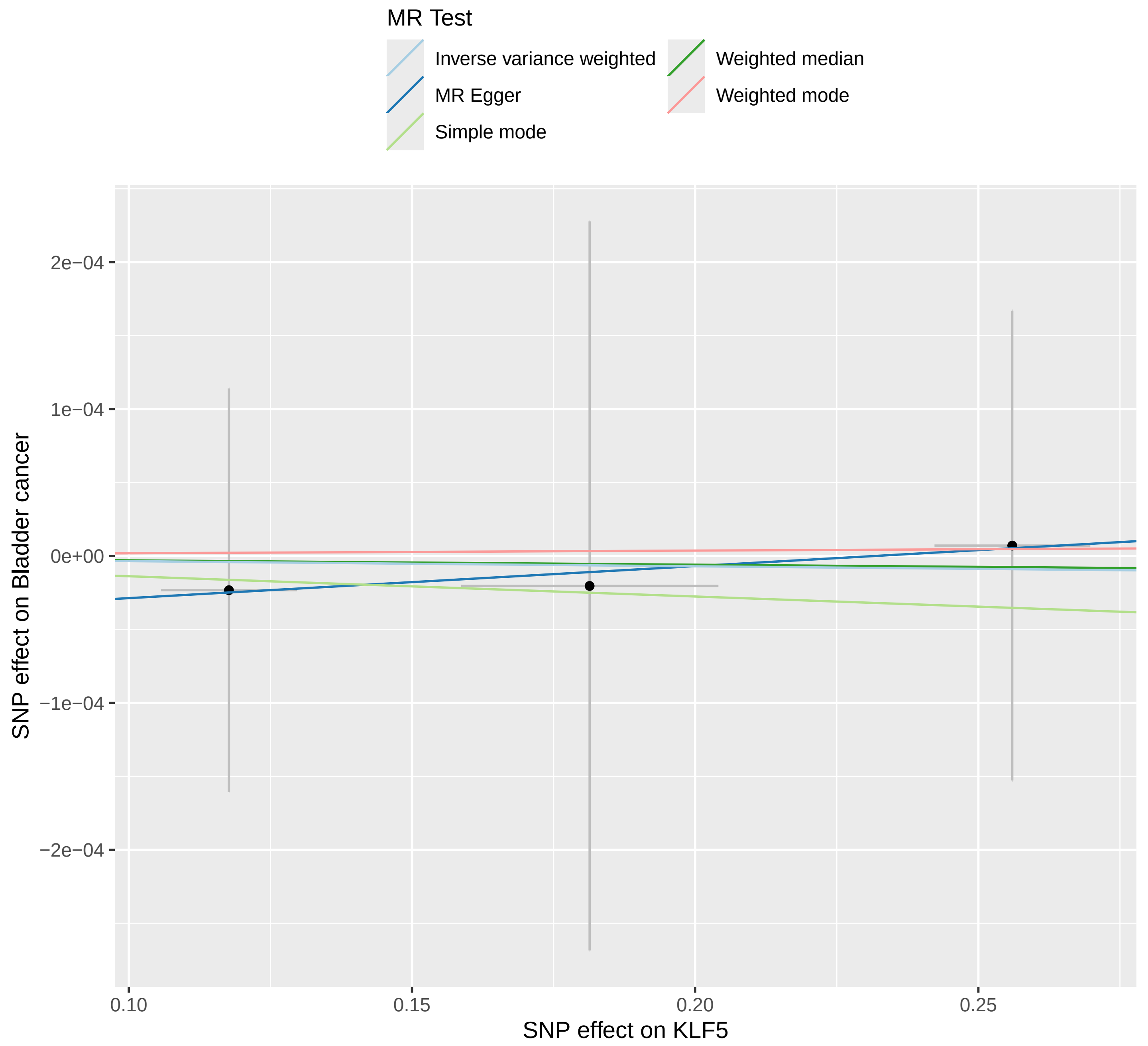
MR Test
Inverse variance weighted
MR Egger
Simple mode
Weighted median
Weighted mode
2e−04
1e−04
0e+00
−1e−04
−2e−04
SNP effect on Bladder cancer
0.10
0.15
0.20
0.25
SNP effect on KLF5

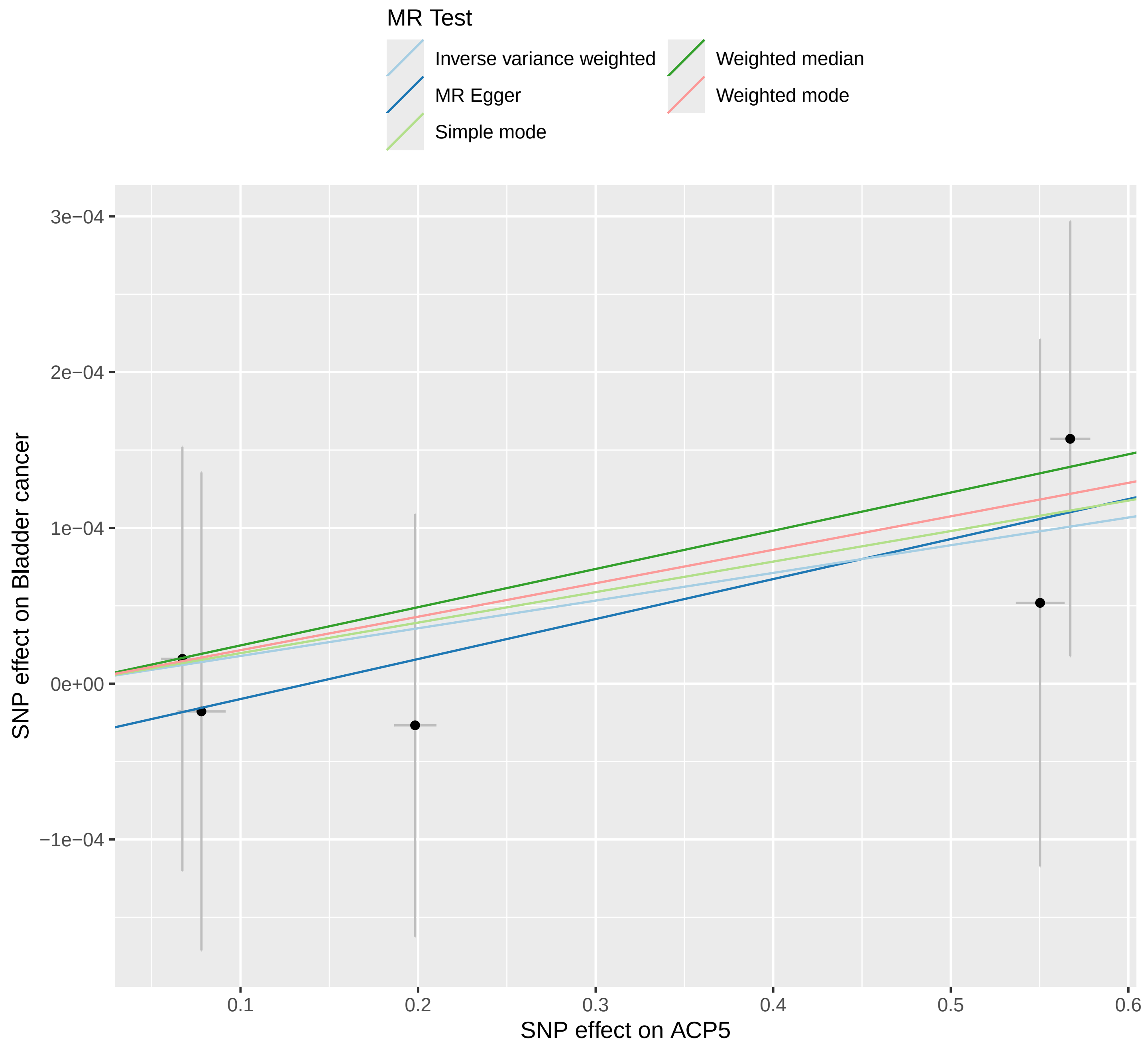

MR Test
Inverse variance weighted
MR Egger
Simple mode
Weighted median
Weighted mode
3e−04
2e−04
1e−04
0e+00
−1e−04
SNP effect on Bladder cancer
0.1
0.2
0.3
0.4
0.5
0.6
SNP effect on ACP5

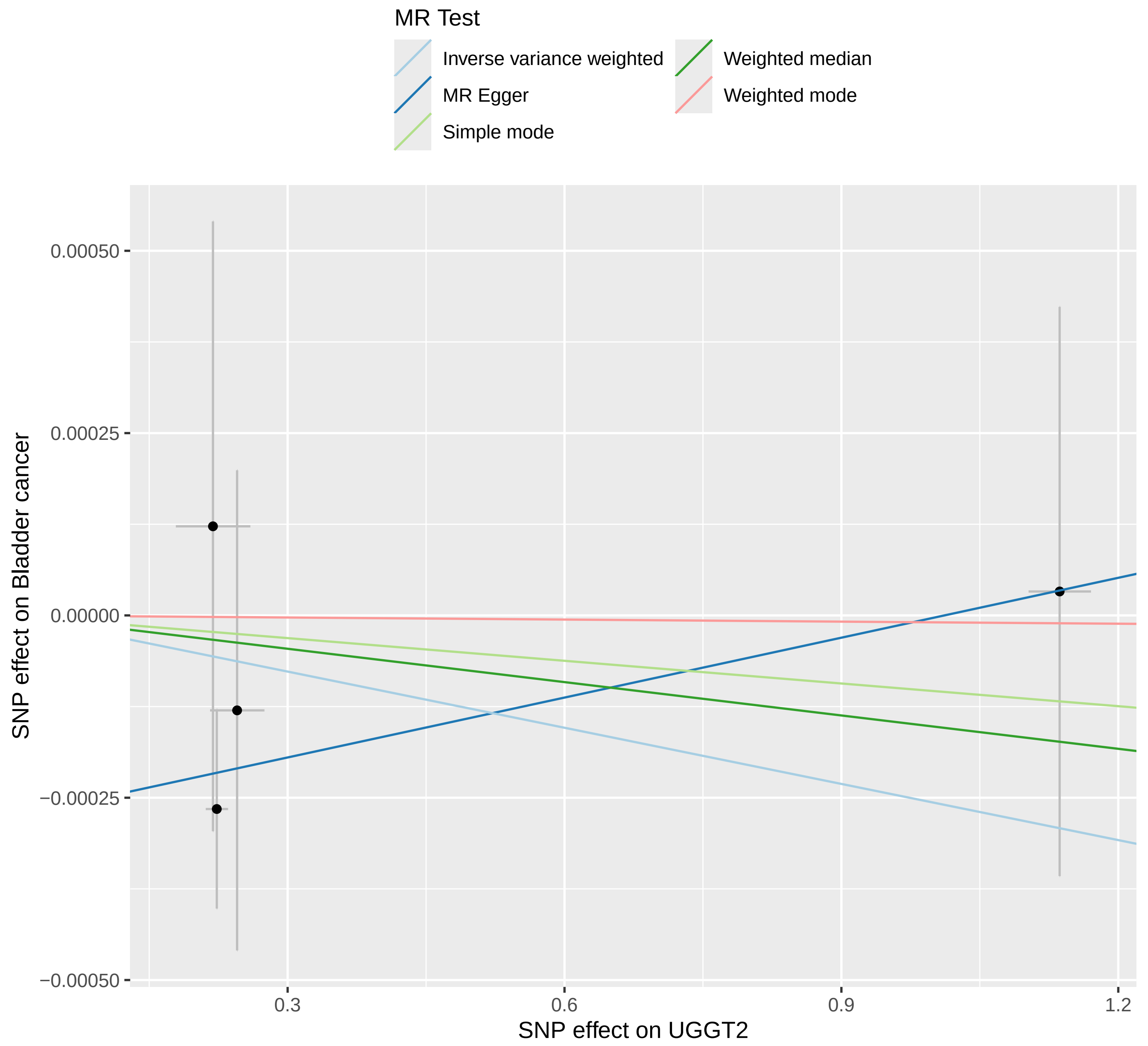

MR Test
Inverse variance weighted
MR Egger
Simple mode
Weighted median
Weighted mode
SNP effect on Bladder cancer
0.00050
0.00025
0.00000
−0.00025
−0.00050
0.3
0.6
0.9
1.2
SNP effect on UGGT2

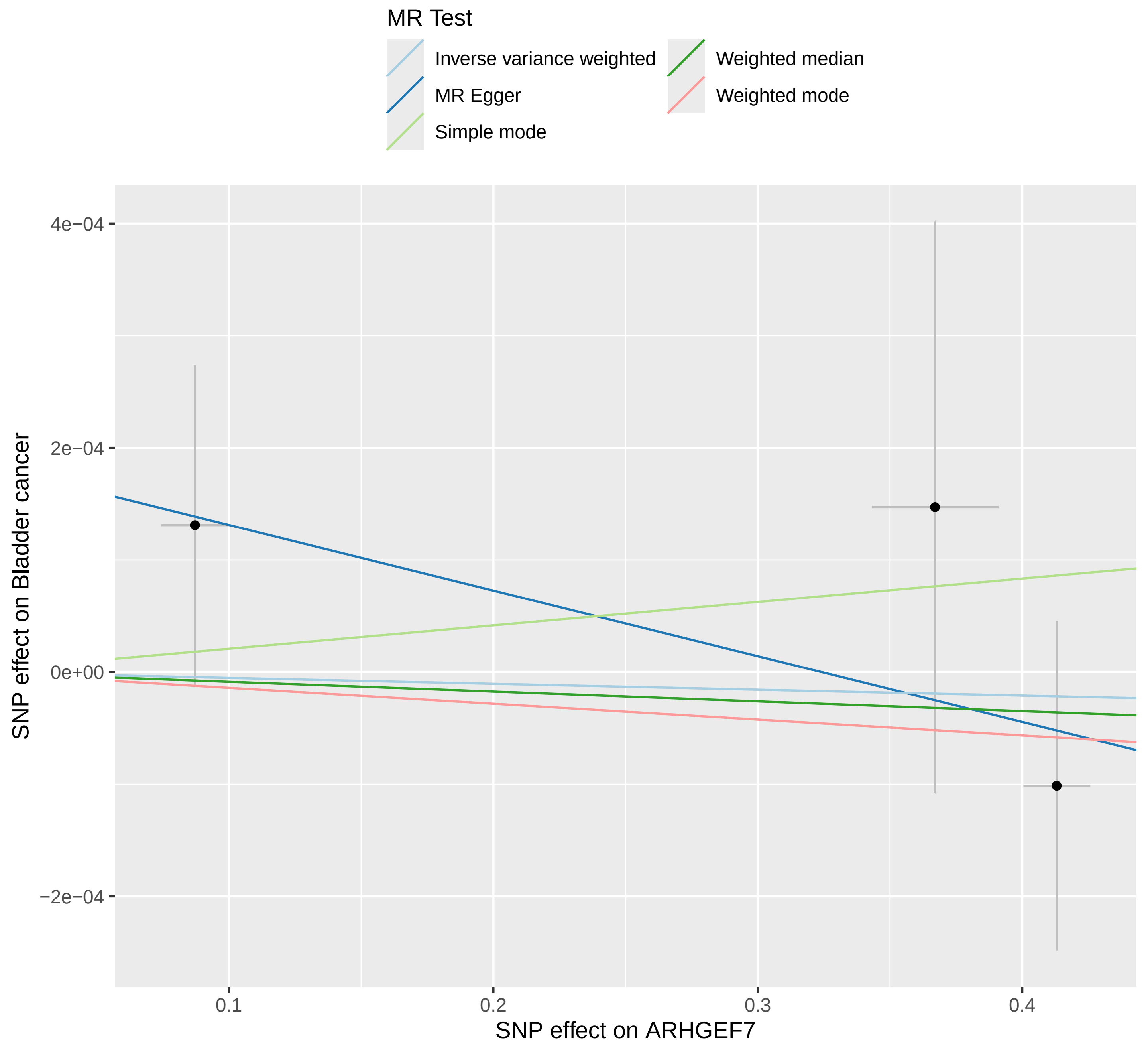

MR Test
Inverse variance weighted
MR Egger
Simple mode
Weighted median
Weighted mode
4e−04
2e−04
0e+00
−2e−04
SNP effect on Bladder cancer
0.1
0.2
0.3
0.4
SNP effect on ARHGEF7

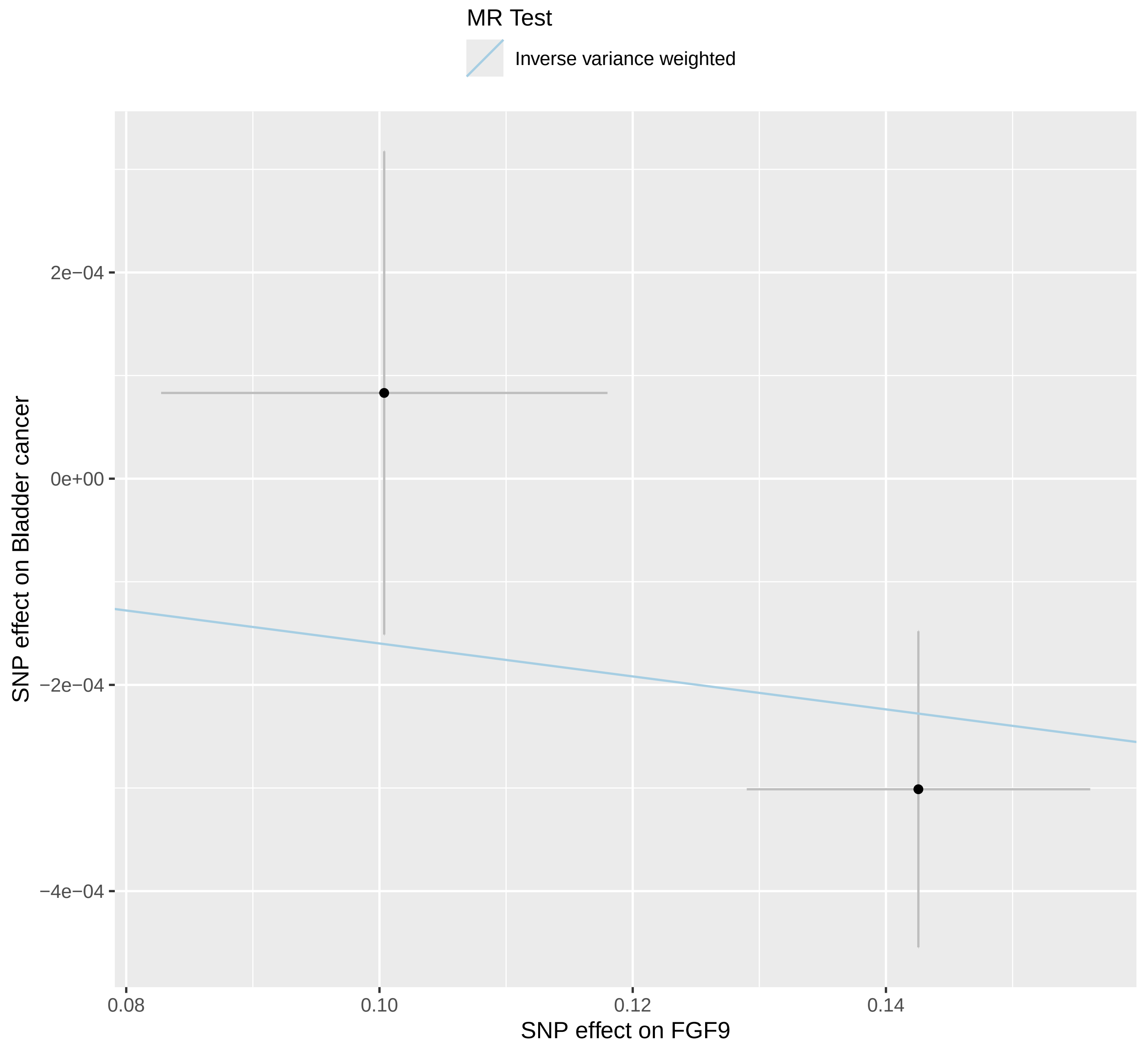
MR Test
Inverse variance weighted
2e−04
0e+00
−2e−04
−4e−04
0.08
0.10
0.12
0.14
SNP effect on FGF9
SNP effect on Bladder cancer

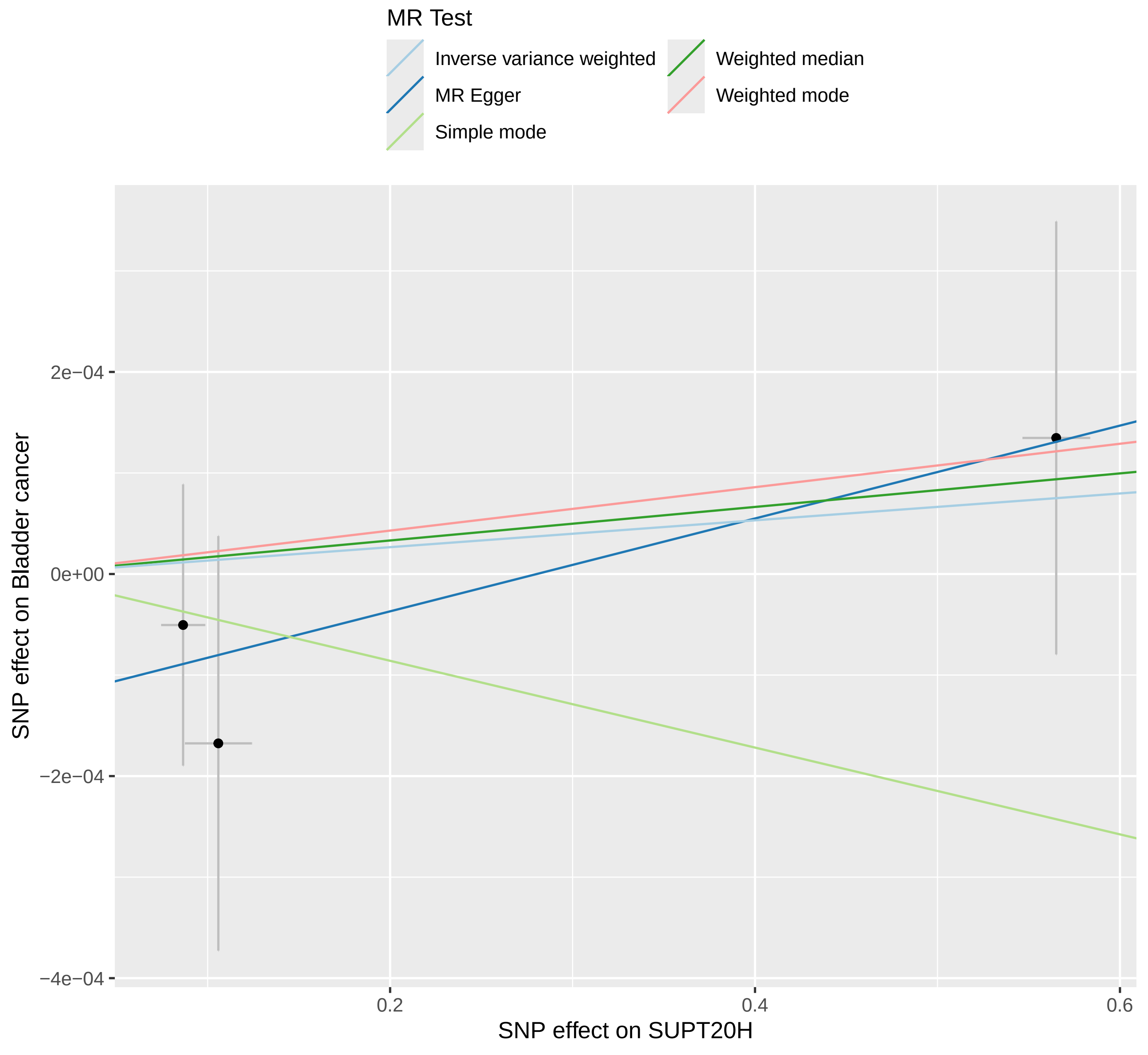

MR Test
Inverse variance weighted
MR Egger
Simple mode
Weighted median
Weighted mode
2e−04
0e+00
−2e−04
−4e−04
0.2
0.4
0.6
SNP effect on Bladder cancer
SNP effect on SUPT20H

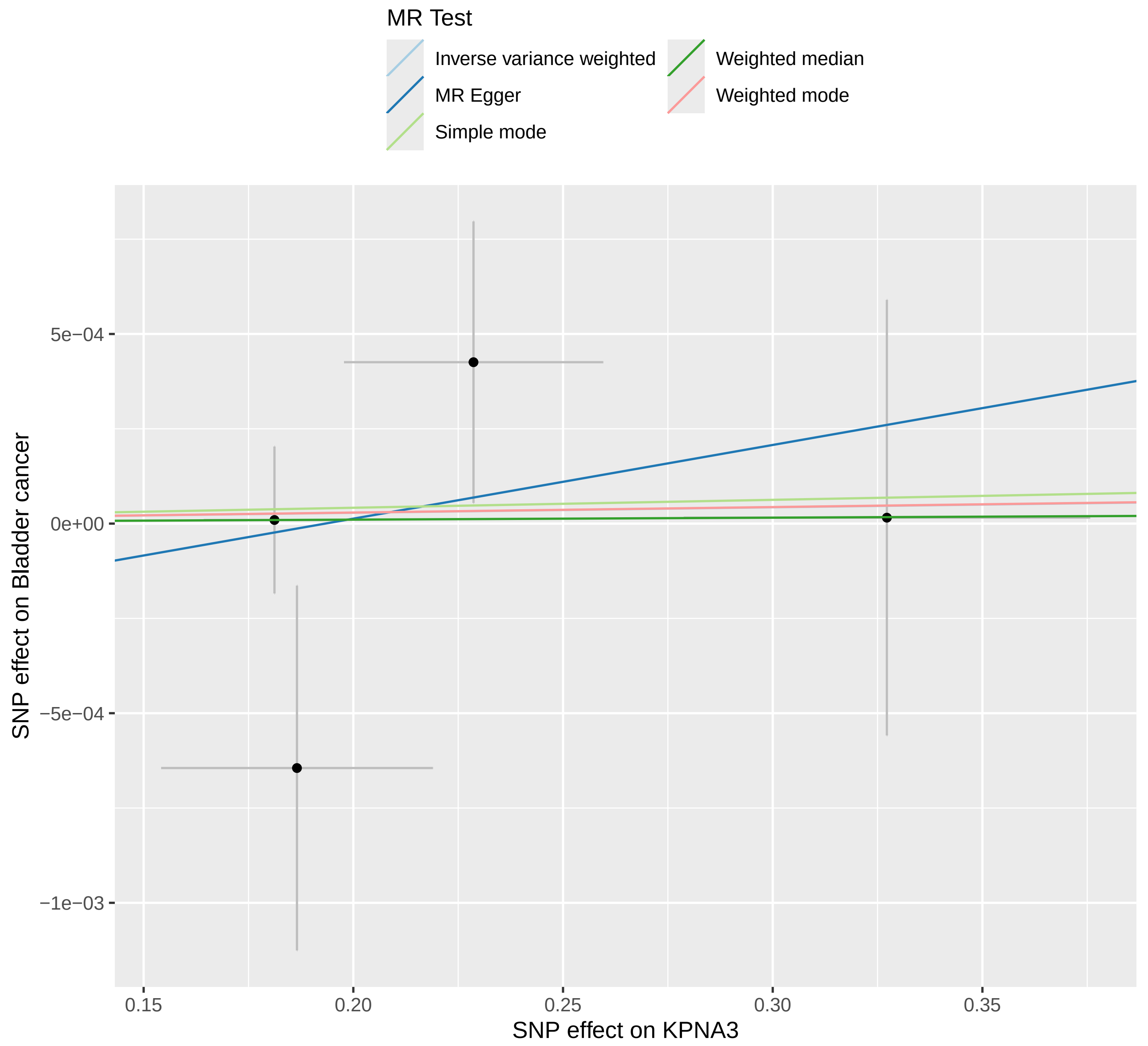

MR Test
Inverse variance weighted
MR Egger
Simple mode
Weighted median
Weighted mode
5e−04
0e+00
−5e−04
−1e−03
0.15
0.20
0.25
0.30
0.35
SNP effect on KPNA3
SNP effect on Bladder cancer

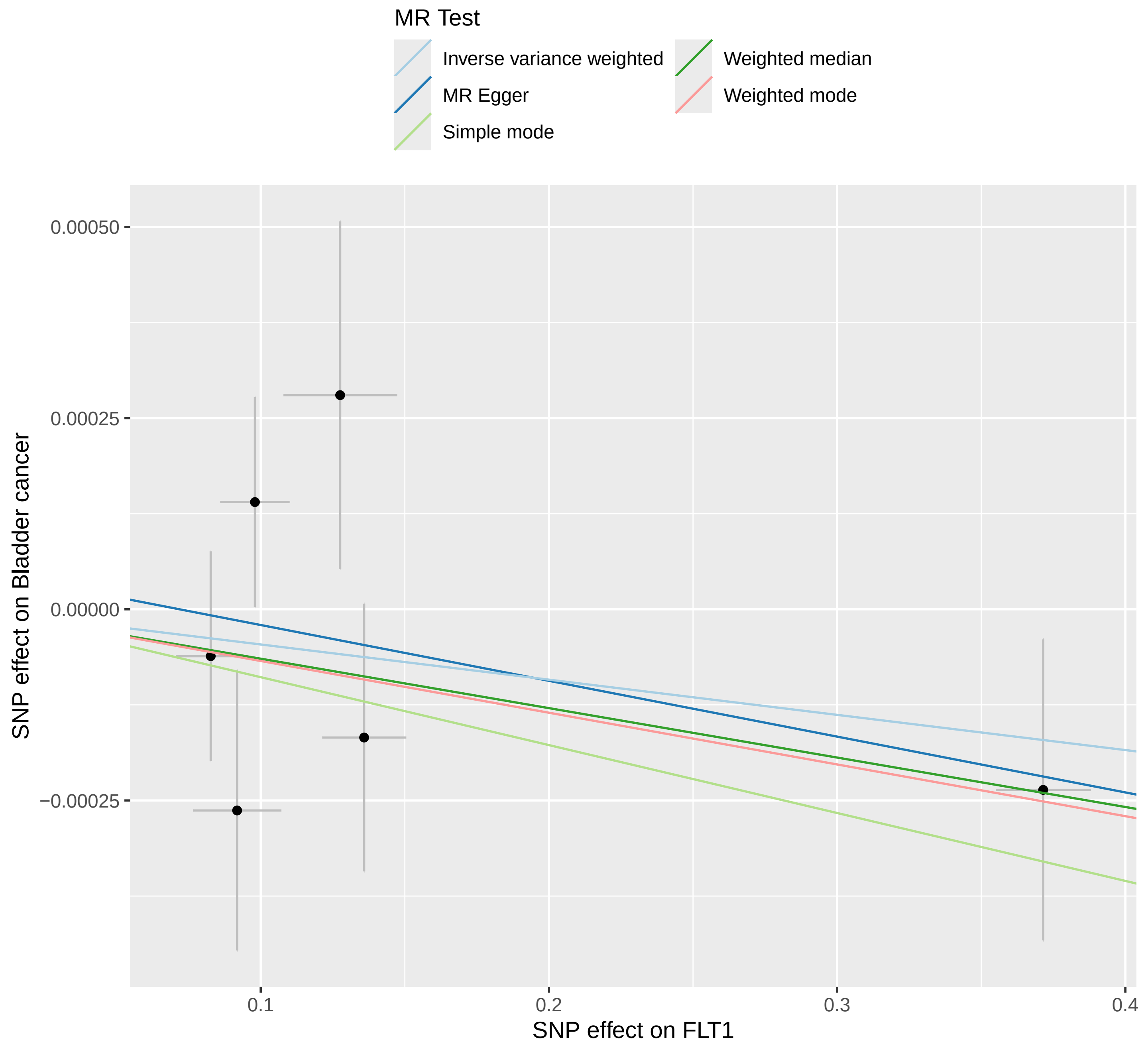
MR Test
Inverse variance weighted
MR Egger
Simple mode
Weighted median
Weighted mode
0.00050
0.00025
0.00000
−0.00025
SNP effect on Bladder cancer
0.1
0.2
0.3
0.4
SNP effect on FLT1

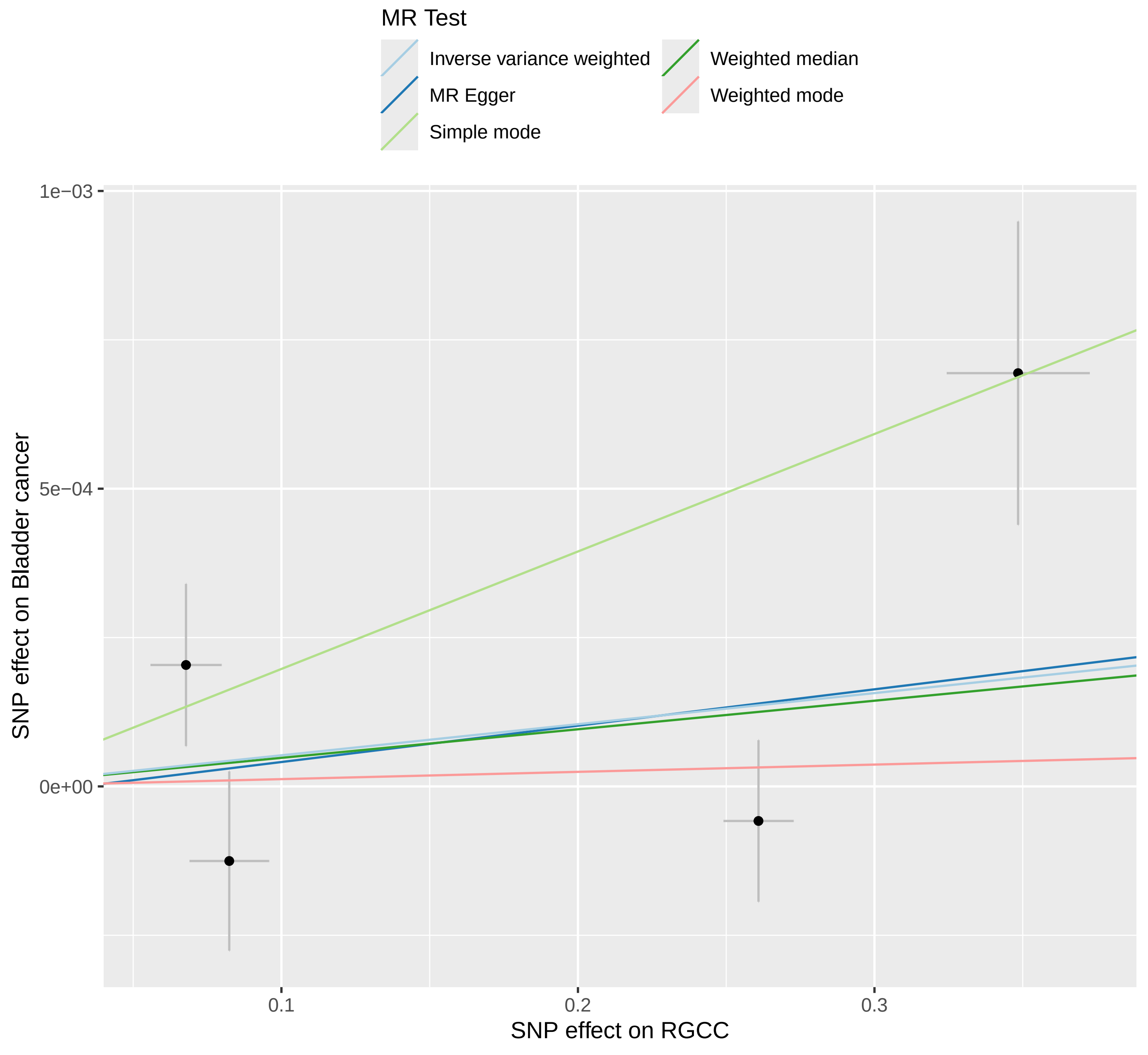
MR Test
Inverse variance weighted
MR Egger
Simple mode
Weighted median
Weighted mode
1e−03
5e−04
0e+00
SNP effect on Bladder cancer
0.1
0.2
0.3
SNP effect on RGCC

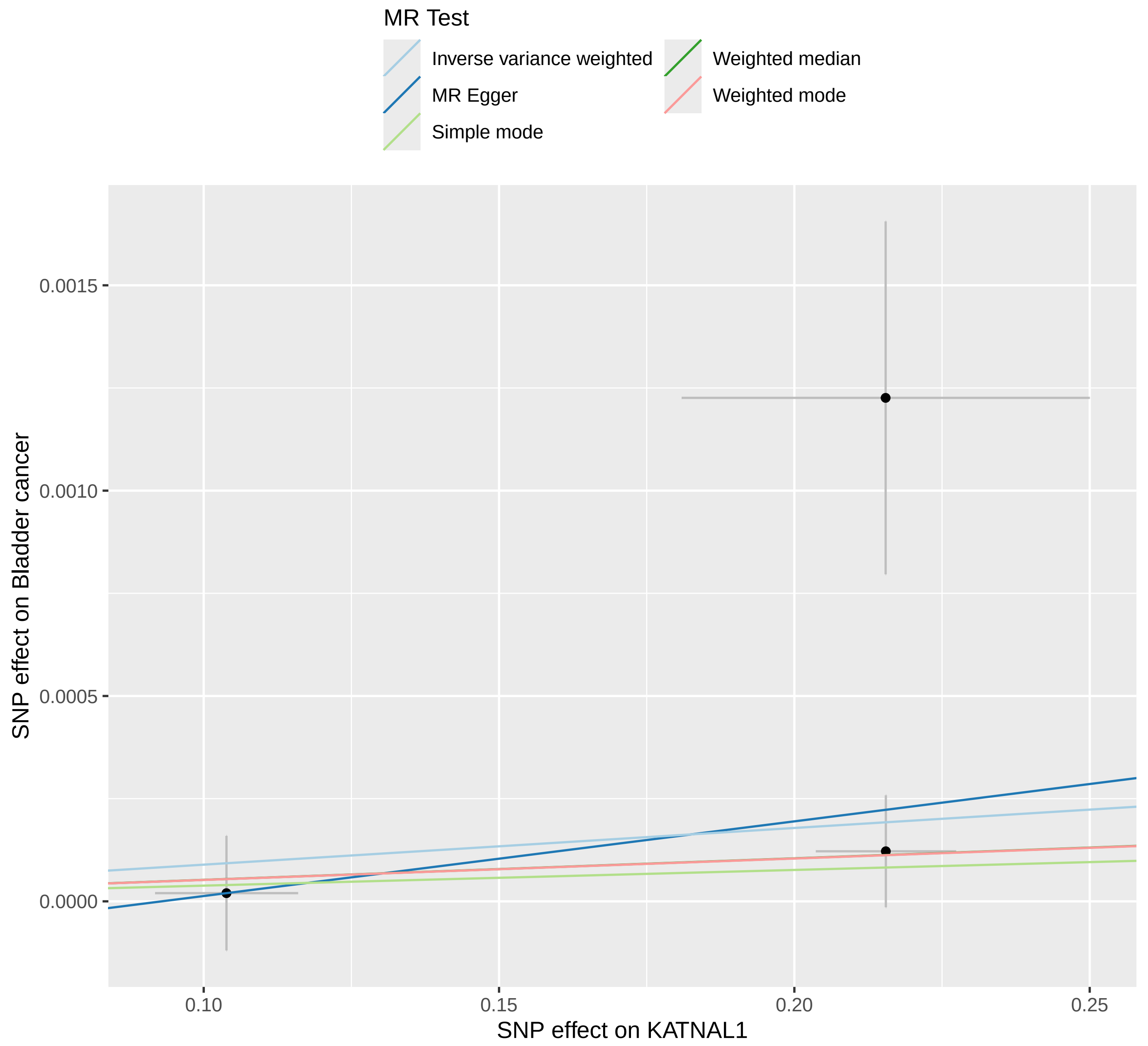
MR Test
Inverse variance weighted
MR Egger
Simple mode
Weighted median
Weighted mode
SNP effect on Bladder cancer
0.0015
0.0010
0.0005
0.0000
0.10
0.15
0.20
0.25
SNP effect on KATNAL1

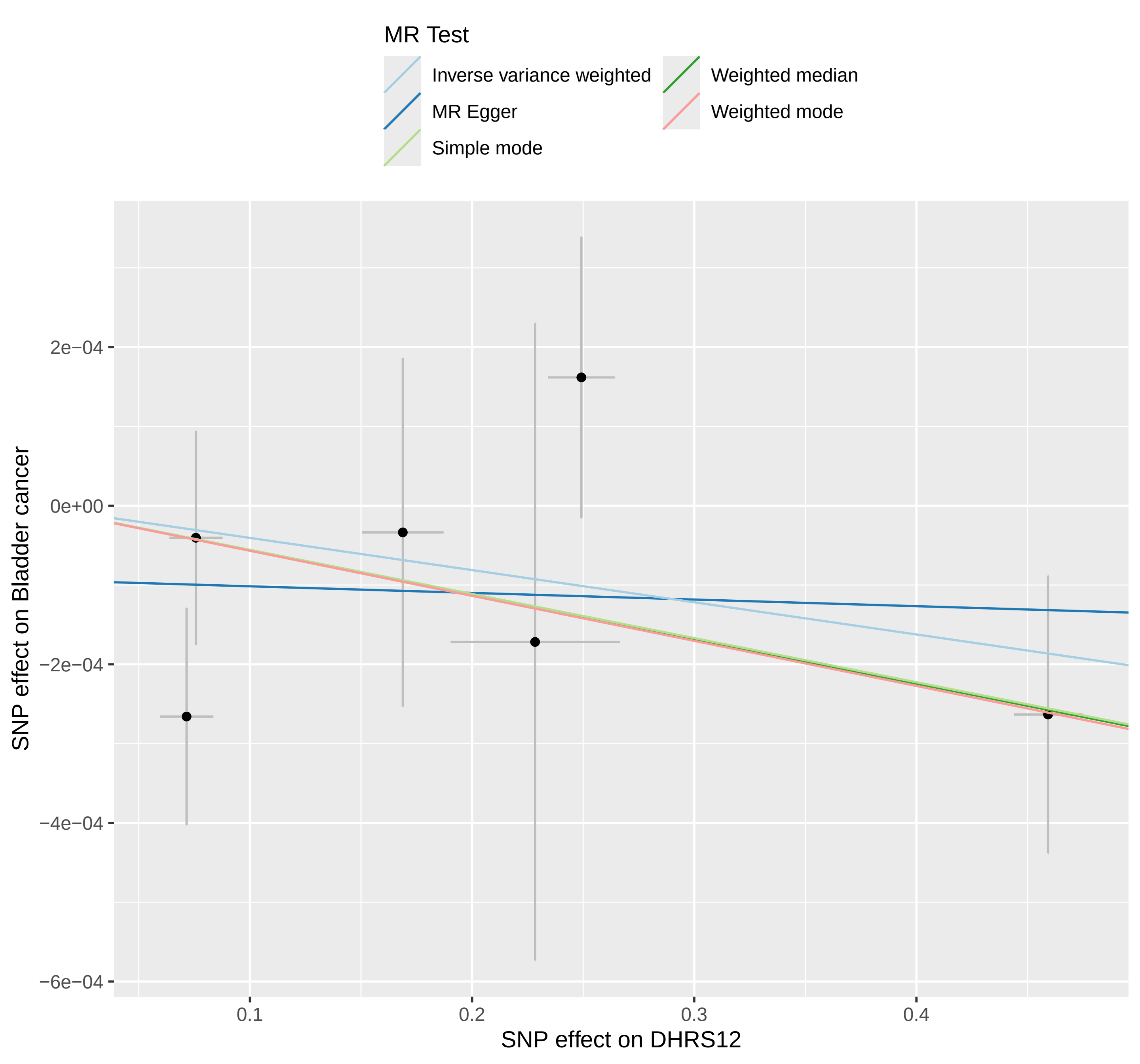

MR Test
Inverse variance weighted
MR Egger
Simple mode
Weighted median
Weighted mode
2e−04
0e+00
−2e−04
−4e−04
−6e−04
SNP effect on Bladder cancer
0.1
0.2
0.3
0.4
SNP effect on DHRS12

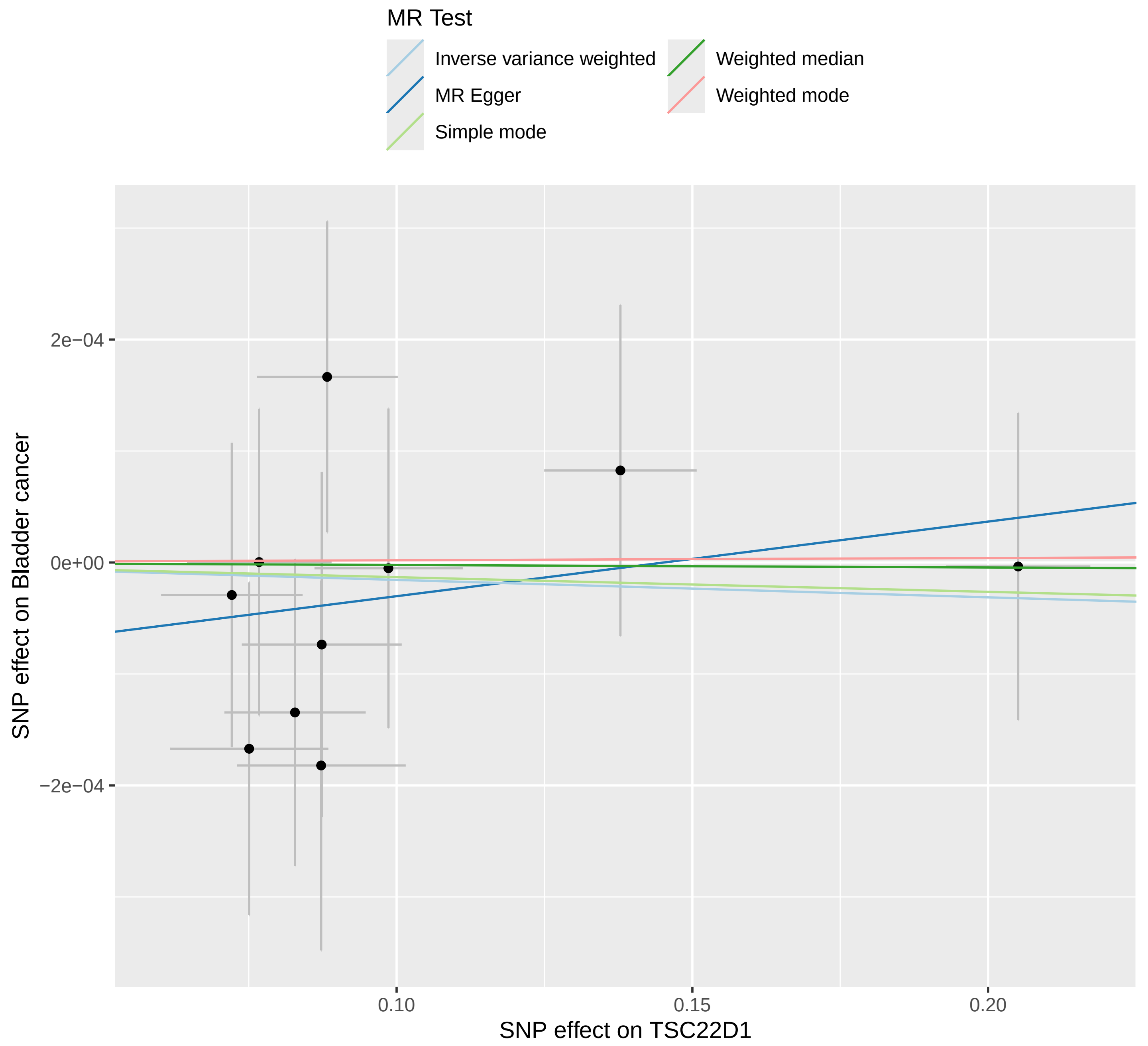

MR Test
Inverse variance weighted
MR Egger
Simple mode
Weighted median
Weighted mode
2e−04
0e+00
−2e−04
0.10
0.15
0.20
SNP effect on TSC22D1
SNP effect on Bladder cancer

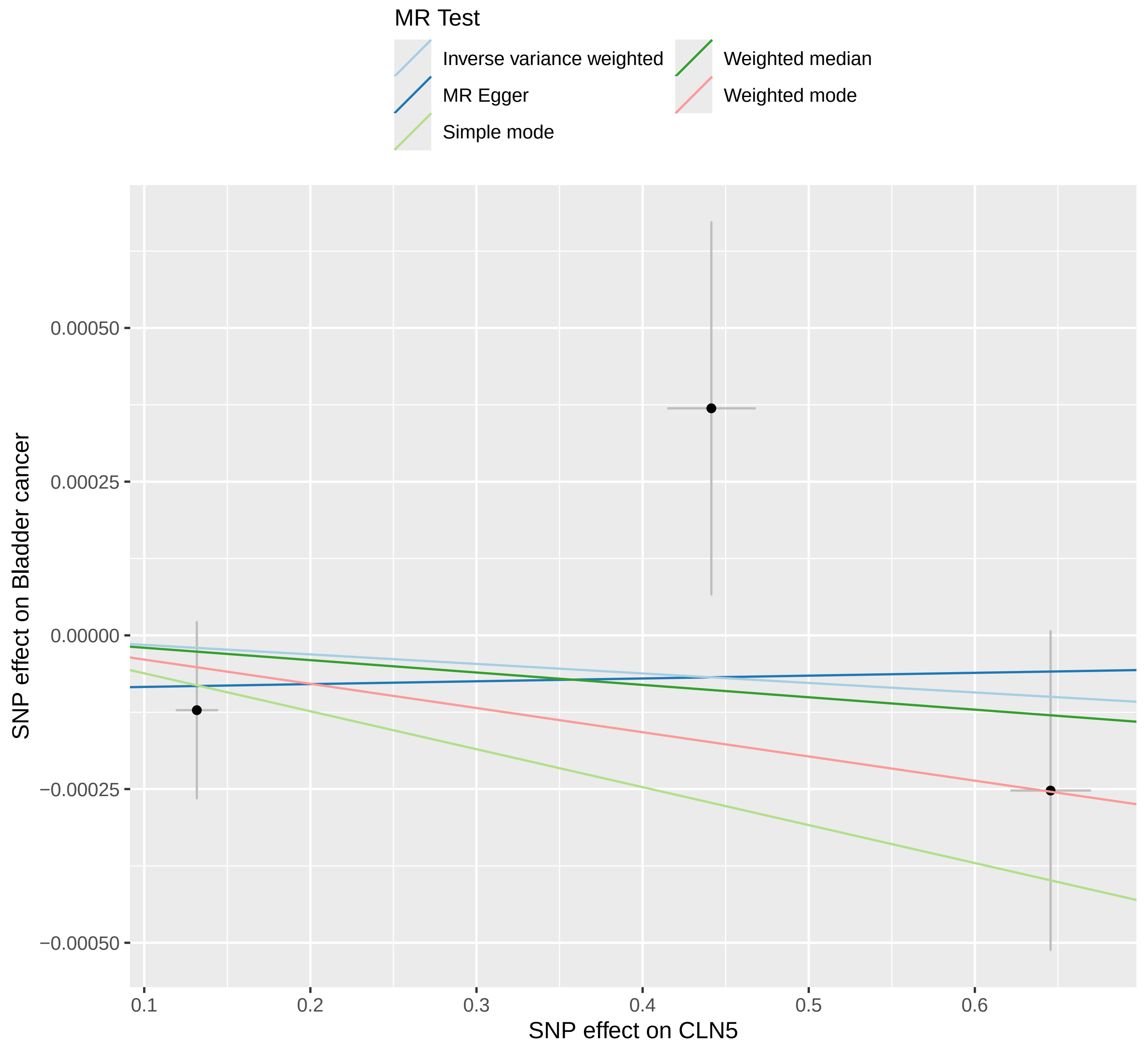

MR Test
Inverse variance weighted
MR Egger
Simple mode
Weighted median
Weighted mode
0.00050
0.00025
0.00000
−0.00025
−0.00050
SNP effect on Bladder cancer
0.1
0.2
0.3
0.4
0.5
0.6
SNP effect on CLN5

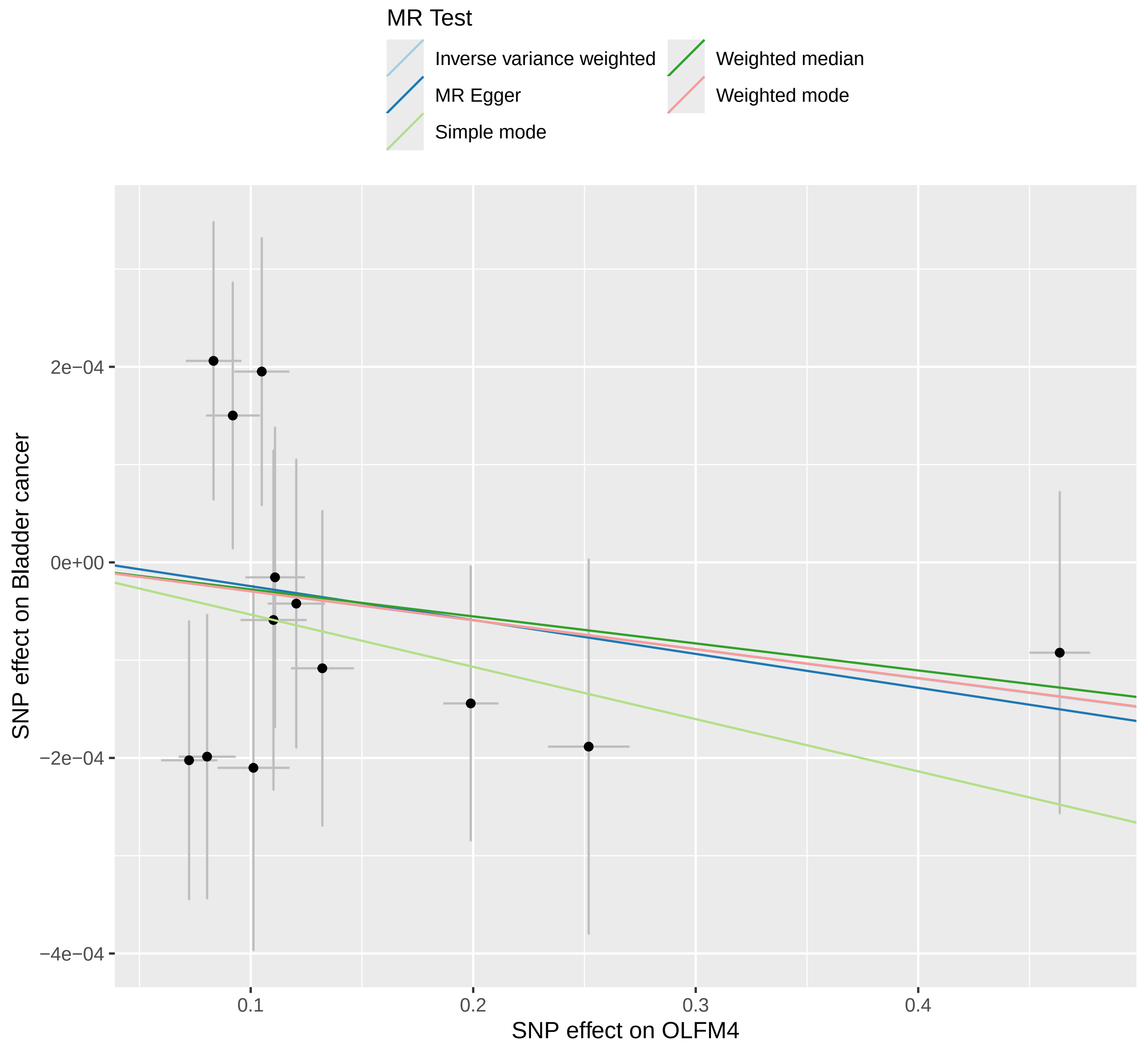
MR Test
Inverse variance weighted
MR Egger
Simple mode
Weighted median
Weighted mode
2e−04
0e+00
−2e−04
−4e−04
SNP effect on Bladder cancer
0.1
0.2
0.3
0.4
SNP effect on OLFM4

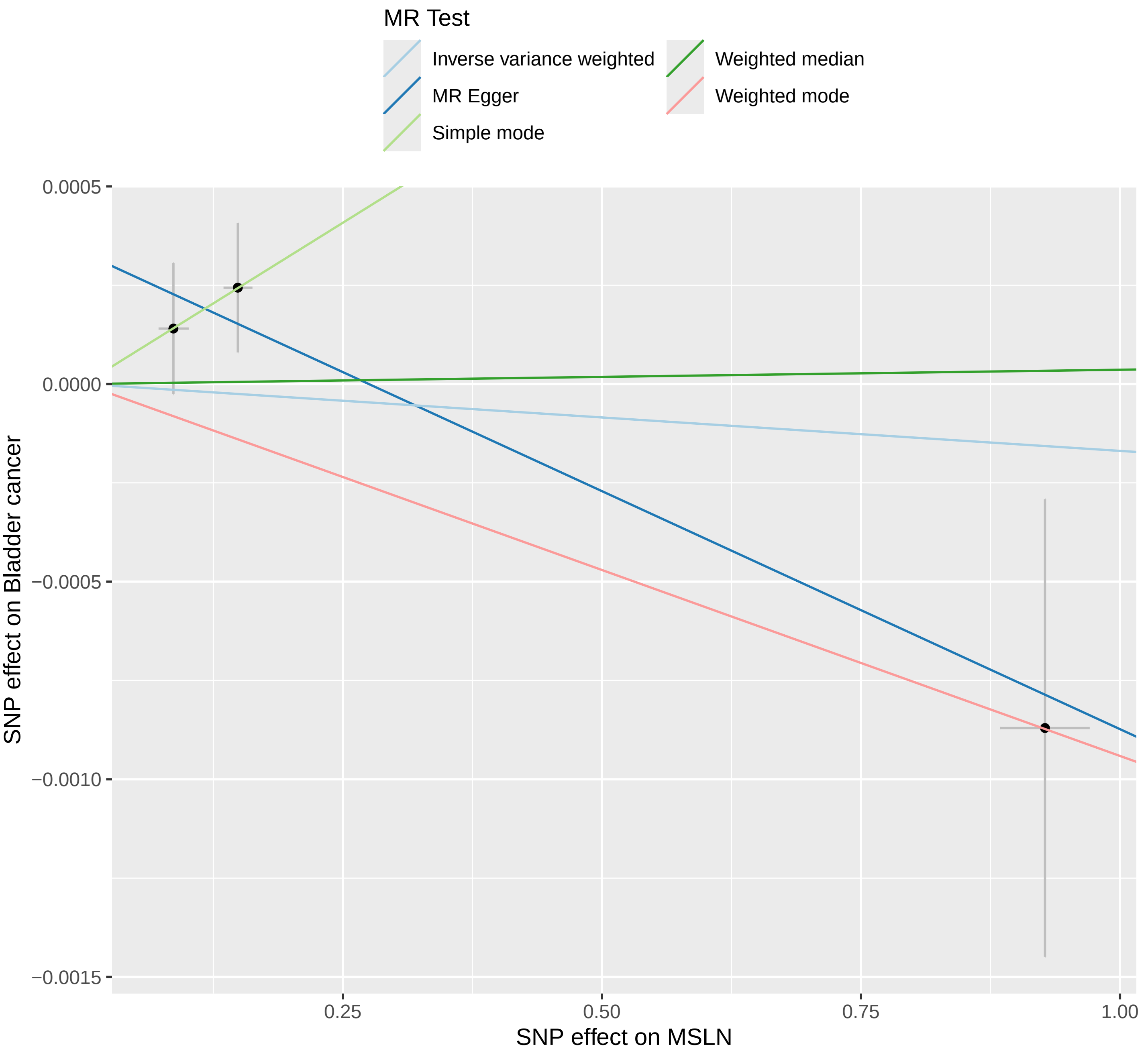

MR Test
Inverse variance weighted
MR Egger
Simple mode
Weighted median
Weighted mode
0.0005
0.0000
−0.0005
−0.0010
−0.0015
SNP effect on Bladder cancer
0.25
0.50
0.75
1.00
SNP effect on MSLN

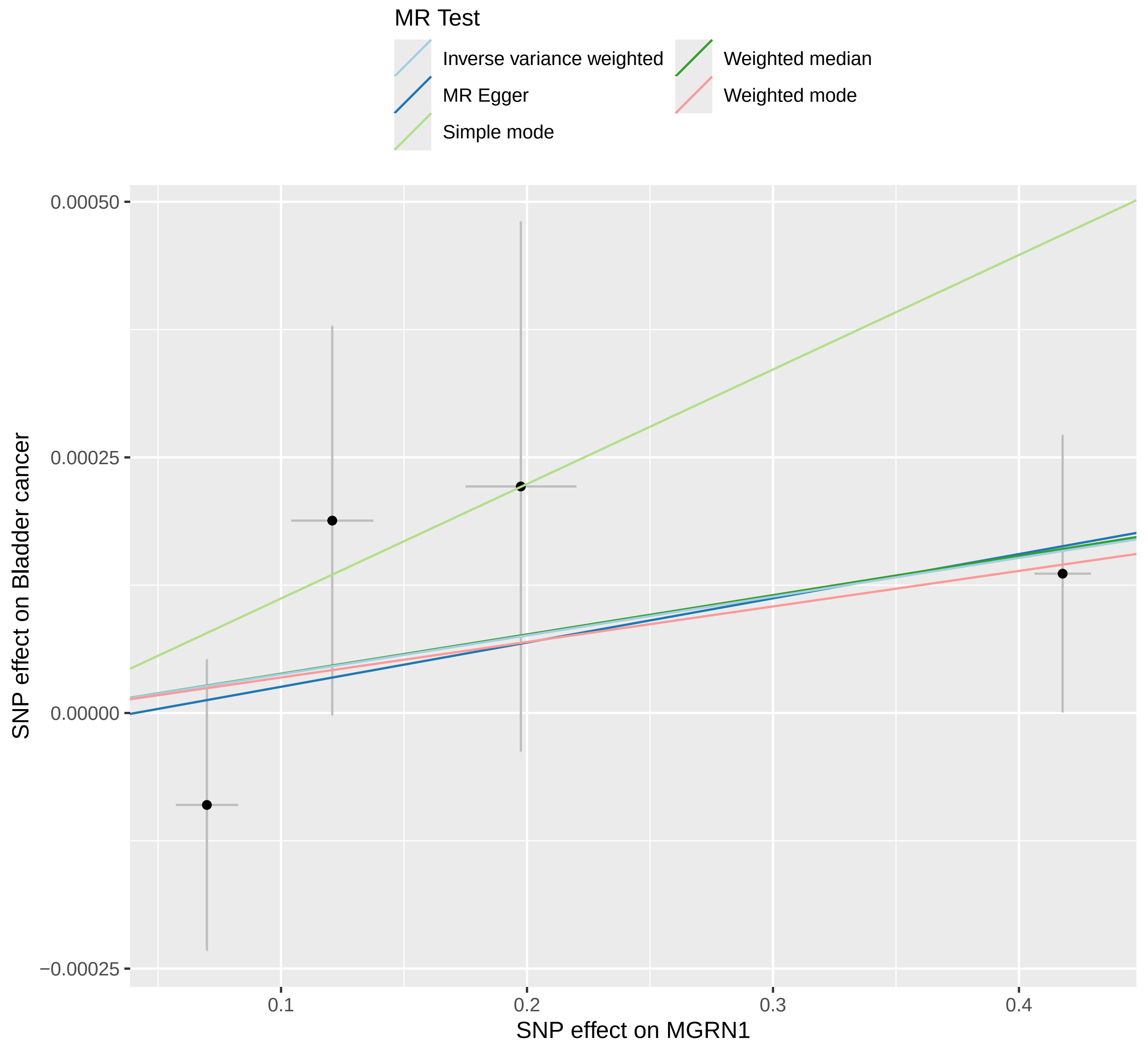

MR Test
Inverse variance weighted
MR Egger
Simple mode
Weighted median
Weighted mode
0.00050
0.00025
0.00000
−0.00025
0.1
0.2
0.3
0.4
SNP effect on MGRN1
SNP effect on Bladder cancer

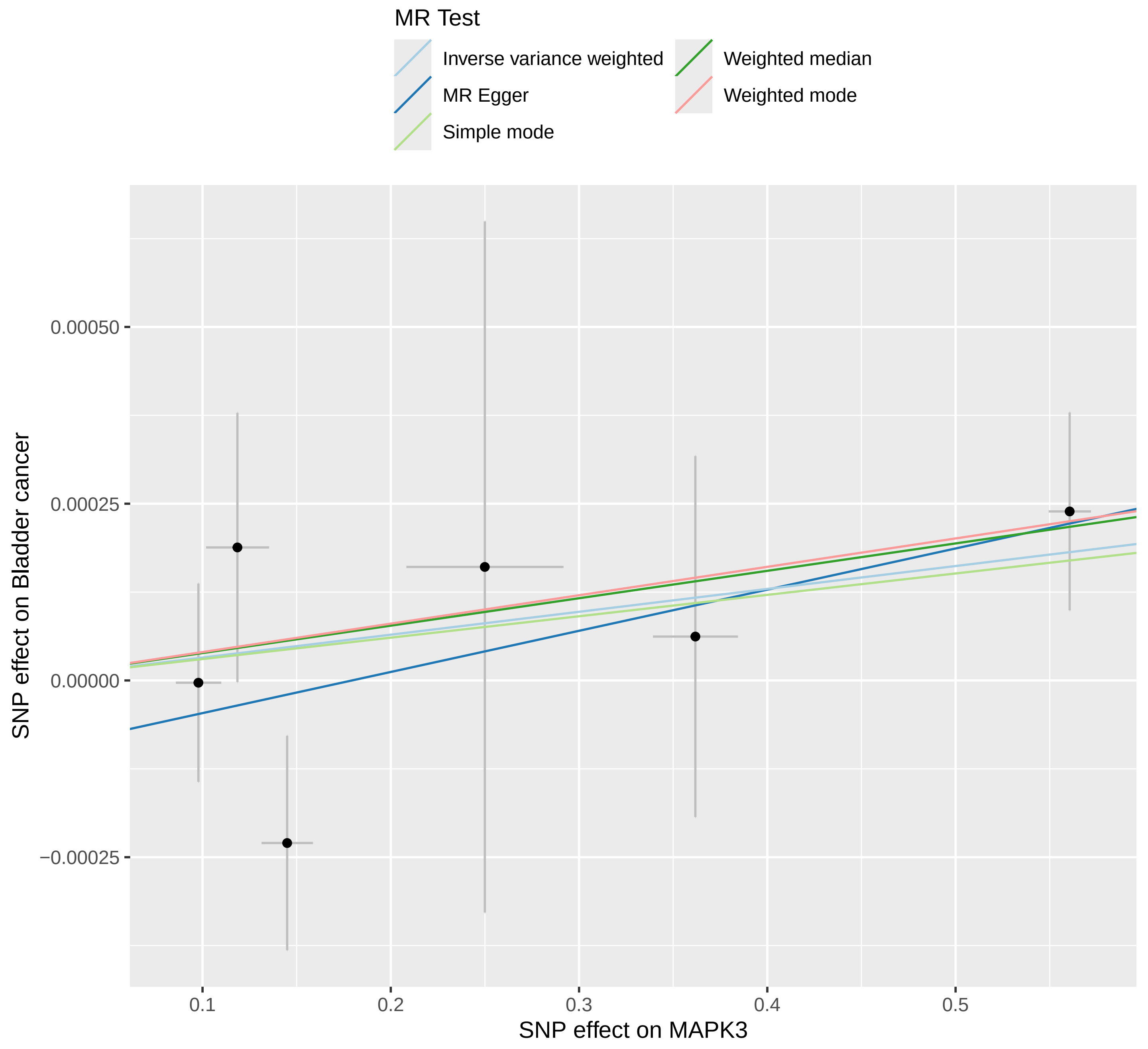
MR Test
Inverse variance weighted
MR Egger
Simple mode
Weighted median
Weighted mode
0.00050
0.00025
0.00000
−0.00025
SNP effect on Bladder cancer
0.1
0.2
0.3
0.4
0.5
SNP effect on MAPK3

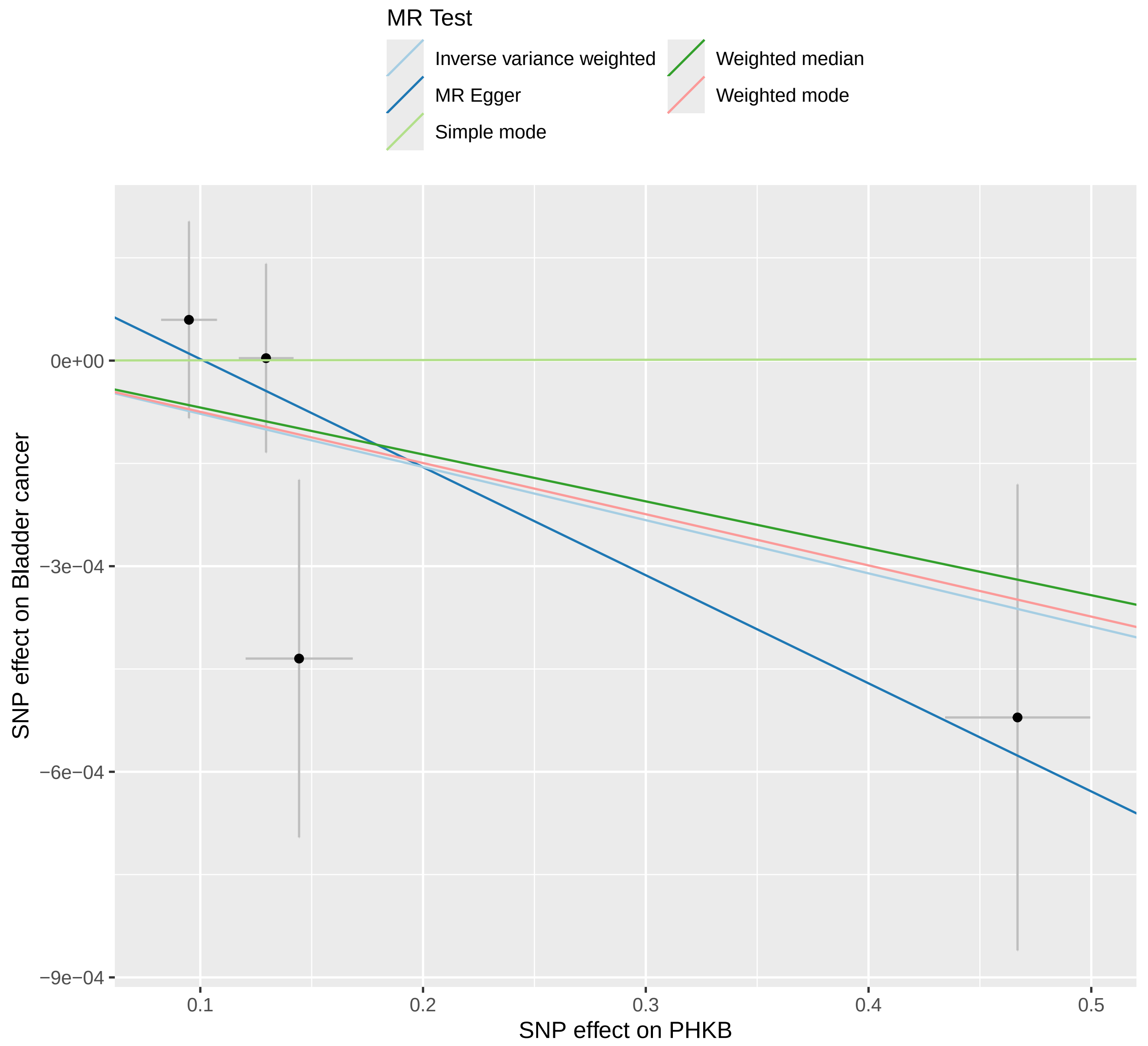

MR Test
Inverse variance weighted
MR Egger
Simple mode
Weighted median
Weighted mode
0e+00
−3e−04
−6e−04
−9e−04
SNP effect on Bladder cancer
0.1
0.2
0.3
0.4
0.5
SNP effect on PHKB

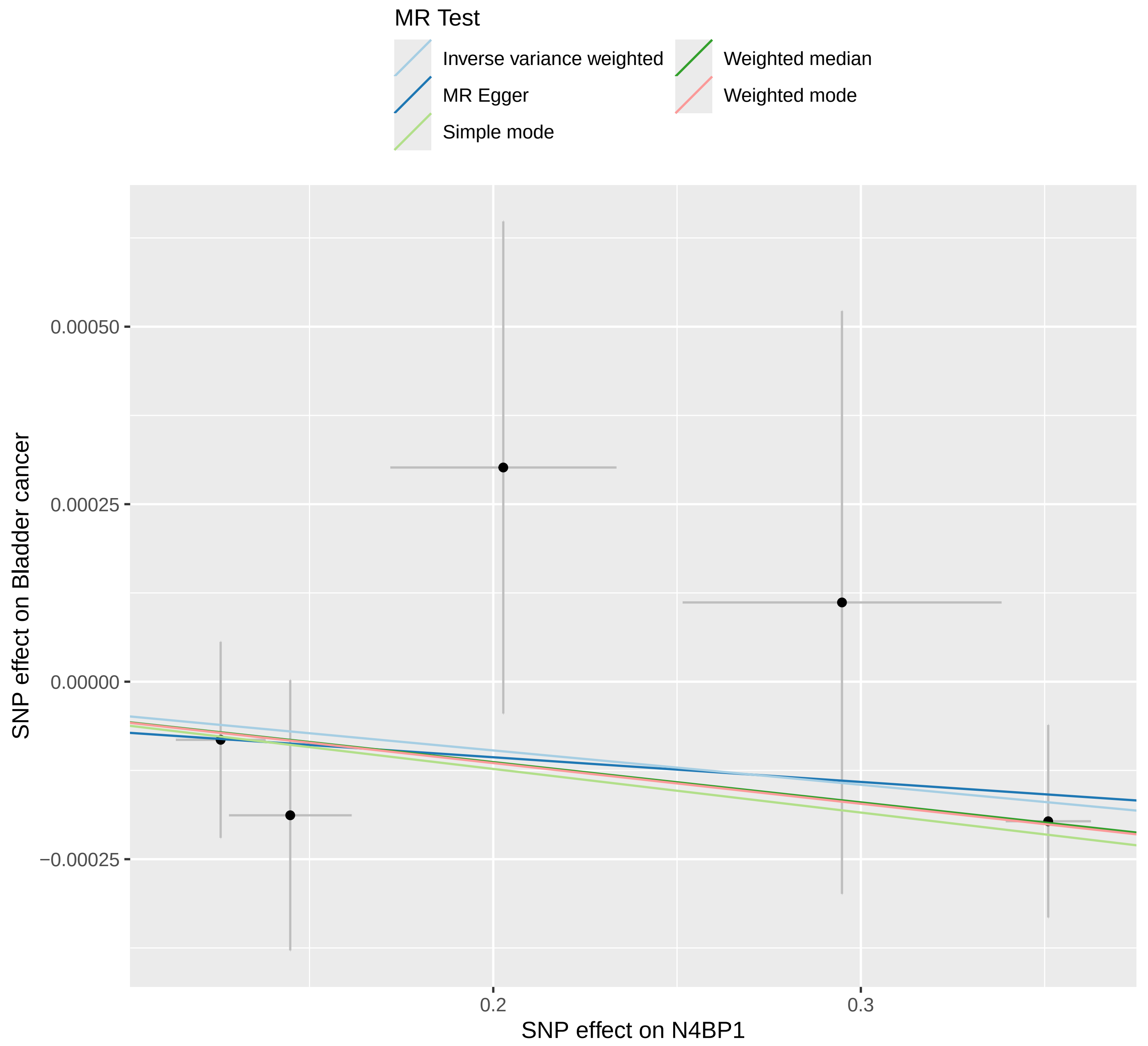

MR Test
Inverse variance weighted
MR Egger
Simple mode
Weighted median
Weighted mode
0.00050
0.00025
0.00000
−0.00025
SNP effect on Bladder cancer
0.2
0.3
SNP effect on N4BP1

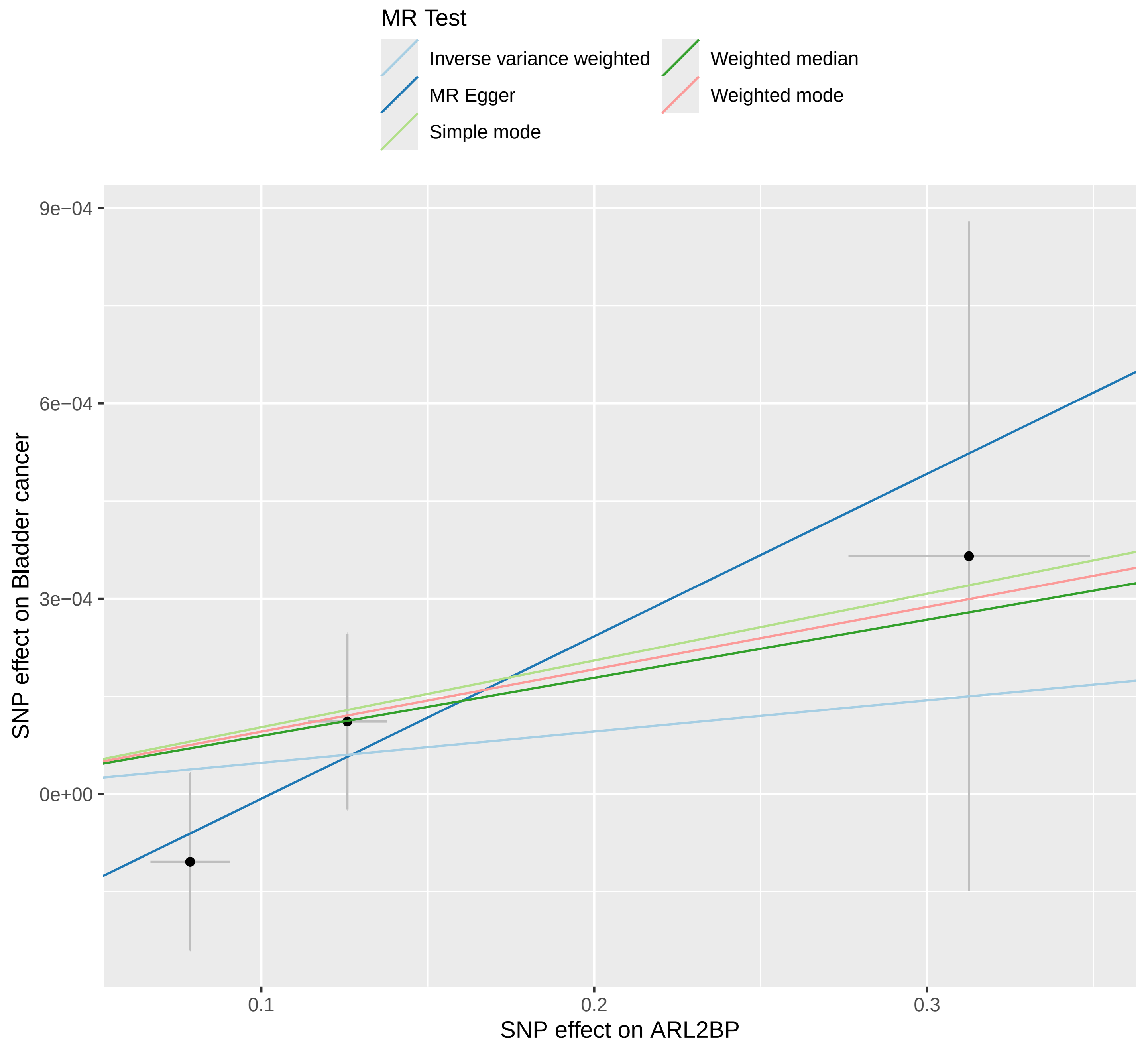
MR Test
Inverse variance weighted
MR Egger
Simple mode
Weighted median
Weighted mode
9e−04
6e−04
3e−04
0e+00
SNP effect on Bladder cancer
0.1
0.2
0.3
SNP effect on ARL2BP

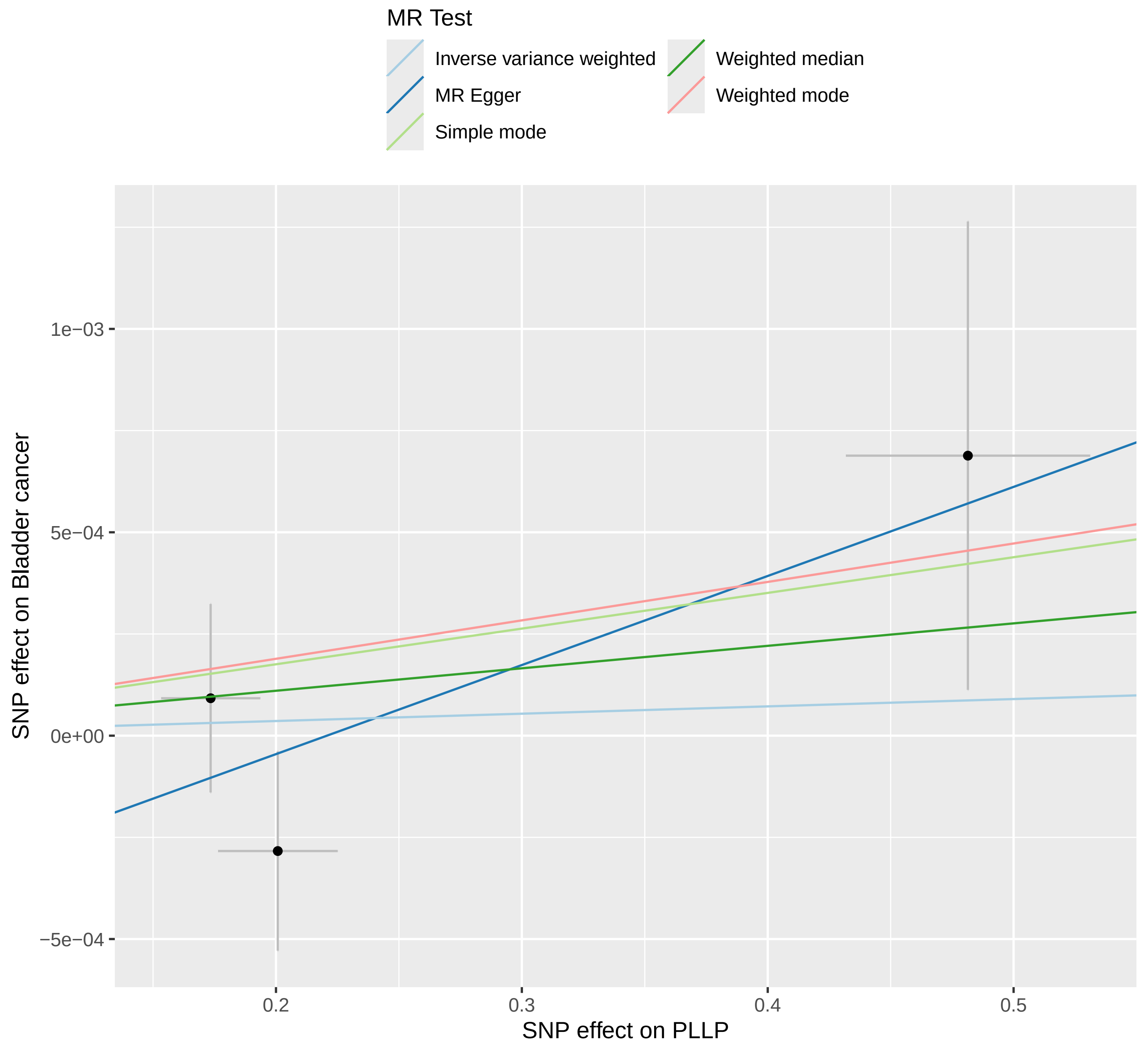

MR Test
Inverse variance weighted
MR Egger
Simple mode
Weighted median
Weighted mode
1e−03
5e−04
0e+00
−5e−04
SNP effect on Bladder cancer
0.2
0.3
0.4
0.5
SNP effect on PLLP

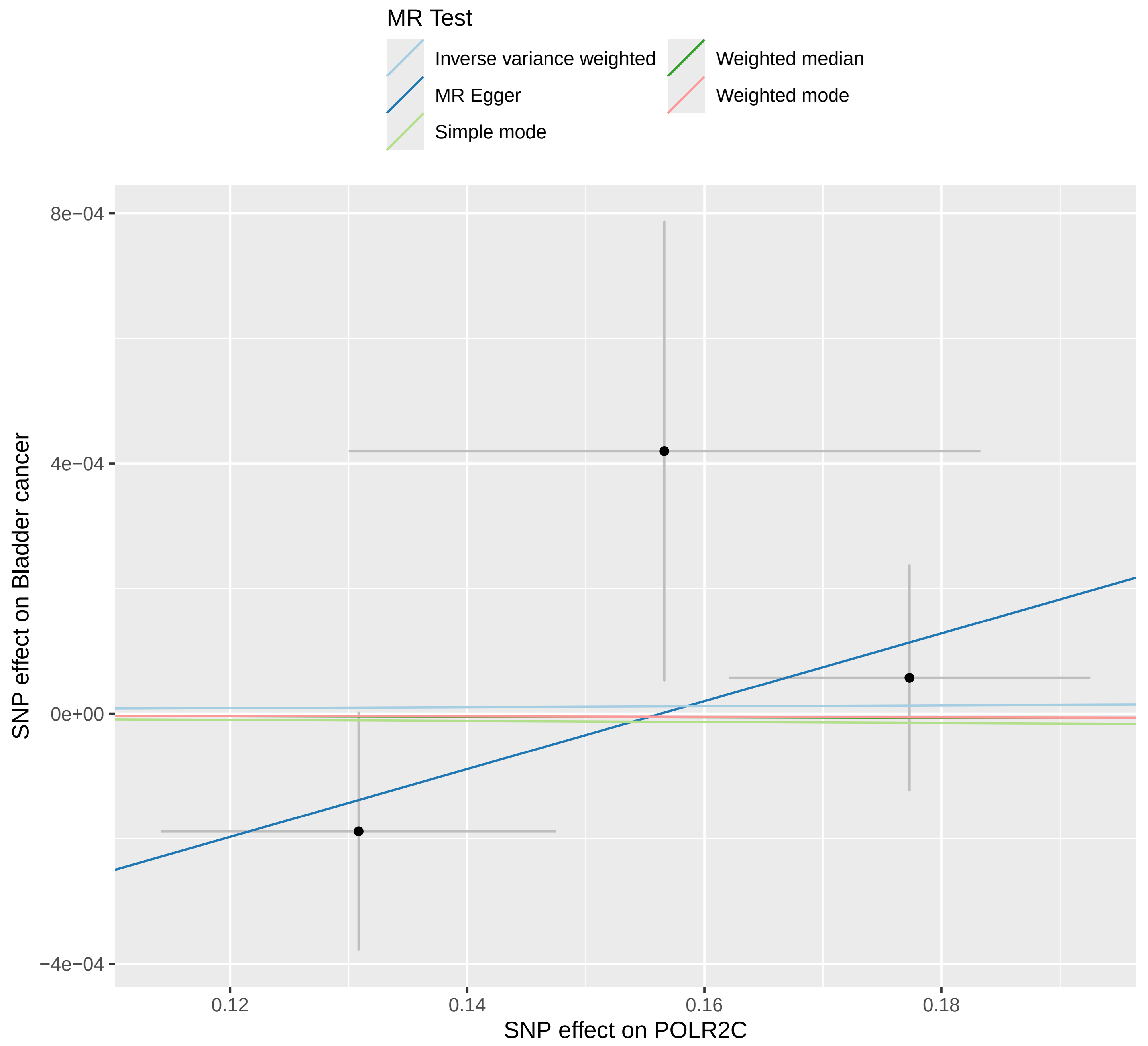

MR Test
Inverse variance weighted
MR Egger
Simple mode
Weighted median
Weighted mode
8e−04
4e−04
0e+00
−4e−04
0.12
0.14
0.16
0.18
SNP effect on POLR2C
SNP effect on Bladder cancer

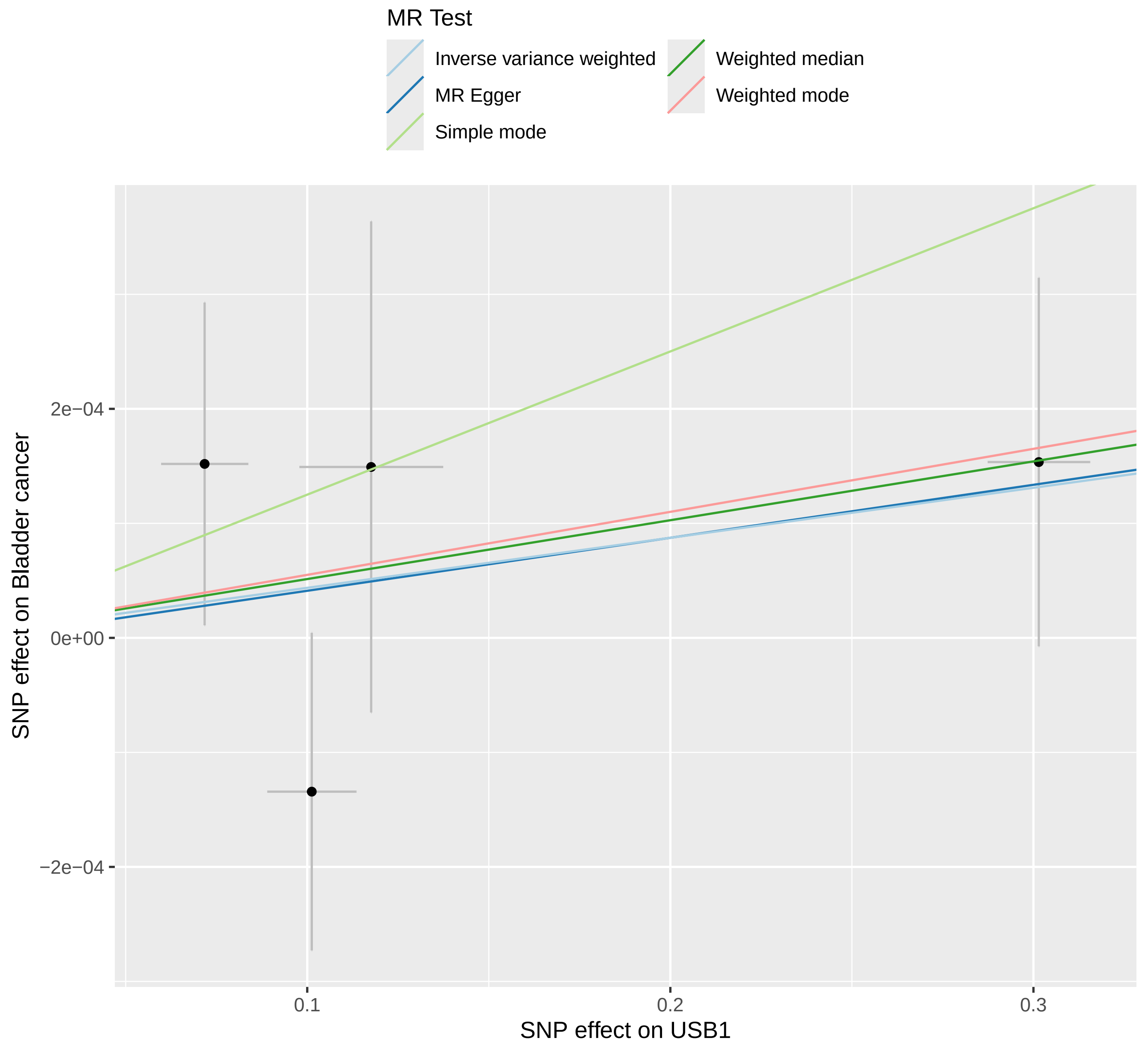

MR Test
Inverse variance weighted
MR Egger
Simple mode
Weighted median
Weighted mode
2e−04
0e+00
−2e−04
SNP effect on Bladder cancer
0.1
0.2
0.3
SNP effect on USB1

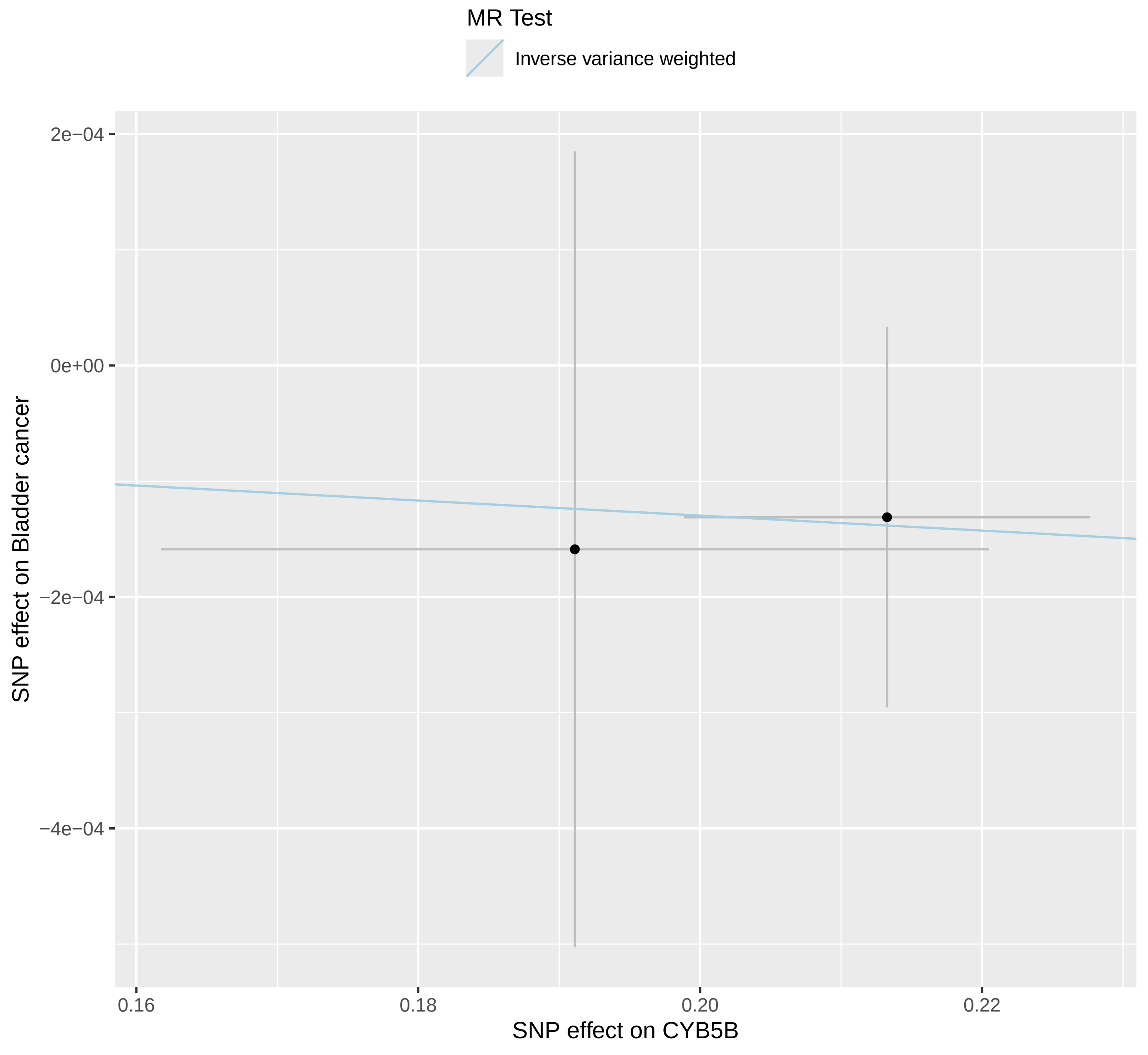
MR Test
Inverse variance weighted
2e−04
0e+00
−2e−04
−4e−04
SNP effect on Bladder cancer
0.16
0.18
0.20
0.22
SNP effect on CYB5B

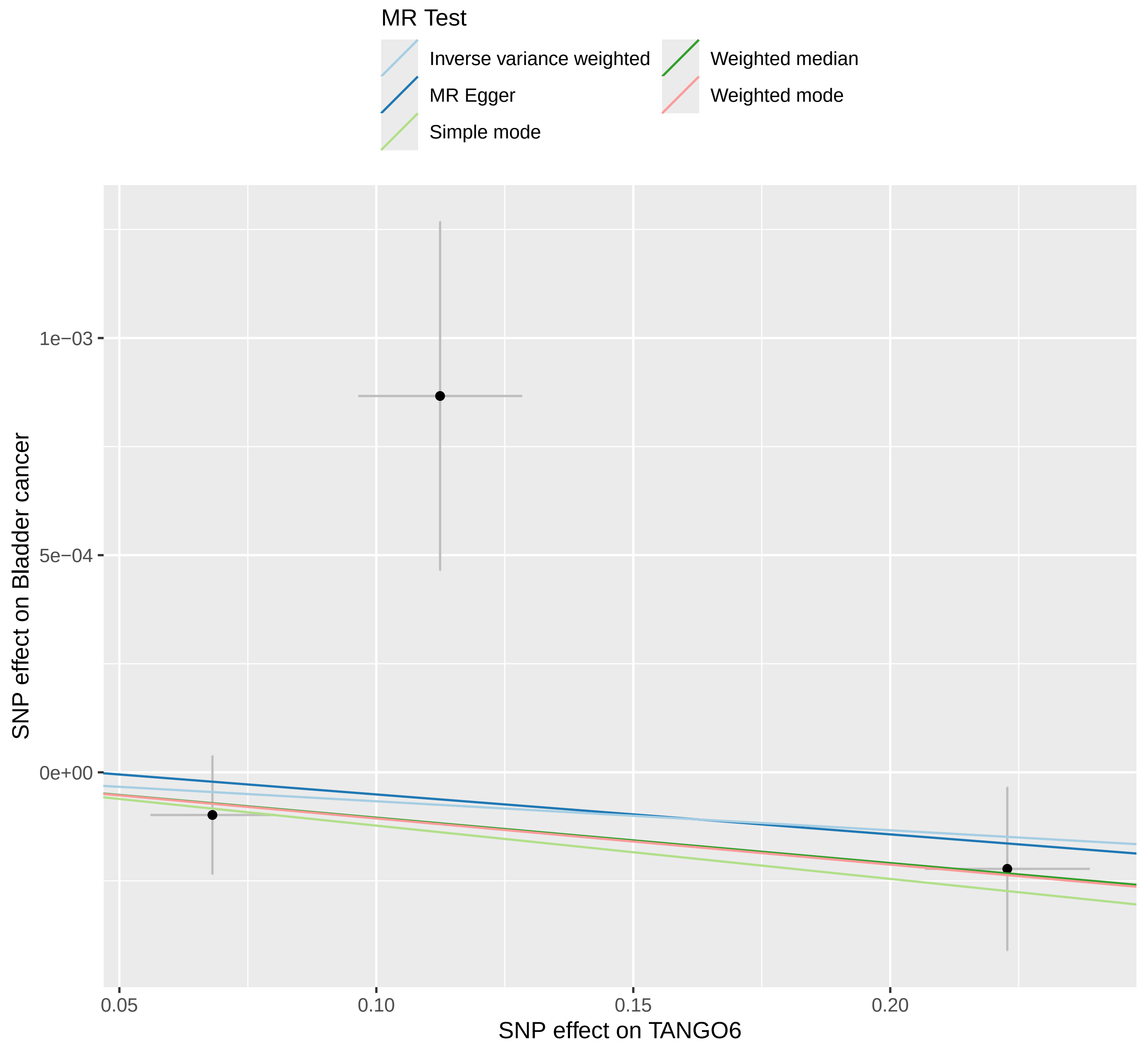
MR Test
Inverse variance weighted
MR Egger
Simple mode
Weighted median
Weighted mode
1e−03
5e−04
0e+00
0.05
0.10
0.15
0.20
SNP effect on Bladder cancer
SNP effect on TANGO6

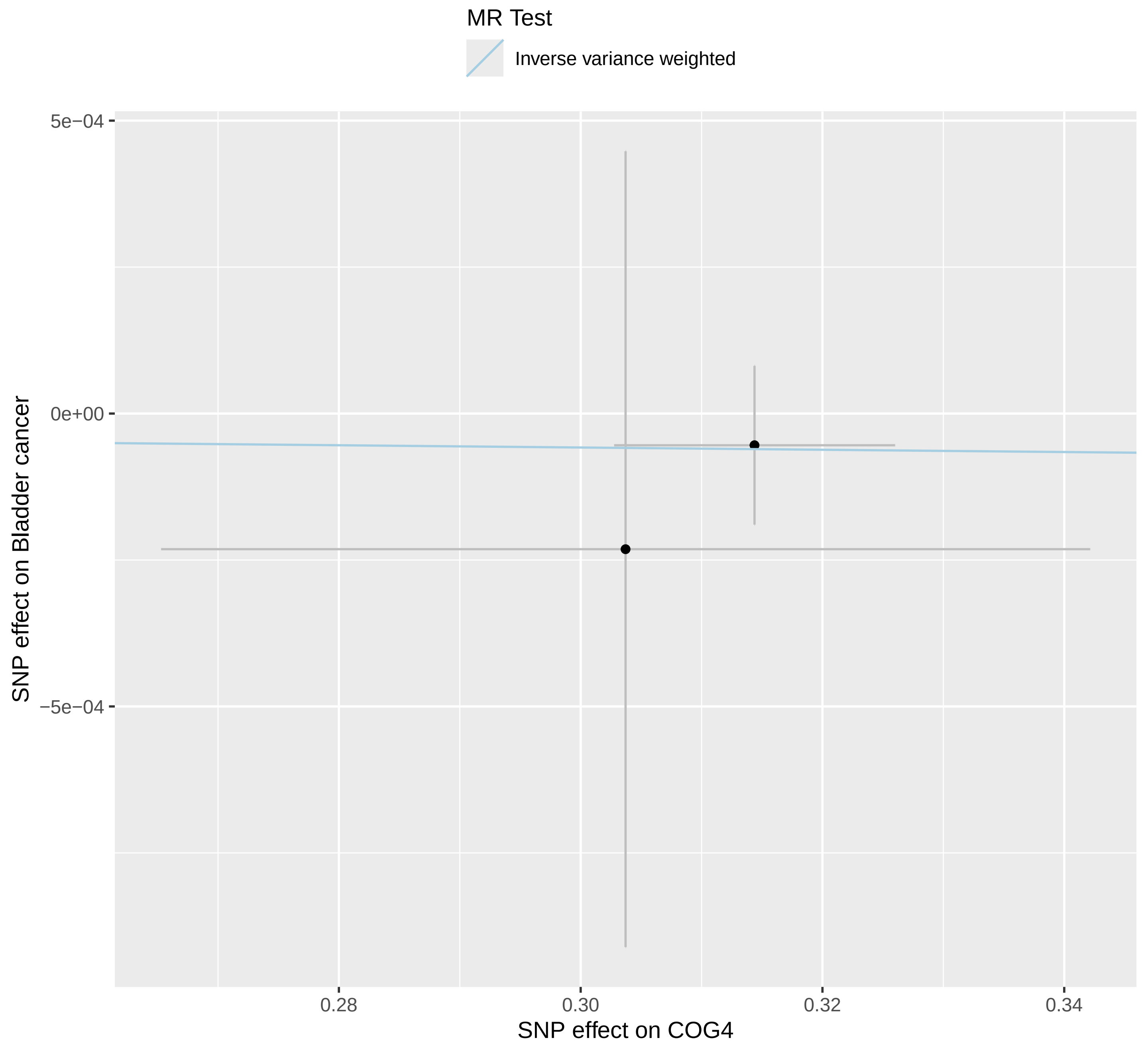

MR Test
Inverse variance weighted
5e−04
0e+00
−5e−04
SNP effect on Bladder cancer
0.28
0.30
0.32
0.34
SNP effect on COG4

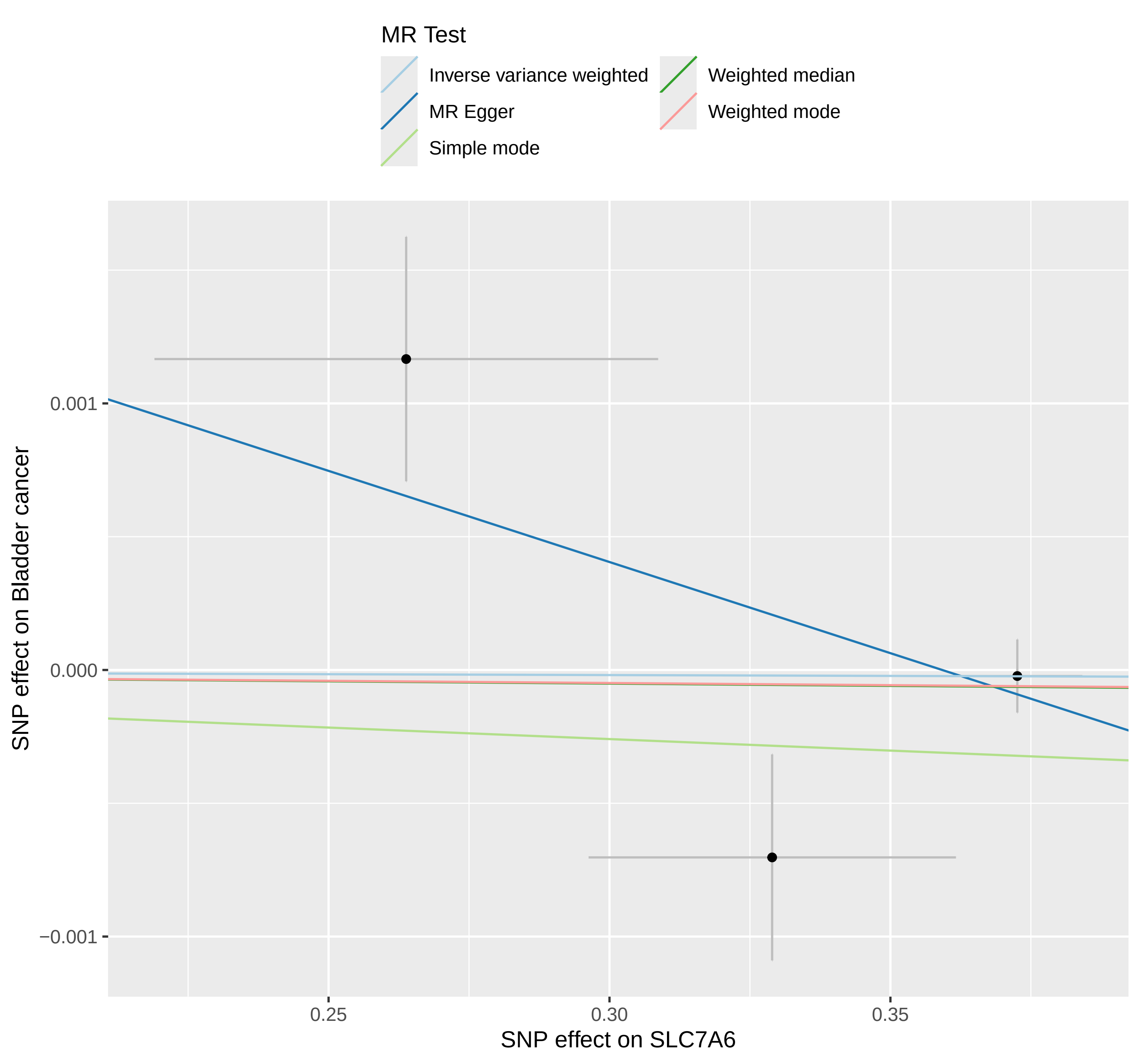

MR Test
Inverse variance weighted
MR Egger
Simple mode
Weighted median
Weighted mode
0.001
0.000
−0.001
SNP effect on Bladder cancer
0.25
0.30
0.35
SNP effect on SLC7A6

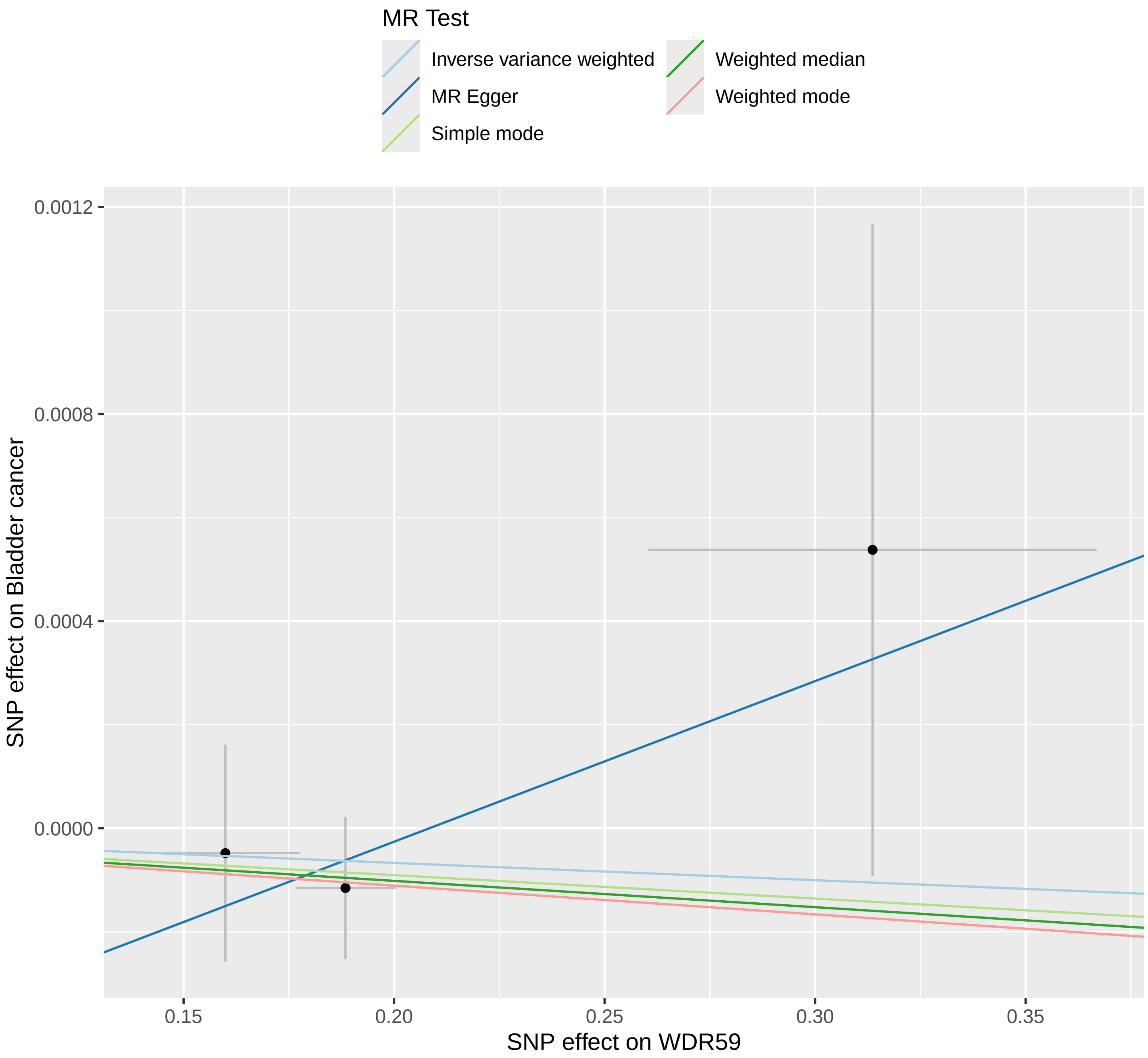

MR Test
Inverse variance weighted
MR Egger
Simple mode
Weighted median
Weighted mode
0.0012
0.0008
0.0004
0.0000
SNP effect on Bladder cancer
0.15
0.20
0.25
0.30
0.35
SNP effect on WDR59

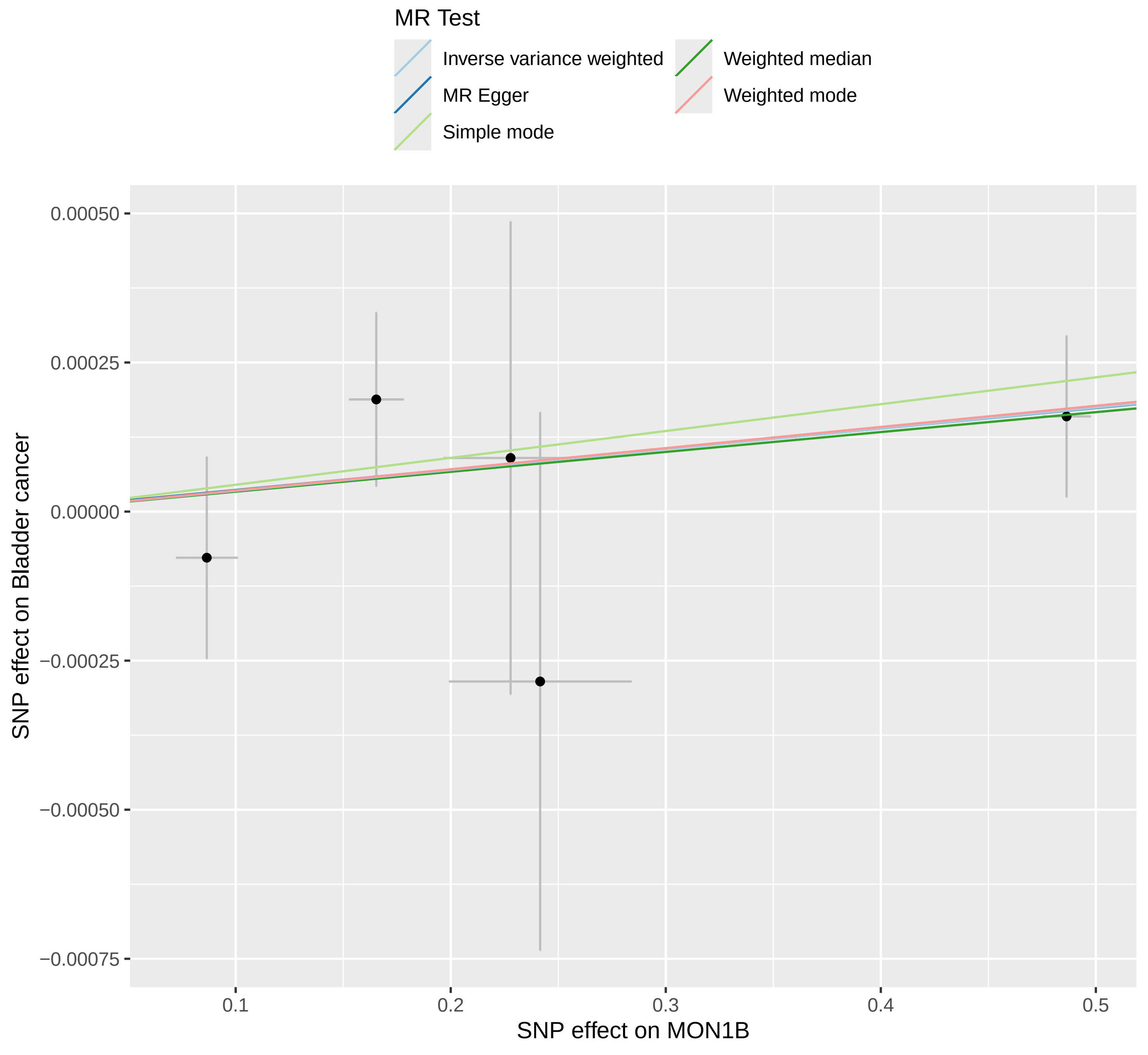

MR Test
Inverse variance weighted
MR Egger
Simple mode
Weighted median
Weighted mode
0.00050
0.00025
0.00000
−0.00025
−0.00050
−0.00075
SNP effect on Bladder cancer
0.1
0.2
0.3
0.4
0.5
SNP effect on MON1B

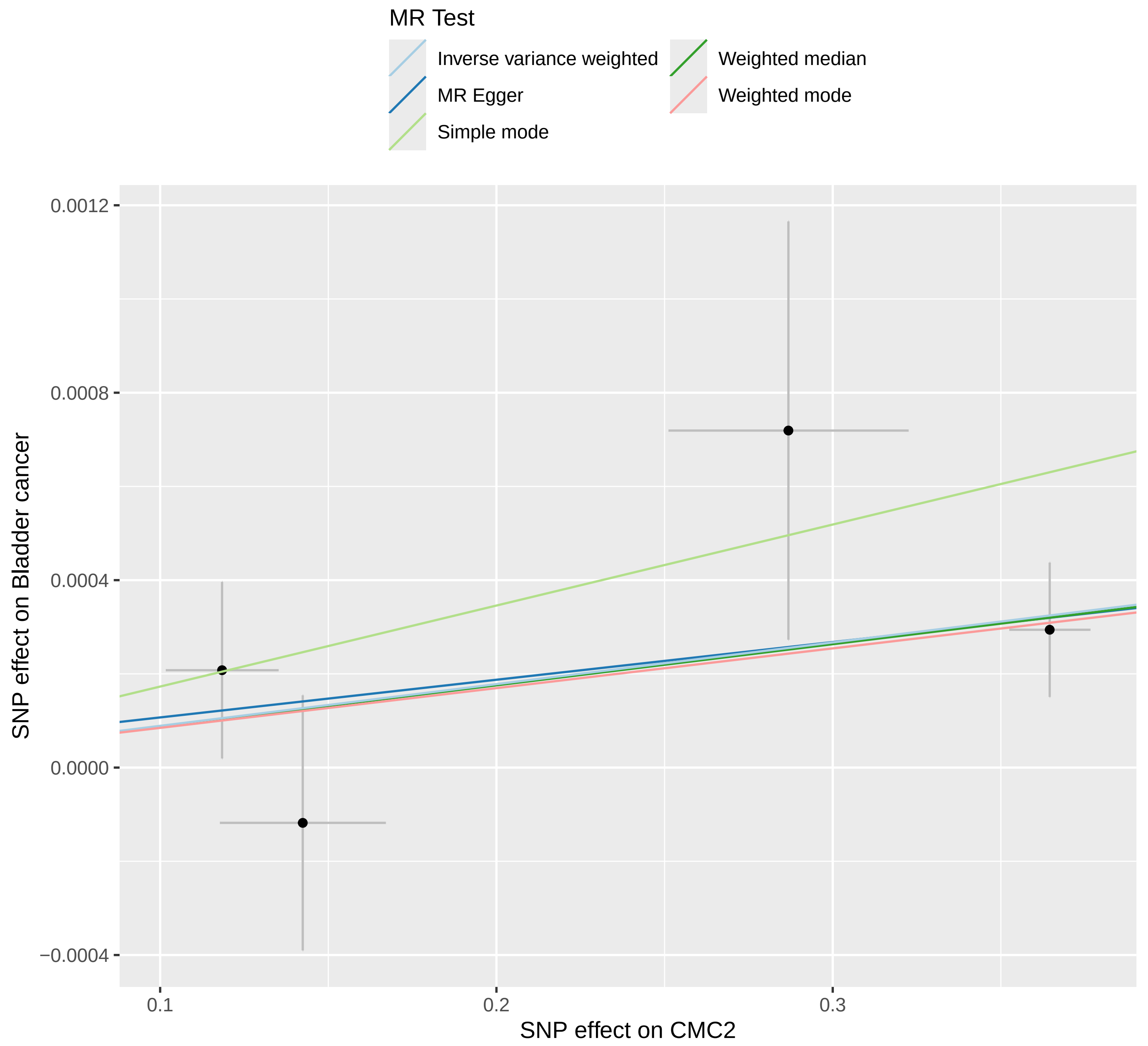
MR Test
Inverse variance weighted
MR Egger
Simple mode
Weighted median
Weighted mode
0.0012
0.0008
0.0004
0.0000
−0.0004
SNP effect on Bladder cancer
0.1
0.2
0.3
SNP effect on CMC2

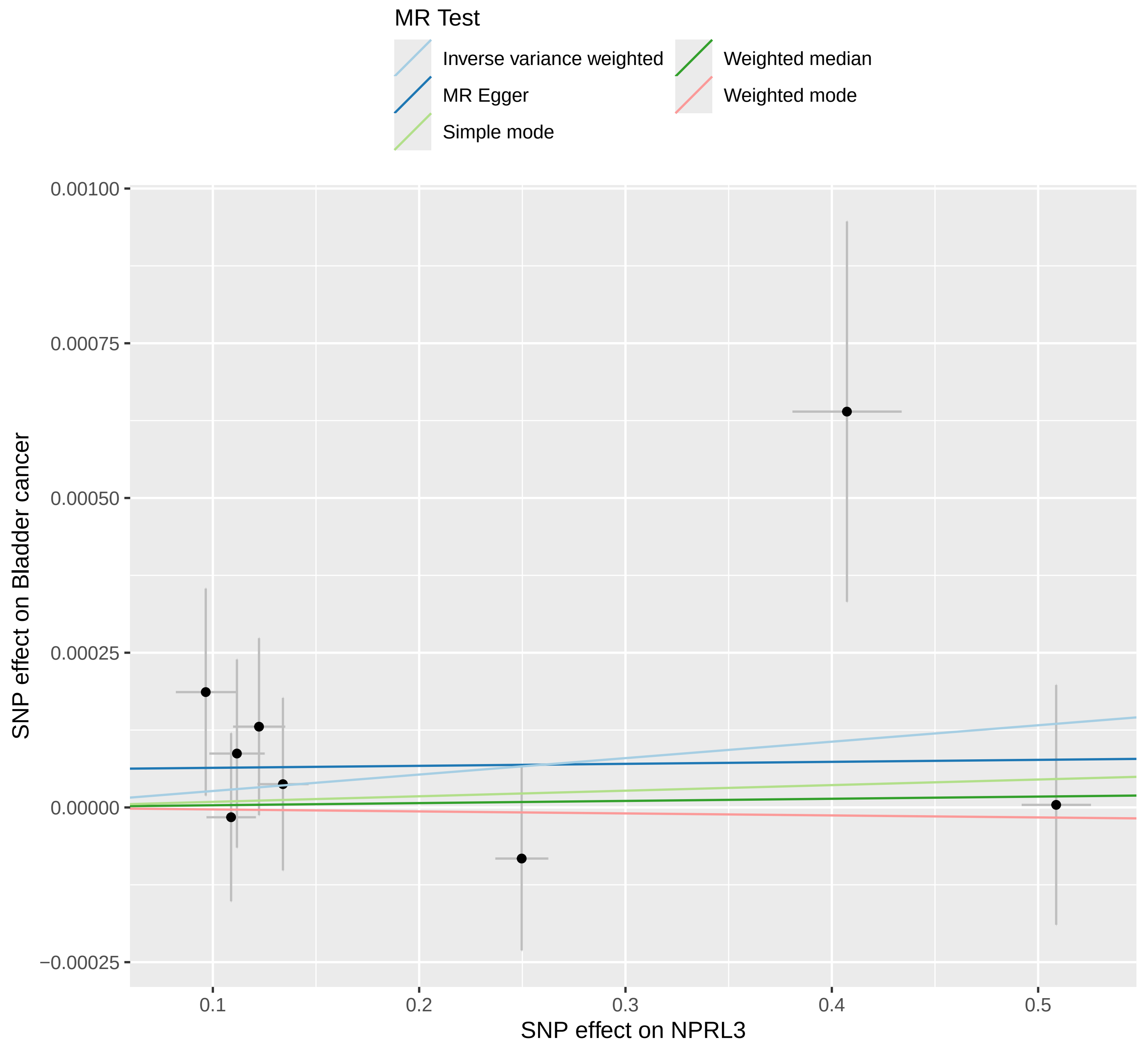
MR Test
Inverse variance weighted
MR Egger
Simple mode
Weighted median
Weighted mode
0.00100
0.00075
0.00050
0.00025
0.00000
−0.00025
SNP effect on Bladder cancer
0.1
0.2
0.3
0.4
0.5
SNP effect on NPRL3

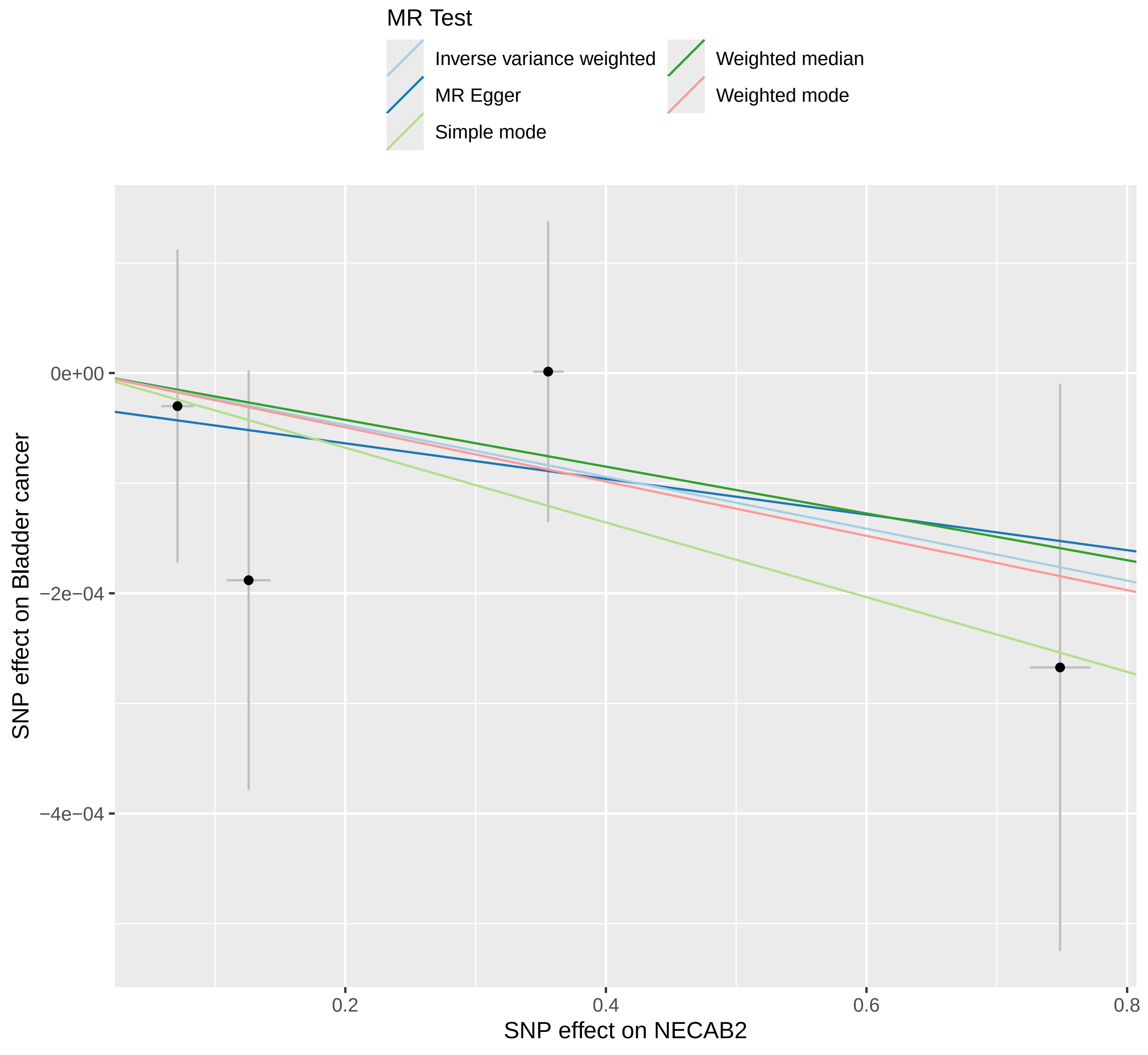

MR Test
Inverse variance weighted
MR Egger
Simple mode
Weighted median
Weighted mode
0e+00
−2e−04
−4e−04
0.2
0.4
0.6
0.8
SNP effect on NECAB2
SNP effect on Bladder cancer

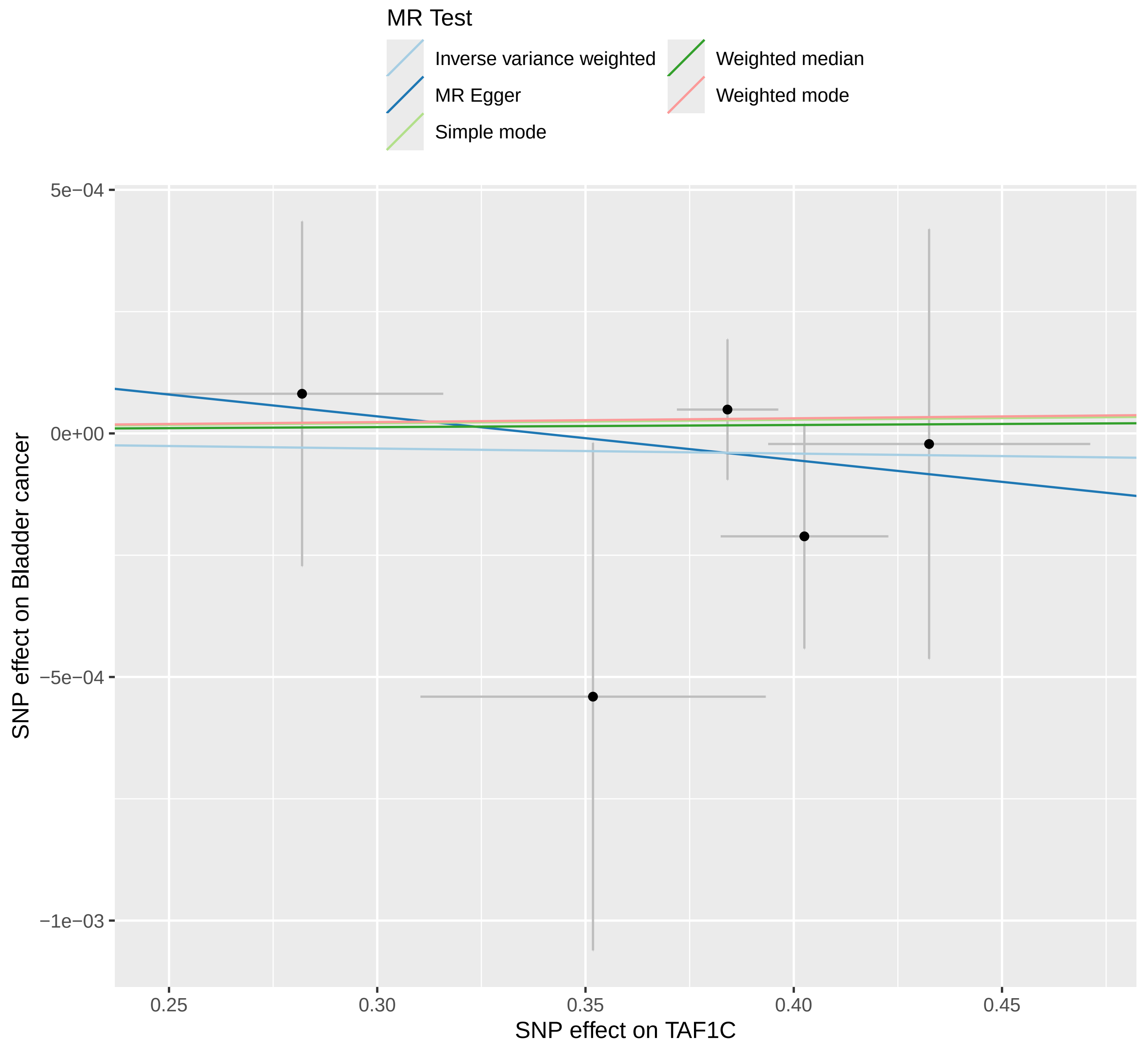
MR Test
Inverse variance weighted
MR Egger
Simple mode
Weighted median
Weighted mode
5e−04
0e+00
−5e−04
−1e−03
0.25
0.30
0.35
0.40
0.45
SNP effect on Bladder cancer
SNP effect on TAF1C

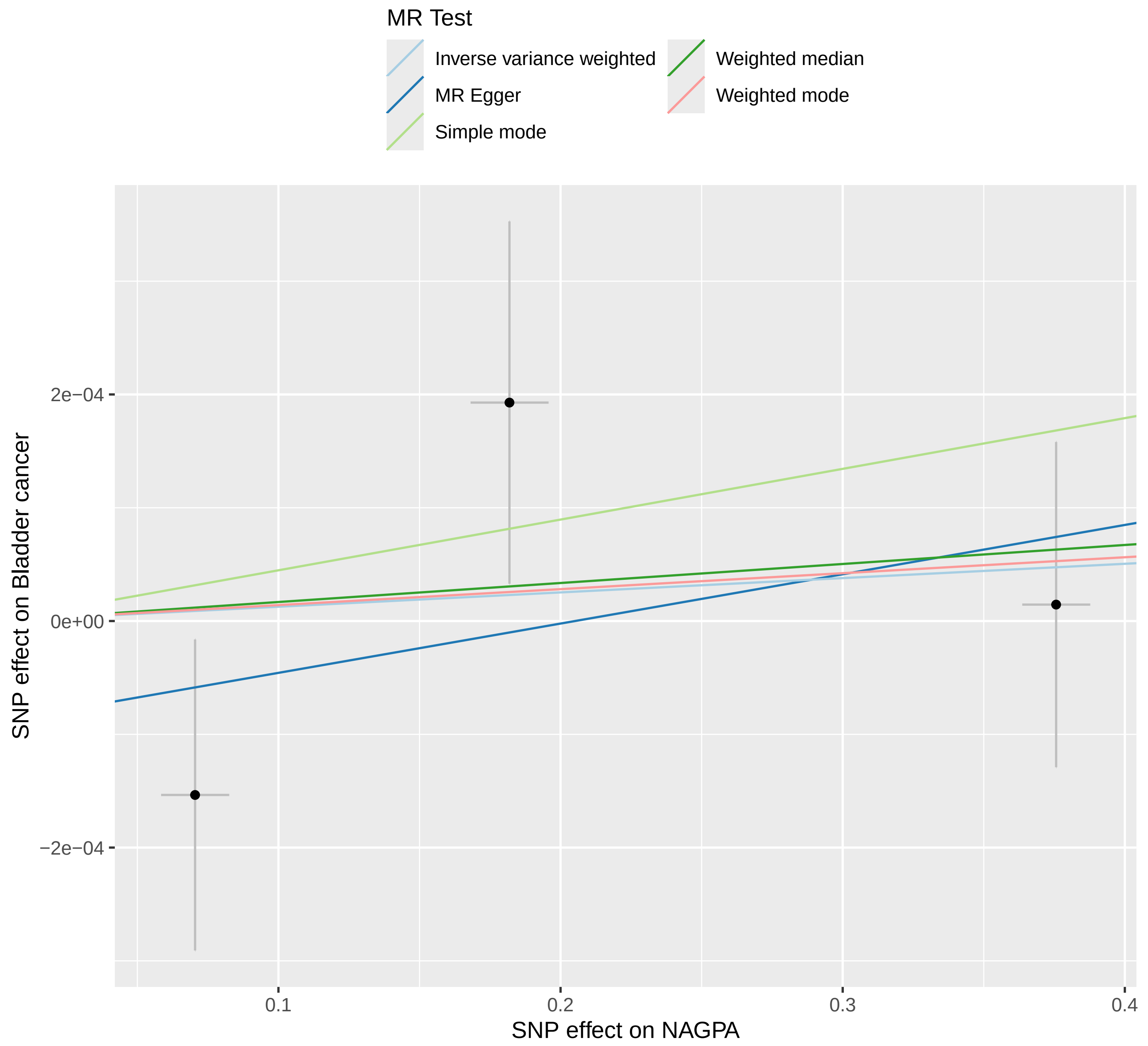
MR Test
Inverse variance weighted
MR Egger
Simple mode
Weighted median
Weighted mode
2e−04
0e+00
−2e−04
SNP effect on Bladder cancer
0.1
0.2
0.3
0.4
SNP effect on NAGPA

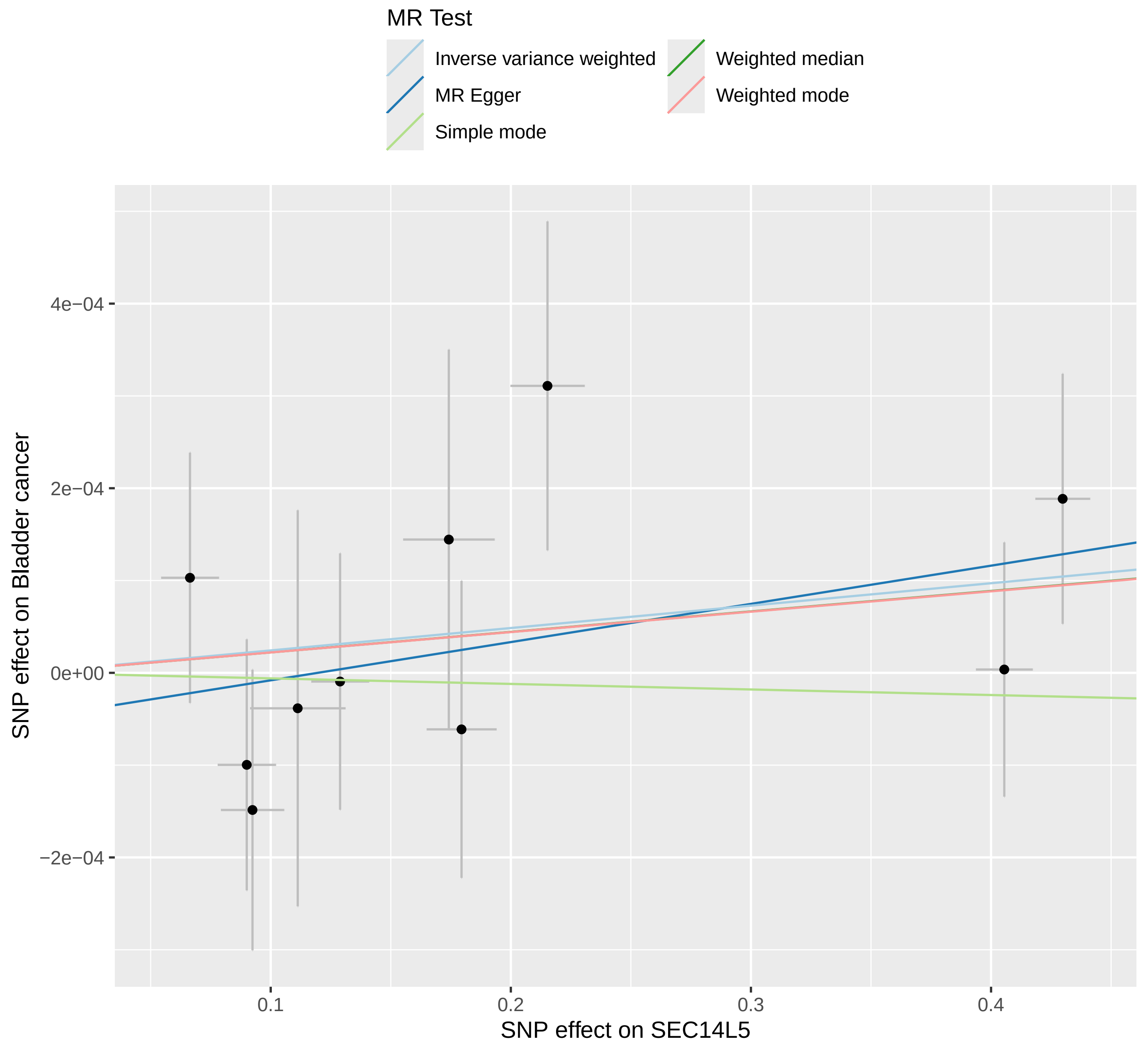

MR Test
Inverse variance weighted
MR Egger
Simple mode
Weighted median
Weighted mode
4e−04
2e−04
0e+00
−2e−04
0.1
0.2
0.3
0.4
SNP effect on SEC14L5
SNP effect on Bladder cancer

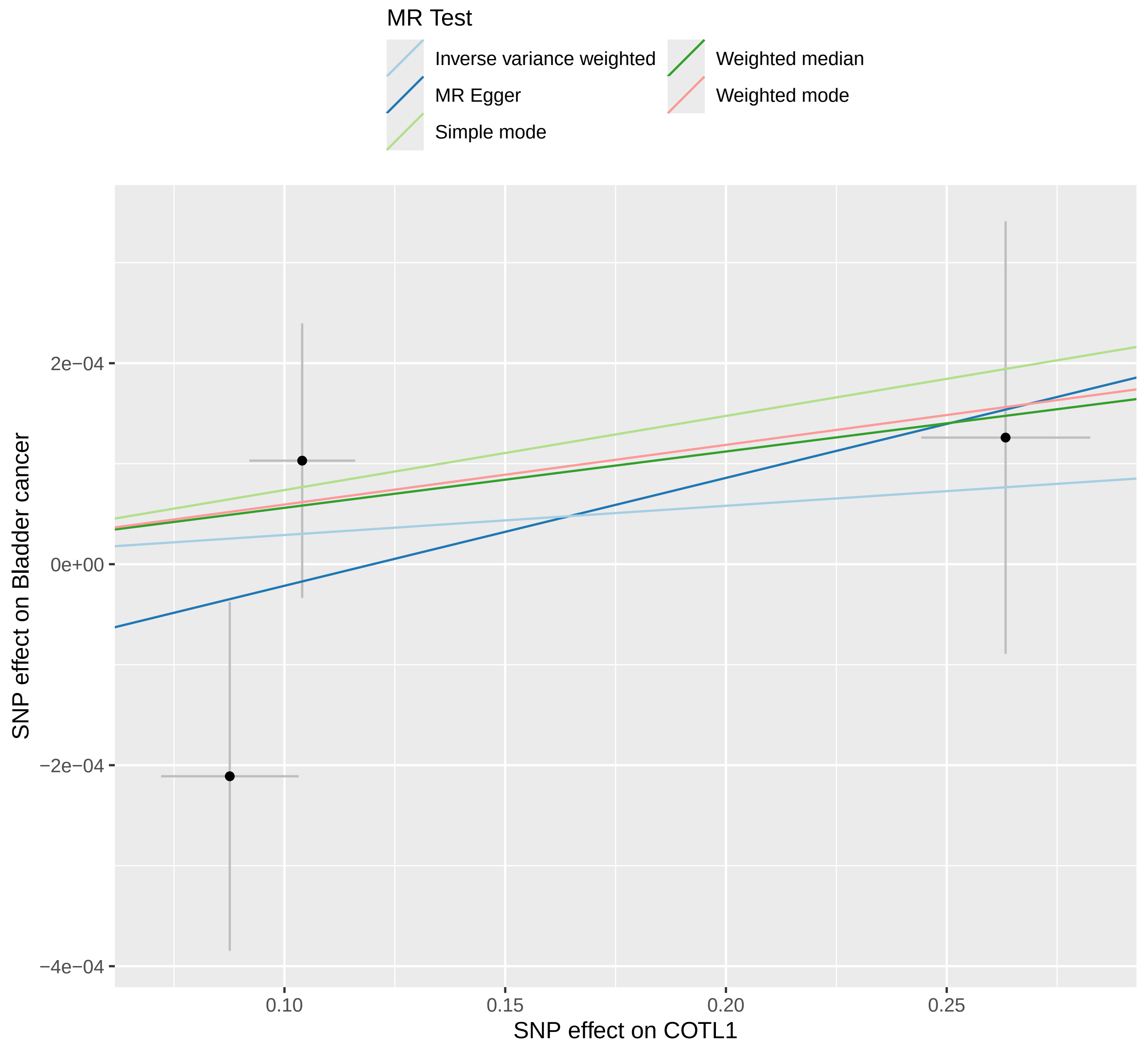

MR Test
Inverse variance weighted
MR Egger
Simple mode
Weighted median
Weighted mode
2e−04
0e+00
−2e−04
−4e−04
SNP effect on Bladder cancer
0.10
0.15
0.20
0.25
SNP effect on COTL1

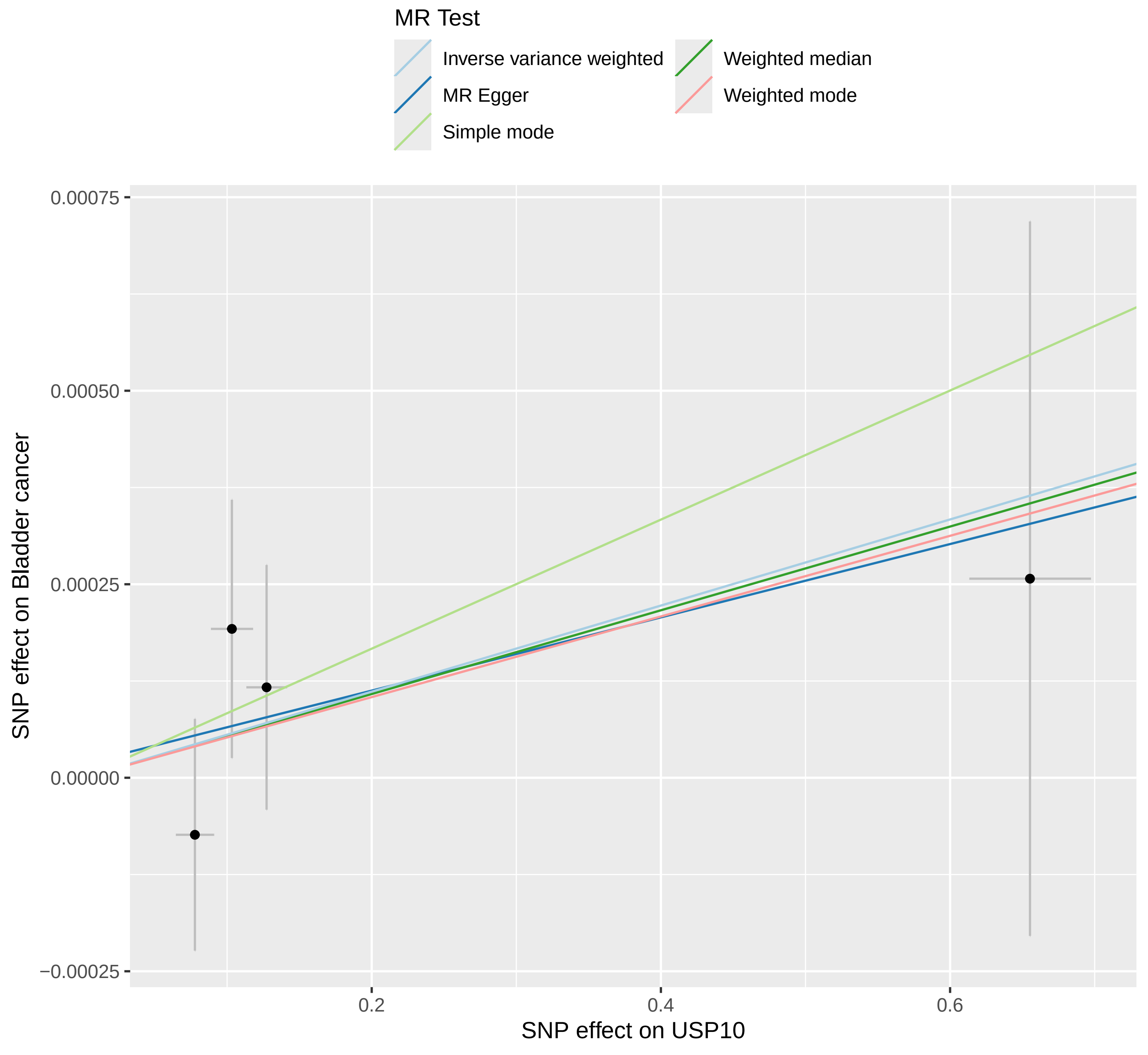

MR Test
Inverse variance weighted
MR Egger
Simple mode
Weighted median
Weighted mode
0.00075
0.00050
0.00025
0.00000
−0.00025
SNP effect on Bladder cancer
0.2
0.4
0.6
SNP effect on USP10

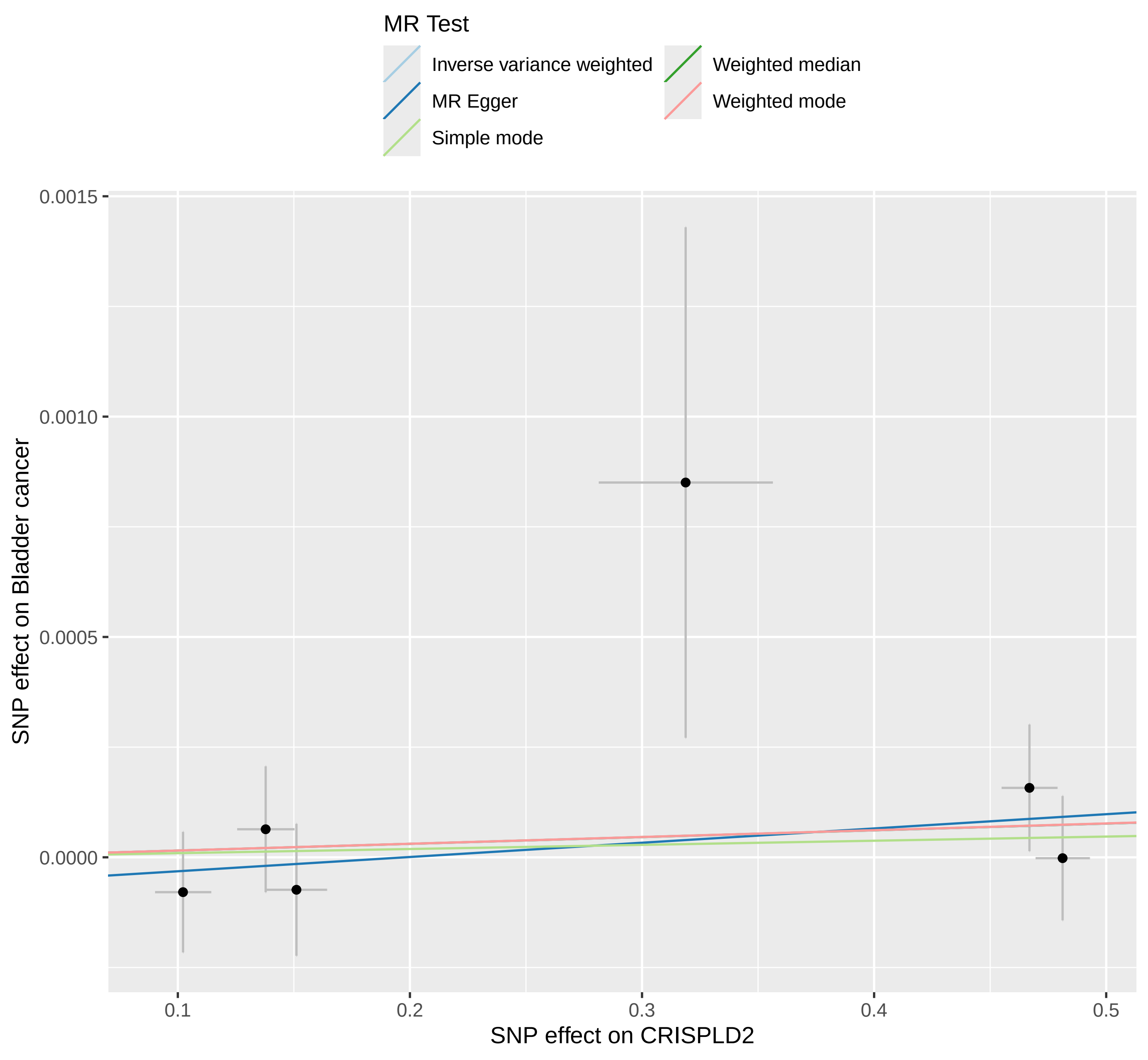

MR Test
Inverse variance weighted
MR Egger
Simple mode
Weighted median
Weighted mode
0.0015
0.0010
0.0005
0.0000
0.1
0.2
0.3
0.4
0.5
SNP effect on Bladder cancer
SNP effect on CRISPLD2

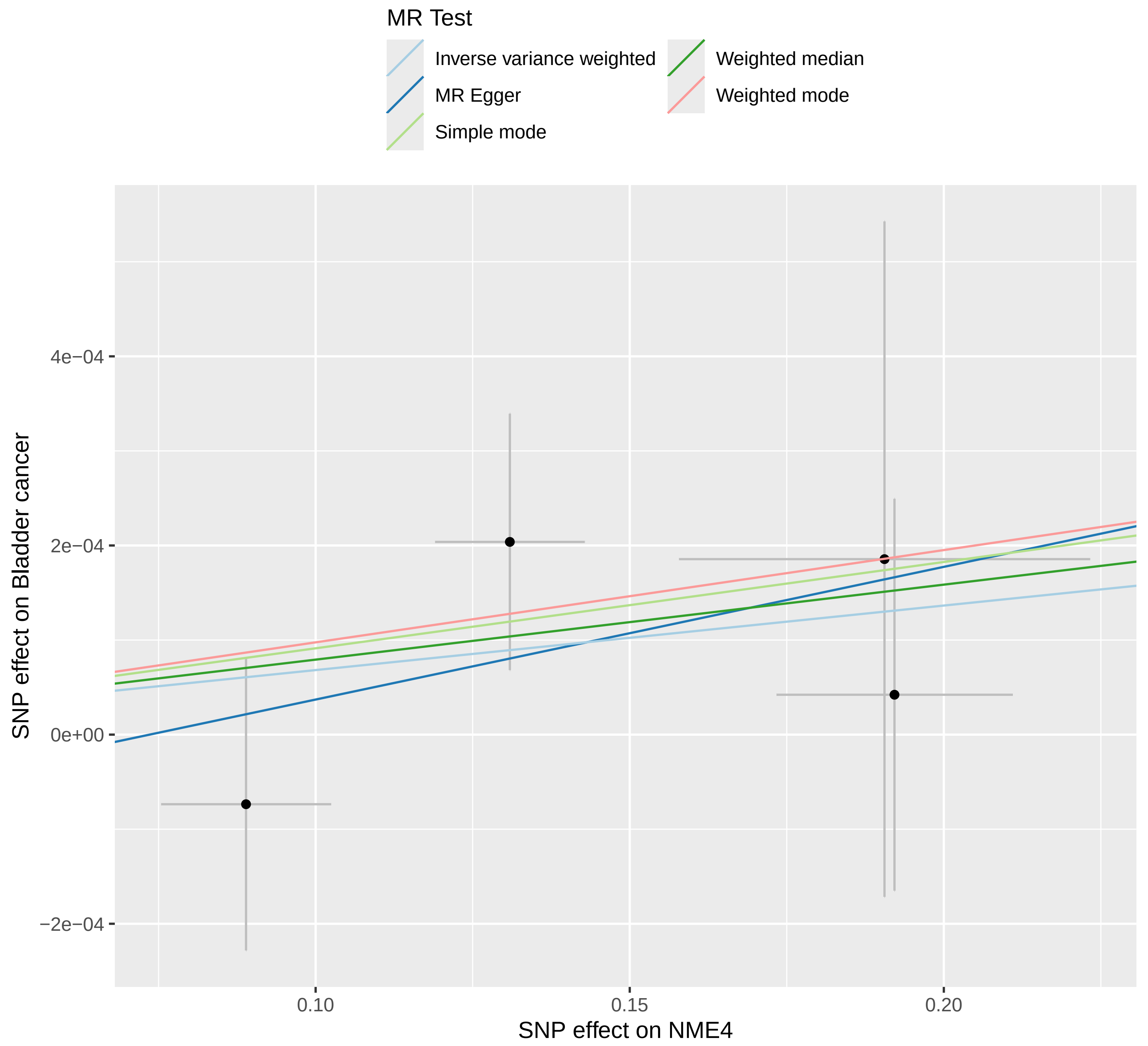
MR Test
Inverse variance weighted
MR Egger
Simple mode
Weighted median
Weighted mode
SNP effect on Bladder cancer
4e−04
2e−04
0e+00
−2e−04
0.10
0.15
0.20
SNP effect on NME4

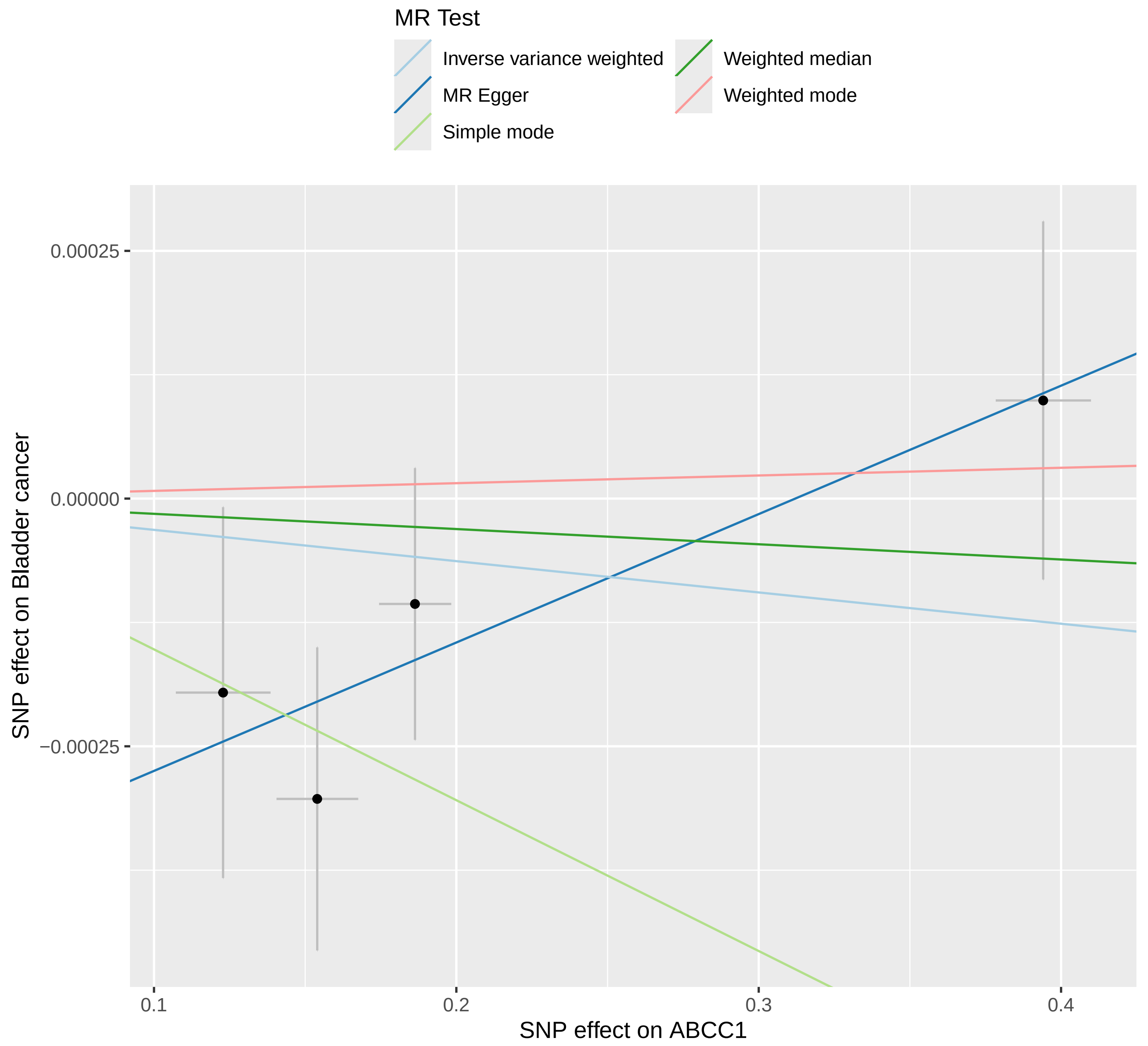
MR Test
Inverse variance weighted
MR Egger
Simple mode
Weighted median
Weighted mode
0.00025
0.00000
−0.00025
0.1
0.2
0.3
0.4
SNP effect on ABCC1
SNP effect on Bladder cancer

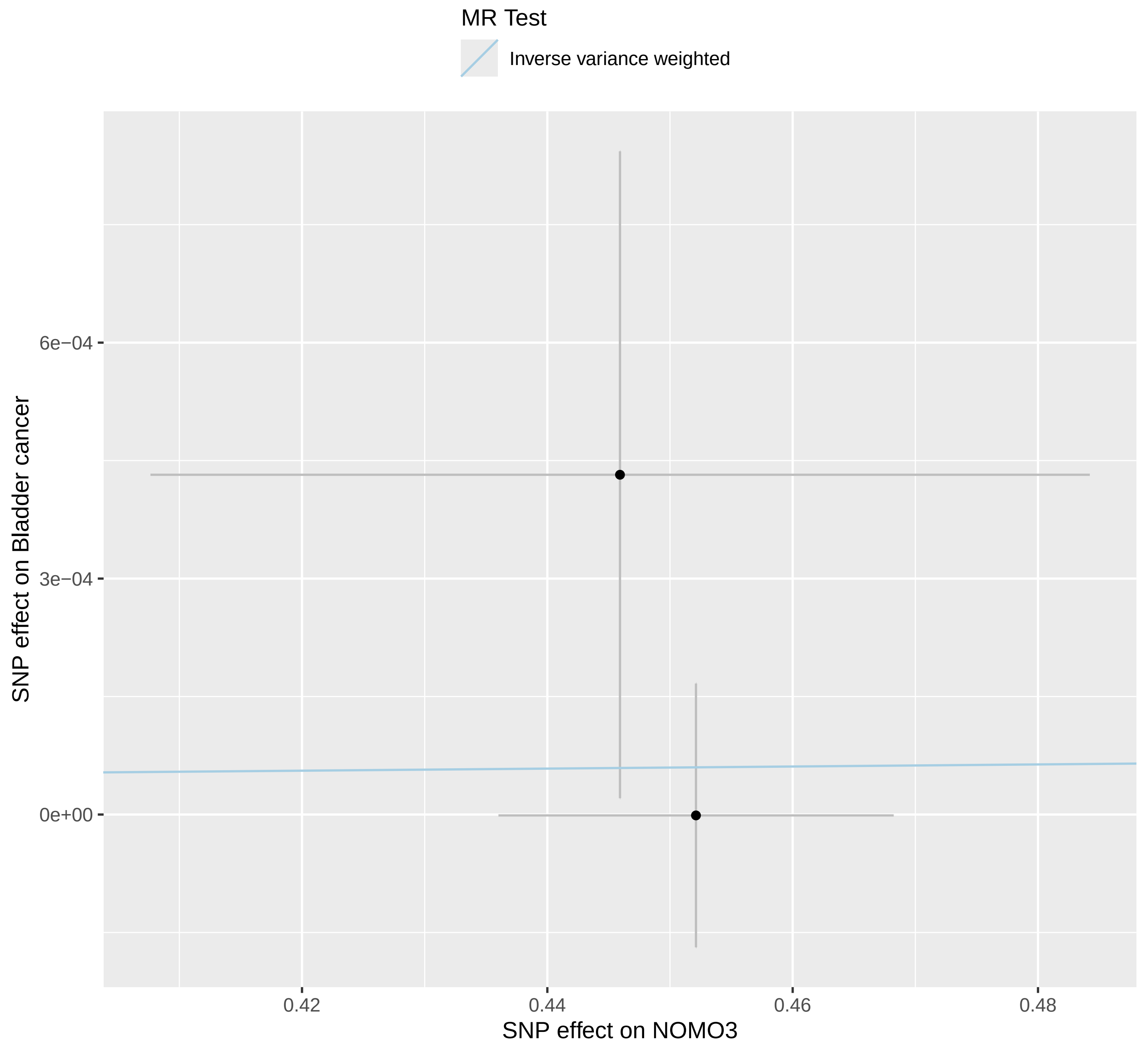
MR Test
Inverse variance weighted
6e−04
3e−04
0e+00
SNP effect on Bladder cancer
0.42
0.44
0.46
0.48
SNP effect on NOMO3

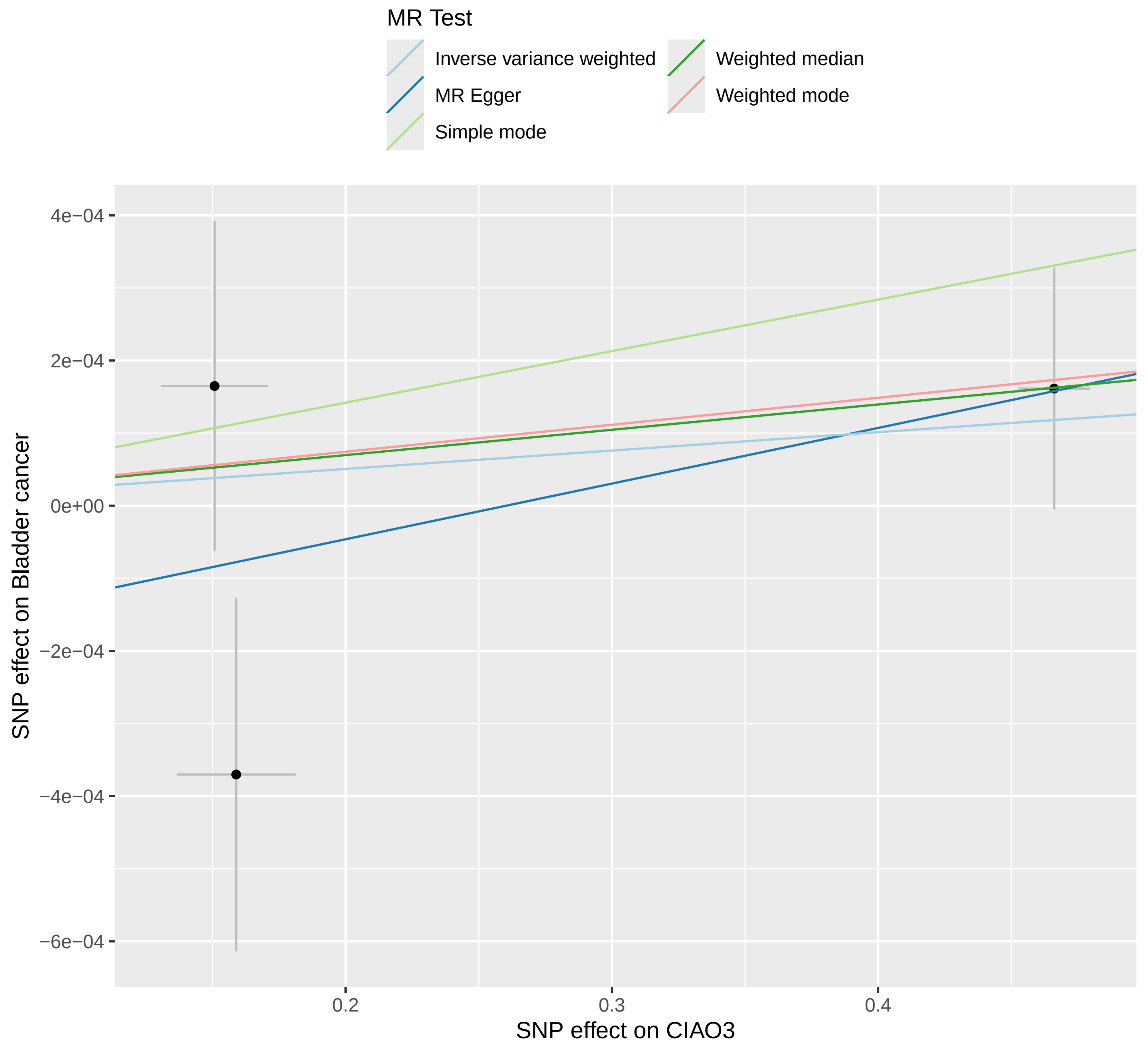
MR Test
Inverse variance weighted
MR Egger
Simple mode
Weighted median
Weighted mode
4e−04
2e−04
0e+00
−2e−04
−4e−04
−6e−04
0.2
0.3
0.4
SNP effect on Bladder cancer
SNP effect on CIAO3

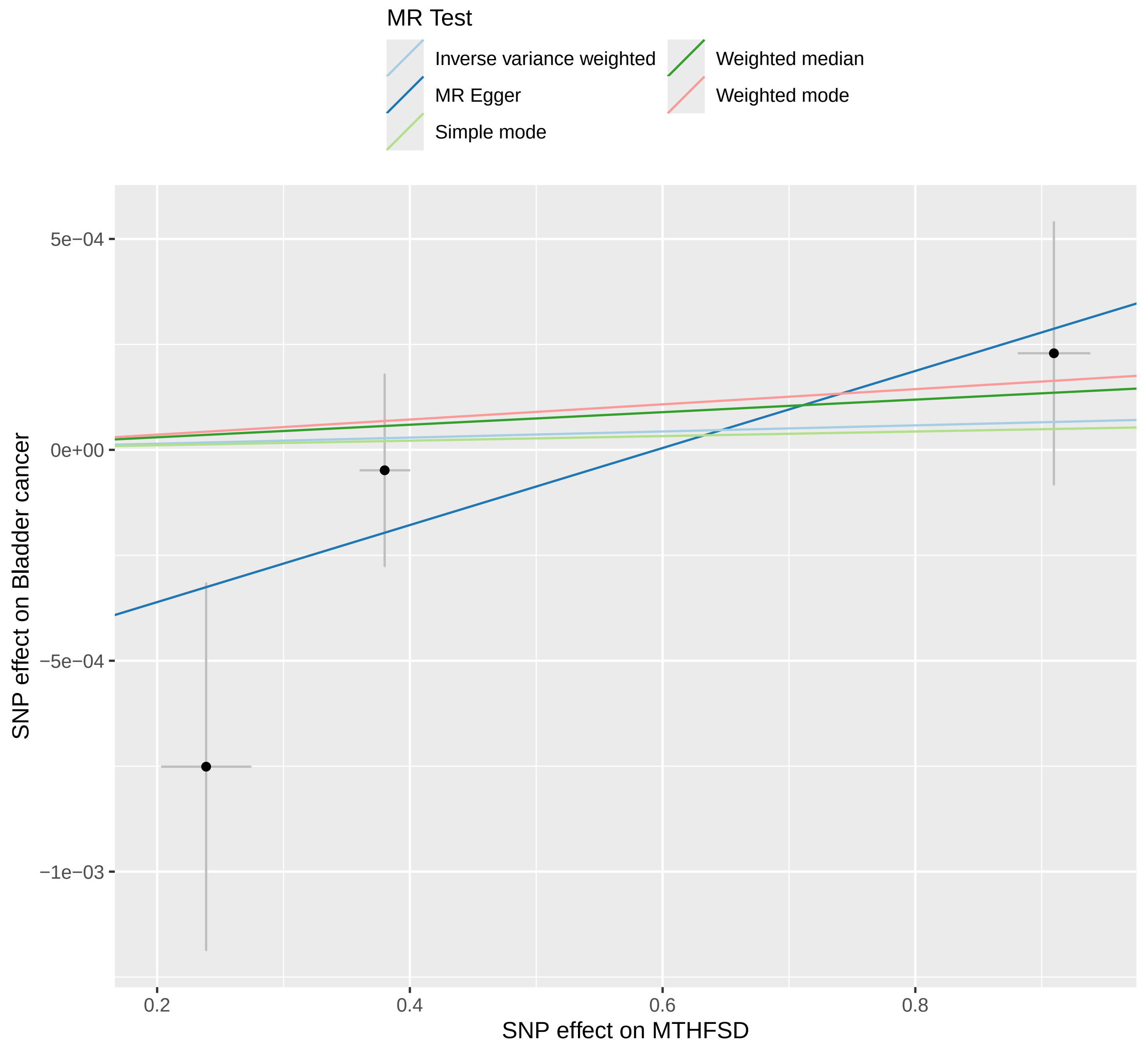
MR Test
Inverse variance weighted
MR Egger
Simple mode
Weighted median
Weighted mode
5e−04
0e+00
−5e−04
−1e−03
SNP effect on Bladder cancer
0.2
0.4
0.6
0.8
SNP effect on MTHFSD

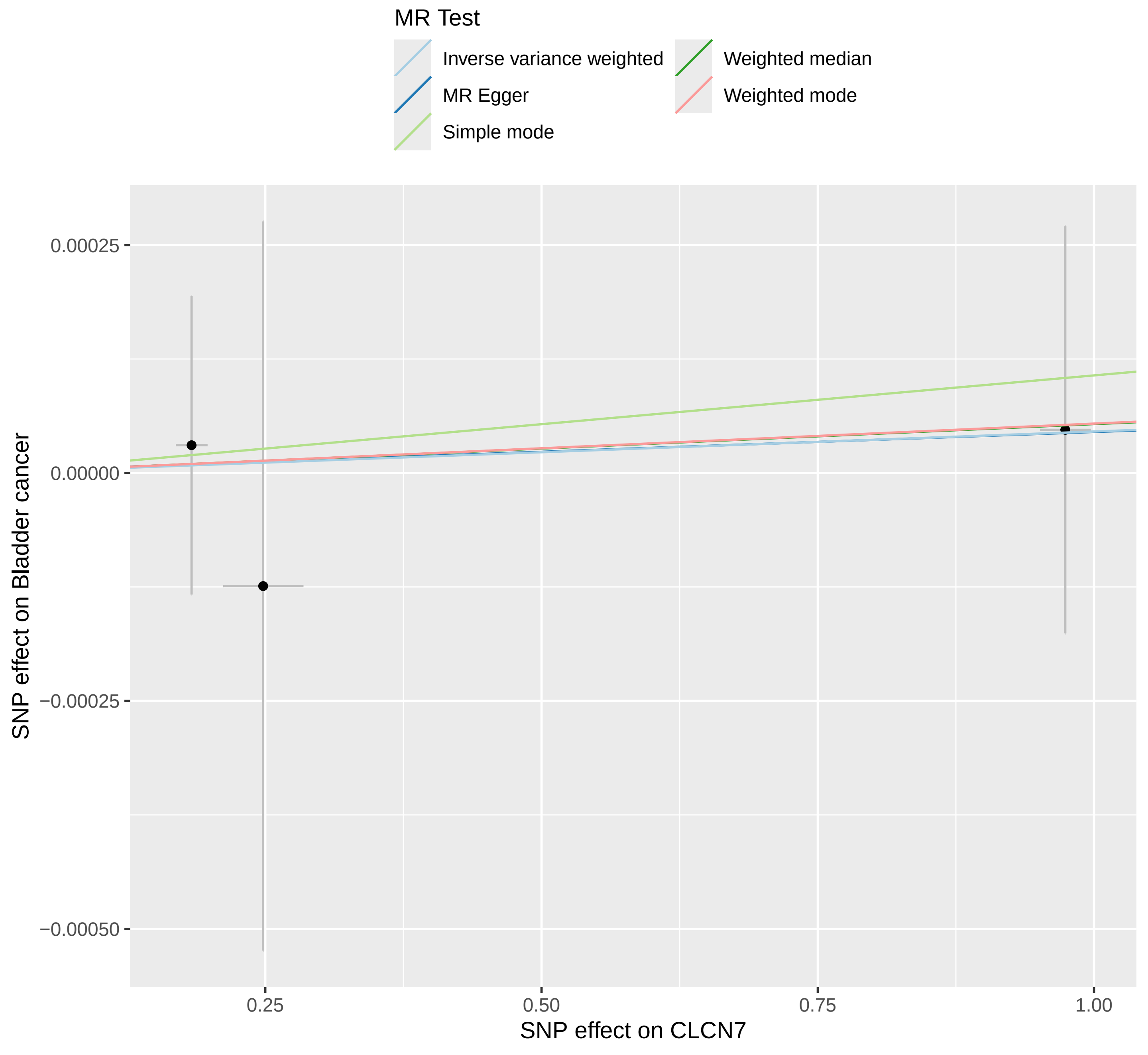

MR Test
Inverse variance weighted
MR Egger
Simple mode
Weighted median
Weighted mode
0.00025
0.00000
−0.00025
−0.00050
SNP effect on Bladder cancer
0.25
0.50
0.75
1.00
SNP effect on CLCN7

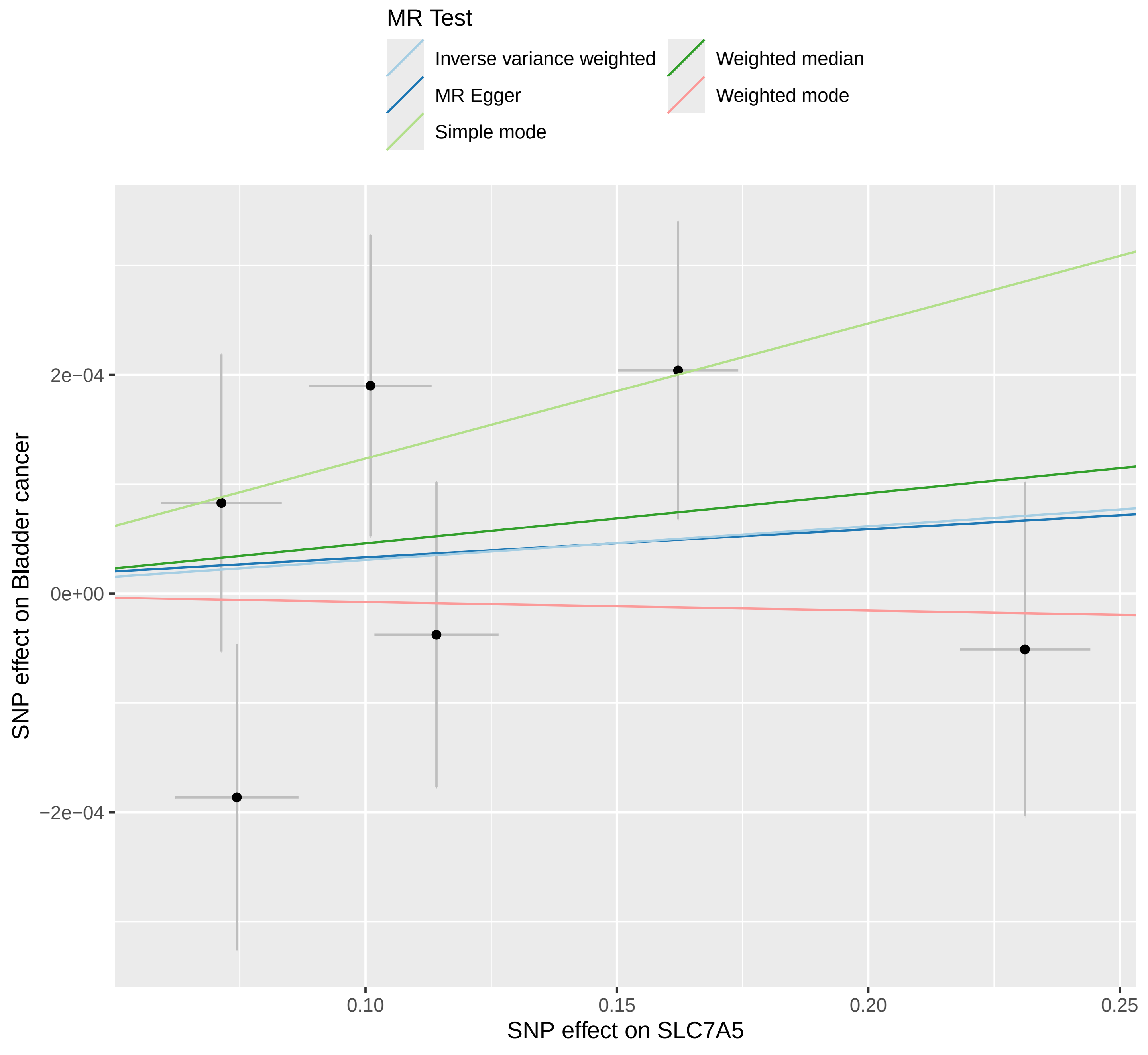
MR Test
Inverse variance weighted
MR Egger
Simple mode
Weighted median
Weighted mode
2e−04
0e+00
−2e−04
0.10
0.15
0.20
0.25
SNP effect on SLC7A5
SNP effect on Bladder cancer

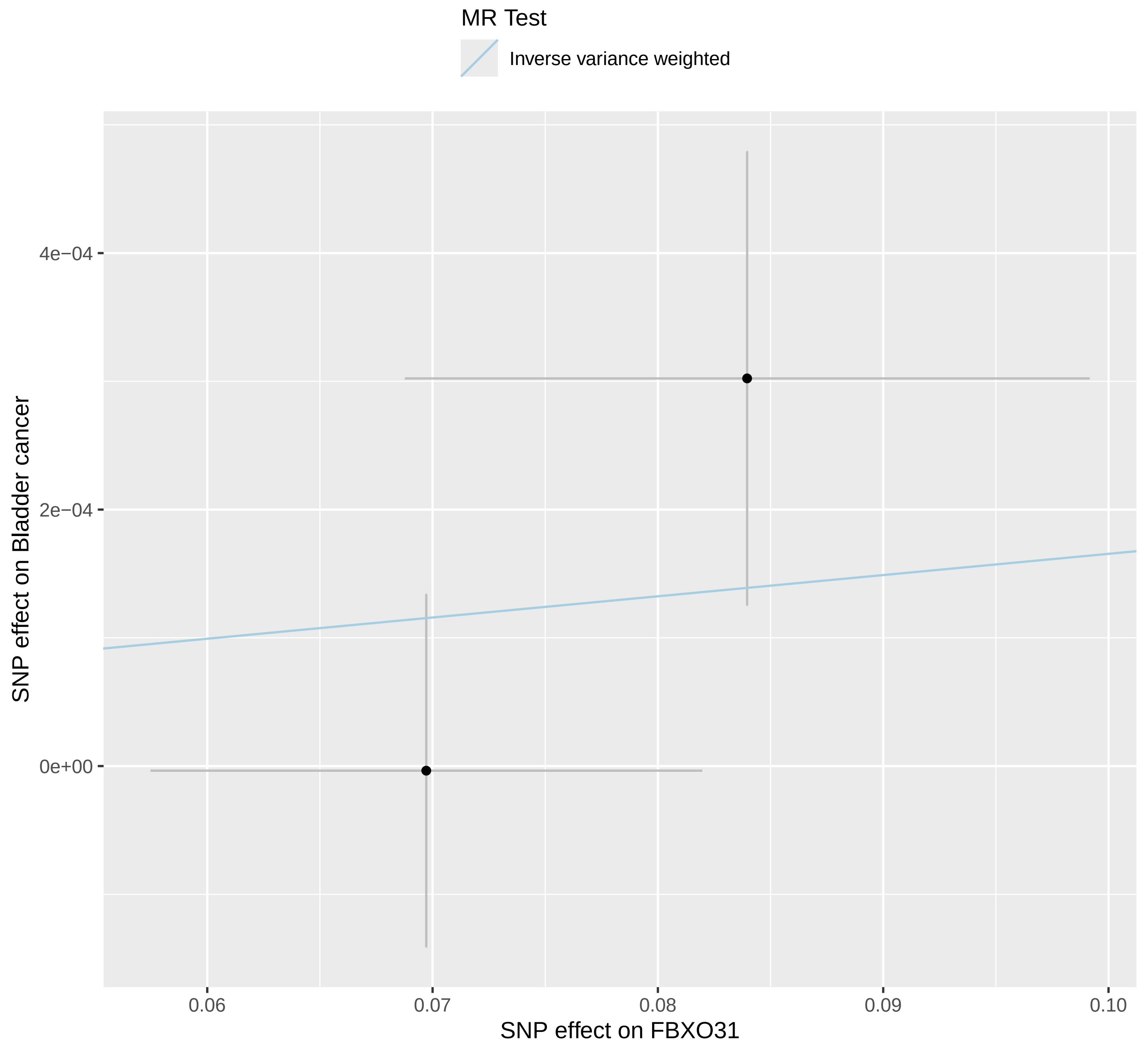
MR Test
Inverse variance weighted
4e−04
2e−04
0e+00
SNP effect on Bladder cancer
0.06
0.07
0.08
0.09
0.10
SNP effect on FBXO31

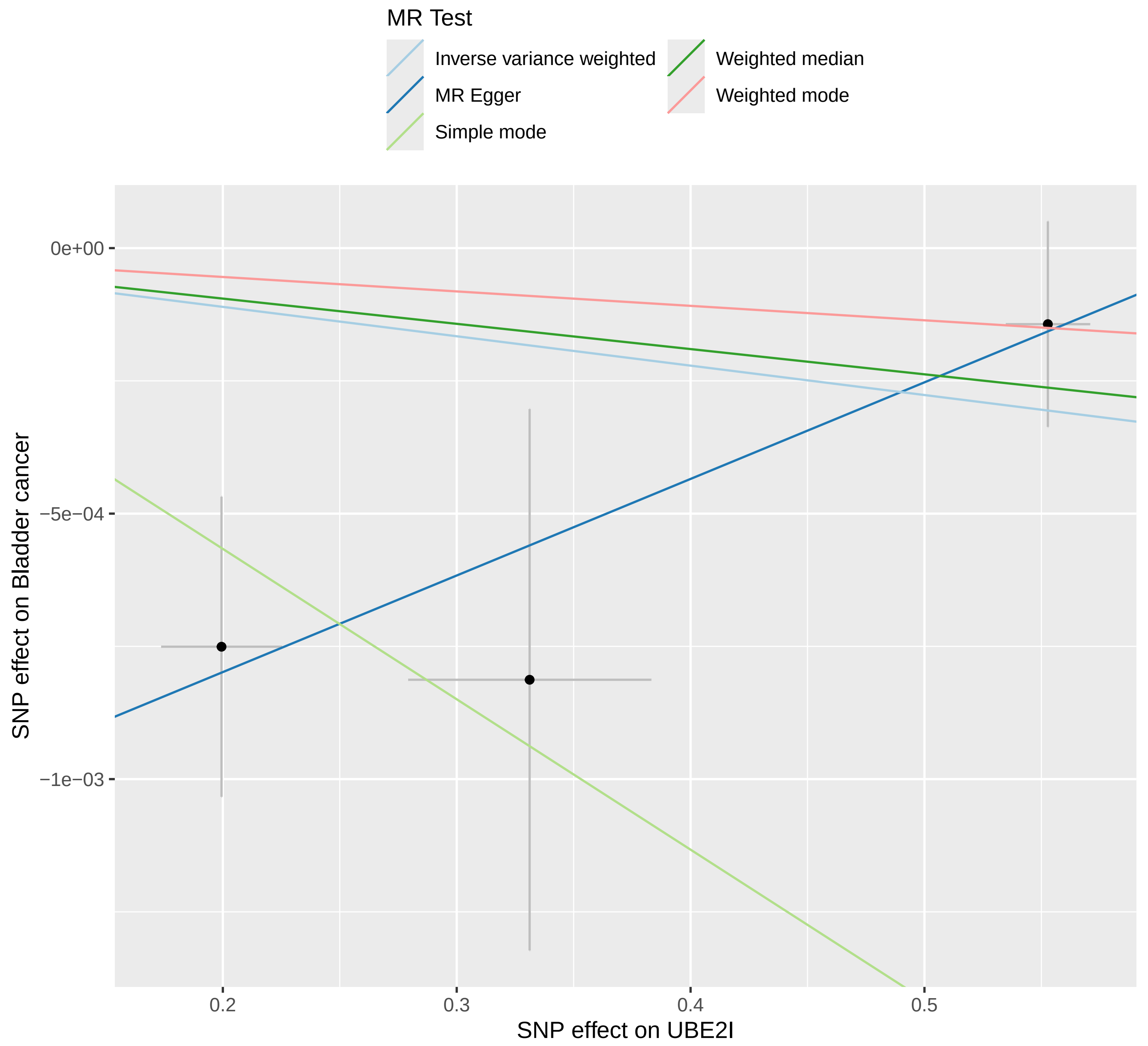
MR Test
Inverse variance weighted
MR Egger
Simple mode
Weighted median
Weighted mode
0e+00
−5e−04
−1e−03
SNP effect on Bladder cancer
0.2
0.3
0.4
0.5
SNP effect on UBE2I

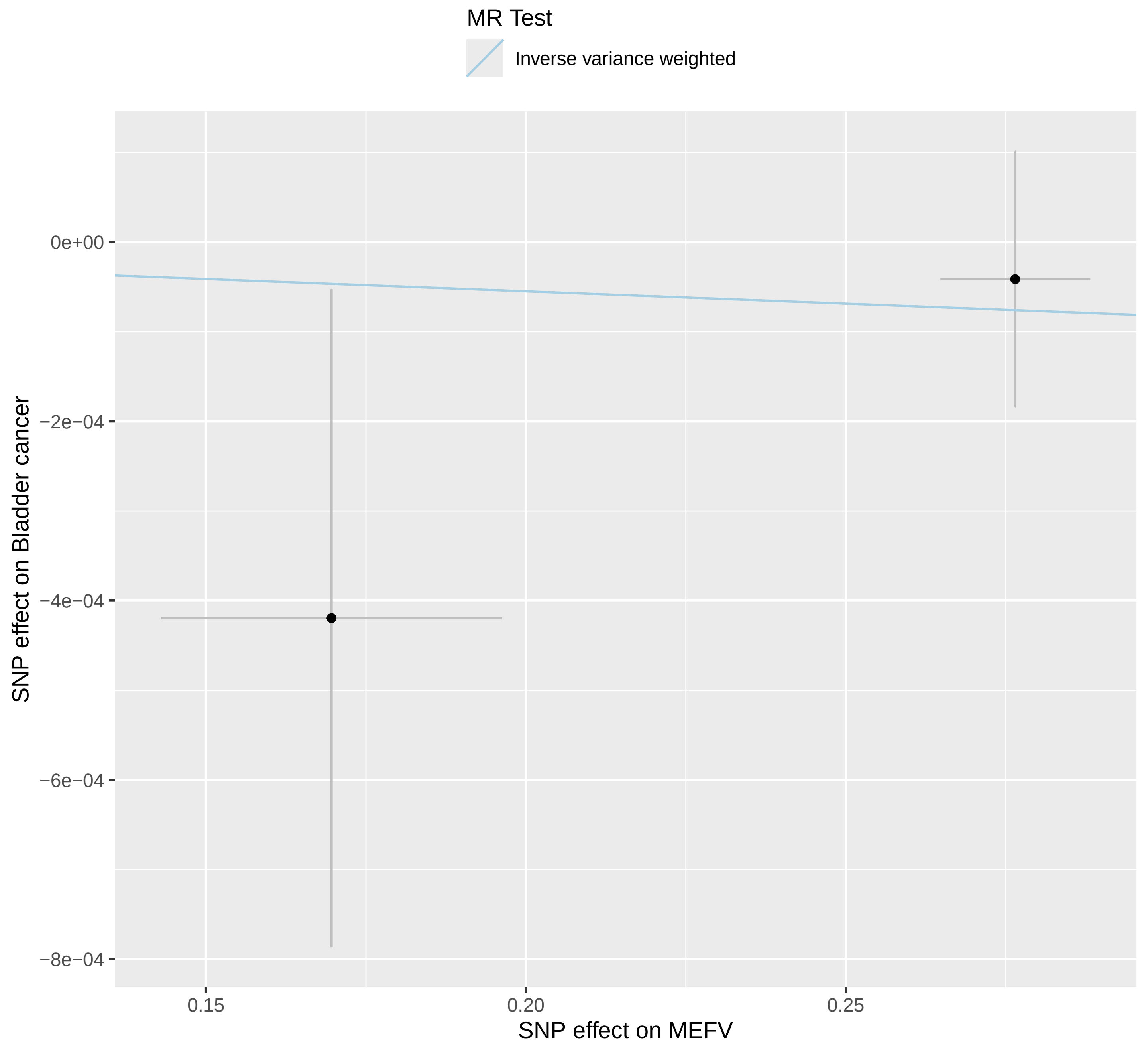

MR Test
Inverse variance weighted
0e+00
−2e−04
−4e−04
−6e−04
−8e−04
0.15
0.20
0.25
SNP effect on MEFV
SNP effect on Bladder cancer

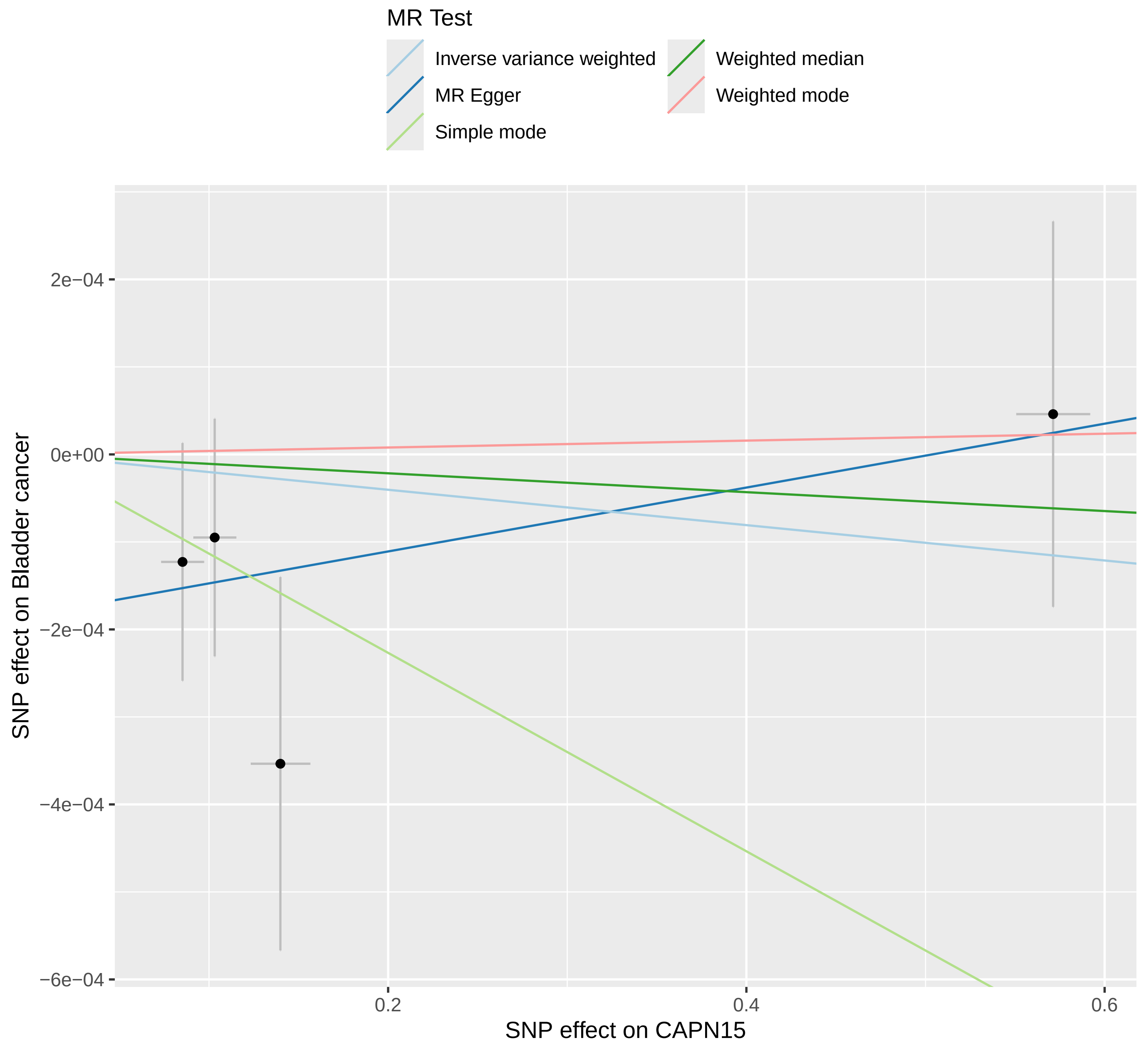

MR Test
Inverse variance weighted
MR Egger
Simple mode
Weighted median
Weighted mode
2e−04
0e+00
−2e−04
−4e−04
−6e−04
0.2
0.4
0.6
SNP effect on Bladder cancer
SNP effect on CAPN15

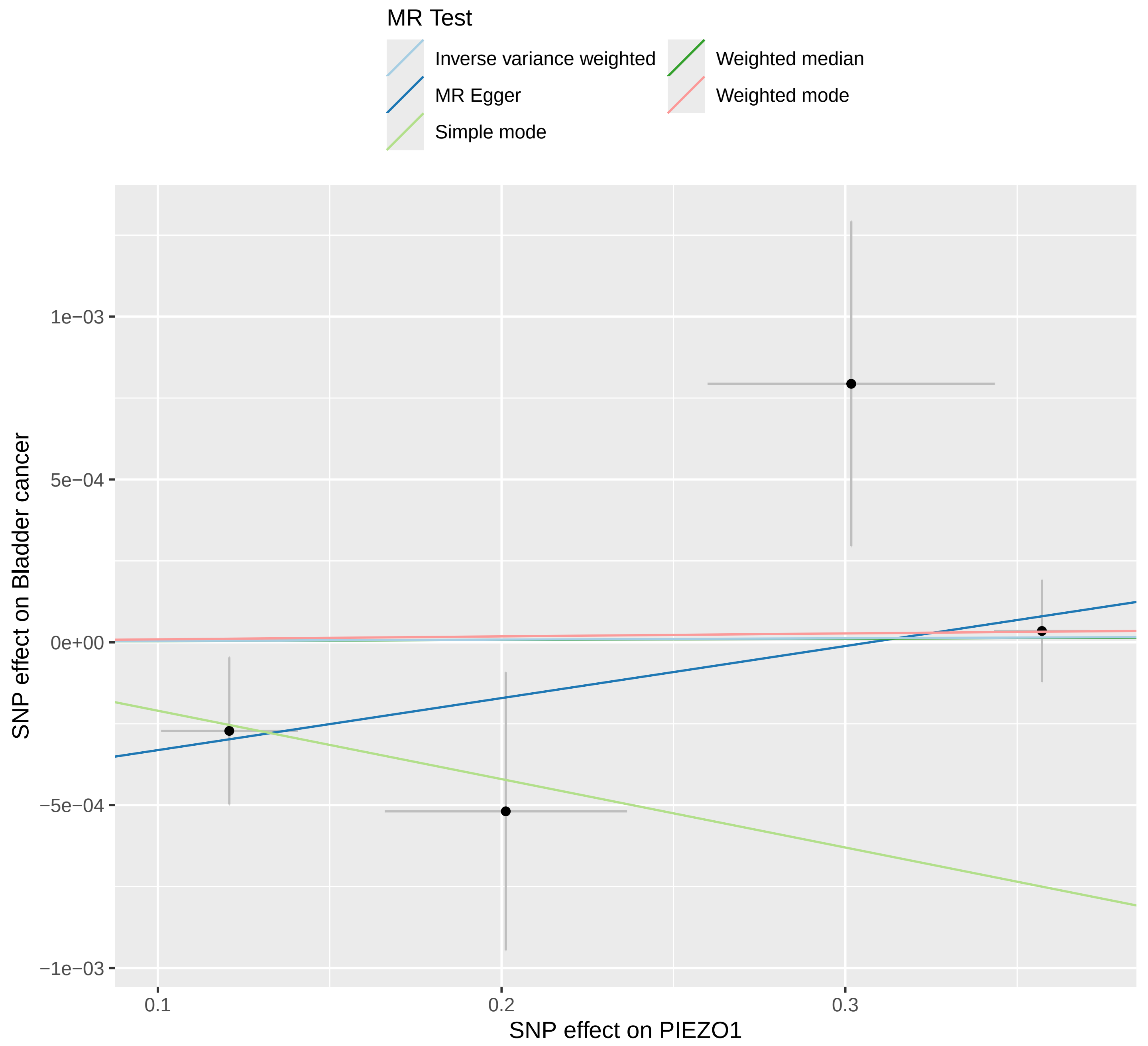

MR Test
Inverse variance weighted
MR Egger
Simple mode
Weighted median
Weighted mode
1e−03
5e−04
0e+00
−5e−04
−1e−03
0.1
0.2
0.3
SNP effect on PIEZO1
SNP effect on Bladder cancer

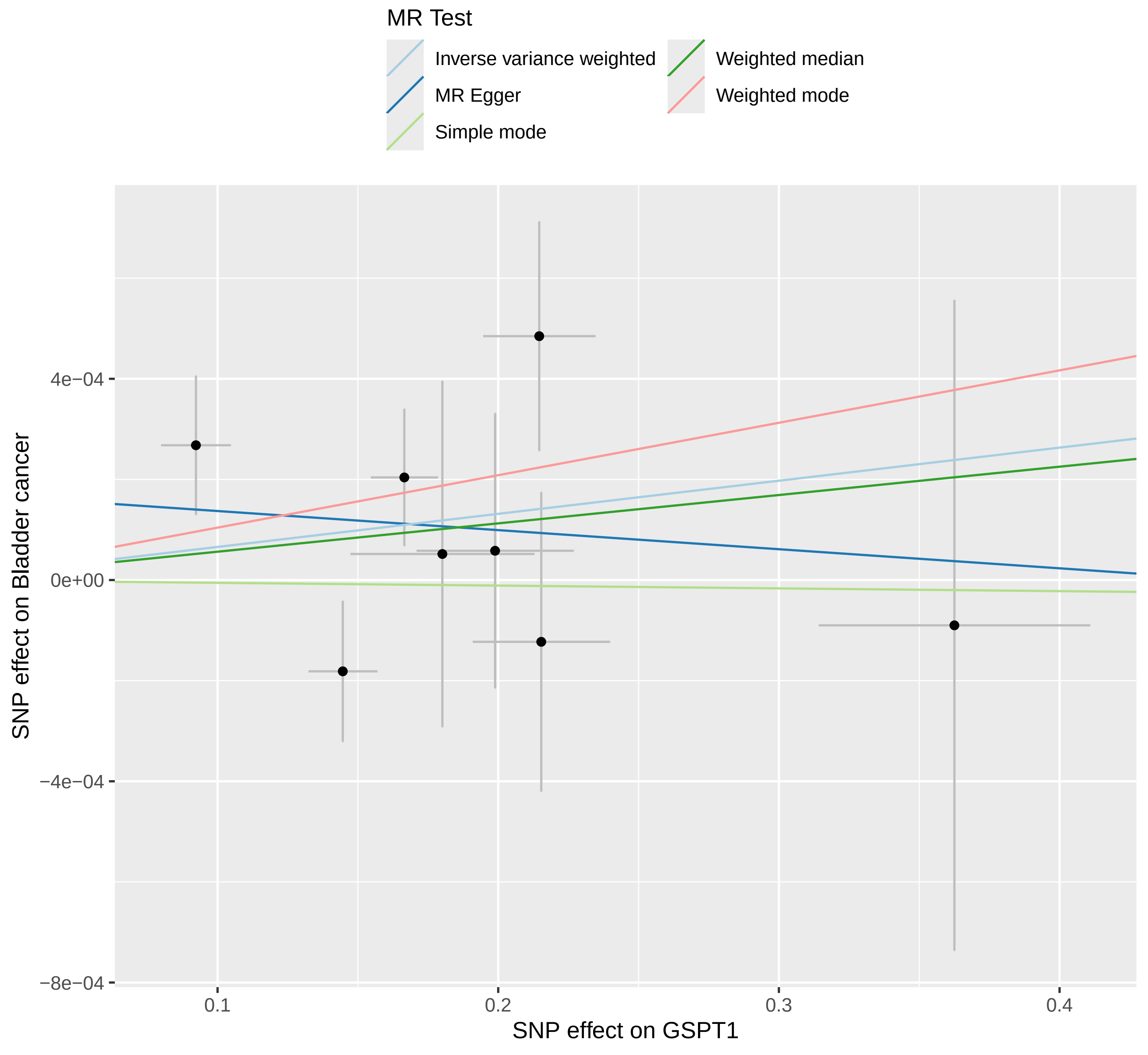
MR Test
Inverse variance weighted
MR Egger
Simple mode
Weighted median
Weighted mode
4e−04
0e+00
−4e−04
−8e−04
SNP effect on Bladder cancer
0.1
0.2
0.3
0.4
SNP effect on GSPT1

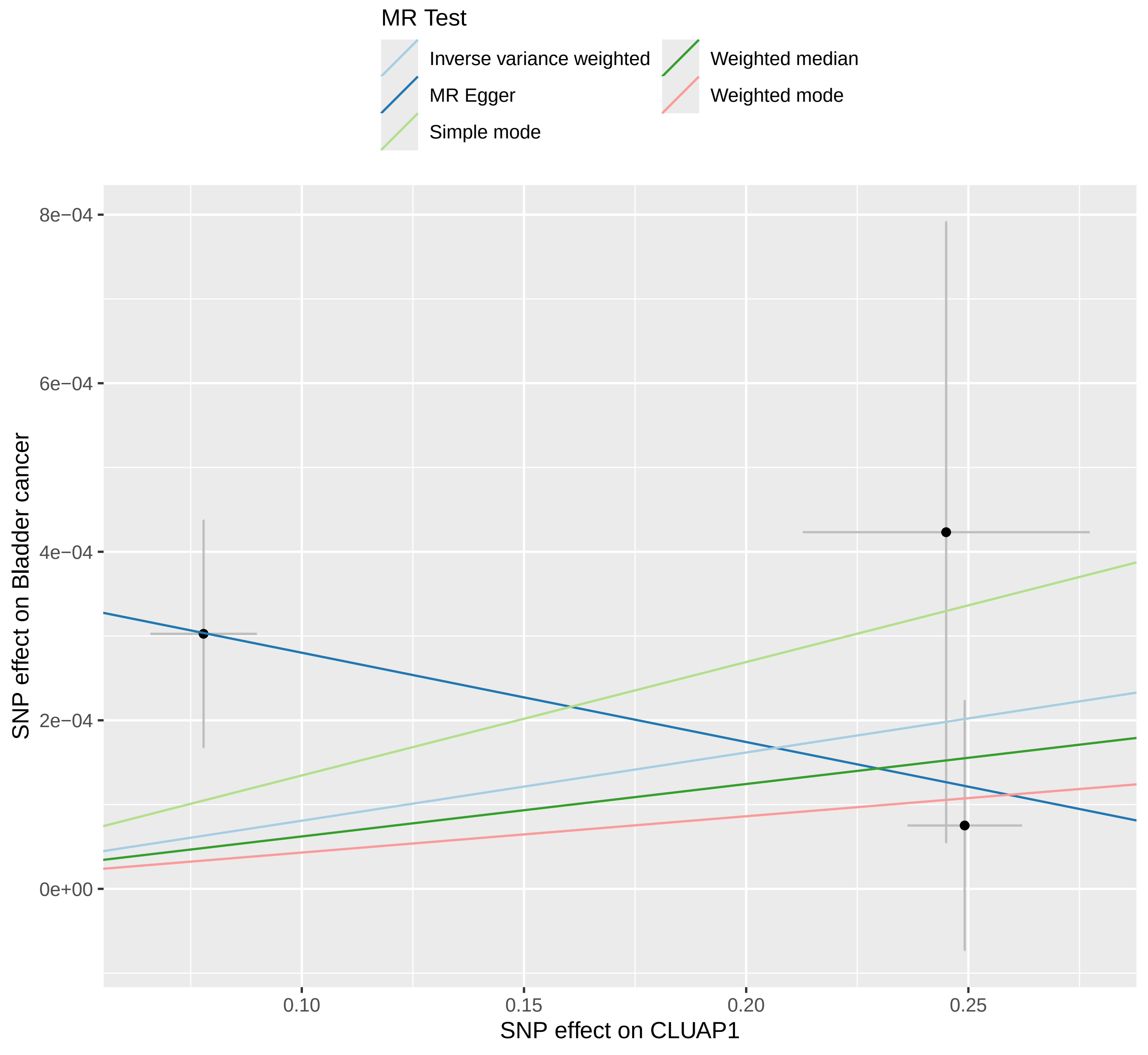

MR Test
Inverse variance weighted
MR Egger
Simple mode
Weighted median
Weighted mode
8e−04
6e−04
4e−04
2e−04
0e+00
SNP effect on Bladder cancer
0.10
0.15
0.20
0.25
SNP effect on CLUAP1

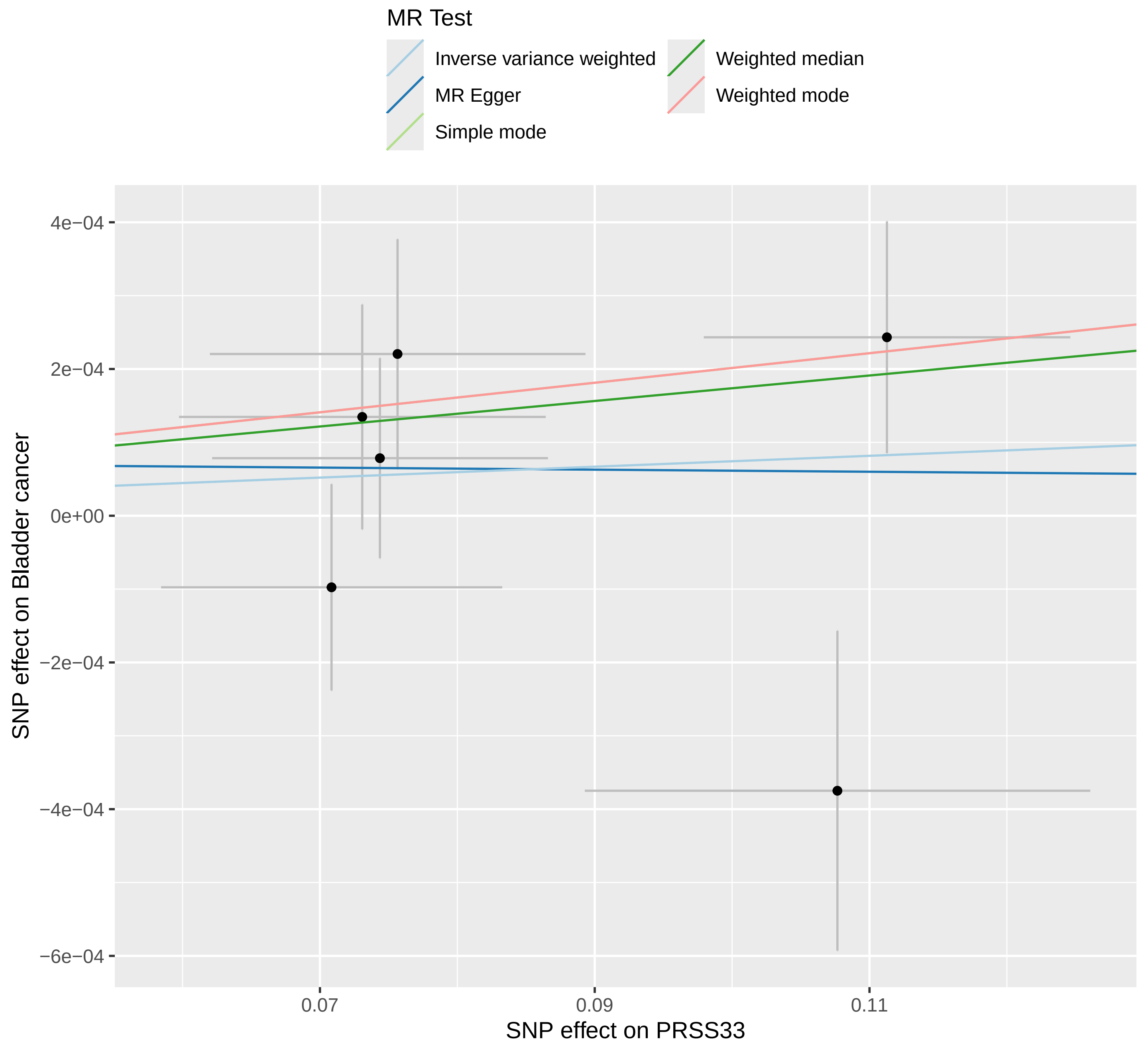
MR Test
Inverse variance weighted
MR Egger
Simple mode
Weighted median
Weighted mode
4e−04
2e−04
0e+00
−2e−04
−4e−04
−6e−04
SNP effect on Bladder cancer
0.07
0.09
0.11
SNP effect on PRSS33

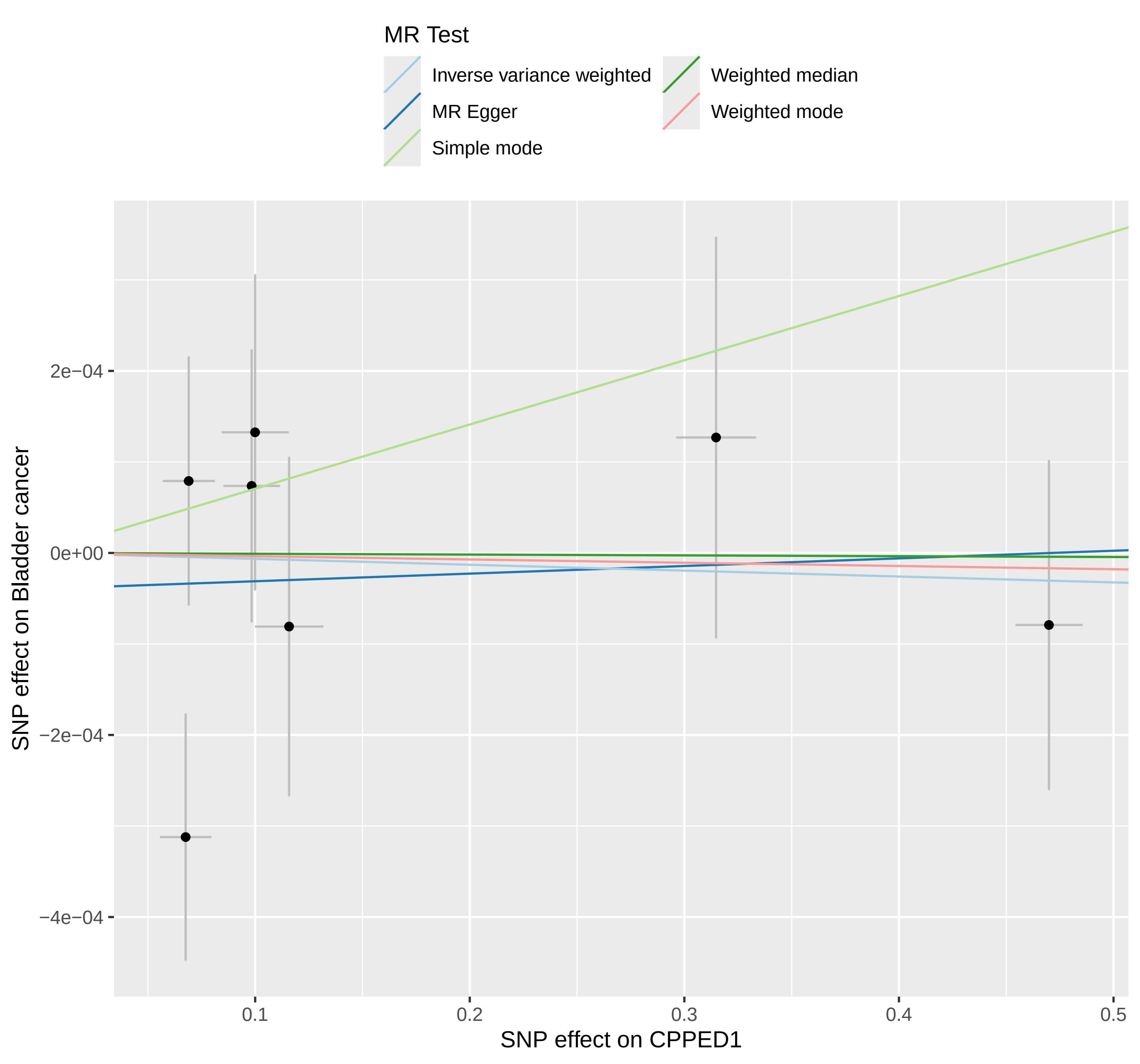

MR Test
Inverse variance weighted
MR Egger
Simple mode
Weighted median
Weighted mode
2e−04
0e+00
−2e−04
−4e−04
SNP effect on Bladder cancer
0.1
0.2
0.3
0.4
0.5
SNP effect on CPPED1

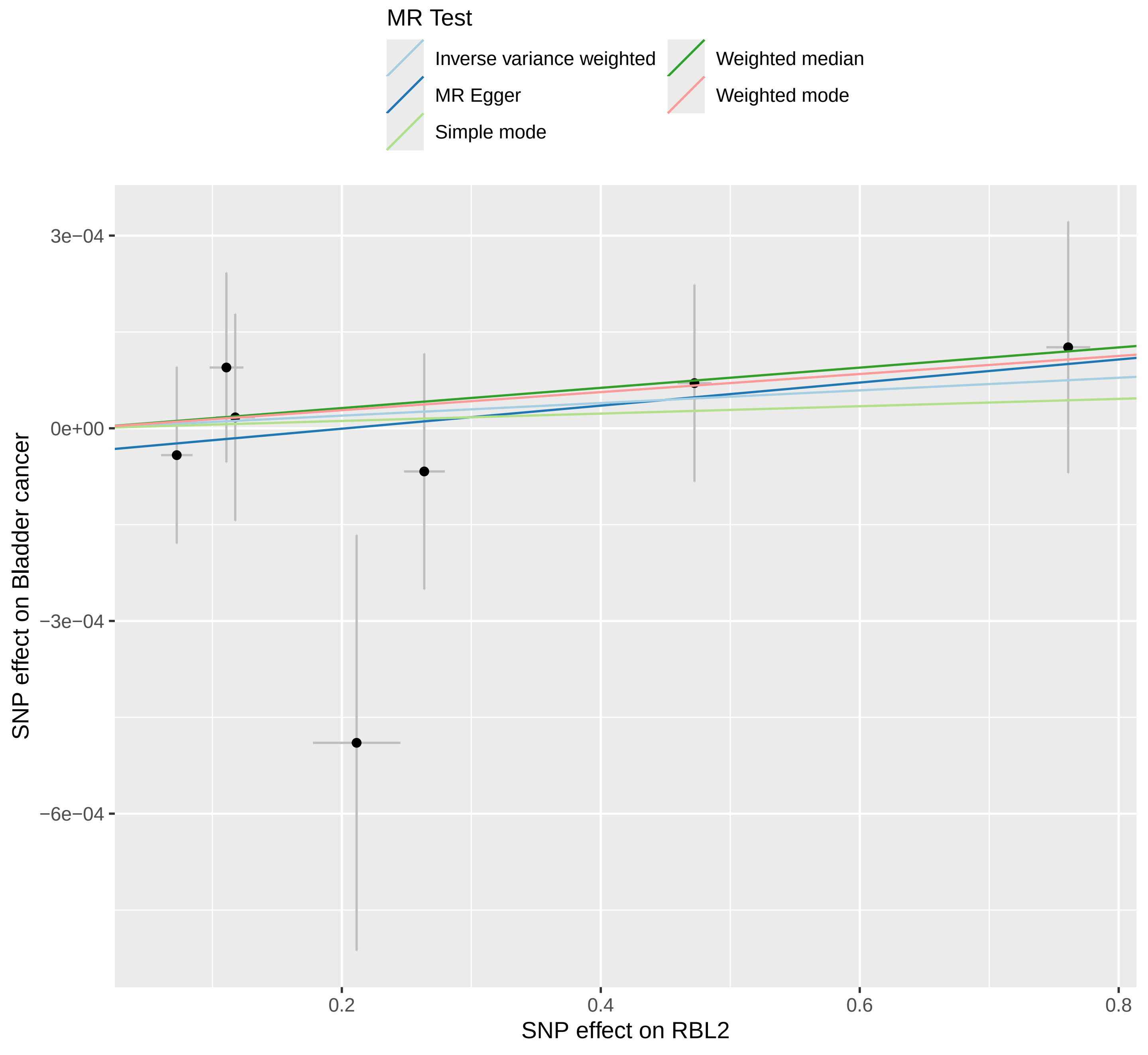

MR Test
Inverse variance weighted
MR Egger
Simple mode
Weighted median
Weighted mode
3e−04
0e+00
−3e−04
−6e−04
SNP effect on Bladder cancer
0.2
0.4
0.6
0.8
SNP effect on RBL2

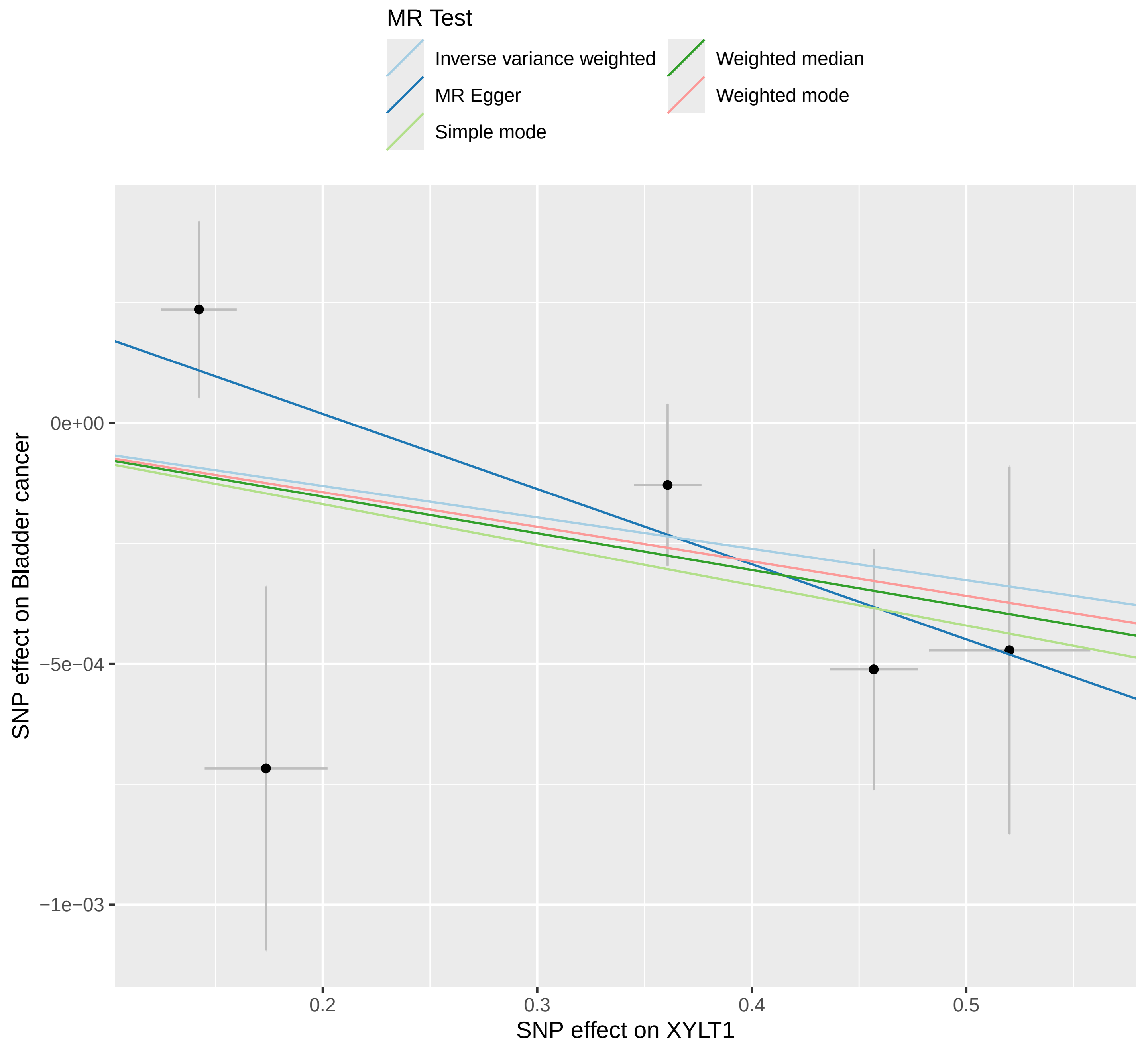
MR Test
Inverse variance weighted
MR Egger
Simple mode
Weighted median
Weighted mode
0e+00
−5e−04
−1e−03
0.2
0.3
0.4
0.5
SNP effect on Bladder cancer
SNP effect on XYLT1

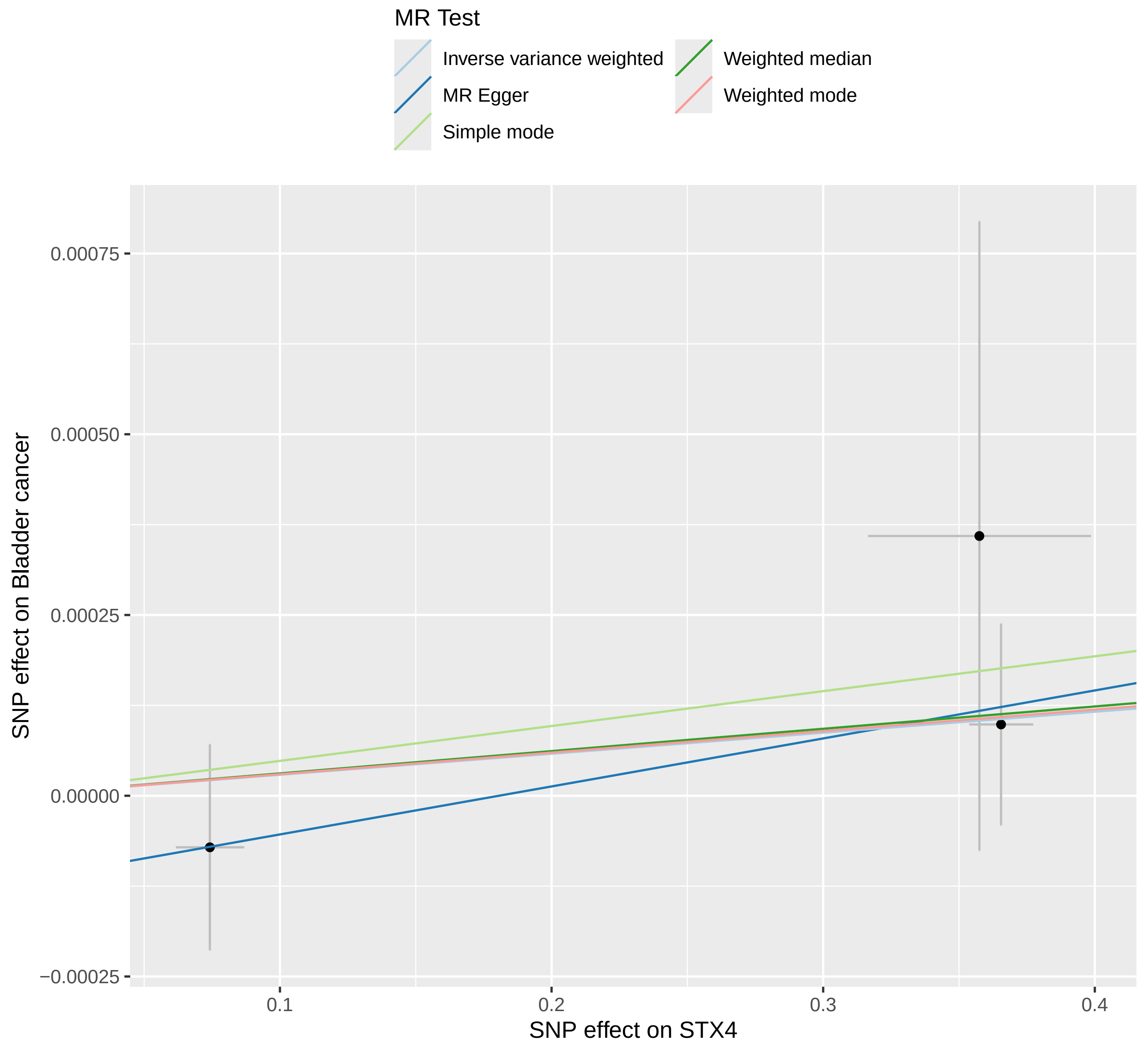
MR Test
Inverse variance weighted
MR Egger
Simple mode
Weighted median
Weighted mode
0.00075
0.00050
0.00025
0.00000
−0.00025
SNP effect on Bladder cancer
0.1
0.2
0.3
0.4
SNP effect on STX4

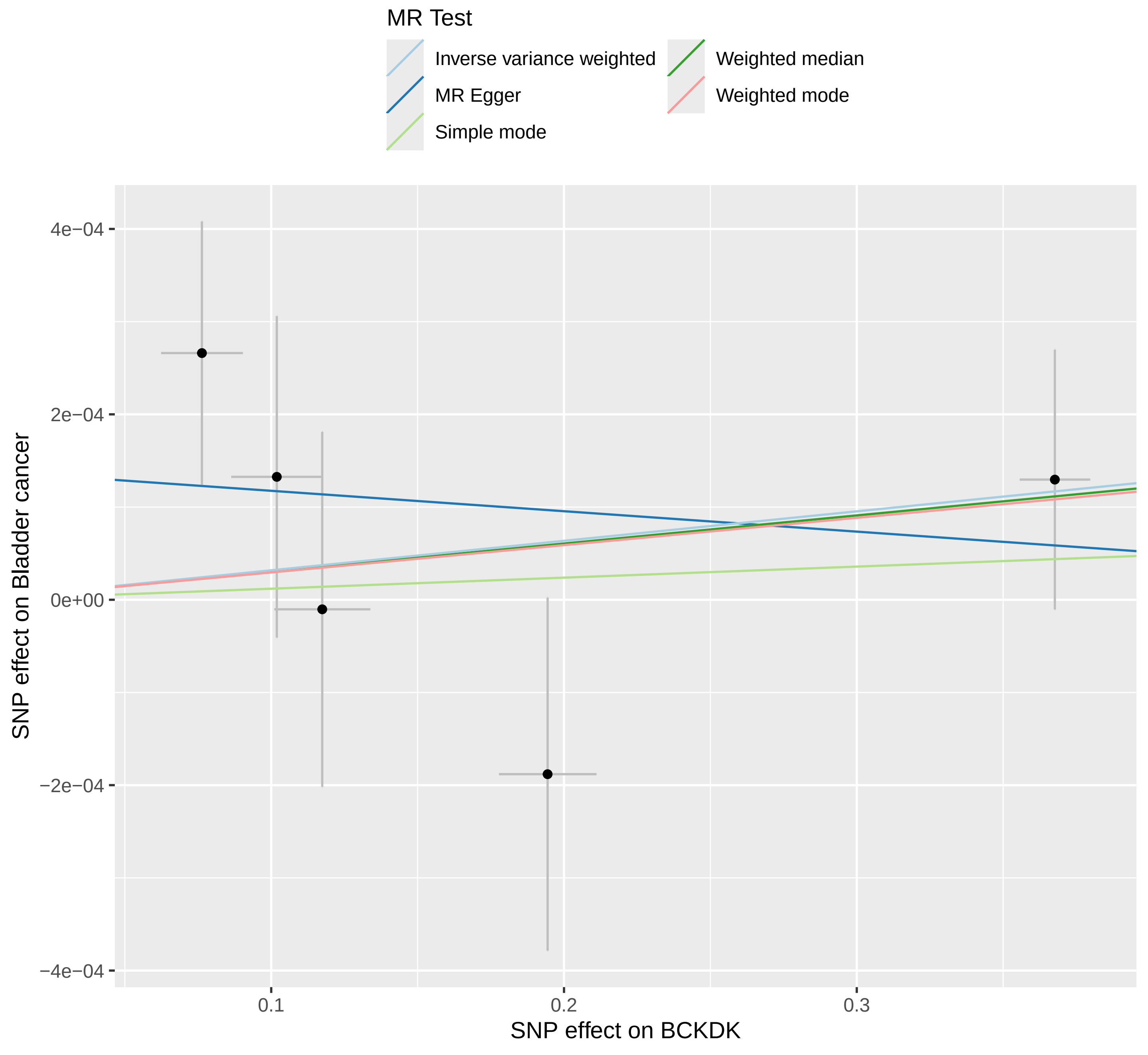
MR Test
Inverse variance weighted
MR Egger
Simple mode
Weighted median
Weighted mode
4e−04
2e−04
0e+00
−2e−04
−4e−04
SNP effect on Bladder cancer
0.1
0.2
0.3
SNP effect on BCKDK

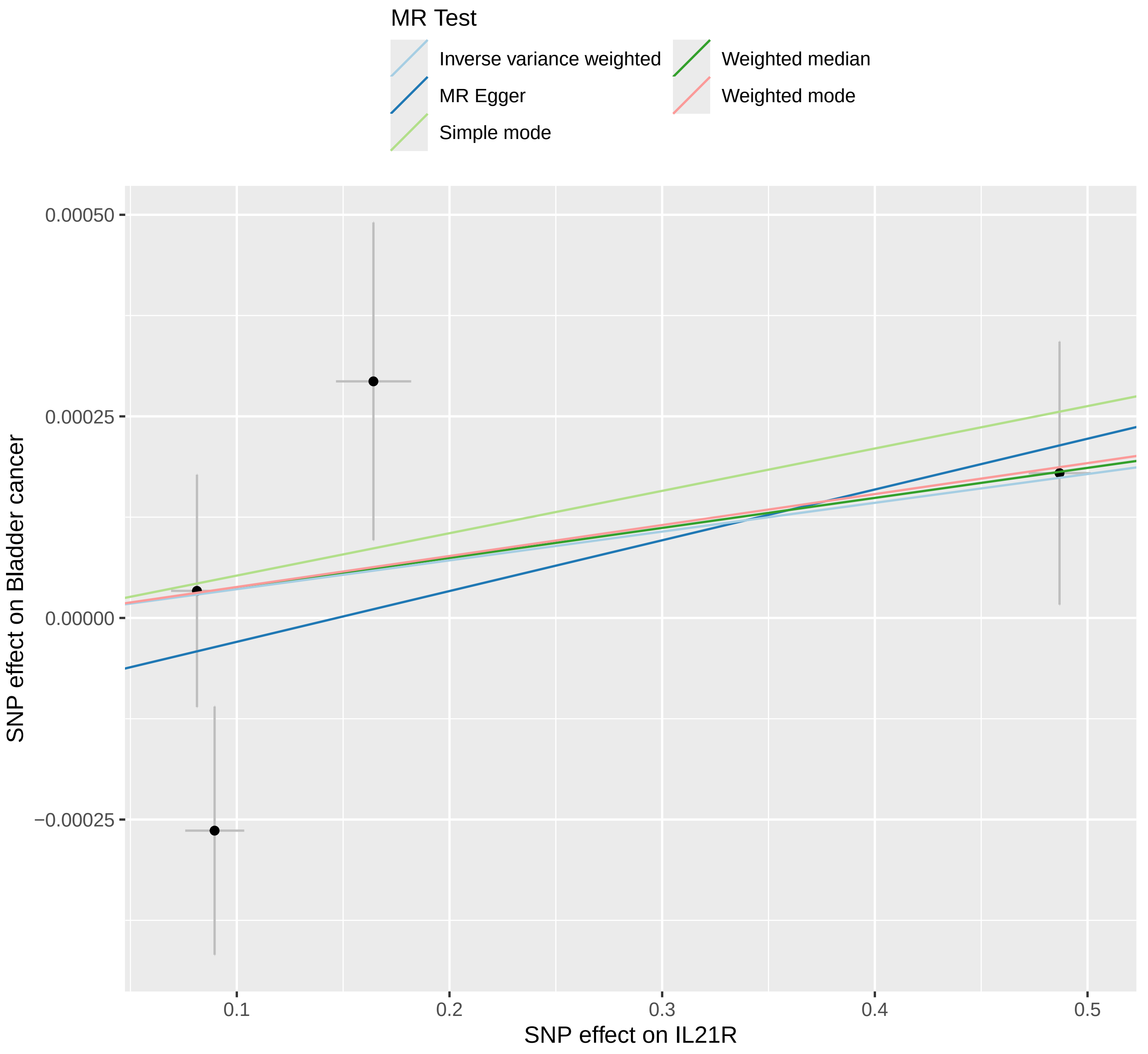
MR Test
Inverse variance weighted
MR Egger
Simple mode
Weighted median
Weighted mode
0.00050
0.00025
0.00000
−0.00025
SNP effect on Bladder cancer
0.1
0.2
0.3
0.4
0.5
SNP effect on IL21R

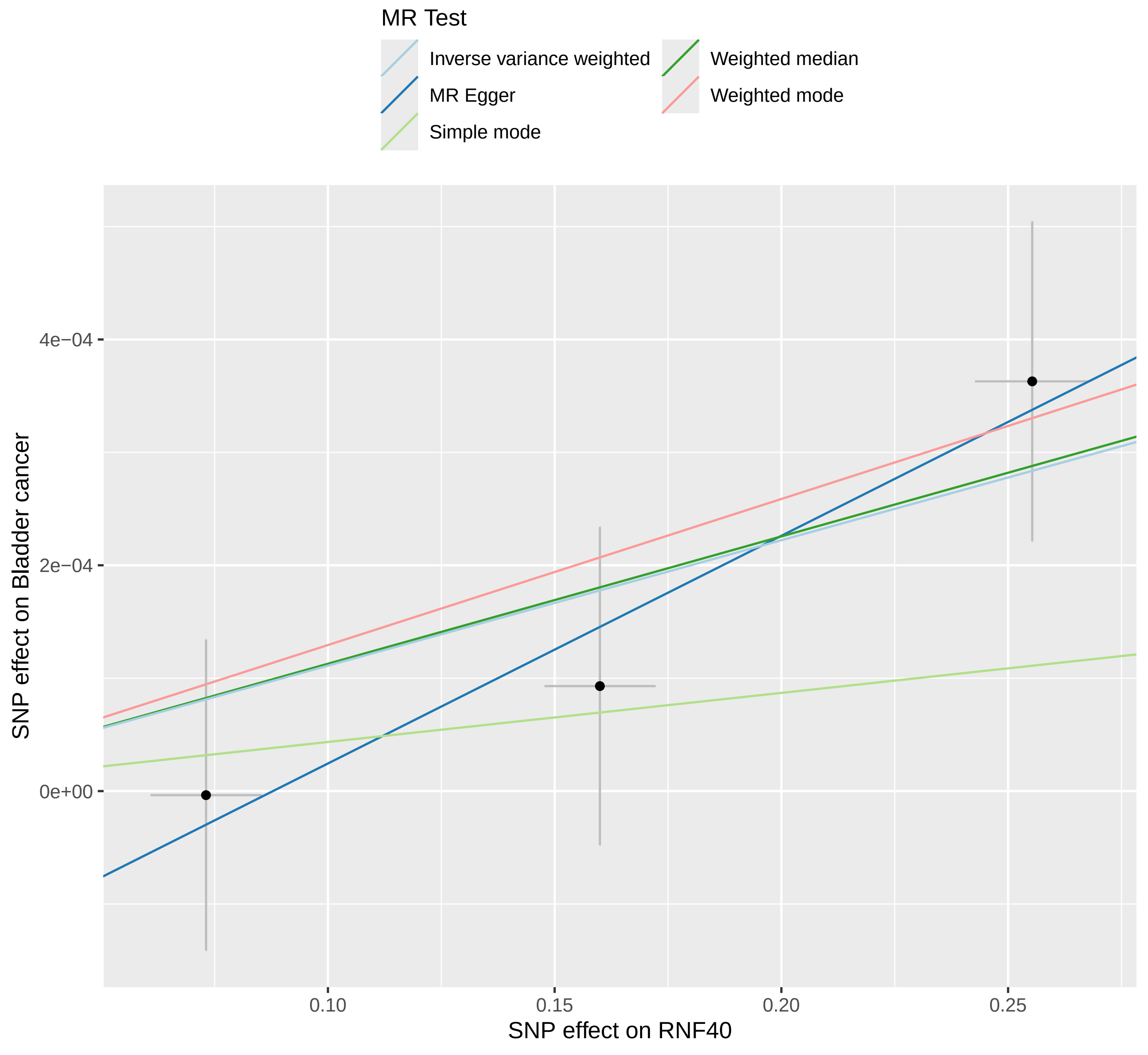
MR Test
Inverse variance weighted
MR Egger
Simple mode
Weighted median
Weighted mode
4e−04
2e−04
0e+00
0.10
0.15
0.20
0.25
SNP effect on Bladder cancer
SNP effect on RNF40

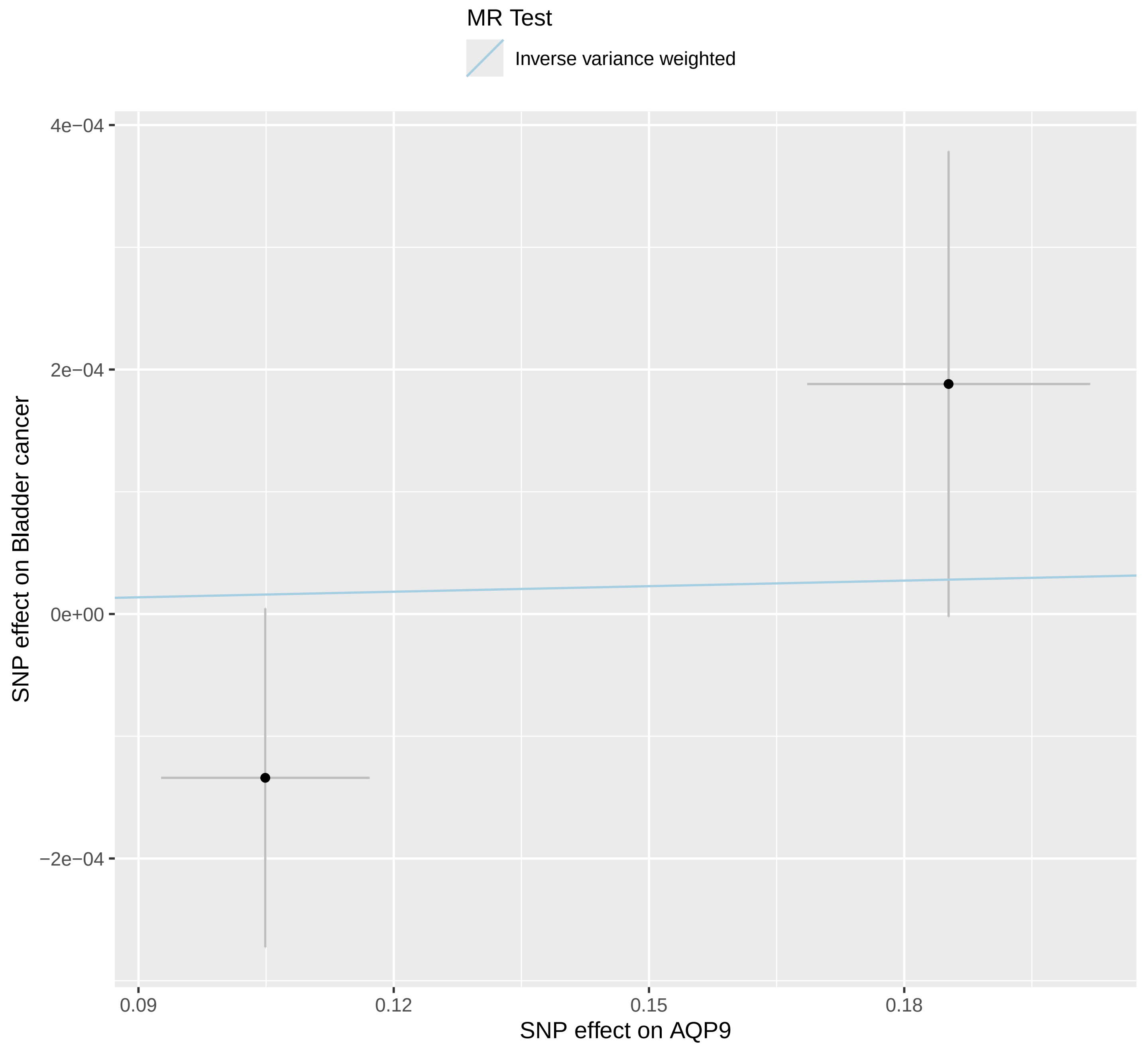
MR Test
Inverse variance weighted
4e−04
2e−04
0e+00
−2e−04
0.09
0.12
0.15
0.18
SNP effect on Bladder cancer
SNP effect on AQP9

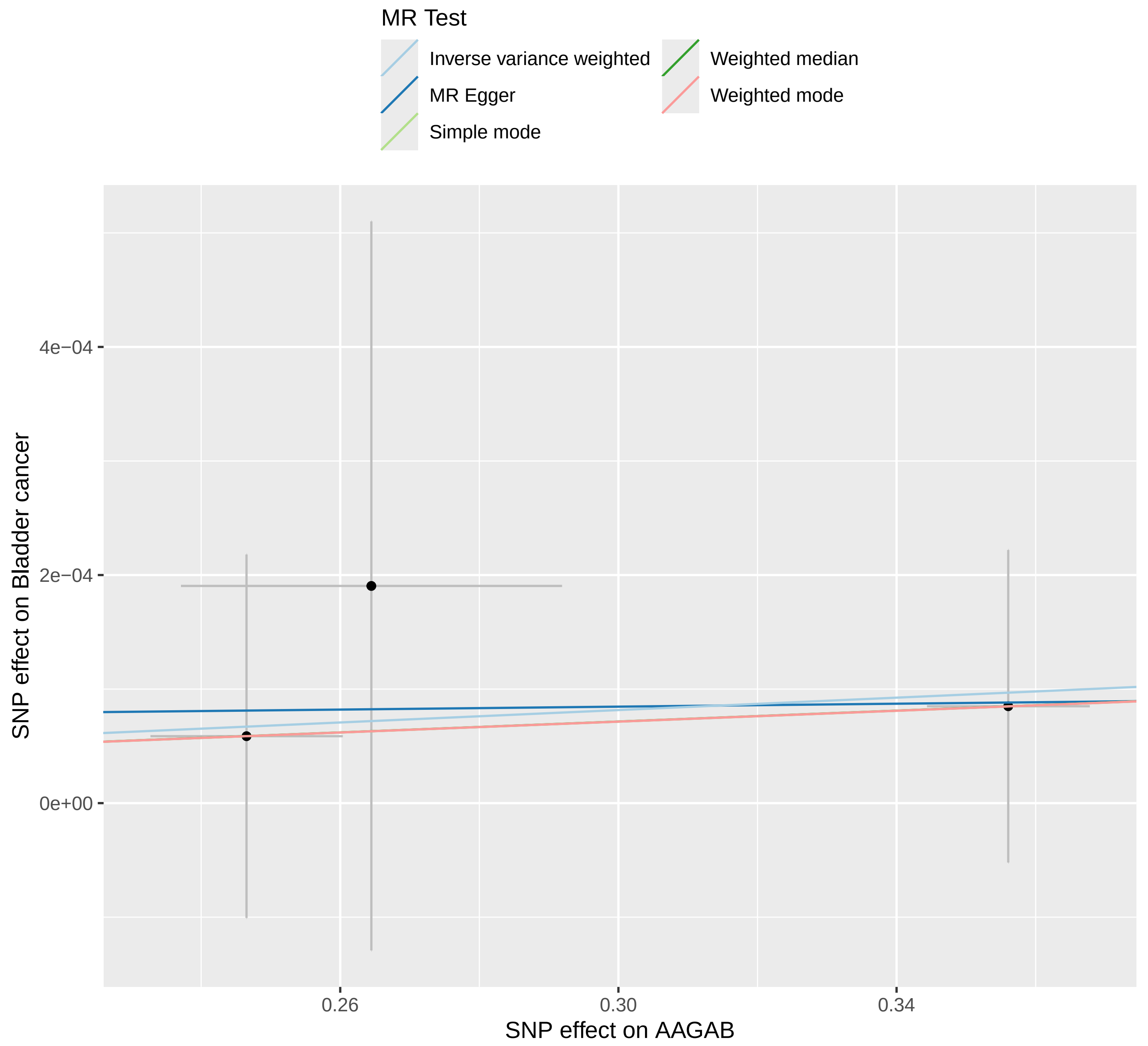

MR Test
Inverse variance weighted
MR Egger
Simple mode
Weighted median
Weighted mode
4e−04
2e−04
0e+00
SNP effect on Bladder cancer
0.26
0.30
0.34
SNP effect on AAGAB

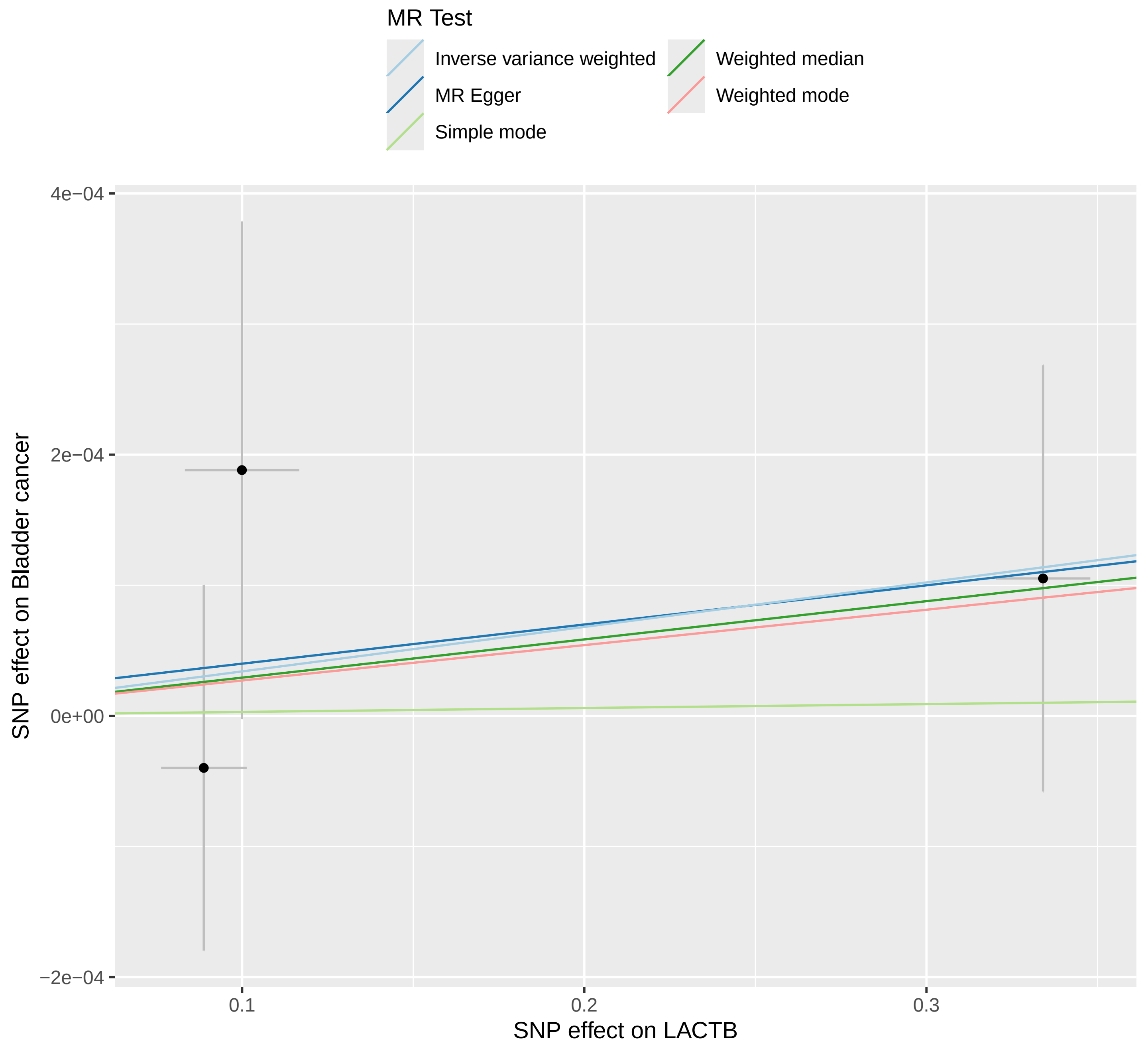

MR Test
Inverse variance weighted
MR Egger
Simple mode
Weighted median
Weighted mode
4e−04
2e−04
0e+00
−2e−04
0.1
0.2
0.3
SNP effect on Bladder cancer
SNP effect on LACTB

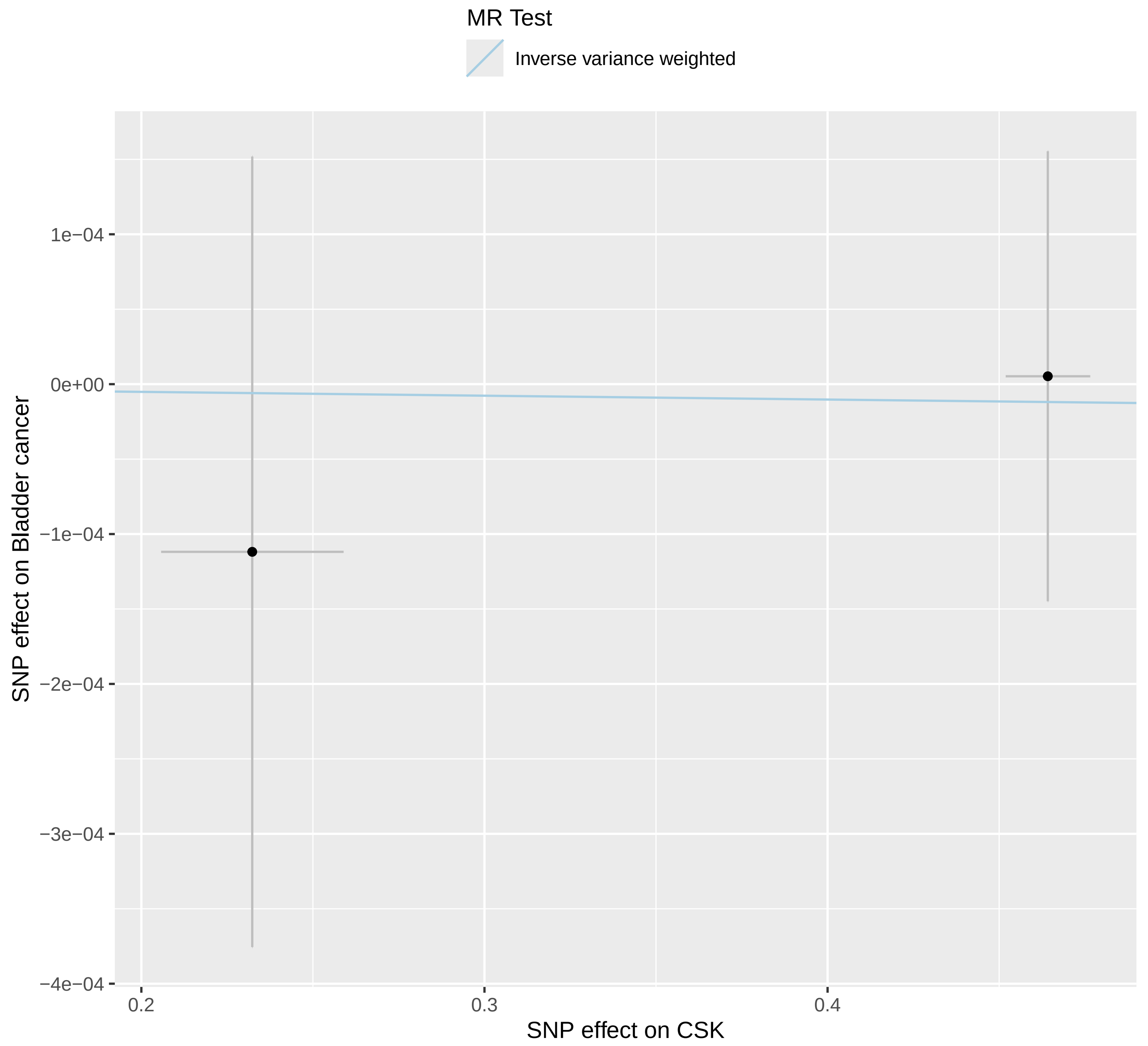
MR Test
Inverse variance weighted
1e−04
0e+00
−1e−04
−2e−04
−3e−04
−4e−04
SNP effect on Bladder cancer
0.2
0.3
0.4
SNP effect on CSK

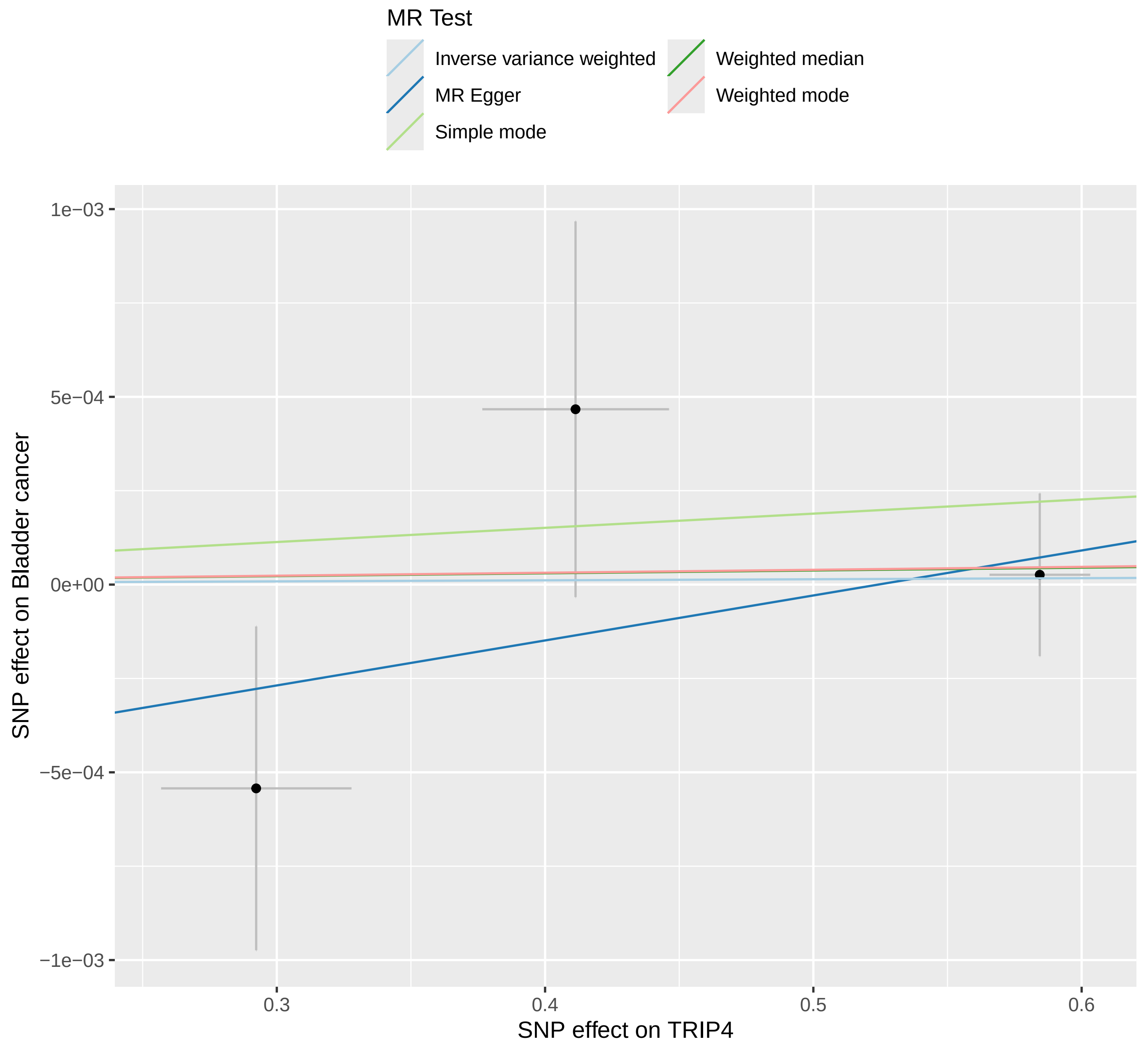
MR Test
Inverse variance weighted
MR Egger
Simple mode
Weighted median
Weighted mode
1e−03
5e−04
0e+00
−5e−04
−1e−03
SNP effect on Bladder cancer
0.3
0.4
0.5
0.6
SNP effect on TRIP4

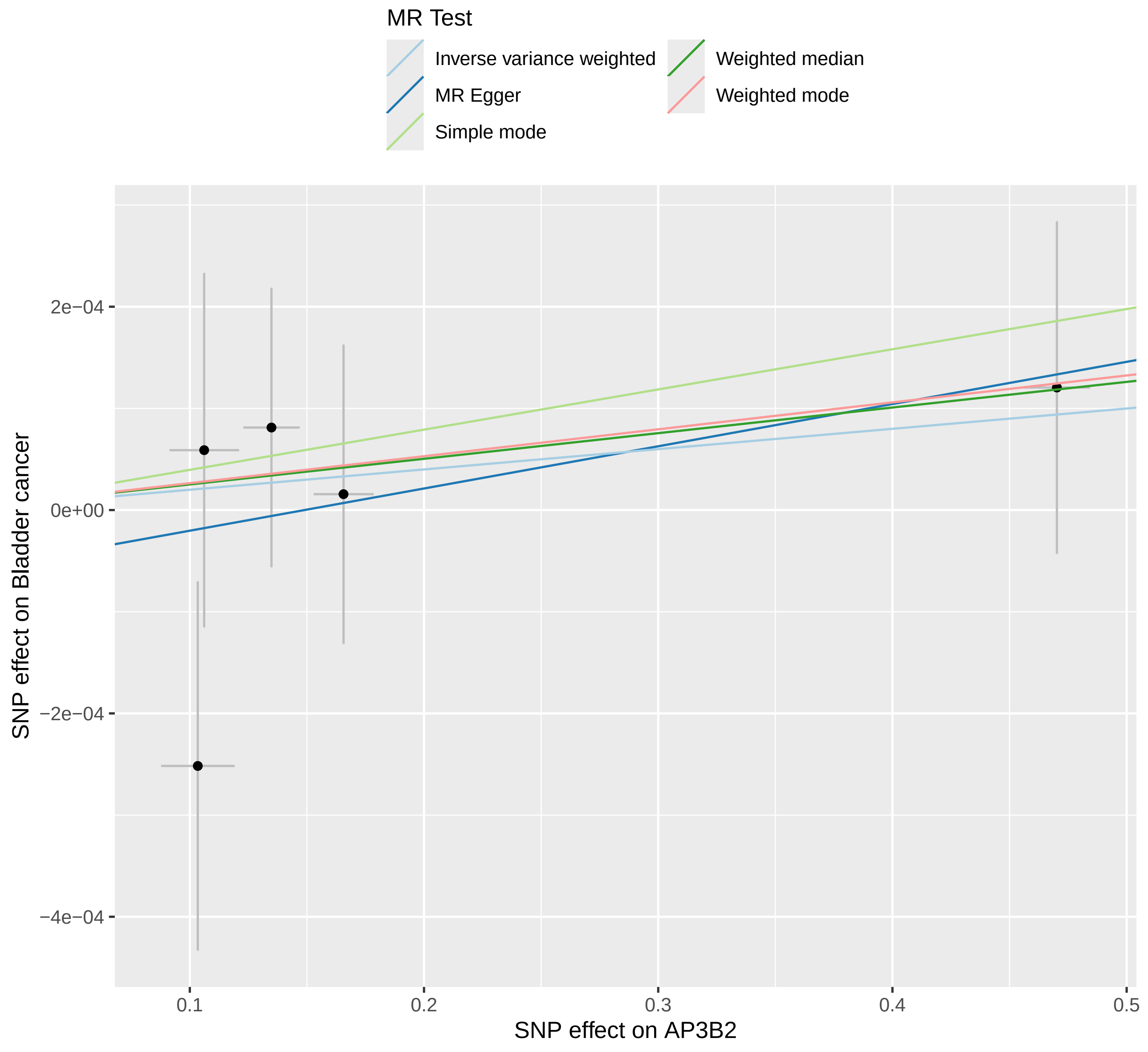
MR Test
Inverse variance weighted
MR Egger
Simple mode
Weighted median
Weighted mode
SNP effect on Bladder cancer
2e−04
0e+00
−2e−04
−4e−04
0.1
0.2
0.3
0.4
0.5
SNP effect on AP3B2

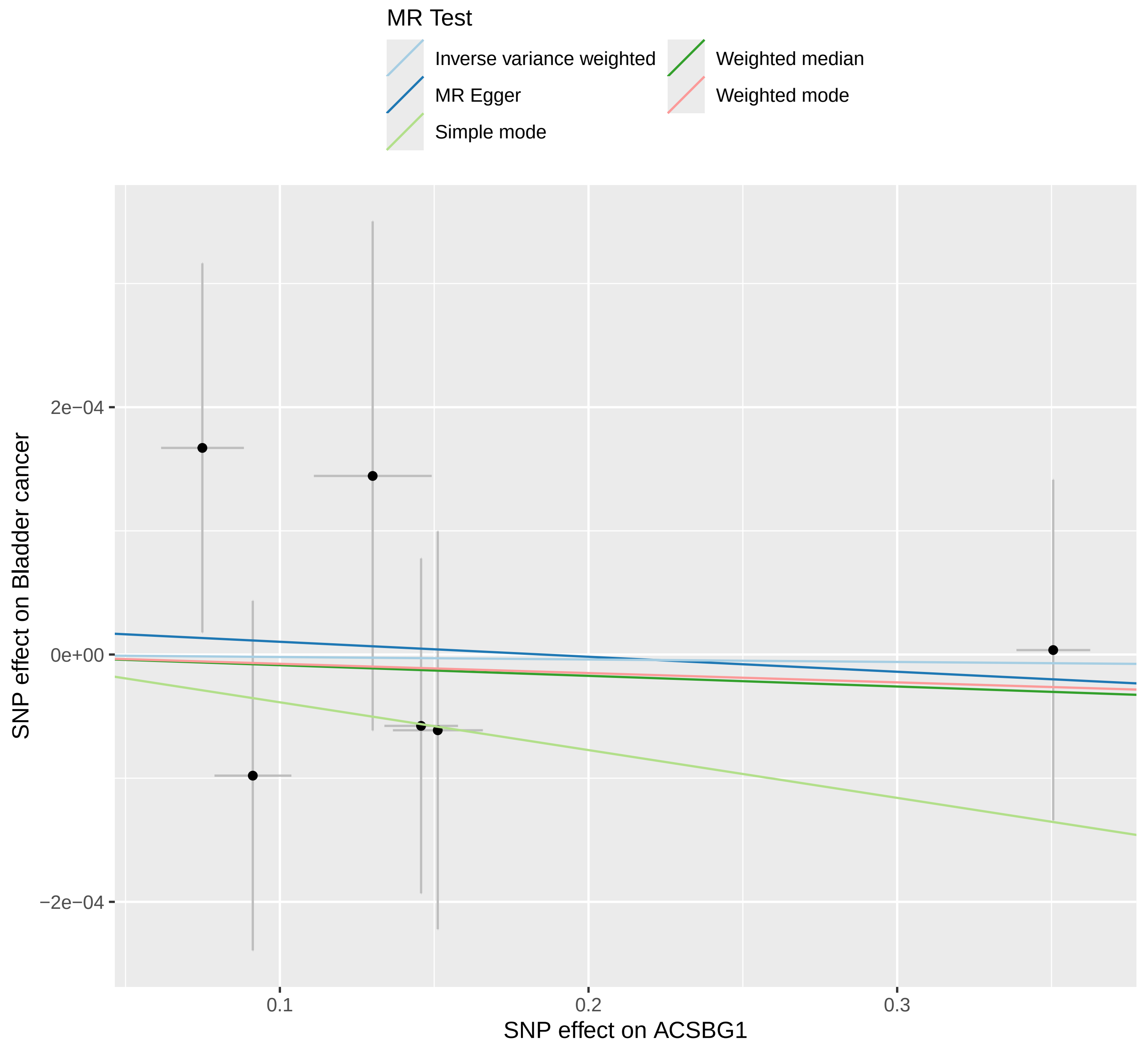

MR Test
Inverse variance weighted
MR Egger
Simple mode
Weighted median
Weighted mode
2e−04
0e+00
−2e−04
SNP effect on Bladder cancer
0.1
0.2
0.3
SNP effect on ACSBG1

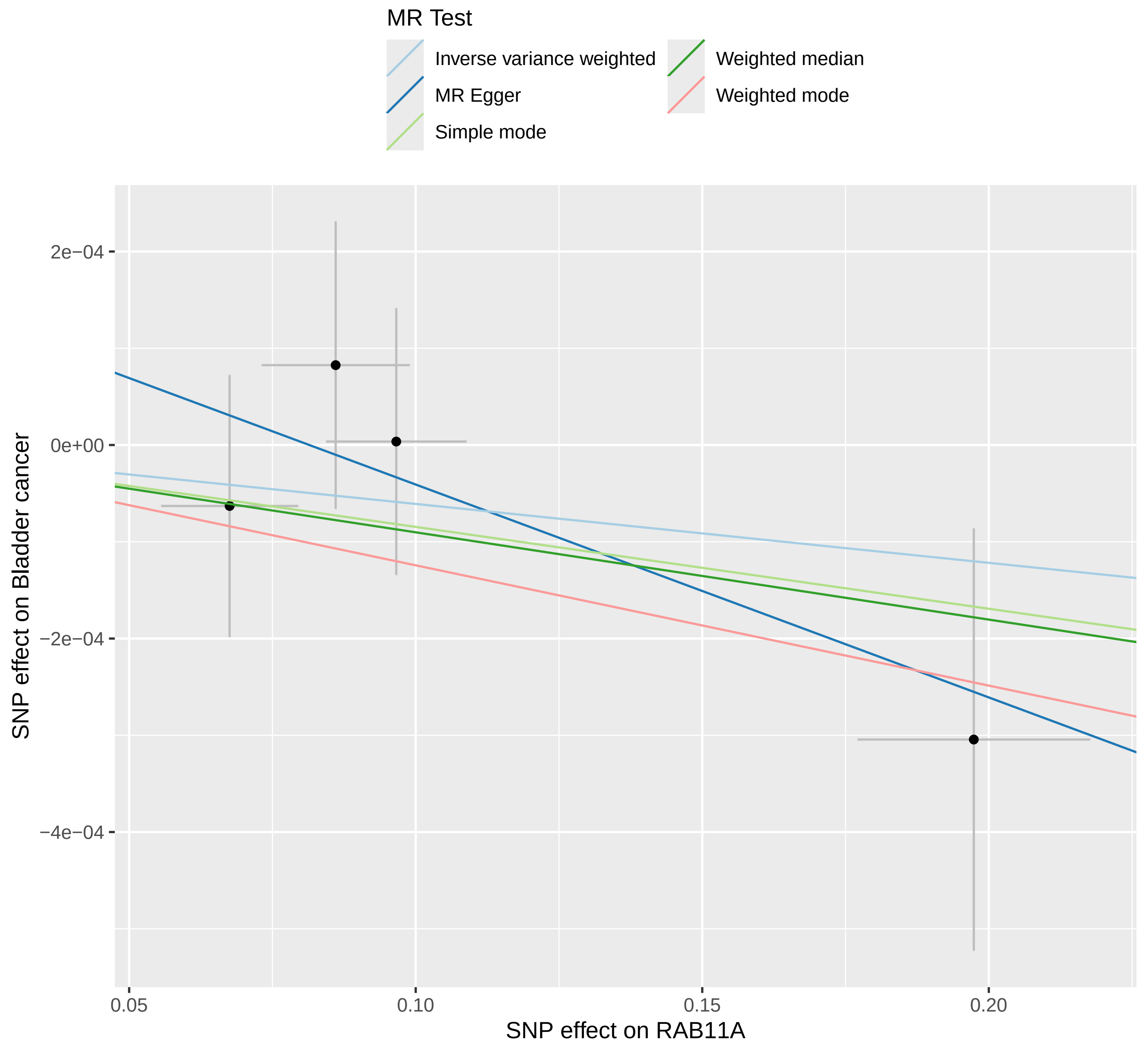

MR Test
Inverse variance weighted
MR Egger
Simple mode
Weighted median
Weighted mode
2e−04
0e+00
−2e−04
−4e−04
SNP effect on Bladder cancer
0.05
0.10
0.15
0.20
SNP effect on RAB11A

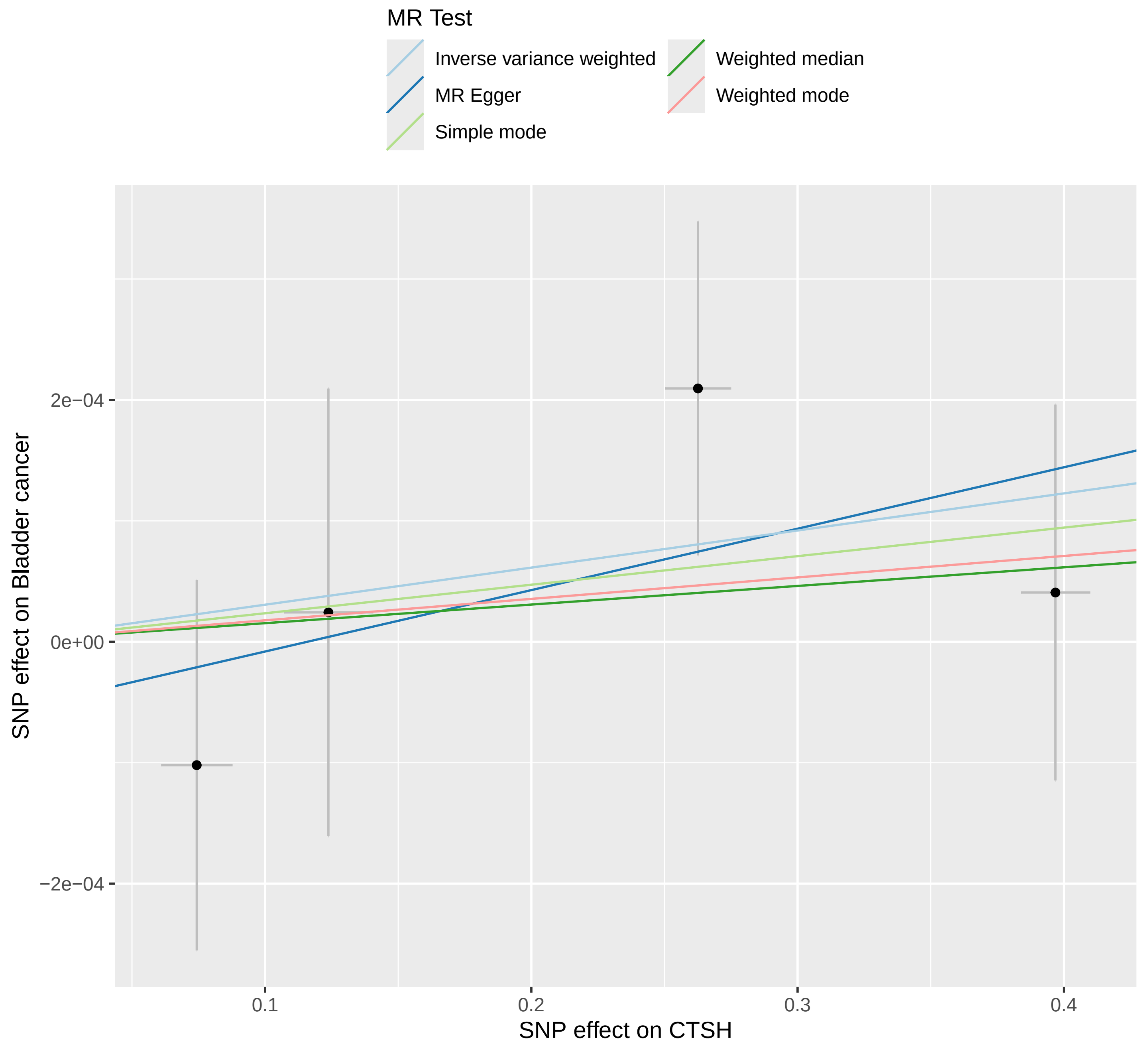

MR Test
Inverse variance weighted
MR Egger
Simple mode
Weighted median
Weighted mode
2e−04
0e+00
−2e−04
SNP effect on Bladder cancer
0.1
0.2
0.3
0.4
SNP effect on CTSH

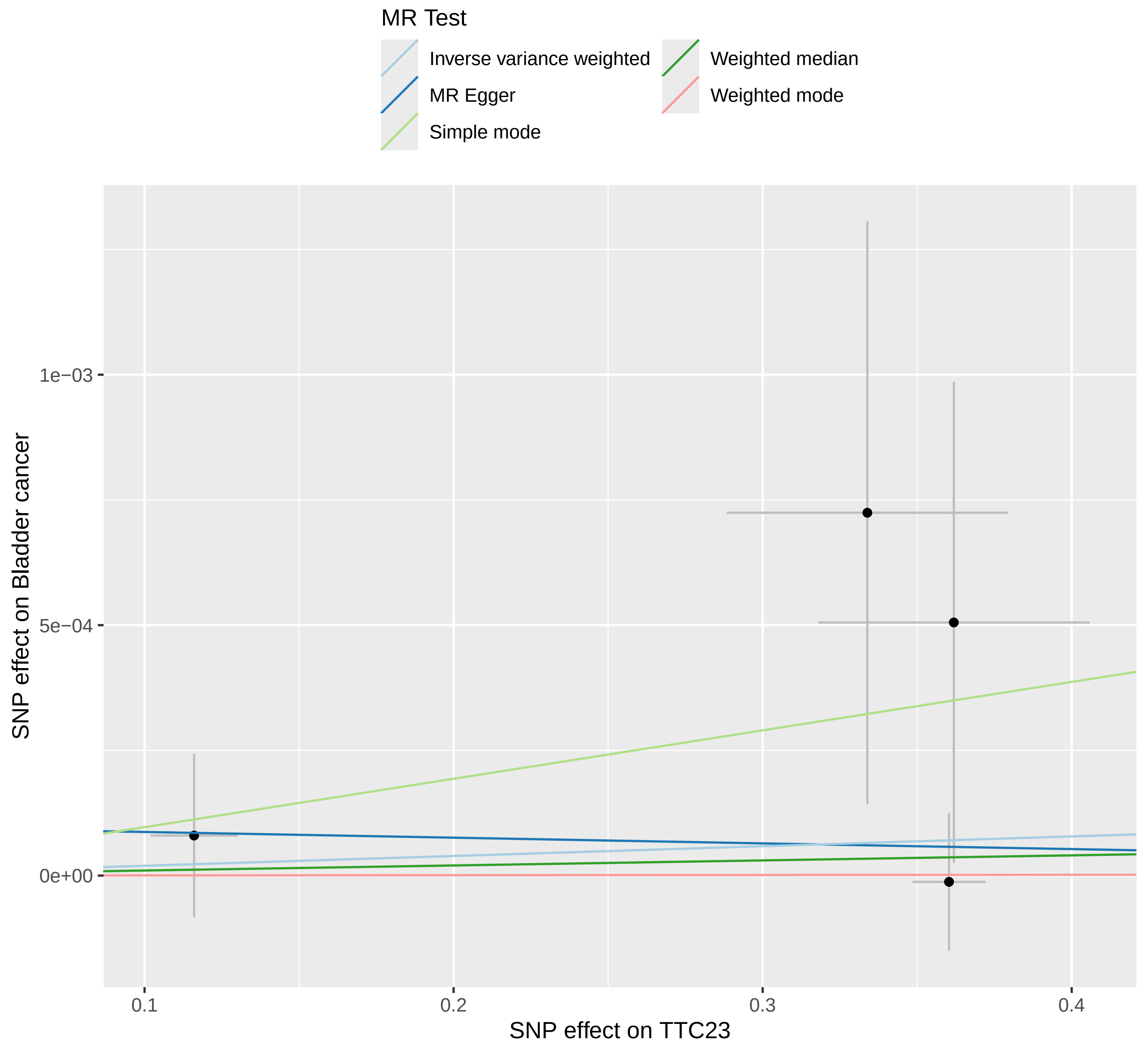
MR Test
Inverse variance weighted
MR Egger
Simple mode
Weighted median
Weighted mode
1e−03
5e−04
0e+00
SNP effect on Bladder cancer
0.1
0.2
0.3
0.4
SNP effect on TTC23

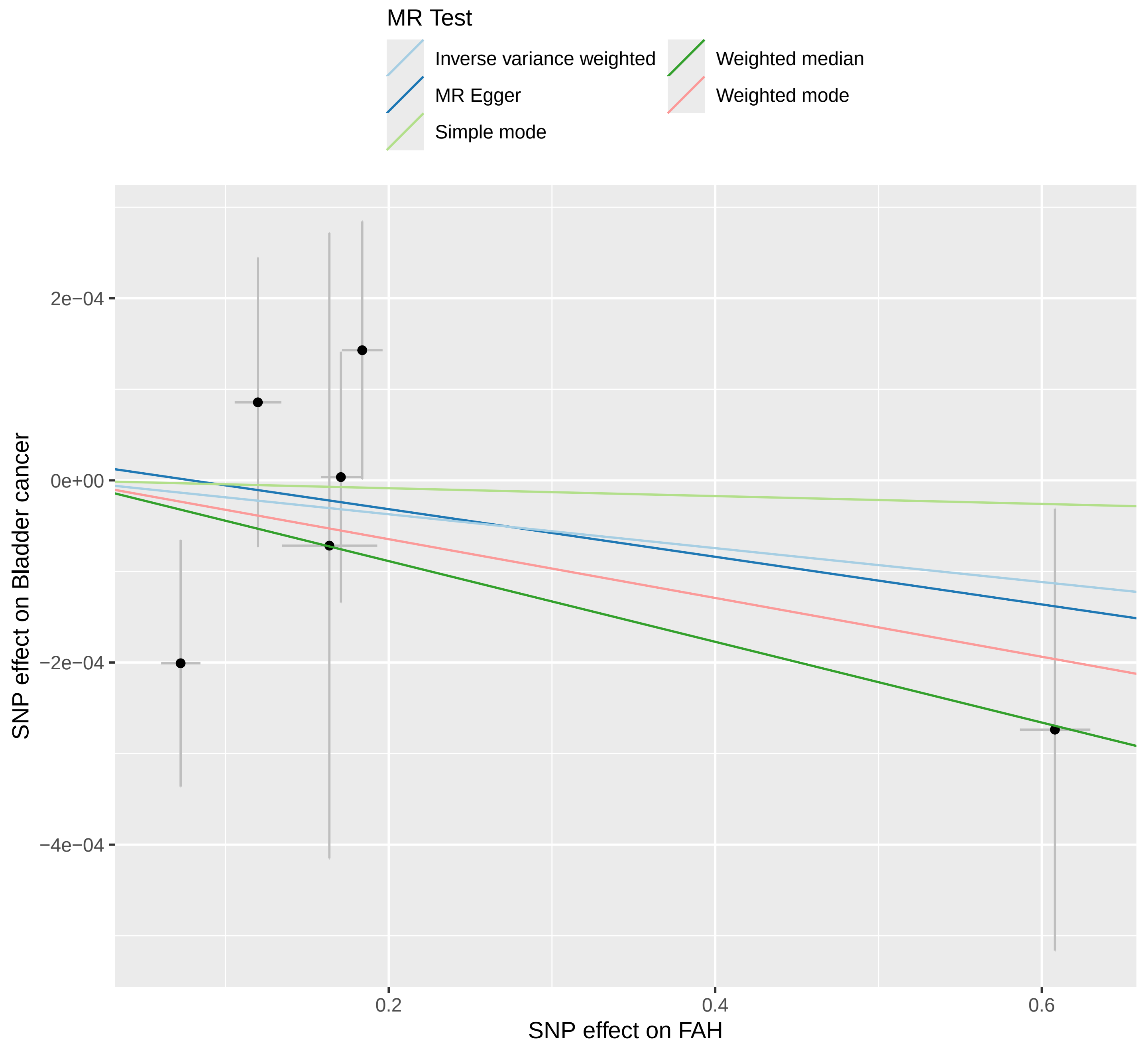

MR Test
Inverse variance weighted
MR Egger
Simple mode
Weighted median
Weighted mode
2e−04
0e+00
−2e−04
−4e−04
SNP effect on Bladder cancer
0.2
0.4
0.6
SNP effect on FAH

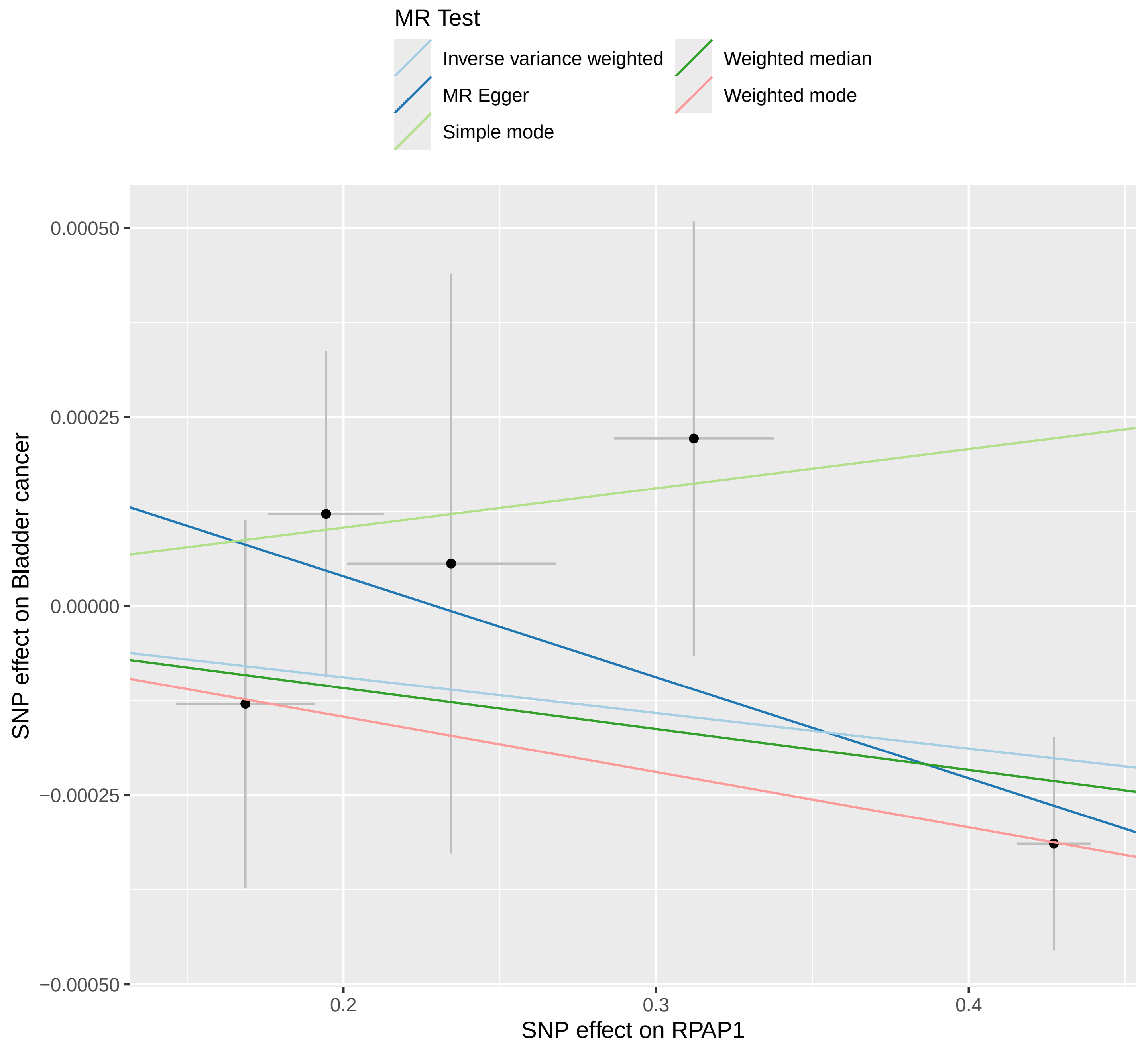
MR Test
Inverse variance weighted
MR Egger
Simple mode
Weighted median
Weighted mode
0.00050
0.00025
0.00000
−0.00025
−0.00050
SNP effect on Bladder cancer
0.2
0.3
0.4
SNP effect on RPAP1

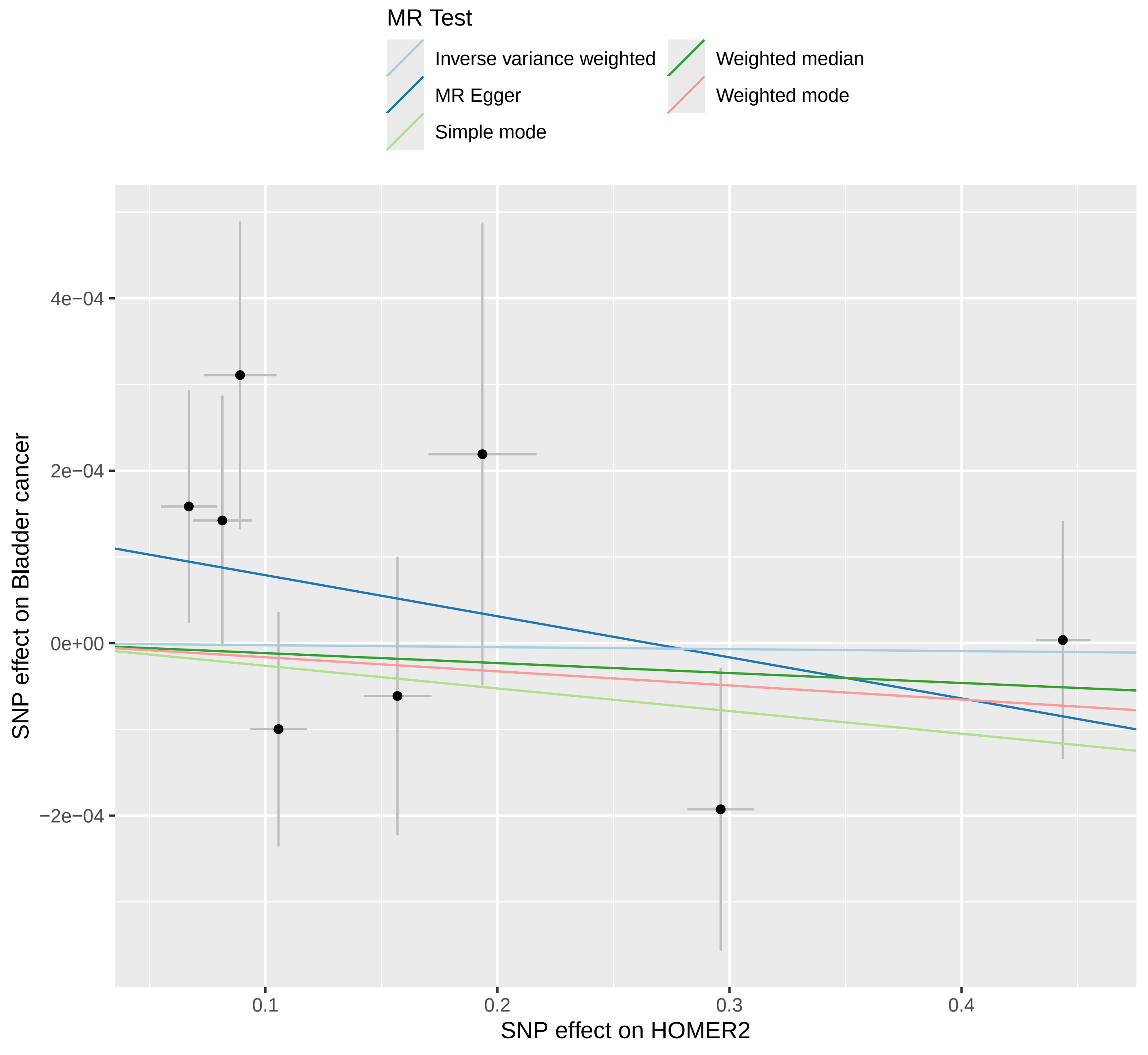

MR Test
Inverse variance weighted
MR Egger
Simple mode
Weighted median
Weighted mode
4e−04
2e−04
0e+00
−2e−04
0.1
0.2
0.3
0.4
SNP effect on Bladder cancer
SNP effect on HOMER2

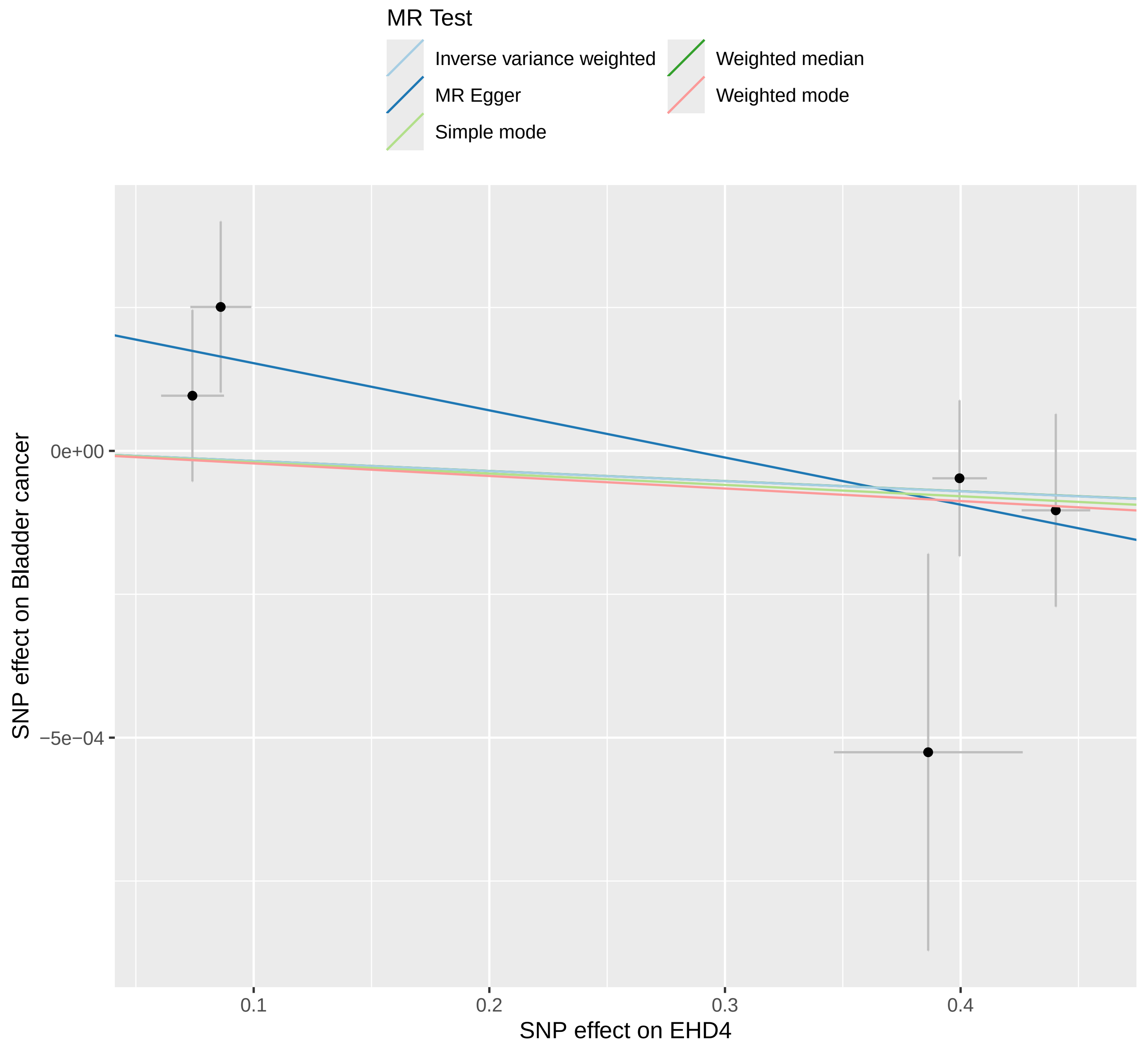
MR Test
Inverse variance weighted
MR Egger
Simple mode
Weighted median
Weighted mode
0e+00
−5e−04
0.1
0.2
0.3
0.4
SNP effect on EHD4
SNP effect on Bladder cancer

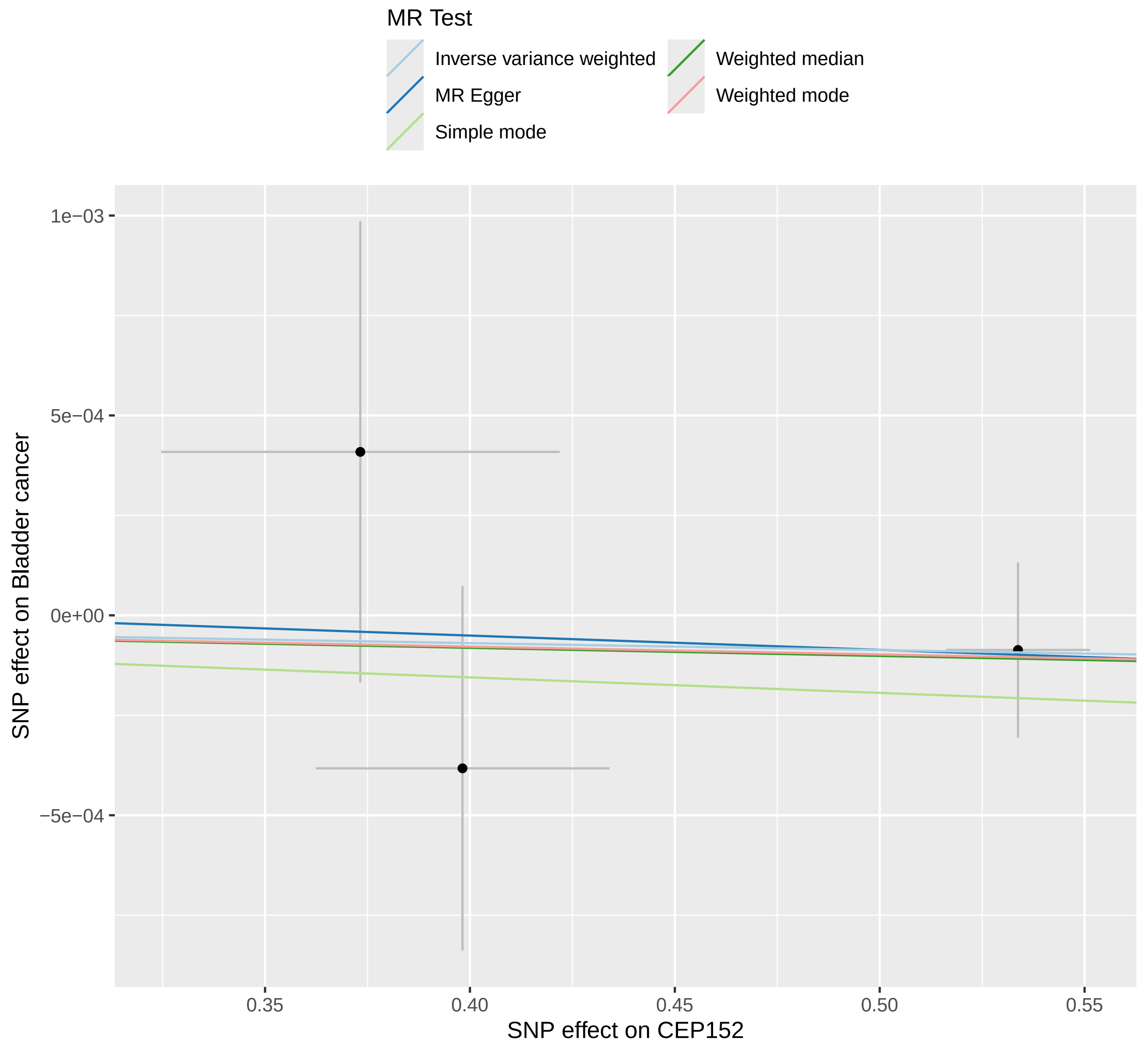

MR Test
Inverse variance weighted
MR Egger
Simple mode
Weighted median
Weighted mode
1e−03
5e−04
0e+00
−5e−04
SNP effect on Bladder cancer
0.35
0.40
0.45
0.50
0.55
SNP effect on CEP152

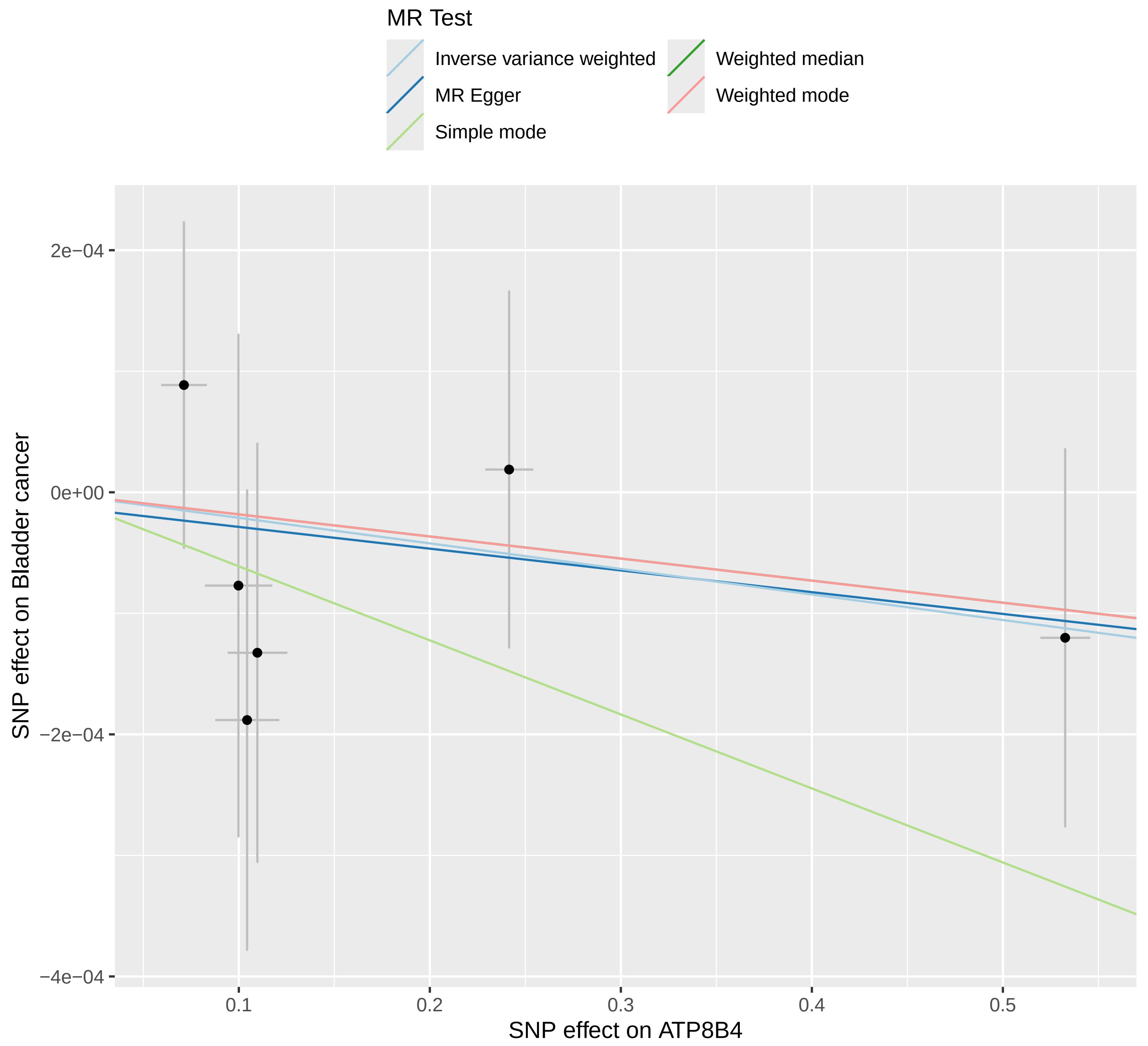

MR Test
Inverse variance weighted
MR Egger
Simple mode
Weighted median
Weighted mode
2e−04
0e+00
−2e−04
−4e−04
SNP effect on Bladder cancer
0.1
0.2
0.3
0.4
0.5
SNP effect on ATP8B4

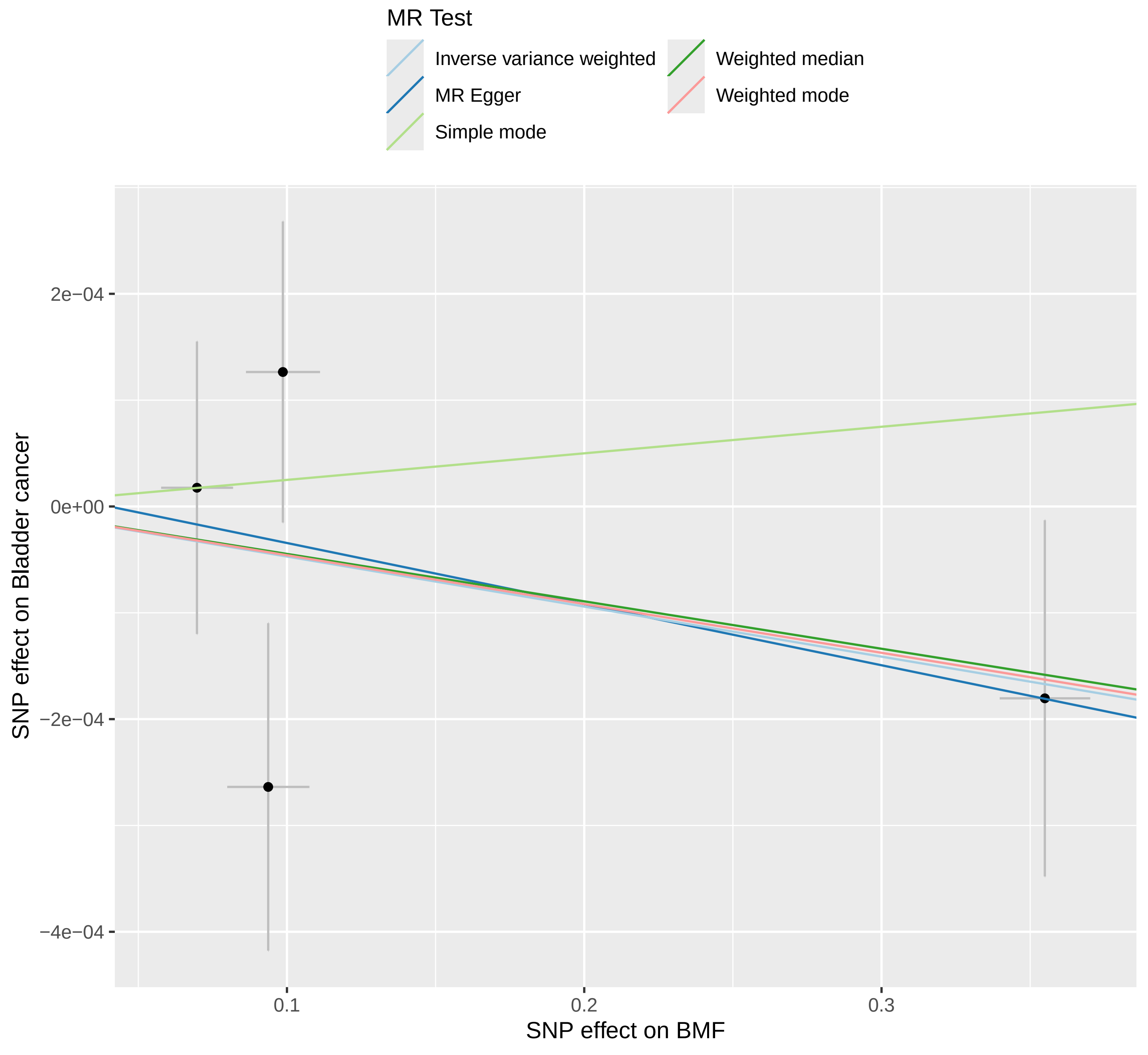
MR Test
Inverse variance weighted
MR Egger
Simple mode
Weighted median
Weighted mode
2e−04
0e+00
−2e−04
−4e−04
0.1
0.2
0.3
SNP effect on BMF
SNP effect on Bladder cancer

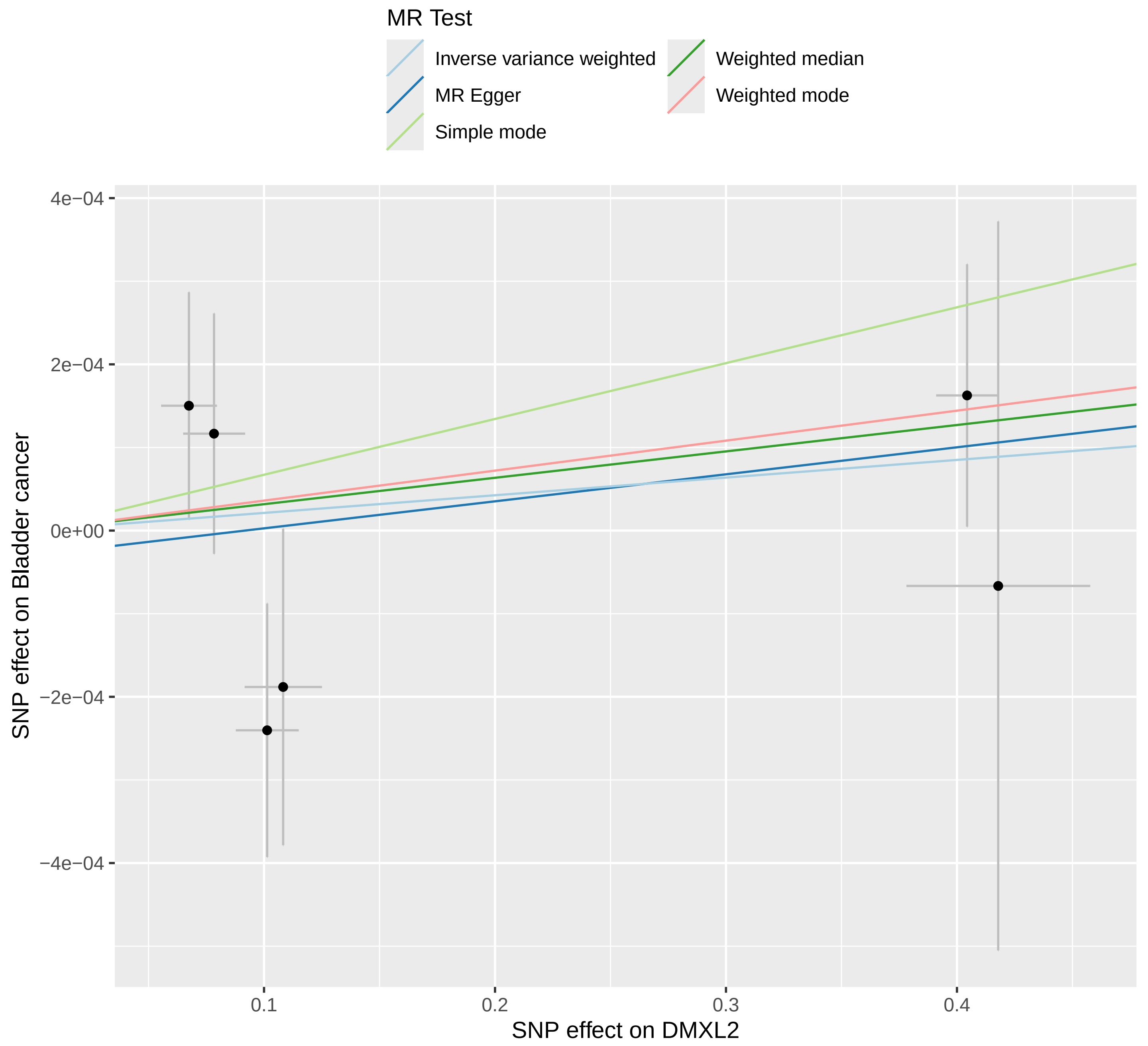
MR Test
Inverse variance weighted
MR Egger
Simple mode
Weighted median
Weighted mode
4e−04
2e−04
0e+00
−2e−04
−4e−04
0.1
0.2
0.3
0.4
SNP effect on Bladder cancer
SNP effect on DMXL2

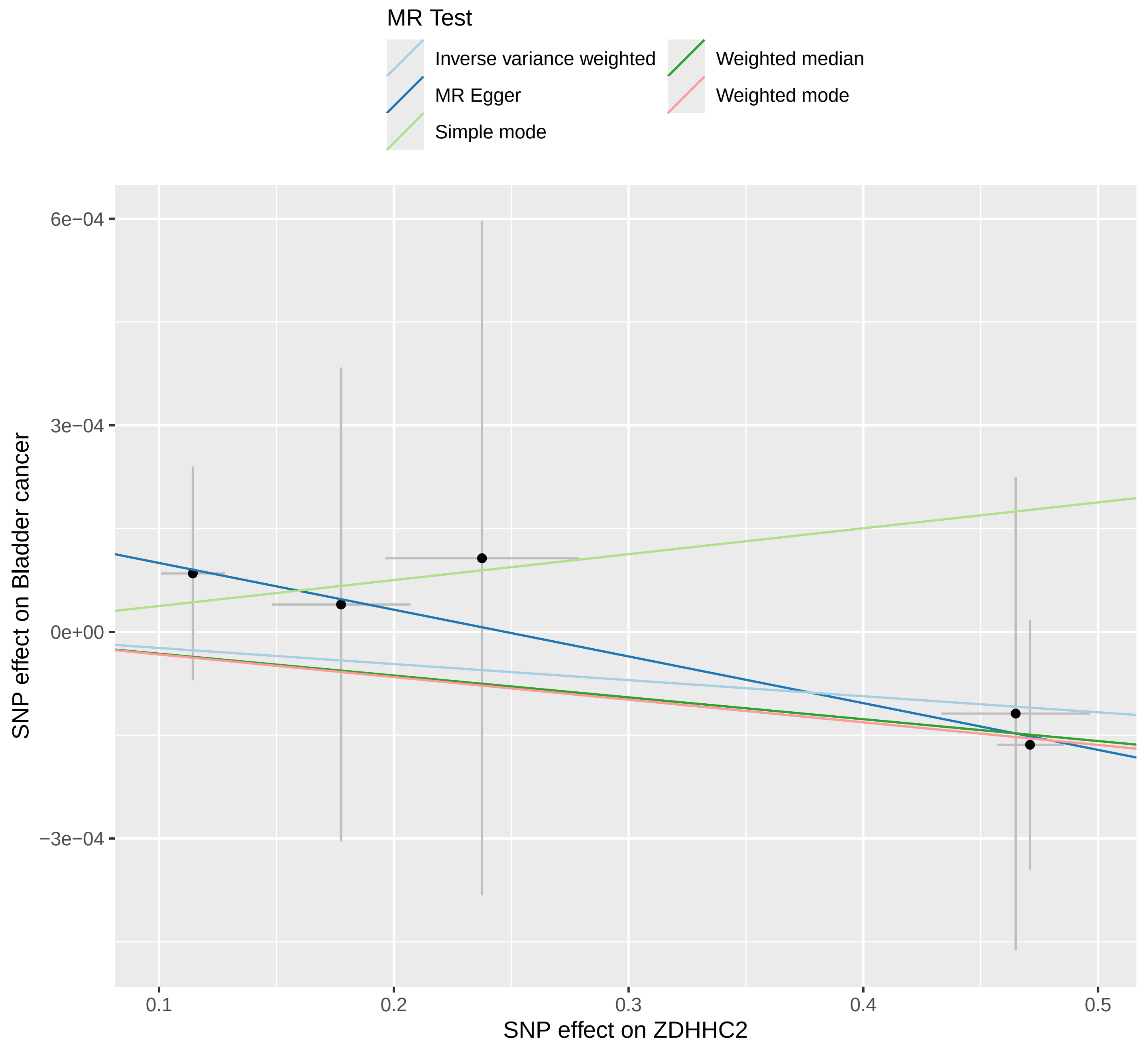

MR Test
Inverse variance weighted
MR Egger
Simple mode
Weighted median
Weighted mode
6e−04
3e−04
0e+00
−3e−04
0.1
0.2
0.3
0.4
0.5
SNP effect on Bladder cancer
SNP effect on ZDHHC2

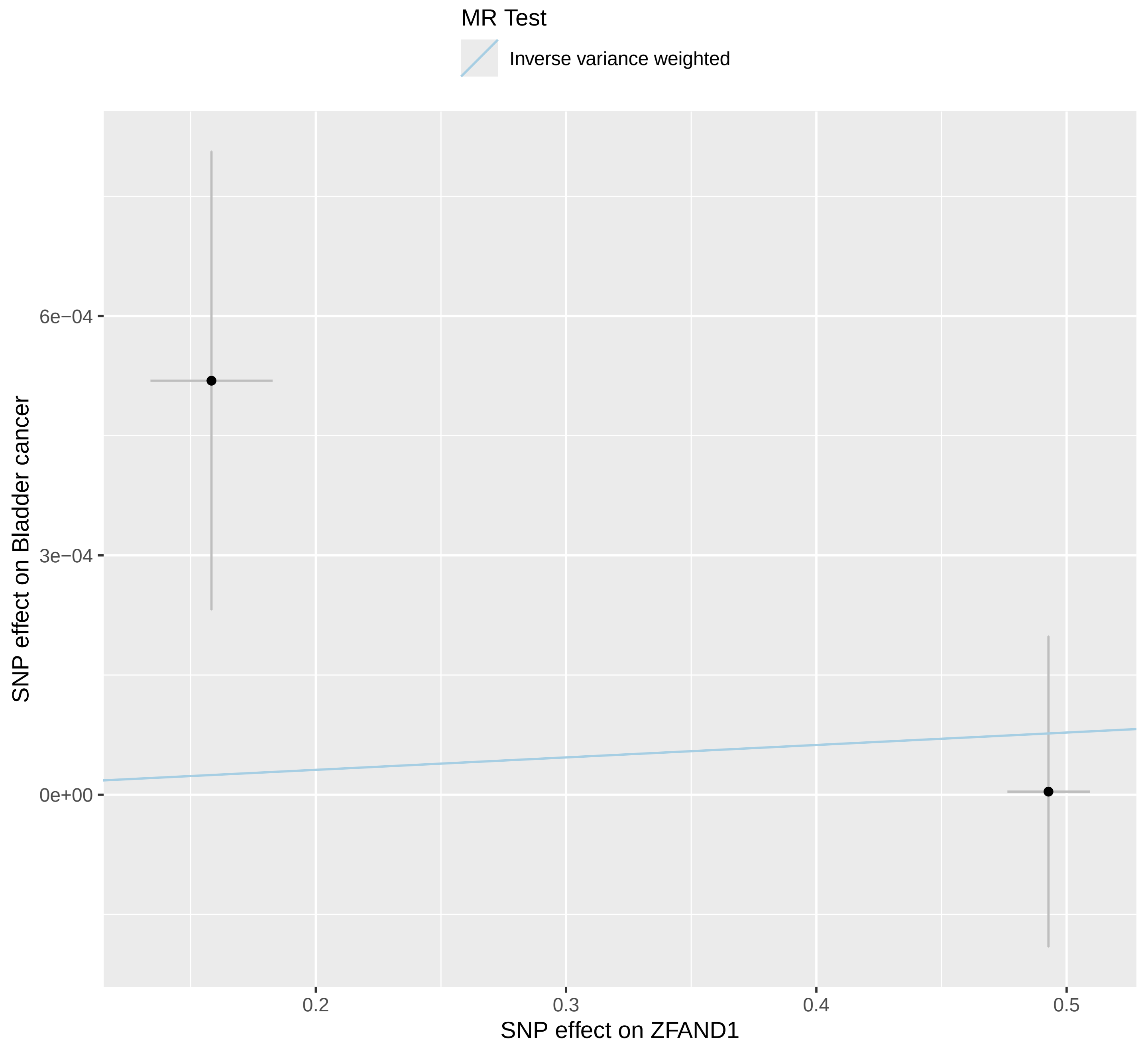

MR Test
Inverse variance weighted
6e−04
3e−04
0e+00
SNP effect on Bladder cancer
0.2
0.3
0.4
0.5
SNP effect on ZFAND1

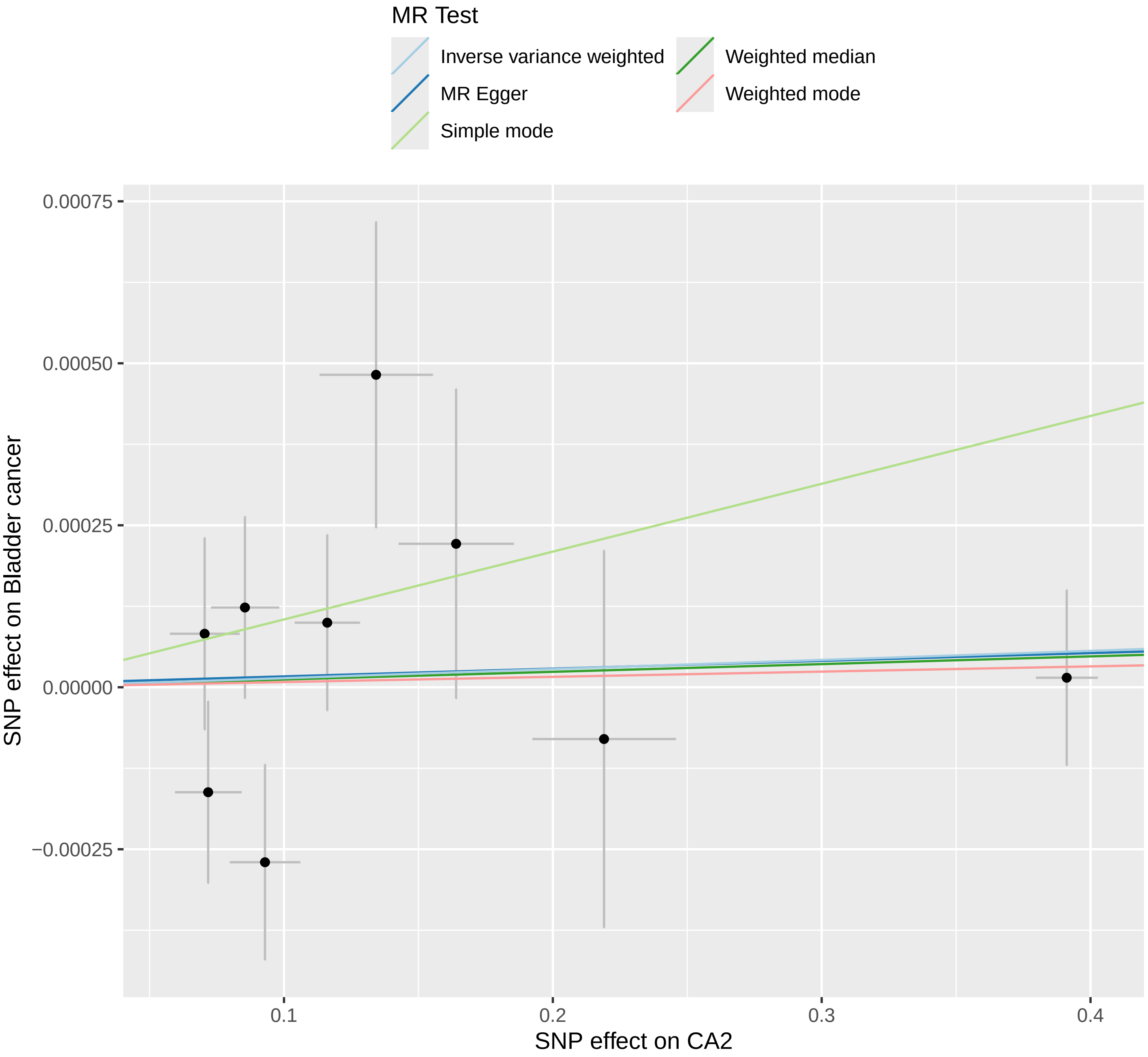

MR Test
Inverse variance weighted
MR Egger
Simple mode
Weighted median
Weighted mode
0.00075
0.00050
0.00025
0.00000
−0.00025
SNP effect on Bladder cancer
0.1
0.2
0.3
0.4
SNP effect on CA2

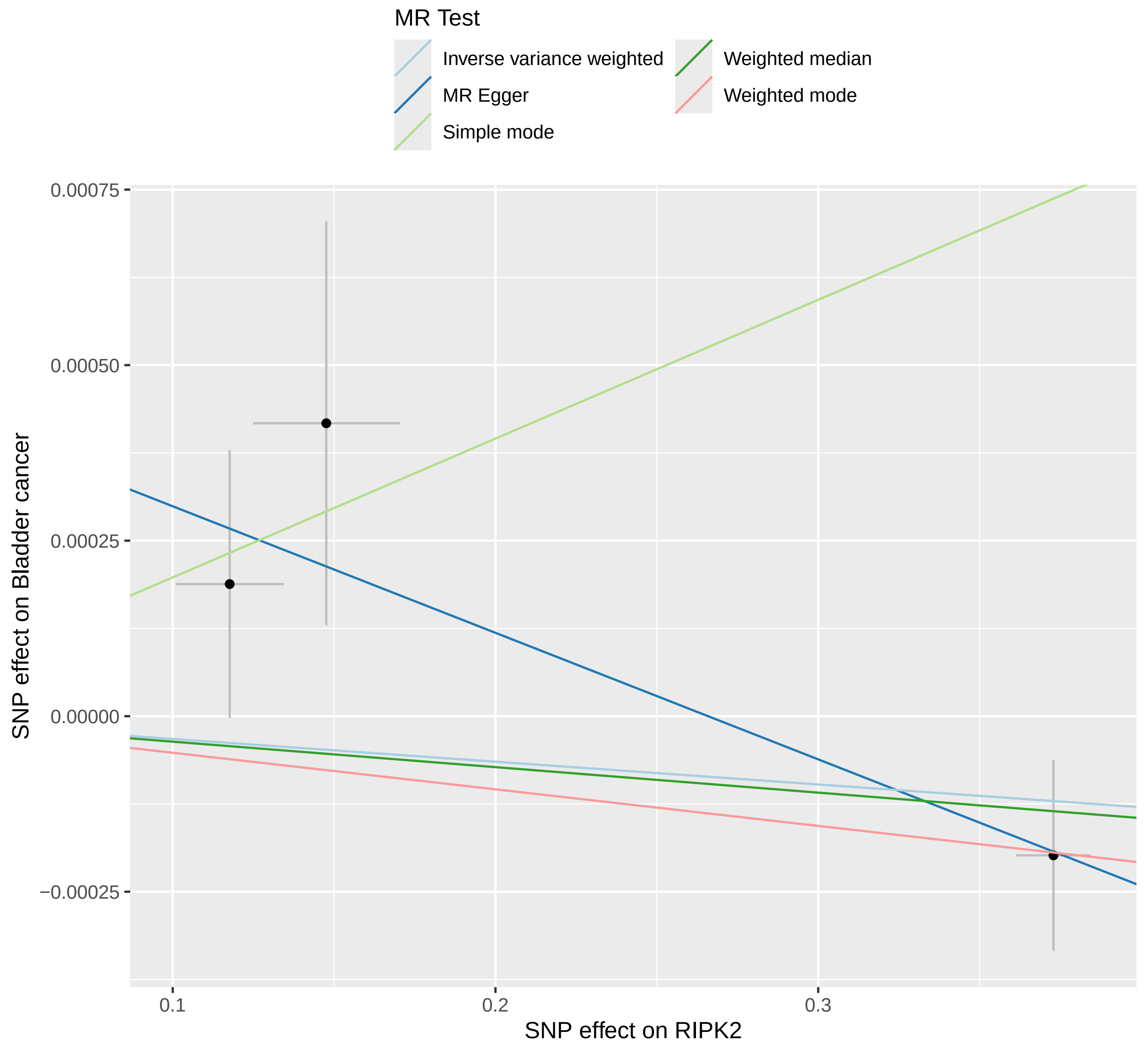
MR Test
Inverse variance weighted
MR Egger
Simple mode
Weighted median
Weighted mode
0.00075
0.00050
0.00025
0.00000
−0.00025
0.1
0.2
0.3
SNP effect on Bladder cancer
SNP effect on RIPK2

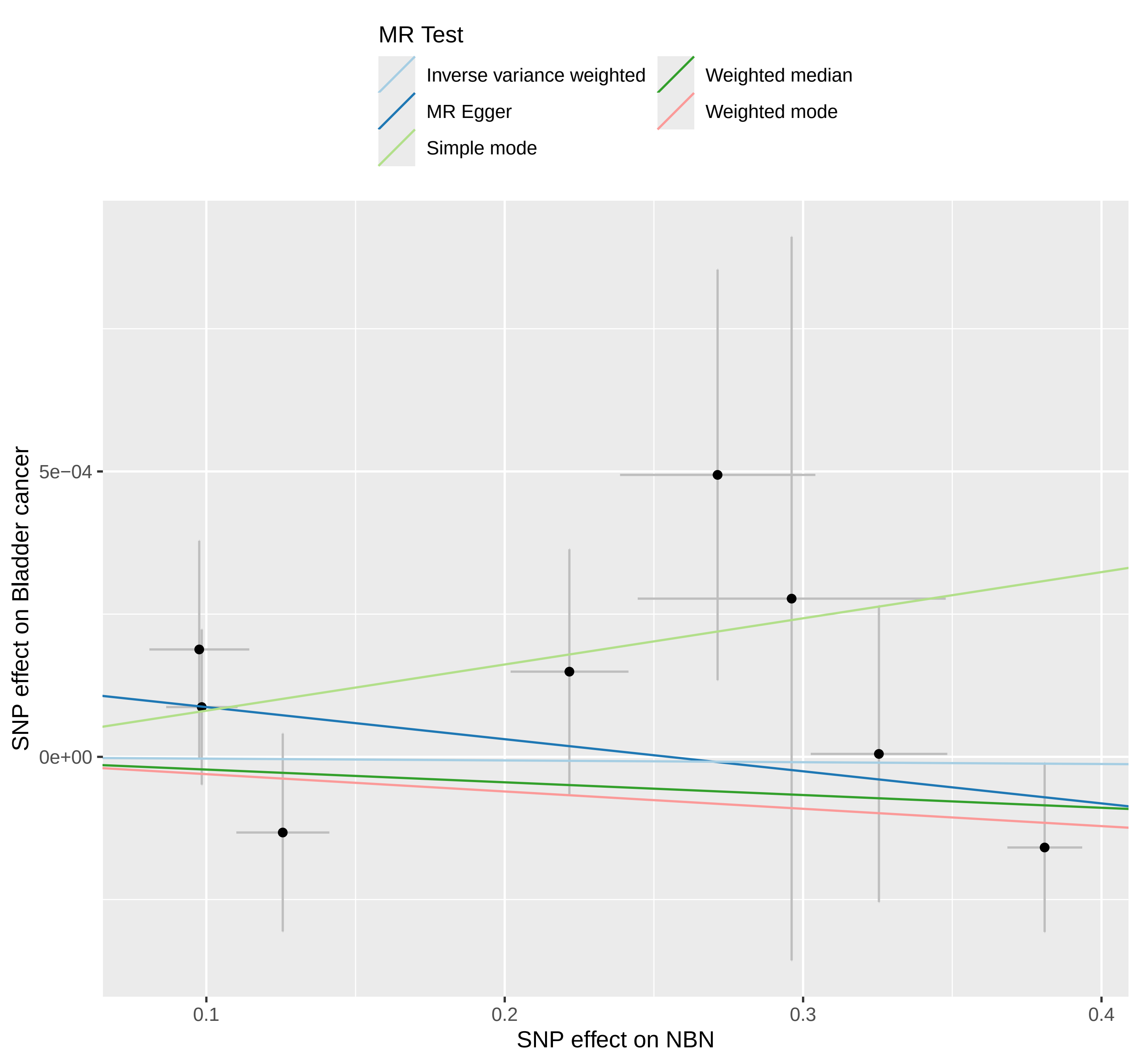
MR Test
Inverse variance weighted
MR Egger
Simple mode
Weighted median
Weighted mode
SNP effect on Bladder cancer
5e−04
0e+00
0.1
0.2
0.3
0.4
SNP effect on NBN

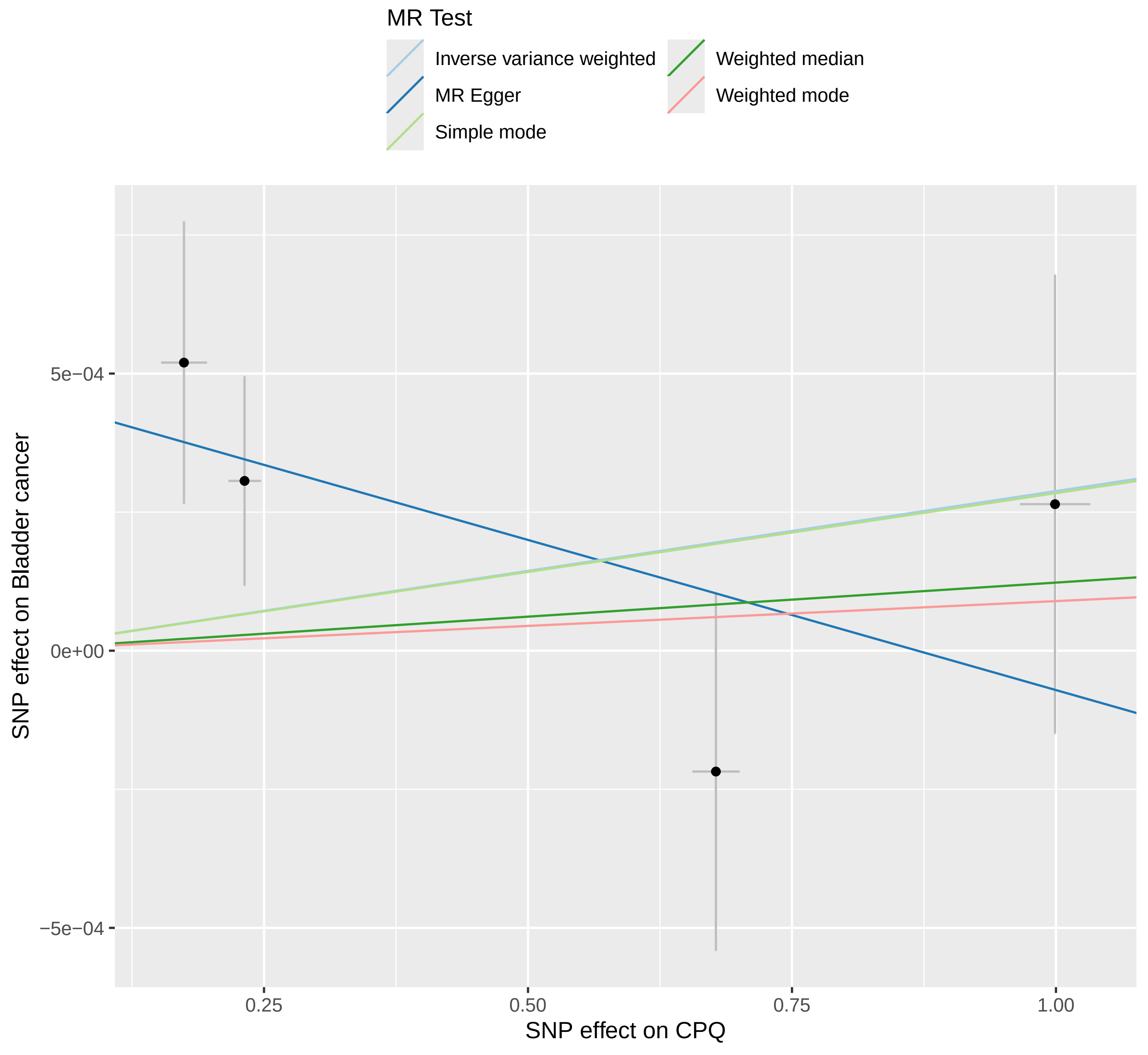

MR Test
Inverse variance weighted
MR Egger
Simple mode
Weighted median
Weighted mode
5e−04
0e+00
−5e−04
0.25
0.50
0.75
1.00
SNP effect on CPQ
SNP effect on Bladder cancer

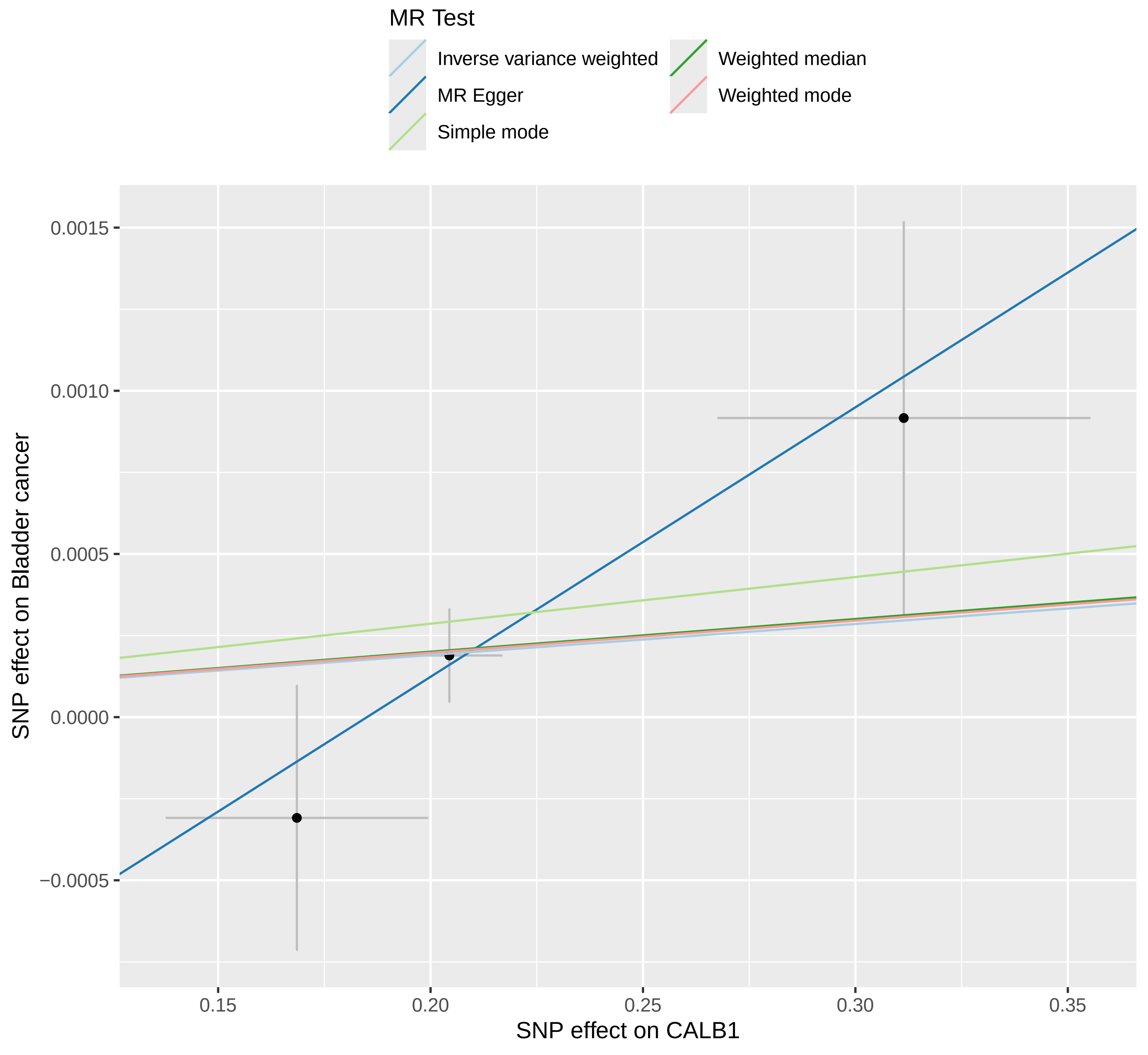
MR Test
Inverse variance weighted
MR Egger
Simple mode
Weighted median
Weighted mode
0.0015
0.0010
0.0005
0.0000
−0.0005
SNP effect on Bladder cancer
0.15
0.20
0.25
0.30
0.35
SNP effect on CALB1

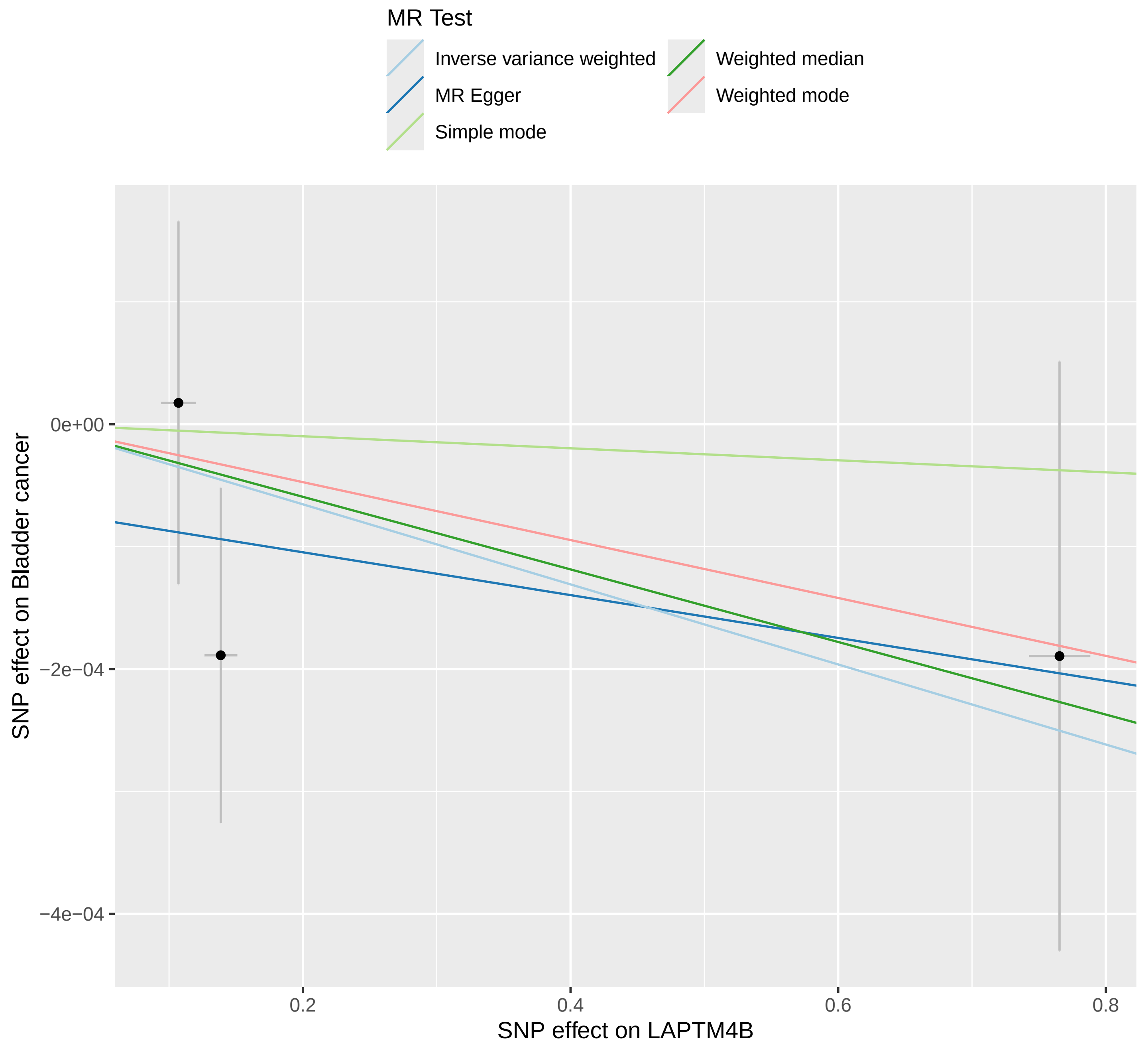
MR Test
Inverse variance weighted
MR Egger
Simple mode
Weighted median
Weighted mode
0e+00
−2e−04
−4e−04
SNP effect on Bladder cancer
0.2
0.4
0.6
0.8
SNP effect on LAPTM4B

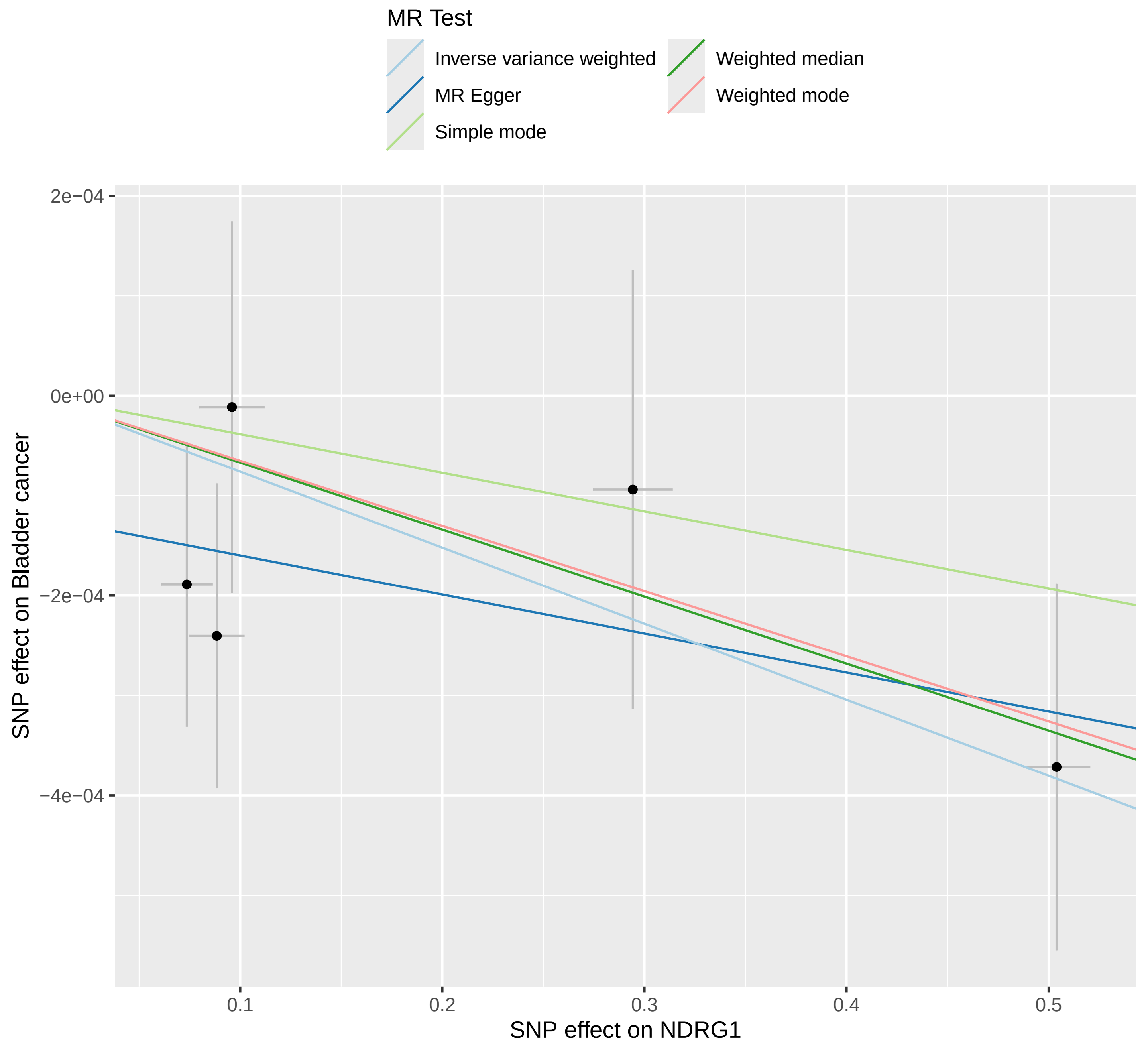

MR Test
Inverse variance weighted
MR Egger
Simple mode
Weighted median
Weighted mode
2e−04
0e+00
−2e−04
−4e−04
SNP effect on Bladder cancer
0.1
0.2
0.3
0.4
0.5
SNP effect on NDRG1

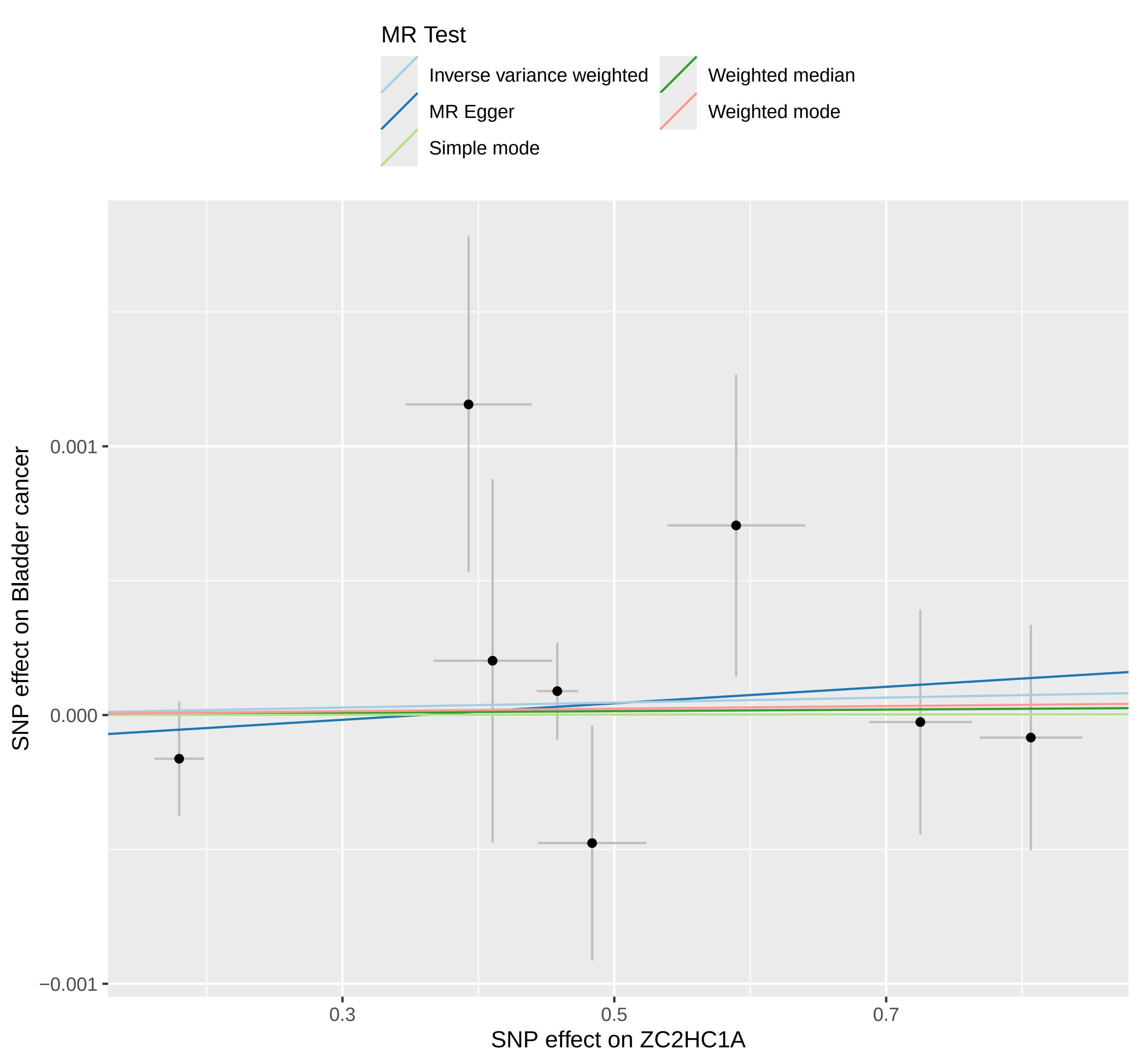
MR Test
Inverse variance weighted
MR Egger
Simple mode
Weighted median
Weighted mode
SNP effect on Bladder cancer
0.001
0.000
−0.001
0.3
0.5
0.7
SNP effect on ZC2HC1A

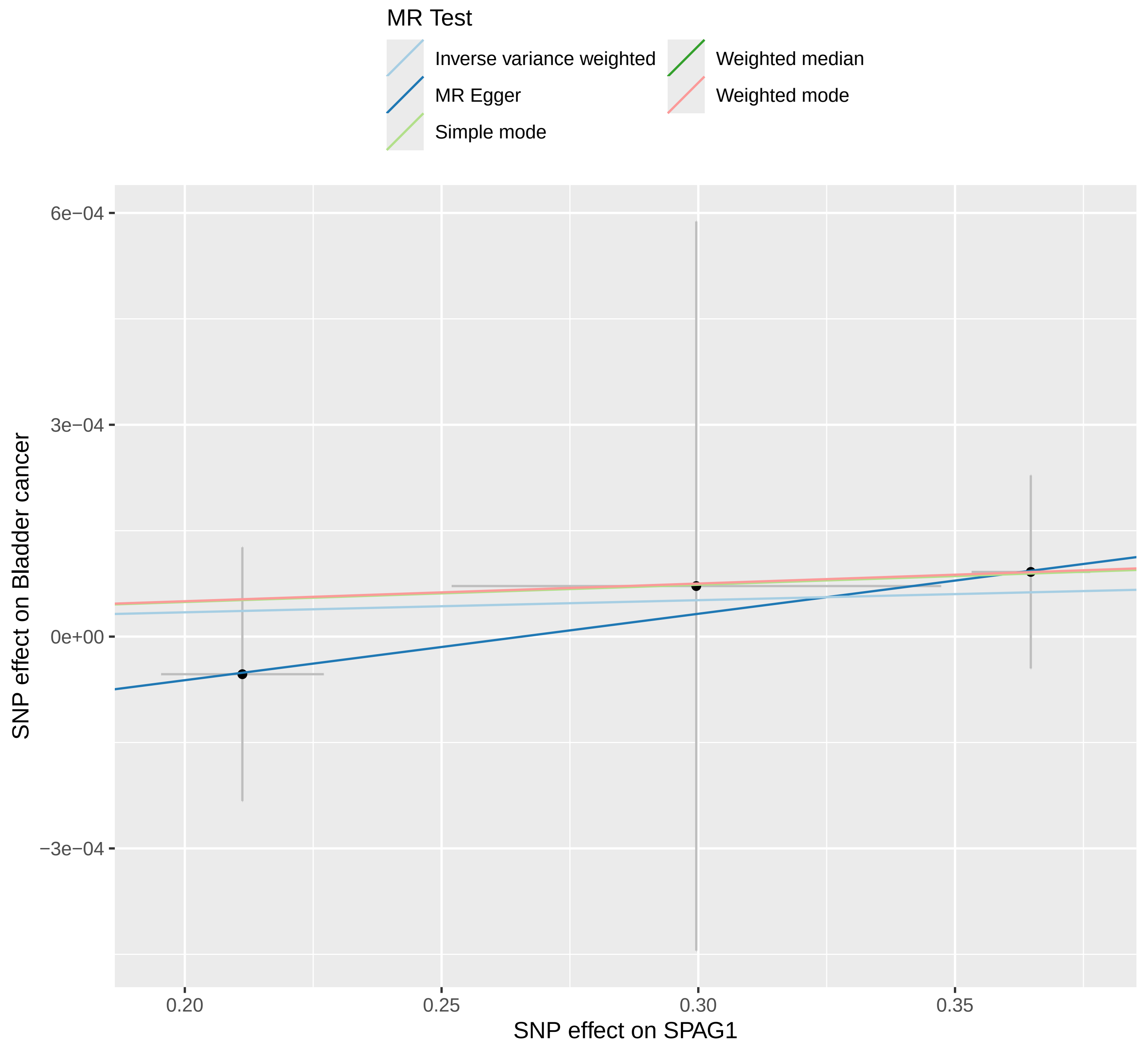

MR Test
Inverse variance weighted
MR Egger
Simple mode
Weighted median
Weighted mode
6e−04
3e−04
0e+00
−3e−04
0.20
0.25
0.30
0.35
SNP effect on SPAG1
SNP effect on Bladder cancer

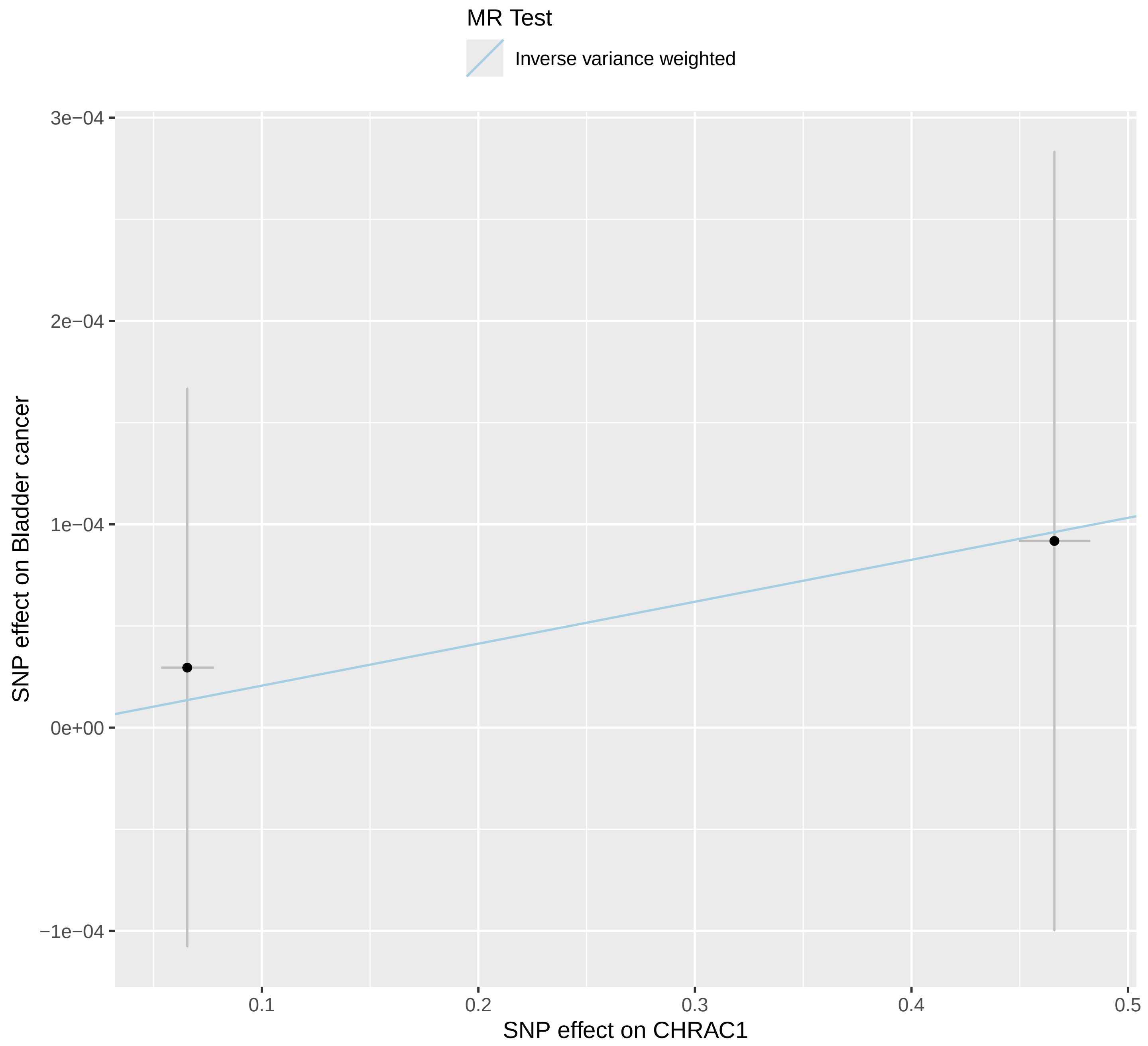

MR Test
Inverse variance weighted
3e−04
2e−04
1e−04
0e+00
−1e−04
0.1
0.2
0.3
0.4
0.5
SNP effect on CHRAC1
SNP effect on Bladder cancer

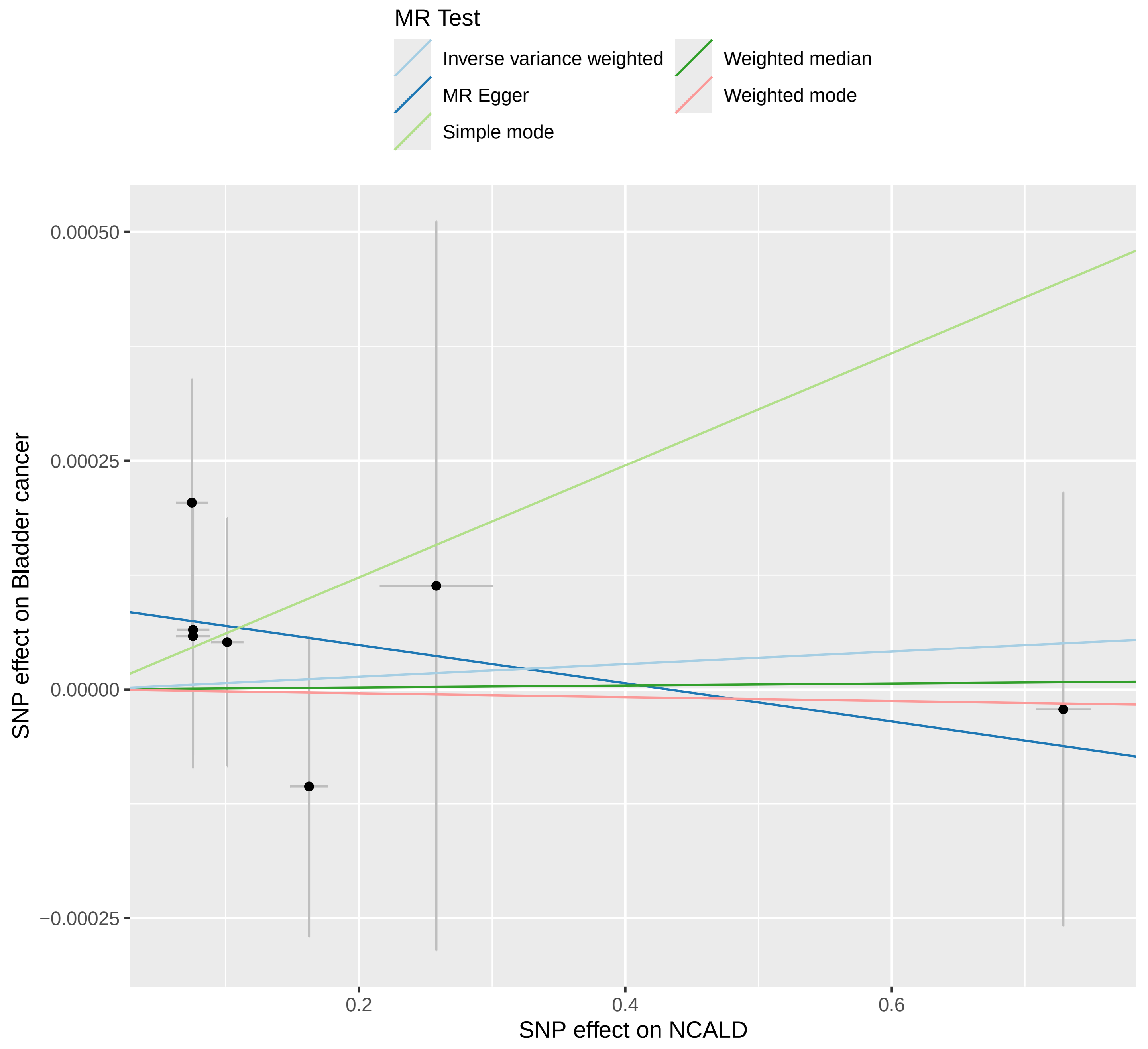

MR Test
Inverse variance weighted
MR Egger
Simple mode
Weighted median
Weighted mode
0.00050
0.00025
0.00000
−0.00025
SNP effect on Bladder cancer
0.2
0.4
0.6
SNP effect on NCALD

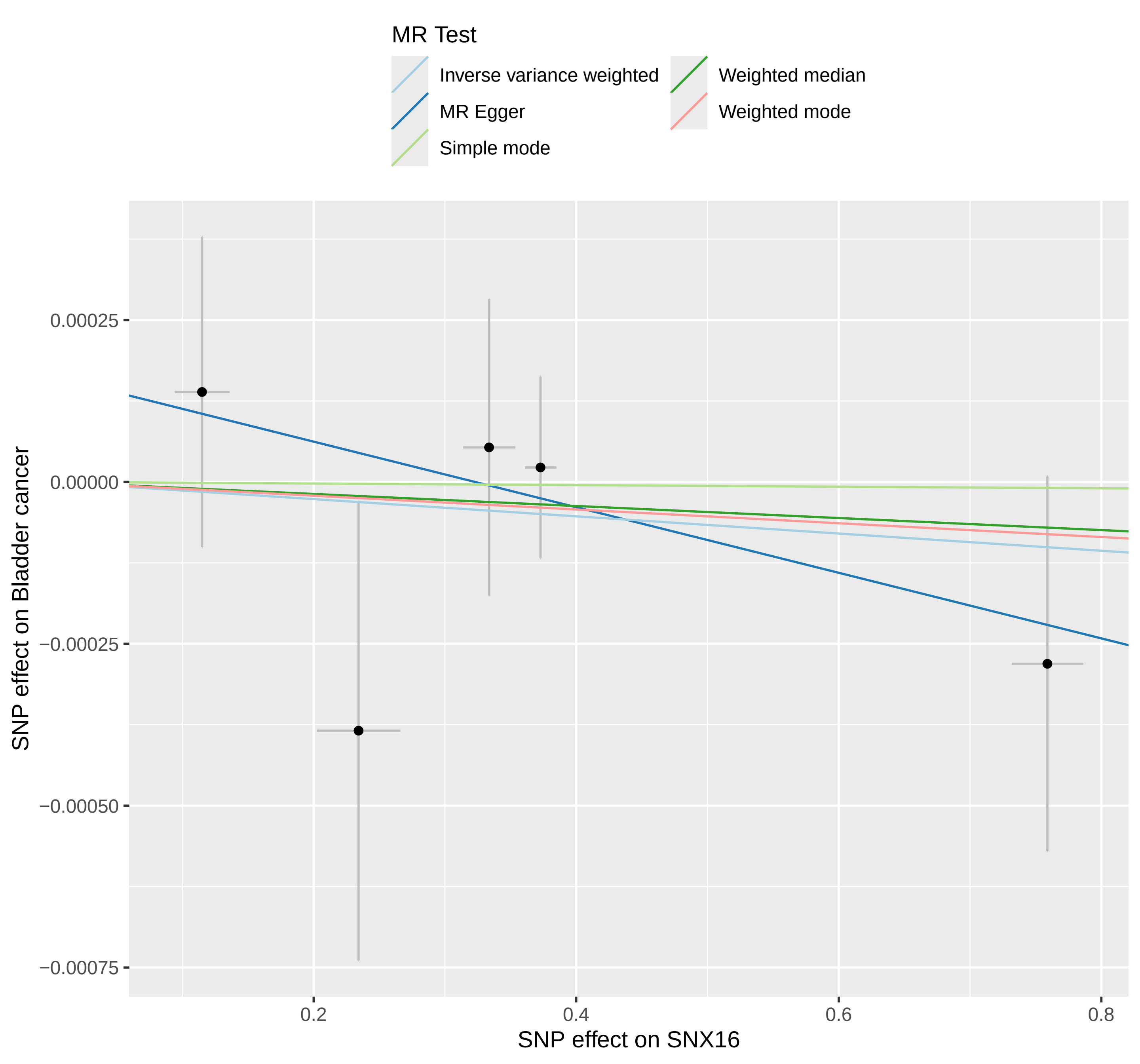
MR Test
Inverse variance weighted
MR Egger
Simple mode
Weighted median
Weighted mode
0.00025
0.00000
−0.00025
−0.00050
−0.00075
SNP effect on Bladder cancer
0.2
0.4
0.6
0.8
SNP effect on SNX16

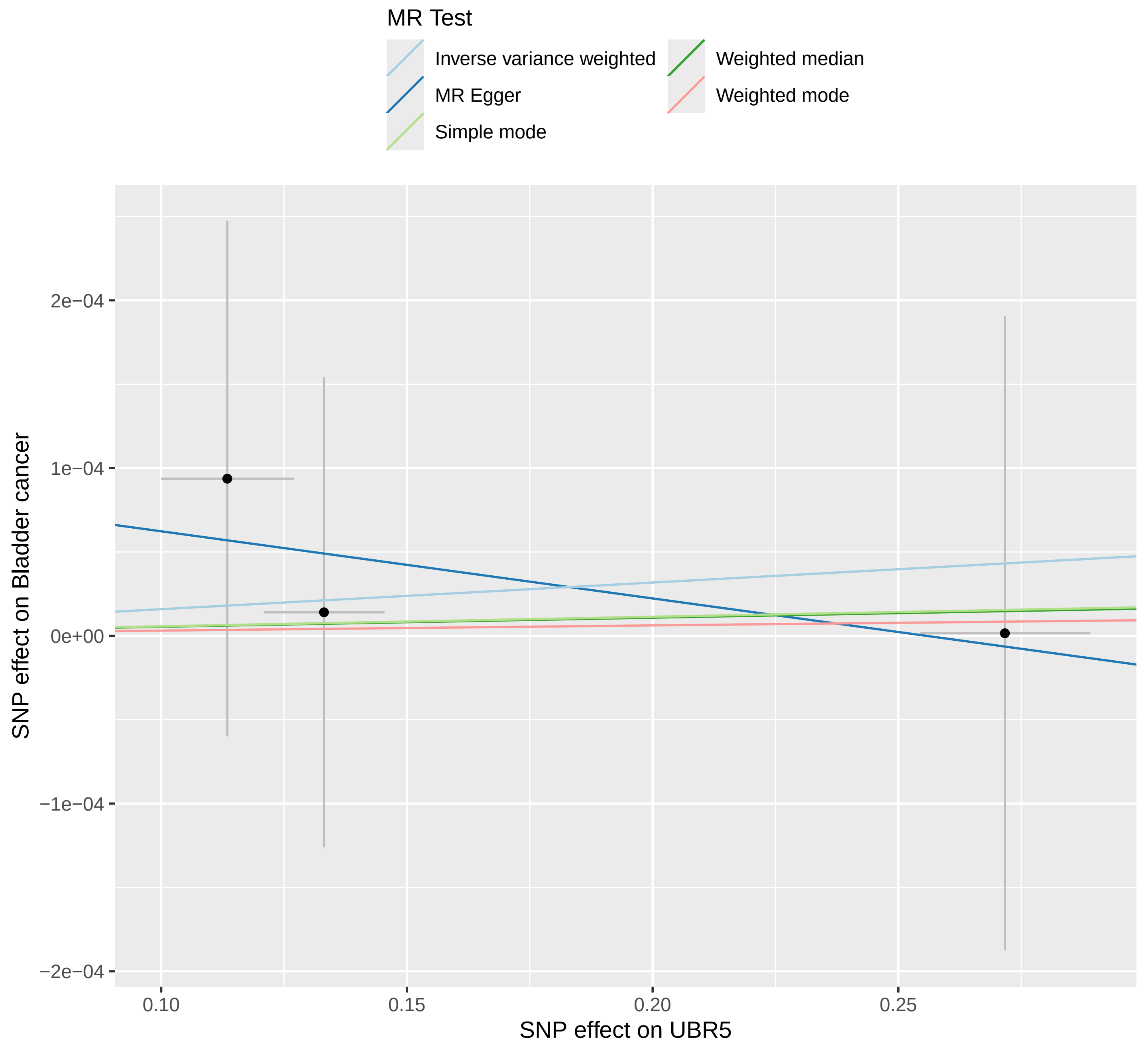
MR Test
Inverse variance weighted
MR Egger
Simple mode
Weighted median
Weighted mode
2e−04
1e−04
0e+00
−1e−04
−2e−04
SNP effect on Bladder cancer
0.10
0.15
0.20
0.25
SNP effect on UBR5

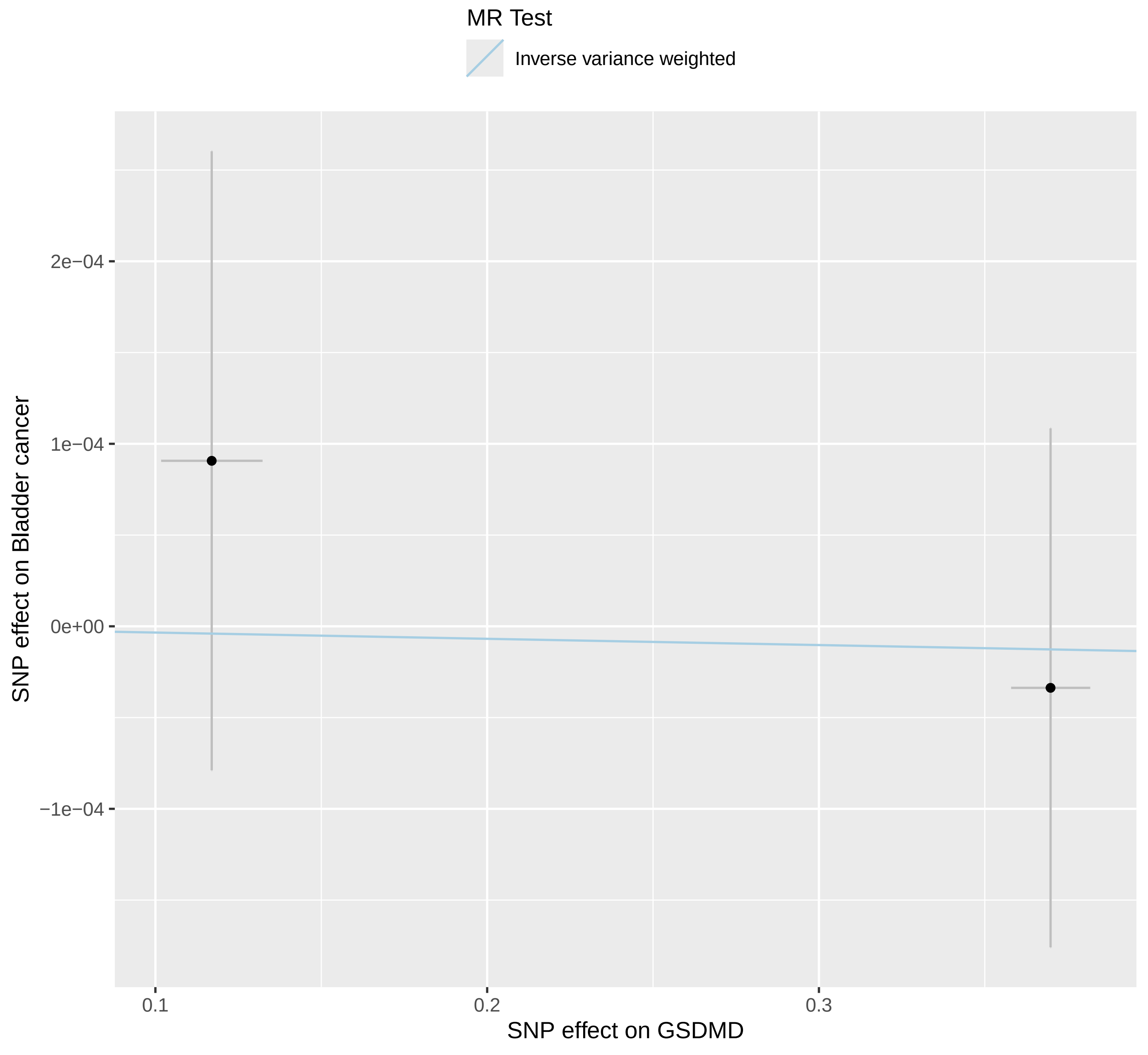
MR Test
Inverse variance weighted
2e−04
1e−04
0e+00
−1e−04
0.1
0.2
0.3
SNP effect on GSDMD
SNP effect on Bladder cancer

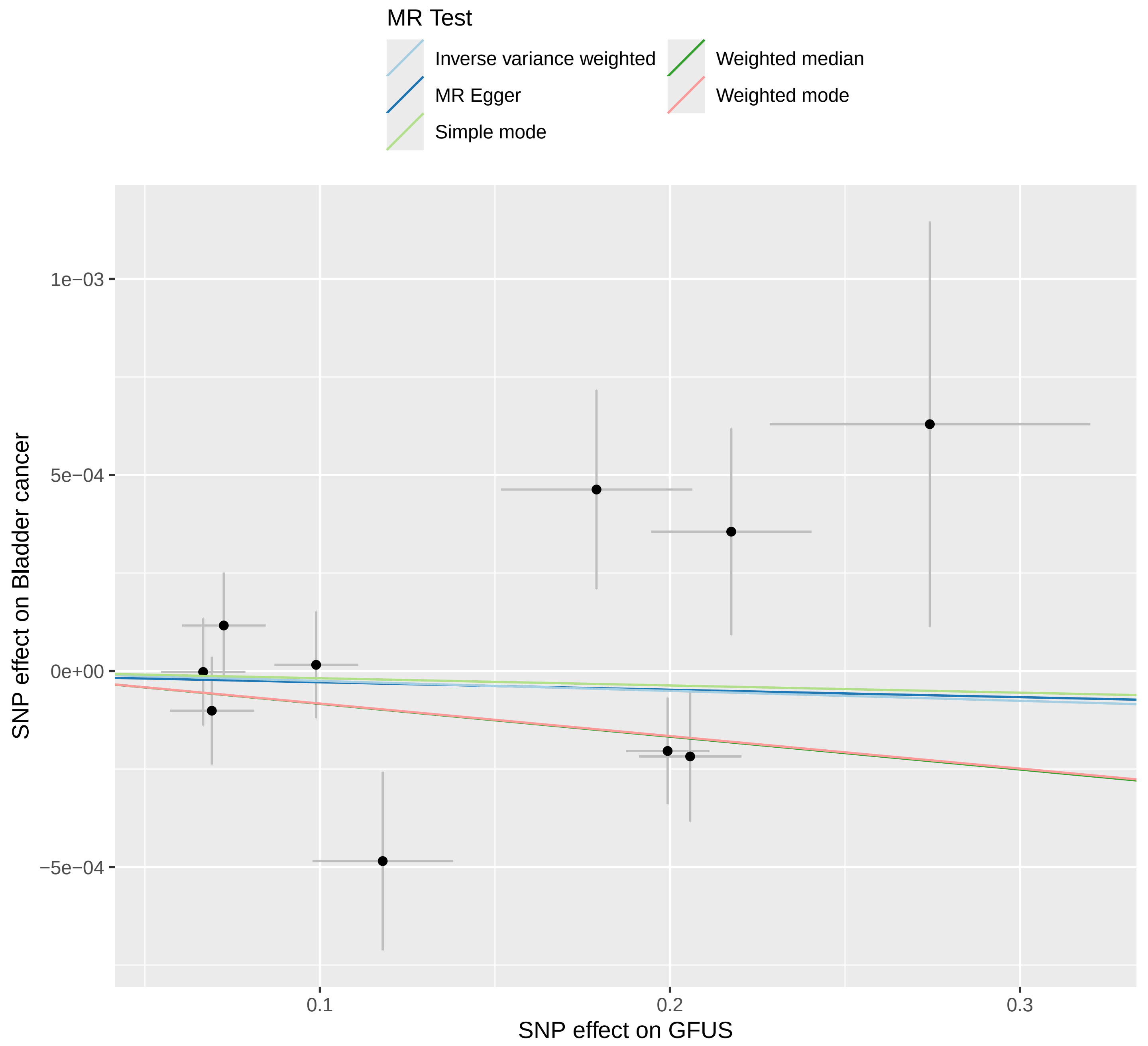
MR Test
Inverse variance weighted
MR Egger
Simple mode
Weighted median
Weighted mode
1e−03
5e−04
0e+00
−5e−04
0.1
0.2
0.3
SNP effect on Bladder cancer
SNP effect on GFUS

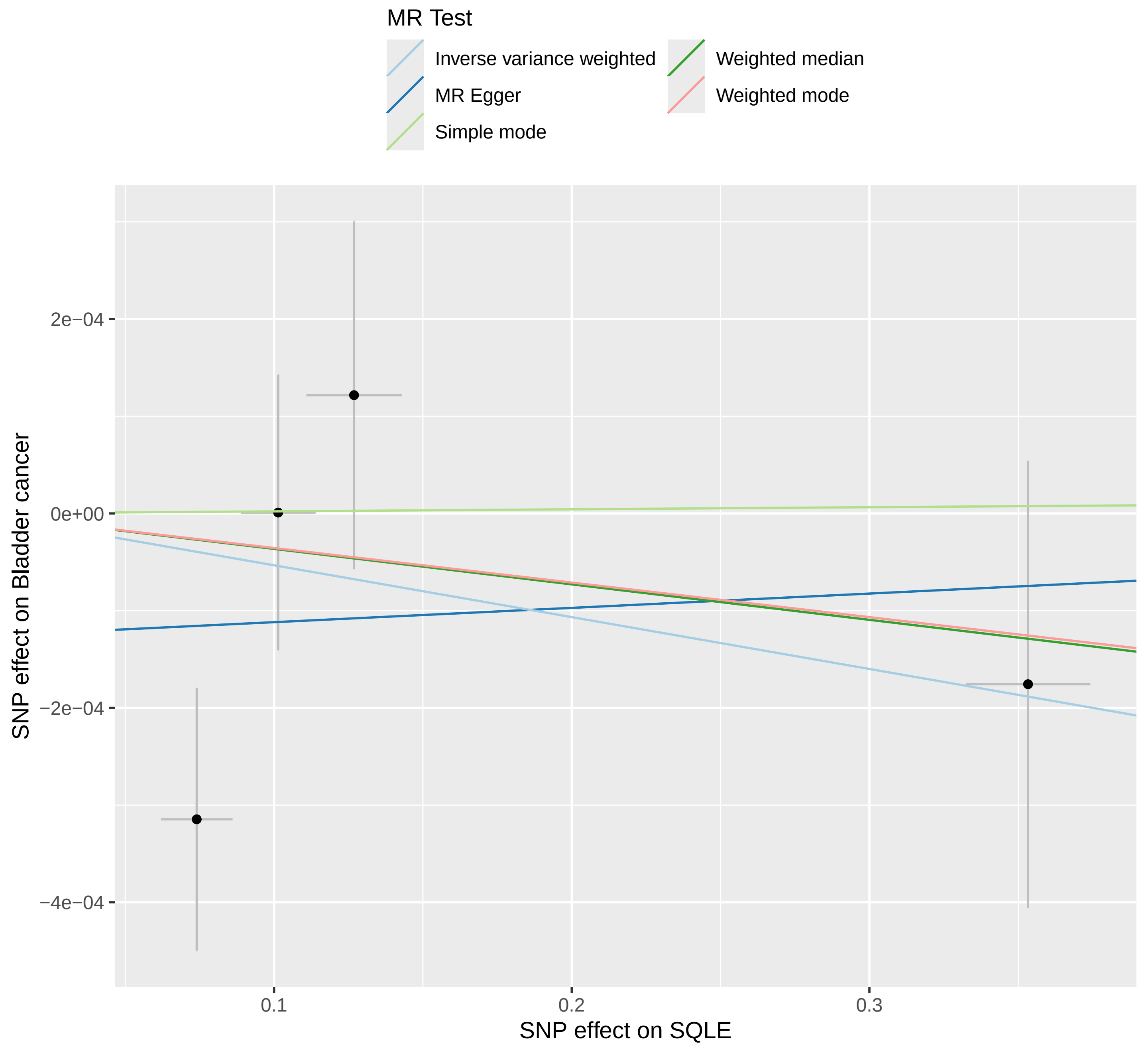
MR Test
Inverse variance weighted
MR Egger
Simple mode
Weighted median
Weighted mode
2e−04
0e+00
−2e−04
−4e−04
SNP effect on Bladder cancer
0.1
0.2
0.3
SNP effect on SQLE

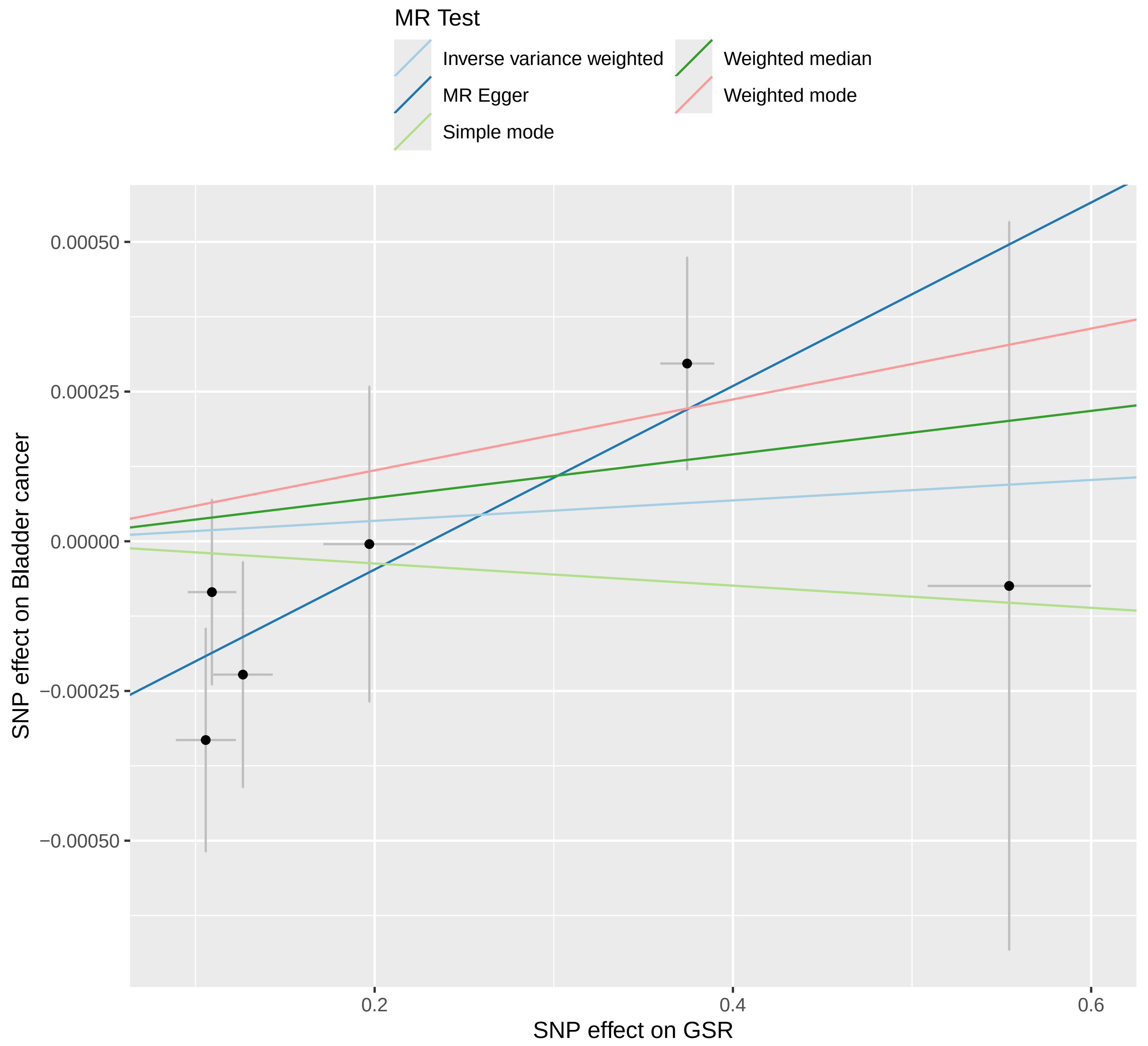
MR Test
Inverse variance weighted
MR Egger
Simple mode
Weighted median
Weighted mode
0.00050
0.00025
0.00000
−0.00025
−0.00050
SNP effect on Bladder cancer
0.2
0.4
0.6
SNP effect on GSR

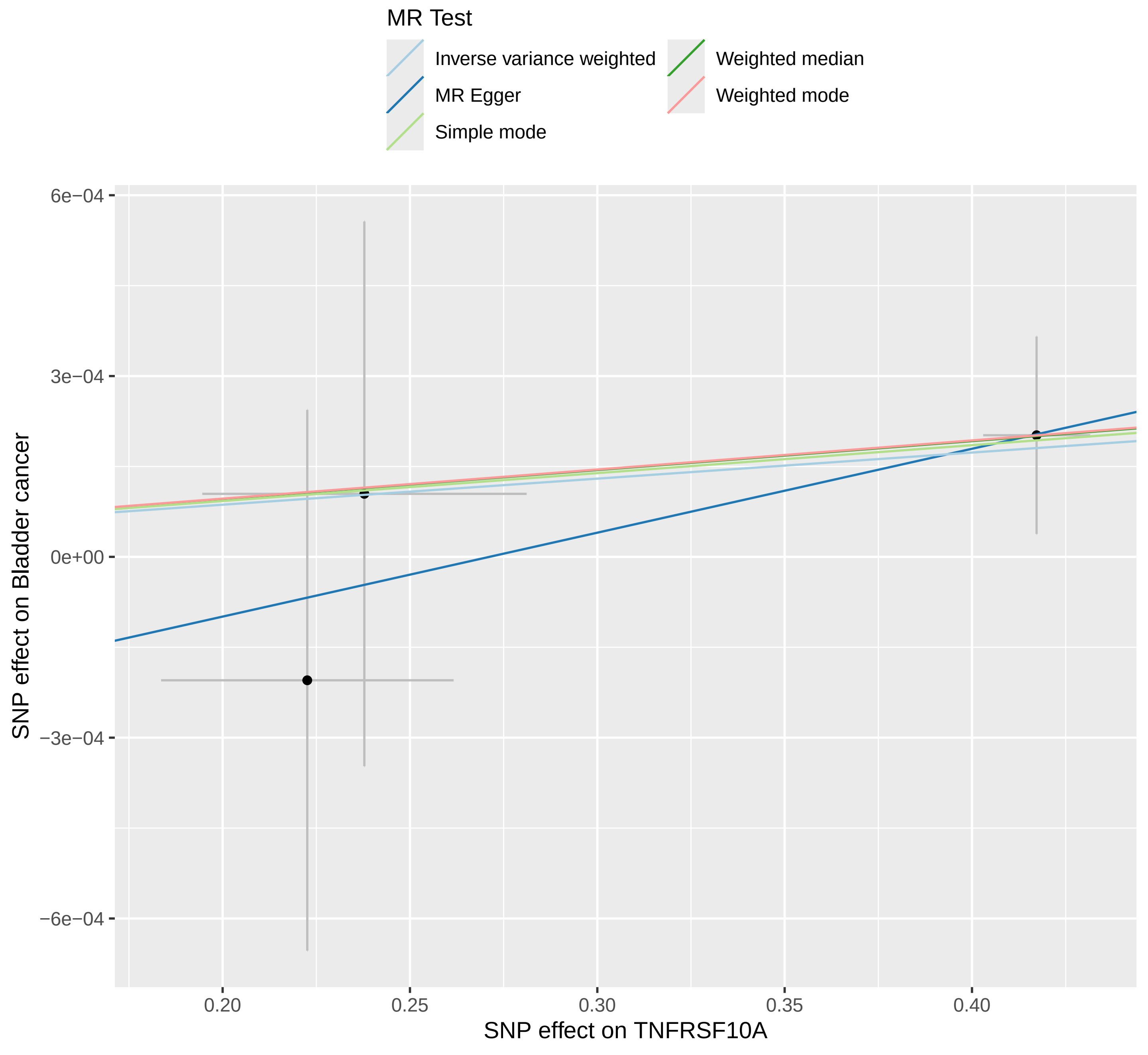

MR Test
Inverse variance weighted
MR Egger
Simple mode
Weighted median
Weighted mode
6e−04
3e−04
0e+00
−3e−04
−6e−04
0.20
0.25
0.30
0.35
0.40
SNP effect on TNFRSF10A
SNP effect on Bladder cancer

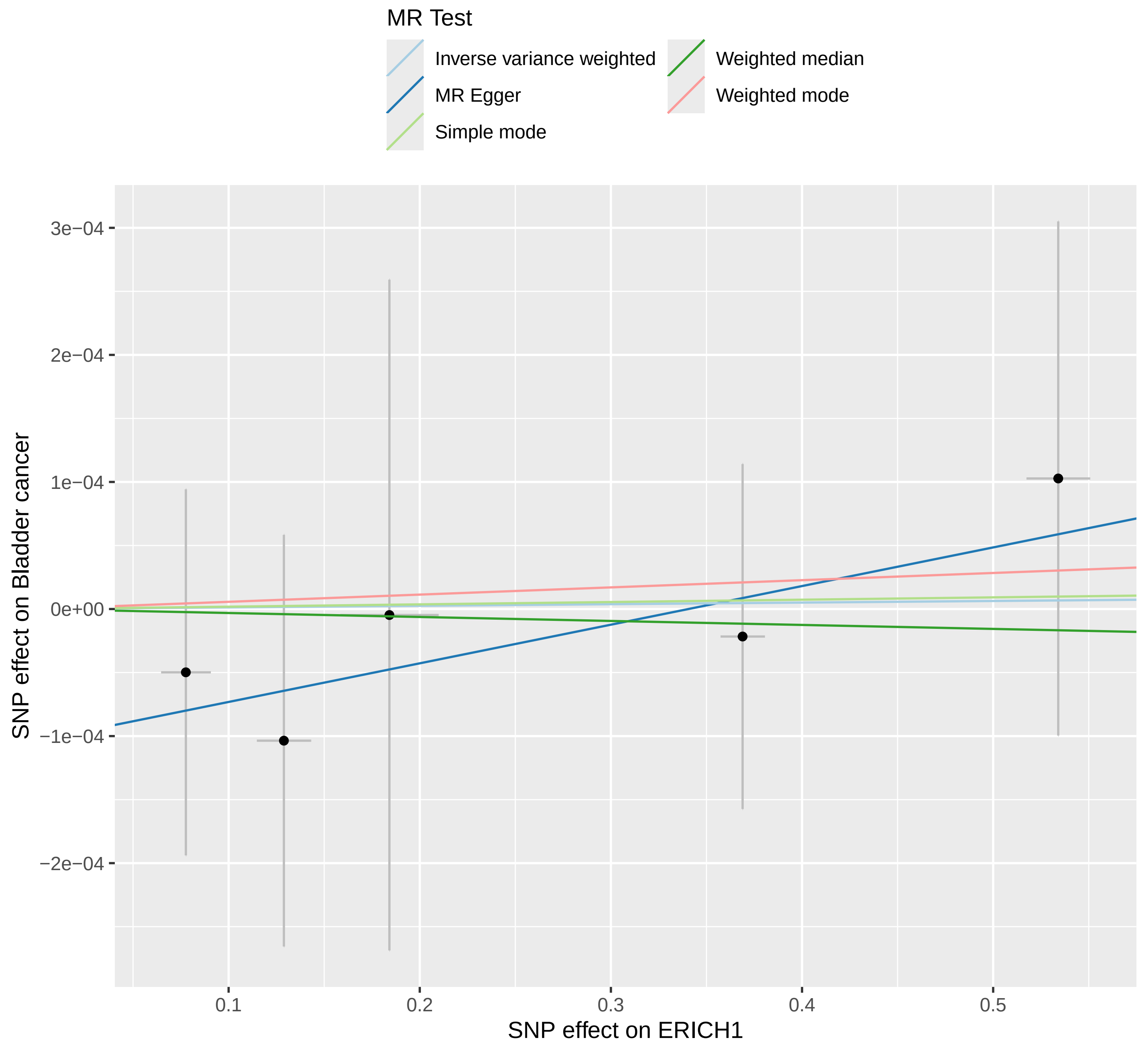
MR Test
Inverse variance weighted
MR Egger
Simple mode
Weighted median
Weighted mode
3e−04
2e−04
1e−04
0e+00
−1e−04
−2e−04
SNP effect on Bladder cancer
0.1
0.2
0.3
0.4
0.5
SNP effect on ERICH1

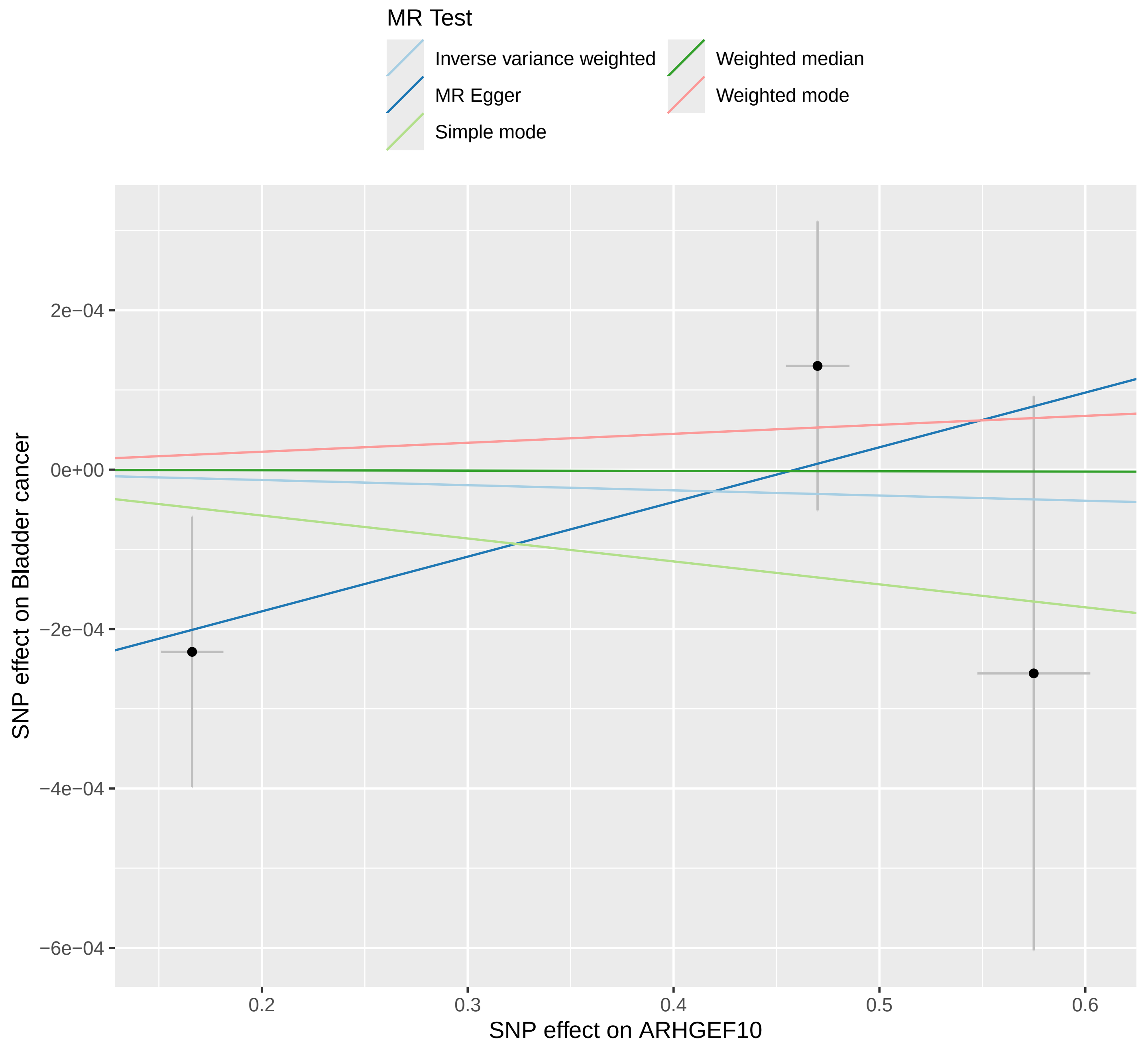

MR Test
Inverse variance weighted
MR Egger
Simple mode
Weighted median
Weighted mode
2e−04
0e+00
−2e−04
−4e−04
−6e−04
SNP effect on Bladder cancer
0.2
0.3
0.4
0.5
0.6
SNP effect on ARHGEF10

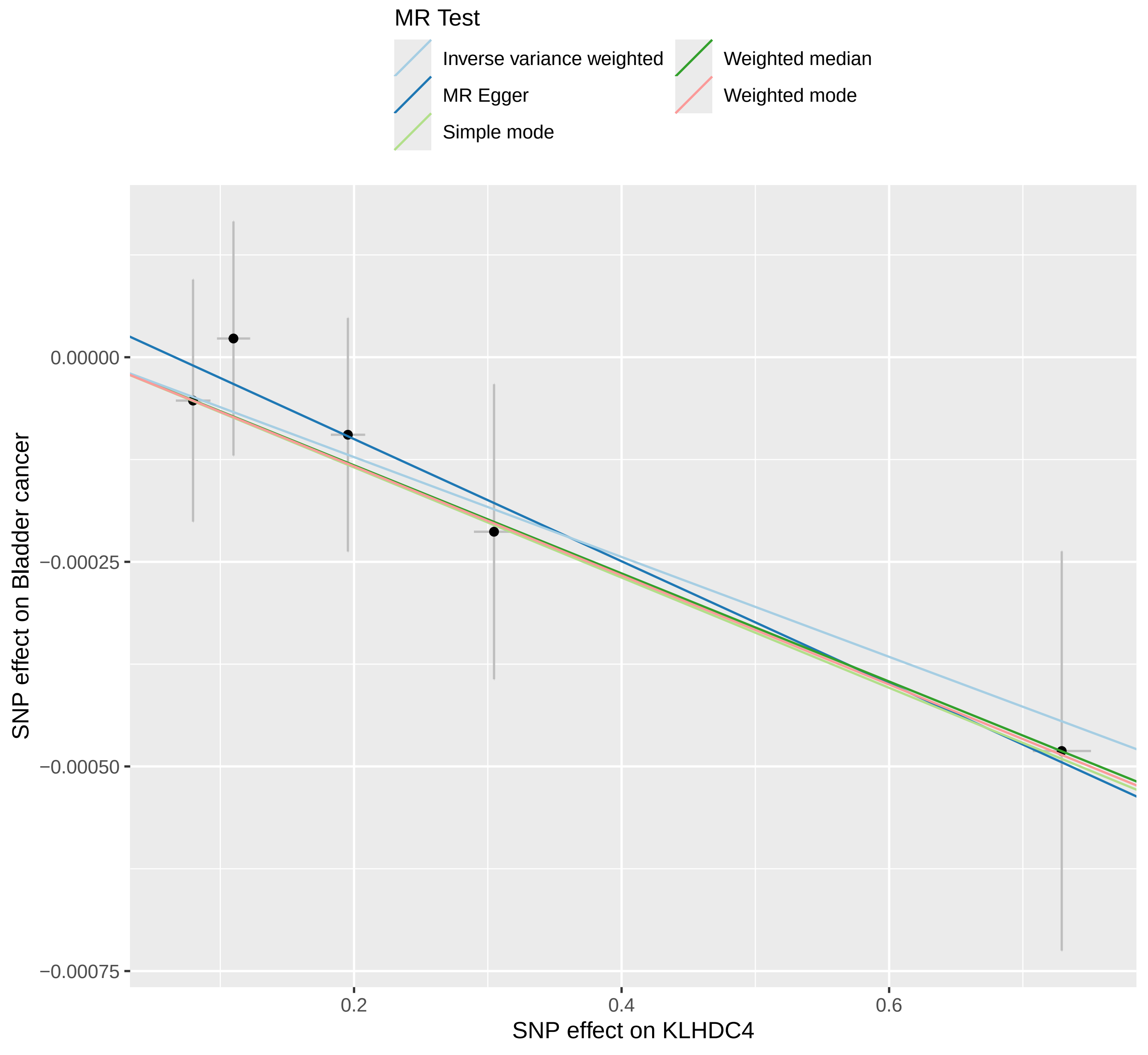
MR Test
Inverse variance weighted
MR Egger
Simple mode
Weighted median
Weighted mode
0.00000
−0.00025
−0.00050
−0.00075
SNP effect on Bladder cancer
0.2
0.4
0.6
SNP effect on KLHDC4

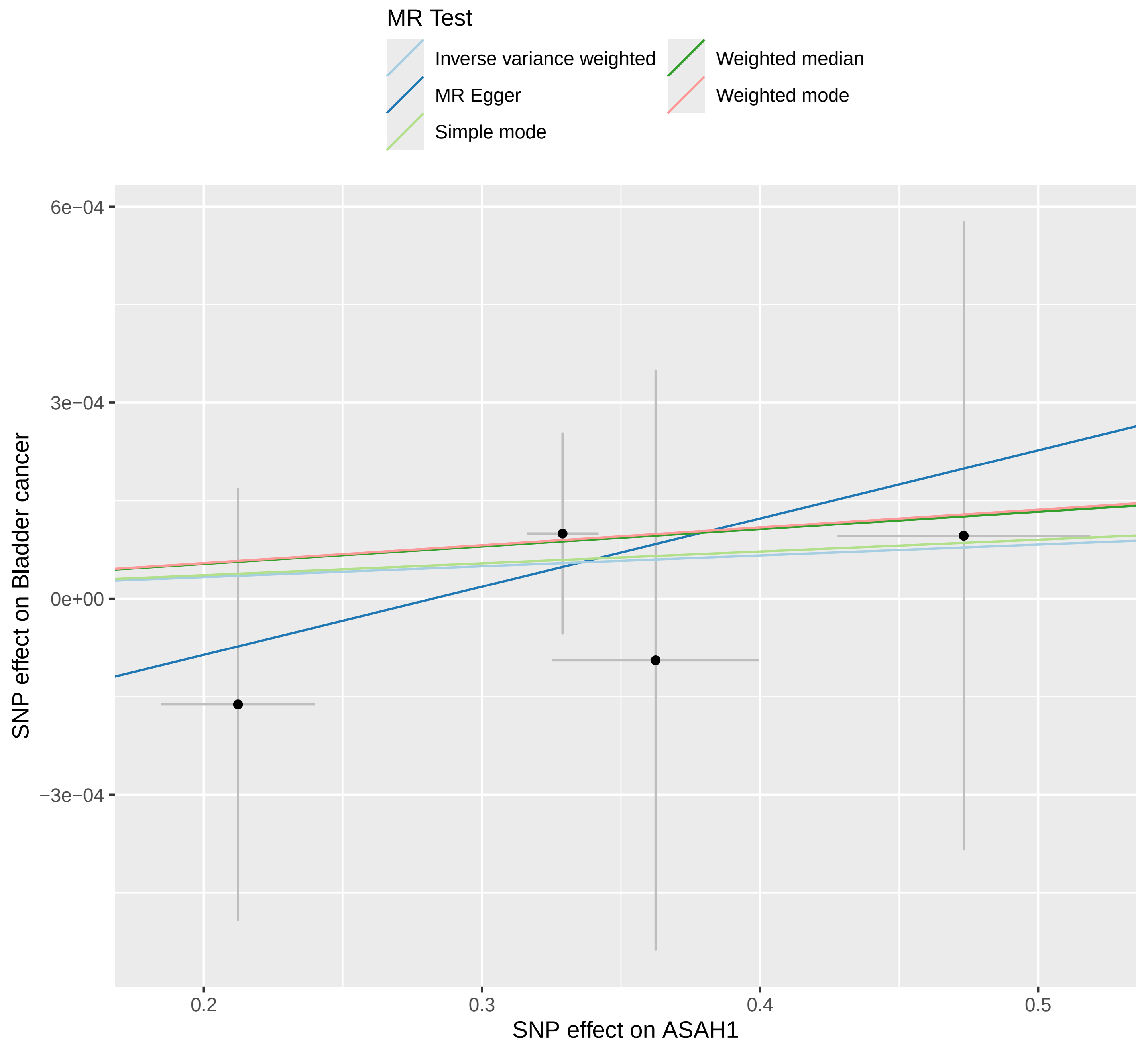

MR Test
Inverse variance weighted
MR Egger
Simple mode
Weighted median
Weighted mode
6e−04
3e−04
0e+00
−3e−04
0.2
0.3
0.4
0.5
SNP effect on ASAH1
SNP effect on Bladder cancer

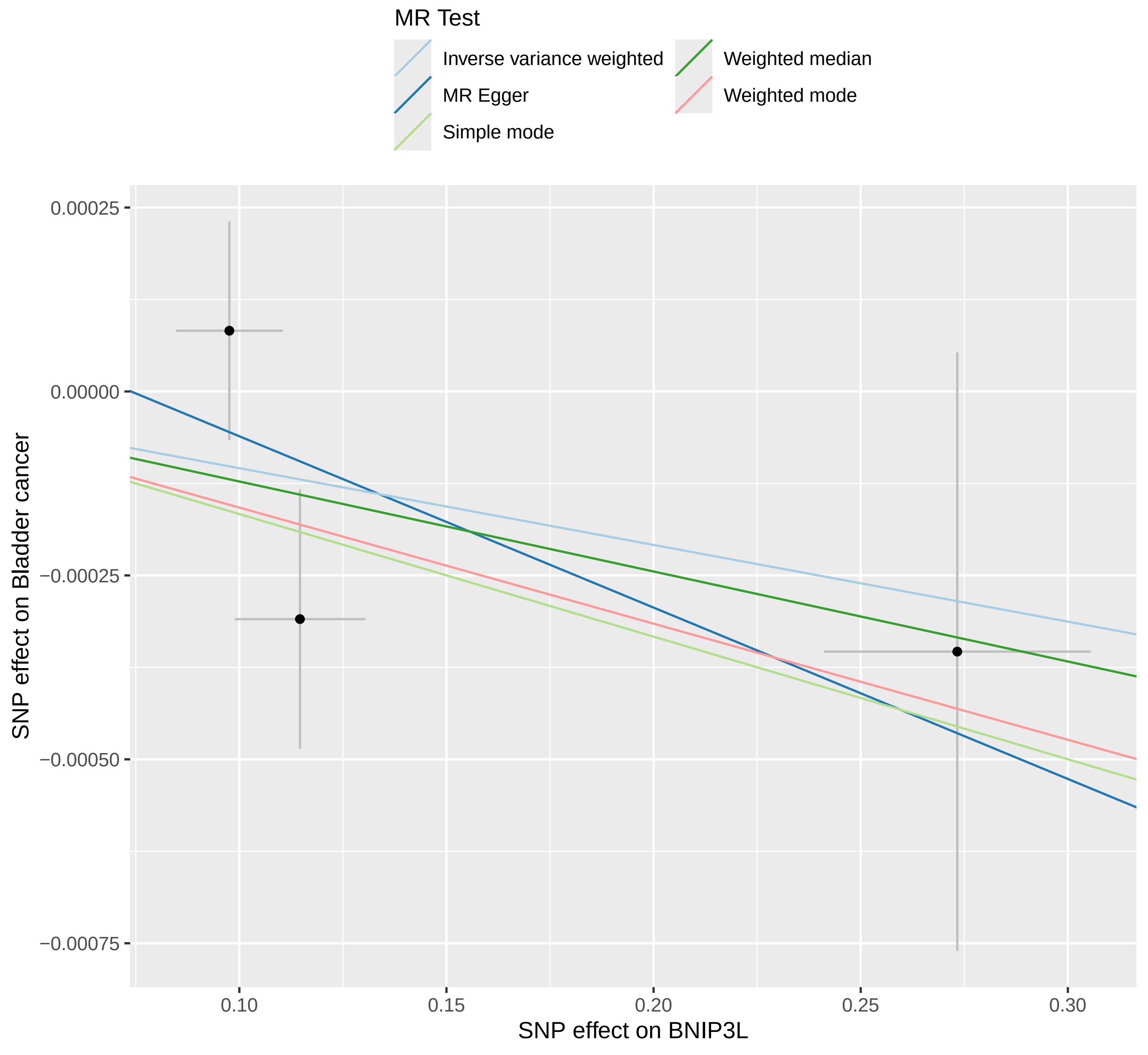
MR Test
Inverse variance weighted
MR Egger
Simple mode
Weighted median
Weighted mode
0.00025
0.00000
−0.00025
−0.00050
−0.00075
SNP effect on Bladder cancer
0.10
0.15
0.20
0.25
0.30
SNP effect on BNIP3L

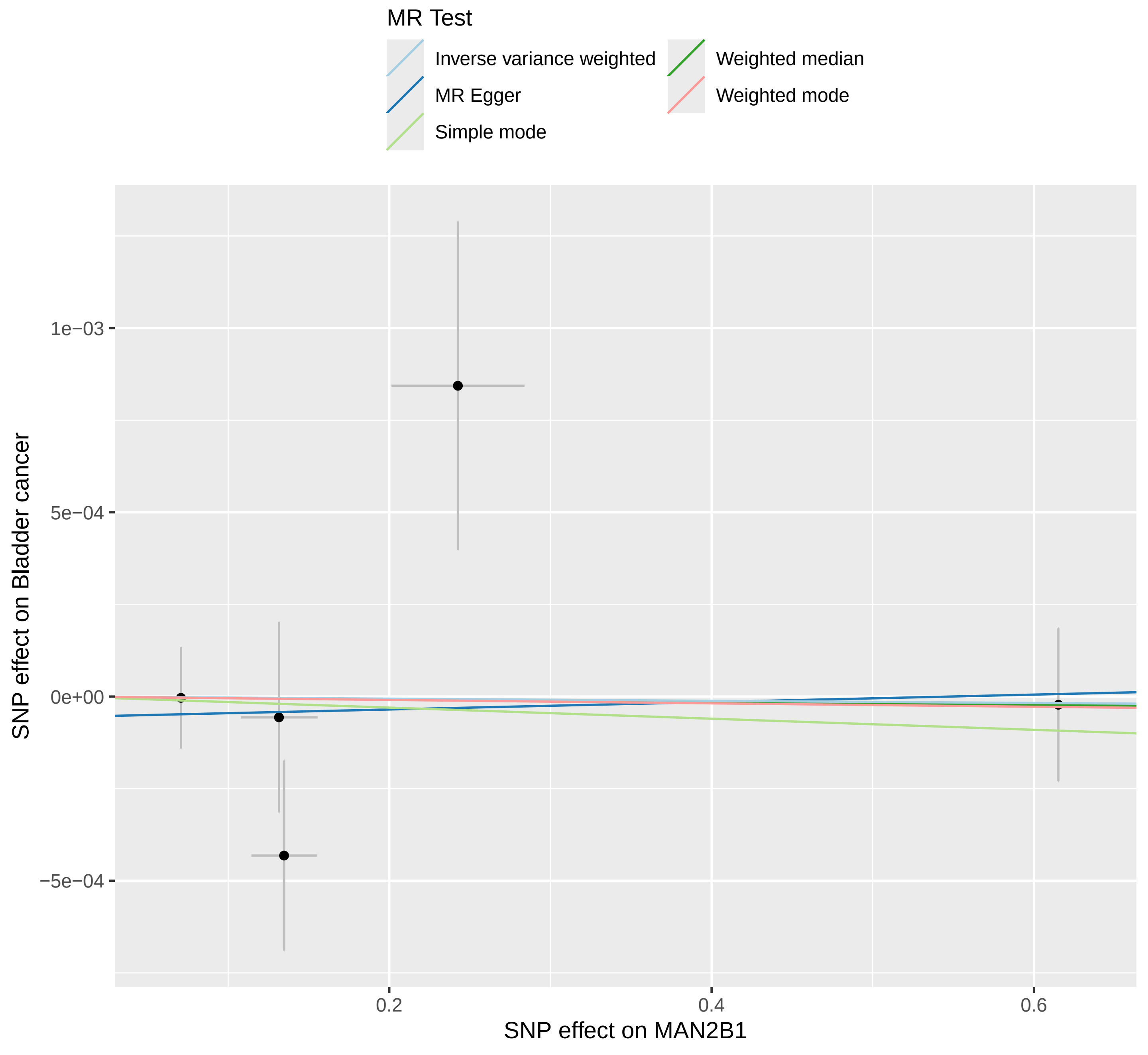

MR Test
Inverse variance weighted
MR Egger
Simple mode
Weighted median
Weighted mode
1e−03
5e−04
0e+00
−5e−04
0.2
0.4
0.6
SNP effect on MAN2B1
SNP effect on Bladder cancer

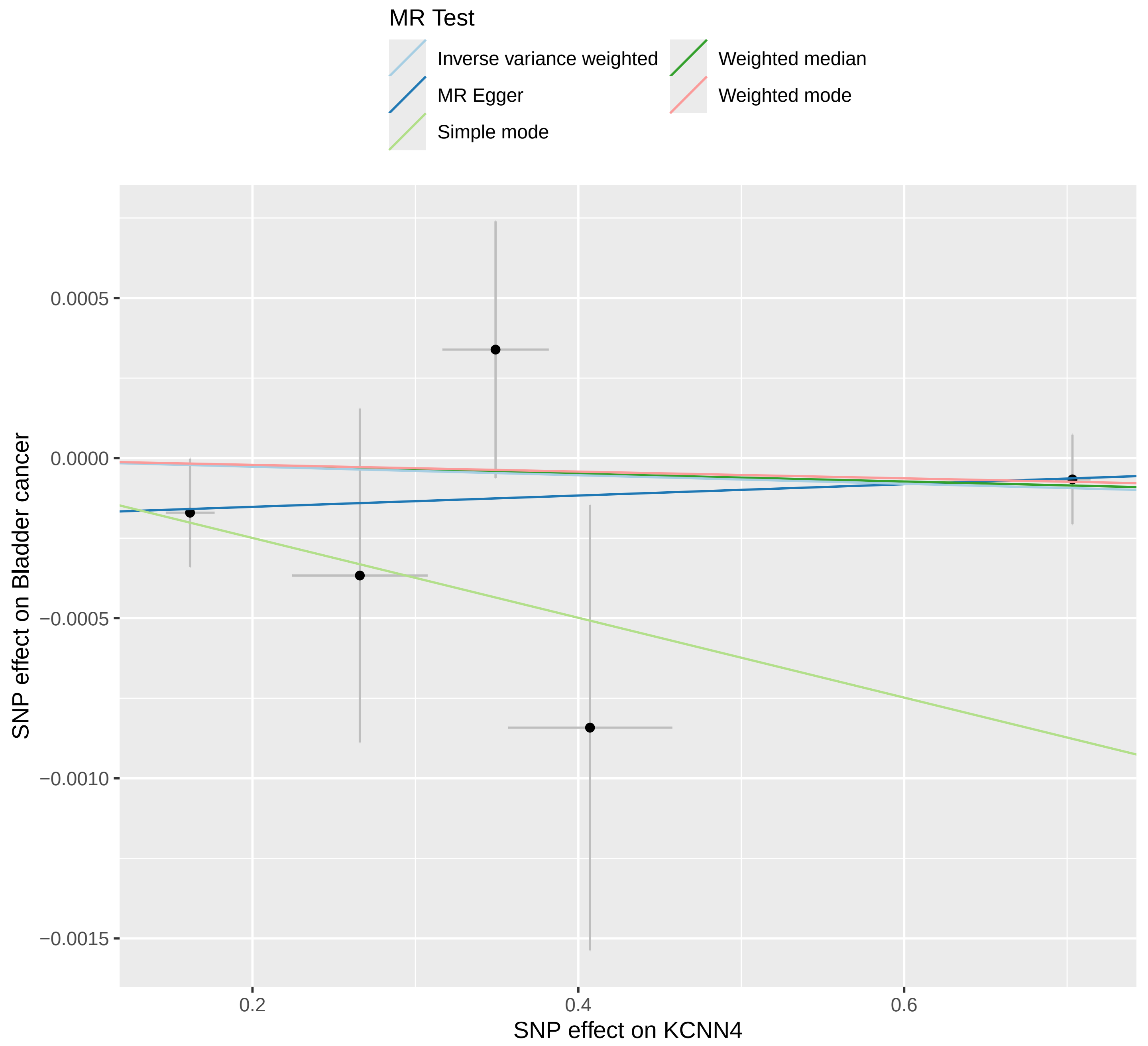

MR Test
Inverse variance weighted
MR Egger
Simple mode
Weighted median
Weighted mode
0.0005
0.0000
−0.0005
−0.0010
−0.0015
SNP effect on Bladder cancer
0.2
0.4
0.6
SNP effect on KCNN4

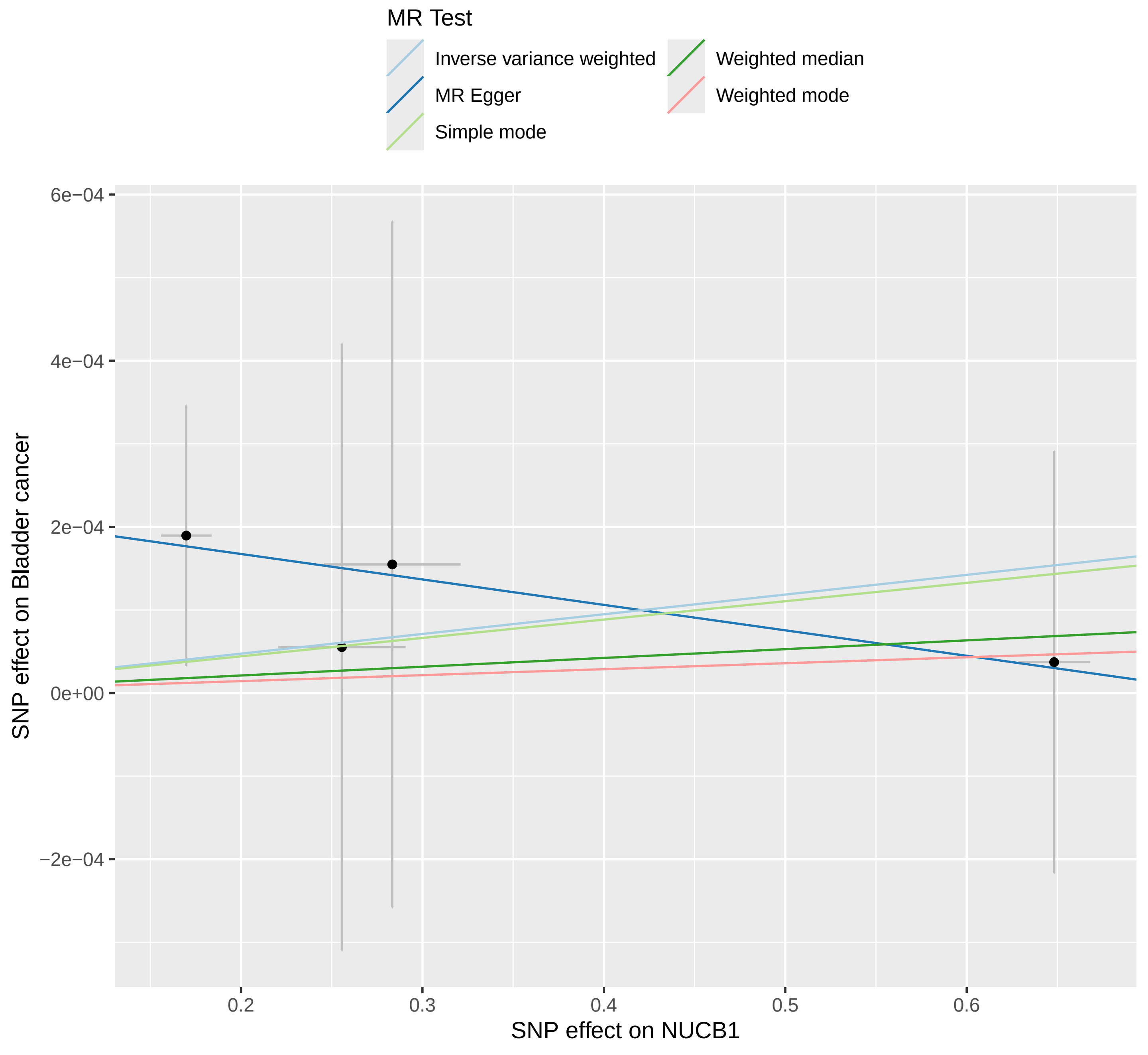

MR Test
Inverse variance weighted
MR Egger
Simple mode
Weighted median
Weighted mode
6e−04
4e−04
2e−04
0e+00
−2e−04
0.2
0.3
0.4
0.5
0.6
SNP effect on Bladder cancer
SNP effect on NUCB1

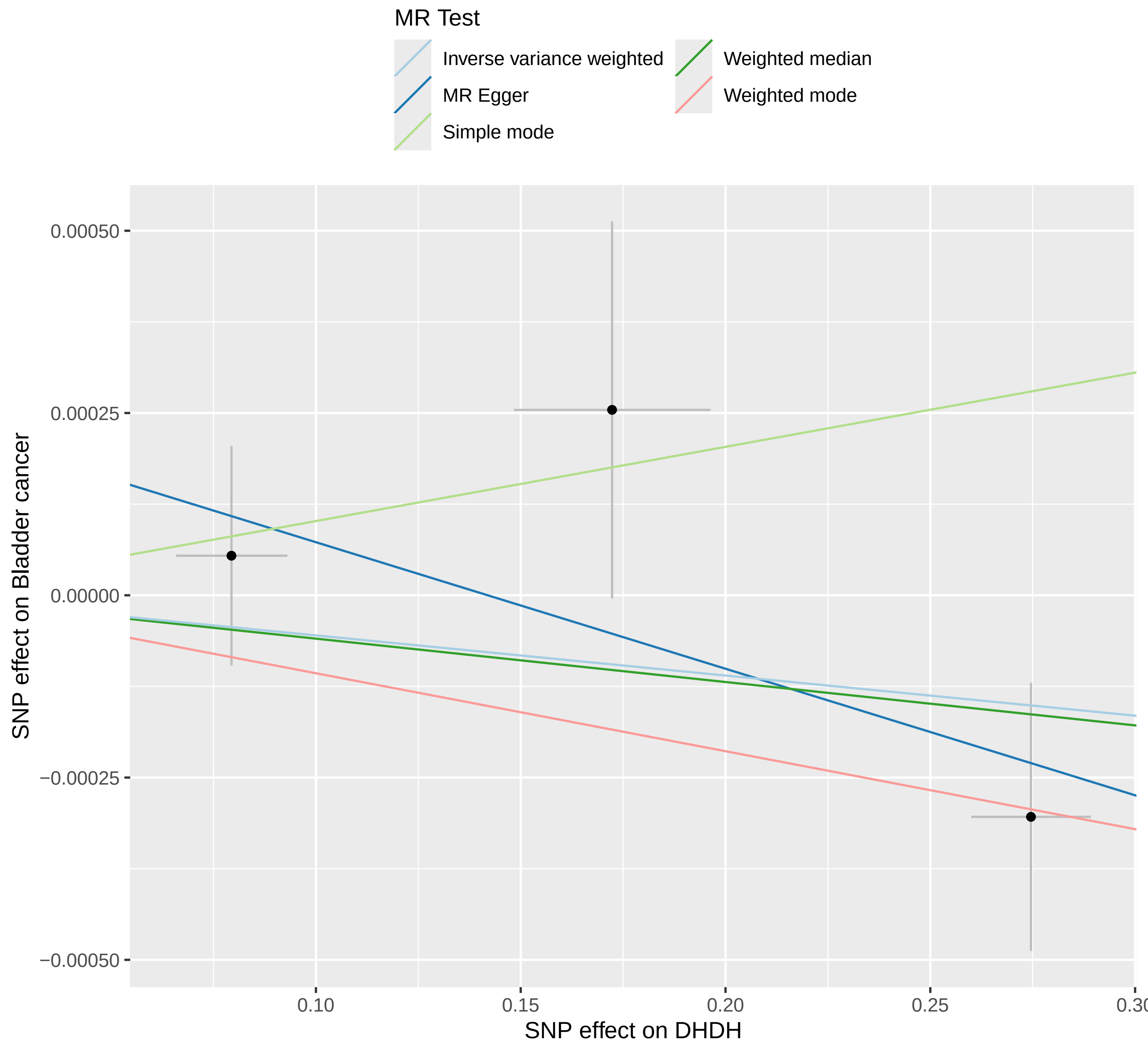
MR Test
Inverse variance weighted
MR Egger
Simple mode
Weighted median
Weighted mode
0.00050
0.00025
0.00000
−0.00025
−0.00050
SNP effect on Bladder cancer
0.10
0.15
0.20
0.25
0.30
SNP effect on DHDH

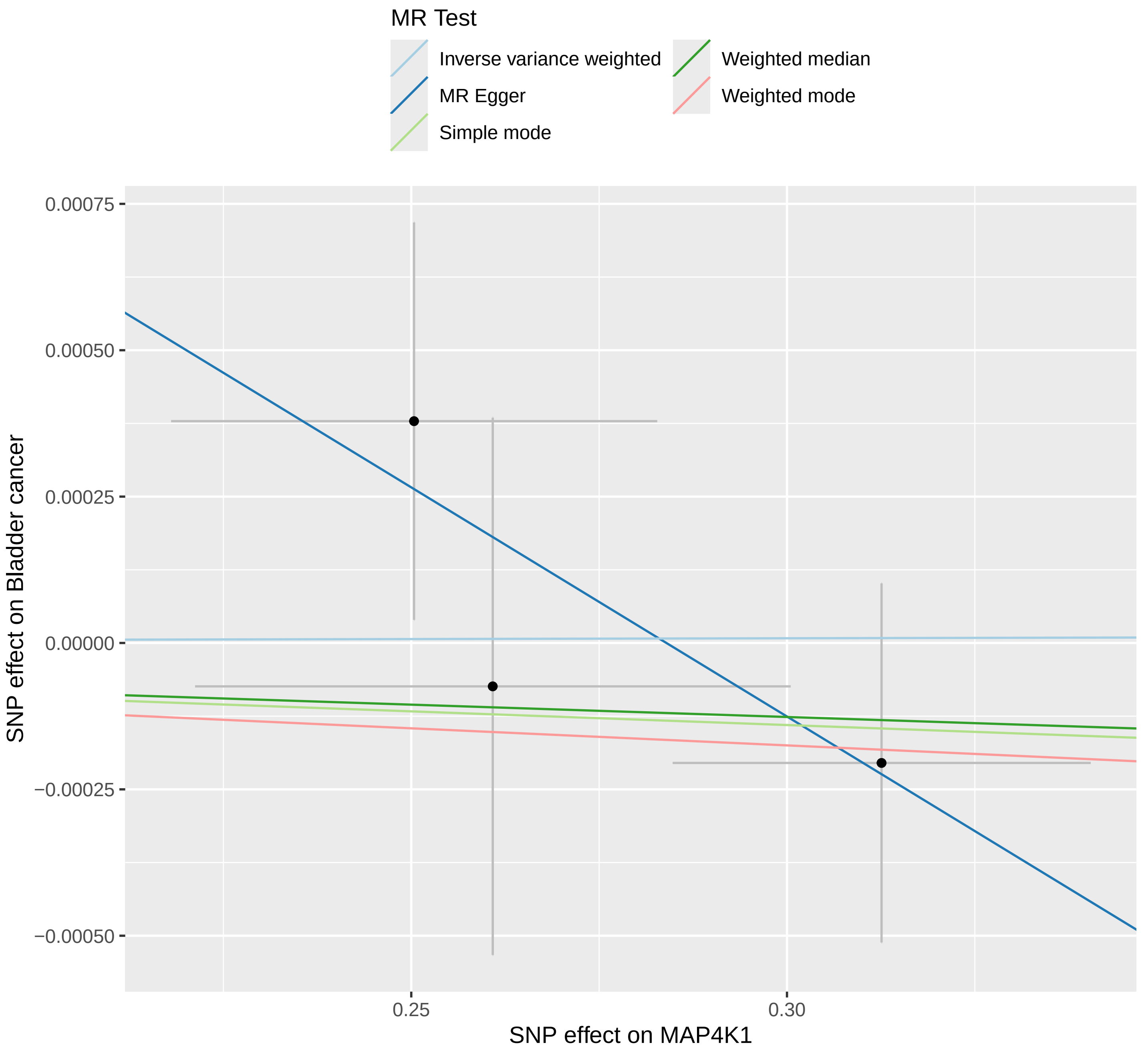
MR Test
Inverse variance weighted
MR Egger
Simple mode
Weighted median
Weighted mode
0.00075
0.00050
0.00025
0.00000
−0.00025
−0.00050
0.25
0.30
SNP effect on MAP4K1
SNP effect on Bladder cancer

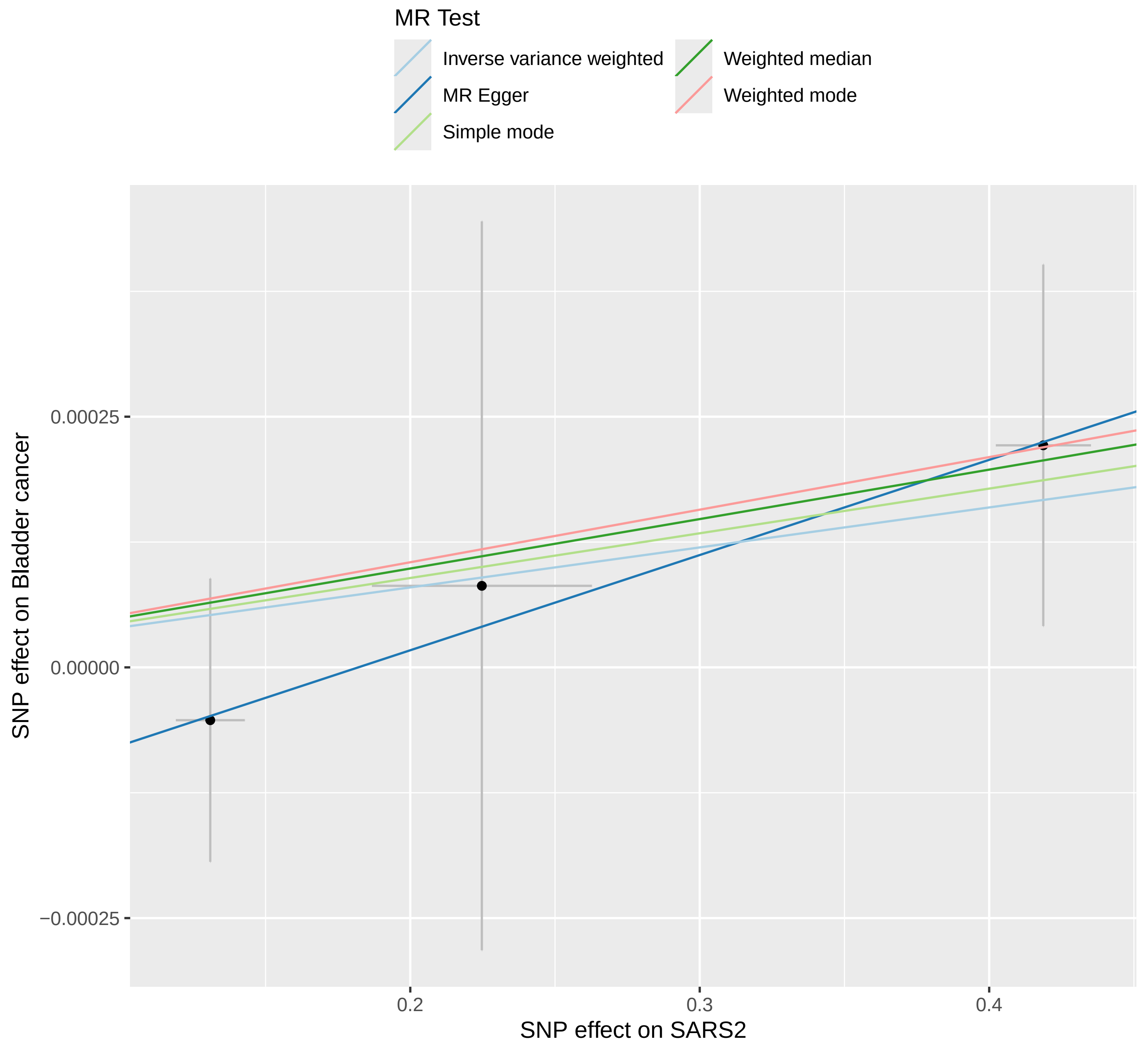
MR Test
Inverse variance weighted
MR Egger
Simple mode
Weighted median
Weighted mode
0.00025
0.00000
−0.00025
SNP effect on Bladder cancer
0.2
0.3
0.4
SNP effect on SARS2

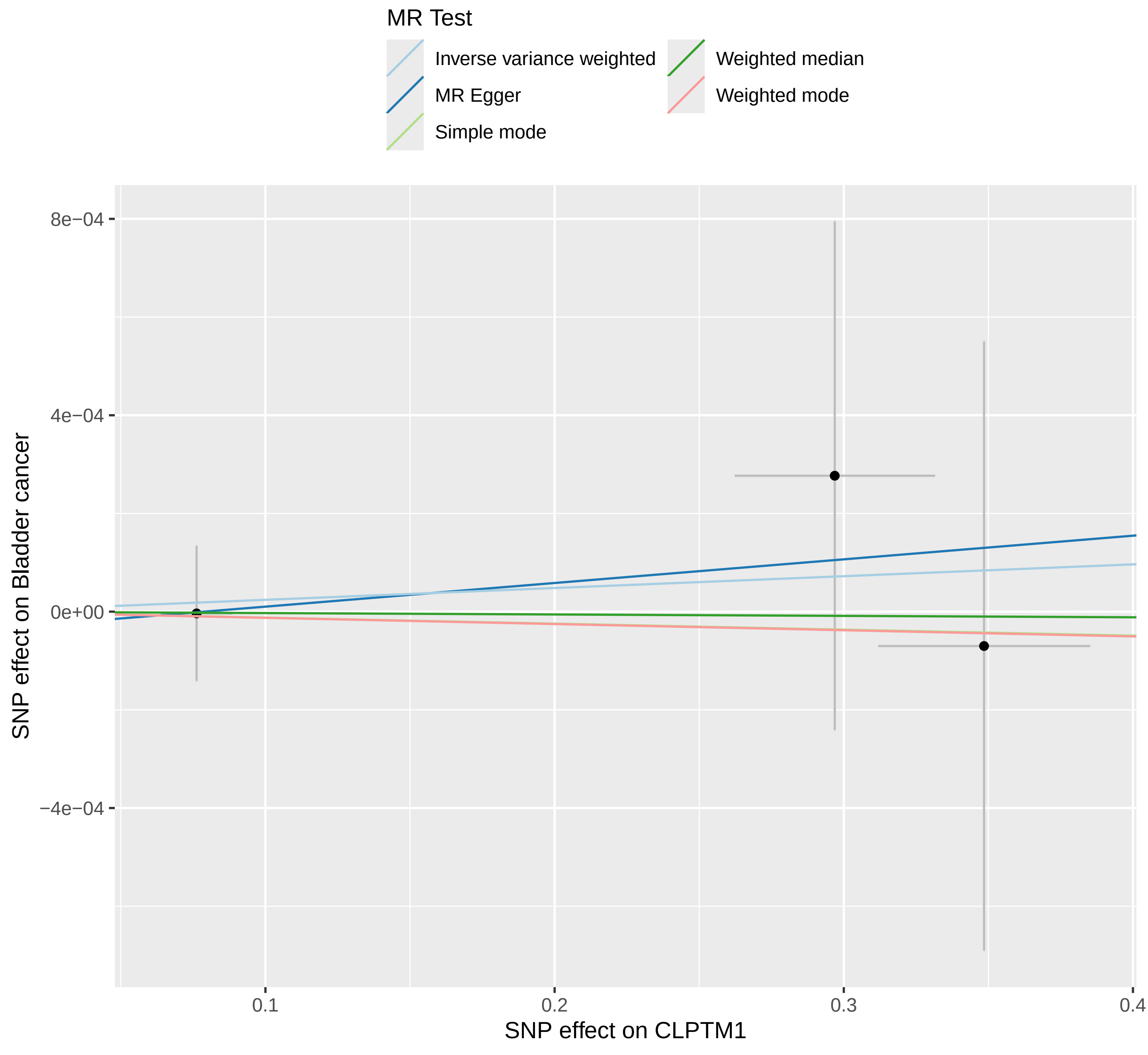
MR Test
Inverse variance weighted
MR Egger
Simple mode
Weighted median
Weighted mode
8e−04
4e−04
0e+00
−4e−04
SNP effect on Bladder cancer
0.1
0.2
0.3
0.4
SNP effect on CLPTM1

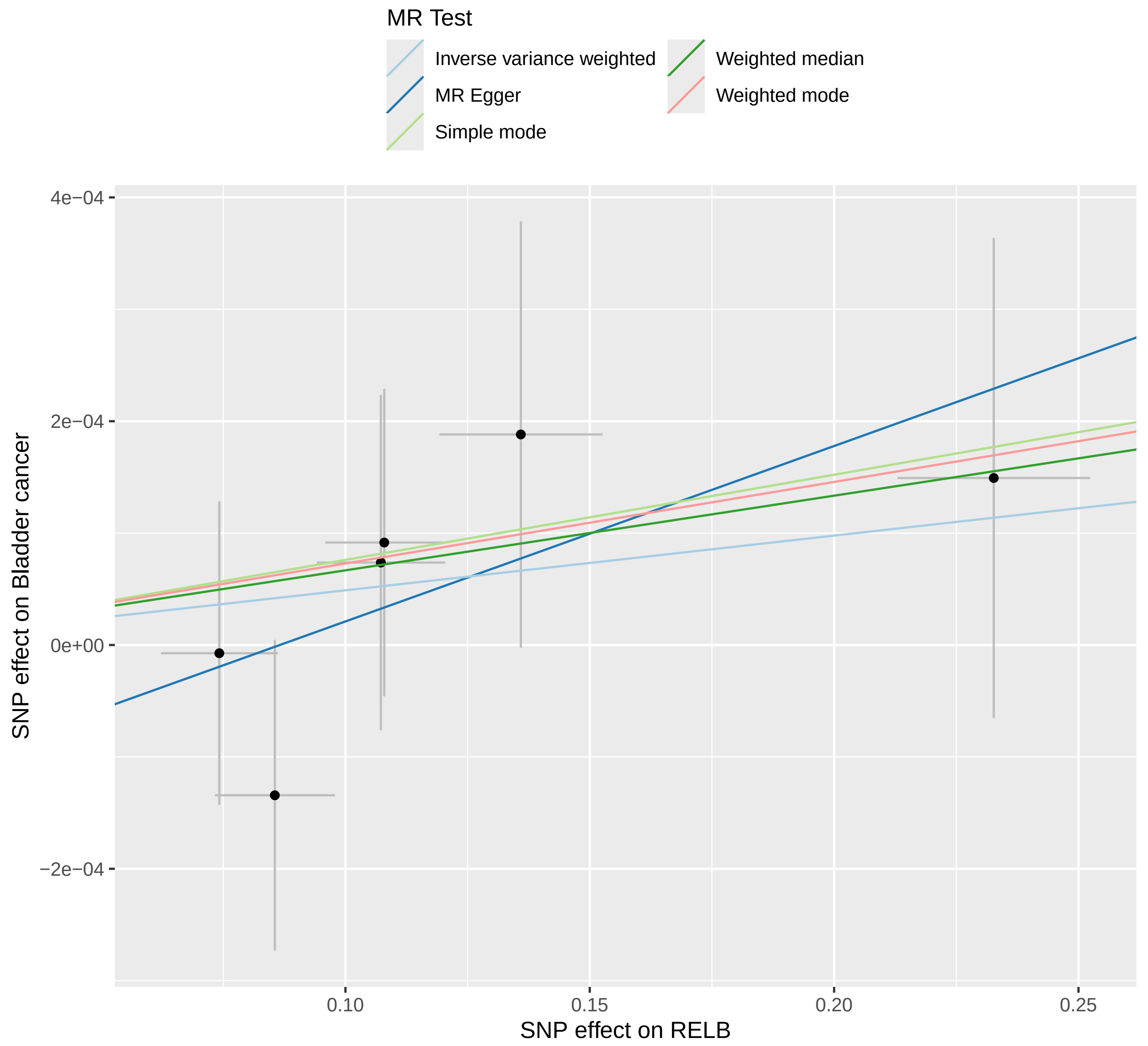
MR Test
Inverse variance weighted
MR Egger
Simple mode
Weighted median
Weighted mode
4e−04
2e−04
0e+00
−2e−04
0.10
0.15
0.20
0.25
SNP effect on RELB
SNP effect on Bladder cancer

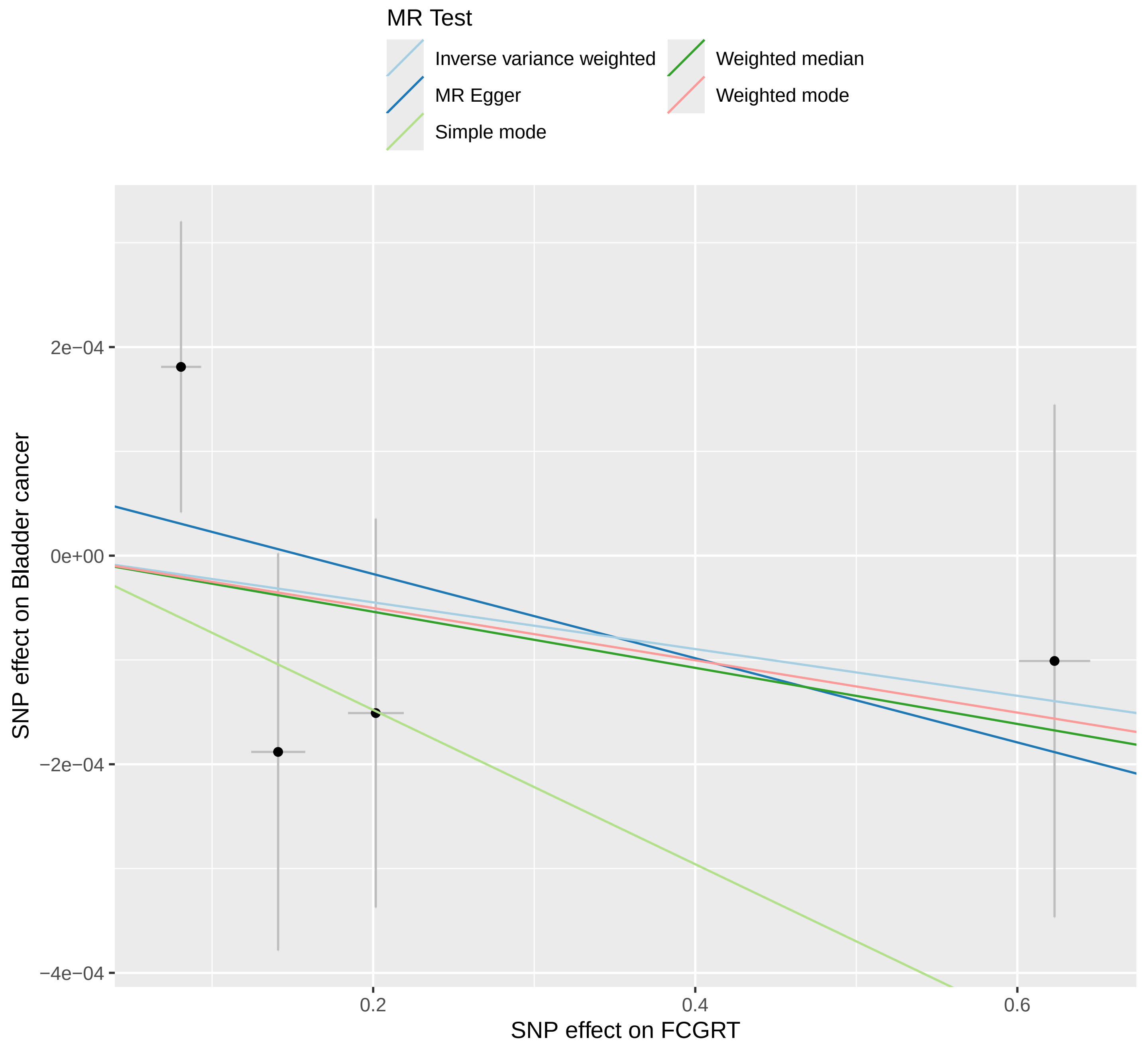

MR Test
Inverse variance weighted
MR Egger
Simple mode
Weighted median
Weighted mode
2e−04
0e+00
−2e−04
−4e−04
0.2
0.4
0.6
SNP effect on Bladder cancer
SNP effect on FCGRT

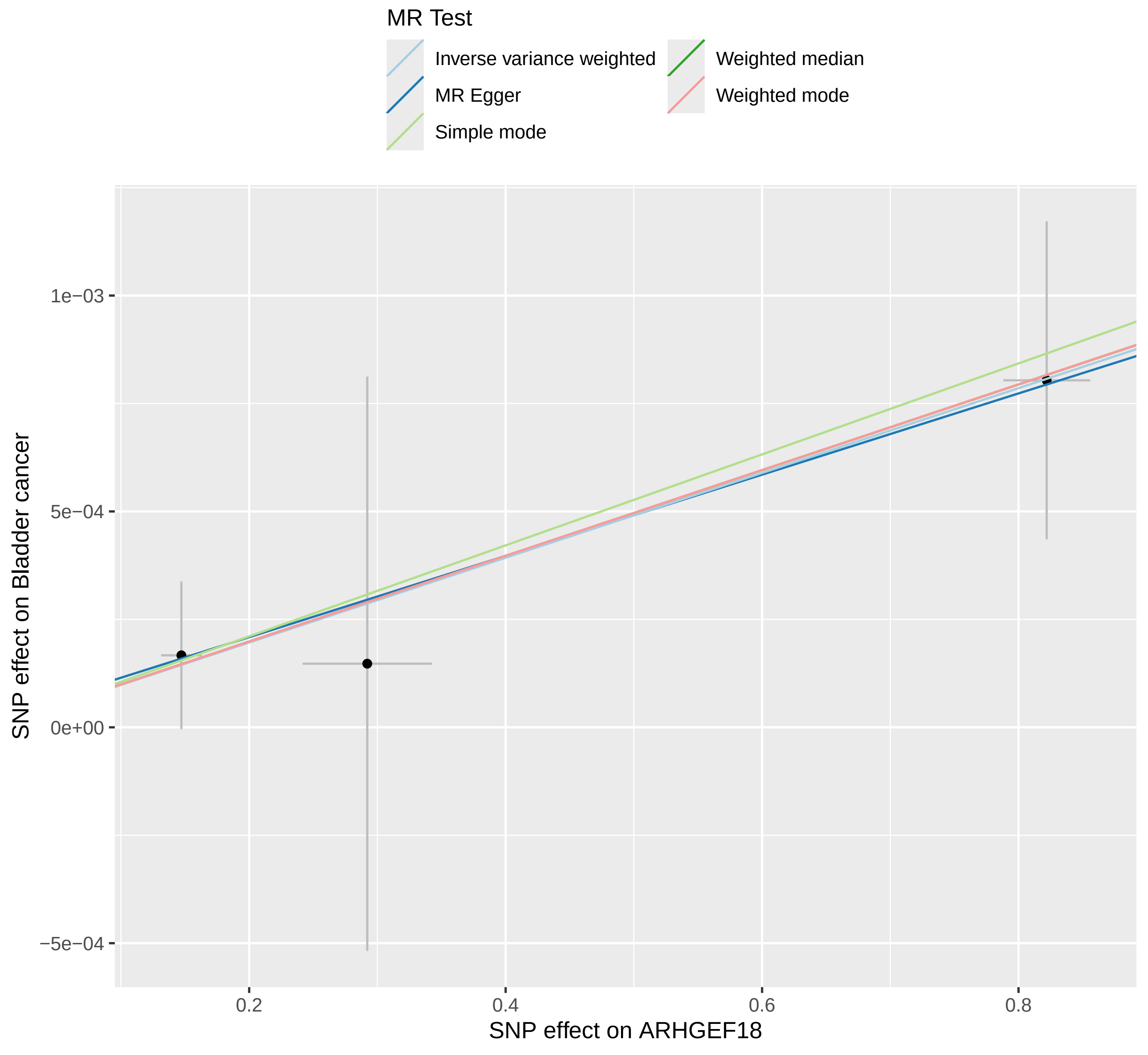

MR Test
Inverse variance weighted
MR Egger
Simple mode
Weighted median
Weighted mode
1e−03
5e−04
0e+00
−5e−04
0.2
0.4
0.6
0.8
SNP effect on ARHGEF18
SNP effect on Bladder cancer

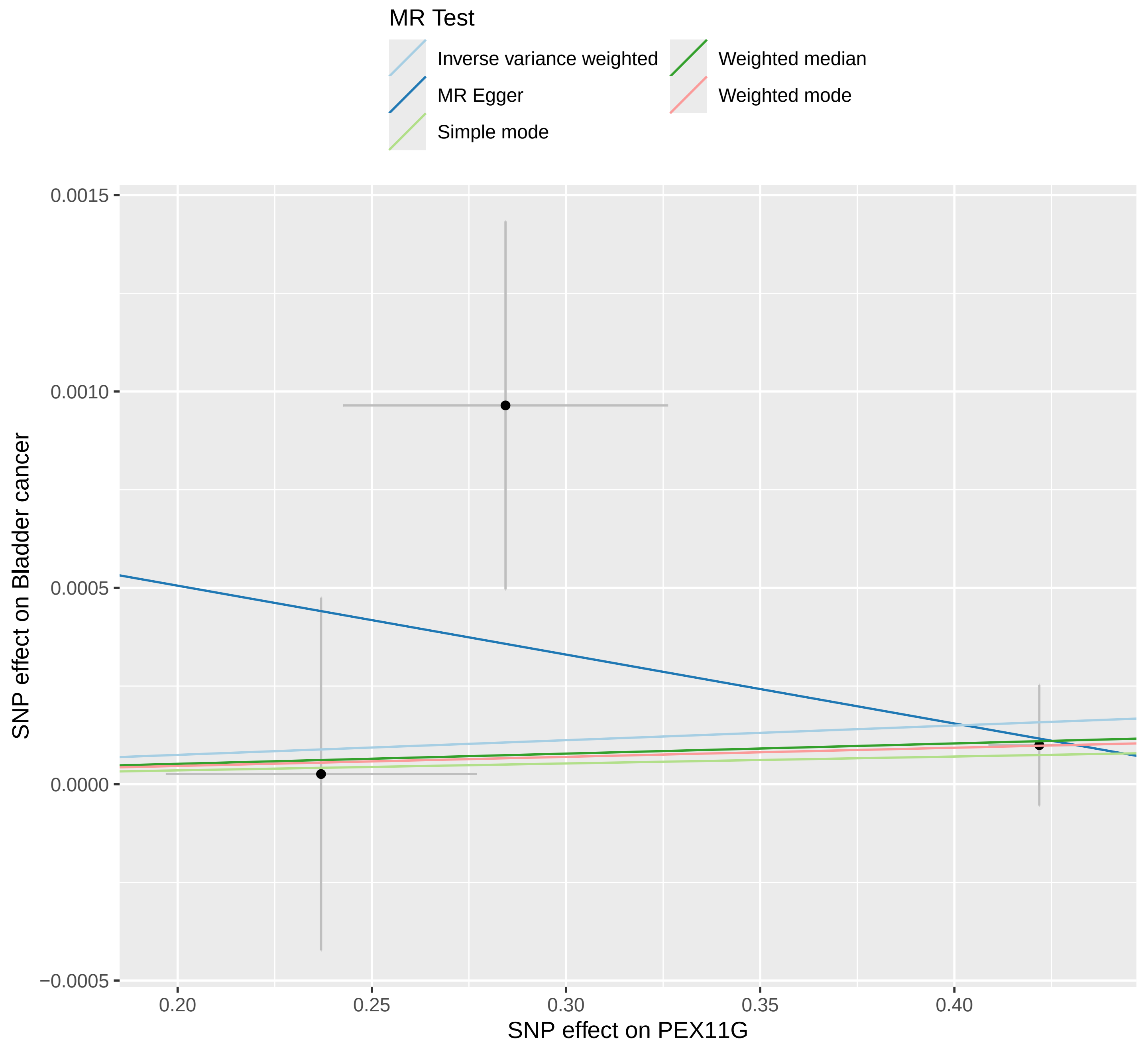

MR Test
Inverse variance weighted
MR Egger
Simple mode
Weighted median
Weighted mode
0.0015
0.0010
0.0005
0.0000
−0.0005
SNP effect on Bladder cancer
0.20
0.25
0.30
0.35
0.40
SNP effect on PEX11G

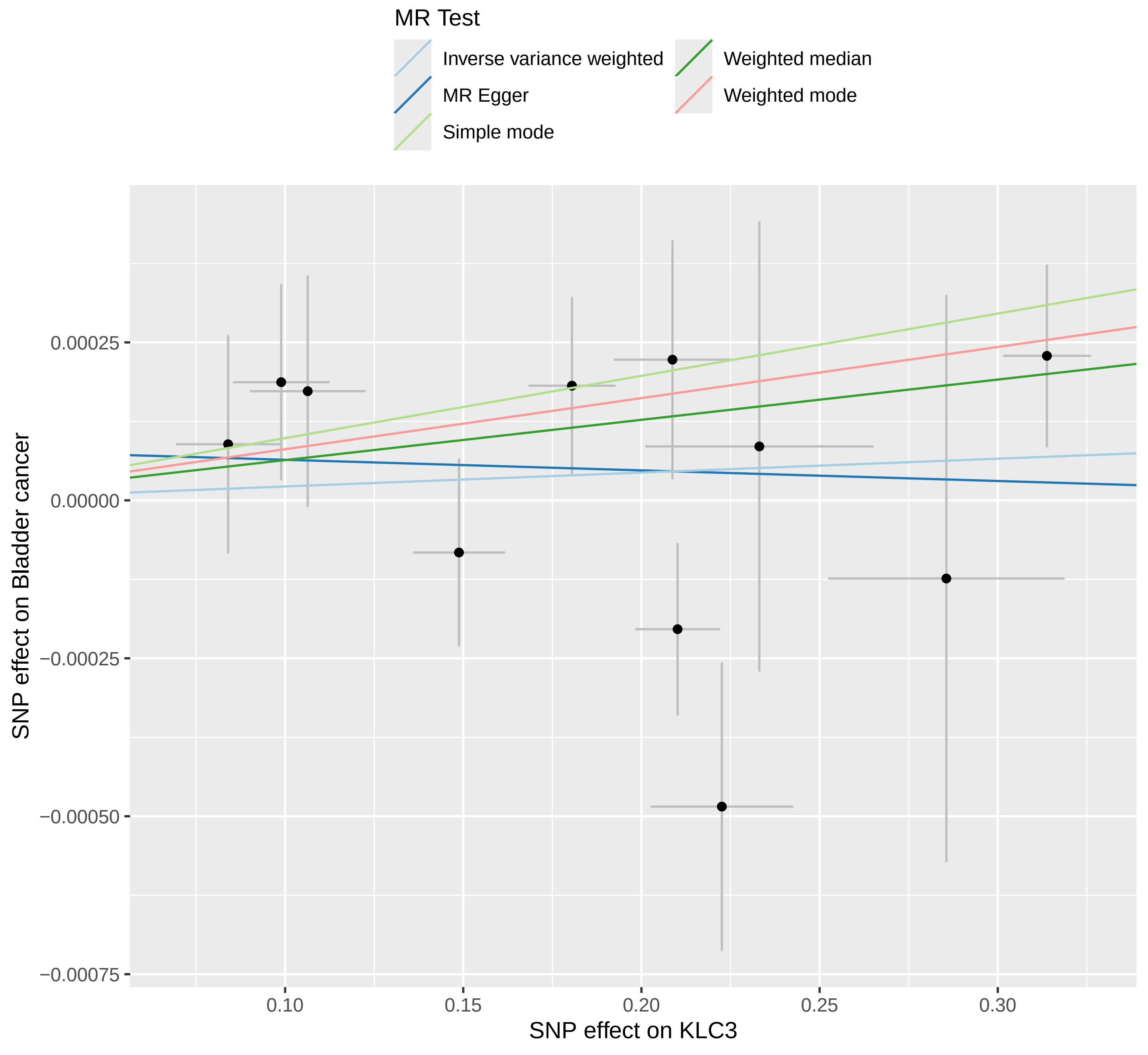
MR Test
Inverse variance weighted
MR Egger
Simple mode
Weighted median
Weighted mode
0.00025
0.00000
−0.00025
−0.00050
−0.00075
SNP effect on Bladder cancer
0.10
0.15
0.20
0.25
0.30
SNP effect on KLC3

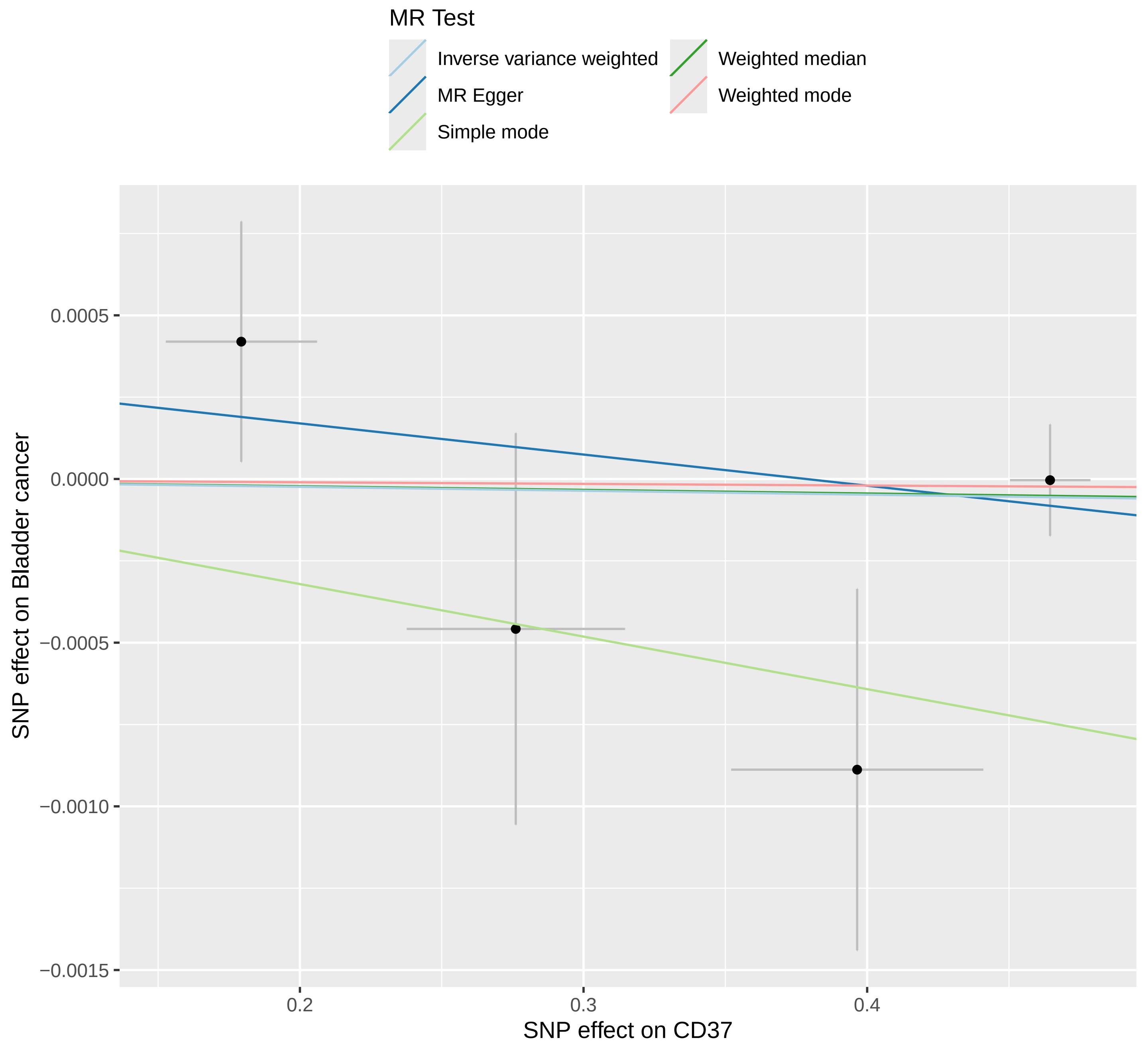

MR Test
Inverse variance weighted
MR Egger
Simple mode
Weighted median
Weighted mode
0.0005
0.0000
−0.0005
−0.0010
−0.0015
SNP effect on Bladder cancer
0.2
0.3
0.4
SNP effect on CD37

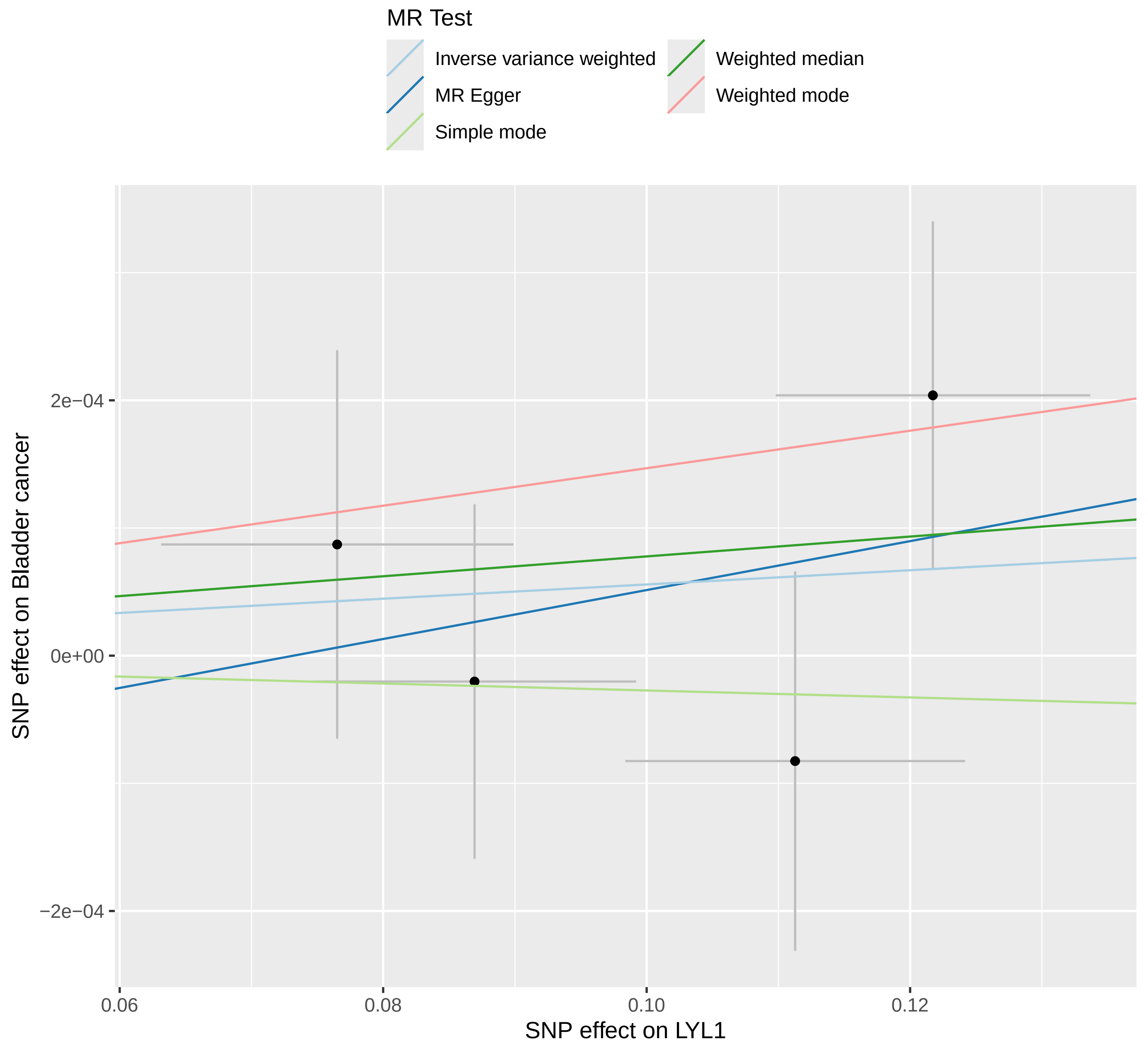

MR Test
Inverse variance weighted
MR Egger
Simple mode
Weighted median
Weighted mode
2e−04
0e+00
−2e−04
SNP effect on Bladder cancer
0.06
0.08
0.10
0.12
SNP effect on LYL1

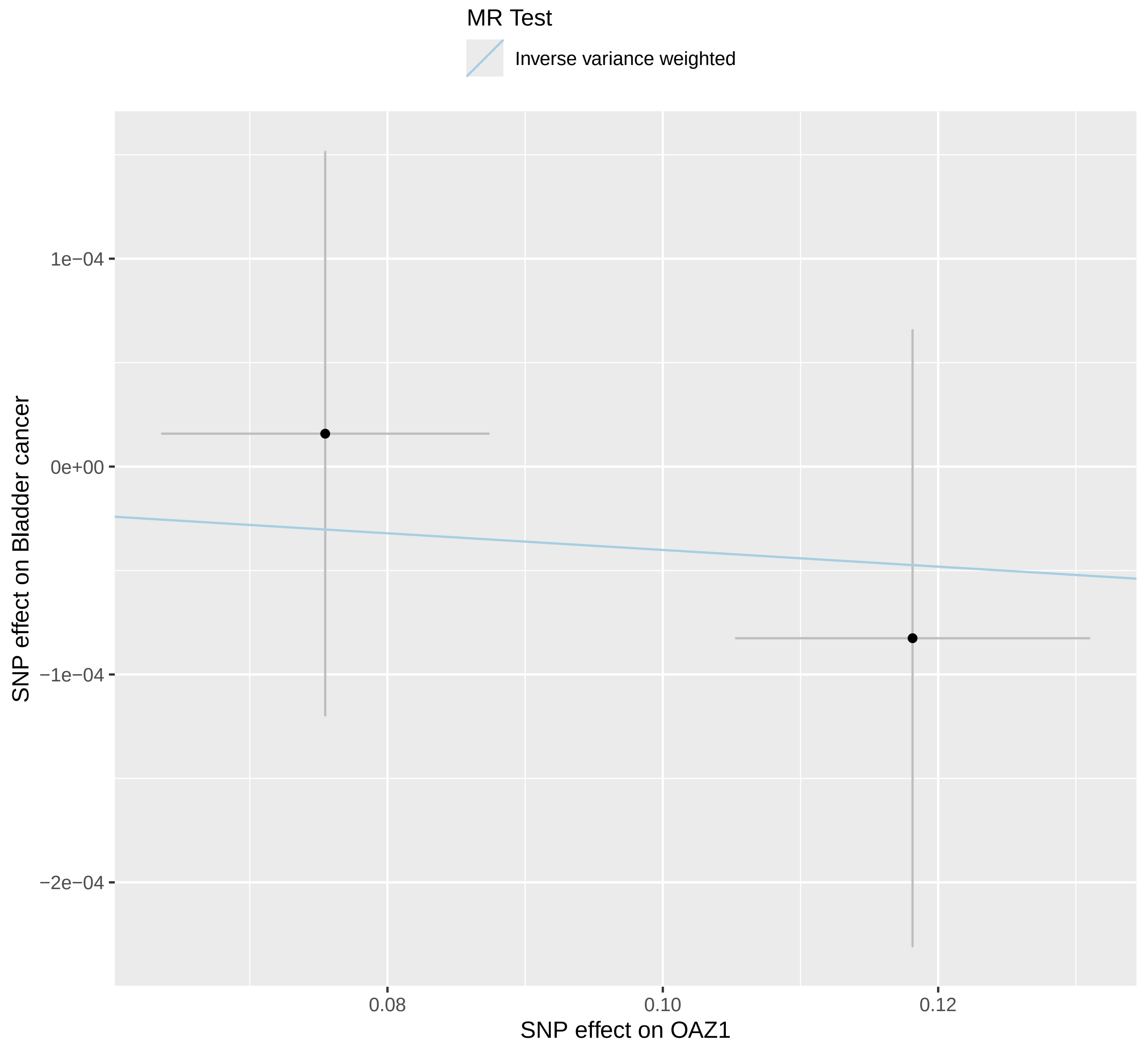

MR Test
Inverse variance weighted
1e−04
0e+00
−1e−04
−2e−04
0.08
0.10
0.12
SNP effect on OAZ1
SNP effect on Bladder cancer

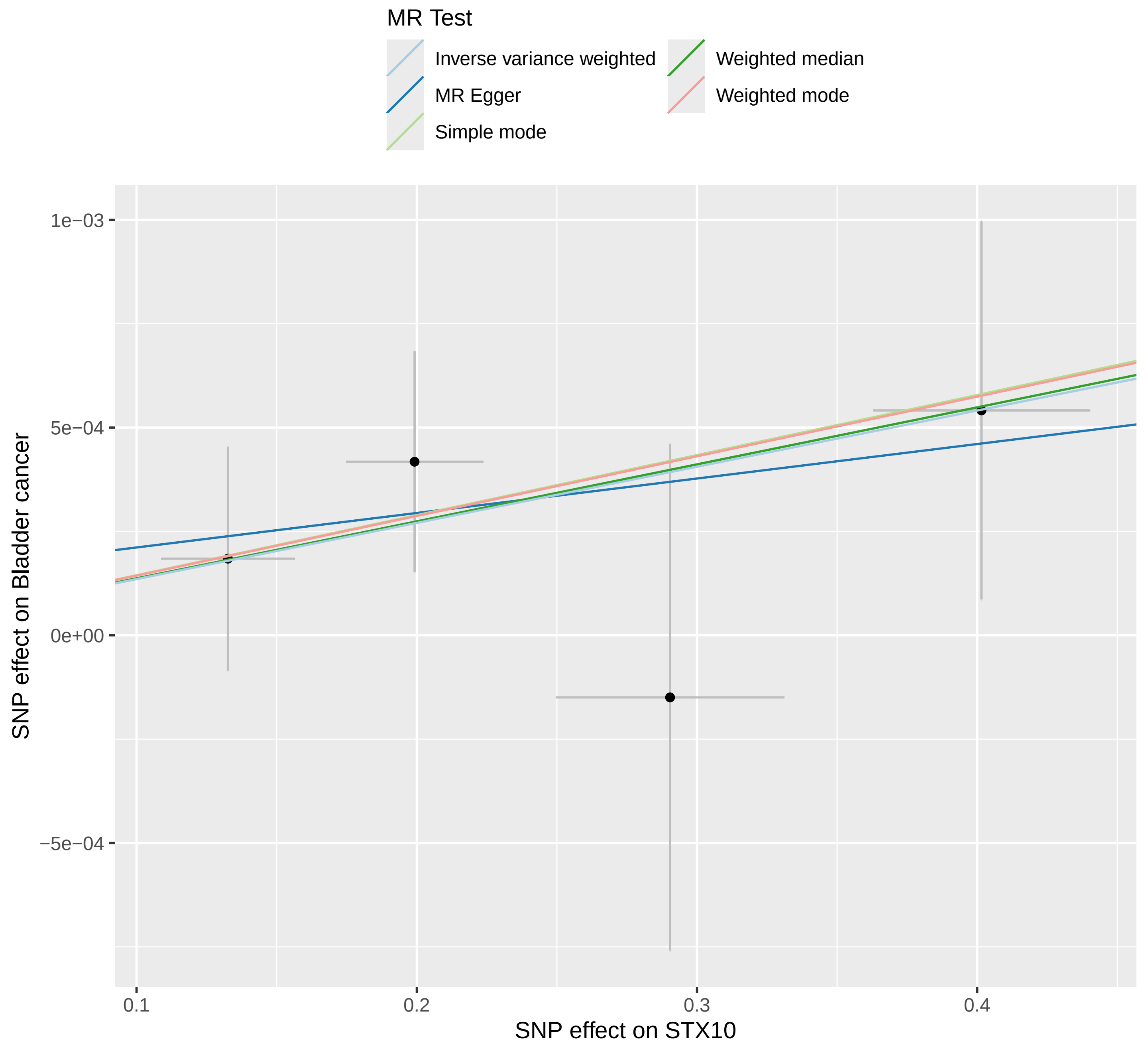

MR Test
Inverse variance weighted
MR Egger
Simple mode
Weighted median
Weighted mode
1e−03
5e−04
0e+00
−5e−04
0.1
0.2
0.3
0.4
SNP effect on STX10
SNP effect on Bladder cancer

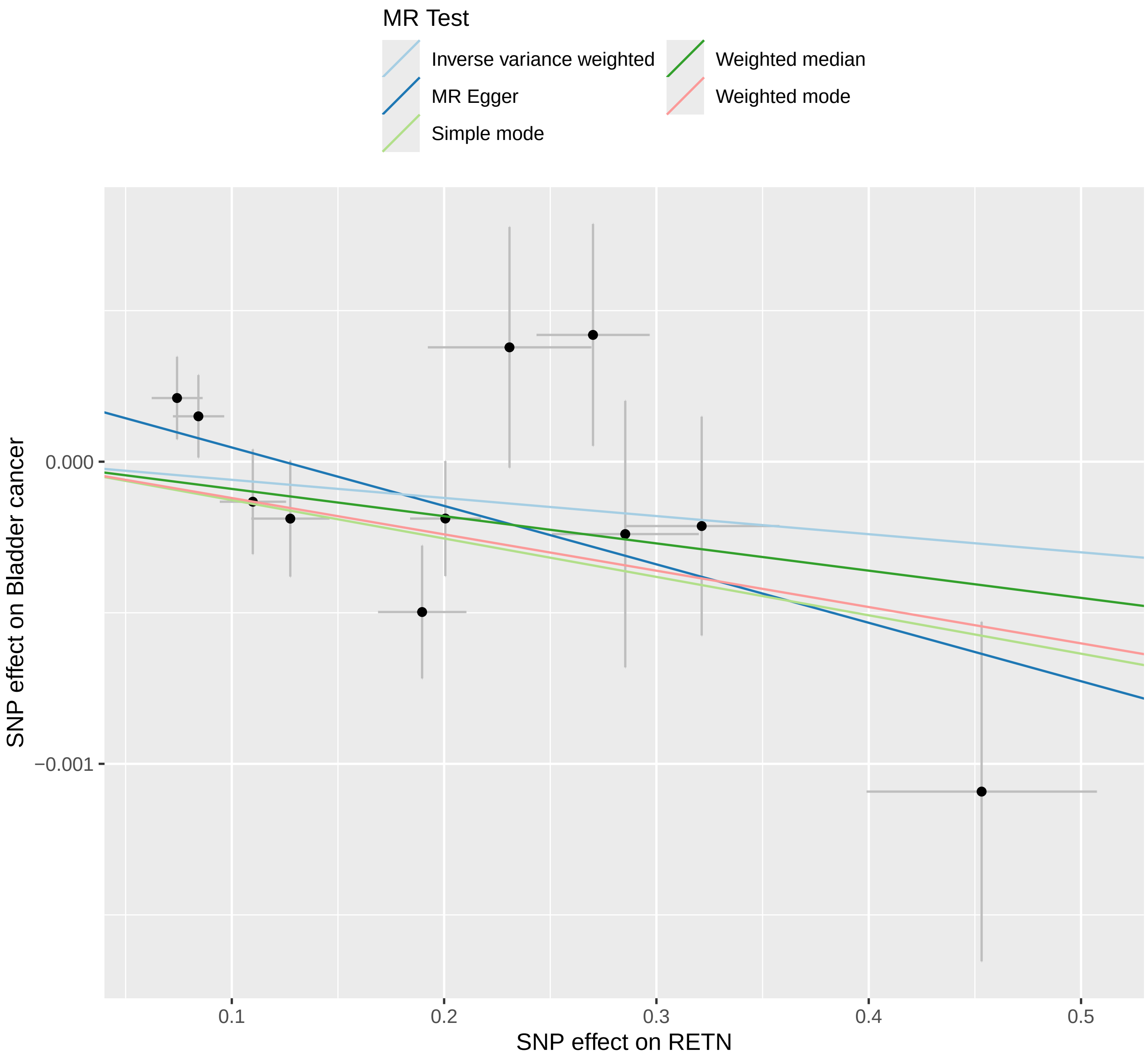

MR Test
Inverse variance weighted
MR Egger
Simple mode
Weighted median
Weighted mode
SNP effect on Bladder cancer
0.000
−0.001
0.1
0.2
0.3
0.4
0.5
SNP effect on RETN

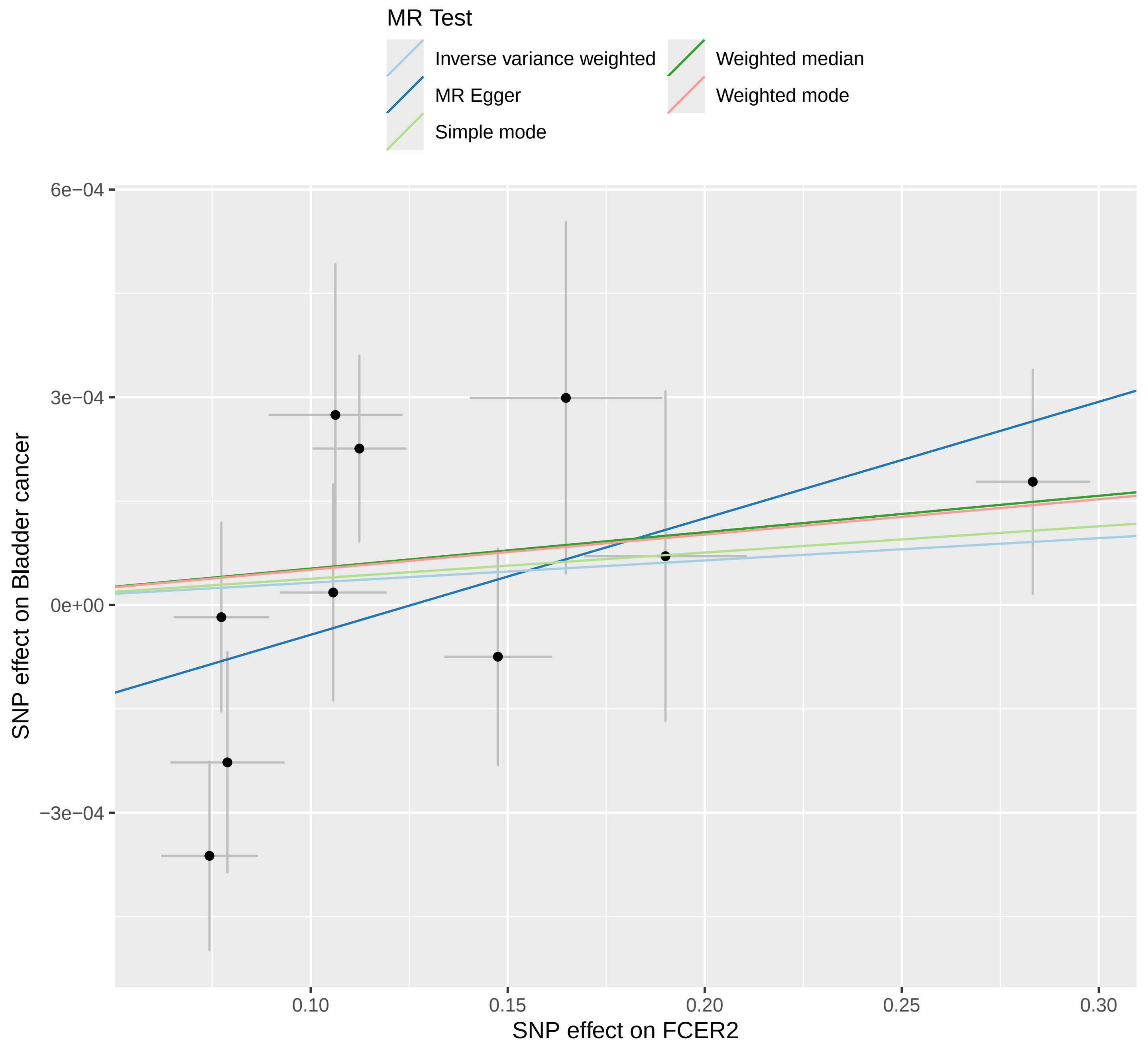

MR Test
Inverse variance weighted
MR Egger
Simple mode
Weighted median
Weighted mode
6e−04
3e−04
0e+00
−3e−04
0.10
0.15
0.20
0.25
0.30
SNP effect on Bladder cancer
SNP effect on FCER2

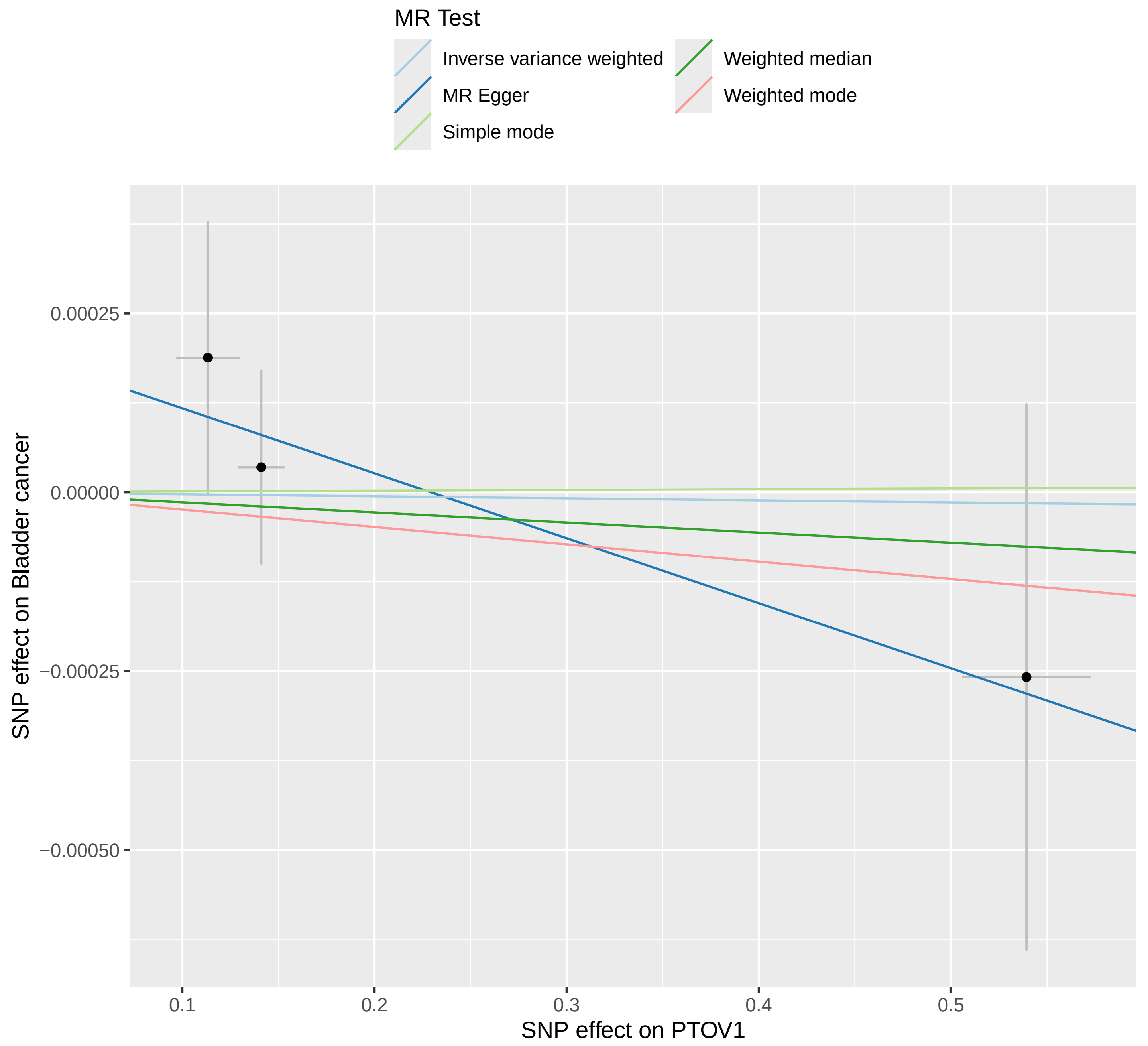
MR Test
Inverse variance weighted
MR Egger
Simple mode
Weighted median
Weighted mode
0.00025
0.00000
−0.00025
−0.00050
SNP effect on Bladder cancer
0.1
0.2
0.3
0.4
0.5
SNP effect on PTOV1

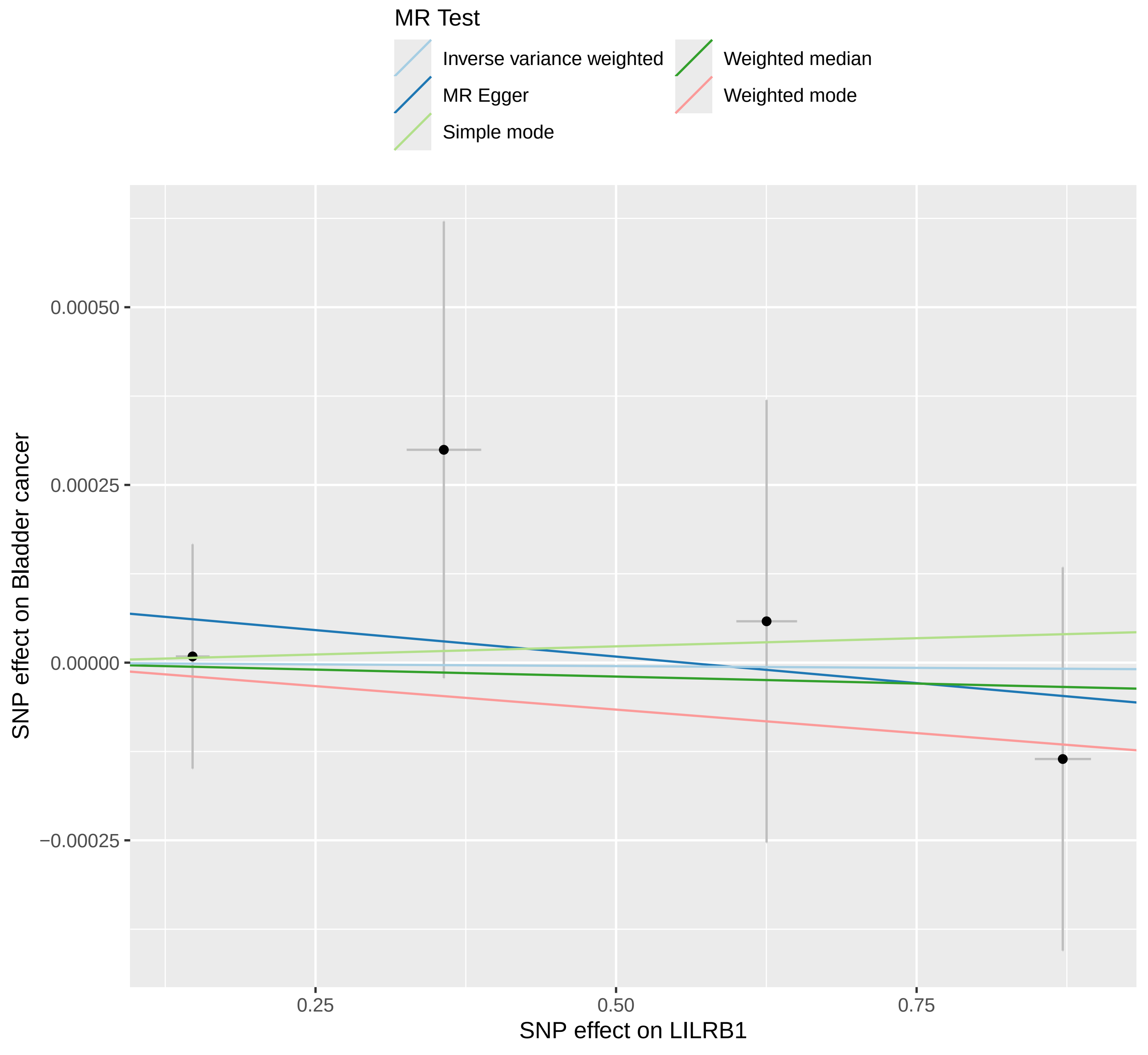

MR Test
Inverse variance weighted
MR Egger
Simple mode
Weighted median
Weighted mode
0.00050
0.00025
0.00000
−0.00025
SNP effect on Bladder cancer
0.25
0.50
0.75
SNP effect on LILRB1

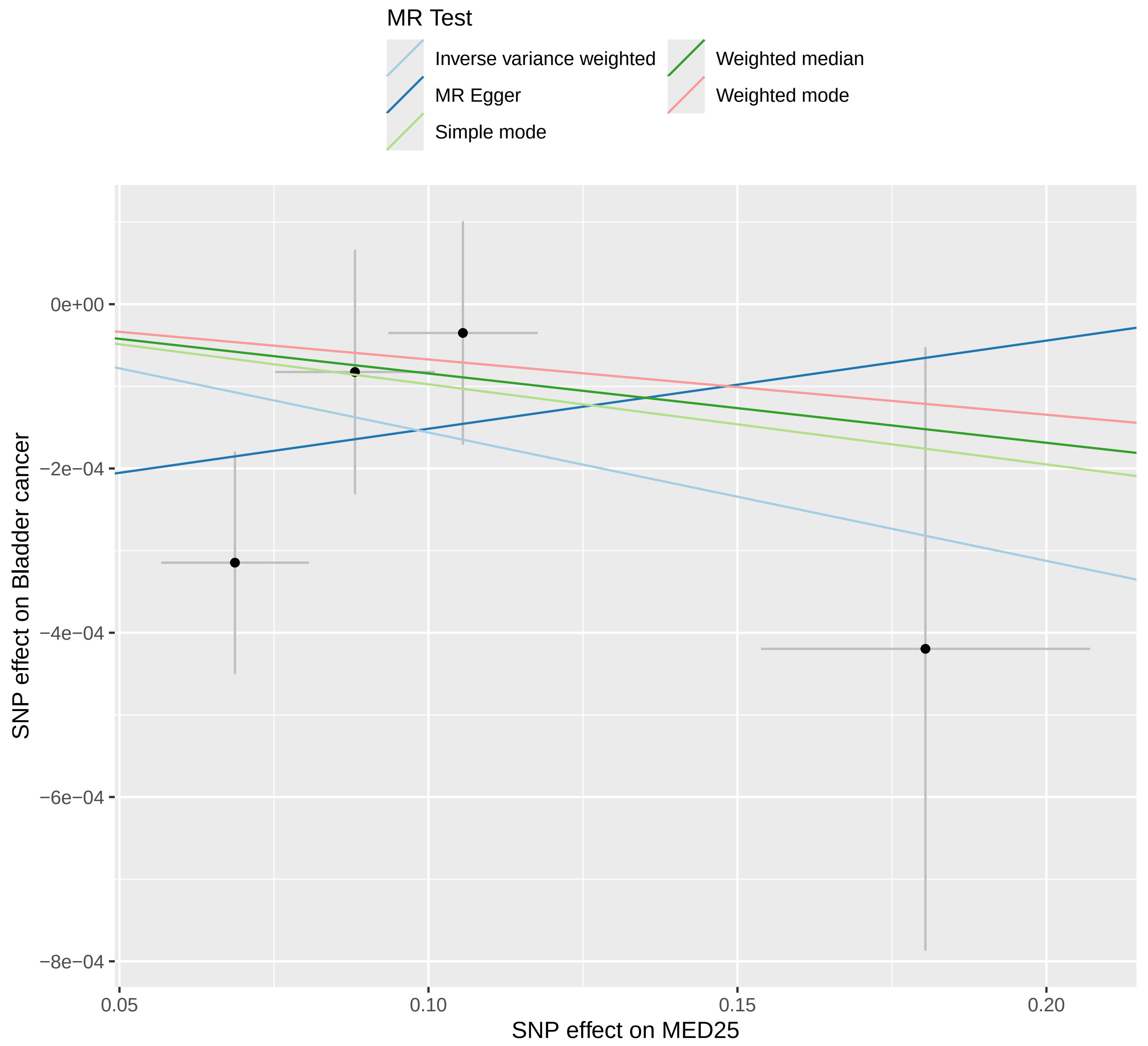
MR Test
Inverse variance weighted
MR Egger
Simple mode
Weighted median
Weighted mode
0e+00
−2e−04
−4e−04
−6e−04
−8e−04
0.05
0.10
0.15
0.20
SNP effect on Bladder cancer
SNP effect on MED25

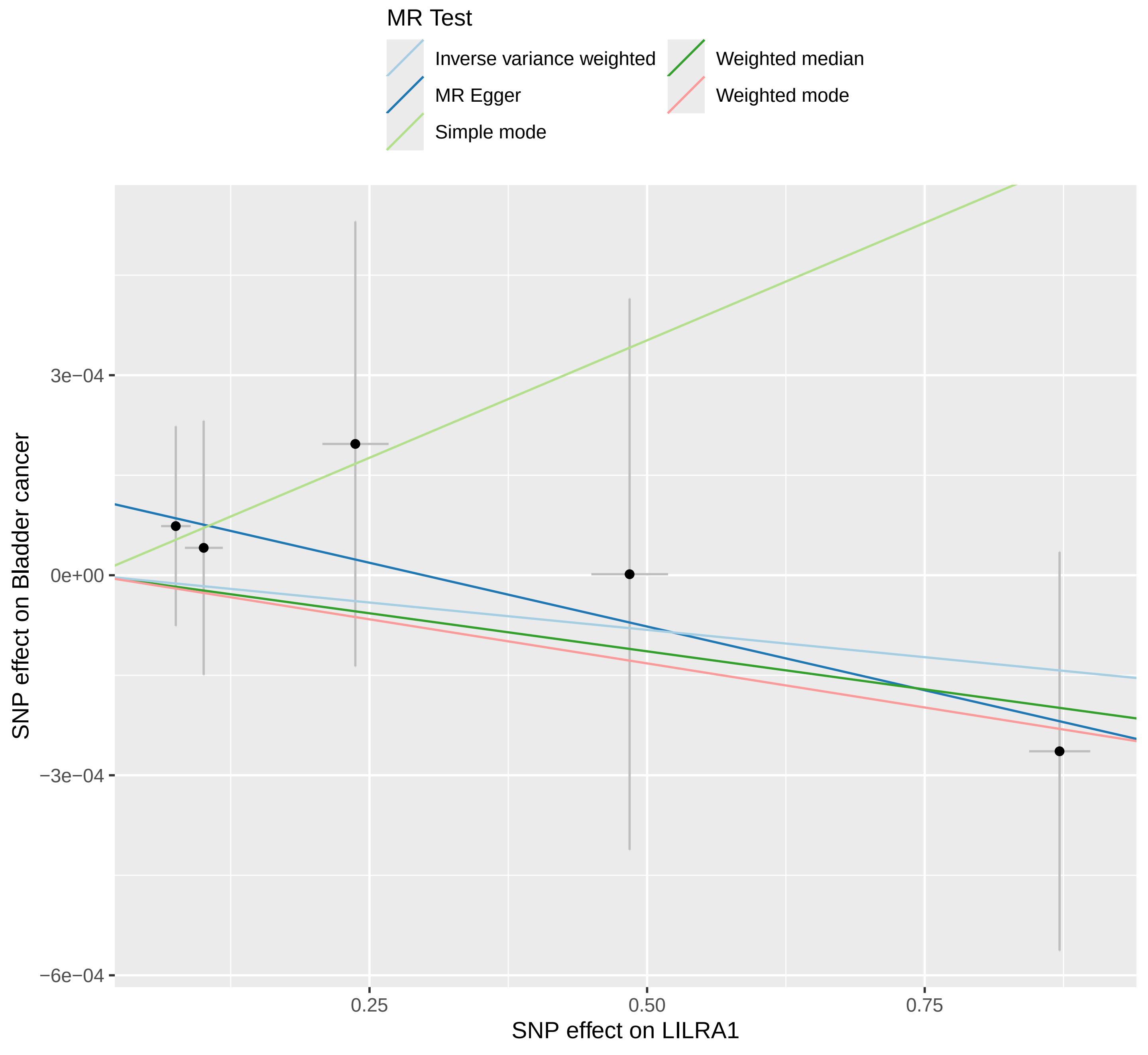

MR Test
Inverse variance weighted
MR Egger
Simple mode
Weighted median
Weighted mode
3e−04
0e+00
−3e−04
−6e−04
SNP effect on Bladder cancer
0.25
0.50
0.75
SNP effect on LILRA1

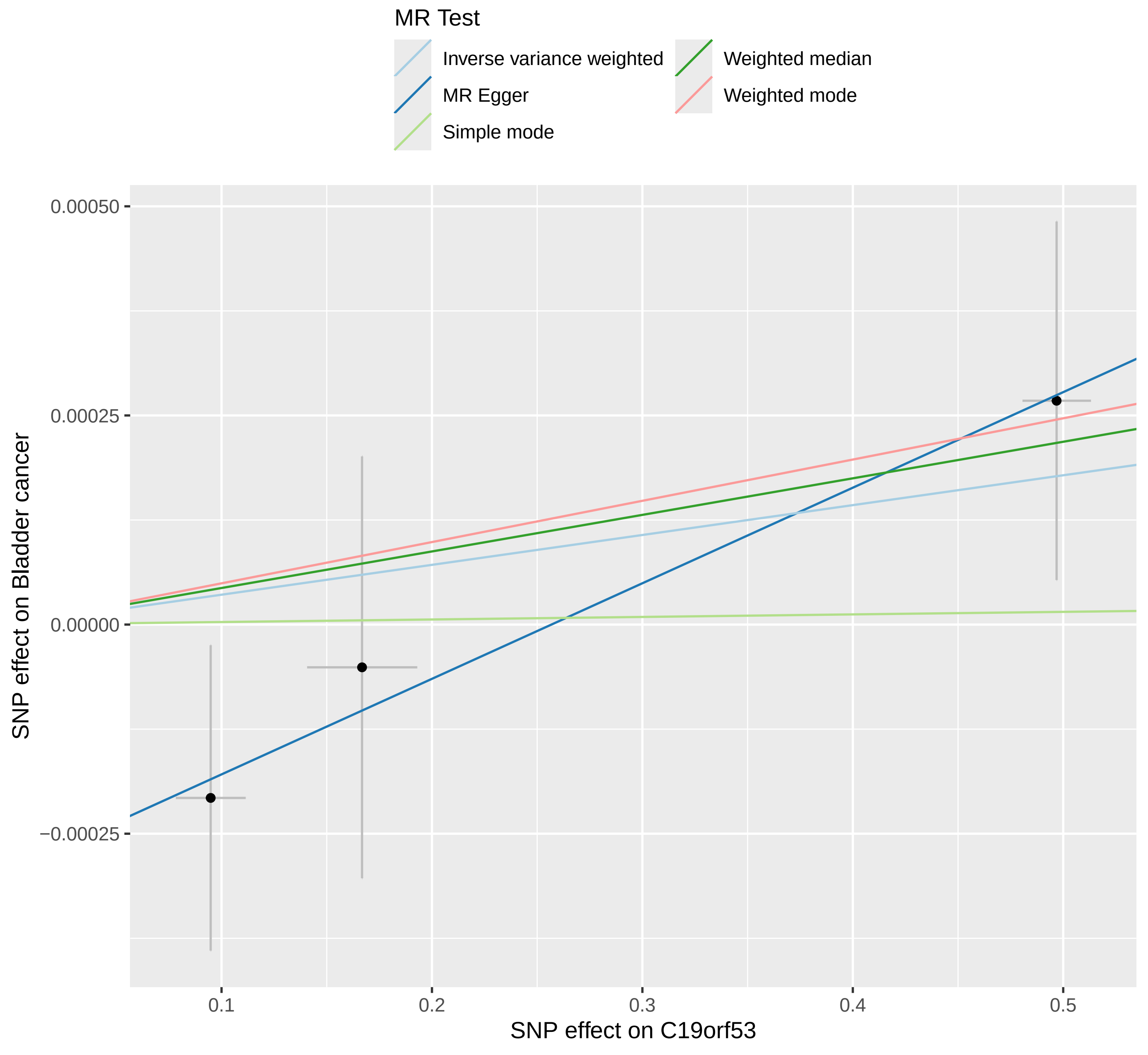
MR Test
Inverse variance weighted
MR Egger
Simple mode
Weighted median
Weighted mode
0.00050
0.00025
0.00000
−0.00025
0.1
0.2
0.3
0.4
0.5
SNP effect on Bladder cancer
SNP effect on C19orf53

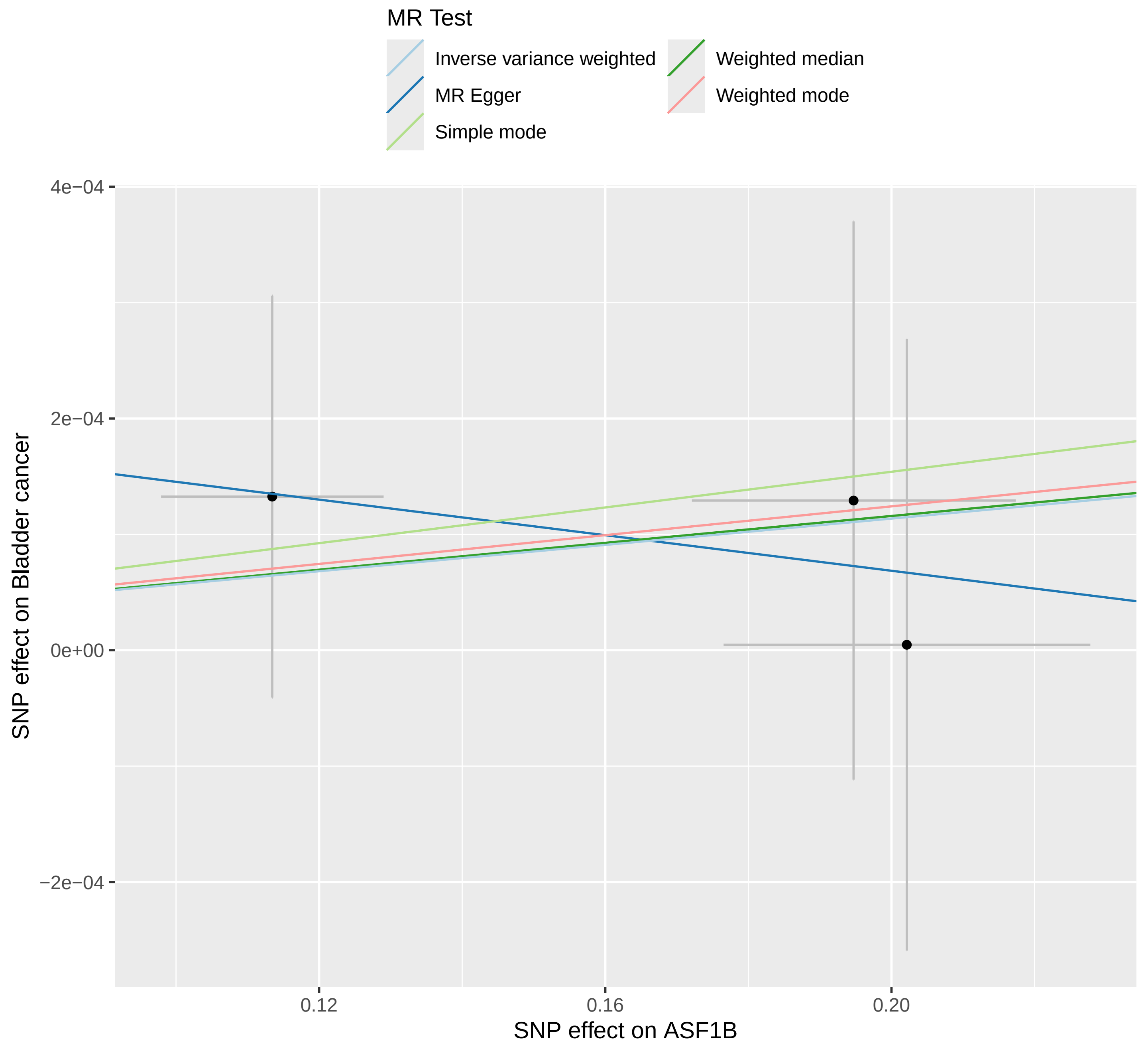

MR Test
Inverse variance weighted
MR Egger
Simple mode
Weighted median
Weighted mode
4e−04
2e−04
0e+00
−2e−04
SNP effect on Bladder cancer
0.12
0.16
0.20
SNP effect on ASF1B

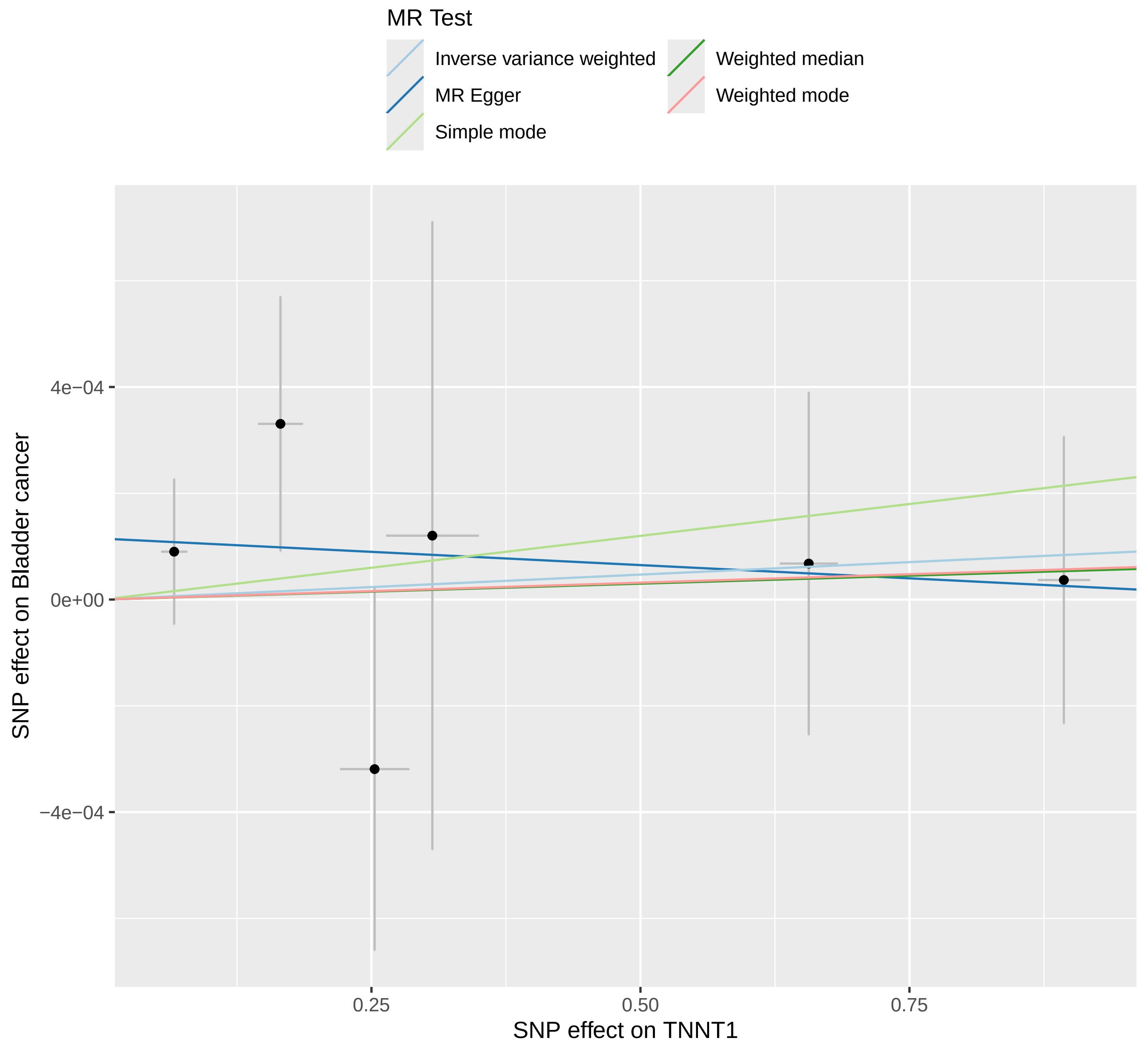

MR Test
Inverse variance weighted
MR Egger
Simple mode
Weighted median
Weighted mode
4e−04
0e+00
−4e−04
0.25
0.50
0.75
SNP effect on Bladder cancer
SNP effect on TNNT1

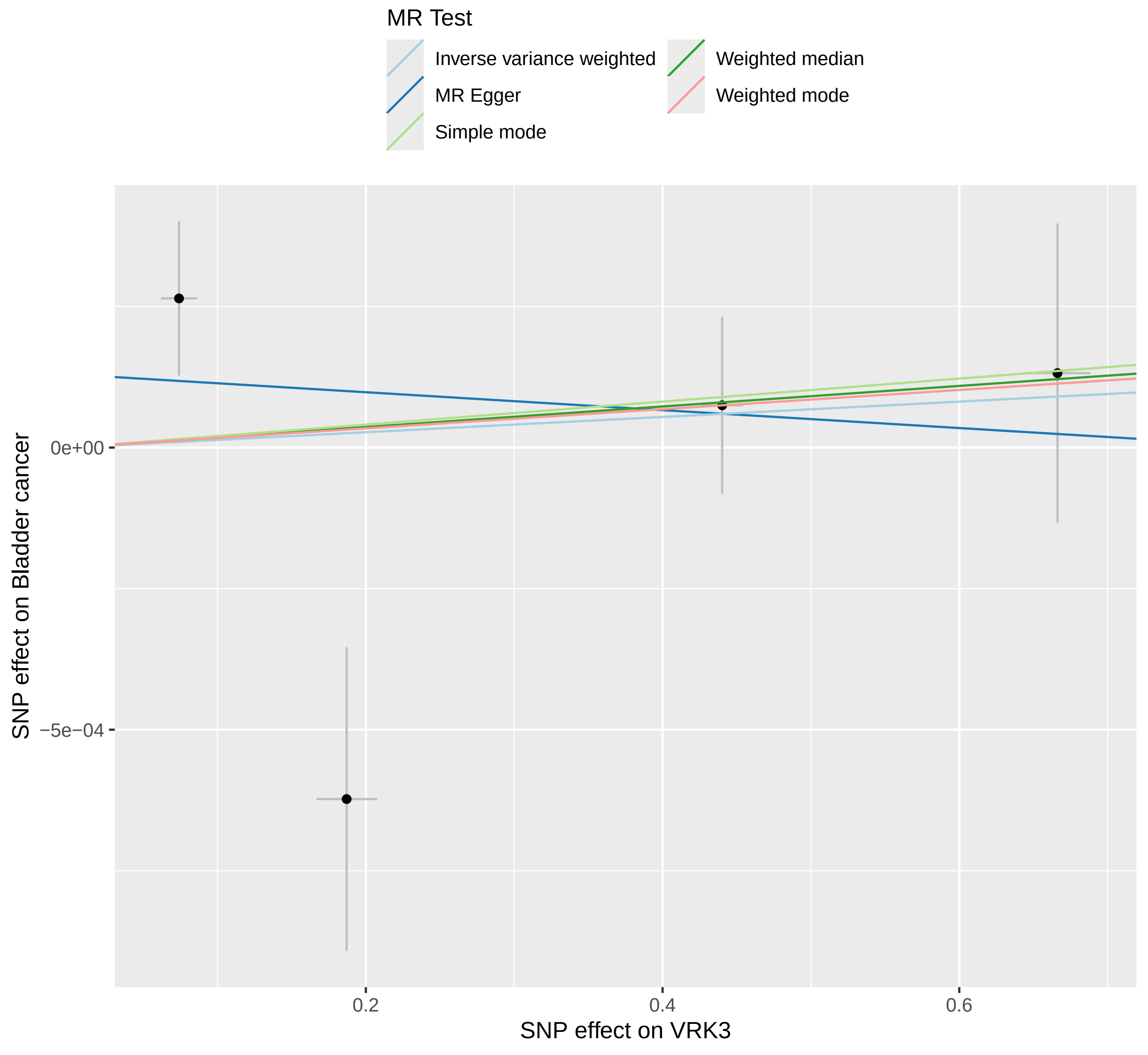
MR Test
Inverse variance weighted
MR Egger
Simple mode
Weighted median
Weighted mode
SNP effect on Bladder cancer
0e+00
−5e−04
0.2
0.4
0.6
SNP effect on VRK3

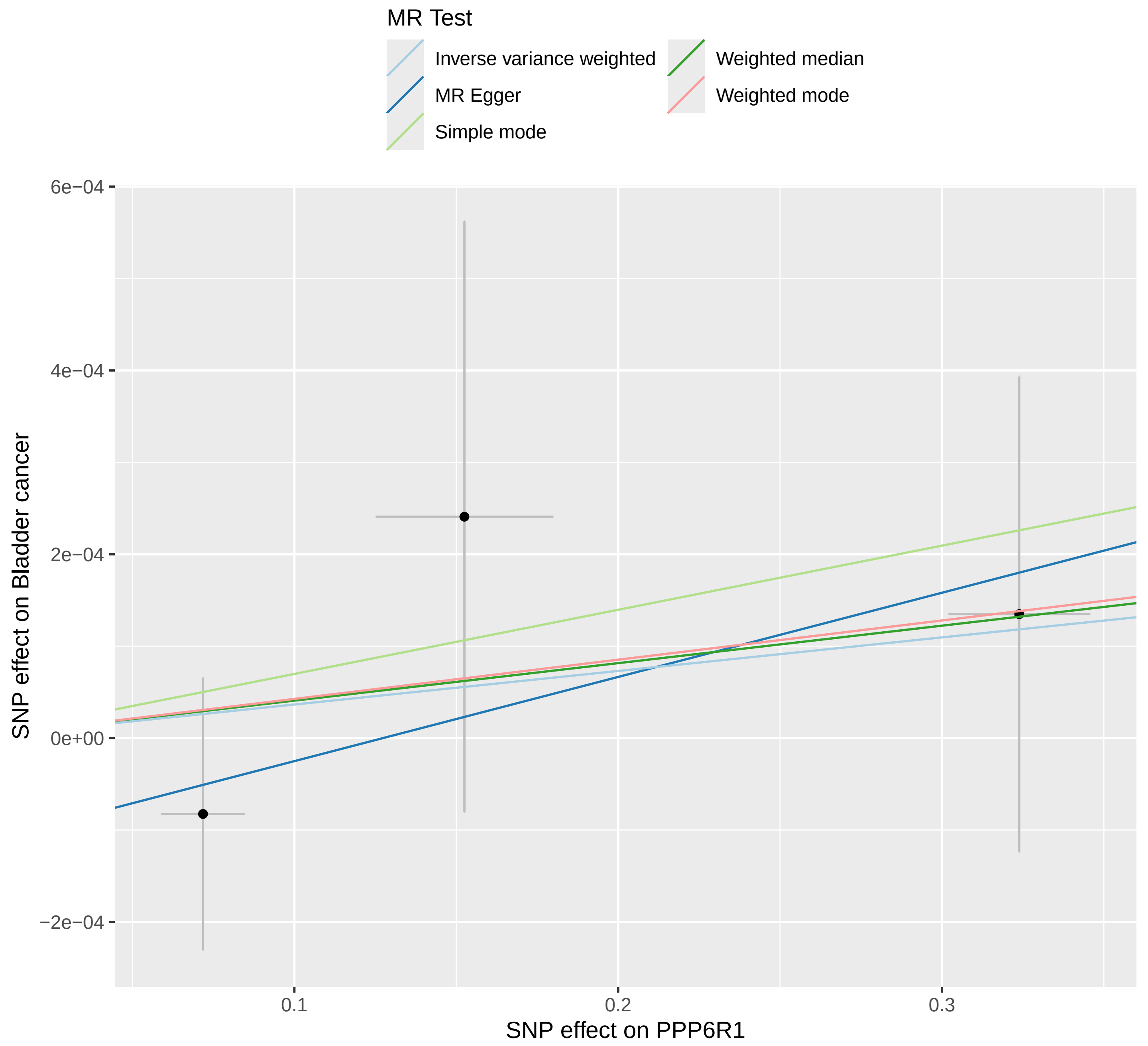
MR Test
Inverse variance weighted
MR Egger
Simple mode
Weighted median
Weighted mode
6e−04
4e−04
2e−04
0e+00
−2e−04
0.1
0.2
0.3
SNP effect on Bladder cancer
SNP effect on PPP6R1

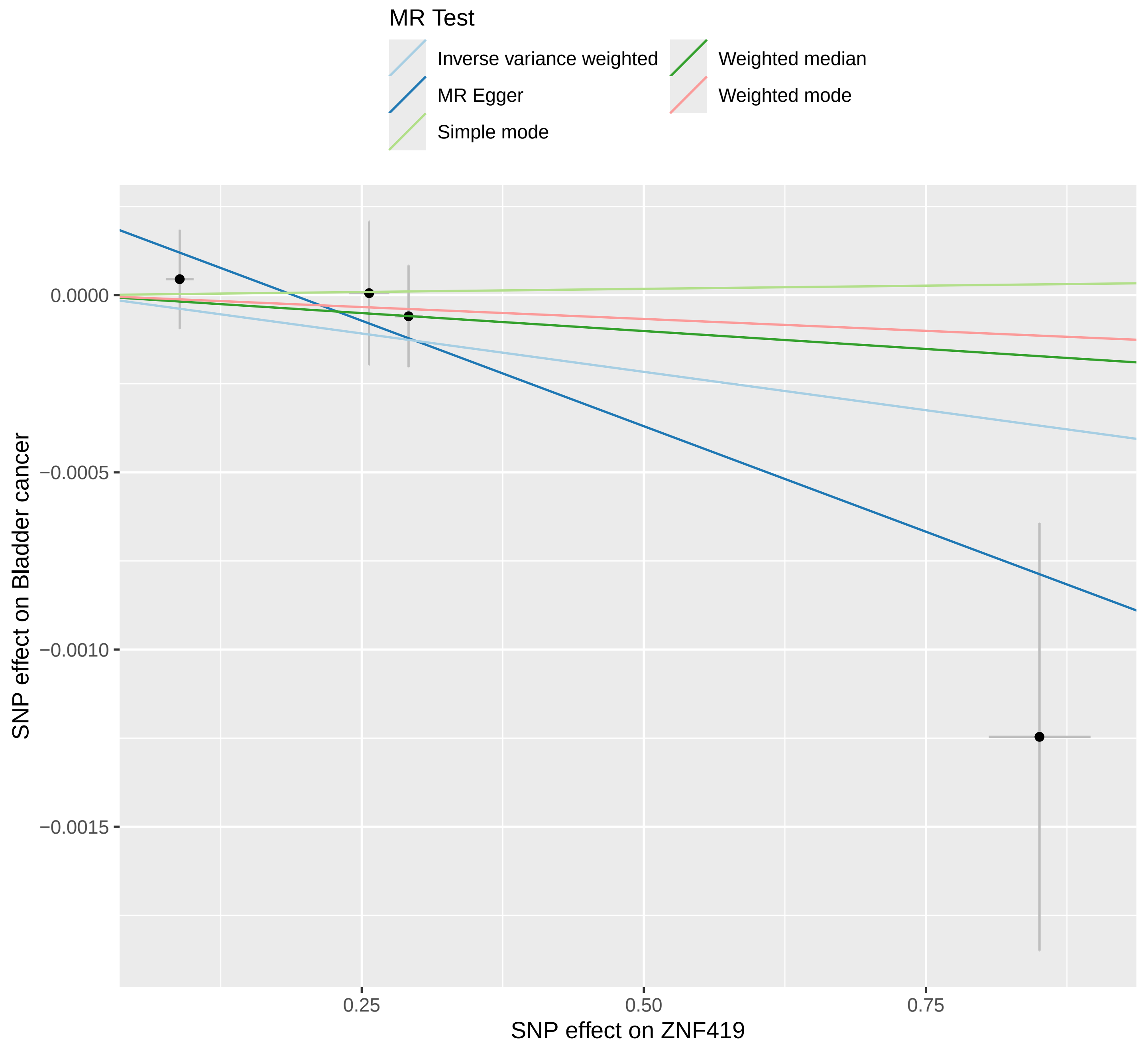

MR Test
Inverse variance weighted
MR Egger
Simple mode
Weighted median
Weighted mode
0.0000
−0.0005
−0.0010
−0.0015
SNP effect on Bladder cancer
0.25
0.50
0.75
SNP effect on ZNF419

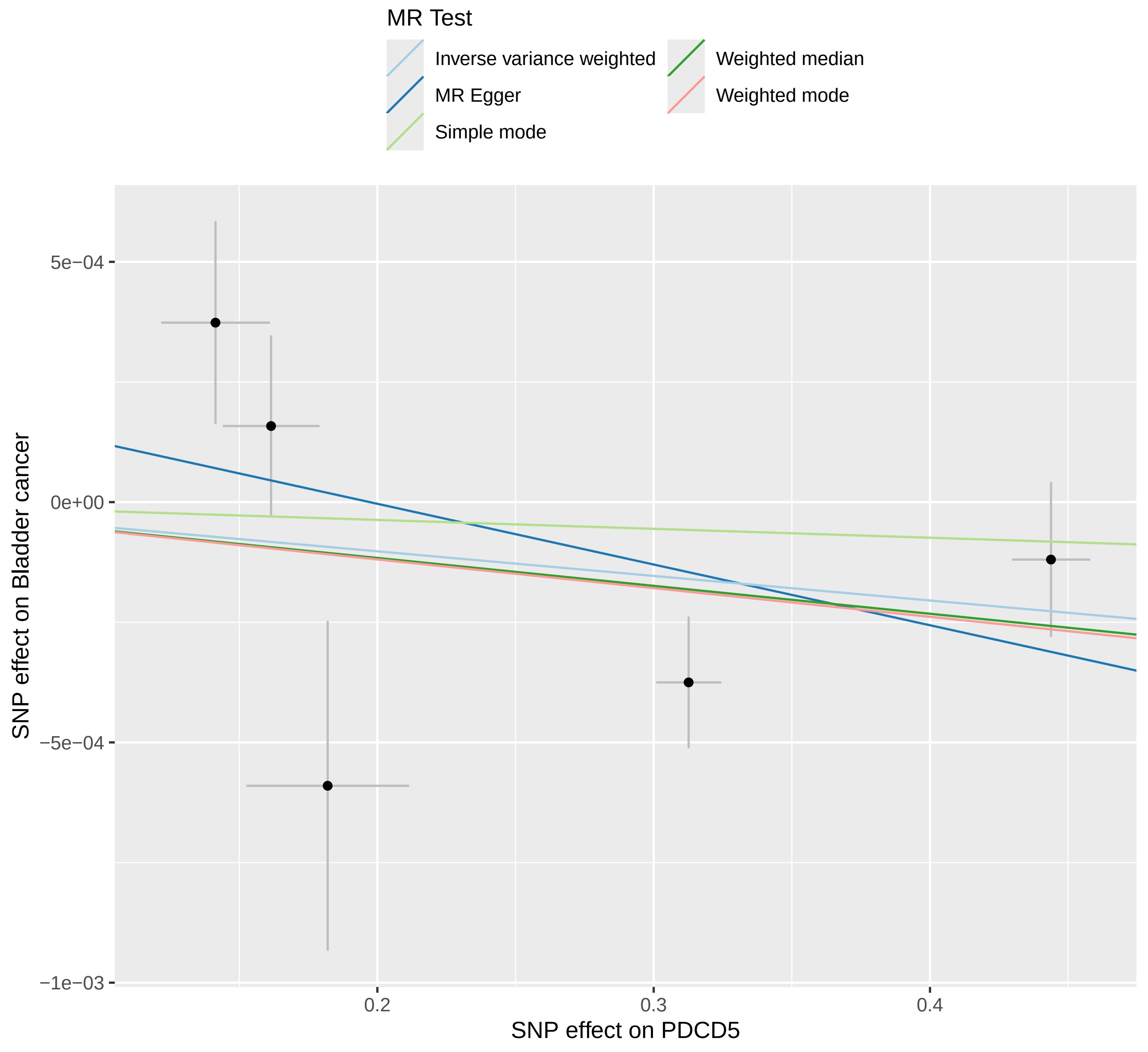
MR Test
Inverse variance weighted
MR Egger
Simple mode
Weighted median
Weighted mode
5e−04
0e+00
−5e−04
−1e−03
SNP effect on Bladder cancer
0.2
0.3
0.4
SNP effect on PDCD5

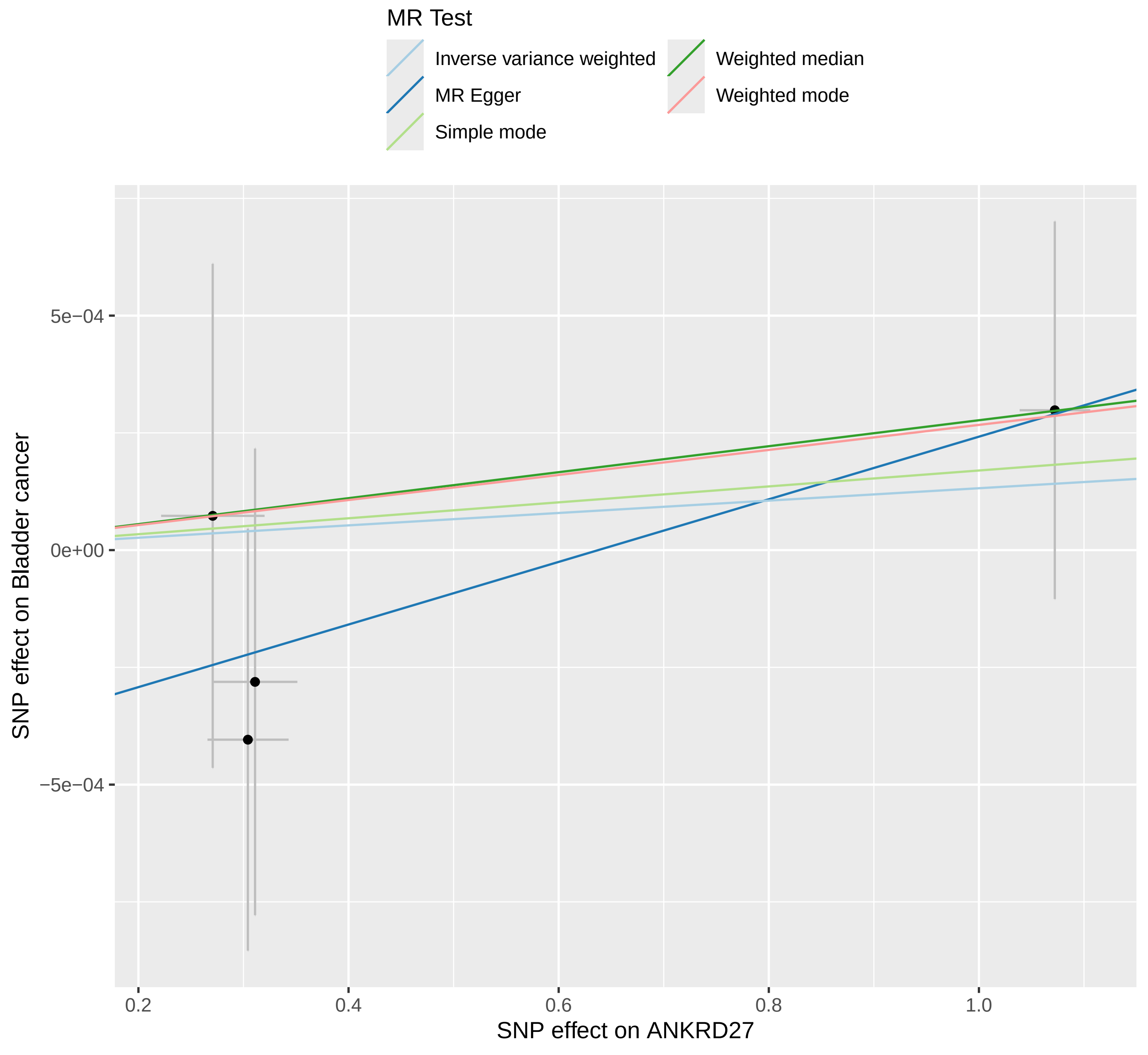

MR Test
Inverse variance weighted
MR Egger
Simple mode
Weighted median
Weighted mode
5e−04
0e+00
−5e−04
SNP effect on Bladder cancer
0.2
0.4
0.6
0.8
1.0
SNP effect on ANKRD27

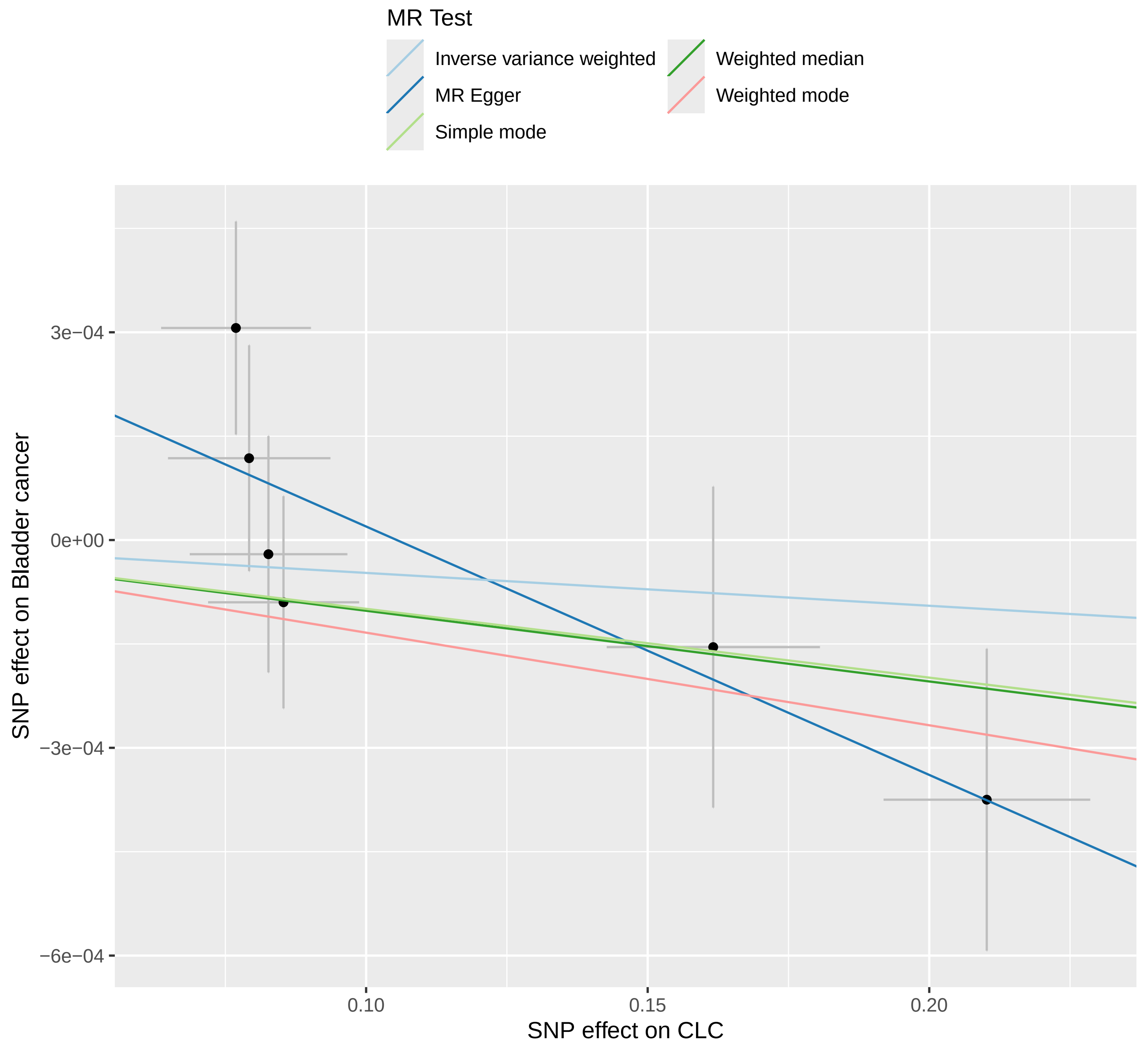
MR Test
Inverse variance weighted
MR Egger
Simple mode
Weighted median
Weighted mode
3e−04
0e+00
−3e−04
−6e−04
SNP effect on Bladder cancer
0.10
0.15
0.20
SNP effect on CLC

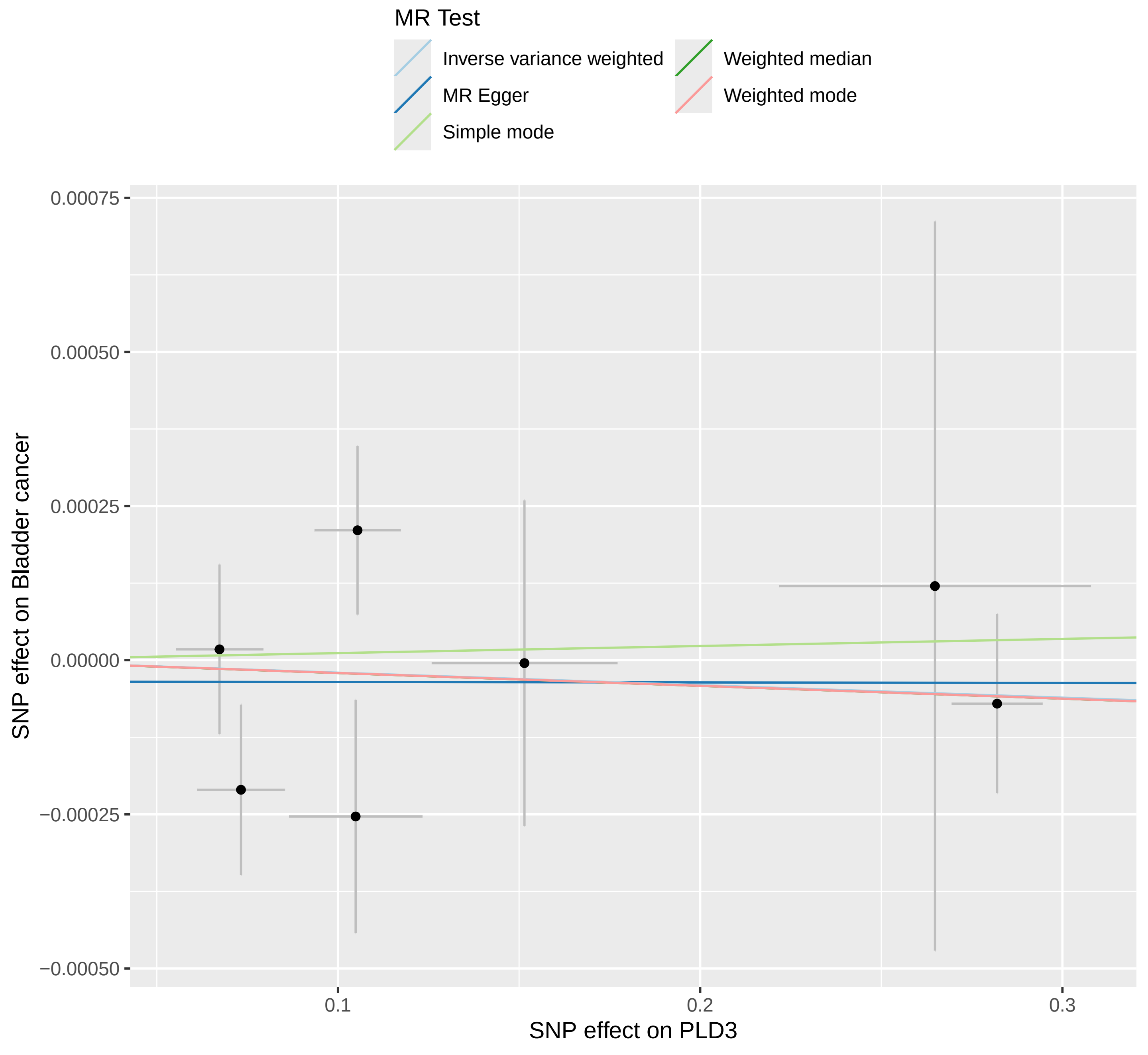

MR Test
Inverse variance weighted
MR Egger
Simple mode
Weighted median
Weighted mode
0.00075
0.00050
0.00025
0.00000
−0.00025
−0.00050
SNP effect on Bladder cancer
0.1
0.2
0.3
SNP effect on PLD3

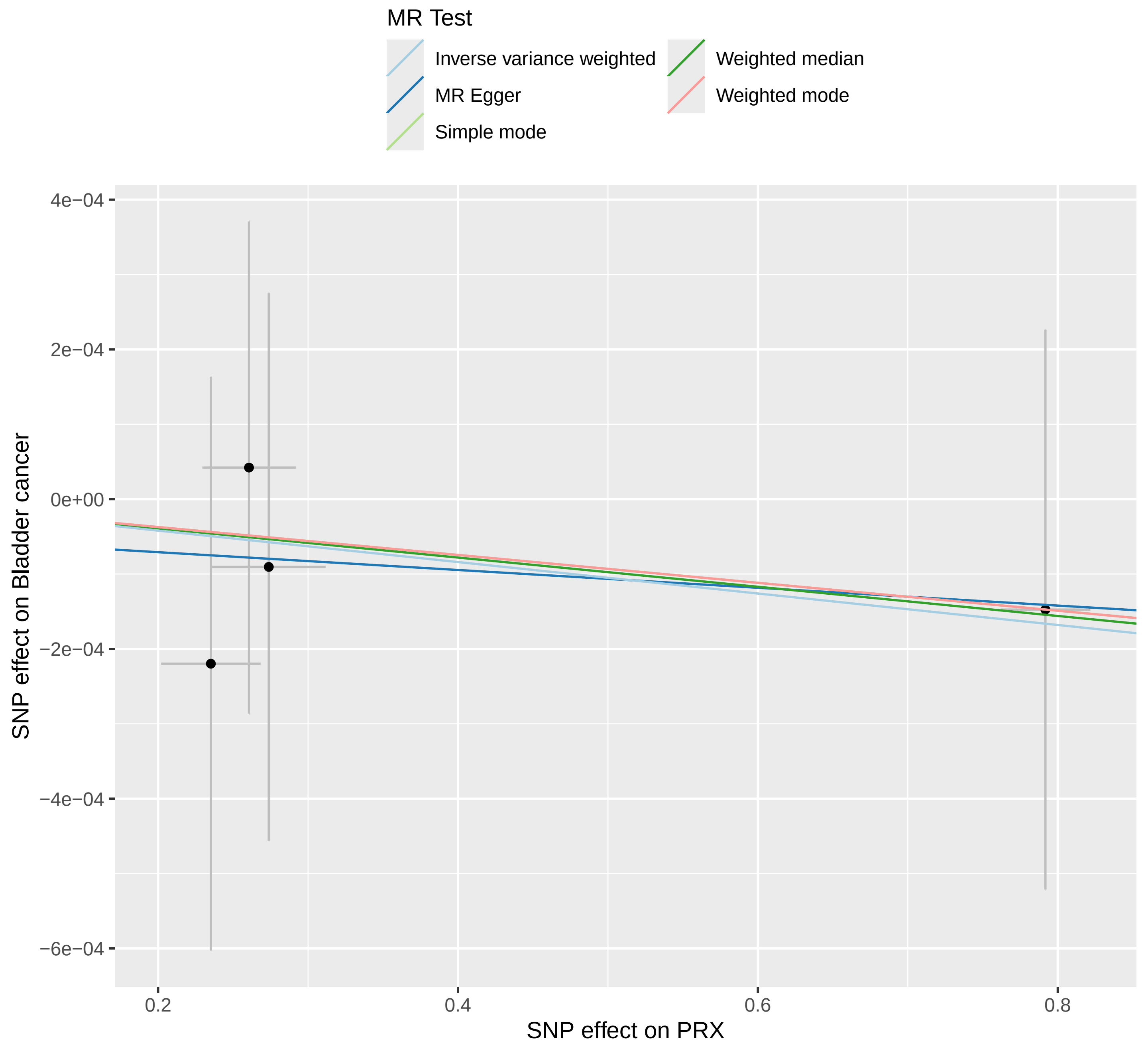

MR Test
Inverse variance weighted
MR Egger
Simple mode
Weighted median
Weighted mode
4e−04
2e−04
0e+00
−2e−04
−4e−04
−6e−04
SNP effect on Bladder cancer
0.2
0.4
0.6
0.8
SNP effect on PRX

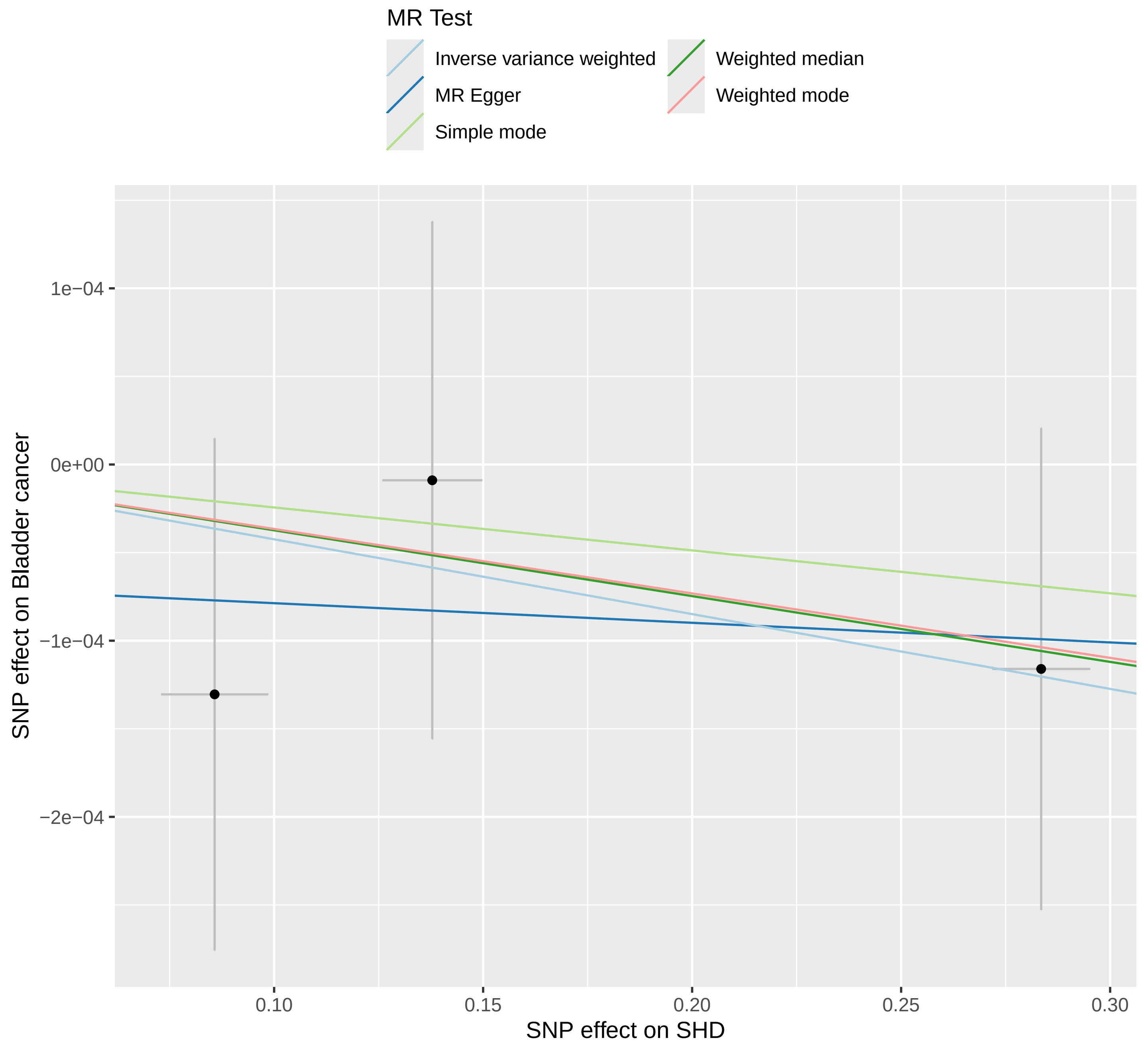

MR Test
Inverse variance weighted
MR Egger
Simple mode
Weighted median
Weighted mode
1e−04
0e+00
−1e−04
−2e−04
SNP effect on Bladder cancer
0.10
0.15
0.20
0.25
0.30
SNP effect on SHD

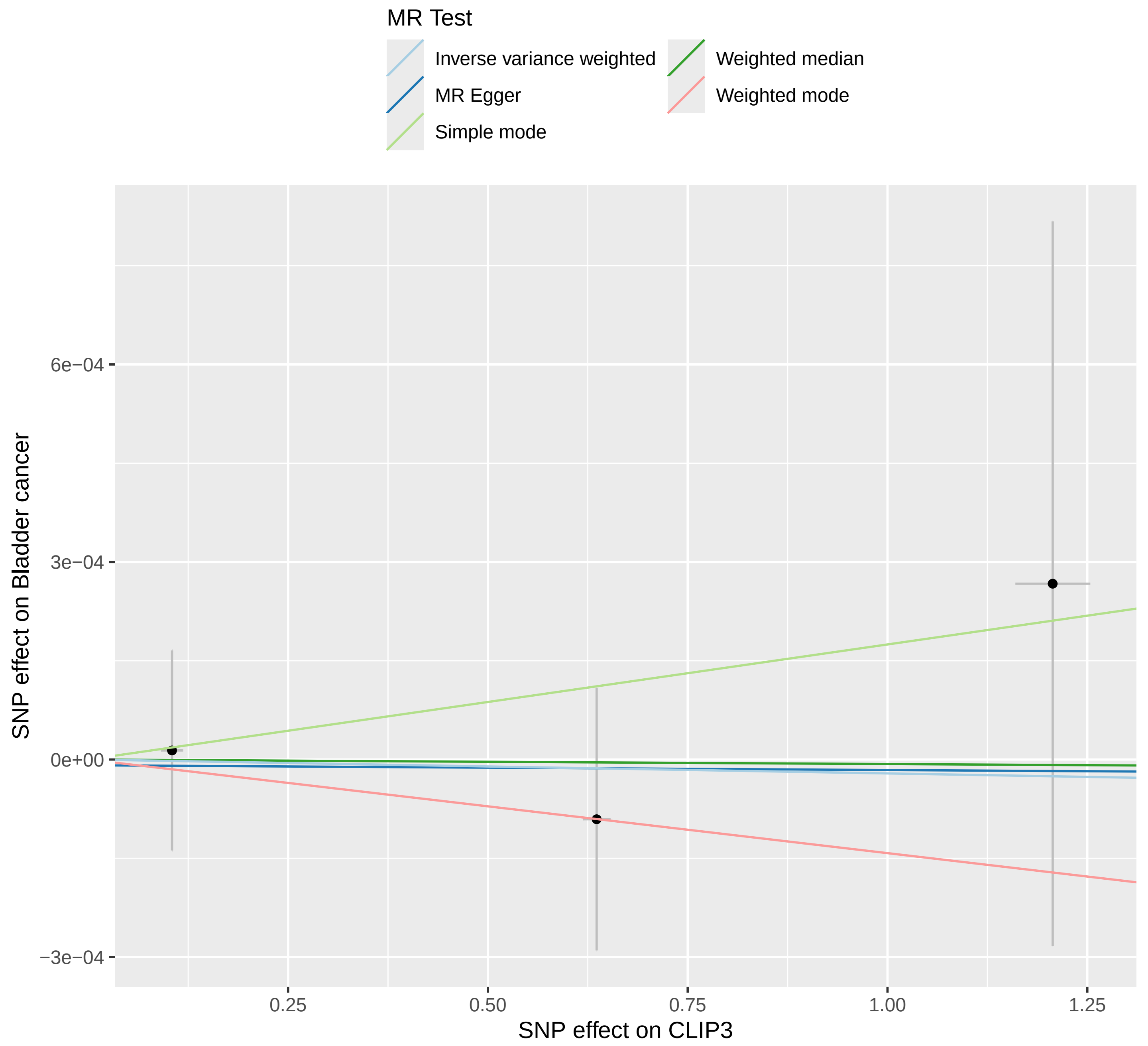

MR Test
Inverse variance weighted
MR Egger
Simple mode
Weighted median
Weighted mode
6e−04
3e−04
0e+00
−3e−04
SNP effect on Bladder cancer
0.25
0.50
0.75
1.00
1.25
SNP effect on CLIP3

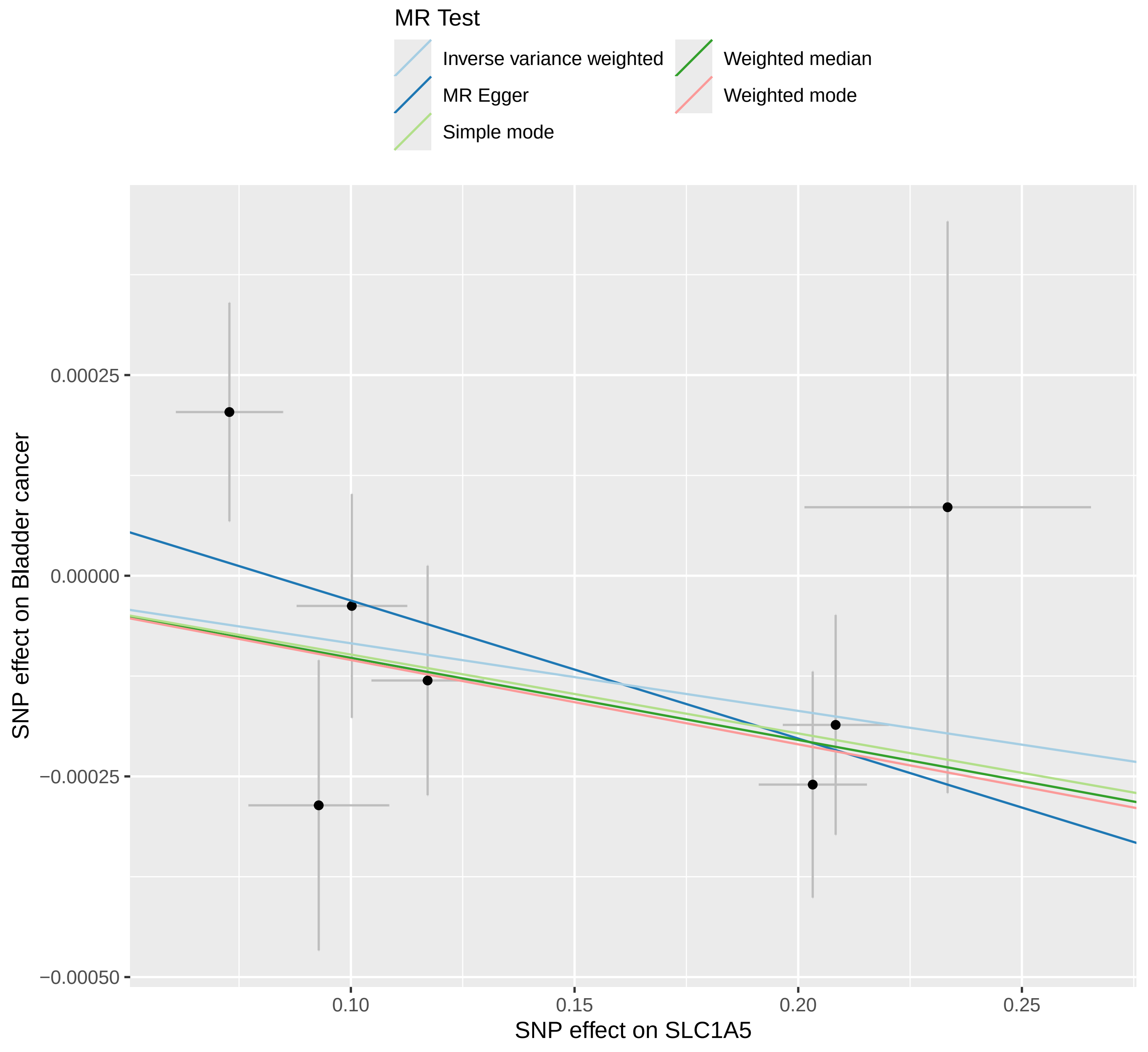
MR Test
Inverse variance weighted
MR Egger
Simple mode
Weighted median
Weighted mode
0.00025
0.00000
−0.00025
−0.00050
SNP effect on Bladder cancer
0.10
0.15
0.20
0.25
SNP effect on SLC1A5

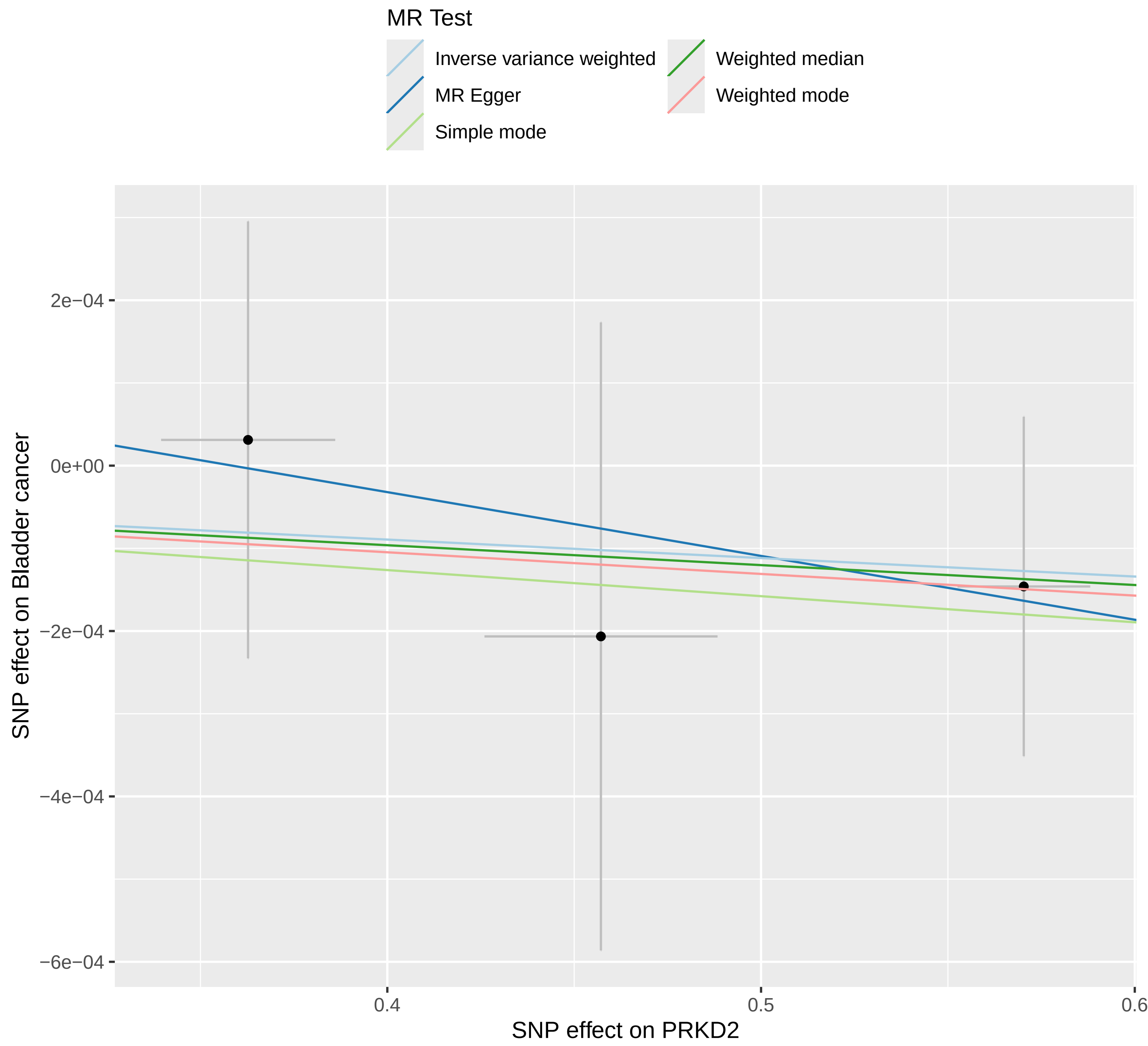

MR Test
Inverse variance weighted
MR Egger
Simple mode
Weighted median
Weighted mode
2e−04
0e+00
−2e−04
−4e−04
−6e−04
SNP effect on Bladder cancer
0.4
0.5
0.6
SNP effect on PRKD2

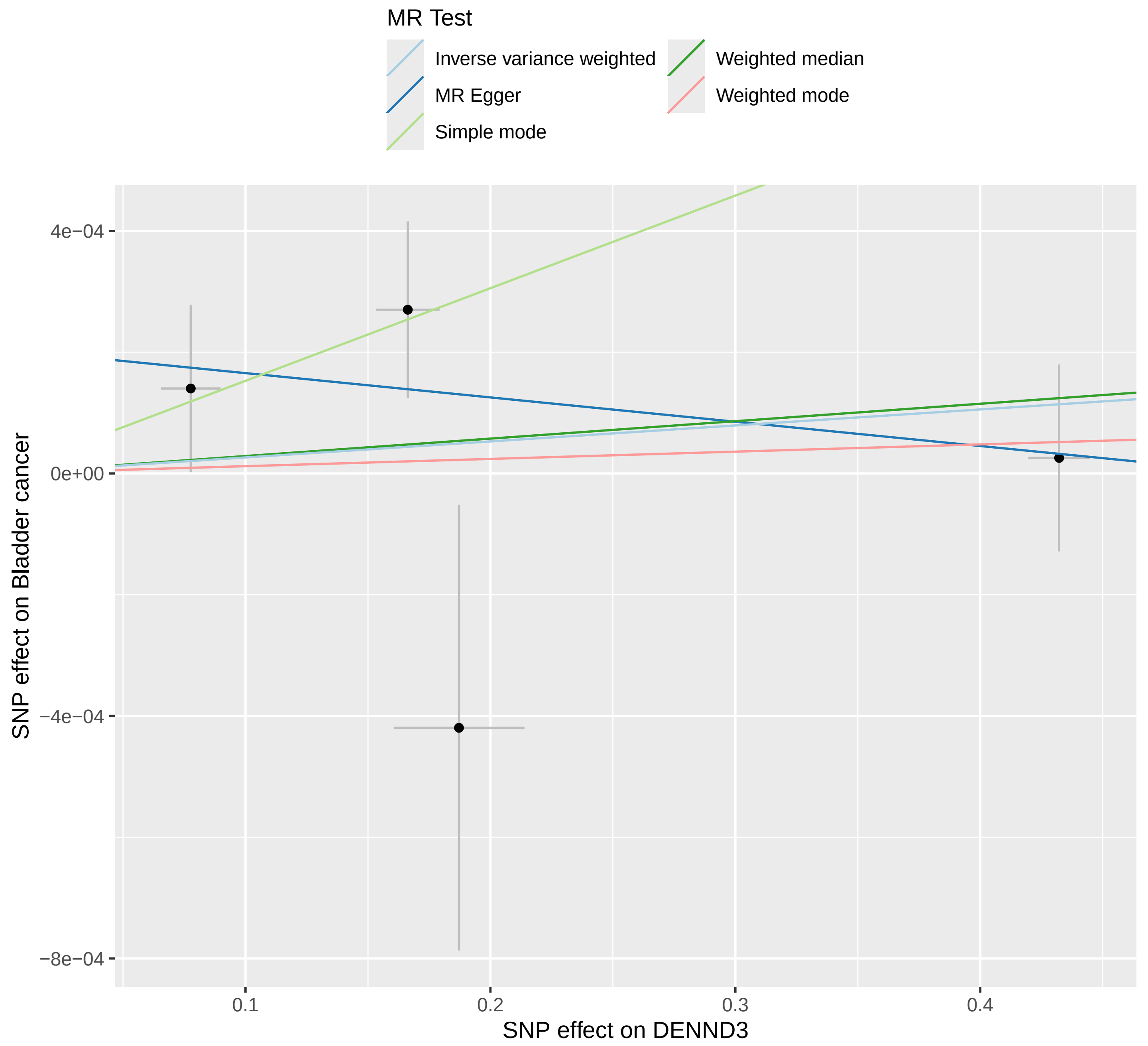

MR Test
Inverse variance weighted
MR Egger
Simple mode
Weighted median
Weighted mode
4e−04
0e+00
−4e−04
−8e−04
SNP effect on Bladder cancer
0.1
0.2
0.3
0.4
SNP effect on DENND3

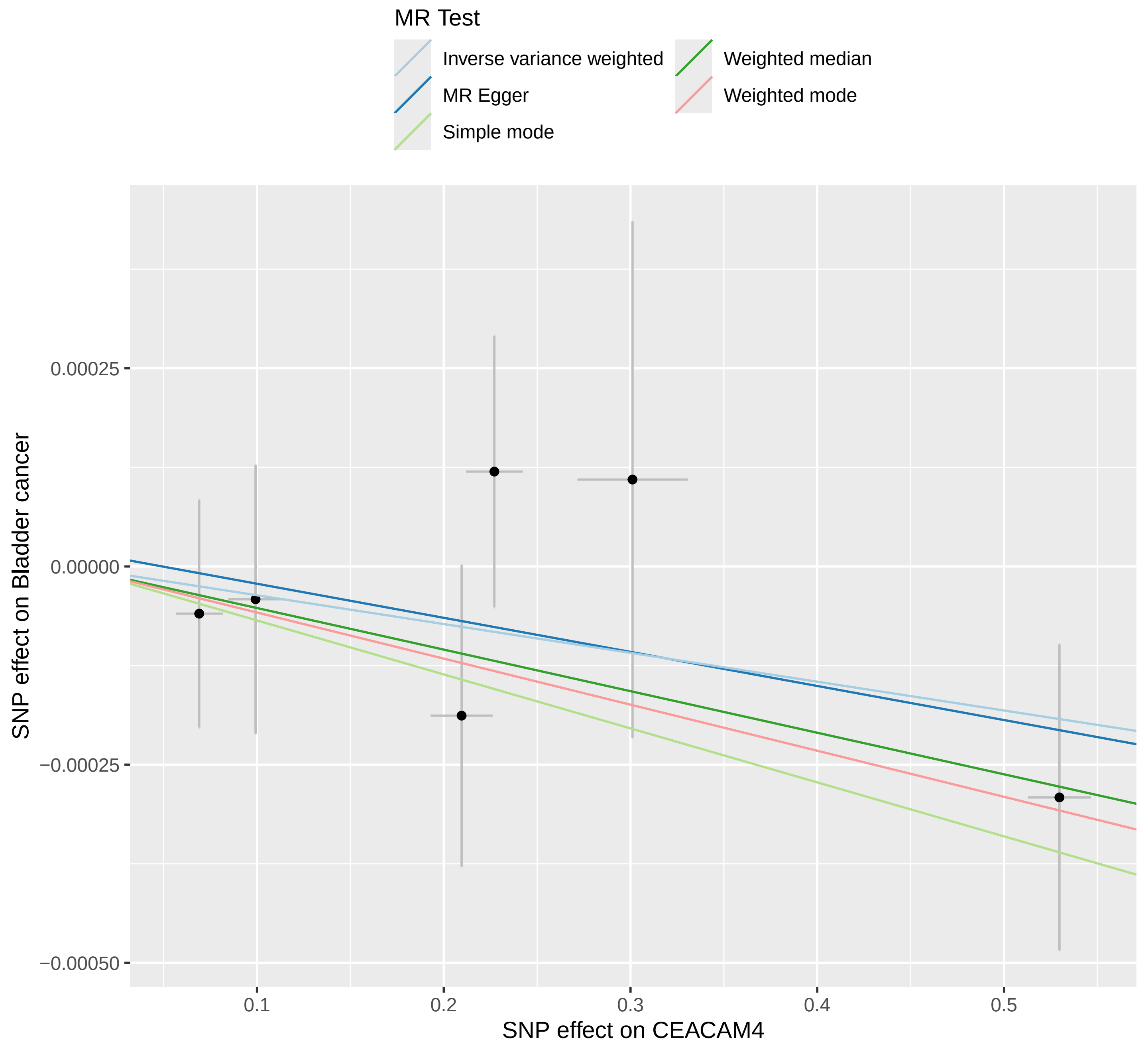
MR Test
Inverse variance weighted
MR Egger
Simple mode
Weighted median
Weighted mode
0.00025
0.00000
−0.00025
−0.00050
SNP effect on Bladder cancer
0.1
0.2
0.3
0.4
0.5
SNP effect on CEACAM4

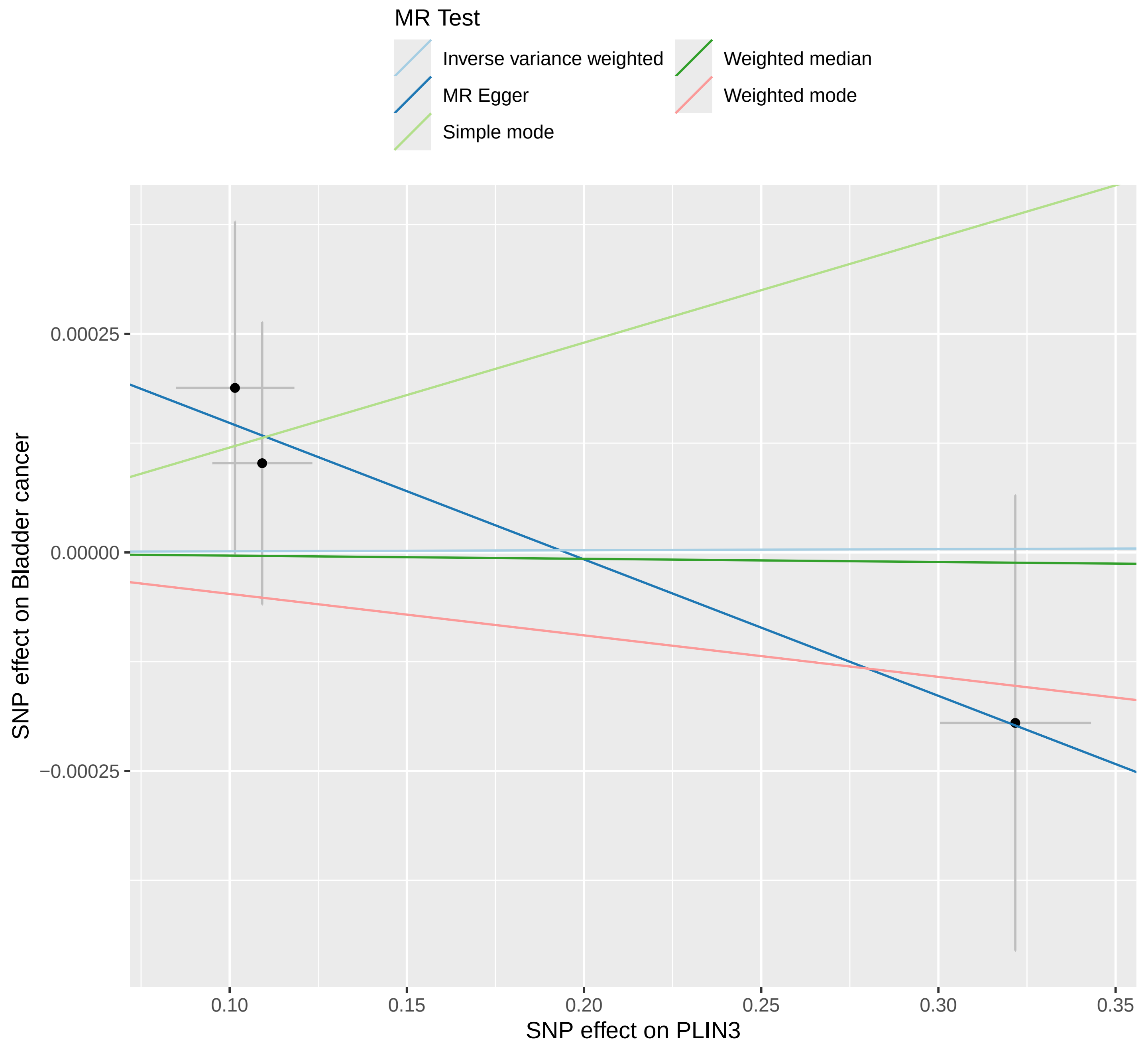
MR Test
Inverse variance weighted
MR Egger
Simple mode
Weighted median
Weighted mode
0.00025
0.00000
−0.00025
0.10
0.15
0.20
0.25
0.30
0.35
SNP effect on PLIN3
SNP effect on Bladder cancer

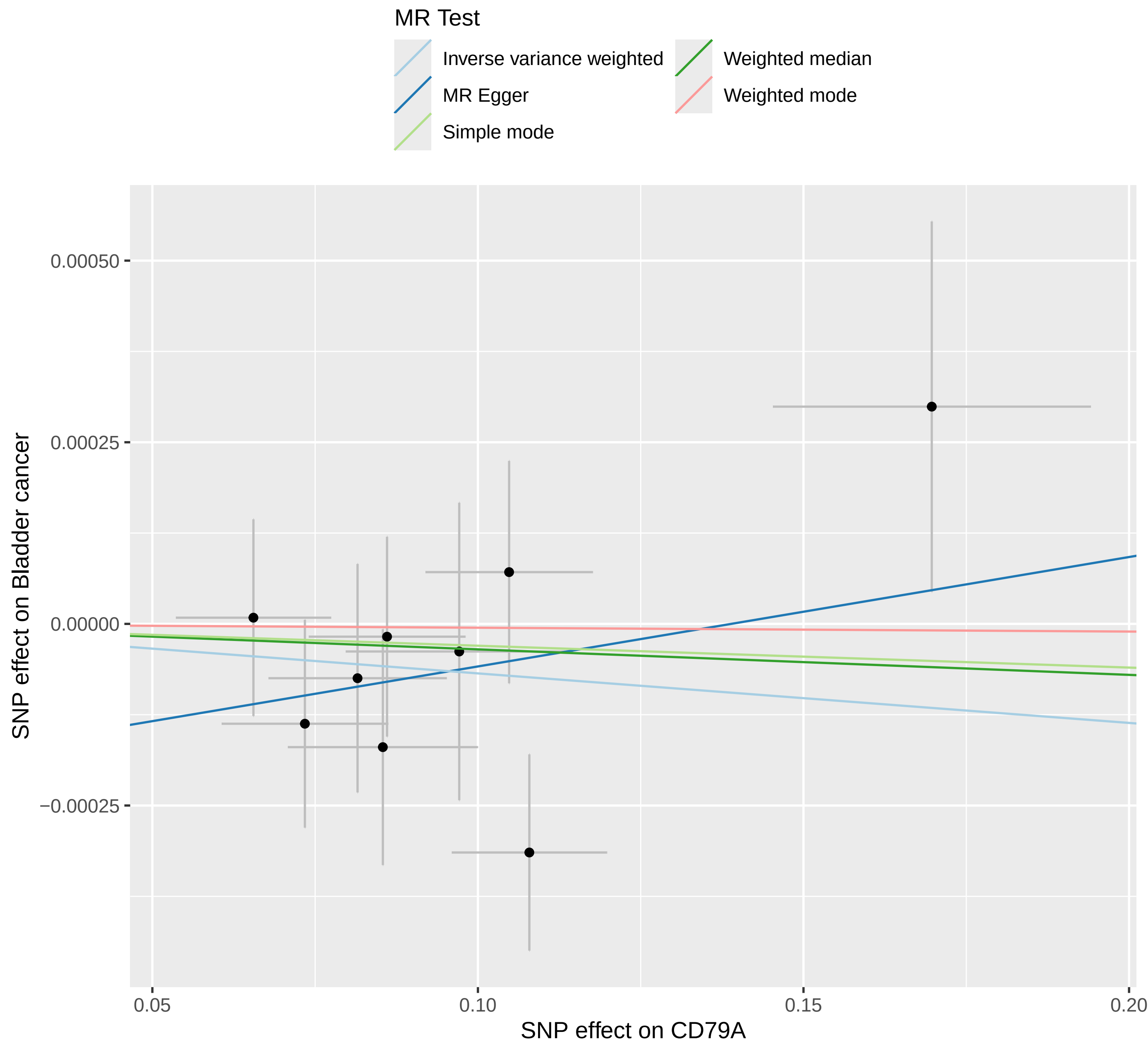
MR Test
Inverse variance weighted
MR Egger
Simple mode
Weighted median
Weighted mode
0.00050
0.00025
0.00000
−0.00025
SNP effect on Bladder cancer
0.05
0.10
0.15
0.20
SNP effect on CD79A

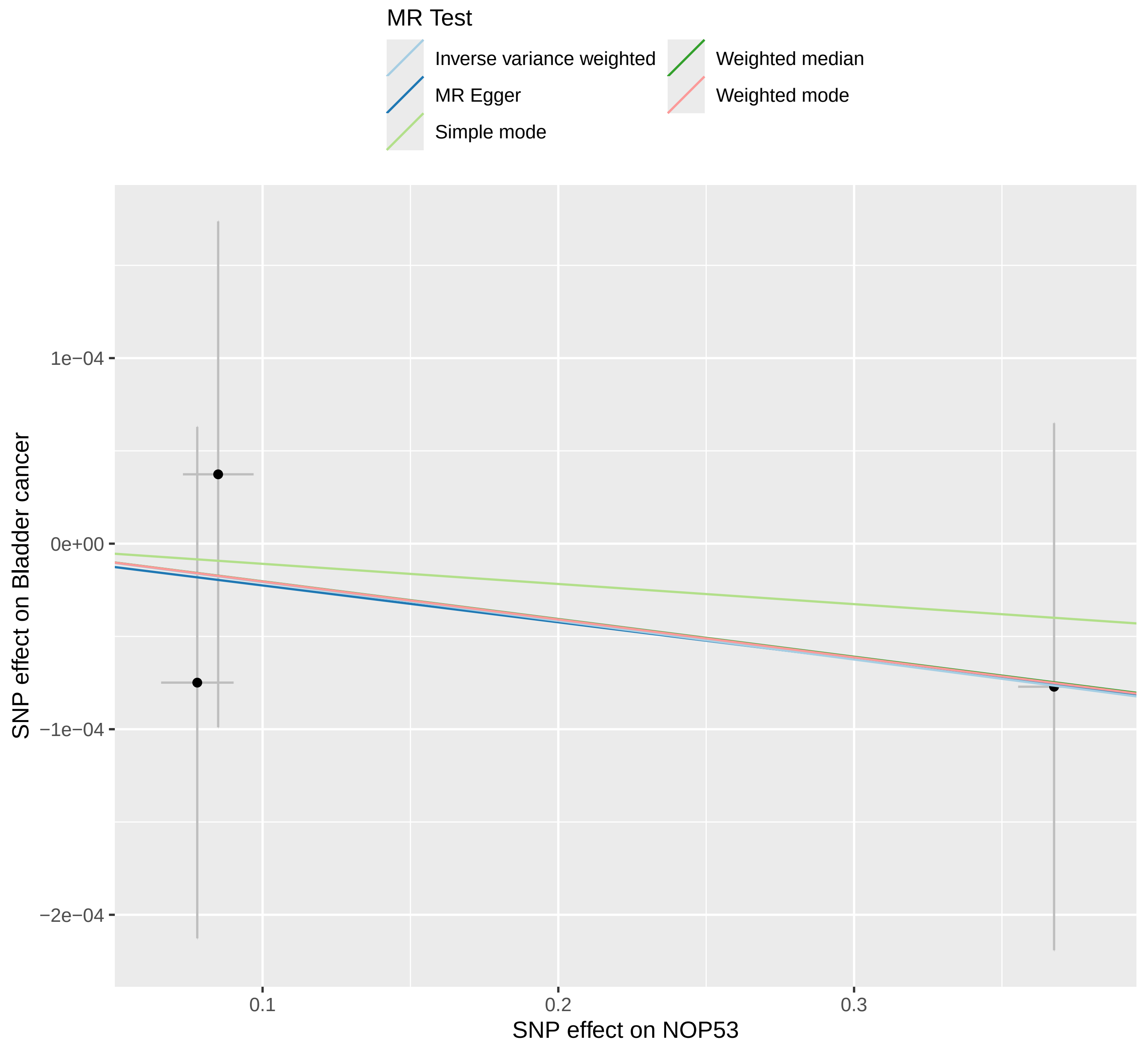
MR Test
Inverse variance weighted
MR Egger
Simple mode
Weighted median
Weighted mode
1e−04
0e+00
−1e−04
−2e−04
SNP effect on Bladder cancer
0.1
0.2
0.3
SNP effect on NOP53

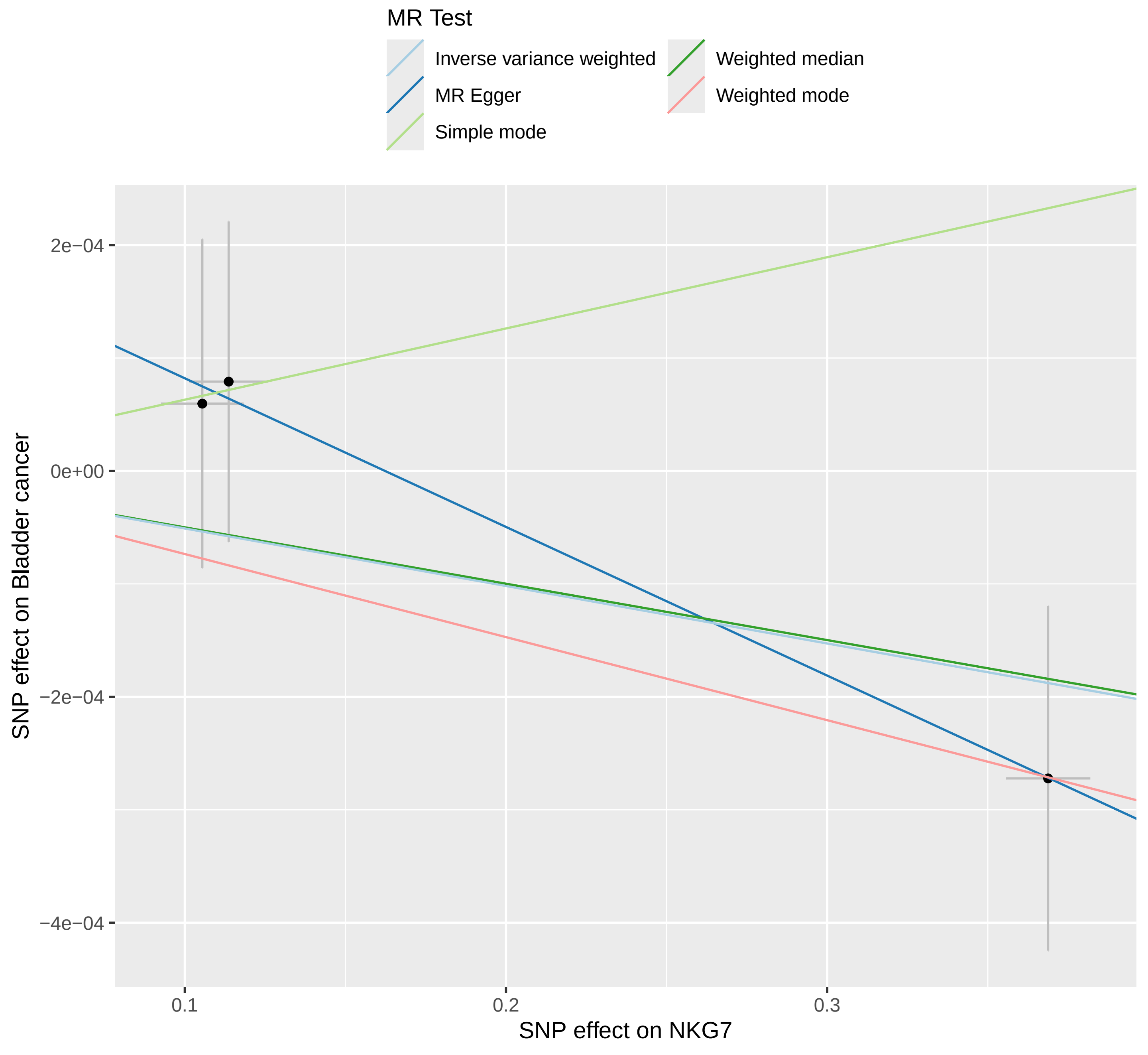

MR Test
Inverse variance weighted
MR Egger
Simple mode
Weighted median
Weighted mode
2e−04
0e+00
−2e−04
−4e−04
0.1
0.2
0.3
SNP effect on Bladder cancer
SNP effect on NKG7

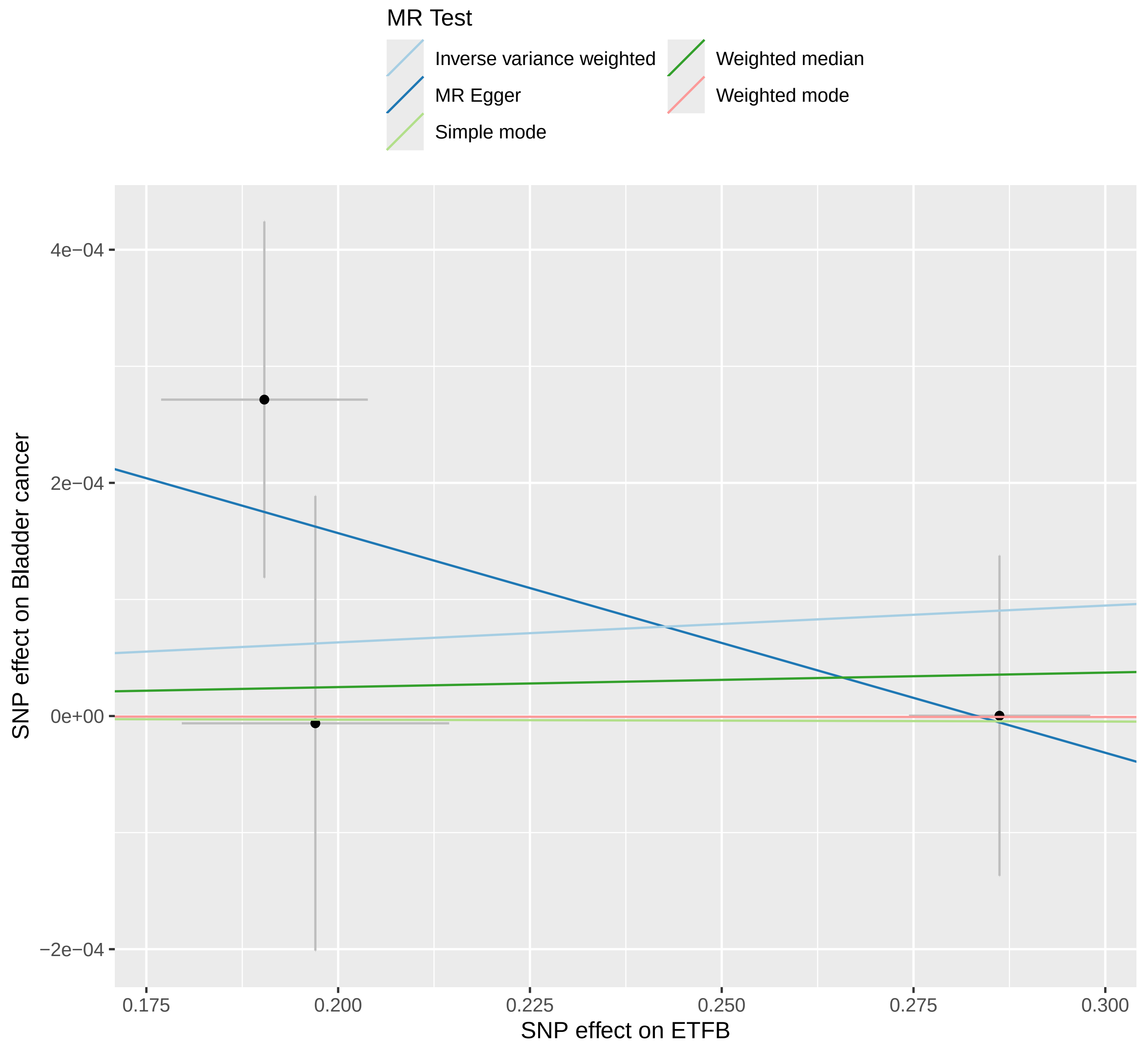
MR Test
Inverse variance weighted
MR Egger
Simple mode
Weighted median
Weighted mode
SNP effect on Bladder cancer
4e−04
2e−04
0e+00
−2e−04
0.175
0.200
0.225
0.250
0.275
0.300
SNP effect on ETFB

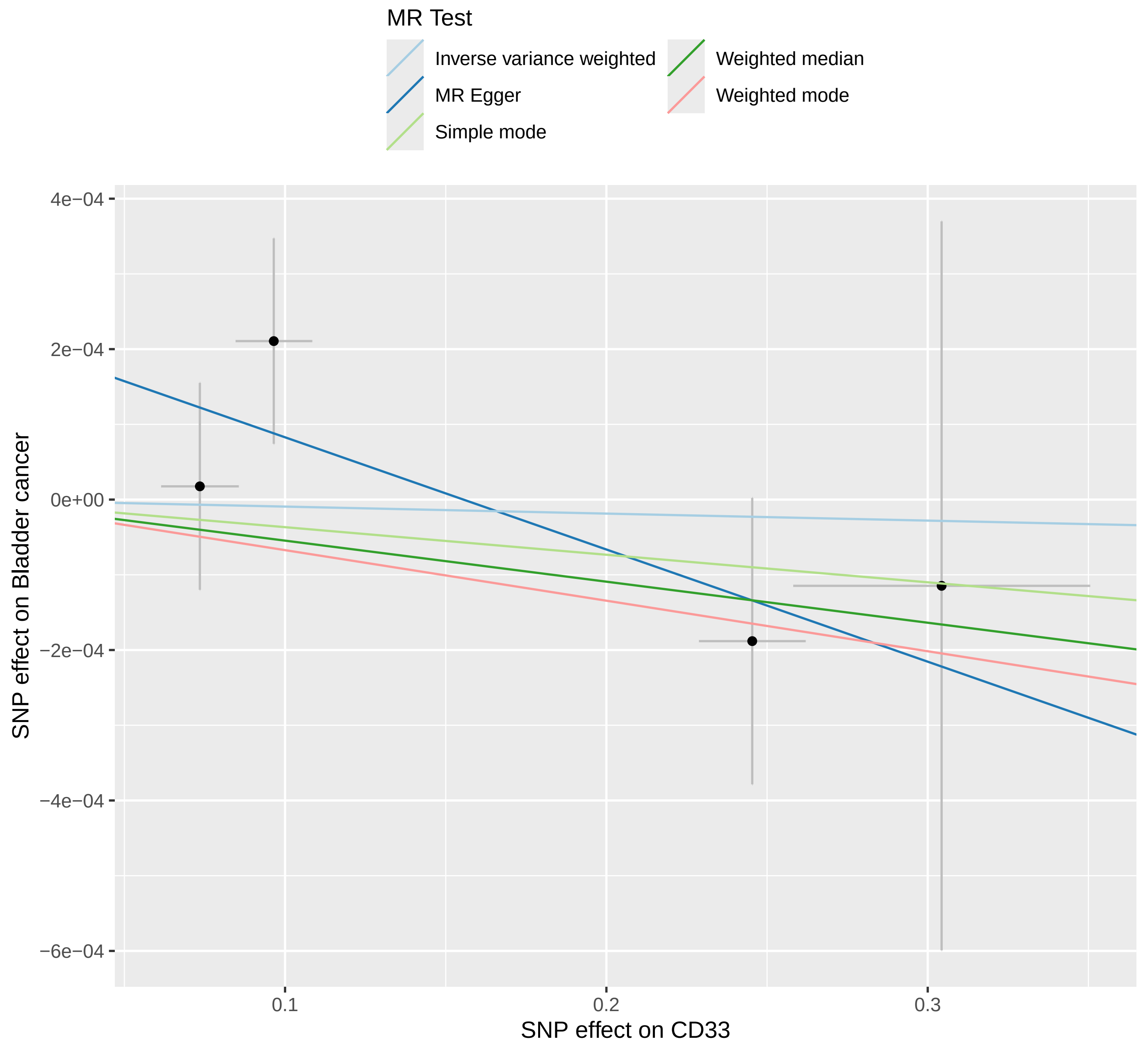
MR Test
Inverse variance weighted
MR Egger
Simple mode
Weighted median
Weighted mode
4e−04
2e−04
0e+00
−2e−04
−4e−04
−6e−04
SNP effect on Bladder cancer
0.1
0.2
0.3
SNP effect on CD33

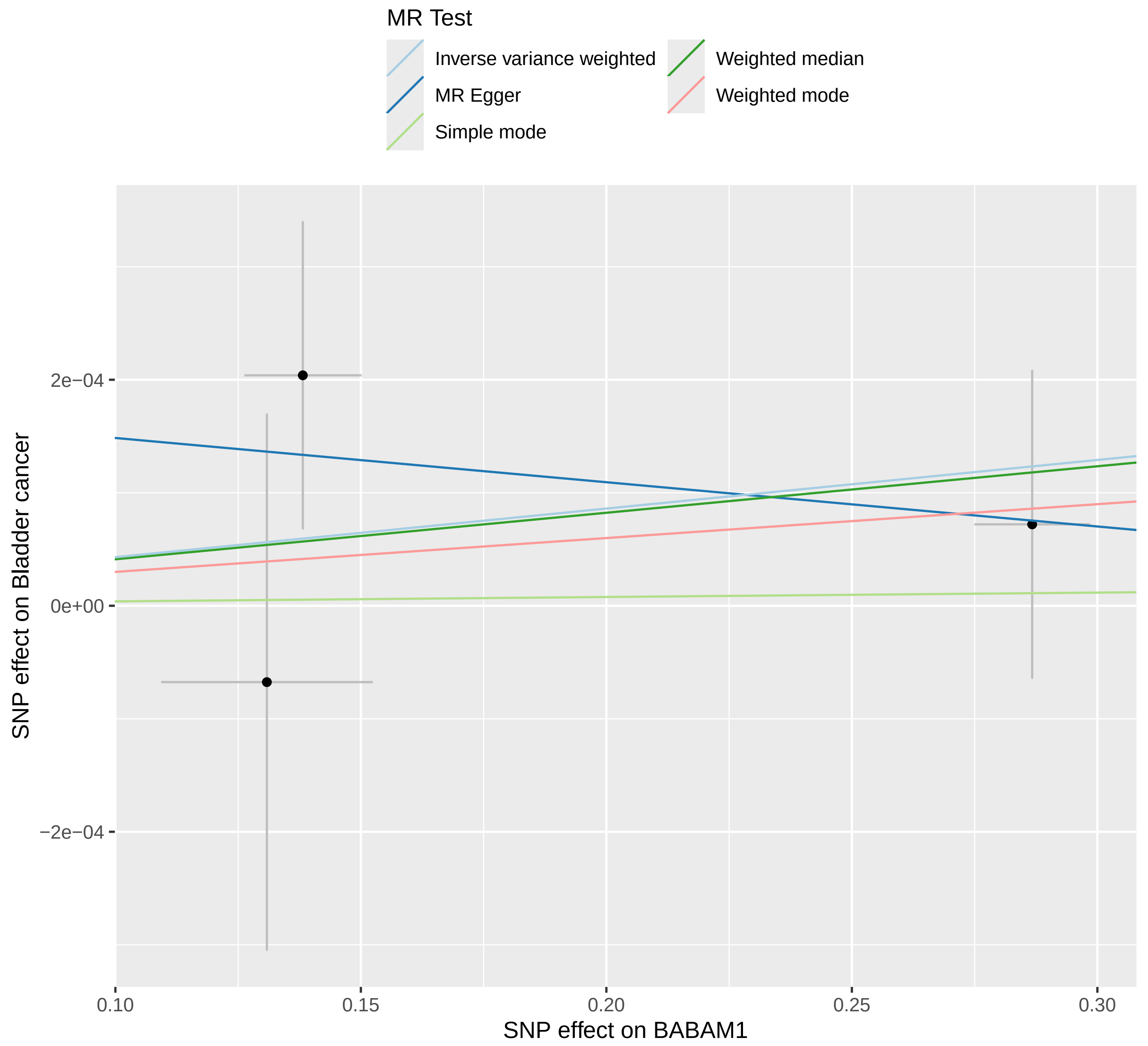

MR Test
Inverse variance weighted
MR Egger
Simple mode
Weighted median
Weighted mode
2e−04
0e+00
−2e−04
SNP effect on Bladder cancer
0.10
0.15
0.20
0.25
0.30
SNP effect on BABAM1

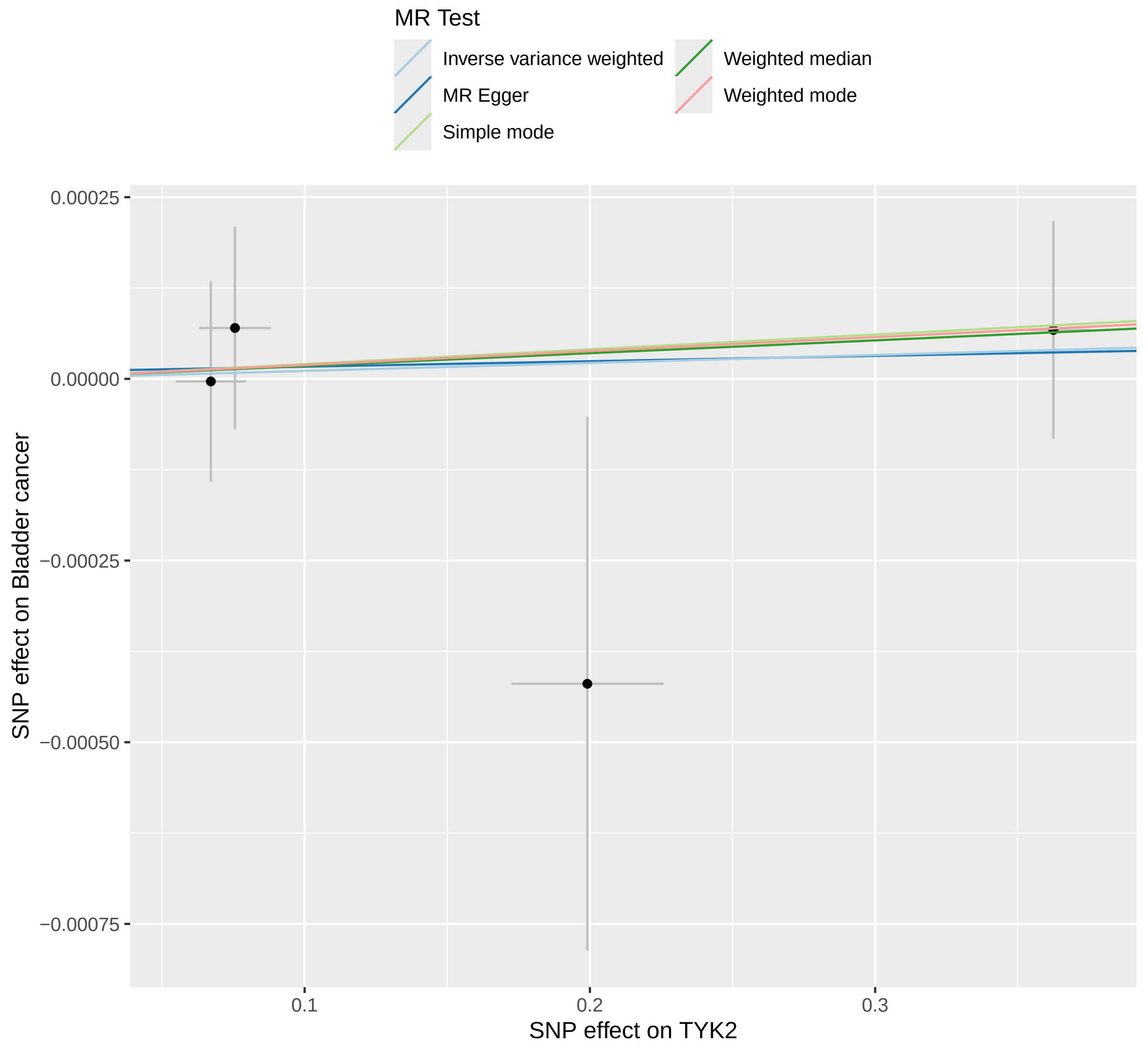

MR Test
Inverse variance weighted
MR Egger
Simple mode
Weighted median
Weighted mode
0.00025
0.00000
−0.00025
−0.00050
−0.00075
SNP effect on Bladder cancer
0.1
0.2
0.3
SNP effect on TYK2

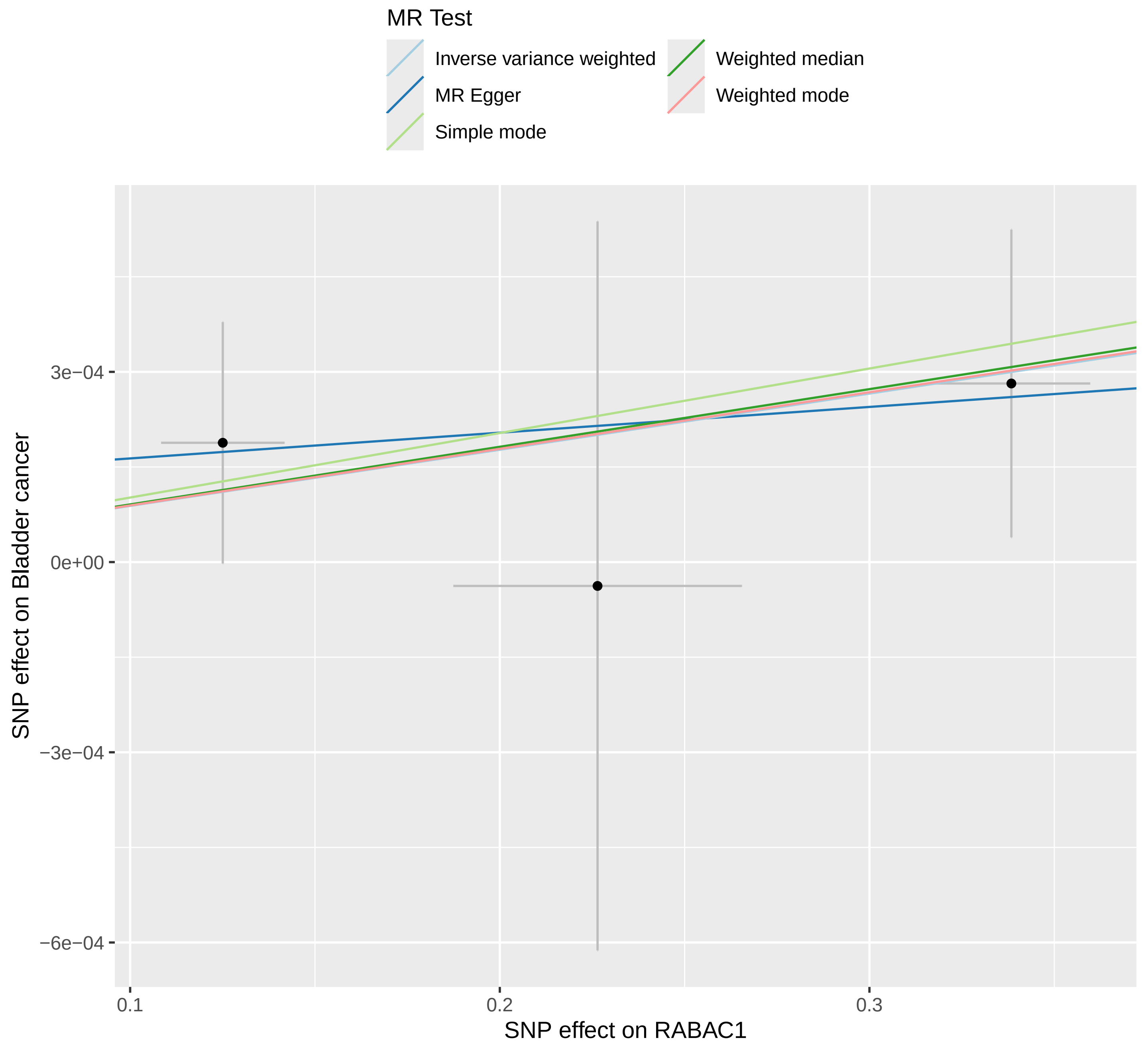

MR Test
Inverse variance weighted
MR Egger
Simple mode
Weighted median
Weighted mode
3e−04
0e+00
−3e−04
−6e−04
SNP effect on Bladder cancer
0.1
0.2
0.3
SNP effect on RABAC1

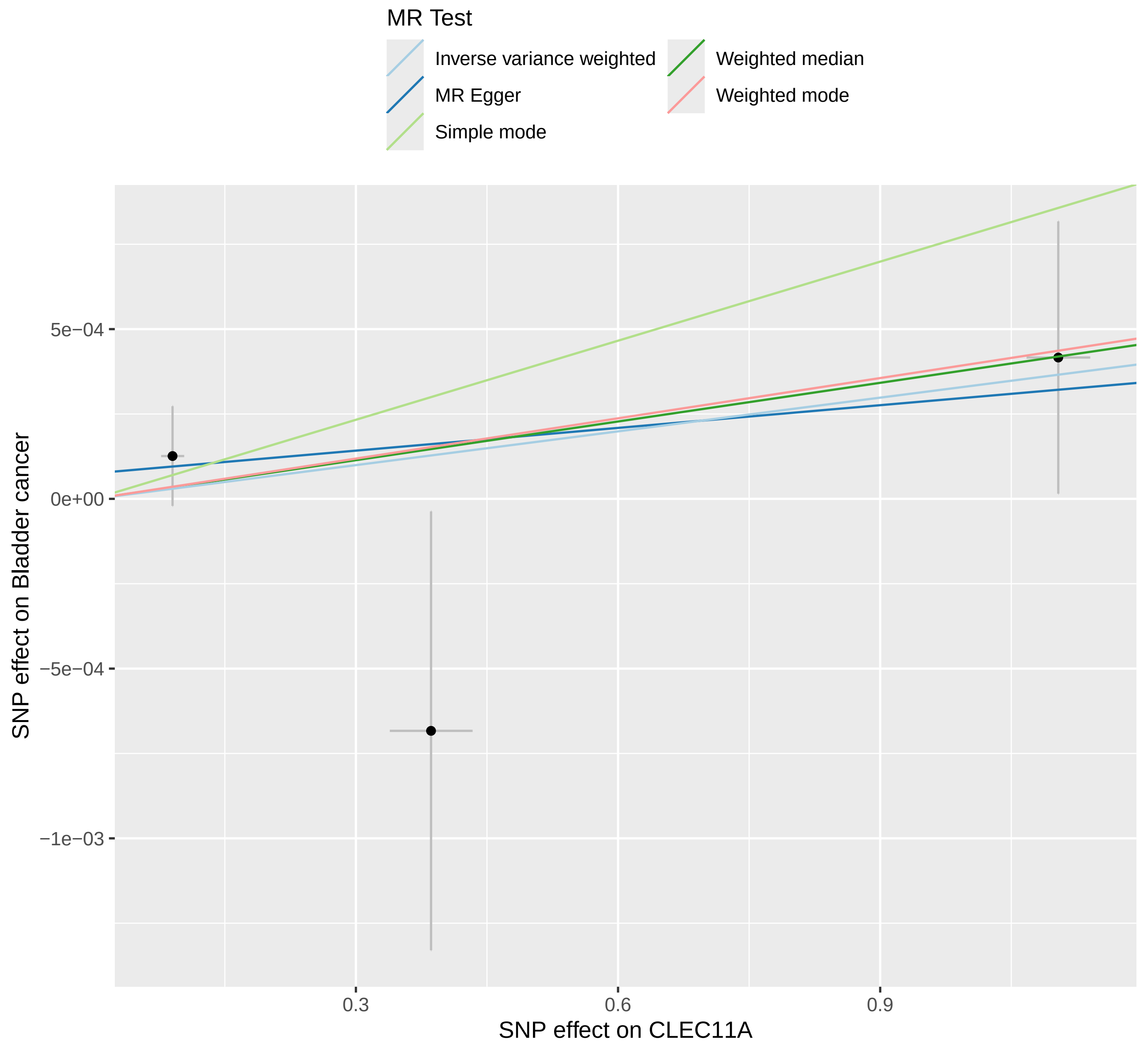

MR Test
Inverse variance weighted
MR Egger
Simple mode
Weighted median
Weighted mode
5e−04
0e+00
−5e−04
−1e−03
SNP effect on Bladder cancer
0.3
0.6
0.9
SNP effect on CLEC11A

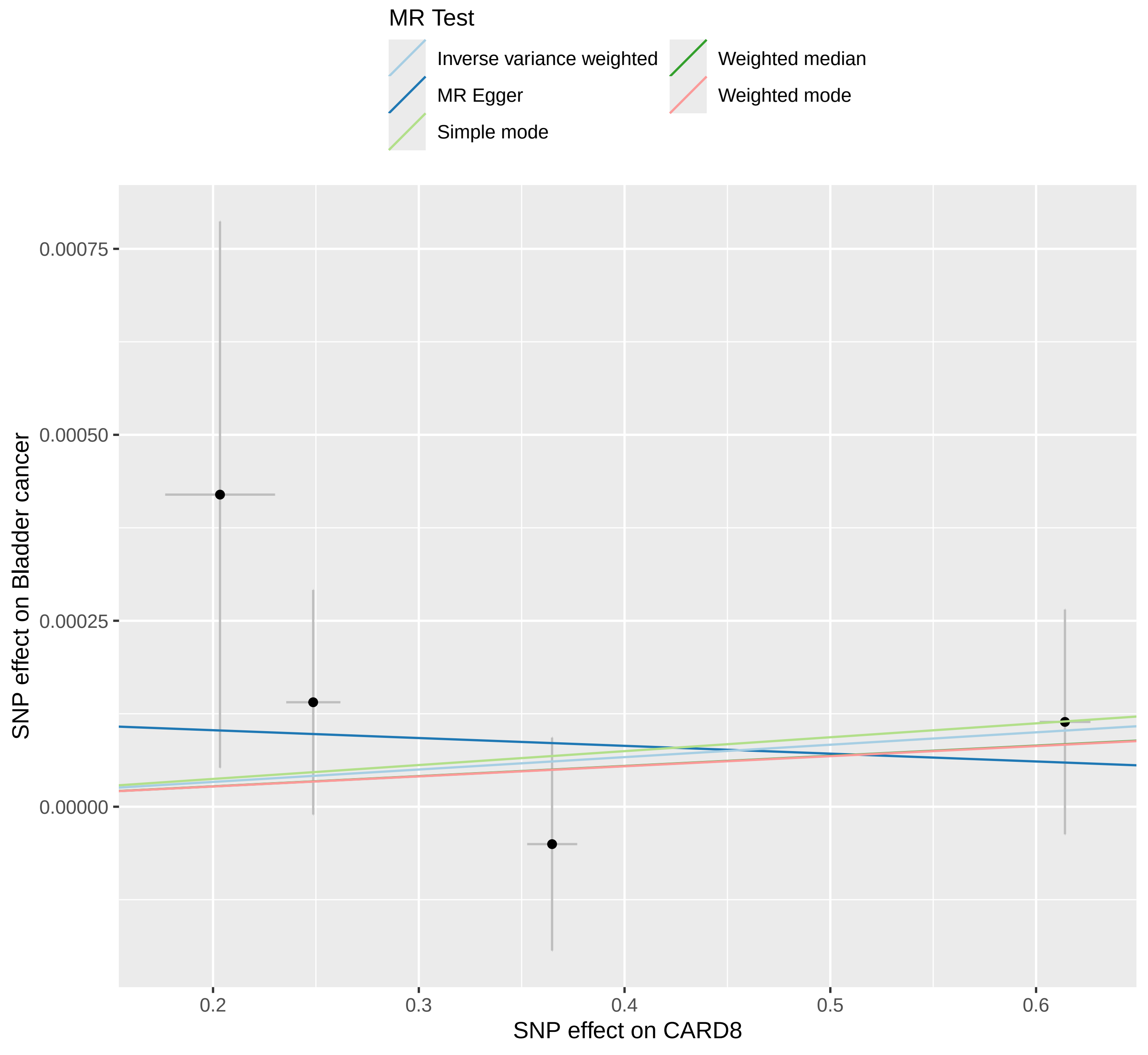

MR Test
Inverse variance weighted
MR Egger
Simple mode
Weighted median
Weighted mode
0.00075
0.00050
0.00025
0.00000
SNP effect on Bladder cancer
0.2
0.3
0.4
0.5
0.6
SNP effect on CARD8

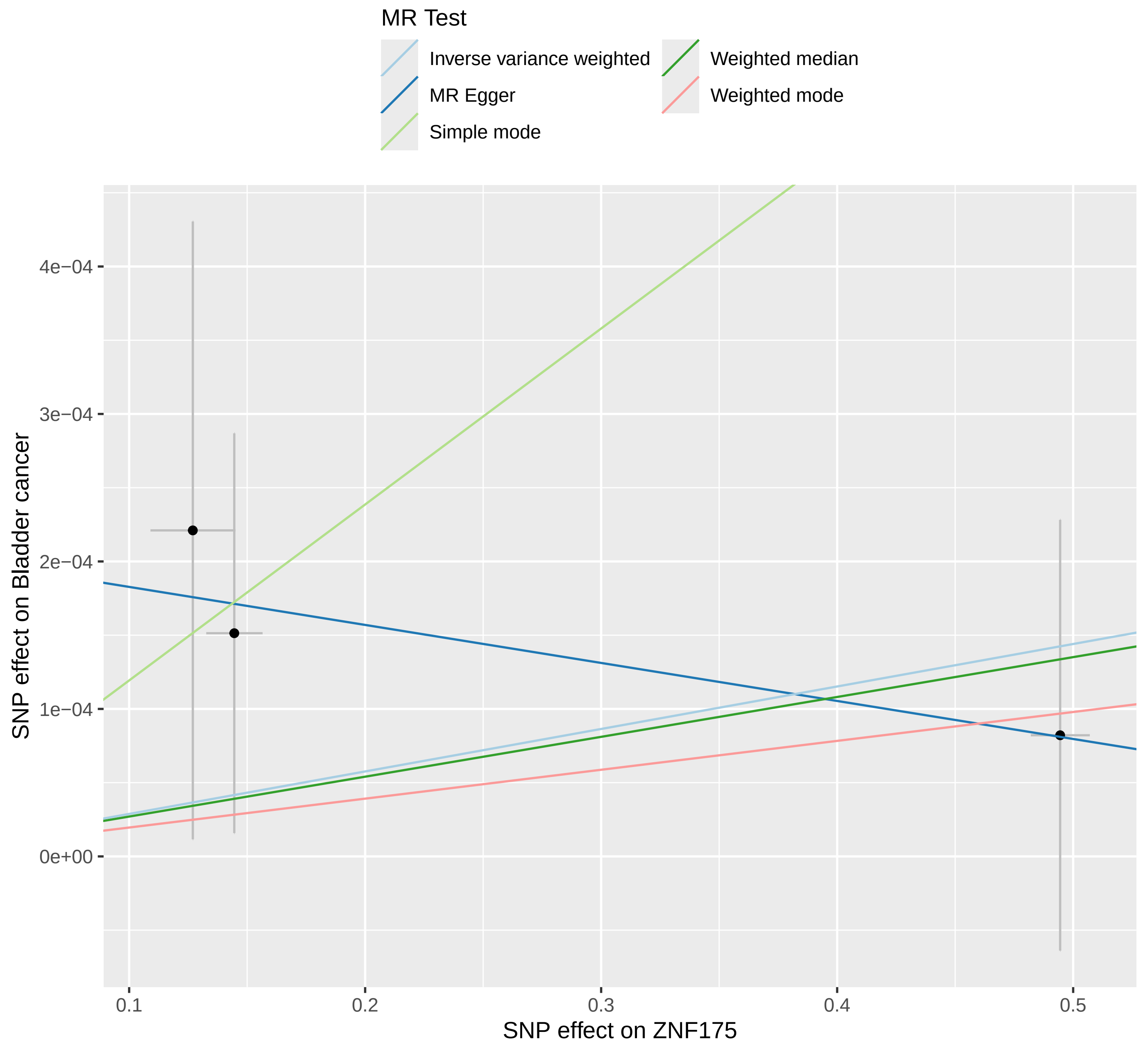

MR Test
Inverse variance weighted
MR Egger
Simple mode
Weighted median
Weighted mode
4e−04
3e−04
2e−04
1e−04
0e+00
SNP effect on Bladder cancer
0.1
0.2
0.3
0.4
0.5
SNP effect on ZNF175

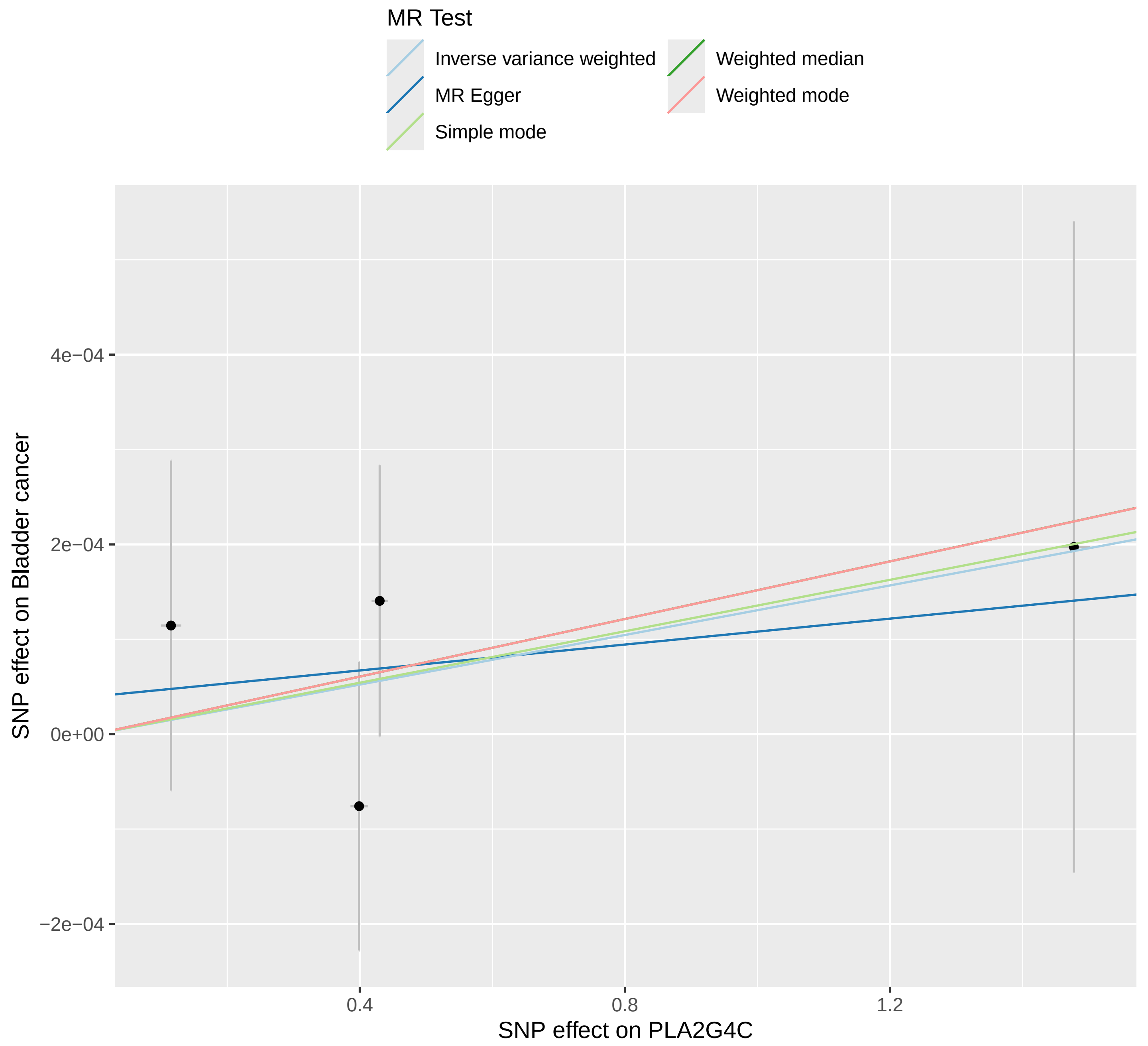
MR Test
Inverse variance weighted
MR Egger
Simple mode
Weighted median
Weighted mode
4e−04
2e−04
0e+00
−2e−04
0.4
0.8
1.2
SNP effect on Bladder cancer
SNP effect on PLA2G4C

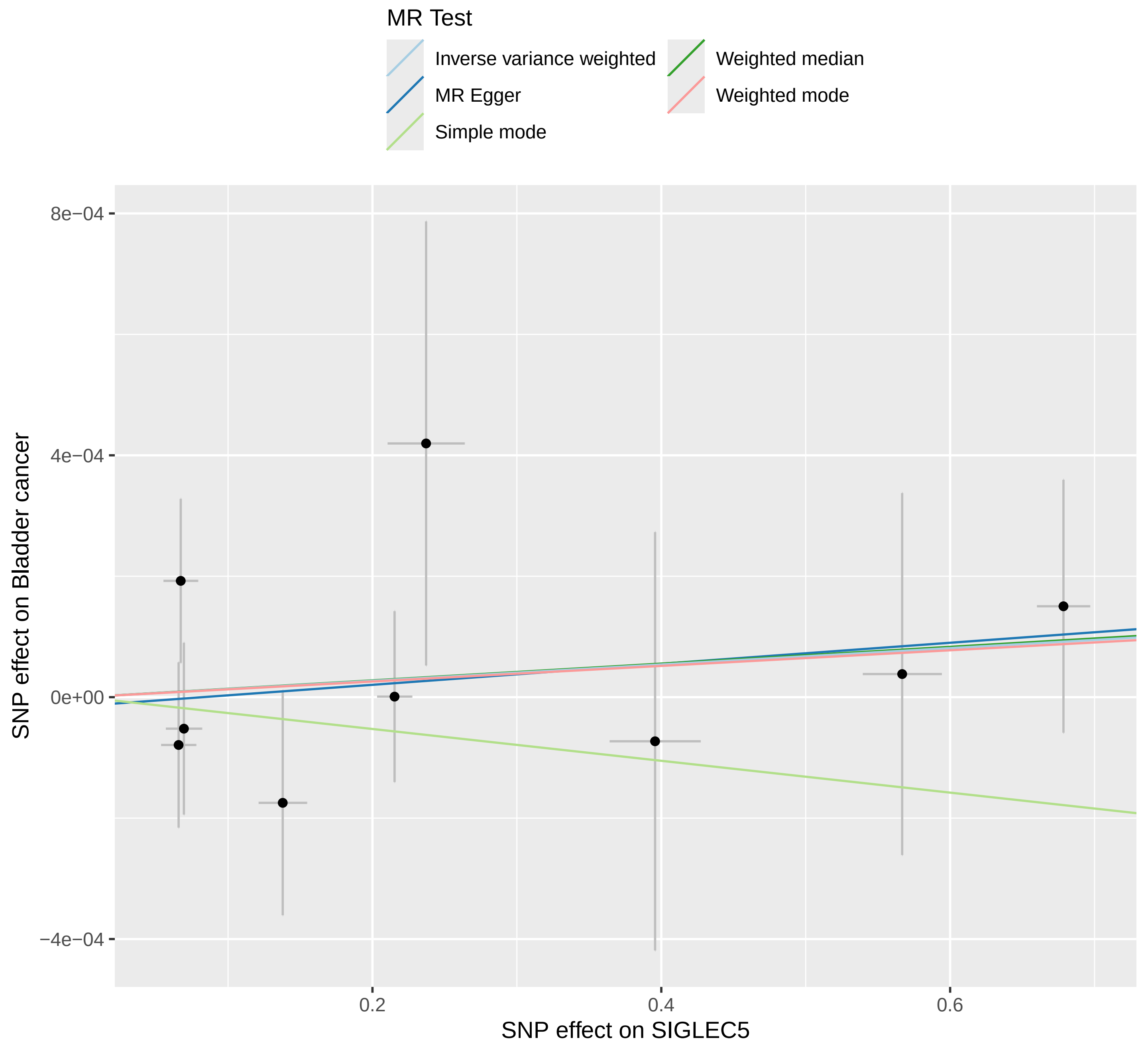
MR Test
Inverse variance weighted
MR Egger
Simple mode
Weighted median
Weighted mode
8e−04
4e−04
0e+00
−4e−04
0.2
0.4
0.6
SNP effect on Bladder cancer
SNP effect on SIGLEC5

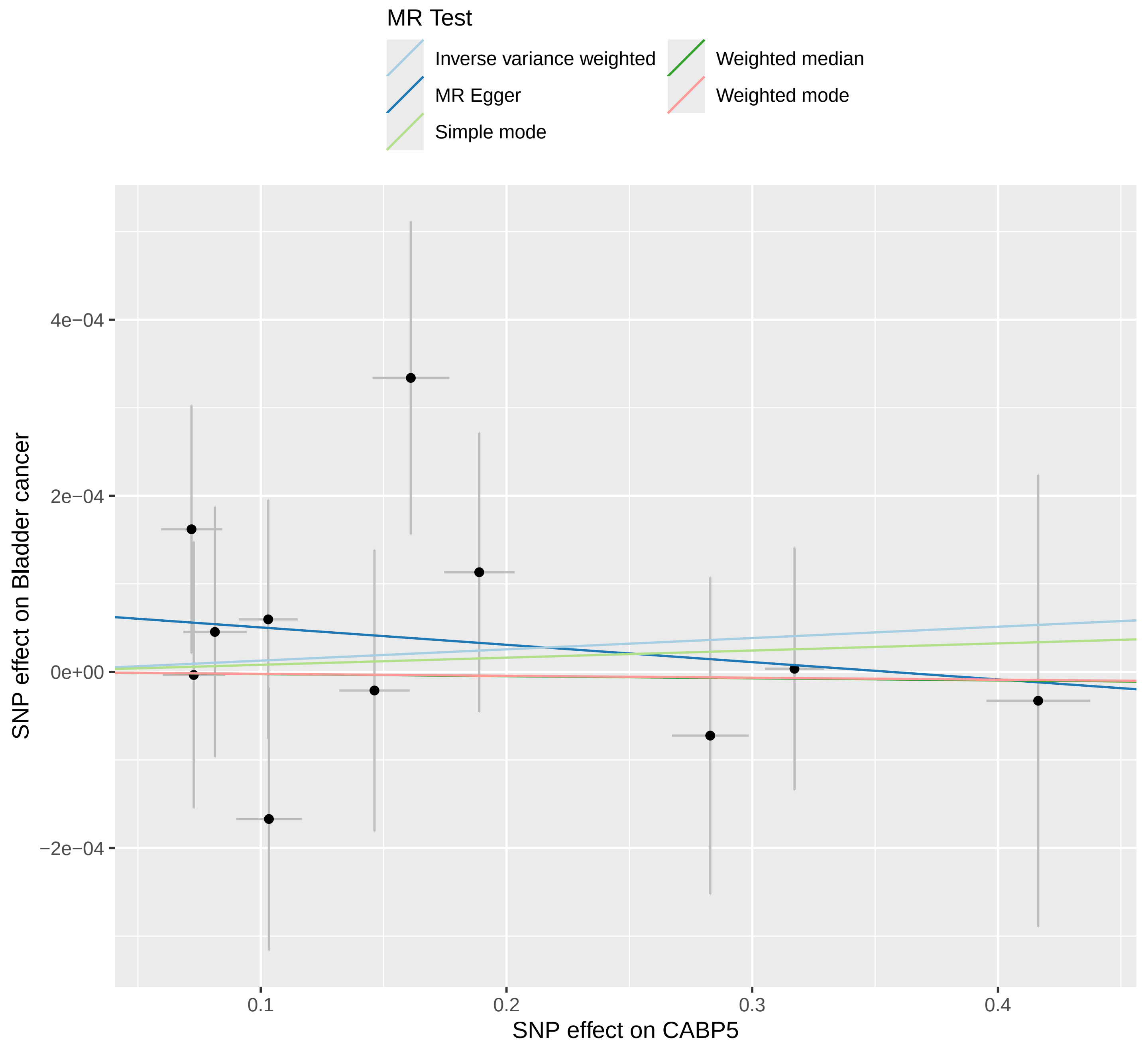
MR Test
Inverse variance weighted
MR Egger
Simple mode
Weighted median
Weighted mode
4e−04
2e−04
0e+00
−2e−04
0.1
0.2
0.3
0.4
SNP effect on Bladder cancer
SNP effect on CABP5

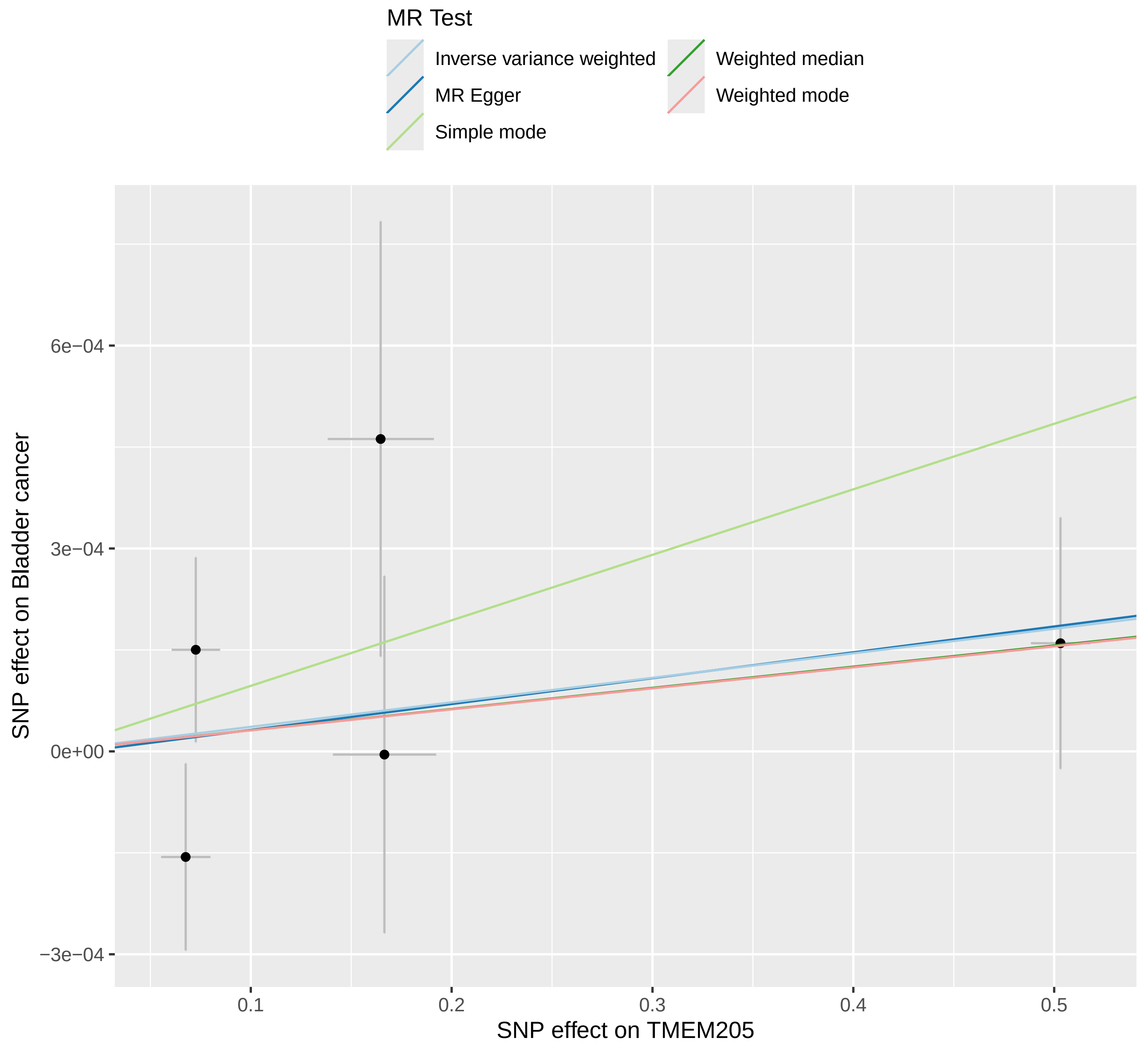
MR Test
Inverse variance weighted
MR Egger
Simple mode
Weighted median
Weighted mode
6e−04
3e−04
0e+00
−3e−04
0.1
0.2
0.3
0.4
0.5
SNP effect on Bladder cancer
SNP effect on TMEM205

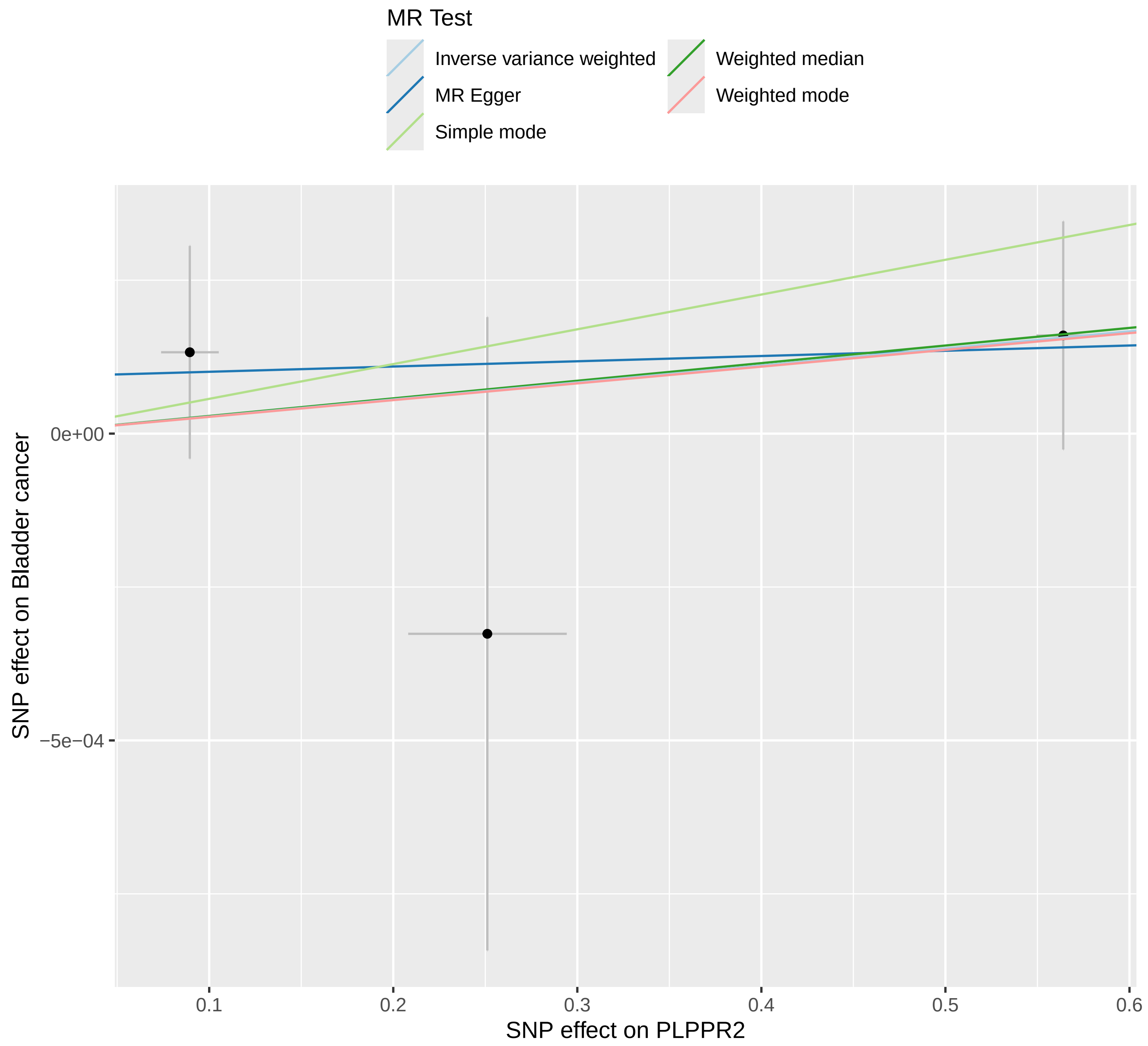

MR Test
Inverse variance weighted
MR Egger
Simple mode
Weighted median
Weighted mode
0e+00
−5e−04
SNP effect on Bladder cancer
0.1
0.2
0.3
0.4
0.5
0.6
SNP effect on PLPPR2

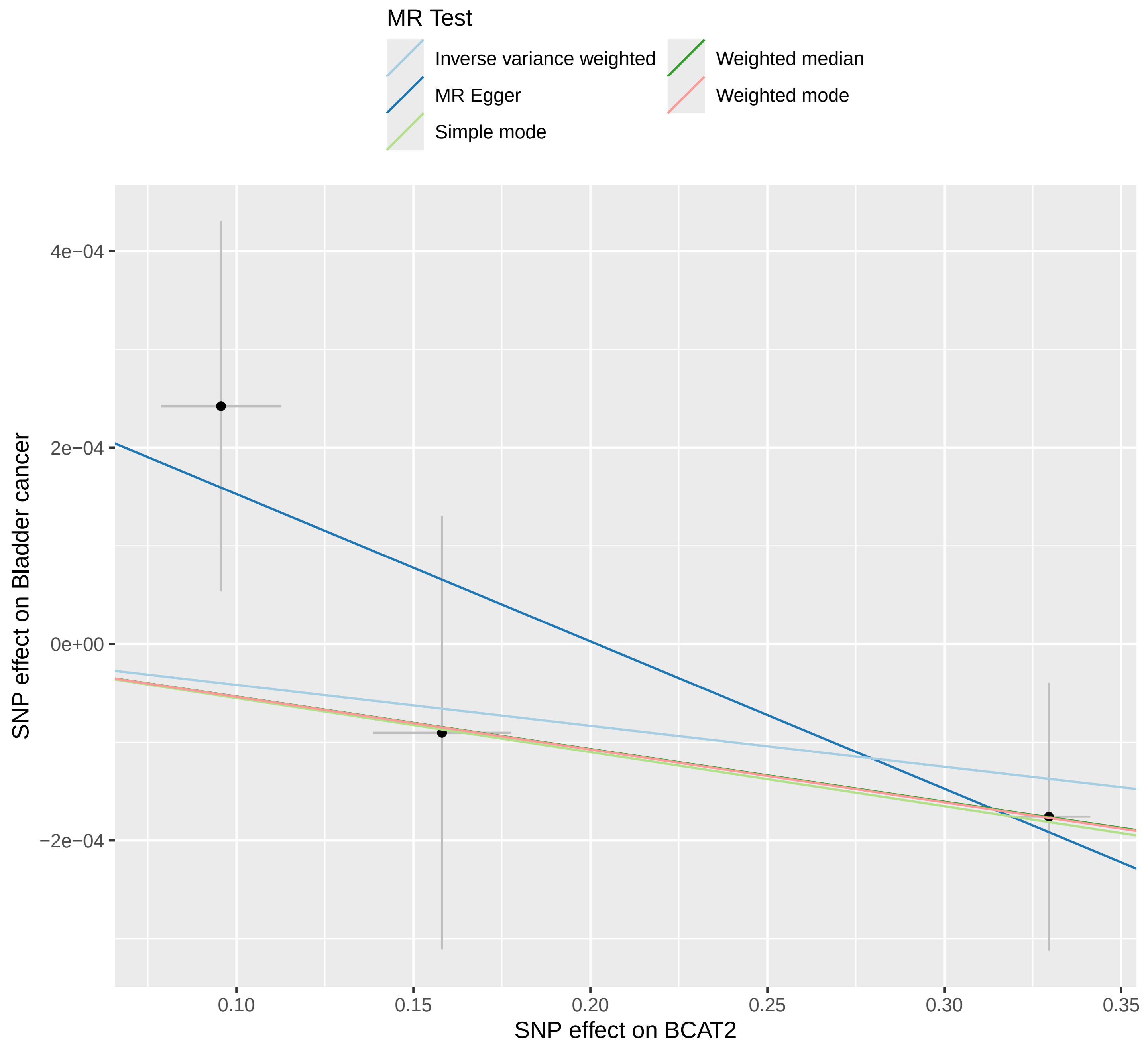
MR Test
Inverse variance weighted
MR Egger
Simple mode
Weighted median
Weighted mode
4e−04
2e−04
0e+00
−2e−04
SNP effect on Bladder cancer
0.10
0.15
0.20
0.25
0.30
0.35
SNP effect on BCAT2

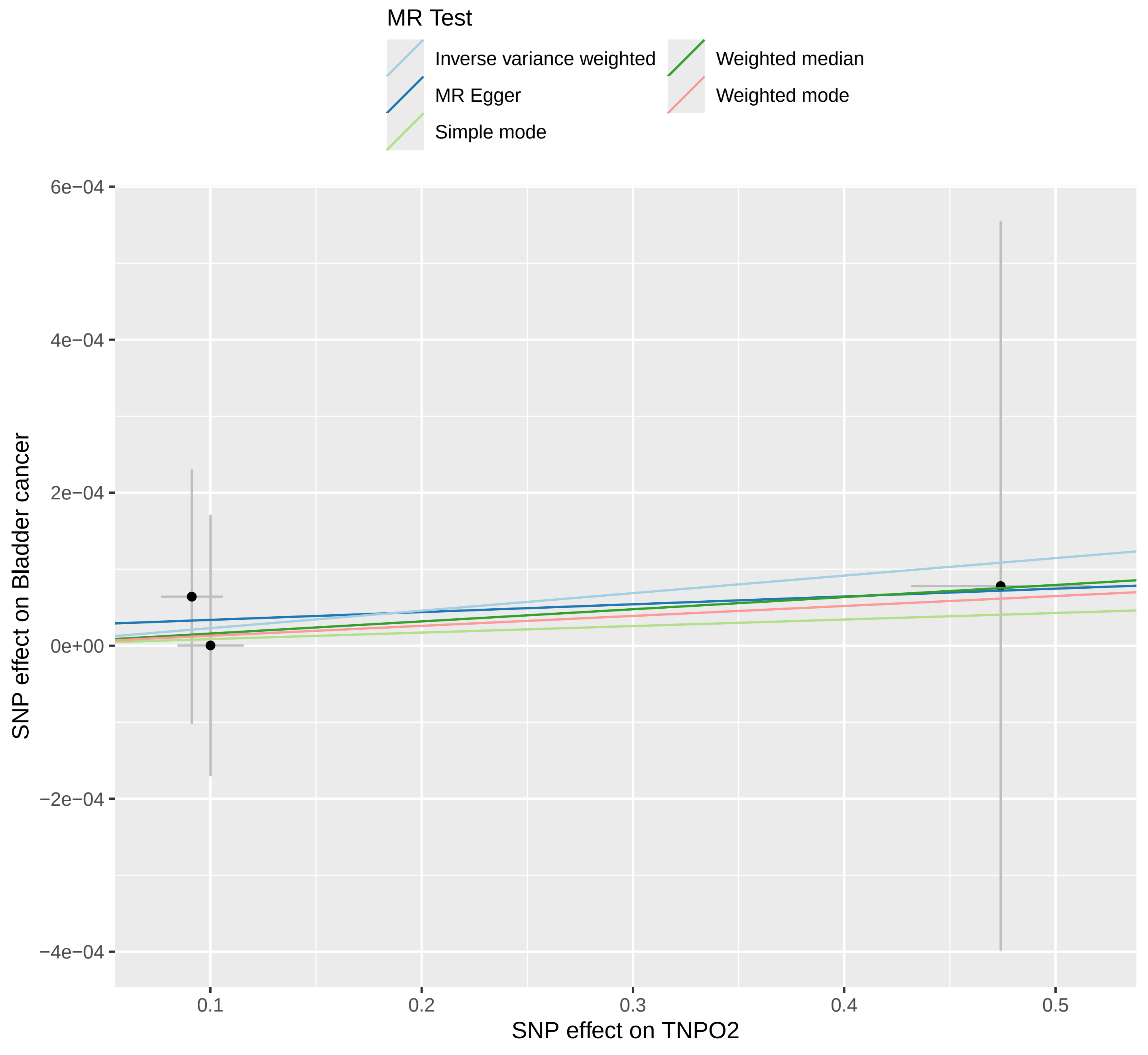

MR Test
Inverse variance weighted
MR Egger
Simple mode
Weighted median
Weighted mode
6e−04
4e−04
2e−04
0e+00
−2e−04
−4e−04
SNP effect on Bladder cancer
0.1
0.2
0.3
0.4
0.5
SNP effect on TNPO2

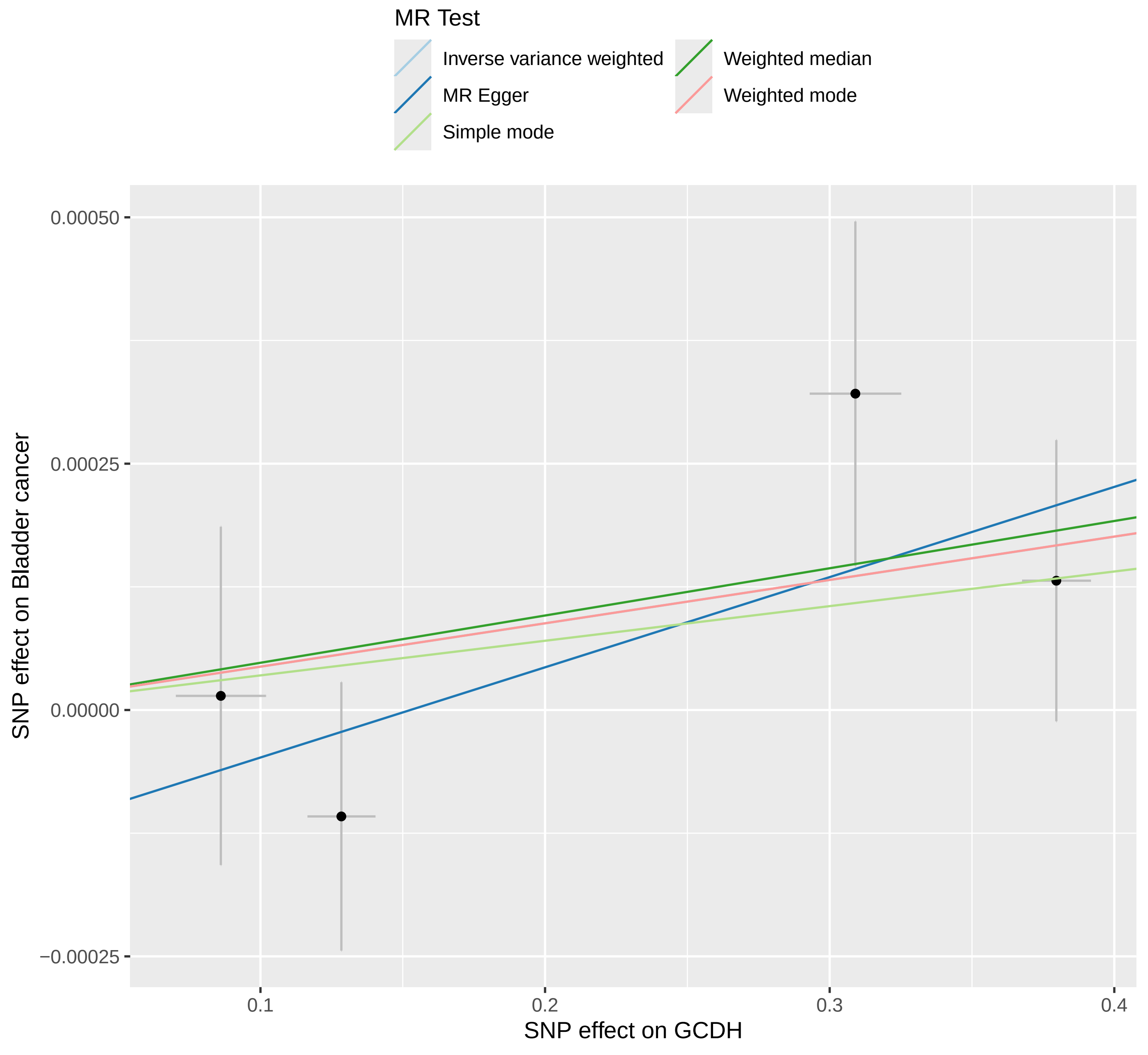

MR Test
Inverse variance weighted
MR Egger
Simple mode
Weighted median
Weighted mode
0.00050
0.00025
0.00000
−0.00025
0.1
0.2
0.3
0.4
SNP effect on GCDH
SNP effect on Bladder cancer

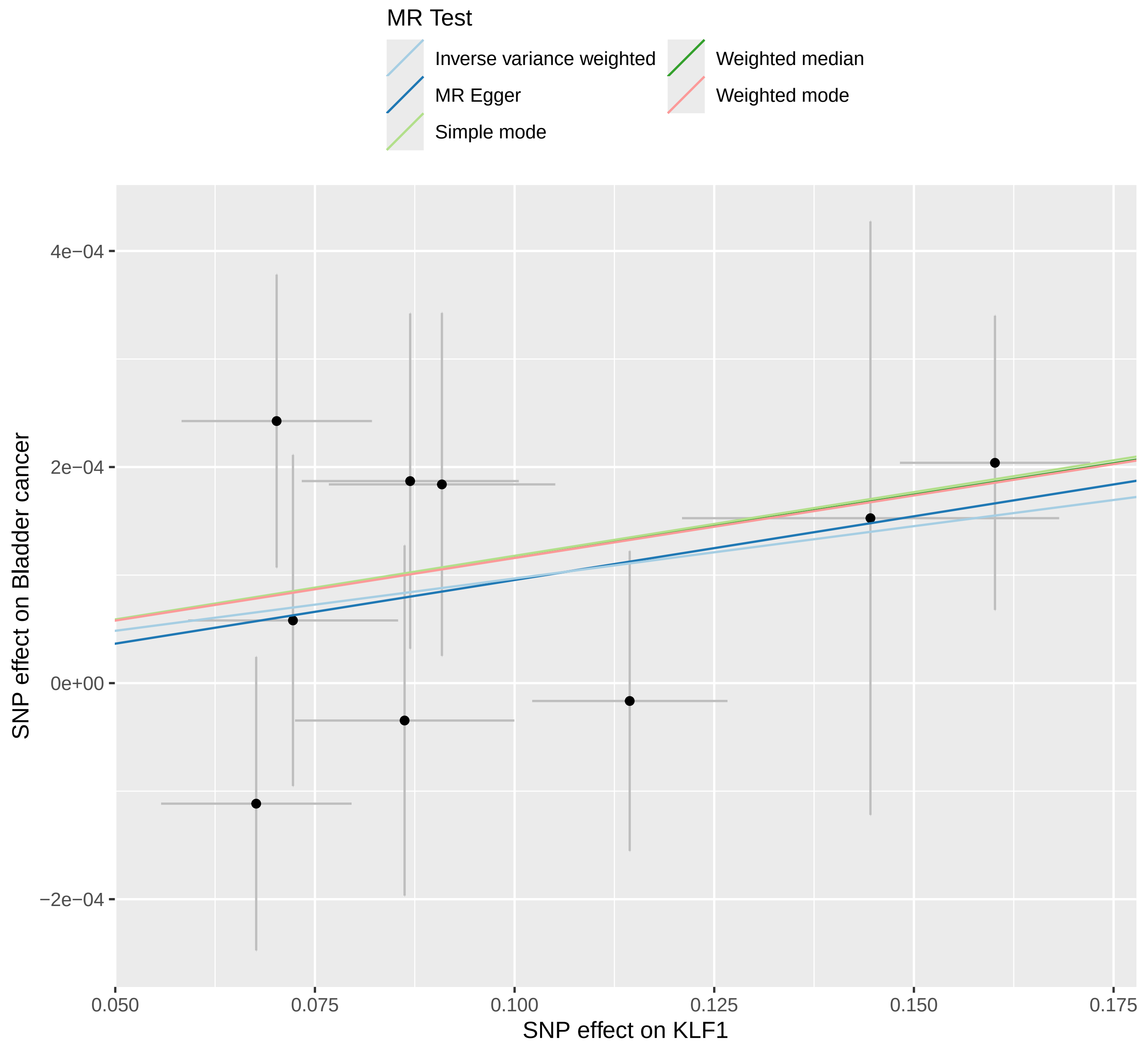

MR Test
Inverse variance weighted
MR Egger
Simple mode
Weighted median
Weighted mode
4e−04
2e−04
0e+00
−2e−04
SNP effect on Bladder cancer
0.050
0.075
0.100
0.125
0.150
0.175
SNP effect on KLF1

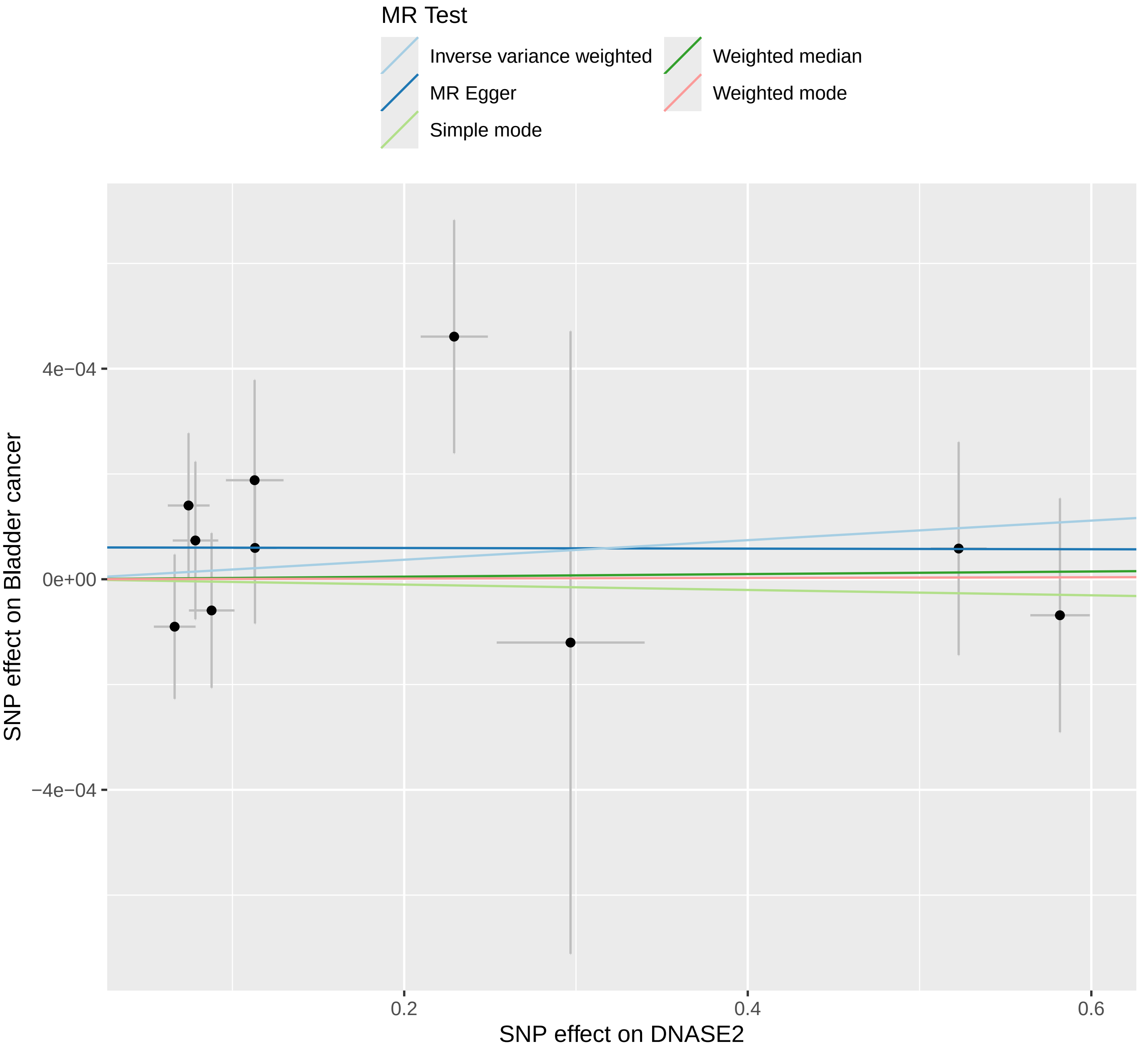
MR Test
Inverse variance weighted
MR Egger
Simple mode
Weighted median
Weighted mode
4e−04
0e+00
−4e−04
0.2
0.4
0.6
SNP effect on Bladder cancer
SNP effect on DNASE2

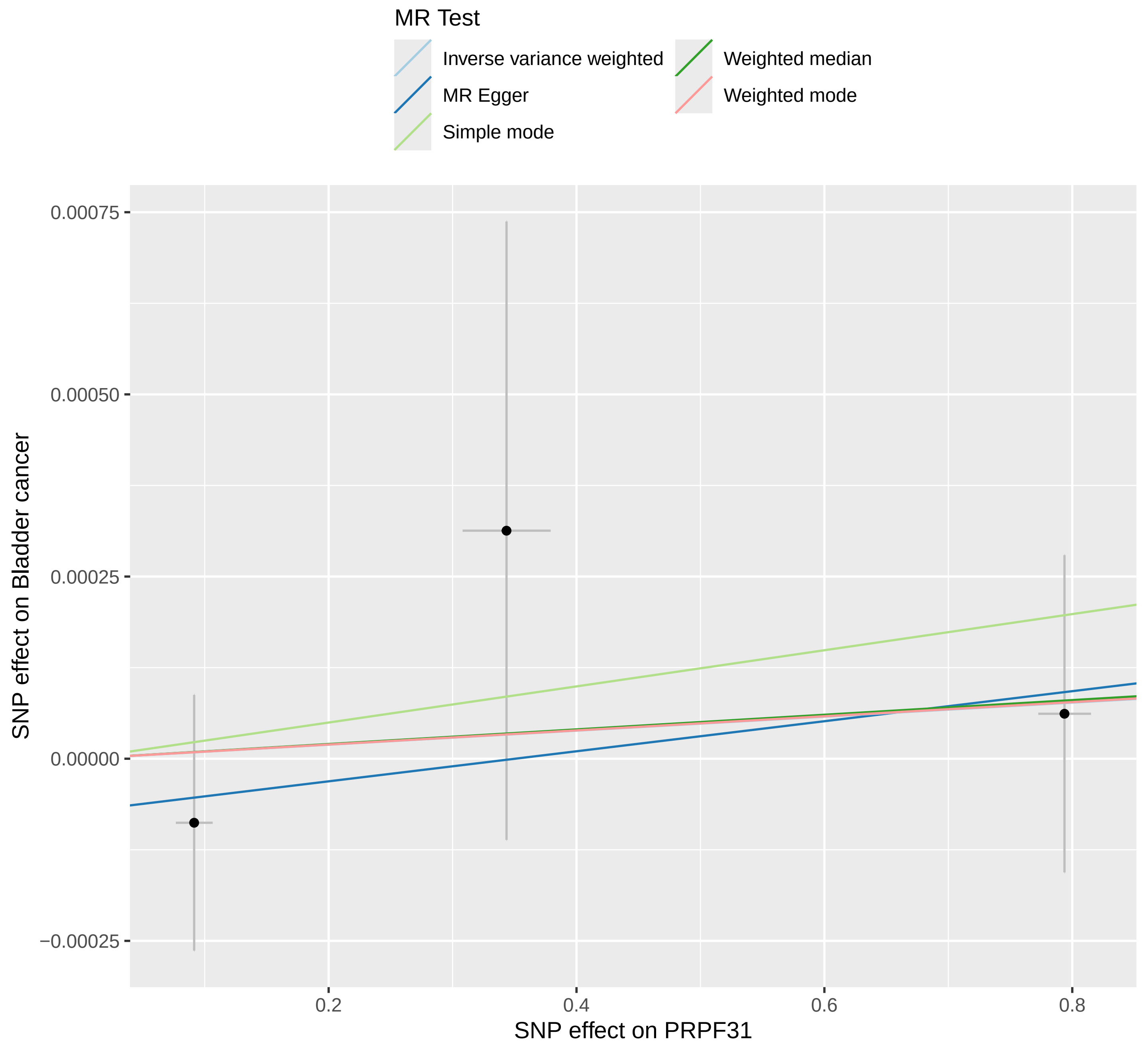
MR Test
Inverse variance weighted
MR Egger
Simple mode
Weighted median
Weighted mode
0.00075
0.00050
0.00025
0.00000
−0.00025
SNP effect on Bladder cancer
0.2
0.4
0.6
0.8
SNP effect on PRPF31

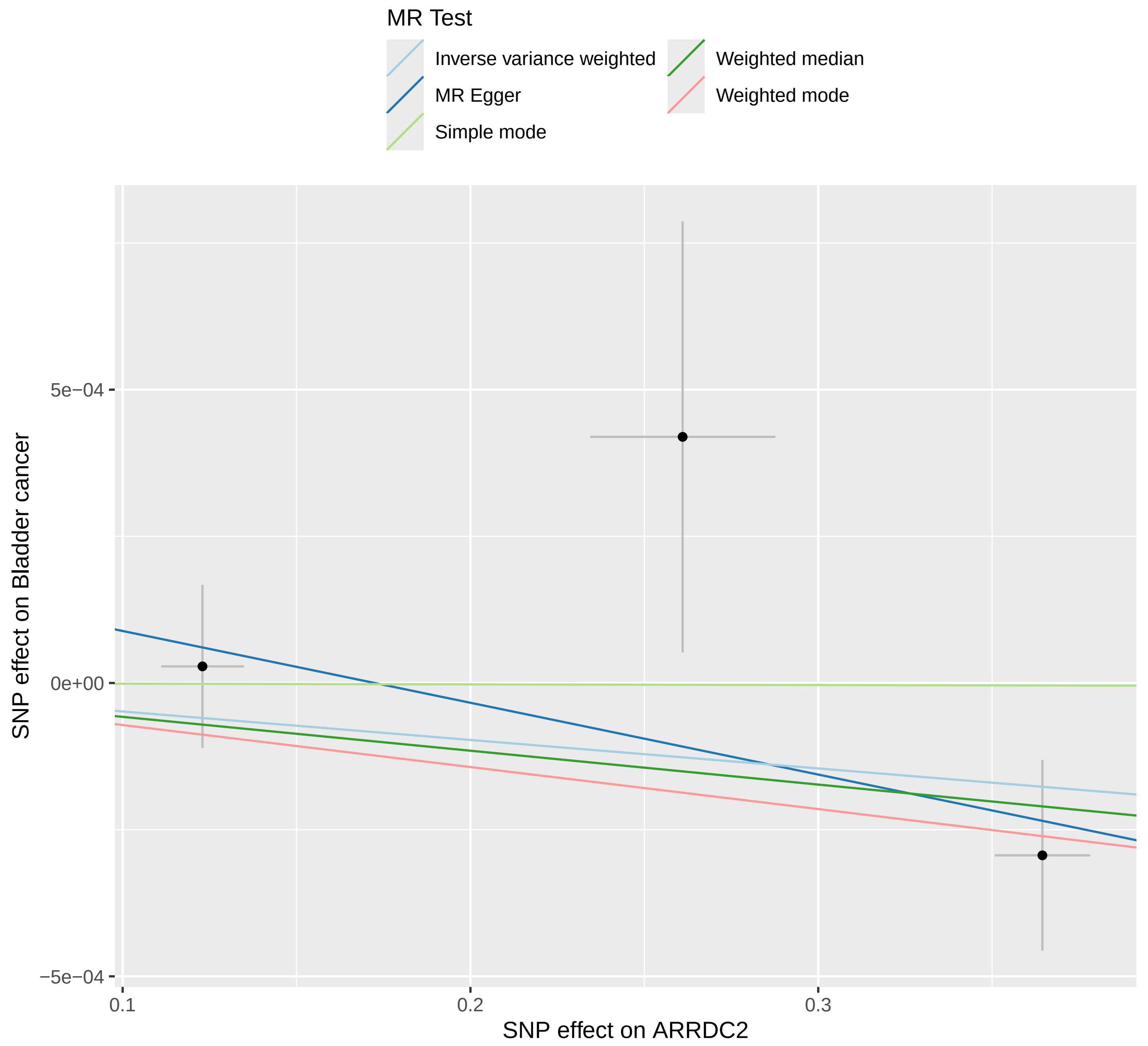

MR Test
Inverse variance weighted
MR Egger
Simple mode
Weighted median
Weighted mode
5e−04
0e+00
−5e−04
0.1
0.2
0.3
SNP effect on Bladder cancer
SNP effect on ARRDC2

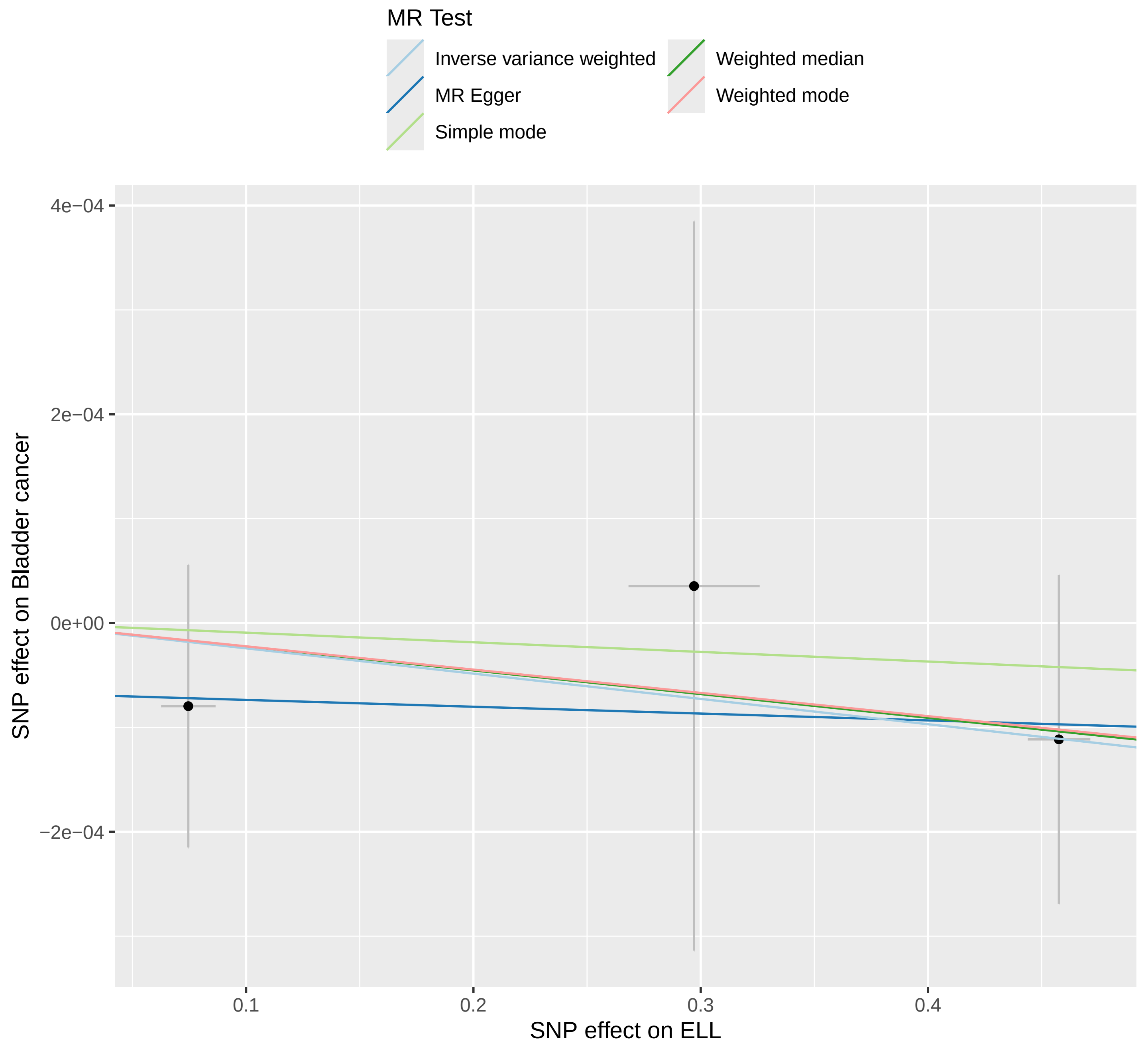

MR Test
Inverse variance weighted
MR Egger
Simple mode
Weighted median
Weighted mode
4e−04
2e−04
0e+00
−2e−04
0.1
0.2
0.3
0.4
SNP effect on Bladder cancer
SNP effect on ELL

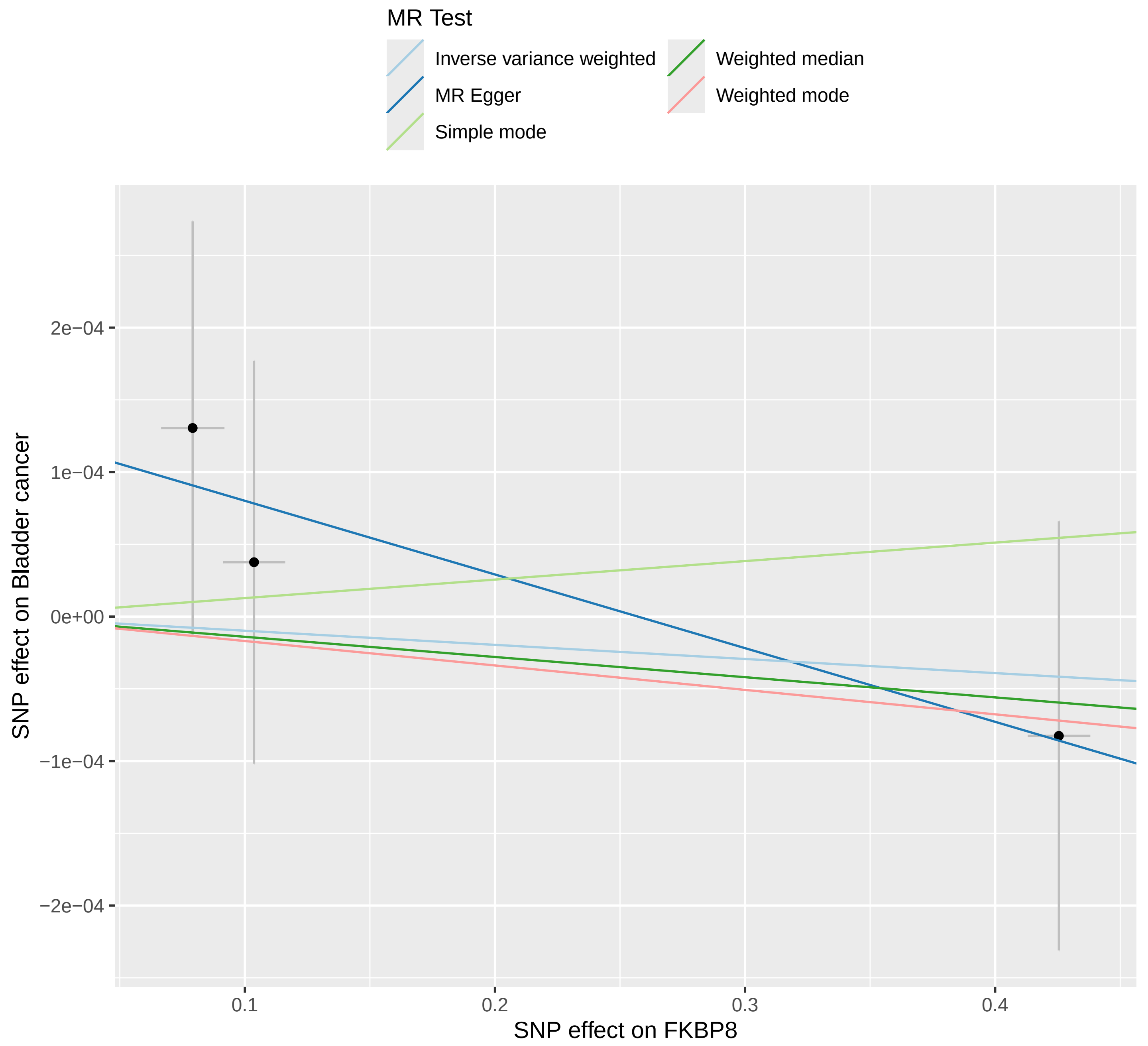
MR Test
Inverse variance weighted
MR Egger
Simple mode
Weighted median
Weighted mode
2e−04
1e−04
0e+00
−1e−04
−2e−04
0.1
0.2
0.3
0.4
SNP effect on Bladder cancer
SNP effect on FKBP8

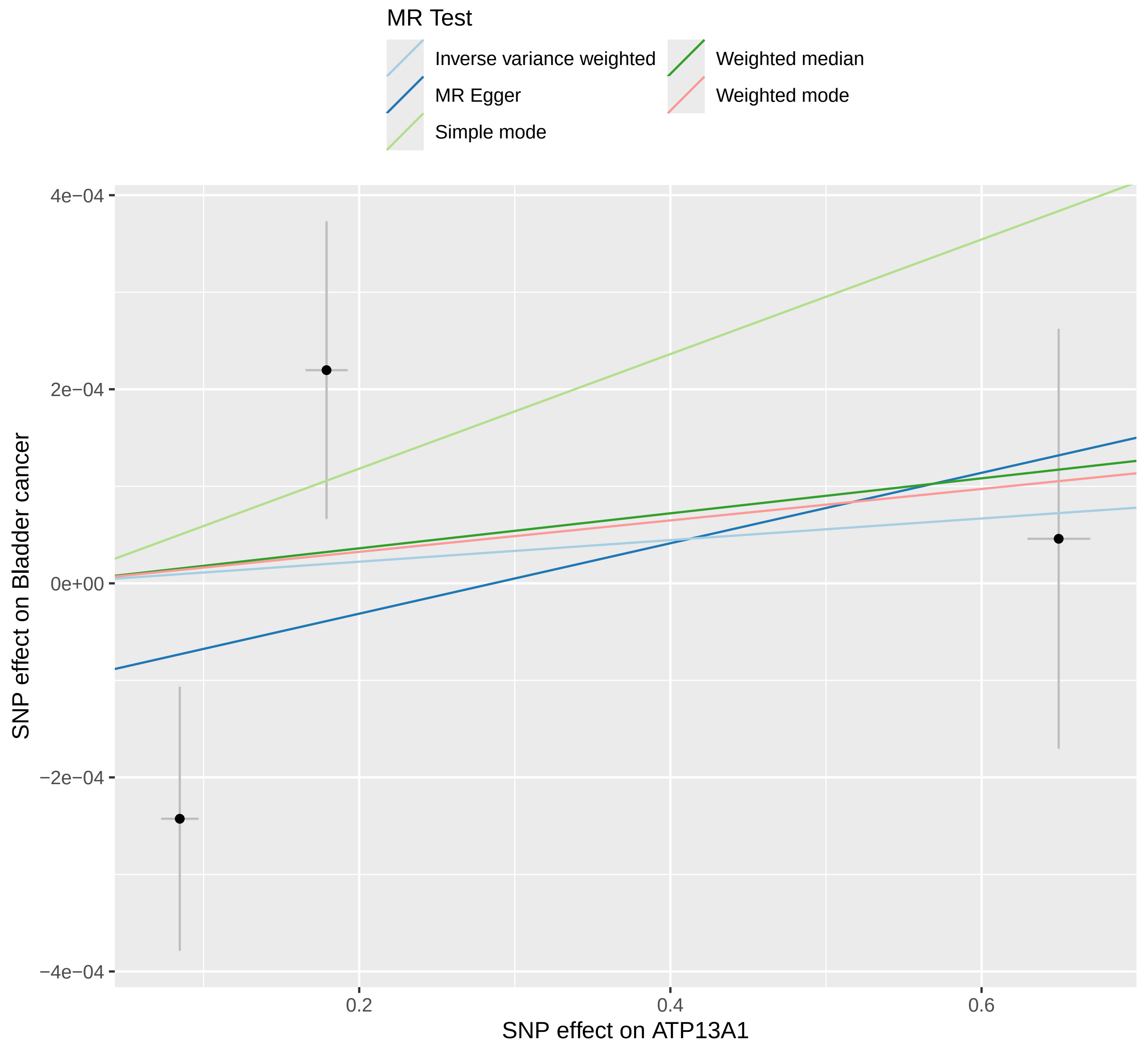

MR Test
Inverse variance weighted
MR Egger
Simple mode
Weighted median
Weighted mode
4e−04
2e−04
0e+00
−2e−04
−4e−04
0.2
0.4
0.6
SNP effect on ATP13A1
SNP effect on Bladder cancer

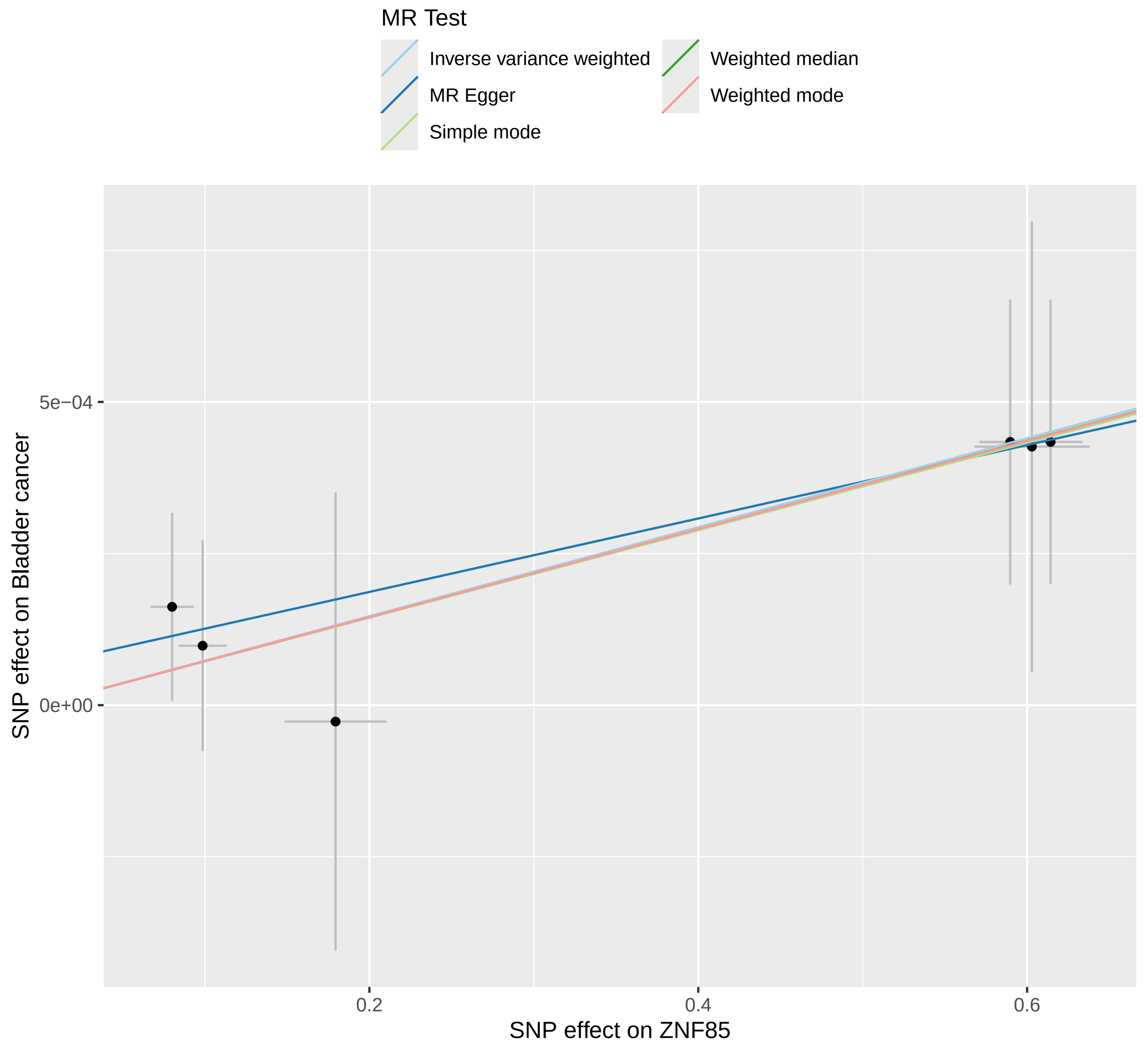

MR Test
Inverse variance weighted
MR Egger
Simple mode
Weighted median
Weighted mode
5e−04
0e+00
0.2
0.4
0.6
SNP effect on ZNF85
SNP effect on Bladder cancer

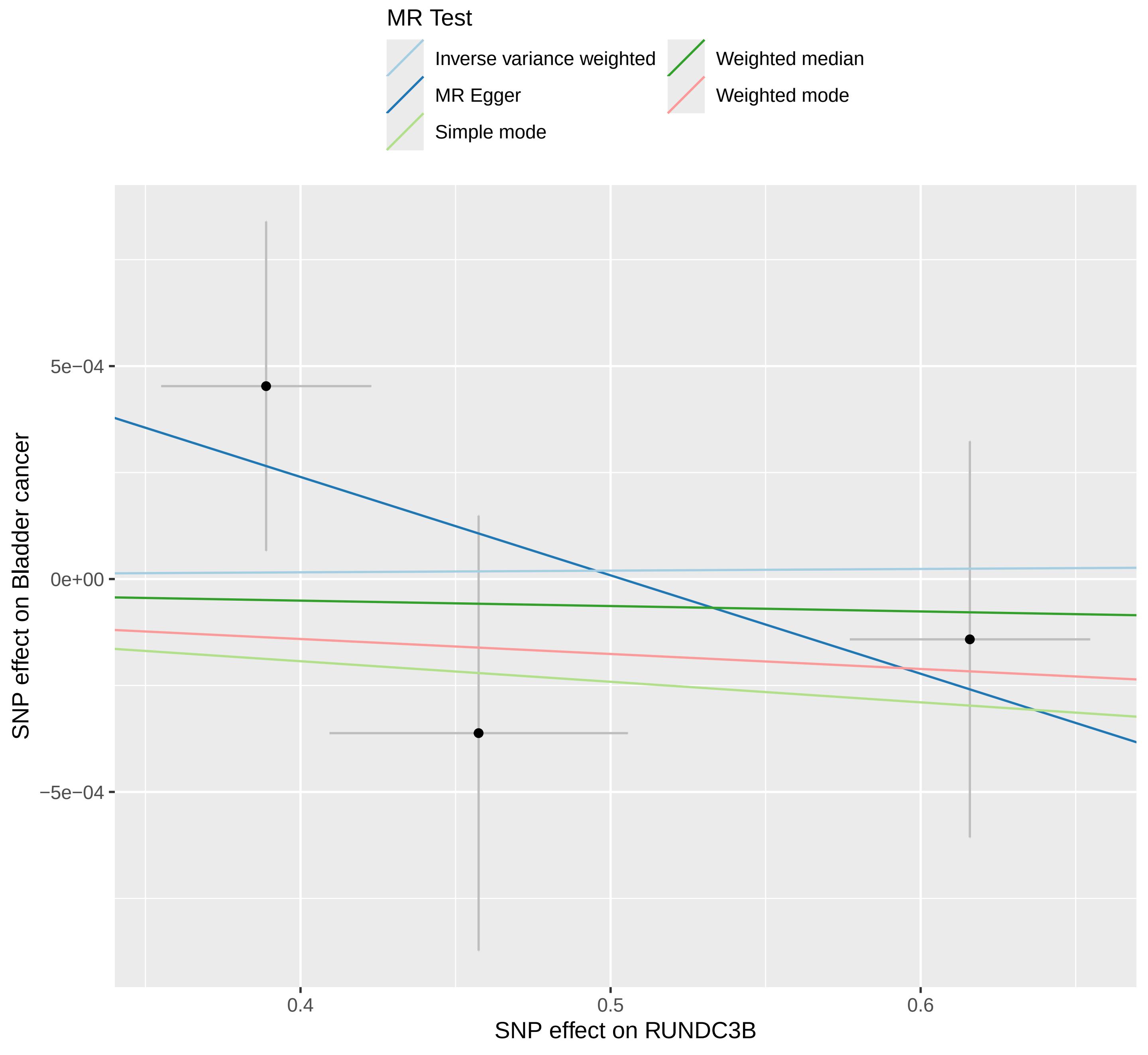
MR Test
Inverse variance weighted
MR Egger
Simple mode
Weighted median
Weighted mode
5e−04
0e+00
−5e−04
0.4
0.5
0.6
SNP effect on Bladder cancer
SNP effect on RUNDC3B

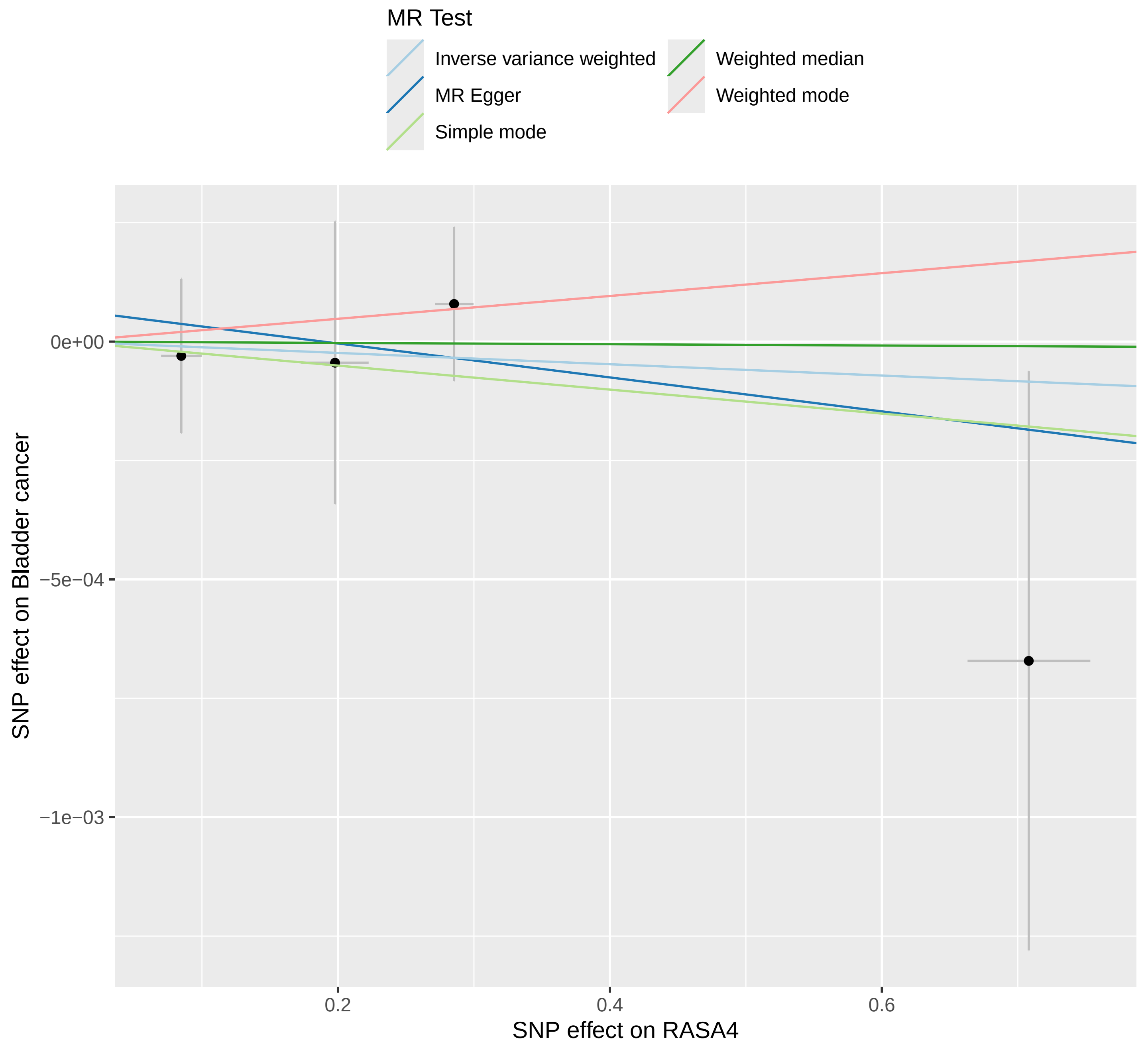

MR Test
Inverse variance weighted
MR Egger
Simple mode
Weighted median
Weighted mode
0e+00
−5e−04
−1e−03
0.2
0.4
0.6
SNP effect on Bladder cancer
SNP effect on RASA4

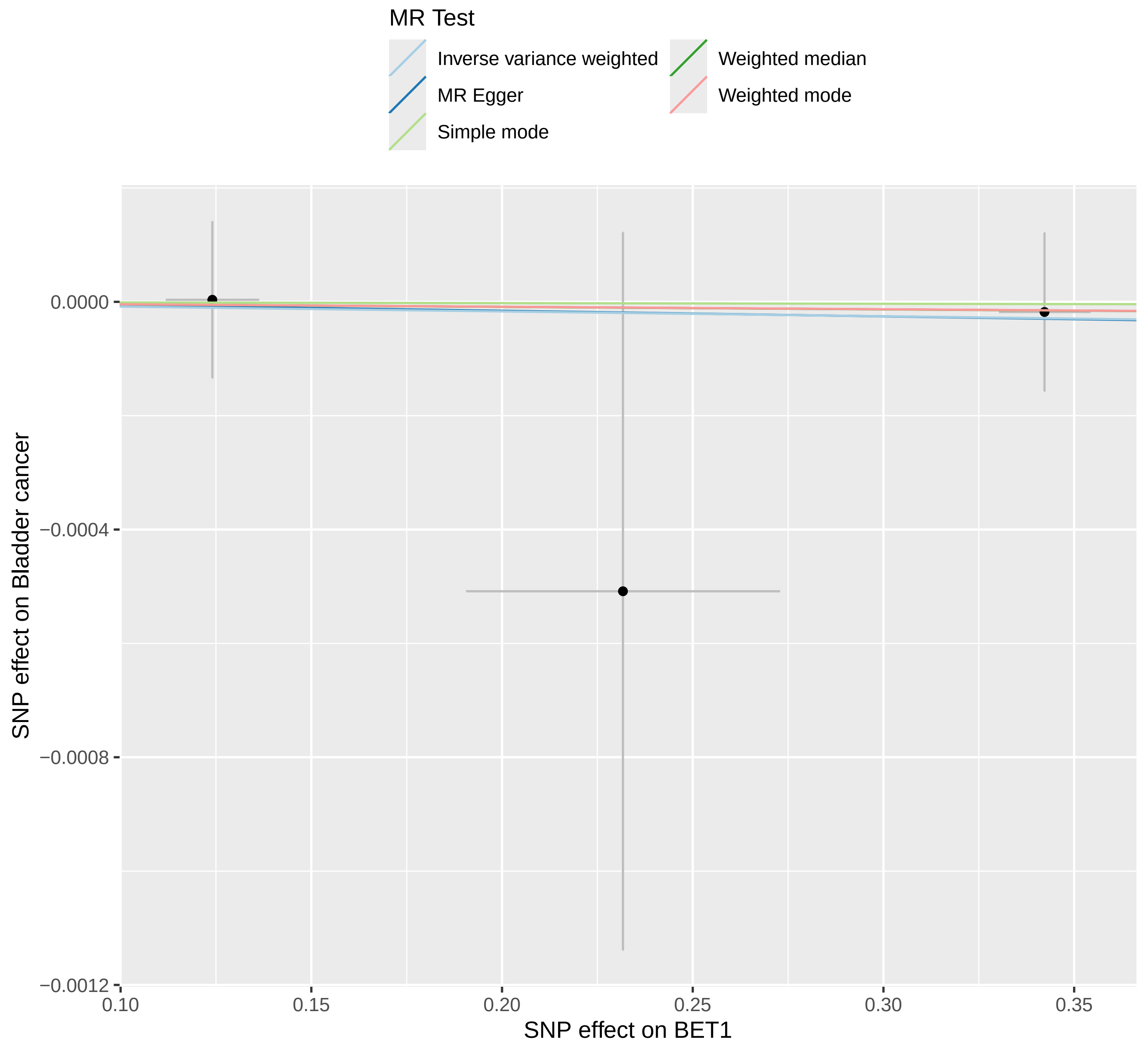

MR Test
Inverse variance weighted
MR Egger
Simple mode
Weighted median
Weighted mode
0.0000
−0.0004
−0.0008
−0.0012
0.10
0.15
0.20
0.25
0.30
0.35
SNP effect on Bladder cancer
SNP effect on BET1

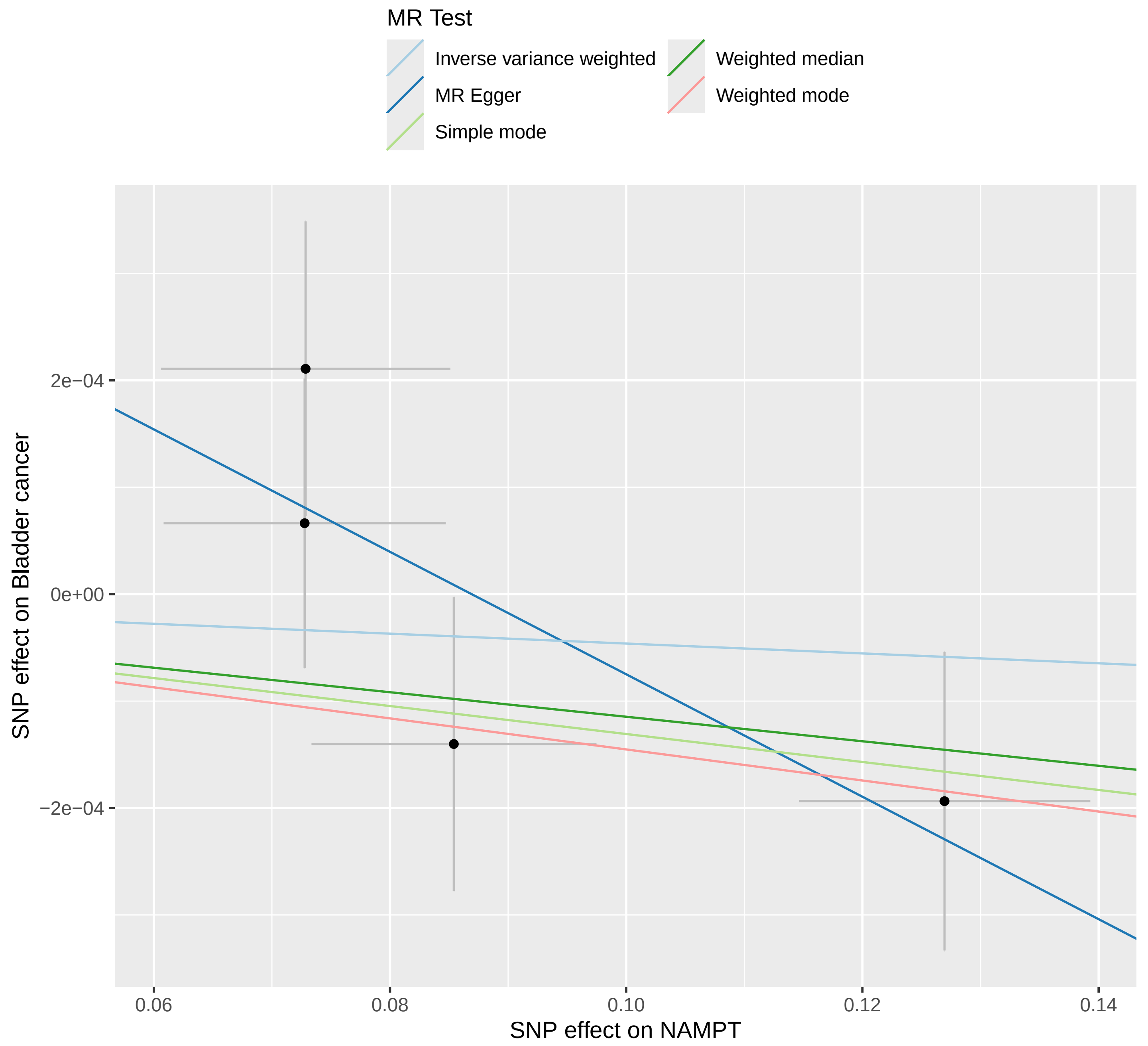

MR Test
Inverse variance weighted
MR Egger
Simple mode
Weighted median
Weighted mode
2e−04
0e+00
−2e−04
SNP effect on Bladder cancer
0.06
0.08
0.10
0.12
0.14
SNP effect on NAMPT

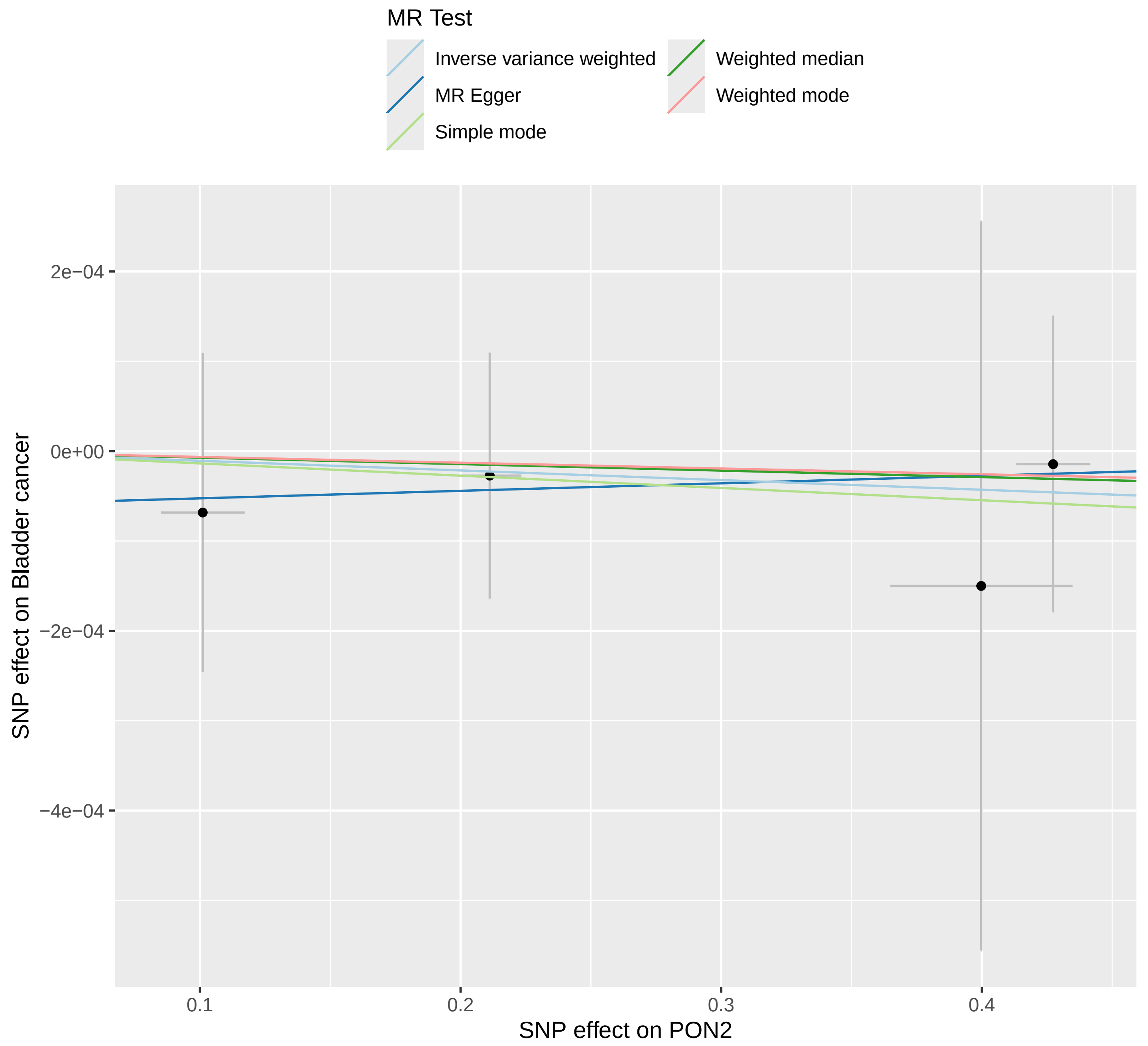

MR Test
Inverse variance weighted
MR Egger
Simple mode
Weighted median
Weighted mode
2e−04
0e+00
−2e−04
−4e−04
SNP effect on Bladder cancer
0.1
0.2
0.3
0.4
SNP effect on PON2

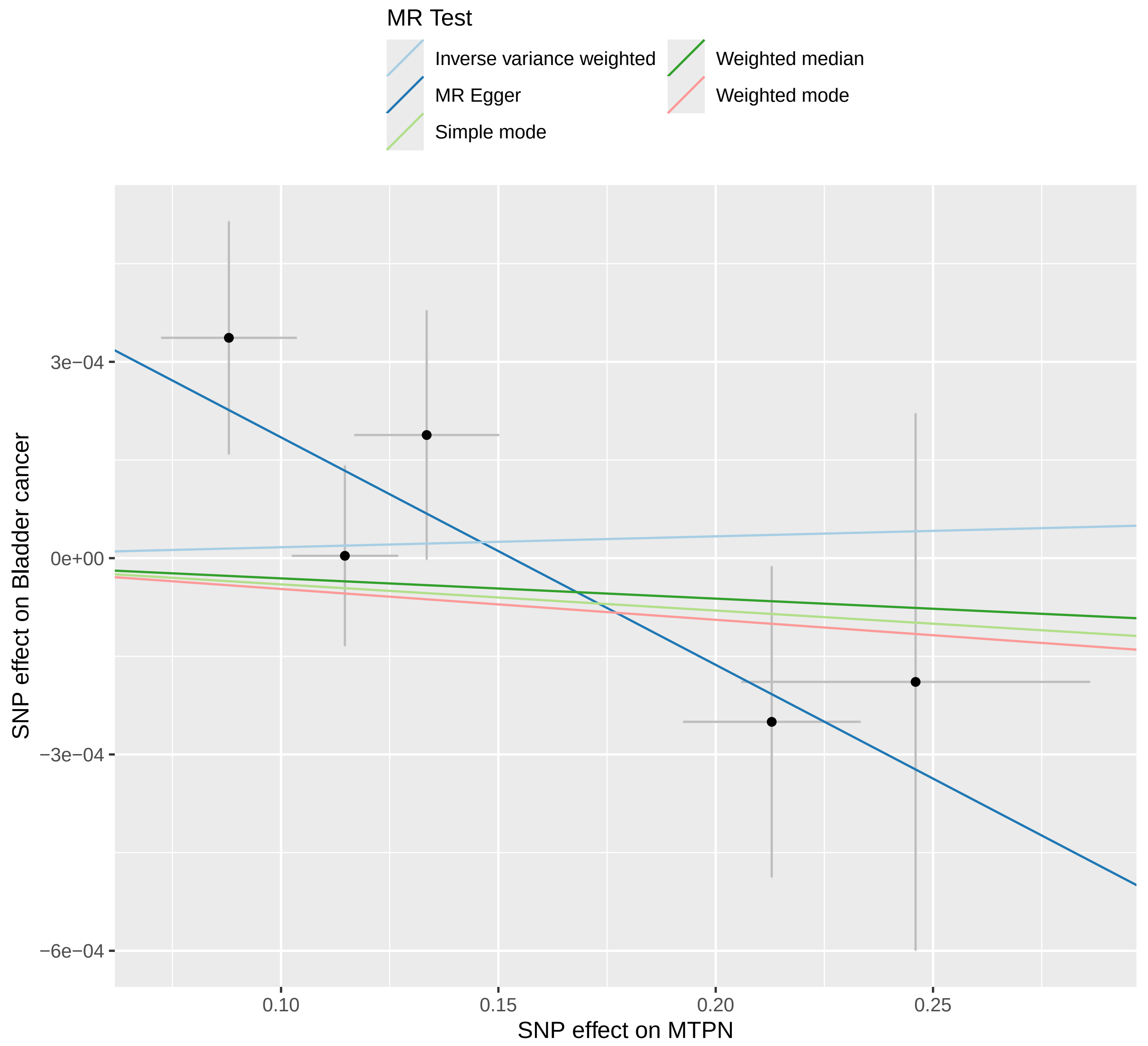
MR Test
Inverse variance weighted
MR Egger
Simple mode
Weighted median
Weighted mode
3e−04
0e+00
−3e−04
−6e−04
0.10
0.15
0.20
0.25
SNP effect on MTPN
SNP effect on Bladder cancer

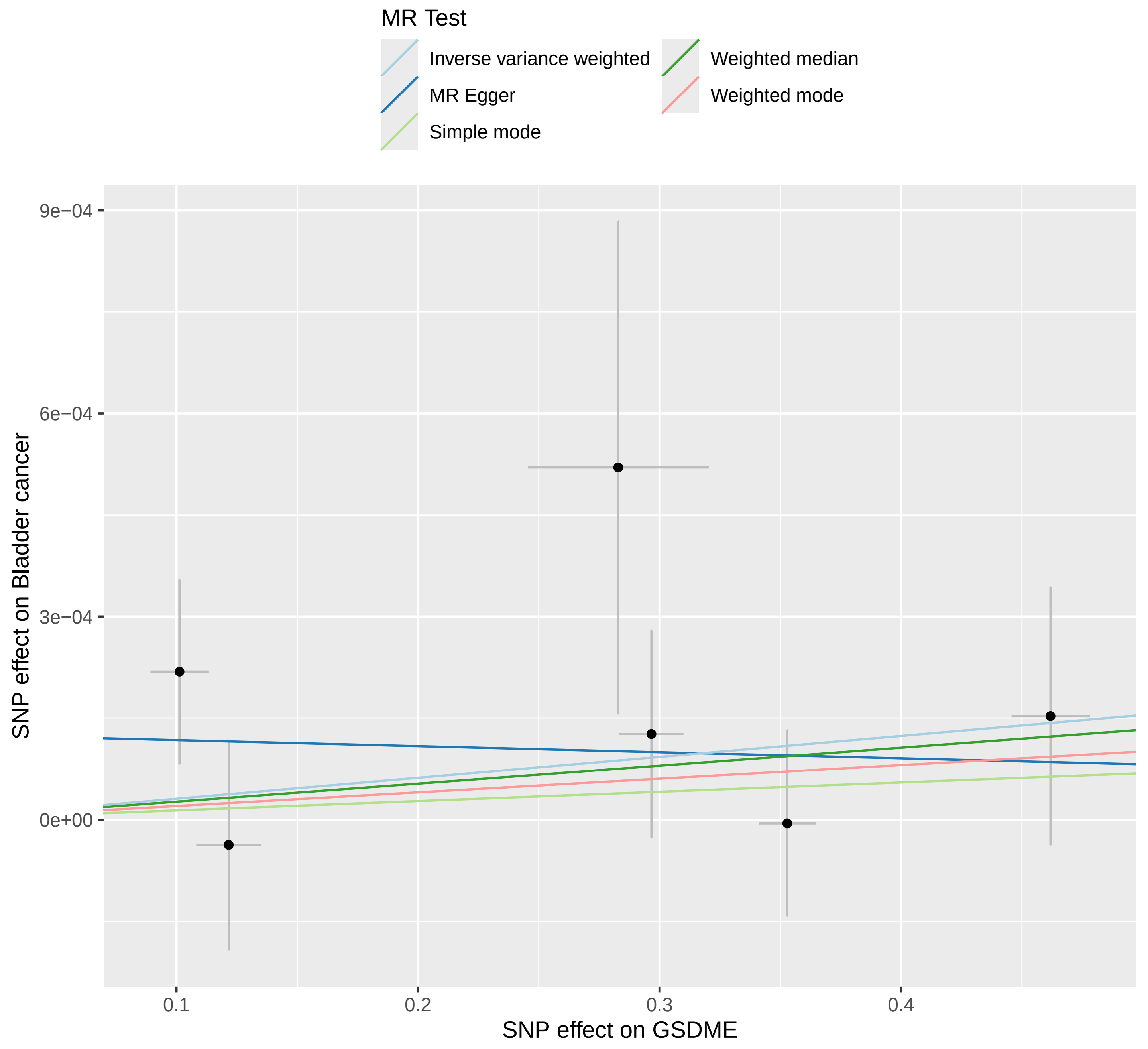

MR Test
Inverse variance weighted
MR Egger
Simple mode
Weighted median
Weighted mode
9e−04
6e−04
3e−04
0e+00
SNP effect on Bladder cancer
0.1
0.2
0.3
0.4
SNP effect on GSDME

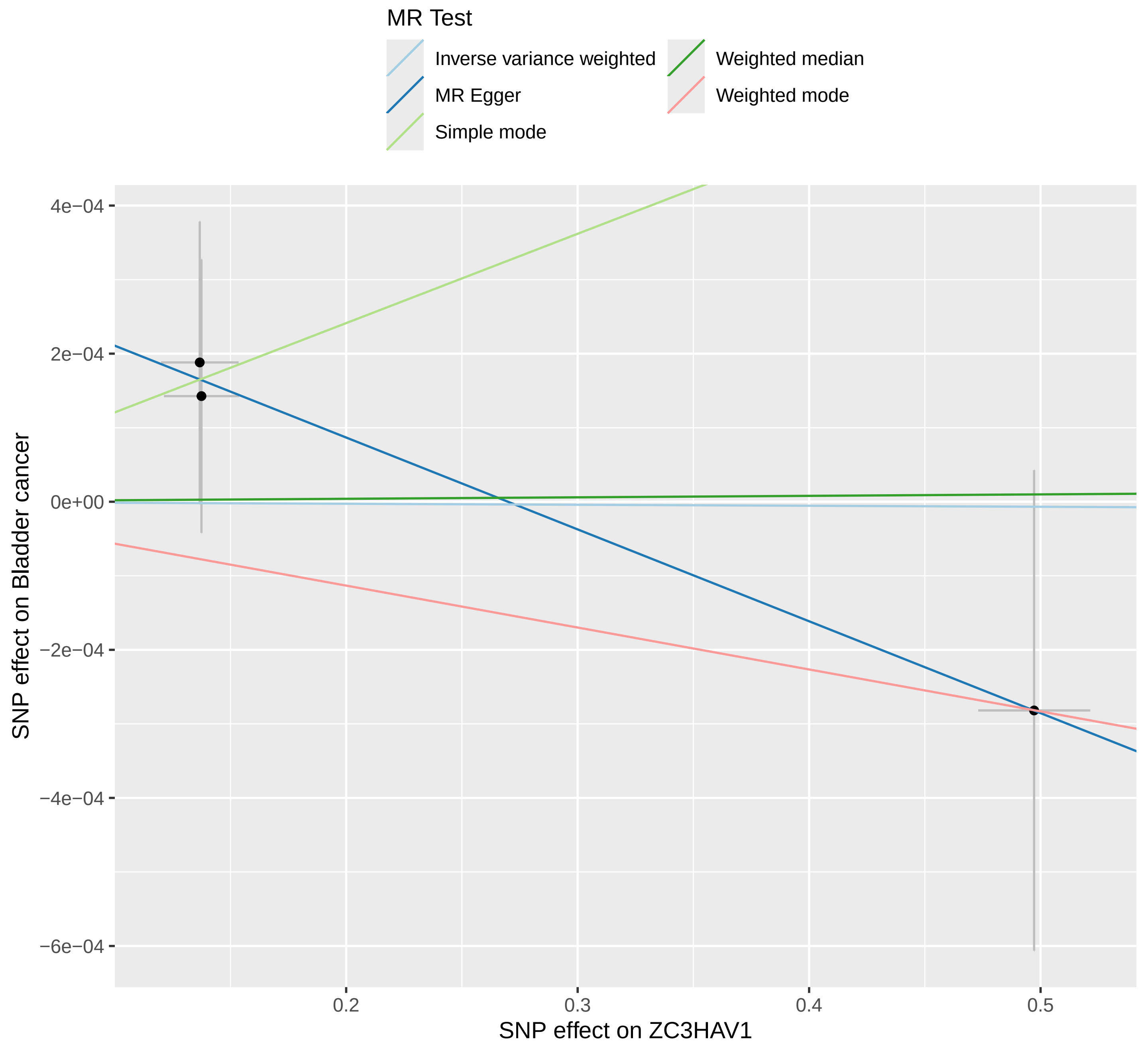
MR Test
Inverse variance weighted
MR Egger
Simple mode
Weighted median
Weighted mode
4e−04
2e−04
0e+00
−2e−04
−4e−04
−6e−04
0.2
0.3
0.4
0.5
SNP effect on ZC3HAV1
SNP effect on Bladder cancer

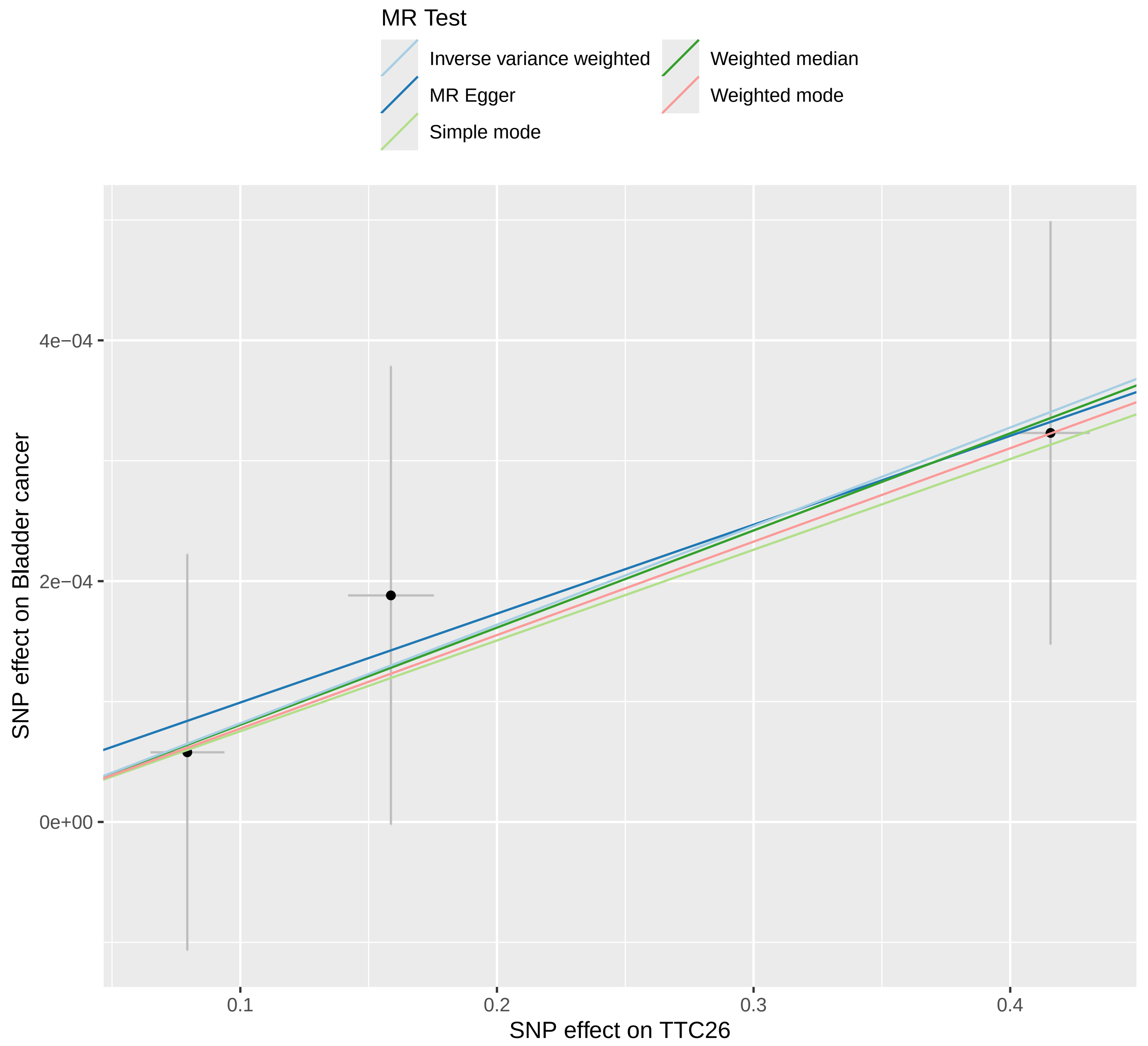

MR Test
Inverse variance weighted
MR Egger
Simple mode
Weighted median
Weighted mode
4e−04
2e−04
0e+00
0.1
0.2
0.3
0.4
SNP effect on Bladder cancer
SNP effect on TTC26

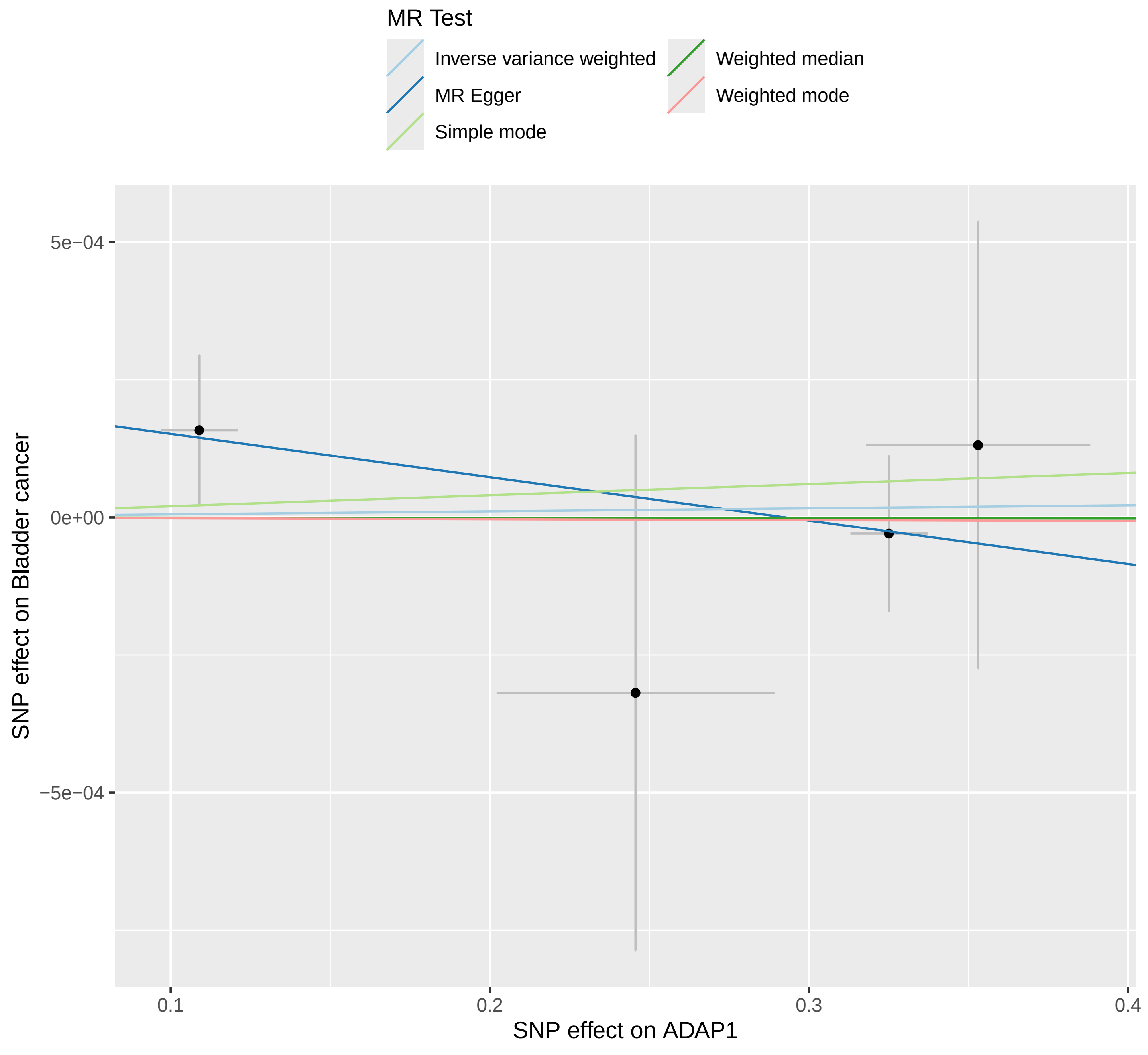

MR Test
Inverse variance weighted
MR Egger
Simple mode
Weighted median
Weighted mode
5e−04
0e+00
−5e−04
0.1
0.2
0.3
0.4
SNP effect on Bladder cancer
SNP effect on ADAP1

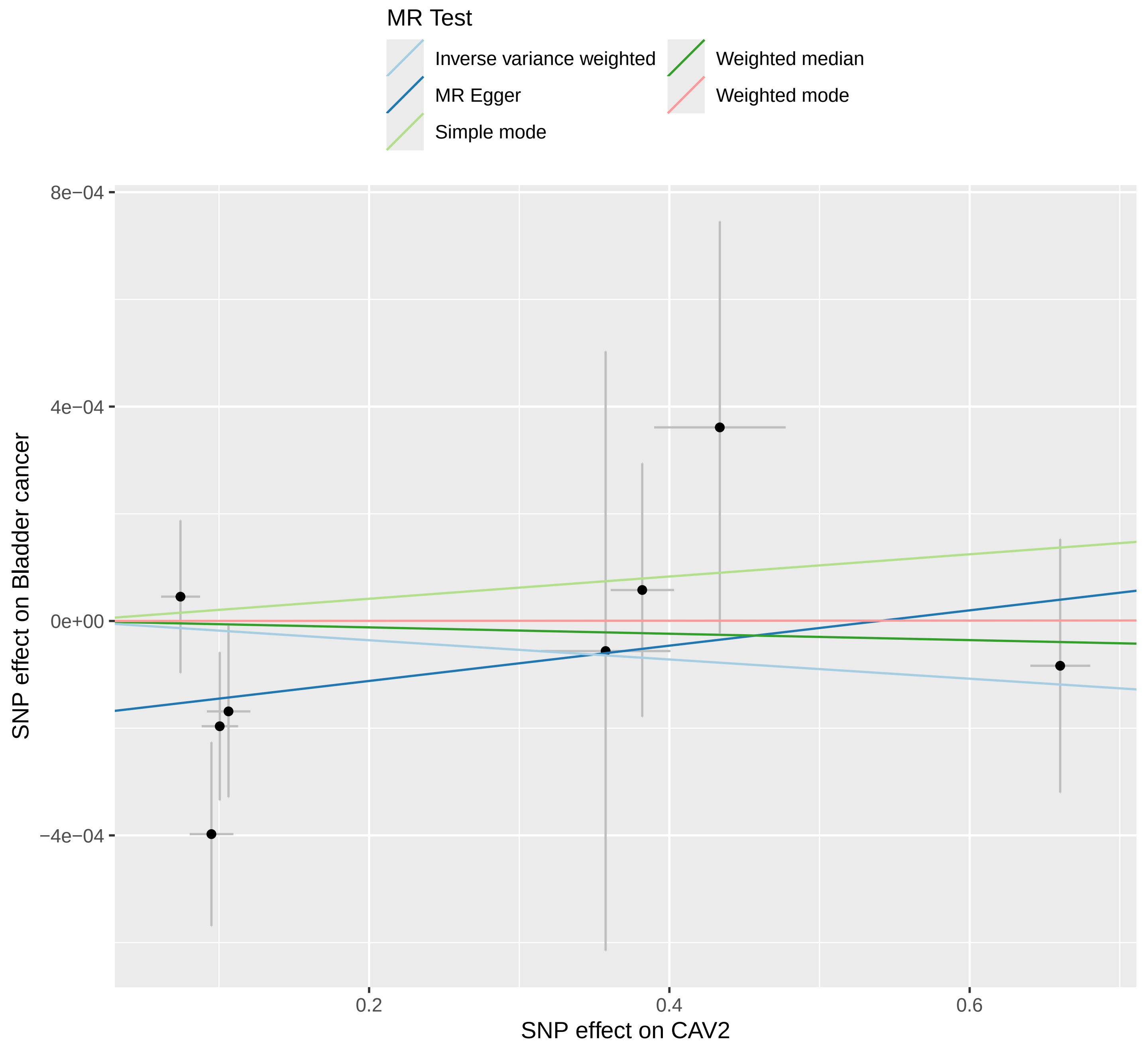
MR Test
Inverse variance weighted
MR Egger
Simple mode
Weighted median
Weighted mode
8e−04
4e−04
0e+00
−4e−04
SNP effect on Bladder cancer
0.2
0.4
0.6
SNP effect on CAV2

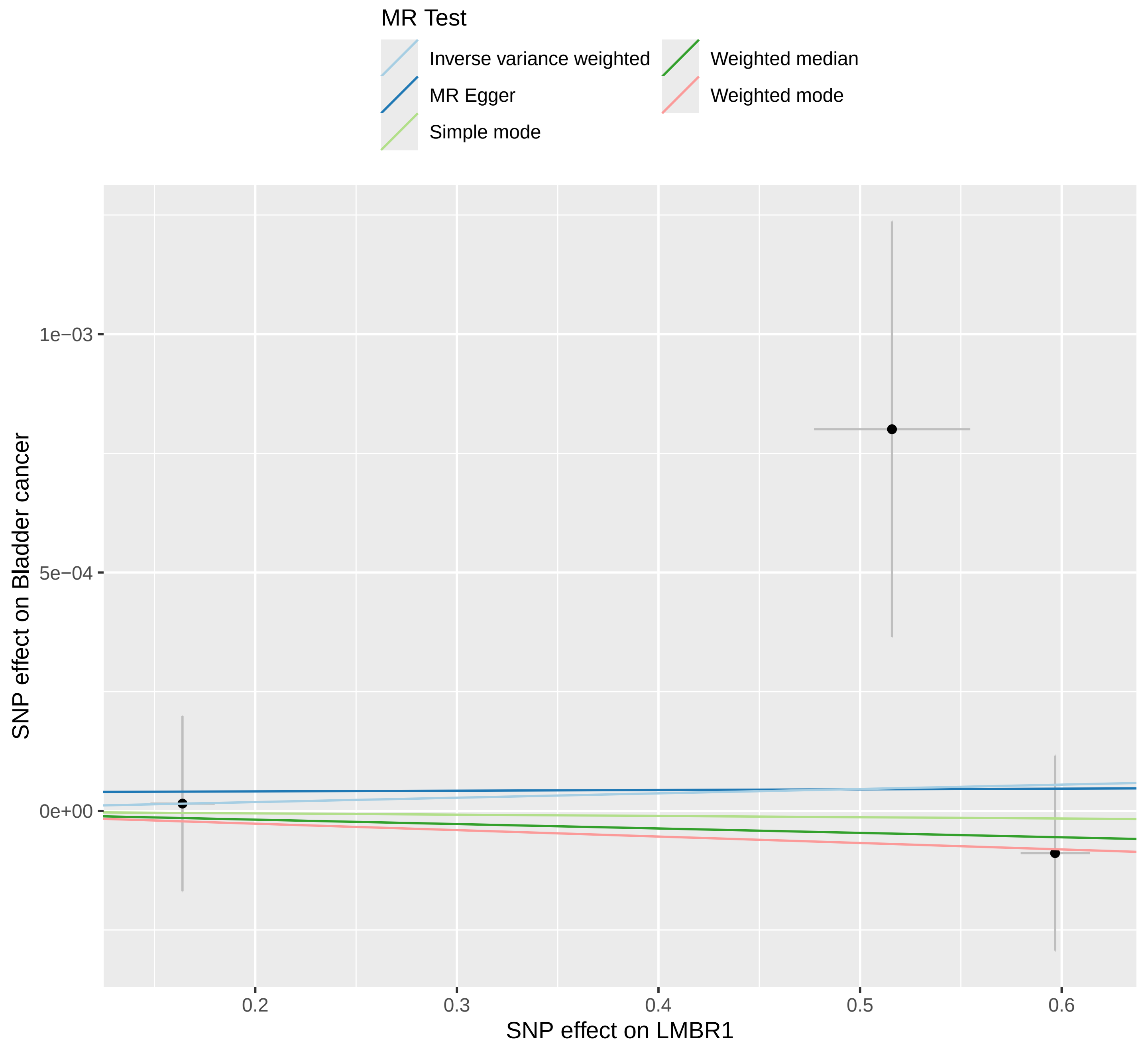

MR Test
Inverse variance weighted
MR Egger
Simple mode
Weighted median
Weighted mode
1e−03
5e−04
0e+00
0.2
0.3
0.4
0.5
0.6
SNP effect on LMBR1
SNP effect on Bladder cancer

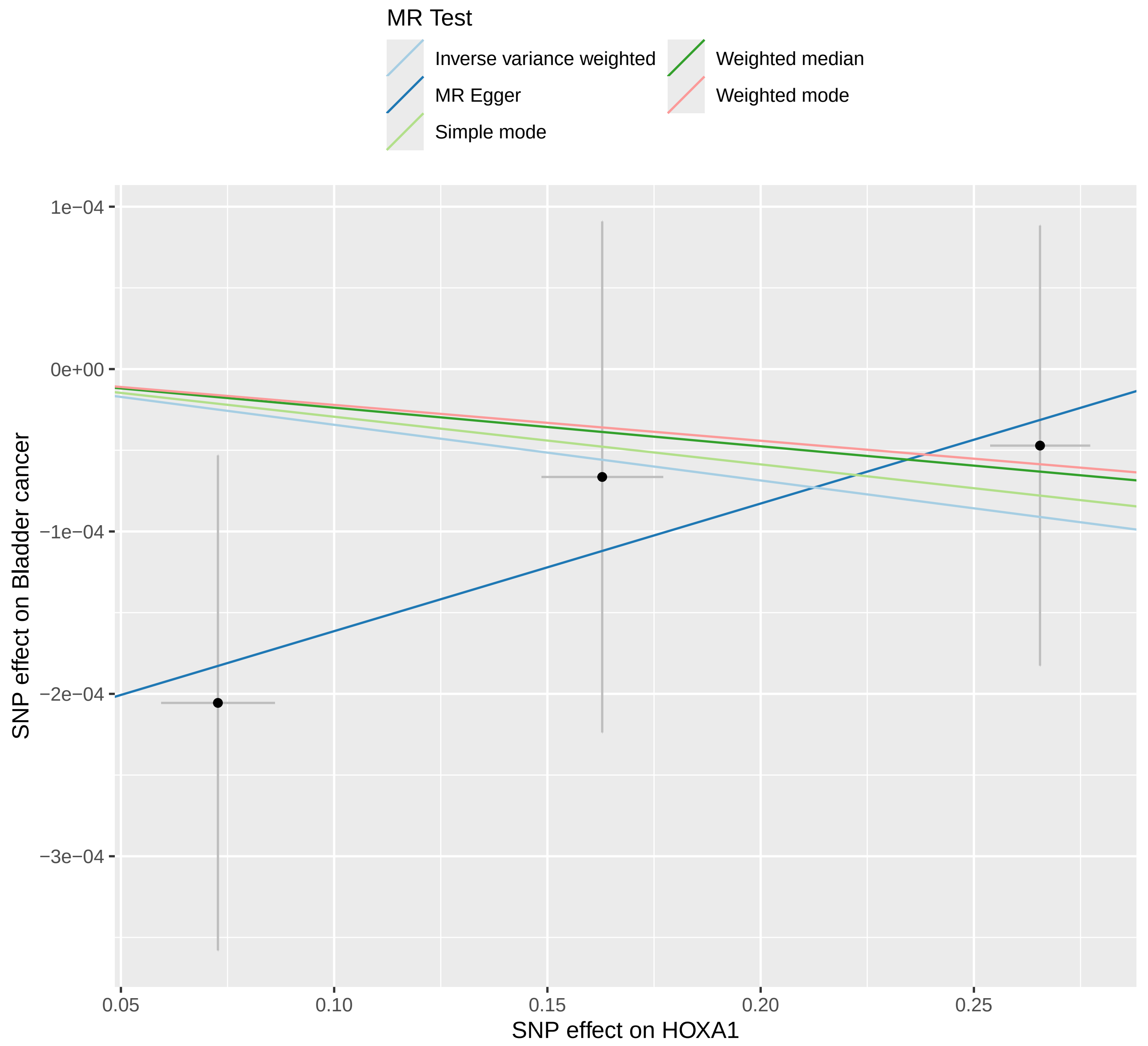

MR Test
Inverse variance weighted
MR Egger
Simple mode
Weighted median
Weighted mode
1e−04
0e+00
−1e−04
−2e−04
−3e−04
SNP effect on Bladder cancer
0.05
0.10
0.15
0.20
0.25
SNP effect on HOXA1

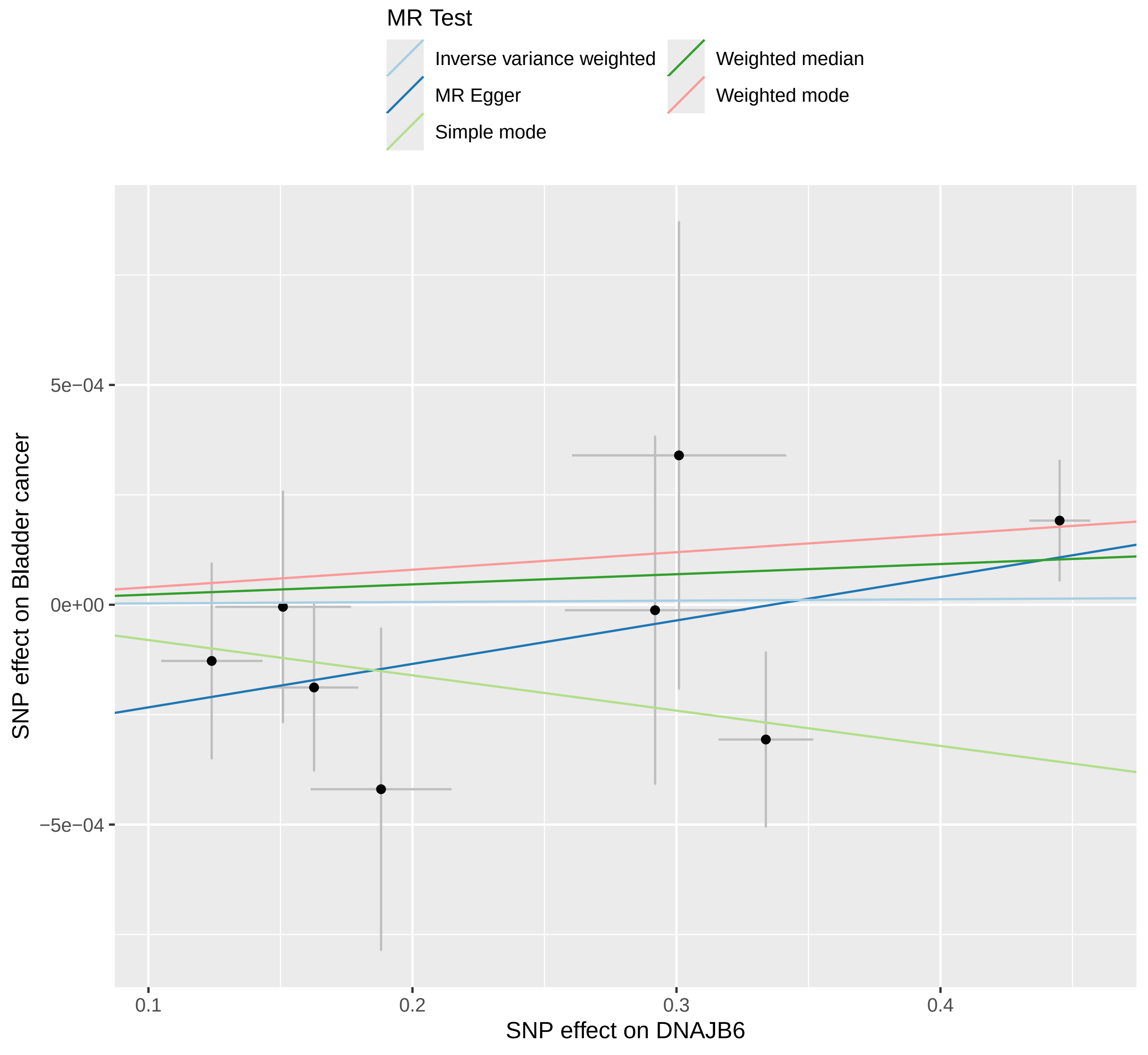

MR Test
Inverse variance weighted
MR Egger
Simple mode
Weighted median
Weighted mode
5e−04
0e+00
−5e−04
SNP effect on Bladder cancer
0.1
0.2
0.3
0.4
SNP effect on DNAJB6

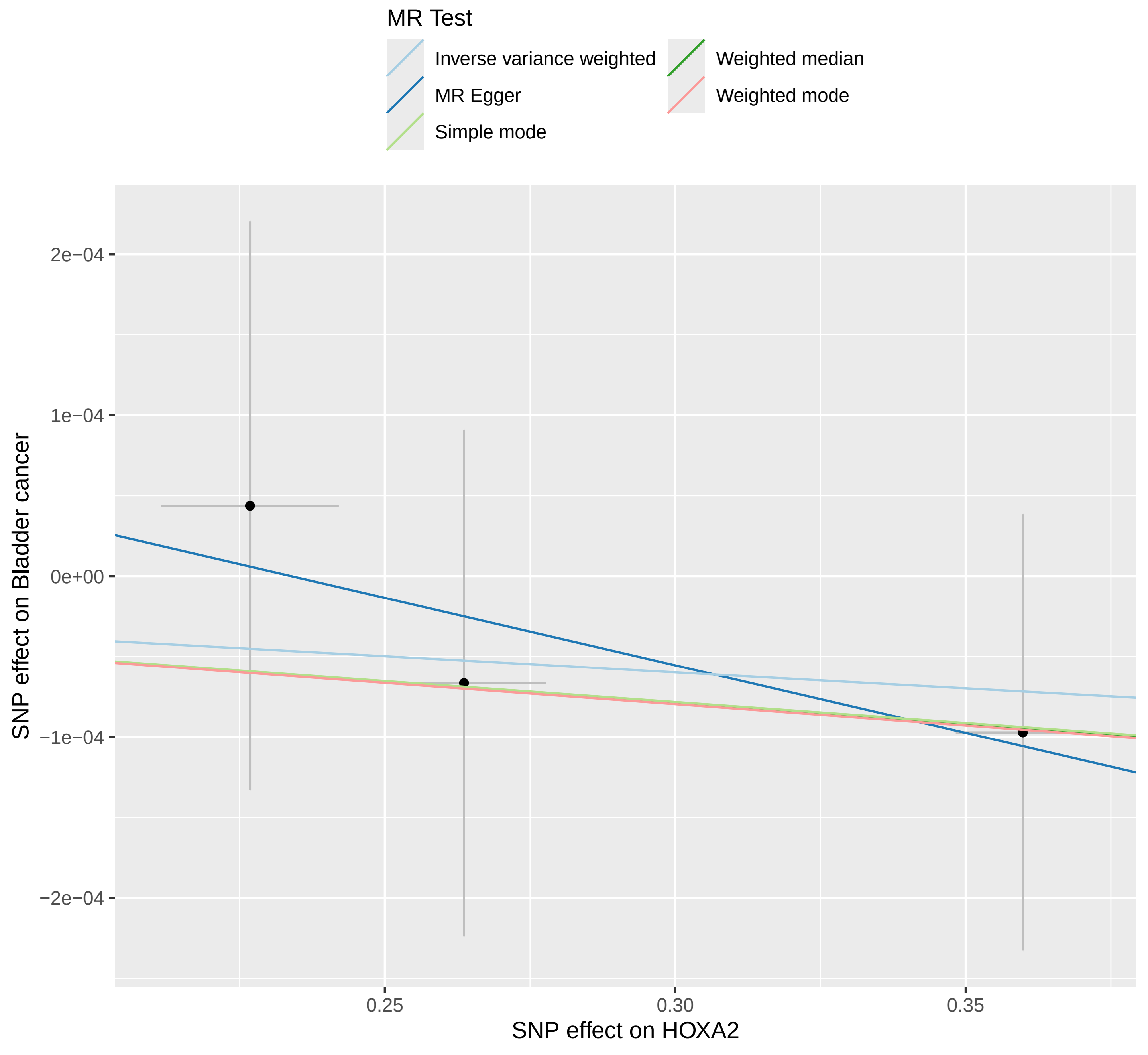
MR Test
Inverse variance weighted
MR Egger
Simple mode
Weighted median
Weighted mode
2e−04
1e−04
0e+00
−1e−04
−2e−04
SNP effect on Bladder cancer
0.25
0.30
0.35
SNP effect on HOXA2

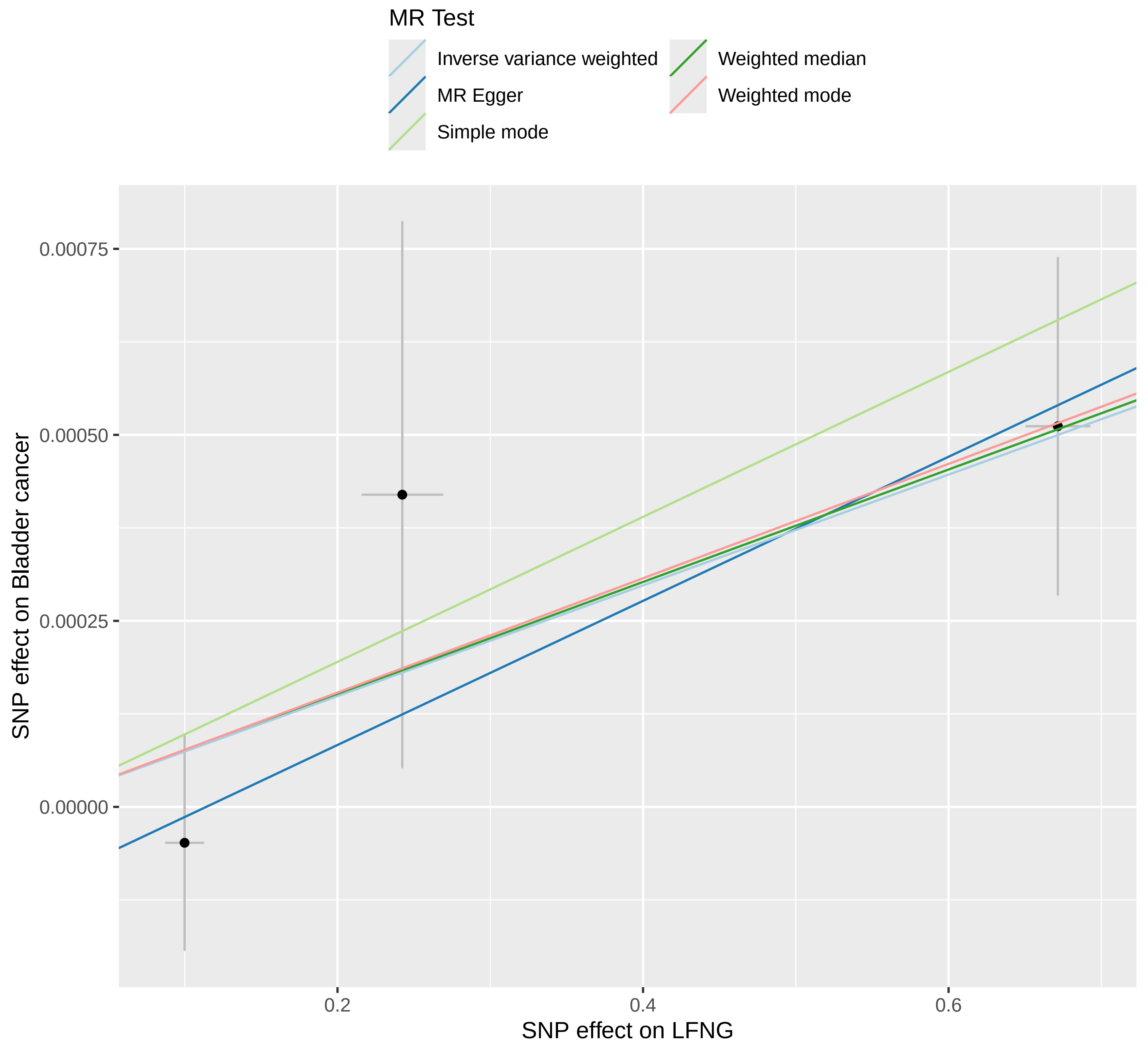
MR Test
Inverse variance weighted
MR Egger
Simple mode
Weighted median
Weighted mode
0.00075
0.00050
0.00025
0.00000
SNP effect on Bladder cancer
0.2
0.4
0.6
SNP effect on LFNG

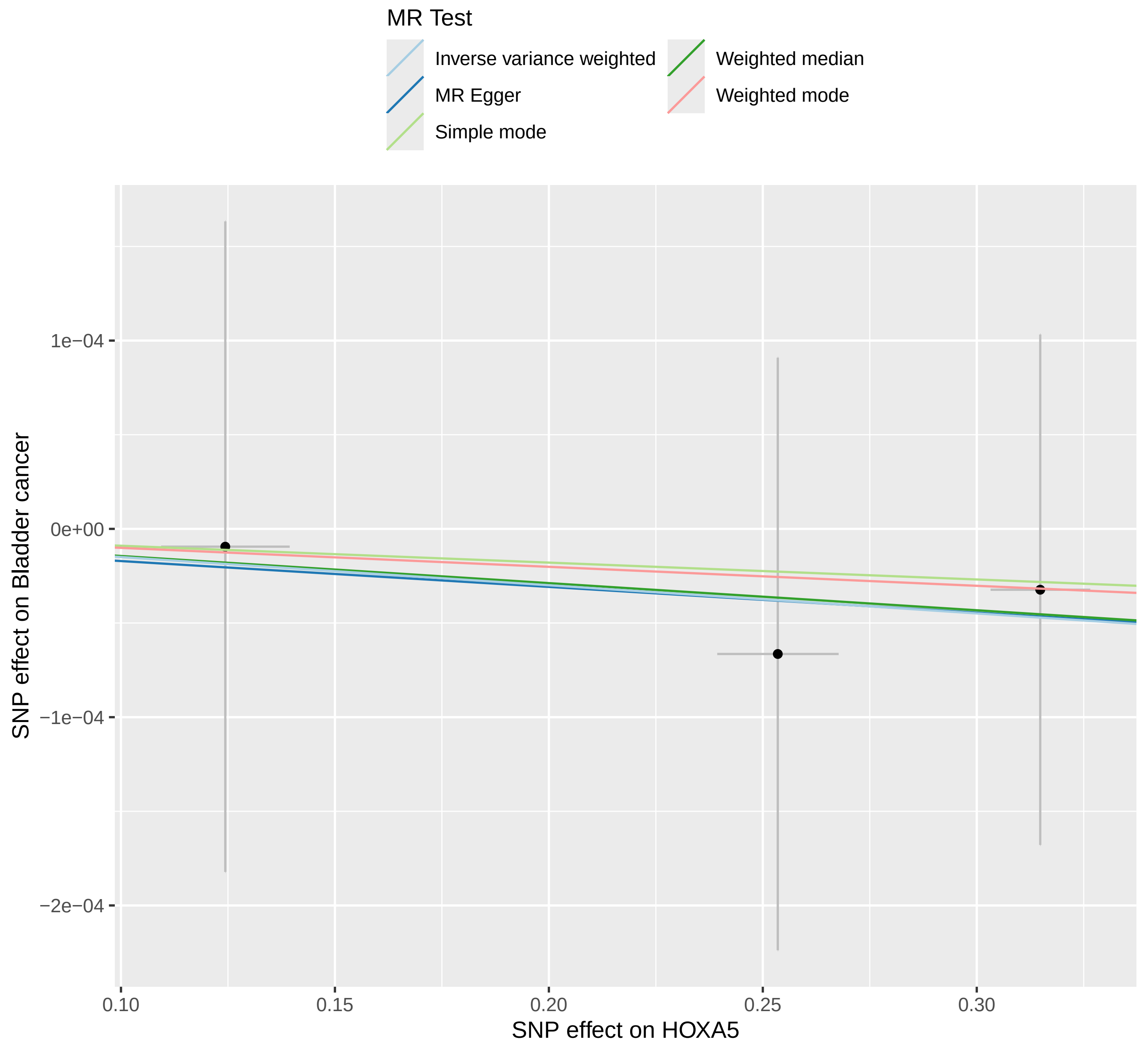
MR Test
Inverse variance weighted
MR Egger
Simple mode
Weighted median
Weighted mode
SNP effect on Bladder cancer
1e−04
0e+00
−1e−04
−2e−04
0.10
0.15
0.20
0.25
0.30
SNP effect on HOXA5

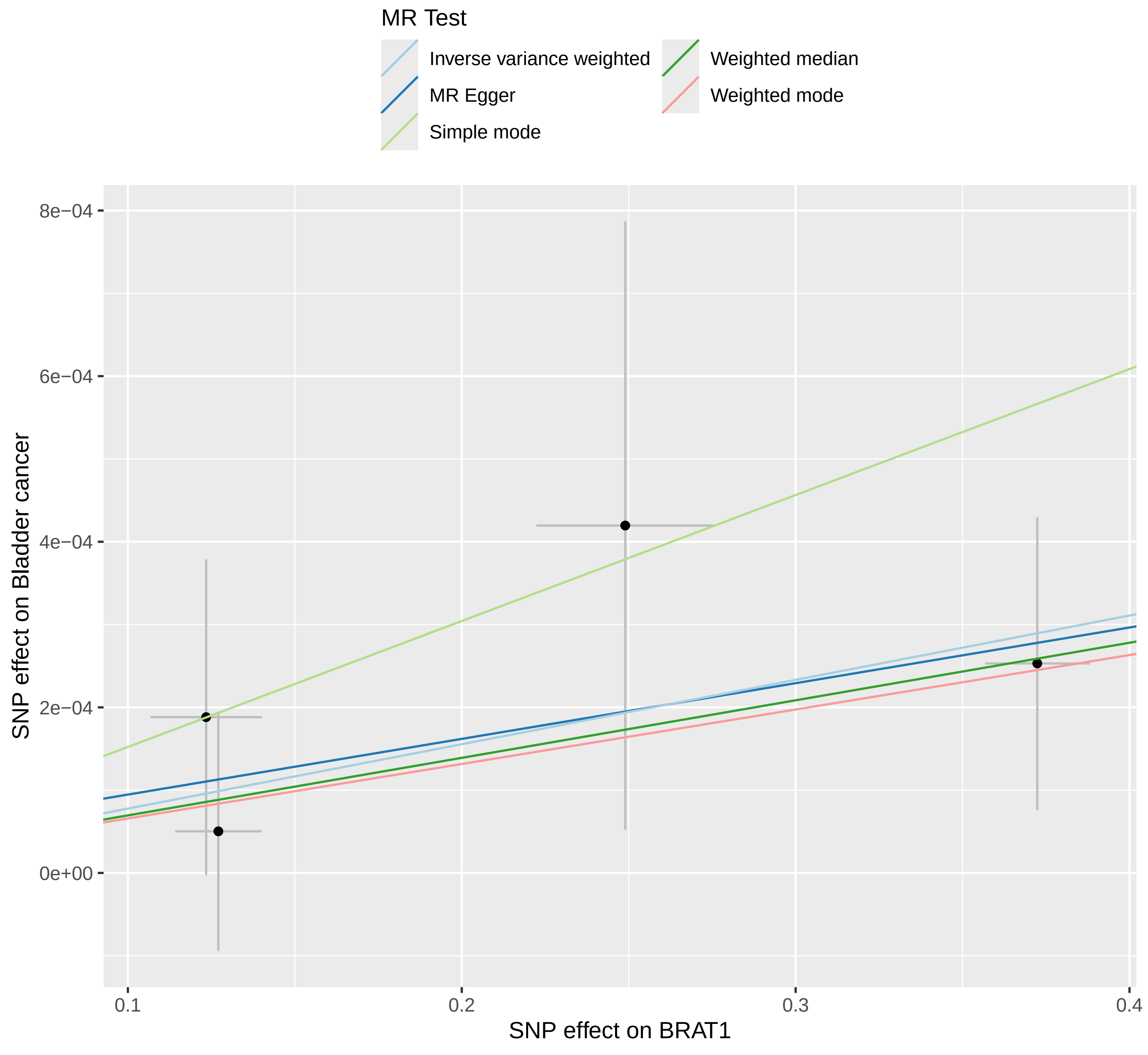
MR Test
Inverse variance weighted
MR Egger
Simple mode
Weighted median
Weighted mode
8e−04
6e−04
4e−04
2e−04
0e+00
SNP effect on Bladder cancer
0.1
0.2
0.3
0.4
SNP effect on BRAT1

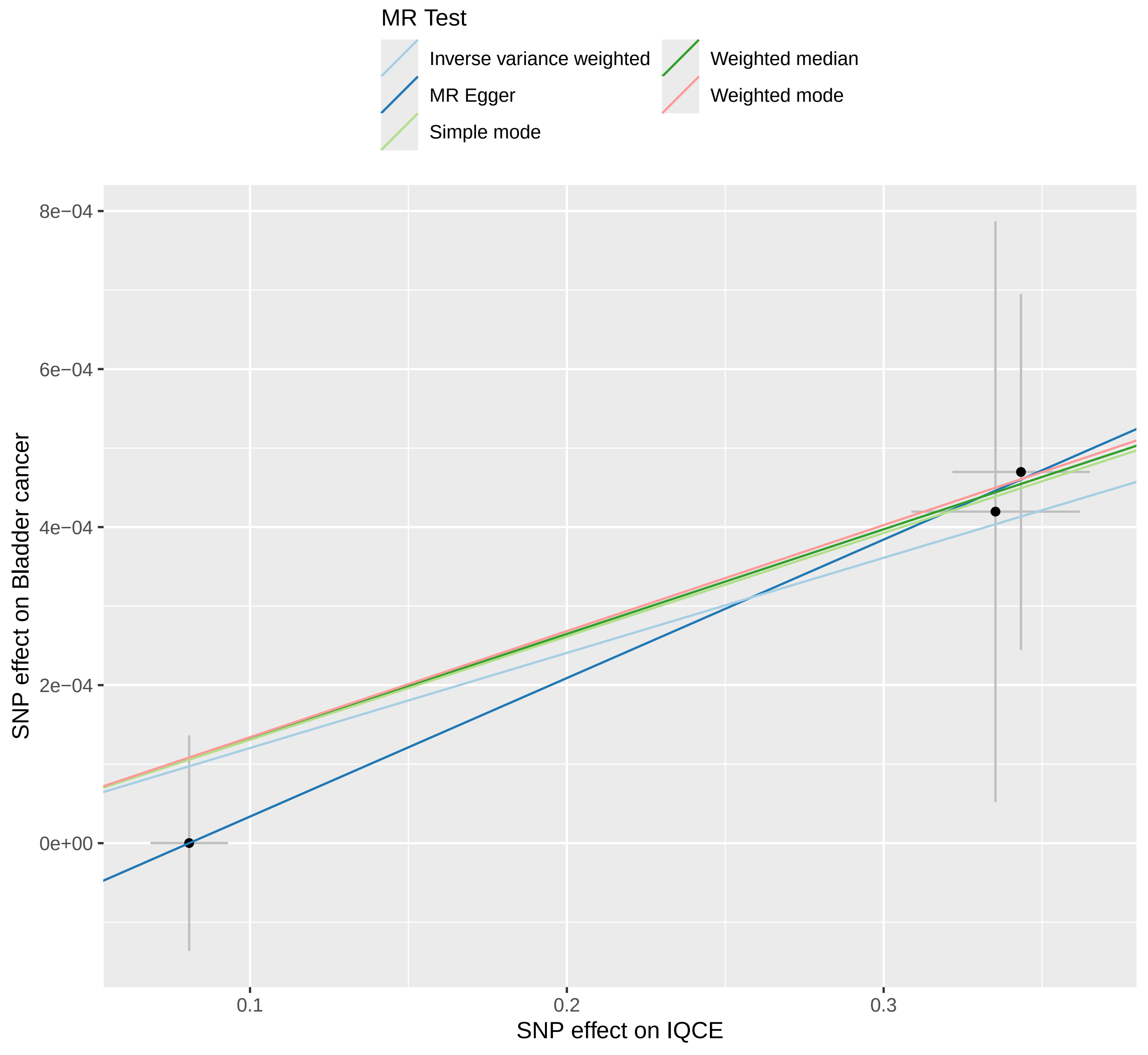

MR Test
Inverse variance weighted
MR Egger
Simple mode
Weighted median
Weighted mode
8e−04
6e−04
4e−04
2e−04
0e+00
SNP effect on Bladder cancer
0.1
0.2
0.3
SNP effect on IQCE

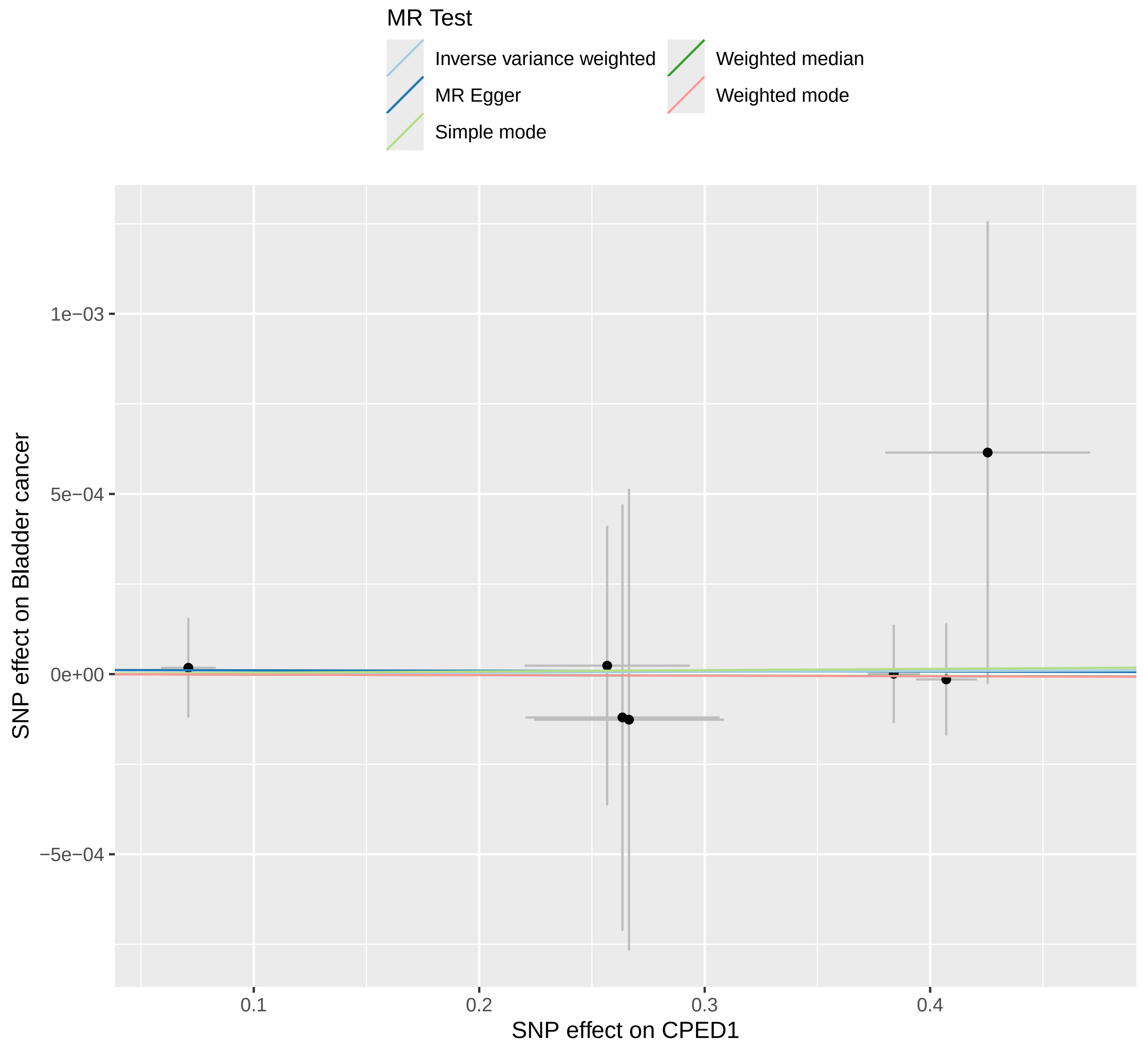

MR Test
Inverse variance weighted
MR Egger
Simple mode
Weighted median
Weighted mode
1e−03
5e−04
0e+00
−5e−04
0.1
0.2
0.3
0.4
SNP effect on CPED1
SNP effect on Bladder cancer

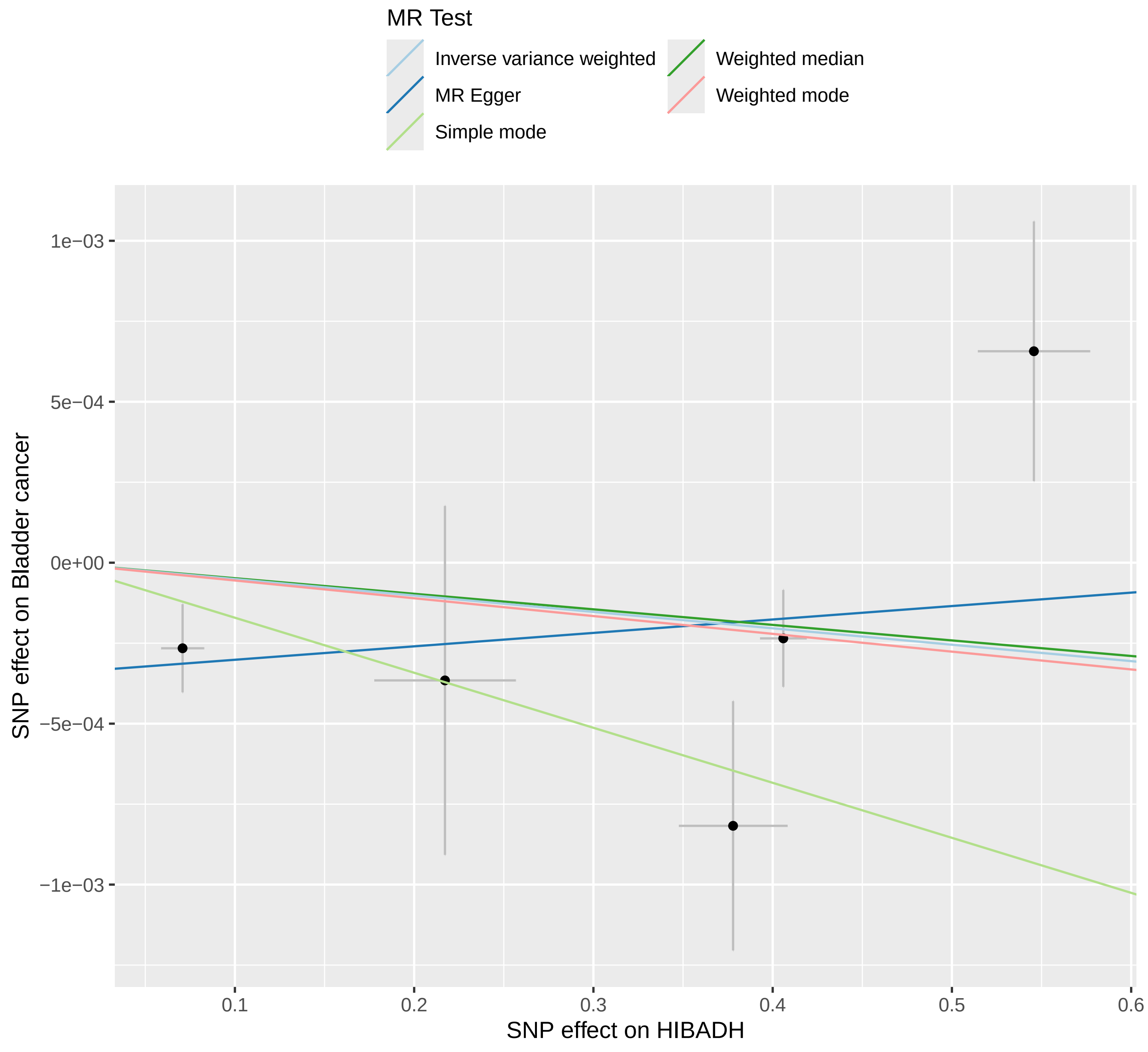
MR Test
Inverse variance weighted
MR Egger
Simple mode
Weighted median
Weighted mode
1e−03
5e−04
0e+00
−5e−04
−1e−03
SNP effect on Bladder cancer
0.1
0.2
0.3
0.4
0.5
0.6
SNP effect on HIBADH

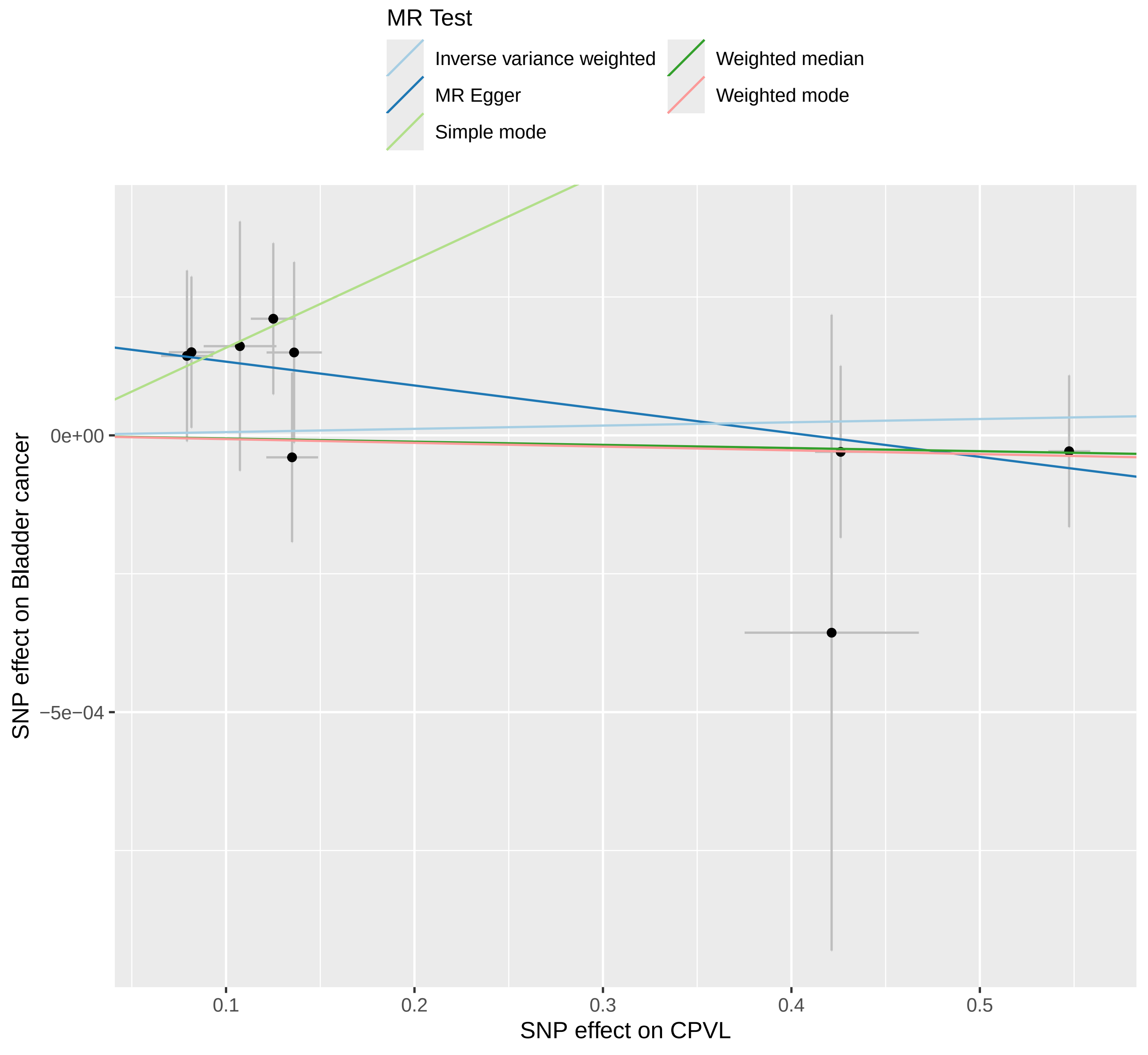
MR Test
Inverse variance weighted
MR Egger
Simple mode
Weighted median
Weighted mode
0e+00
−5e−04
0.1
0.2
0.3
0.4
0.5
SNP effect on CPVL
SNP effect on Bladder cancer

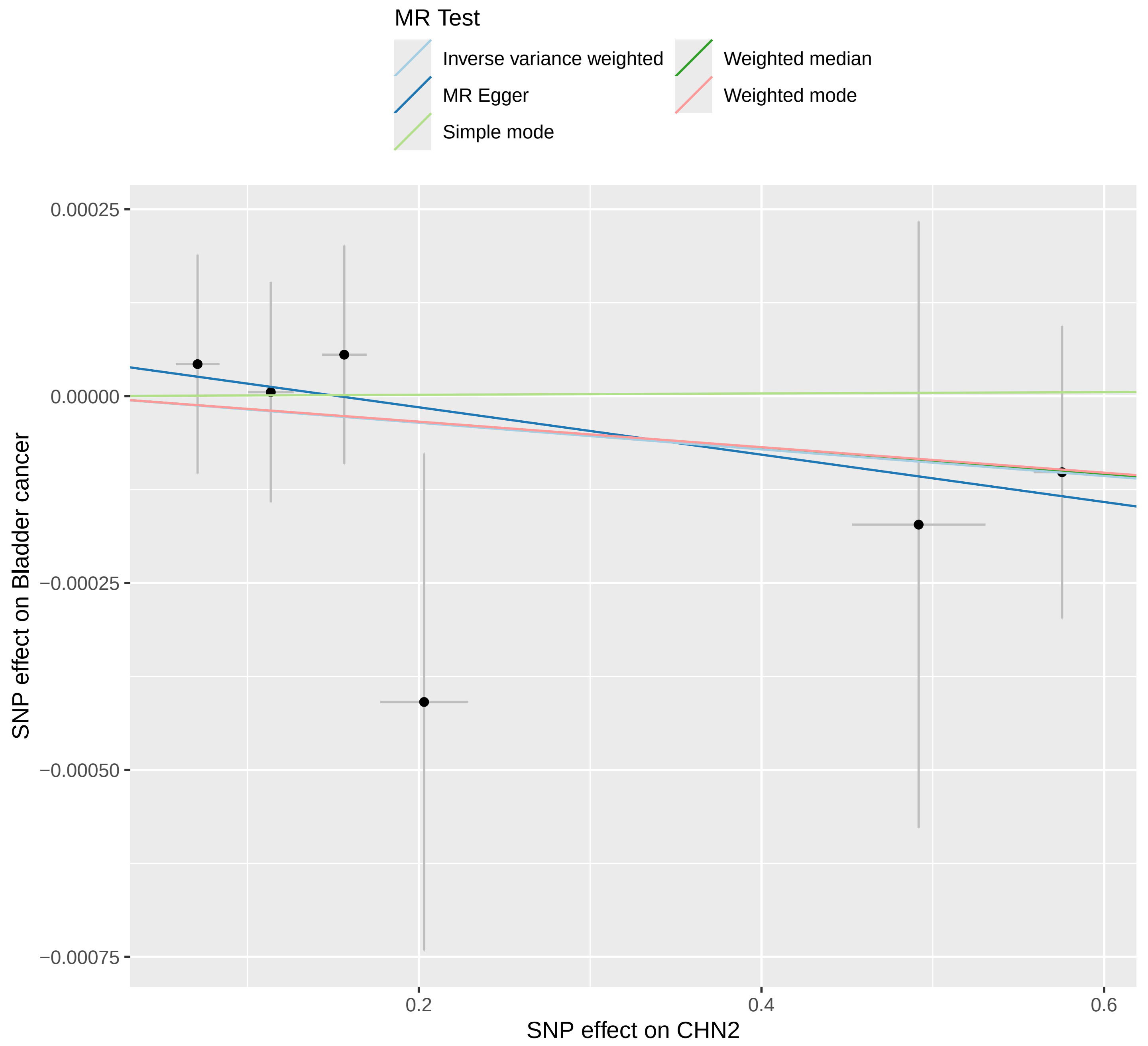

MR Test
Inverse variance weighted
MR Egger
Simple mode
Weighted median
Weighted mode
0.00025
0.00000
−0.00025
−0.00050
−0.00075
SNP effect on Bladder cancer
0.2
0.4
0.6
SNP effect on CHN2
