# Supplementary material for: Integrative multi-omics and single-cell transcriptomics reveal ARHGEF12 driving chemoresistance in bladder cancer
Source: Hereditas. 2025 Nov 27;162:234. doi: 10.1186/s41065-025-00606-1 (PMC12661753; doi:10.1186/s41065-025-00606-1)
Supplement: Supplementary file 1 — Supplementary Material 1. [file 41065_2025_606_MOESM1_ESM.zip › Supplementary1/Supplementary - MR/eQTL-MR/MRpic/SLBP.scatter_plot.pdf]

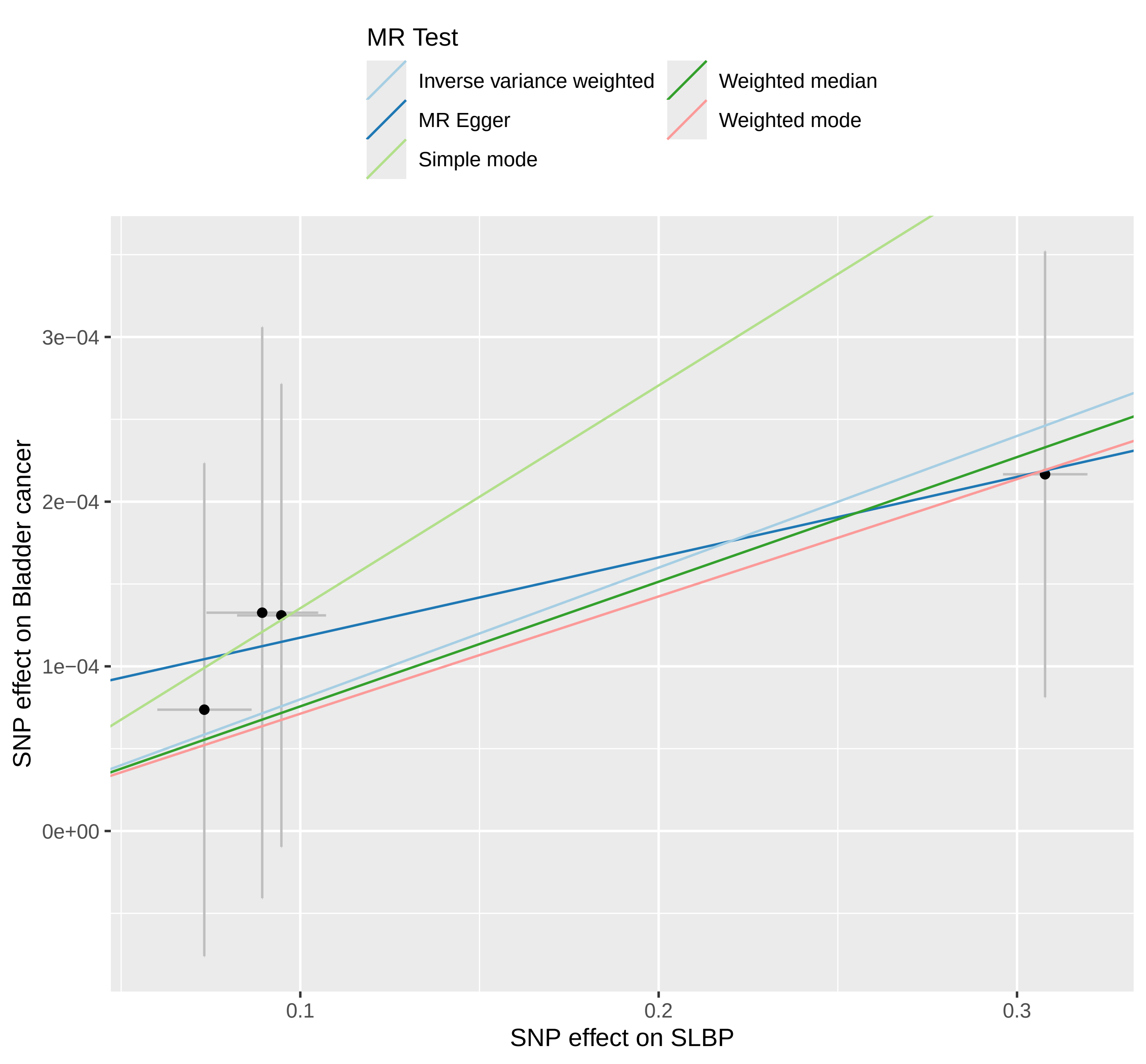
MR Test
Inverse variance weighted
MR Egger
Simple mode
Weighted median
Weighted mode
SNP effect on Bladder cancer
3e−04
2e−04
1e−04
0e+00
0.1
0.2
0.3
SNP effect on SLBP
