# Supplementary material for: Integrative multi-omics and single-cell transcriptomics reveal ARHGEF12 driving chemoresistance in bladder cancer
Source: Hereditas. 2025 Nov 27;162:234. doi: 10.1186/s41065-025-00606-1 (PMC12661753; doi:10.1186/s41065-025-00606-1)
Supplement: Supplementary file 1 — Supplementary Material 1. [file 41065_2025_606_MOESM1_ESM.zip › Supplementary1/Supplementary - MR/eQTL-MR/MRpic/SLK.forest.pdf]

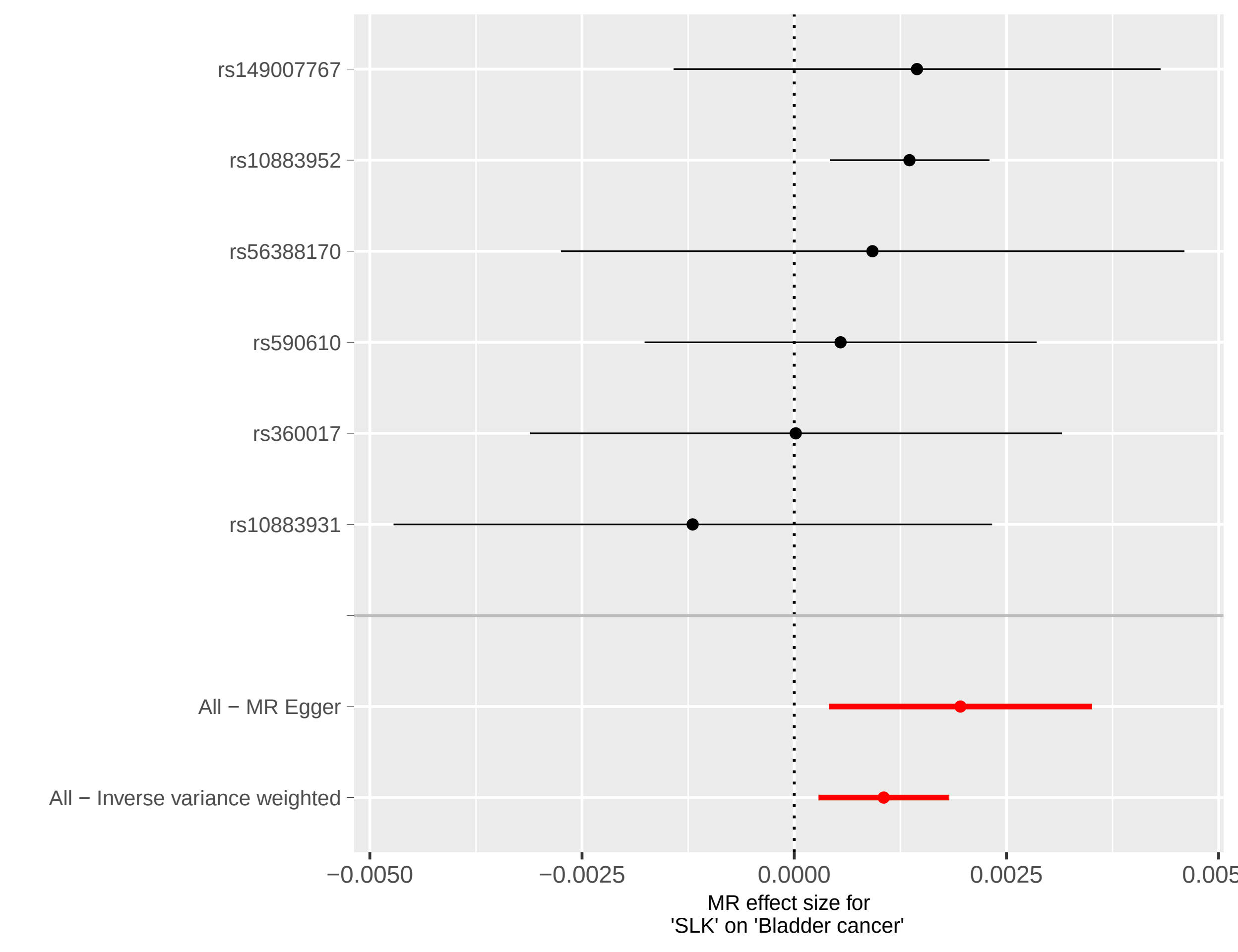
rs149007767
rs10883952
rs56388170
rs590610
rs360017
rs10883931
All − MR Egger
All − Inverse variance weighted
−0.0050
−0.0025
0.0000
0.0025
0.005
MR effect size for
'SLK' on 'Bladder cancer'
