# Supplementary material for: Integrative multi-omics and single-cell transcriptomics reveal ARHGEF12 driving chemoresistance in bladder cancer
Source: Hereditas. 2025 Nov 27;162:234. doi: 10.1186/s41065-025-00606-1 (PMC12661753; doi:10.1186/s41065-025-00606-1)
Supplement: Supplementary file 1 — Supplementary Material 1. [file 41065_2025_606_MOESM1_ESM.zip › Supplementary1/Supplementary - MR/eQTL-MR/MRpic/SPDYC.leaveoneout.pdf]

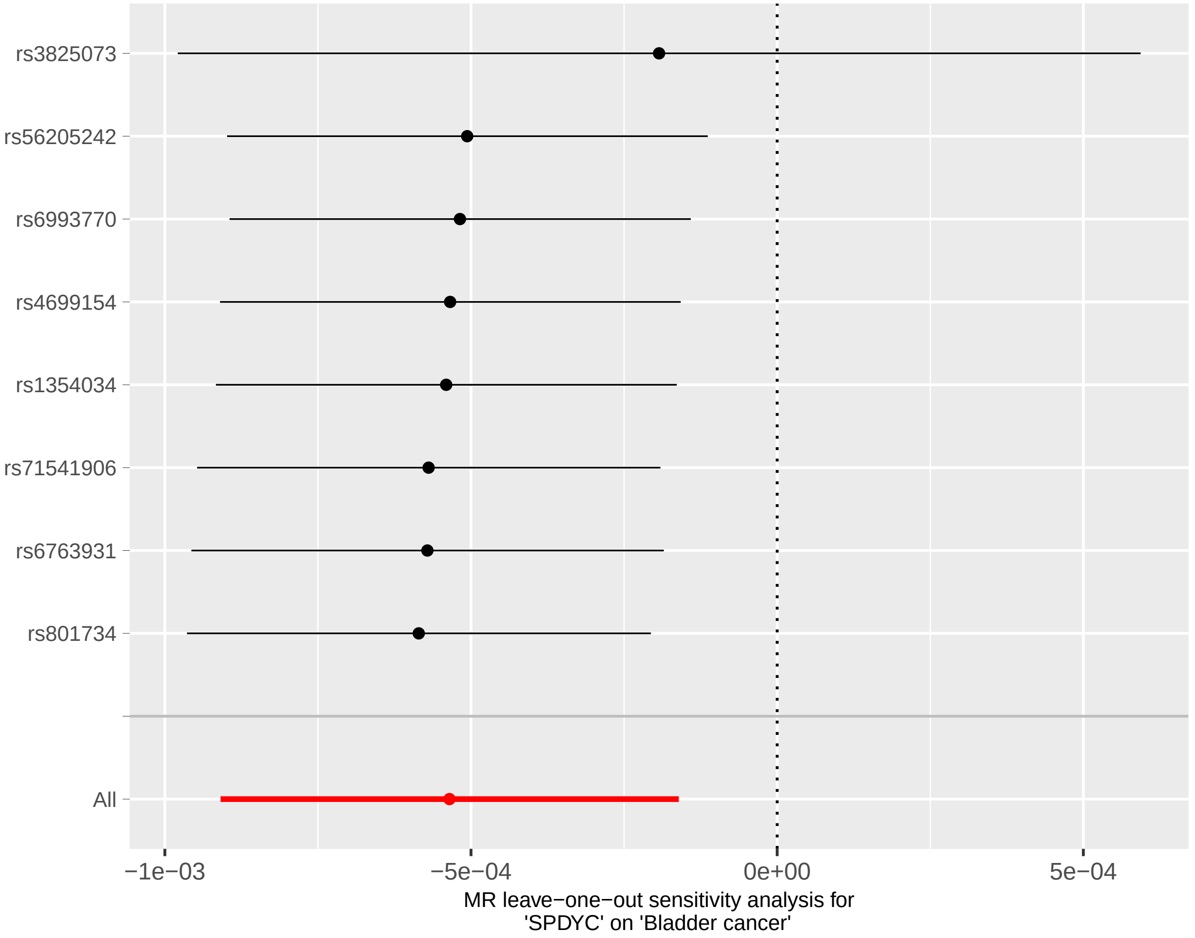
rs3825073
rs56205242
rs6993770
rs4699154
rs1354034
rs71541906
rs6763931
rs801734
All
−1e−03
−5e−04
0e+00
5e−04
MR leave−one−out sensitivity analysis for
'SPDYC' on 'Bladder cancer'
