# Supplementary material for: Integrative multi-omics and single-cell transcriptomics reveal ARHGEF12 driving chemoresistance in bladder cancer
Source: Hereditas. 2025 Nov 27;162:234. doi: 10.1186/s41065-025-00606-1 (PMC12661753; doi:10.1186/s41065-025-00606-1)
Supplement: Supplementary file 1 — Supplementary Material 1. [file 41065_2025_606_MOESM1_ESM.zip › Supplementary1/Supplementary - MR/eQTL-MR/MRpic/TBC1D9.leaveoneout.pdf]

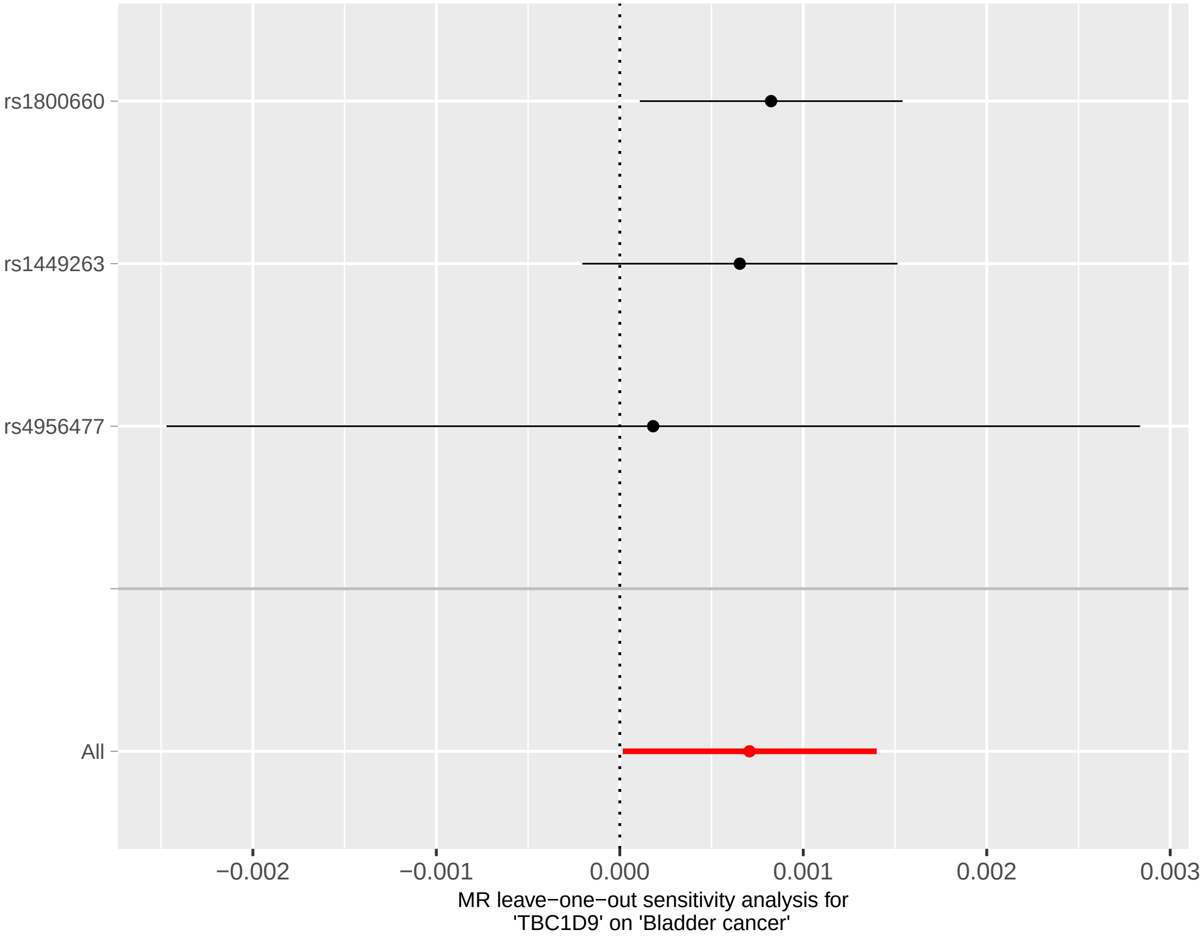

rs1800660
rs1449263
rs4956477
All
−0.002
−0.001
0.000
0.001
0.002
0.003
MR leave−one−out sensitivity analysis for
'TBC1D9' on 'Bladder cancer'
