# Supplementary material for: Integrative multi-omics and single-cell transcriptomics reveal ARHGEF12 driving chemoresistance in bladder cancer
Source: Hereditas. 2025 Nov 27;162:234. doi: 10.1186/s41065-025-00606-1 (PMC12661753; doi:10.1186/s41065-025-00606-1)
Supplement: Supplementary file 1 — Supplementary Material 1. [file 41065_2025_606_MOESM1_ESM.zip › Supplementary1/Supplementary - MR/eQTL-MR/MRpic/TC2N.leaveoneout.pdf]

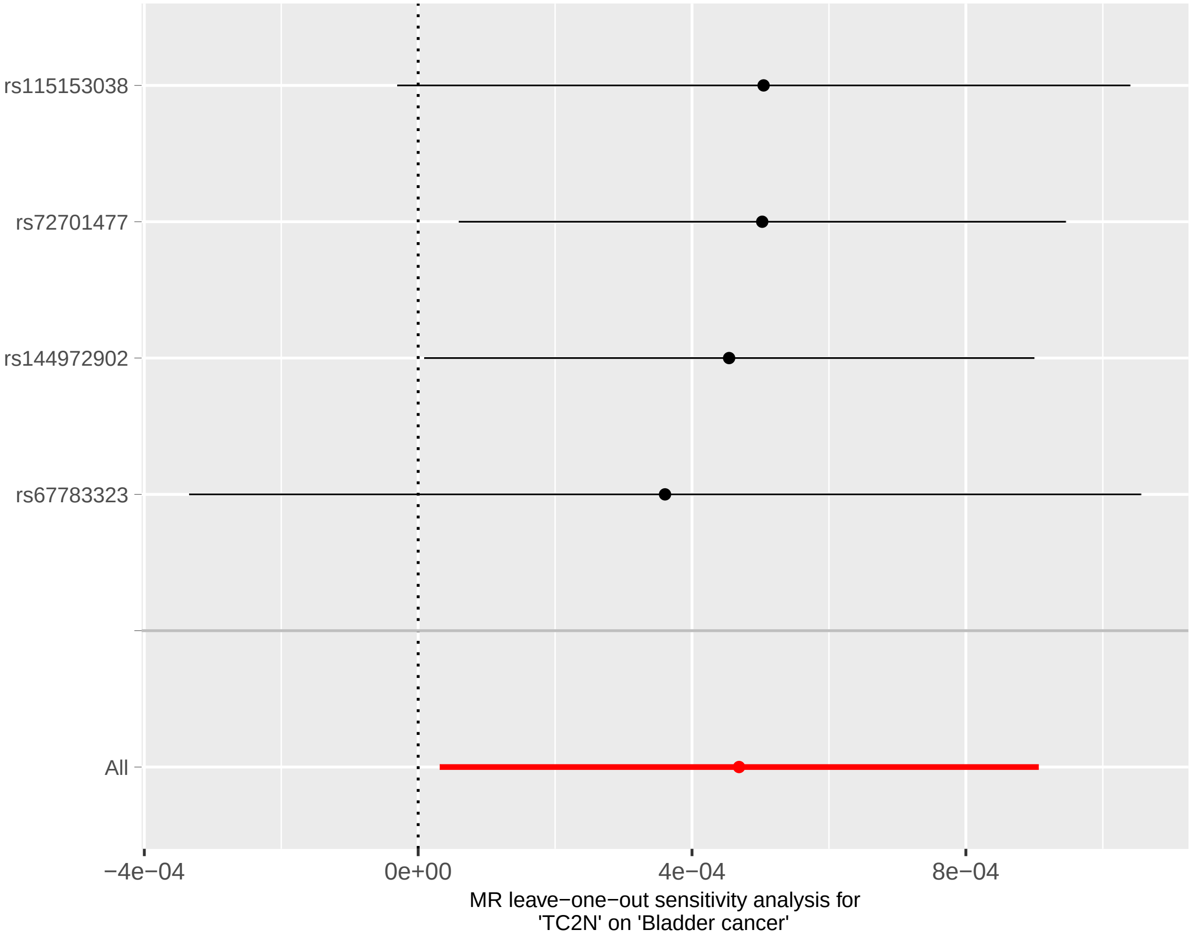
rs115153038
rs72701477
rs144972902
rs67783323
All
−4e−04
0e+00
4e−04
8e−04
MR leave−one−out sensitivity analysis for
'TC2N' on 'Bladder cancer'
