# Supplementary material for: Integrative multi-omics and single-cell transcriptomics reveal ARHGEF12 driving chemoresistance in bladder cancer
Source: Hereditas. 2025 Nov 27;162:234. doi: 10.1186/s41065-025-00606-1 (PMC12661753; doi:10.1186/s41065-025-00606-1)

Fig. 6. The Role of ARHGEF12 in Bladder Cancer Drug Resistance Development.

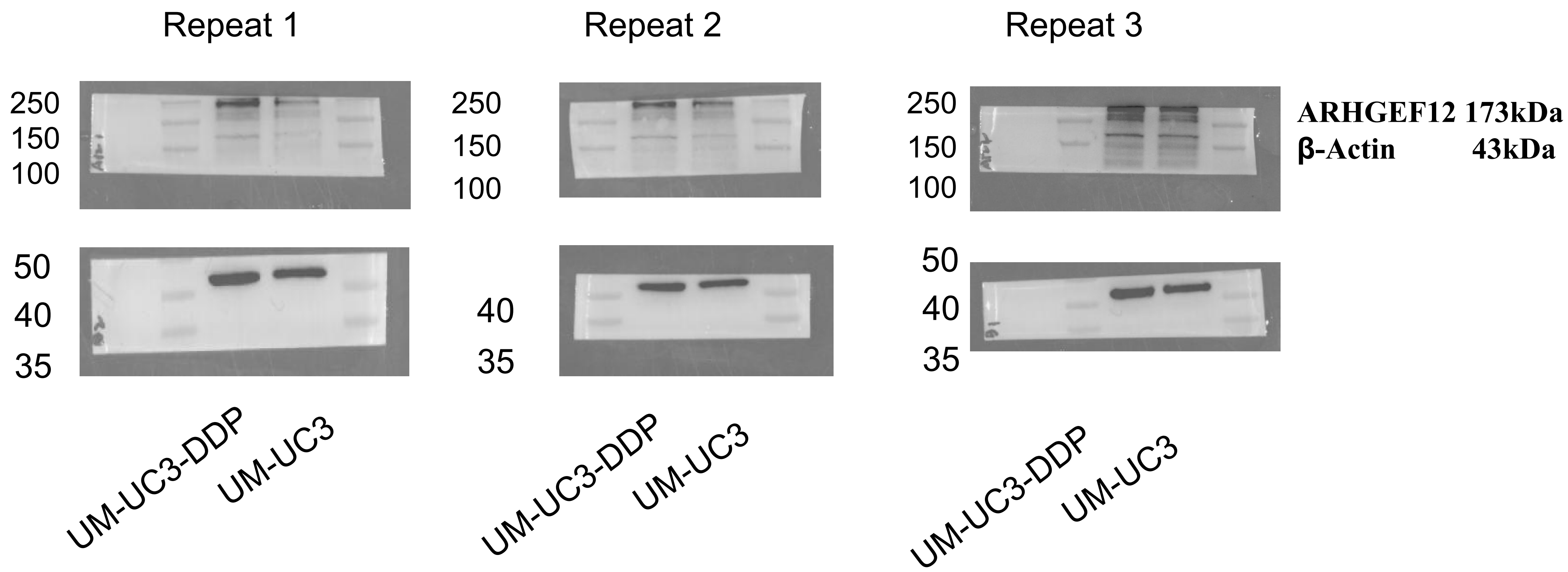

Fig. 6. The Role of ARHGEF12 in Bladder Cancer Drug Resistance Development.

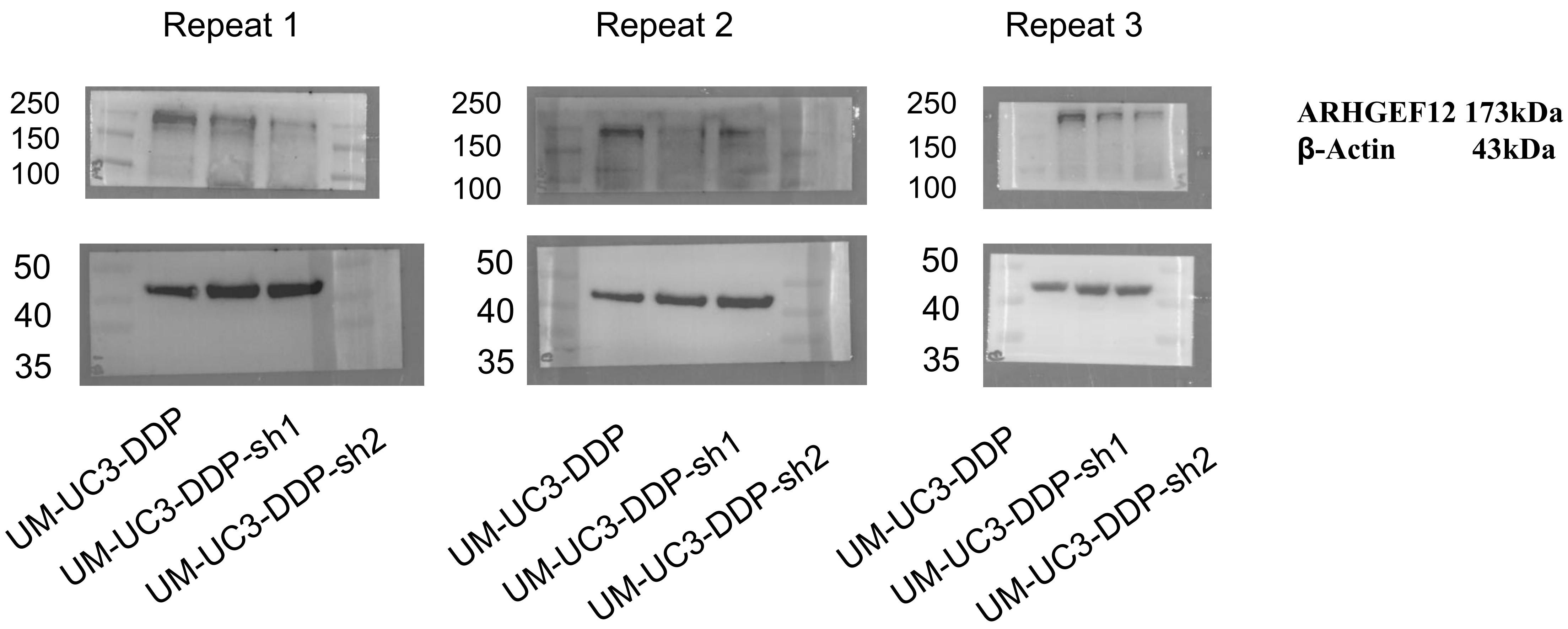

Fig.7. ARHGEF12 promotes cisplatin resistance in BLCA via RhoA/ROCK-mediated activation of the PI3K/Akt pathway.

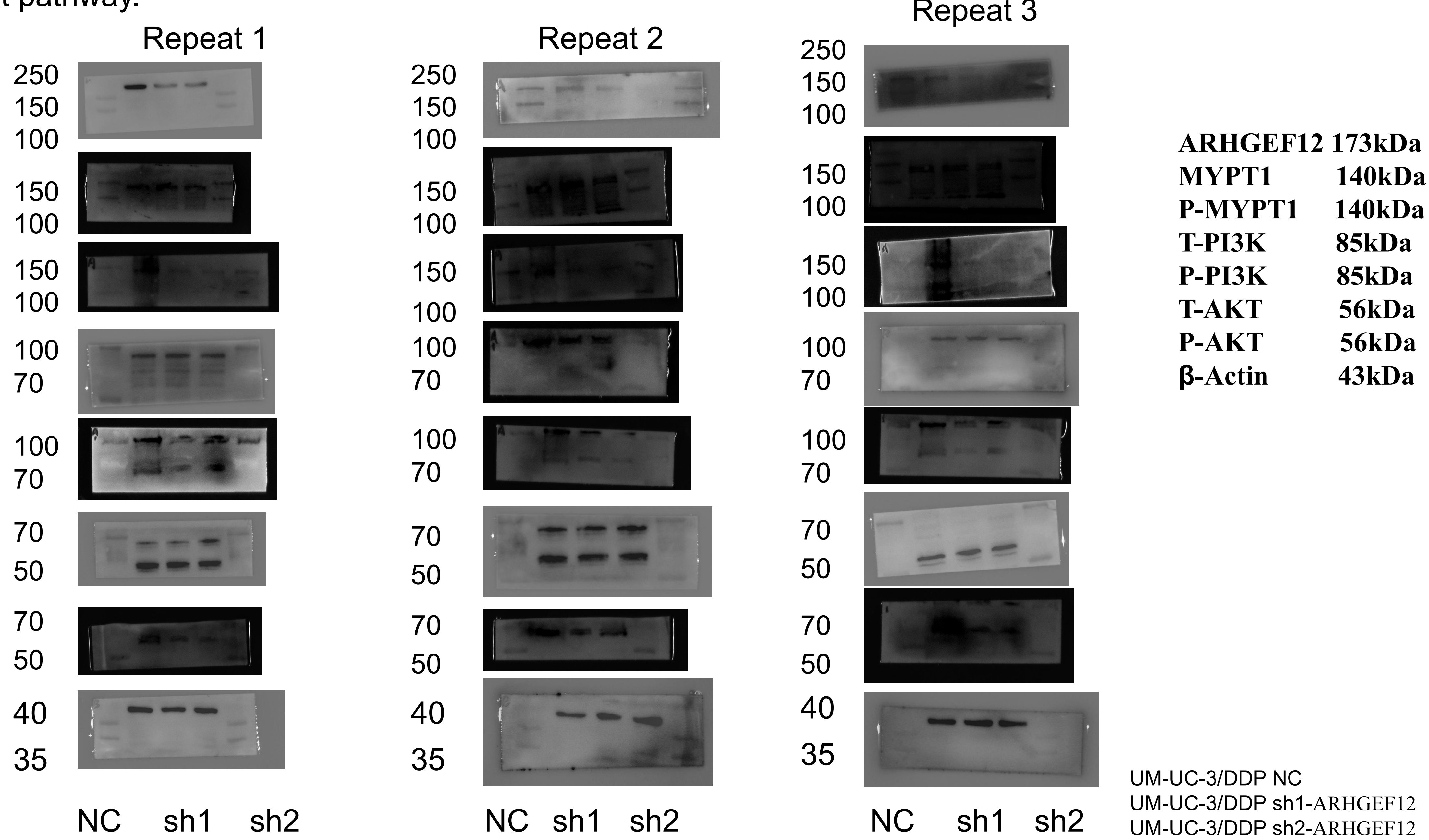

Fig.7. ARHGEF12 promotes cisplatin resistance in BLCA via RhoA/ROCK-mediated activation of the PI3K/Akt pathway.

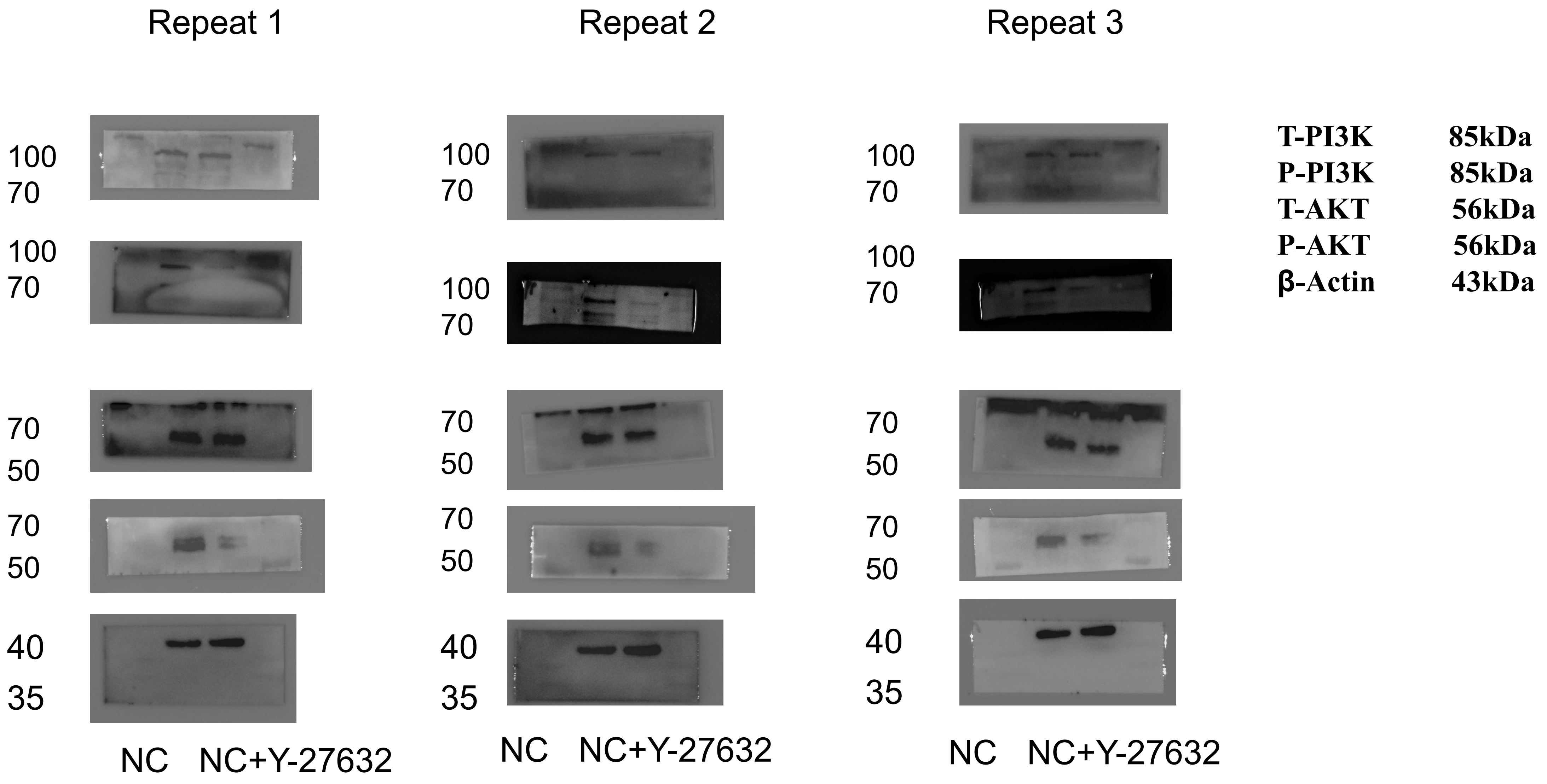

Supplement: Supplementary file 1 — Supplementary Material 1. [file 41065_2025_606_MOESM1_ESM.zip › Supplementary1/Supplementary_WB/wb.pdf]
