# Supplementary material for: Integrative multi-omics and single-cell transcriptomics reveal ARHGEF12 driving chemoresistance in bladder cancer
Source: Hereditas. 2025 Nov 27;162:234. doi: 10.1186/s41065-025-00606-1 (PMC12661753; doi:10.1186/s41065-025-00606-1)
Supplement: Supplementary file 1 — Supplementary Material 1. [file 41065_2025_606_MOESM1_ESM.zip › Supplementary/supplementary.docx]

1. ***ARHGEF12*-Related Immune Infiltration and Somatic Mutations**

GSEA analysis showed that *ARHGEF12* is enriched in multiple immune-related pathways, pointing to a possible role in immune regulation that warrants further investigation. To explore this, we divided BLCA samples based on the median *ARHGEF12* expression and applied the CIBERSORT algorithm to TCGA-BLCA transcriptomic data. This allowed us to assess immune cell infiltration levels and determine how *ARHGEF12* expression correlates with the distribution of 22 immune cell subtypes in BLCA tissues. The analysis revealed that high *ARHGEF12* expression is significantly associated with increased infiltration of resting mast cells (Fig. S1A). Correlation analysis between *ARHGEF12* expression and immune cell types showed positive associations with resting CD4 memory T cells and resting mast cells, while a negative association was observed with memory B cells (Fig. S1B–E). Given the relevance of immunotherapy in BLCA, we also analyzed the relationship between *ARHGEF12* expression and immune checkpoint molecules. As shown in Fig. S1F–G, *ARHGEF12* expression correlated with several immune regulatory targets: *TNFSF15* (r = 0.30, *p* = 6.47e-10), *CD276* (r = 0.187, *p* < 0.001), *TNFRSF4* (r = -0.276, *p* = 1.40e-08), *NRP1* (r = 0.241, *p* = 8.71e-07), *CD160* (r = 0.327, *p* = 1.25e-11), and *LAG3* (r = -0.169, *p* < 0.001). These results suggest that *ARHGEF12* expression may influence immune microenvironment composition and could be relevant to immunotherapeutic strategies in BLCA.

In a cohort of 407 BLCA samples, we identified somatic mutations associated with *ARHGEF12* expression levels. The waterfall plot (Fig. S1H) shows that 288 patients (70.8%) harbored somatic mutations. The top 15 mutated genes and their mutation types are marked in distinct colors. Notably, patients in the high *ARHGEF12* expression group had a significantly higher mutation frequency in the top 10 BLCA-associated genes, including *TP53*, *RB1*, *FLG*, *DST*, *PCNT*, *VPS13B*, *NRXN1*, *CMYA5*, *PEG3*, and *SF3B1*, compared to those in the low-expression group.

**Fig. S1.** *ARHGEF12-Associated Immune Infiltration and Somatic Mutations.***(A)** Box plot illustrates the differences in immune cell infiltration abundance between the high-expression and low-expression *ARHGEF12* groups. **(B)** Lollipop chart depicts the correlation between *ARHGEF12* expression and immune cell subtypes.**(C-E)** *ARHGEF12* expression is positively correlated with T cells CD4 memory resting and Mast cells resting, while it is negatively correlated with memory B cells**(F-G)** Correlation between *ARHGEF12* expression and immune checkpoint genes.**(H)** *ARHGEF12*-related somatic mutations.
